# A Complex Gene Network Mediated by Ethylene Signal Transduction TFs Defines the Flower Induction and Differentiation in *Olea europaea* L.

**DOI:** 10.3390/genes12040545

**Published:** 2021-04-09

**Authors:** Amelia Salimonti, Ivano Forgione, Tiziana Maria Sirangelo, Guglielmo Puccio, Antonio Mauceri, Francesco Mercati, Francesco Sunseri, Fabrizio Carbone

**Affiliations:** 1 Research Centre for Olive, Citrus and Tree Fruit, Council for Agricultural Research and Economics (CREA), 87036 Rende, Italy; amelia.salimonti@crea.gov.it (A.S.); ivanoforgione@gmail.com (I.F.); tizianasirangelo@gmail.com (T.M.S.); 2 Department of Agricultural, Food and Forest Sciences, University of Palermo, 90133 Palermo, Italy; gugpuccio@gmail.com; 3 Department of Agriculture, University Mediterranea of Reggio Calabria, 89124 Reggio Calabria, Italy; antonio.mauceri87@gmail.com (A.M.); francesco.sunseri@unirc.it (F.S.); 4 Institute of Biosciences and BioResources (IBBR), National Research Council of Italy (CNR), 90129 Palermo, Italy; francesco.mercati@ibbr.cnr.it

**Keywords:** *Olea europaea*, flowering, alternate bearing, NGS, lateral bud, transcriptome profiling

## Abstract

The olive tree (*Olea europaea* L.) is a typical Mediterranean crop, important for olive and oil production. The high tendency to bear fruits in an uneven manner, defined as irregular or alternate bearing, results in a significant economic impact for the high losses in olives and oil production. Buds from heavy loaded (‘ON’) and unloaded (‘OFF’) branches of a unique olive tree were collected in July and the next March to compare the transcriptomic profiles and get deep insight into the molecular mechanisms regulating floral induction and differentiation. A wide set of DEGs related to ethylene TFs and to hormonal, sugar, and phenylpropanoid pathways was identified in buds collected from ‘OFF’ branches. These genes could directly and indirectly modulate different pathways, suggesting their key role during the lateral bud transition to flowering stage. Interestingly, several genes related to the flowering process appeared as over-expressed in buds from March ‘OFF’ branches and they could address the buds towards flower differentiation. By this approach, interesting candidate genes related to the switch from vegetative to reproductive stages were detected and analyzed. The functional analysis of these genes will provide tools for developing breeding programs to obtain olive trees characterized by more constant productivity over the years.

## 1. Introduction

The olive tree (*O. europaea* L.), which belongs to *Oleaceae*, is an evergreen plant native and is largely cultivated in the Mediterranean Basin. The cultivated forms have been introduced into many areas worldwide [1] as they are one of the most economically important fruit crops and there are many nutritional and health benefits of olive fruits and the derived oil. In addition to these agricultural and dietary qualities, the olive tree frequently exhibits a high tendency to bear fruits in an uneven manner, resulting in a significant economic impact, as oil olives are an industry-dependent commodity [2,3,4]. Alternate bearing also reported as biennial or “irregular bearing” is widespread in many fruit trees such as pistachio, apple, citrus, olive, and mango [5]. Alternate bearing is mainly related to the main stages of fruiting, as flower bud differentiation, fruit set and abscission, and growth. Thus, this syndrome is firstly triggered by a first-year intensity of blooming of heavy yield (ON-year) followed by hardly any flowering and light yield in second year (OFF-year) [5,6]. Alternate bearing determines harvest yield variation in olive tree depending on genetic and physiological factors, as well as environment influence. To decrease the amount of yield variation, several modifications in agronomic practices are being applied [3]. Three main factors involved in the alternate bearing phenomenon have been proposed: the flowering-site limitation, with competition between vegetative and reproductive organs proposed to influence the periodicity [7]; the level of endogenous plant growth regulators, since differences in certain hormones have been reported, with balances between these hormones being considered as key regulators of the alternate bearing [8]; and finally, the carbohydrate storage control, it being observed that the storage of nutrients during the “OFF” year is used for reproductive growth the following year [7,9]. Thus, the research has been focused firstly on the early reproductive process, which is under the tight control of a complex genetic network [10]. Several studies on physiological (e.g., [11]), molecular systematic [12,13], and molecular genetics/genomics [14,15] aspects of olive have been reported. Further genetic studies involving the molecular mechanism of fruit set, fruit development, fruit detachment, and alternate bearing in olive have not been widely reported though there are reports on various aspects of alternate bearing such as endogenous and environmental factors [1,16,17]. To date there are few studies about the complex gene network that would be activated during the transition to flower buds in fruit trees [18,19,20]. Nowadays, the next-generation sequencing (NGS) and other high-throughput sequencing approaches present intriguing chances for life sciences, by improving the efficiency and speed of gene discovery [21,22]. Nowadays, the sequencing of transcriptome using NGS (RNAseq) is one of the most widespread approaches able to offer relevant information on the putative function of genes expressed in the fruit and of potential relevance in regulating metabolic pathways deputed to its development and maturation in olive tree [15,23,24]. Further, the sequencing, assembly, and annotation of olive genome should provide a valuable resource for new insight into the genetic control of physiological and developmental processes and pivotal phenotypic complex traits of olive tree [25,26,27]. Very recently, a differential transcriptomic analysis between ‘ON’ and ‘OFF’ buds and leaves revealed the involvement of the metabolism of carbohydrates, polyamines, phytohormones, and polyphenol oxidase correlated to antioxidant system in the alternate bearing in olive tree [28]. Here, we lead to identify differential expressed genes during the switch from the vegetative to the reproductive phase of the lateral buds in the olive cv. Leccino. The aim of our study is to highlight the differences in transcripts abundance between buds collected from heavy loaded branches (‘ON’) and unloaded branches (‘OFF’), in both July and March. A wide set of over-expressed DEGs related to hormonal, sugar, and phenylpropanoid pathways was identified in buds collected from ‘OFF’ branches. More interestingly, ethylene-related transcription factors (TF) seem to act as triggers for the flower meristem fate in the lateral buds in July. Afterwards, during the flower differentiation in March, a genes network appeared over-expressed including TFs of the constans gene family, previously reported to be linked to flower biology, as well as N-related genes of the *NRT1/PTR* family directly or indirectly involved in buds switch from vegetative to reproductive stage.

## 2. Materials and Methods

### 2.1. Plant Material and Sample Collection

Plant material was collected from an olive (*O. europaea* L. subsp. *europaea* var. *europaea*) tree, cultivar Leccino, grown in open field, in an olive grove planted in the early 90s, at the experimental farm of the CREA-Research Centre of Olive, Fruit and Citrus Crops in Rende (Cosenza), Italy, (latitude 39°21′57″ N, longitude 16°13′44″ E). Before sampling, heavy loaded (‘ON’) and unloaded (‘OFF’) branches, evenly distributed in the entire tree crown, were identified and labelled. For each branch, lateral buds were collected, at the same time of the day, at two times; July 2018 (before the stone hardening stage) and March 2019, corresponding to the flower induction and bud differentiation, respectively. The bud pools from at least three loaded and three unloaded branches and at different time samplings were immediately frozen in liquid nitrogen and stored briefly at −80 °C, until RNA extraction.

### 2.2. RNA Purification and Quantification

Each of three bud pools (about 70–100 buds) was ground with mortar and pestle in liquid nitrogen and total RNA extractions were performed from each pool using the RNeasy Plant Mini Kit (Qiagen, Hilden, Germany), according to the manufacturer’s instructions.

Total RNA was treated using Invitrogen™ TURBO DNA-free™ Kit (Thermo Fisher Scientific, Waltham, MA, USA) to remove DNA contamination. The nucleic acid purity was analyzed by Thermo Scientific™ NanoDrop™ 2000/2000 c Spectrophotometer (Thermo Fisher Scientific, Waltham, MA, USA) and samples with 260/280 and 260/230 nm absorbance ratios greater than 1.8 nm were used for following experiments. RNA integrity was measured on a 2100 Bioanalyzer Instrument (Agilent Technologies, Santa Clara, CA, USA) through RNA 6000 Nano Kit (Agilent Technologies, Santa Clara, CA, USA) while RNA quantification was performed using Invitrogen™ Qubit™ RNA HS Assay Kit (Thermo Fisher Scientific, Waltham, MA, USA) on Invitrogen™ Qubit™ 4 Fluorometer (Thermo Fisher Scientific, Waltham, MA, USA).

### 2.3. Library Construction and RNA Sequencing

RNA was used for library preparation through the TruSeq^®^ Stranded mRNA Library Prep (Illumina^®^, San Diego, CA, USA) according to the manufacturer’s protocol. Complementary DNA (cDNA) libraries were paired-end (PE) sequenced with reads of 150 bp using the NextSeq 500 Instrument (Illumina^®^, San Diego, CA, USA). The raw reads were archived in the NCBI SRA database (Accession number: PRJNA674067).

### 2.4. Bioinformatics Analysis

Raw reads were processed following pipeline illustrated as a flow diagram in Figure S1 in Supplementary Materials. Briefly, quality control checks of raw sequence data coming from Illumina sequencing were performed through FastQC [29]. The TruSeq adaptors were removed, and the low-quality regions were trimmed (Phred cut-off 20), excluding reads with final length less than 35 bp, by using Trimmomatic [30]. The reads were aligned on the olive tree genome (cultivar Farga release Oe6, [25]), through the functions align and featureCounts in Rsubread Bioconductor package [31].

They were then filtered by expression, ruling out any transcript with an abundance less than 10, normalized through TMM (Trimmed Mean of M Values) and finally analyzed for differential expression using quasi-likelihood methods in EdgeR Bioconductor package [32]. ‘ON’ and ‘OFF’ samples were compared among the same time sampling (March or July), only genes showing a Fold Change (FC) greater than 2 and with an FDR adjusted *p*-value less than of 0.05, were considered as differential expressed (DEGs).

Venn diagrams were generated by the web-tool [33] and the GO terms enrichment analysis was performed through “goana” function in the Limma Bioconductor package [34] using the hyper- geometric test (Fisher’s exact test). GO terms with a false discovery rate (FDR) less than 0.05 were then taken into account for depicting GO terms plots by using ggplot2 R package [35].

The most significant genes obtained from EdgeR quasi-likelihood analysis were further analyzed in terms of exon differential usage through DEXSeq Bioconductor package [36], to highlight differences in isoforms expression per locus among different tissues and conditions.

Finally, networks were visualized in the open access platform Cytoscape v. 3.8.0 [37] and CoExpNetViz plugin [38] was used to analyze and predict complex DE gene networks.

### 2.5. qRT-PCR for Transcriptomic Data Validation

Total RNA was retro-transcripted employing oligo-d(T) and Invitrogen™ SuperScript™ III Reverse Transcriptase (Thermo Fisher Scientific, Waltham, MA, USA) according to the manufacturer’s instructions. cDNA was used as template for qRT-PCR reactions performed on Applied Biosystems™ 7500 fast instrument (Thermo Fisher Scientific, Waltham, MA, USA) using Applied Biosystems™ Power SYBR™ Green PCR Master Mix (2X) (Thermo Fisher Scientific, Waltham, MA, USA). Three independent biological replicates were used for each cDNA and all reactions were run in triplicate in 96-well reaction plates. PCR conditions were: one cycle at 95 °C for 20 s, followed by 40 cycles of 95 °C for 10 s and 58 °C for 30 s. At the end of the PCR, to confirm the presence of a unique amplicon, the melting curve was evaluated and a single peak in every reaction was observed. Relative template abundance was quantified using the standard curve method [39] and the *Elongation Factor 1-α* (*EF1A*) [40] was used as reference gene for expression normalization. This gene is already being used in our previous studies [23,24] and results on the same samples of this study are comparable to those using *Clathrin adaptor complex medium subunit* (*CLATHRIN*) and *Ubiquitin-conjugating enzyme* (*BC1*) [40] (Figure S2). PCR efficiency was estimated using six-point, 10-fold, diluted standard curves. Means from three independent replicates were subjected to SEM calculation, Student’s t test using PAST software [41].

Among DEGs, six genes were selected for transcriptomic data validation. The primers were designed in the exon region closest to the 3′ UTR of each gene, using the Primer3 software web version 4.1.0 (Table S1).

## 3. Results

The later buds were collected at two stages alongside flower development, in July and the following March, when the flower induction as well as lateral buds break and the flowering occur, respectively.

**Figure 1:**
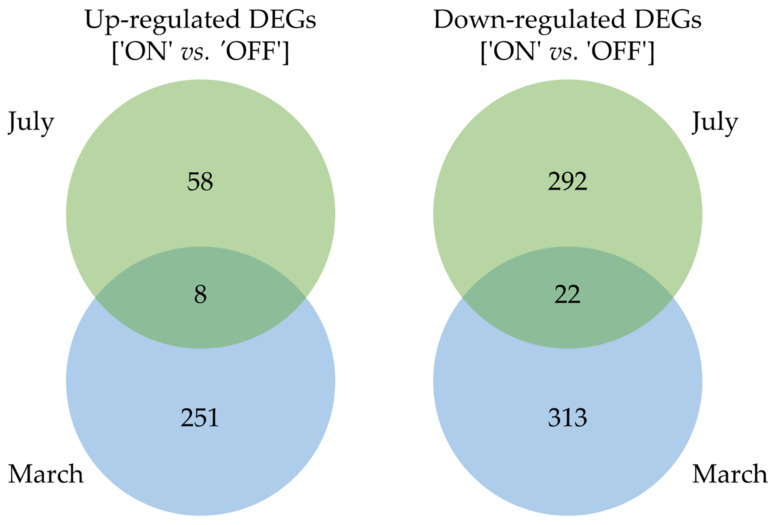
Venn diagram of up- and down-regulated DEGs extracted from ‘ON’ vs ‘OFF’ branches comparison, at two significant stages for olive flower biology, July and March.

The high-quality filtered reads were mapped to the assembled olive reference genome (cv. Farga [25]) by using quasi-likelihood methods in edgeR Bioconductor package [32] to identify the differentially expressed genes (DEGs) between lateral buds sampled from ‘ON’ and ‘OFF’ branches at both stages. The total average mapping rate was 95.24% as 349,739,459 out of 367,201,161 olive buds read pairs (Table 1). Transcriptome profiling revealed 380 and 594 DEGs (fold change ≥ 2 and FDR adjusted *p*-value less than of 0.05) between ‘ON’ and ‘OFF’ branches lateral buds sampled in July and March, respectively. Venn diagram underlined up- and down-regulated DEGs between ‘ON’ and ‘OFF’ branches for each sampling (Figure 1). An increased number of DEGs between ‘ON’ and ‘OFF’ branches in buds sampled in March compared to July was observed (Figure 1). In July, 66 (17.4%) and 314 (82.6%) out of 380 DEGs resulted in being significantly (fold change ≥ 2 and FDR adjusted *p*-value less than of 0.05) up- and down-regulated in the ‘ON’ vs. ‘OFF’ comparison, respectively (Figure 1). In March, 259 (43.6%) and 335 (56.4%) out of 594 detected DEGs were significantly up- and down-regulated in the ON vs. OFF comparison, respectively (Figure 1).

Interestingly, the least number of up-regulated DEGs in the ‘ON’ branches lateral buds was observed in July then in March, suggesting a lower transcriptional activity (17.4% vs. 43.6%). By contrast, a very low number of common DEGs in the July vs. March comparison was observed (eight up- and twenty-two down-regulated), according to the different phenological stages (Figure 1).

### 3.1. Gene Ontology Classification of Differentially Expressed Genes

**Figure 2:**
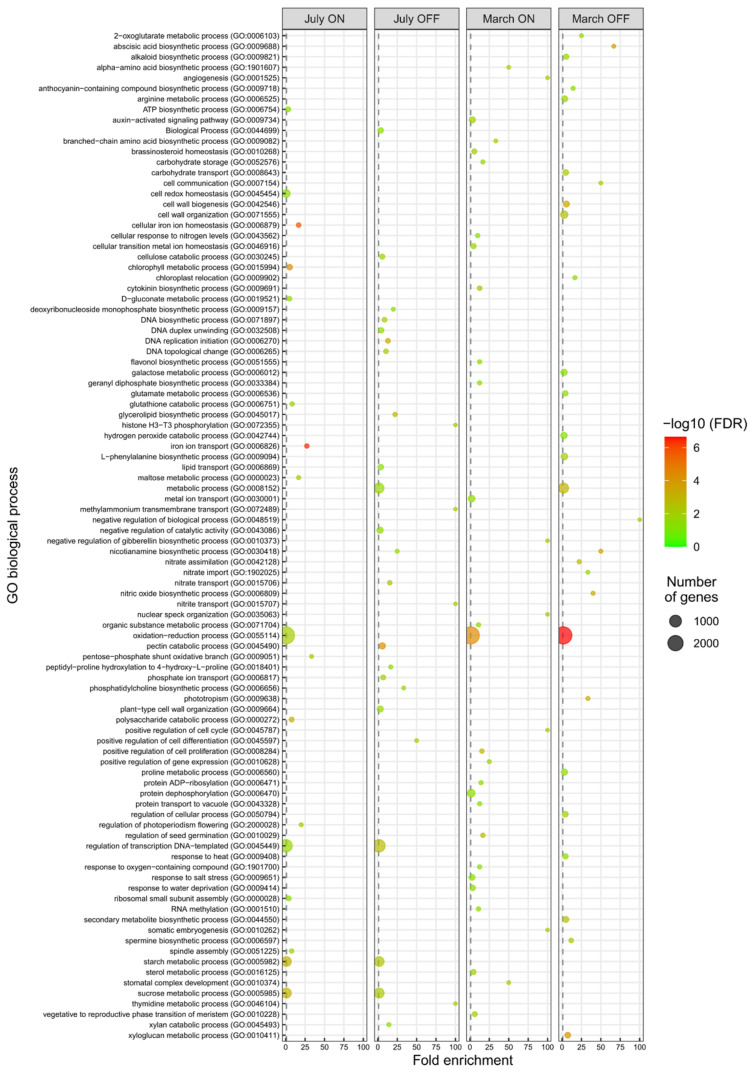
Comparison of biological Gene Ontology (GO) enrichment of DEGs in buds sampled from ‘ON’ branches compared to buds sampled from ‘OFF’ branches, at two significant stages for olive flower biology, July and March. The most representative and significant biological processes are represented and are sorted by fold enrichment. The dot size indicates the number of DEGs associated with the process and the dot color indicates the significance of the enrichment (−log10 (FDR-corrected *p*-values)). The vertical grey dashed line represents a fold enrichment of 1.

**Figure 3:**
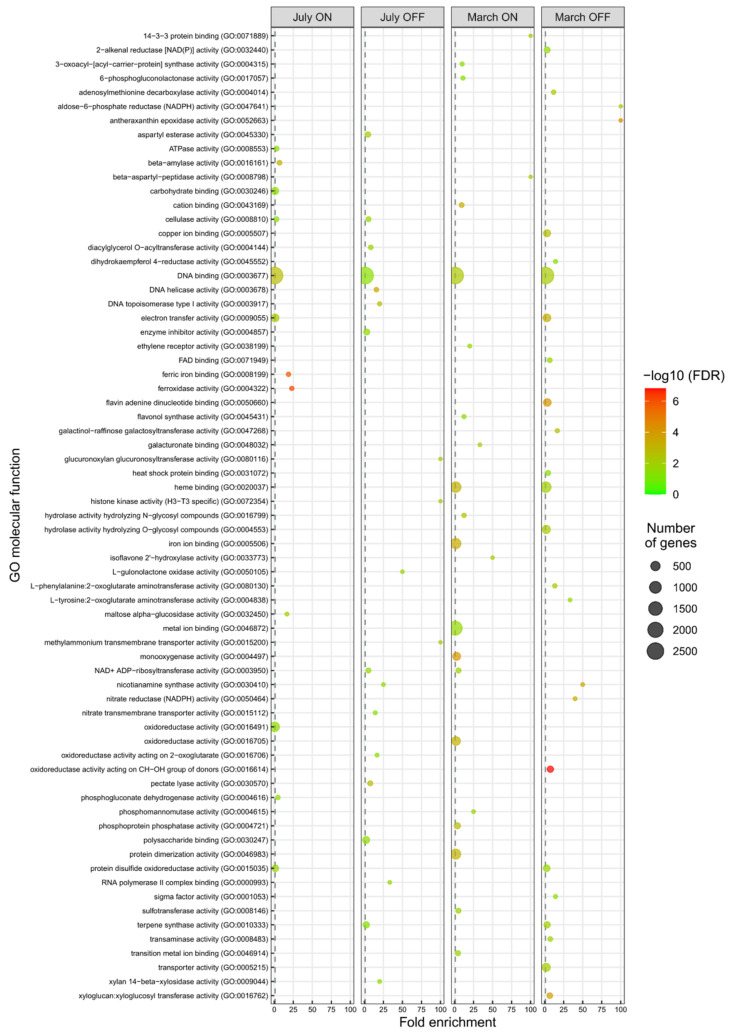
Comparison of molecular Gene Ontology (GO) enrichment of DEGs in buds sampled from ‘ON’ branches compared to buds sampled from ‘OFF’ branches, at two significant stages for olive flower biology, July and March. The most representative and significant molecular functions are represented and are sorted by fold enrichment. The dot size indicates the number of DEGs associated with the function and the dot color indicates the significance of the enrichment (−log10 (FDR-corrected *p*-values)). The vertical grey dashed line represents a fold enrichment of 1.

Gene ontology (GO) classification of the DEGs identified in each pairwise comparison (‘ON’ vs. ‘OFF’ branches) and sampling month (July vs. March) was carried out (Figure 2 and Figure 3 and Figure S3). Test for gene ontology (GO) terms over-representation was generated by goana function in the limma Bioconductor package by using the hyper- geometric test (Fisher’s exact test) with a false discovery rate (FDR) cutoff less than 0.05 [34].

Many enriched GO categories were identified, 97 in the Biological Process, 70 Molecular Function, and 26 among the Cellular Component (Figure 2 and Figure 3 and Figure S3). In agreement with their least number of up-regulated DEGs, July ‘ON’ samples showed the fewest over-represented GO categories of both Biological Process and Molecular Function.

Significant DEG groups were identified between ‘ON’ and ‘OFF’ branch lateral buds, in at least one sampling month, and in two out of four experimental samples, among which pathways and/or metabolites/hormones putatively involved in the bud transition from vegetative to flowering stage were included.

In both ‘OFF’ samples (July and March), Cell Wall metabolism GO terms were over-represented. In detail, GO: 0005618 in Component Cellular and eight GO terms in Biological Process (cell wall biogenesis, GO: 0042546; cell wall organization, GO: 0071555; xyloglucan metabolic process, GO: 0010411; cellulose catabolic process, GO: 0030245; pectin catabolic process, GO: 0045490; negative regulation of catalytic activity, GO: 0043086; plant-type cell wall organization, GO: 0009664; xylan catabolic process, GO: 0045493). Furthermore, six categories of Molecular Function (hydrolase activity, GO: 0004553; transporter activity, GO: 0005215; xyloglucan: xyloglucosyl transferase activity, GO: 0016762; pectate lyase activity, GO: 0030570; cellulase activity, GO: 0008810; aspartyl esterase activity, GO: 0045330; xylan 14-β xylosidase activity, GO: 0009044) appeared enriched in unloaded (‘OFF’) branches in July as well as in March (Table 2).

Noteworthy, even if not among the most represented, the GO terms included in the metabolic pathway of nitrate/nitrite transport were over-represented in unloaded (‘OFF’) branches, both in July and March (nitrate assimilation, GO: 0042128; nitrate import, GO: 1902025; nitrate transport, GO: 0015706; nitric oxide biosynthetic process, GO: 0006809 of Biological Process. Nitrate reductase (NADPH) activity, GO: 0050464; nitrate transmembrane transporter activity, GO: 0015112; nitrite transport, GO: 0015707 of Molecular Function) (Table 2).

A significant number of DEGs related to carbohydrate and phenylpropanoid metabolic/transport pathways was identified in March ‘OFF’ sample (Table 2). Two categories of Molecular Function (aldose-6-phosphate reductase (NADPH) activity, GO: 0047641; galactinol-raffinose galactosyltransferase activity, GO: 0047268) and Biological Process (carbohydrate transport, GO: 0008643; galactose metabolic process, GO: 0006012) among carbohydrates were also found. Finally, two categories of Biological Process (anthocyanin-containing compound biosynthetic process, GO: 0009718; secondary metabolite biosynthetic process, GO: 0044550) and one category of Molecular Function (dihydrokaempferol 4-reductase activity, GO: 0045552), related to phenylpropanoid pathway, were enriched in unloaded branches in March.

Significant differences in DEGs related to plant photosynthesis/plastidial activity, between ‘OFF’ and ‘ON’ samples, were found, mainly in March (Table 2). In detail, Cellular Component (Chloroplast thylakoid membrane, GO: 0009535), Molecular Function (sigma factor activity, GO: 0001053) and two categories of Biological Process (chloroplast relocation, GO: 0009902; phototropism, GO: 0009638) showed a high significance in the down-regulated DEGs in March ‘OFF’ sample. More interestingly, a category of Molecular Function (oxidoreductase activity acting on 2-oxoglutarate, GO: 0016706) related to ethylene-forming was over-represented in July ‘OFF’ sample. In detail, 15 genes in the lateral buds of unloaded (‘OFF’) compared to heavy loaded (‘ON’) branches resulted in being over-expressed in July (Table 2). The *1-aminocyclopropane-1-carboxylate oxidase homolog 1-like* is involved in ethylene biosynthesis, while the other 14 DEGs were TFs, as confirmed by the over-represented GO categories related to transcription modulation and DNA replication. Indeed, deoxyribonucleoside monophosphate biosynthetic process, GO: 0009157; DNA biosynthetic process, GO: 0071897; DNA duplex unwinding, GO: 0032508; DNA replication initiation, GO: 0006270 DNA topological change, GO: 0006265; regulation of transcription DNA-templated, GO: 0045449 among Biological Process categories and DNA binding, GO: 0003677; DNA helicase activity, GO: 0003678; DNA topoisomerase type I activity, GO: 0003917; histone kinase activity (H3-T3_specific), GO: 0072354 and RNA polymerase II complex binding, GO: 0000993 of Molecular Function resulted over-represented in July ‘OFF’ sample. Among the TFs, 13 were members of the *ethylene response factor (ERF)/APETALA2 (AP2)* superfamily. Two appeared to be of particular interest: an *ethylene-responsive transcription factor ERF086* (OE6A116298), ortholog of *Arabidopsis PUCHI*, and *AP2-like ethylene-responsive transcription factor AIL6*, ortholog of *Arabidopsis AINTEGUMENTA-LIKE 6/PLETHORA 3*, represented by two isoforms (OE6A055915 and OE6A007177) (Table 2).

Two DEGs with a putative key role in delaying the flowering process were identified in both ‘ON’ samples. *AP2 ERF and B3 domain-containing transcription factor RAV1-like* (OE6A096297), ortholog of *Arabidopsis TEMPRANILLO 1/ethylene response DNA binding factor 1*, which encodes for a member of *RAV* TF family involved in ethylene signaling, and the *ethylene receptor2 (ETR2)* (OE6A052171) [42,43].

Finally, only one GO term related to the gibberellins, which are hormones usually involved in flowering, although still with an unclear role in fruit-trees, was found over-represented in March ‘ON’ branch lateral buds (negative regulation of gibberellin biosynthetic process, GO: 0010373) (Table 2).

### 3.2. Functional Data Mining of the Differential Transcriptome

**Figure 4:**
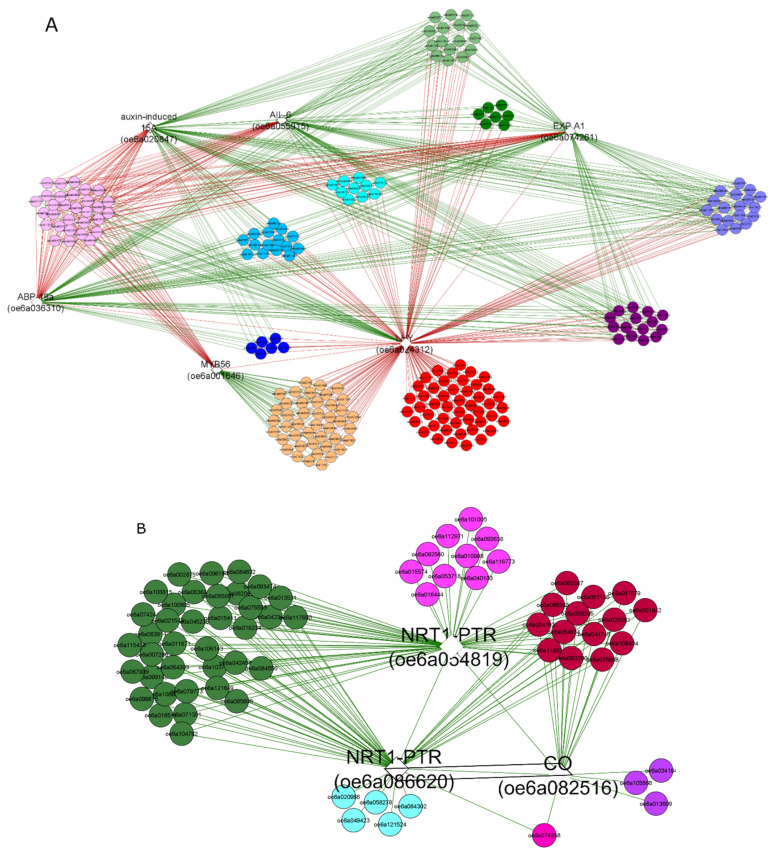
Two gene-gene association networks created in Cytoscape using CoExpNetViz plugin. Network 1 with six bait DEGs (**A**) and network 2 with three bait DEGs (**B**). The green and red edges highlights correlation and anti-correlation, respectively.

To predict co-expression networks between DEGs by using Cytoscape software, nine bait genes among those gene families detected by GO enriched categories, were chosen. In the networks, green and red edges indicated correlated and anti-related genes, respectively; the main anti-correlations concerned DEGs with two different time samples, July and March (Figure 4 and Table 2 and Table S2). Six bait genes, *expansin A1* (OE6A074261), *LHY-like isoform X1* (OE6A024312), *transcription factor myb56* (OE6A001646), *auxin-binding ABP19a-like* (OE6A036310), *auxin-induced 15A-like* (OE6A020847), and *AP2-like ethylene-responsive transcription factor AIL6* (OE6A055915) were co-expressed together with their correlated or anti-related genes (Figure 4A).

In detail, the network highlighted that the *expansin A1* expression triggered different gene clusters expression and in particular many genes involved in DNA replication and cell cycle regulation, lipid and carbohydrate metabolisms, and some other genes involved in cell wall metabolism. *Expansin A1* appeared also co-expressed with *auxin-induced 15A-like, auxin-binding ABP19a-like,* and *AP2-like ethylene-responsive transcription factor AIL6*. Interestingly, *auxin-binding ABP19a-like* (OE6A036310), ortholog of *Arabidopsis AtGER3/GLP3*, encoded a germin-like protein that follows the circadian rhythm [44], while *auxin-induced 15A-like* (OE6A020847), ortholog of *Arabidopsis SAUR50* [45], is an auxin-related gene expressed during germination. Furthermore, other genes involved in the auxin signalling and transport (OE6A102373, OE6A112705, OE6A086941) resulted in being over-expressed in July ‘OFF’. Moreover, the network highlighted a negative correlation between *expansin A1* expression and *LHY*, with different time samples, in July ‘OFF’ *expansin A1* and *LHY* up- and down-regulated, and vice versa in March ‘OFF’.

An interesting anti-relation was found between *LHY* (OE6A024312), a key gene in the circadian clock, and *myb56* (OE6A001646), a negative flowering regulator [46], more expressed in March ‘ON’ compared to ‘OFF’ lateral buds. Several TFs belonging to *B box zing finger constans-like (COL)* gene family [47] were detected in March buds, when flower bud differentiation in olive occurs [48]. LHY showed a negative correlation with zinc finger *CONSTANS-LIKE 6* (OE6A043940), *zinc finger CONSTANS-LIKE 10-like isoform X1* (OE6A111642), and *zinc finger CONSTANS-LIKE 9-like* (OE6A061348); *myb56* showed a positive correlation with *zinc finger CONSTANS-LIKE 16-like* (OE6A061639). In detail, *COL16* and two *COL9* isoforms, reported as flowering inhibitors [49,50], were over-expressed in March ‘ON’ compared to March ‘OFF’ lateral buds. Moreover, a *COL6* isoform was found to be higher expressed in ‘ON’ samples in both months, while another isoform resulted in being over-expressed only at March ‘ON’ sample (Table 2 and Table S2). To date, the *COL6* putative role in flowering is not reported; our results showed an opposite expression pattern of *constans (CO)* gene isoforms compared to the other members of *COL* family, resulting in being more expressed in March ‘OFF’ compared to ‘ON’ lateral buds (Table 2 and Table S2). Anti-correlations between *myb56* and *gibberellin 2-β-dioxygenase 2-like* (OE6A120203) as well as *abscisic acid 8 -hydroxylase 2* (OE6A091606) were detected, confirming a putative, albeit controversial, role of gibberellins and abscisic acid in the olive tree flower biology.

The other gene network was defined by using as baits a *constans (CO) gene* isoform (OE6A082516), one of the major flowering inducers [51,52], and two *NRT1-PTR* nitrate transporter members (OE6A086620 and OE6A054819) (Figure 4B). Seven out of eight nitrate-related DEGs detected in March ‘OFF’ belong to the *NRT1* family, among them a *NRT1/PTR family-like* (OE6A047446) gene, ortholog of *Arabidopsis* dual-affinity nitrate transporter *NRT1.1 (CHL1)*, appeared to be of particular interest [53]. *CHL1* was reported to affect flowering time, interacting with the target *FT* in the *FLC*-dependent flowering pathway [54]. Thus, these three baits and their co-expressed genes in March ‘OFF’ suggested their role in the determination of the flower meristem (Figure 4B).

A member of *MYB transcription factor* family, *early flowering MYB (EFM)*, which would be involved in the regulation of the florigen *FT* expression in a dose-dependent manner in the leaf vasculature, appeared as an important joining link for determining the reproduction stage throughout plant responses to light and temperature [55]. Moreover, *NRT1/PTR* gene (OE6A086620) was also co-expressed with the TF *MYB86* (OE6A020966), involved in transcriptional regulation of nitrogen metabolism [56].

In both co-expression networks, genes related to photoperiod-dependent flowering pathway [52,57,58] as well as some genes involved in the photosynthetic metabolism and the phenylpropanoids pathway were included (Figure 4) [15,23]. Among them, several TF family members and DNA binding proteins (GO: 0003677) were putatively related to photoperiod-dependent flowering pathway (Table 2 and Table S2). The genes encoding for DNA binding proteins were found to be more expressed in March ‘OFF’ compared to ‘ON’ samples. In particular, two different *LHY-like* (OE6A024312 and OE6A037580) isoforms with a key role in the central circadian clock [59] and a *MYB-like* transcription factor *REVEILLE 8* (OE6A052015) transcript [60] were detected (Table 2 and Table S2). A similar expression pattern was also observed for three *UV resistance locus 8* (OE6A062062, OE6A106023, OE6A036299) isoforms, a UV-B light photoreceptor that mediates UV-B light responses in plants [61], and three *cycling dof* TF (OE6A104771, OE6A021342, OE6A085809) isoforms, for which a role in *Arabidopsis* flowering regulation was reported through a co-regulation of miR156 and miR172 [62]. In the co-expression network analysis, *UVR8* (OE6A062062) and *CDF2* (OE6A085809) expression were triggered by *NRT1-PTR* bait genes and they were also co-expressed with *CONSTANS* (OE6A082516) (Figure 4B).

### 3.3. Differential Expression of mRNA Isoforms

The differential expression of mRNA isoforms analysis was performed by the DEXSeq software, an R Bioconductor package widely used in differential exons expression analysis [36]. This was able to confirm DEGs isolated by edgeR quasi-likelihood approach and to identify the specific isoform per locus really expressed in different conditions, among the most interesting genes (Figures S4 and S5).

**Figure 5:**
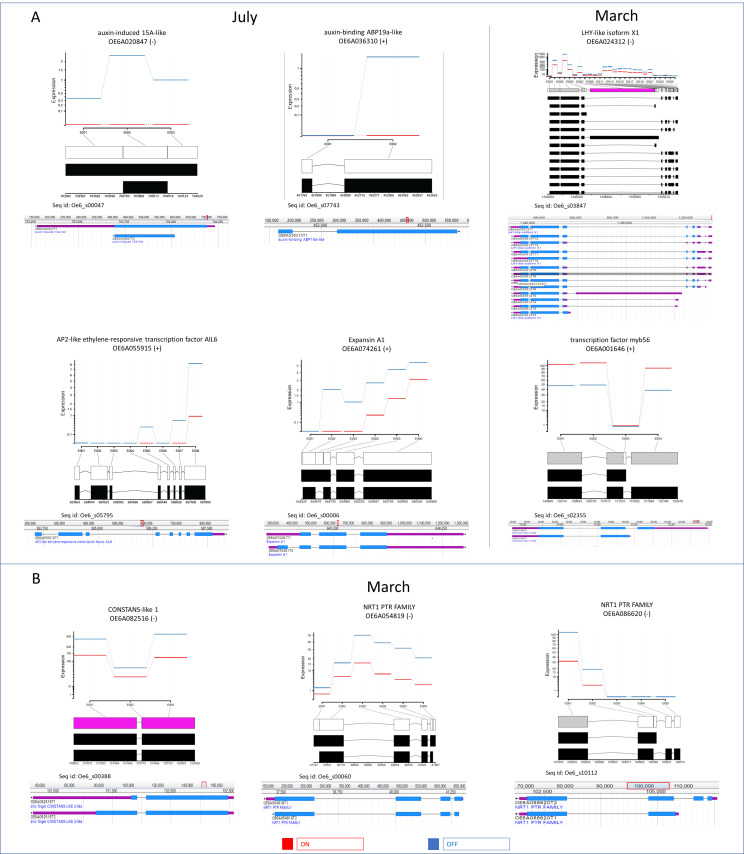
DEXseq representation of significant exon change of six bait DEGs from network 1 (**A**) and network 2 (**B**). Shown in red and in blue are the expression level of buds sampled from ‘ON’ and ‘OFF’, respectively. For each gene, feature tracks of annotated transcripts with genomic coordinates directly related to the reference genome were reported.

Interestingly, DEXSeq analysis was also able to elucidate the isoforms per locus really expressed among those annotated in olive genome (Figures S4 and S5). Two out of six bait genes (Figure 4A) showed a unique main isoform per locus annotated on olive genome and they both exhibited different expression along the whole transcript gene length with only one and three exons transcribed, respectively (OE6A055915 and OE6A036310, Figure 5A). Among the genes related to photoperiod-dependent flowering pathway included in the same network, the specific 5′-end of *myb56* (OE6A001646) T1 transcript portion was very low expressed compared to T2, while *LHY-like isoform X1* (OE6A024312) showed six isoforms more transcribed than the others (Figure 5A).

Moreover, *expansin A1* (OE6A074261) T2 transcript seems to be more active than the others, by contrast, both annotated transcripts of *auxin-induced 15A-like gene* were co-expressed (Figure 5A). Most of the 43 genes correlated with *myb56* (OE6A001646) and were anti-related to *LHY-like isoform X1* (OE6A024312), which showed a unique main isoform per locus annotated in olive genome (Figure 5A and Figure S5). Remarkably, six genes showed a unique transcript expressed in our samples among those annotated in the draft genome (OE6A085498, OE6A043896, OE6A064437, OE6A036706, OE6A100922, OE6A086809; Figure 5A and Figure S5).

The three bait genes, *CO* (OE6A082516) and two *NRT1-PTR* (OE6A054819, OE6A086620) (Figure 4B), showed two isoforms per locus annotated in the olive genome, but one transcript seemed more expressed compared to the other (Figure 5B). Half of the genes co-regulated with these three bait genes (Figure 4B) showed a unique main isoform per locus, while four expressed at least two transcripts among those annotated in draft genome (OE6A081679, OE6A081156, OE6A044745, OE6A054834; Figure 5B and Figure S5).

### 3.4. Validation of RNA-Seq Expressions of Selected Genes by Real-Time qRT-PCR

**Figure 6:**
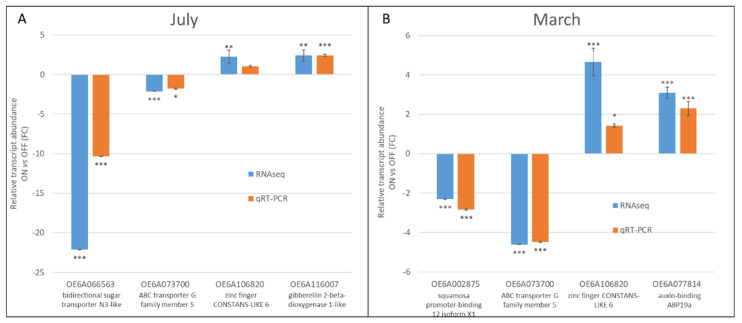
qRT-PCR validation of the differentially expressed genes selected. The data indicate the mean value of Fold Change (FC) expression of genes in July (**A**) and March (**B**) between ‘ON’ and ‘OFF’ samples. For each gene, the blue bar indicates the FC expression determined by RNAseq; the orange bar indicates the FC expression determined by qRT-PCR. The analyses were performed as triplicates and the error bars indicate the standard error of the mean (s.e.m.). Statistical significance of the expression between ‘ON’ and ‘OFF’ samples is indicated (* *p* < 0.05; ** *p* < 0.01; *** *p* < 0.001).

To verify the reliability of RNA-seq data, the expressions of six key genes were analyzed throughout qRT-PCR. We selected one gene over-expressed in both July and March ‘OFF’ (*ABC transporter G family member 5*, OE6A073700), as well another over-expressed in both July and March ‘ON’ (*zinc finger CONSTANS-LIKE 6*, OE6A106820), and two couples of genes higher expressed in July ‘OFF’ (*bidirectional sugar transporter N3-like*, OE6A066563) and ‘ON’ (*gibberellin 2-β-dioxygenase 1-like*, OE6A116007) and March ‘OFF’ (*squamosa promoter-binding 12 isoform X1*, OE6A002875) and ‘ON’ (*auxin-binding ABP19a*, OE6A077814), respectively. At least one DEG from all four samples were included, allowing us to further evaluate the RNA-seq dataset quality. The results suggested that gene expression patterns between RNA-seq and qRT-PCR showed a similar trend, confirming the accuracy of the transcriptomic analysis (Figure 6).

## 4. Discussion

A distinctive olive tree bio-agronomic trait is the alternate bearing. This complex trait is controlled by genetic and environmental factors; thus, each cultivar shows gradualness in the alternate bearing behavior. Among others, the cv. Leccino exhibits a medium alternate-bearing tendency. There are still few studies on the molecular network that would underlie flower induction and differentiation in olive lateral bud meristems, which are directly involved in alternate bearing. Recently, a whole transcriptome sequencing in the olive cv. Conservalia highlighted the molecular mechanisms behind this phenomenon at flower induction [28]. Here, we analyzed the different transcriptomic profiles of lateral buds from both loaded (‘ON’) and unloaded (‘OFF’) branches in the same olive tree in July (flower induction) and the following March (flower differentiation); it is useful to highlight a relationship between flower induction and differentiation in terms of DEGs. In particular, we selected a tree over 30 years old, about 5 m high, and with an equally wide canopy, with a high variability between branches due to exposure to light and size of branches and with a concurrent presence of both ‘ON’ and ‘OFF’ branches. The experimental design should reduce environmental and genetic variables that could strongly affect the outcome. Similar studies are largely descriptive and the results were difficult to interpret when functional studies were lacking. However, our strategy aims to better promote the focus on differential expressed genes that could link flower induction and differentiation. In our opinion, this approach might justify the lower number of DEG between ‘ON’ and ‘OFF’ compared to those observed by Dastkar and colleagues [28].

**Figure 7:**
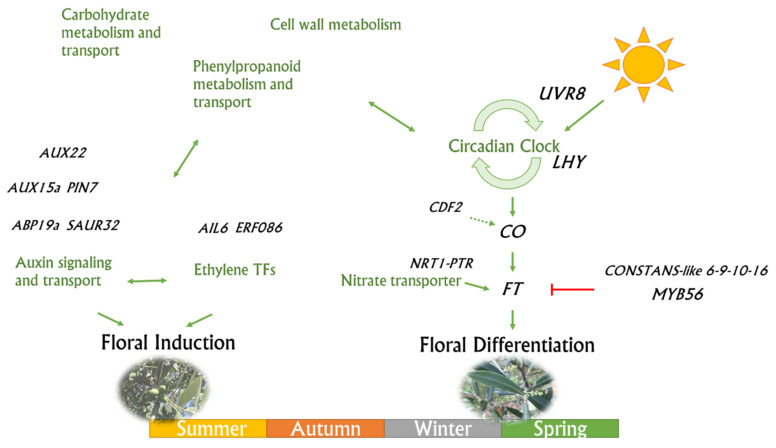
A schematic model summarizing the genes and pathways involved in the flowering induction and differentiation stages of olive lateral buds. Carbohydrates and phenylpropanoids would promote both induction and flower differentiation. Some ethylene transcription factors would interact with some genes active in auxin signaling and transport during the flower induction stage. Circadian clock genes would promote the *constans* florigen gene, which in turn would promote the FT gene, directly involved in flower differentiation. The dashed arrow indicates the different roles identified between *Olea* and *Arabidopsis* for the *CDF2* gene. The *NRT1/PTR* gene would also play a role in promoting the *FT* gene expression. On the other hand, *constans-like 6*, *9*, *10*, *16*, and *myb56* would have inhibitory effects on the *FT* gene and therefore on flower differentiation.

Several TFs and genes belonging to different pathways, directly and/or indirectly involved in the bud meristems transition from both vegetative to the reproductive stage and flower induction to differentiation were identified (Figure 7) [16,63,64]. DEGs analysis sustained a consistent flower induction in July on unloaded branches, together with a higher crop yield in the following year, triggering the alternate bearing.

### 4.1. Carbohydrate Metabolism/Transport and Phenylpropanoid Genes

Our analyses also highlighted a significant up-regulation of carbohydrate metabolic/transport function in July and March ‘OFF’ samples (‘ON’-buds for return bloom) (Table 2). A relationship between carbohydrates metabolism and the biennial bearing in olive tree has already been reported [28,65,66]. Indeed, carbohydrate availability fluctuation was suggested as a key factor triggering alternate bearing by the inhibition of flower buds that occur when carbohydrate storage is reduced, which is usually recorded after a high crop production [7,67]. A higher nutrients utilization rate by olive trees in the ‘OFF’ year was also observed, confirming that the regulation of their levels plays an important role in the alternate bearing [66]. Moreover, significant changes in the transcriptional profile of genes related to both sugar signaling and cell wall metabolism during the transition from vegetative to the reproductive bud stage were reported in other species [68,69]. A differential expression of genes related to cell wall metabolism, metabolism/transport of carbohydrates, as well as fatty acid metabolism between leaves from ‘ON’ and ‘OFF’ years was observed in our experiment as previously observed [66]. Many genes belonging to the photosynthetic pathways, light reactions, and photorespiration were significantly induced on March ‘OFF’ samples as well (Table 2).

In addition, many genes from the phenylpropanoid pathway resulted in being over-expressed in March ‘OFF’ samples (Table 2). The phenylpropanoids over-produced in stressful conditions are reported to be synthesized in response to photo-assimilated excess, which could occur under Long Day (LD) condition [70]. An increased expression level of the transcripts related to flavonoid biosynthesis in the ‘OFF’ compared to ‘ON’ year leaves was reported in the olive tree [28,66,71].

All these results agreed with the findings on *Citrus reticulate* [72], sustaining that buds and fruits compete for resources and nutrients in ‘ON’ branches, but not in ‘OFF’ ones where the buds tend to accumulate photo-assimilated and storage molecules. These observations did not agree with those previously observed in olive, probably due to the only sampling time [28].

Recently, the *flowering locus T (FT)* was reported to induce the transcription of *SWEET10*, a bidirectional sucrose transporter, suggesting that the *FT*-signaling pathway activates the transcription of a sucrose uptake/efflux carrier during the flower differentiation in *Arabidopsis* [73]. In agreement, an over-expression of *bidirectional sugar transporter SWEET12-like*, ortholog of *Arabidopsis SWEET11*, was found in lateral buds from March ‘OFF’ olive branches (Table 2). Otherwise, a slight up-regulation of *FT* transcript levels was observed in March ‘OFF’ samples, albeit not significant, in accordance to previous reports in citrus and olive tree [74,75].

### 4.2. Ethylene Signal Transduction TFs

At earliest, the *ethylene response factor (ERF)/apetala2 (AP2)* transcription factor superfamily was characterized for its function and involvement in the response to biotic and abiotic stress [76,77]. In addition, a role of these TFs is also known in plant development, from seed germination to flowering until the fruit ripening [78,79,80,81,82,83], as well as into photo-perception regulated by the circadian rhythm [84]. More interestingly, 13 over-expressed genes in July ‘OFF’ were *ERF* TF family members: *ERF086*, ortholog of *Arabidopsis PUCHI*, and two isoforms of the *AP2-like ethylene-responsive transcription factor AIL6 gene*, ortholog of *Arabidopsis aintegumenta-like 6/ pletora 3* (Table 2). Indeed, both *PUCHI* and *aintegumenta-like6/ pletora 3* showed a key role in determining the identity of the bud flower meristem as well as defining the flower organ initiation [85,86]. In *Arabidopsis*, *PUCHI* expression during the flower meristem identity process appeared transcriptionally regulated by auxins [87], which in turn regulated their levels acting on the polar auxin transport and would also promote the over-expression of *LEAFY*, one of the genes that showed a key role in the determination of the flower meristem identity. Likewise, *aintegumenta-like 6/pletora 3* would play a pivotal role on flower meristem identity, following auxin stimulation by acting directly on the meristem or indirectly by inducing the *LEAFY* expression [85]. The functional observations in Arabidopsis allowed us to hypothesize a role of these ethylene-related genes as inducers of the transition from the vegetative to the flowering stage in the lateral buds of unloaded (‘OFF’) branches in July, corresponding to the flower induction in olive. Noteworthy, both *ERF086* and *AIL6* seemed regulated by the auxins and related DEGs, involved in signaling and transport; in July ‘OFF’ sample, *LEAFY* gene showed also a similar trend, although not significant.

Interestingly, a member of the auxin efflux facilitators *PIN* protein family, as well as two genes belonging to the *SAUR* family, whose expression could be regulated by TFs involved in plant development, such as *LEAFY, AP1, AP2, SEP3, and SOC1,* were up-regulated in July ‘OFF’ sample, as already reported [88] (Table 2). Stortenbeker and Berner [45] reported the complex regulation of the *SAUR* genes via environmental- (e.g., light and warm temperature), developmental- and clock-controlled pathways at both transcriptional and post-transcriptional levels. Finally, an auxin-binding *ABP19a-like*, ortholog of *Arabidopsis AtGER3/GLP3*, encoding a germin-like protein, characterized by elements involved in circadian regulation of the gene expression [44], was similarly up-regulated (Table 2). Noteworthy, an over-expression of the same gene was also detected in flower buds both in *Eucalyptus* [89] and *Arabidopsis* [90].

Auxin involvement in the olive flower induction was confirmed by *AP2-like ethylene-responsive transcription factor AIL6* over-expression in July ‘OFF’ sample (Table 2). This ortholog gene of *Arabidopsis AINTEGUMENTA-LIKE 6/PLETHORA 3* should trigger the flower induction as already reported in *Arabidopsis* [85,86].

The potential functional role of the AP2 ERF and B3 domain-containing transcription factor RAV1-like gene up-regulated in July ‘ON’ appeared also of interest. Indeed, the RAV1 ortholog of Arabidopsis ethylene response DNA binding factor 1/tempranillo 1 contains the AP2 and B3 binding domains, which are transcriptional regulators involved in the ethylene signaling [43]. The RAV1 over-expression caused late flowering throughout the flowering locus T (FT) repression as well as the gibberellin biosynthesis, that in turn could elongate the juvenile stage in Arabidopsis [43].

A putative key role in olive flower biology seems to be played by *ethylene receptor2 (ETR2)*, which is over-expressed in March ‘ON’ samples. Indeed, the *ETR2* over-expression in transgenic rice reduced the ethylene sensitivity and delayed the flowering transition, throughout a putative up-regulation of *gigantea* and *terminal flower 1/centroradialis homolog (RCN1),* causing a flowering delay [42]. The over-expression of these two last genes, together with *ETR2*, in the lateral buds from ‘ON’ branches at both July and March, allowed us to speculate about their role in the lack of flower transition on the heavy loaded (‘ON’) branches.

More interestingly, we observed a key role of ethylene-related TFs for providing a signal to switch from the vegetative to the reproductive bud stages. These evidences find foundation in a highly significant accumulation of sugars and storage molecules in the early stages of flower induction of unloaded branches, as above mentioned, resulting also in a fine regulation of key genes before the spring flowering, as discussed below.

### 4.3. Photo-Perception and Flowering Control Genes

Furthermore, a putative role of nitrogen (N) that could regulate the flowering time in plants, regardless of the light, was strongly taken into account as previously reported in *Arabidopsis* [91,92,93,94]. Interestingly, a *NRT1.1/CHL1* ortholog, member of *NRT1/PTR* family, acting as plant nitrate sensor [95] but also flowering inducer under limited N-availability [54,96], resulted in up-regulation in March ‘OFF’ lateral buds (Table 2). More interestingly, *NRT1.1* flowering inducer role, regardless of the photoperiod and *FLC*-dependent flowering pathway, was recently reported [54,96]. *FLC* is known as a flowering transition repressor gene, which in turn would negatively influence the *FT* target gene, which plays a key role in flowering transition. Overall, *NRT1.1* seems to play a key role in signal transduction for regulating flowering, a role of *NRT1/PTR* family in the auxin, ABA, GA, and potassium, as well as nitrate transport, has been recently hypothesized [97,98,99,100,101].

Furthermore, a relationship between the *NRT1/PTR* family (recently recalled *NPF*) and the flowering-time genes (*FcFE, FcFT*) was speculated by a *NPF1.2* role in the *FcFT* regulation, which in turn triggers *FcFE* transcription in response to nitrate signaling [102]. Finally, the co-expression of *NRT1 PTR* (OE6A086620) isoform and the TF early flowering *MYB (EFM),* probably involved in the regulation of *FT* gene expression in a dosage-dependent manner, was reported [55]. Remarkably, *EFM* and another member of *NRT1/PTR* family (OE6A054819) were co-expressed with a *CONSTANS-like 1 isoform* (OE6A082516) in lateral buds of March ‘OFF’ branches, supporting the role of nitrate transporters in the determination of the olive flower meristem (Table 2).

The differential expression of several genes related to olive tree flowering differentiation drawn from our transcriptomic profiles, sustained a taken-home message that lateral buds from March ‘OFF’ samples could result in their full flowering fate. Fine regulation of genome-wide expression profiles in these lateral buds suggested that flower differentiation is occurring, leading truly to load branches in the following year.

The higher expression in March ‘OFF’ of two putative zinc finger protein *CONSTANS*, ortholog of the *Arabidopsis CO* gene, fully supported our hypothesis throughout the *CO* direct binding of the *flowering locus T (FT)* promoter for its transcription leading to induced flowering in *Arabidopsis* [103,104] (Table 2). Moreover, *CO* as well as *gigantea (GI), FT*, *late elongated hypocotyl (LHY), and cryptochrome2 (CRY2)* also promote flowering under LD light [57,105]. Indeed, co mutants showed a flowering delay under LD [51,105], while plants over-expressing *CO* showed an earlier flowering compared to the wild type [106]. Concurrently, the plant circadian clock modulates the genes expression also involved in flowering [58]. In this framework, the *CO* differential higher expression that we found in the lateral buds of March ‘OFF’ may be responsible for the crosstalk between flowering time control and the circadian clock. In agreement to the LD flowering time model proposed by Suarez-Lopez et al. [57], the different *LHY* isoforms more expressed in March ‘OFF’ may influence *CO* mRNA abundance.

By contrast, a group of genes encoding for TFs known for their negative role in flowering differentiation appeared up-regulated in March ‘ON’ compared to March ‘OFF’, confirming our findings (Table 2). Indeed, transgenic rice over-expressing *constans-like 16 (COL16)* resulted in late flowering in both Long (LD) and Short Day (SD) conditions [50]. Likewise, *COL9* over-expression in *Arabidopsis* delayed flowering, showing an opposite role to *CO* [49]. In addition, the more expressed *myb56* in the March ‘ON’ lateral buds resulted in a negative regulator of *FT* through the direct control of its expression as already reported in Arabidopsis [46] (Table 2). Thus, our data are consistent with these studies, supporting the hypothesis that *COL16, COL9,* and *myb56* functioned as flowering inhibitors in March ‘ON’ olive lateral buds. The co-expression network analysis highlighted the anti-relation between *myb56* and *gibberellin 2-β-dioxygenase 2-like* as well as *abscisic acid 8 -hydroxylase 2*. The role of these hormones in flower differentiation appears still controversial, in some cases ABA is considered a repressor of flowering [107], while in others a positive regulator of TFs involved in flower differentiation operating together with the gibberellins determined the photoperiod-mediated expression of *FT* [108,109].

Interestingly, the three *cycling dof factor (CDF2)* isoforms more expressed in March ‘OFF’ lateral buds might have a different role in olive compared to *Arabidopsis* (Table 2). Indeed, *AtCDF2-like* as well as the maize ortholog showed a negative control on flowering extending the juvenile plant stage, as a transcriptional repressor of *CO* [110,111,112,113]. Otherwise, a new pathway for regulating the flowering through *CDF2* and miRNAs was reported, regardless of *CO* [62]. In our study, miRNA expressions were not evaluated, so we are not able to elucidate these differences; therefore, *OeCDF2* involvement in flower differentiation by an alternative strategy compared to the orthologues in *Arabidopsis* and maize appeared conceivable, although it requires further investigation.

## 5. Conclusions

Our study provided new insights into olive flower biology in the framework of the alternate bearing, with a particular focus on the molecular mechanisms underlying both flower induction, differentiation, and their relationship. Thus, we were able to identify DEGs between buds from ‘ON’ and ‘OFF’ branches as well as the genes trend along flower induction and differentiation by co-expression networks. 

As a first instance, many genes related to the metabolic and transport pathways of carbohydrates, lipids, and phenylpropanoids, as well as those linked to cell wall metabolism in the buds from unloaded (‘OFF’) branches, appeared differentially higher expressed, both in July and March, as partially previously described [28]. Interestingly, we further observed a similar expression trend, albeit only in March, of several genes related to photo-perception, photorespiration, and circadian clock (Table 2).

Nevertheless, the induction of some ethylene-related TFs, together with auxin in ‘OFF’ branches in July, would affect key genes involved in the first stage of the flower meristem identity in the lateral buds in July. Afterwards, a complex genes network would be activated starting from the over-expression of some other TFs belonging to the *constans* family as well as nitrate-related genes (*NRT1/PTR family*); in turn, they are higher expressed in ‘OFF’ branches in March and directly or indirectly involved in the switch from the vegetative to the reproductive bud stages, during flower differentiation (Table 2).

Altogether, our results suggest that the induction of ethylene-related TFs could provide a signal for the switch from the vegetative to the reproductive bud stages. This hypothesis finds foundation in the accumulation of sugars and storage molecules in the unloaded branches at the early stages of flower induction as well as a regulation of key flowering genes before the spring, when the same ‘OFF’ branches resulted ready for floral differentiation (Figure 7). 

This report on differential transcriptomic profiles from loaded (‘ON’) and unloaded (‘OFF’) branches of a unique olive tree was powerful in identifying flower biology key genes involved in the alternate bearing, both at flower induction and differentiation. The expression level of these genes will be assessed on a wide set of cultivars, showing a different tendency to alternate bearing for deepening insight into the molecular mechanisms of this important bio-agronomic trait in olive trees. These further analyses will furnish a definitive panel of genes and pathways to be manipulated for preventing alternate bearing in olive trees.

## Figures and Tables

**Table 1:** Summary of read filtering and mapping processes.

Sample	Raw Reads	HQ Filtered Reads	HQ Filtered Reads %	EdgeR Quasi-Likelihood		DEXSeq	
				Mapped Reads	Mapped Reads %	Mapped Reads	Mapped Reads %
July_ON_I	40,593,053	31,569,903	77.77	29,714,448	94.12	25,315,943	80.19
July_ON_II	42,108,562	33,459,722	79.46	31,458,822	94.02	27,065,303	80.89
July_ON_III	26,150,715	20,443,071	78.17	19,201,031	93.92	16,352,358	79.99
July_OFF_I	33,840,148	25,637,894	75.76	24,124,474	94.10	20,388,017	79.52
July_OFF_II	33,269,865	25,739,109	77.36	24,244,597	94.19	20,809,948	80.85
July_OFF_III	30,811,350	24,090,568	78.19	22,582,687	93.74	19,165,647	79.56
March_ON_I	33,180,348	25,694,796	77.44	24,725,540	96.23	20,284,894	78.95
March_ON_II	36,289,476	28,672,112	79.01	27,511,974	95.95	22,778,612	79.45
March_ON_III	44,132,732	34,442,821	78.04	33,109,833	96.13	27,411,604	79.59
March_OFF_I	35,659,418	27,479,198	77.06	26,466,879	96.32	21,998,929	80.06
March_OFF_II	46,213,740	36,374,337	78.71	35,027,996	96.30	28,777,594	79.12
March_OFF_III	70,730,485	53,597,630	75.78	51,571,178	96.22	42,877,173	80.00
Total	472,979,892	367,201,161	77.64	349,739,459	95.24	293,226,022	79.85

**Table 2:** Summary of key DEGs between ‘ON’ and ‘OFF’ buds, which might be responsible and involved in flower induction (July) and differentiation (March). FC: Fold change; FDR: adjusted *p*-value.

	Gene_ID	Annotation	Gene Ontology Terms	FC	*p*-Value	FDR
July						
**Ethylene** **transcription factor**	OE6A116298	ethylene-responsive transcription factor ERF086	DNA binding (GO: 0003677); DNA-binding transcription factor activity (GO: 0003700); transcription regulator complex (GO: 0005667); regulation of transcription, DNA-templated (GO: 0006355); flower development (GO: 0009908)	−2.12	6.10 × 10^−3^	3.87 × 10^−2^
	OE6A055915	AP2-like ethylene-responsive transcription factor AIL6	DNA binding (GO: 0003677); DNA-binding transcription factor activity (GO: 0003700); transcription regulator complex (GO: 0005667); regulation of transcription, DNA-templated (GO: 0006355)	−5.67	2.34 × 10^−5^	4.37 × 10^−4^
	OE6A007177	AP2-like ethylene-responsive transcription factor AIL6	DNA binding (GO: 0003677); DNA-binding transcription factor activity (GO: 0003700); transcription regulator complex (GO: 0005667); regulation of transcription, DNA-templated (GO: 0006355)	−4.67	1.01 × 10^−4^	1.48 × 10^−3^
	OE6A096297	AP2 ERF and B3 domain-containing transcription factor RAV1-like	DNA binding (GO: 0003677); DNA-binding transcription factor activity (GO: 0003700); nucleus (GO: 0005634); transcription regulator complex (GO: 0005667); regulation of transcription, DNA-templated (GO: 0006355)	2.01	6.98 × 10^−5^	1.08 × 10^−3^
**Auxin binding and transport**	OE6A036310	auxin-binding ABP19a-like	cell wall (GO: 0005618); auxin-activated signaling pathway (GO: 0009734); manganese ion binding (GO: 0030145); nutrient reservoir activity (GO: 0045735); apoplast (GO: 0048046)	−4.83	2.48 × 10^−3^	1.94 × 10^−2^
	OE6A020847	auxin-induced 15A-like	response to auxin (GO: 0009733); integral component of membrane (GO: 0016021)	−25.06	1.21 × 10^−3^	1.10 × 10^−2^
	OE6A102373	auxin-induced AUX22-like	protein binding (GO: 0005515); regulation of cellular process (GO: 0050794)	−3.09	6.34 × 10^−3^	3.97 × 10^−2^
	OE6A086941	auxin-responsive SAUR32-like	response to auxin (GO: 0009733)	−4.18	9.82 × 10^−6^	2.10 × 10^−4^
	OE6A112705	PIN-LIKES 7	integral component of membrane (GO: 0016021); transmembrane transport (GO: 0055085)	−2.77	6.58 × 10^−3^	4.09 × 10^−2^
**Metabolism and transport of** **carbohydrates**	OE6A090116	β-galactosidase-like	β-galactosidase activity (GO: 0004565); cell wall (GO: 0005618); vacuole (GO: 0005773); galactose metabolic process (GO: 0006012); glycosaminoglycan catabolic process (GO: 0006027); glycosphingolipid metabolic process (GO: 0006687); β-galactosidase complex (GO: 0009341); carbohydrate binding (GO: 0030246); glycerolipid metabolic process (GO: 0046486)	−2.02	1.72 × 10^−3^	1.46 × 10^−2^
	OE6A066563	bidirectional sugar transporter N3-like	carbohydrate transport (GO: 0008643); integral component of membrane (GO: 0016021); sugar transmembrane transporter activity (GO: 0051119)	−22.11	5.11 × 10^−33^	1.87 × 10^−28^
	OE6A090057	stachyose synthase	galactose metabolic process (GO: 0006012); galactinol-raffinose galactosyltransferase activity (GO: 0047268)	−3.03	8.25 × 10^−7^	2.63 × 10^−5^
	OE6A012151	β-D-xylosidase 1-like	starch metabolic process (GO: 0005982); sucrose metabolic process (GO: 0005985); xylan 1,4-β-xylosidase activity (GO: 0009044); nucleotide metabolic process (GO: 0009117); plant-type cell wall (GO: 0009505); arabinan catabolic process (GO: 0031222); xylan catabolic process (GO: 0045493); α-L-arabinofuranosidase activity (GO: 0046556)	−2.50	9.08 × 10^−6^	1.97 × 10^−4^
	OE6A077201	β-fructofuranosidase	sucrose α-glucosidase activity (GO: 0004575); starch metabolic process (GO: 0005982); sucrose metabolic process (GO: 0005985); integral component of membrane (GO: 0016021); glucosidase II complex (GO: 0017177)	−2.36	7.86 × 10^−4^	7.87 × 10^−3^
**Cell wall** **metabolism**	OE6A074261	expansin A1	extracellular region (GO: 0005576); cell wall (GO: 0005618); plant-type cell wall organization (GO: 0009664); membrane (GO: 0016020)	−2.86	3.20 × 10^−4^	3.81 × 10^−3^
	OE6A056682	glucan endo-1	starch metabolic process (GO: 0005982); sucrose metabolic process (GO: 0005985); polysaccharide binding (GO: 0030247); glucan endo-1,3-β-D-glucosidase activity (GO: 0042973); anchored component of plasma membrane (GO: 0046658)	−19.66	6.80 × 10^−5^	1.06 × 10^−3^
	OE6A060375	expansin-A5-like isoform X2	extracellular region (GO: 0005576); cell wall (GO: 0005618); plant-type cell wall organization (GO: 0009664); unidimensional cell growth (GO: 0009826); membrane (GO: 0016020); primary root development (GO: 0080022)	−2.56	1.59 × 10^−8^	8.72 × 10^−7^
	OE6A105425	expansin-A10 isoform X1	extracellular region (GO: 0005576); cell wall (GO: 0005618); plant-type cell wall organization (GO: 0009664); membrane (GO: 0016020)	−4.26	2.07 × 10^−10^	2.06 × 10^−8^
	OE6A118180	glucan endo-1	hydrolase activity, hydrolyzing O-glycosyl compounds (GO: 0004553); carbohydrate metabolic process (GO: 0005975); starch metabolic process (GO: 0005982); sucrose metabolic process (GO: 0005985); polysaccharide binding (GO: 0030247); glucan endo-1,3-β-D-glucosidase activity (GO: 0042973); anchored component of plasma membrane (GO: 0046658)	−2.45	6.39 × 10^−20^	8.98 × 10^−17^
	OE6A021910	xyloglucan glycosyltransferase 4	starch metabolic process (GO: 0005982); sucrose metabolic process (GO: 0005985); UDP-glucose metabolic process (GO: 0006011); integral component of membrane (GO: 0016021); cellulose synthase (UDP-forming) activity (GO: 0016760); cellulose biosynthetic process (GO: 0030244)	−2.23	3.25 × 10^−4^	3.86 × 10^−3^
	OE6A046955	expansin-B3-like	extracellular region (GO: 0005576); sexual reproduction (GO: 0019953)	−3.01	1.26 × 10^−3^	1.14 × 10^−2^
	OE6A048399	wall-associated receptor kinase 3-like	polysaccharide binding (GO: 0030247)	−2.72	2.64 × 10^−3^	2.04 × 10^−2^
	OE6A021910	xyloglucan glycosyltransferase 4	starch metabolic process (GO: 0005982); sucrose metabolic process (GO: 0005985); UDP-glucose metabolic process (GO: 0006011); integral component of membrane (GO: 0016021); cellulose synthase (UDP-forming) activity (GO: 0016760); cellulose biosynthetic process (GO: 0030244)	−2.23	3.25 × 10^−4^	3.86 × 10^−3^
**Metabolism and transport of** **phenylpropanoids**	OE6A055711	pelargonidin 3-O-(6-caffeoylglucoside) 5-O-(6-O-malonylglucoside) 4 -malonyltransferase-like	transferase activity, transferring acyl groups other than amino-acyl groups (GO: 0016747)	−2.16	7.52 × 10^−4^	7.58 × 10^−3^
	OE6A120332	anthocyanidin 3-O-glucosyltransferase 5-like	metabolic process (GO: 0008152); transferase activity, transferring hexosyl groups (GO: 0016758)	−7.17	3.01 × 10^−10^	2.84 × 10^−8^
	OE6A053282	anthocyanidin 3-O-glucosyltransferase 2-like	metabolic process (GO: 0008152); integral component of membrane (GO: 0016021); transferase activity, transferring hexosyl groups (GO: 0016758)	−2.15	6.11 × 10^−15^	2.63 × 10^−12^
	OE6A106148	2-hydroxyisoflavanone dehydratase-like	metabolic process (GO: 0008152); hydrolase activity (GO: 0016787)	−2.08	1.28 × 10^−7^	5.31 × 10^−6^
	OE6A085084	(-)-isopiperitenol (-)-carveol dehydrogenase	oxidoreductase activity (GO: 0016491)	−2.17	8.70 × 10^−12^	1.30 × 10^−9^
	OE6A072403	terpene synthase 10-like	magnesium ion binding (GO: 0000287); metabolic process (GO: 0008152); terpene synthase activity (GO: 0010333)	−3.74	1.80 × 10^−12^	3.20 × 10^−10^
**March**						
**Ethylene** **signalling**	OE6A052171	ethylene receptor2	phosphorelay sensor kinase activity (GO: 0000155); protein binding (GO: 0005515); endoplasmic reticulum membrane (GO: 0005789); protein histidine kinase complex (GO: 0009365); negative regulation of ethylene-activated signaling pathway (GO: 0010105); integral component of membrane (GO: 0016021); peptidyl-histidine phosphorylation (GO: 0018106); ethylene receptor activity (GO: 0038199); ethylene binding (GO: 0051740)	2.66	5.85 × 10^−17^	1.13 × 10^−14^
**Photoperception and flowering** **control**	OE6A024312	LHY like isoform X1	DNA binding (GO: 0003677); regulation of transcription, DNA-templated (GO: 0006355); negative regulation of biological process (GO: 0048519)	−2.60	1.84 × 10^−29^	1.20 × 10^−25^
	OE6A037580	LHY-like isoform X1	DNA binding (GO: 0003677); regulation of transcription, DNA-templated (GO: 0006355)	−5.83	2.88 × 10^−38^	1.05 × 10^−33^
	OE6A001646	transcription factor myb56	DNA binding (GO: 0003677)	2.25	7.05 × 10^−12^	3.79 × 10^−10^
	OE6A020966	transcription factor myb86	DNA binding (GO: 0003677)	−2.10	1.17 × 10^−3^	5.52 × 10^−3^
	OE6A052015	MYB-like transcription factor REVEILLE 8	DNA binding (GO: 0003677); nucleus (GO: 0005634); regulation of transcription, DNA-templated (GO: 0006355)	−2.12	1.10 × 10^−20^	6.37 × 10^−18^
	OE6A062062	UV resistance locus 8	NA	−2.06	1.49 × 10^−24^	2.48 × 10^−21^
	OE6A106023	UV resistance locus 8	NA	−2.97	1.56 × 10^−16^	2.81 × 10^−14^
	OE6A036299	UV resistance locus 8	NA	−2.61	1.97 × 10^−29^	1.20 × 10^−25^
	OE6A104771	cyclic dof factor 1-like	DNA binding (GO: 0003677); regulation of transcription, DNA-templated (GO: 0006355)	−3.98	1.79 × 10^−17^	3.89 × 10^−15^
	OE6A021342	cyclic dof factor 1-like	DNA binding (GO: 0003677); regulation of transcription, DNA-templated (GO: 0006355)	−2.22	4.30 × 10^−13^	3.18 × 10^−11^
	OE6A085809	cyclic dof factor 2-like	DNA binding (GO: 0003677); regulation of transcription, DNA-templated (GO: 0006355)	−2.31	1.42 × 10^−16^	2.57 × 10^−14^
	OE6A085809	cyclic dof factor 2-like	DNA binding (GO: 0003677); regulation of transcription, DNA-templated (GO: 0006355)	−2.31	1.42 × 10^−16^	2.57 × 10^−14^
	OE6A062062	ultraviolet-B receptor UVR8	NA	−2.06	1.49 × 10^−24^	2.48 × 10^−21^
	OE6A103537	flowering locus T	phosphatidylethanolamine binding (GO: 0008429); regulation of flower development (GO: 0009909); photoperiodism, flowering (GO: 0048573)	−1.48	3.96 × 10^−4^	2.26 × 10^−3^
	OE6A043940	zinc finger CONSTANS-LIKE 6	protein binding (GO: 0005515)	3.67	4.24 × 10^−6^	4.52 × 10^−5^
	OE6A111642	zinc finger CONSTANS-LIKE 10-like isoform X1	protein binding (GO: 0005515); intracellular anatomical structure (GO: 0005622); zinc ion binding (GO: 0008270)	2.47	1.29 × 10^−10^	4.93 × 10^−9^
	OE6A061348	zinc finger CONSTANS-LIKE 9-like	protein binding (GO: 0005515); intracellular anatomical structure (GO: 0005622); zinc ion binding (GO: 0008270)	3.70	5.65 × 10^−18^	1.48 × 10^−15^
	OE6A061639	zinc finger CONSTANS-LIKE 16-like	protein binding (GO: 0005515); intracellular anatomical structure (GO: 0005622); zinc ion binding (GO: 0008270)	2.25	1.25 × 10^−10^	4.81 × 10^−9^
	OE6A082516	zinc finger CONSTANS-LIKE 2-like	protein binding (GO: 0005515); intracellular anatomical structure (GO: 0005622); nucleus (GO: 0005634); zinc ion binding (GO: 0008270); response to light stimulus (GO: 0009416); regulation of flower development (GO: 0009909)	−2.37	4.09 × 10^−19^	1.47 × 10^−16^
**Gibberellin** **metabolism**	OE6A120203	gibberellin 2-β-dioxygenase 2-like	2-oxoglutarate-dependent dioxygenase activity (GO: 0016706); metal ion binding (GO: 0046872); obsolete oxidation-reduction process (GO: 0055114); organic substance metabolic process (GO: 0071704)	2.44	1.36 × 10^−7^	2.27 × 10^−6^
**Abscisic acid** **metabolism**	OE6A091606	abscisic acid 8-hydroxylase 2	monooxygenase activity (GO: 0004497); iron ion binding (GO: 0005506);integral component of membrane (GO: 0016021);oxidoreductase activity, acting on paired donors, with incorporation or reduction of molecular oxygen (GO: 0016705);heme binding (GO: 0020037);obsolete oxidation-reduction process (GO: 0055114)	2.23	2.71 × 10^−4^	1.64 × 10^−3^
**Nitrate** **transporter**	OE6A086620	NRT1-PTR nitrate transporter	transporter activity (GO: 0005215); transport (GO: 0006810); integral component of membrane (GO: 0016021)	−3.44	6.27 × 10^−15^	7.91 × 10^−13^
	OE6A054819	NRT1-PTR nitrate transporter	transporter activity (GO: 0005215); transport (GO: 0006810); integral component of membrane (GO: 0016021)	−3.73	4.08 × 10^−15^	5.41 × 10^−13^
	OE6A047446	NRT1/PTR family-like	transporter activity (GO: 0005215); oligopeptide transport (GO: 0006857); integral component of membrane (GO: 0016021)	−2.69	1.44 × 10^−5^	1.32 × 10^−4^
**Metabolism and transport of** **carbohydrates**	OE6A012642	bidirectional sugar transporter SWEET12-like	plasma membrane (GO: 0005886); carbohydrate transport (GO: 0008643); integral component of membrane (GO: 0016021)	−2.31	9.01 × 10^−3^	2.94 × 10^−2^
	OE6A006122	UDP-galactose UDP-glucose transporter 4	carbohydrate transport (GO: 0008643); integral component of membrane (GO: 0016021); integral component of Golgi membrane (GO: 0030173); integral component of endoplasmic reticulum membrane (GO: 0030176); 3′-phosphoadenosine 5′-phosphosulfate transmembrane transporter activity (GO: 0046964); transmembrane transport (GO: 0055085); 3′-phospho-5′-adenylyl sulfate transmembrane transport (GO: 1902559)	−2.12	4.02 × 10^−21^	2.82 × 10^−18^
	OE6A058275	probable sucrose-phosphate synthase 2	Golgi apparatus (GO: 0005794); starch metabolic process (GO: 0005982); sucrose metabolic process (GO: 0005985); sucrose biosynthetic process (GO: 0005986); L-phenylalanine metabolic process (GO: 0006558); tyrosine metabolic process (GO: 0006570); sucrose synthase activity (GO: 0016157); sucrose-phosphate synthase activity (GO: 0046524); glutamine N-phenylacetyltransferase activity (GO: 0047947)	−2.16	2.96 × 10^−14^	3.09 × 10^−12^
	OE6A071301	UDP-glucose 6-dehydrogenase 1-like	UDP-glucose 6-dehydrogenase activity (GO: 0003979); starch metabolic process (GO: 0005982); sucrose metabolic process (GO: 0005985); nucleotide metabolic process (GO: 0009117); NAD binding (GO: 0051287); obsolete oxidation-reduction process (GO: 0055114)	−3.18	8.59 × 10^−9^	1.98 × 10^−7^
	OE6A084556	β-galactosidase 16-like	β-galactosidase activity (GO: 0004565); cell wall (GO: 0005618); vacuole (GO: 0005773); galactose metabolic process (GO: 0006012); glycosaminoglycan catabolic process (GO: 0006027); glycosphingolipid metabolic process (GO: 0006687); β-galactosidase complex (GO: 0009341); carbohydrate binding (GO: 0030246); glycerolipid metabolic process (GO: 0046486)	−2.32	9.87 × 10^−21^	5.92 × 10^−18^
	OE6A061003	fructose-1	fructose catabolic process (GO: 0006001); mannose metabolic process (GO: 0006013);gluconeogenesis (GO: 0006094); glycolytic process (GO: 0006096); pentose-phosphate shunt (GO: 0006098); chloroplast (GO: 0009507); carbon utilization (GO: 0015976); dephosphorylation (GO: 0016311); reductive pentose-phosphate cycle (GO: 0019253); 2-alkenal reductase [NAD(P) + ] activity (GO: 0032440); fructose 1,6-bisphosphate 1-phosphatase activity (GO: 0042132); metal ion binding (GO: 0046872); obsolete oxidation-reduction process (GO: 0055114)	−2.05	6.82 × 10^−5^	5.07 × 10^−4^
	OE6A019327	NDR1 HIN1 26	membrane (GO: 0016020)	−2.06	5.99 × 10^−3^	2.12 × 10^−2^
	OE6A085951	stachyose synthase-like	galactose metabolic process (GO: 0006012); galactinol-raffinose galactosyltransferase activity (GO: 0047268)	−3.48	4.88 × 10^−6^	5.12 × 10^−5^
	OE6A035536	NDR1 HIN1 26	integral component of membrane (GO: 0016021)	−2.04	7.25 × 10^−3^	2.48 × 10^−2^
	OE6A035740	O-fucosyltransferase 19-like	cytoplasm (GO: 0005737); integral component of membrane (GO: 0016021)	−2.75	1.23 × 10^−7^	2.07 × 10^−6^
	OE6A055193	NADP-dependent D-sorbitol-6-phosphate dehydrogenase	aldose-6-phosphate reductase (NADPH) activity (GO: 0047641); obsolete oxidation-reduction process (GO: 0055114)	−2.76	6.69 × 10^−31^	8.15 × 10^−27^
**Cell wall** **metabolism**	OE6A100291	xyloglucan endotransglycosylase hydrolase	hydrolase activity, hydrolyzing O-glycosyl compounds (GO: 0004553); cell wall (GO: 0005618); xyloglucan metabolic process (GO: 0010411); xyloglucan:xyloglucosyl transferase activity (GO: 0016762); cell wall biogenesis (GO: 0042546); apoplast (GO: 0048046); cell wall organization (GO: 0071555)	−2.19	4.00 × 10^−20^	1.93 × 10^−17^
	OE6A117660	xyloglucan endotransglucosylase hydrolase 2-like	hydrolase activity, hydrolyzing O-glycosyl compounds (GO: 0004553); cell wall (GO: 0005618); xyloglucan metabolic process (GO: 0010411); xyloglucan:xyloglucosyl transferase activity (GO: 0016762); cell wall biogenesis (GO: 0042546); apoplast (GO: 0048046)	−2.45	4.33 × 10^−7^	6.26 × 10^−6^
	OE6A112971	Glucan 1	hydrolase activity, hydrolyzing O-glycosyl compounds (GO: 0004553); carbohydrate metabolic process (GO: 0005975)	−2.93	1.64 × 10^−25^	3.34 × 10^−22^
	OE6A053366	probable pectate lyase 5	pectate lyase activity (GO: 0030570); pectin catabolic process (GO: 0045490); metal ion binding (GO: 0046872)	−2.19	4.88 × 10^−19^	1.72 × 10^−16^
	OE6A014742	β-glucosidase-like	hydrolase activity, hydrolyzing O-glycosyl compounds (GO: 0004553); carbohydrate metabolic process (GO: 0005975)	−2.28	3.24 × 10^−6^	3.58 × 10^−5^
	OE6A100291	xyloglucan endotransglycosylase hydrolase	hydrolase activity, hydrolyzing O-glycosyl compounds (GO: 0004553); cell wall (GO: 0005618); xyloglucan metabolic process (GO: 0010411); xyloglucan:xyloglucosyl transferase activity (GO: 0016762); cell wall biogenesis (GO: 0042546); apoplast (GO: 0048046); cell wall organization (GO: 0071555)	−2.19	4.00 × 10^−20^	1.93 × 10^−17^
	OE6A117660	xyloglucan endotransglucosylase hydrolase 2-like	hydrolase activity, hydrolyzing O-glycosyl compounds (GO: 0004553); cell wall (GO: 0005618); xyloglucan metabolic process (GO: 0010411); xyloglucan:xyloglucosyl transferase activity (GO: 0016762); cell wall biogenesis (GO: 0042546); apoplast (GO: 0048046)	−2.45	4.33 × 10^−7^	6.26 × 10^−6^
	OE6A024144	cellulose synthase G3	starch metabolic process (GO: 0005982); sucrose metabolic process (GO: 0005985); UDP-glucose metabolic process (GO: 0006011); integral component of membrane (GO: 0016021); cellulose synthase (UDP-forming) activity (GO: 0016760); cellulose biosynthetic process (GO: 0030244); cell wall organization (GO: 0071555)	−3.03	2.31 × 10^−5^	1.97 × 10^−4^
	OE6A058805	endoglucanase 12	starch metabolic process (GO: 0005982); sucrose metabolic process (GO: 0005985); cellulase activity (GO: 0008810); integral component of membrane (GO: 0016021); cellulose catabolic process (GO: 0030245)	−2.15	1.92 × 10^−3^	8.31 × 10^−3^
	OE6A104762	probable xyloglucan endotransglucosylase hydrolase 23	hydrolase activity, hydrolyzing O-glycosyl compounds (GO: 0004553); cell wall (GO: 0005618); xyloglucan metabolic process (GO: 0010411); xyloglucan:xyloglucosyl transferase activity (GO: 0016762); cell wall biogenesis (GO: 0042546); apoplast (GO: 0048046); cell wall organization (GO: 0071555)	−2.00	8.11 × 10^−10^	2.51 × 10^−8^
	OE6A076685	phospho-2-dehydro-3-deoxyheptonate aldolase 2	tryptophan biosynthetic process (GO: 0000162); 3-deoxy-7-phosphoheptulonate synthase activity (GO: 0003849); tyrosine biosynthetic process (GO: 0006571); L-phenylalanine biosynthetic process (GO: 0009094); chloroplast thylakoid (GO: 0009534)	−2.46	2.56 × 10^−5^	2.16 × 10^−4^
**Metabolism and transport of phenylpropanoids**	OE6A074244	cannabidiolic acid synthase-like	oxidoreductase activity, acting on CH-OH group of donors (GO: 0016614); flavin adenine dinucleotide binding (GO: 0050660); obsolete oxidation-reduction process (GO: 0055114)	−2.14	1.90 × 10^−10^	6.93 × 10^−9^
	OE6A080485	α-farnesene synthase	magnesium ion binding (GO: 0000287); metabolic process (GO: 0008152); terpene synthase activity (GO: 0010333)	−2.02	1.24 × 10^−19^	5.14 × 10^−17^
	OE6A038167	secoisolariciresinol dehydrogenase-like	oxidoreductase activity (GO: 0016491)	−2.19	3.09 × 10^−20^	1.57 × 10^−17^
	OE6A025381	zeaxanthin epoxidase	protein binding (GO: 0005515); response to heat (GO: 0009408); response to water deprivation (GO: 0009414); chloroplast thylakoid membrane (GO: 0009535); zeaxanthin epoxidase [overall] activity (GO: 0009540); abscisic acid biosynthetic process (GO: 0009688); xanthophyll biosynthetic process (GO: 0016123); chloroplast membrane (GO: 0031969); secondary metabolite biosynthetic process (GO: 0044550); zeaxanthin epoxidase activity (GO: 0052662); antheraxanthin epoxidase activity (GO: 0052663); obsolete oxidation-reduction process (GO: 0055114)	−2.03	3.39 × 10^−8^	6.73 × 10^−7^
	OE6A057061	secoisolariciresinol dehydrogenase-like	oxidoreductase activity (GO: 0016491)	−2.16	4.25 × 10^−19^	1.51 × 10^−16^
	OE6A097656	(-)-germacrene D synthase	magnesium ion binding (GO: 0000287); metabolic process (GO: 0008152); terpene synthase activity (GO: 0010333)	−2.12	3.86 × 10^−20^	1.89 × 10^−17^
	OE6A015761	secoisolariciresinol dehydrogenase-like	oxidoreductase activity (GO: 0016491)	−2.39	5.77 × 10^−14^	5.54 × 10^−12^
	OE6A104708	myrcene synthase	magnesium ion binding (GO: 0000287); metabolic process (GO: 0008152); terpene synthase activity (GO: 0010333)	−2.51	5.87 × 10^−3^	2.08 × 10^−2^
	OE6A081156	flavone synthase II	iron ion binding (GO: 0005506); integral component of membrane (GO: 0016021); oxidoreductase activity, acting on paired donors, with incorporation or reduction of molecular oxygen, NAD(P)H as one donor, and incorporation of one atom of oxygen (GO: 0016709); heme binding (GO: 0020037); secondary metabolite biosynthetic process (GO: 0044550); obsolete oxidation-reduction process (GO: 0055114)	−2.03	3.54 × 10^−17^	7.14 × 10^−15^
	OE6A093474	2-hydroxyisoflavanone dehydratase-like	metabolic process (GO: 0008152); hydrolase activity (GO: 0016787)	−2.38	8.61 × 10^−17^	1.59 × 10^−14^
	OE6A063869	diihydroflavonol 4-reductase	anthocyanin-containing compound biosynthetic process (GO: 0009718); integral component of membrane (GO: 0016021); dihydrokaempferol 4-reductase activity (GO: 0045552); obsolete coenzyme binding (GO: 0050662); obsolete oxidation-reduction process (GO: 0055114)	−2.21	3.38 × 10^−3^	1.32 × 10^−2^
	OE6A096251	pelargonidin 3-O-(6-caffeoylglucoside) 5-O-(6-O-malonylglucoside) 4 -malonyltransferase-like	transferase activity, transferring acyl groups other than amino-acyl groups (GO: 0016747)	−2.35	9.44 × 10^−9^	2.15 × 10^−7^
	OE6A022660	α-farnesene synthase	magnesium ion binding (GO: 0000287); metabolic process (GO: 0008152); terpene synthase activity (GO: 0010333)	−2.19	1.58 × 10^−19^	6.41 × 10^−17^

## Data Availability

The raw reads were archived in the NCBI SRA database under accession number PRJNA674067.

## Supplementary Materials

The following are available online at https://www.mdpi.com/article/10.3390/genes12040545/s1, Figure S1: Flow diagram of pipeline, Figure S2: qRT-PCR of reference genes for expression normalization, Figure S3: Gene Ontology categories identified among the Cellular component, Figure S4: Gene isoforms per locus detected in July by DEXSeq analysis, Figure S5: Gene isoforms per locus detected in March by DEXSeq analysis, Table S1: List of real-time qRT-PCR primers, Table S2: List of DEGs in July and March.
