# Supplementary figures and images for: A Complex Gene Network Mediated by Ethylene Signal Transduction TFs Defines the Flower Induction and Differentiation in Olea europaea L
Source: Genes (Basel). 2021 Apr 9;12(4):545. doi: 10.3390/genes12040545 (PMC8070190; doi:10.3390/genes12040545)

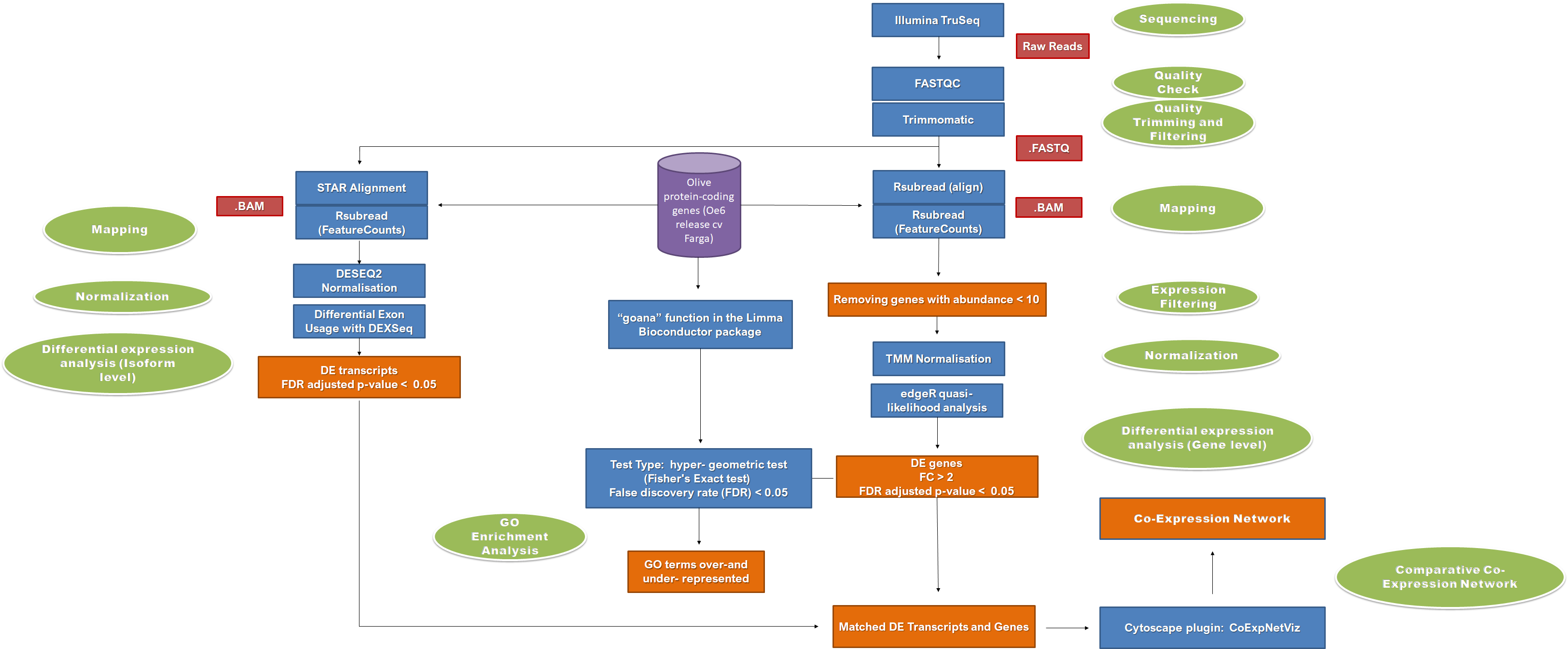

Supplement: Supplementary file 1 [file genes-12-00545-s001.zip › SupplementaryData/FigureS1.tif]

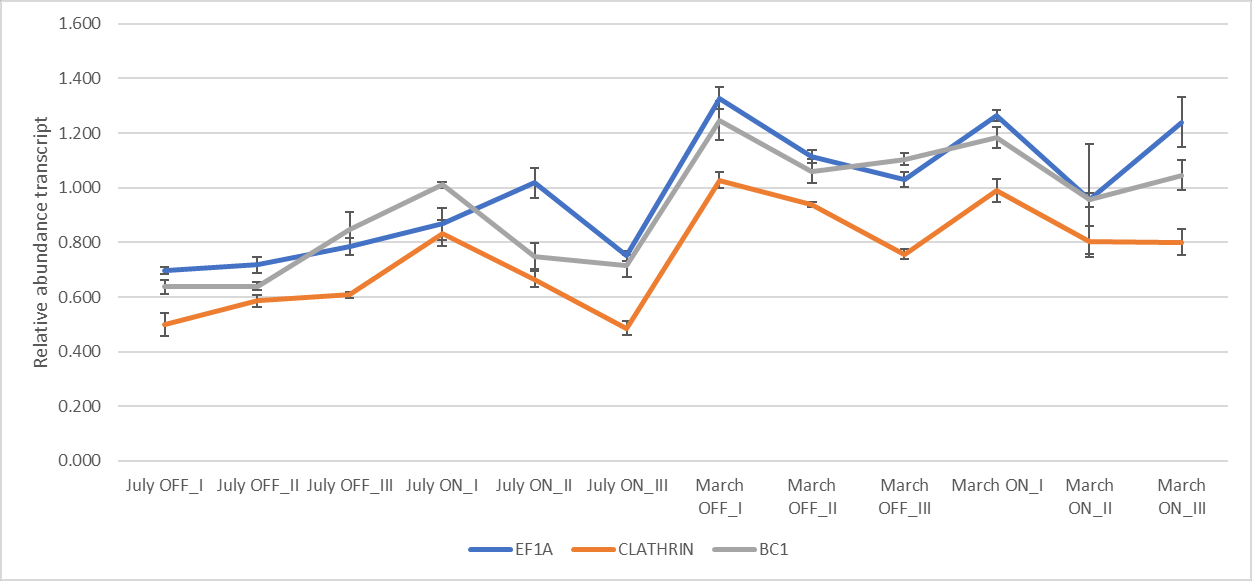

Supplement: Supplementary file 1 [file genes-12-00545-s001.zip › SupplementaryData/FigureS2.tif]

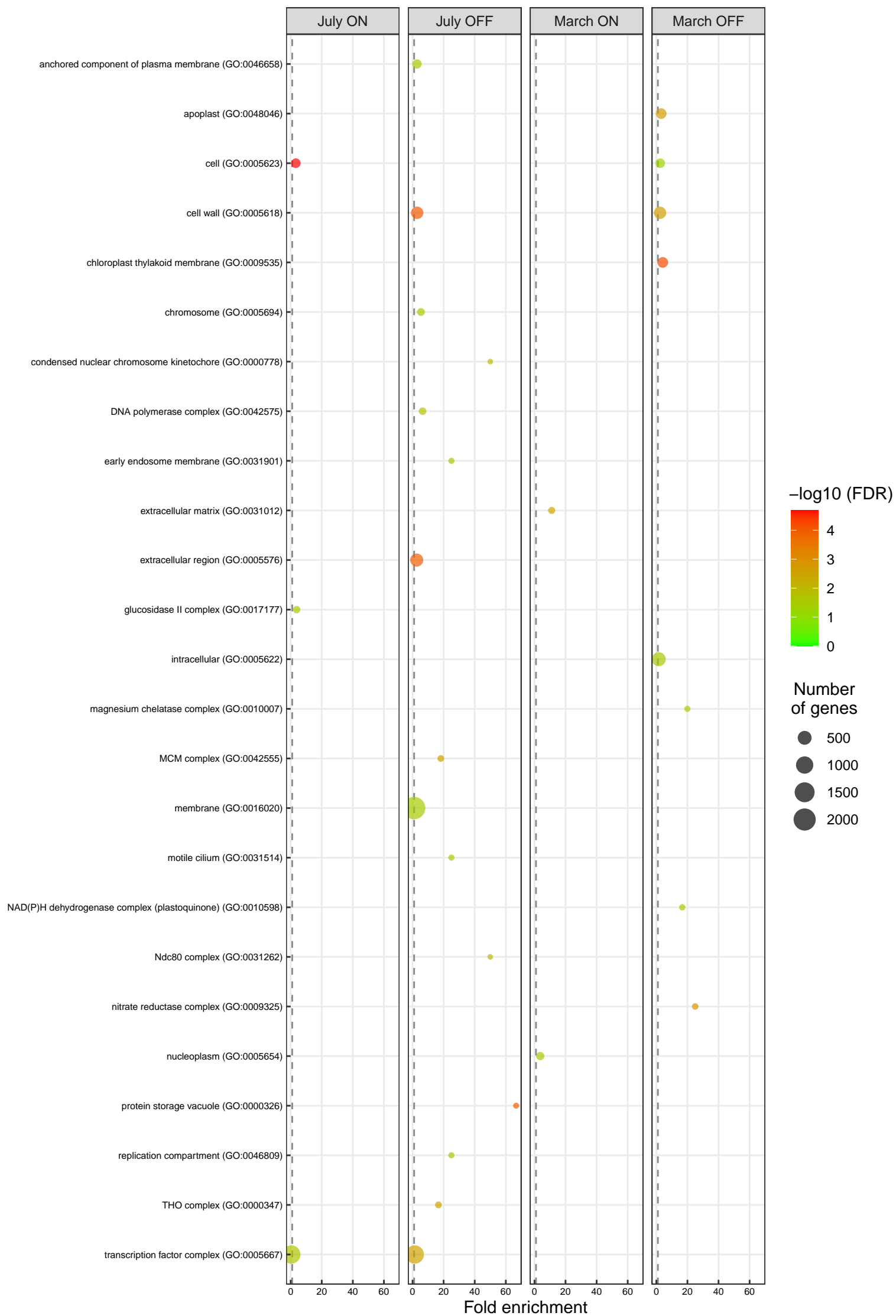

Supplement: Supplementary file 1 [file genes-12-00545-s001.zip › SupplementaryData/FigureS3.pdf]
