# Supplementary material for: A Complex Gene Network Mediated by Ethylene Signal Transduction TFs Defines the Flower Induction and Differentiation in Olea europaea L
Source: Genes (Basel). 2021 Apr 9;12(4):545. doi: 10.3390/genes12040545 (PMC8070190; doi:10.3390/genes12040545)
Supplement: Supplementary file 1 [file FigureS4.pdf › SupplementaryData/FigureS4.pdf]

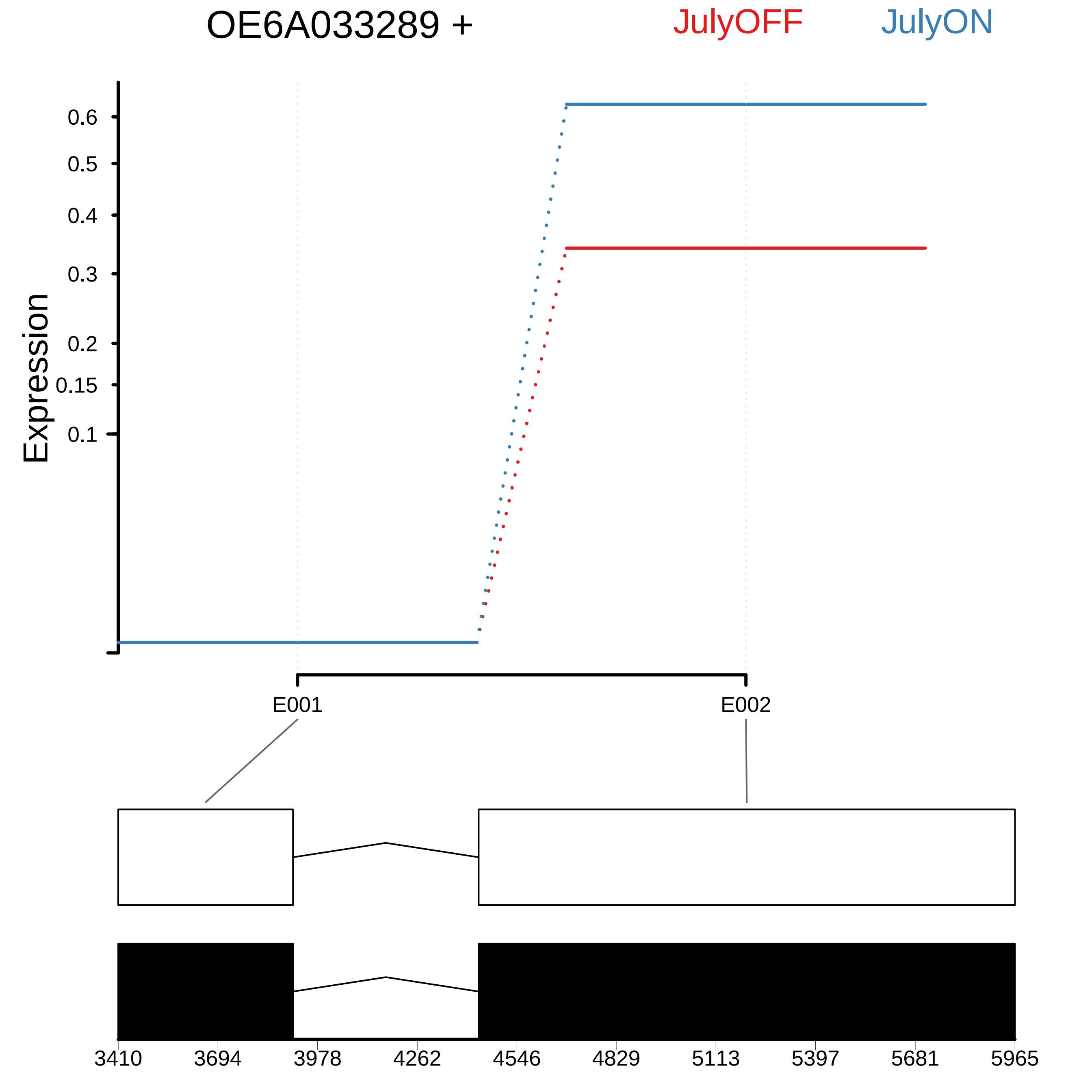

OE6A033289 +
JulyOFF
JulyON
Expression
0.6
0.5
0.4
0.3
0.2
0.15
0.1
E001
E002
3410
3694
3978
4262
4546
4829
5113
5397
5681
5965

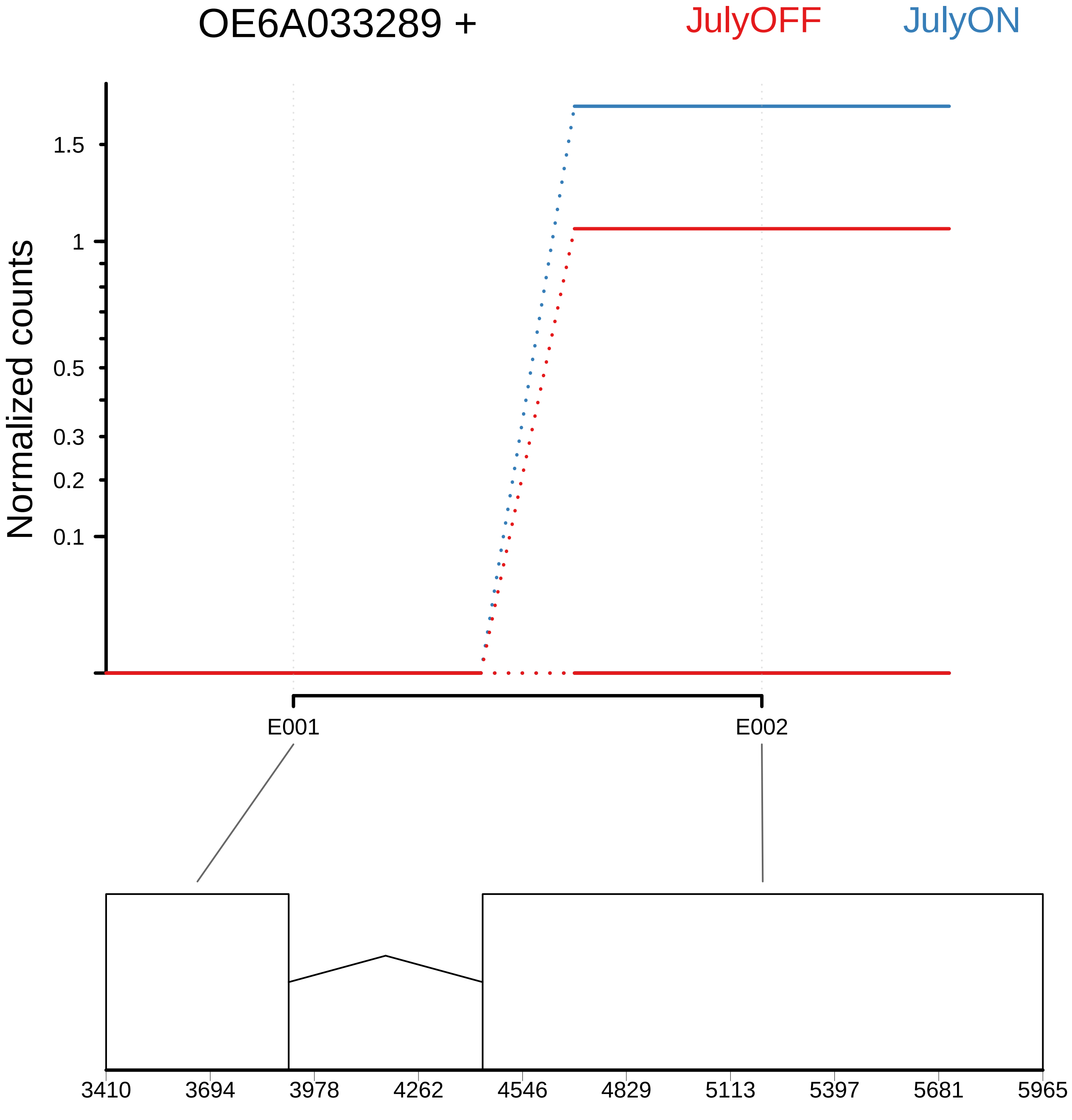
OE6A033289 +
JulyOFF
JulyON
Normalized counts
1.5
1
0.5
0.3
0.2
0.1
E001
E002
3410
3694
3978
4262
4546
4829
5113
5397
5681
5965

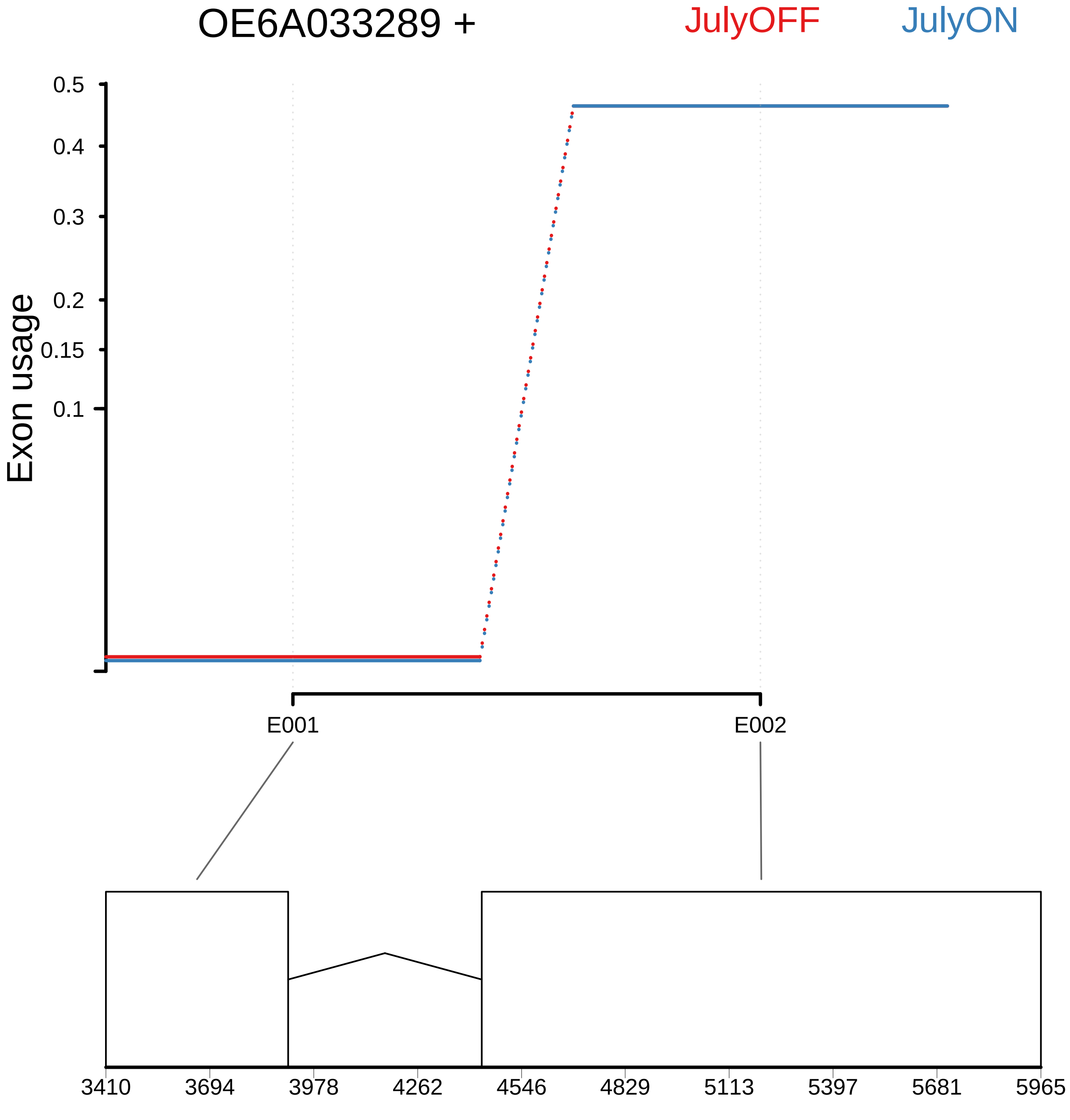

OE6A033289 +
JulyOFF
JulyON
Exon usage
0.5
0.4
0.3
0.2
0.15
0.1
E001
E002
3410
3694
3978
4262
4546
4829
5113
5397
5681
5965

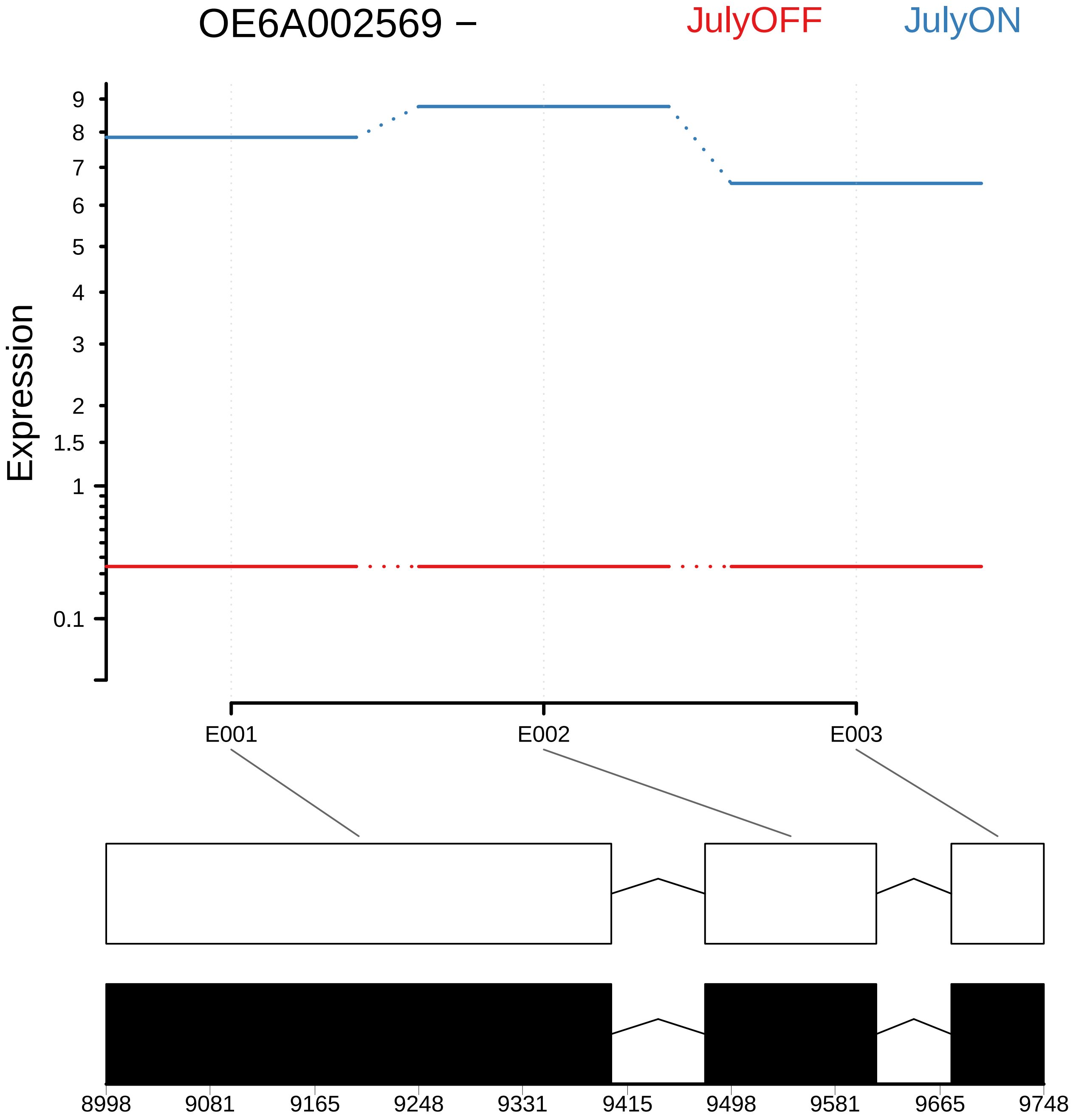

OE6A002569 –
JulyOFF
JulyON
Expression
9
8
7
6
5
4
3
2
1.5
1
0.1
E001
E002
E003
8998
9081
9165
9248
9331
9415
9498
9581
9665
9748

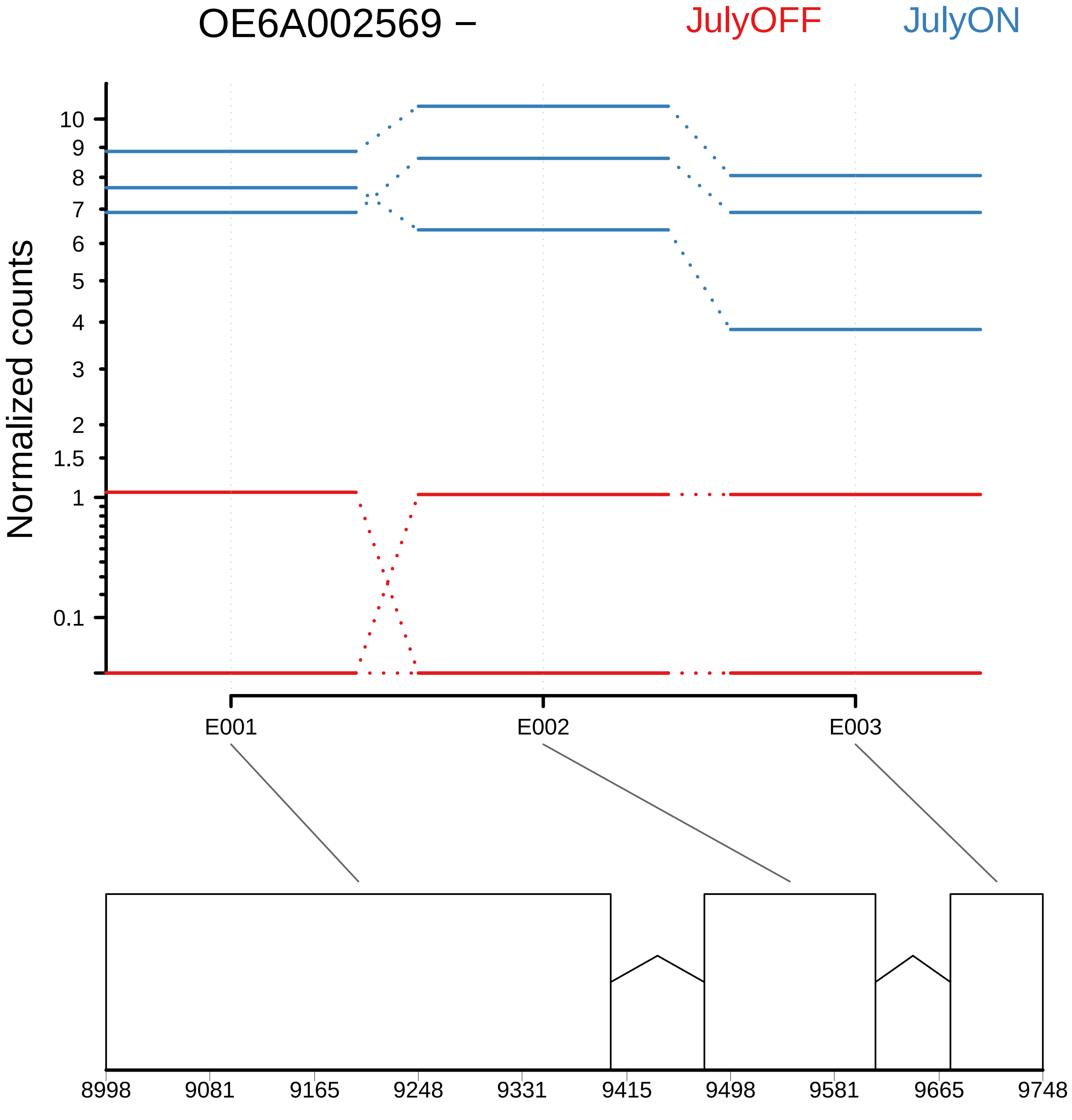
OE6A002569 −
JulyOFF
JulyON
Normalized counts
10
9
8
7
6
5
4
3
2
1.5
1
0.1
E001
E002
E003
8998
9081
9165
9248
9331
9415
9498
9581
9665
9748

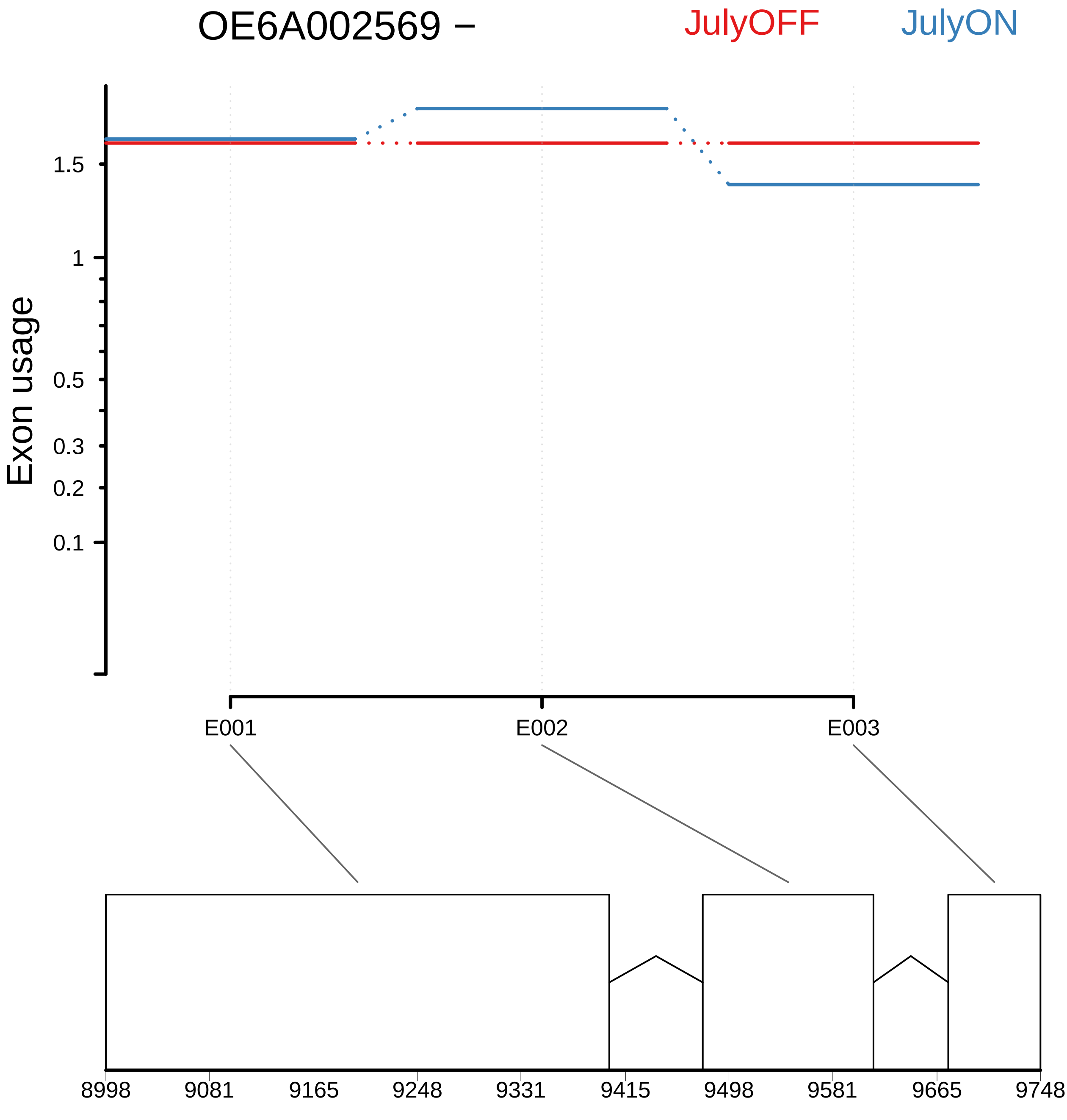

OE6A002569 −
JulyOFF
JulyON
Exon usage
1.5
1
0.5
0.3
0.2
0.1
E001
E002
E003
8998
9081
9165
9248
9331
9415
9498
9581
9665
9748

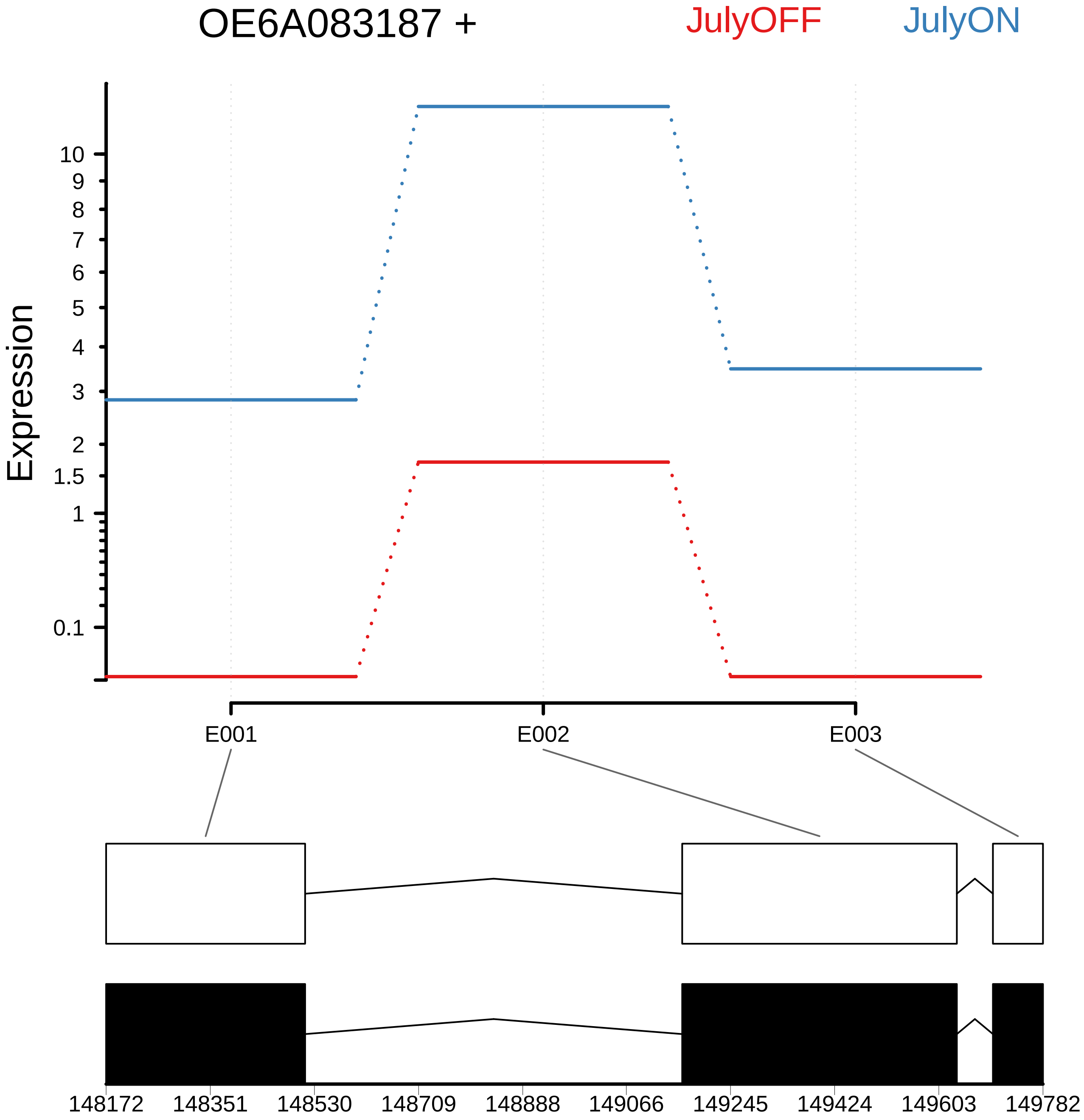
OE6A083187 +
JulyOFF
JulyON
Expression
10
9
8
7
6
5
4
3
2
1.5
1
0.1
E001
E002
E003
148172
148351
148530
148709
148888
149066
149245
149424
149603
149782

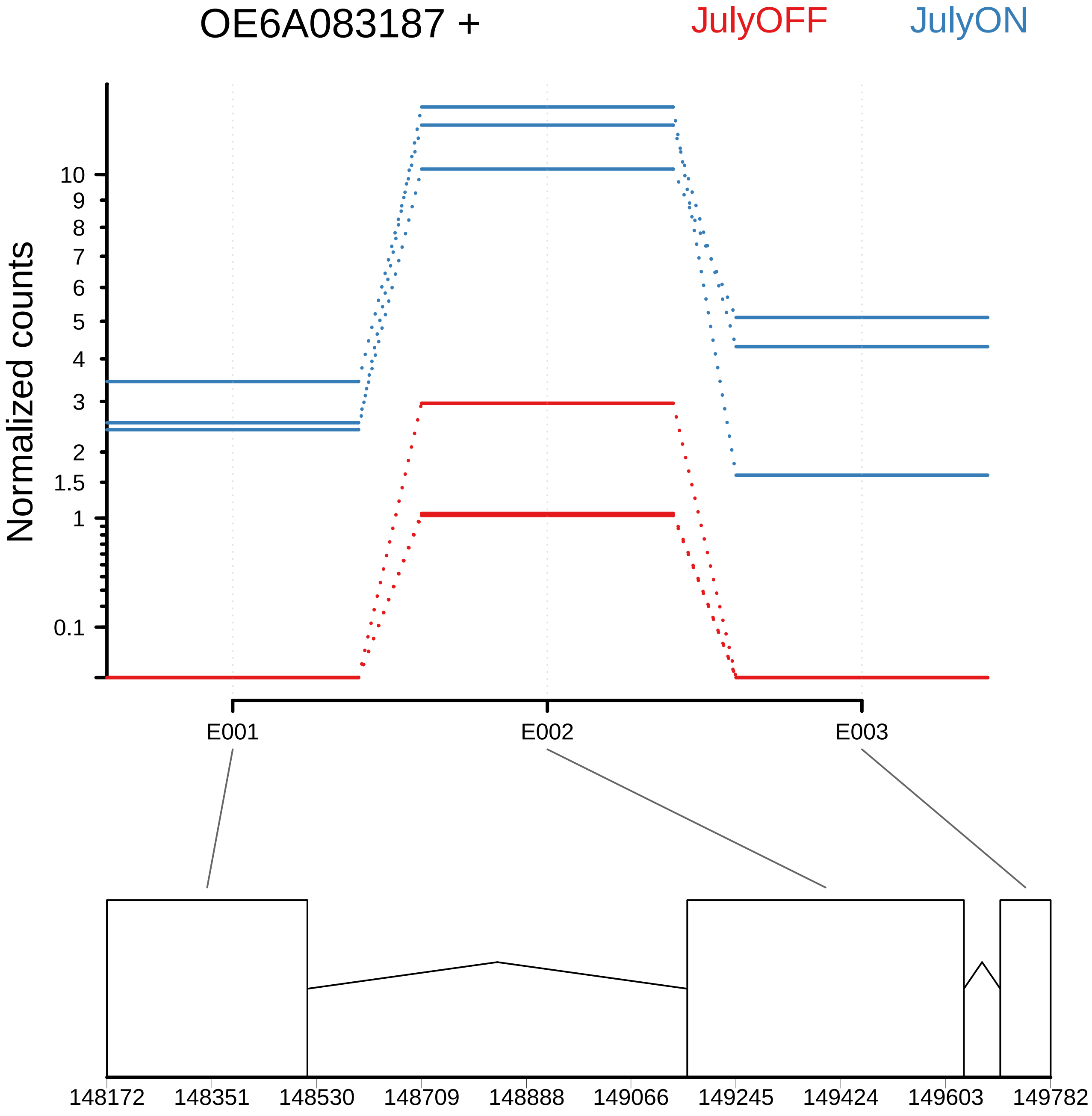

OE6A083187 +
JulyOFF
JulyON
Normalized counts
10
9
8
7
6
5
4
3
2
1.5
1
0.1
E001
E002
E003
148172
148351
148530
148709
148888
149066
149245
149424
149603
149782

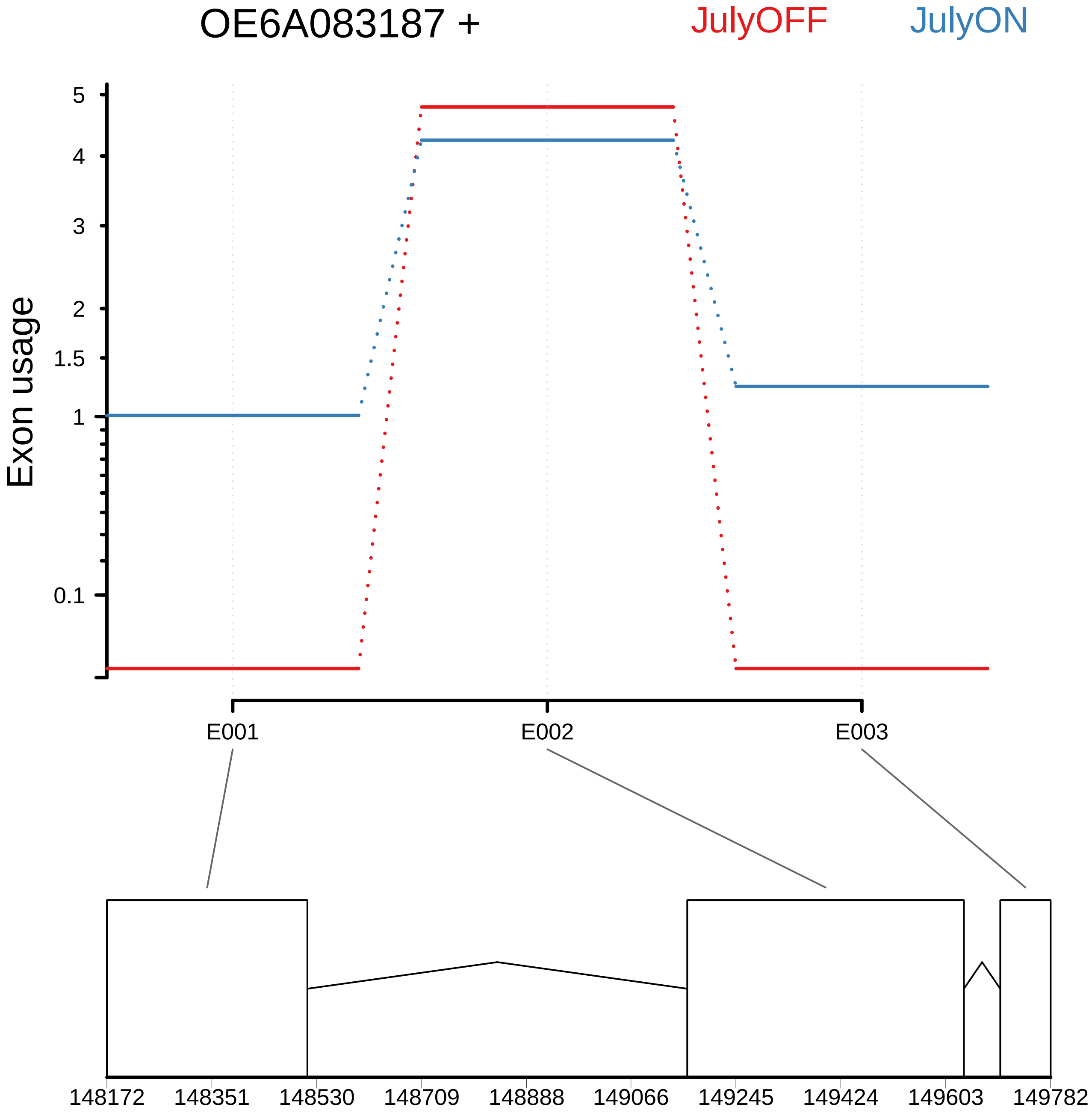

OE6A083187 +
JulyOFF
JulyON
Exon usage
5
4
3
2
1.5
1
0.1
E001
E002
E003
148172
148351
148530
148709
148888
149066
149245
149424
149603
149782

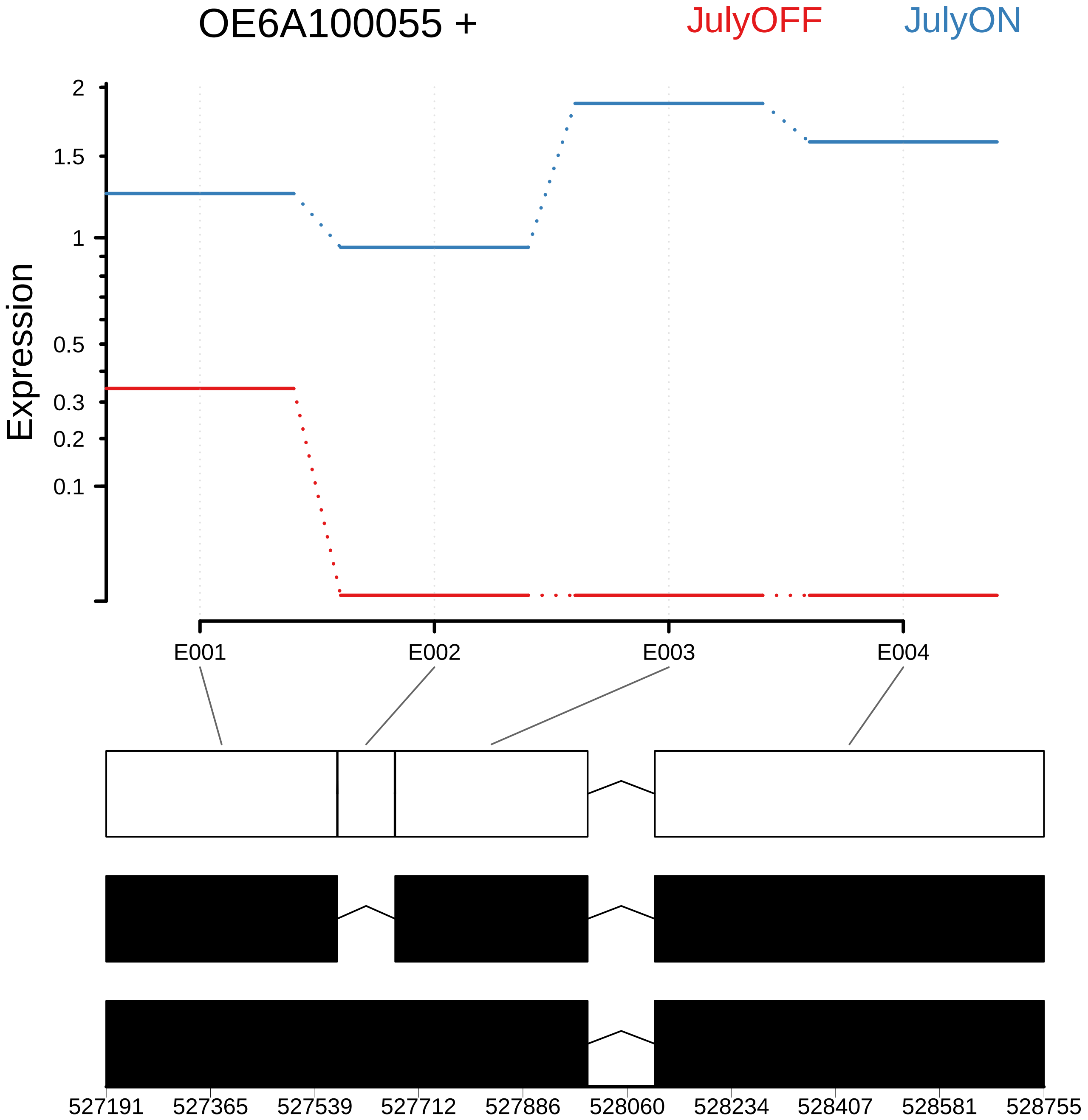

OE6A100055 +
JullyOFF
JulyON
Expression
2
1.5
1
0.5
0.3
0.2
0.1
E001
E002
E003
E004
527191
527365
527539
527712
527886
528060
528234
528407
528581
528755

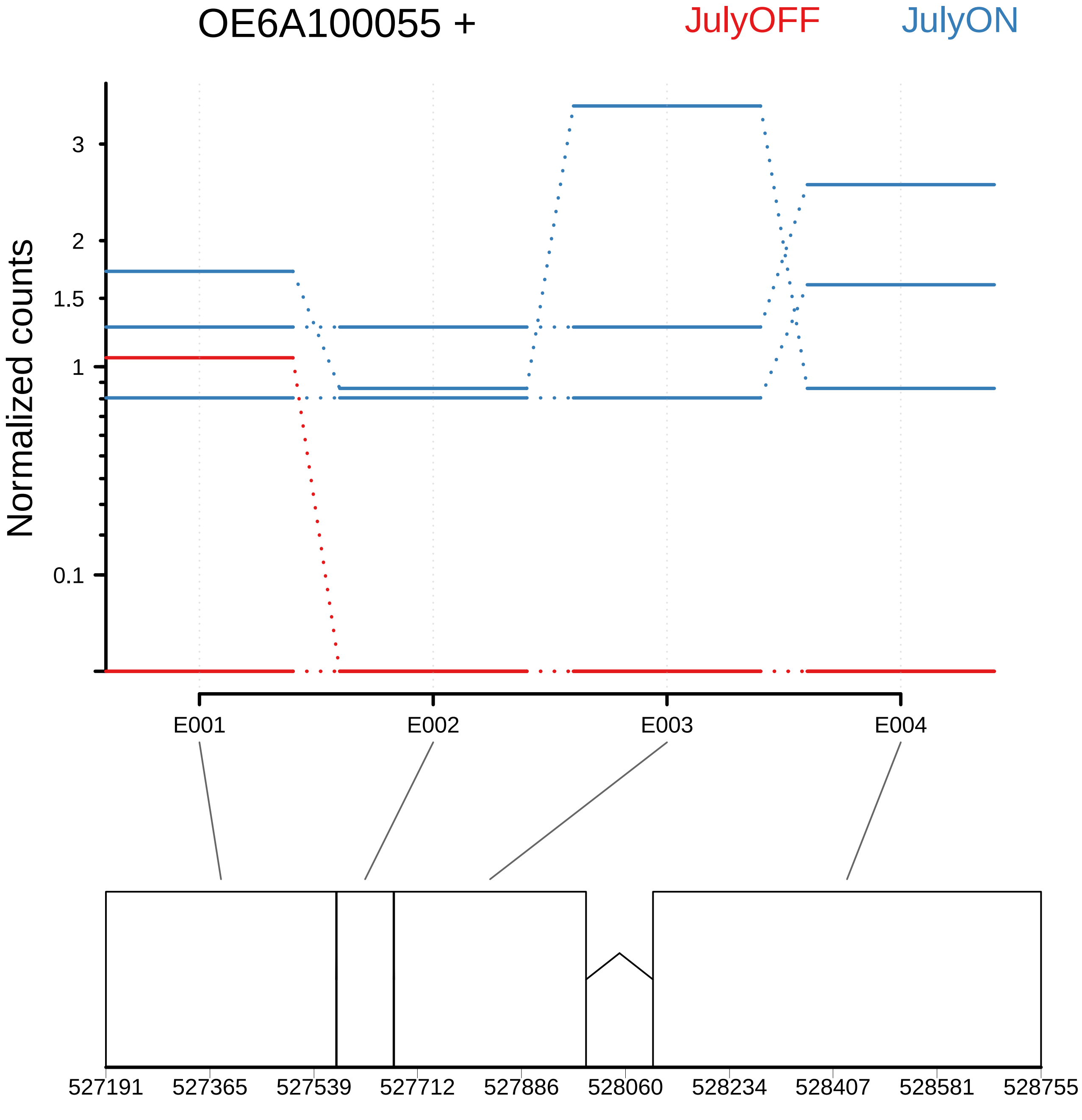

OE6A100055 +
JulyOFF
JulyON
Normalized counts
3
2
1.5
1
0.1
E001
E002
E003
E004
527191
527365
527539
527712
527886
528060
528234
528407
528581
528755

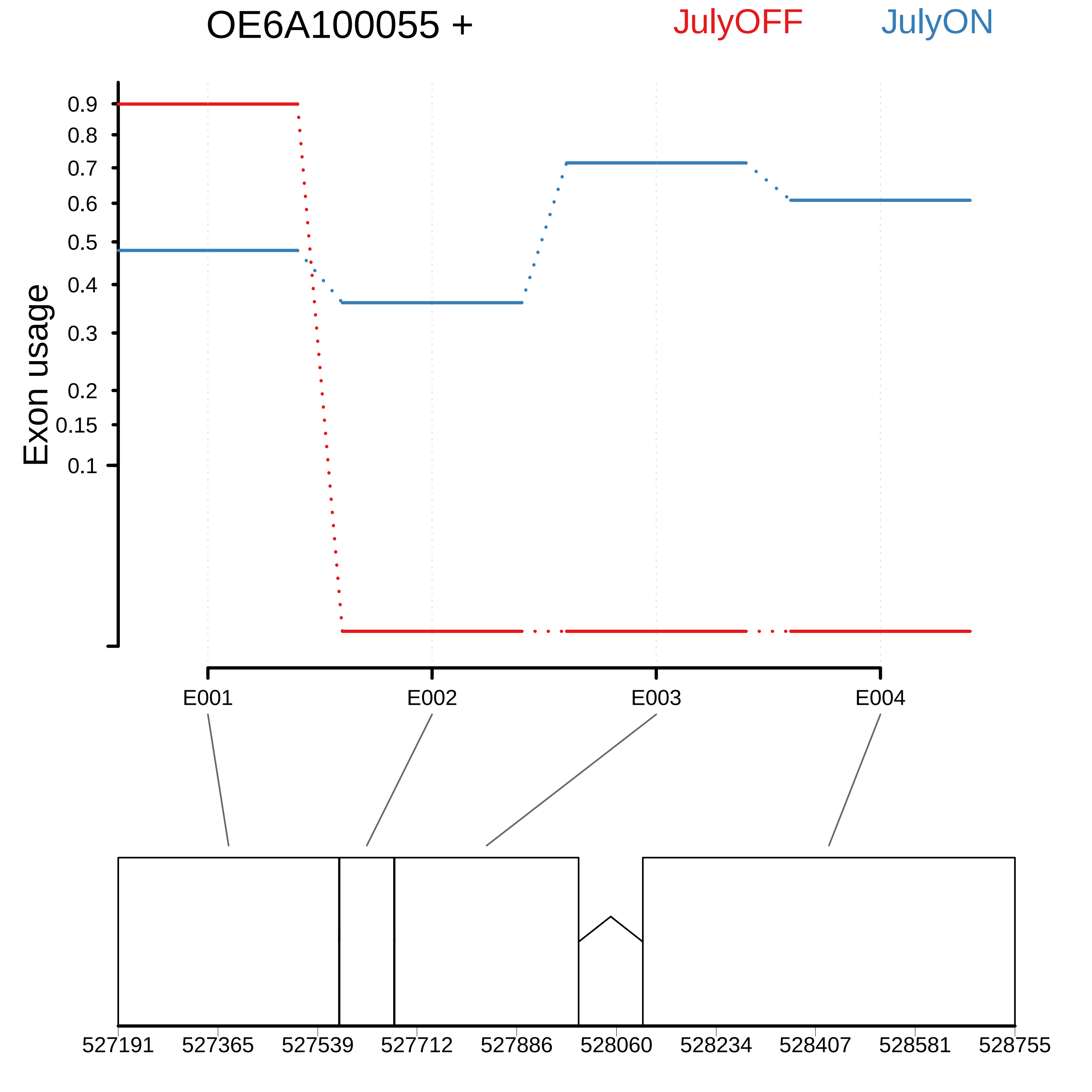

OE6A100055 +
JulyOFF
JulyON
Exon usage
0.9
0.8
0.7
0.6
0.5
0.4
0.3
0.2
0.15
0.1
E001
E002
E003
E004
527191
527365
527539
527712
527886
528060
528234
528407
528581
528755

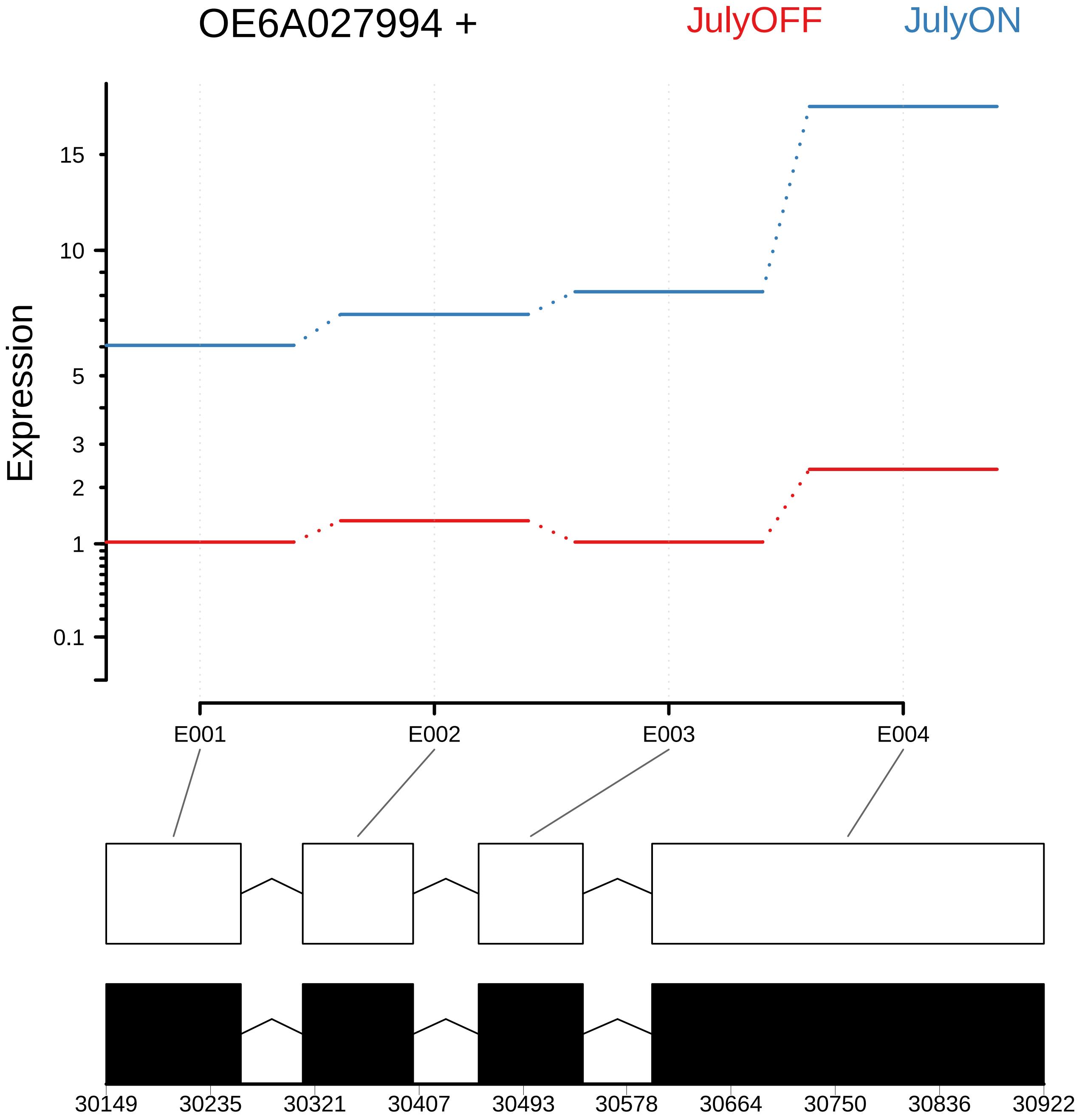

OE6A027994 +
JulyOFF
JulyON
Expression
15
10
5
3
2
1
0.1
E001
E002
E003
E004
30149
30235
30321
30407
30493
30578
30664
30750
30836
30922

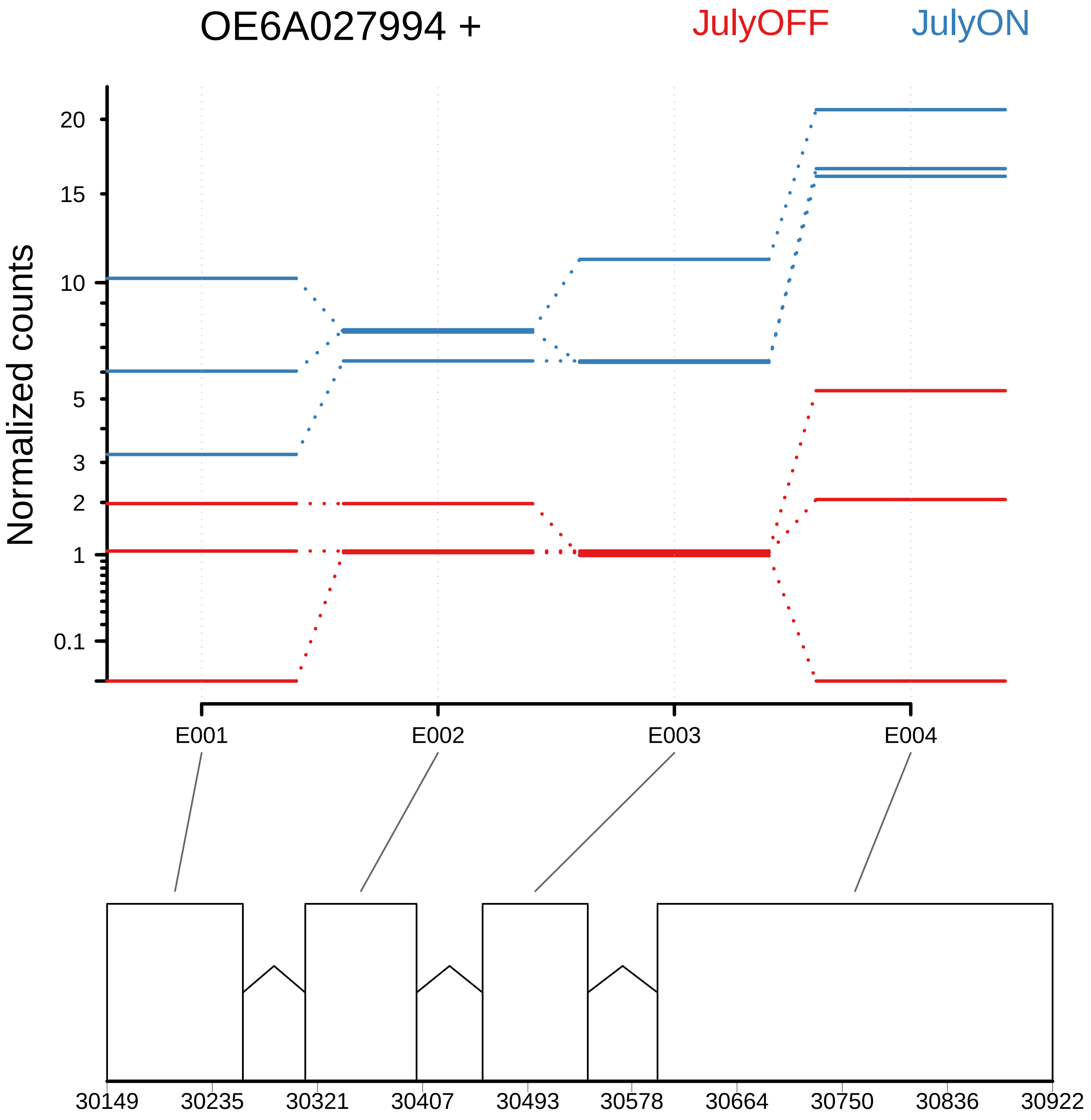

OE6A027994 +
JulyOFF
JulyON
Normalized counts
20
15
10
5
3
2
1
0.1
E001
E002
E003
E004
30149
30235
30321
30407
30493
30578
30664
30750
30836
30922

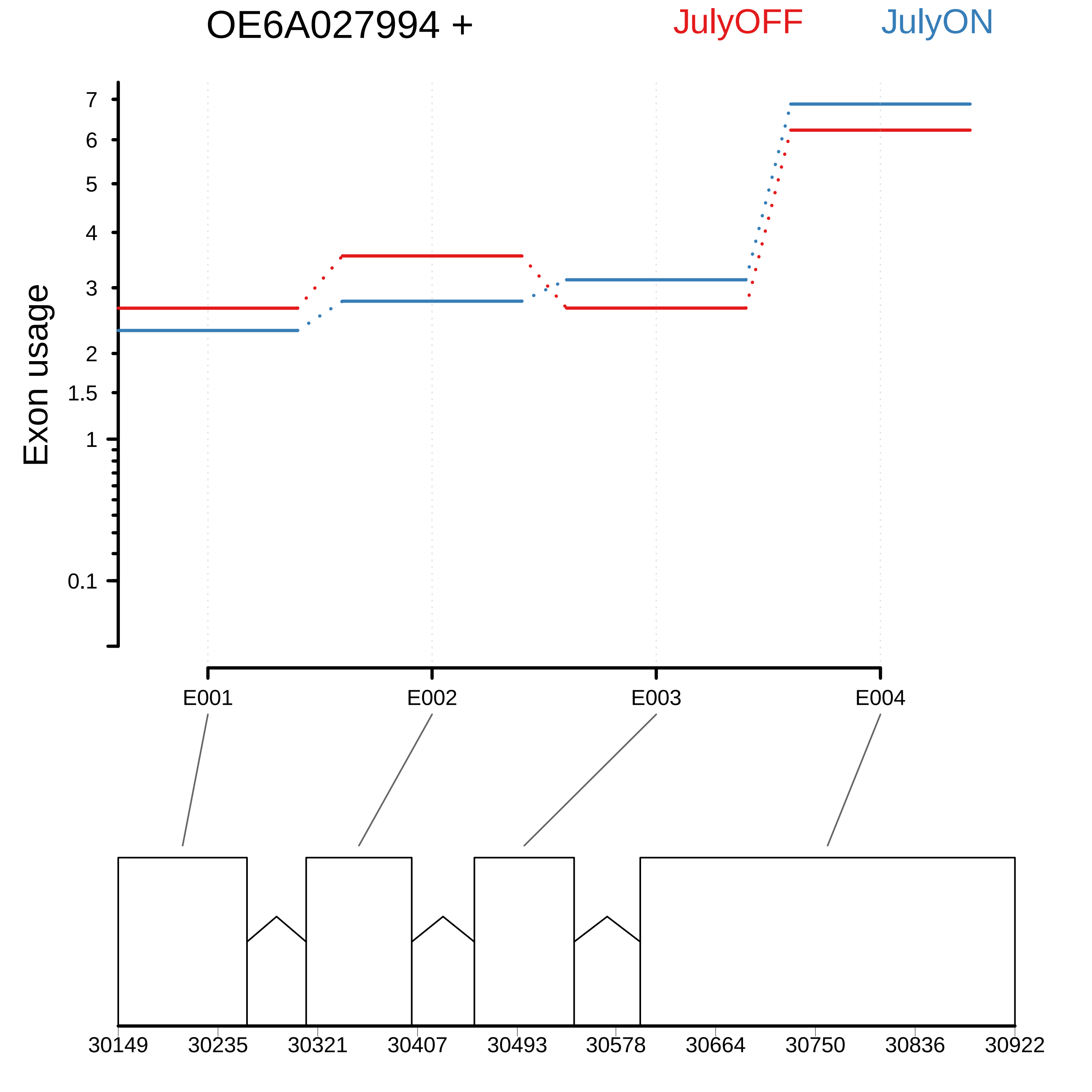
OE6A027994 +
JulyOFF
JulyON
Exon usage
7
6
5
4
3
2
1.5
1
0.1
E001
E002
E003
E004
30149
30235
30321
30407
30493
30578
30664
30750
30836
30922

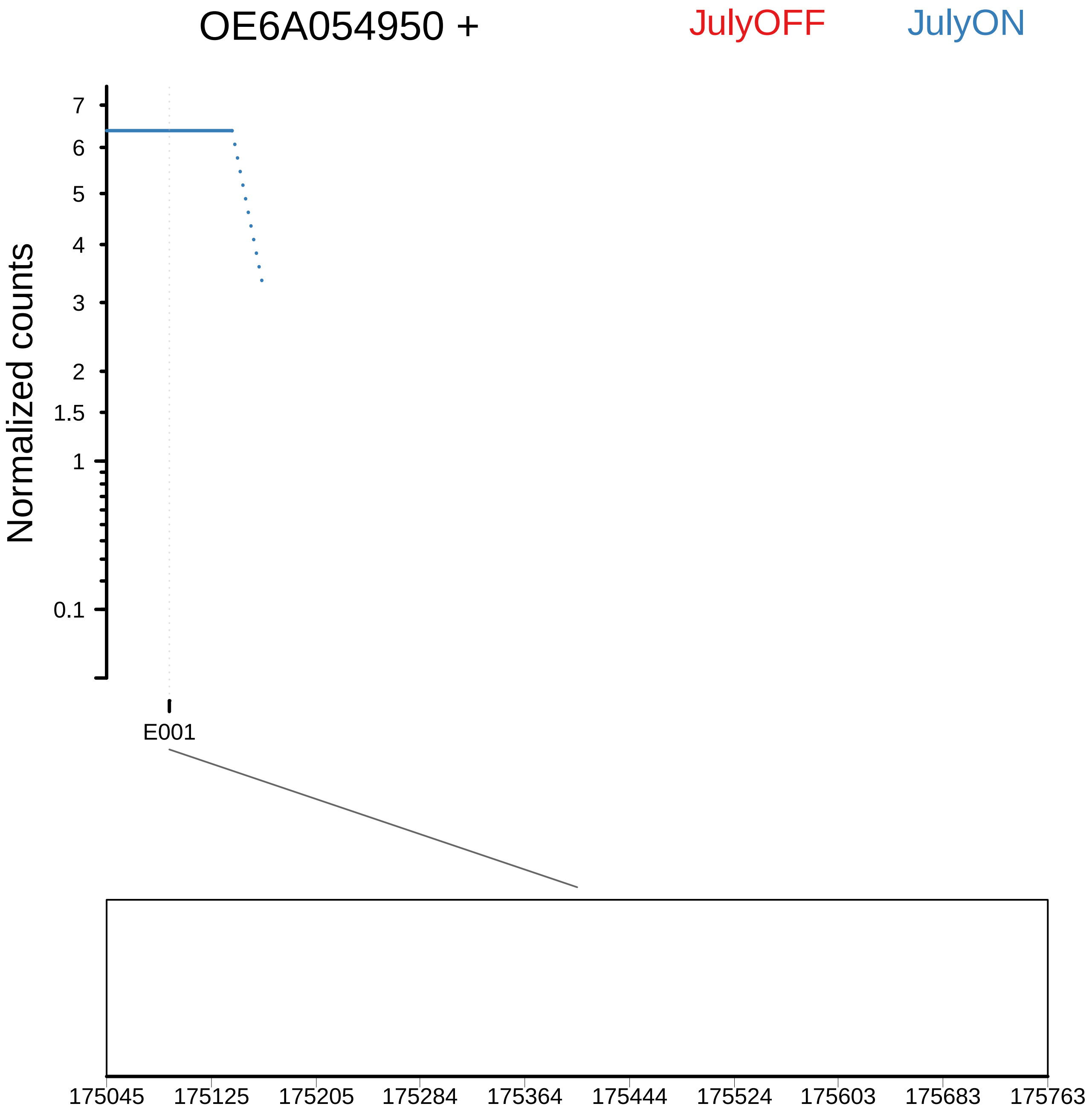
OE6A054950 +
JulyOFF
JulyON
Normalized counts
7
6
5
4
3
2
1.5
1
0.1
E001
175045
175125
175205
175284
175364
175444
175524
175603
175683
175763

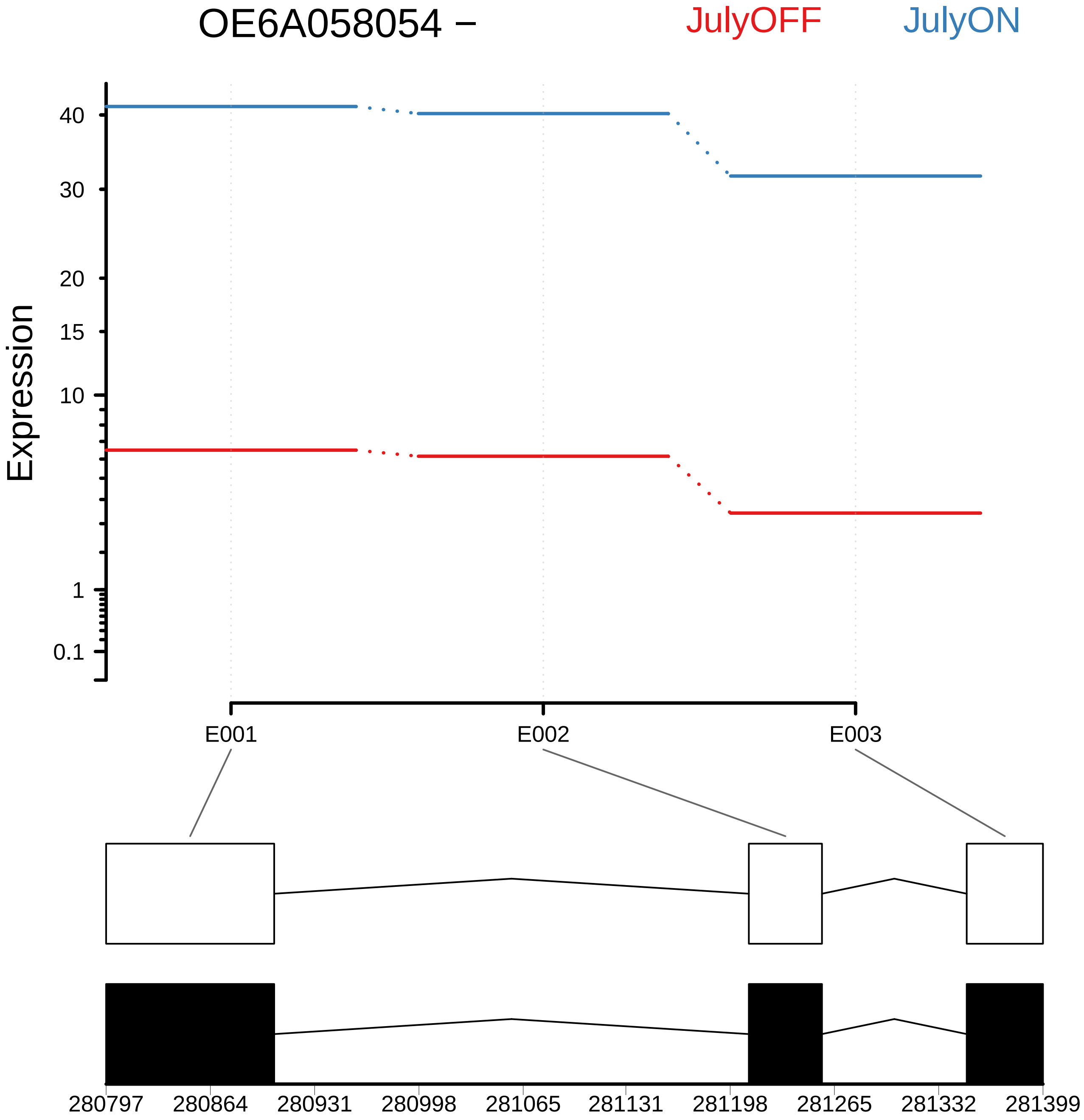

OE6A058054 −
JulyOFF
JulyON
Expression
40
30
20
15
10
1
0.1
E001
E002
E003
280797
280864
280931
280998
281065
281131
281198
281265
281332
281399

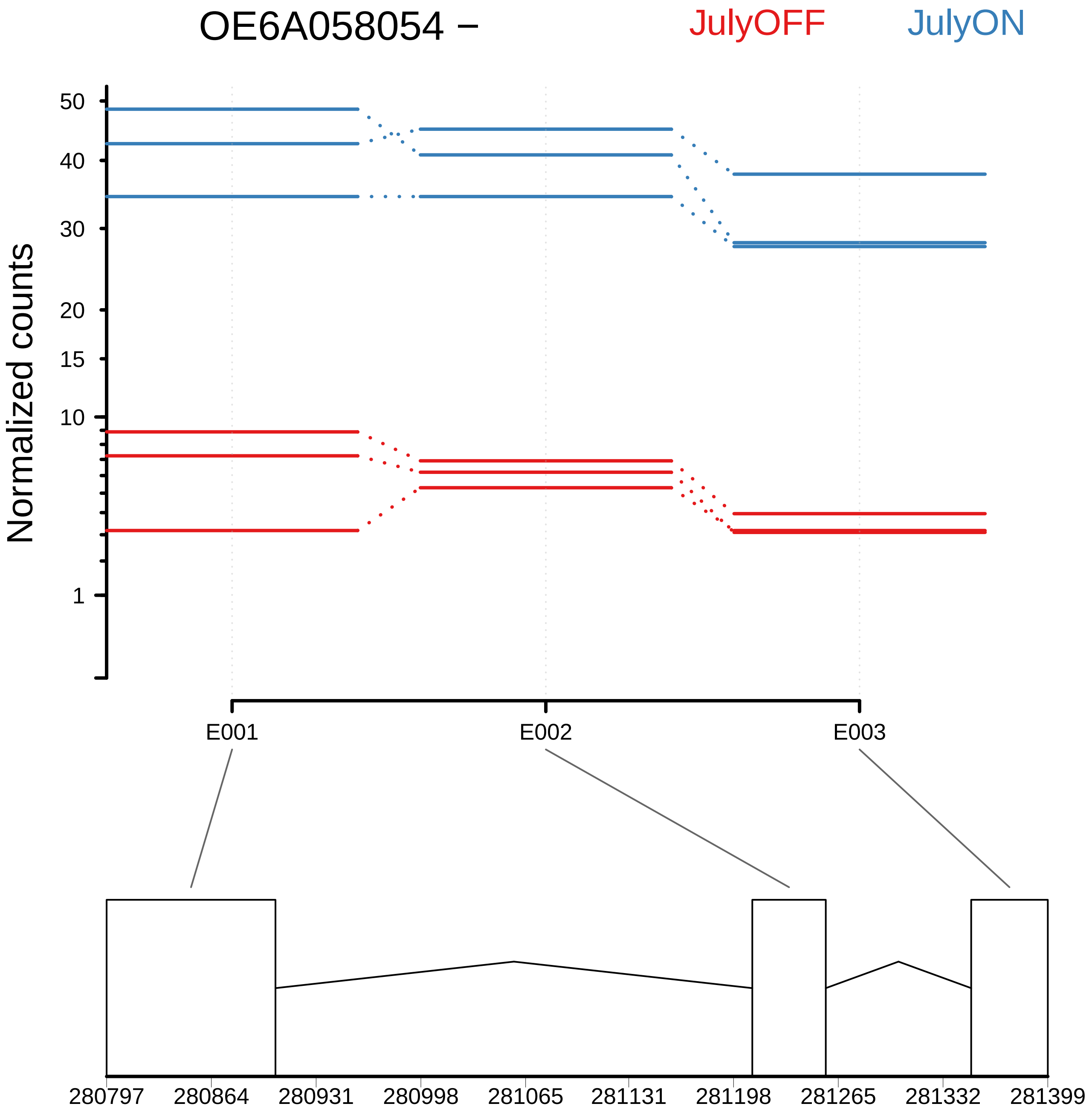

OE6A058054 −
JulyOFF
JulyON
Normalized counts
50
40
30
20
15
10
1
E001
E002
E003
280797
280864
280931
280998
281065
281131
281198
281265
281332
281399

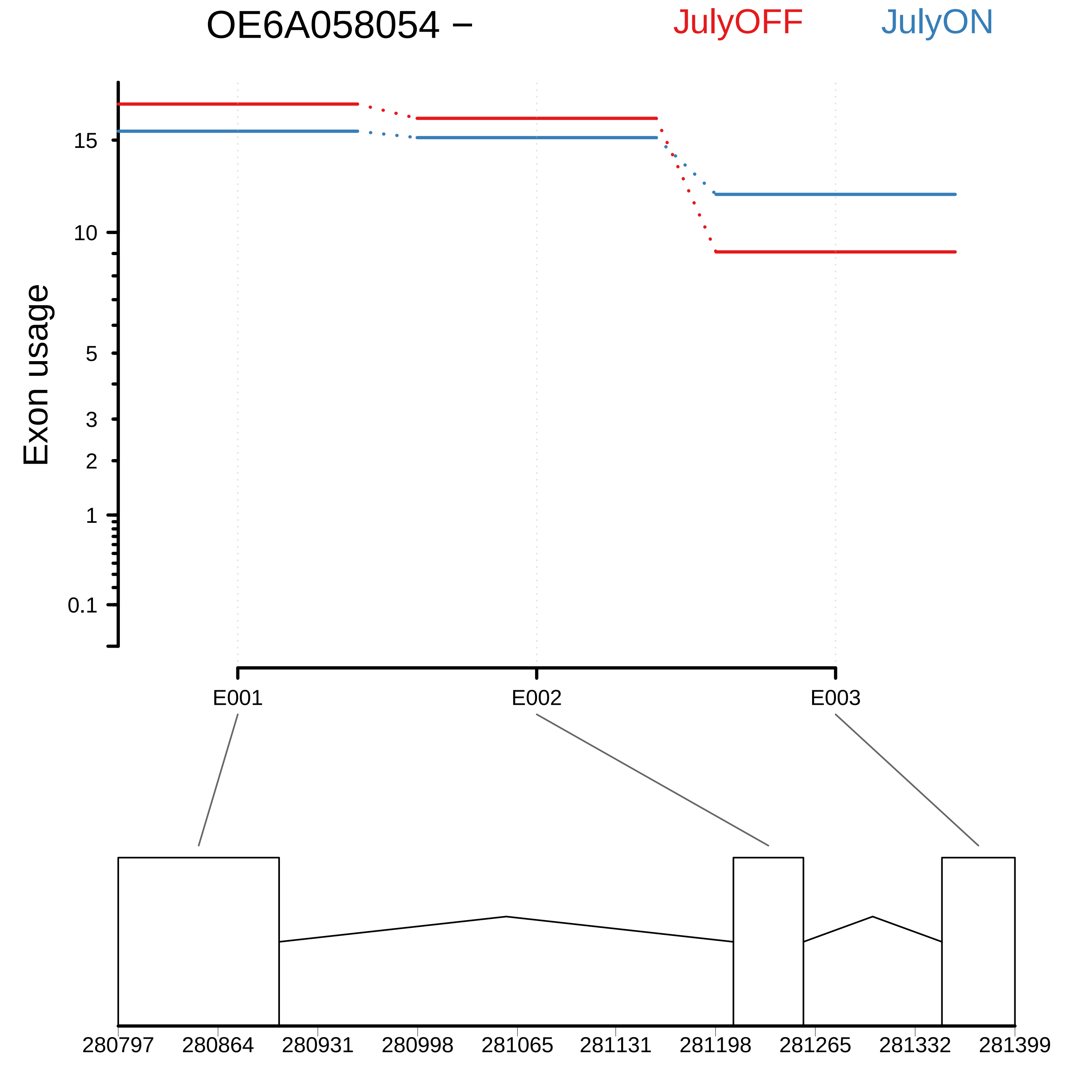
OE6A058054 −
JulyOFF
JulyON
Exon usage
15
10
5
3
2
1
0.1
E001
E002
E003
280797
280864
280931
280998
281065
281131
281198
281265
281332
281399

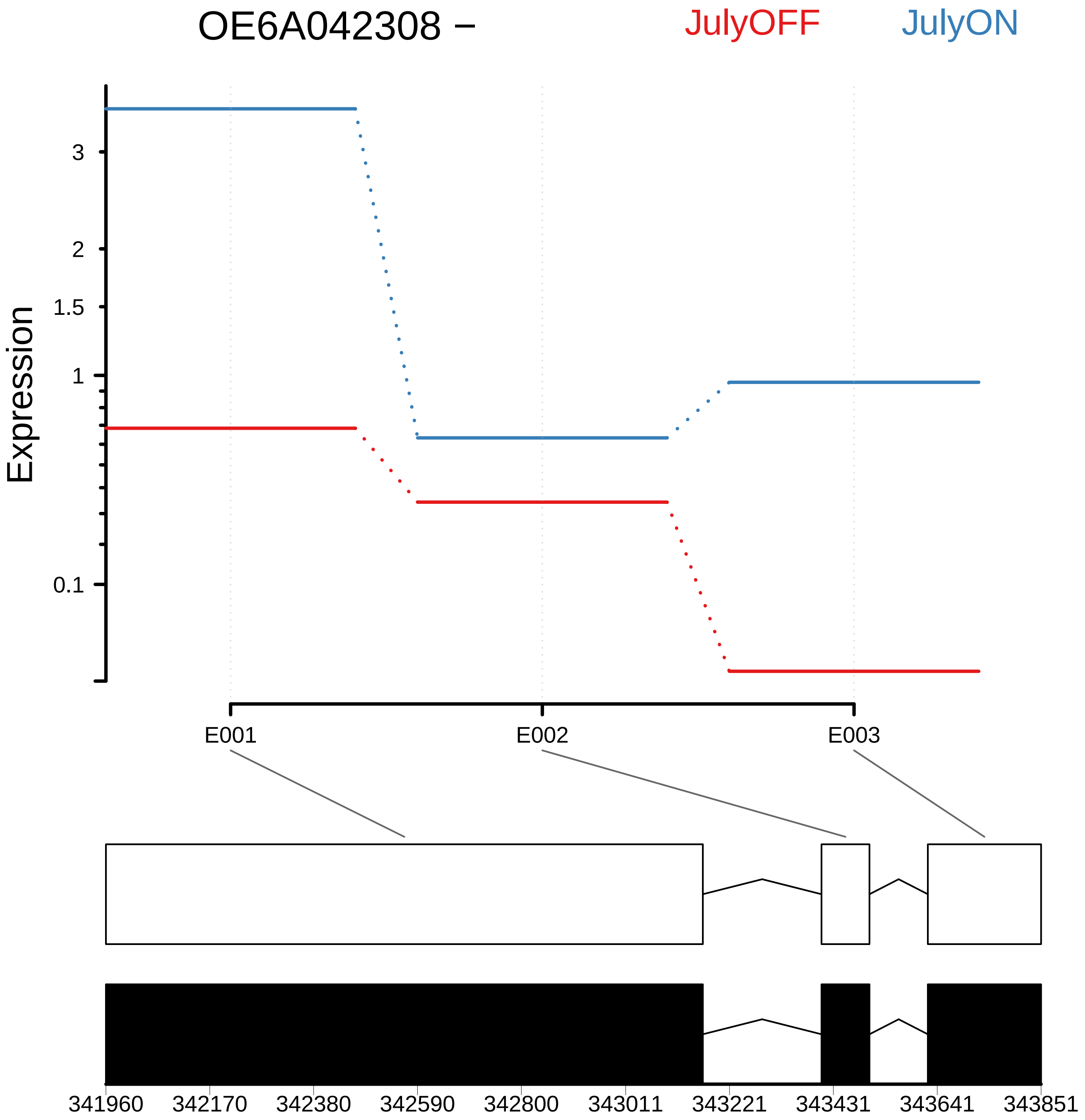
OE6A042308 −
JulyOFF
JulyON
Expression
3
2
1.5
1
0.1
E001
E002
E003
341960
342170
342380
342590
342800
343011
343221
343431
343641
343851

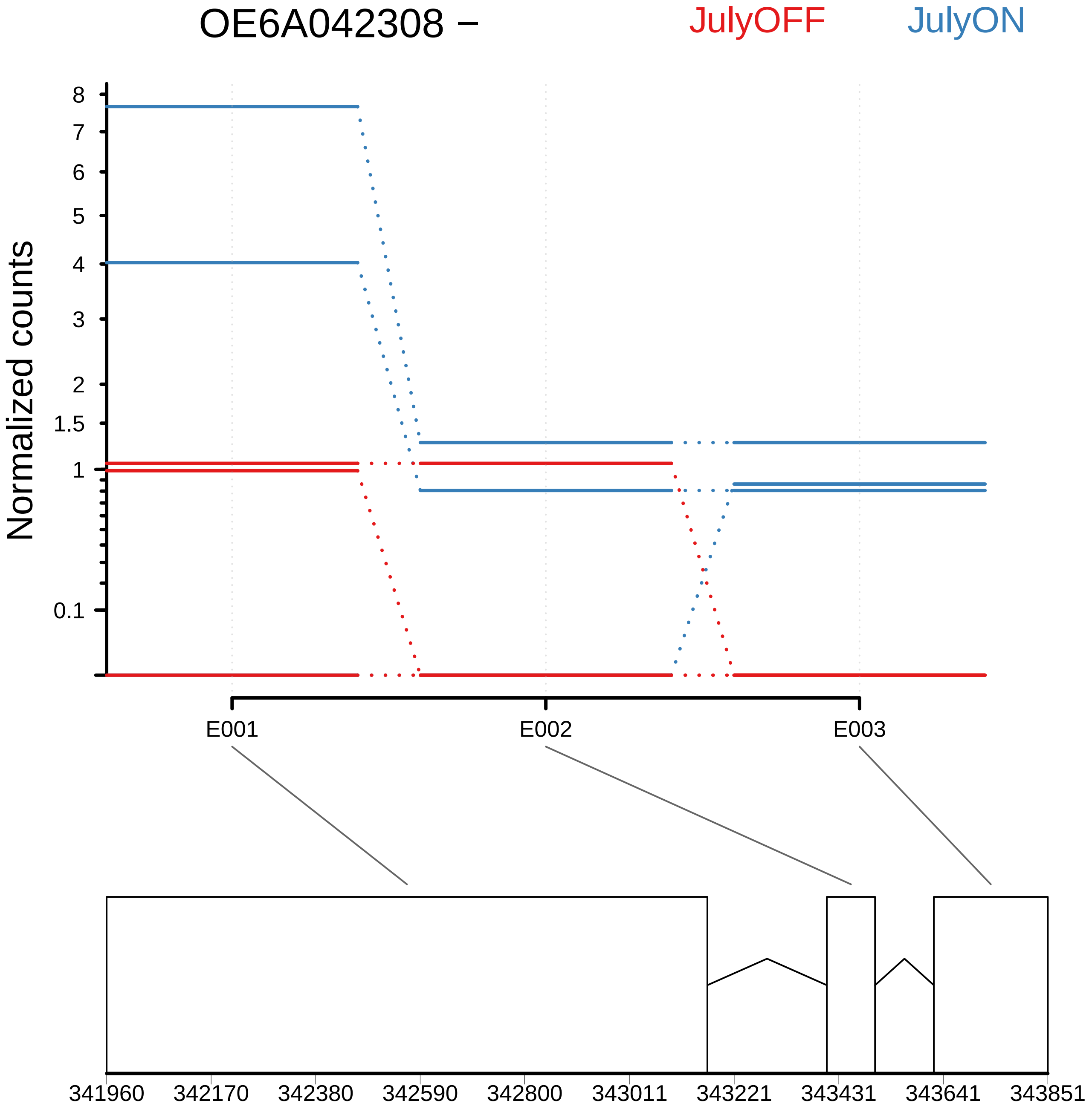

OE6A042308 −
JulyOFF
JulyON
Normalized counts
8
7
6
5
4
3
2
1.5
1
0.1
E001
E002
E003
341960
342170
342380
342590
342800
343011
343221
343431
343641
343851

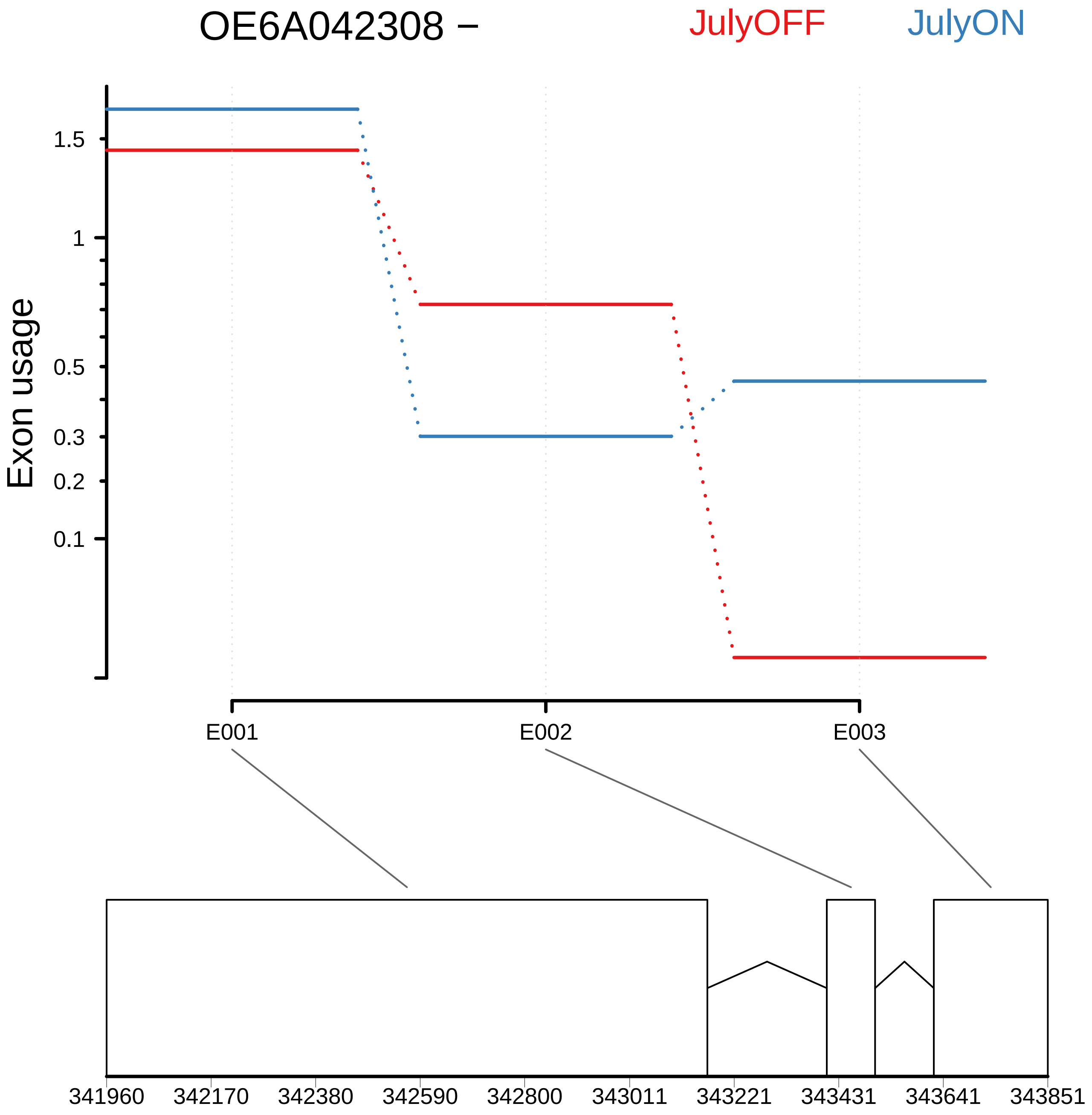
OE6A042308 −
JulyOFF
JulyON
Exon usage
1.5
1
0.5
0.3
0.2
0.1
E001
E002
E003
341960
342170
342380
342590
342800
343011
343221
343431
343641
343851

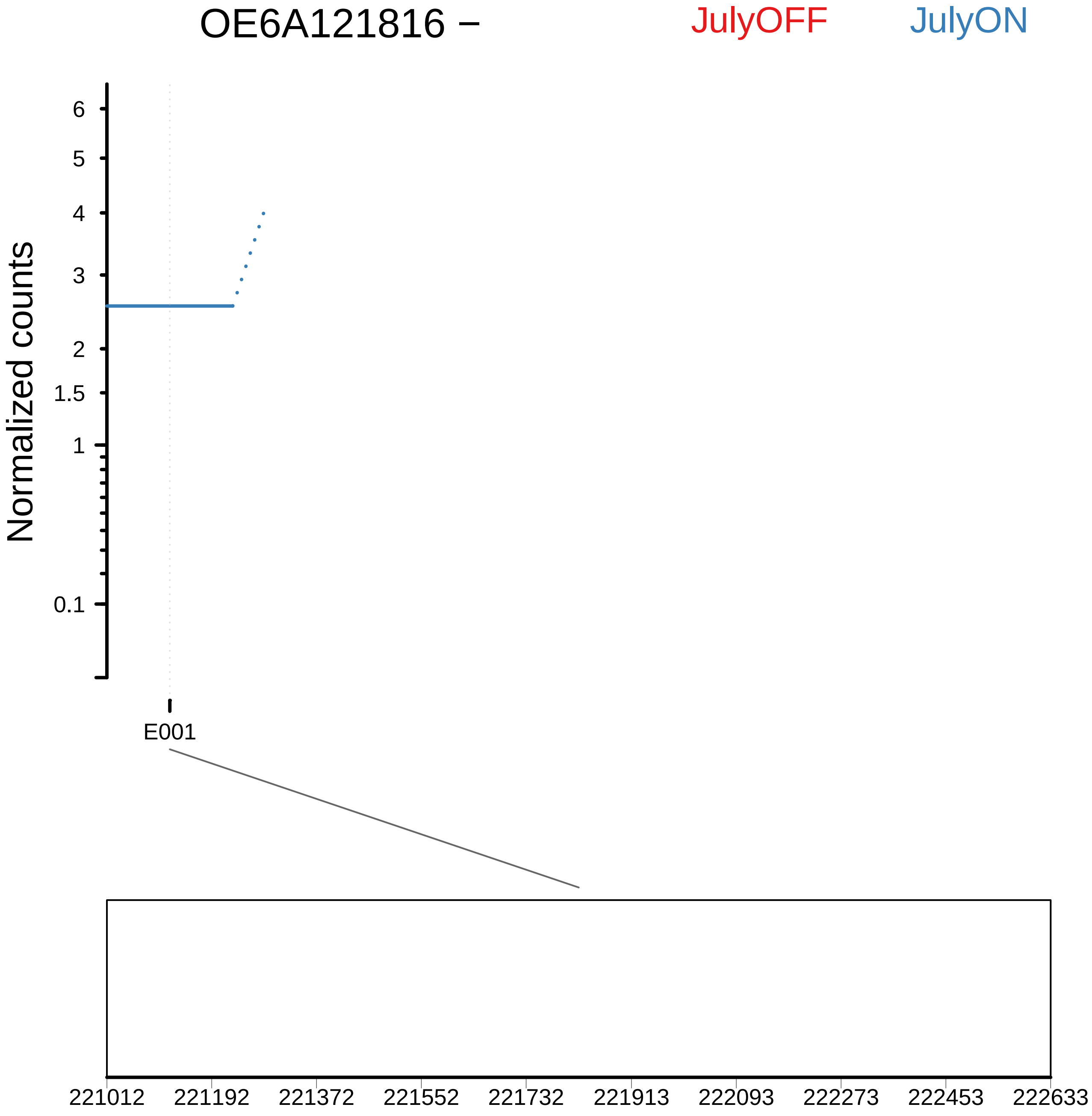

OE6A121816 −
JulyOFF
JulyON
Normalized counts
6
5
4
3
2
1.5
1
0.1
E001
221012
221192
221372
221552
221732
221913
222093
222273
222453
222633

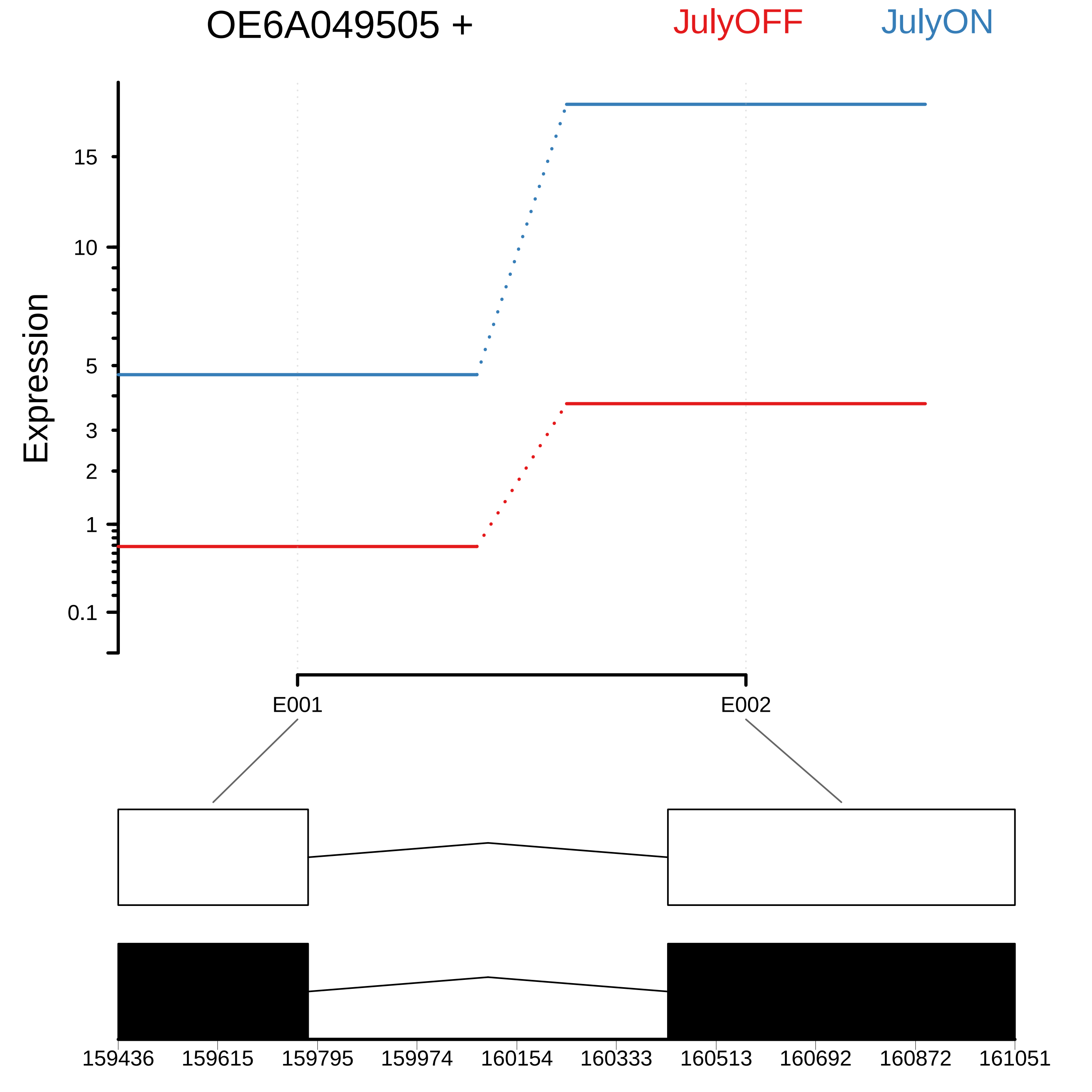

OE6A049505 +
JulyOFF
JulyON
Expression
15
10
5
3
2
1
0.1
E001
E002
159436
159615
159795
159974
160154
160333
160513
160692
160872
161051

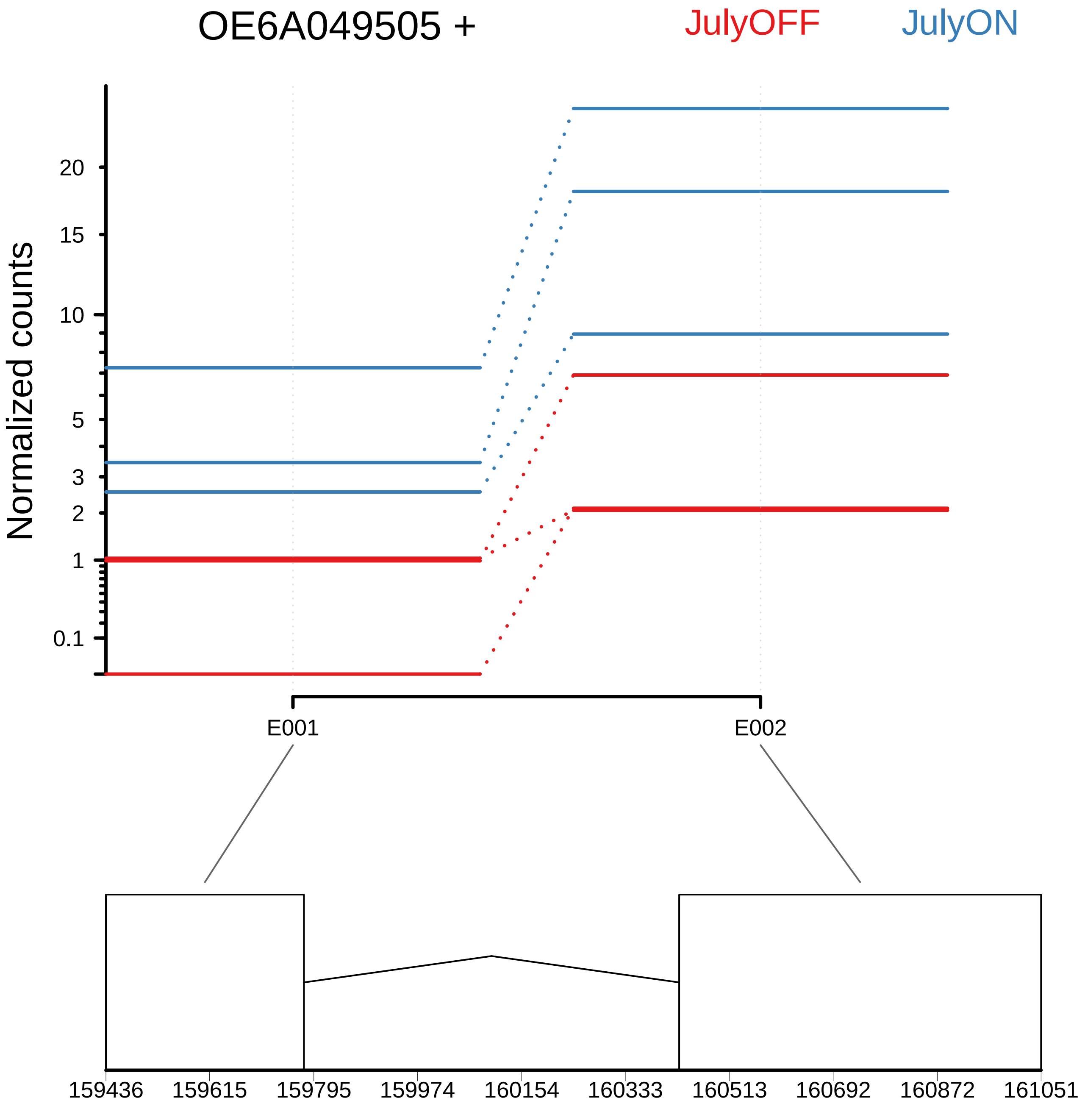

OE6A049505 +
JulyOFF
JulyON
Normalized counts
20
15
10
5
3
2
1
0.1
E001
E002
159436
159615
159795
159974
160154
160333
160513
160692
160872
161051

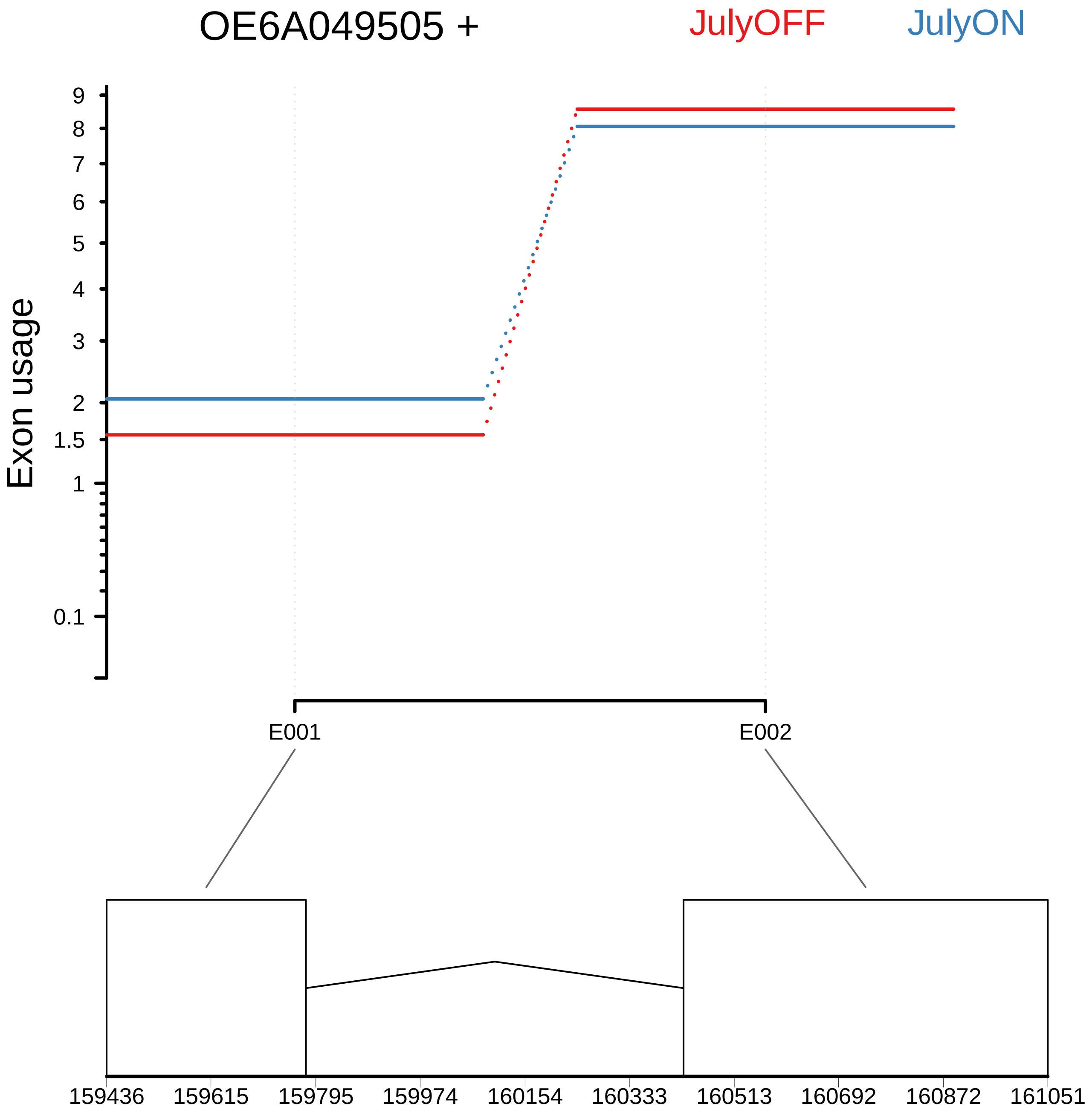

OE6A049505 +
JulyOFF
JulyON
Exon usage
9
8
7
6
5
4
3
2
1.5
1
0.1
E001
E002
159436
159615
159795
159974
160154
160333
160513
160692
160872
161051

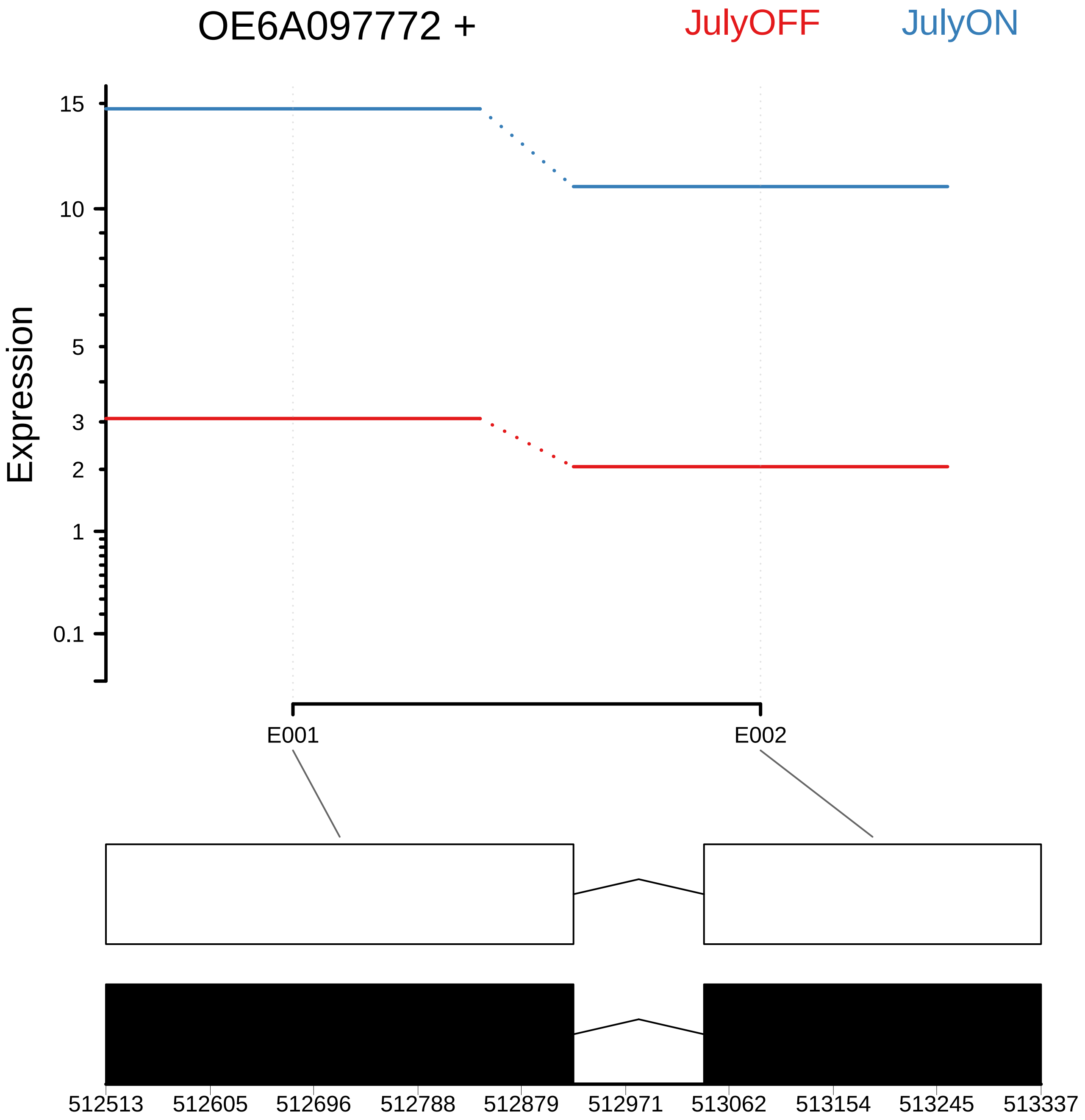

OE6A097772 +
JulyOFF
JulyON
Expression
15
10
5
3
2
1
0.1
E001
E002
512513
512605
512696
512788
512879
512971
513062
513154
513245
513337

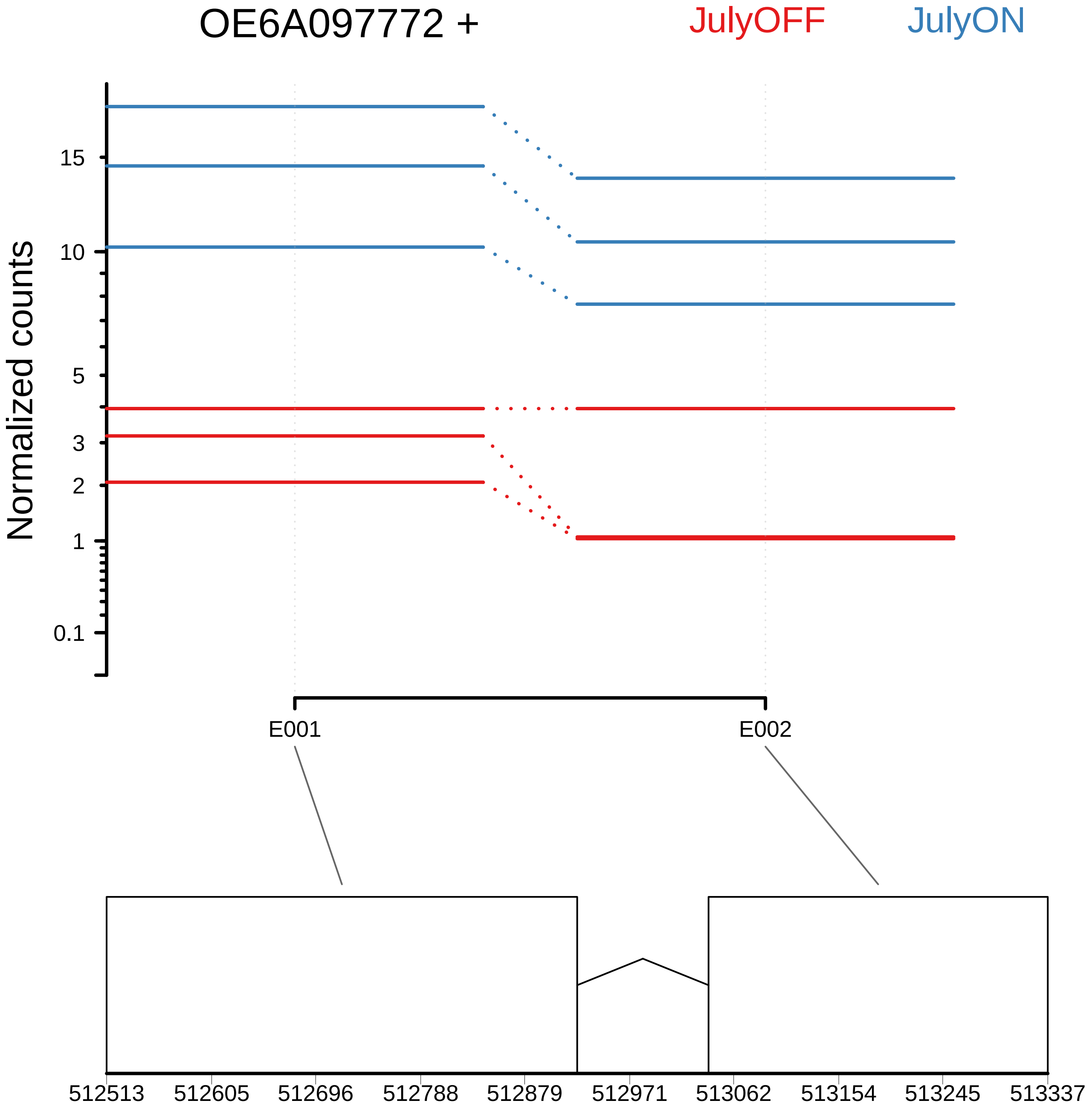
OE6A097772 +
JulyOFF
JulyON
Normalized counts
15
10
5
3
2
1
0.1
E001
E002
512513
512605
512696
512788
512879
512971
513062
513154
513245
513337

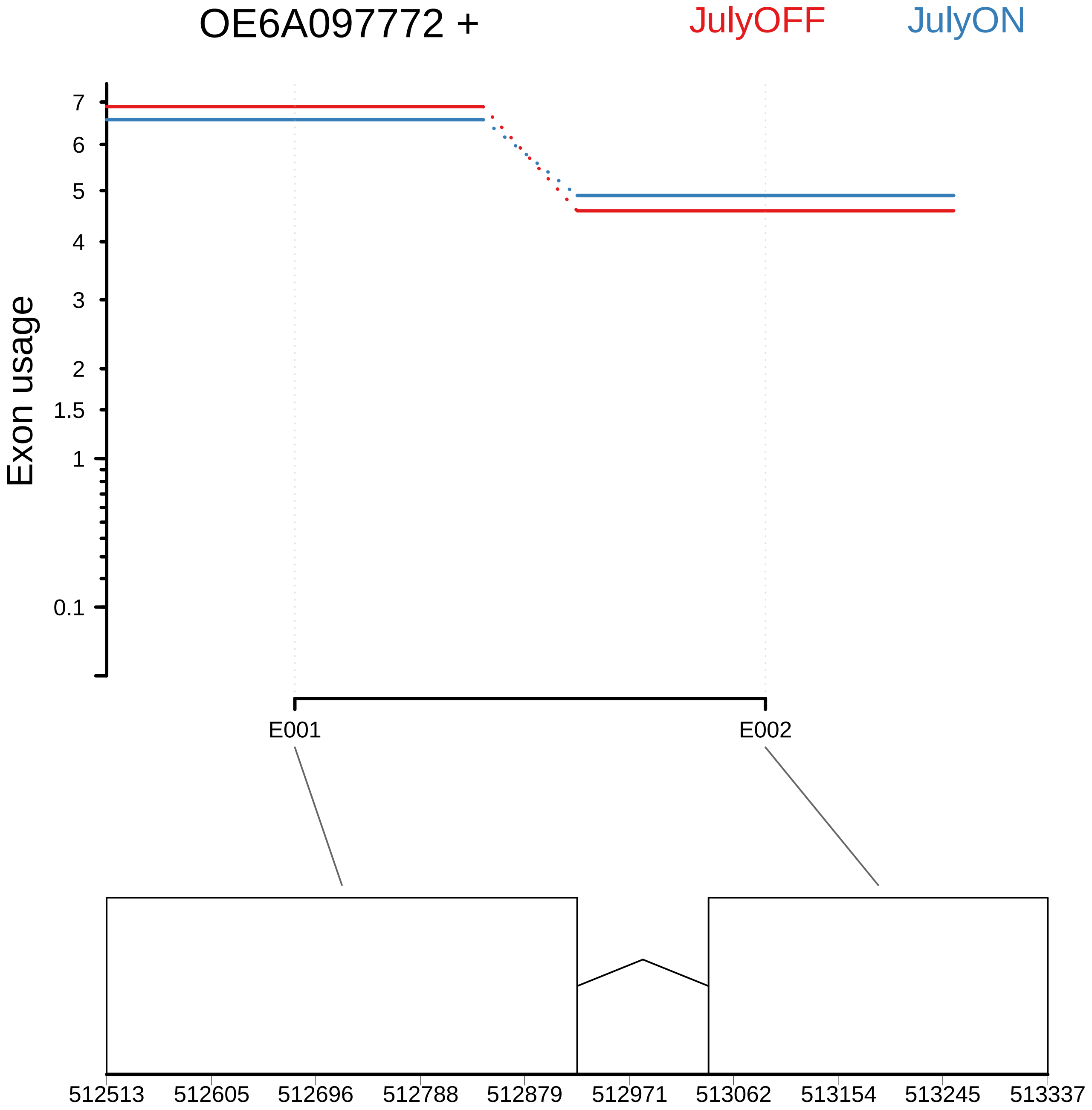
OE6A097772 +
JulyOFF
JulyON
Exon usage
7
6
5
4
3
2
1.5
1
0.1
E001
E002
512513
512605
512696
512788
512879
512971
513062
513154
513245
513337

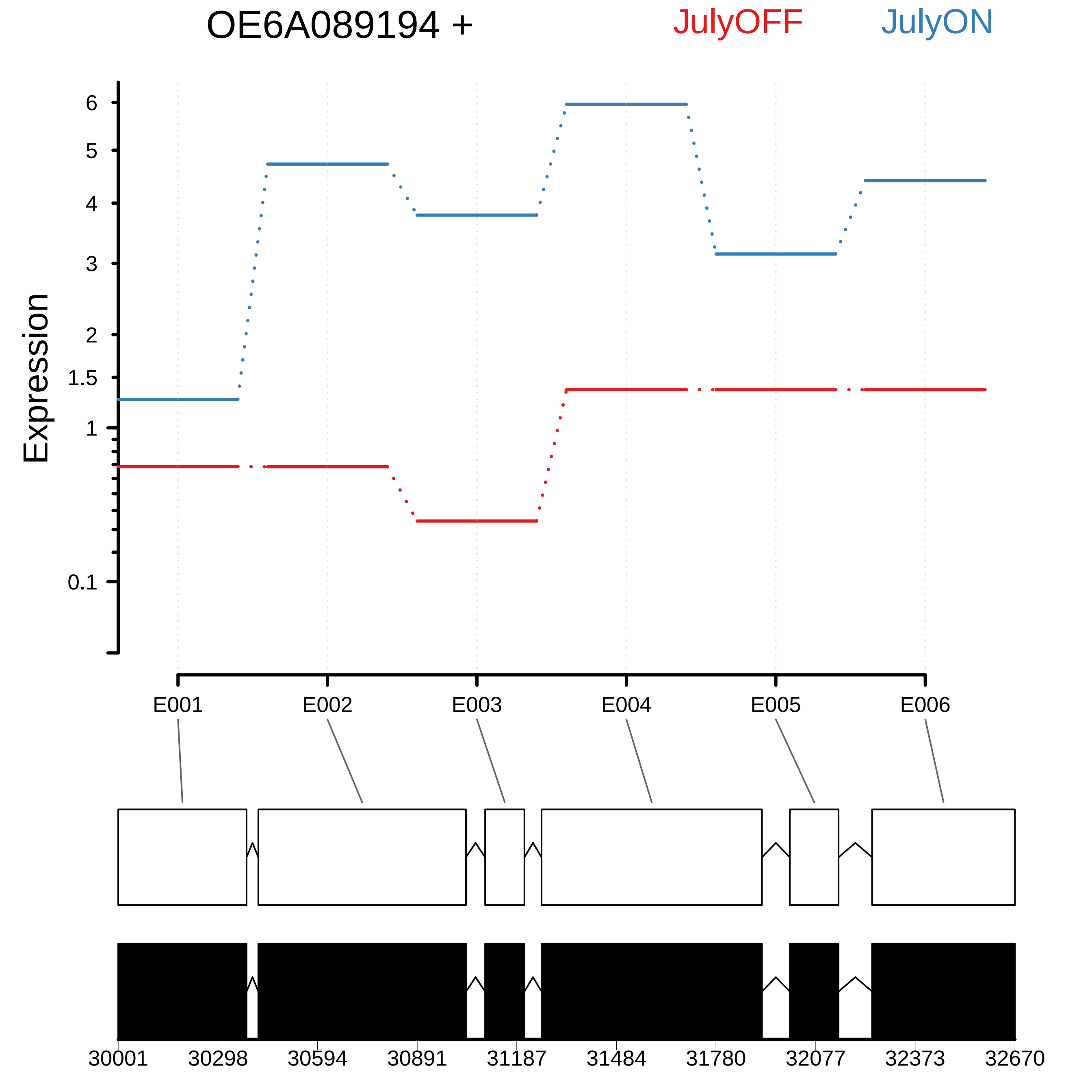
OE6A089194 +
JulyOFF
JulyON
Expression
6
5
4
3
2
1.5
1
0.1
E001
E002
E003
E004
E005
E006
30001
30298
30594
30891
31187
31484
31780
32077
32373
32670

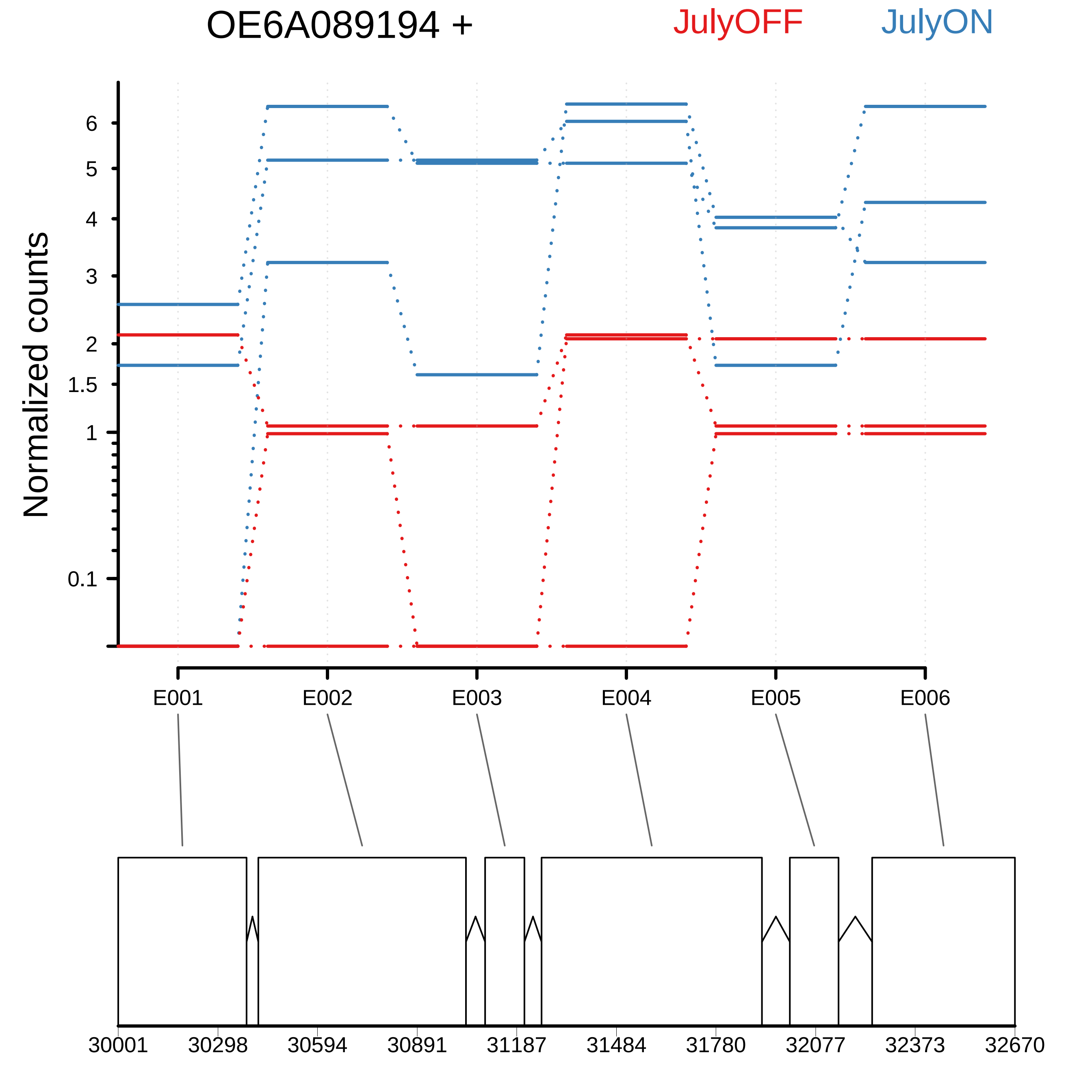
OE6A089194 +
JulyOFF
JulyON
Normalized counts
6
5
4
3
2
1.5
1
0.1
E001
E002
E003
E004
E005
E006
30001
30298
30594
30891
31187
31484
31780
32077
32373
32670

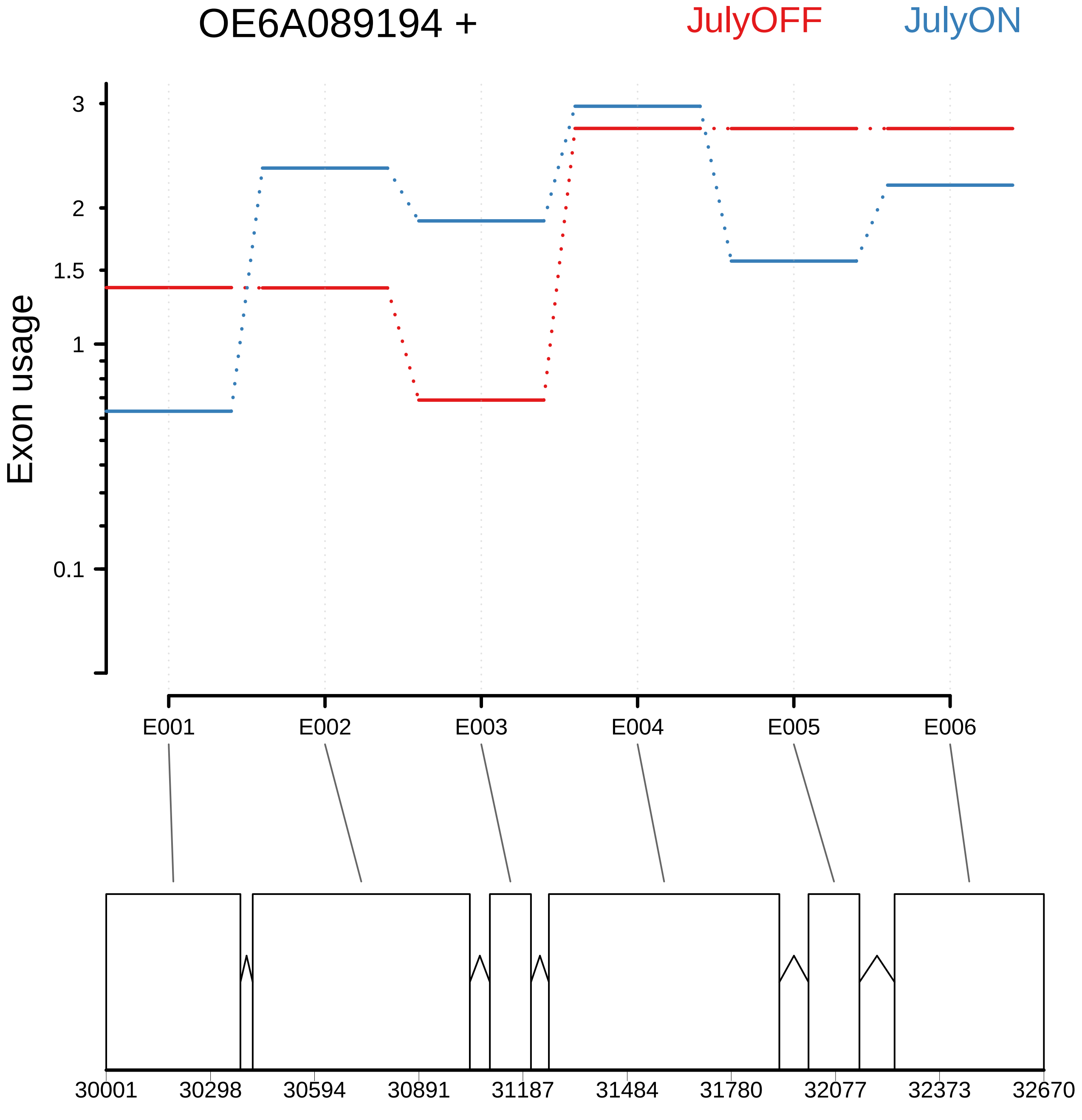

OE6A089194 +
JulyOFF
JulyON
Exon usage
3
2
1.5
1
0.1
E001
E002
E003
E004
E005
E006
30001
30298
30594
30891
31187
31484
31780
32077
32373
32670

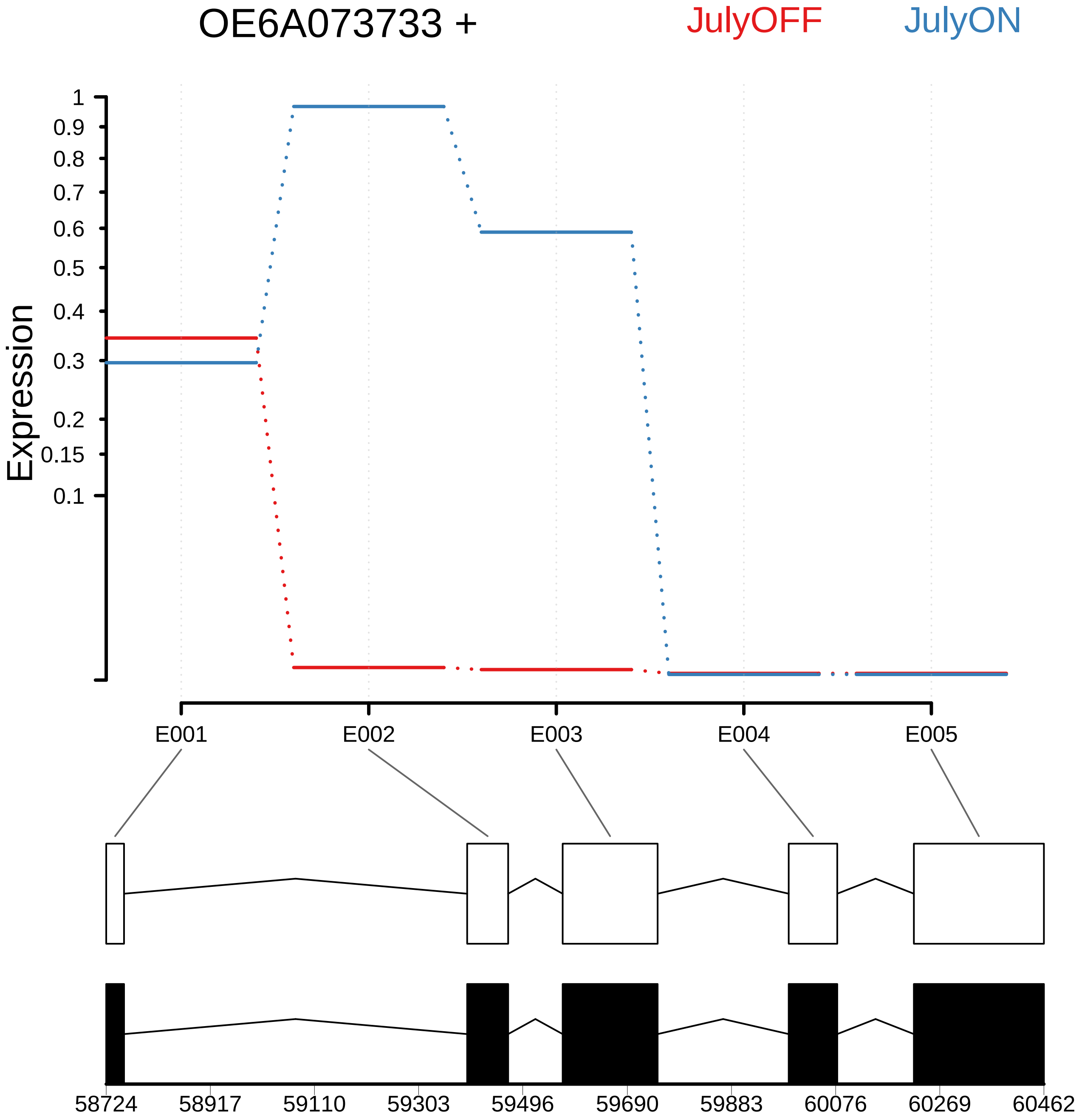

OE6A073733 +
JulyOFF
JulyON
Expression
1
0.9
0.8
0.7
0.6
0.5
0.4
0.3
0.2
0.15
0.1
E001
E002
E003
E004
E005
58724
58917
59110
59303
59496
59690
59883
60076
60269
60462

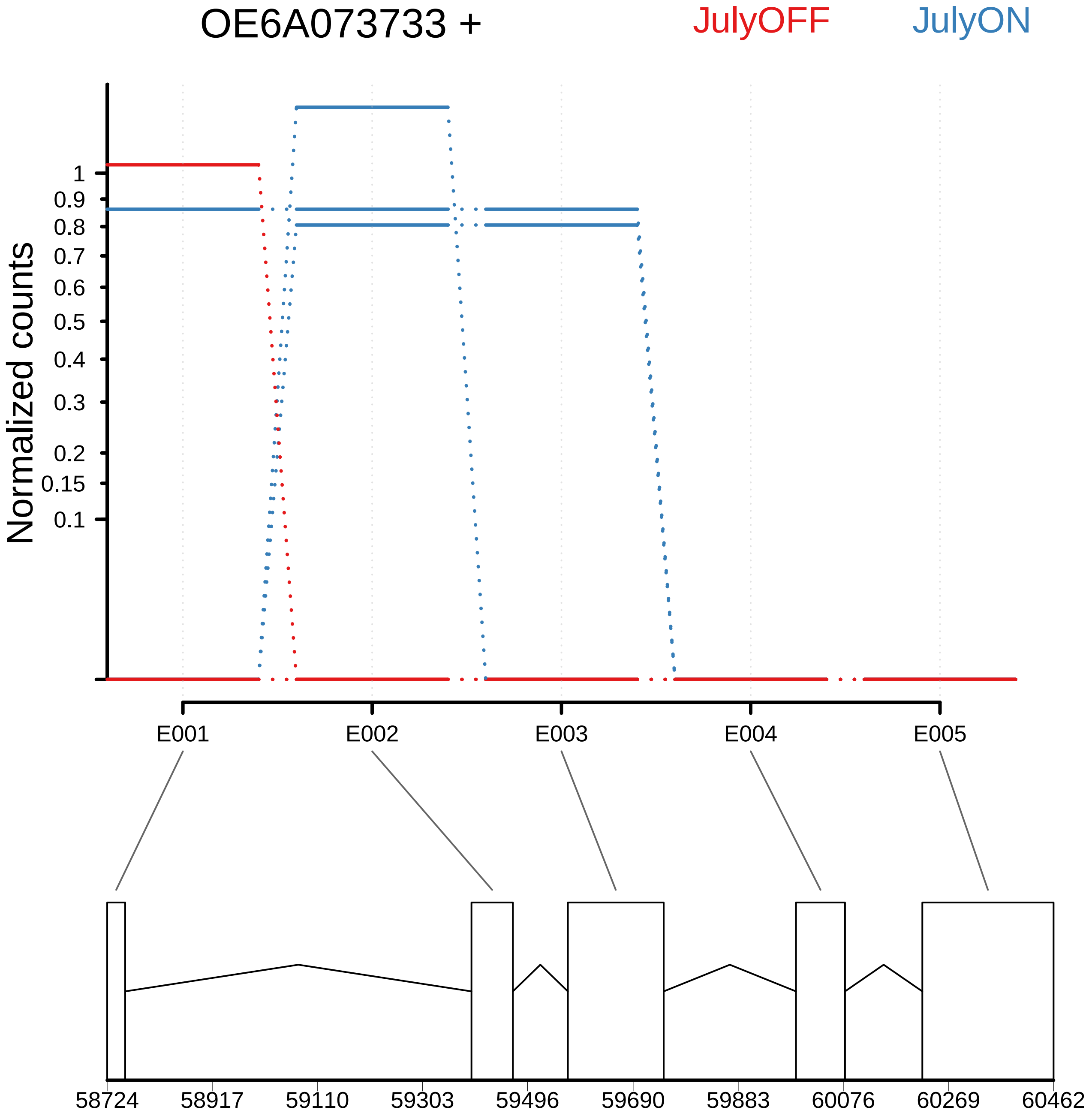

OE6A073733 +
JulyOFF
JulyON
Normalized counts
1
0.9
0.8
0.7
0.6
0.5
0.4
0.3
0.2
0.15
0.1
E001
E002
E003
E004
E005
58724
58917
59110
59303
59496
59690
59883
60076
60269
60462

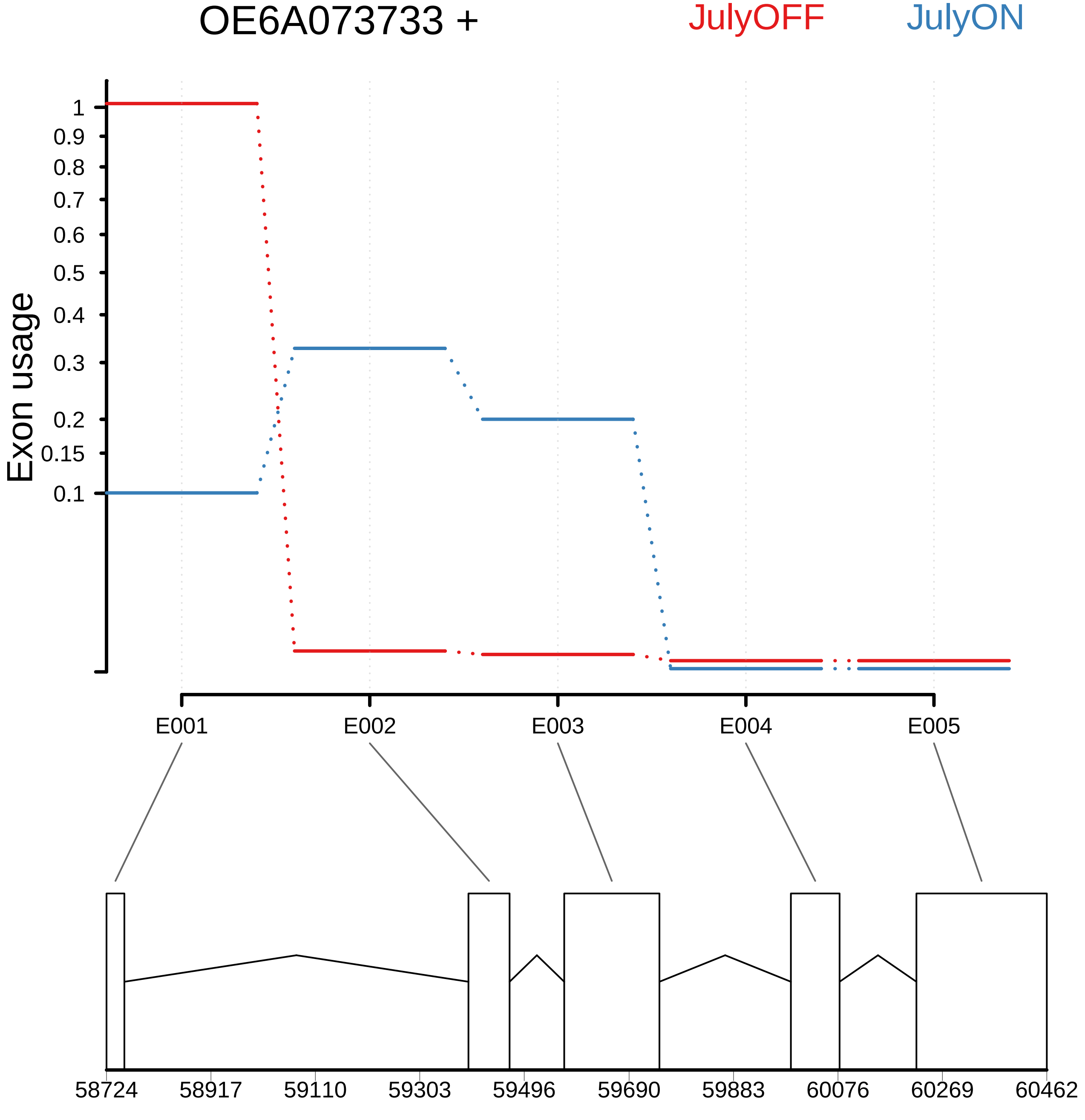
OE6A073733 +
JulyOFF
JulyON
Exon usage
1
0.9
0.8
0.7
0.6
0.5
0.4
0.3
0.2
0.15
0.1
E001
E002
E003
E004
E005
58724
58917
59110
59303
59496
59690
59883
60076
60269
60462

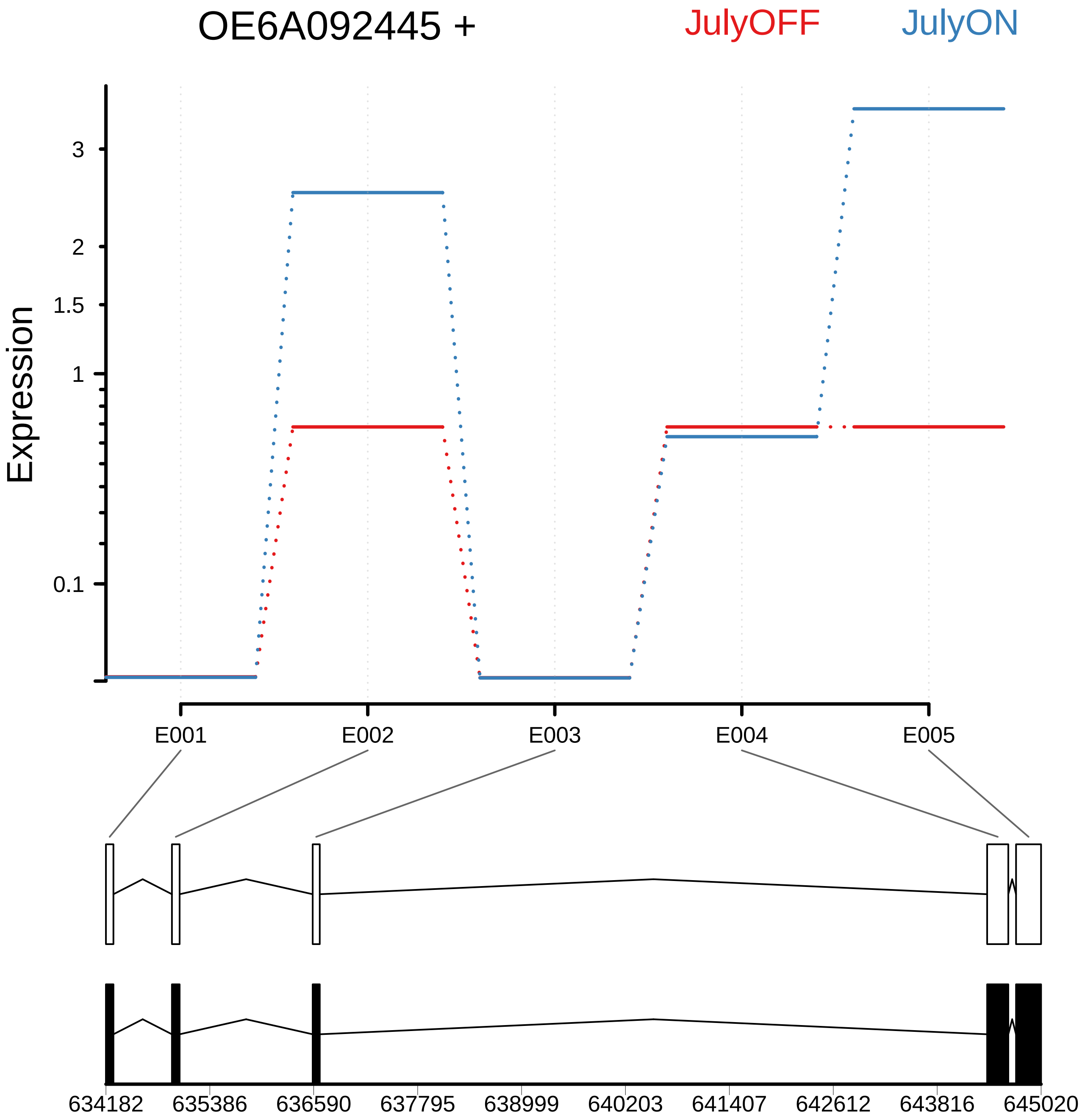

OE6A092445 +
JulyOFF
JulyON
Expression
3
2
1.5
1
0.1
E001
E002
E003
E004
E005
634182
635386
636590
637795
638999
640203
641407
642612
643816
645020

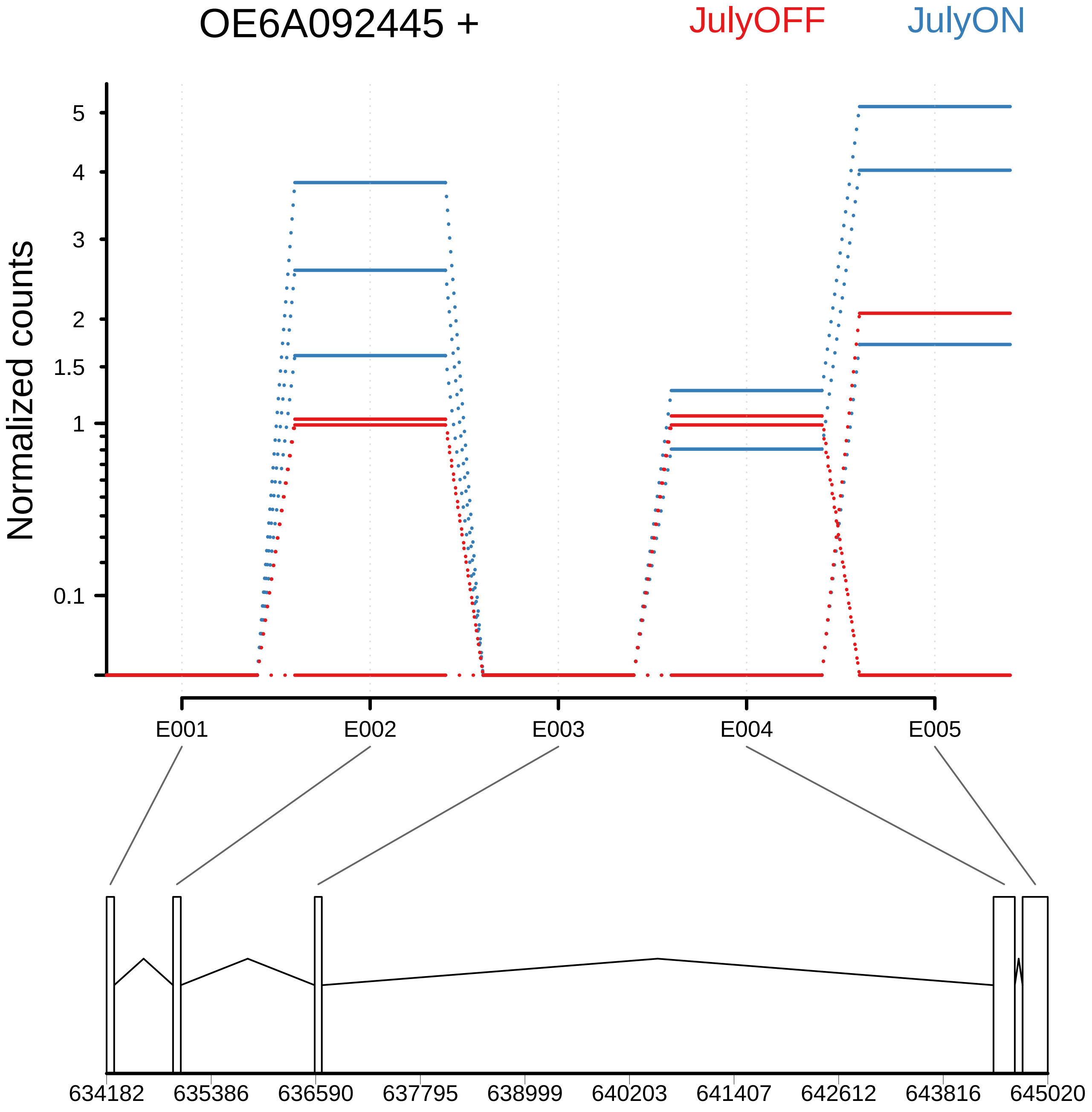

OE6A092445 +
JulyOFF
JulyON
Normalized counts
5
4
3
2
1.5
1
0.1
E001
E002
E003
E004
E005
634182
635386
636590
637795
638999
640203
641407
642612
643816
645020

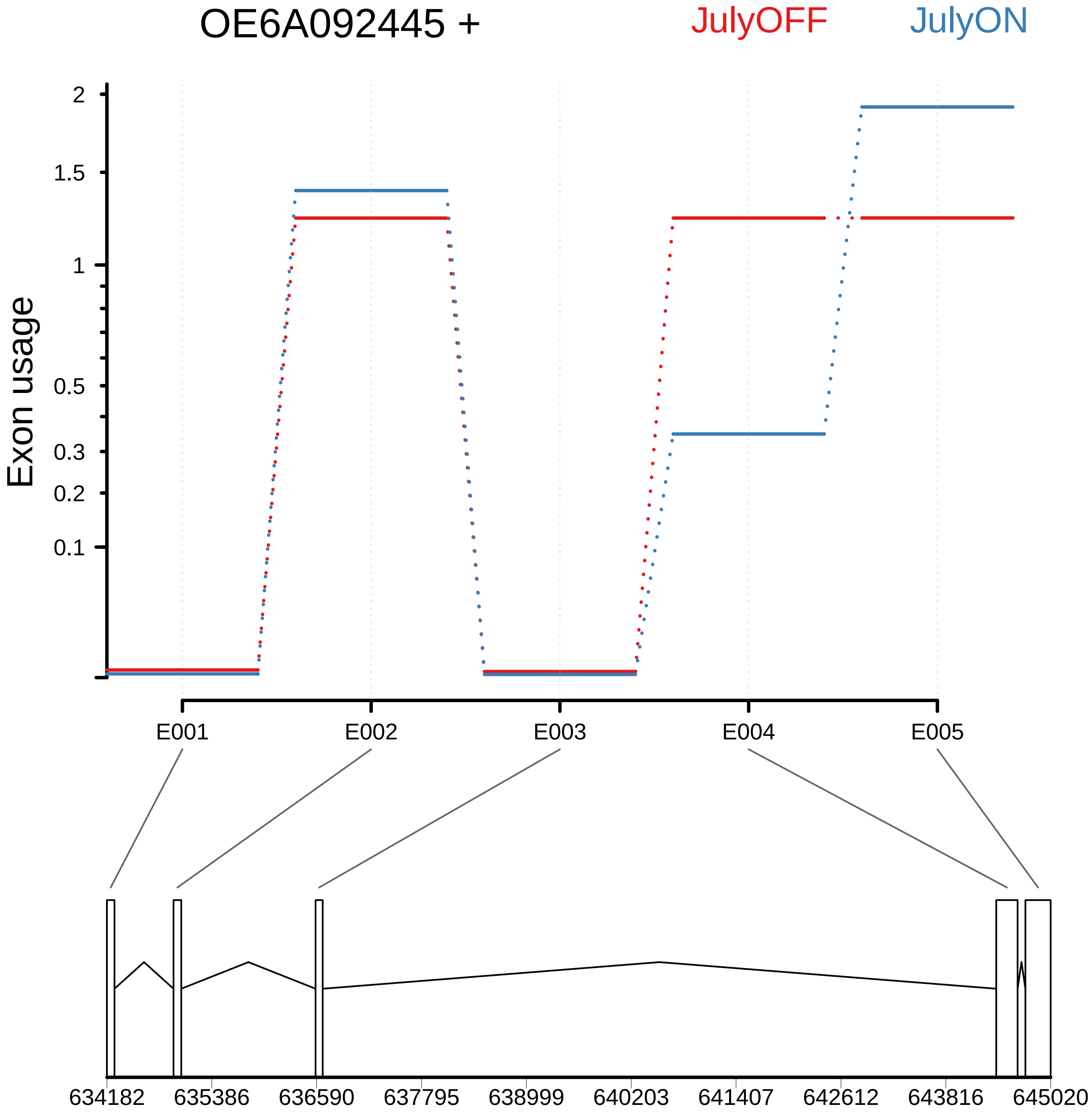

OE6A092445 +
JulyOFF
JulyON
Exon usage
2
1.5
1
0.5
0.3
0.2
0.1
E001
E002
E003
E004
E005
634182
635386
636590
637795
638999
640203
641407
642612
643816
645020

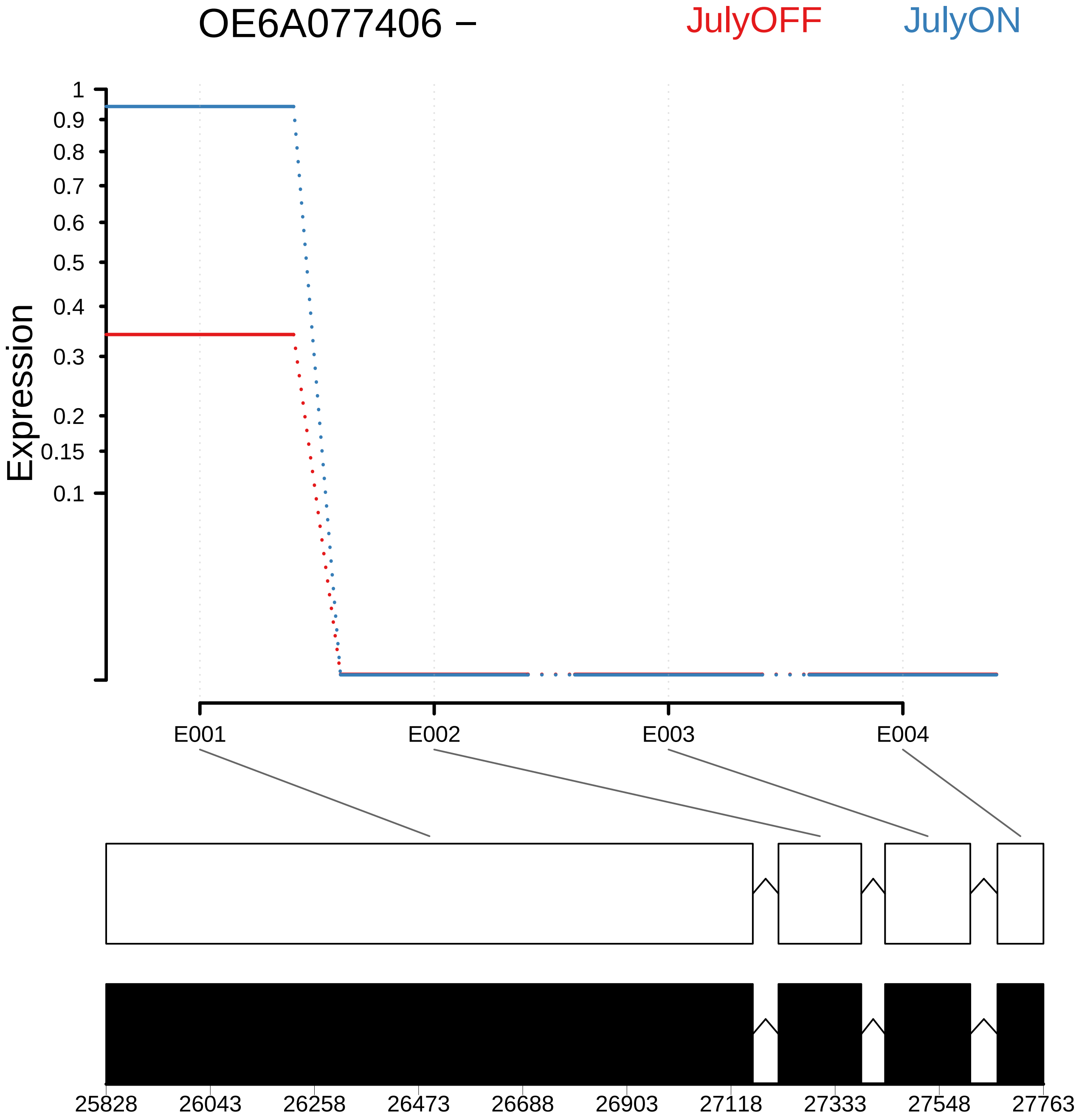

OE6A077406 –
JulyOFF
JulyON
Expression
1
0.9
0.8
0.7
0.6
0.5
0.4
0.3
0.2
0.15
0.1
E001
E002
E003
E004
25828
26043
26258
26473
26688
26903
27118
27333
27548
27763

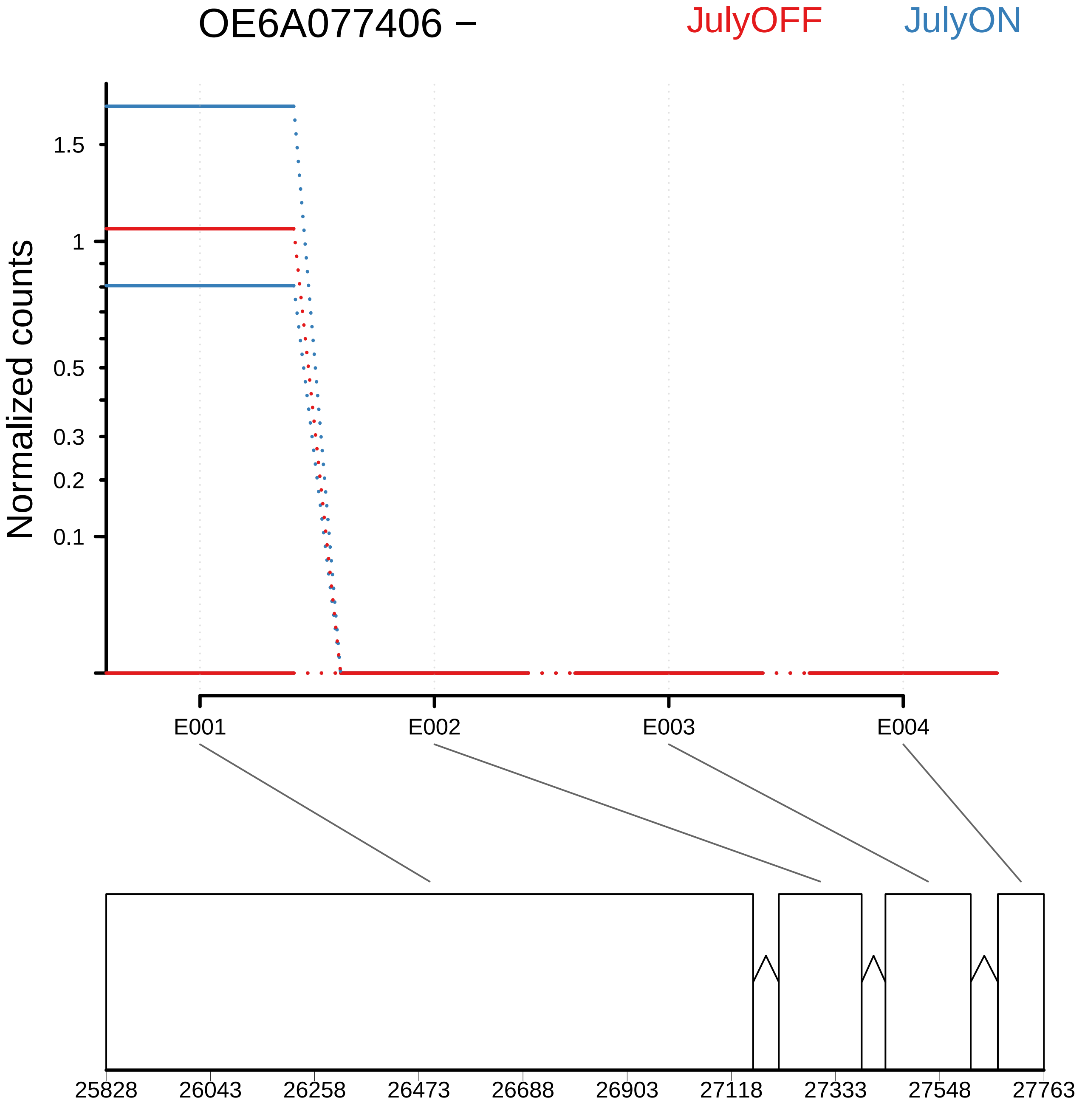

OE6A077406 −
JulyOFF
JulyON
Normalized counts
1.5
1
0.5
0.3
0.2
0.1
E001
E002
E003
E004
25828
26043
26258
26473
26688
26903
27118
27333
27548
27763

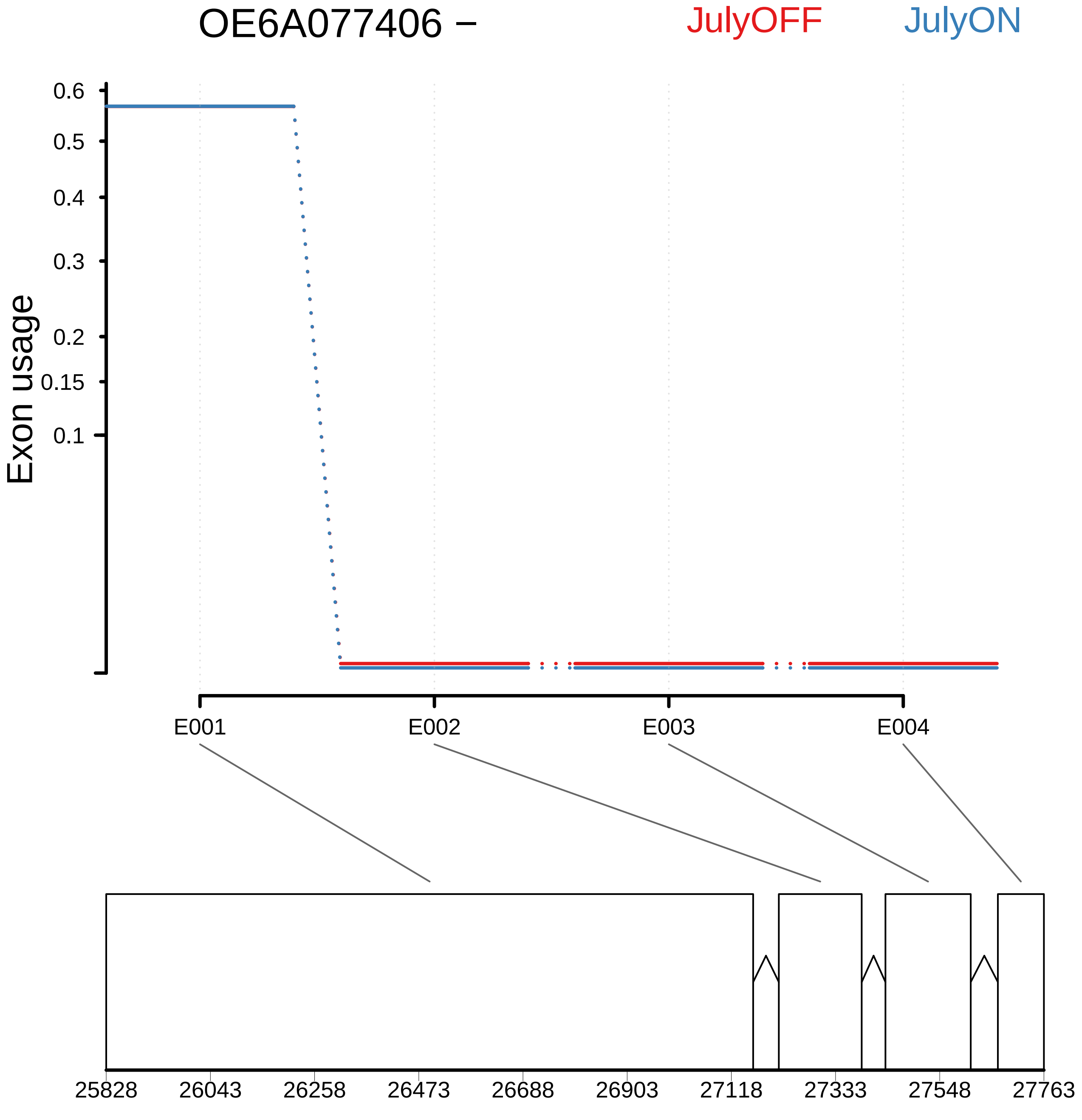

OE6A077406 −
JulyOFF
JulyON
Exon usage
0.6
0.5
0.4
0.3
0.2
0.15
0.1
E001
E002
E003
E004
25828
26043
26258
26473
26688
26903
27118
27333
27548
27763

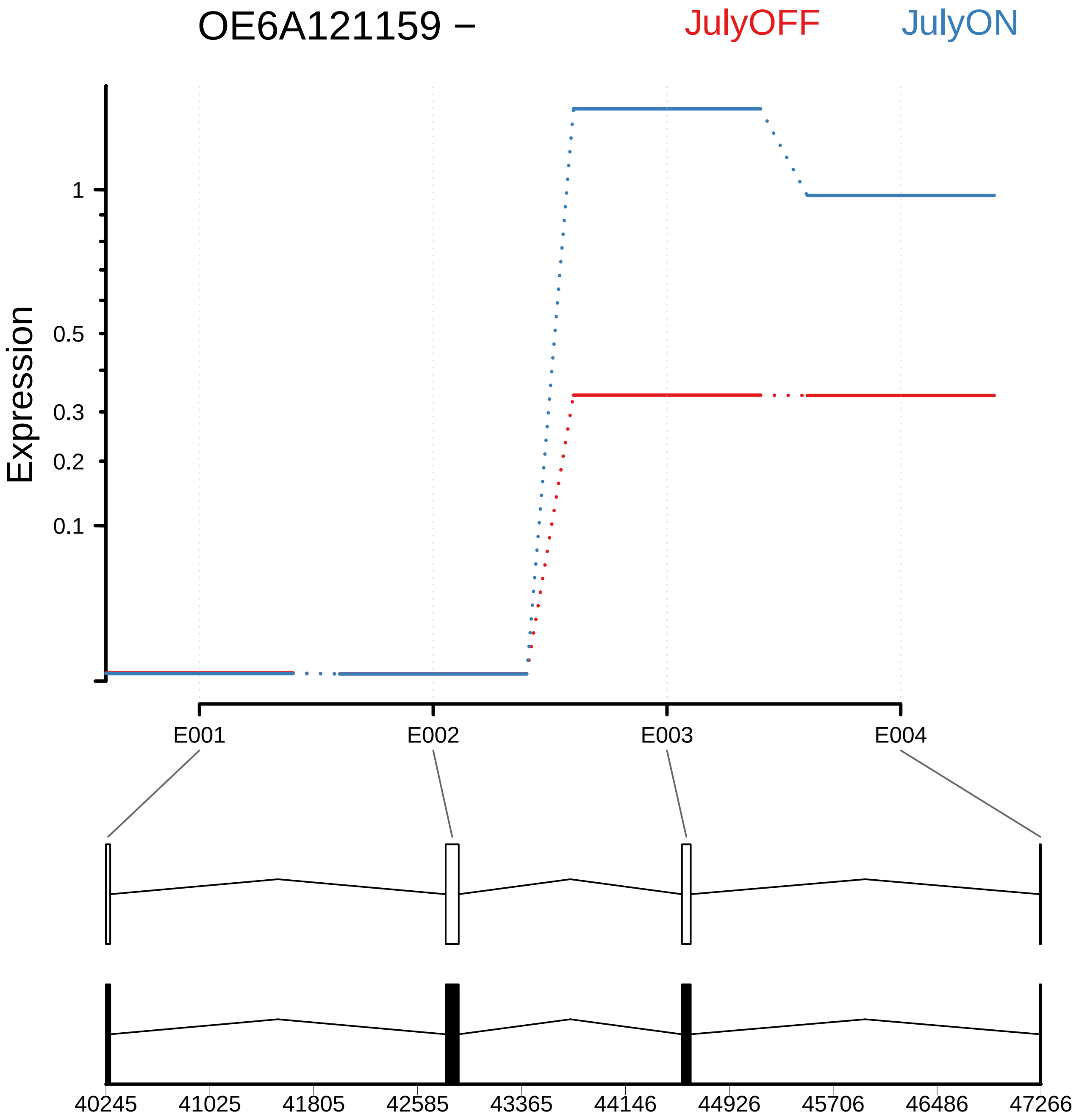

OE6A121159 −
JulyOFF
JulyON
Expression
1
0.5
0.3
0.2
0.1
E001
E002
E003
E004
40245
41025
41805
42585
43365
44146
44926
45706
46486
47266

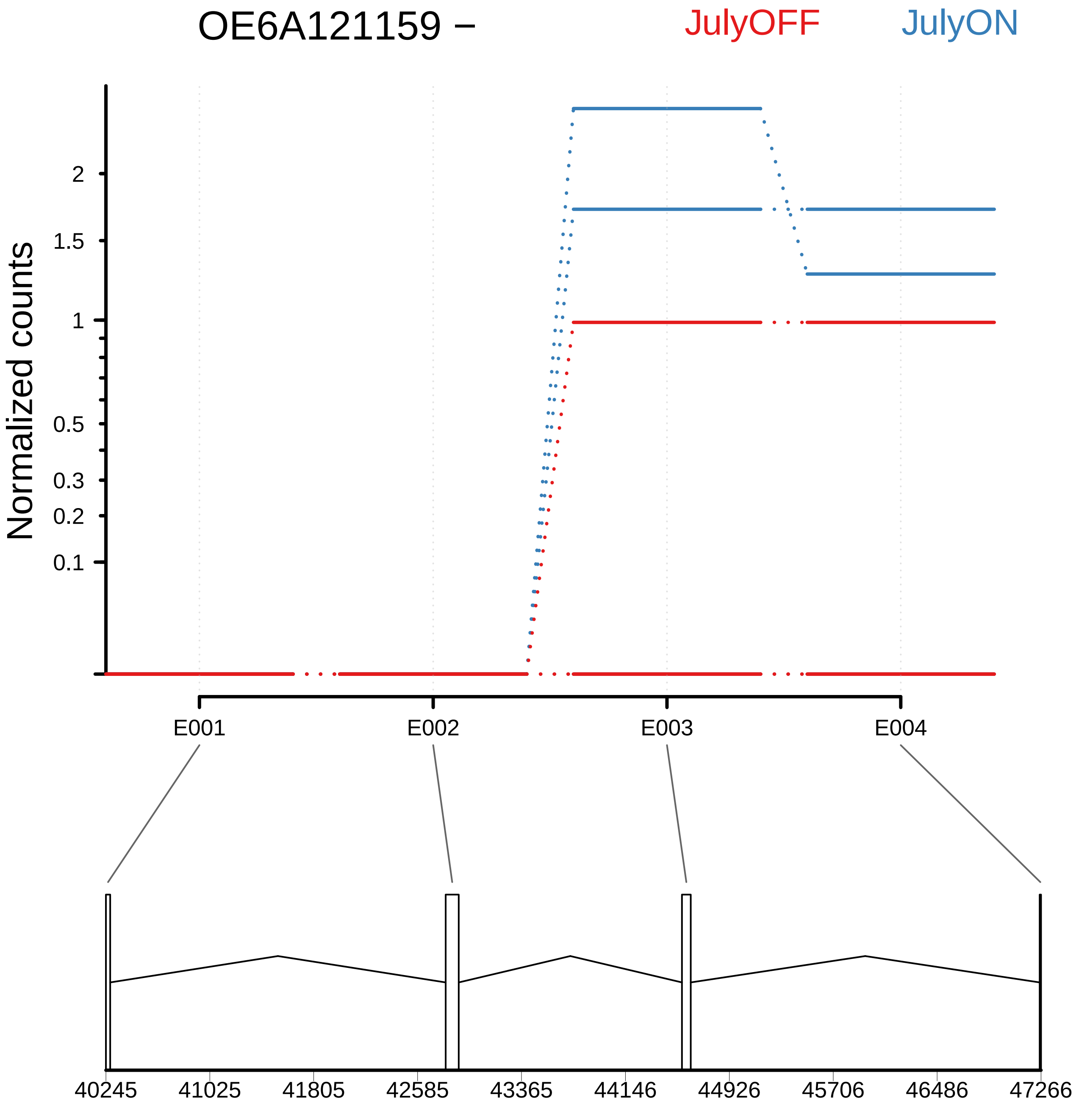

OE6A121159 −
JulyOFF
JulyON
Normalized counts
2
1.5
1
0.5
0.3
0.2
0.1
E001
E002
E003
E004
40245
41025
41805
42585
43365
44146
44926
45706
46486
47266

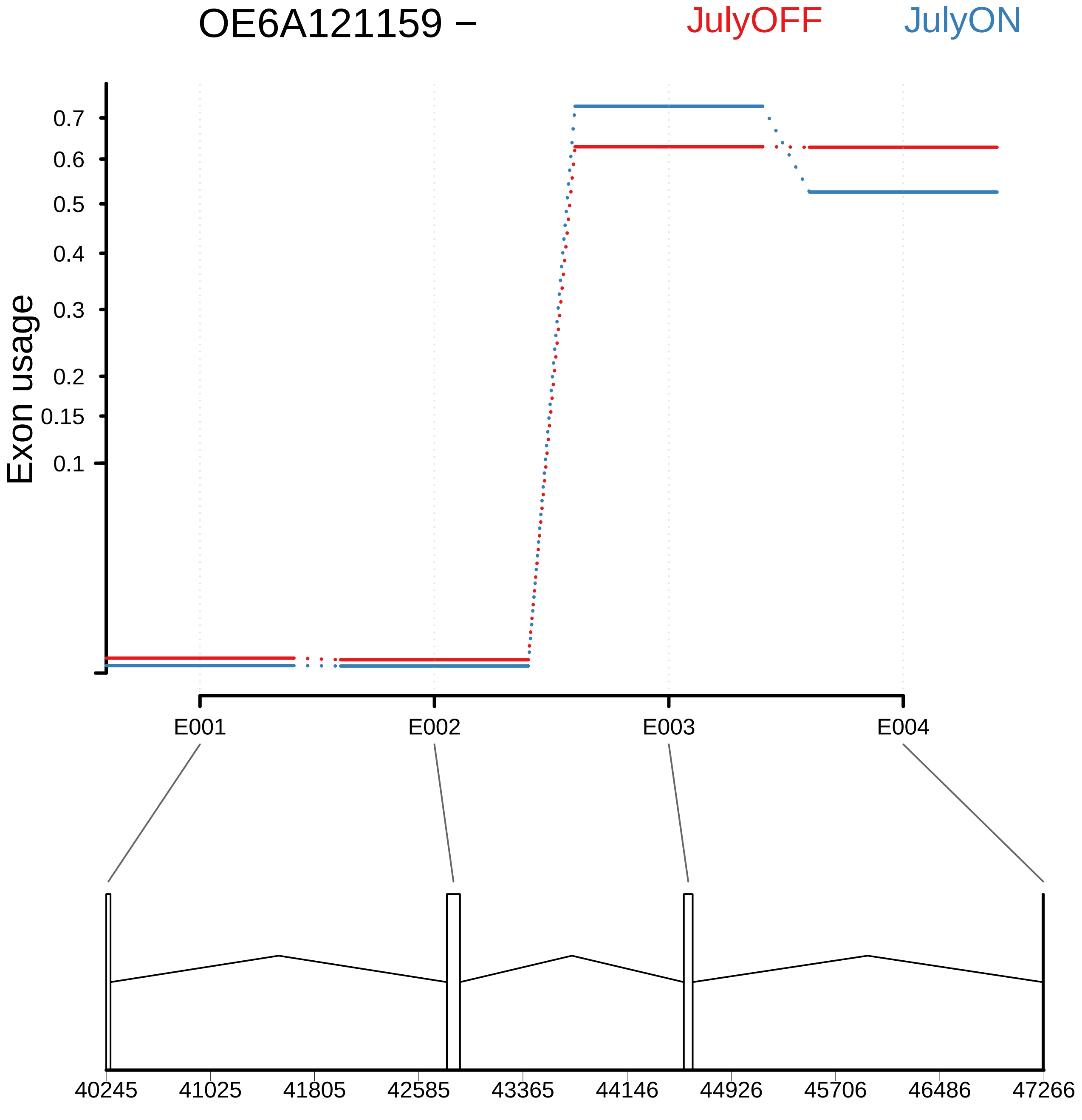
OE6A121159 −
JulyOFF
JulyON
Exon usage
0.7
0.6
0.5
0.4
0.3
0.2
0.15
0.1
E001
E002
E003
E004
40245
41025
41805
42585
43365
44146
44926
45706
46486
47266

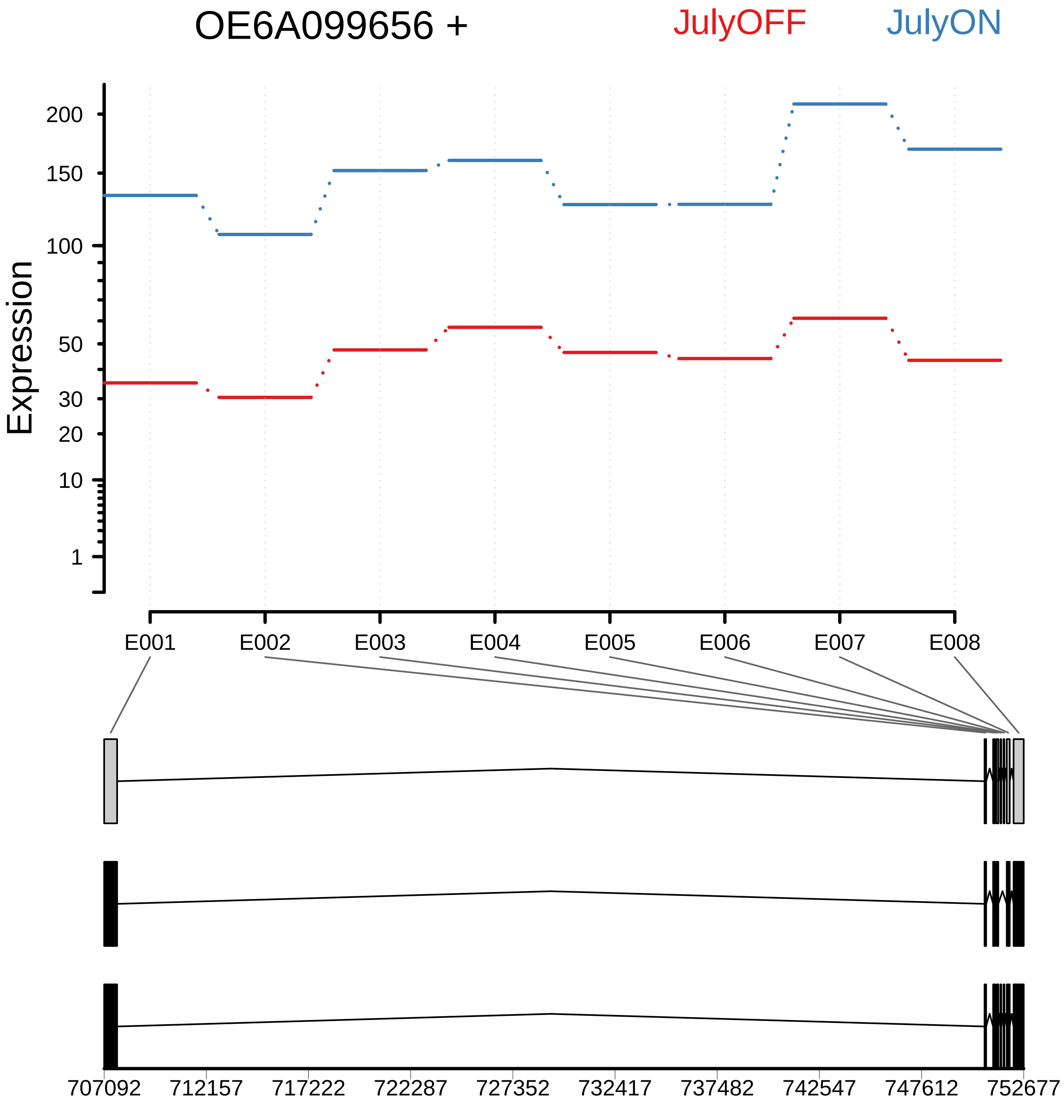
OE6A099656 +
JulyOFF
JulyON
Expression
200
150
100
50
30
20
10
1
E001
E002
E003
E004
E005
E006
E007
E008
707092
712157
717222
722287
727352
732417
737482
742547
747612
752677

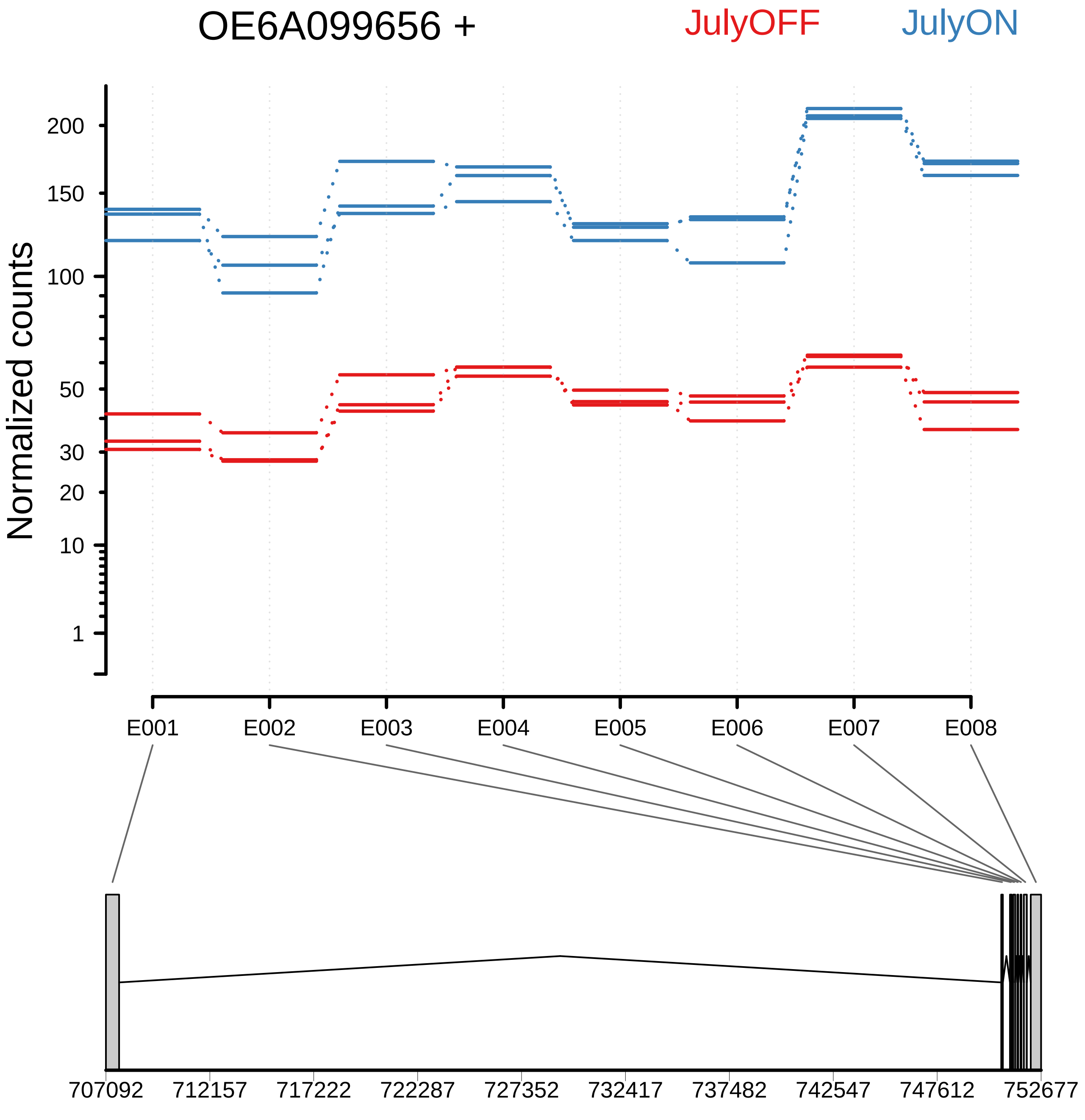
OE6A099656 +
JulyOFF
JulyON
Normalized counts
200
150
100
50
30
20
10
1
E001
E002
E003
E004
E005
E006
E007
E008
707092
712157
717222
722287
727352
732417
737482
742547
747612
752677

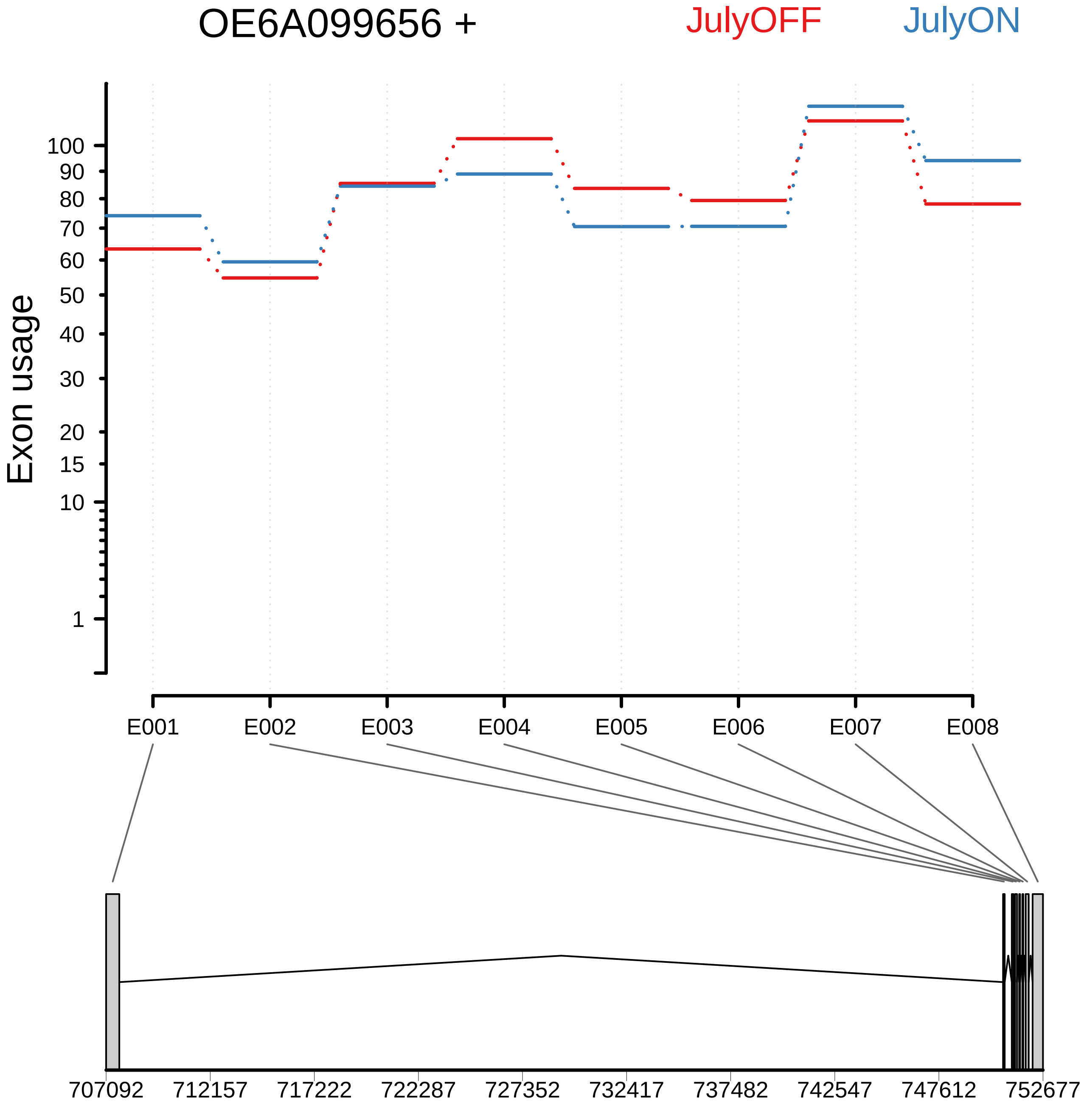

OE6A099656 +
JulyOFF
JulyON
Exon usage
100
90
80
70
60
50
40
30
20
15
10
1
E001
E002
E003
E004
E005
E006
E007
E008
707092
712157
717222
722287
727352
732417
737482
742547
747612
752677

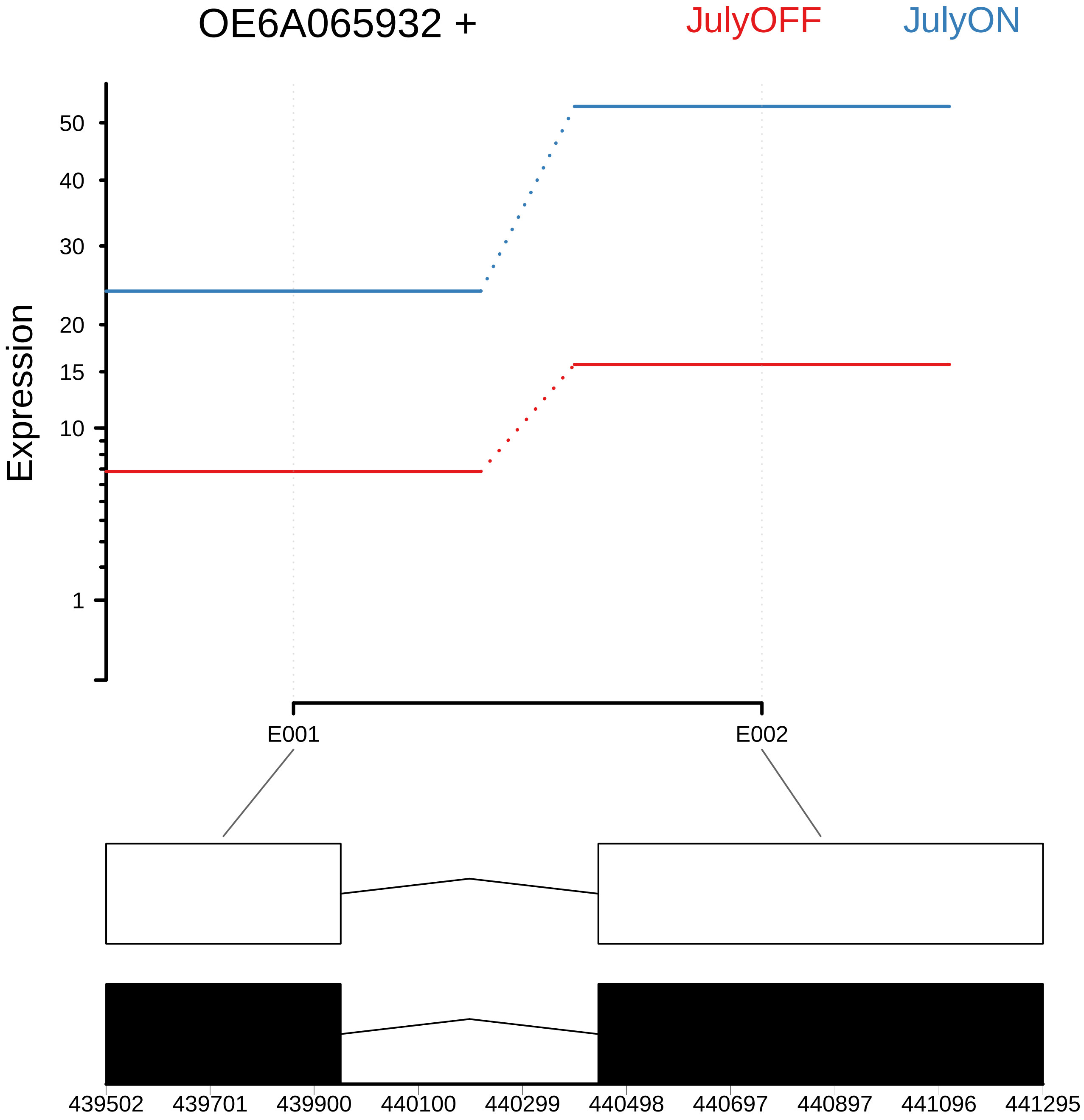
OE6A065932 +
JulyOFF
JulyON
Expression
50
40
30
20
15
10
1
E001
E002
439502
439701
439900
440100
440299
440498
440697
440897
441096
441295

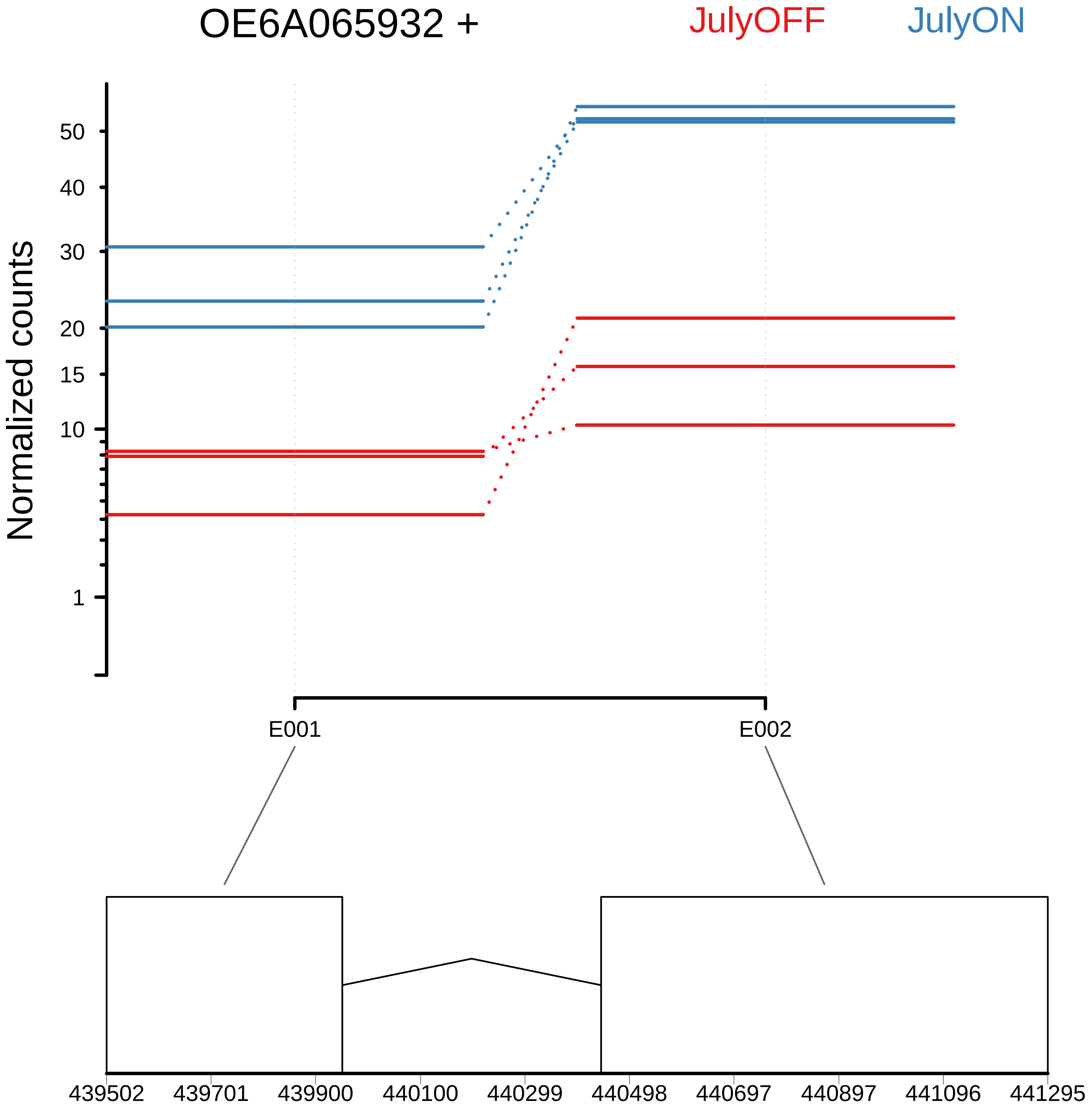

OE6A065932 +
JulyOFF
JulyON
Normalized counts
50
40
30
20
15
10
1
E001
E002
439502
439701
439900
440100
440299
440498
440697
440897
441096
441295

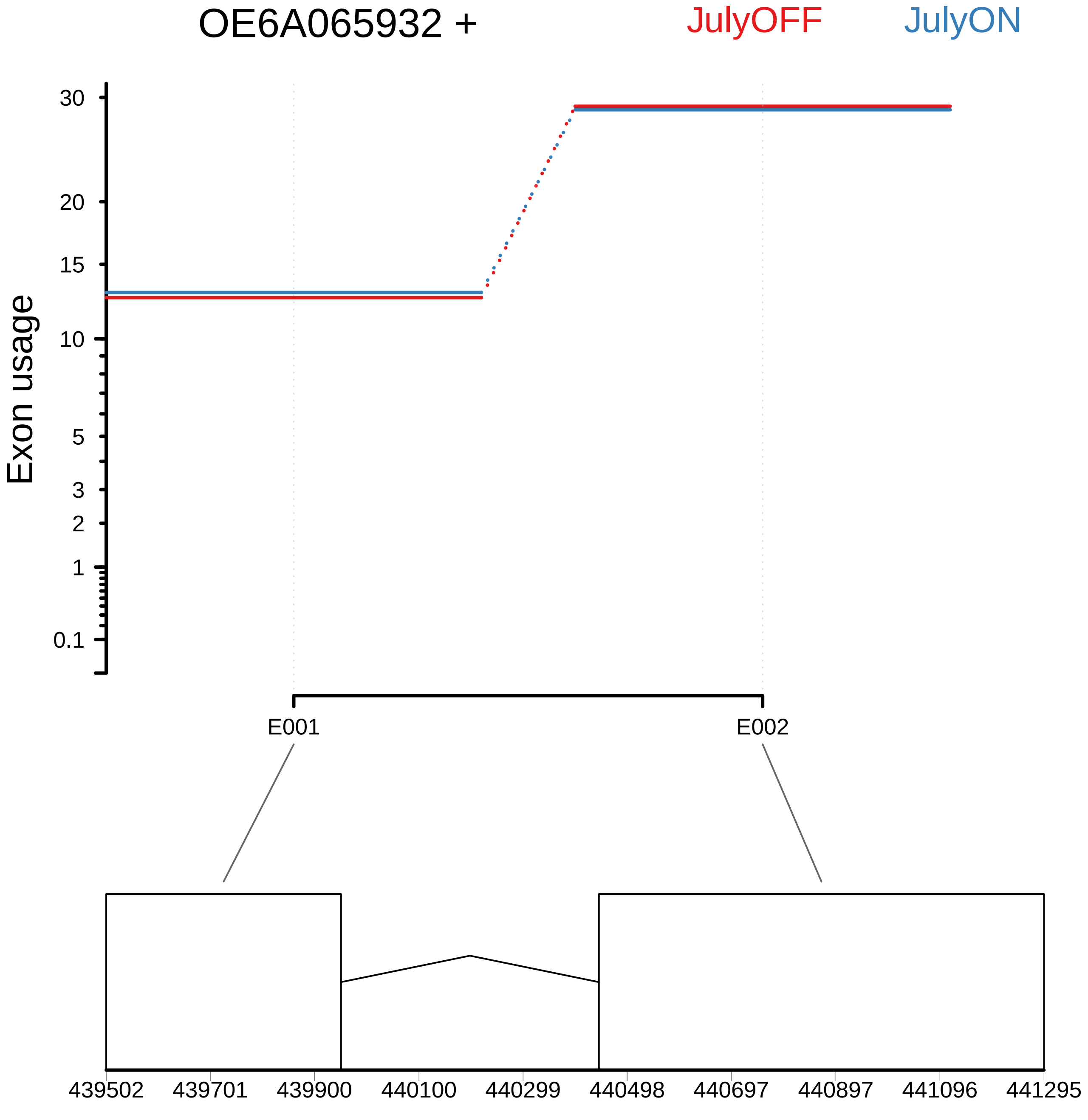
OE6A065932 +
JulyOFF
JulyON
Exon usage
30
20
15
10
5
3
2
1
0.1
E001
E002
439502
439701
439900
440100
440299
440498
440697
440897
441096
441295

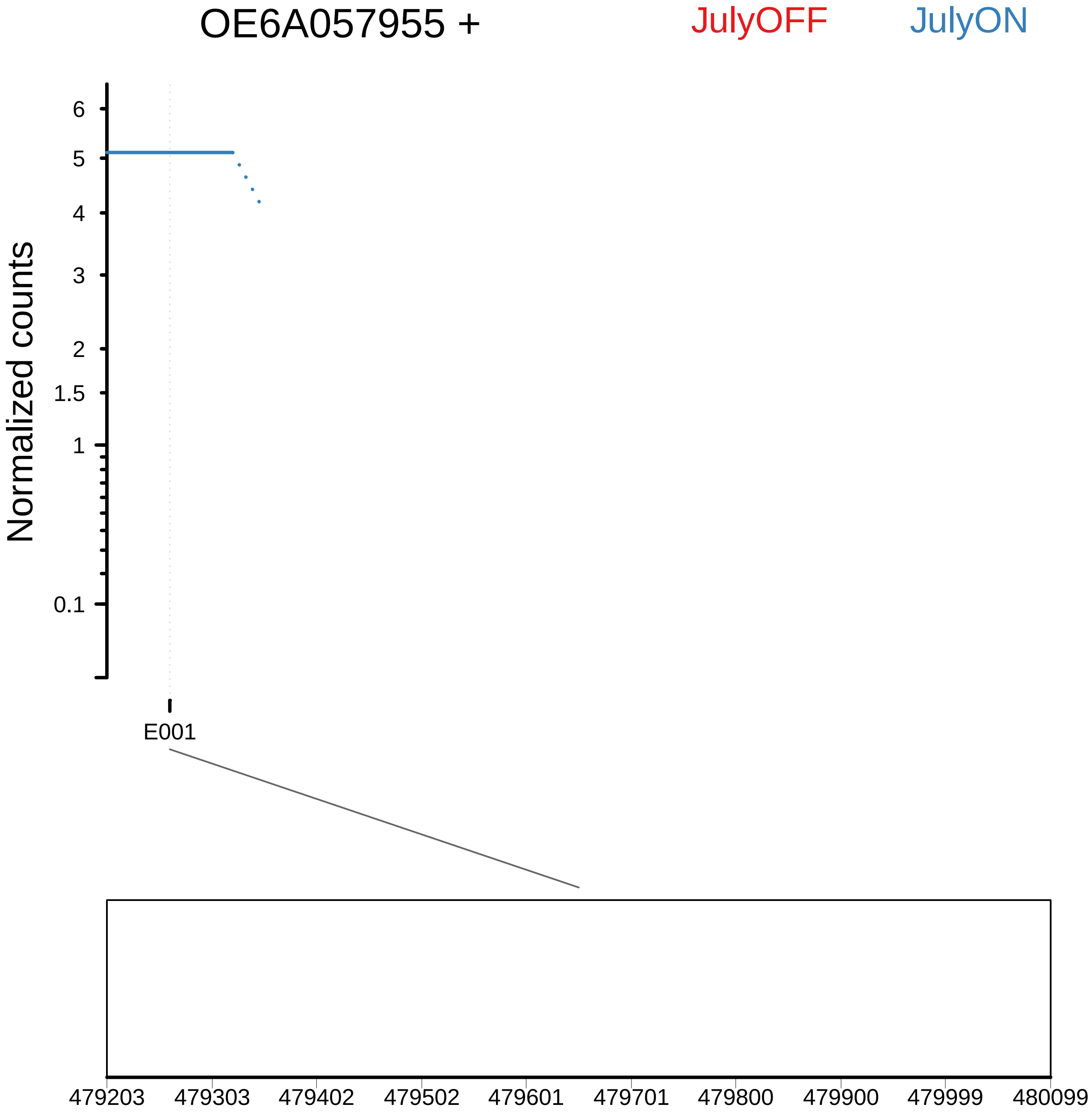
OE6A057955 +
JulyOFF
JulyON
Normalized counts
6
5
4
3
2
1.5
1
0.1
E001
479203
479303
479402
479502
479601
479701
479800
479900
479999
480099

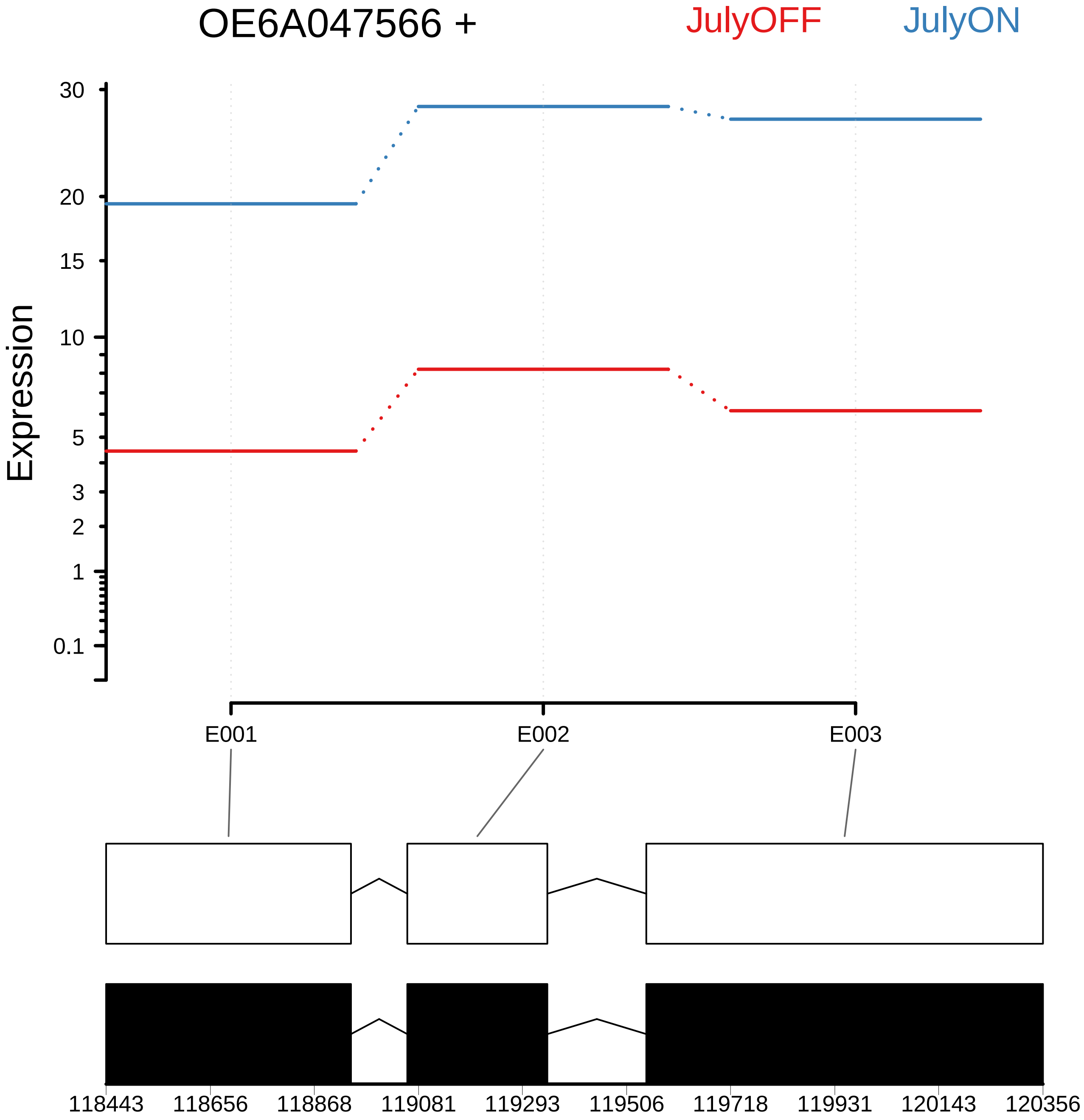
OE6A047566 +
JulyOFF
JulyON
Expression
30
20
15
10
5
3
2
1
0.1
E001
E002
E003
118443
118656
118868
119081
119293
119506
119718
119931
120143
120356

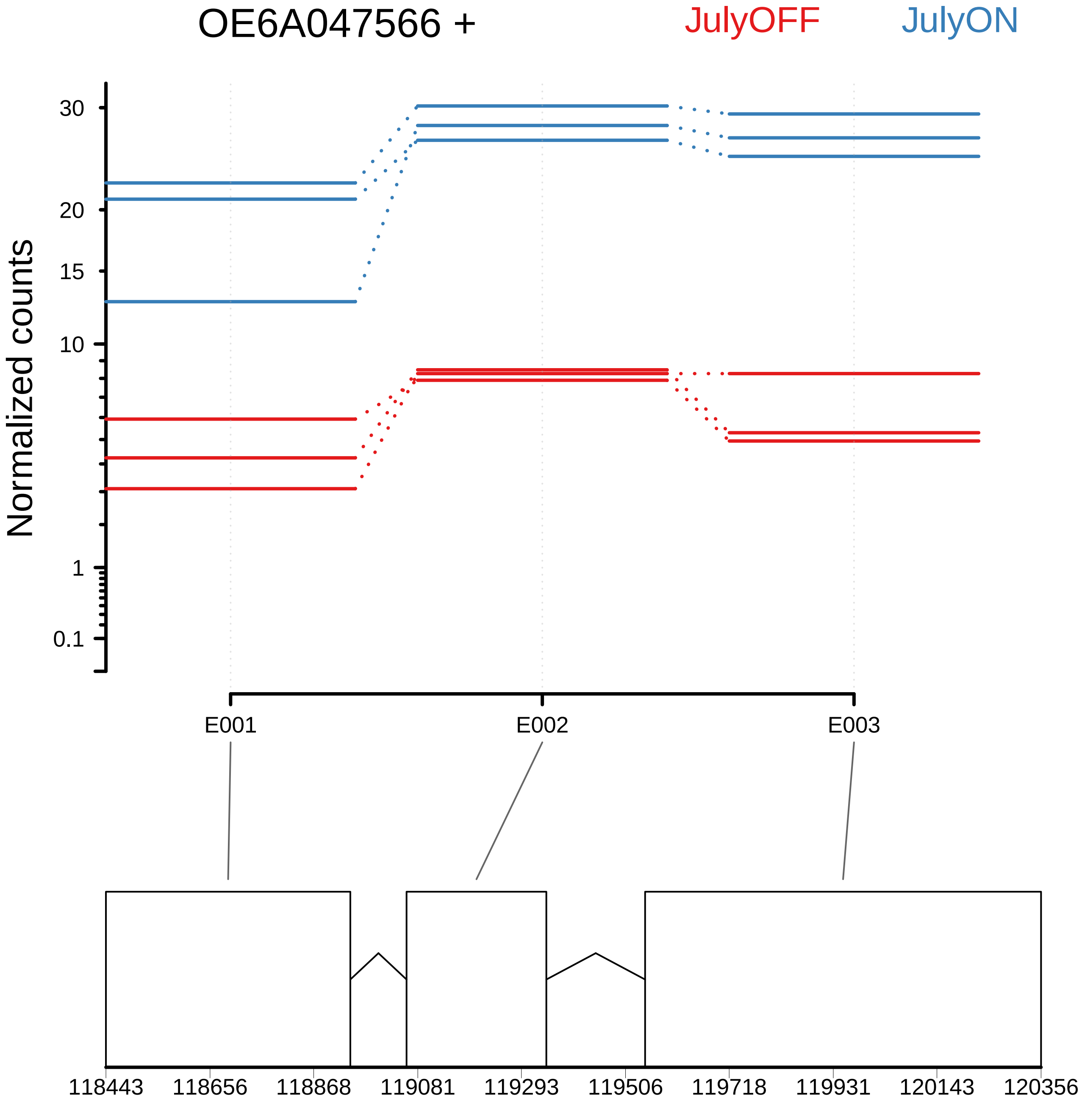

OE6A047566 +
JulyOFF
JulyON
Normalized counts
30
20
15
10
1
0.1
E001
E002
E003
118443
118656
118868
119081
119293
119506
119718
119931
120143
120356

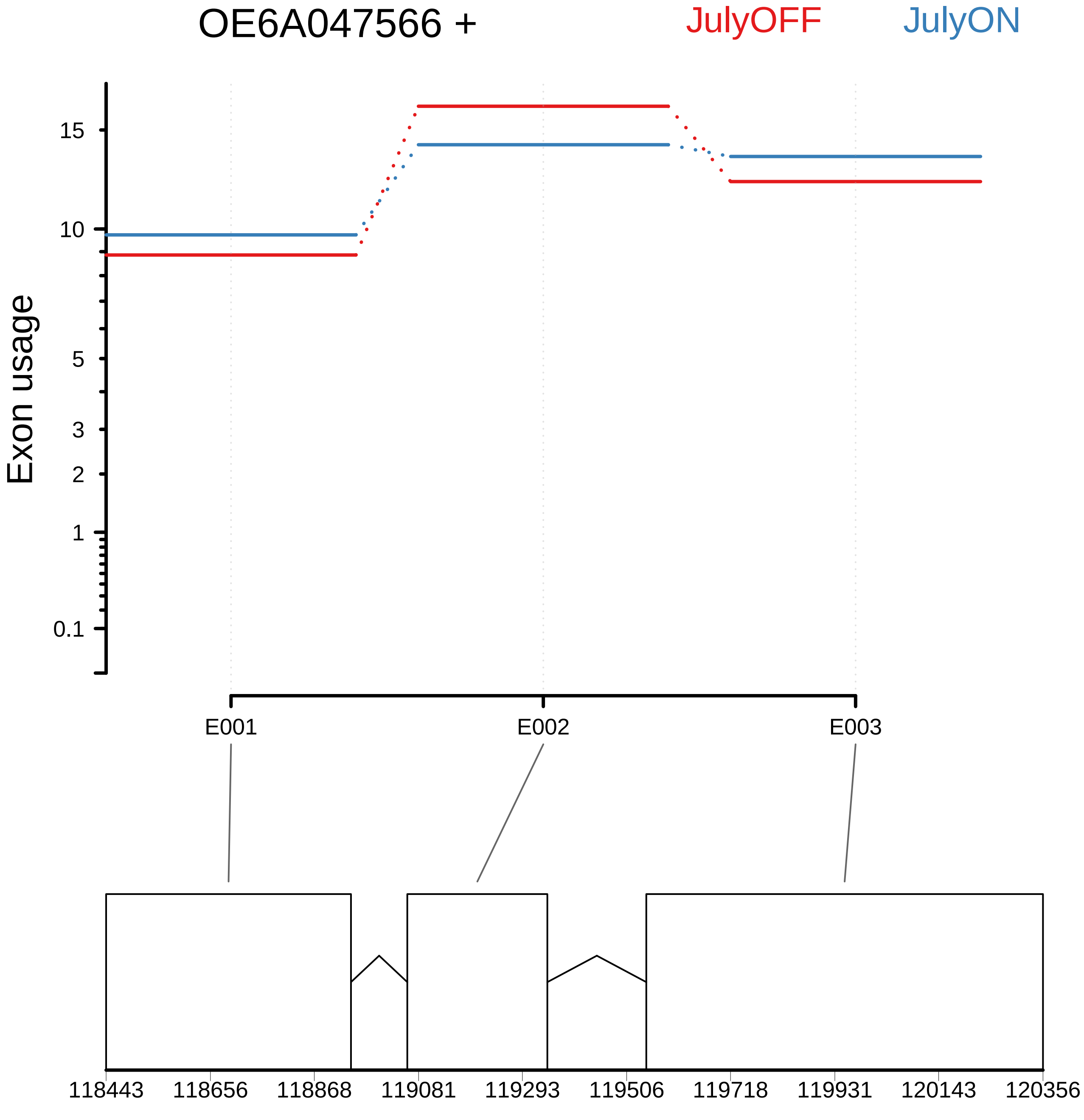
OE6A047566 +
JulyOFF
JulyON
Exon usage
15
10
5
3
2
1
0.1
E001
E002
E003
118443
118656
118868
119081
119293
119506
119718
119931
120143
120356

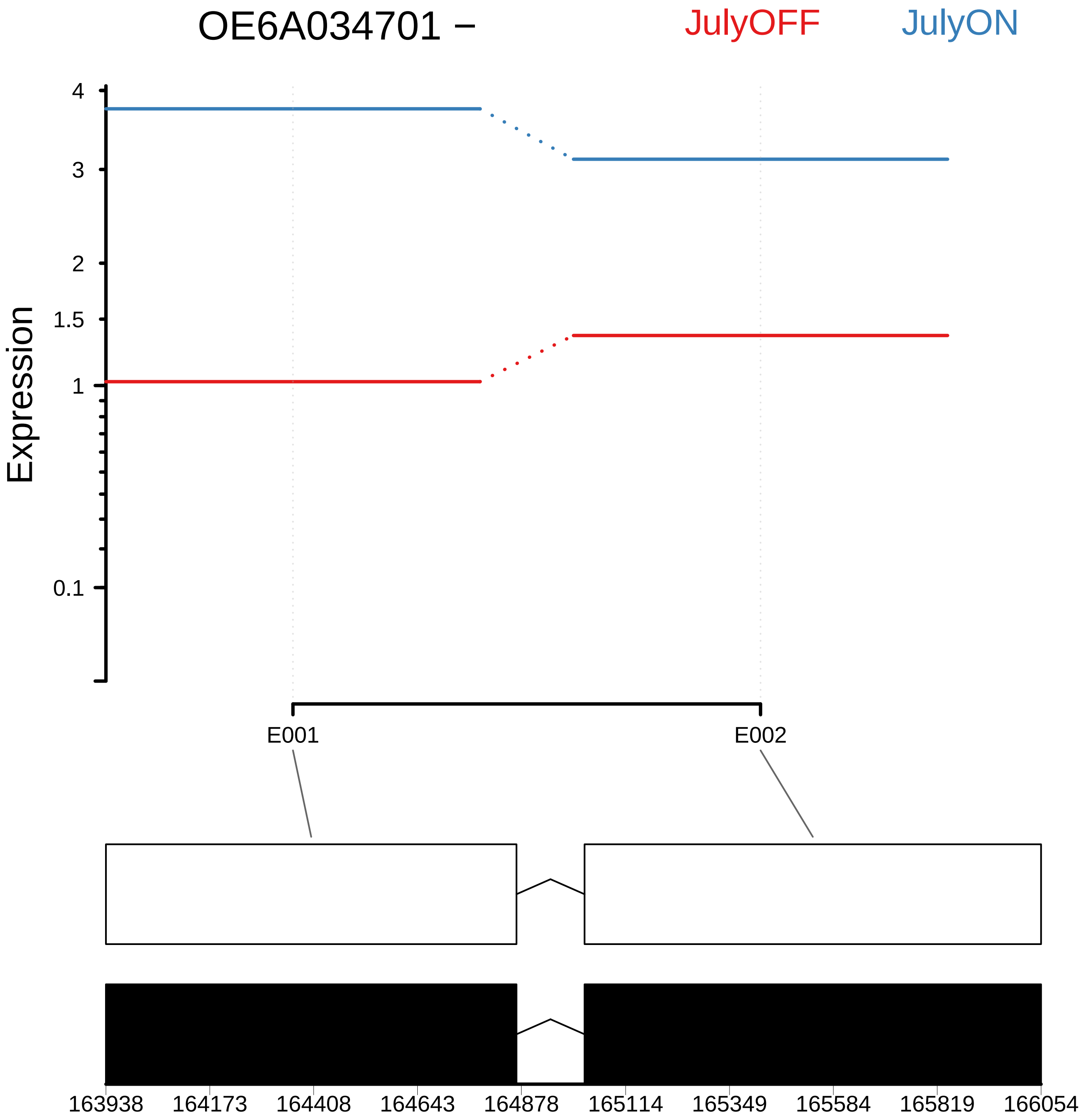

OE6A034701 −
JulyOFF
JulyON
Expression
4
3
2
1.5
1
0.1
E001
E002
163938
164173
164408
164643
164878
165114
165349
165584
165819
166054

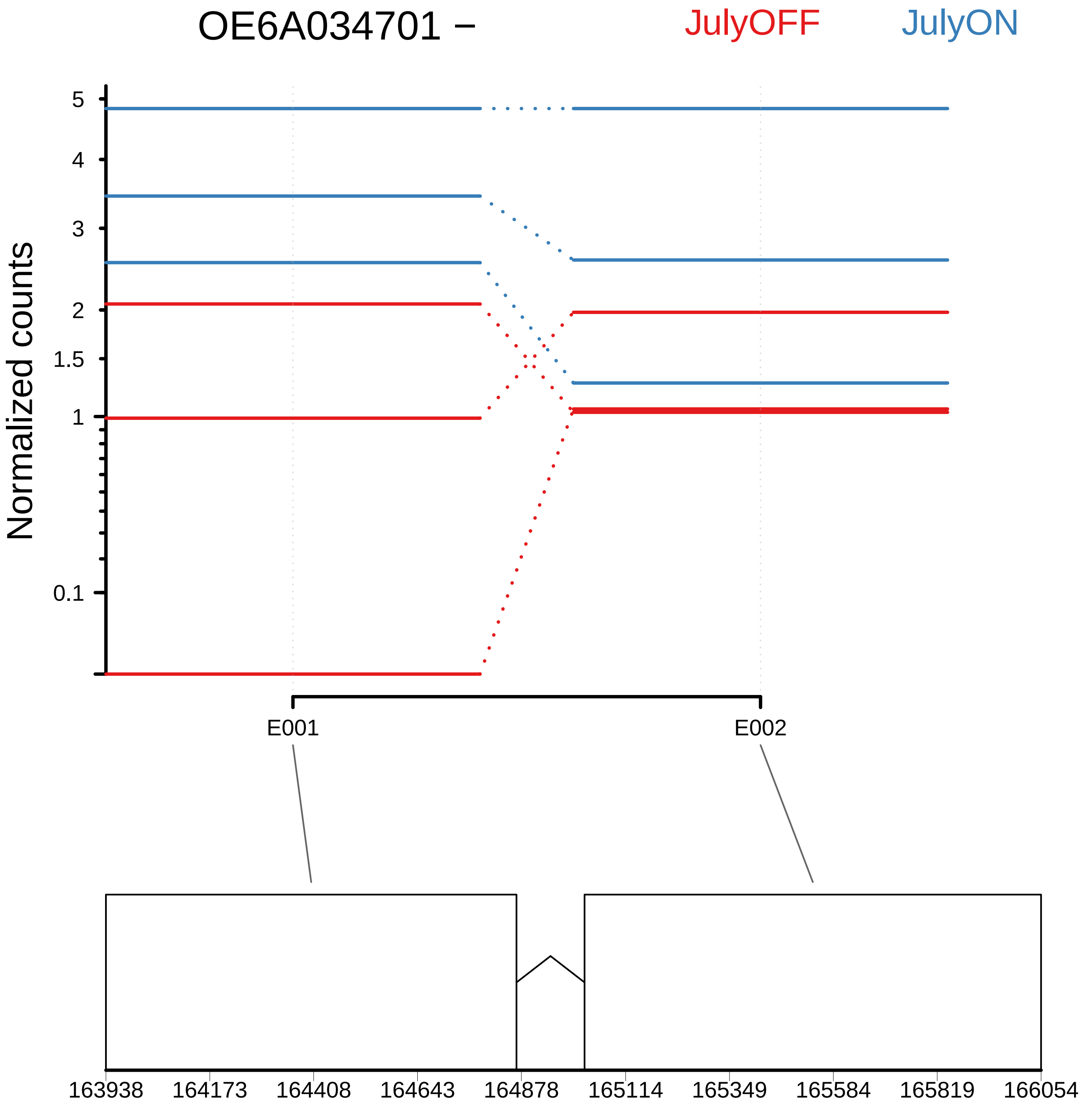

OE6A034701 −
JulyOFF
JulyON
Normalized counts
5
4
3
2
1.5
1
0.1
E001
E002
163938
164173
164408
164643
164878
165114
165349
165584
165819
166054

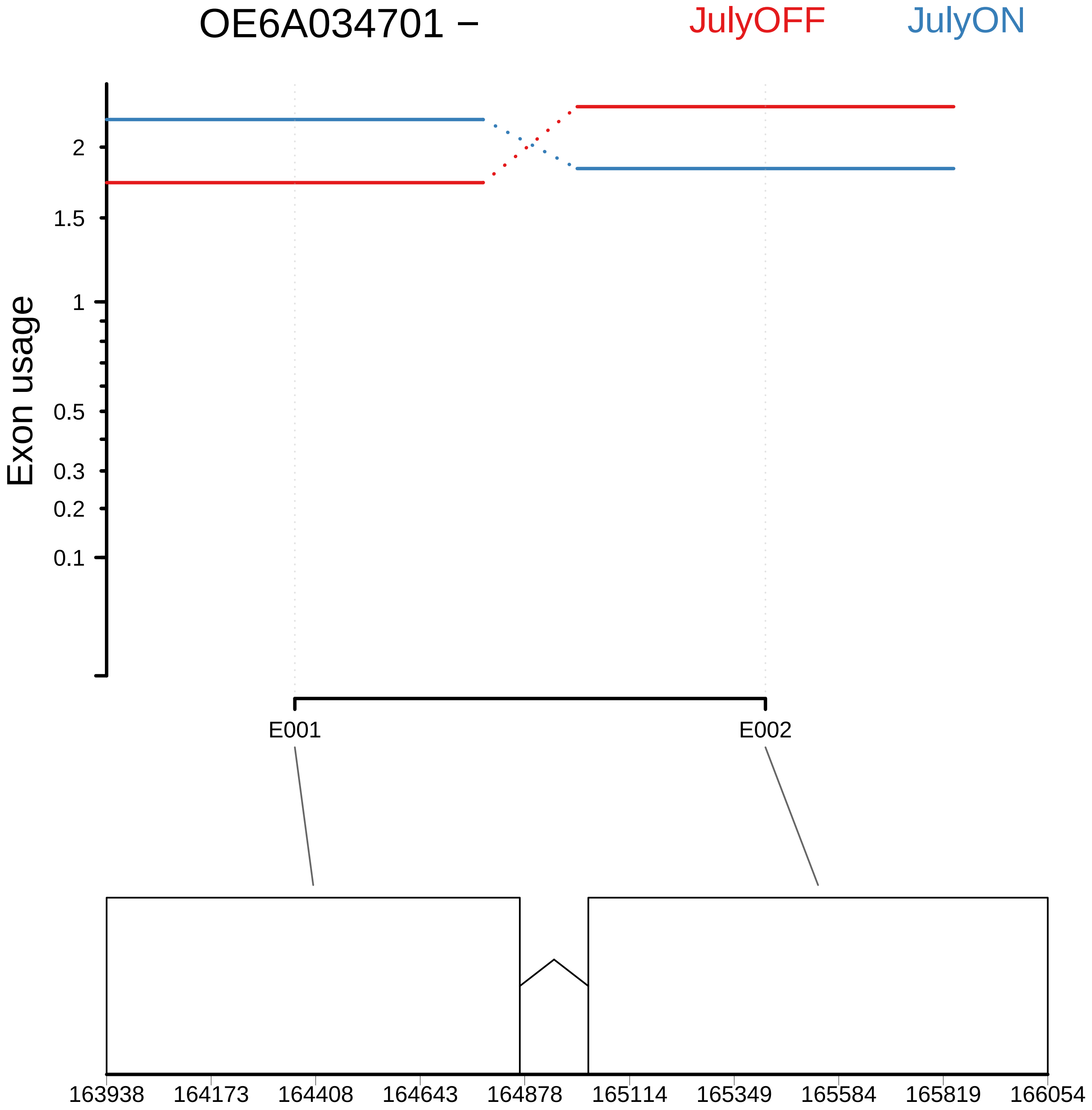
OE6A034701 −
JulyOFF
JulyON
Exon usage
2
1.5
1
0.5
0.3
0.2
0.1
E001
E002
163938
164173
164408
164643
164878
165114
165349
165584
165819
166054

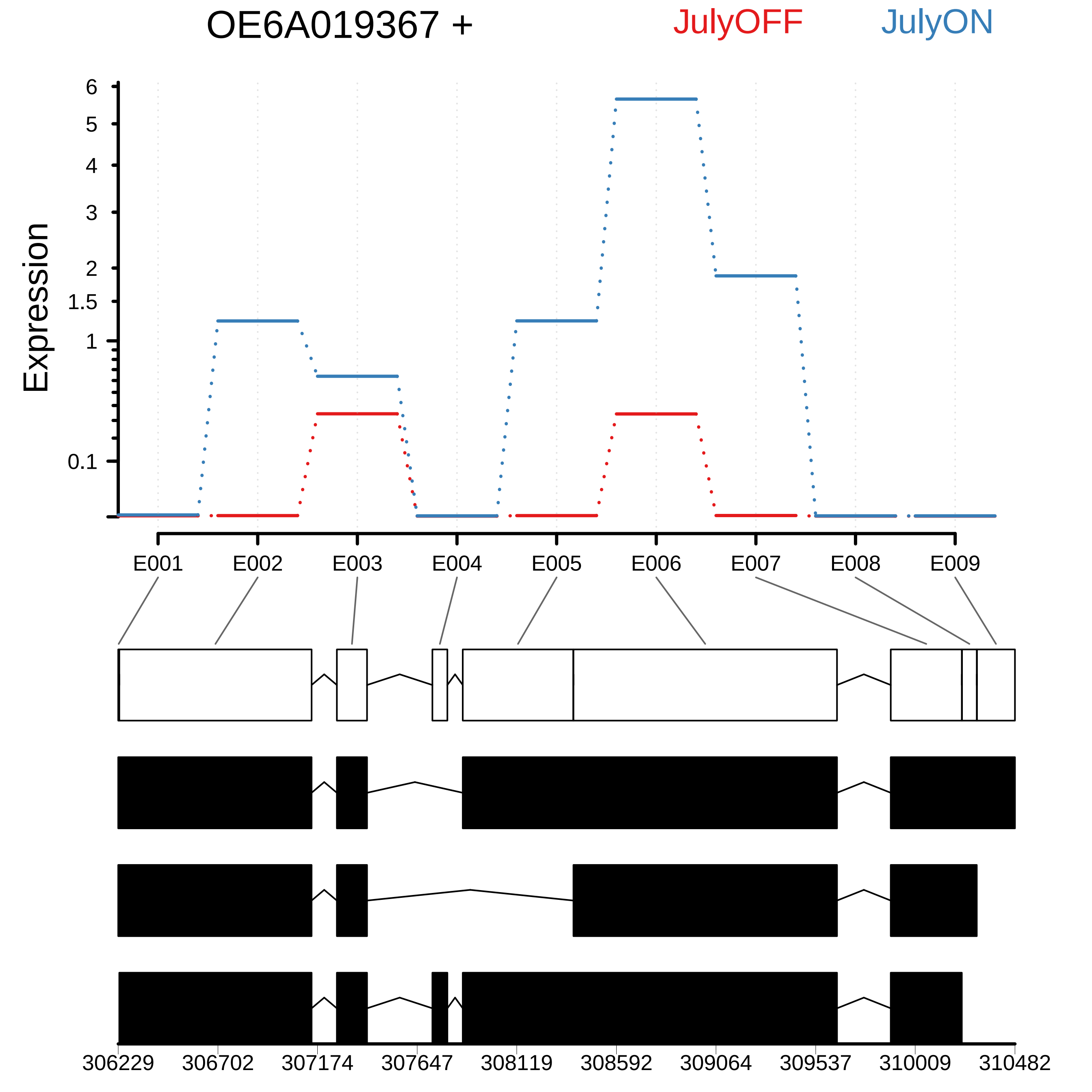
OE6A019367 +
JulyOFF
JulyON
Expression
6
5
4
3
2
1.5
1
0.1
E001
E002
E003
E004
E005
E006
E007
E008
E009
306229
306702
307174
307647
308119
308592
309064
309537
310009
310482

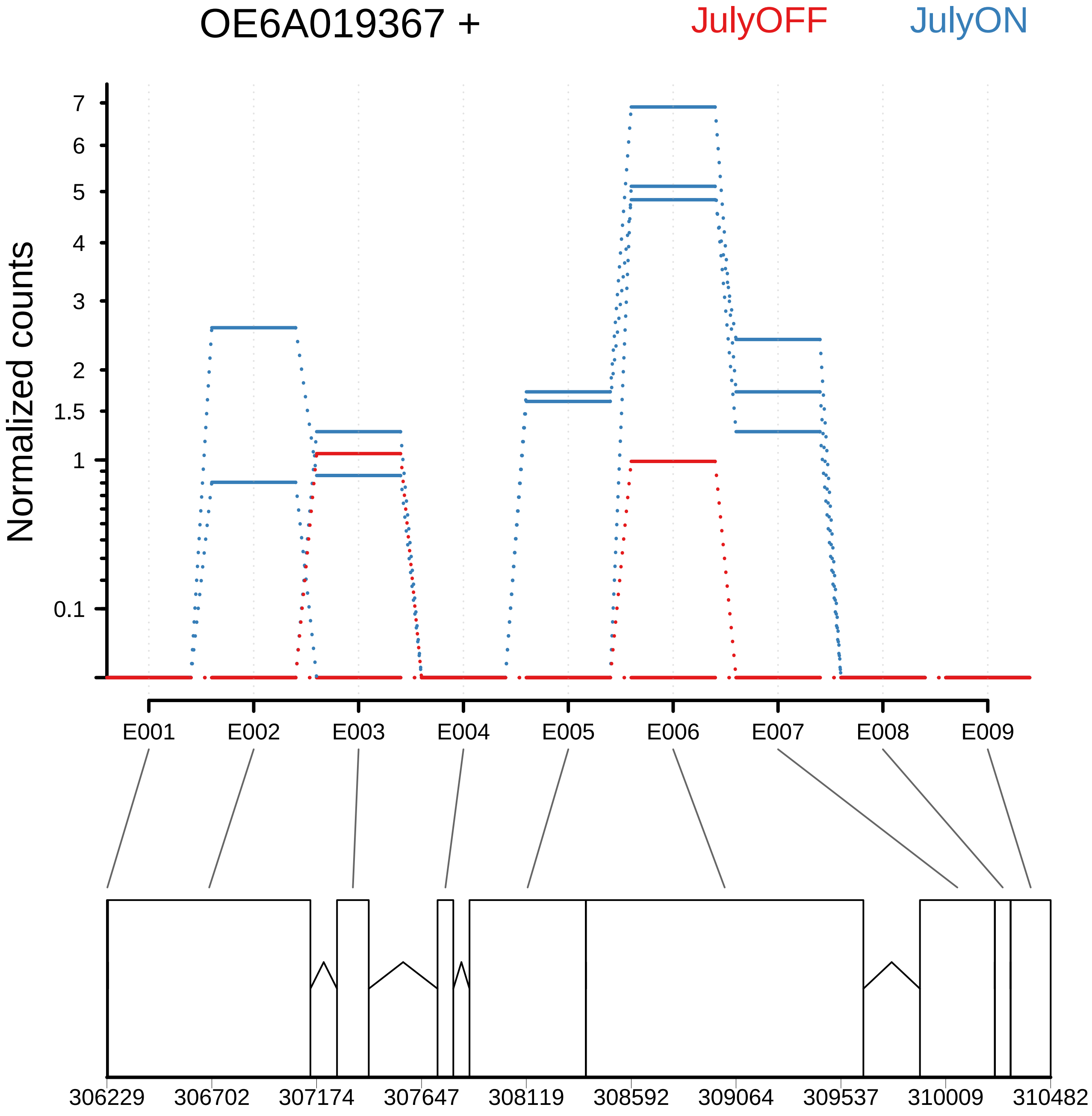
OE6A019367 +
JulyOFF
JulyON
Normalized counts
7
6
5
4
3
2
1.5
1
0.1
E001
E002
E003
E004
E005
E006
E007
E008
E009
306229
306702
307174
307647
308119
308592
309064
309537
310009
310482

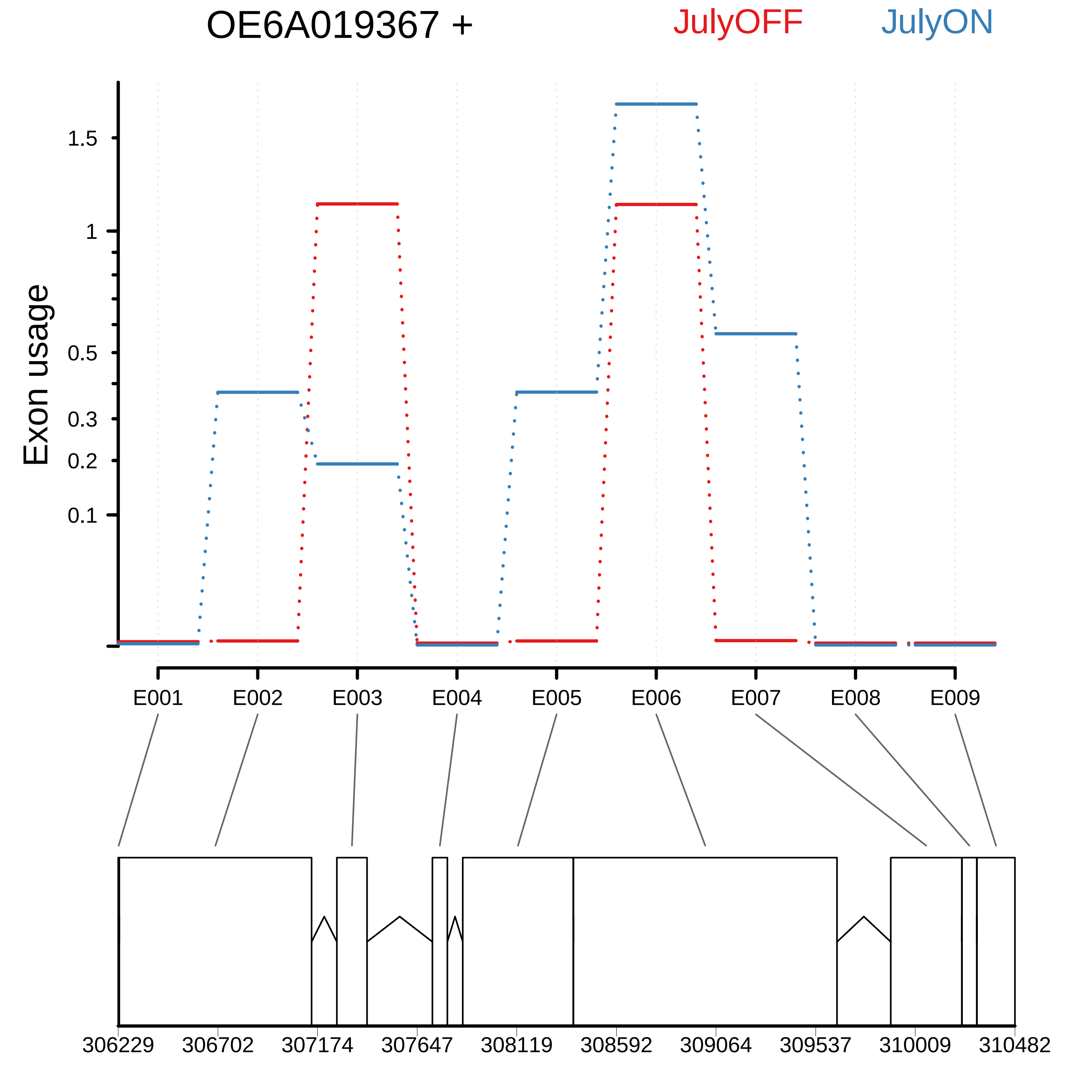

OE6A019367 +
JulyOFF
JulyON
Exon usage
1.5
1
0.5
0.3
0.2
0.1
E001
E002
E003
E004
E005
E006
E007
E008
E009
306229
306702
307174
307647
308119
308592
309064
309537
310009
310482

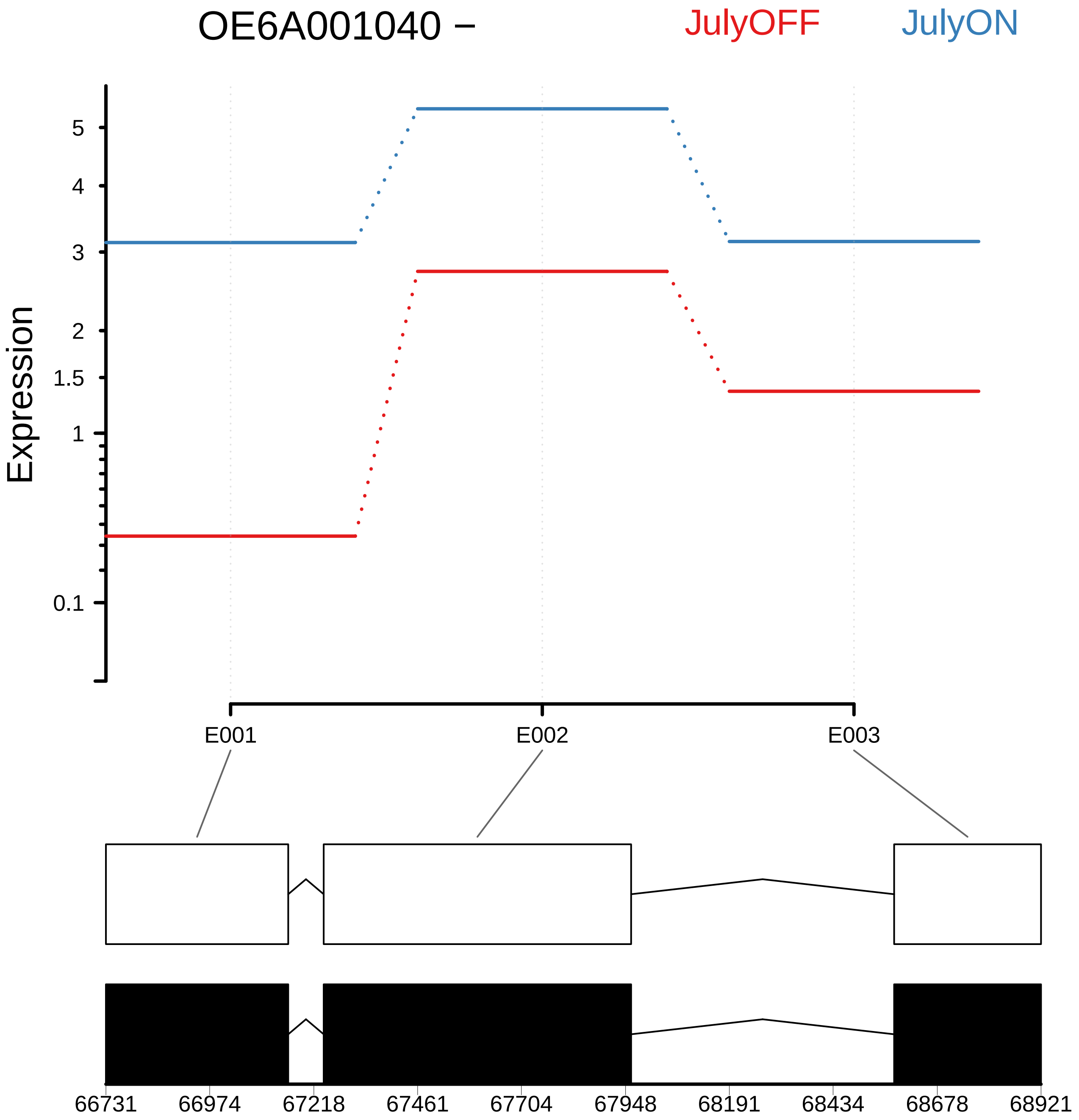
OE6A001040 −
JulyOFF
JulyON
Expression
5
4
3
2
1.5
1
0.1
E001
E002
E003
66731
66974
67218
67461
67704
67948
68191
68434
68678
68921

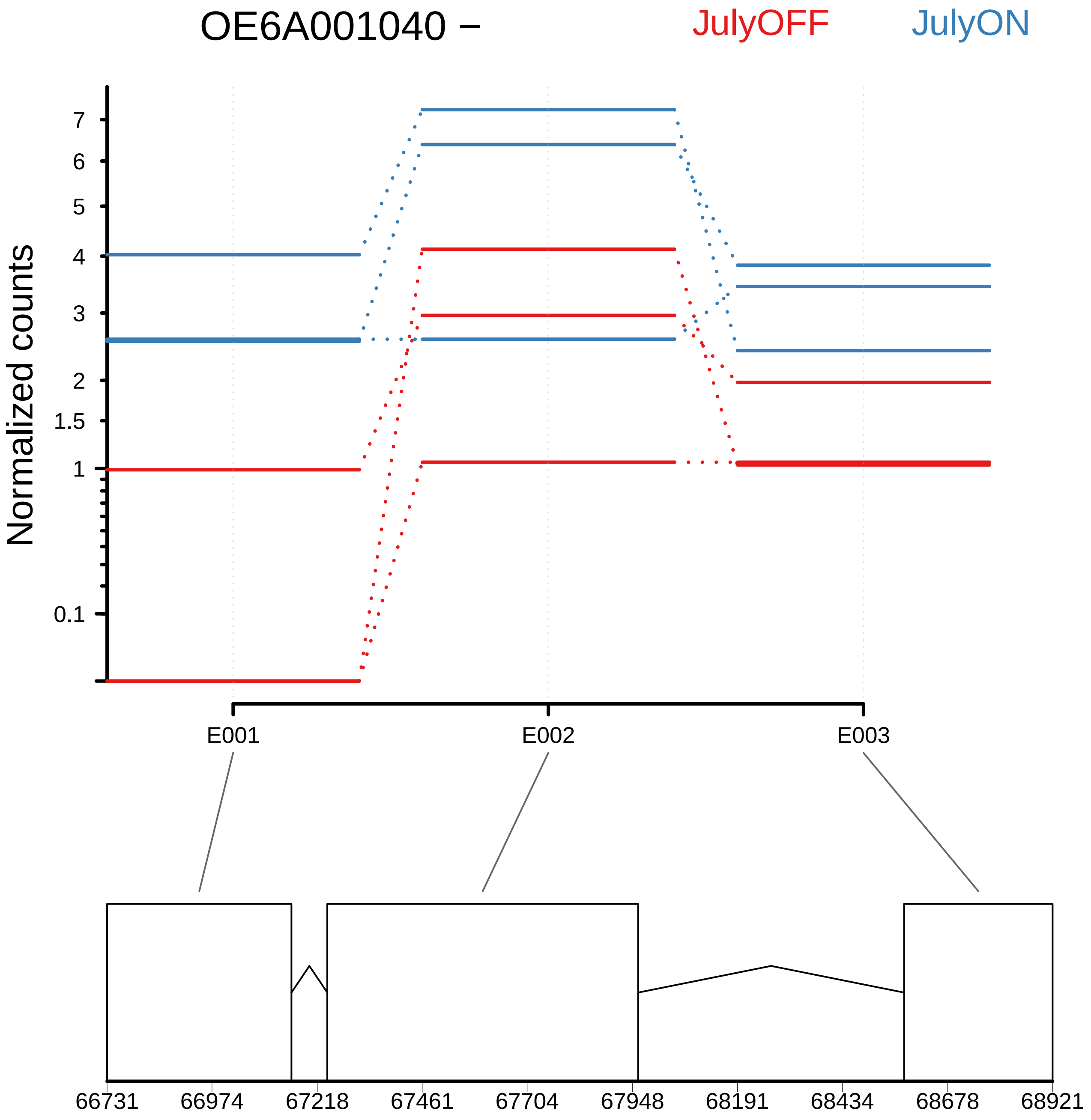
OE6A001040 −
JulyOFF
JulyON
Normalized counts
7
6
5
4
3
2
1.5
1
0.1
E001
E002
E003
66731
66974
67218
67461
67704
67948
68191
68434
68678
68921

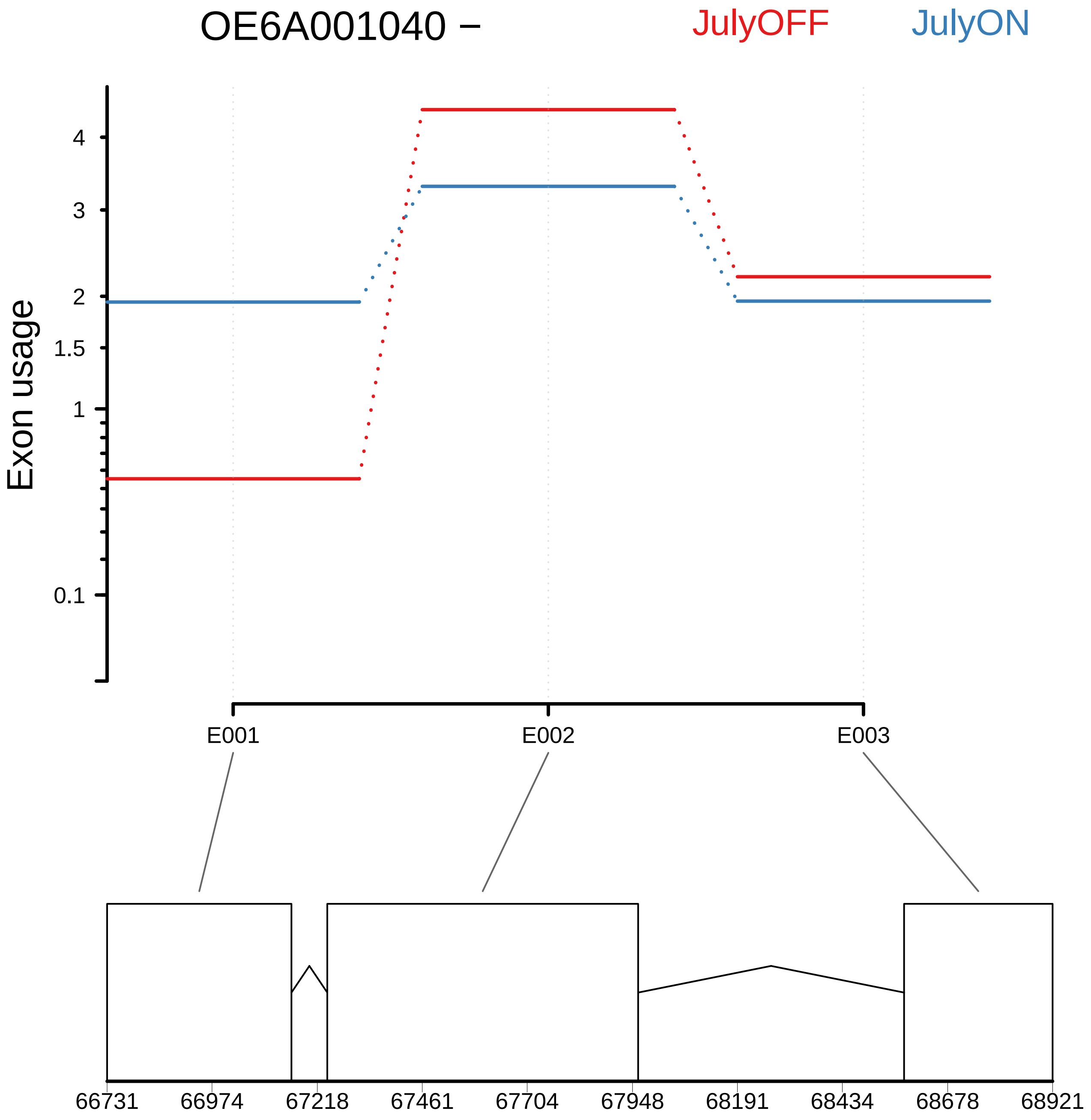

OE6A001040 −
JulyOFF
JulyON
Exon usage
4
3
2
1.5
1
0.1
E001
E002
E003
66731
66974
67218
67461
67704
67948
68191
68434
68678
68921

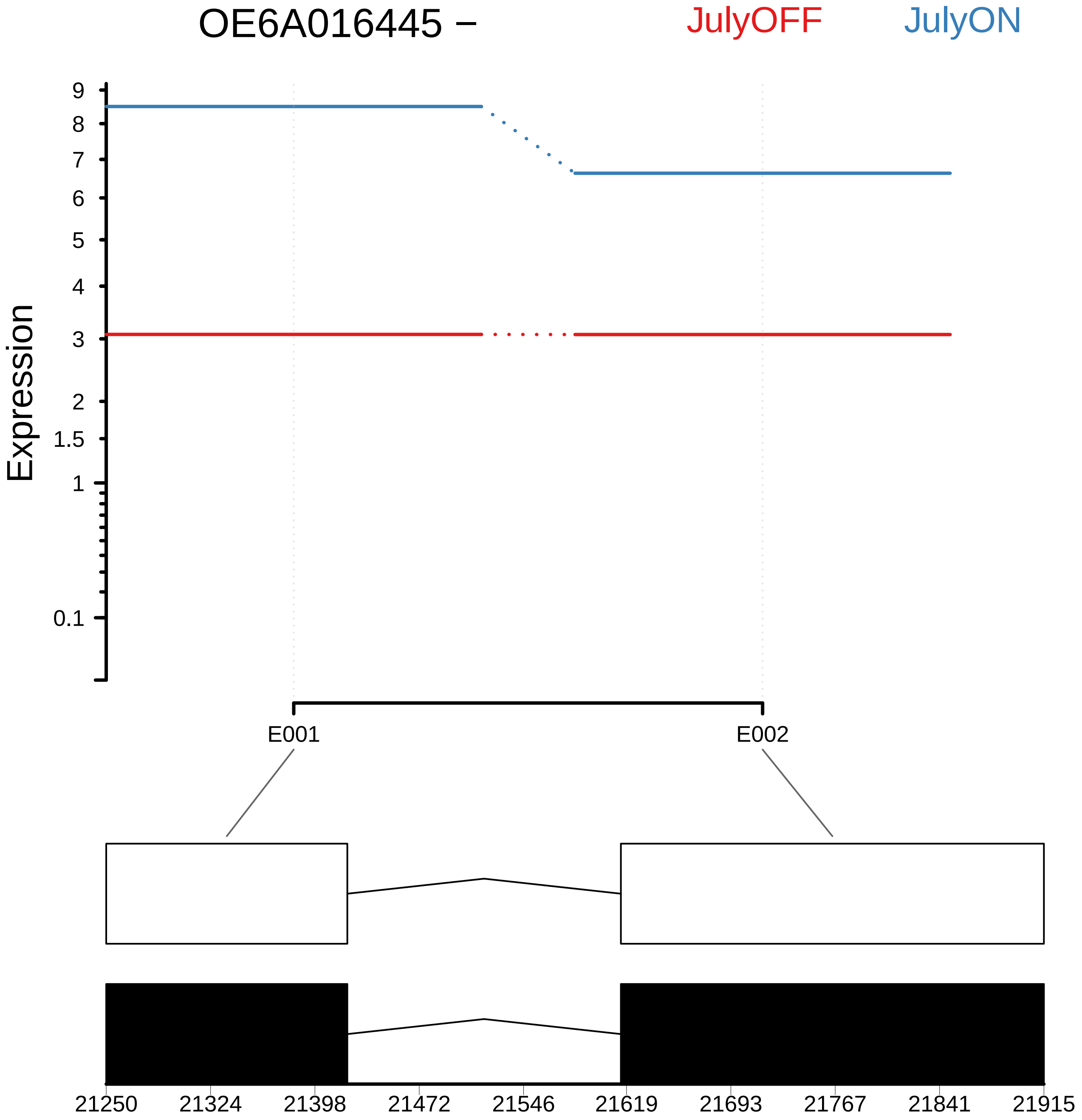

OE6A016445 −
JulyOFF
JulyON
Expression
9
8
7
6
5
4
3
2
1.5
1
0.1
E001
E002
21250
21324
21398
21472
21546
21619
21693
21767
21841
21915

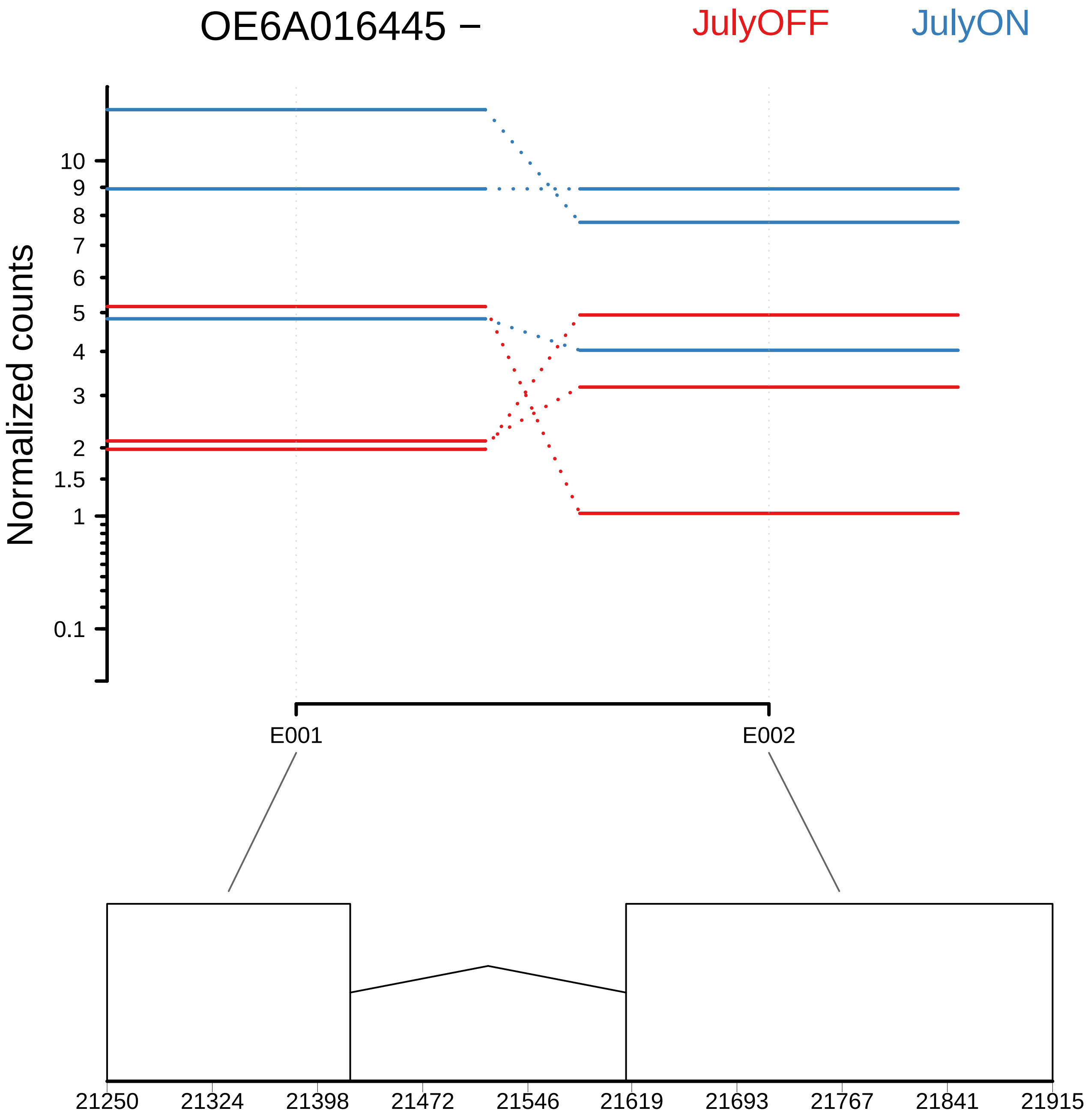

OE6A016445 −
JulyOFF
JulyON
Normalized counts
10
9
8
7
6
5
4
3
2
1.5
1
0.1
E001
E002
21250
21324
21398
21472
21546
21619
21693
21767
21841
21915

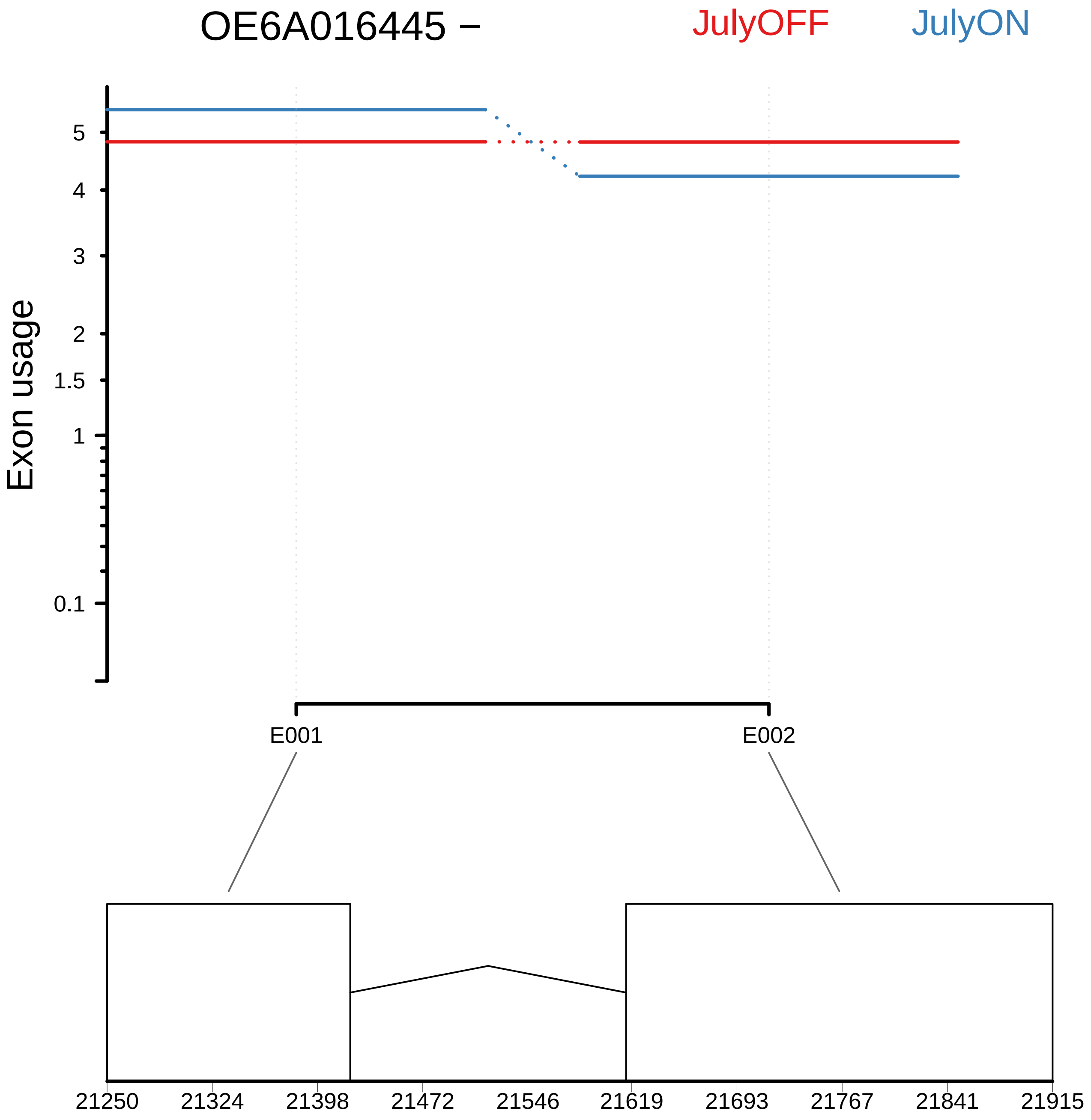

OE6A016445 −
JulyOFF
JulyON
Exon usage
5
4
3
2
1.5
1
0.1
E001
E002
21250
21324
21398
21472
21546
21619
21693
21767
21841
21915

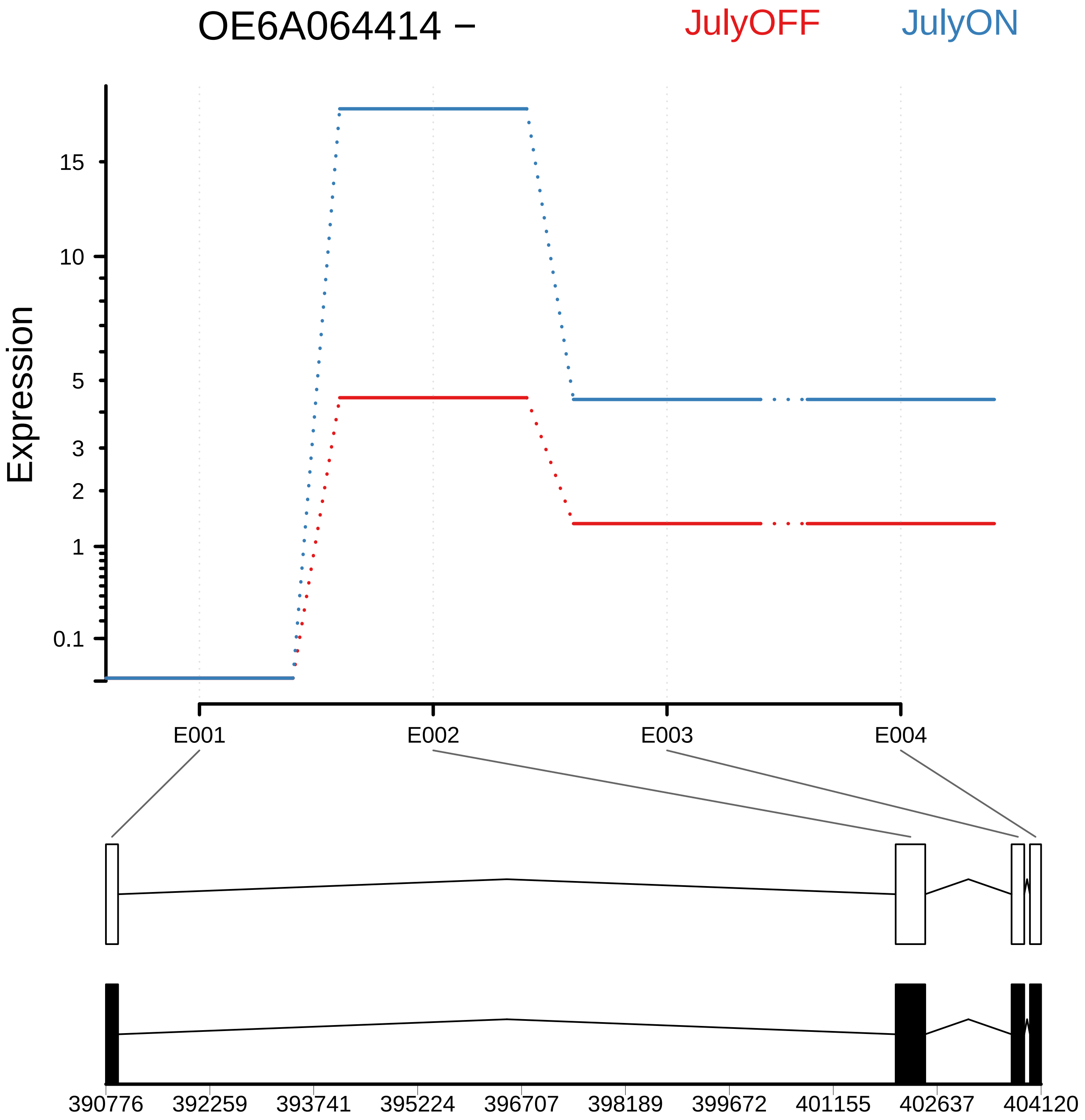
OE6A064414 −
JulyOFF
JulyON
Expression
15
10
5
3
2
1
0.1
E001
E002
E003
E004
390776
392259
393741
395224
396707
398189
399672
401155
402637
404120

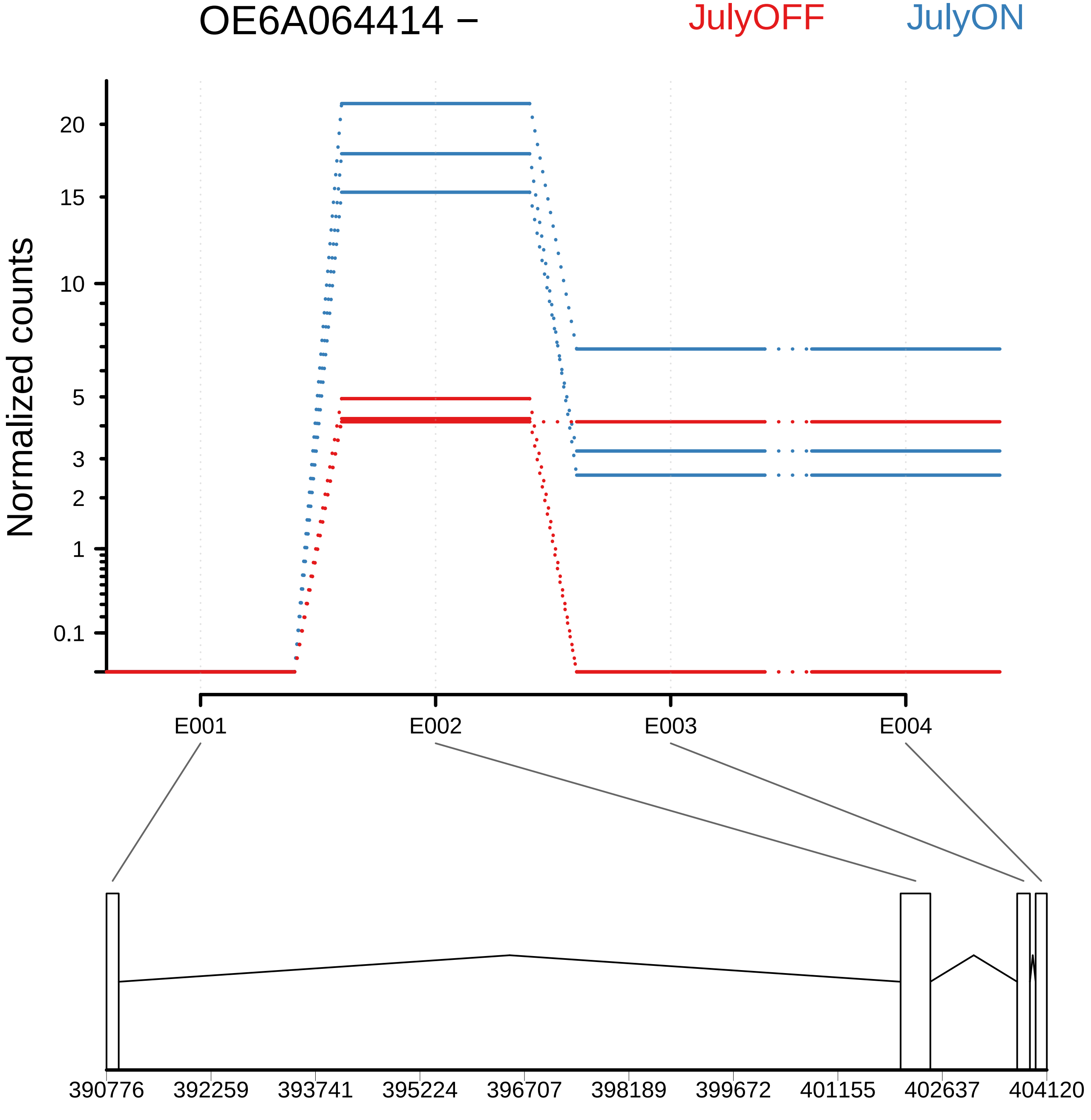

OE6A064414 −
JulyOFF
JulyON
Normalized counts
20
15
10
5
3
2
1
0.1
E001
E002
E003
E004
390776
392259
393741
395224
396707
398189
399672
401155
402637
404120

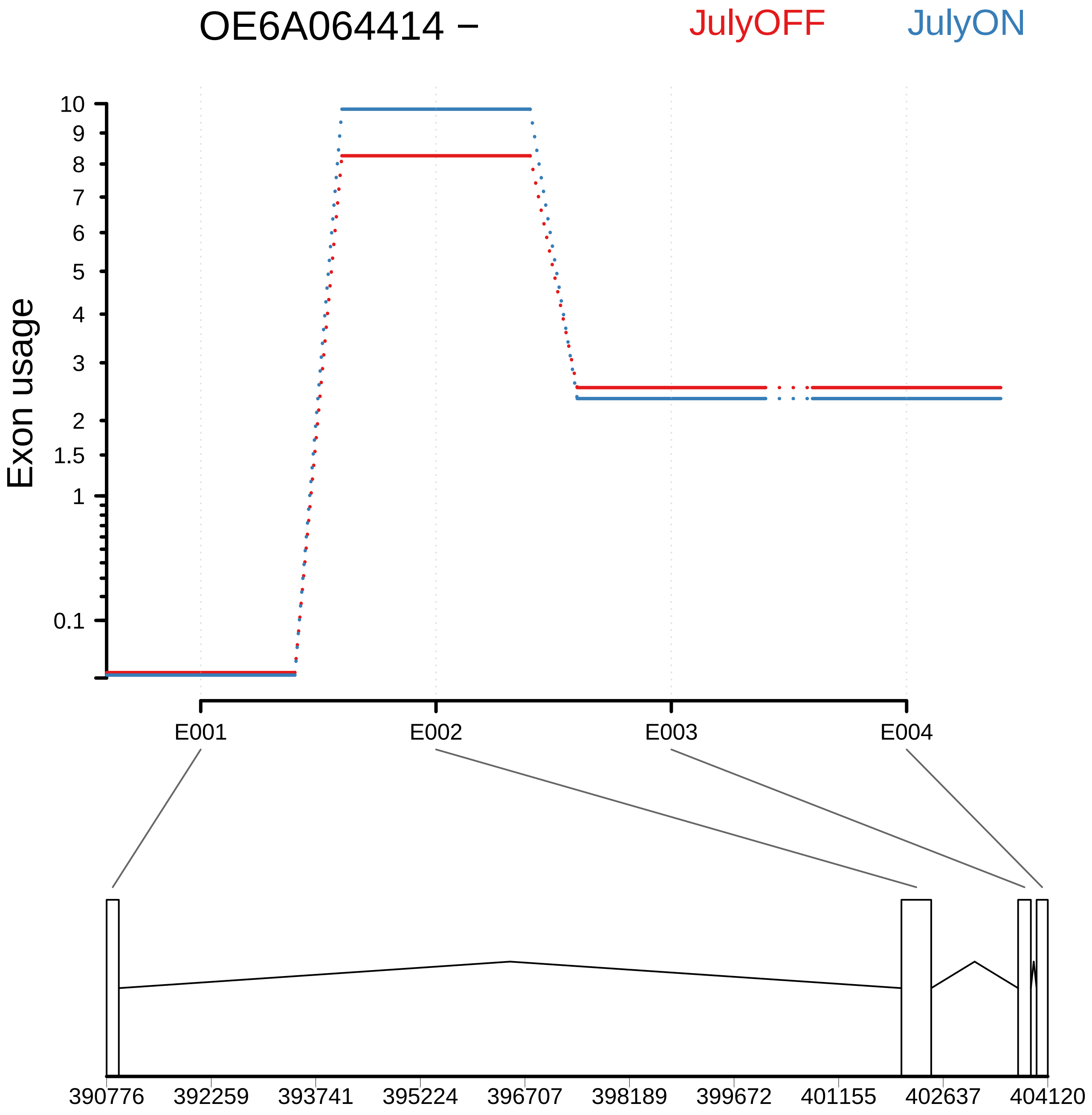

OE6A064414 −
JulyOFF
JulyON
Exon usage
10
9
8
7
6
5
4
3
2
1.5
1
0.1
E001
E002
E003
E004
390776
392259
393741
395224
396707
398189
399672
401155
402637
404120

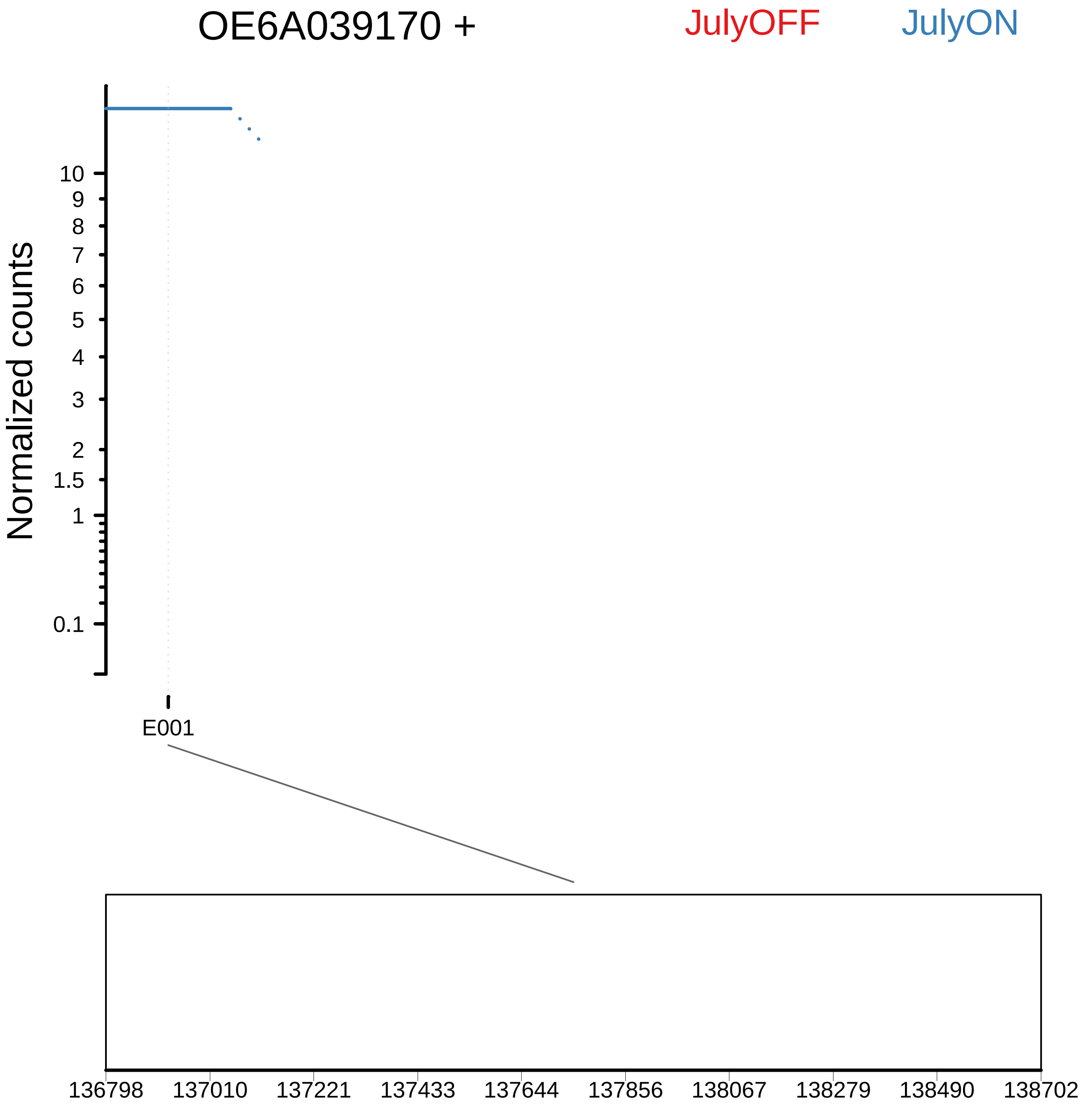

OE6A039170 +
JulyOFF
JulyON
Normalized counts
10
9
8
7
6
5
4
3
2
1.5
1
0.1
E001
136798
137010
137221
137433
137644
137856
138067
138279
138490
138702

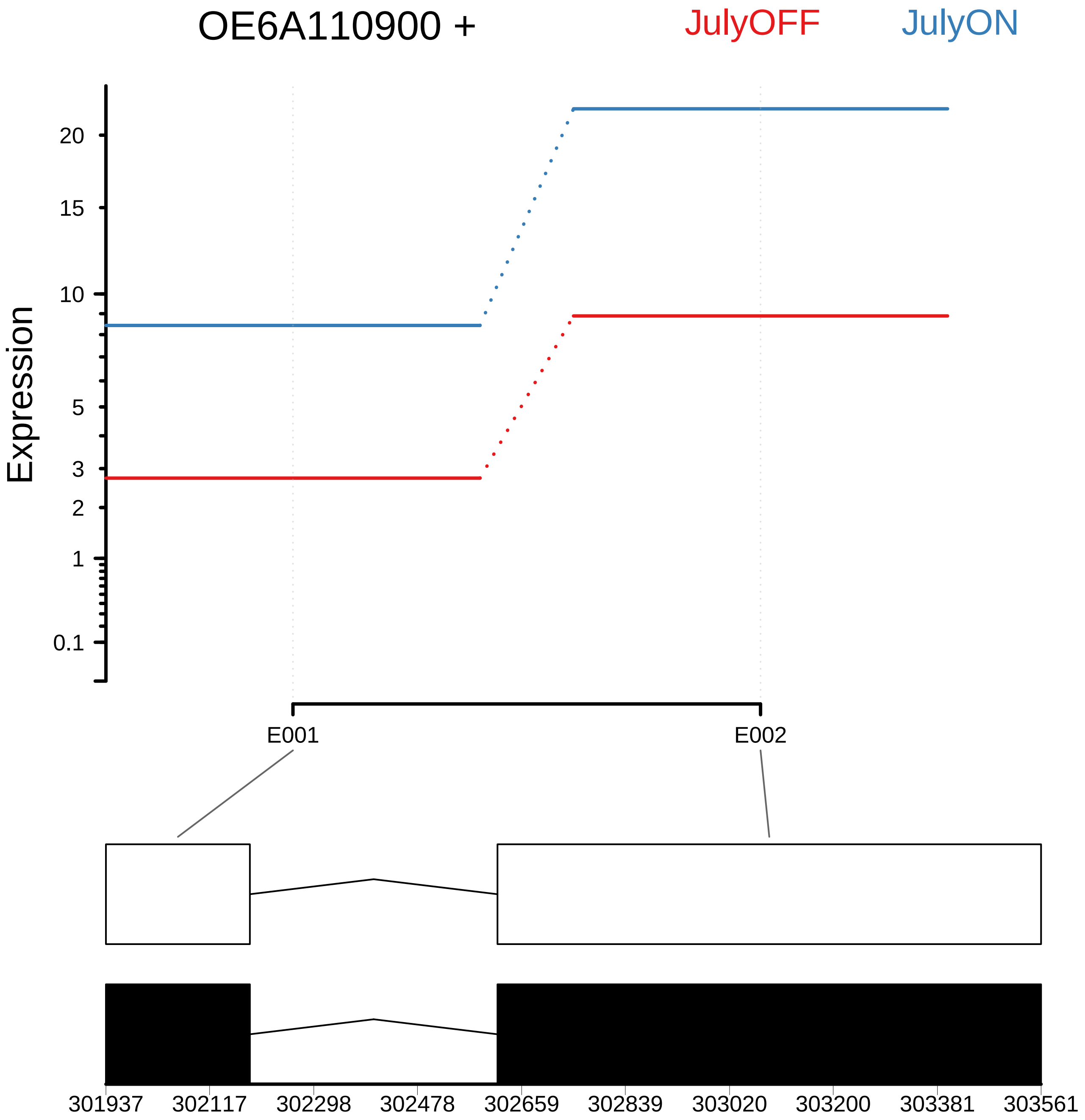
OE6A110900 +
JulyOFF
JulyON
Expression
20
15
10
5
3
2
1
0.1
E001
E002
301937
302117
302298
302478
302659
302839
303020
303200
303381
303561

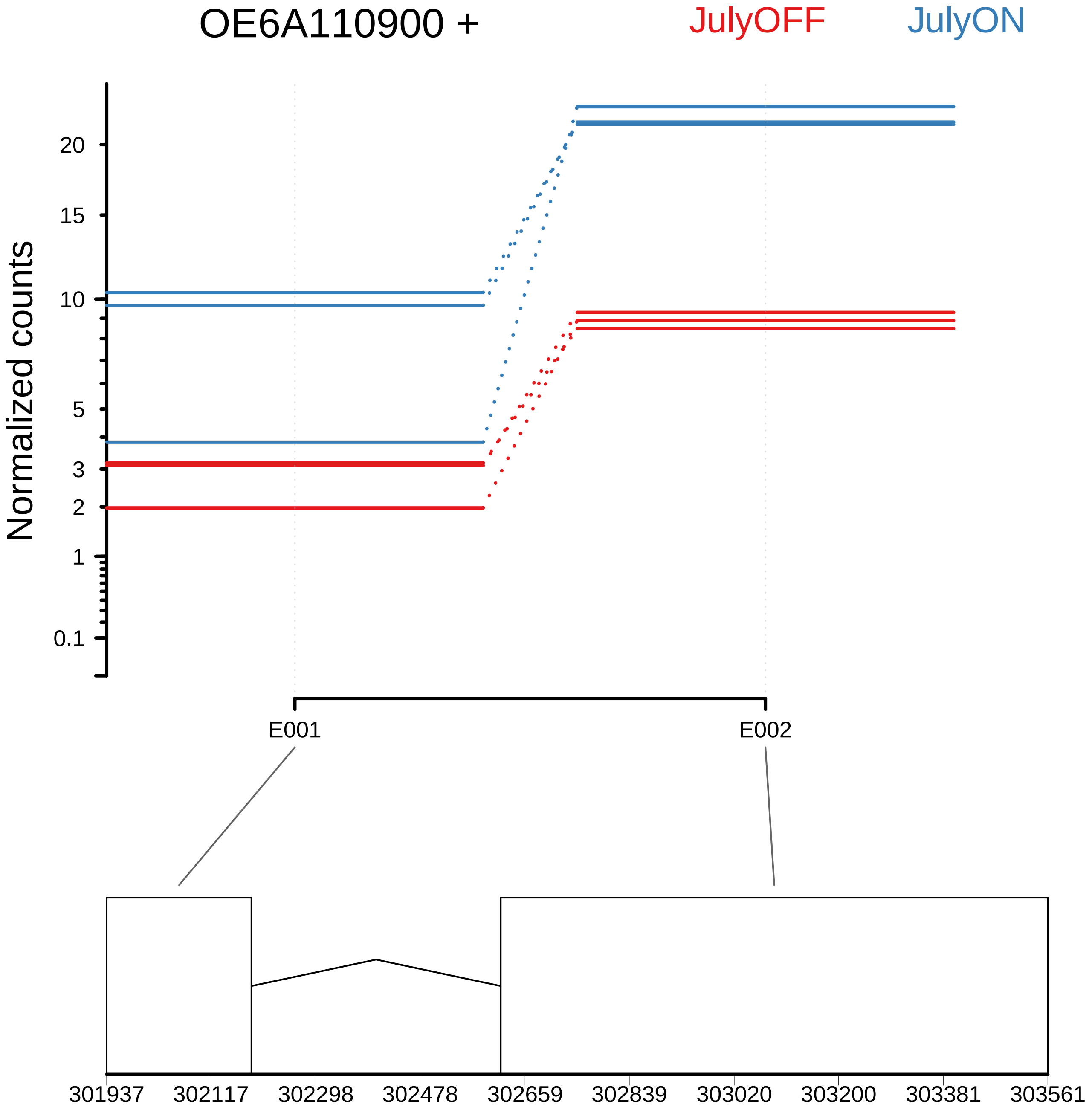
OE6A110900 +
JulyOFF
JulyON
Normalized counts
20
15
10
5
3
2
1
0.1
E001
E002
301937
302117
302298
302478
302659
302839
303020
303200
303381
303561

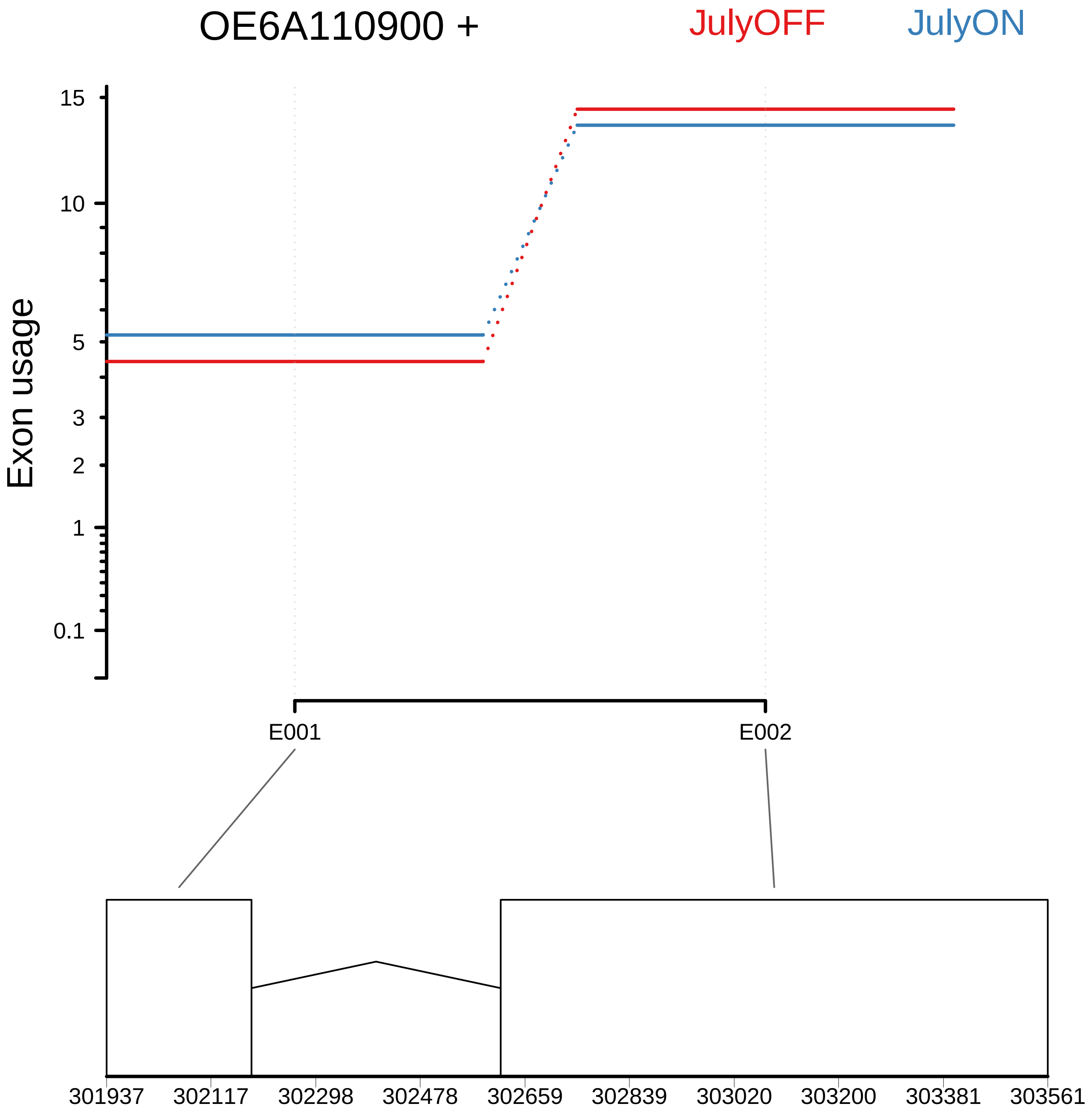
OE6A110900 +
JulyOFF
JulyON
Exon usage
15
10
5
3
2
1
0.1
E001
E002
301937
302117
302298
302478
302659
302839
303020
303200
303381
303561

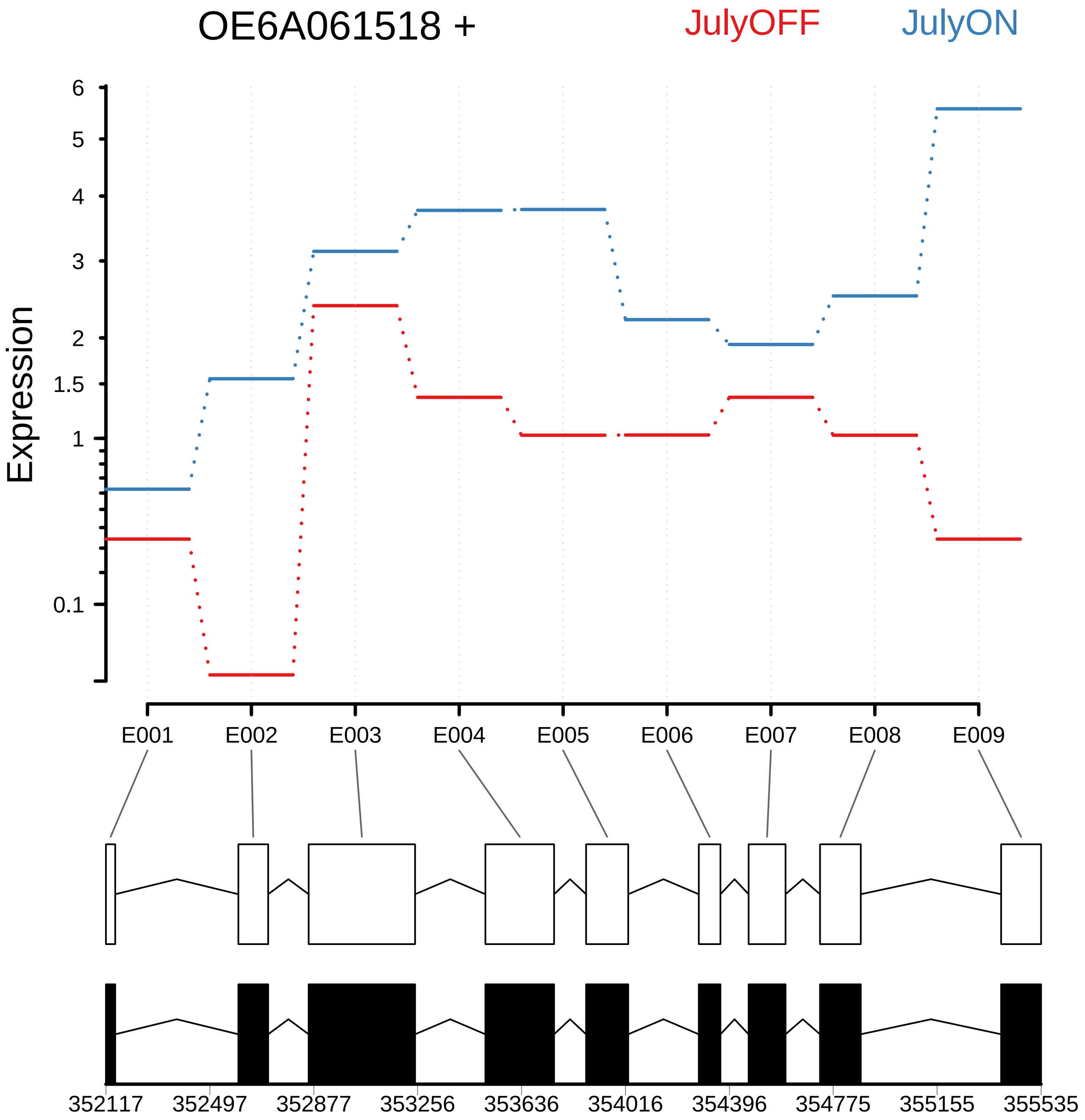
OE6A061518 +
JulyOFF
JulyON
Expression
6
5
4
3
2
1.5
1
0.1
E001
E002
E003
E004
E005
E006
E007
E008
E009
352117
352497
352877
353256
353636
354016
354396
354775
355155
355535

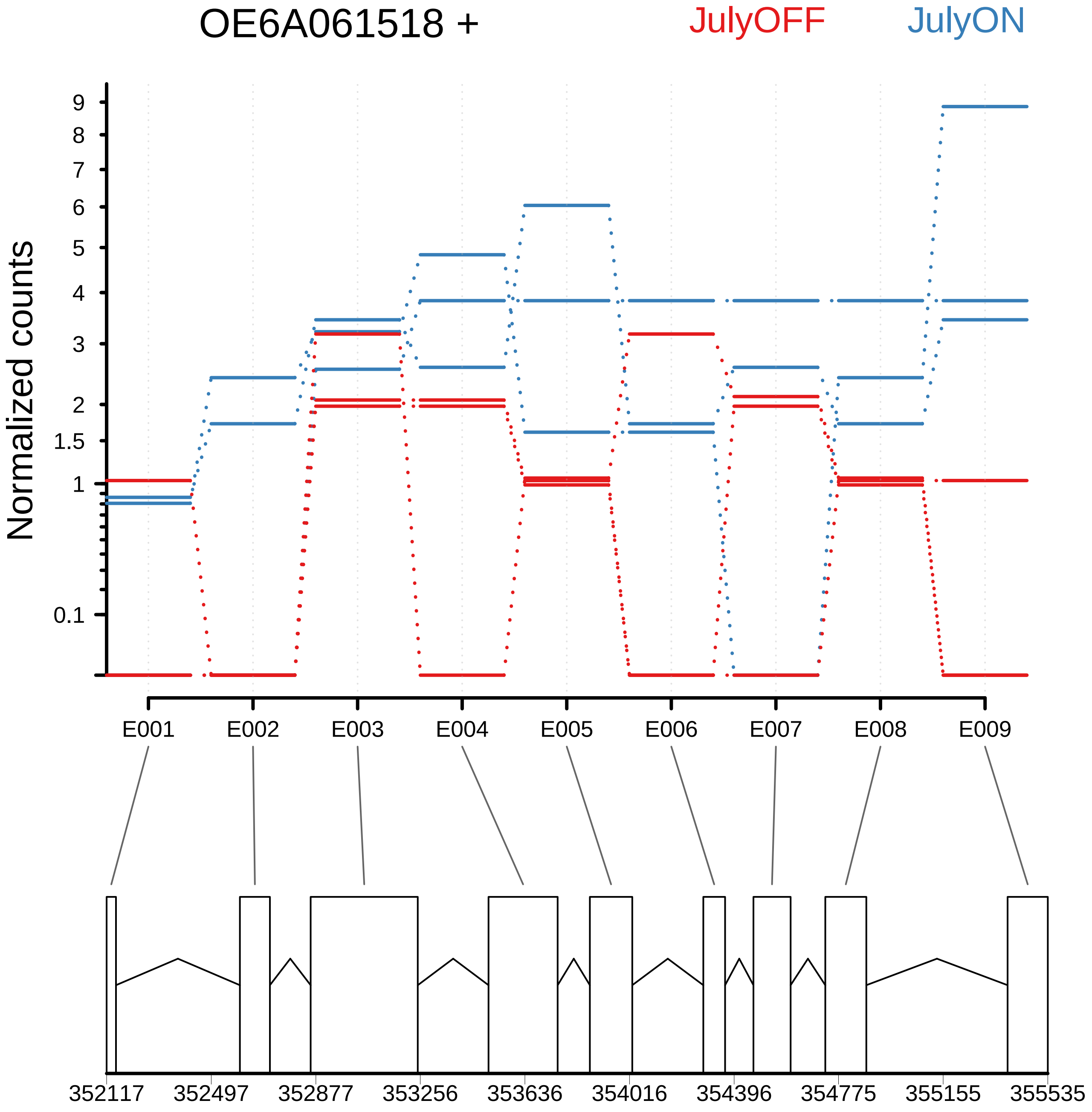

OE6A061518 +
JulyOFF
JulyON
Normalized counts
9
8
7
6
5
4
3
2
1.5
1
0.1
E001
E002
E003
E004
E005
E006
E007
E008
E009
352117
352497
352877
353256
353636
354016
354396
354775
355155
355535

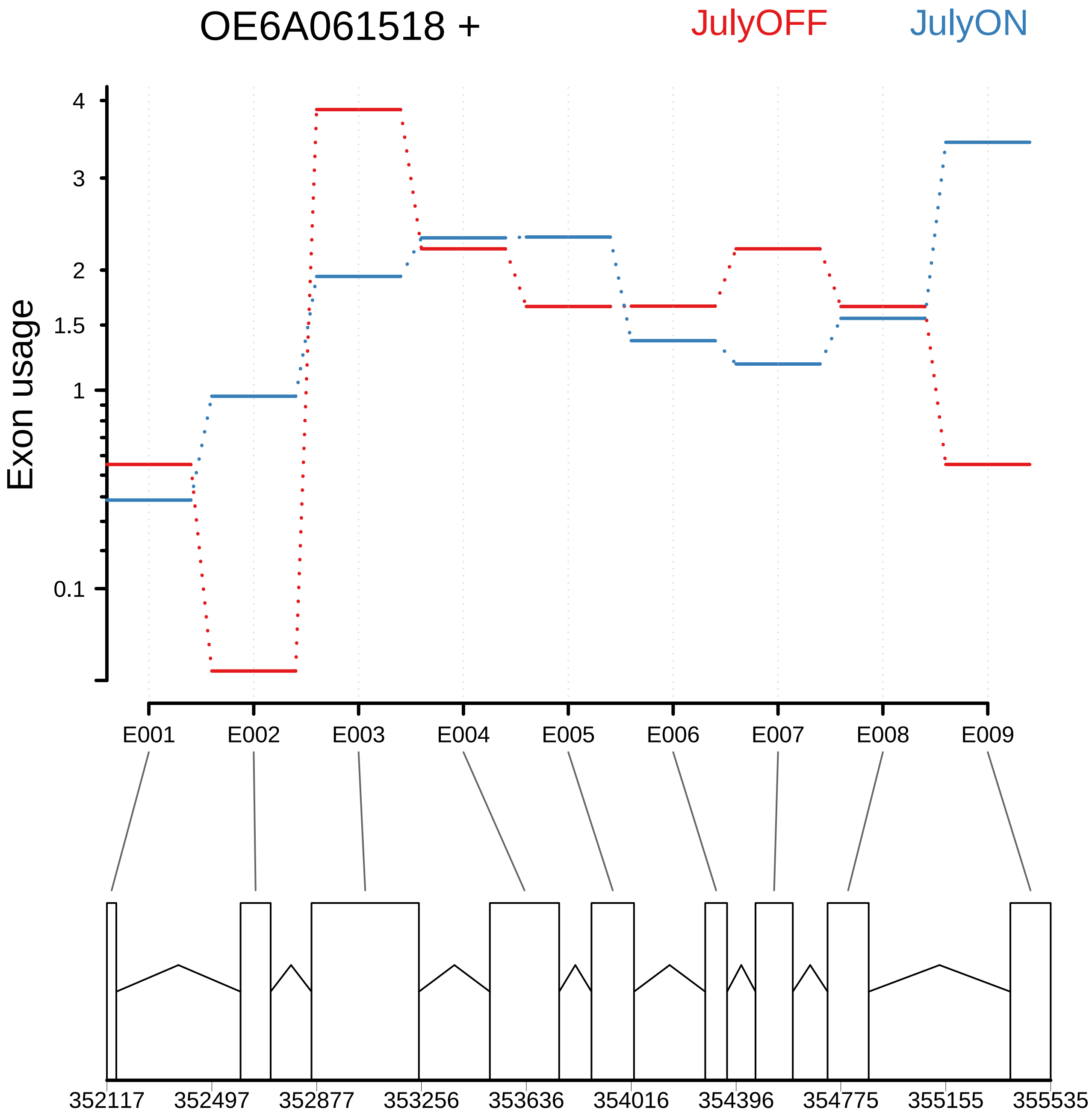

OE6A061518 +
JulyOFF
JulyON
Exon usage
4
3
2
1.5
1
0.1
E001
E002
E003
E004
E005
E006
E007
E008
E009
352117
352497
352877
353256
353636
354016
354396
354775
355155
355535

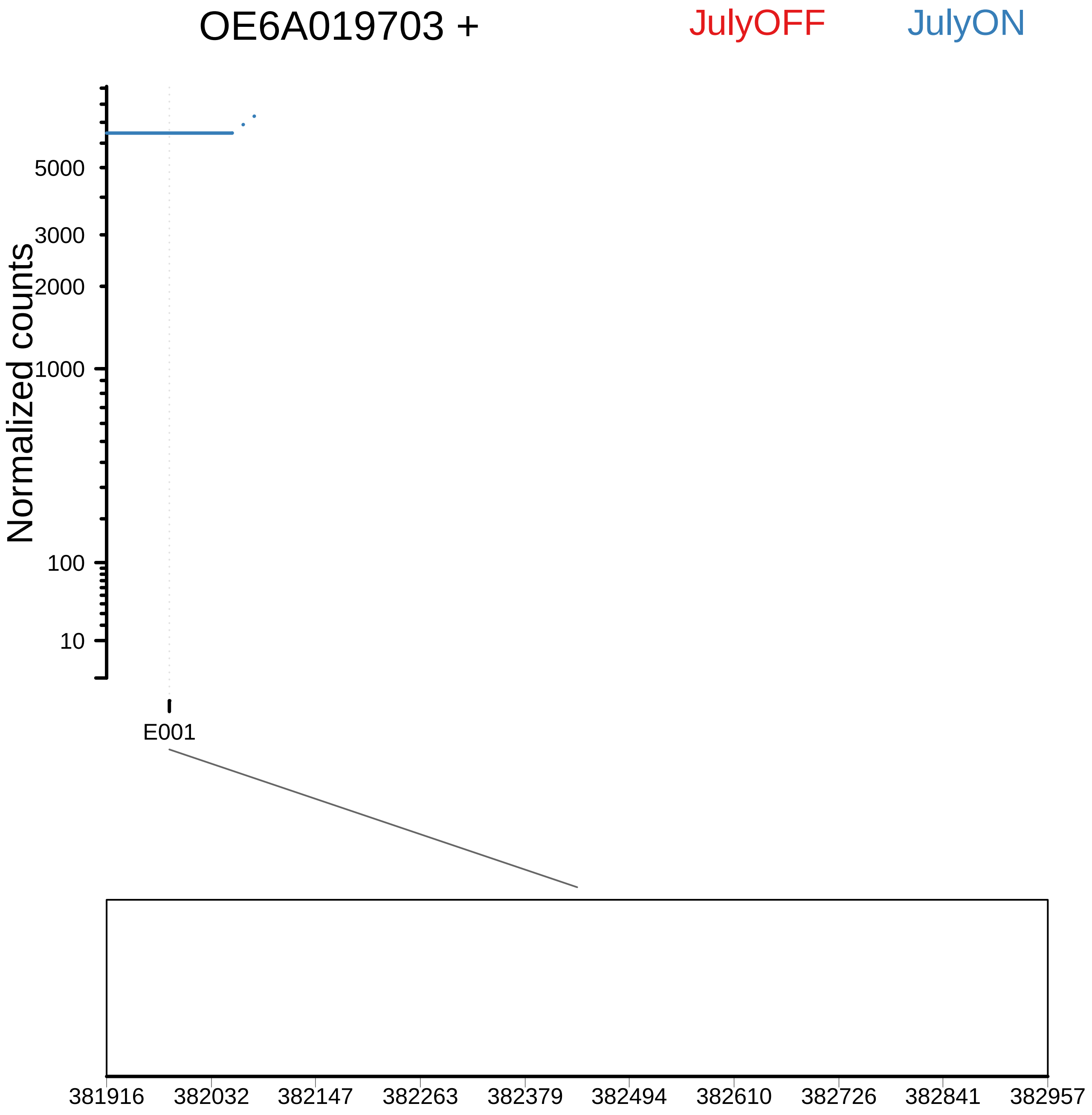
OE6A019703 +
JulyOFF
JulyON
Normalized counts
5000
3000
2000
1000
100
10
E001
381916
382032
382147
382263
382379
382494
382610
382726
382841
382957

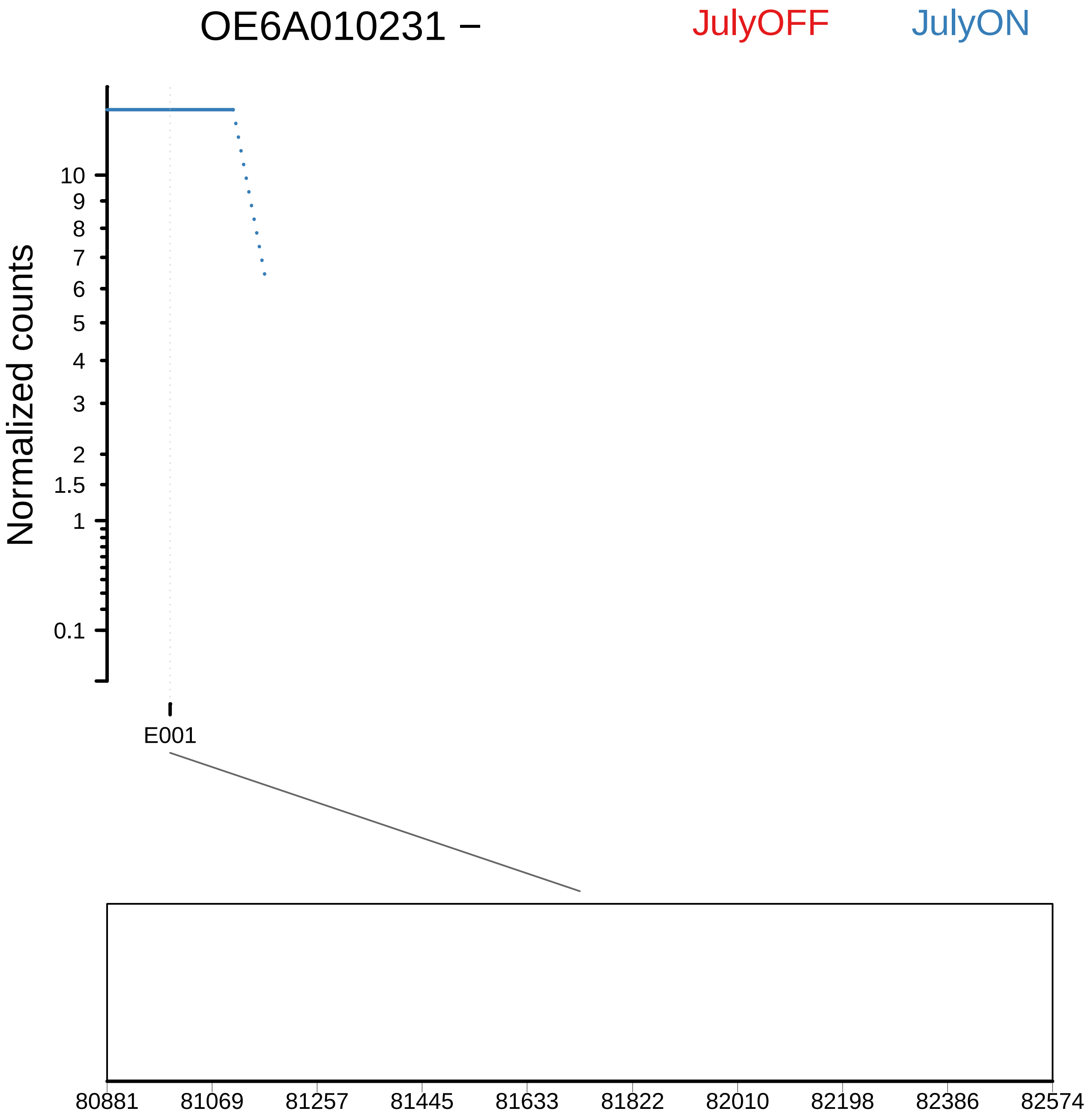

OE6A010231 −
JulyOFF
JulyON
Normalized counts
10
9
8
7
6
5
4
3
2
1.5
1
0.1
E001
80881
81069
81257
81445
81633
81822
82010
82198
82386
82574

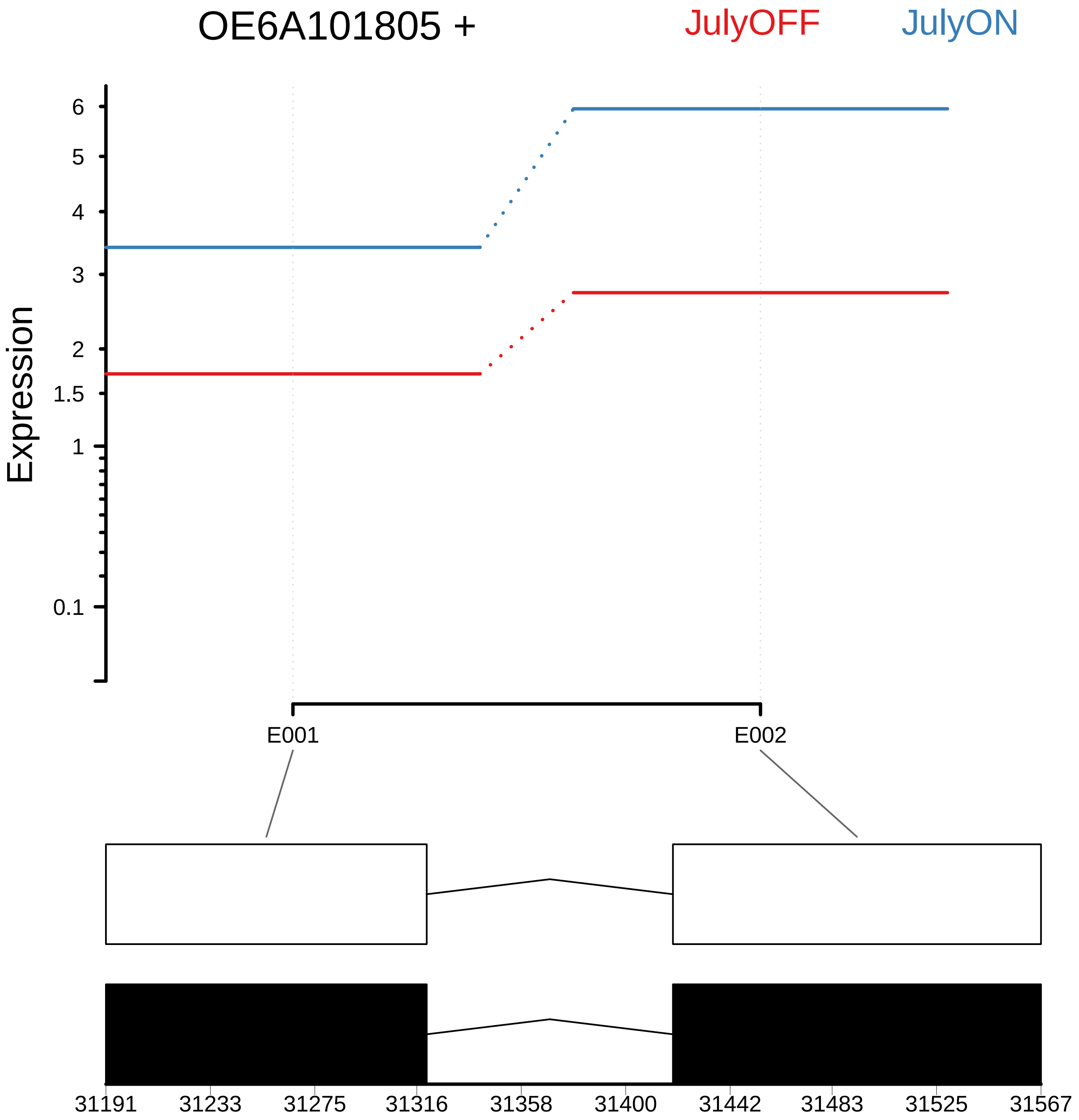
OE6A101805 +
JulyOFF
JulyON
Expression
6
5
4
3
2
1.5
1
0.1
E001
E002
31191
31233
31275
31316
31358
31400
31442
31483
31525
31567

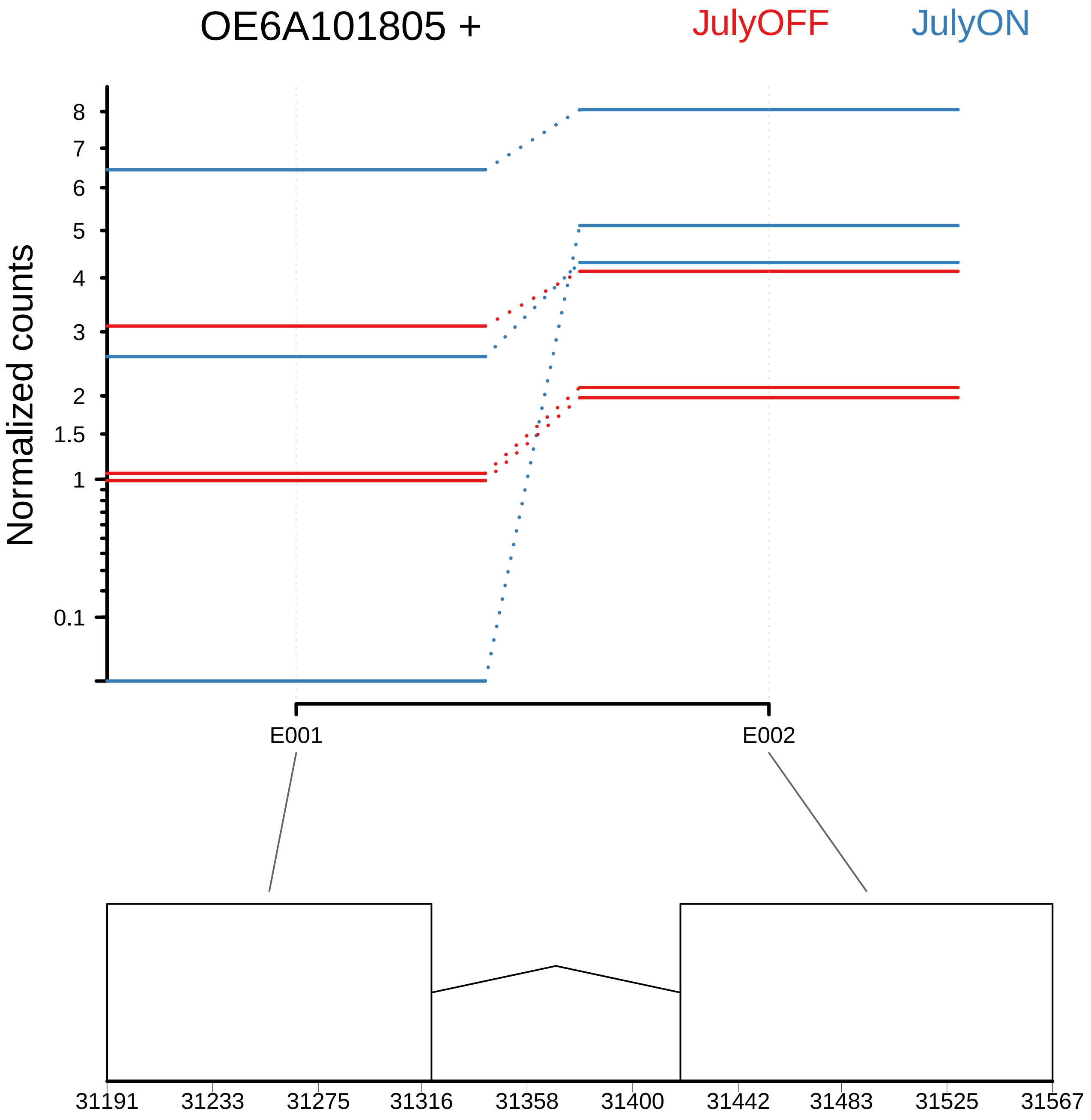

OE6A101805 +
JulyOFF
JulyON
Normalized counts
8
7
6
5
4
3
2
1.5
1
0.1
E001
E002
31191
31233
31275
31316
31358
31400
31442
31483
31525
31567

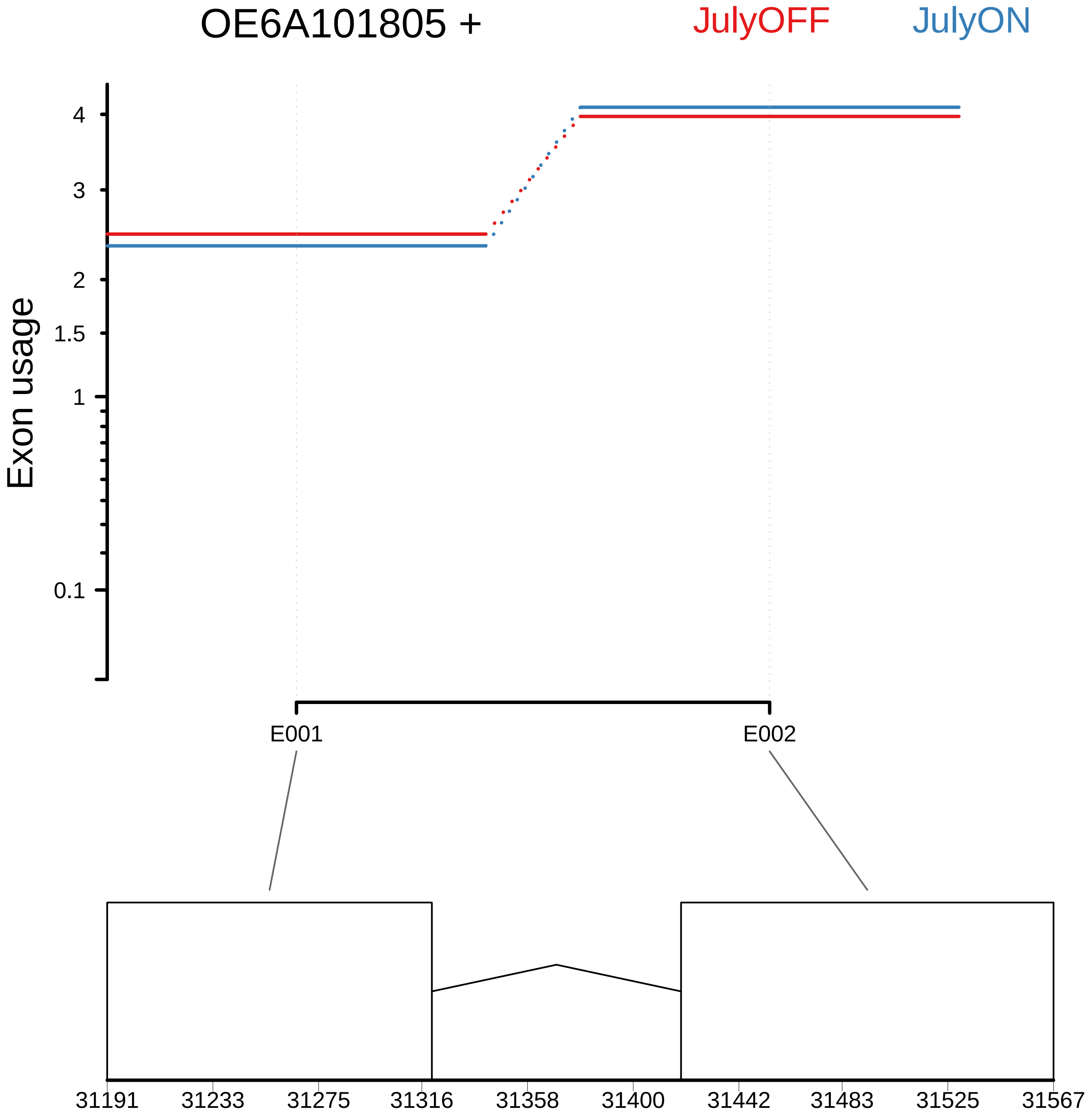

OE6A101805 +
JulyOFF
JulyON
Exon usage
4
3
2
1.5
1
0.1
E001
E002
31191
31233
31275
31316
31358
31400
31442
31483
31525
31567

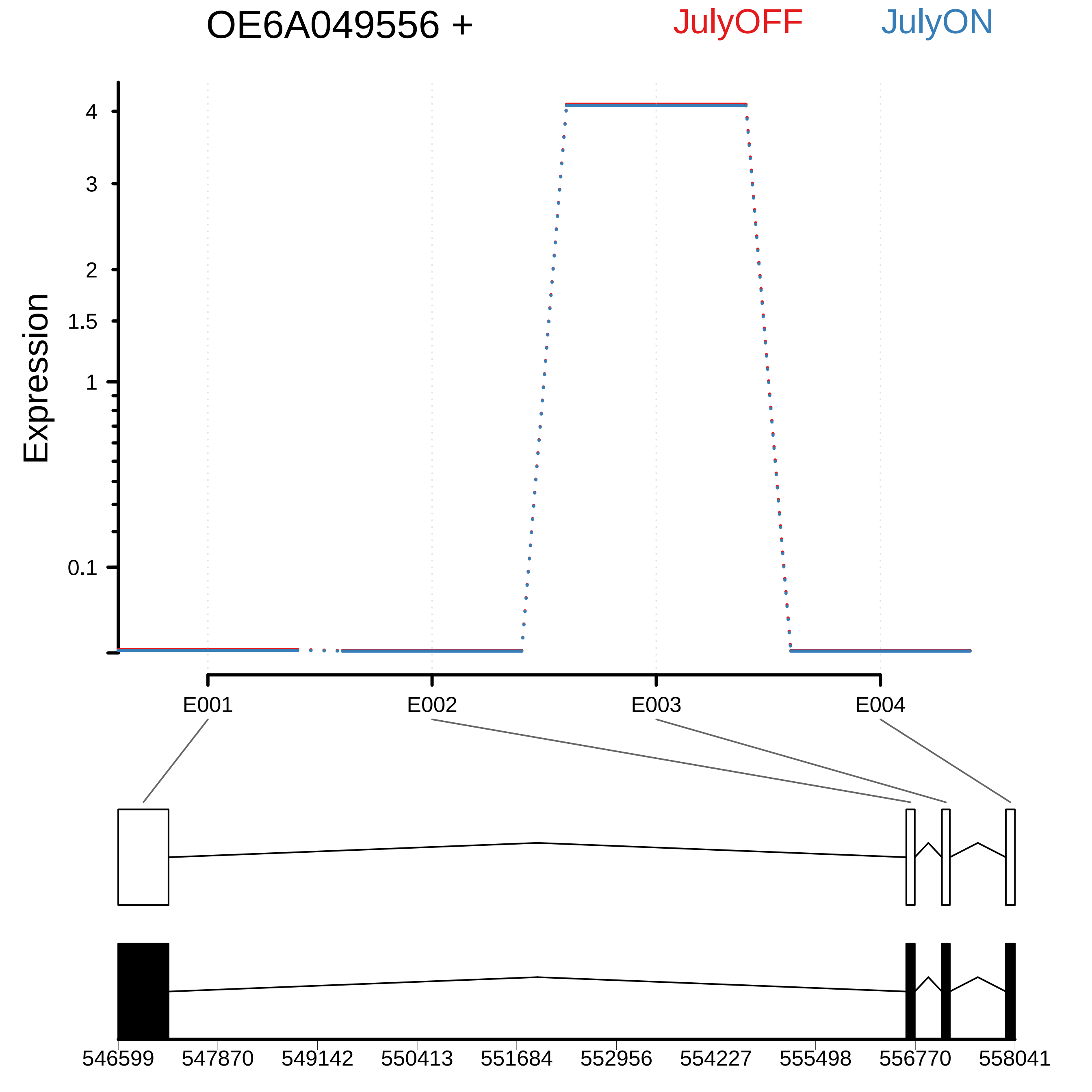

OE6A049556 +
JulyOFF
JulyON
Expression
4
3
2
1.5
1
0.1
E001
E002
E003
E004
546599
547870
549142
550413
551684
552956
554227
555498
556770
558041

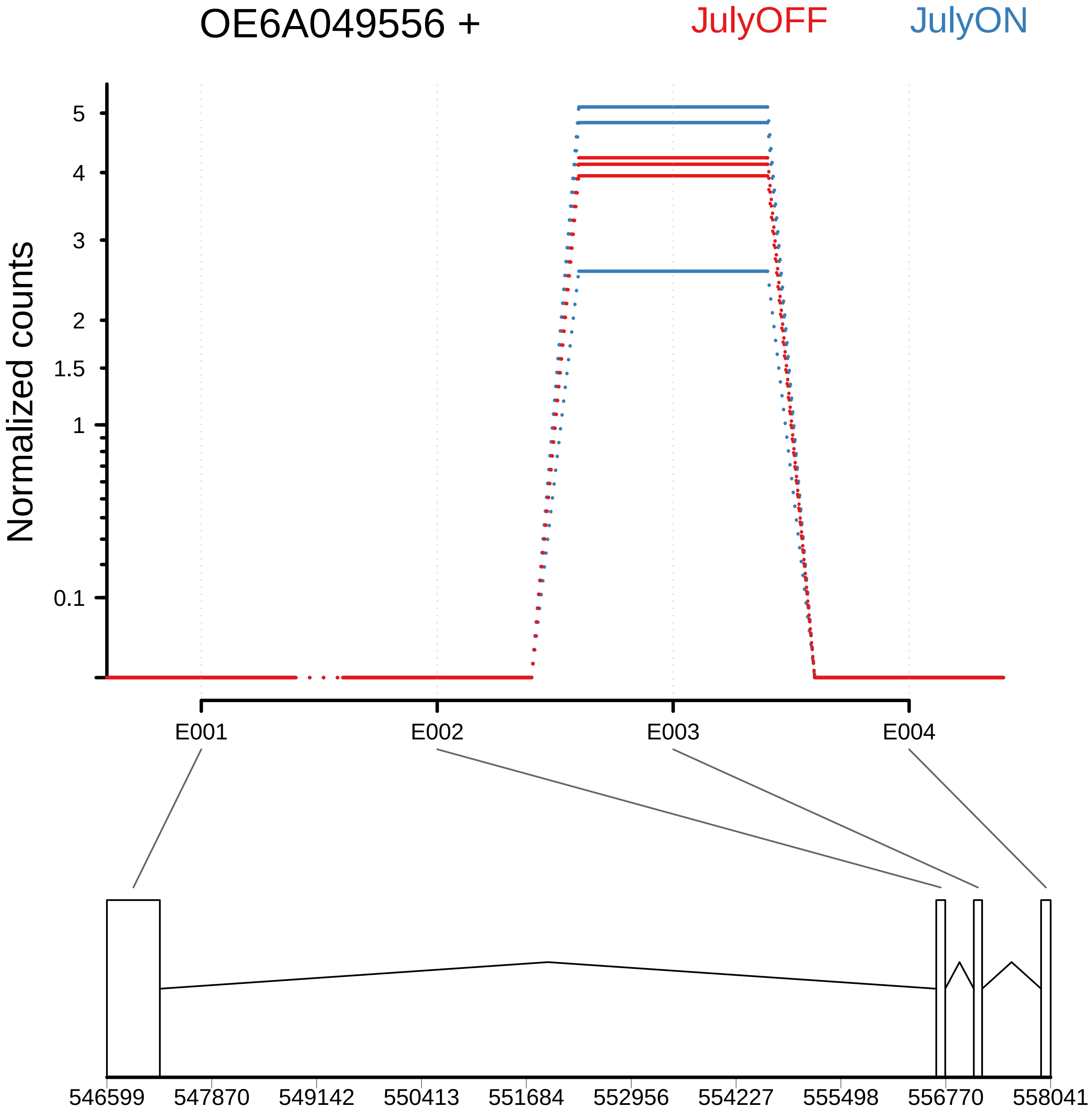

OE6A049556 +
JulyOFF
JulyON
Normalized counts
5
4
3
2
1.5
1
0.1
E001
E002
E003
E004
546599
547870
549142
550413
551684
552956
554227
555498
556770
558041

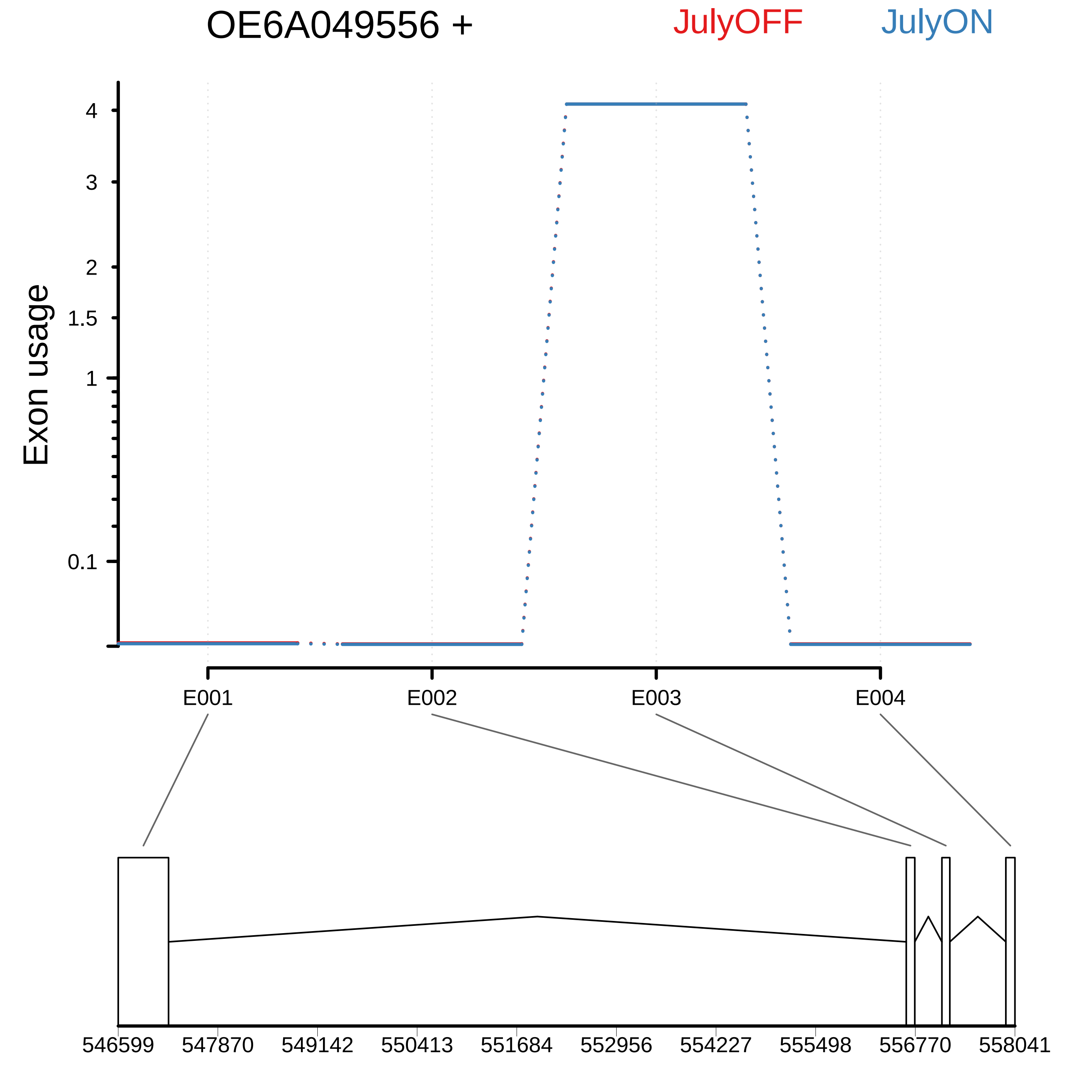
OE6A049556 +
JulyOFF
JulyON
Exon usage
4
3
2
1.5
1
0.1
E001
E002
E003
E004
546599
547870
549142
550413
551684
552956
554227
555498
556770
558041

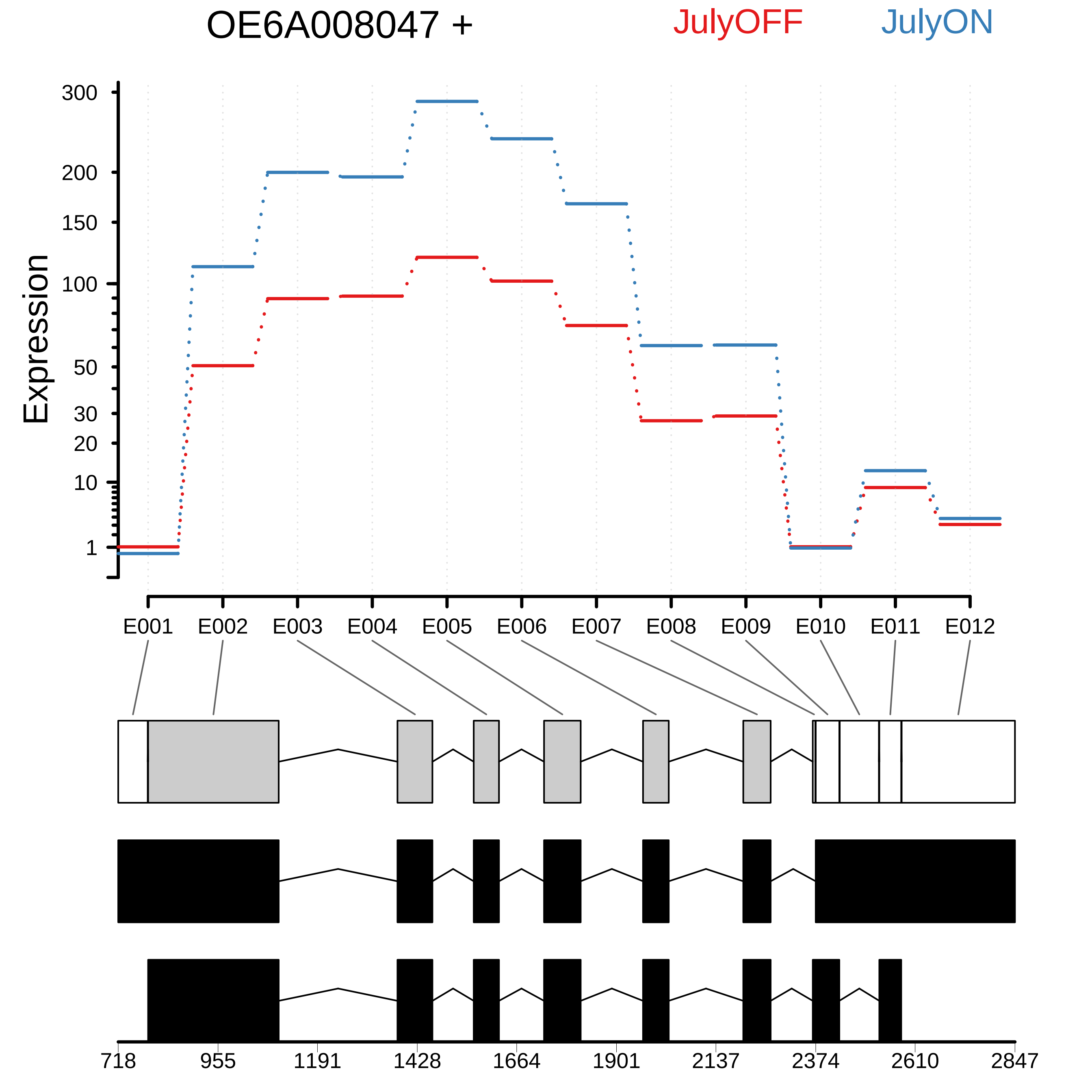
OE6A008047 +
JulyOFF
JulyON
Expression
300
200
150
100
50
30
20
10
1
E001
E002
E003
E004
E005
E006
E007
E008
E009
E010
E011
E012
718
955
1191
1428
1664
1901
2137
2374
2610
2847

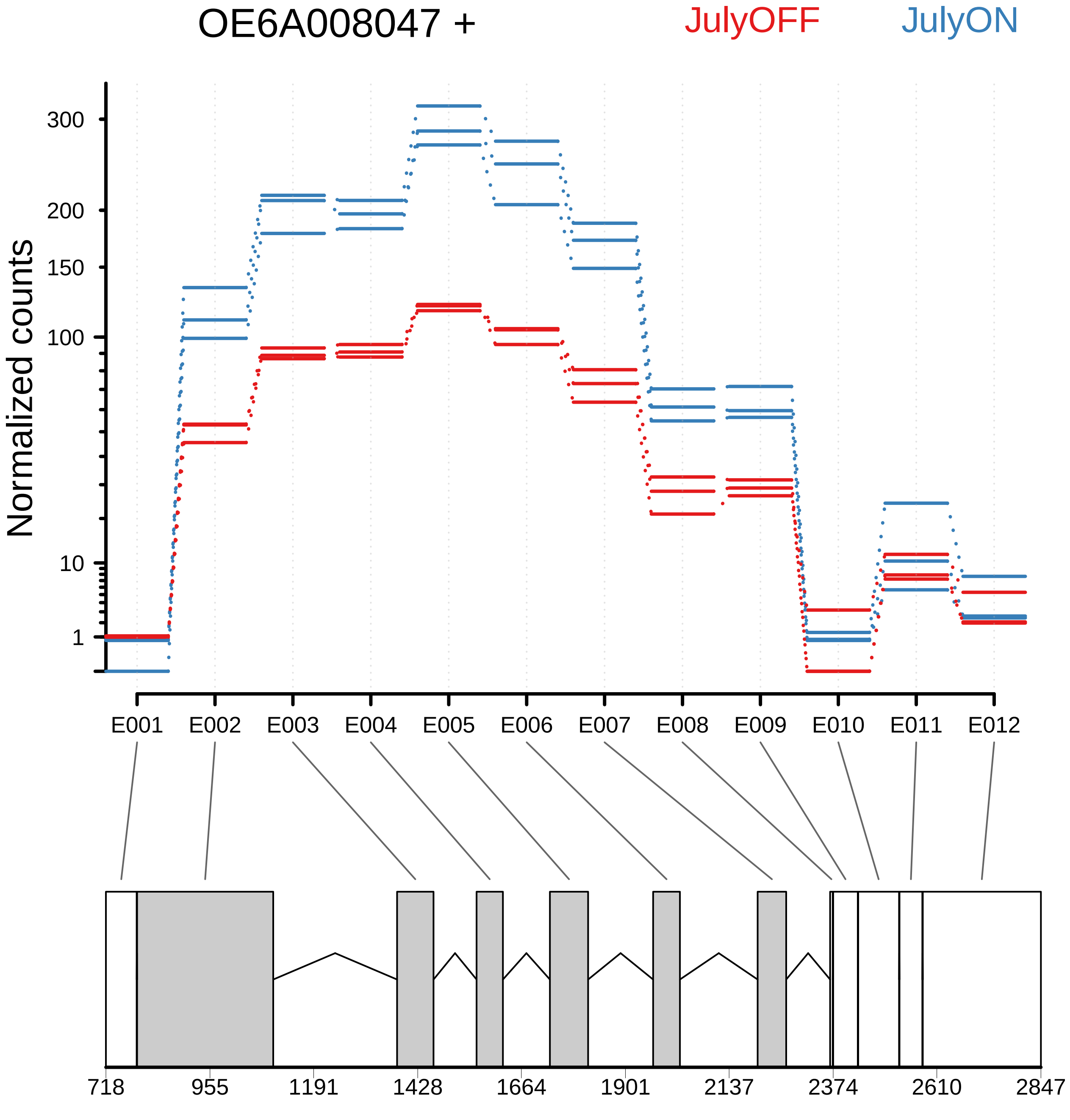

OE6A008047 +
JulyOFF
JulyON
Normalized counts
300
200
150
100
10
1
E001
E002
E003
E004
E005
E006
E007
E008
E009
E010
E011
E012
718
955
1191
1428
1664
1901
2137
2374
2610
2847

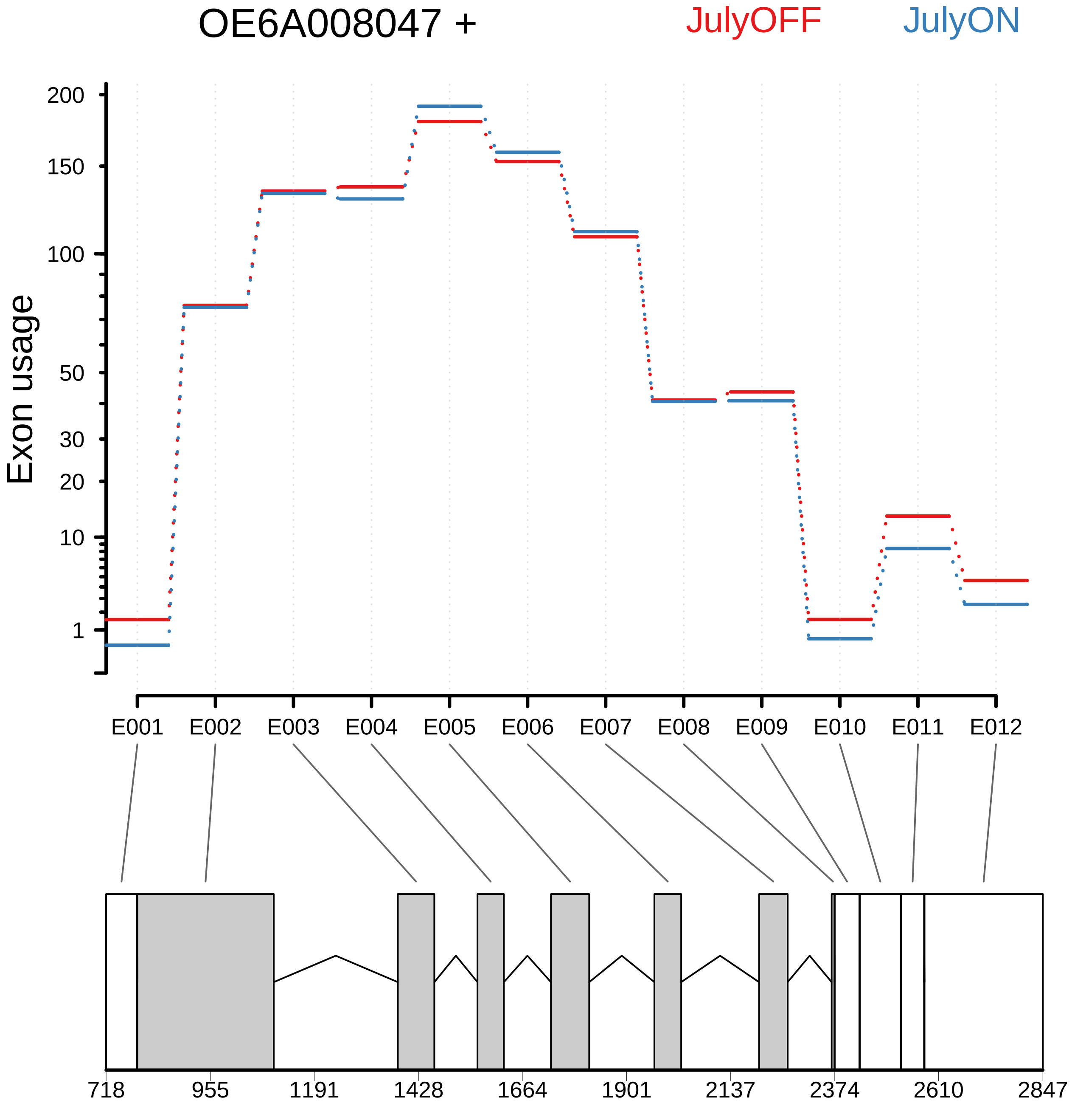

OE6A008047 +
JulyOFF
JulyON
Exon usage
200
150
100
50
30
20
10
1
E001
E002
E003
E004
E005
E006
E007
E008
E009
E010
E011
E012
718
955
1191
1428
1664
1901
2137
2374
2610
2847

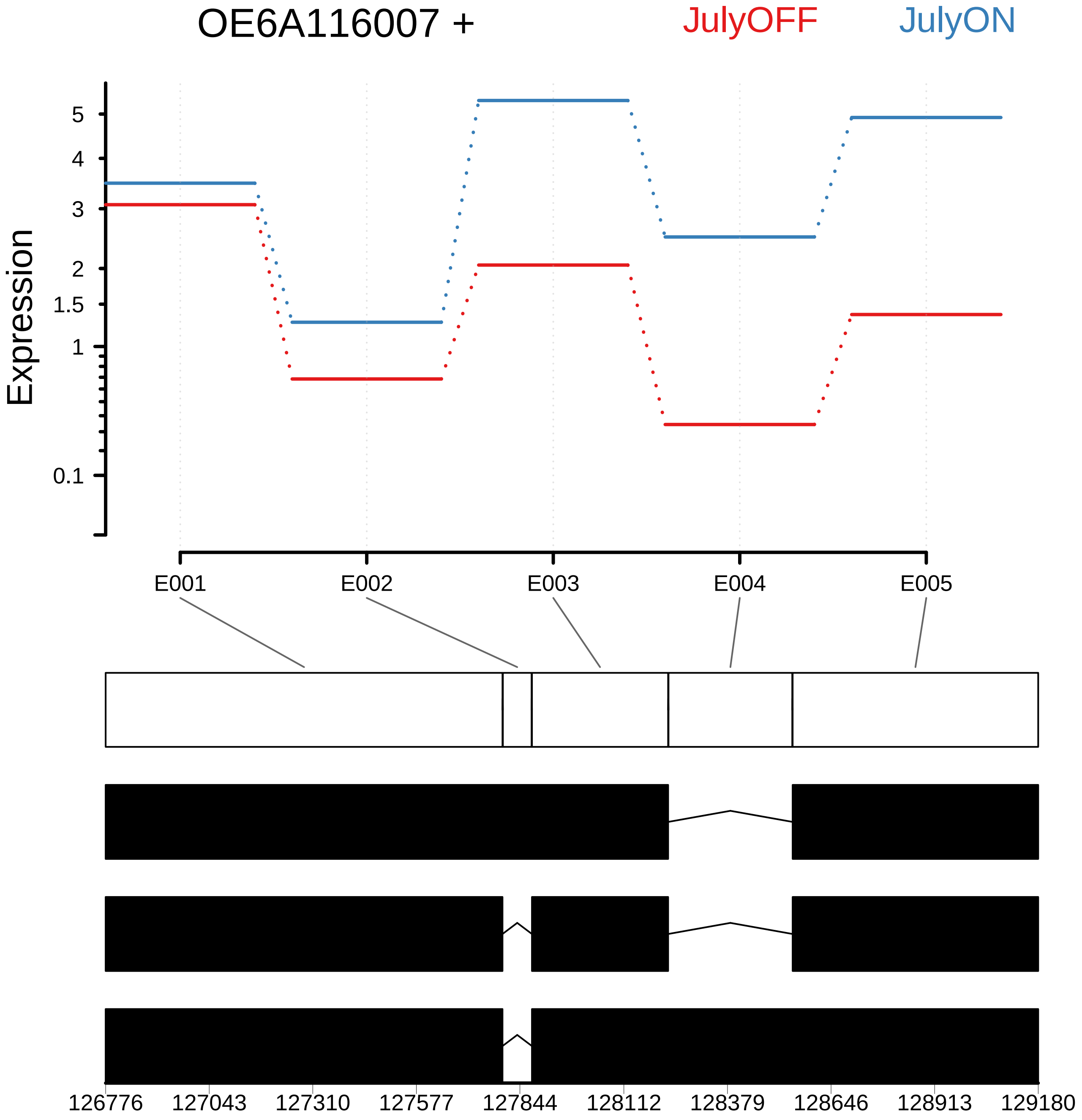
OE6A116007 +
JulyOFF
JulyON
Expression
5
4
3
2
1.5
1
0.1
E001
E002
E003
E004
E005
126776
127043
127310
127577
127844
128112
128379
128646
128913
129180

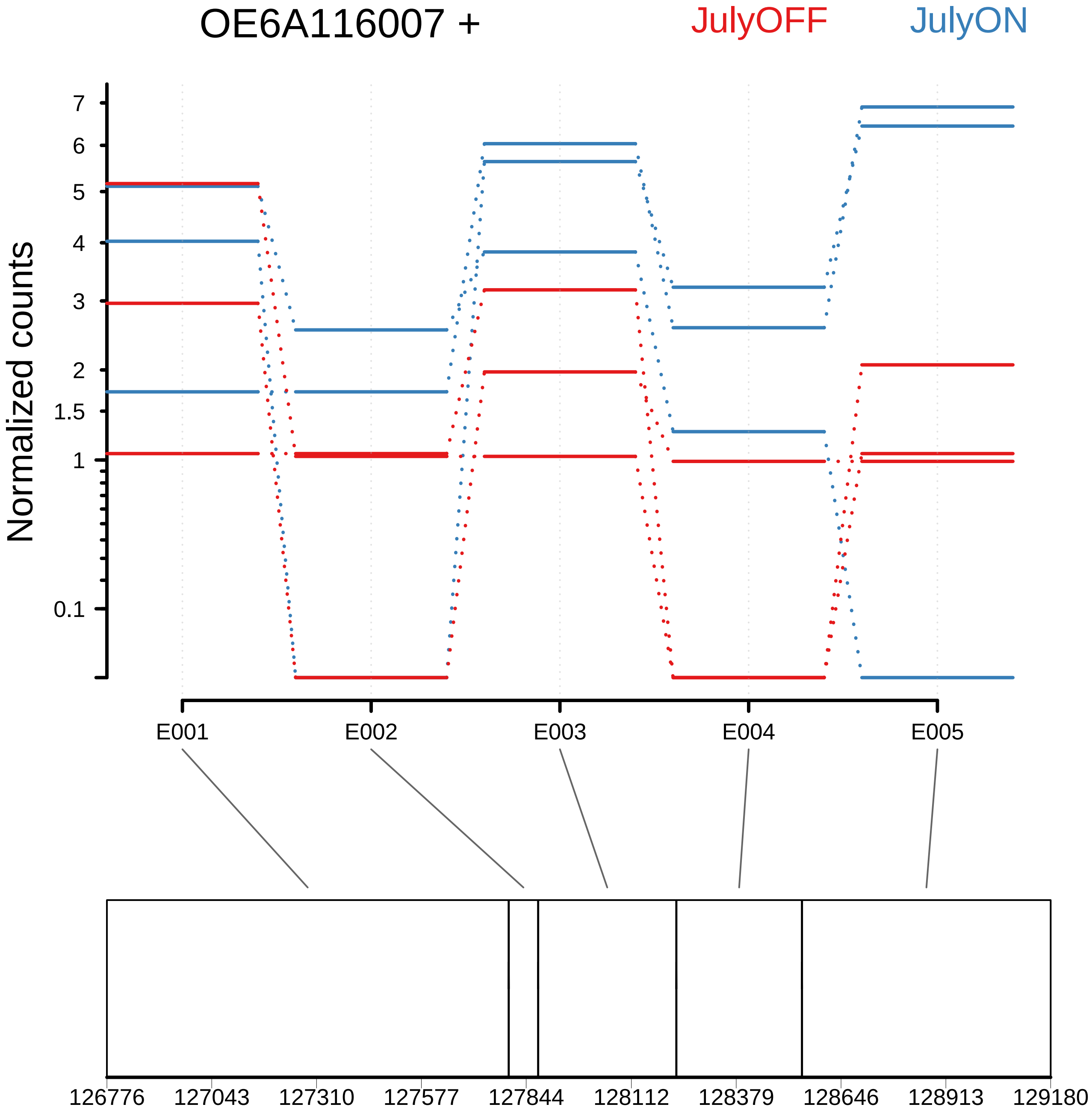

OE6A116007 +
JulyOFF
JulyON
Normalized counts
7
6
5
4
3
2
1.5
1
0.1
E001
E002
E003
E004
E005
126776
127043
127310
127577
127844
128112
128379
128646
128913
129180

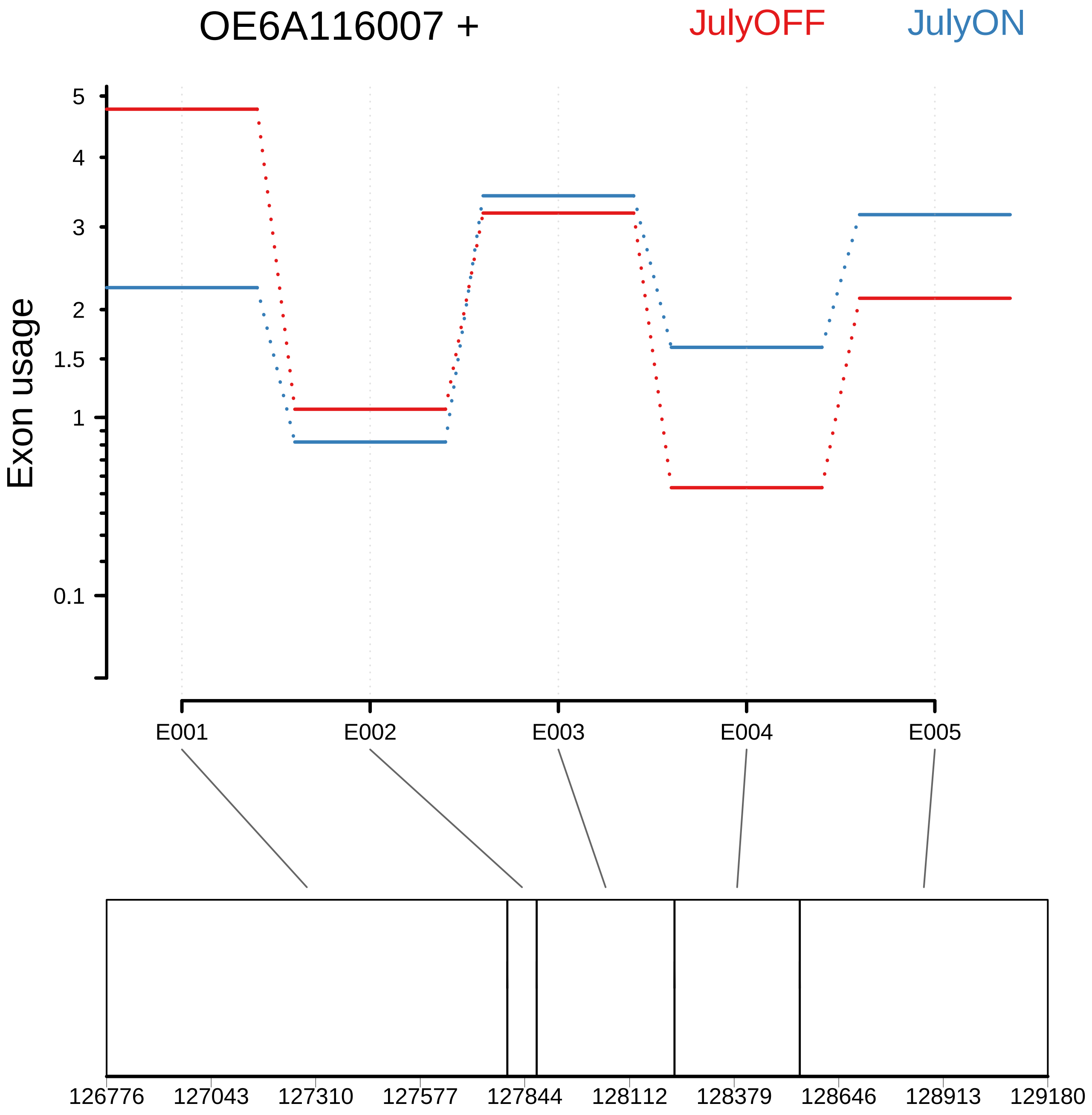
OE6A116007 +
JulyOFF
JulyON
Exon usage
5
4
3
2
1.5
1
0.1
E001
E002
E003
E004
E005
126776
127043
127310
127577
127844
128112
128379
128646
128913
129180

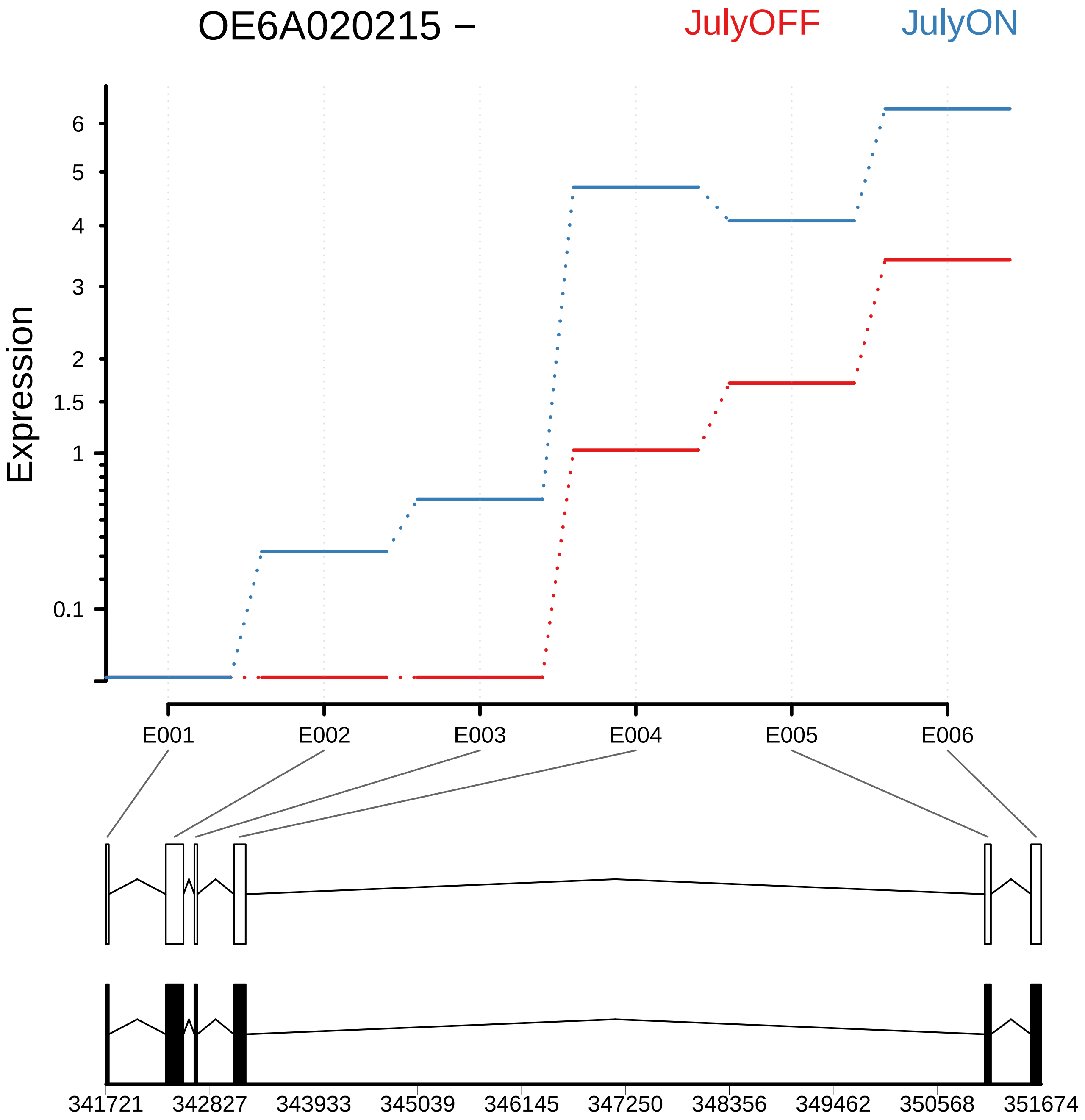
OE6A020215 −
JulyOFF
JulyON
Expression
6
5
4
3
2
1.5
1
0.1
E001
E002
E003
E004
E005
E006
341721
342827
343933
345039
346145
347250
348356
349462
350568
351674

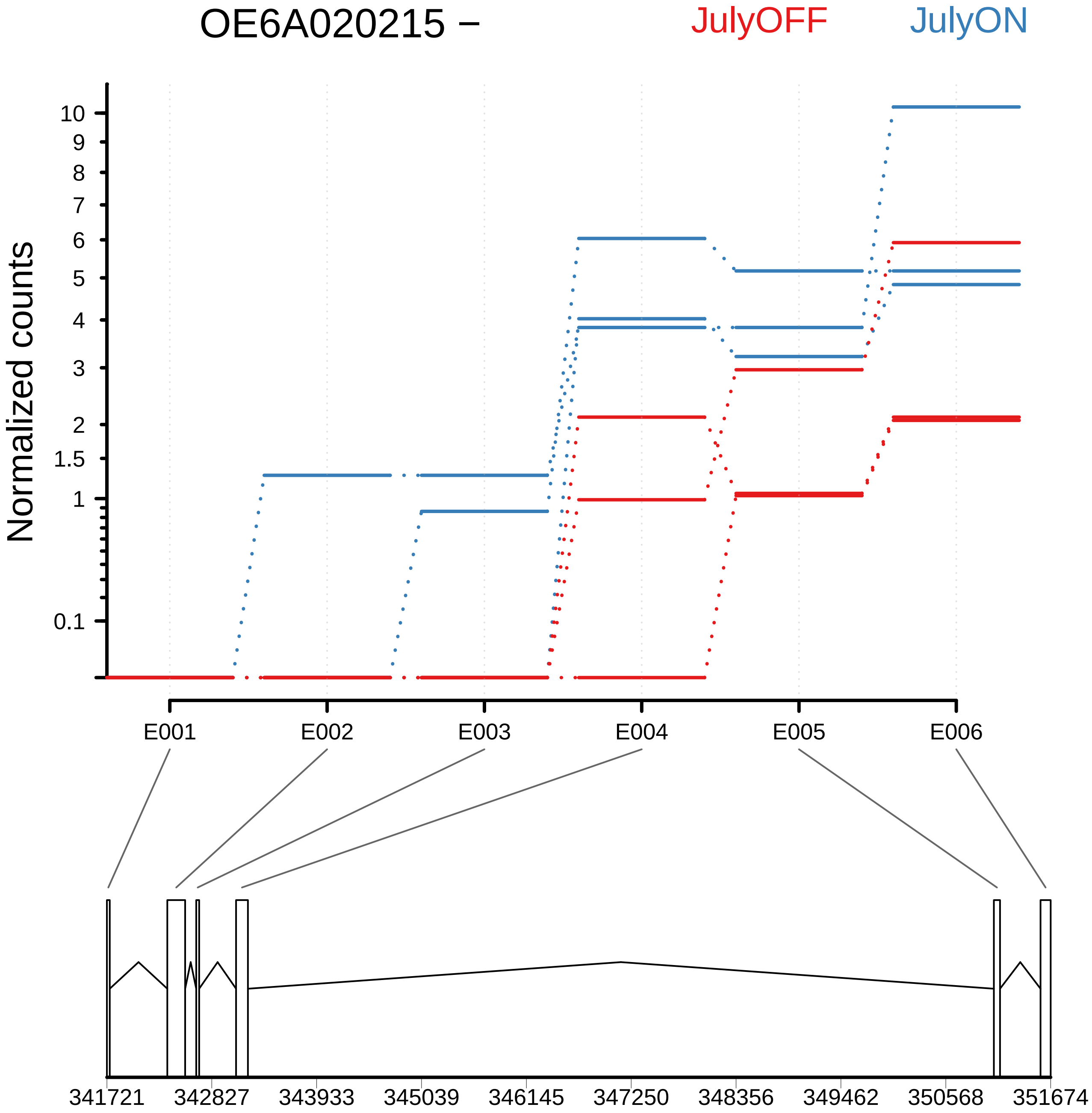

OE6A020215 −
JulyOFF
JulyON
Normalized counts
10
9
8
7
6
5
4
3
2
1.5
1
0.1
E001
E002
E003
E004
E005
E006
341721
342827
343933
345039
346145
347250
348356
349462
350568
351674

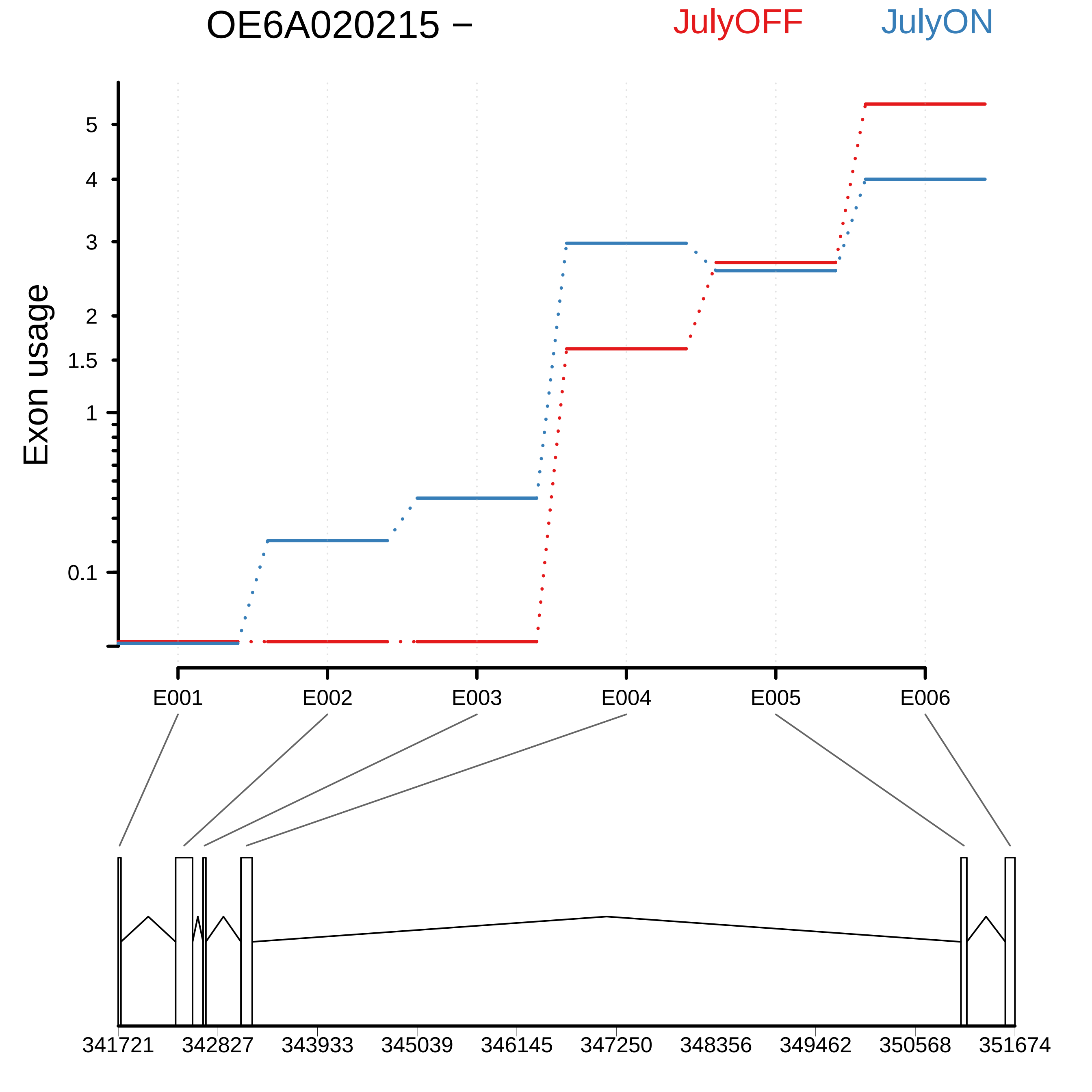
OE6A020215 −
JulyOFF
JulyON
Exon usage
5
4
3
2
1.5
1
0.1
E001
E002
E003
E004
E005
E006
341721
342827
343933
345039
346145
347250
348356
349462
350568
351674

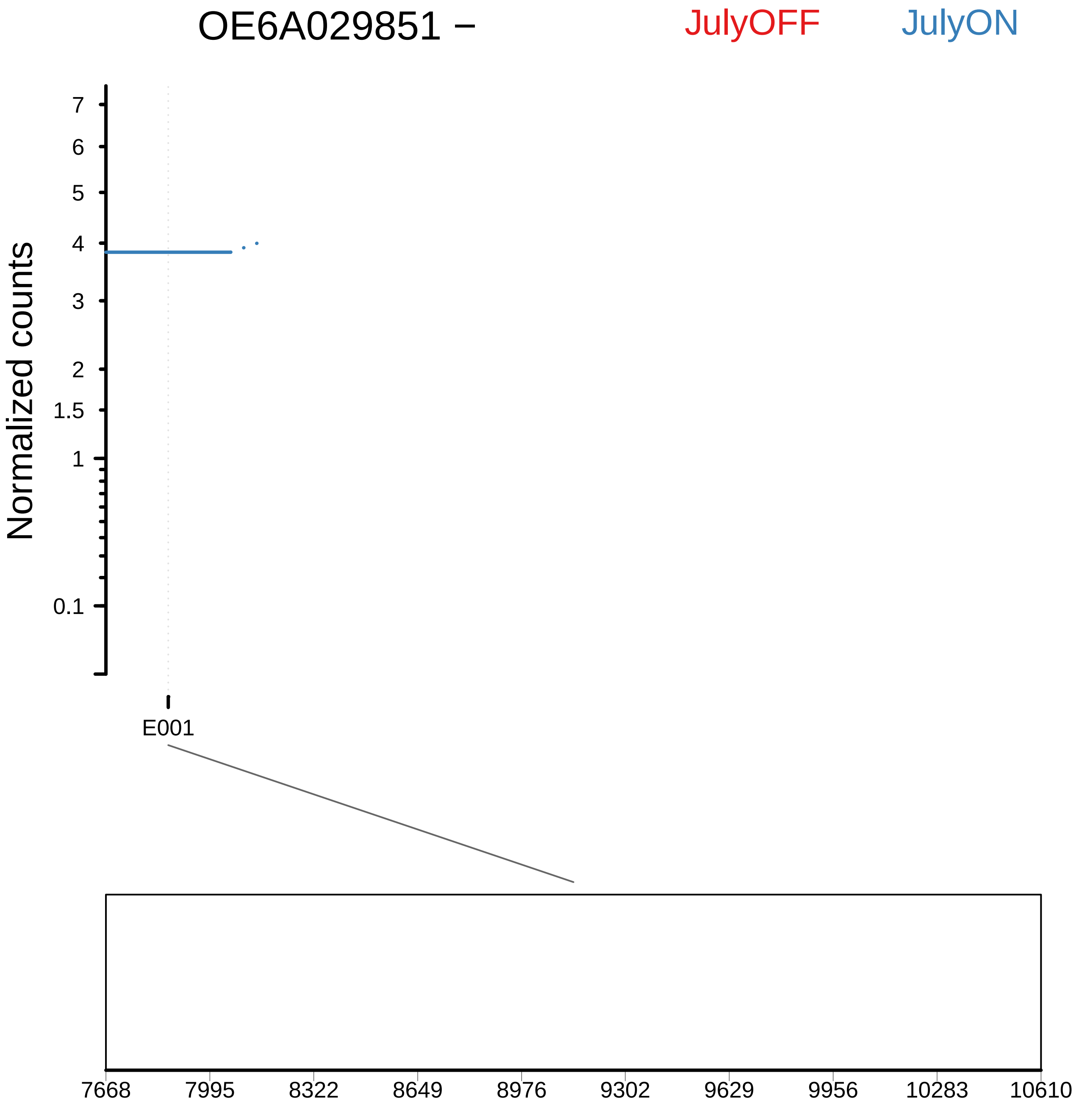

OE6A029851 −
JulyOFF
JulyON
Normalized counts
7
6
5
4
3
2
1.5
1
0.1
E001
7668
7995
8322
8649
8976
9302
9629
9956
10283
10610

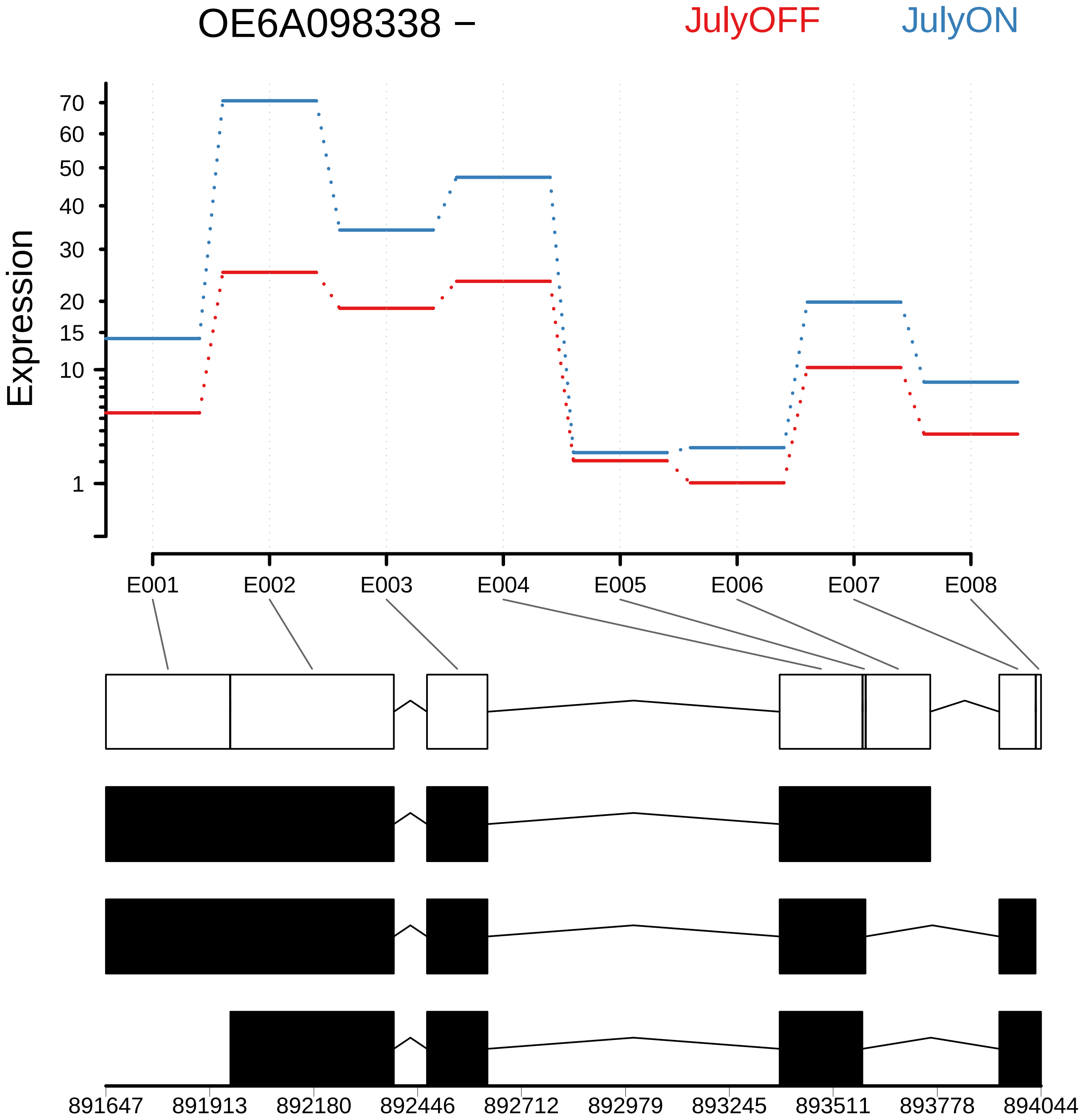

OE6A098338 –
JulyOFF
JulyON
Expression
70
60
50
40
30
20
15
10
1
E001
E002
E003
E004
E005
E006
E007
E008
891647
891913
892180
892446
892712
892979
893245
893511
893778
894044

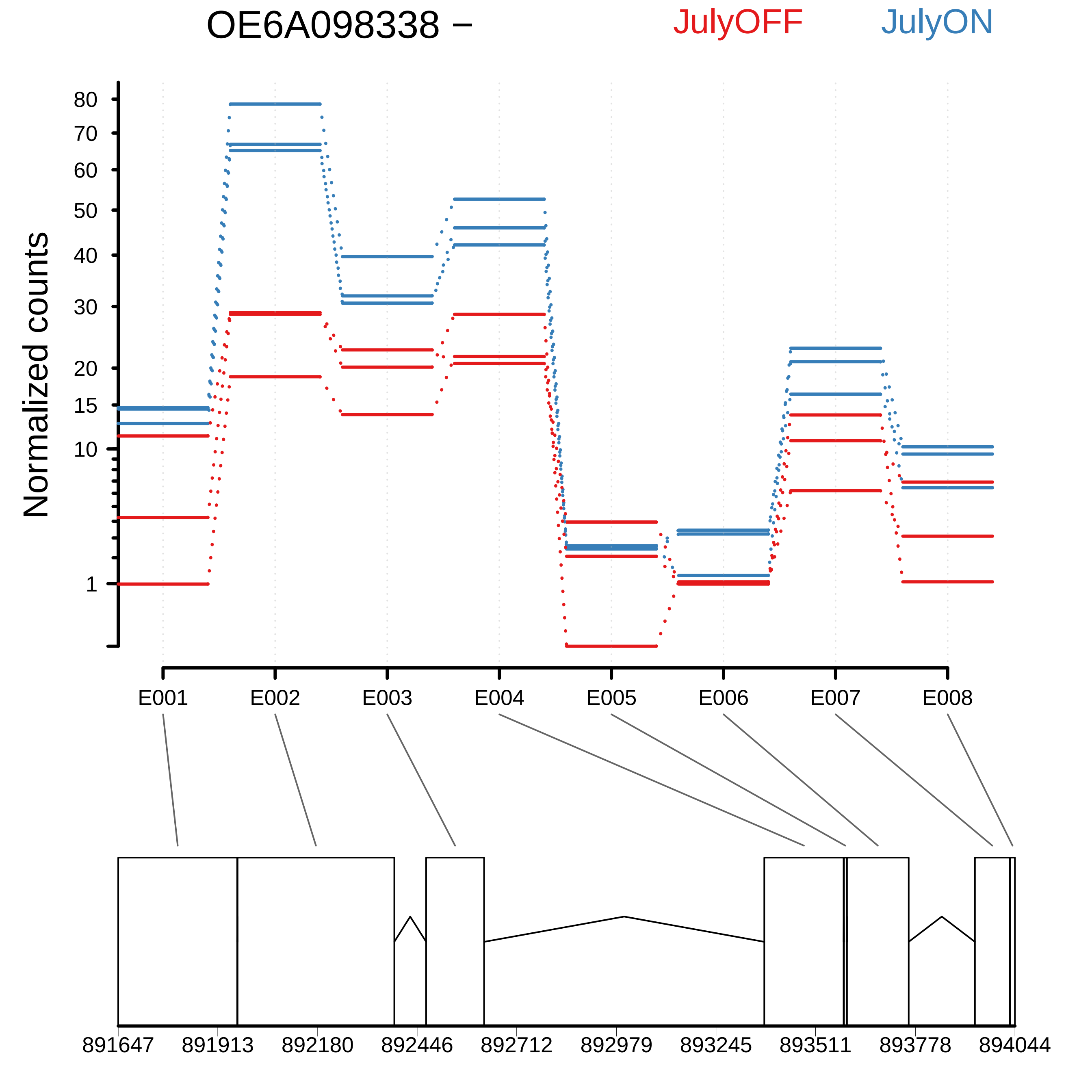
OE6A098338 –
JulyOFF
JulyON
Normalized counts
80
70
60
50
40
30
20
15
10
1
E001
E002
E003
E004
E005
E006
E007
E008
891647
891913
892180
892446
892712
892979
893245
893511
893778
894044

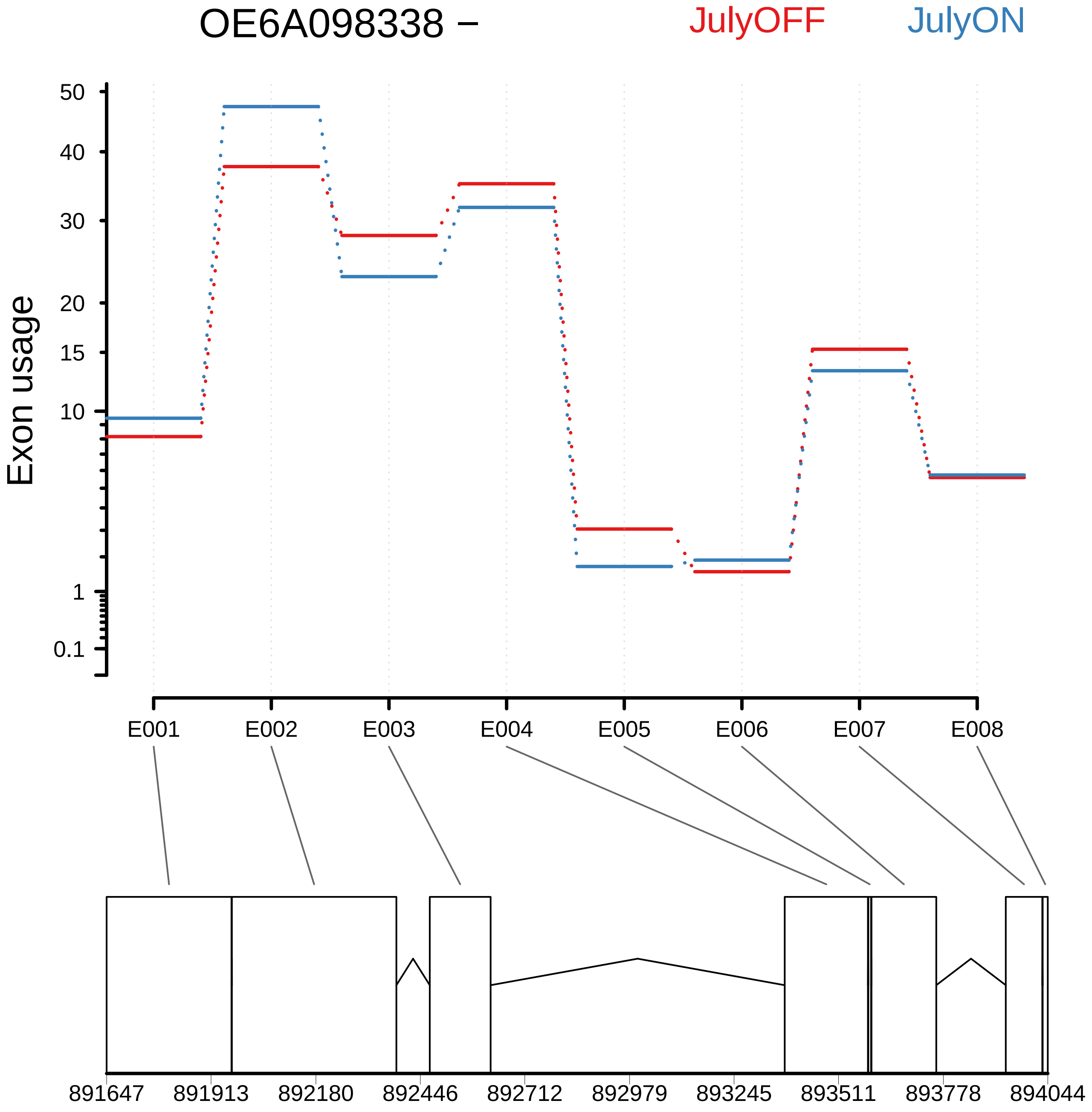
OE6A098338 −
JulyOFF
JulyON
Exon usage
50
40
30
20
15
10
1
0.1
E001
E002
E003
E004
E005
E006
E007
E008
891647
891913
892180
892446
892712
892979
893245
893511
893778
894044

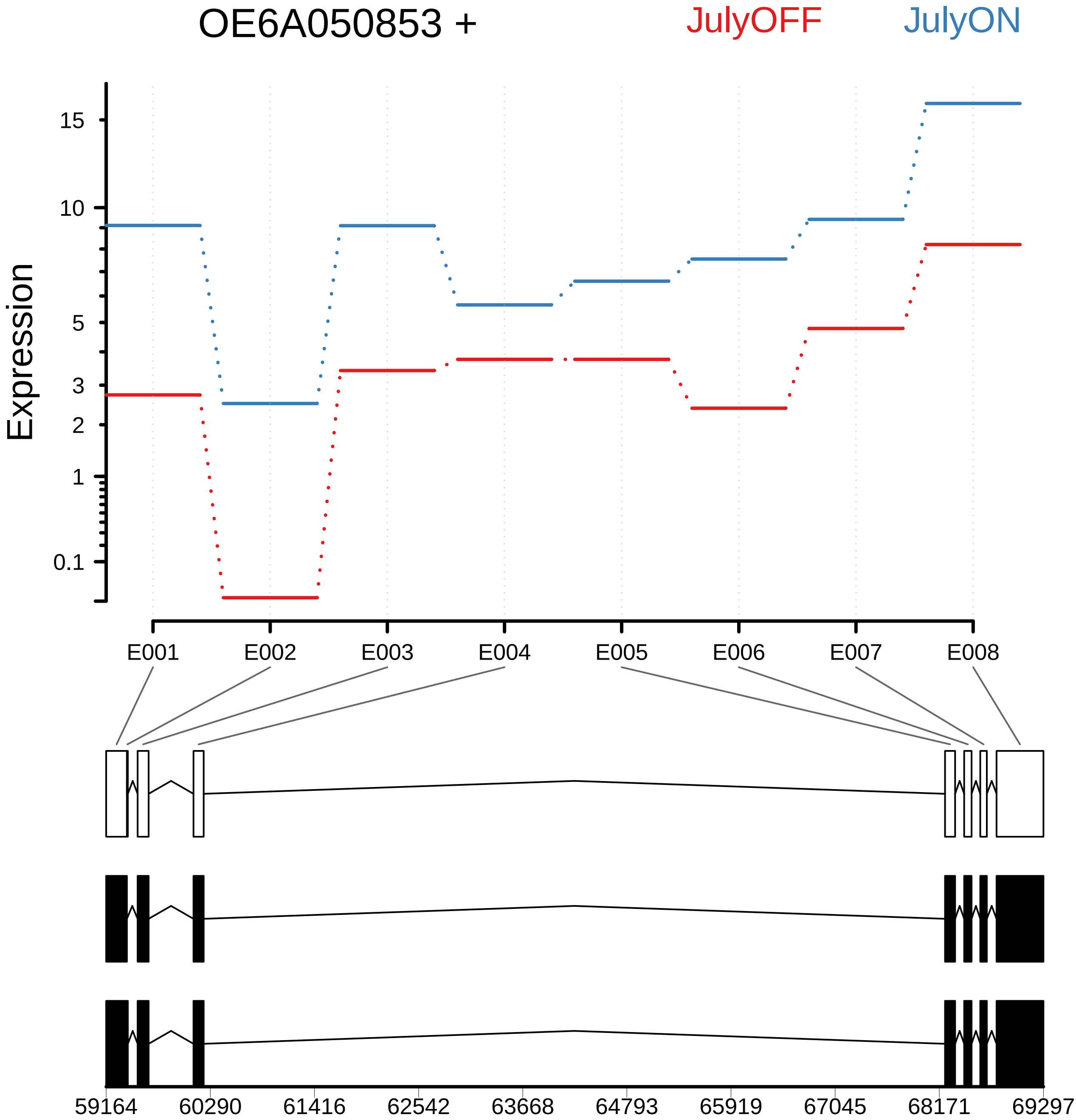

OE6A050853 +
JulyOFF
JulyON
Expression
15
10
5
3
2
1
0.1
E001
E002
E003
E004
E005
E006
E007
E008
59164
60290
61416
62542
63668
64793
65919
67045
68171
69297

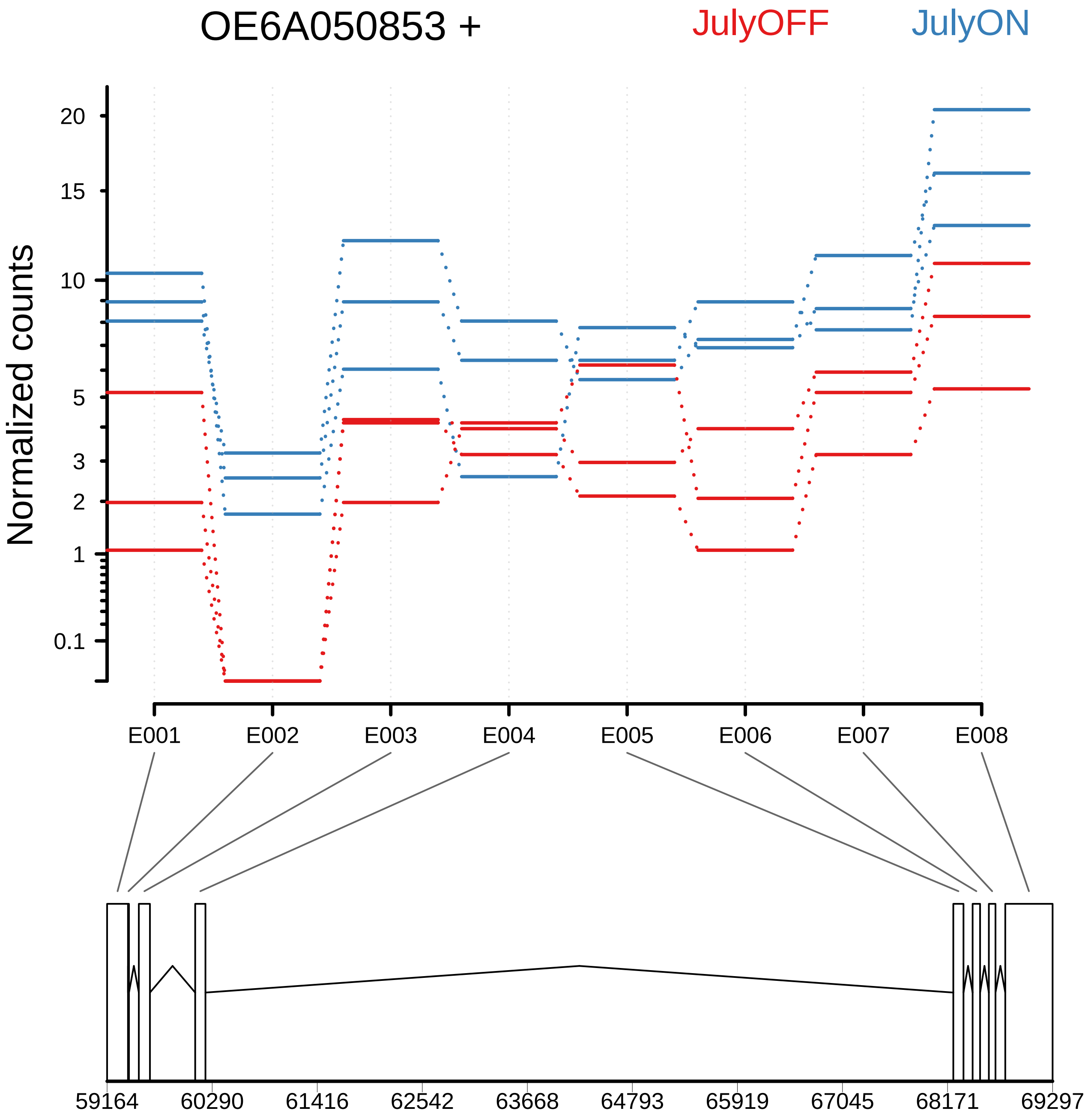

OE6A050853 +
JulyOFF
JulyON
Normalized counts
20
15
10
5
3
2
1
0.1
E001
E002
E003
E004
E005
E006
E007
E008
59164
60290
61416
62542
63668
64793
65919
67045
68171
69297

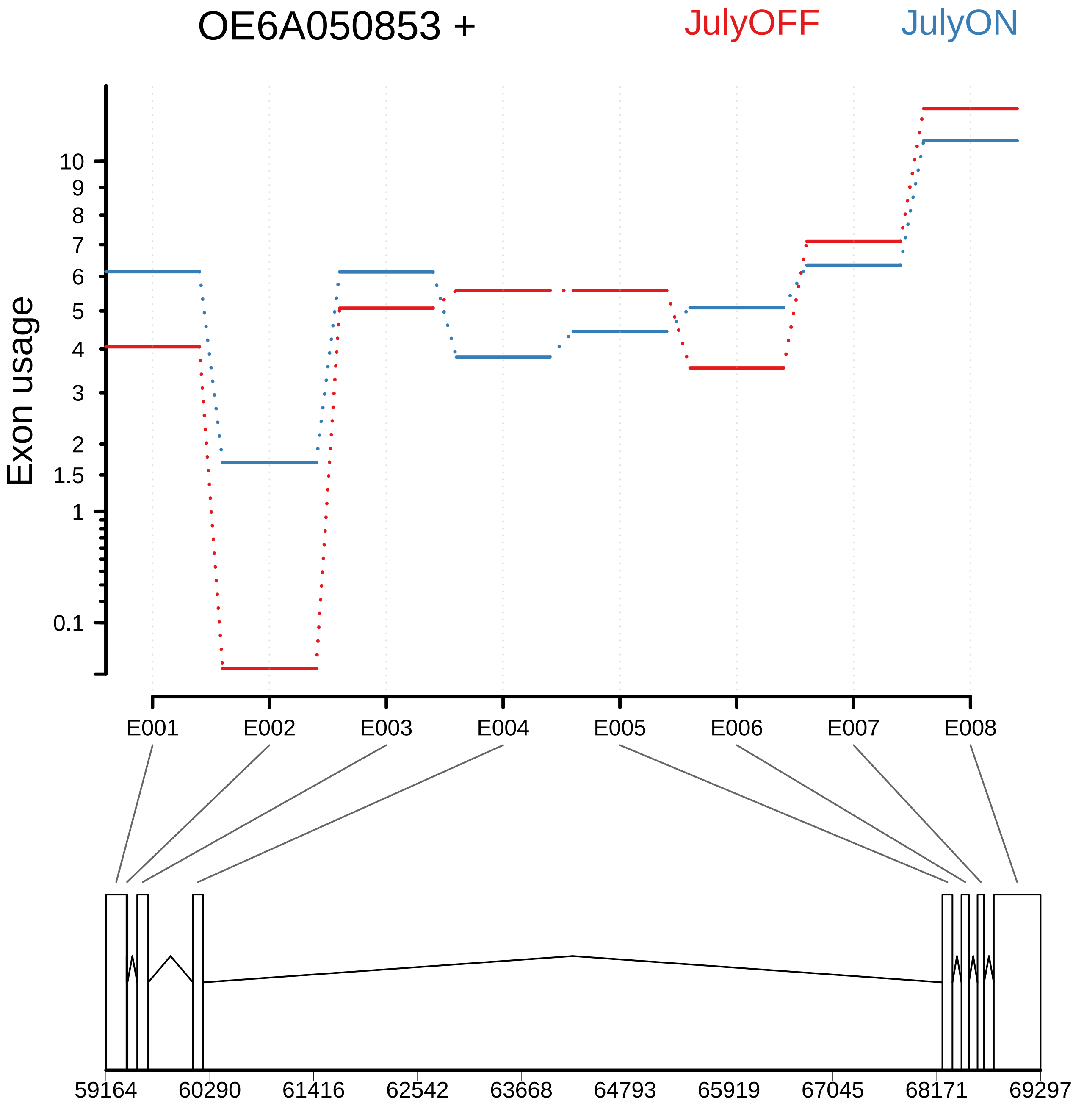
OE6A050853 +
JulyOFF
JulyON
Exon usage
10
9
8
7
6
5
4
3
2
1.5
1
0.1
E001
E002
E003
E004
E005
E006
E007
E008
59164
60290
61416
62542
63668
64793
65919
67045
68171
69297

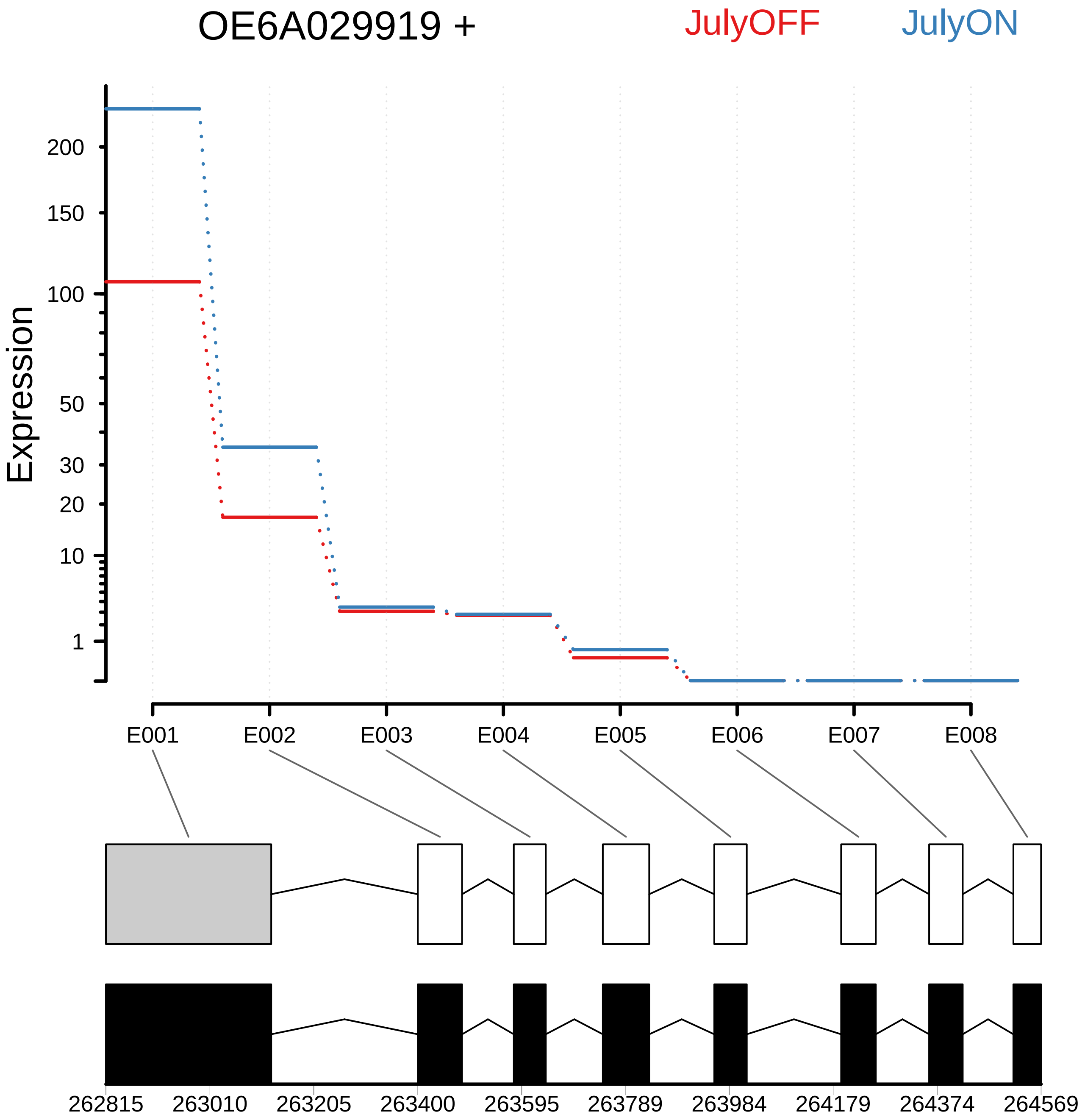
OE6A029919 +
JulyOFF
JulyON
Expression
200
150
100
50
30
20
10
1
E001
E002
E003
E004
E005
E006
E007
E008
262815
263010
263205
263400
263595
263789
263984
264179
264374
264569

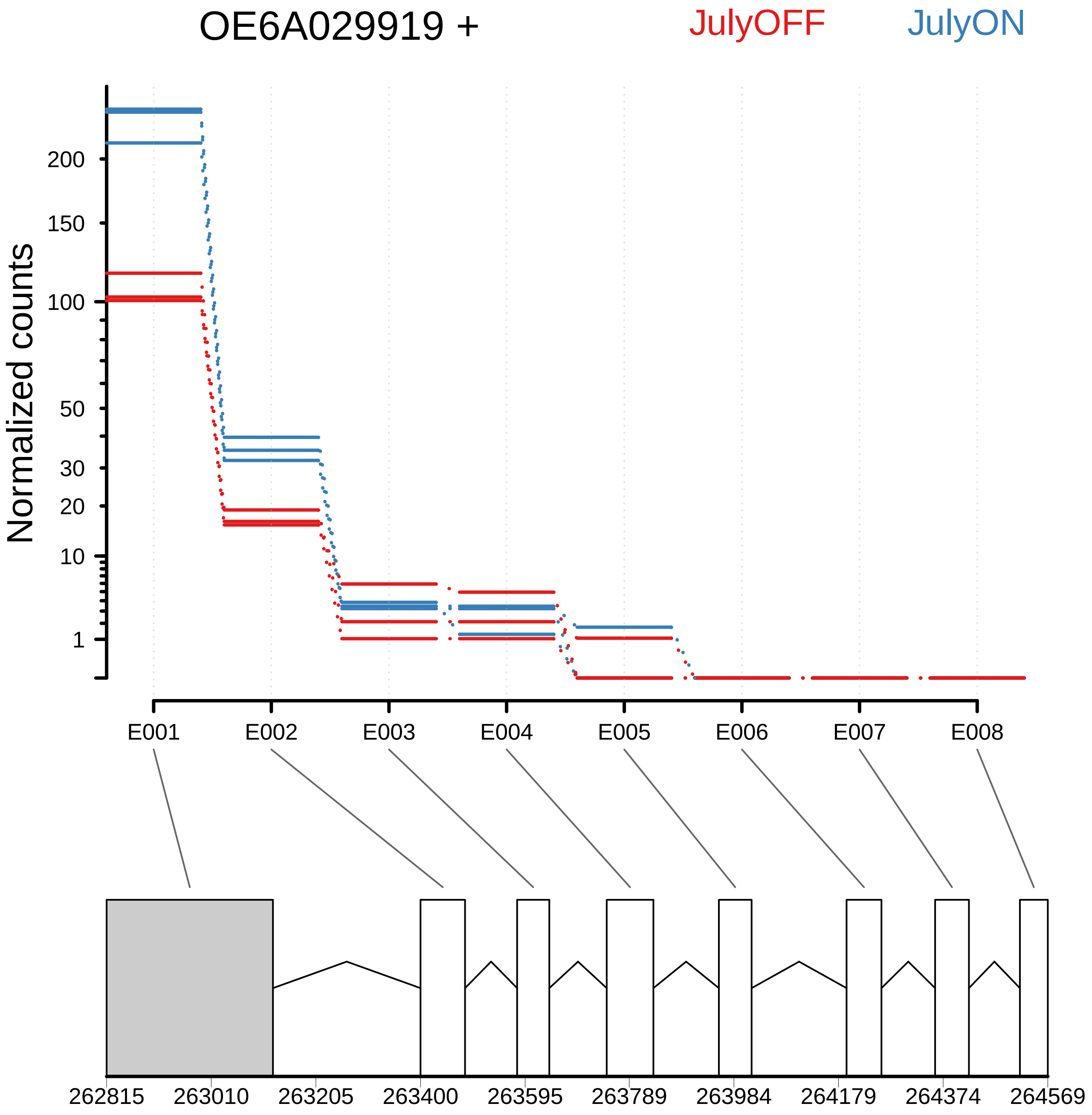

OE6A029919 +
JulyOFF
JulyON
Normalized counts
200
150
100
50
30
20
10
1
E001
E002
E003
E004
E005
E006
E007
E008
262815
263010
263205
263400
263595
263789
263984
264179
264374
264569

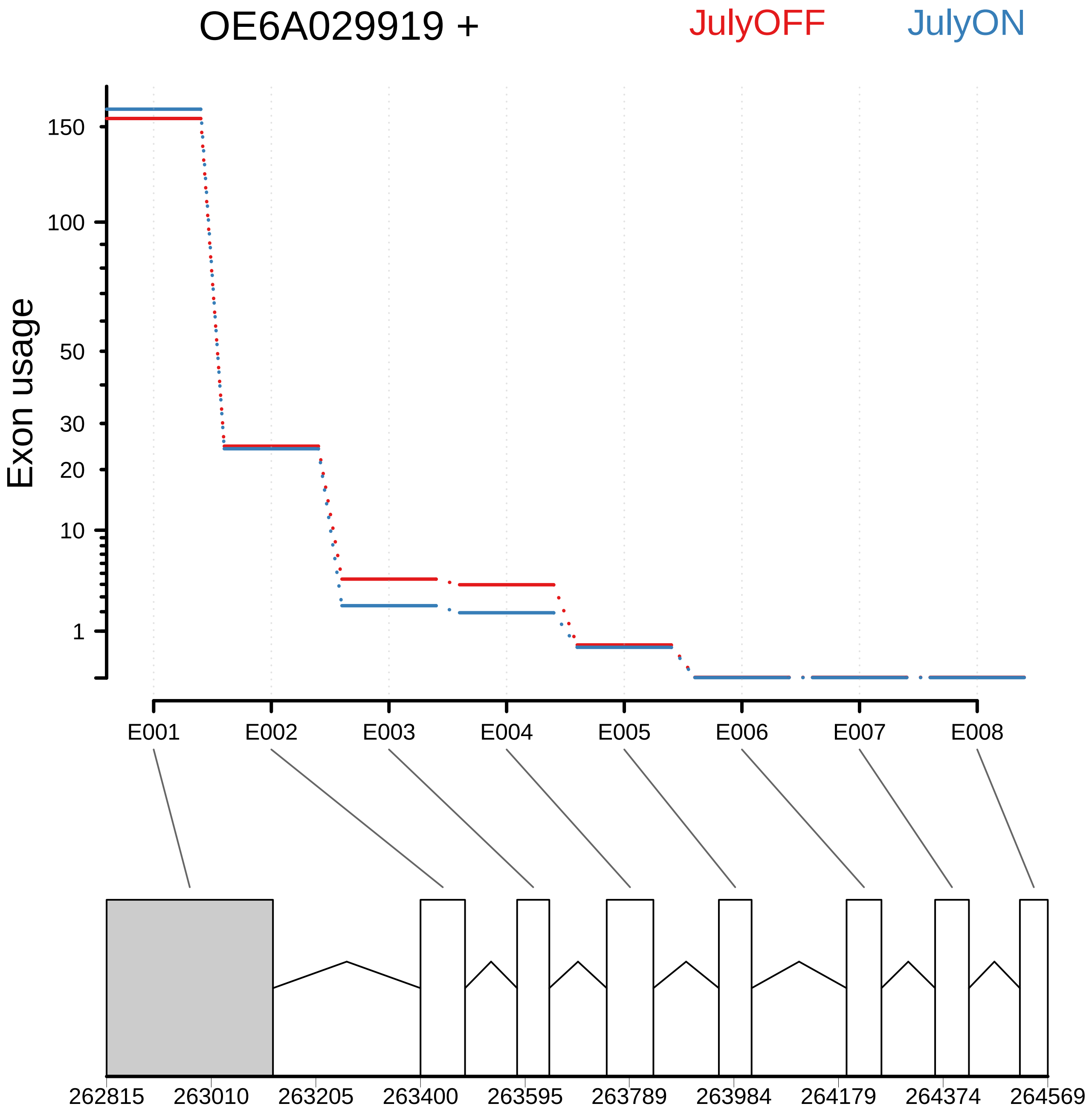

OE6A029919 +
JulyOFF
JulyON
Exon usage
150
100
50
30
20
10
1
E001
E002
E003
E004
E005
E006
E007
E008
262815
263010
263205
263400
263595
263789
263984
264179
264374
264569

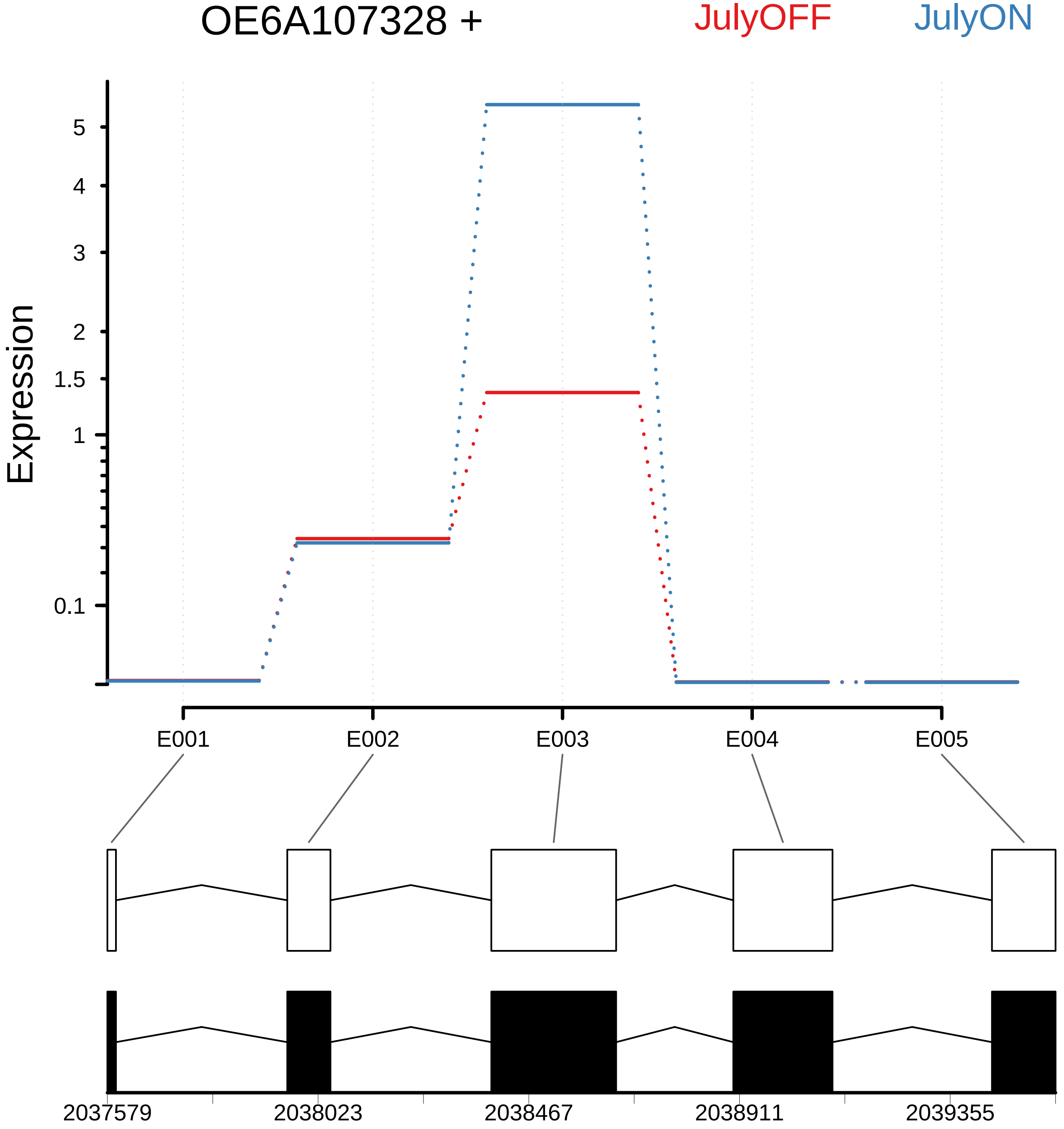

OE6A107328 +
JulyOFF
JulyON
Expression
5
4
3
2
1.5
1
0.1
E001
E002
E003
E004
E005
2037579
2038023
2038467
2038911
2039355

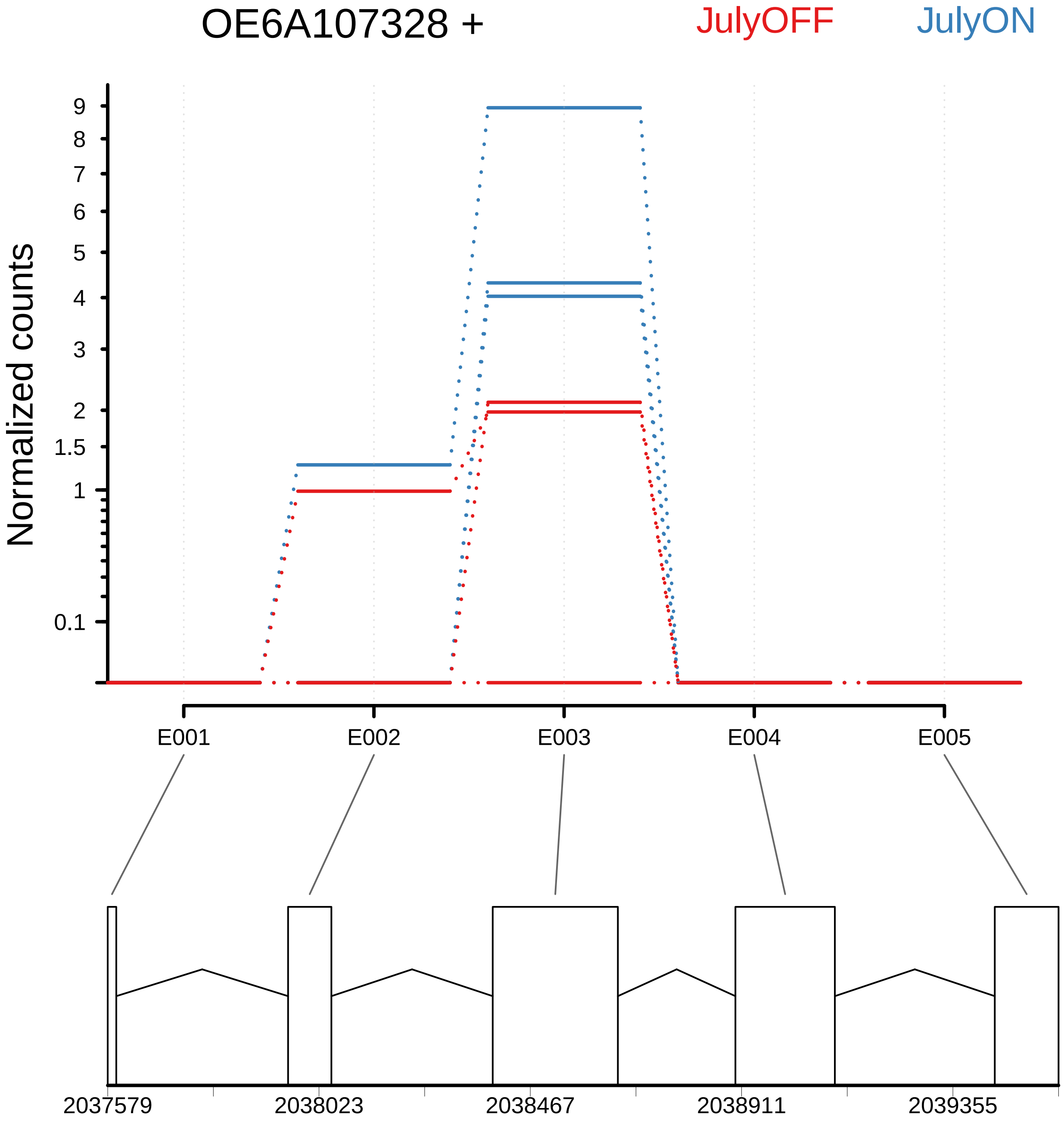

OE6A107328 +
JulyOFF
JulyON
Normalized counts
9
8
7
6
5
4
3
2
1.5
1
0.1
E001
E002
E003
E004
E005
2037579
2038023
2038467
2038911
2039355

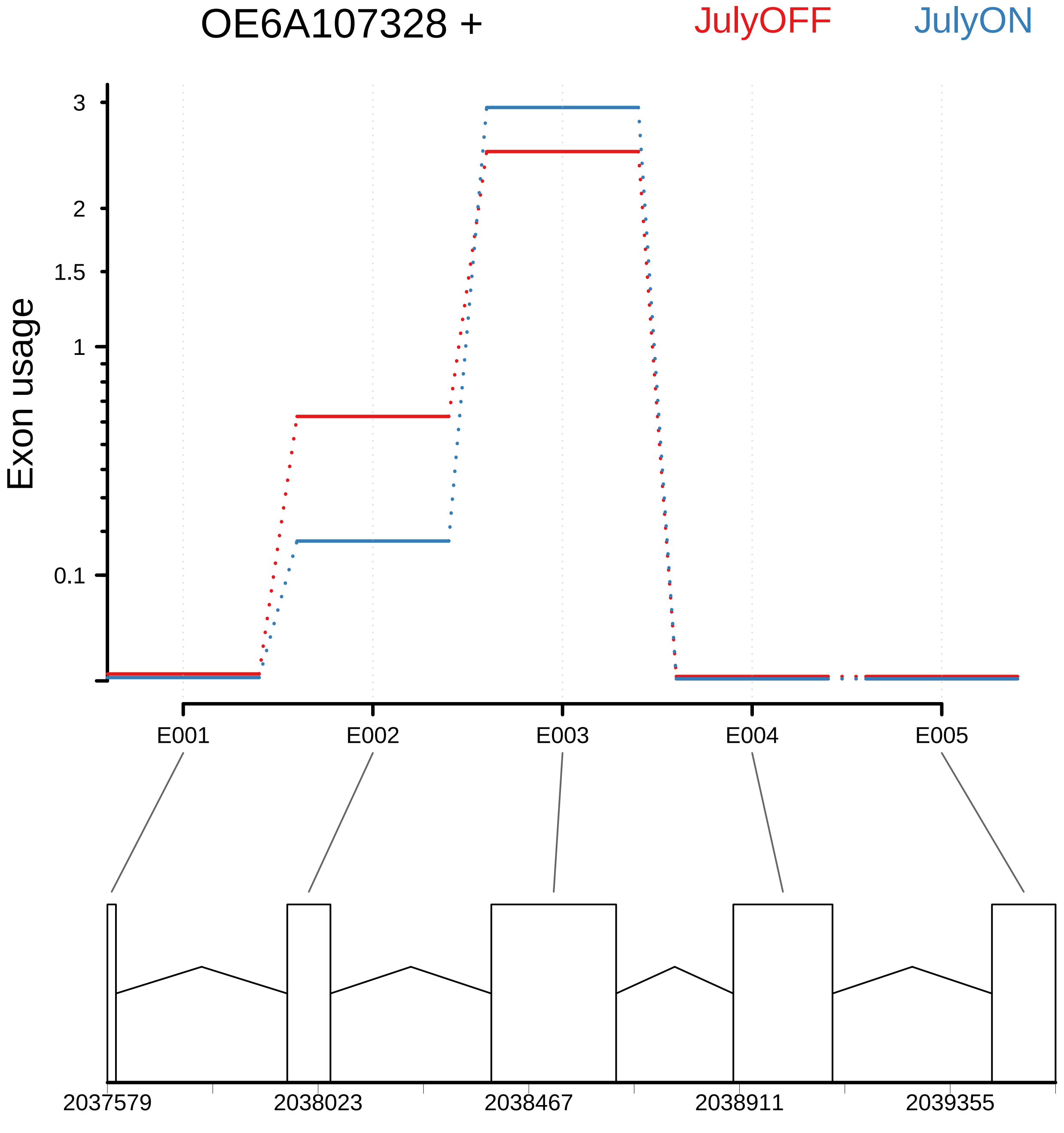

OE6A107328 +
JulyOFF
JulyON
Exon usage
3
2
1.5
1
0.1
E001
E002
E003
E004
E005
2037579
2038023
2038467
2038911
2039355

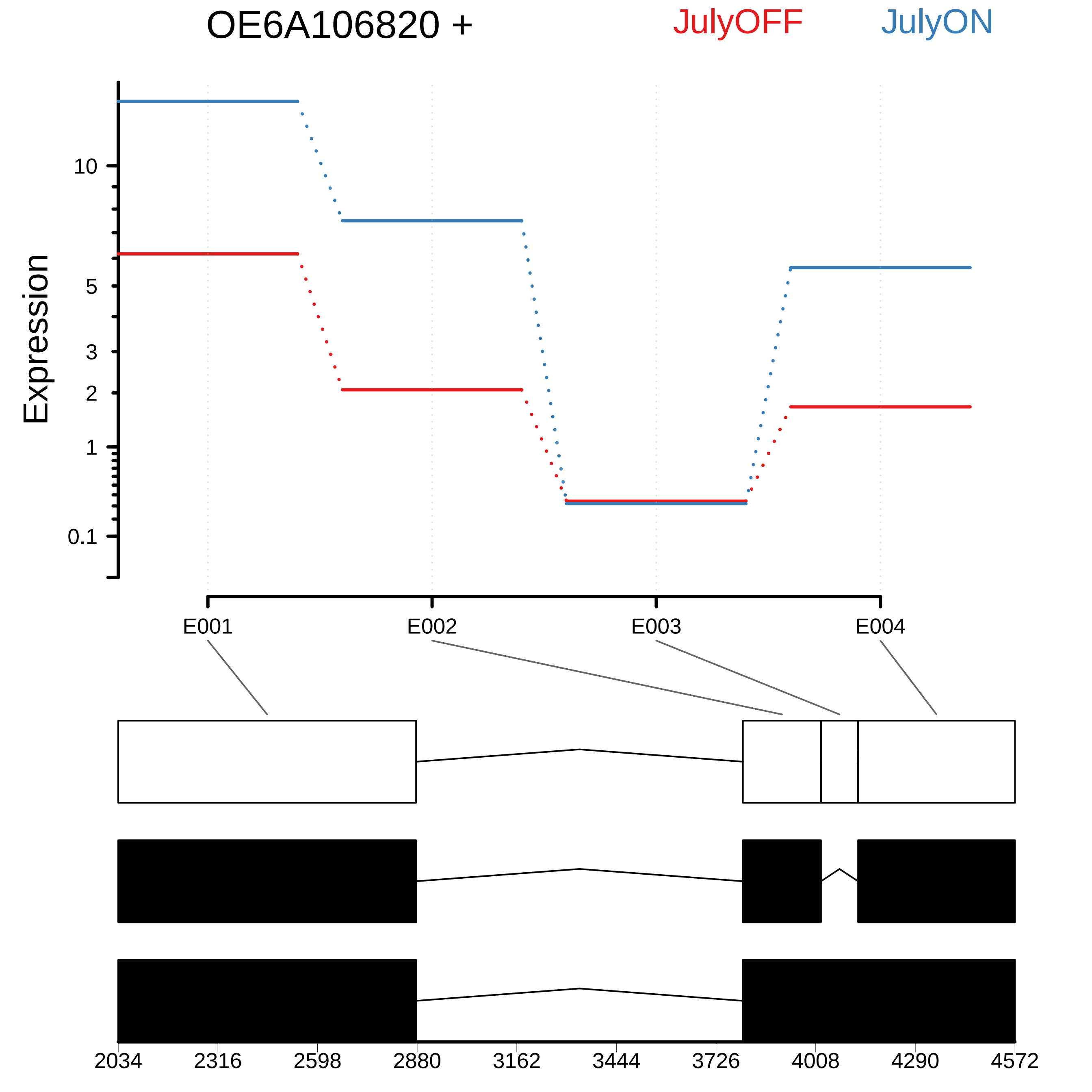
OE6A106820 +
JulyOFF
JulyON
Expression
10
5
3
2
1
0.1
E001
E002
E003
E004
2034
2316
2598
2880
3162
3444
3726
4008
4290
4572

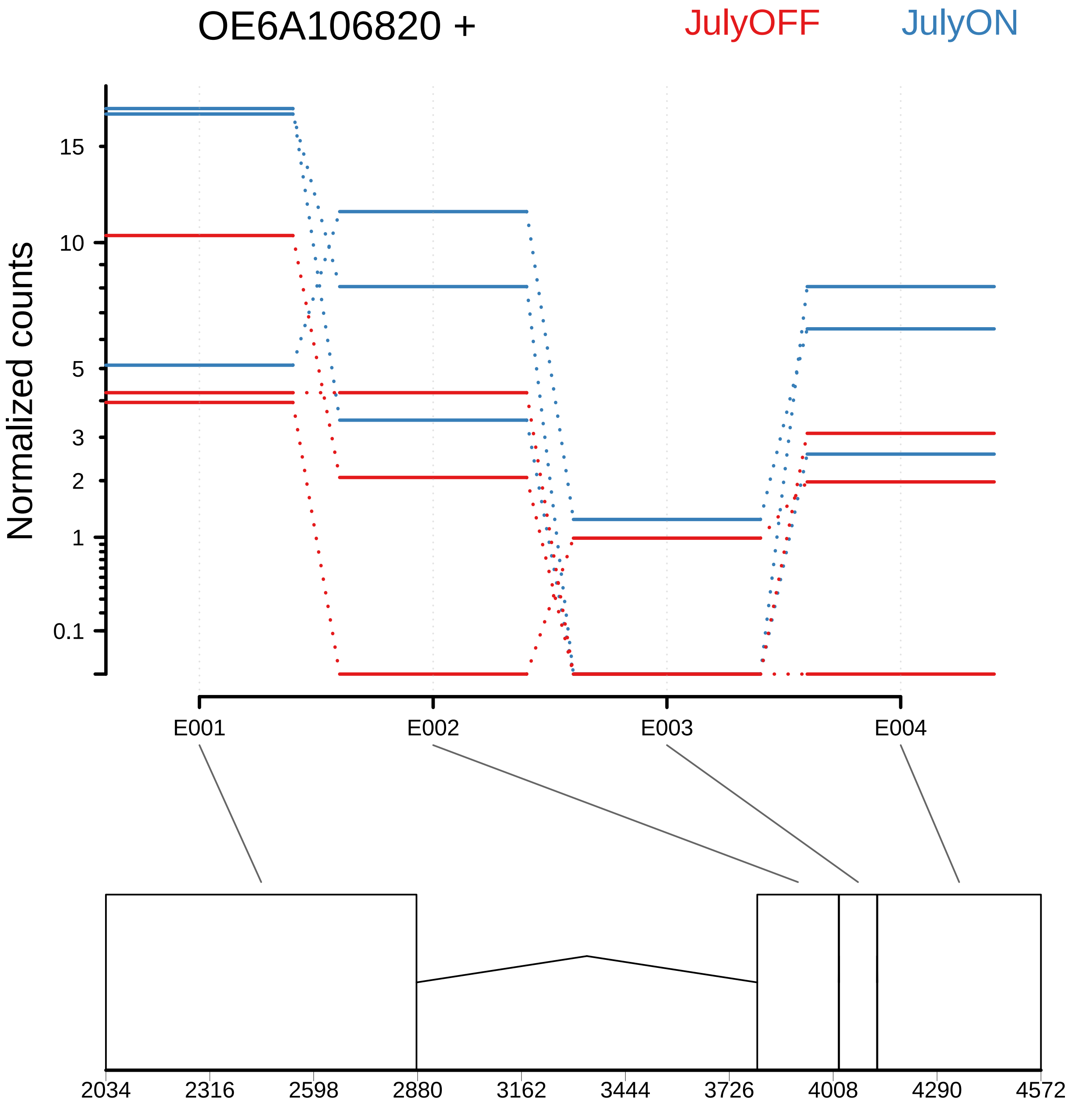
OE6A106820 +
JulyOFF
JulyON
Normalized counts
15
10
5
3
2
1
0.1
E001
E002
E003
E004
2034
2316
2598
2880
3162
3444
3726
4008
4290
4572

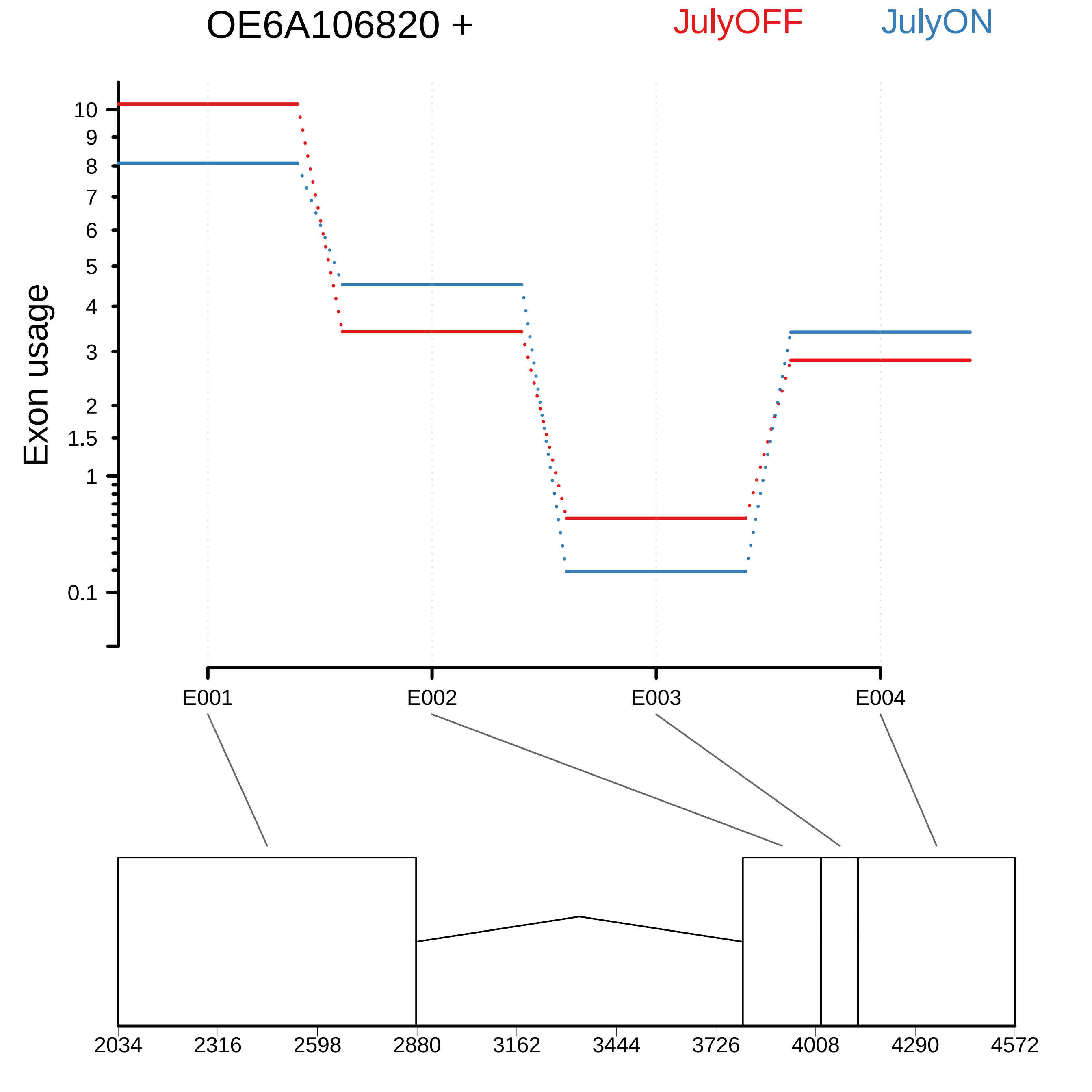

OE6A106820 +
JulyOFF
JulyON
Exon usage
10
9
8
7
6
5
4
3
2
1.5
1
0.1
E001
E002
E003
E004
2034
2316
2598
2880
3162
3444
3726
4008
4290
4572

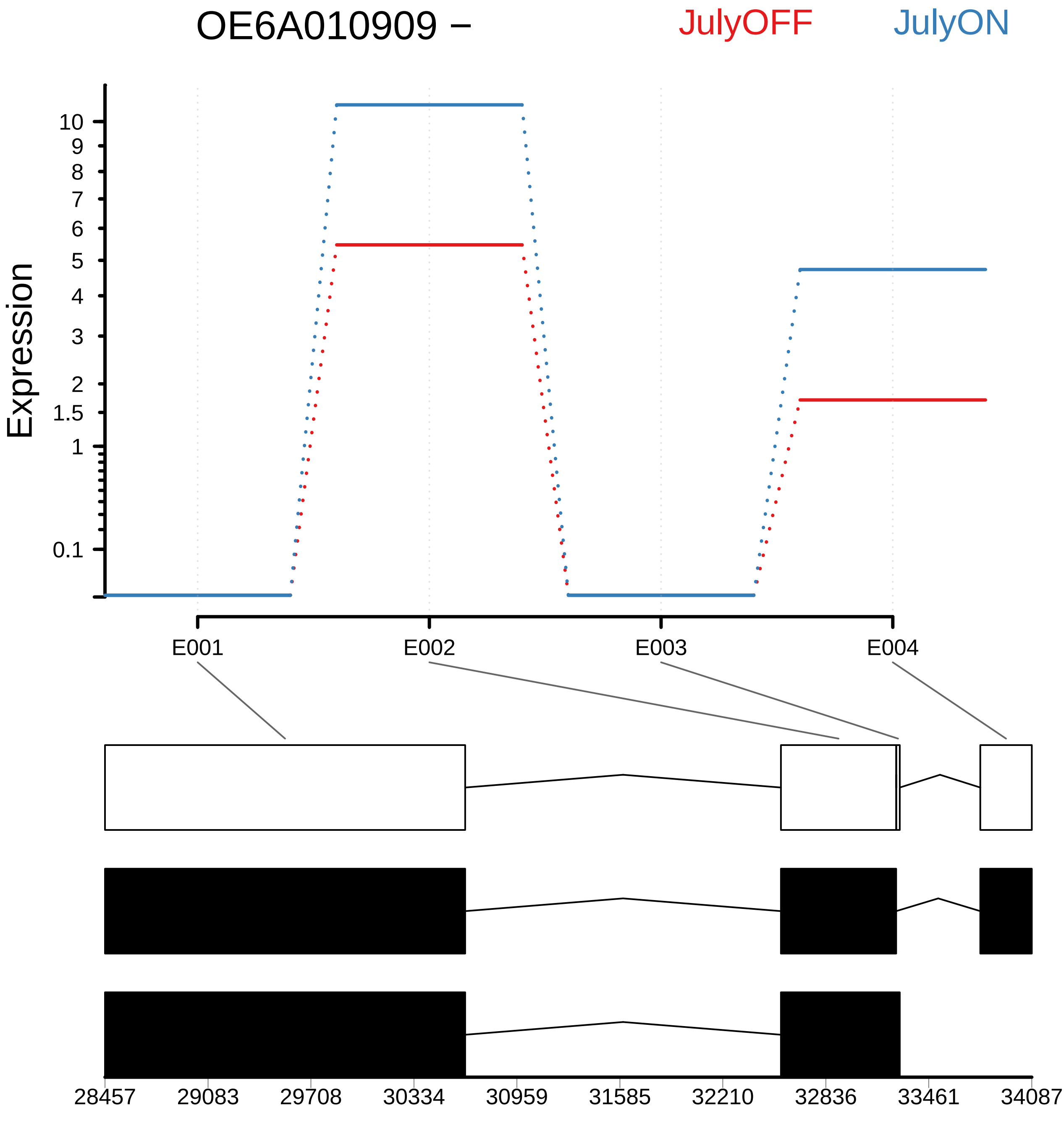
OE6A010909 –
JulyOFF
JulyON
Expression
10
9
8
7
6
5
4
3
2
1.5
1
0.1
E001
E002
E003
E004
28457
29083
29708
30334
30959
31585
32210
32836
33461
34087

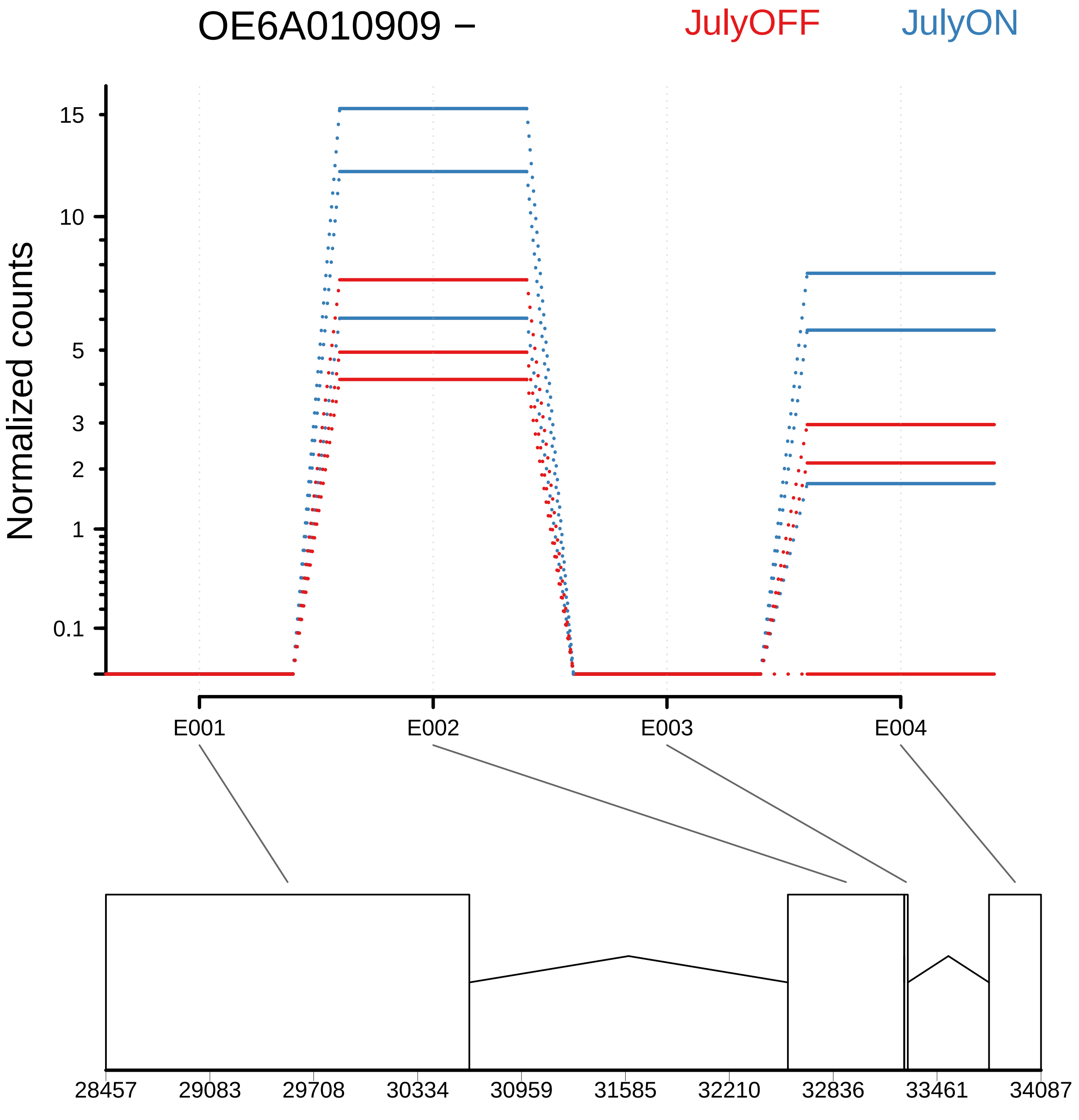
OE6A010909 –
JulyOFF
JulyON
Normalized counts
15
10
5
3
2
1
0.1
E001
E002
E003
E004
28457
29083
29708
30334
30959
31585
32210
32836
33461
34087

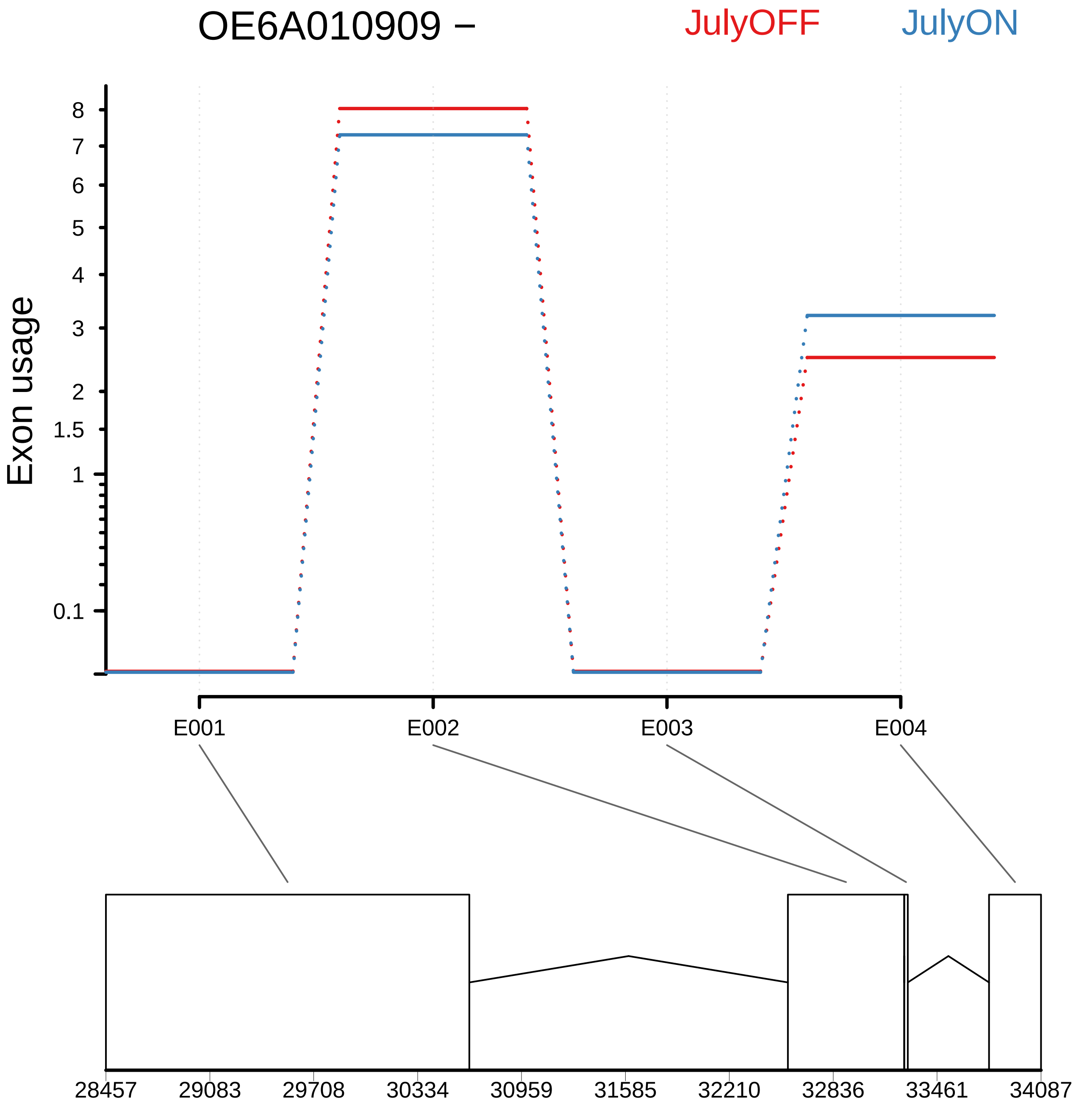
OE6A010909 −
JulyOFF
JulyON
Exon usage
8
7
6
5
4
3
2
1.5
1
0.1
E001
E002
E003
E004
28457
29083
29708
30334
30959
31585
32210
32836
33461
34087

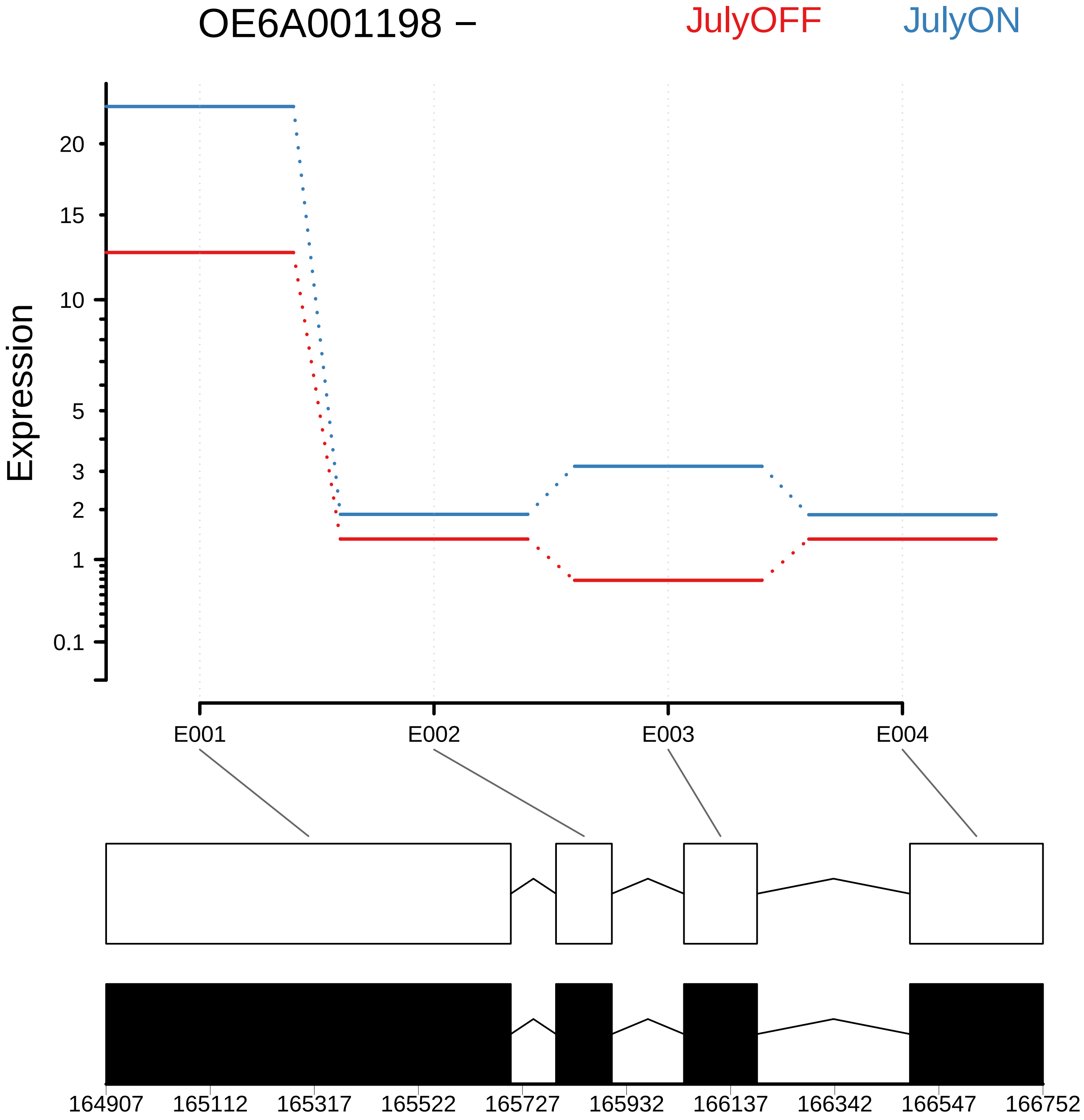

OE6A001198 –
JulyOFF
JulyON
Expression
20
15
10
5
3
2
1
0.1
E001
E002
E003
E004
164907
165112
165317
165522
165727
165932
166137
166342
166547
166752

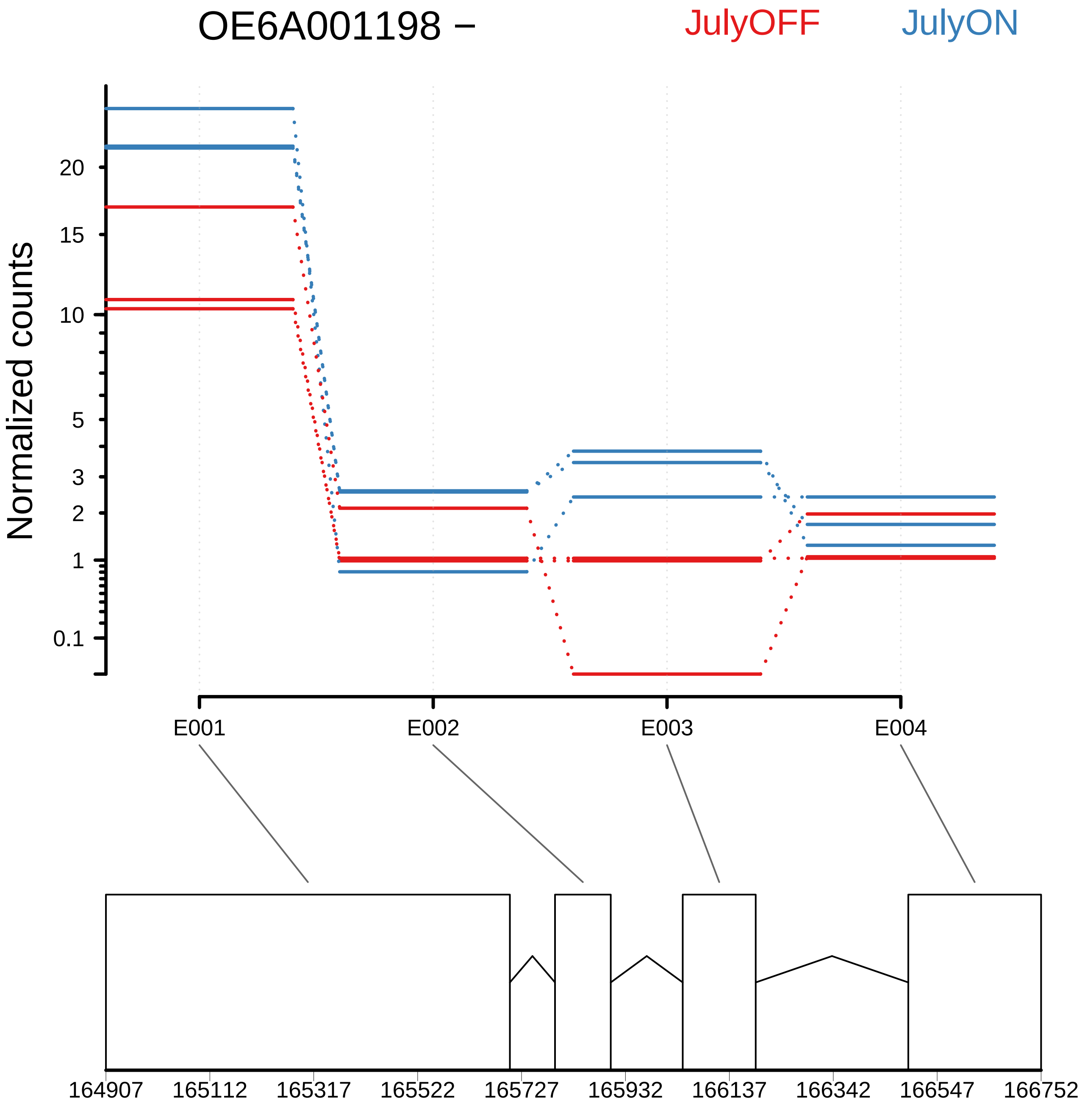

OE6A001198 –
JulyOFF
JulyON
Normalized counts
20
15
10
5
3
2
1
0.1
E001
E002
E003
E004
164907
165112
165317
165522
165727
165932
166137
166342
166547
166752

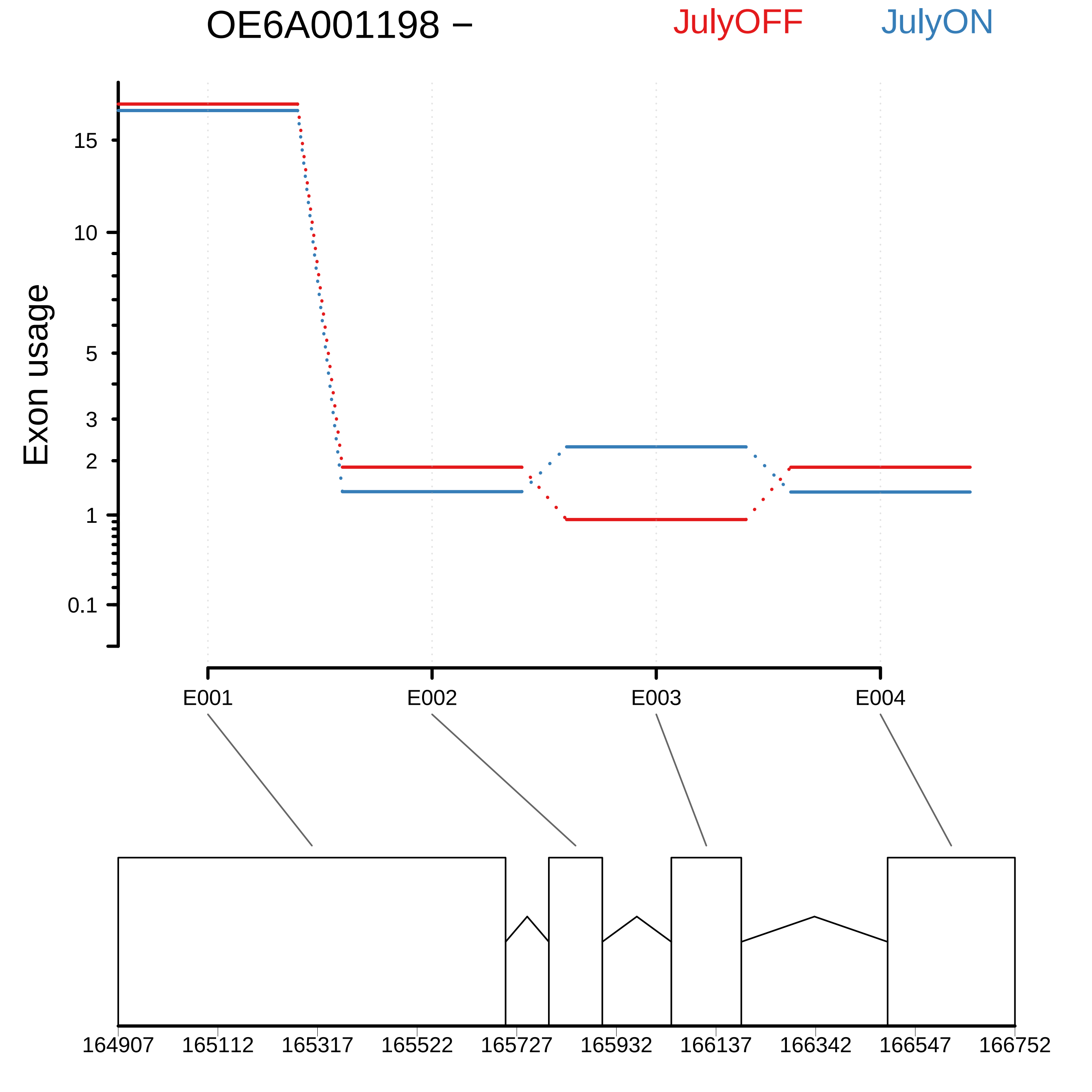

OE6A001198 −
JulyOFF
JulyON
Exon usage
15
10
5
3
2
1
0.1
E001
E002
E003
E004
164907
165112
165317
165522
165727
165932
166137
166342
166547
166752

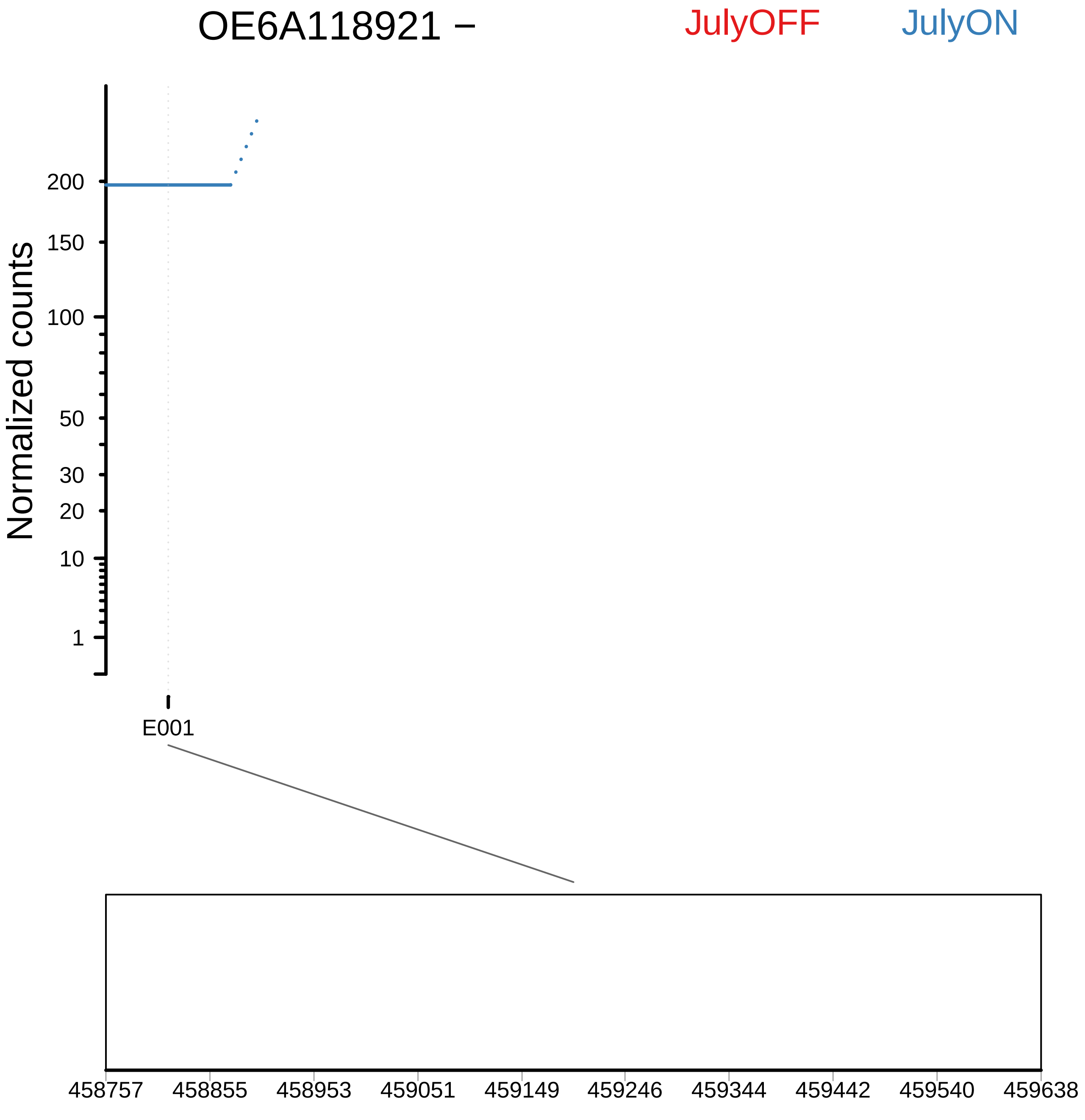
OE6A118921 −
JulyOFF
JulyON
Normalized counts
200
150
100
50
30
20
10
1
E001
458757
458855
458953
459051
459149
459246
459344
459442
459540
459638

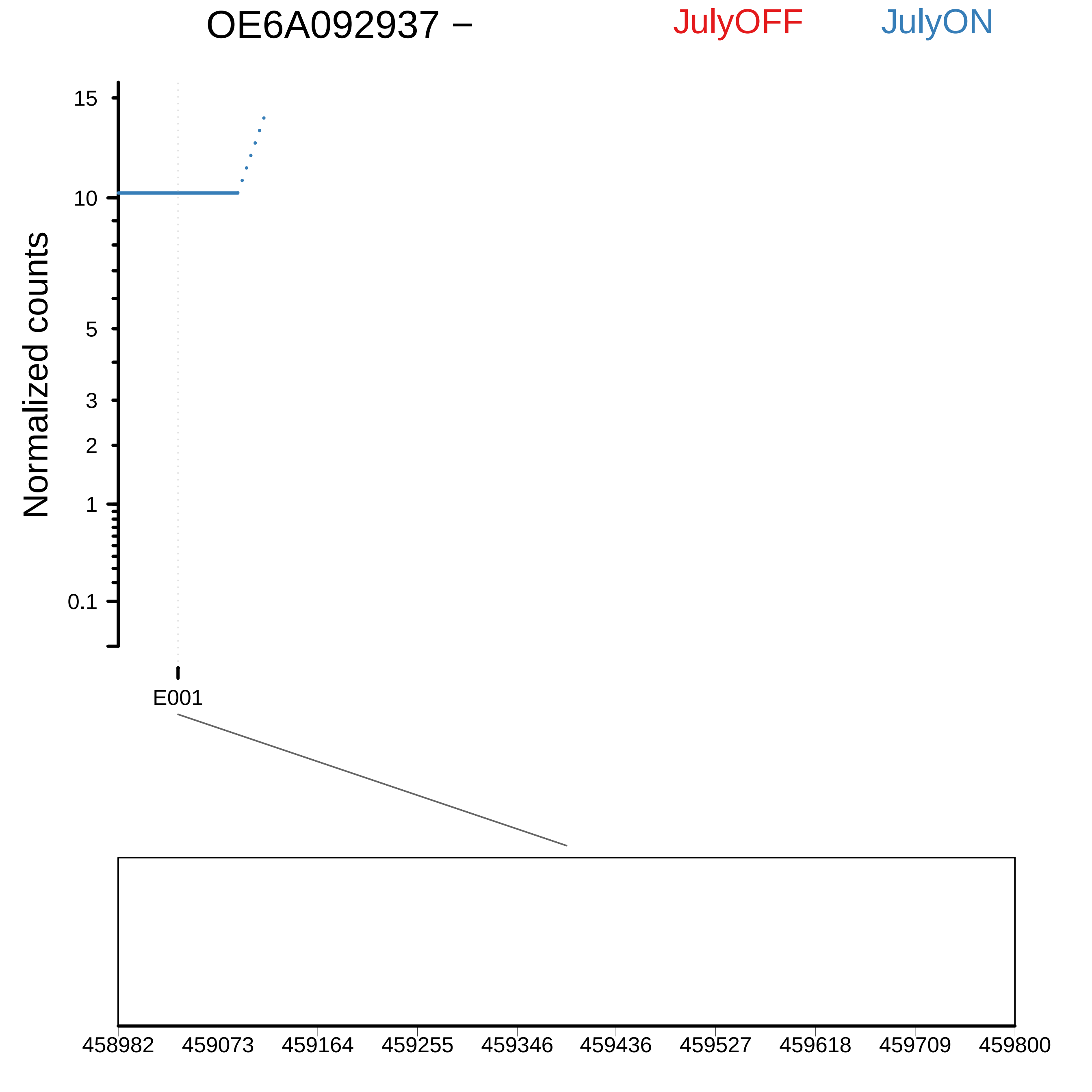

OE6A092937 −
JulyOFF
JulyON
Normalized counts
15
10
5
3
2
1
0.1
E001
458982
459073
459164
459255
459346
459436
459527
459618
459709
459800

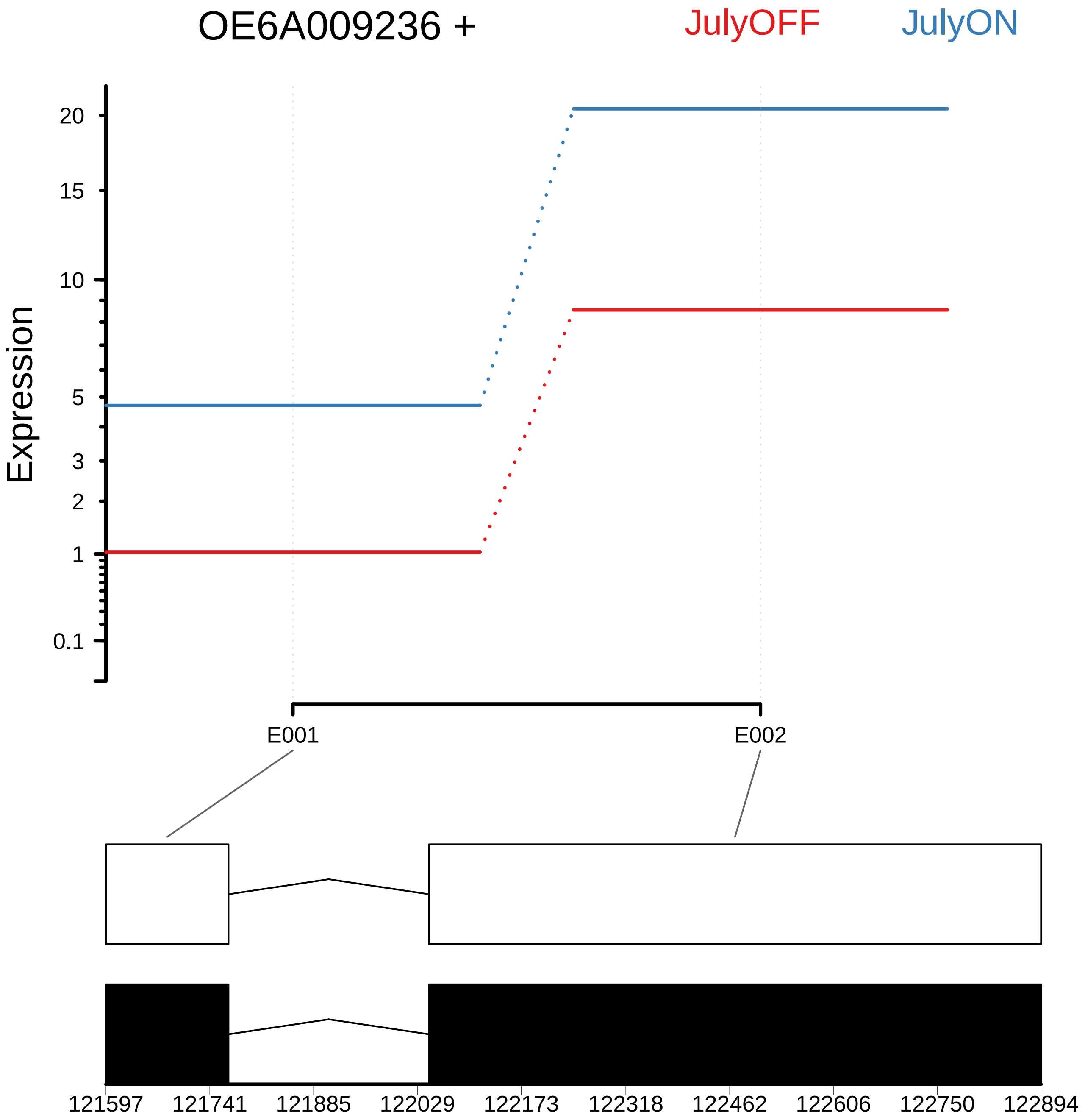

OE6A009236 +
JulyOFF
JulyON
Expression
20
15
10
5
3
2
1
0.1
E001
E002
121597
121741
121885
122029
122173
122318
122462
122606
122750
122894

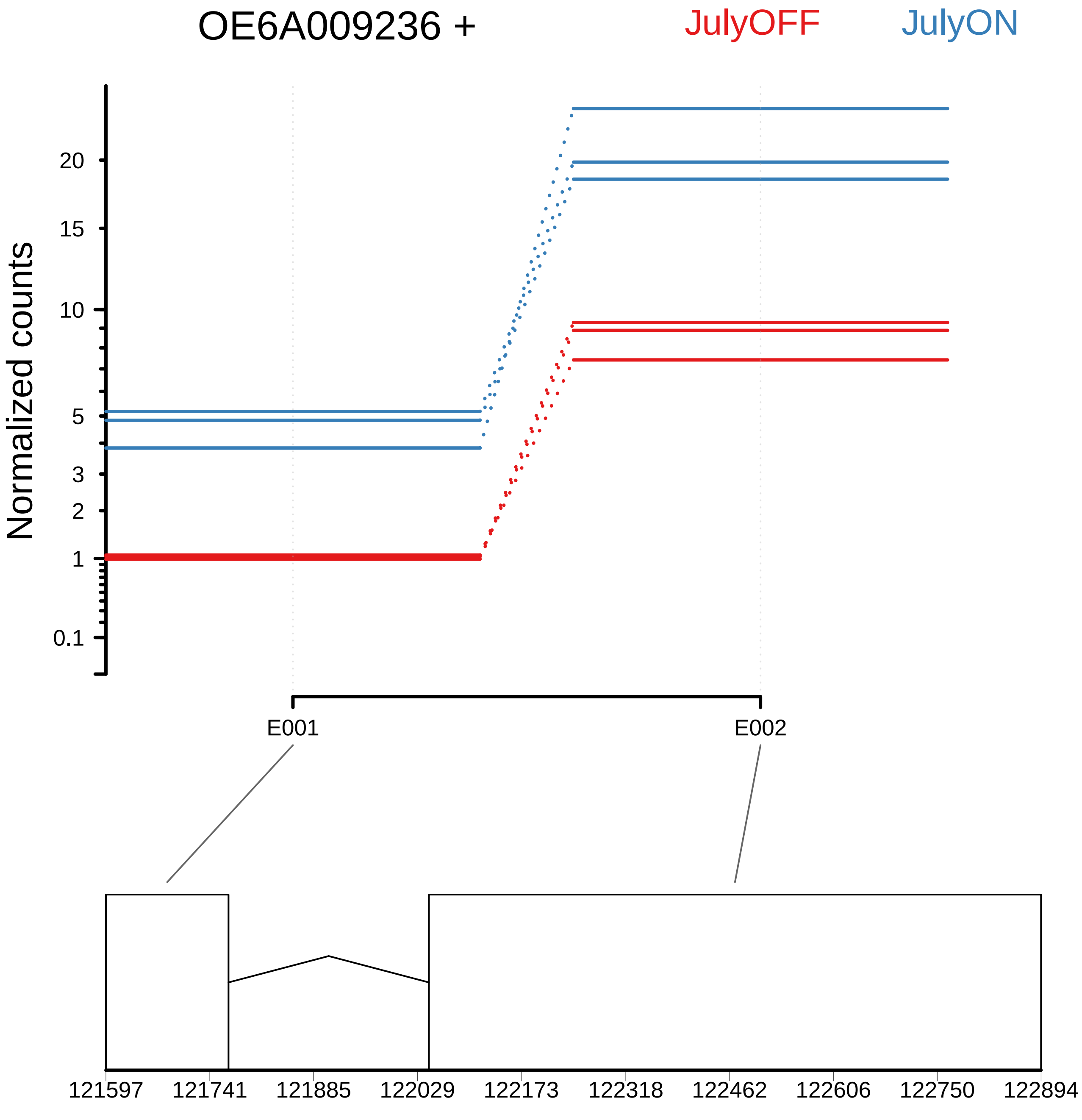

OE6A009236 +
JulyOFF
JulyON
Normalized counts
20
15
10
5
3
2
1
0.1
E001
E002
121597
121741
121885
122029
122173
122318
122462
122606
122750
122894

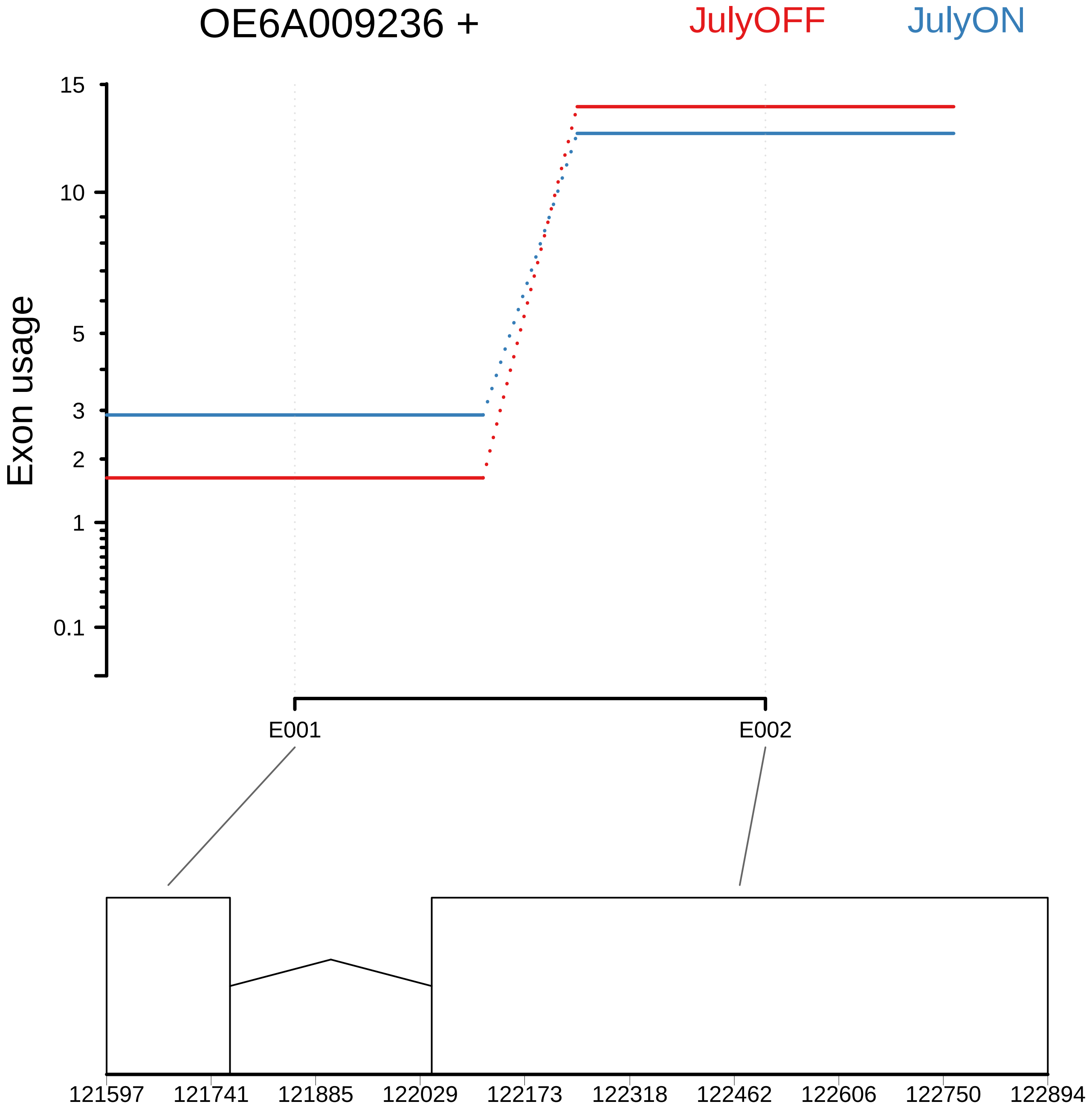
OE6A009236 +
JulyOFF
JulyON
Exon usage
15
10
5
3
2
1
0.1
E001
E002
121597
121741
121885
122029
122173
122318
122462
122606
122750
122894

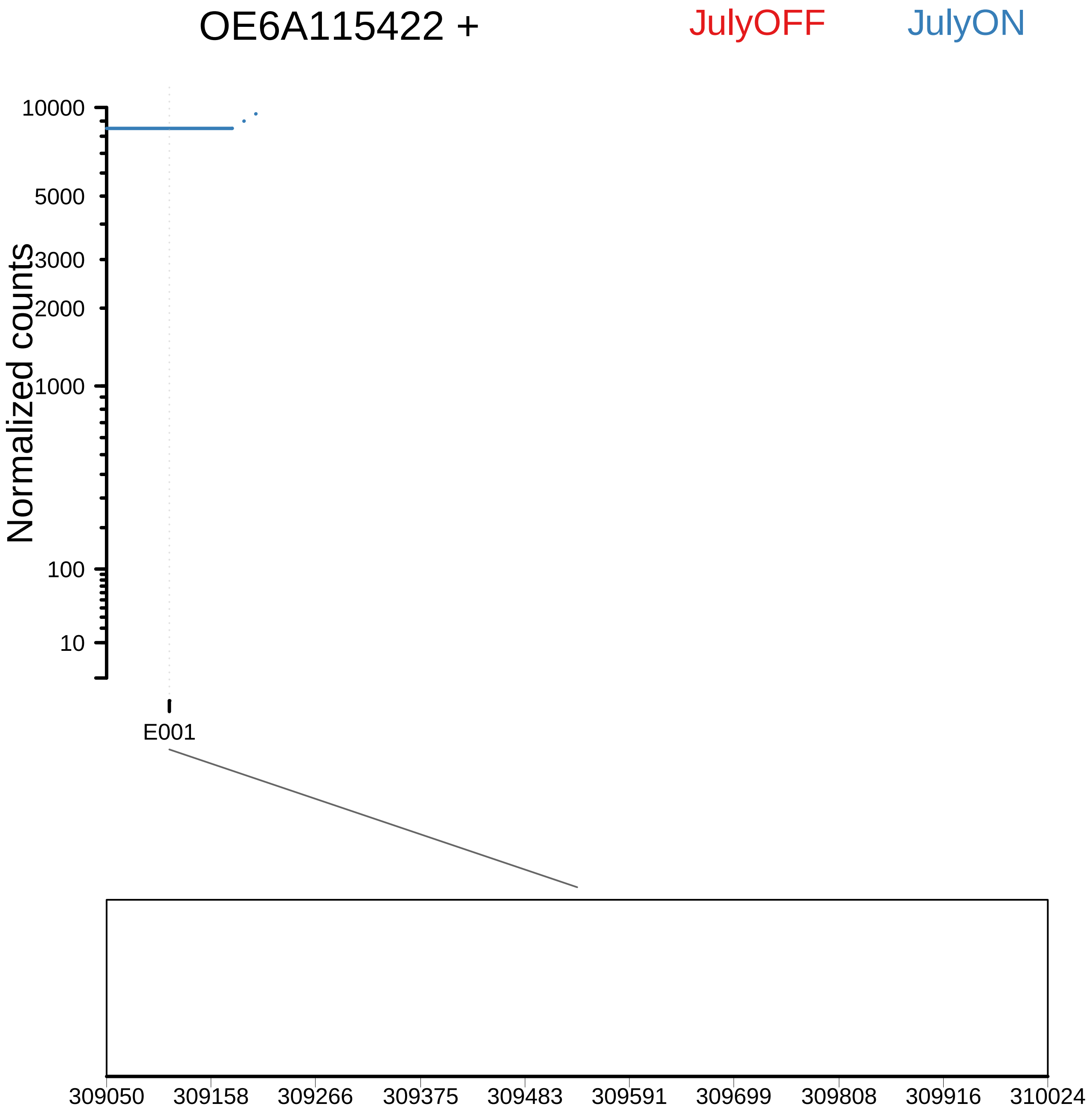
OE6A115422 +
JulyOFF
JulyON
Normalized counts
10000
5000
3000
2000
1000
100
10
E001
309050
309158
309266
309375
309483
309591
309699
309808
309916
310024

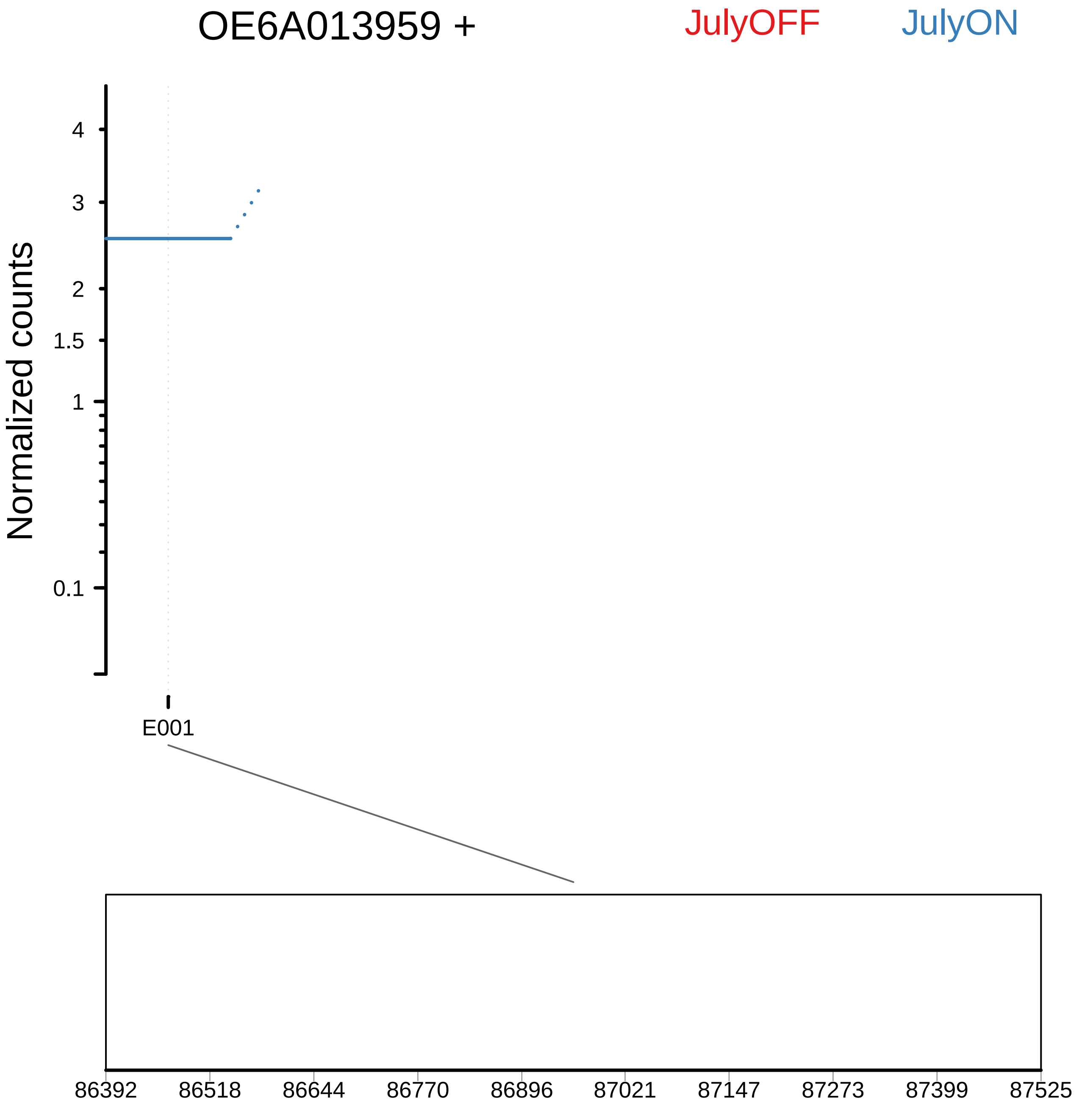
OE6A013959 +
JulyOFF
JulyON
Normalized counts
4
3
2
1.5
1
0.1
E001
86392
86518
86644
86770
86896
87021
87147
87273
87399
87525

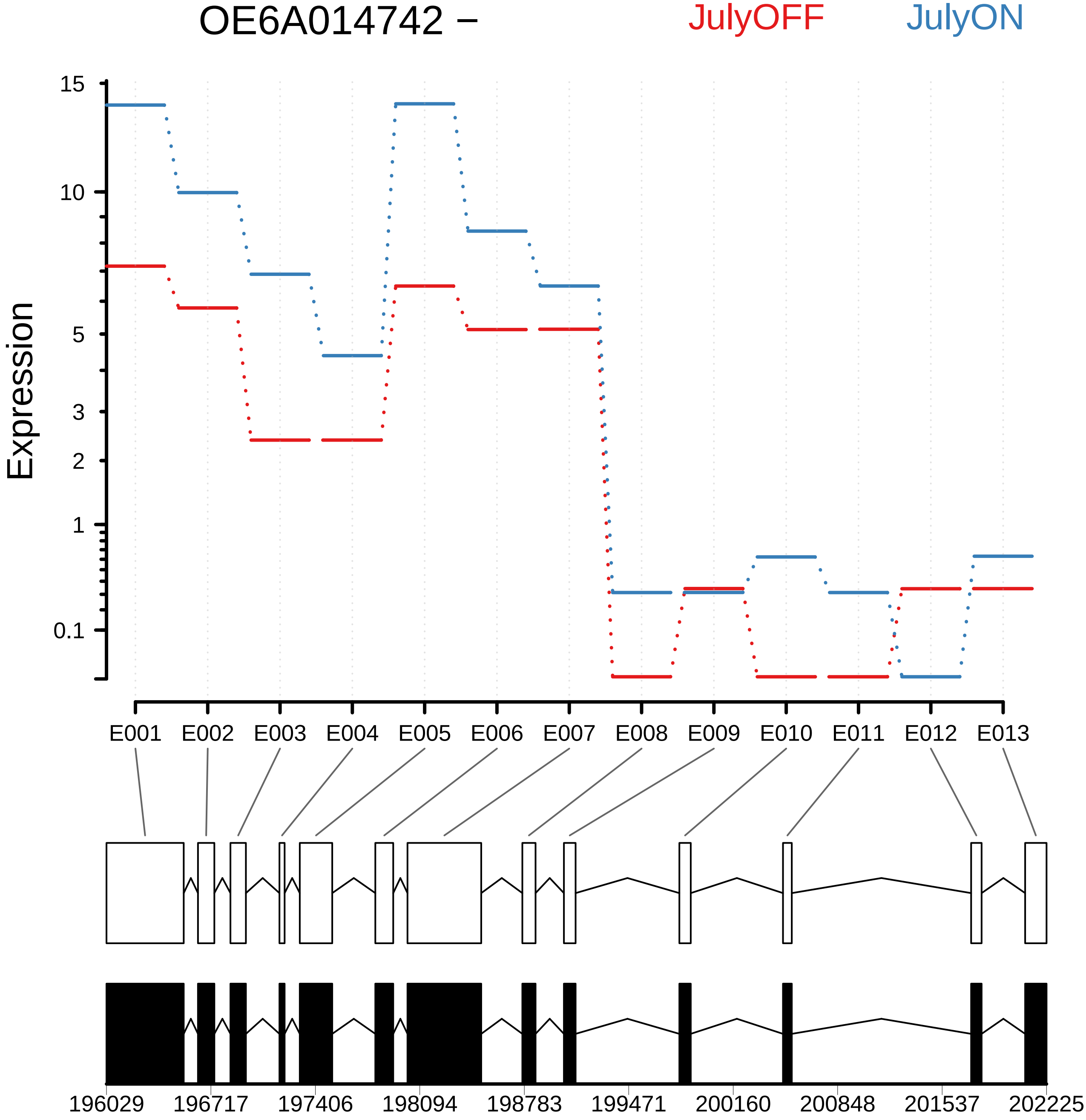

OE6A014742 –
JulyOFF
JulyON
Expression
15
10
5
3
2
1
0.1
E001
E002
E003
E004
E005
E006
E007
E008
E009
E010
E011
E012
E013
196029
196717
197406
198094
198783
199471
200160
200848
201537
202225

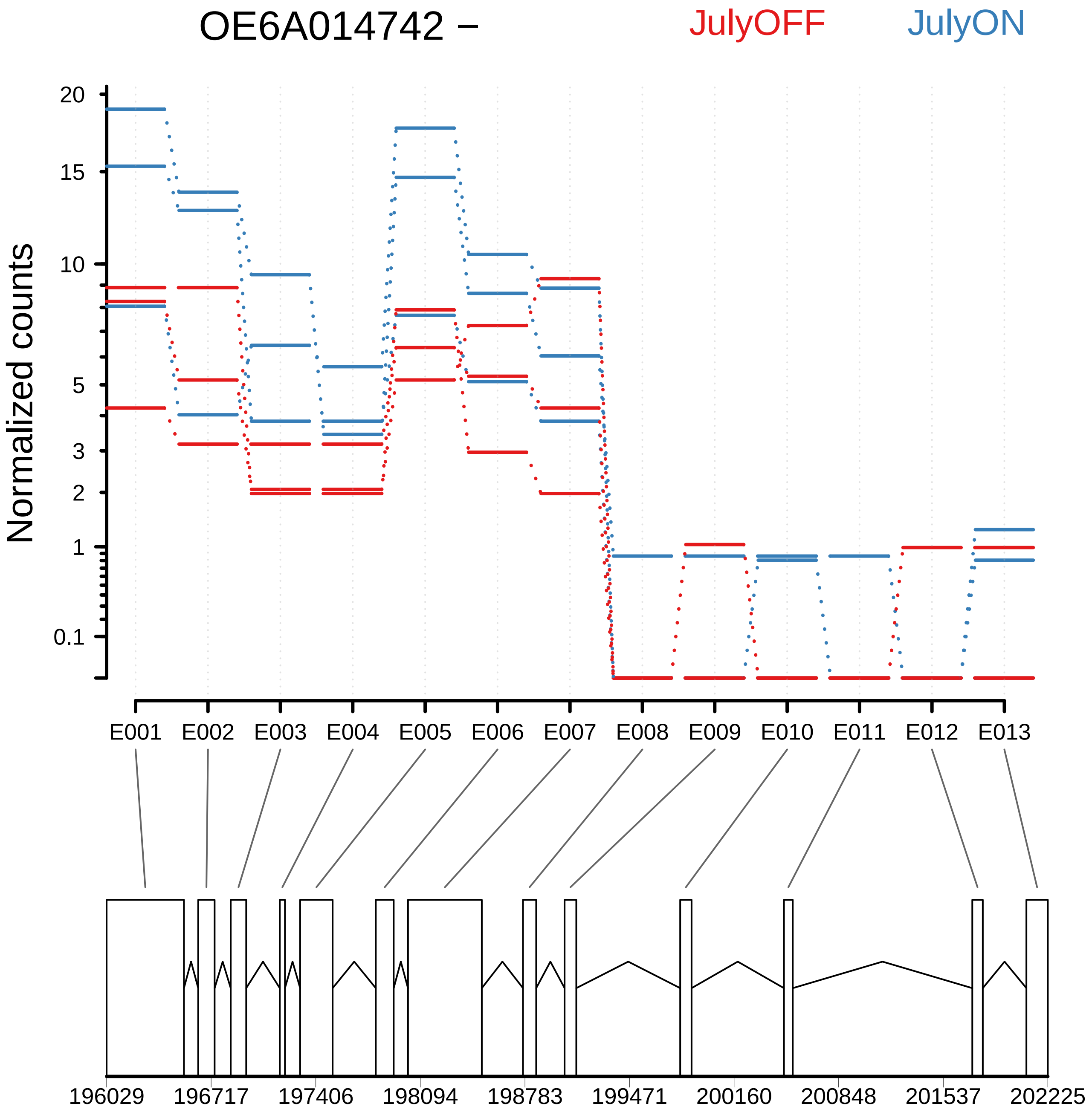

OE6A014742 −
JulyOFF
JulyON
Normalized counts
20
15
10
5
3
2
1
0.1
E001
E002
E003
E004
E005
E006
E007
E008
E009
E010
E011
E012
E013
196029
196717
197406
198094
198783
199471
200160
200848
201537
202225

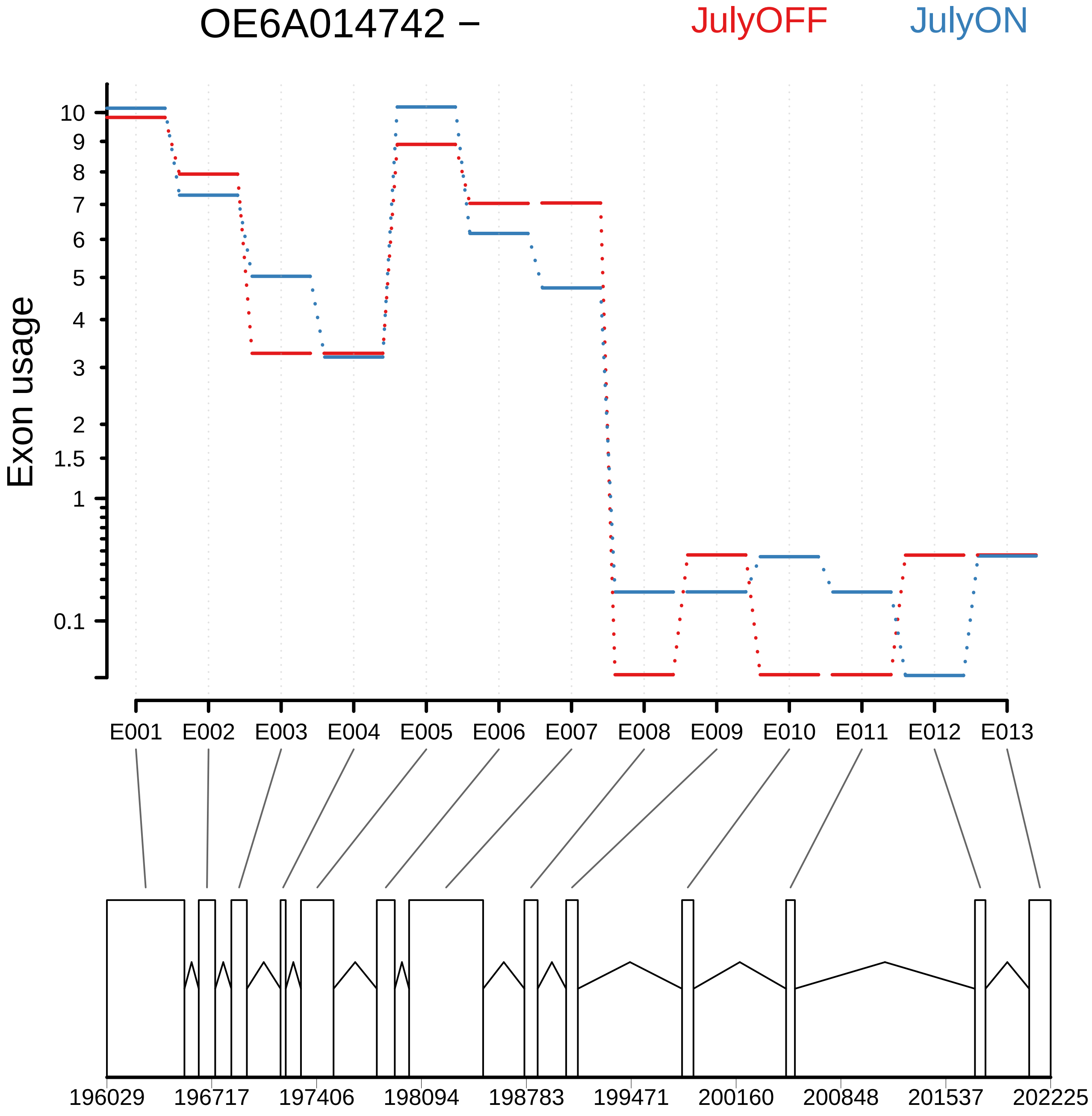
OE6A014742 −
JulyOFF
JulyON
Exon usage
10
9
8
7
6
5
4
3
2
1.5
1
0.1
E001
E002
E003
E004
E005
E006
E007
E008
E009
E010
E011
E012
E013
196029
196717
197406
198094
198783
199471
200160
200848
201537
202225

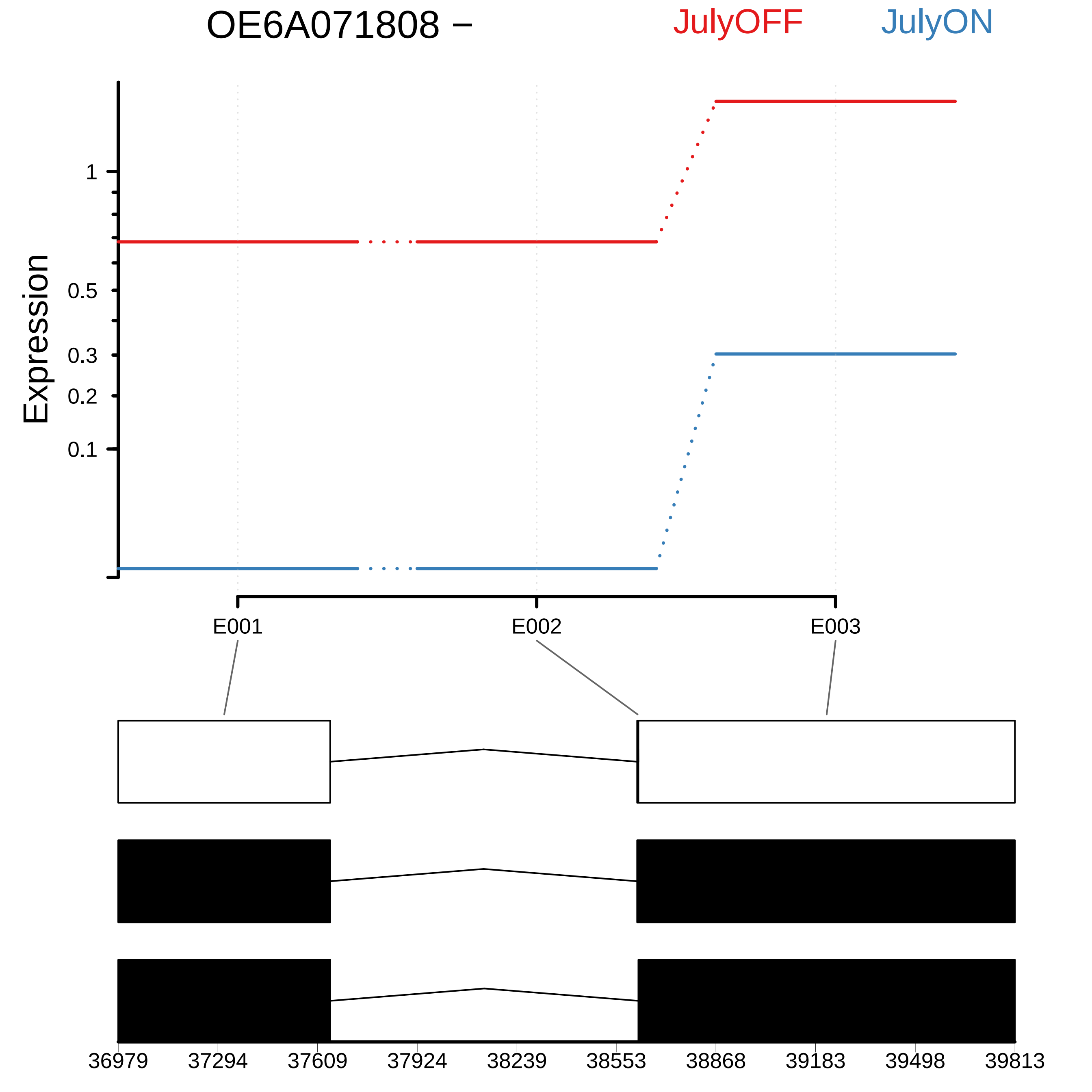
OE6A071808 −
JulyOFF
JulyON
Expression
1
0.5
0.3
0.2
0.1
E001
E002
E003
36979
37294
37609
37924
38239
38553
38868
39183
39498
39813

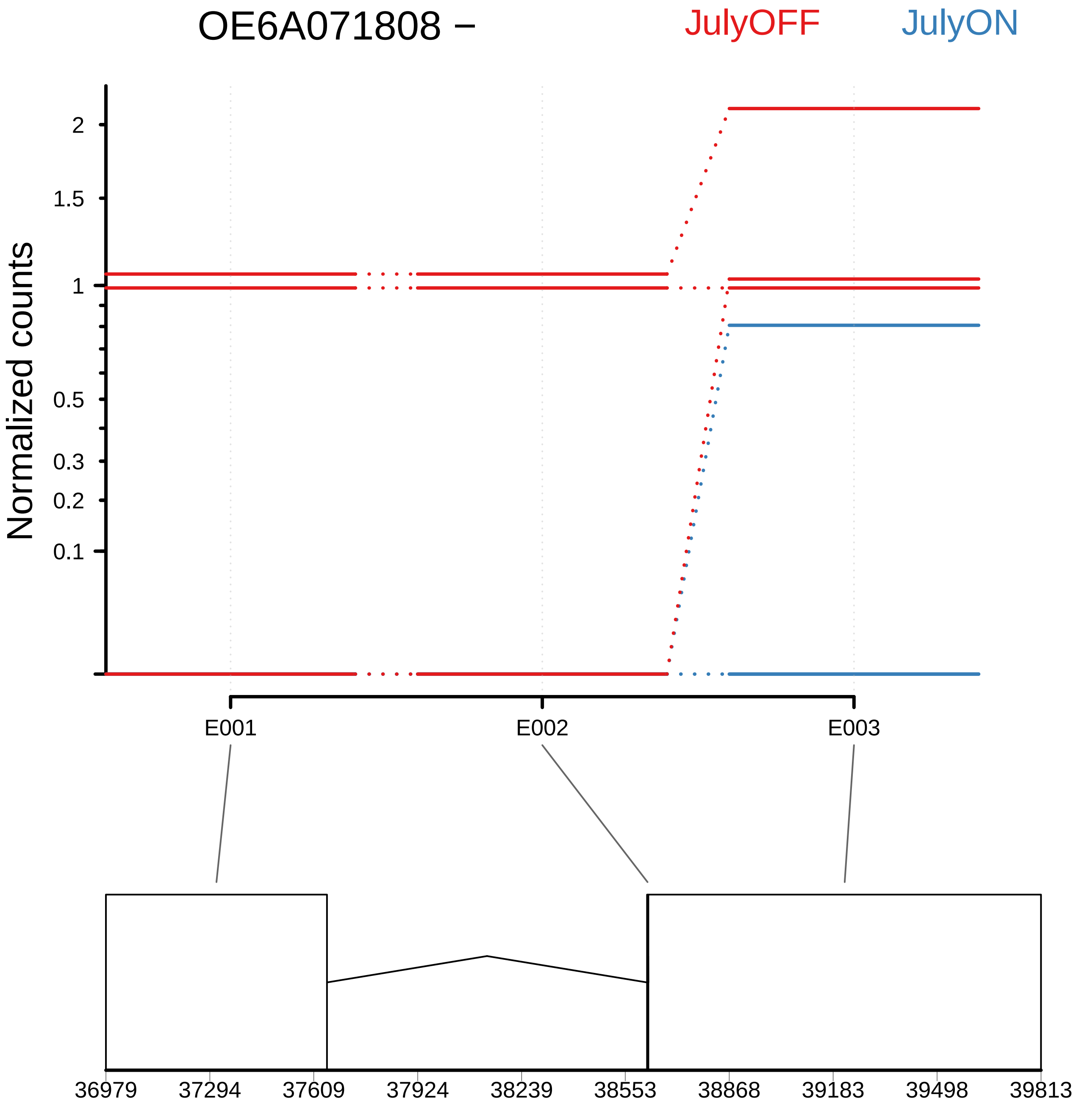

OE6A071808 −
JulyOFF
JulyON
Normalized counts
2
1.5
1
0.5
0.3
0.2
0.1
E001
E002
E003
36979
37294
37609
37924
38239
38553
38868
39183
39498
39813

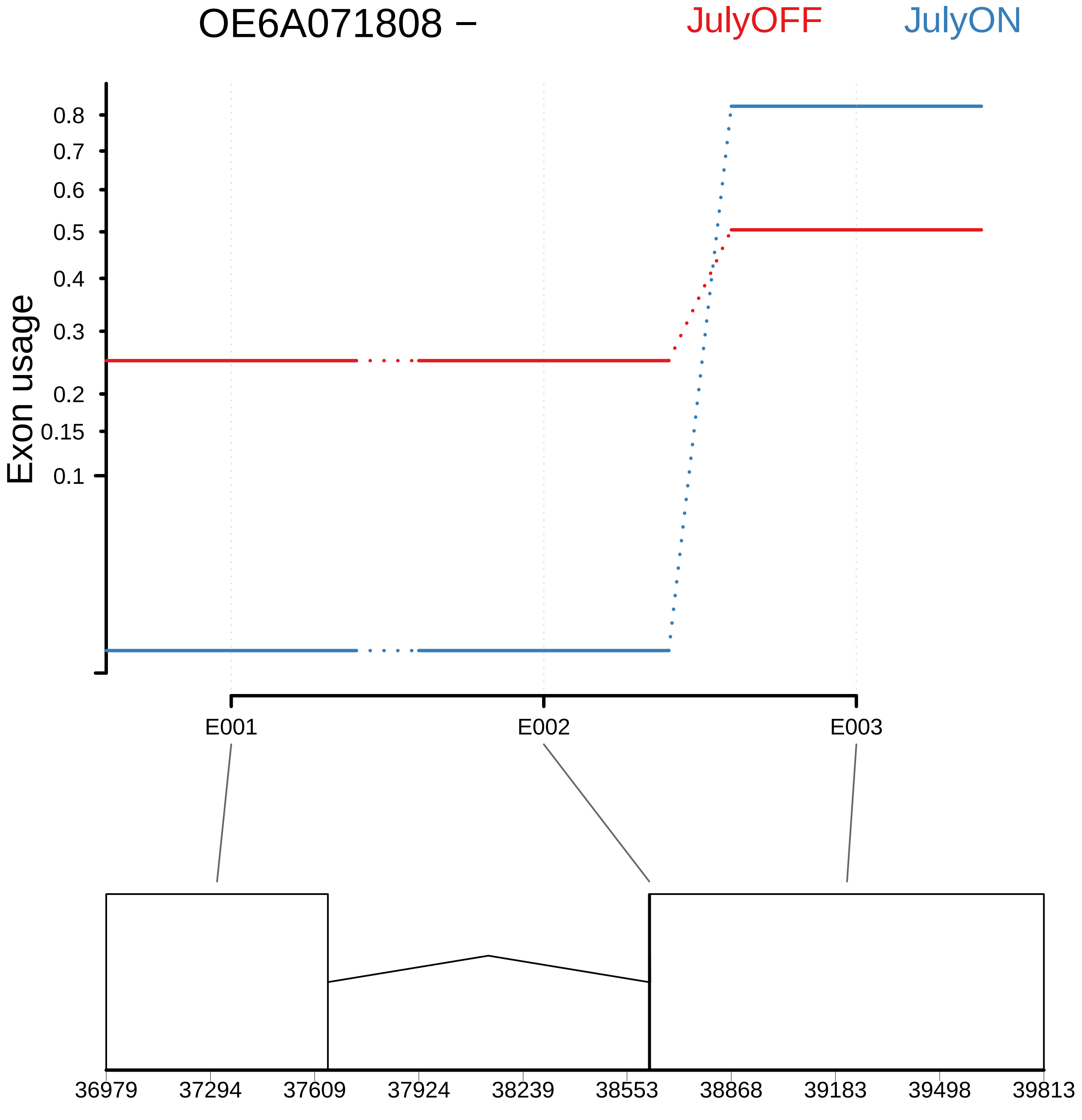
OE6A071808 −
JulyOFF
JulyON
Exon usage
0.8
0.7
0.6
0.5
0.4
0.3
0.2
0.15
0.1
E001
E002
E003
36979
37294
37609
37924
38239
38553
38868
39183
39498
39813

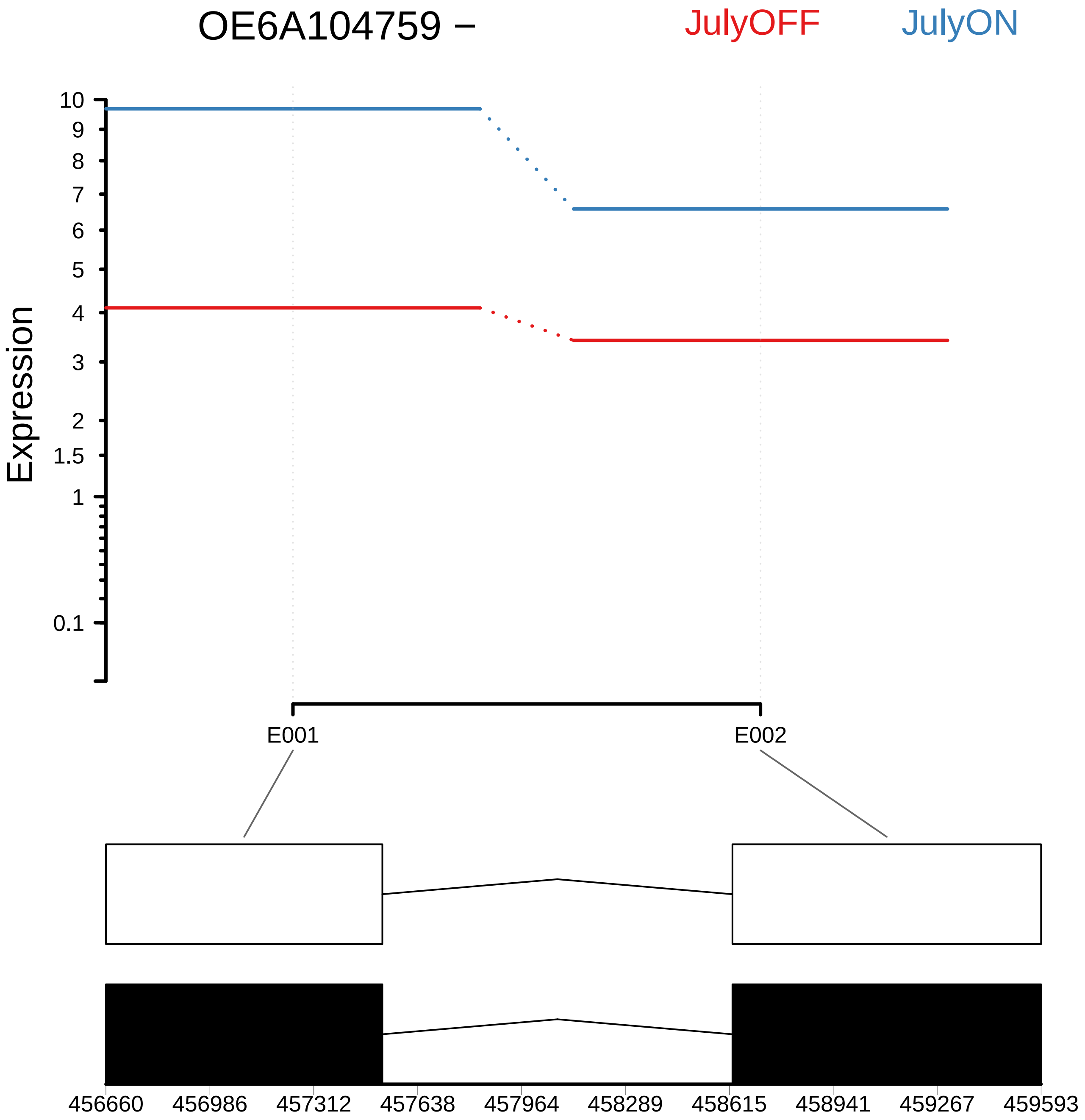

OE6A104759 −
JulyOFF
JulyON
Expression
10
9
8
7
6
5
4
3
2
1.5
1
0.1
E001
E002
456660
456986
457312
457638
457964
458289
458615
458941
459267
459593

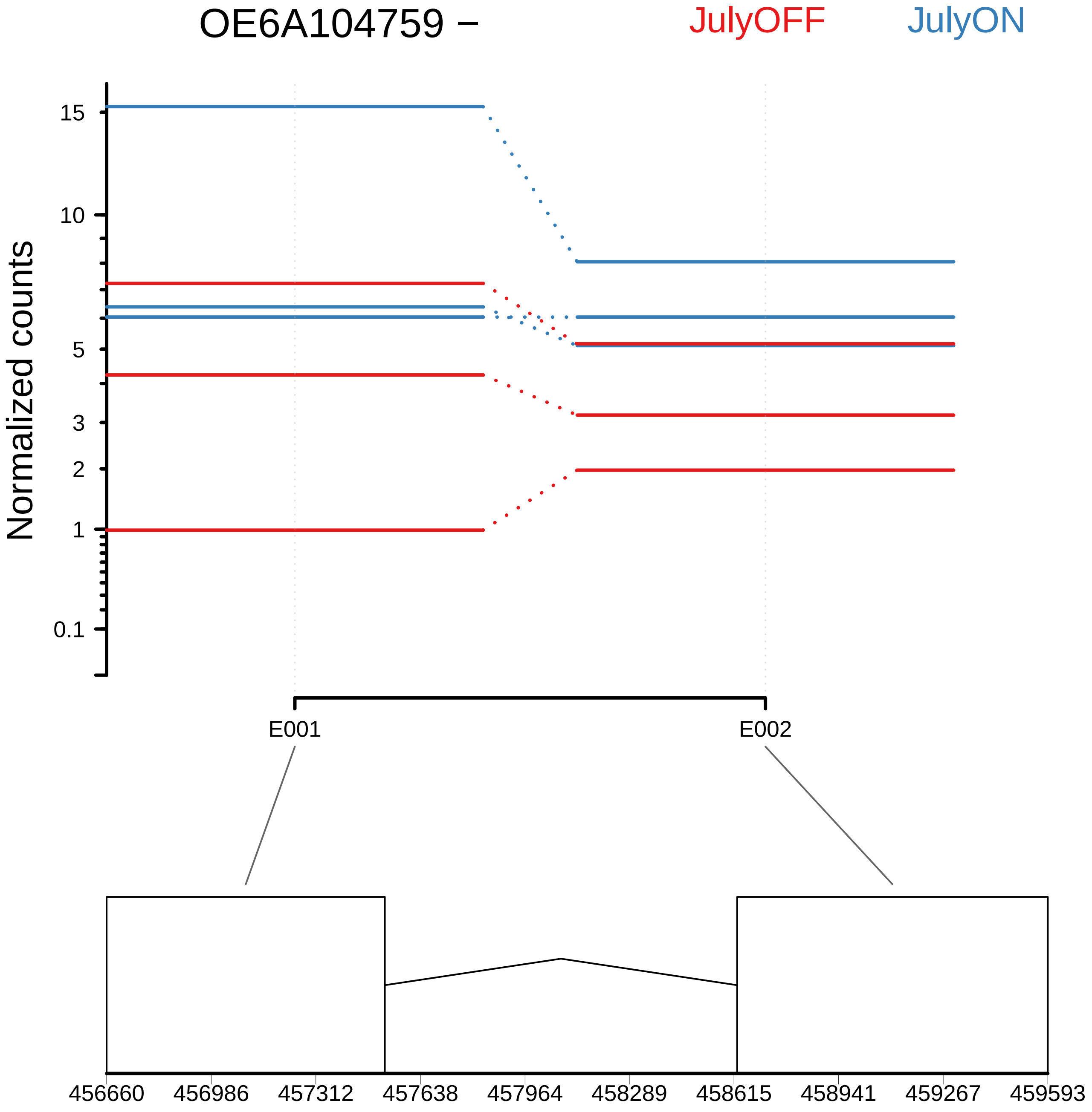

OE6A104759 −
JulyOFF
JulyON
Normalized counts
15
10
5
3
2
1
0.1
E001
E002
456660
456986
457312
457638
457964
458289
458615
458941
459267
459593

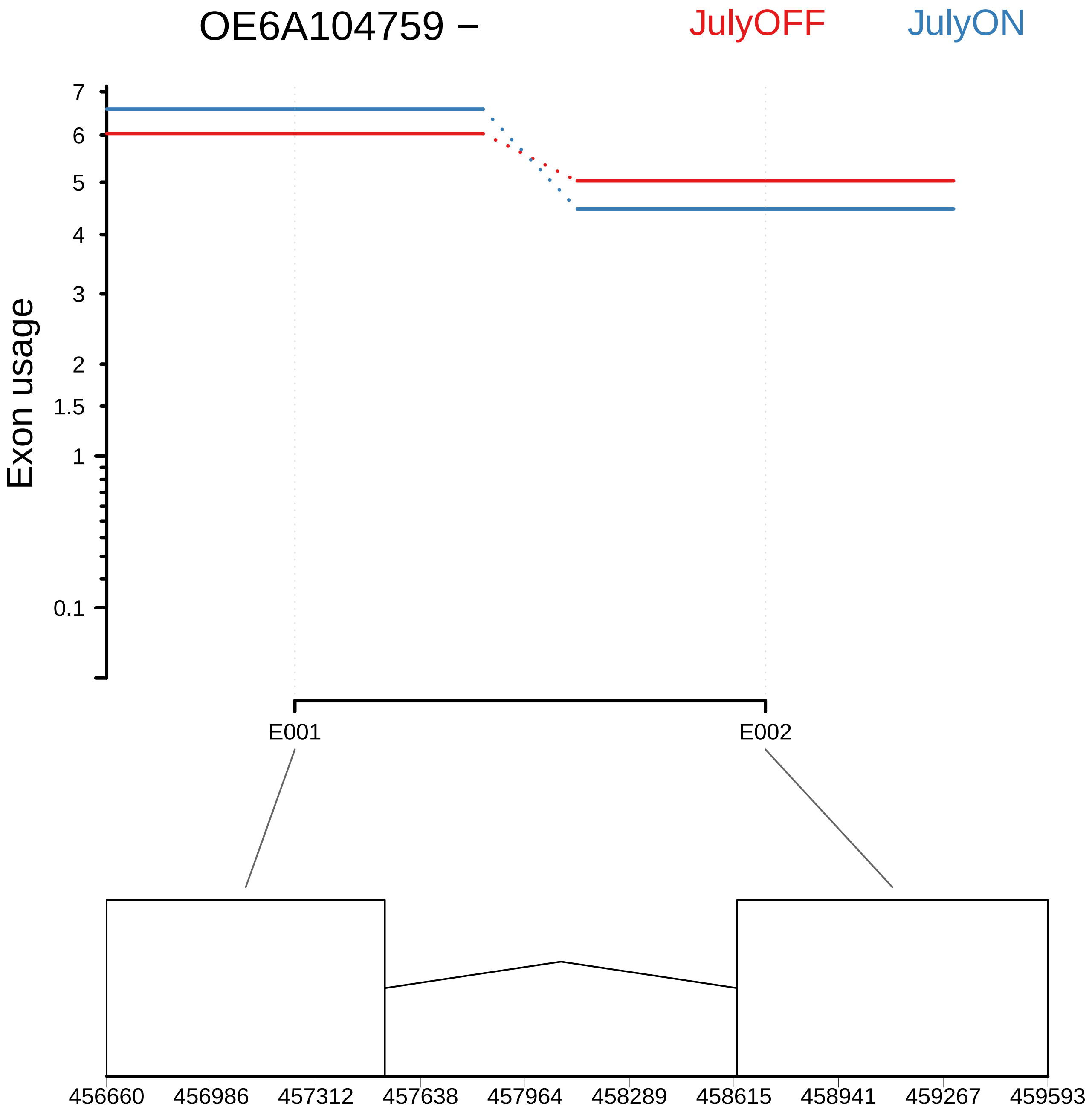

OE6A104759 −
JulyOFF
JulyON
Exon usage
7
6
5
4
3
2
1.5
1
0.1
E001
E002
456660
456986
457312
457638
457964
458289
458615
458941
459267
459593

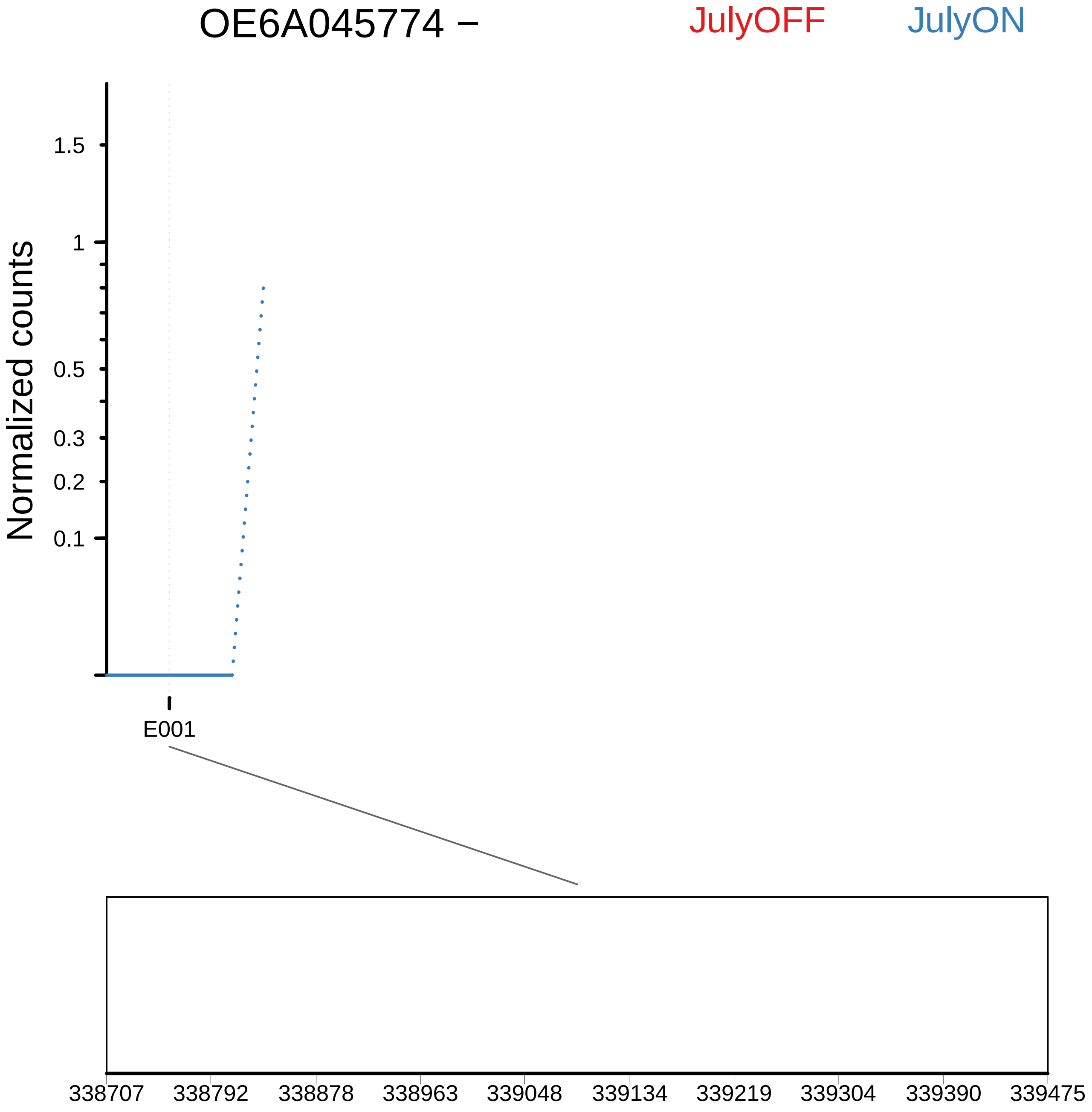

OE6A045774 −
JulyOFF
JulyON
Normalized counts
1.5
1
0.5
0.3
0.2
0.1
E001
338707
338792
338878
338963
339048
339134
339219
339304
339390
339475

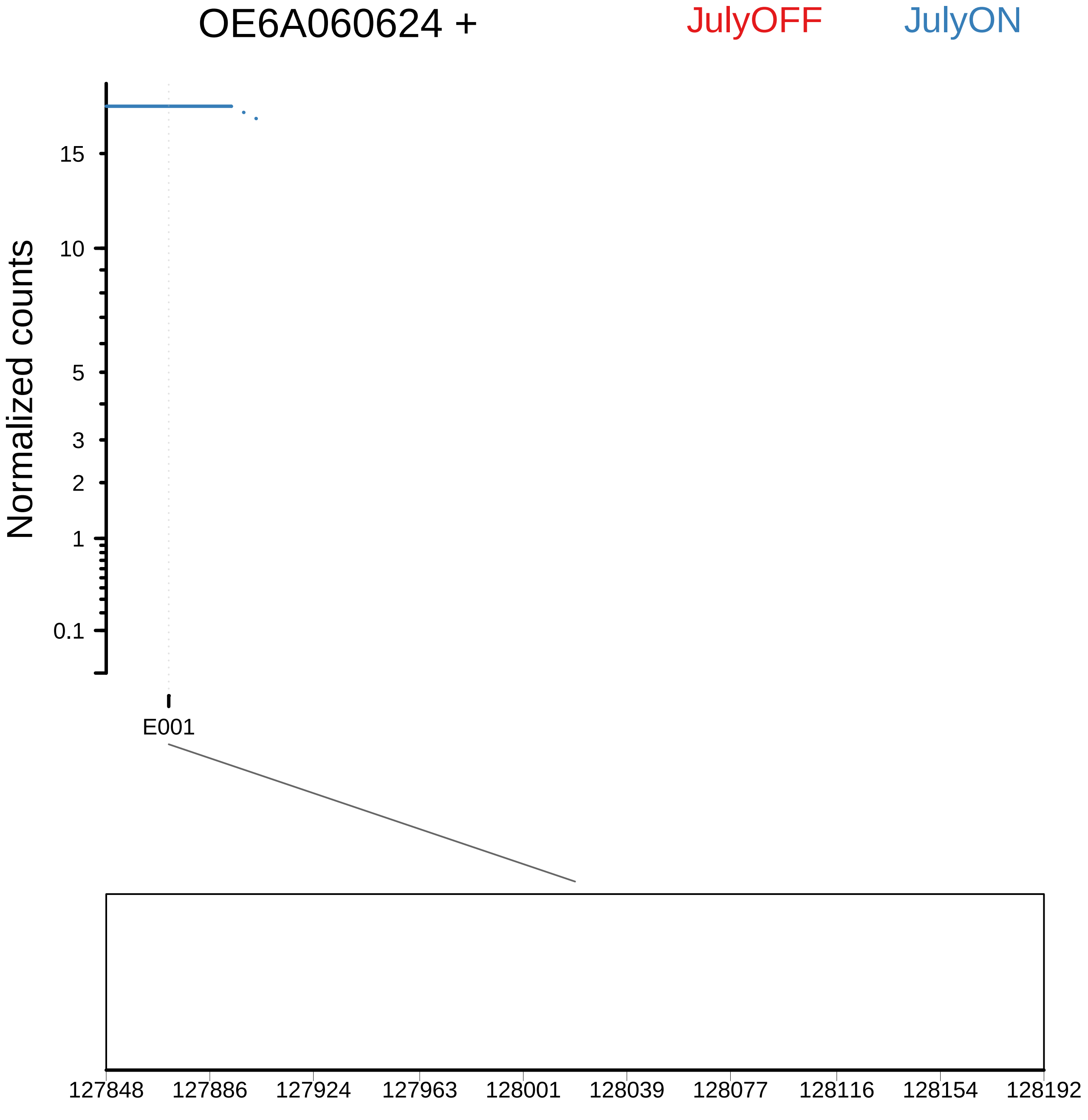
OE6A060624 +
JulyOFF
JulyON
Normalized counts
15
10
5
3
2
1
0.1
E001
127848
127886
127924
127963
128001
128039
128077
128116
128154
128192

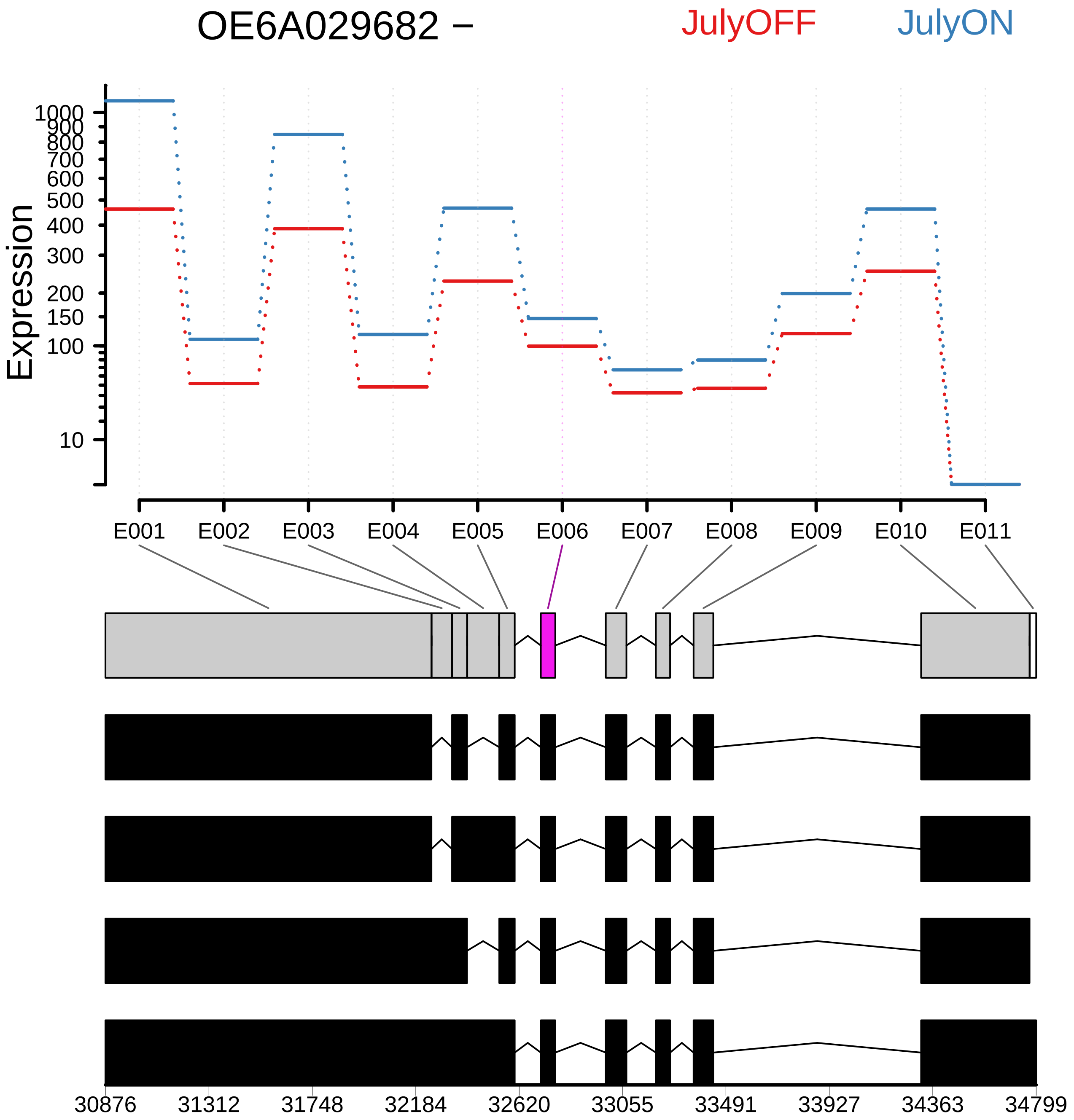

OE6A029682 –
JulyOFF
JulyON
Expression
1000
900
800
700
600
500
400
300
200
150
100
10
E001
E002
E003
E004
E005
E006
E007
E008
E009
E010
E011
30876
31312
31748
32184
32620
33055
33491
33927
34363
34799

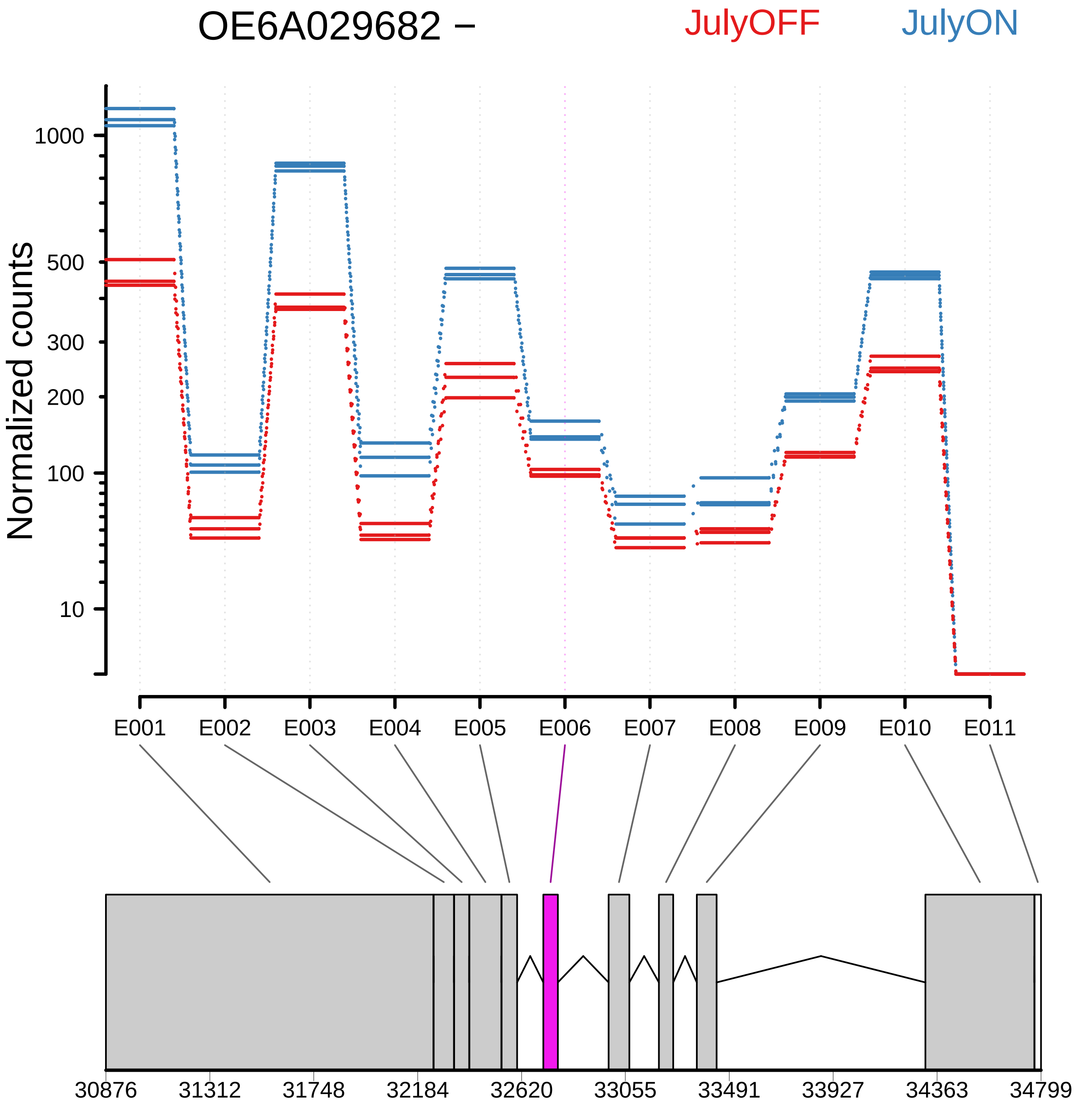
OE6A029682 –
JulyOFF
JulyON
Normalized counts
1000
500
300
200
100
10
E001
E002
E003
E004
E005
E006
E007
E008
E009
E010
E011
30876
31312
31748
32184
32620
33055
33491
33927
34363
34799

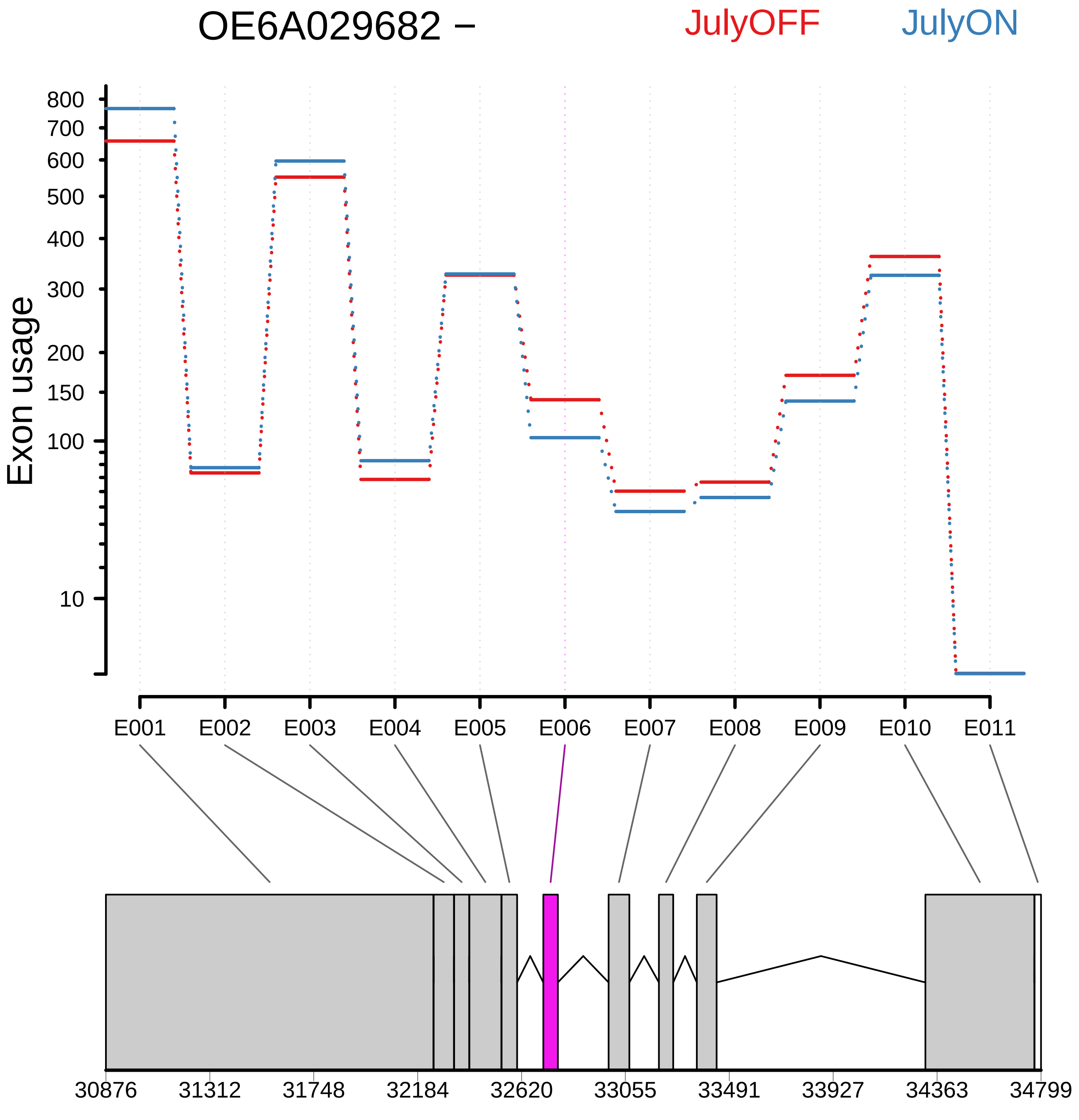

OE6A029682 −
JulyOFF
JulyON
Exon usage
800
700
600
500
400
300
200
150
100
10
E001
E002
E003
E004
E005
E006
E007
E008
E009
E010
E011
30876
31312
31748
32184
32620
33055
33491
33927
34363
34799

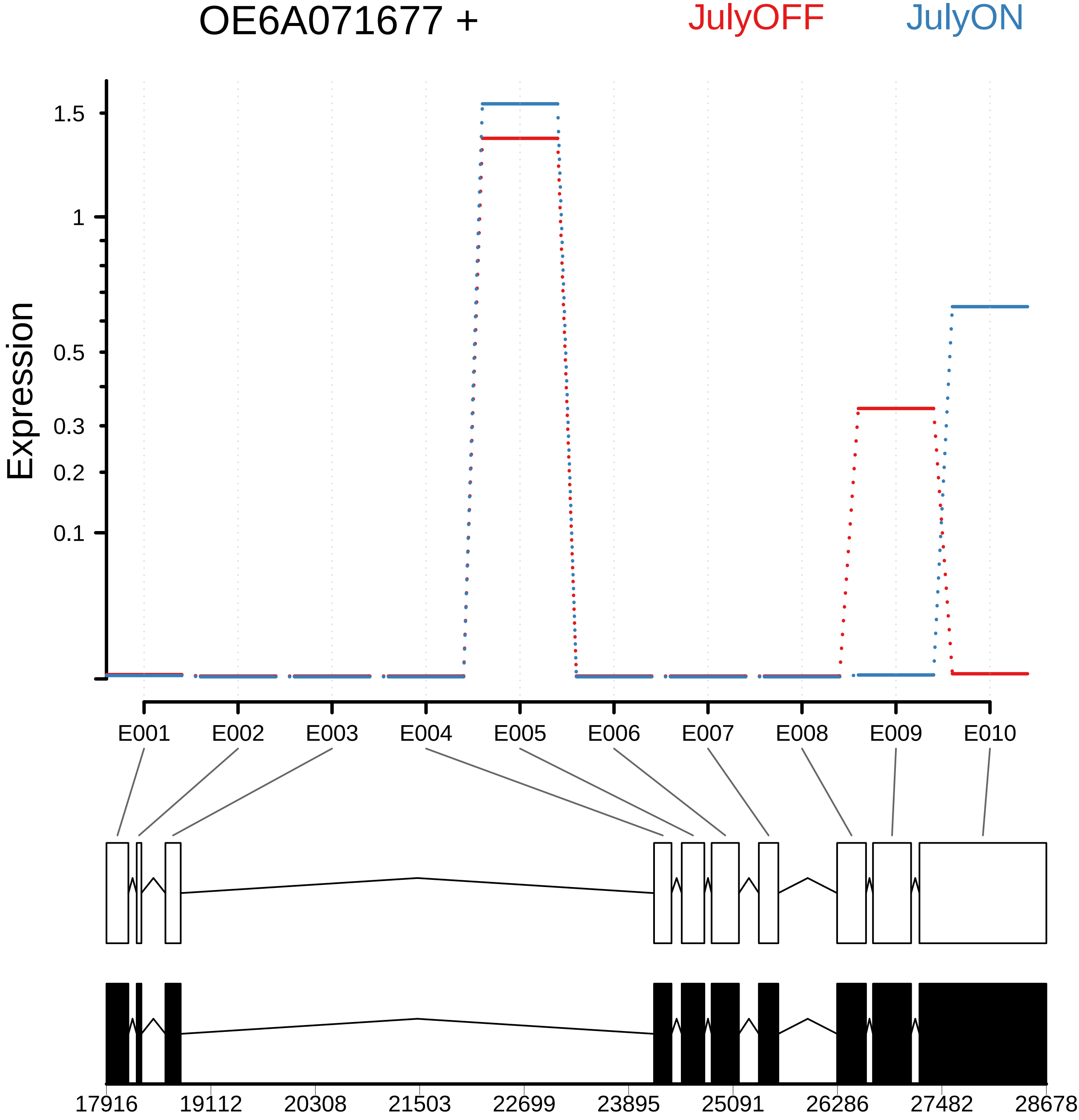

OE6A071677 +
JulyOFF
JulyON
Expression
1.5
1
0.5
0.3
0.2
0.1
E001
E002
E003
E004
E005
E006
E007
E008
E009
E010
17916
19112
20308
21503
22699
23895
25091
26286
27482
28678

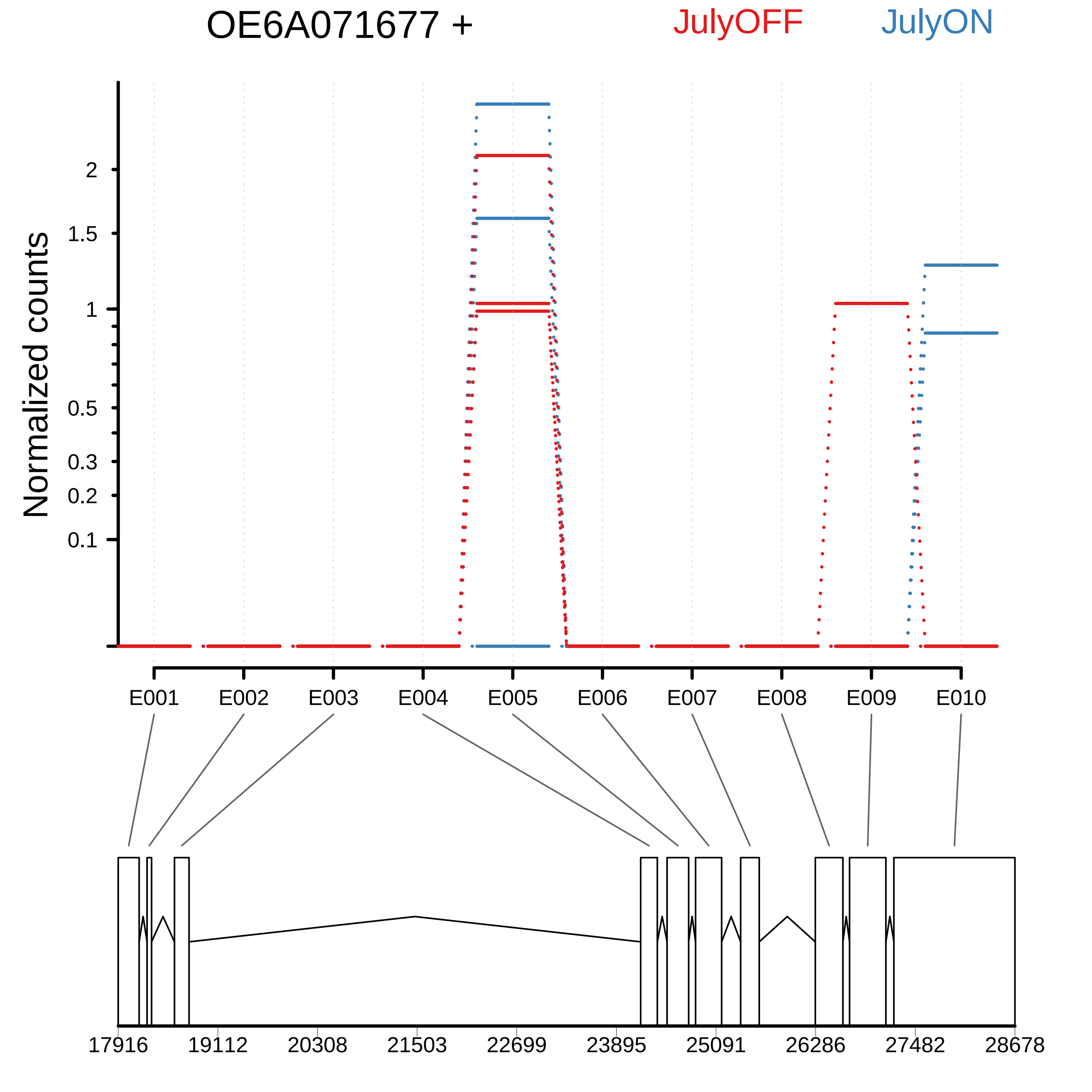

OE6A071677 +
JulyOFF
JulyON
Normalized counts
2
1.5
1
0.5
0.3
0.2
0.1
E001
E002
E003
E004
E005
E006
E007
E008
E009
E010
17916
19112
20308
21503
22699
23895
25091
26286
27482
28678

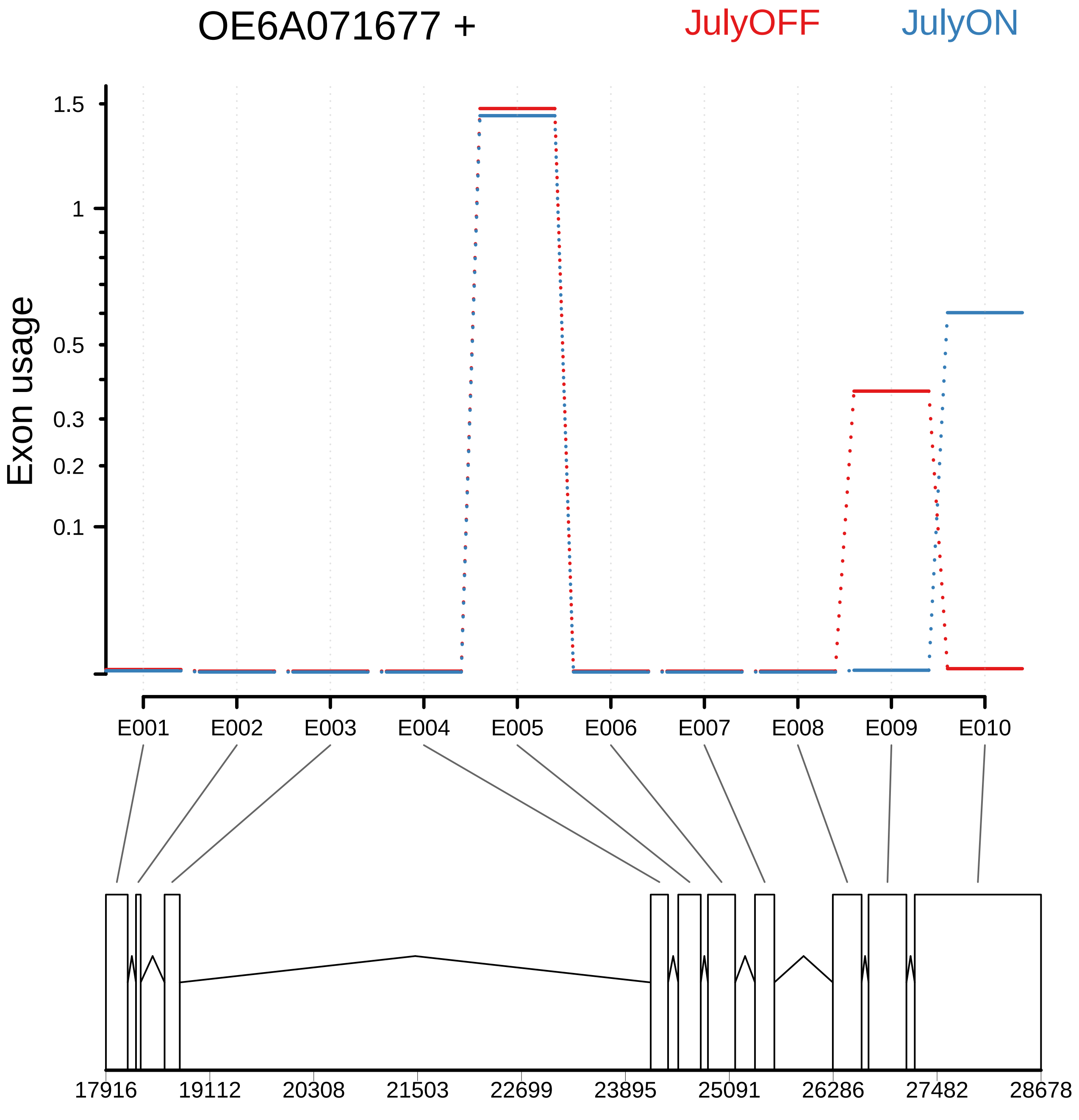
OE6A071677 +
JulyOFF
JulyON
Exon usage
1.5
1
0.5
0.3
0.2
0.1
E001
E002
E003
E004
E005
E006
E007
E008
E009
E010
17916
19112
20308
21503
22699
23895
25091
26286
27482
28678

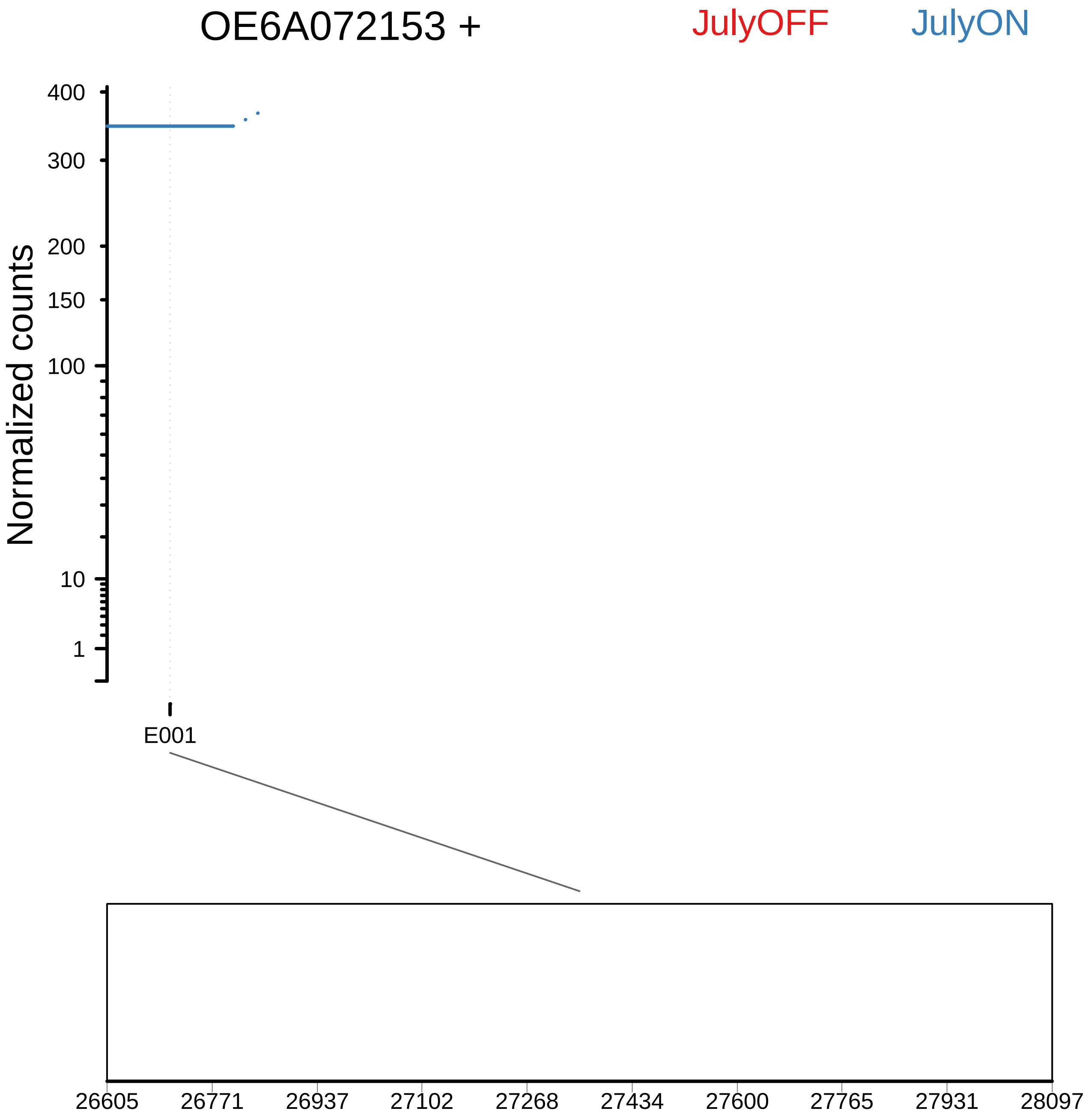
OE6A072153 +
JulyOFF
JulyON
Normalized counts
400
300
200
150
100
10
1
E001
26605
26771
26937
27102
27268
27434
27600
27765
27931
28097

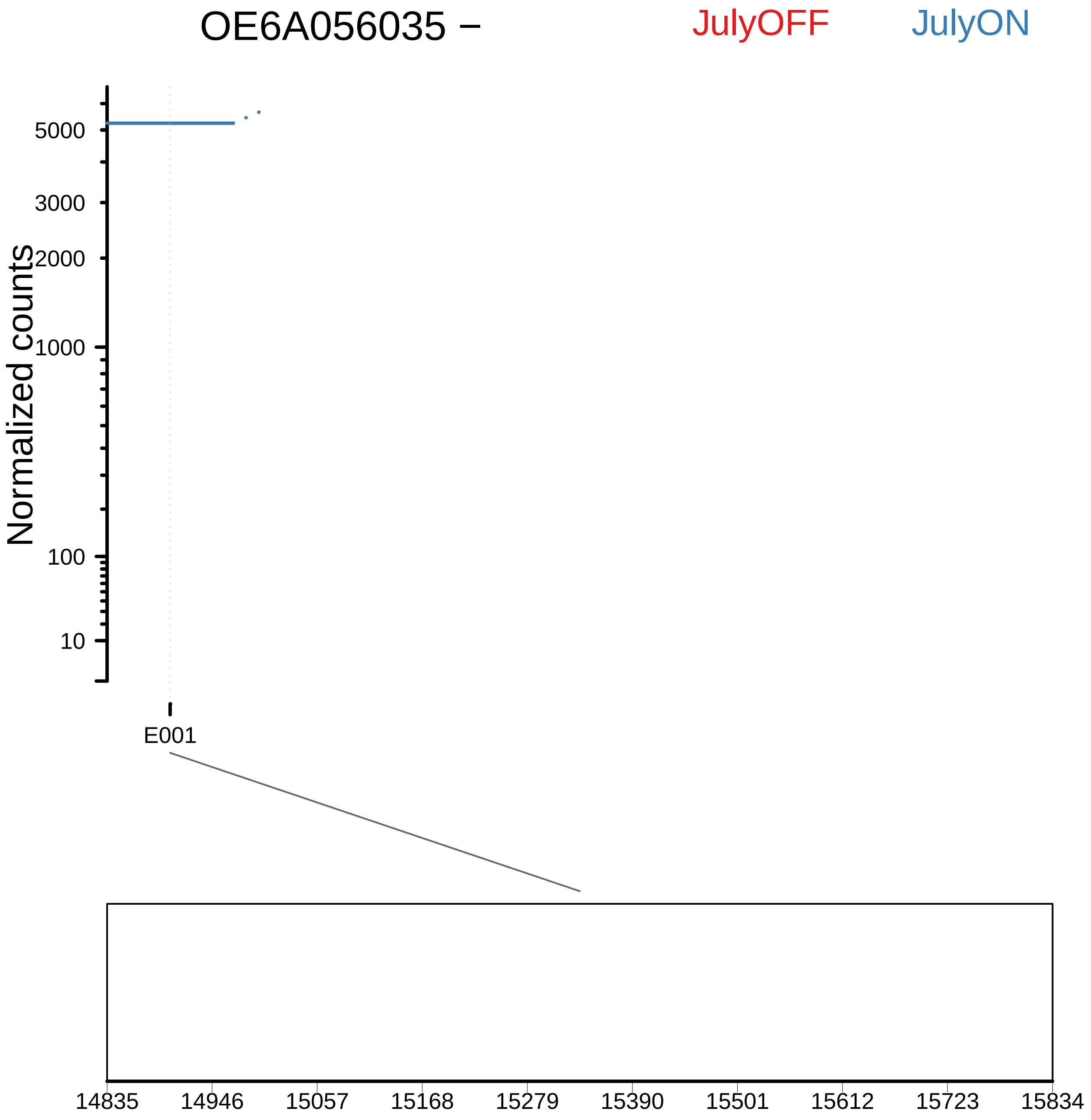
OE6A056035 −
JulyOFF
JulyON
Normalized counts
5000
3000
2000
1000
100
10
E001
14835
14946
15057
15168
15279
15390
15501
15612
15723
15834

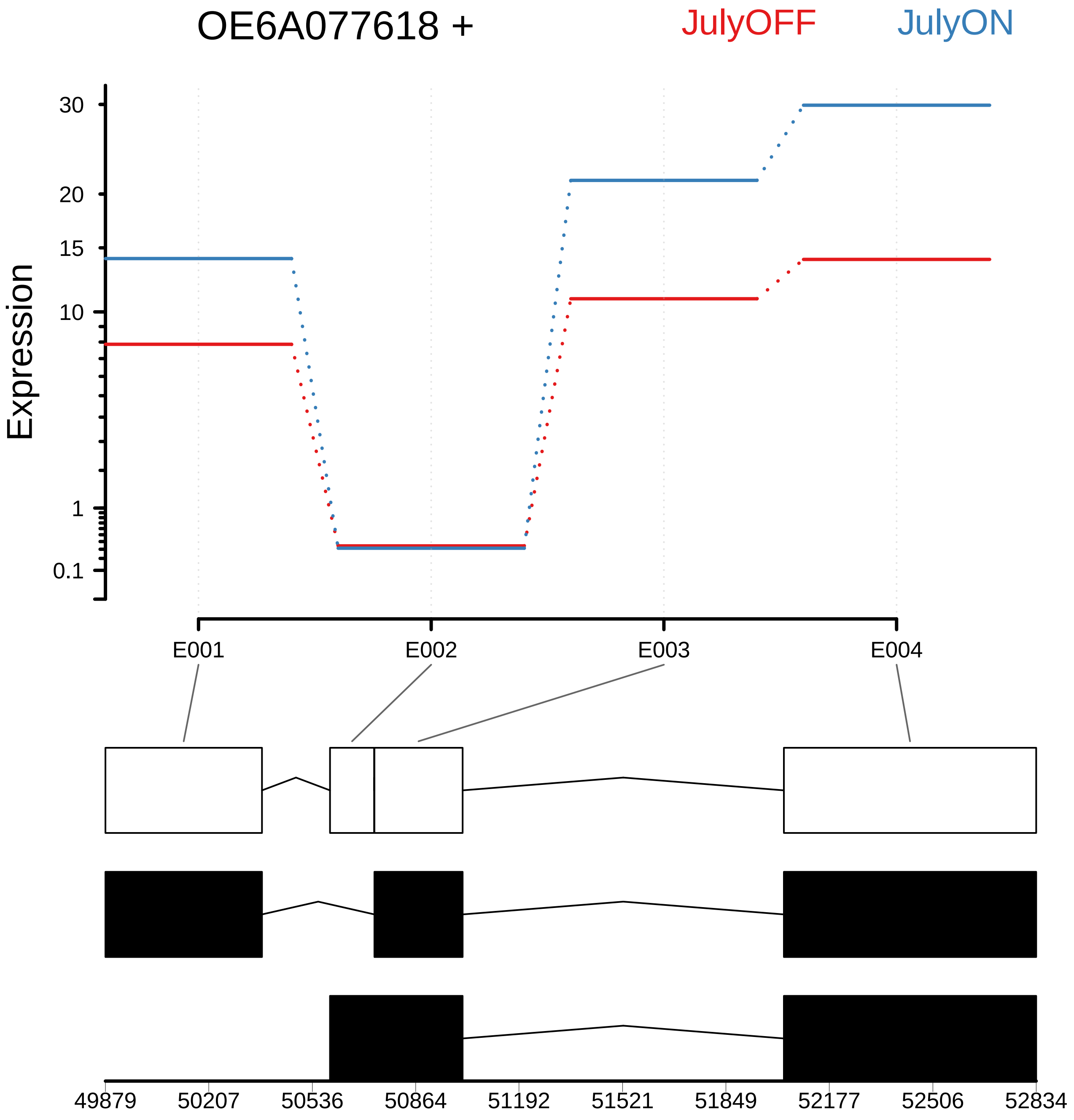

OE6A077618 +
JulyOFF
JulyON
Expression
30
20
15
10
1
0.1
E001
E002
E003
E004
49879
50207
50536
50864
51192
51521
51849
52177
52506
52834

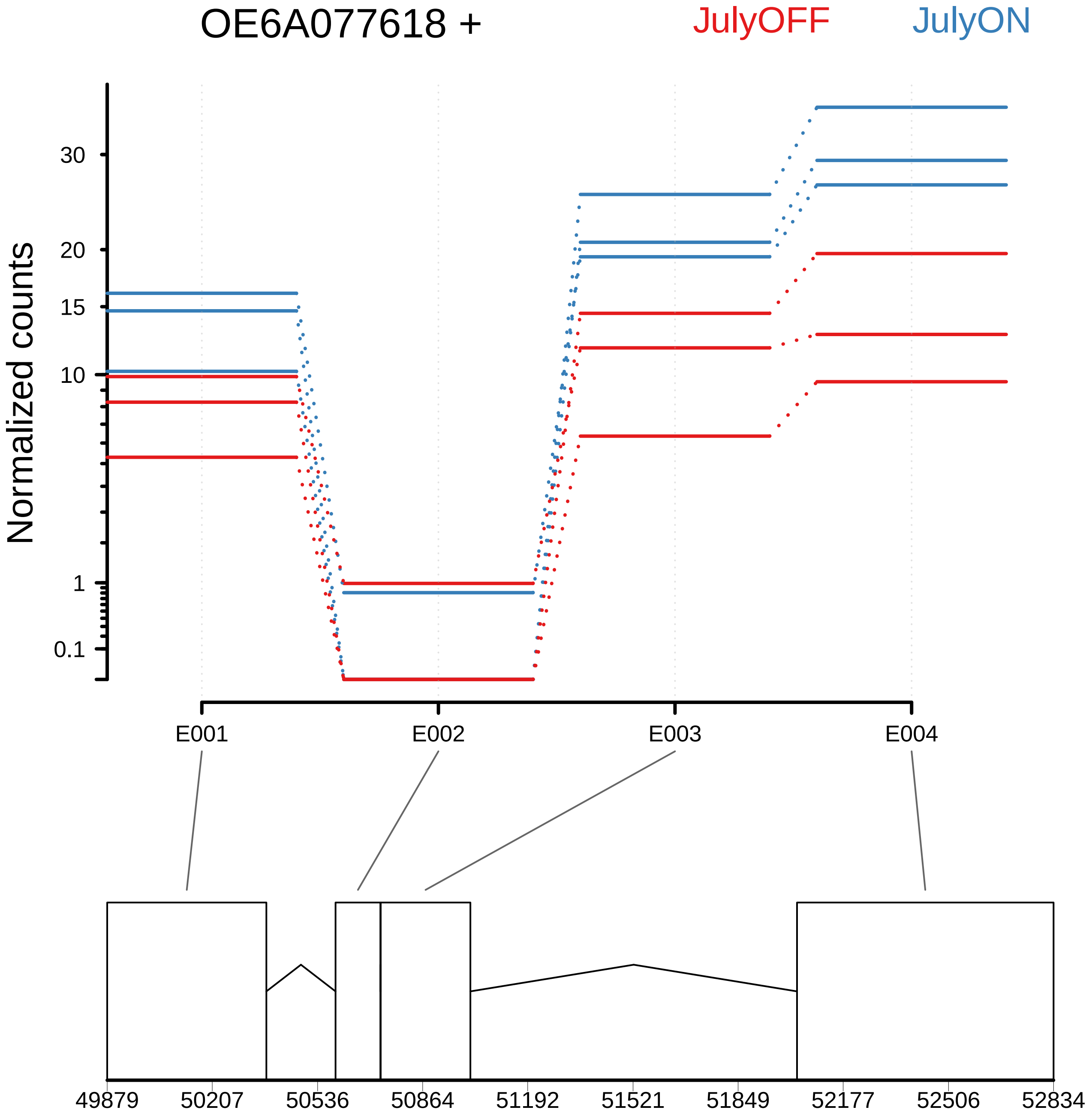
OE6A077618 +
JulyOFF
JulyON
Normalized counts
30
20
15
10
1
0.1
E001
E002
E003
E004
49879
50207
50536
50864
51192
51521
51849
52177
52506
52834

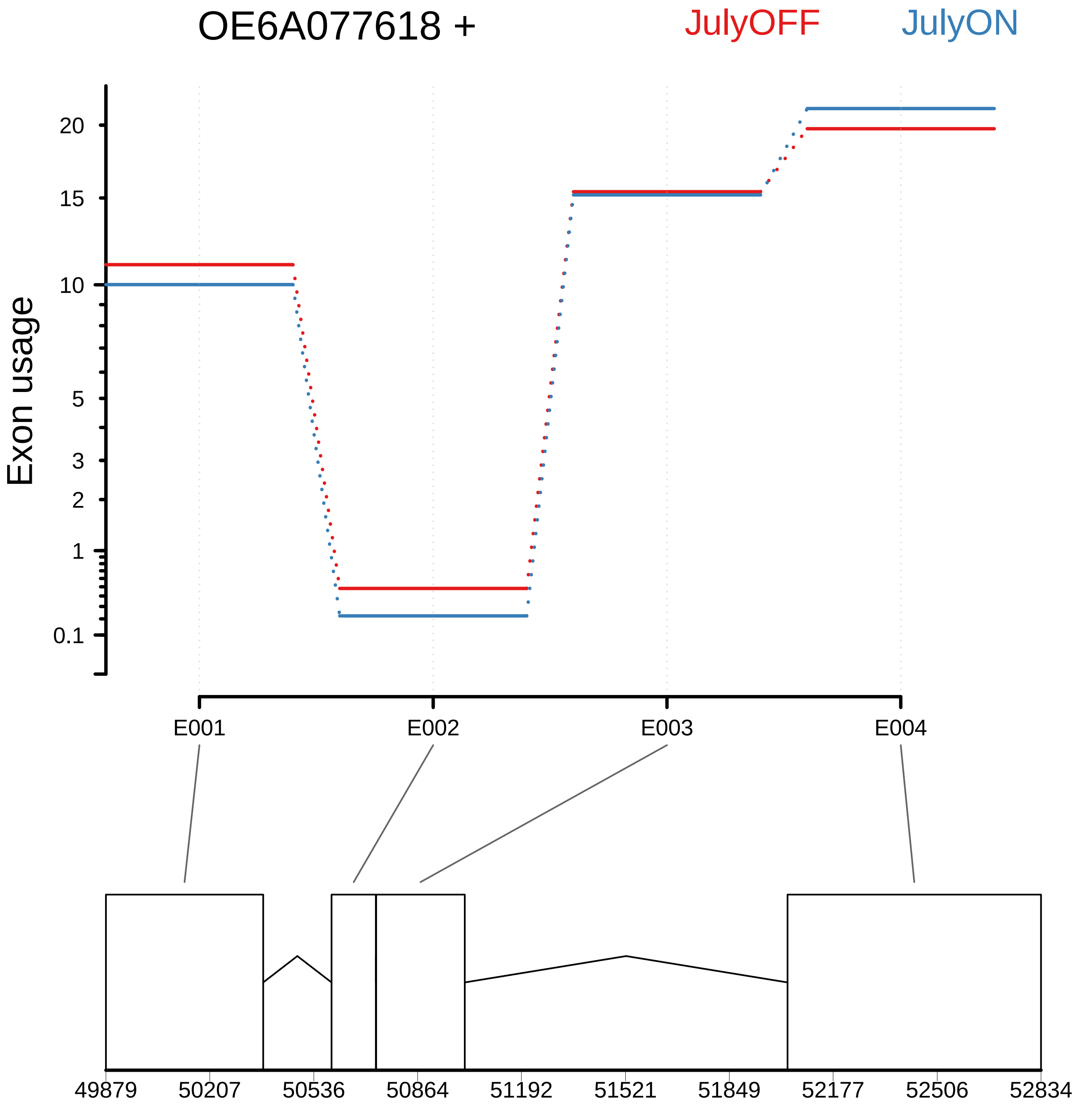

OE6A077618 +
JulyOFF
JulyON
Exon usage
20
15
10
5
3
2
1
0.1
E001
E002
E003
E004
49879
50207
50536
50864
51192
51521
51849
52177
52506
52834

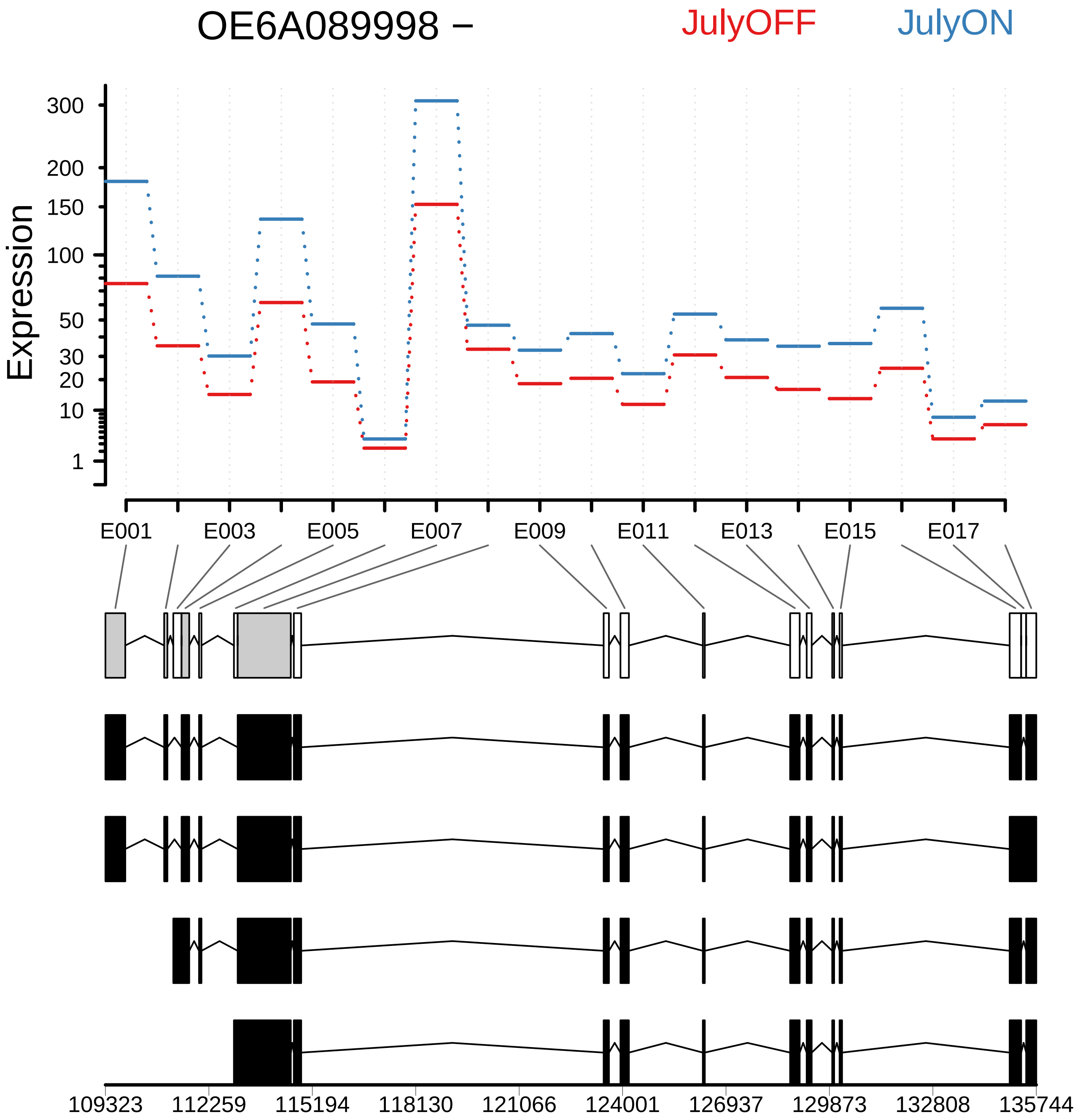

OE6A089998 –
JulyOFF
JulyON
Expression
300
200
150
100
50
30
20
10
1
E001
E003
E005
E007
E009
E011
E013
E015
E017
109323
112259
115194
118130
121066
124001
126937
129873
132808
135744

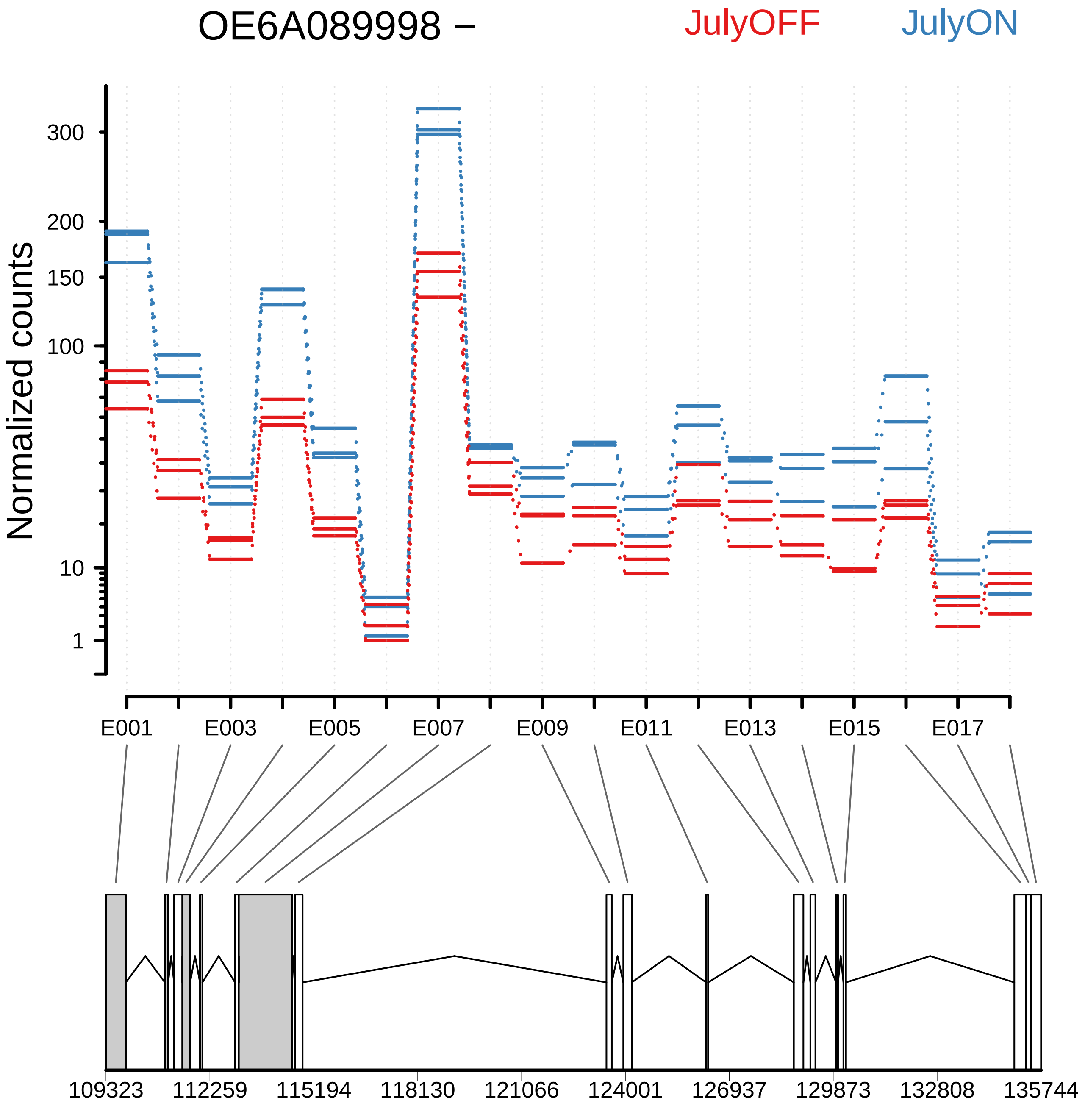
OE6A089998 –
JulyOFF
JulyON
Normalized counts
300
200
150
100
10
1
E001
E003
E005
E007
E009
E011
E013
E015
E017
109323
112259
115194
118130
121066
124001
126937
129873
132808
135744

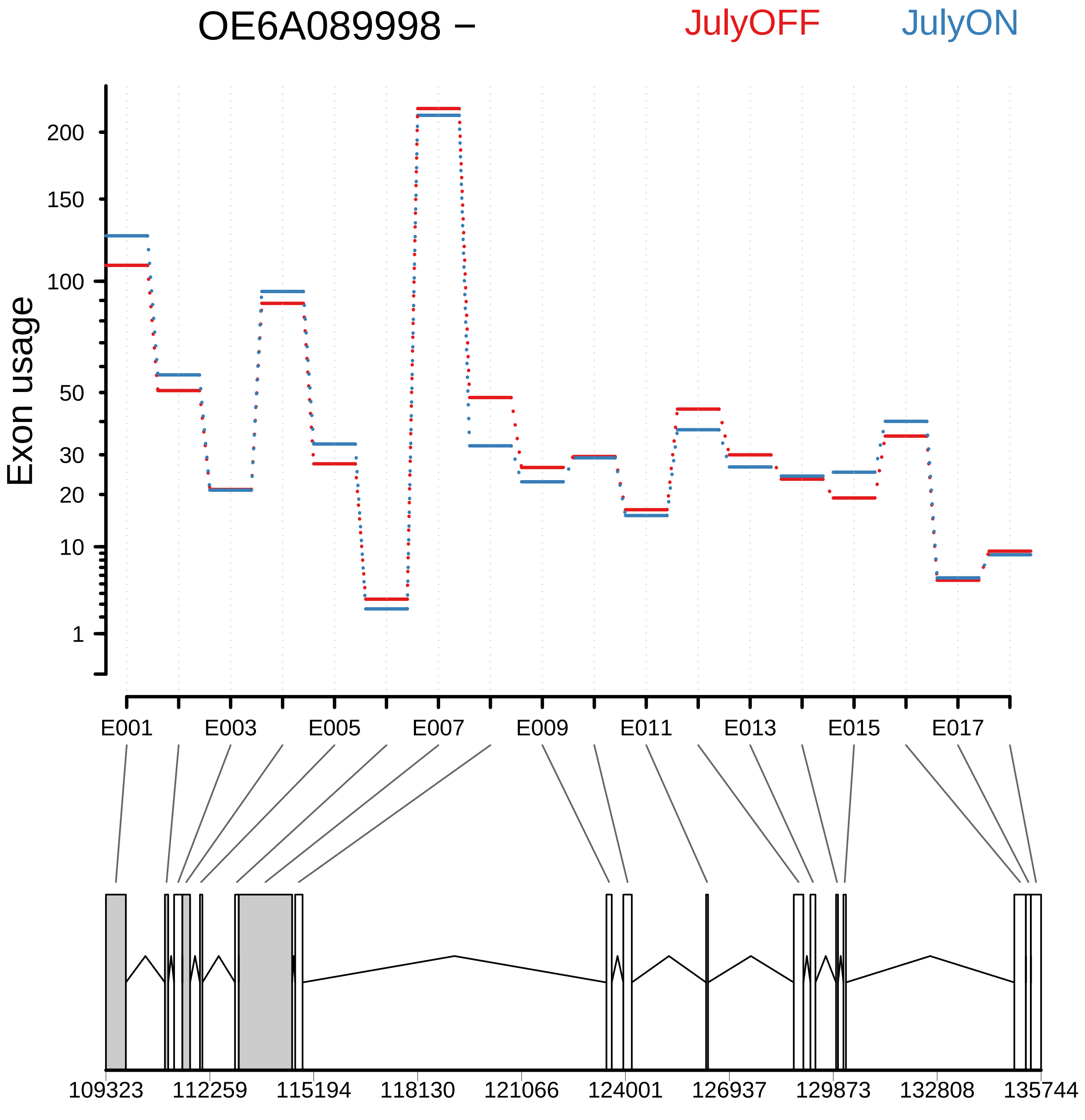

OE6A089998 –
JulyOFF
JulyON
Exon usage
200
150
100
50
30
20
10
1
E001
E003
E005
E007
E009
E011
E013
E015
E017
109323
112259
115194
118130
121066
124001
126937
129873
132808
135744

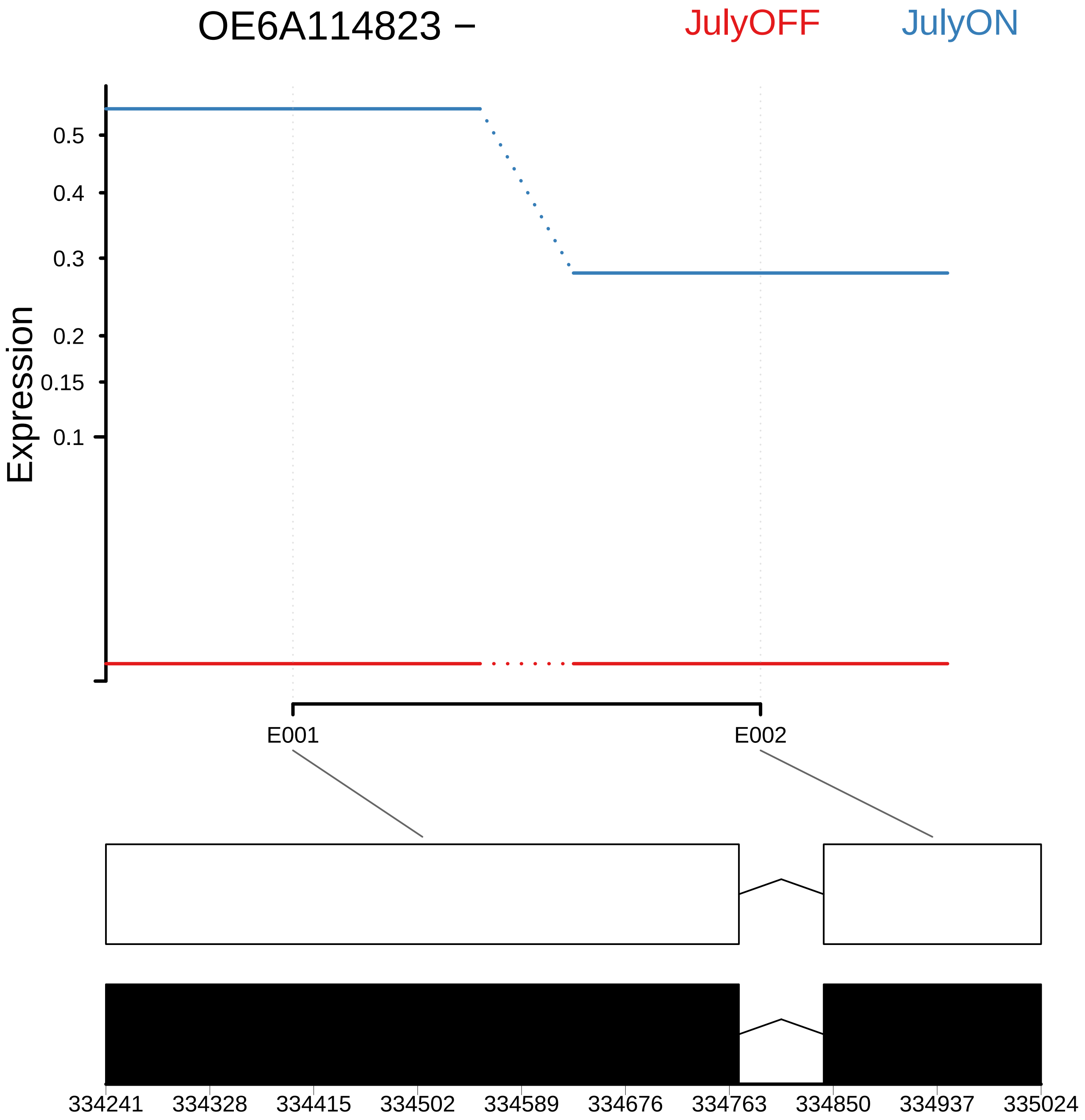

OE6A114823 −
JulyOFF
JulyON
Expression
0.5
0.4
0.3
0.2
0.15
0.1
E001
E002
334241
334328
334415
334502
334589
334676
334763
334850
334937
335024

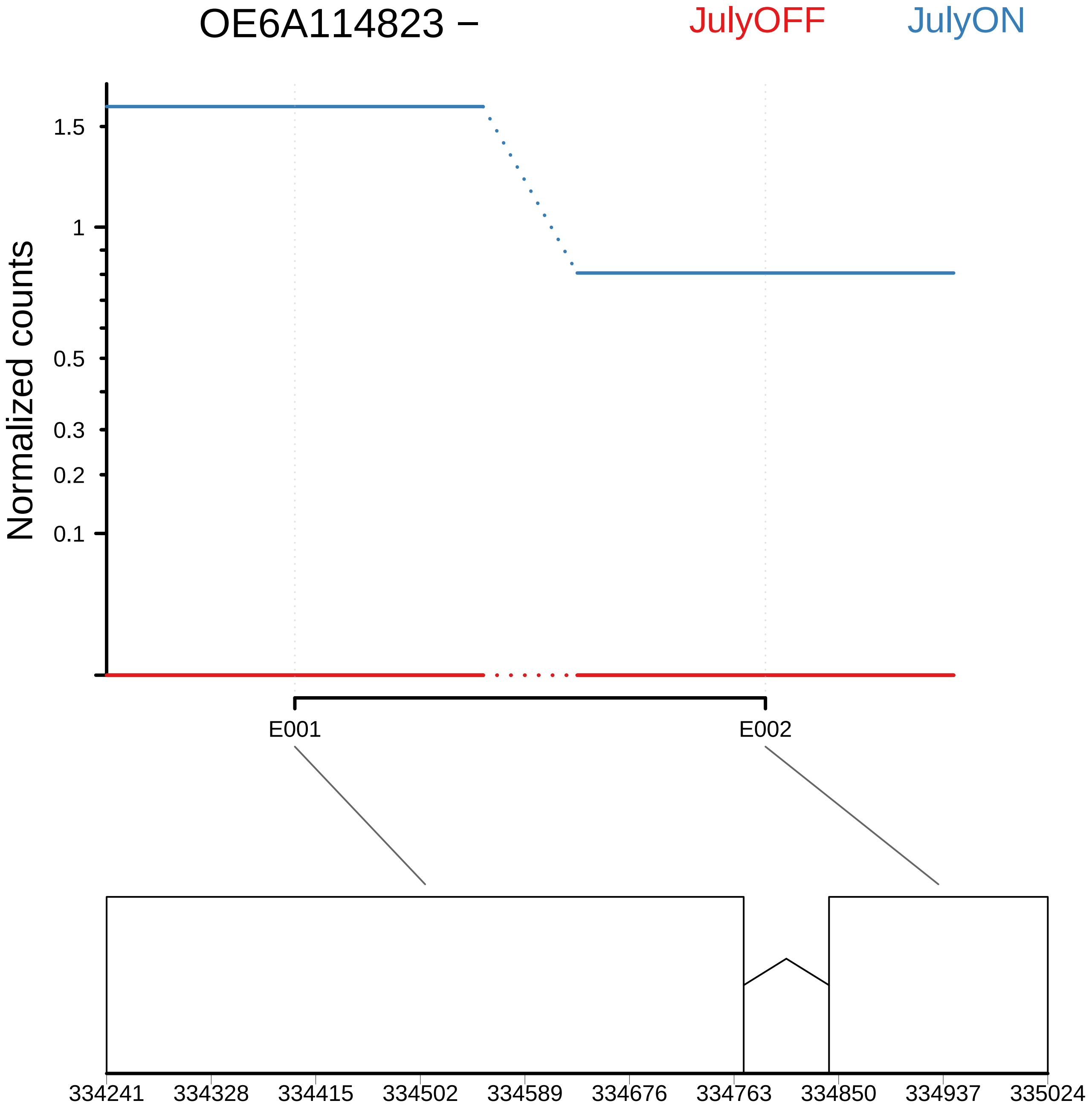

OE6A114823 −
JulyOFF
JulyON
Normalized counts
1.5
1
0.5
0.3
0.2
0.1
E001
E002
334241
334328
334415
334502
334589
334676
334763
334850
334937
335024

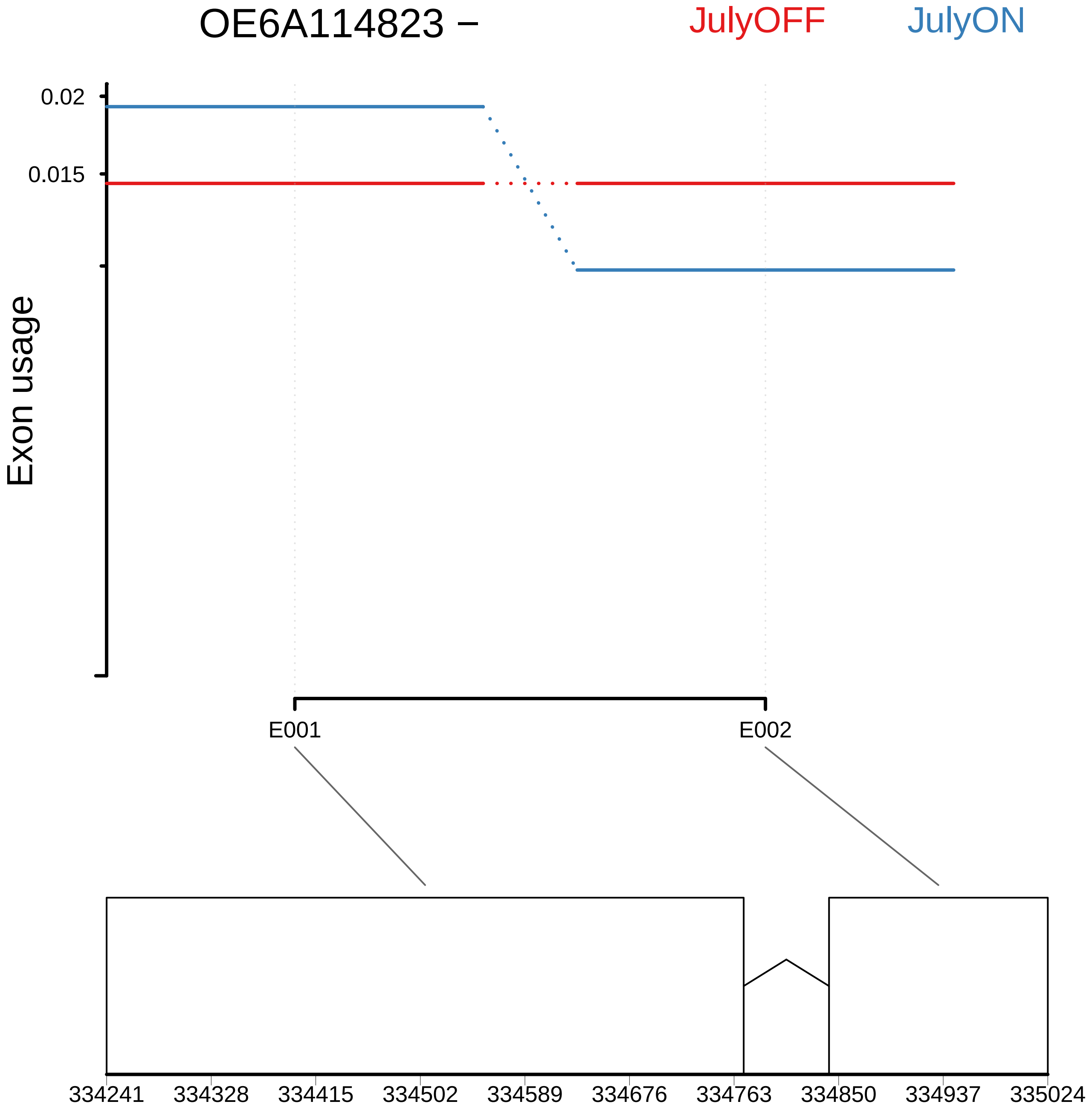
OE6A114823 −
JulyOFF
JulyON
Exon usage
0.02
0.015
E001
E002
334241
334328
334415
334502
334589
334676
334763
334850
334937
335024

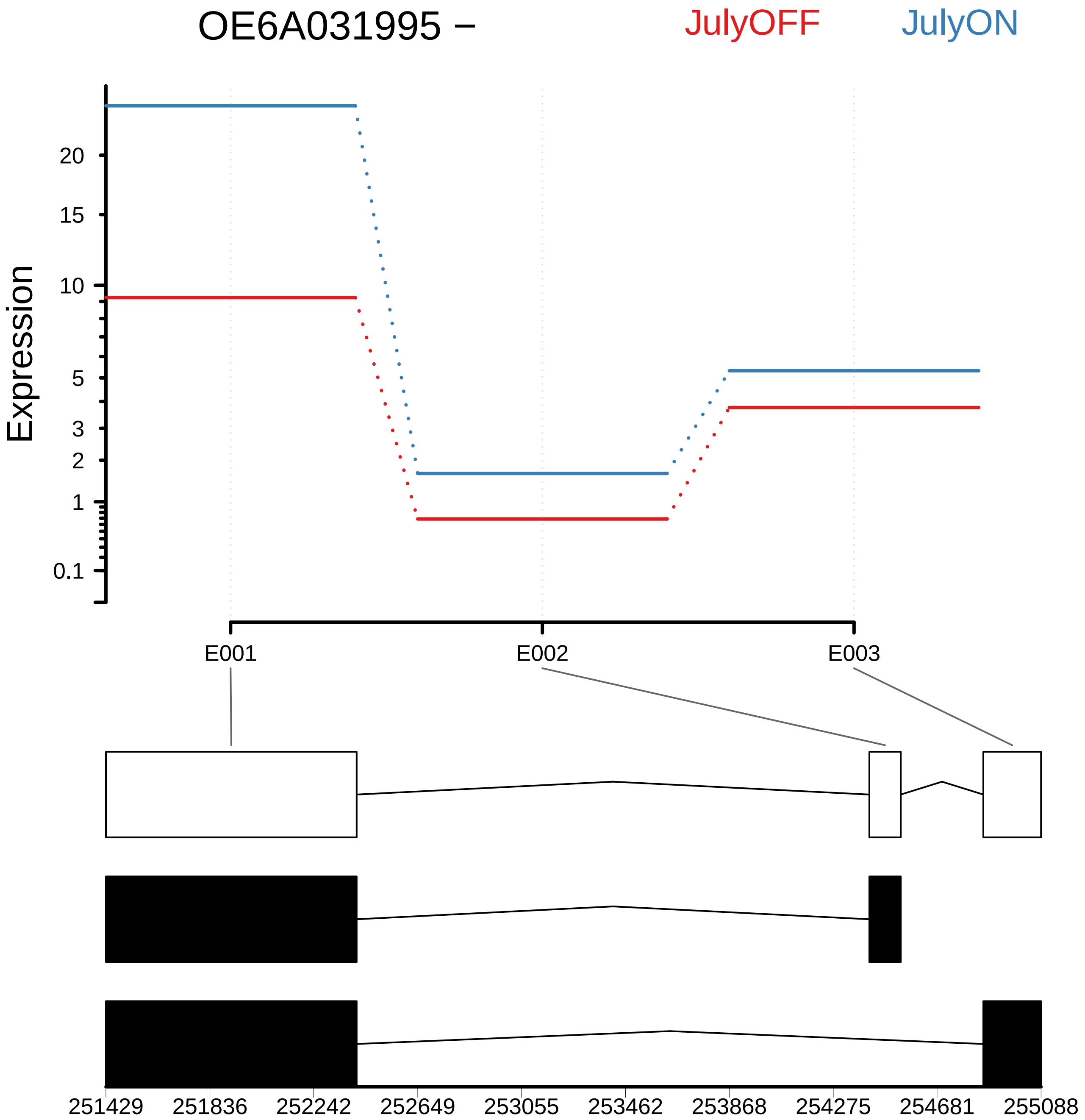

OE6A031995 –
JulyOFF
JulyON
Expression
20
15
10
5
3
2
1
0.1
E001
E002
E003
251429
251836
252242
252649
253055
253462
253868
254275
254681
255088

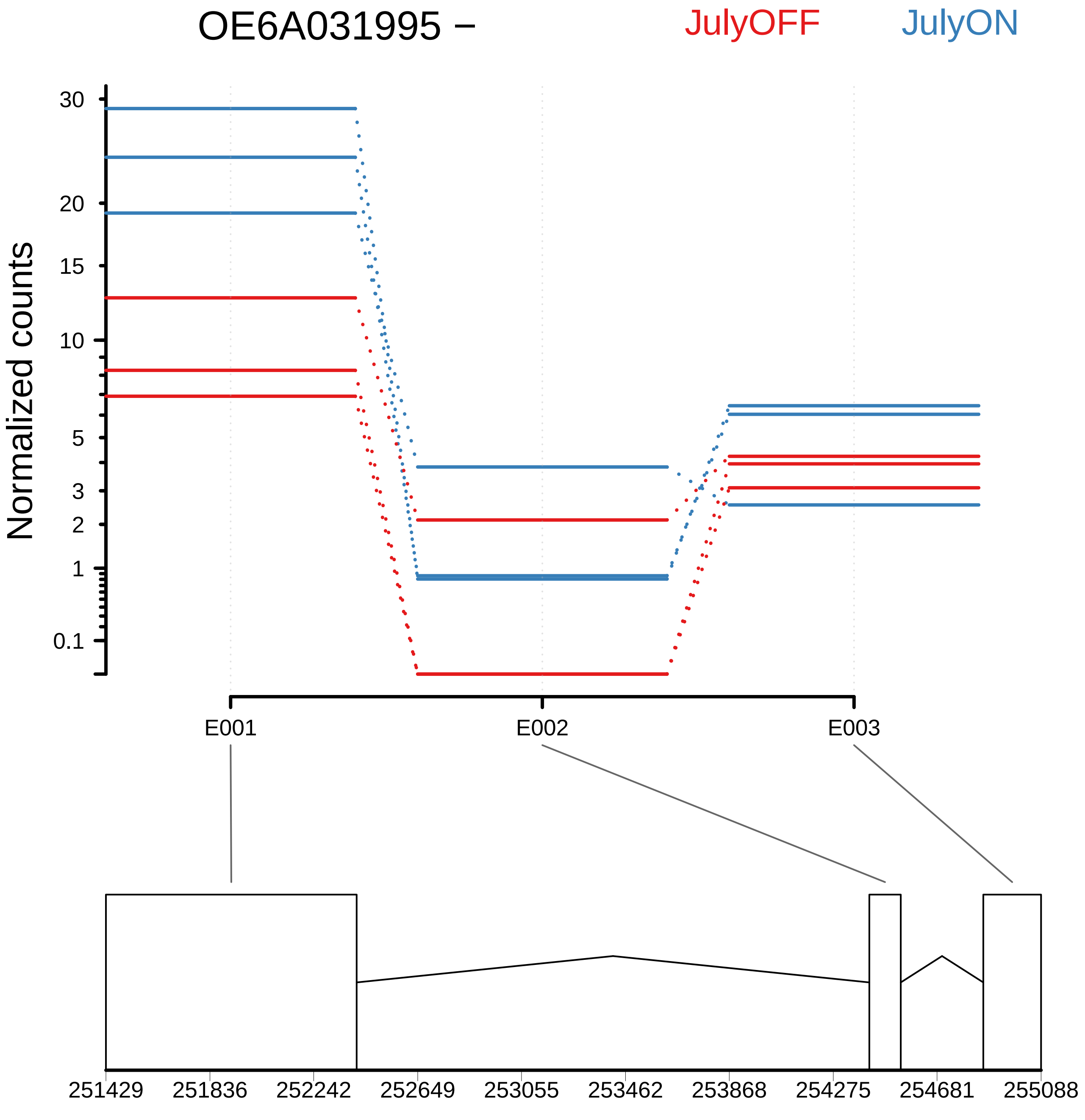

OE6A031995 −
JulyOFF
JulyON
Normalized counts
30
20
15
10
5
3
2
1
0.1
E001
E002
E003
251429
251836
252242
252649
253055
253462
253868
254275
254681
255088

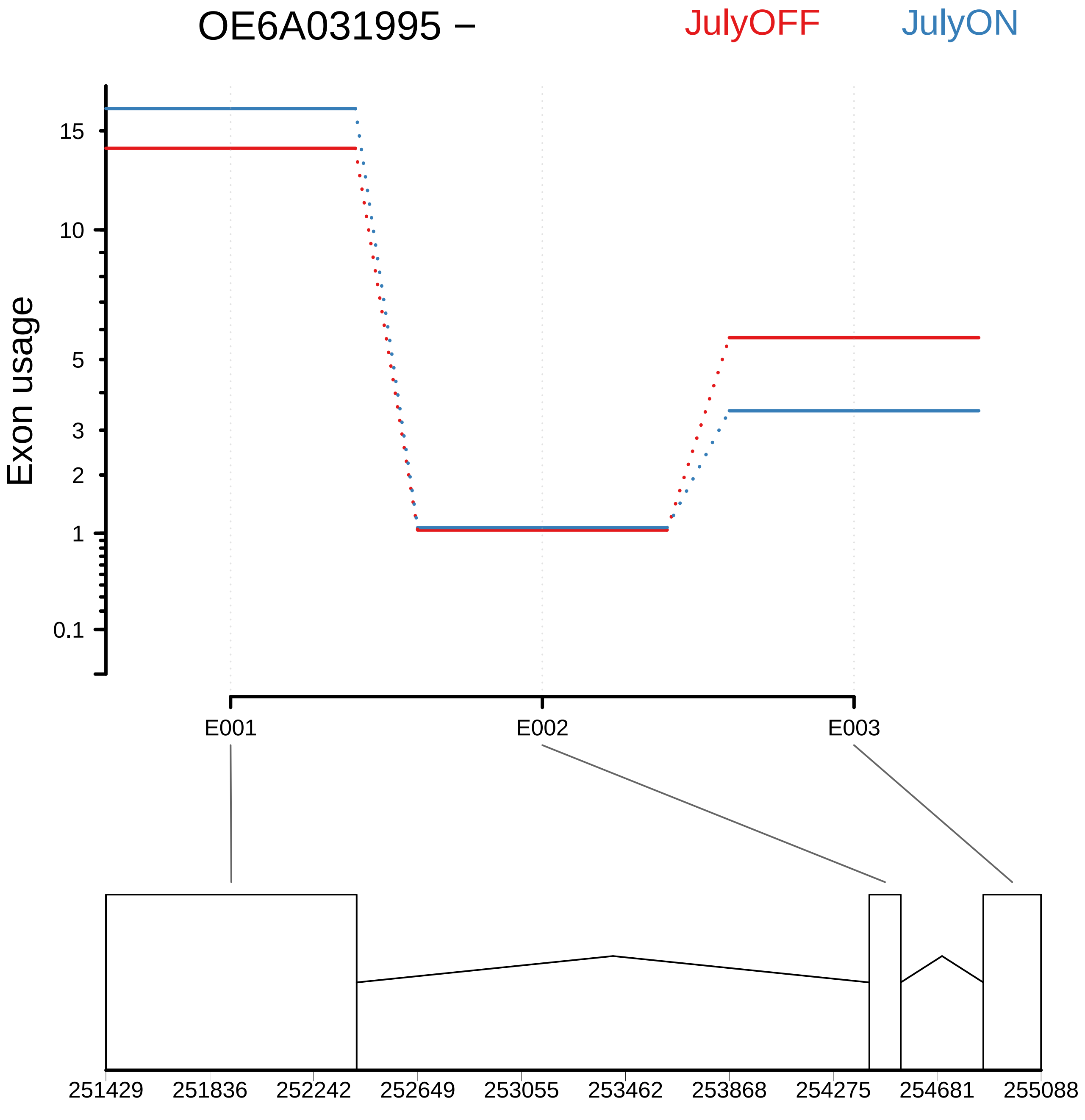
OE6A031995 −
JulyOFF
JulyON
Exon usage
15
10
5
3
2
1
0.1
E001
E002
E003
251429
251836
252242
252649
253055
253462
253868
254275
254681
255088

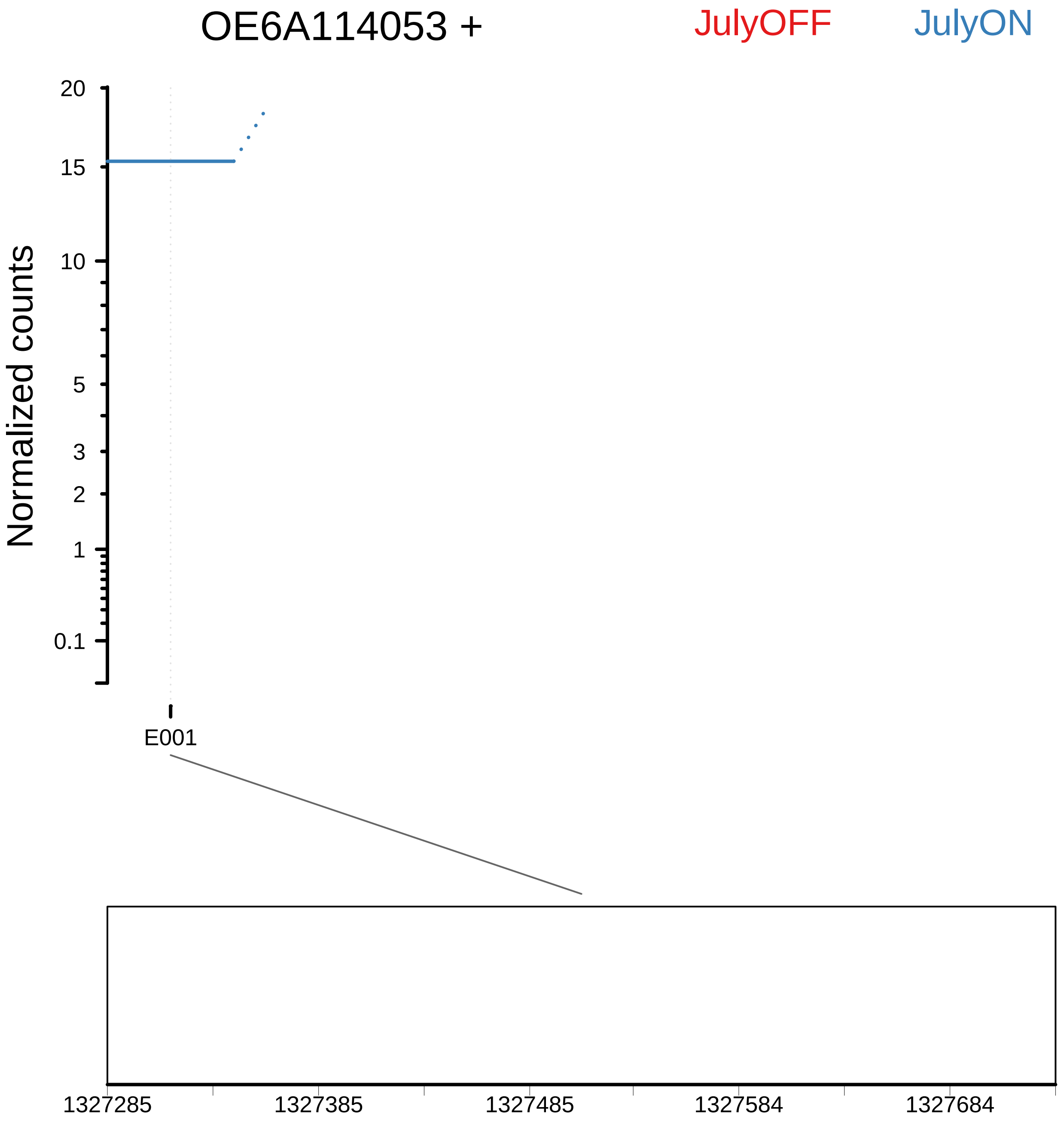
OE6A114053 +
JulyOFF
JulyON
Normalized counts
20
15
10
5
3
2
1
0.1
E001
1327285
1327385
1327485
1327584
1327684

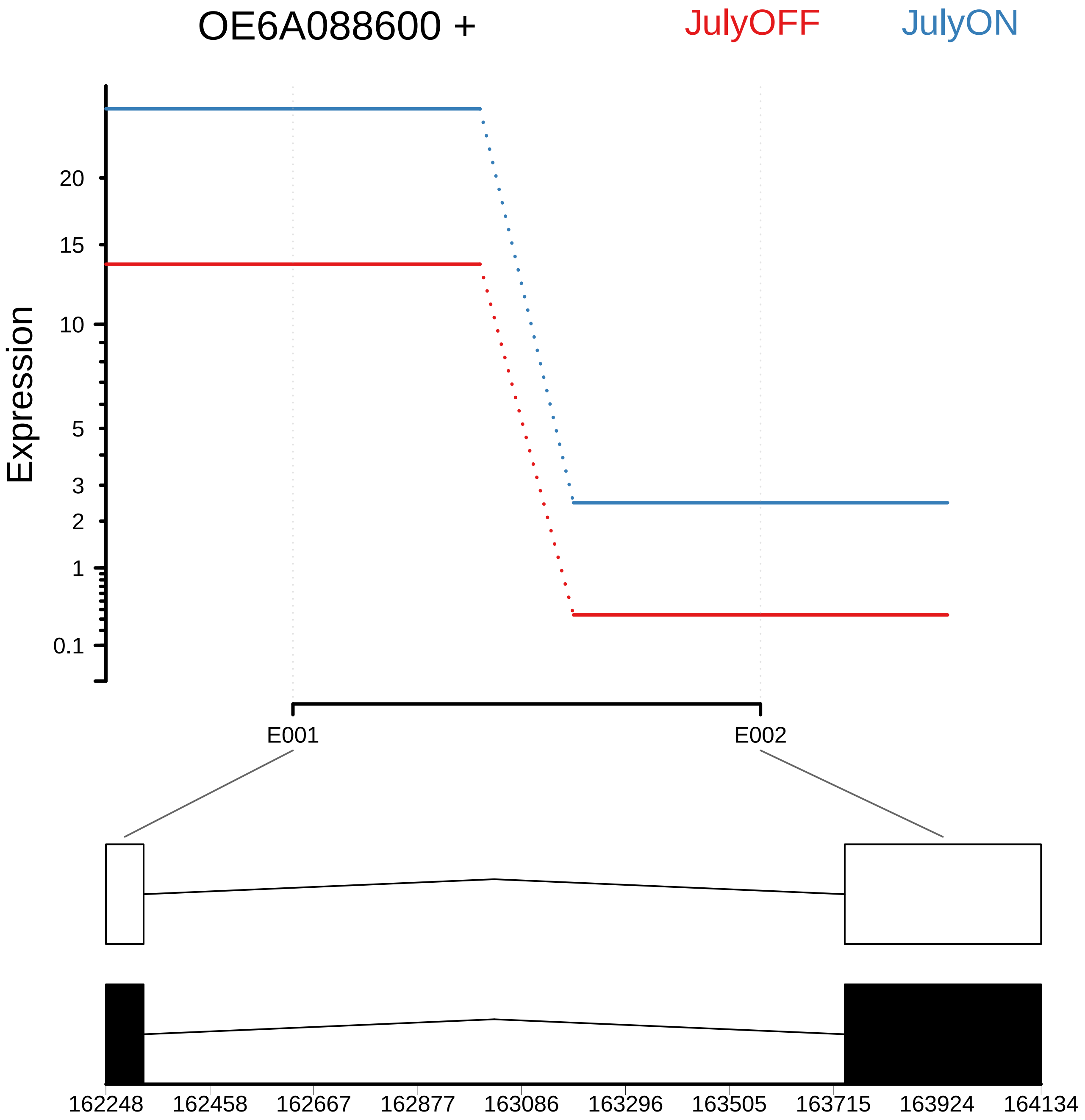

OE6A088600 +
JulyOFF
JulyON
Expression
20
15
10
5
3
2
1
0.1
E001
E002
162248
162458
162667
162877
163086
163296
163505
163715
163924
164134

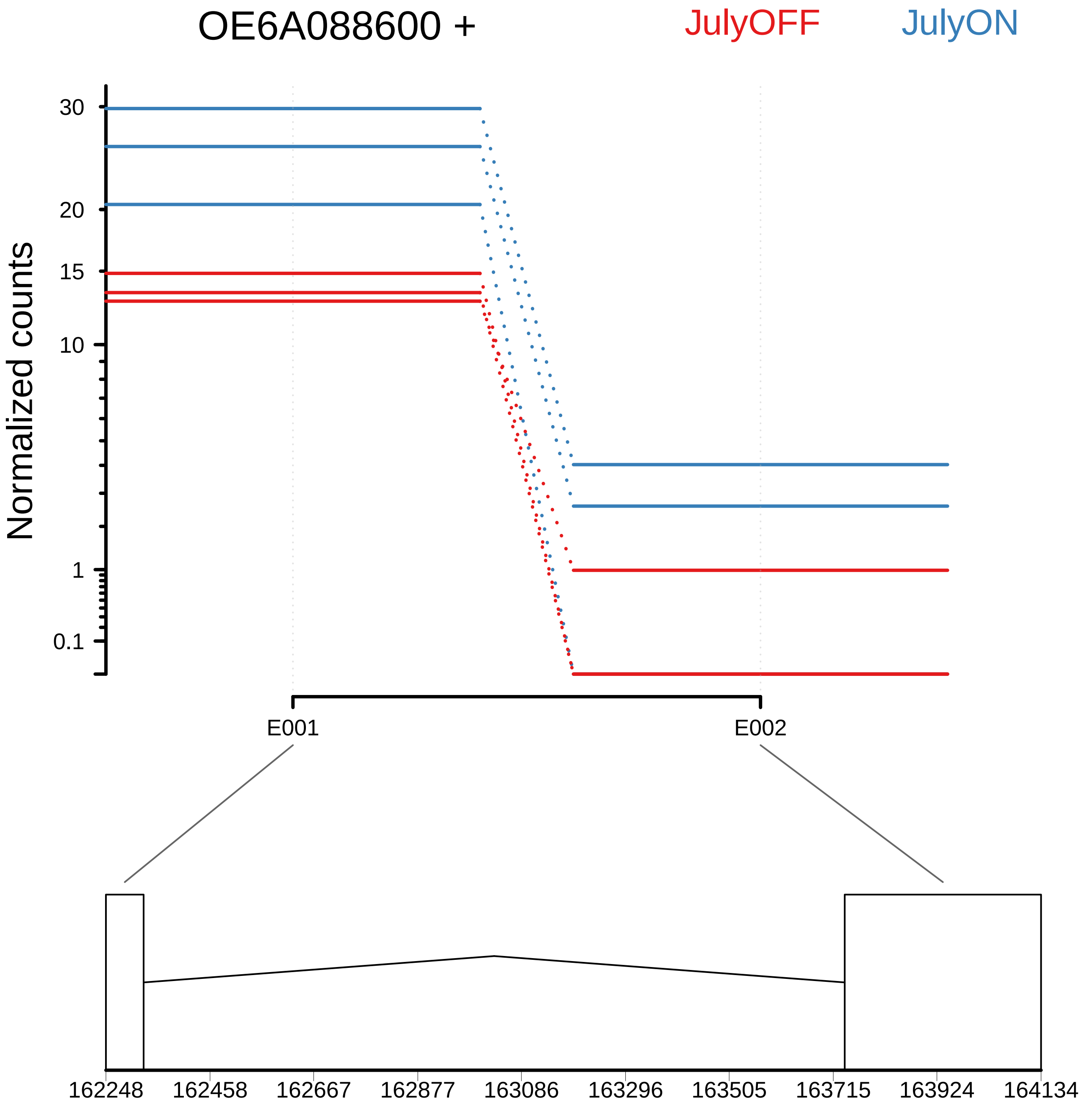
OE6A088600 +
JulyOFF
JulyON
Normalized counts
30
20
15
10
1
0.1
E001
E002
162248
162458
162667
162877
163086
163296
163505
163715
163924
164134

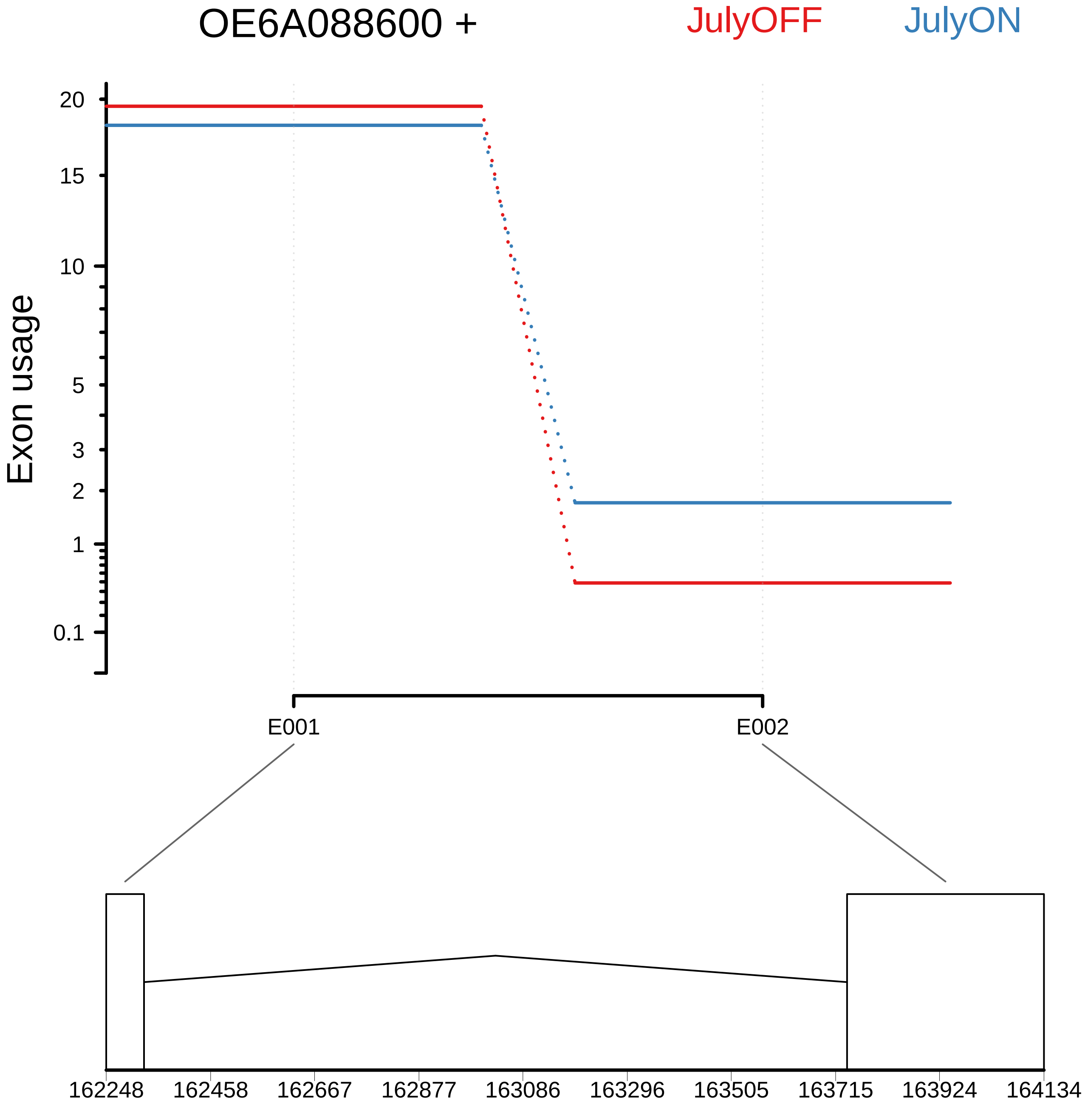
OE6A088600 +
JulyOFF
JulyON
Exon usage
20
15
10
5
3
2
1
0.1
E001
E002
162248
162458
162667
162877
163086
163296
163505
163715
163924
164134

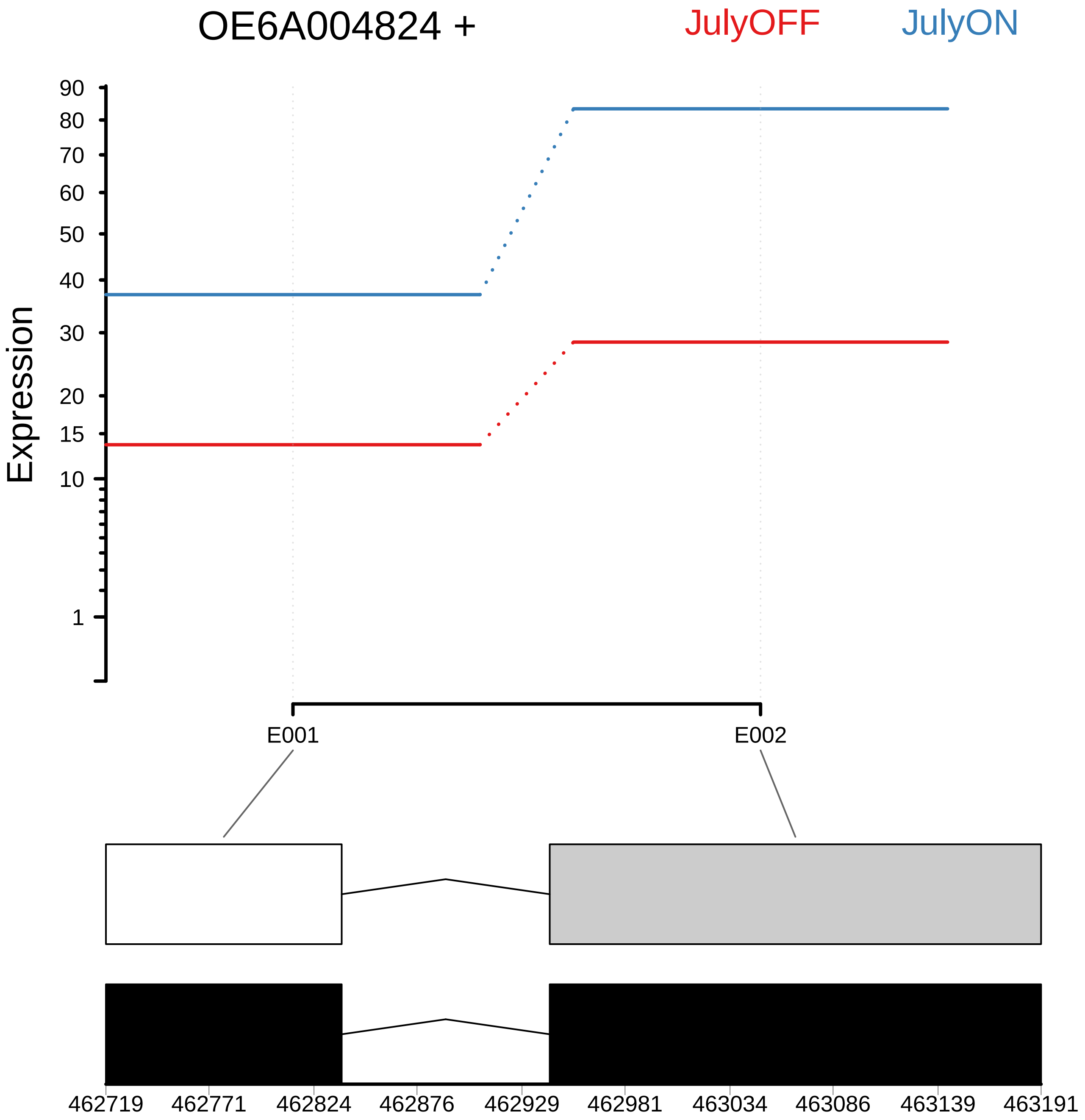
OE6A004824 +
JulyOFF
JulyON
Expression
90
80
70
60
50
40
30
20
15
10
1
E001
E002
462719
462771
462824
462876
462929
462981
463034
463086
463139
463191

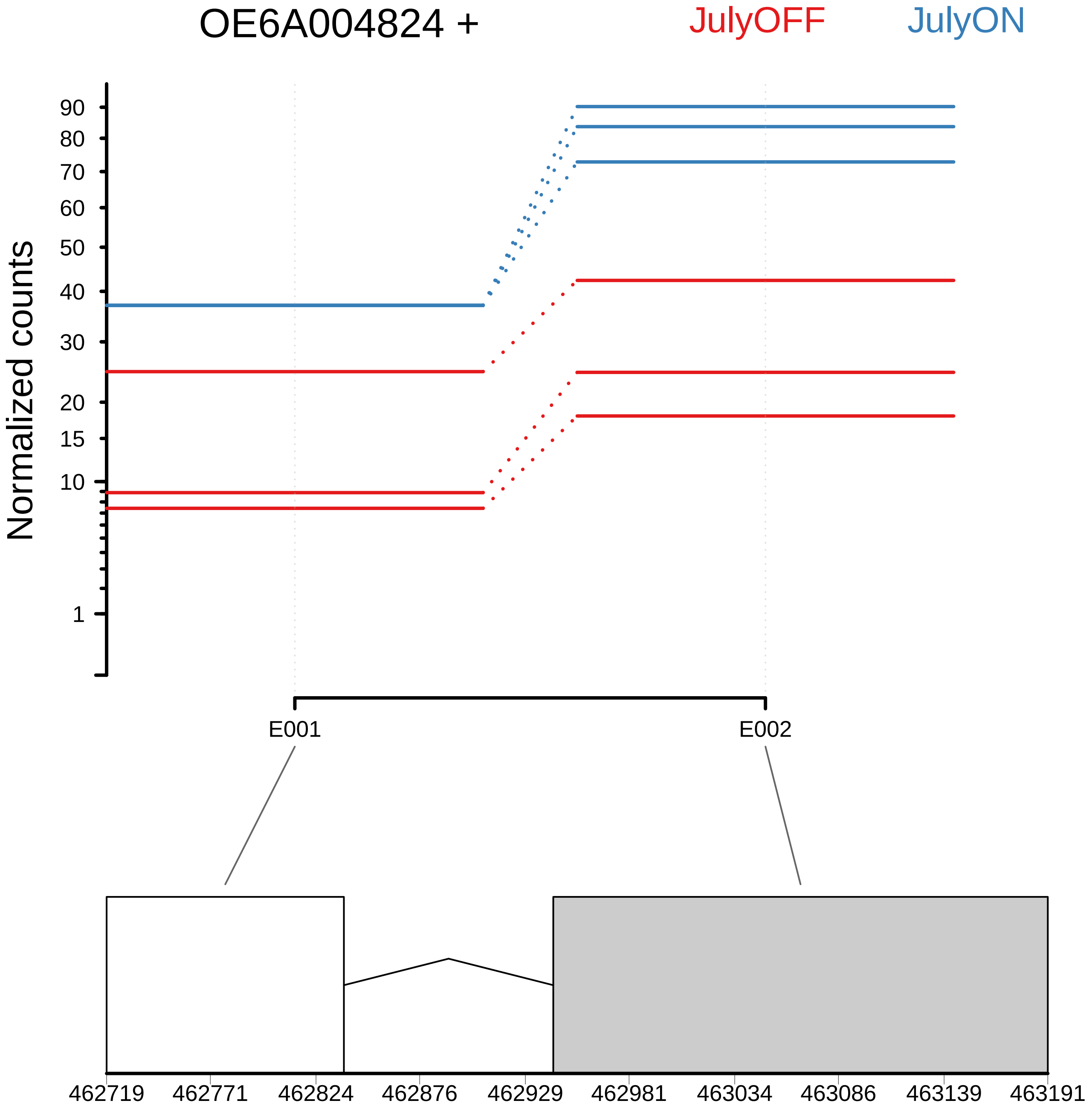

OE6A004824 +
JulyOFF
JulyON
Normalized counts
90
80
70
60
50
40
30
20
15
10
1
E001
E002
462719
462771
462824
462876
462929
462981
463034
463086
463139
463191

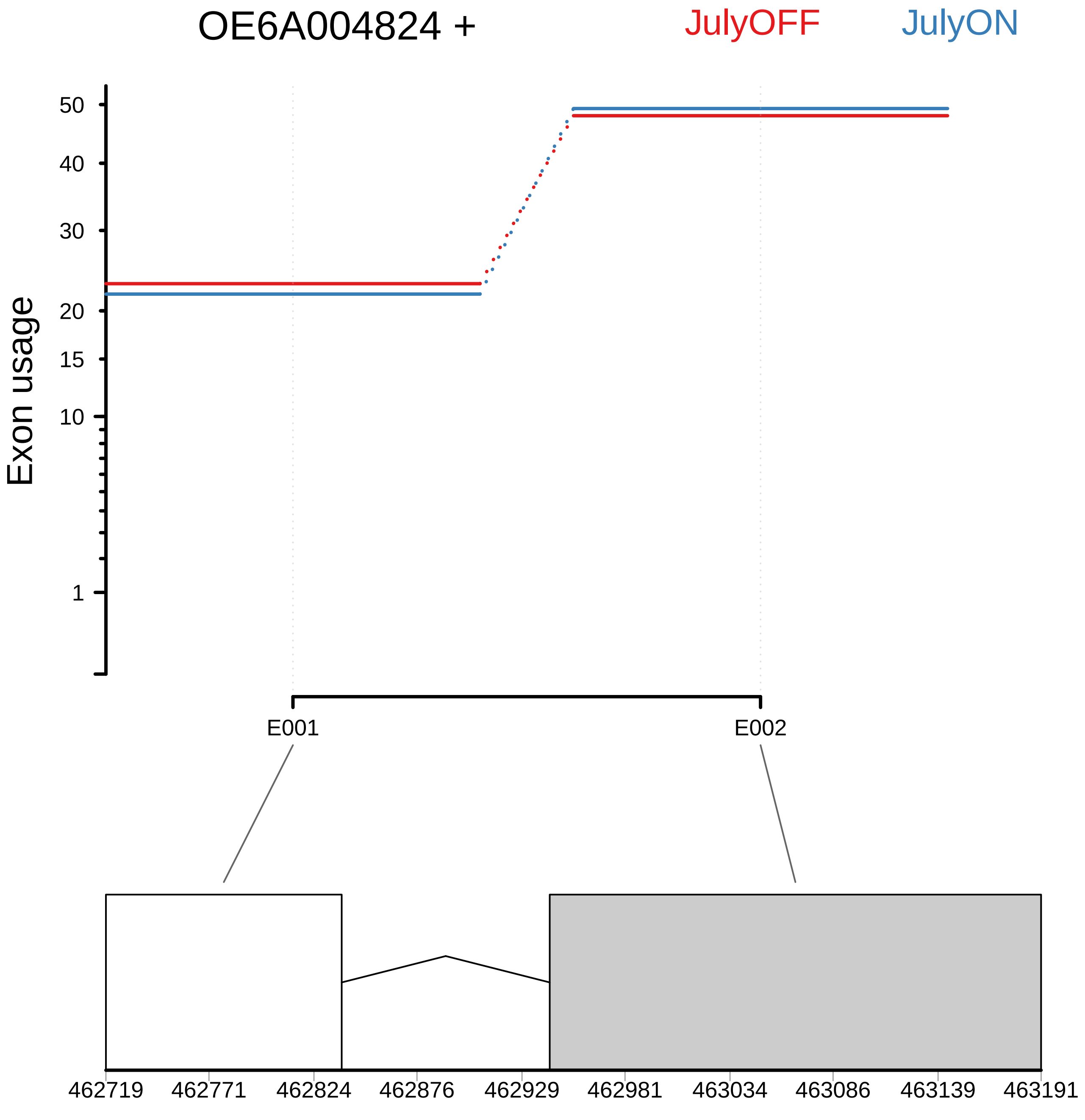
OE6A004824 +
JulyOFF
JulyON
Exon usage
50
40
30
20
15
10
1
E001
E002
462719
462771
462824
462876
462929
462981
463034
463086
463139
463191

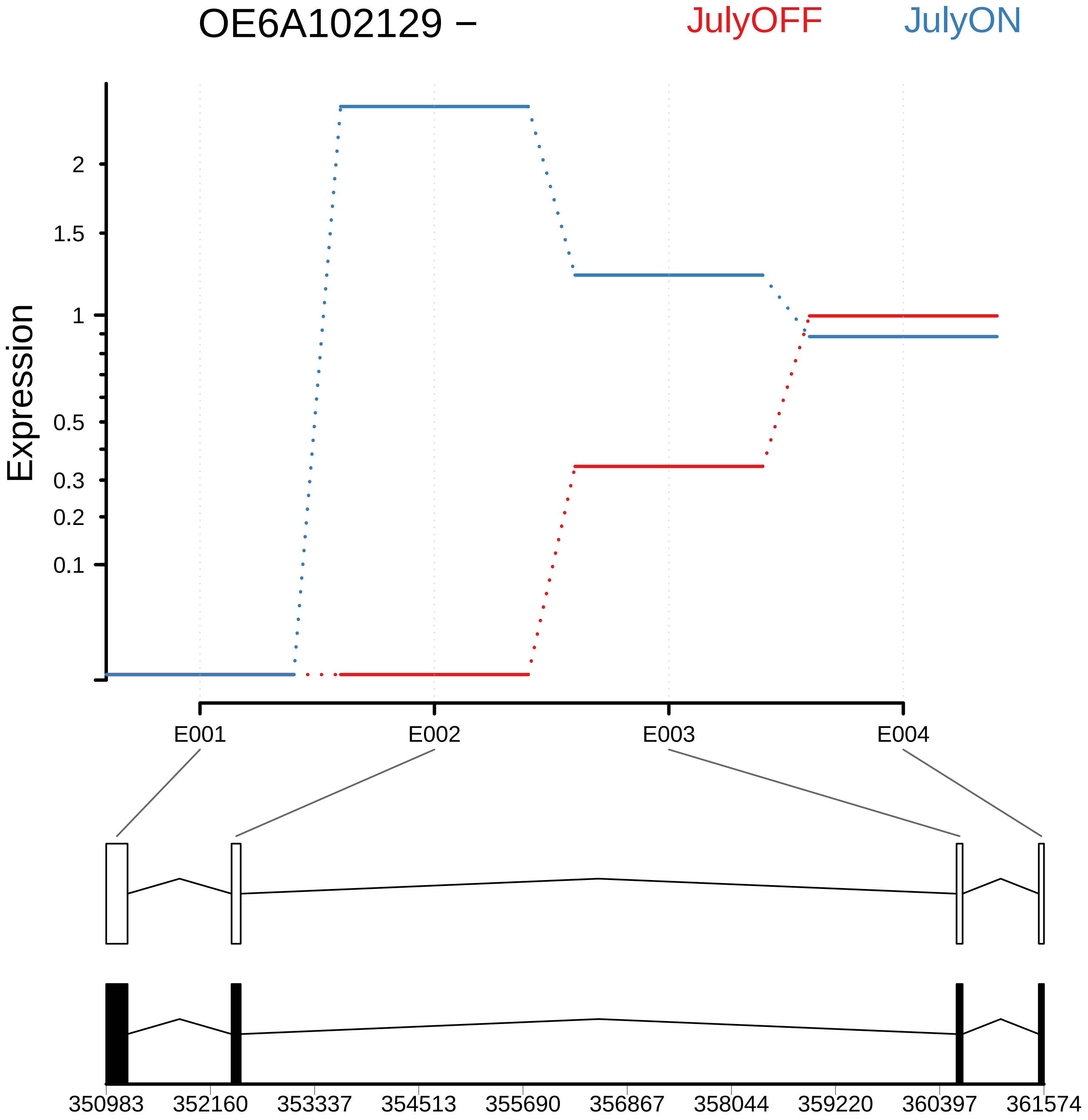

OE6A102129 −
JulyOFF
JulyON
Expression
2
1.5
1
0.5
0.3
0.2
0.1
E001
E002
E003
E004
350983
352160
353337
354513
355690
356867
358044
359220
360397
361574

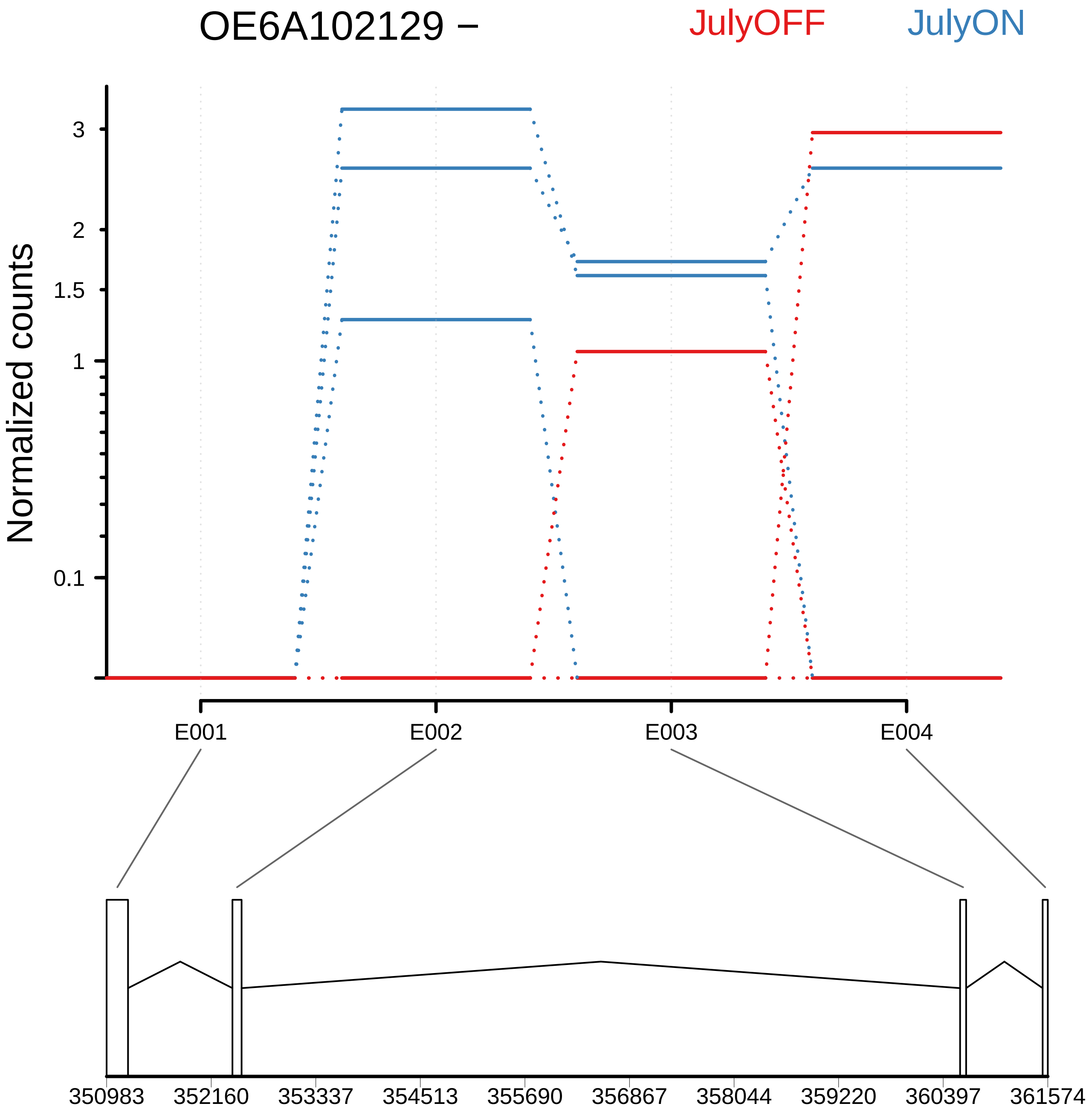

OE6A102129 −
JulyOFF
JulyON
Normalized counts
3
2
1.5
1
0.1
E001
E002
E003
E004
350983
352160
353337
354513
355690
356867
358044
359220
360397
361574

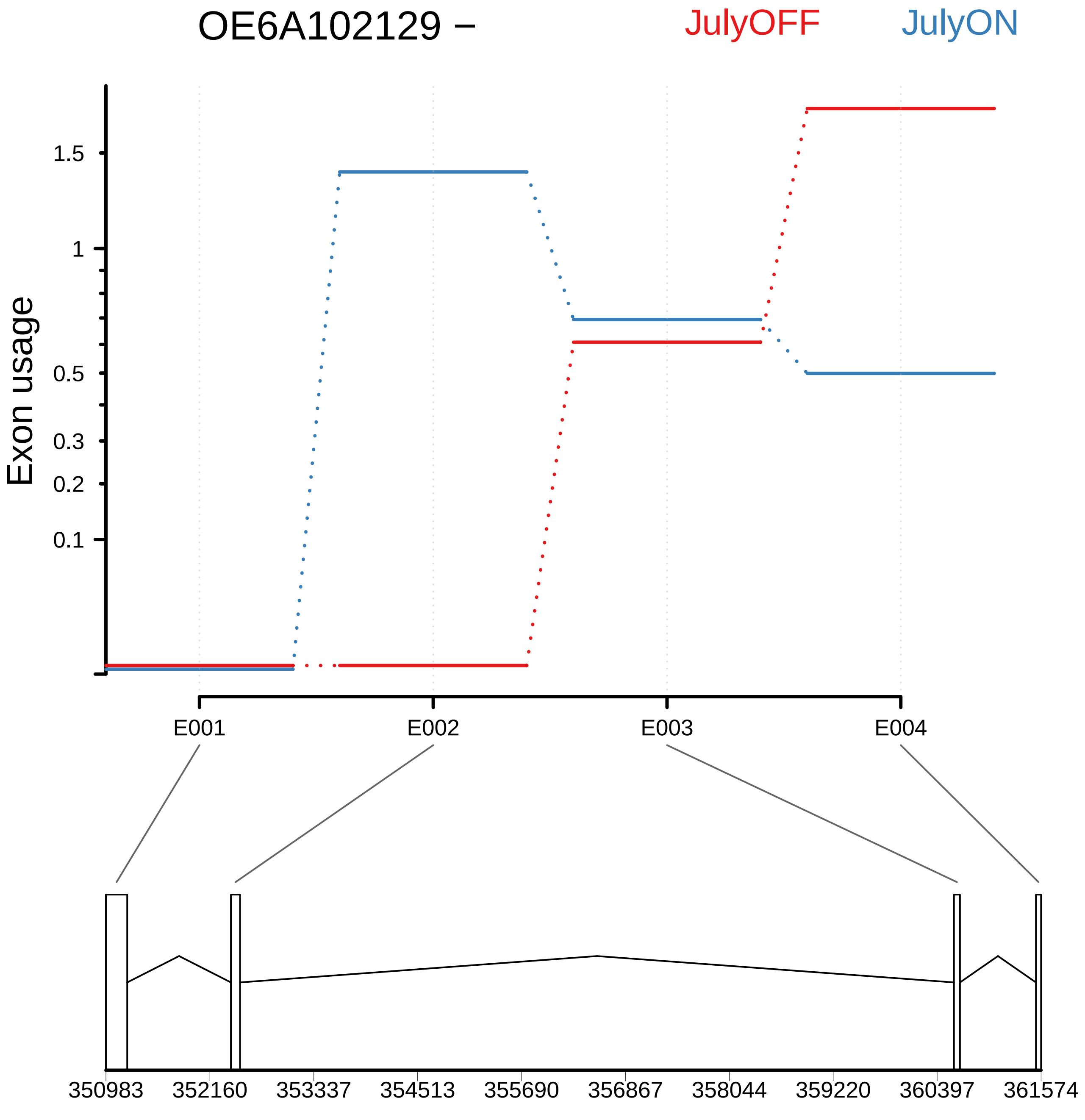

OE6A102129 −
JulyOFF
JulyON
Exon usage
1.5
1
0.5
0.3
0.2
0.1
E001
E002
E003
E004
350983
352160
353337
354513
355690
356867
358044
359220
360397
361574

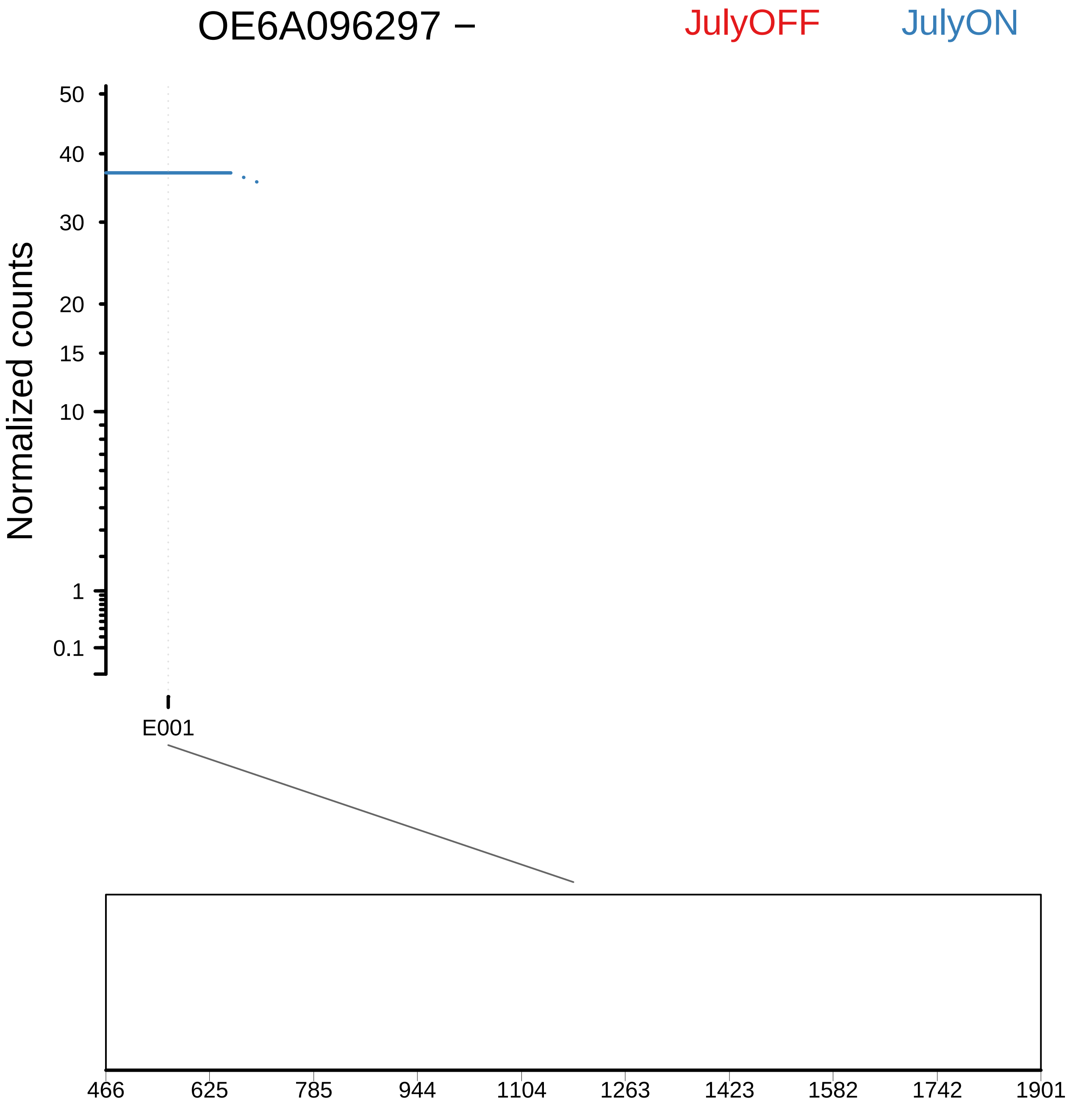

OE6A096297 −
JulyOFF
JulyON
Normalized counts
50
40
30
20
15
10
1
0.1
E001
466
625
785
944
1104
1263
1423
1582
1742
1901

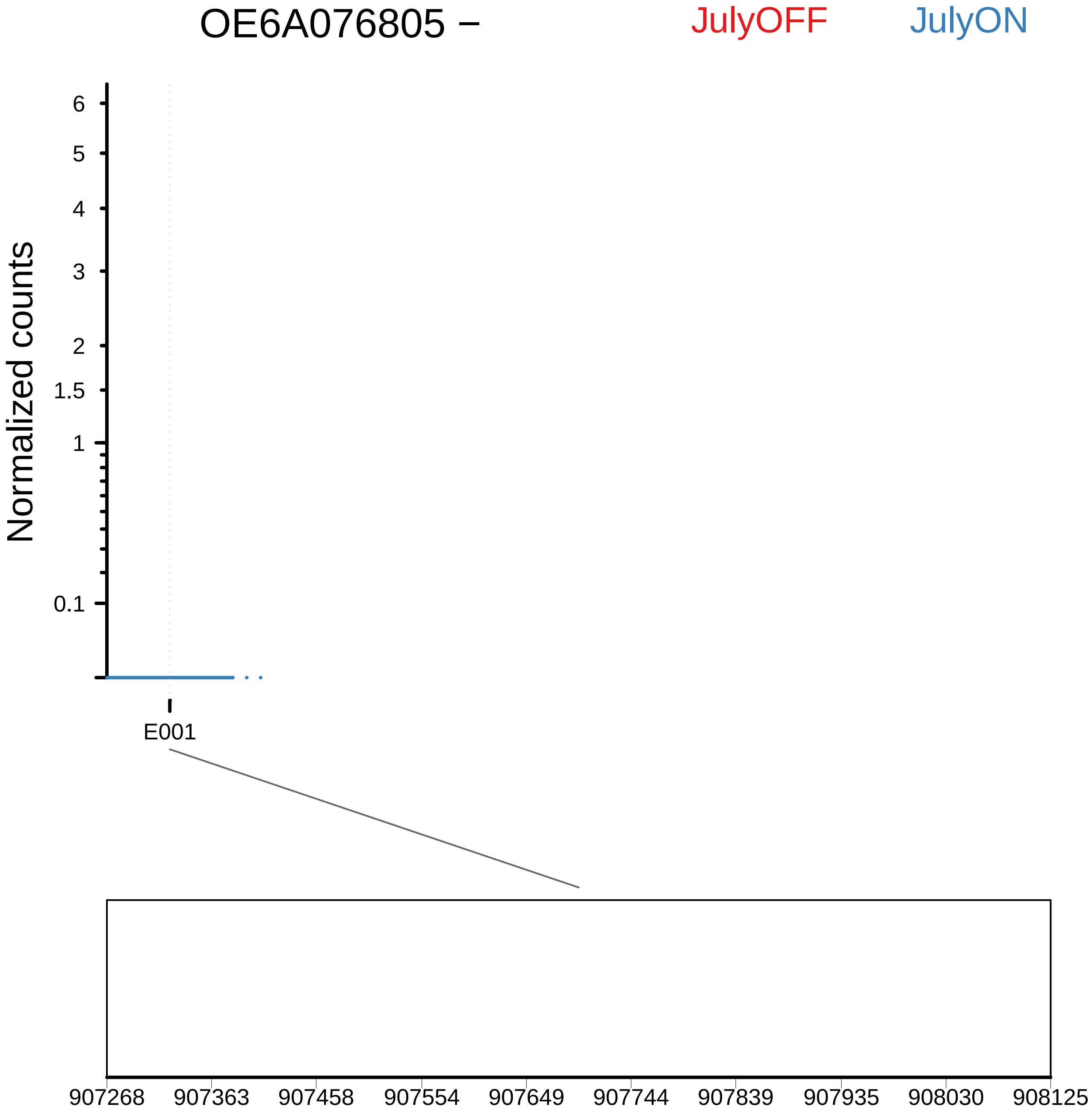

OE6A076805 −
JulyOFF
JulyON
Normalized counts
6
5
4
3
2
1.5
1
0.1
E001
907268
907363
907458
907554
907649
907744
907839
907935
908030
908125

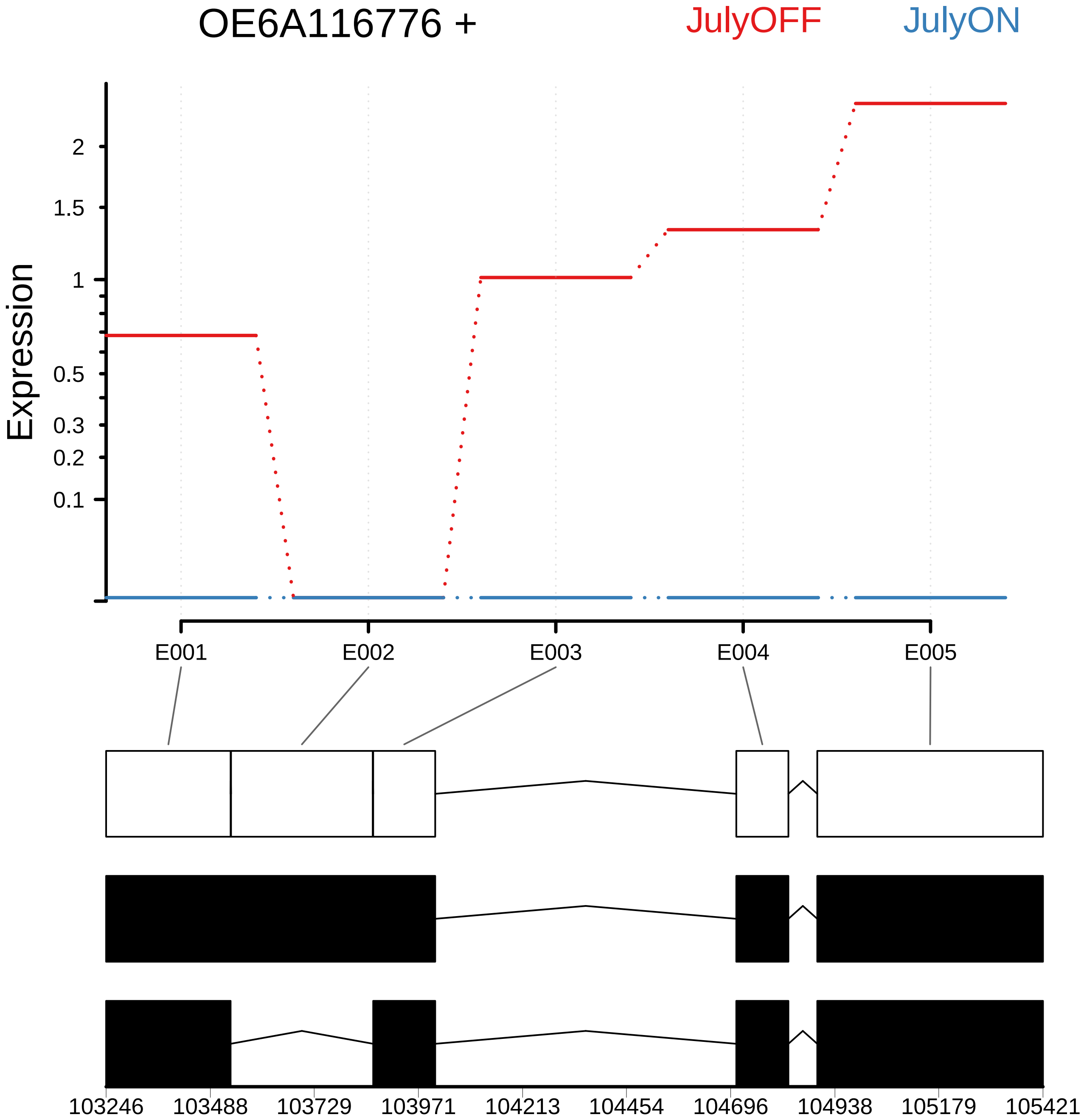

OE6A116776 +
JulyOFF
JulyON
Expression
2
1.5
1
0.5
0.3
0.2
0.1
E001
E002
E003
E004
E005
103246
103488
103729
103971
104213
104454
104696
104938
105179
105421

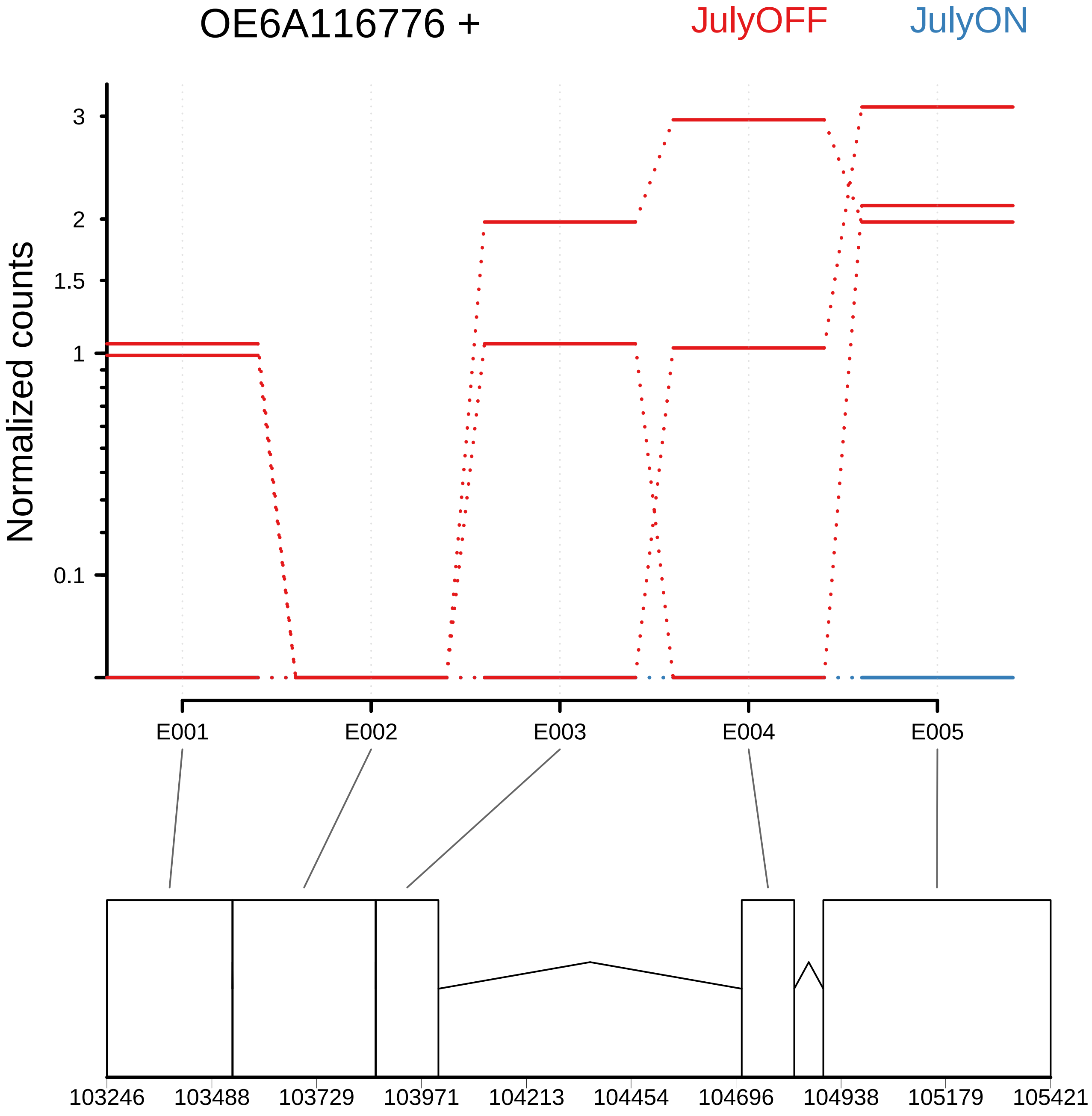

OE6A116776 +
JulyOFF
JulyON
Normalized counts
3
2
1.5
1
0.1
E001
E002
E003
E004
E005
103246
103488
103729
103971
104213
104454
104696
104938
105179
105421

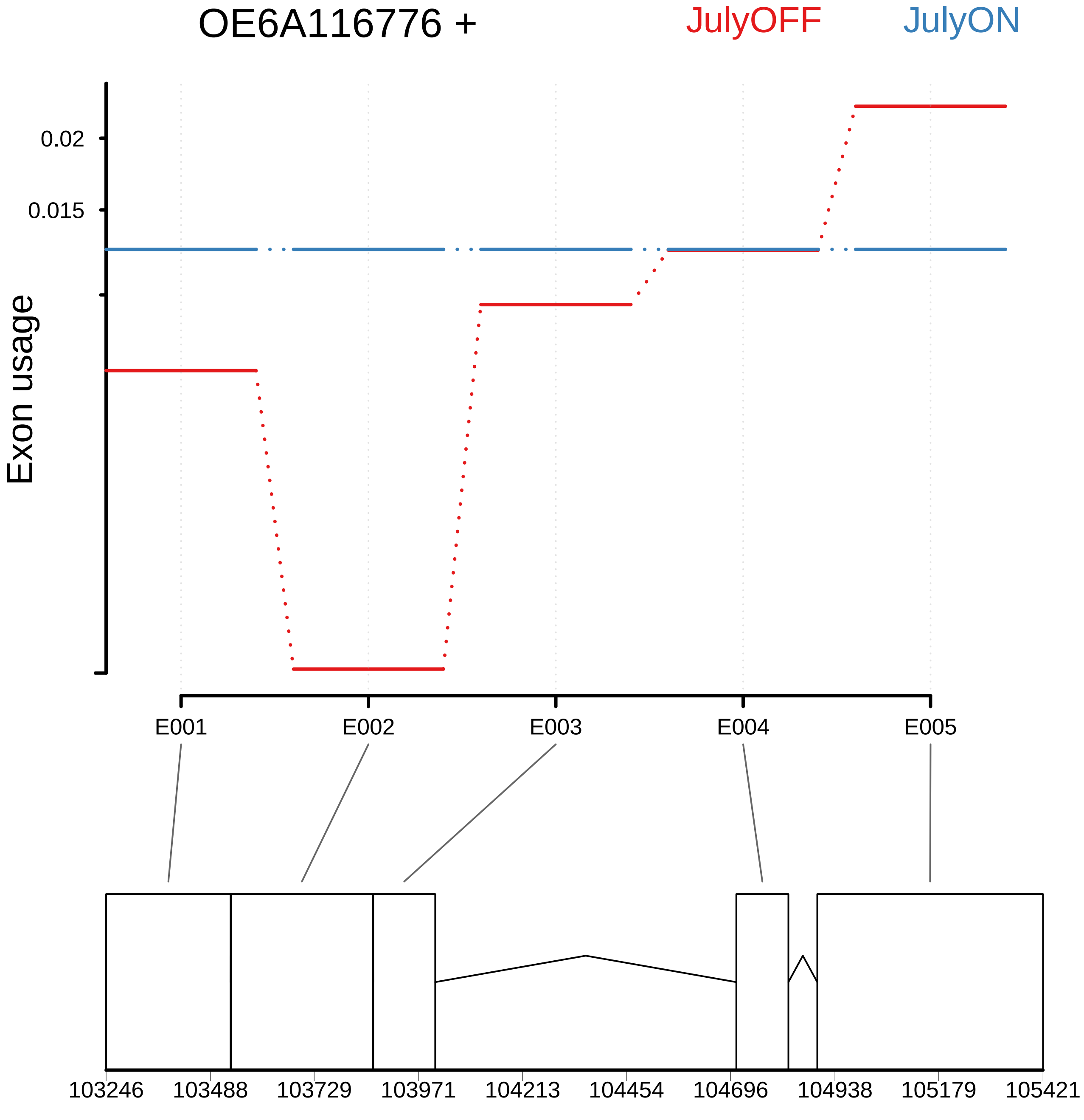

OE6A116776 +
JulyOFF
JulyON
Exon usage
0.02
0.015
E001
E002
E003
E004
E005
103246
103488
103729
103971
104213
104454
104696
104938
105179
105421

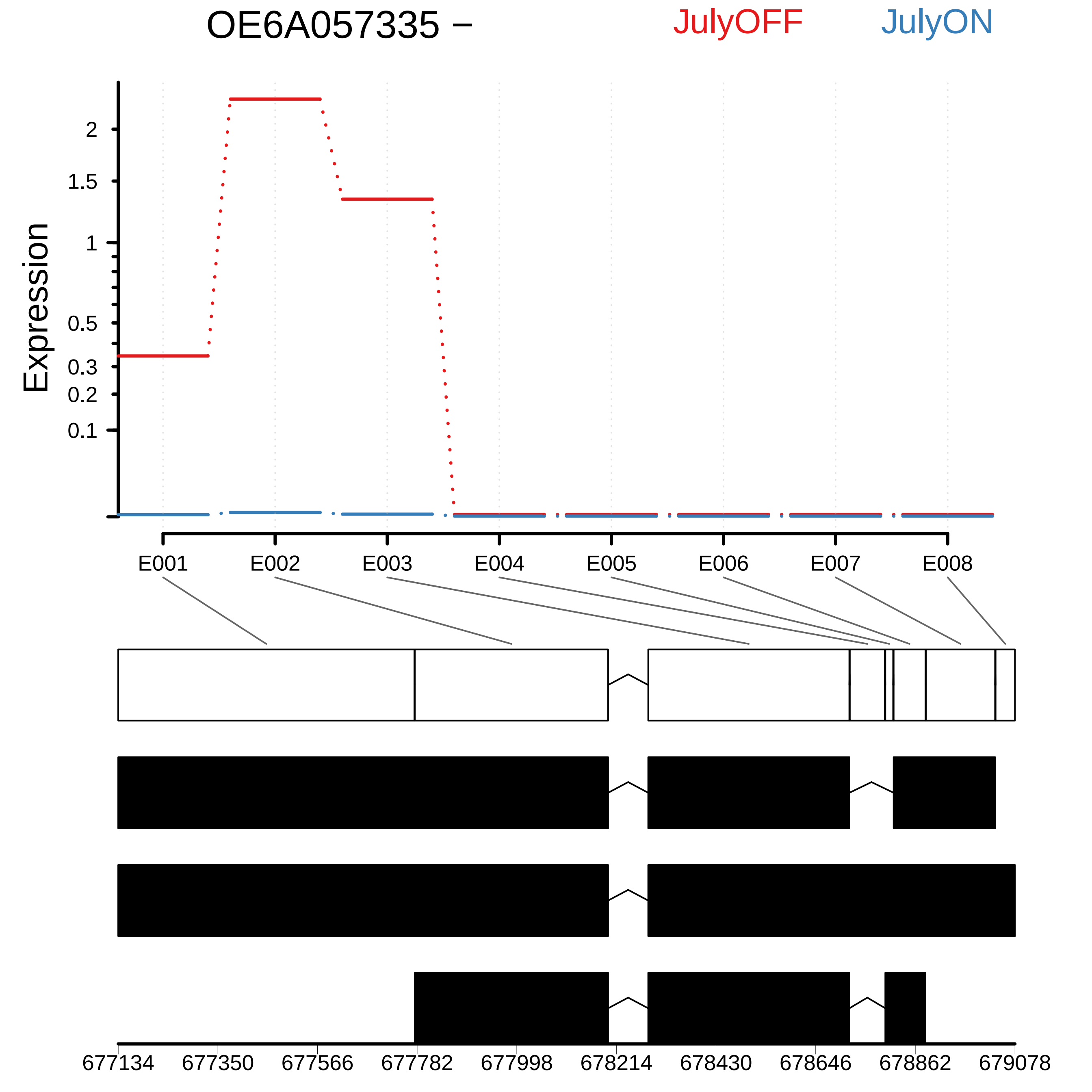
OE6A057335 –
JulyOFF
JulyON
Expression
2
1.5
1
0.5
0.3
0.2
0.1
E001
E002
E003
E004
E005
E006
E007
E008
677134
677350
677566
677782
677998
678214
678430
678646
678862
679078

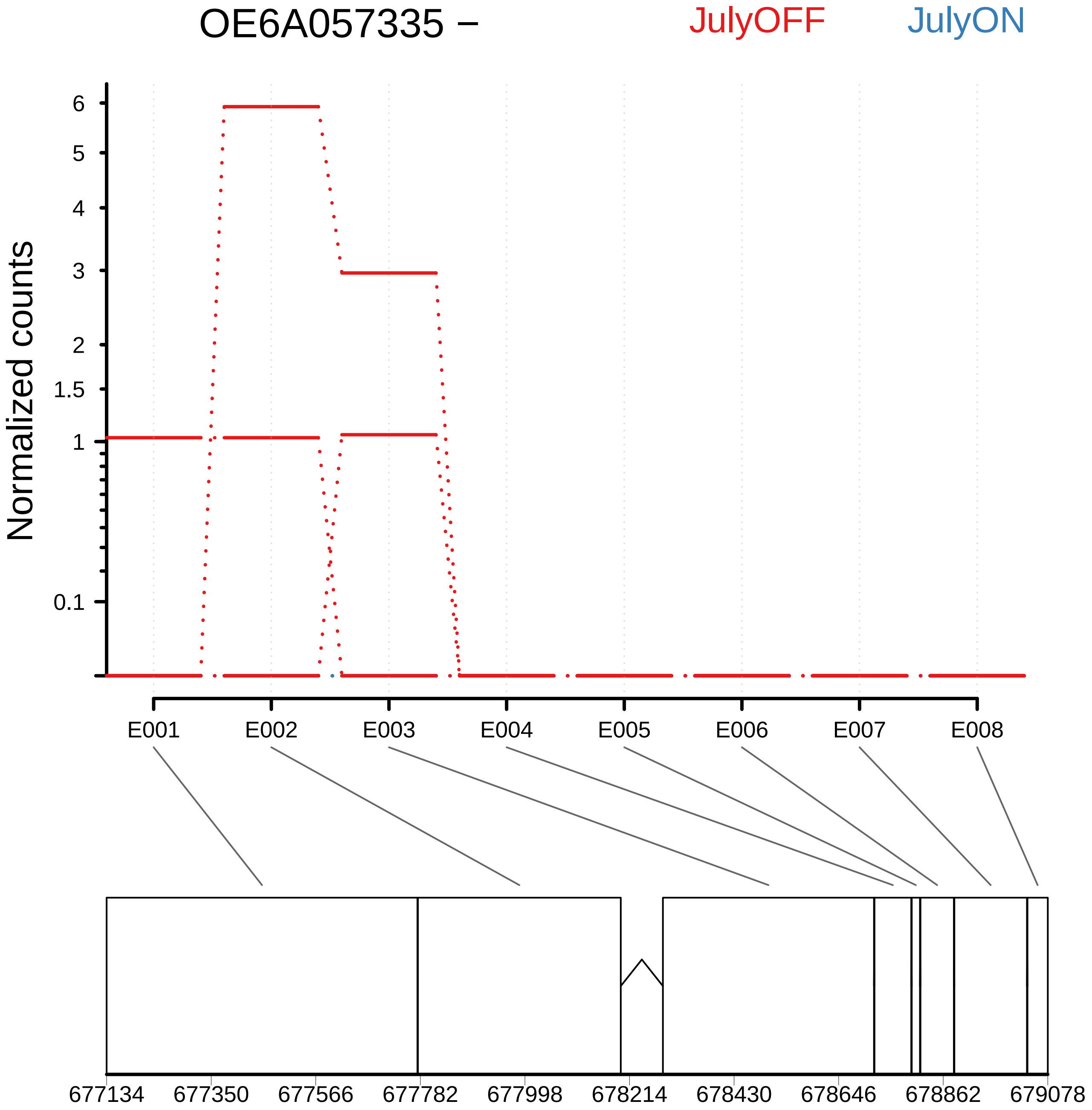

OE6A057335 −
JulyOFF
JulyON
Normalized counts
6
5
4
3
2
1.5
1
0.1
E001
E002
E003
E004
E005
E006
E007
E008
677134
677350
677566
677782
677998
678214
678430
678646
678862
679078

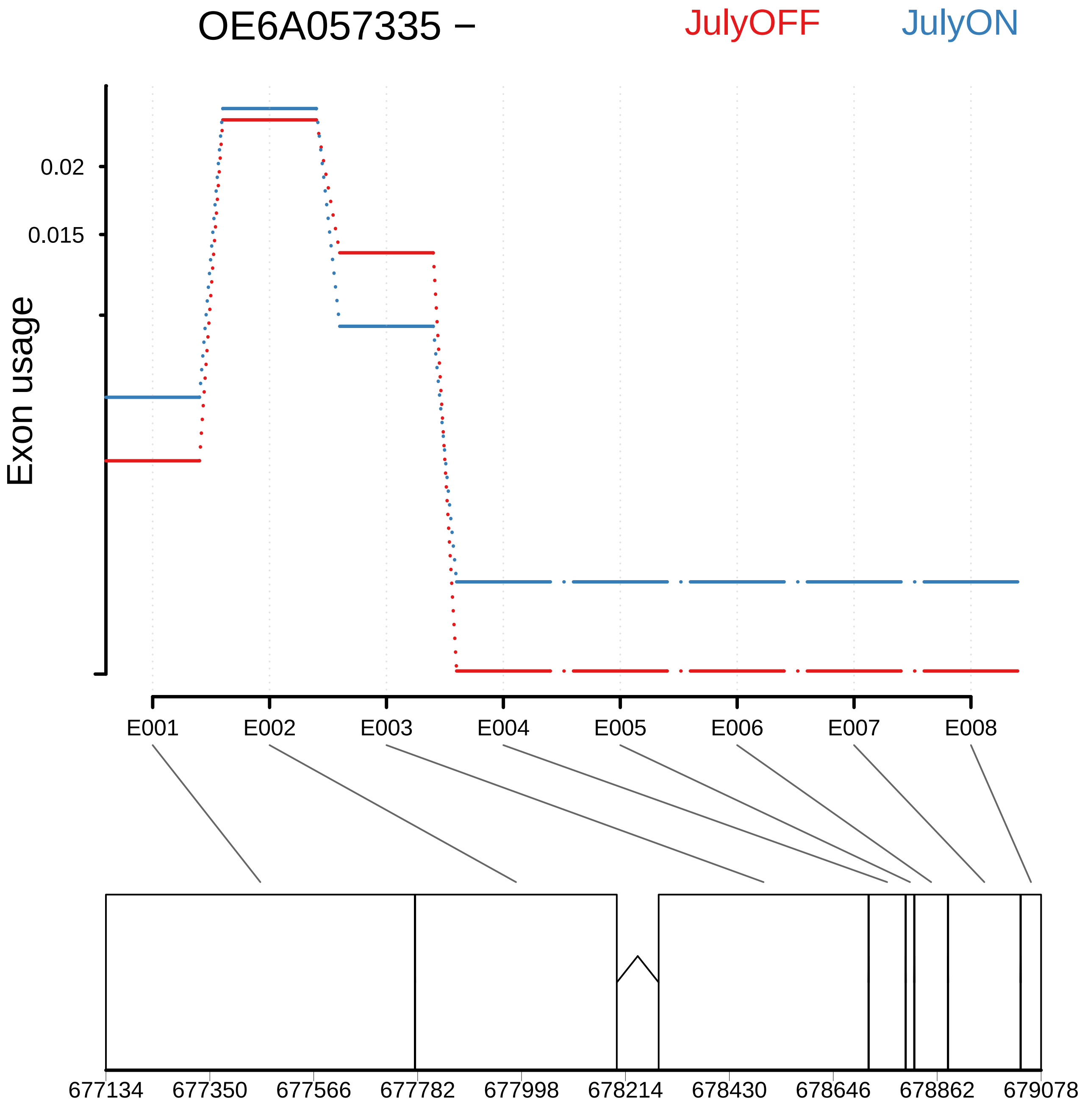
OE6A057335 −
JulyOFF
JulyON
Exon usage
0.02
0.015
E001
E002
E003
E004
E005
E006
E007
E008
677134
677350
677566
677782
677998
678214
678430
678646
678862
679078

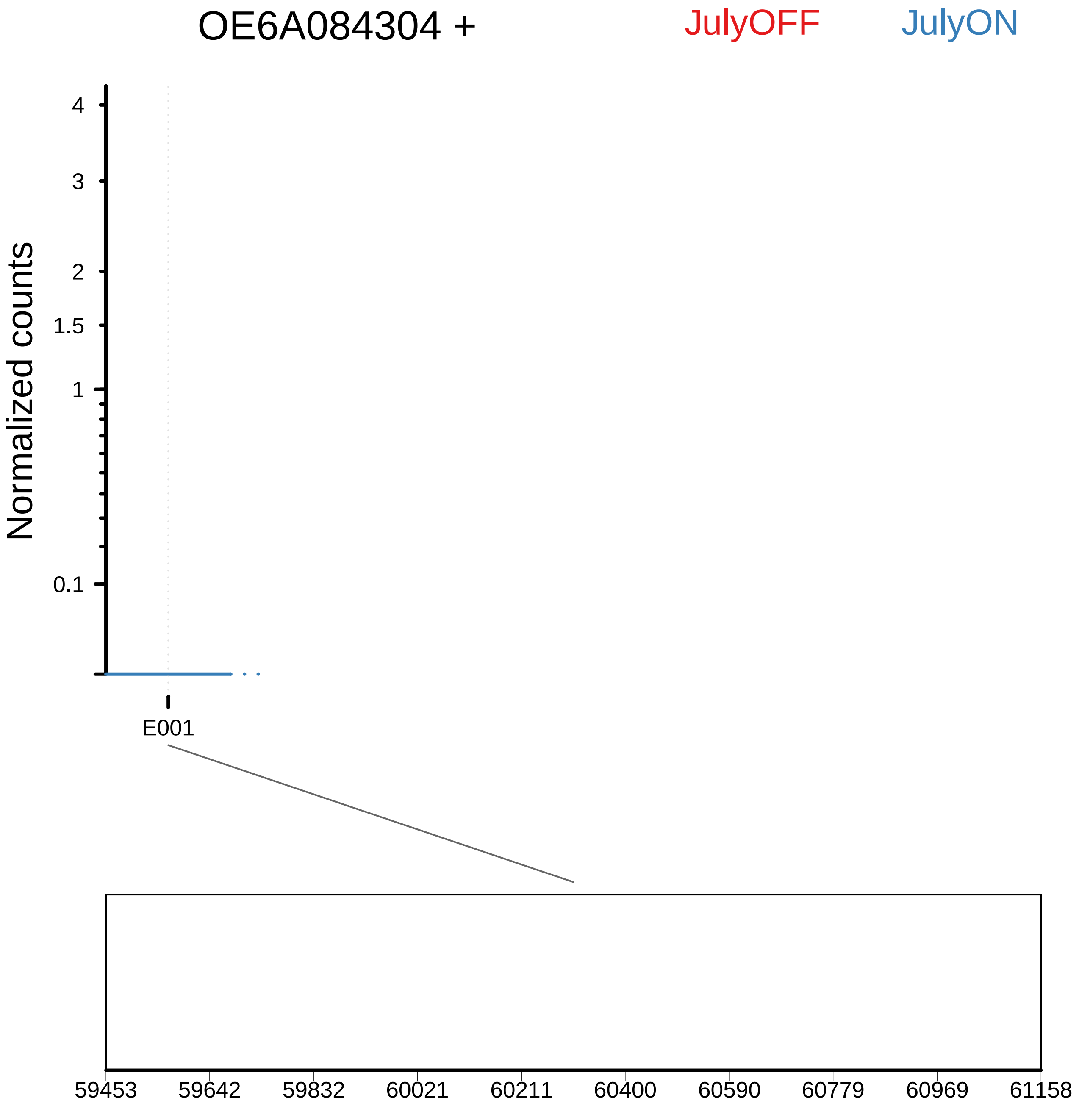

OE6A084304 +
JulyOFF
JulyON
Normalized counts
4
3
2
1.5
1
0.1
E001
59453
59642
59832
60021
60211
60400
60590
60779
60969
61158

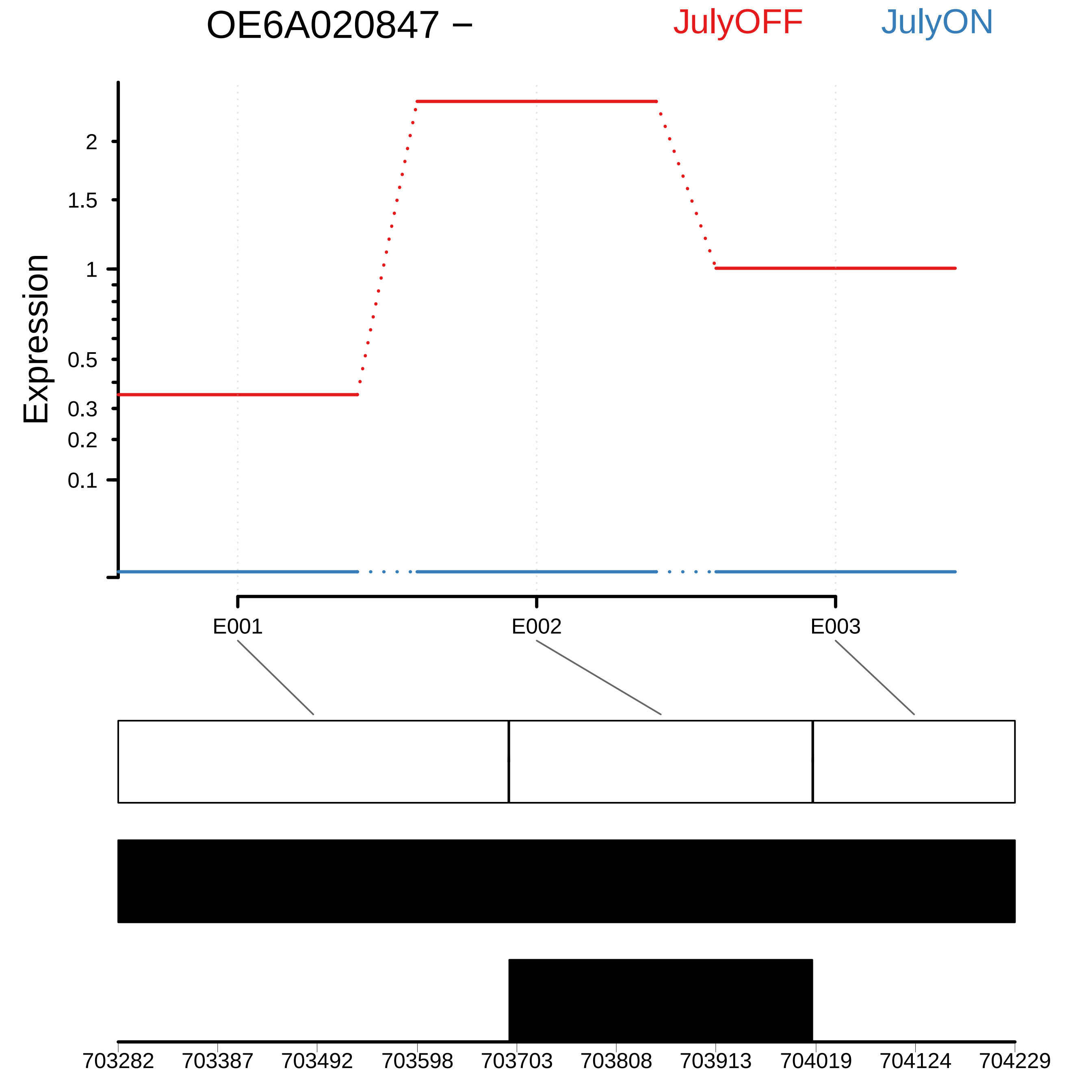

OE6A020847 −
JulyOFF
JulyON
Expression
2
1.5
1
0.5
0.3
0.2
0.1
E001
E002
E003
703282
703387
703492
703598
703703
703808
703913
704019
704124
704229

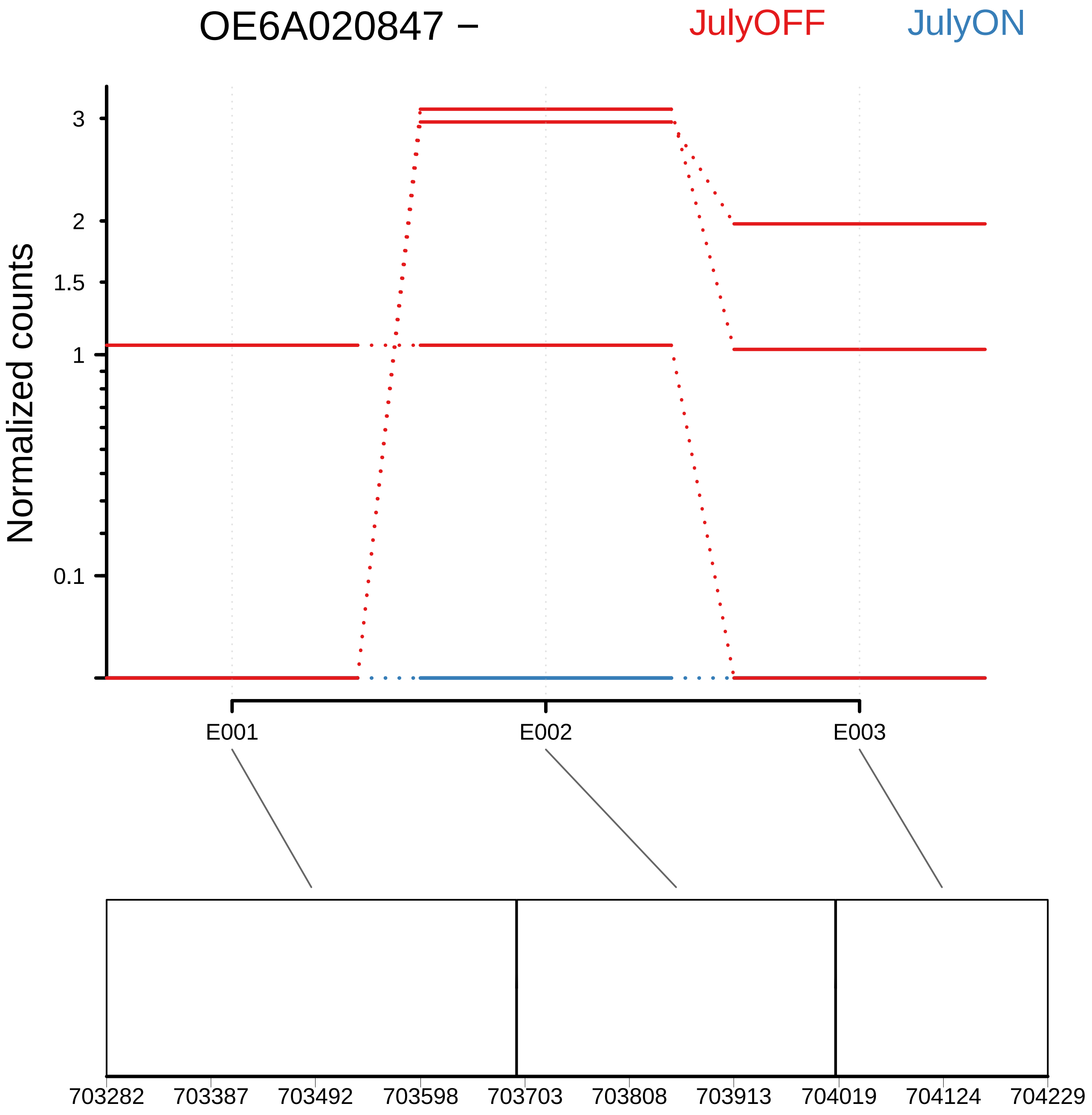
OE6A020847 −
JulyOFF
JulyON
Normalized counts
3
2
1.5
1
0.1
E001
E002
E003
703282
703387
703492
703598
703703
703808
703913
704019
704124
704229

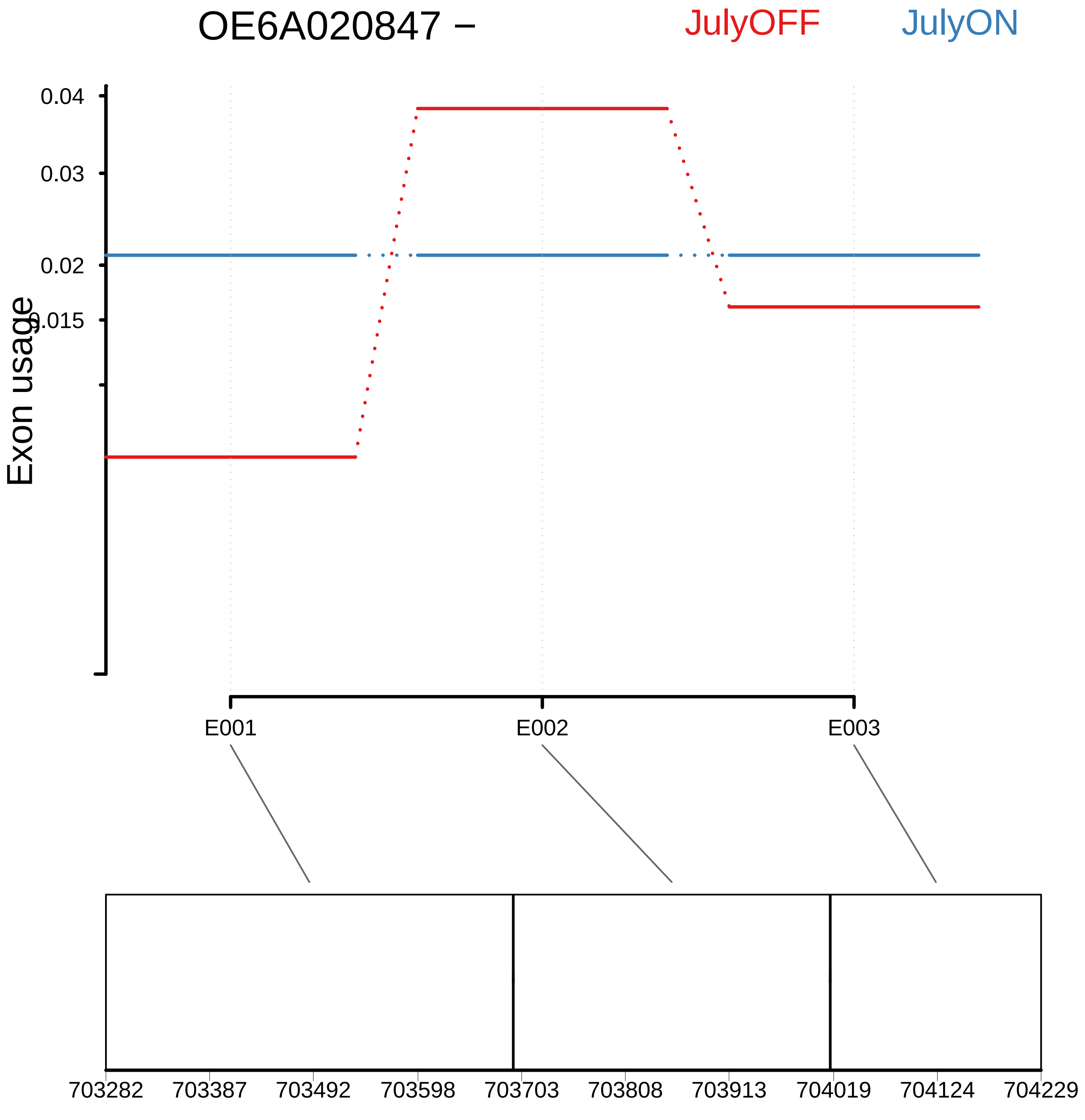

OE6A020847 −
JulyOFF
JulyON
Exon usage
0.04
0.03
0.02
0.015
E001
E002
E003
703282
703387
703492
703598
703703
703808
703913
704019
704124
704229

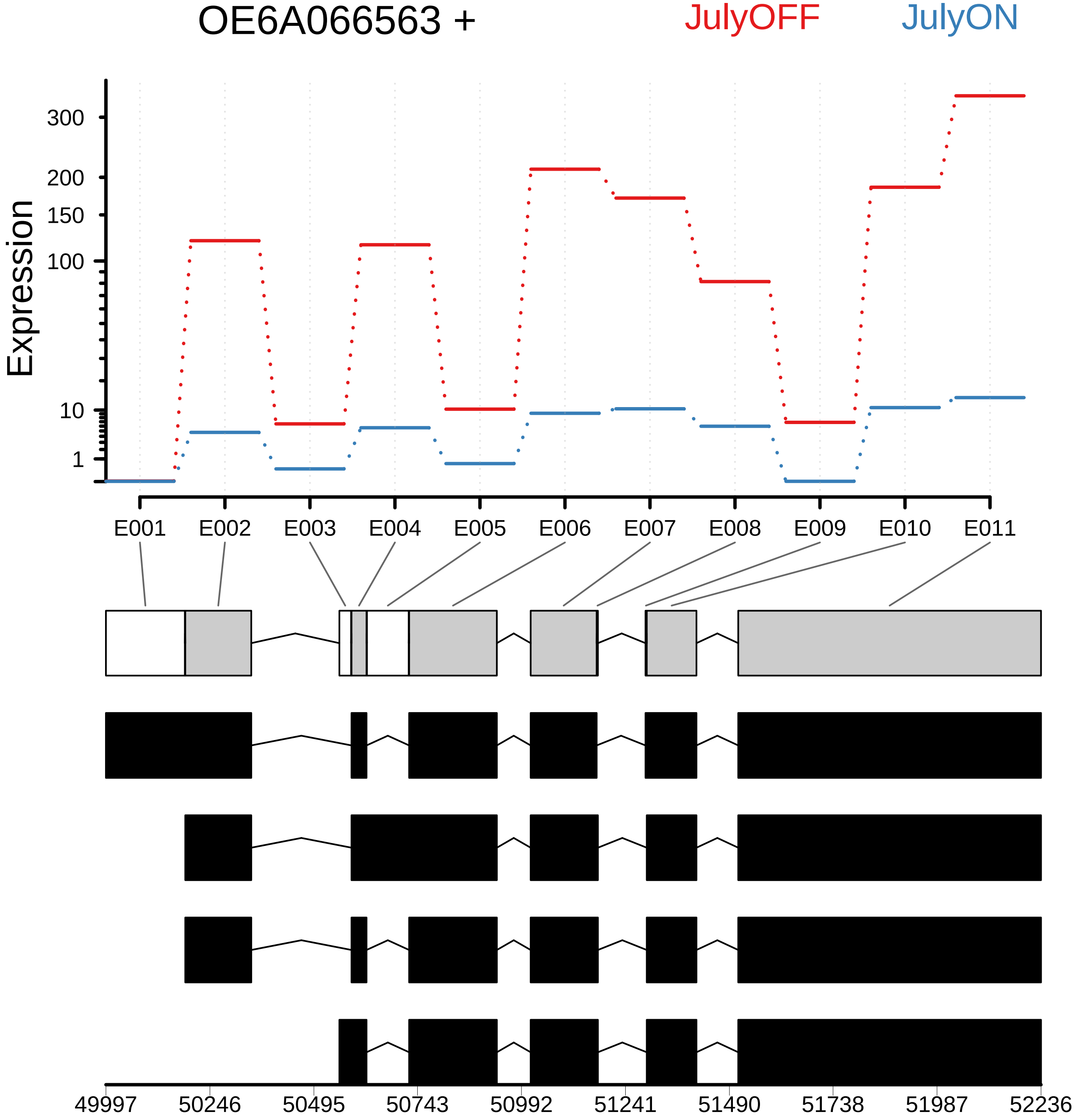

OE6A066563 +
JulyOFF
JulyON
Expression
300
200
150
100
10
1
E001
E002
E003
E004
E005
E006
E007
E008
E009
E010
E011
49997
50246
50495
50743
50992
51241
51490
51738
51987
52236

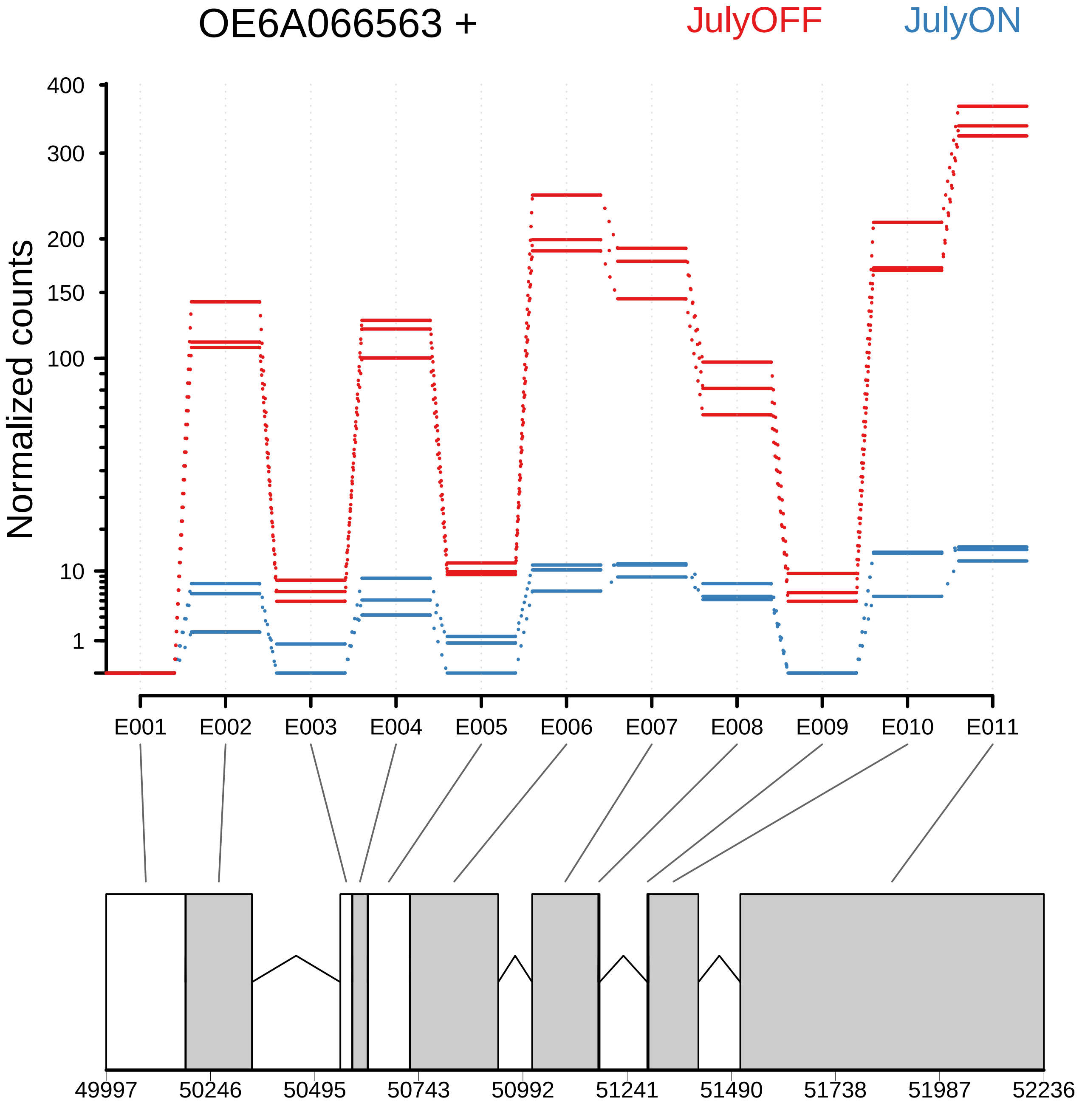

OE6A066563 +
JulyOFF
JulyON
Normalized counts
400
300
200
150
100
10
1
E001
E002
E003
E004
E005
E006
E007
E008
E009
E010
E011
49997
50246
50495
50743
50992
51241
51490
51738
51987
52236

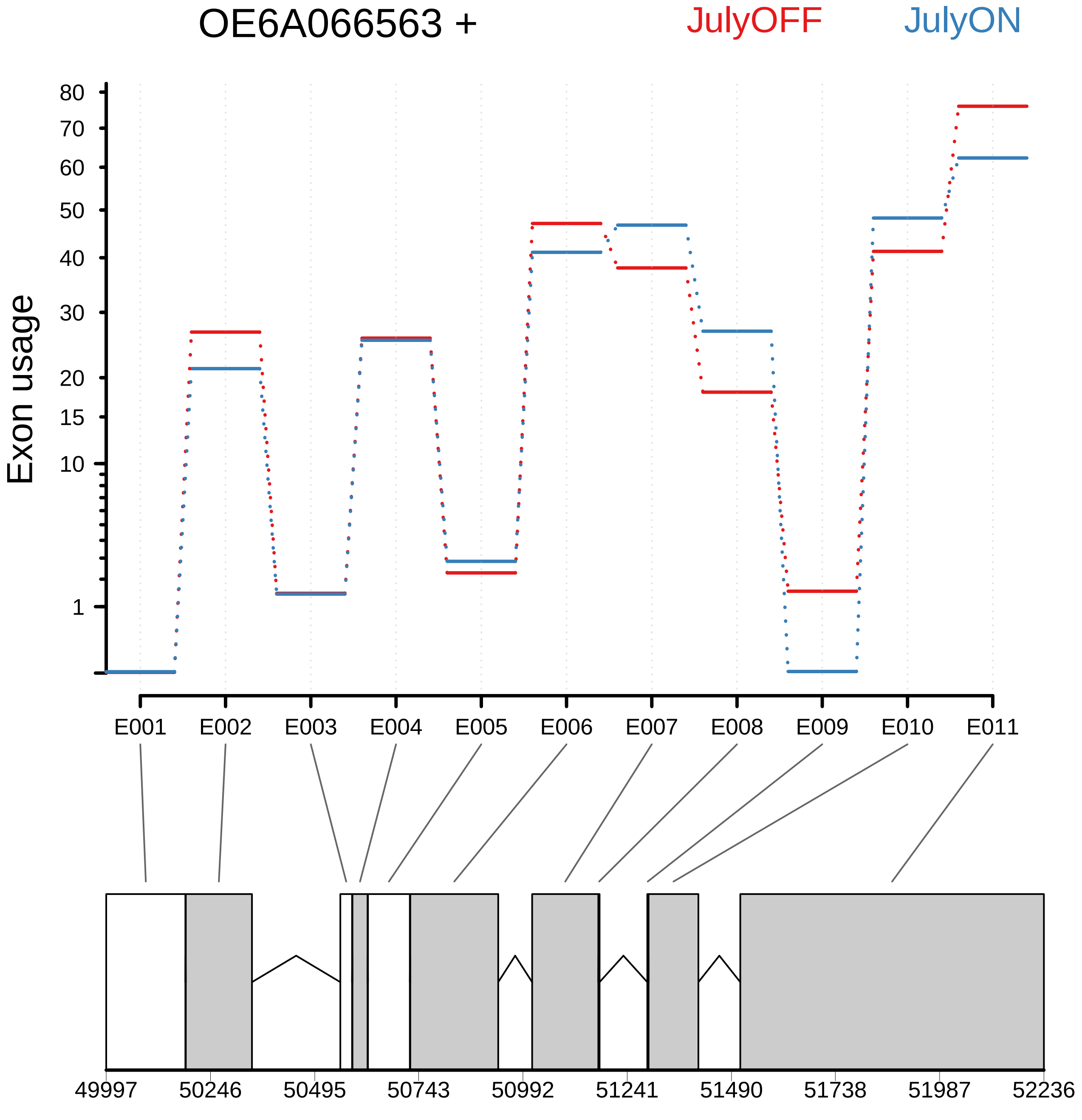
OE6A066563 +
JulyOFF
JulyON
Exon usage
80
70
60
50
40
30
20
15
10
1
E001
E002
E003
E004
E005
E006
E007
E008
E009
E010
E011
49997
50246
50495
50743
50992
51241
51490
51738
51987
52236

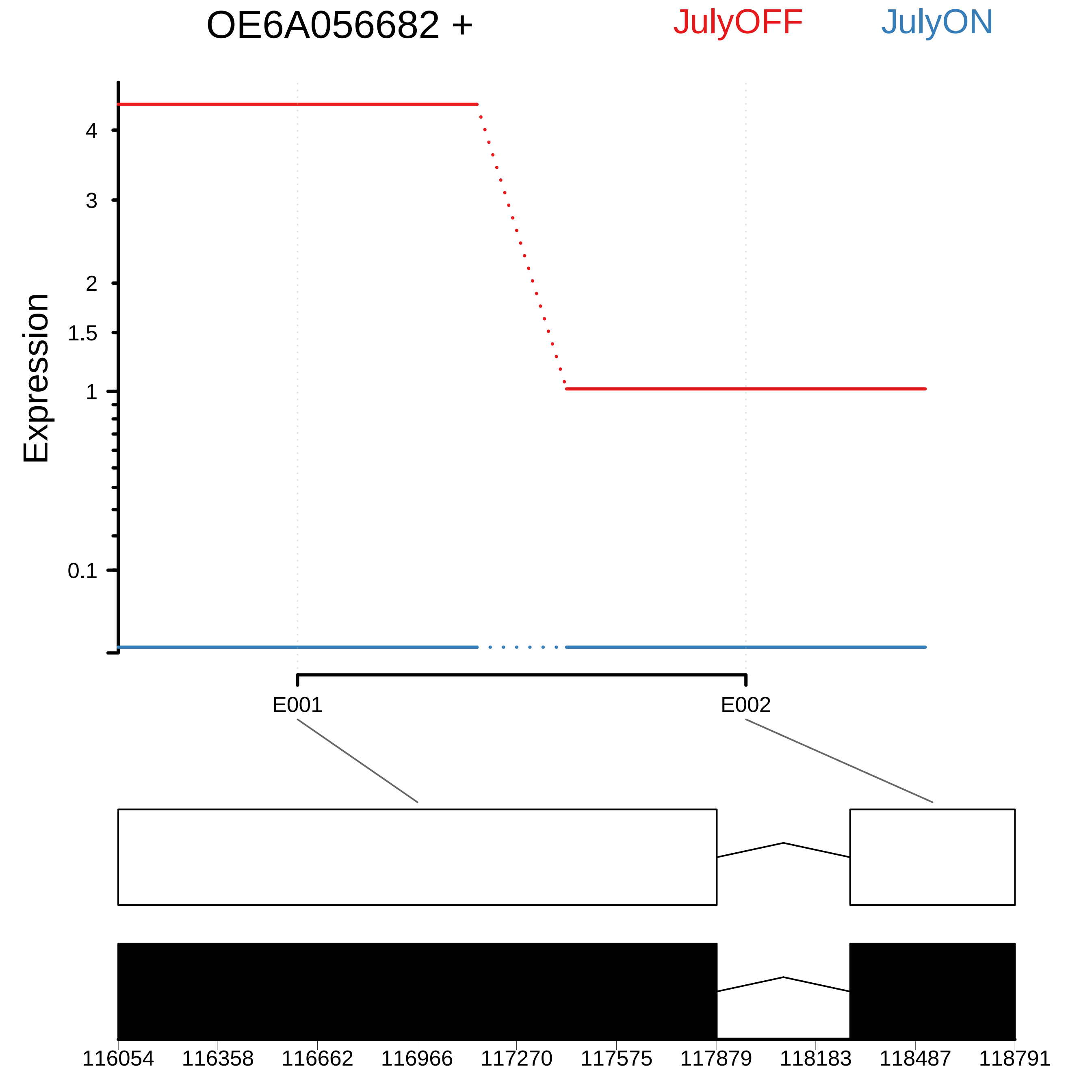

OE6A056682 +
JulyOFF
JulyON
Expression
4
3
2
1.5
1
0.1
E001
E002
116054
116358
116662
116966
117270
117575
117879
118183
118487
118791

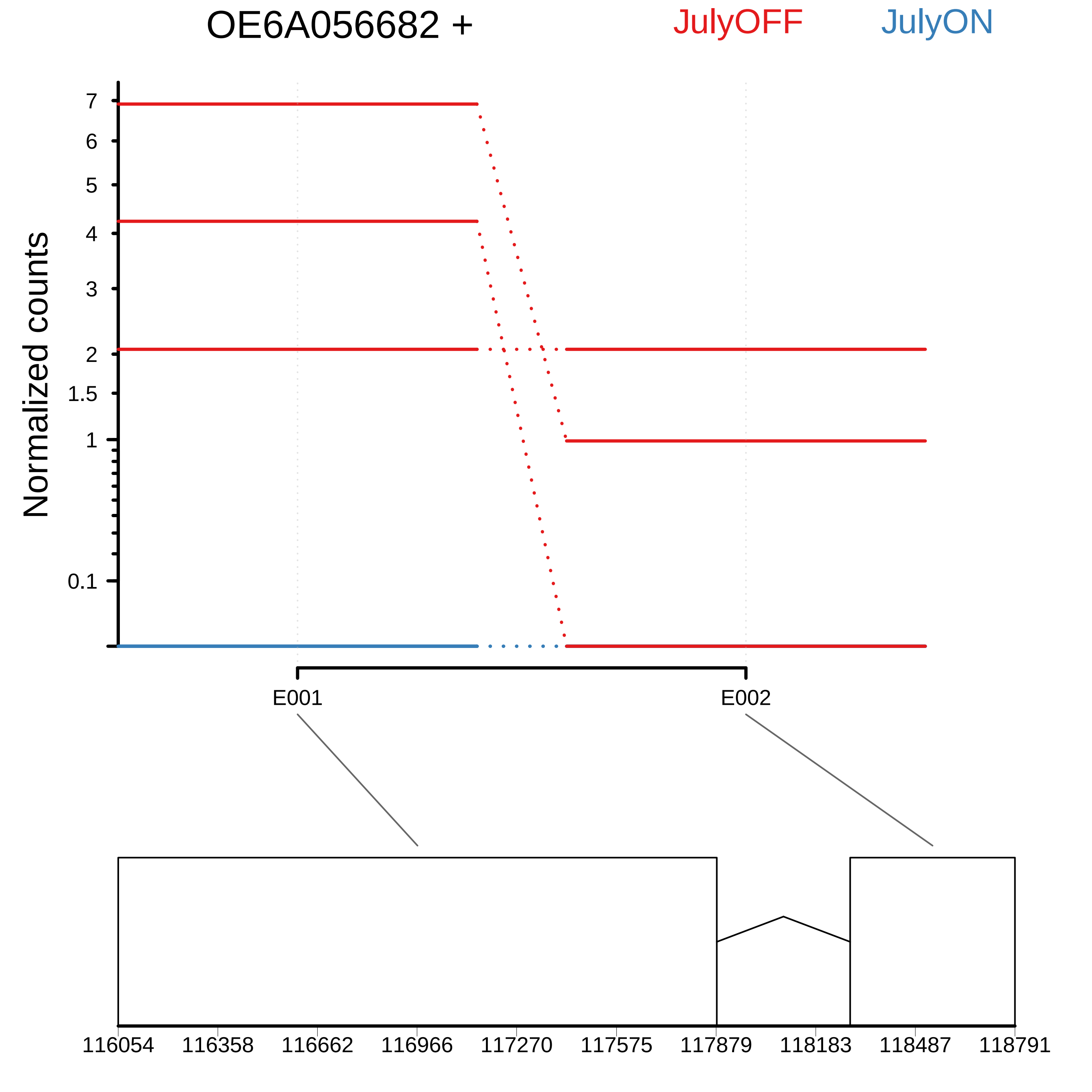

OE6A056682 +
JulyOFF
JulyON
Normalized counts
7
6
5
4
3
2
1.5
1
0.1
E001
E002
116054
116358
116662
116966
117270
117575
117879
118183
118487
118791

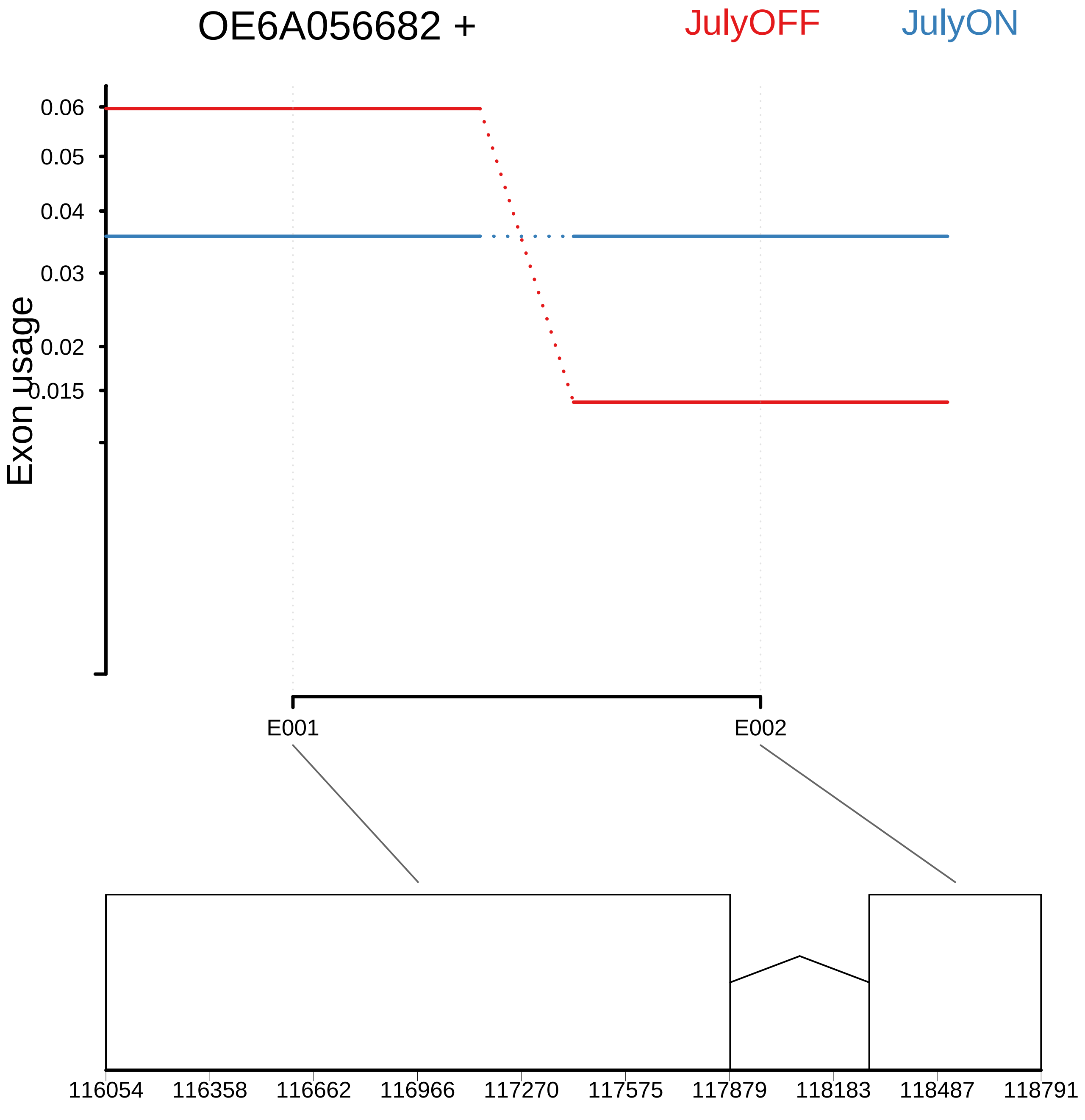
OE6A056682 +
JulyOFF
JulyON
Exon usage
0.06
0.05
0.04
0.03
0.02
0.015
E001
E002
116054
116358
116662
116966
117270
117575
117879
118183
118487
118791

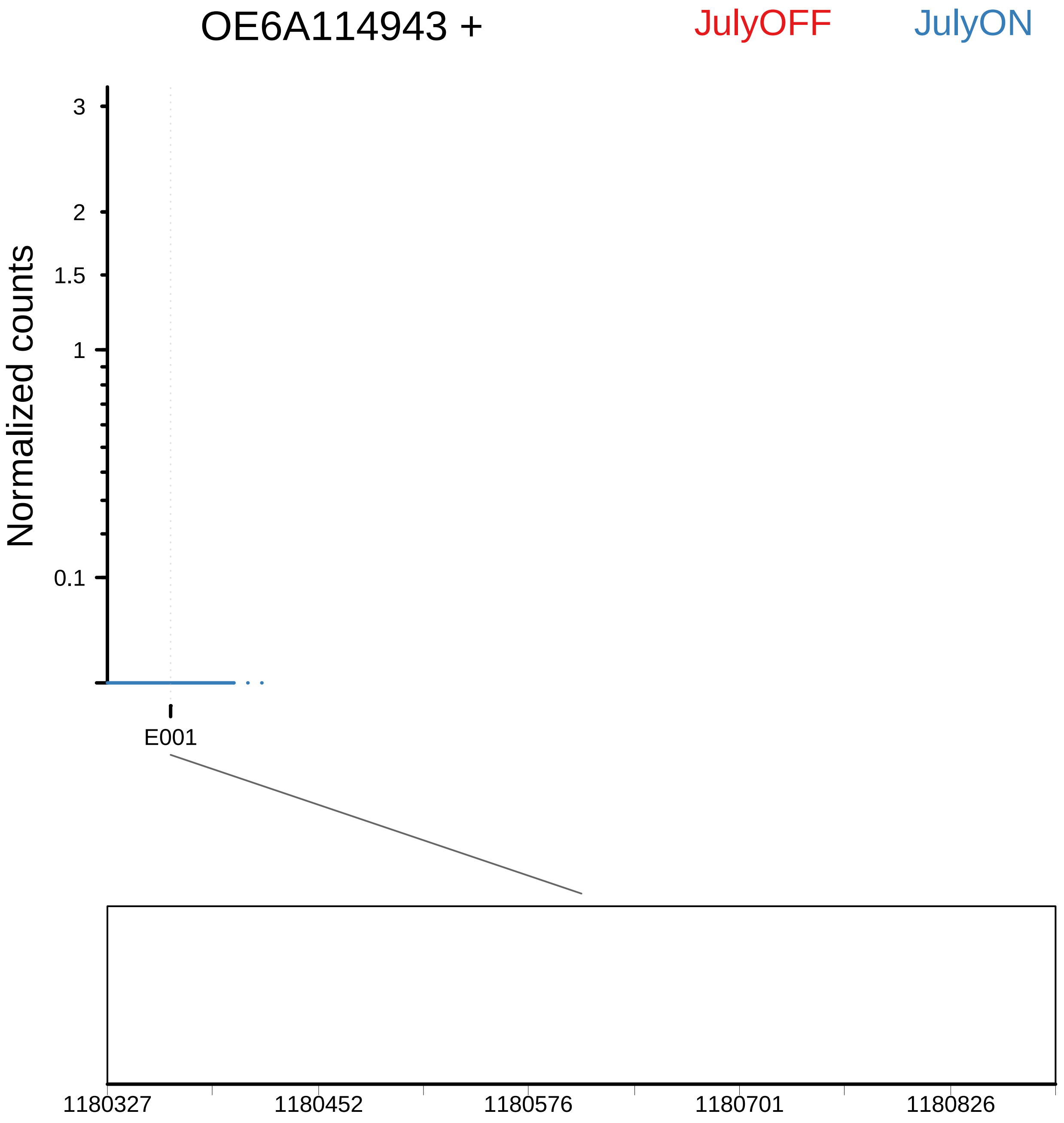
OE6A114943 +
JulyOFF
JulyON
Normalized counts
3
2
1.5
1
0.1
E001
1180327
1180452
1180576
1180701
1180826

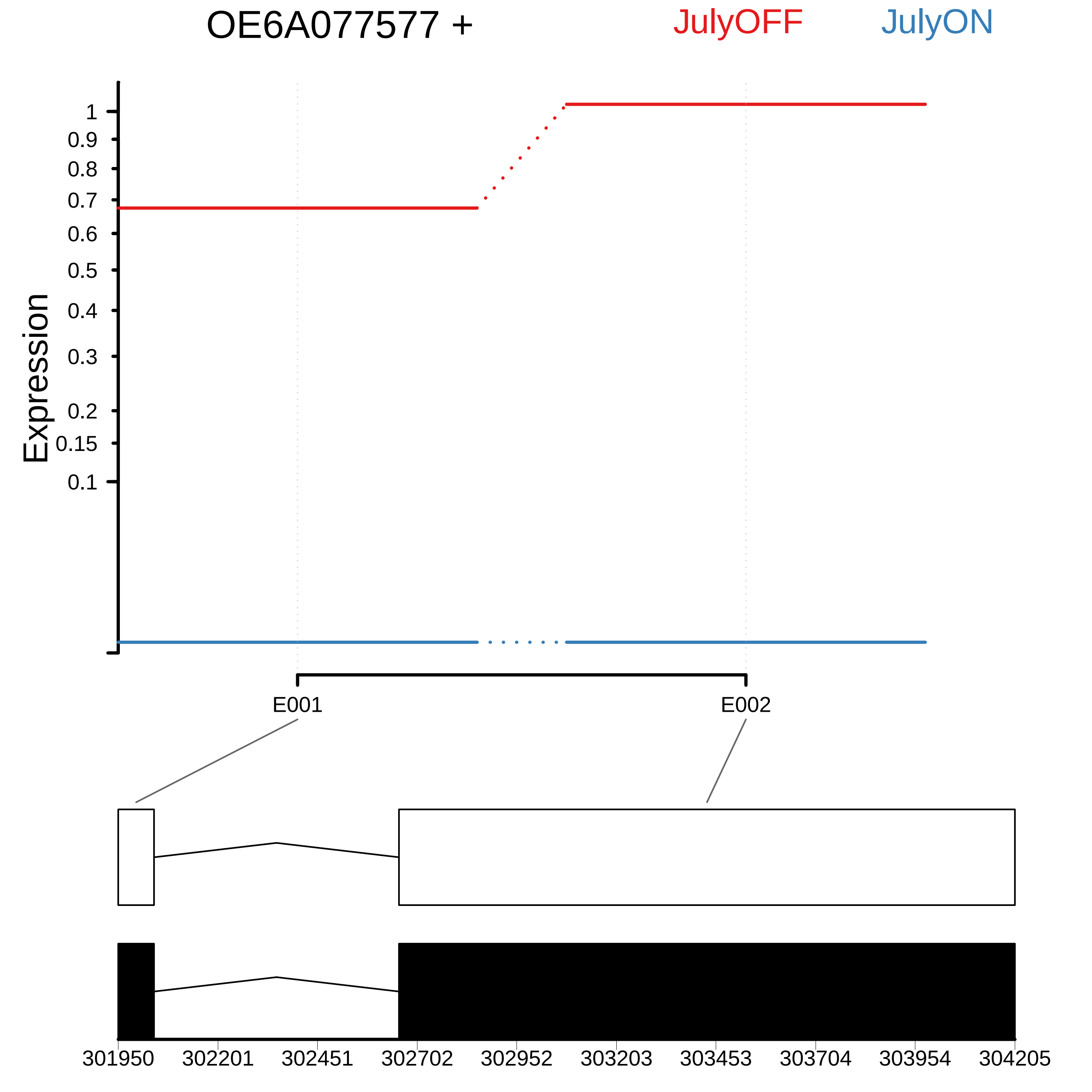

OE6A077577 +
JulyOFF
JulyON
Expression
1
0.9
0.8
0.7
0.6
0.5
0.4
0.3
0.2
0.15
0.1
E001
E002
301950
302201
302451
302702
302952
303203
303453
303704
303954
304205

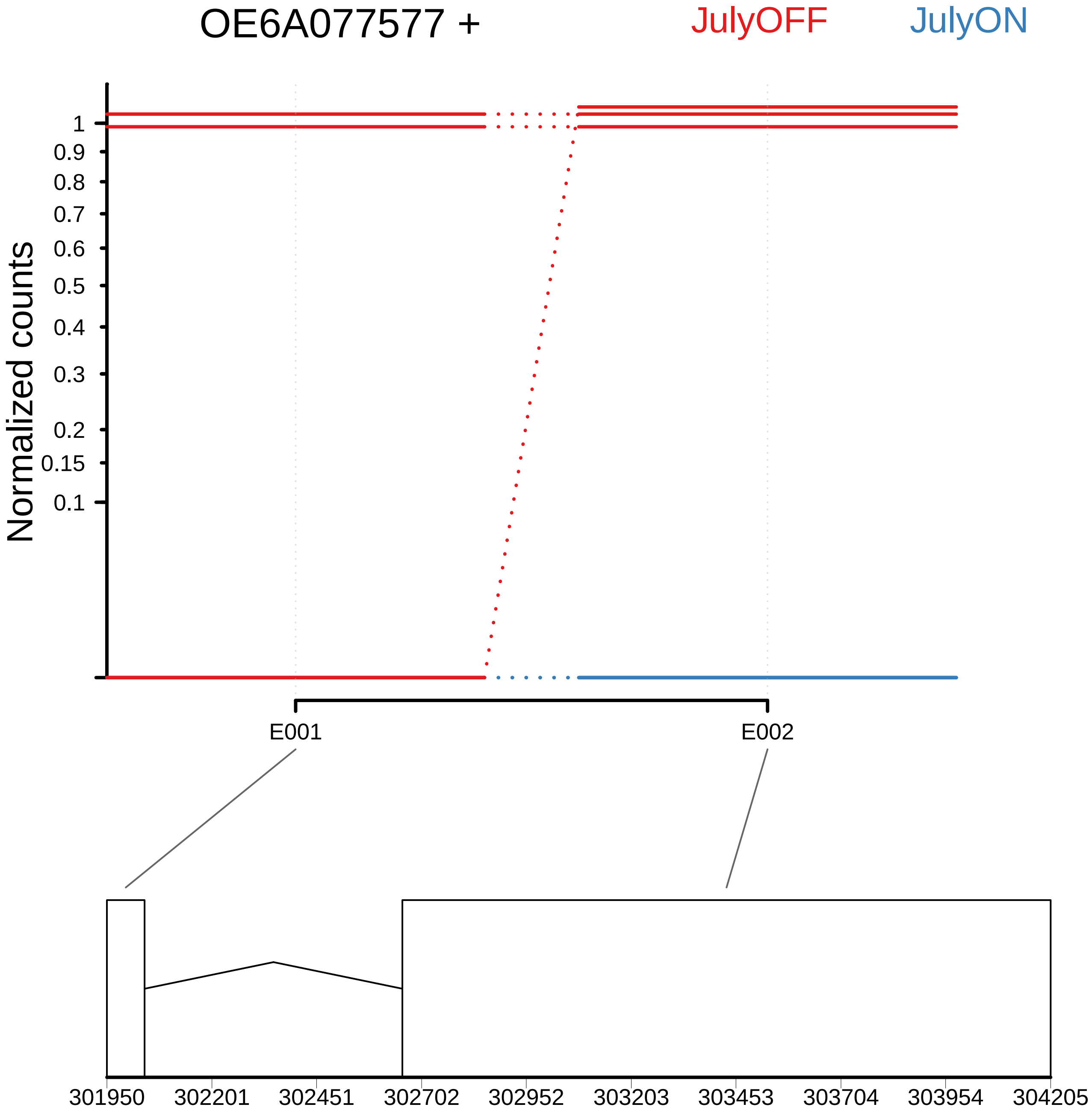

OE6A077577 +
JulyOFF
JulyON
Normalized counts
1
0.9
0.8
0.7
0.6
0.5
0.4
0.3
0.2
0.15
0.1
E001
E002
301950
302201
302451
302702
302952
303203
303453
303704
303954
304205

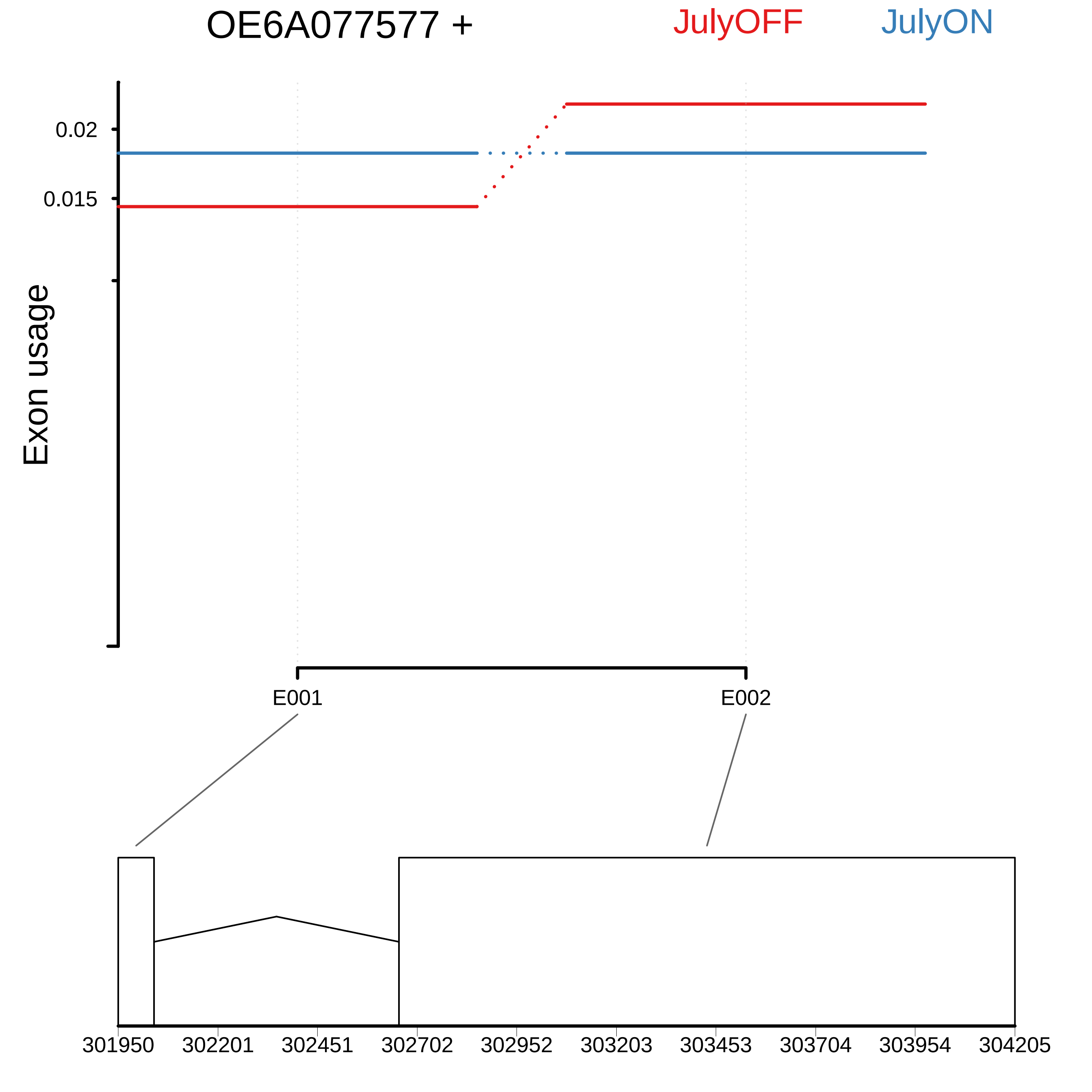
OE6A077577 +
JulyOFF
JulyON
0.02
0.015
Exon usage
E001
E002
301950
302201
302451
302702
302952
303203
303453
303704
303954
304205

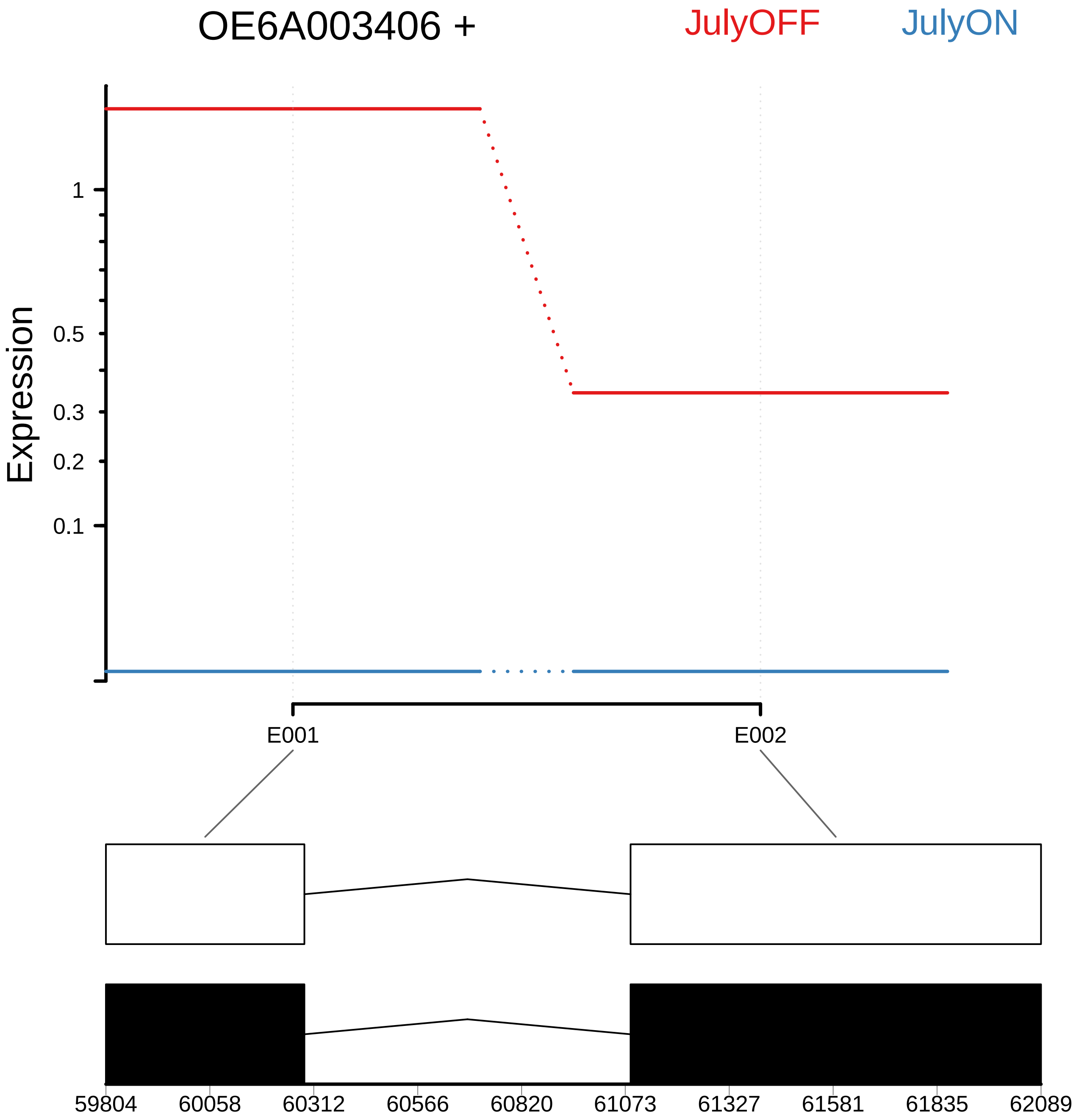
OE6A003406 +
JulyOFF
JulyON
Expression
1
0.5
0.3
0.2
0.1
E001
E002
59804
60058
60312
60566
60820
61073
61327
61581
61835
62089

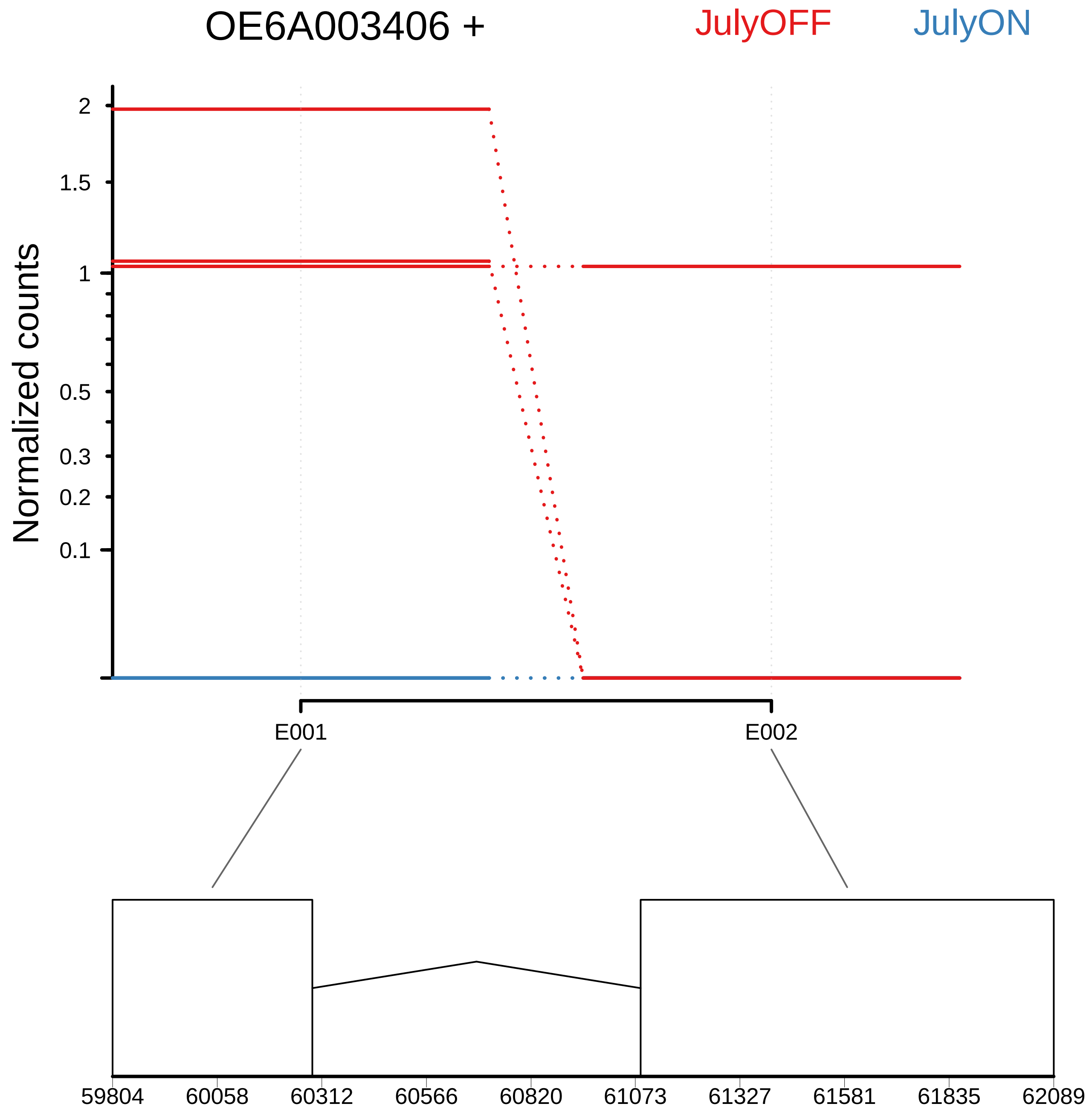

OE6A003406 +
JulyOFF
JulyON
Normalized counts
2
1.5
1
0.5
0.3
0.2
0.1
E001
E002
59804
60058
60312
60566
60820
61073
61327
61581
61835
62089

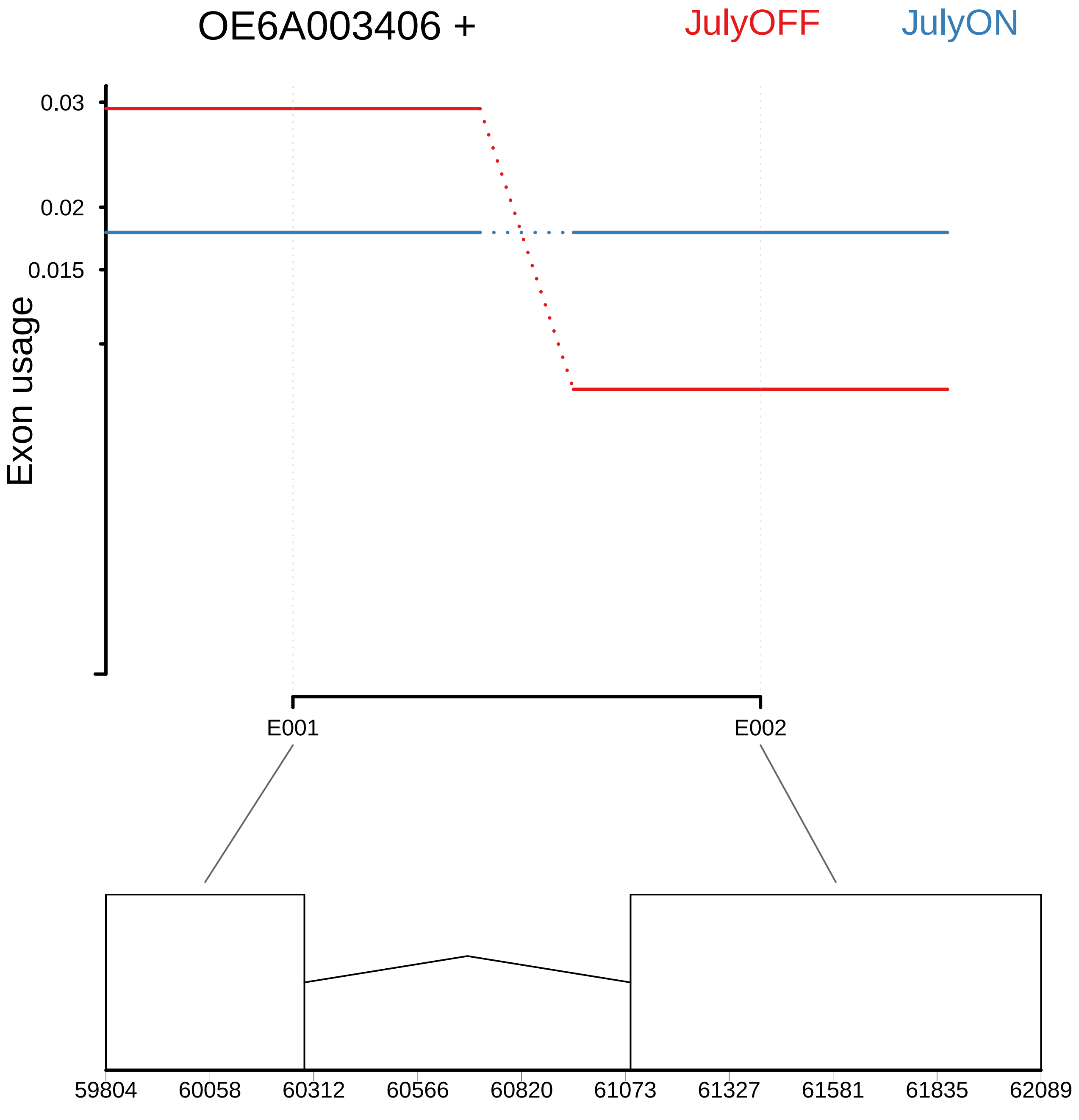
OE6A003406 +
JulyOFF
JulyON
0.03
0.02
0.015
Exon usage
E001
E002
59804
60058
60312
60566
60820
61073
61327
61581
61835
62089

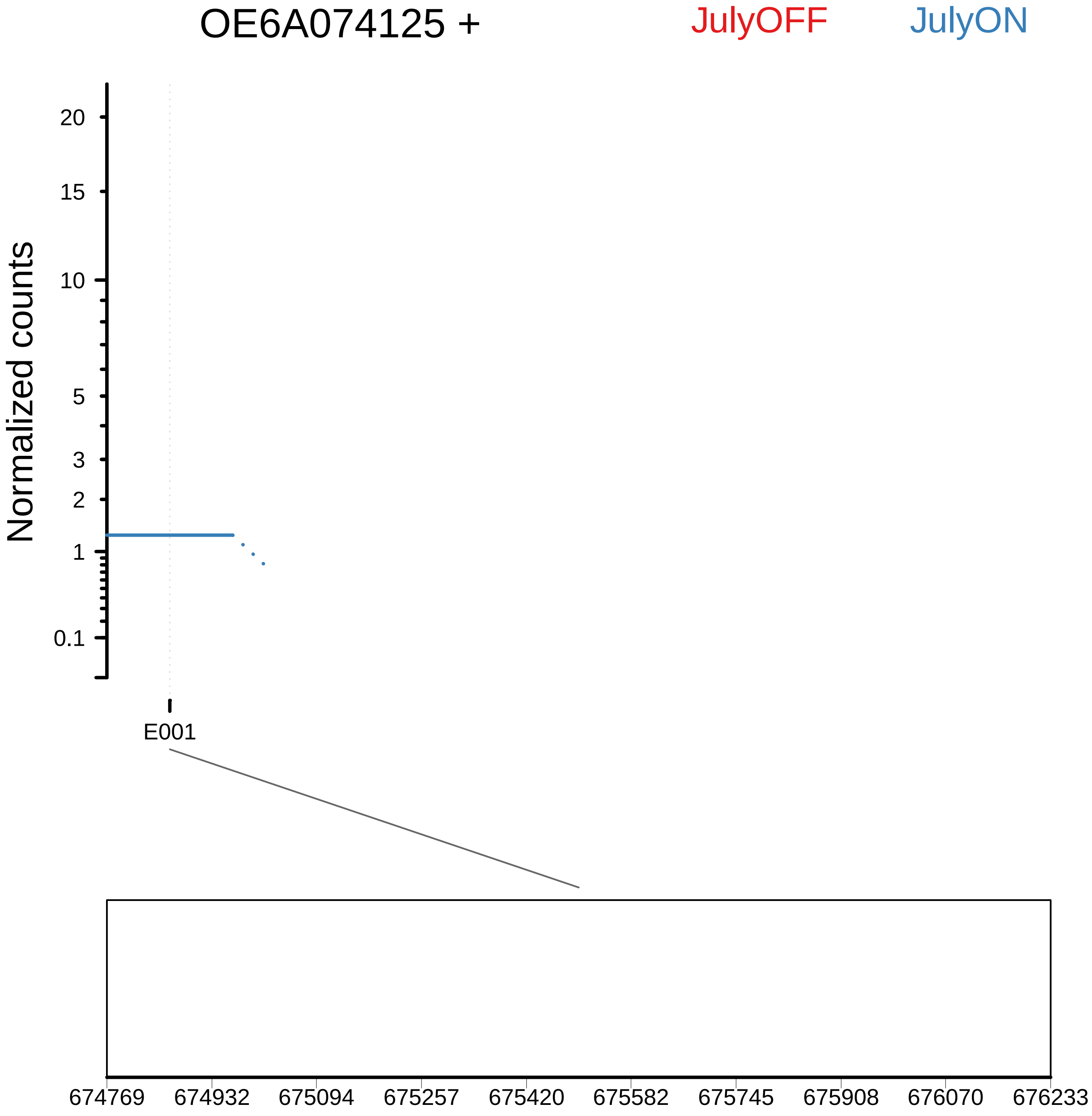
OE6A074125 +
JulyOFF
JulyON
Normalized counts
20
15
10
5
3
2
1
0.1
E001
674769
674932
675094
675257
675420
675582
675745
675908
676070
676233

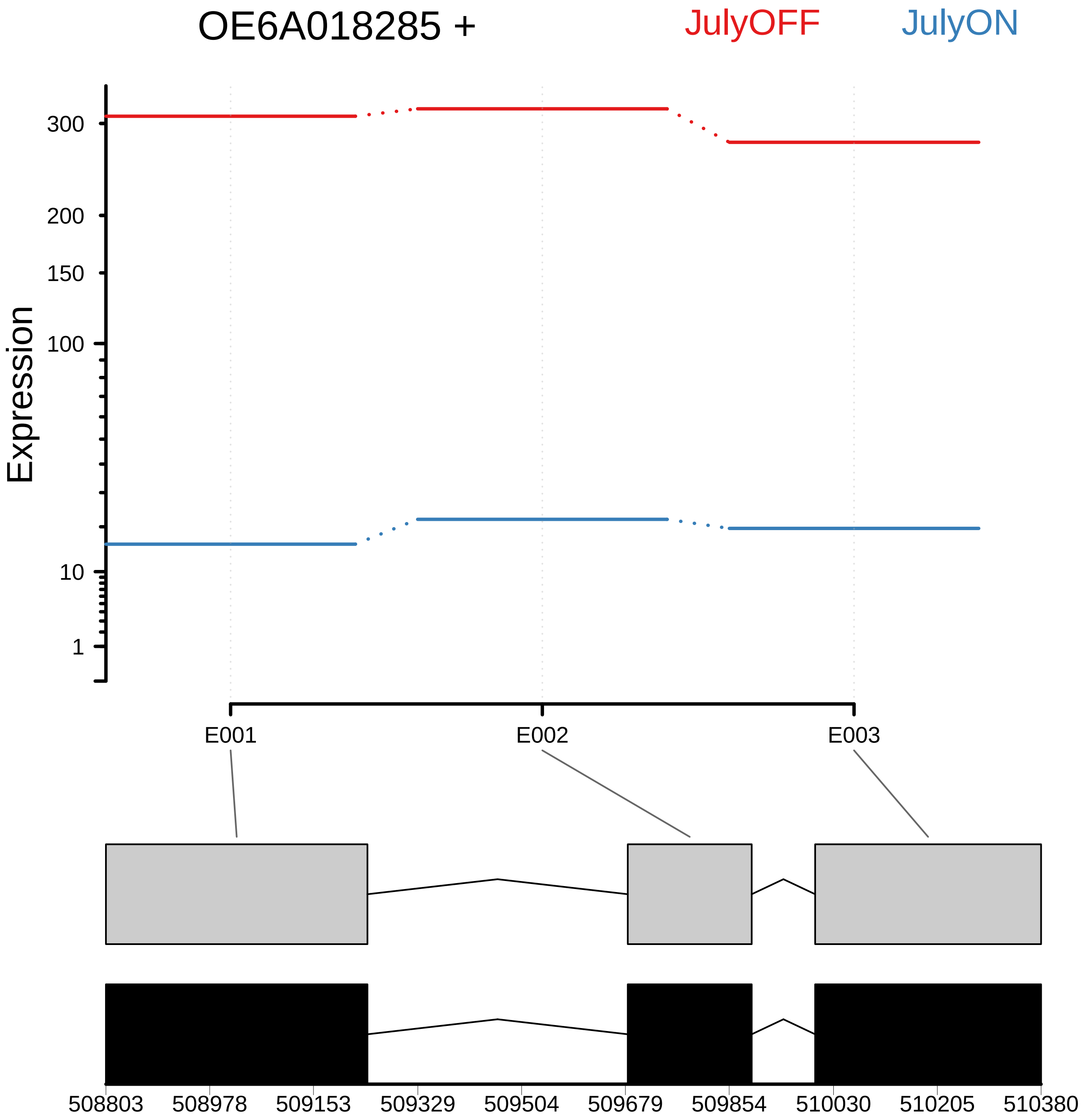
OE6A018285 +
JulyOFF
JulyON
Expression
300
200
150
100
10
1
E001
E002
E003
508803
508978
509153
509329
509504
509679
509854
510030
510205
510380

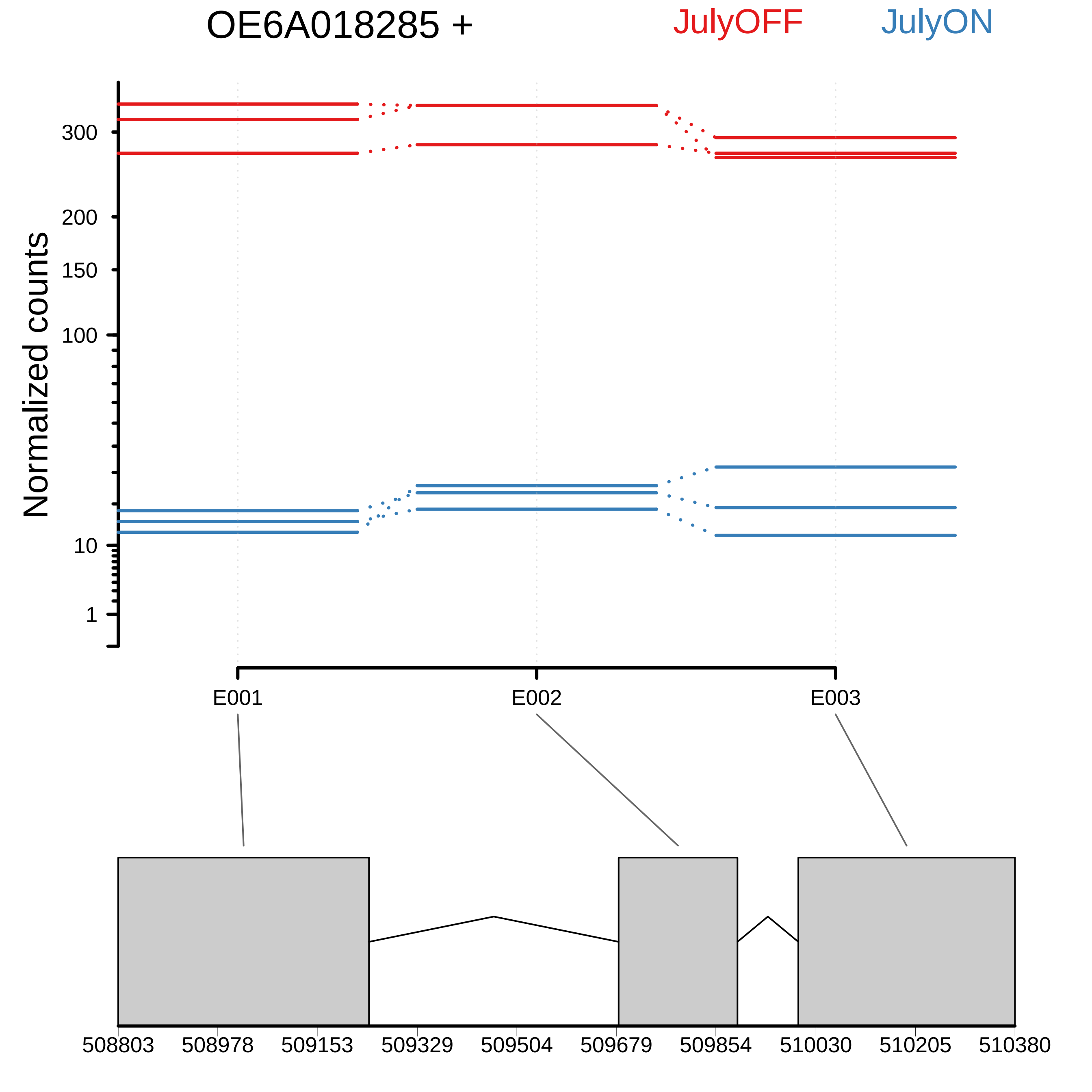

OE6A018285 +
JulyOFF
JulyON
Normalized counts
300
200
150
100
10
1
E001
E002
E003
508803
508978
509153
509329
509504
509679
509854
510030
510205
510380

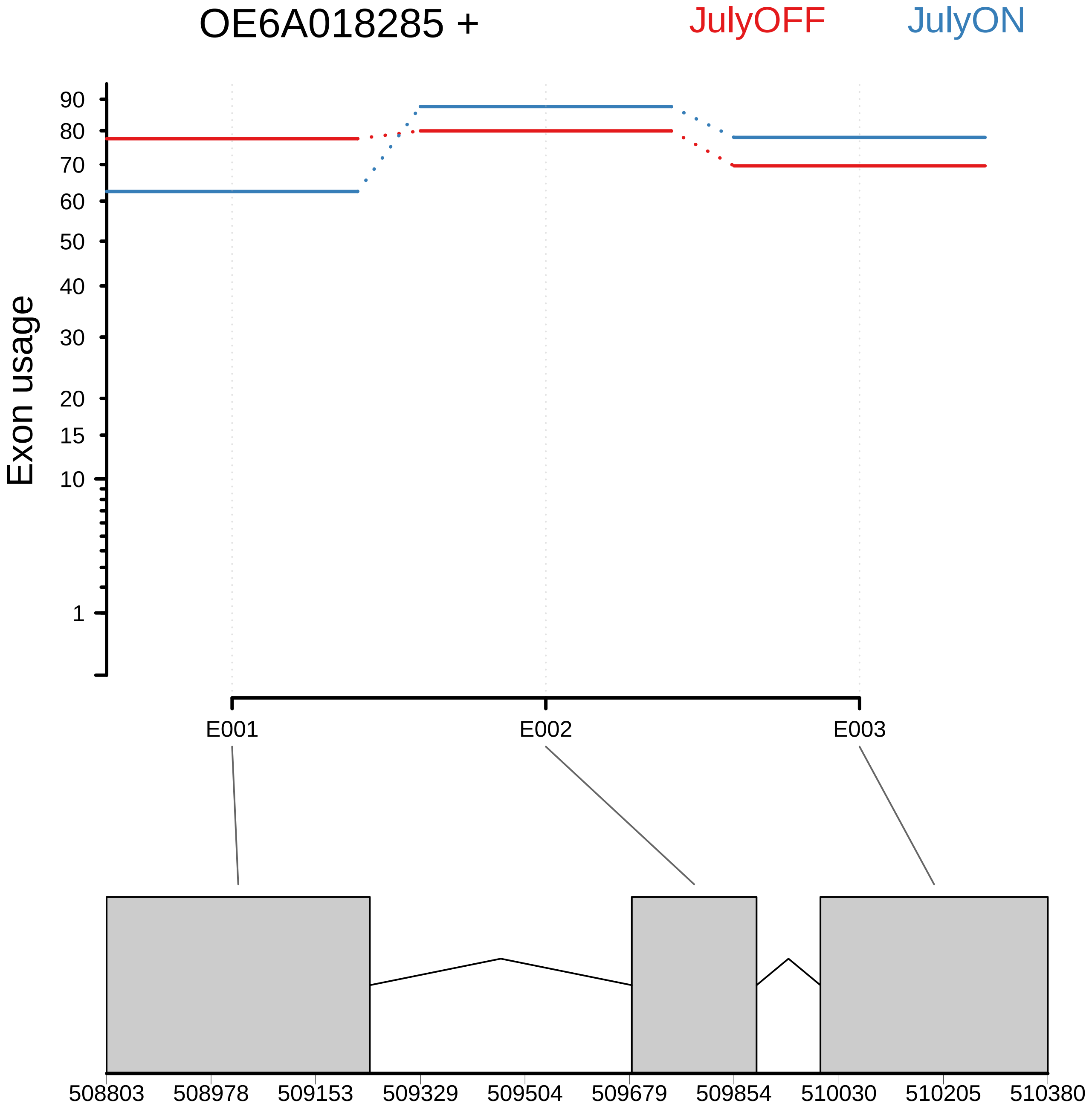

OE6A018285 +
JulyOFF
JulyON
Exon usage
90
80
70
60
50
40
30
20
15
10
1
E001
E002
E003
508803
508978
509153
509329
509504
509679
509854
510030
510205
510380

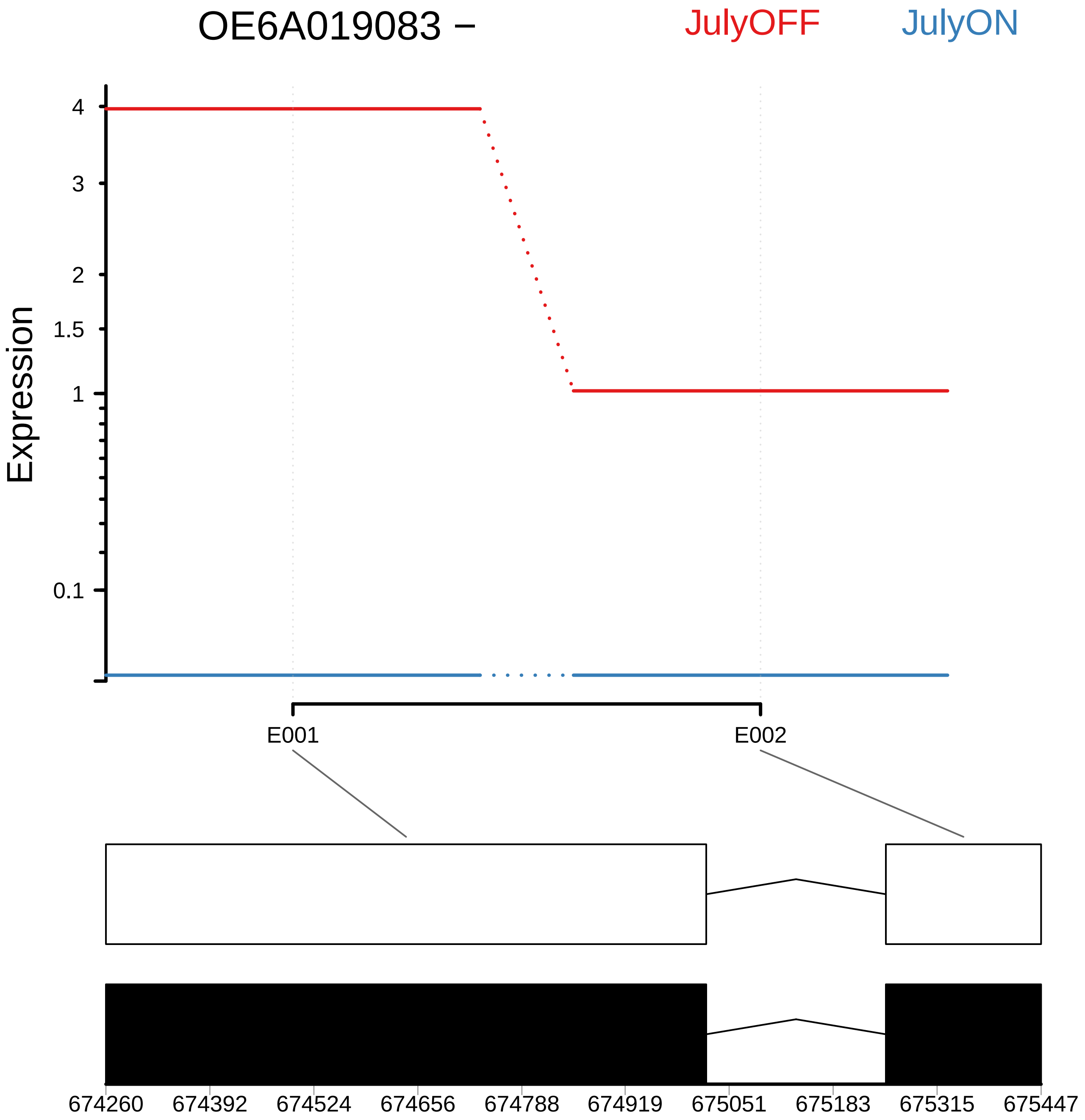

OE6A019083 −
JulyOFF
JulyON
Expression
4
3
2
1.5
1
0.1
E001
E002
674260
674392
674524
674656
674788
674919
675051
675183
675315
675447

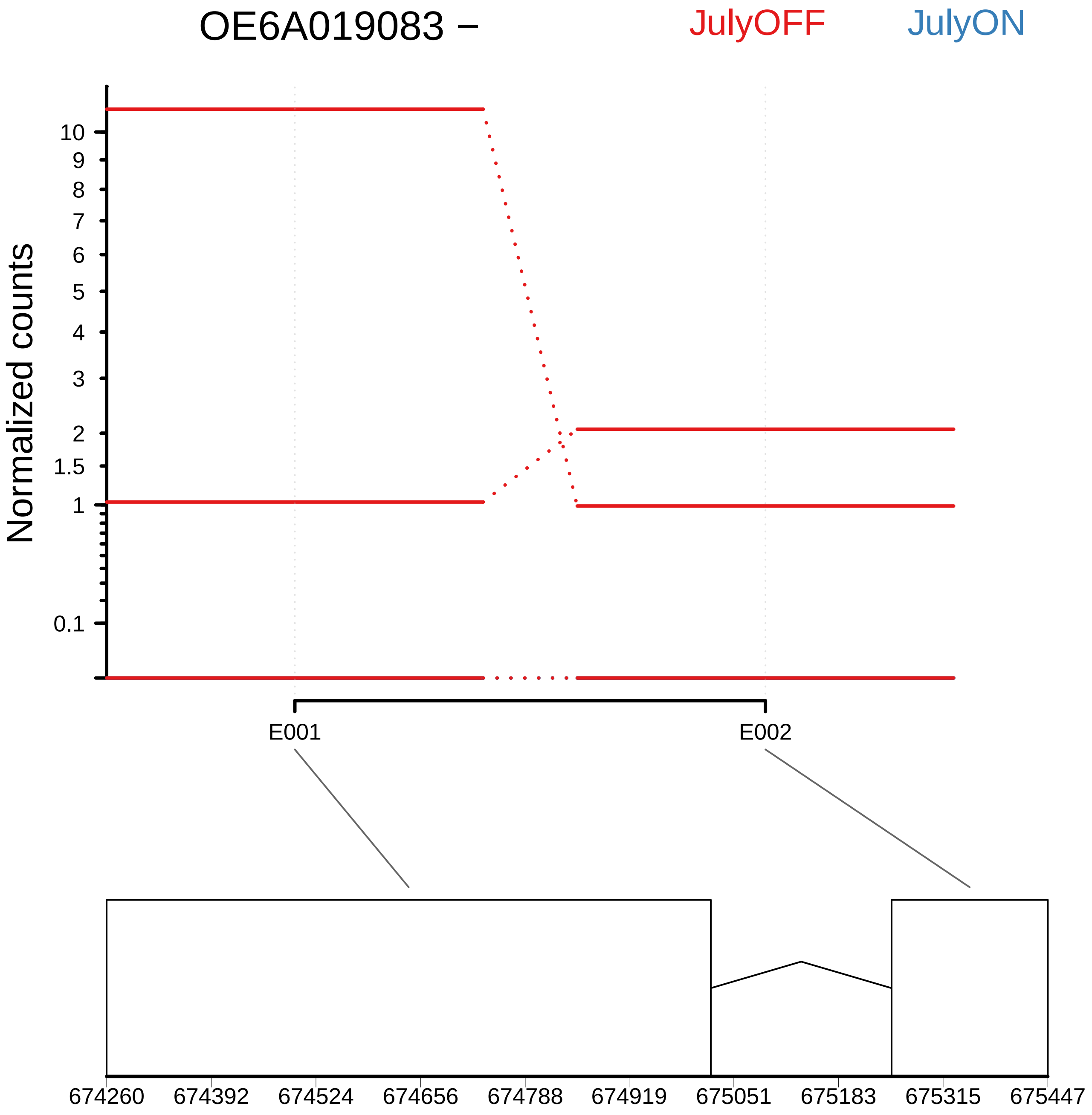

OE6A019083 −
JulyOFF
JulyON
Normalized counts
10
9
8
7
6
5
4
3
2
1.5
1
0.1
E001
E002
674260
674392
674524
674656
674788
674919
675051
675183
675315
675447

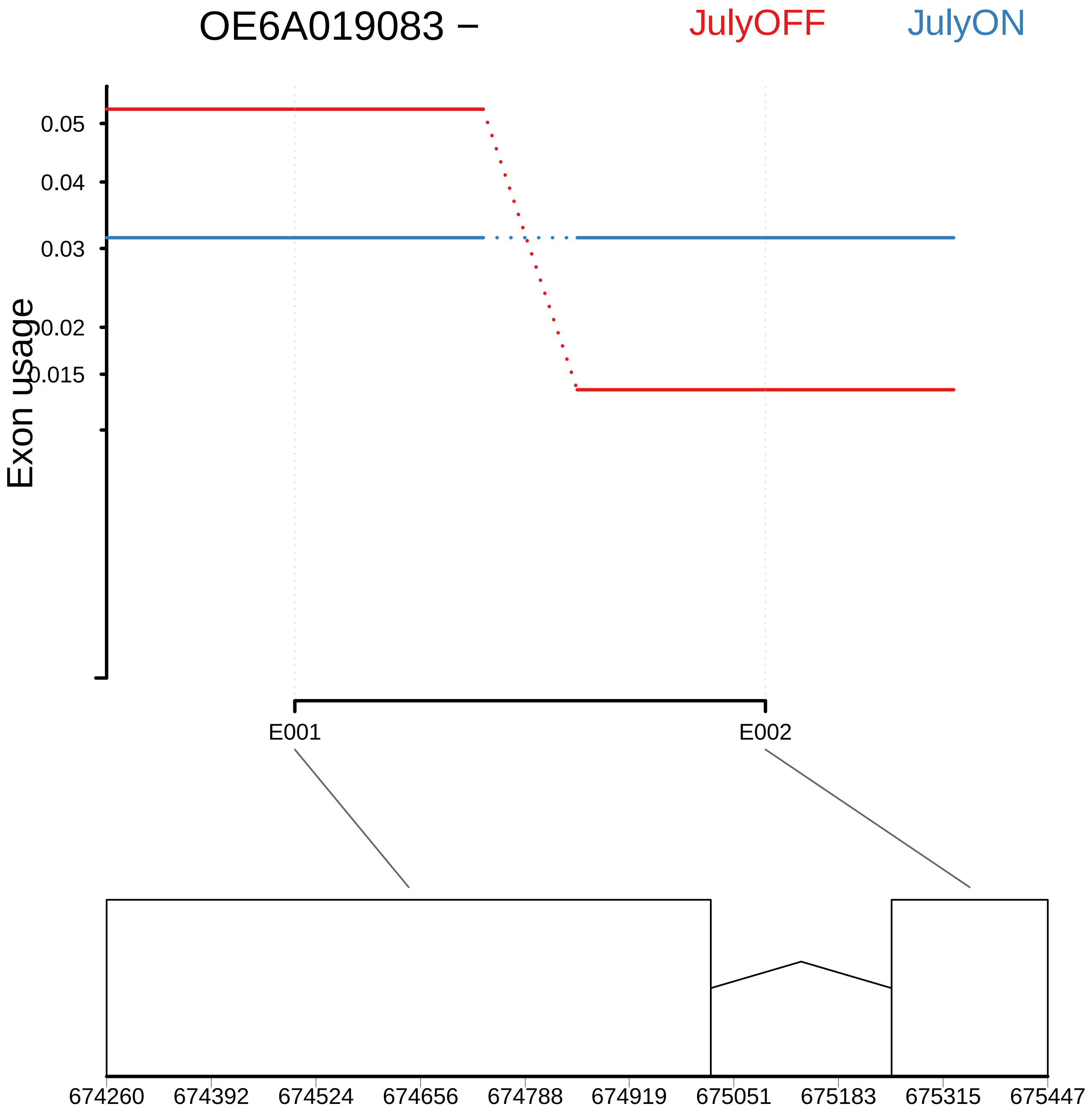
OE6A019083 −
JulyOFF
JulyON
Exon usage
0.05
0.04
0.03
0.02
0.015
E001
E002
674260
674392
674524
674656
674788
674919
675051
675183
675315
675447

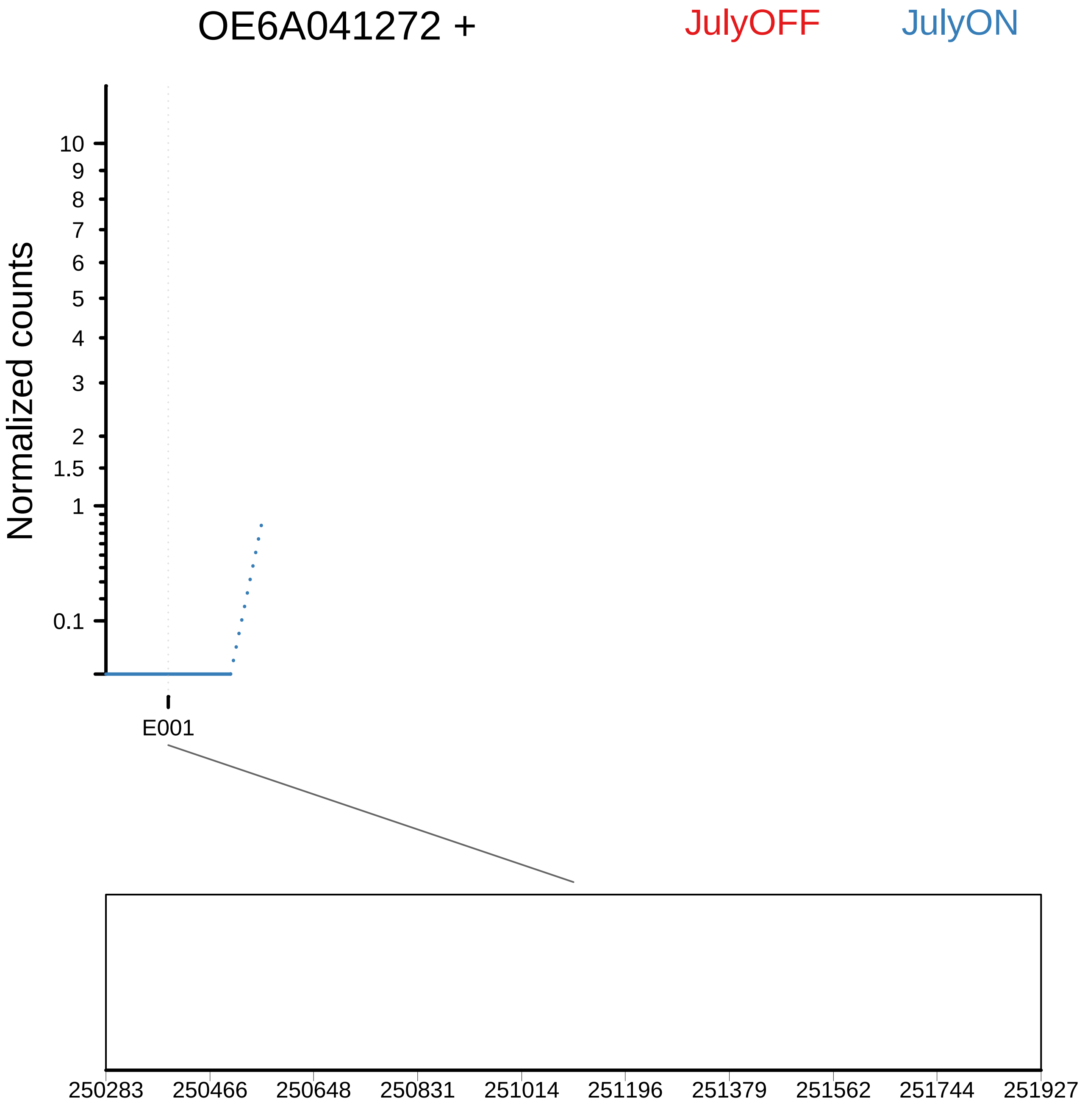

OE6A041272 +
JulyOFF
JulyON
Normalized counts
10
9
8
7
6
5
4
3
2
1.5
1
0.1
E001
250283
250466
250648
250831
251014
251196
251379
251562
251744
251927

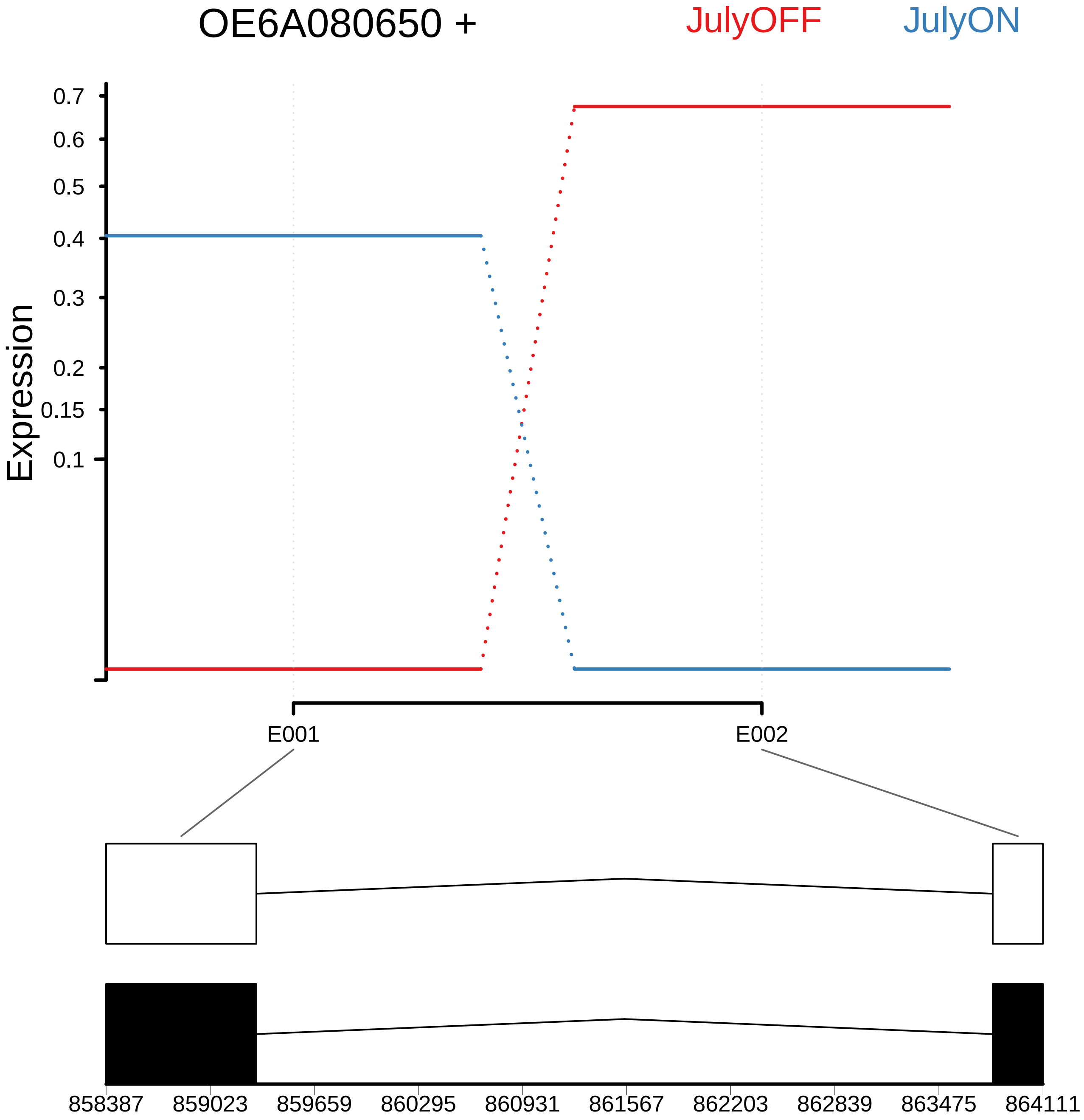
OE6A080650 +
JulyOFF
JulyON
Expression
0.7
0.6
0.5
0.4
0.3
0.2
0.15
0.1
E001
E002
858387
859023
859659
860295
860931
861567
862203
862839
863475
864111

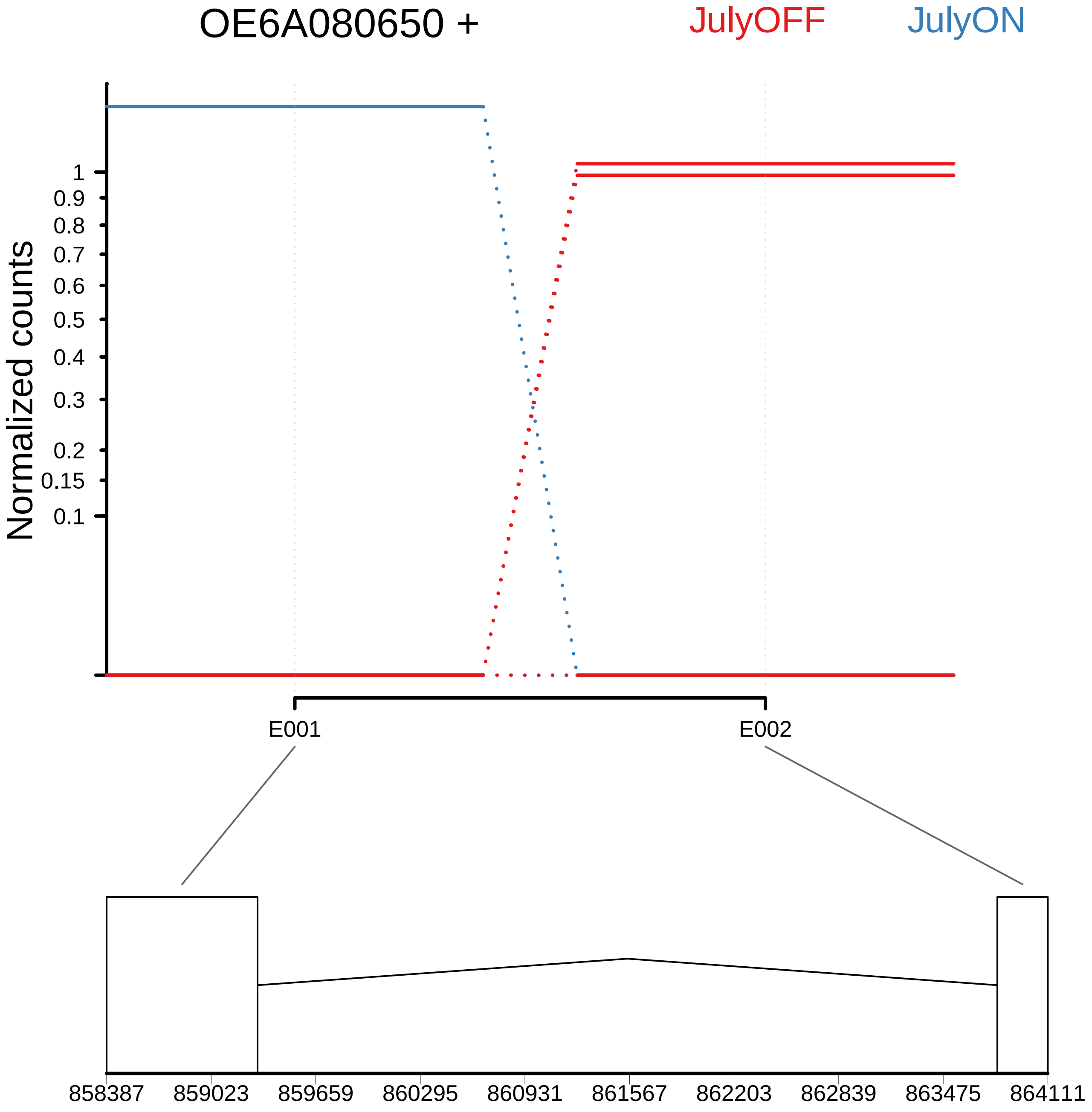
OE6A080650 +
JulyOFF
JulyON
Normalized counts
1
0.9
0.8
0.7
0.6
0.5
0.4
0.3
0.2
0.15
0.1
E001
E002
858387
859023
859659
860295
860931
861567
862203
862839
863475
864111

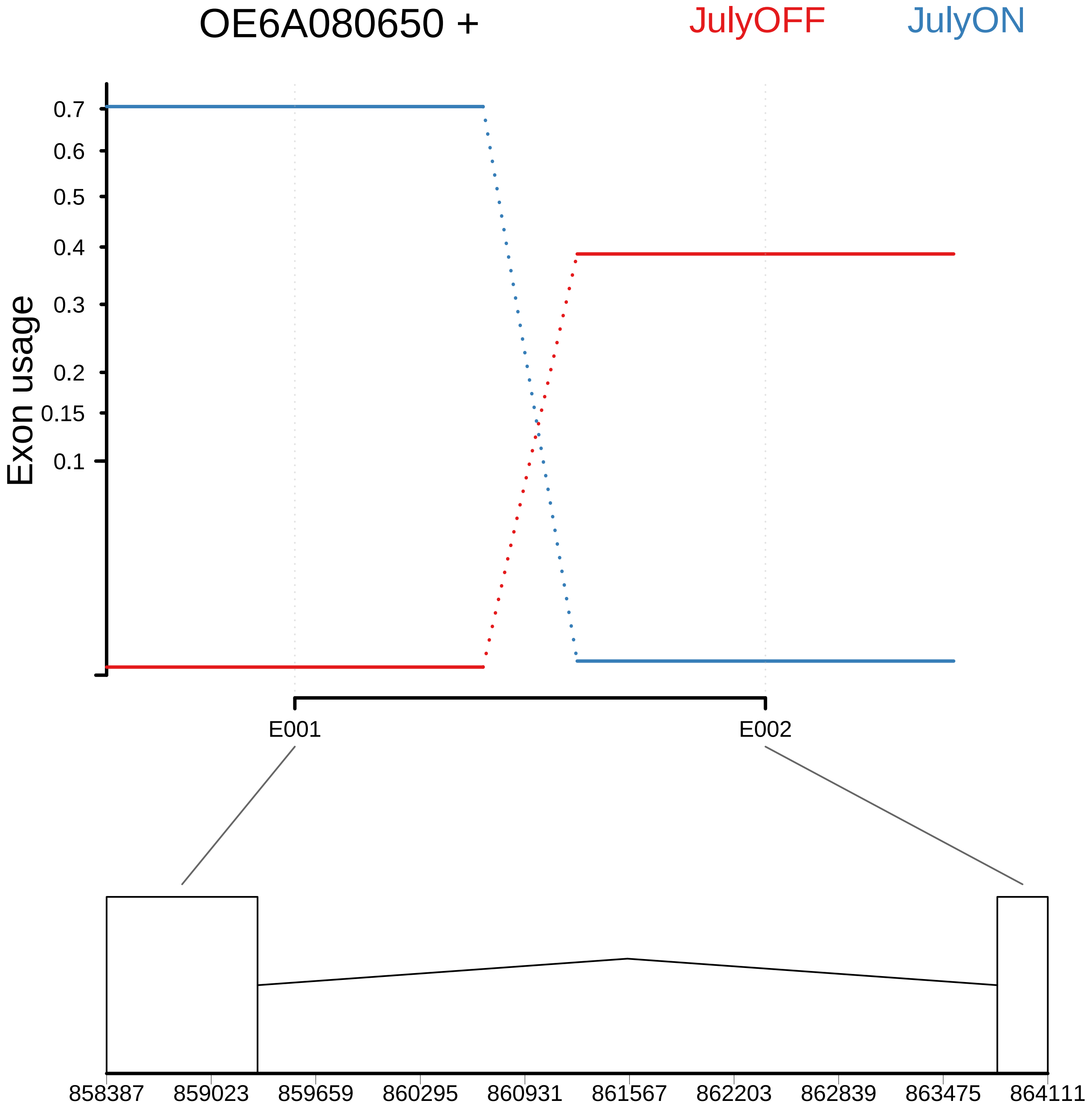
OE6A080650 +
JulyOFF
JulyON
Exon usage
0.7
0.6
0.5
0.4
0.3
0.2
0.15
0.1
E001
E002
858387
859023
859659
860295
860931
861567
862203
862839
863475
864111

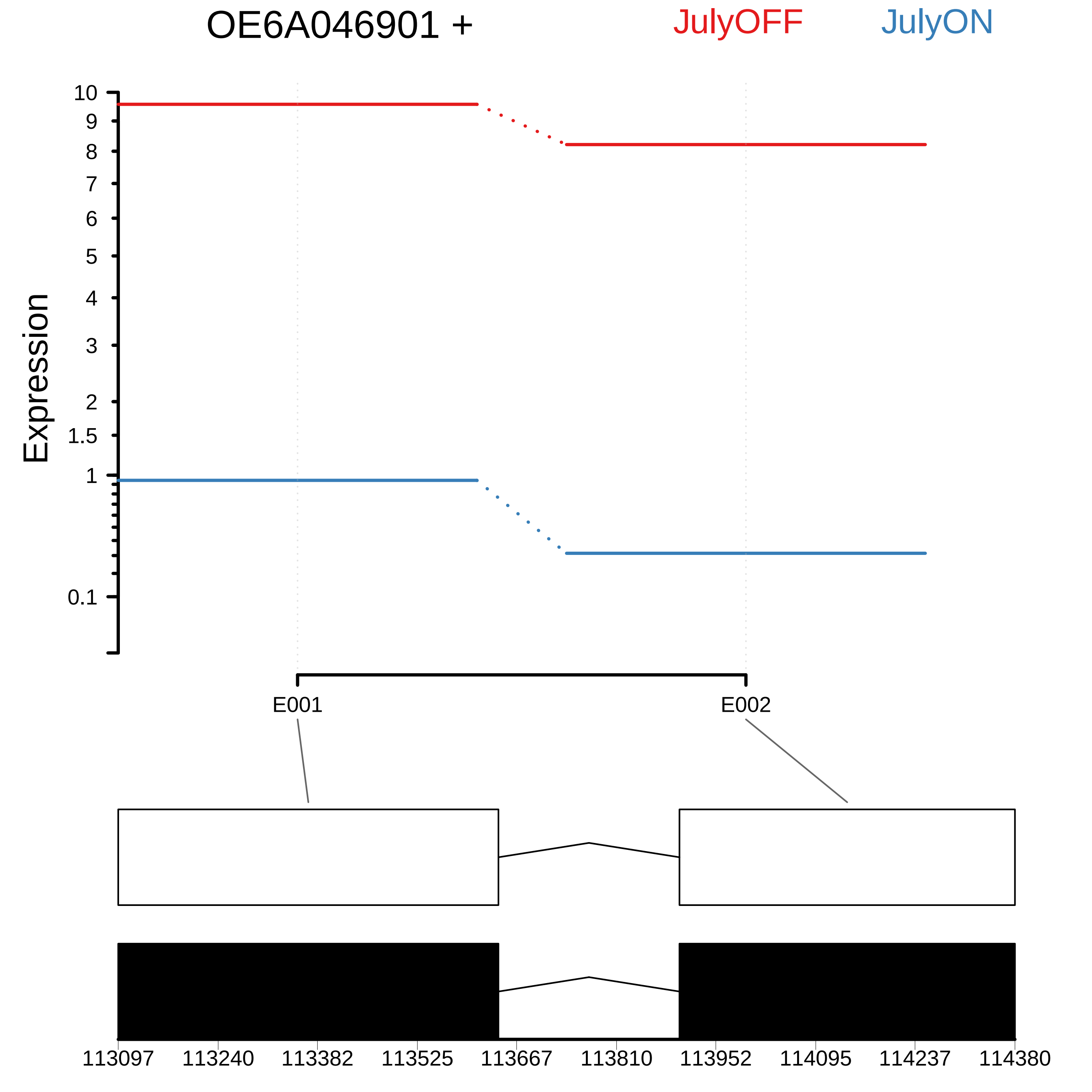

OE6A046901 +
JulyOFF
JulyON
Expression
10
9
8
7
6
5
4
3
2
1.5
1
0.1
E001
E002
113097
113240
113382
113525
113667
113810
113952
114095
114237
114380

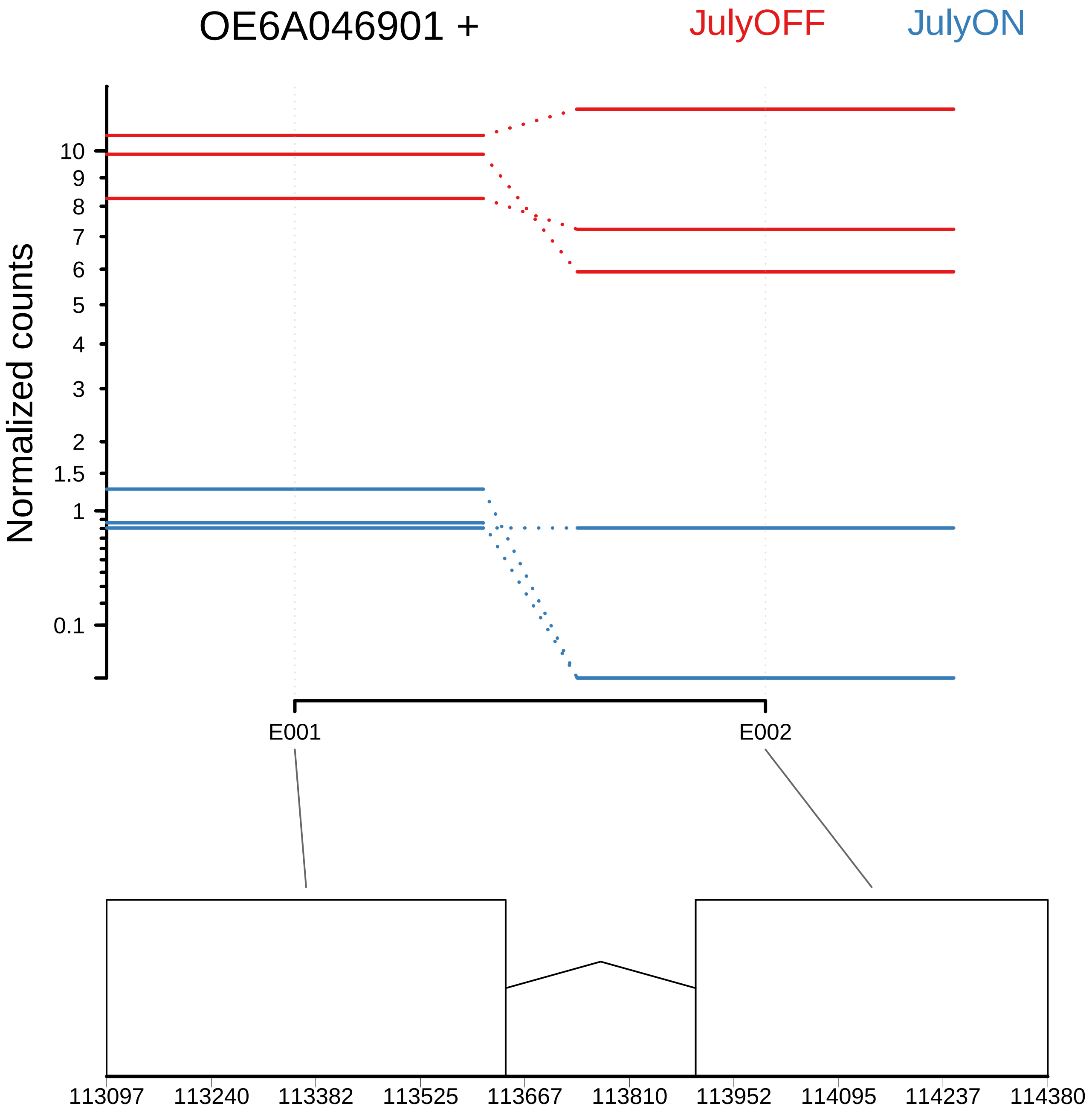
OE6A046901 +
JulyOFF
JulyON
Normalized counts
10
9
8
7
6
5
4
3
2
1.5
1
0.1
E001
E002
113097
113240
113382
113525
113667
113810
113952
114095
114237
114380

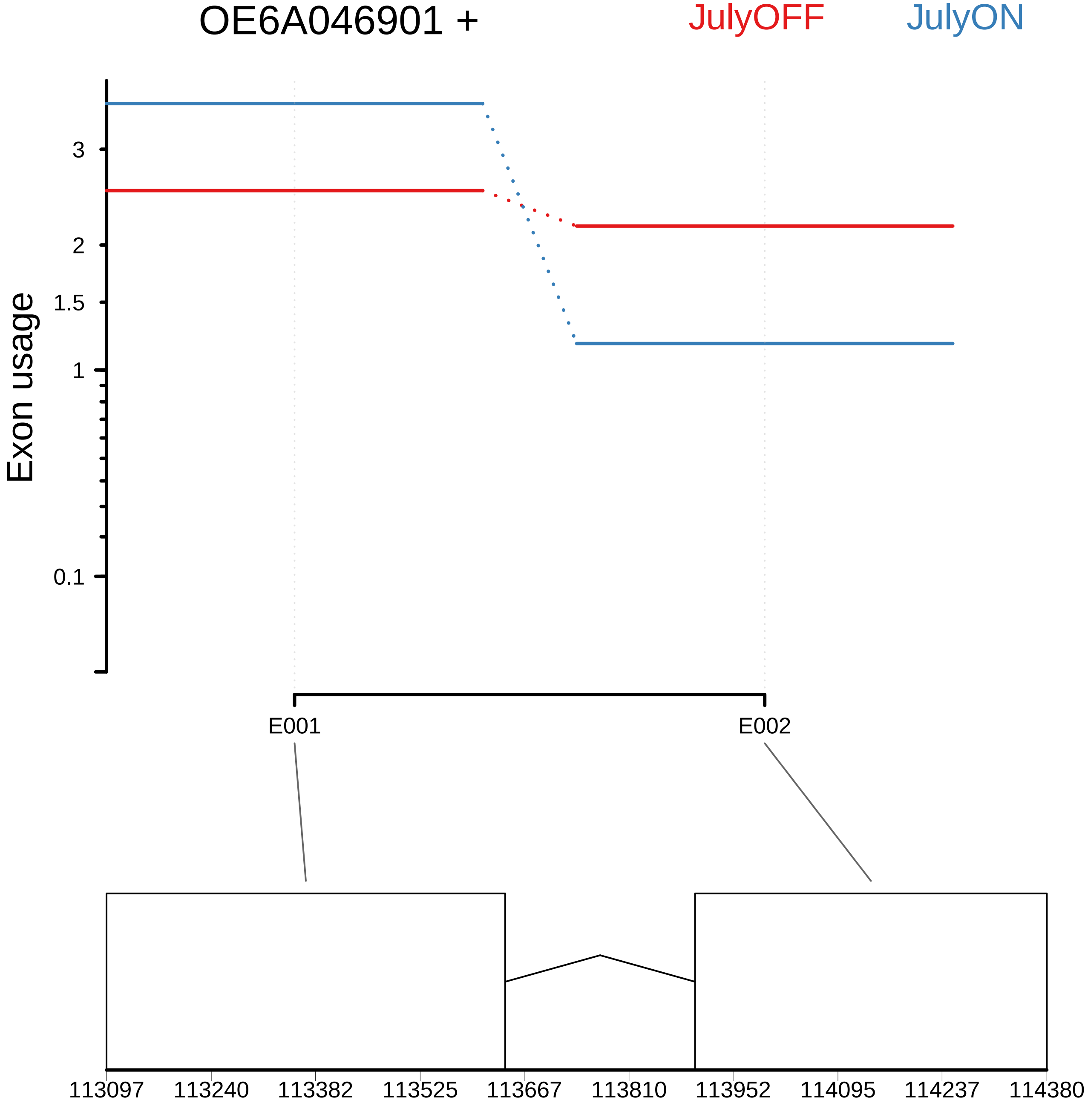

OE6A046901 +
JulyOFF
JulyON
Exon usage
3
2
1.5
1
0.1
E001
E002
113097
113240
113382
113525
113667
113810
113952
114095
114237
114380

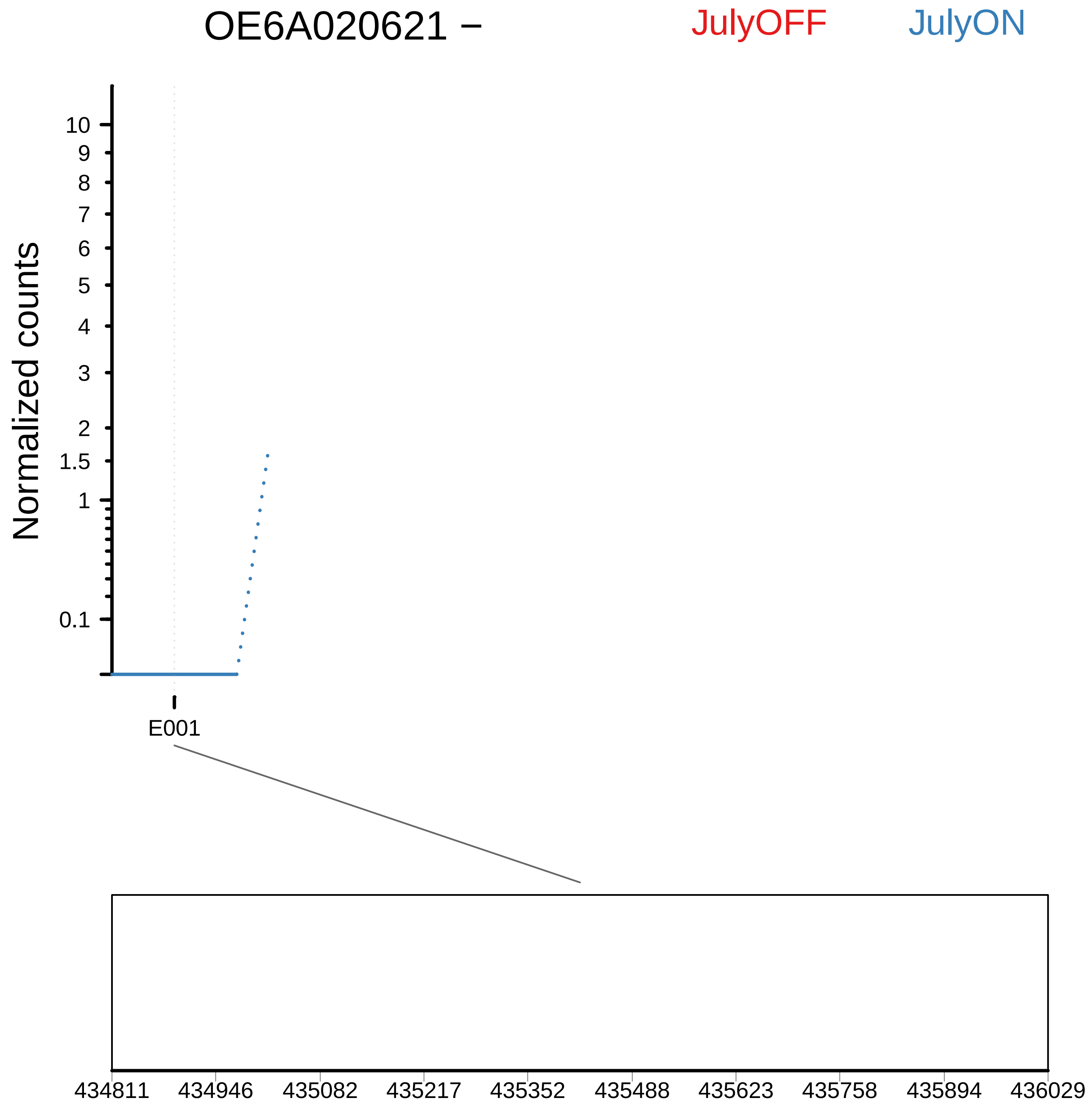
OE6A020621 −
JulyOFF
JulyON
Normalized counts
10
9
8
7
6
5
4
3
2
1.5
1
0.1
E001
434811
434946
435082
435217
435352
435488
435623
435758
435894
436029

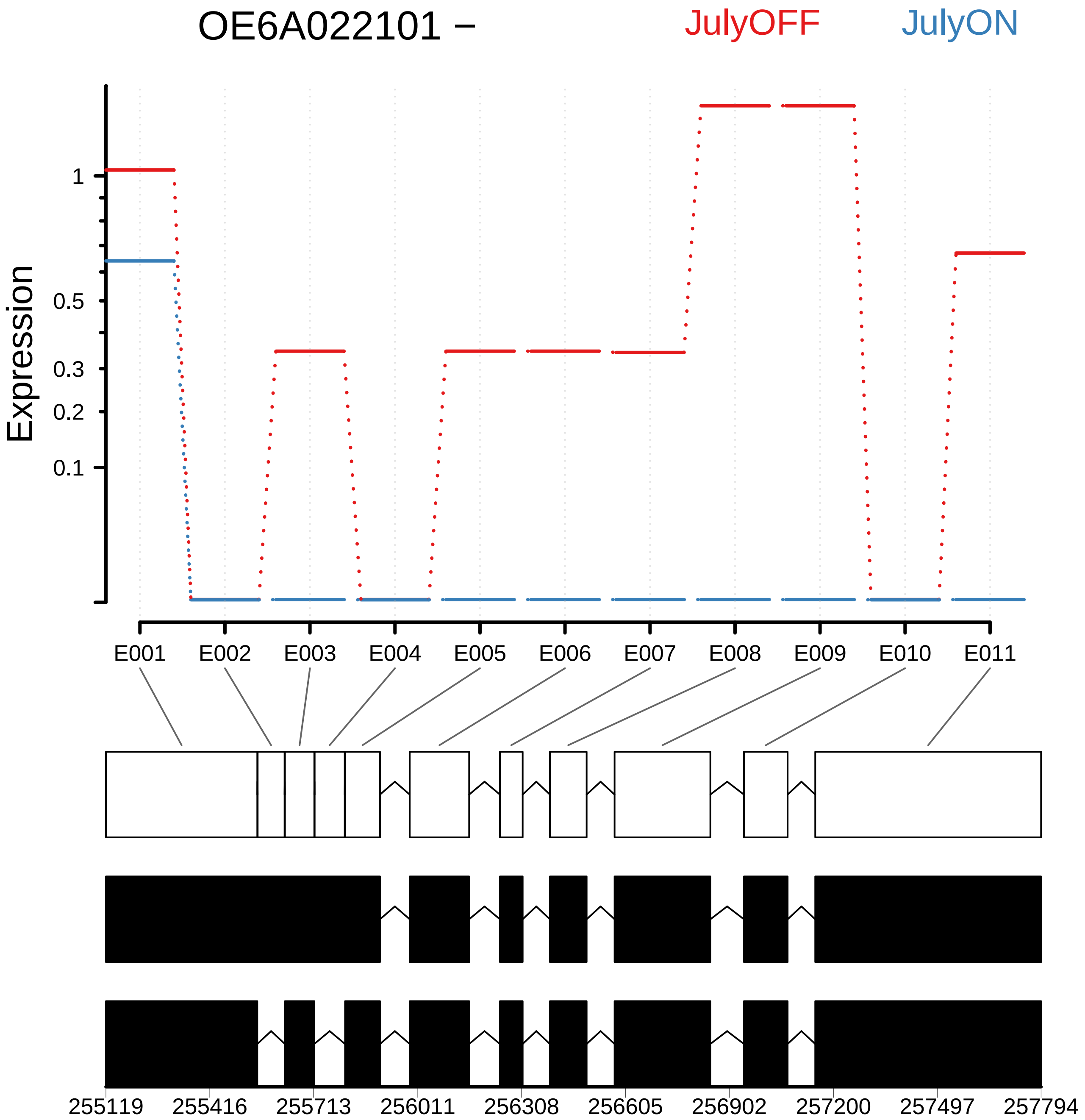

OE6A022101 −
JulyOFF
JulyON
Expression
1
0.5
0.3
0.2
0.1
E001
E002
E003
E004
E005
E006
E007
E008
E009
E010
E011
255119
255416
255713
256011
256308
256605
256902
257200
257497
257794

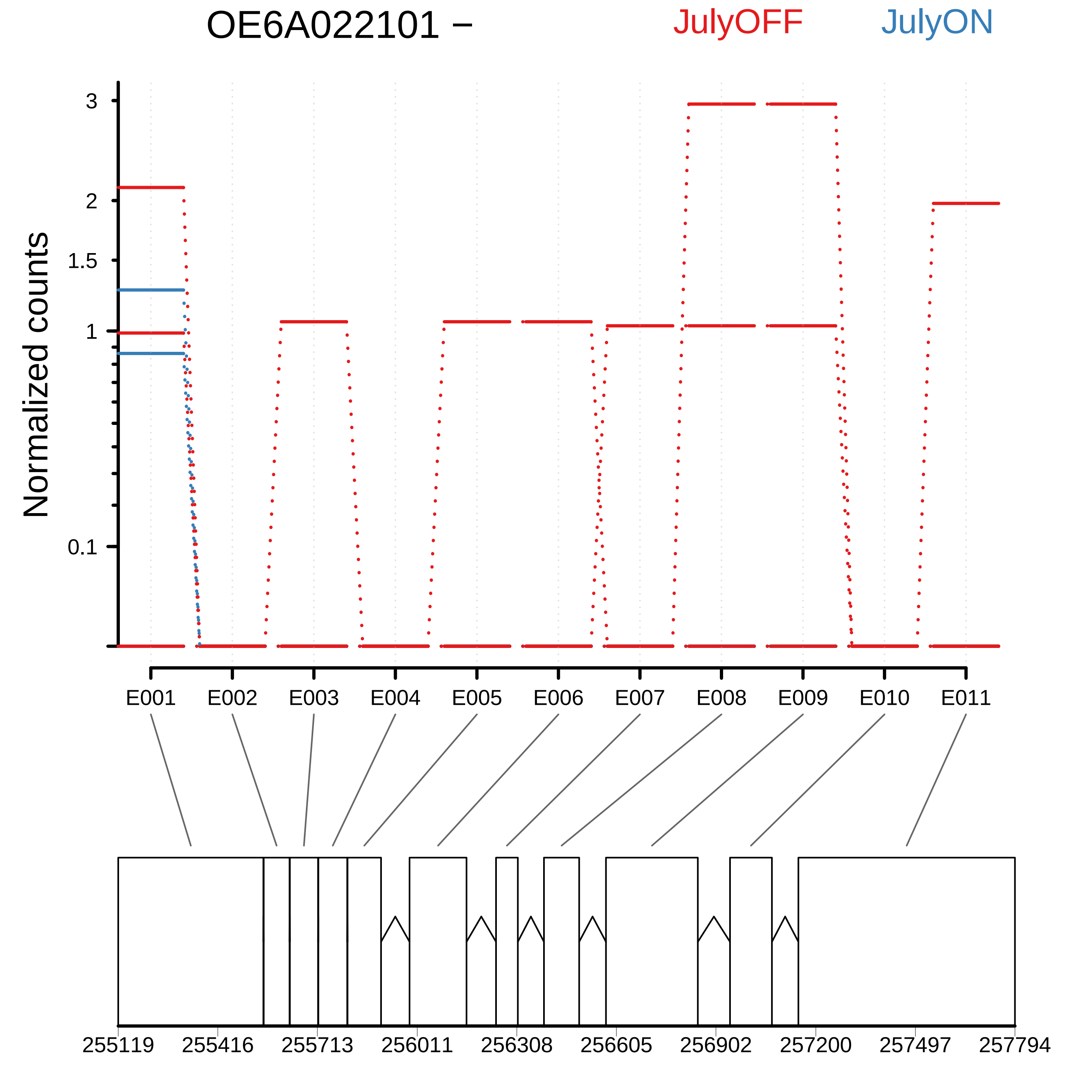

OE6A022101 −
JulyOFF
JulyON
Normalized counts
3
2
1.5
1
0.1
E001
E002
E003
E004
E005
E006
E007
E008
E009
E010
E011
255119
255416
255713
256011
256308
256605
256902
257200
257497
257794

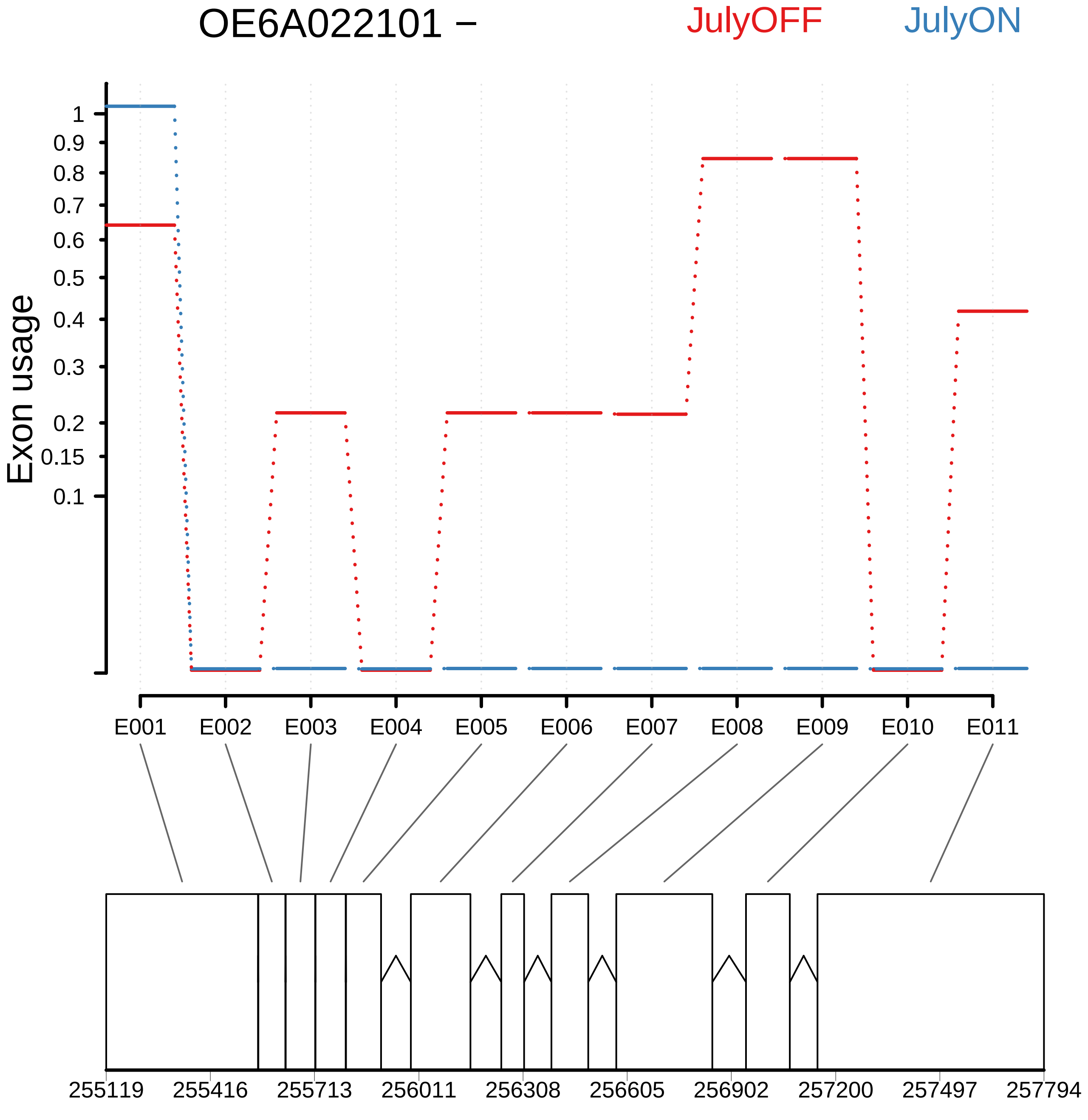

OE6A022101 −
JulyOFF
JulyON
Exon usage
1
0.9
0.8
0.7
0.6
0.5
0.4
0.3
0.2
0.15
0.1
E001
E002
E003
E004
E005
E006
E007
E008
E009
E010
E011
255119
255416
255713
256011
256308
256605
256902
257200
257497
257794

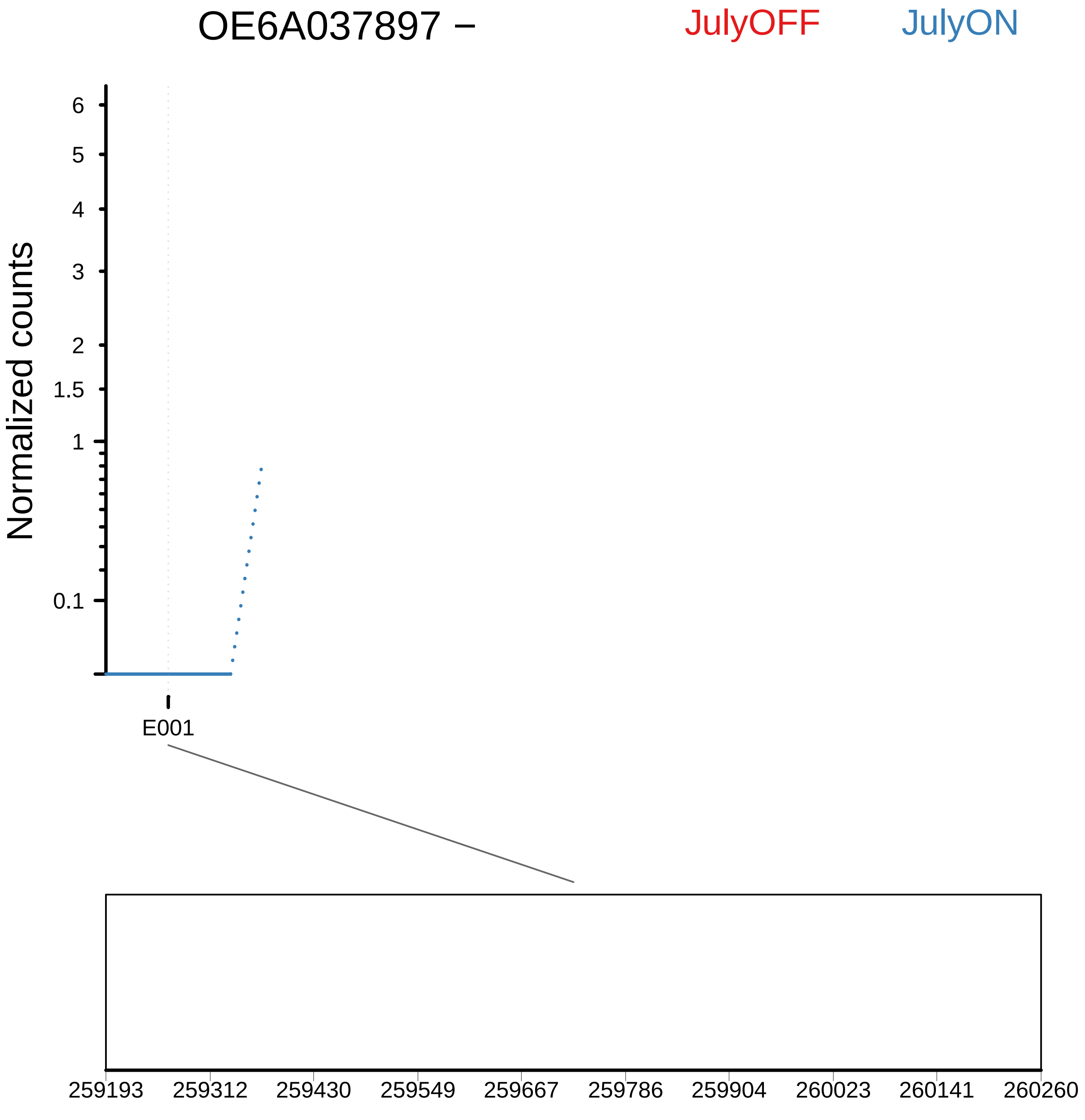
OE6A037897 −
JulyOFF
JulyON
Normalized counts
6
5
4
3
2
1.5
1
0.1
E001
259193
259312
259430
259549
259667
259786
259904
260023
260141
260260

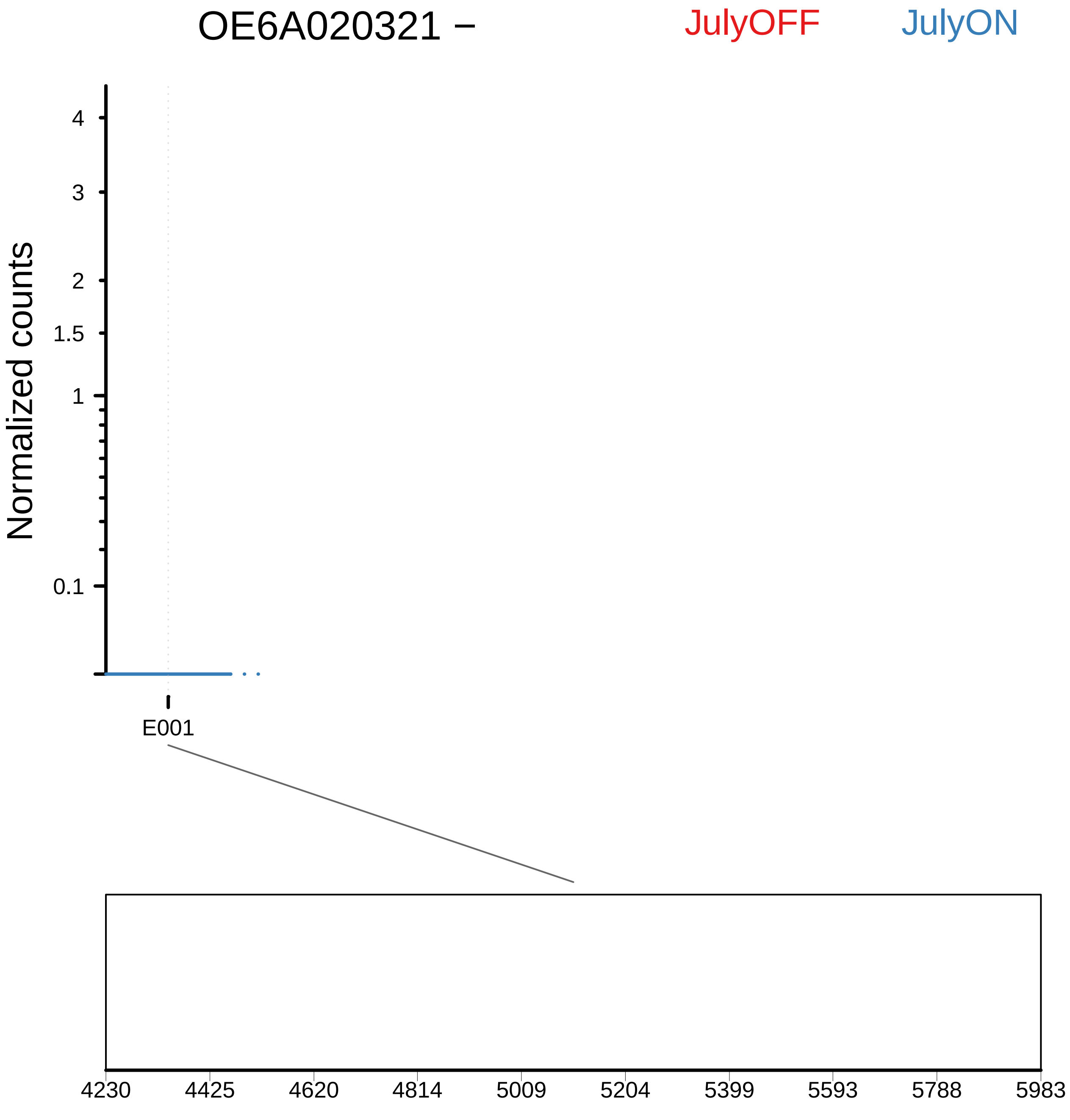

OE6A020321 −
JulyOFF
JulyON
Normalized counts
4
3
2
1.5
1
0.1
E001
4230
4425
4620
4814
5009
5204
5399
5593
5788
5983

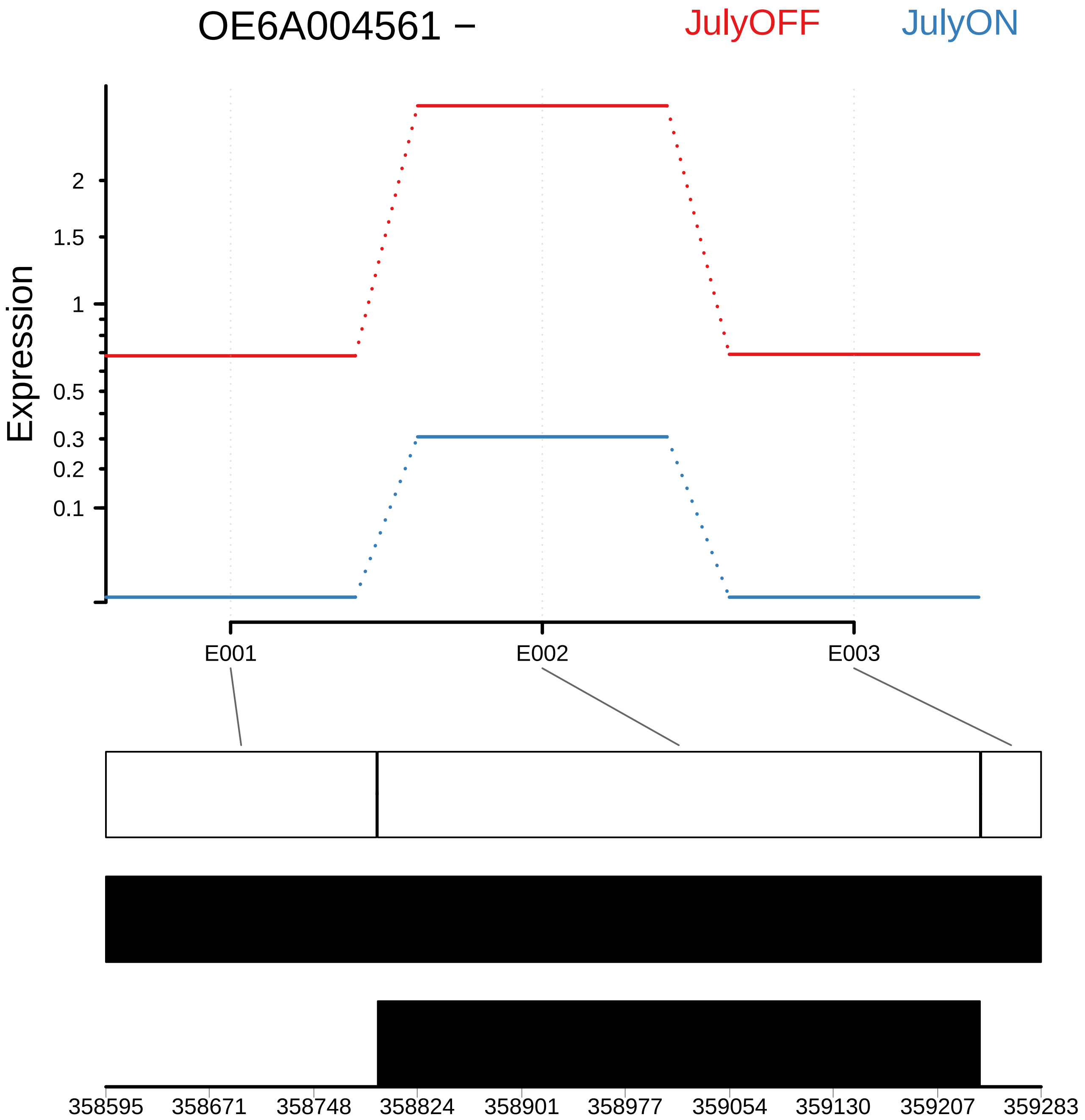

OE6A004561 −
JulyOFF
JulyON
Expression
2
1.5
1
0.5
0.3
0.2
0.1
E001
E002
E003
358595
358671
358748
358824
358901
358977
359054
359130
359207
359283

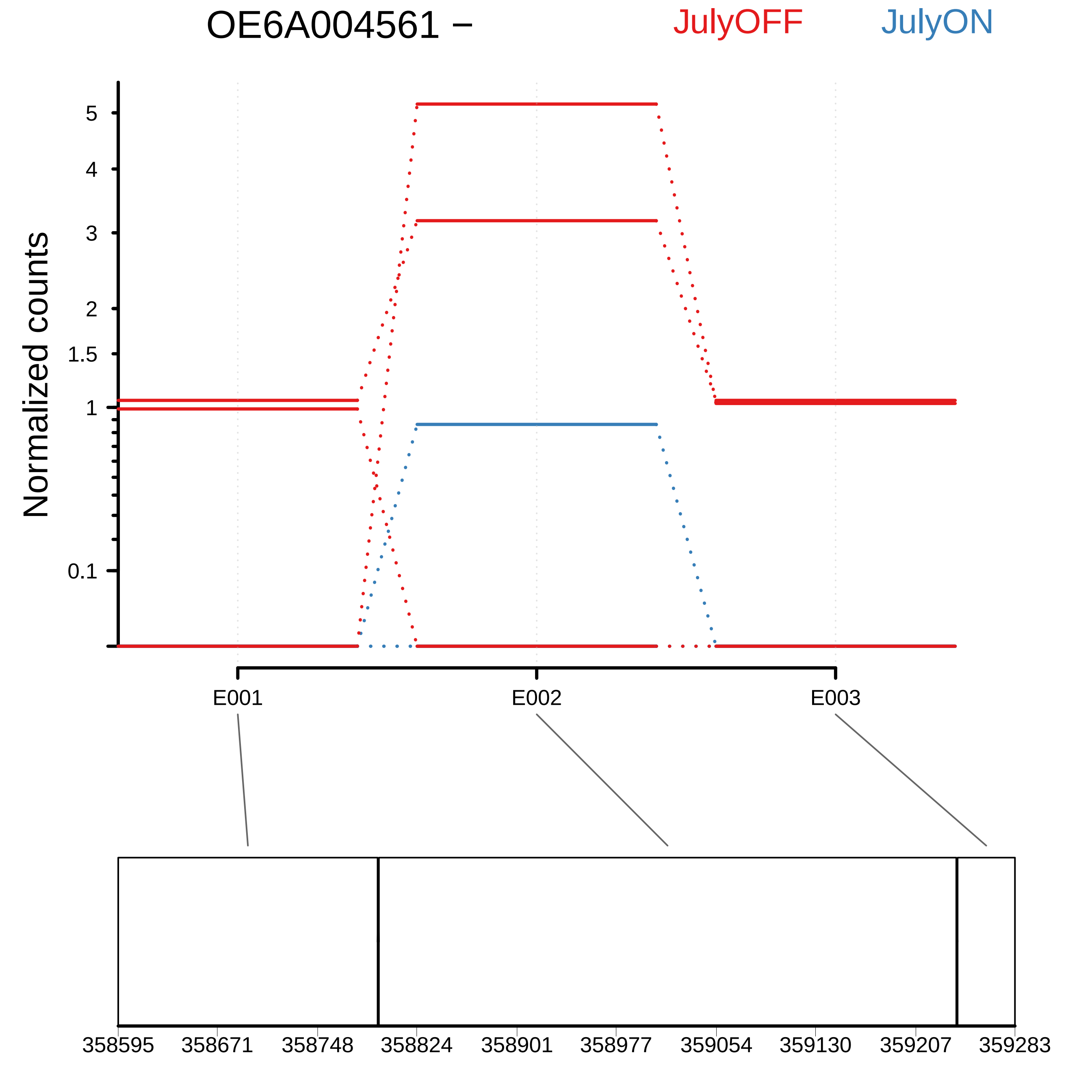

OE6A004561 −
JulyOFF
JulyON
Normalized counts
5
4
3
2
1.5
1
0.1
E001
E002
E003
358595
358671
358748
358824
358901
358977
359054
359130
359207
359283

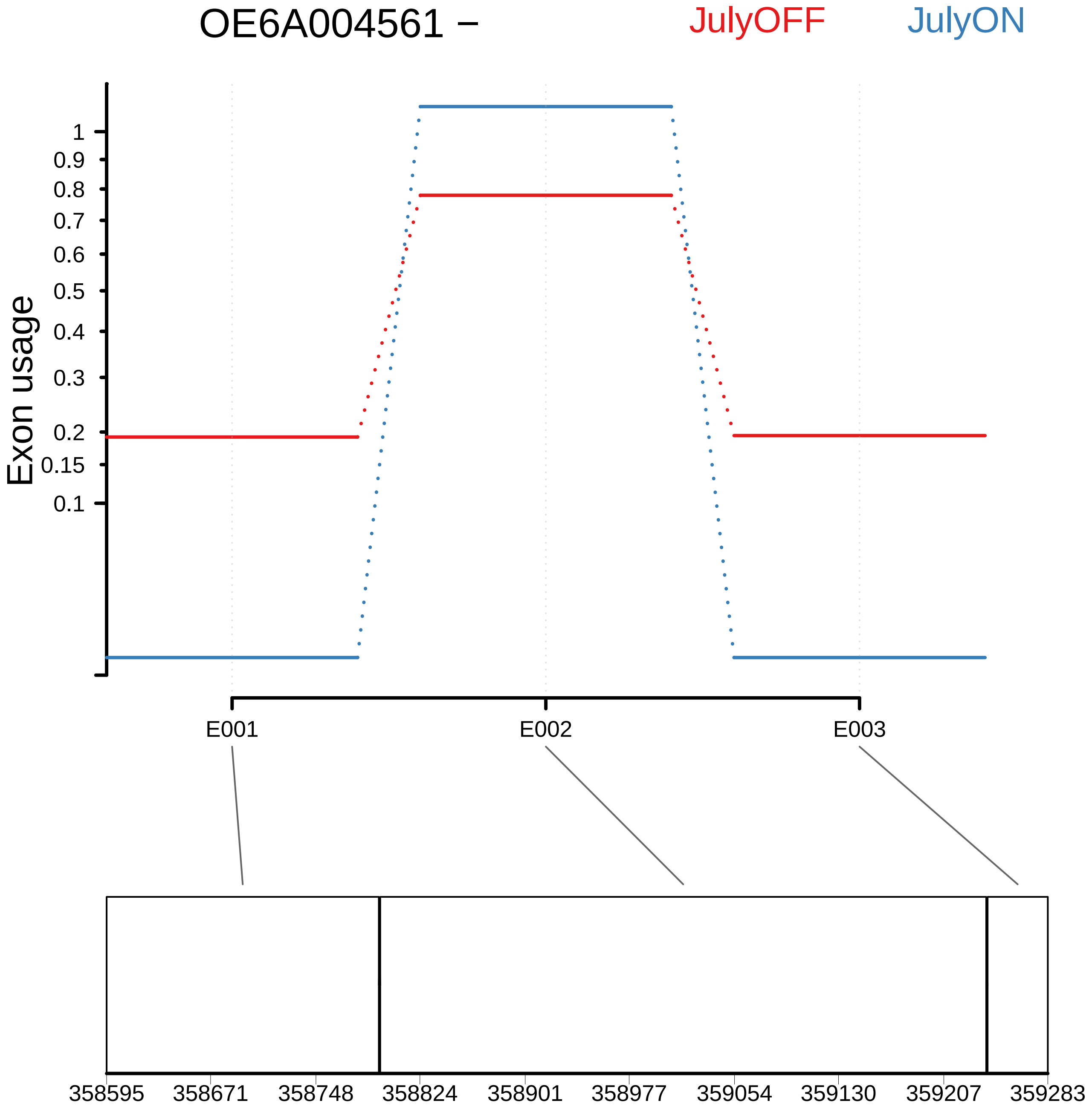

OE6A004561 −
JulyOFF
JulyON
Exon usage
1
0.9
0.8
0.7
0.6
0.5
0.4
0.3
0.2
0.15
0.1
E001
E002
E003
358595
358671
358748
358824
358901
358977
359054
359130
359207
359283

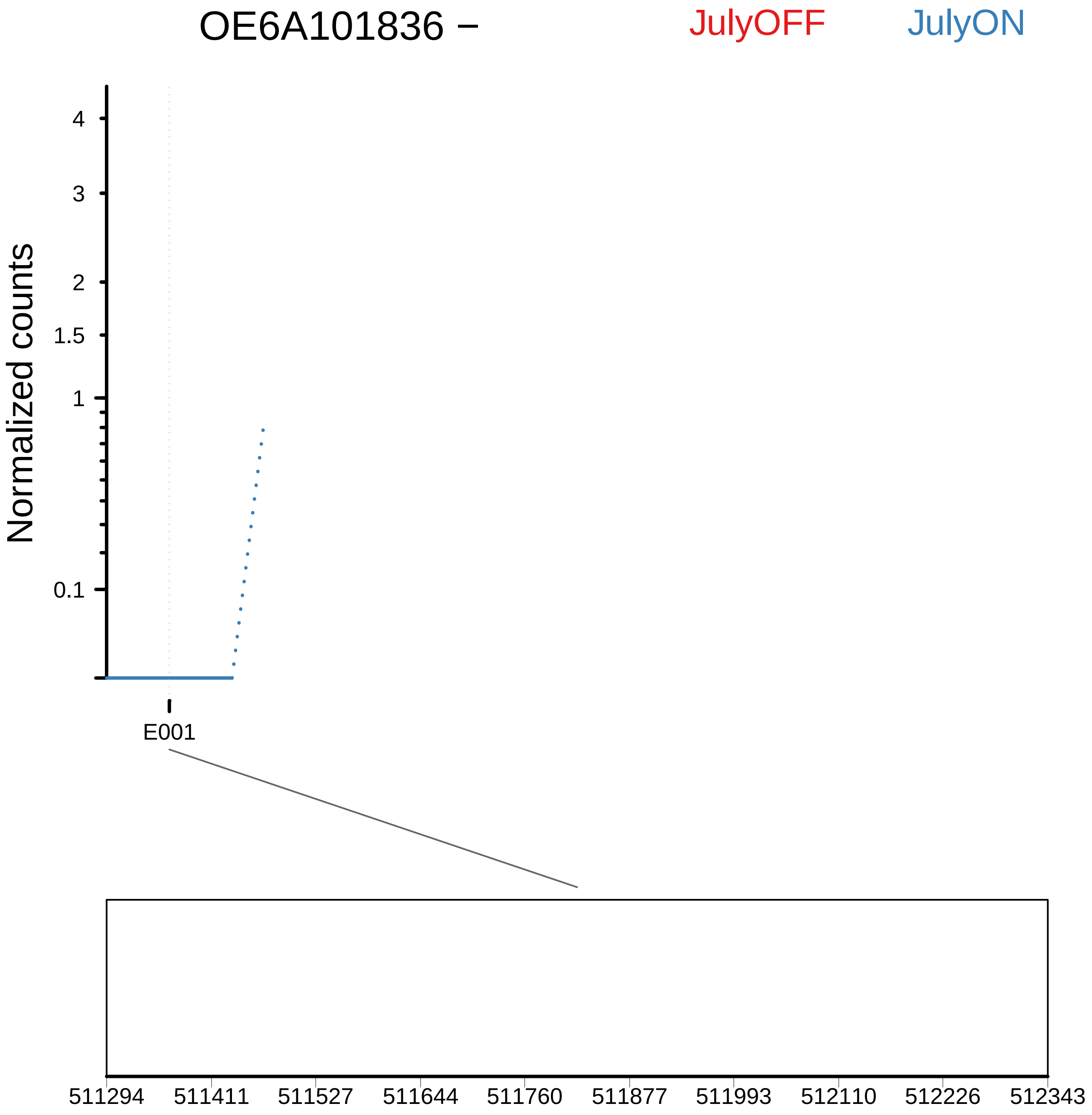
OE6A101836 −
JulyOFF
JulyON
Normalized counts
4
3
2
1.5
1
0.1
E001
511294
511411
511527
511644
511760
511877
511993
512110
512226
512343

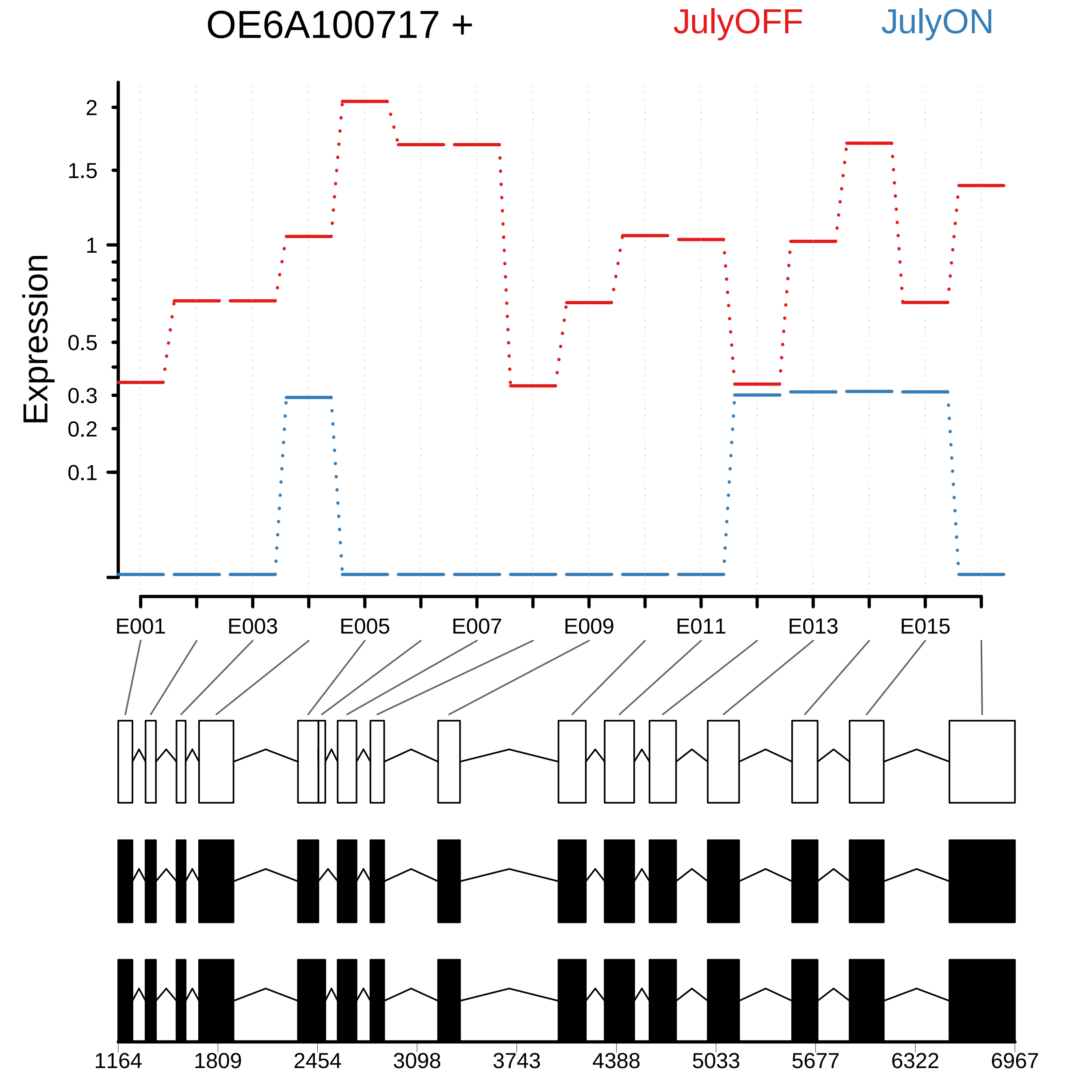
OE6A100717 +
JulyOFF
JulyON
Expression
2
1.5
1
0.5
0.3
0.2
0.1
E001
E003
E005
E007
E009
E011
E013
E015
1164
1809
2454
3098
3743
4388
5033
5677
6322
6967

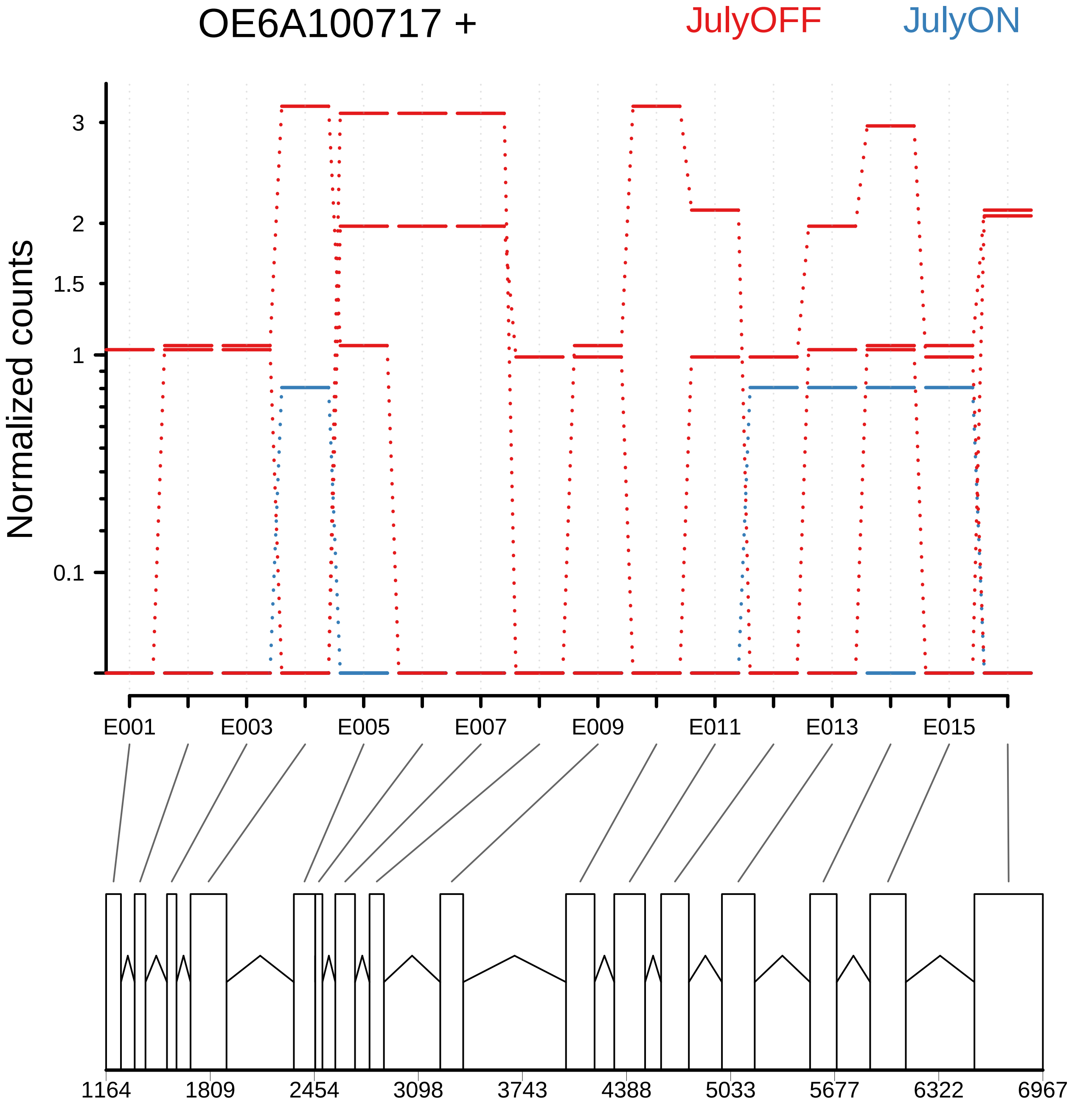

OE6A100717 +
JulyOFF
JulyON
Normalized counts
3
2
1.5
1
0.1
E001
E003
E005
E007
E009
E011
E013
E015
1164
1809
2454
3098
3743
4388
5033
5677
6322
6967

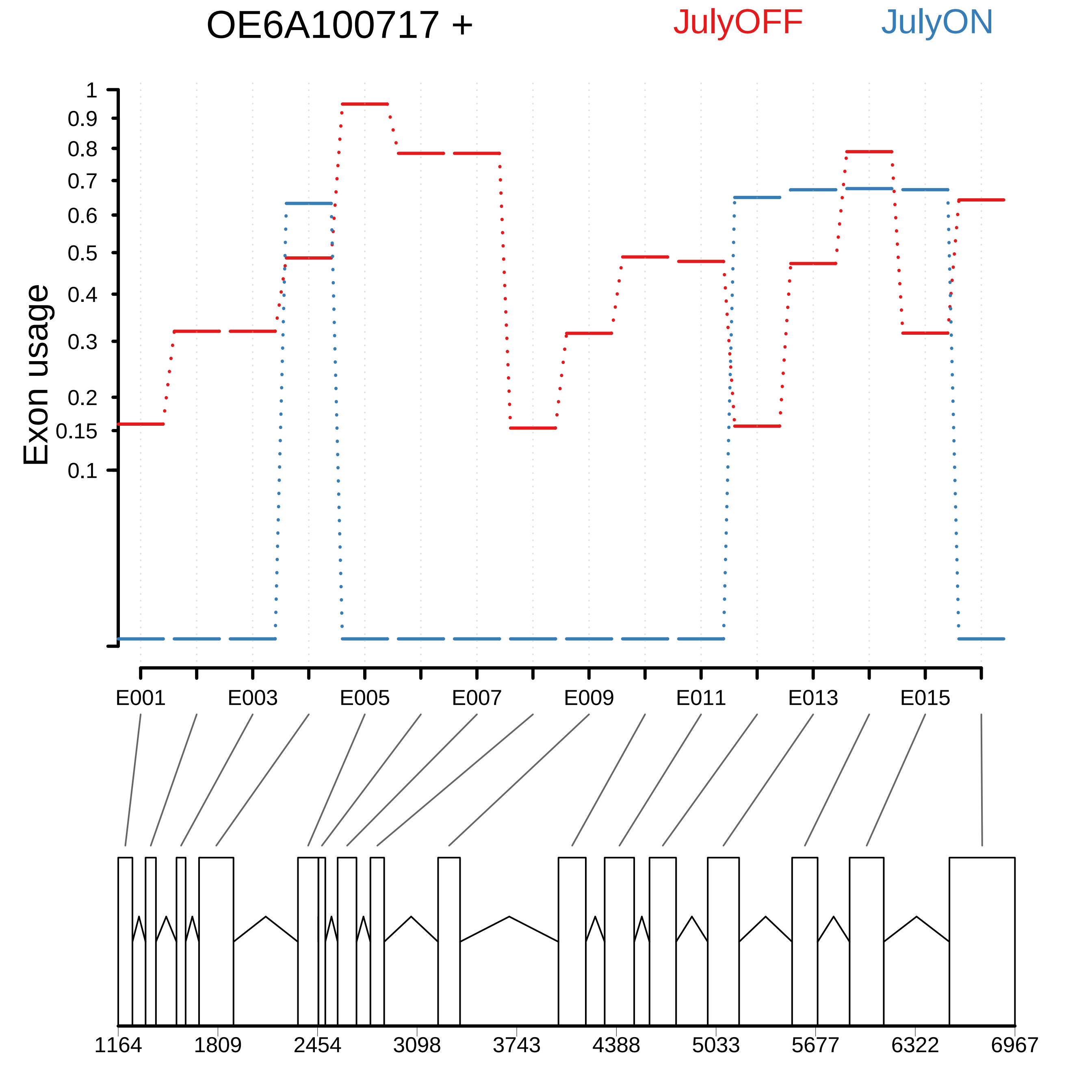
OE6A100717 +
JulyOFF
JulyON
Exon usage
1
0.9
0.8
0.7
0.6
0.5
0.4
0.3
0.2
0.15
0.1
E001
E003
E005
E007
E009
E011
E013
E015
1164
1809
2454
3098
3743
4388
5033
5677
6322
6967

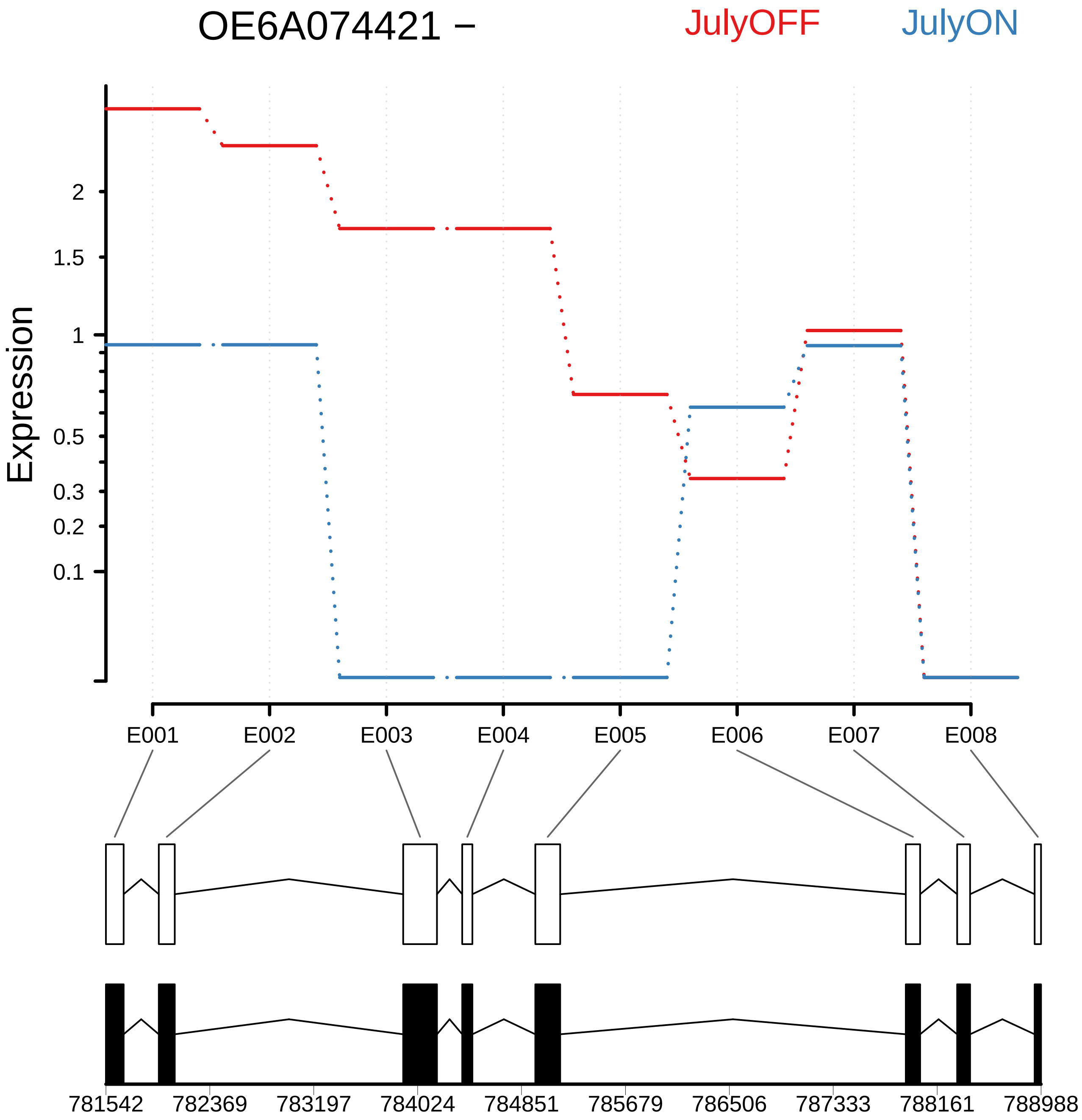
OE6A074421 –
JulyOFF
JulyON
Expression
2
1.5
1
0.5
0.3
0.2
0.1
E001
E002
E003
E004
E005
E006
E007
E008
781542
782369
783197
784024
784851
785679
786506
787333
788161
788988

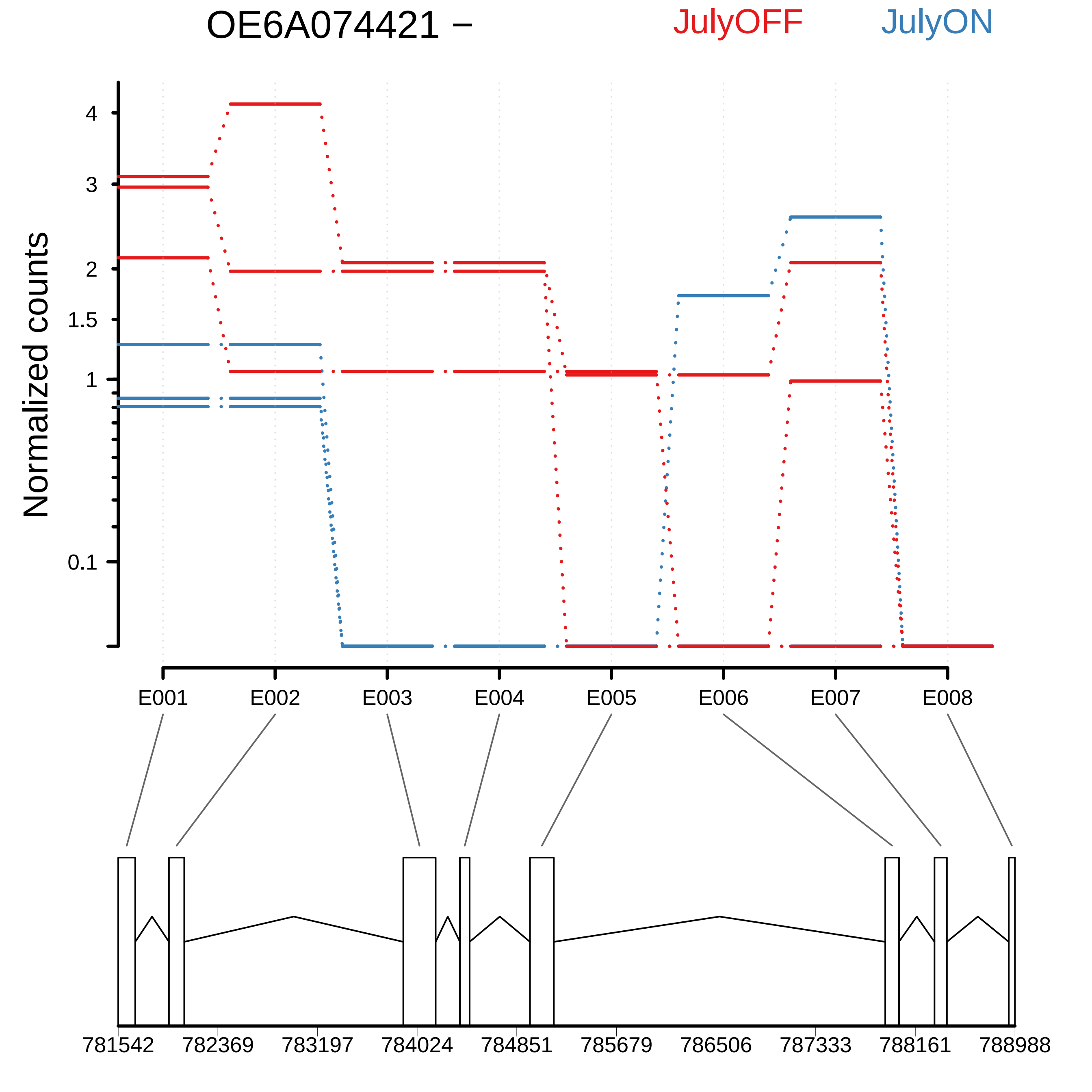
OE6A074421 −
JulyOFF
JulyON
Normalized counts
4
3
2
1.5
1
0.1
E001
E002
E003
E004
E005
E006
E007
E008
781542
782369
783197
784024
784851
785679
786506
787333
788161
788988

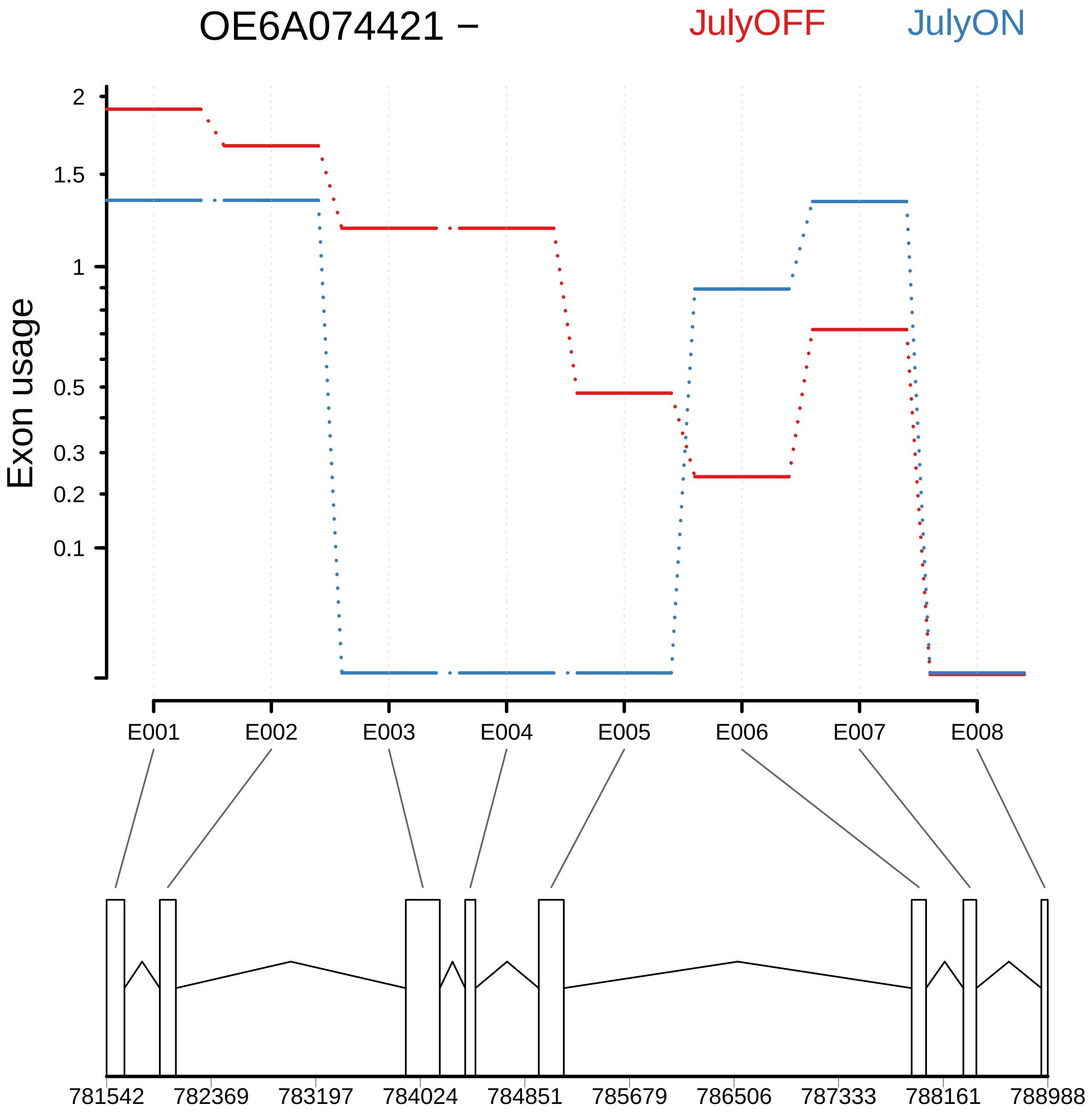
OE6A074421 –
JulyOFF
JulyON
Exon usage
2
1.5
1
0.5
0.3
0.2
0.1
E001
E002
E003
E004
E005
E006
E007
E008
781542
782369
783197
784024
784851
785679
786506
787333
788161
788988

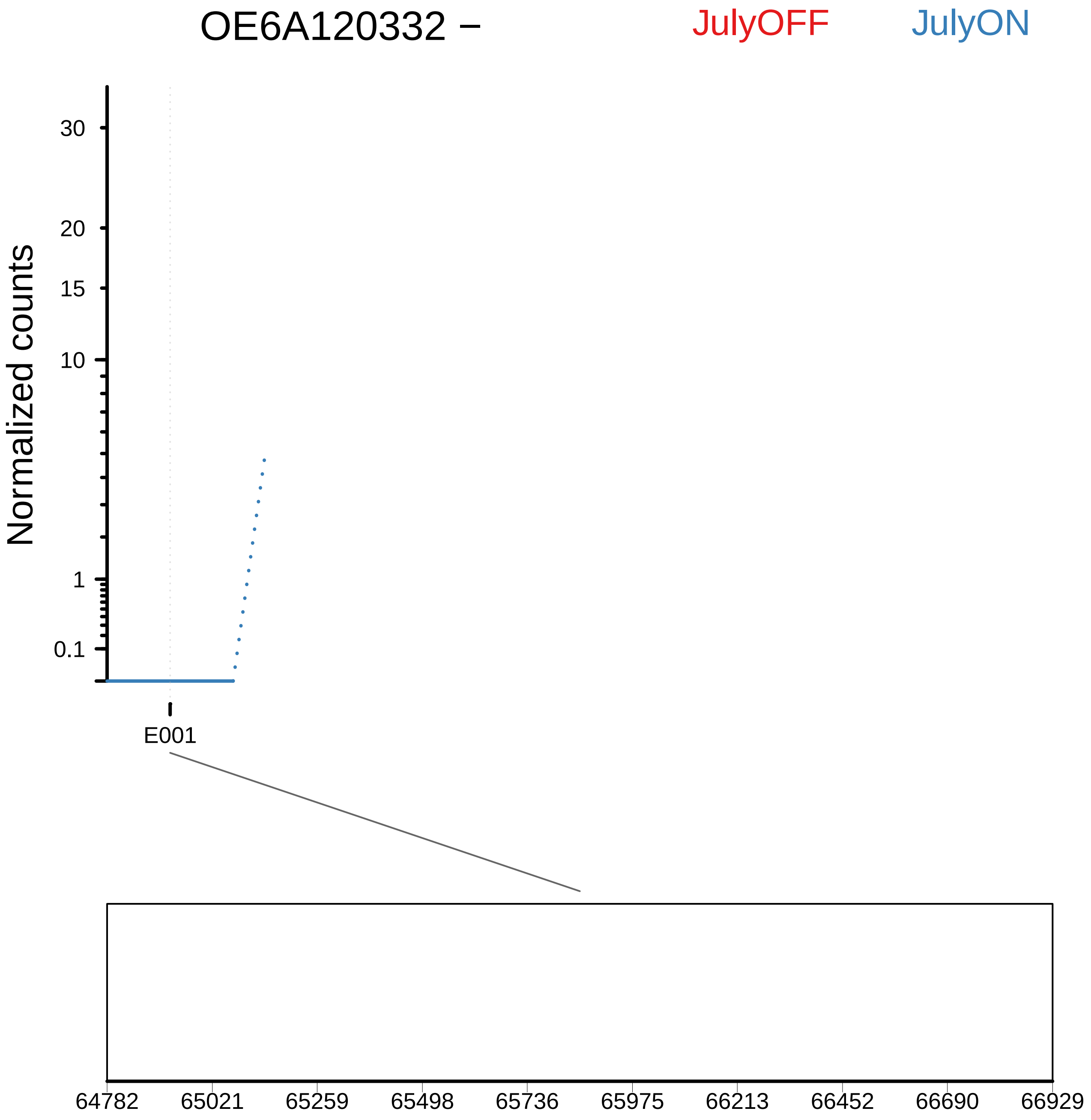
OE6A120332 −
JulyOFF
JulyON
Normalized counts
30
20
15
10
1
0.1
E001
64782
65021
65259
65498
65736
65975
66213
66452
66690
66929

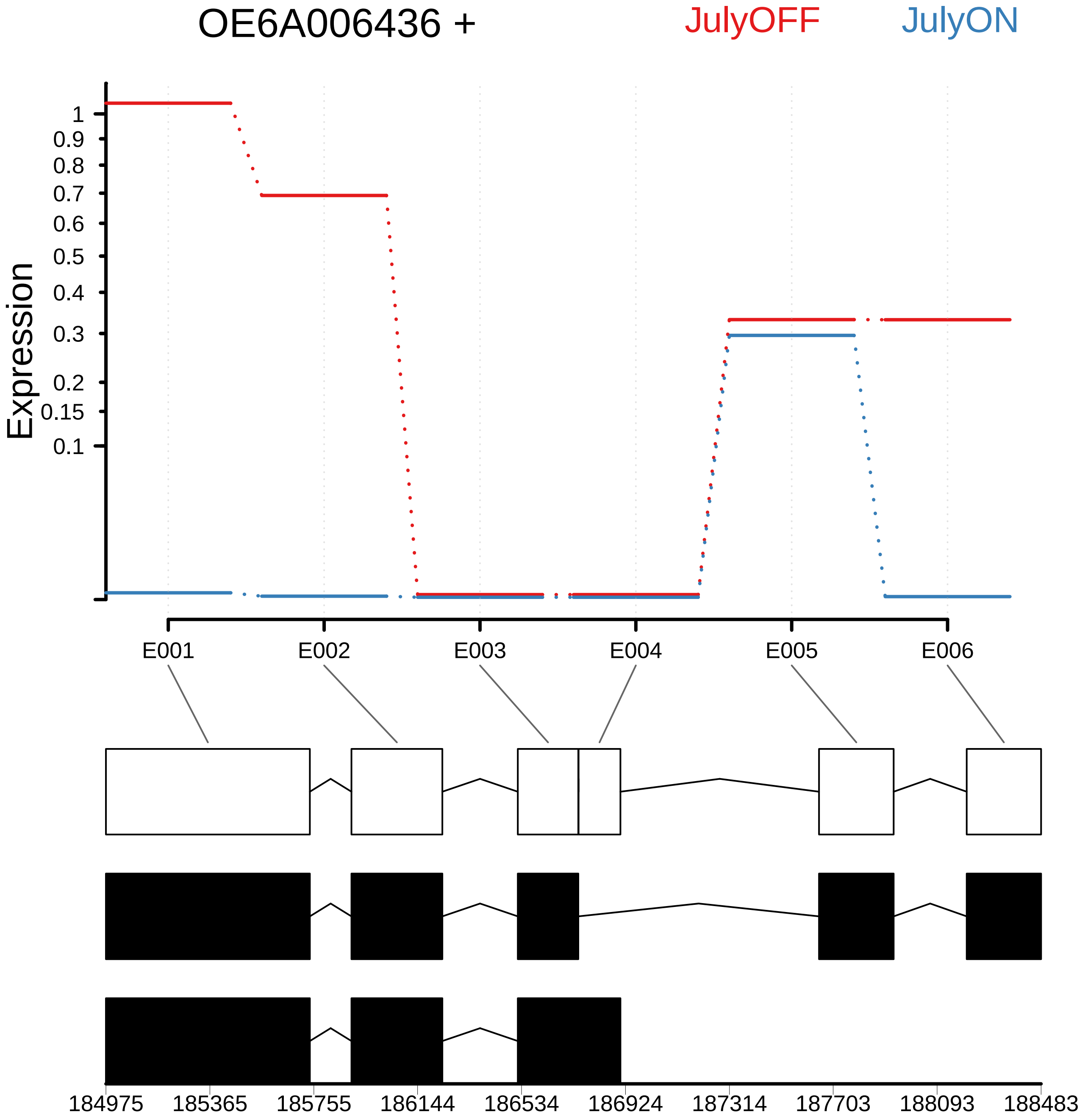

OE6A006436 +
JulyOFF
JulyON
Expression
1
0.9
0.8
0.7
0.6
0.5
0.4
0.3
0.2
0.15
0.1
E001
E002
E003
E004
E005
E006
184975
185365
185755
186144
186534
186924
187314
187703
188093
188483

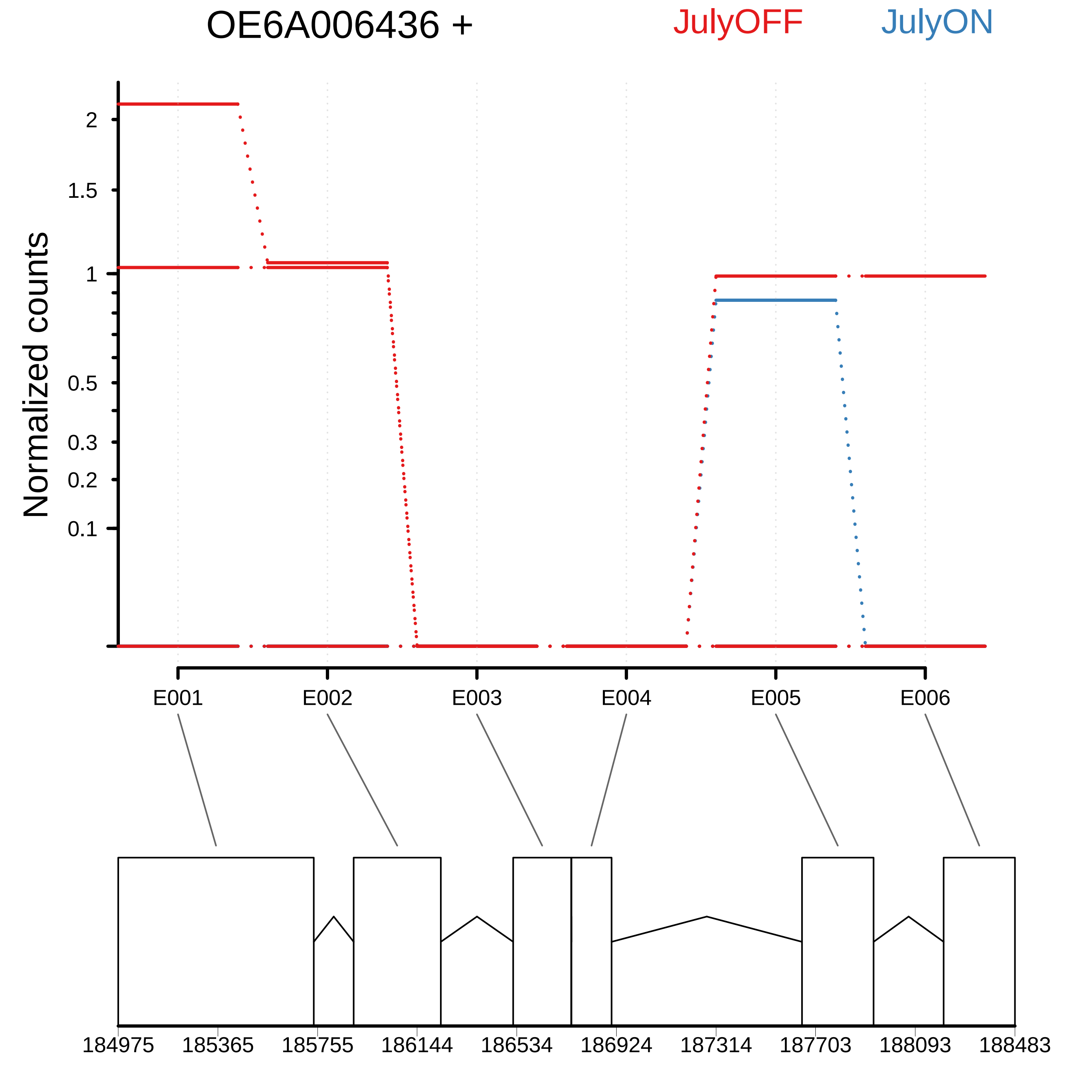
OE6A006436 +
JulyOFF
JulyON
Normalized counts
2
1.5
1
0.5
0.3
0.2
0.1
E001
E002
E003
E004
E005
E006
184975
185365
185755
186144
186534
186924
187314
187703
188093
188483

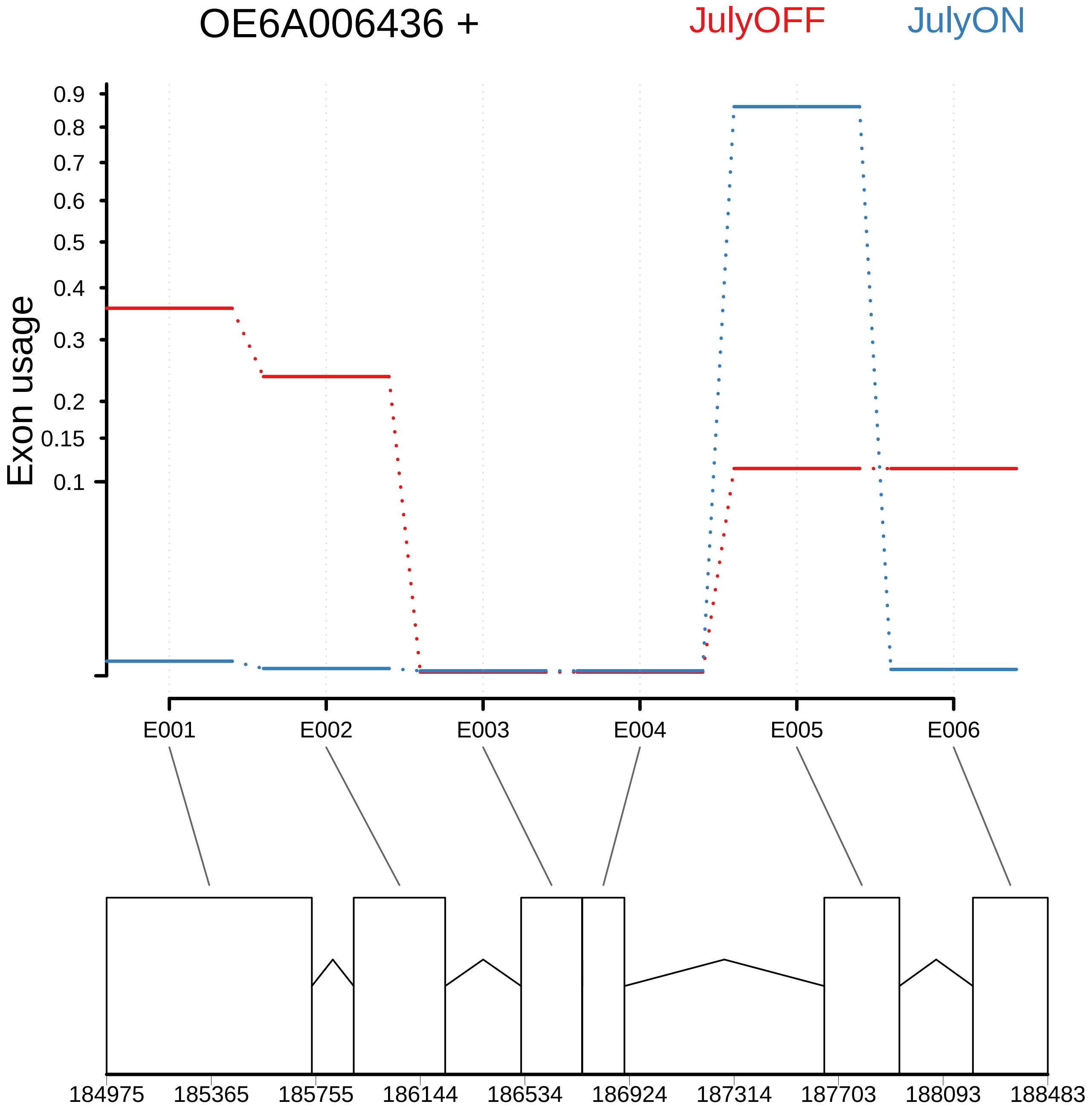

OE6A006436 +
JulyOFF
JulyON
Exon usage
0.9
0.8
0.7
0.6
0.5
0.4
0.3
0.2
0.15
0.1
E001
E002
E003
E004
E005
E006
184975
185365
185755
186144
186534
186924
187314
187703
188093
188483

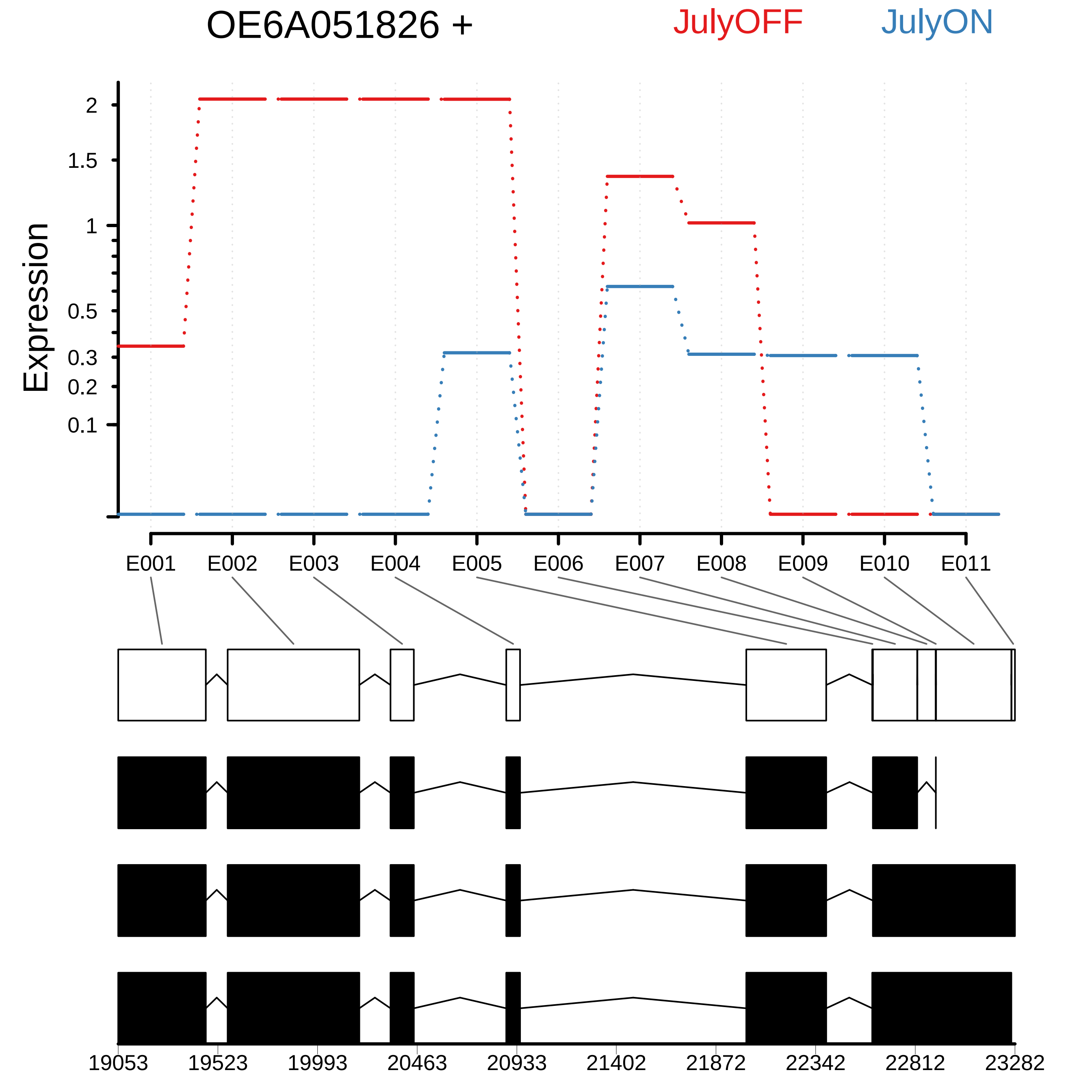
OE6A051826 +
JulyOFF
JulyON
Expression
2
1.5
1
0.5
0.3
0.2
0.1
E001
E002
E003
E004
E005
E006
E007
E008
E009
E010
E011
19053
19523
19993
20463
20933
21402
21872
22342
22812
23282

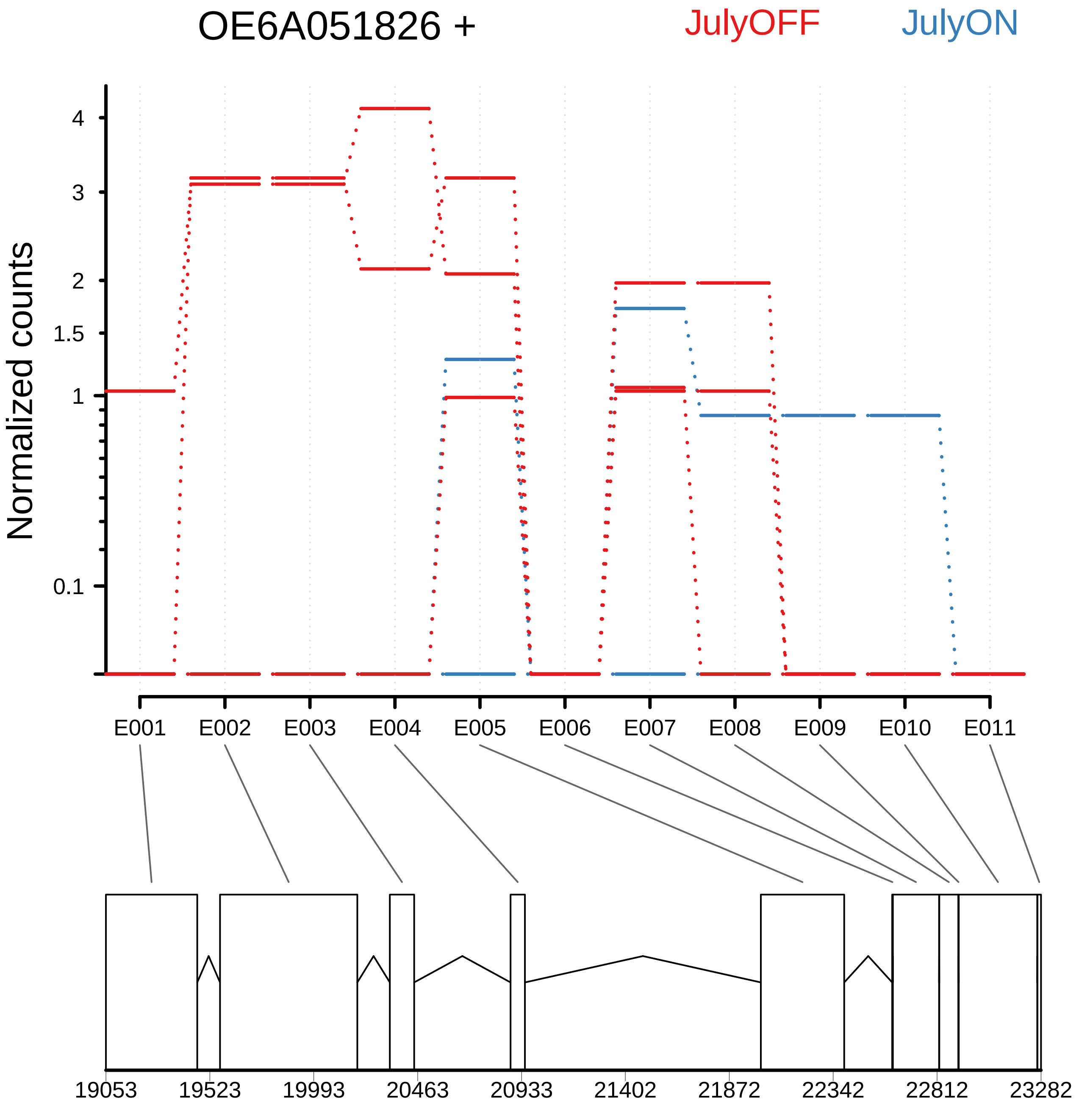

OE6A051826 +
JulyOFF
JulyON
Normalized counts
4
3
2
1.5
1
0.1
E001
E002
E003
E004
E005
E006
E007
E008
E009
E010
E011
19053
19523
19993
20463
20933
21402
21872
22342
22812
23282

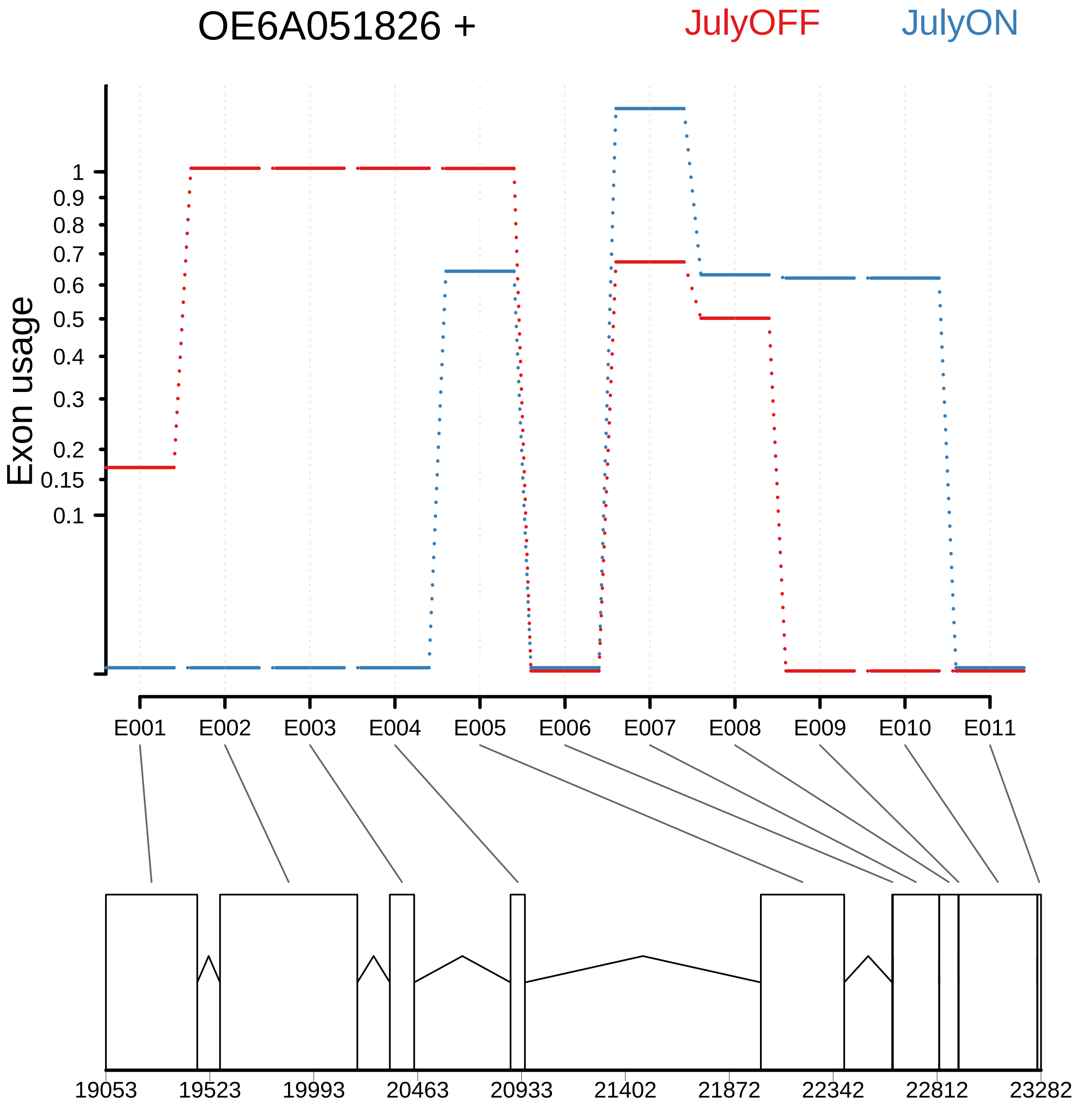
OE6A051826 +
JulyOFF
JulyON
Exon usage
1
0.9
0.8
0.7
0.6
0.5
0.4
0.3
0.2
0.15
0.1
E001
E002
E003
E004
E005
E006
E007
E008
E009
E010
E011
19053
19523
19993
20463
20933
21402
21872
22342
22812
23282

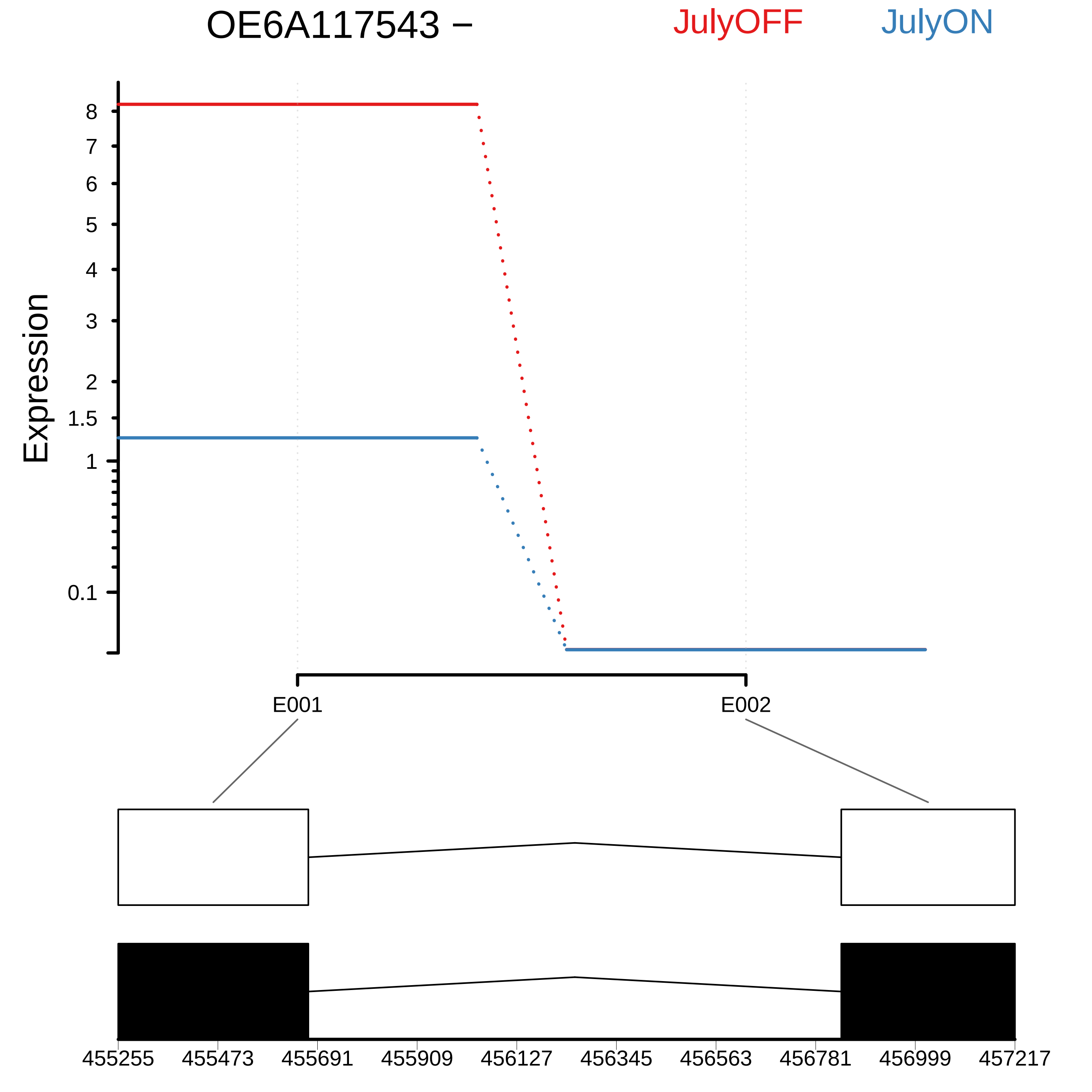
OE6A117543 −
JulyOFF
JulyON
Expression
8
7
6
5
4
3
2
1.5
1
0.1
E001
E002
455255
455473
455691
455909
456127
456345
456563
456781
456999
457217

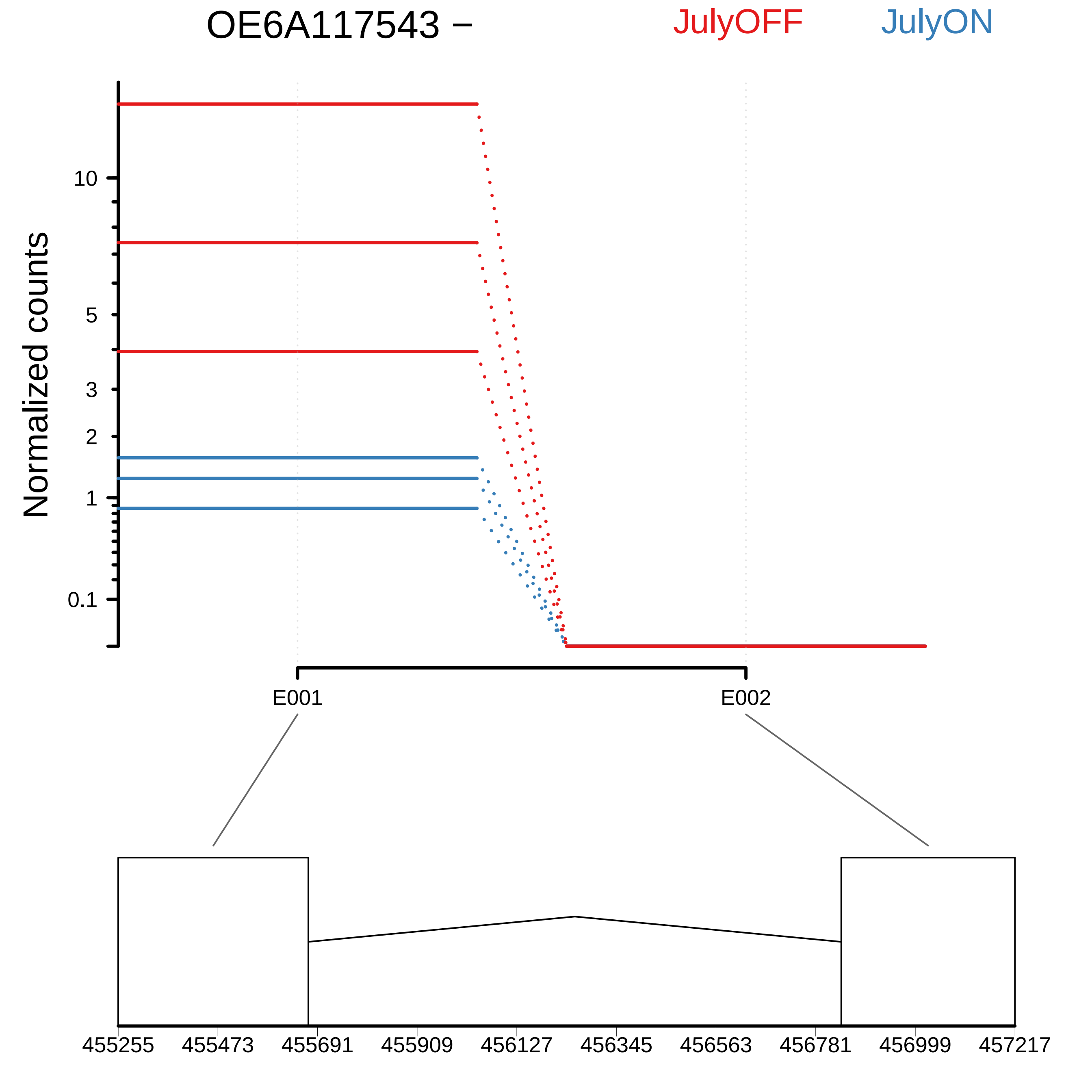

OE6A117543 −
JulyOFF
JulyON
Normalized counts
10
5
3
2
1
0.1
E001
E002
455255
455473
455691
455909
456127
456345
456563
456781
456999
457217

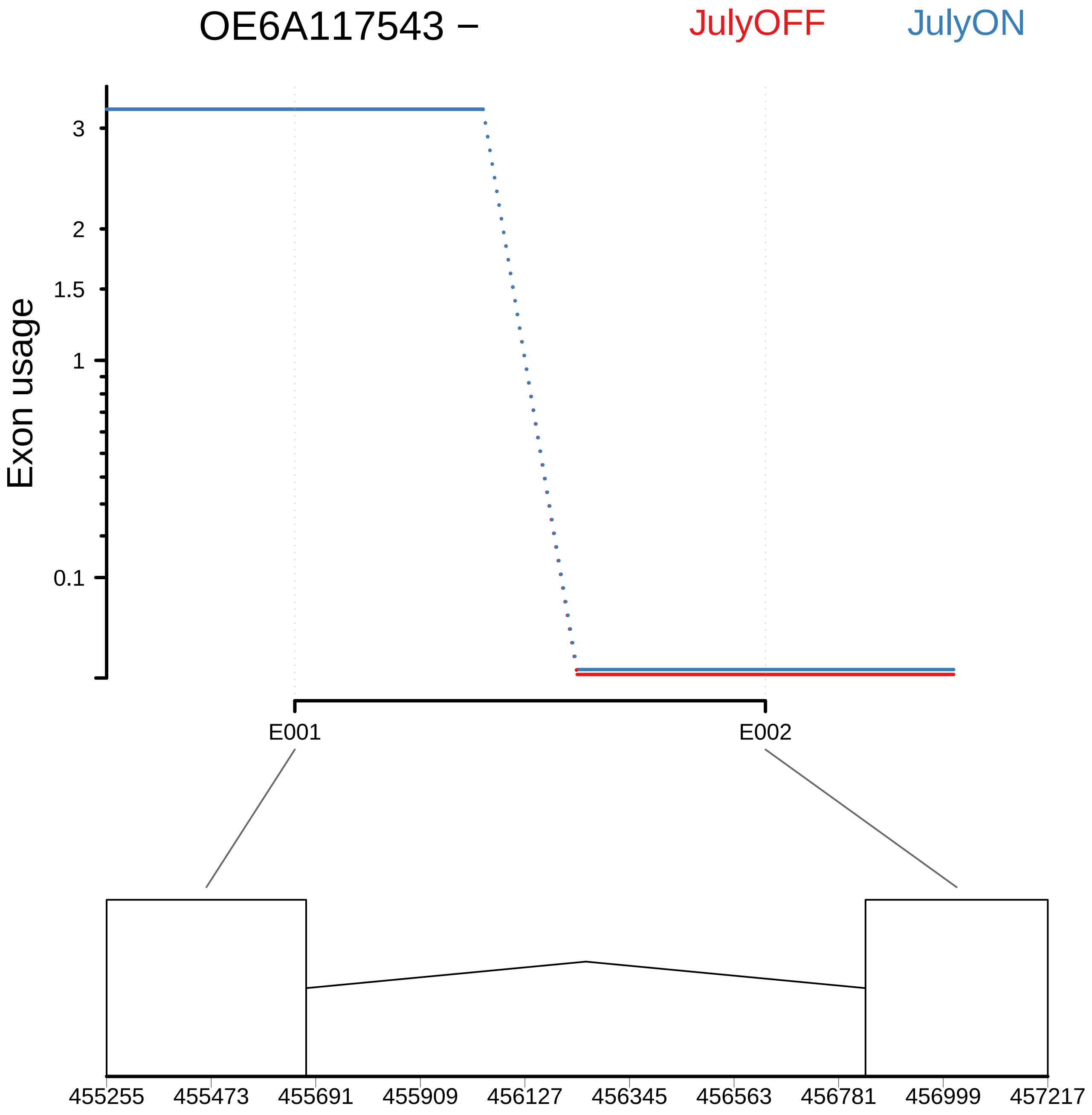
OE6A117543 −
JulyOFF
JulyON
Exon usage
3
2
1.5
1
0.1
E001
E002
455255
455473
455691
455909
456127
456345
456563
456781
456999
457217

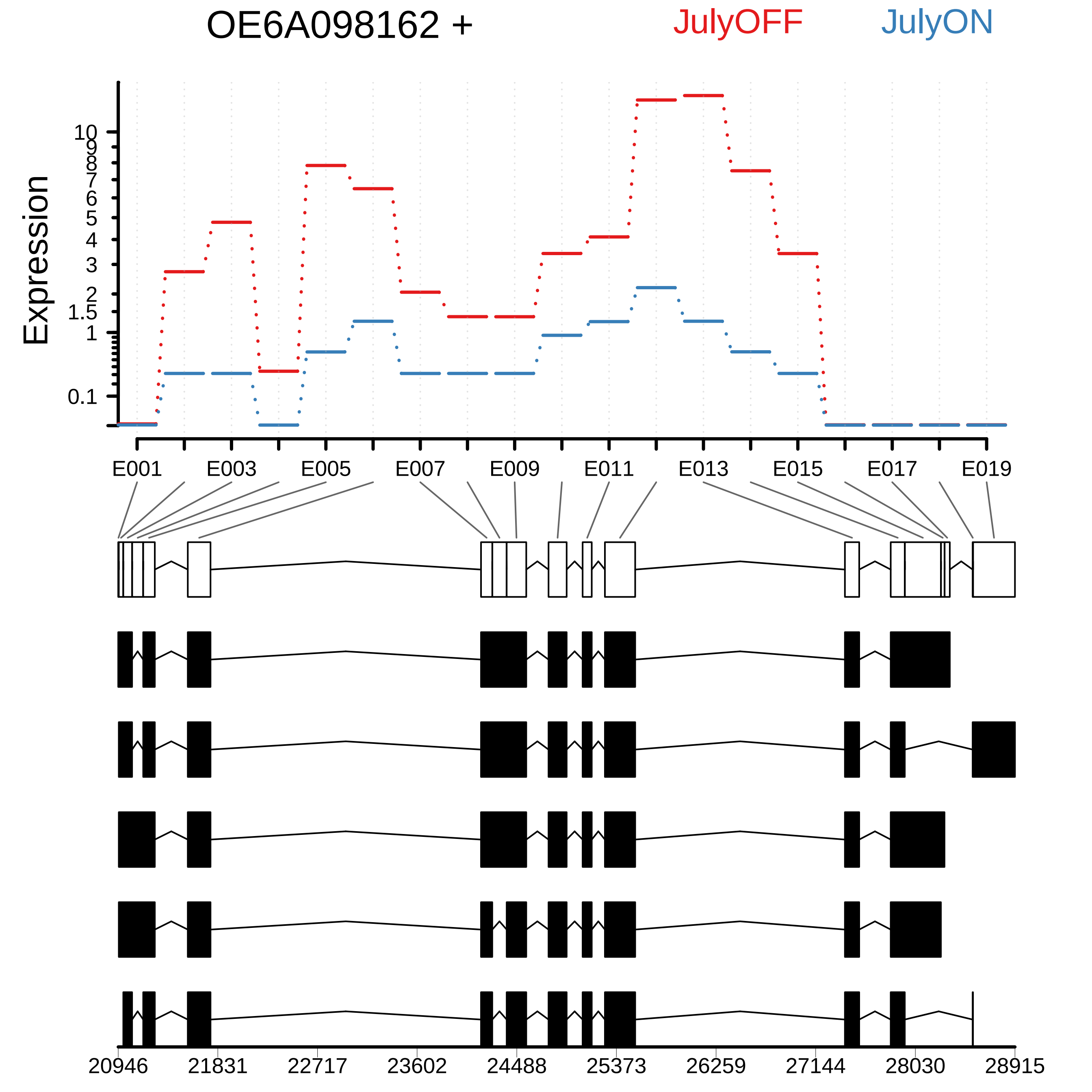

OE6A098162 +
JulyOFF
JulyON
Expression
10
9
8
7
6
5
4
3
2
1.5
1
0.1
E001
E003
E005
E007
E009
E011
E013
E015
E017
E019
20946
21831
22717
23602
24488
25373
26259
27144
28030
28915

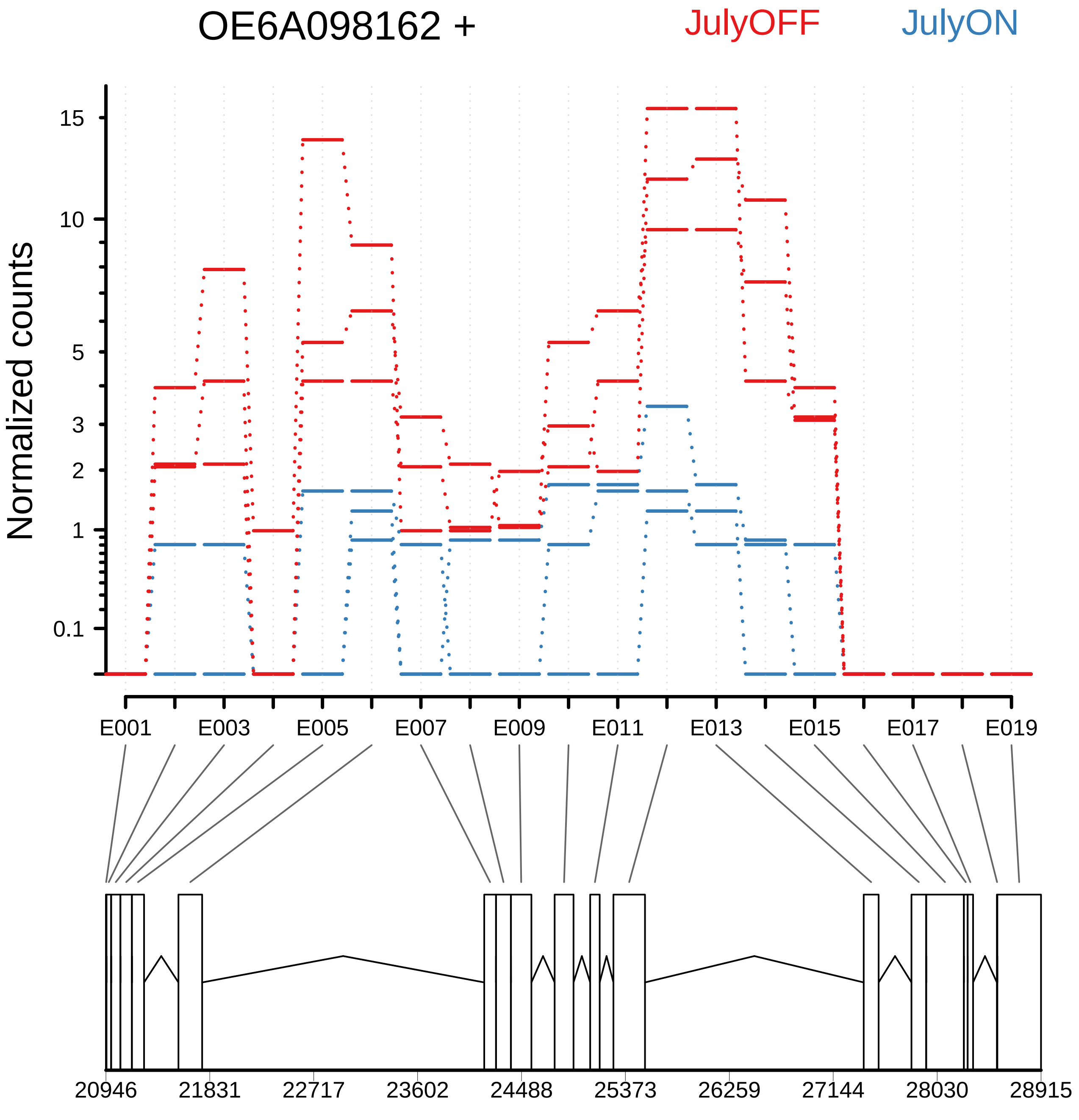

OE6A098162 +
JulyOFF
JulyON
Normalized counts
15
10
5
3
2
1
0.1
E001
E003
E005
E007
E009
E011
E013
E015
E017
E019
20946
21831
22717
23602
24488
25373
26259
27144
28030
28915

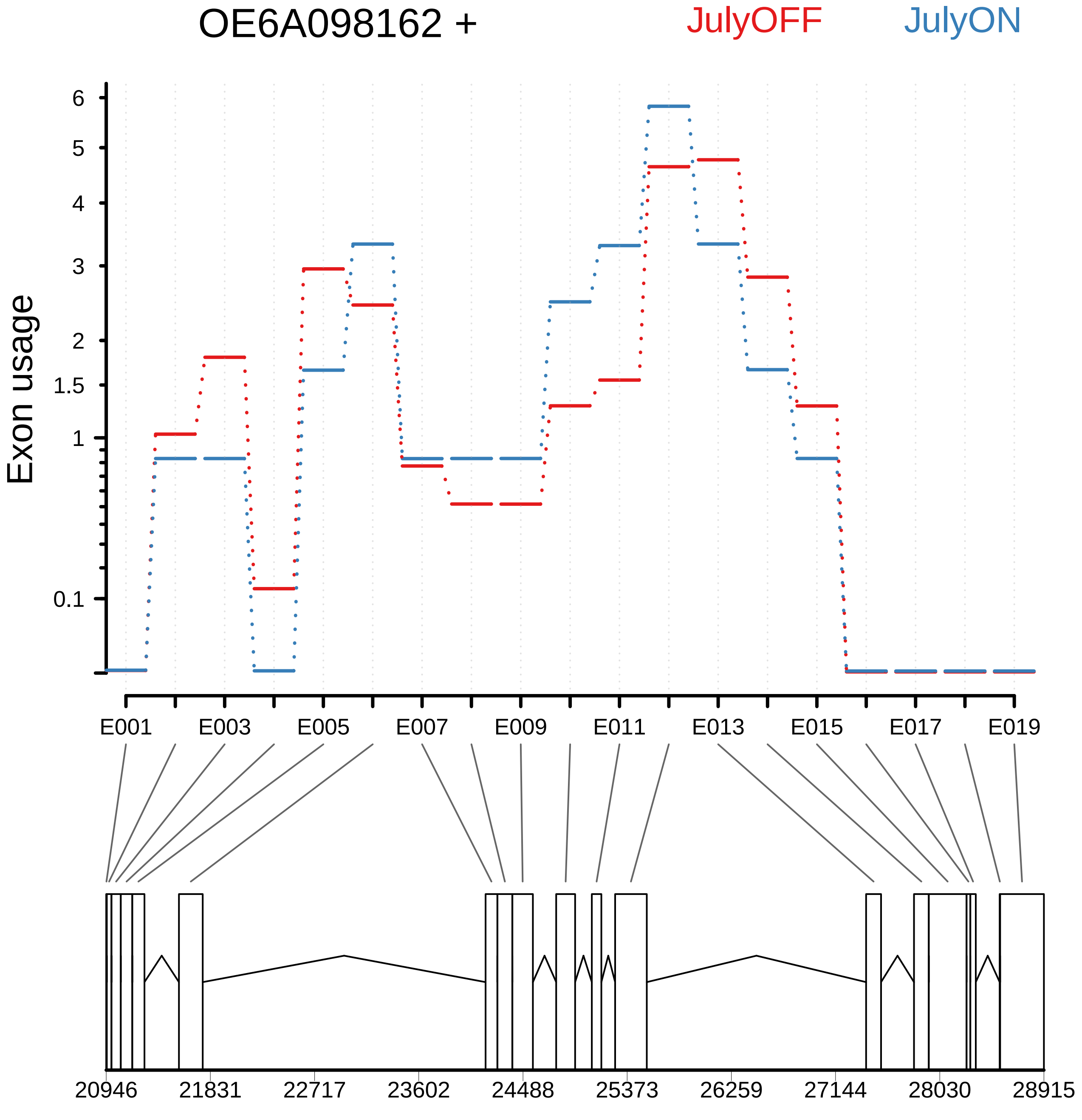
OE6A098162 +
JulyOFF
JulyON
Exon usage
6
5
4
3
2
1.5
1
0.1
E001
E003
E005
E007
E009
E011
E013
E015
E017
E019
20946
21831
22717
23602
24488
25373
26259
27144
28030
28915

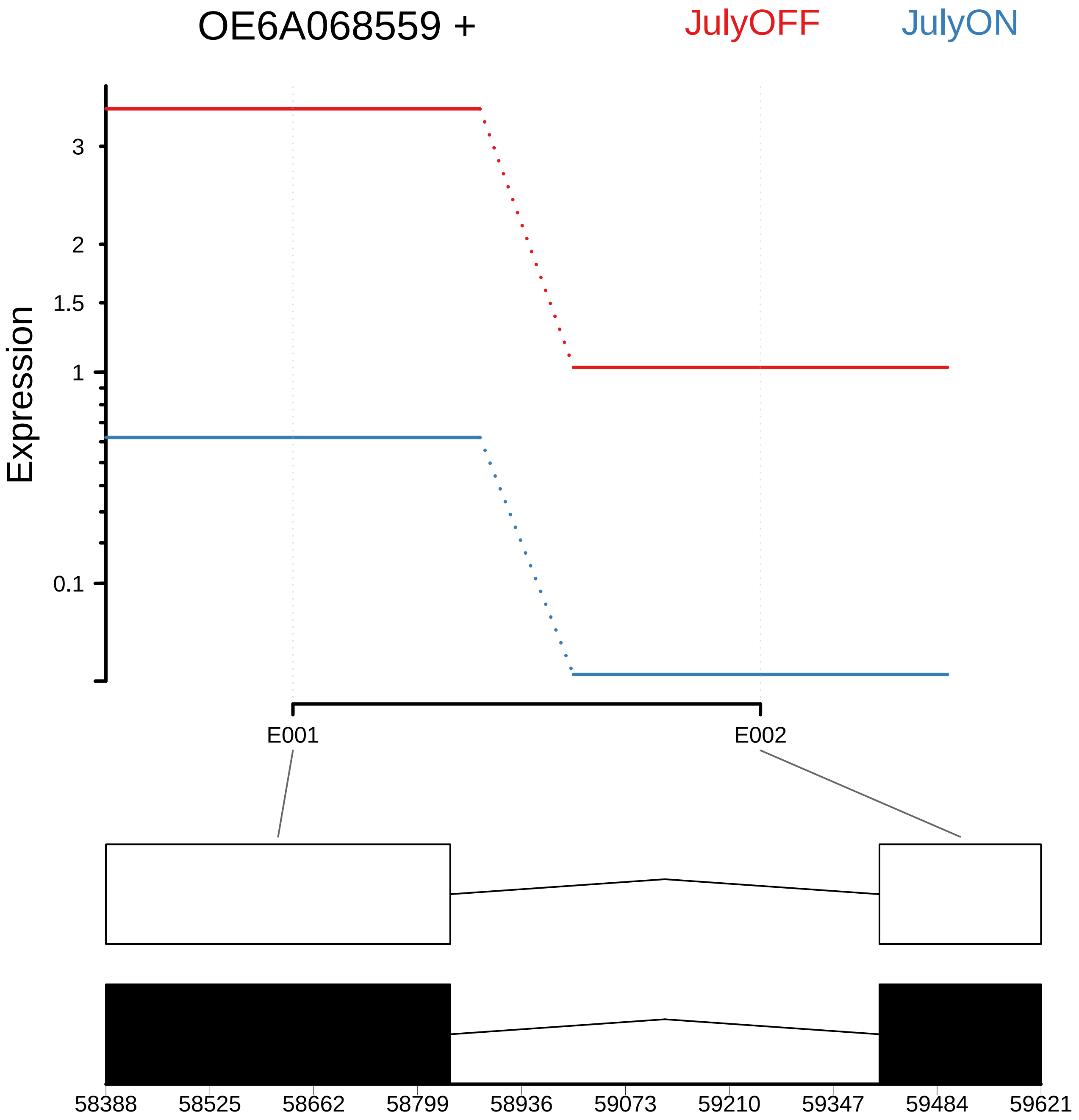

OE6A068559 +
JulyOFF
JulyON
Expression
3
2
1.5
1
0.1
E001
E002
58388
58525
58662
58799
58936
59073
59210
59347
59484
59621

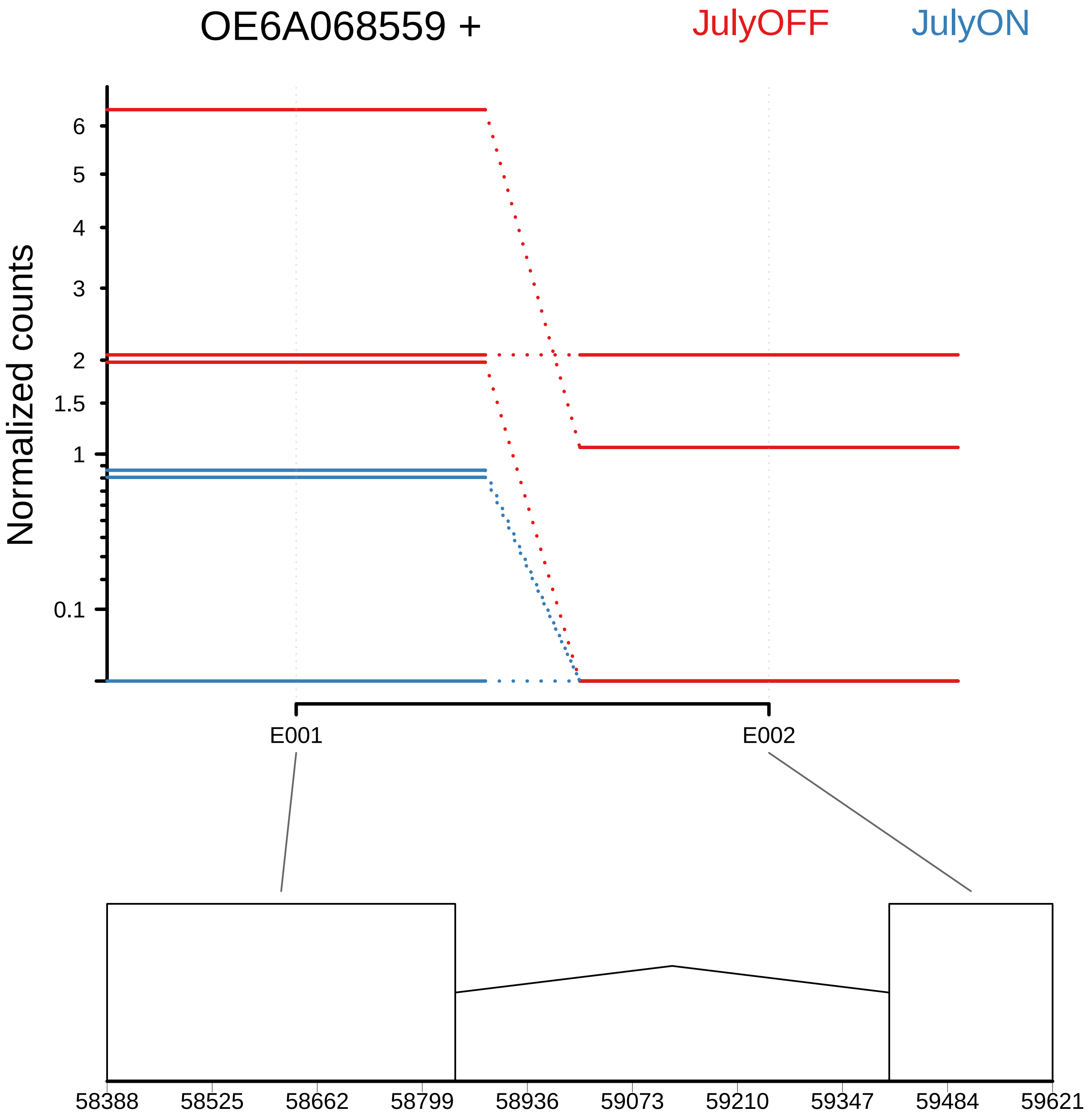

OE6A068559 +
JulyOFF
JulyON
Normalized counts
6
5
4
3
2
1.5
1
0.1
E001
E002
58388
58525
58662
58799
58936
59073
59210
59347
59484
59621

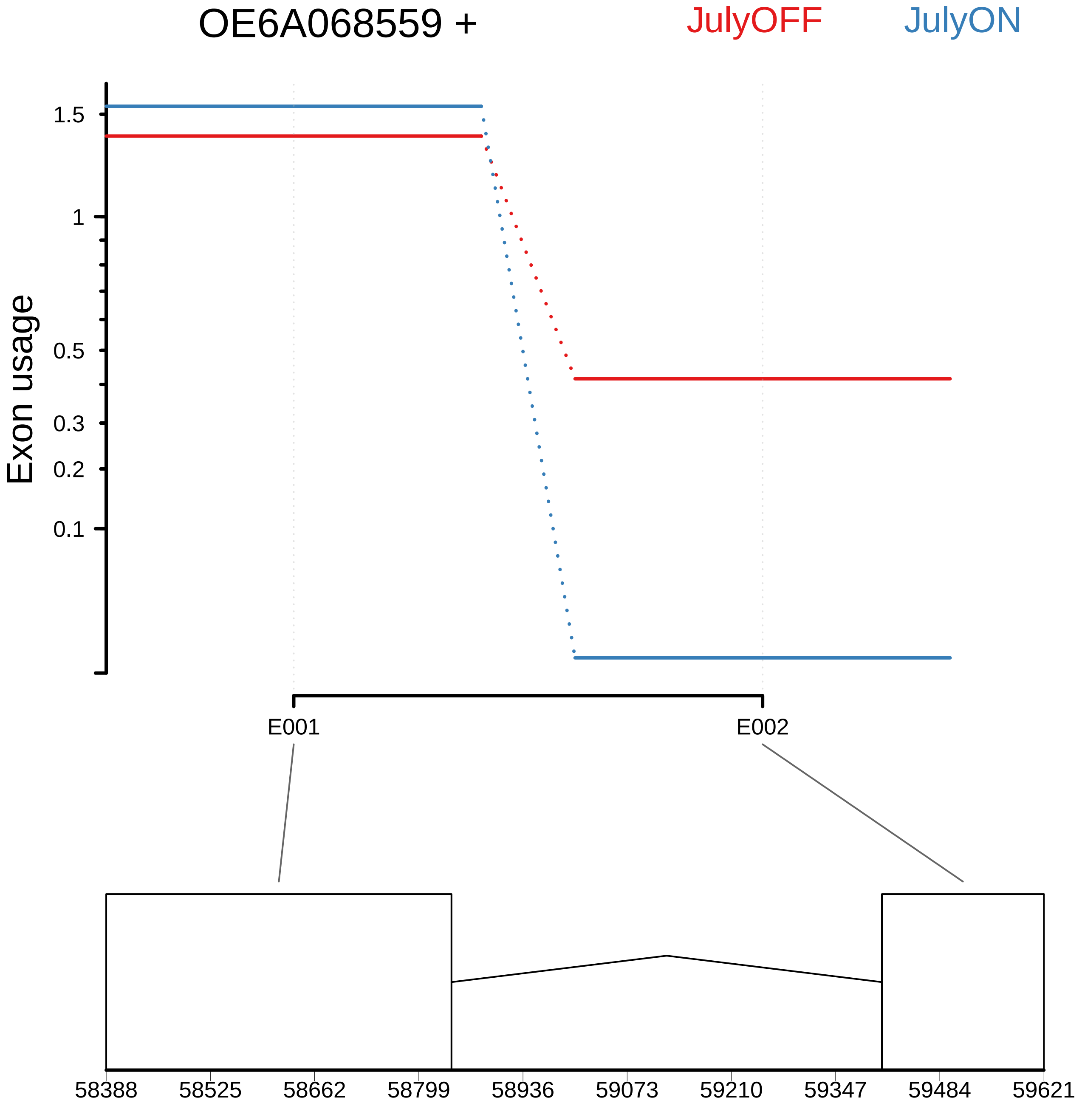
OE6A068559 +
JulyOFF
JulyON
Exon usage
1.5
1
0.5
0.3
0.2
0.1
E001
E002
58388
58525
58662
58799
58936
59073
59210
59347
59484
59621

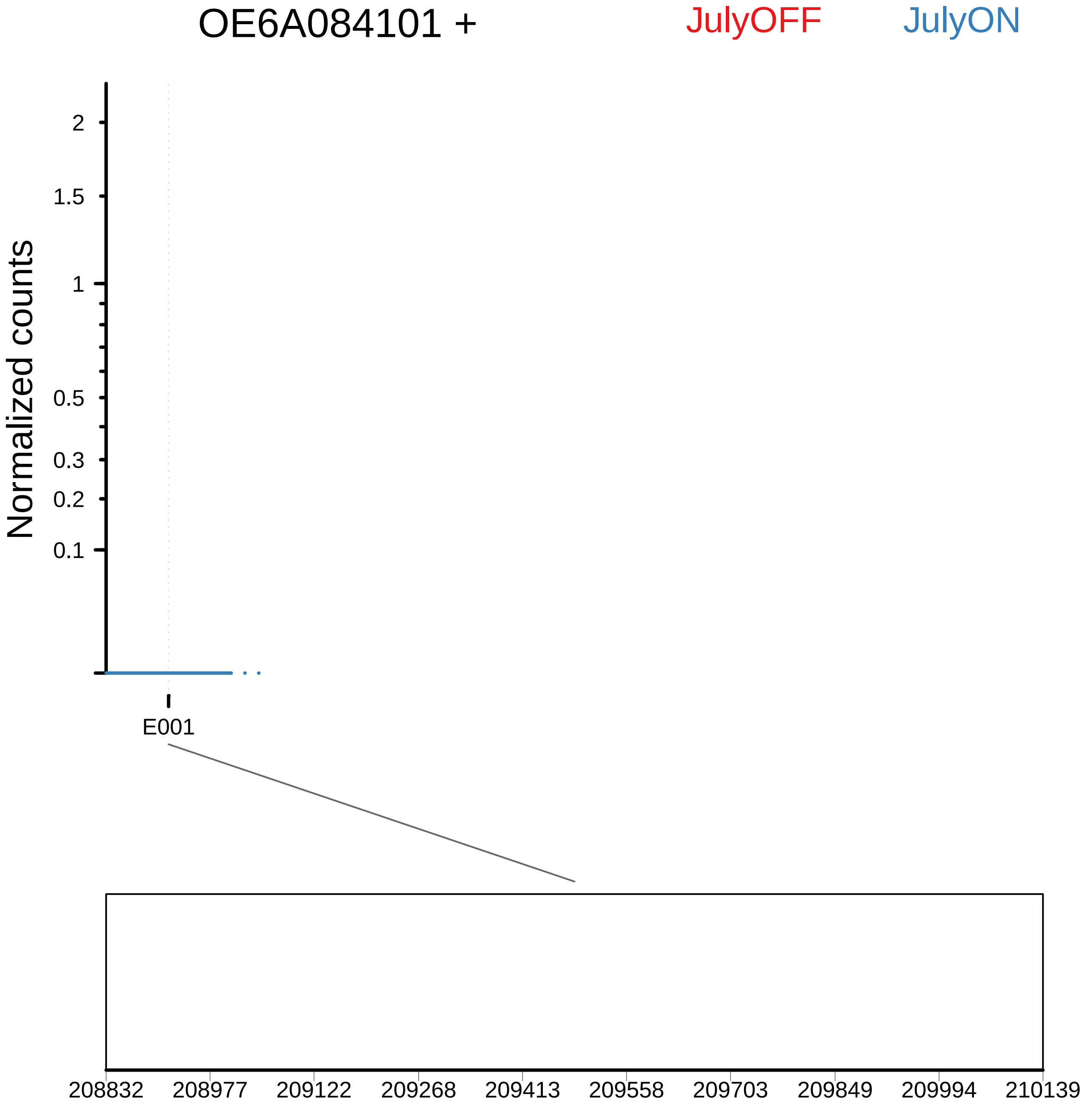

OE6A084101 +
JulyOFF
JulyON
Normalized counts
2
1.5
1
0.5
0.3
0.2
0.1
E001
208832
208977
209122
209268
209413
209558
209703
209849
209994
210139

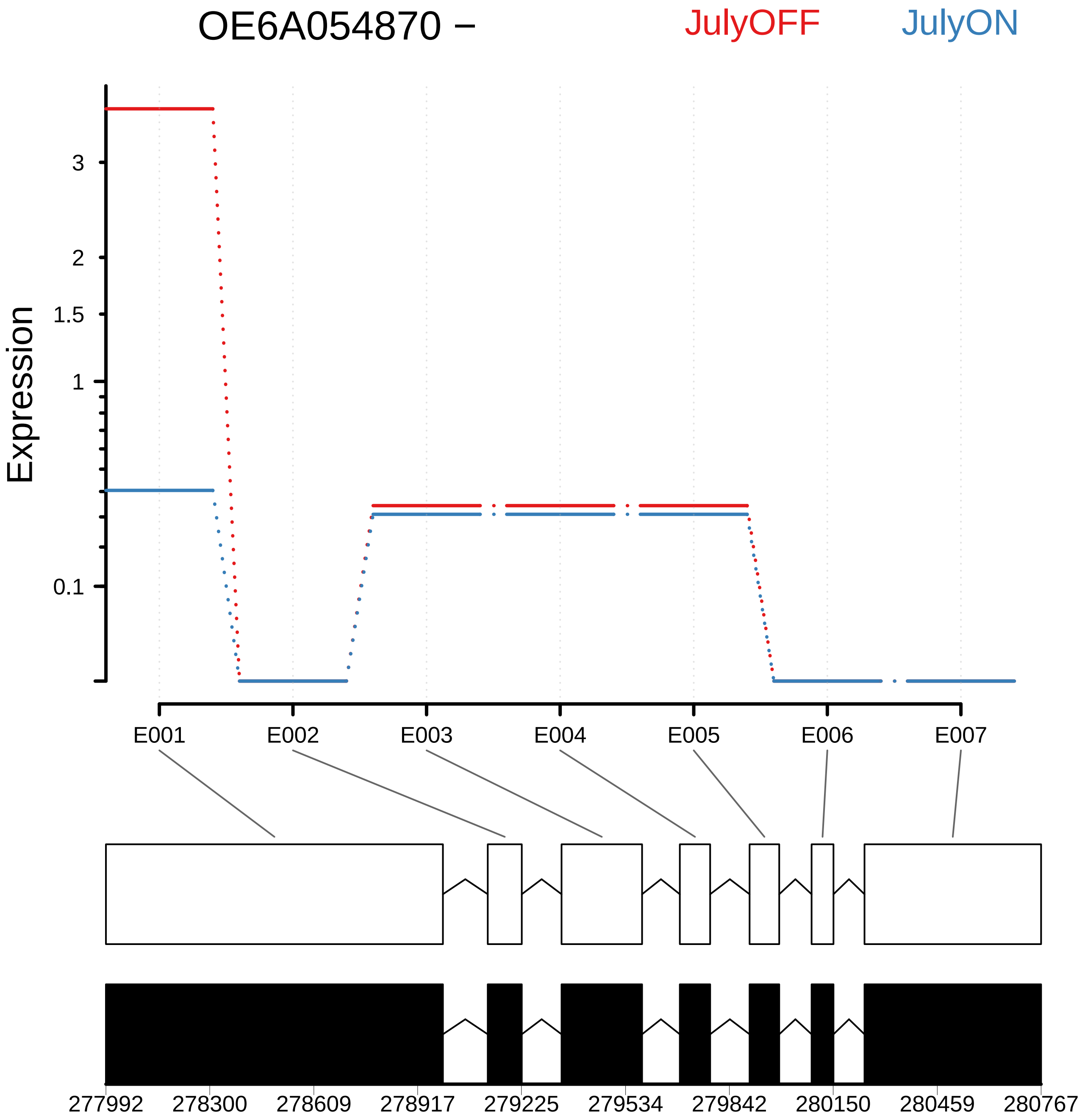

OE6A054870 −
JulyOFF
JulyON
Expression
3
2
1.5
1
0.1
E001
E002
E003
E004
E005
E006
E007
277992
278300
278609
278917
279225
279534
279842
280150
280459
280767

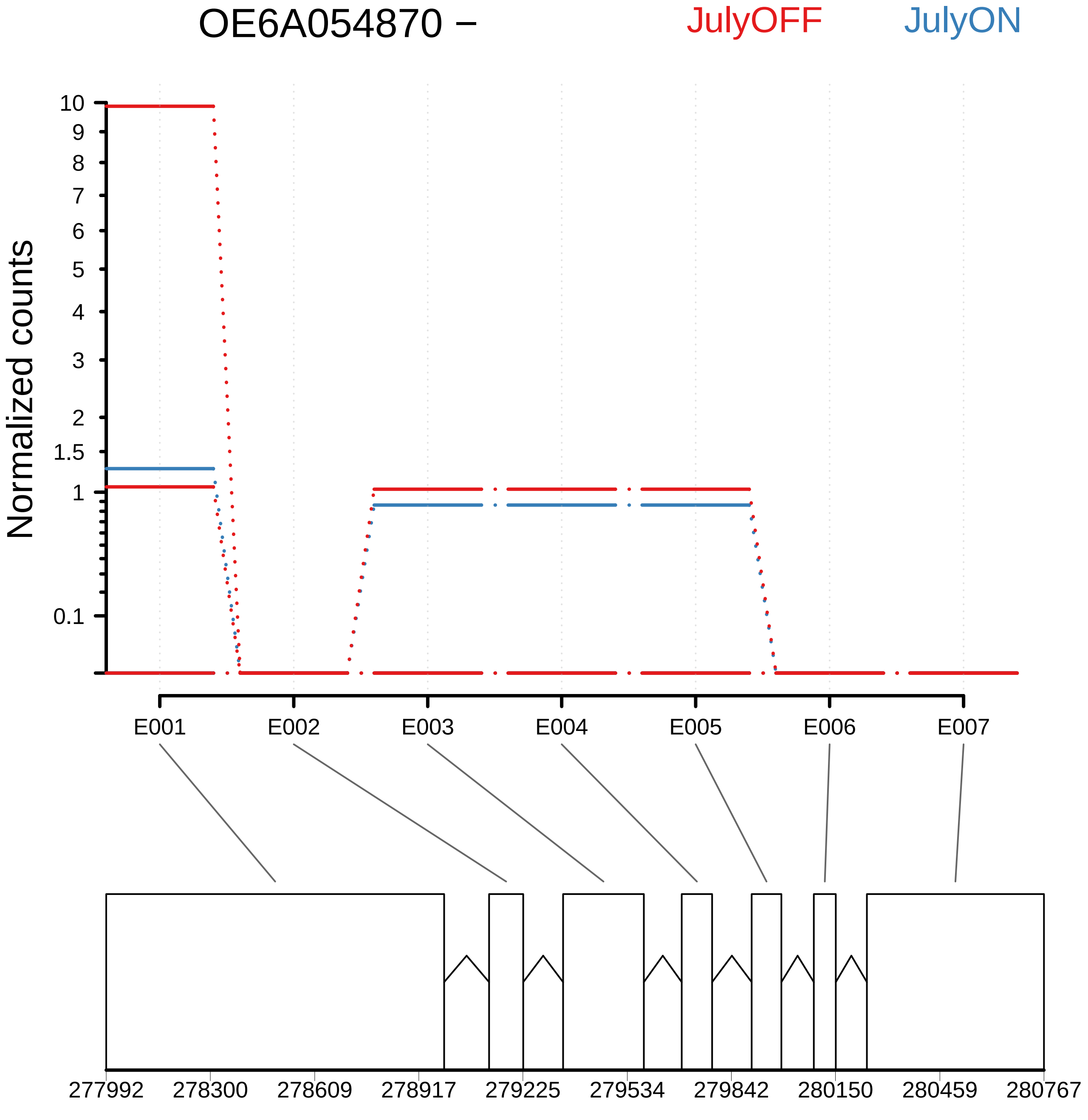

OE6A054870 −
JulyOFF
JulyON
Normalized counts
10
9
8
7
6
5
4
3
2
1.5
1
0.1
E001
E002
E003
E004
E005
E006
E007
277992
278300
278609
278917
279225
279534
279842
280150
280459
280767

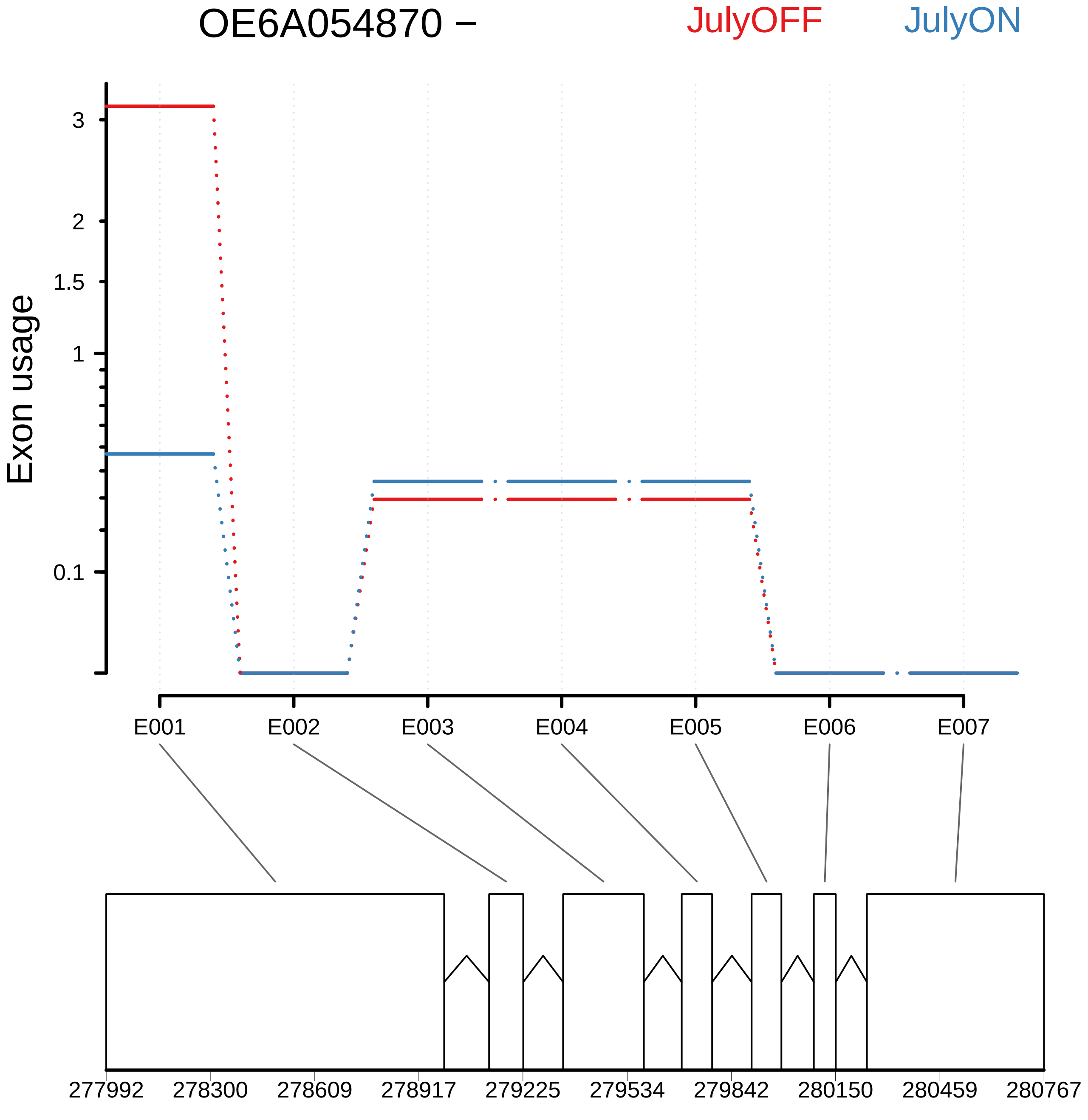
OE6A054870 −
JulyOFF
JulyON
Exon usage
3
2
1.5
1
0.1
E001
E002
E003
E004
E005
E006
E007
277992
278300
278609
278917
279225
279534
279842
280150
280459
280767

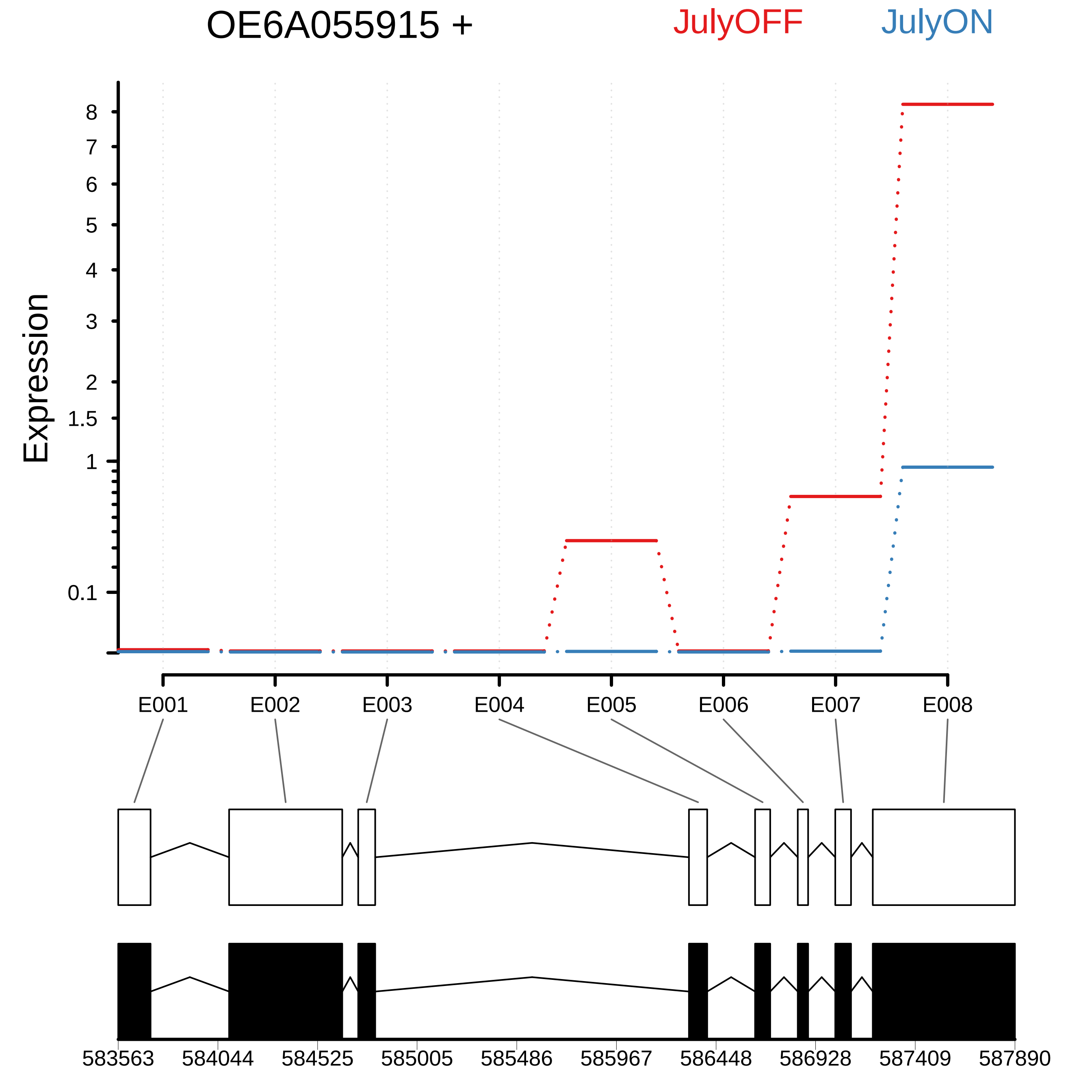

OE6A055915 +
JulyOFF
JulyON
Expression
8
7
6
5
4
3
2
1.5
1
0.1
E001
E002
E003
E004
E005
E006
E007
E008
583563
584044
584525
585005
585486
585967
586448
586928
587409
587890

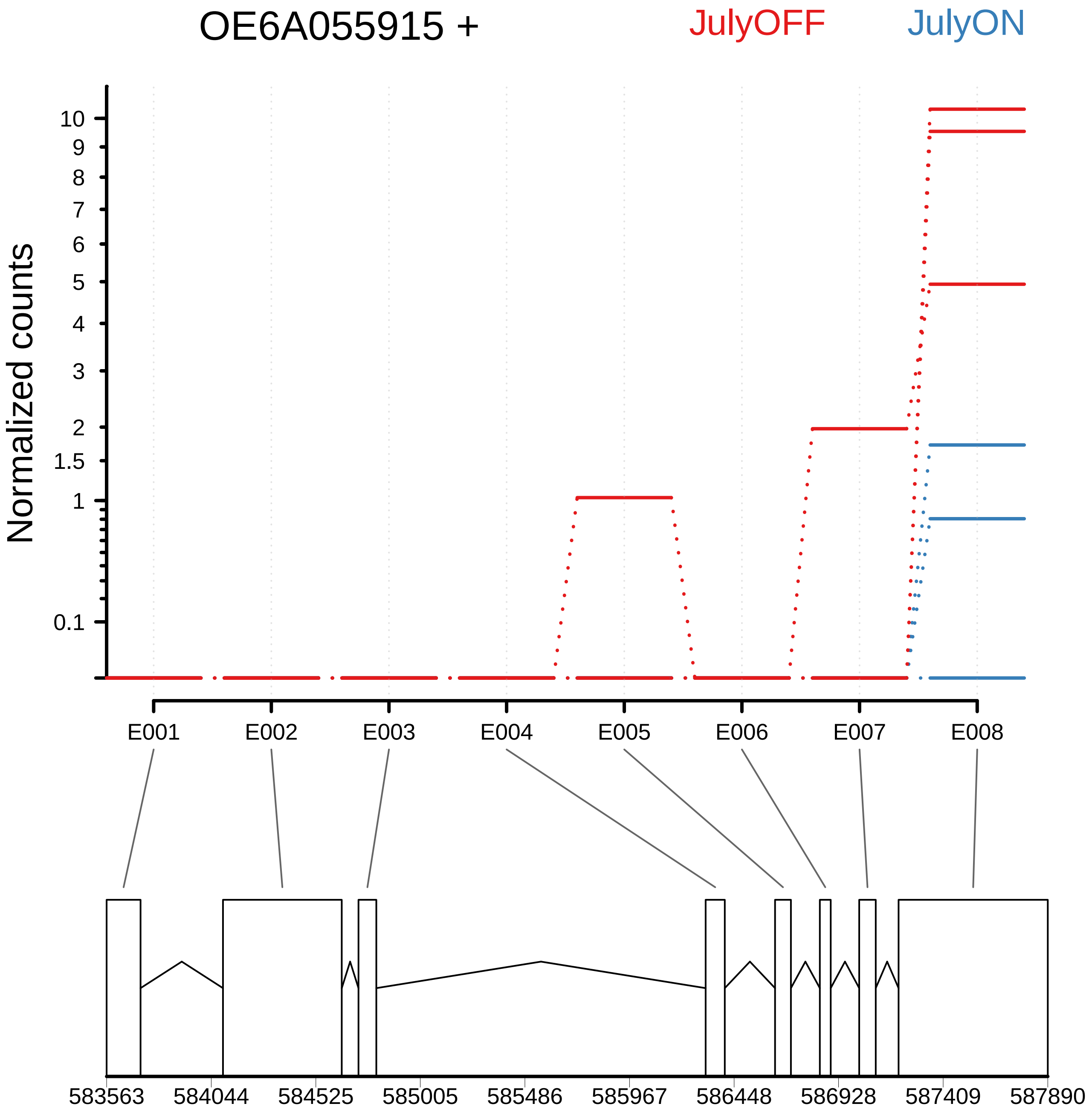
OE6A055915 +
JulyOFF
JulyON
Normalized counts
10
9
8
7
6
5
4
3
2
1.5
1
0.1
E001
E002
E003
E004
E005
E006
E007
E008
583563
584044
584525
585005
585486
585967
586448
586928
587409
587890

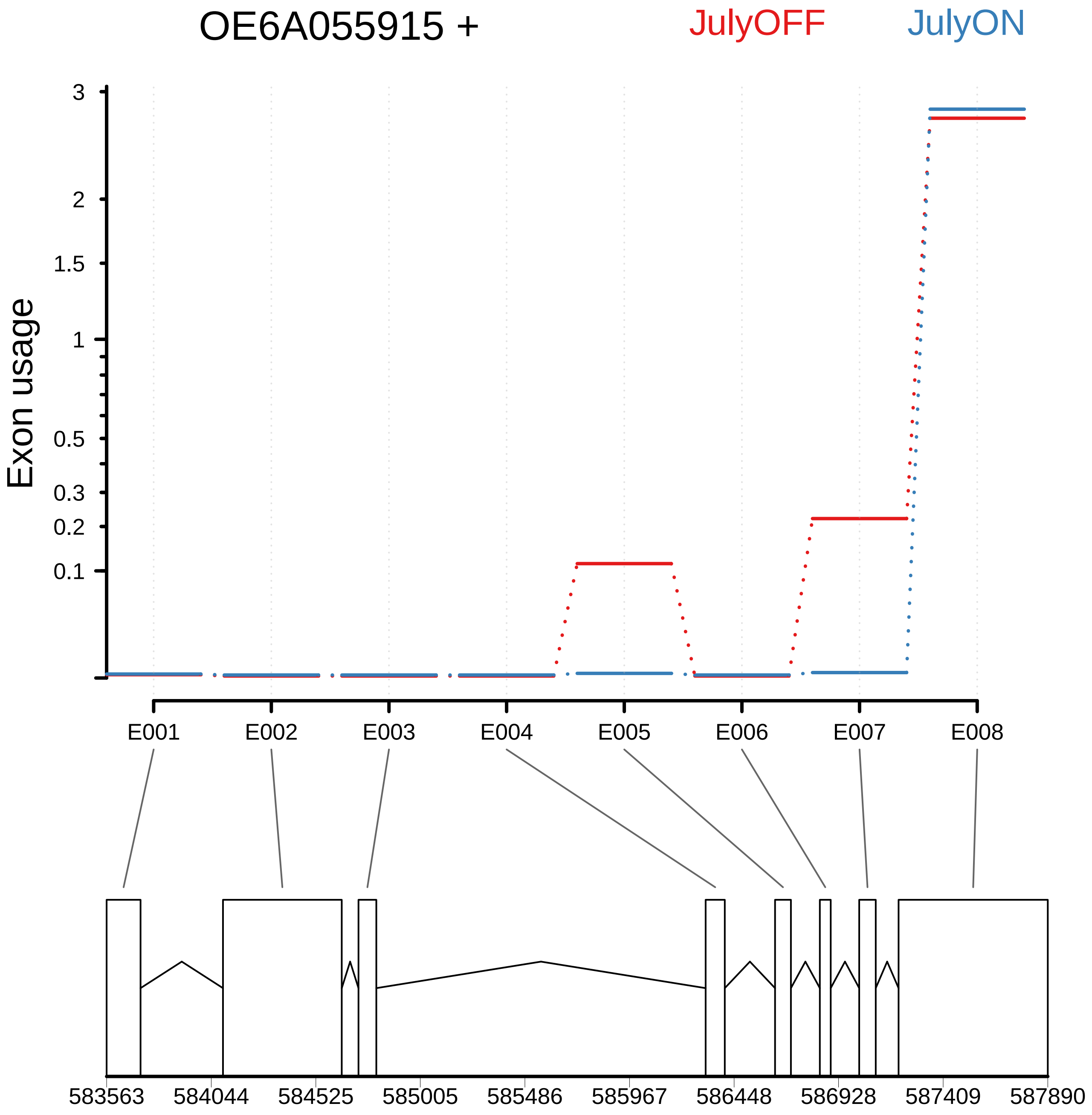

OE6A055915 +
JulyOFF
JulyON
Exon usage
3
2
1.5
1
0.5
0.3
0.2
0.1
E001
E002
E003
E004
E005
E006
E007
E008
583563
584044
584525
585005
585486
585967
586448
586928
587409
587890

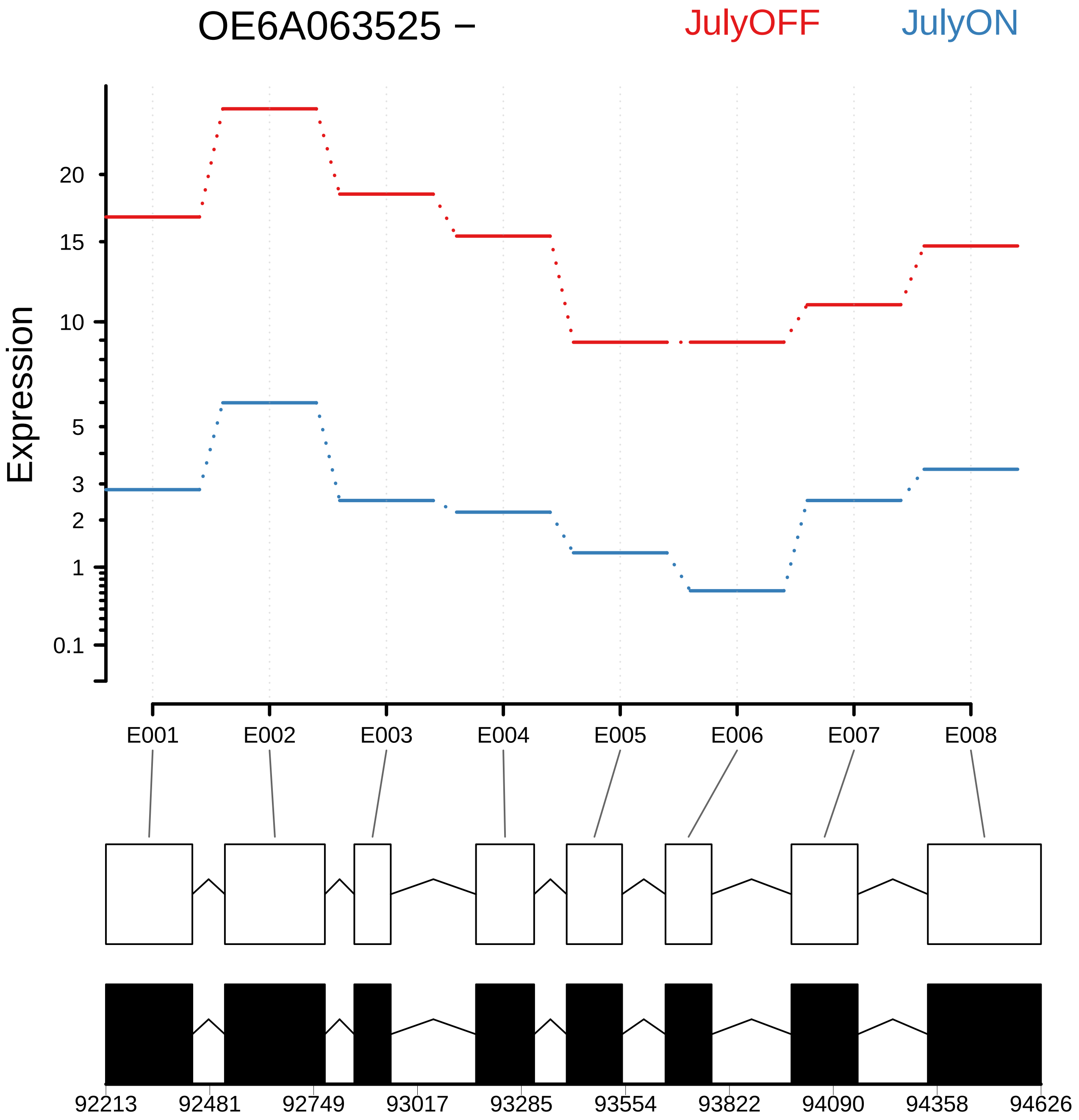
OE6A063525 −
JulyOFF
JulyON
Expression
20
15
10
5
3
2
1
0.1
E001
E002
E003
E004
E005
E006
E007
E008
92213
92481
92749
93017
93285
93554
93822
94090
94358
94626

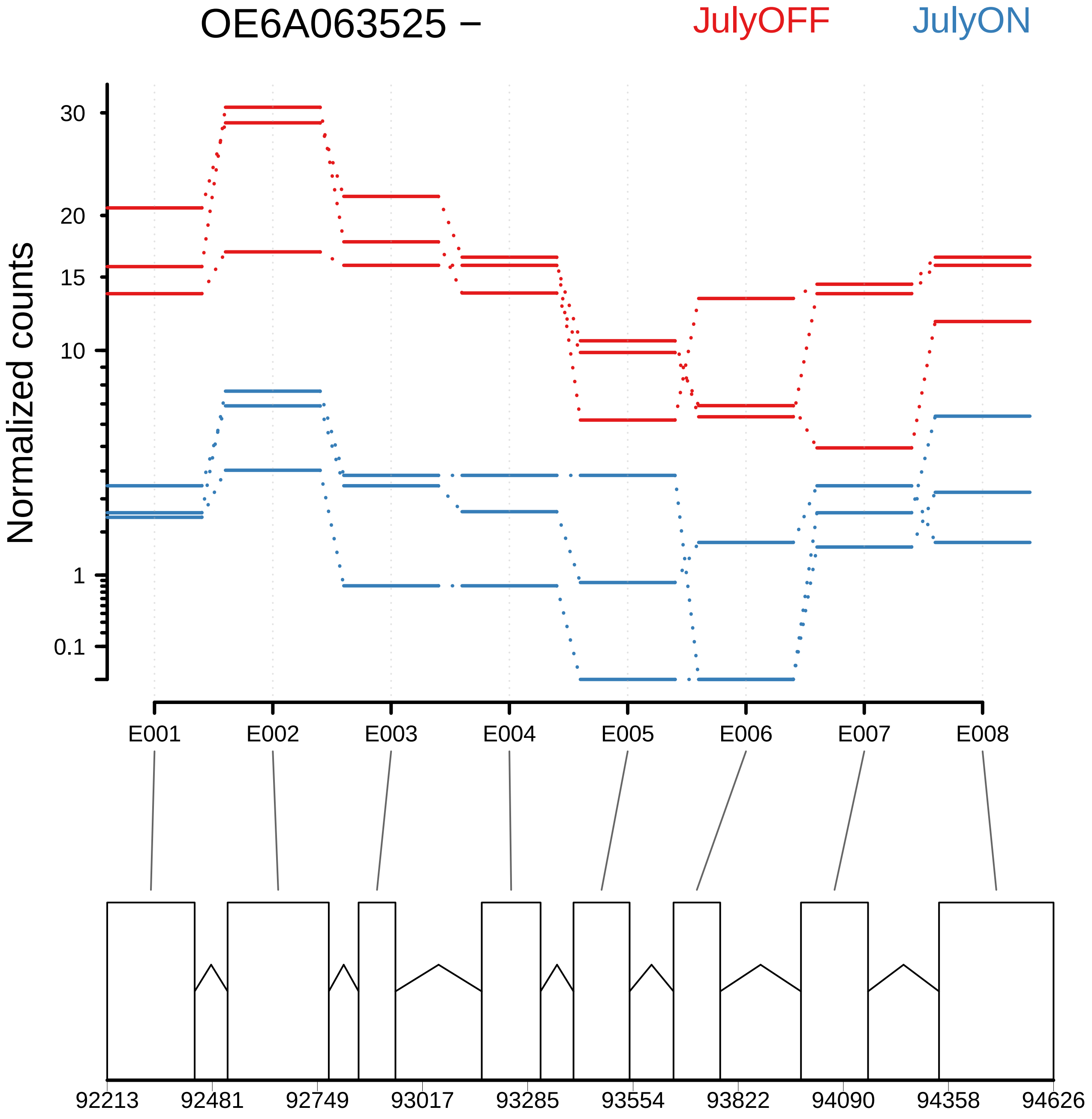

OE6A063525 −
JulyOFF
JulyON
Normalized counts
30
20
15
10
1
0.1
E001
E002
E003
E004
E005
E006
E007
E008
92213
92481
92749
93017
93285
93554
93822
94090
94358
94626

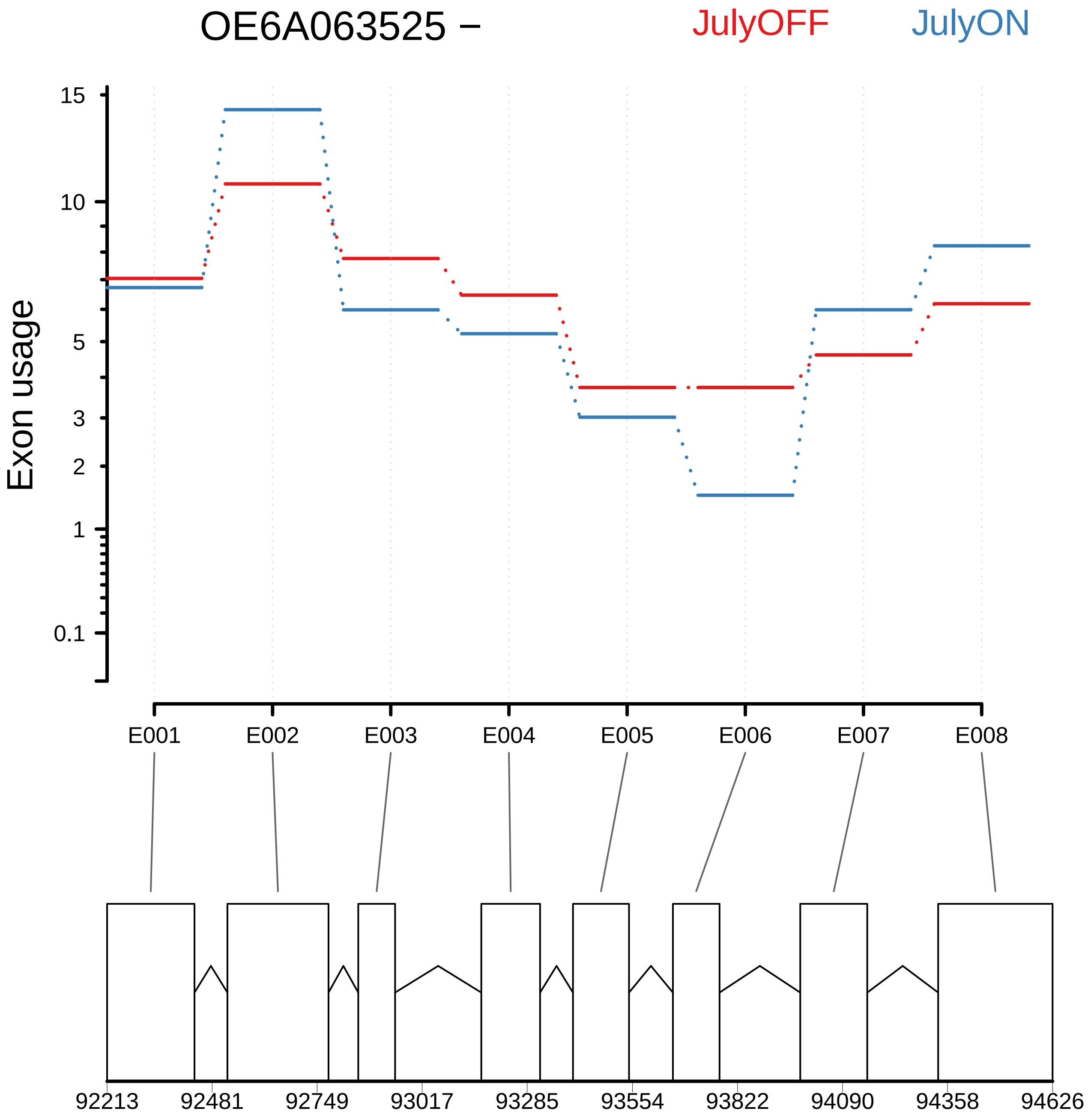

OE6A063525 −
JulyOFF
JulyON
Exon usage
15
10
5
3
2
1
0.1
E001
E002
E003
E004
E005
E006
E007
E008
92213
92481
92749
93017
93285
93554
93822
94090
94358
94626

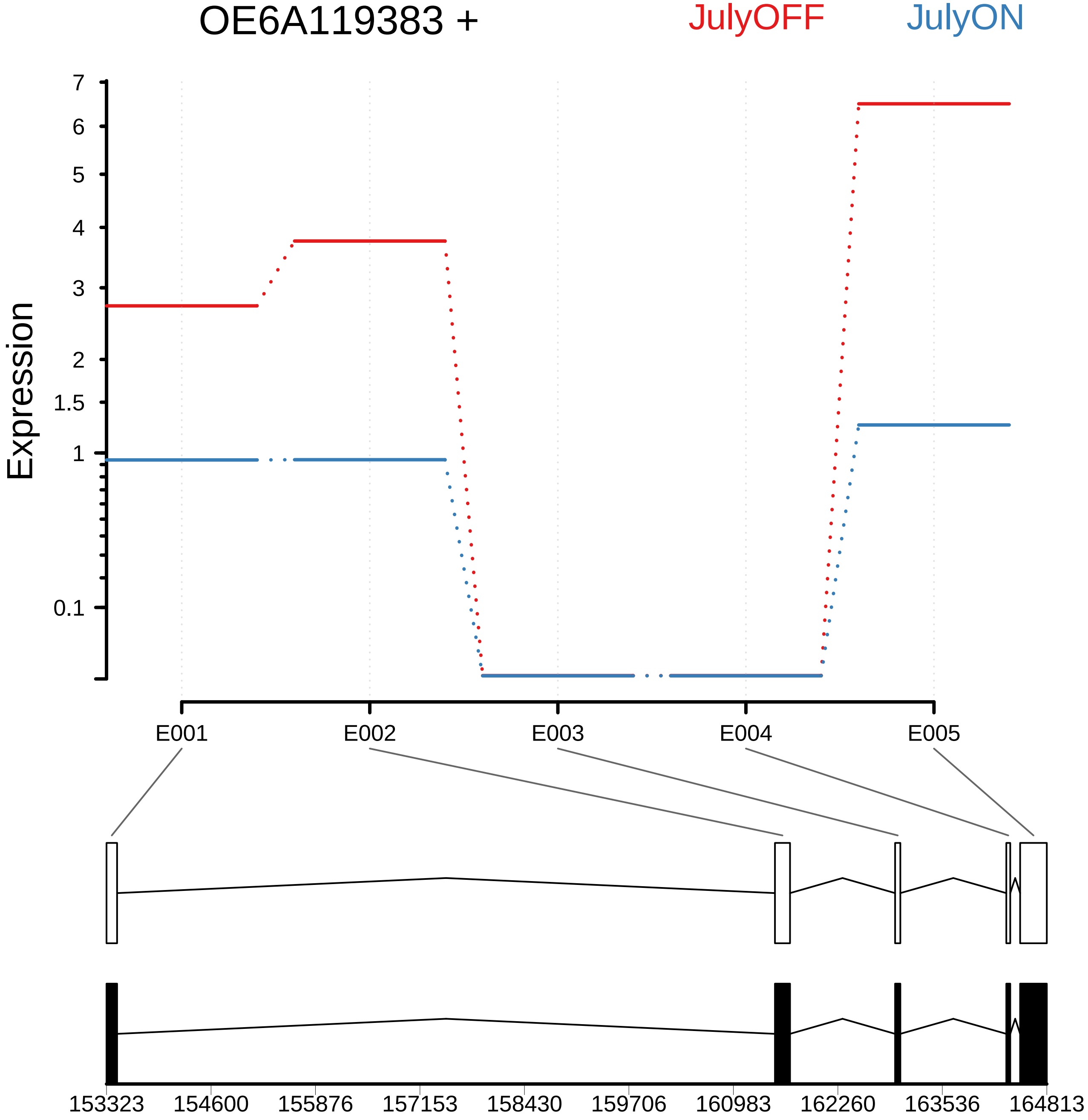

OE6A119383 +
JulyOFF
JulyON
Expression
7
6
5
4
3
2
1.5
1
0.1
E001
E002
E003
E004
E005
153323
154600
155876
157153
158430
159706
160983
162260
163536
164813

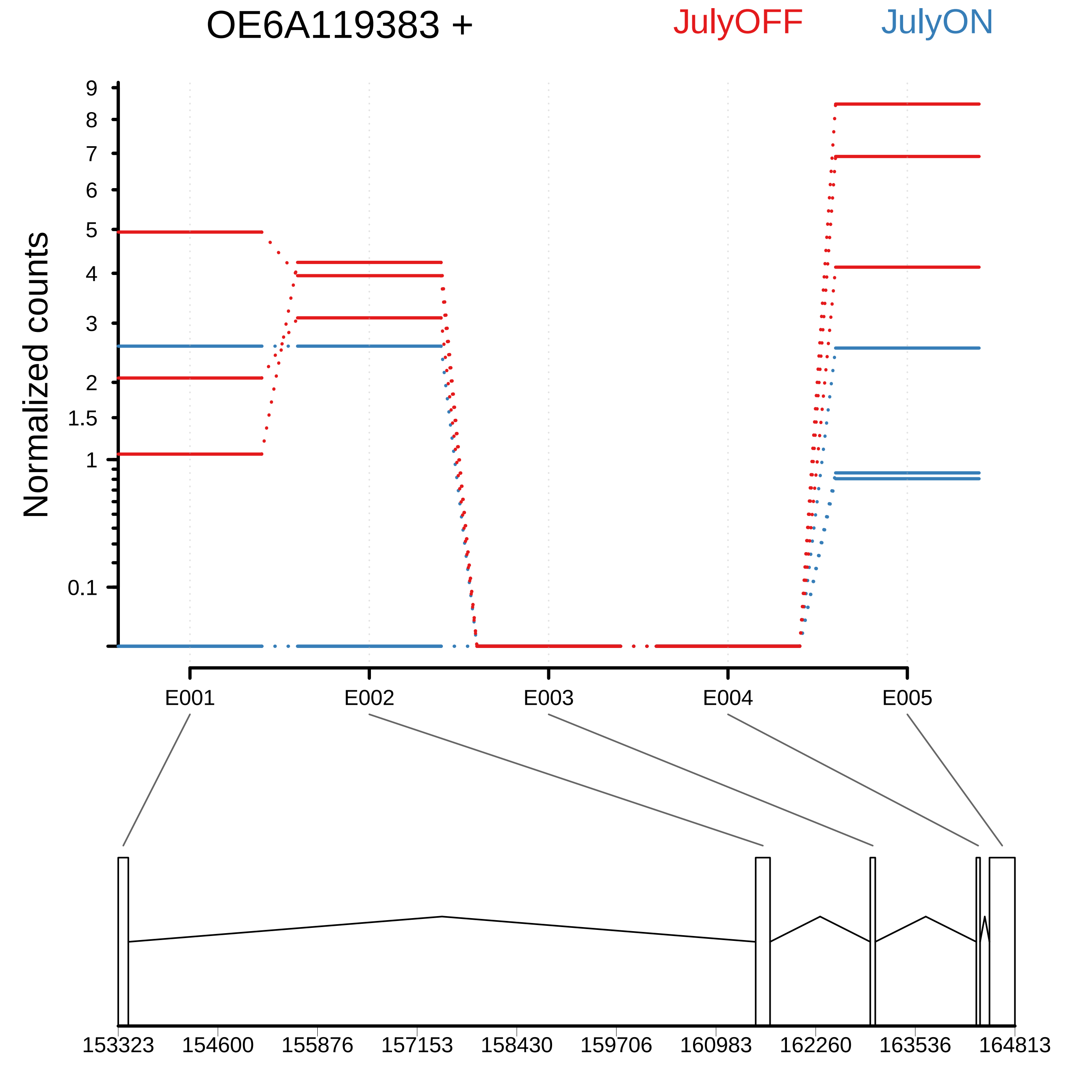

OE6A119383 +
JulyOFF
JulyON
Normalized counts
9
8
7
6
5
4
3
2
1.5
1
0.1
E001
E002
E003
E004
E005
153323
154600
155876
157153
158430
159706
160983
162260
163536
164813

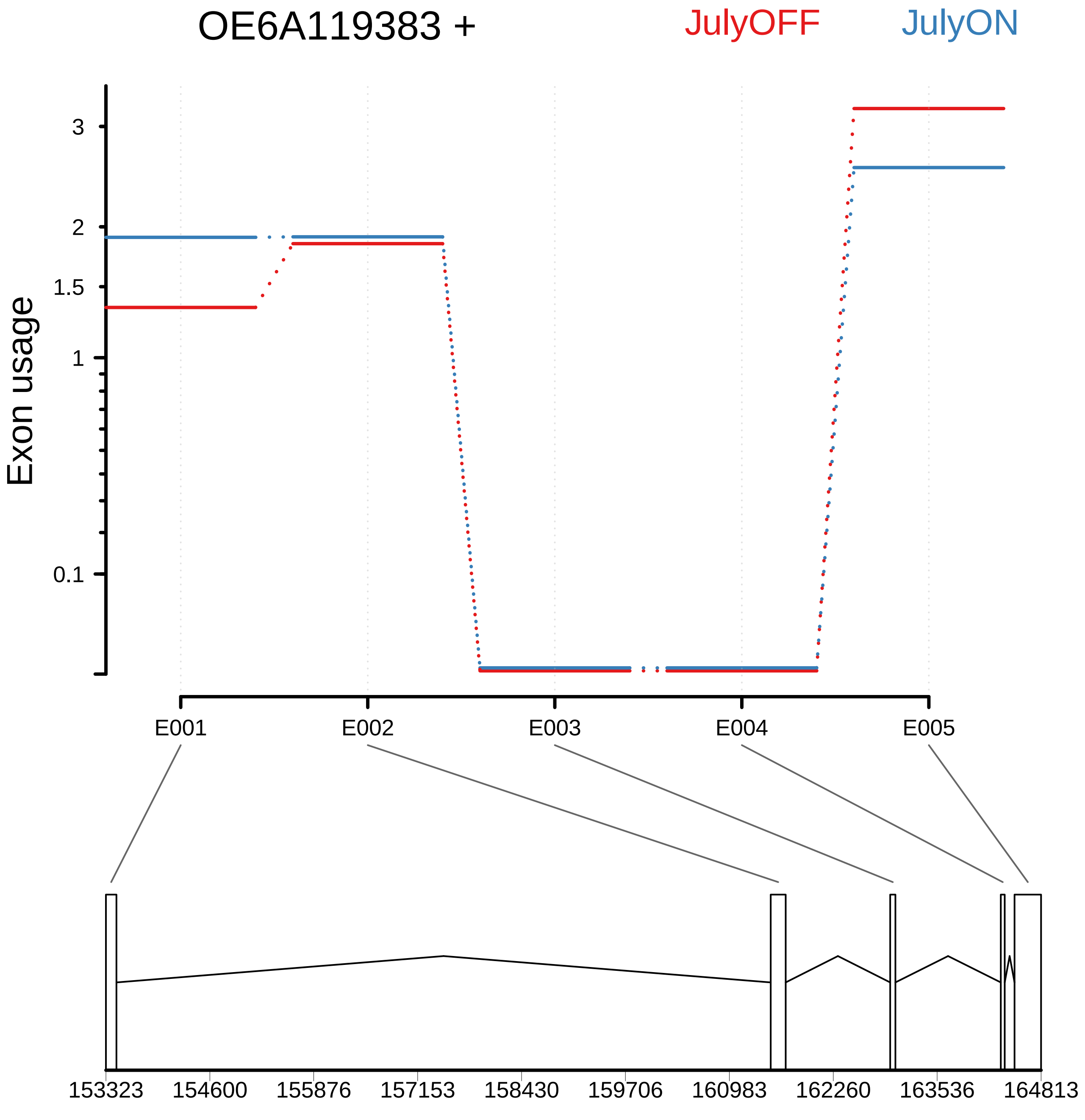

OE6A119383 +
JulyOFF
JulyON
Exon usage
3
2
1.5
1
0.1
E001
E002
E003
E004
E005
153323
154600
155876
157153
158430
159706
160983
162260
163536
164813

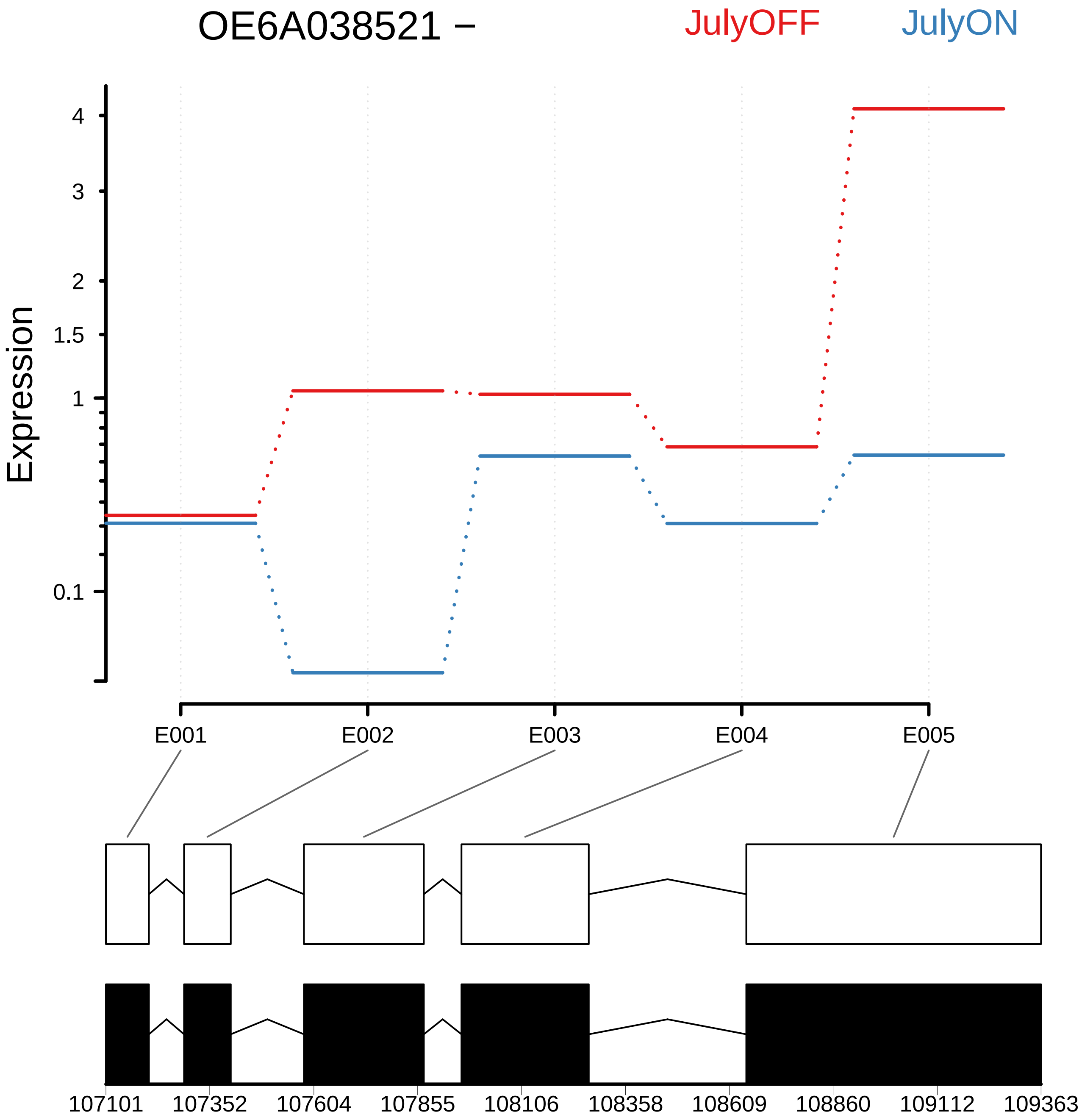
OE6A038521 −
JulyOFF
JulyON
Expression
4
3
2
1.5
1
0.1
E001
E002
E003
E004
E005
107101
107352
107604
107855
108106
108358
108609
108860
109112
109363

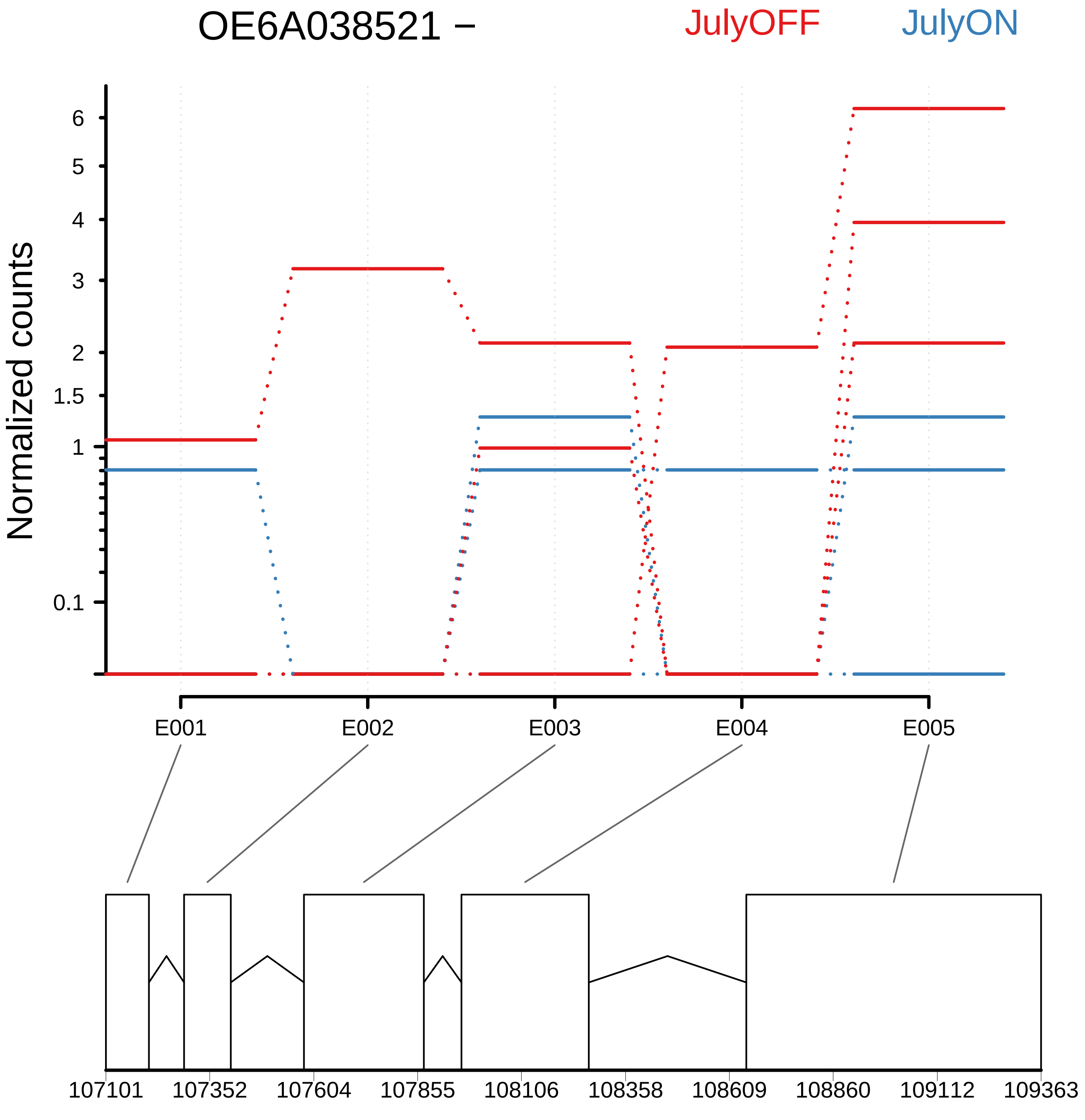
OE6A038521 −
JulyOFF
JulyON
Normalized counts
6
5
4
3
2
1.5
1
0.1
E001
E002
E003
E004
E005
107101
107352
107604
107855
108106
108358
108609
108860
109112
109363

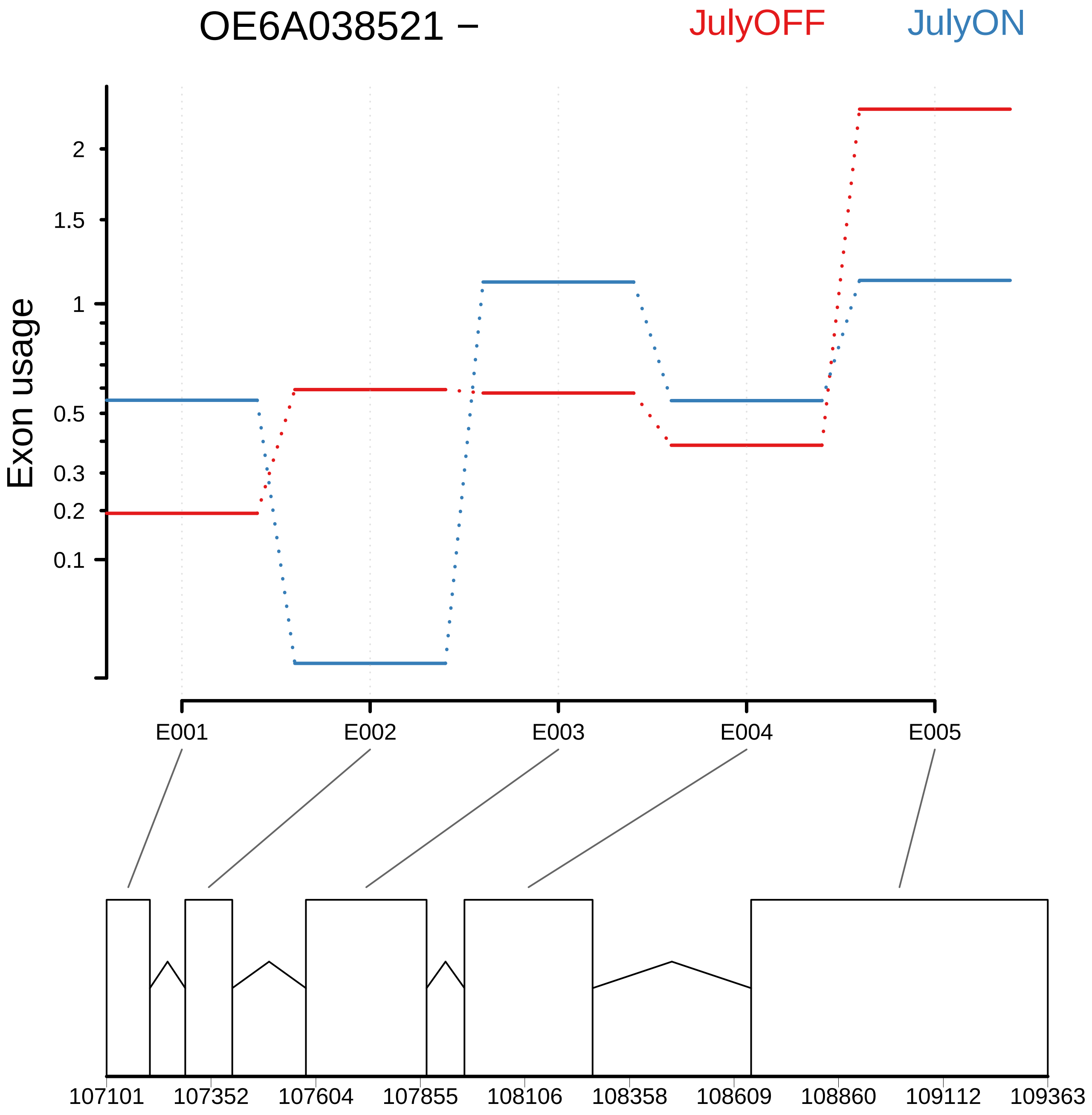
OE6A038521 −
JulyOFF
JulyON
Exon usage
2
1.5
1
0.5
0.3
0.2
0.1
E001
E002
E003
E004
E005
107101
107352
107604
107855
108106
108358
108609
108860
109112
109363

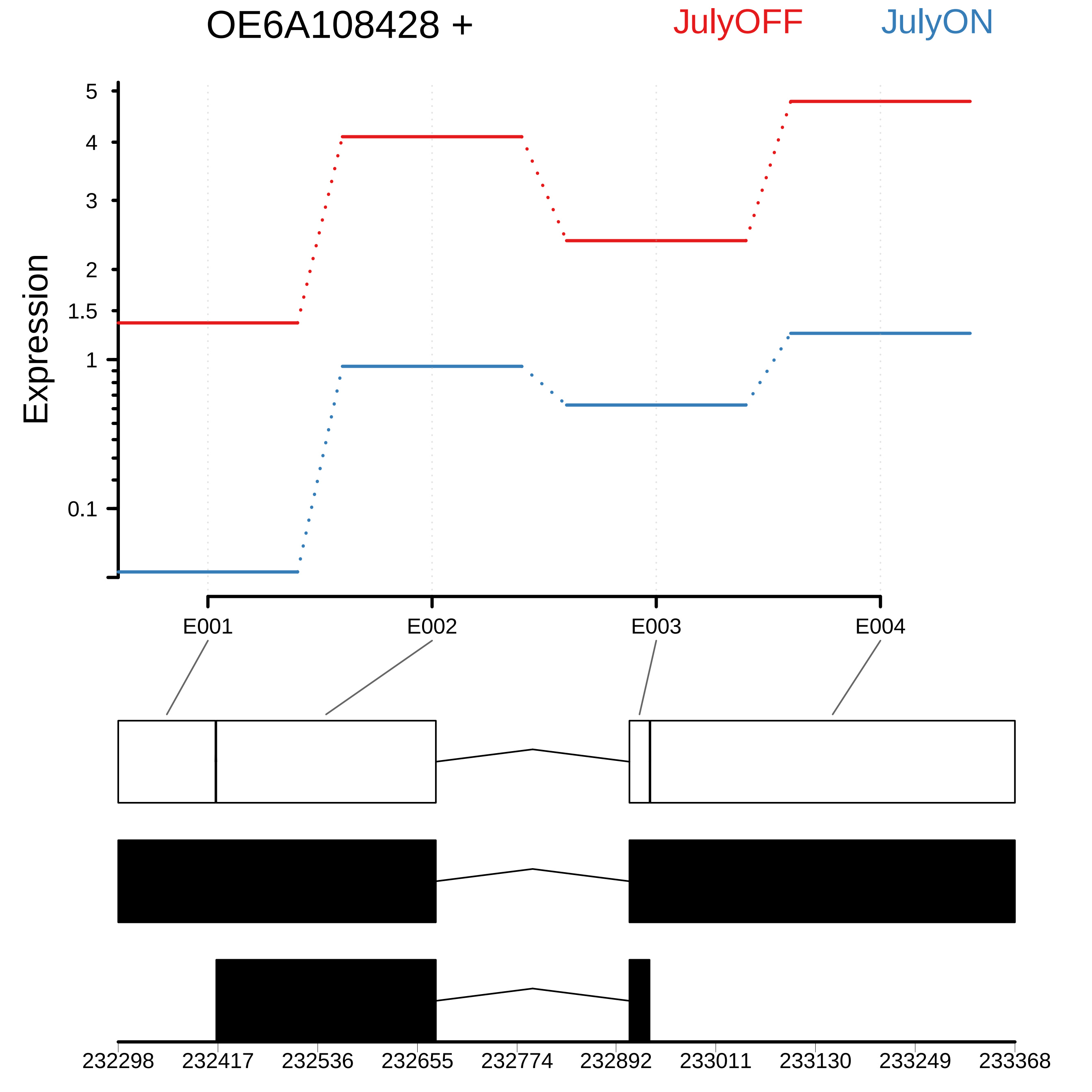
OE6A108428 +
JulyOFF
JulyON
Expression
5
4
3
2
1.5
1
0.1
E001
E002
E003
E004
232298
232417
232536
232655
232774
232892
233011
233130
233249
233368

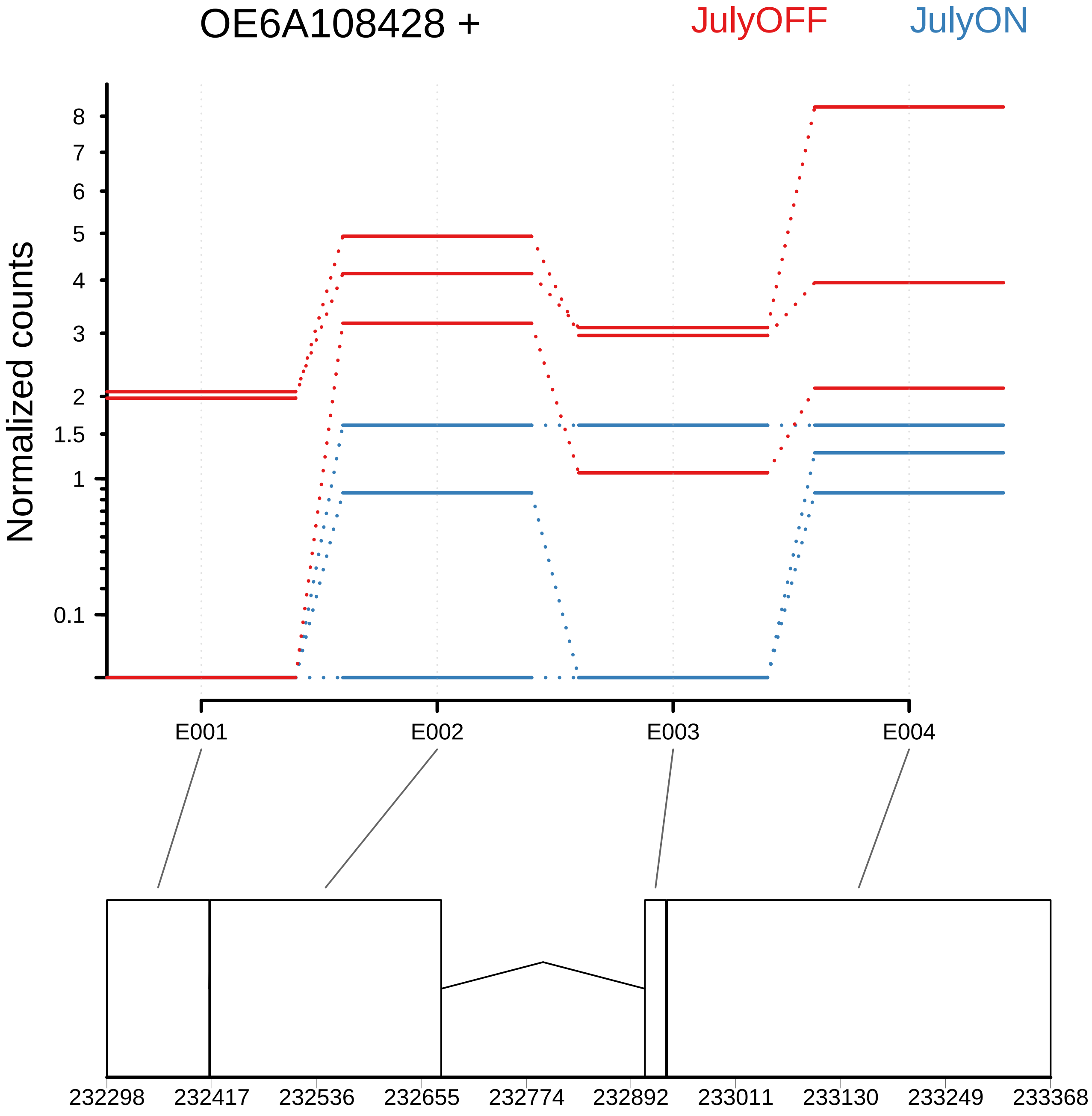
OE6A108428 +
JulyOFF
JulyON
Normalized counts
8
7
6
5
4
3
2
1.5
1
0.1
E001
E002
E003
E004
232298
232417
232536
232655
232774
232892
233011
233130
233249
233368

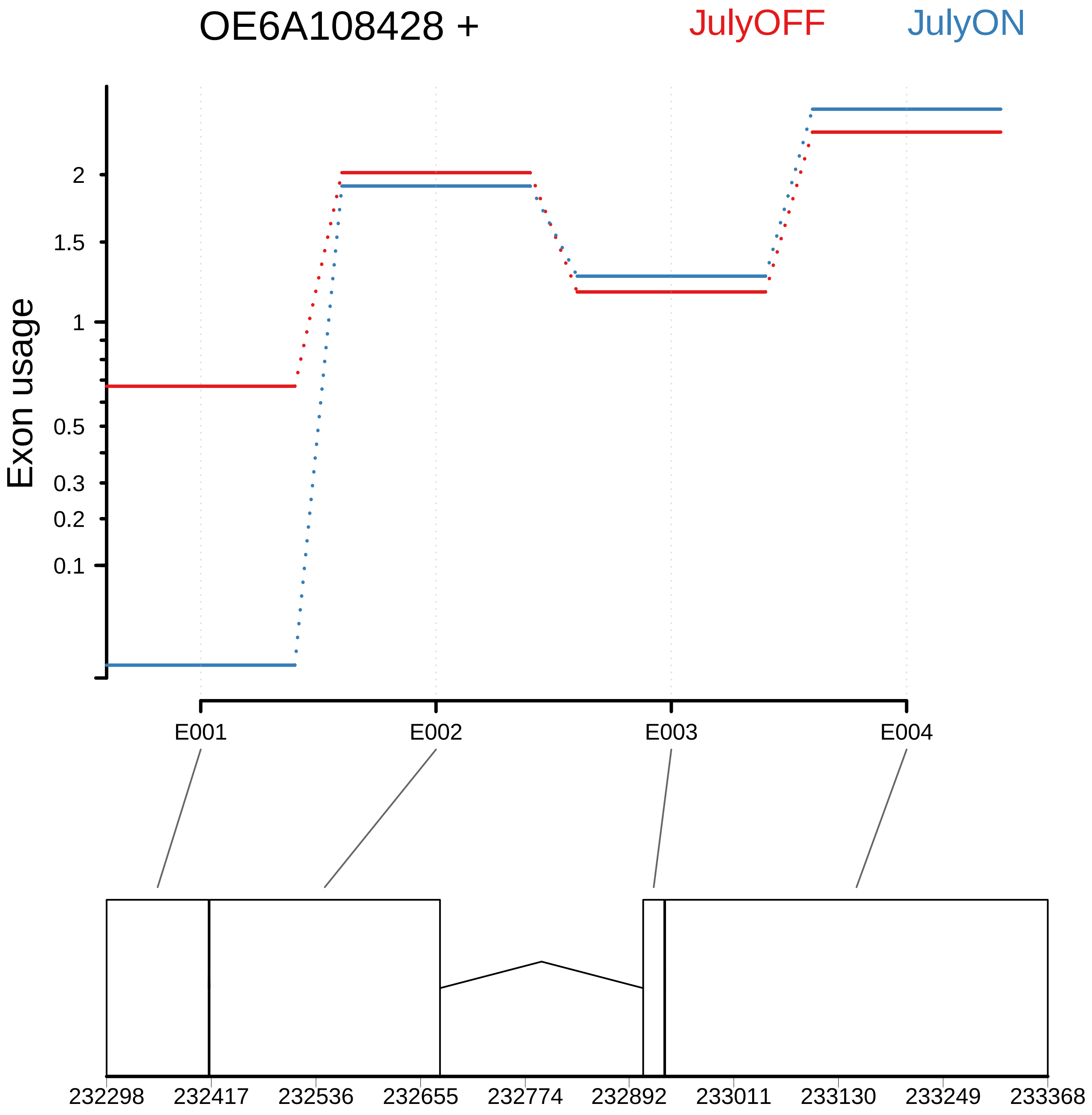

OE6A108428 +
JulyOFF
JulyON
Exon usage
2
1.5
1
0.5
0.3
0.2
0.1
E001
E002
E003
E004
232298
232417
232536
232655
232774
232892
233011
233130
233249
233368

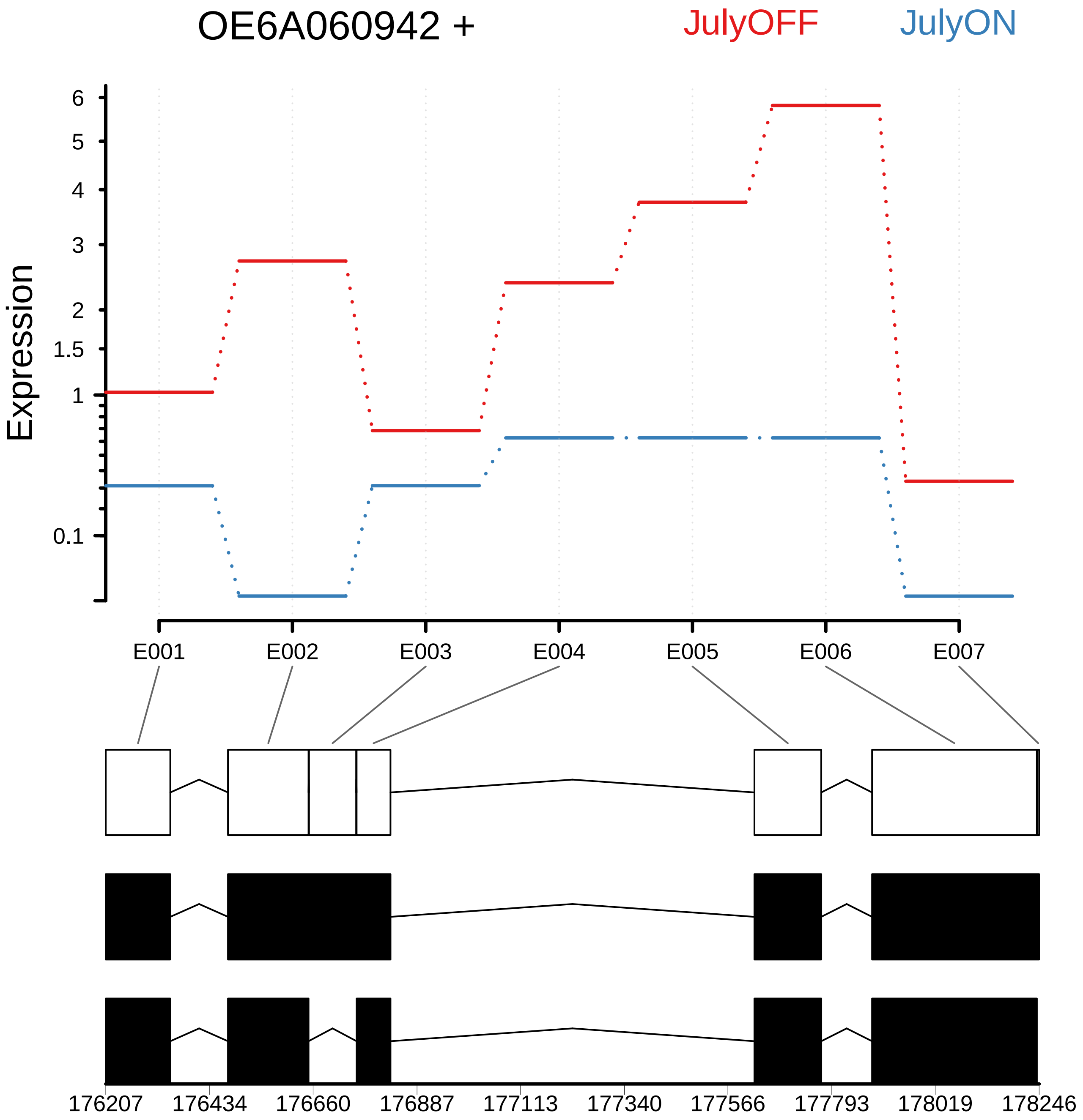

OE6A060942 +
JulyOFF
JulyON
Expression
6
5
4
3
2
1.5
1
0.1
E001
E002
E003
E004
E005
E006
E007
176207
176434
176660
176887
177113
177340
177566
177793
178019
178246

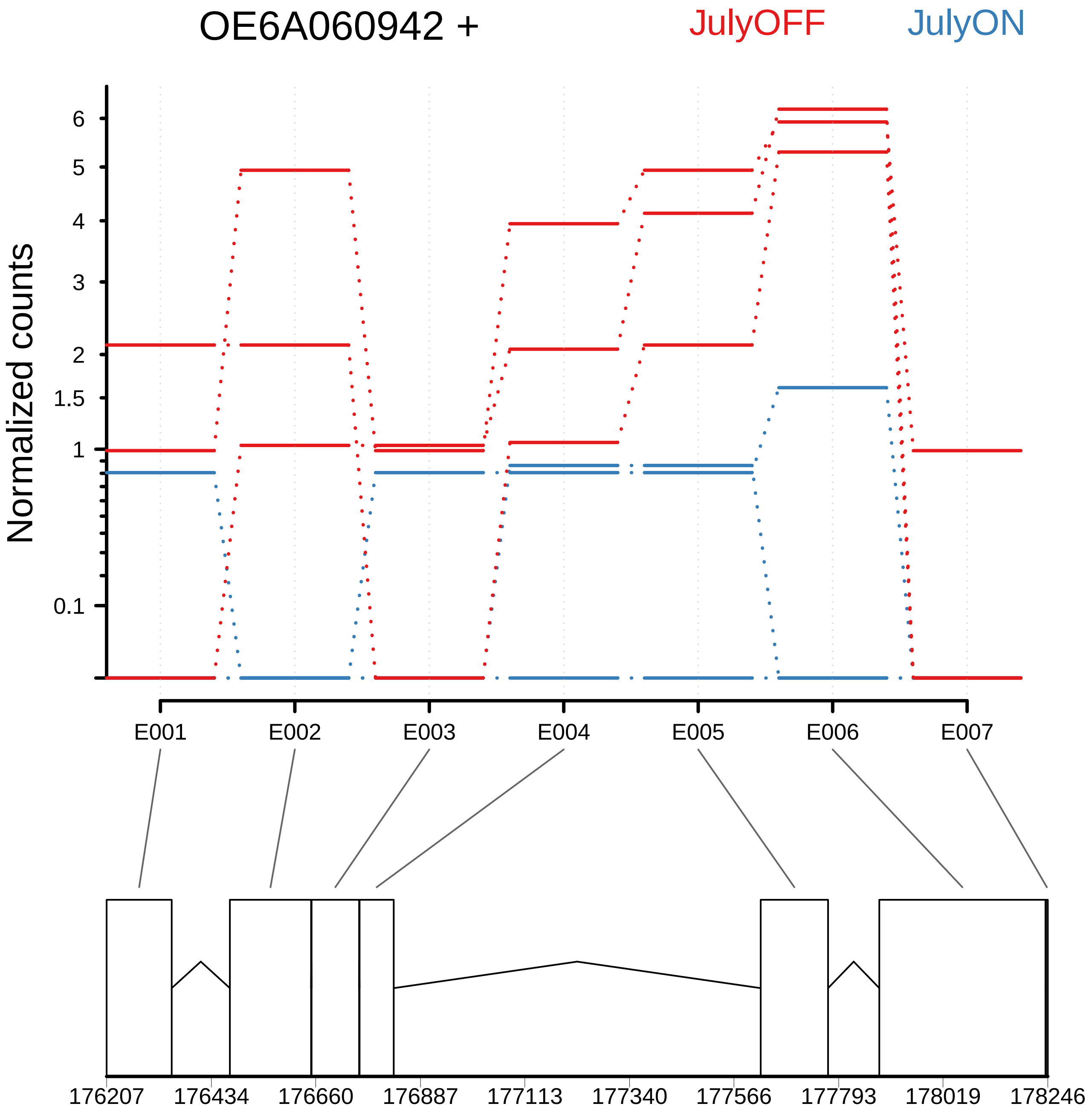

OE6A060942 +
JulyOFF
JulyON
Normalized counts
6
5
4
3
2
1.5
1
0.1
E001
E002
E003
E004
E005
E006
E007
176207
176434
176660
176887
177113
177340
177566
177793
178019
178246

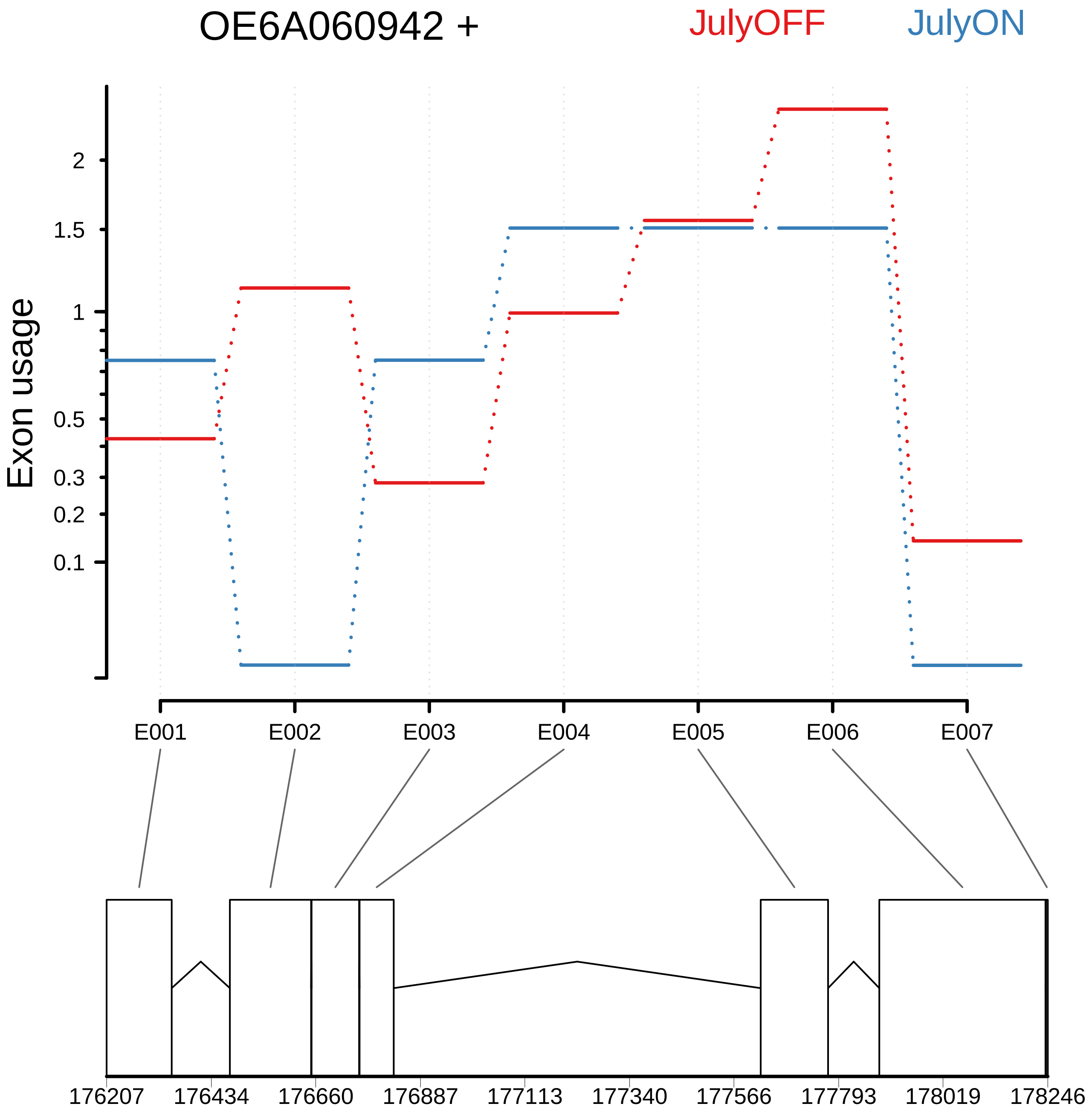

OE6A060942 +
JulyOFF
JulyON
Exon usage
2
1.5
1
0.5
0.3
0.2
0.1
E001
E002
E003
E004
E005
E006
E007
176207
176434
176660
176887
177113
177340
177566
177793
178019
178246

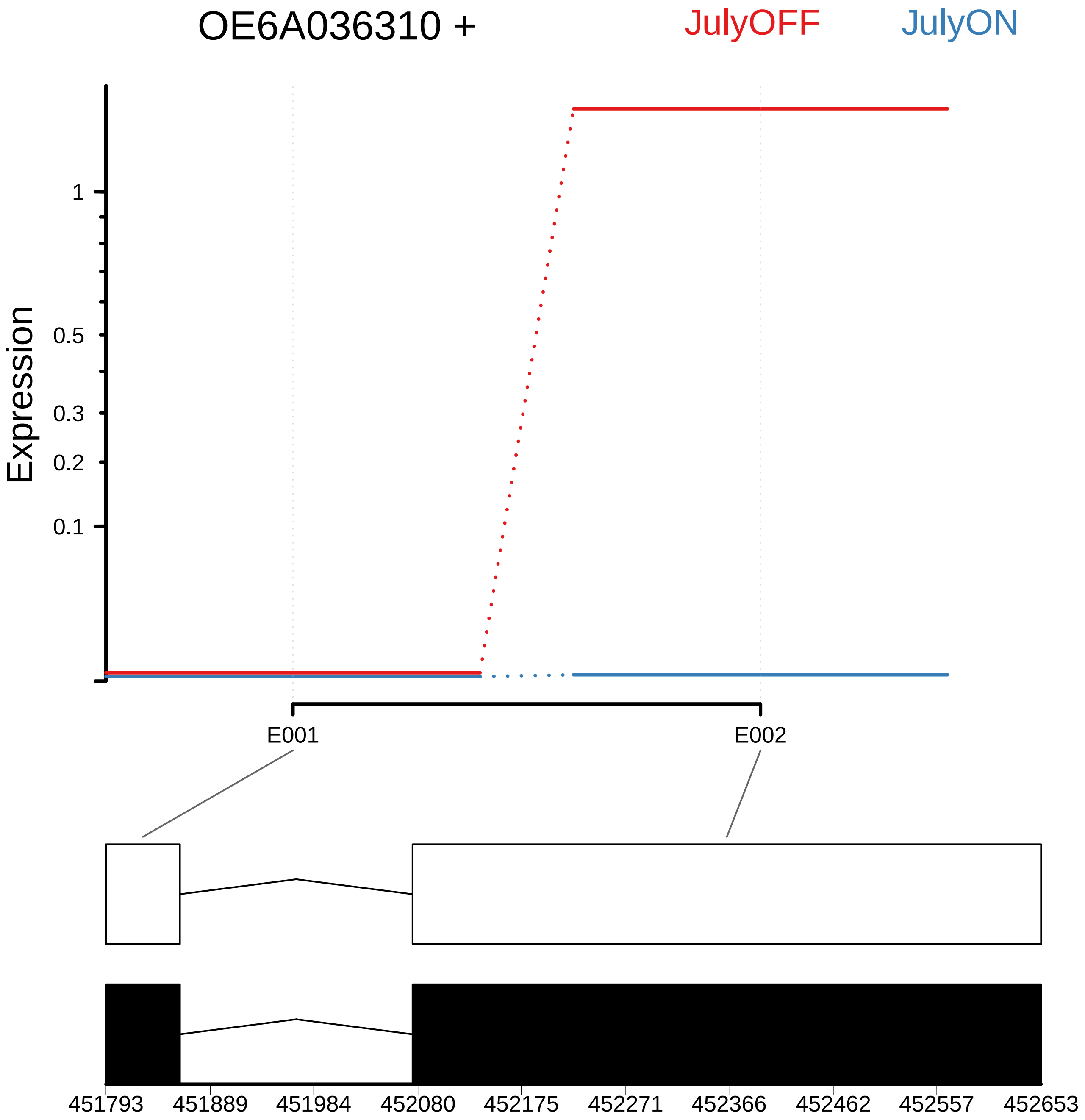

OE6A036310 +
JulyOFF
JulyON
Expression
1
0.5
0.3
0.2
0.1
E001
E002
451793
451889
451984
452080
452175
452271
452366
452462
452557
452653

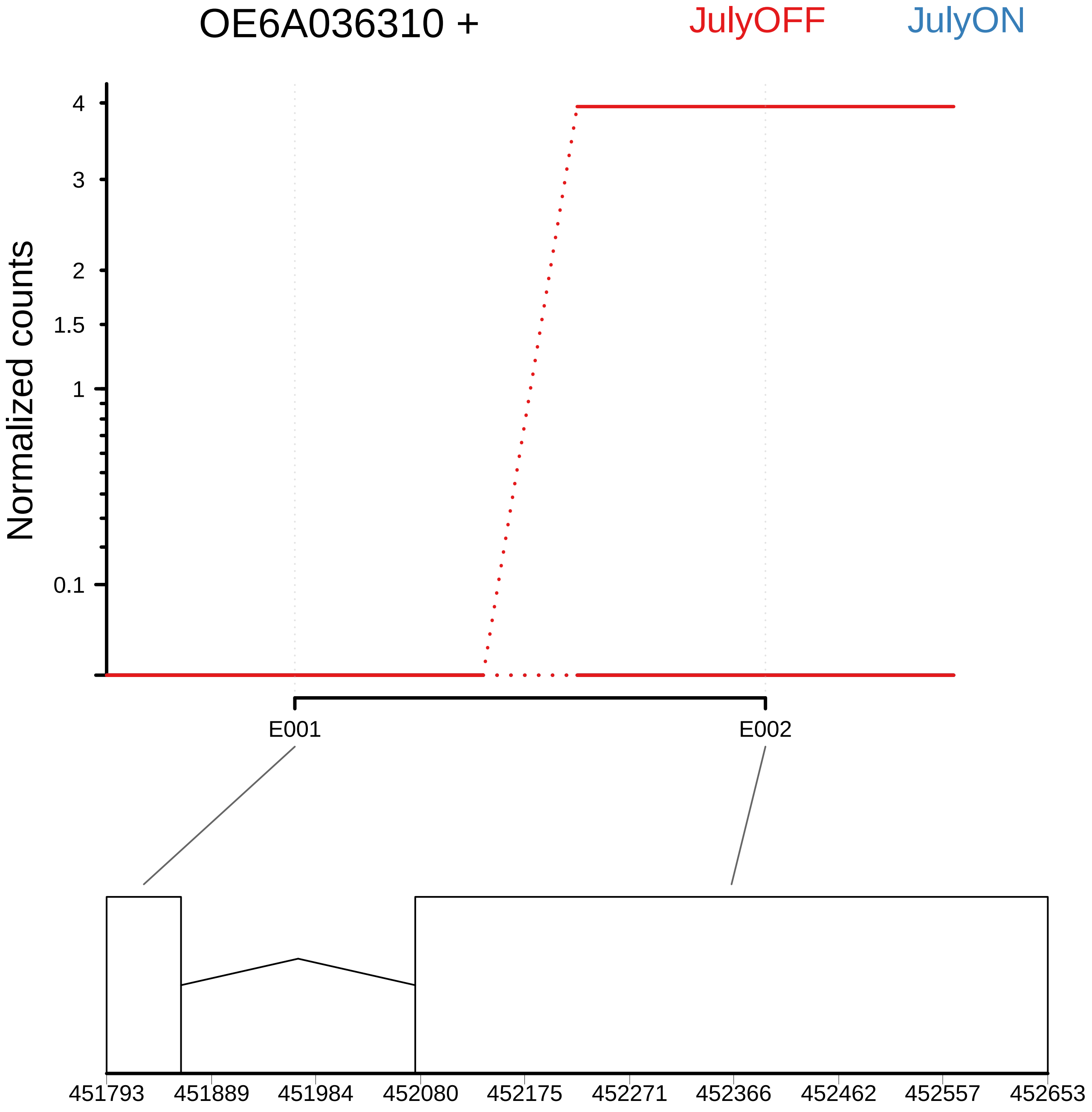

OE6A036310 +
JulyOFF
JulyON
Normalized counts
4
3
2
1.5
1
0.1
E001
E002
451793
451889
451984
452080
452175
452271
452366
452462
452557
452653

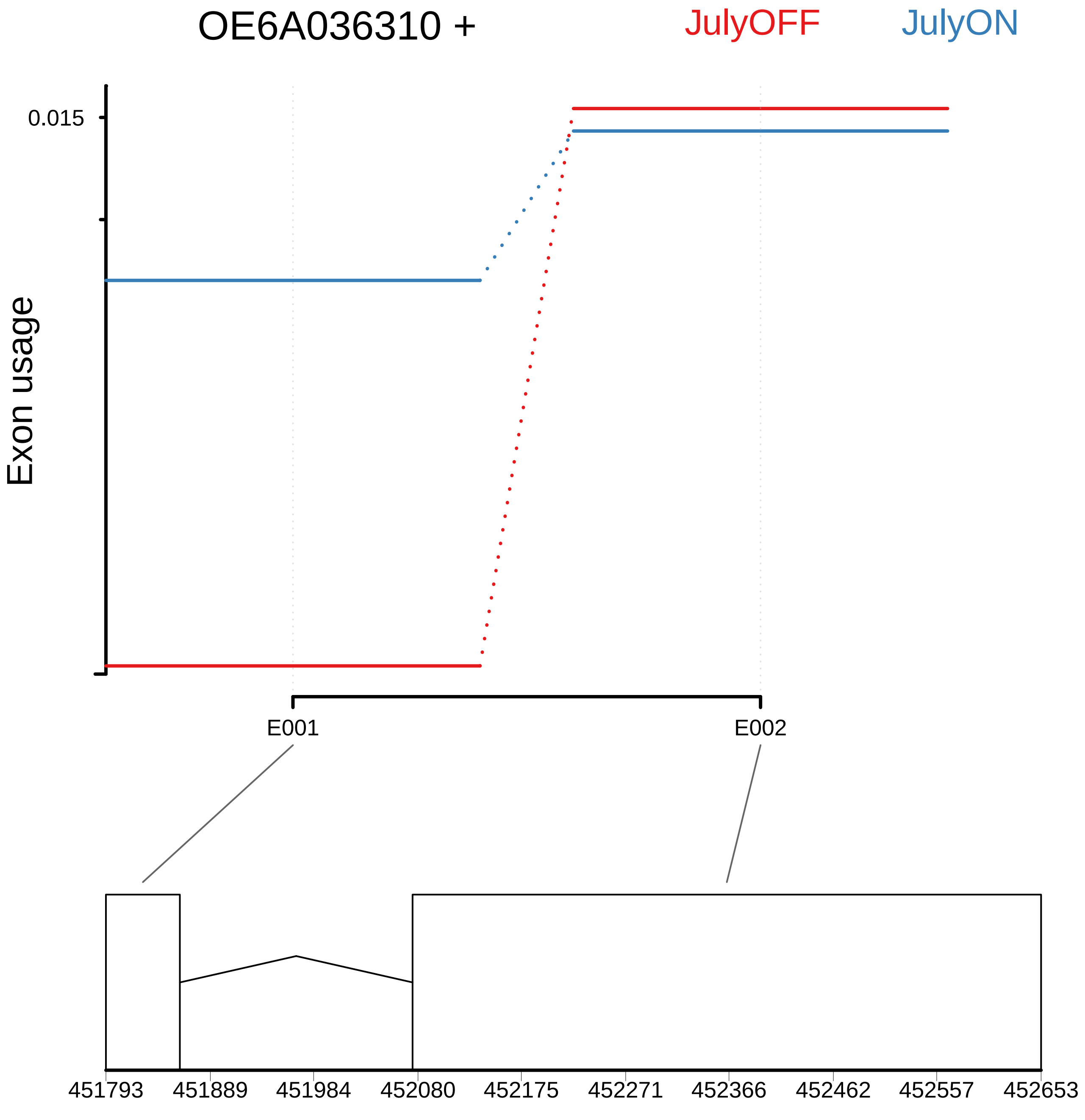
OE6A036310 +
JulyOFF
JulyON
0.015
Exon usage
E001
E002
451793
451889
451984
452080
452175
452271
452366
452462
452557
452653

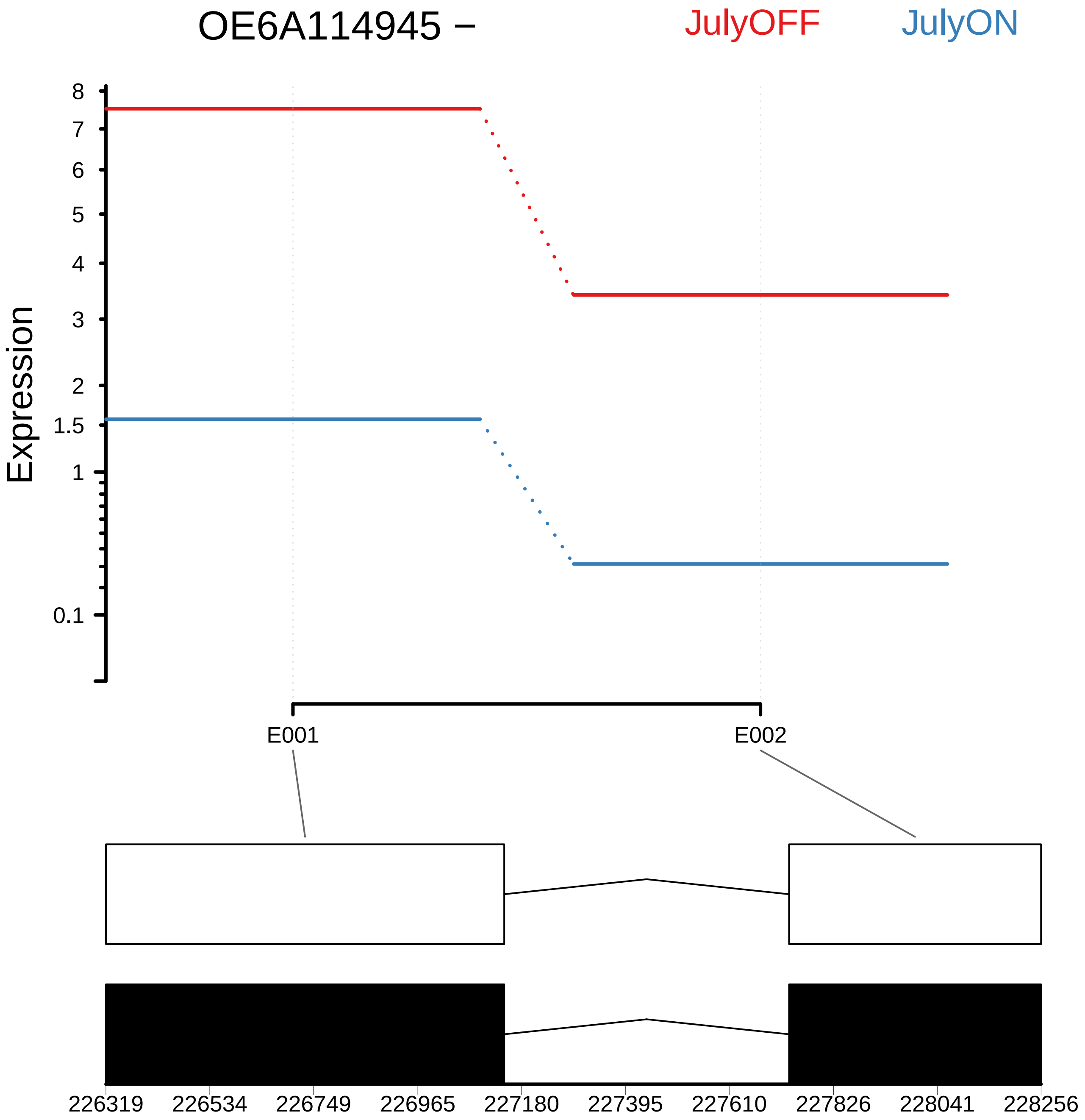
OE6A114945 −
JulyOFF
JulyON
Expression
8
7
6
5
4
3
2
1.5
1
0.1
E001
E002
226319
226534
226749
226965
227180
227395
227610
227826
228041
228256

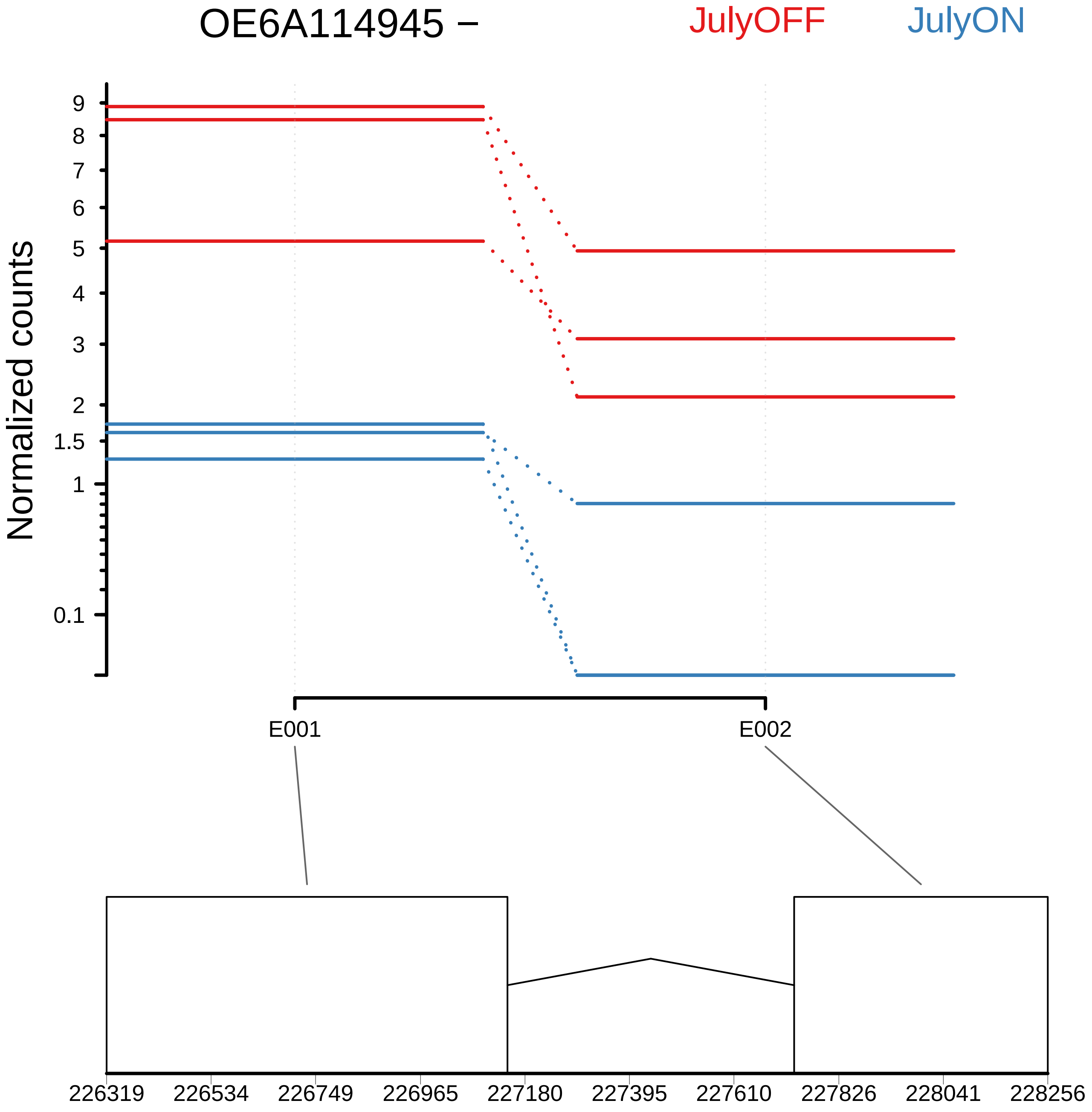

OE6A114945 −
JulyOFF
JulyON
Normalized counts
9
8
7
6
5
4
3
2
1.5
1
0.1
E001
E002
226319
226534
226749
226965
227180
227395
227610
227826
228041
228256

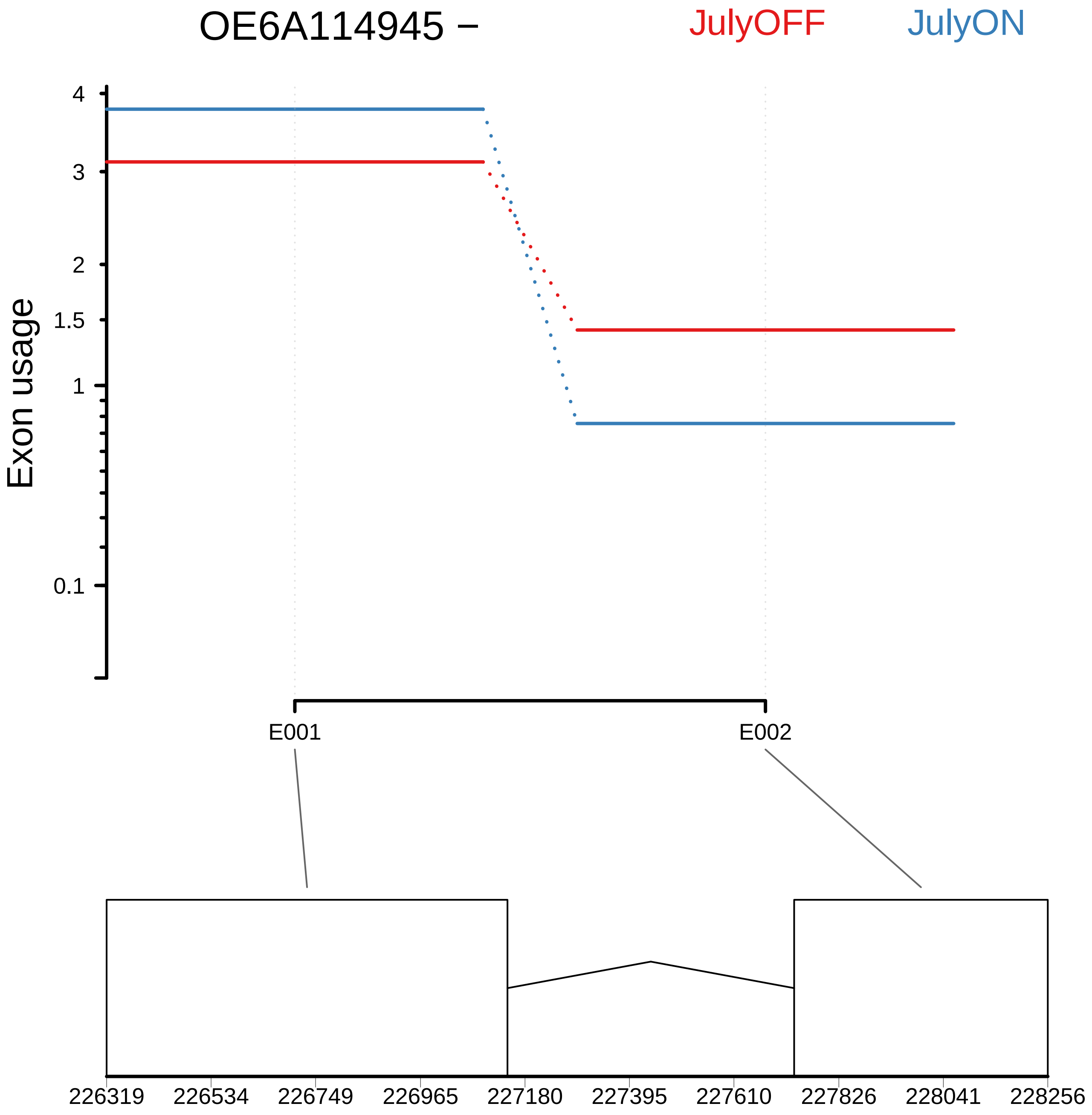
OE6A114945 −
JulyOFF
JulyON
Exon usage
4
3
2
1.5
1
0.1
E001
E002
226319
226534
226749
226965
227180
227395
227610
227826
228041
228256

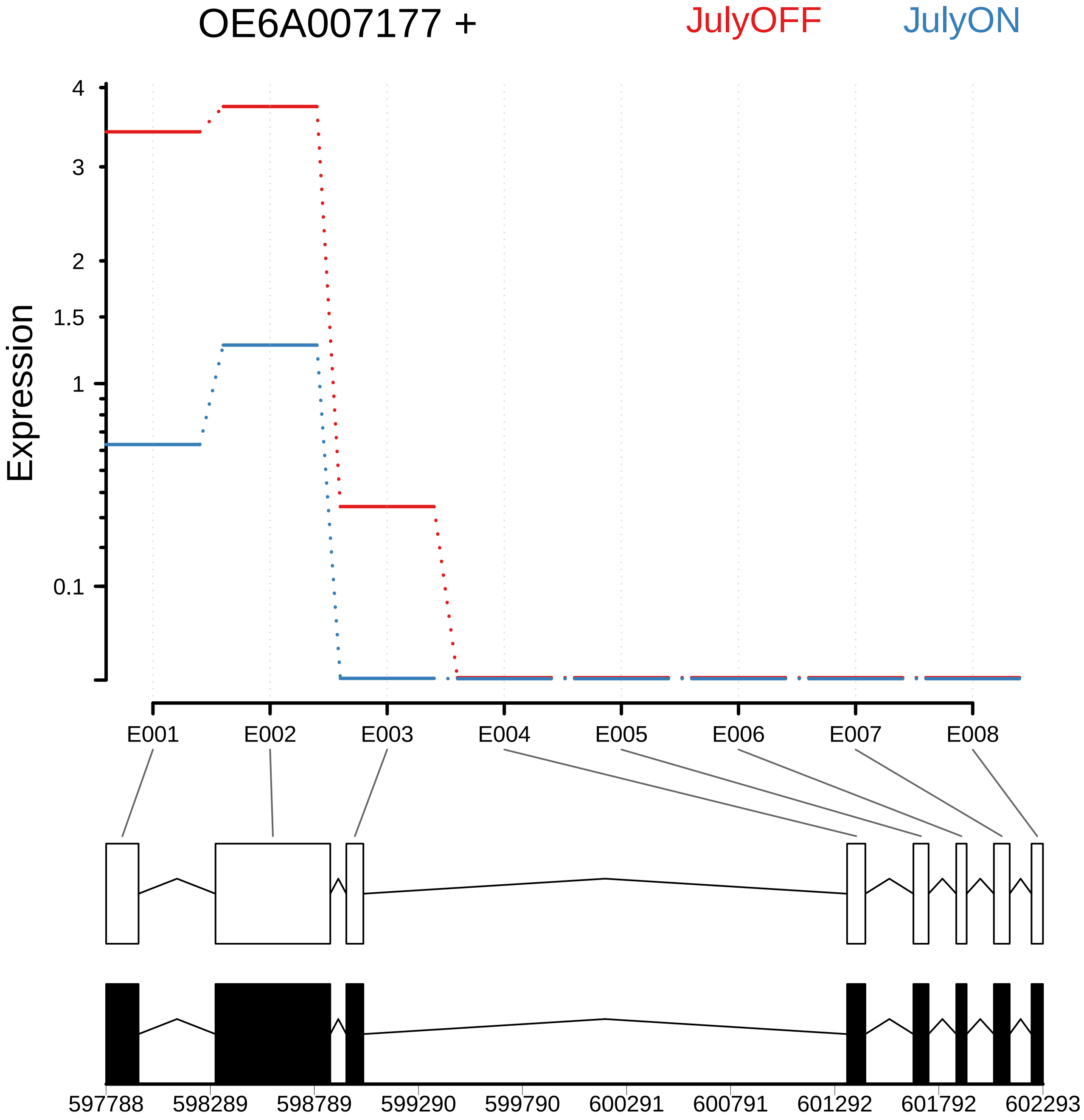
OE6A007177 +
JulyOFF
JulyON
Expression
4
3
2
1.5
1
0.1
E001
E002
E003
E004
E005
E006
E007
E008
597788
598289
598789
599290
599790
600291
600791
601292
601792
602293

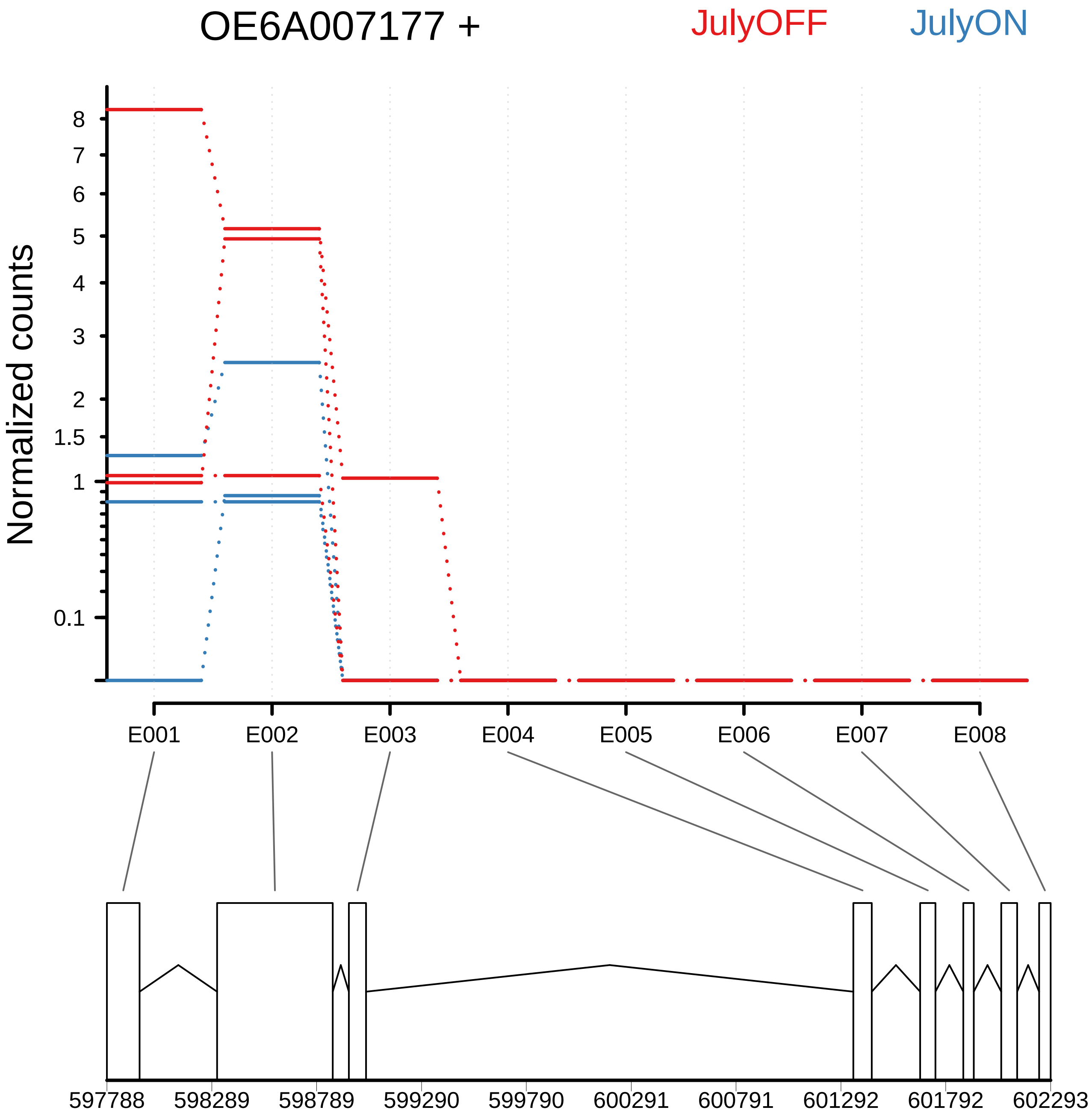
OE6A007177 +
JulyOFF
JulyON
Normalized counts
8
7
6
5
4
3
2
1.5
1
0.1
E001
E002
E003
E004
E005
E006
E007
E008
597788
598289
598789
599290
599790
600291
600791
601292
601792
602293

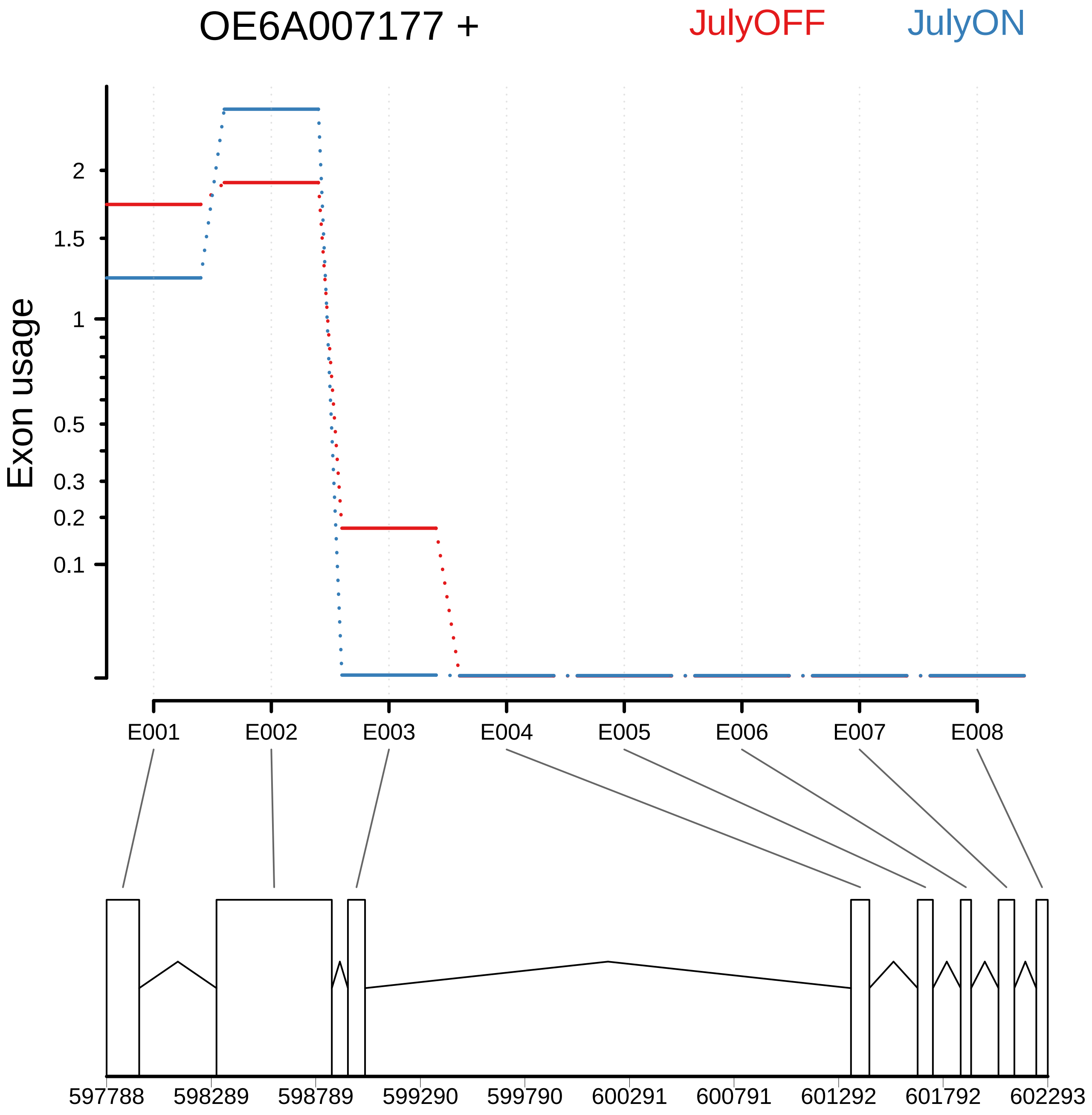
OE6A007177 +
JulyOFF
JulyON
Exon usage
2
1.5
1
0.5
0.3
0.2
0.1
E001
E002
E003
E004
E005
E006
E007
E008
597788
598289
598789
599290
599790
600291
600791
601292
601792
602293

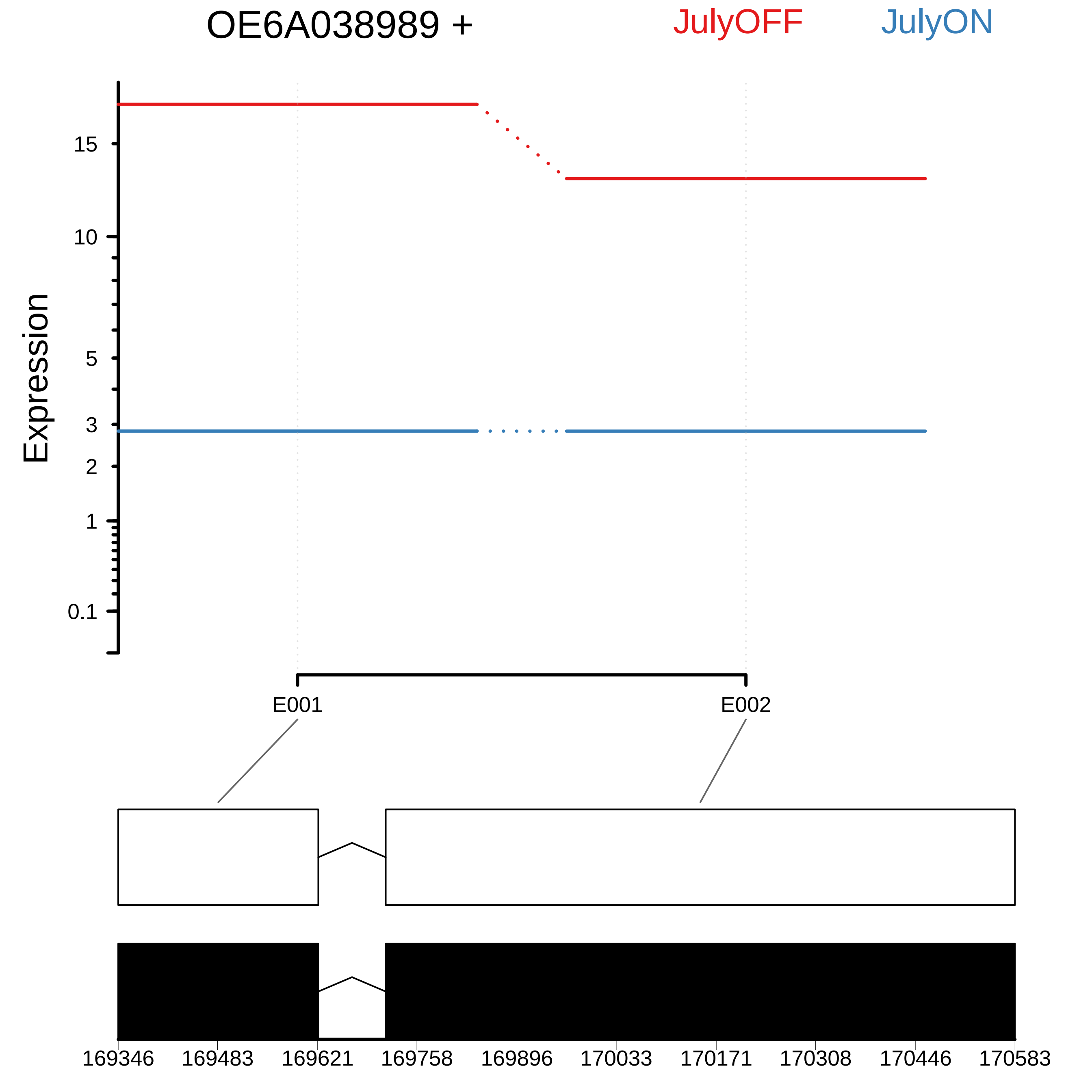
OE6A038989 +
JulyOFF
JulyON
Expression
15
10
5
3
2
1
0.1
E001
E002
169346
169483
169621
169758
169896
170033
170171
170308
170446
170583

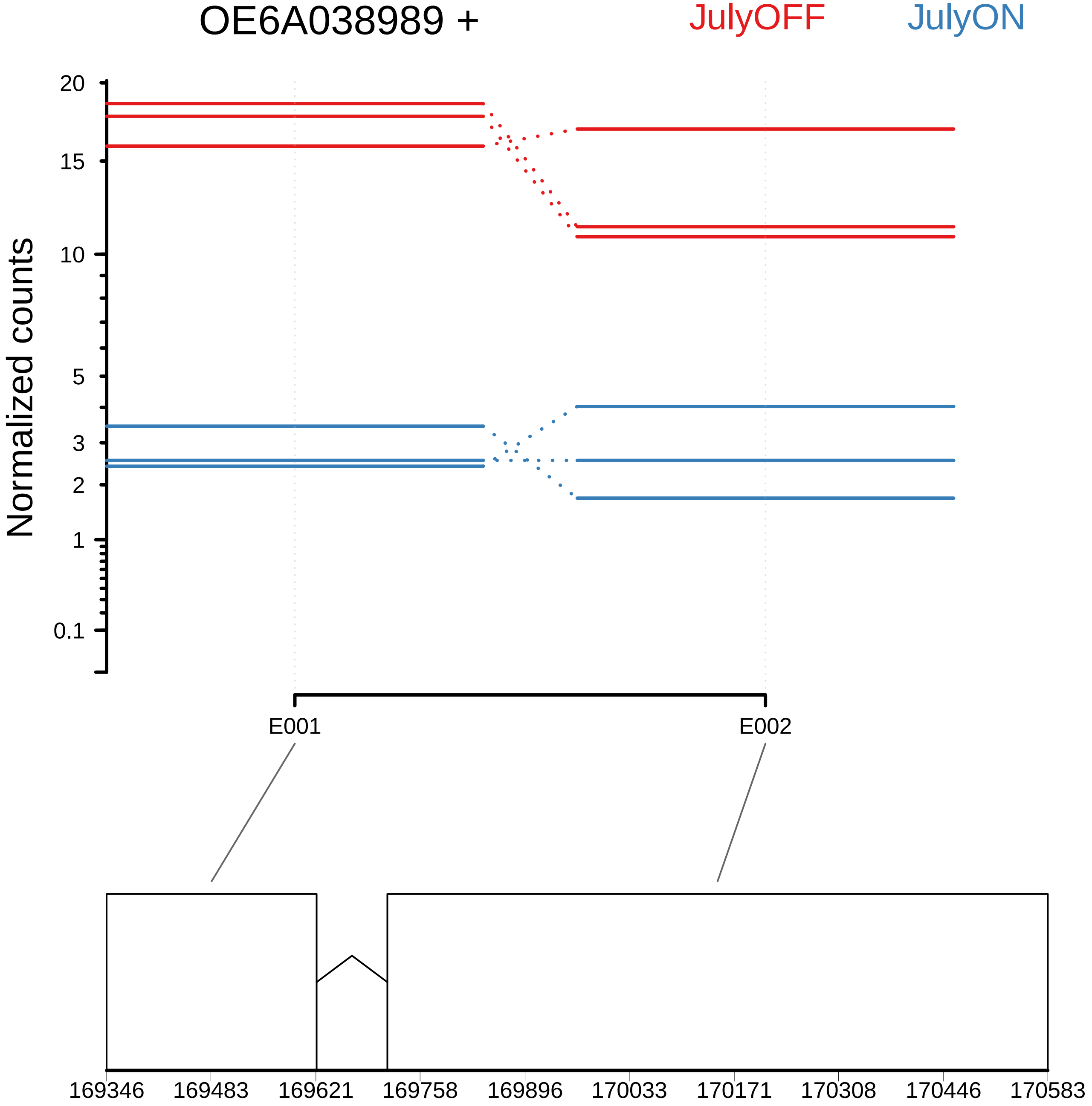
OE6A038989 +
JulyOFF
JulyON
Normalized counts
20
15
10
5
3
2
1
0.1
E001
E002
169346
169483
169621
169758
169896
170033
170171
170308
170446
170583

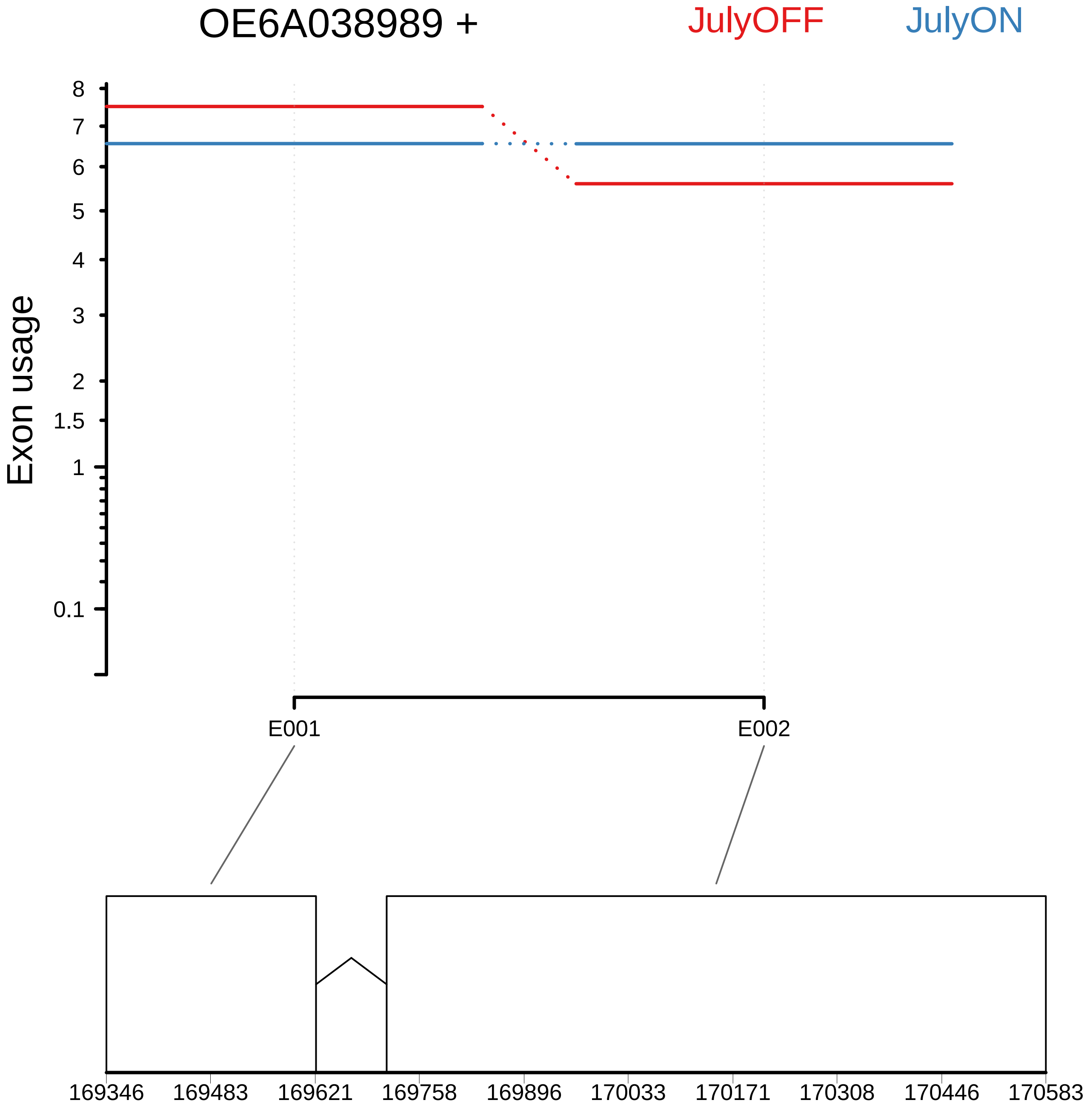
OE6A038989 +
JulyOFF
JulyON
Exon usage
8
7
6
5
4
3
2
1.5
1
0.1
E001
E002
169346
169483
169621
169758
169896
170033
170171
170308
170446
170583

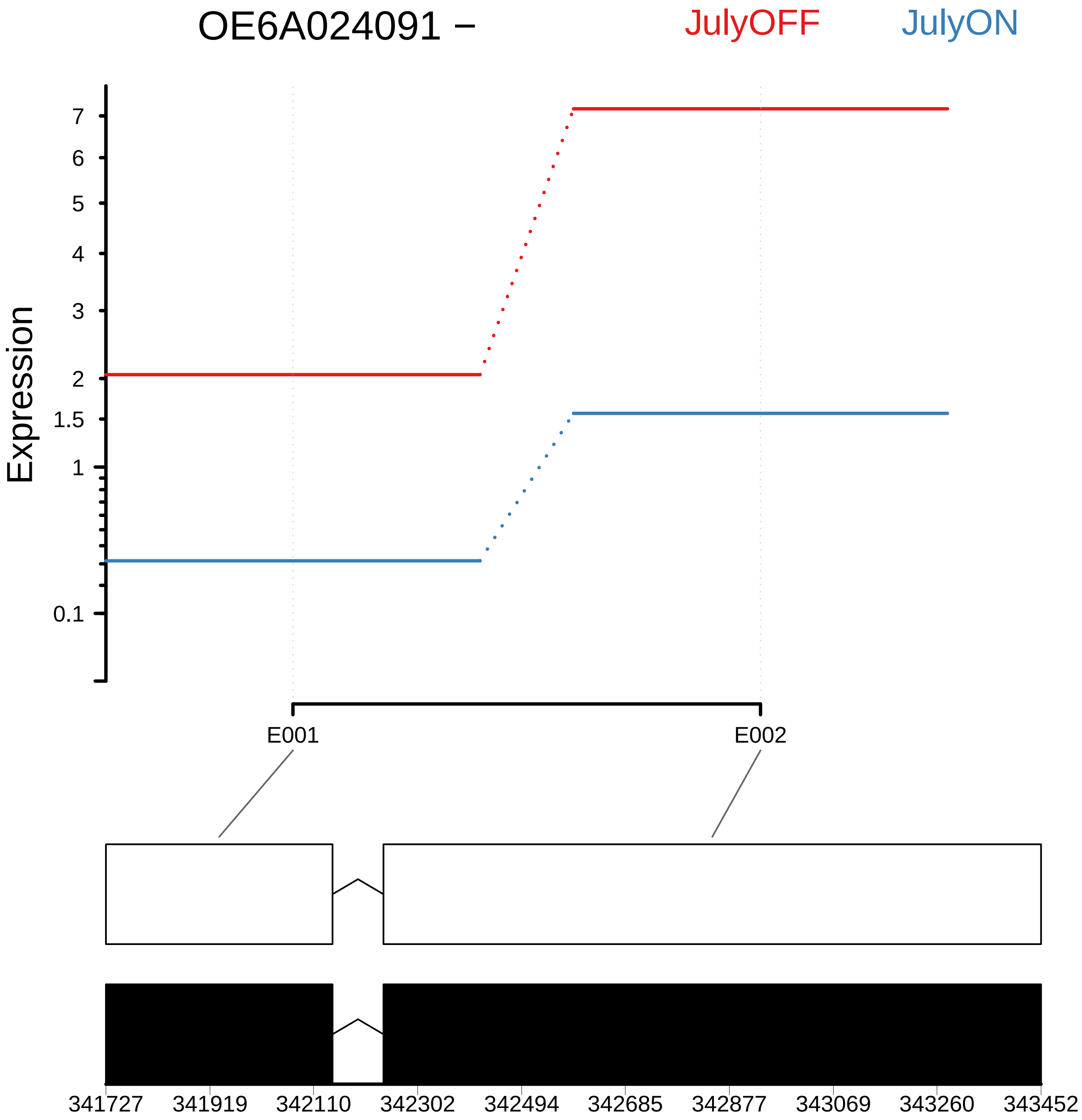

OE6A024091 −
JulyOFF
JulyON
Expression
7
6
5
4
3
2
1.5
1
0.1
E001
E002
341727
341919
342110
342302
342494
342685
342877
343069
343260
343452

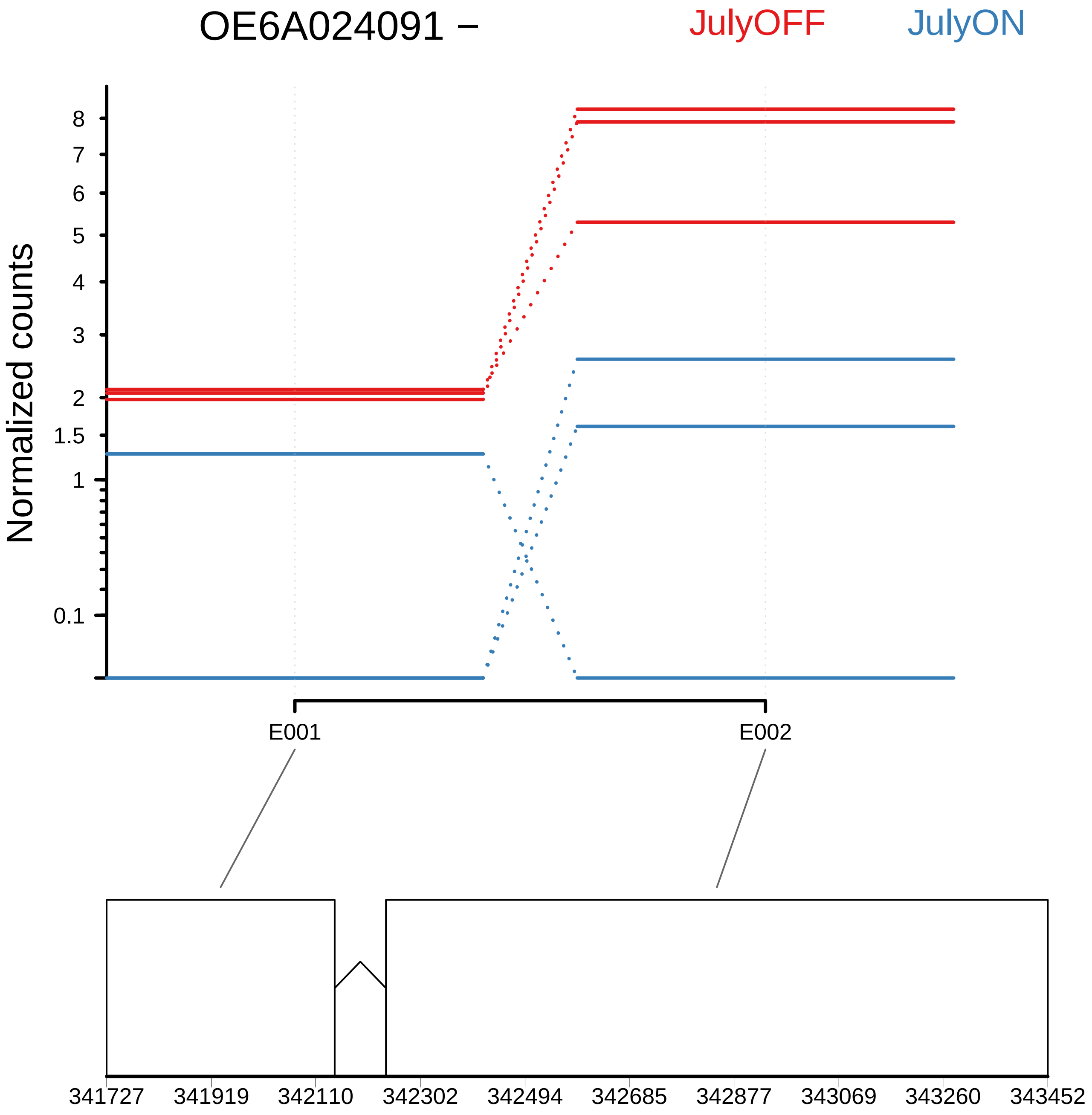

OE6A024091 −
JulyOFF
JulyON
Normalized counts
8
7
6
5
4
3
2
1.5
1
0.1
E001
E002
341727
341919
342110
342302
342494
342685
342877
343069
343260
343452

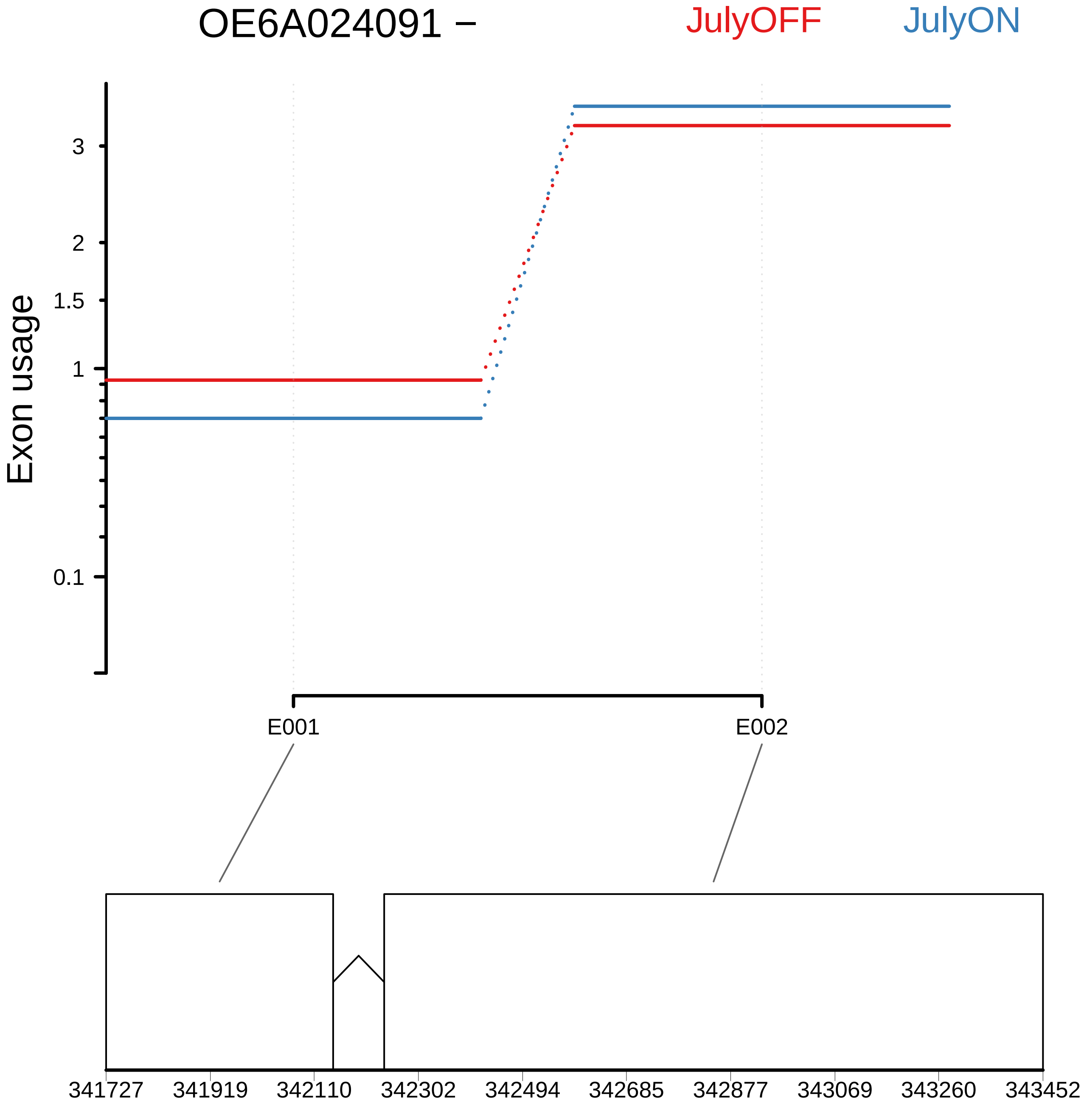

OE6A024091 −
JulyOFF
JulyON
Exon usage
3
2
1.5
1
0.1
E001
E002
341727
341919
342110
342302
342494
342685
342877
343069
343260
343452

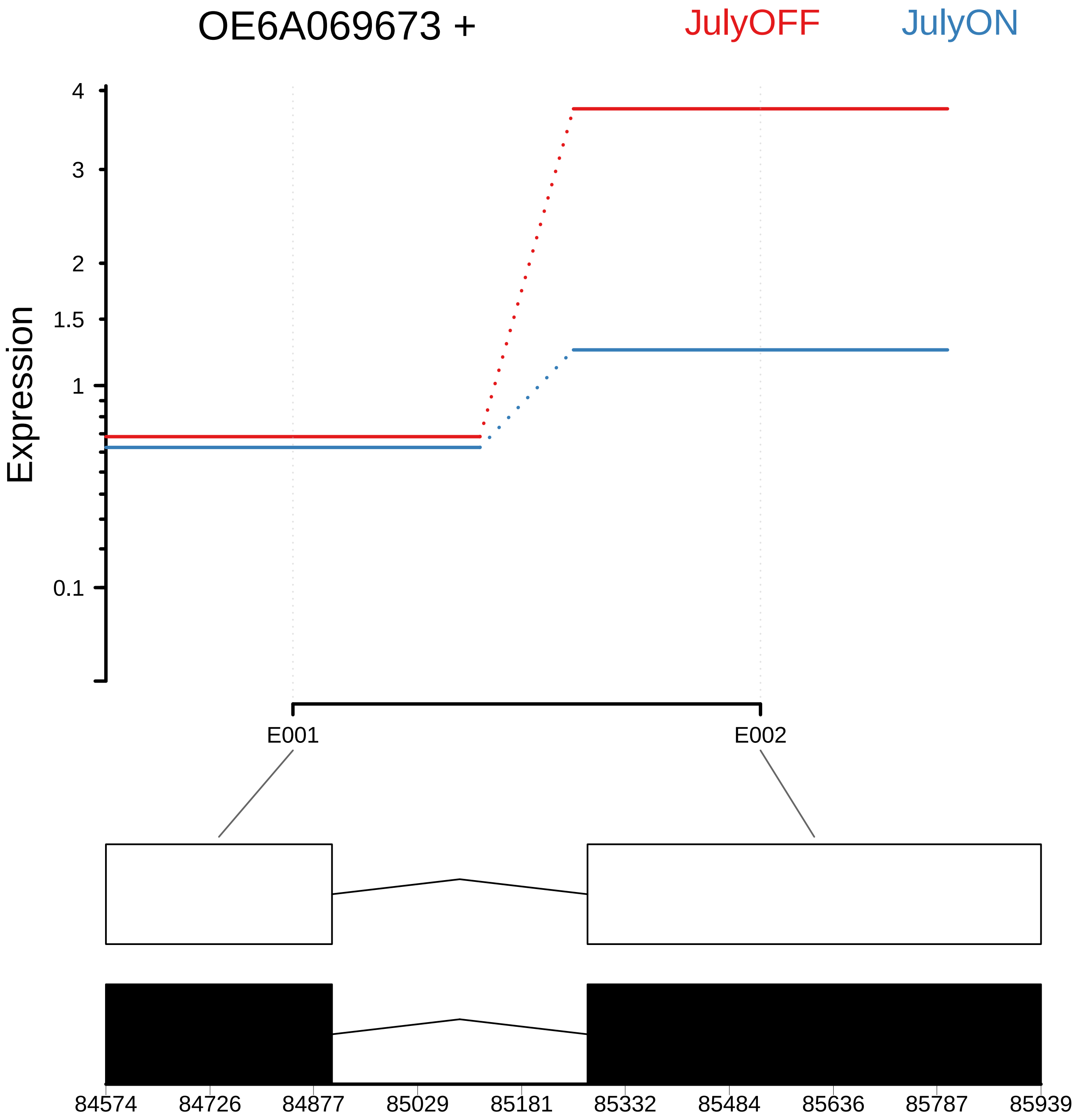
OE6A069673 +
JulyOFF
JulyON
Expression
4
3
2
1.5
1
0.1
E001
E002
84574
84726
84877
85029
85181
85332
85484
85636
85787
85939

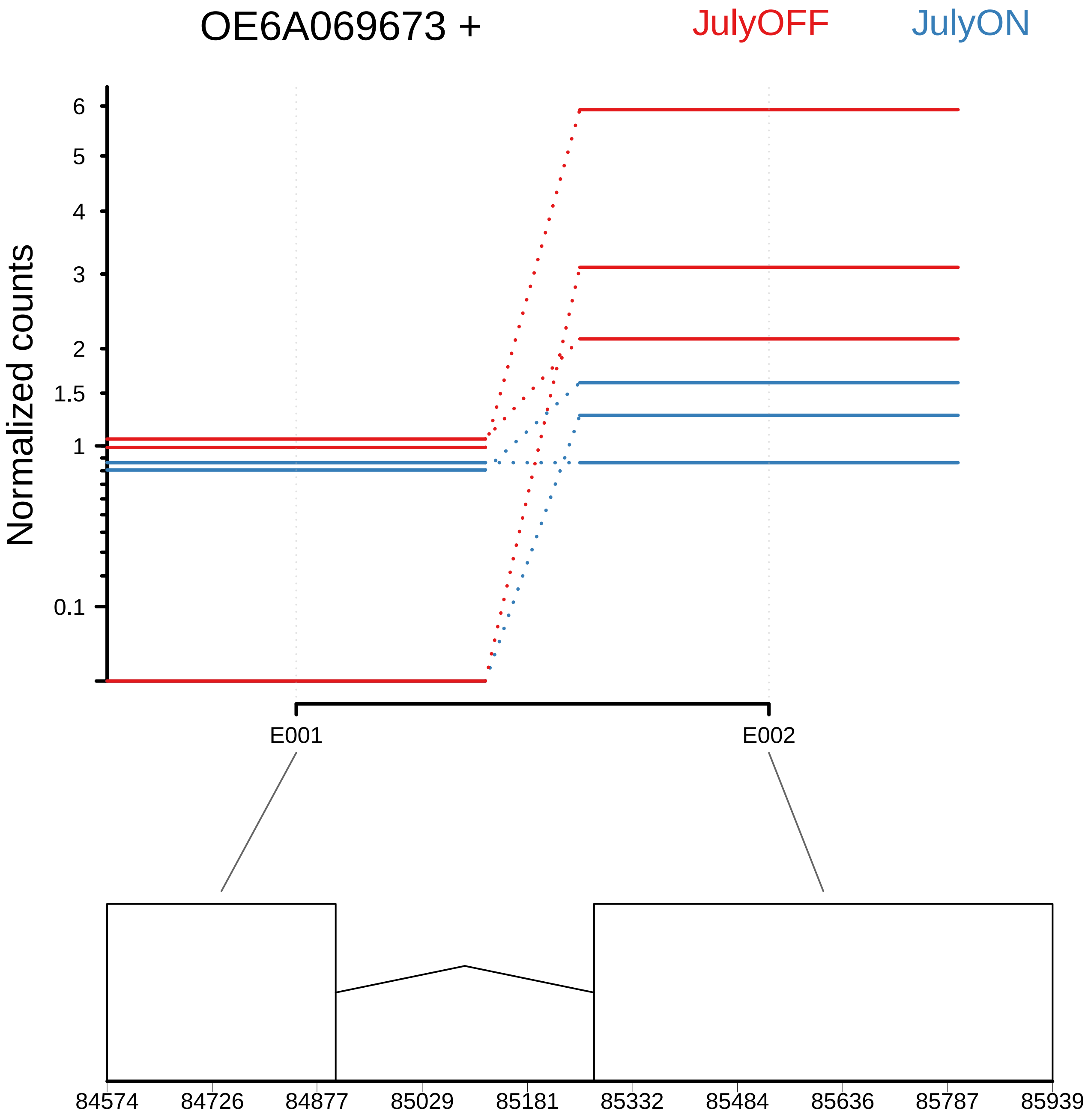

OE6A069673 +
JulyOFF
JulyON
Normalized counts
6
5
4
3
2
1.5
1
0.1
E001
E002
84574
84726
84877
85029
85181
85332
85484
85636
85787
85939

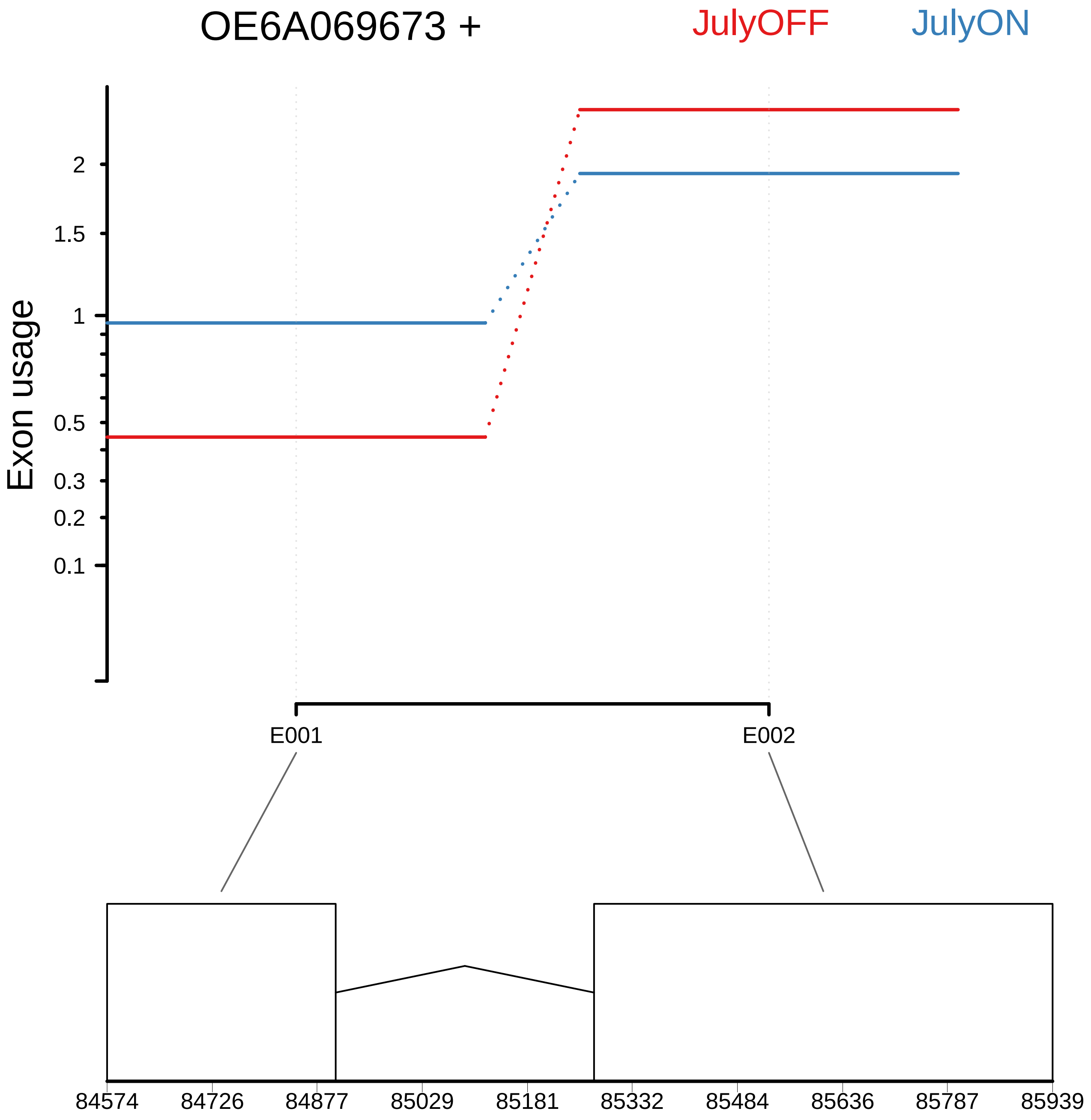
OE6A069673 +
JulyOFF
JulyON
Exon usage
2
1.5
1
0.5
0.3
0.2
0.1
E001
E002
84574
84726
84877
85029
85181
85332
85484
85636
85787
85939

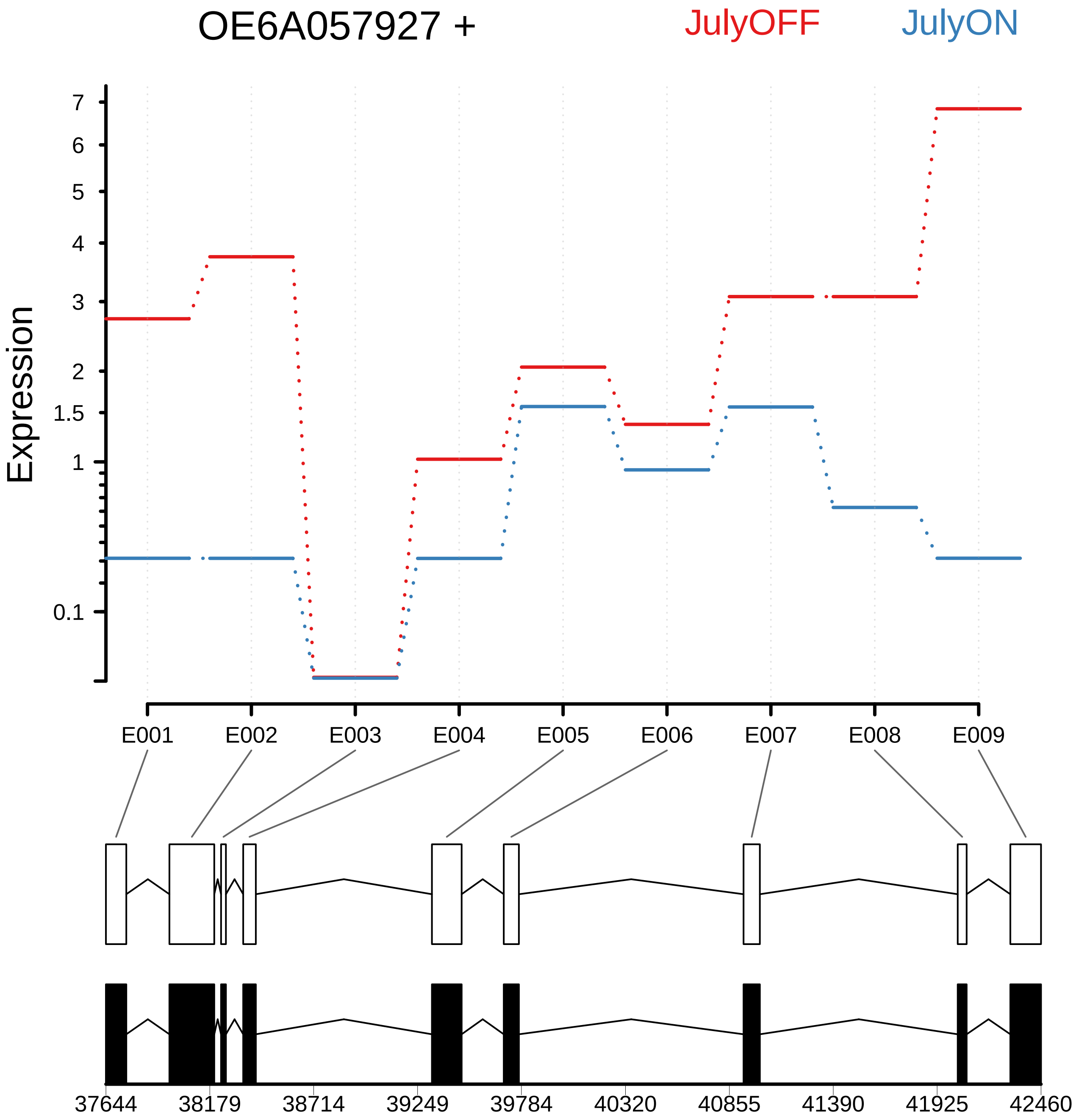

OE6A057927 +
JulyOFF
JulyON
Expression
7
6
5
4
3
2
1.5
1
0.1
E001
E002
E003
E004
E005
E006
E007
E008
E009
37644
38179
38714
39249
39784
40320
40855
41390
41925
42460

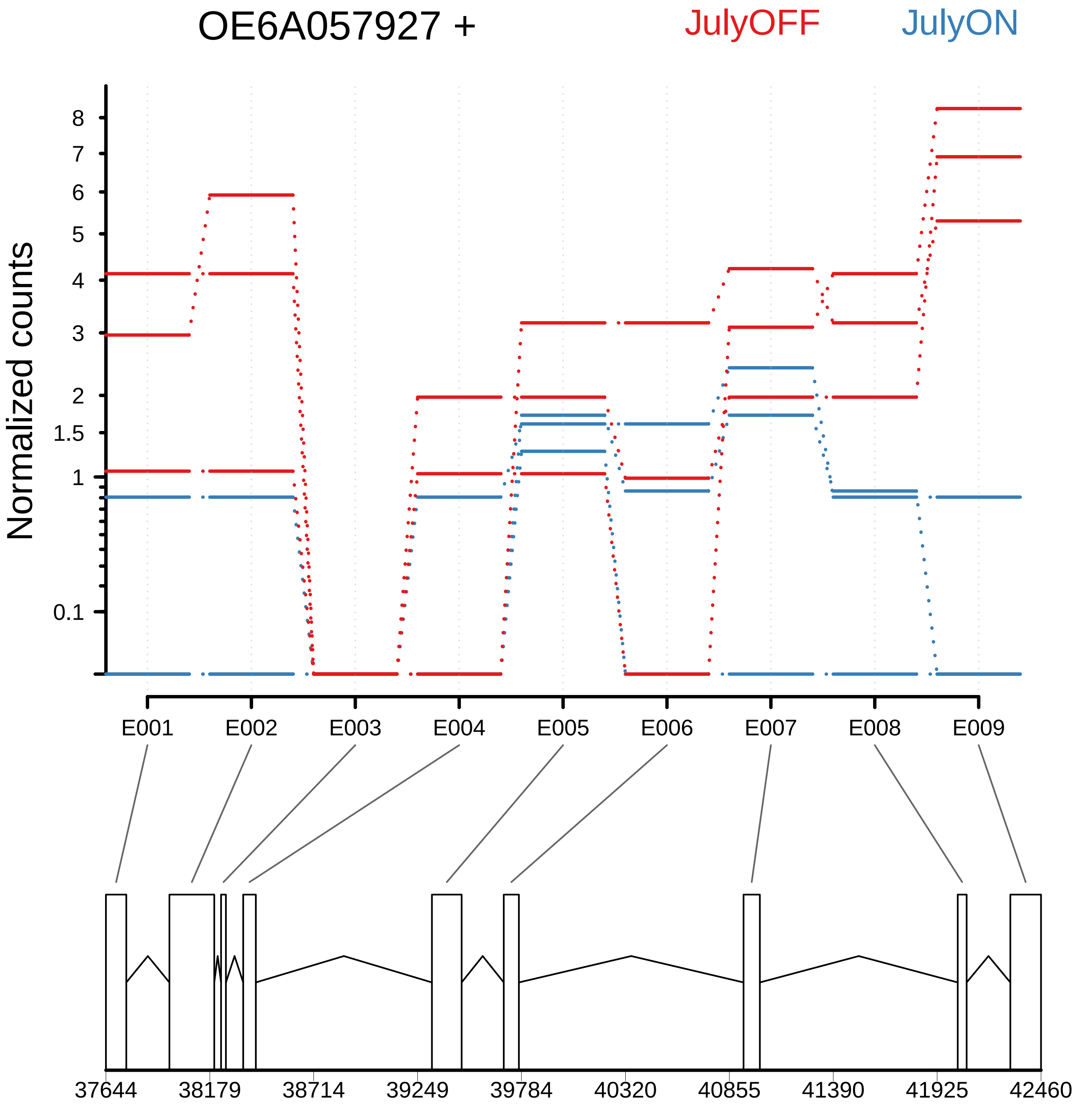

OE6A057927 +
JulyOFF
JulyON
Normalized counts
8
7
6
5
4
3
2
1.5
1
0.1
E001
E002
E003
E004
E005
E006
E007
E008
E009
37644
38179
38714
39249
39784
40320
40855
41390
41925
42460

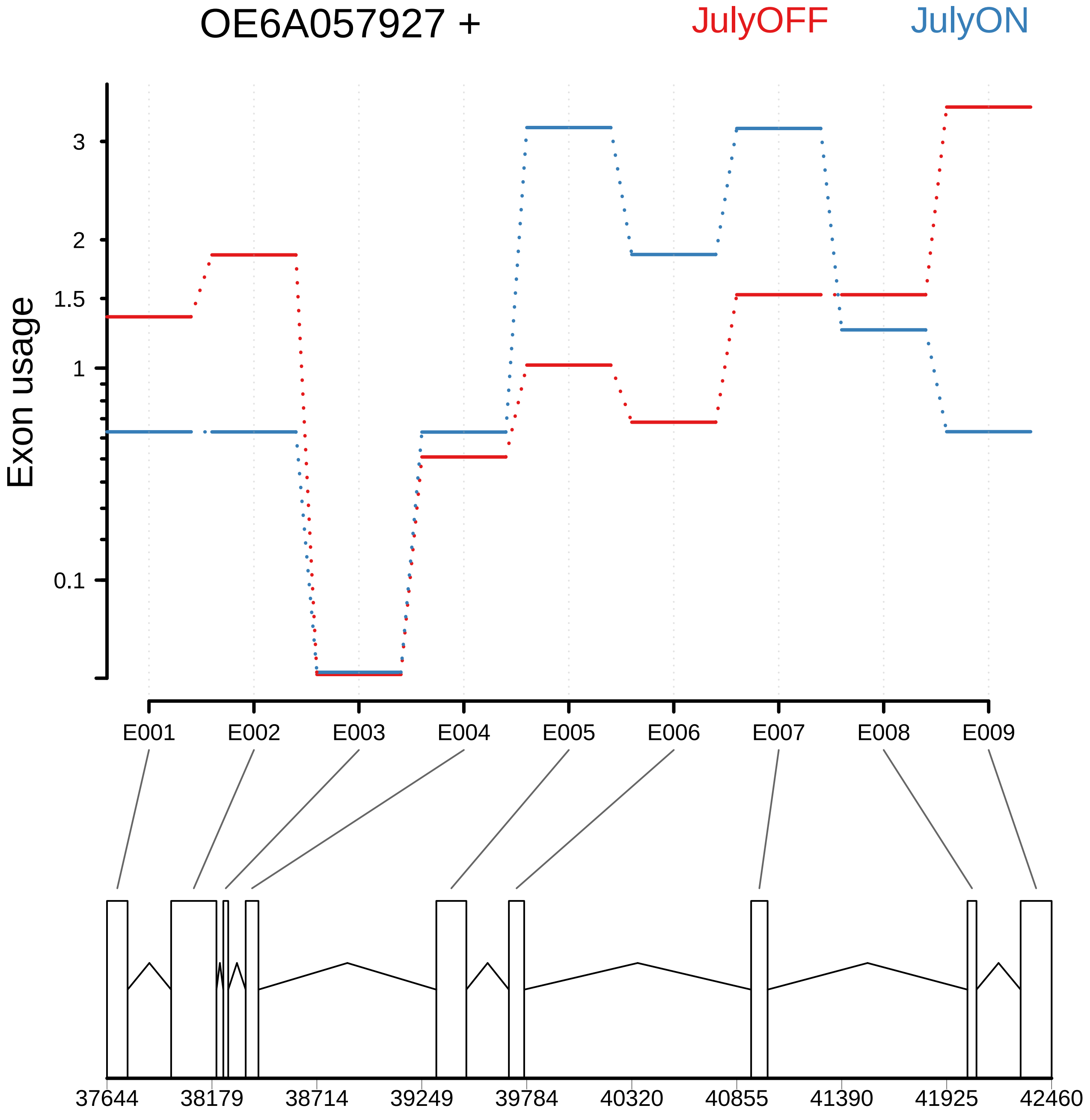
OE6A057927 +
JulyOFF
JulyON
Exon usage
3
2
1.5
1
0.1
E001
E002
E003
E004
E005
E006
E007
E008
E009
37644
38179
38714
39249
39784
40320
40855
41390
41925
42460

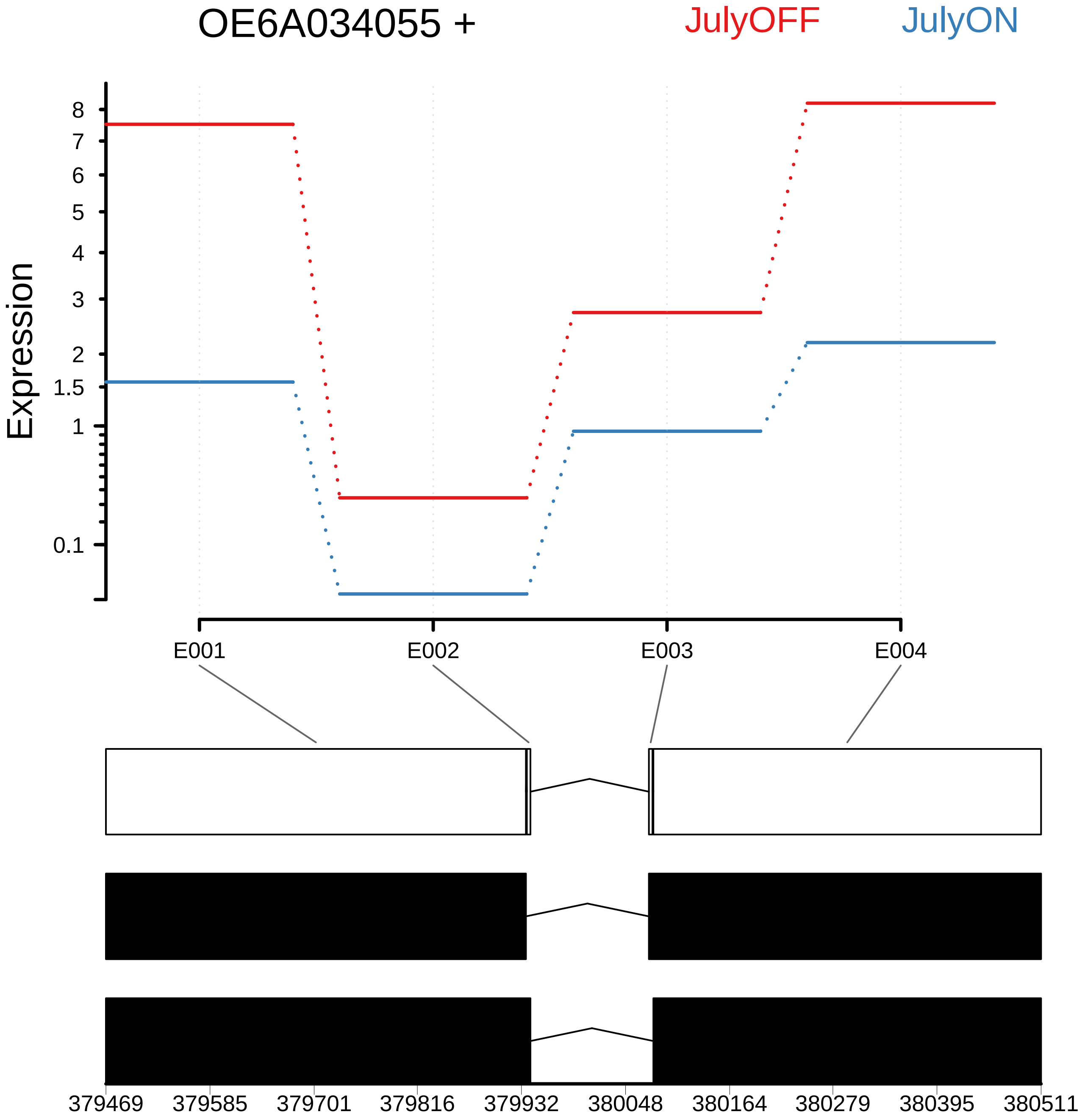
OE6A034055 +
JulyOFF
JulyON
Expression
8
7
6
5
4
3
2
1.5
1
0.1
E001
E002
E003
E004
379469
379585
379701
379816
379932
380048
380164
380279
380395
380511

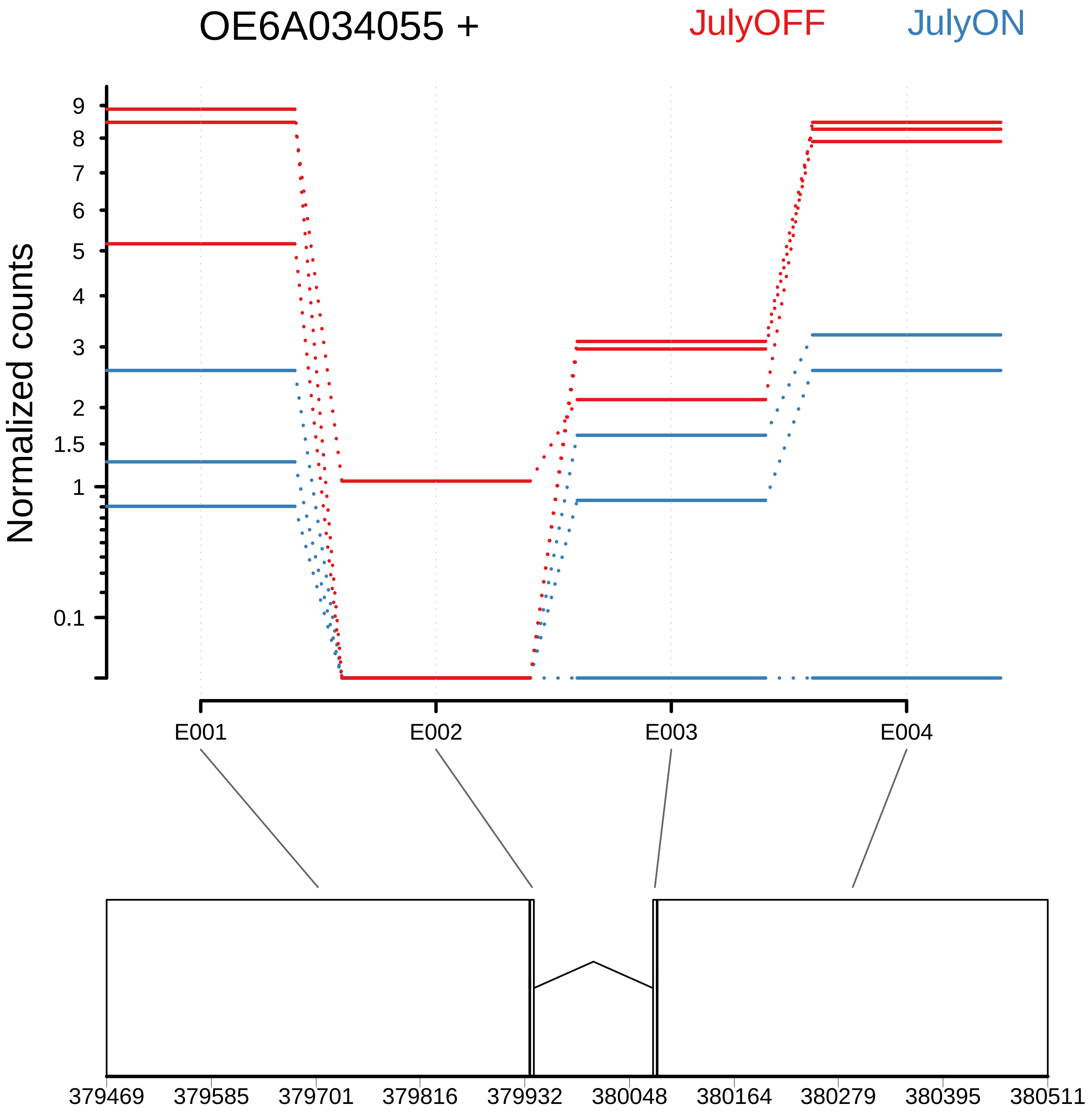

OE6A034055 +
JulyOFF
JulyON
Normalized counts
9
8
7
6
5
4
3
2
1.5
1
0.1
E001
E002
E003
E004
379469
379585
379701
379816
379932
380048
380164
380279
380395
380511

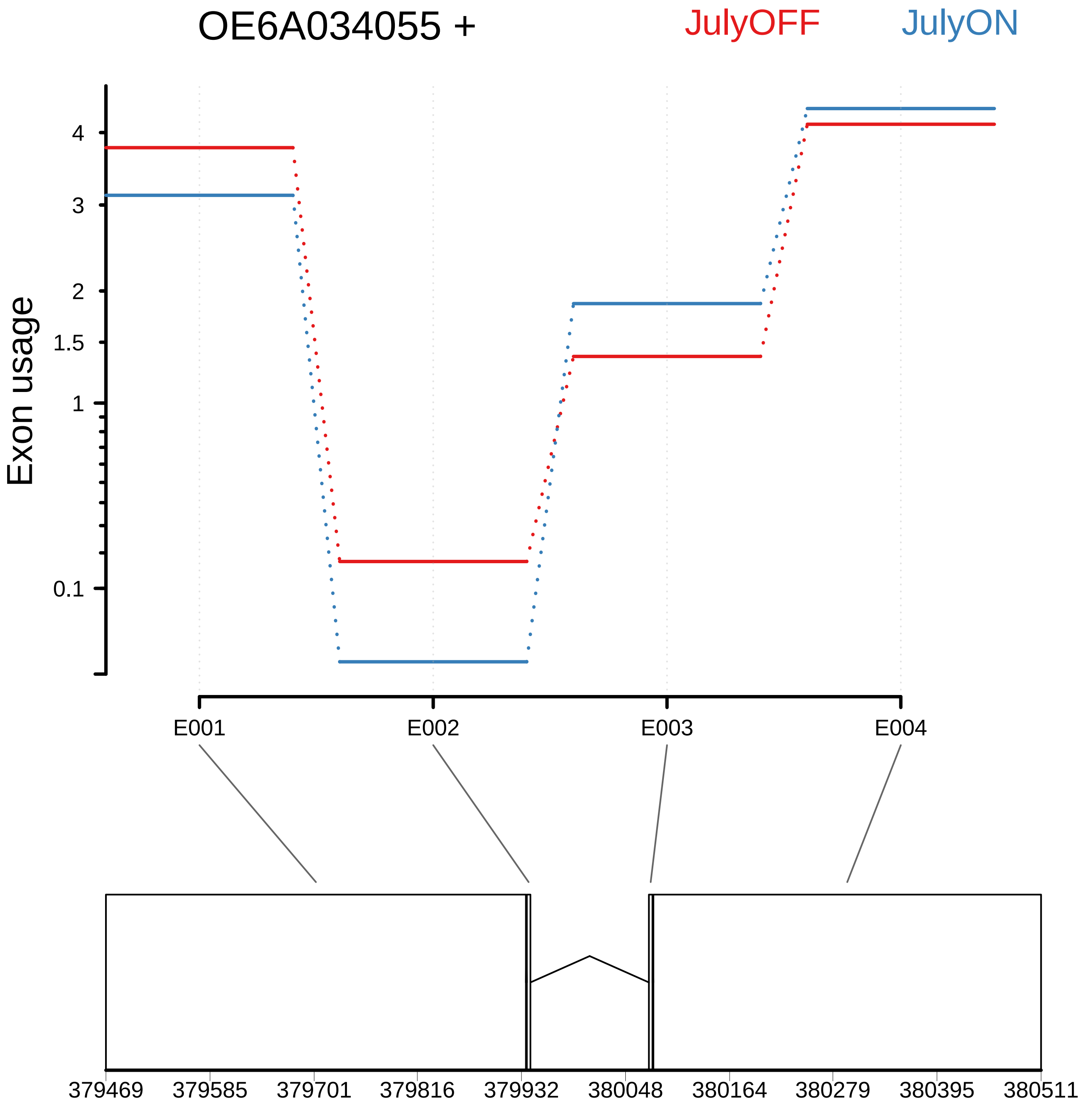
OE6A034055 +
JulyOFF
JulyON
Exon usage
4
3
2
1.5
1
0.1
E001
E002
E003
E004
379469
379585
379701
379816
379932
380048
380164
380279
380395
380511

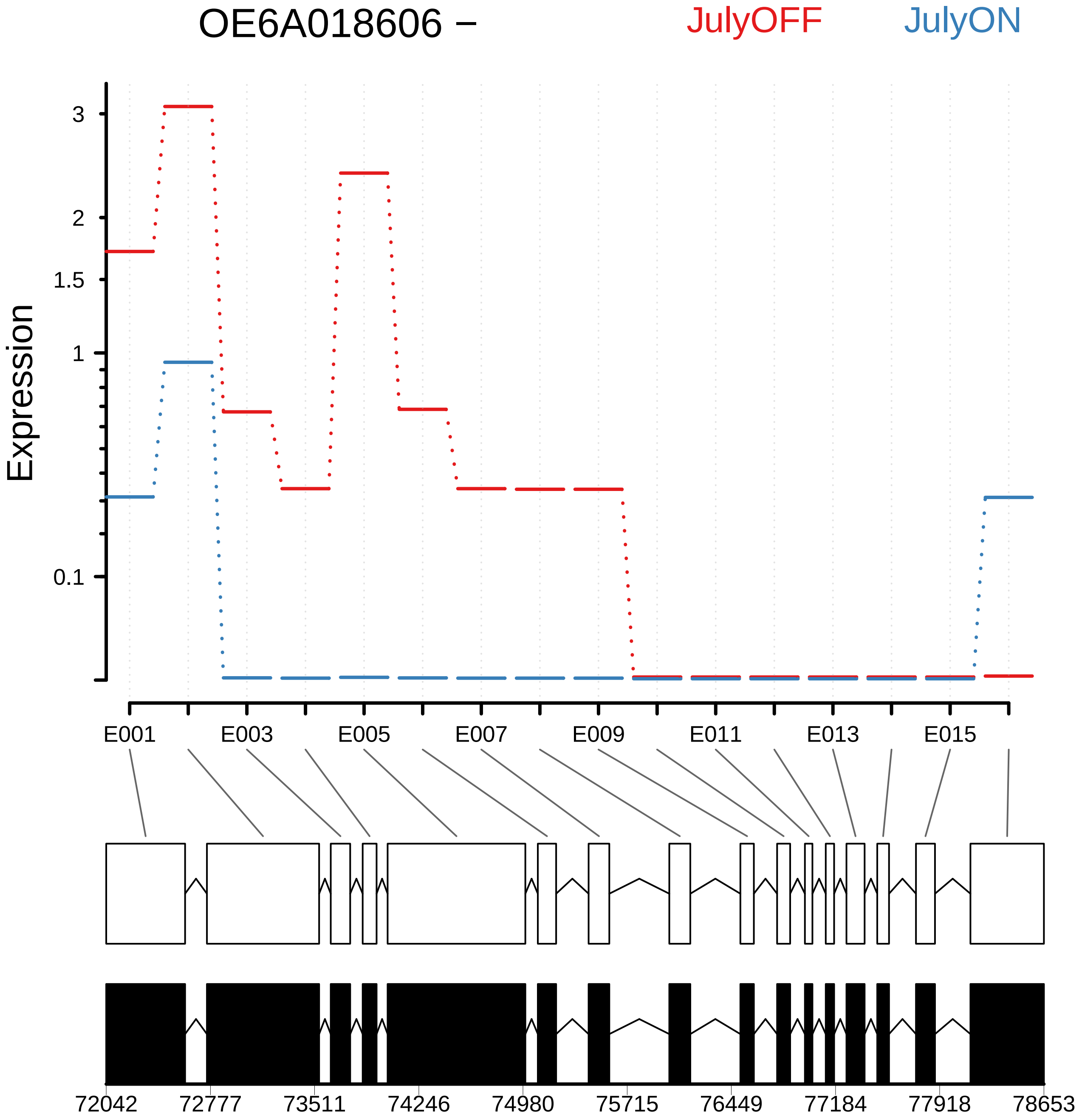

OE6A018606 –
JulyOFF
JulyON
Expression
3
2
1.5
1
0.1
E001
E003
E005
E007
E009
E011
E013
E015
72042
72777
73511
74246
74980
75715
76449
77184
77918
78653

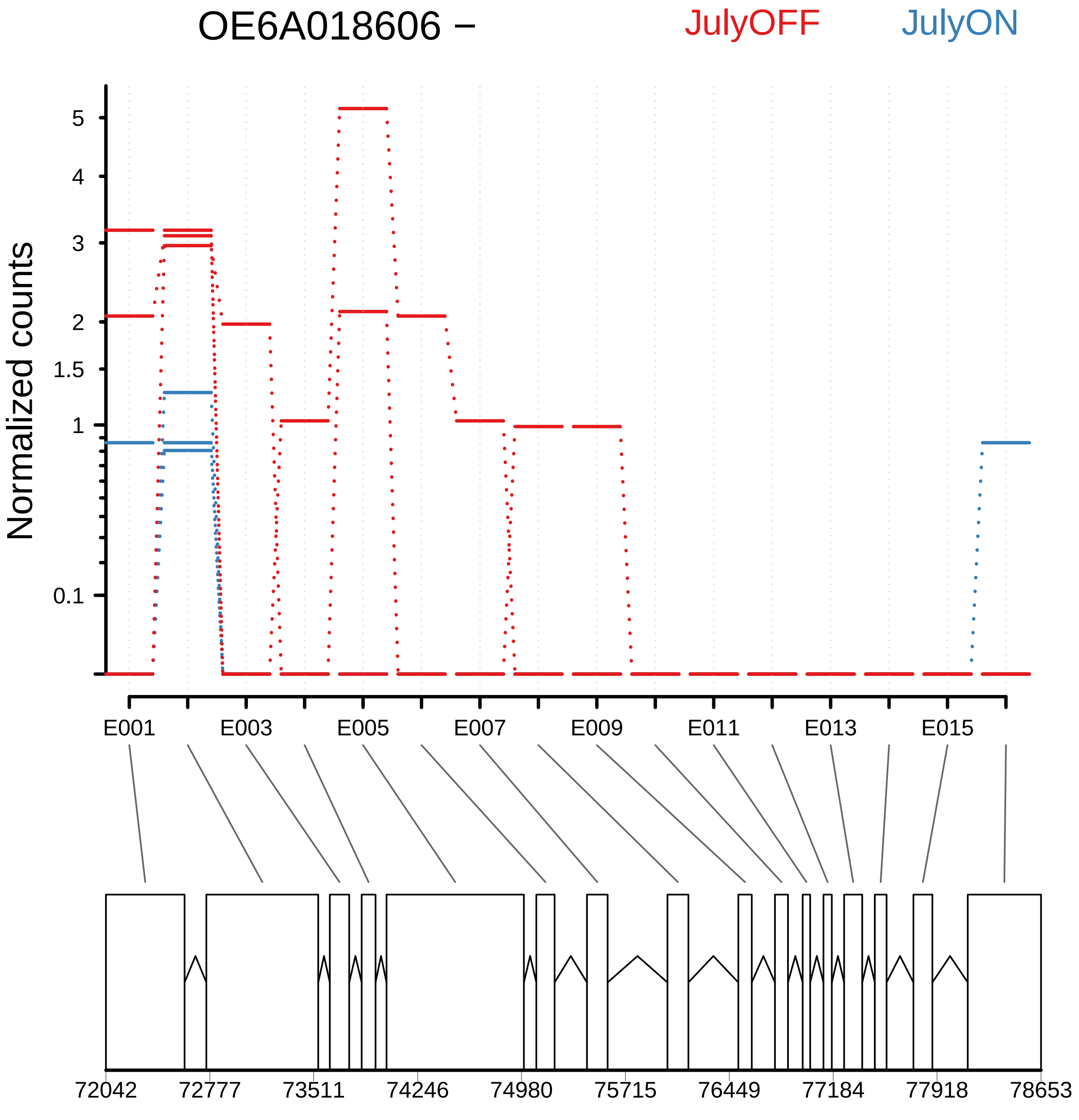

OE6A018606 −
JulyOFF
JulyON
Normalized counts
5
4
3
2
1.5
1
0.1
E001
E003
E005
E007
E009
E011
E013
E015
72042
72777
73511
74246
74980
75715
76449
77184
77918
78653

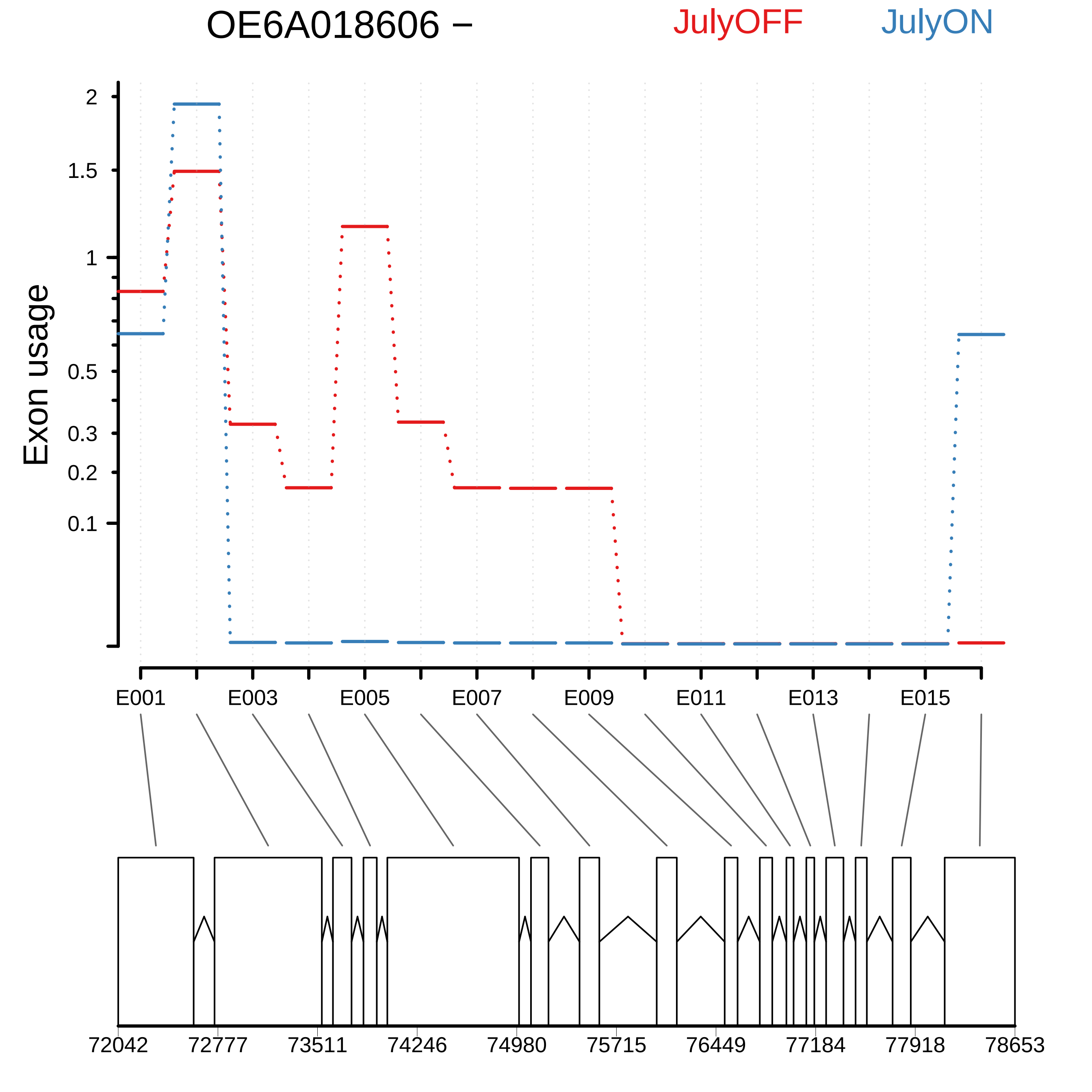
OE6A018606 −
JulyOFF
JulyON
Exon usage
2
1.5
1
0.5
0.3
0.2
0.1
E001
E003
E005
E007
E009
E011
E013
E015
72042
72777
73511
74246
74980
75715
76449
77184
77918
78653

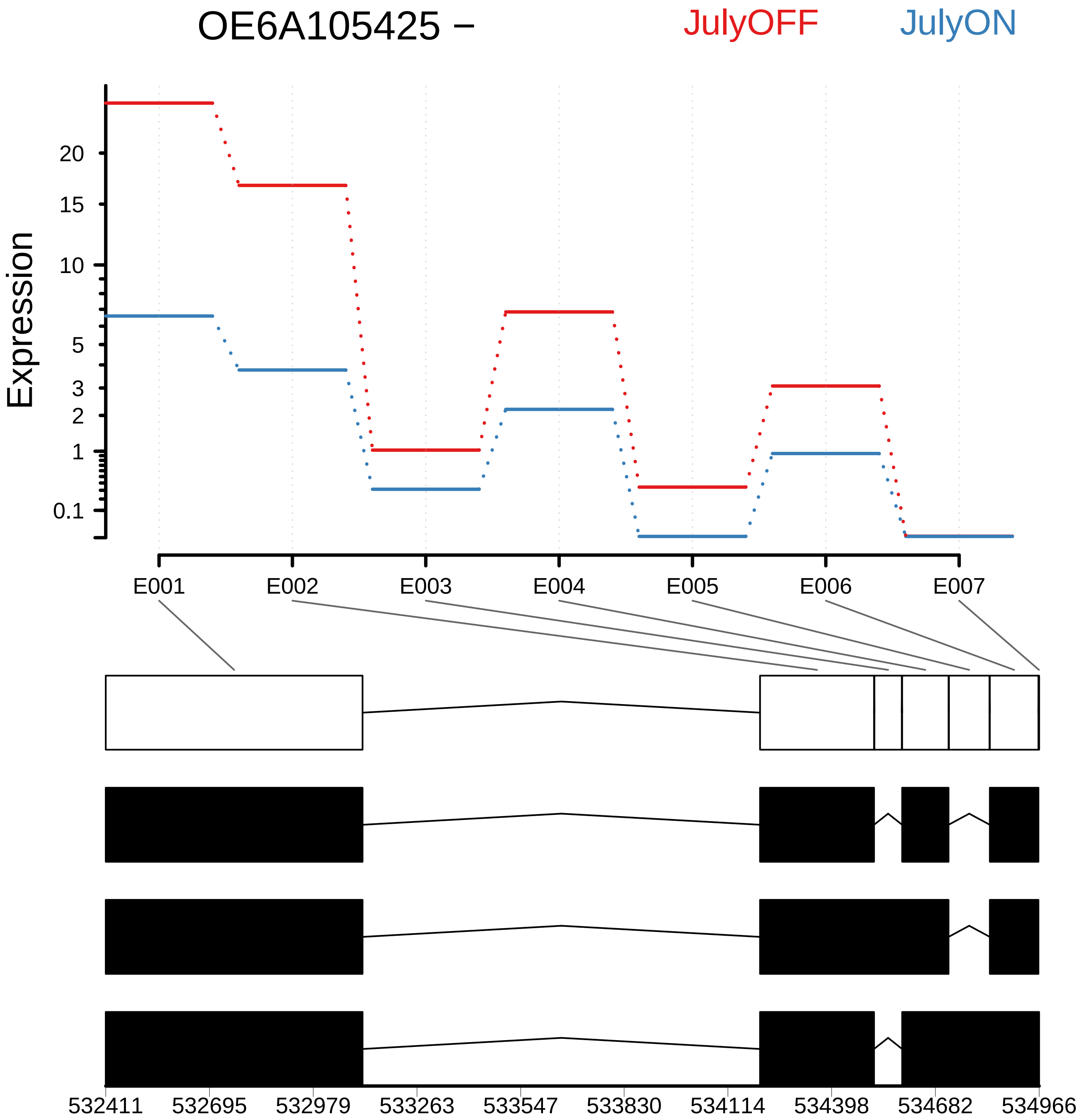
OE6A105425 –
JulyOFF
JulyON
Expression
20
15
10
5
3
2
1
0.1
E001
E002
E003
E004
E005
E006
E007
532411
532695
532979
533263
533547
533830
534114
534398
534682
534966

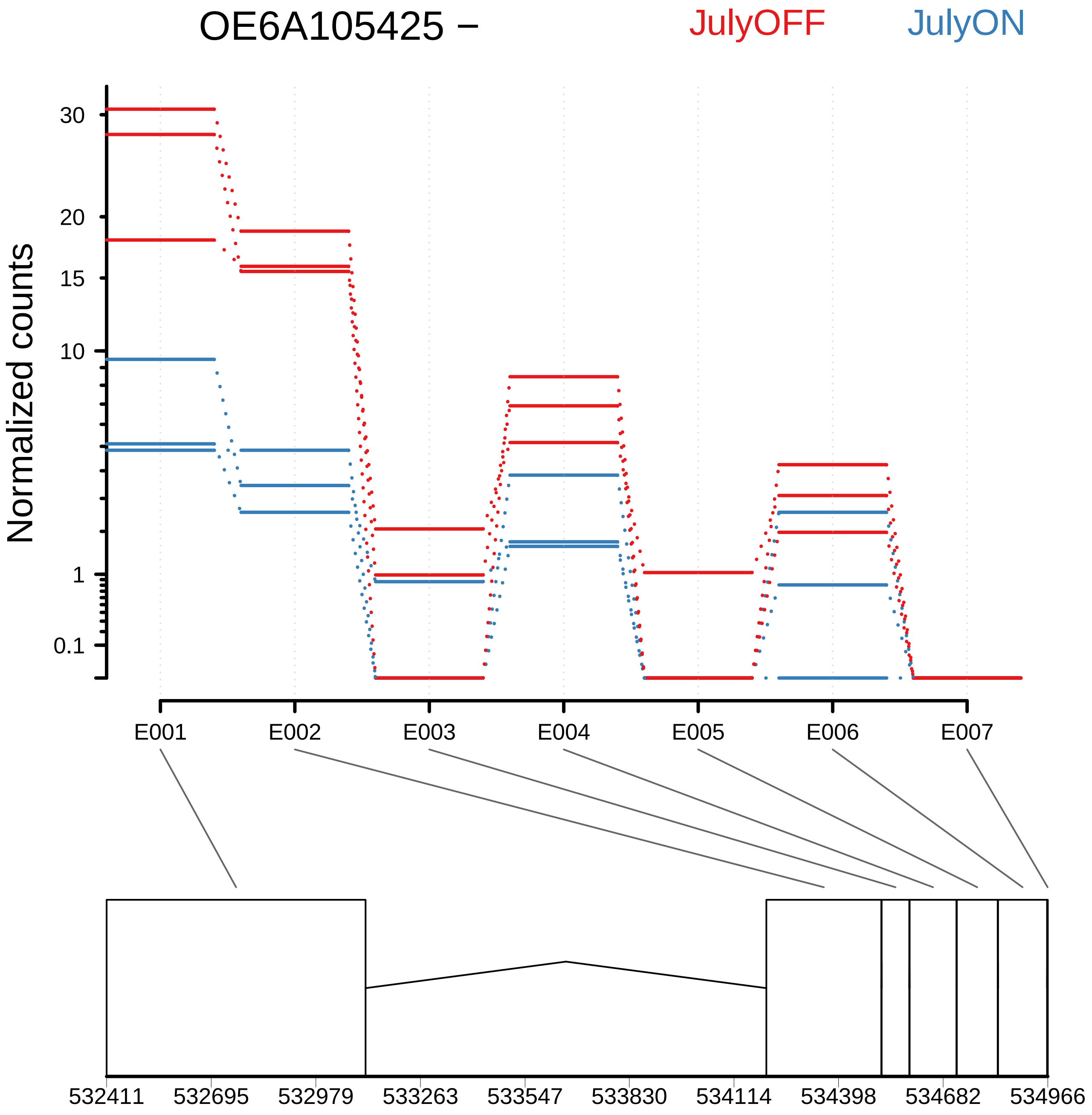
OE6A105425 −
JulyOFF
JulyON
Normalized counts
30
20
15
10
1
0.1
E001
E002
E003
E004
E005
E006
E007
532411
532695
532979
533263
533547
533830
534114
534398
534682
534966

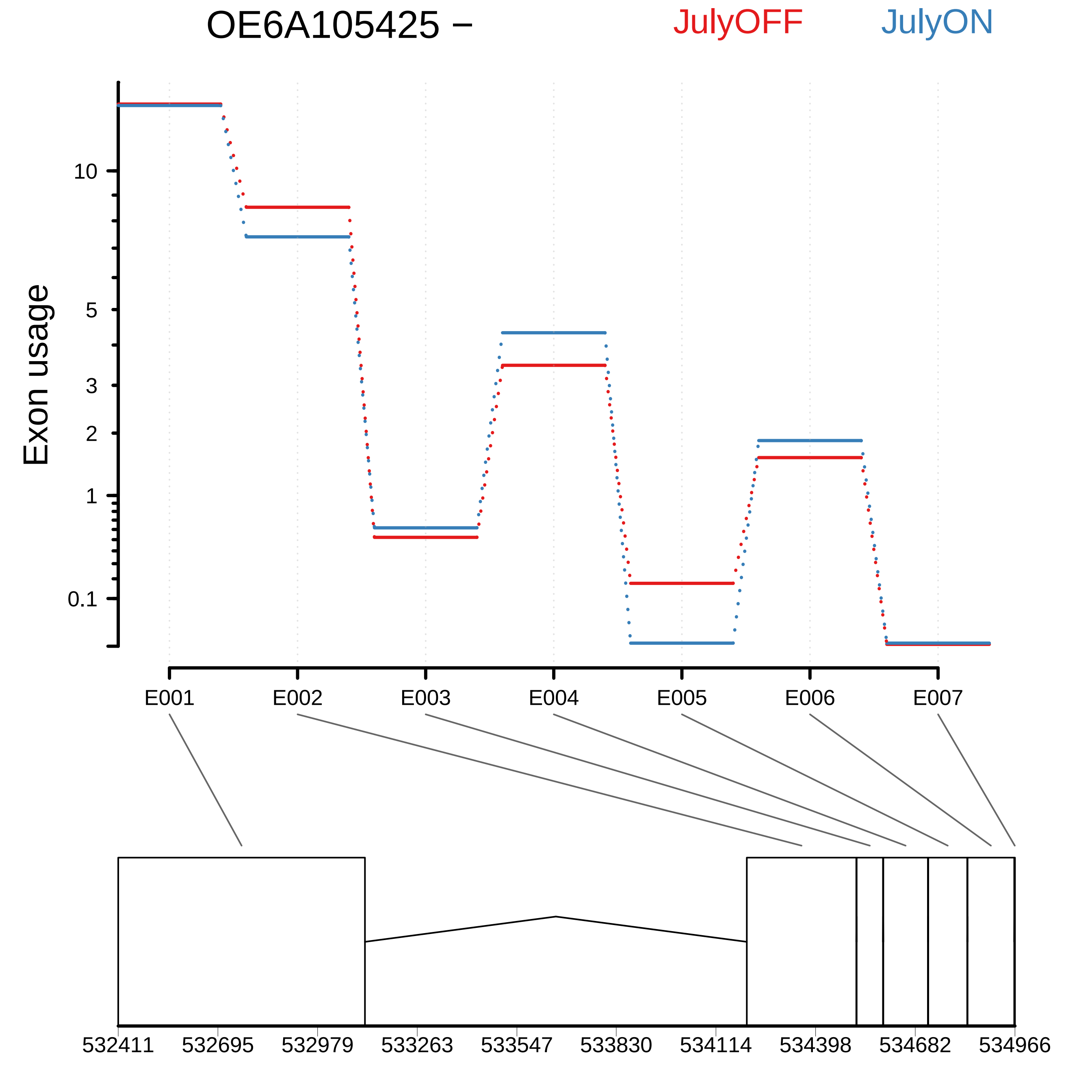
OE6A105425 −
JulyOFF
JulyON
Exon usage
10
5
3
2
1
0.1
E001
E002
E003
E004
E005
E006
E007
532411
532695
532979
533263
533547
533830
534114
534398
534682
534966

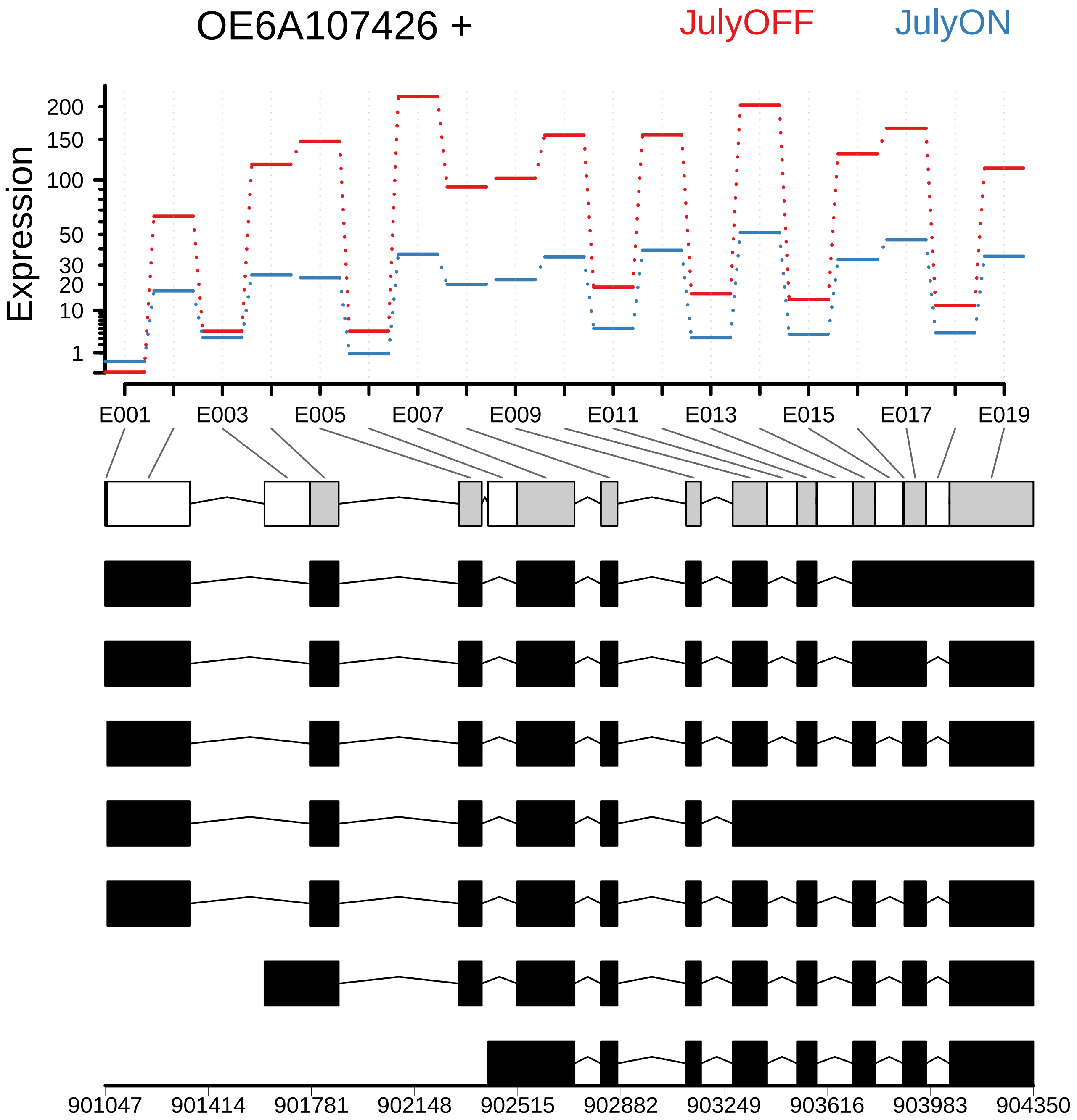
OE6A107426 +
JulyOFF
JulyON
Expression
200
150
100
50
30
20
10
1
E001
E003
E005
E007
E009
E011
E013
E015
E017
E019
901047
901414
901781
902148
902515
902882
903249
903616
903983
904350

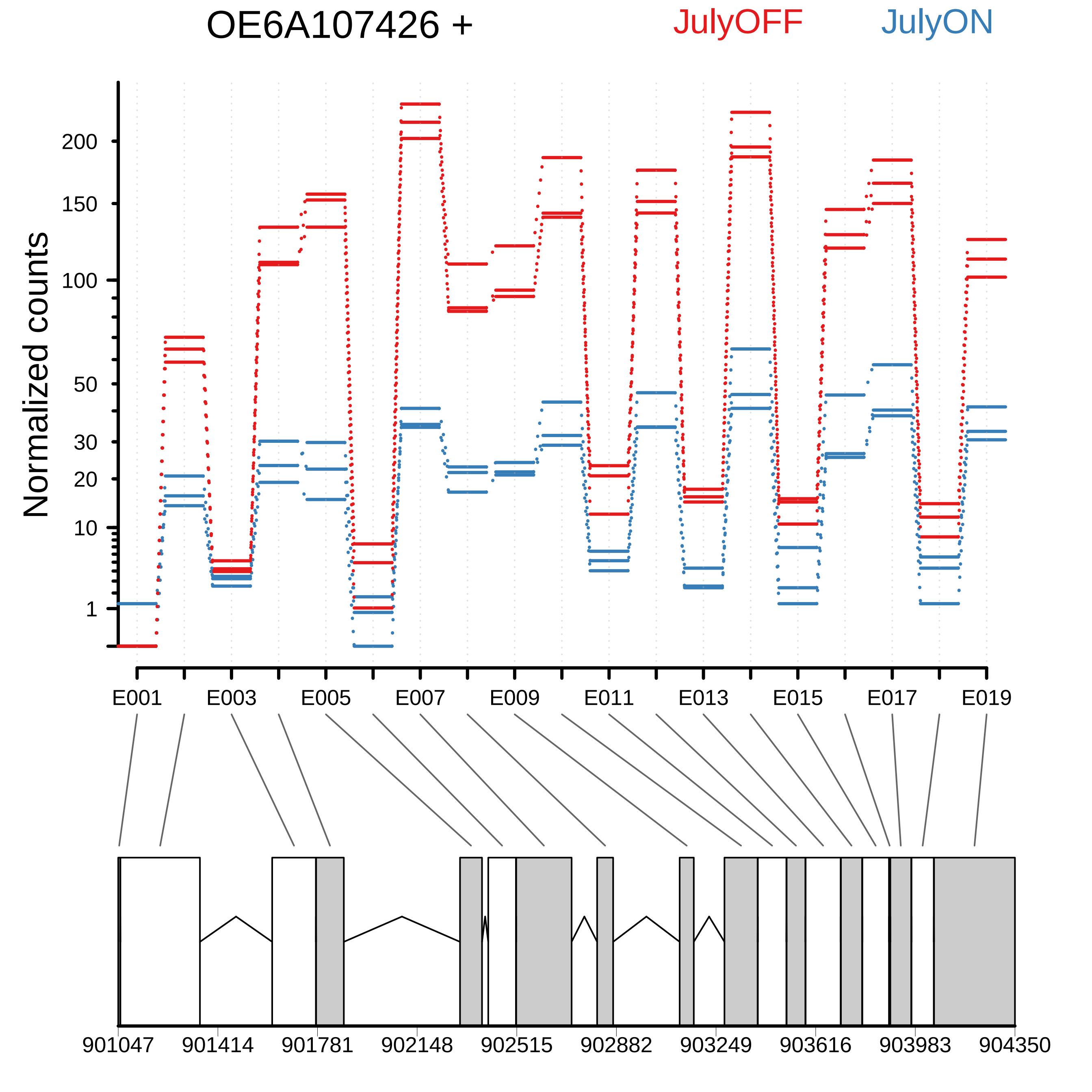

OE6A107426 +
JulyOFF
JulyON
Normalized counts
200
150
100
50
30
20
10
1
E001
E003
E005
E007
E009
E011
E013
E015
E017
E019
901047
901414
901781
902148
902515
902882
903249
903616
903983
904350

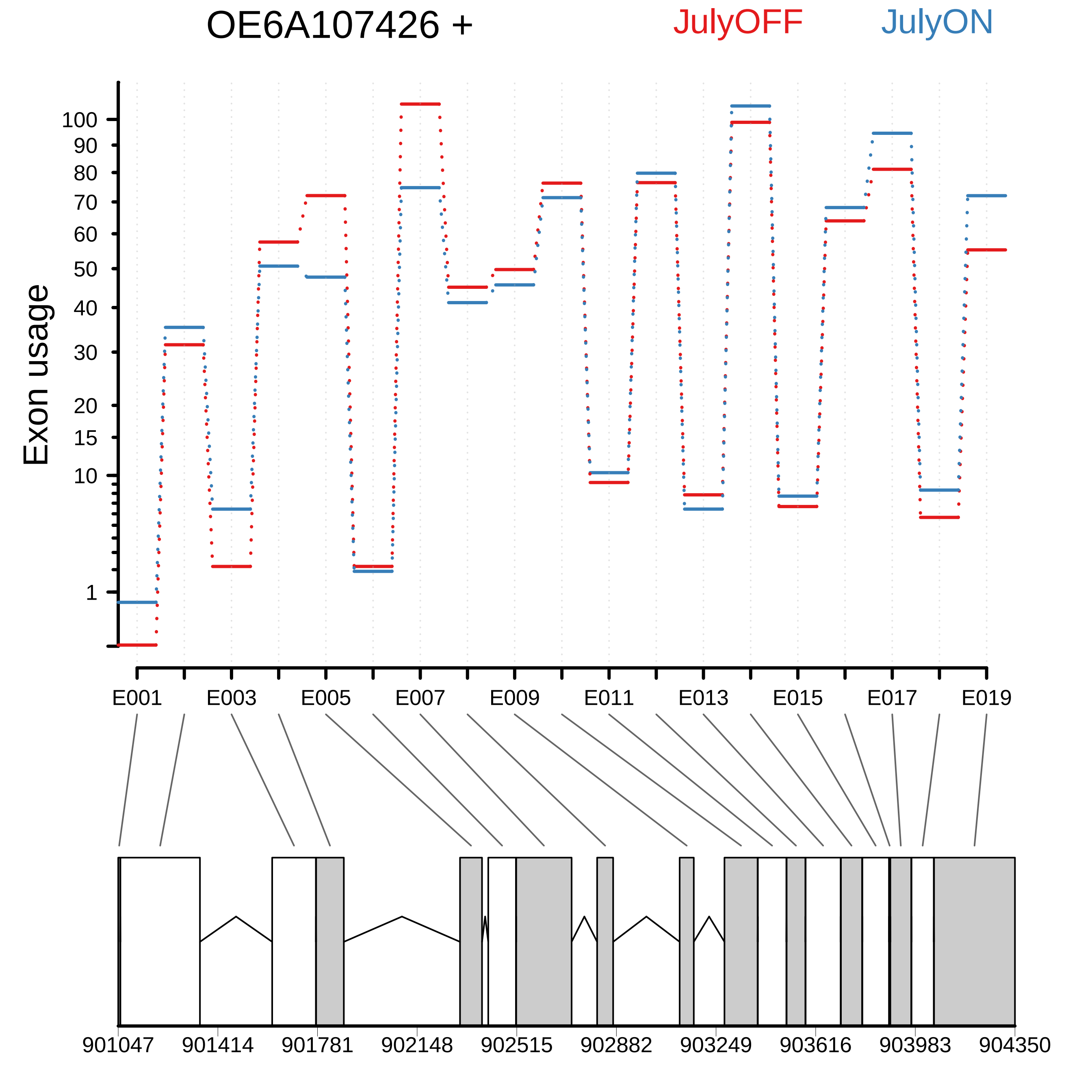

OE6A107426 +
JulyOFF
JulyON
Exon usage
100
90
80
70
60
50
40
30
20
15
10
1
E001
E003
E005
E007
E009
E011
E013
E015
E017
E019
901047
901414
901781
902148
902515
902882
903249
903616
903983
904350

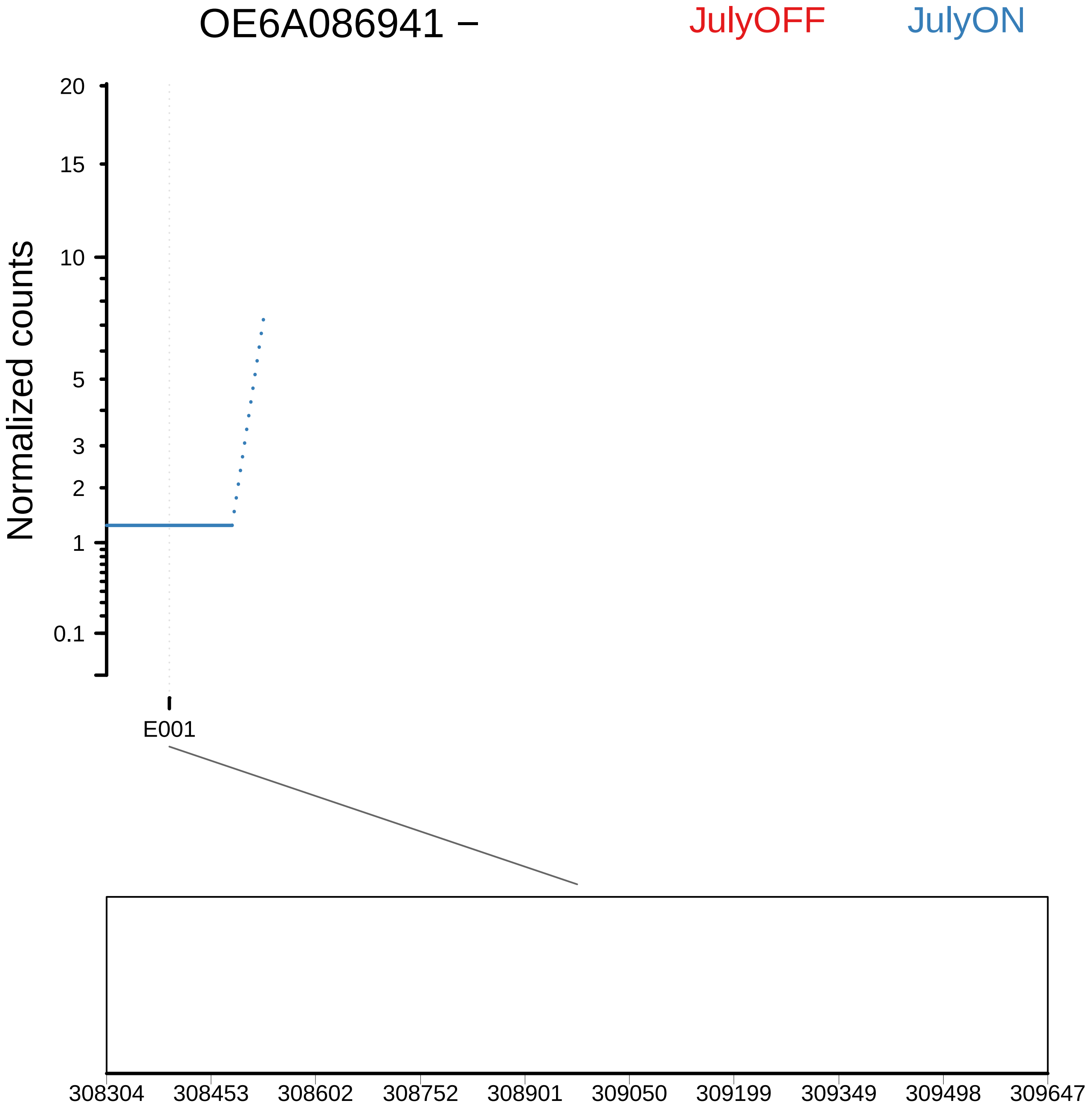

OE6A086941 −
JulyOFF
JulyON
Normalized counts
20
15
10
5
3
2
1
0.1
E001
308304
308453
308602
308752
308901
309050
309199
309349
309498
309647

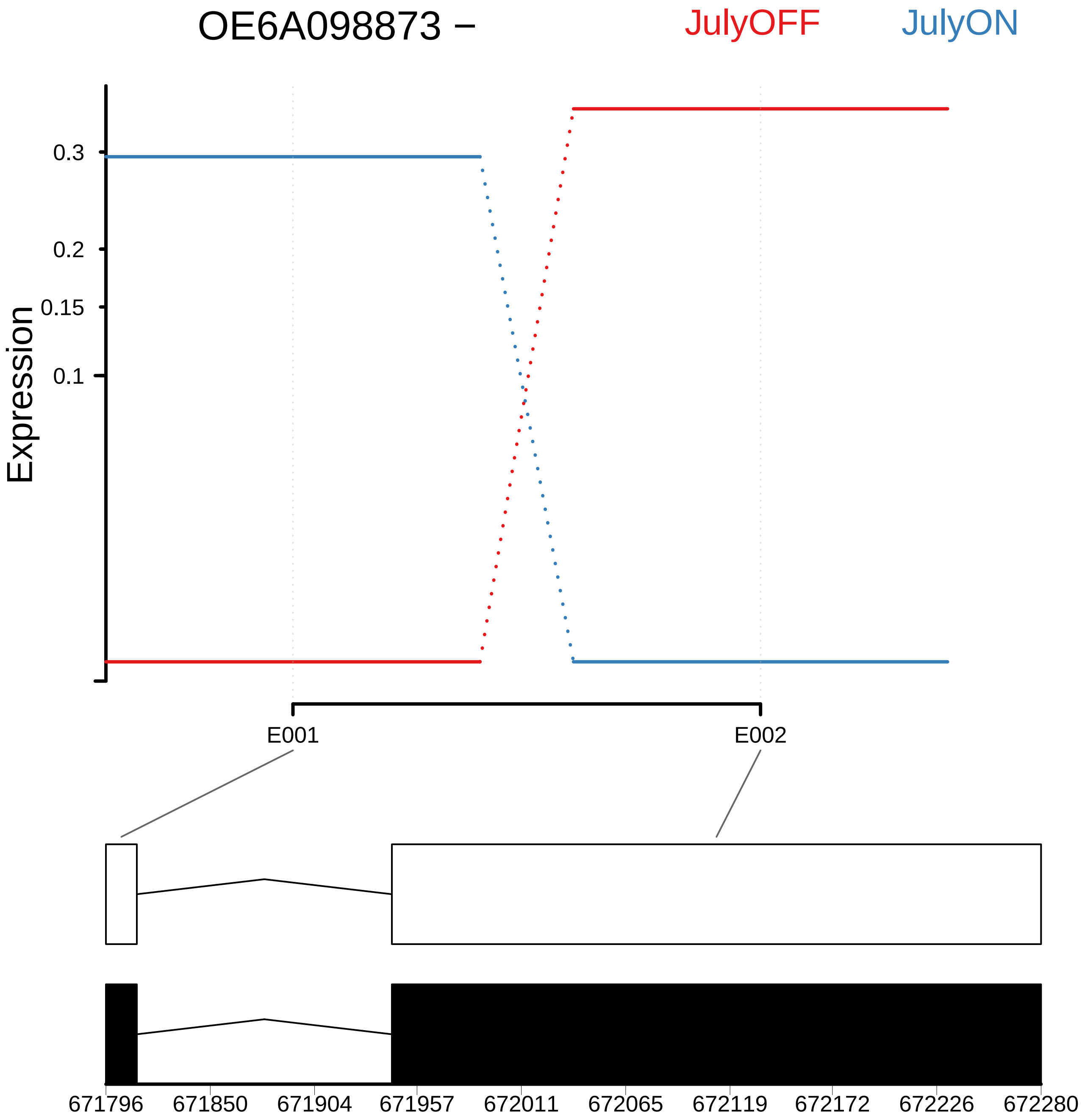

OE6A098873 −
JulyOFF
JulyON
Expression
0.3
0.2
0.15
0.1
E001
E002
671796
671850
671904
671957
672011
672065
672119
672172
672226
672280

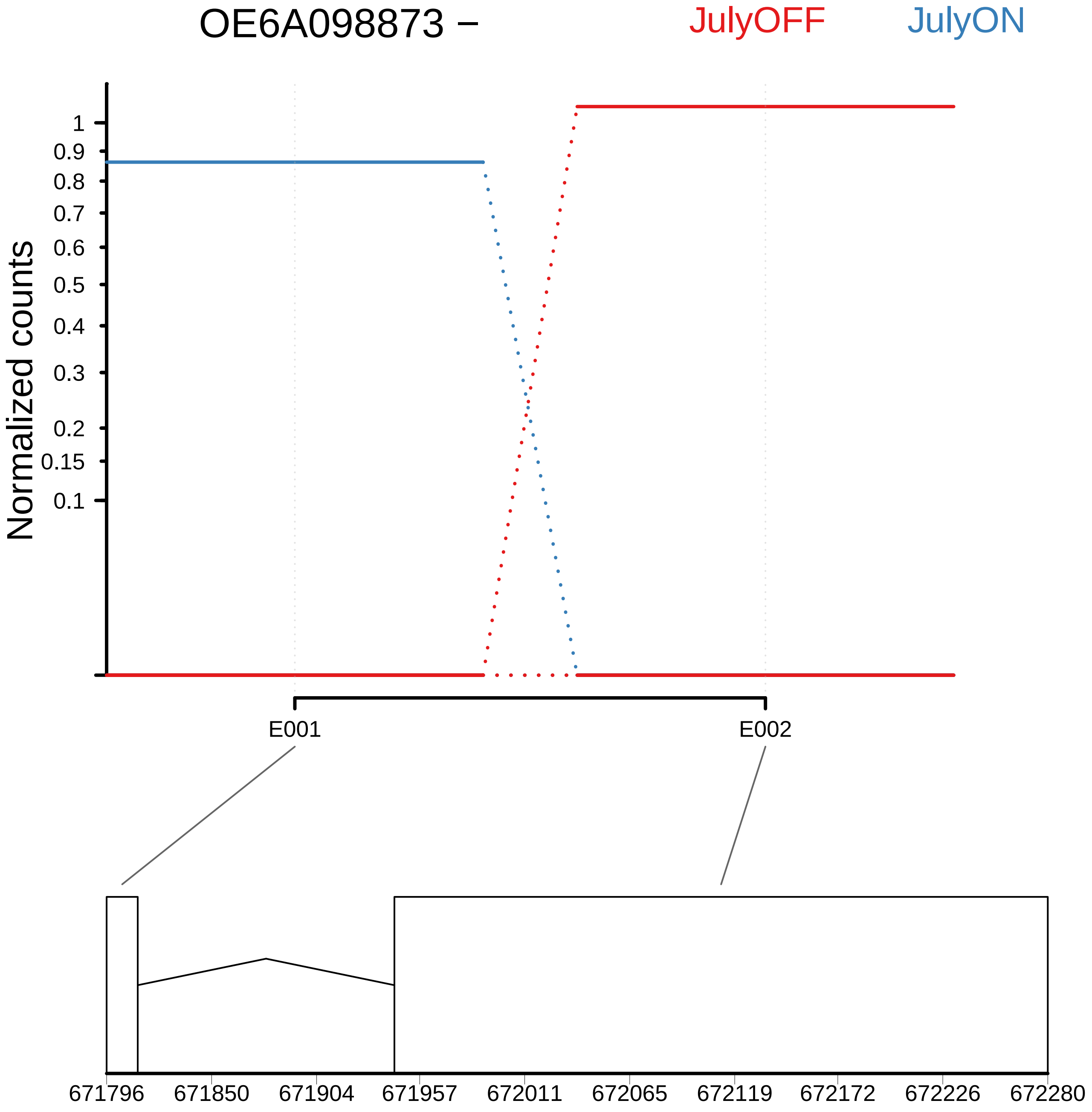

OE6A098873 −
JulyOFF
JulyON
Normalized counts
1
0.9
0.8
0.7
0.6
0.5
0.4
0.3
0.2
0.15
0.1
E001
E002
671796
671850
671904
671957
672011
672065
672119
672172
672226
672280

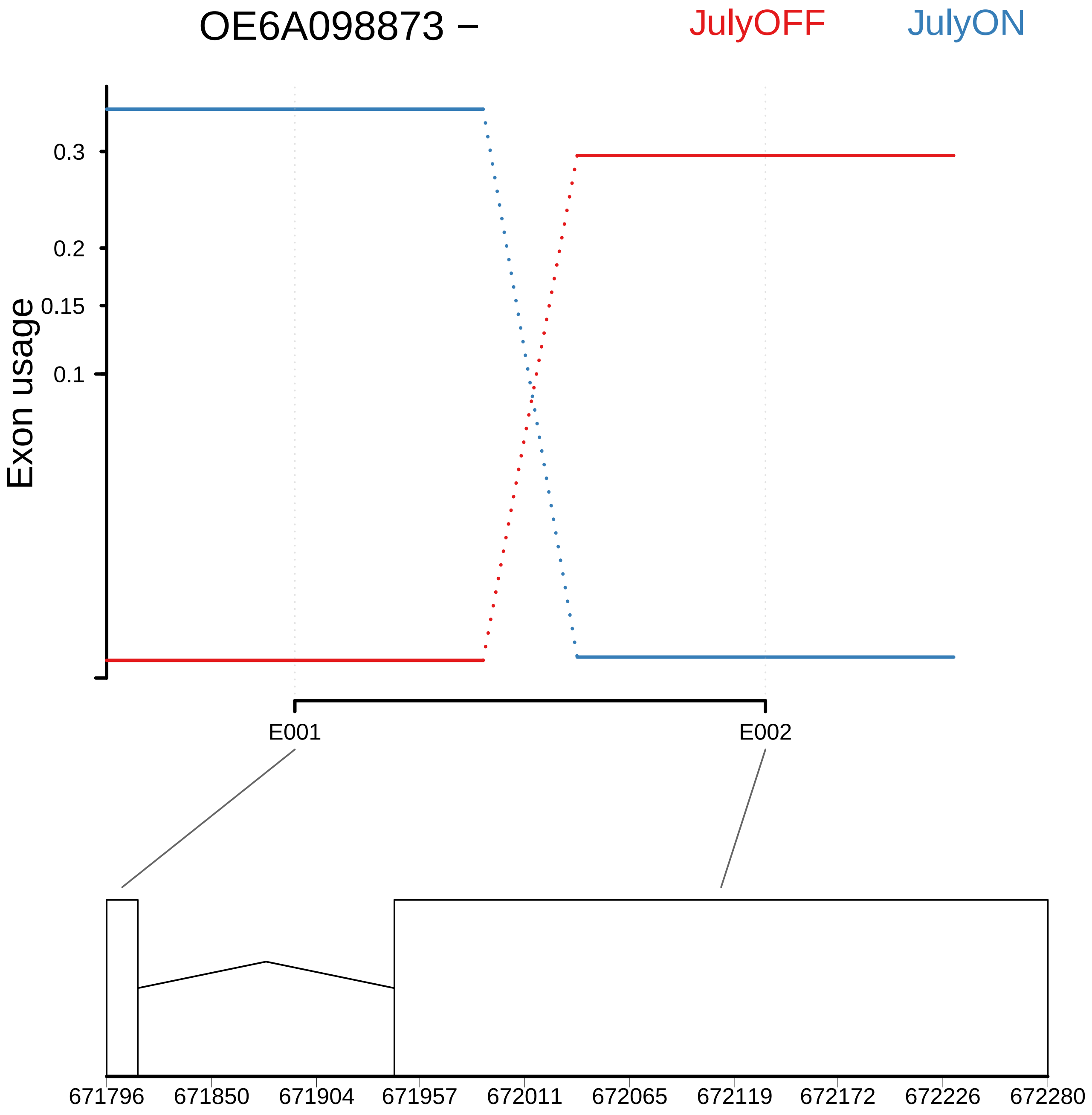

OE6A098873 −
JulyOFF
JulyON
Exon usage
0.3
0.2
0.15
0.1
E001
E002
671796
671850
671904
671957
672011
672065
672119
672172
672226
672280

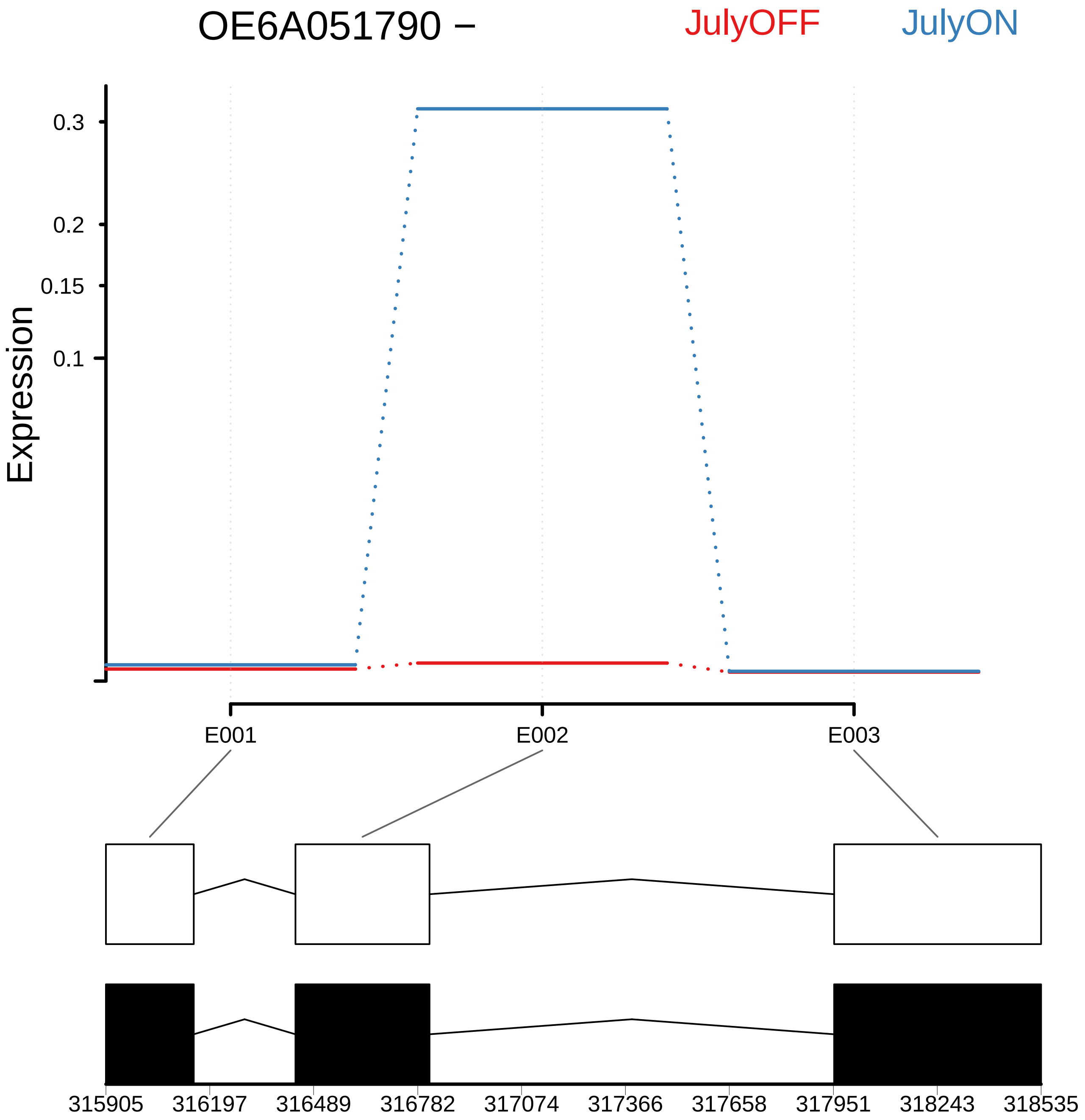

OE6A051790 −
JulyOFF
JulyON
Expression
0.3
0.2
0.15
0.1
E001
E002
E003
315905
316197
316489
316782
317074
317366
317658
317951
318243
318535

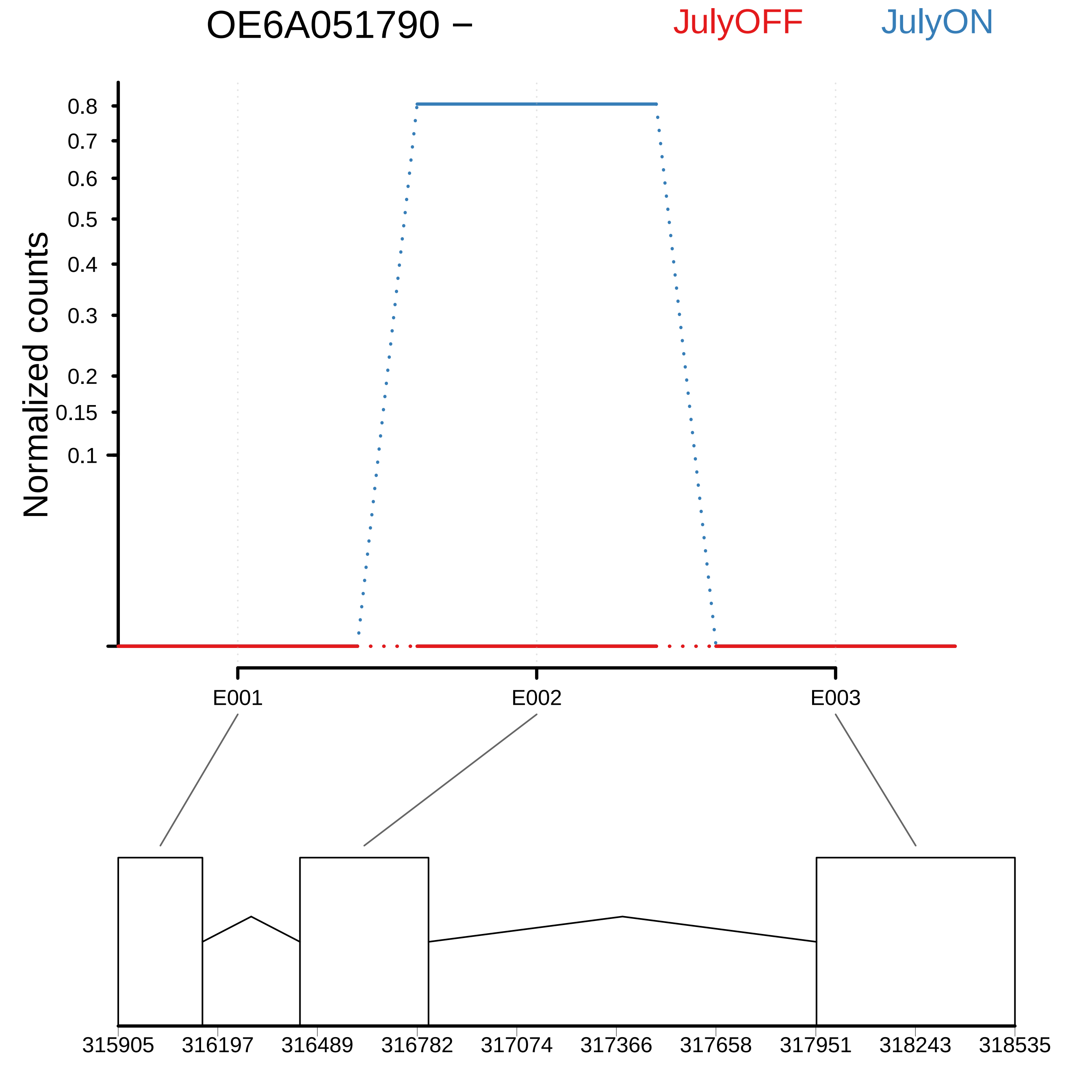

OE6A051790 −
JulyOFF
JulyON
Normalized counts
0.8
0.7
0.6
0.5
0.4
0.3
0.2
0.15
0.1
E001
E002
E003
315905
316197
316489
316782
317074
317366
317658
317951
318243
318535

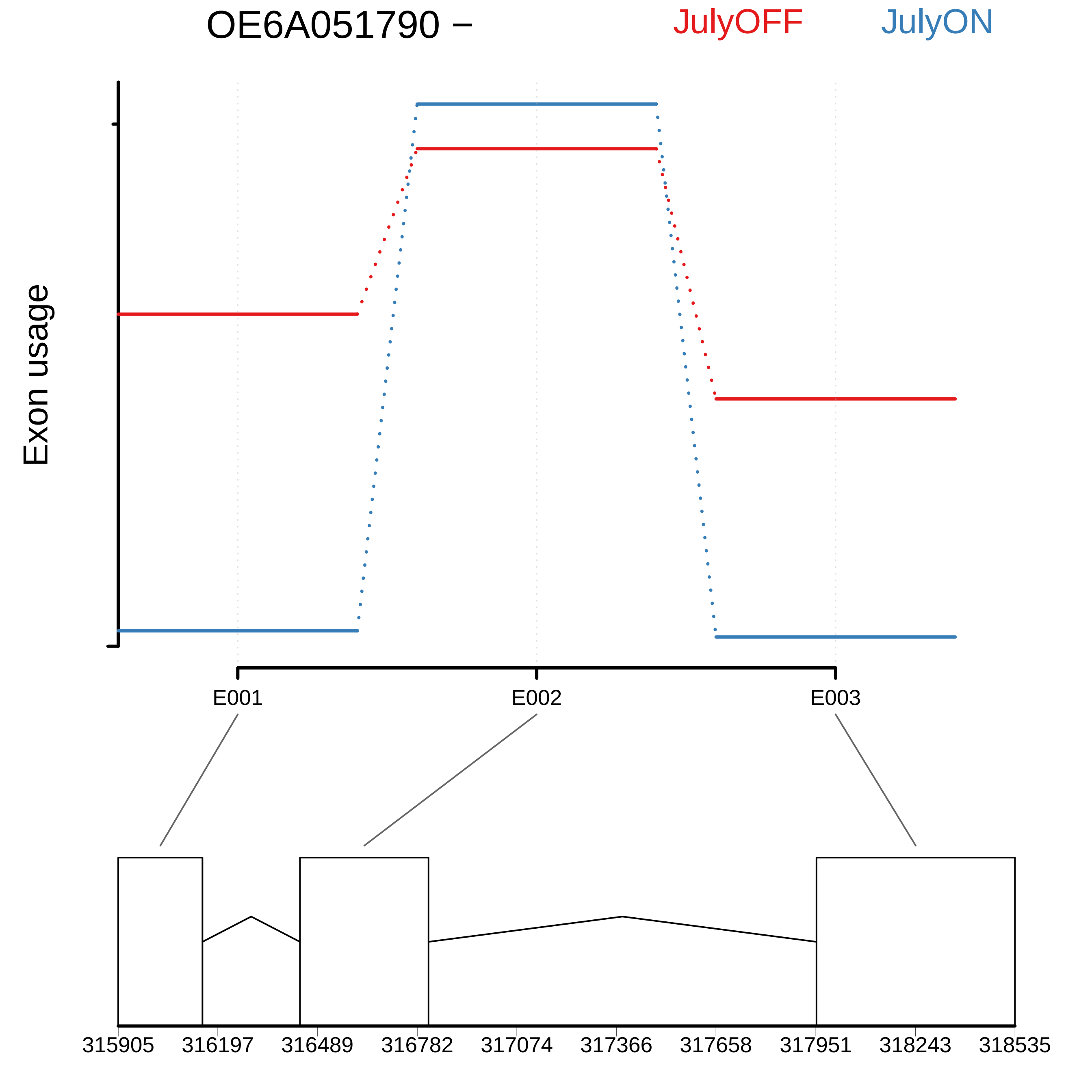

OE6A051790 −
JulyOFF
JulyON
Exon usage
E001
E002
E003
315905
316197
316489
316782
317074
317366
317658
317951
318243
318535

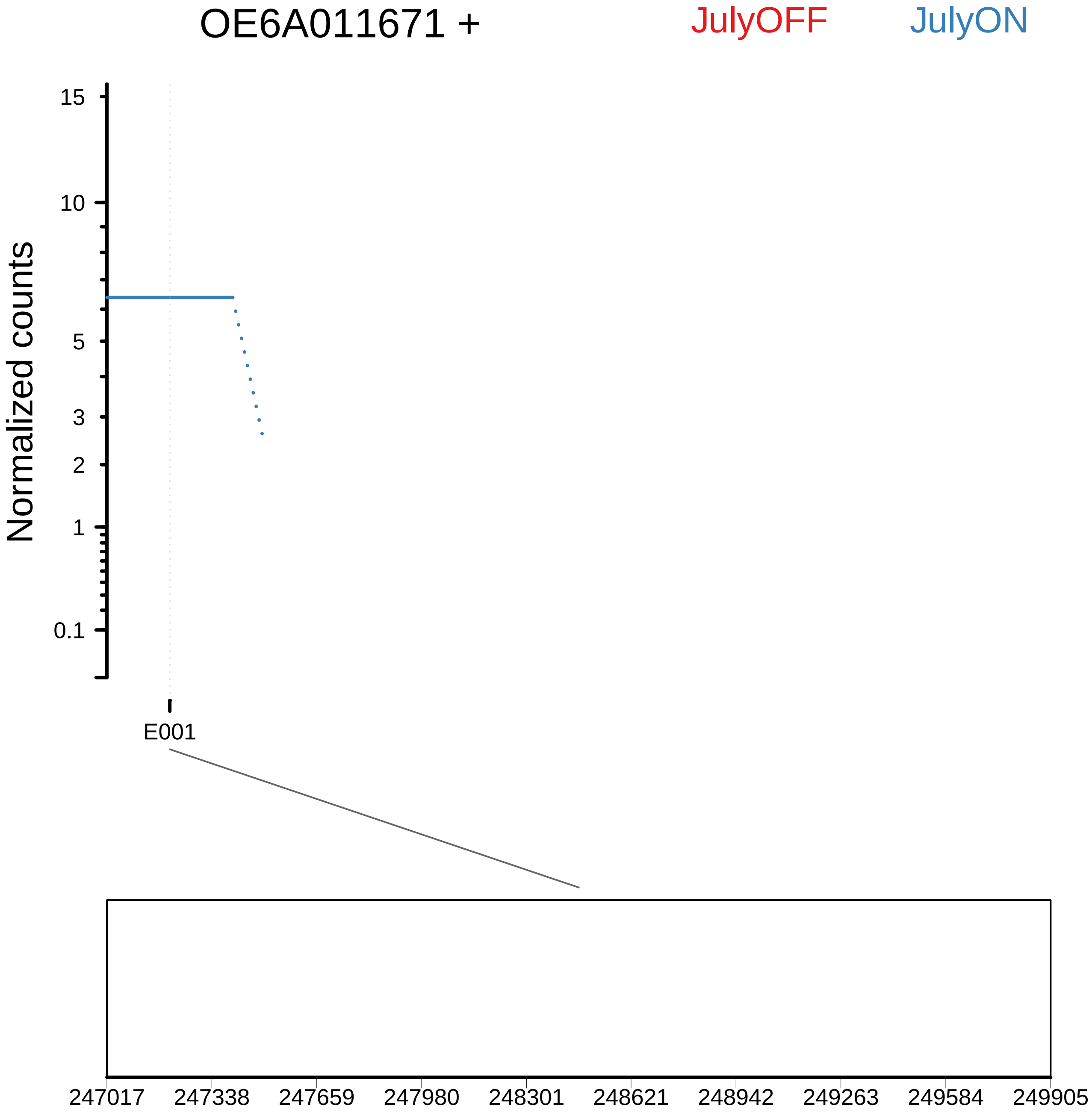

OE6A011671 +
JulyOFF
JulyON
Normalized counts
15
10
5
3
2
1
0.1
E001
247017
247338
247659
247980
248301
248621
248942
249263
249584
249905

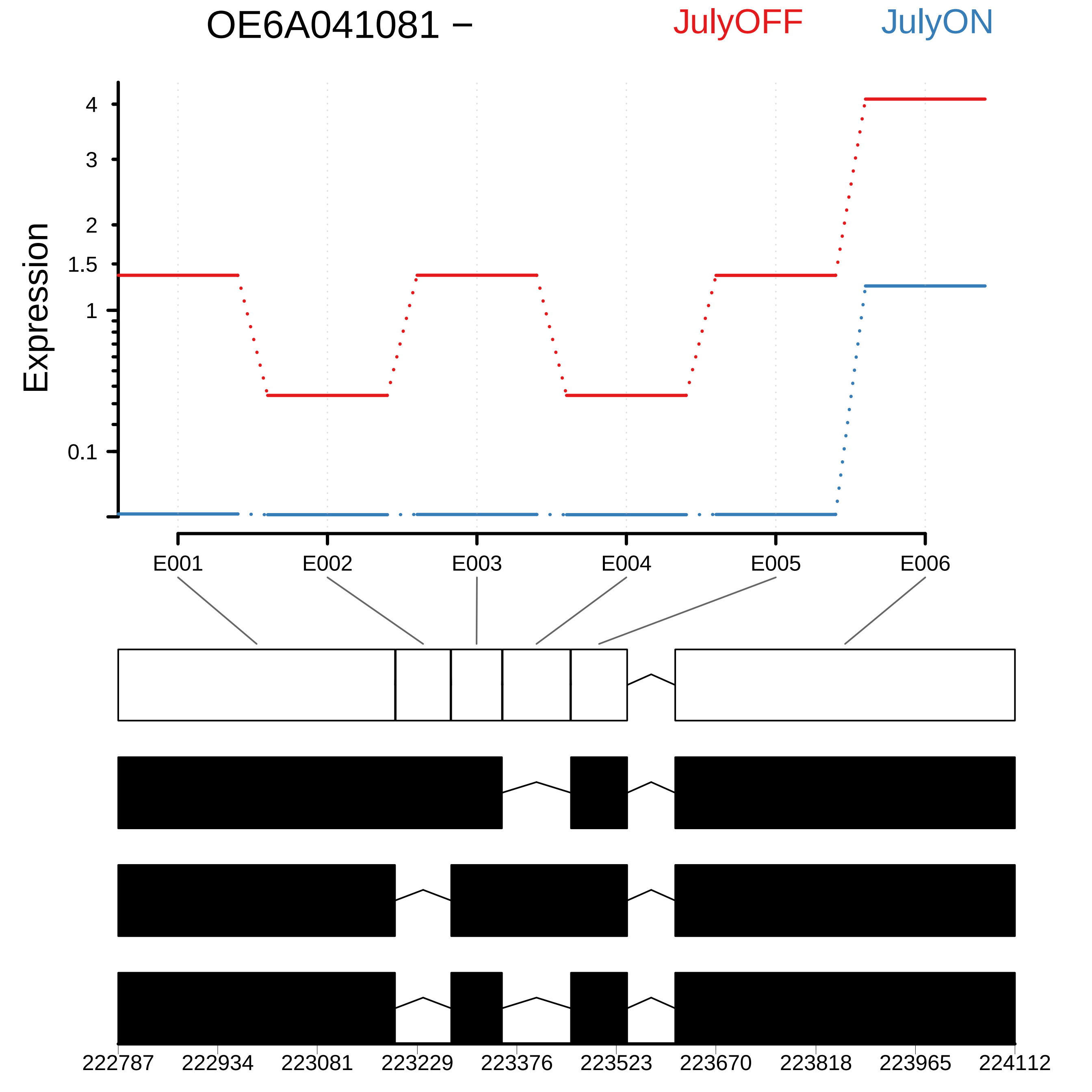

OE6A041081 –
JulyOFF
JulyON
Expression
4
3
2
1.5
1
0.1
E001
E002
E003
E004
E005
E006
222787
222934
223081
223229
223376
223523
223670
223818
223965
224112

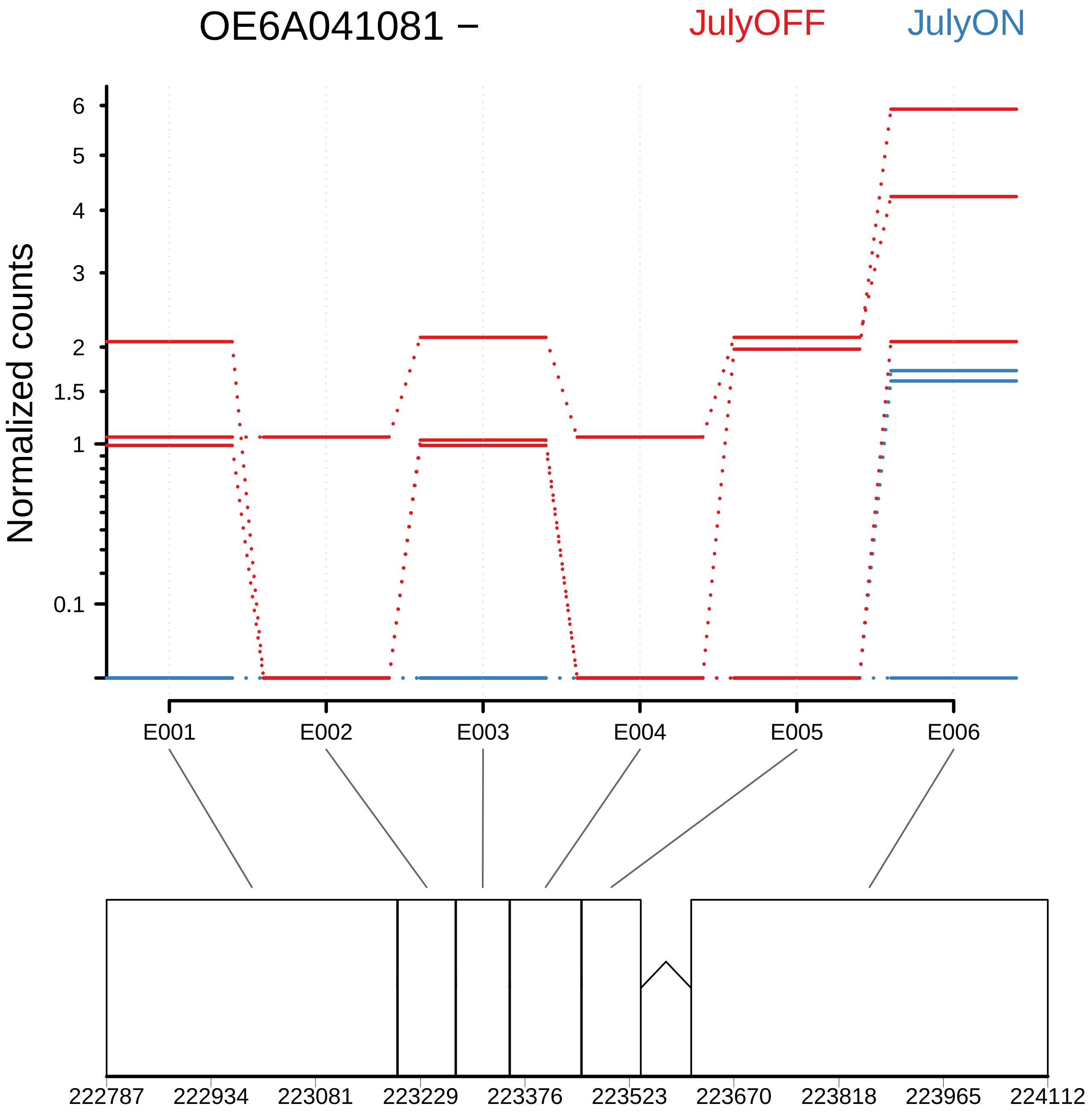
OE6A041081 −
JulyOFF
JulyON
Normalized counts
6
5
4
3
2
1.5
1
0.1
E001
E002
E003
E004
E005
E006
222787
222934
223081
223229
223376
223523
223670
223818
223965
224112

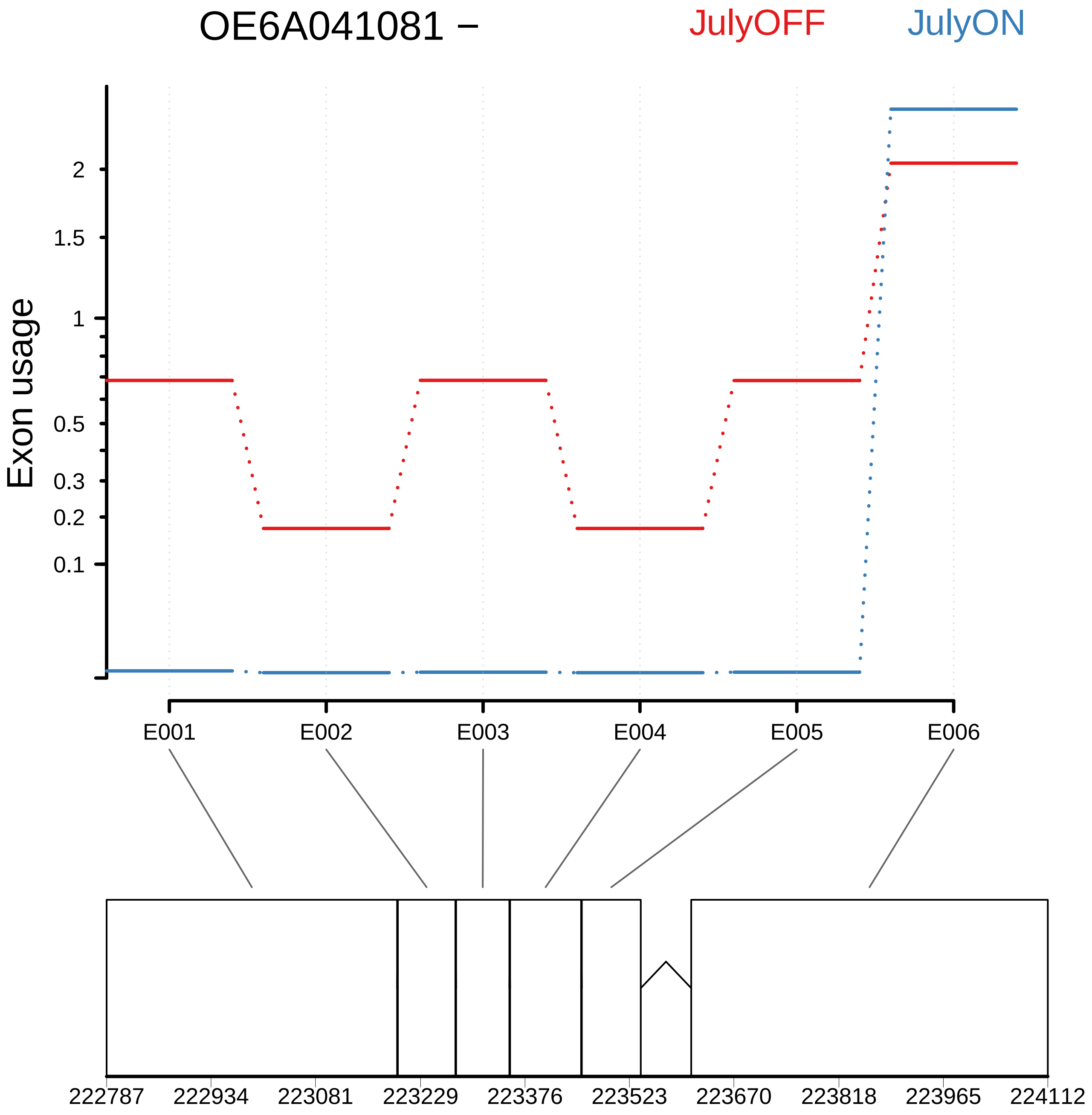
OE6A041081 −
JulyOFF
JulyON
Exon usage
2
1.5
1
0.5
0.3
0.2
0.1
E001
E002
E003
E004
E005
E006
222787
222934
223081
223229
223376
223523
223670
223818
223965
224112

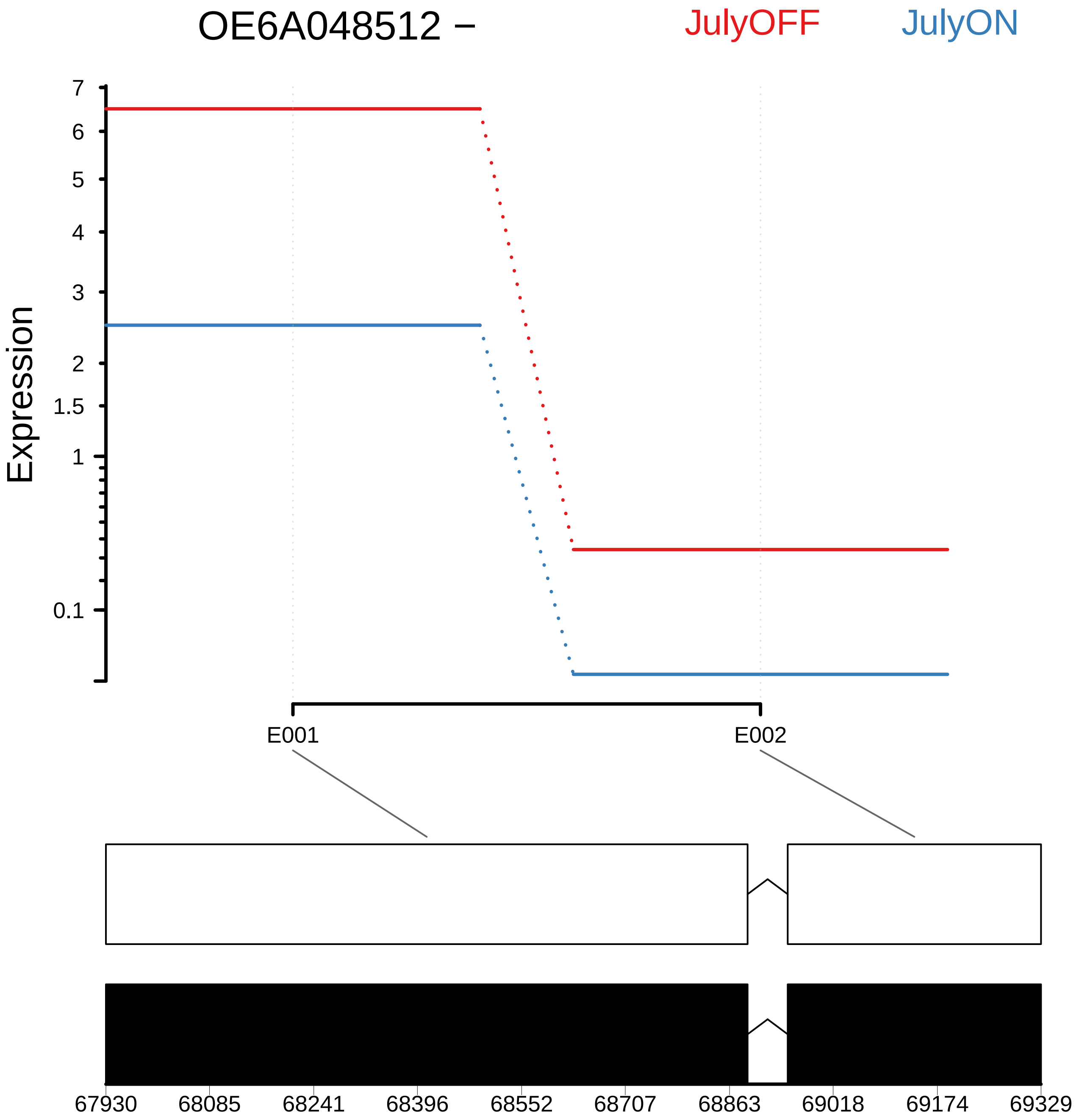

OE6A048512 −
JulyOFF
JulyON
Expression
7
6
5
4
3
2
1.5
1
0.1
E001
E002
67930
68085
68241
68396
68552
68707
68863
69018
69174
69329

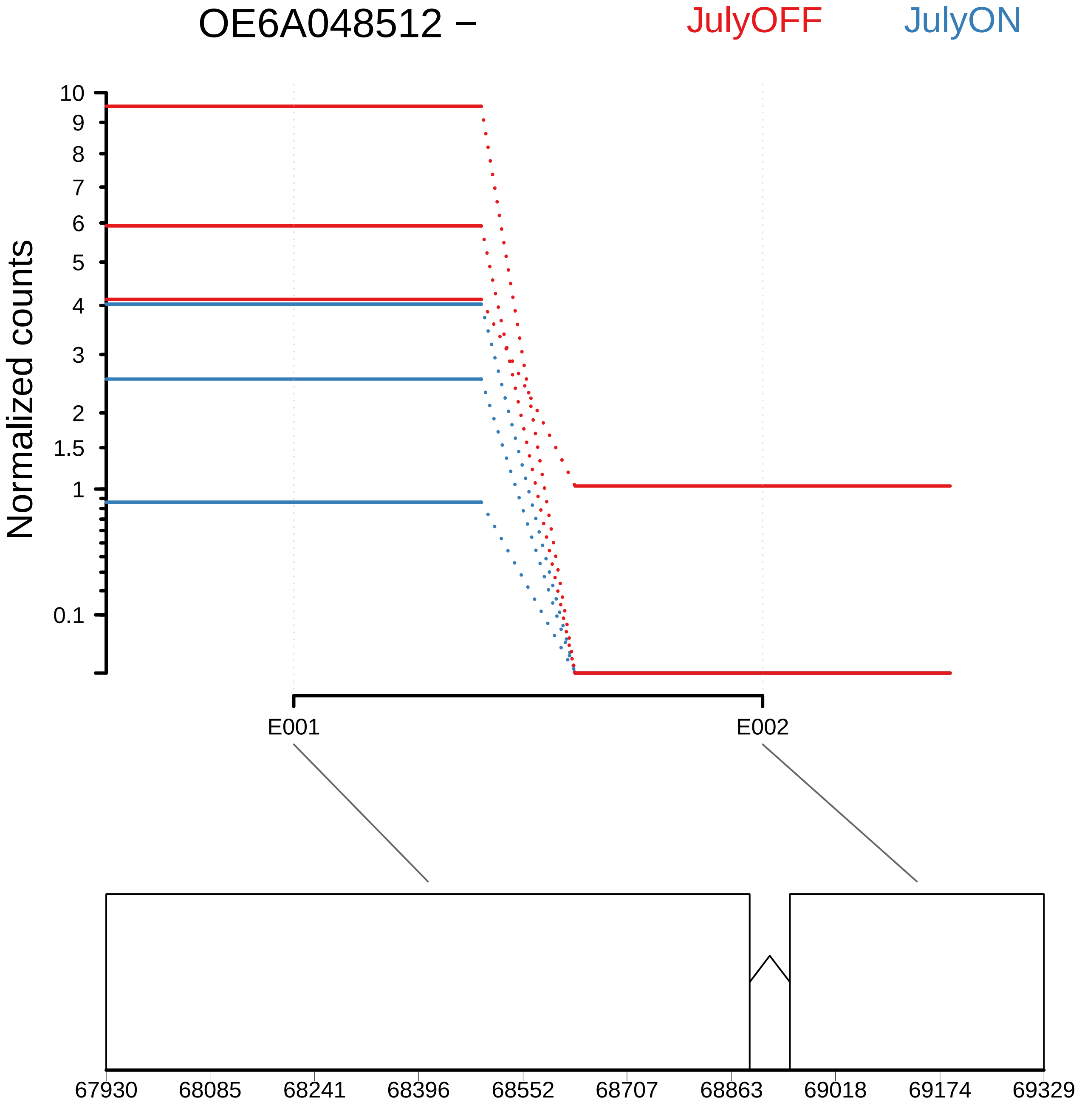

OE6A048512 −
JulyOFF
JulyON
Normalized counts
10
9
8
7
6
5
4
3
2
1.5
1
0.1
E001
E002
67930
68085
68241
68396
68552
68707
68863
69018
69174
69329

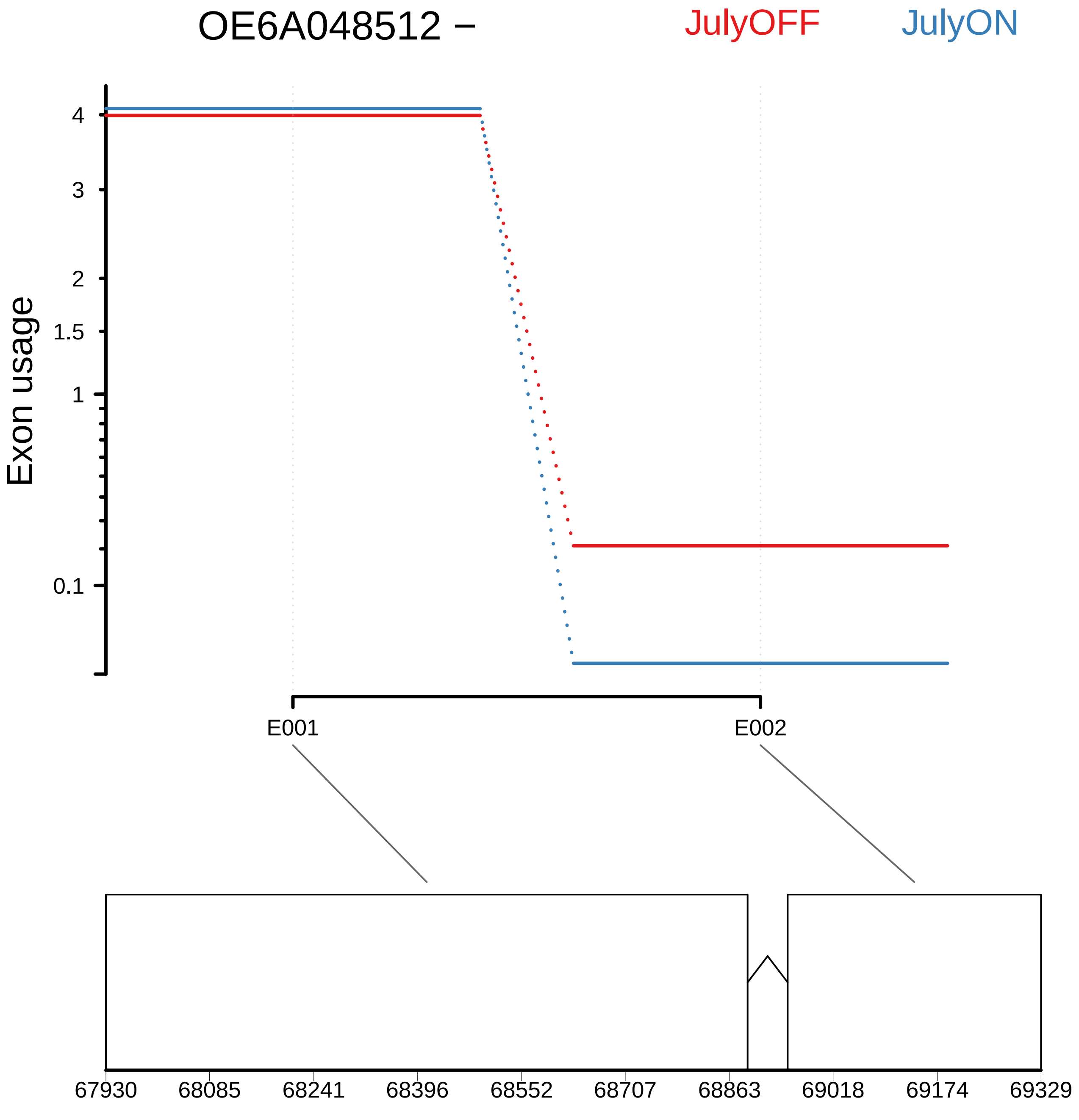
OE6A048512 −
JulyOFF
JulyON
Exon usage
4
3
2
1.5
1
0.1
E001
E002
67930
68085
68241
68396
68552
68707
68863
69018
69174
69329

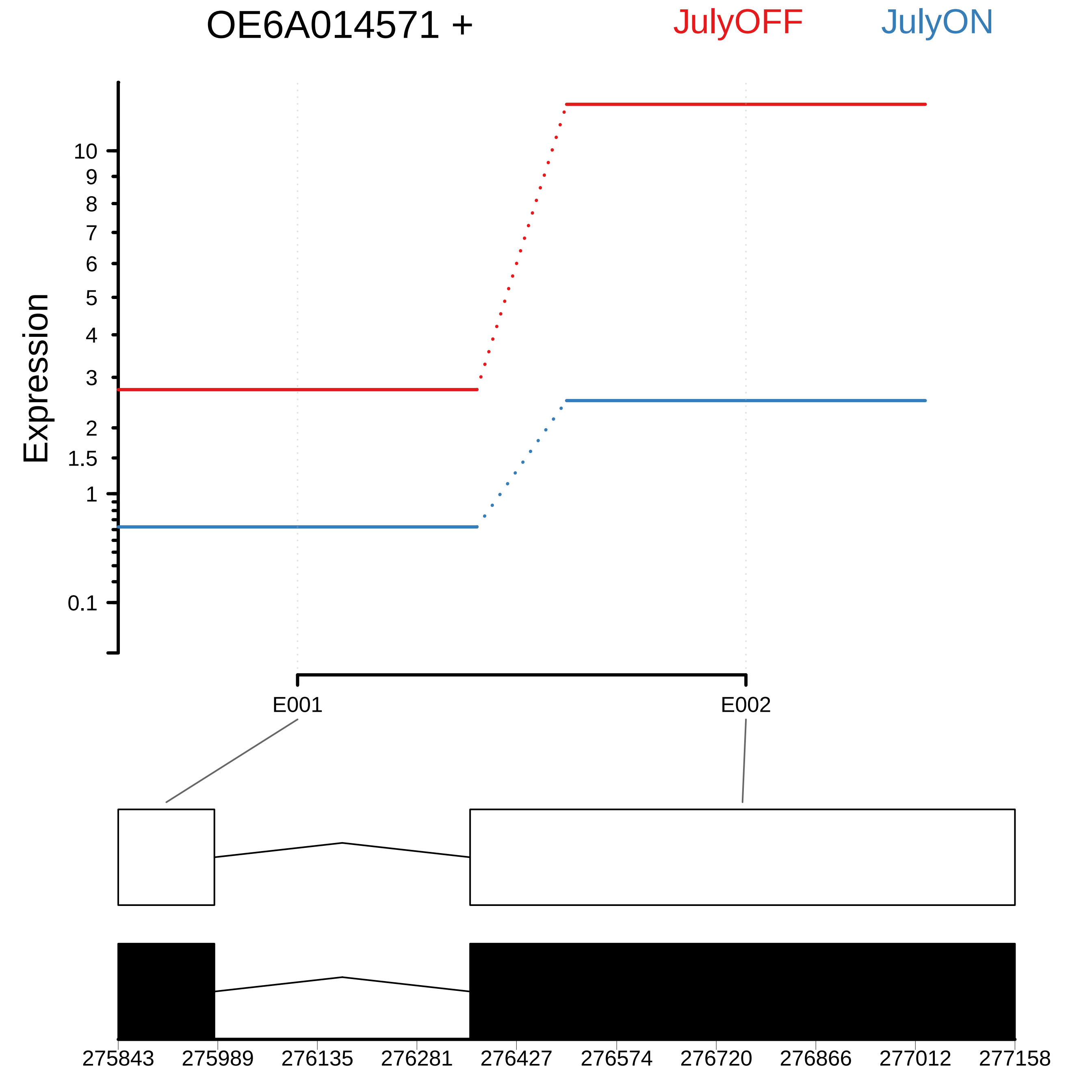
OE6A014571 +
JulyOFF
JulyON
Expression
10
9
8
7
6
5
4
3
2
1.5
1
0.1
E001
E002
275843
275989
276135
276281
276427
276574
276720
276866
277012
277158

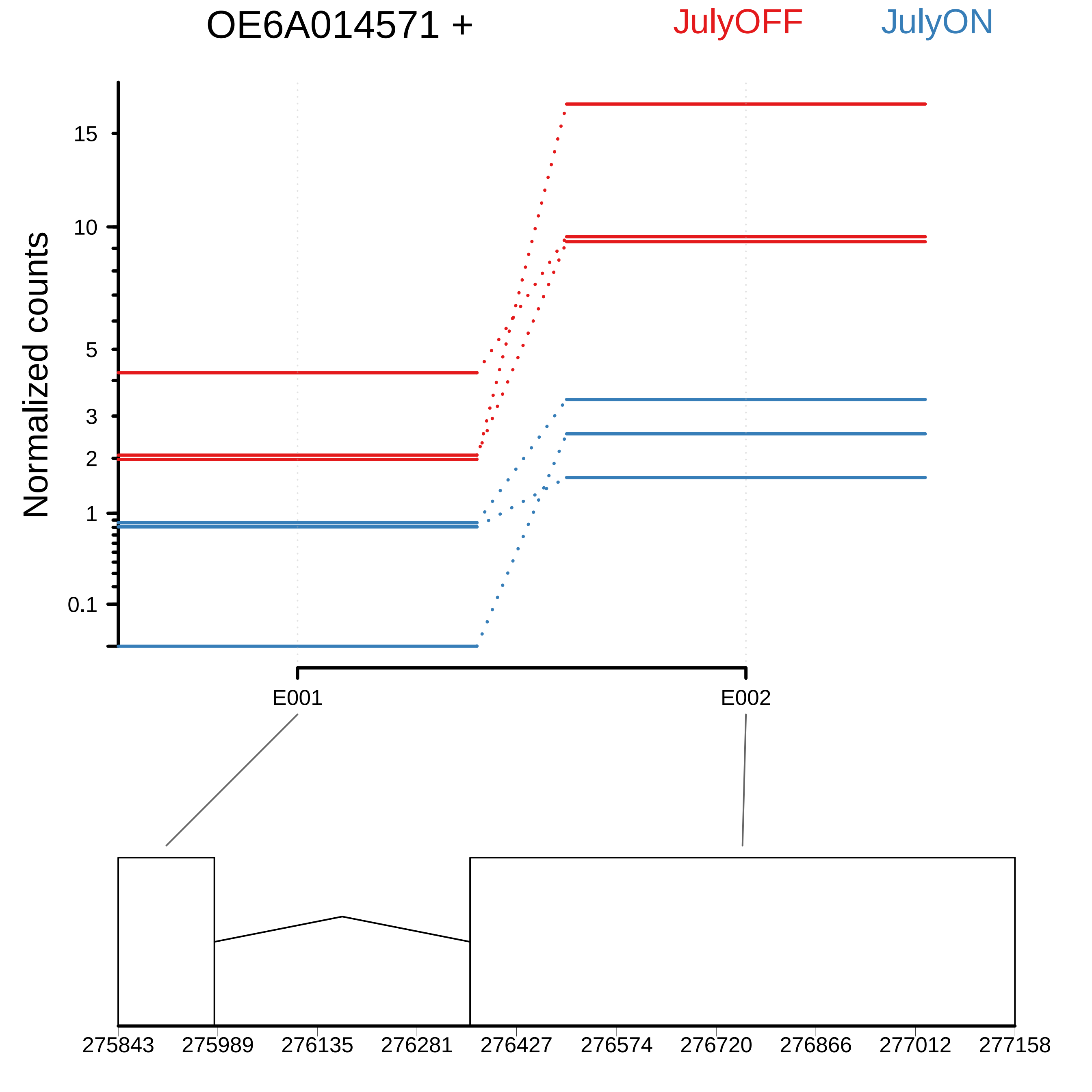
OE6A014571 +
JulyOFF
JulyON
Normalized counts
15
10
5
3
2
1
0.1
E001
E002
275843
275989
276135
276281
276427
276574
276720
276866
277012
277158

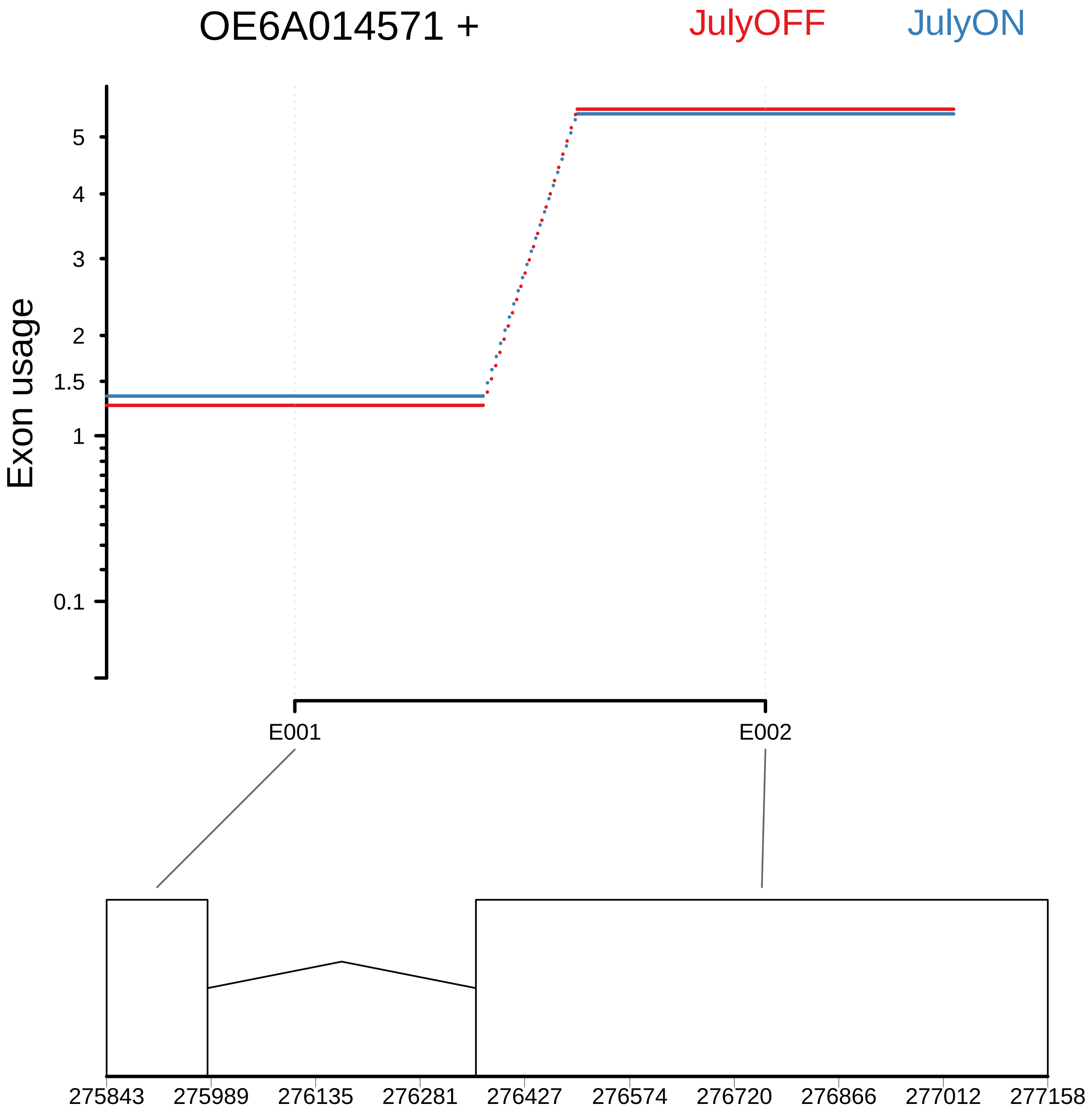
OE6A014571 +
JulyOFF
JulyON
Exon usage
5
4
3
2
1.5
1
0.1
E001
E002
275843
275989
276135
276281
276427
276574
276720
276866
277012
277158

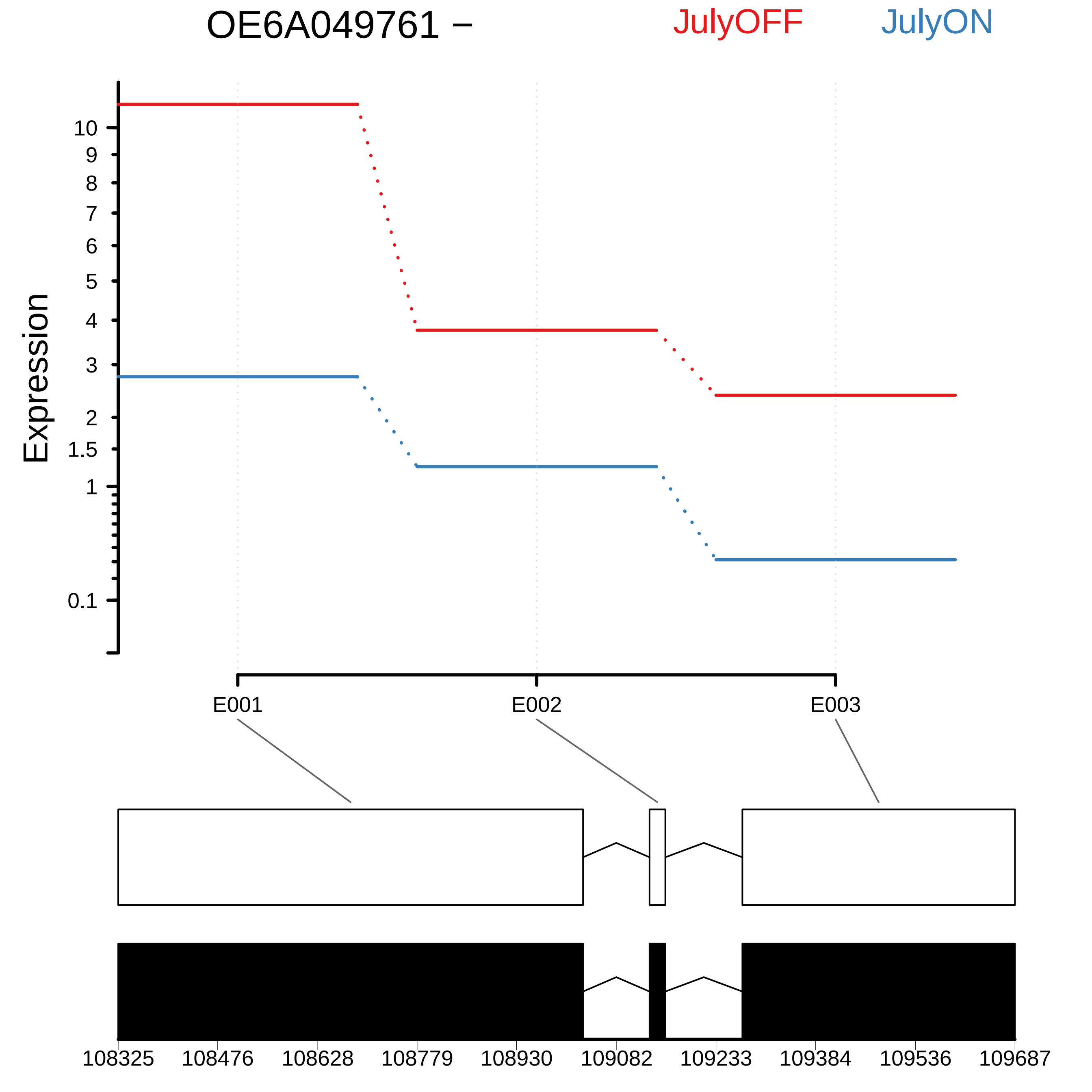
OE6A049761 –
JulyOFF
JulyON
Expression
10
9
8
7
6
5
4
3
2
1.5
1
0.1
E001
E002
E003
108325
108476
108628
108779
108930
109082
109233
109384
109536
109687

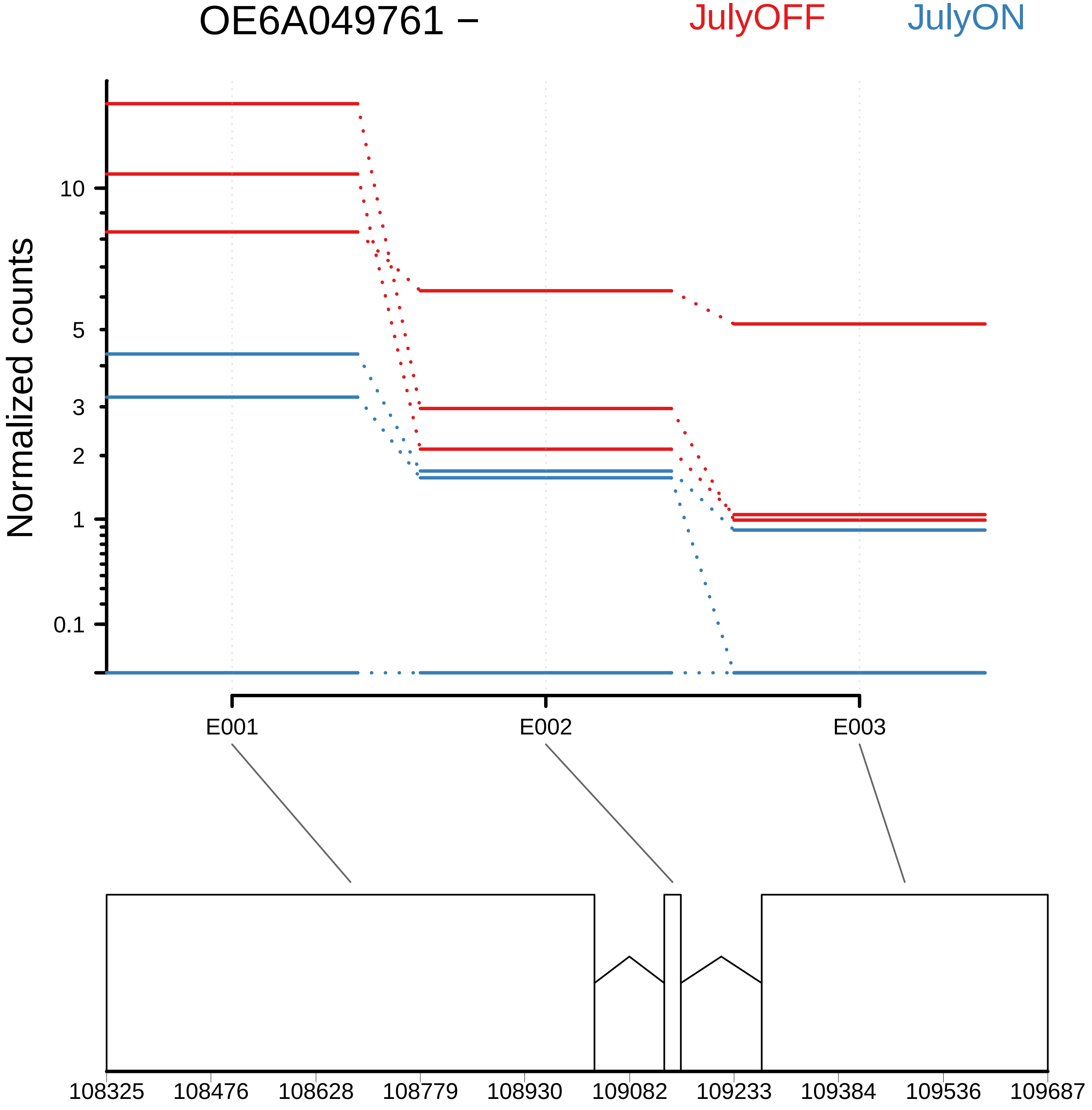
OE6A049761 –
JulyOFF
JulyON
Normalized counts
10
5
3
2
1
0.1
E001
E002
E003
108325
108476
108628
108779
108930
109082
109233
109384
109536
109687

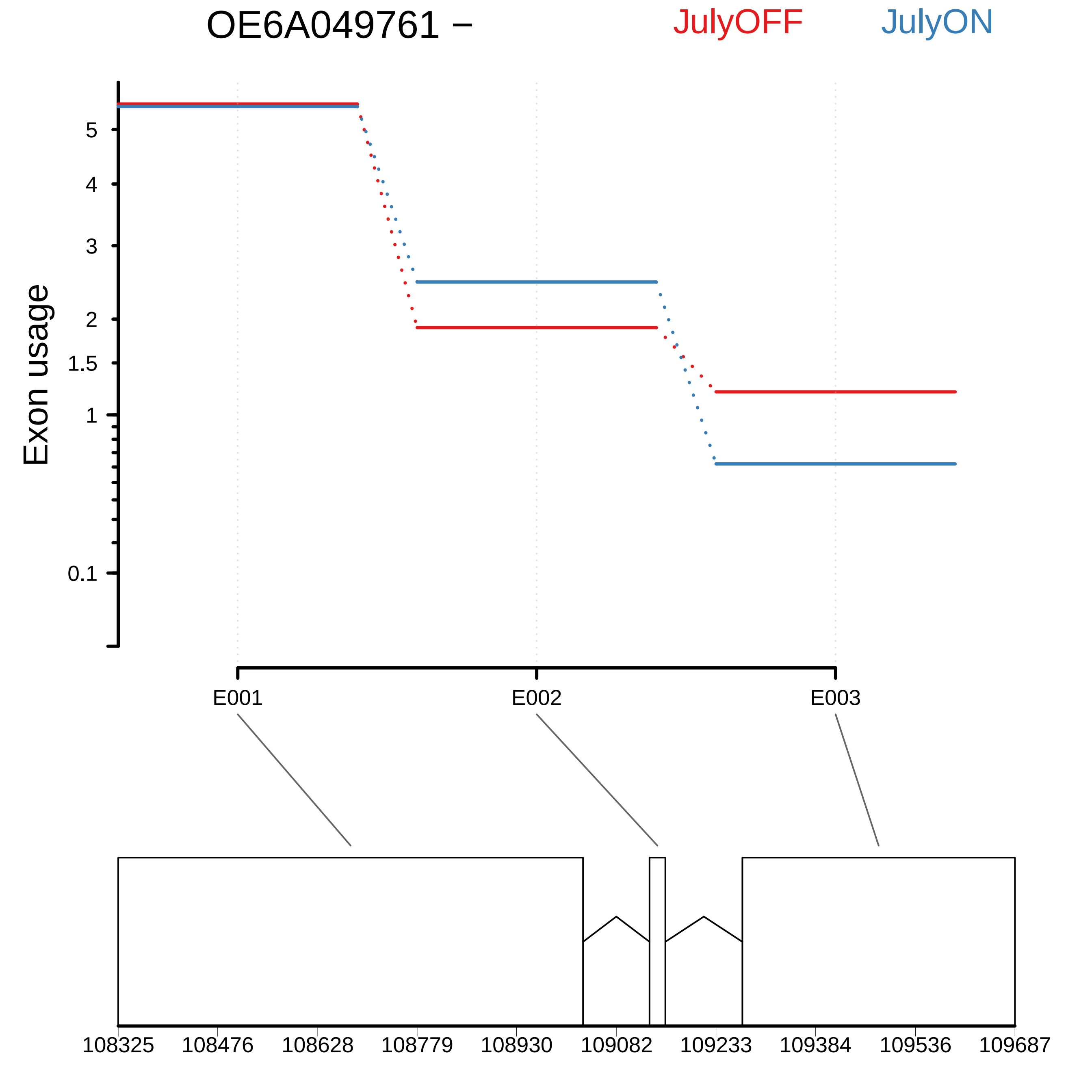

OE6A049761 −
JulyOFF
JulyON
Exon usage
5
4
3
2
1.5
1
0.1
E001
E002
E003
108325
108476
108628
108779
108930
109082
109233
109384
109536
109687

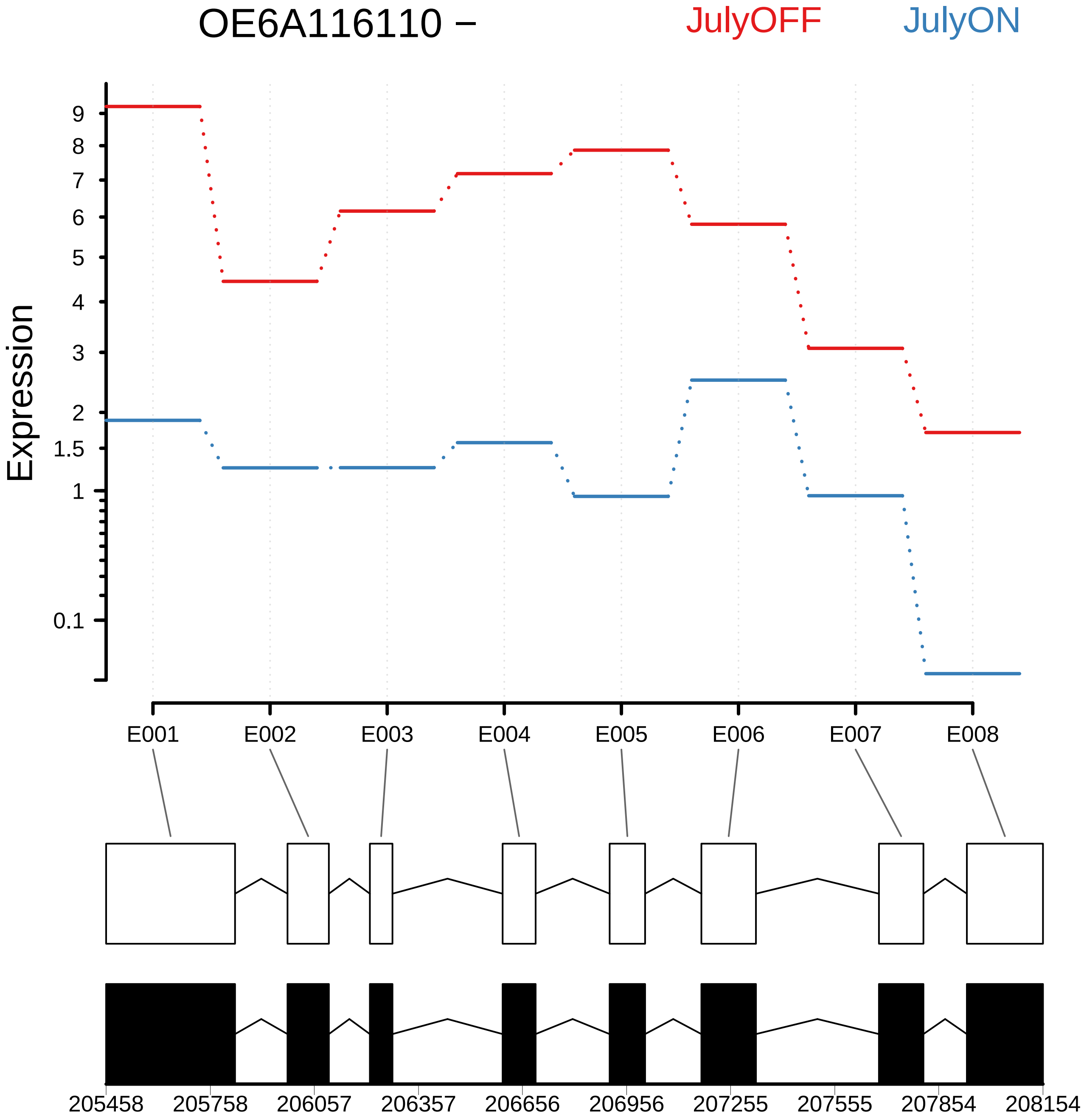
OE6A116110 −
JulyOFF
JulyON
Expression
9
8
7
6
5
4
3
2
1.5
1
0.1
E001
E002
E003
E004
E005
E006
E007
E008
205458
205758
206057
206357
206656
206956
207255
207555
207854
208154

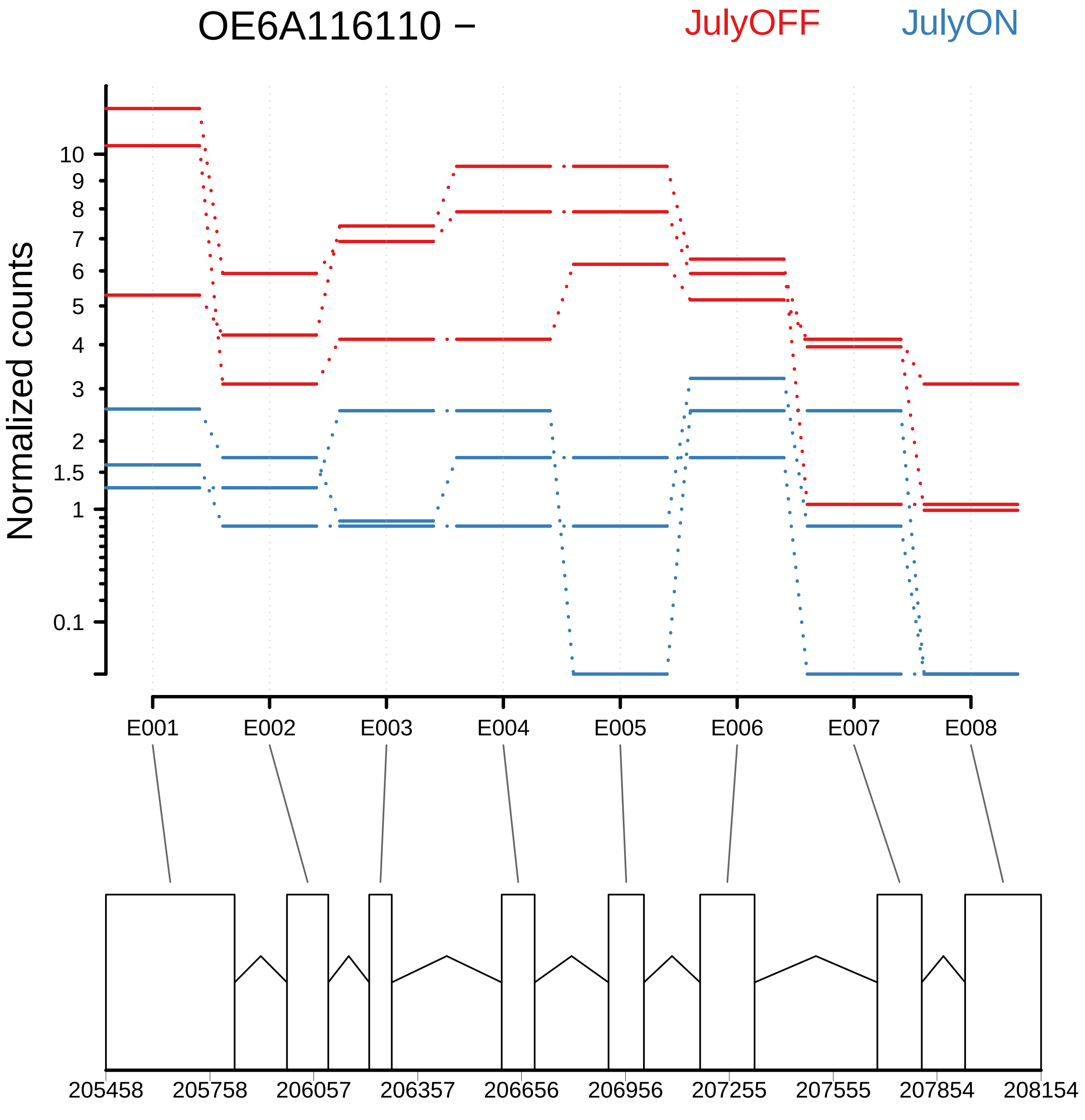

OE6A116110 −
JulyOFF
JulyON
Normalized counts
10
9
8
7
6
5
4
3
2
1.5
1
0.1
E001
E002
E003
E004
E005
E006
E007
E008
205458
205758
206057
206357
206656
206956
207255
207555
207854
208154

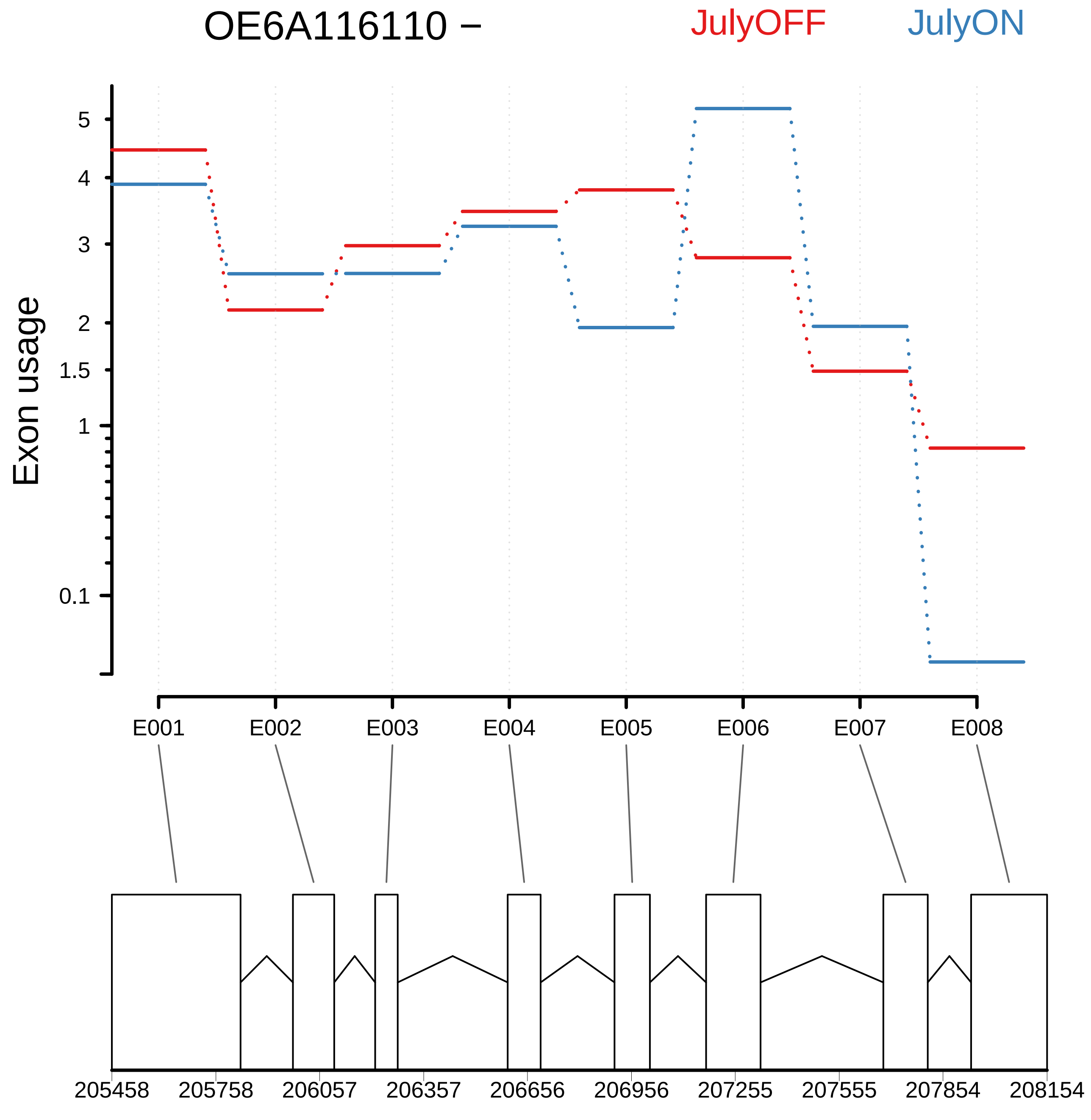

OE6A116110 −
JulyOFF
JulyON
Exon usage
5
4
3
2
1.5
1
0.1
E001
E002
E003
E004
E005
E006
E007
E008
205458
205758
206057
206357
206656
206956
207255
207555
207854
208154

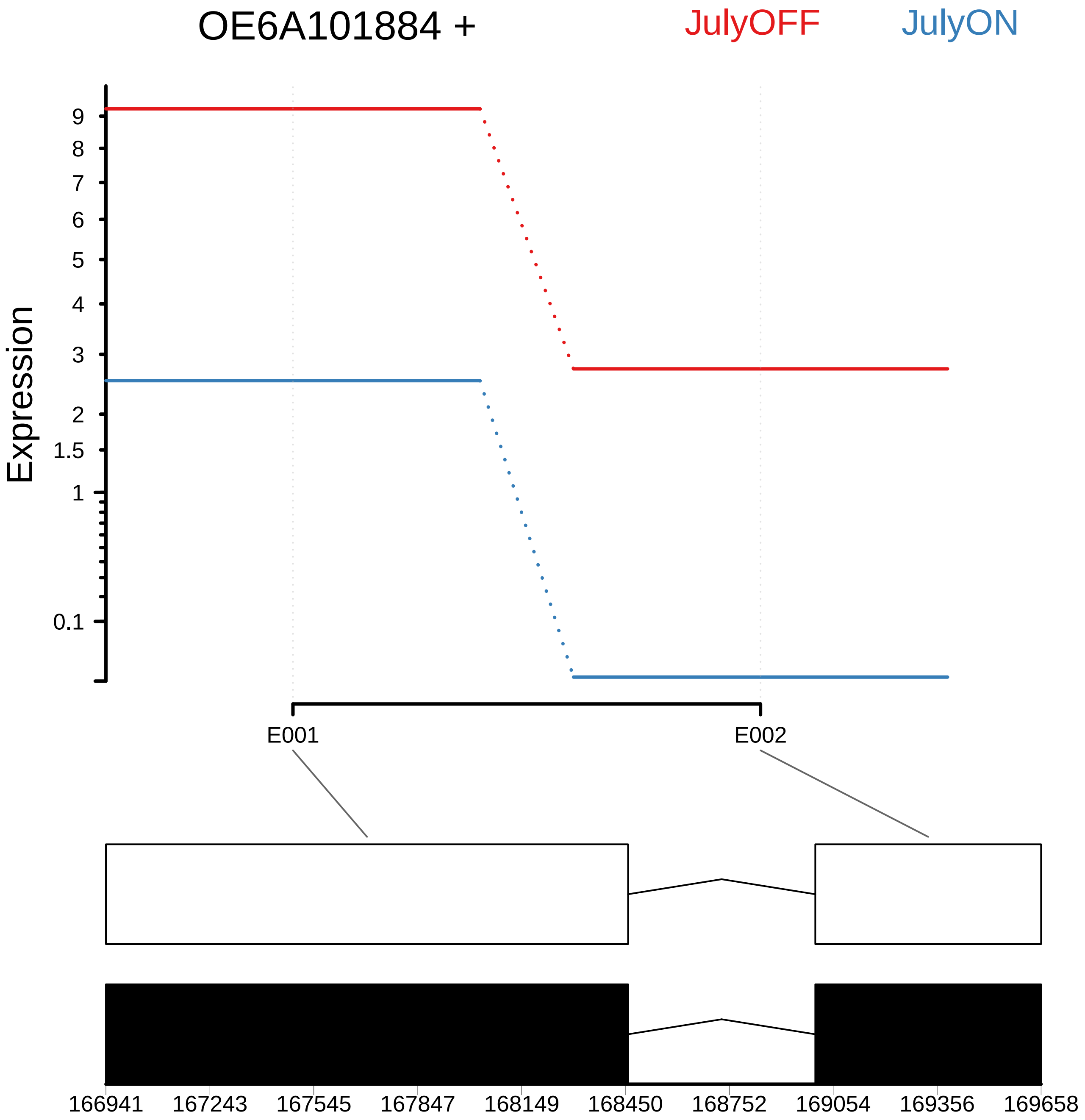

OE6A101884 +
JulyOFF
JulyON
Expression
9
8
7
6
5
4
3
2
1.5
1
0.1
E001
E002
166941
167243
167545
167847
168149
168450
168752
169054
169356
169658

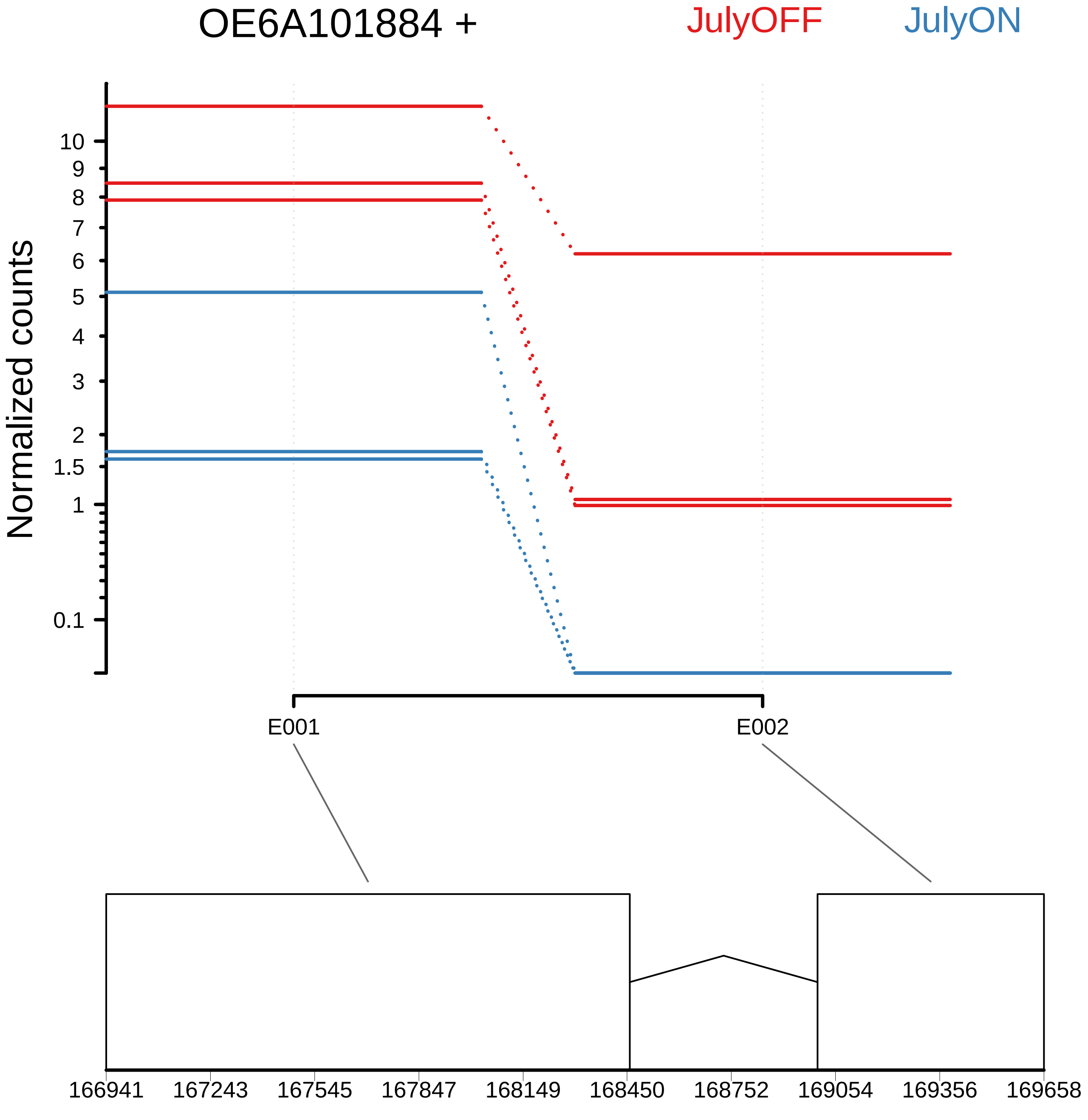
OE6A101884 +
JulyOFF
JulyON
Normalized counts
10
9
8
7
6
5
4
3
2
1.5
1
0.1
E001
E002
166941
167243
167545
167847
168149
168450
168752
169054
169356
169658

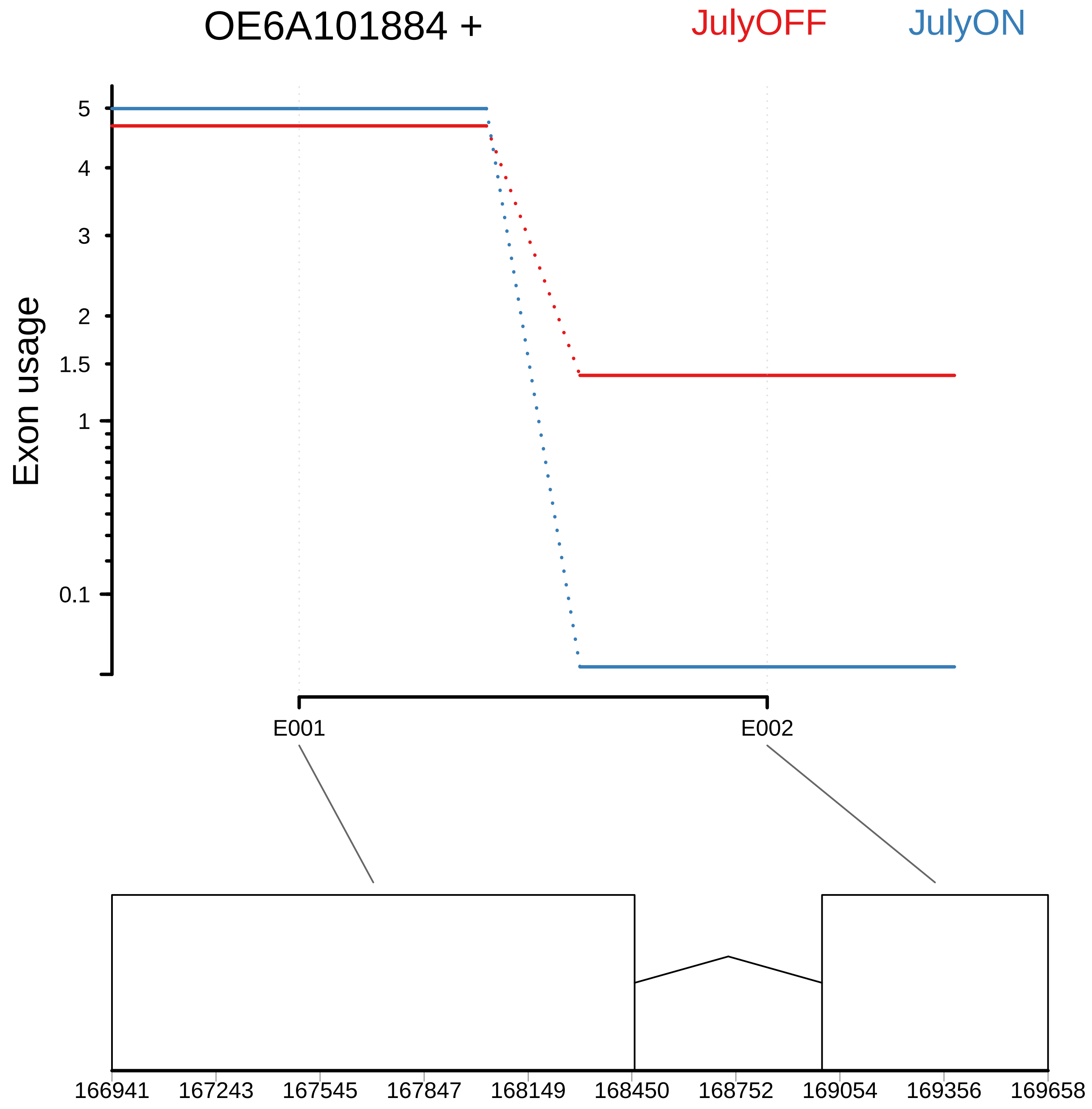

OE6A101884 +
JulyOFF
JulyON
Exon usage
5
4
3
2
1.5
1
0.1
E001
E002
166941
167243
167545
167847
168149
168450
168752
169054
169356
169658

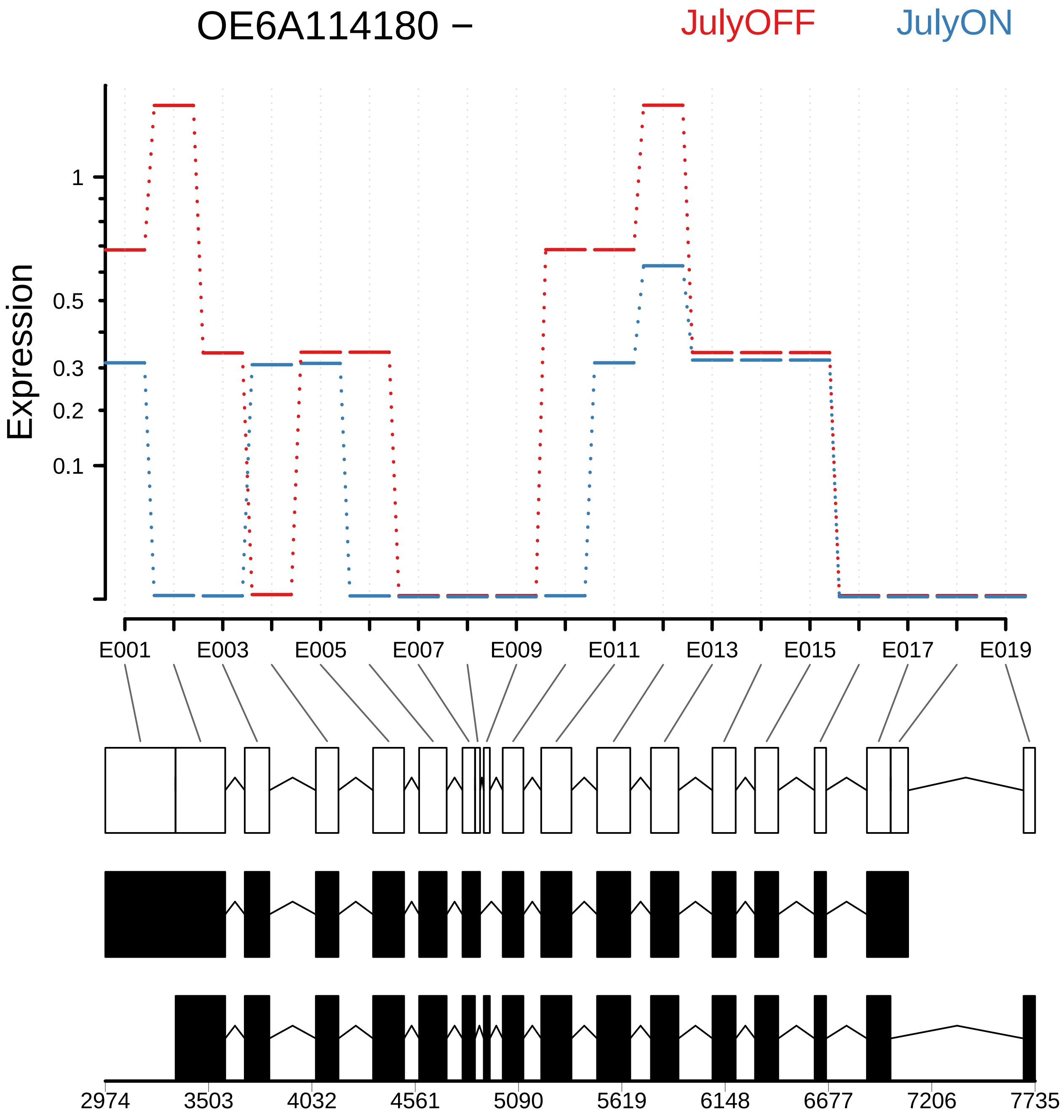

OE6A114180 −
JulyOFF
JulyON
Expression
1
0.5
0.3
0.2
0.1
E001
E003
E005
E007
E009
E011
E013
E015
E017
E019
2974
3503
4032
4561
5090
5619
6148
6677
7206
7735

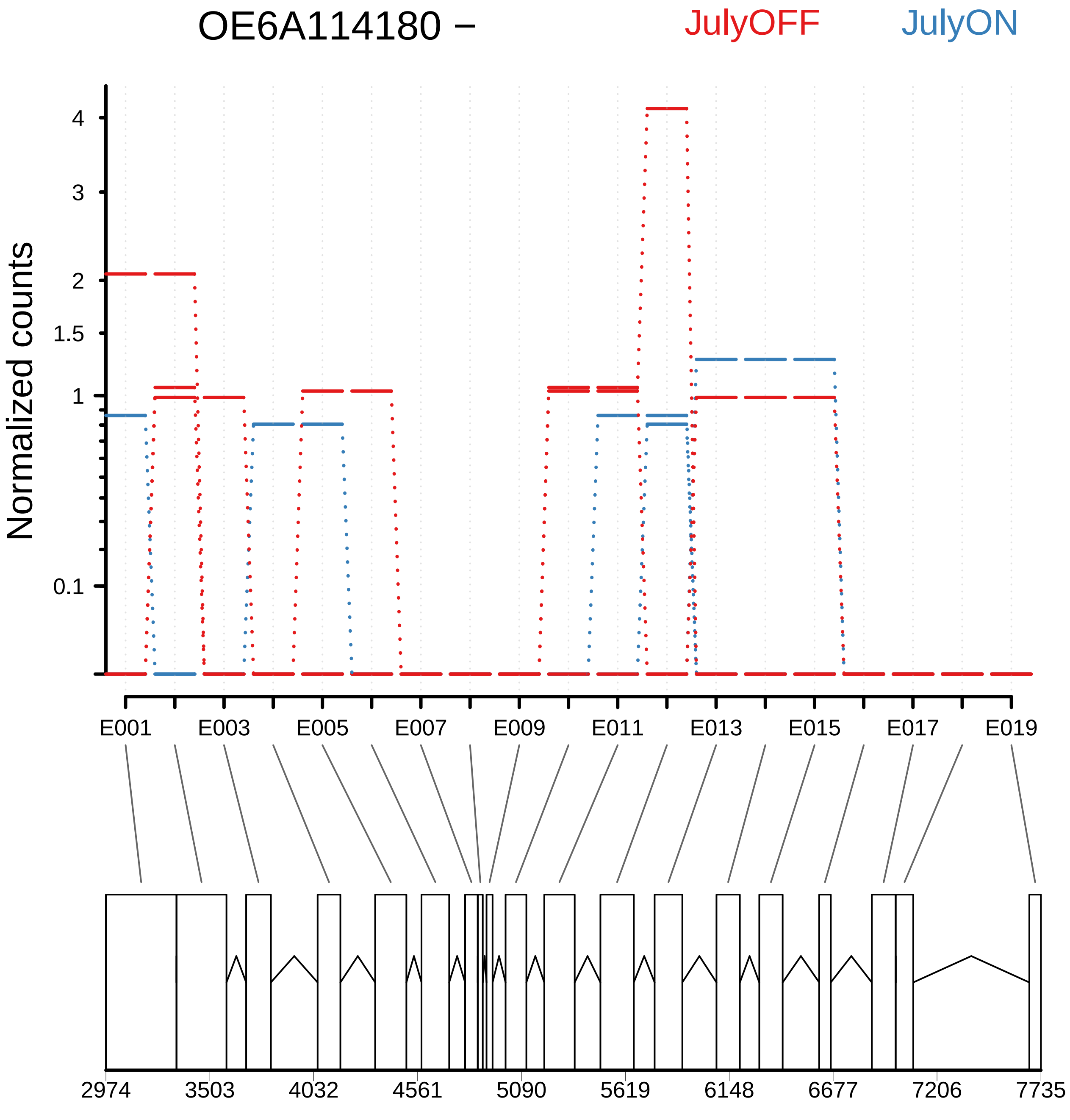

OE6A114180 −
JulyOFF
JulyON
Normalized counts
4
3
2
1.5
1
0.1
E001
E003
E005
E007
E009
E011
E013
E015
E017
E019
2974
3503
4032
4561
5090
5619
6148
6677
7206
7735

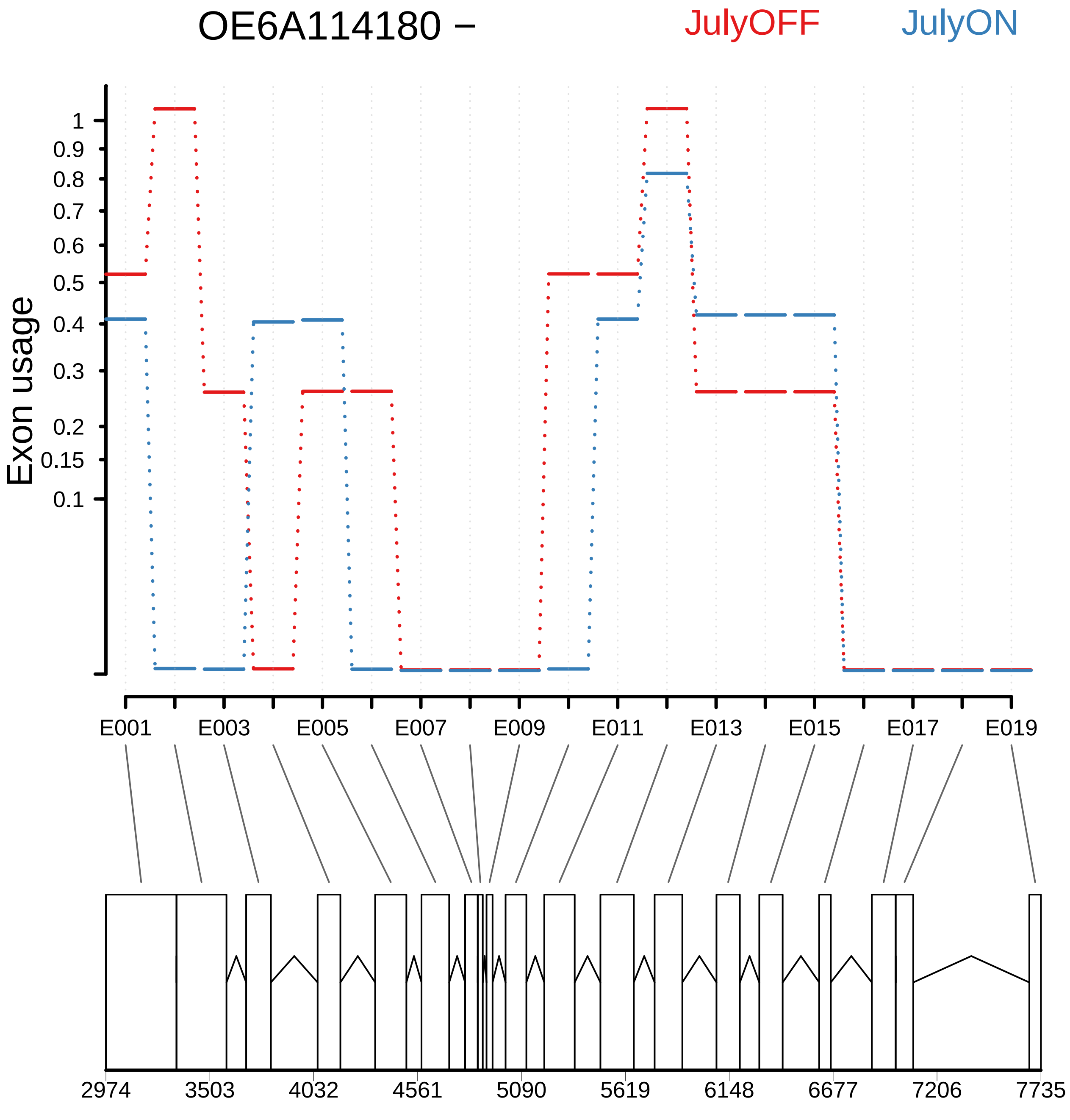

OE6A114180 −
JulyOFF
JulyON
Exon usage
1
0.9
0.8
0.7
0.6
0.5
0.4
0.3
0.2
0.15
0.1
E001
E003
E005
E007
E009
E011
E013
E015
E017
E019
2974
3503
4032
4561
5090
5619
6148
6677
7206
7735

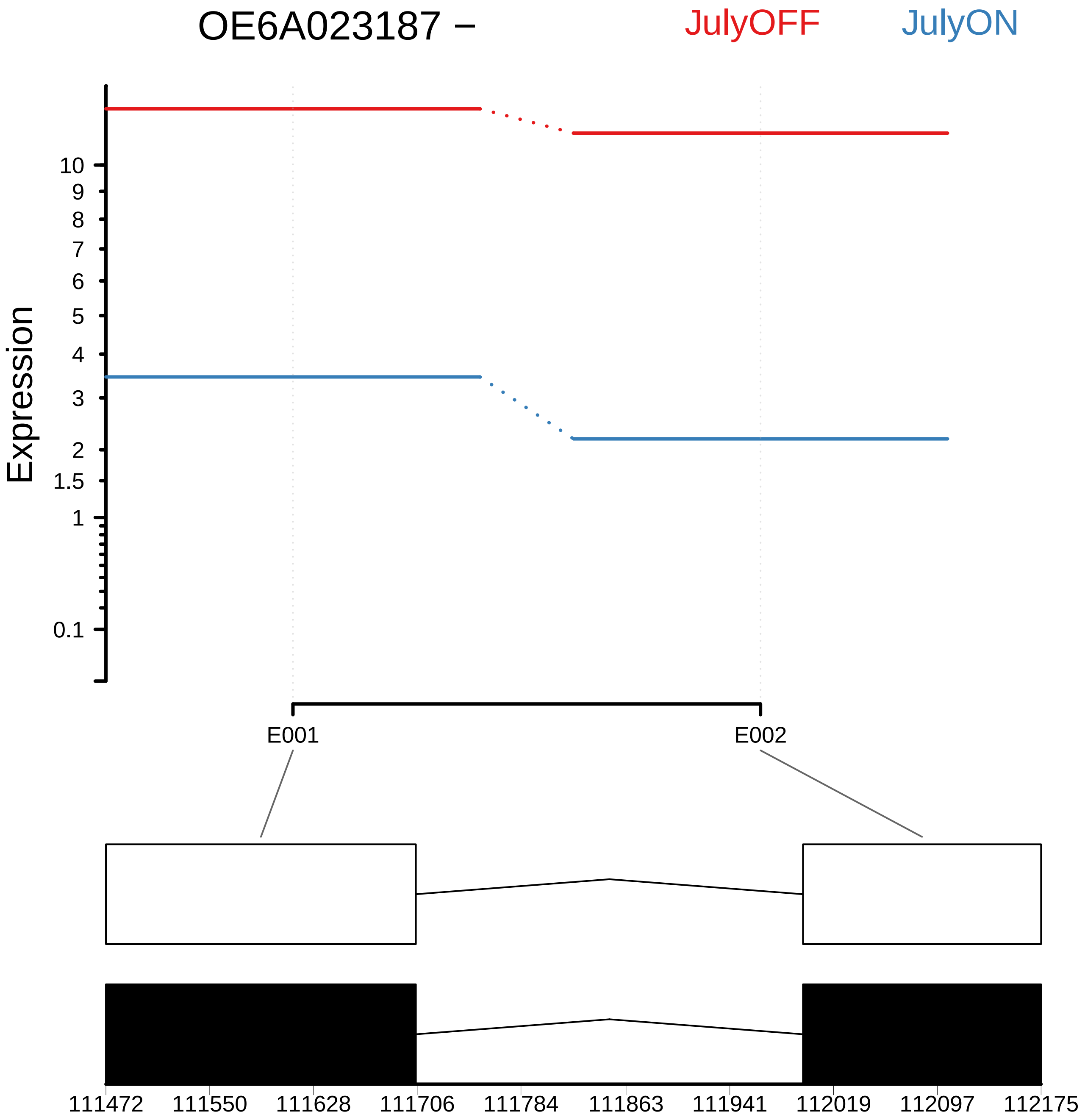

OE6A023187 −
JulyOFF
JulyON
Expression
10
9
8
7
6
5
4
3
2
1.5
1
0.1
E001
E002
111472
111550
111628
111706
111784
111863
111941
112019
112097
112175

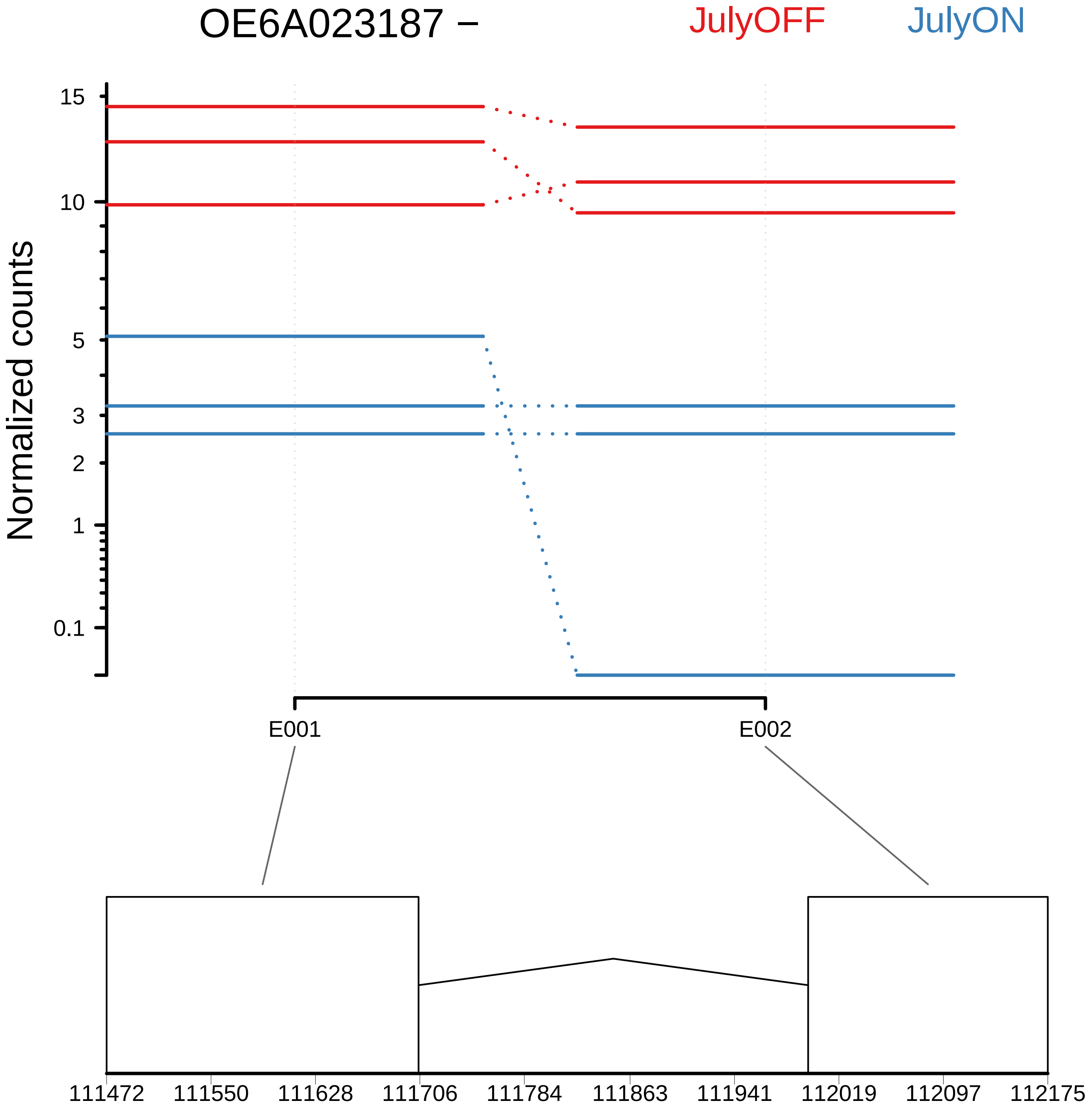
OE6A023187 −
JulyOFF
JulyON
Normalized counts
15
10
5
3
2
1
0.1
E001
E002
111472
111550
111628
111706
111784
111863
111941
112019
112097
112175

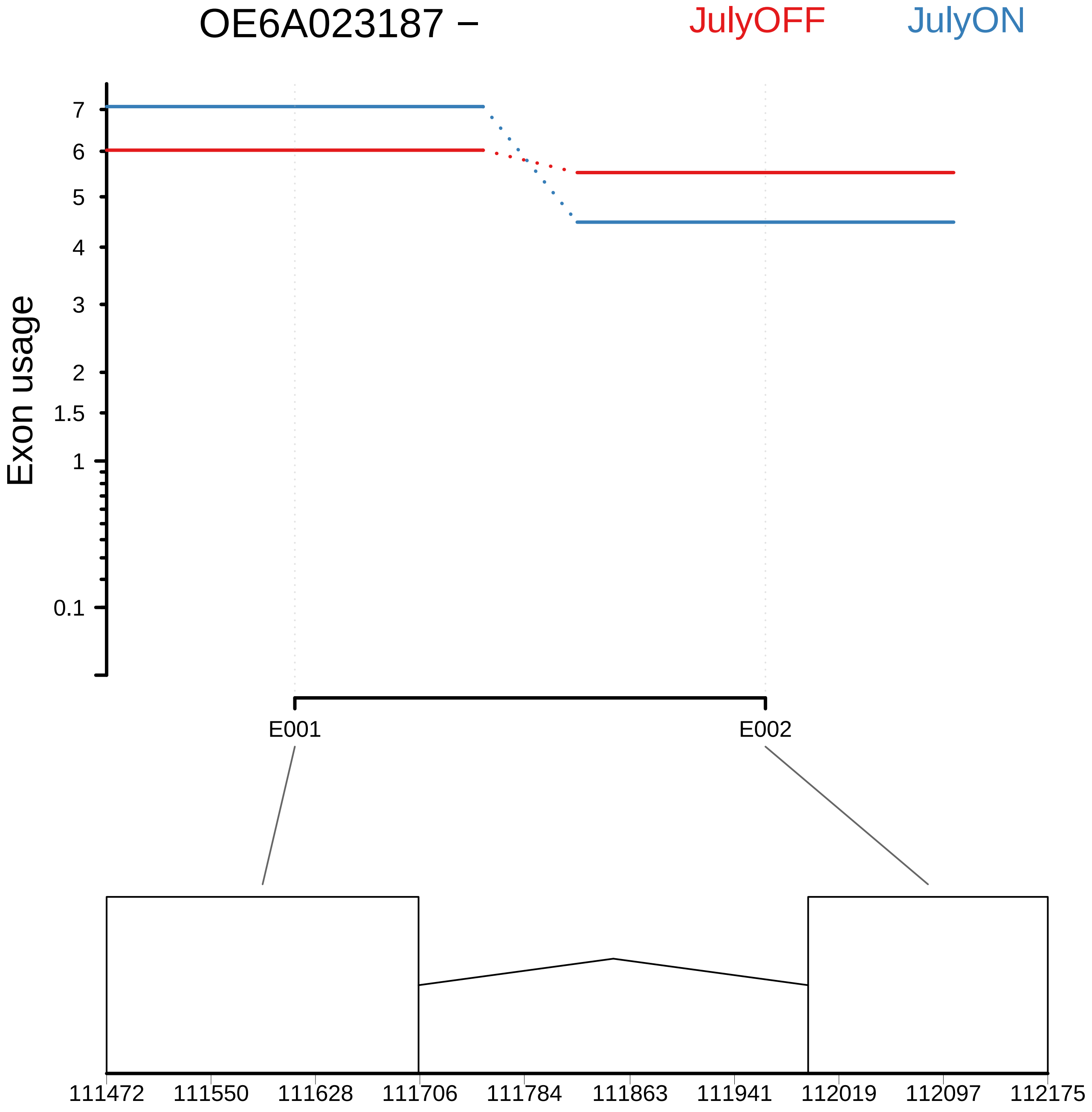
OE6A023187 −
JulyOFF
JulyON
Exon usage
7
6
5
4
3
2
1.5
1
0.1
E001
E002
111472
111550
111628
111706
111784
111863
111941
112019
112097
112175

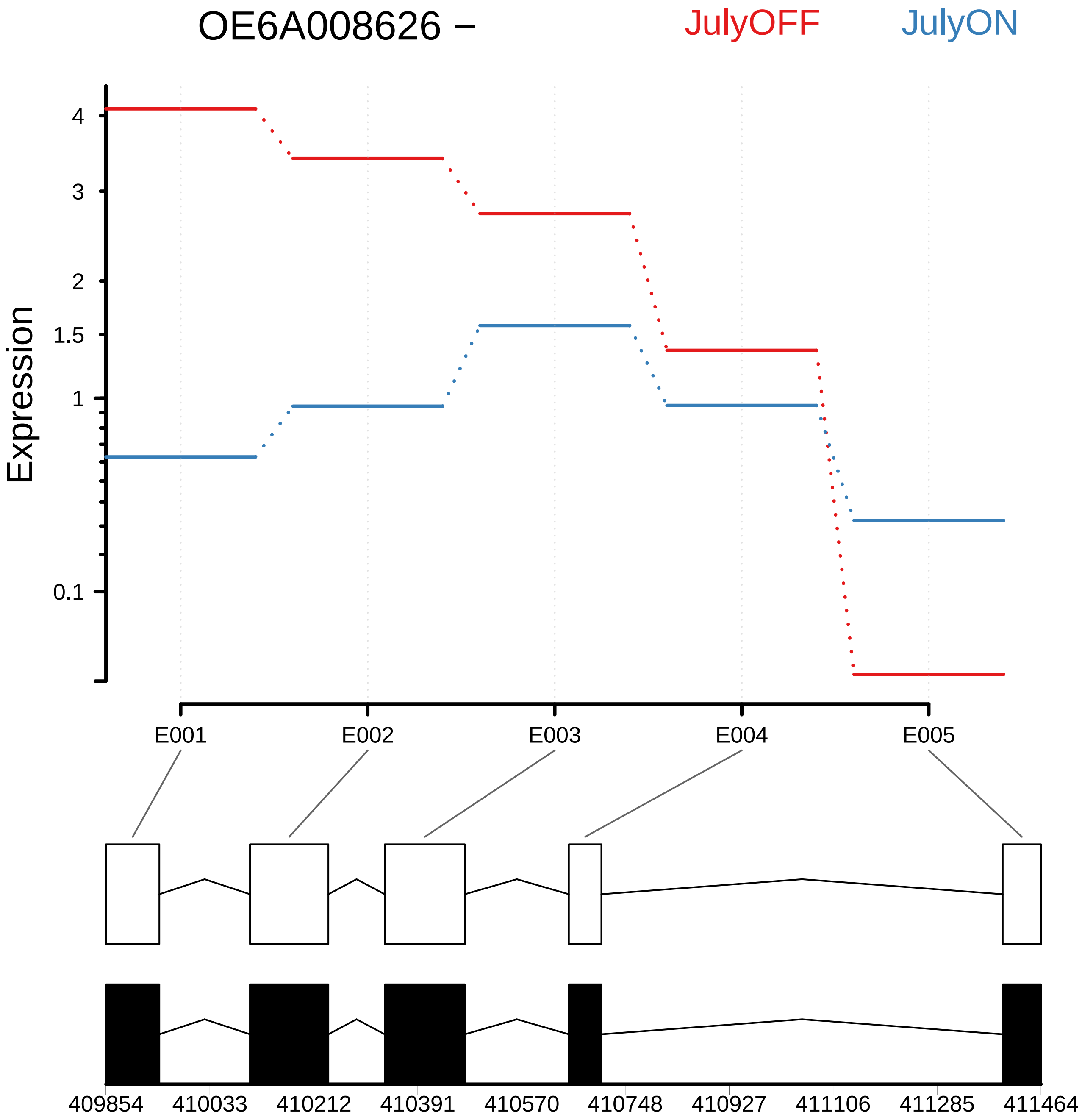
OE6A008626 −
JulyOFF
JulyON
Expression
4
3
2
1.5
1
0.1
E001
E002
E003
E004
E005
409854
410033
410212
410391
410570
410748
410927
411106
411285
411464

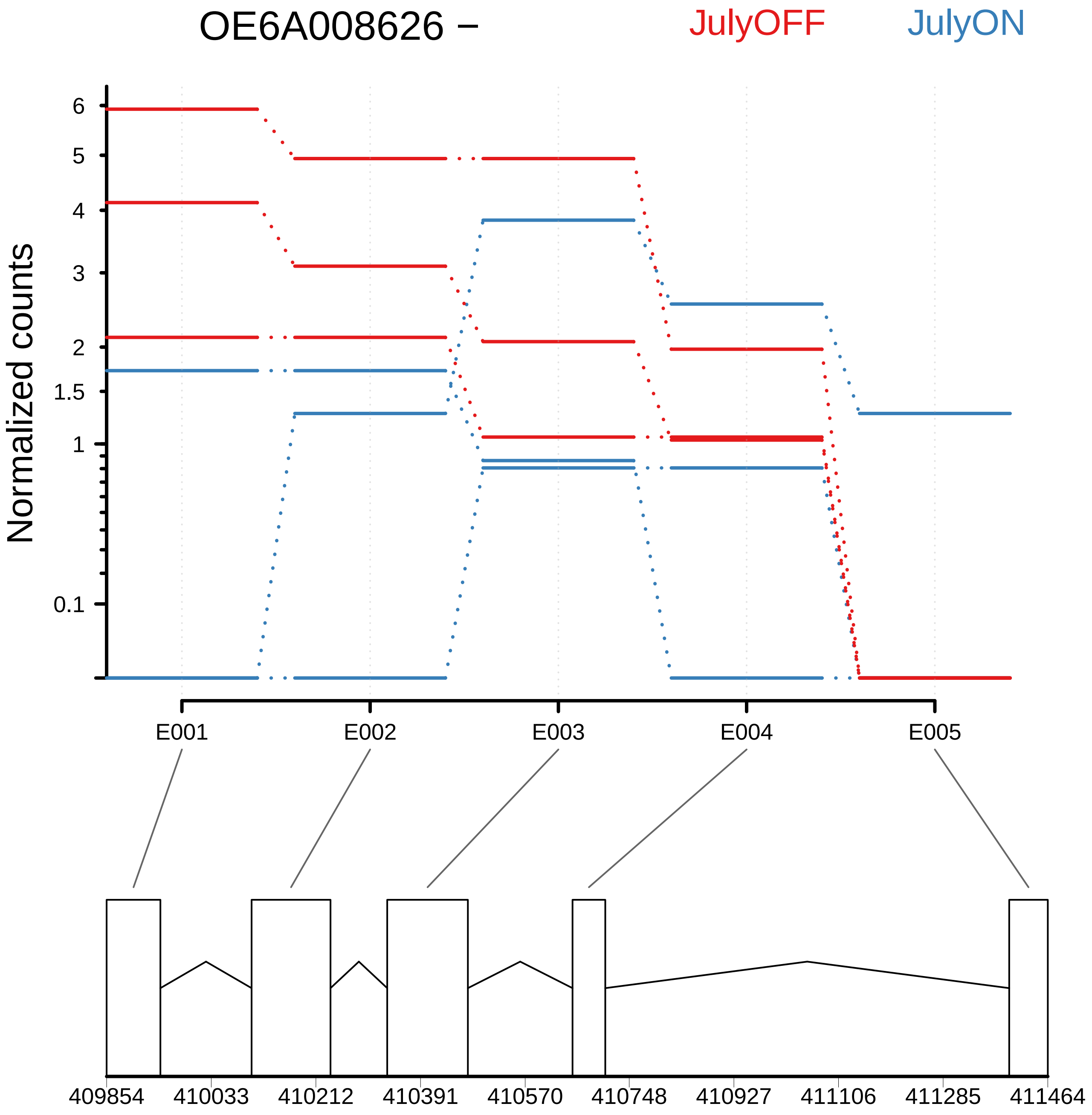

OE6A008626 −
JulyOFF
JulyON
Normalized counts
6
5
4
3
2
1.5
1
0.1
E001
E002
E003
E004
E005
409854
410033
410212
410391
410570
410748
410927
411106
411285
411464

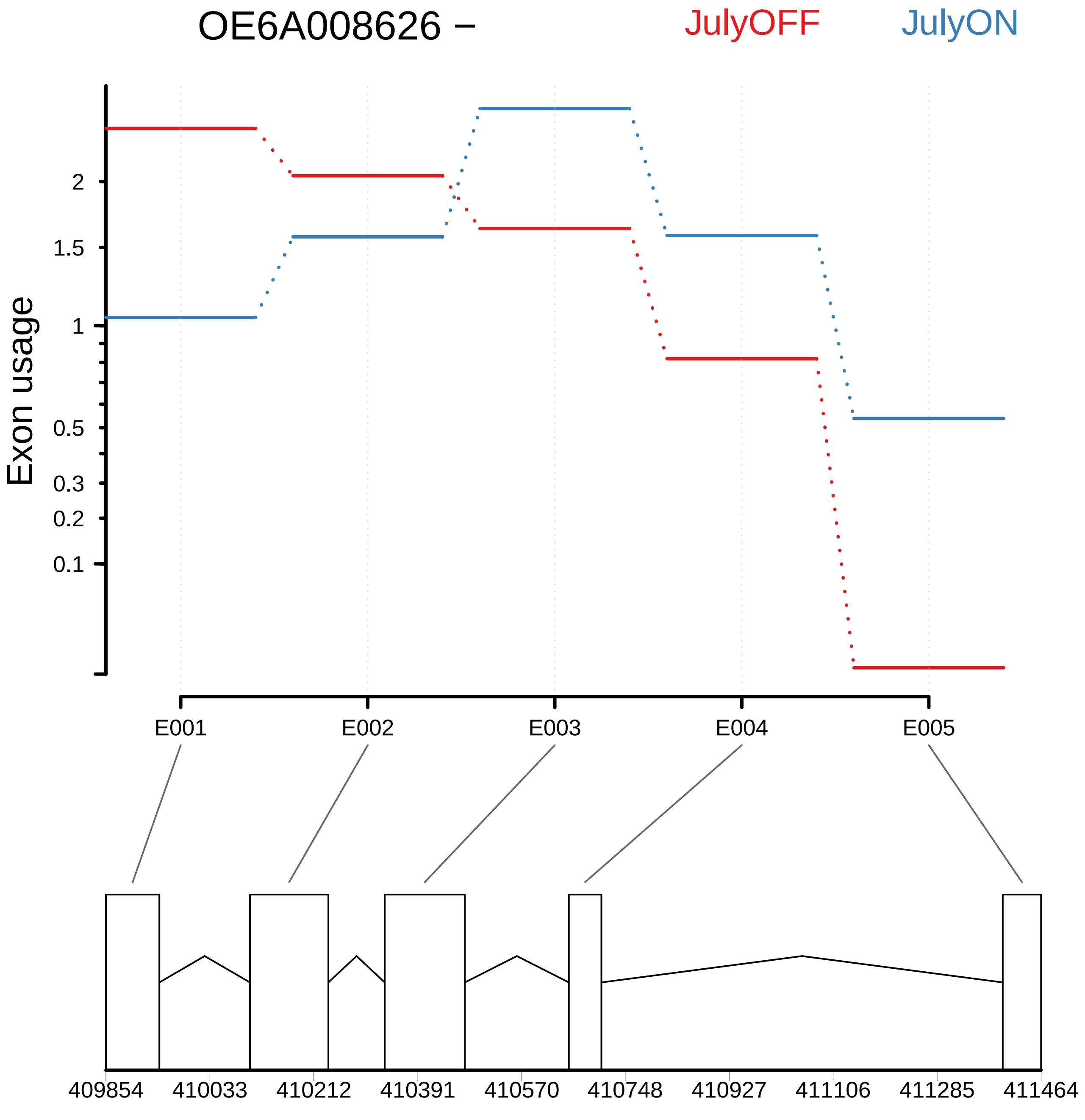

OE6A008626 −
JulyOFF
JulyON
Exon usage
2
1.5
1
0.5
0.3
0.2
0.1
E001
E002
E003
E004
E005
409854
410033
410212
410391
410570
410748
410927
411106
411285
411464

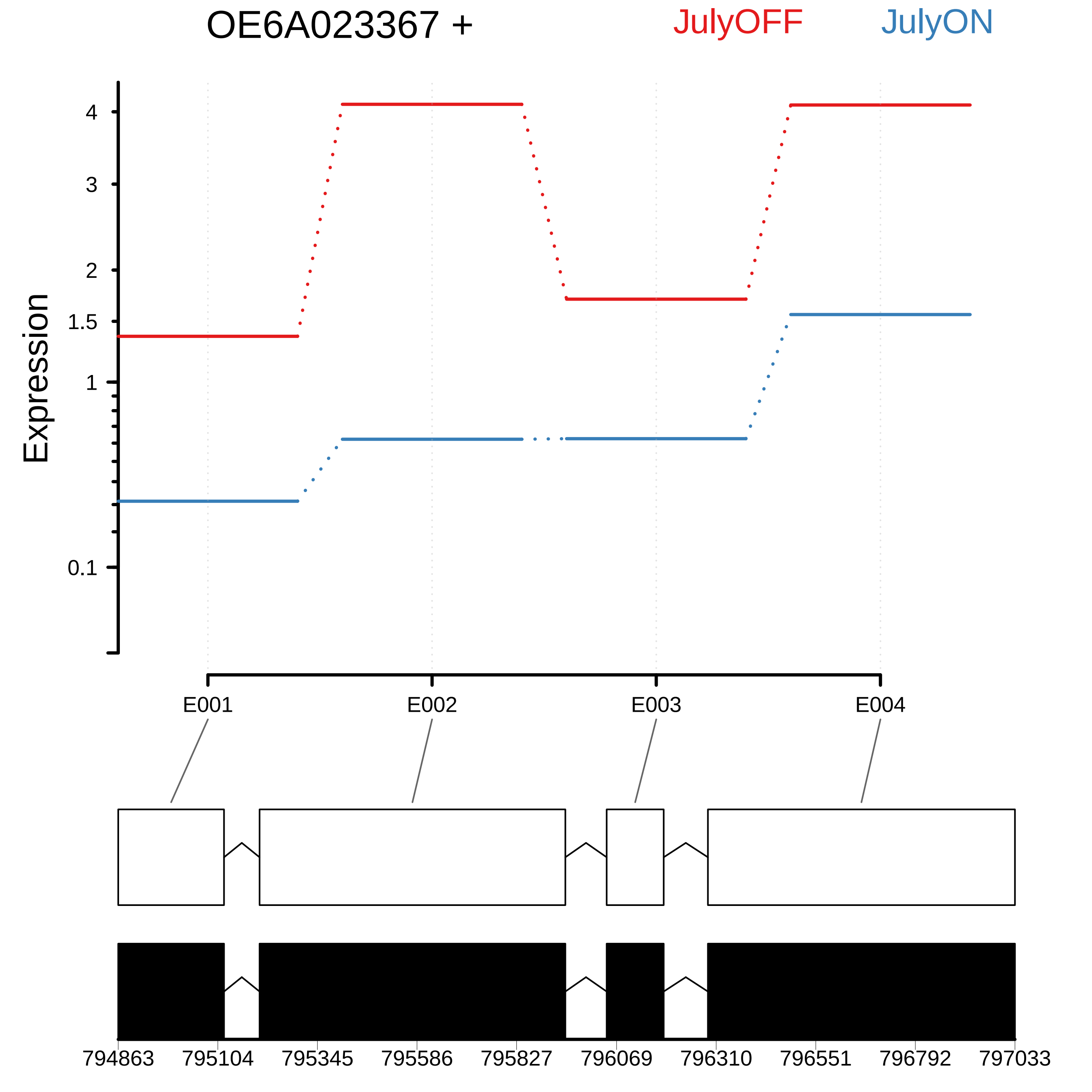
OE6A023367 +
JulyOFF
JulyON
Expression
4
3
2
1.5
1
0.1
E001
E002
E003
E004
794863
795104
795345
795586
795827
796069
796310
796551
796792
797033

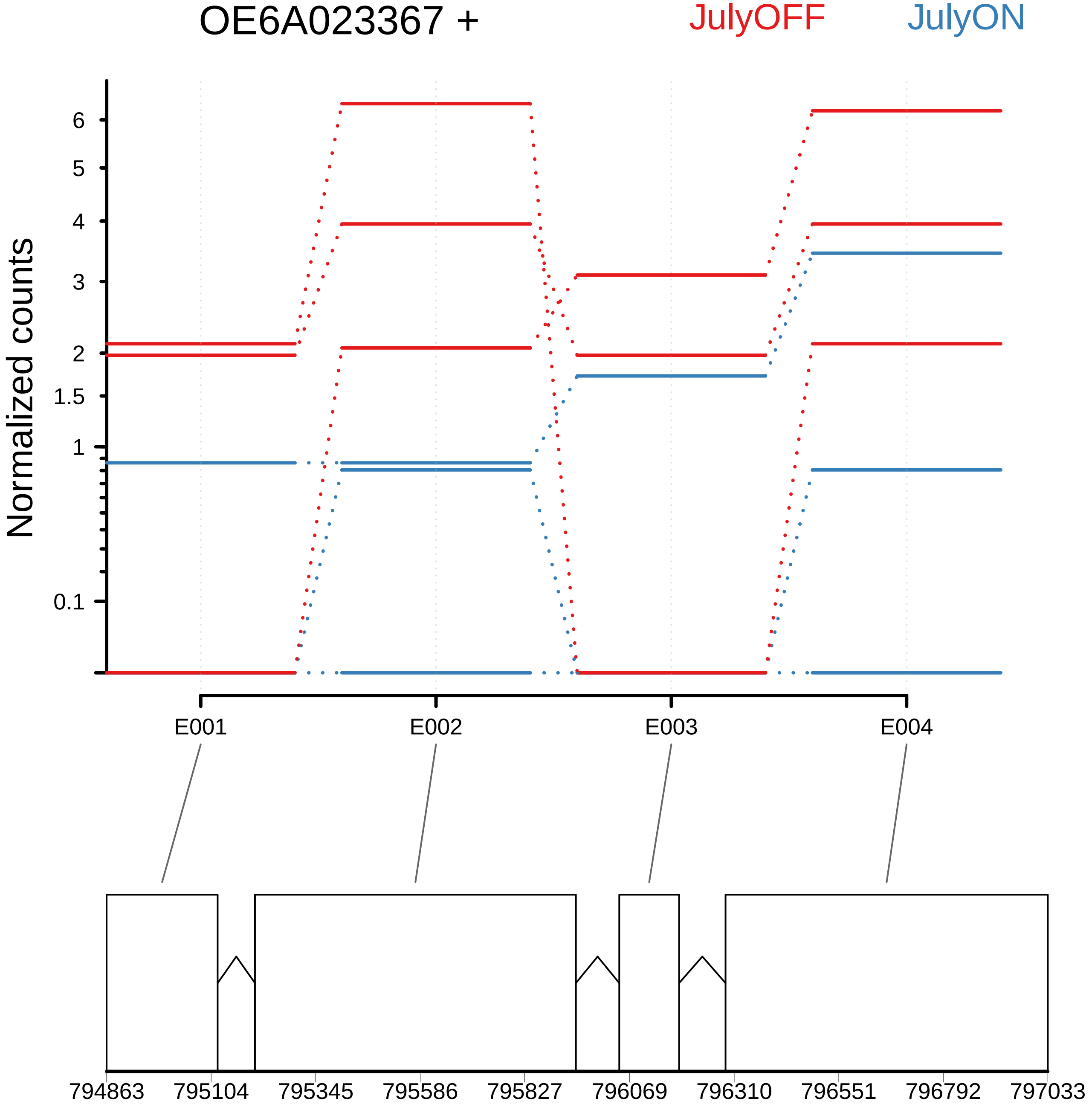

OE6A023367 +
JulyOFF
JulyON
Normalized counts
6
5
4
3
2
1.5
1
0.1
E001
E002
E003
E004
794863
795104
795345
795586
795827
796069
796310
796551
796792
797033

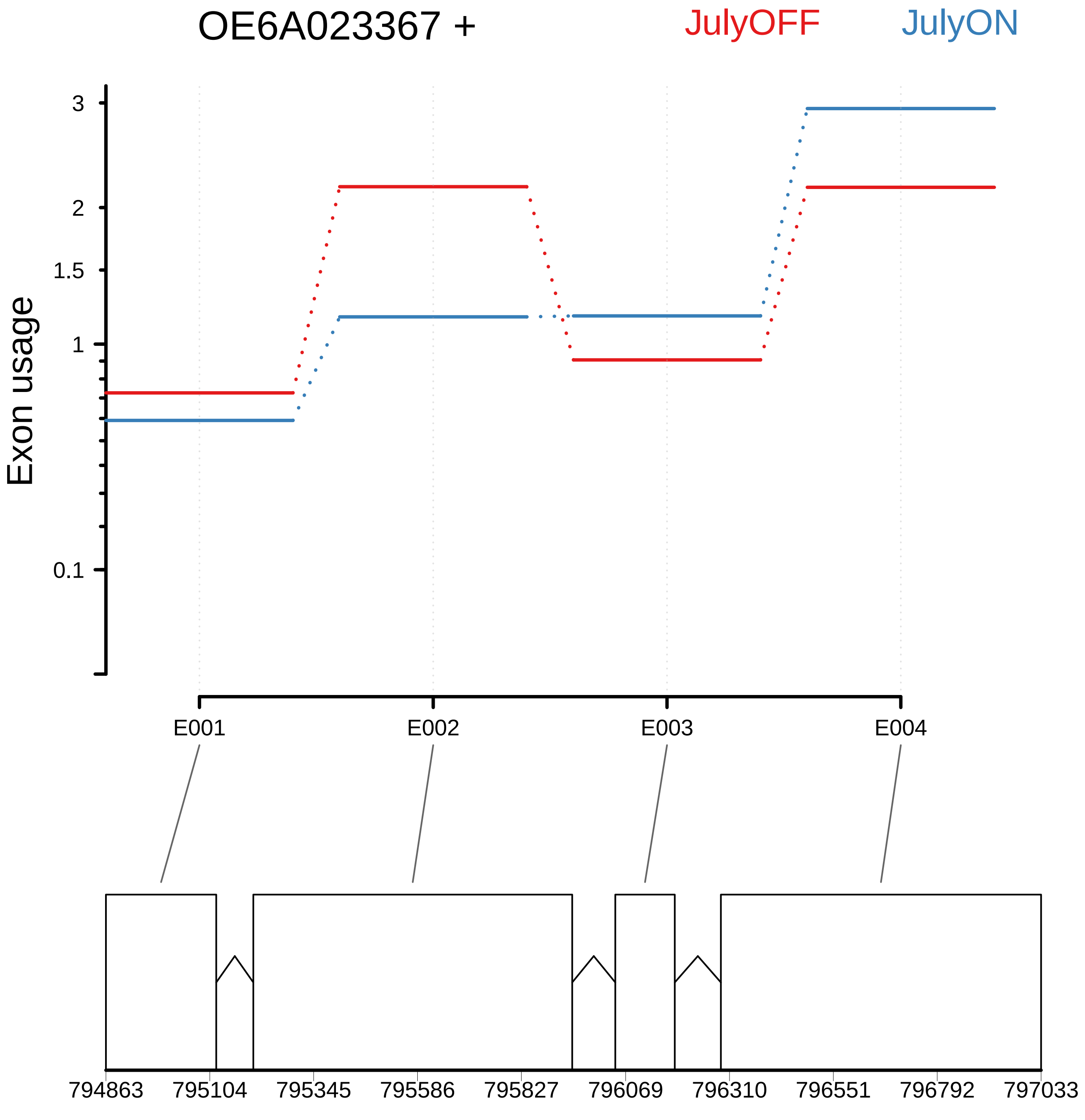
OE6A023367 +
JulyOFF
JulyON
Exon usage
3
2
1.5
1
0.1
E001
E002
E003
E004
794863
795104
795345
795586
795827
796069
796310
796551
796792
797033

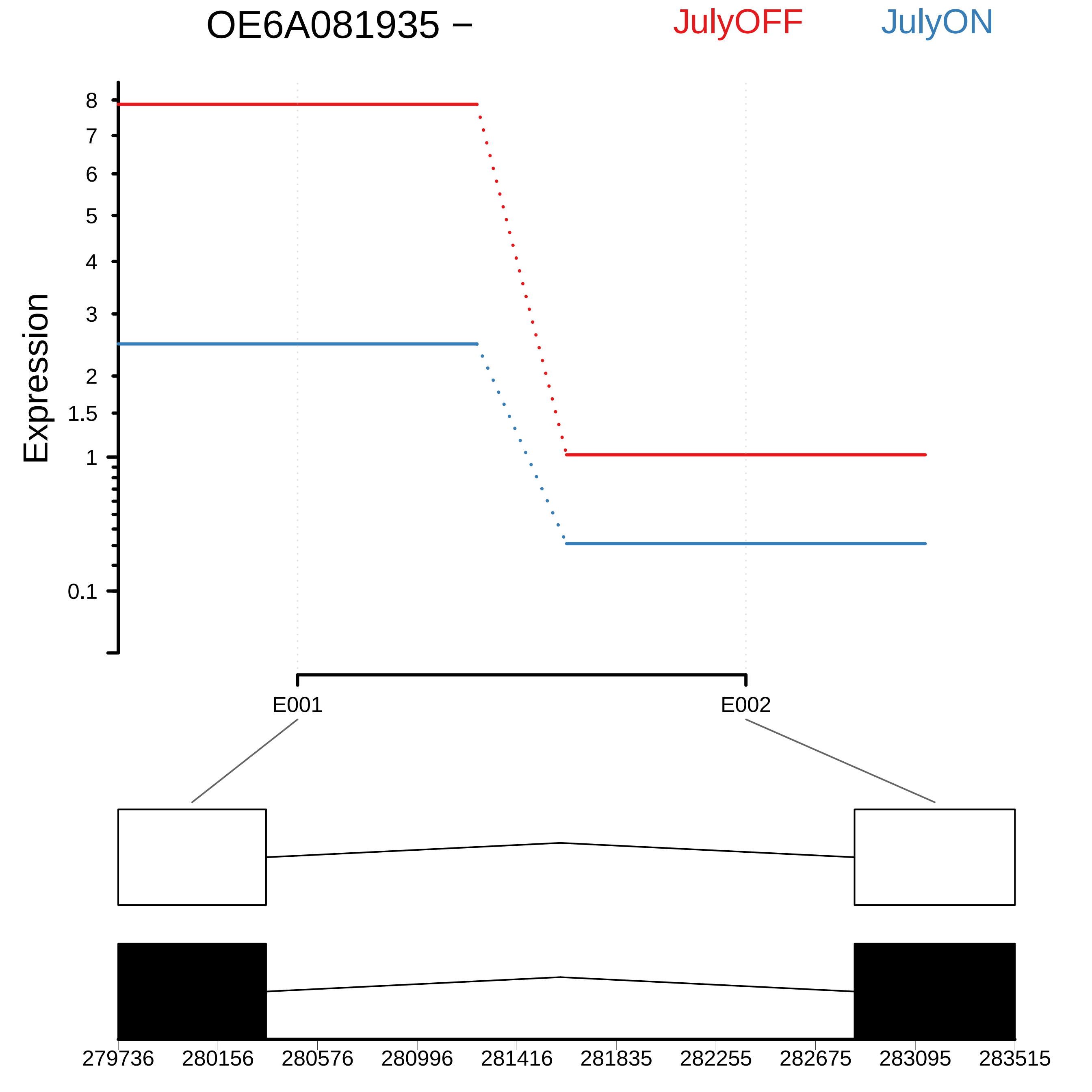

OE6A081935 −
JulyOFF
JulyON
Expression
8
7
6
5
4
3
2
1.5
1
0.1
E001
E002
279736
280156
280576
280996
281416
281835
282255
282675
283095
283515

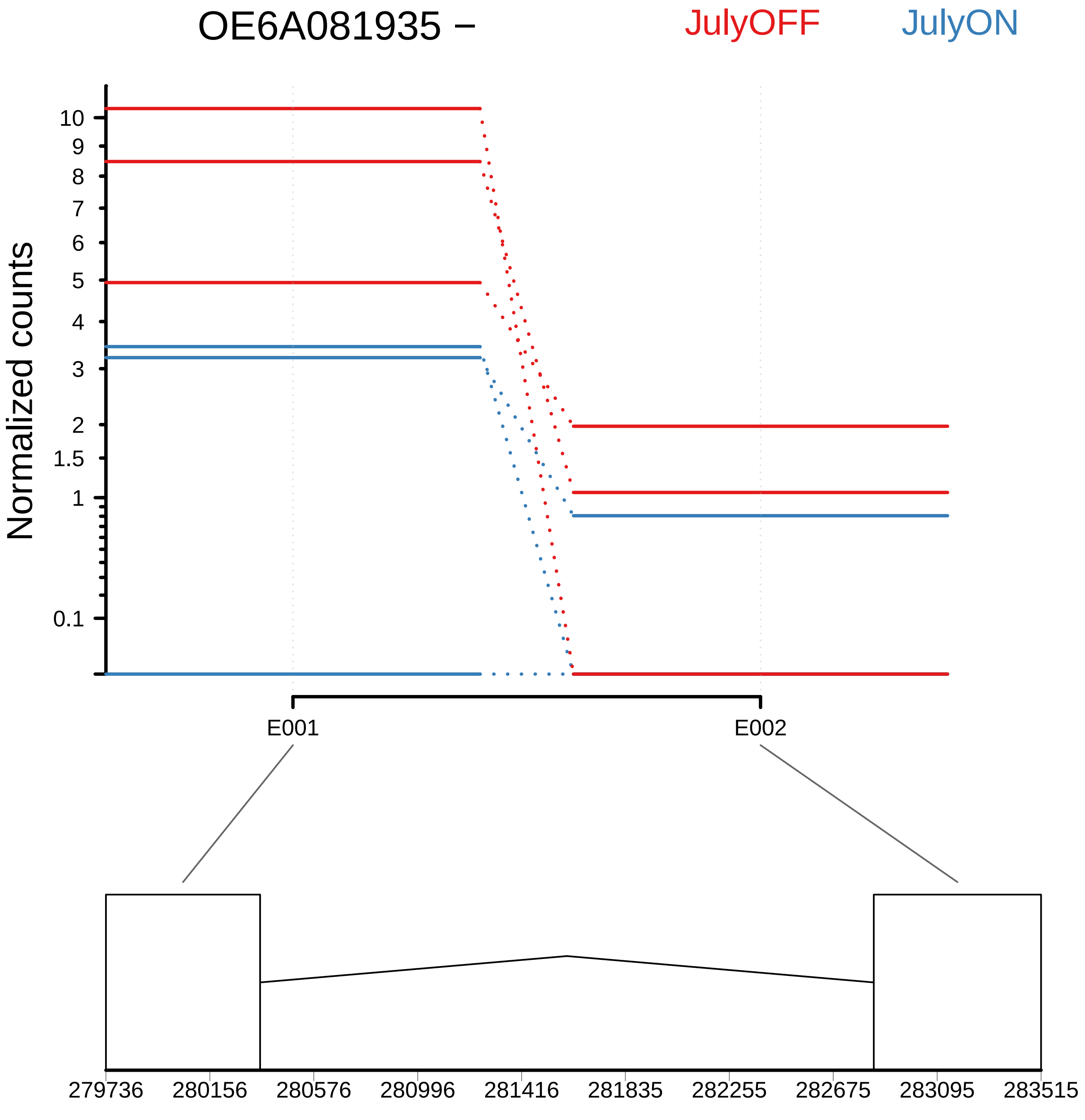

OE6A081935 −
JulyOFF
JulyON
Normalized counts
10
9
8
7
6
5
4
3
2
1.5
1
0.1
E001
E002
279736
280156
280576
280996
281416
281835
282255
282675
283095
283515

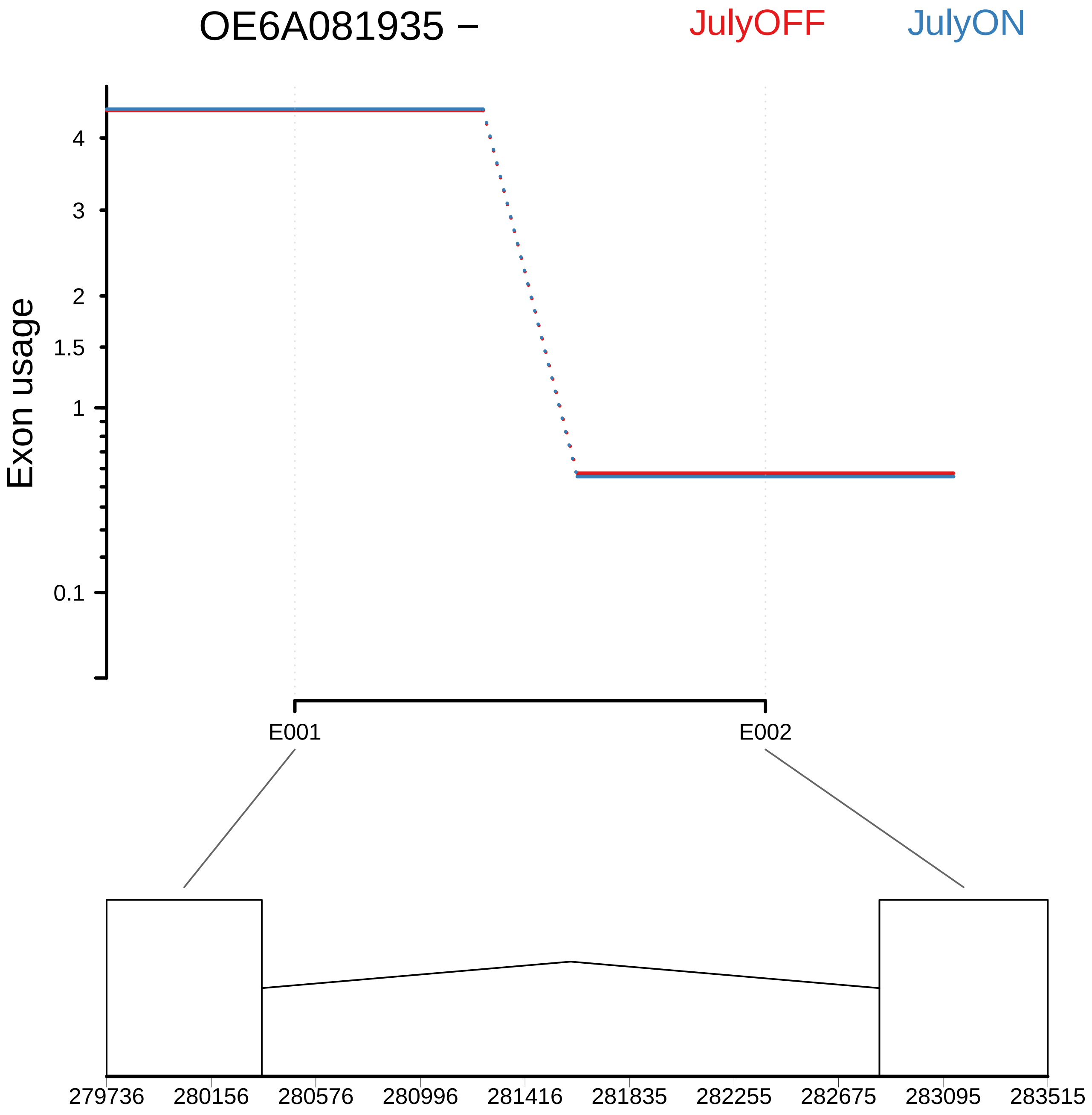
OE6A081935 −
JulyOFF
JulyON
Exon usage
4
3
2
1.5
1
0.1
E001
E002
279736
280156
280576
280996
281416
281835
282255
282675
283095
283515

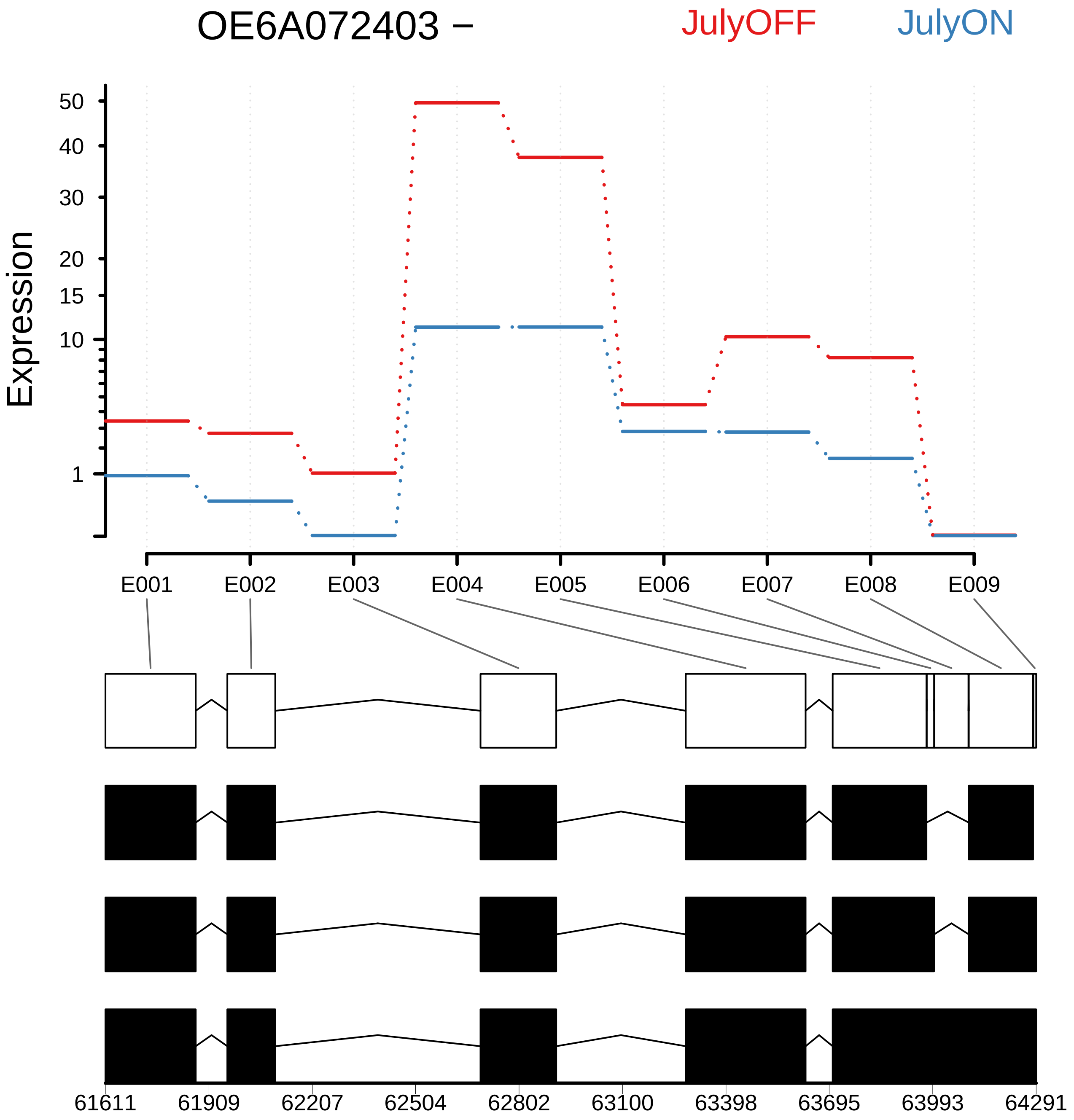
OE6A072403 −
JulyOFF
JulyON
Expression
50
40
30
20
15
10
1
E001
E002
E003
E004
E005
E006
E007
E008
E009
61611
61909
62207
62504
62802
63100
63398
63695
63993
64291

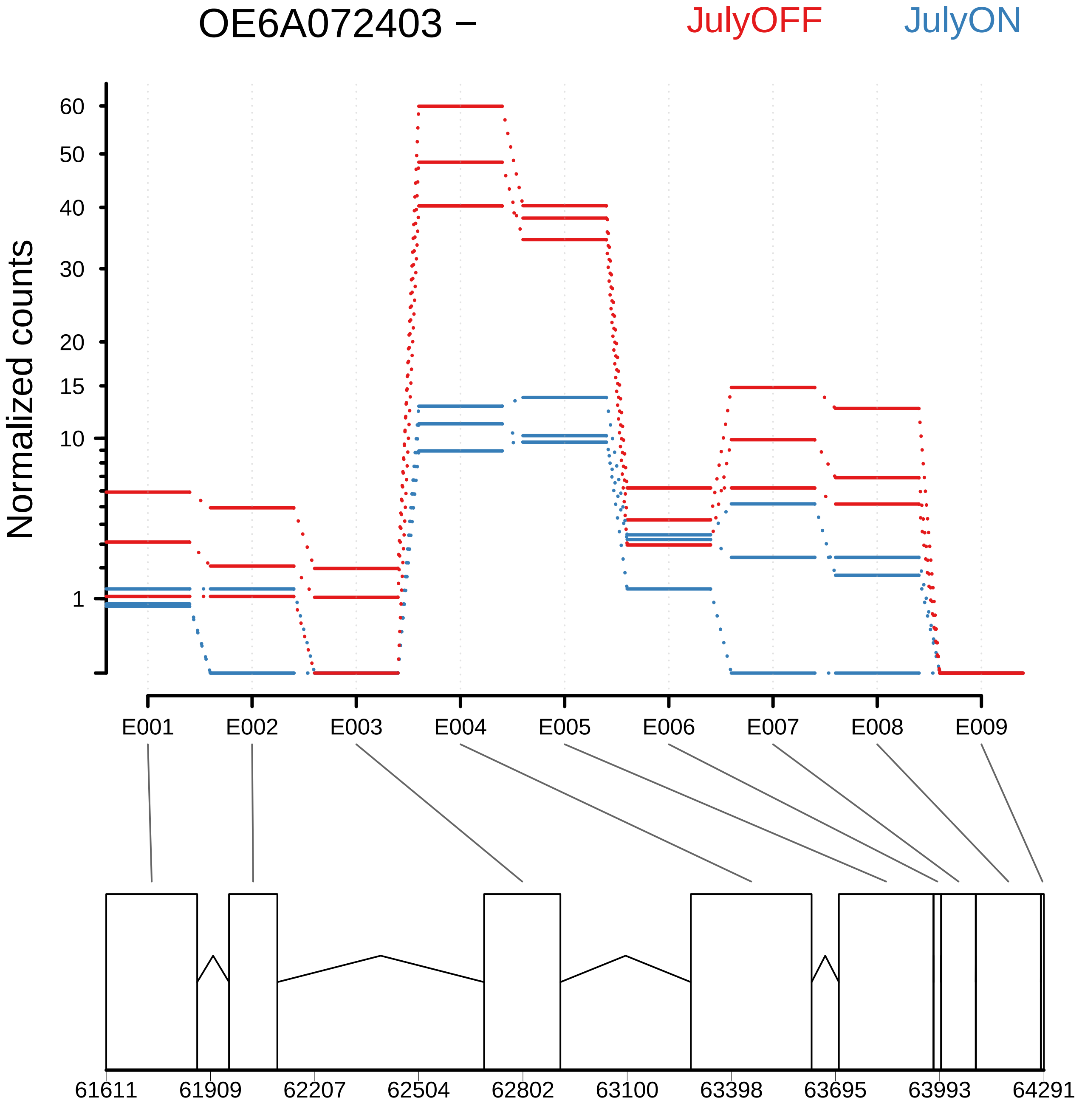

OE6A072403 −
JulyOFF
JulyON
Normalized counts
60
50
40
30
20
15
10
1
E001
E002
E003
E004
E005
E006
E007
E008
E009
61611
61909
62207
62504
62802
63100
63398
63695
63993
64291

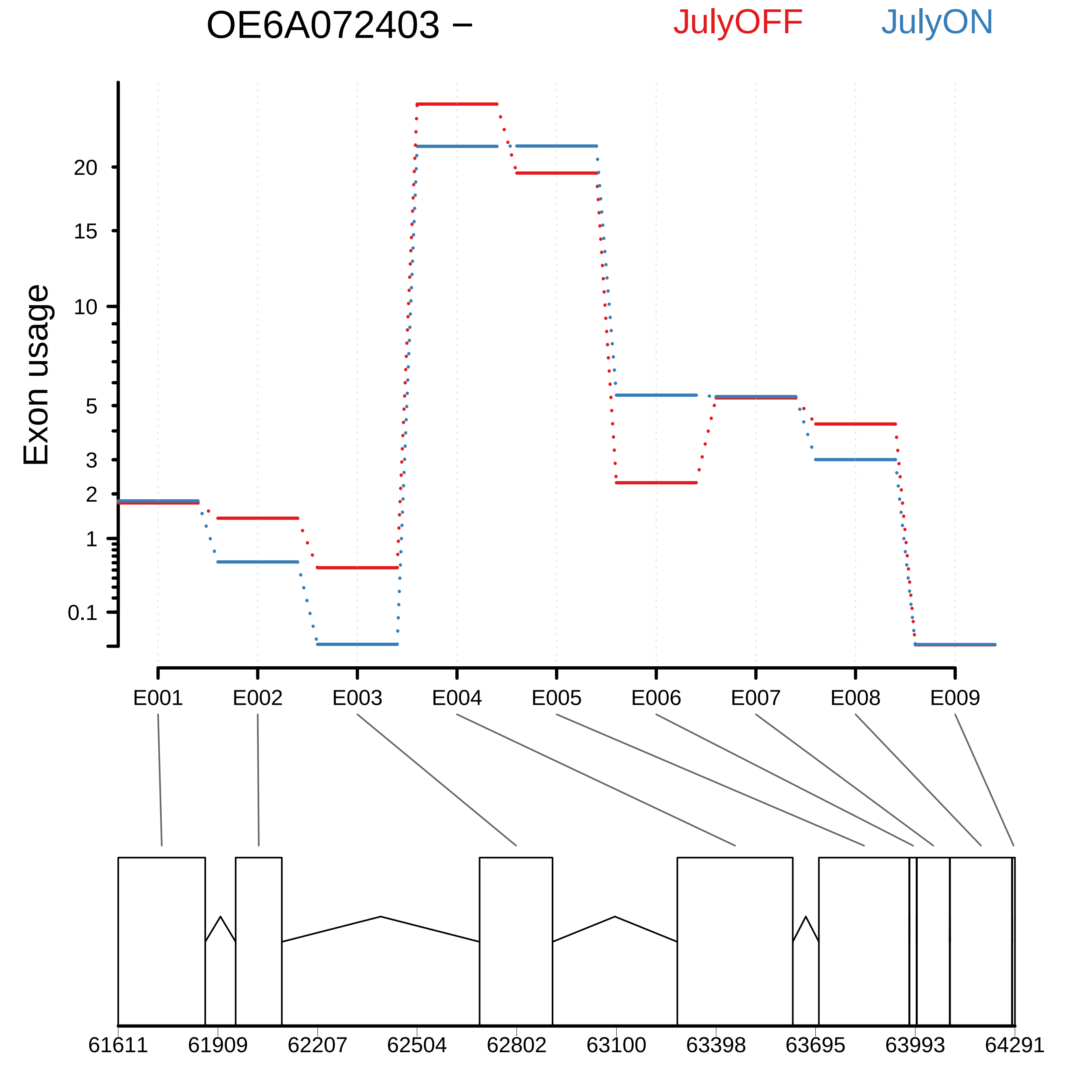
OE6A072403 −
JulyOFF
JulyON
Exon usage
20
15
10
5
3
2
1
0.1
E001
E002
E003
E004
E005
E006
E007
E008
E009
61611
61909
62207
62504
62802
63100
63398
63695
63993
64291

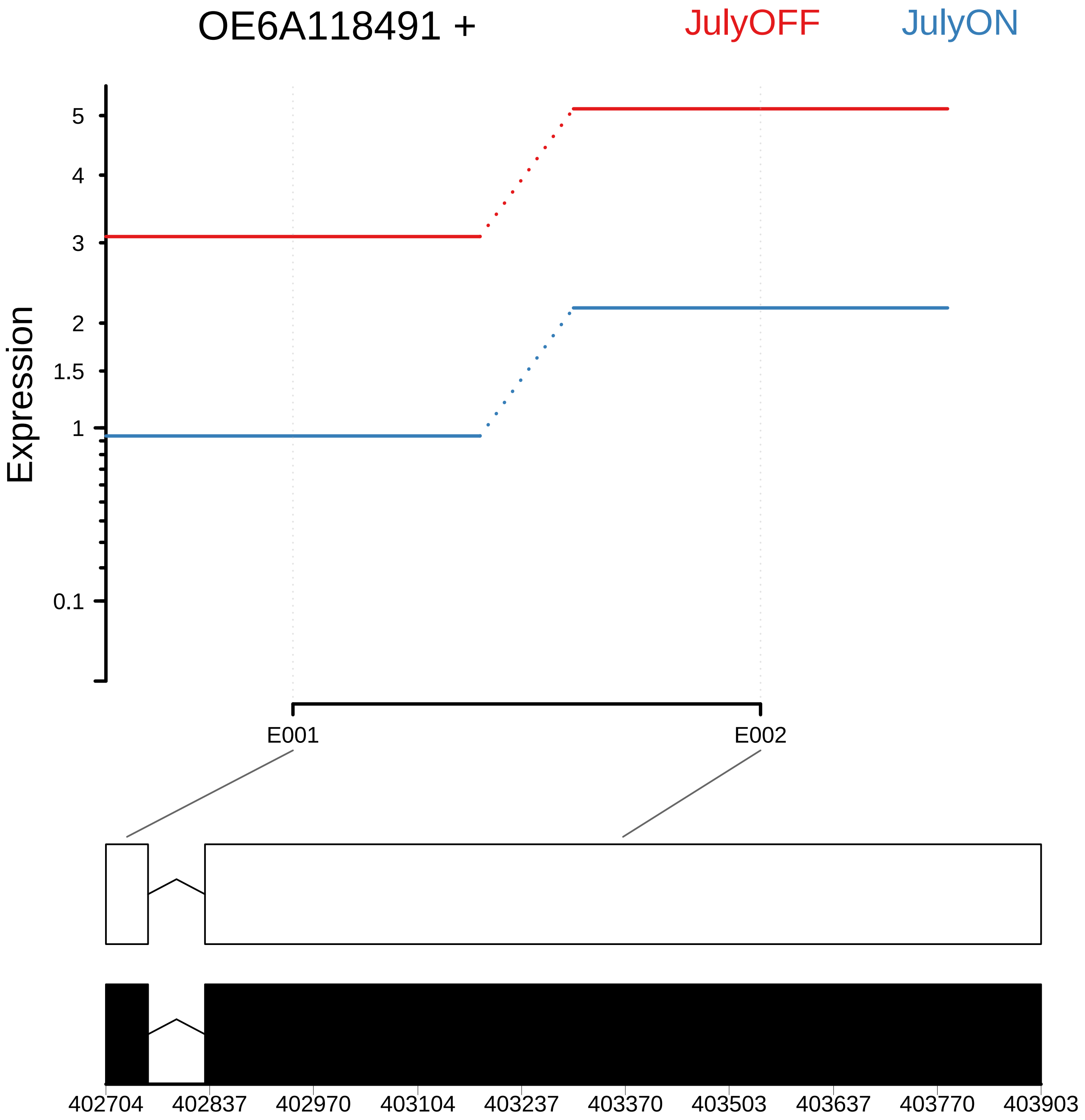

OE6A118491 +
JulyOFF
JulyON
Expression
5
4
3
2
1.5
1
0.1
E001
E002
402704
402837
402970
403104
403237
403370
403503
403637
403770
403903

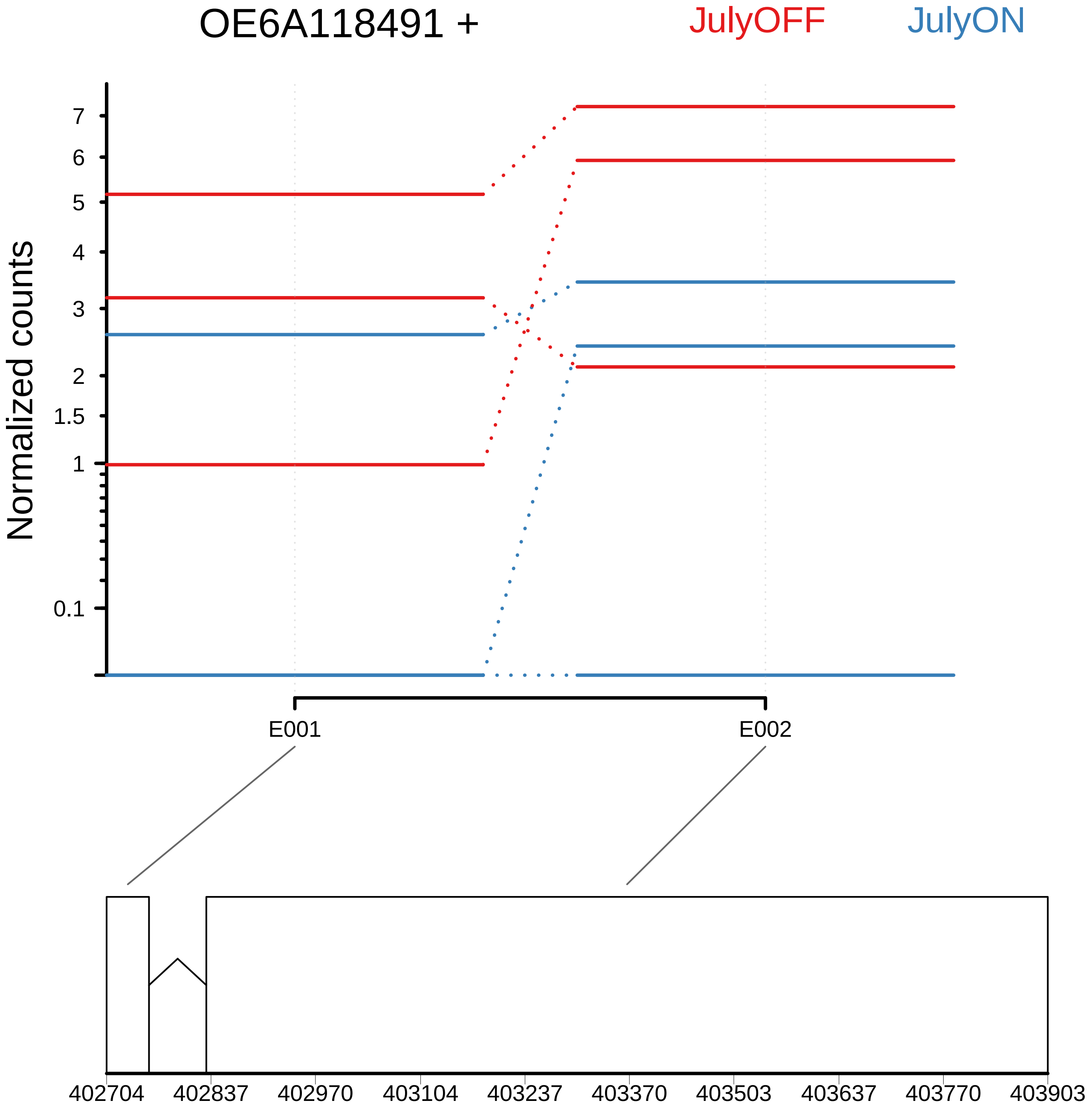

OE6A118491 +
JulyOFF
JulyON
Normalized counts
7
6
5
4
3
2
1.5
1
0.1
E001
E002
402704
402837
402970
403104
403237
403370
403503
403637
403770
403903

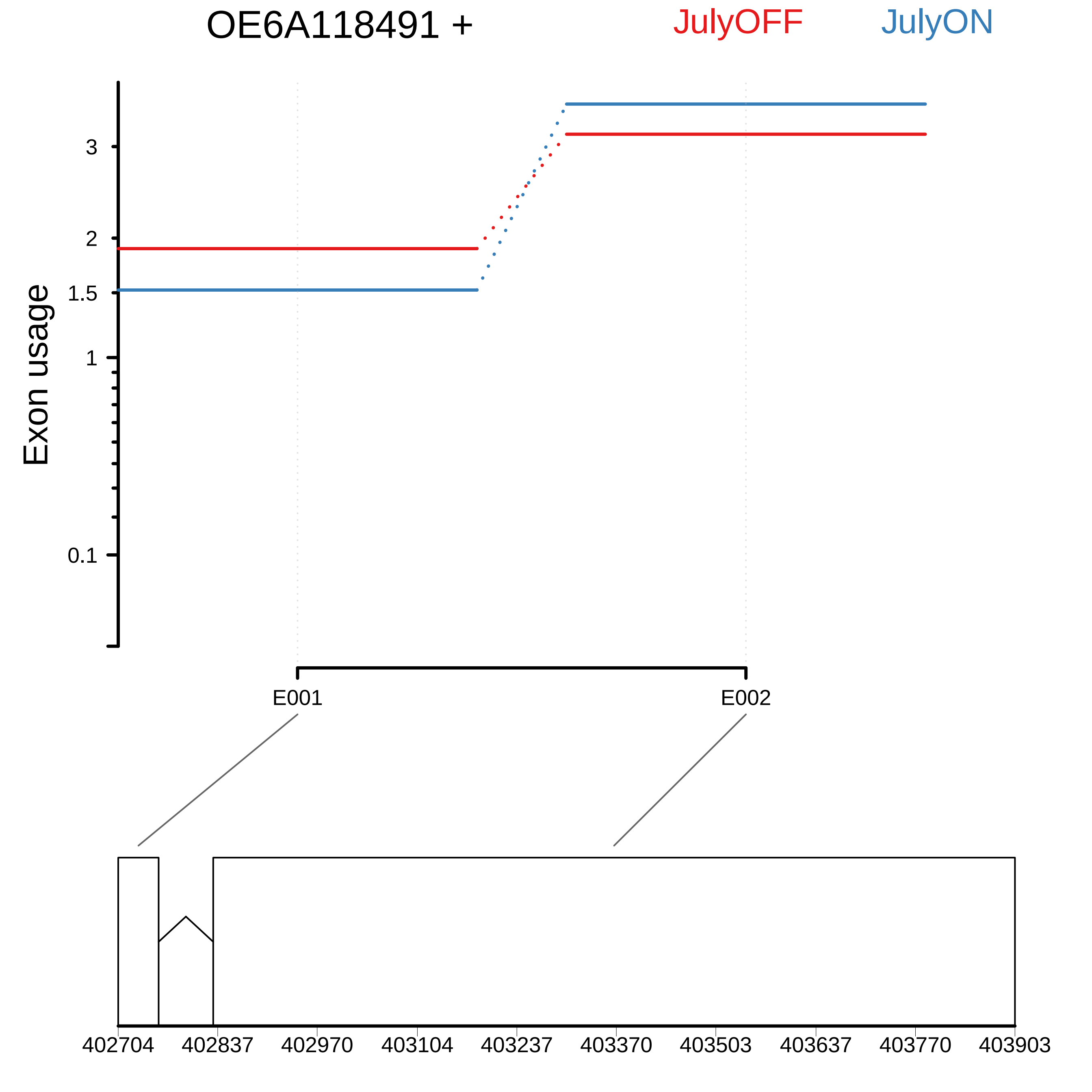
OE6A118491 +
JulyOFF
JulyON
Exon usage
3
2
1.5
1
0.1
E001
E002
402704
402837
402970
403104
403237
403370
403503
403637
403770
403903

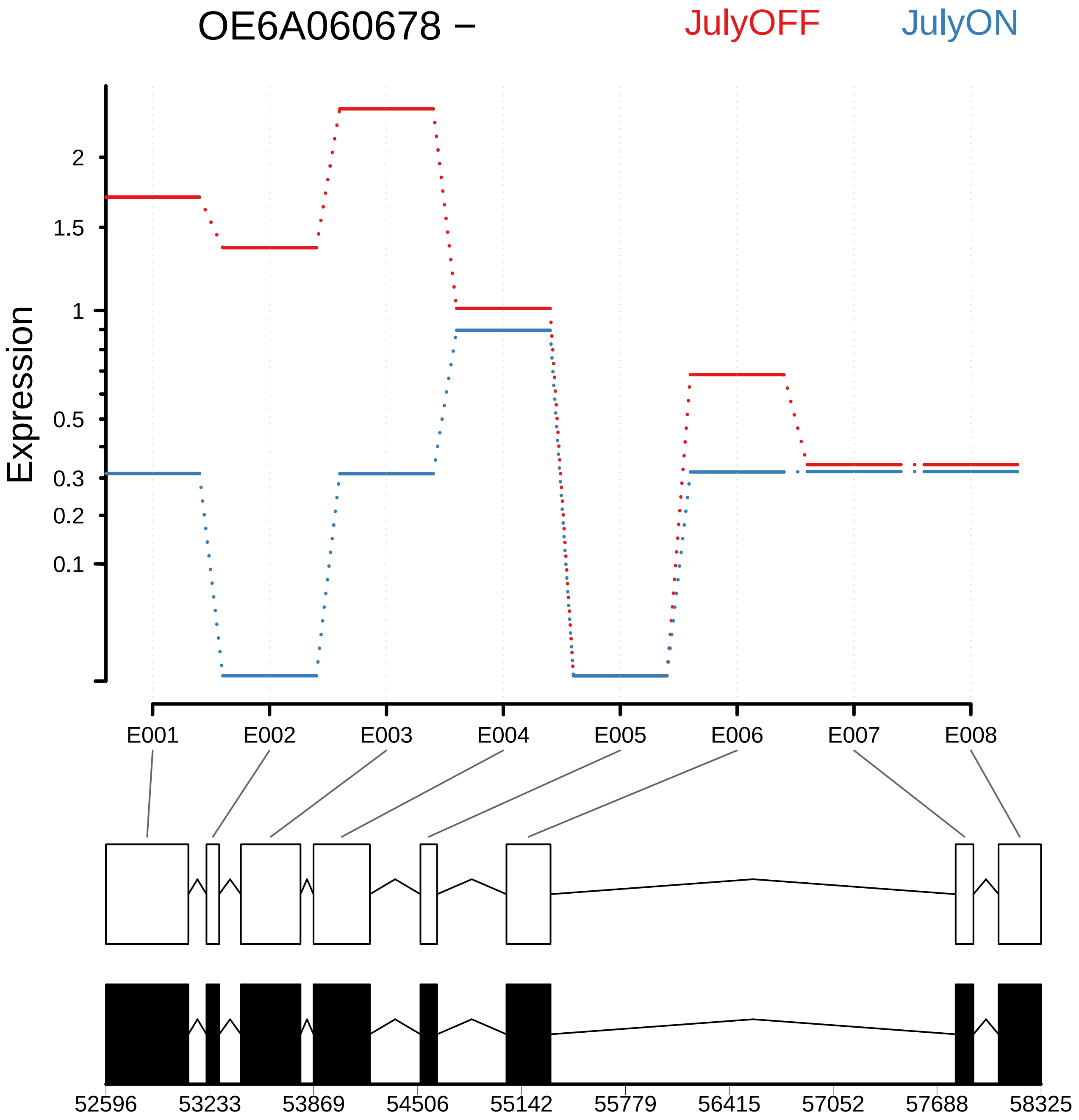

OE6A060678 −
JulyOFF
JulyON
Expression
2
1.5
1
0.5
0.3
0.2
0.1
E001
E002
E003
E004
E005
E006
E007
E008
52596
53233
53869
54506
55142
55779
56415
57052
57688
58325

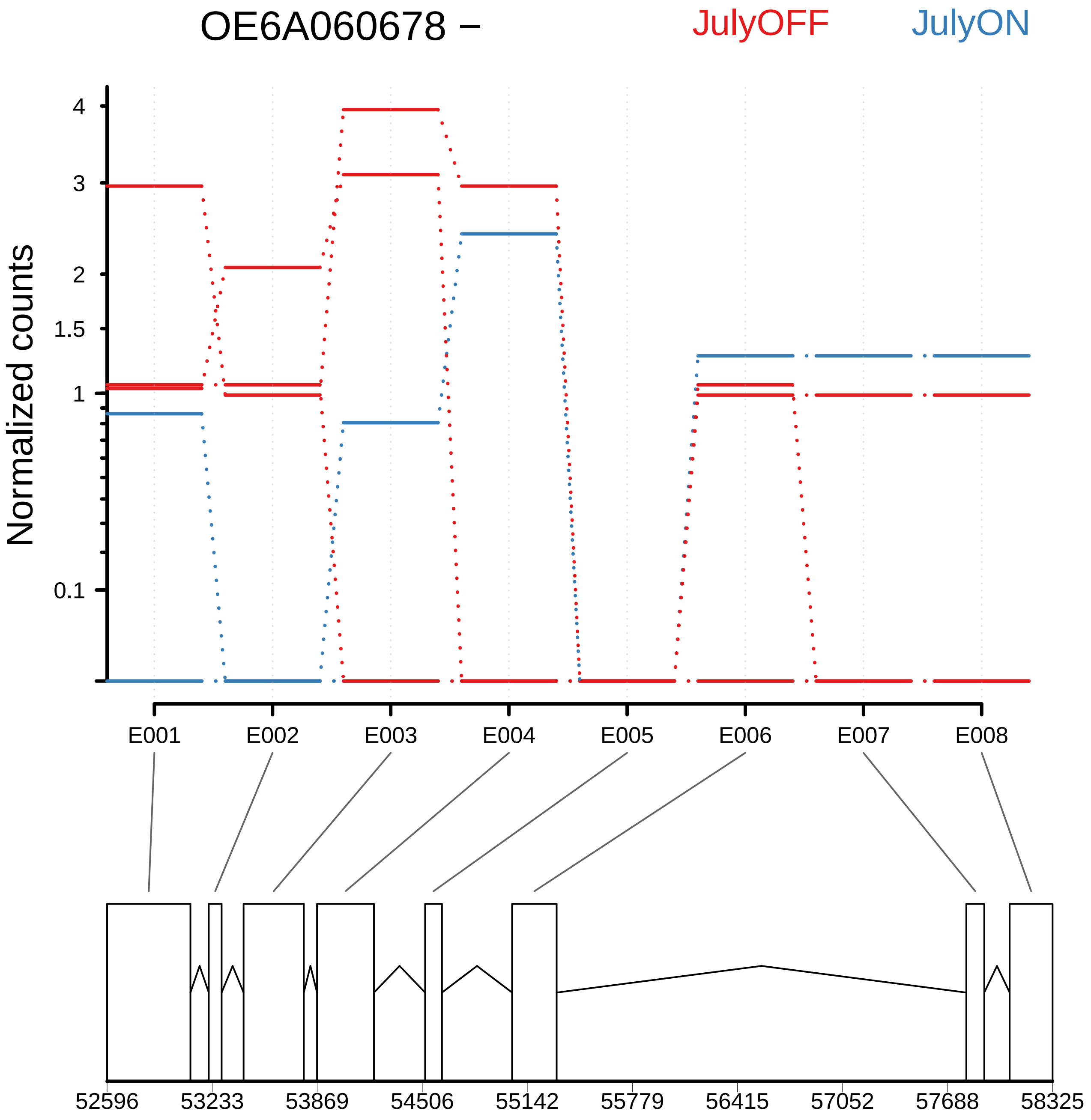
OE6A060678 −
JulyOFF
JulyON
Normalized counts
4
3
2
1.5
1
0.1
E001
E002
E003
E004
E005
E006
E007
E008
52596
53233
53869
54506
55142
55779
56415
57052
57688
58325

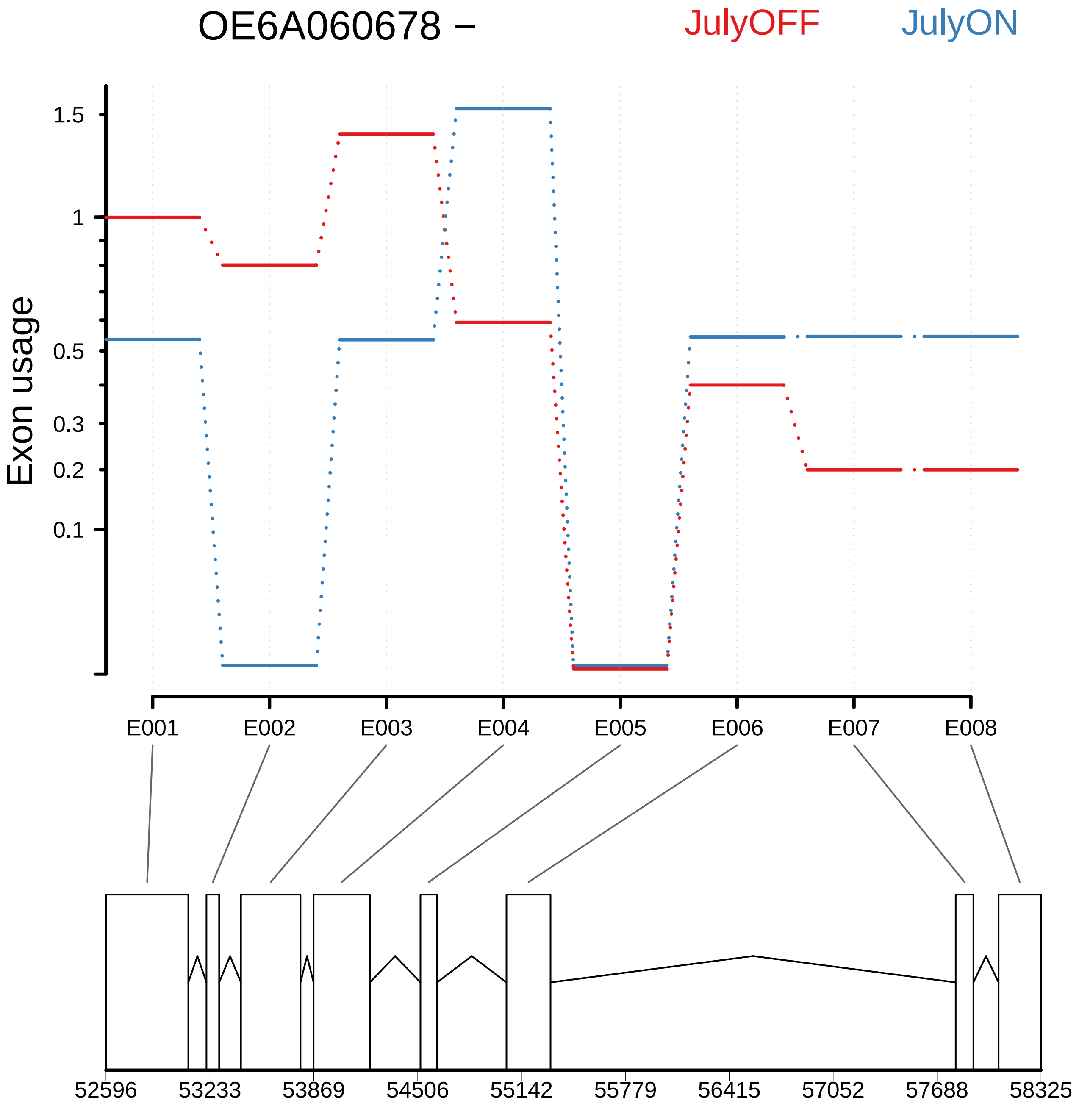

OE6A060678 –
JulyOFF
JulyON
Exon usage
1.5
1
0.5
0.3
0.2
0.1
E001
E002
E003
E004
E005
E006
E007
E008
52596
53233
53869
54506
55142
55779
56415
57052
57688
58325

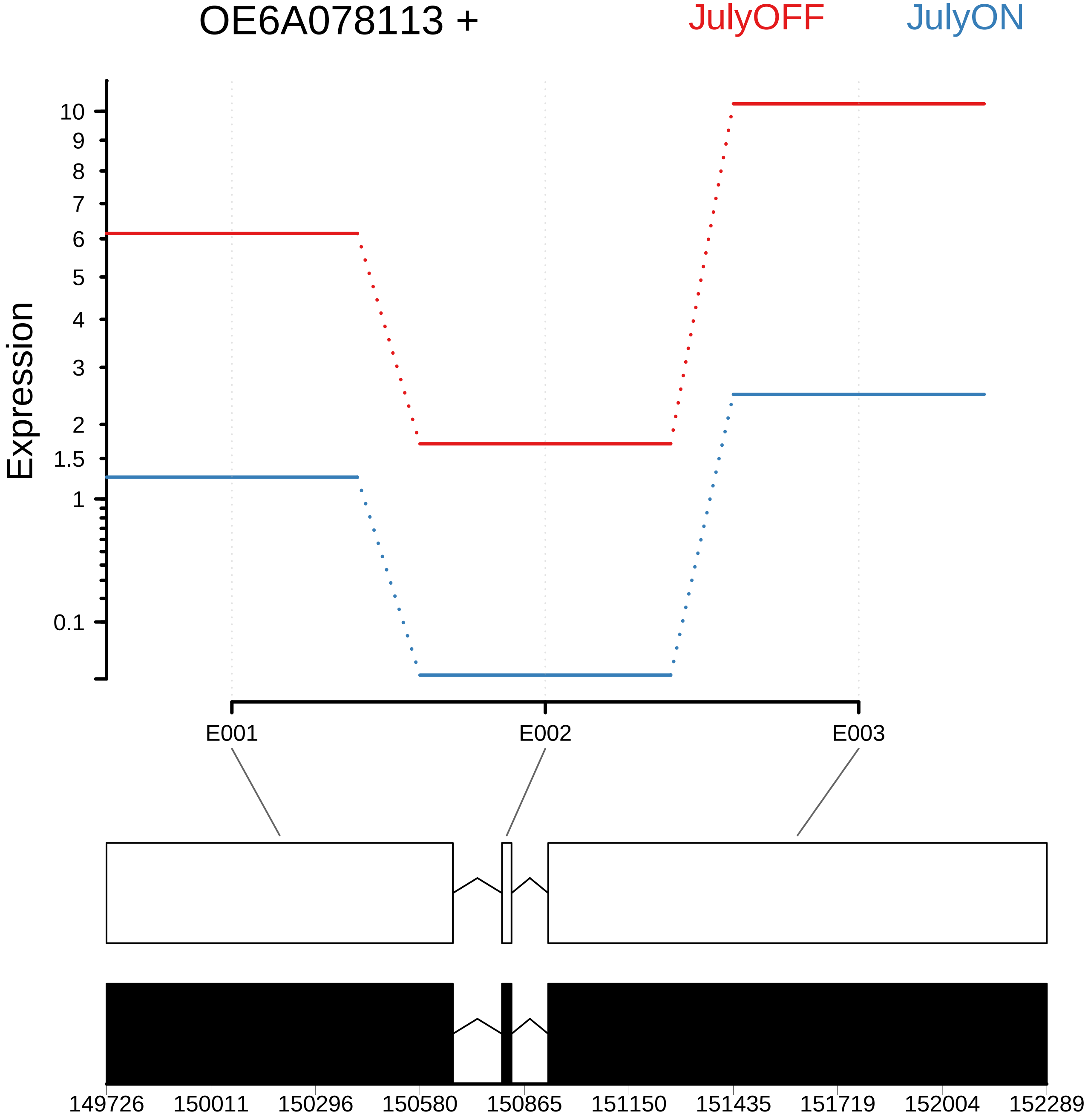
OE6A078113 +
JulyOFF
JulyON
Expression
10
9
8
7
6
5
4
3
2
1.5
1
0.1
E001
E002
E003
149726
150011
150296
150580
150865
151150
151435
151719
152004
152289

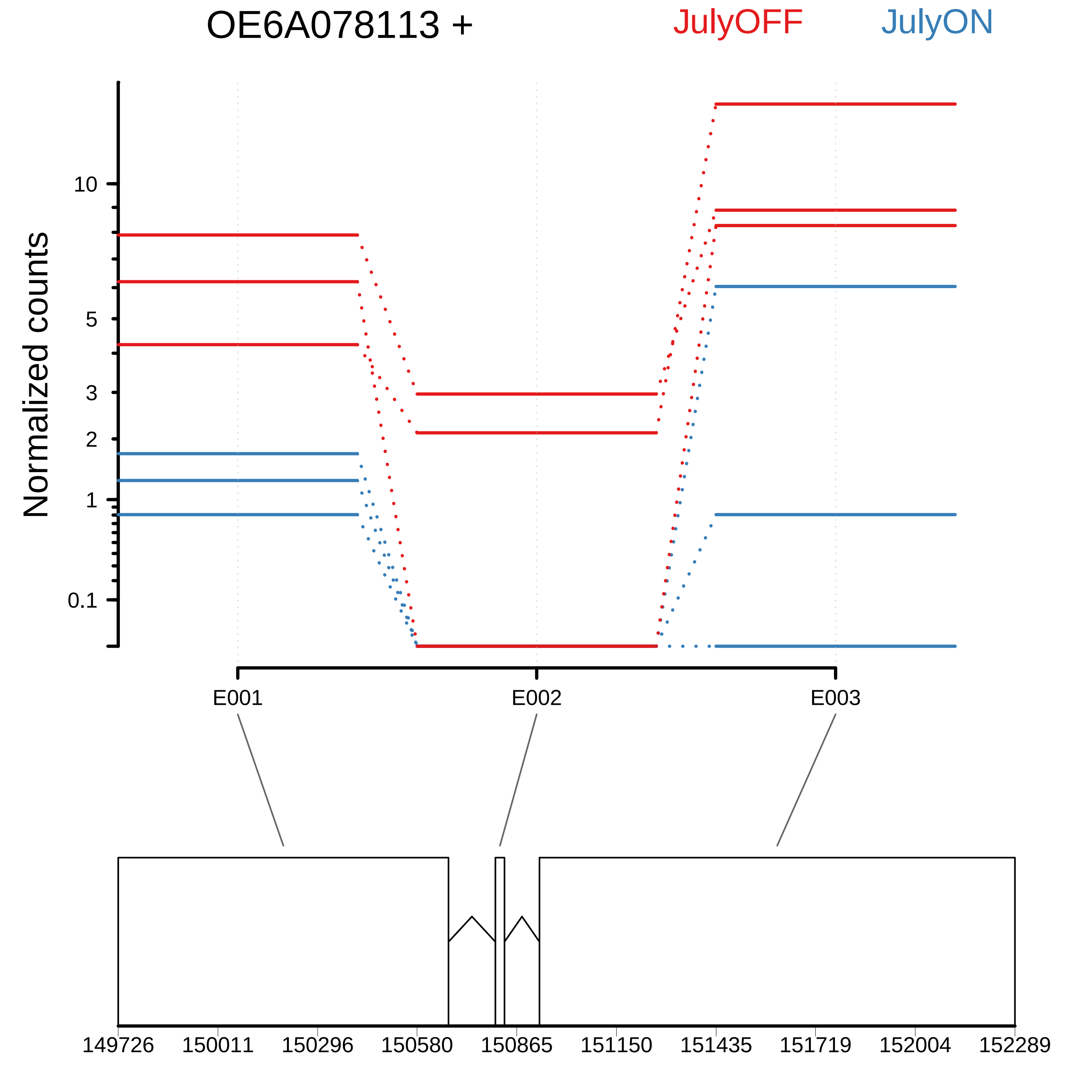

OE6A078113 +
JulyOFF
JulyON
Normalized counts
10
5
3
2
1
0.1
E001
E002
E003
149726
150011
150296
150580
150865
151150
151435
151719
152004
152289

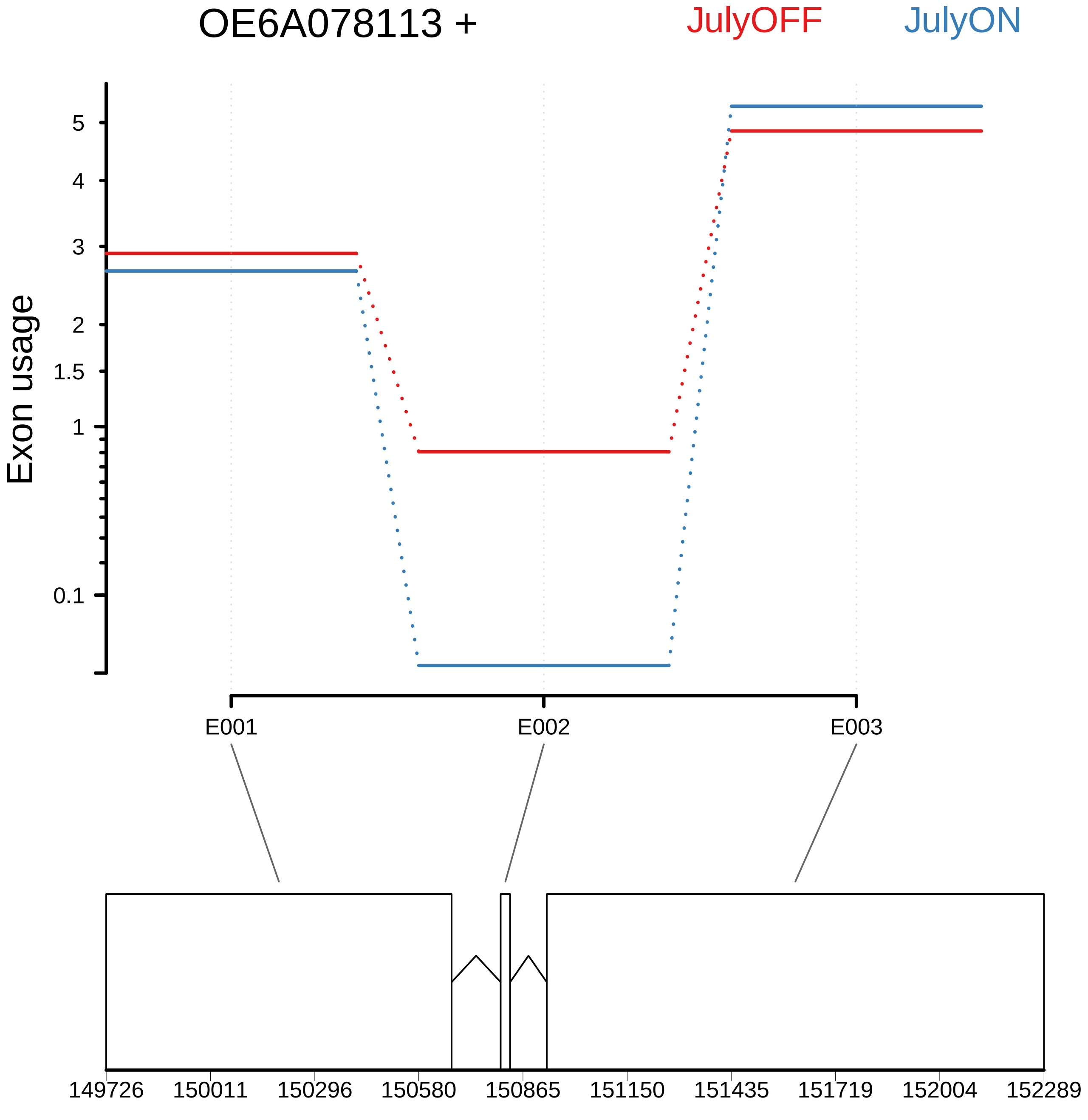
OE6A078113 +
JulyOFF
JulyON
Exon usage
5
4
3
2
1.5
1
0.1
E001
E002
E003
149726
150011
150296
150580
150865
151150
151435
151719
152004
152289

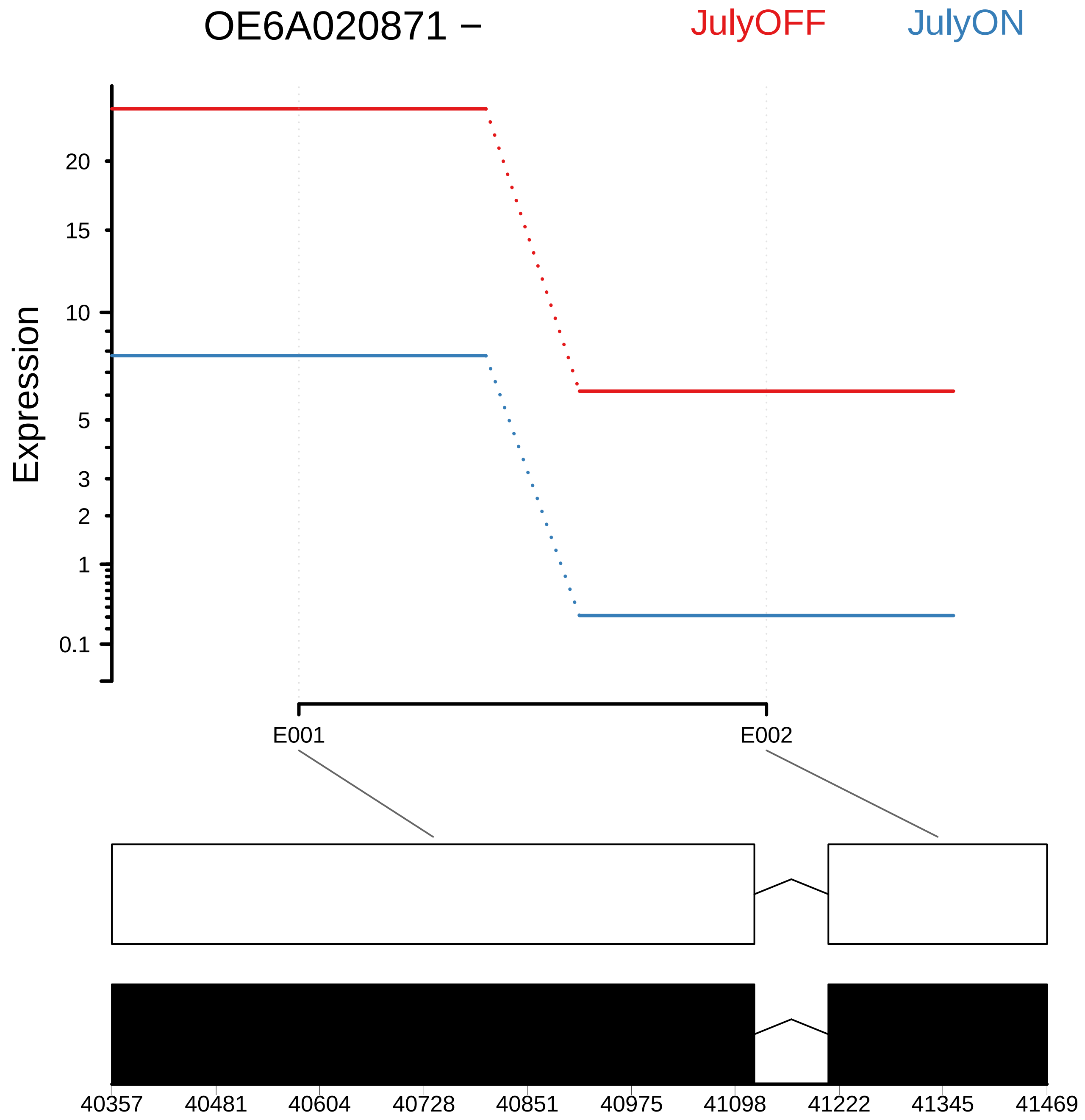

OE6A020871 −
JulyOFF
JulyON
Expression
20
15
10
5
3
2
1
0.1
E001
E002
40357
40481
40604
40728
40851
40975
41098
41222
41345
41469

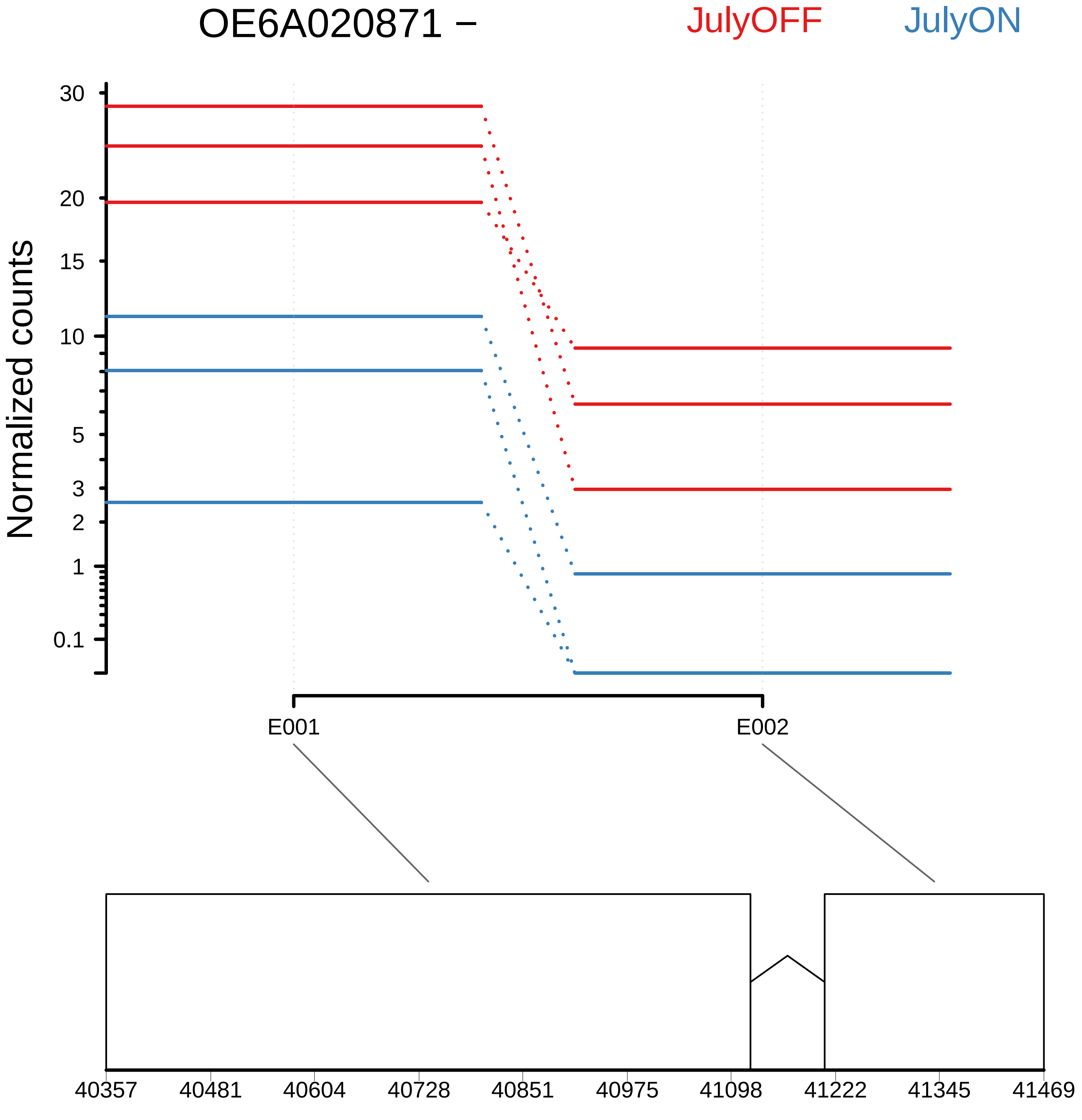

OE6A020871 −
JulyOFF
JulyON
Normalized counts
30
20
15
10
5
3
2
1
0.1
E001
E002
40357
40481
40604
40728
40851
40975
41098
41222
41345
41469

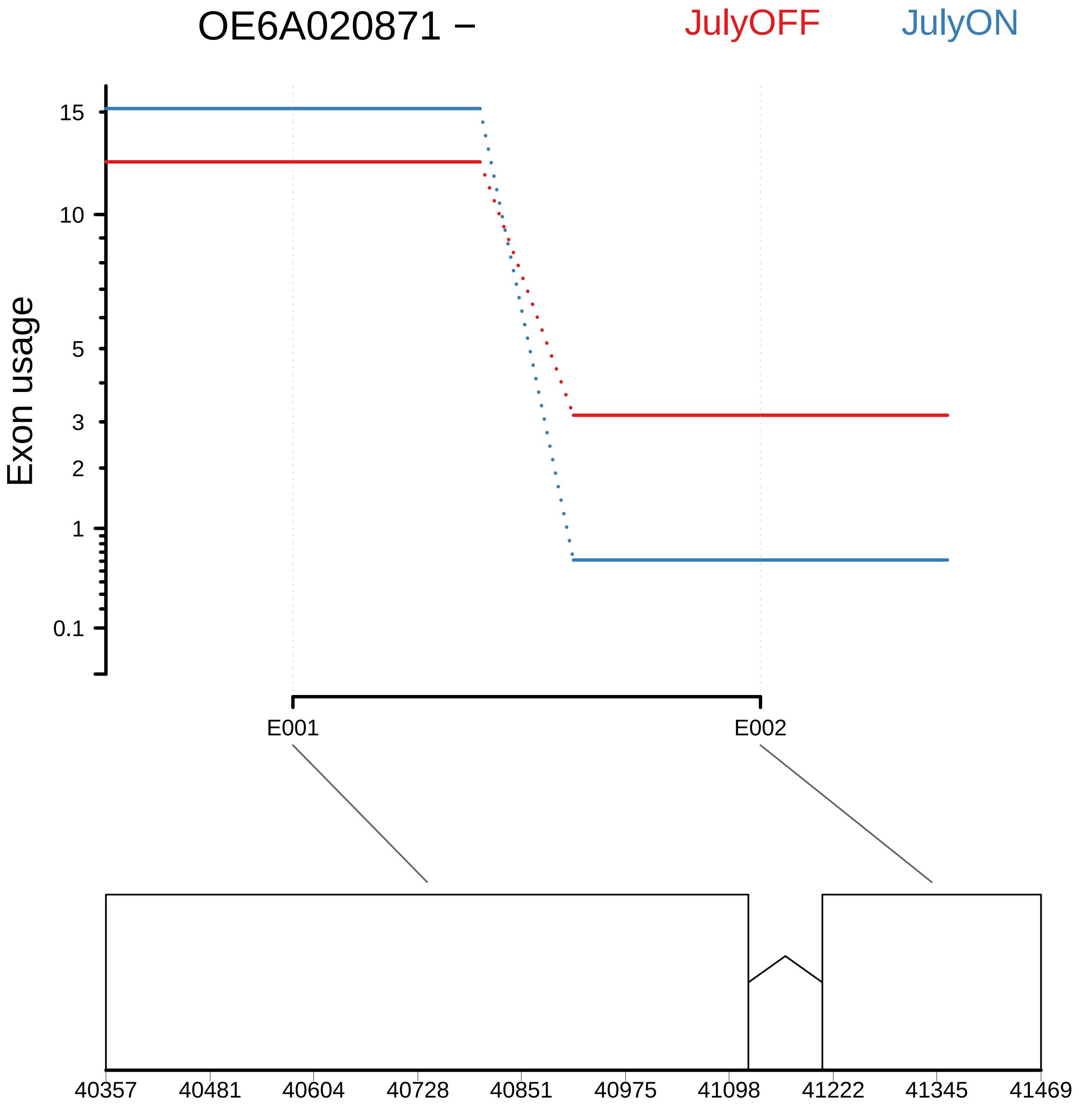

OE6A020871 −
JulyOFF
JulyON
Exon usage
15
10
5
3
2
1
0.1
E001
E002
40357
40481
40604
40728
40851
40975
41098
41222
41345
41469

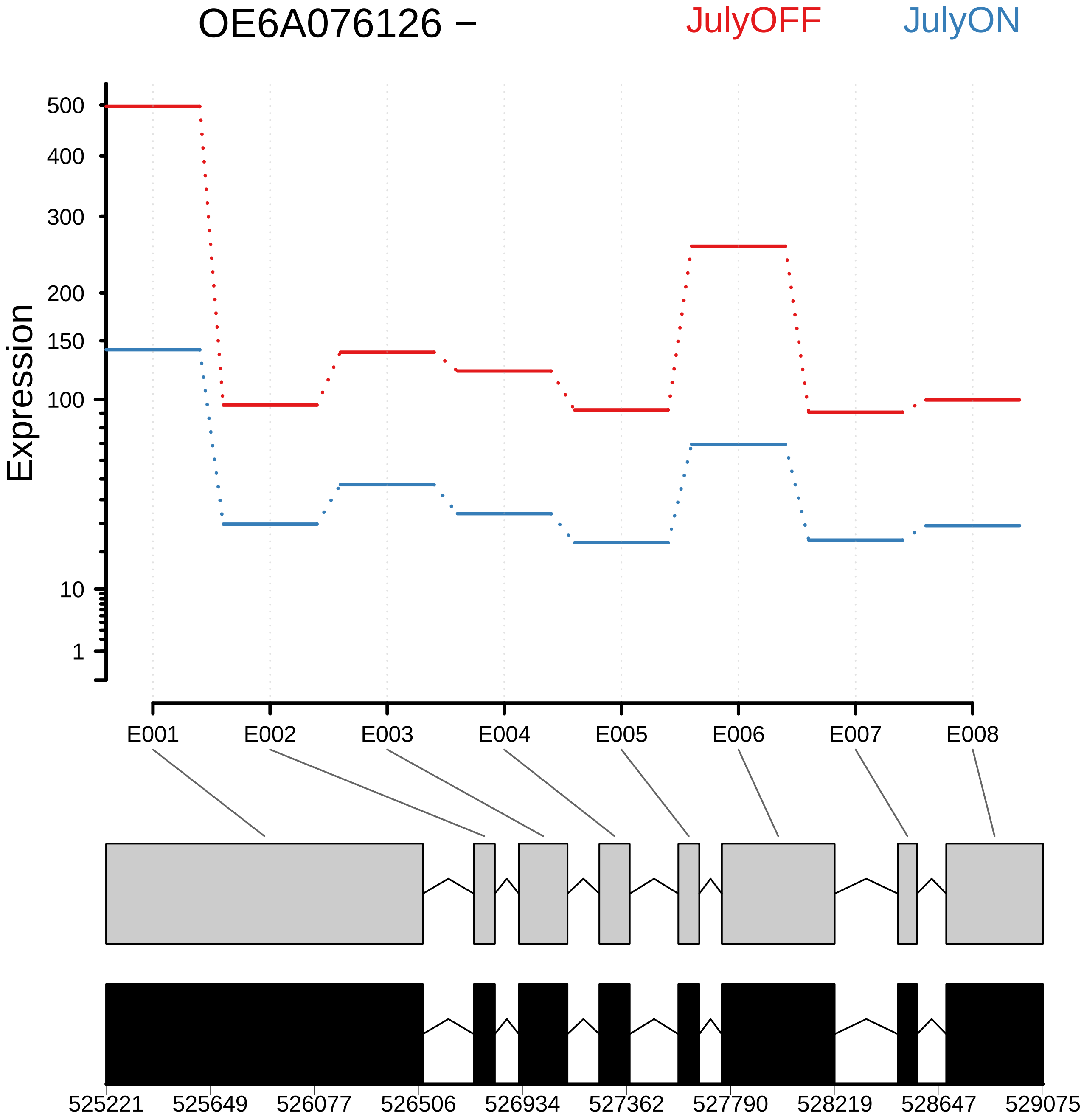

OE6A076126 −
JulyOFF
JulyON
Expression
500
400
300
200
150
100
10
1
E001
E002
E003
E004
E005
E006
E007
E008
525221
525649
526077
526506
526934
527362
527790
528219
528647
529075

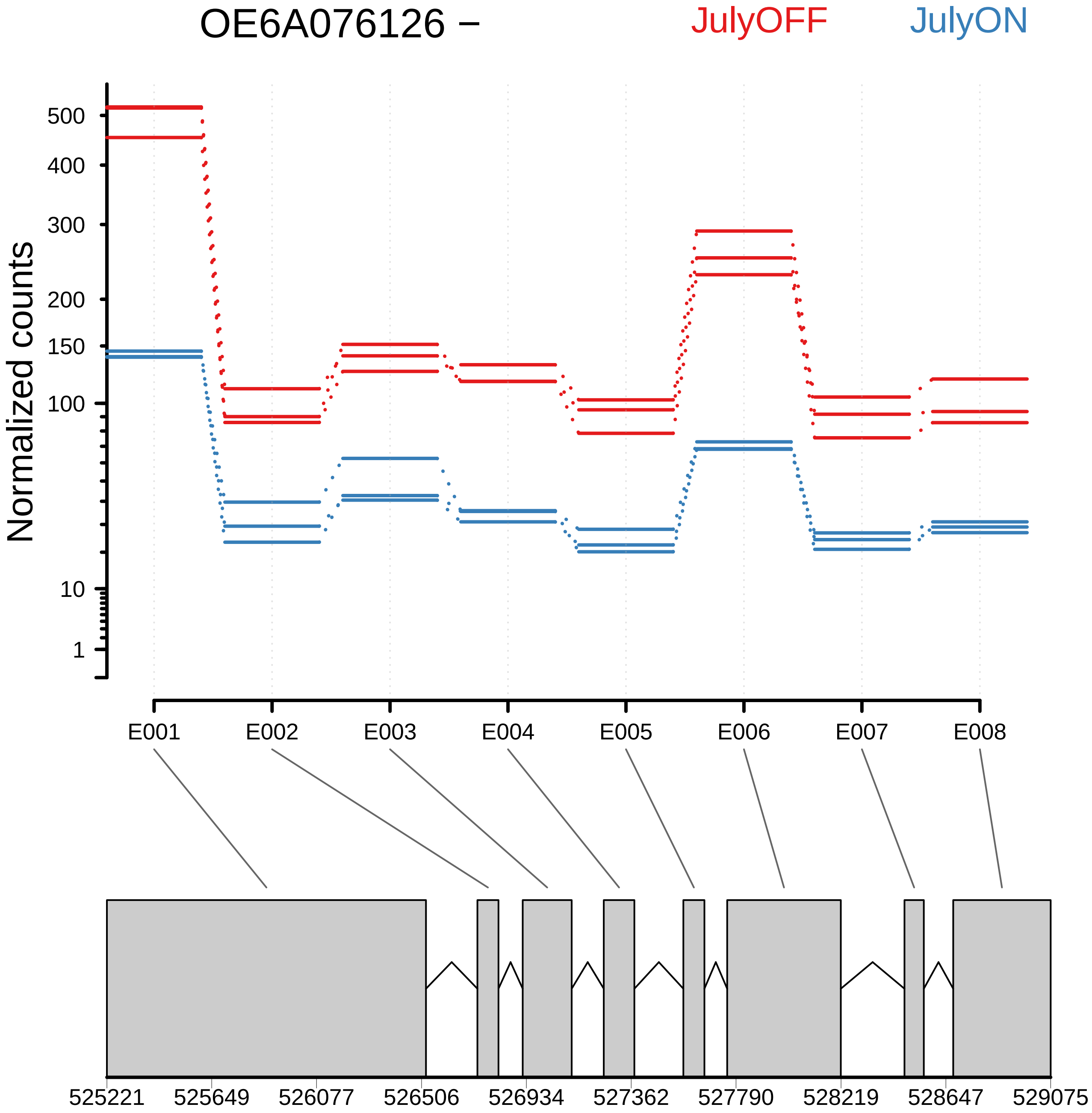

OE6A076126 −
JulyOFF
JulyON
Normalized counts
500
400
300
200
150
100
10
1
E001
E002
E003
E004
E005
E006
E007
E008
525221
525649
526077
526506
526934
527362
527790
528219
528647
529075

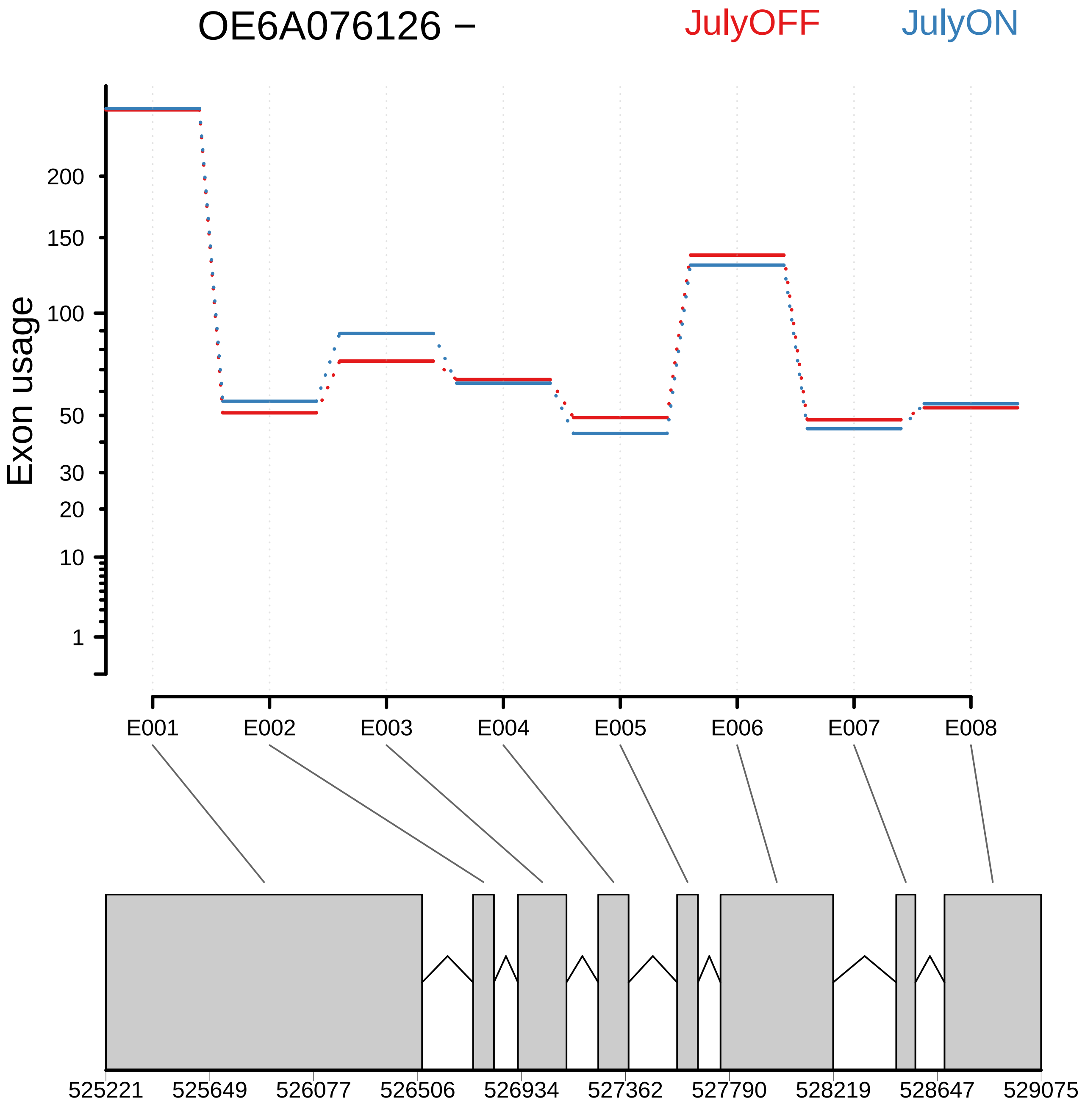
OE6A076126 −
JulyOFF
JulyON
Exon usage
200
150
100
50
30
20
10
1
E001
E002
E003
E004
E005
E006
E007
E008
525221
525649
526077
526506
526934
527362
527790
528219
528647
529075

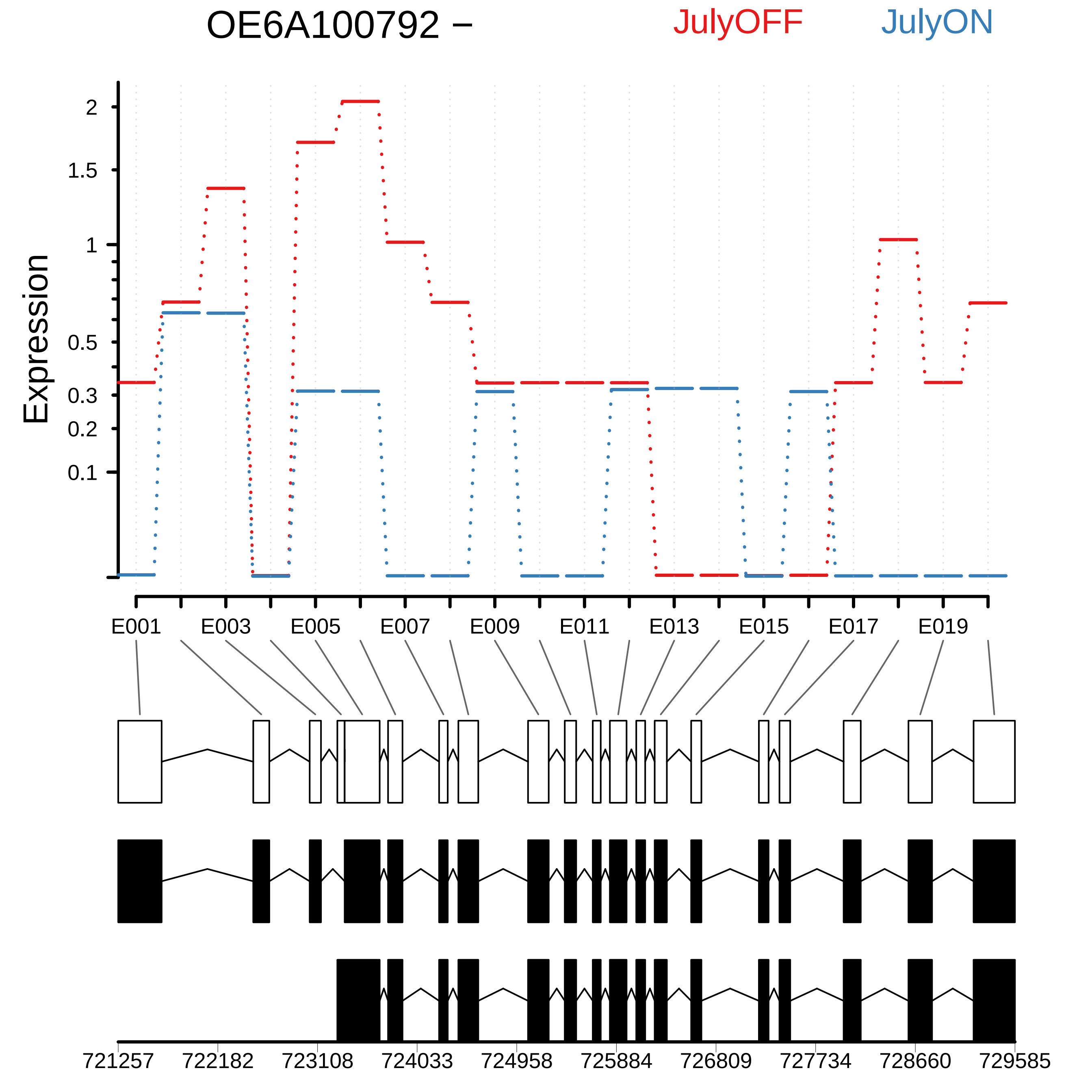

OE6A100792 −
JulyOFF
JulyON
Expression
2
1.5
1
0.5
0.3
0.2
0.1
E001
E003
E005
E007
E009
E011
E013
E015
E017
E019
721257
722182
723108
724033
724958
725884
726809
727734
728660
729585

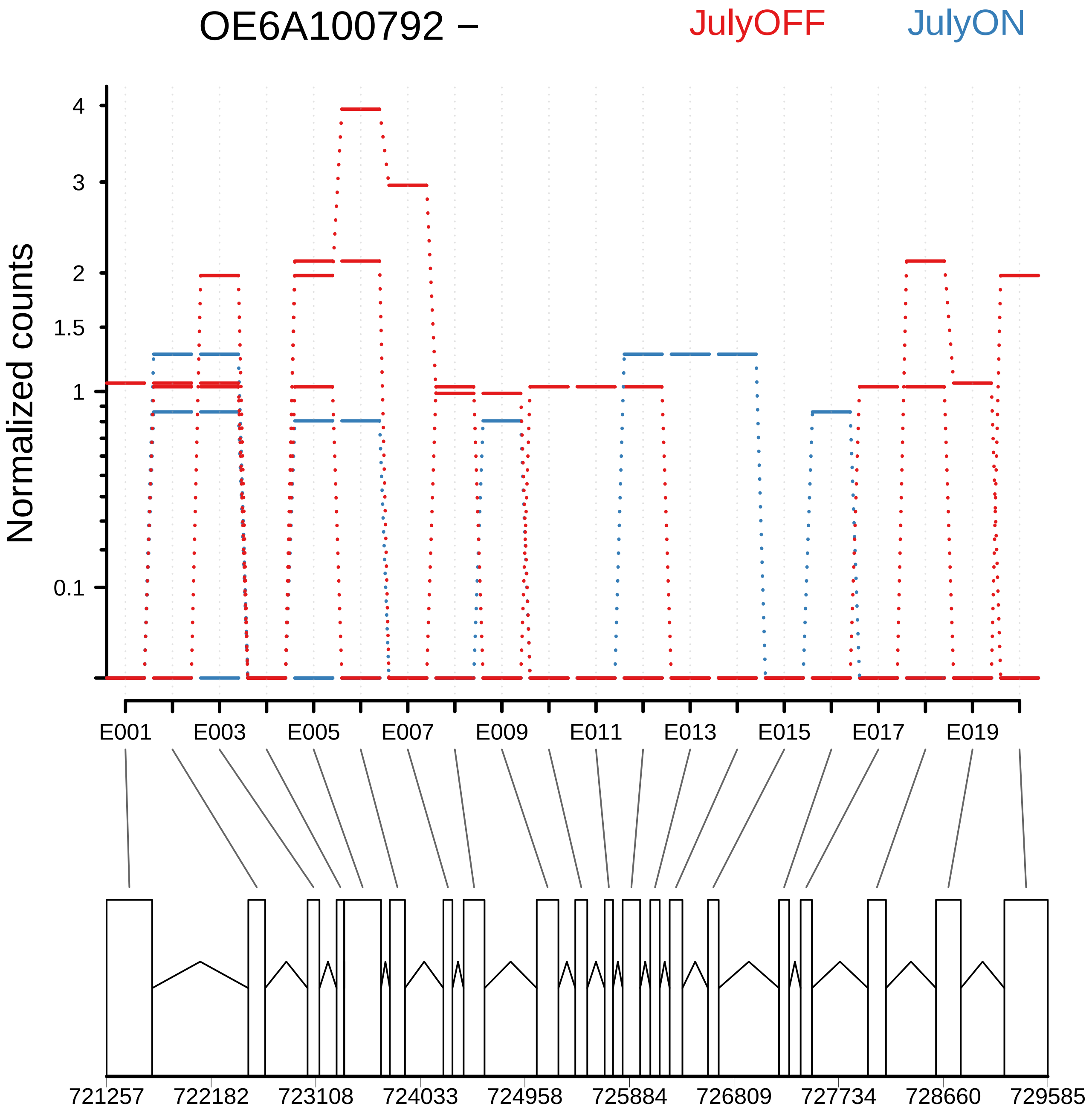

OE6A100792 −
JulyOFF
JulyON
Normalized counts
4
3
2
1.5
1
0.1
E001
E003
E005
E007
E009
E011
E013
E015
E017
E019
721257
722182
723108
724033
724958
725884
726809
727734
728660
729585

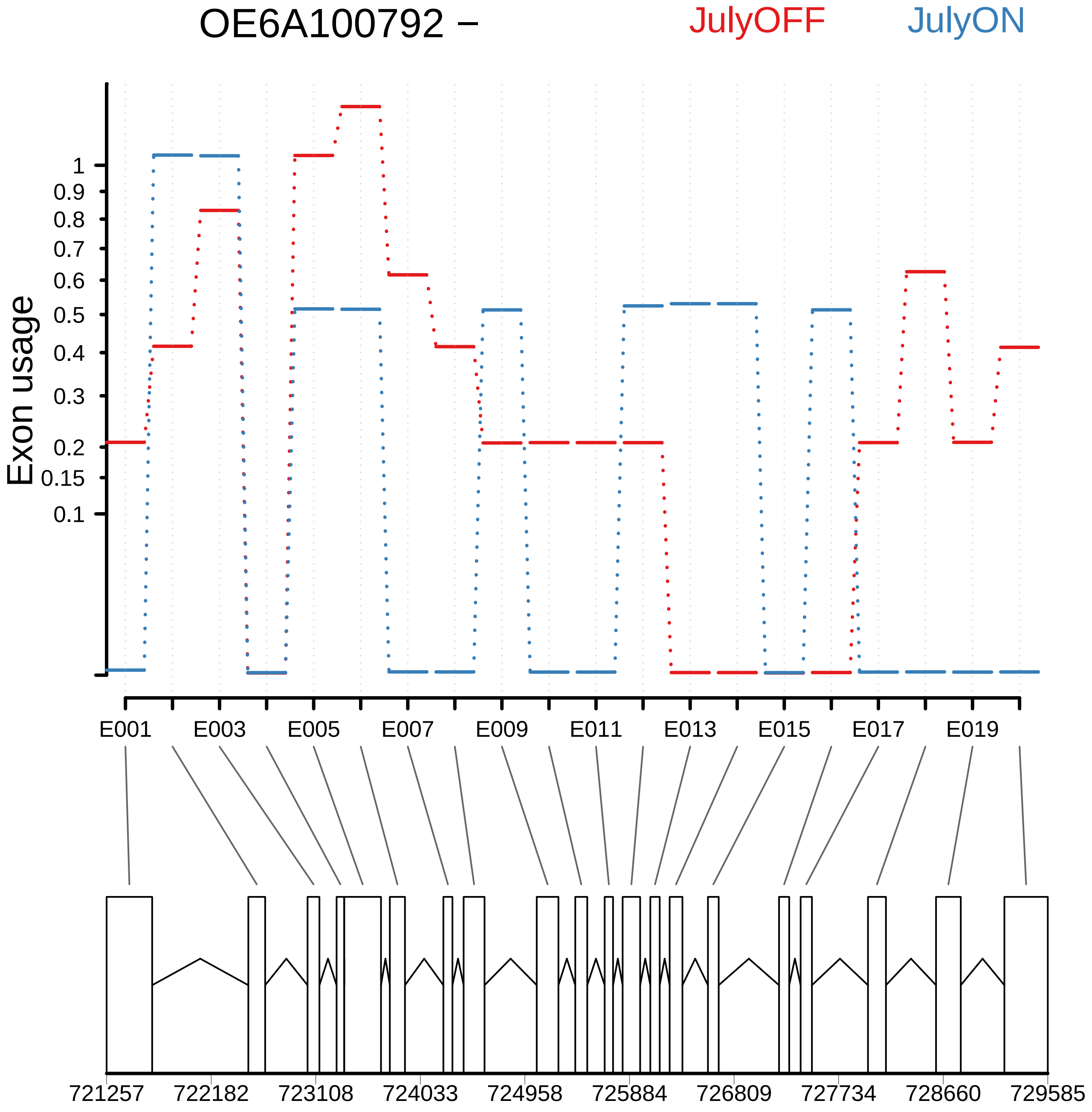
OE6A100792 −
JulyOFF
JulyON
Exon usage
1
0.9
0.8
0.7
0.6
0.5
0.4
0.3
0.2
0.15
0.1
E001
E003
E005
E007
E009
E011
E013
E015
E017
E019
721257
722182
723108
724033
724958
725884
726809
727734
728660
729585

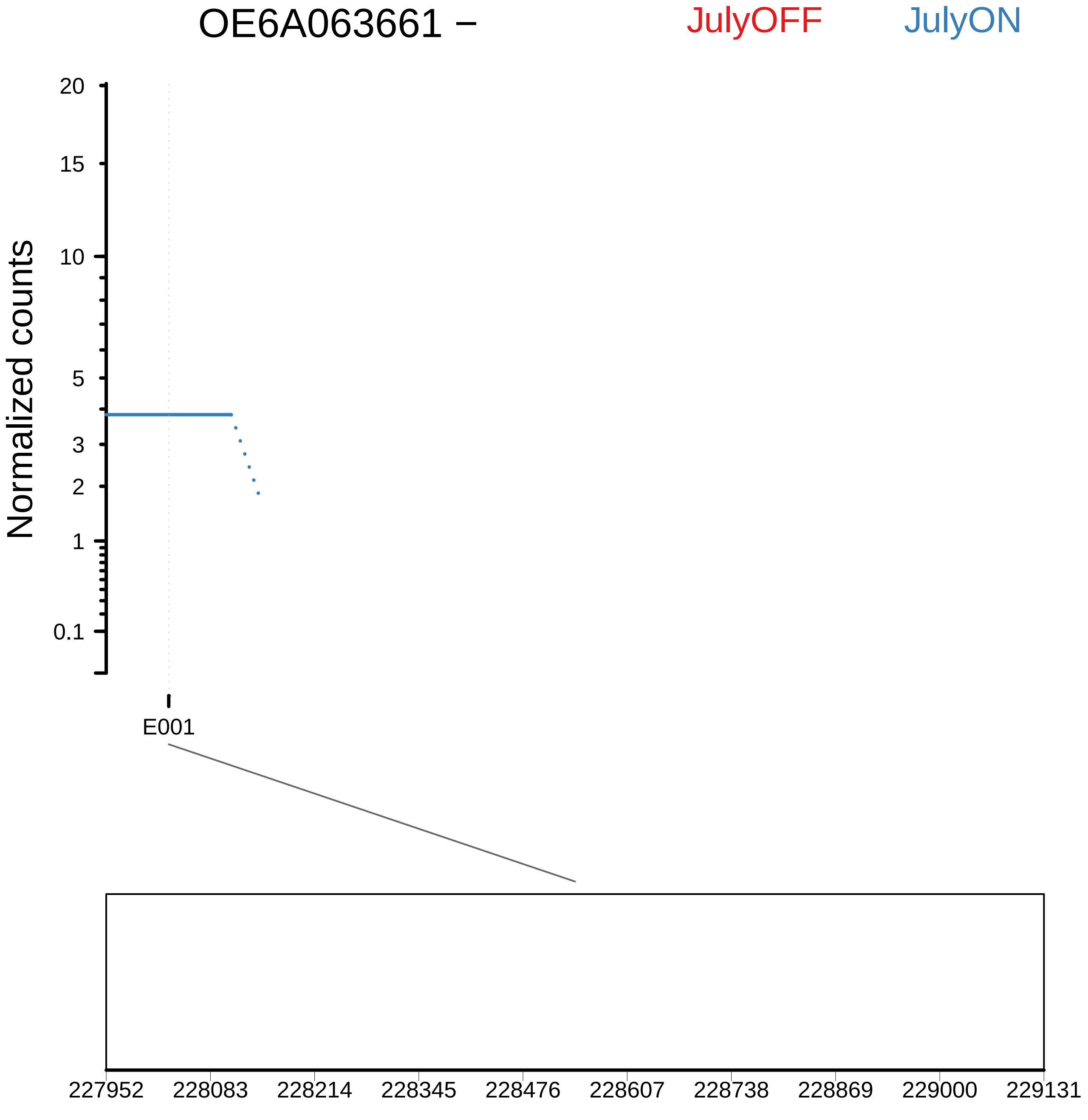

OE6A063661 −
JulyOFF
JulyON
Normalized counts
20
15
10
5
3
2
1
0.1
E001
227952
228083
228214
228345
228476
228607
228738
228869
229000
229131

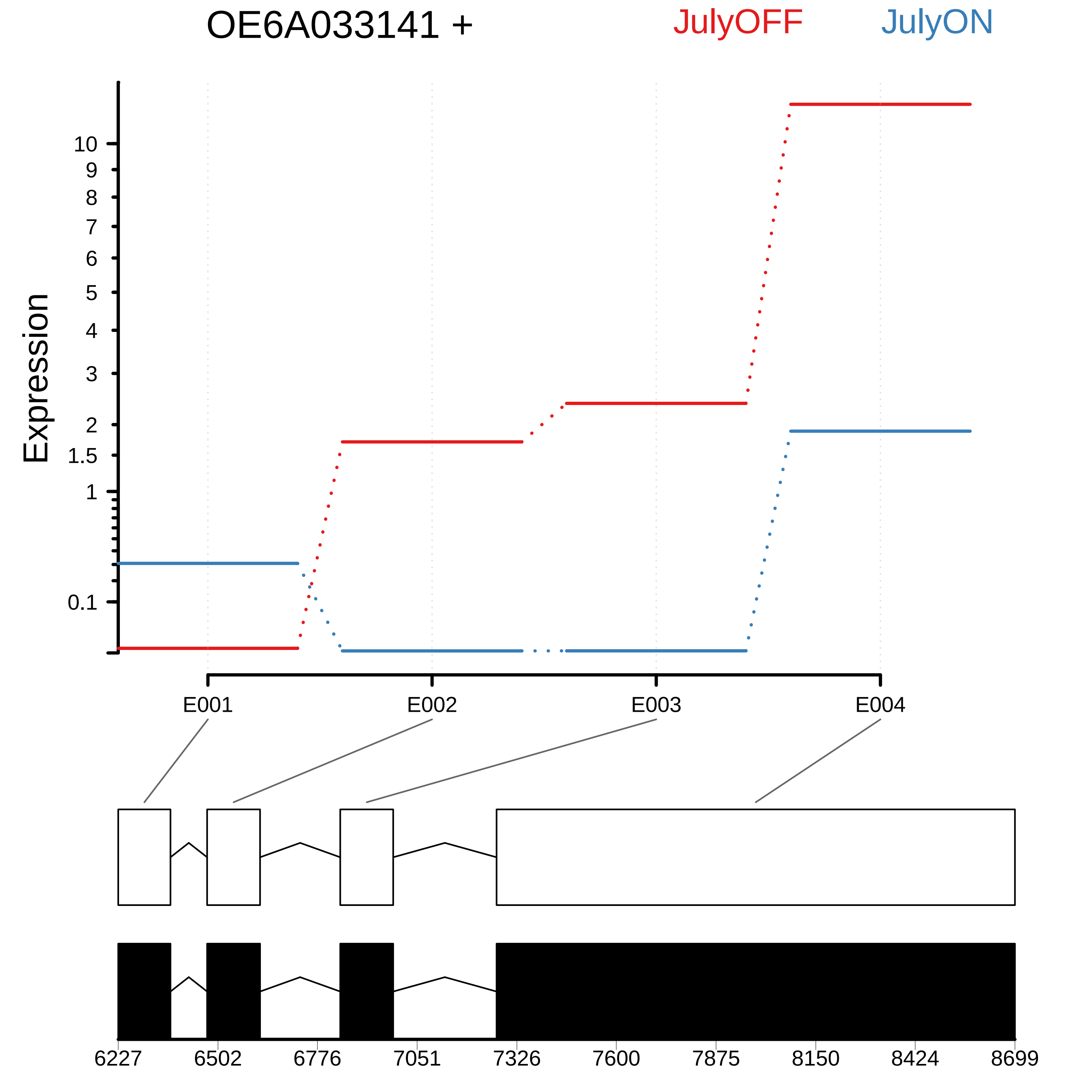

OE6A033141 +
JulyOFF
JulyON
Expression
10
9
8
7
6
5
4
3
2
1.5
1
0.1
E001
E002
E003
E004
6227
6502
6776
7051
7326
7600
7875
8150
8424
8699

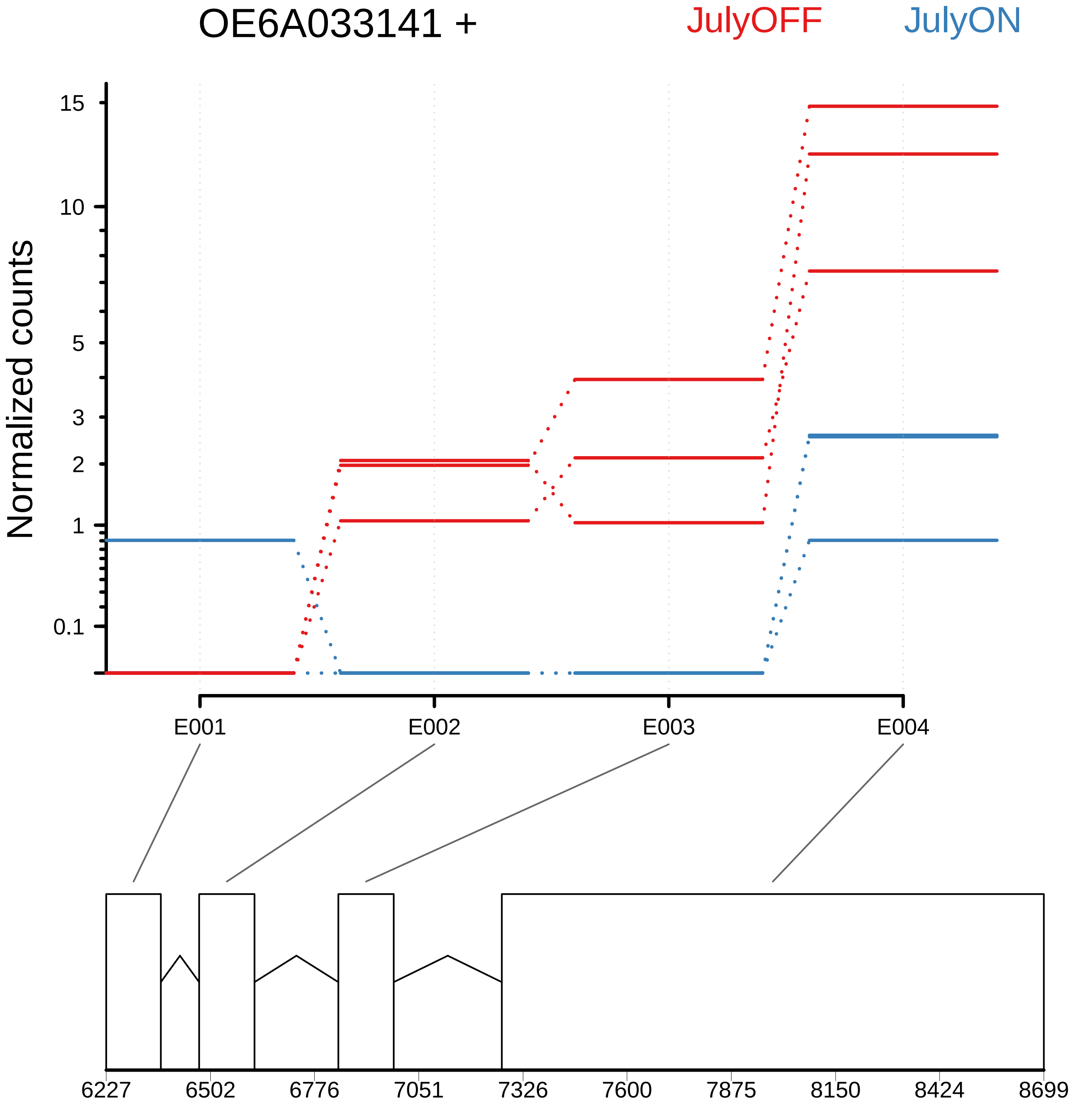

OE6A033141 +
JulyOFF
JulyON
Normalized counts
15
10
5
3
2
1
0.1
E001
E002
E003
E004
6227
6502
6776
7051
7326
7600
7875
8150
8424
8699

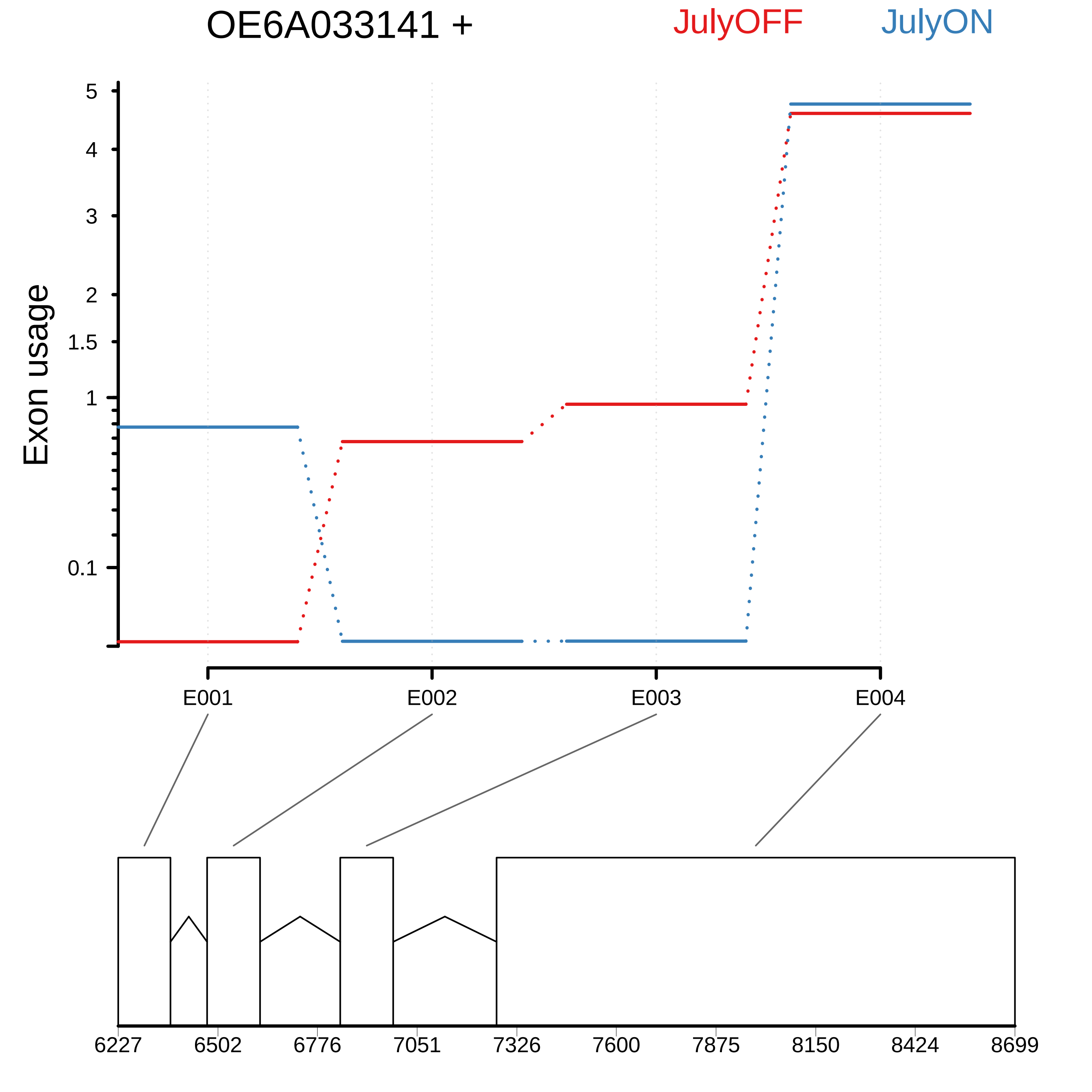

OE6A033141 +
JulyOFF
JulyON
Exon usage
5
4
3
2
1.5
1
0.1
E001
E002
E003
E004
6227
6502
6776
7051
7326
7600
7875
8150
8424
8699

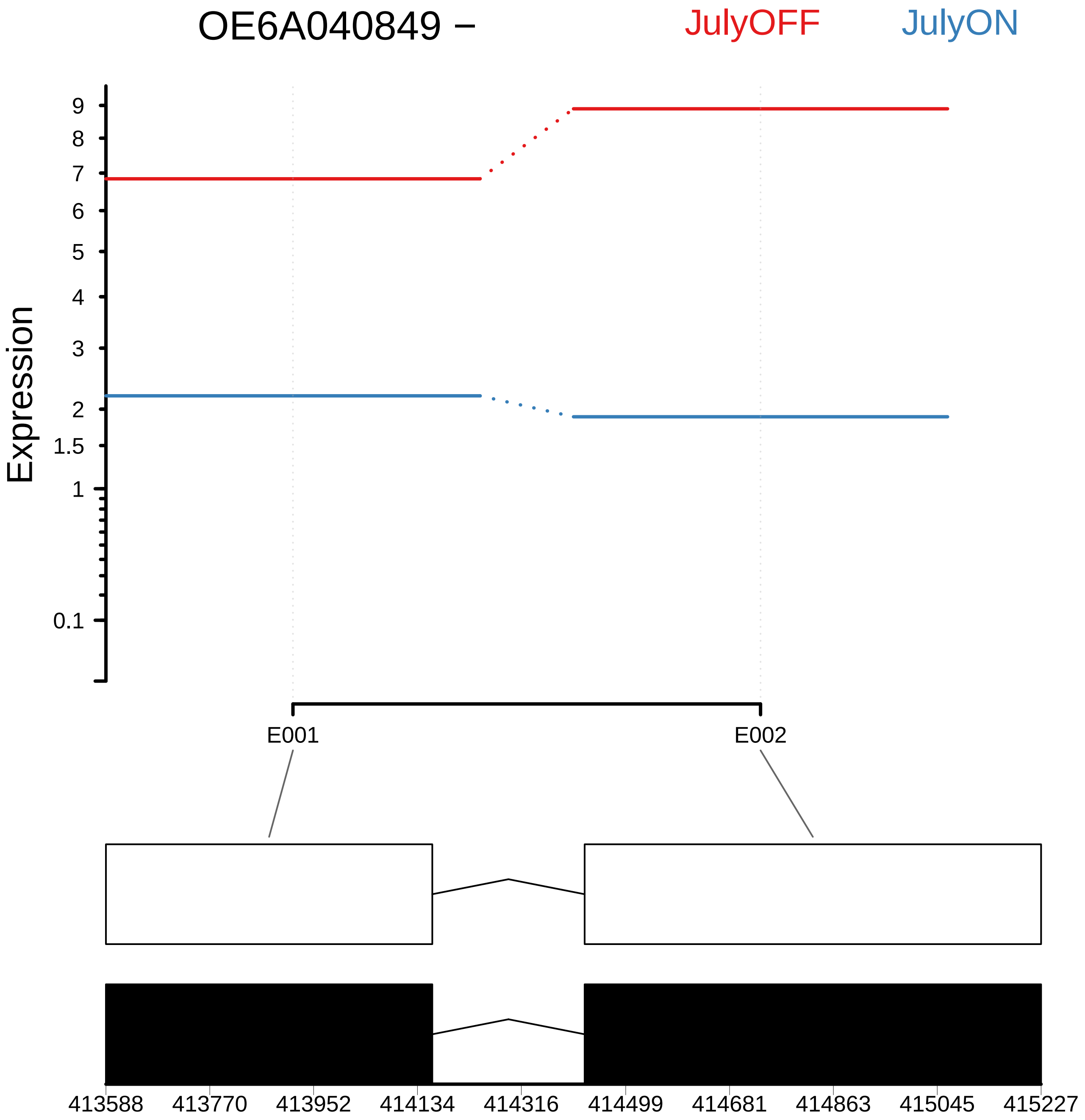

OE6A040849 −
JulyOFF
JulyON
Expression
9
8
7
6
5
4
3
2
1.5
1
0.1
E001
E002
413588
413770
413952
414134
414316
414499
414681
414863
415045
415227

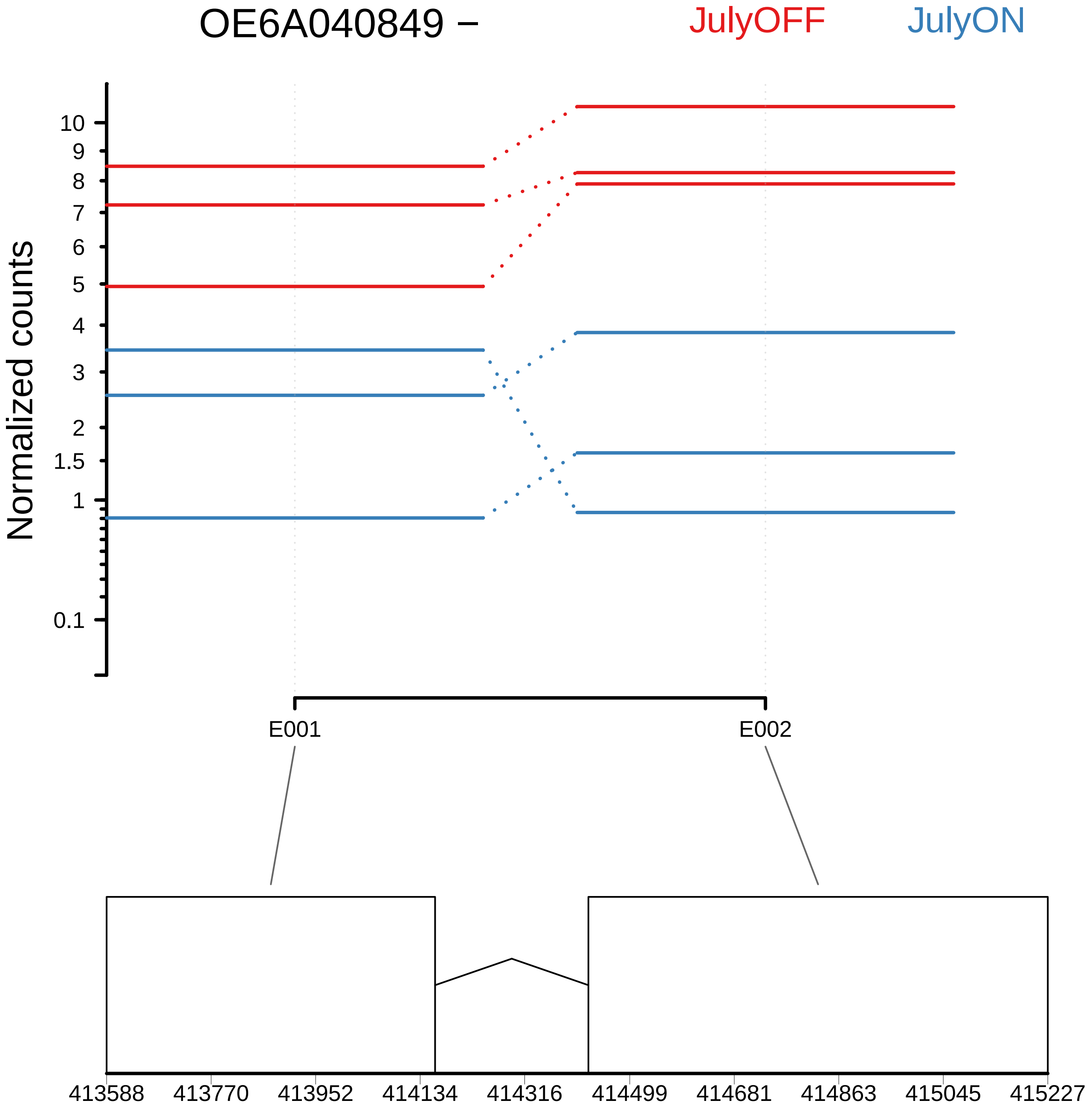
OE6A040849 −
JulyOFF
JulyON
Normalized counts
10
9
8
7
6
5
4
3
2
1.5
1
0.1
E001
E002
413588
413770
413952
414134
414316
414499
414681
414863
415045
415227

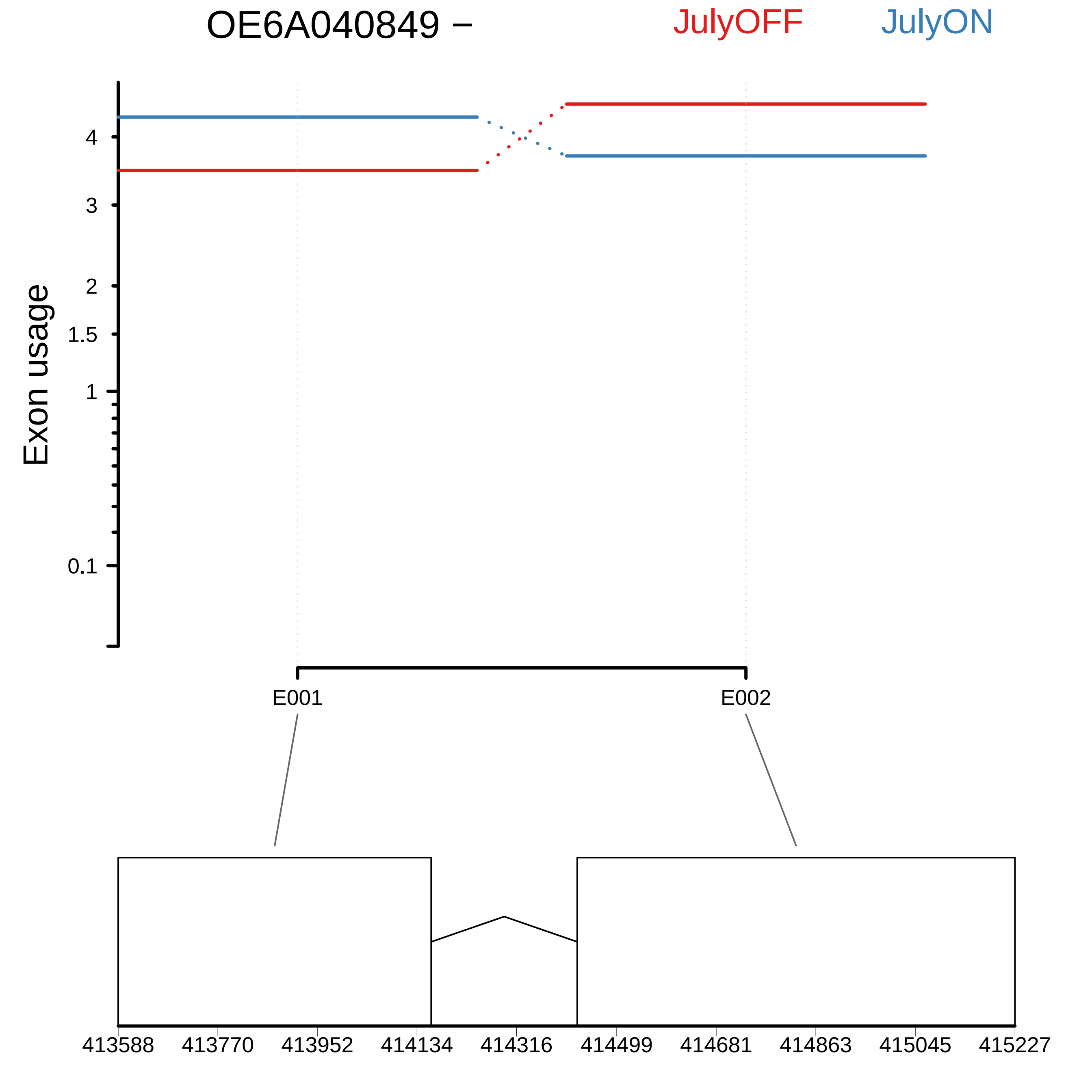

OE6A040849 −
JulyOFF
JulyON
Exon usage
4
3
2
1.5
1
0.1
E001
E002
413588
413770
413952
414134
414316
414499
414681
414863
415045
415227

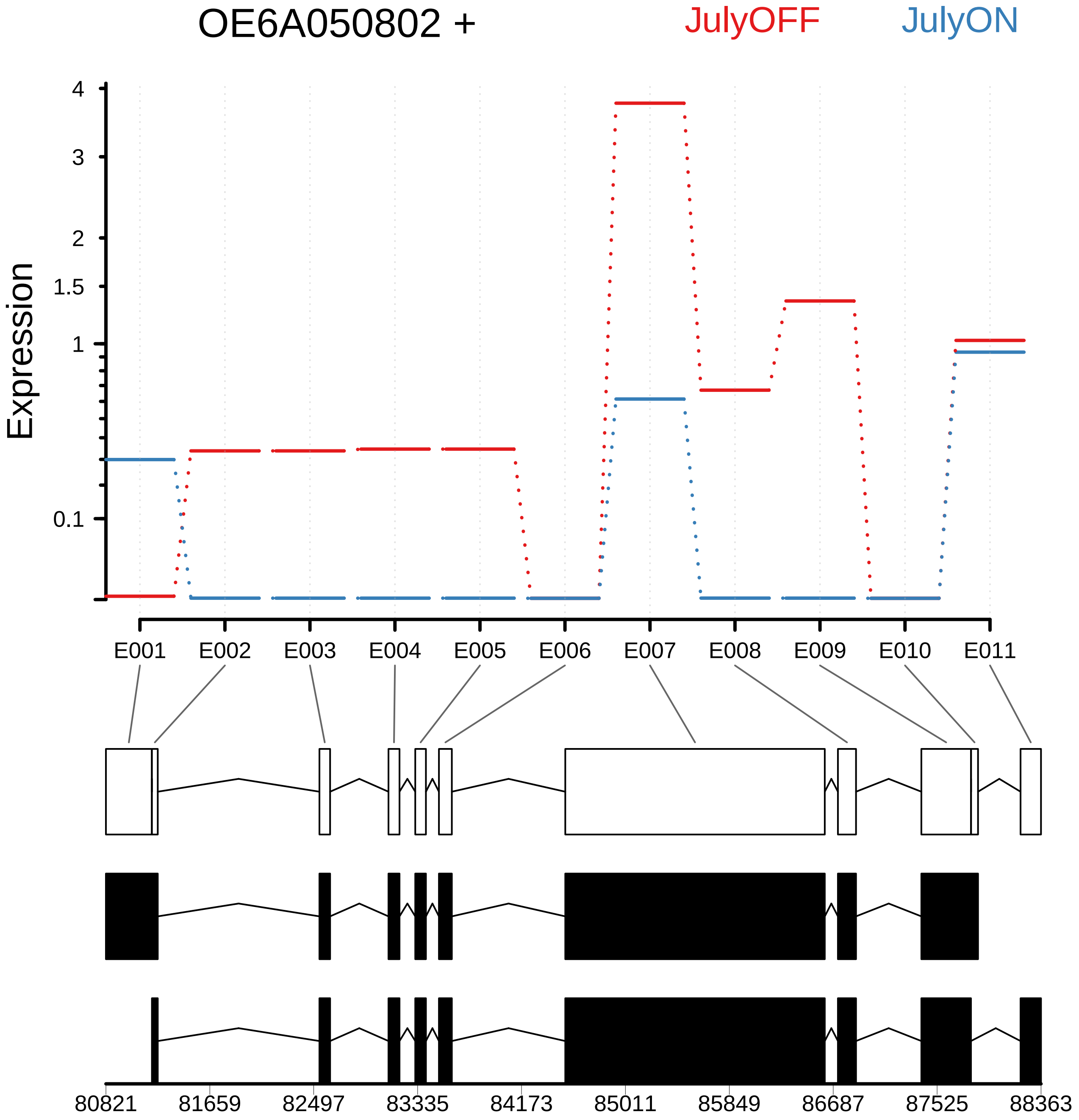

OE6A050802 +
JulyOFF
JulyON
Expression
4
3
2
1.5
1
0.1
E001
E002
E003
E004
E005
E006
E007
E008
E009
E010
E011
80821
81659
82497
83335
84173
85011
85849
86687
87525
88363

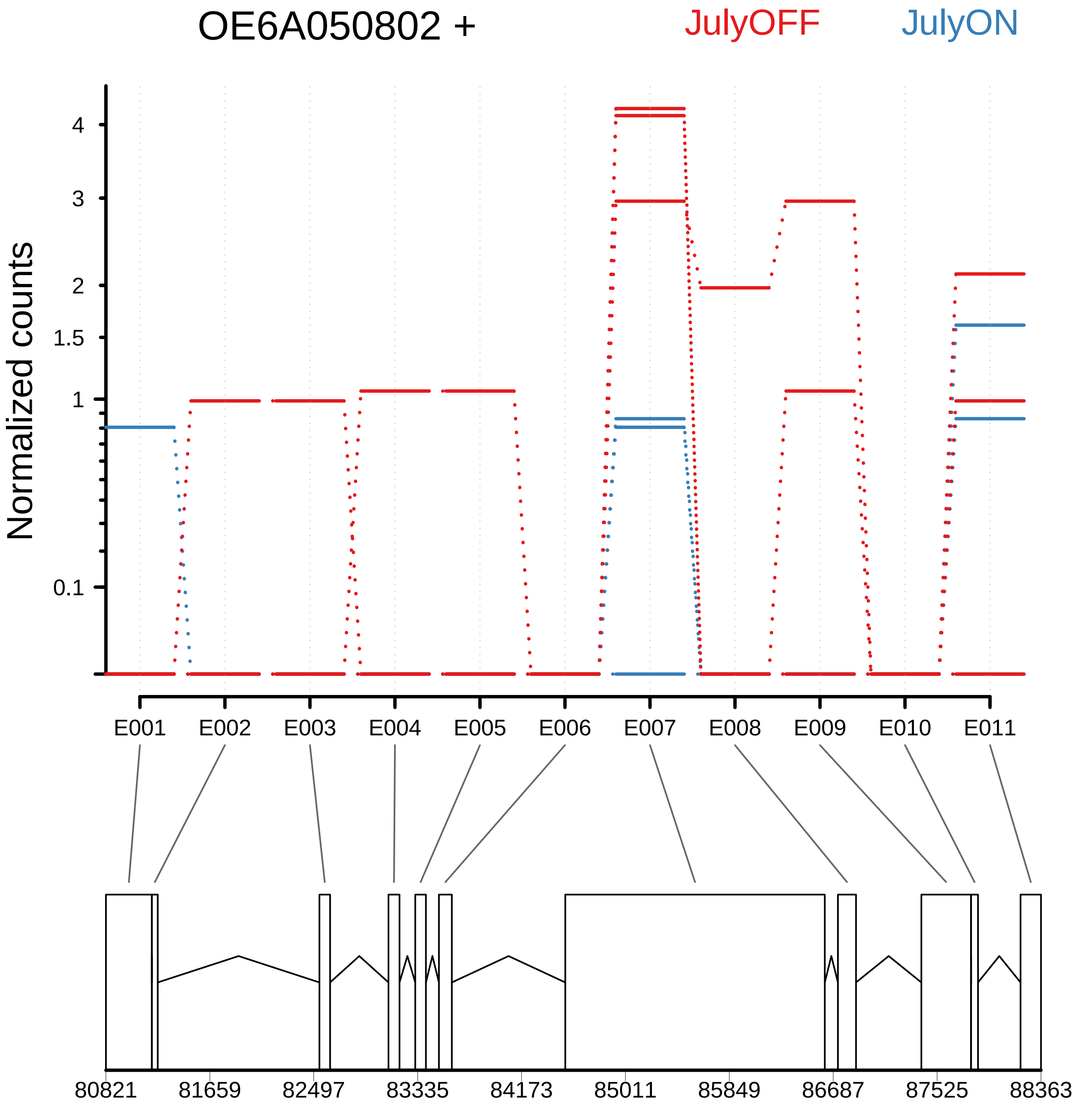
OE6A050802 +
JulyOFF
JulyON
Normalized counts
4
3
2
1.5
1
0.1
E001
E002
E003
E004
E005
E006
E007
E008
E009
E010
E011
80821
81659
82497
83335
84173
85011
85849
86687
87525
88363

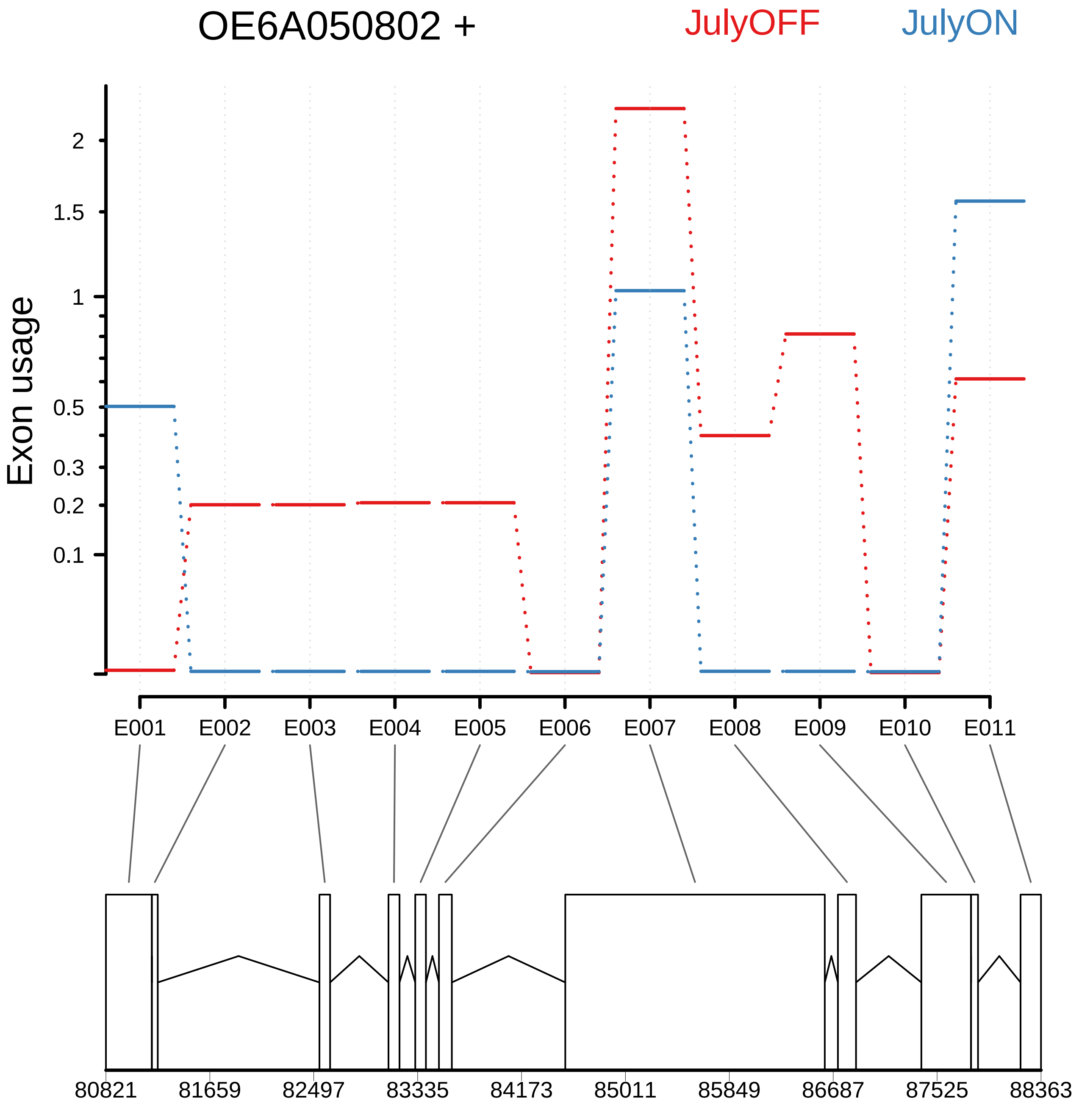
OE6A050802 +
JulyOFF
JulyON
Exon usage
2
1.5
1
0.5
0.3
0.2
0.1
E001
E002
E003
E004
E005
E006
E007
E008
E009
E010
E011
80821
81659
82497
83335
84173
85011
85849
86687
87525
88363

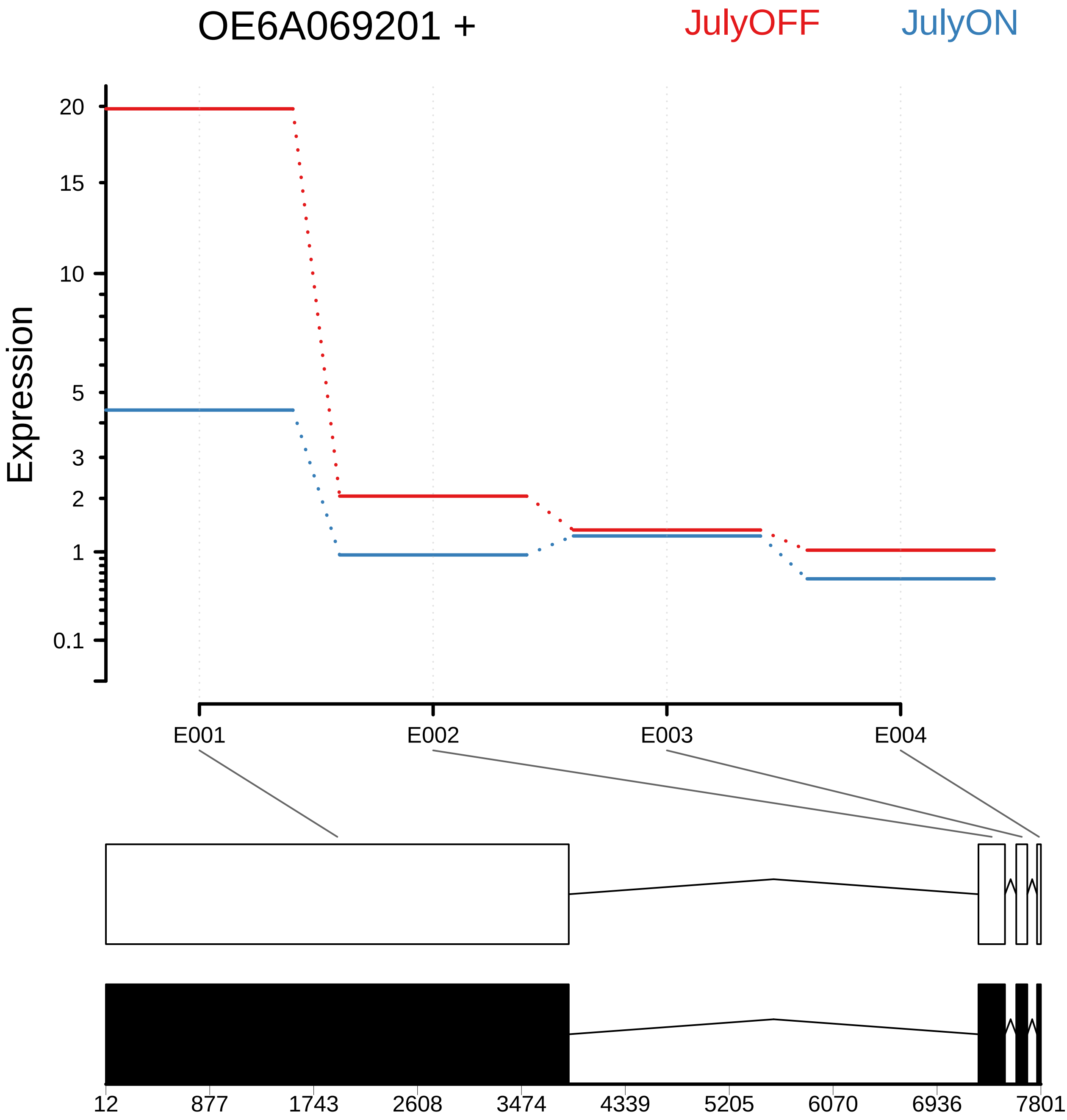
OE6A069201 +
JulyOFF
JulyON
Expression
20
15
10
5
3
2
1
0.1
E001
E002
E003
E004
12
877
1743
2608
3474
4339
5205
6070
6936
7801

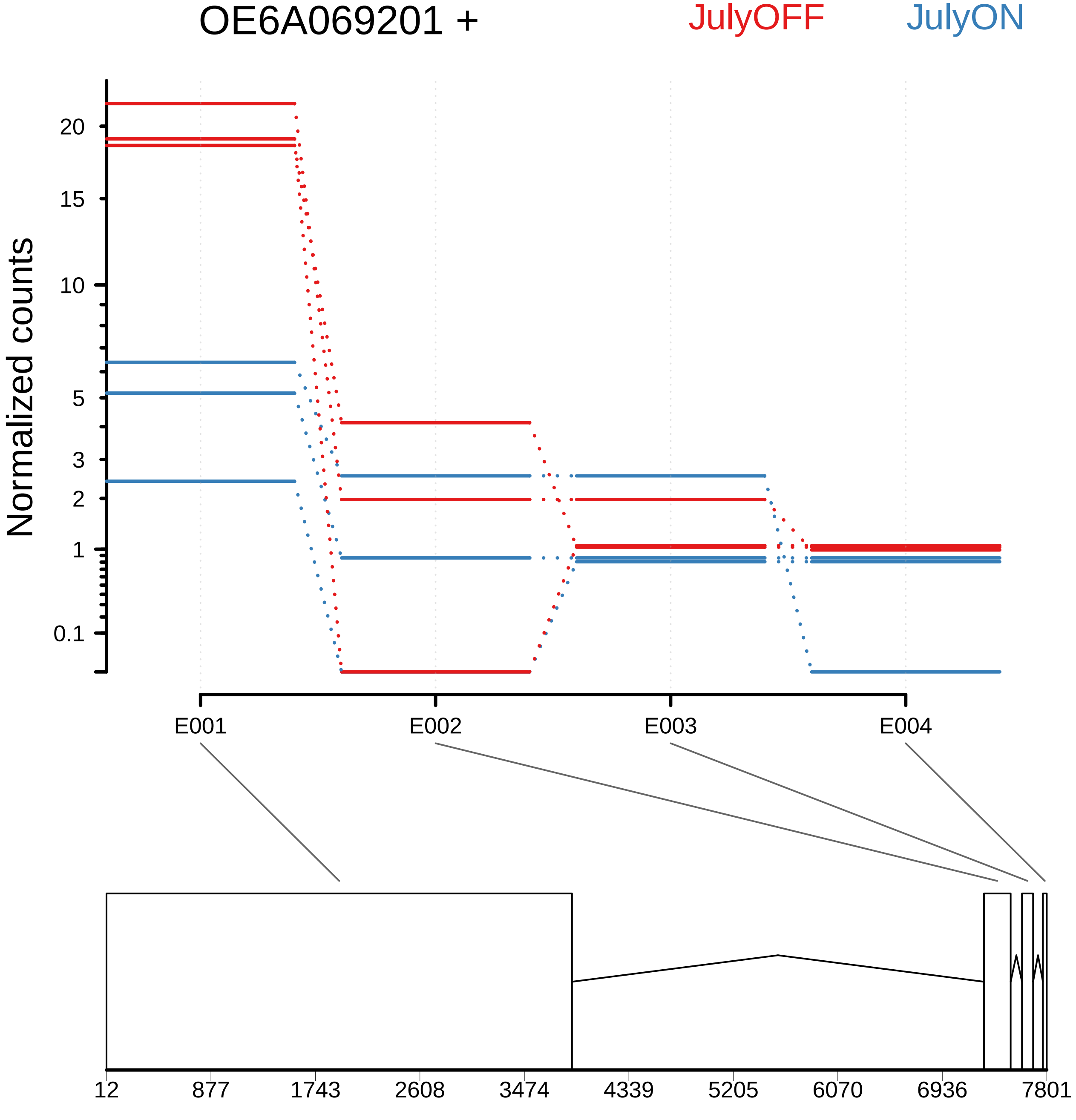

OE6A069201 +
JulyOFF
JulyON
Normalized counts
20
15
10
5
3
2
1
0.1
E001
E002
E003
E004
12
877
1743
2608
3474
4339
5205
6070
6936
7801

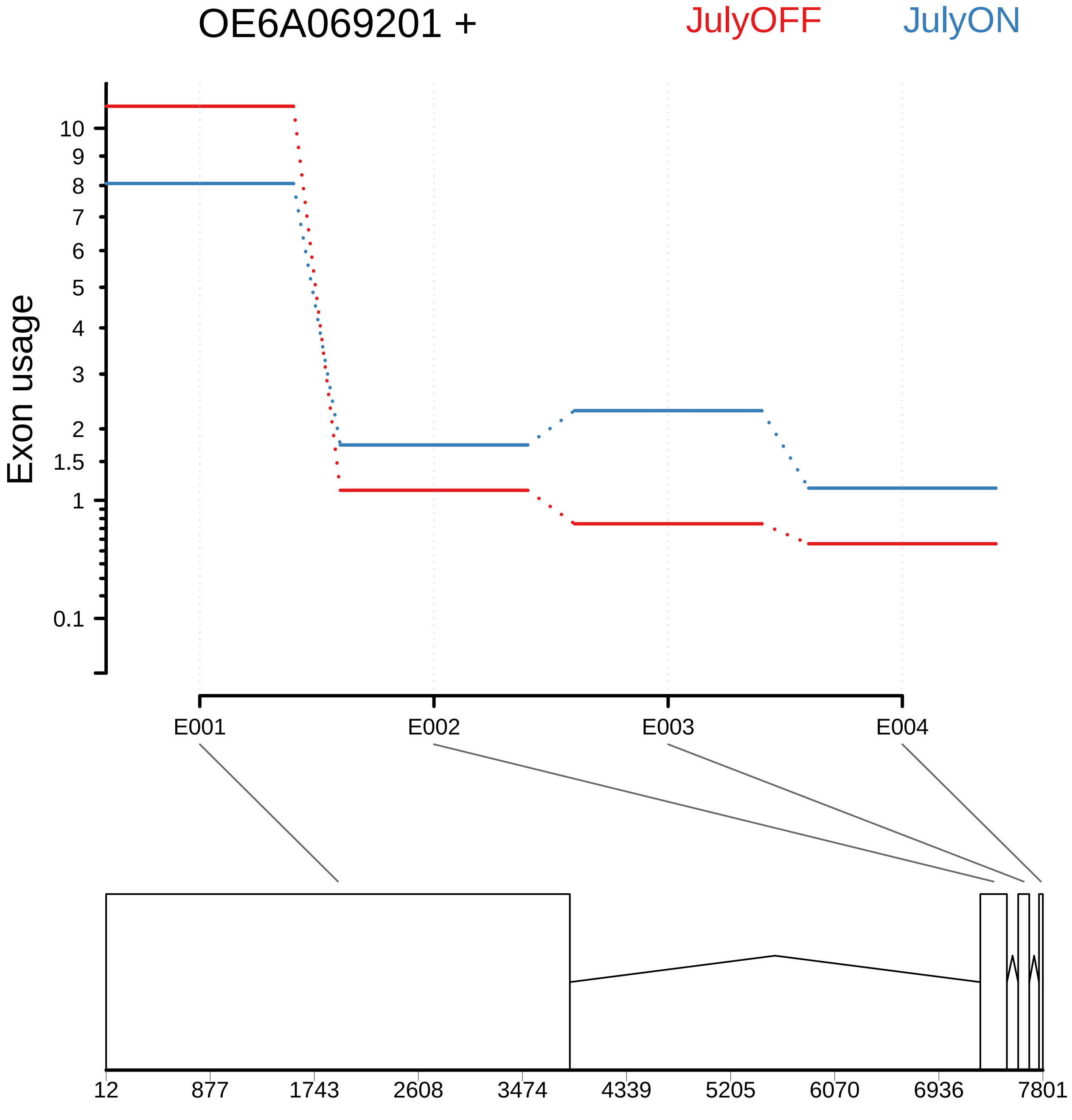
OE6A069201 +
JulyOFF
JulyON
Exon usage
10
9
8
7
6
5
4
3
2
1.5
1
0.1
E001
E002
E003
E004
12
877
1743
2608
3474
4339
5205
6070
6936
7801

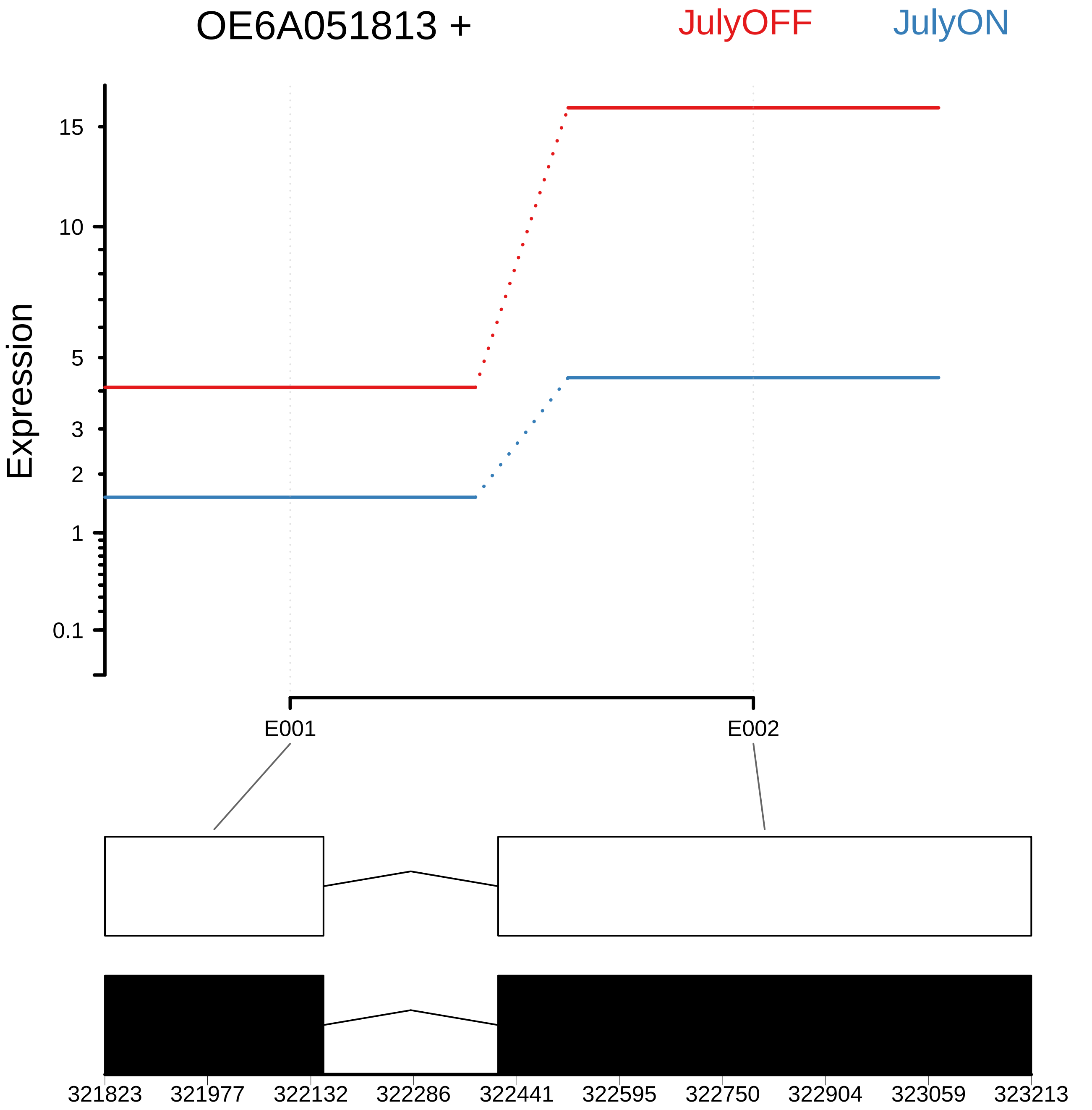

OE6A051813 +
JulyOFF
JulyON
Expression
15
10
5
3
2
1
0.1
E001
E002
321823
321977
322132
322286
322441
322595
322750
322904
323059
323213

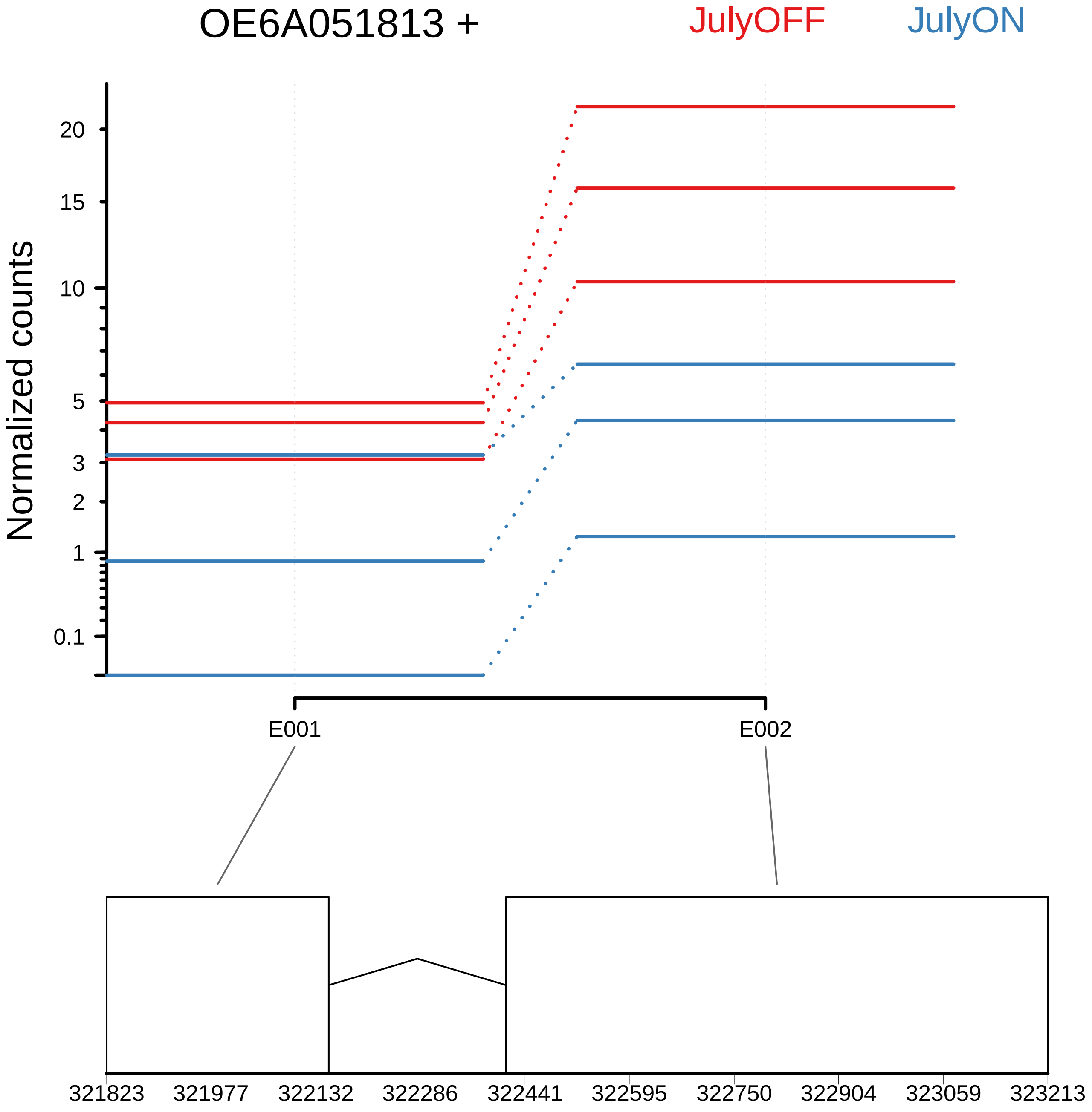
OE6A051813 +
JulyOFF
JulyON
Normalized counts
20
15
10
5
3
2
1
0.1
E001
E002
321823
321977
322132
322286
322441
322595
322750
322904
323059
323213

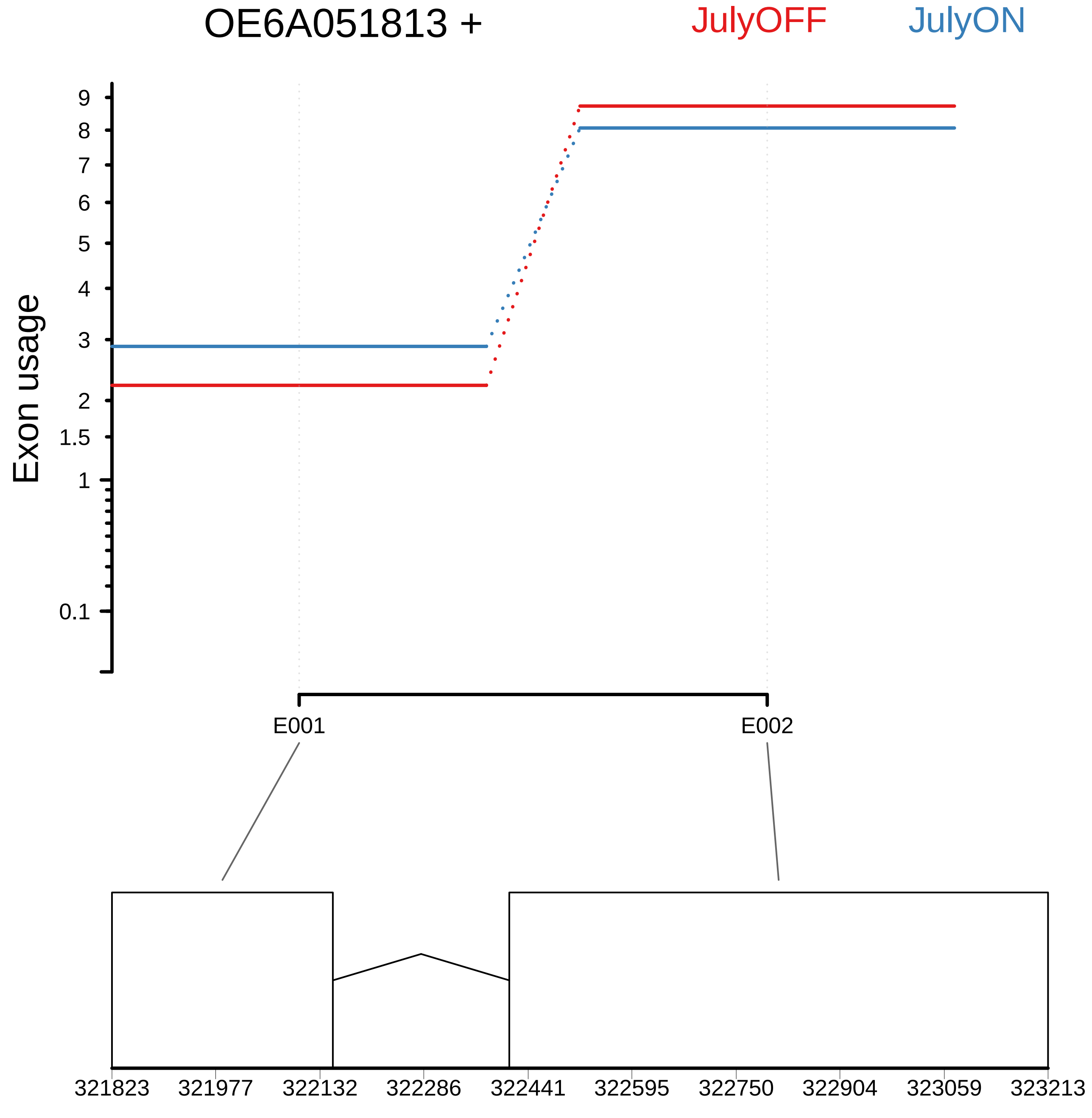

OE6A051813 +
JulyOFF
JulyON
Exon usage
9
8
7
6
5
4
3
2
1.5
1
0.1
E001
E002
321823
321977
322132
322286
322441
322595
322750
322904
323059
323213

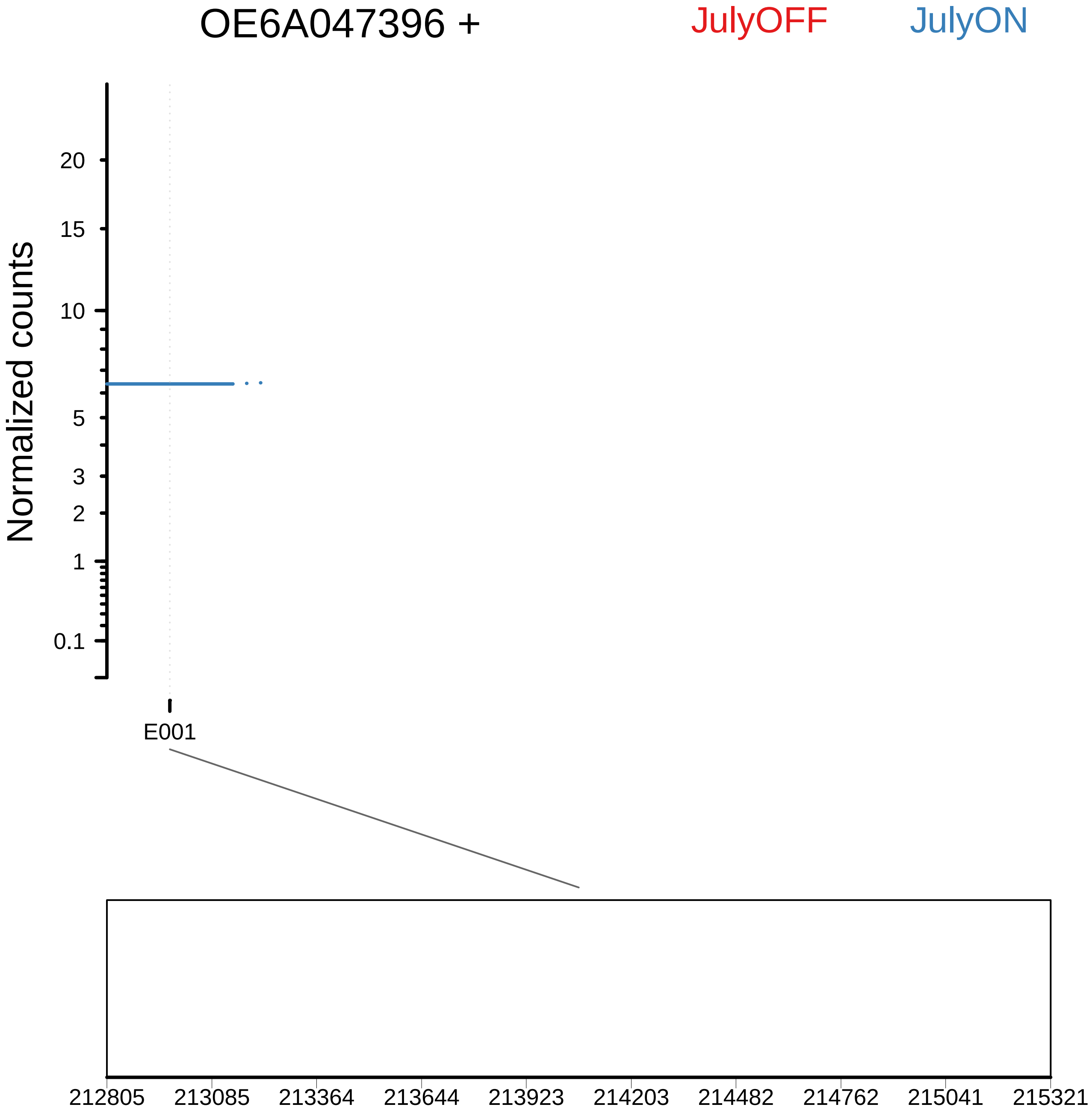

OE6A047396 +
JulyOFF
JulyON
Normalized counts
20
15
10
5
3
2
1
0.1
E001
212805
213085
213364
213644
213923
214203
214482
214762
215041
215321

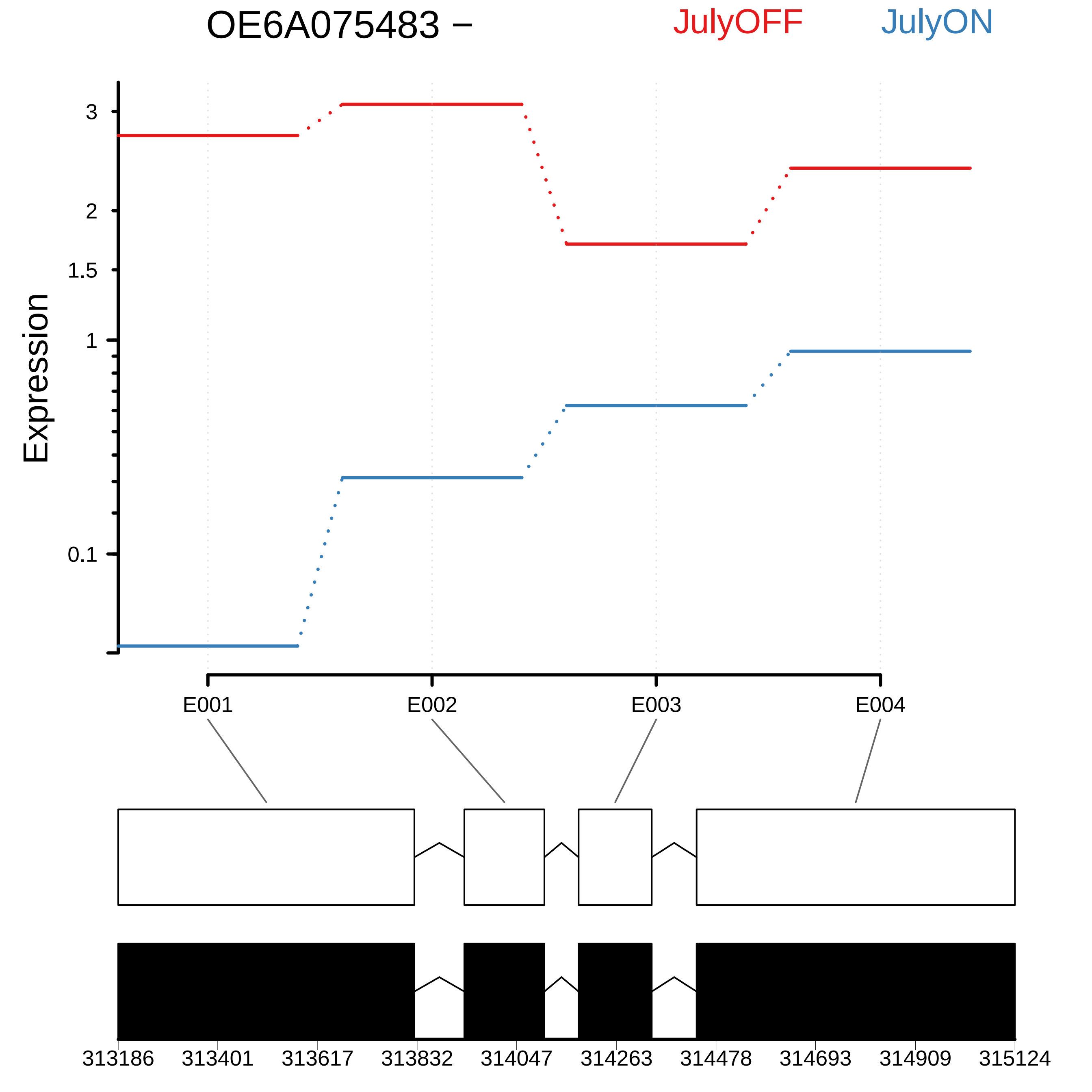

OE6A075483 –
JulyOFF
JulyON
Expression
3
2
1.5
1
0.1
E001
E002
E003
E004
313186
313401
313617
313832
314047
314263
314478
314693
314909
315124

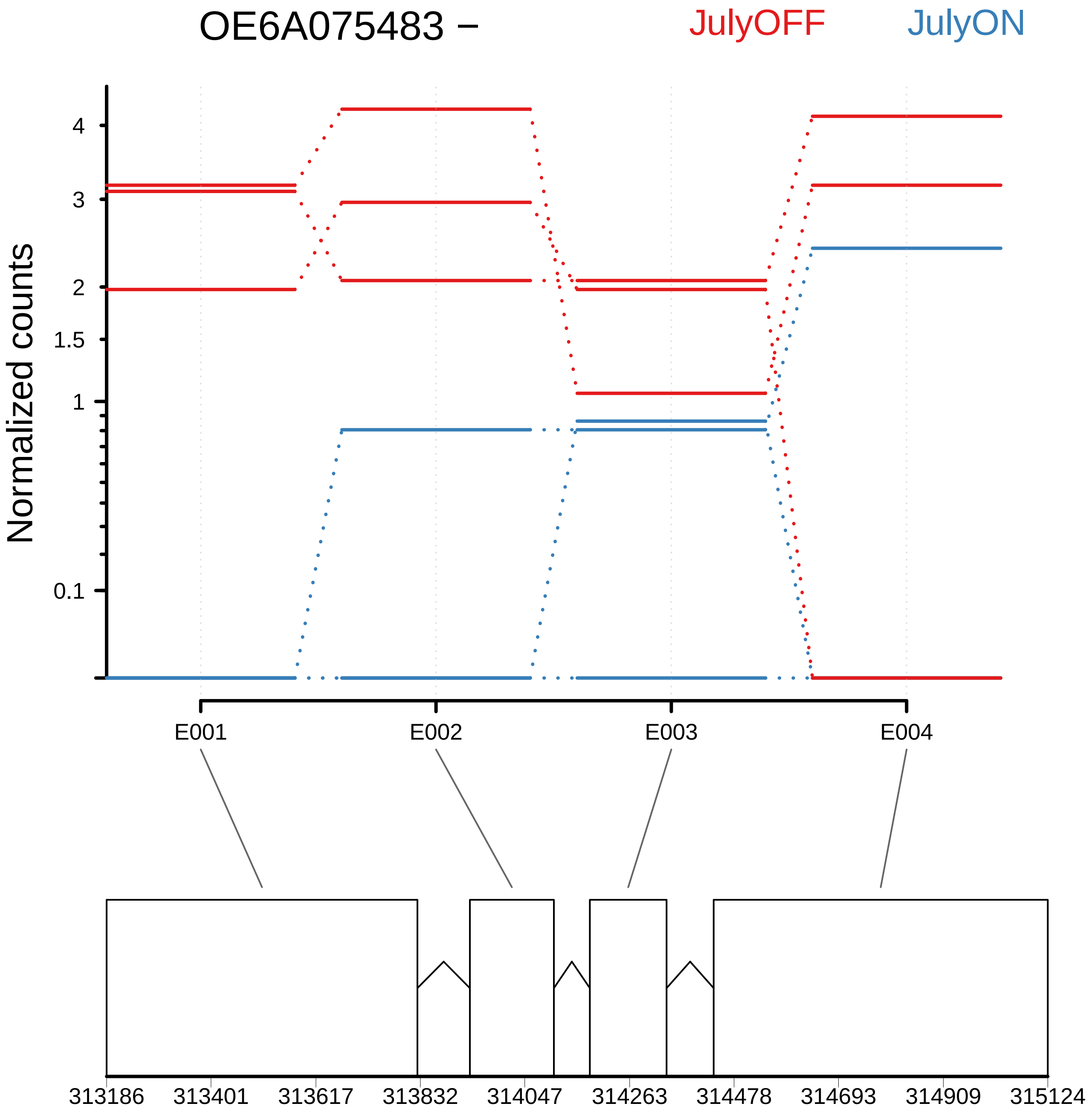

OE6A075483 −
JulyOFF
JulyON
Normalized counts
4
3
2
1.5
1
0.1
E001
E002
E003
E004
313186
313401
313617
313832
314047
314263
314478
314693
314909
315124

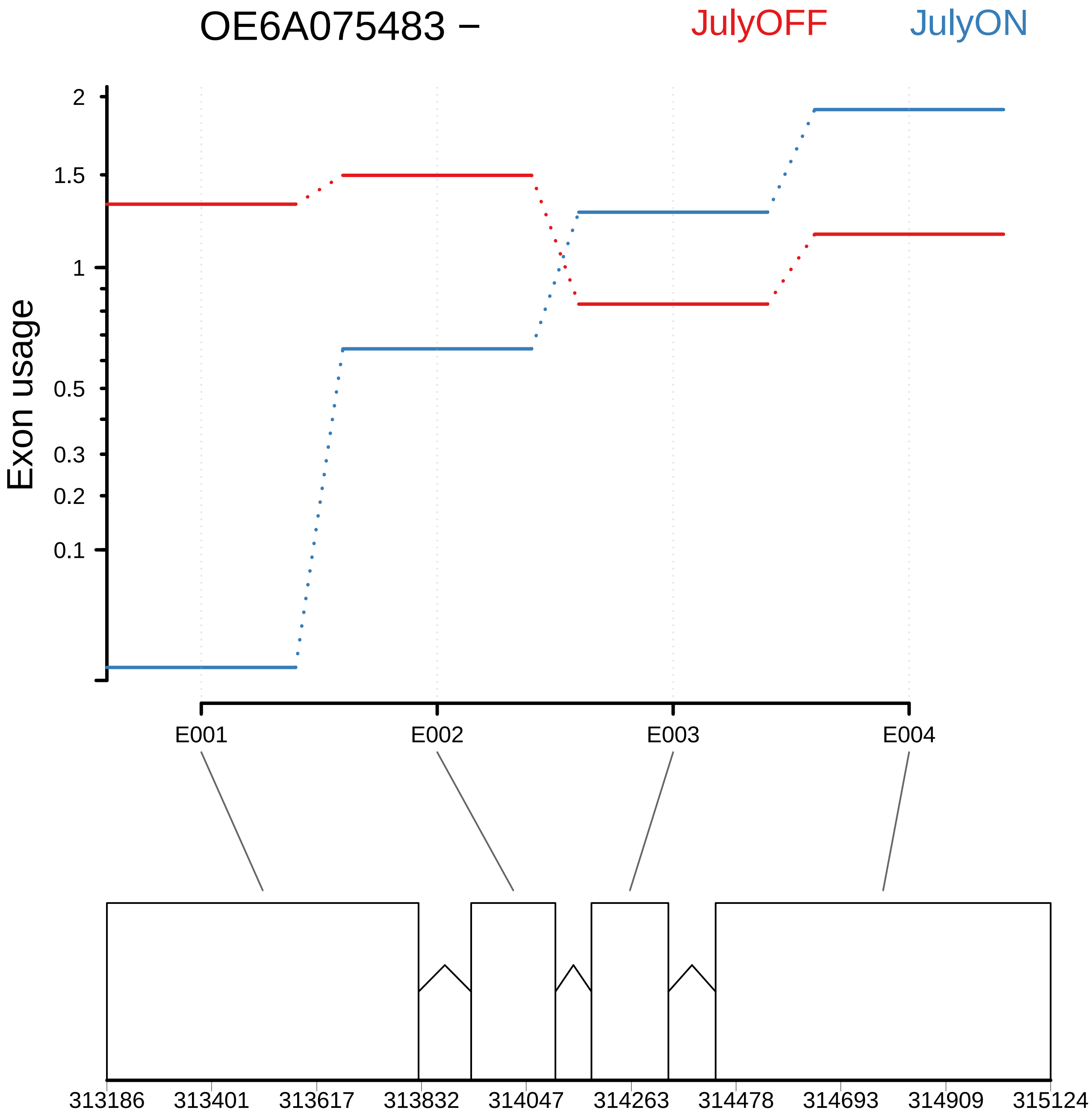
OE6A075483 −
JulyOFF
JulyON
Exon usage
2
1.5
1
0.5
0.3
0.2
0.1
E001
E002
E003
E004
313186
313401
313617
313832
314047
314263
314478
314693
314909
315124

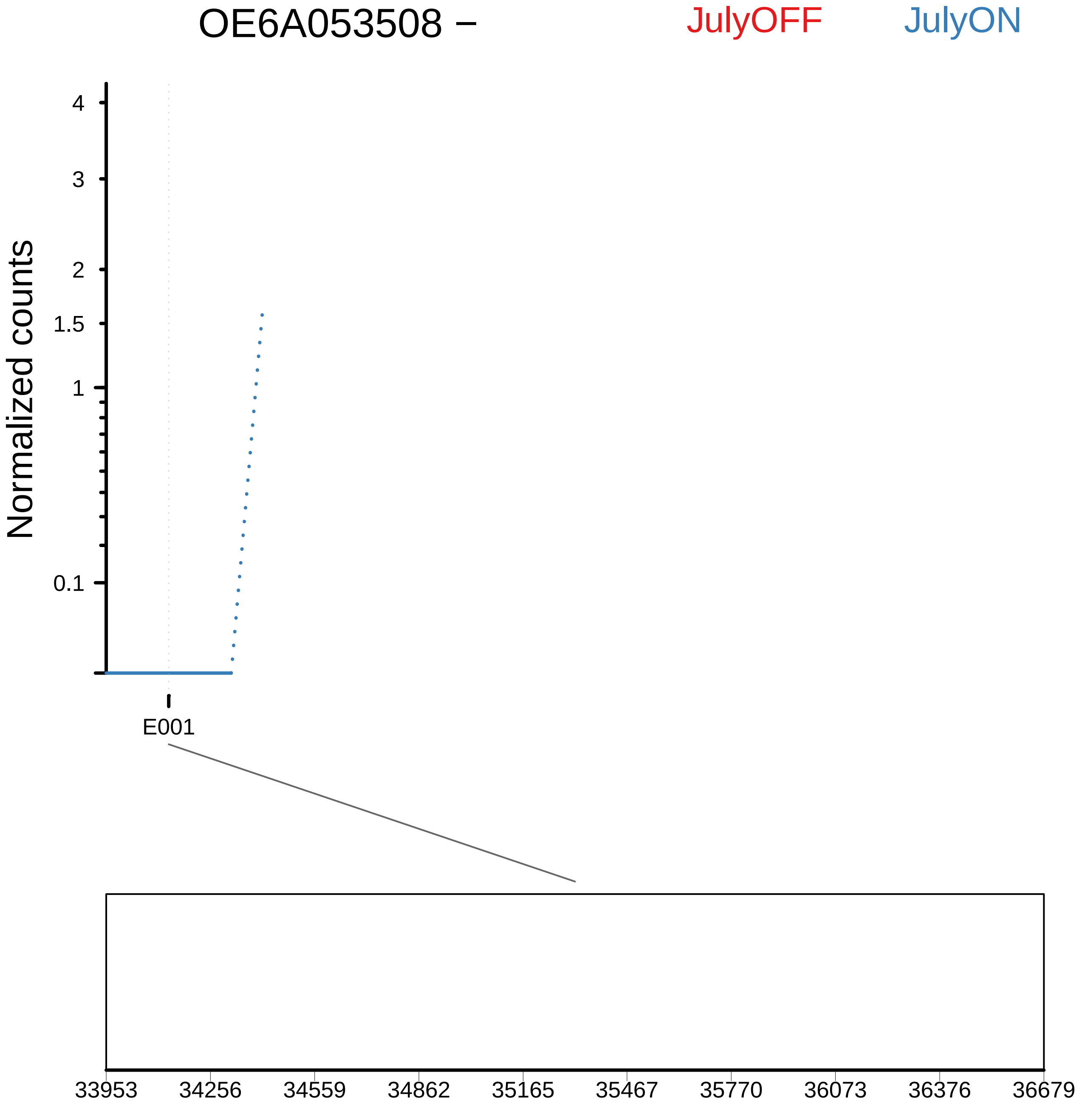

OE6A053508 −
JulyOFF
JulyON
Normalized counts
4
3
2
1.5
1
0.1
E001
33953
34256
34559
34862
35165
35467
35770
36073
36376
36679

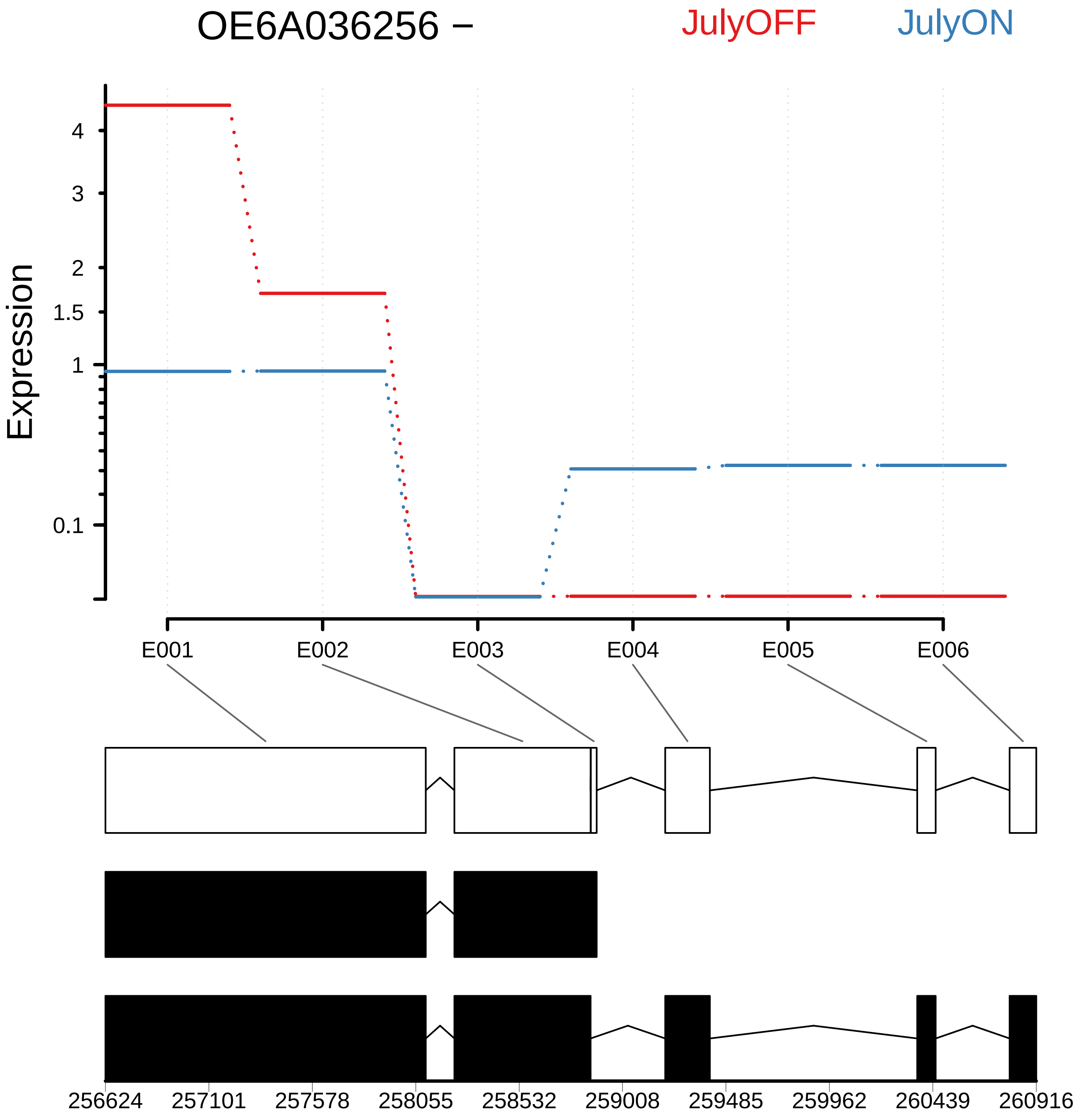

OE6A036256 −
JulyOFF
JulyON
Expression
4
3
2
1.5
1
0.1
E001
E002
E003
E004
E005
E006
256624
257101
257578
258055
258532
259008
259485
259962
260439
260916

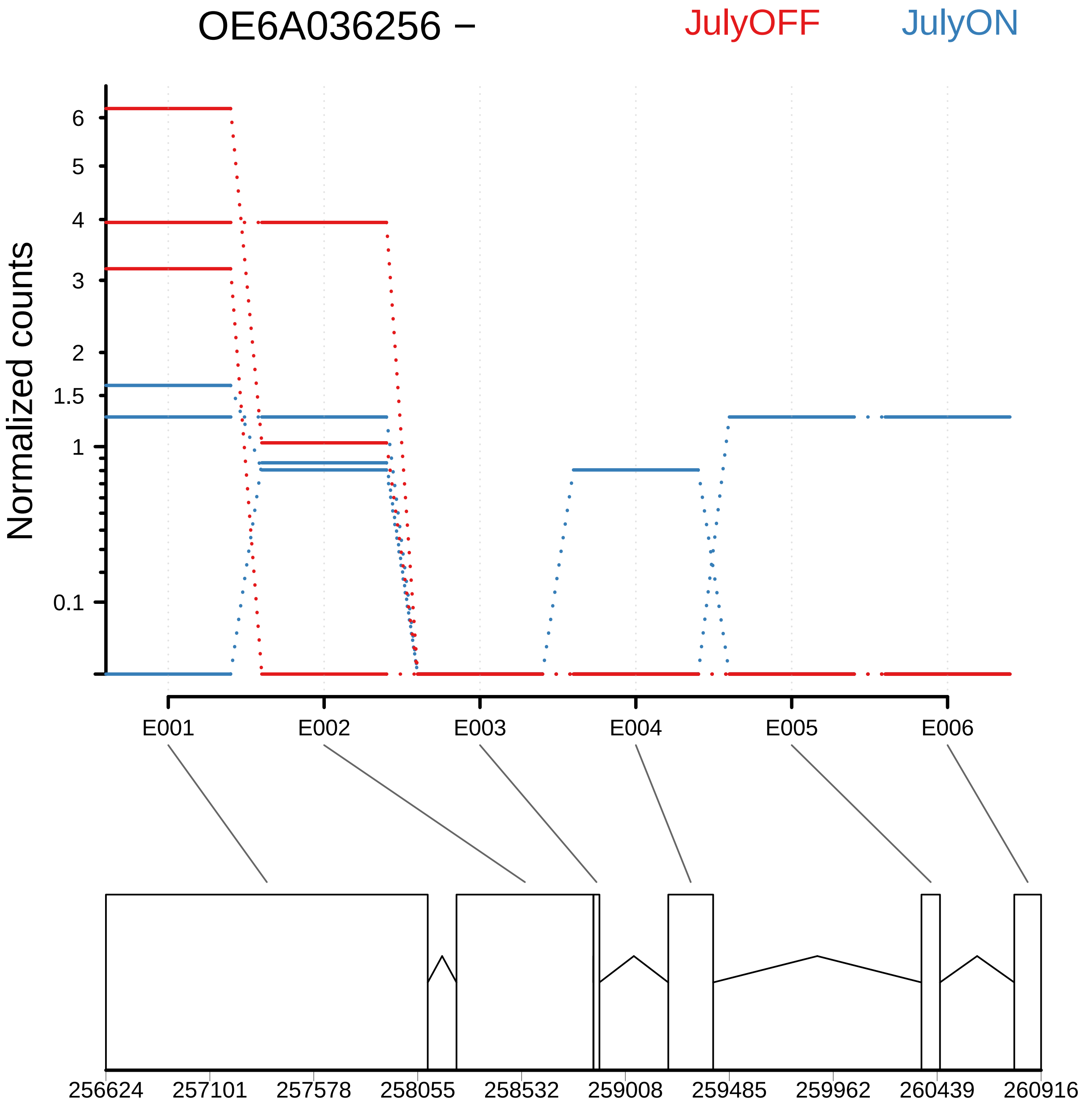
OE6A036256 −
JulyOFF
JulyON
Normalized counts
6
5
4
3
2
1.5
1
0.1
E001
E002
E003
E004
E005
E006
256624
257101
257578
258055
258532
259008
259485
259962
260439
260916

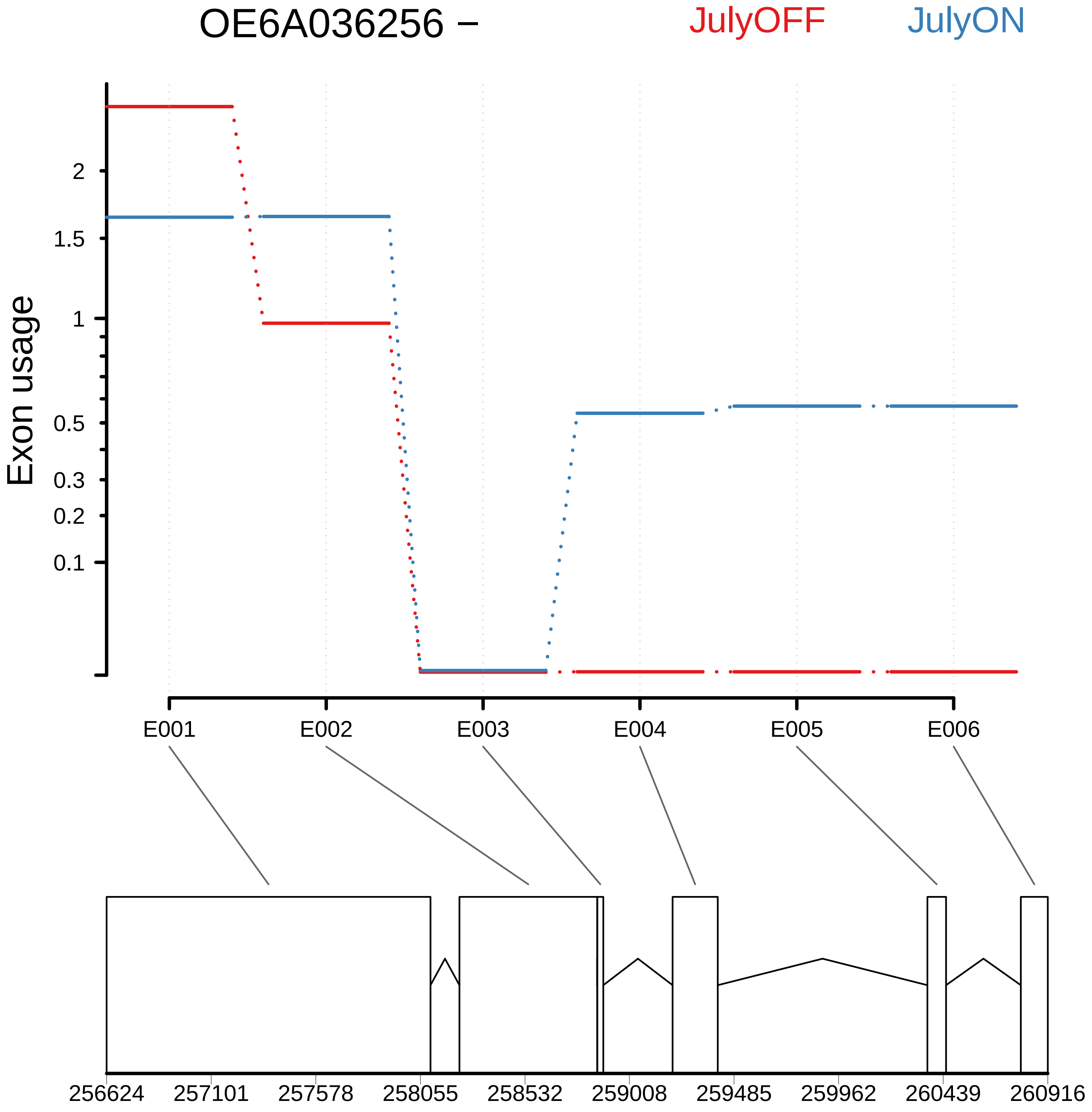

OE6A036256 −
JulyOFF
JulyON
Exon usage
2
1.5
1
0.5
0.3
0.2
0.1
E001
E002
E003
E004
E005
E006
256624
257101
257578
258055
258532
259008
259485
259962
260439
260916

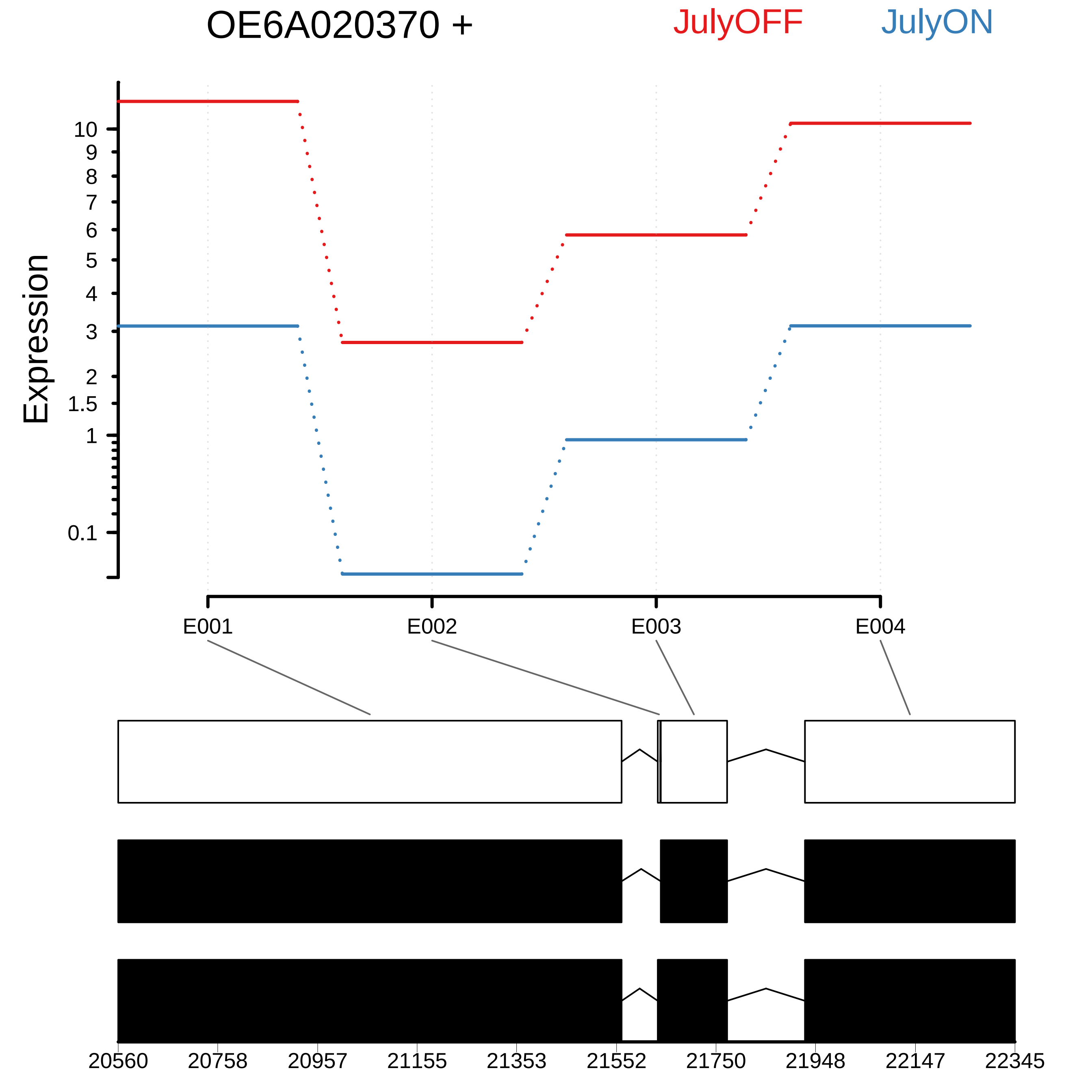
OE6A020370 +
JulyOFF
JulyON
Expression
10
9
8
7
6
5
4
3
2
1.5
1
0.1
E001
E002
E003
E004
20560
20758
20957
21155
21353
21552
21750
21948
22147
22345

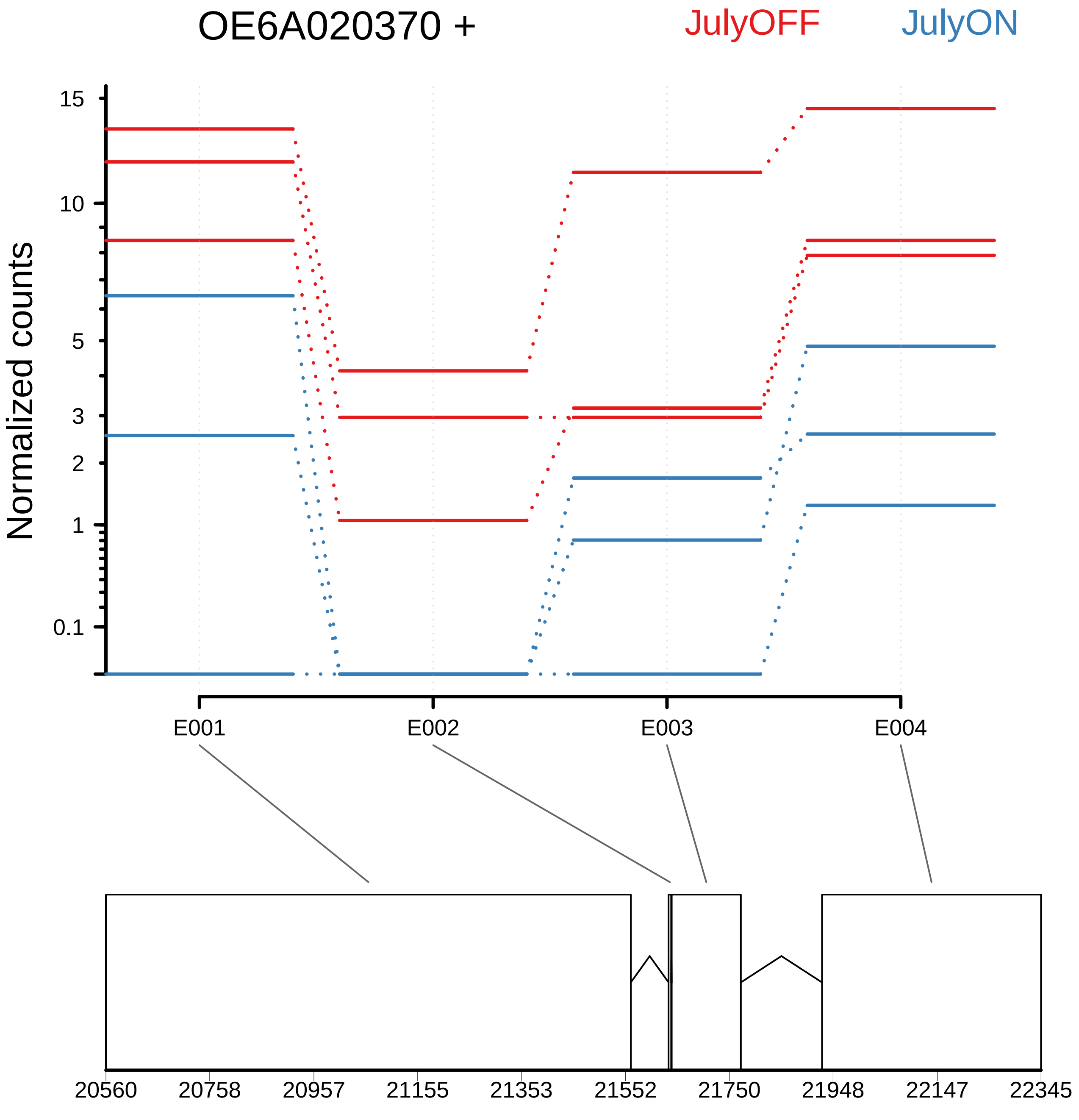
OE6A020370 +
JulyOFF
JulyON
Normalized counts
15
10
5
3
2
1
0.1
E001
E002
E003
E004
20560
20758
20957
21155
21353
21552
21750
21948
22147
22345

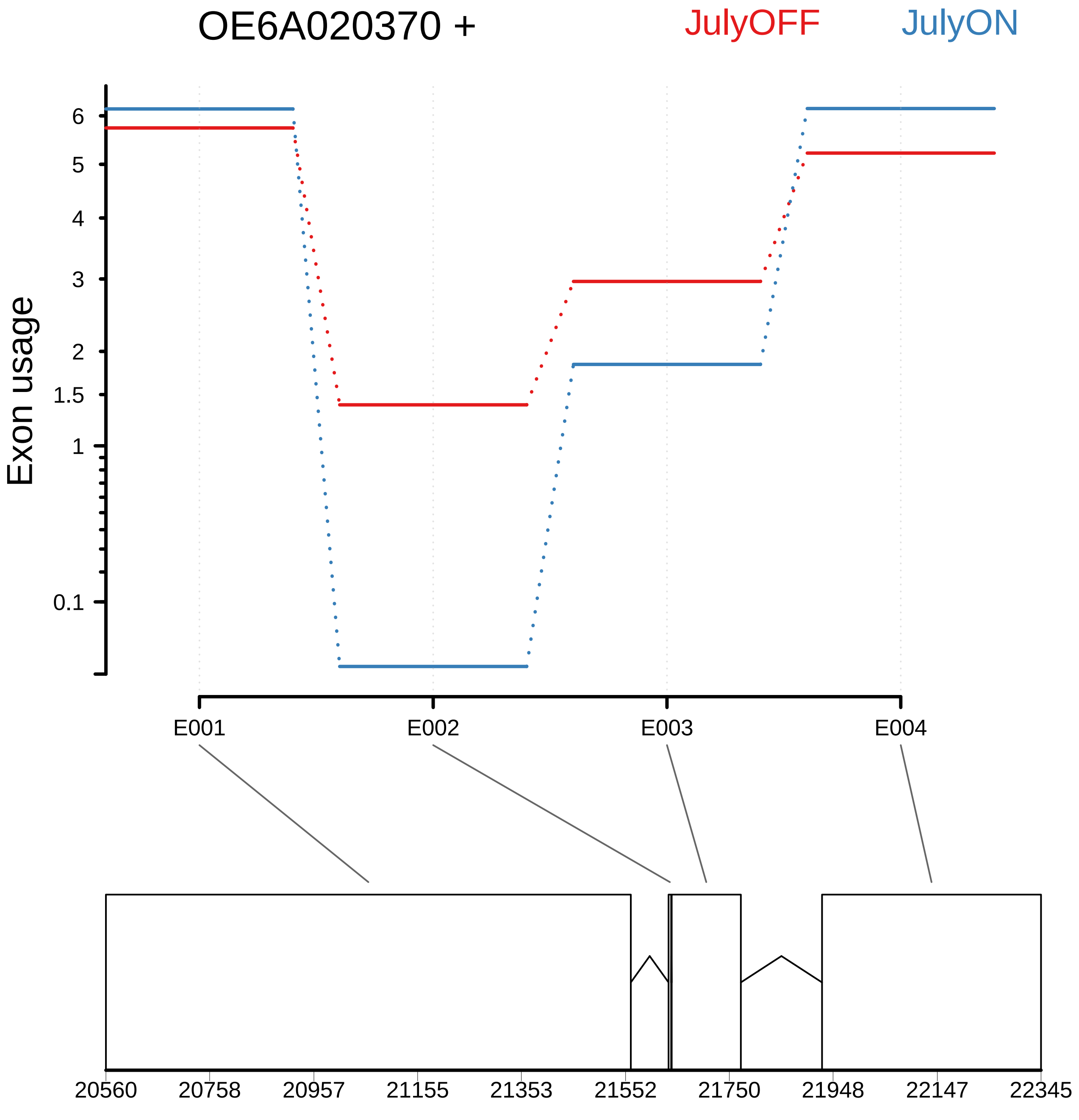
OE6A020370 +
JulyOFF
JulyON
Exon usage
6
5
4
3
2
1.5
1
0.1
E001
E002
E003
E004
20560
20758
20957
21155
21353
21552
21750
21948
22147
22345

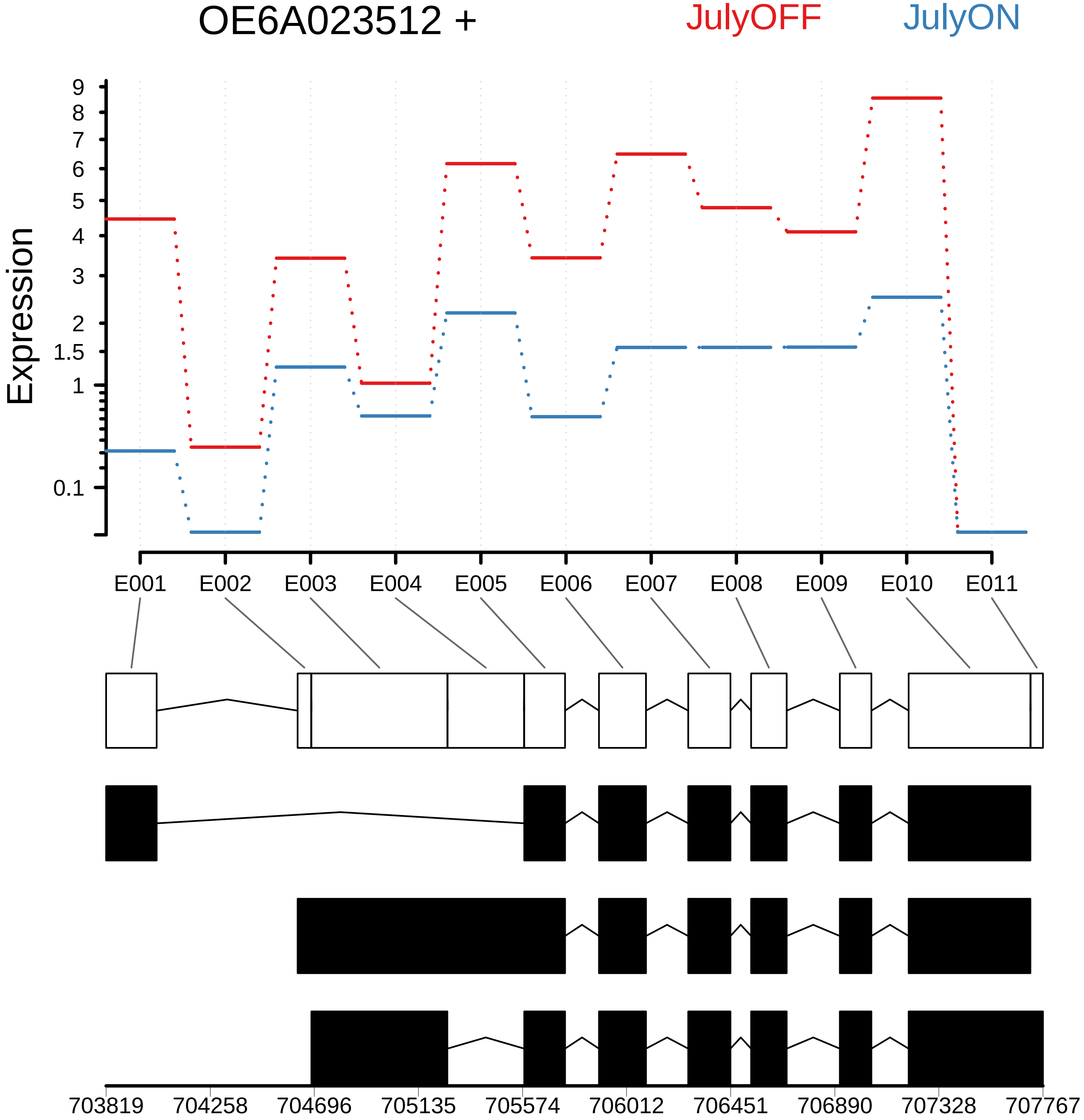

OE6A023512 +
JulyOFF
JulyON
Expression
9
8
7
6
5
4
3
2
1.5
1
0.1
E001
E002
E003
E004
E005
E006
E007
E008
E009
E010
E011
703819
704258
704696
705135
705574
706012
706451
706890
707328
707767

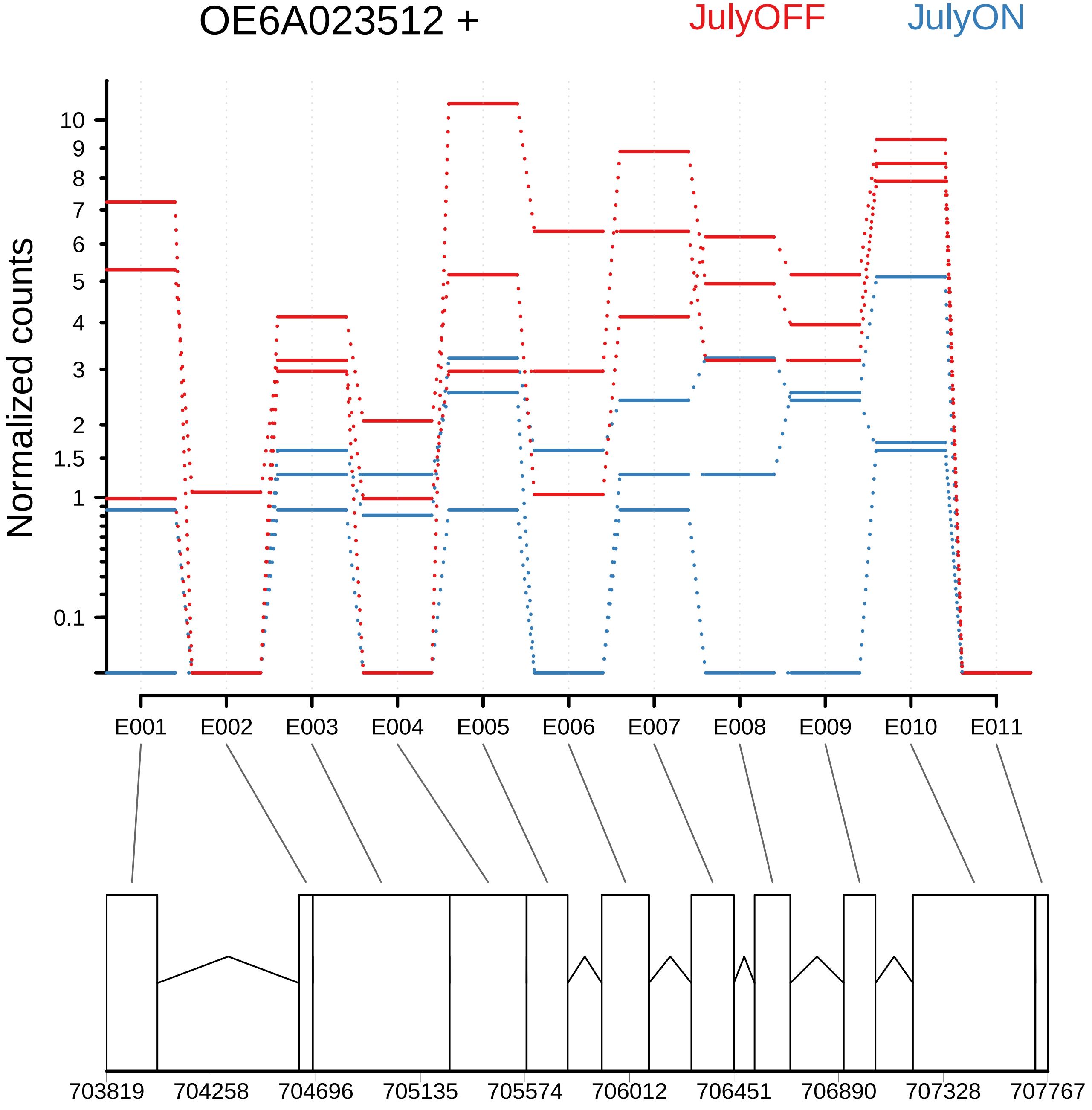

OE6A023512 +
JulyOFF
JulyON
Normalized counts
10
9
8
7
6
5
4
3
2
1.5
1
0.1
E001
E002
E003
E004
E005
E006
E007
E008
E009
E010
E011
703819
704258
704696
705135
705574
706012
706451
706890
707328
707767

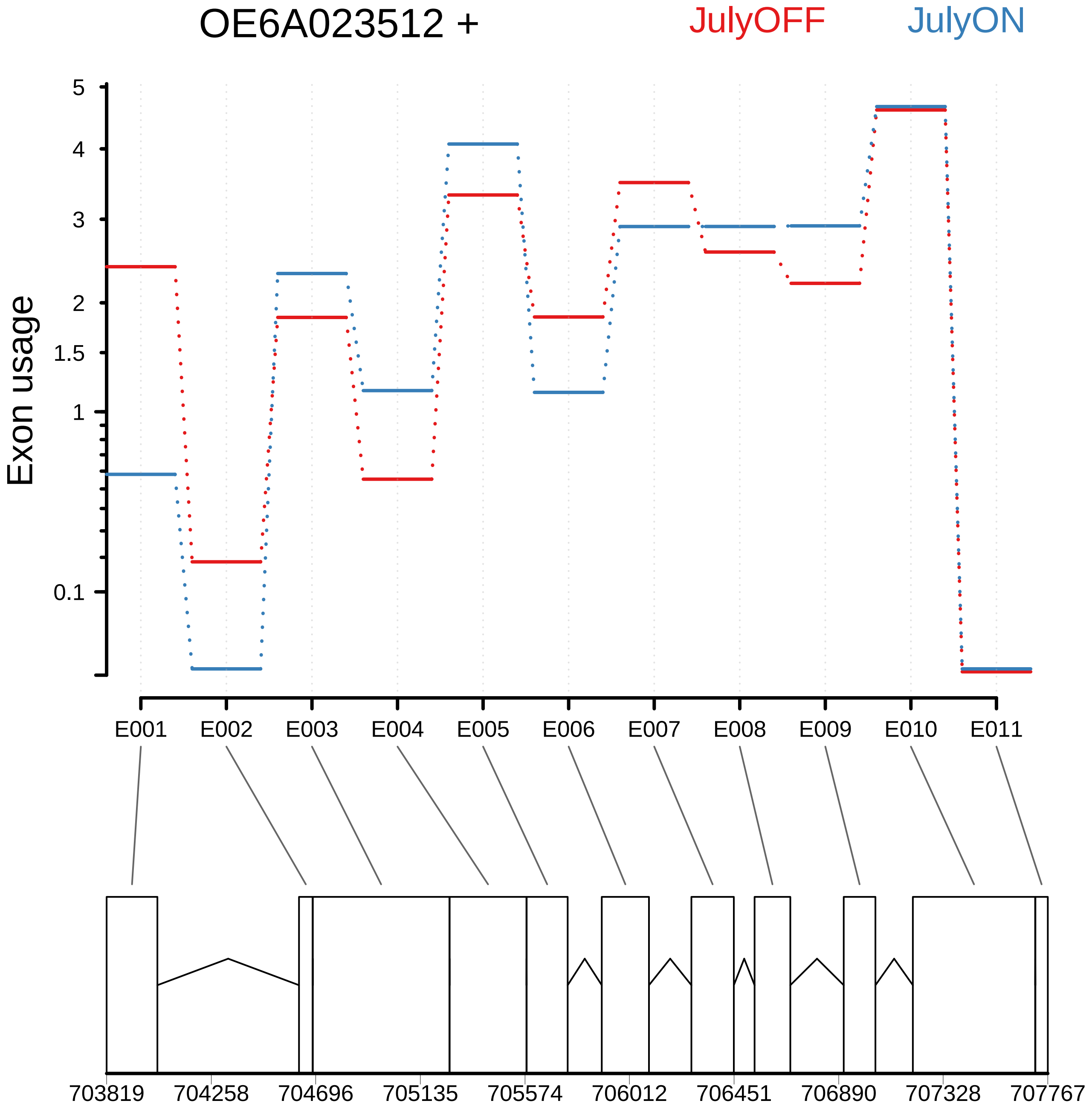
OE6A023512 +
JulyOFF
JulyON
Exon usage
5
4
3
2
1.5
1
0.1
E001
E002
E003
E004
E005
E006
E007
E008
E009
E010
E011
703819
704258
704696
705135
705574
706012
706451
706890
707328
707767

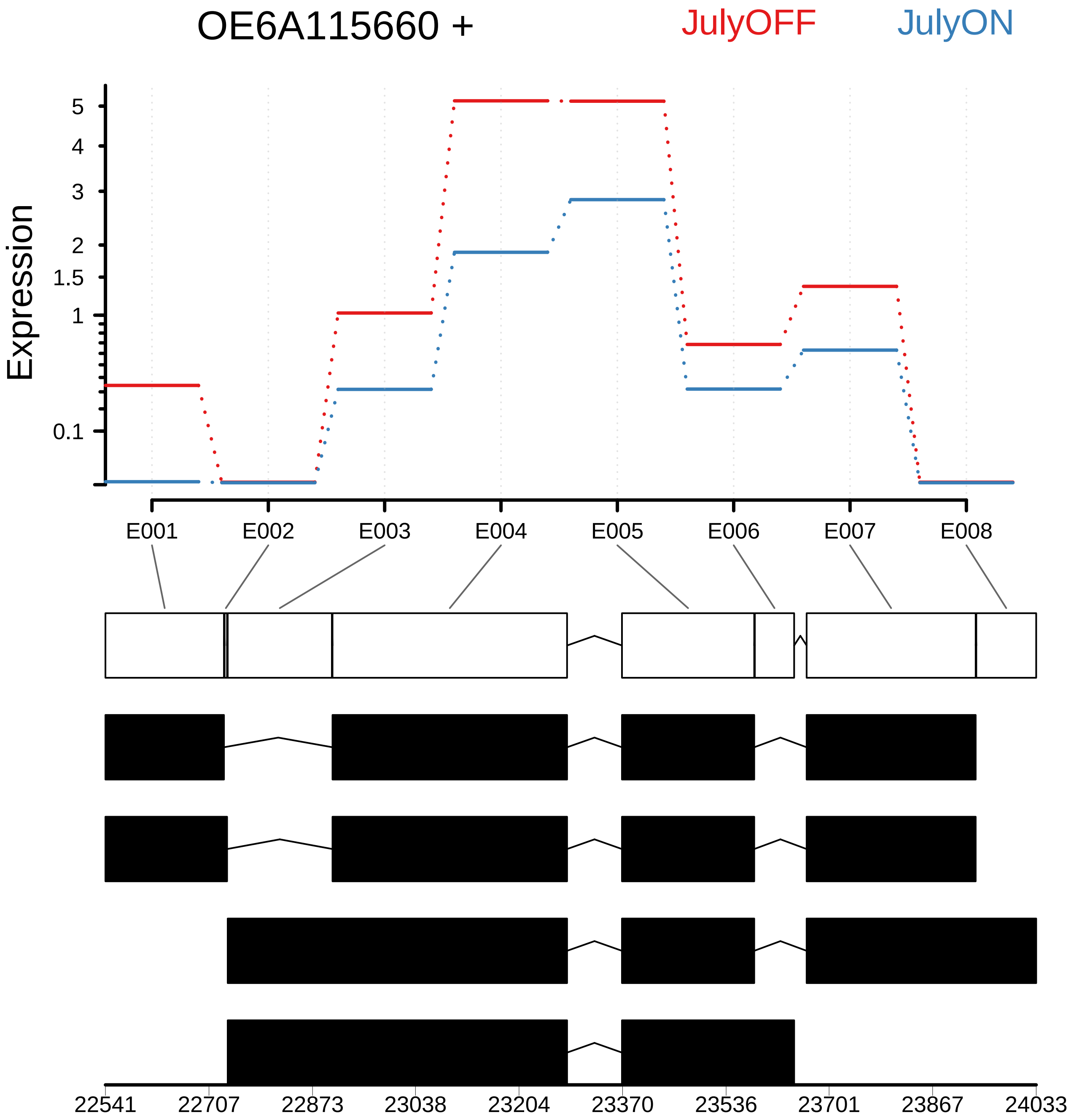

OE6A115660 +
JulyOFF
JulyON
Expression
5
4
3
2
1.5
1
0.1
E001
E002
E003
E004
E005
E006
E007
E008
22541
22707
22873
23038
23204
23370
23536
23701
23867
24033

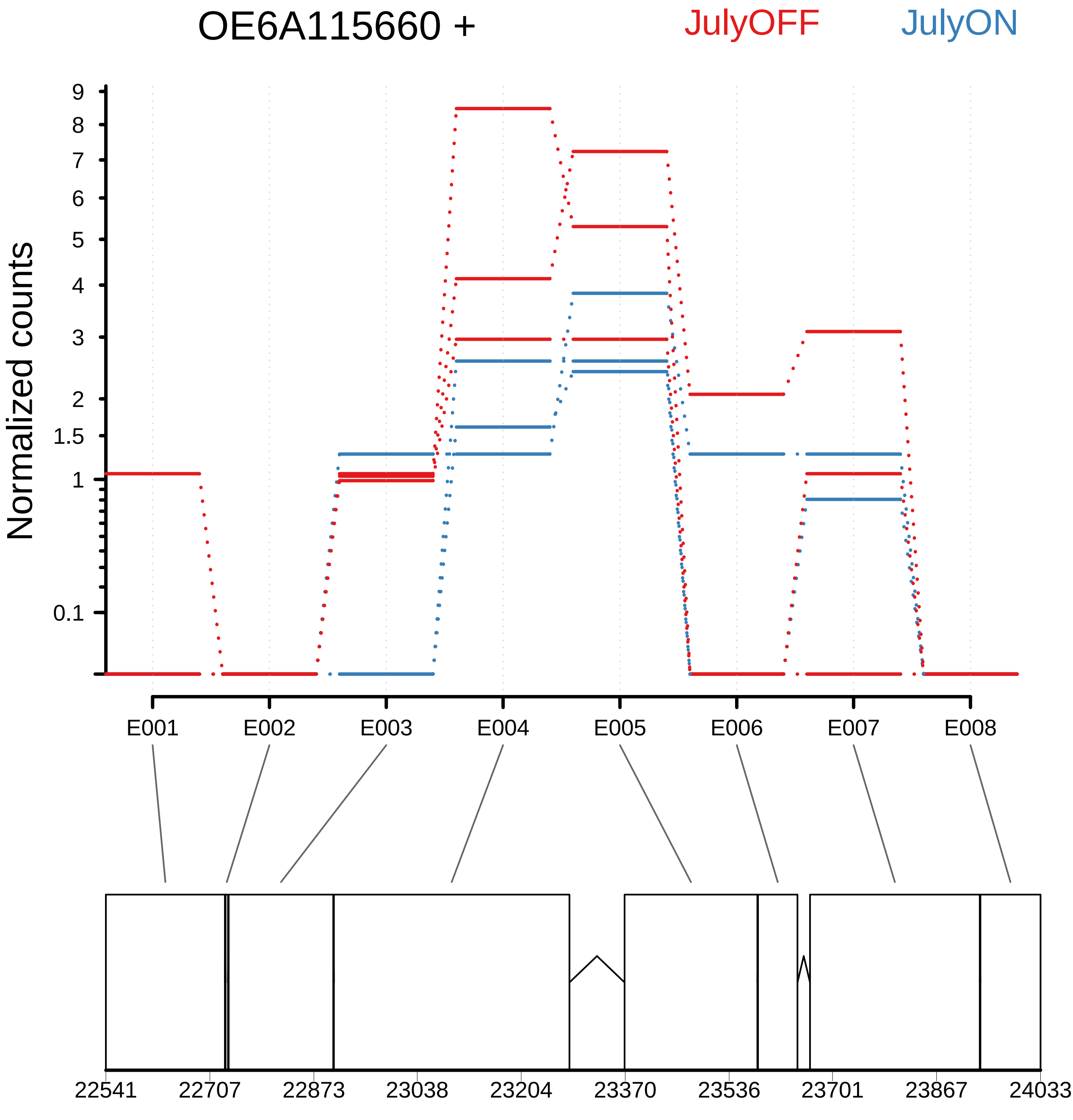

OE6A115660 +
JulyOFF
JulyON
Normalized counts
9
8
7
6
5
4
3
2
1.5
1
0.1
E001
E002
E003
E004
E005
E006
E007
E008
22541
22707
22873
23038
23204
23370
23536
23701
23867
24033

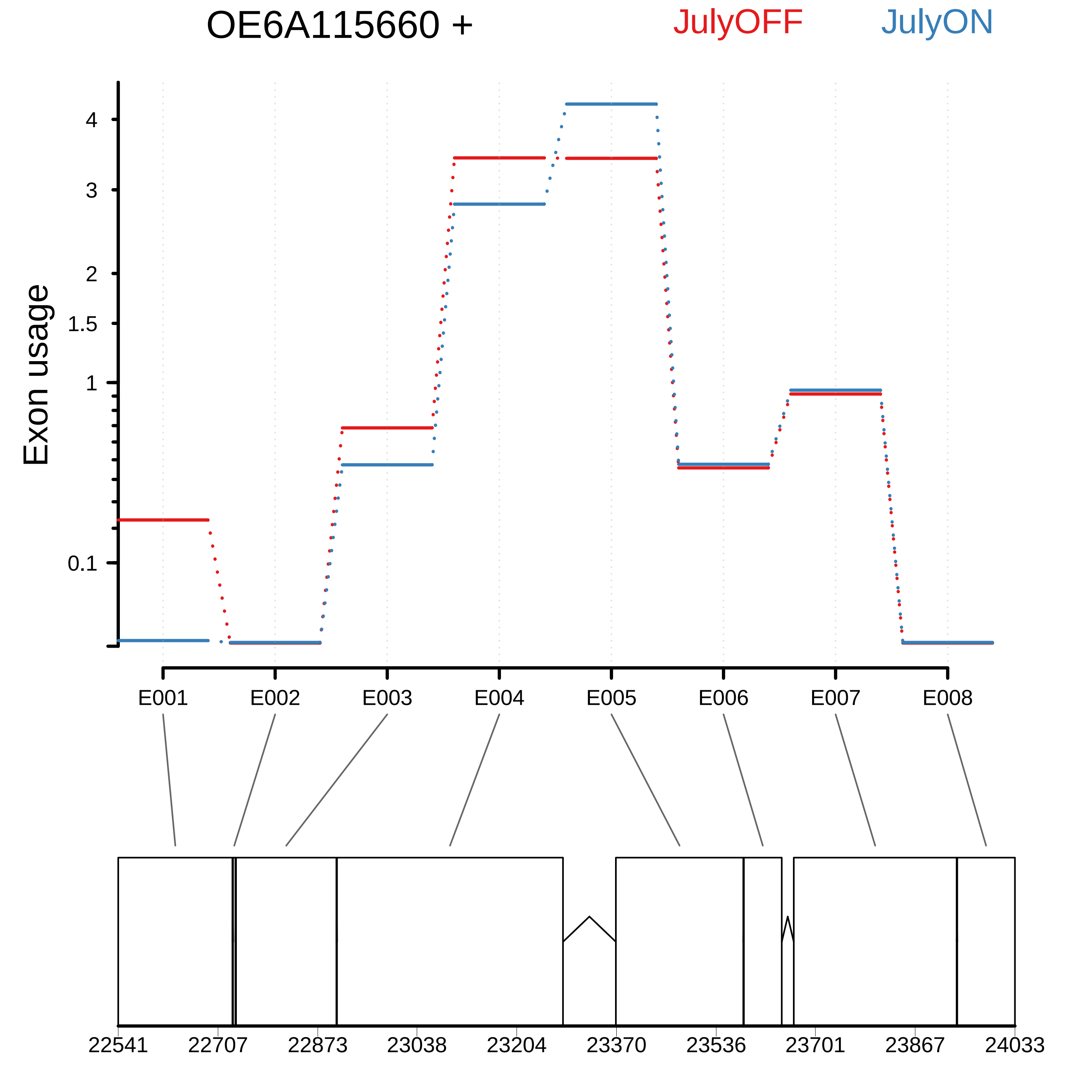

OE6A115660 +
JulyOFF
JulyON
Exon usage
4
3
2
1.5
1
0.1
E001
E002
E003
E004
E005
E006
E007
E008
22541
22707
22873
23038
23204
23370
23536
23701
23867
24033

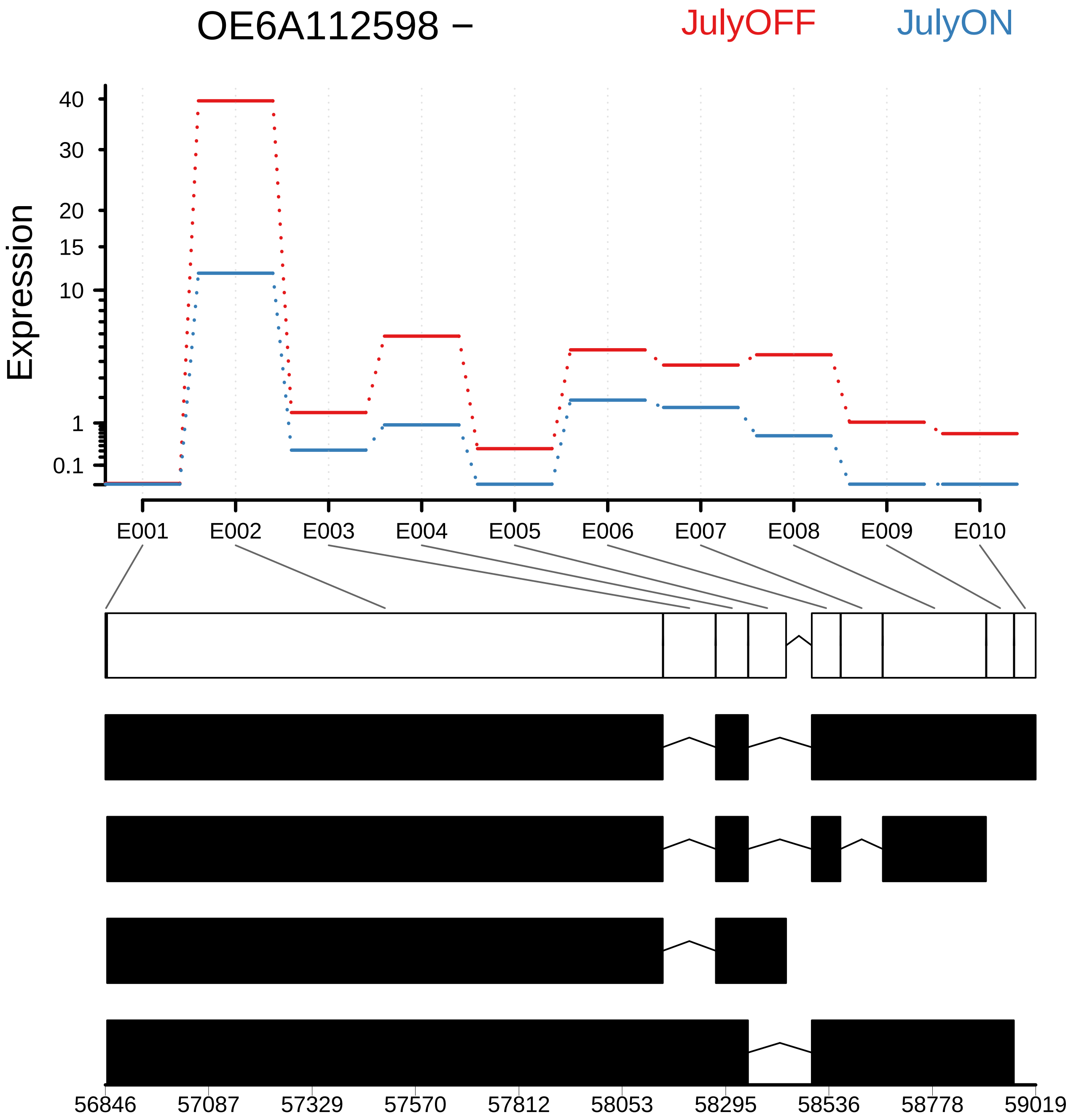

OE6A112598 −
JulyOFF
JulyON
Expression
40
30
20
15
10
1
0.1
E001
E002
E003
E004
E005
E006
E007
E008
E009
E010
56846
57087
57329
57570
57812
58053
58295
58536
58778
59019

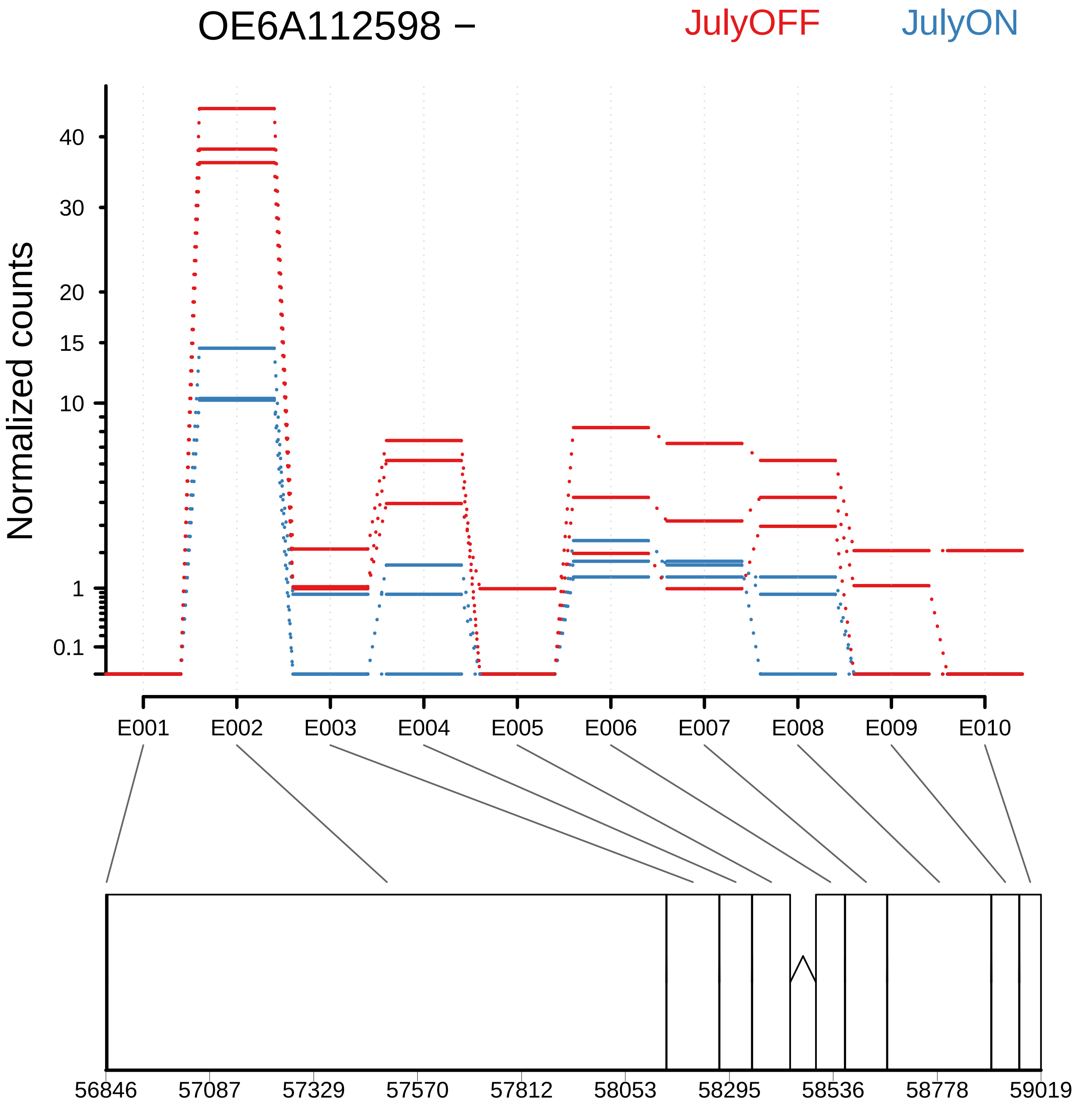
OE6A112598 −
JulyOFF
JulyON
Normalized counts
40
30
20
15
10
1
0.1
E001
E002
E003
E004
E005
E006
E007
E008
E009
E010
56846
57087
57329
57570
57812
58053
58295
58536
58778
59019

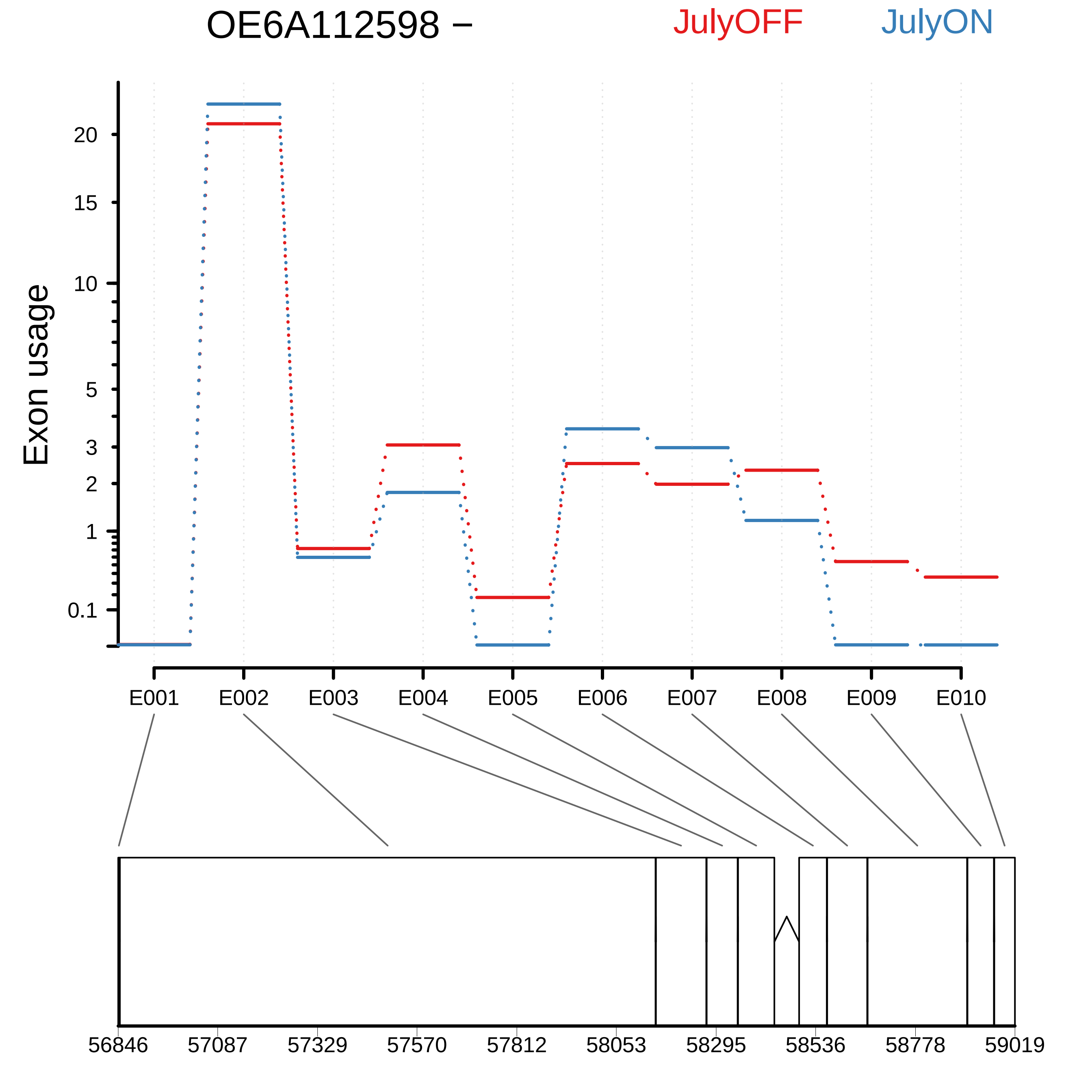
OE6A112598 –
JulyOFF
JulyON
Exon usage
20
15
10
5
3
2
1
0.1
E001
E002
E003
E004
E005
E006
E007
E008
E009
E010
56846
57087
57329
57570
57812
58053
58295
58536
58778
59019

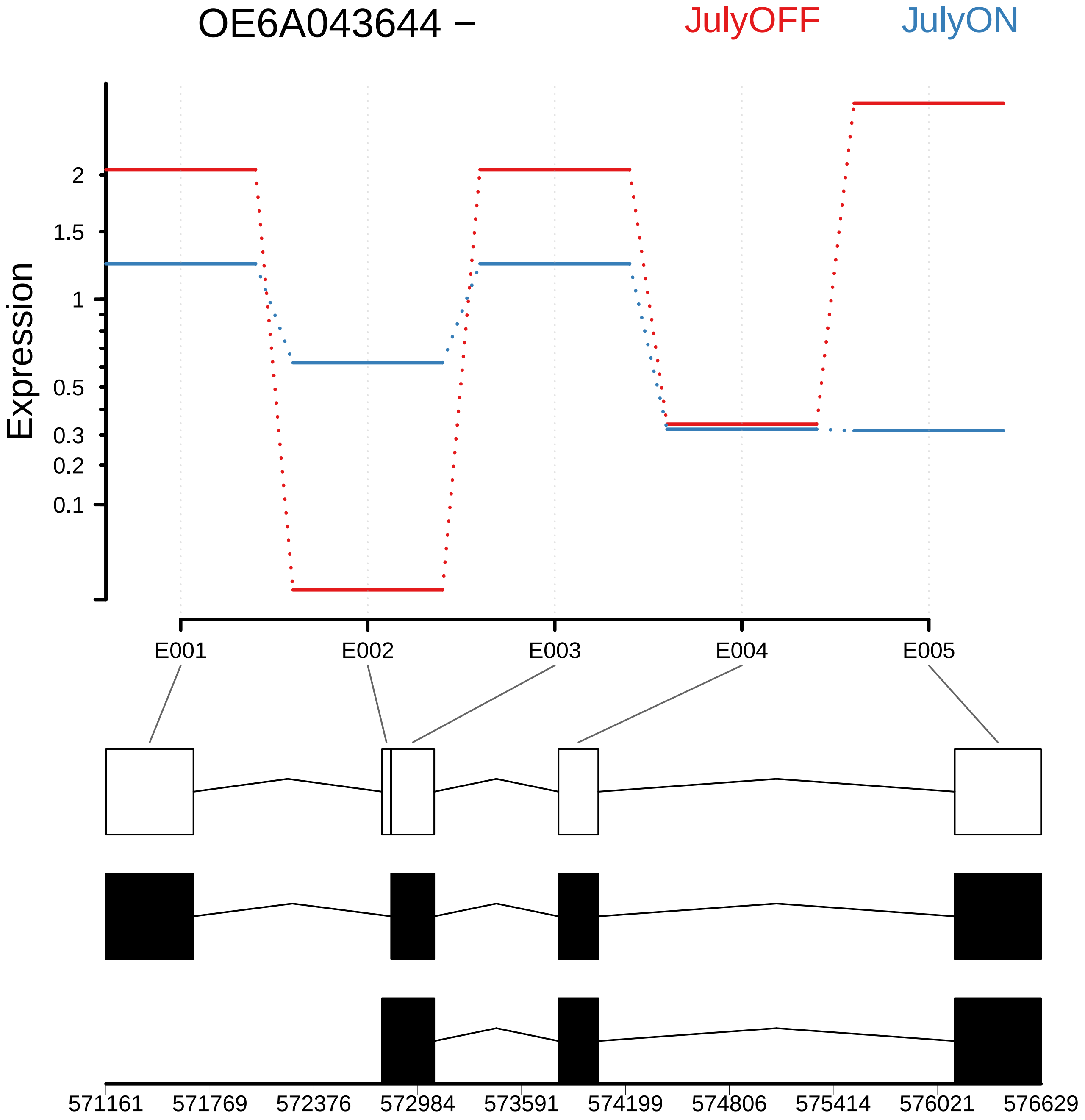

OE6A043644 −
JulyOFF
JulyON
Expression
2
1.5
1
0.5
0.3
0.2
0.1
E001
E002
E003
E004
E005
571161
571769
572376
572984
573591
574199
574806
575414
576021
576629

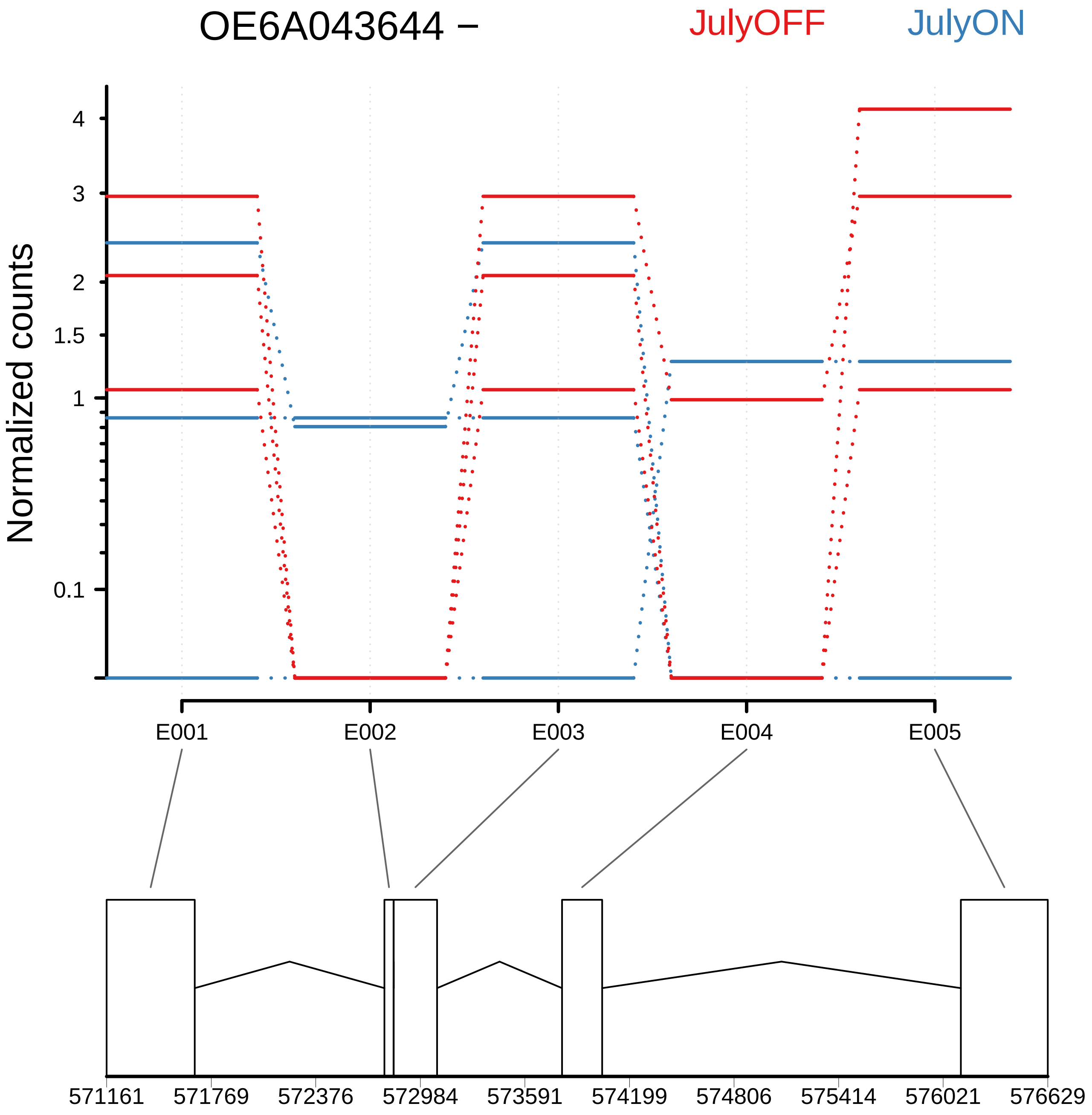

OE6A043644 −
JulyOFF
JulyON
Normalized counts
4
3
2
1.5
1
0.1
E001
E002
E003
E004
E005
571161
571769
572376
572984
573591
574199
574806
575414
576021
576629

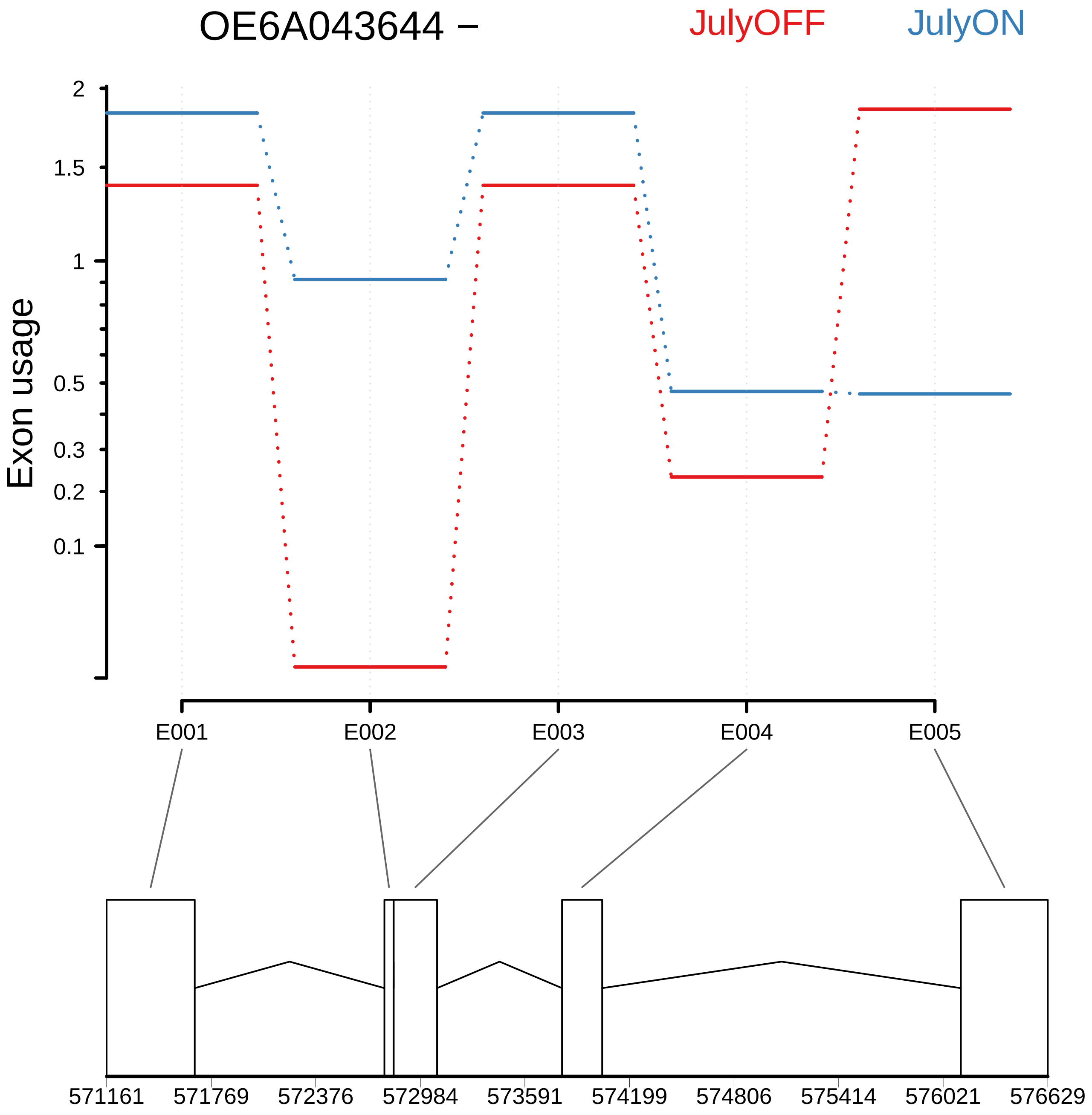

OE6A043644 −
JulyOFF
JulyON
Exon usage
2
1.5
1
0.5
0.3
0.2
0.1
E001
E002
E003
E004
E005
571161
571769
572376
572984
573591
574199
574806
575414
576021
576629

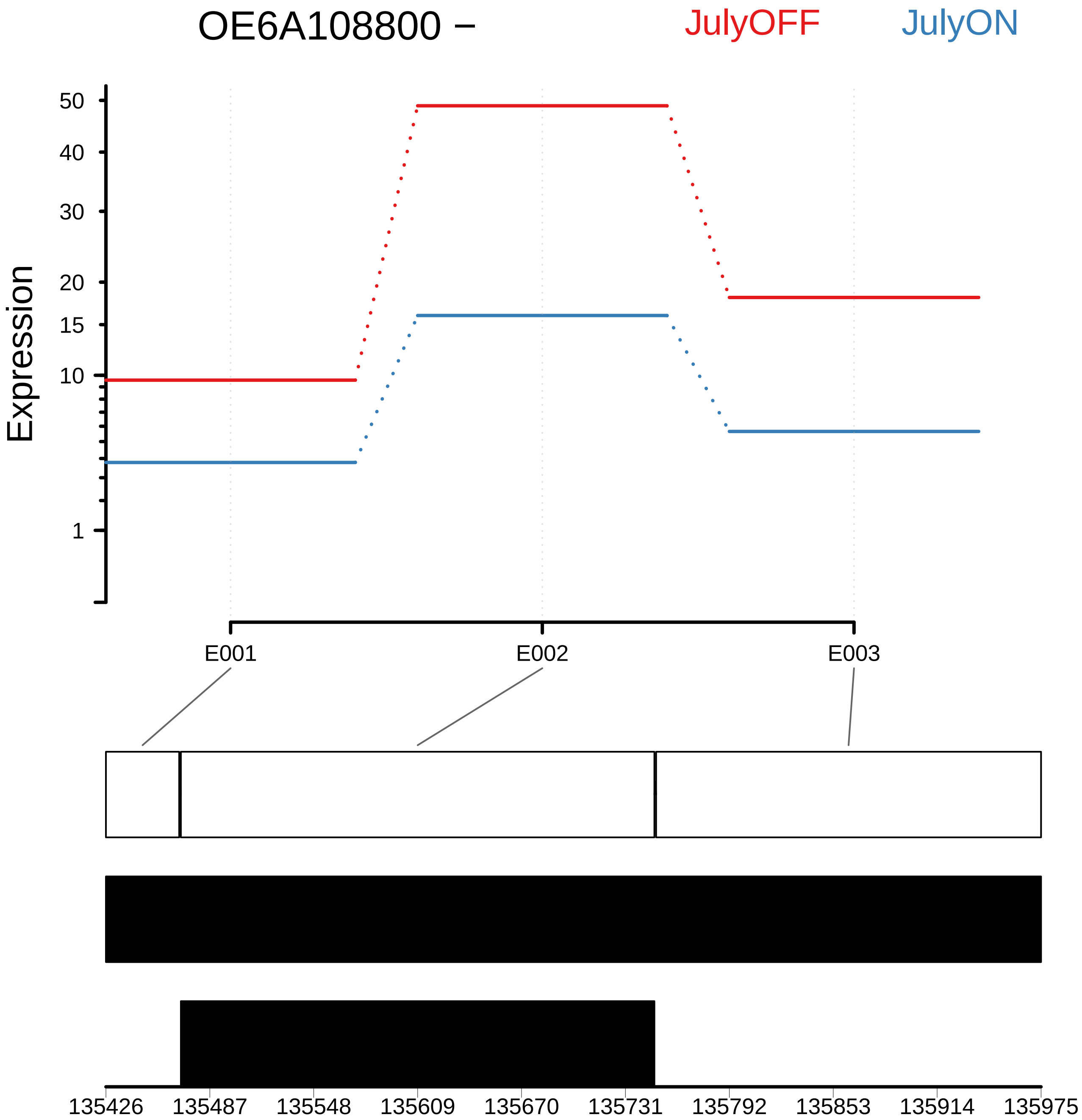

OE6A108800 −
JulyOFF
JulyON
Expression
50
40
30
20
15
10
1
E001
E002
E003
135426
135487
135548
135609
135670
135731
135792
135853
135914
135975

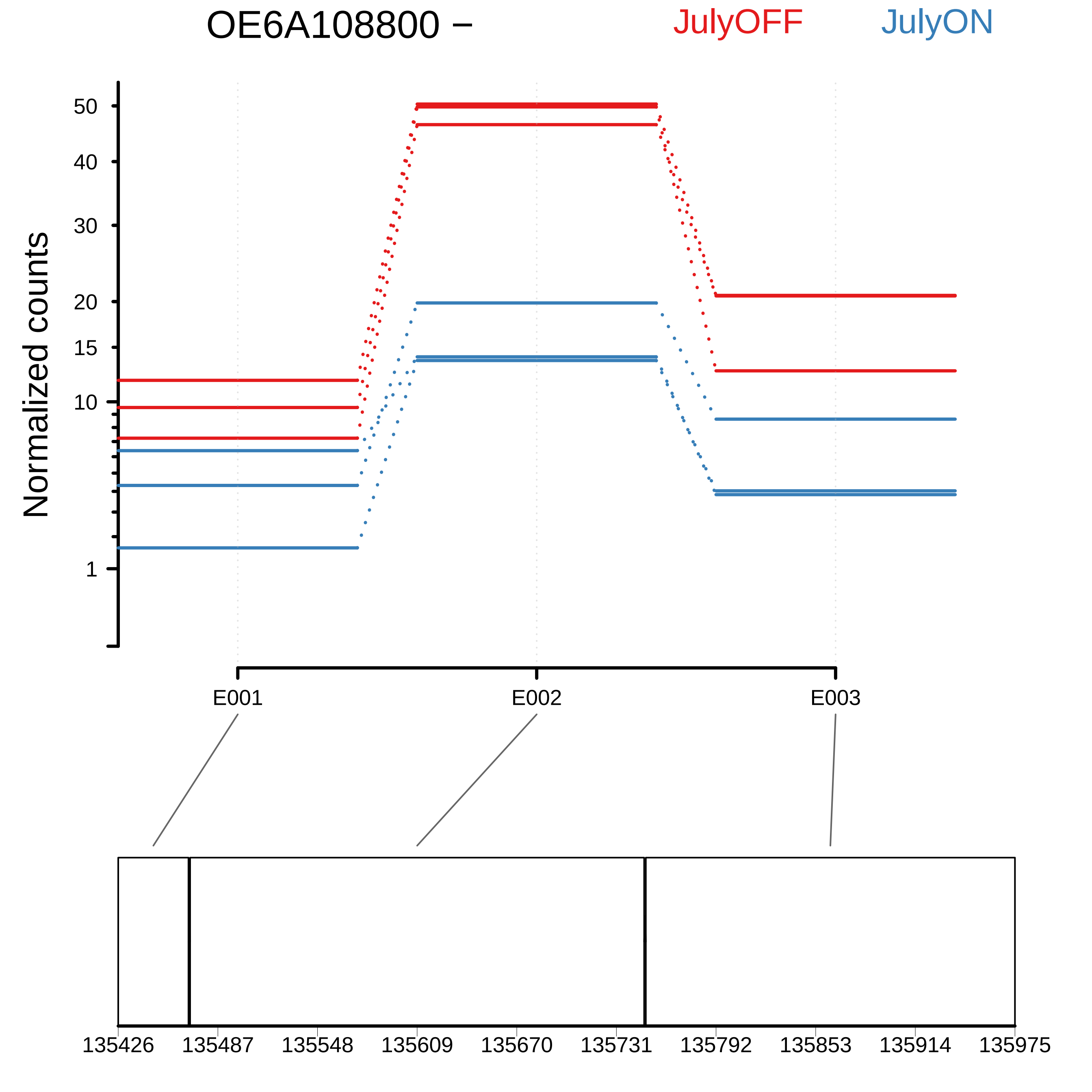

OE6A108800 −
JulyOFF
JulyON
Normalized counts
50
40
30
20
15
10
1
E001
E002
E003
135426
135487
135548
135609
135670
135731
135792
135853
135914
135975

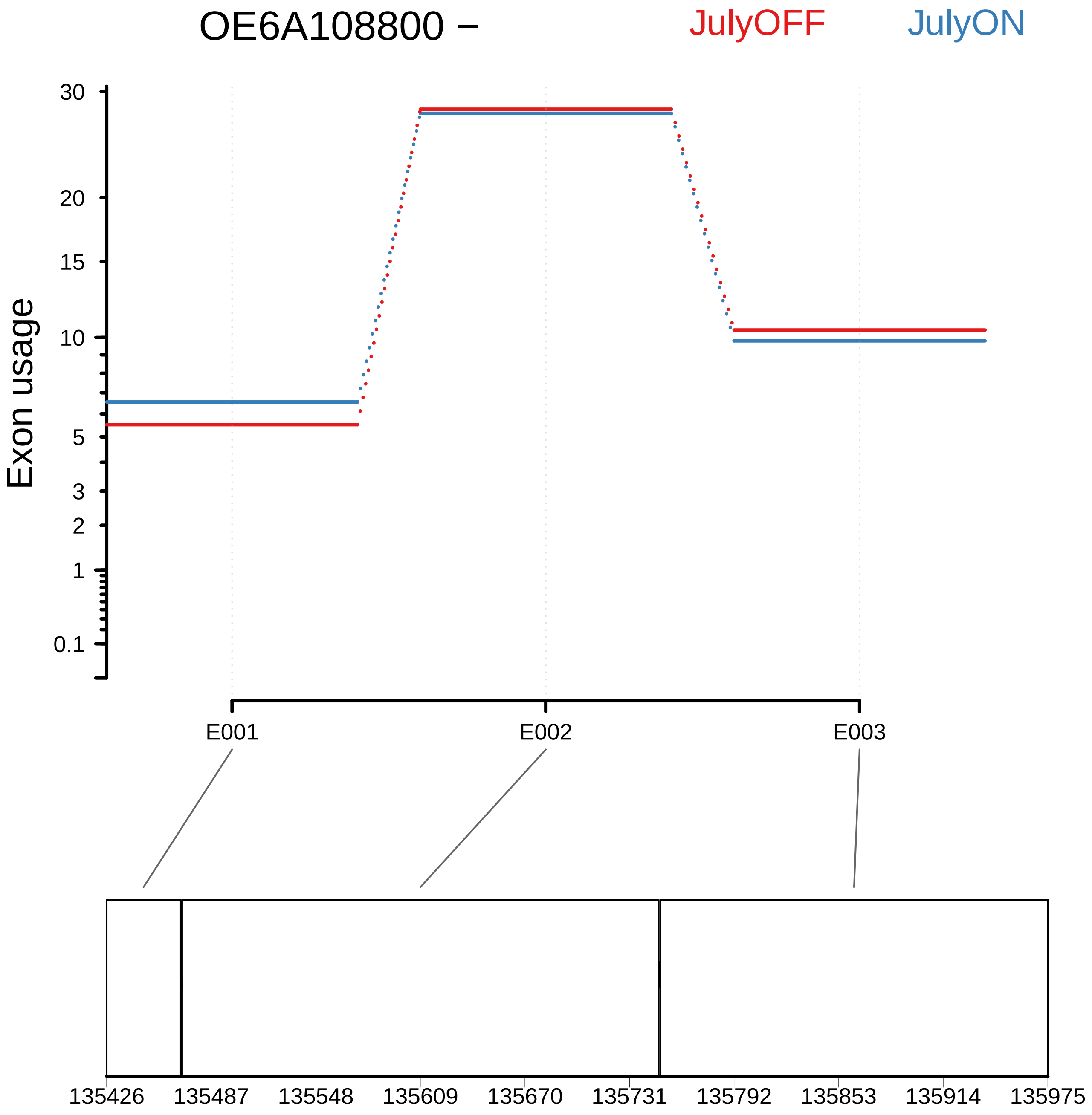
OE6A108800 −
JulyOFF
JulyON
Exon usage
30
20
15
10
5
3
2
1
0.1
E001
E002
E003
135426
135487
135548
135609
135670
135731
135792
135853
135914
135975

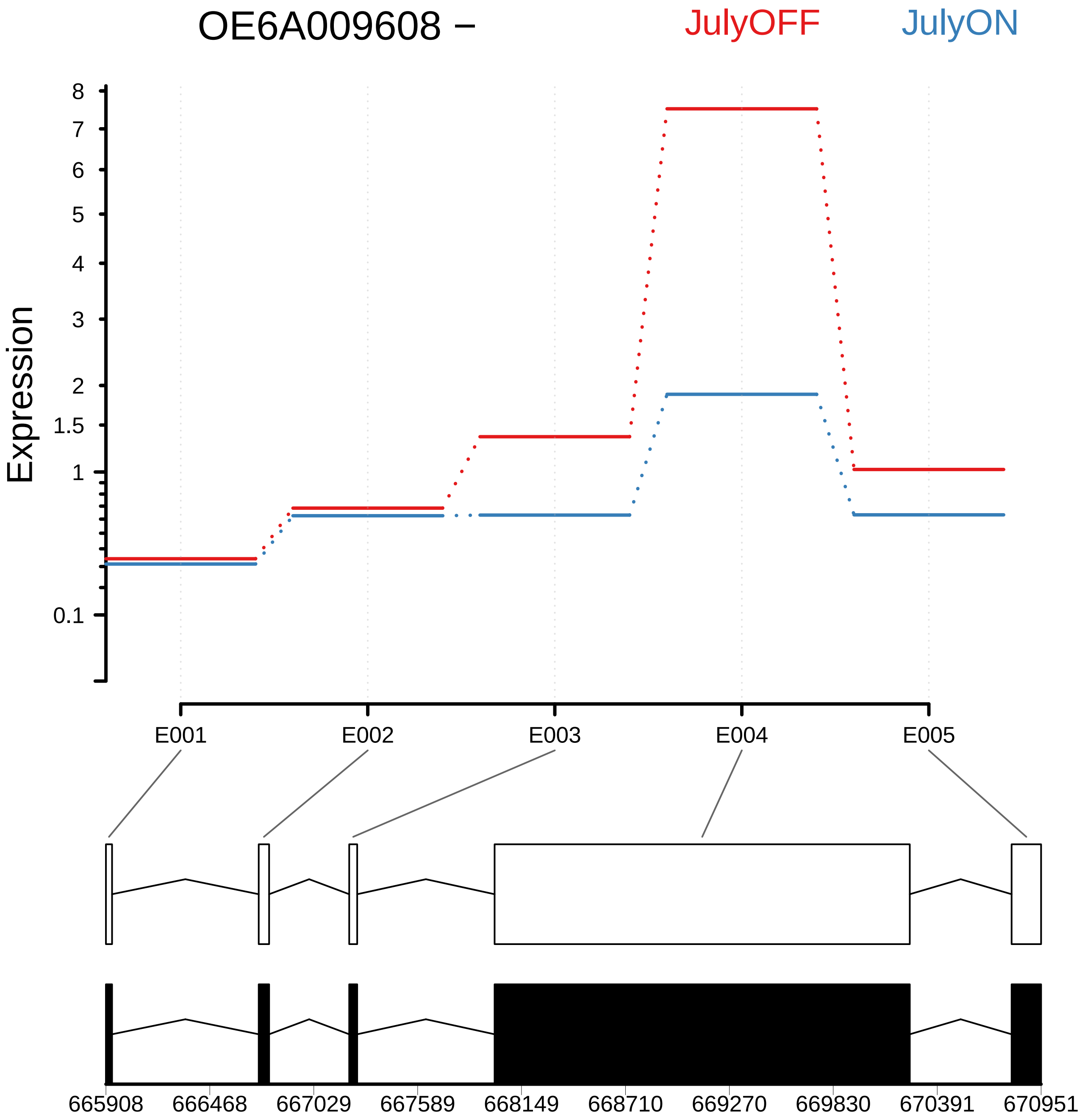
OE6A009608 –
JulyOFF
JulyON
Expression
8
7
6
5
4
3
2
1.5
1
0.1
E001
E002
E003
E004
E005
665908
666468
667029
667589
668149
668710
669270
669830
670391
670951

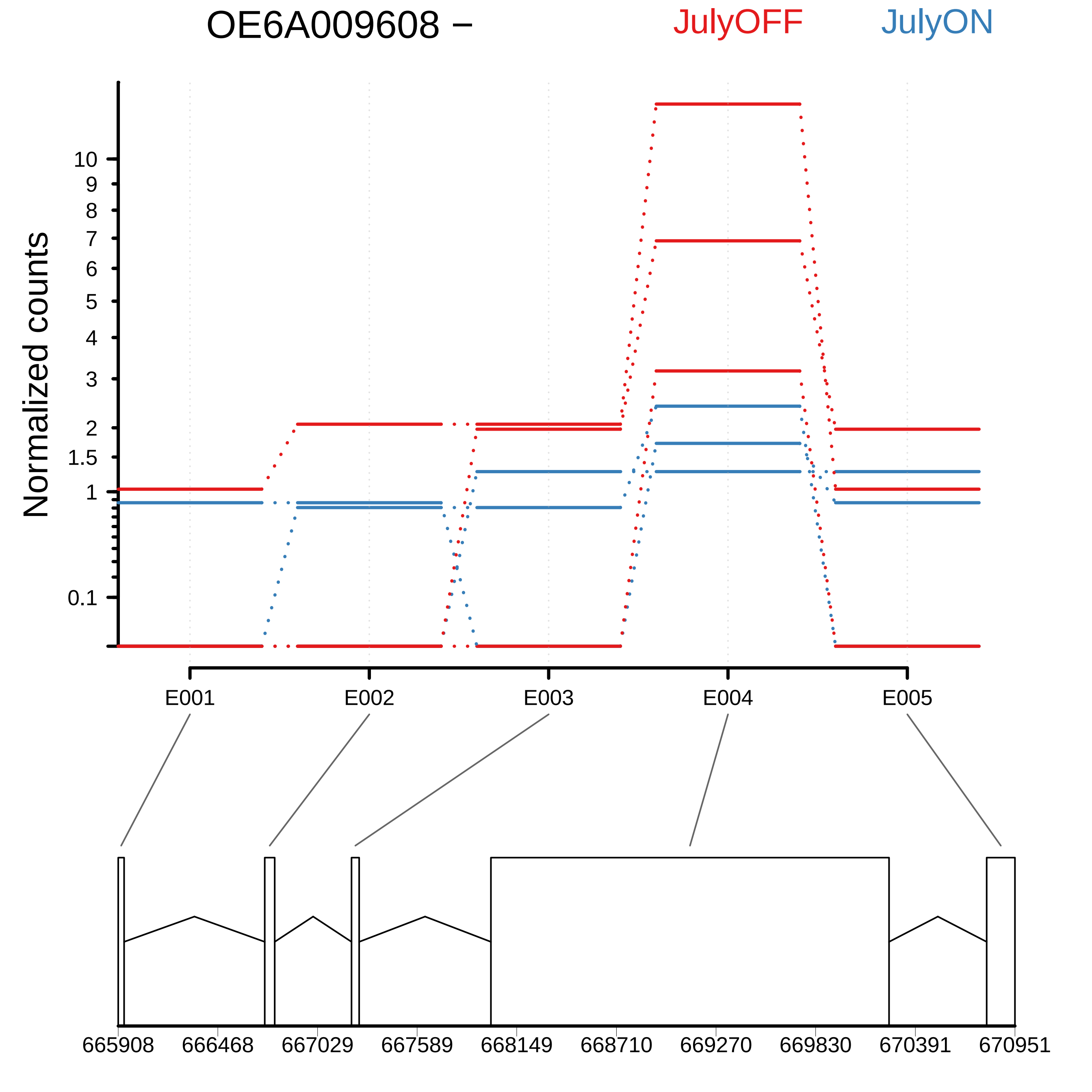

OE6A009608 −
JulyOFF
JulyON
Normalized counts
10
9
8
7
6
5
4
3
2
1.5
1
0.1
E001
E002
E003
E004
E005
665908
666468
667029
667589
668149
668710
669270
669830
670391
670951

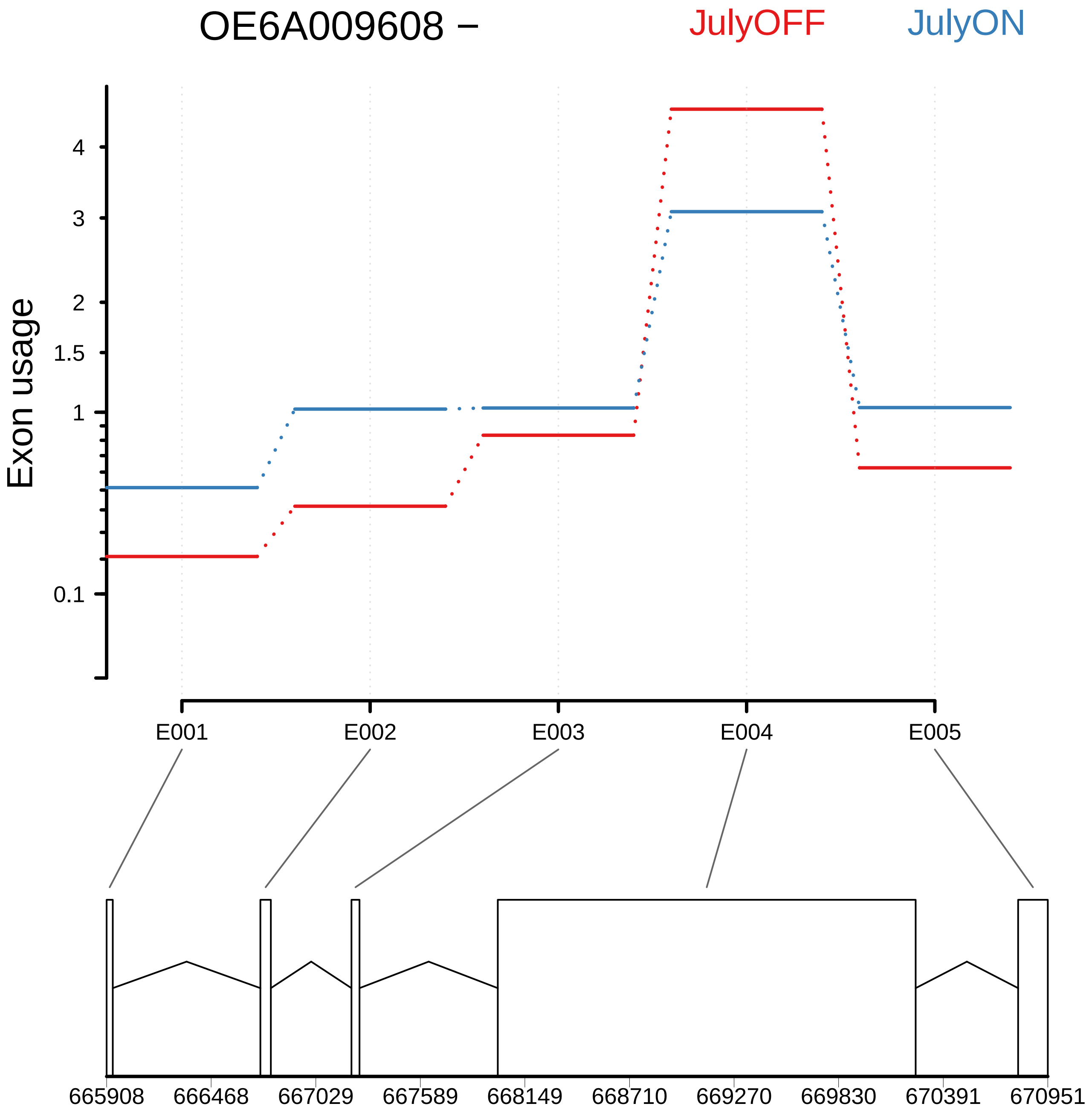

OE6A009608 −
JulyOFF
JulyON
Exon usage
4
3
2
1.5
1
0.1
E001
E002
E003
E004
E005
665908
666468
667029
667589
668149
668710
669270
669830
670391
670951

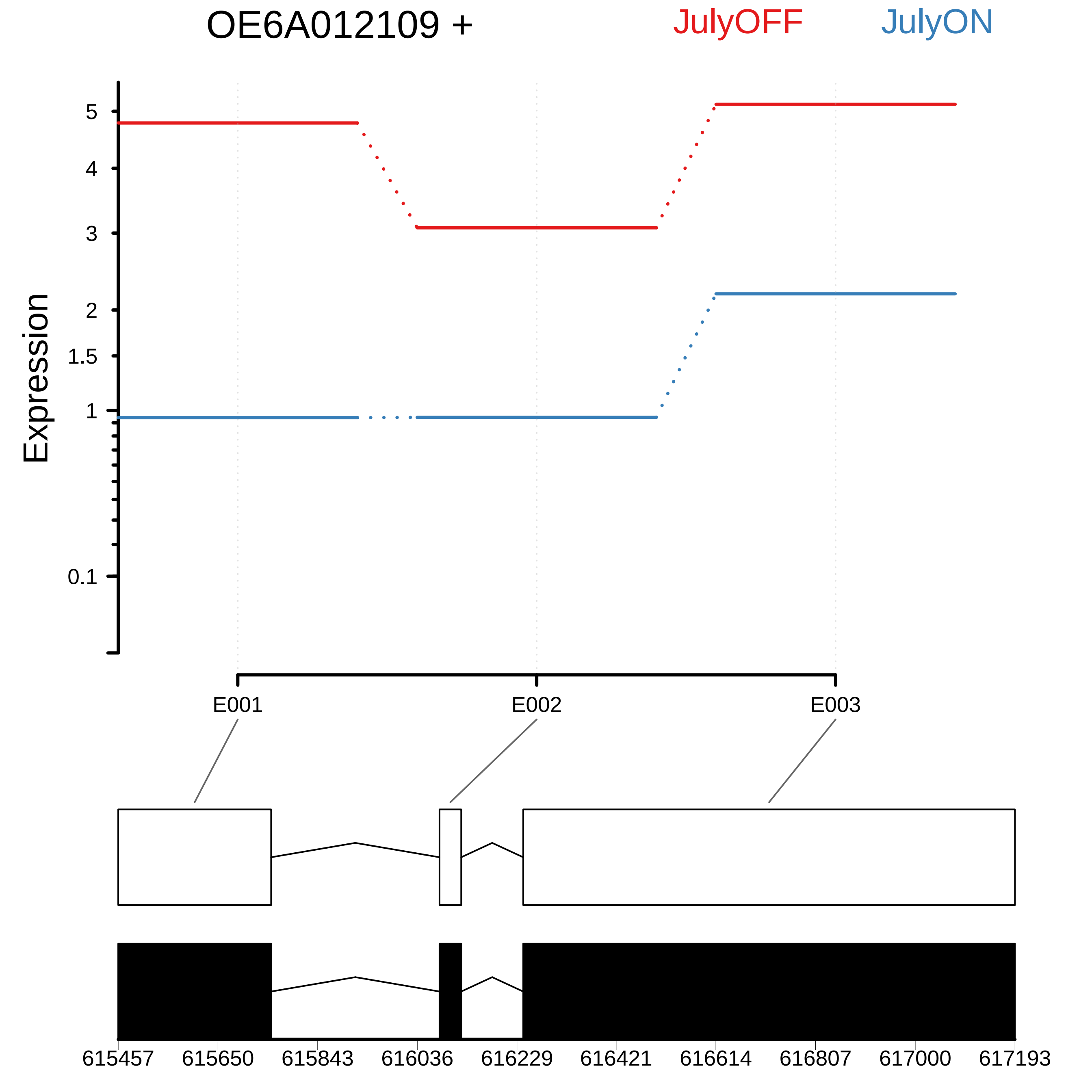

OE6A012109 +
JulyOFF
JulyON
Expression
5
4
3
2
1.5
1
0.1
E001
E002
E003
615457
615650
615843
616036
616229
616421
616614
616807
617000
617193

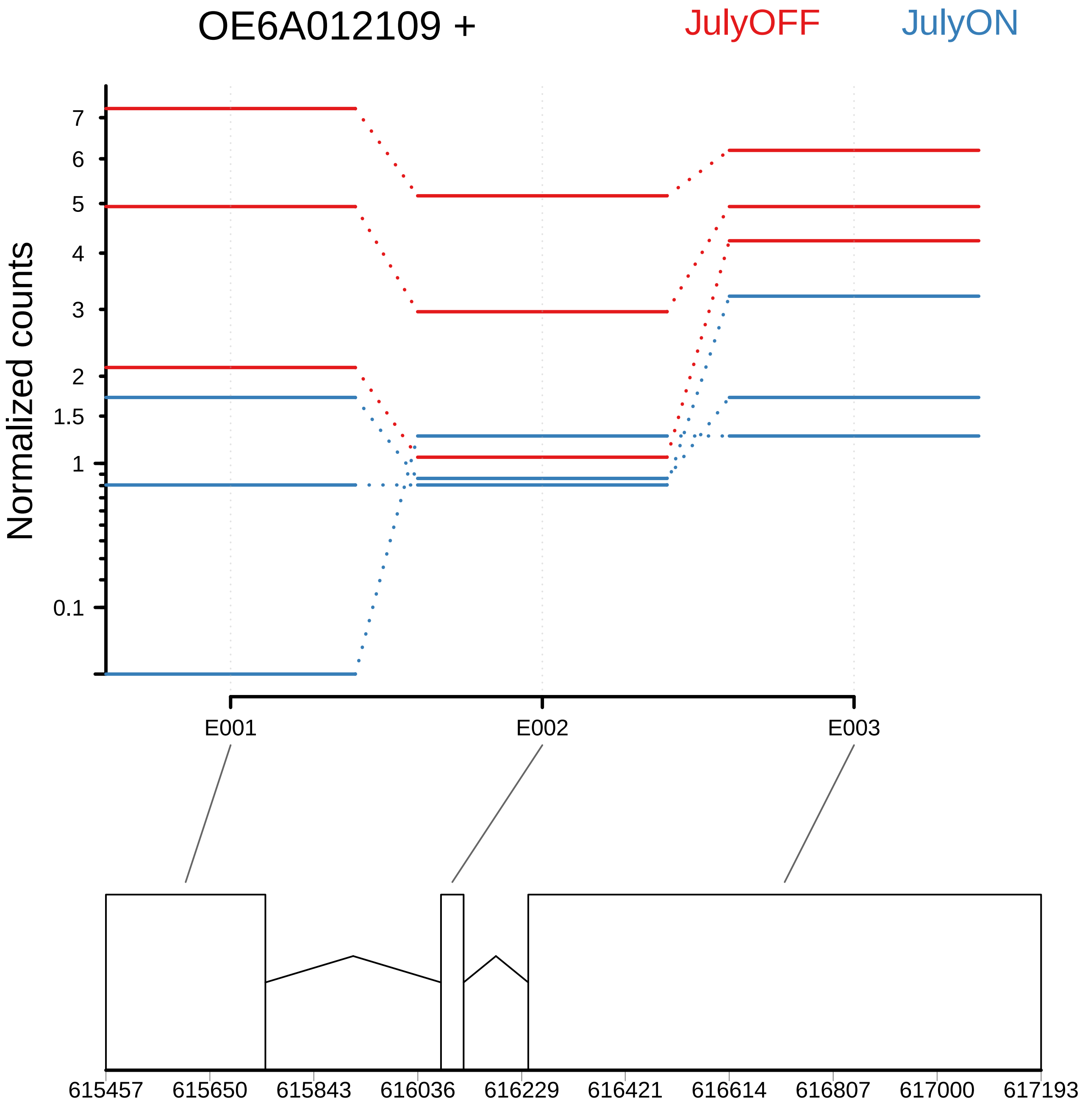

OE6A012109 +
JulyOFF
JulyON
Normalized counts
7
6
5
4
3
2
1.5
1
0.1
E001
E002
E003
615457
615650
615843
616036
616229
616421
616614
616807
617000
617193

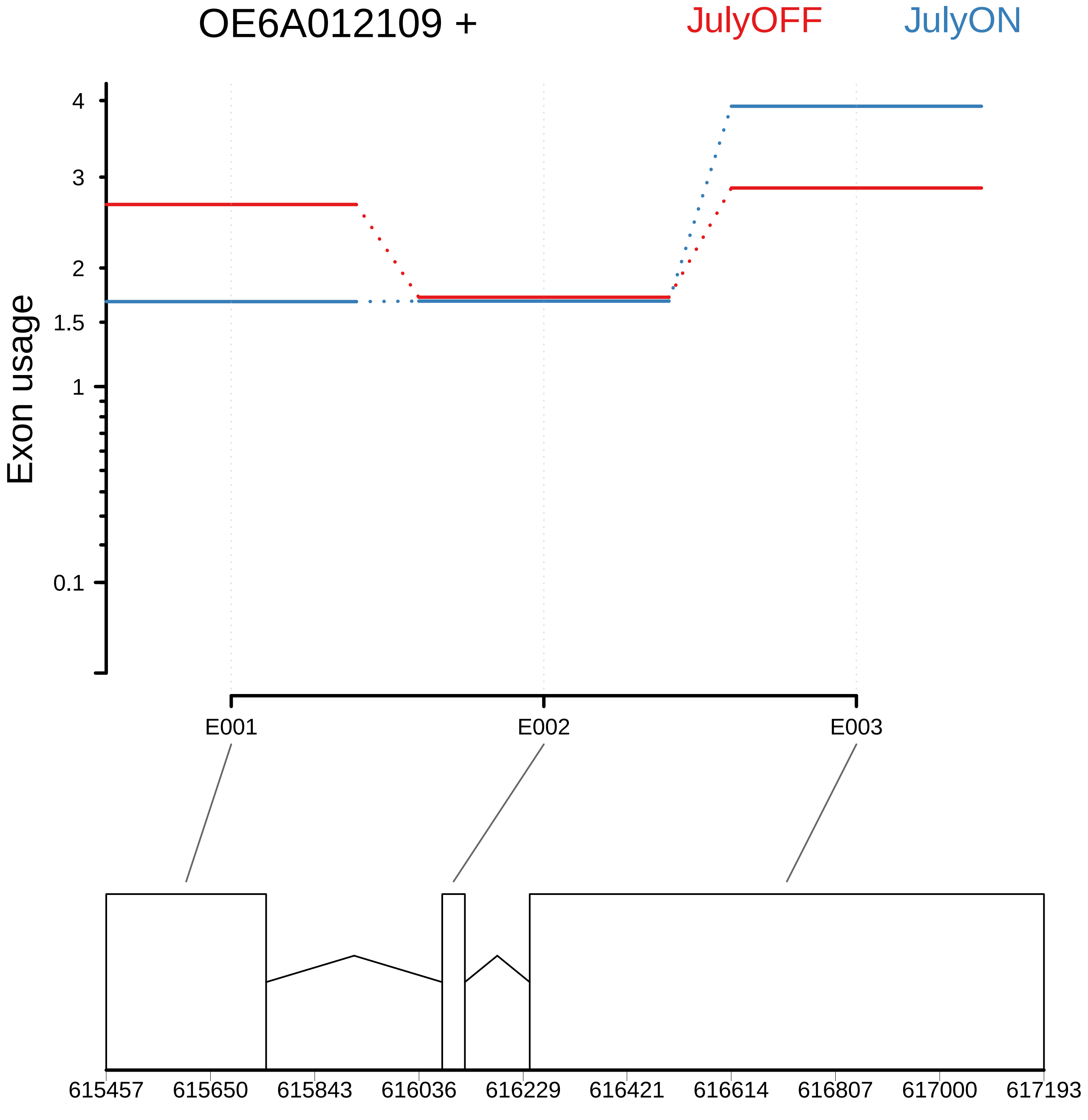

OE6A012109 +
JulyOFF
JulyON
Exon usage
4
3
2
1.5
1
0.1
E001
E002
E003
615457
615650
615843
616036
616229
616421
616614
616807
617000
617193

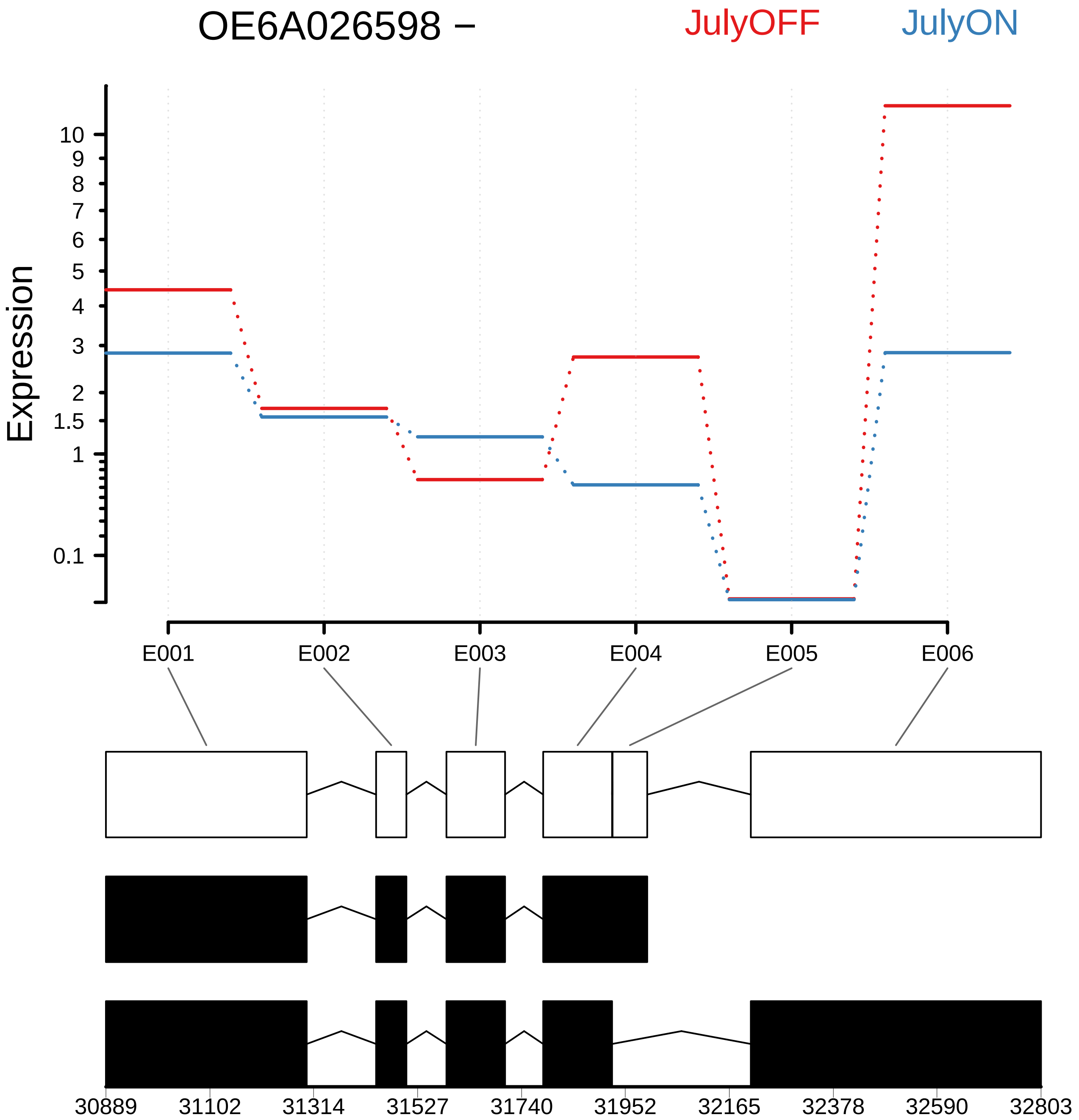

OE6A026598 –
JulyOFF
JulyON
Expression
10
9
8
7
6
5
4
3
2
1.5
1
0.1
E001
E002
E003
E004
E005
E006
30889
31102
31314
31527
31740
31952
32165
32378
32590
32803

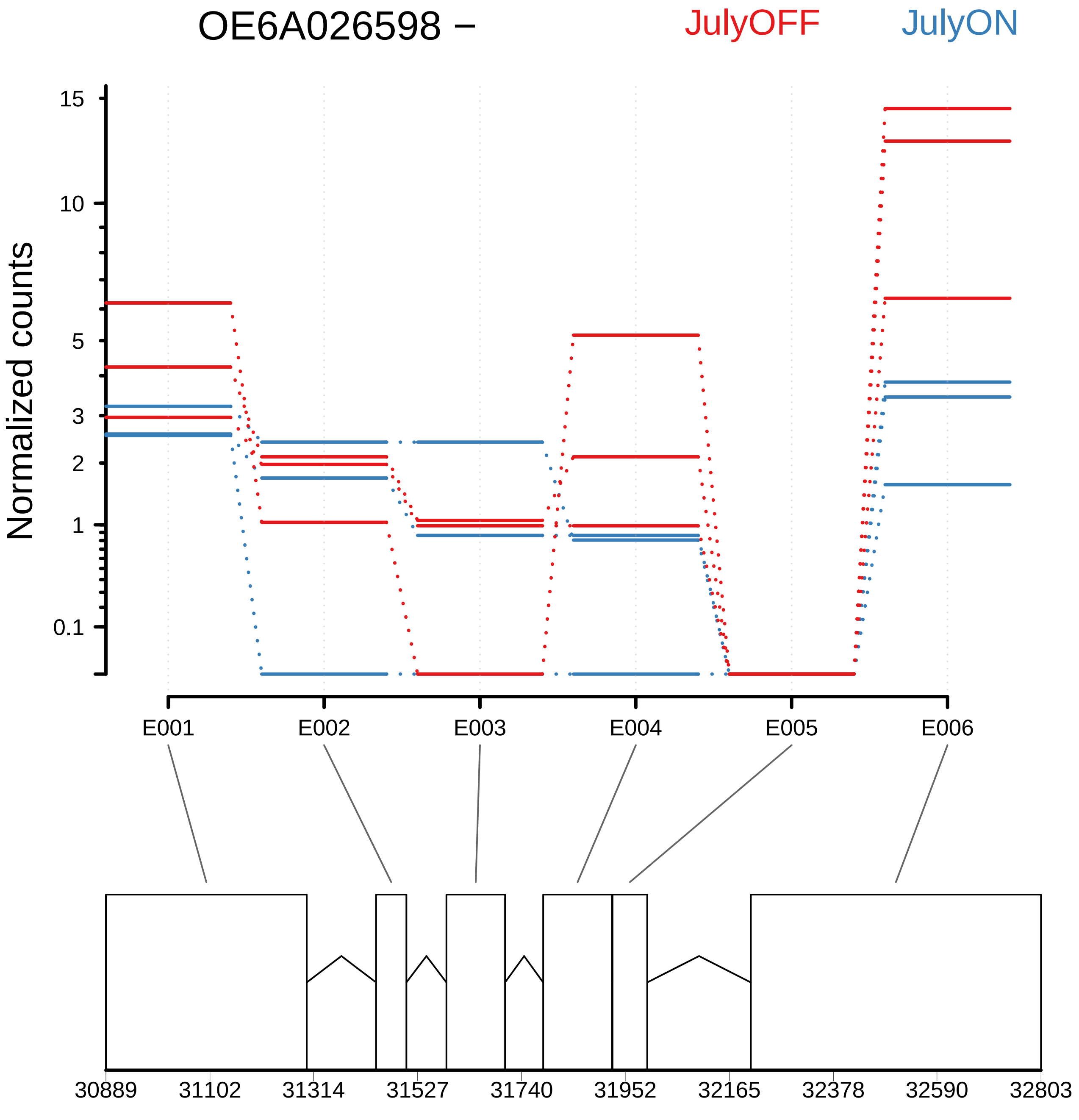

OE6A026598 –
JulyOFF
JulyON
Normalized counts
15
10
5
3
2
1
0.1
E001
E002
E003
E004
E005
E006
30889
31102
31314
31527
31740
31952
32165
32378
32590
32803

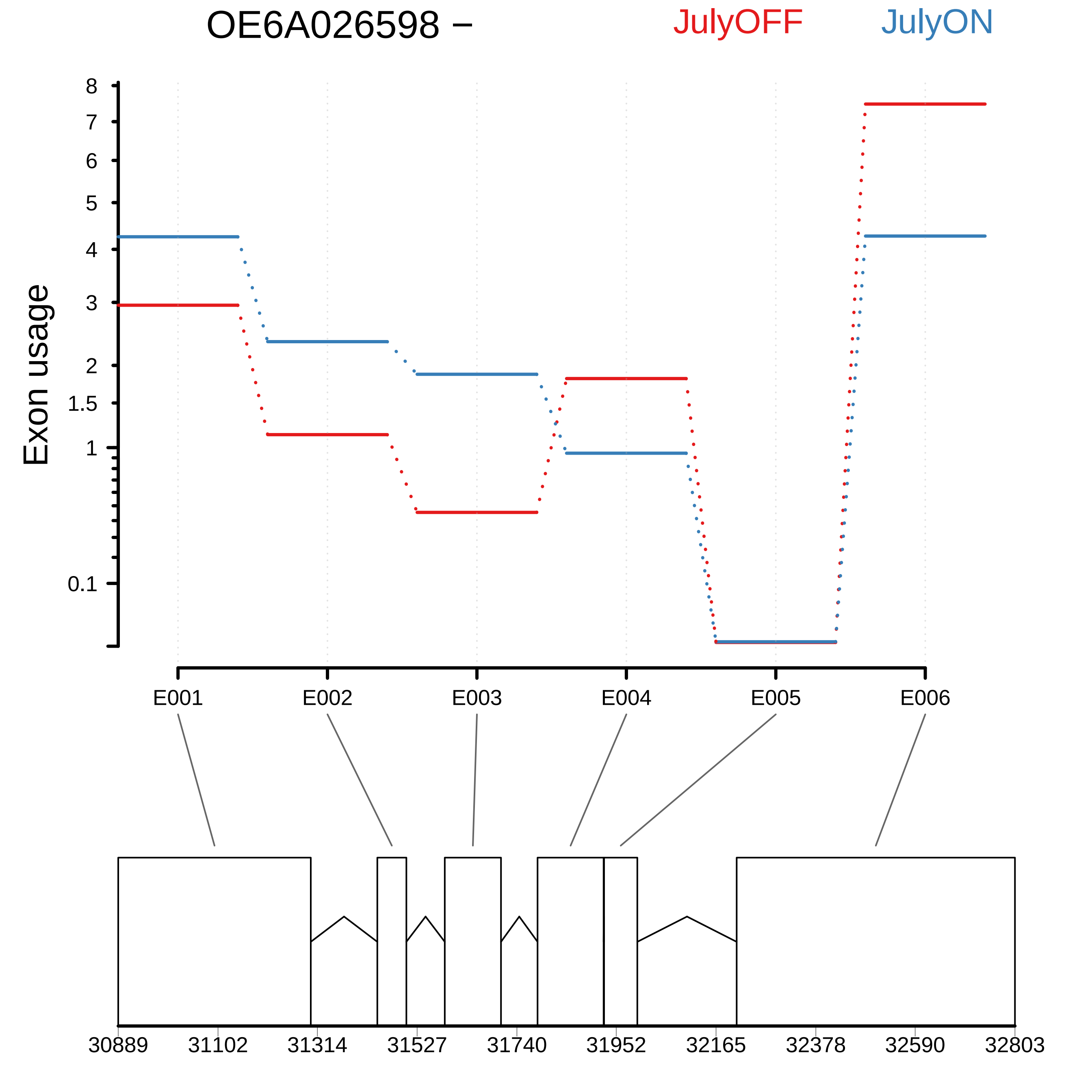
OE6A026598 –
JulyOFF
JulyON
Exon usage
8
7
6
5
4
3
2
1.5
1
0.1
E001
E002
E003
E004
E005
E006
30889
31102
31314
31527
31740
31952
32165
32378
32590
32803

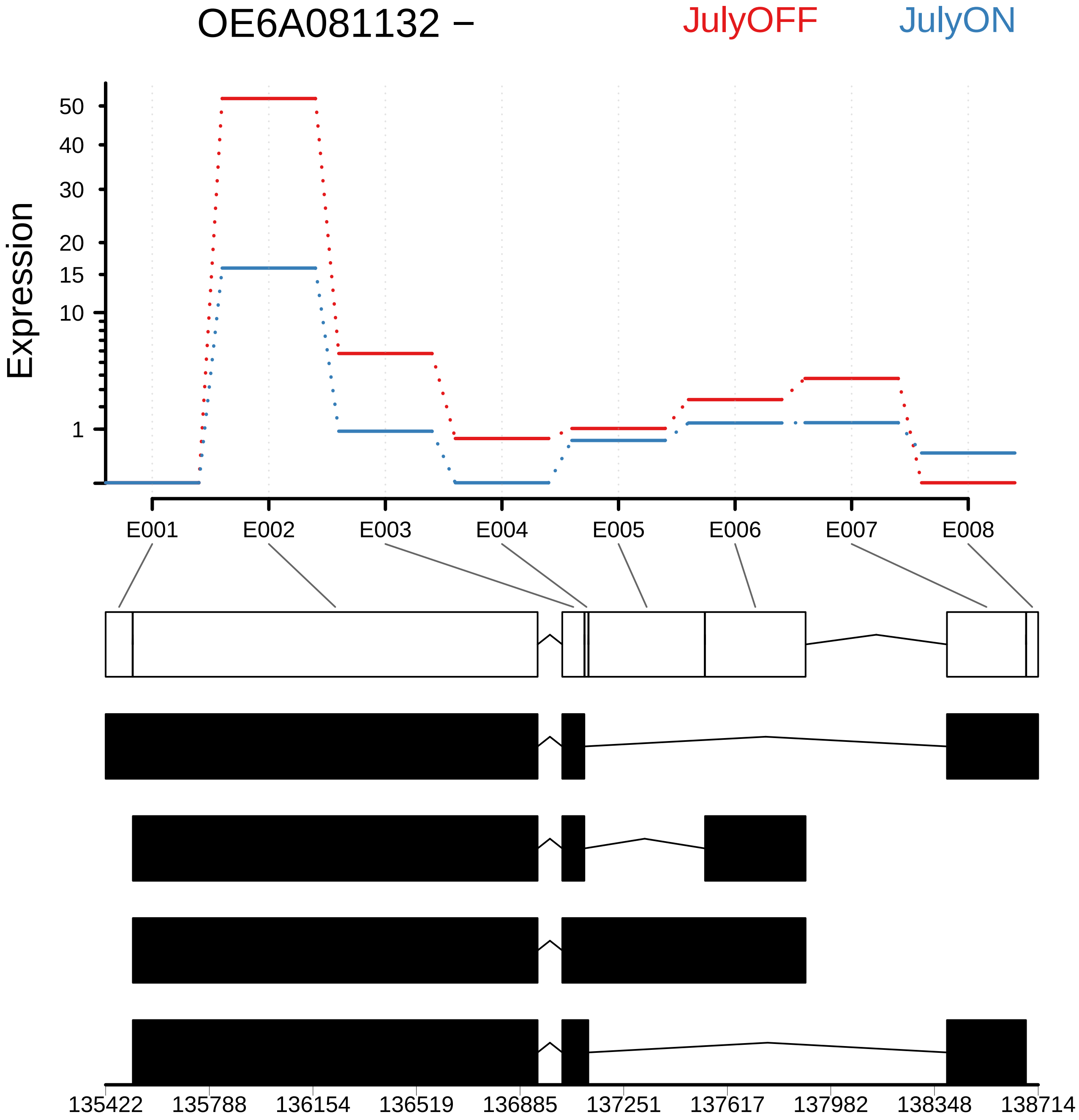

OE6A081132 –
JulyOFF
JulyON
Expression
50
40
30
20
15
10
1
E001
E002
E003
E004
E005
E006
E007
E008
135422
135788
136154
136519
136885
137251
137617
137982
138348
138714

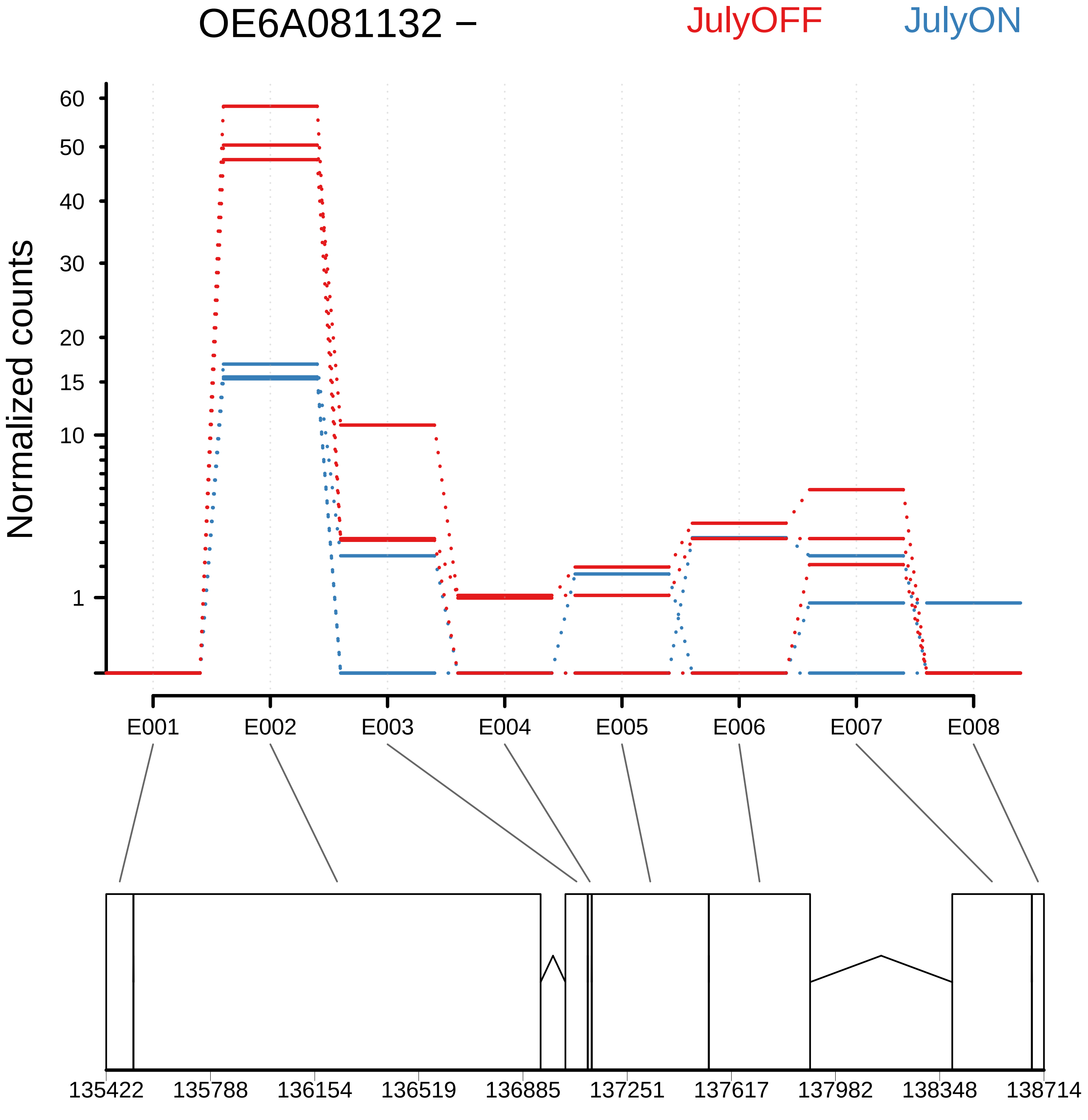

OE6A081132 −
JulyOFF
JulyON
Normalized counts
60
50
40
30
20
15
10
1
E001
E002
E003
E004
E005
E006
E007
E008
135422
135788
136154
136519
136885
137251
137617
137982
138348
138714

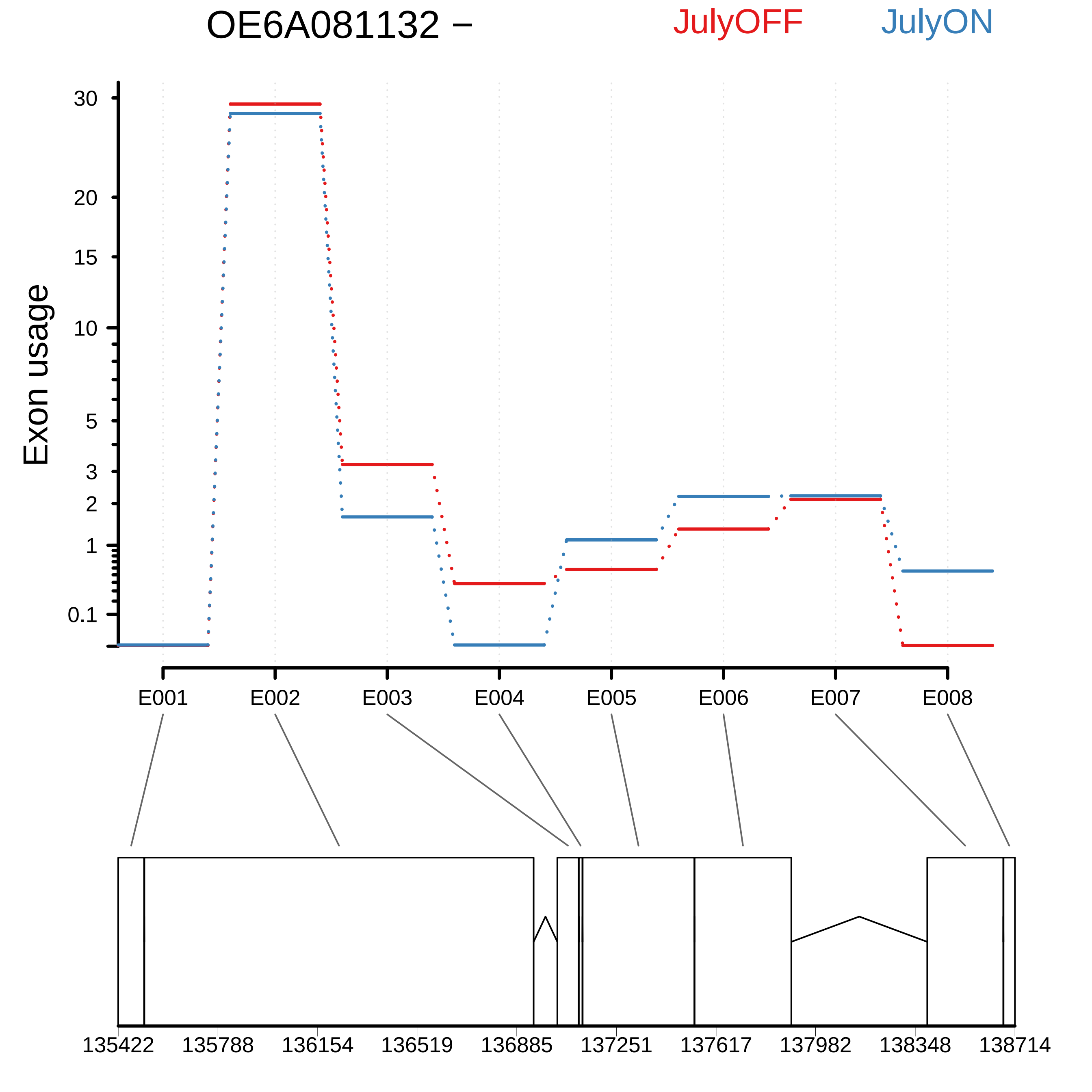

OE6A081132 −
JulyOFF
JulyON
Exon usage
30
20
15
10
5
3
2
1
0.1
E001
E002
E003
E004
E005
E006
E007
E008
135422
135788
136154
136519
136885
137251
137617
137982
138348
138714

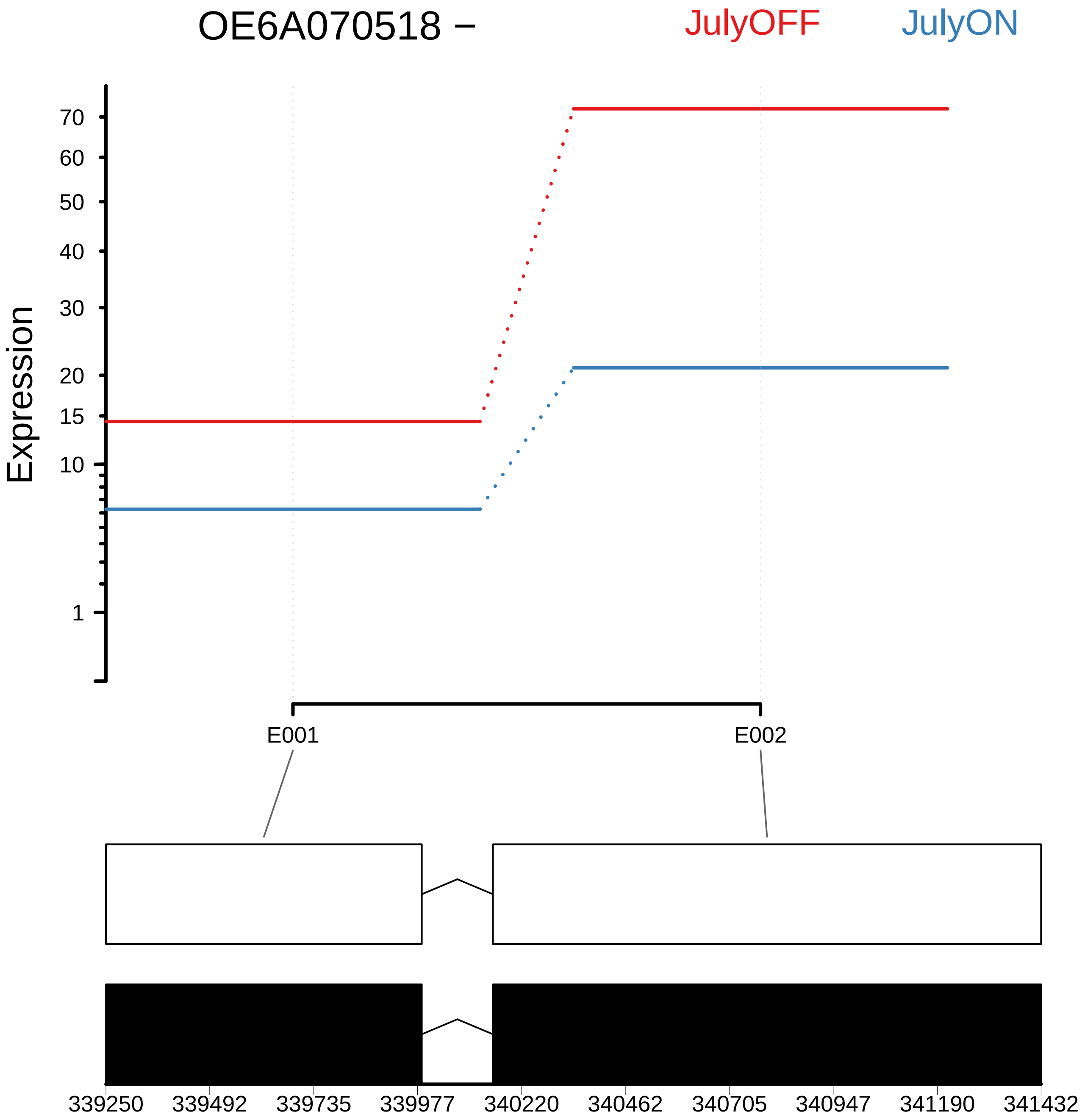

OE6A070518 −
JulyOFF
JulyON
Expression
70
60
50
40
30
20
15
10
1
E001
E002
339250
339492
339735
339977
340220
340462
340705
340947
341190
341432

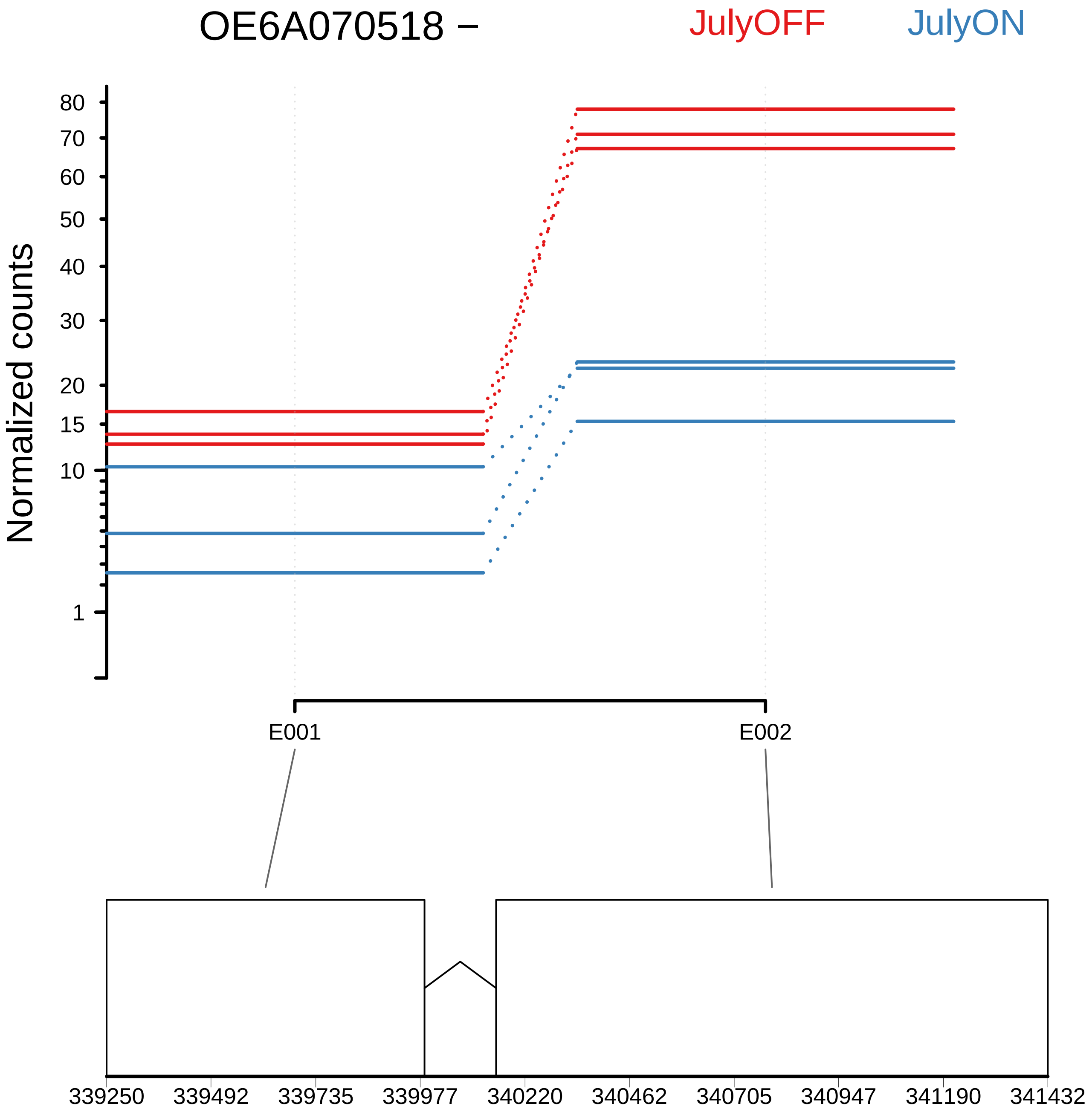
OE6A070518 −
JulyOFF
JulyON
Normalized counts
80
70
60
50
40
30
20
15
10
1
E001
E002
339250
339492
339735
339977
340220
340462
340705
340947
341190
341432

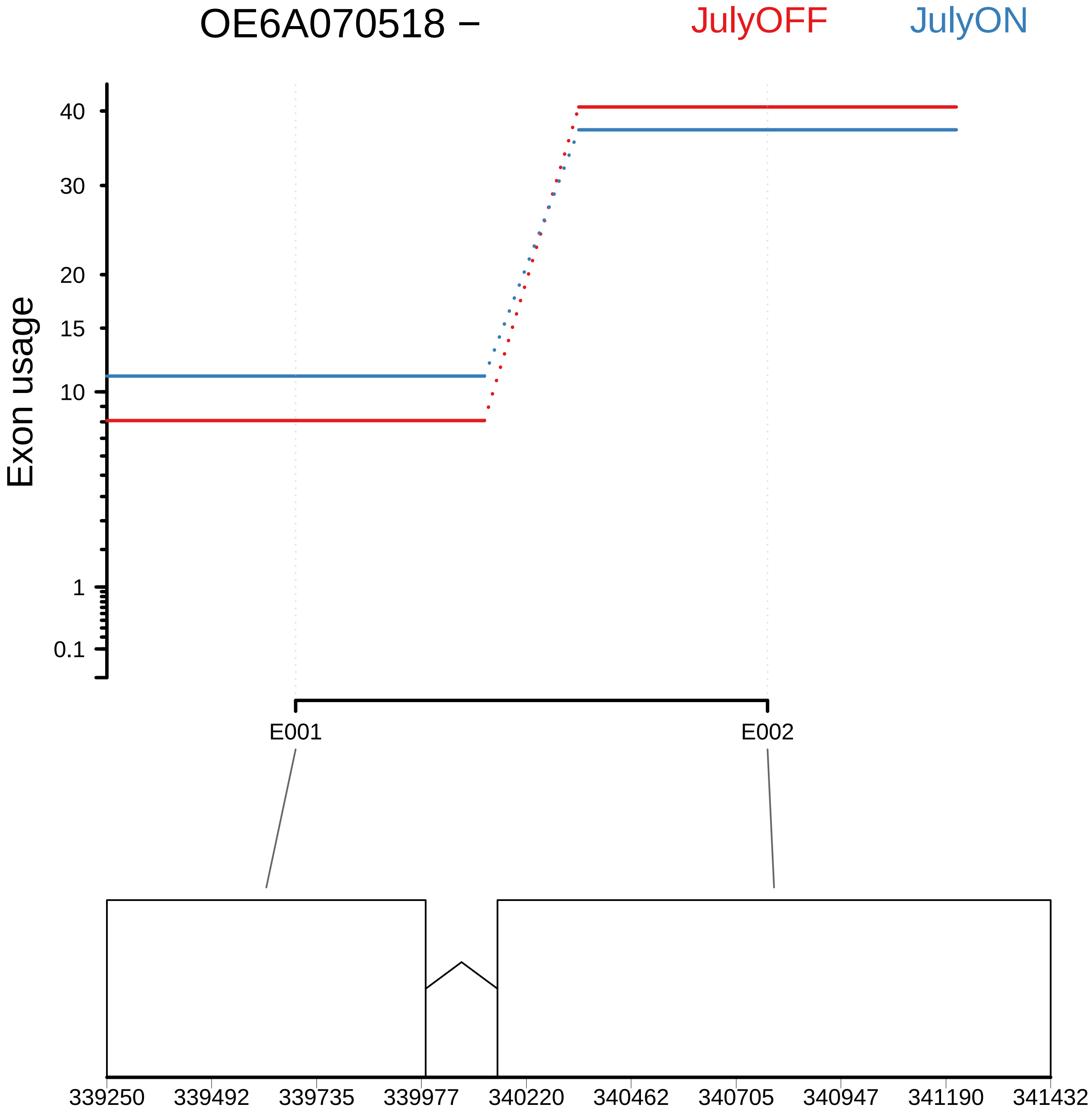
OE6A070518 −
JulyOFF
JulyON
Exon usage
40
30
20
15
10
1
0.1
E001
E002
339250
339492
339735
339977
340220
340462
340705
340947
341190
341432

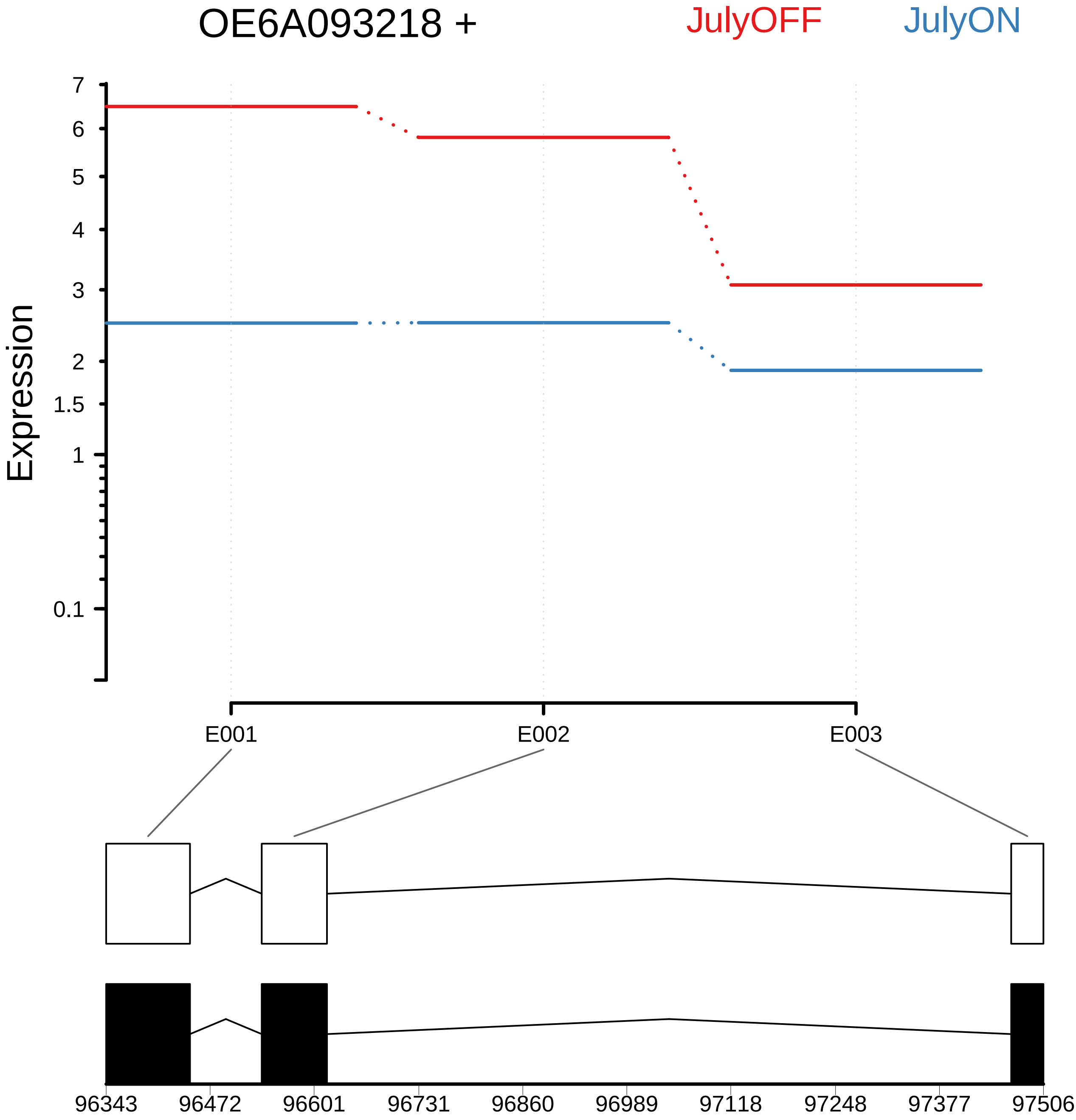
OE6A093218 +
JulyOFF
JulyON
Expression
7
6
5
4
3
2
1.5
1
0.1
E001
E002
E003
96343
96472
96601
96731
96860
96989
97118
97248
97377
97506

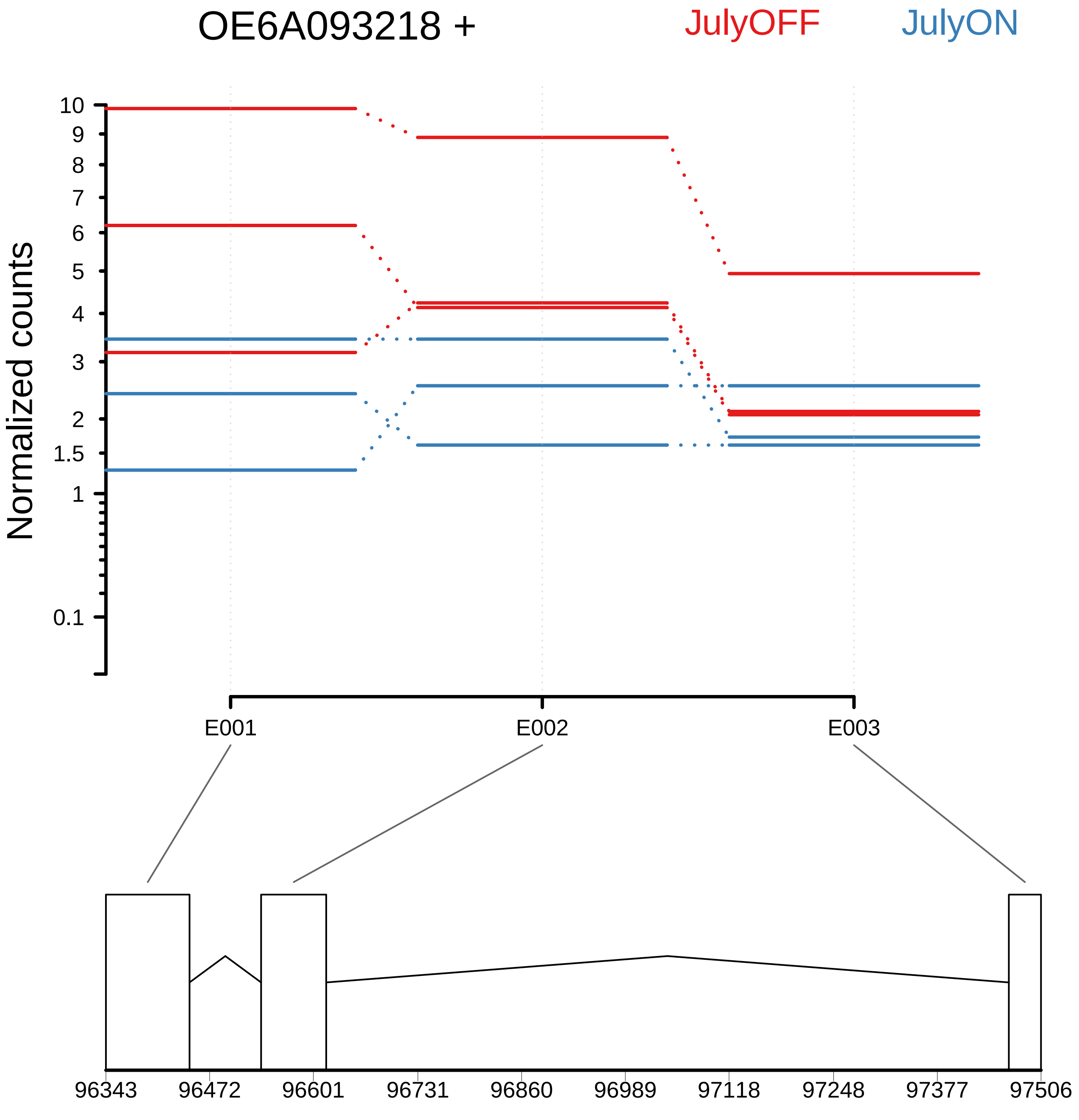

OE6A093218 +
JulyOFF
JulyON
Normalized counts
10
9
8
7
6
5
4
3
2
1.5
1
0.1
E001
E002
E003
96343
96472
96601
96731
96860
96989
97118
97248
97377
97506

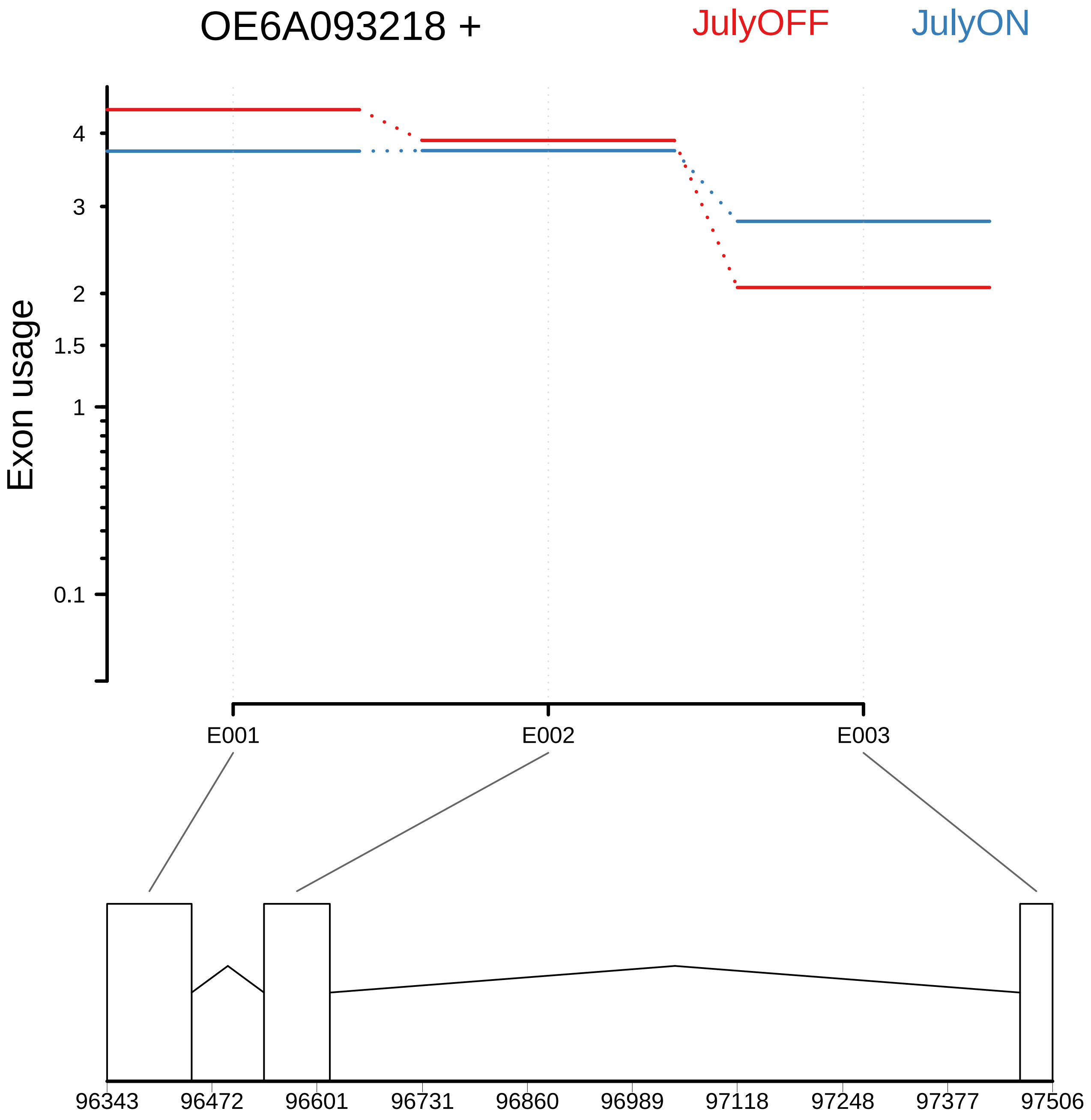
OE6A093218 +
JulyOFF
JulyON
Exon usage
4
3
2
1.5
1
0.1
E001
E002
E003
96343
96472
96601
96731
96860
96989
97118
97248
97377
97506

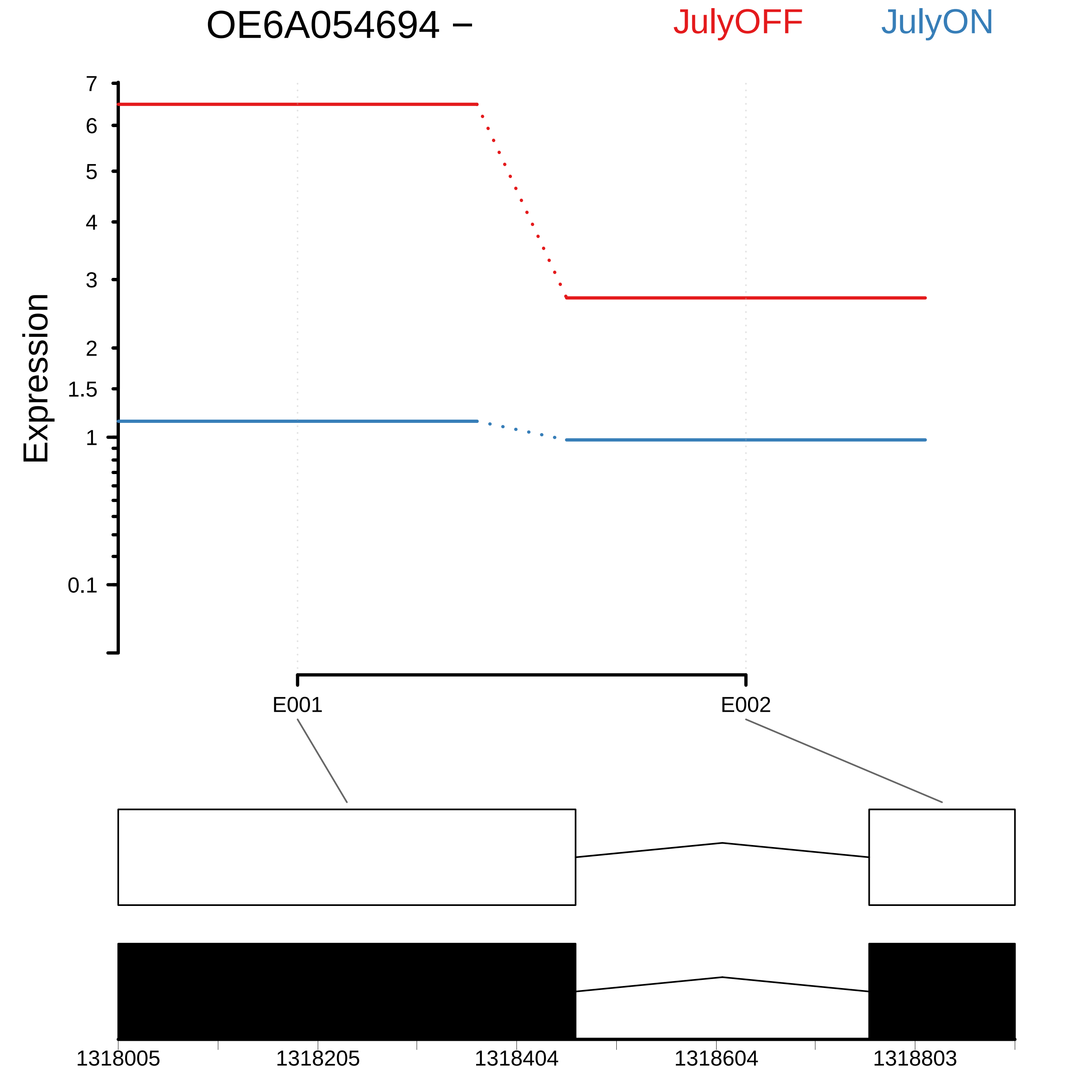
OE6A054694 −
JulyOFF
JulyON
Expression
7
6
5
4
3
2
1.5
1
0.1
E001
E002
1318005
1318205
1318404
1318604
1318803

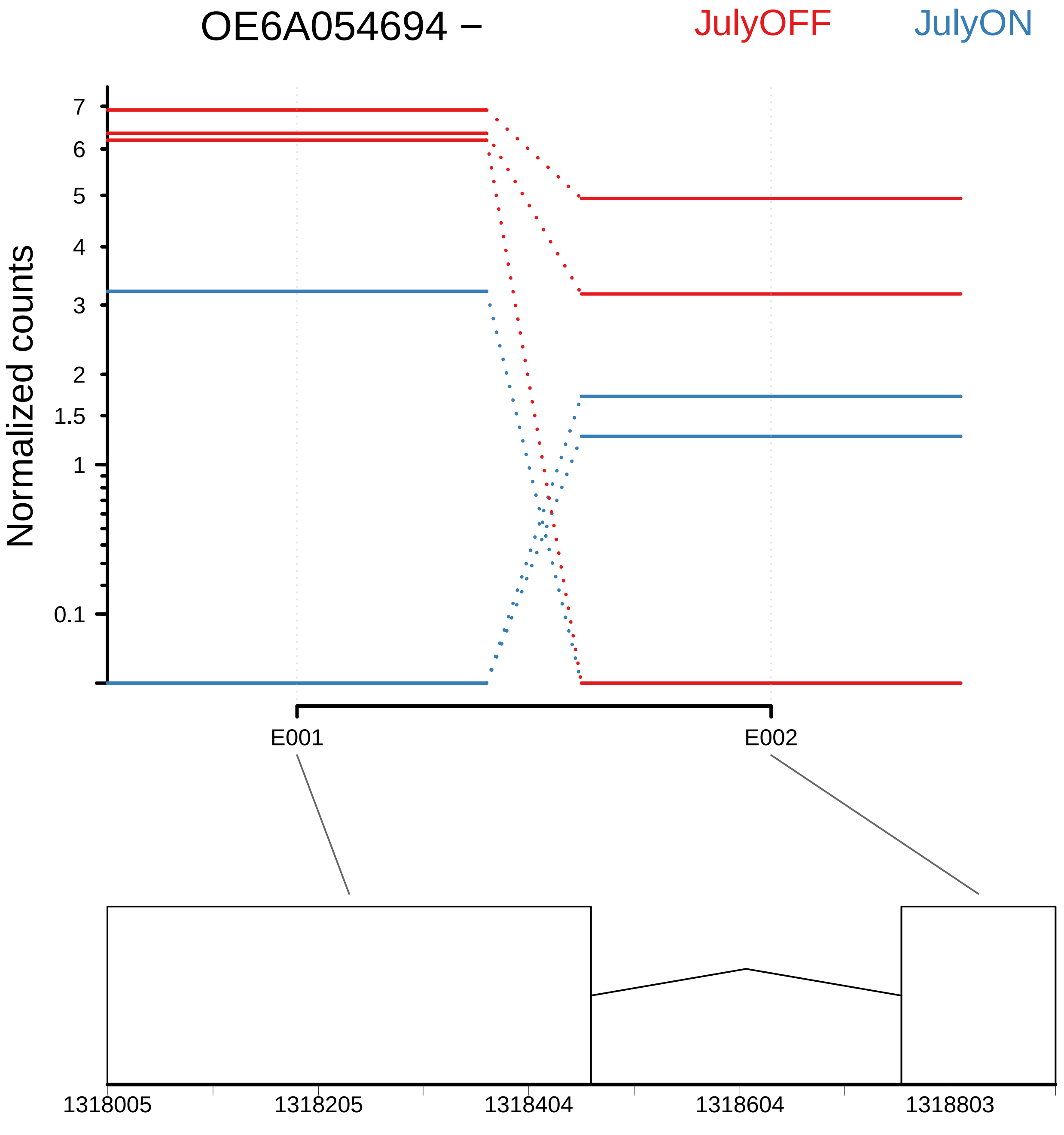
OE6A054694 −
JulyOFF
JulyON
Normalized counts
7
6
5
4
3
2
1.5
1
0.1
E001
E002
1318005
1318205
1318404
1318604
1318803

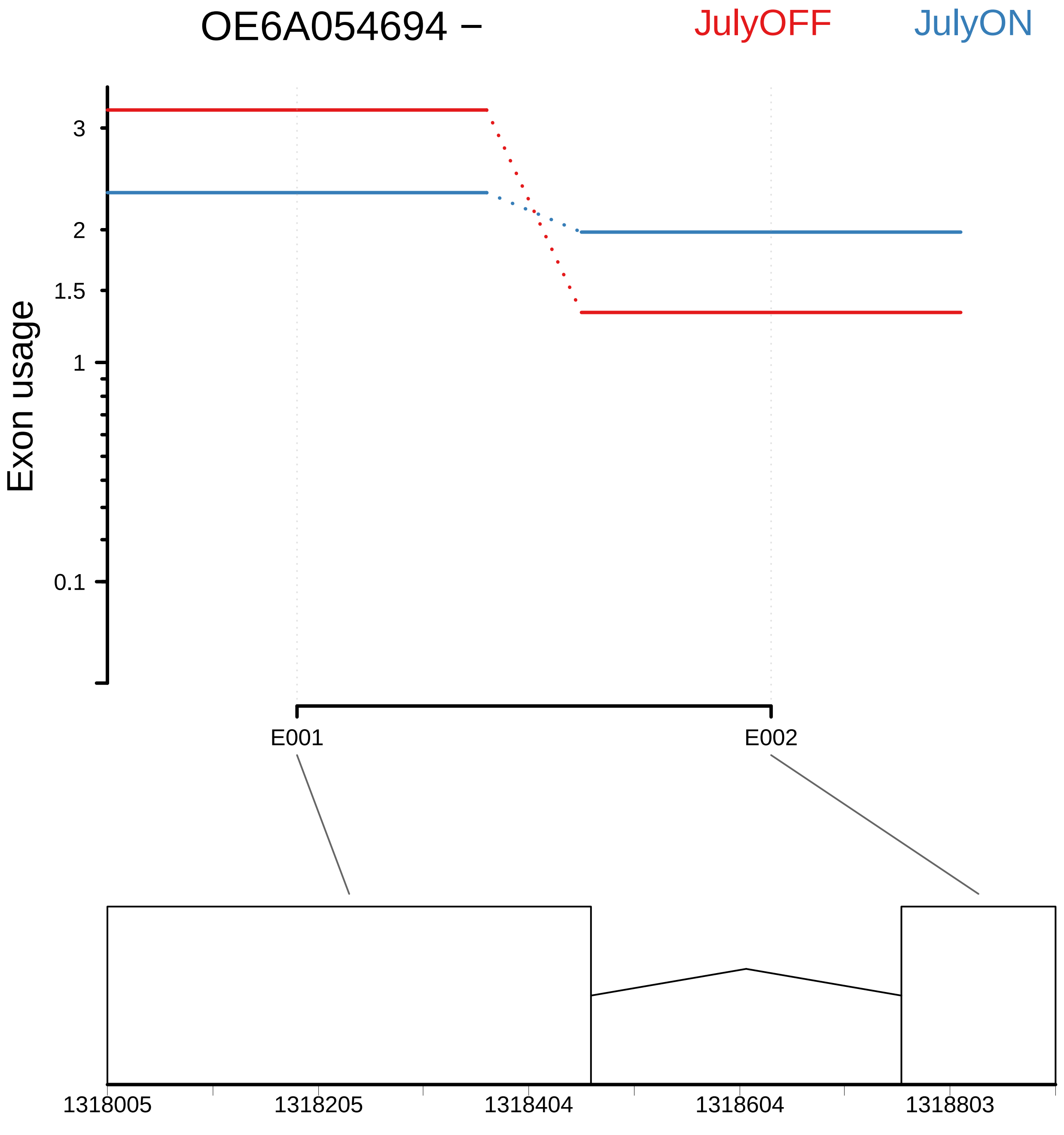

OE6A054694 −
JulyOFF
JulyON
Exon usage
3
2
1.5
1
0.1
E001
E002
1318005
1318205
1318404
1318604
1318803

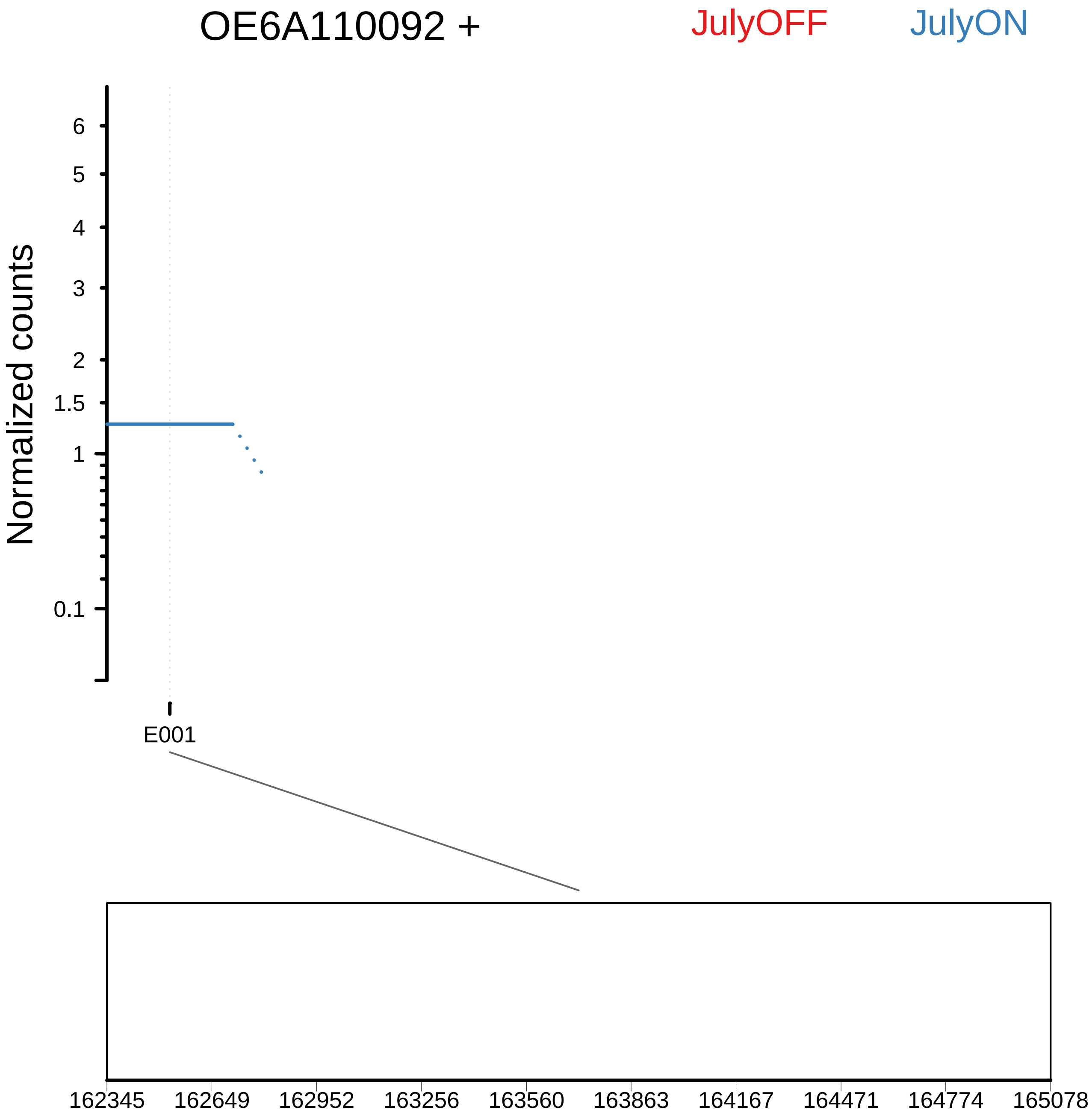

OE6A110092 +
JulyOFF
JulyON
Normalized counts
6
5
4
3
2
1.5
1
0.1
E001
162345
162649
162952
163256
163560
163863
164167
164471
164774
165078

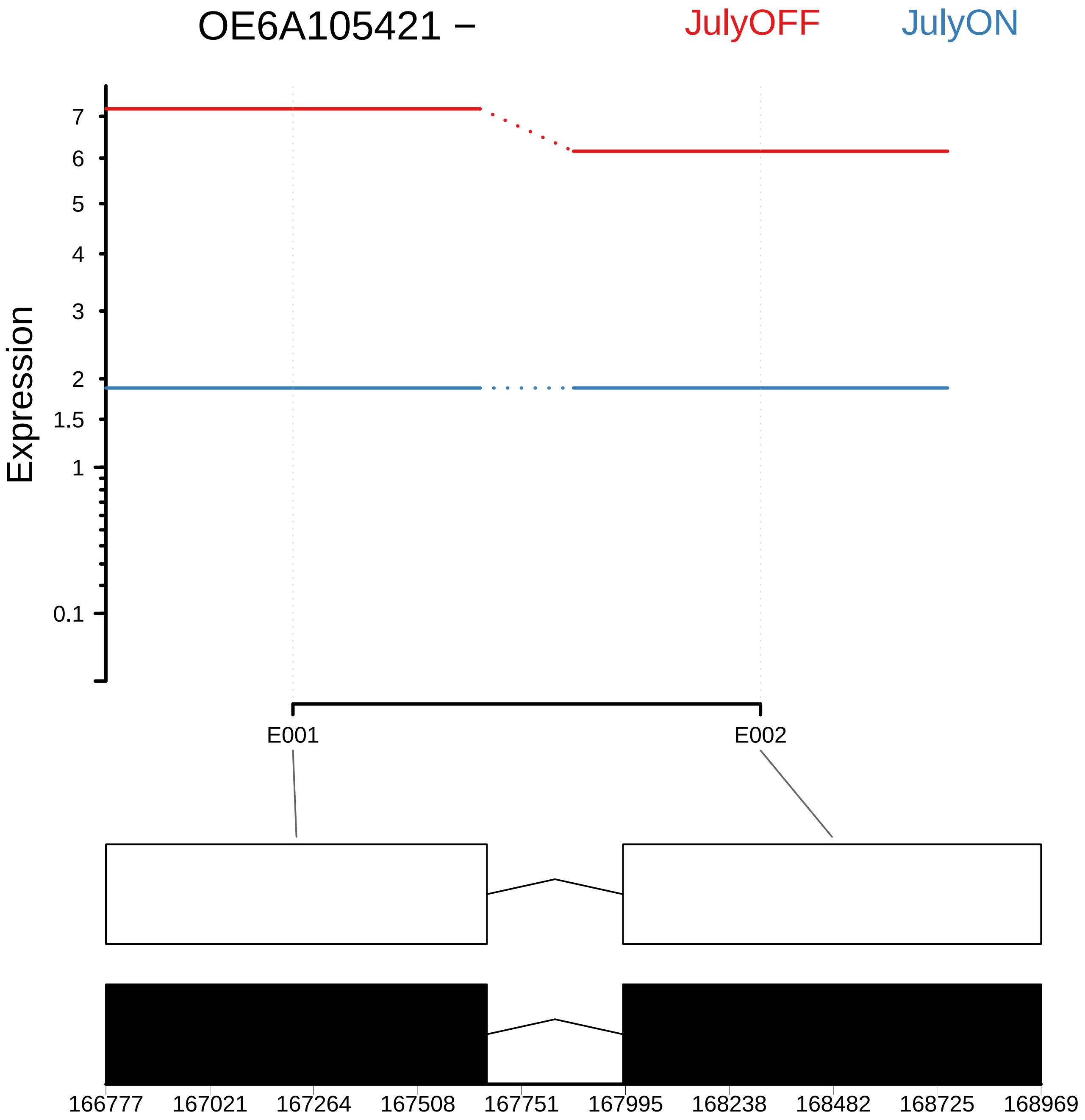

OE6A105421 −
JulyOFF
JulyON
Expression
7
6
5
4
3
2
1.5
1
0.1
E001
E002
166777
167021
167264
167508
167751
167995
168238
168482
168725
168969

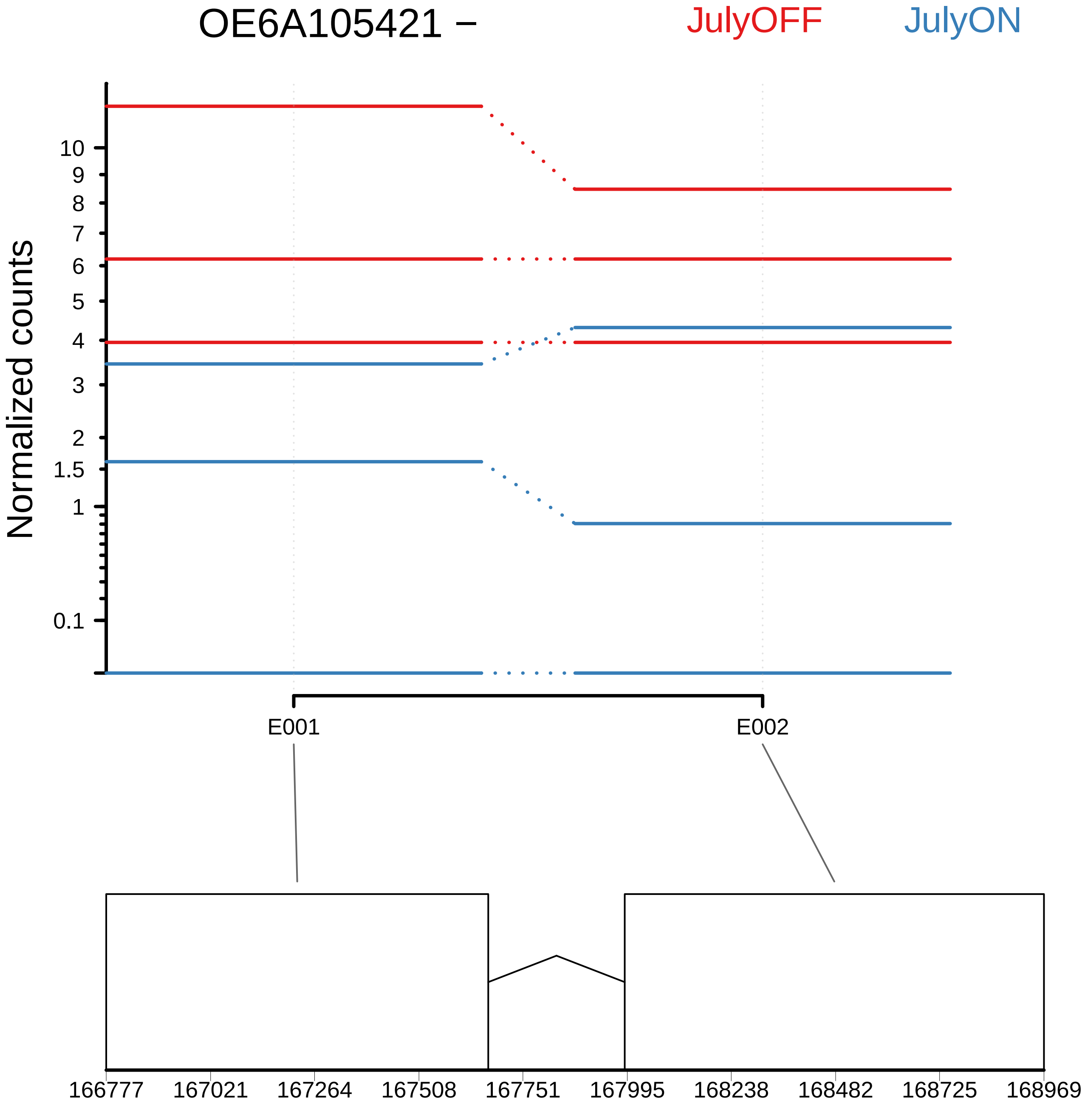

OE6A105421 −
JulyOFF
JulyON
Normalized counts
10
9
8
7
6
5
4
3
2
1.5
1
0.1
E001
E002
166777
167021
167264
167508
167751
167995
168238
168482
168725
168969

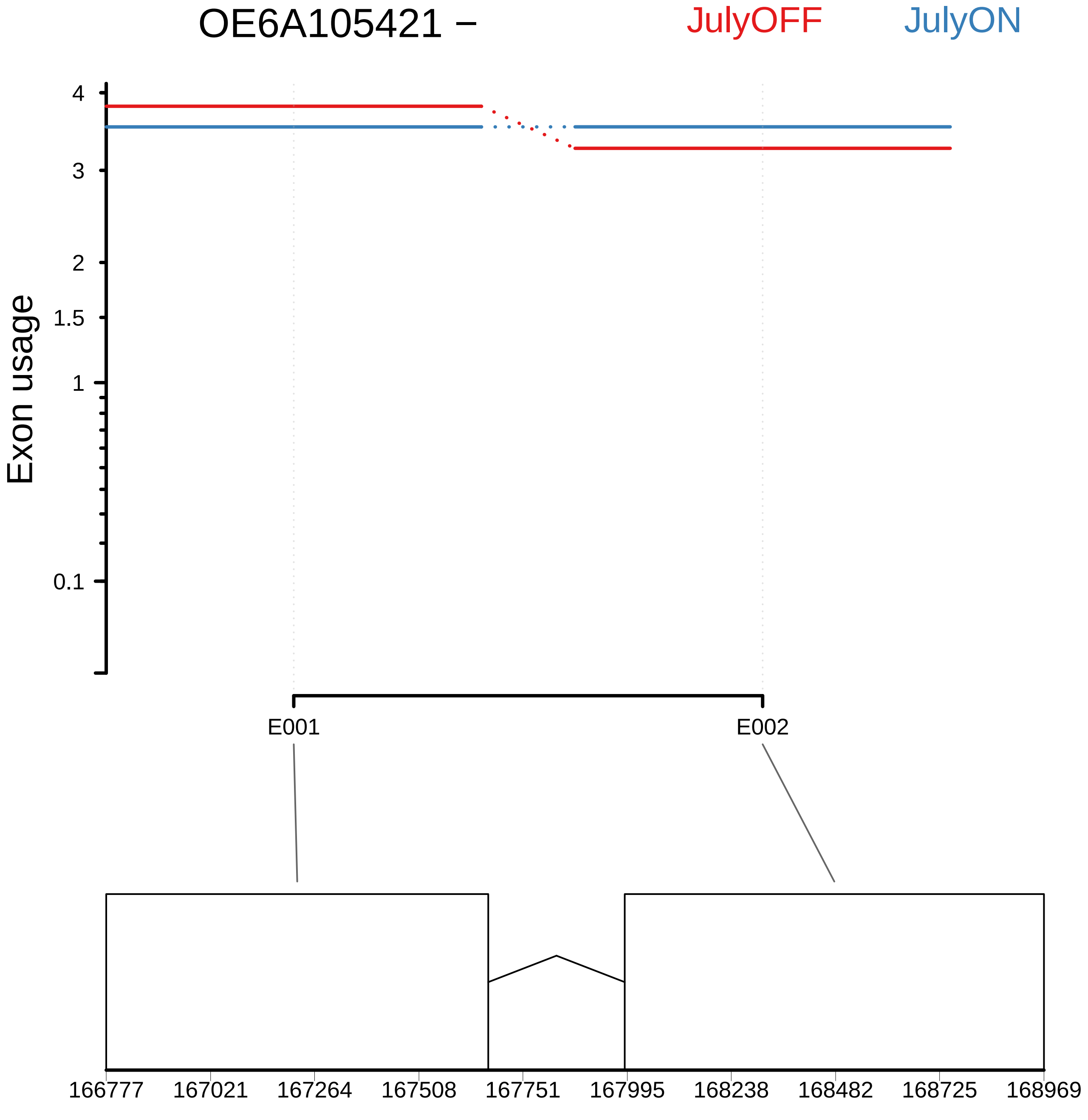
OE6A105421 −
JulyOFF
JulyON
Exon usage
4
3
2
1.5
1
0.1
E001
E002
166777
167021
167264
167508
167751
167995
168238
168482
168725
168969

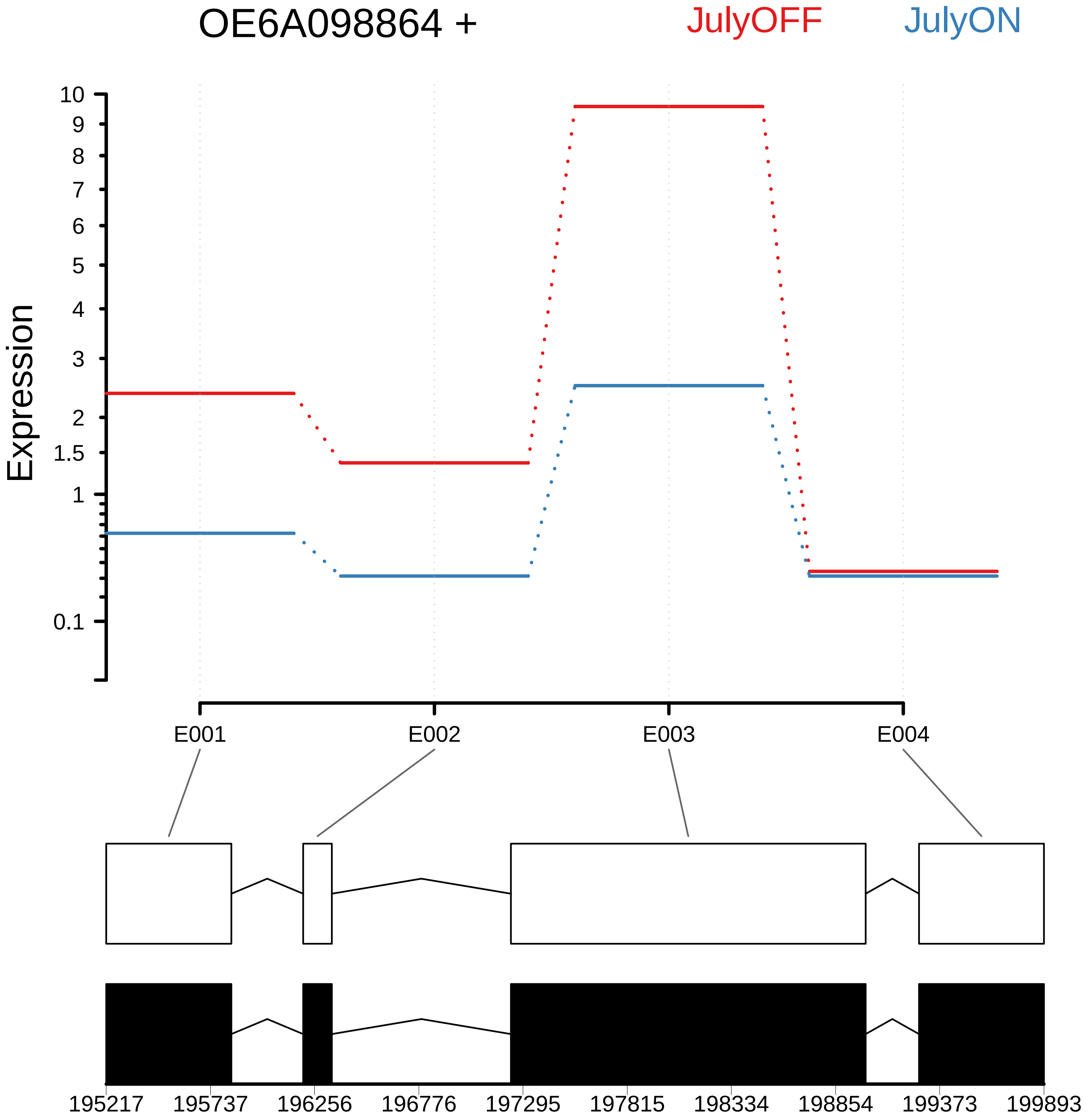
OE6A098864 +
JulyOFF
JulyON
Expression
10
9
8
7
6
5
4
3
2
1.5
1
0.1
E001
E002
E003
E004
195217
195737
196256
196776
197295
197815
198334
198854
199373
199893

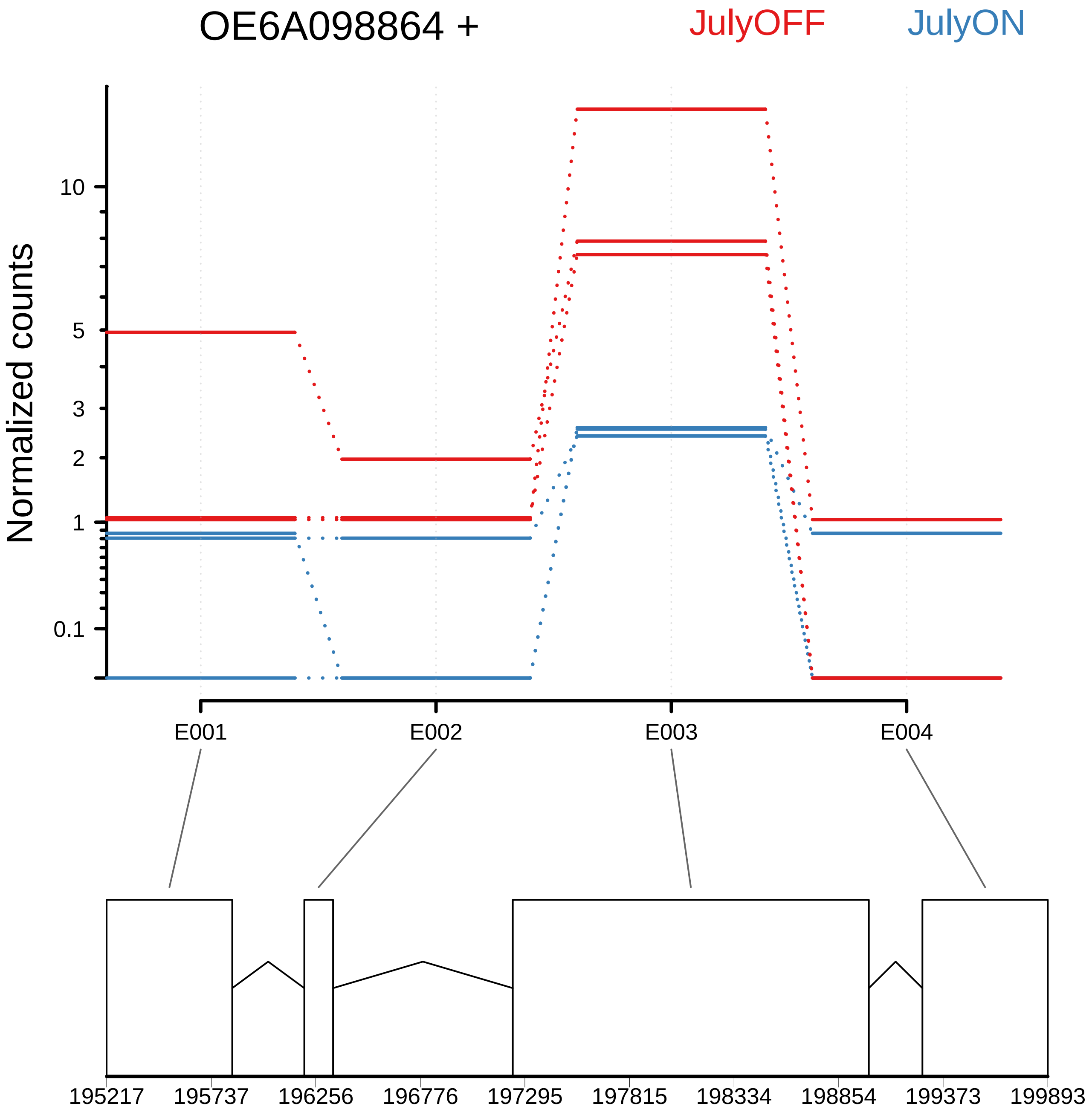
OE6A098864 +
JulyOFF
JulyON
Normalized counts
10
5
3
2
1
0.1
E001
E002
E003
E004
195217
195737
196256
196776
197295
197815
198334
198854
199373
199893

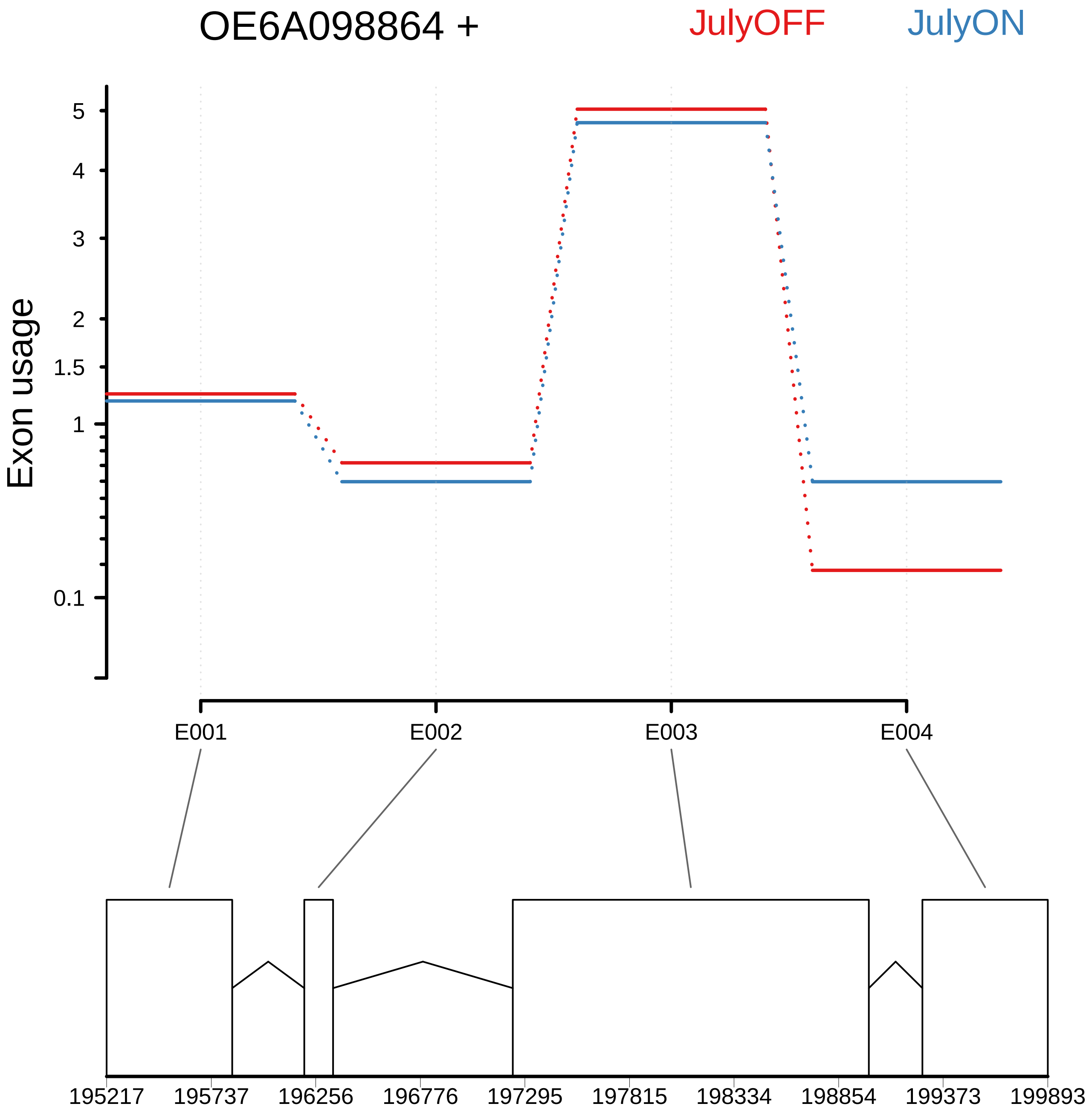
OE6A098864 +
JulyOFF
JulyON
Exon usage
5
4
3
2
1.5
1
0.1
E001
E002
E003
E004
195217
195737
196256
196776
197295
197815
198334
198854
199373
199893

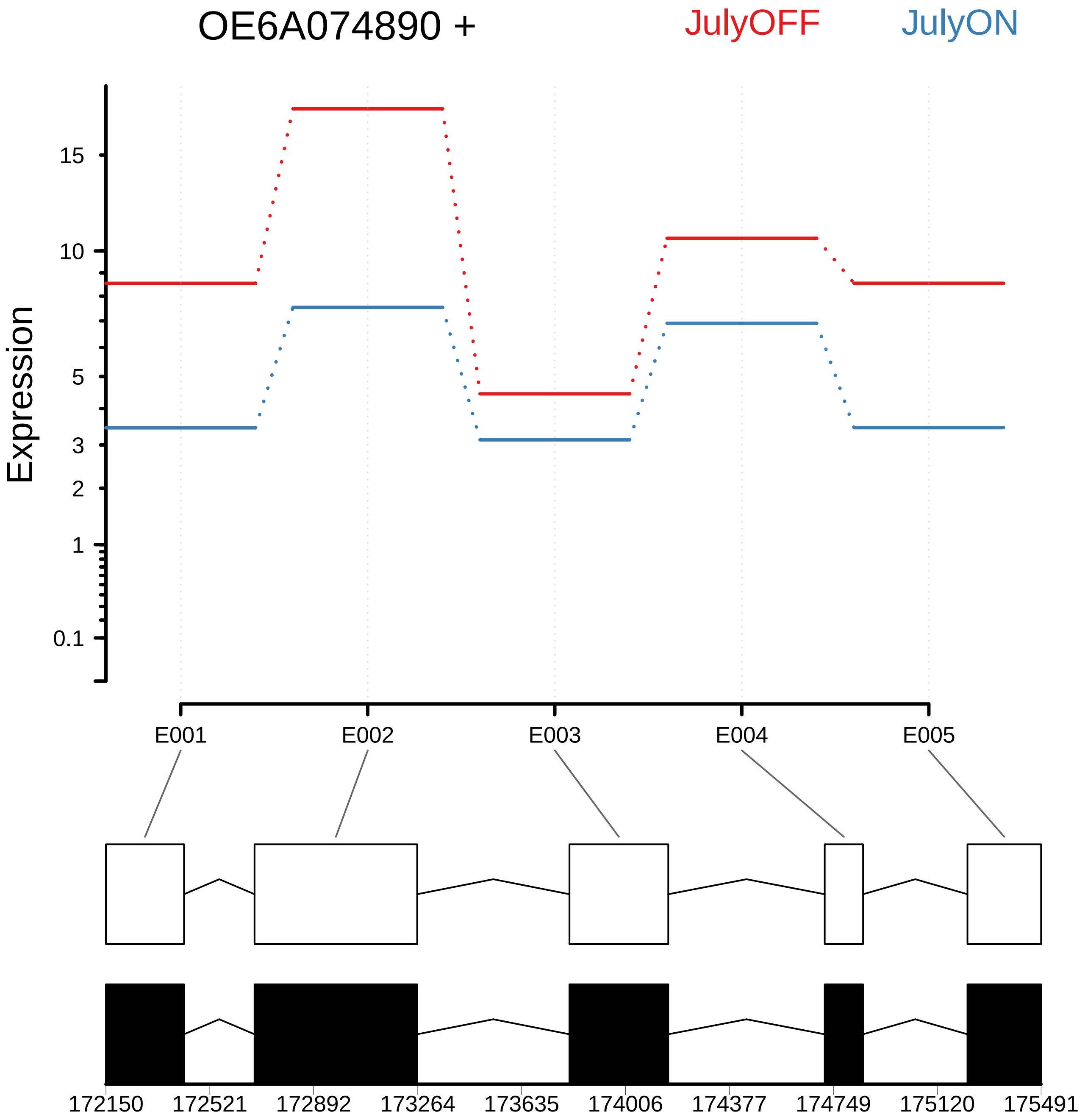

OE6A074890 +
JulyOFF
JulyON
Expression
15
10
5
3
2
1
0.1
E001
E002
E003
E004
E005
172150
172521
172892
173264
173635
174006
174377
174749
175120
175491

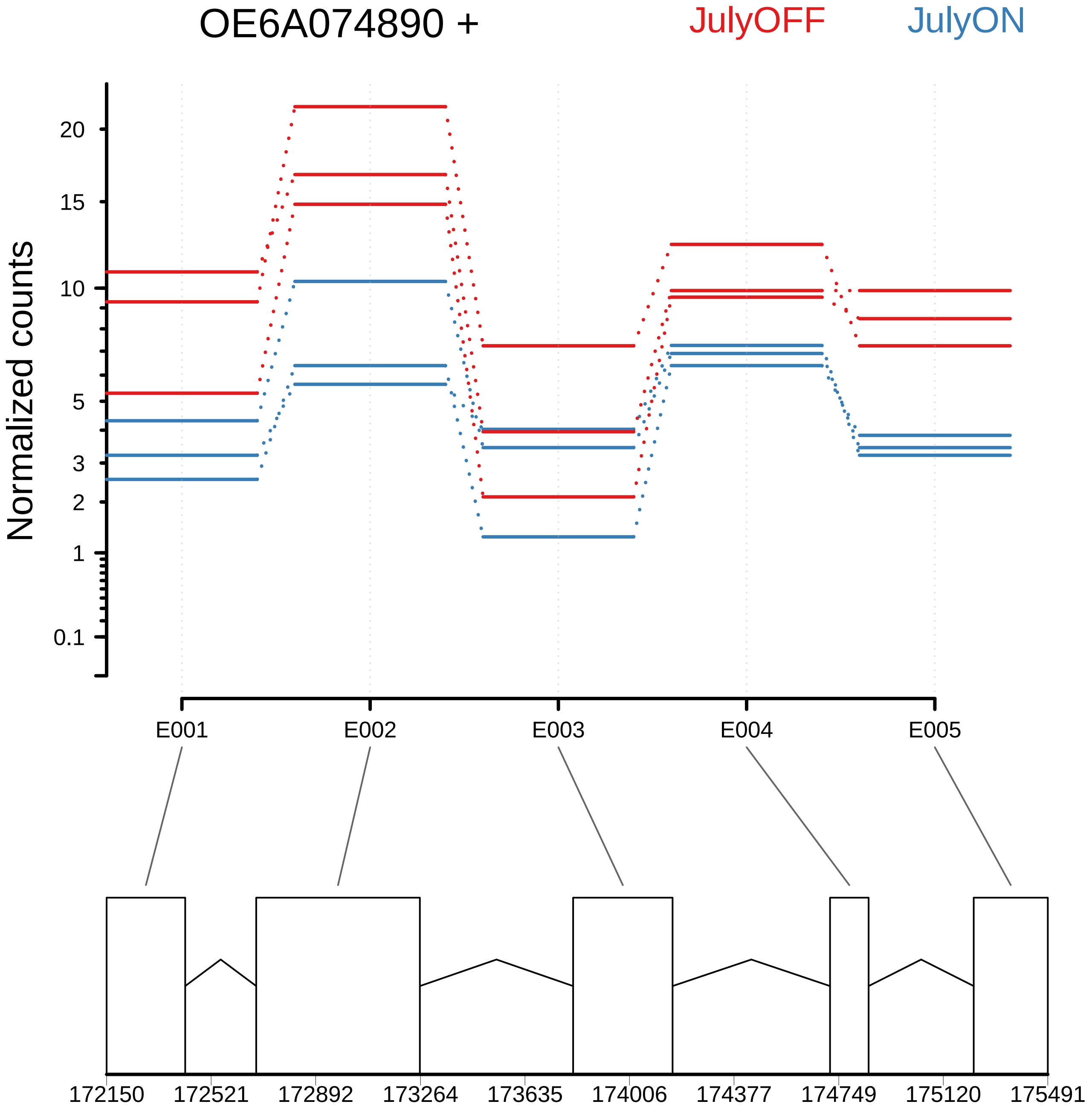

OE6A074890 +
JulyOFF
JulyON
Normalized counts
20
15
10
5
3
2
1
0.1
E001
E002
E003
E004
E005
172150
172521
172892
173264
173635
174006
174377
174749
175120
175491

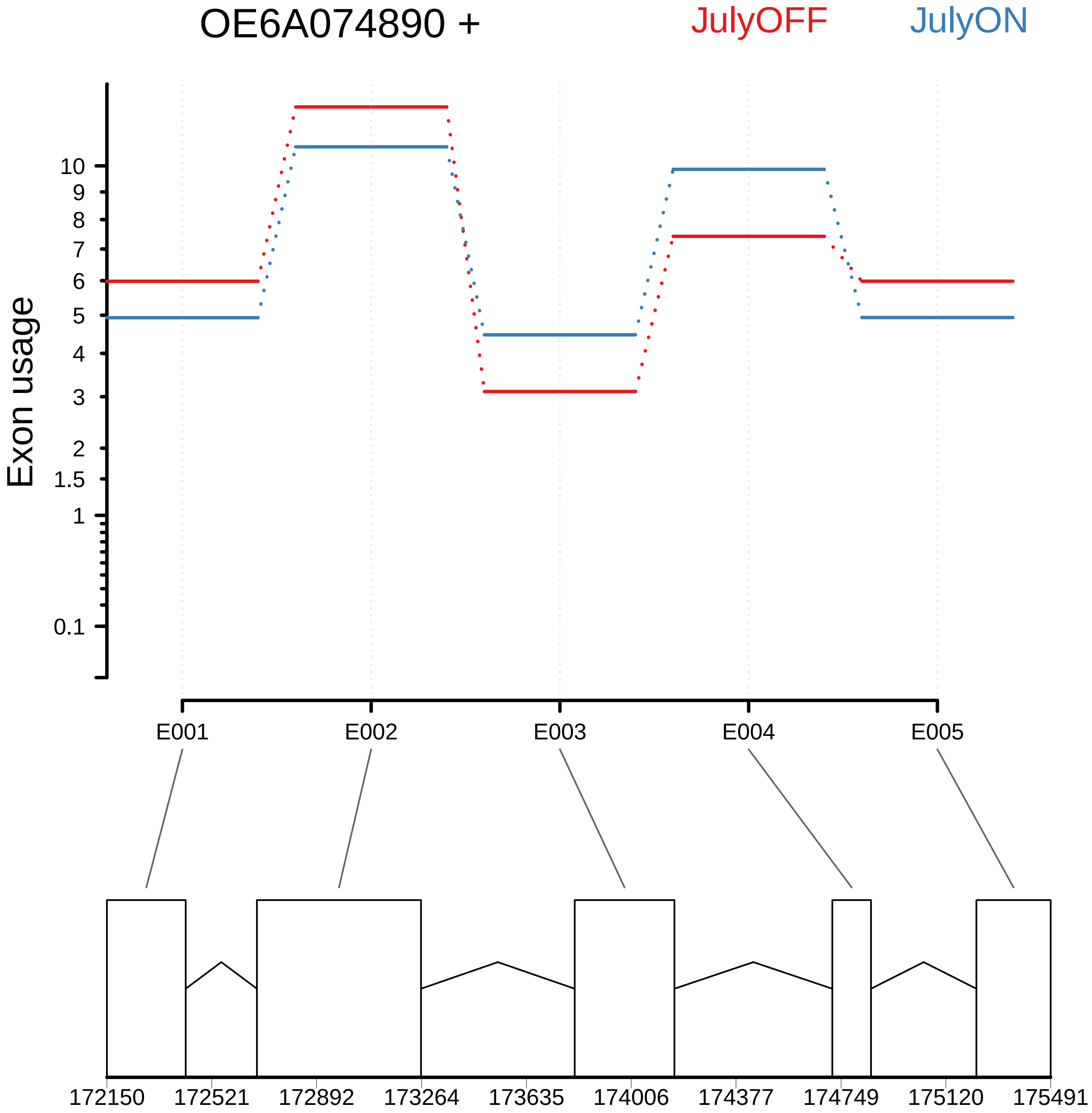
OE6A074890 +
JulyOFF
JulyON
Exon usage
10
9
8
7
6
5
4
3
2
1.5
1
0.1
E001
E002
E003
E004
E005
172150
172521
172892
173264
173635
174006
174377
174749
175120
175491

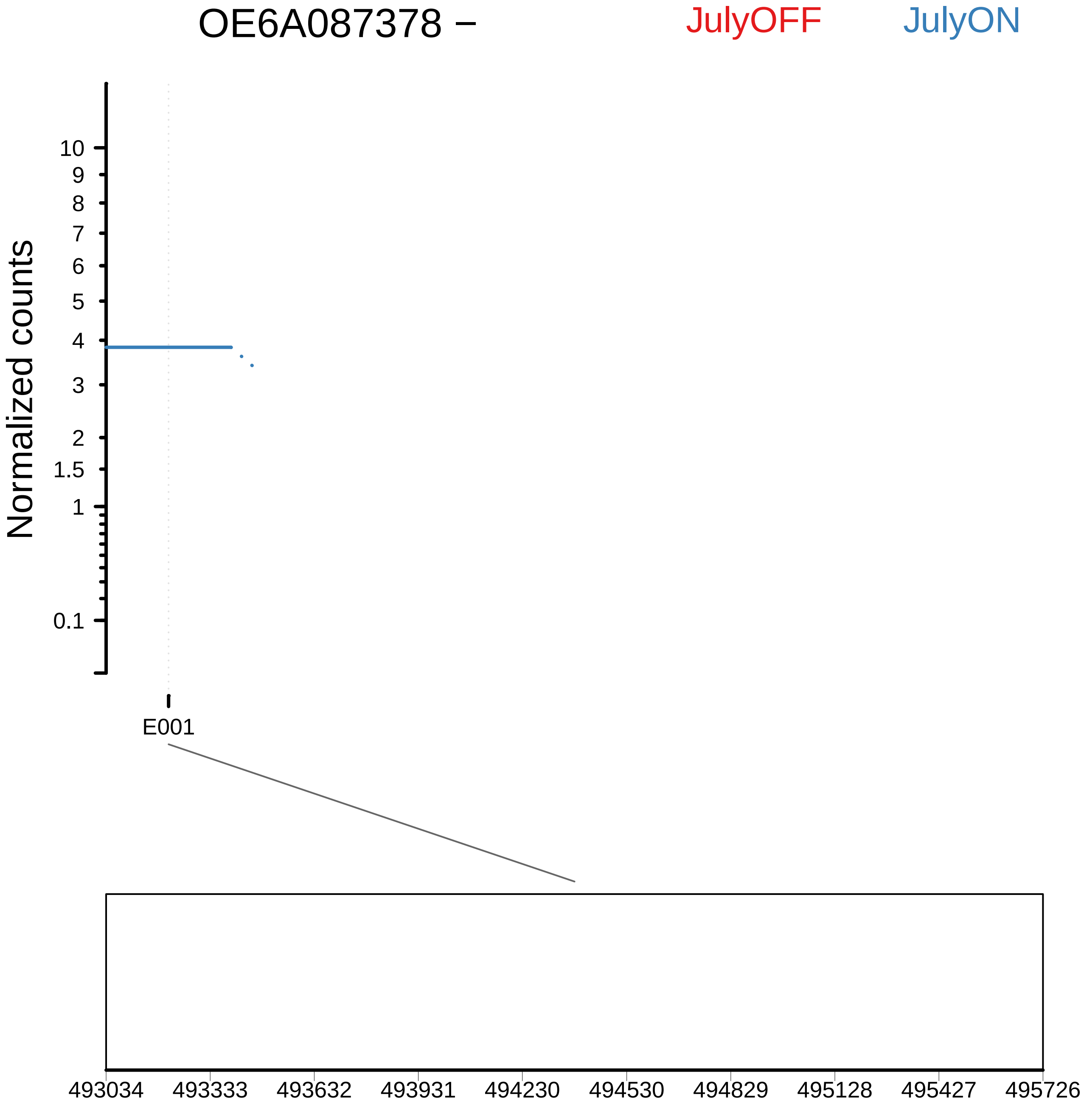
OE6A087378 −
JulyOFF
JulyON
Normalized counts
10
9
8
7
6
5
4
3
2
1.5
1
0.1
E001
493034
493333
493632
493931
494230
494530
494829
495128
495427
495726

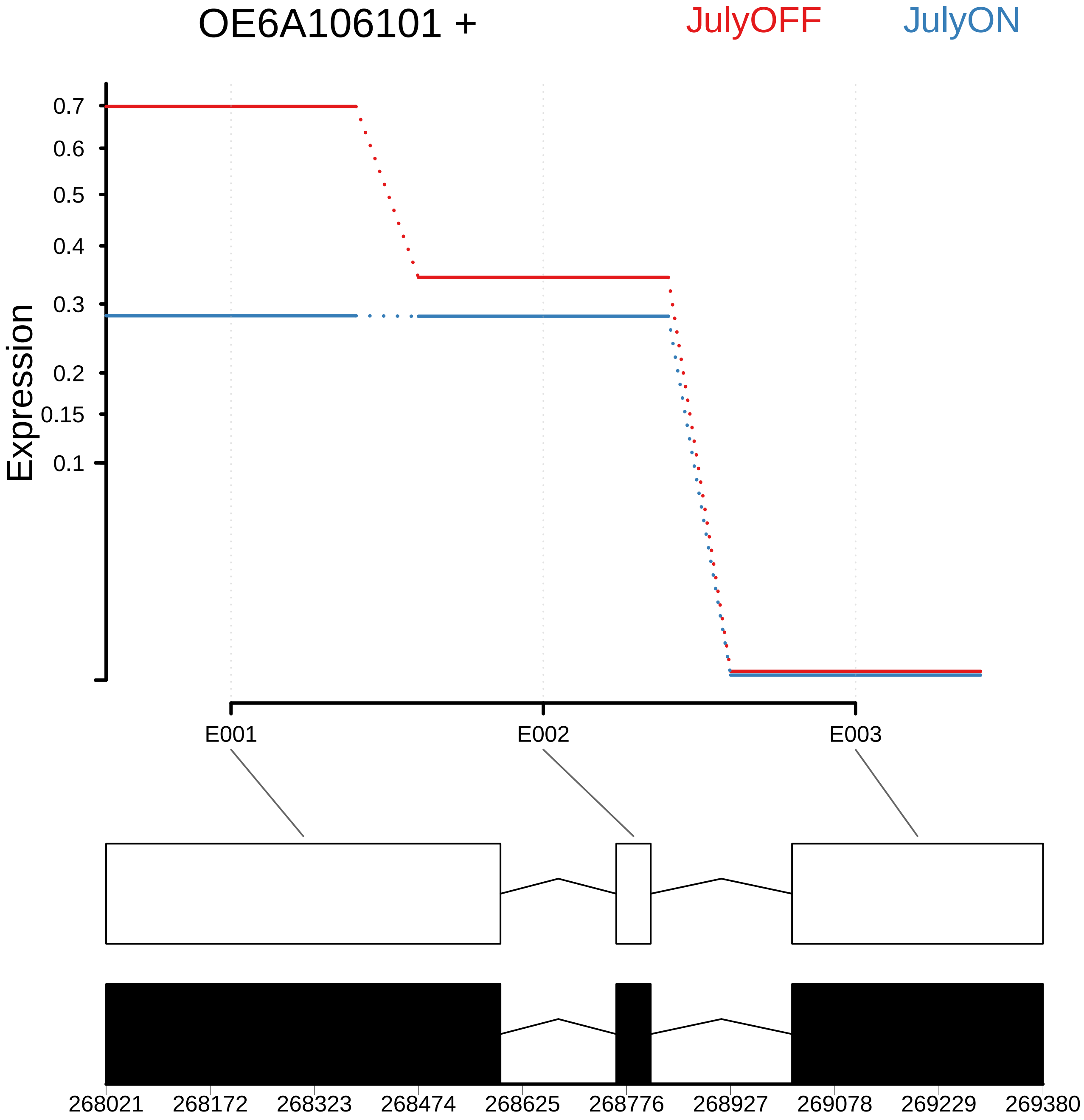
OE6A106101 +
JulyOFF
JulyON
Expression
0.7
0.6
0.5
0.4
0.3
0.2
0.15
0.1
E001
E002
E003
268021
268172
268323
268474
268625
268776
268927
269078
269229
269380

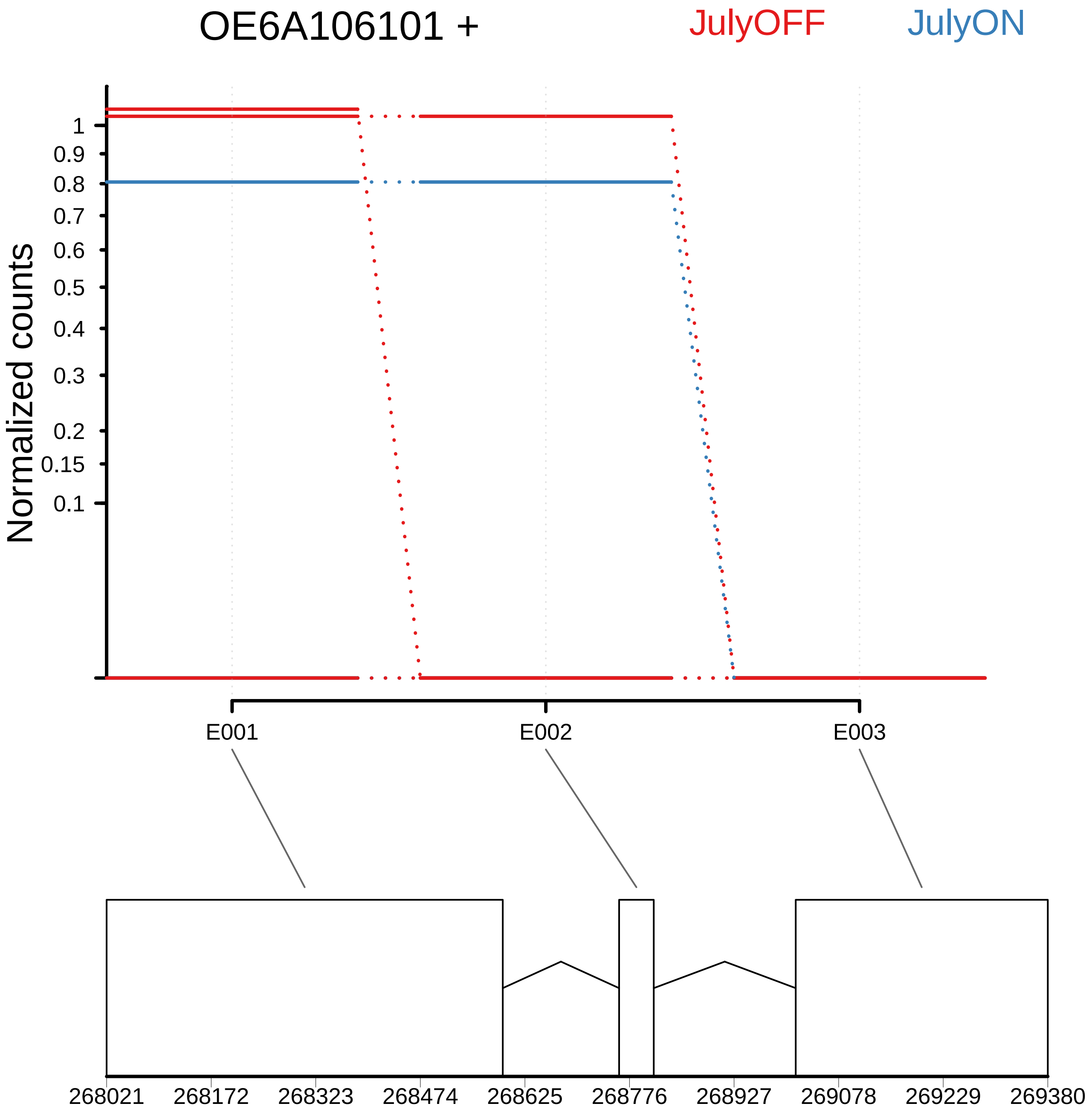
OE6A106101 +
JulyOFF
JulyON
Normalized counts
1
0.9
0.8
0.7
0.6
0.5
0.4
0.3
0.2
0.15
0.1
E001
E002
E003
268021
268172
268323
268474
268625
268776
268927
269078
269229
269380

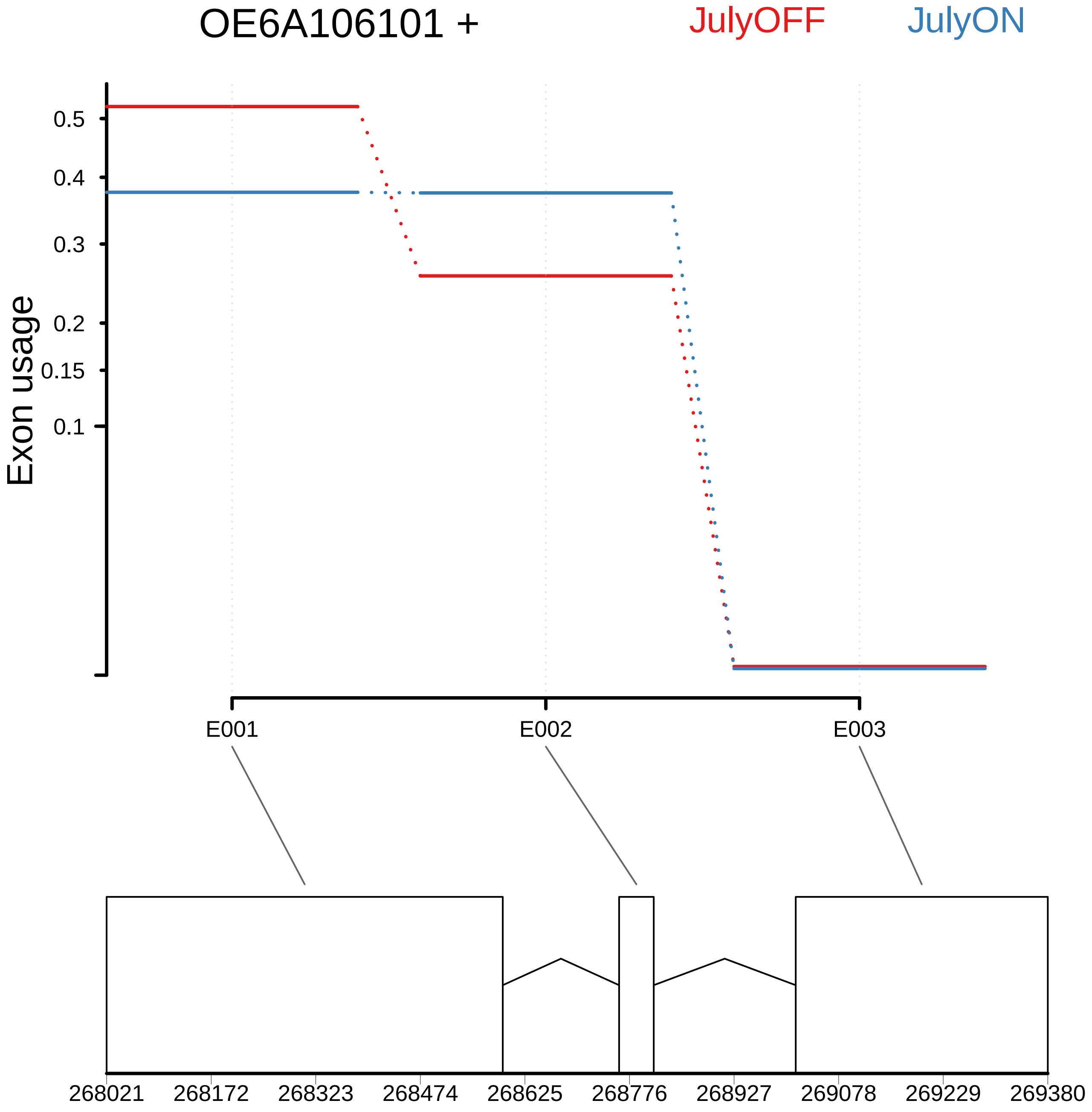
OE6A106101 +
JulyOFF
JulyON
Exon usage
0.5
0.4
0.3
0.2
0.15
0.1
E001
E002
E003
268021
268172
268323
268474
268625
268776
268927
269078
269229
269380

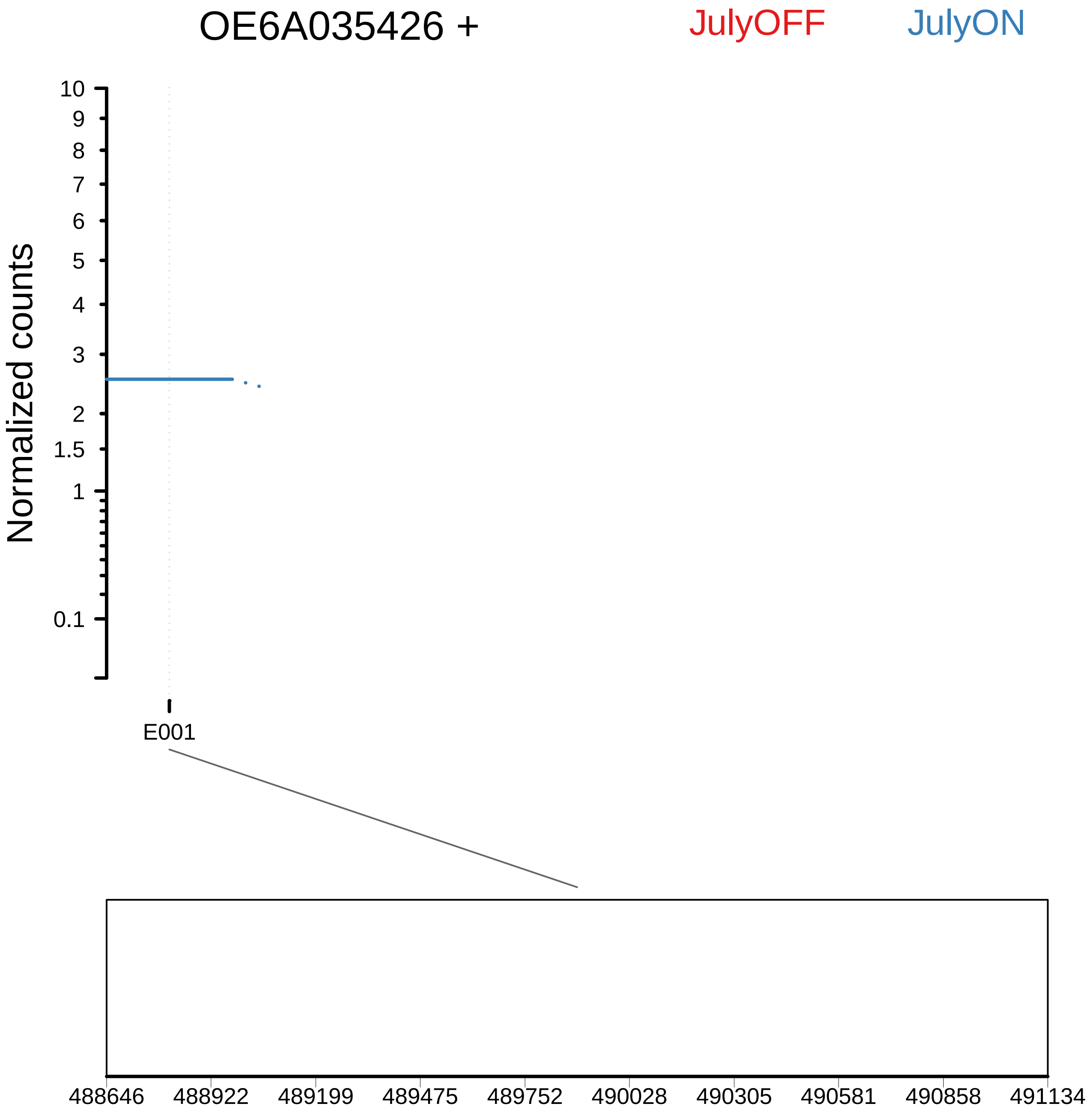
OE6A035426 +
JulyOFF
JulyON
Normalized counts
10
9
8
7
6
5
4
3
2
1.5
1
0.1
E001
488646
488922
489199
489475
489752
490028
490305
490581
490858
491134

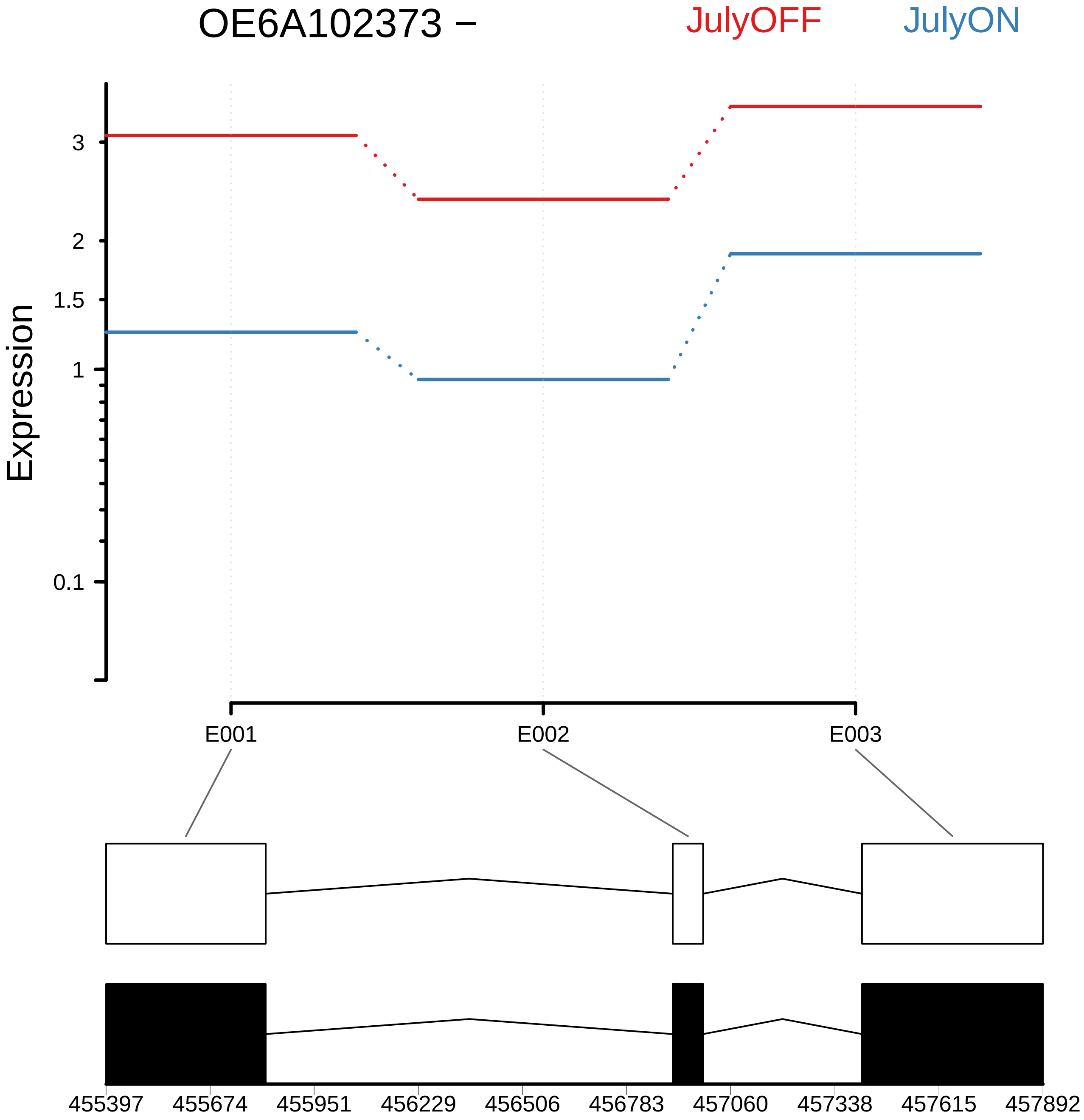
OE6A102373 −
JulyOFF
JulyON
Expression
3
2
1.5
1
0.1
E001
E002
E003
455397
455674
455951
456229
456506
456783
457060
457338
457615
457892

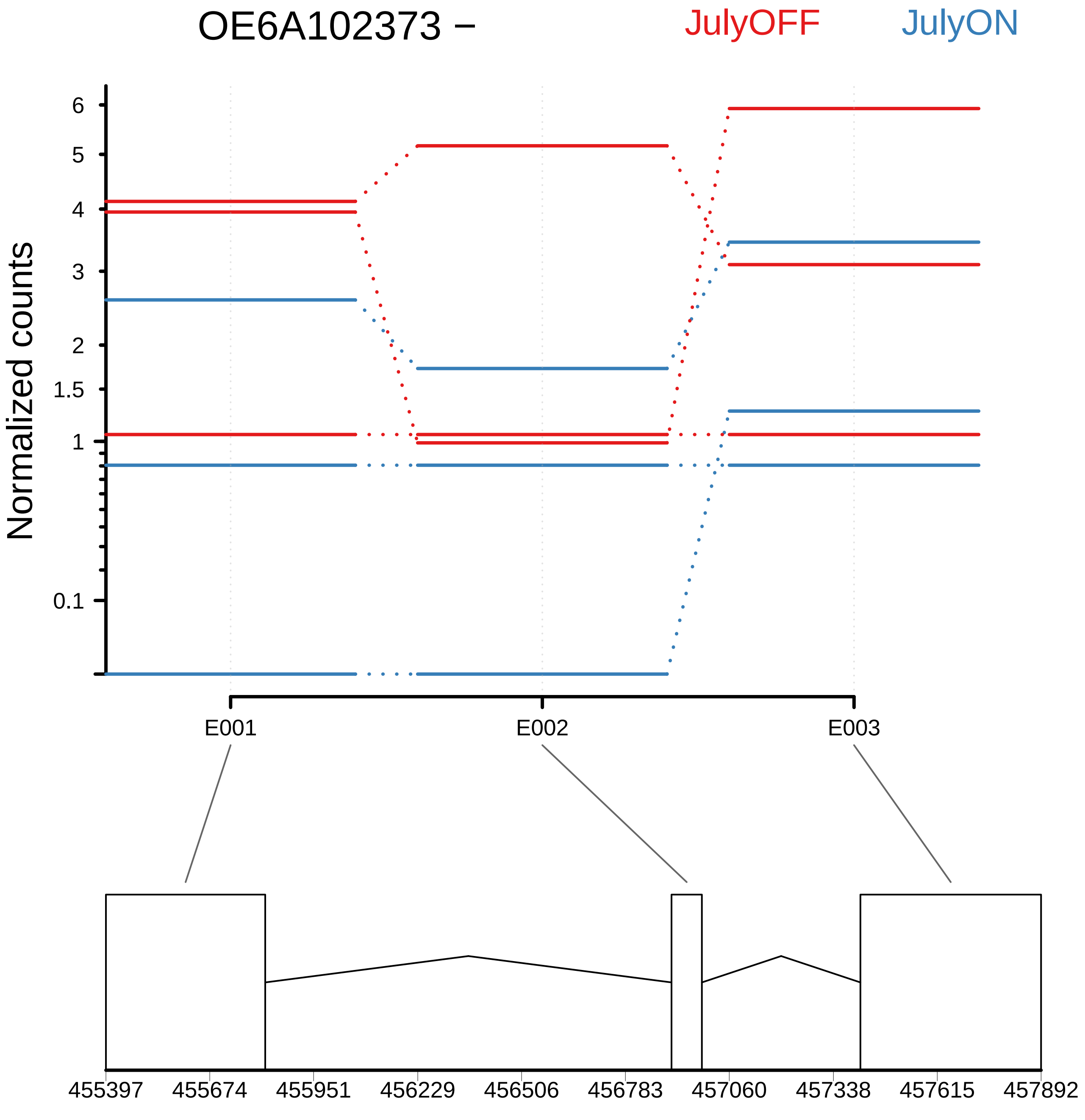

OE6A102373 −
JulyOFF
JulyON
Normalized counts
6
5
4
3
2
1.5
1
0.1
E001
E002
E003
455397
455674
455951
456229
456506
456783
457060
457338
457615
457892

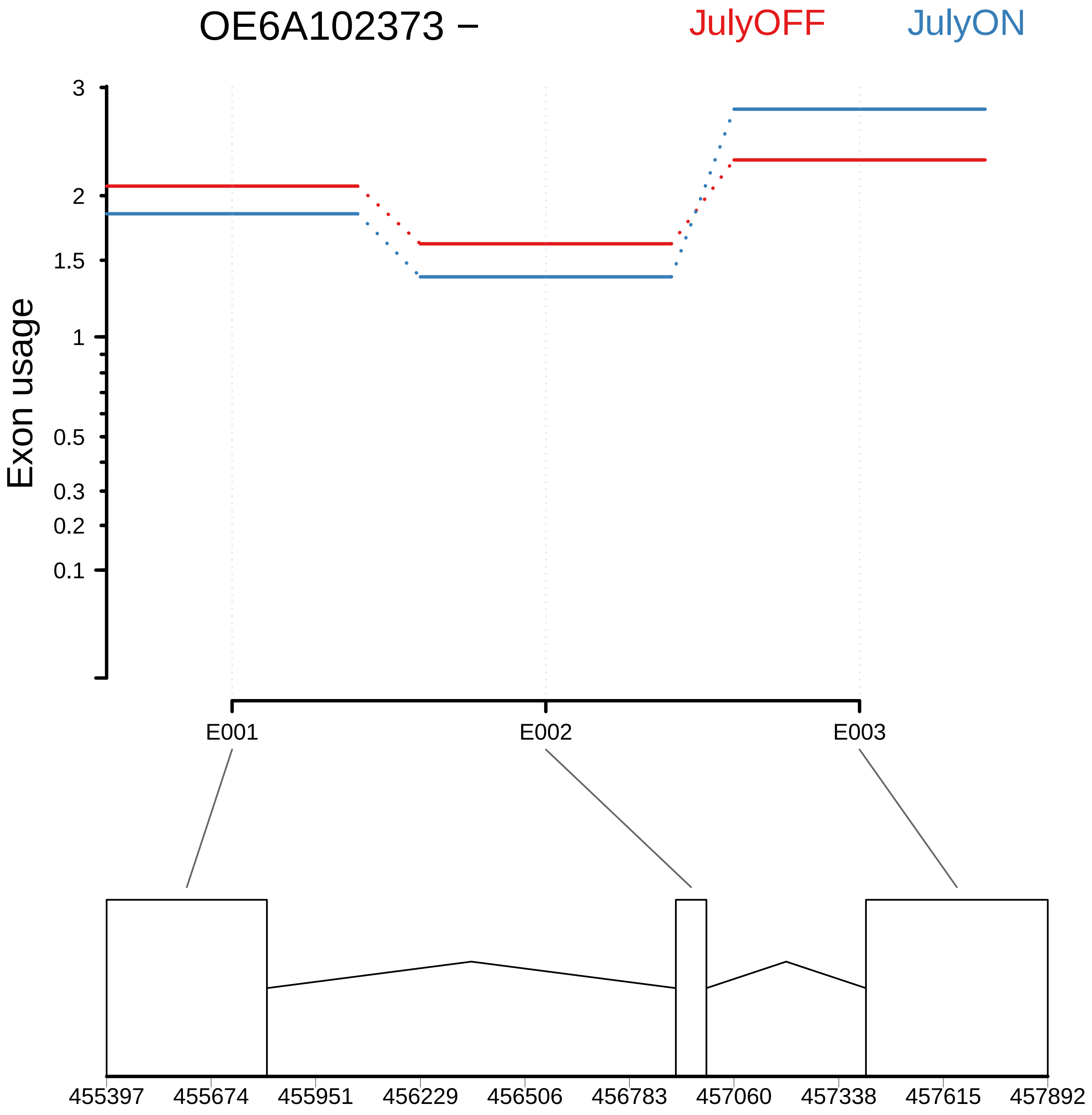
OE6A102373 −
JulyOFF
JulyON
Exon usage
3
2
1.5
1
0.5
0.3
0.2
0.1
E001
E002
E003
455397
455674
455951
456229
456506
456783
457060
457338
457615
457892

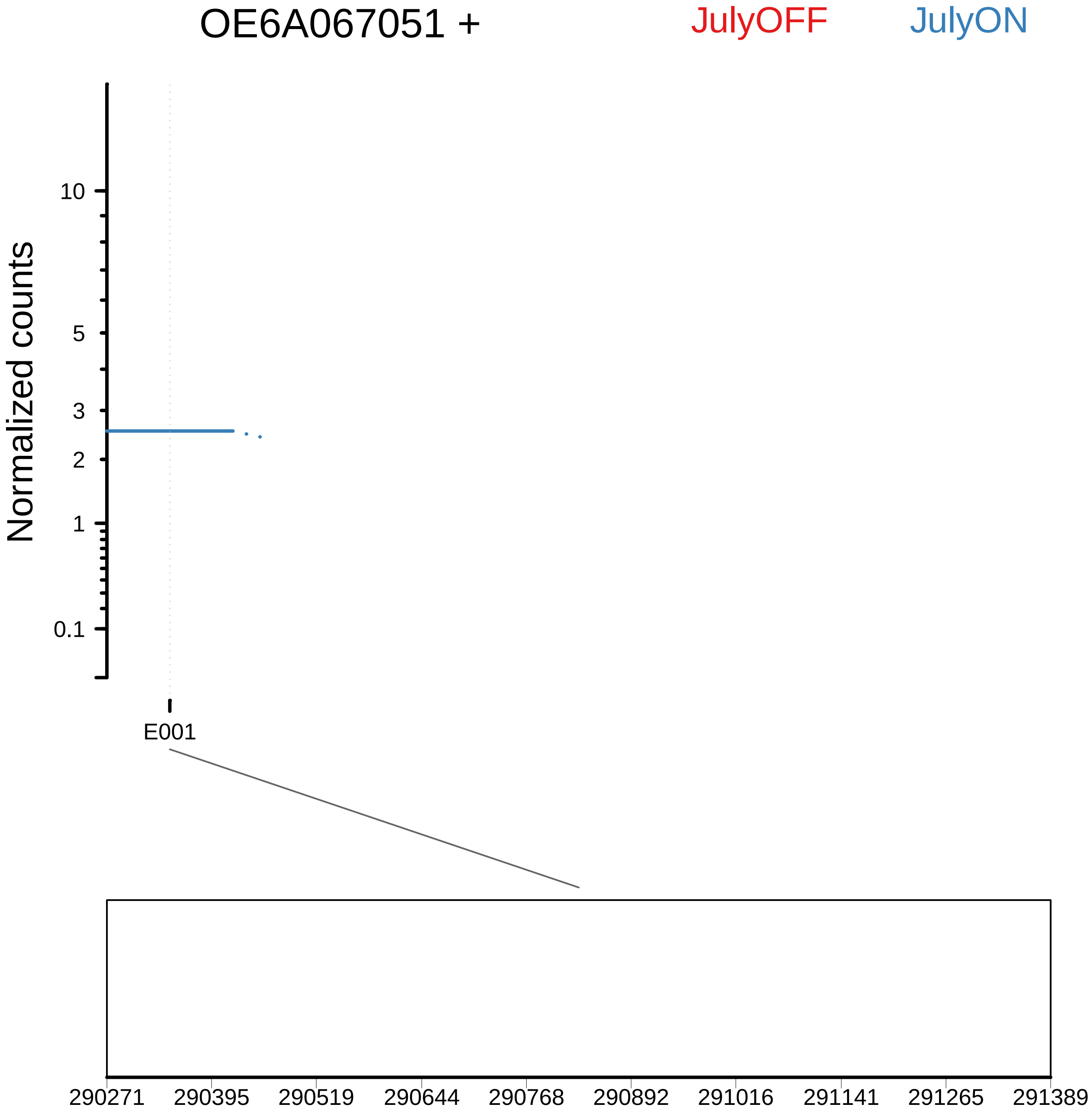
OE6A067051 +
JulyOFF
JulyON
Normalized counts
10
5
3
2
1
0.1
E001
290271
290395
290519
290644
290768
290892
291016
291141
291265
291389

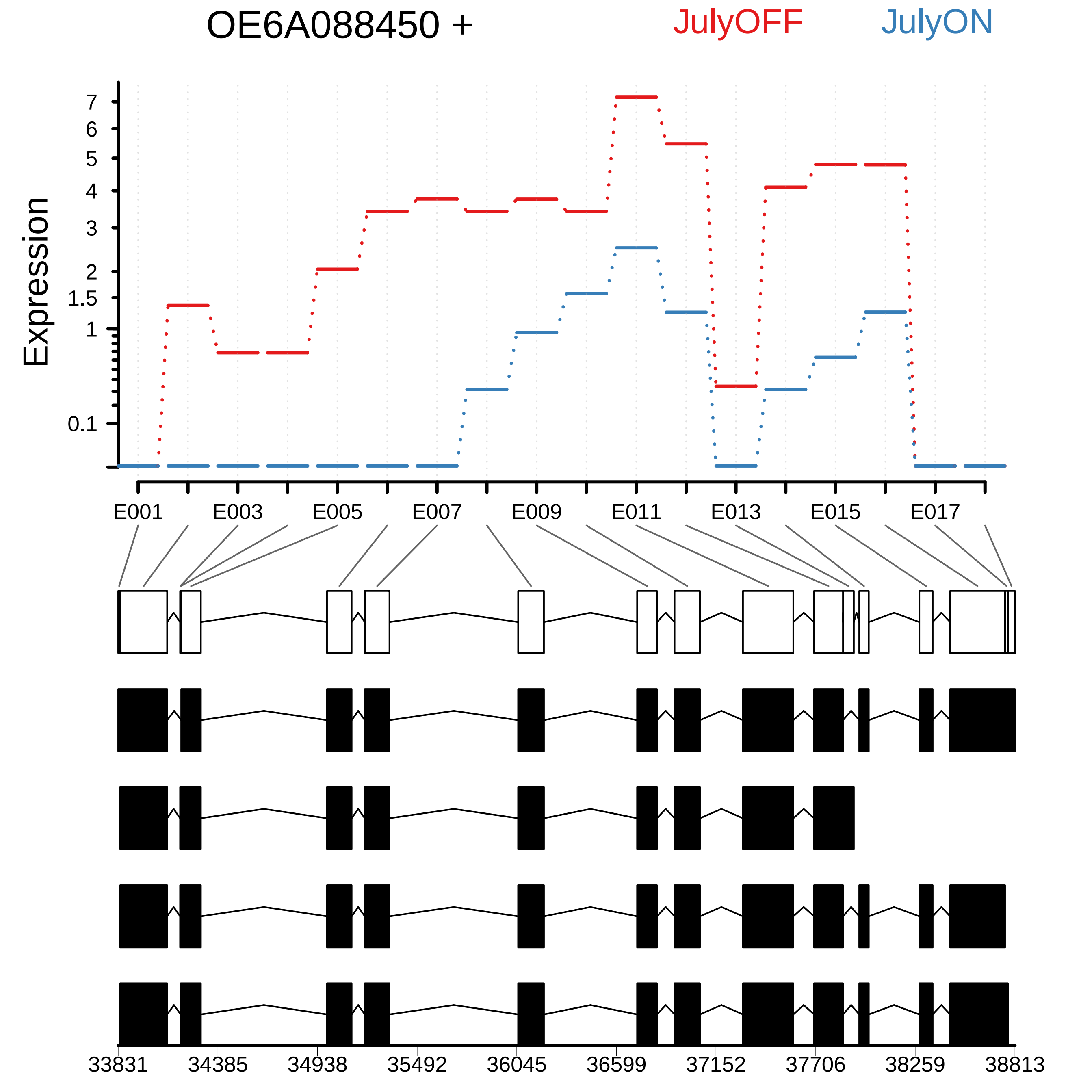

OE6A088450 +
JulyOFF
JulyON
Expression
7
6
5
4
3
2
1.5
1
0.1
E001
E003
E005
E007
E009
E011
E013
E015
E017
33831
34385
34938
35492
36045
36599
37152
37706
38259
38813

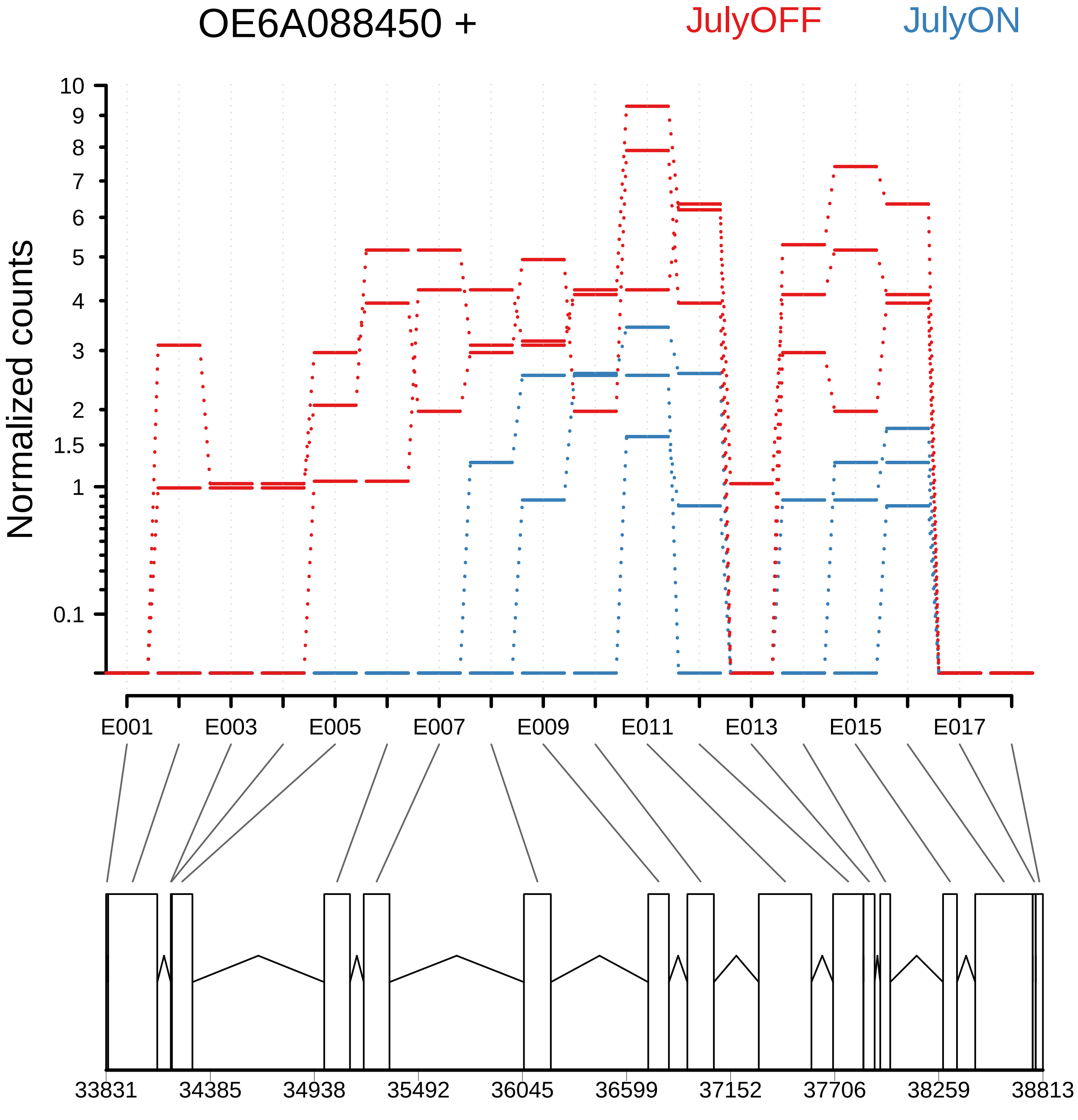

OE6A088450 +
JulyOFF
JulyON
Normalized counts
10
9
8
7
6
5
4
3
2
1.5
1
0.1
E001
E003
E005
E007
E009
E011
E013
E015
E017
33831
34385
34938
35492
36045
36599
37152
37706
38259
38813

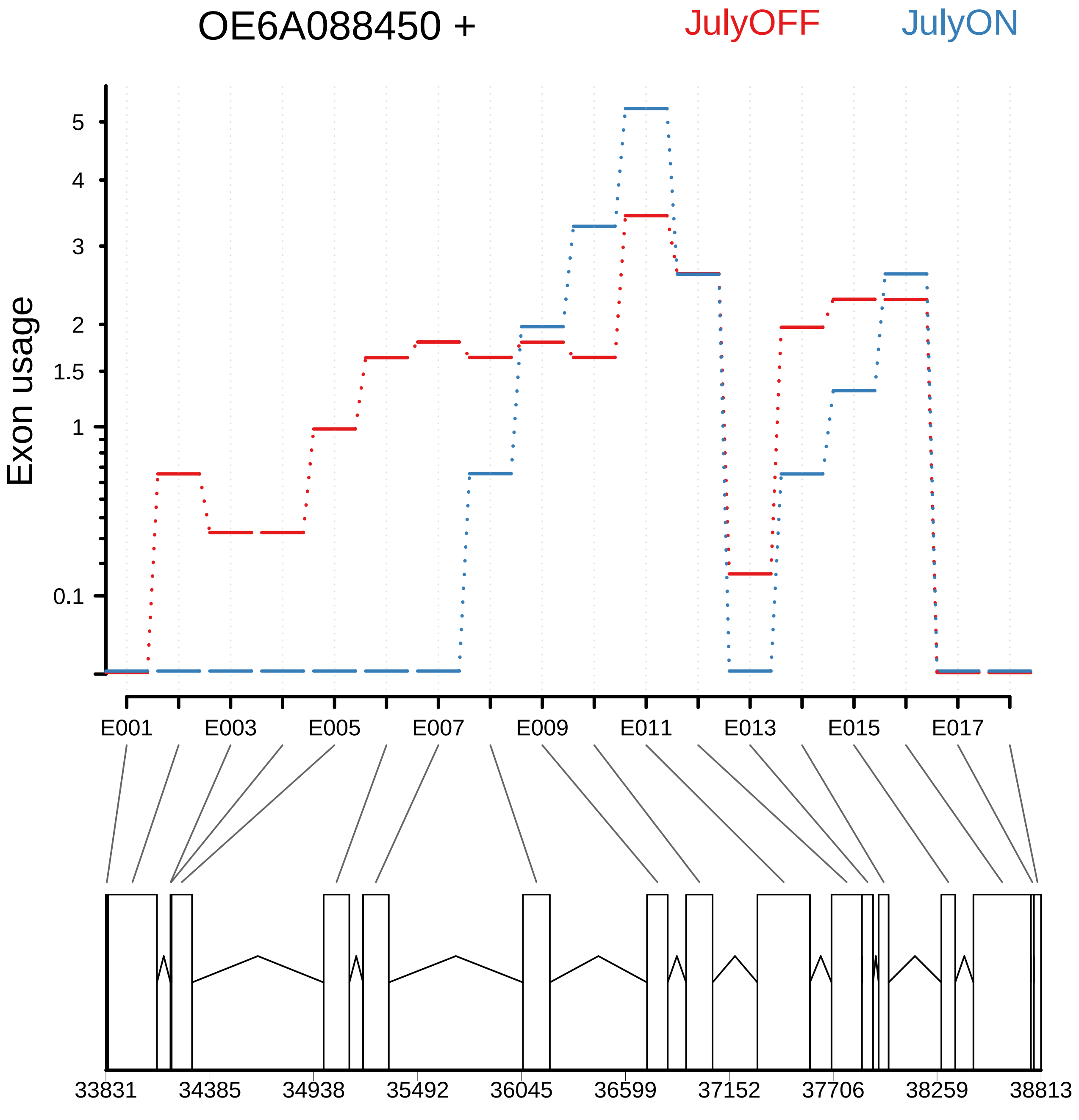
OE6A088450 +
JulyOFF
JulyON
Exon usage
5
4
3
2
1.5
1
0.1
E001
E003
E005
E007
E009
E011
E013
E015
E017
33831
34385
34938
35492
36045
36599
37152
37706
38259
38813

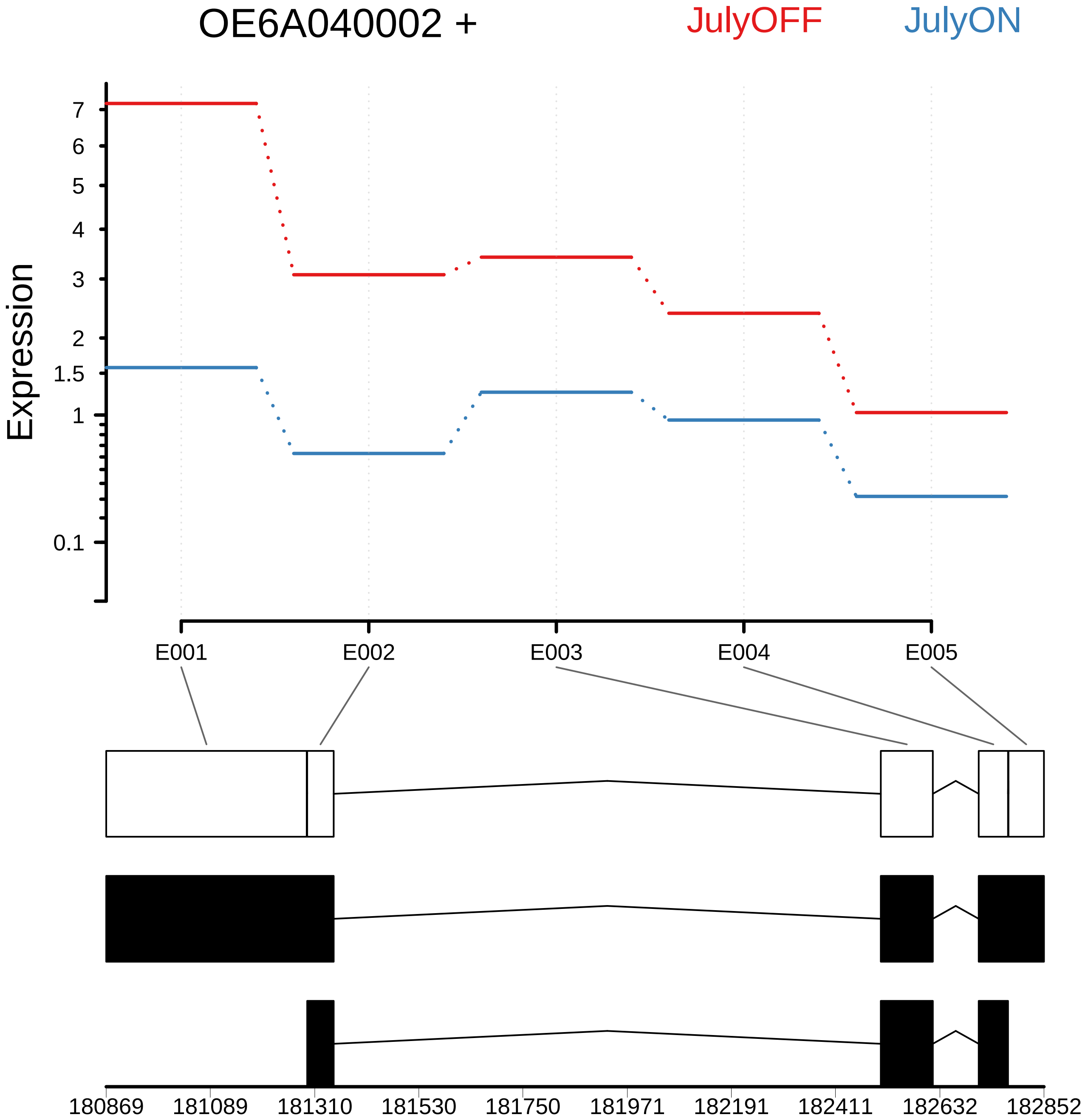
OE6A040002 +
JulyOFF
JulyON
Expression
7
6
5
4
3
2
1.5
1
0.1
E001
E002
E003
E004
E005
180869
181089
181310
181530
181750
181971
182191
182411
182632
182852

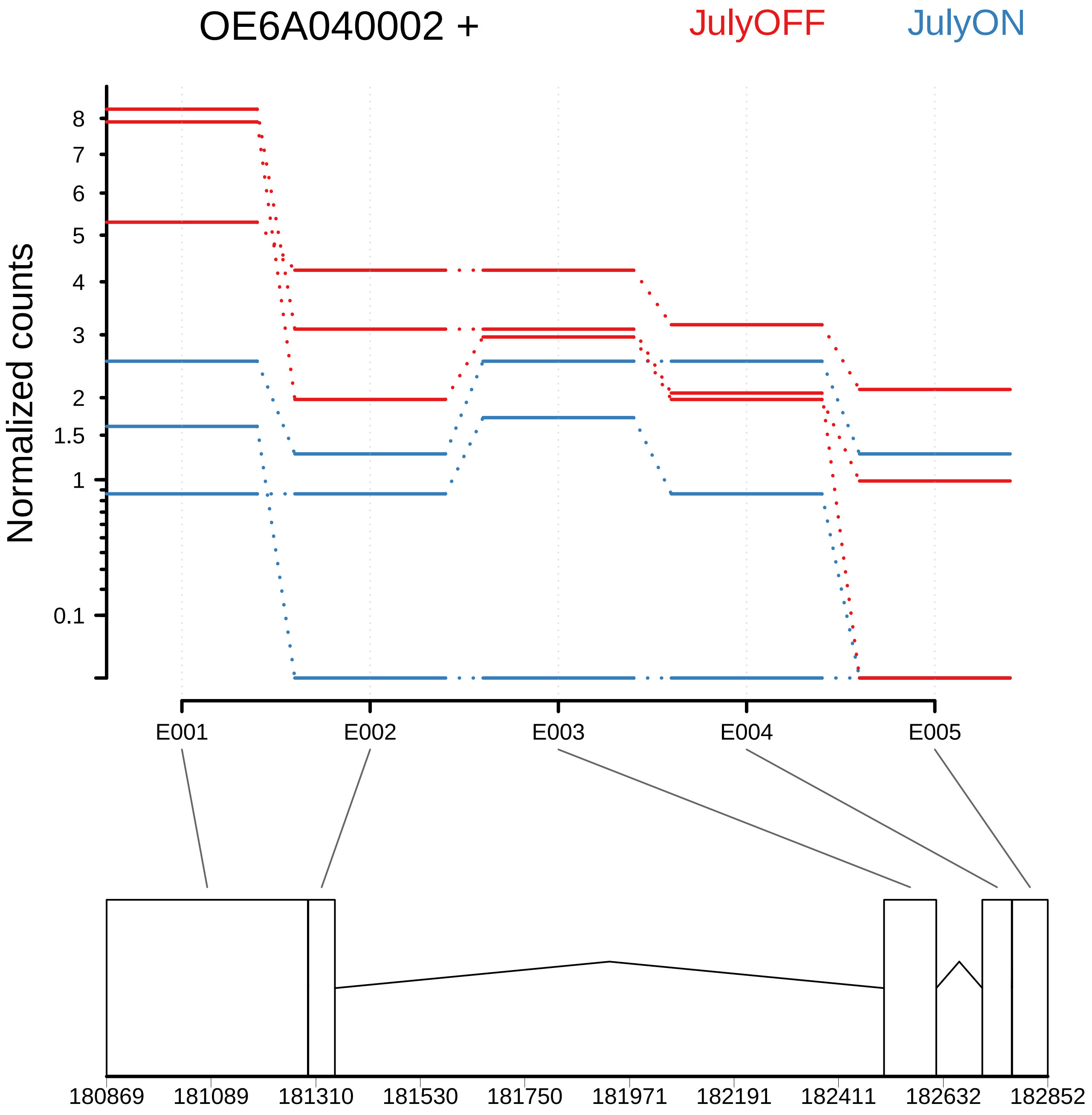

OE6A040002 +
JulyOFF
JulyON
Normalized counts
8
7
6
5
4
3
2
1.5
1
0.1
E001
E002
E003
E004
E005
180869
181089
181310
181530
181750
181971
182191
182411
182632
182852

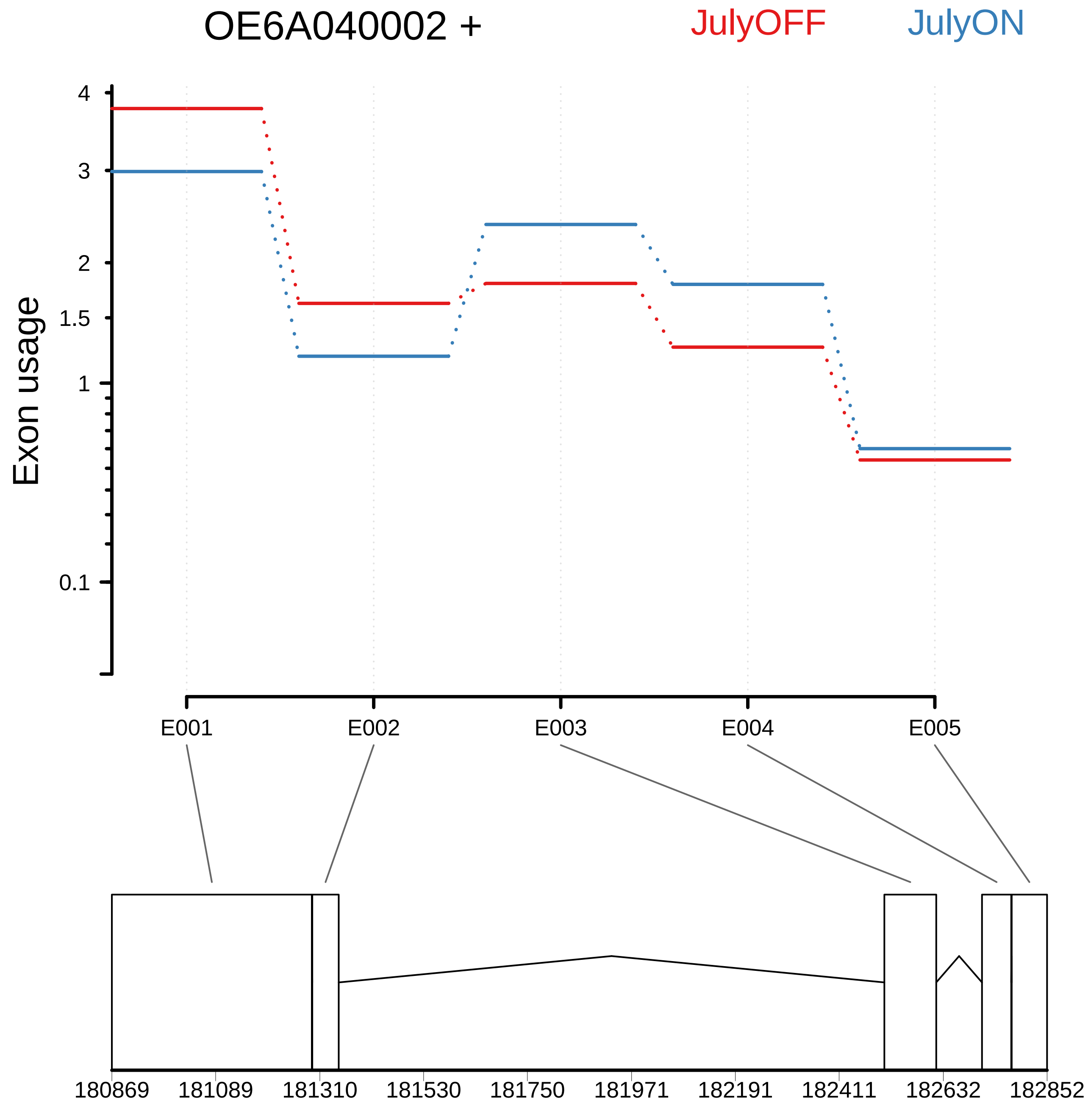

OE6A040002 +
JulyOFF
JulyON
Exon usage
4
3
2
1.5
1
0.1
E001
E002
E003
E004
E005
180869
181089
181310
181530
181750
181971
182191
182411
182632
182852

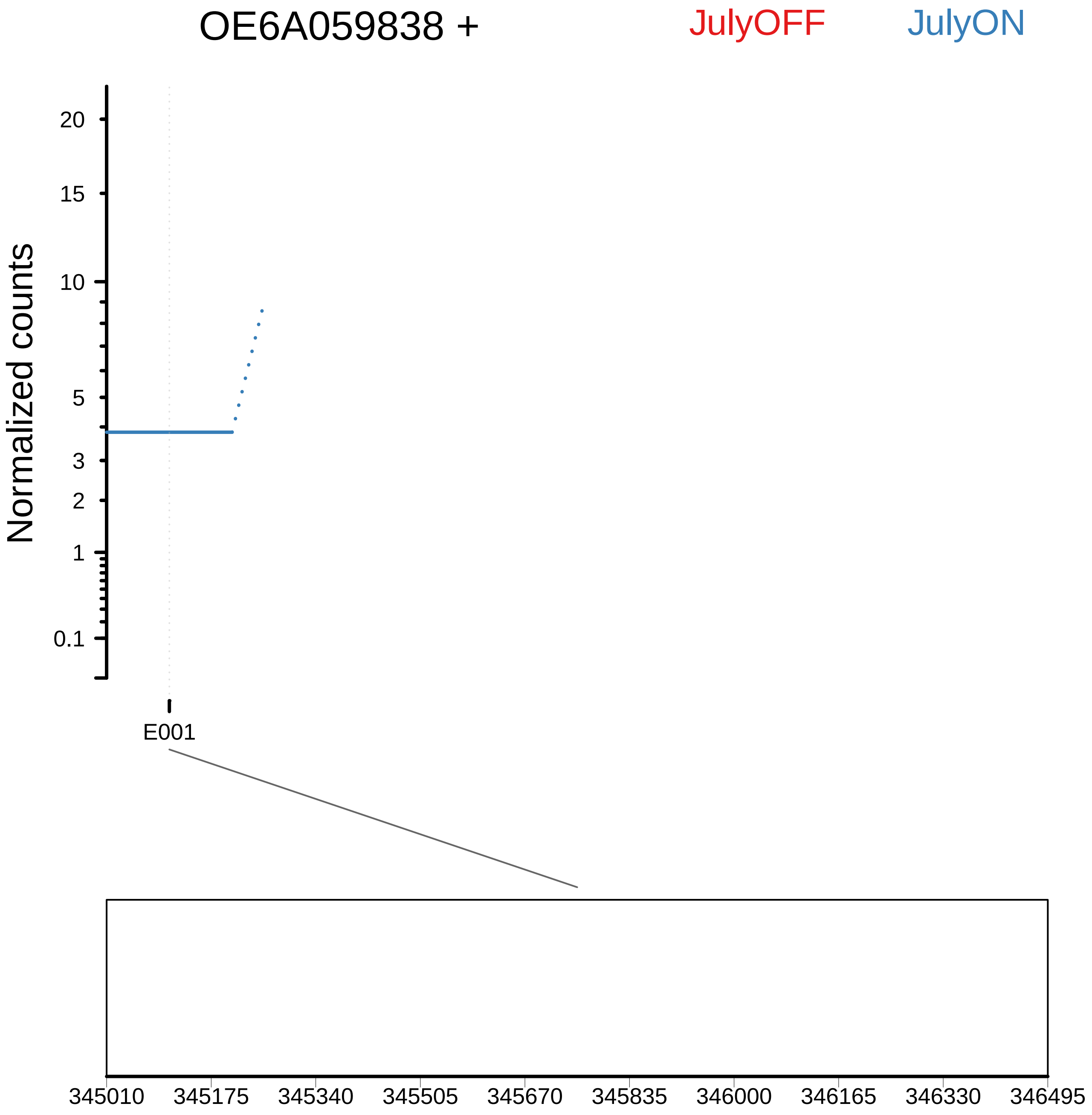
OE6A059838 +
JulyOFF
JulyON
Normalized counts
20
15
10
5
3
2
1
0.1
E001
345010
345175
345340
345505
345670
345835
346000
346165
346330
346495

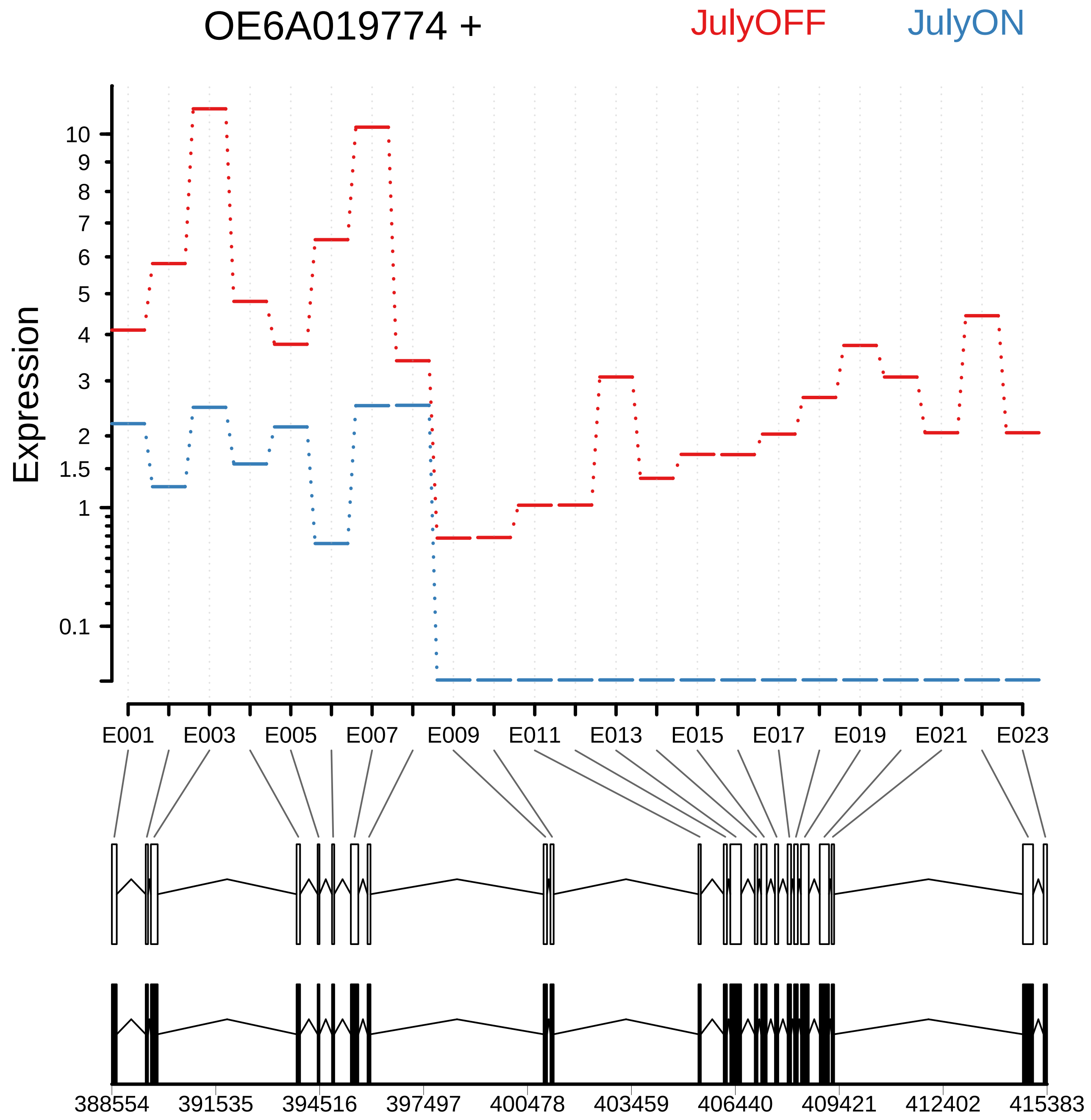

OE6A019774 +
JulyOFF
JulyON
Expression
10
9
8
7
6
5
4
3
2
1.5
1
0.1
E001
E003
E005
E007
E009
E011
E013
E015
E017
E019
E021
E023
388554
391535
394516
397497
400478
403459
406440
409421
412402
415383

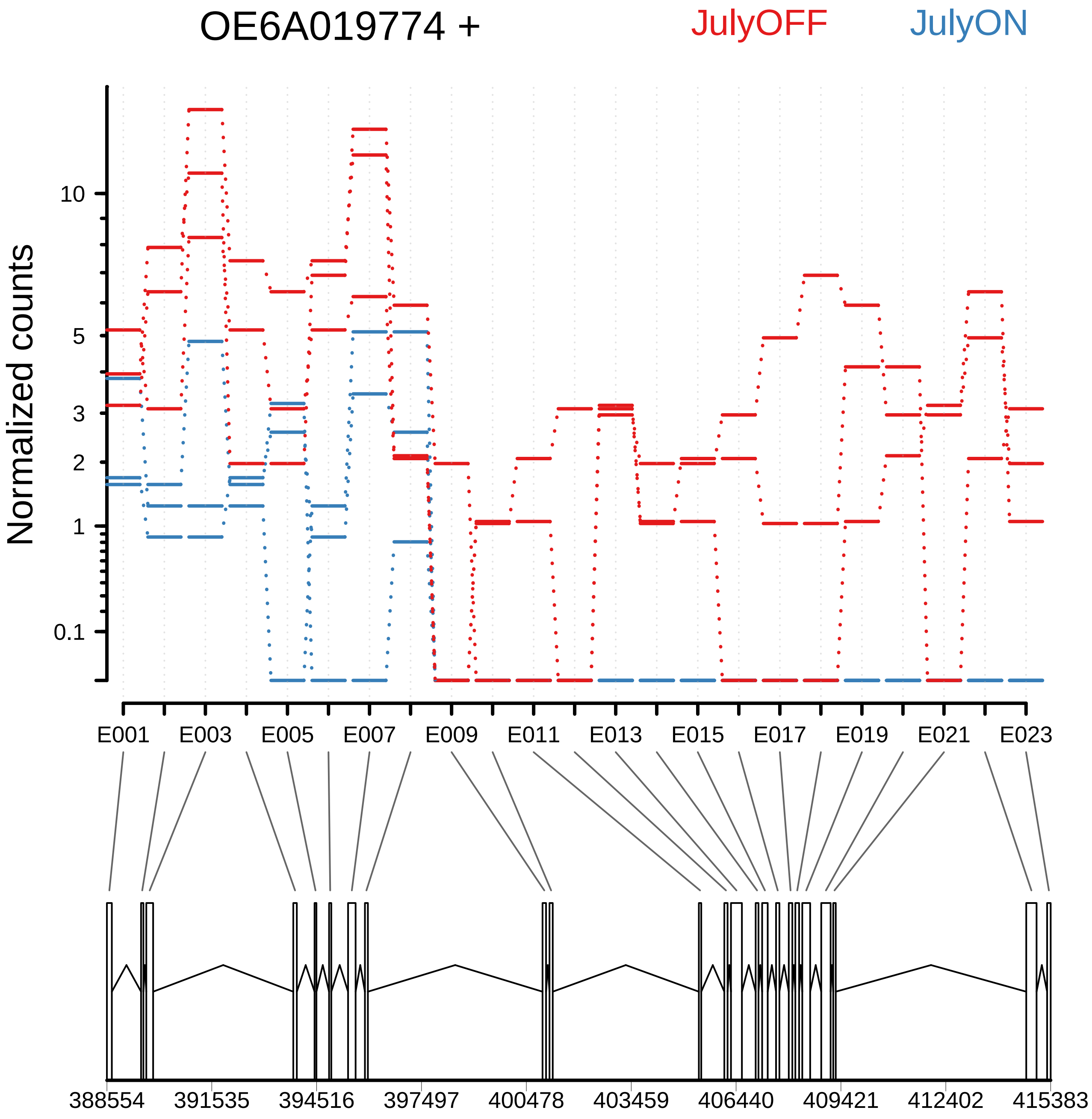

OE6A019774 +
JulyOFF
JulyON
Normalized counts
10
5
3
2
1
0.1
E001
E003
E005
E007
E009
E011
E013
E015
E017
E019
E021
E023
388554
391535
394516
397497
400478
403459
406440
409421
412402
415383

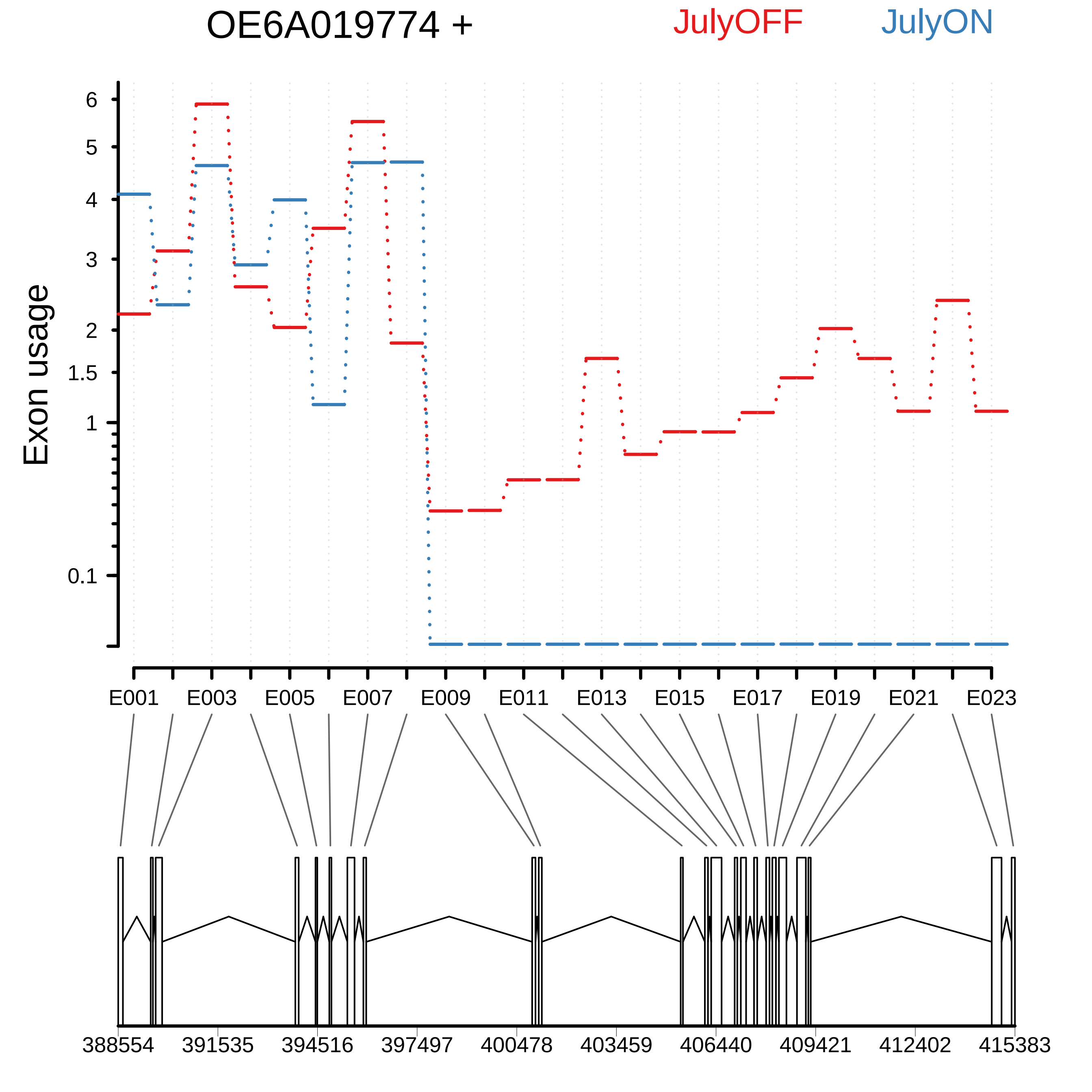

OE6A019774 +
JulyOFF
JulyON
Exon usage
6
5
4
3
2
1.5
1
0.1
E001
E003
E005
E007
E009
E011
E013
E015
E017
E019
E021
E023
388554
391535
394516
397497
400478
403459
406440
409421
412402
415383

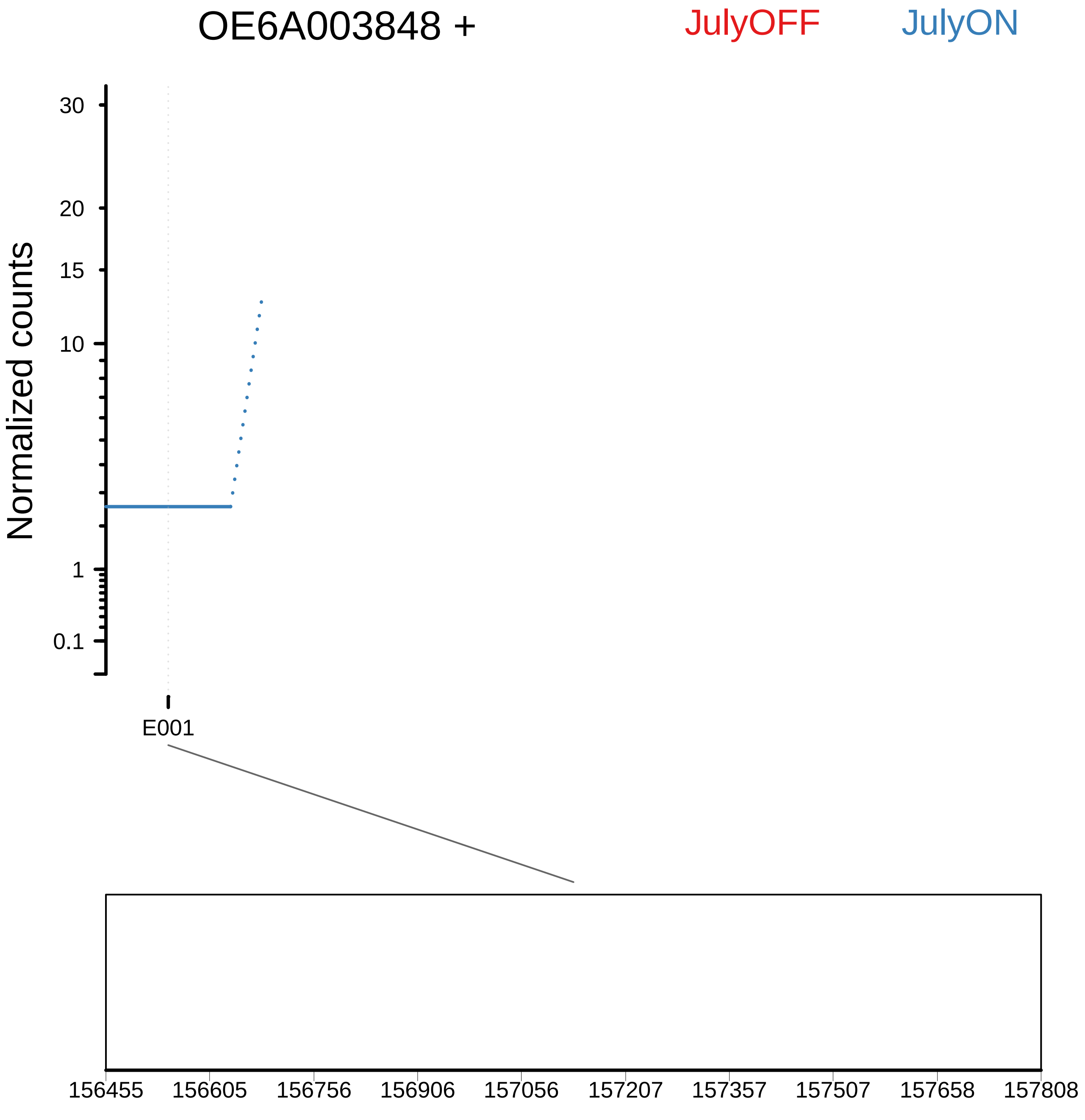
OE6A003848 +
JulyOFF
JulyON
Normalized counts
30
20
15
10
1
0.1
E001
156455
156605
156756
156906
157056
157207
157357
157507
157658
157808

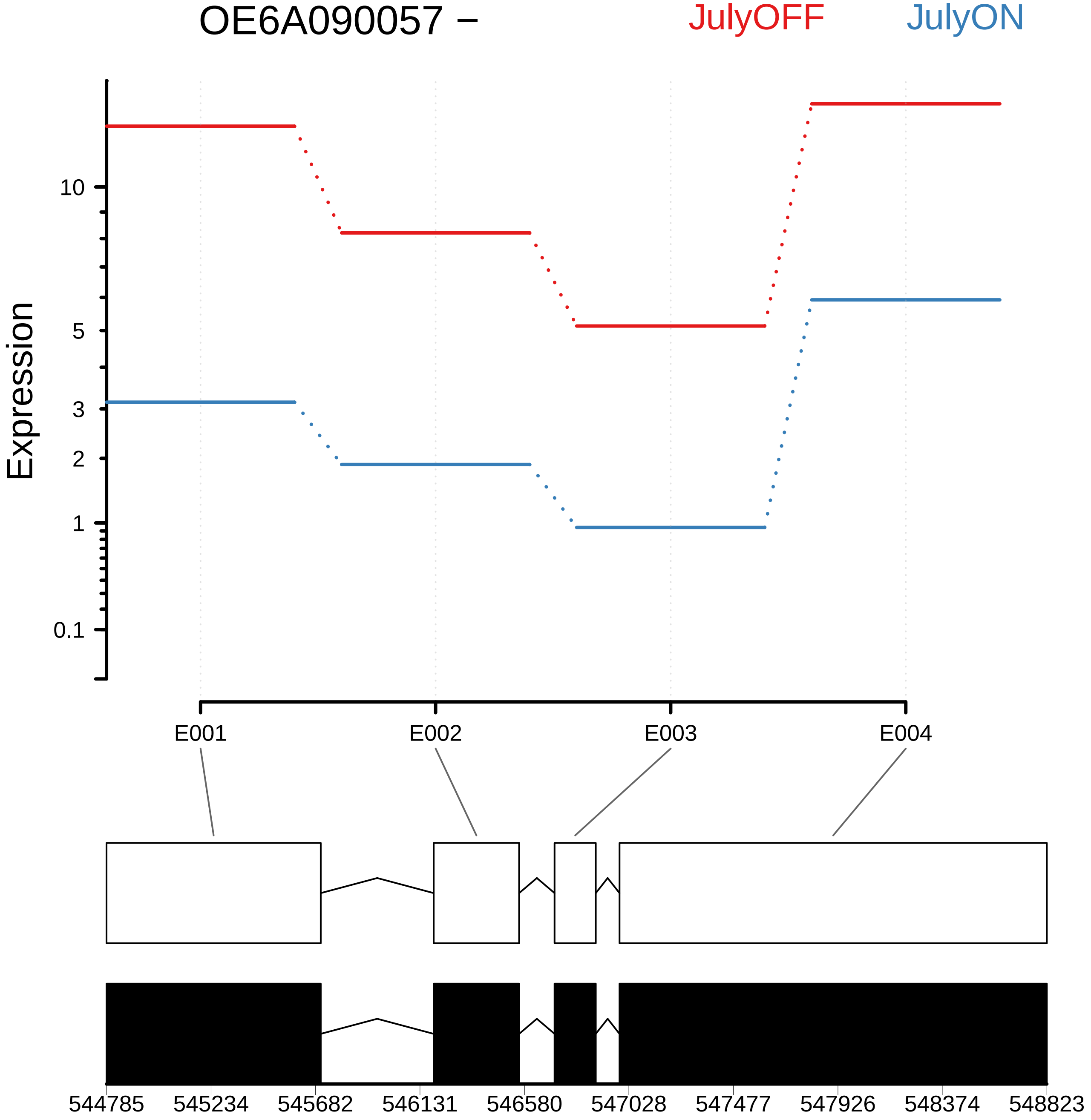

OE6A090057 −
JulyOFF
JulyON
Expression
10
5
3
2
1
0.1
E001
E002
E003
E004
544785
545234
545682
546131
546580
547028
547477
547926
548374
548823

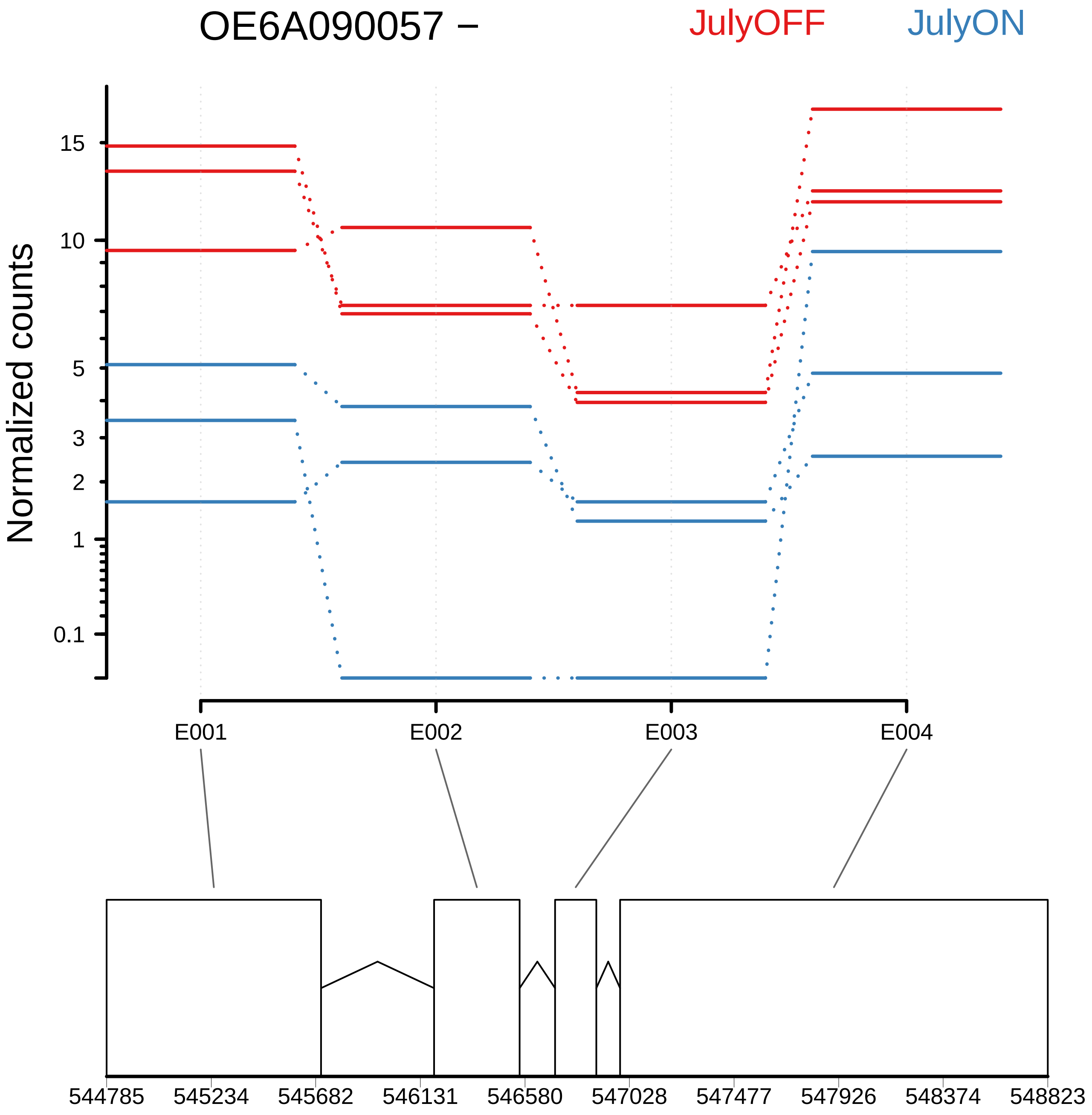
OE6A090057 −
JulyOFF
JulyON
Normalized counts
15
10
5
3
2
1
0.1
E001
E002
E003
E004
544785
545234
545682
546131
546580
547028
547477
547926
548374
548823

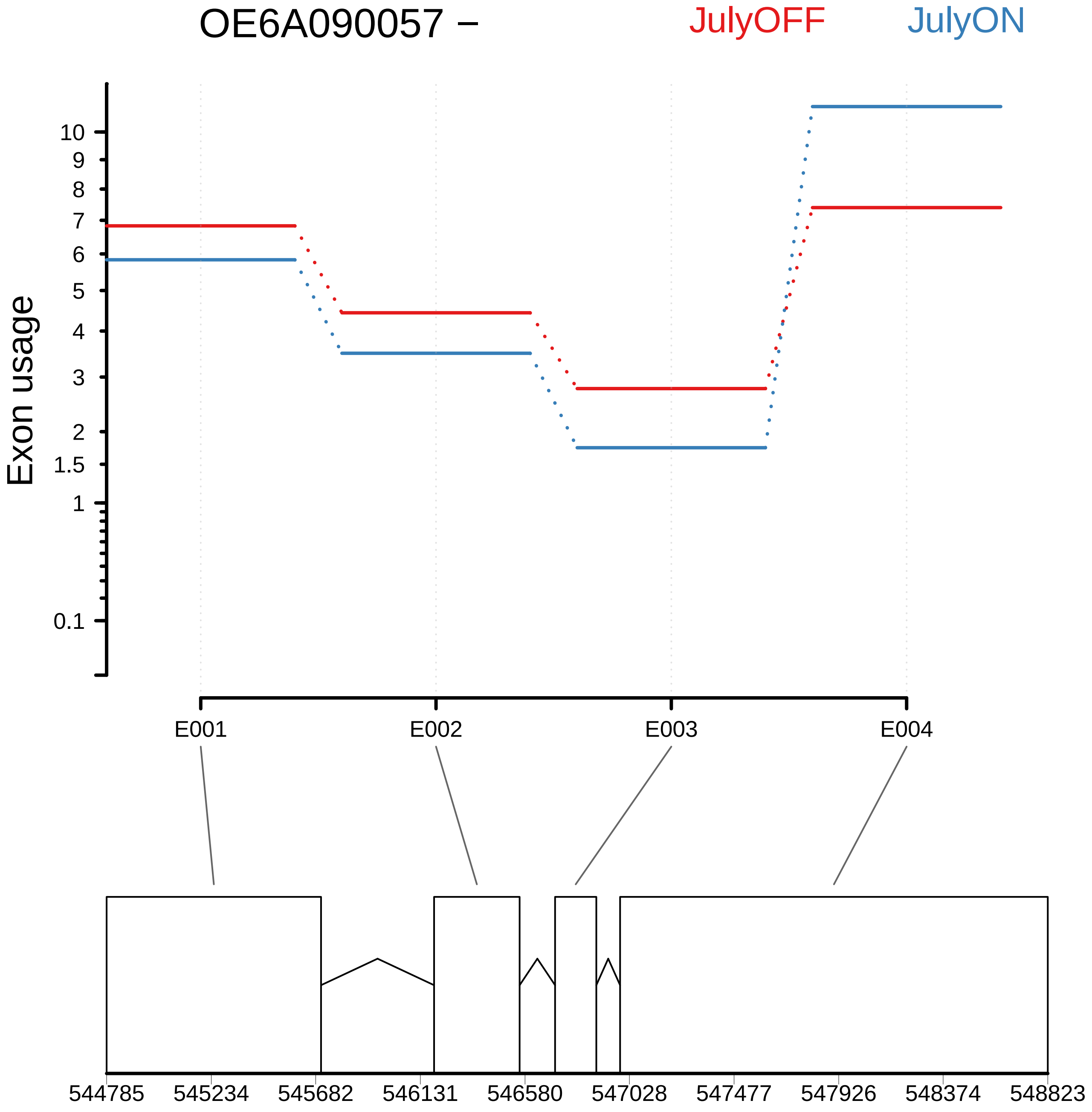

OE6A090057 −
JulyOFF
JulyON
Exon usage
10
9
8
7
6
5
4
3
2
1.5
1
0.1
E001
E002
E003
E004
544785
545234
545682
546131
546580
547028
547477
547926
548374
548823

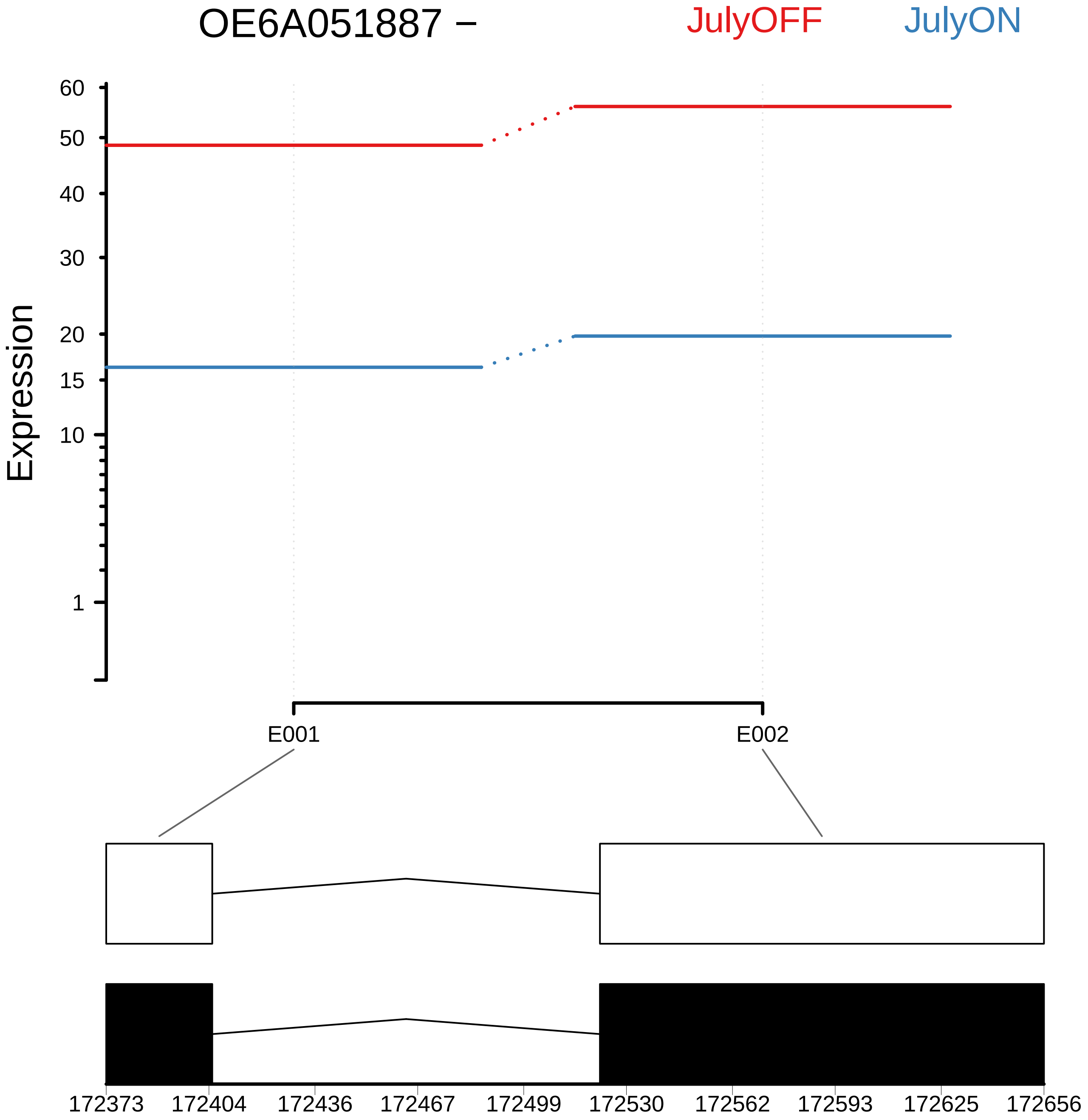

OE6A051887 −
JulyOFF
JulyON
Expression
60
50
40
30
20
15
10
1
E001
E002
172373
172404
172436
172467
172499
172530
172562
172593
172625
172656

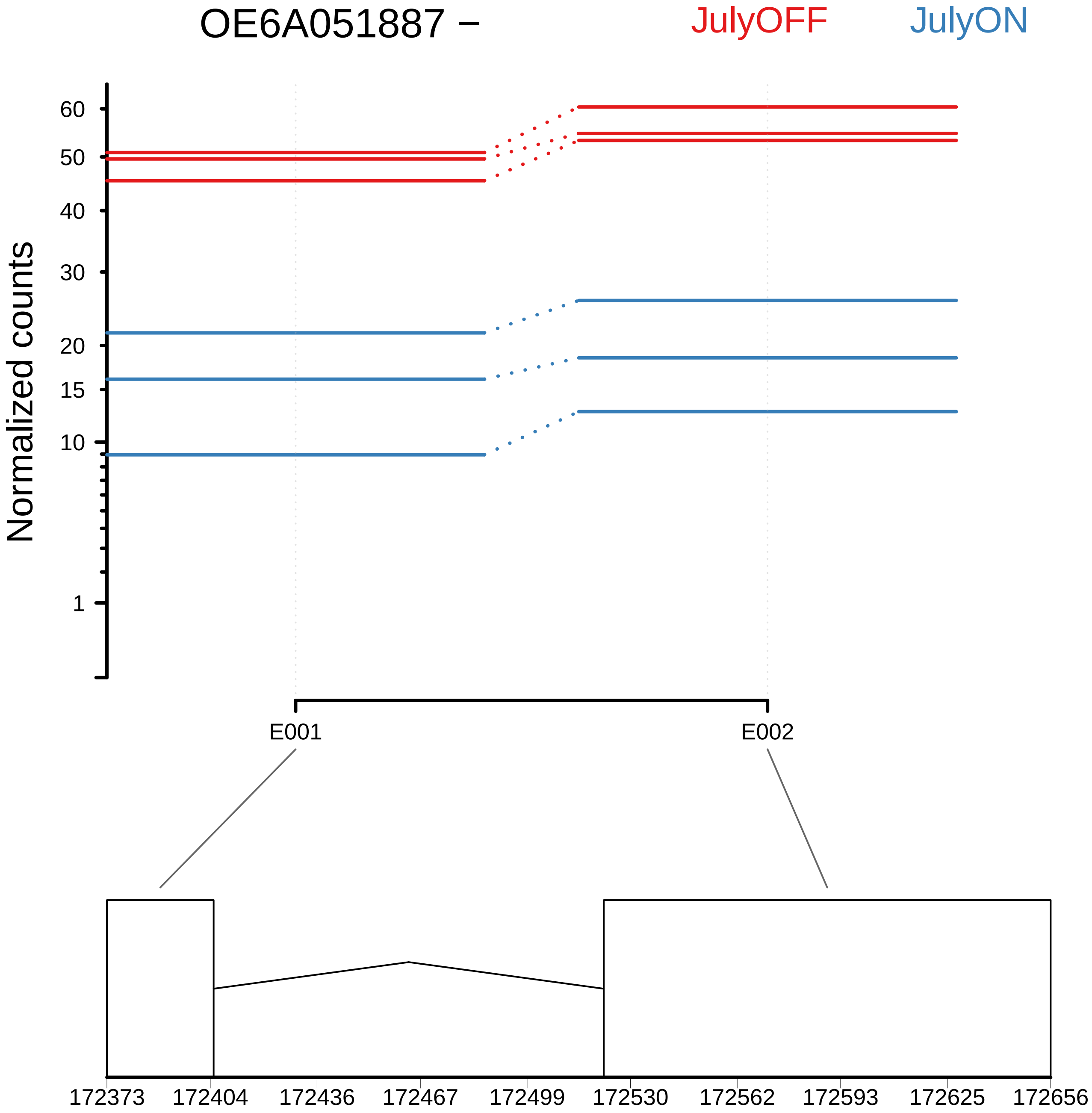

OE6A051887 −
JulyOFF
JulyON
Normalized counts
60
50
40
30
20
15
10
1
E001
E002
172373
172404
172436
172467
172499
172530
172562
172593
172625
172656

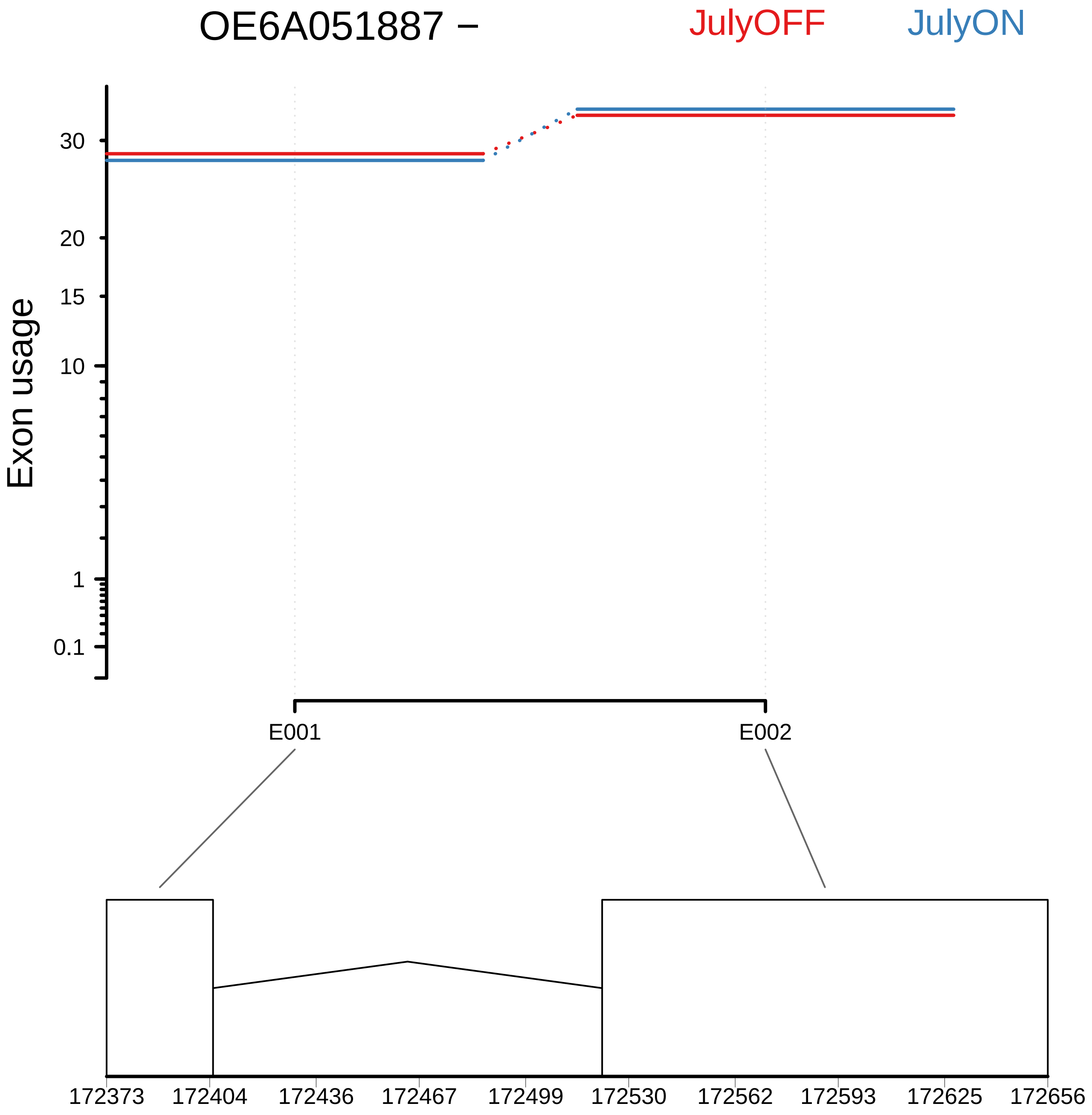

OE6A051887 −
JulyOFF
JulyON
Exon usage
30
20
15
10
1
0.1
E001
E002
172373
172404
172436
172467
172499
172530
172562
172593
172625
172656

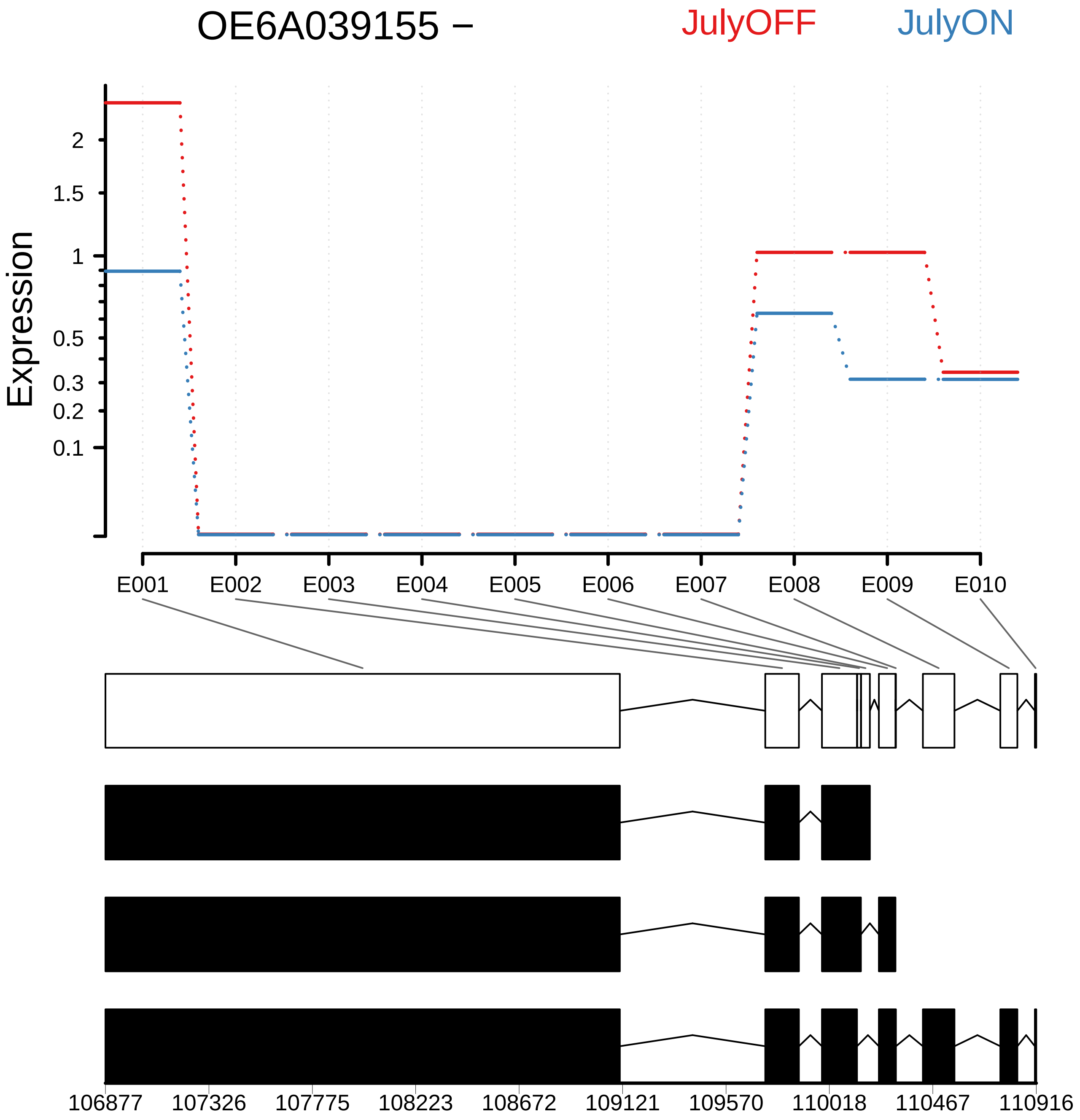

OE6A039155 −
JulyOFF
JulyON
Expression
2
1.5
1
0.5
0.3
0.2
0.1
E001
E002
E003
E004
E005
E006
E007
E008
E009
E010
106877
107326
107775
108223
108672
109121
109570
110018
110467
110916

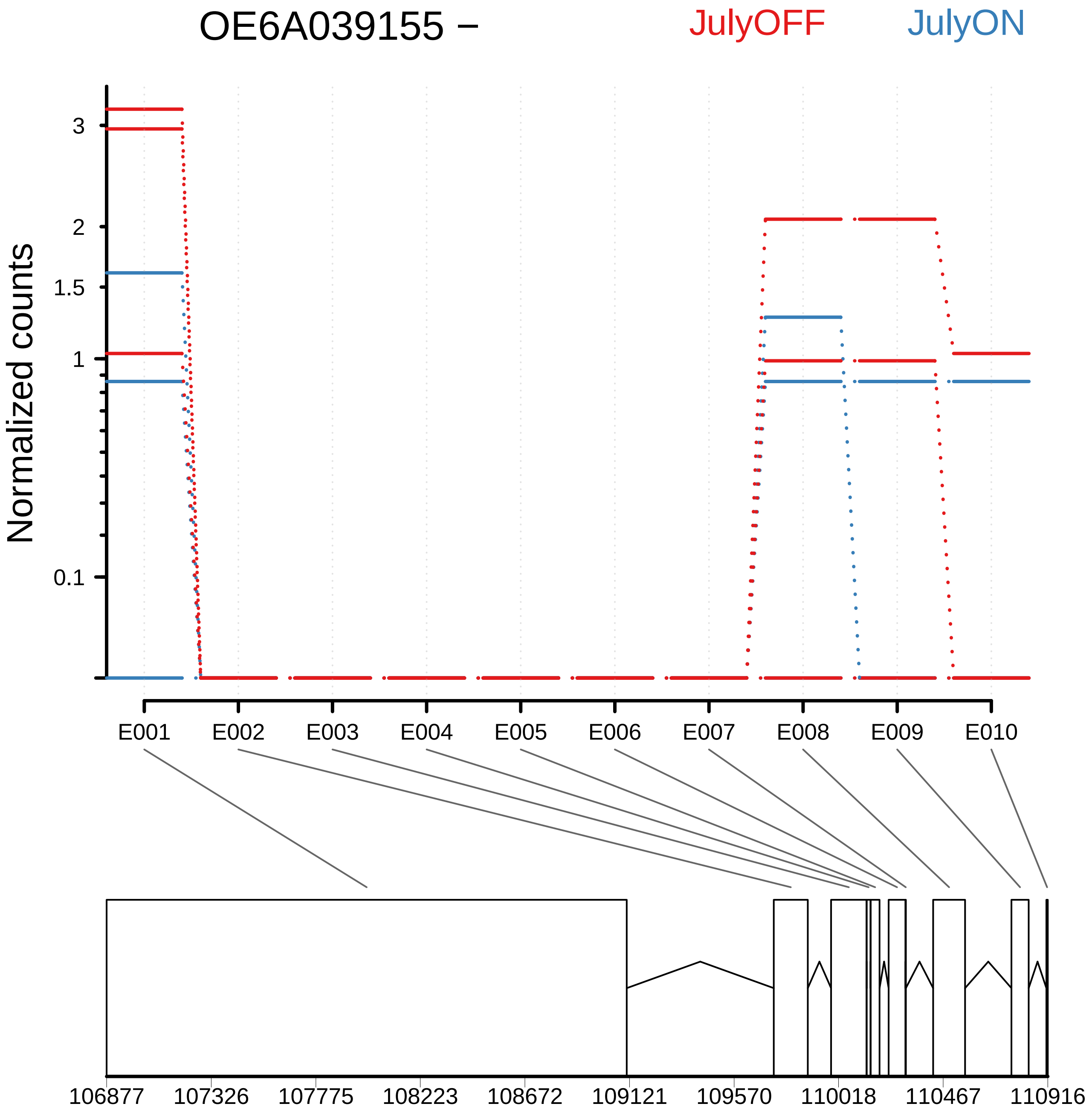

OE6A039155 −
JulyOFF
JulyON
Normalized counts
3
2
1.5
1
0.1
E001
E002
E003
E004
E005
E006
E007
E008
E009
E010
106877
107326
107775
108223
108672
109121
109570
110018
110467
110916

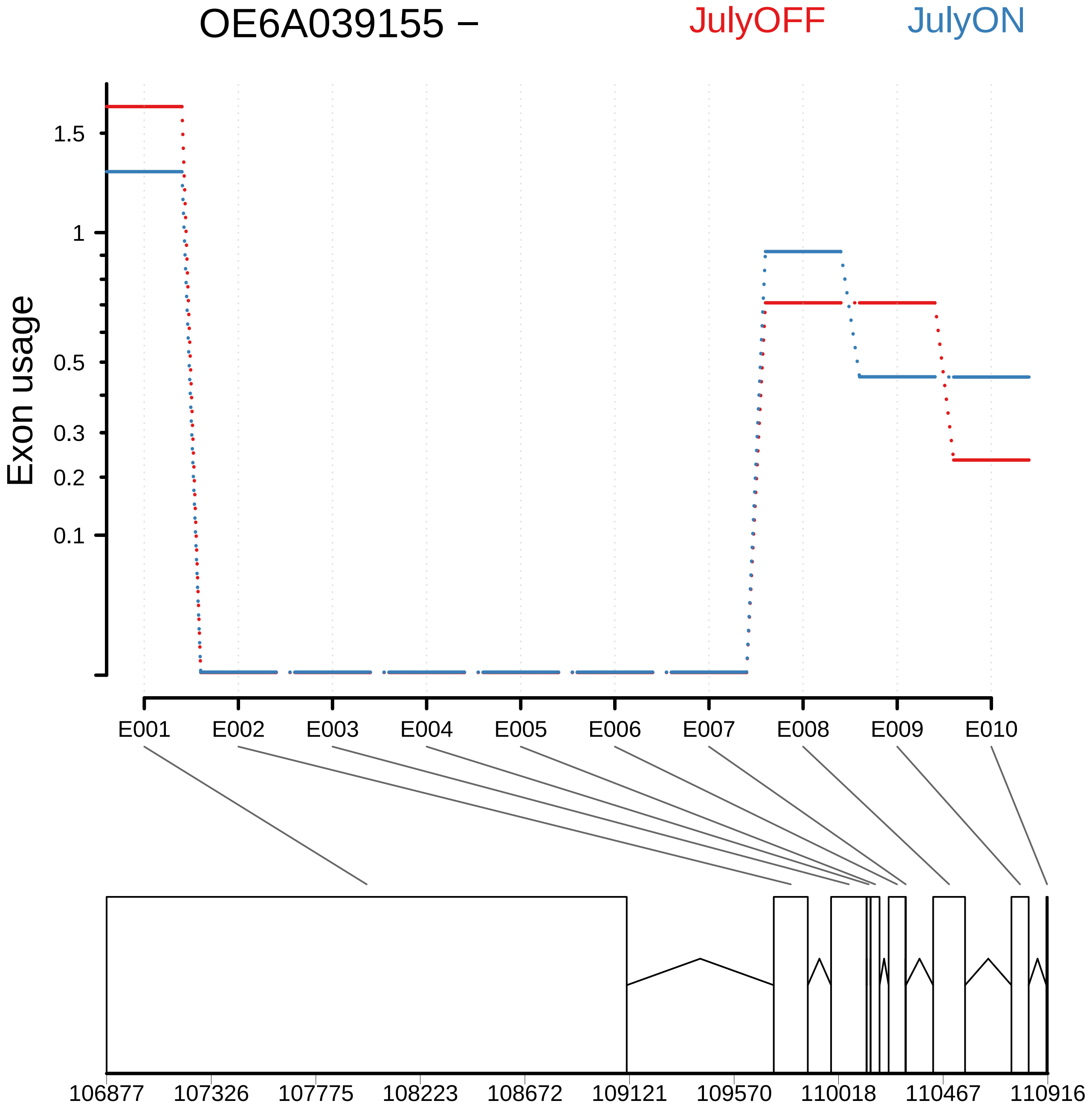

OE6A039155 −
JulyOFF
JulyON
Exon usage
1.5
1
0.5
0.3
0.2
0.1
E001
E002
E003
E004
E005
E006
E007
E008
E009
E010
106877
107326
107775
108223
108672
109121
109570
110018
110467
110916

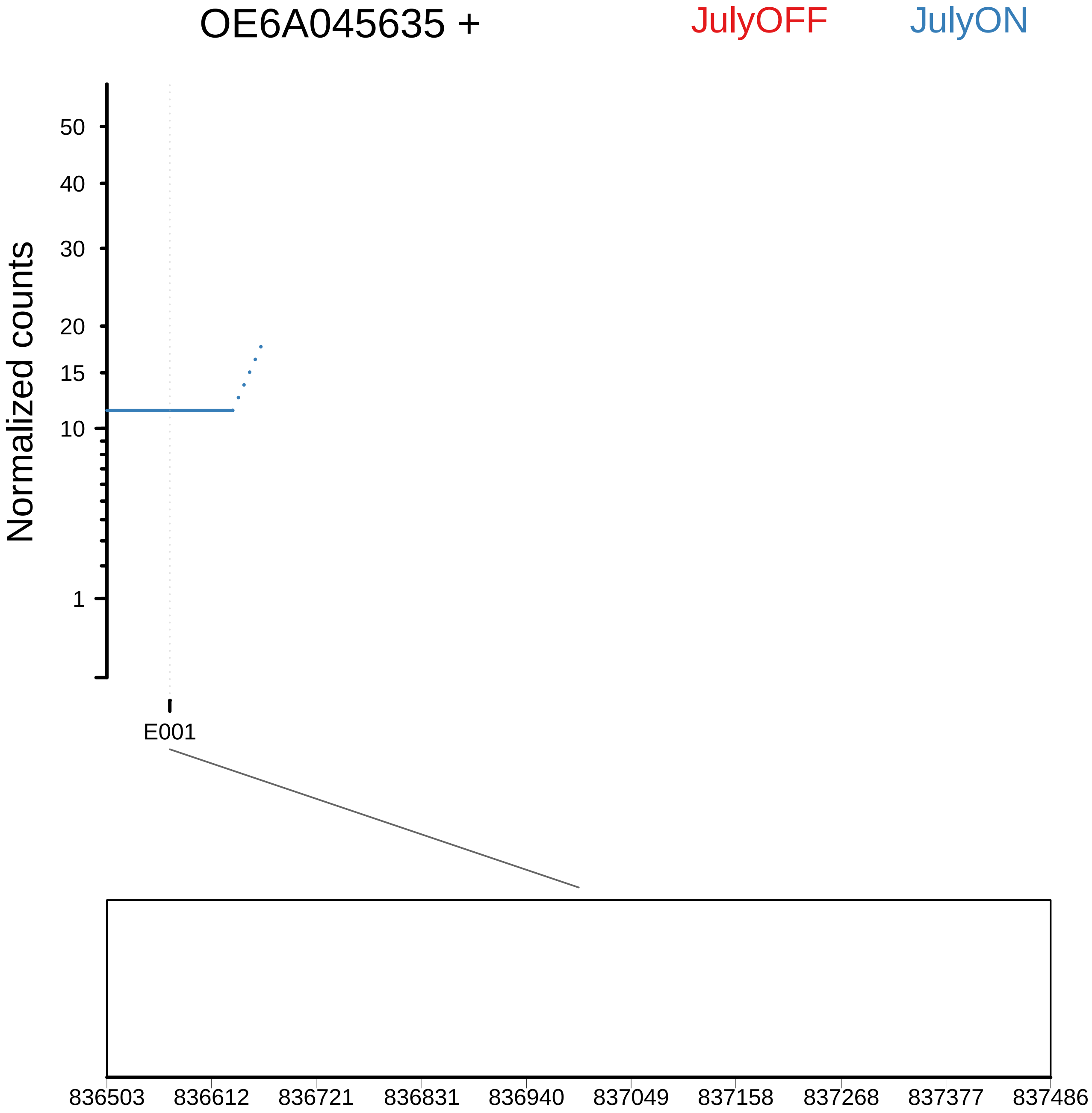

OE6A045635 +
JulyOFF
JulyON
Normalized counts
50
40
30
20
15
10
1
E001
836503
836612
836721
836831
836940
837049
837158
837268
837377
837486

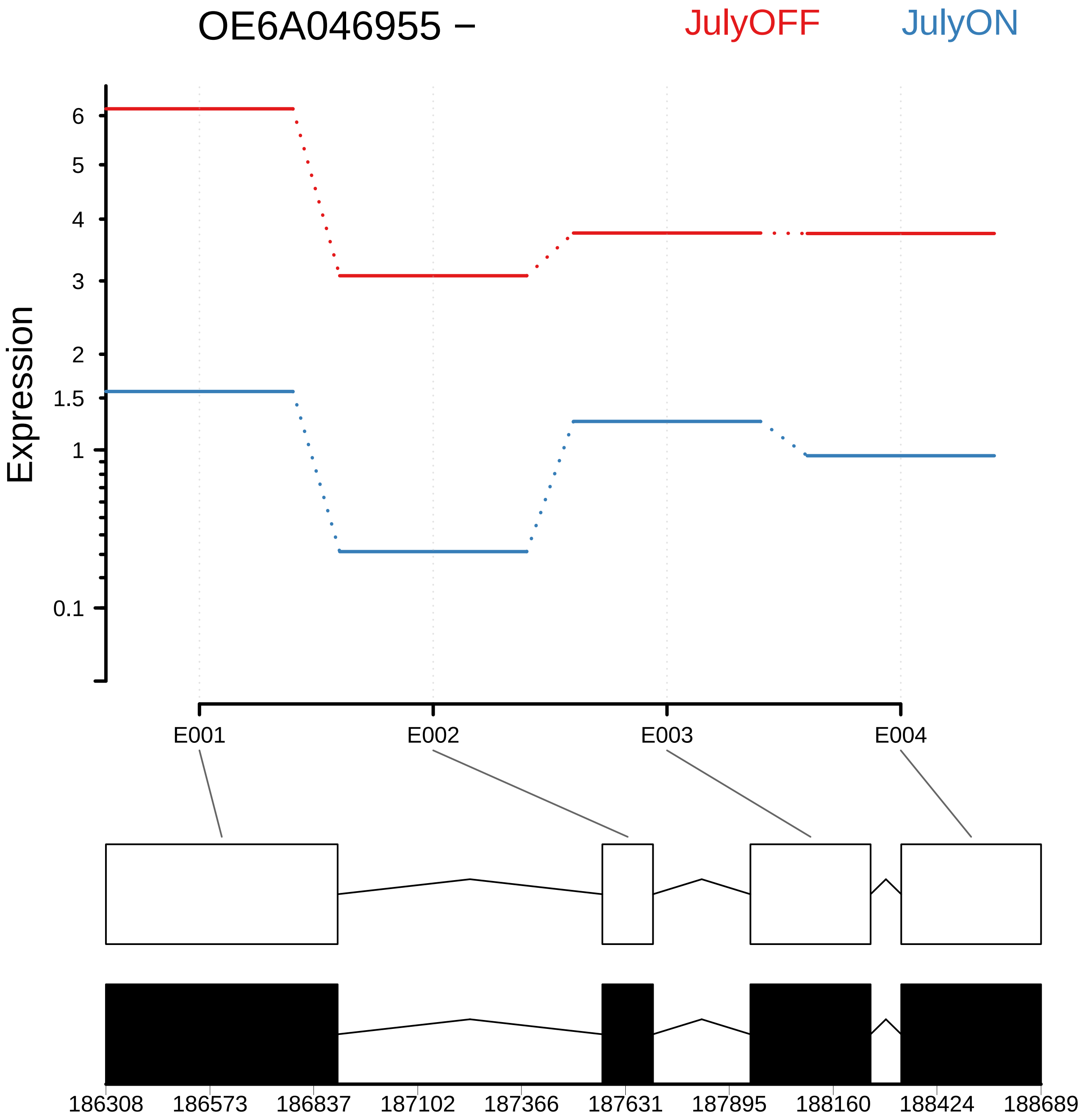

OE6A046955 −
JulyOFF
JulyON
Expression
6
5
4
3
2
1.5
1
0.1
E001
E002
E003
E004
186308
186573
186837
187102
187366
187631
187895
188160
188424
188689

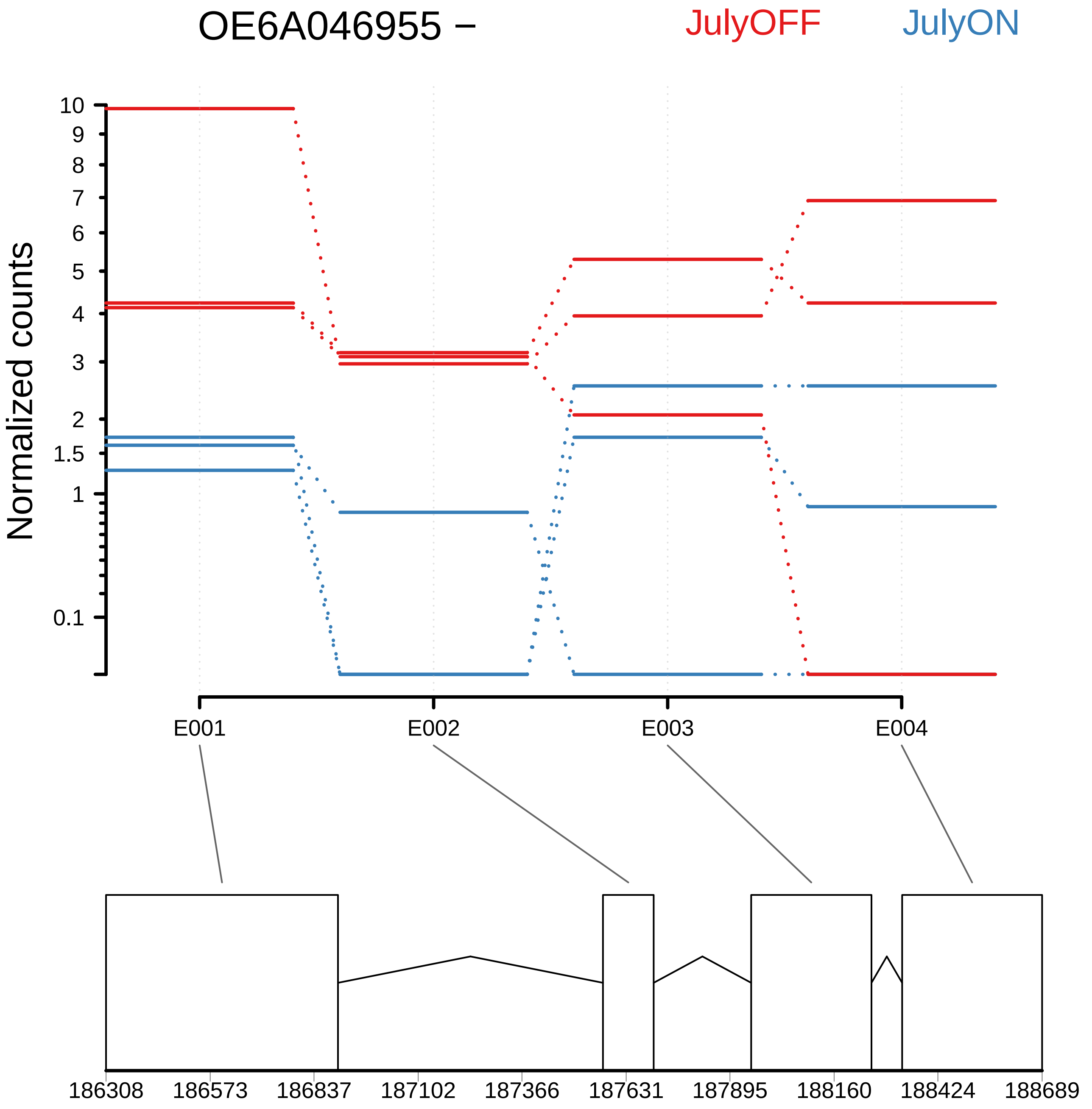

OE6A046955 –
JulyOFF
JulyON
Normalized counts
10
9
8
7
6
5
4
3
2
1.5
1
0.1
E001
E002
E003
E004
186308
186573
186837
187102
187366
187631
187895
188160
188424
188689

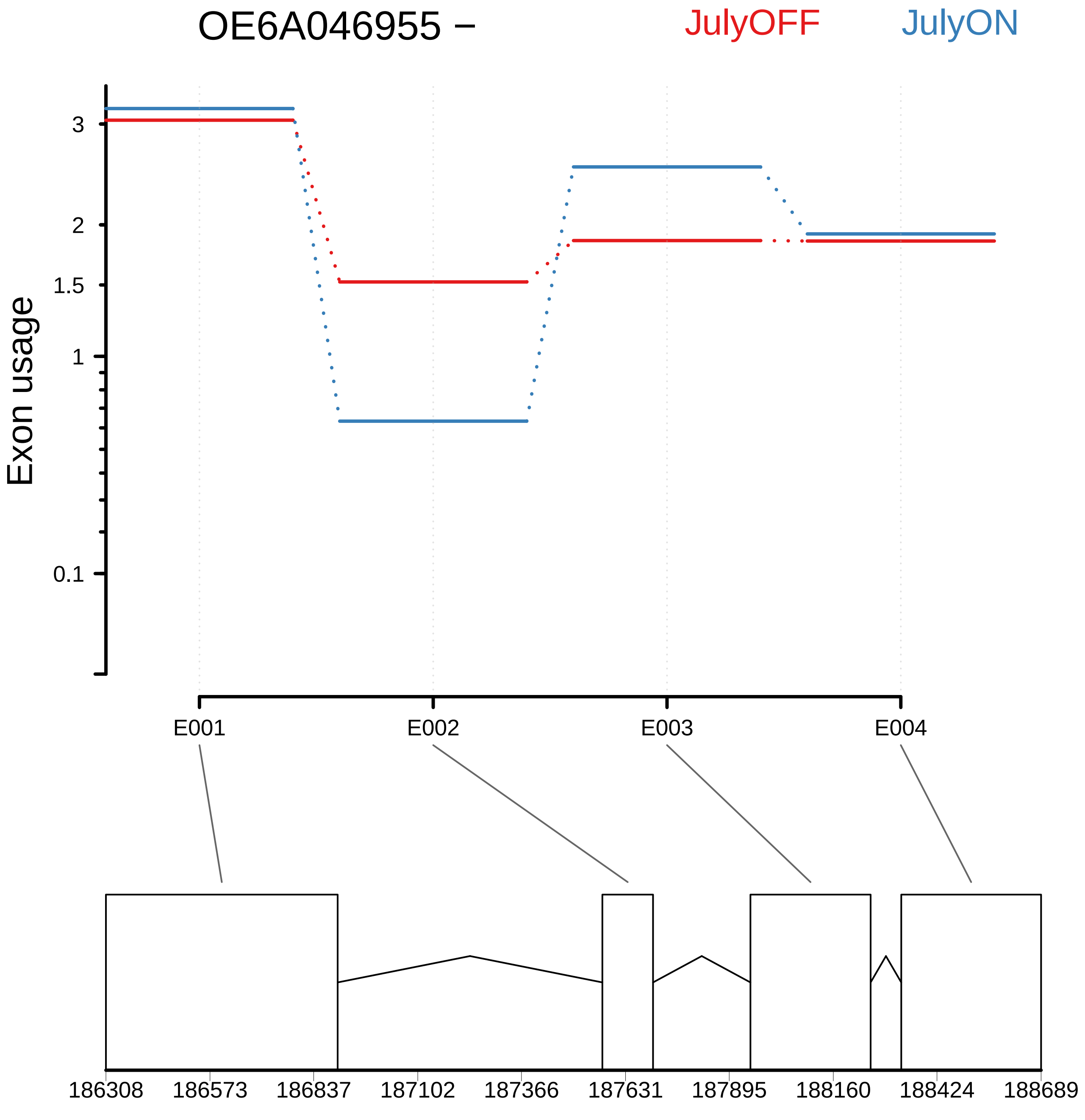

OE6A046955 –
JulyOFF
JulyON
Exon usage
3
2
1.5
1
0.1
E001
E002
E003
E004
186308
186573
186837
187102
187366
187631
187895
188160
188424
188689

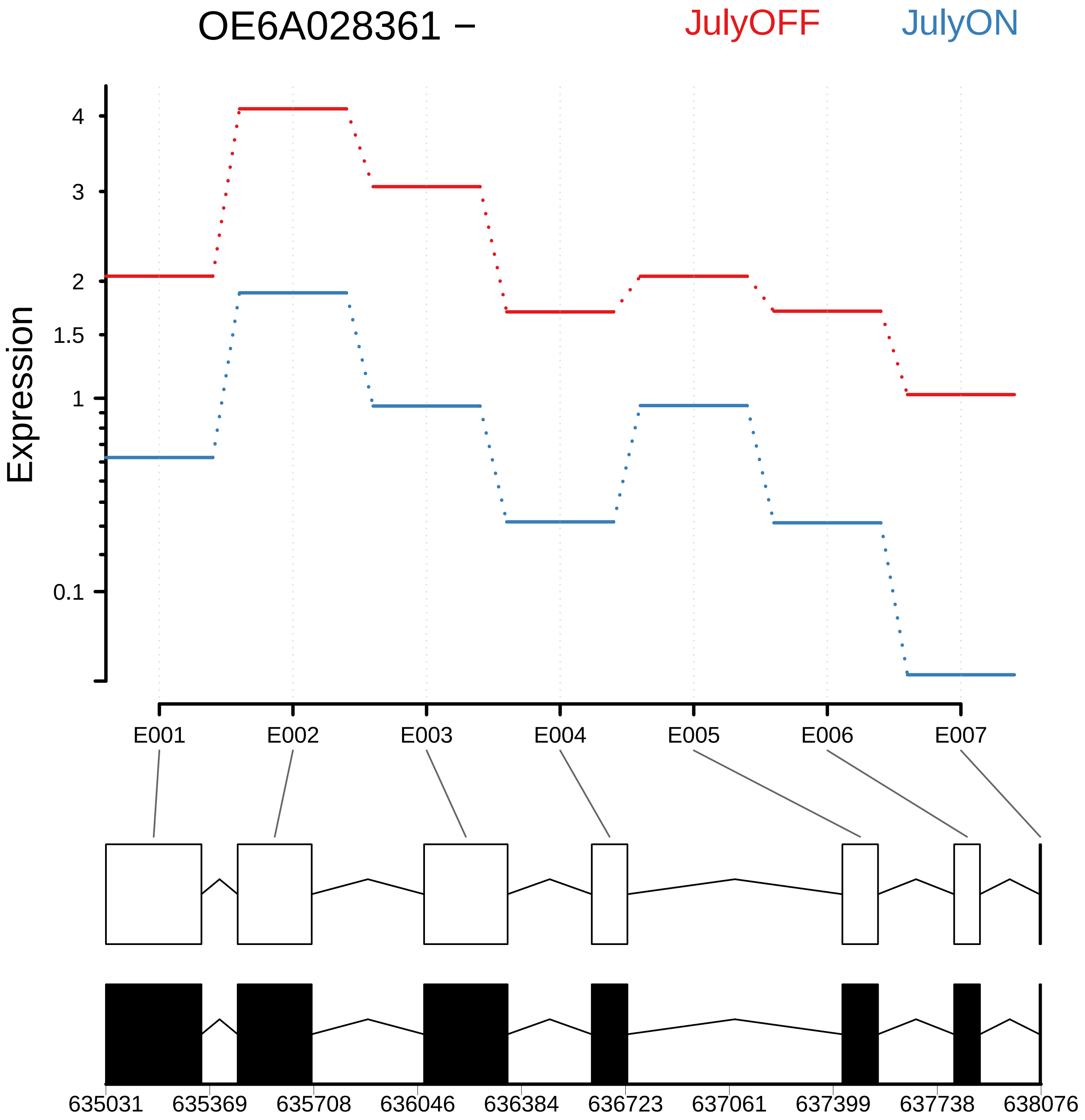

OE6A028361 −
JulyOFF
JulyON
Expression
4
3
2
1.5
1
0.1
E001
E002
E003
E004
E005
E006
E007
635031
635369
635708
636046
636384
636723
637061
637399
637738
638076

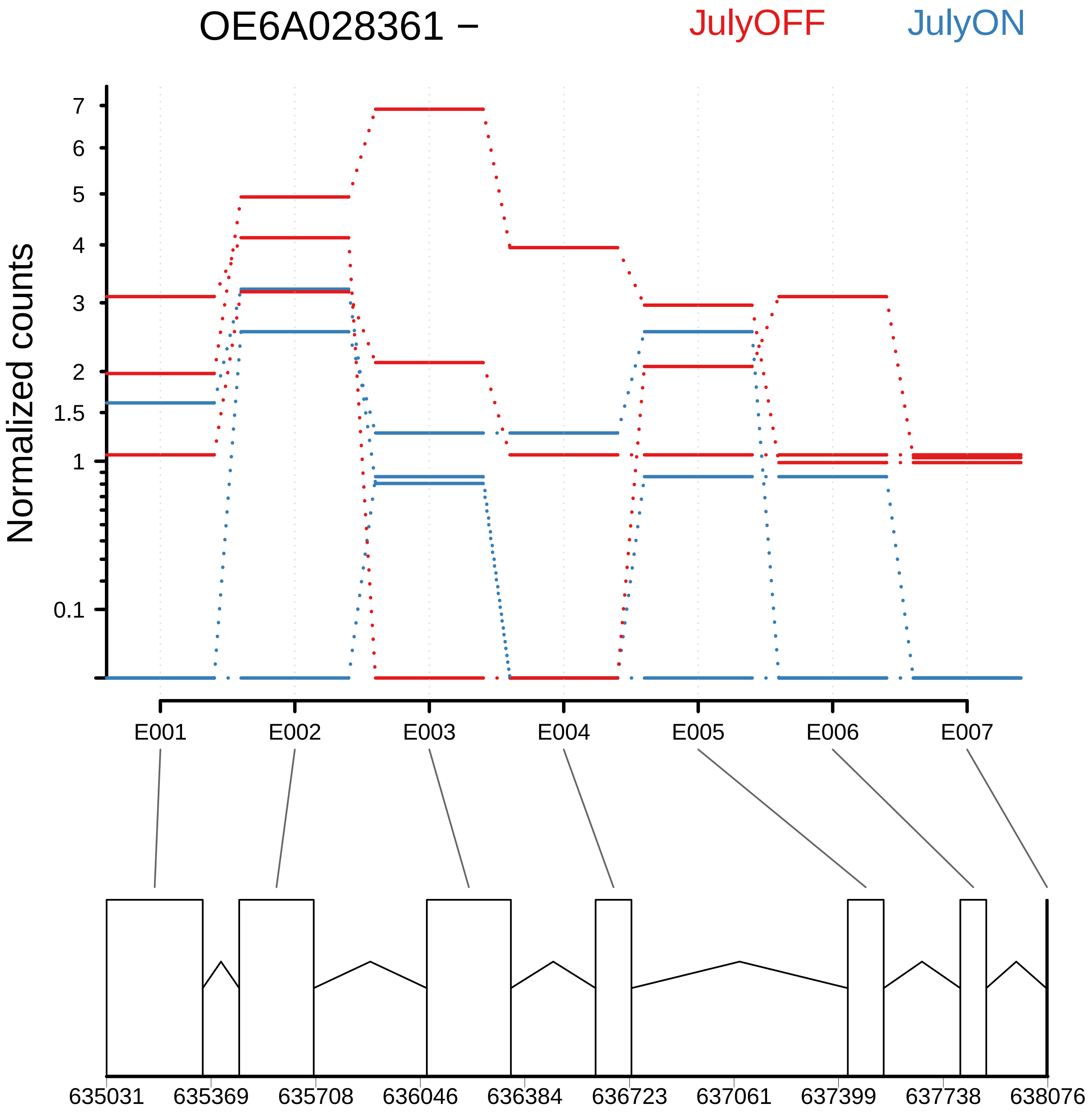

OE6A028361 −
JulyOFF
JulyON
Normalized counts
7
6
5
4
3
2
1.5
1
0.1
E001
E002
E003
E004
E005
E006
E007
635031
635369
635708
636046
636384
636723
637061
637399
637738
638076

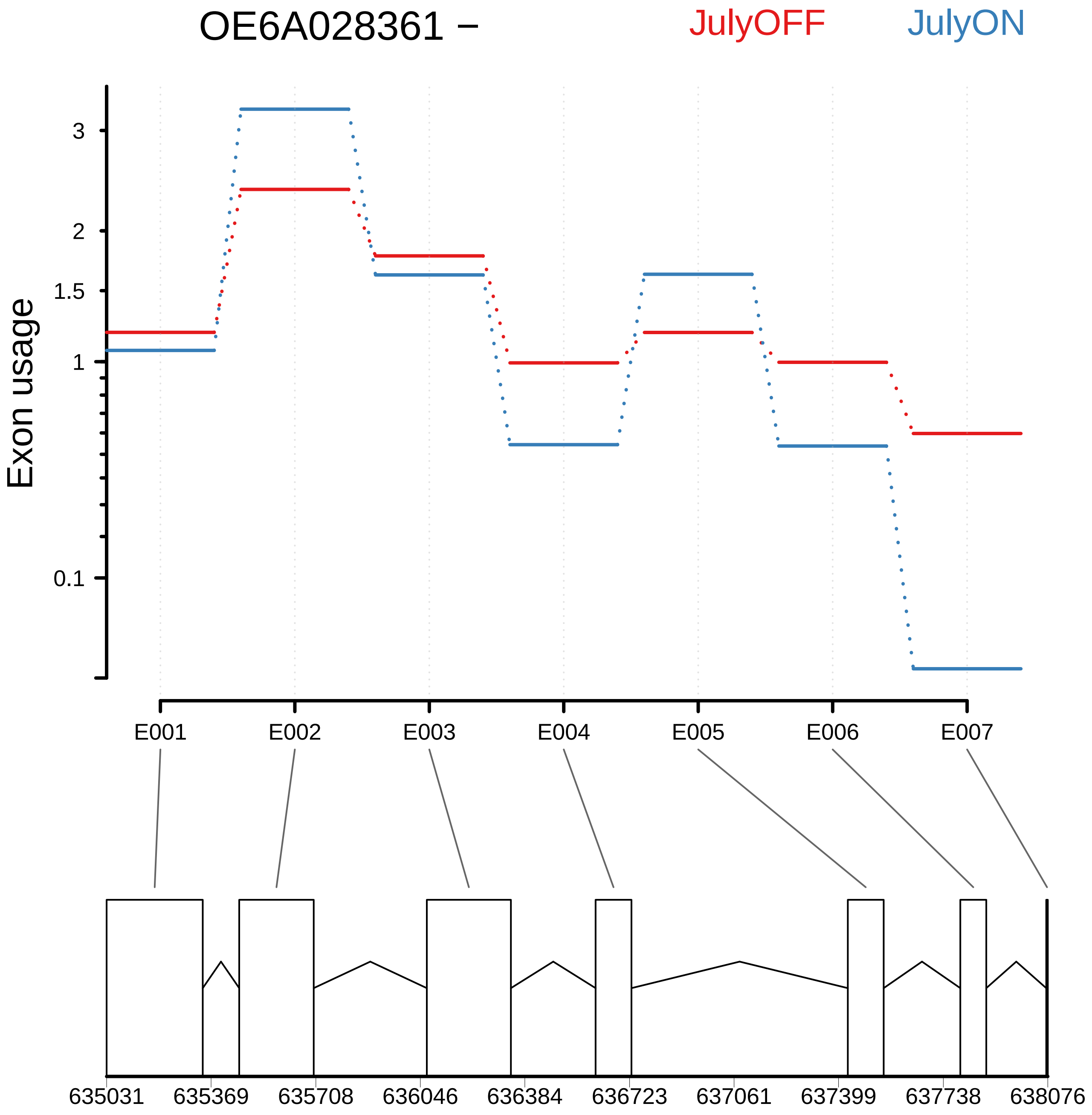
OE6A028361 −
JulyOFF
JulyON
Exon usage
3
2
1.5
1
0.1
E001
E002
E003
E004
E005
E006
E007
635031
635369
635708
636046
636384
636723
637061
637399
637738
638076

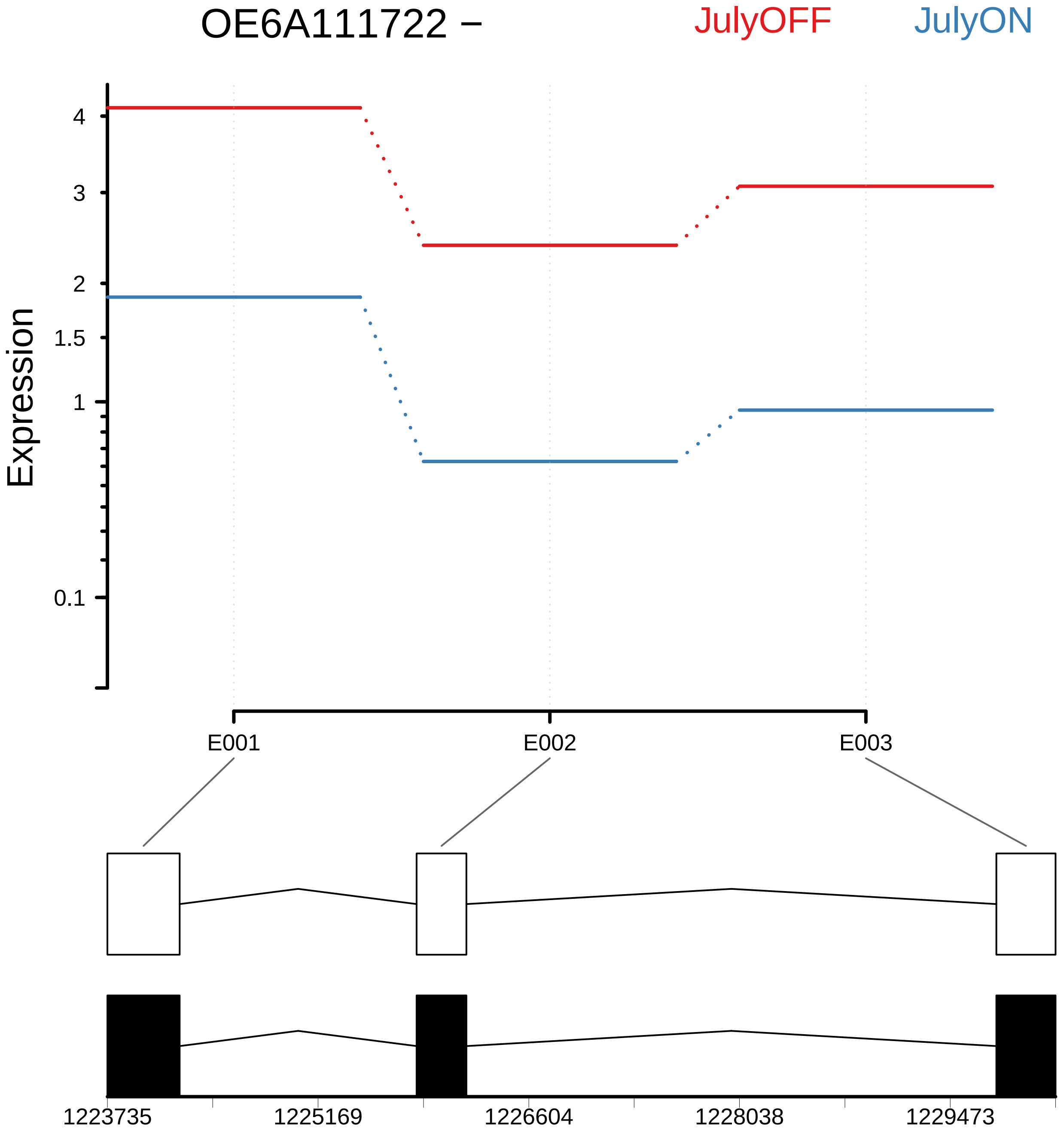
OE6A111722 −
JulyOFF
JulyON
Expression
4
3
2
1.5
1
0.1
E001
E002
E003
1223735
1225169
1226604
1228038
1229473

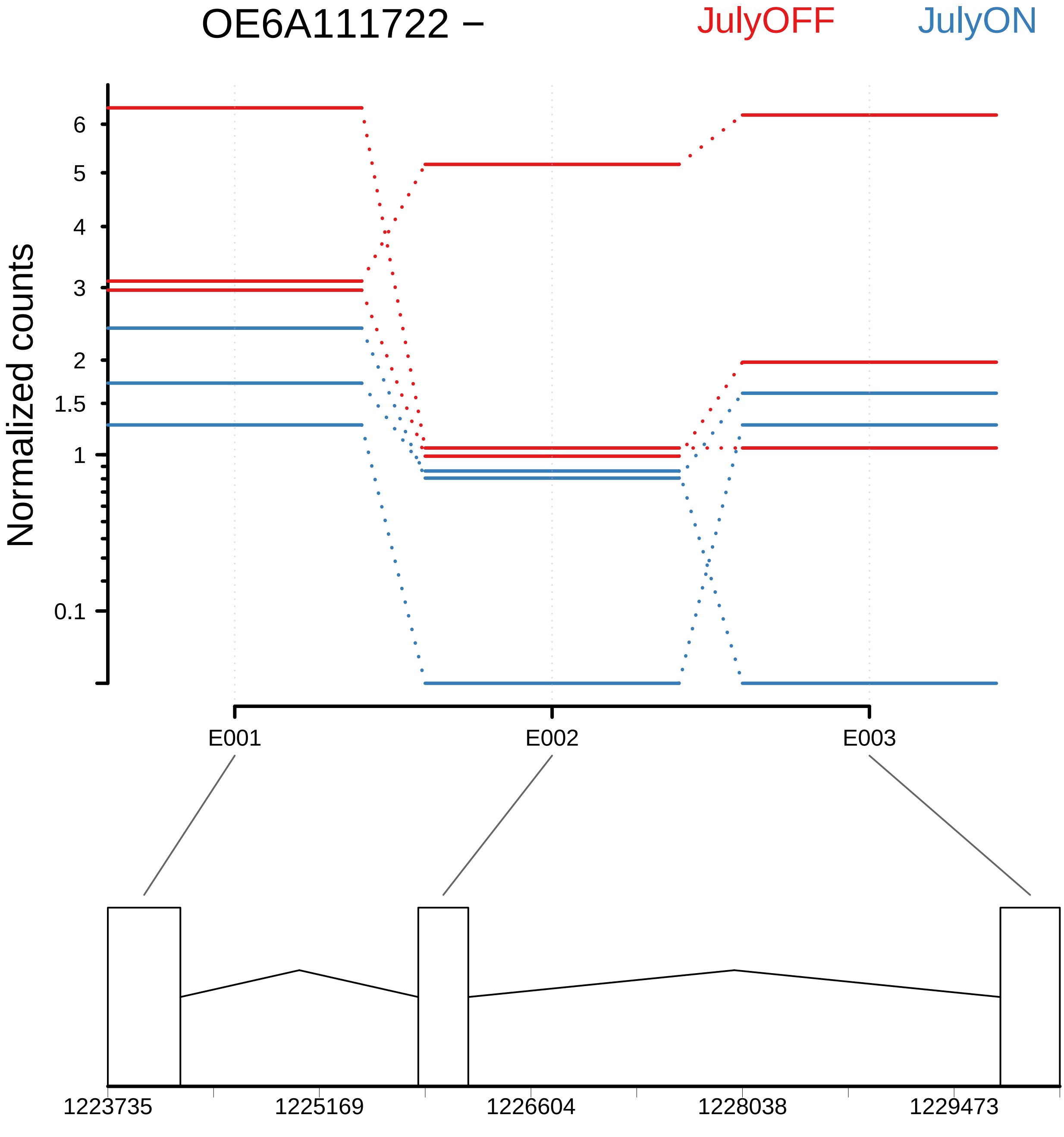
OE6A111722 −
JulyOFF
JulyON
Normalized counts
6
5
4
3
2
1.5
1
0.1
E001
E002
E003
1223735
1225169
1226604
1228038
1229473

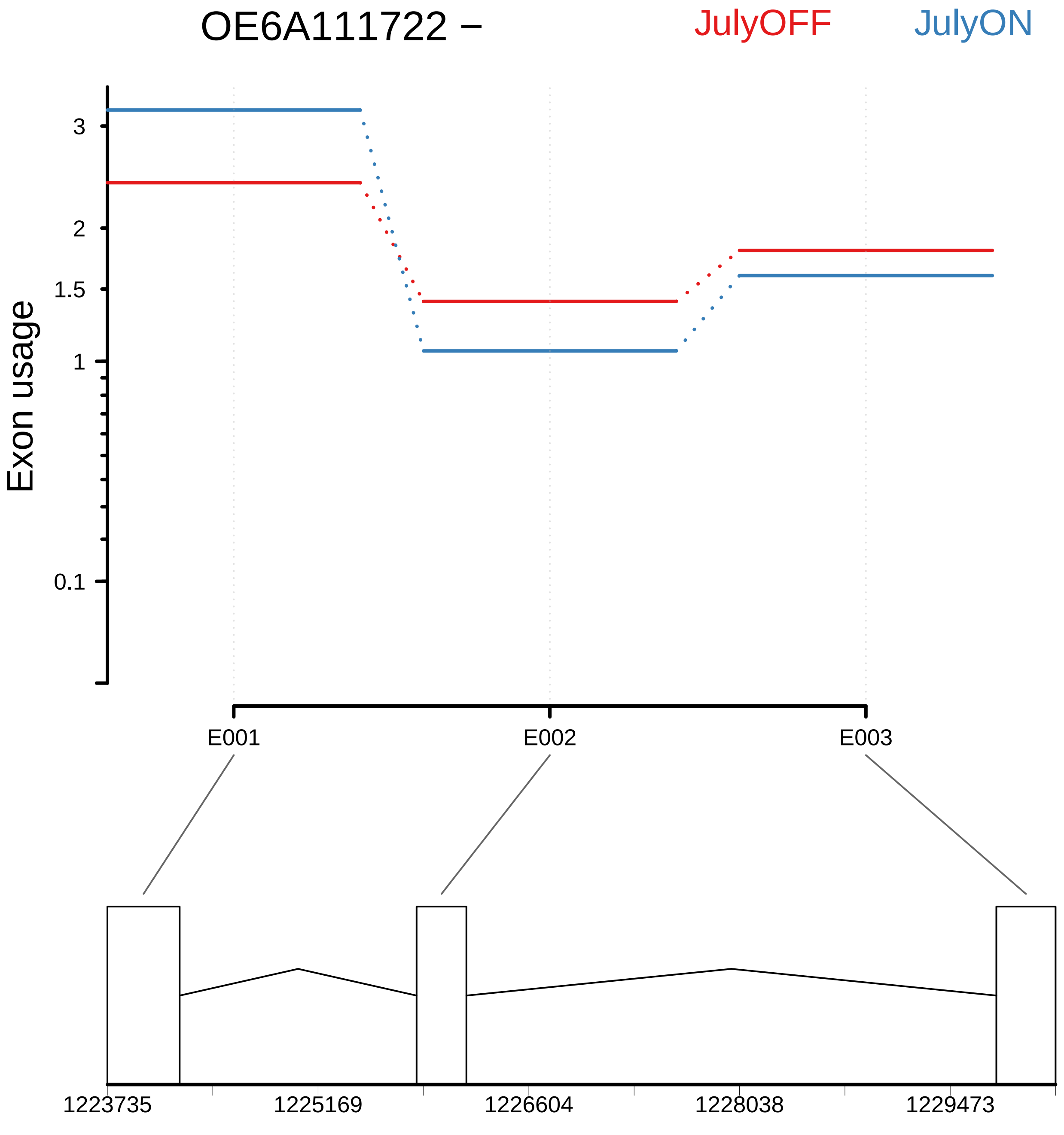

OE6A111722 −
JulyOFF
JulyON
Exon usage
3
2
1.5
1
0.1
E001
E002
E003
1223735
1225169
1226604
1228038
1229473

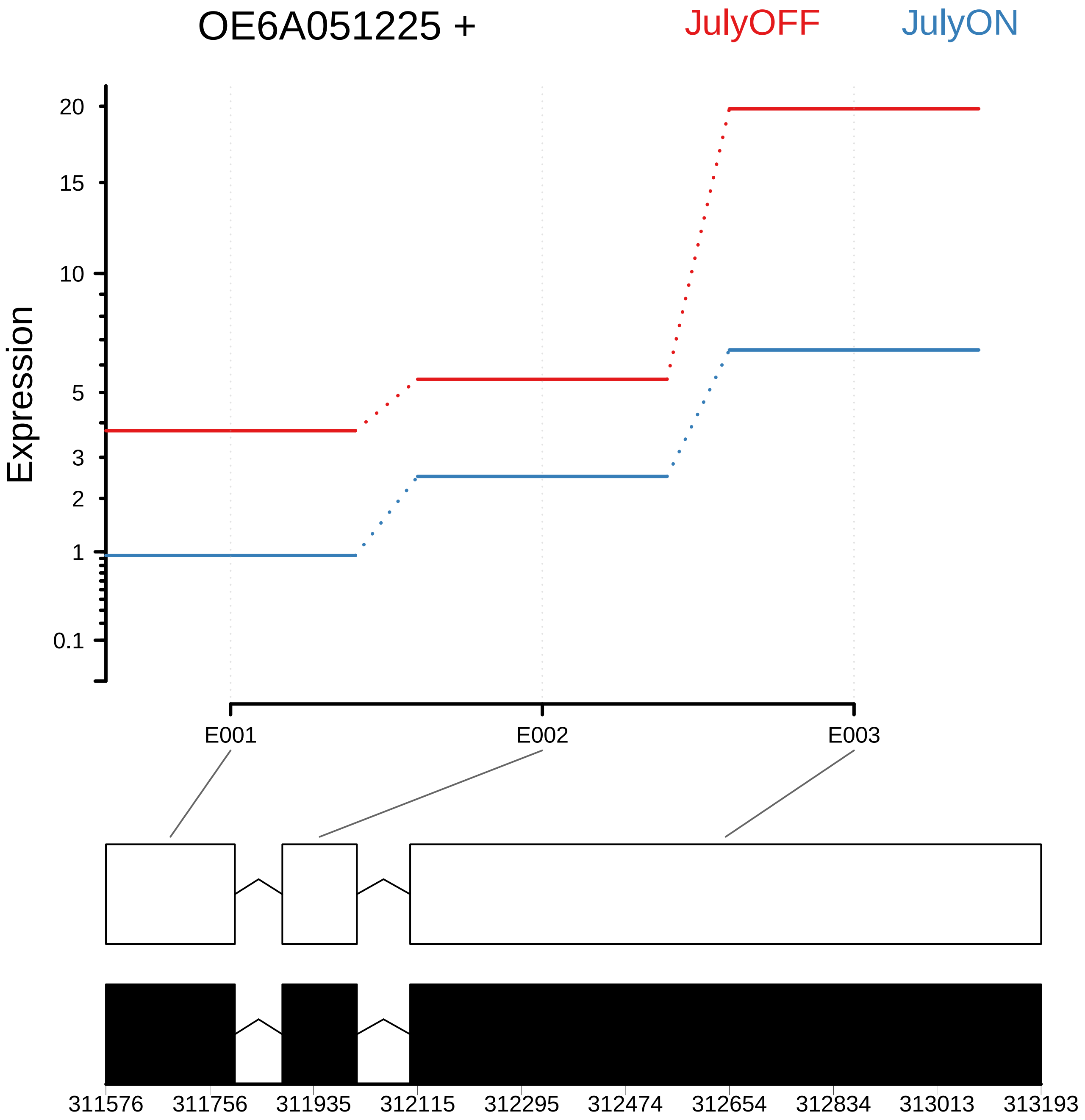
OE6A051225 +
JulyOFF
JulyON
Expression
20
15
10
5
3
2
1
0.1
E001
E002
E003
311576
311756
311935
312115
312295
312474
312654
312834
313013
313193

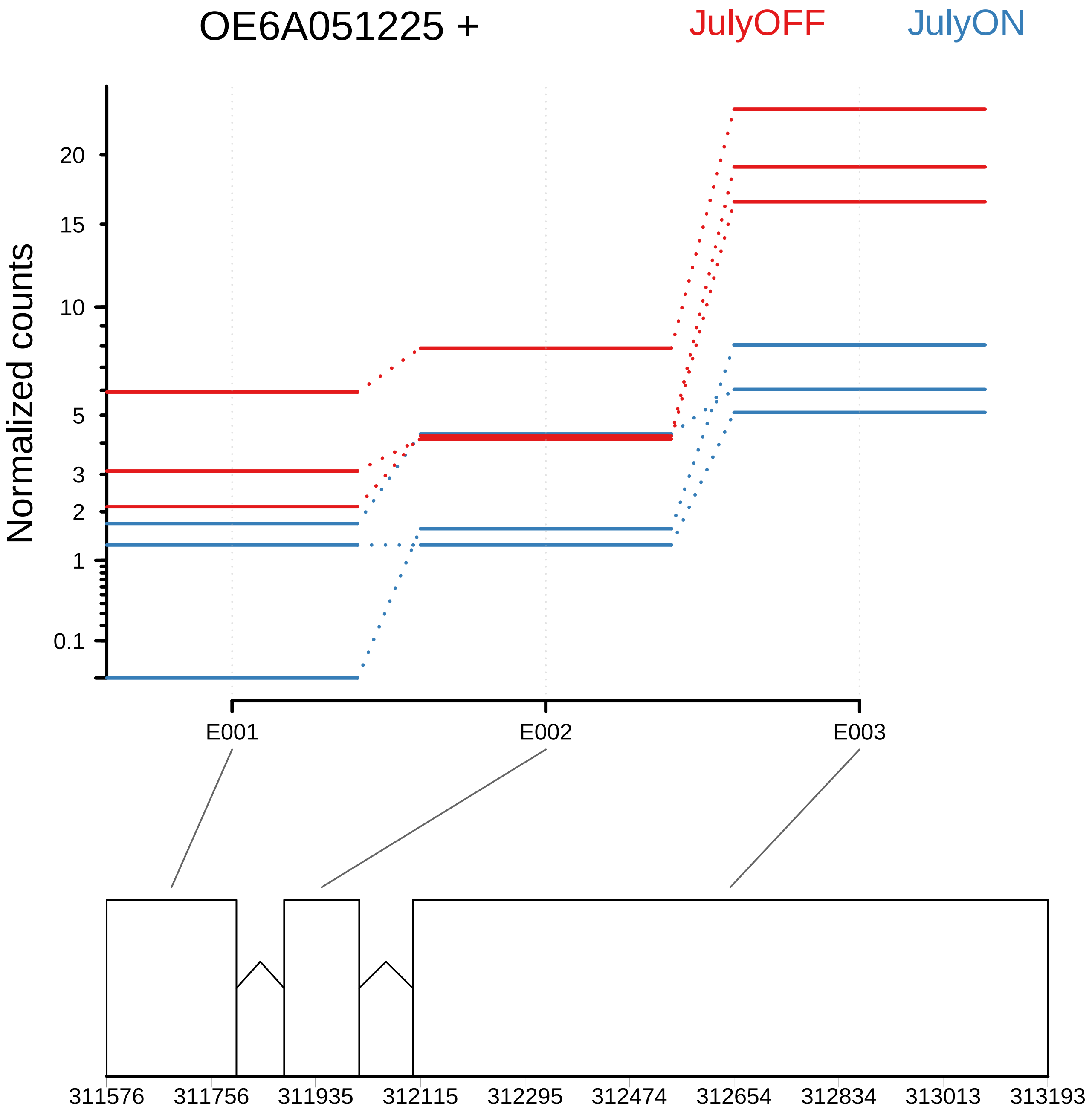
OE6A051225 +
JulyOFF
JulyON
Normalized counts
20
15
10
5
3
2
1
0.1
E001
E002
E003
311576
311756
311935
312115
312295
312474
312654
312834
313013
313193

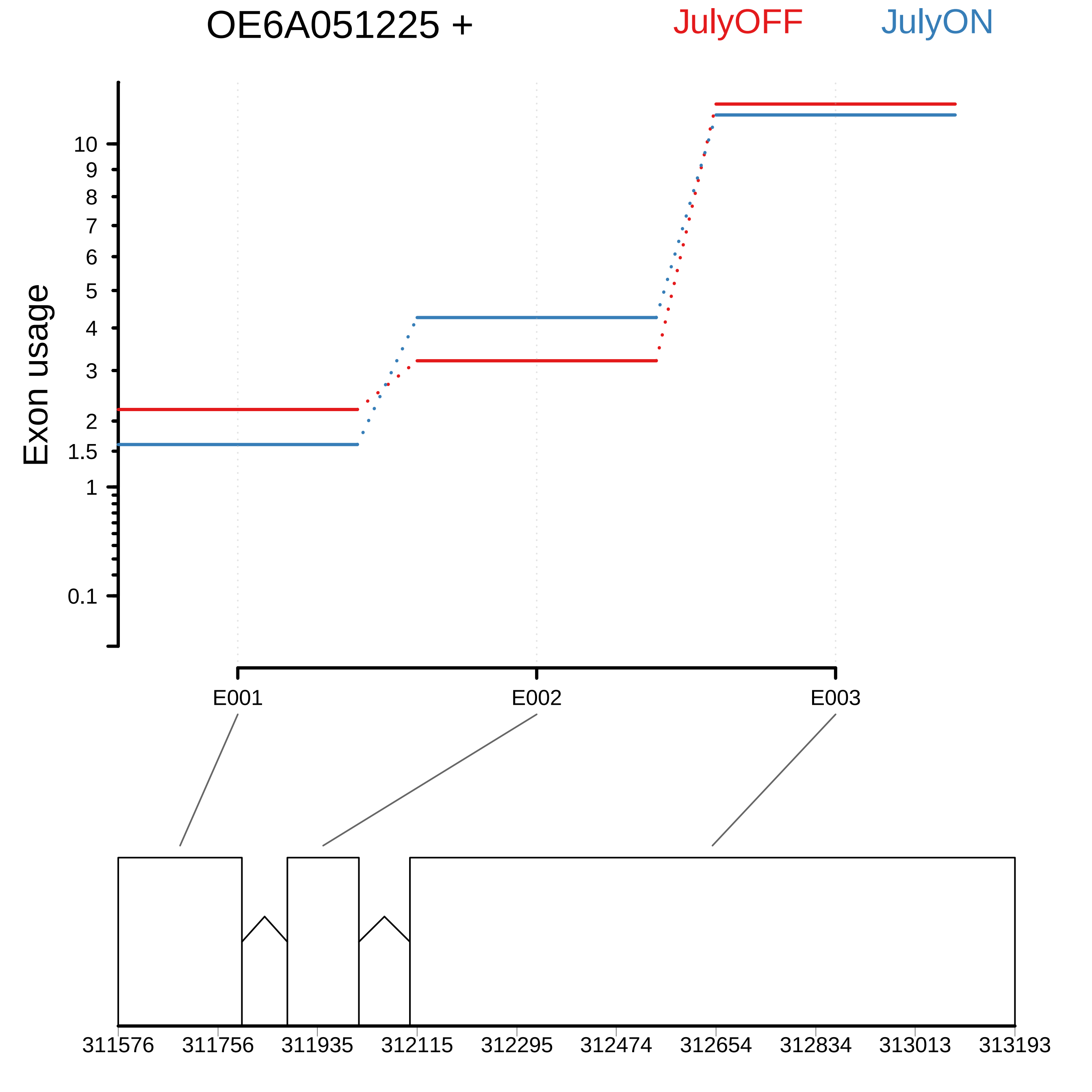

OE6A051225 +
JulyOFF
JulyON
Exon usage
10
9
8
7
6
5
4
3
2
1.5
1
0.1
E001
E002
E003
311576
311756
311935
312115
312295
312474
312654
312834
313013
313193

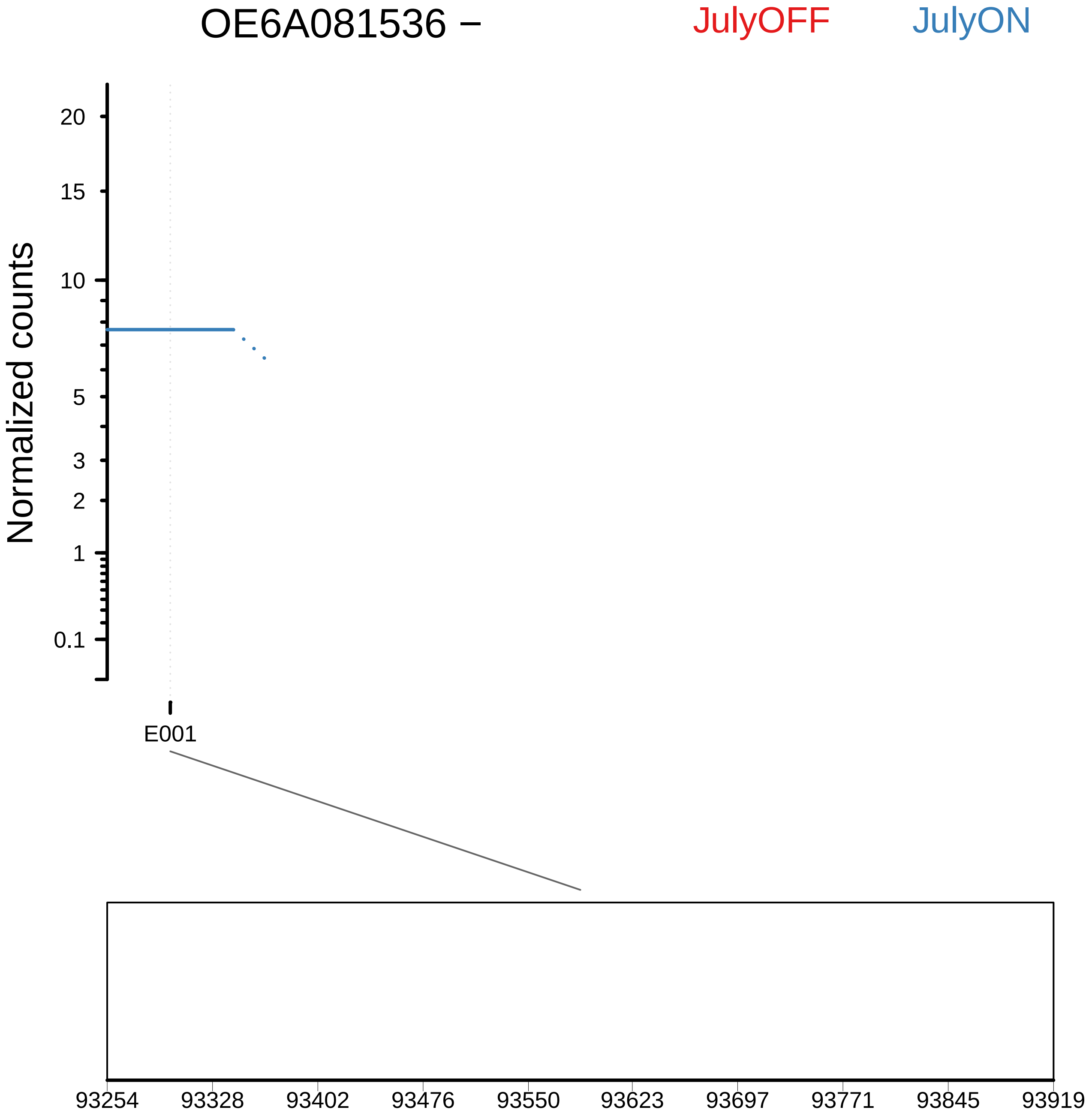
OE6A081536 −
JulyOFF
JulyON
Normalized counts
20
15
10
5
3
2
1
0.1
E001
93254
93328
93402
93476
93550
93623
93697
93771
93845
93919

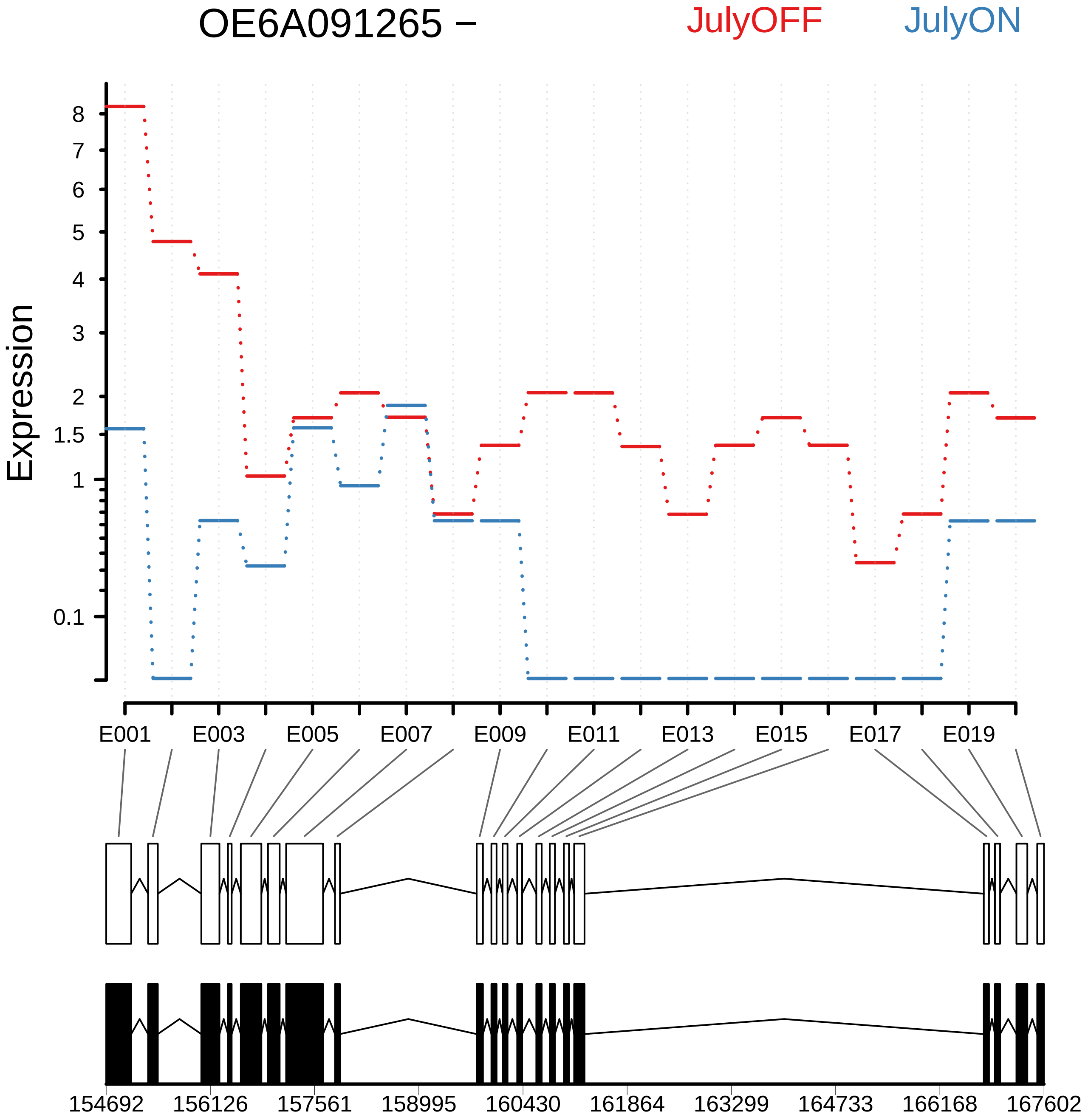

OE6A091265 −
JulyOFF
JulyON
Expression
8
7
6
5
4
3
2
1.5
1
0.1
E001
E003
E005
E007
E009
E011
E013
E015
E017
E019
154692
156126
157561
158995
160430
161864
163299
164733
166168
167602

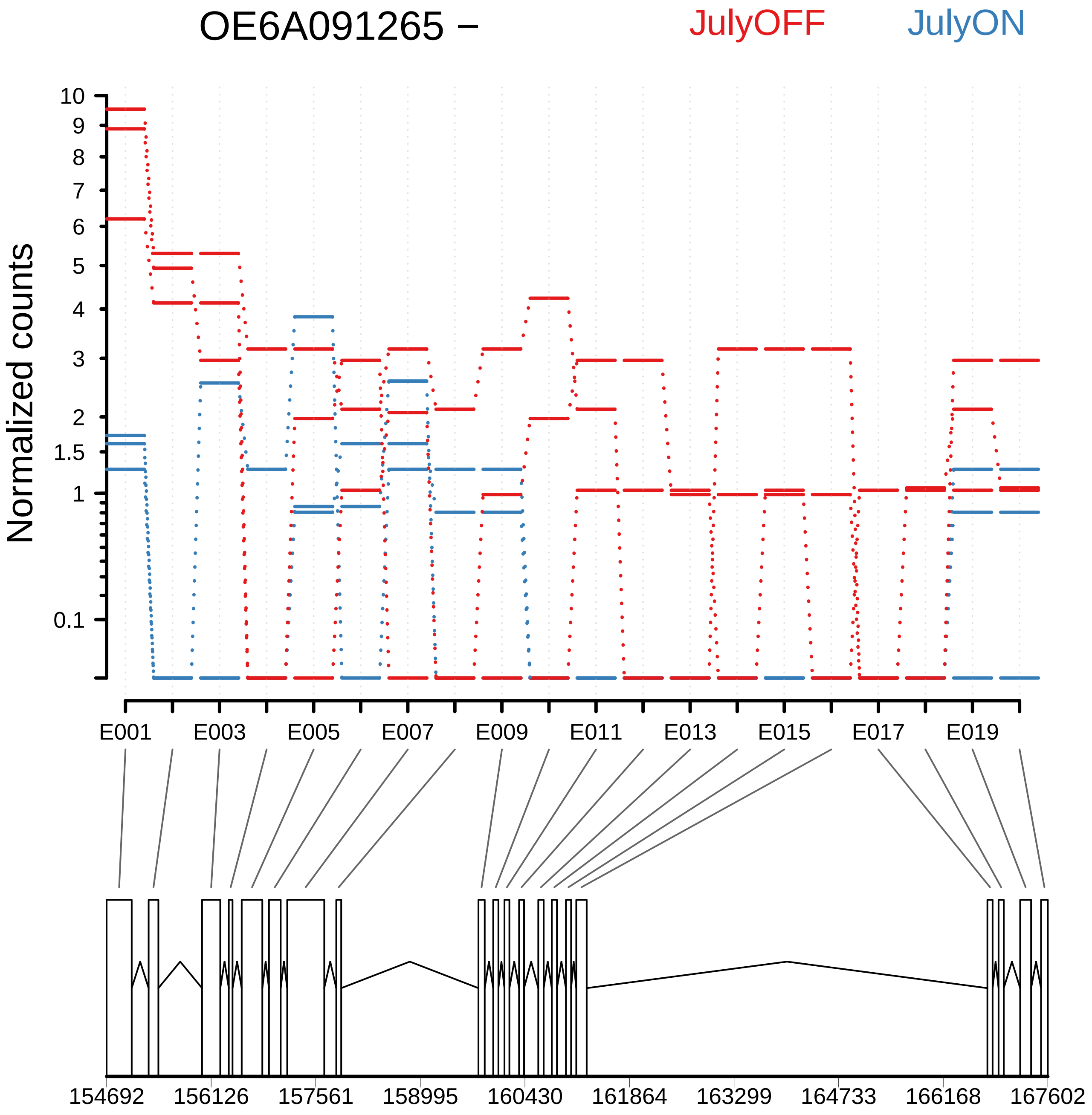
OE6A091265 −
JulyOFF
JulyON
Normalized counts
10
9
8
7
6
5
4
3
2
1.5
1
0.1
E001
E003
E005
E007
E009
E011
E013
E015
E017
E019
154692
156126
157561
158995
160430
161864
163299
164733
166168
167602

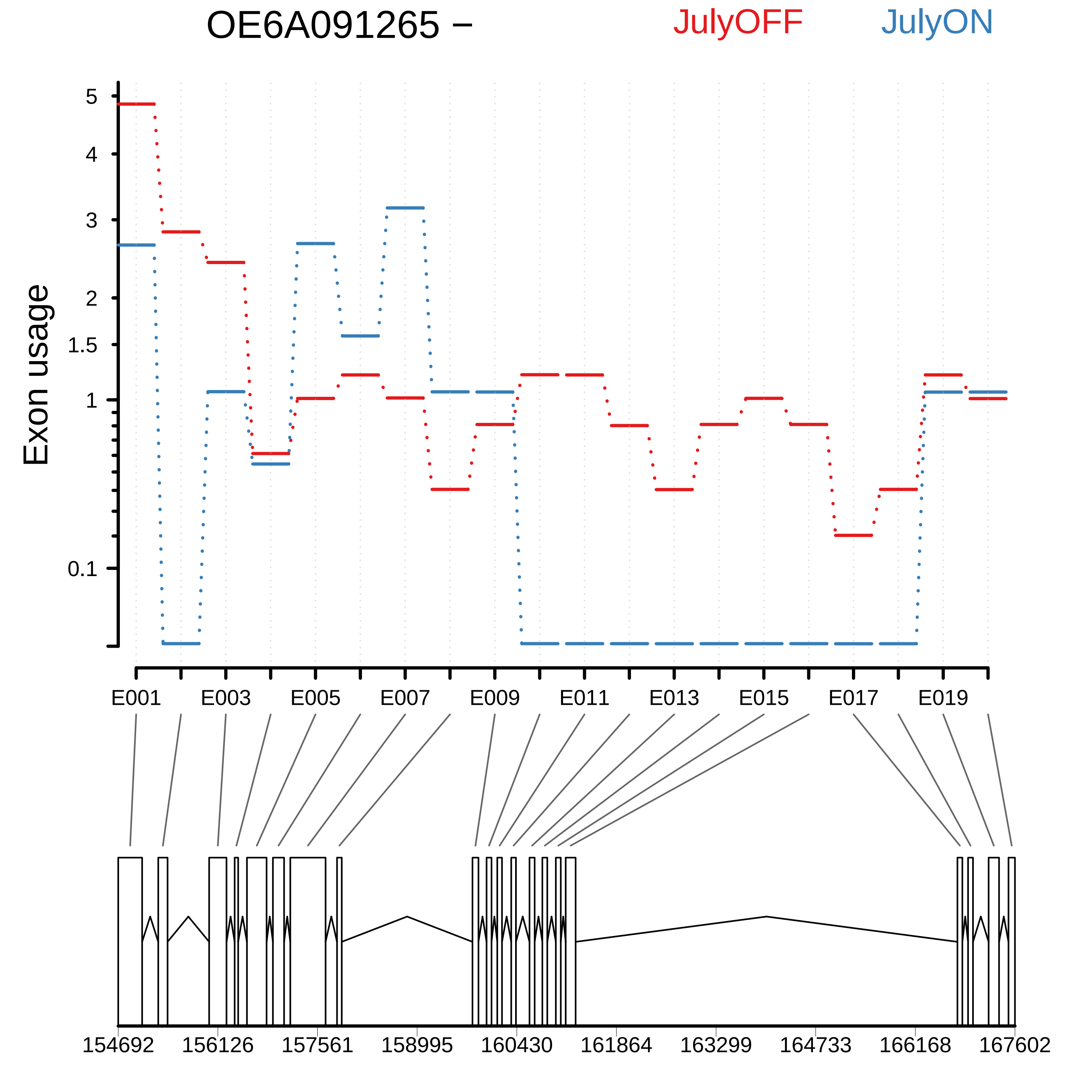

OE6A091265 −
JulyOFF
JulyON
Exon usage
5
4
3
2
1.5
1
0.1
E001
E003
E005
E007
E009
E011
E013
E015
E017
E019
154692
156126
157561
158995
160430
161864
163299
164733
166168
167602

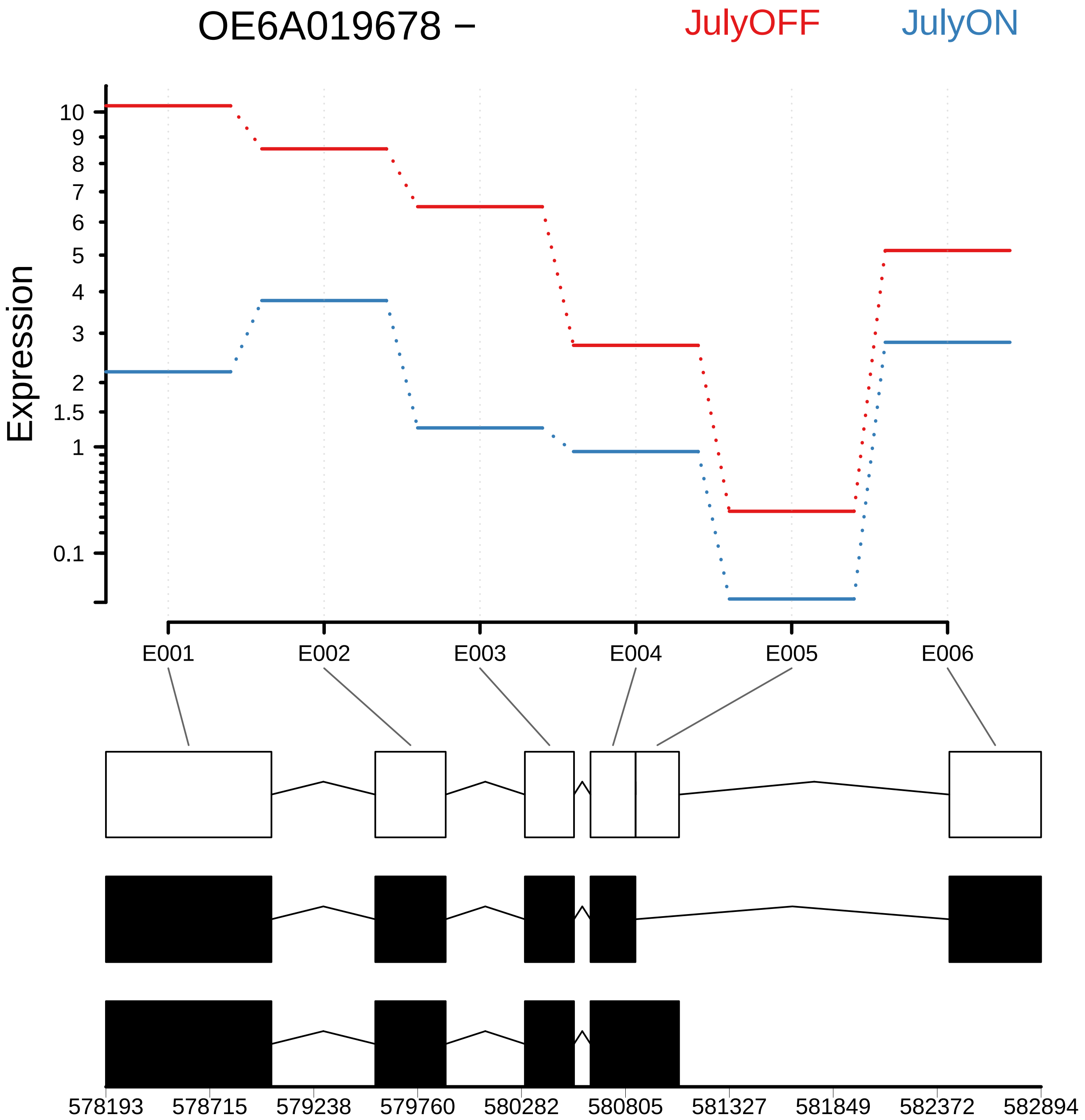

OE6A019678 –
JulyOFF
JulyON
Expression
10
9
8
7
6
5
4
3
2
1.5
1
0.1
E001
E002
E003
E004
E005
E006
578193
578715
579238
579760
580282
580805
581327
581849
582372
582894

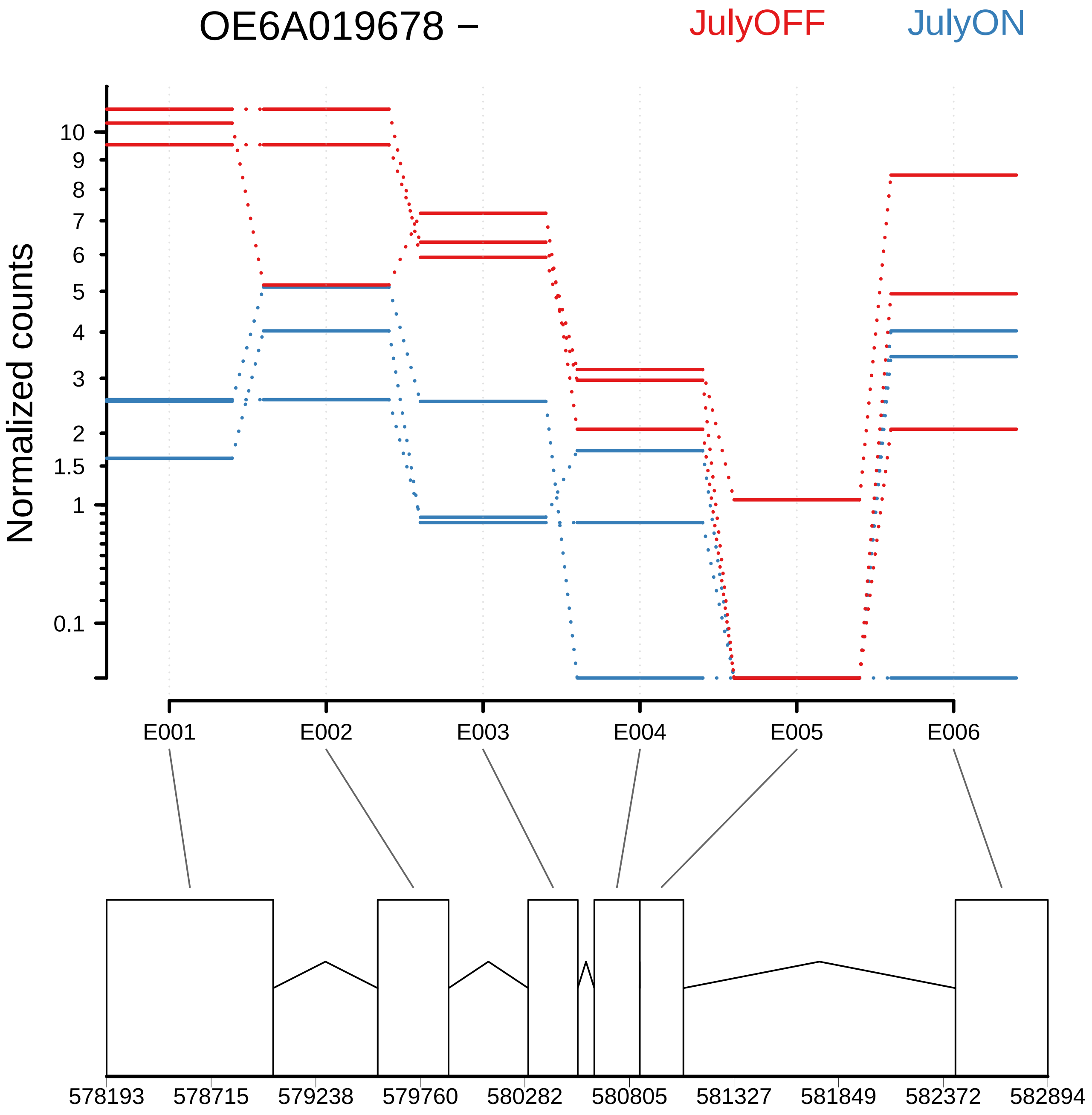

OE6A019678 –
JulyOFF
JulyON
Normalized counts
10
9
8
7
6
5
4
3
2
1.5
1
0.1
E001
E002
E003
E004
E005
E006
578193
578715
579238
579760
580282
580805
581327
581849
582372
582894

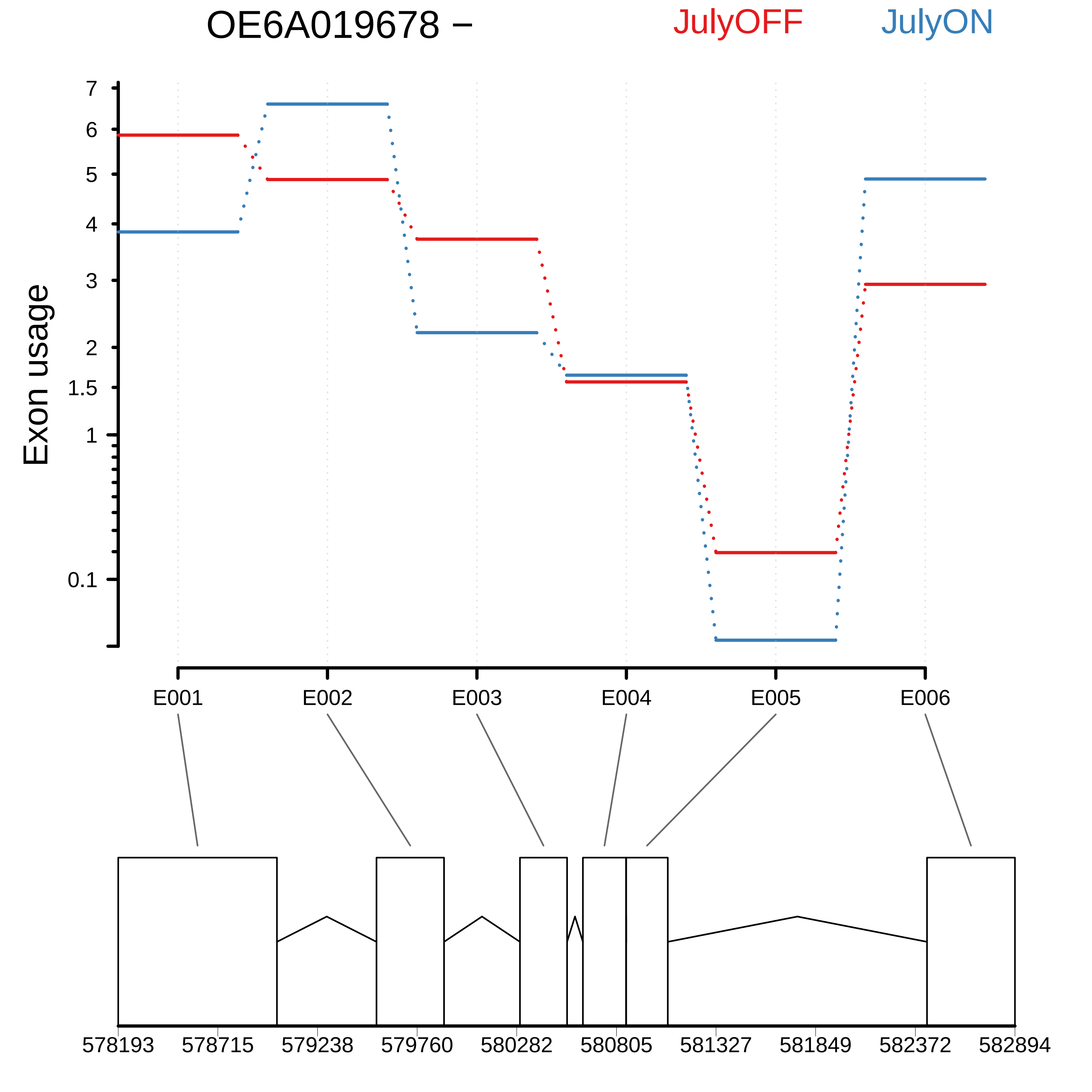

OE6A019678 −
JulyOFF
JulyON
Exon usage
7
6
5
4
3
2
1.5
1
0.1
E001
E002
E003
E004
E005
E006
578193
578715
579238
579760
580282
580805
581327
581849
582372
582894

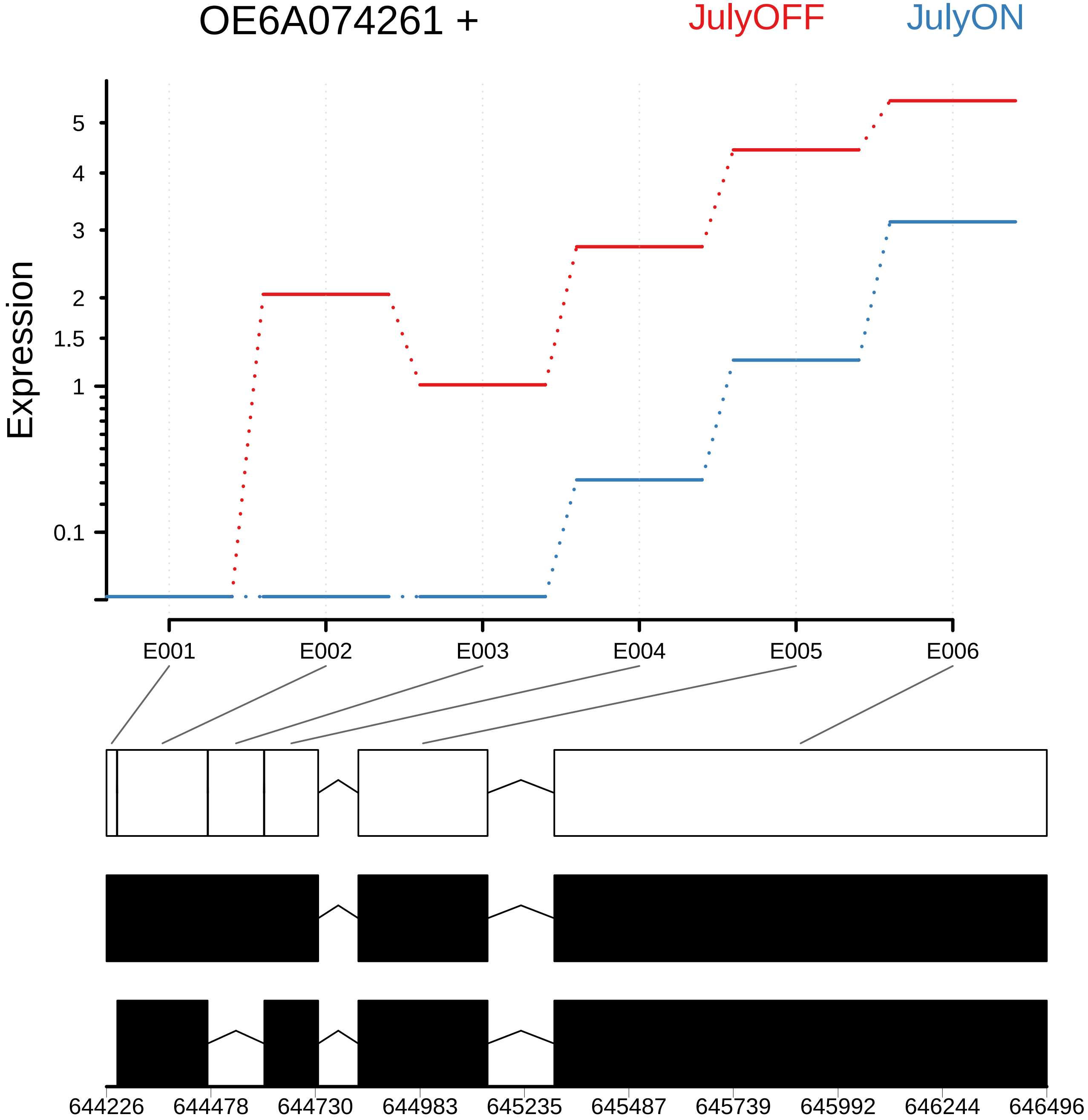
OE6A074261 +
JulyOFF
JulyON
Expression
5
4
3
2
1.5
1
0.1
E001
E002
E003
E004
E005
E006
644226
644478
644730
644983
645235
645487
645739
645992
646244
646496

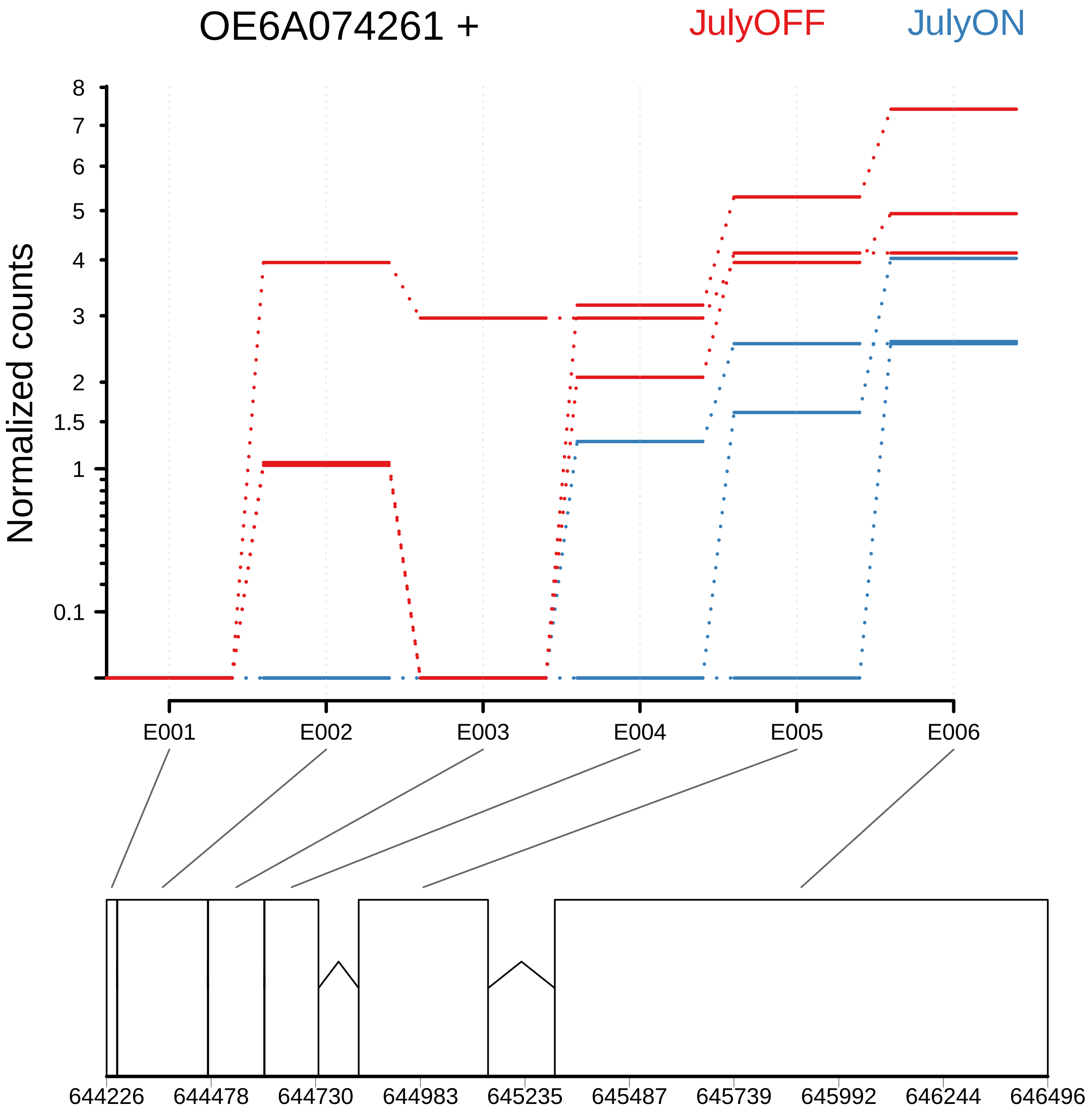

OE6A074261 +
JulyOFF
JulyON
Normalized counts
8
7
6
5
4
3
2
1.5
1
0.1
E001
E002
E003
E004
E005
E006
644226
644478
644730
644983
645235
645487
645739
645992
646244
646496

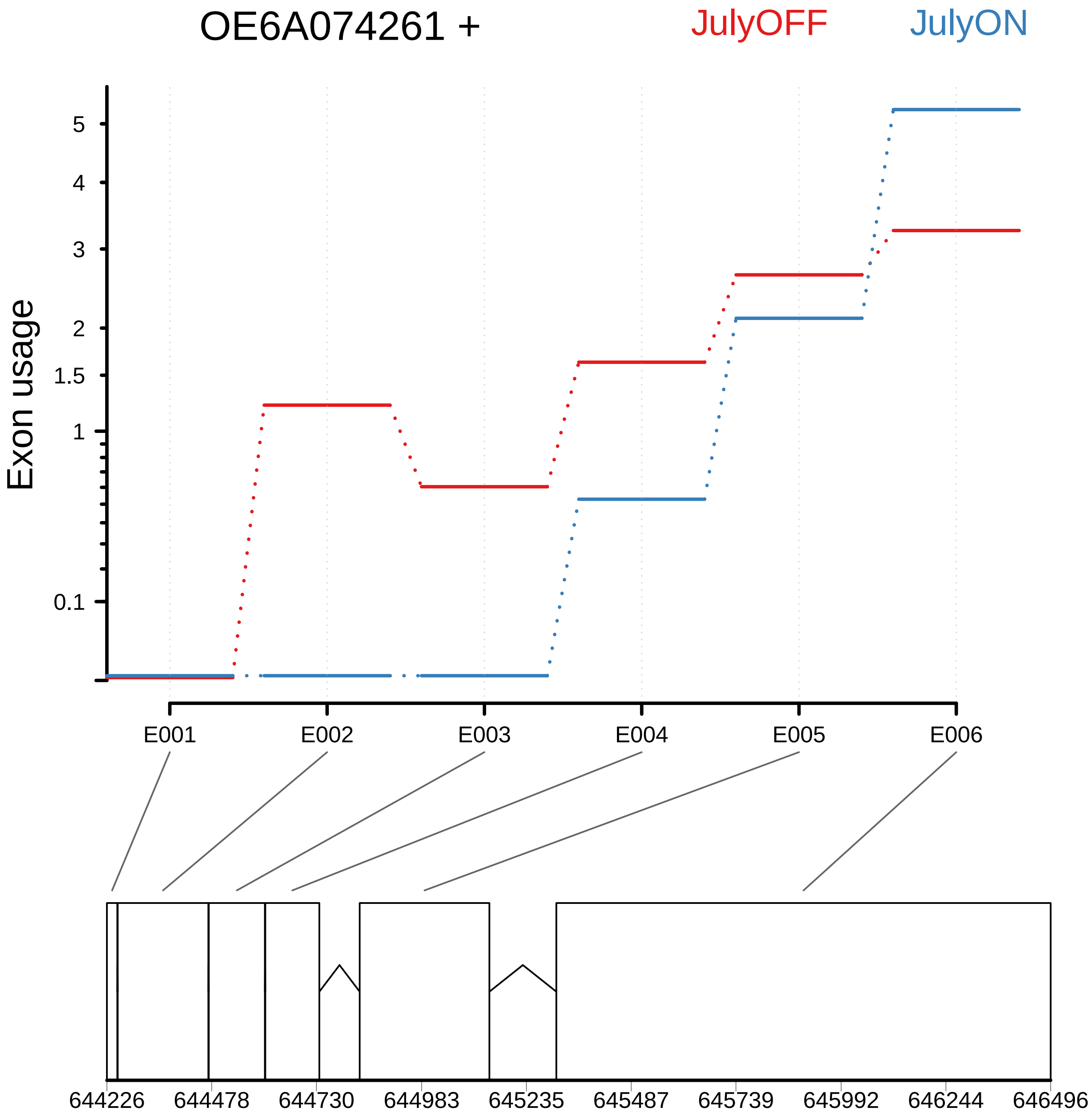

OE6A074261 +
JulyOFF
JulyON
Exon usage
5
4
3
2
1.5
1
0.1
E001
E002
E003
E004
E005
E006
644226
644478
644730
644983
645235
645487
645739
645992
646244
646496

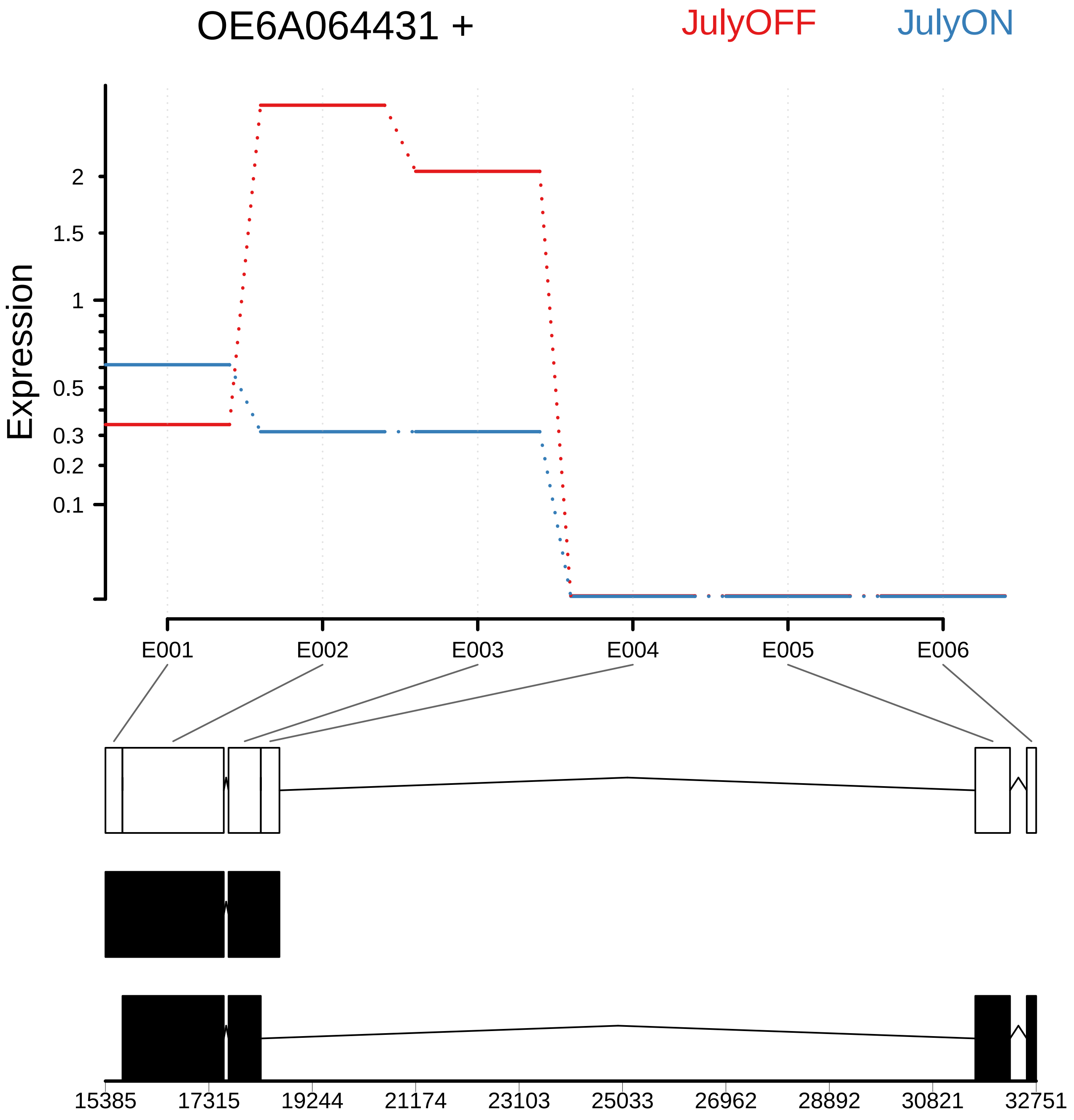
OE6A064431 +
JulyOFF
JulyON
Expression
2
1.5
1
0.5
0.3
0.2
0.1
E001
E002
E003
E004
E005
E006
15385
17315
19244
21174
23103
25033
26962
28892
30821
32751

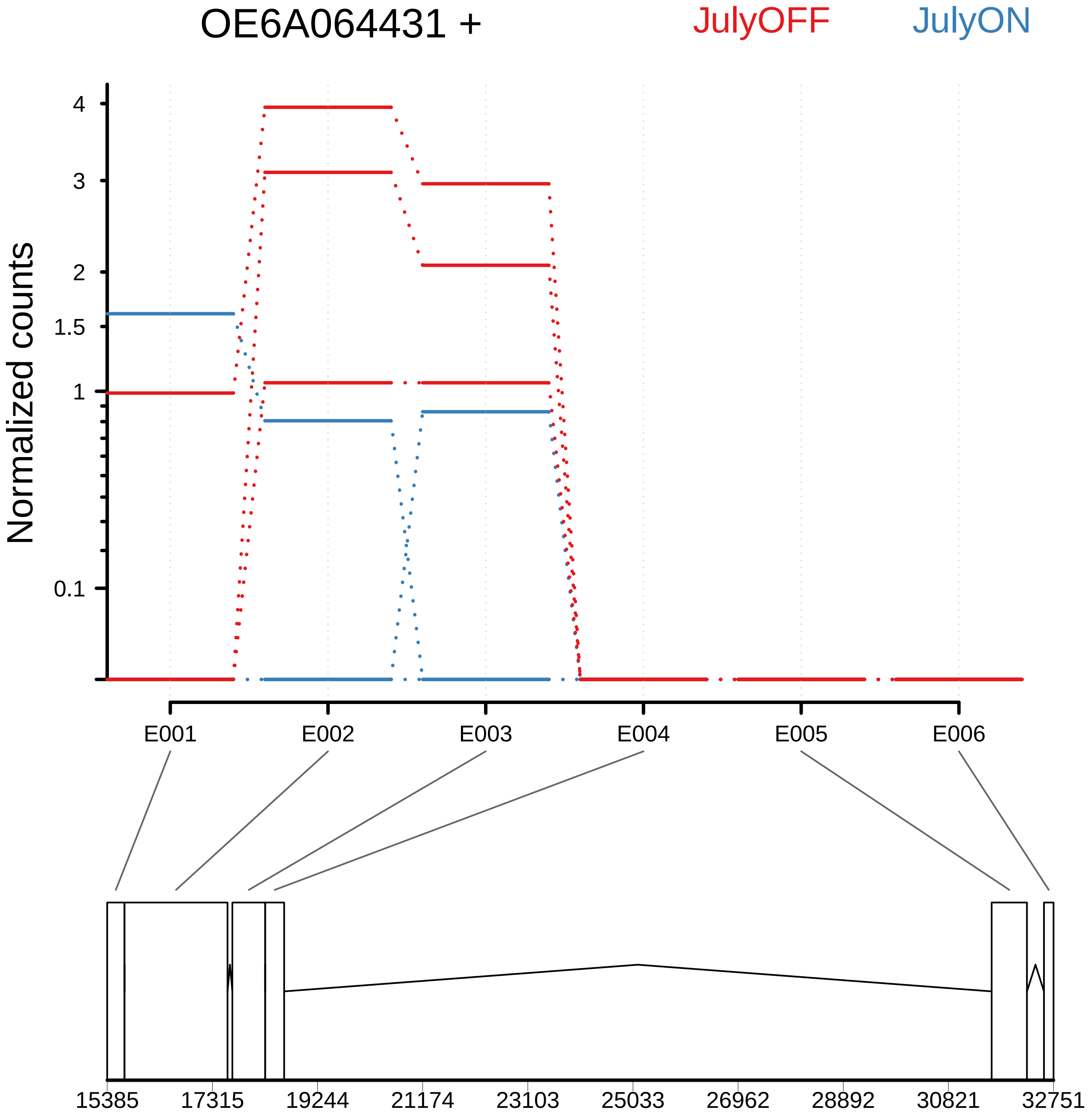

OE6A064431 +
JulyOFF
JulyON
Normalized counts
4
3
2
1.5
1
0.1
E001
E002
E003
E004
E005
E006
15385
17315
19244
21174
23103
25033
26962
28892
30821
32751

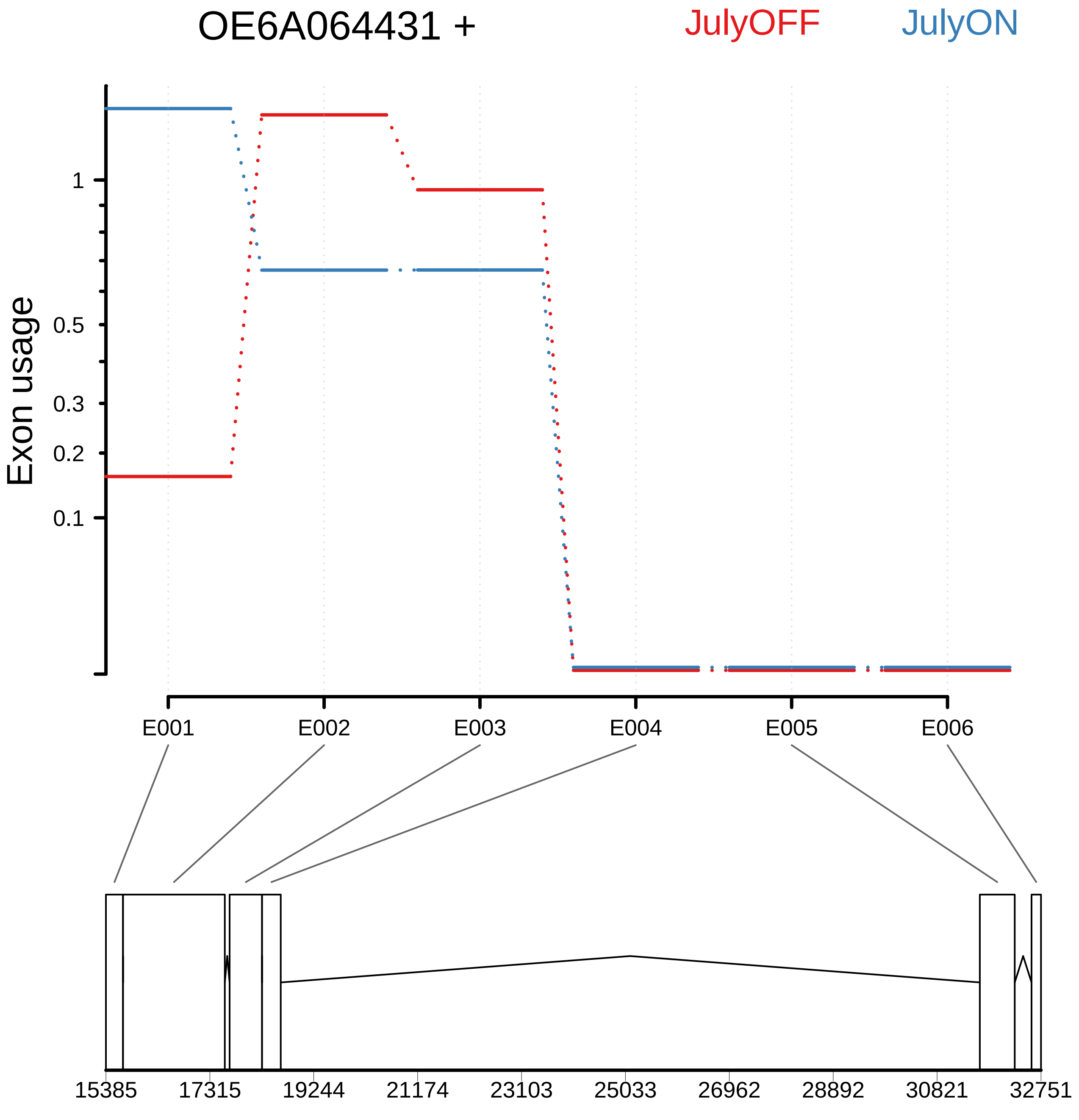

OE6A064431 +
JulyOFF
JulyON
Exon usage
1
0.5
0.3
0.2
0.1
E001
E002
E003
E004
E005
E006
15385
17315
19244
21174
23103
25033
26962
28892
30821
32751

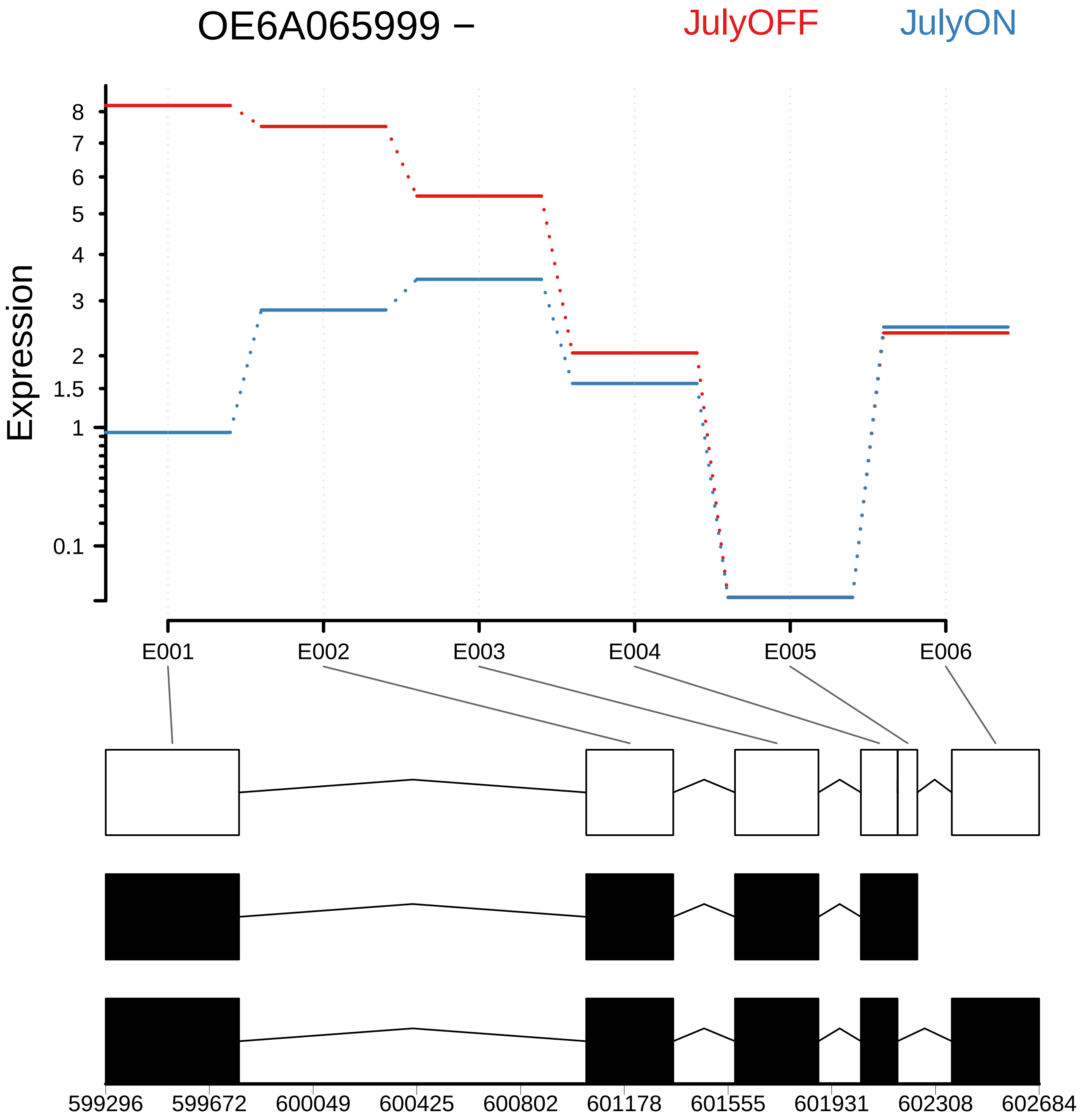

OE6A065999 –
JulyOFF
JulyON
Expression
8
7
6
5
4
3
2
1.5
1
0.1
E001
E002
E003
E004
E005
E006
599296
599672
600049
600425
600802
601178
601555
601931
602308
602684

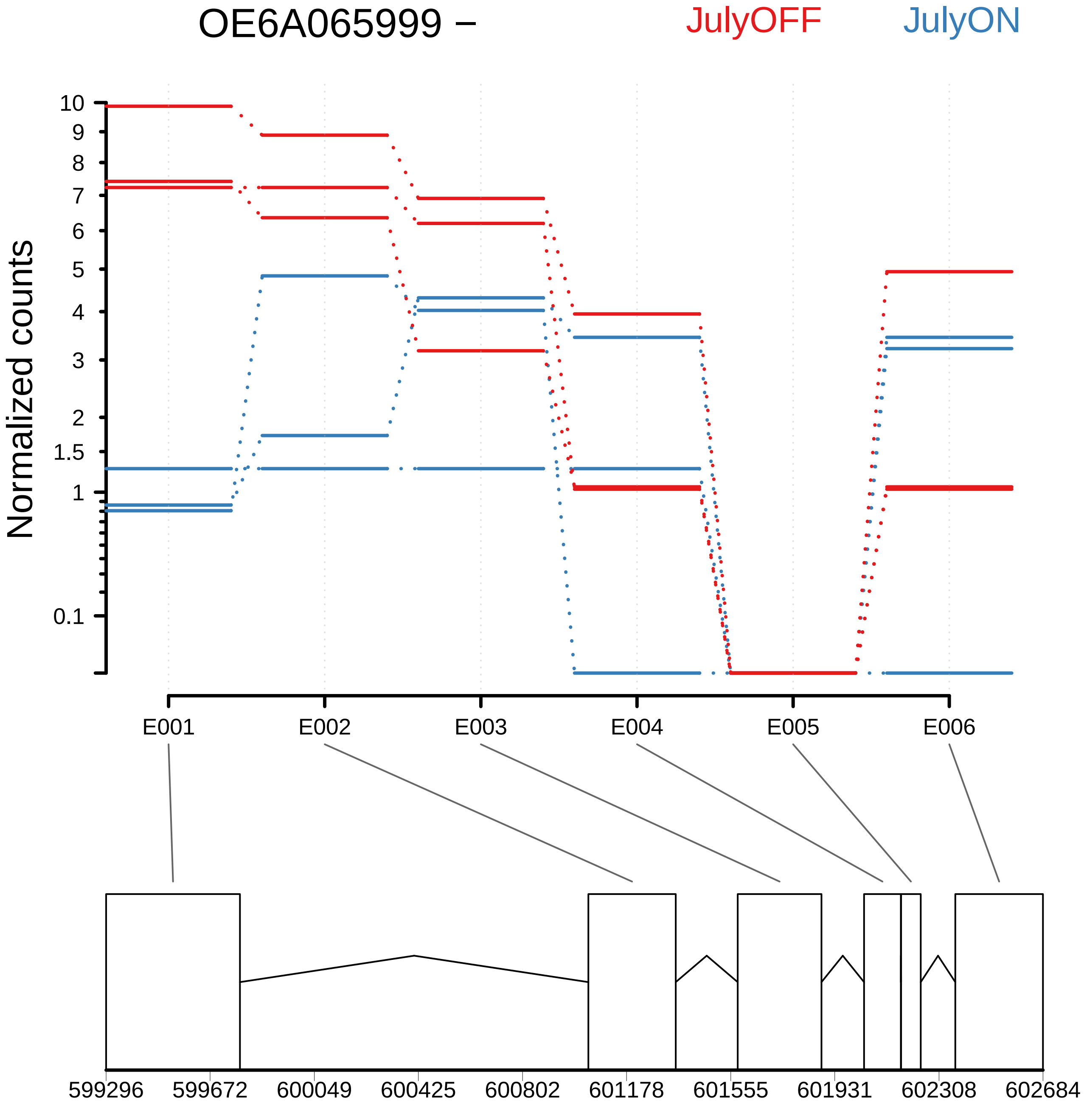

OE6A065999 −
JulyOFF
JulyON
Normalized counts
10
9
8
7
6
5
4
3
2
1.5
1
0.1
E001
E002
E003
E004
E005
E006
599296
599672
600049
600425
600802
601178
601555
601931
602308
602684

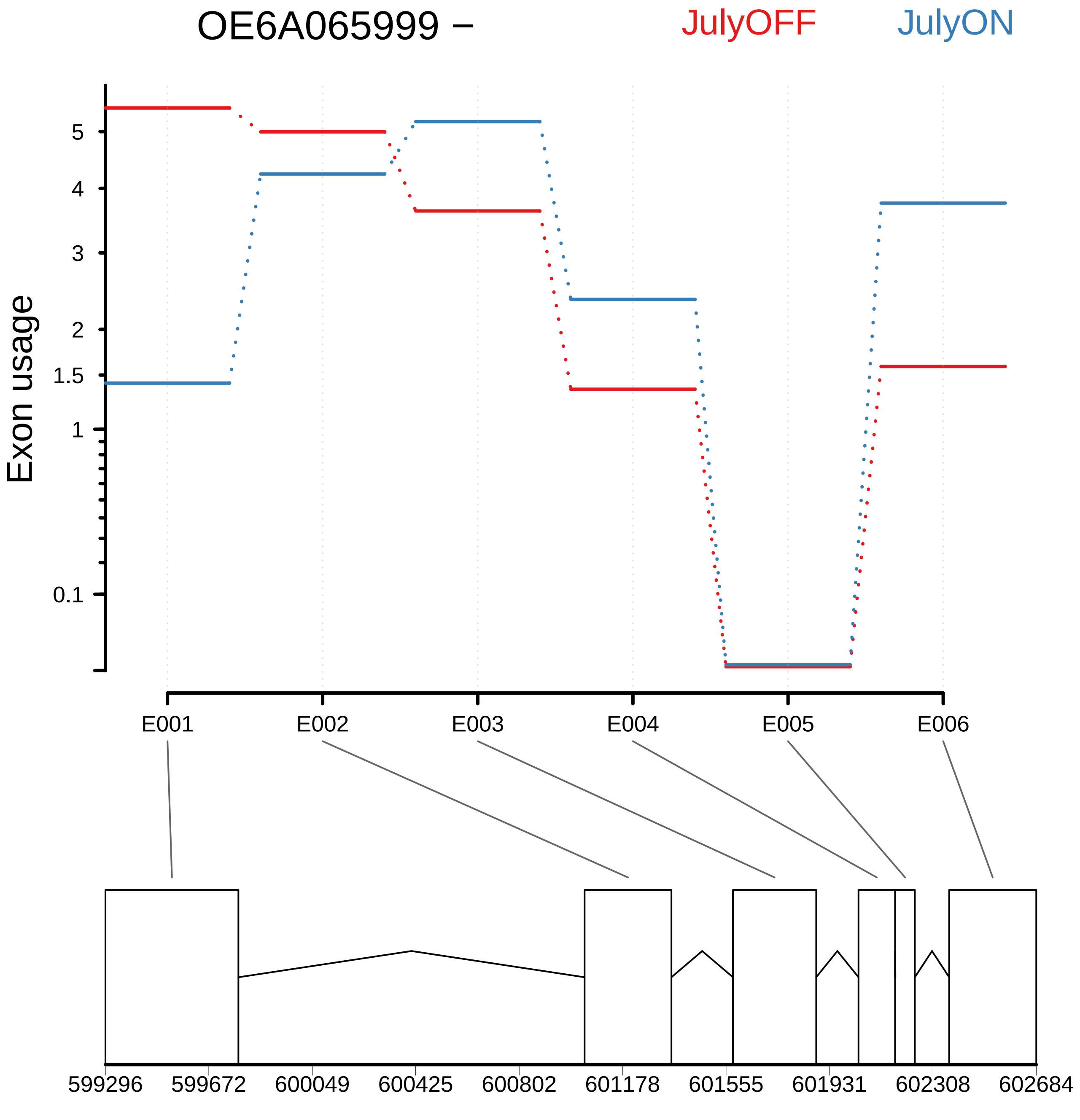

OE6A065999 –
JulyOFF
JulyON
Exon usage
5
4
3
2
1.5
1
0.1
E001
E002
E003
E004
E005
E006
599296
599672
600049
600425
600802
601178
601555
601931
602308
602684

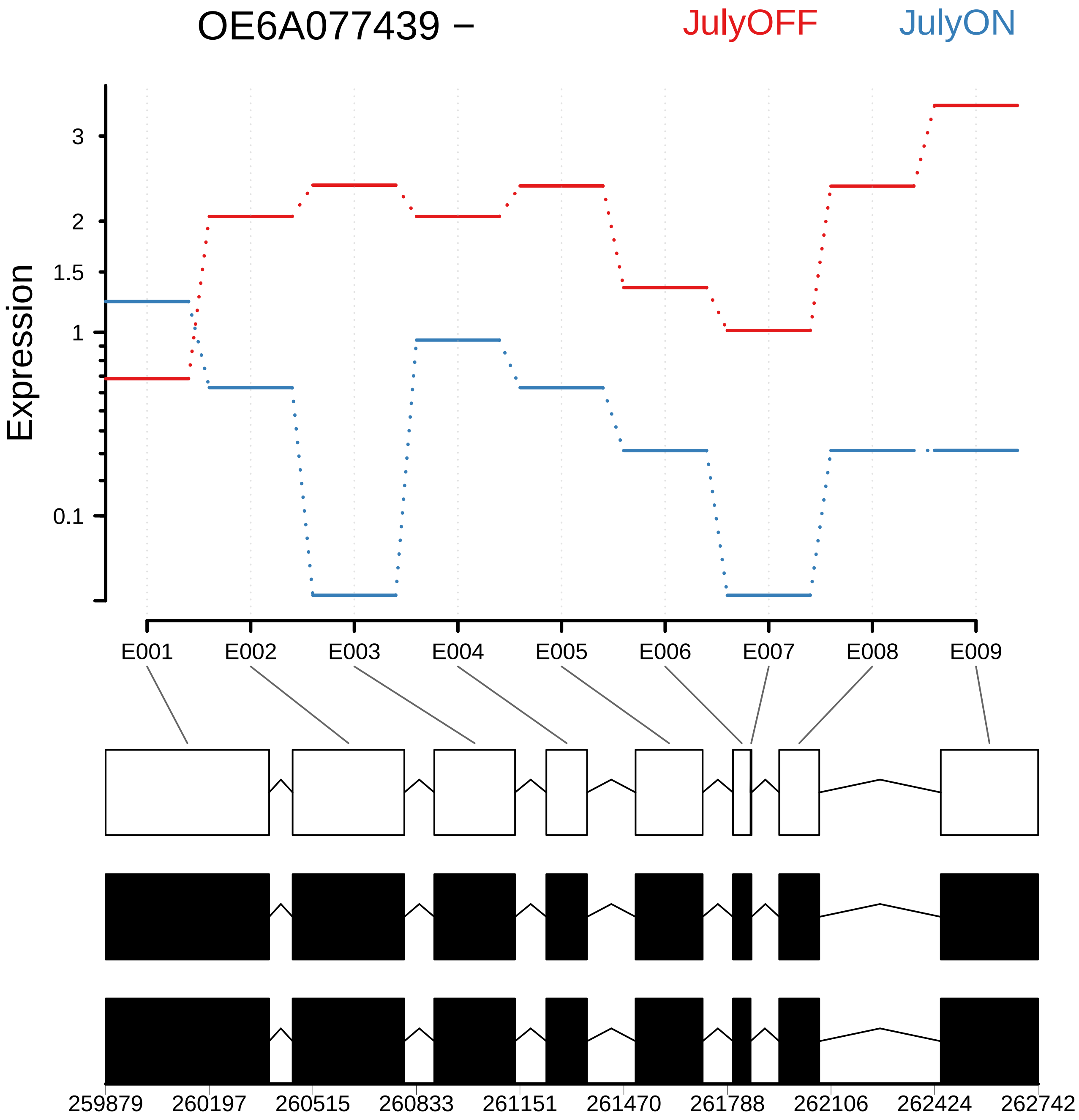

OE6A077439 −
JulyOFF
JulyON
Expression
3
2
1.5
1
0.1
E001
E002
E003
E004
E005
E006
E007
E008
E009
259879
260197
260515
260833
261151
261470
261788
262106
262424
262742

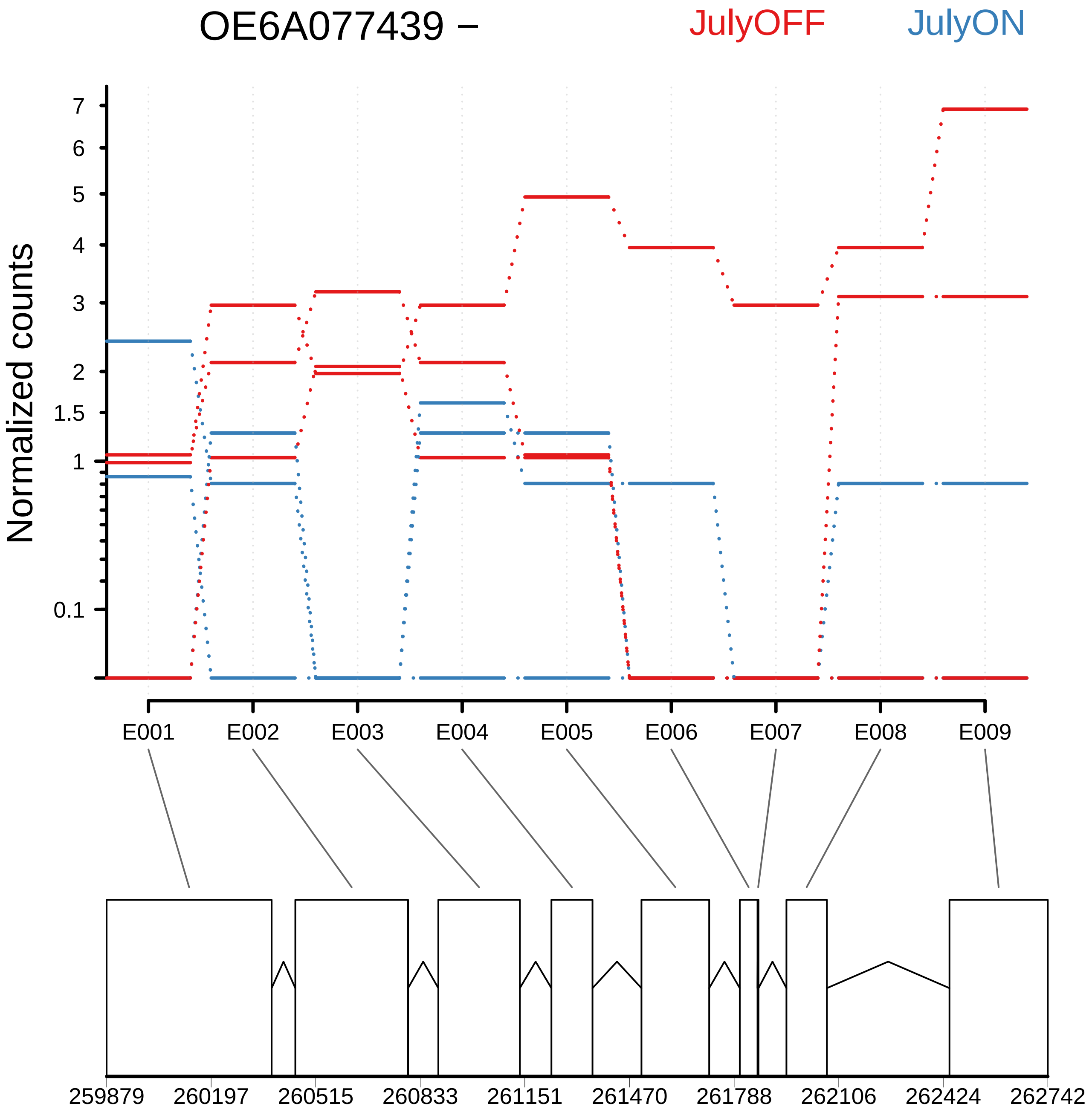
OE6A077439 –
JulyOFF
JulyON
Normalized counts
7
6
5
4
3
2
1.5
1
0.1
E001
E002
E003
E004
E005
E006
E007
E008
E009
259879
260197
260515
260833
261151
261470
261788
262106
262424
262742

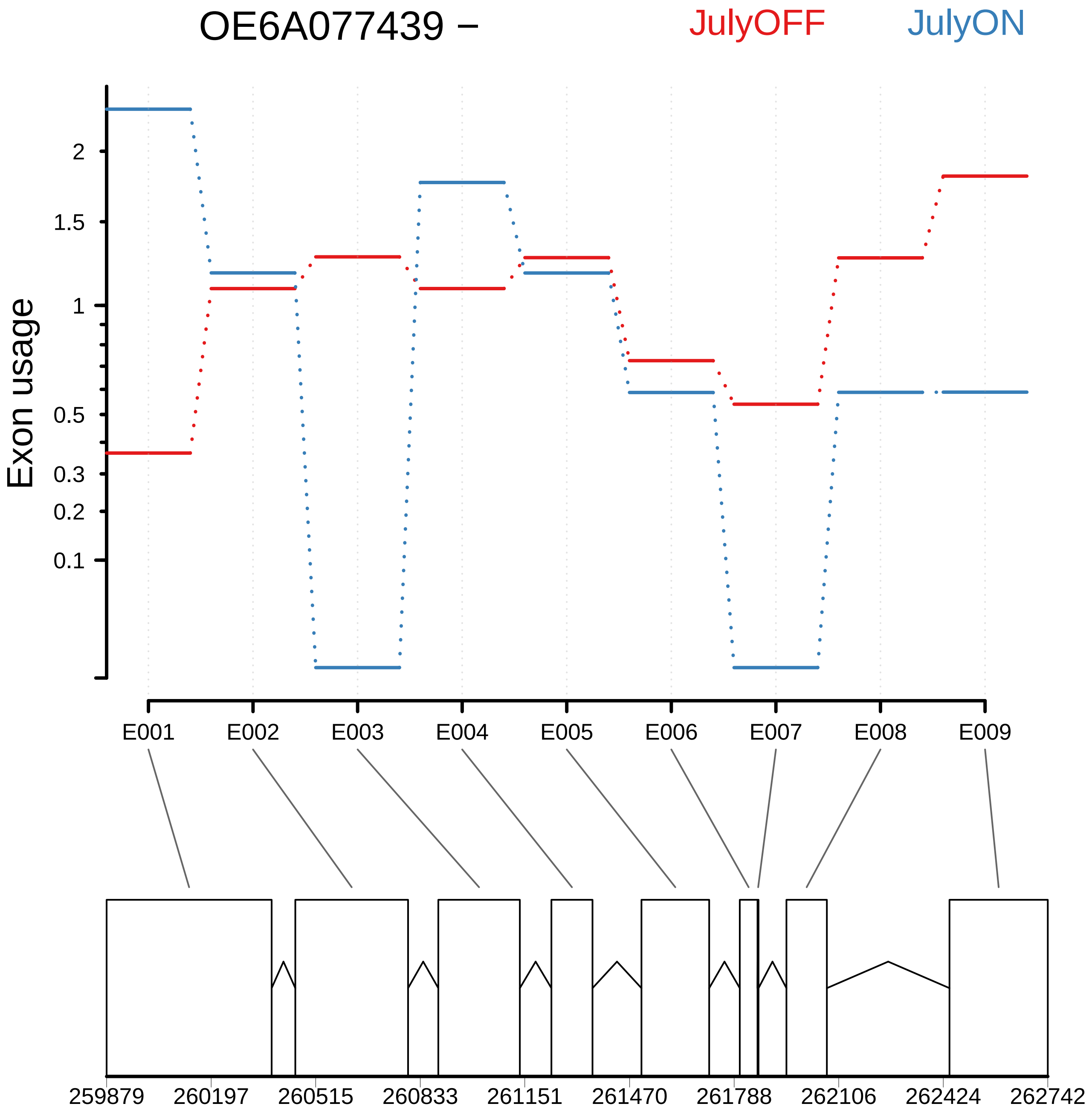
OE6A077439 –
JulyOFF
JulyON
Exon usage
2
1.5
1
0.5
0.3
0.2
0.1
E001
E002
E003
E004
E005
E006
E007
E008
E009
259879
260197
260515
260833
261151
261470
261788
262106
262424
262742

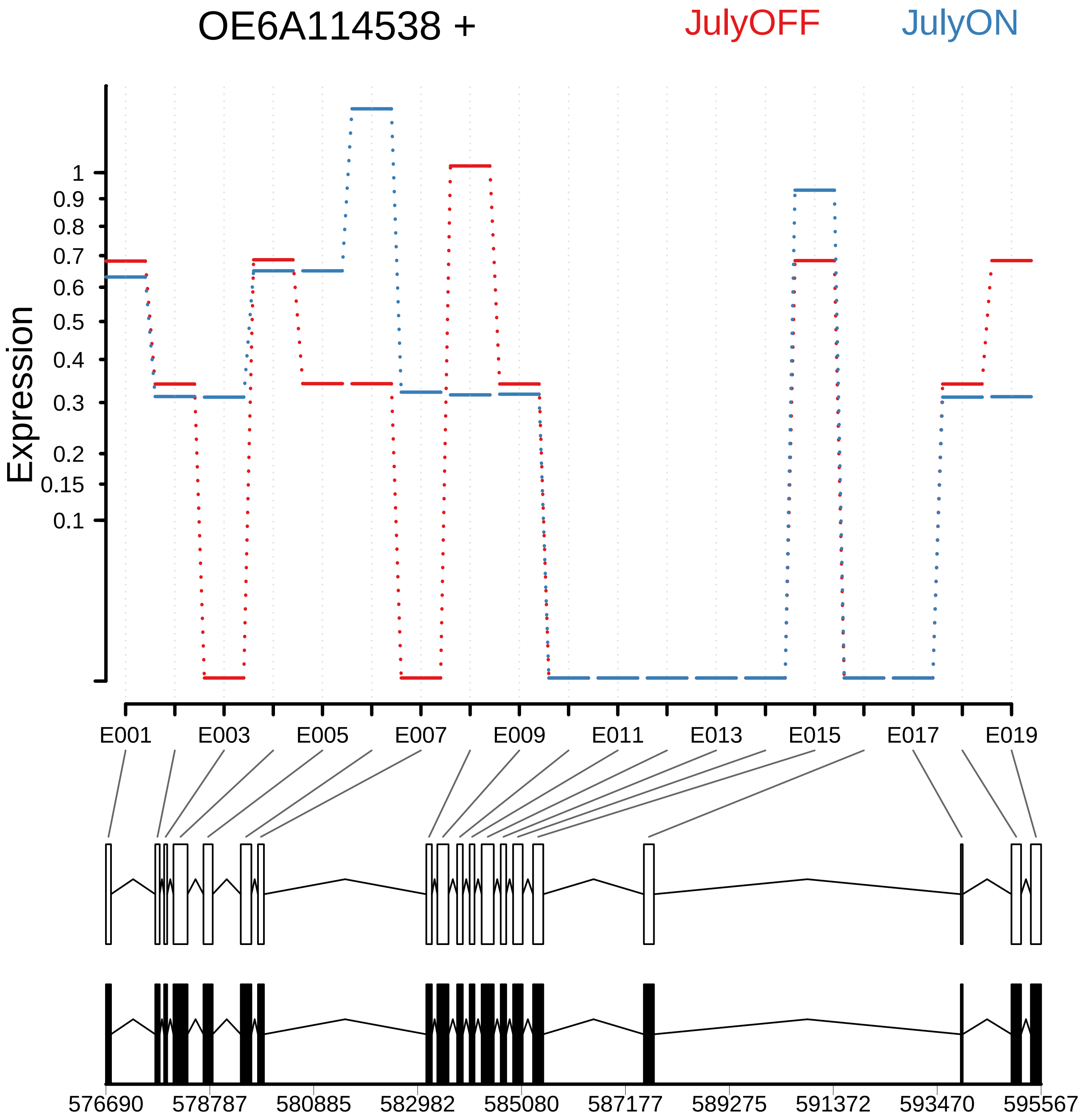
OE6A114538 +
JulyOFF
JulyON
Expression
1
0.9
0.8
0.7
0.6
0.5
0.4
0.3
0.2
0.15
0.1
E001
E003
E005
E007
E009
E011
E013
E015
E017
E019
576690
578787
580885
582982
585080
587177
589275
591372
593470
595567

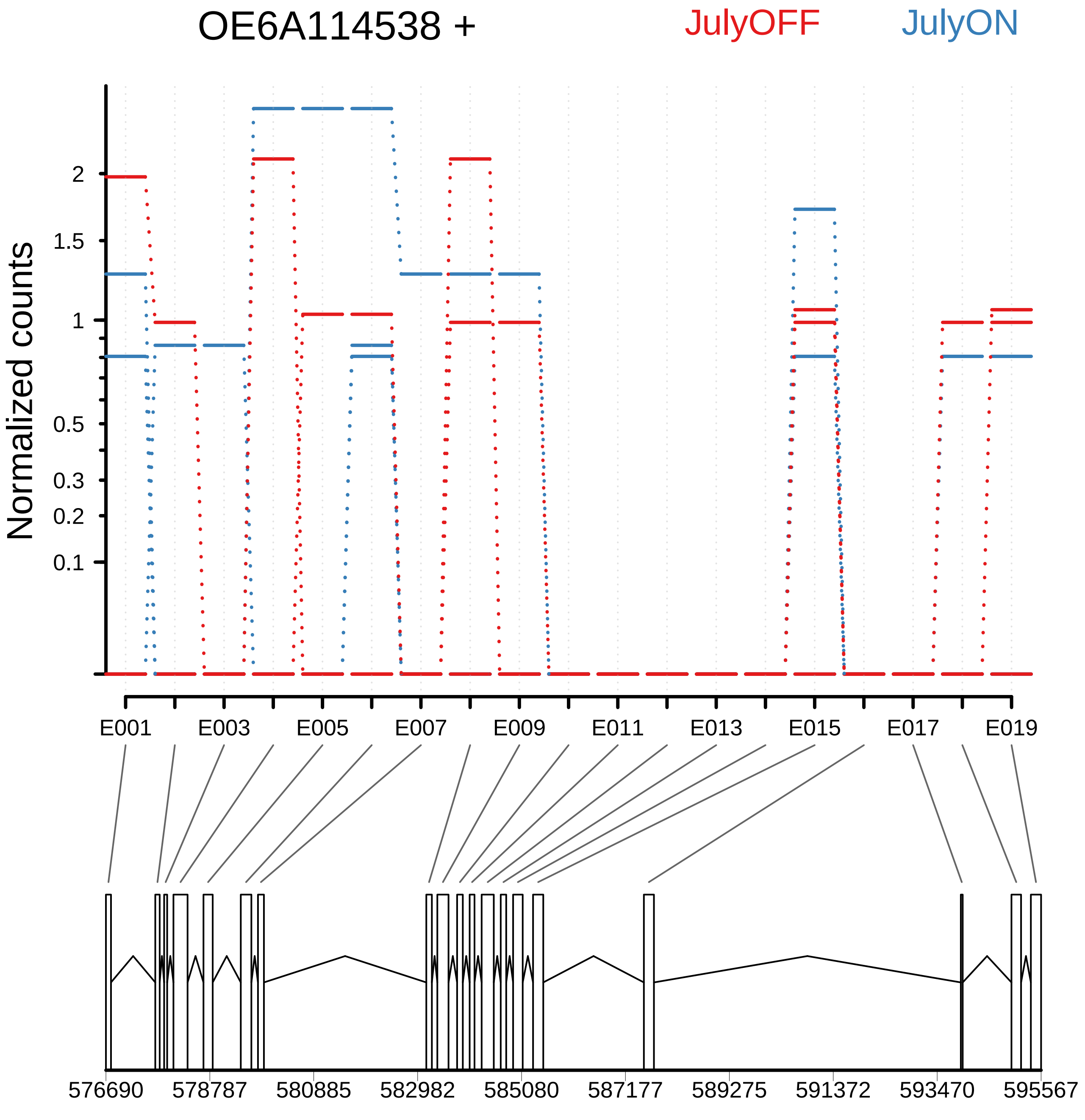

OE6A114538 +
JulyOFF
JulyON
Normalized counts
2
1.5
1
0.5
0.3
0.2
0.1
E001
E003
E005
E007
E009
E011
E013
E015
E017
E019
576690
578787
580885
582982
585080
587177
589275
591372
593470
595567

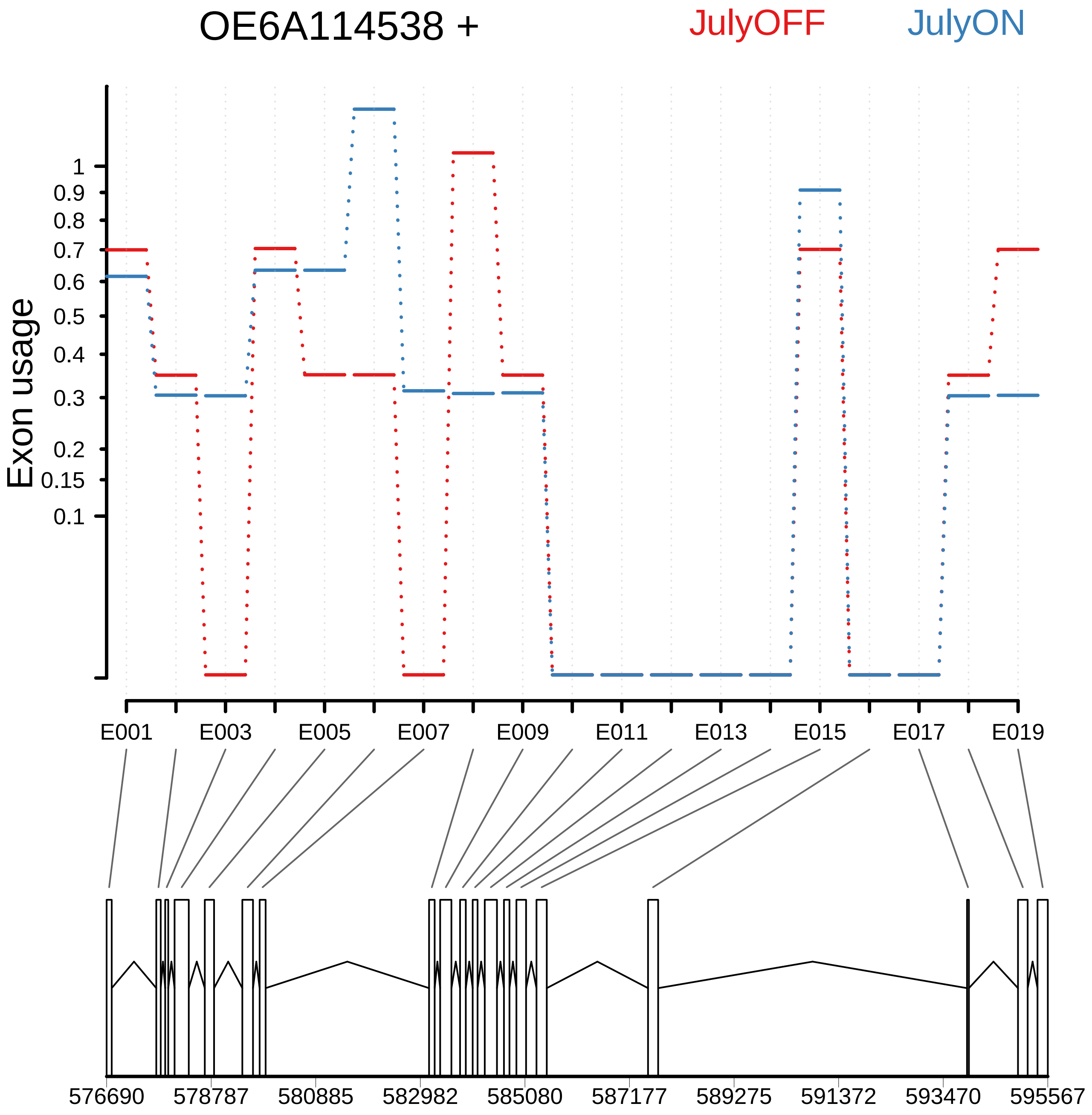

OE6A114538 +
JulyOFF
JulyON
Exon usage
1
0.9
0.8
0.7
0.6
0.5
0.4
0.3
0.2
0.15
0.1
E001
E003
E005
E007
E009
E011
E013
E015
E017
E019
576690
578787
580885
582982
585080
587177
589275
591372
593470
595567

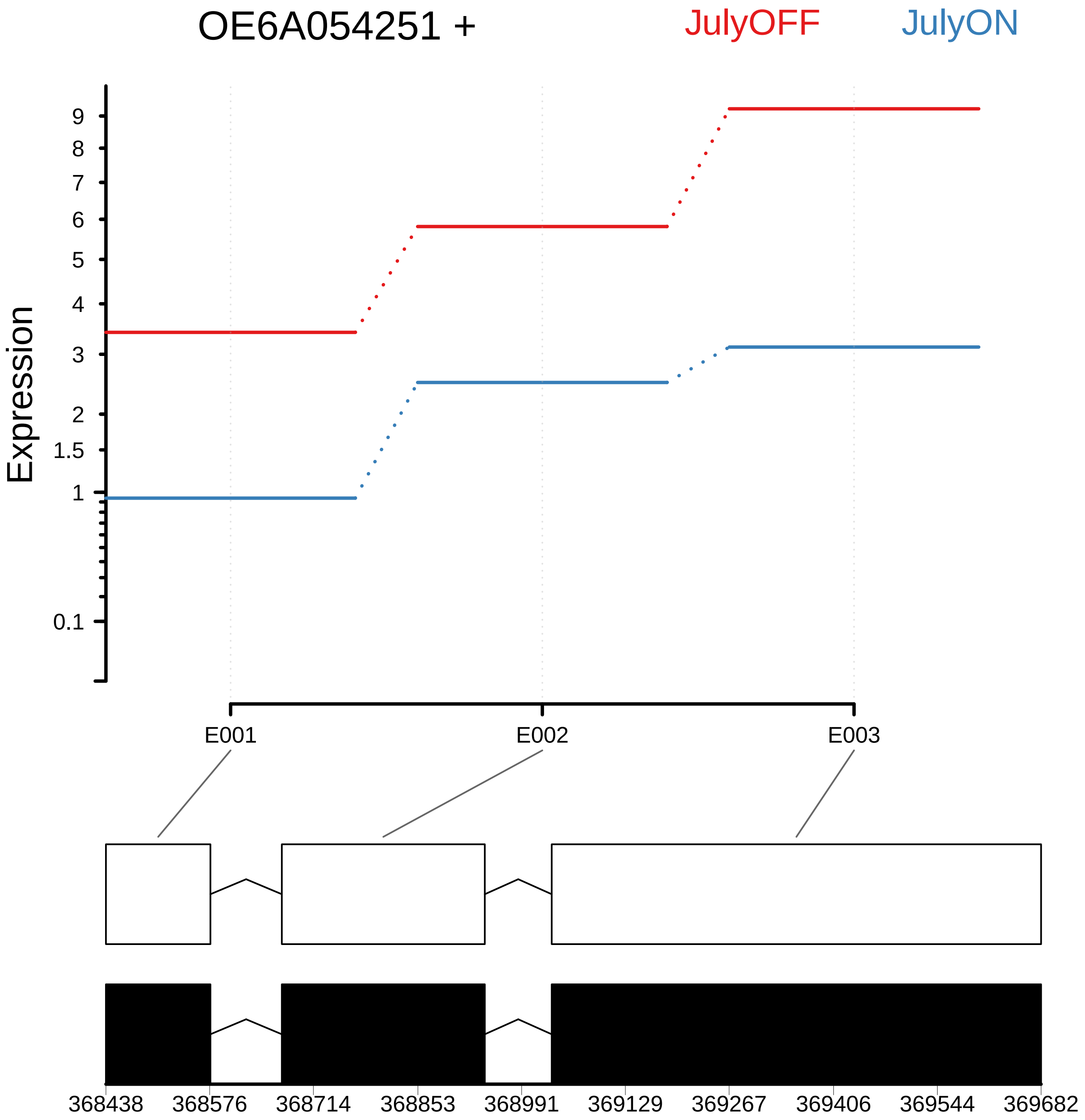
OE6A054251 +
JulyOFF
JulyON
Expression
9
8
7
6
5
4
3
2
1.5
1
0.1
E001
E002
E003
368438
368576
368714
368853
368991
369129
369267
369406
369544
369682

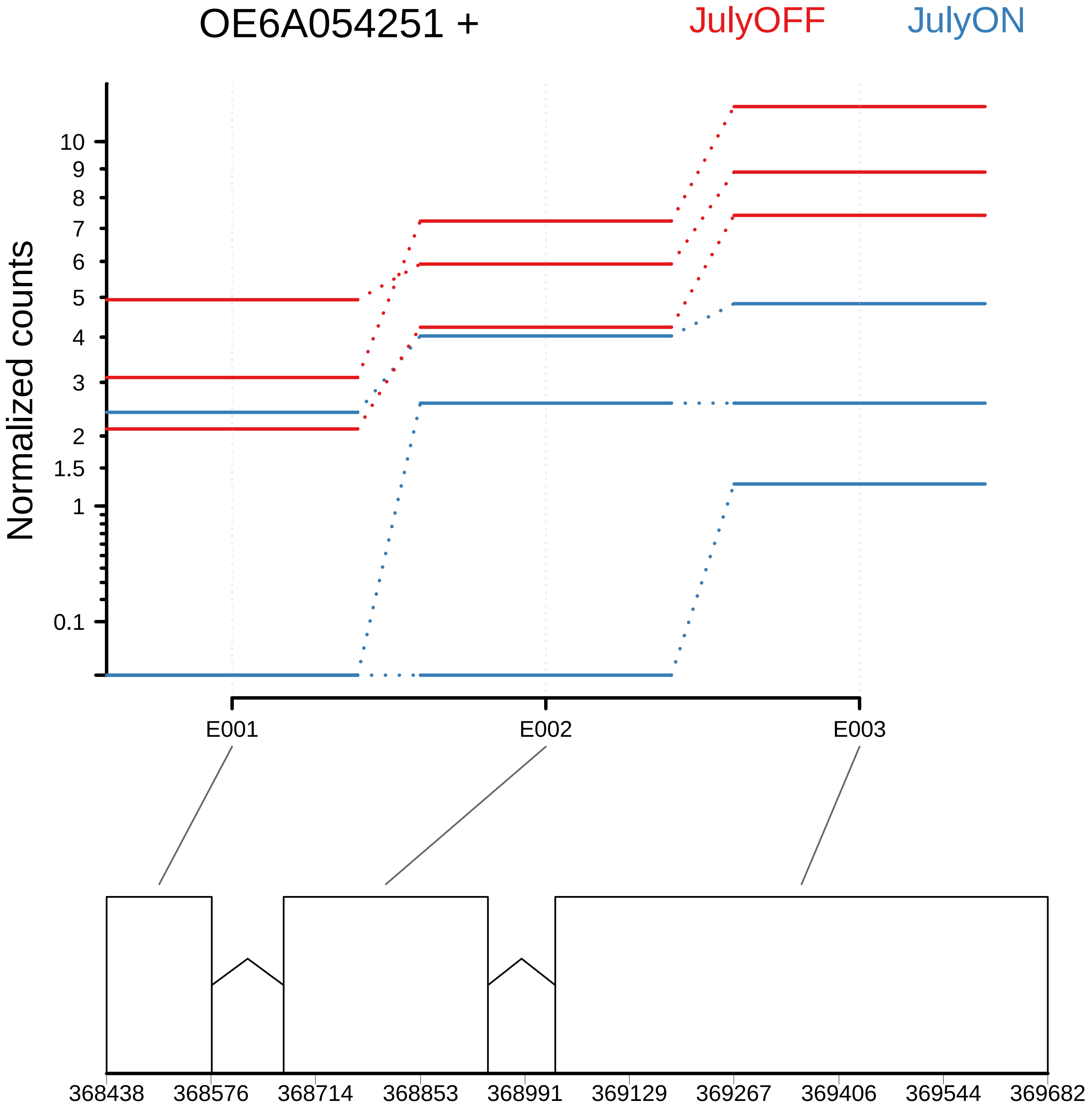
OE6A054251 +
JulyOFF
JulyON
Normalized counts
10
9
8
7
6
5
4
3
2
1.5
1
0.1
E001
E002
E003
368438
368576
368714
368853
368991
369129
369267
369406
369544
369682

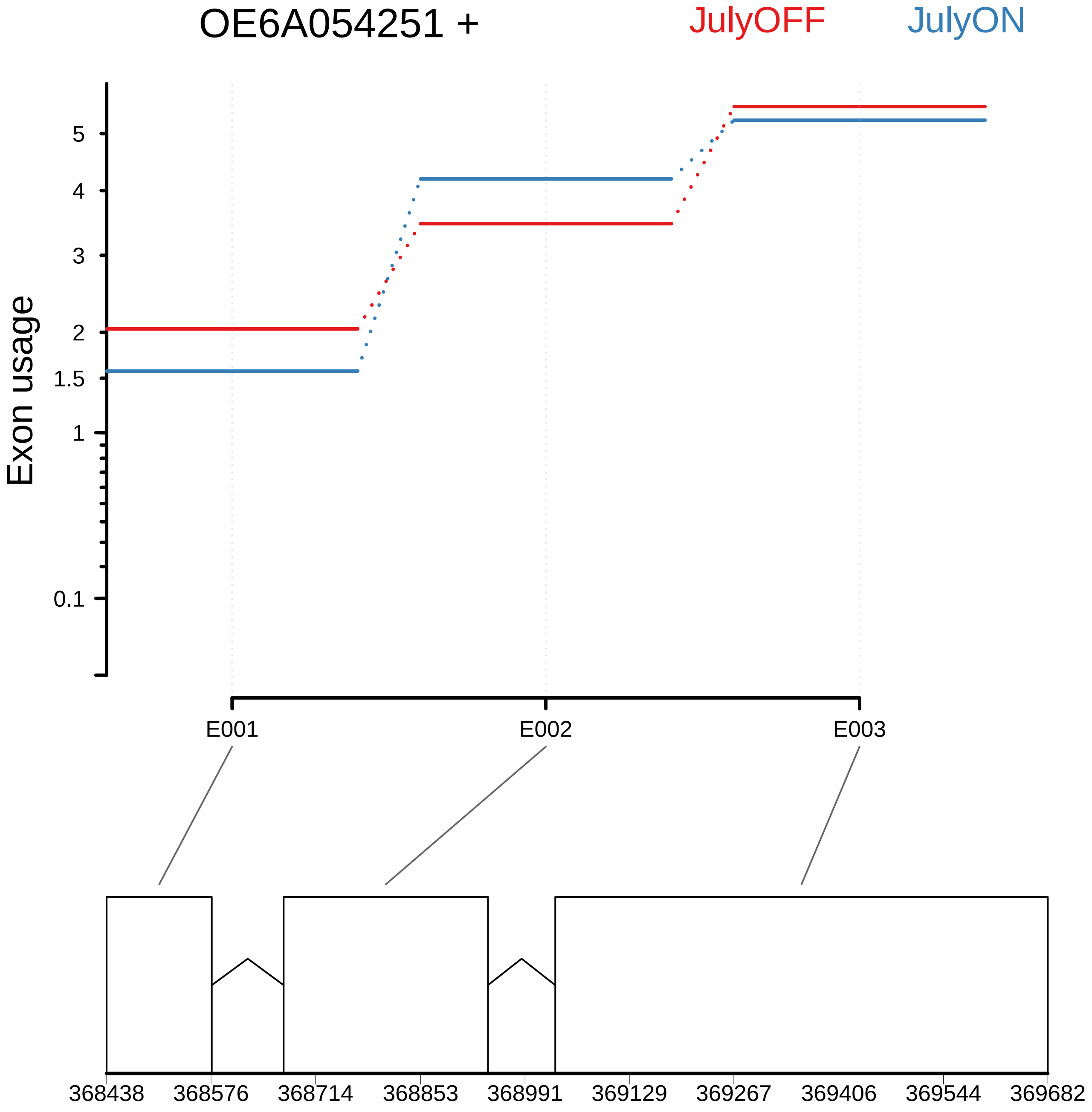
OE6A054251 +
JulyOFF
JulyON
Exon usage
5
4
3
2
1.5
1
0.1
E001
E002
E003
368438
368576
368714
368853
368991
369129
369267
369406
369544
369682

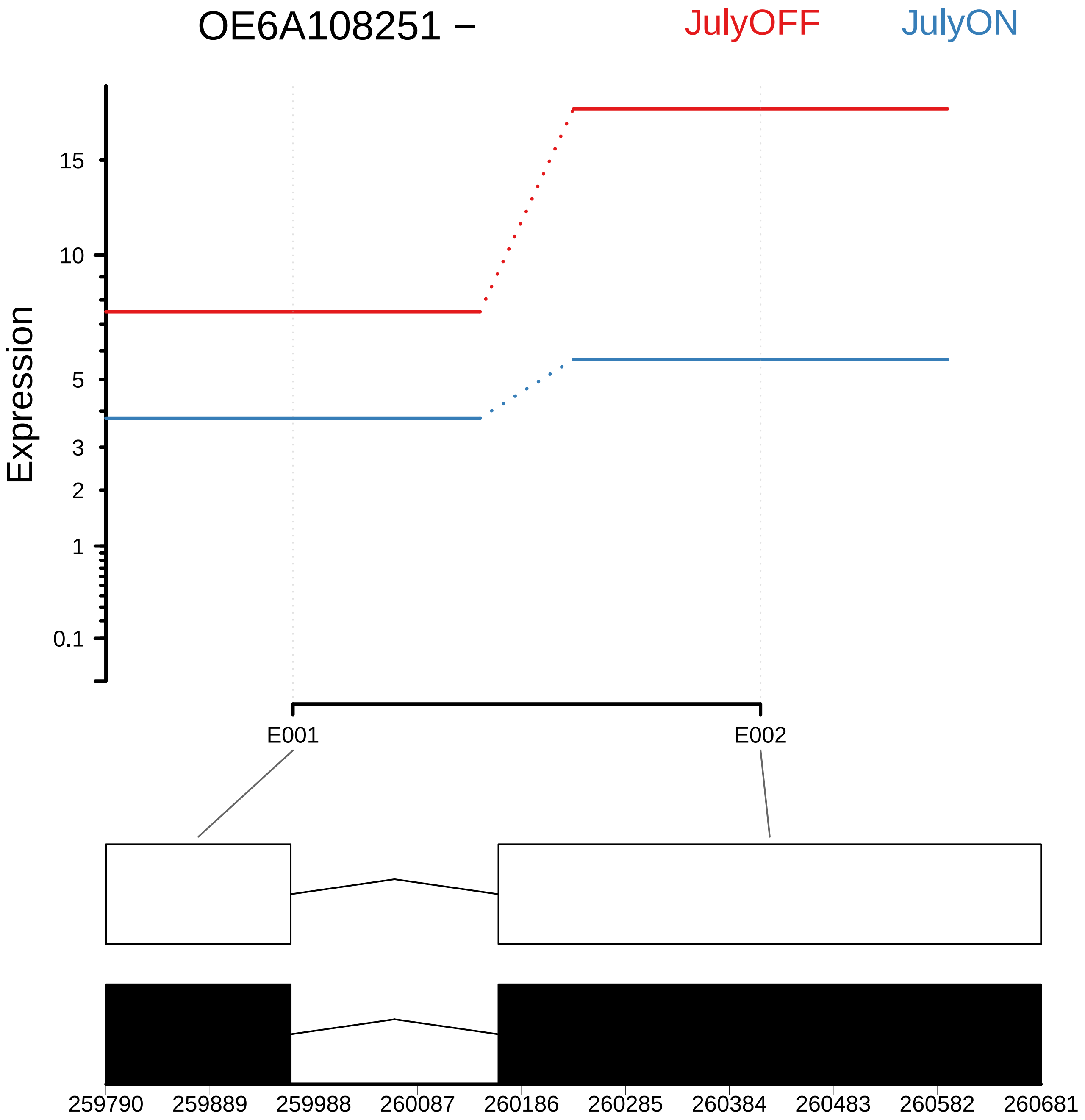

OE6A108251 −
JulyOFF
JulyON
Expression
15
10
5
3
2
1
0.1
E001
E002
259790
259889
259988
260087
260186
260285
260384
260483
260582
260681

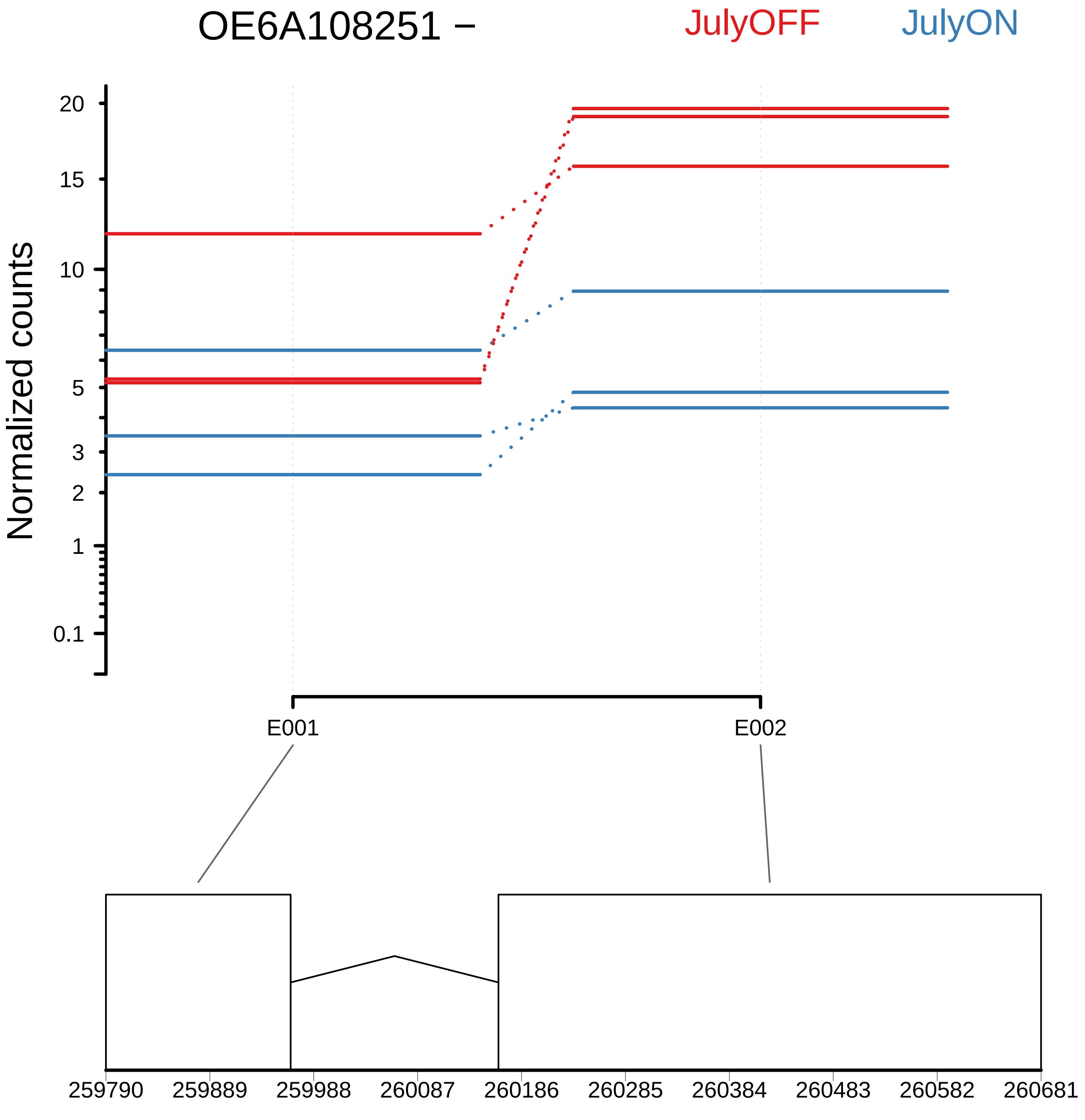

OE6A108251 −
JulyOFF
JulyON
Normalized counts
20
15
10
5
3
2
1
0.1
E001
E002
259790
259889
259988
260087
260186
260285
260384
260483
260582
260681

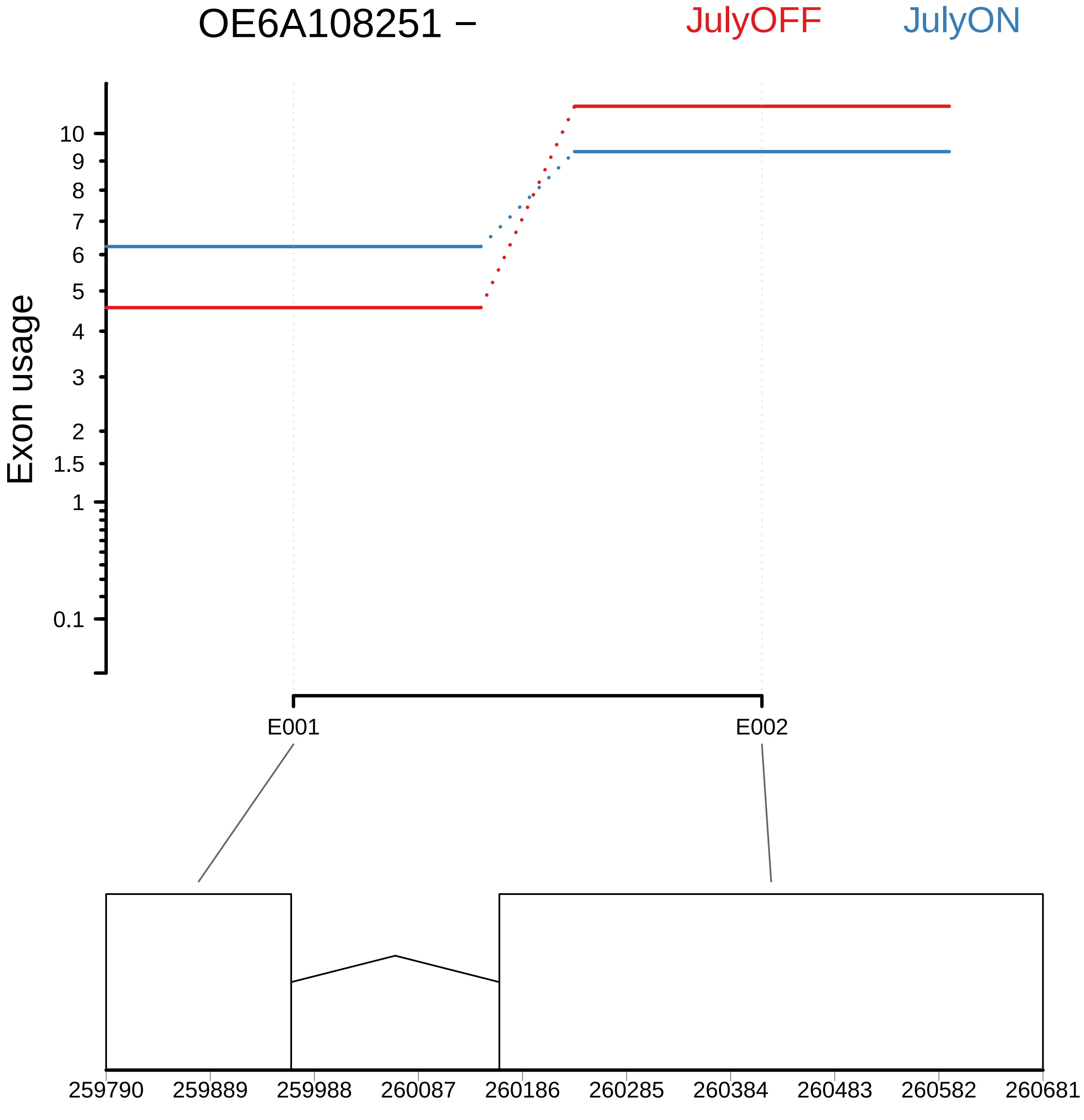
OE6A108251 −
JulyOFF
JulyON
Exon usage
10
9
8
7
6
5
4
3
2
1.5
1
0.1
E001
E002
259790
259889
259988
260087
260186
260285
260384
260483
260582
260681

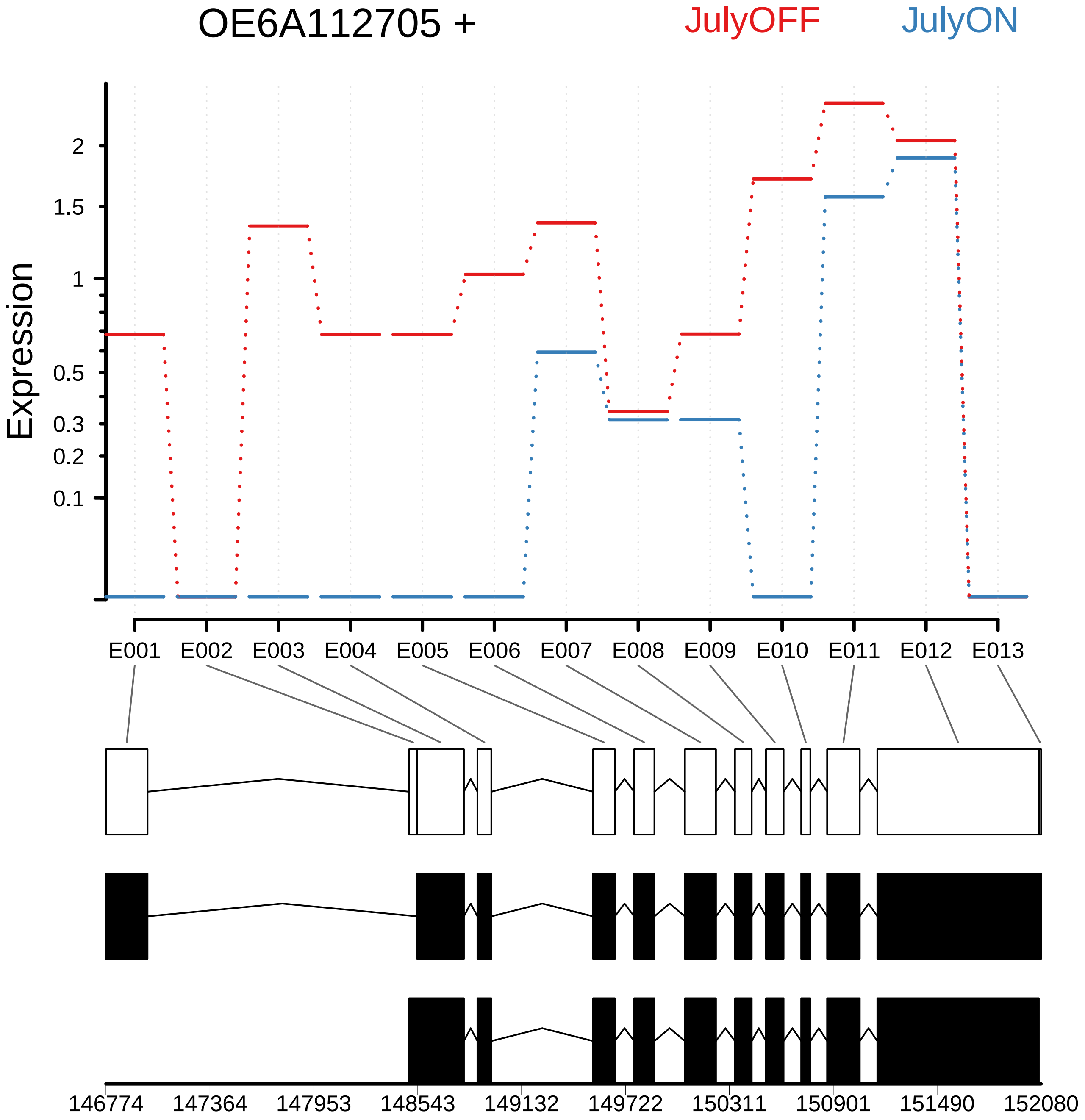

OE6A112705 +
JulyOFF
JulyON
Expression
2
1.5
1
0.5
0.3
0.2
0.1
E001
E002
E003
E004
E005
E006
E007
E008
E009
E010
E011
E012
E013
146774
147364
147953
148543
149132
149722
150311
150901
151490
152080

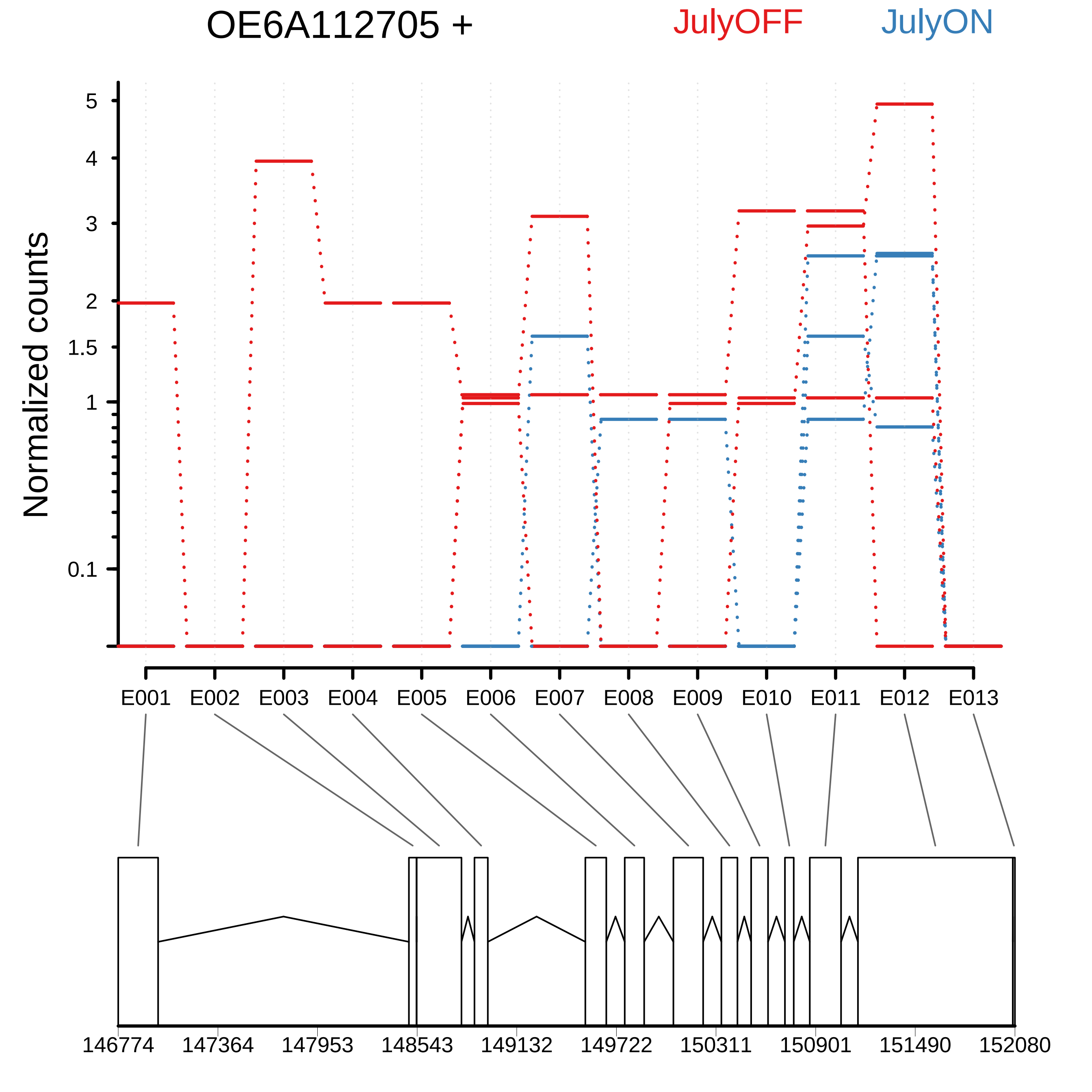

OE6A112705 +
JulyOFF
JulyON
Normalized counts
5
4
3
2
1.5
1
0.1
E001
E002
E003
E004
E005
E006
E007
E008
E009
E010
E011
E012
E013
146774
147364
147953
148543
149132
149722
150311
150901
151490
152080

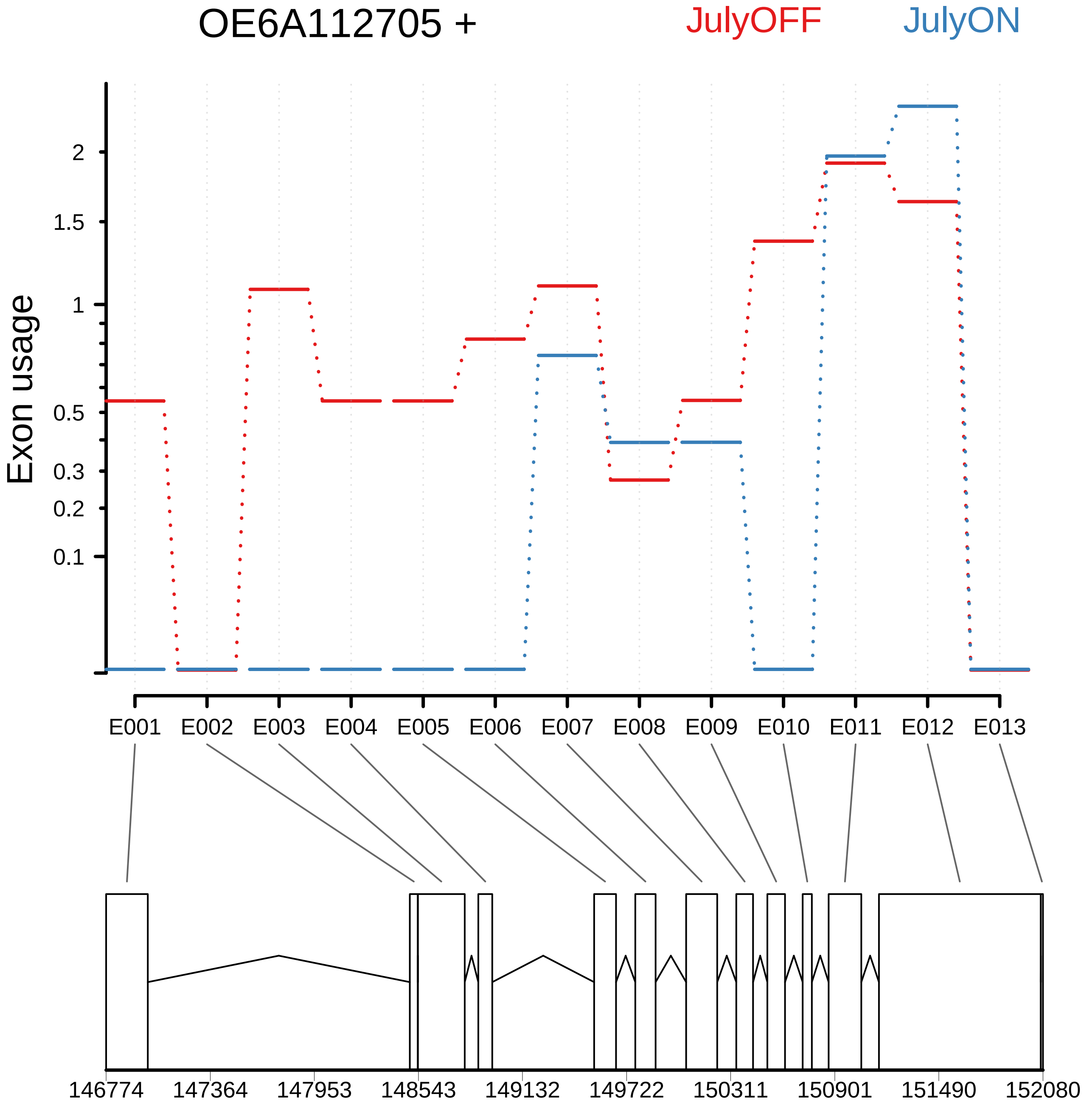
OE6A112705 +
JulyOFF
JulyON
Exon usage
2
1.5
1
0.5
0.3
0.2
0.1
E001
E002
E003
E004
E005
E006
E007
E008
E009
E010
E011
E012
E013
146774
147364
147953
148543
149132
149722
150311
150901
151490
152080

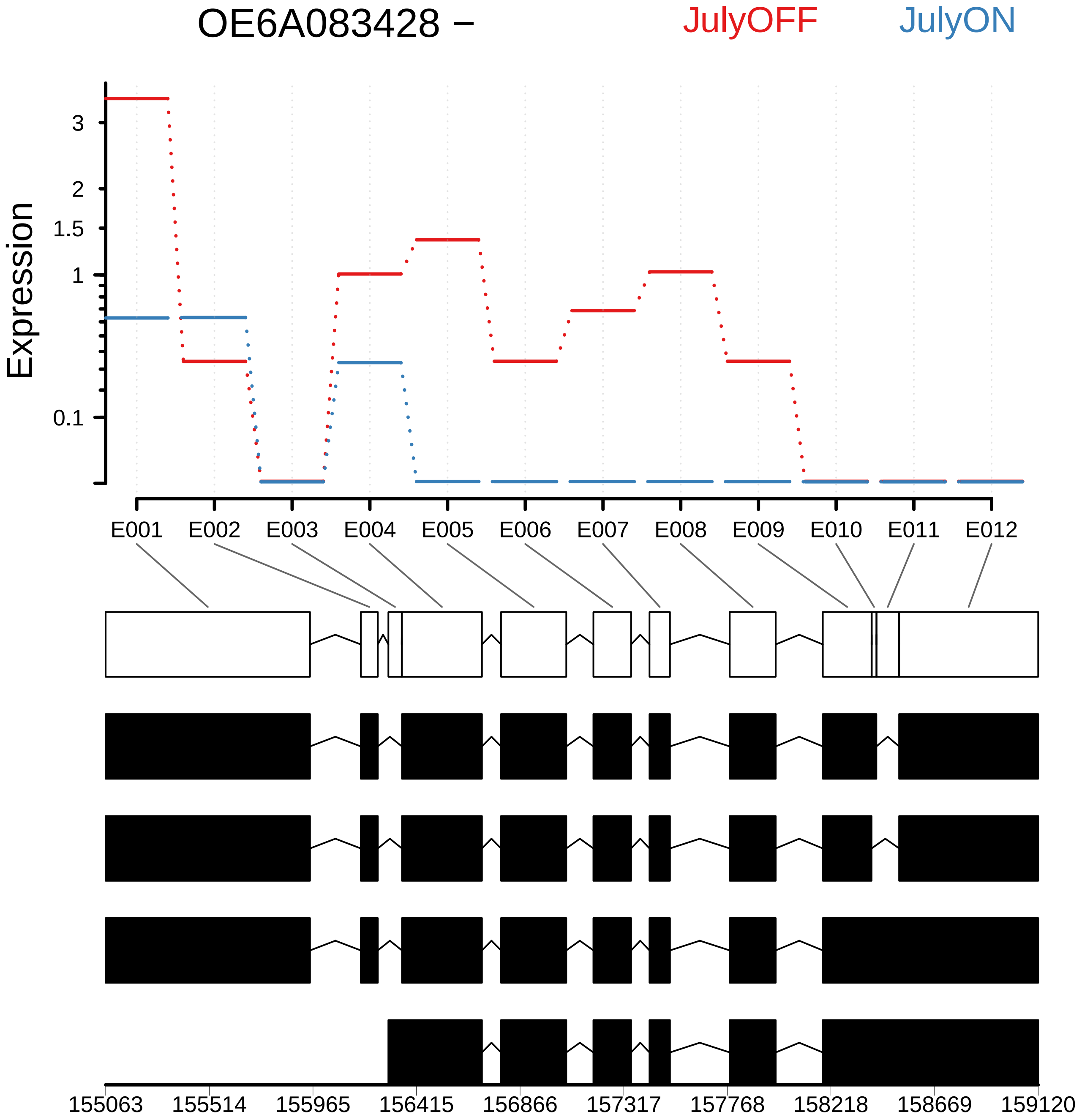

OE6A083428 –
JulyOFF
JulyON
Expression
3
2
1.5
1
0.1
E001
E002
E003
E004
E005
E006
E007
E008
E009
E010
E011
E012
155063
155514
155965
156415
156866
157317
157768
158218
158669
159120

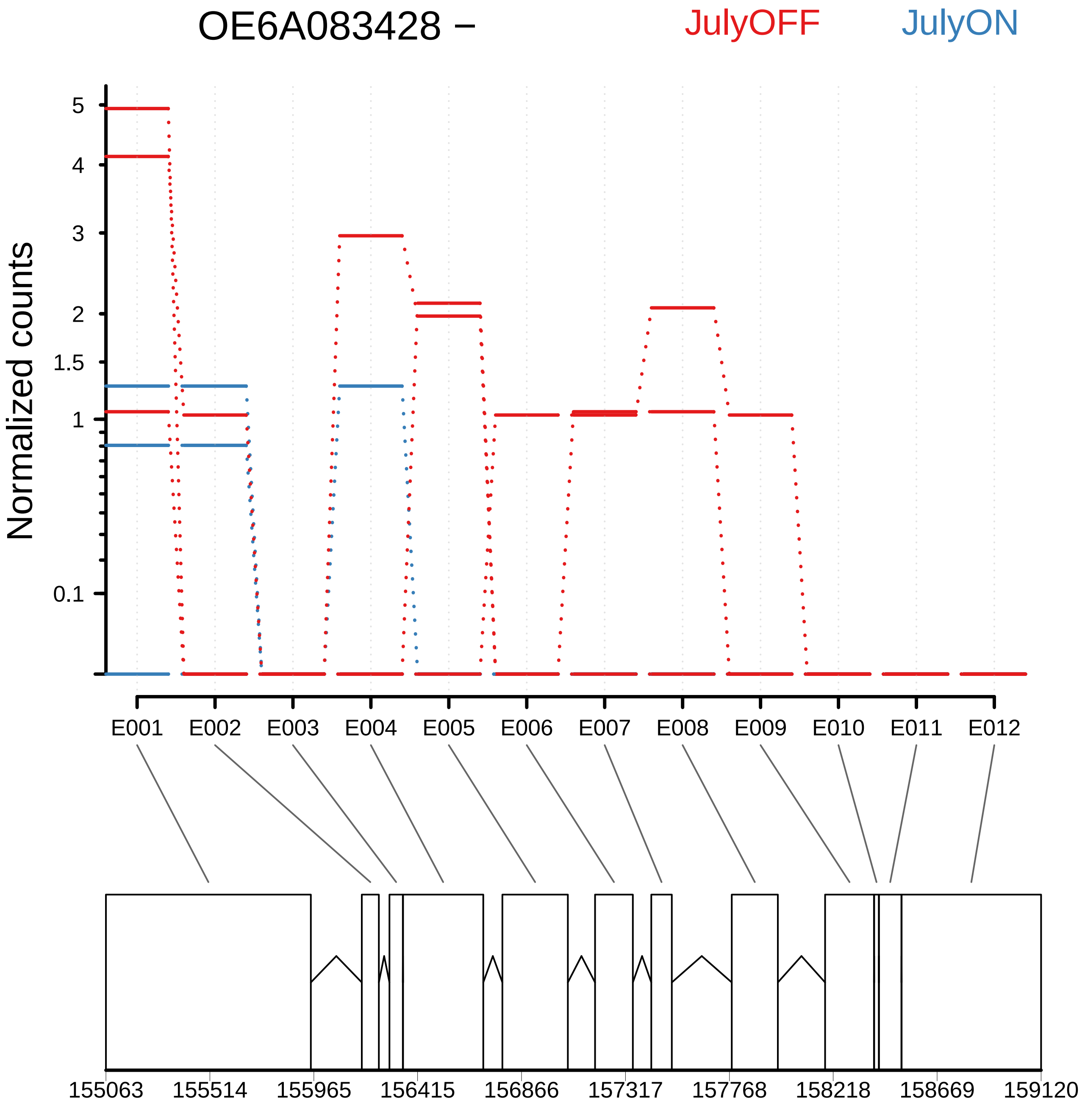

OE6A083428 −
JulyOFF
JulyON
Normalized counts
5
4
3
2
1.5
1
0.1
E001
E002
E003
E004
E005
E006
E007
E008
E009
E010
E011
E012
155063
155514
155965
156415
156866
157317
157768
158218
158669
159120

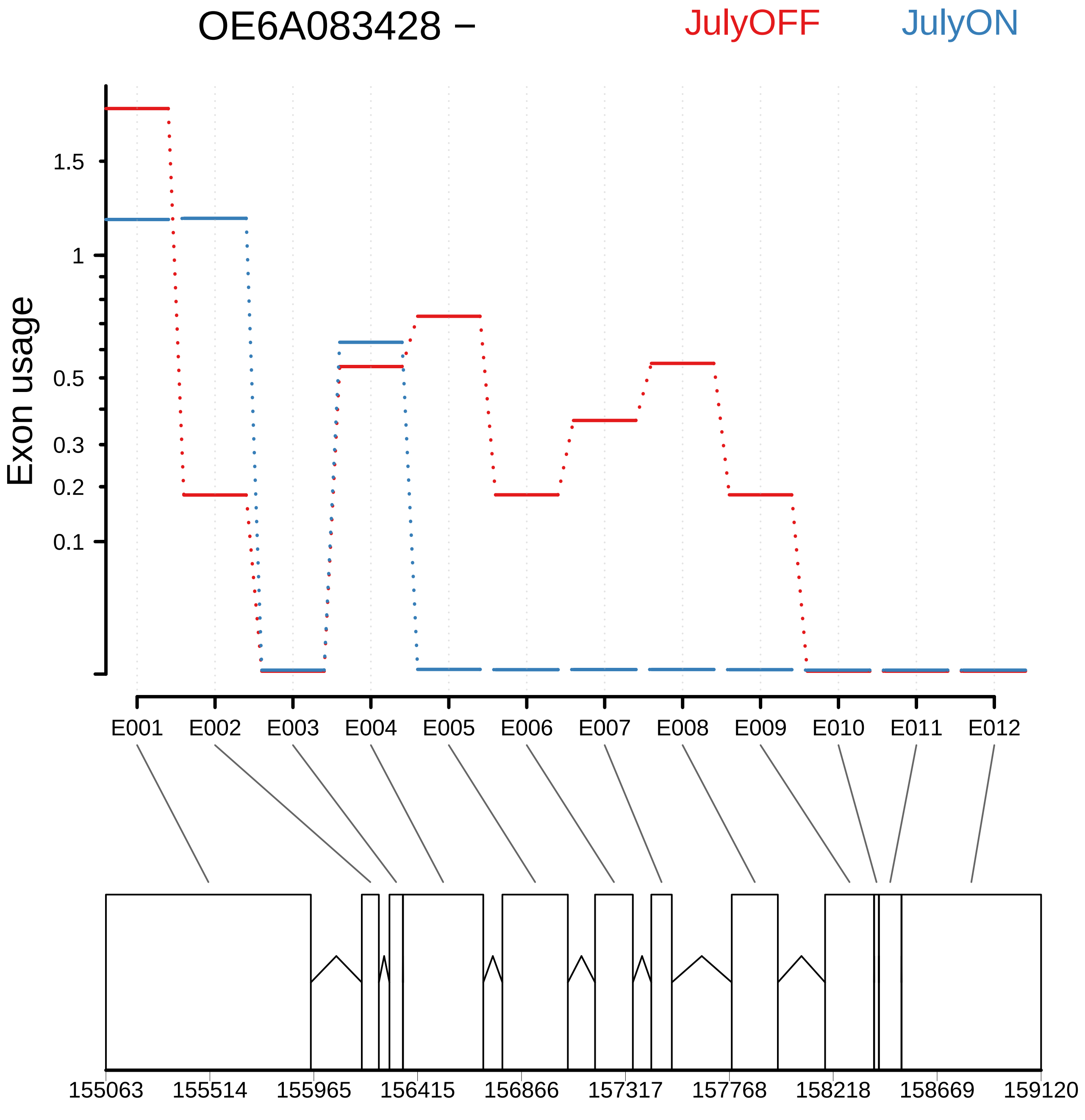

OE6A083428 −
JulyOFF
JulyON
Exon usage
1.5
1
0.5
0.3
0.2
0.1
E001
E002
E003
E004
E005
E006
E007
E008
E009
E010
E011
E012
155063
155514
155965
156415
156866
157317
157768
158218
158669
159120

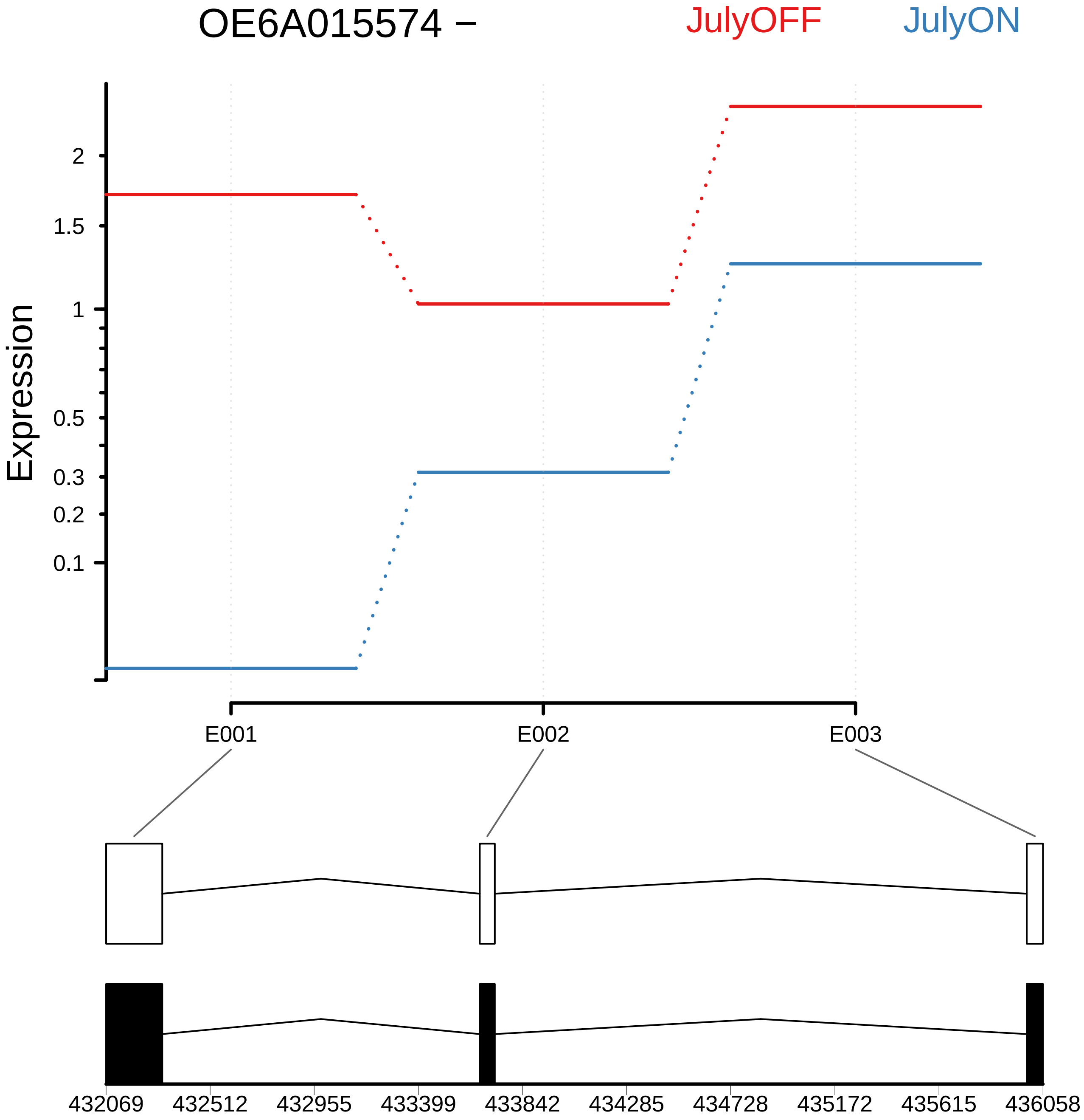
OE6A015574 −
JulyOFF
JulyON
Expression
2
1.5
1
0.5
0.3
0.2
0.1
E001
E002
E003
432069
432512
432955
433399
433842
434285
434728
435172
435615
436058

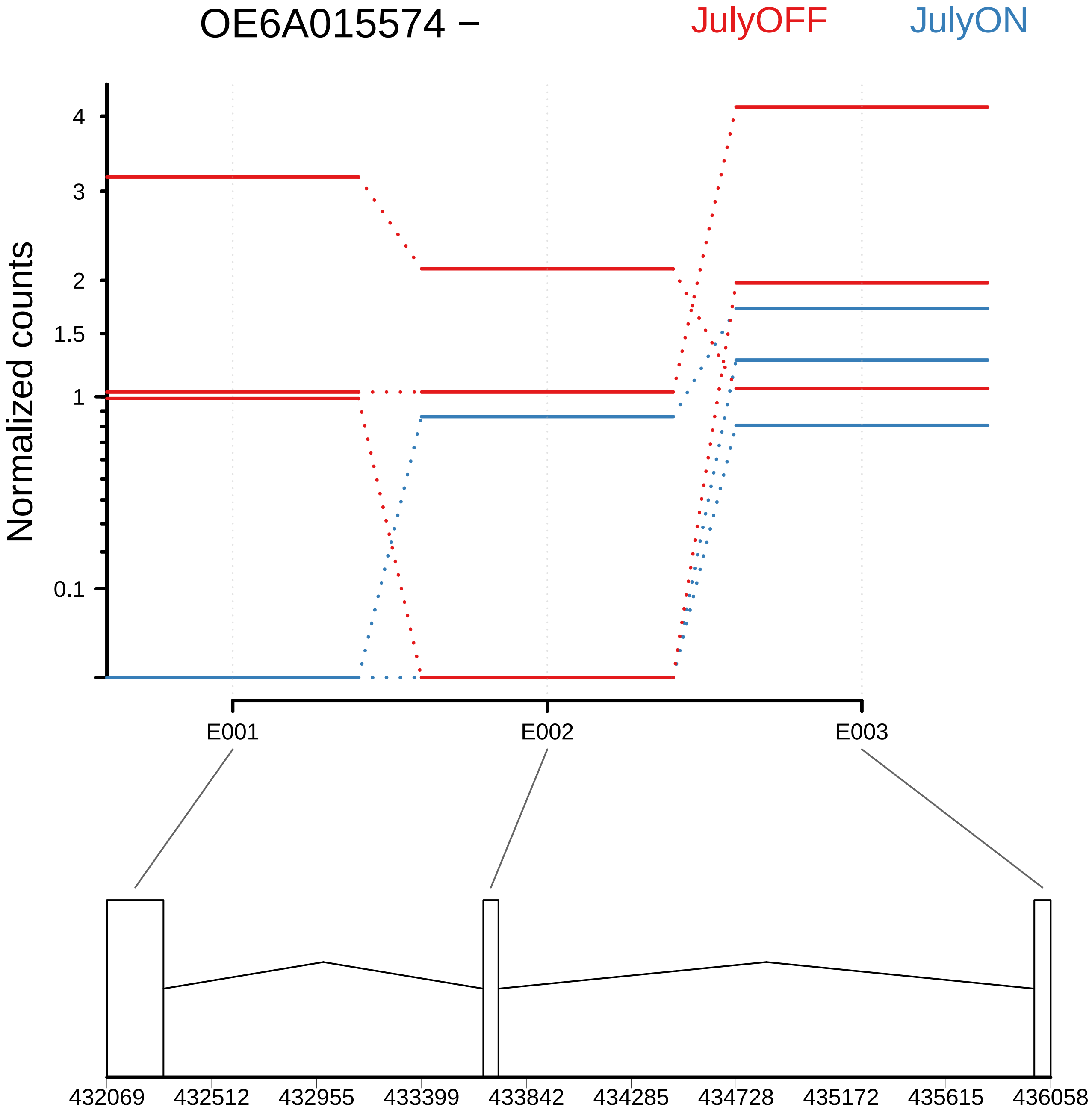

OE6A015574 −
JulyOFF
JulyON
Normalized counts
4
3
2
1.5
1
0.1
E001
E002
E003
432069
432512
432955
433399
433842
434285
434728
435172
435615
436058

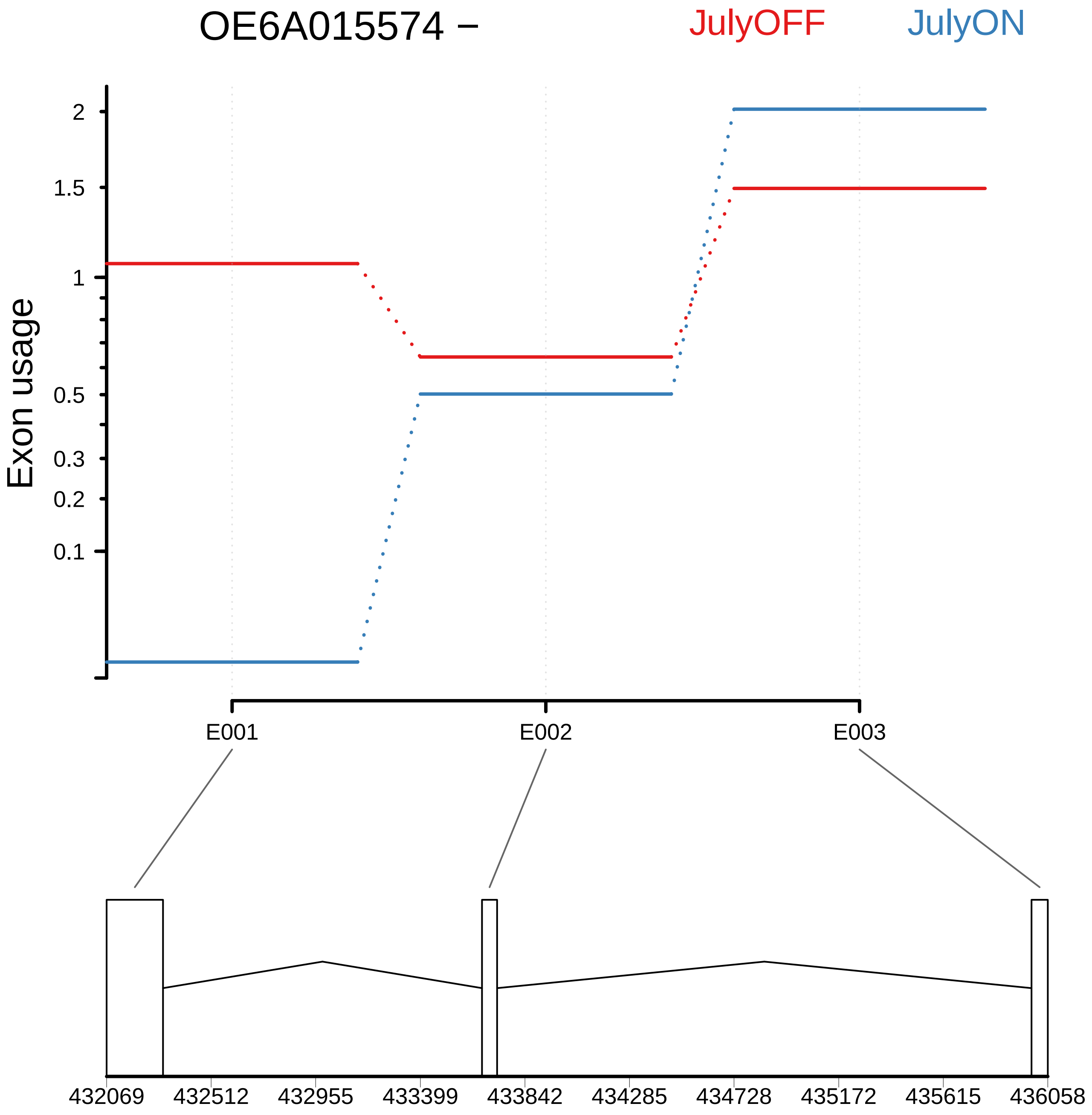
OE6A015574 −
JulyOFF
JulyON
Exon usage
2
1.5
1
0.5
0.3
0.2
0.1
E001
E002
E003
432069
432512
432955
433399
433842
434285
434728
435172
435615
436058

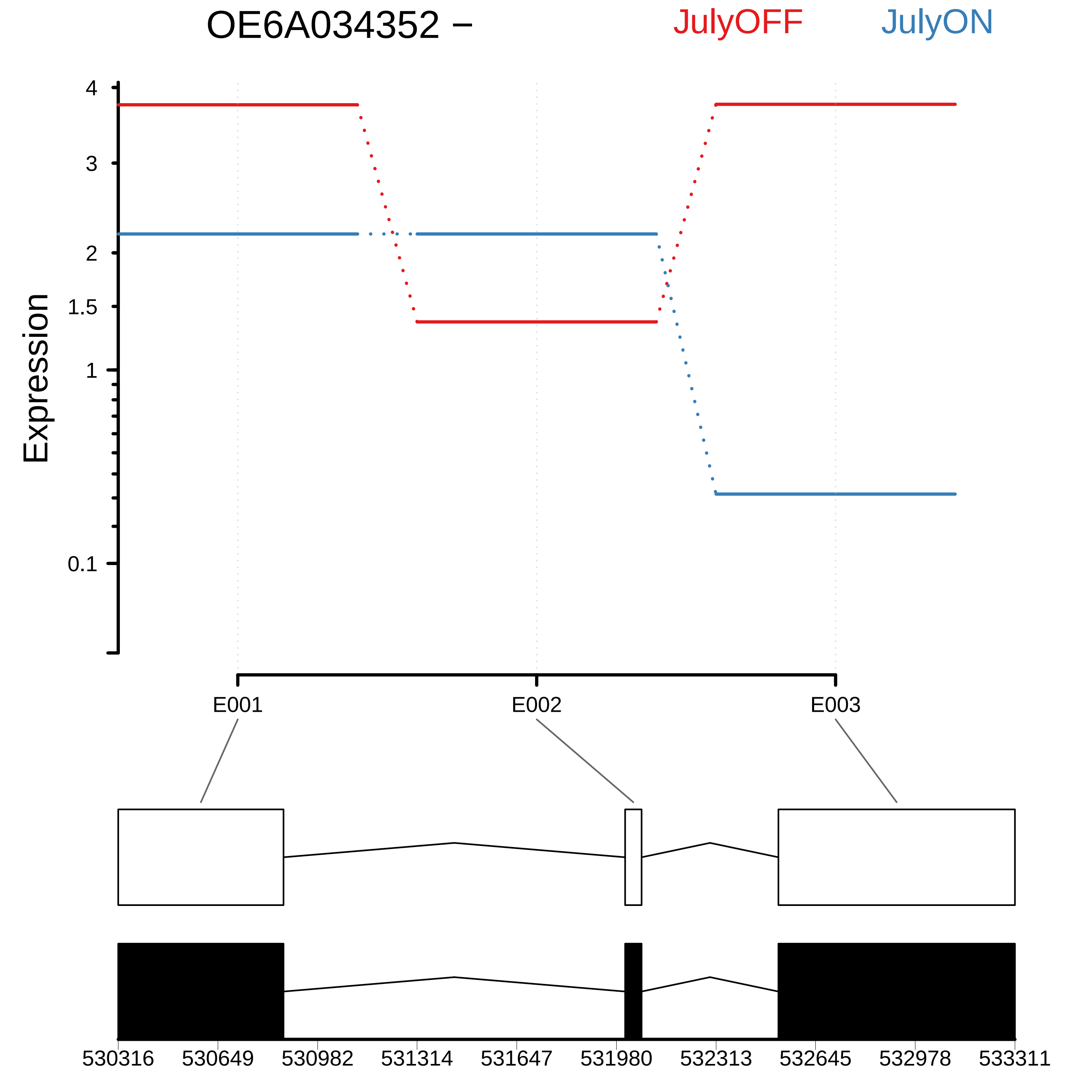

OE6A034352 −
JulyOFF
JulyON
Expression
4
3
2
1.5
1
0.1
E001
E002
E003
530316
530649
530982
531314
531647
531980
532313
532645
532978
533311

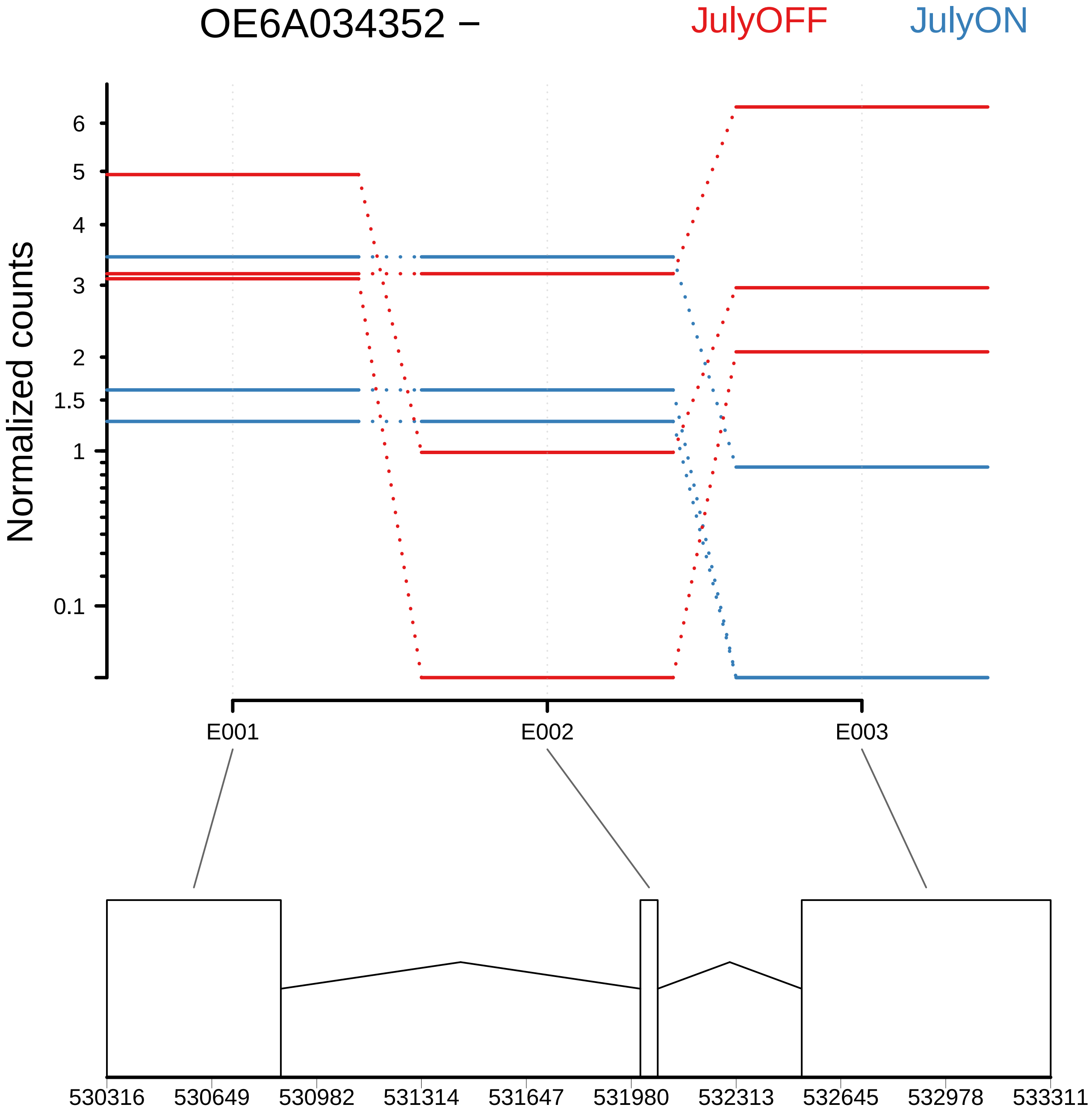

OE6A034352 −
JulyOFF
JulyON
Normalized counts
6
5
4
3
2
1.5
1
0.1
E001
E002
E003
530316
530649
530982
531314
531647
531980
532313
532645
532978
533311

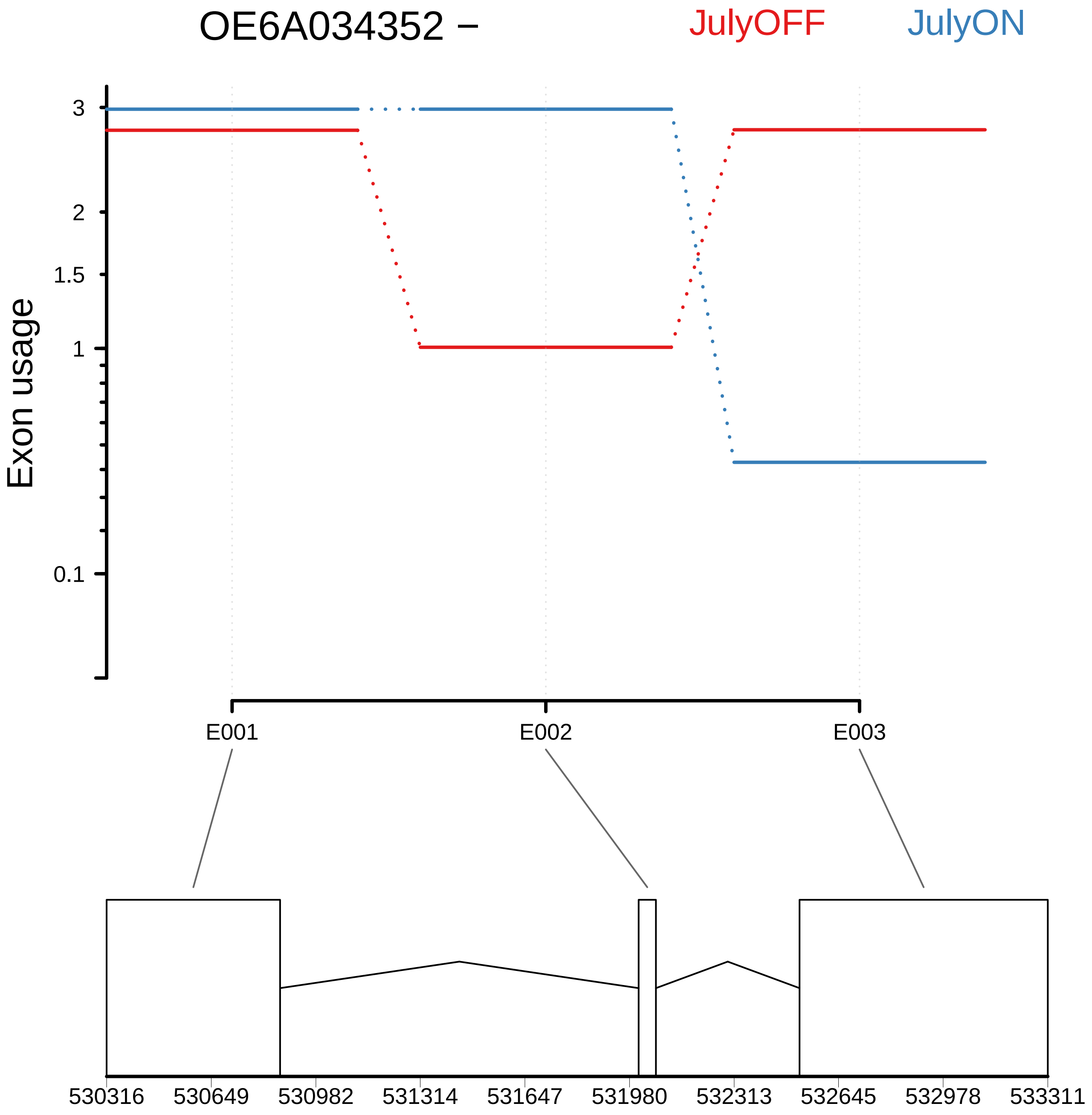

OE6A034352 −
JulyOFF
JulyON
Exon usage
3
2
1.5
1
0.1
E001
E002
E003
530316
530649
530982
531314
531647
531980
532313
532645
532978
533311

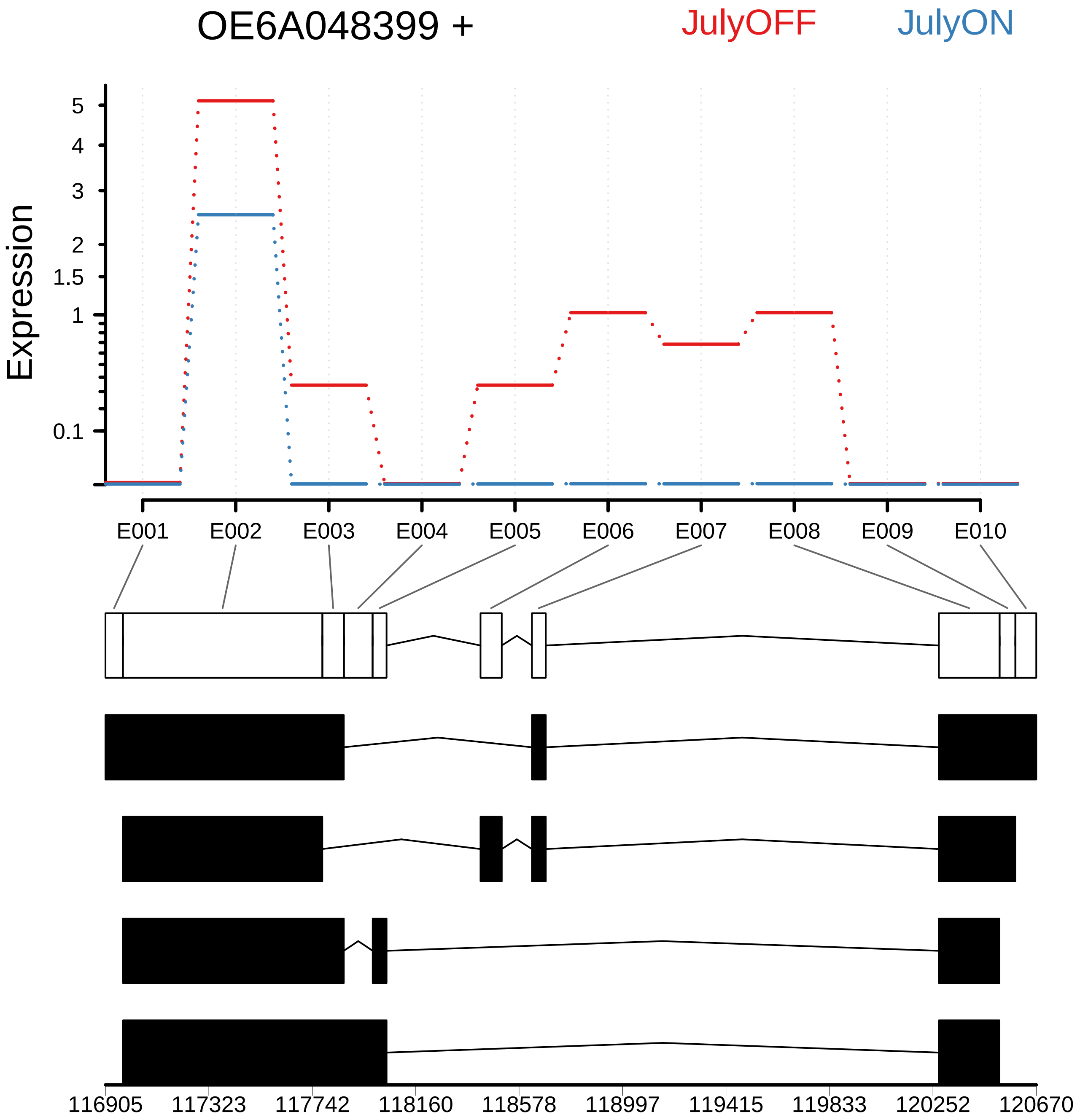

OE6A048399 +
JulyOFF
JulyON
Expression
5
4
3
2
1.5
1
0.1
E001
E002
E003
E004
E005
E006
E007
E008
E009
E010
116905
117323
117742
118160
118578
118997
119415
119833
120252
120670

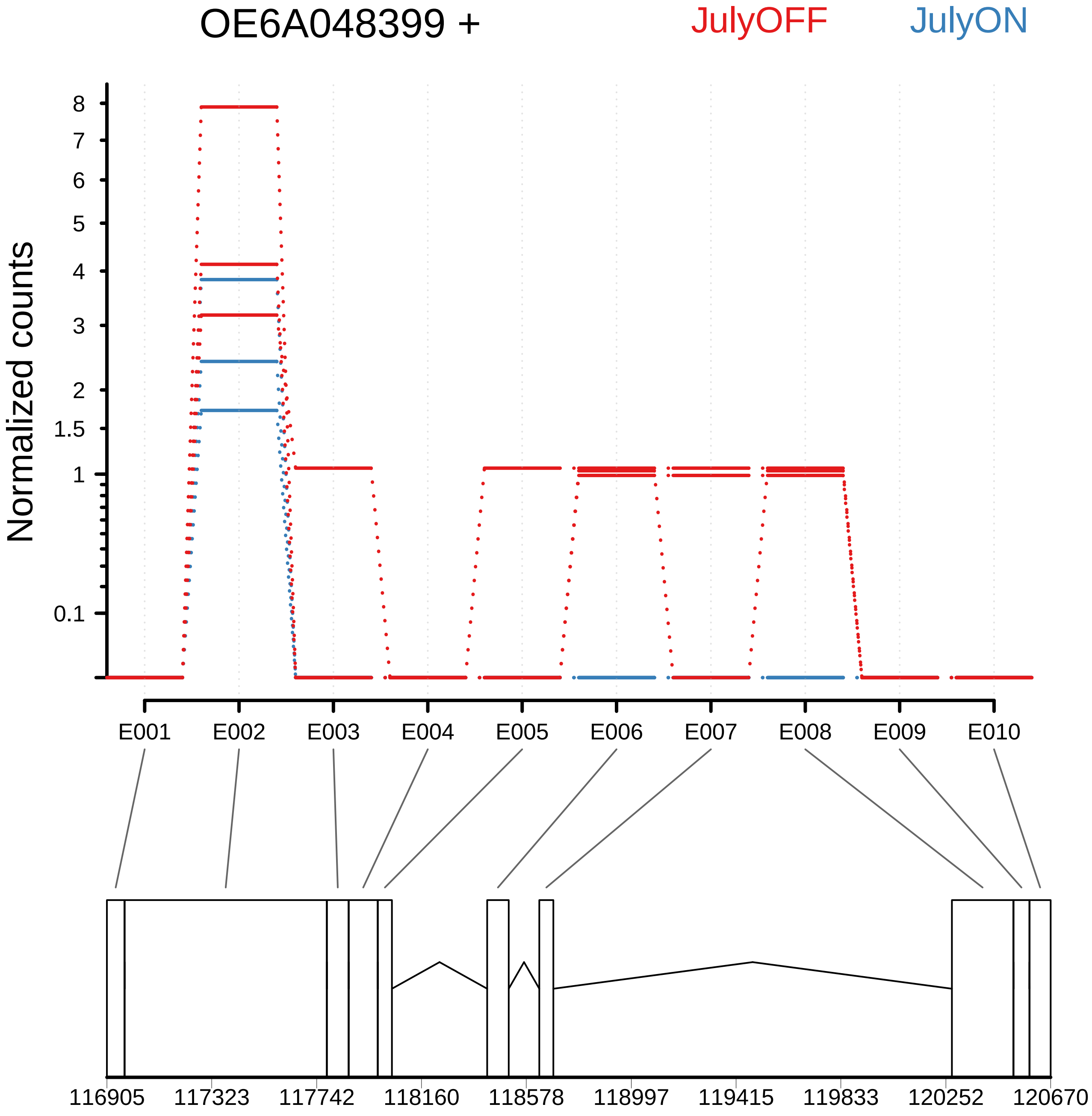

OE6A048399 +
JulyOFF
JulyON
Normalized counts
8
7
6
5
4
3
2
1.5
1
0.1
E001
E002
E003
E004
E005
E006
E007
E008
E009
E010
116905
117323
117742
118160
118578
118997
119415
119833
120252
120670

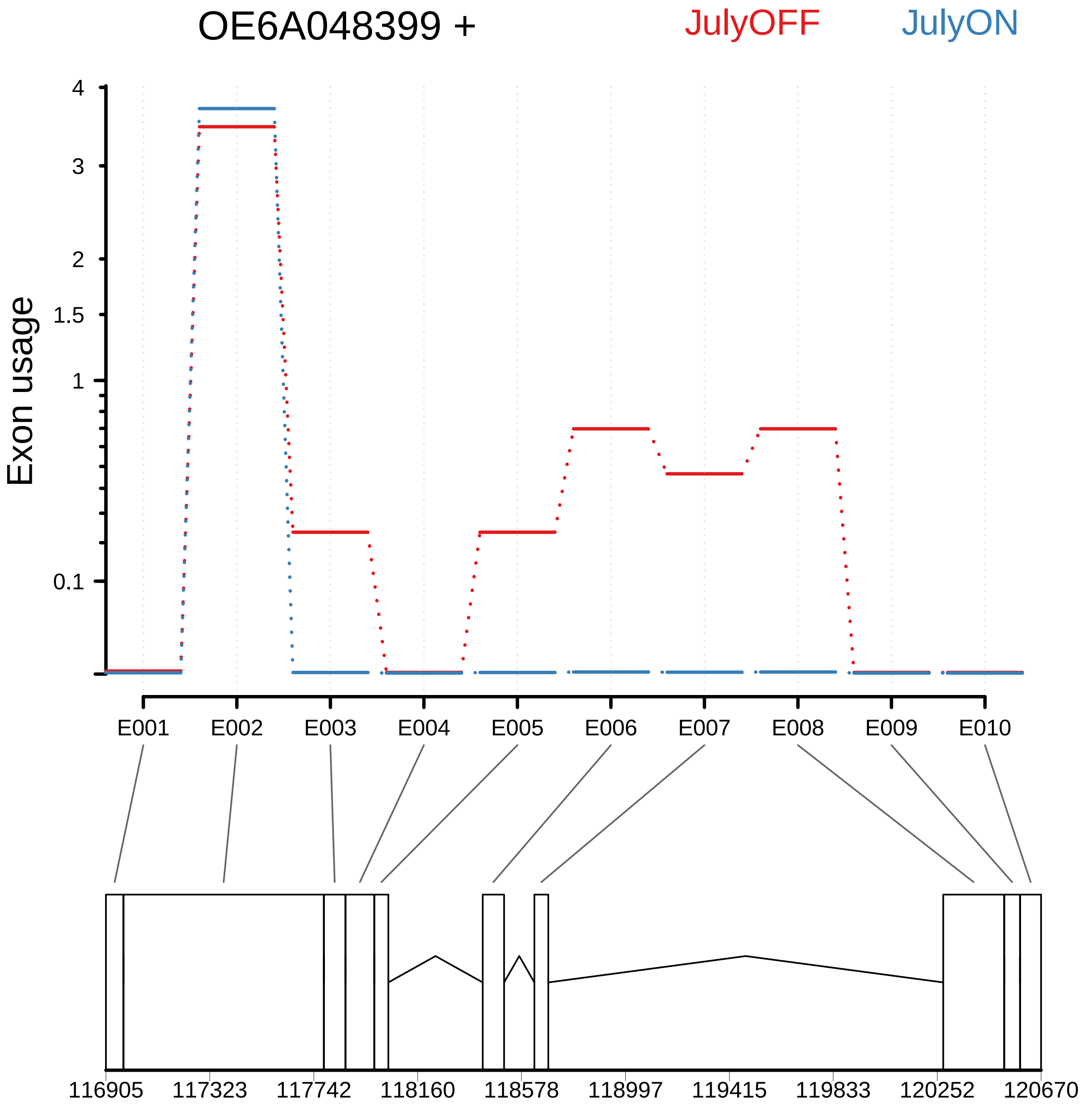
OE6A048399 +
JulyOFF
JulyON
Exon usage
4
3
2
1.5
1
0.1
E001
E002
E003
E004
E005
E006
E007
E008
E009
E010
116905
117323
117742
118160
118578
118997
119415
119833
120252
120670

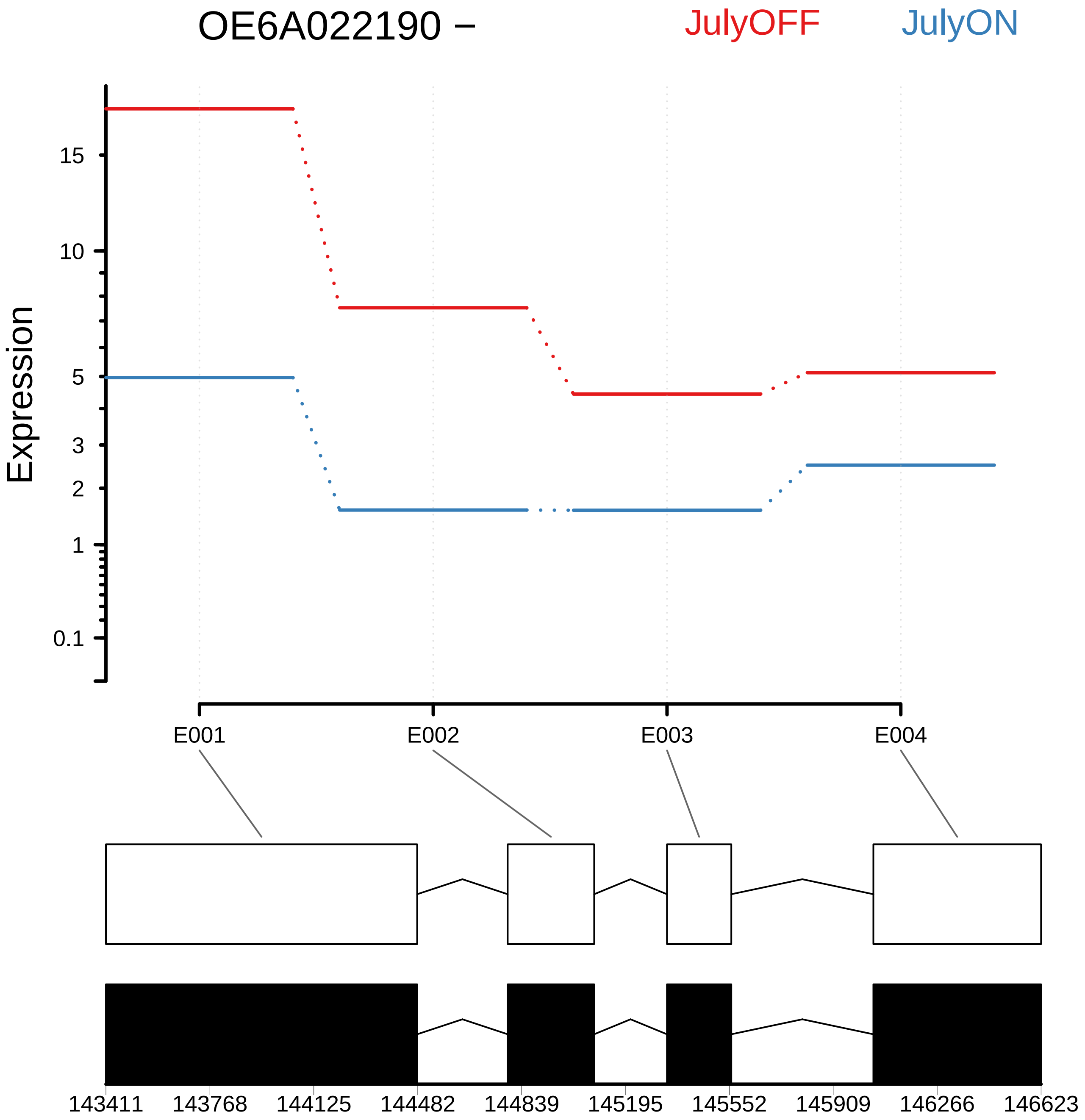
OE6A022190 −
JulyOFF
JulyON
Expression
15
10
5
3
2
1
0.1
E001
E002
E003
E004
143411
143768
144125
144482
144839
145195
145552
145909
146266
146623

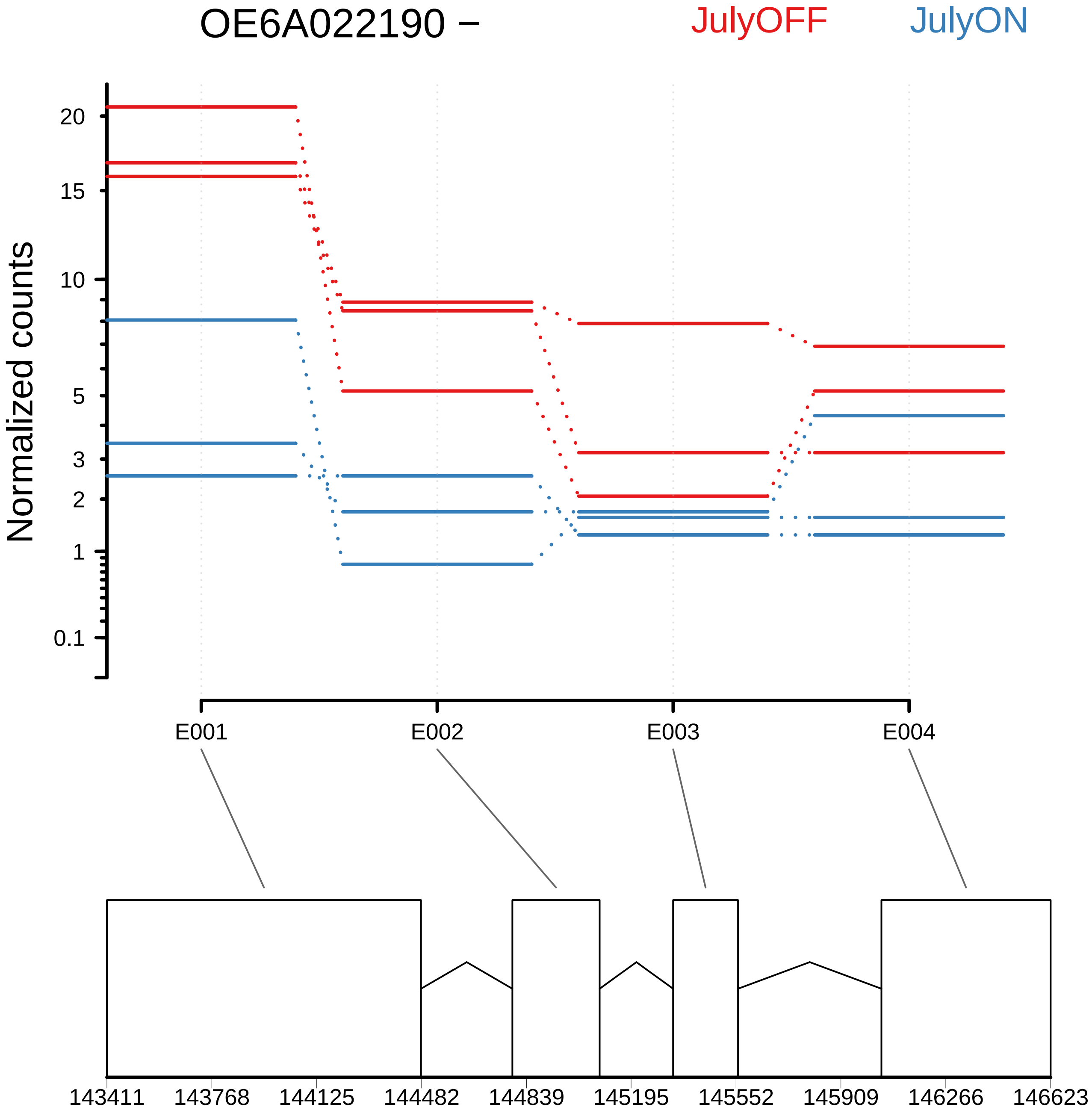

OE6A022190 −
JulyOFF
JulyON
Normalized counts
20
15
10
5
3
2
1
0.1
E001
E002
E003
E004
143411
143768
144125
144482
144839
145195
145552
145909
146266
146623

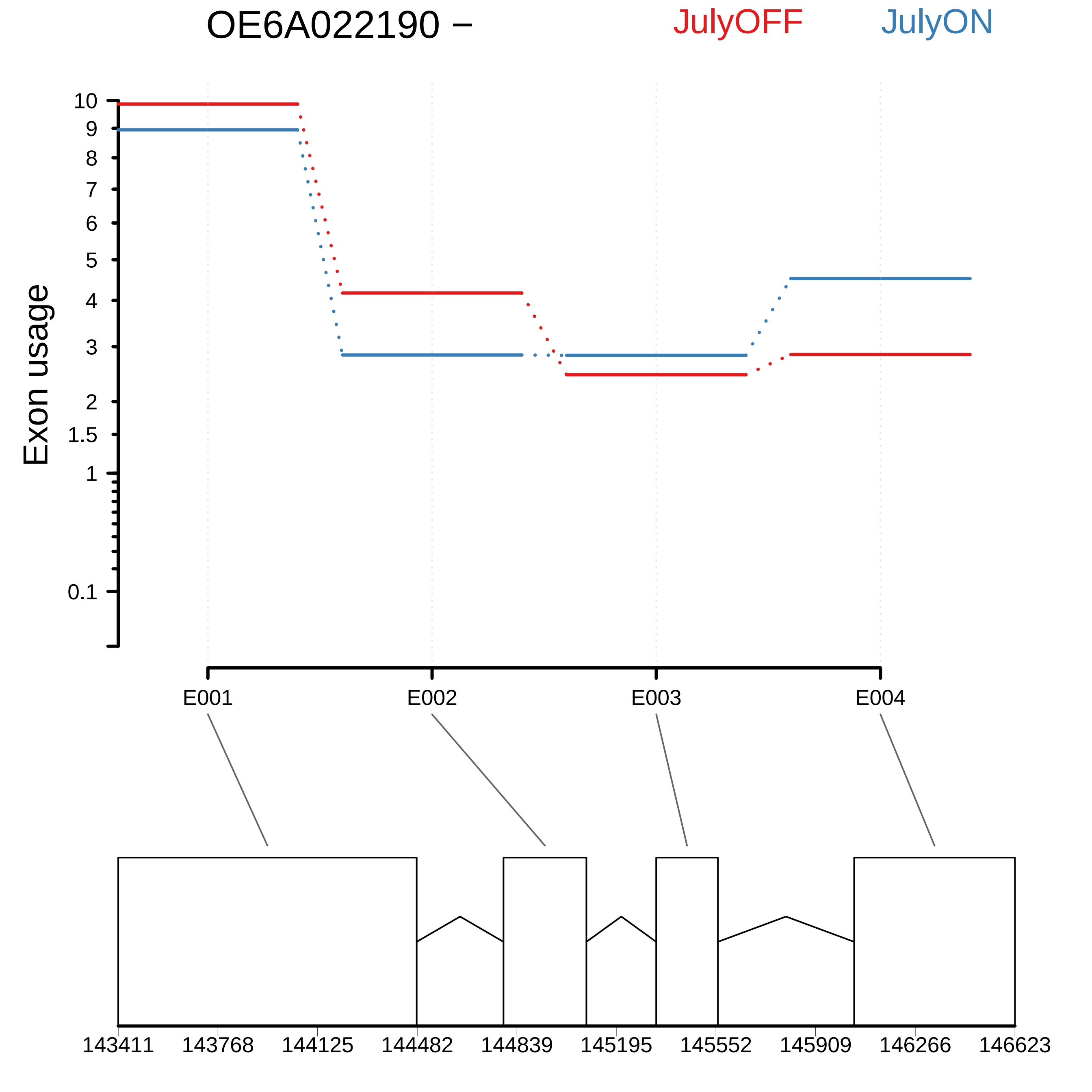

OE6A022190 −
JulyOFF
JulyON
Exon usage
10
9
8
7
6
5
4
3
2
1.5
1
0.1
E001
E002
E003
E004
143411
143768
144125
144482
144839
145195
145552
145909
146266
146623

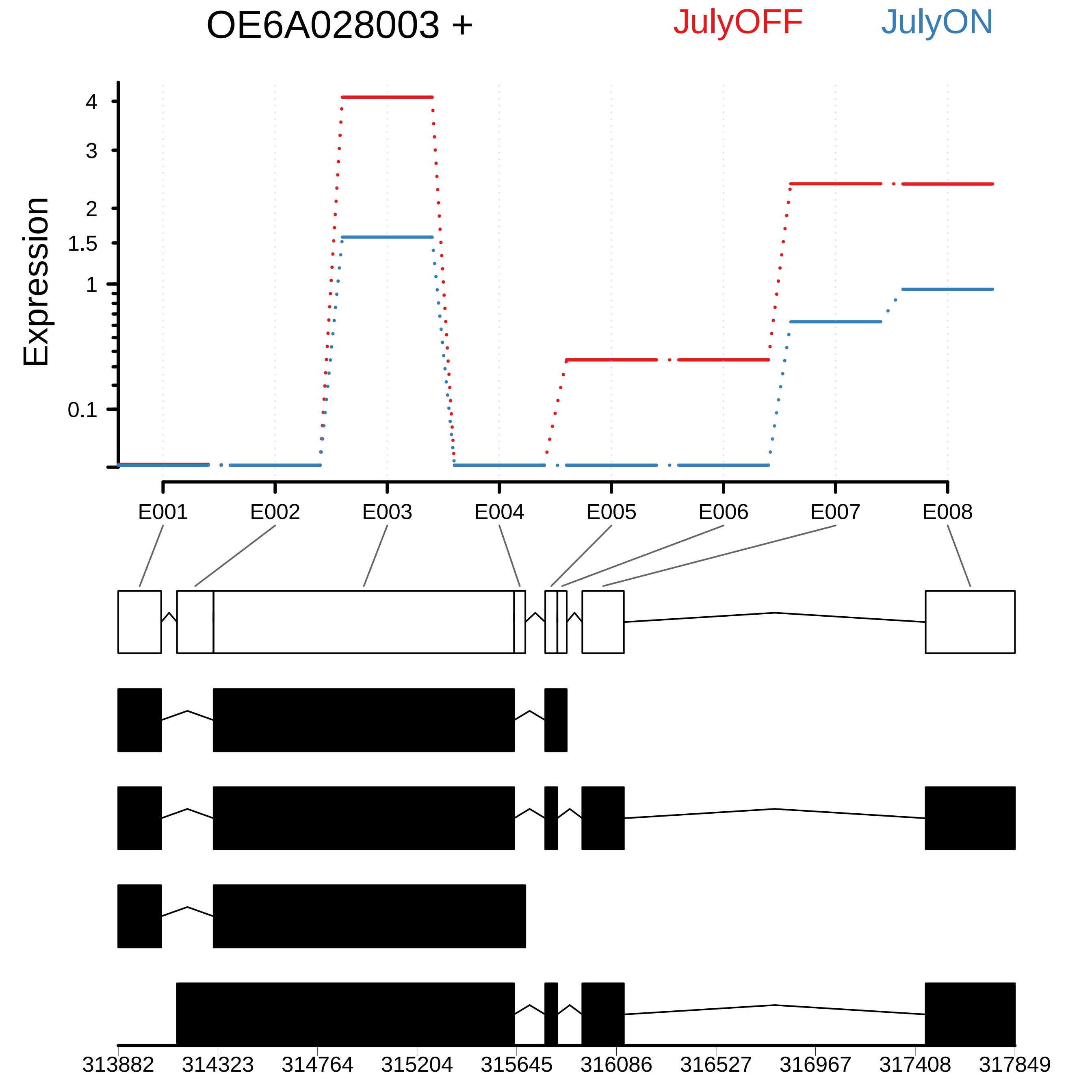
OE6A028003 +
JulyOFF
JulyON
Expression
4
3
2
1.5
1
0.1
E001
E002
E003
E004
E005
E006
E007
E008
313882
314323
314764
315204
315645
316086
316527
316967
317408
317849

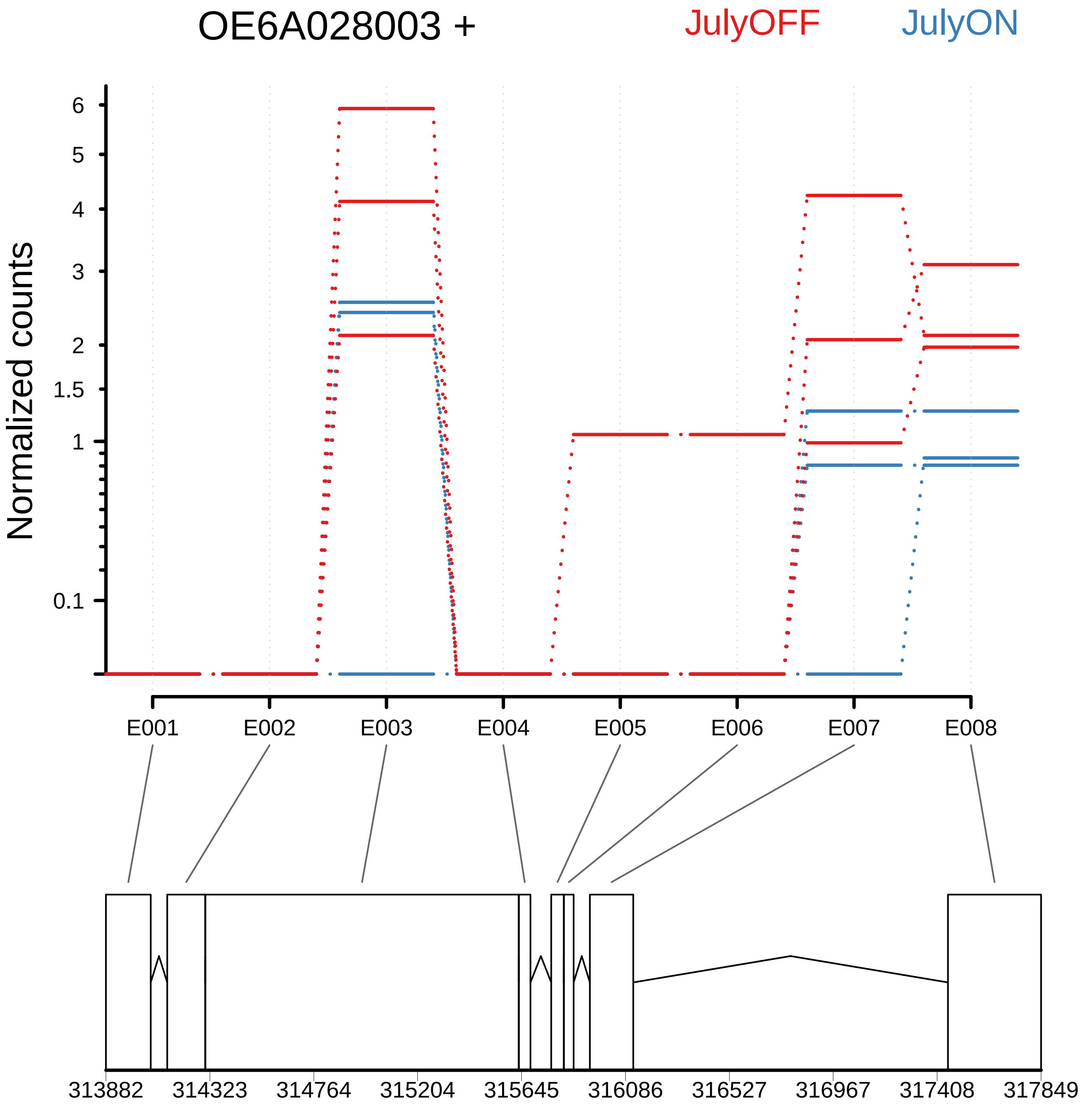

OE6A028003 +
JulyOFF
JulyON
Normalized counts
6
5
4
3
2
1.5
1
0.1
E001
E002
E003
E004
E005
E006
E007
E008
313882
314323
314764
315204
315645
316086
316527
316967
317408
317849

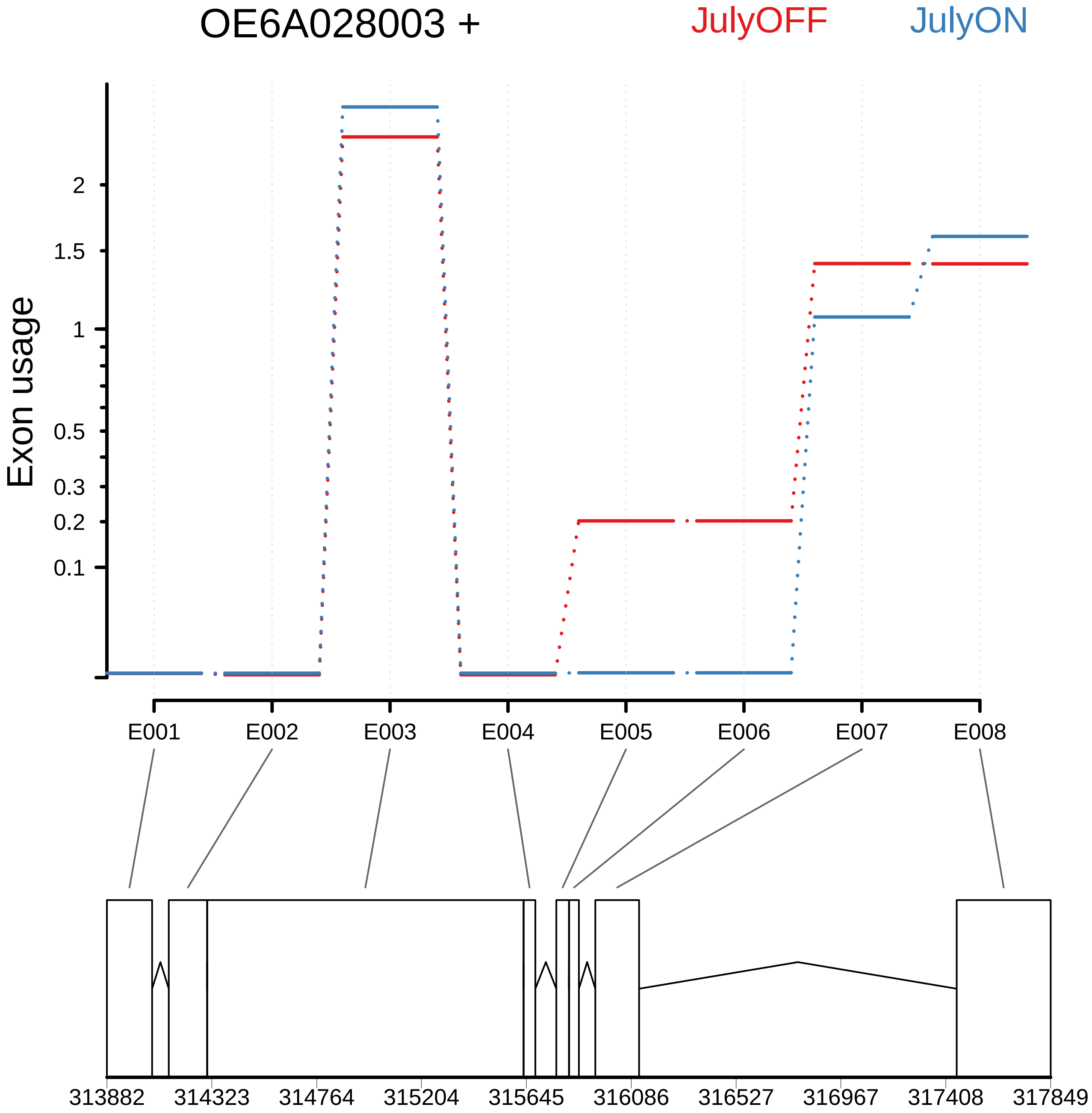

OE6A028003 +
JulyOFF
JulyON
Exon usage
2
1.5
1
0.5
0.3
0.2
0.1
E001
E002
E003
E004
E005
E006
E007
E008
313882
314323
314764
315204
315645
316086
316527
316967
317408
317849

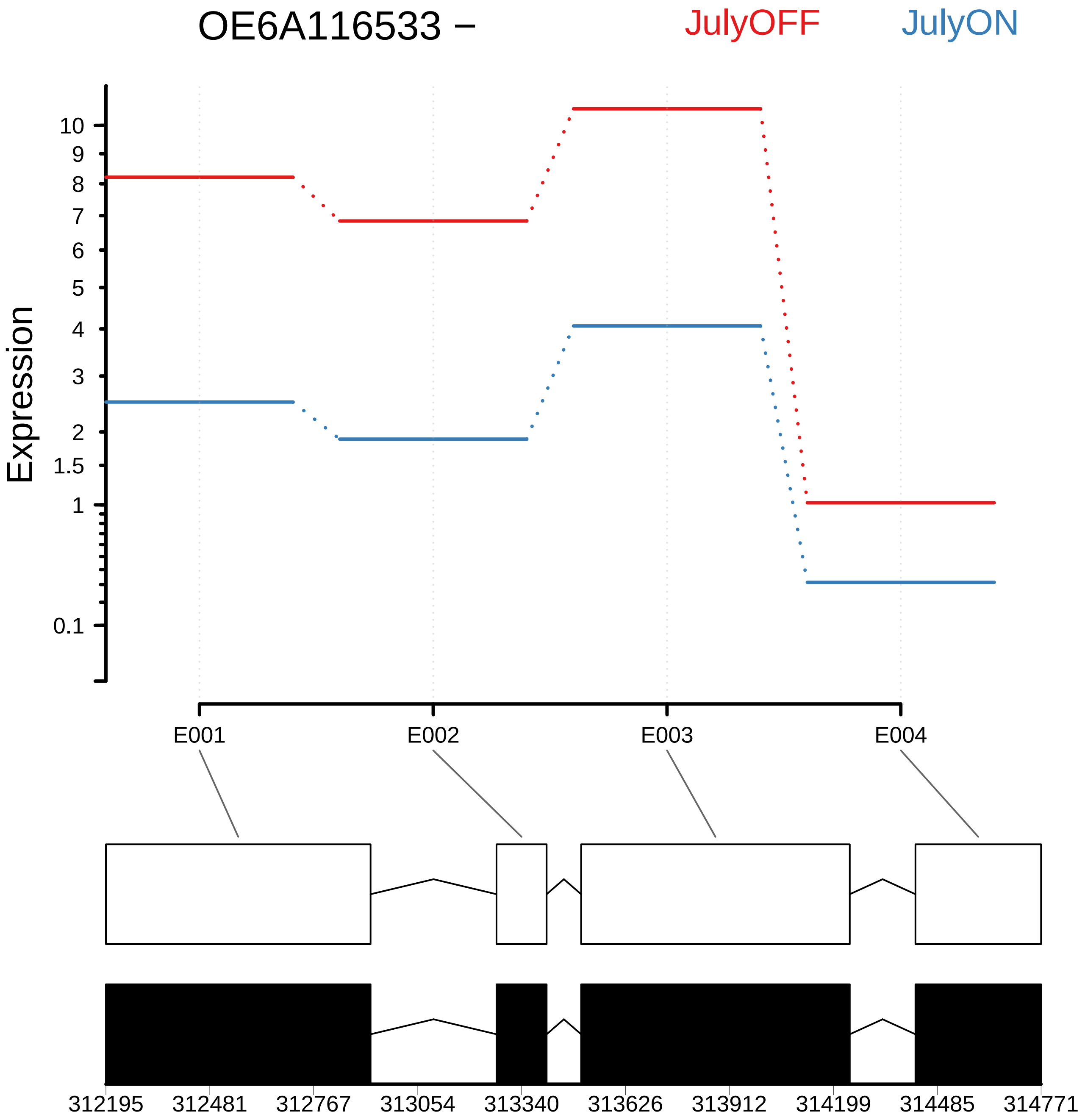
OE6A116533 –
JulyOFF
JulyON
Expression
10
9
8
7
6
5
4
3
2
1.5
1
0.1
E001
E002
E003
E004
312195
312481
312767
313054
313340
313626
313912
314199
314485
314771

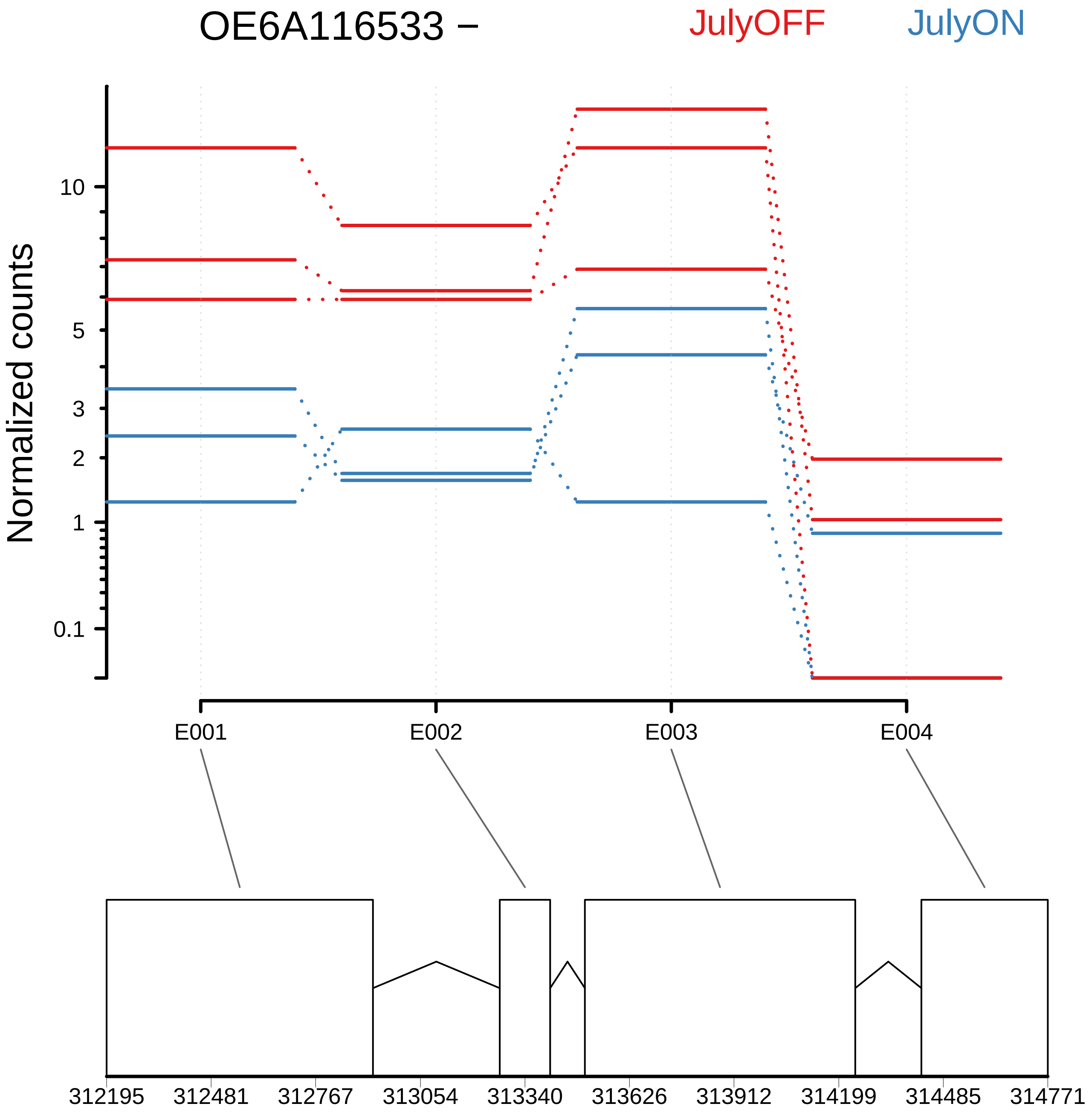
OE6A116533 −
JulyOFF
JulyON
Normalized counts
10
5
3
2
1
0.1
E001
E002
E003
E004
312195
312481
312767
313054
313340
313626
313912
314199
314485
314771

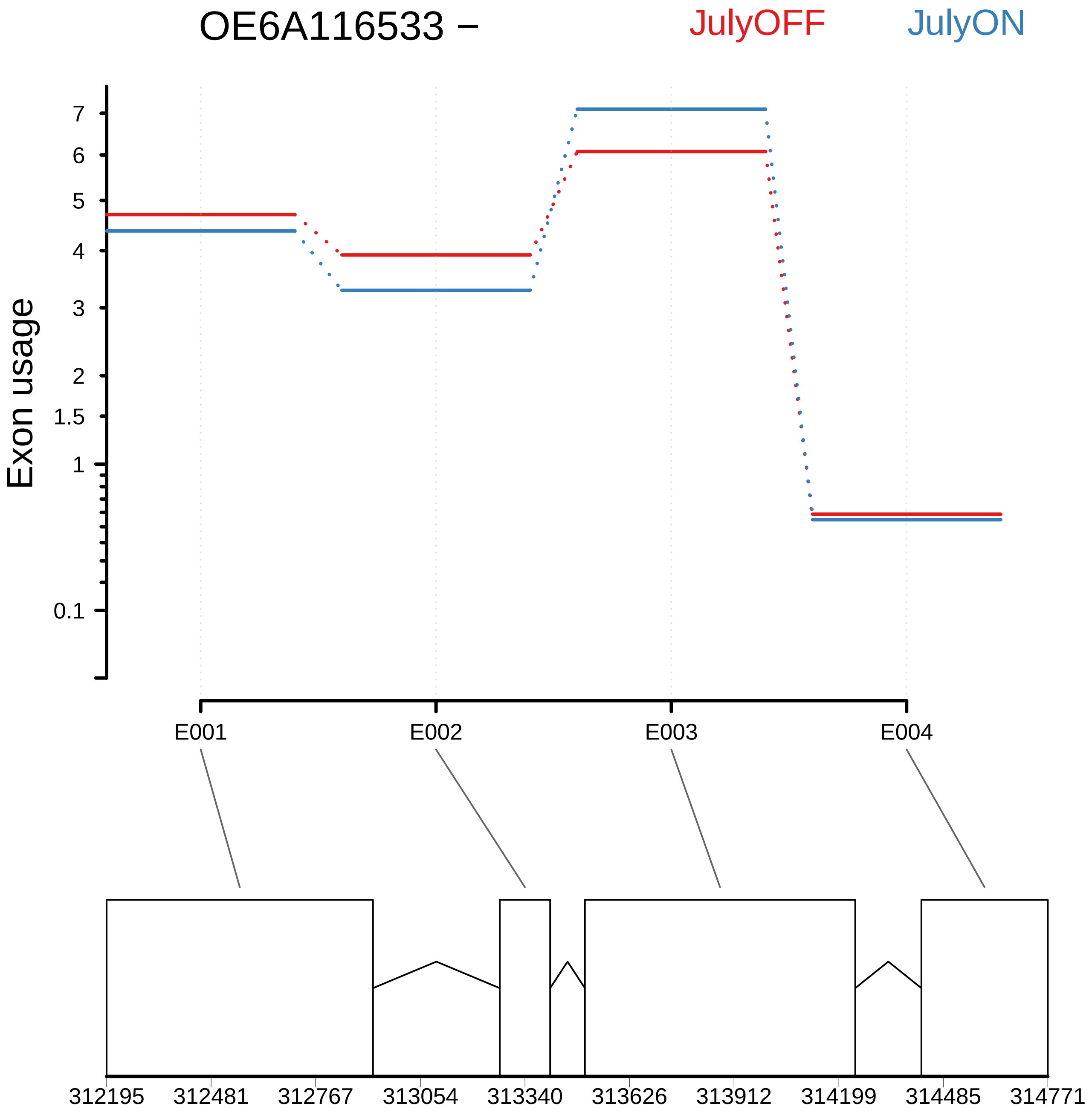
OE6A116533 −
JulyOFF
JulyON
Exon usage
7
6
5
4
3
2
1.5
1
0.1
E001
E002
E003
E004
312195
312481
312767
313054
313340
313626
313912
314199
314485
314771

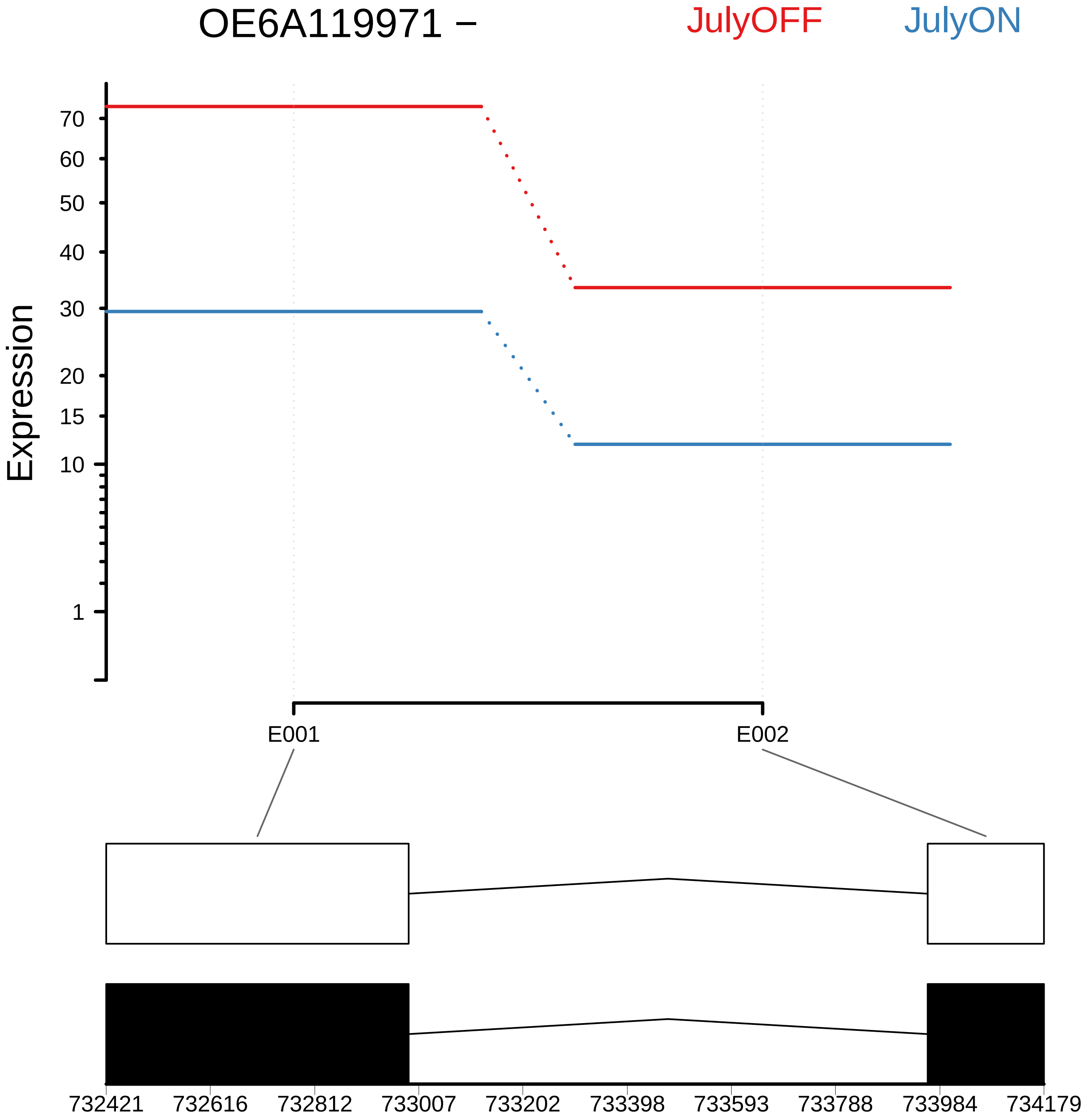
OE6A119971 −
JulyOFF
JulyON
Expression
70
60
50
40
30
20
15
10
1
E001
E002
732421
732616
732812
733007
733202
733398
733593
733788
733984
734179

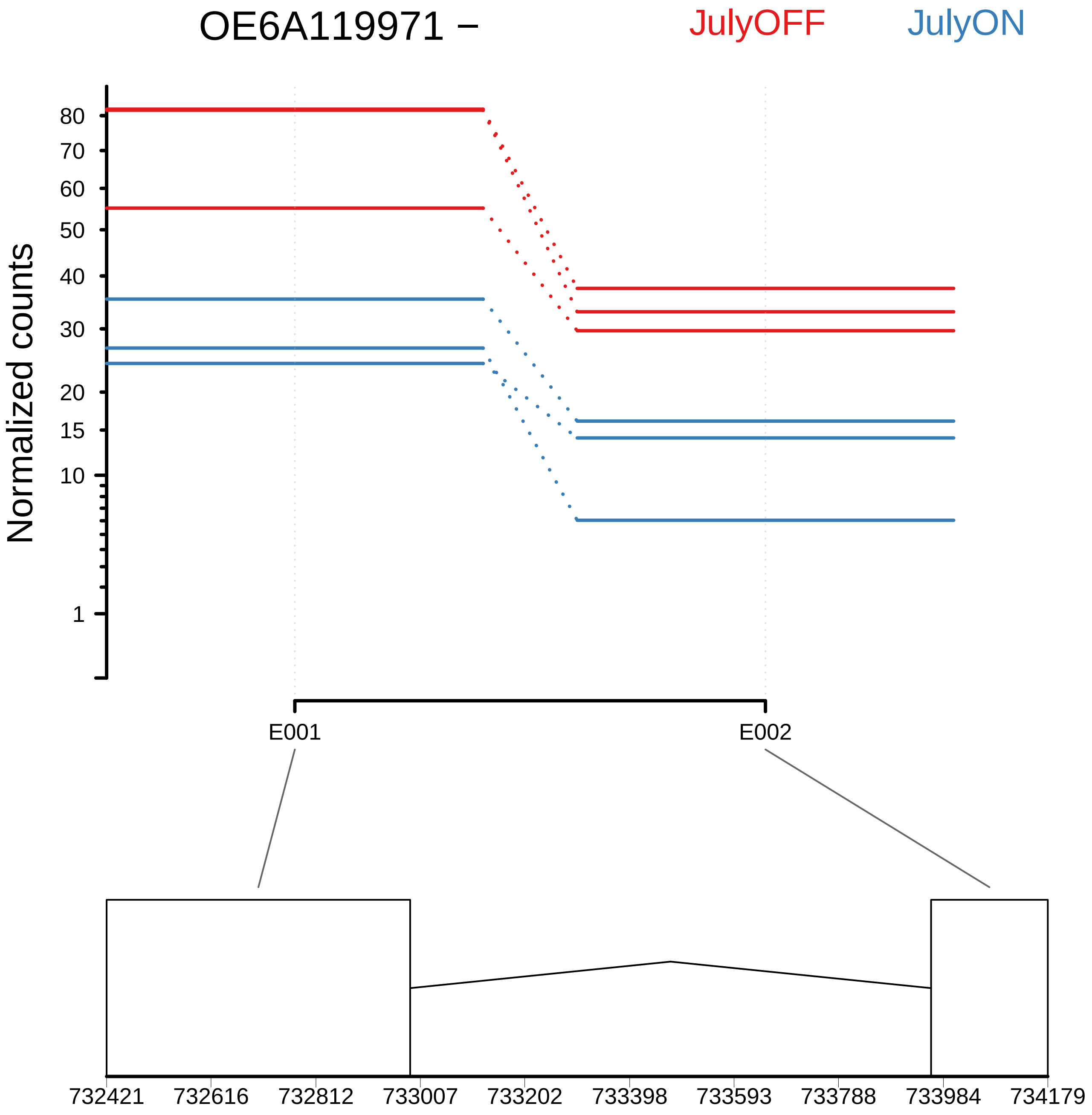
OE6A119971 −
JulyOFF
JulyON
Normalized counts
80
70
60
50
40
30
20
15
10
1
E001
E002
732421
732616
732812
733007
733202
733398
733593
733788
733984
734179

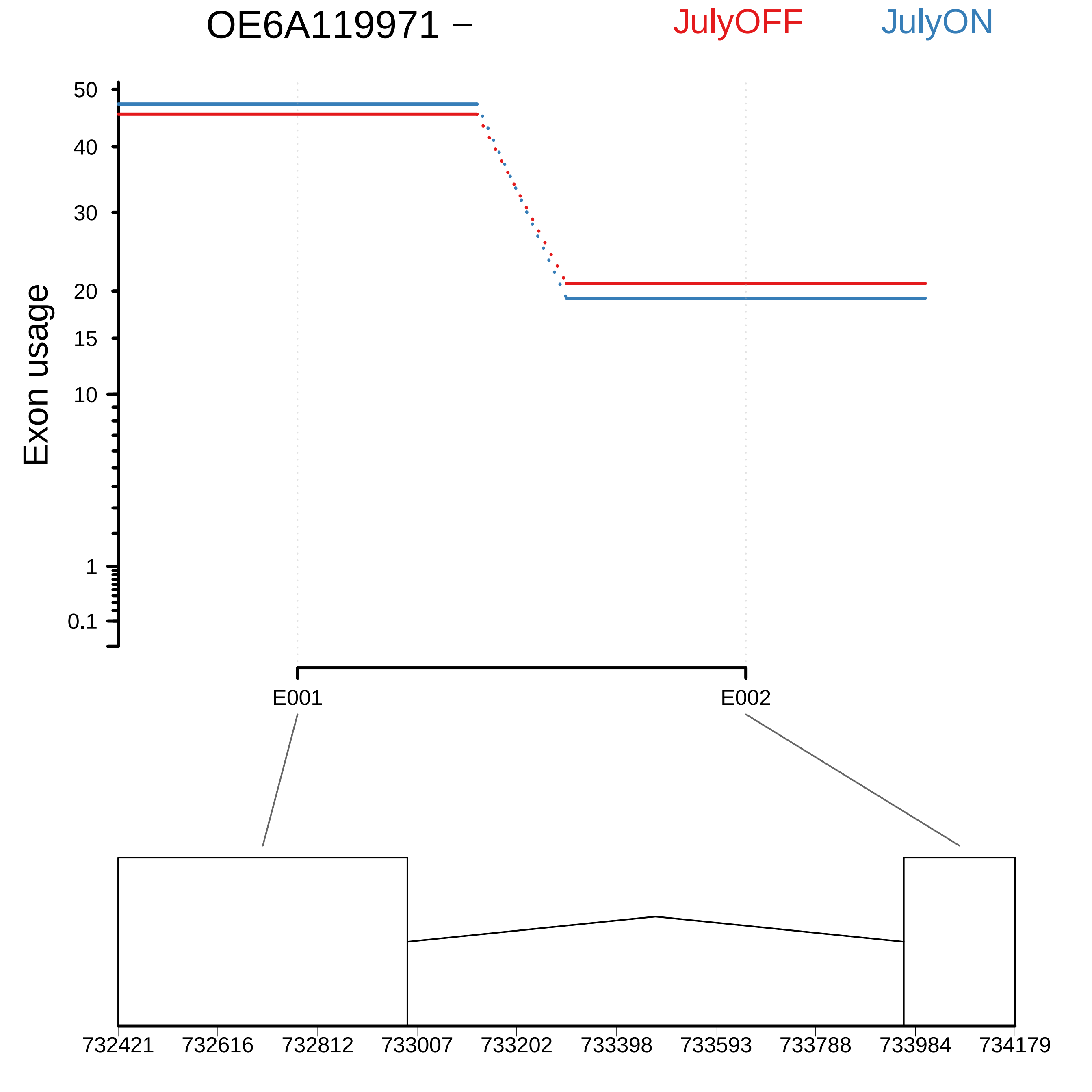
OE6A119971 −
JulyOFF
JulyON
Exon usage
50
40
30
20
15
10
1
0.1
E001
E002
732421
732616
732812
733007
733202
733398
733593
733788
733984
734179

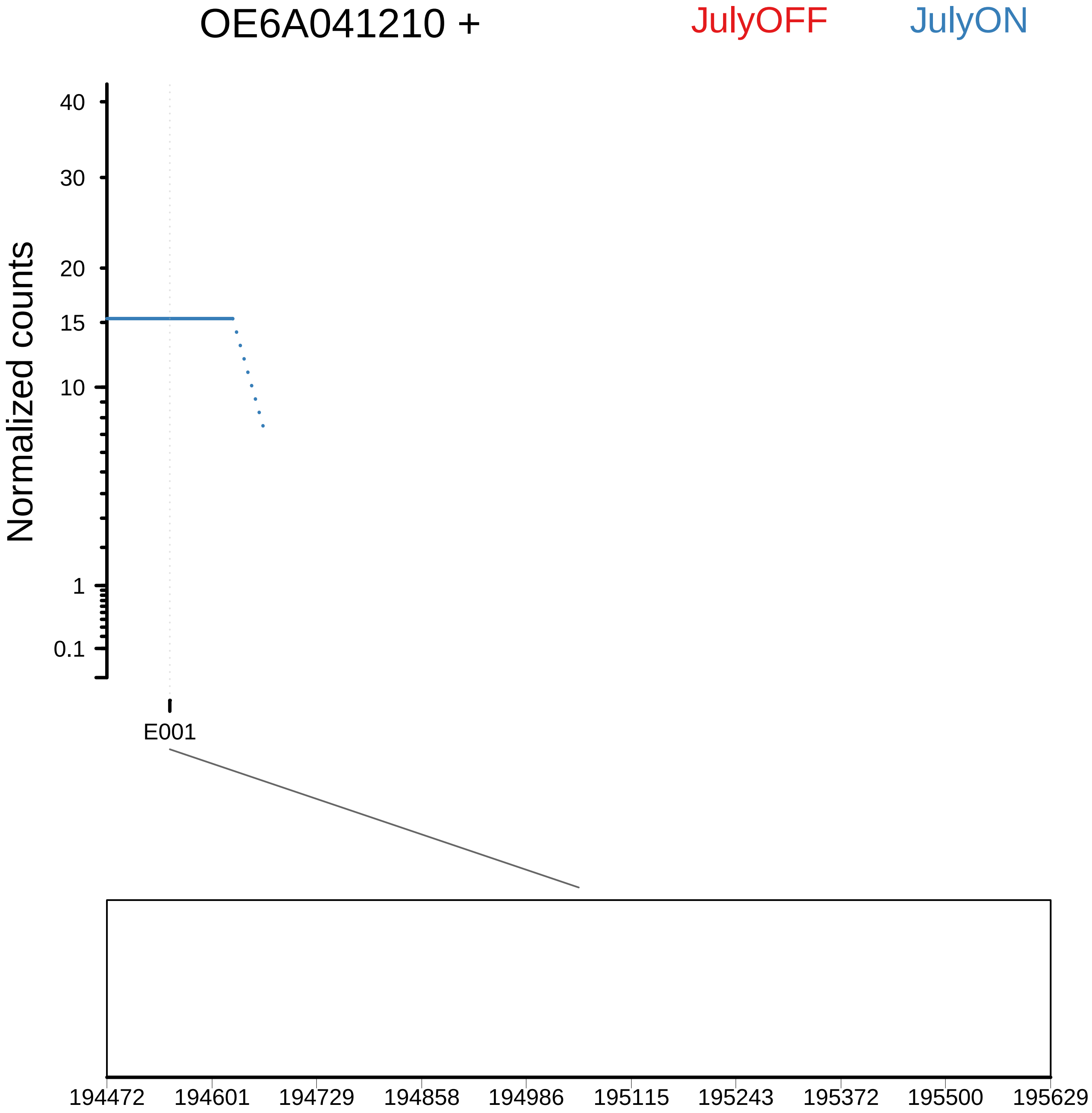
OE6A041210 +
JulyOFF
JulyON
Normalized counts
40
30
20
15
10
1
0.1
E001
194472
194601
194729
194858
194986
195115
195243
195372
195500
195629

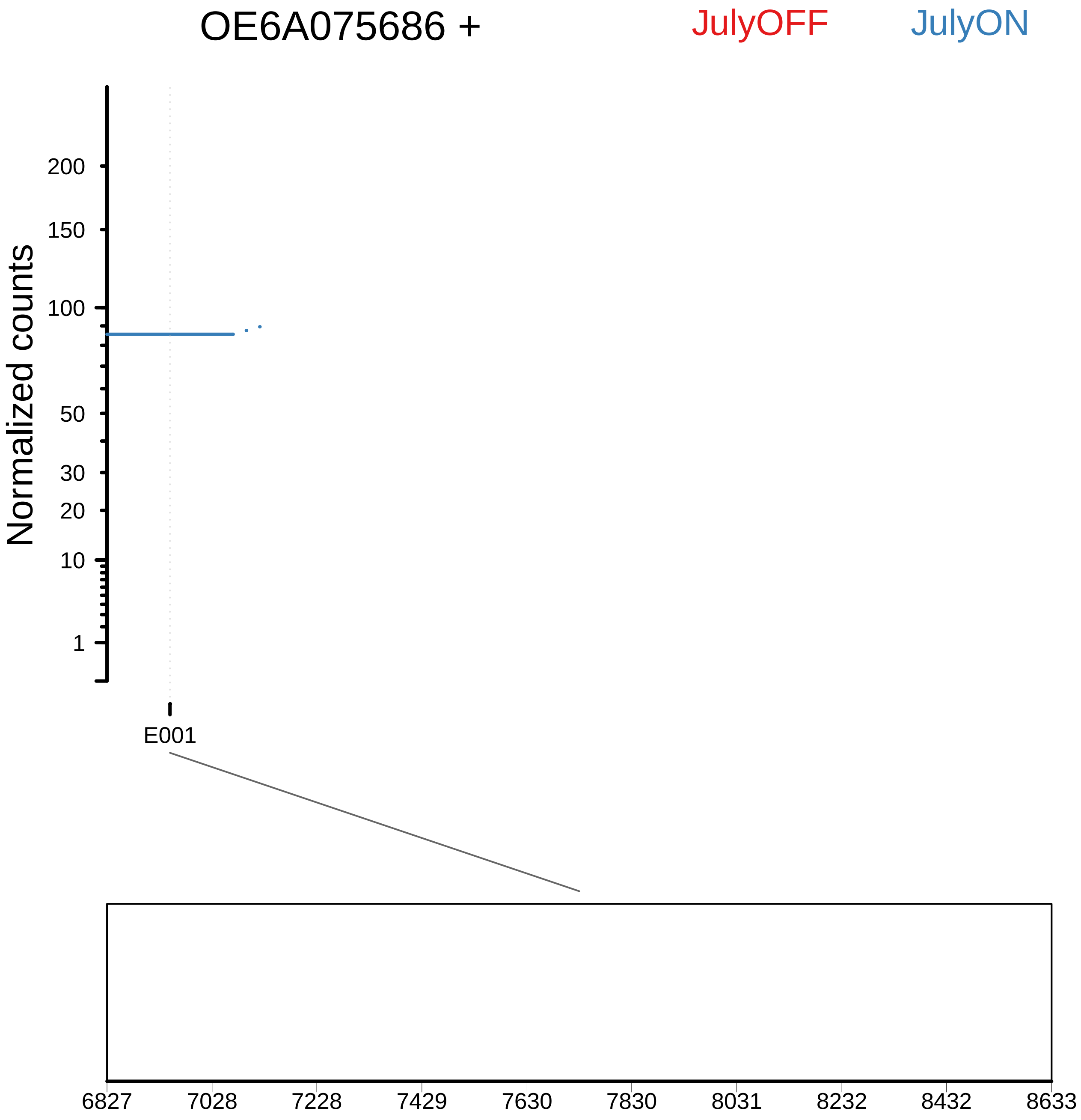

OE6A075686 +
JulyOFF
JulyON
Normalized counts
200
150
100
50
30
20
10
1
E001
6827
7028
7228
7429
7630
7830
8031
8232
8432
8633

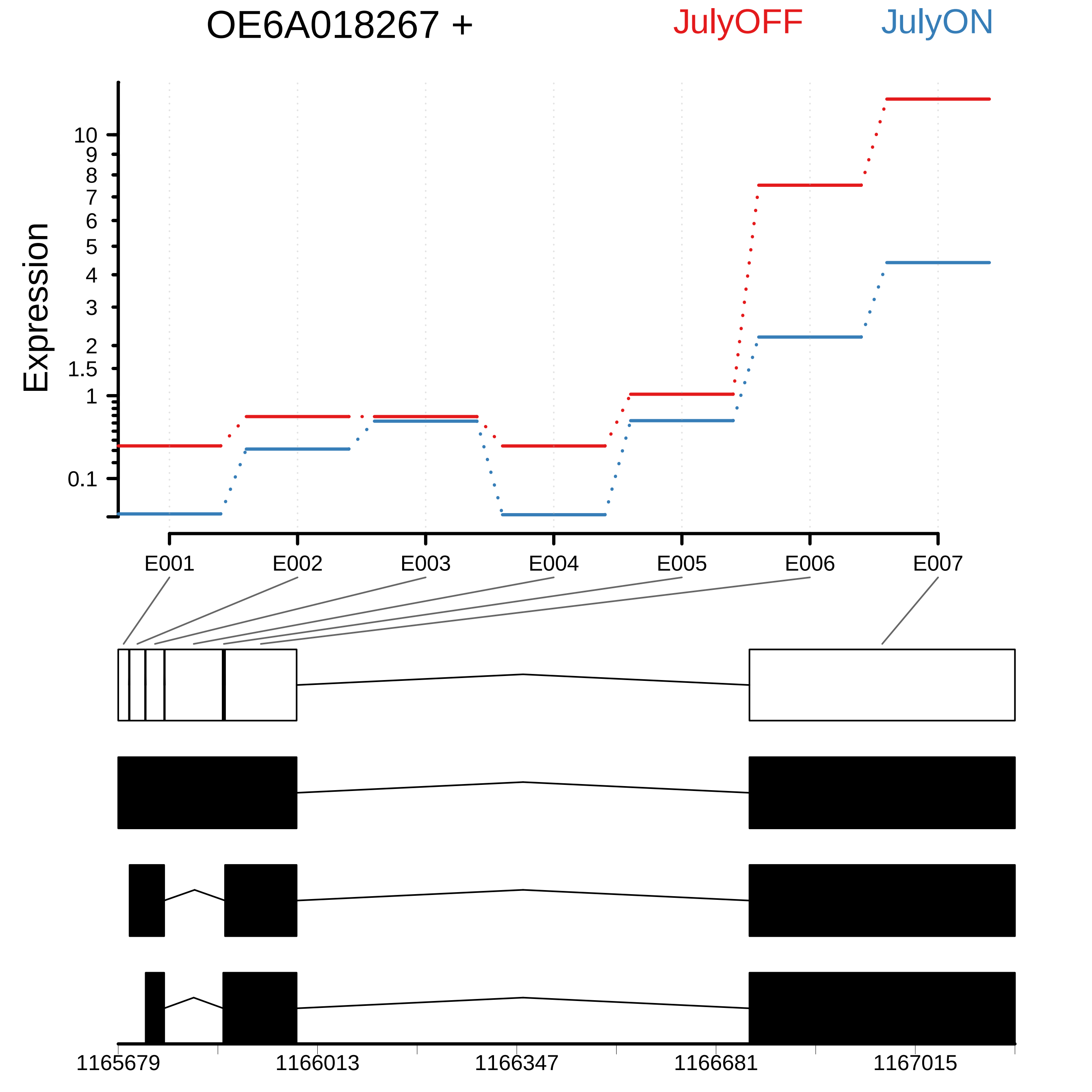

OE6A018267 +
JulyOFF
JulyON
Expression
10
9
8
7
6
5
4
3
2
1.5
1
0.1
E001
E002
E003
E004
E005
E006
E007
1165679
1166013
1166347
1166681
1167015

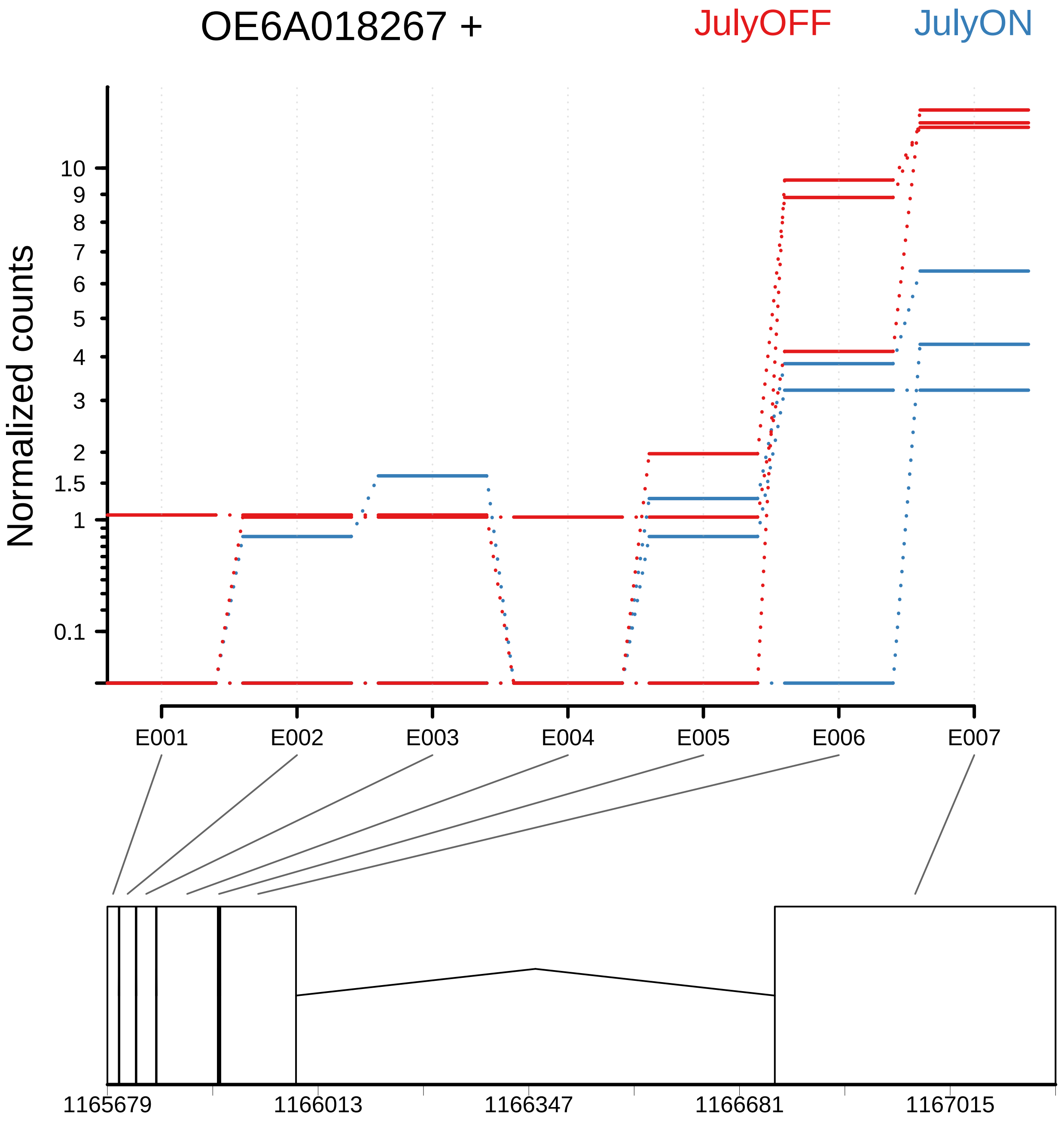
OE6A018267 +
JulyOFF
JulyON
Normalized counts
10
9
8
7
6
5
4
3
2
1.5
1
0.1
E001
E002
E003
E004
E005
E006
E007
1165679
1166013
1166347
1166681
1167015

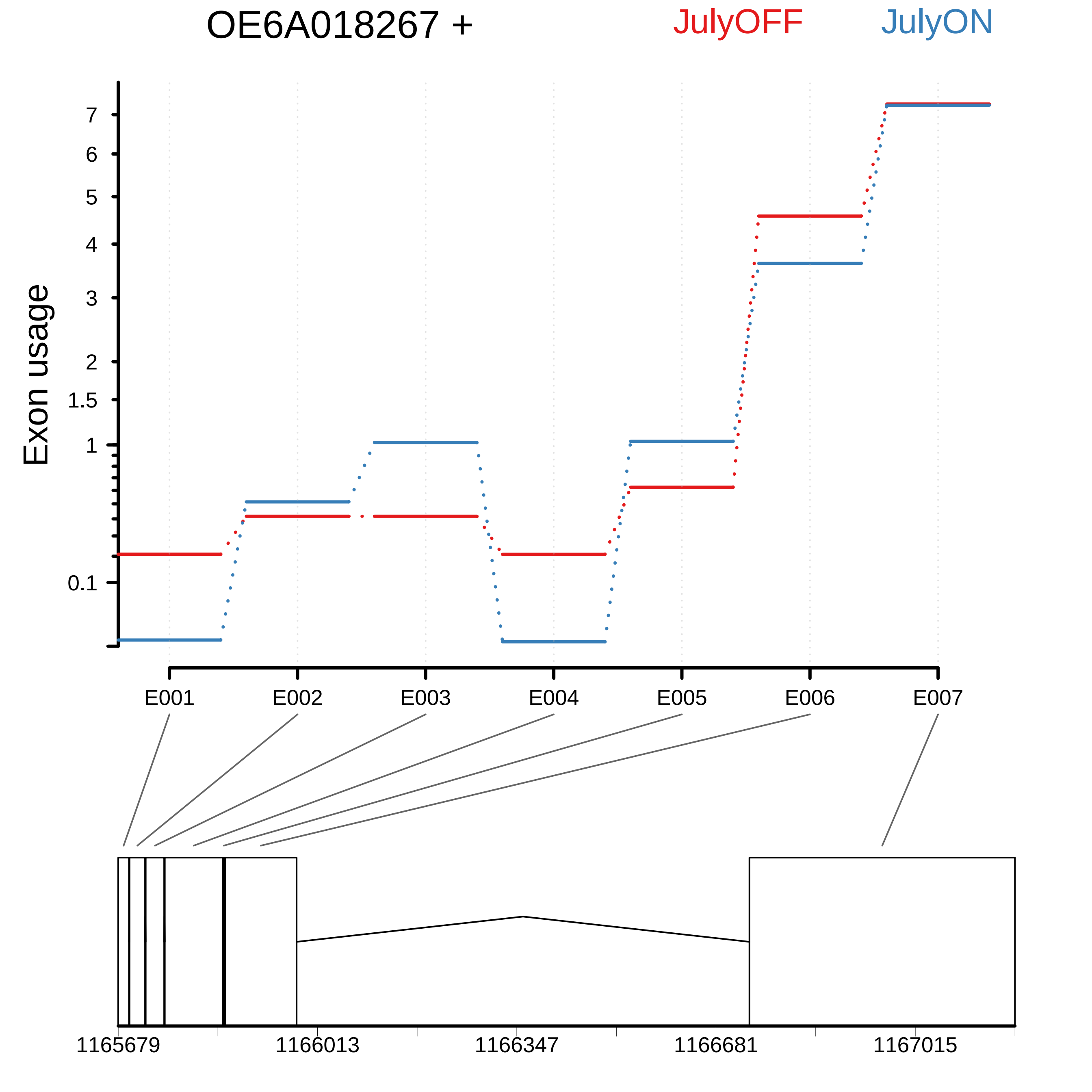
OE6A018267 +
JulyOFF
JulyON
Exon usage
7
6
5
4
3
2
1.5
1
0.1
E001
E002
E003
E004
E005
E006
E007
1165679
1166013
1166347
1166681
1167015

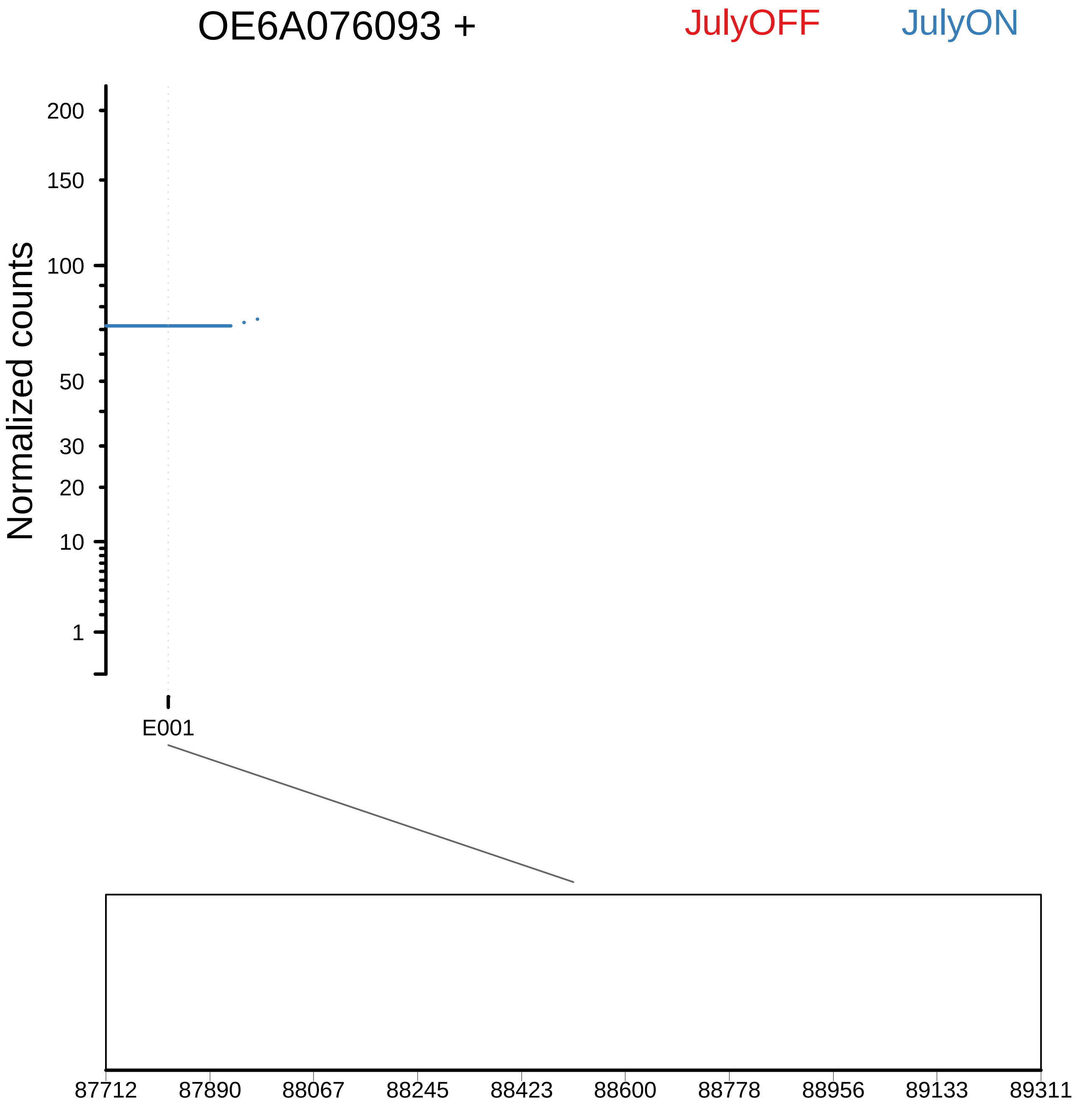
OE6A076093 +
JulyOFF
JulyON
Normalized counts
200
150
100
50
30
20
10
1
E001
87712
87890
88067
88245
88423
88600
88778
88956
89133
89311

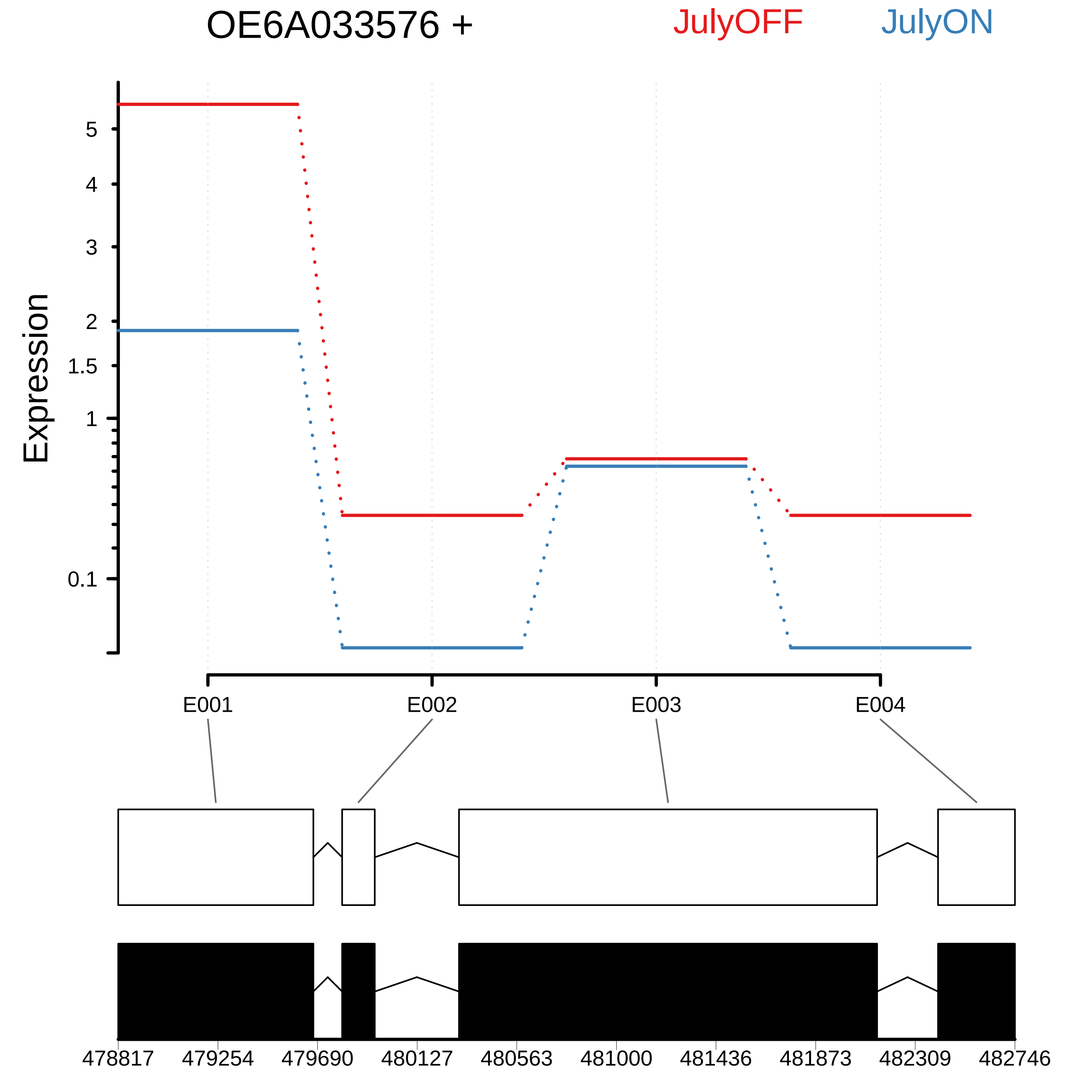
OE6A033576 +
JulyOFF
JulyON
Expression
5
4
3
2
1.5
1
0.1
E001
E002
E003
E004
478817
479254
479690
480127
480563
481000
481436
481873
482309
482746

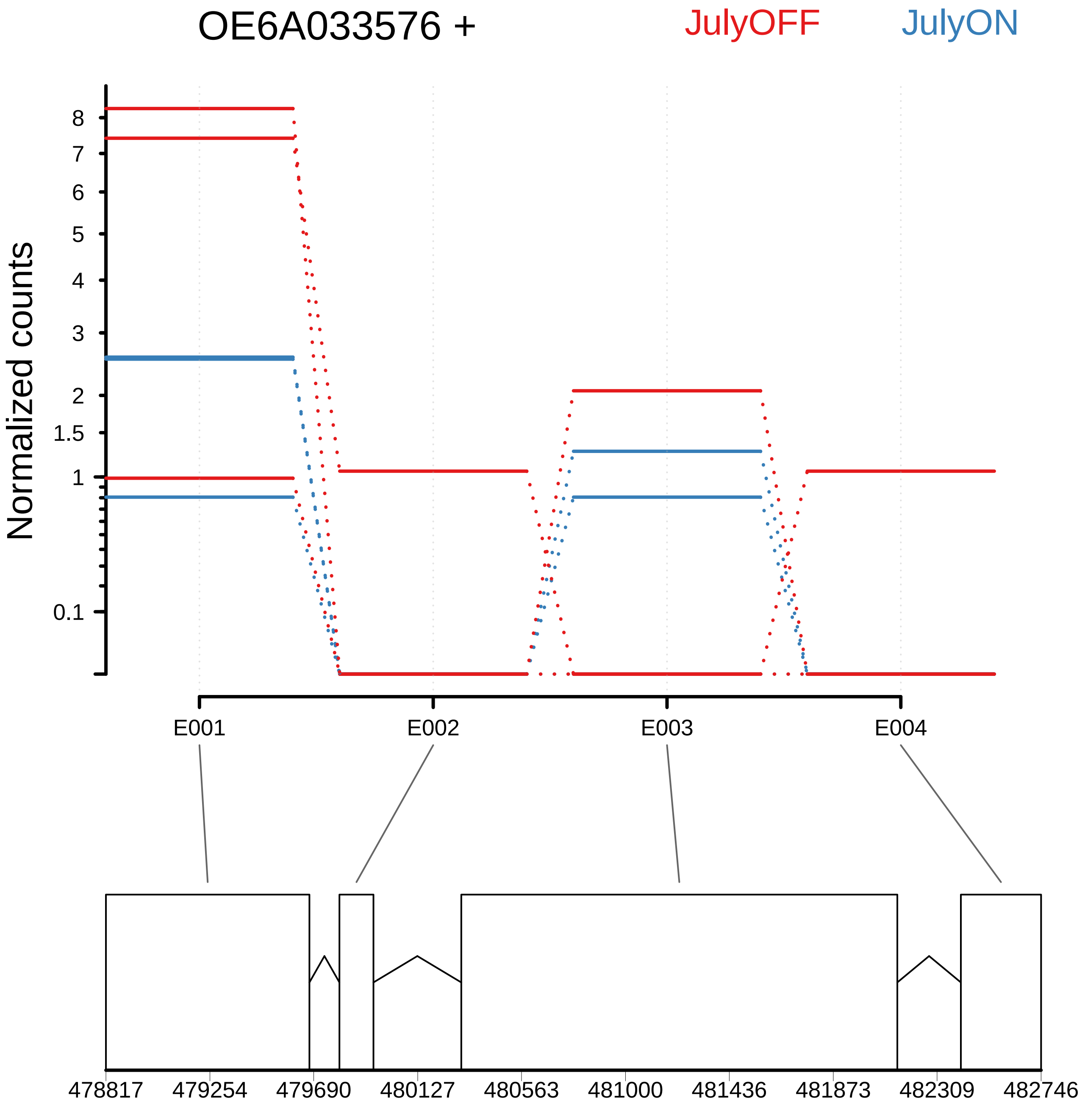
OE6A033576 +
JulyOFF
JulyON
Normalized counts
8
7
6
5
4
3
2
1.5
1
0.1
E001
E002
E003
E004
478817
479254
479690
480127
480563
481000
481436
481873
482309
482746

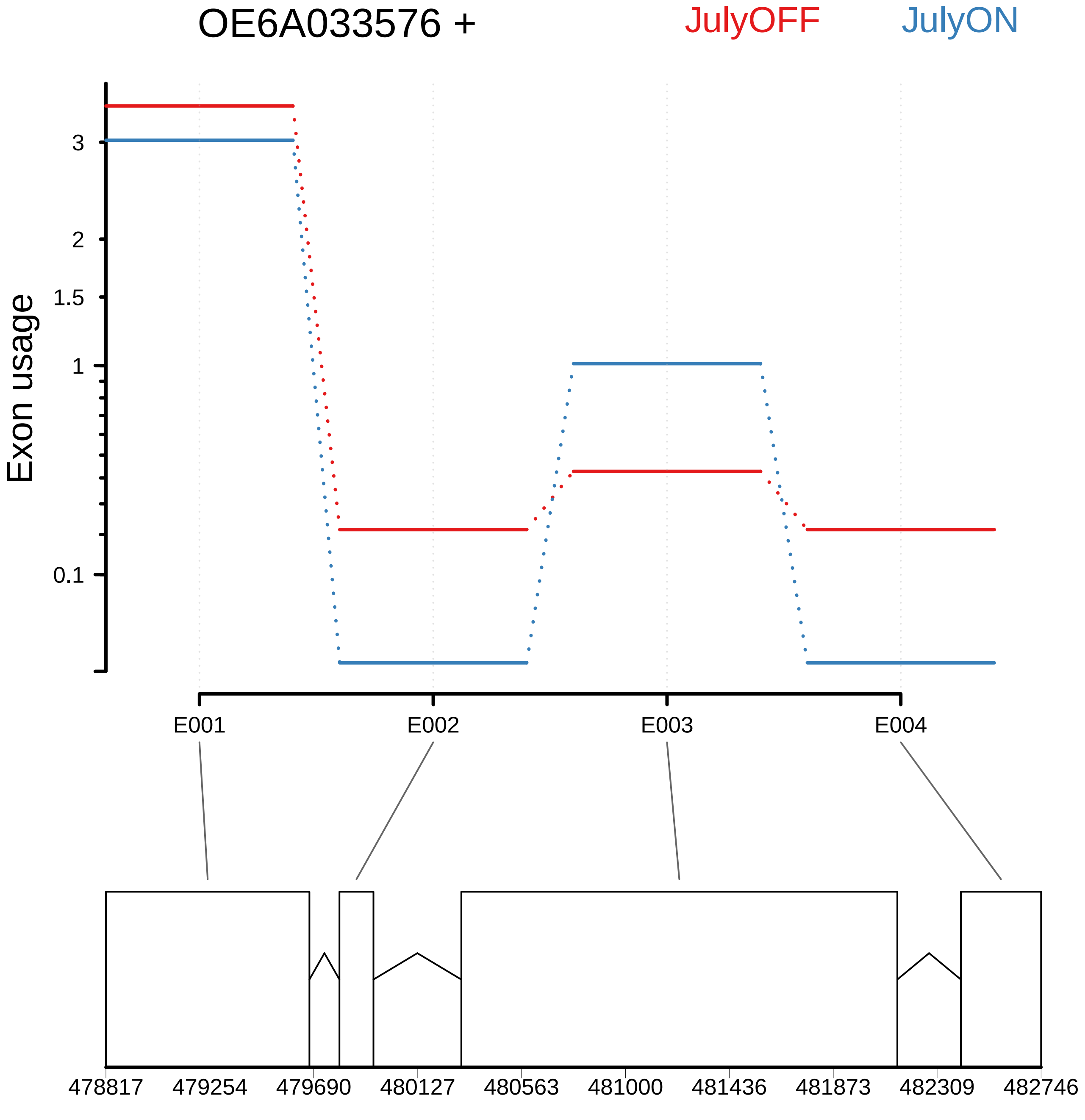
OE6A033576 +
JulyOFF
JulyON
Exon usage
3
2
1.5
1
0.1
E001
E002
E003
E004
478817
479254
479690
480127
480563
481000
481436
481873
482309
482746

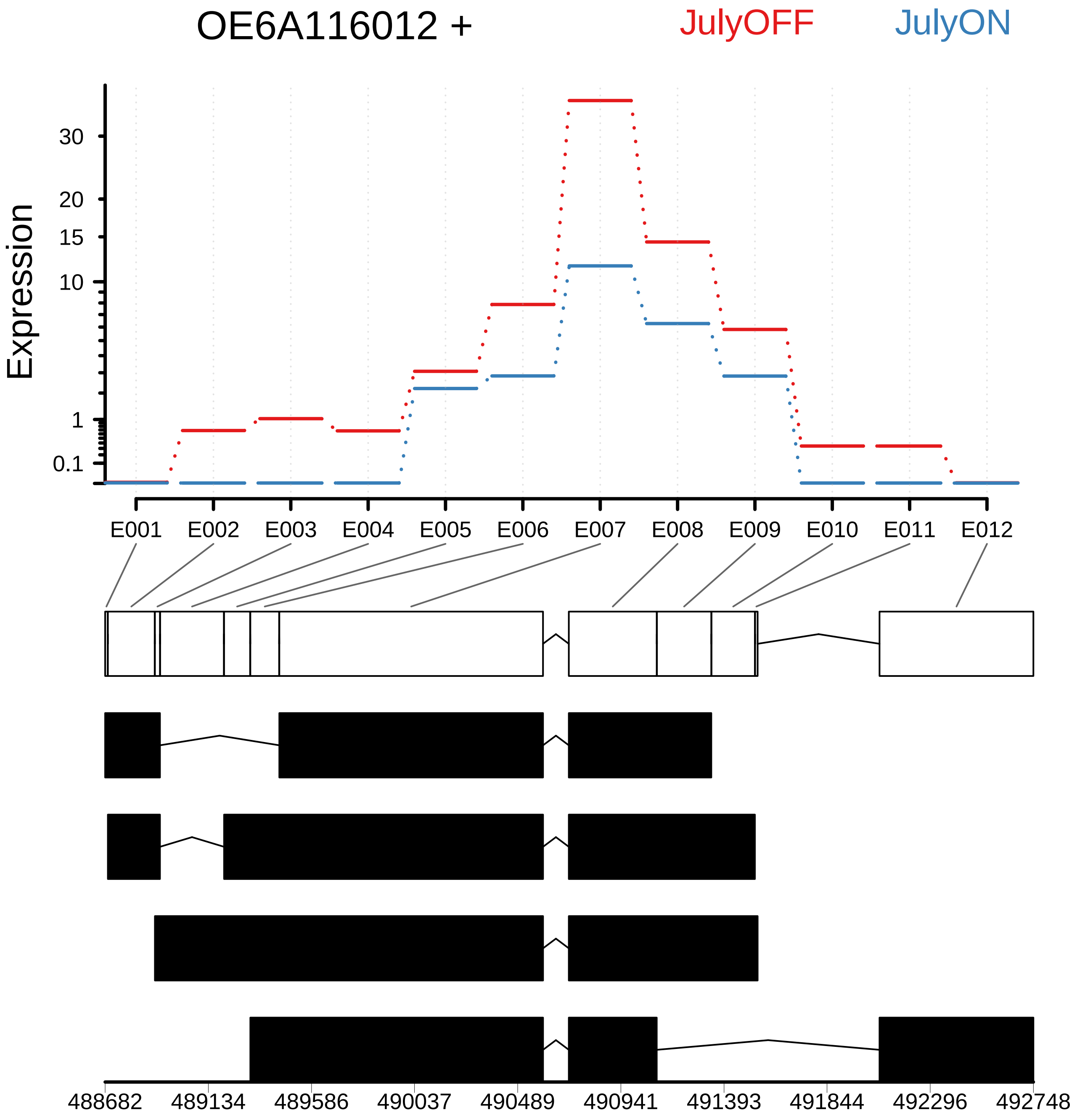
OE6A116012 +
JulyOFF
JulyON
Expression
30
20
15
10
1
0.1
E001
E002
E003
E004
E005
E006
E007
E008
E009
E010
E011
E012
488682
489134
489586
490037
490489
490941
491393
491844
492296
492748

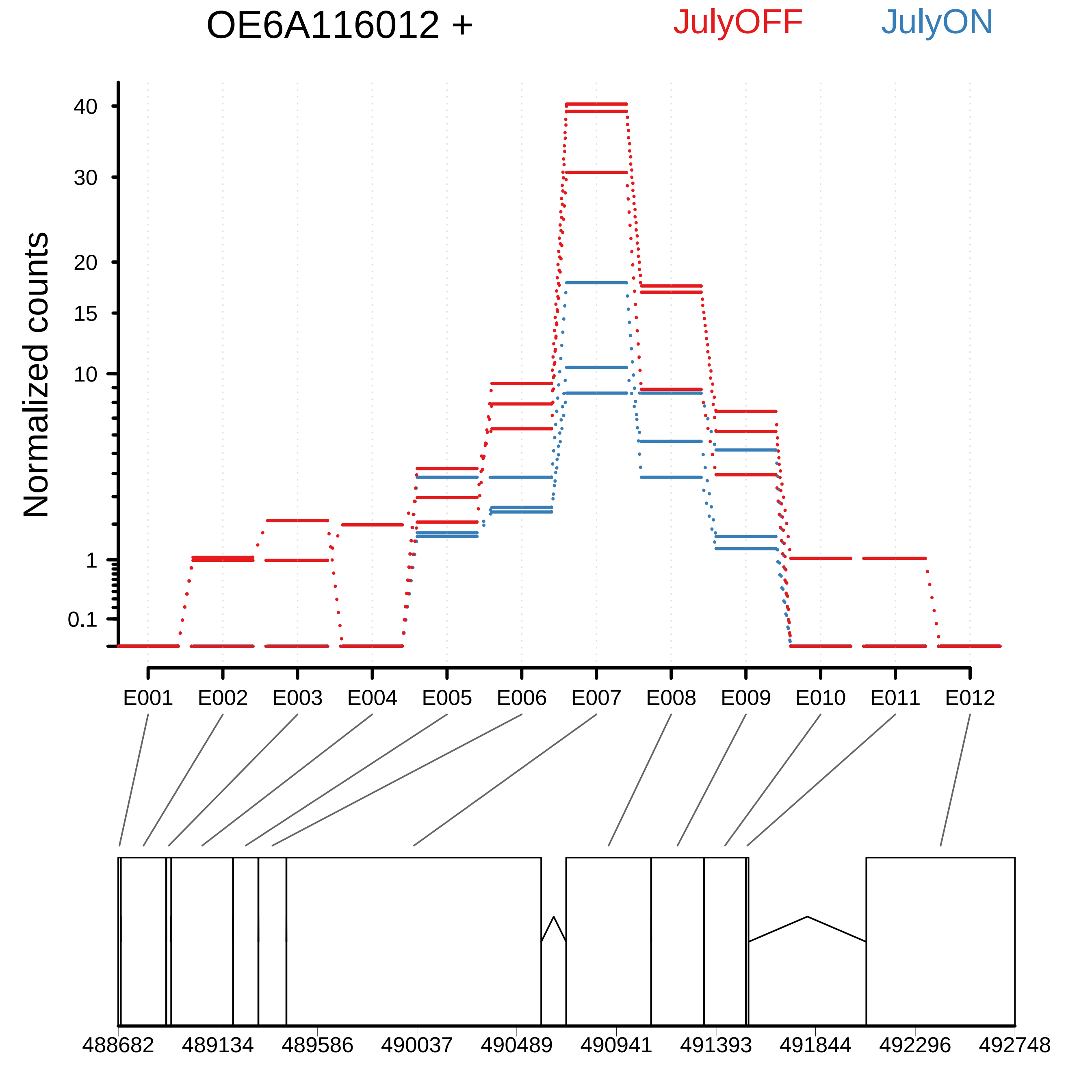
OE6A116012 +
JulyOFF
JulyON
Normalized counts
40
30
20
15
10
1
0.1
E001
E002
E003
E004
E005
E006
E007
E008
E009
E010
E011
E012
488682
489134
489586
490037
490489
490941
491393
491844
492296
492748

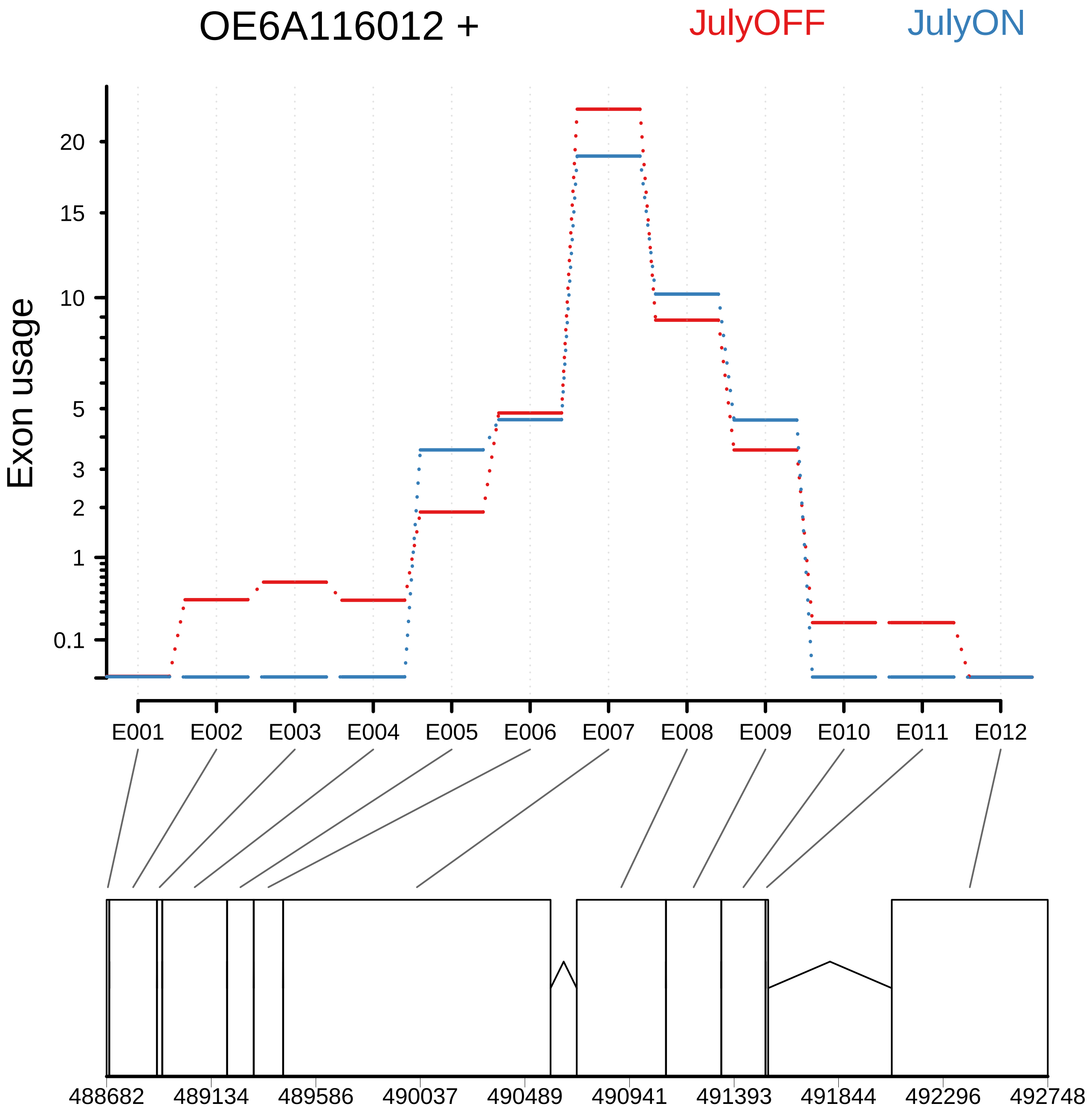

OE6A116012 +
JulyOFF
JulyON
Exon usage
20
15
10
5
3
2
1
0.1
E001
E002
E003
E004
E005
E006
E007
E008
E009
E010
E011
E012
488682
489134
489586
490037
490489
490941
491393
491844
492296
492748

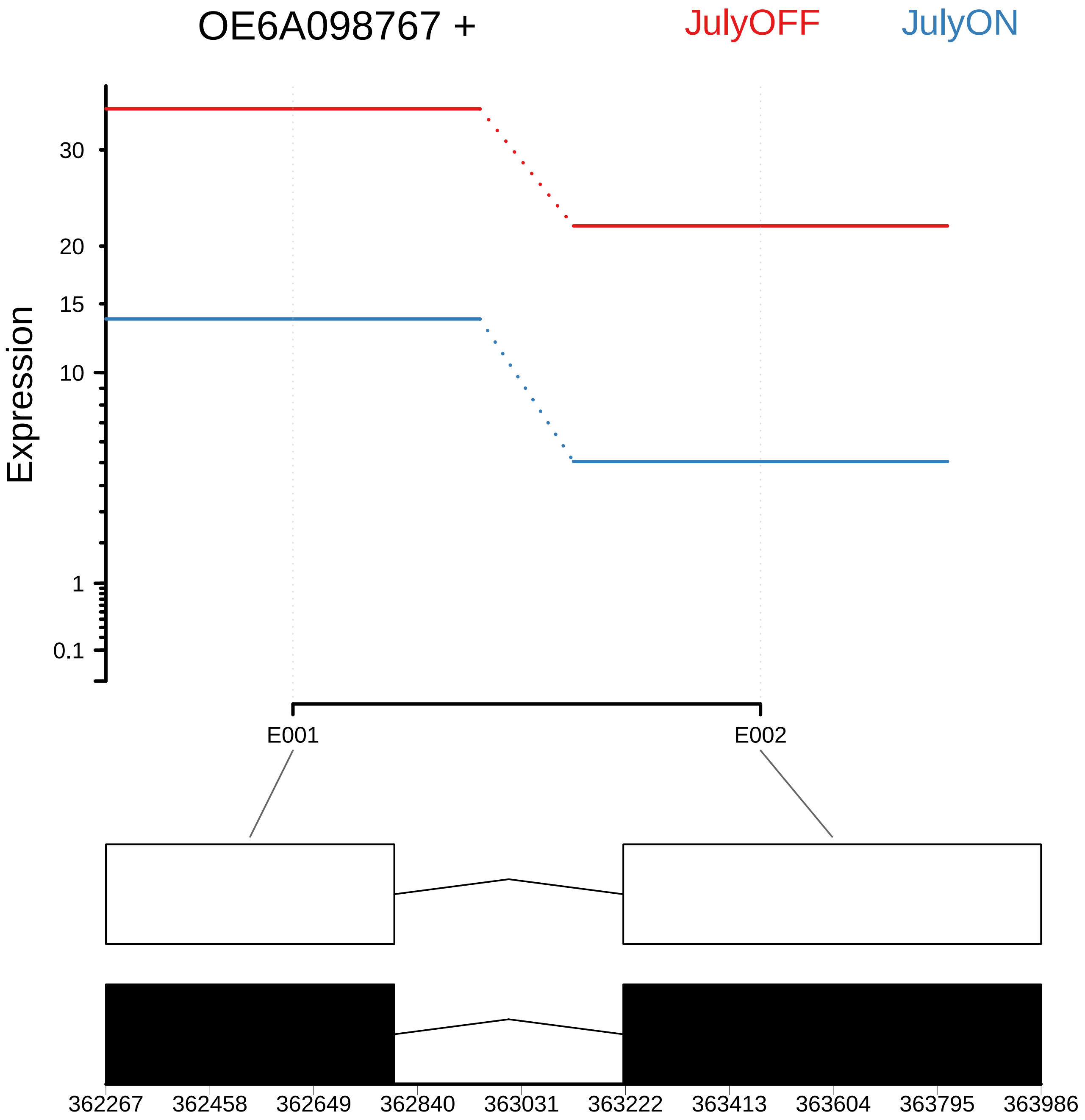

OE6A098767 +
JulyOFF
JulyON
Expression
30
20
15
10
1
0.1
E001
E002
362267
362458
362649
362840
363031
363222
363413
363604
363795
363986

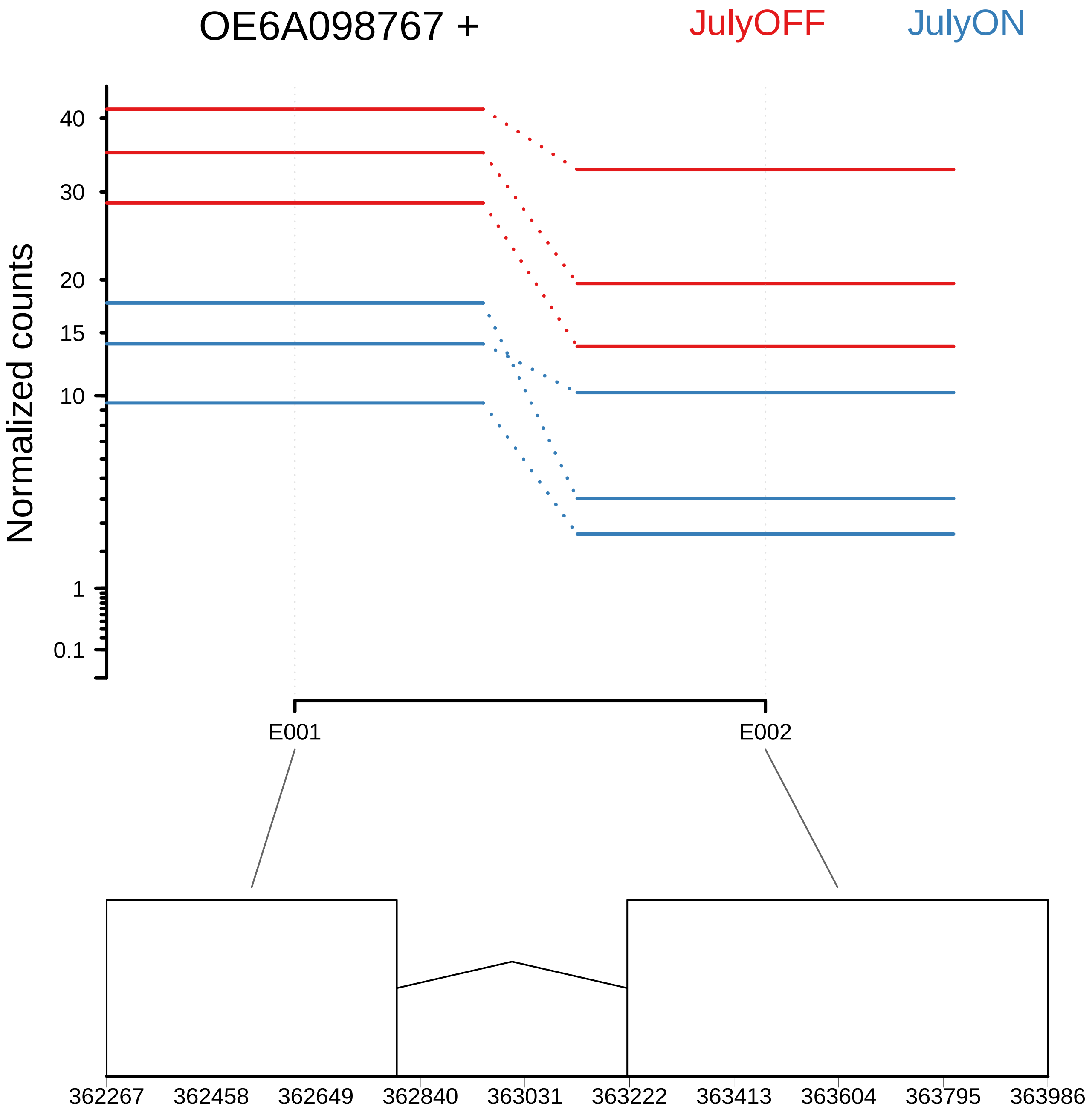

OE6A098767 +
JulyOFF
JulyON
Normalized counts
40
30
20
15
10
1
0.1
E001
E002
362267
362458
362649
362840
363031
363222
363413
363604
363795
363986

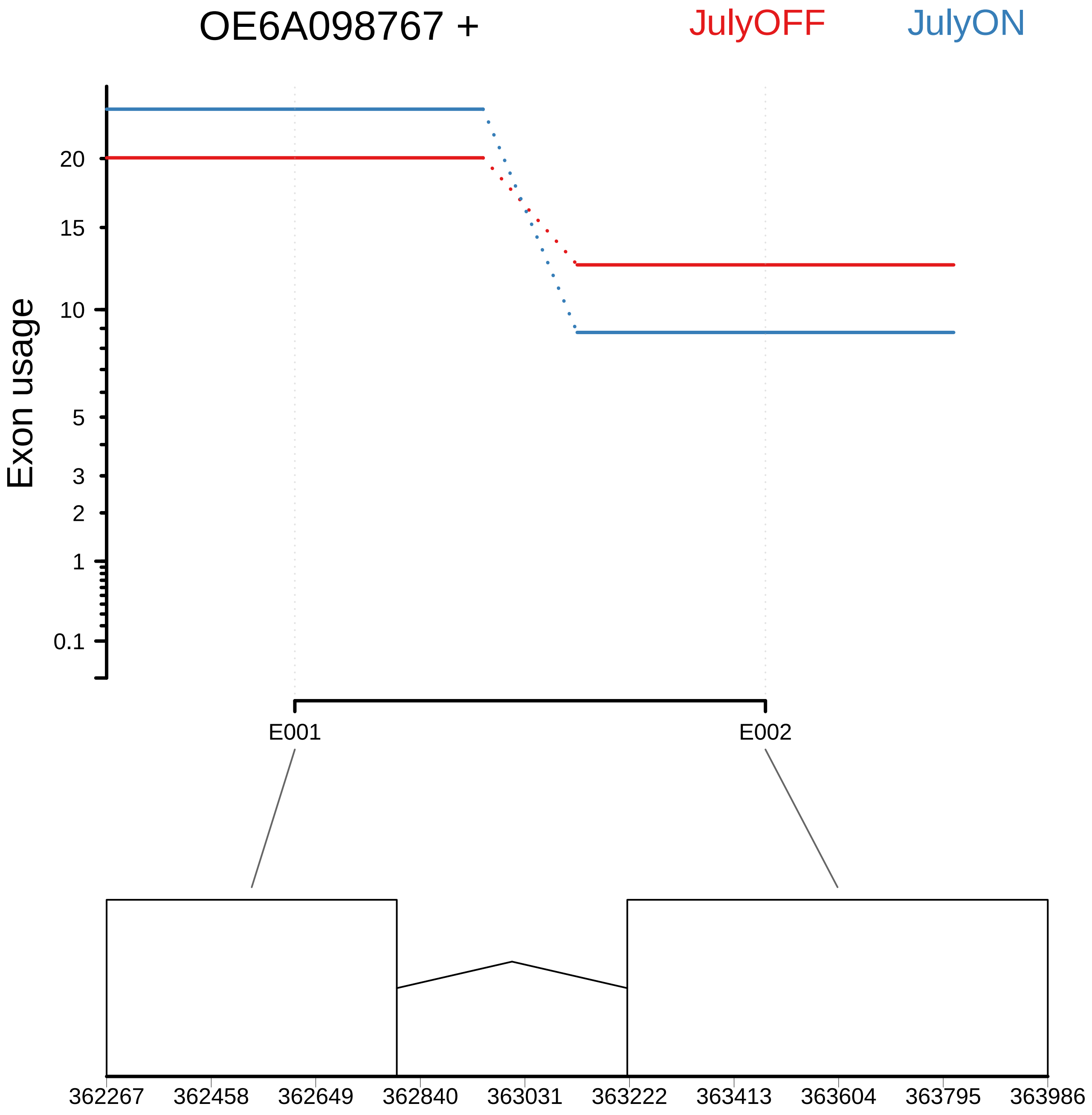
OE6A098767 +
JulyOFF
JulyON
Exon usage
20
15
10
5
3
2
1
0.1
E001
E002
362267
362458
362649
362840
363031
363222
363413
363604
363795
363986

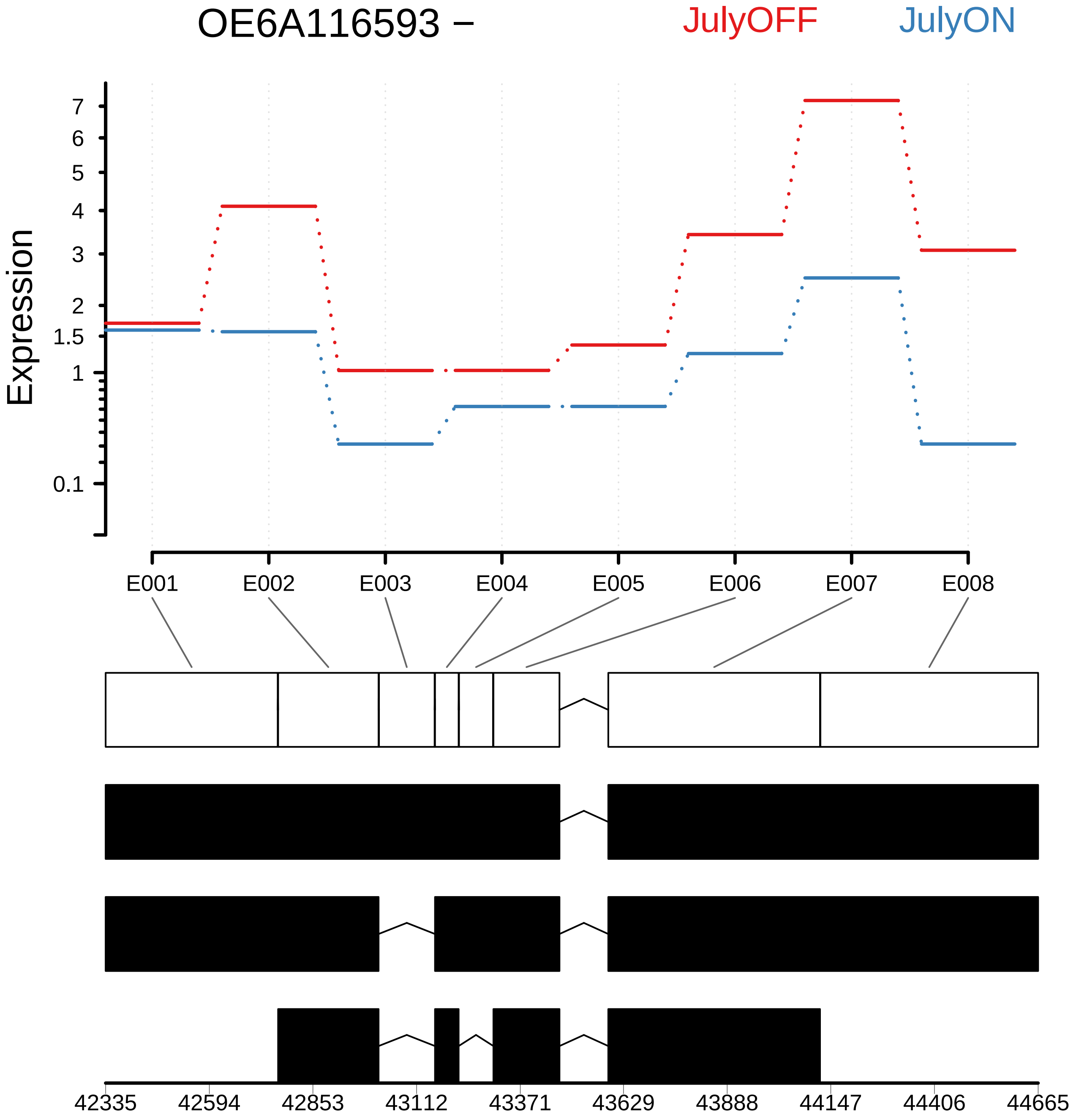

OE6A116593 –
JulyOFF
JulyON
Expression
7
6
5
4
3
2
1.5
1
0.1
E001
E002
E003
E004
E005
E006
E007
E008
42335
42594
42853
43112
43371
43629
43888
44147
44406
44665

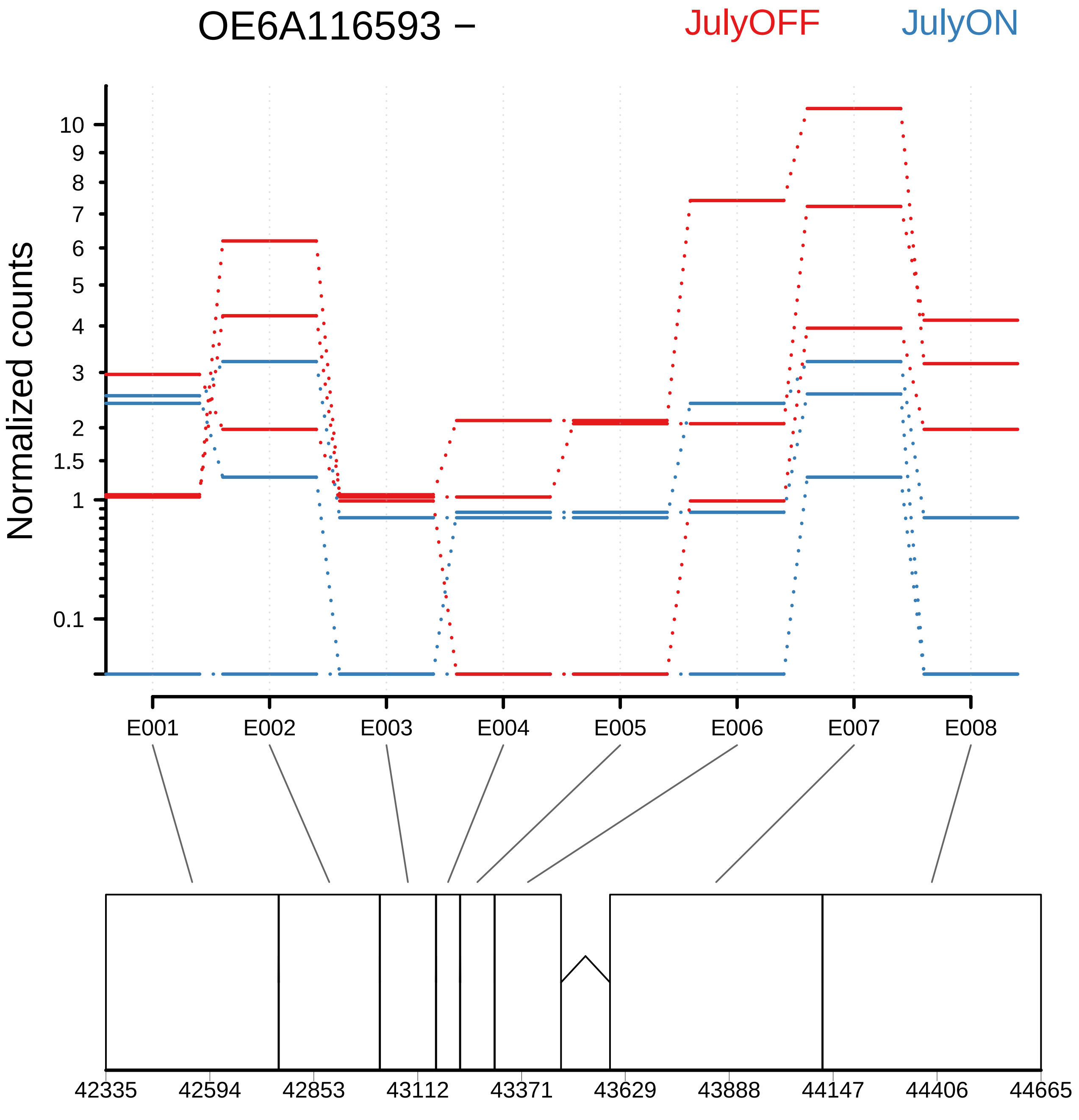

OE6A116593 –
JulyOFF
JulyON
Normalized counts
10
9
8
7
6
5
4
3
2
1.5
1
0.1
E001
E002
E003
E004
E005
E006
E007
E008
42335
42594
42853
43112
43371
43629
43888
44147
44406
44665

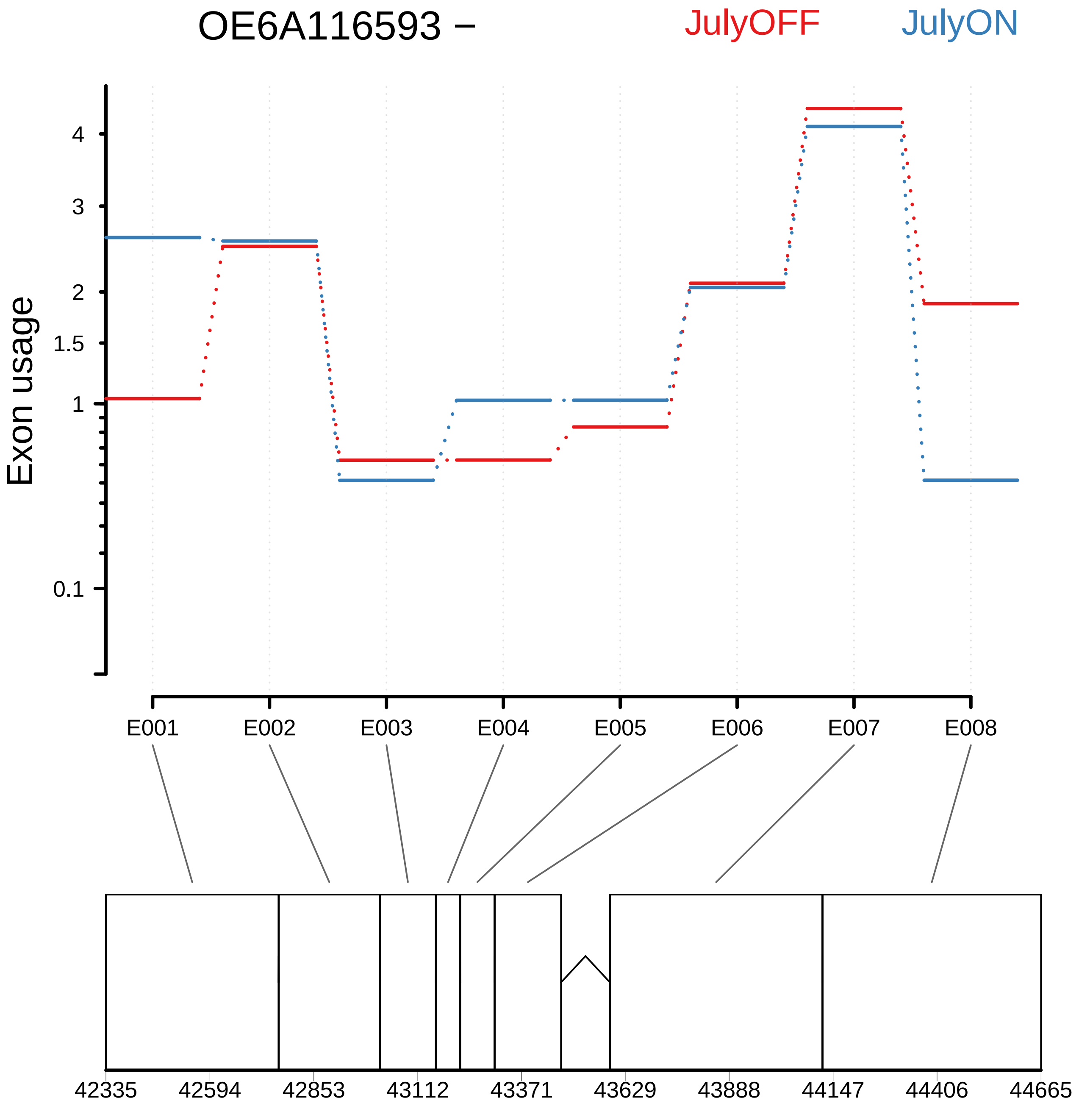

OE6A116593 −
JulyOFF
JulyON
Exon usage
4
3
2
1.5
1
0.1
E001
E002
E003
E004
E005
E006
E007
E008
42335
42594
42853
43112
43371
43629
43888
44147
44406
44665

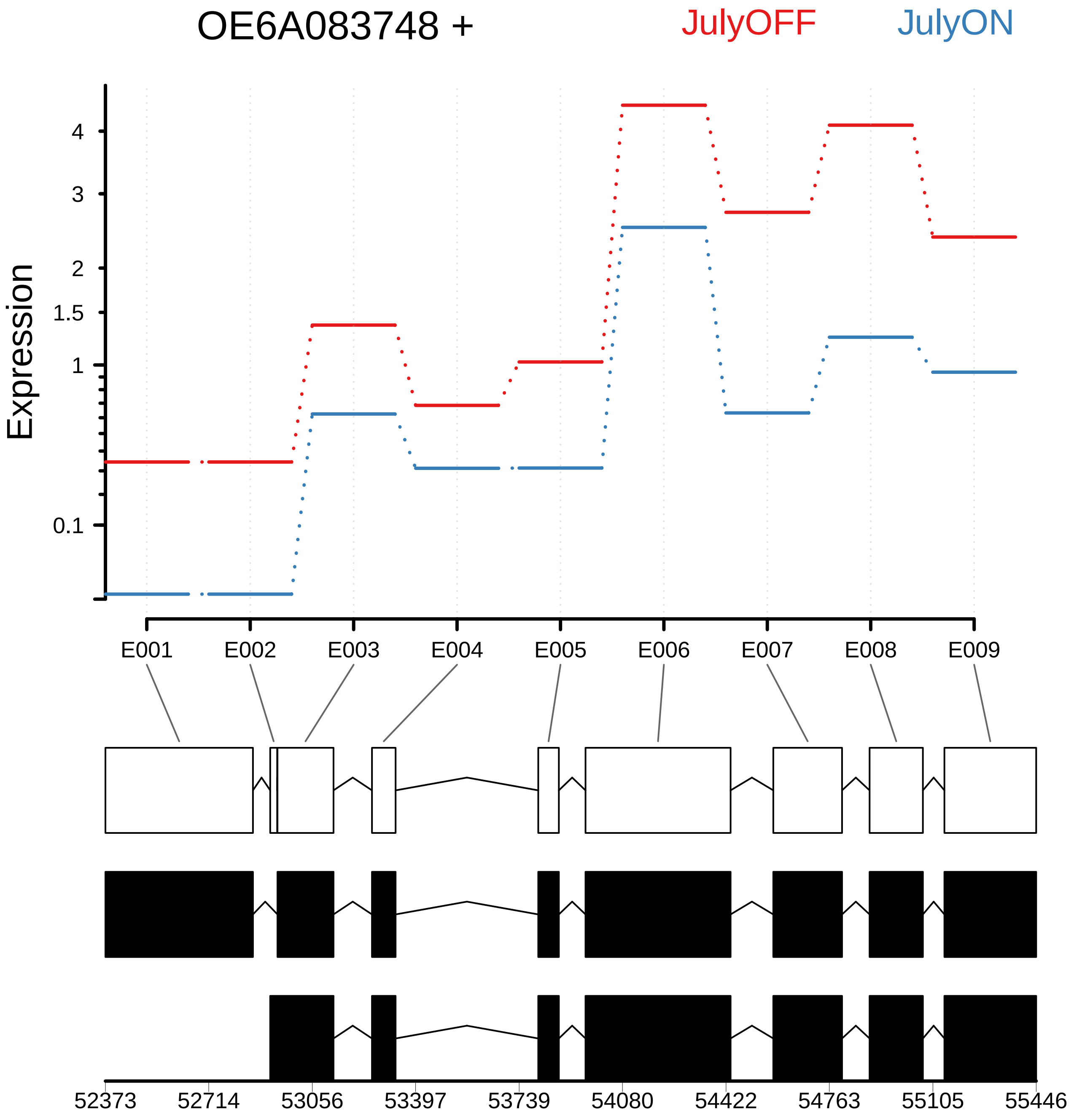
OE6A083748 +
JulyOFF
JulyON
Expression
4
3
2
1.5
1
0.1
E001
E002
E003
E004
E005
E006
E007
E008
E009
52373
52714
53056
53397
53739
54080
54422
54763
55105
55446

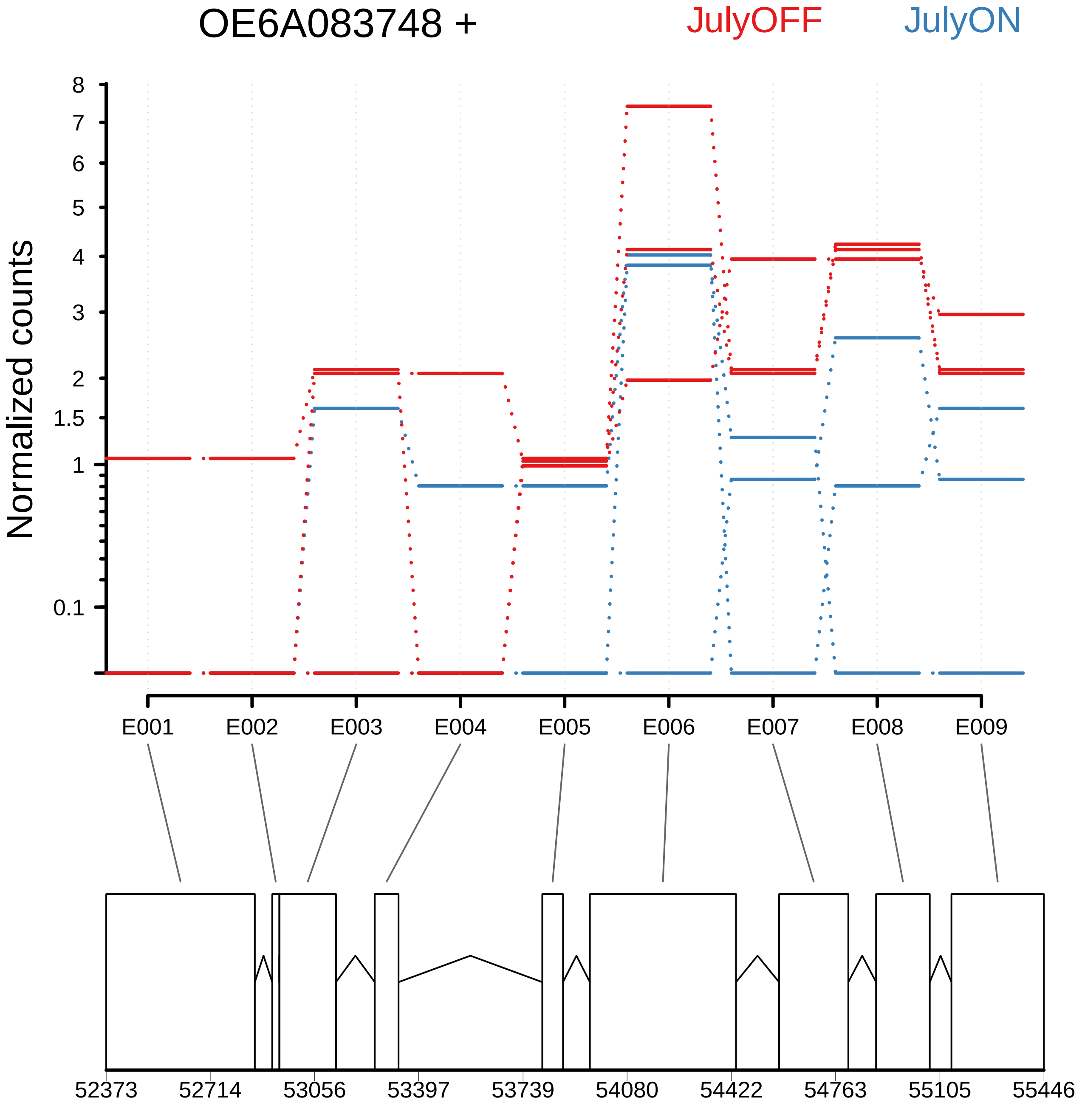

OE6A083748 +
JulyOFF
JulyON
Normalized counts
8
7
6
5
4
3
2
1.5
1
0.1
E001
E002
E003
E004
E005
E006
E007
E008
E009
52373
52714
53056
53397
53739
54080
54422
54763
55105
55446

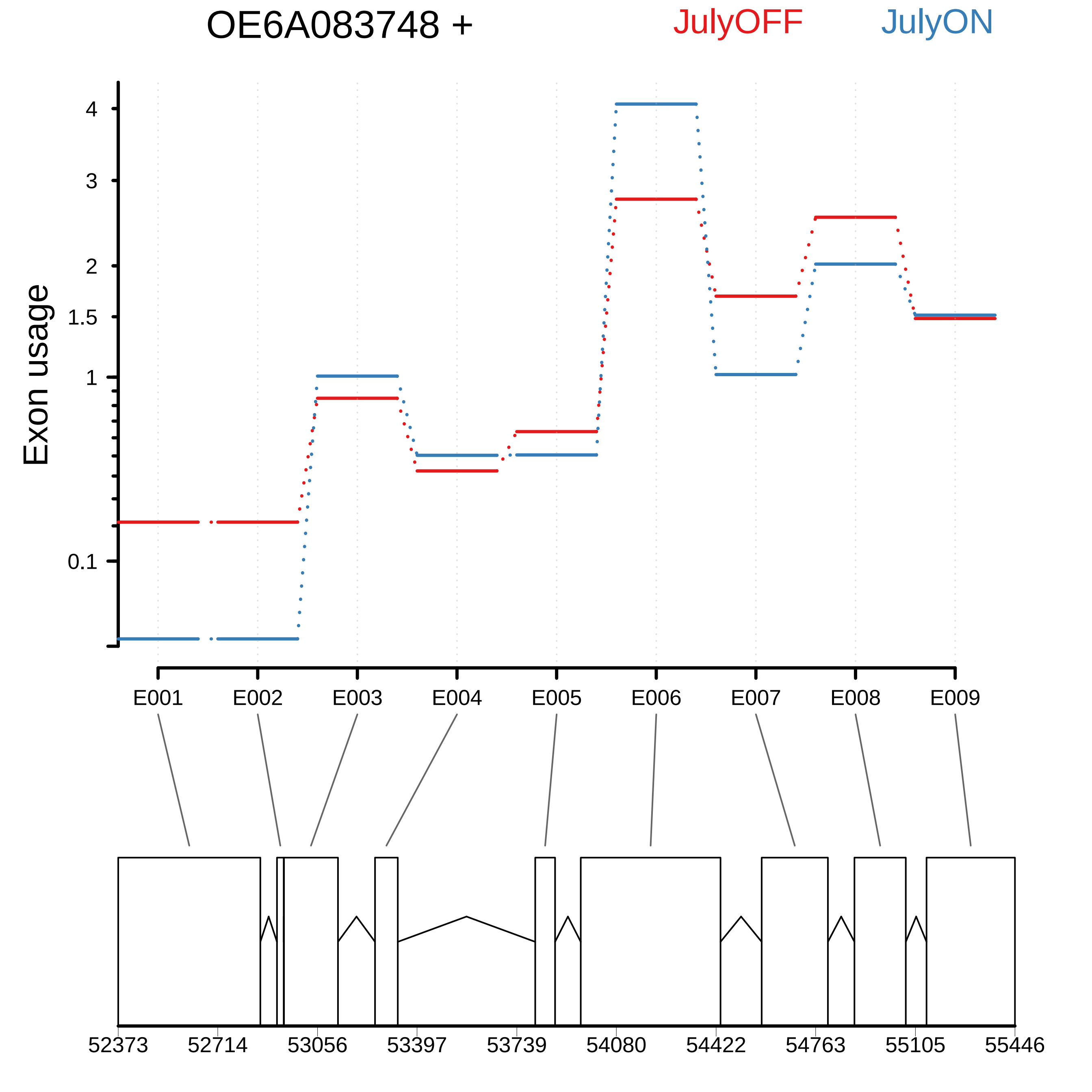
OE6A083748 +
JulyOFF
JulyON
Exon usage
4
3
2
1.5
1
0.1
E001
E002
E003
E004
E005
E006
E007
E008
E009
52373
52714
53056
53397
53739
54080
54422
54763
55105
55446

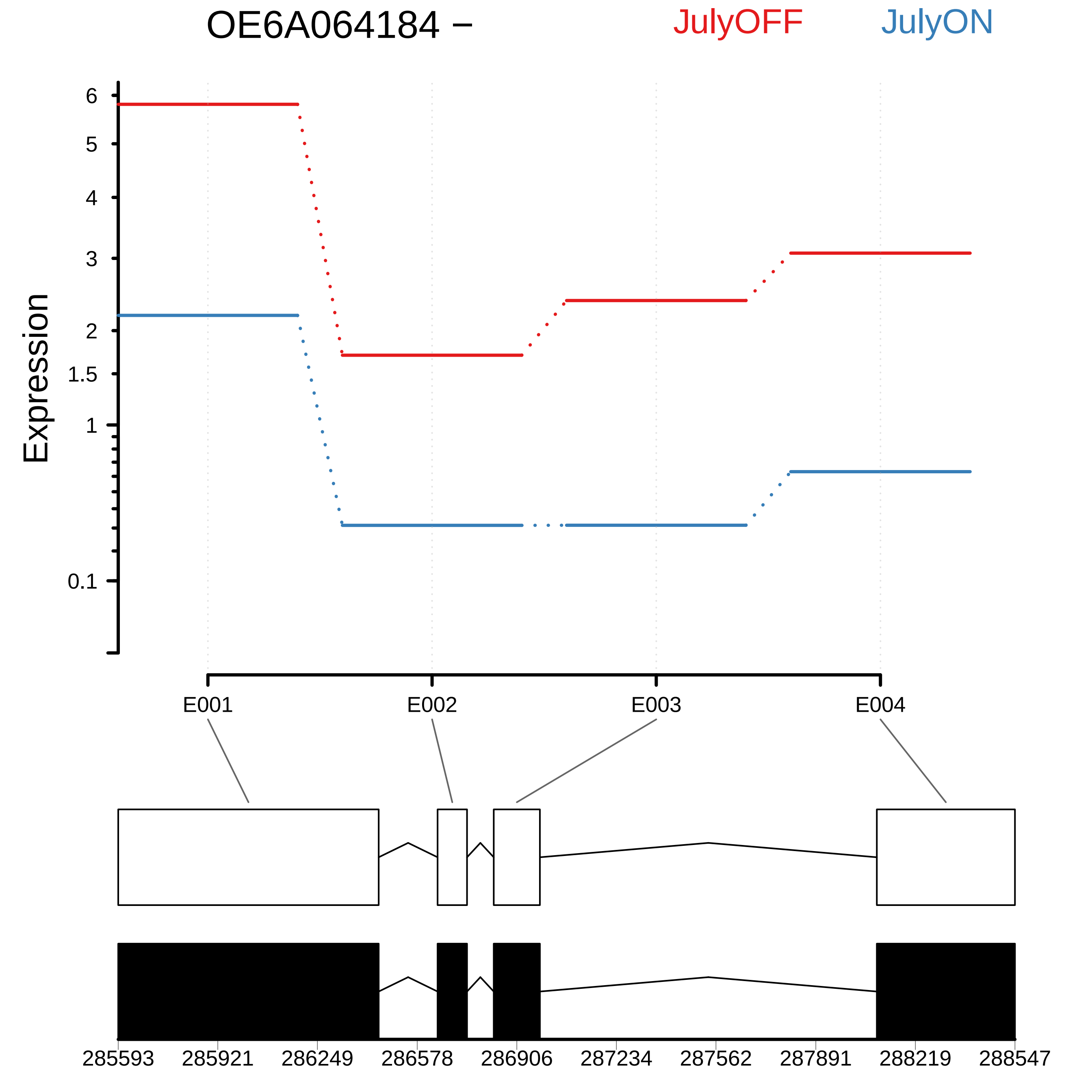

OE6A064184 −
JulyOFF
JulyON
Expression
6
5
4
3
2
1.5
1
0.1
E001
E002
E003
E004
285593
285921
286249
286578
286906
287234
287562
287891
288219
288547

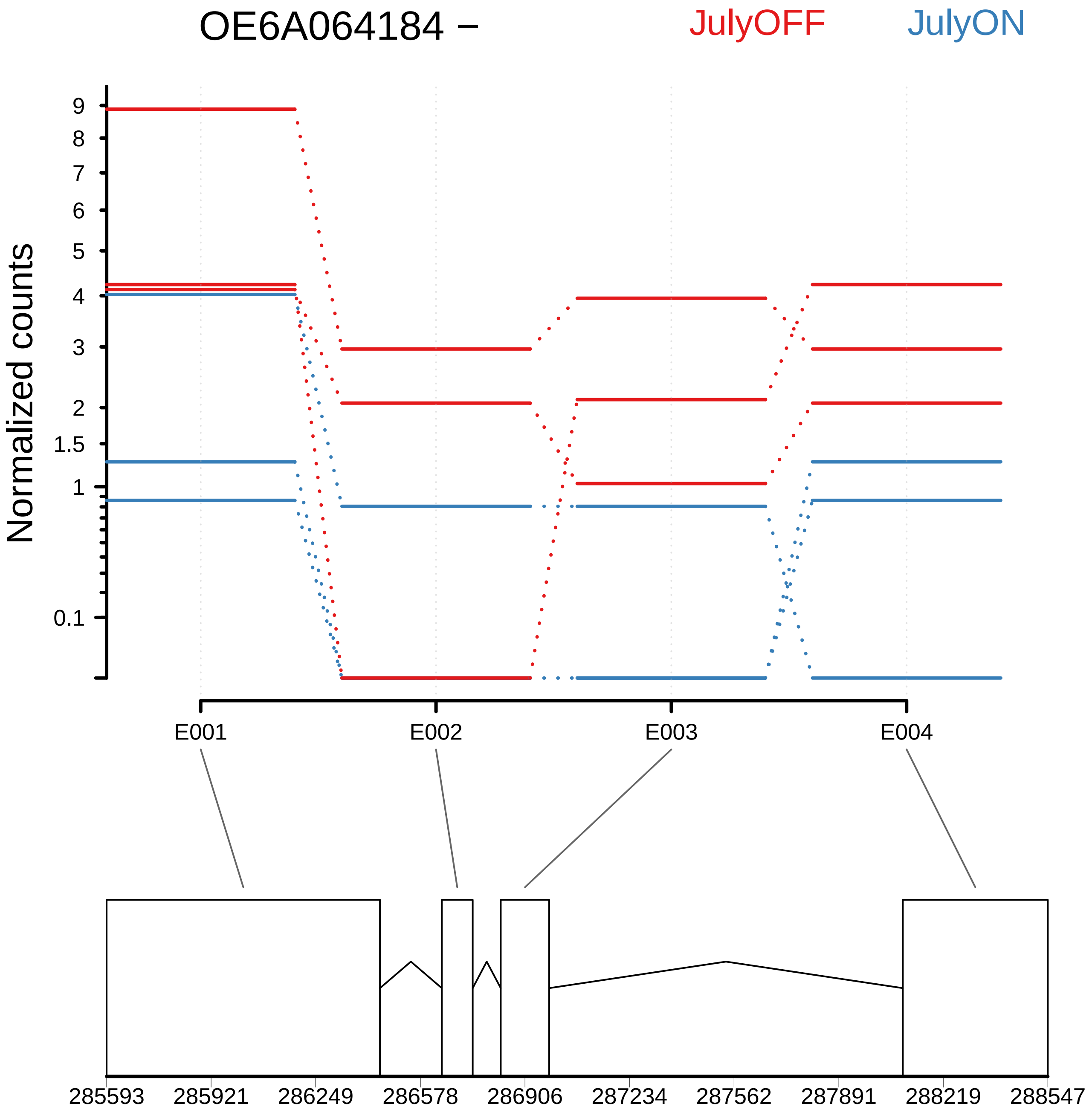

OE6A064184 −
JulyOFF
JulyON
Normalized counts
9
8
7
6
5
4
3
2
1.5
1
0.1
E001
E002
E003
E004
285593
285921
286249
286578
286906
287234
287562
287891
288219
288547

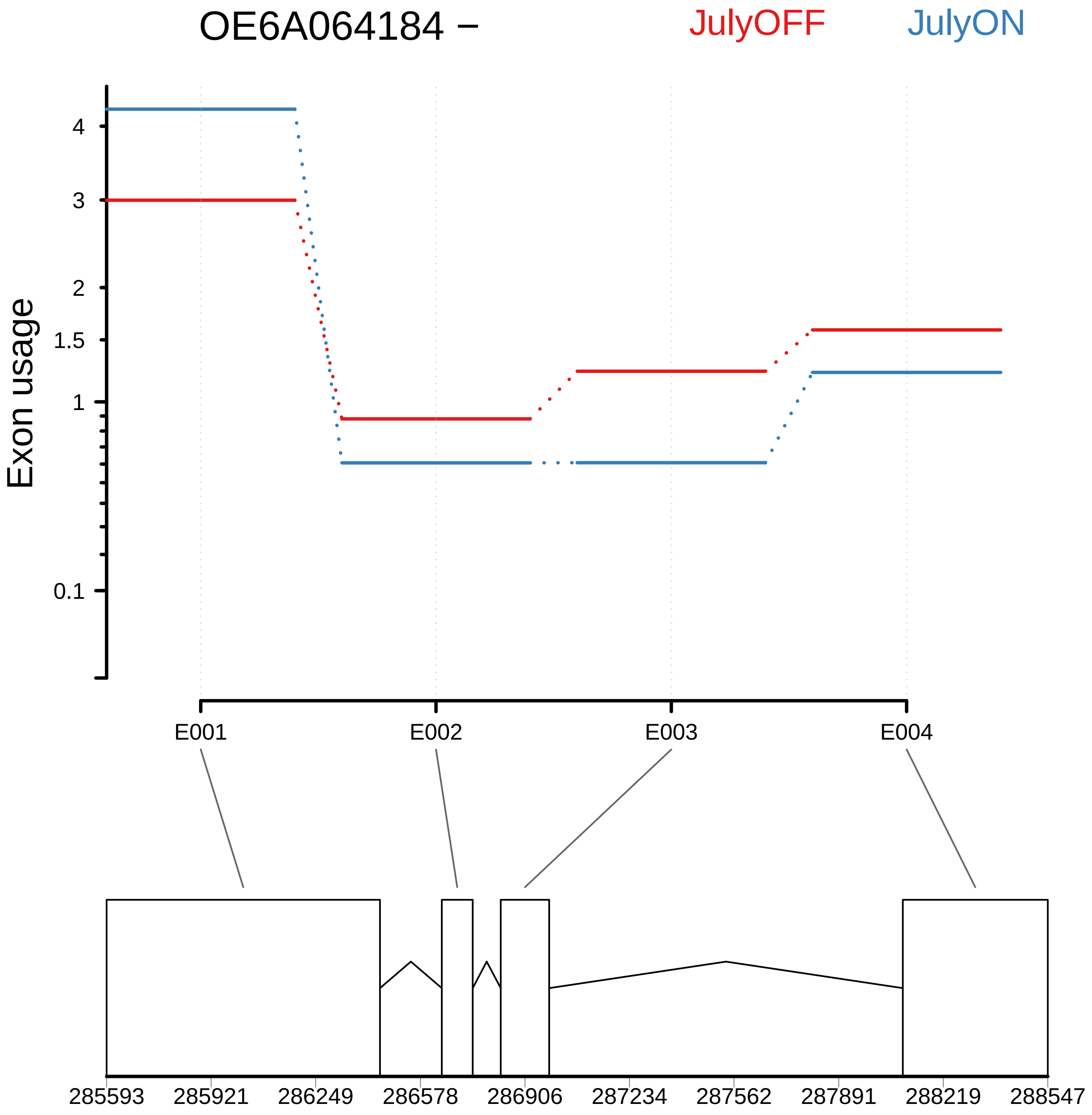

OE6A064184 −
JulyOFF
JulyON
Exon usage
4
3
2
1.5
1
0.1
E001
E002
E003
E004
285593
285921
286249
286578
286906
287234
287562
287891
288219
288547

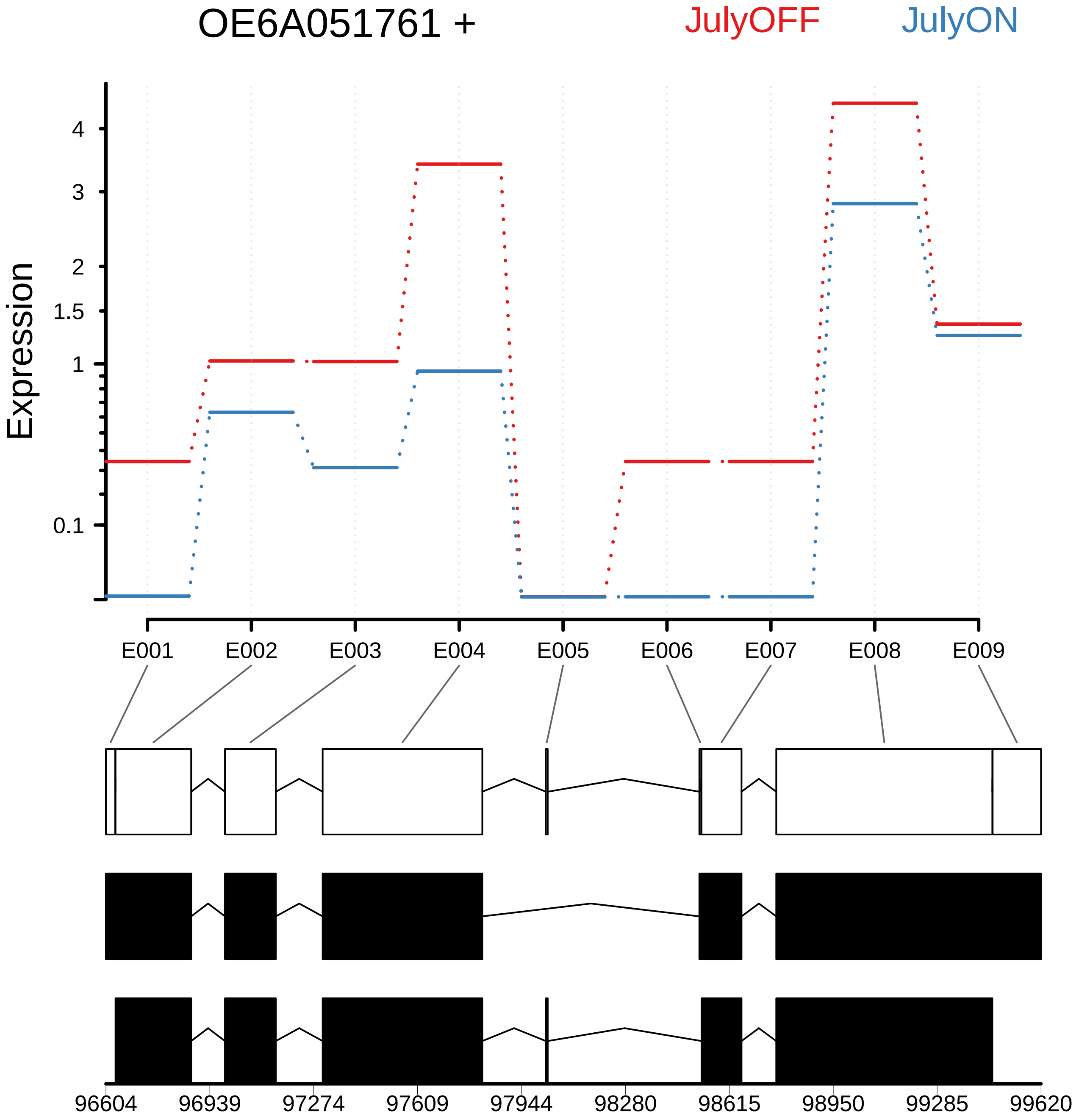

OE6A051761 +
JulyOFF
JulyON
Expression
4
3
2
1.5
1
0.1
E001
E002
E003
E004
E005
E006
E007
E008
E009
96604
96939
97274
97609
97944
98280
98615
98950
99285
99620

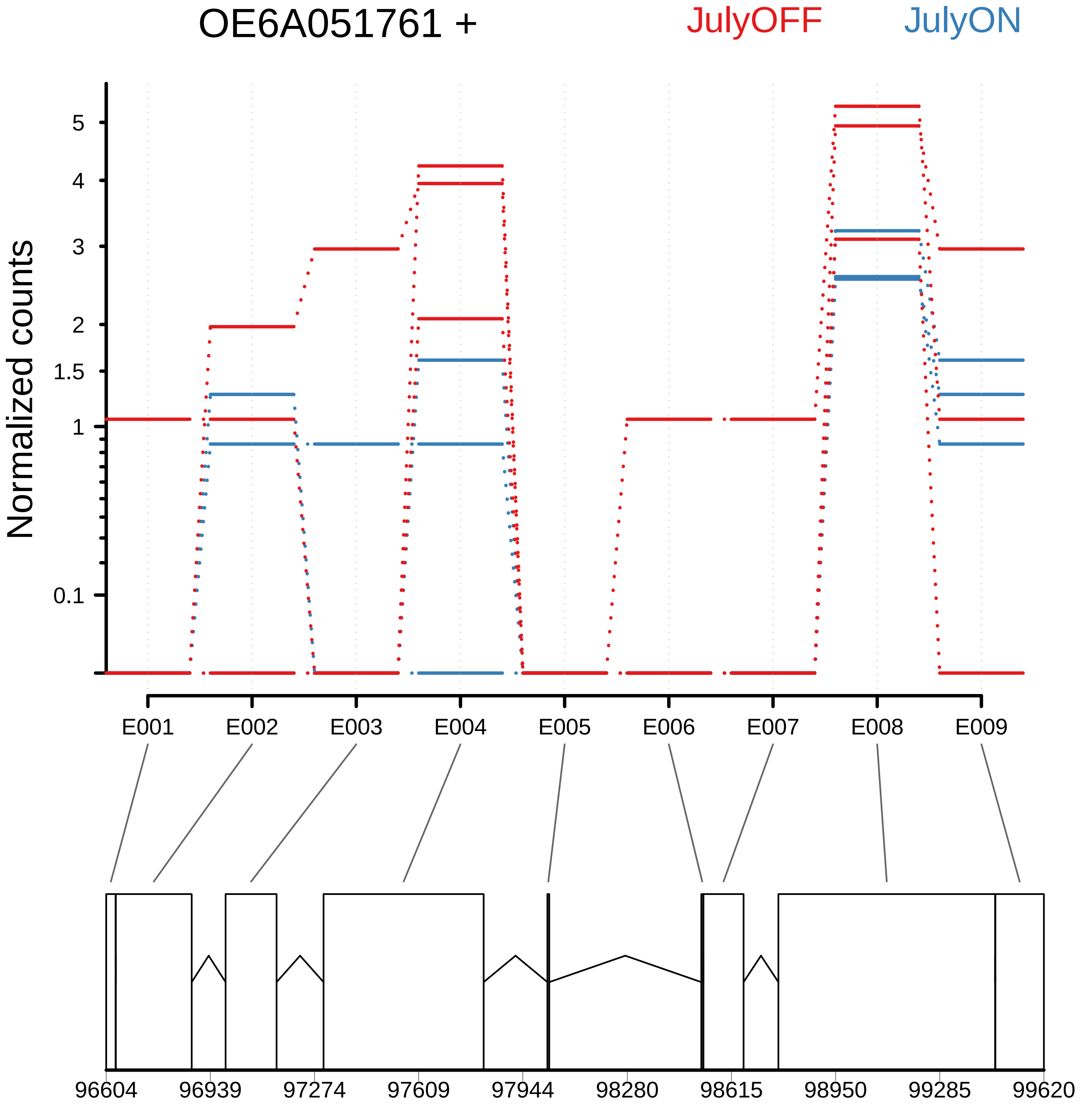
OE6A051761 +
JulyOFF
JulyON
Normalized counts
5
4
3
2
1.5
1
0.1
E001
E002
E003
E004
E005
E006
E007
E008
E009
96604
96939
97274
97609
97944
98280
98615
98950
99285
99620

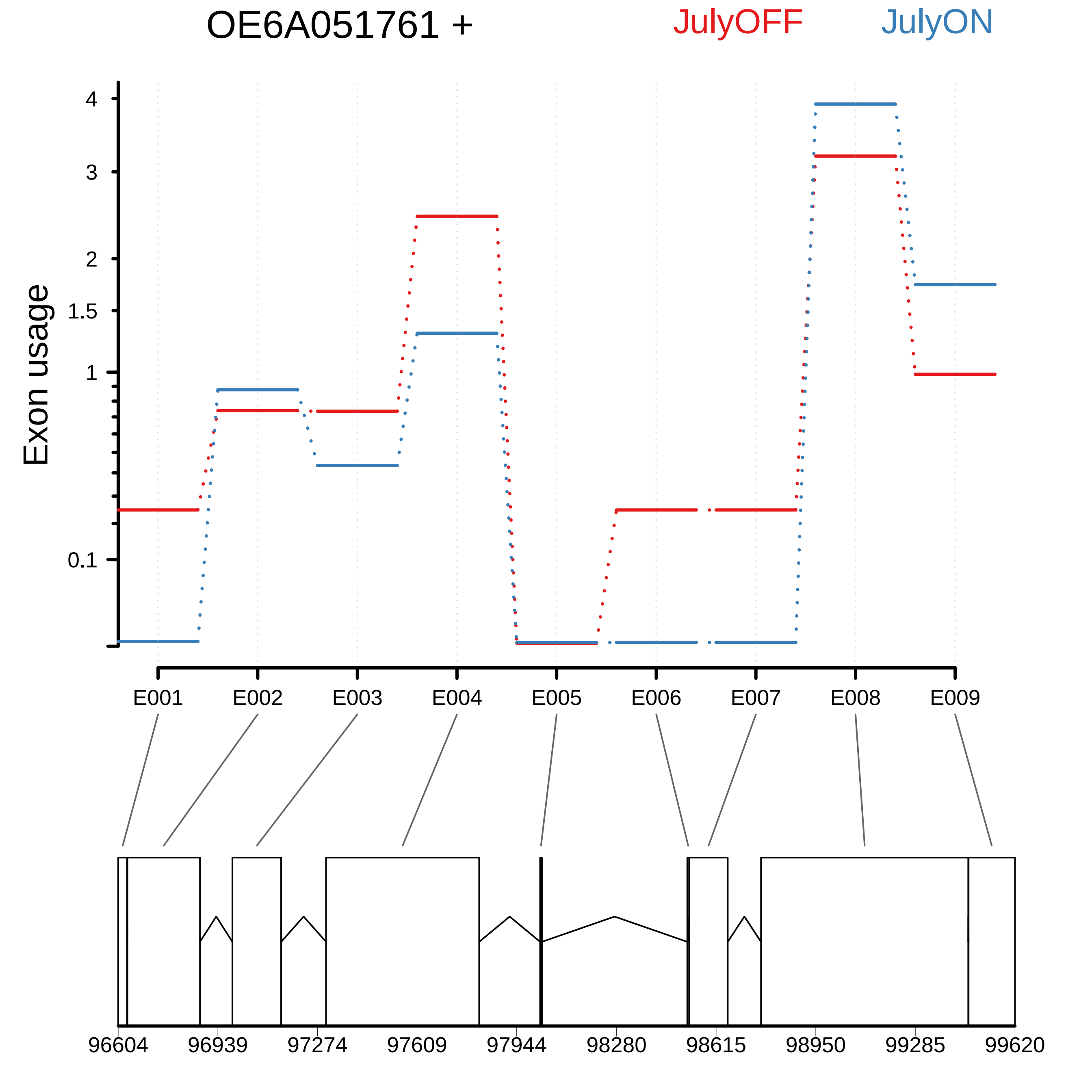

OE6A051761 +
JulyOFF
JulyON
Exon usage
4
3
2
1.5
1
0.1
E001
E002
E003
E004
E005
E006
E007
E008
E009
96604
96939
97274
97609
97944
98280
98615
98950
99285
99620

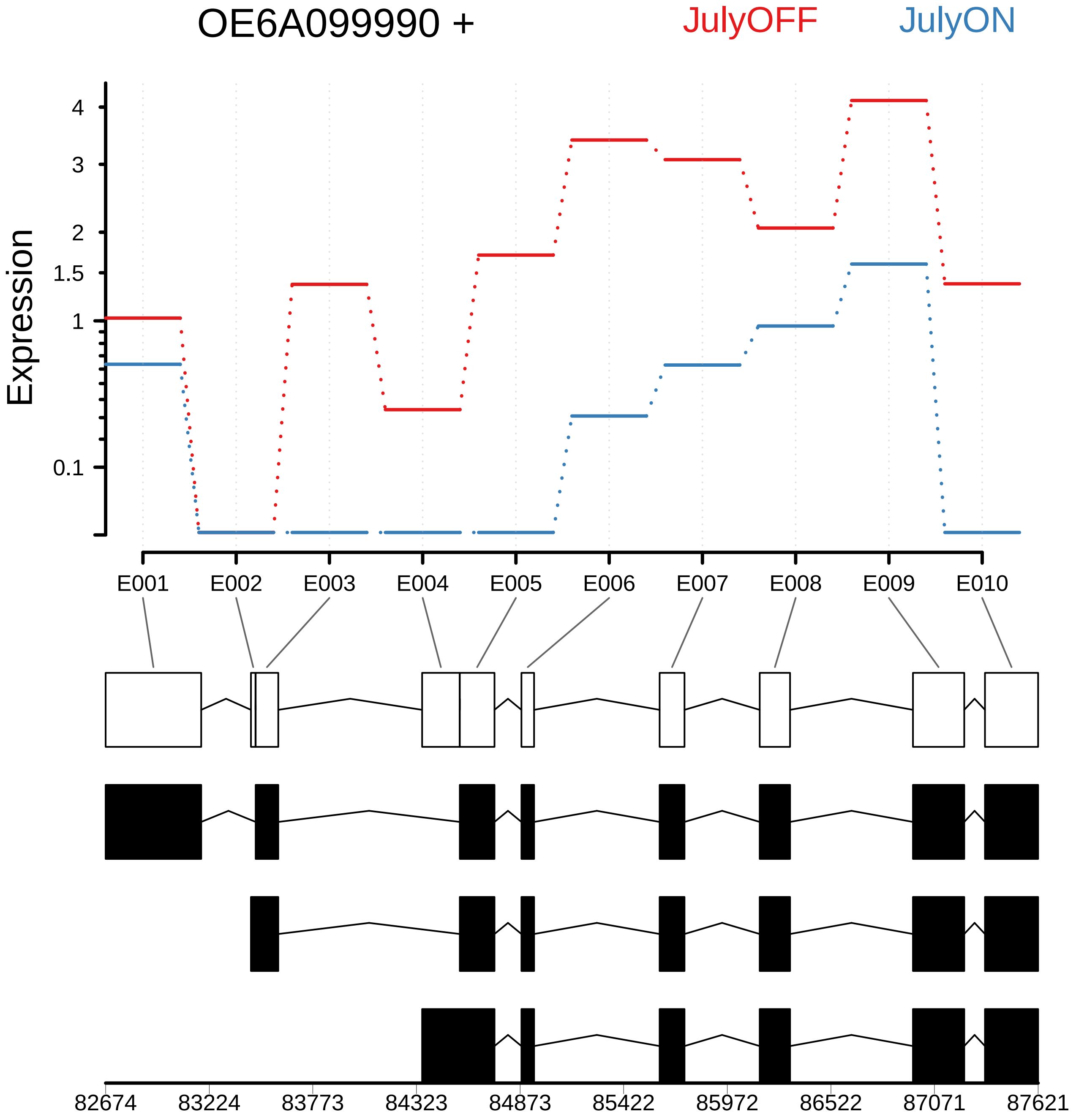

OE6A099990 +
JulyOFF
JulyON
Expression
4
3
2
1.5
1
0.1
E001
E002
E003
E004
E005
E006
E007
E008
E009
E010
82674
83224
83773
84323
84873
85422
85972
86522
87071
87621

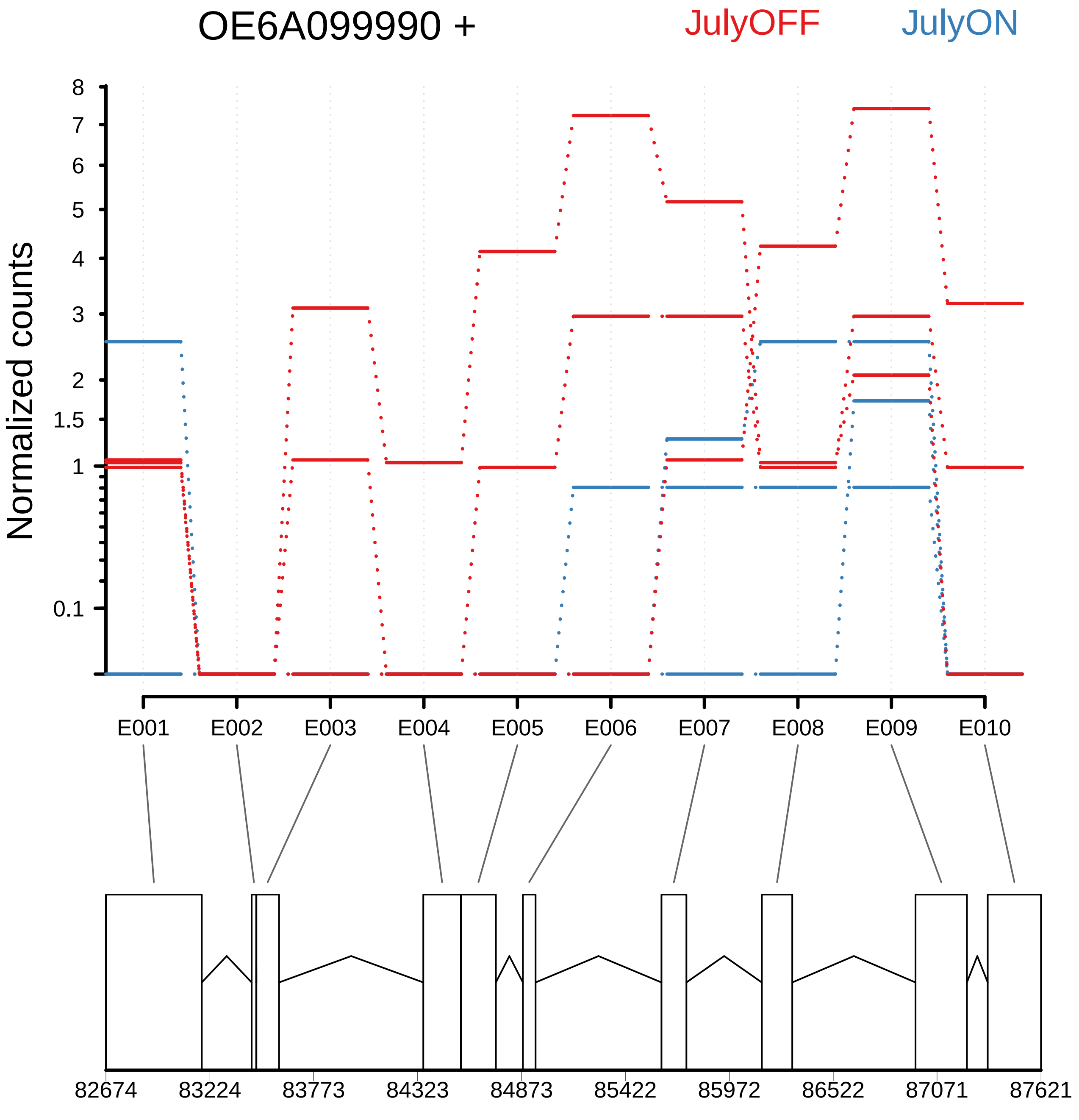
OE6A099990 +
JulyOFF
JulyON
Normalized counts
8
7
6
5
4
3
2
1.5
1
0.1
E001
E002
E003
E004
E005
E006
E007
E008
E009
E010
82674
83224
83773
84323
84873
85422
85972
86522
87071
87621

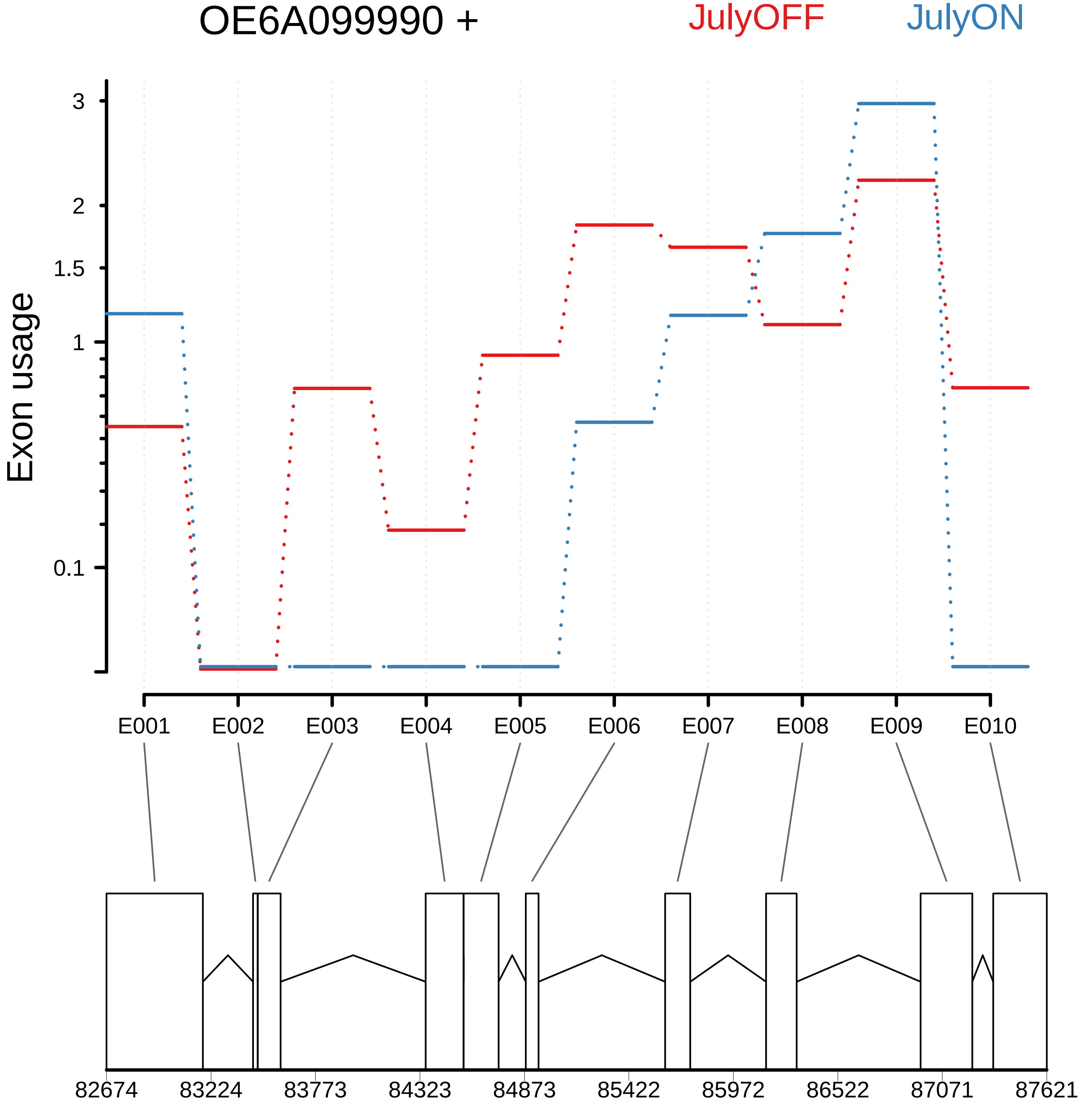
OE6A099990 +
JulyOFF
JulyON
Exon usage
3
2
1.5
1
0.1
E001
E002
E003
E004
E005
E006
E007
E008
E009
E010
82674
83224
83773
84323
84873
85422
85972
86522
87071
87621

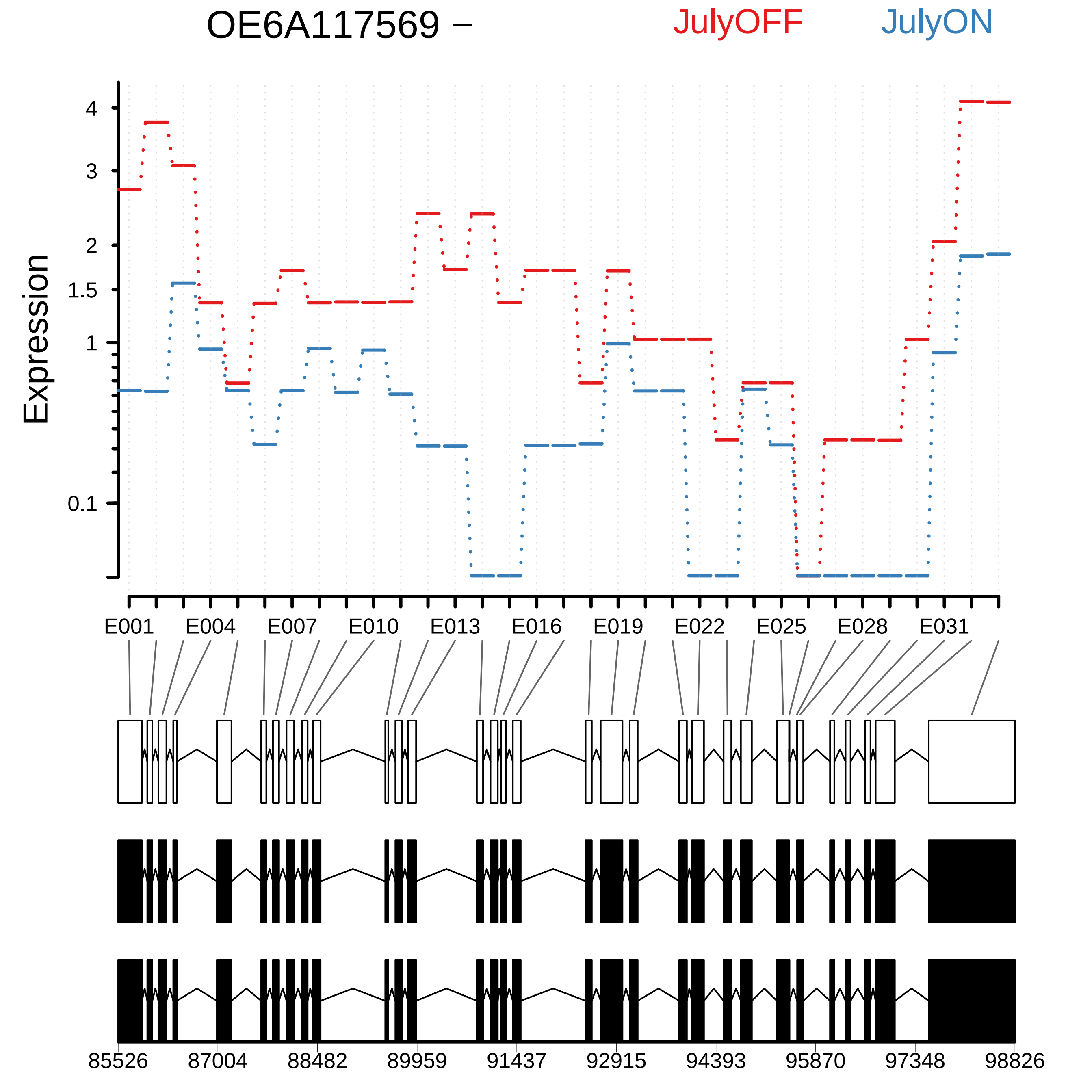

OE6A117569 –
JulyOFF
JulyON
Expression
4
3
2
1.5
1
0.1
E001
E004
E007
E010
E013
E016
E019
E022
E025
E028
E031
85526
87004
88482
89959
91437
92915
94393
95870
97348
98826

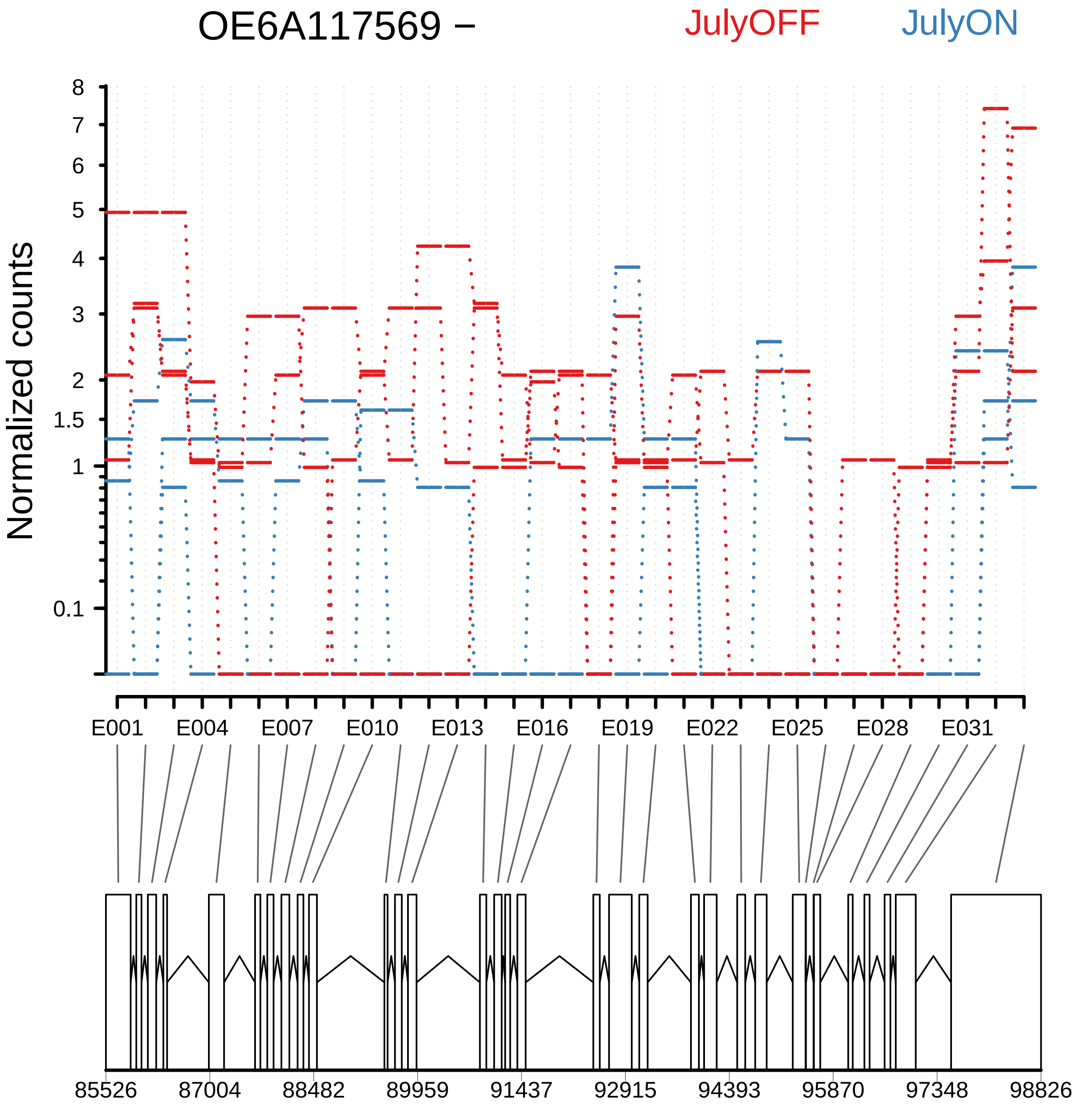
OE6A117569 –
JulyOFF
JulyON
Normalized counts
8
7
6
5
4
3
2
1.5
1
0.1
E001
E004
E007
E010
E013
E016
E019
E022
E025
E028
E031
85526
87004
88482
89959
91437
92915
94393
95870
97348
98826

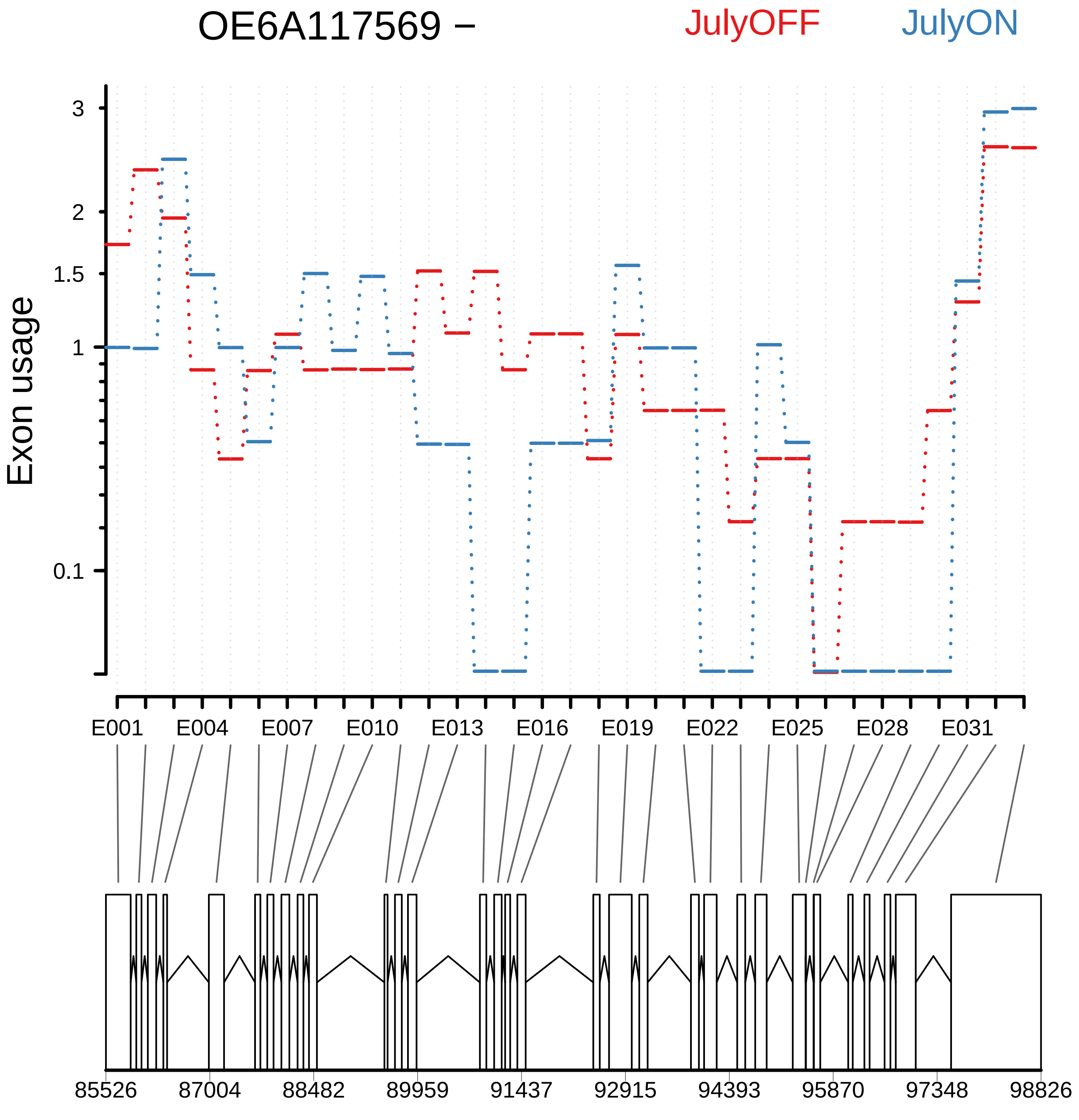

OE6A117569 –
JulyOFF
JulyON
Exon usage
3
2
1.5
1
0.1
E001
E004
E007
E010
E013
E016
E019
E022
E025
E028
E031
85526
87004
88482
89959
91437
92915
94393
95870
97348
98826

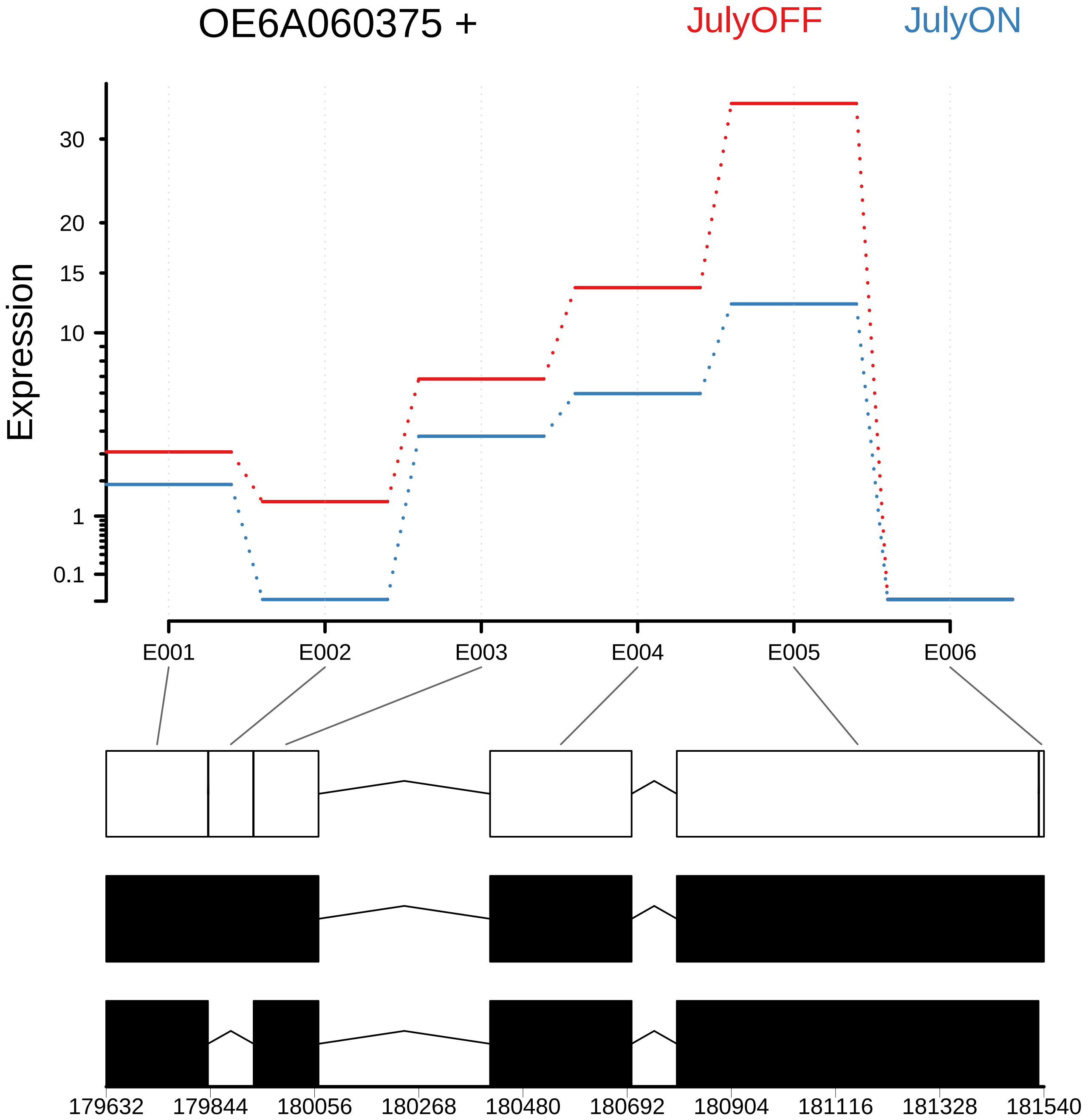
OE6A060375 +
JulyOFF
JulyON
Expression
30
20
15
10
1
0.1
E001
E002
E003
E004
E005
E006
179632
179844
180056
180268
180480
180692
180904
181116
181328
181540

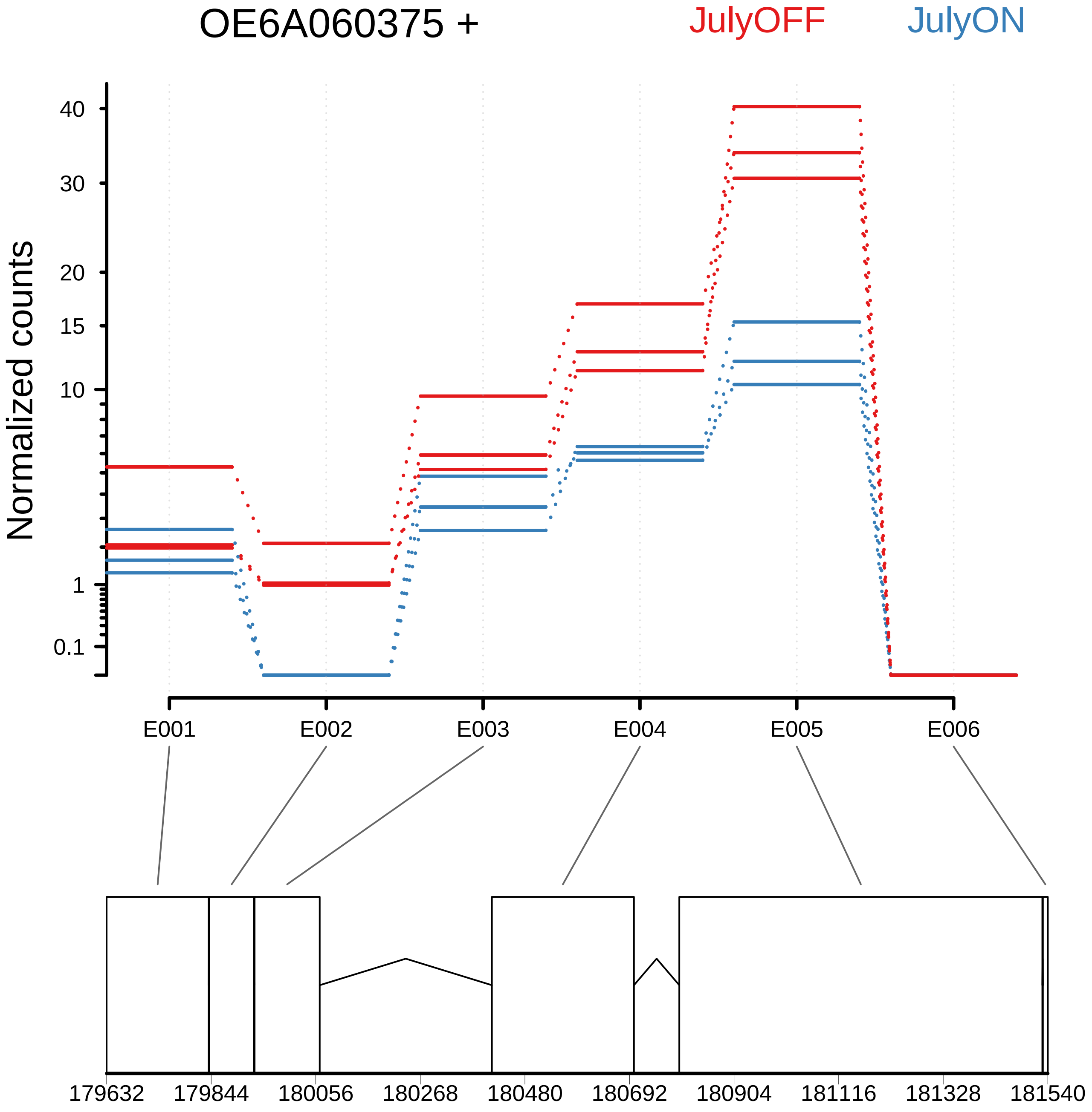

OE6A060375 +
JulyOFF
JulyON
Normalized counts
40
30
20
15
10
1
0.1
E001
E002
E003
E004
E005
E006
179632
179844
180056
180268
180480
180692
180904
181116
181328
181540

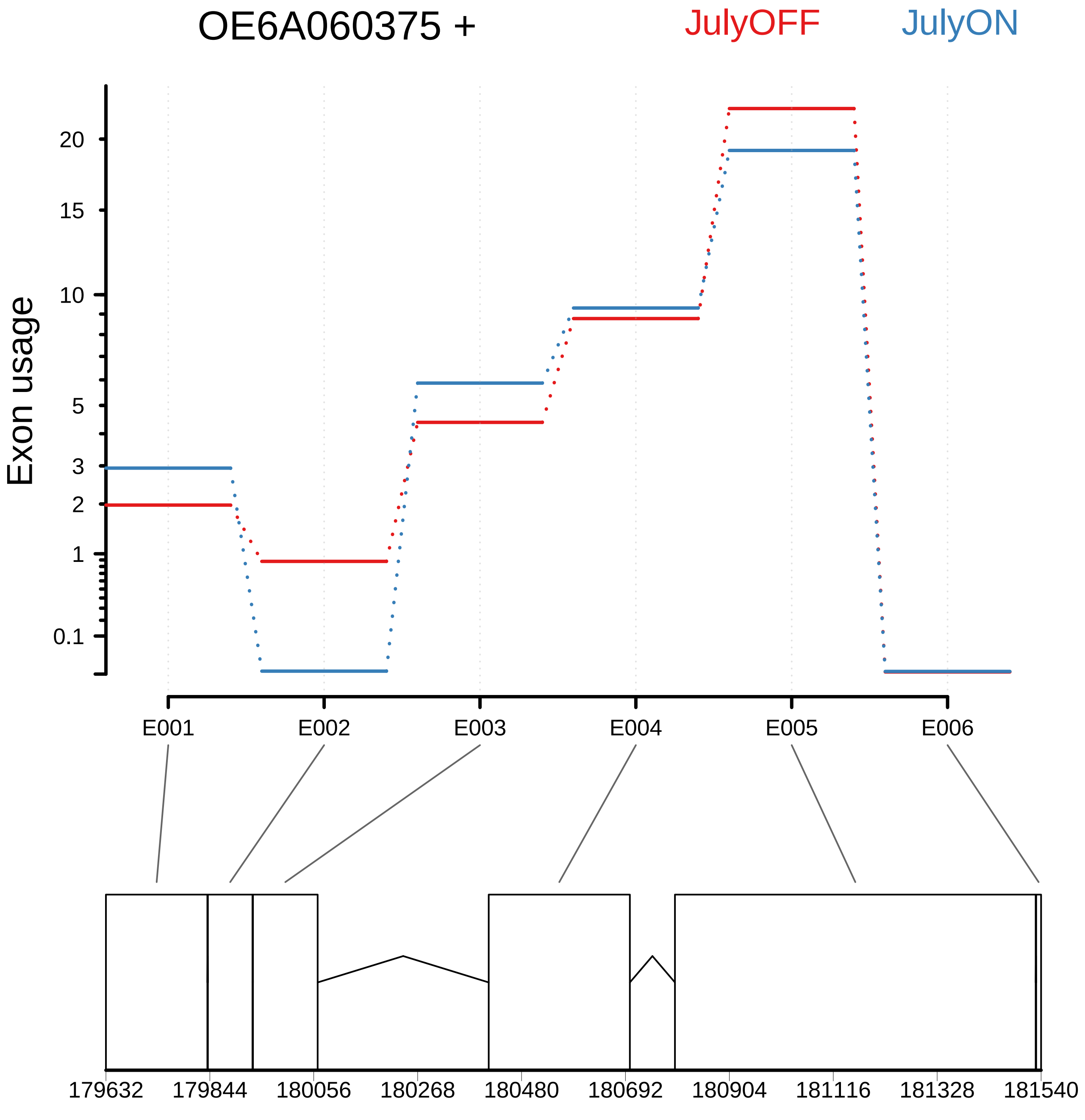
OE6A060375 +
JulyOFF
JulyON
Exon usage
20
15
10
5
3
2
1
0.1
E001
E002
E003
E004
E005
E006
179632
179844
180056
180268
180480
180692
180904
181116
181328
181540

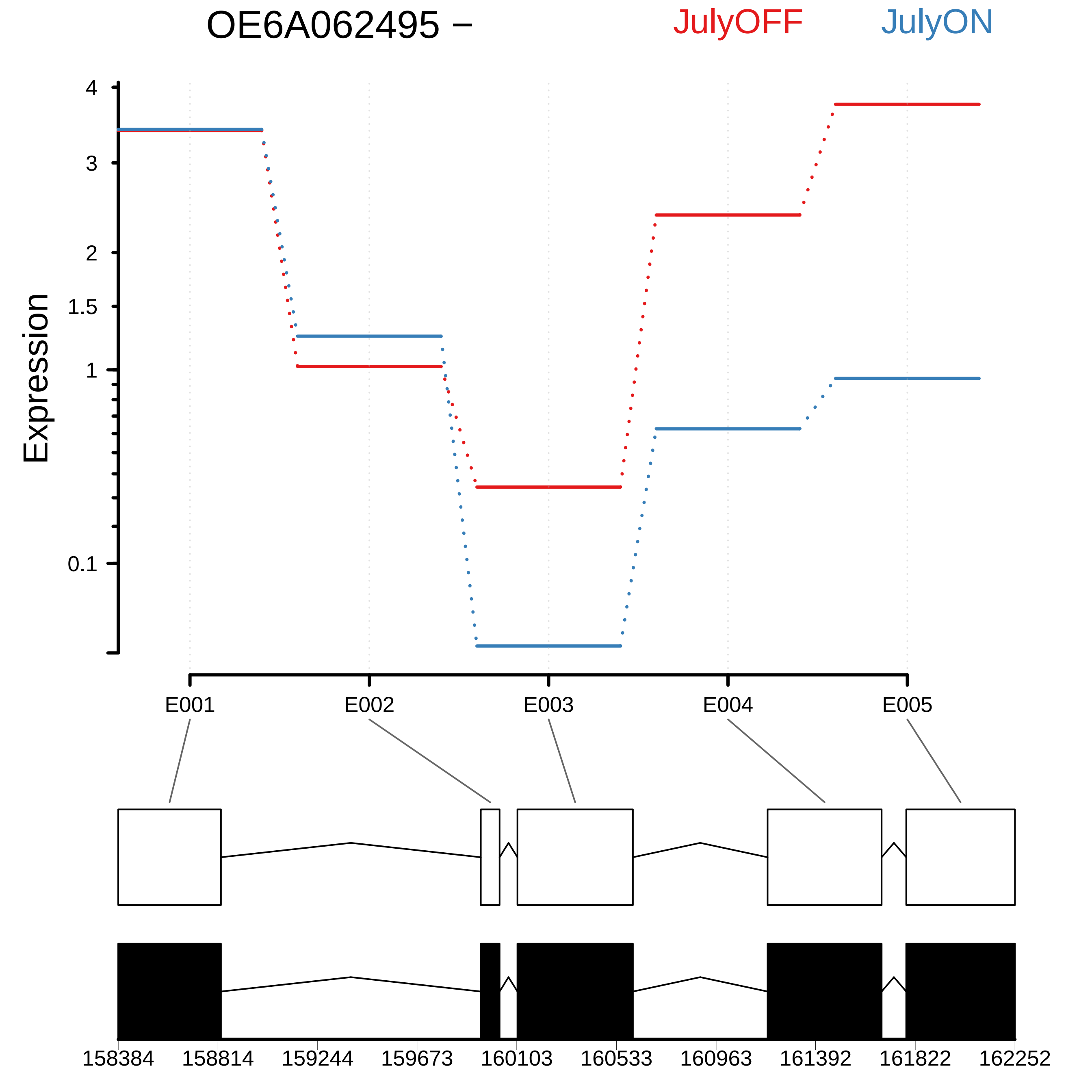

OE6A062495 −
JulyOFF
JulyON
Expression
4
3
2
1.5
1
0.1
E001
E002
E003
E004
E005
158384
158814
159244
159673
160103
160533
160963
161392
161822
162252

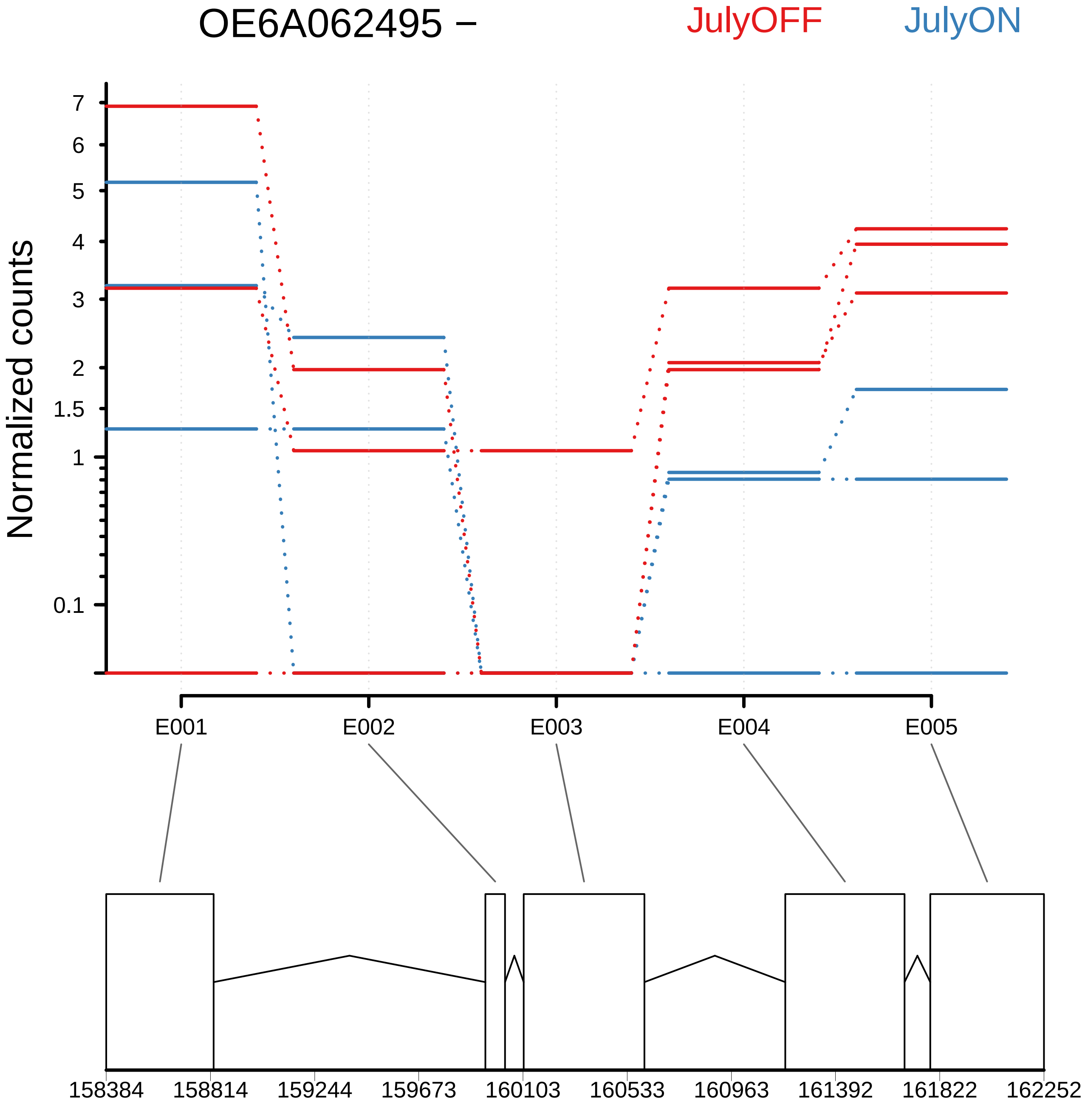

OE6A062495 –
JulyOFF
JulyON
Normalized counts
7
6
5
4
3
2
1.5
1
0.1
E001
E002
E003
E004
E005
158384
158814
159244
159673
160103
160533
160963
161392
161822
162252

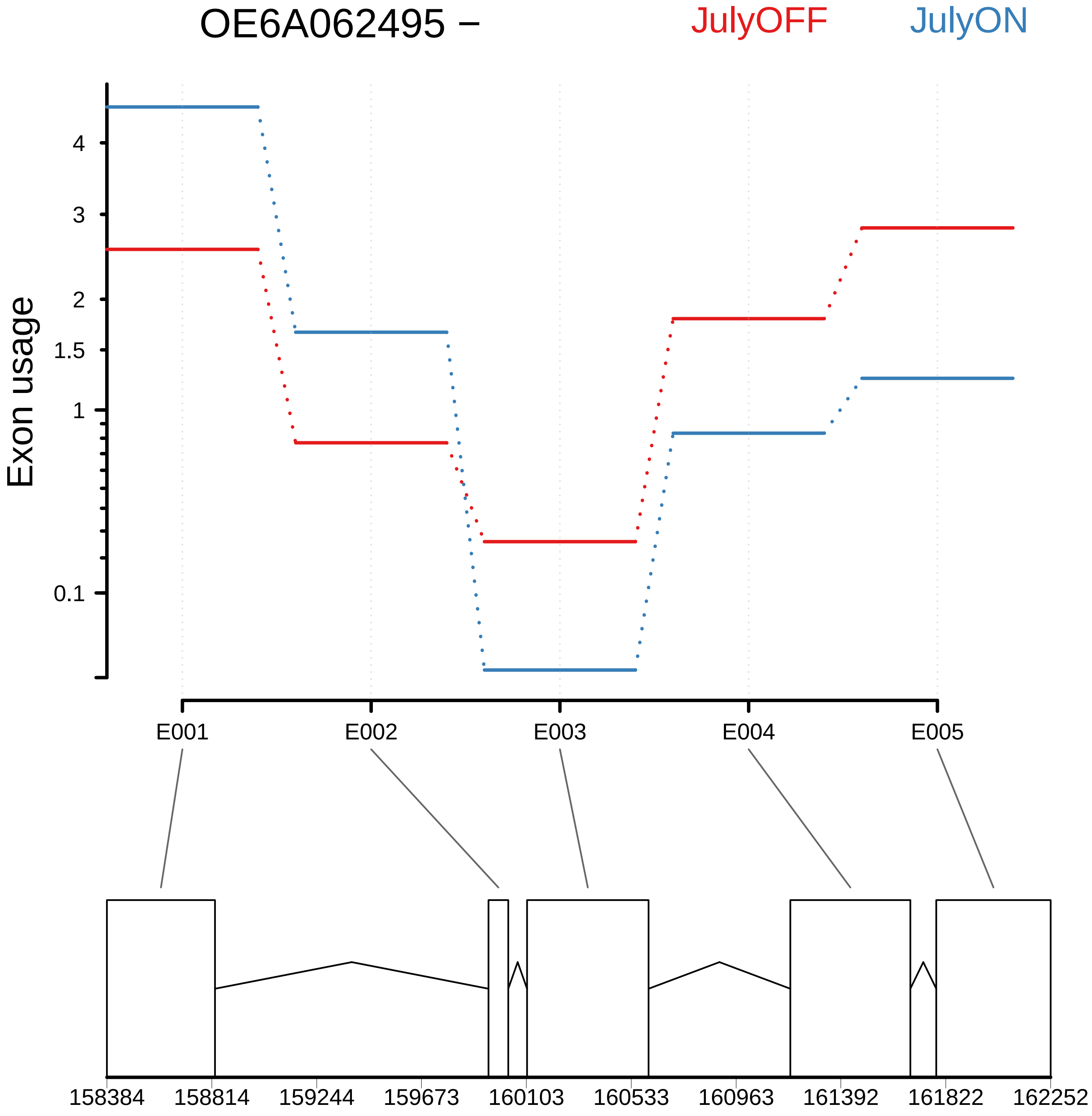

OE6A062495 −
JulyOFF
JulyON
Exon usage
4
3
2
1.5
1
0.1
E001
E002
E003
E004
E005
158384
158814
159244
159673
160103
160533
160963
161392
161822
162252

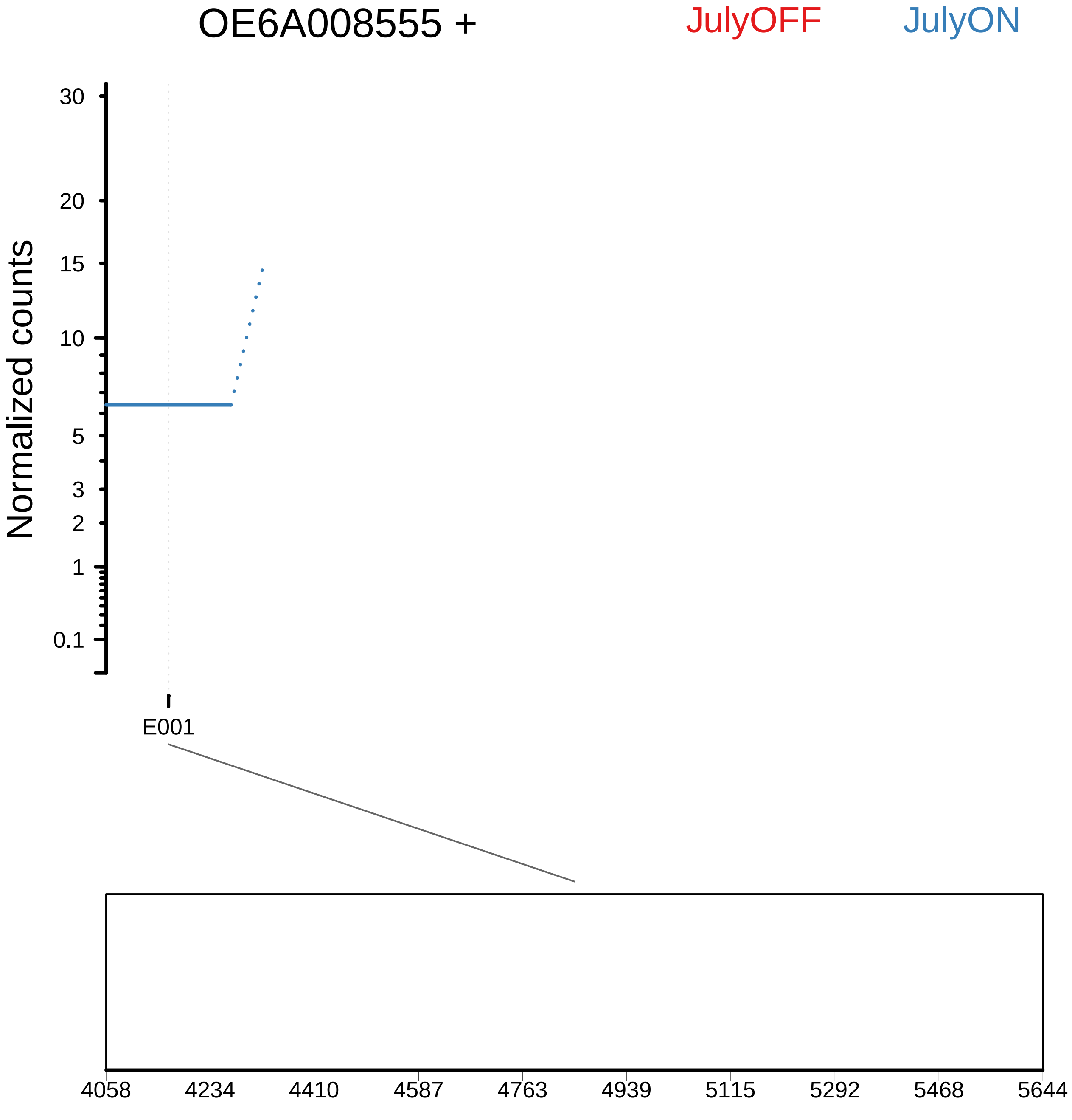

OE6A008555 +
JulyOFF
JulyON
Normalized counts
30
20
15
10
5
3
2
1
0.1
E001
4058
4234
4410
4587
4763
4939
5115
5292
5468
5644

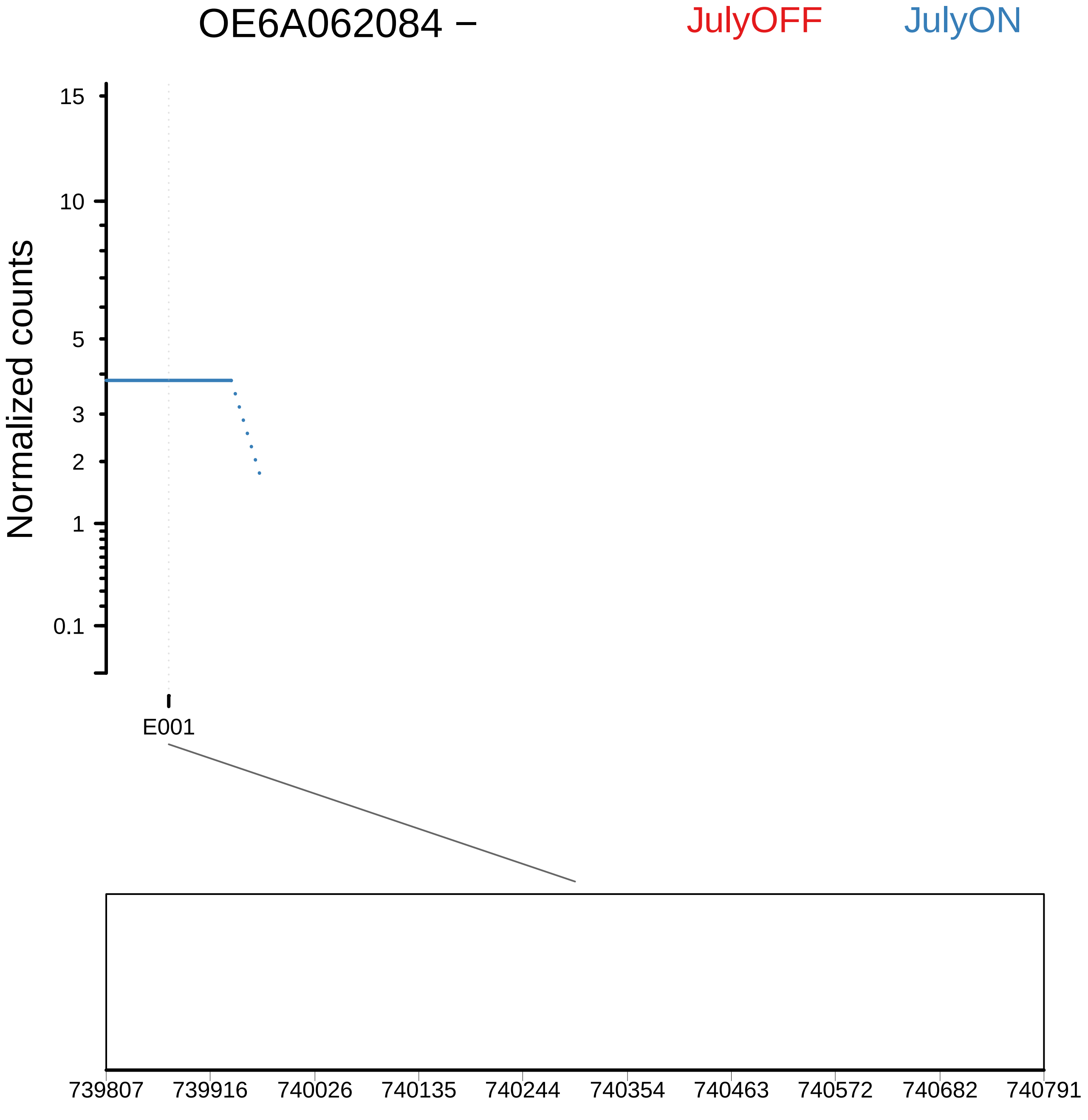

OE6A062084 −
JulyOFF
JulyON
Normalized counts
15
10
5
3
2
1
0.1
E001
739807
739916
740026
740135
740244
740354
740463
740572
740682
740791

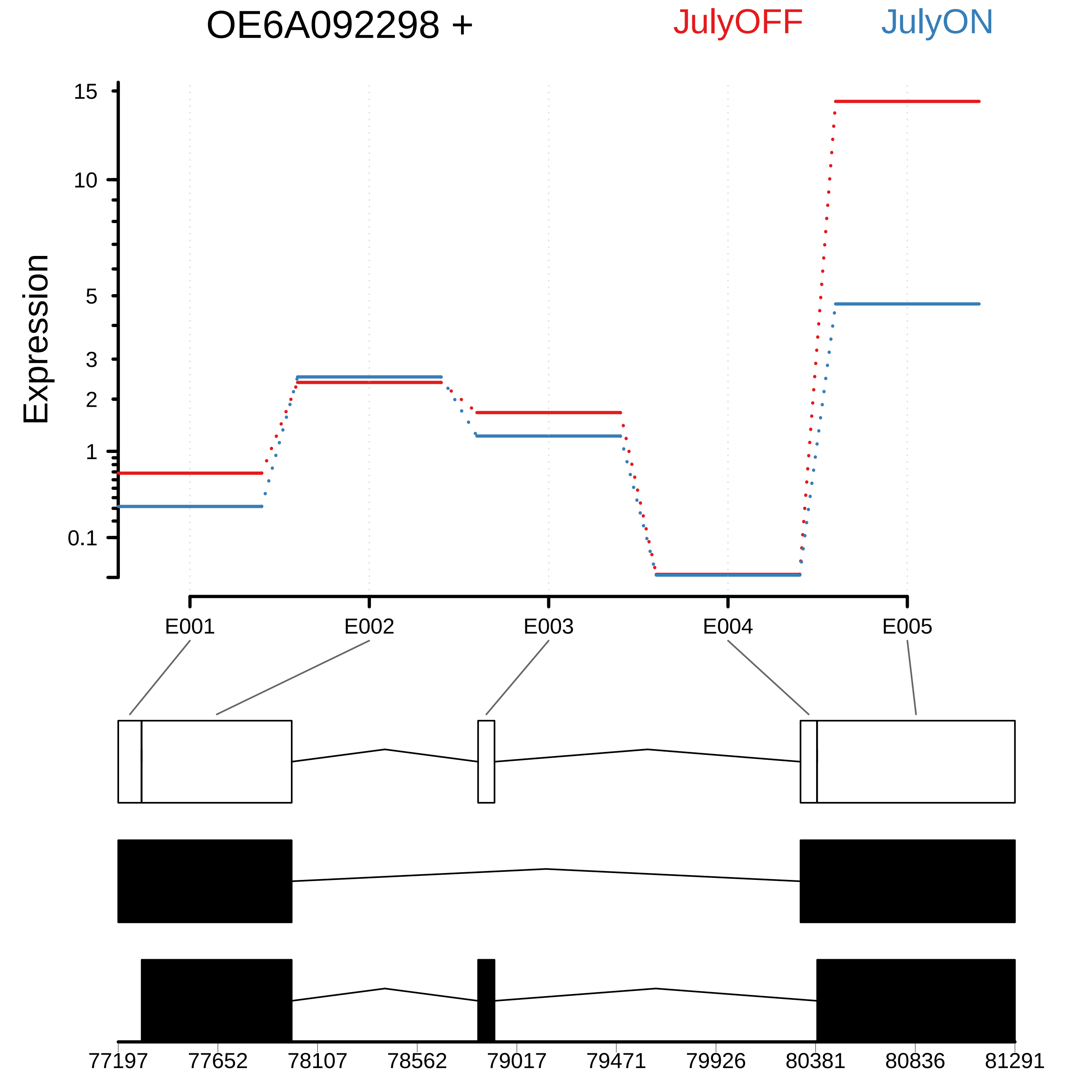

OE6A092298 +
JulyOFF
JulyON
Expression
15
10
5
3
2
1
0.1
E001
E002
E003
E004
E005
77197
77652
78107
78562
79017
79471
79926
80381
80836
81291

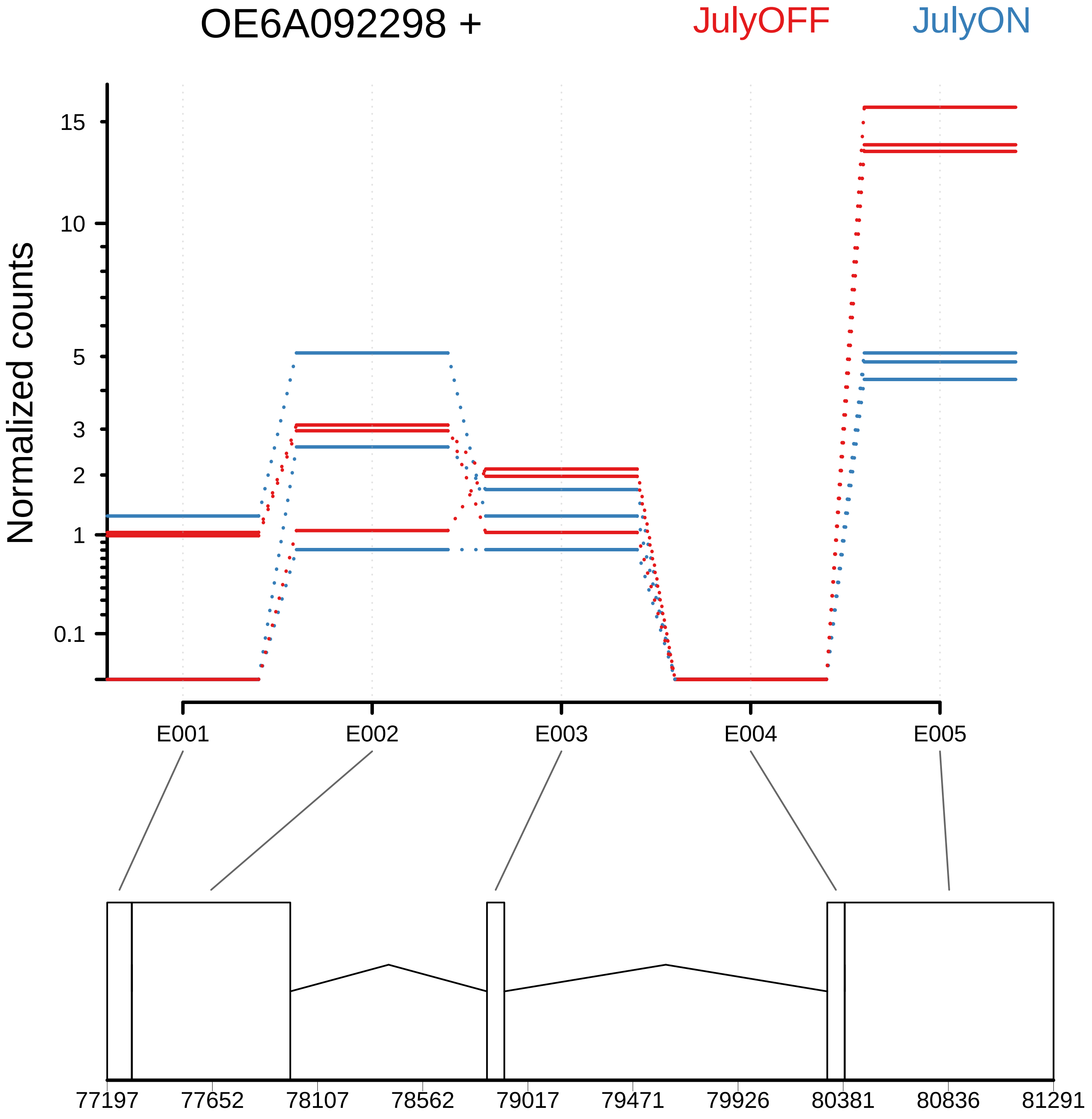
OE6A092298 +
JulyOFF
JulyON
Normalized counts
15
10
5
3
2
1
0.1
E001
E002
E003
E004
E005
77197
77652
78107
78562
79017
79471
79926
80381
80836
81291

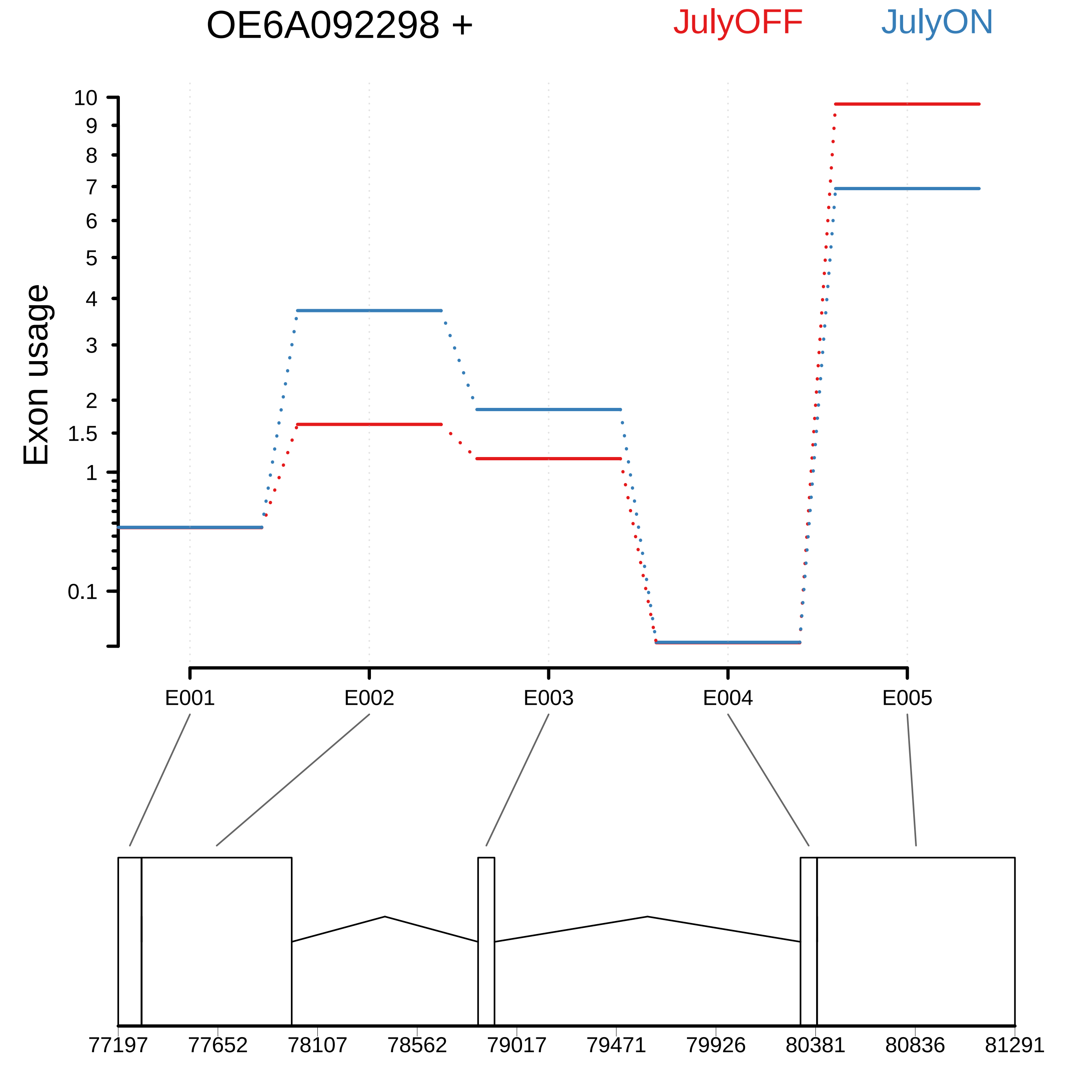
OE6A092298 +
JulyOFF
JulyON
Exon usage
10
9
8
7
6
5
4
3
2
1.5
1
0.1
E001
E002
E003
E004
E005
77197
77652
78107
78562
79017
79471
79926
80381
80836
81291

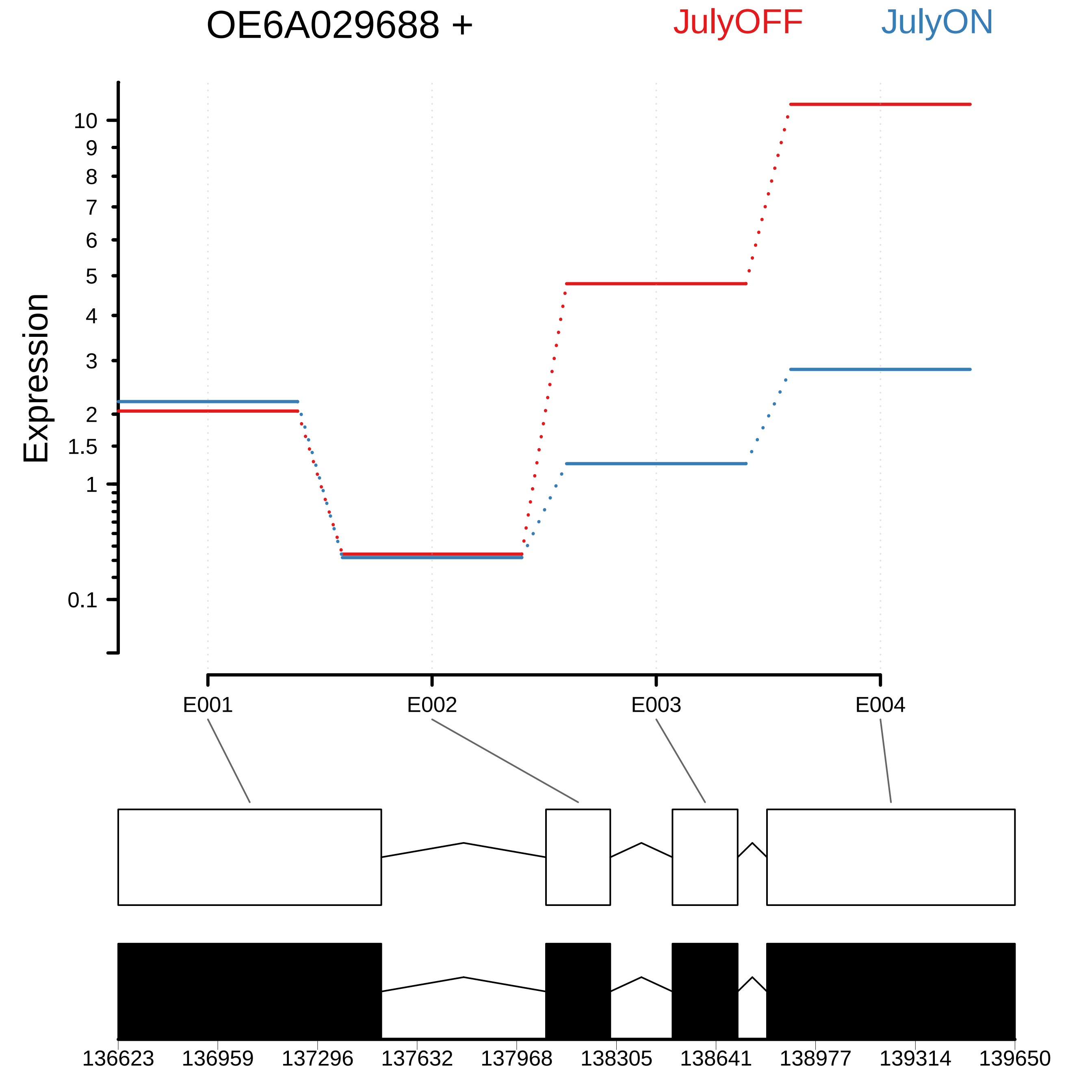
OE6A029688 +
JulyOFF
JulyON
Expression
10
9
8
7
6
5
4
3
2
1.5
1
0.1
E001
E002
E003
E004
136623
136959
137296
137632
137968
138305
138641
138977
139314
139650

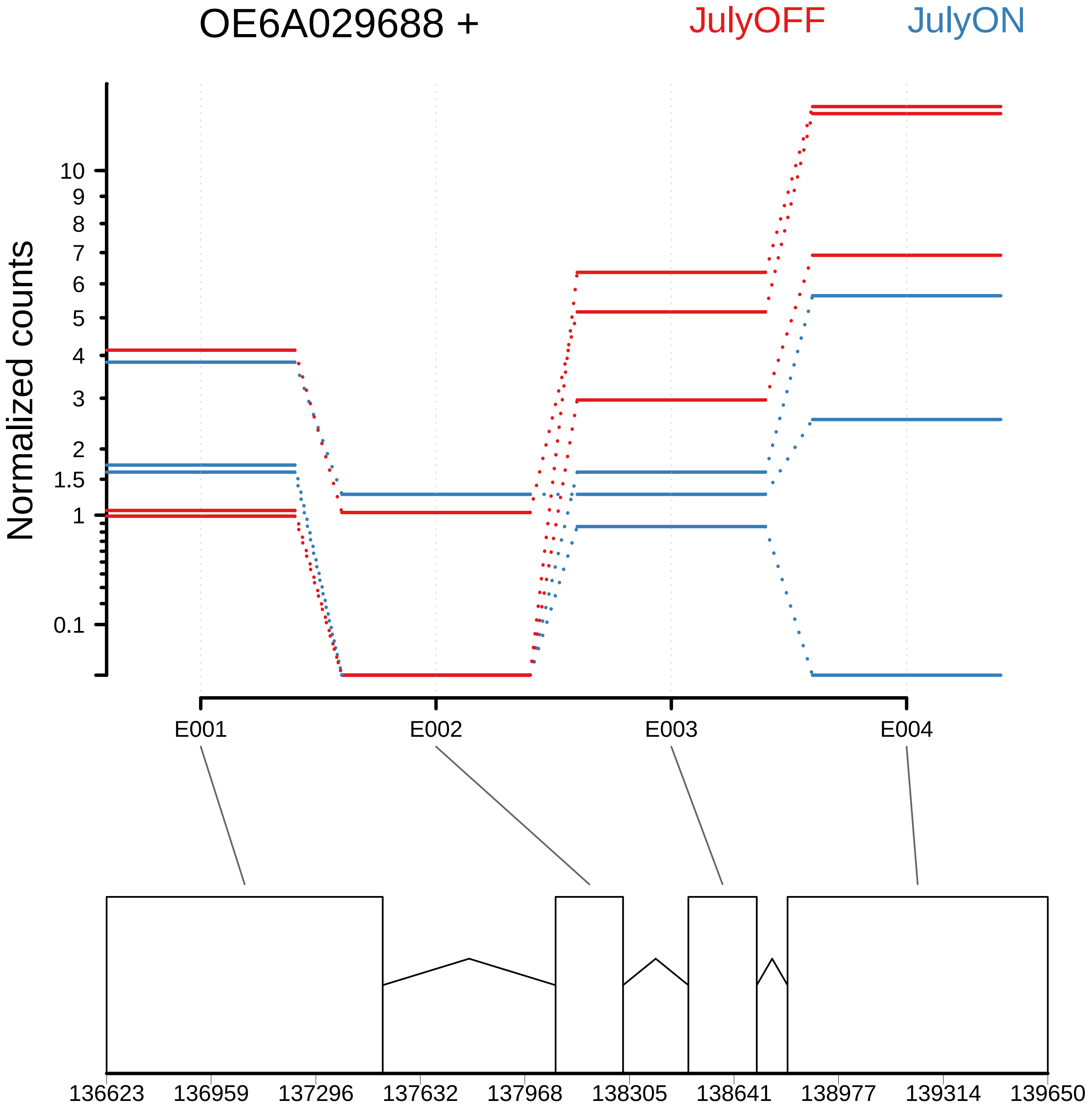
OE6A029688 +
JulyOFF
JulyON
Normalized counts
10
9
8
7
6
5
4
3
2
1.5
1
0.1
E001
E002
E003
E004
136623
136959
137296
137632
137968
138305
138641
138977
139314
139650

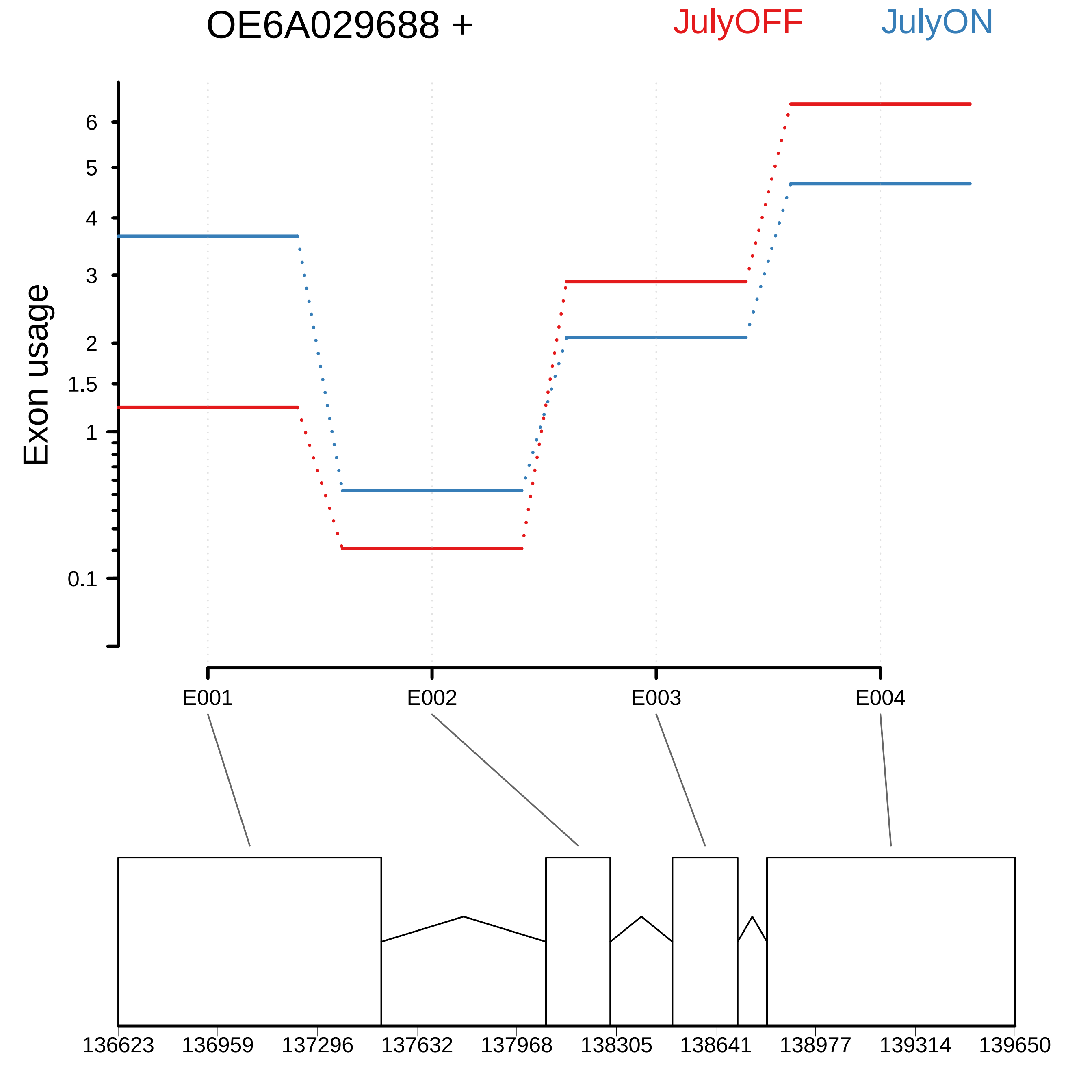

OE6A029688 +
JulyOFF
JulyON
Exon usage
6
5
4
3
2
1.5
1
0.1
E001
E002
E003
E004
136623
136959
137296
137632
137968
138305
138641
138977
139314
139650

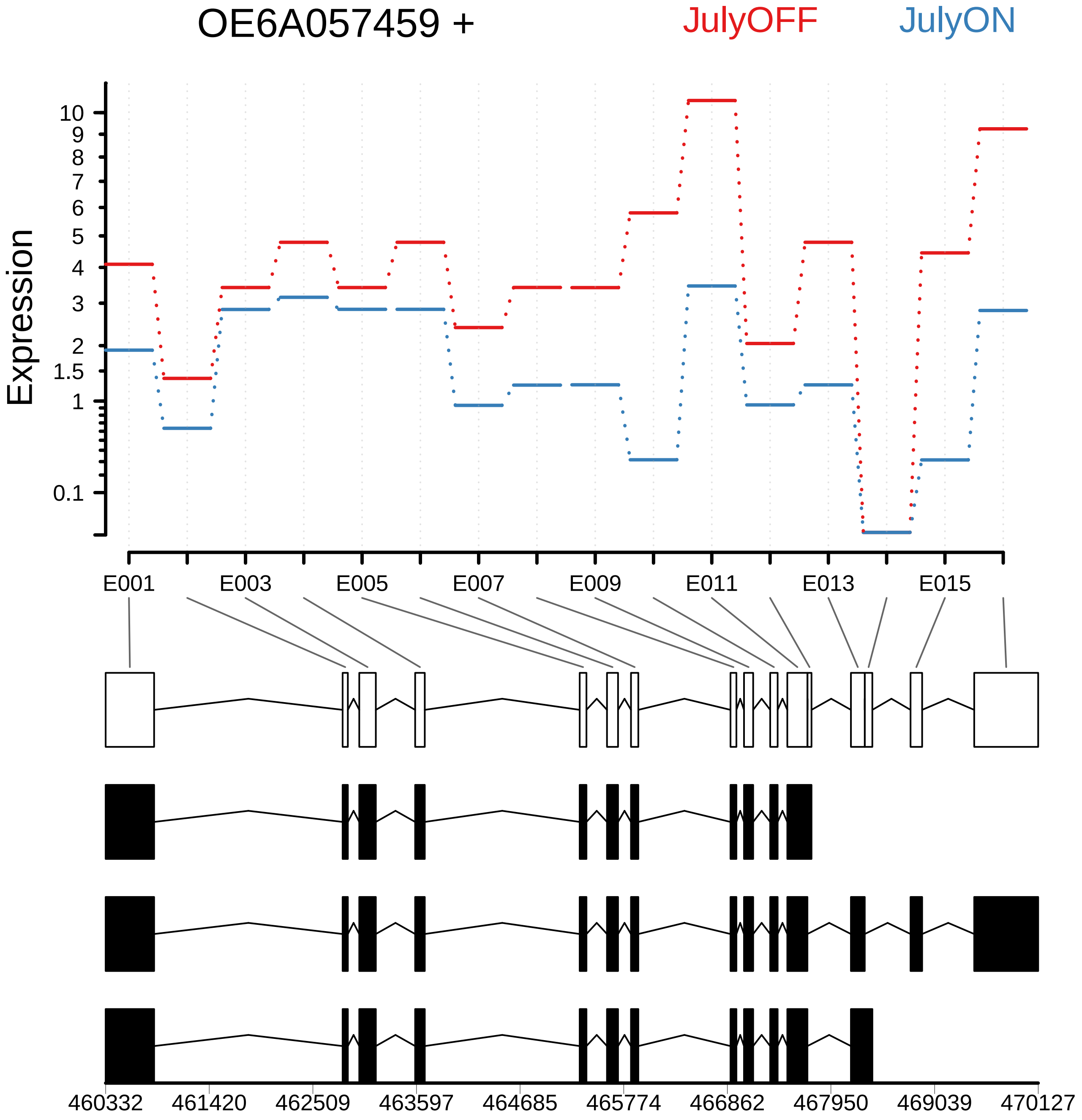

OE6A057459 +
JulyOFF
JulyON
Expression
10
9
8
7
6
5
4
3
2
1.5
1
0.1
E001
E003
E005
E007
E009
E011
E013
E015
460332
461420
462509
463597
464685
465774
466862
467950
469039
470127

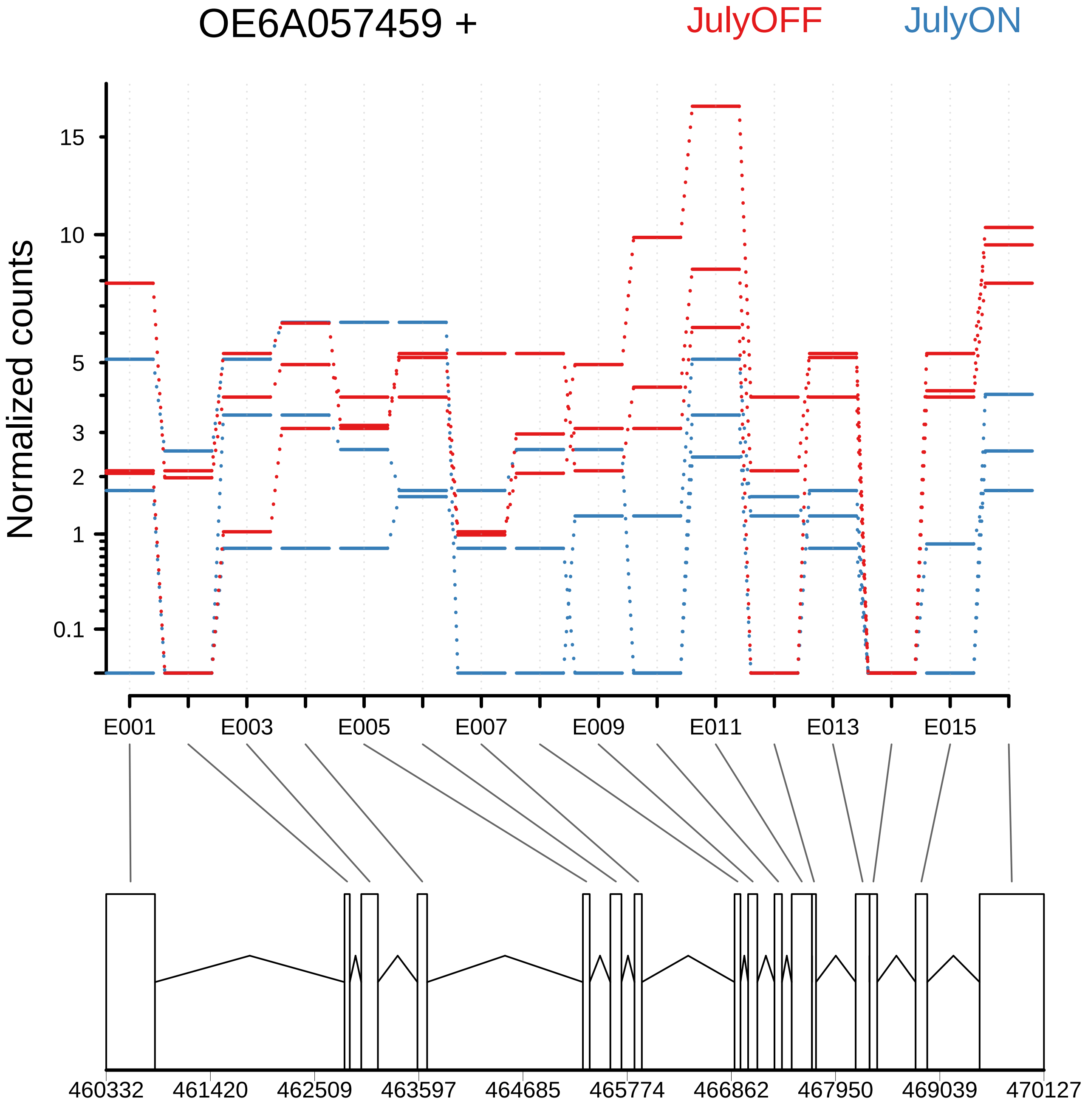

OE6A057459 +
JulyOFF
JulyON
Normalized counts
15
10
5
3
2
1
0.1
E001
E003
E005
E007
E009
E011
E013
E015
460332
461420
462509
463597
464685
465774
466862
467950
469039
470127

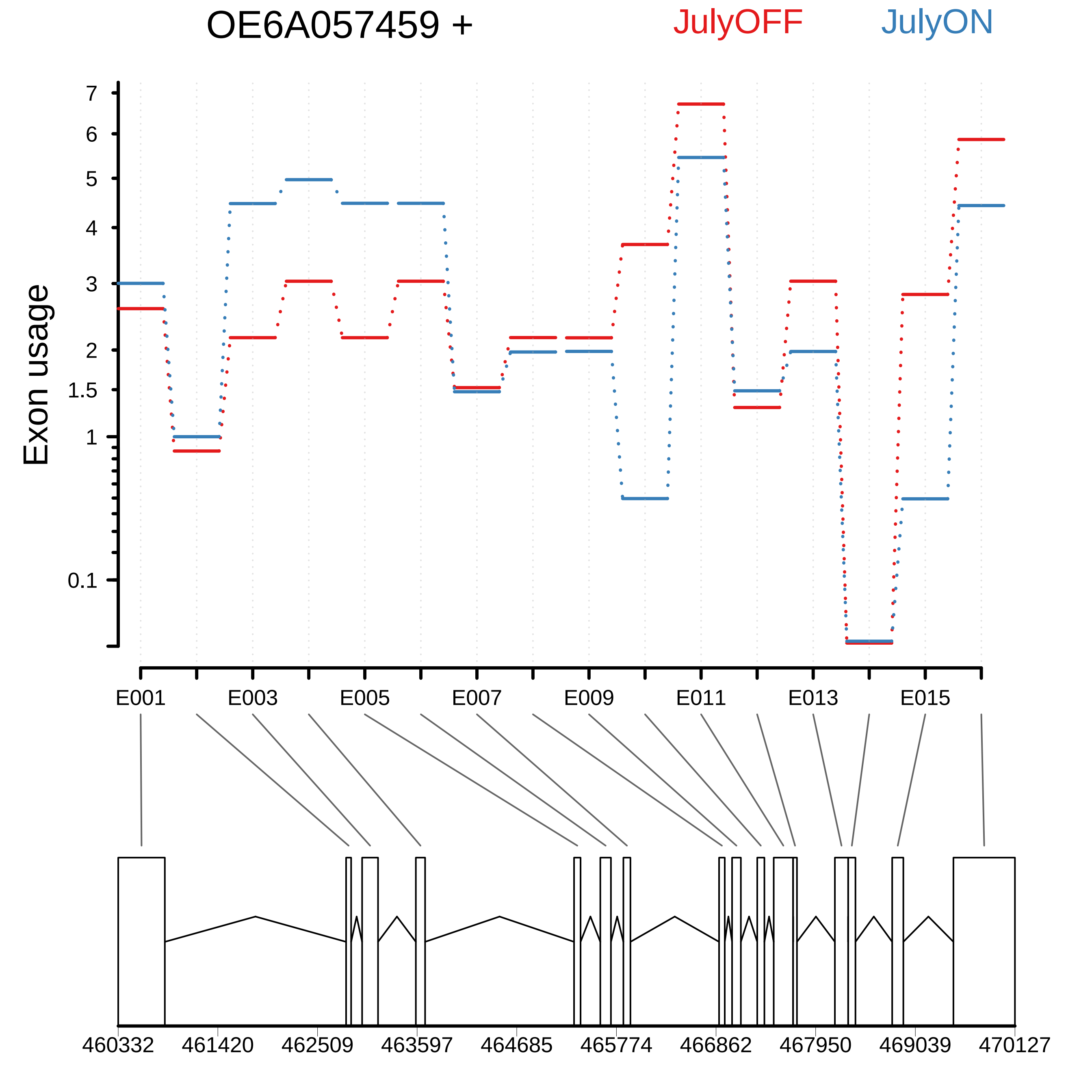

OE6A057459 +
JulyOFF
JulyON
Exon usage
7
6
5
4
3
2
1.5
1
0.1
E001
E003
E005
E007
E009
E011
E013
E015
460332
461420
462509
463597
464685
465774
466862
467950
469039
470127

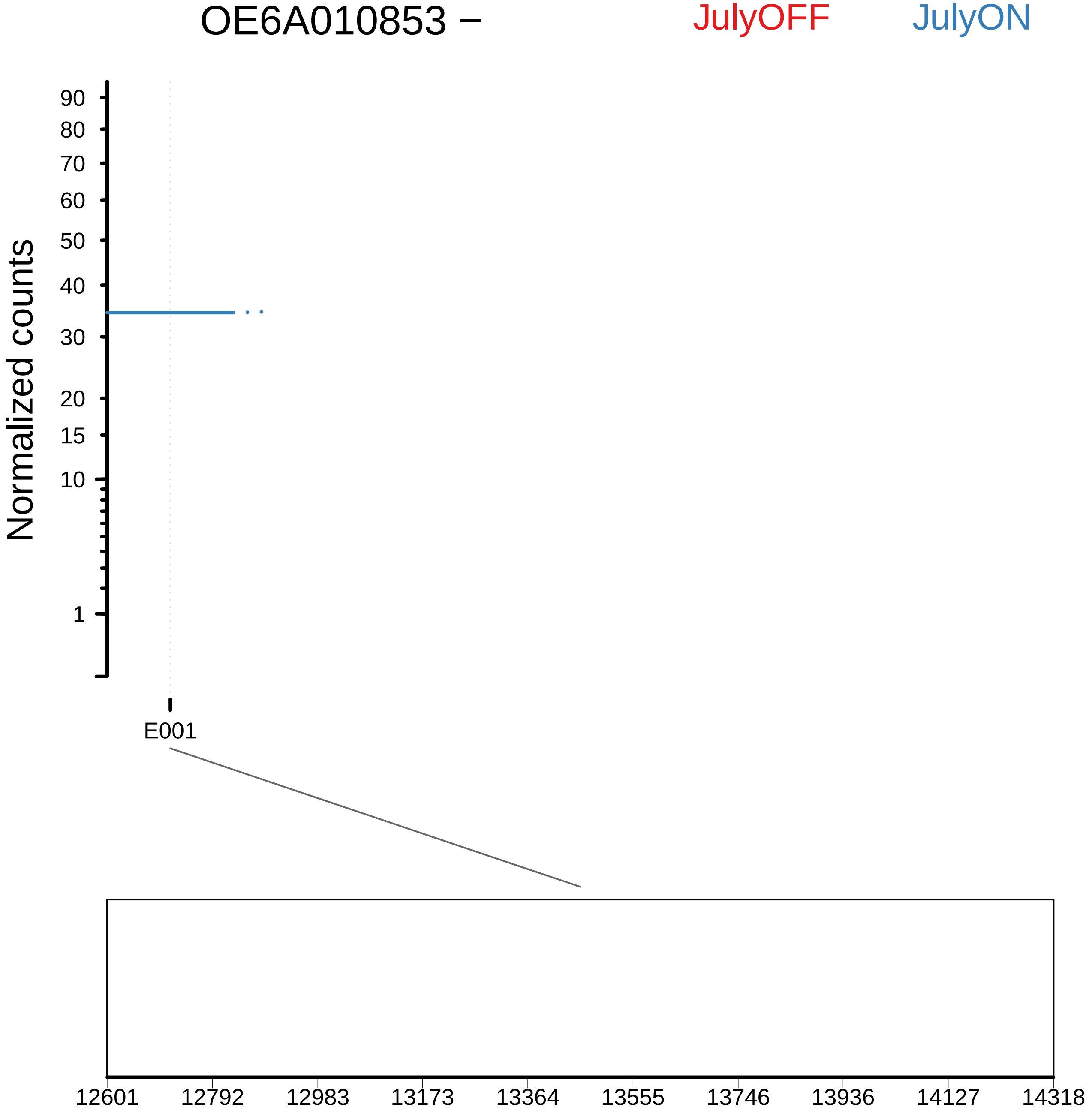
OE6A010853 −
JulyOFF
JulyON
Normalized counts
90
80
70
60
50
40
30
20
15
10
1
E001
12601
12792
12983
13173
13364
13555
13746
13936
14127
14318

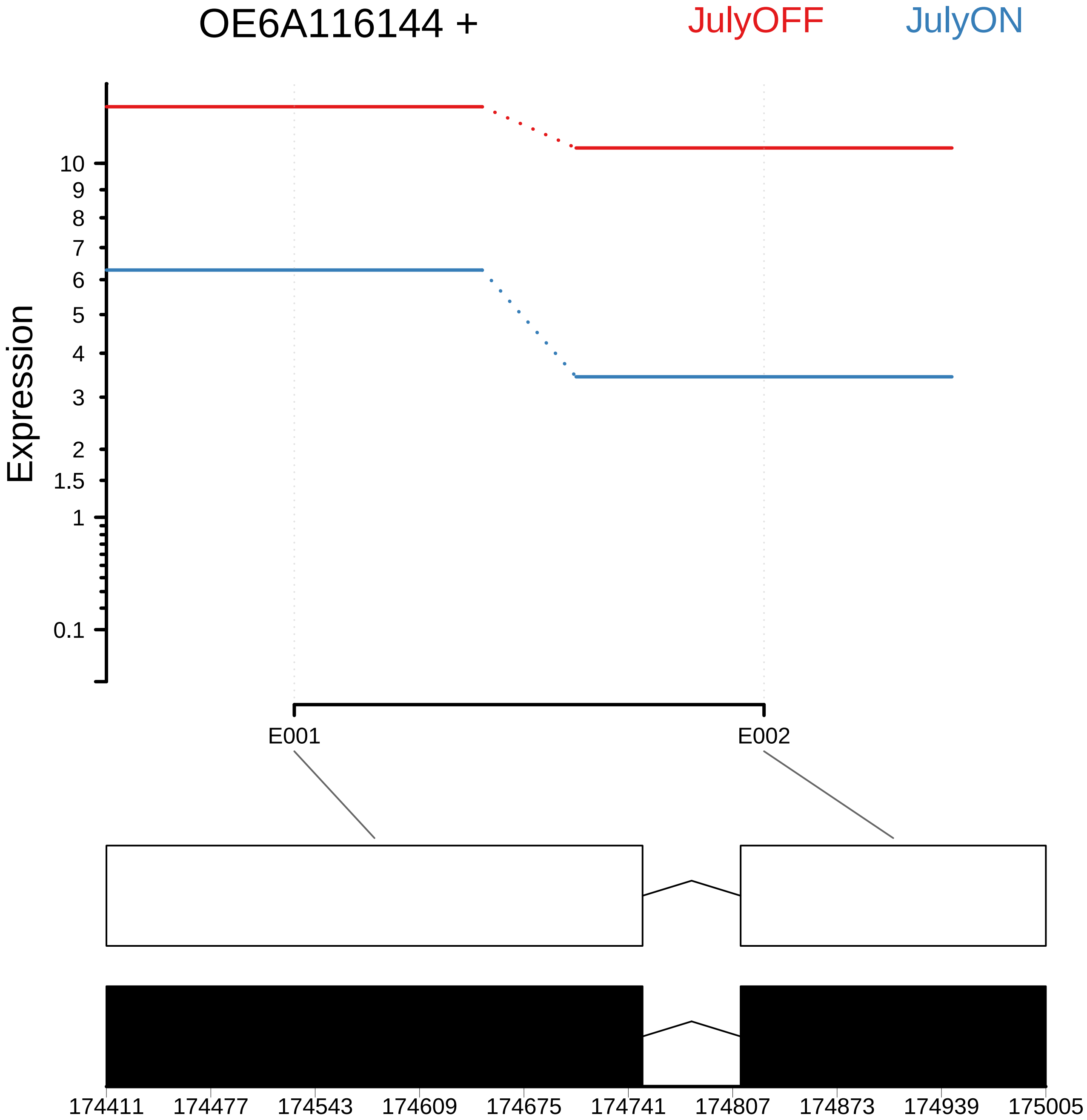

OE6A116144 +
JulyOFF
JulyON
Expression
10
9
8
7
6
5
4
3
2
1.5
1
0.1
E001
E002
174411
174477
174543
174609
174675
174741
174807
174873
174939
175005

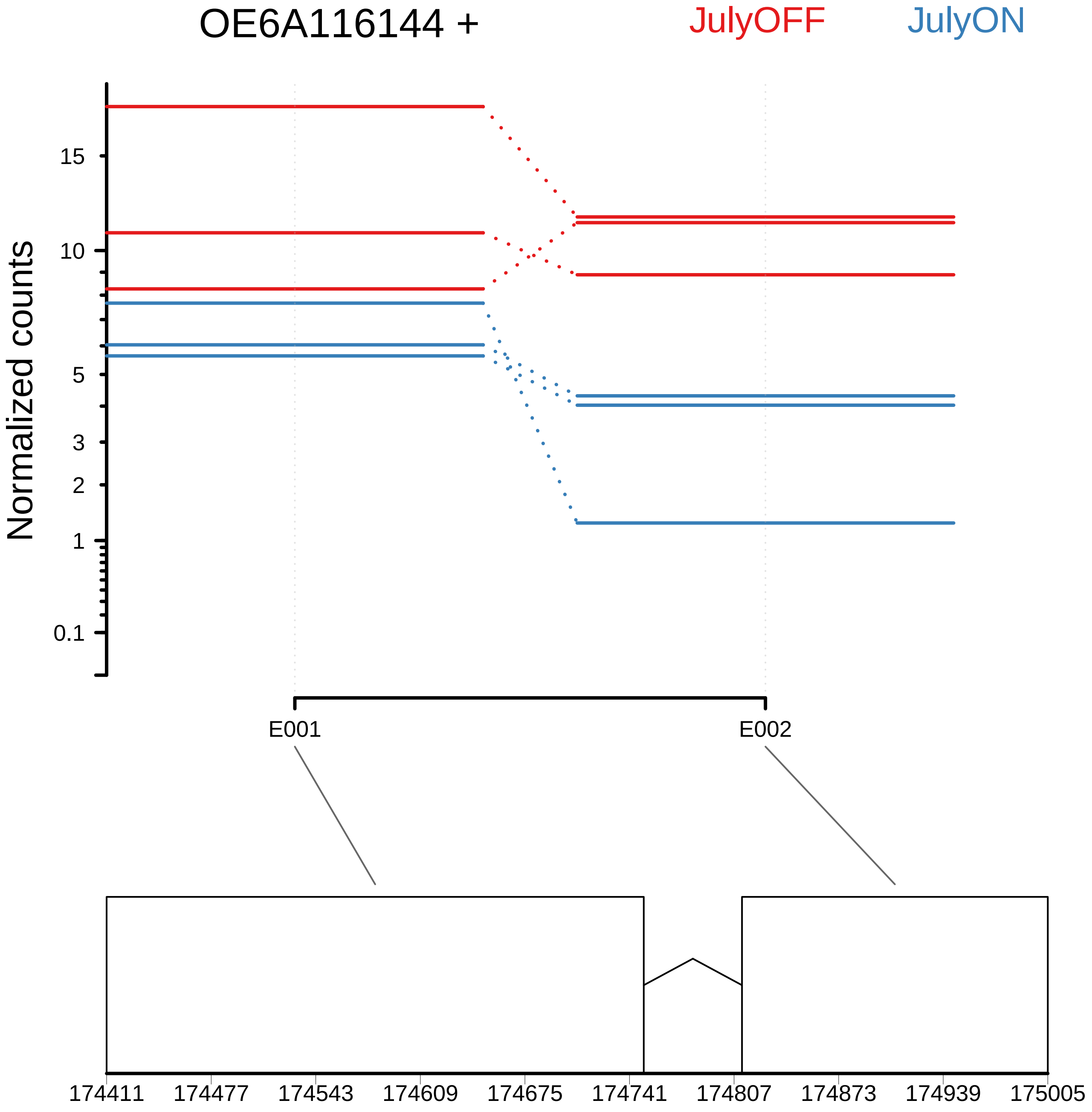

OE6A116144 +
JulyOFF
JulyON
Normalized counts
15
10
5
3
2
1
0.1
E001
E002
174411
174477
174543
174609
174675
174741
174807
174873
174939
175005

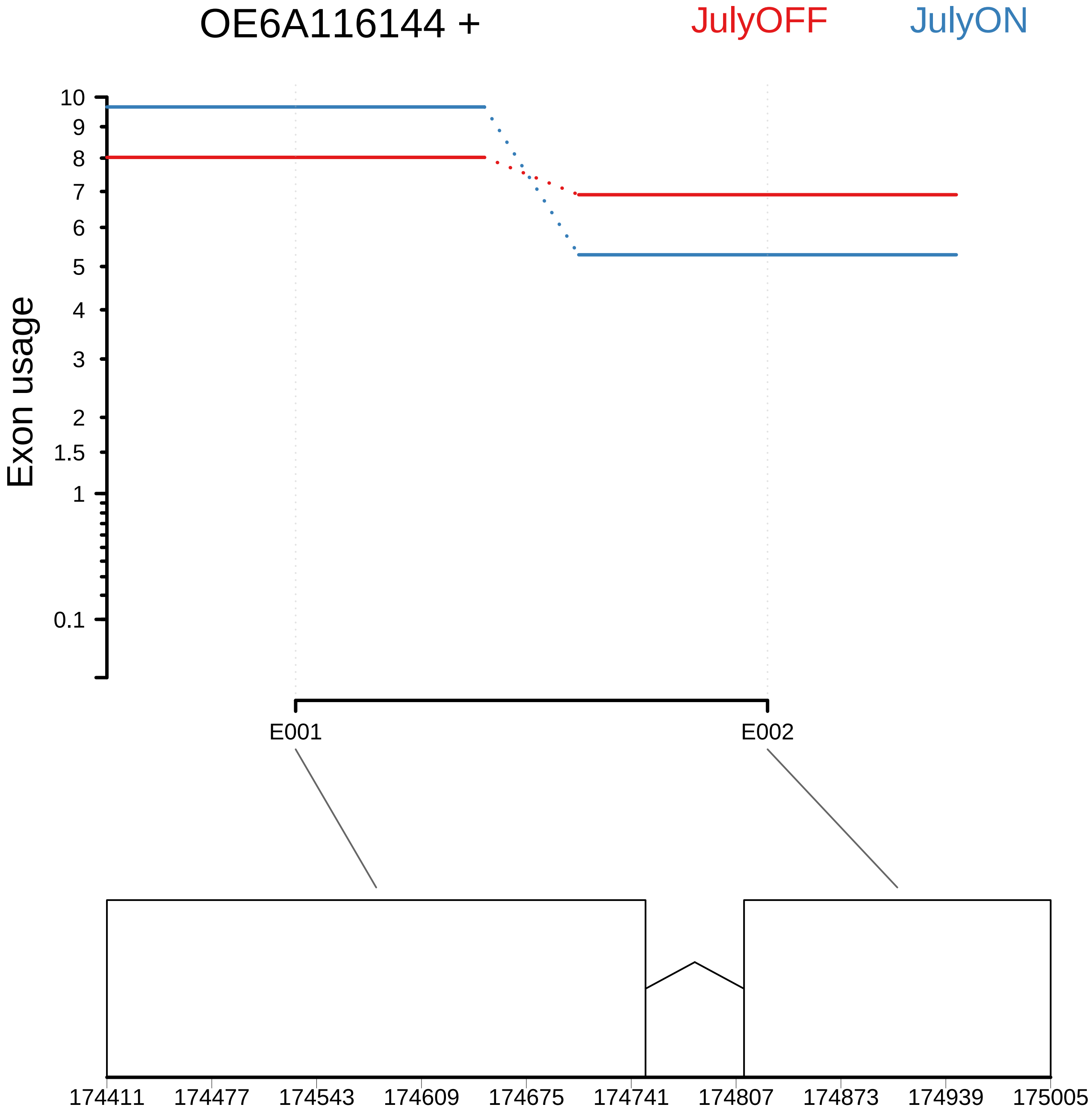

OE6A116144 +
JulyOFF
JulyON
Exon usage
10
9
8
7
6
5
4
3
2
1.5
1
0.1
E001
E002
174411
174477
174543
174609
174675
174741
174807
174873
174939
175005

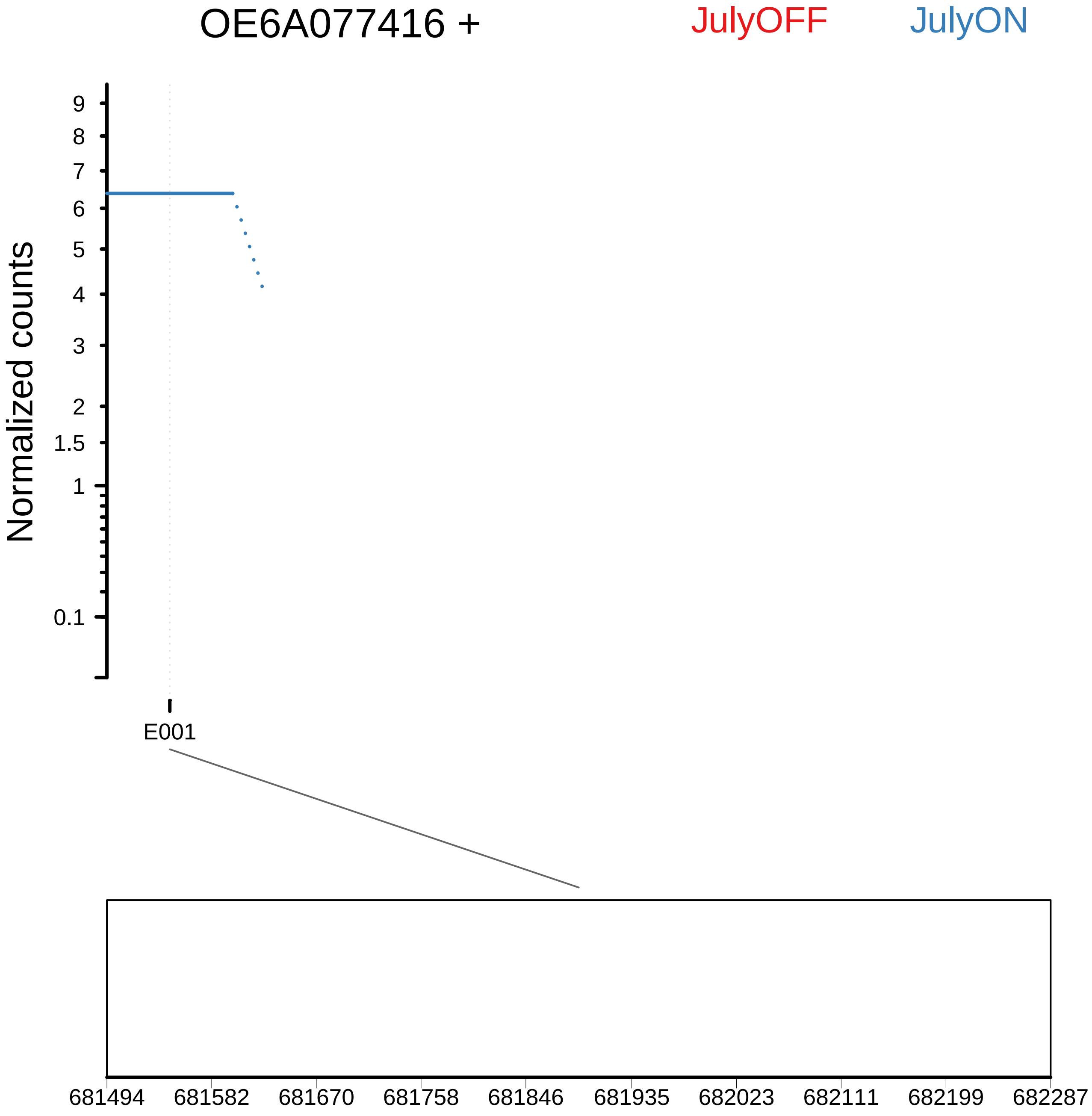

OE6A077416 +
JulyOFF
JulyON
Normalized counts
9
8
7
6
5
4
3
2
1.5
1
0.1
E001
681494
681582
681670
681758
681846
681935
682023
682111
682199
682287

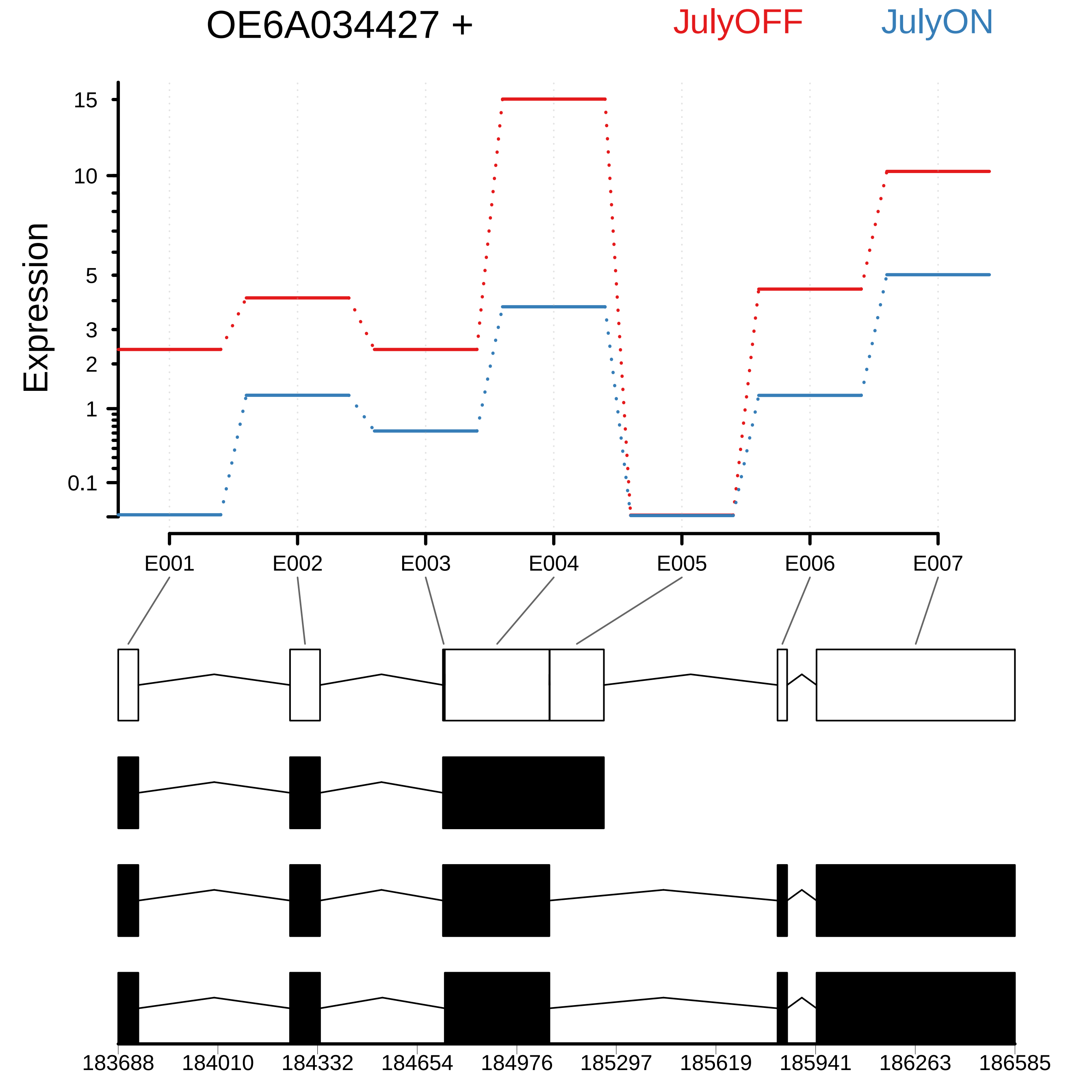

OE6A034427 +
JulyOFF
JulyON
Expression
15
10
5
3
2
1
0.1
E001
E002
E003
E004
E005
E006
E007
183688
184010
184332
184654
184976
185297
185619
185941
186263
186585

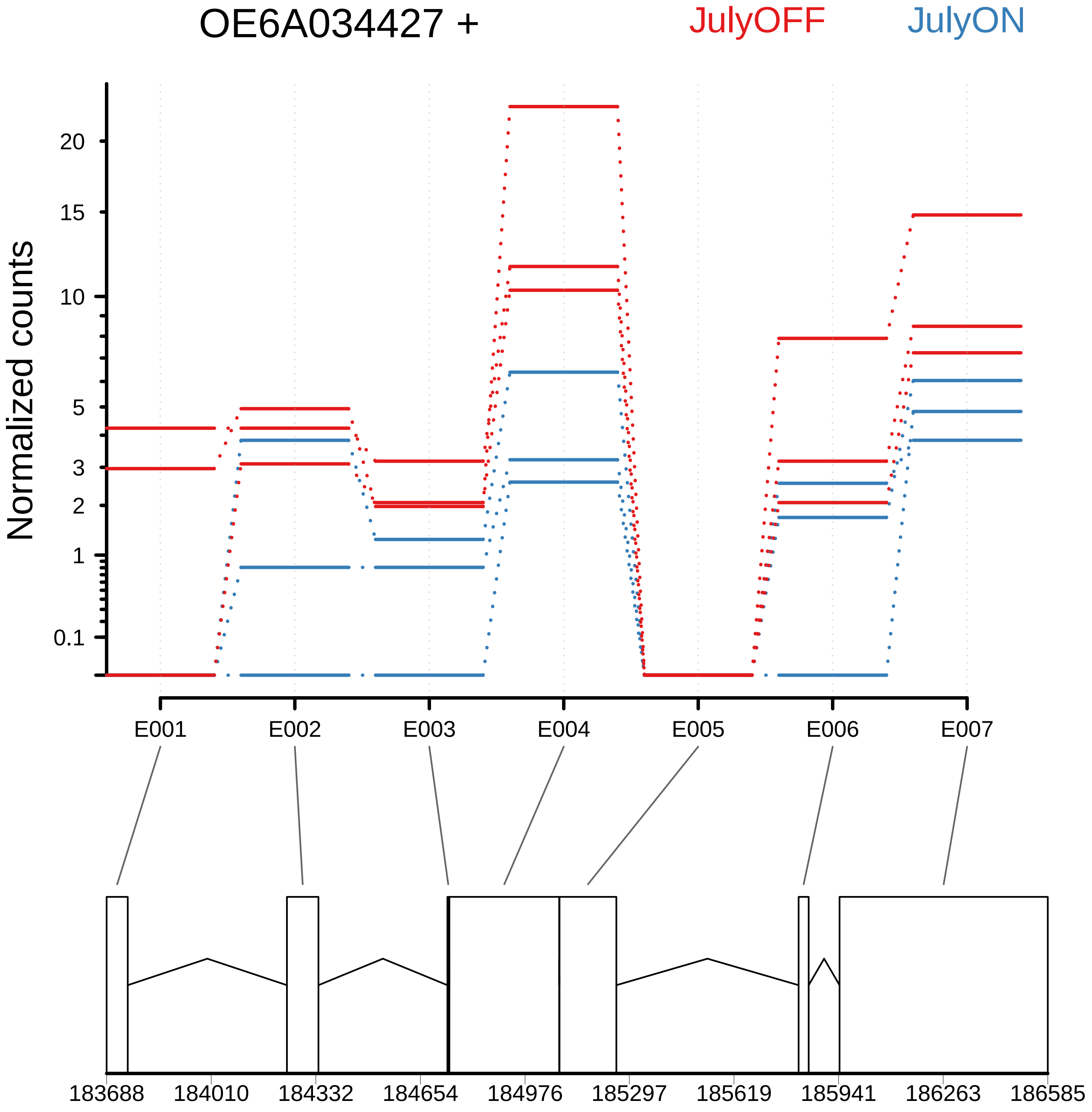

OE6A034427 +
JulyOFF
JulyON
Normalized counts
20
15
10
5
3
2
1
0.1
E001
E002
E003
E004
E005
E006
E007
183688
184010
184332
184654
184976
185297
185619
185941
186263
186585

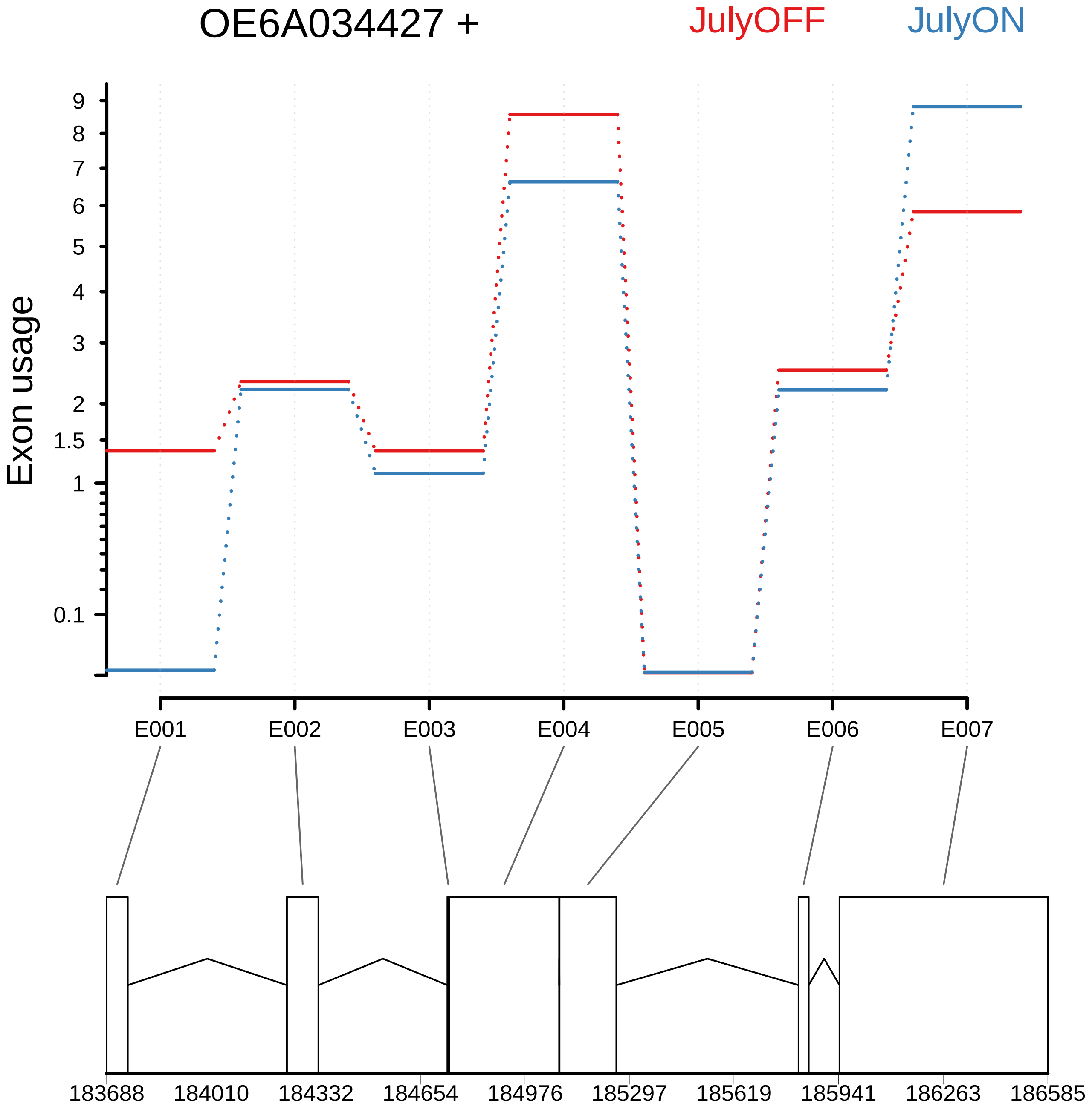
OE6A034427 +
JulyOFF
JulyON
Exon usage
9
8
7
6
5
4
3
2
1.5
1
0.1
E001
E002
E003
E004
E005
E006
E007
183688
184010
184332
184654
184976
185297
185619
185941
186263
186585

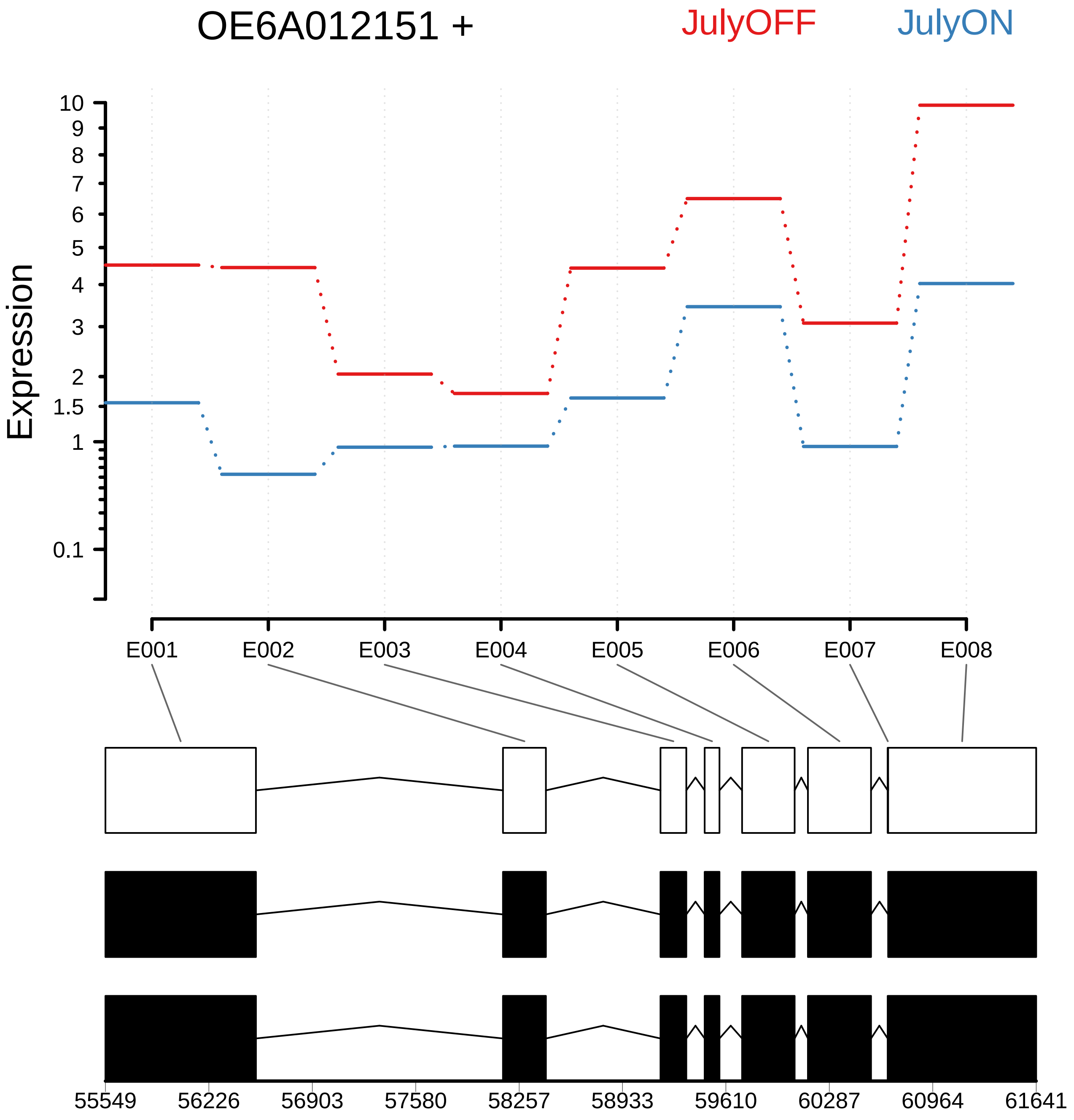
OE6A012151 +
JulyOFF
JulyON
Expression
10
9
8
7
6
5
4
3
2
1.5
1
0.1
E001
E002
E003
E004
E005
E006
E007
E008
55549
56226
56903
57580
58257
58933
59610
60287
60964
61641

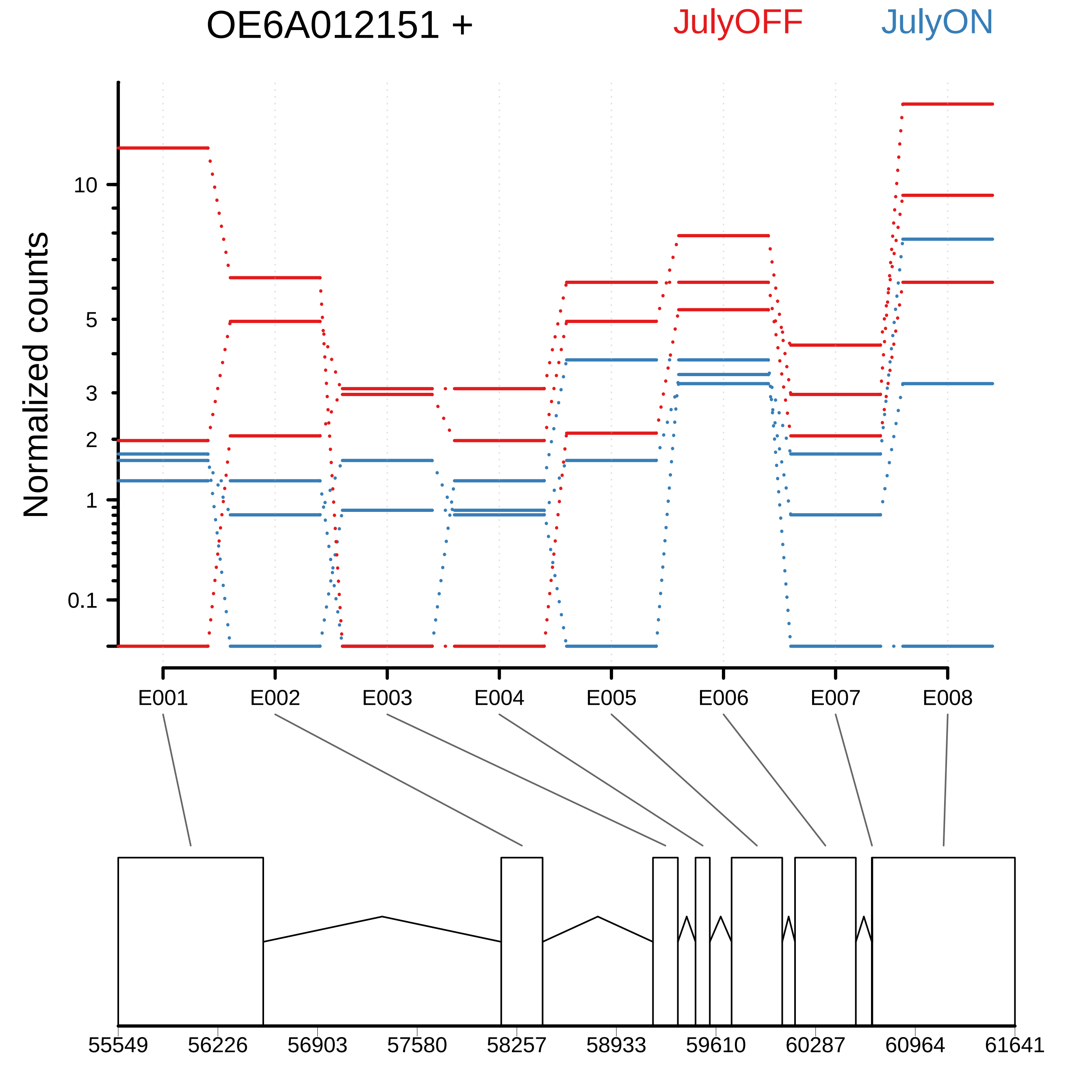
OE6A012151 +
JulyOFF
JulyON
Normalized counts
10
5
3
2
1
0.1
E001
E002
E003
E004
E005
E006
E007
E008
55549
56226
56903
57580
58257
58933
59610
60287
60964
61641

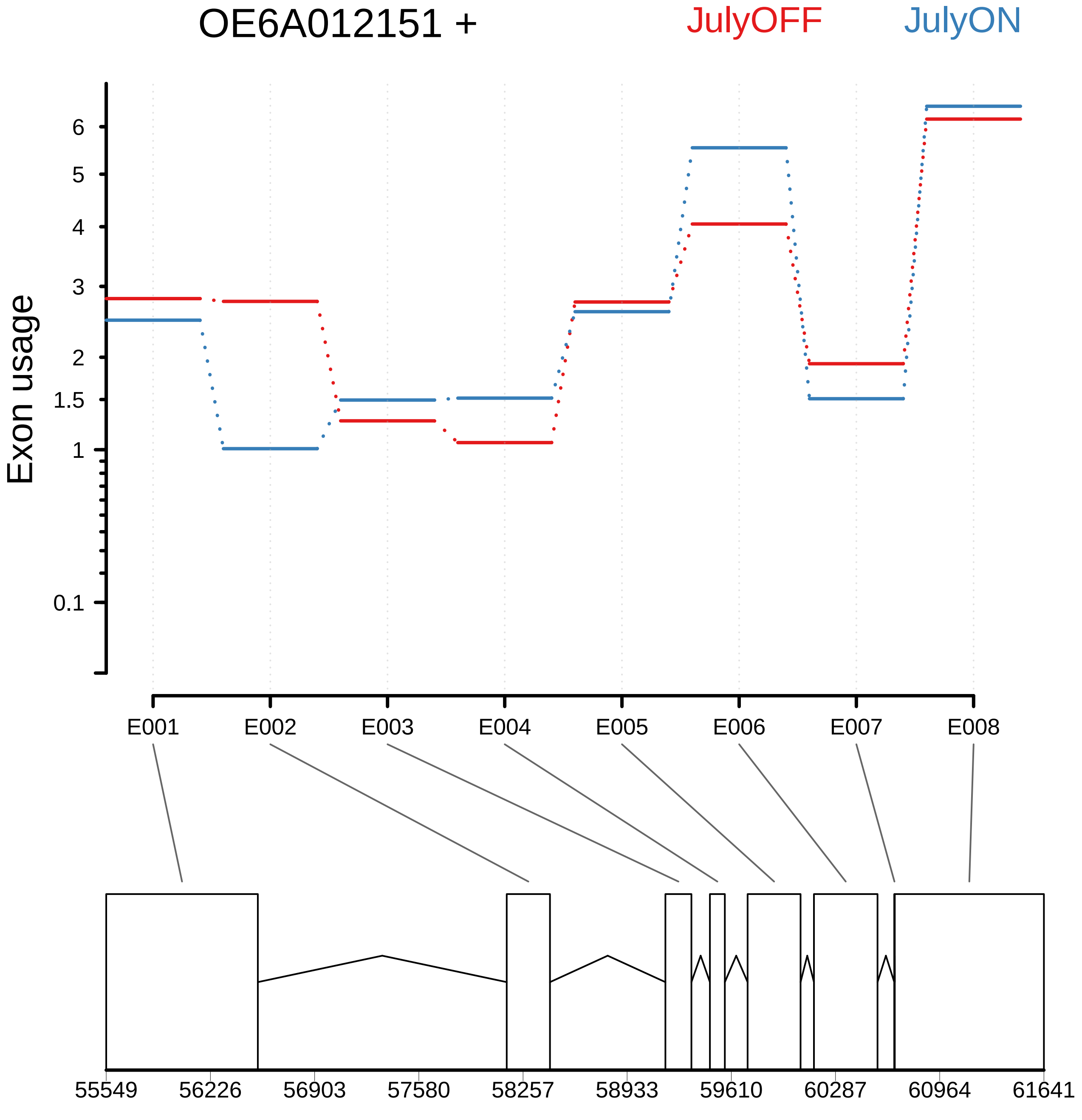

OE6A012151 +
JulyOFF
JulyON
Exon usage
6
5
4
3
2
1.5
1
0.1
E001
E002
E003
E004
E005
E006
E007
E008
55549
56226
56903
57580
58257
58933
59610
60287
60964
61641

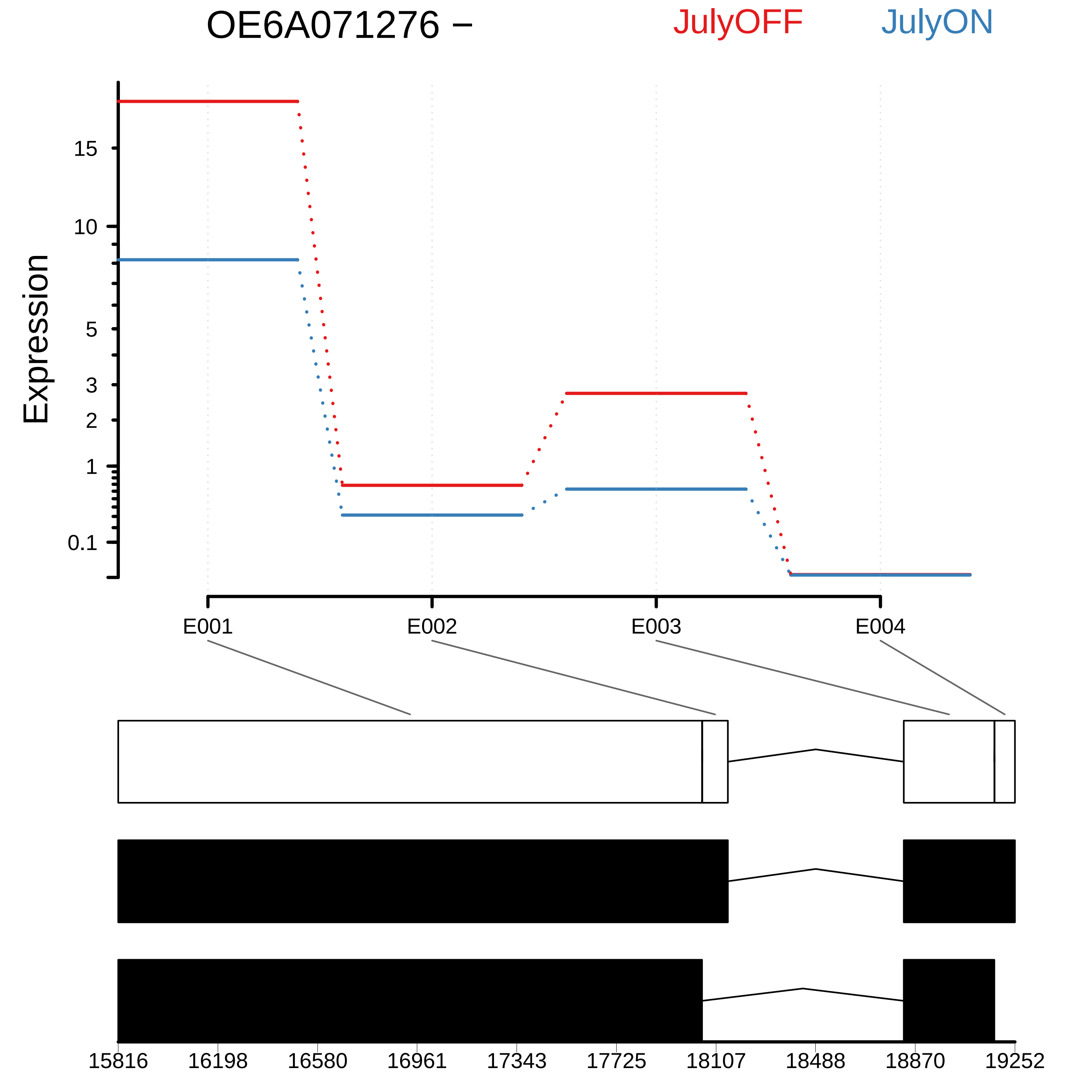

OE6A071276 −
JulyOFF
JulyON
Expression
15
10
5
3
2
1
0.1
E001
E002
E003
E004
15816
16198
16580
16961
17343
17725
18107
18488
18870
19252

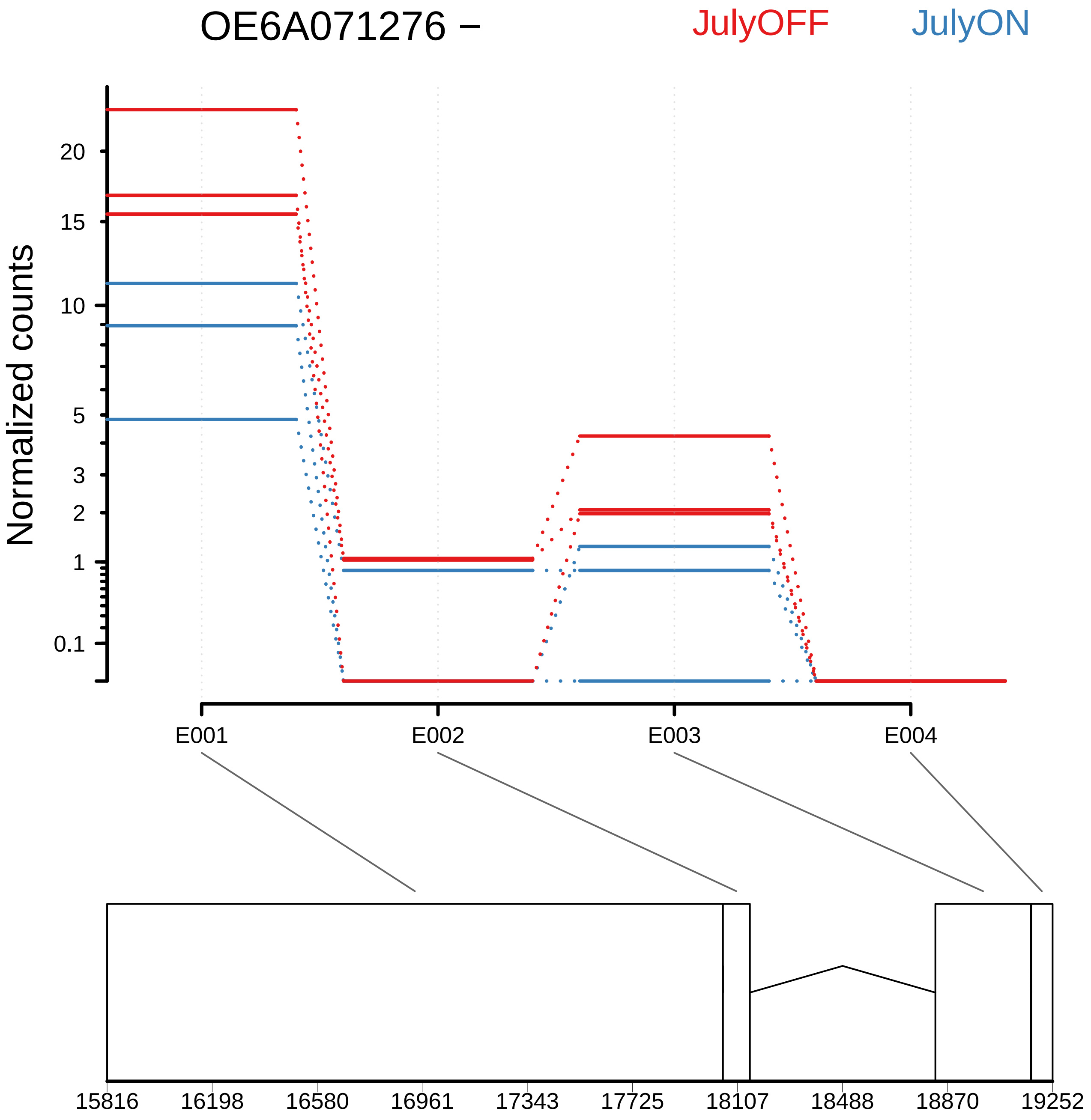

OE6A071276 −
JulyOFF
JulyON
Normalized counts
20
15
10
5
3
2
1
0.1
E001
E002
E003
E004
15816
16198
16580
16961
17343
17725
18107
18488
18870
19252

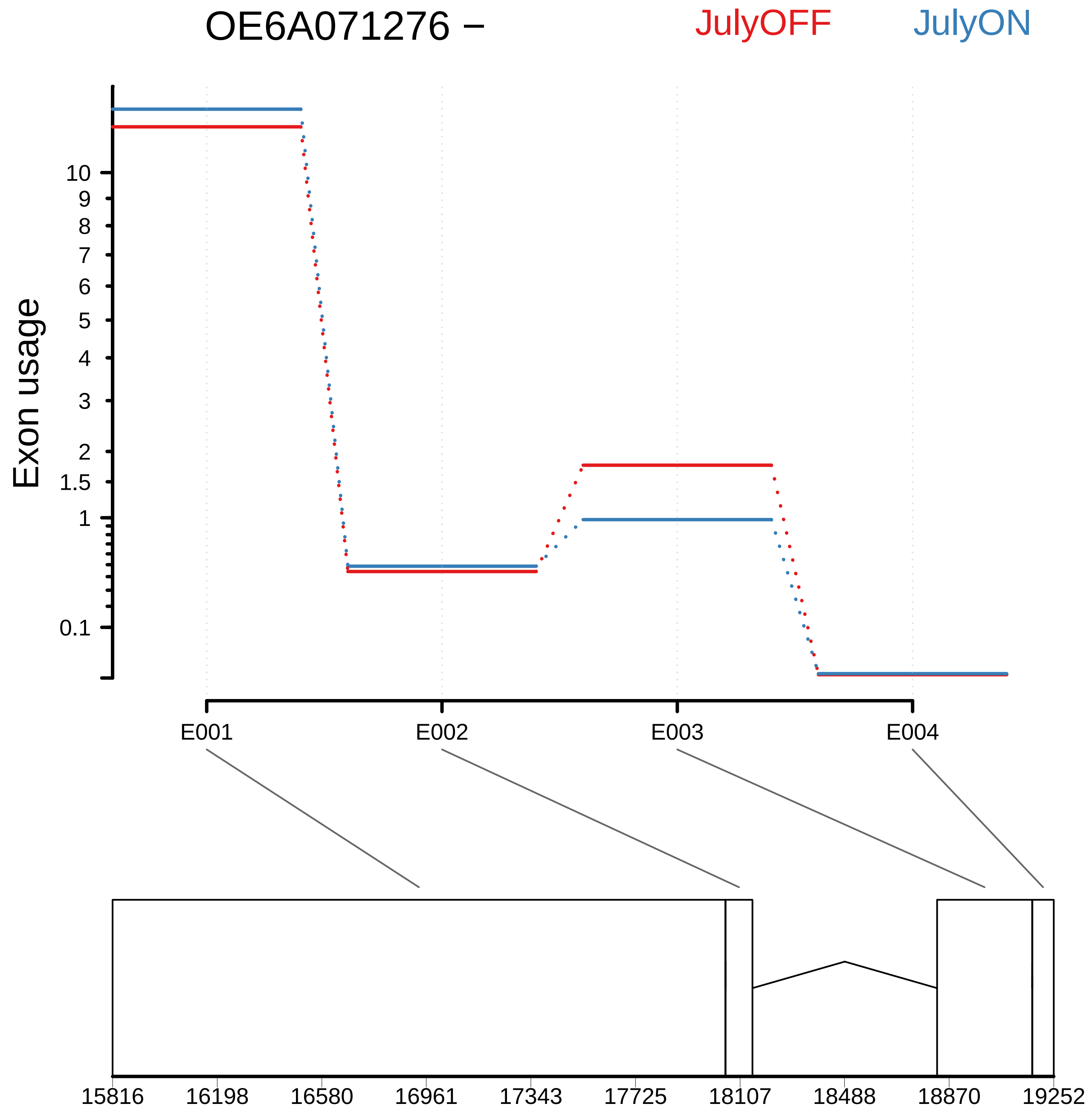

OE6A071276 −
JulyOFF
JulyON
Exon usage
10
9
8
7
6
5
4
3
2
1.5
1
0.1
E001
E002
E003
E004
15816
16198
16580
16961
17343
17725
18107
18488
18870
19252

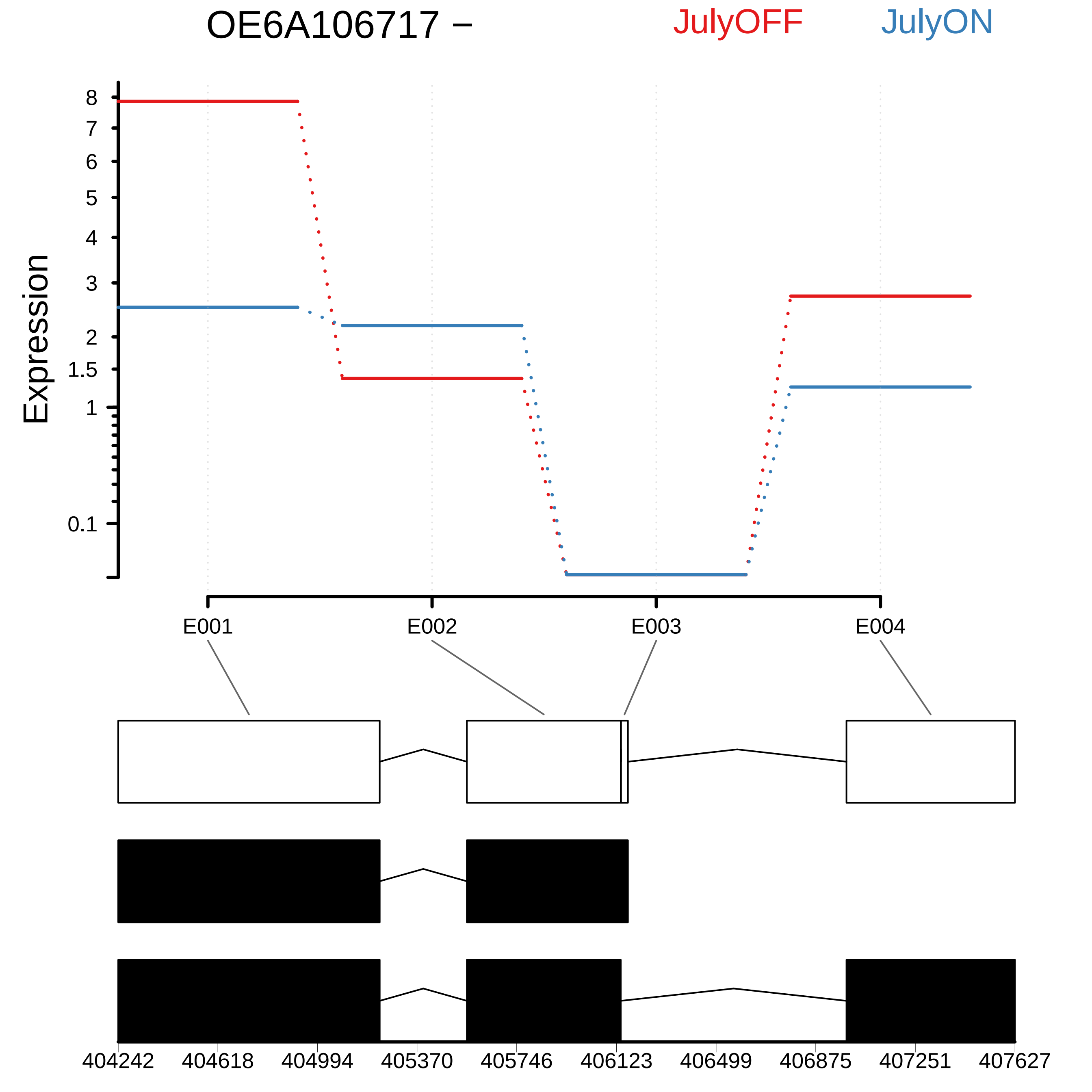

OE6A106717 −
JulyOFF
JulyON
Expression
8
7
6
5
4
3
2
1.5
1
0.1
E001
E002
E003
E004
404242
404618
404994
405370
405746
406123
406499
406875
407251
407627

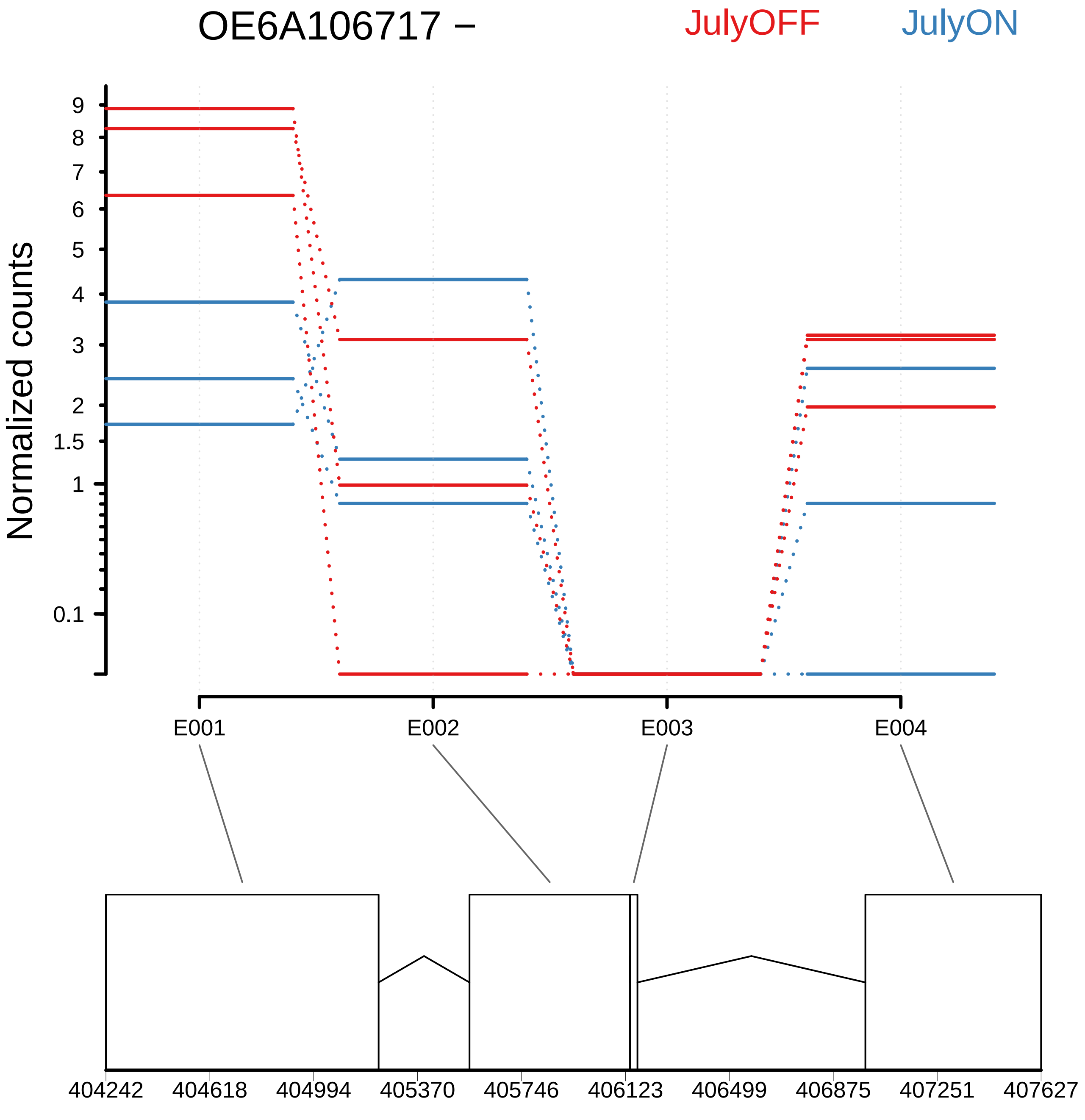

OE6A106717 −
JulyOFF
JulyON
Normalized counts
9
8
7
6
5
4
3
2
1.5
1
0.1
E001
E002
E003
E004
404242
404618
404994
405370
405746
406123
406499
406875
407251
407627

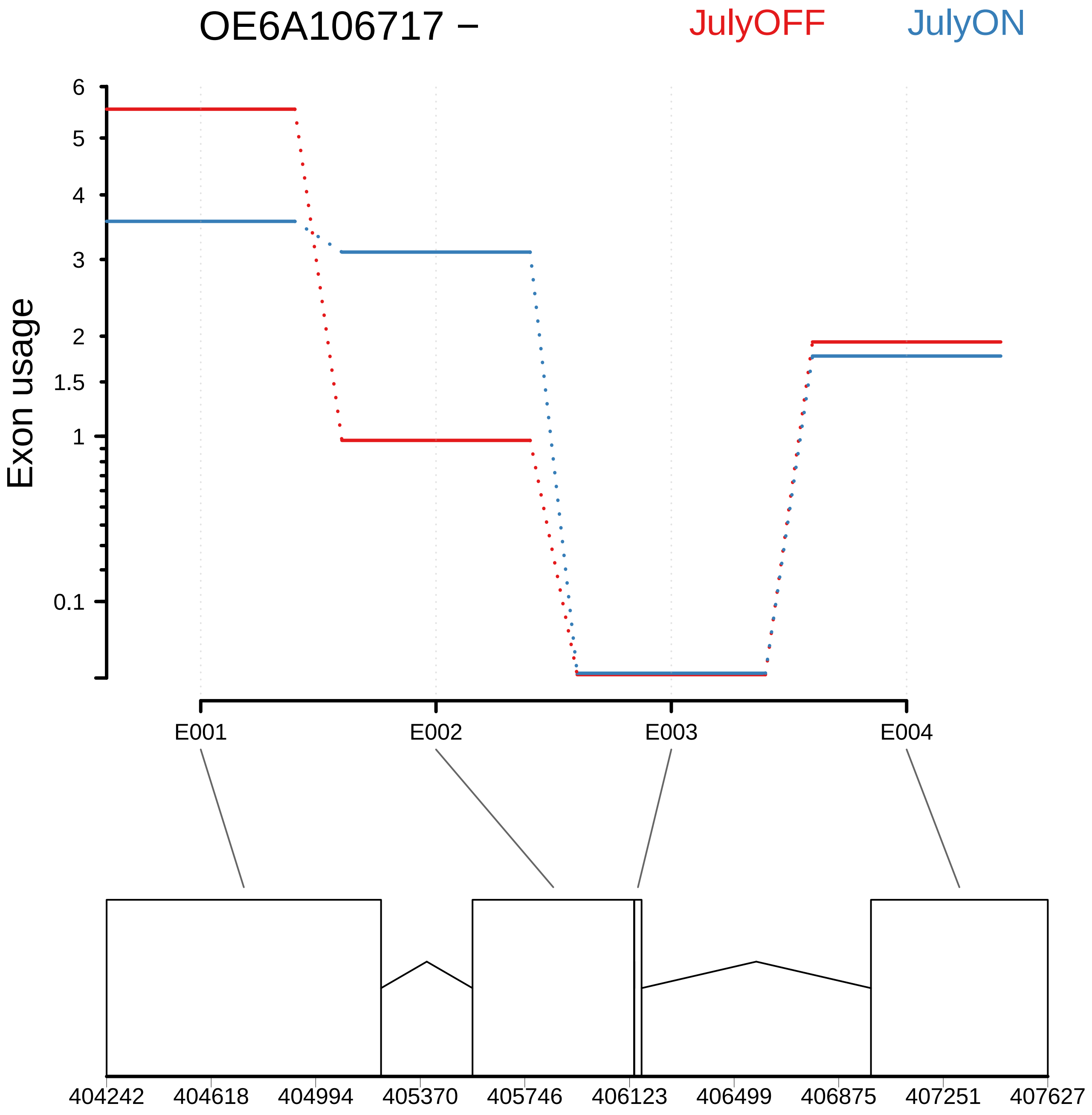
OE6A106717 −
JulyOFF
JulyON
Exon usage
6
5
4
3
2
1.5
1
0.1
E001
E002
E003
E004
404242
404618
404994
405370
405746
406123
406499
406875
407251
407627

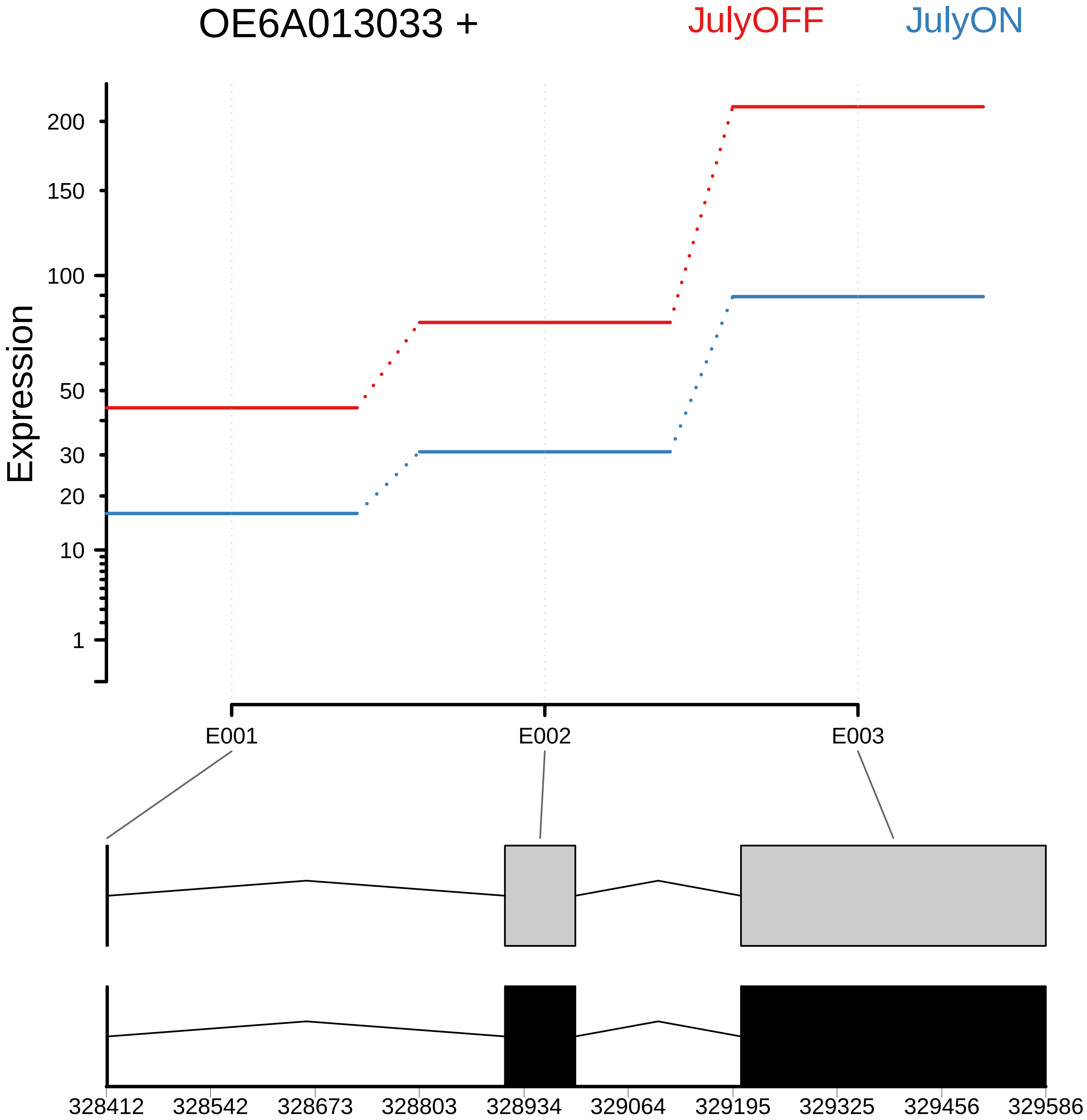
OE6A013033 +
JulyOFF
JulyON
Expression
200
150
100
50
30
20
10
1
E001
E002
E003
328412
328542
328673
328803
328934
329064
329195
329325
329456
329586

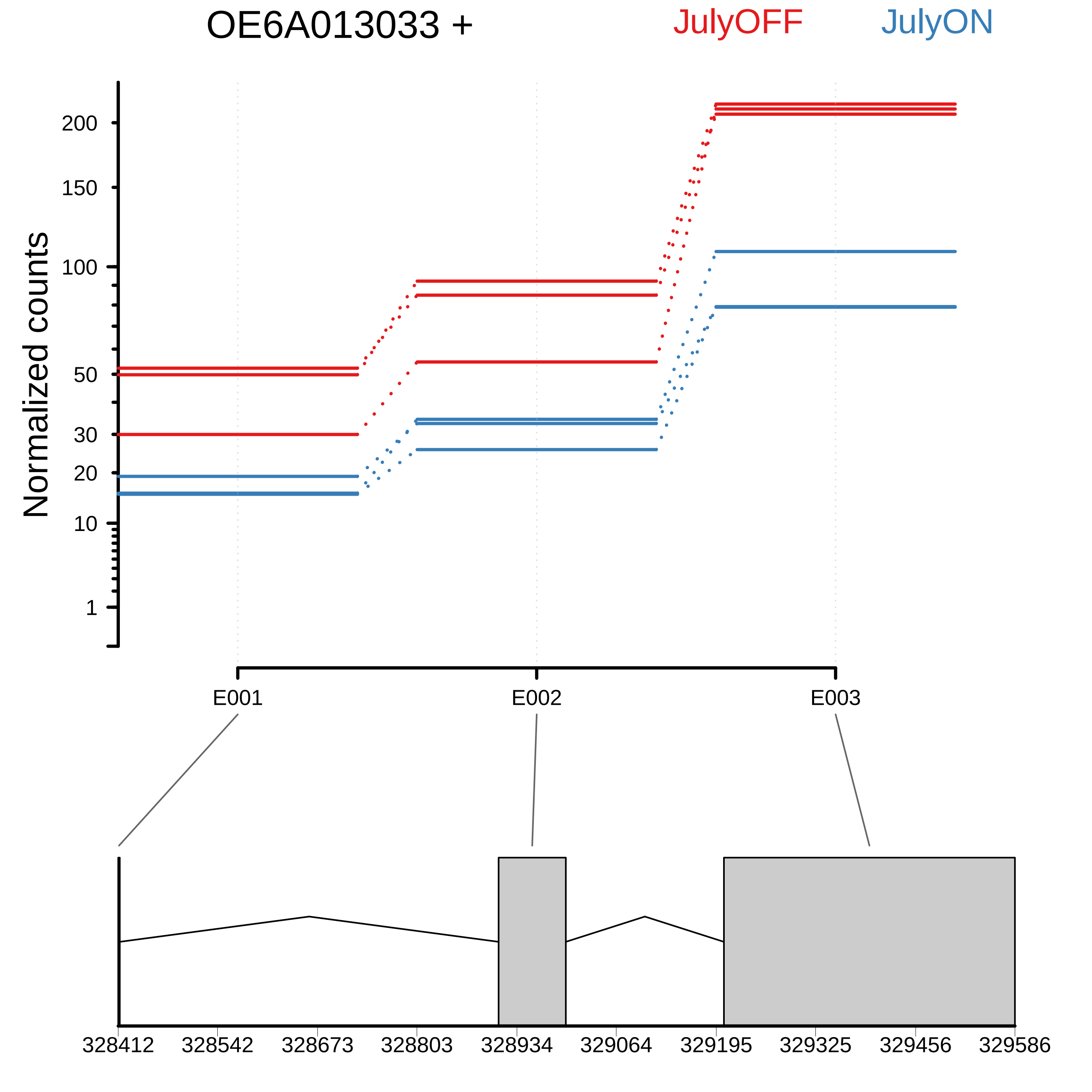

OE6A013033 +
JulyOFF
JulyON
Normalized counts
200
150
100
50
30
20
10
1
E001
E002
E003
328412
328542
328673
328803
328934
329064
329195
329325
329456
329586

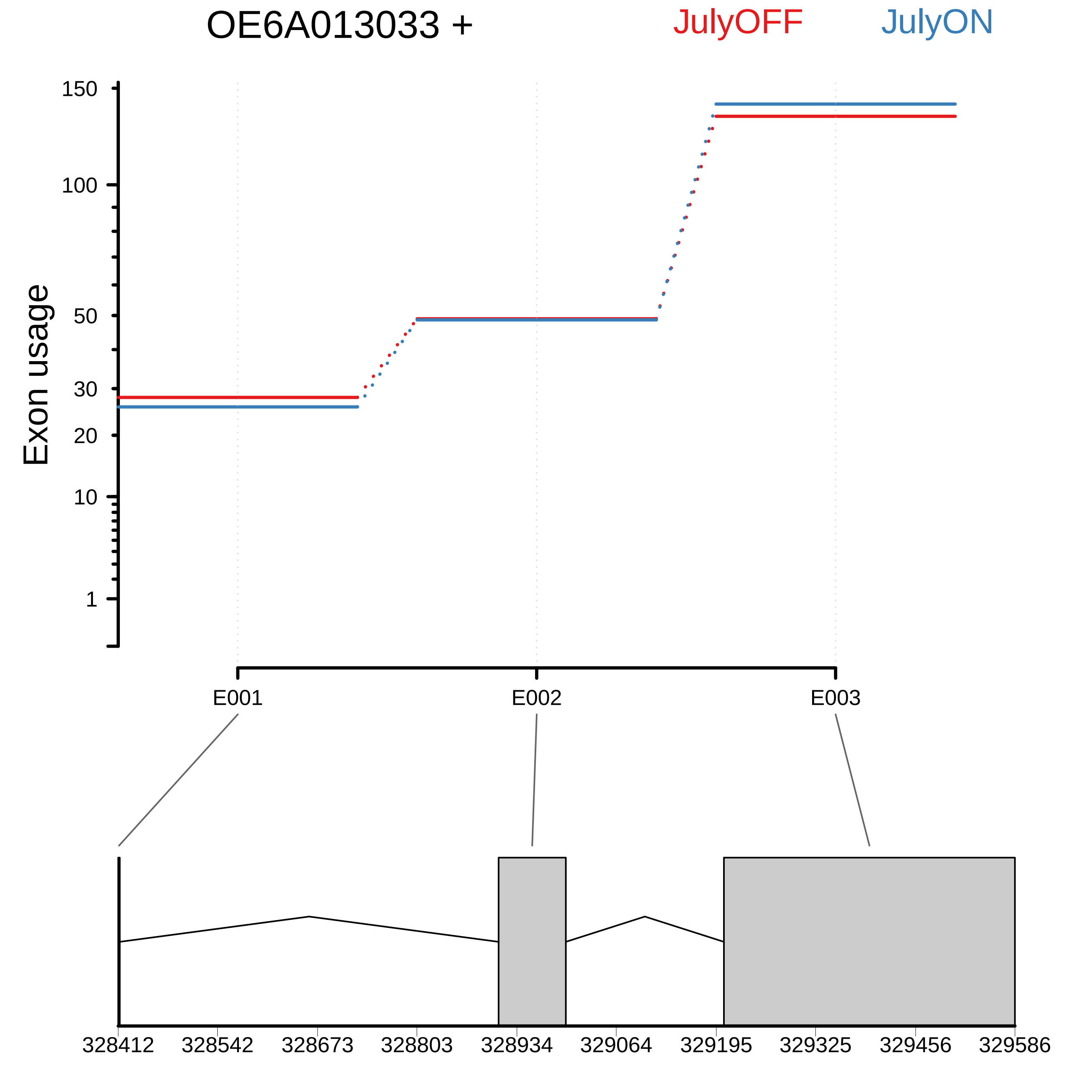

OE6A013033 +
JulyOFF
JulyON
Exon usage
150
100
50
30
20
10
1
E001
E002
E003
328412
328542
328673
328803
328934
329064
329195
329325
329456
329586

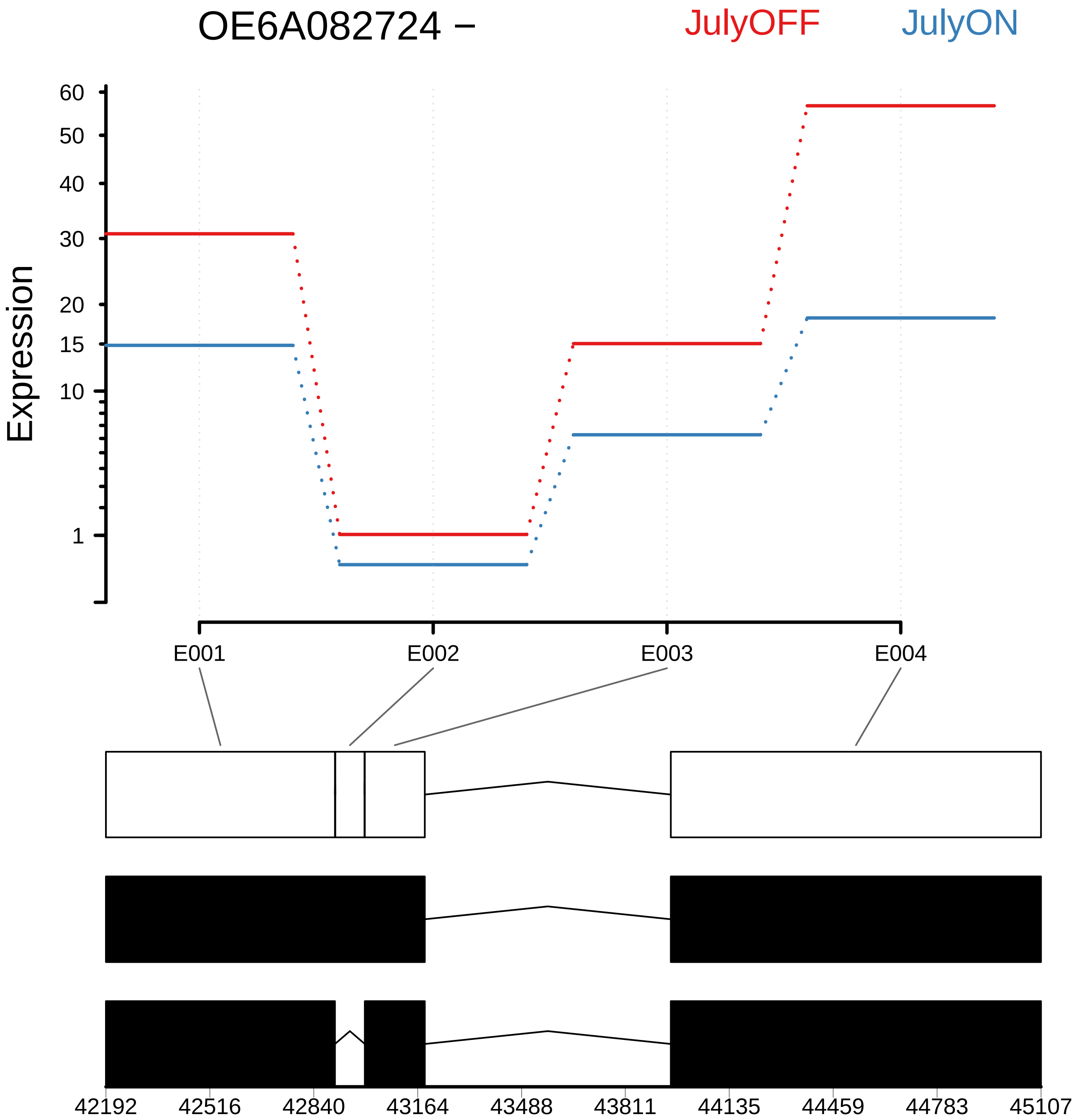
OE6A082724 −
JulyOFF
JulyON
Expression
60
50
40
30
20
15
10
1
E001
E002
E003
E004
42192
42516
42840
43164
43488
43811
44135
44459
44783
45107

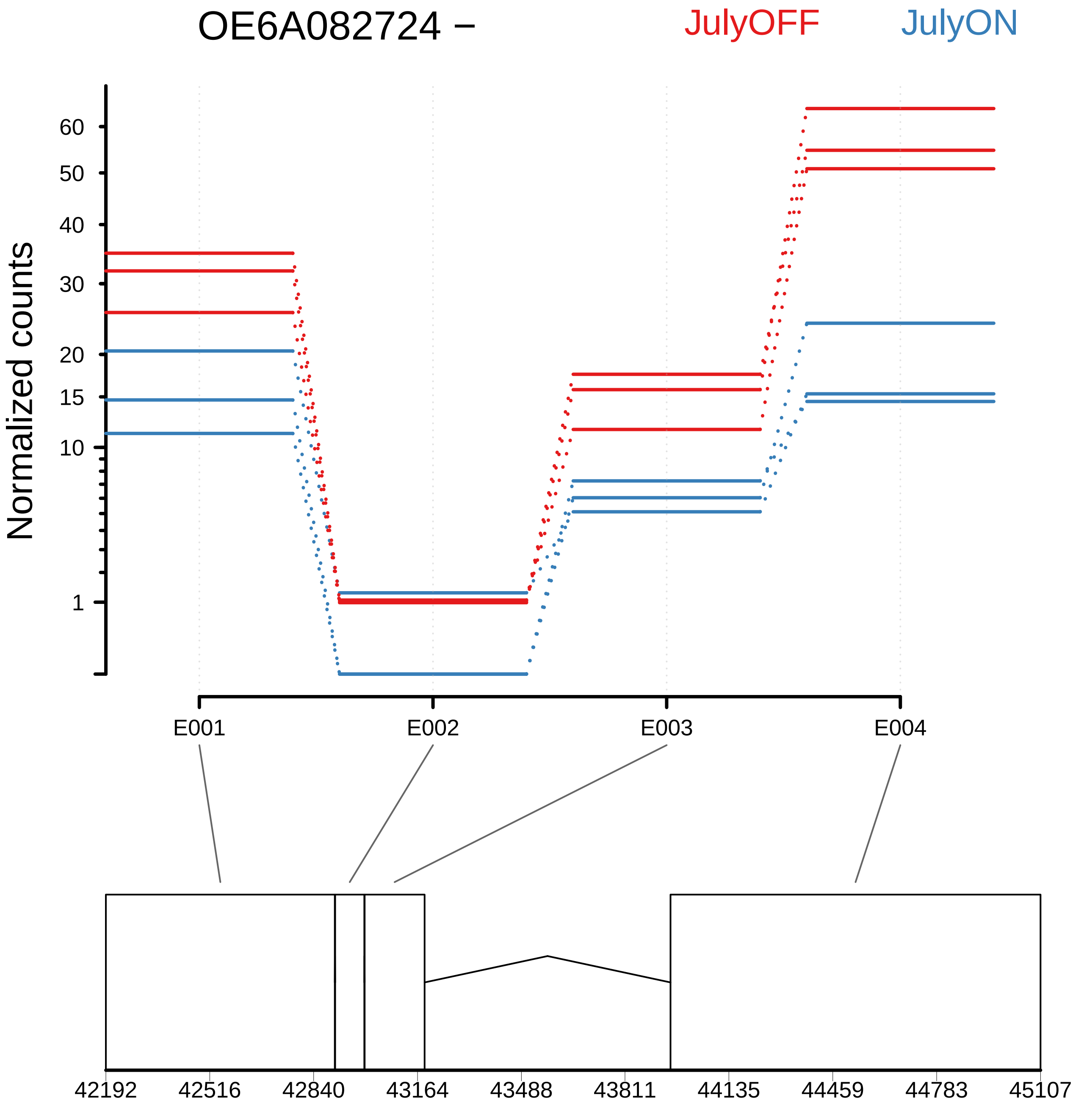
OE6A082724 −
JulyOFF
JulyON
Normalized counts
60
50
40
30
20
15
10
1
E001
E002
E003
E004
42192
42516
42840
43164
43488
43811
44135
44459
44783
45107

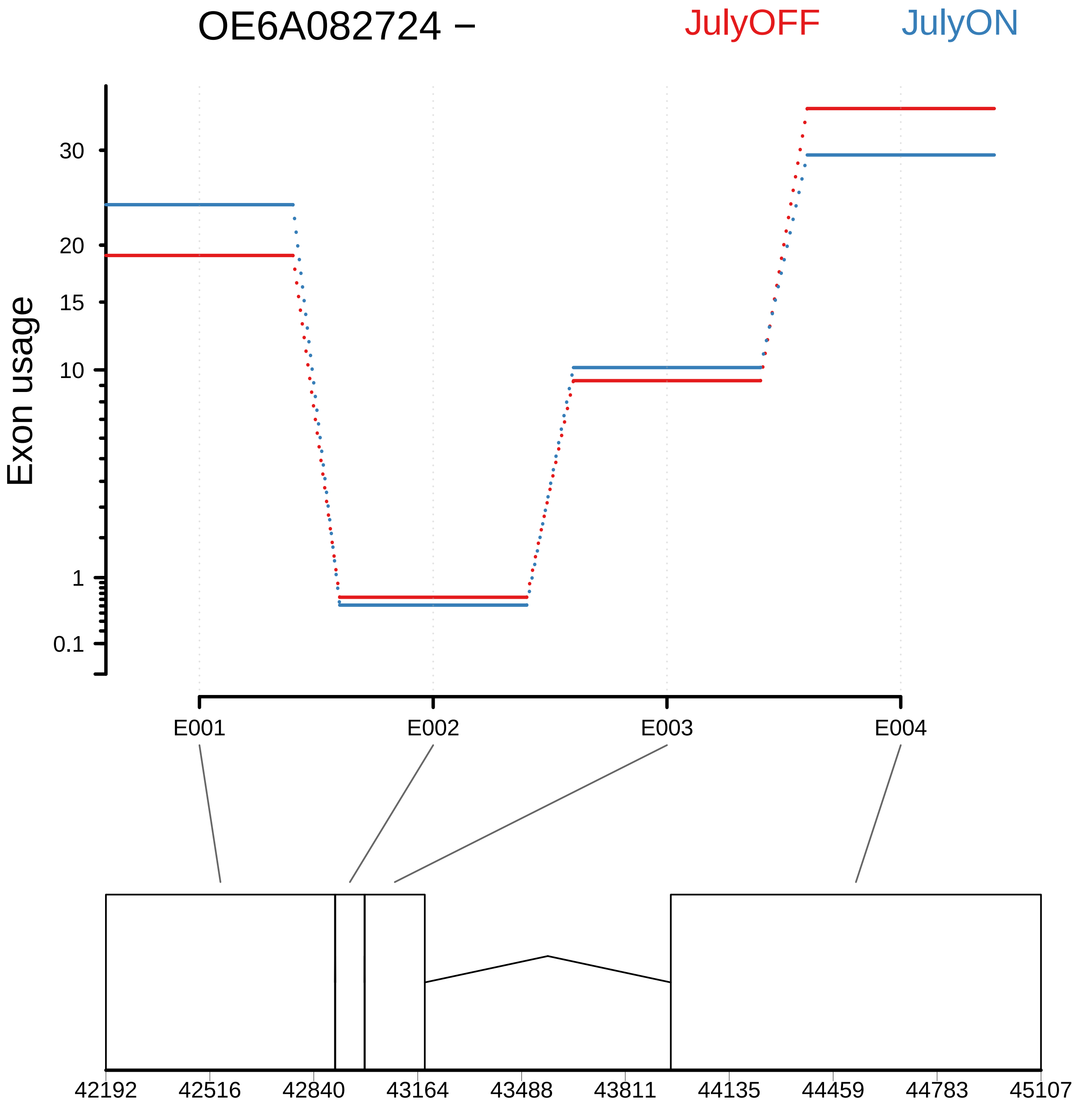
OE6A082724 −
JulyOFF
JulyON
Exon usage
30
20
15
10
1
0.1
E001
E002
E003
E004
42192
42516
42840
43164
43488
43811
44135
44459
44783
45107

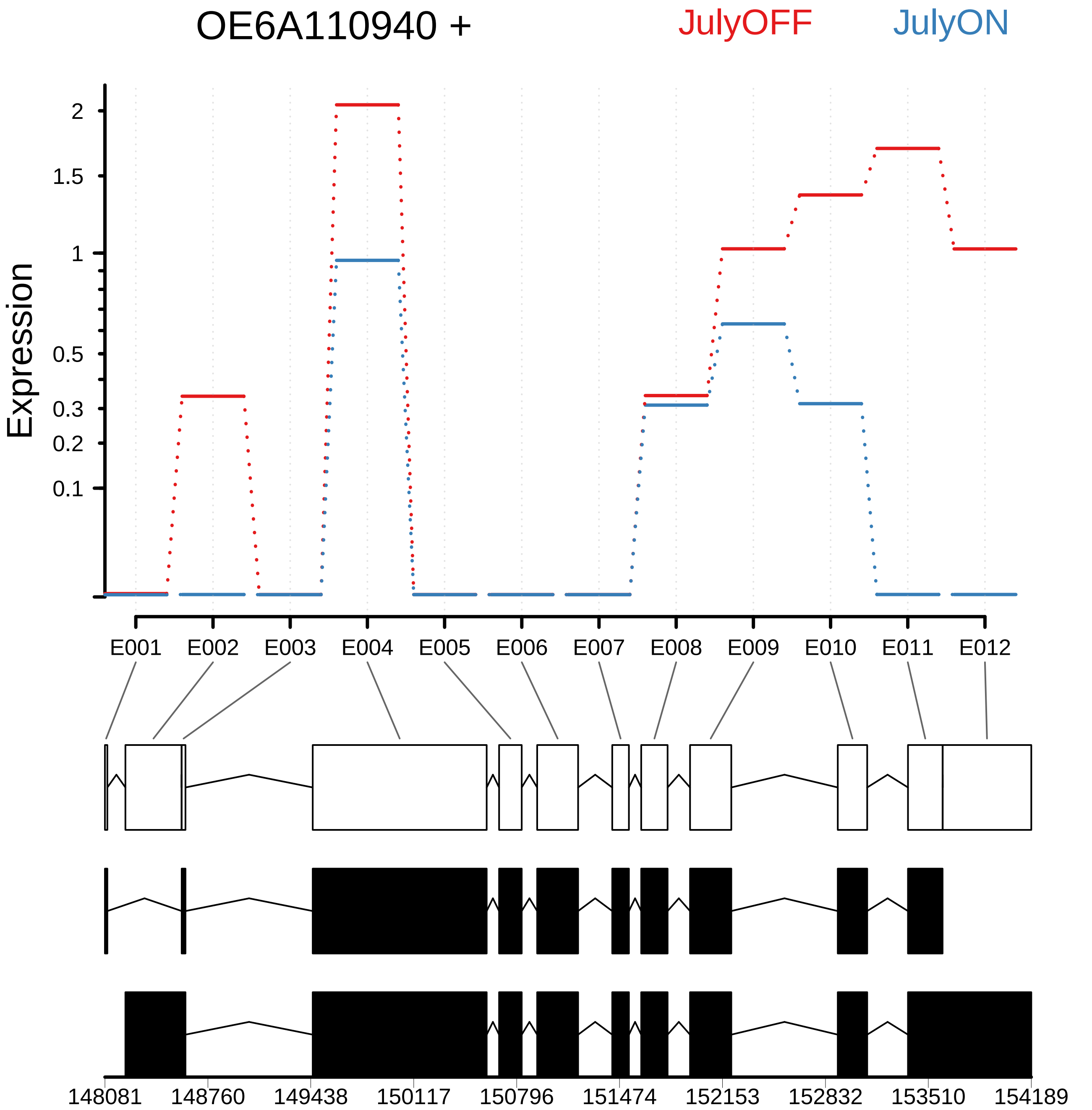

OE6A110940 +
JulyOFF
JulyON
Expression
2
1.5
1
0.5
0.3
0.2
0.1
E001
E002
E003
E004
E005
E006
E007
E008
E009
E010
E011
E012
148081
148760
149438
150117
150796
151474
152153
152832
153510
154189

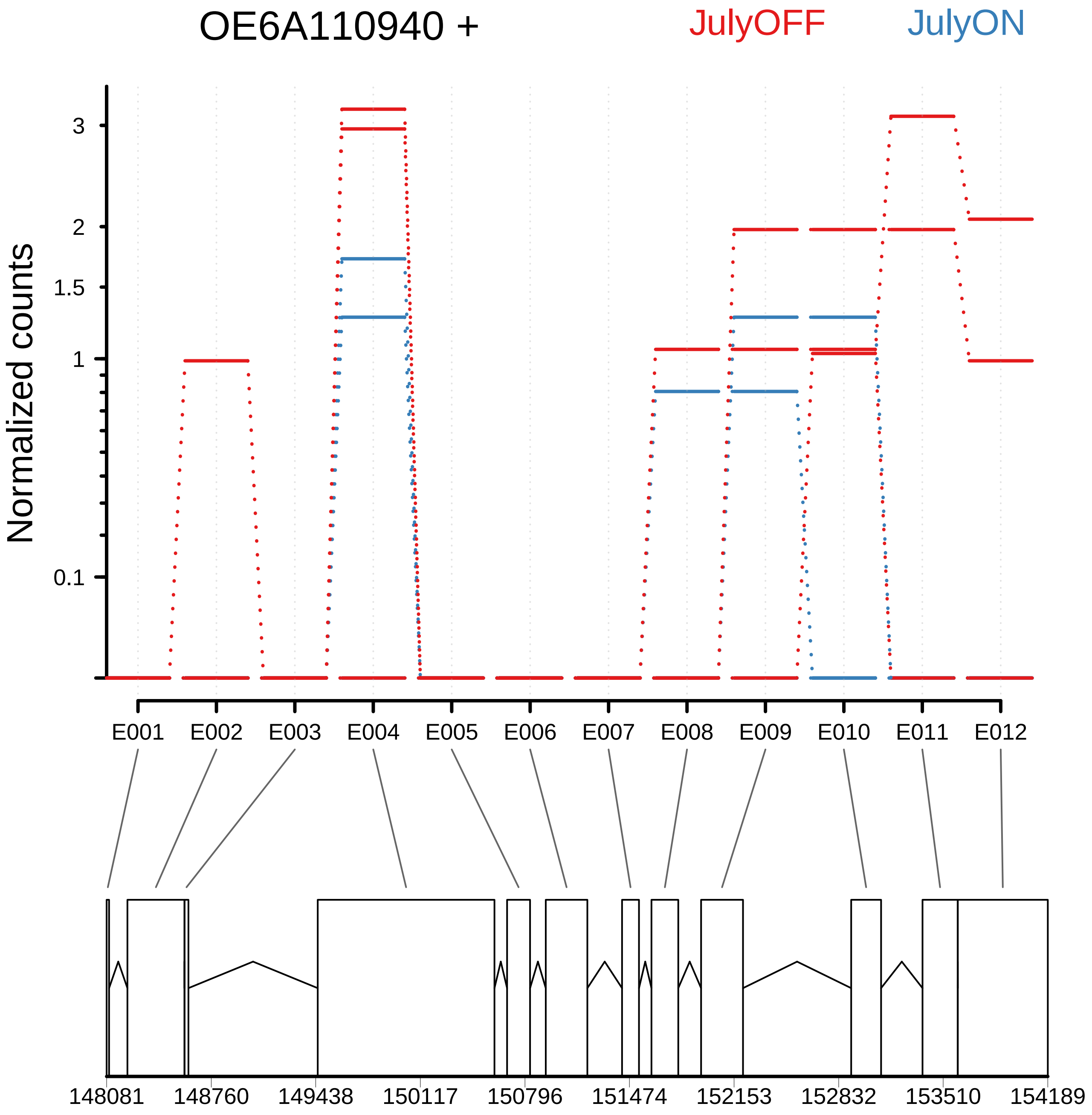

OE6A110940 +
JulyOFF
JulyON
Normalized counts
3
2
1.5
1
0.1
E001
E002
E003
E004
E005
E006
E007
E008
E009
E010
E011
E012
148081
148760
149438
150117
150796
151474
152153
152832
153510
154189

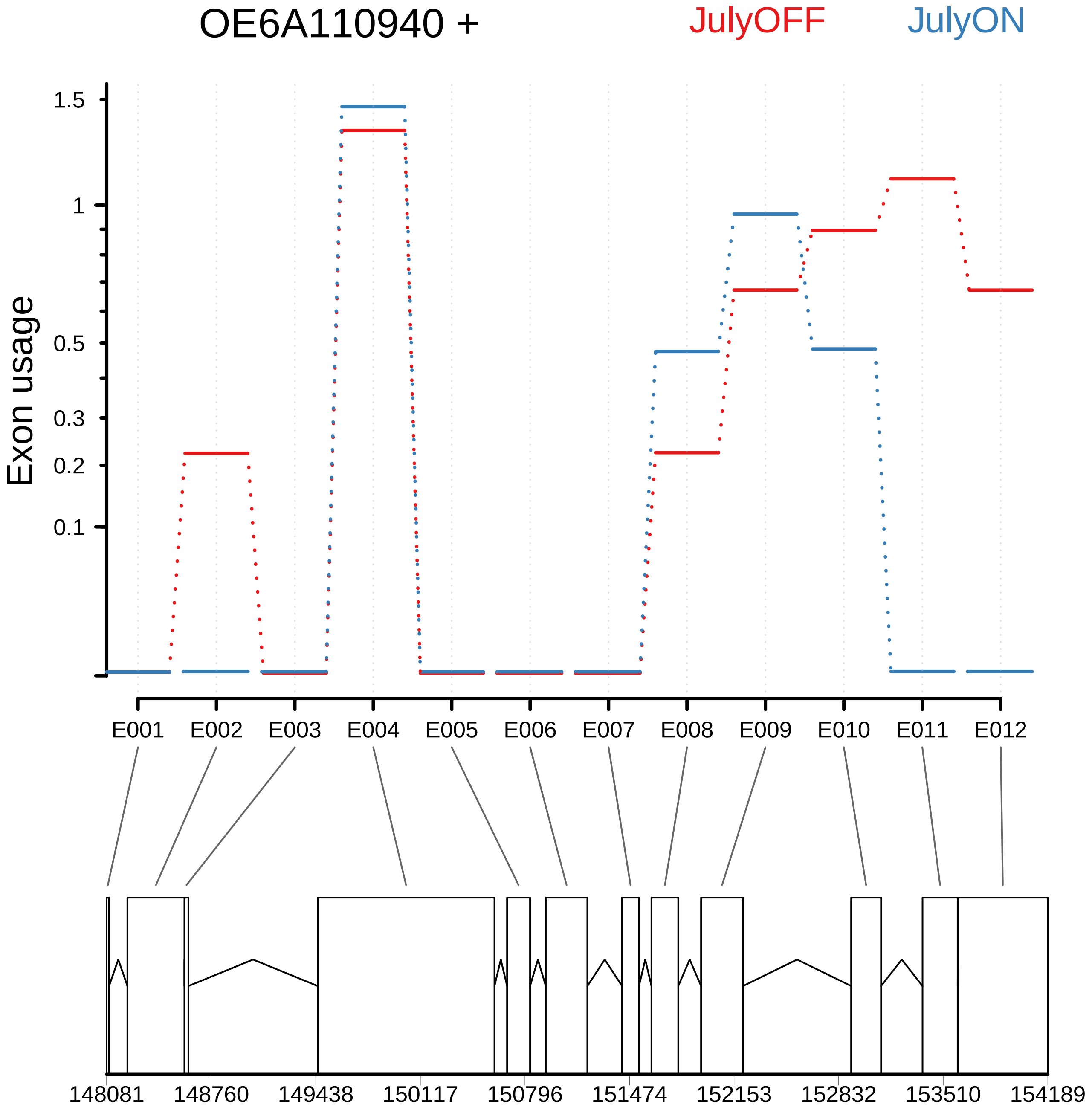
OE6A110940 +
JulyOFF
JulyON
Exon usage
1.5
1
0.5
0.3
0.2
0.1
E001
E002
E003
E004
E005
E006
E007
E008
E009
E010
E011
E012
148081
148760
149438
150117
150796
151474
152153
152832
153510
154189

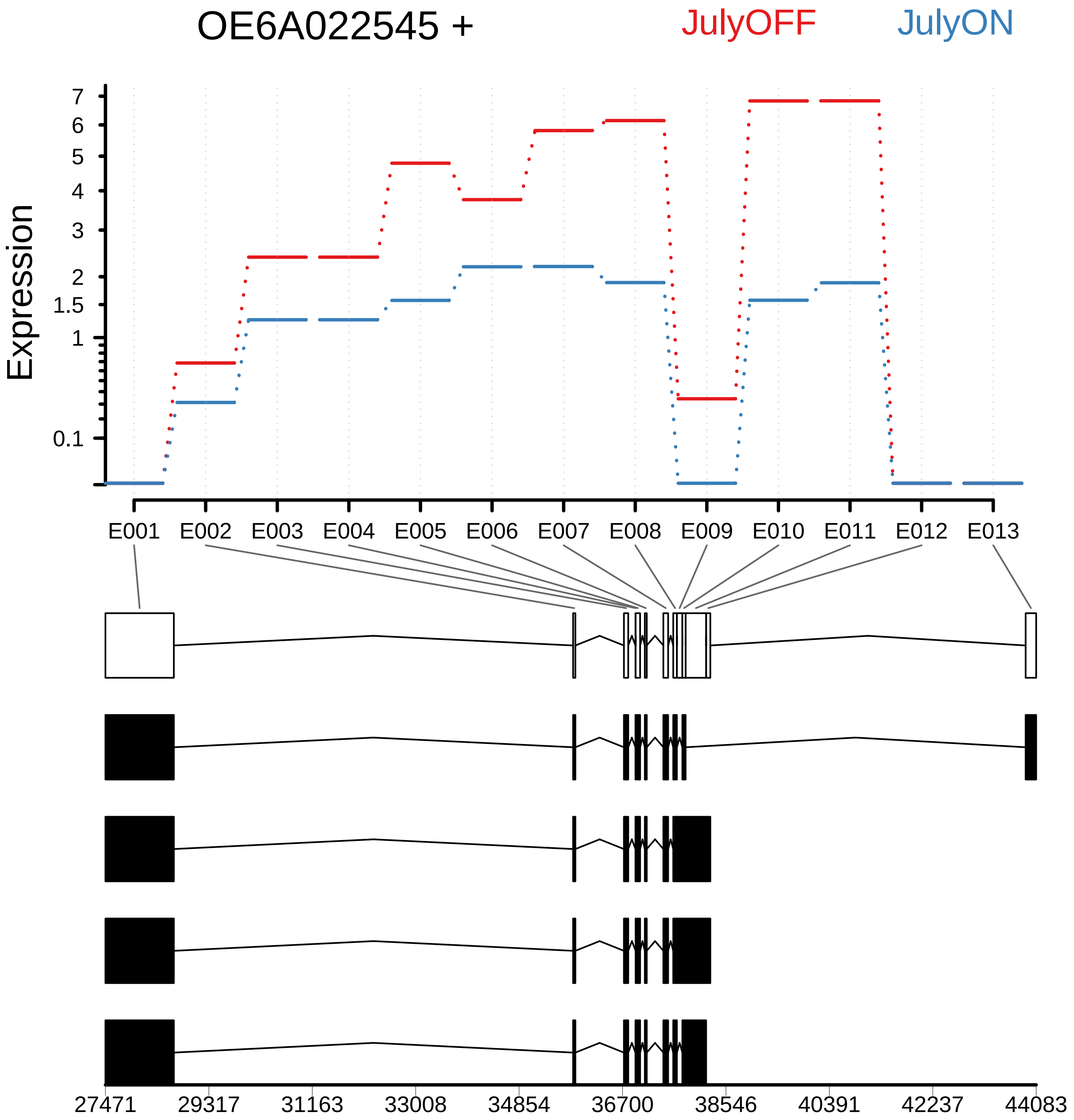

OE6A022545 +
JulyOFF
JulyON
Expression
7
6
5
4
3
2
1.5
1
0.1
E001
E002
E003
E004
E005
E006
E007
E008
E009
E010
E011
E012
E013
27471
29317
31163
33008
34854
36700
38546
40391
42237
44083

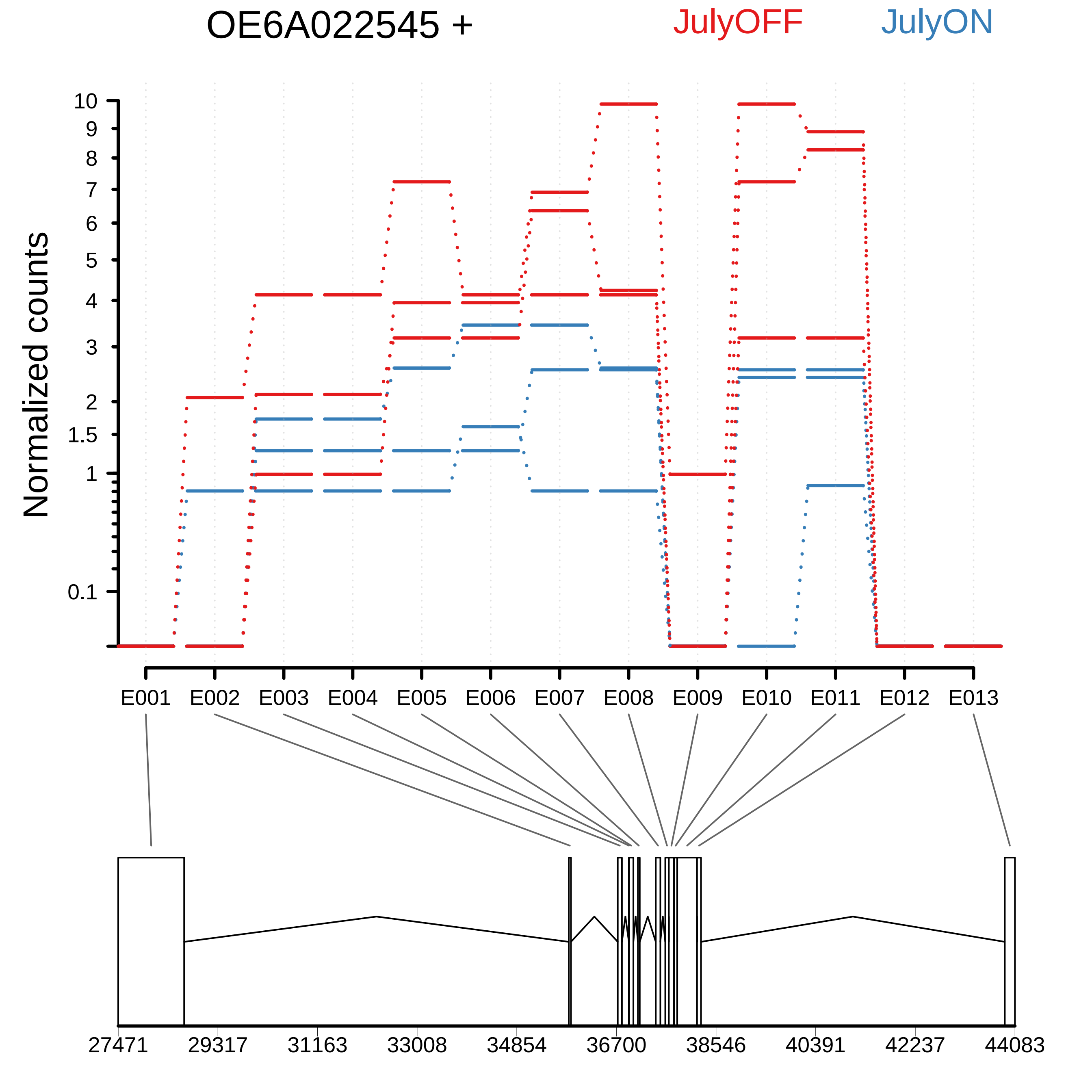
OE6A022545 +
JulyOFF
JulyON
Normalized counts
10
9
8
7
6
5
4
3
2
1.5
1
0.1
E001
E002
E003
E004
E005
E006
E007
E008
E009
E010
E011
E012
E013
27471
29317
31163
33008
34854
36700
38546
40391
42237
44083

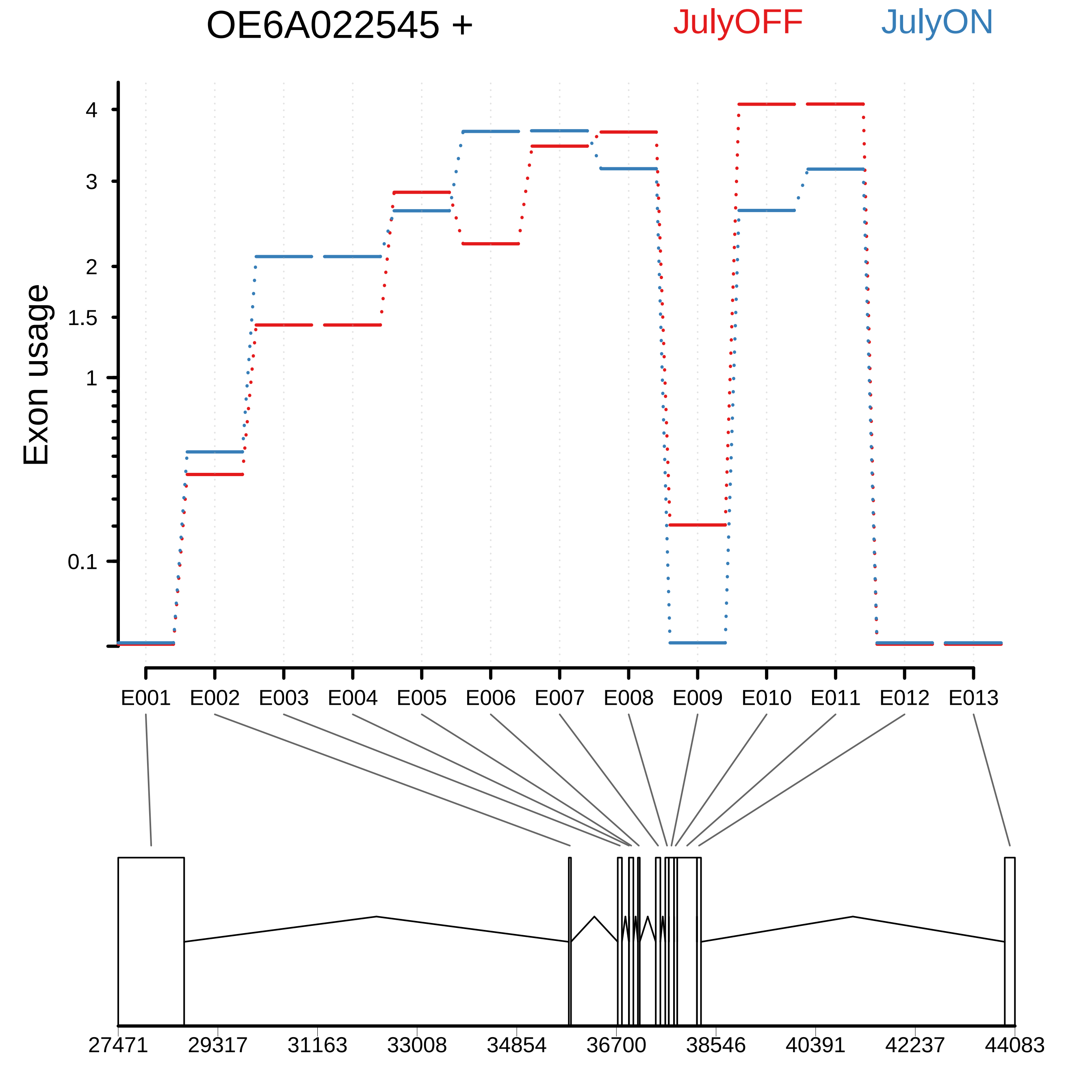

OE6A022545 +
JulyOFF
JulyON
Exon usage
4
3
2
1.5
1
0.1
E001
E002
E003
E004
E005
E006
E007
E008
E009
E010
E011
E012
E013
27471
29317
31163
33008
34854
36700
38546
40391
42237
44083

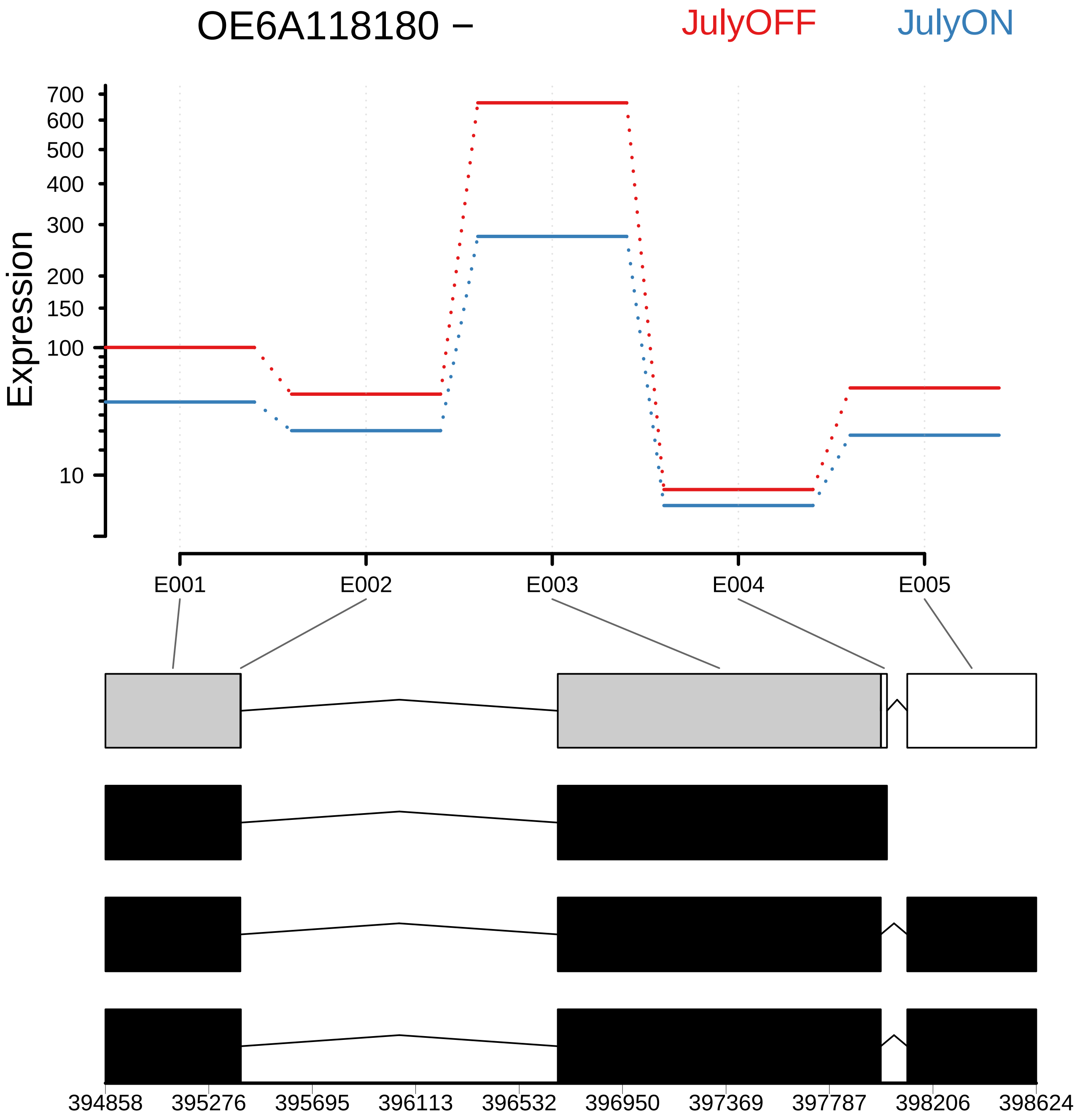

OE6A118180 –
JulyOFF
JulyON
Expression
700
600
500
400
300
200
150
100
10
E001
E002
E003
E004
E005
394858
395276
395695
396113
396532
396950
397369
397787
398206
398624

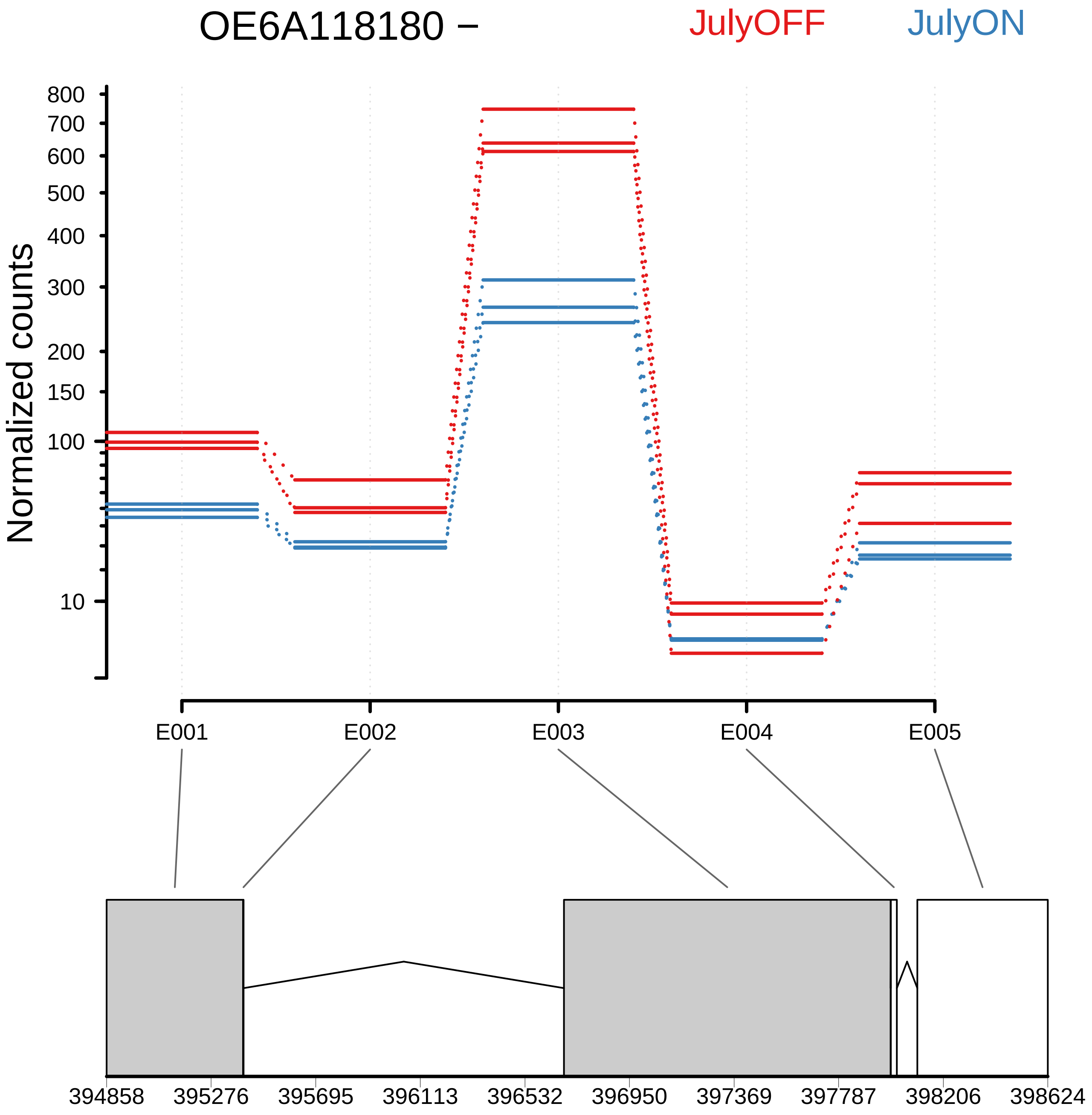

OE6A118180 −
JulyOFF
JulyON
Normalized counts
800
700
600
500
400
300
200
150
100
10
E001
E002
E003
E004
E005
394858
395276
395695
396113
396532
396950
397369
397787
398206
398624

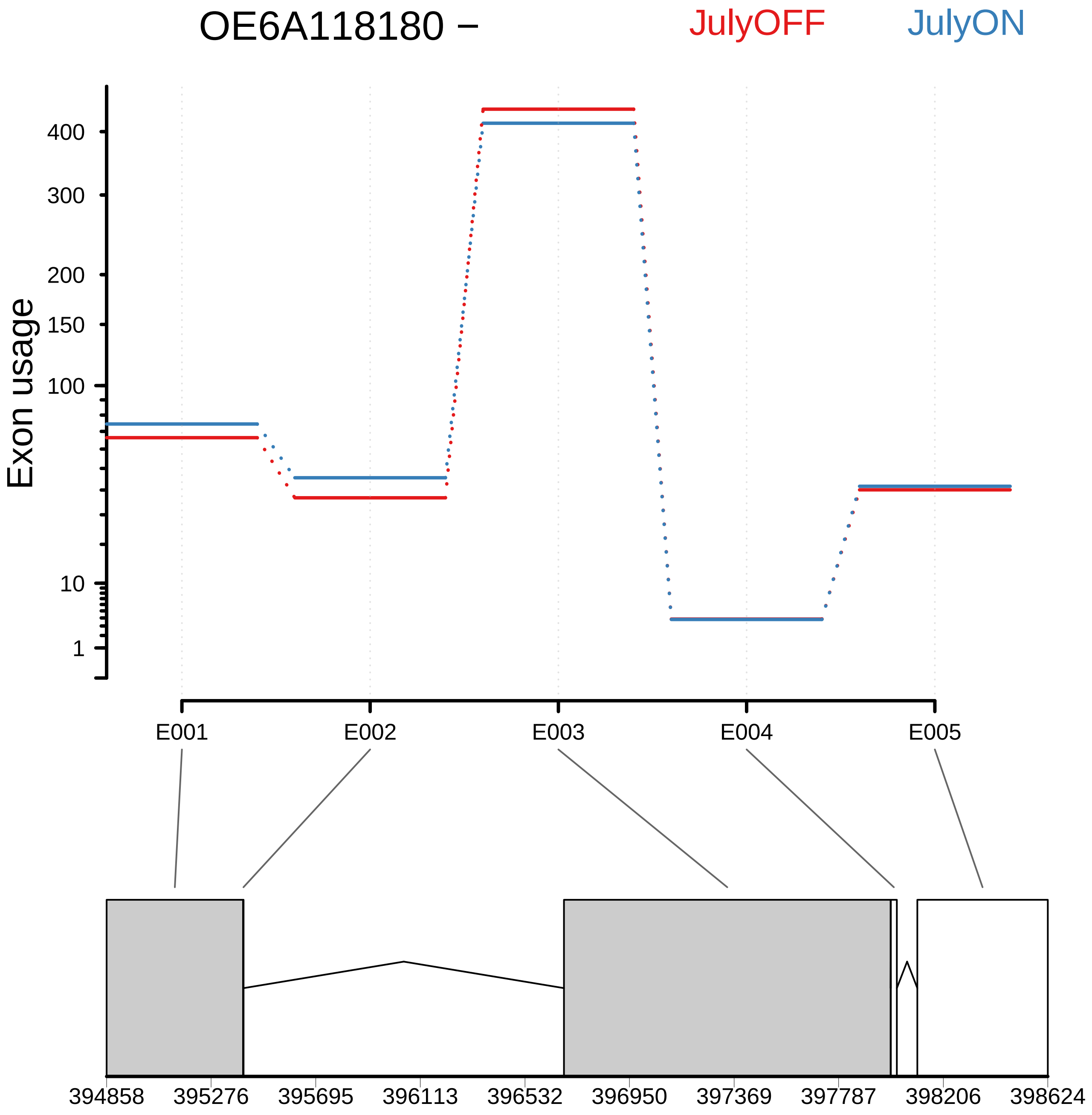
OE6A118180 −
JulyOFF
JulyON
Exon usage
400
300
200
150
100
10
1
E001
E002
E003
E004
E005
394858
395276
395695
396113
396532
396950
397369
397787
398206
398624

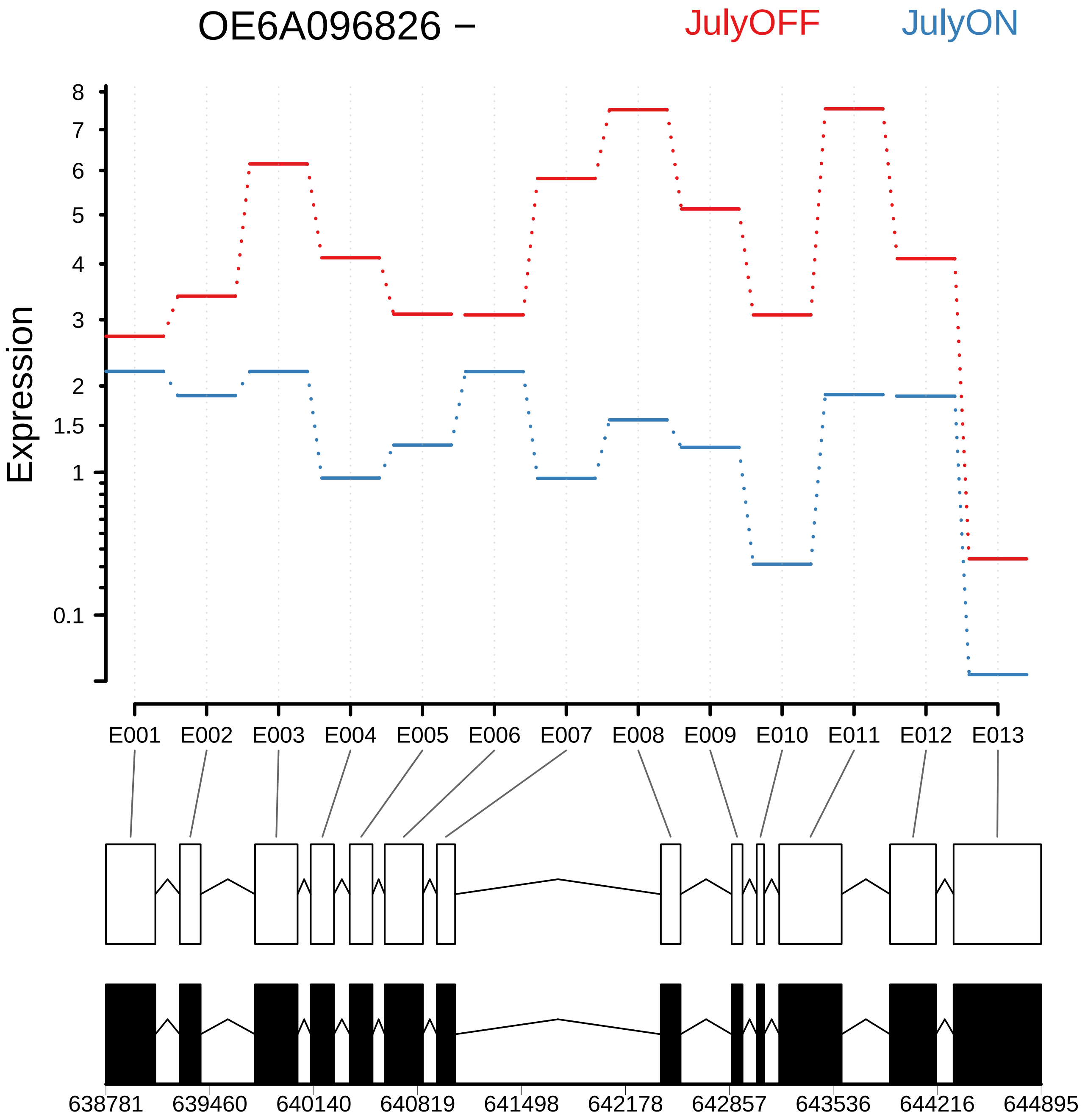

OE6A096826 −
JulyOFF
JulyON
Expression
8
7
6
5
4
3
2
1.5
1
0.1
E001
E002
E003
E004
E005
E006
E007
E008
E009
E010
E011
E012
E013
638781
639460
640140
640819
641498
642178
642857
643536
644216
644895

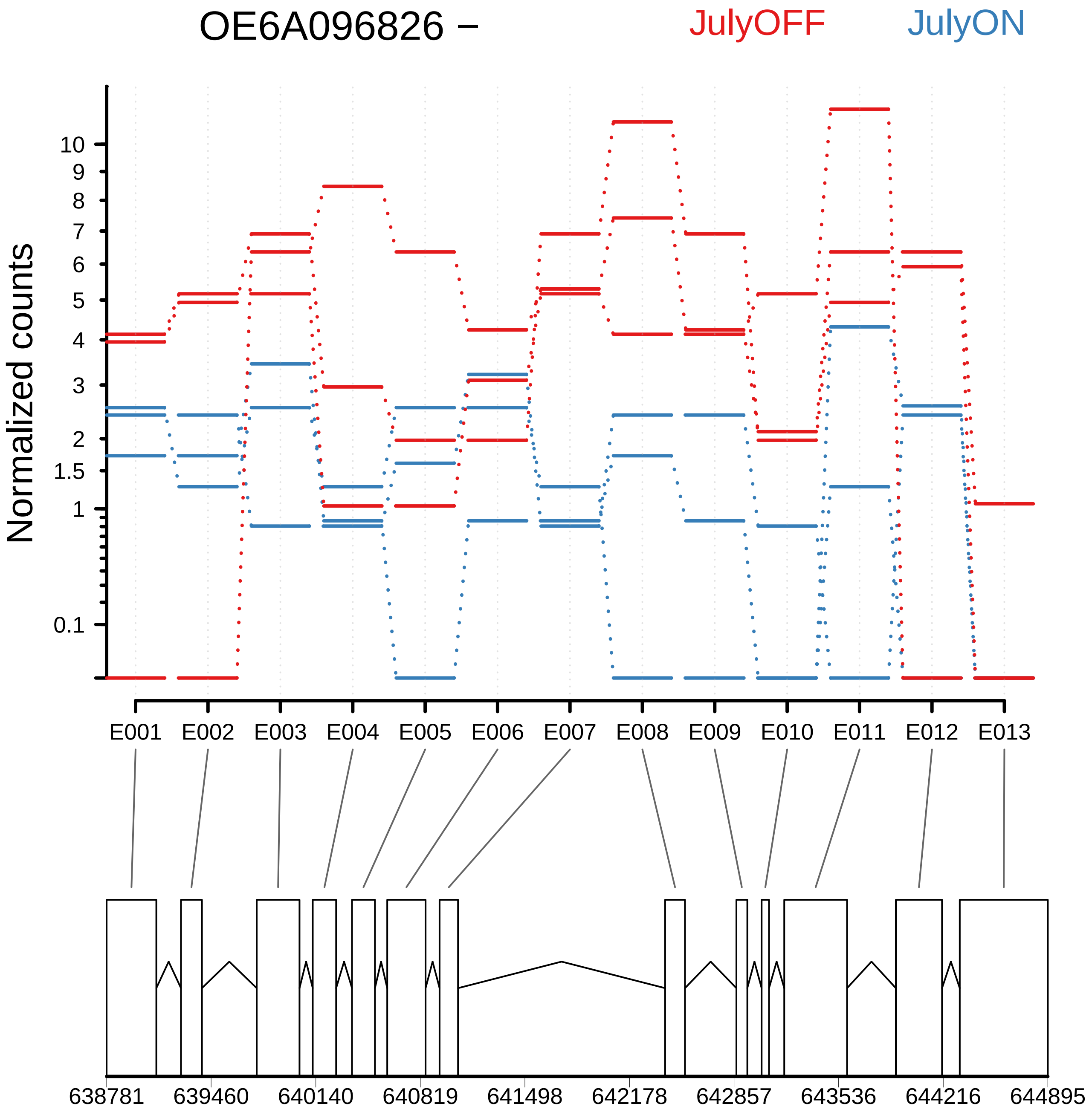
OE6A096826 −
JulyOFF
JulyON
Normalized counts
10
9
8
7
6
5
4
3
2
1.5
1
0.1
E001
E002
E003
E004
E005
E006
E007
E008
E009
E010
E011
E012
E013
638781
639460
640140
640819
641498
642178
642857
643536
644216
644895

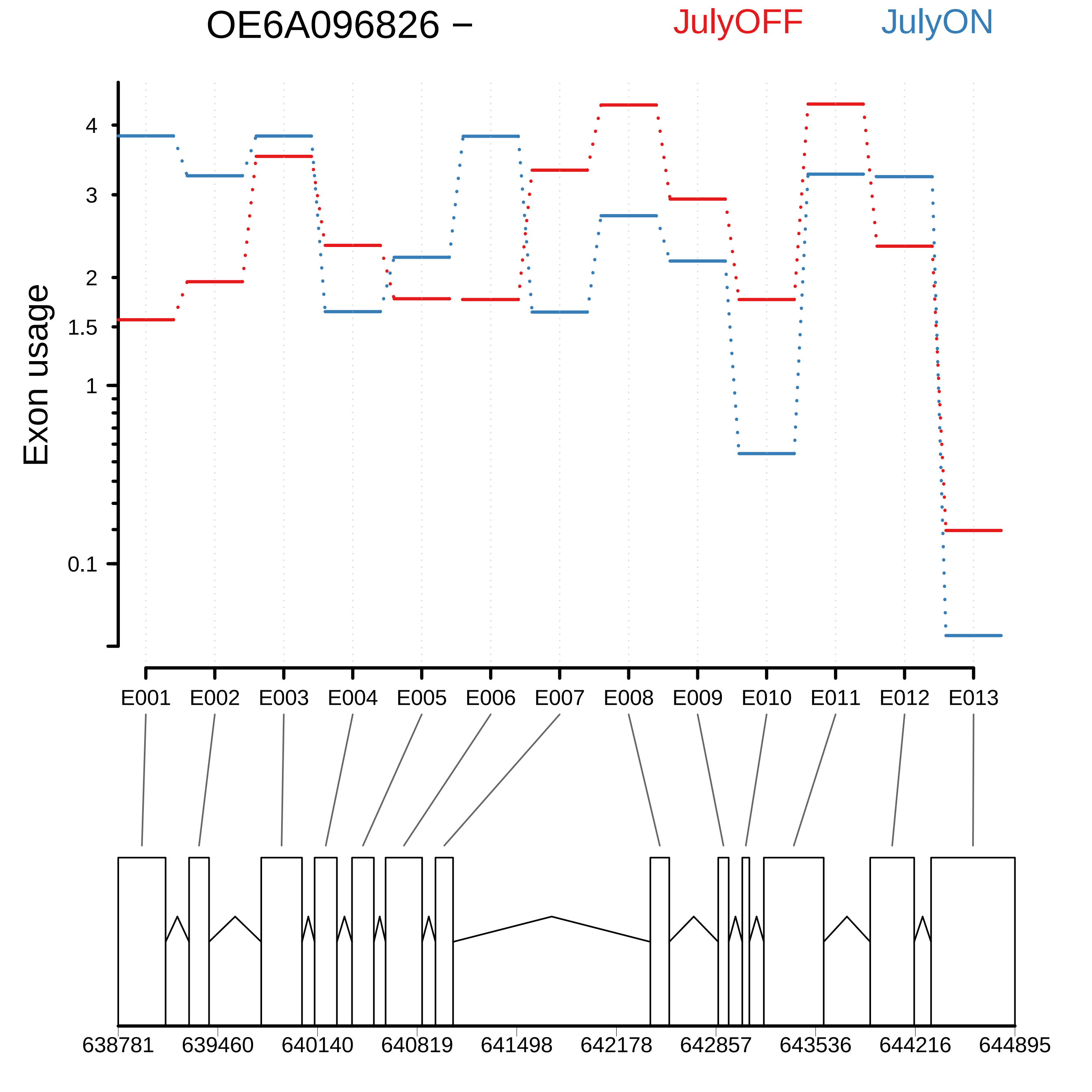
OE6A096826 −
JulyOFF
JulyON
Exon usage
4
3
2
1.5
1
0.1
E001
E002
E003
E004
E005
E006
E007
E008
E009
E010
E011
E012
E013
638781
639460
640140
640819
641498
642178
642857
643536
644216
644895

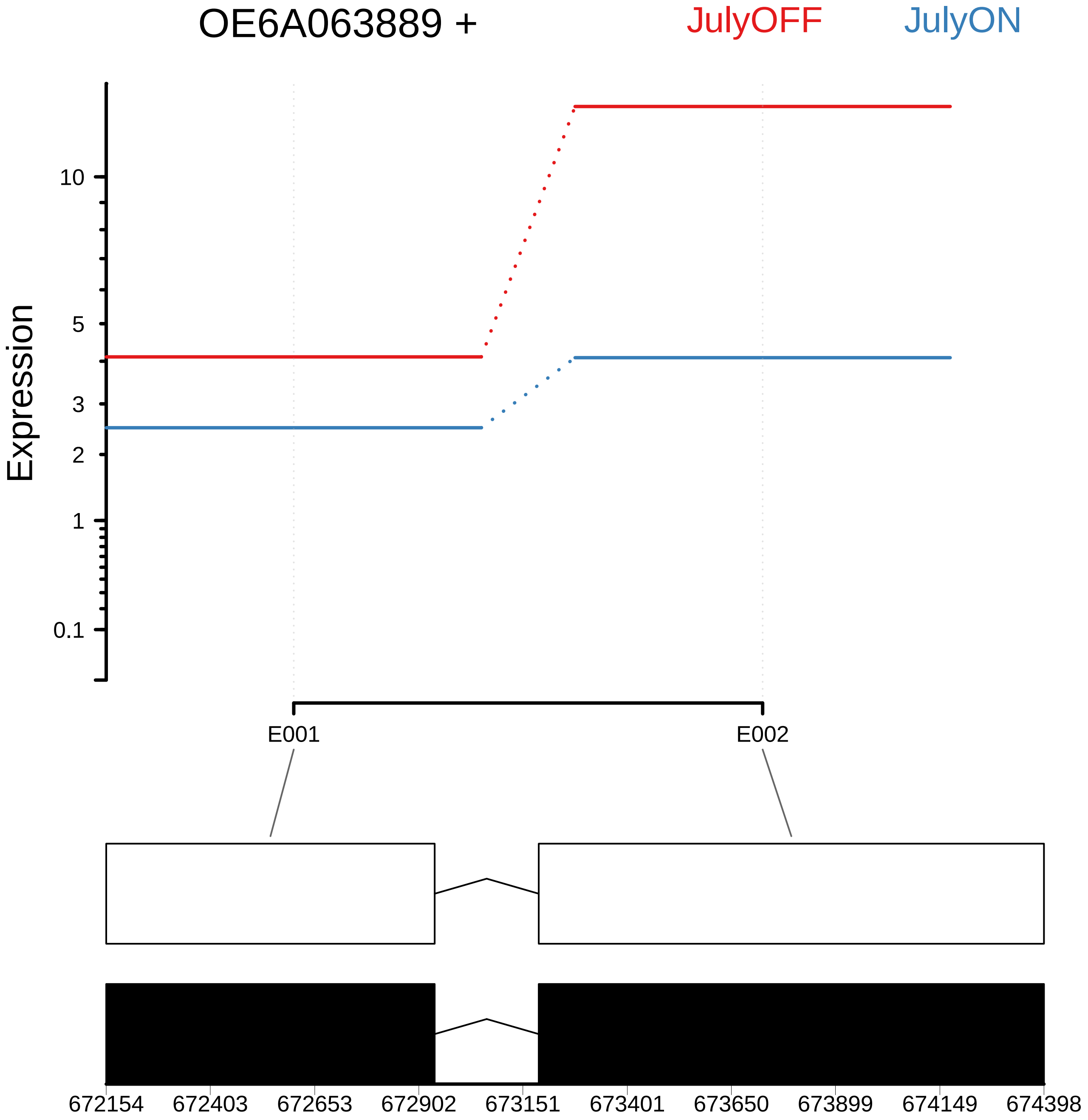

OE6A063889 +
JulyOFF
JulyON
Expression
10
5
3
2
1
0.1
E001
E002
672154
672403
672653
672902
673151
673401
673650
673899
674149
674398

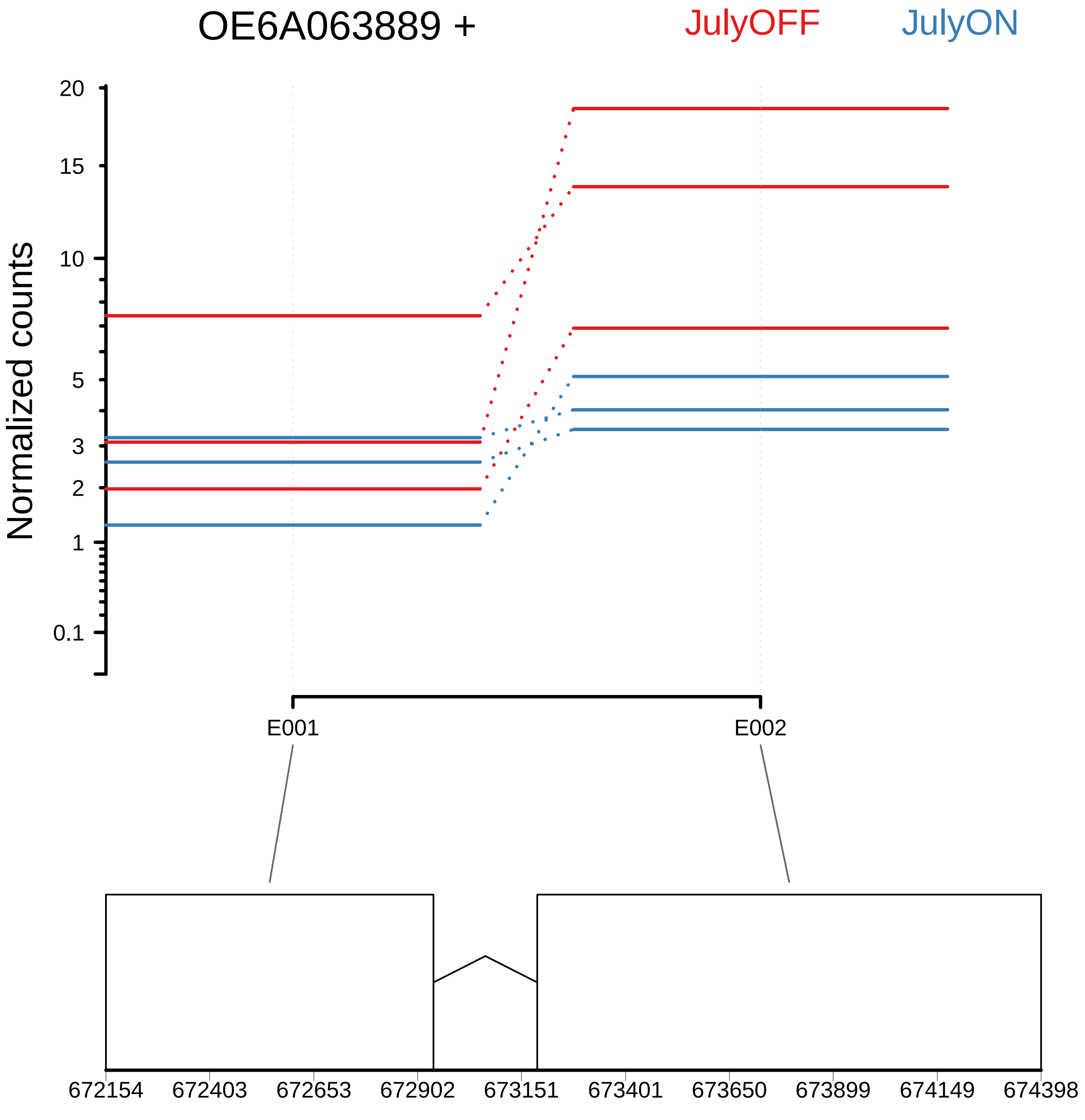

OE6A063889 +
JulyOFF
JulyON
Normalized counts
20
15
10
5
3
2
1
0.1
E001
E002
672154
672403
672653
672902
673151
673401
673650
673899
674149
674398

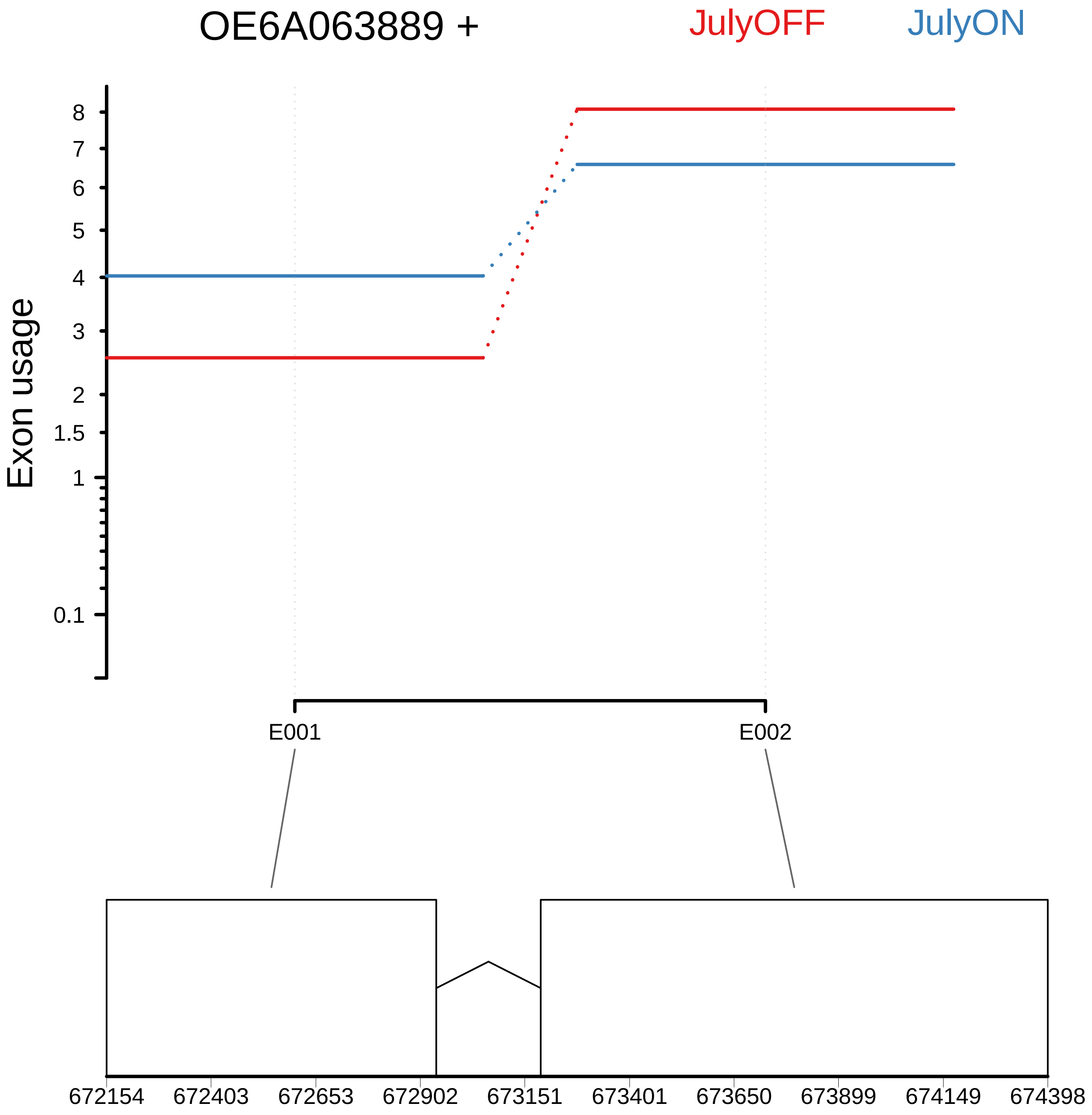

OE6A063889 +
JulyOFF
JulyON
Exon usage
8
7
6
5
4
3
2
1.5
1
0.1
E001
E002
672154
672403
672653
672902
673151
673401
673650
673899
674149
674398

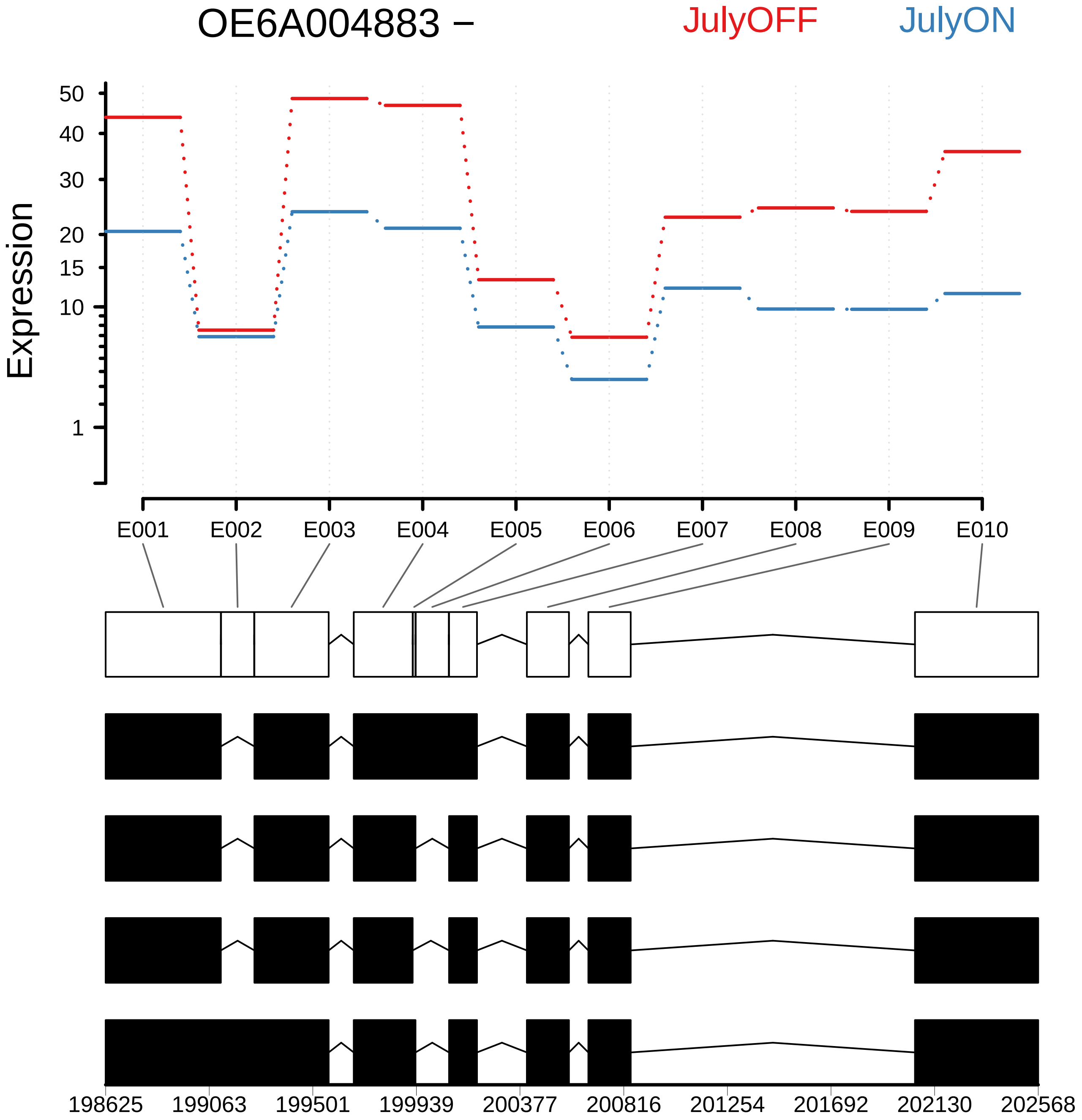

OE6A004883 –
JulyOFF
JulyON
Expression
50
40
30
20
15
10
1
E001
E002
E003
E004
E005
E006
E007
E008
E009
E010
198625
199063
199501
199939
200377
200816
201254
201692
202130
202568

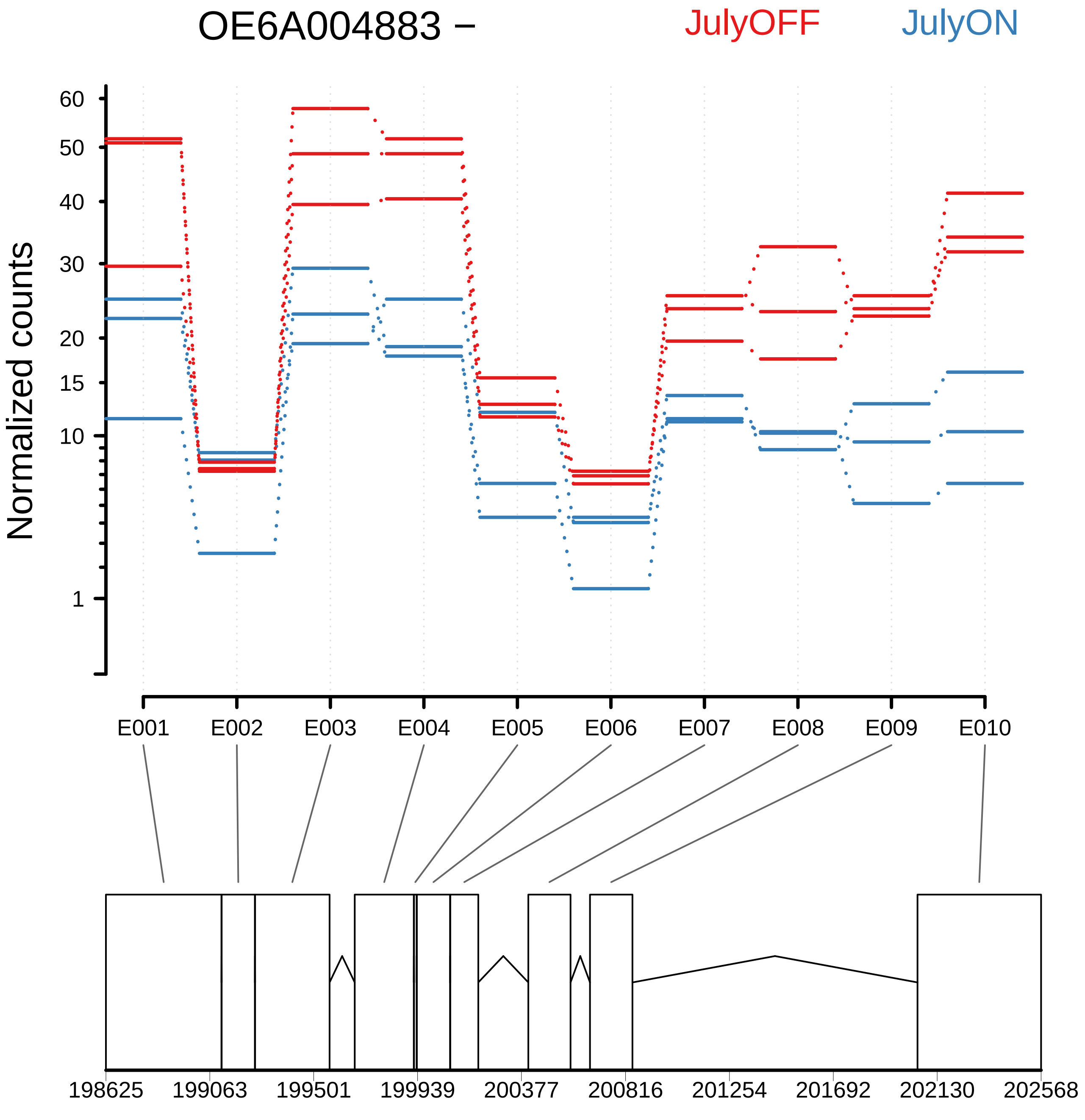
OE6A004883 −
JulyOFF
JulyON
Normalized counts
60
50
40
30
20
15
10
1
E001
E002
E003
E004
E005
E006
E007
E008
E009
E010
198625
199063
199501
199939
200377
200816
201254
201692
202130
202568

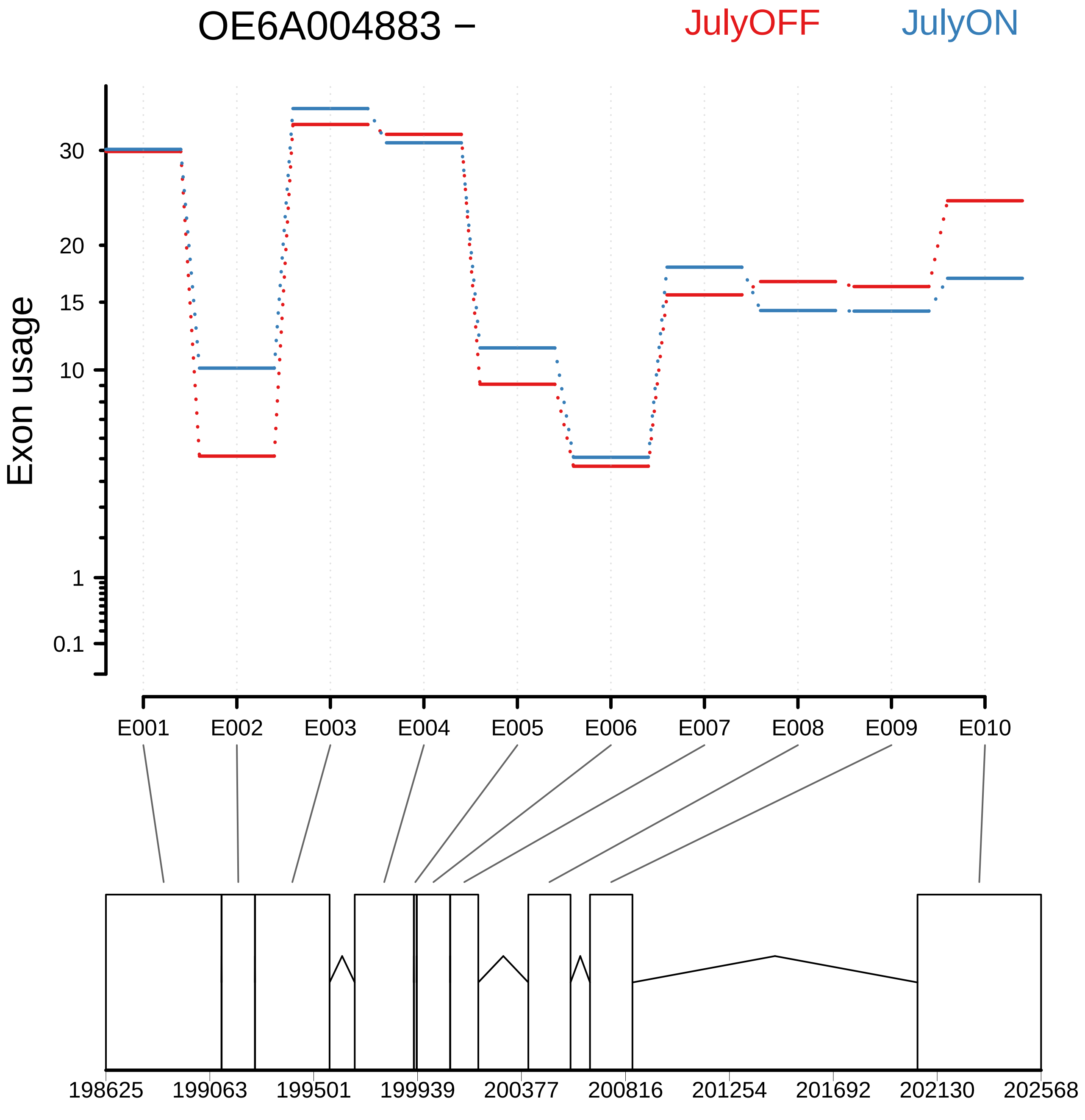

OE6A004883 –
JulyOFF
JulyON
Exon usage
30
20
15
10
1
0.1
E001
E002
E003
E004
E005
E006
E007
E008
E009
E010
198625
199063
199501
199939
200377
200816
201254
201692
202130
202568

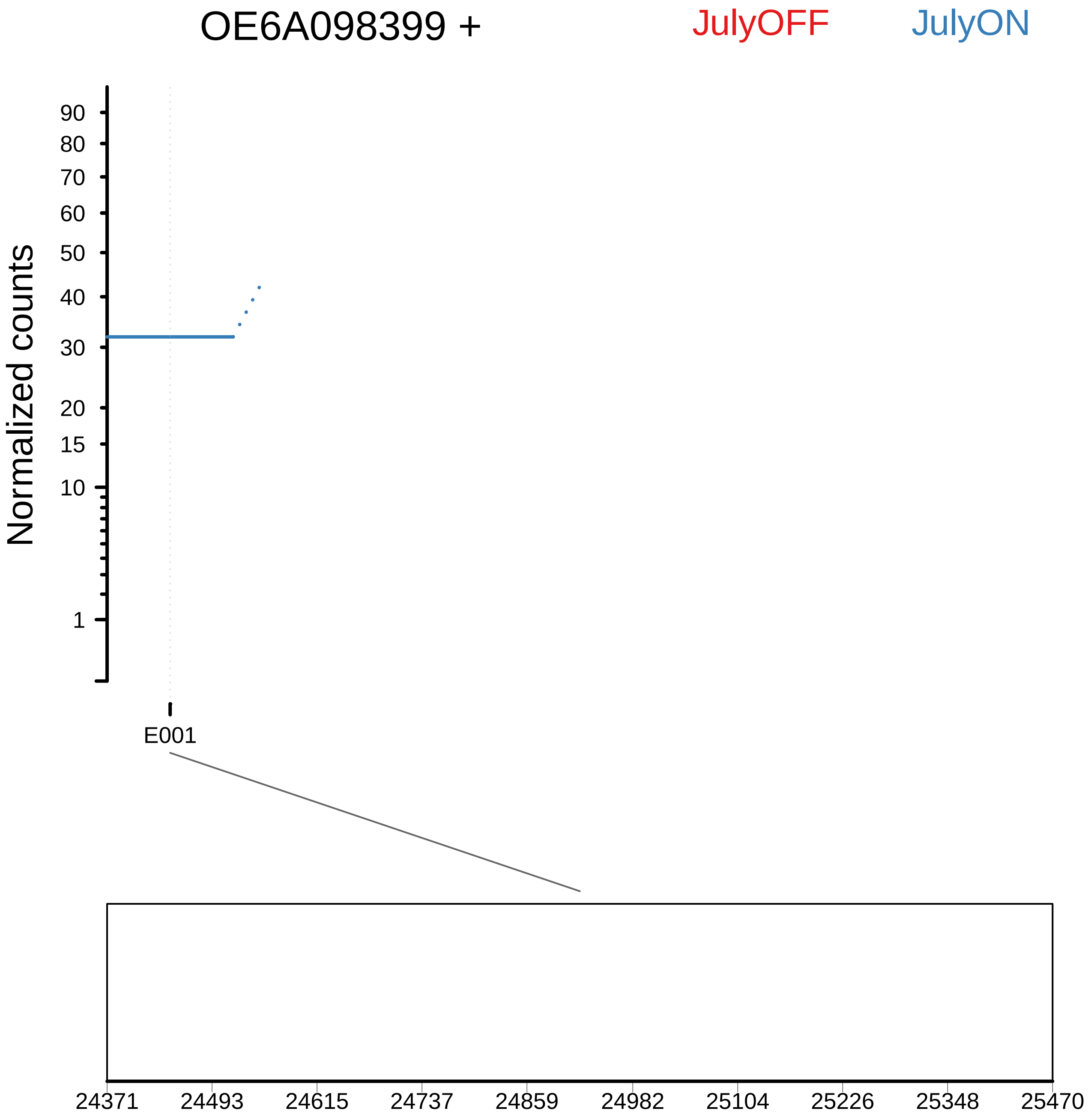

OE6A098399 +
JulyOFF
JulyON
Normalized counts
90
80
70
60
50
40
30
20
15
10
1
E001
24371
24493
24615
24737
24859
24982
25104
25226
25348
25470

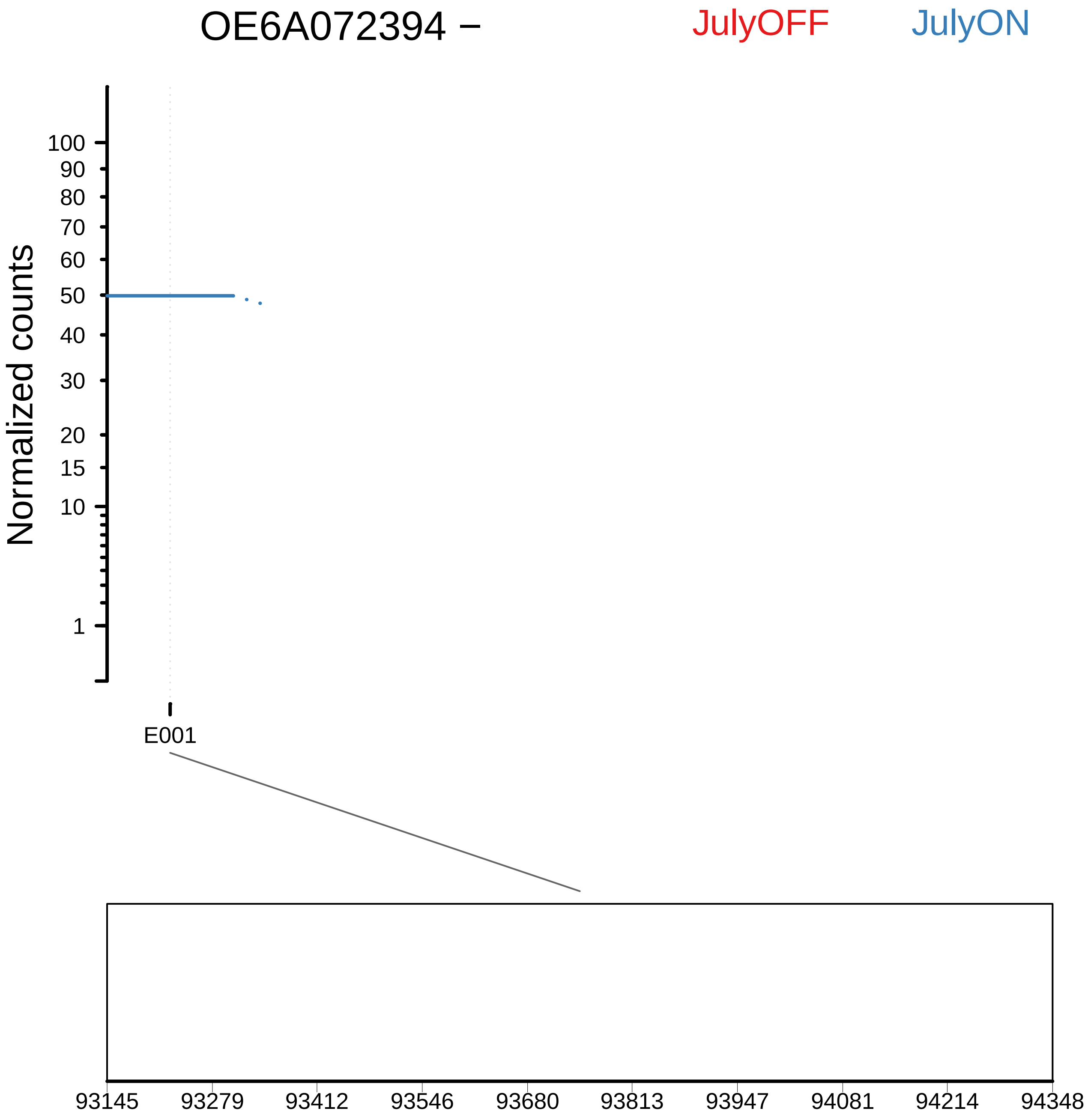

OE6A072394 −
JulyOFF
JulyON
Normalized counts
100
90
80
70
60
50
40
30
20
15
10
1
E001
93145
93279
93412
93546
93680
93813
93947
94081
94214
94348

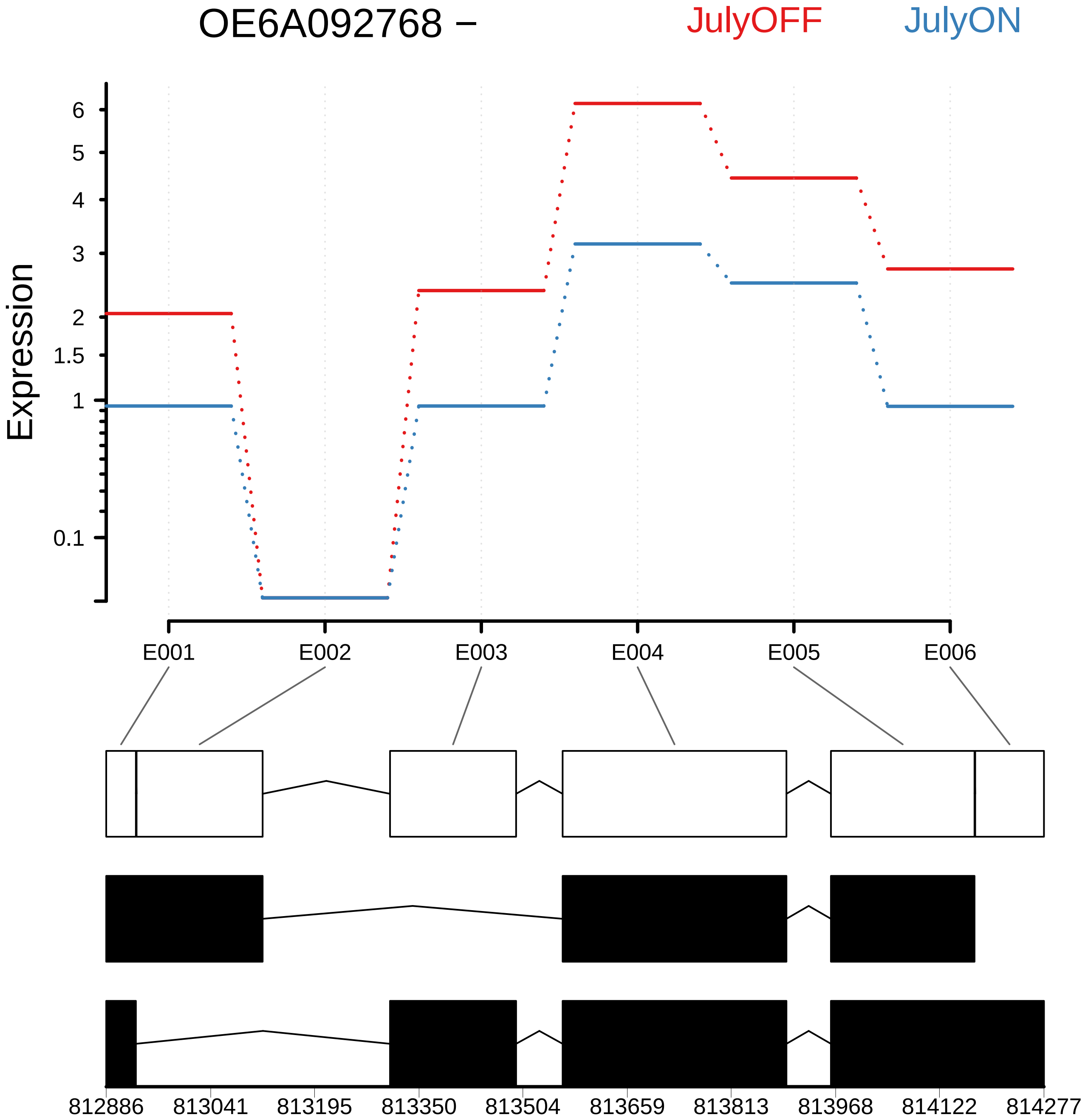

OE6A092768 −
JulyOFF
JulyON
Expression
6
5
4
3
2
1.5
1
0.1
E001
E002
E003
E004
E005
E006
812886
813041
813195
813350
813504
813659
813813
813968
814122
814277

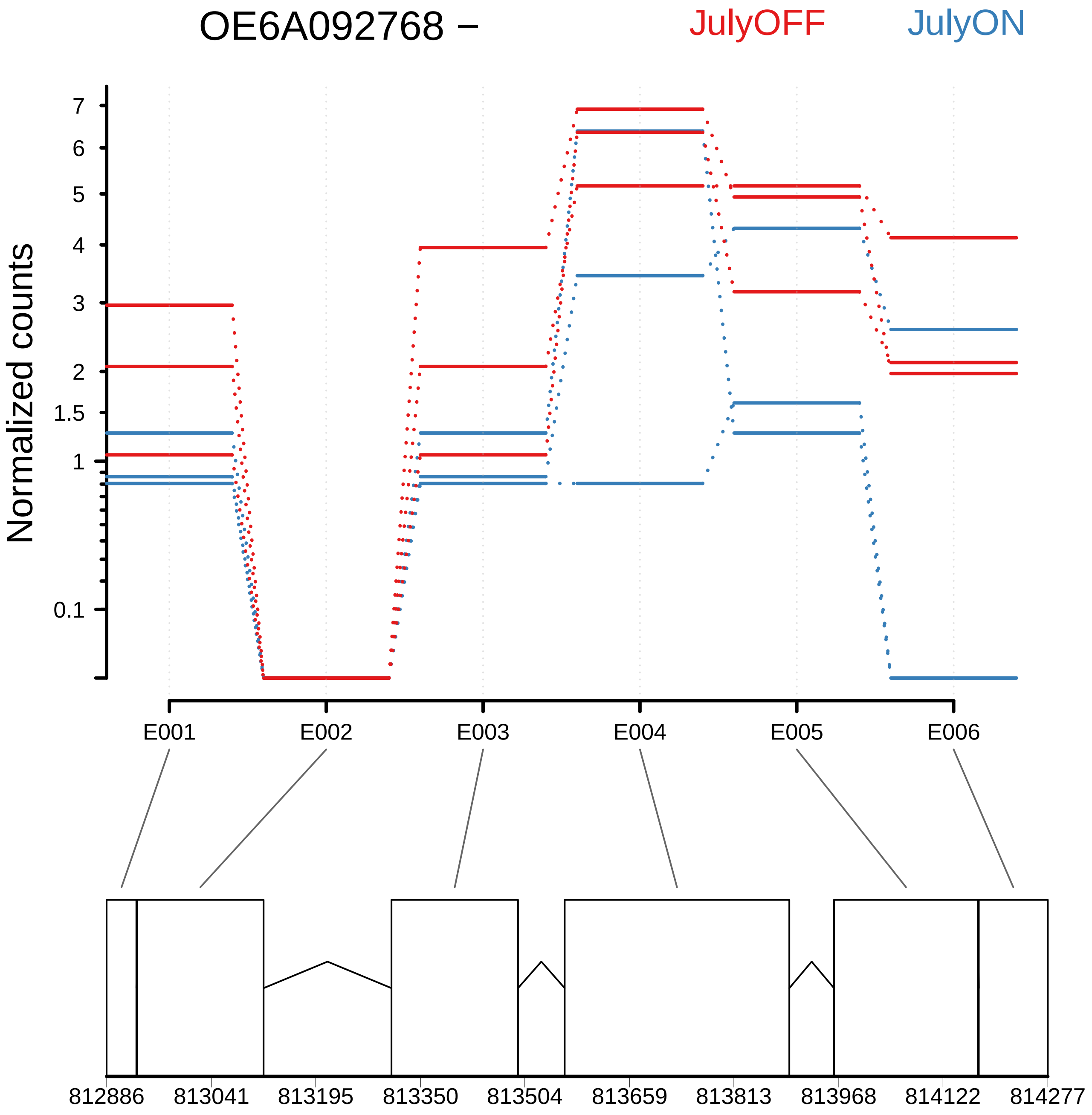

OE6A092768 −
JulyOFF
JulyON
Normalized counts
7
6
5
4
3
2
1.5
1
0.1
E001
E002
E003
E004
E005
E006
812886
813041
813195
813350
813504
813659
813813
813968
814122
814277

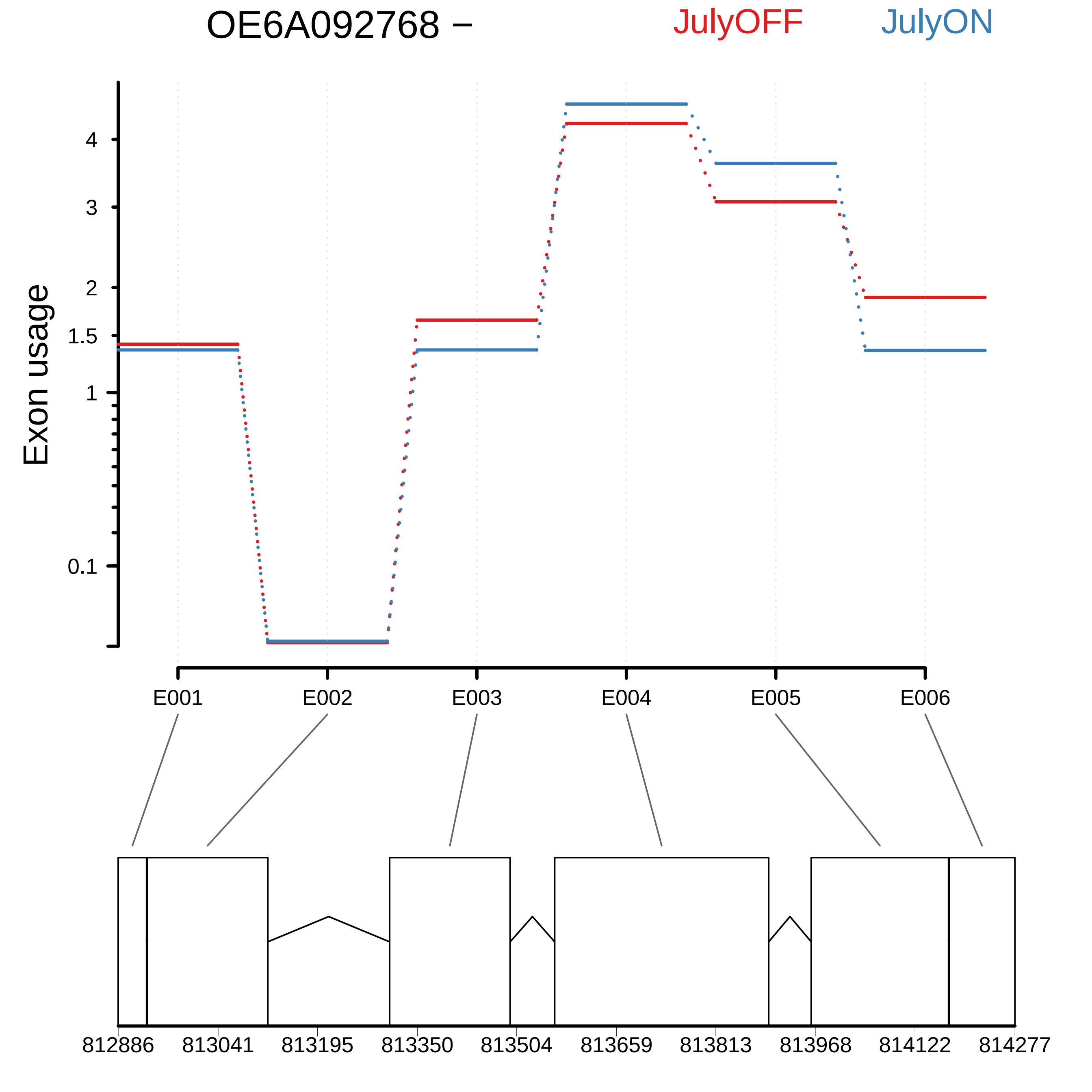

OE6A092768 −
JulyOFF
JulyON
Exon usage
4
3
2
1.5
1
0.1
E001
E002
E003
E004
E005
E006
812886
813041
813195
813350
813504
813659
813813
813968
814122
814277

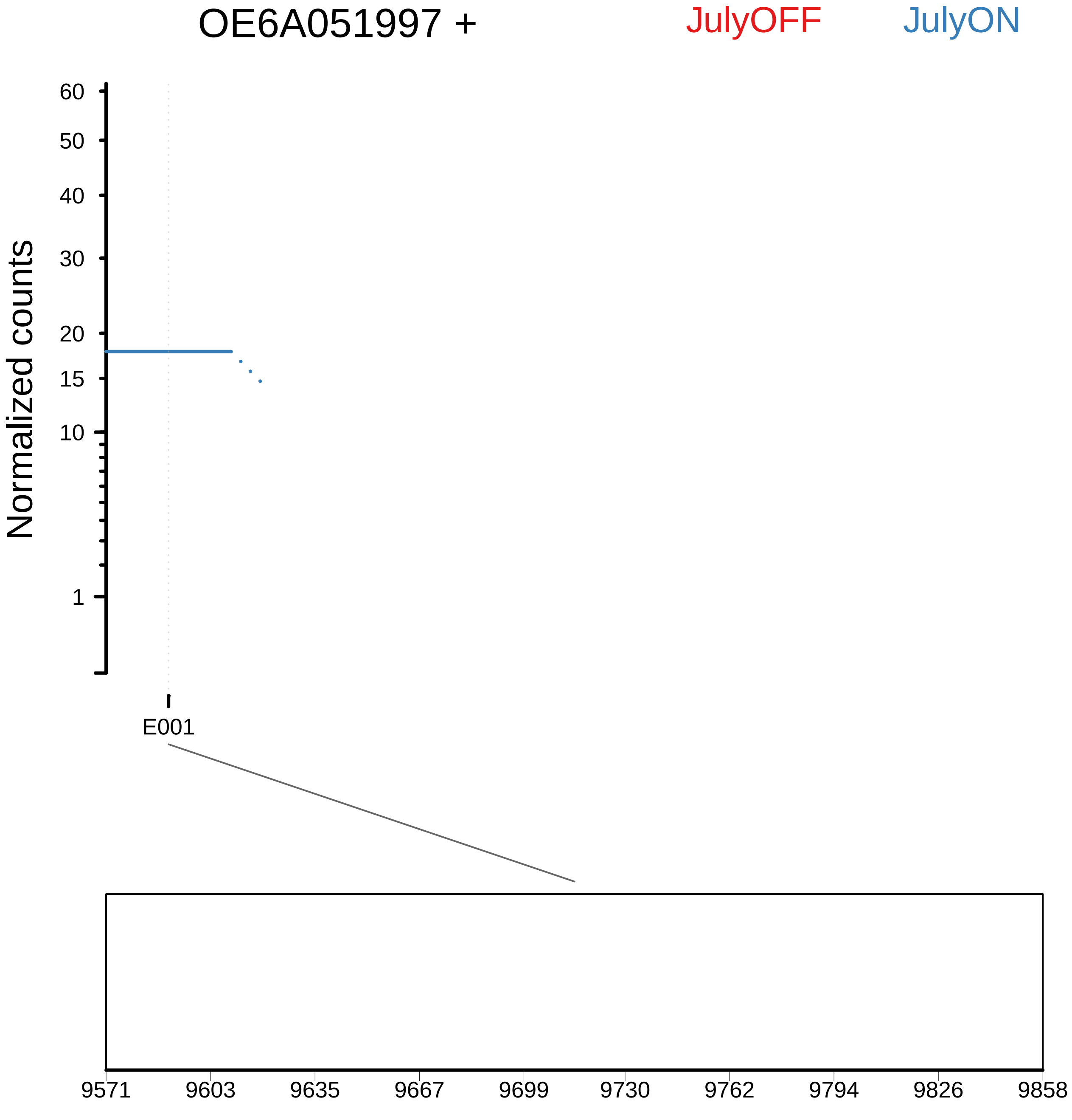

OE6A051997 +
JulyOFF
JulyON
Normalized counts
60
50
40
30
20
15
10
1
E001
9571
9603
9635
9667
9699
9730
9762
9794
9826
9858

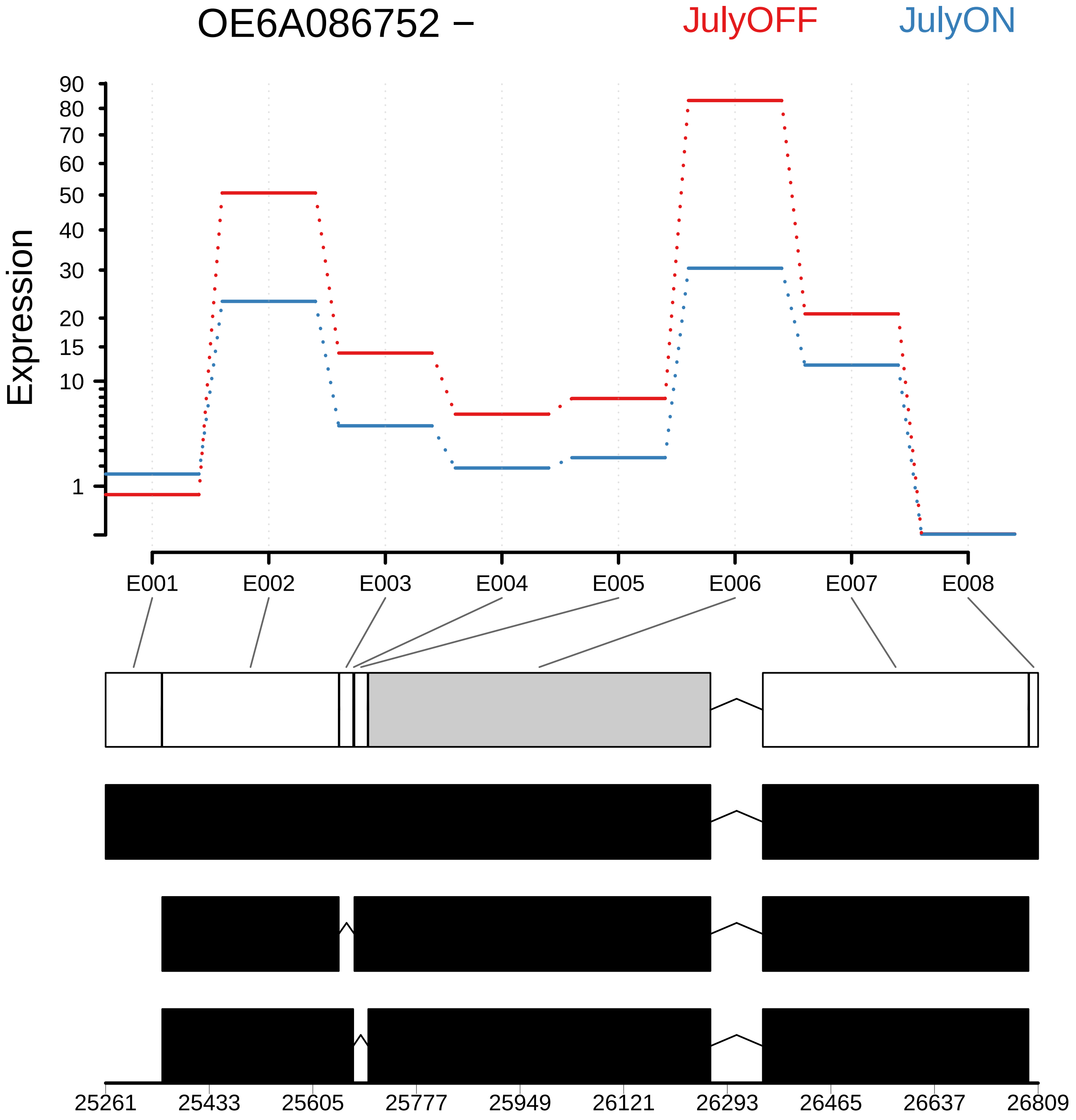

OE6A086752 −
JulyOFF
JulyON
Expression
90
80
70
60
50
40
30
20
15
10
1
E001
E002
E003
E004
E005
E006
E007
E008
25261
25433
25605
25777
25949
26121
26293
26465
26637
26809

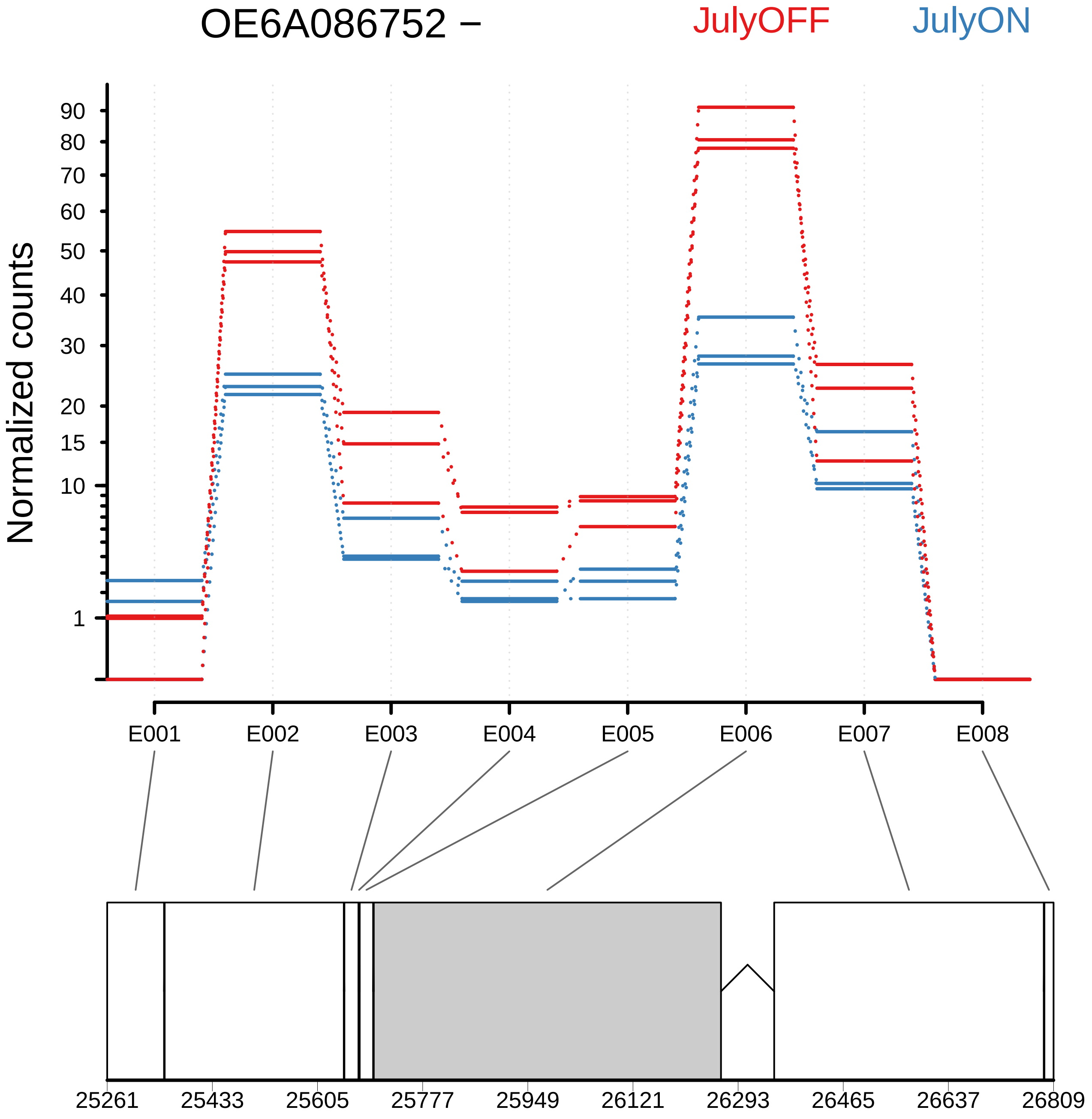

OE6A086752 −
JulyOFF
JulyON
Normalized counts
90
80
70
60
50
40
30
20
15
10
1
E001
E002
E003
E004
E005
E006
E007
E008
25261
25433
25605
25777
25949
26121
26293
26465
26637
26809

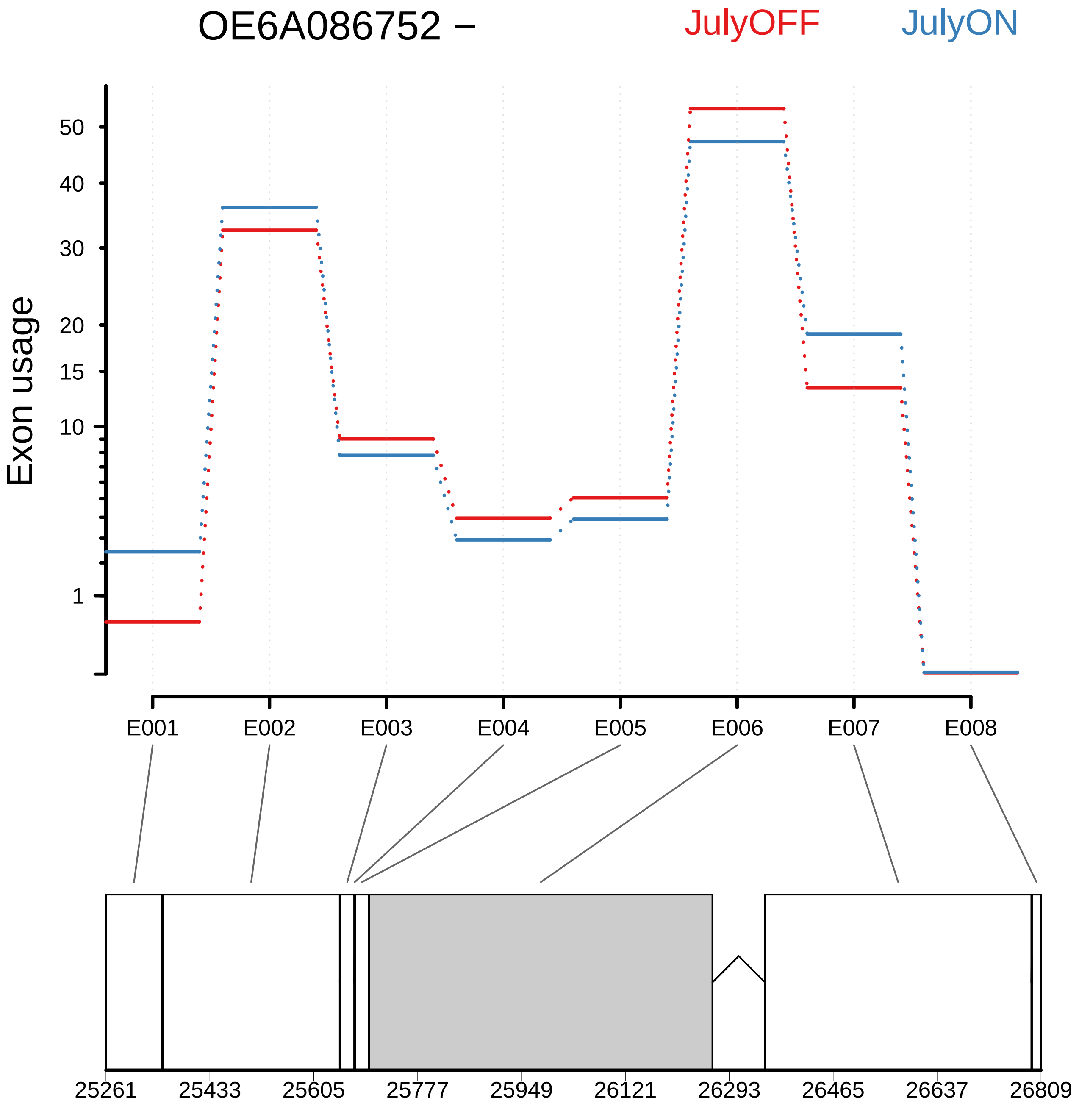

OE6A086752 −
JulyOFF
JulyON
Exon usage
50
40
30
20
15
10
1
E001
E002
E003
E004
E005
E006
E007
E008
25261
25433
25605
25777
25949
26121
26293
26465
26637
26809

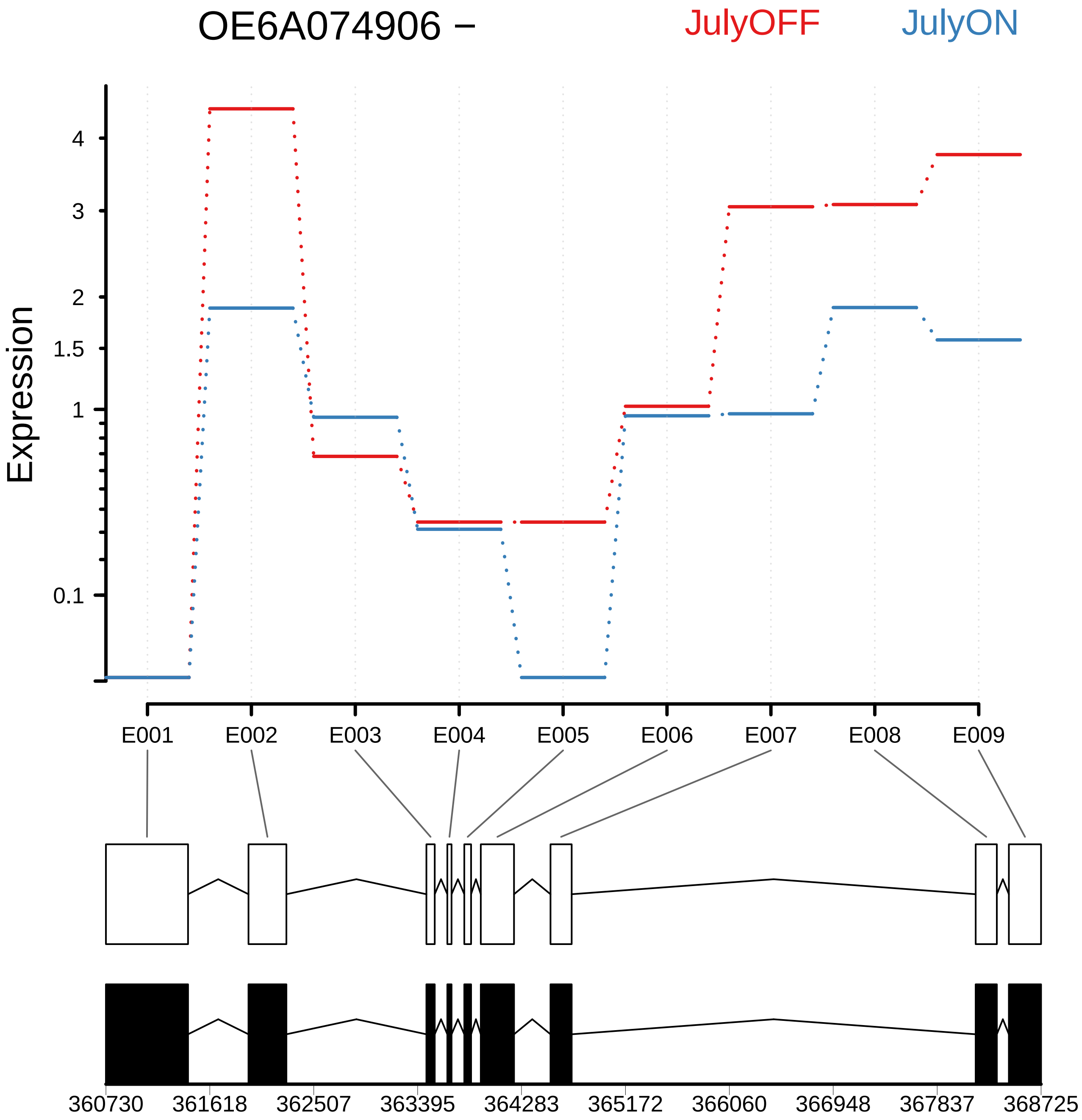
OE6A074906 –
JulyOFF
JulyON
Expression
4
3
2
1.5
1
0.1
E001
E002
E003
E004
E005
E006
E007
E008
E009
360730
361618
362507
363395
364283
365172
366060
366948
367837
368725

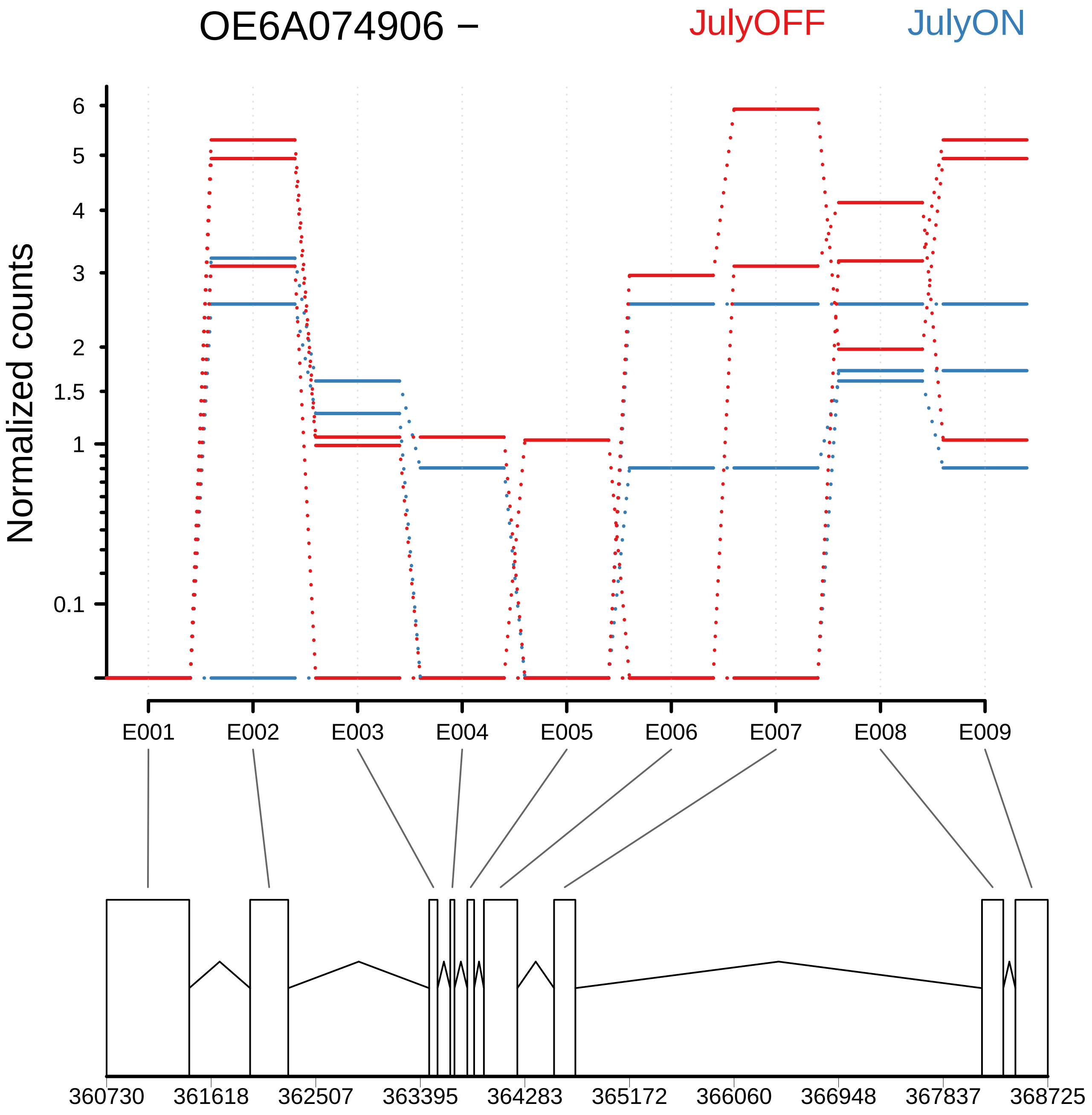

OE6A074906 −
JulyOFF
JulyON
Normalized counts
6
5
4
3
2
1.5
1
0.1
E001
E002
E003
E004
E005
E006
E007
E008
E009
360730
361618
362507
363395
364283
365172
366060
366948
367837
368725

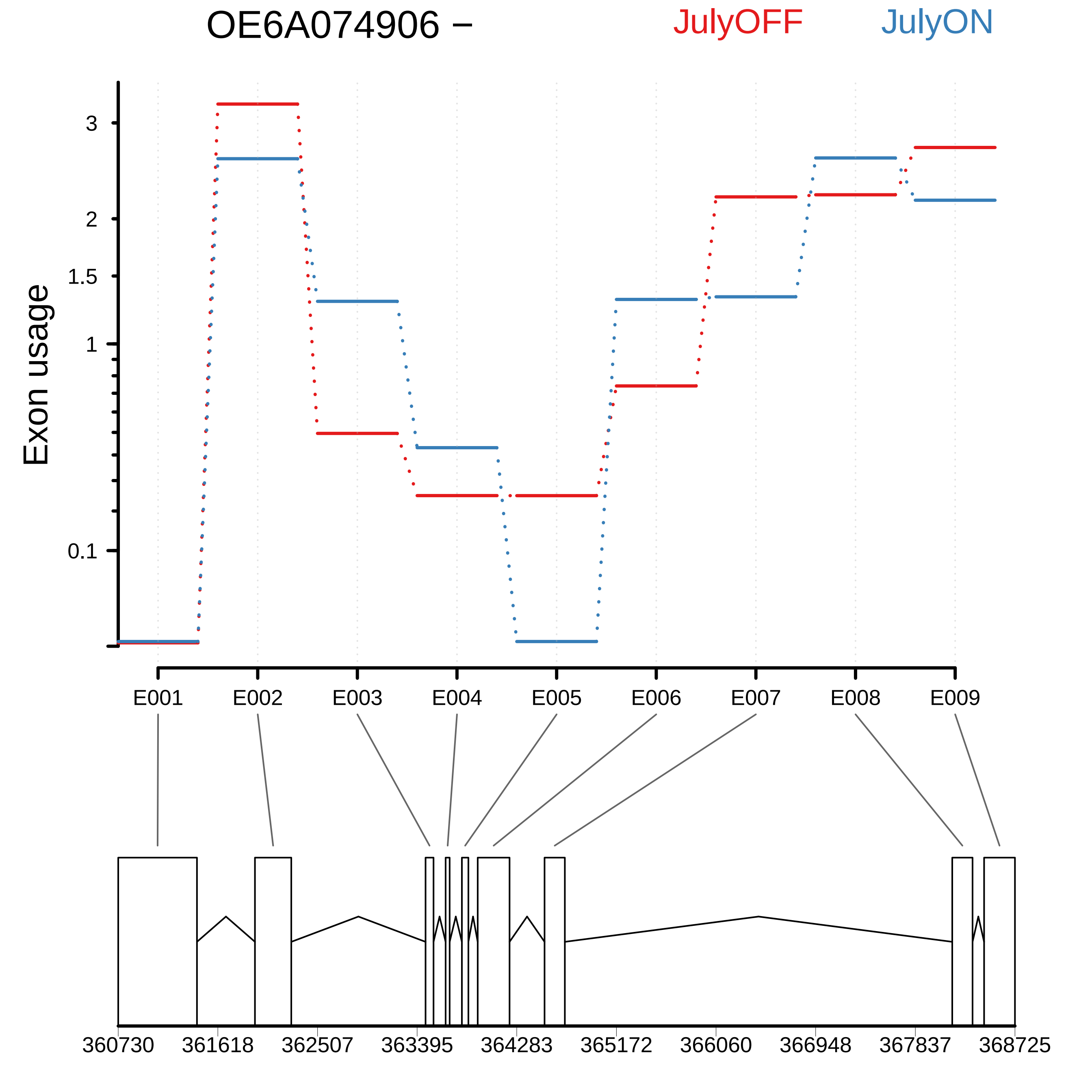

OE6A074906 −
JulyOFF
JulyON
Exon usage
3
2
1.5
1
0.1
E001
E002
E003
E004
E005
E006
E007
E008
E009
360730
361618
362507
363395
364283
365172
366060
366948
367837
368725

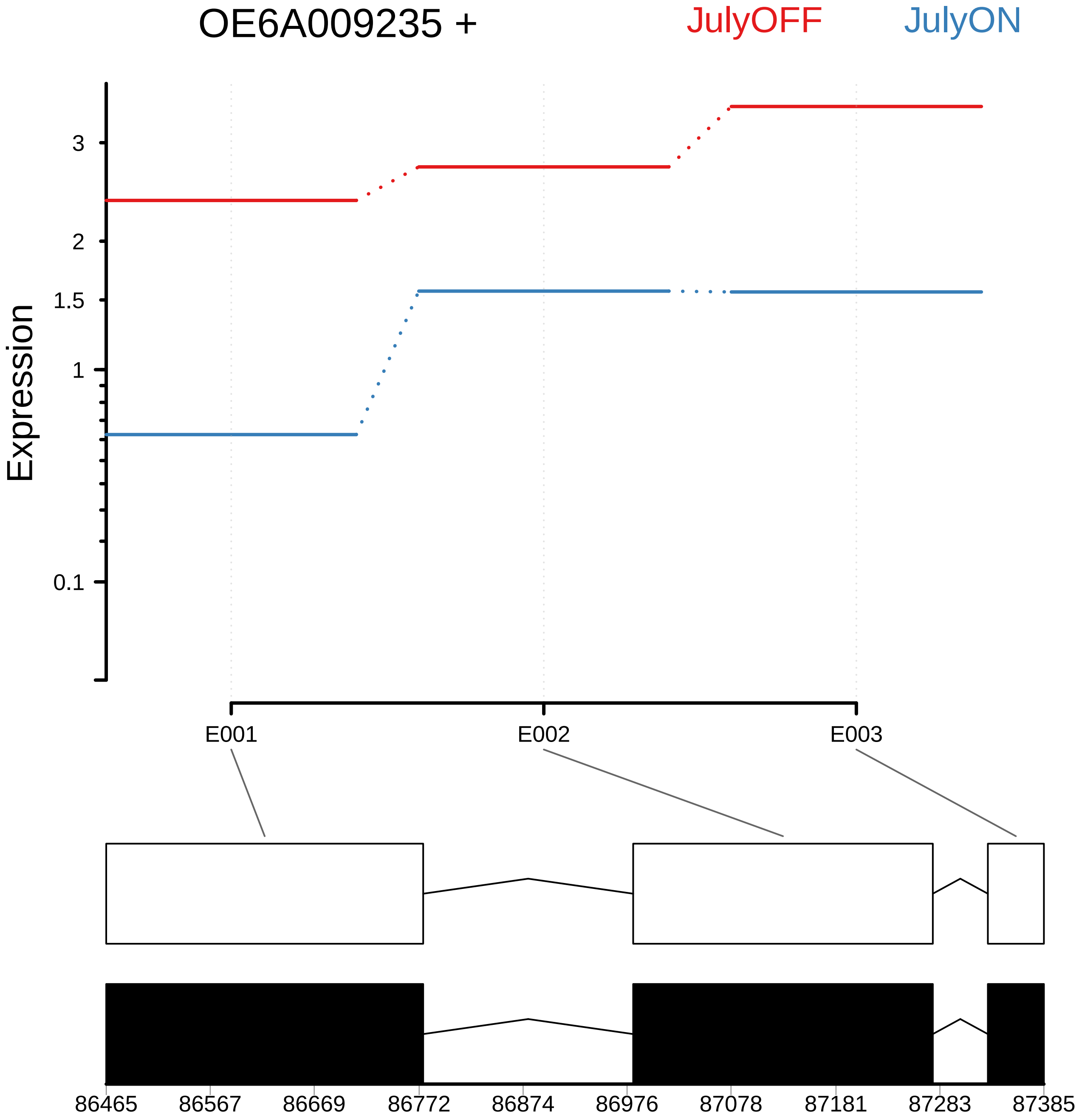
OE6A009235 +
JulyOFF
JulyON
Expression
3
2
1.5
1
0.1
E001
E002
E003
86465
86567
86669
86772
86874
86976
87078
87181
87283
87385

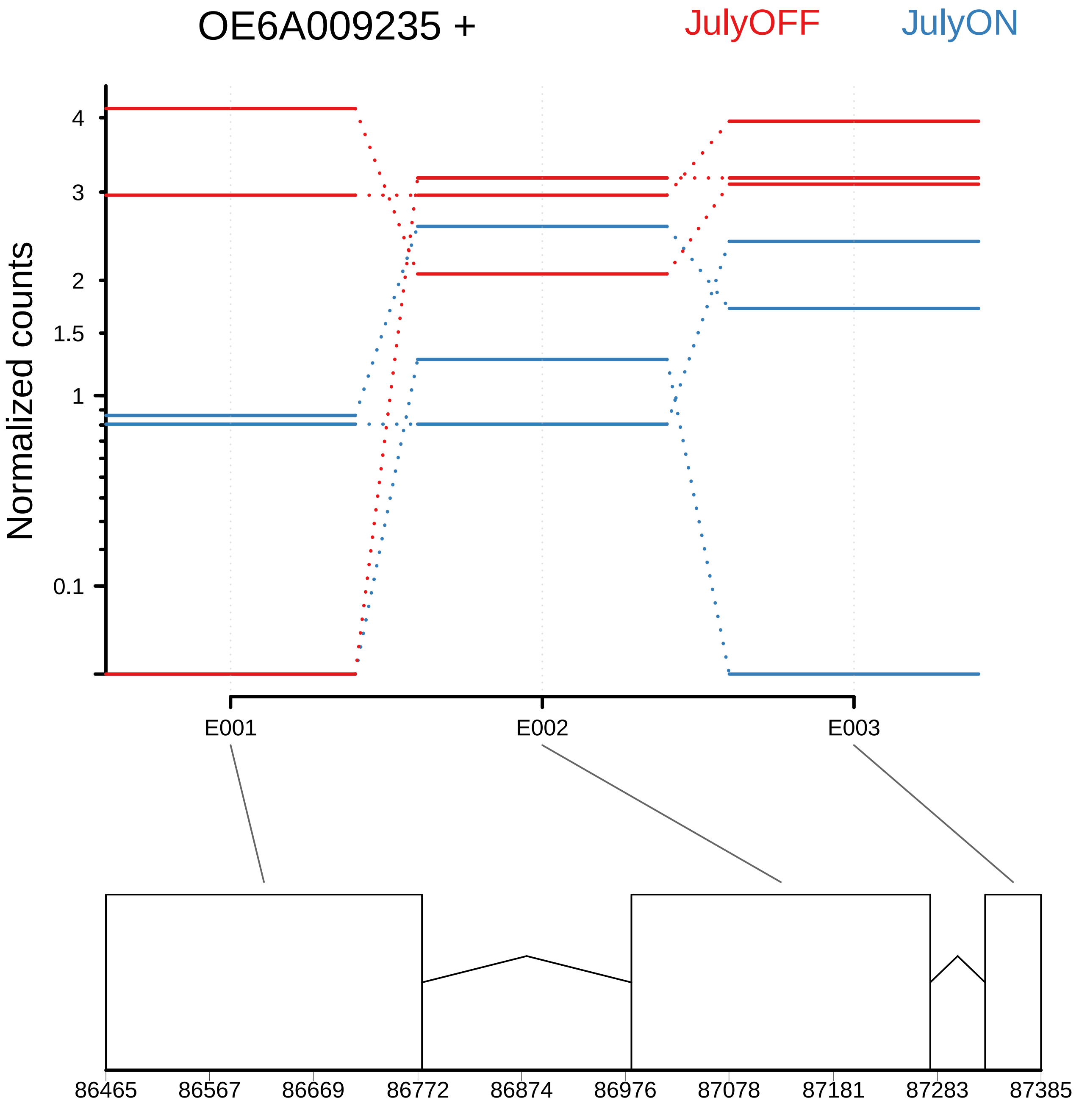

OE6A009235 +
JulyOFF
JulyON
Normalized counts
4
3
2
1.5
1
0.1
E001
E002
E003
86465
86567
86669
86772
86874
86976
87078
87181
87283
87385

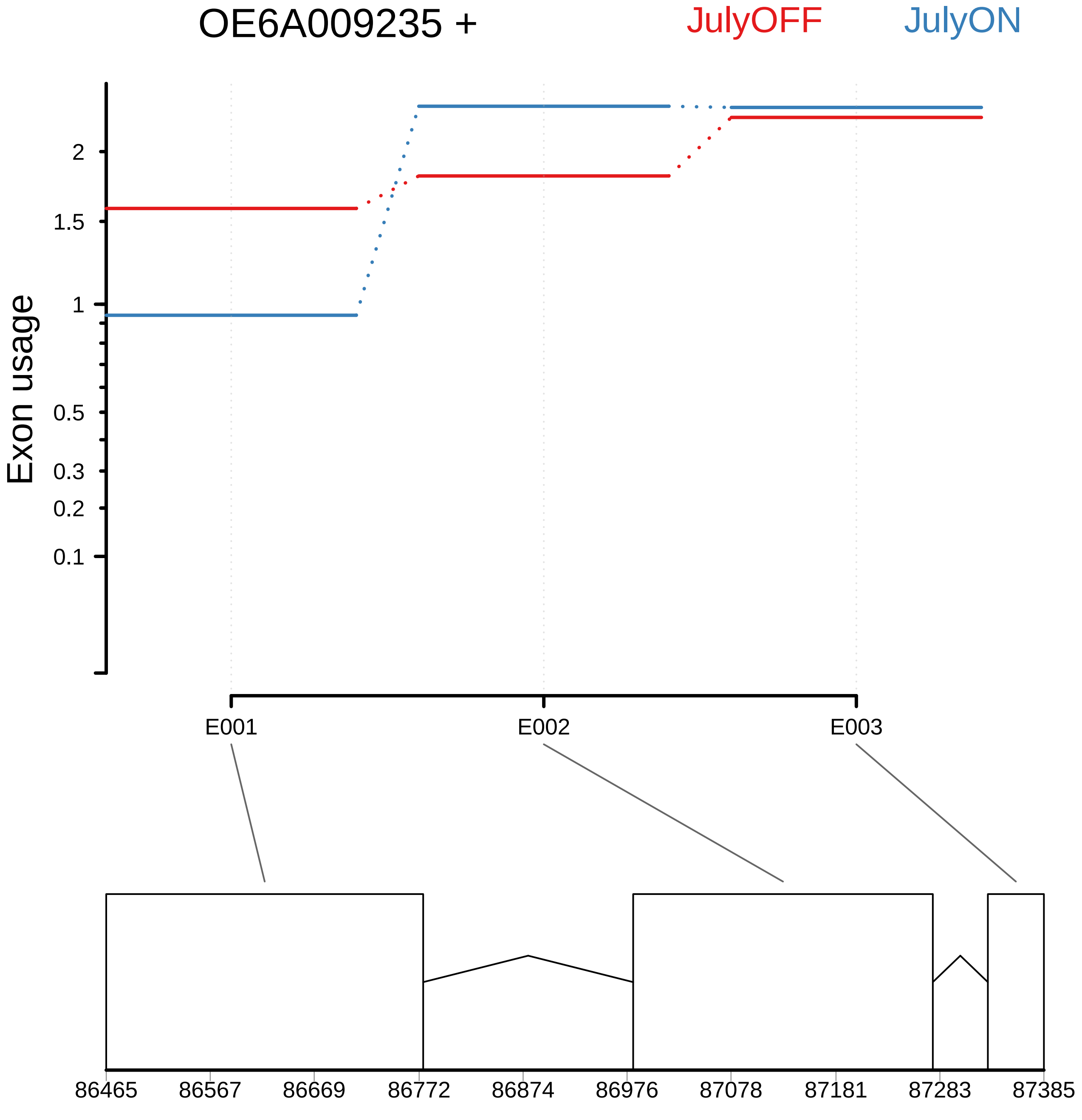

OE6A009235 +
JulyOFF
JulyON
Exon usage
2
1.5
1
0.5
0.3
0.2
0.1
E001
E002
E003
86465
86567
86669
86772
86874
86976
87078
87181
87283
87385

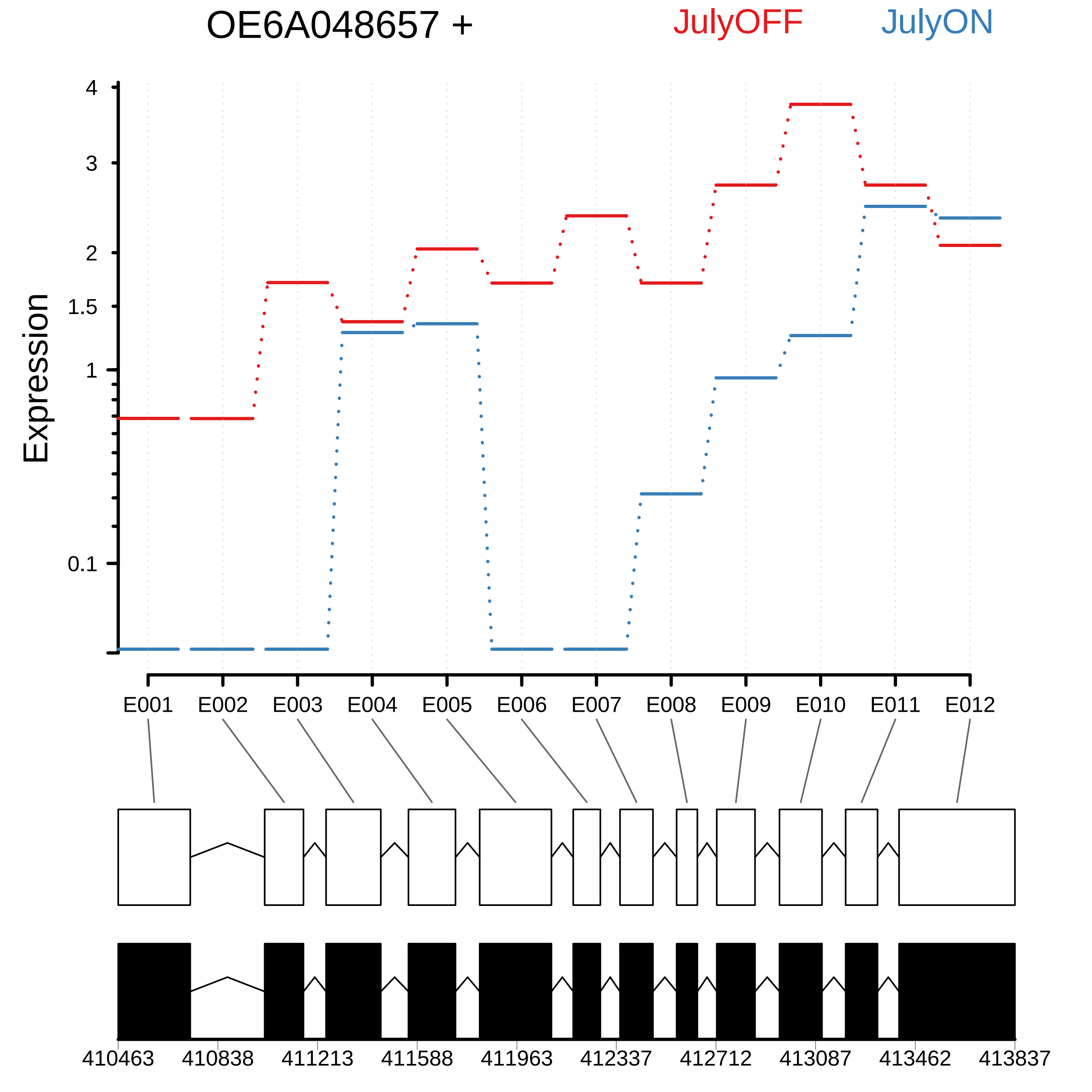
OE6A048657 +
JulyOFF
JulyON
Expression
4
3
2
1.5
1
0.1
E001
E002
E003
E004
E005
E006
E007
E008
E009
E010
E011
E012
410463
410838
411213
411588
411963
412337
412712
413087
413462
413837

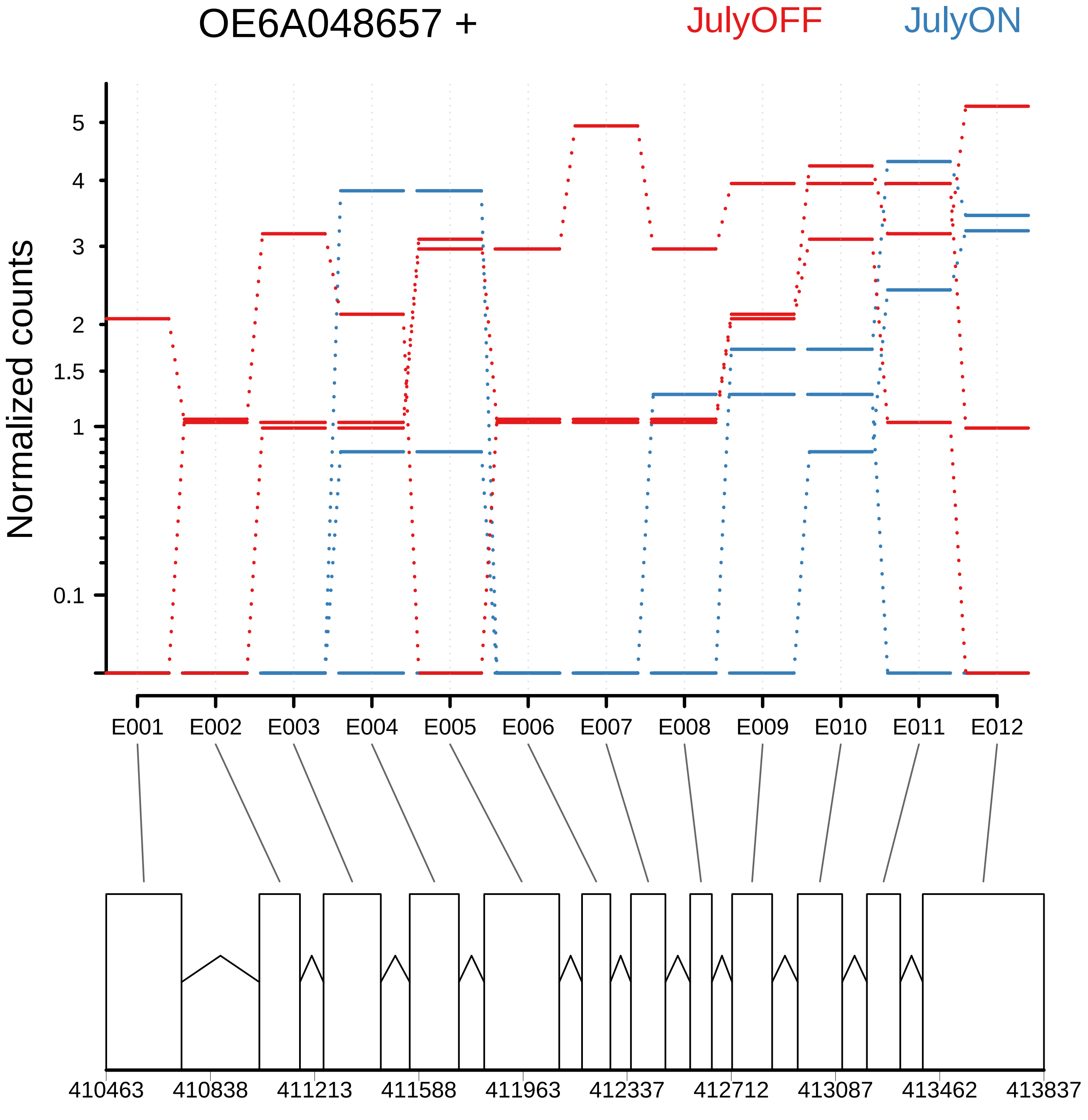

OE6A048657 +
JulyOFF
JulyON
Normalized counts
5
4
3
2
1.5
1
0.1
E001
E002
E003
E004
E005
E006
E007
E008
E009
E010
E011
E012
410463
410838
411213
411588
411963
412337
412712
413087
413462
413837

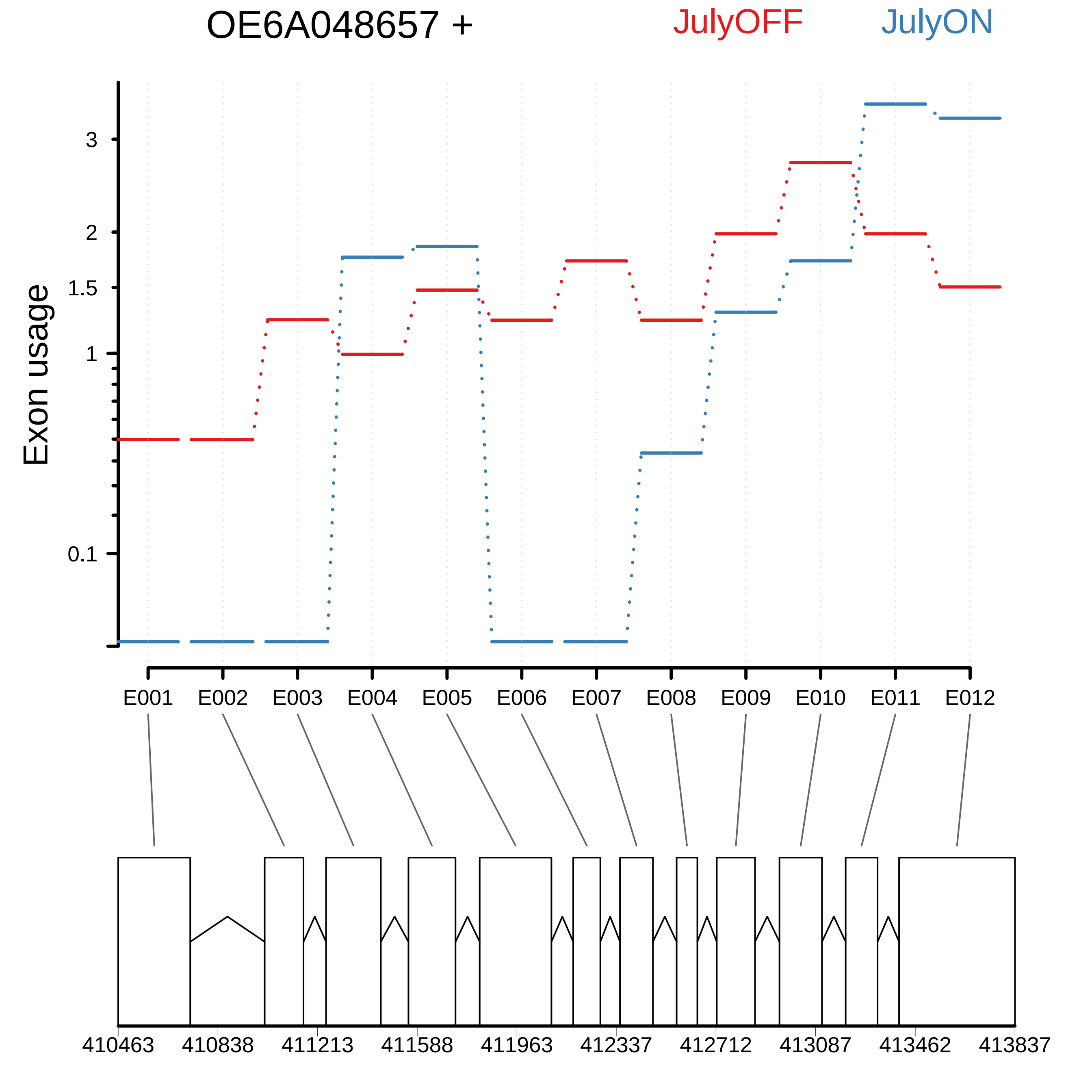

OE6A048657 +
JulyOFF
JulyON
Exon usage
3
2
1.5
1
0.1
E001
E002
E003
E004
E005
E006
E007
E008
E009
E010
E011
E012
410463
410838
411213
411588
411963
412337
412712
413087
413462
413837

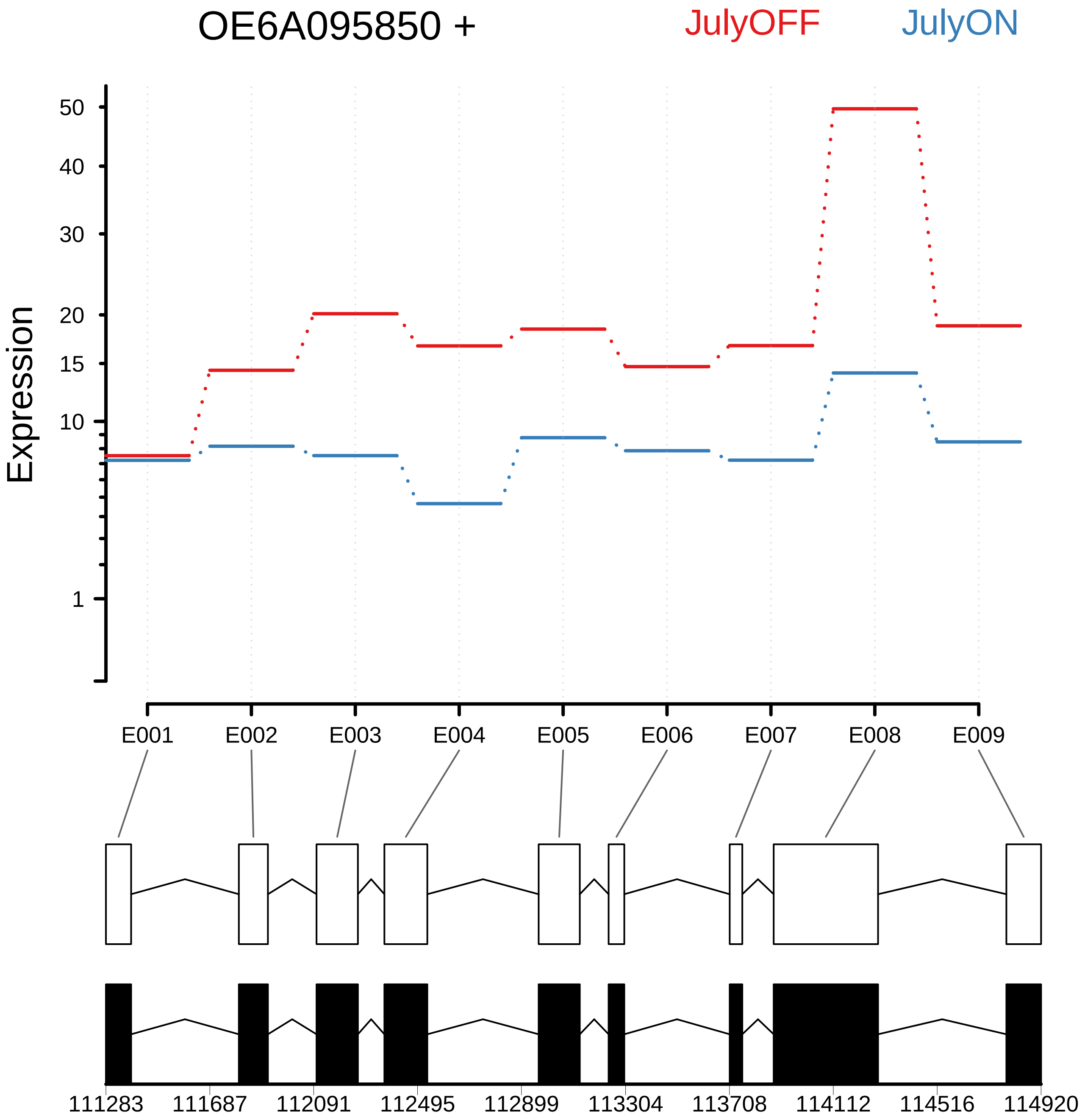

OE6A095850 +
JulyOFF
JulyON
Expression
50
40
30
20
15
10
1
E001
E002
E003
E004
E005
E006
E007
E008
E009
111283
111687
112091
112495
112899
113304
113708
114112
114516
114920

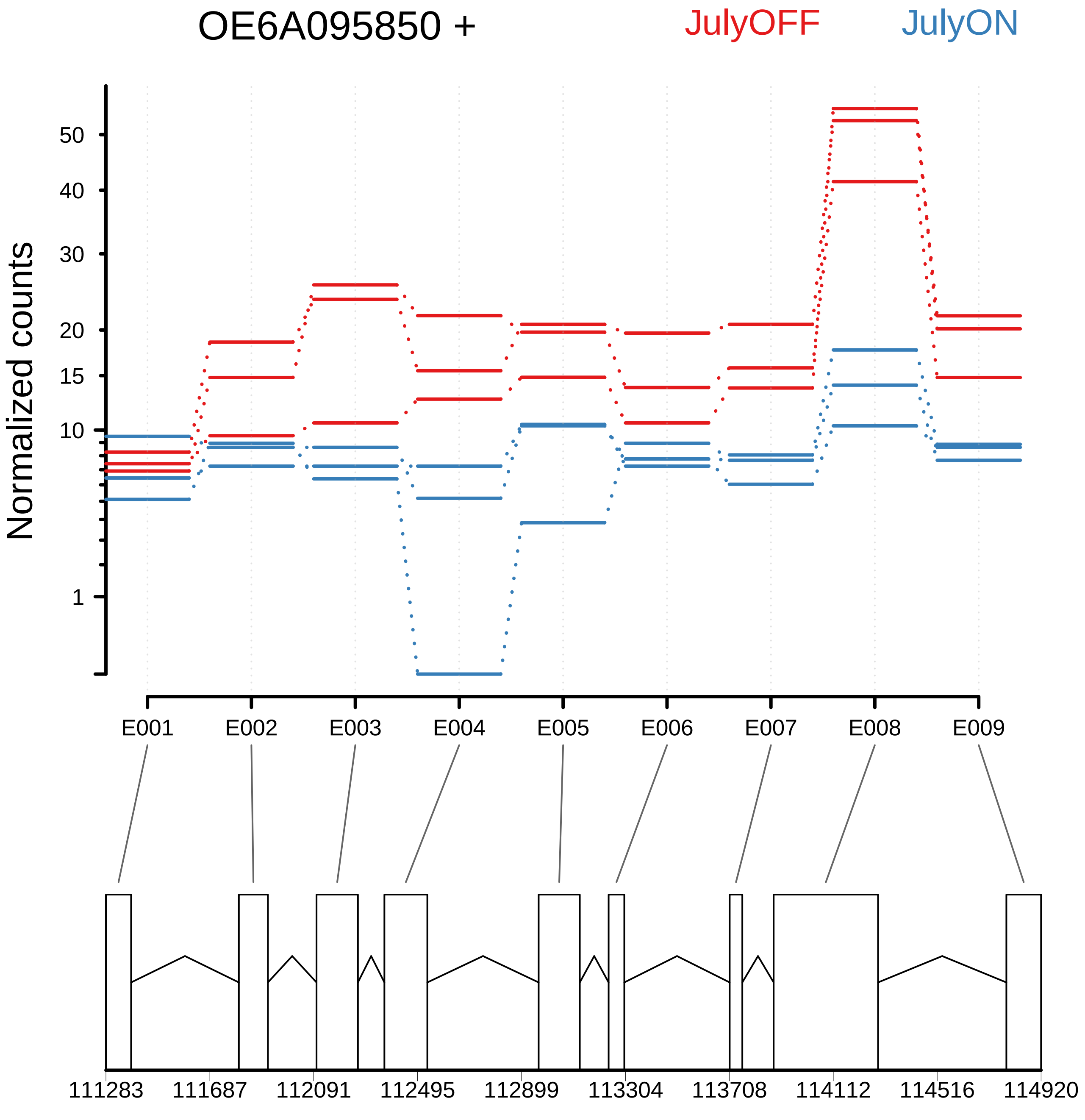

OE6A095850 +
JulyOFF
JulyON
Normalized counts
50
40
30
20
15
10
1
E001
E002
E003
E004
E005
E006
E007
E008
E009
111283
111687
112091
112495
112899
113304
113708
114112
114516
114920

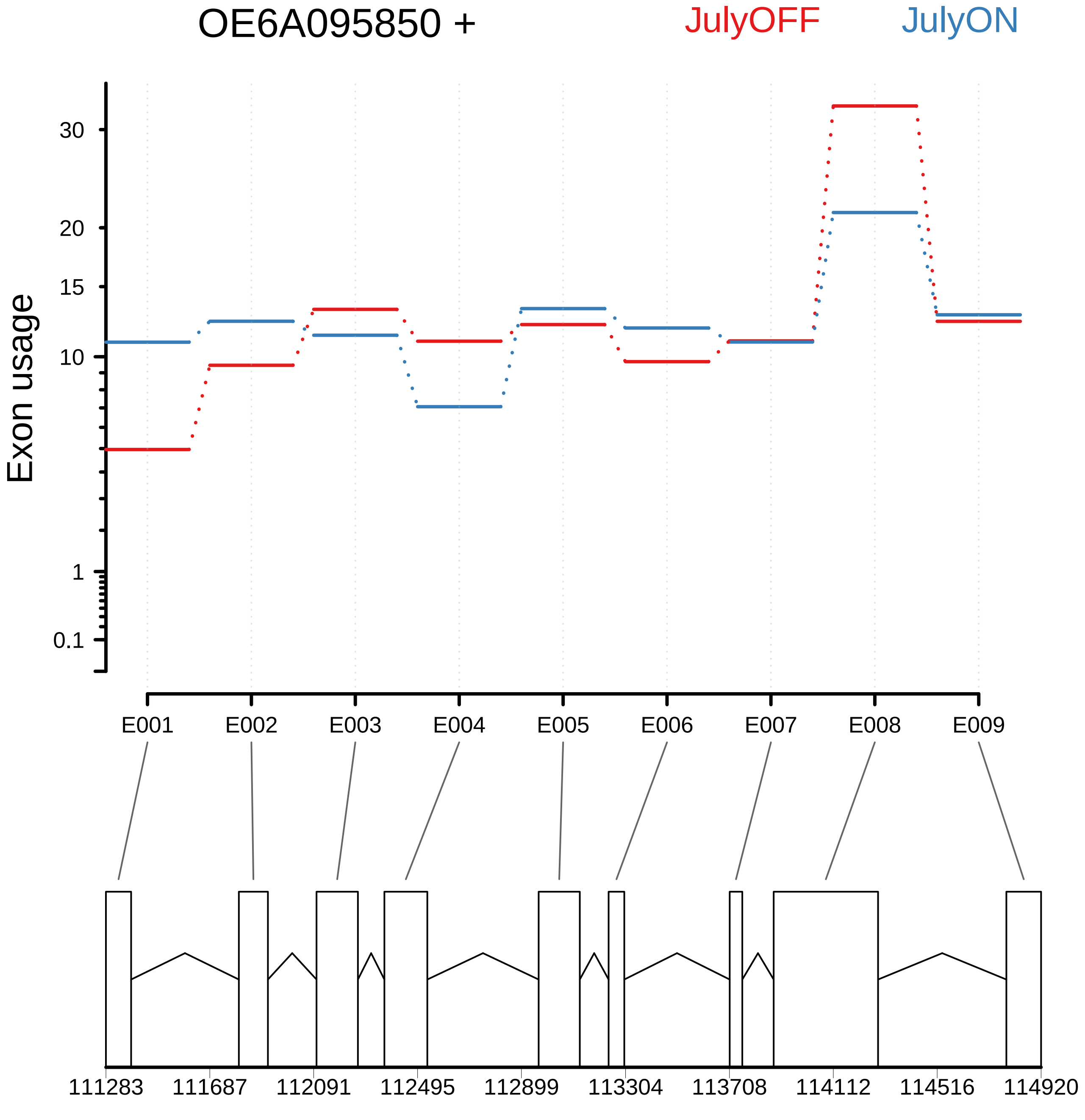

OE6A095850 +
JulyOFF
JulyON
Exon usage
30
20
15
10
1
0.1
E001
E002
E003
E004
E005
E006
E007
E008
E009
111283
111687
112091
112495
112899
113304
113708
114112
114516
114920

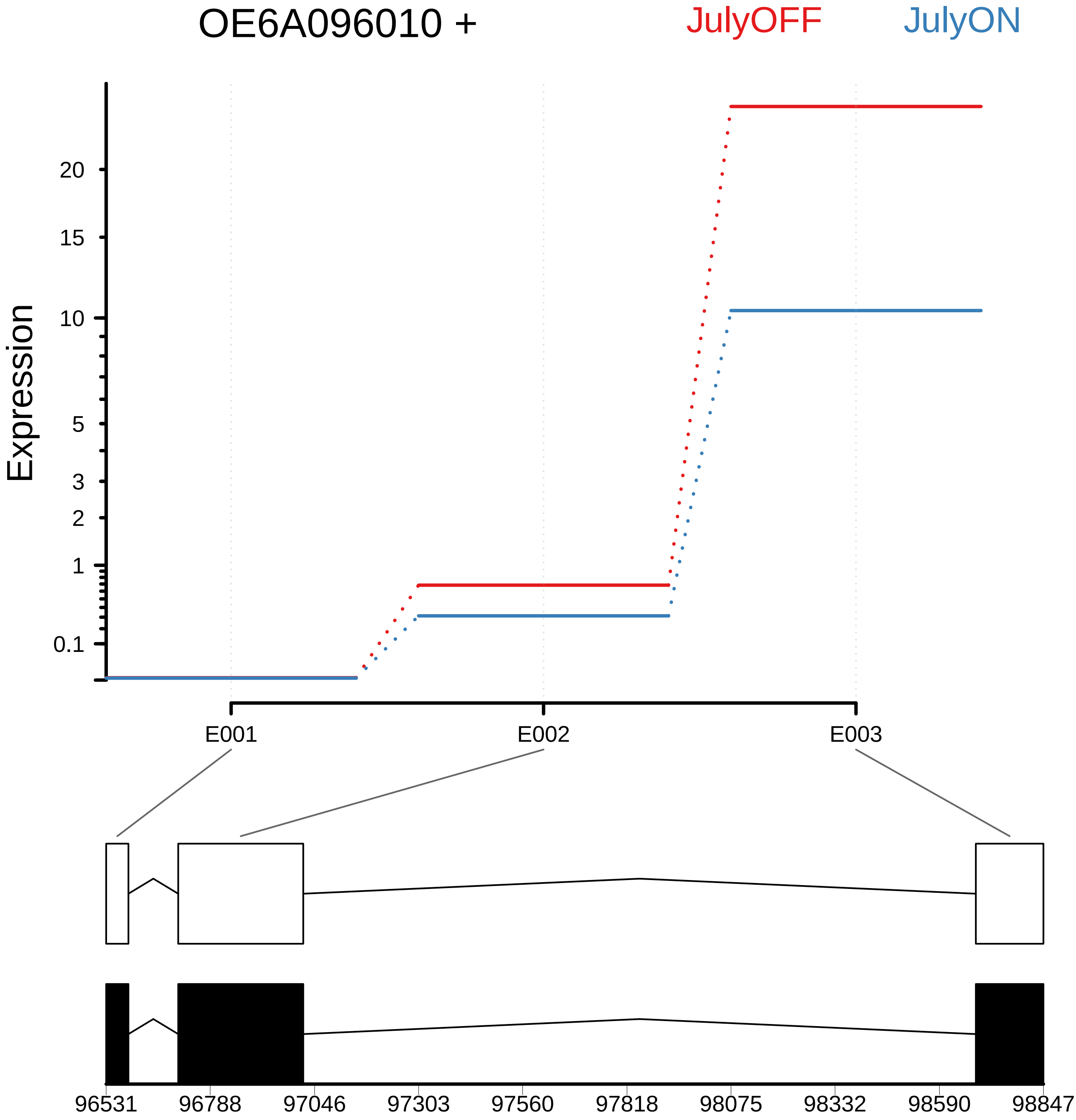

OE6A096010 +
JulyOFF
JulyON
Expression
20
15
10
5
3
2
1
0.1
E001
E002
E003
96531
96788
97046
97303
97560
97818
98075
98332
98590
98847

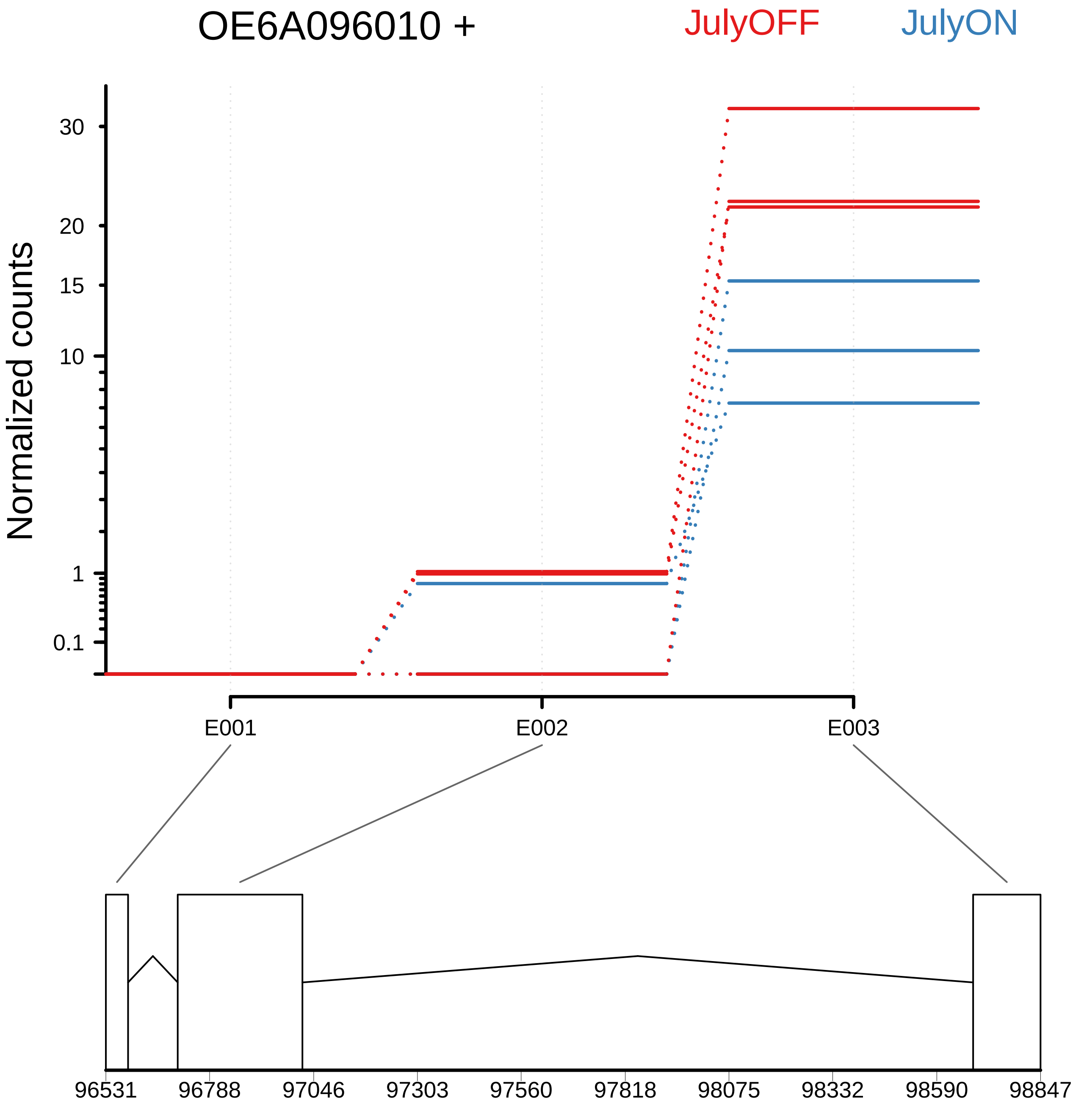

OE6A096010 +
JulyOFF
JulyON
Normalized counts
30
20
15
10
1
0.1
E001
E002
E003
96531
96788
97046
97303
97560
97818
98075
98332
98590
98847

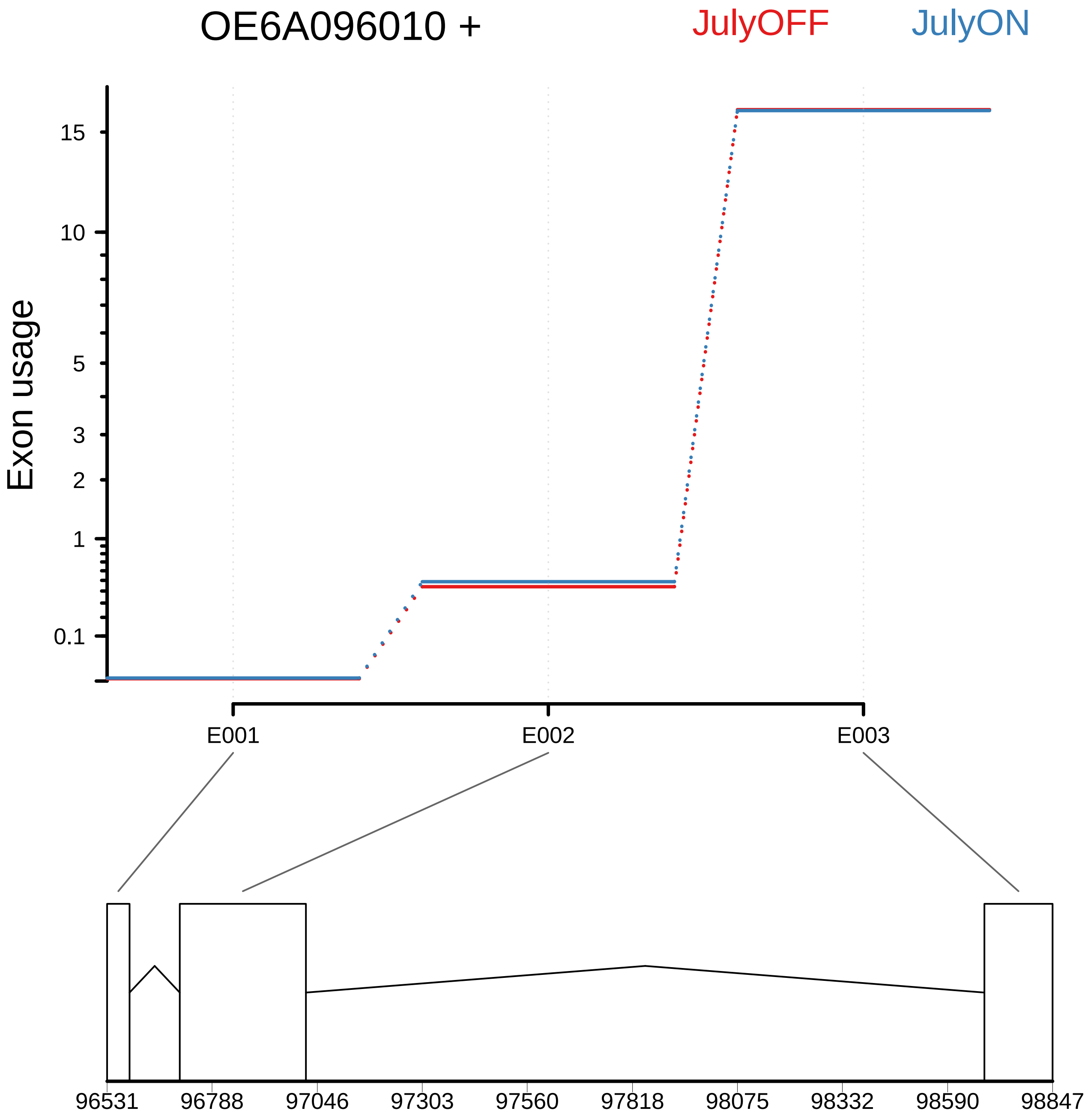
OE6A096010 +
JulyOFF
JulyON
Exon usage
15
10
5
3
2
1
0.1
E001
E002
E003
96531
96788
97046
97303
97560
97818
98075
98332
98590
98847

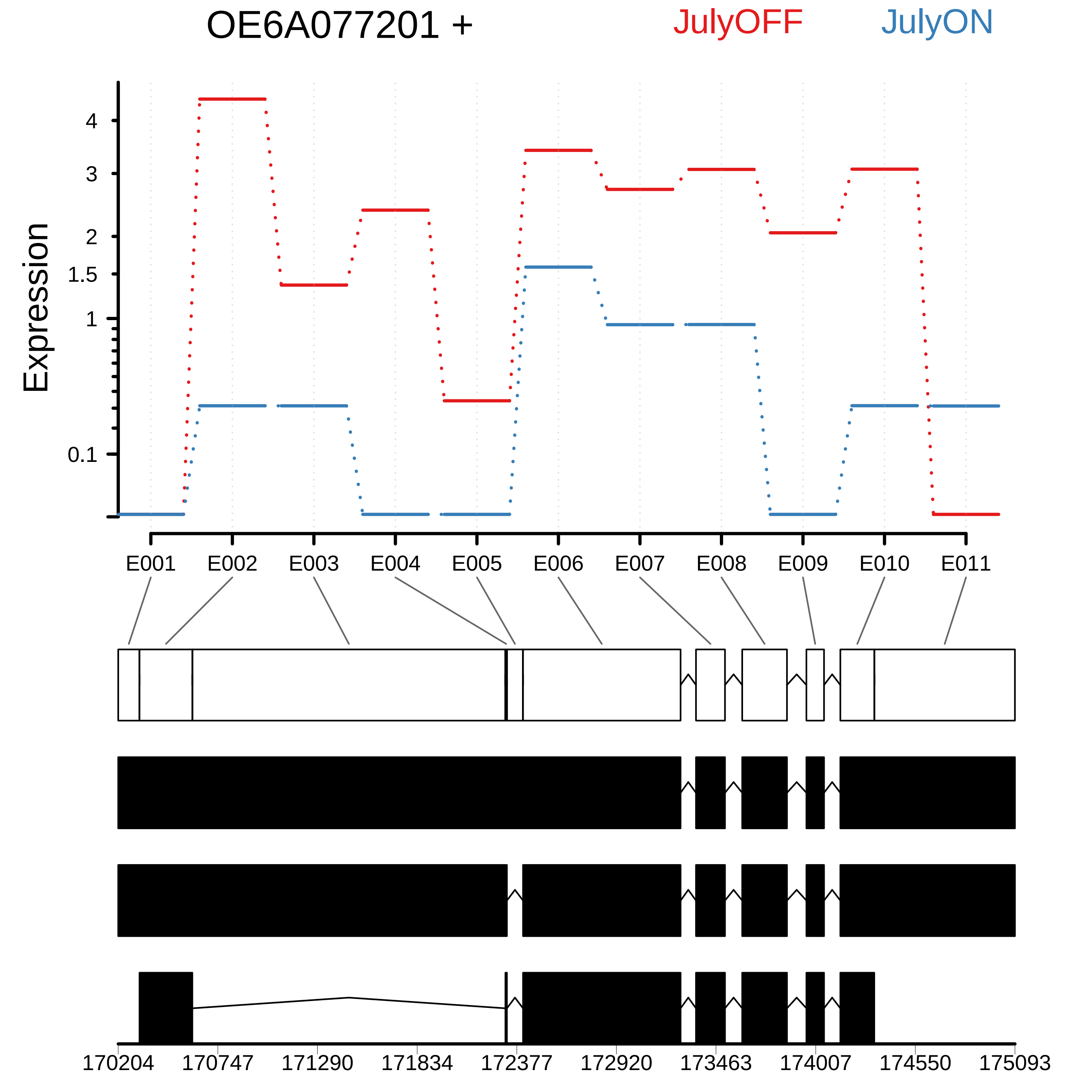
OE6A077201 +
JulyOFF
JulyON
Expression
4
3
2
1.5
1
0.1
E001
E002
E003
E004
E005
E006
E007
E008
E009
E010
E011
170204
170747
171290
171834
172377
172920
173463
174007
174550
175093

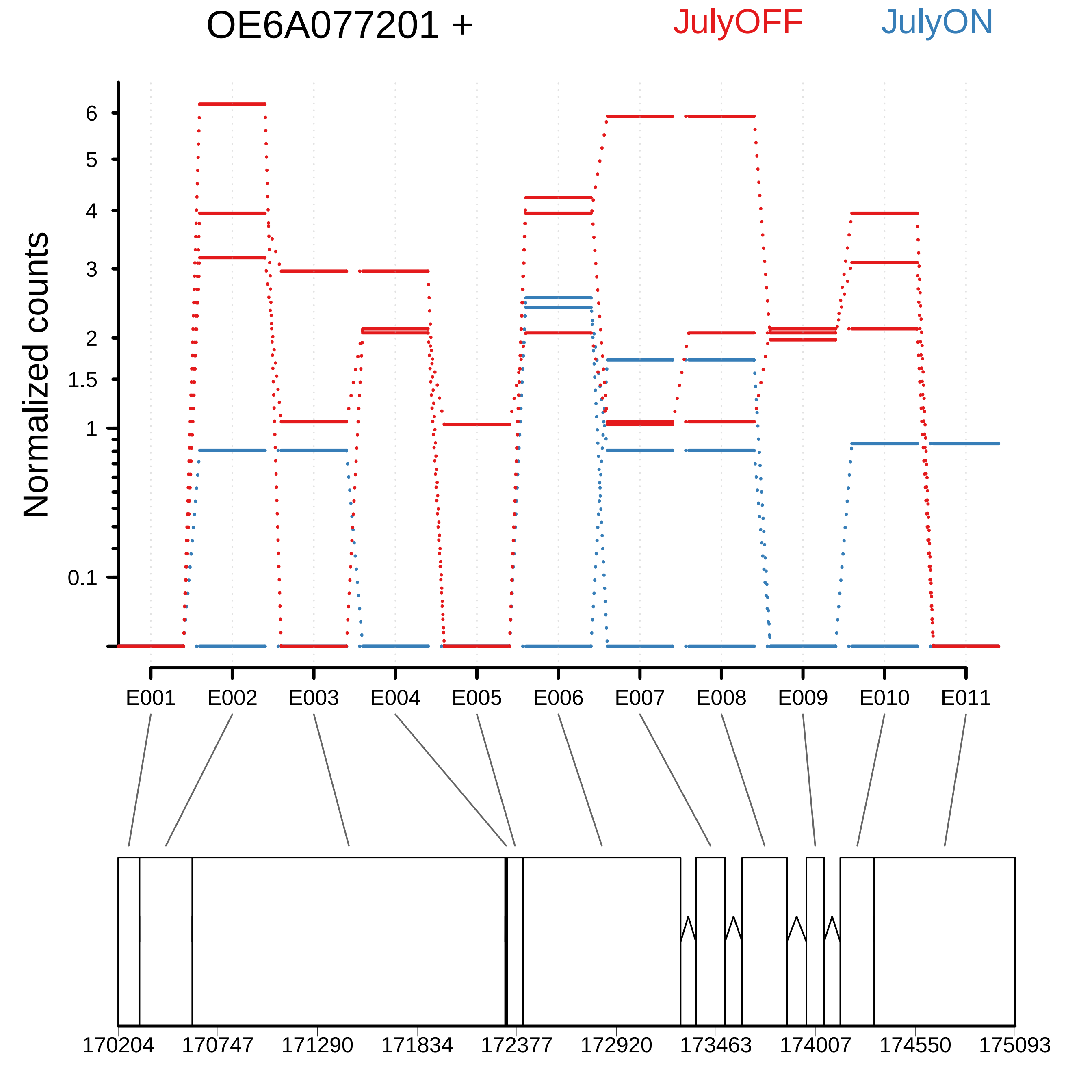
OE6A077201 +
JulyOFF
JulyON
Normalized counts
6
5
4
3
2
1.5
1
0.1
E001
E002
E003
E004
E005
E006
E007
E008
E009
E010
E011
170204
170747
171290
171834
172377
172920
173463
174007
174550
175093

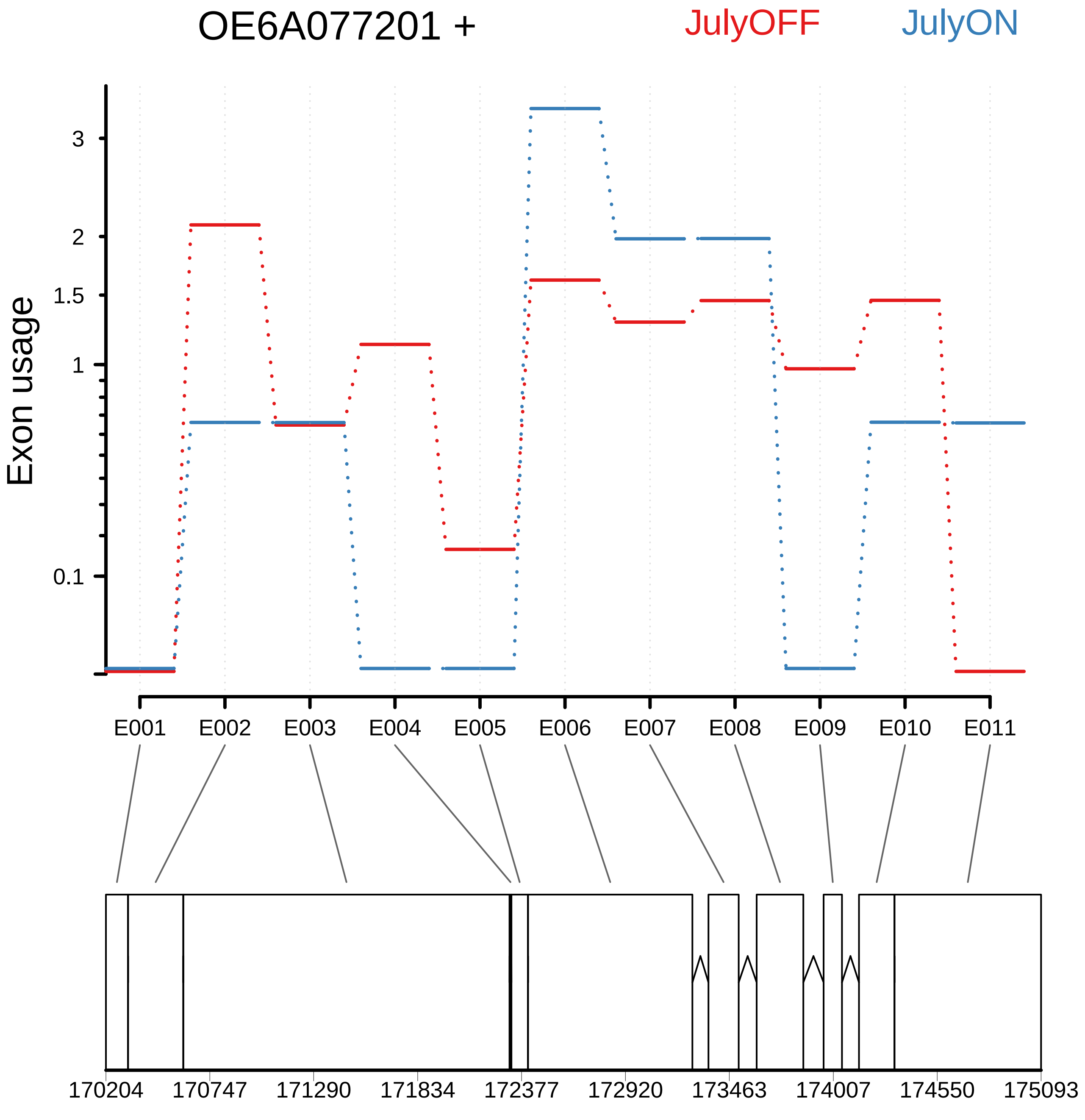

OE6A077201 +
JulyOFF
JulyON
Exon usage
3
2
1.5
1
0.1
E001
E002
E003
E004
E005
E006
E007
E008
E009
E010
E011
170204
170747
171290
171834
172377
172920
173463
174007
174550
175093

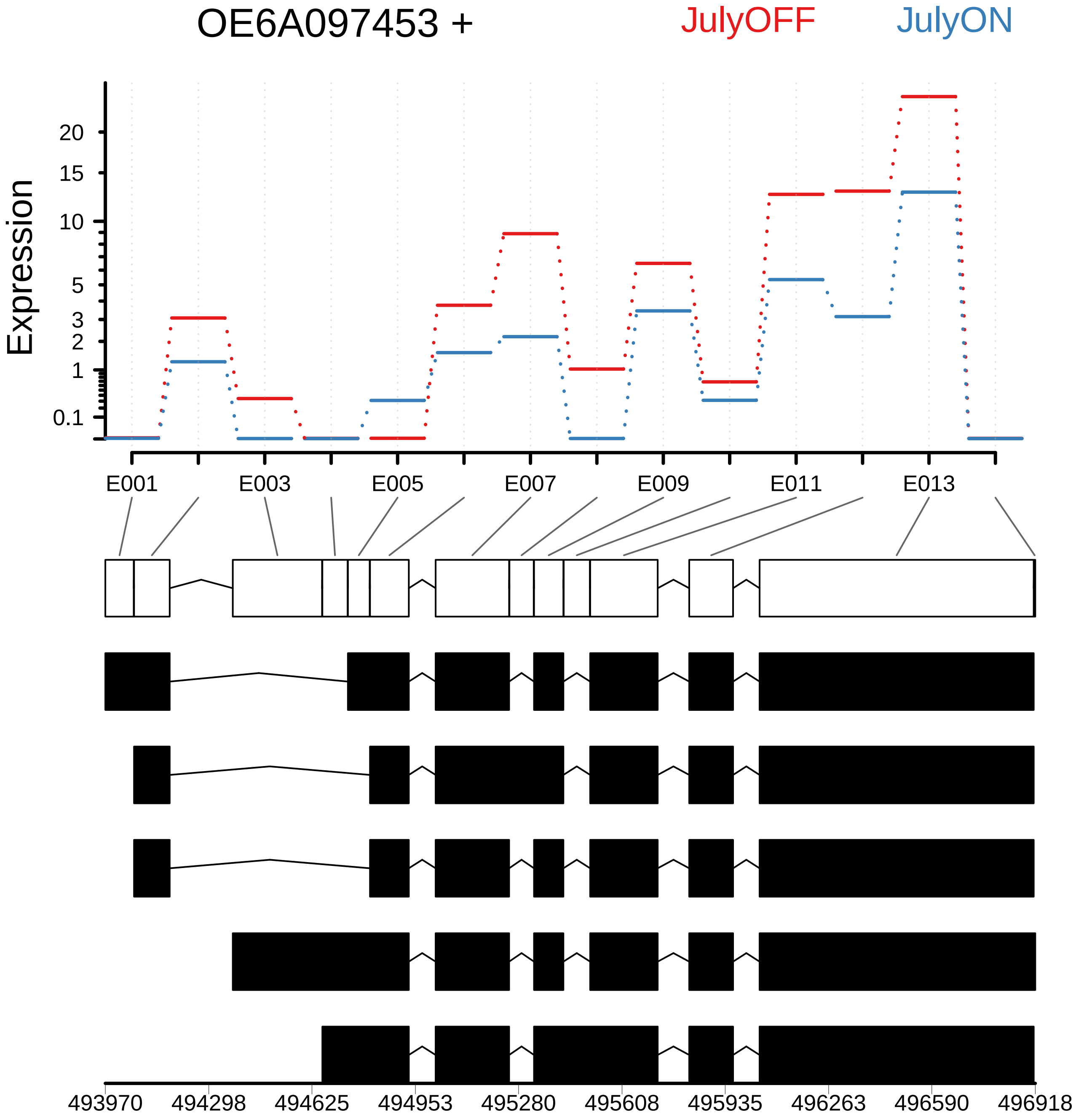
OE6A097453 +
JulyOFF
JulyON
Expression
20
15
10
5
3
2
1
0.1
E001
E003
E005
E007
E009
E011
E013
493970
494298
494625
494953
495280
495608
495935
496263
496590
496918

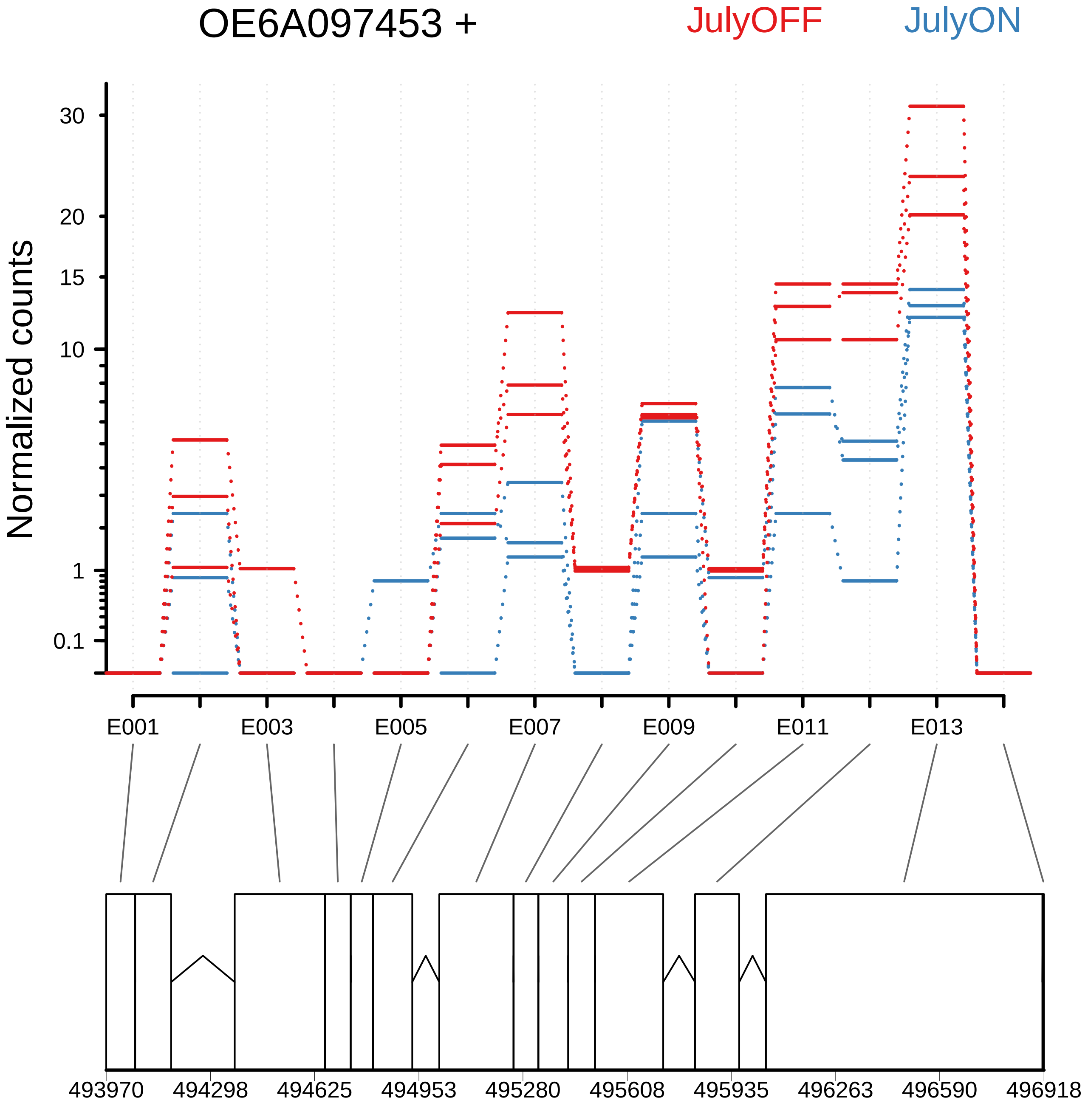

OE6A097453 +
JulyOFF
JulyON
Normalized counts
30
20
15
10
1
0.1
E001
E003
E005
E007
E009
E011
E013
493970
494298
494625
494953
495280
495608
495935
496263
496590
496918

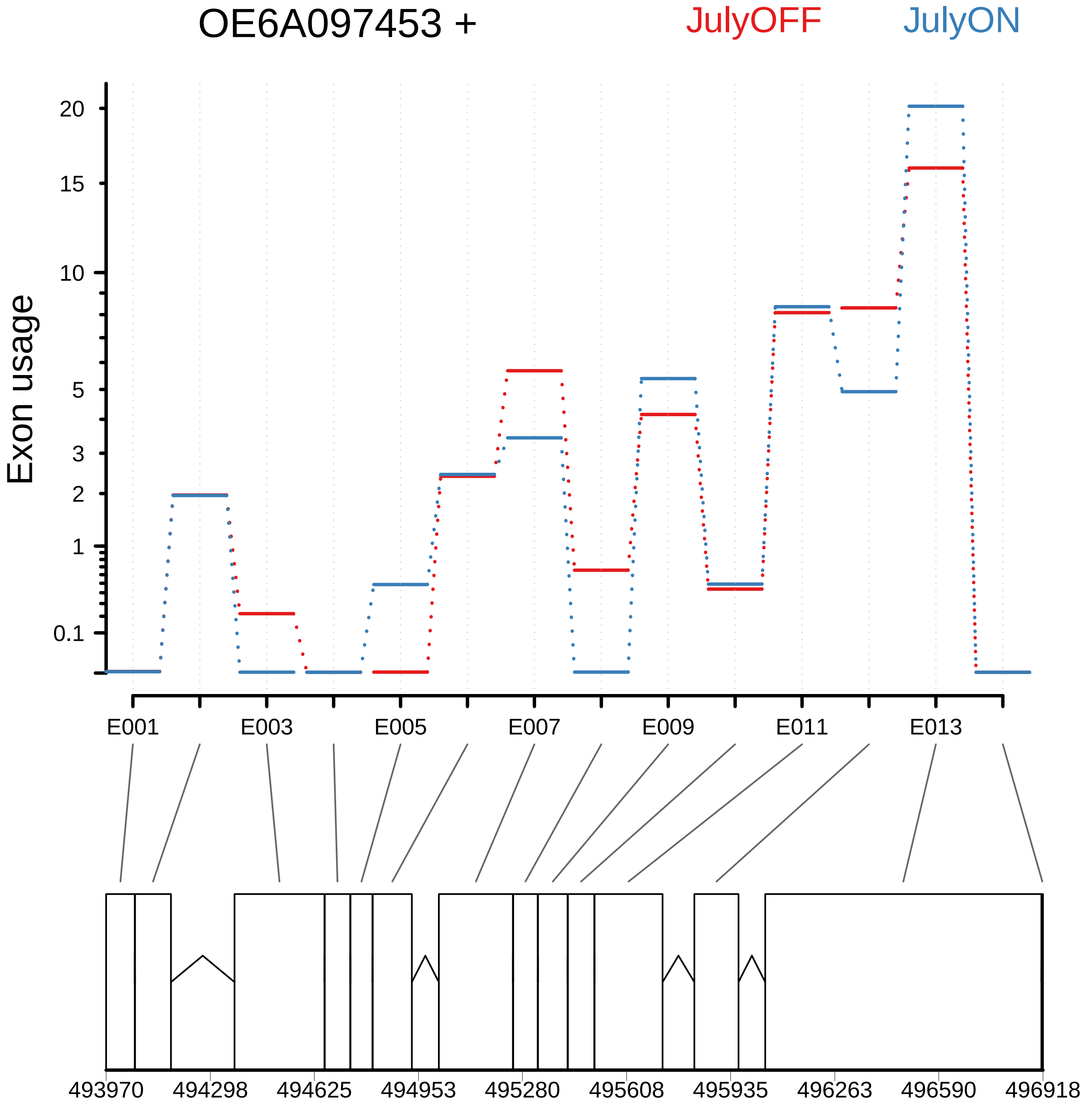

OE6A097453 +
JulyOFF
JulyON
Exon usage
20
15
10
5
3
2
1
0.1
E001
E003
E005
E007
E009
E011
E013
493970
494298
494625
494953
495280
495608
495935
496263
496590
496918

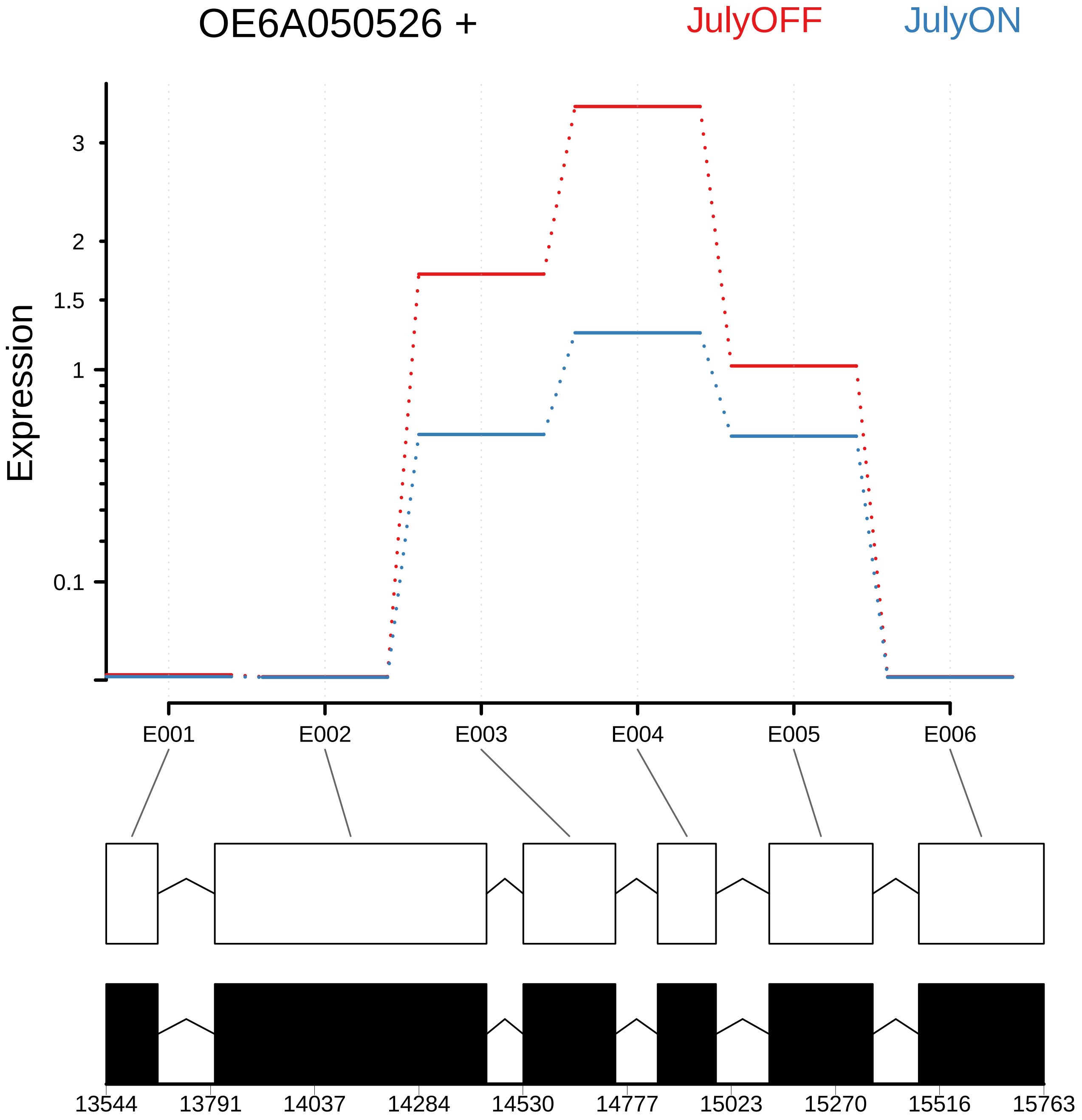
OE6A050526 +
JulyOFF
JulyON
Expression
3
2
1.5
1
0.1
E001
E002
E003
E004
E005
E006
13544
13791
14037
14284
14530
14777
15023
15270
15516
15763

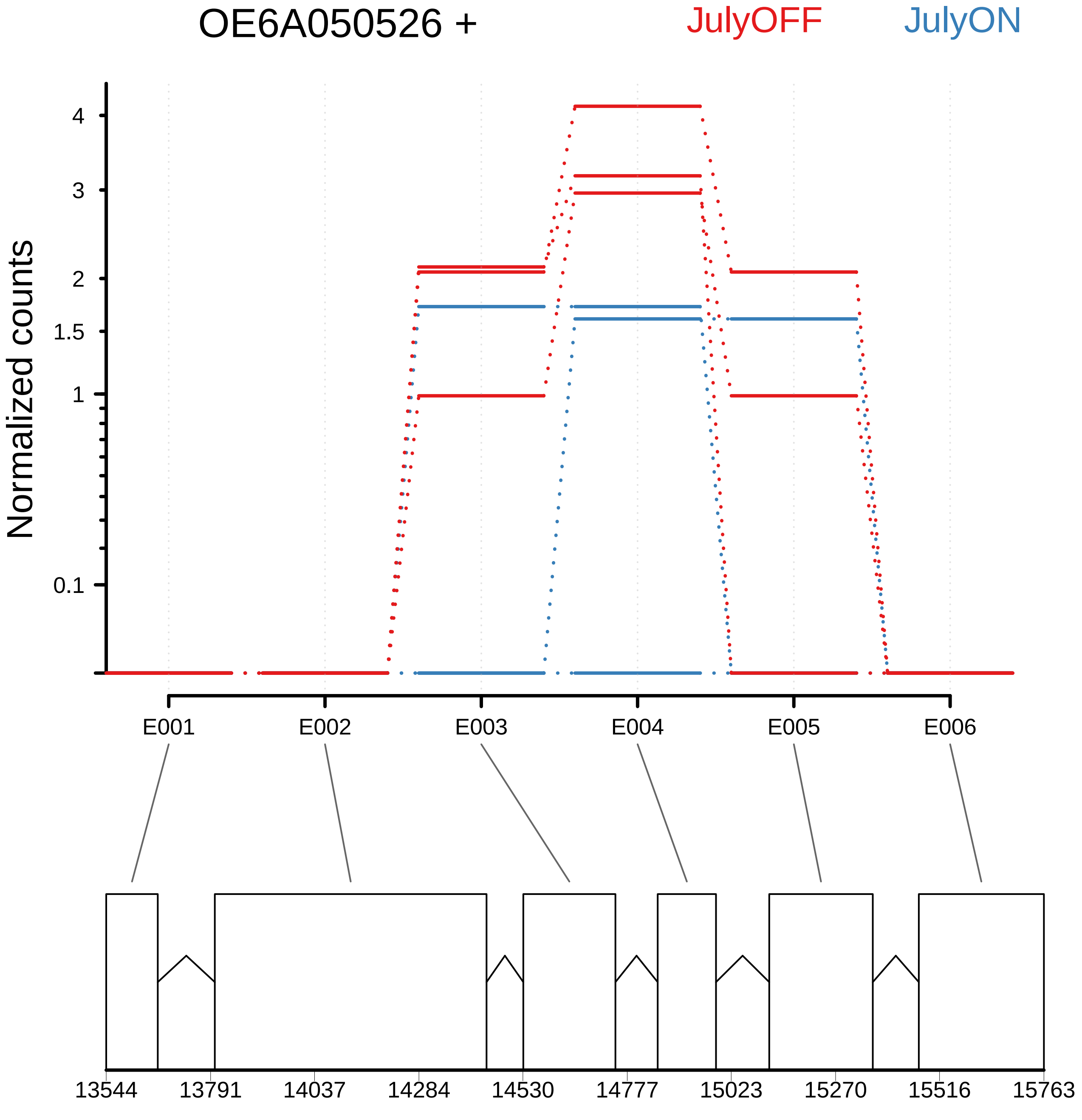

OE6A050526 +
JulyOFF
JulyON
Normalized counts
4
3
2
1.5
1
0.1
E001
E002
E003
E004
E005
E006
13544
13791
14037
14284
14530
14777
15023
15270
15516
15763

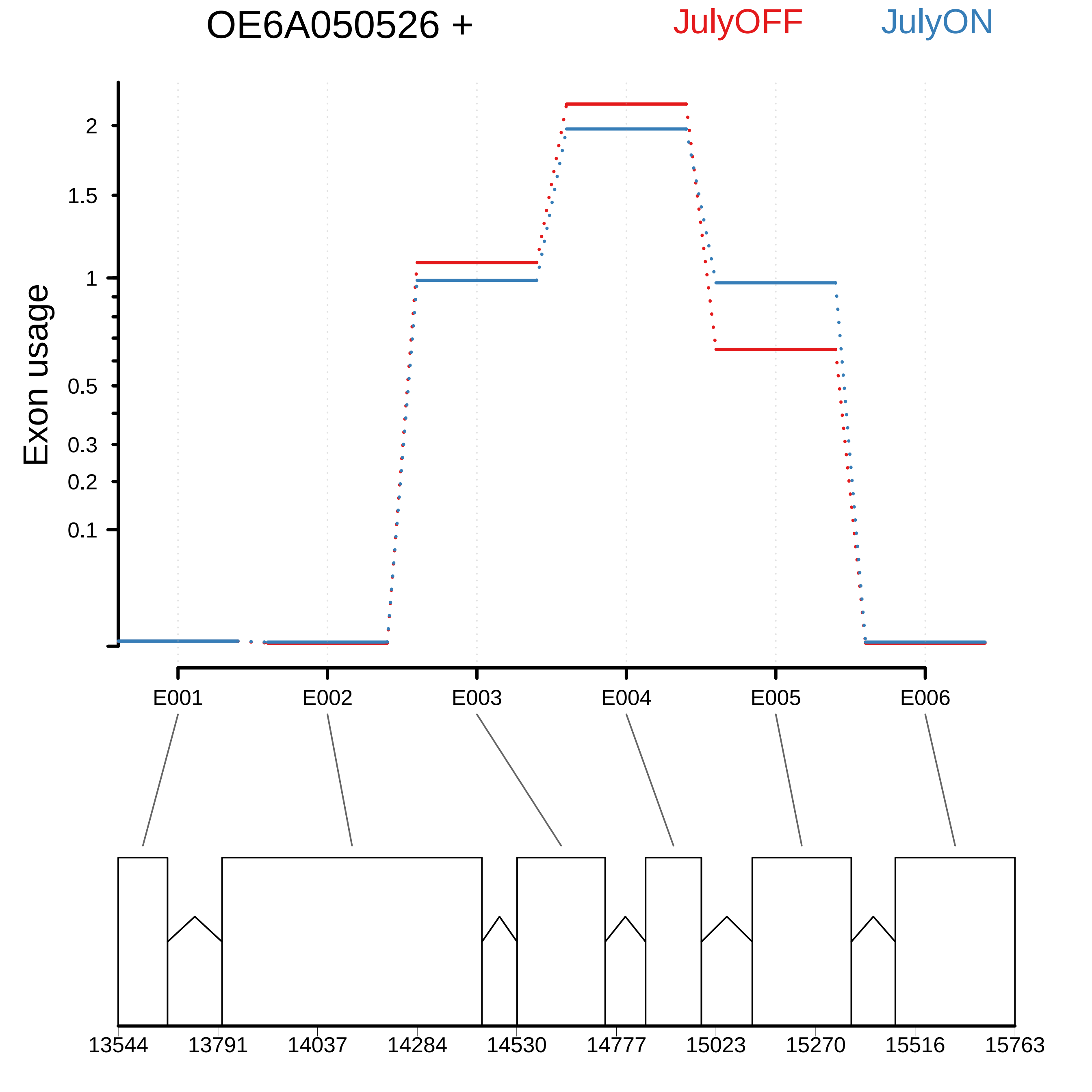

OE6A050526 +
JulyOFF
JulyON
Exon usage
2
1.5
1
0.5
0.3
0.2
0.1
E001
E002
E003
E004
E005
E006
13544
13791
14037
14284
14530
14777
15023
15270
15516
15763

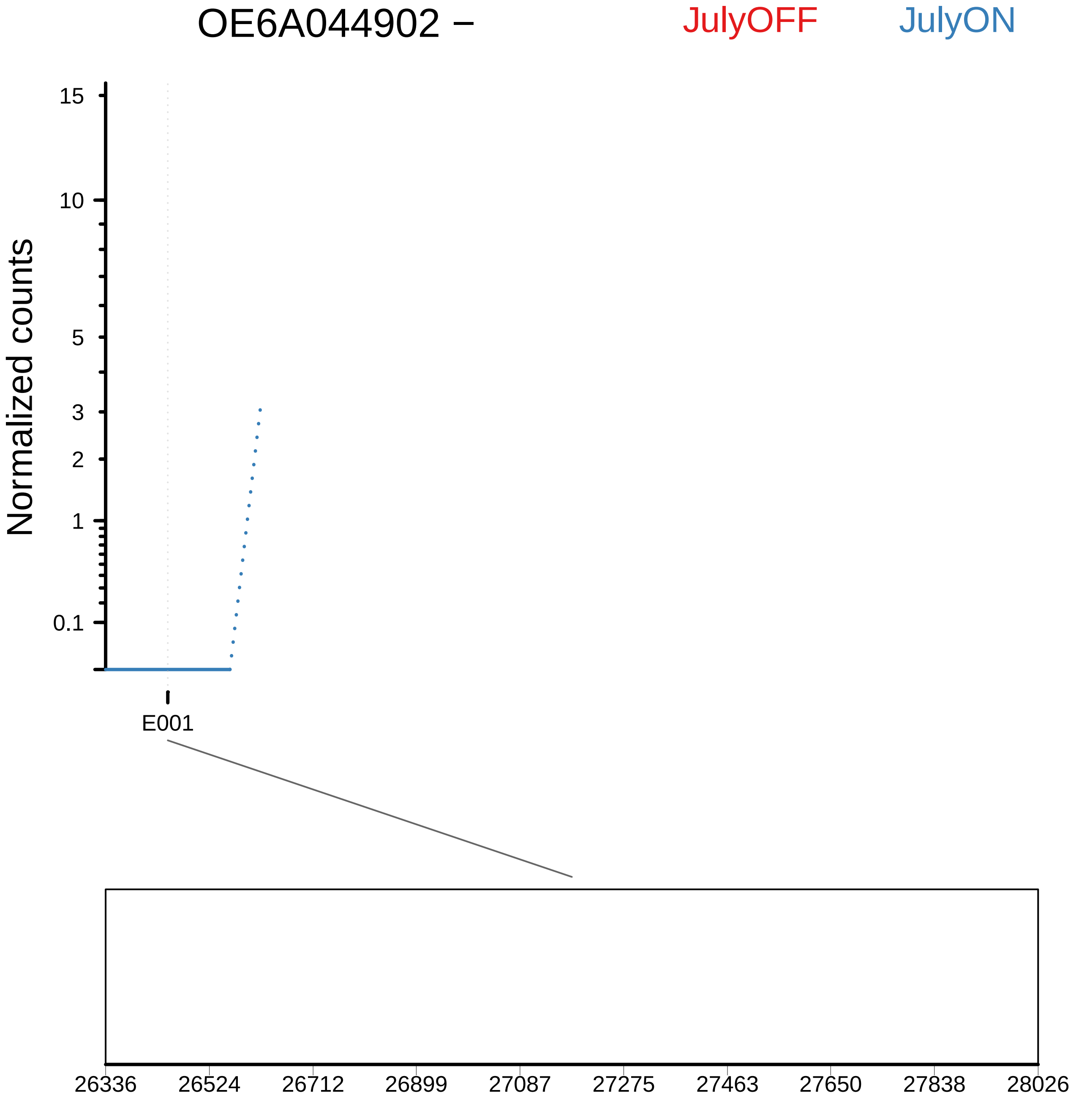
OE6A044902 −
JulyOFF
JulyON
Normalized counts
15
10
5
3
2
1
0.1
E001
26336
26524
26712
26899
27087
27275
27463
27650
27838
28026

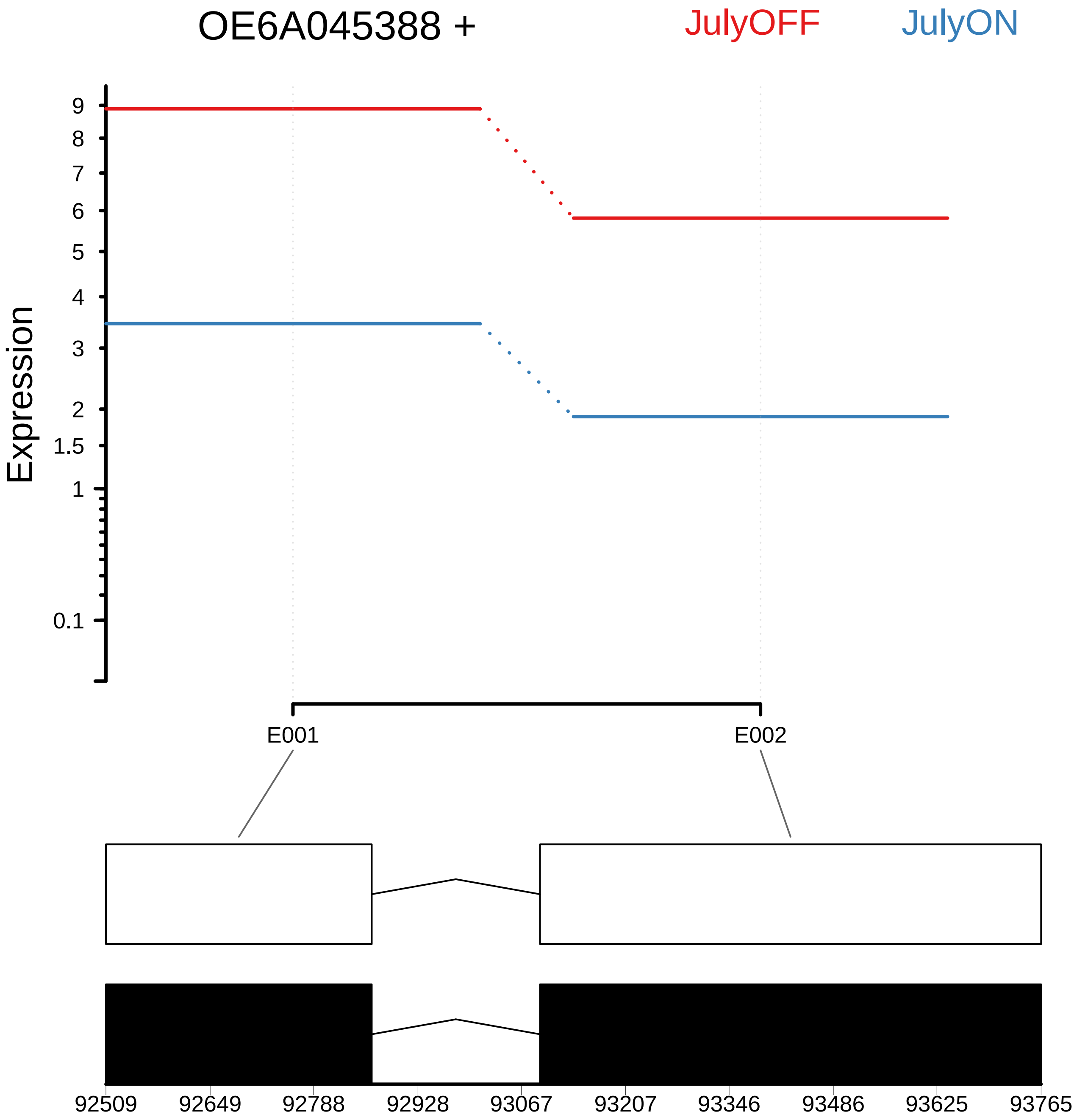
OE6A045388 +
JulyOFF
JulyON
Expression
9
8
7
6
5
4
3
2
1.5
1
0.1
E001
E002
92509
92649
92788
92928
93067
93207
93346
93486
93625
93765

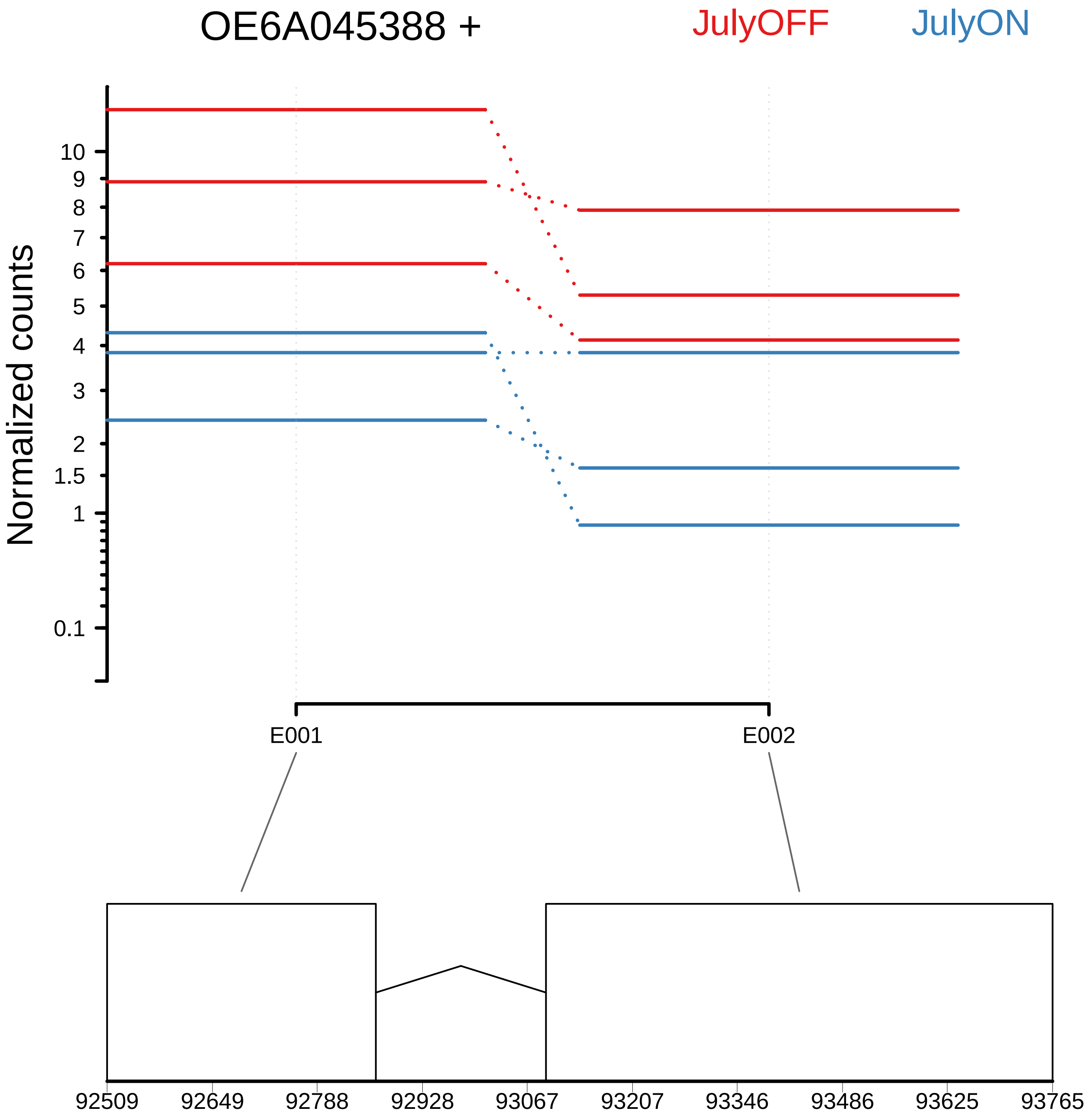

OE6A045388 +
JulyOFF
JulyON
Normalized counts
10
9
8
7
6
5
4
3
2
1.5
1
0.1
E001
E002
92509
92649
92788
92928
93067
93207
93346
93486
93625
93765

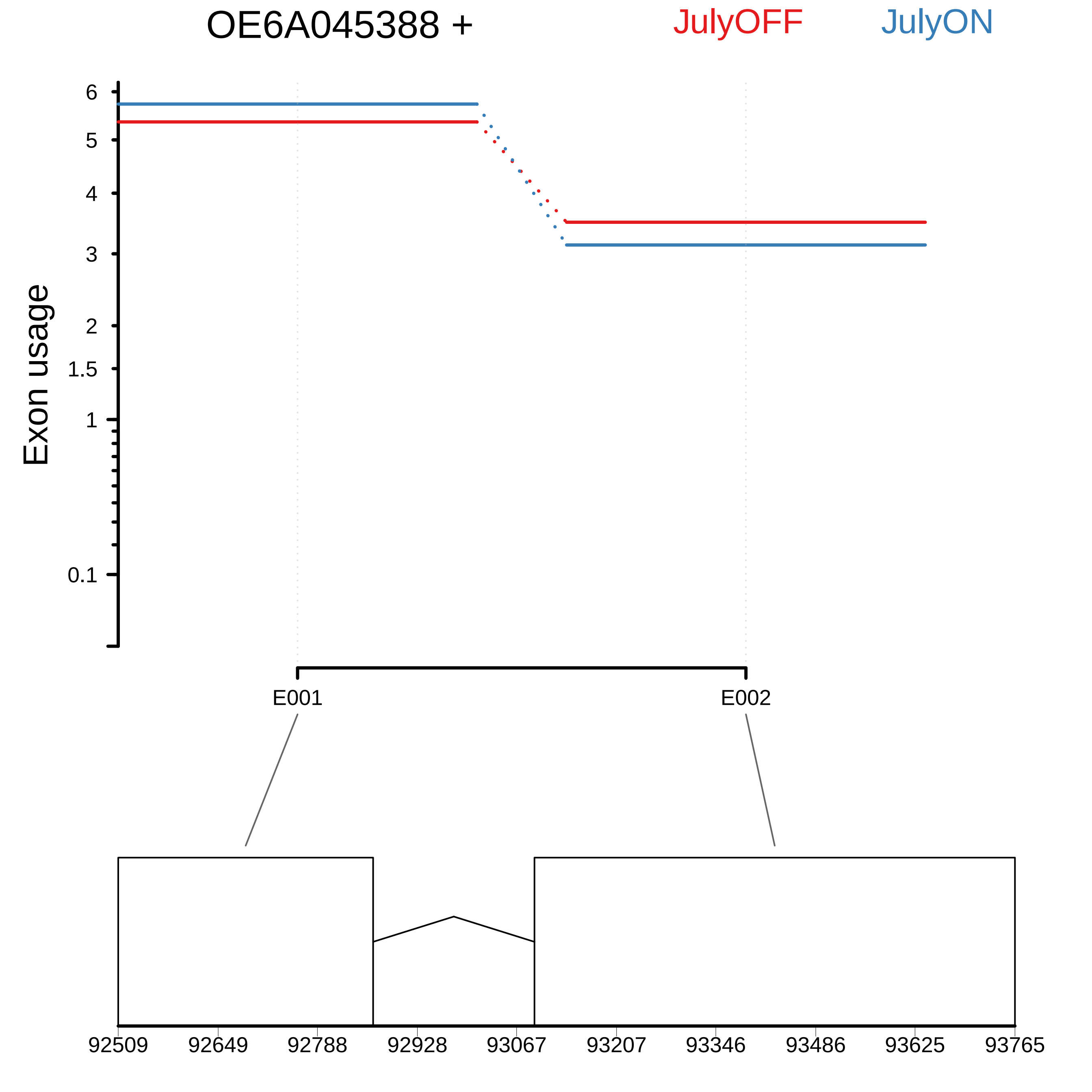

OE6A045388 +
JulyOFF
JulyON
Exon usage
6
5
4
3
2
1.5
1
0.1
E001
E002
92509
92649
92788
92928
93067
93207
93346
93486
93625
93765

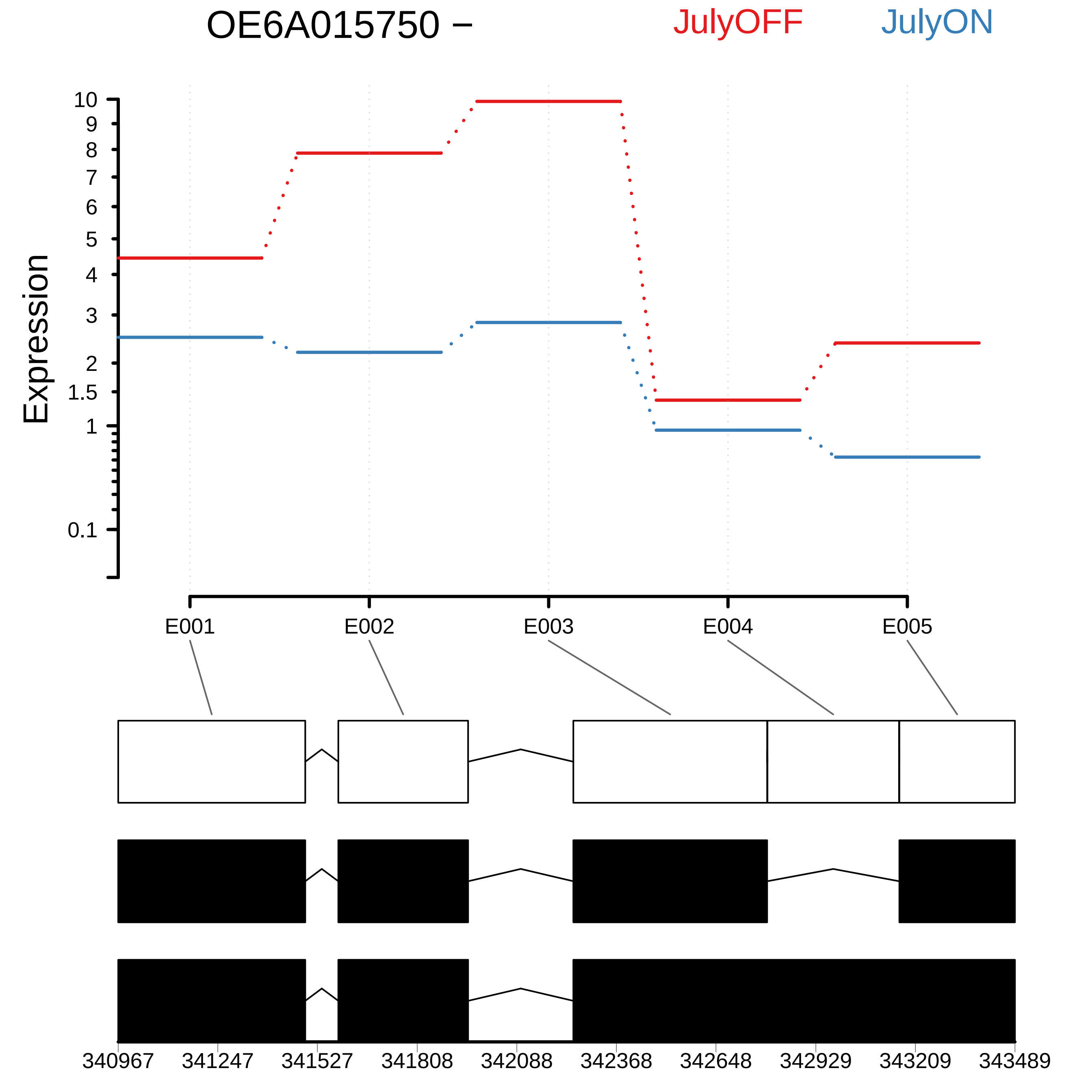
OE6A015750 –
JulyOFF
JulyON
Expression
10
9
8
7
6
5
4
3
2
1.5
1
0.1
E001
E002
E003
E004
E005
340967
341247
341527
341808
342088
342368
342648
342929
343209
343489

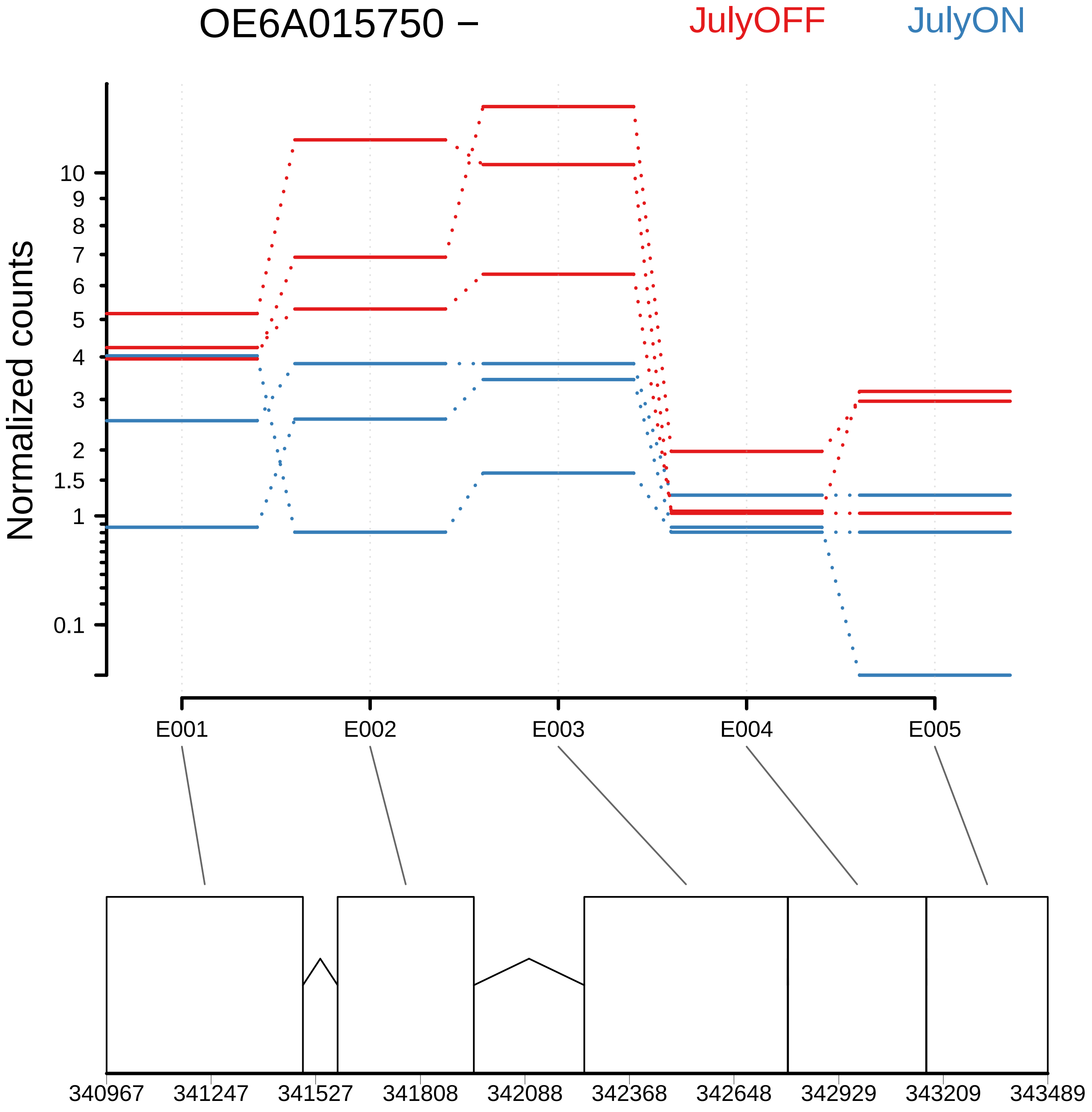
OE6A015750 –
JulyOFF
JulyON
Normalized counts
10
9
8
7
6
5
4
3
2
1.5
1
0.1
E001
E002
E003
E004
E005
340967
341247
341527
341808
342088
342368
342648
342929
343209
343489

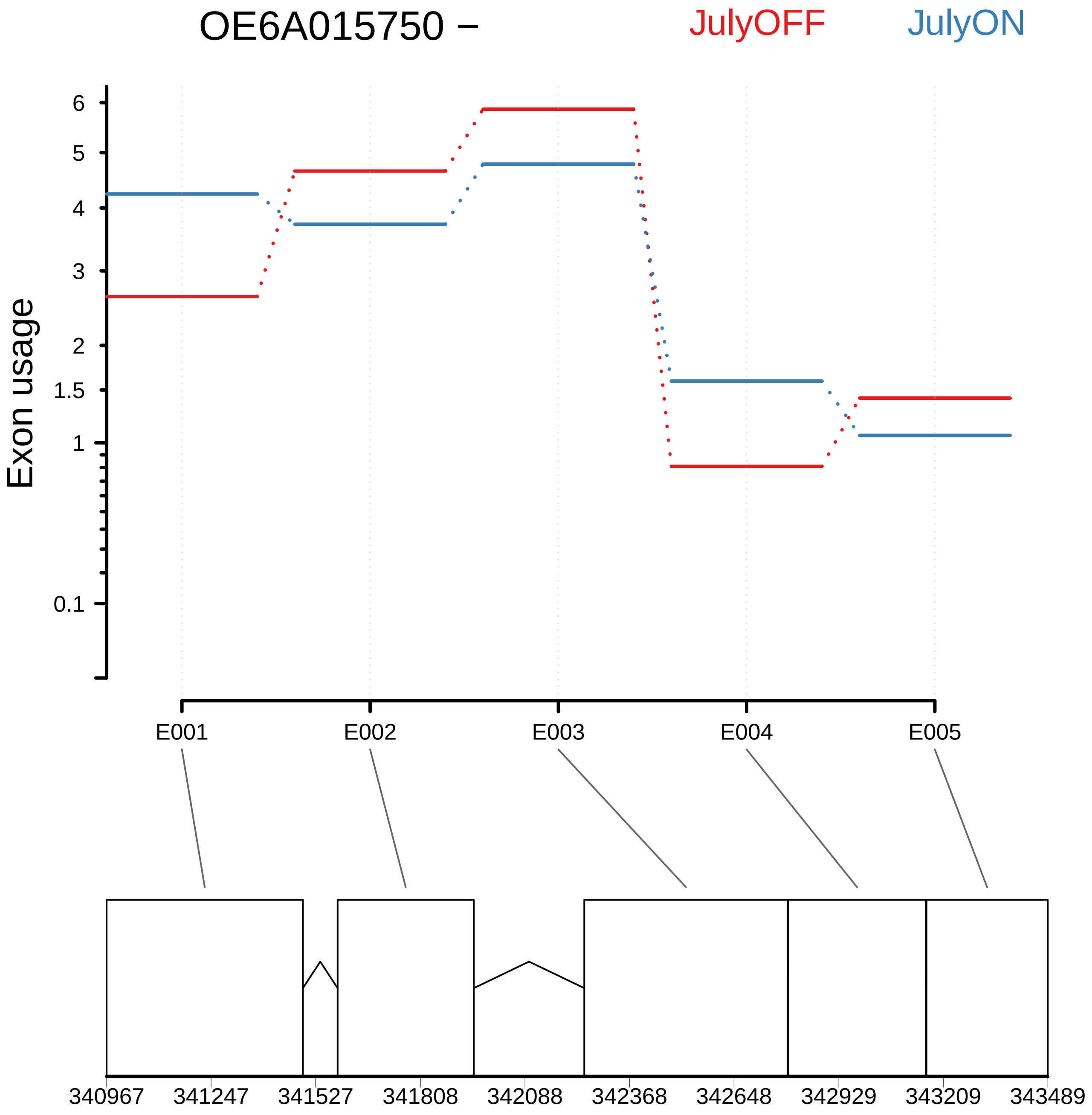
OE6A015750 −
JulyOFF
JulyON
Exon usage
6
5
4
3
2
1.5
1
0.1
E001
E002
E003
E004
E005
340967
341247
341527
341808
342088
342368
342648
342929
343209
343489

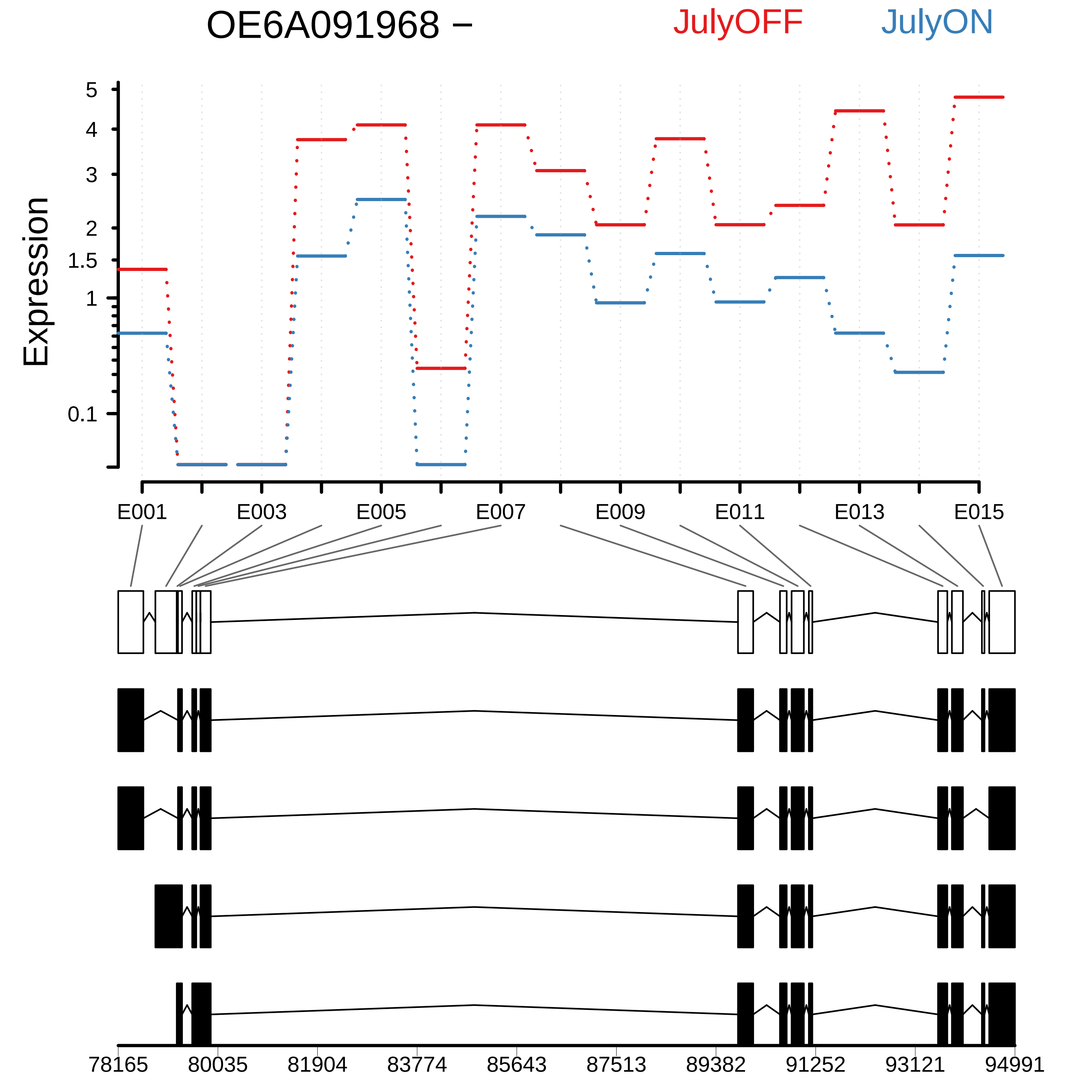

OE6A091968 –
JulyOFF
JulyON
Expression
5
4
3
2
1.5
1
0.1
E001
E003
E005
E007
E009
E011
E013
E015
78165
80035
81904
83774
85643
87513
89382
91252
93121
94991

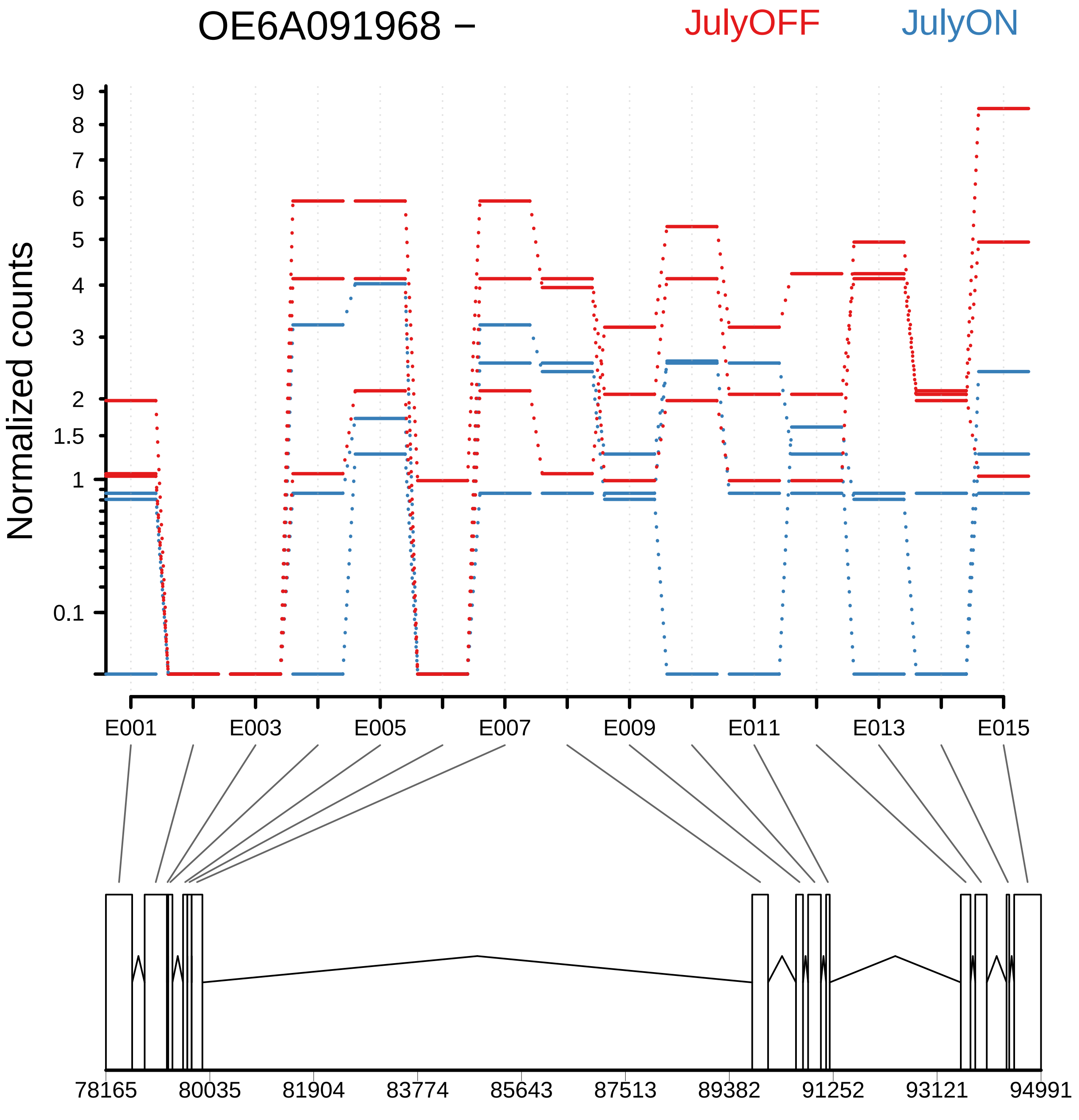

OE6A091968 −
JulyOFF
JulyON
Normalized counts
9
8
7
6
5
4
3
2
1.5
1
0.1
E001
E003
E005
E007
E009
E011
E013
E015
78165
80035
81904
83774
85643
87513
89382
91252
93121
94991

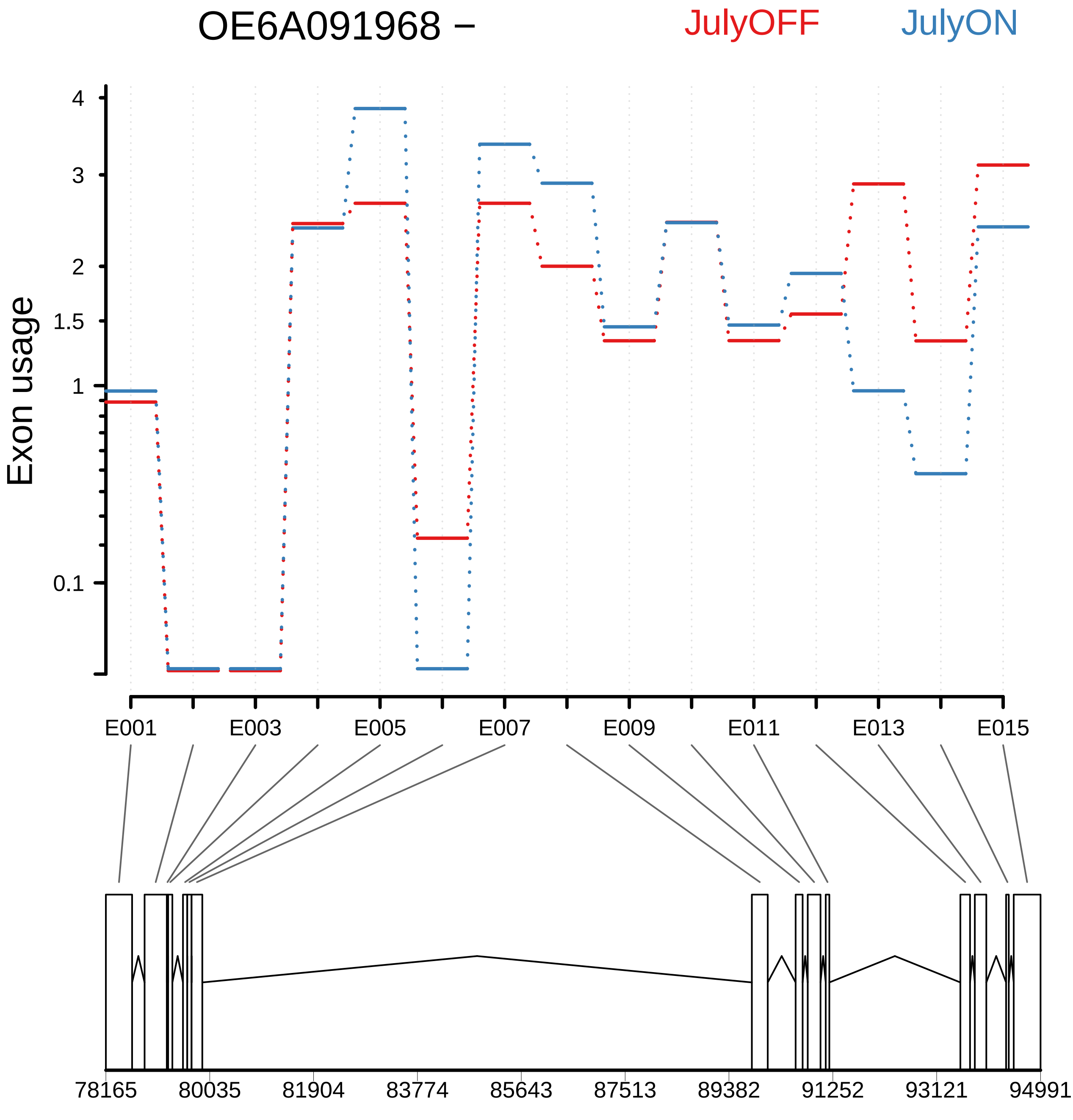

OE6A091968 –
JulyOFF
JulyON
Exon usage
4
3
2
1.5
1
0.1
E001
E003
E005
E007
E009
E011
E013
E015
78165
80035
81904
83774
85643
87513
89382
91252
93121
94991

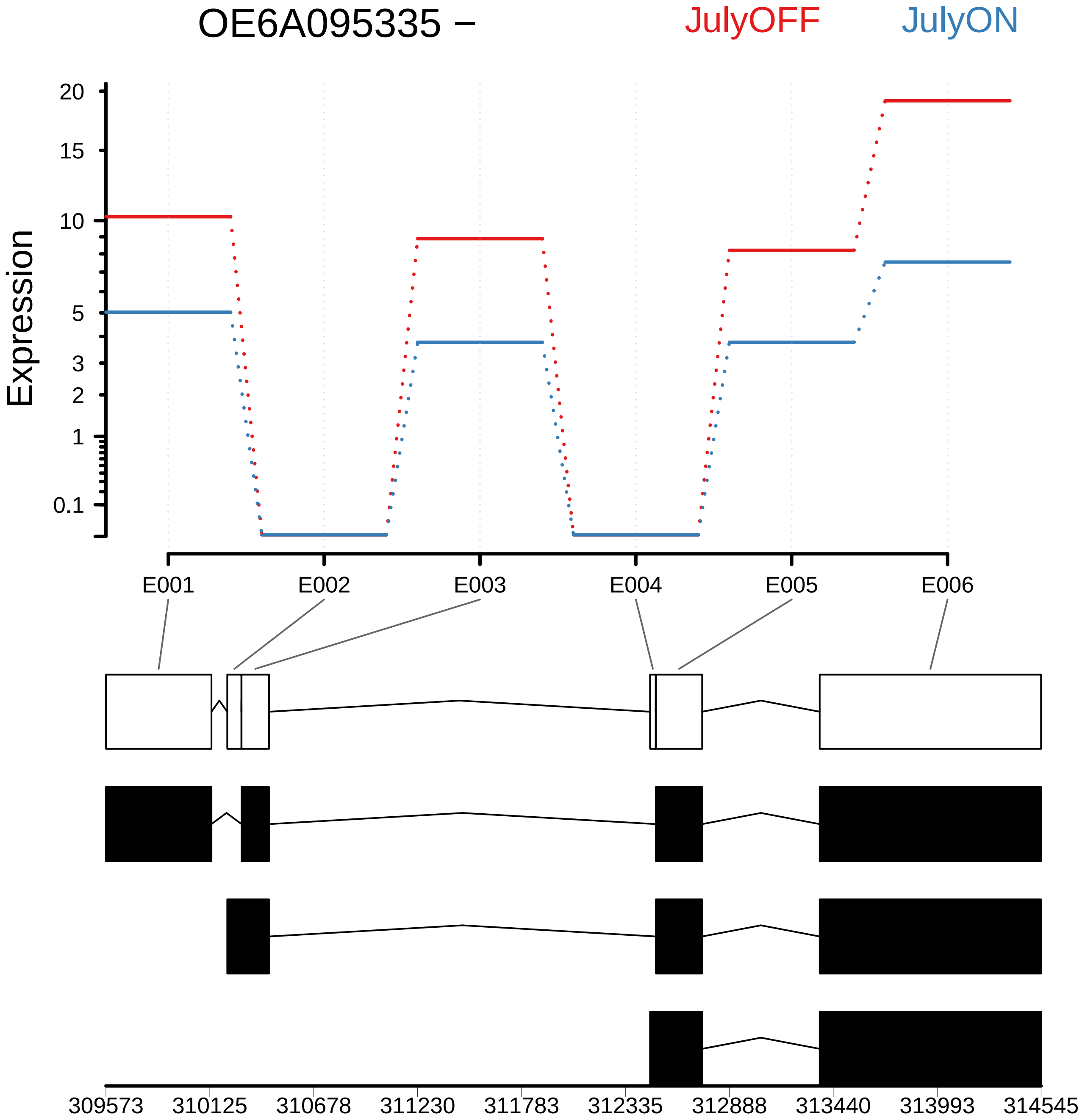
OE6A095335 −
JulyOFF
JulyON
Expression
20
15
10
5
3
2
1
0.1
E001
E002
E003
E004
E005
E006
309573
310125
310678
311230
311783
312335
312888
313440
313993
314545

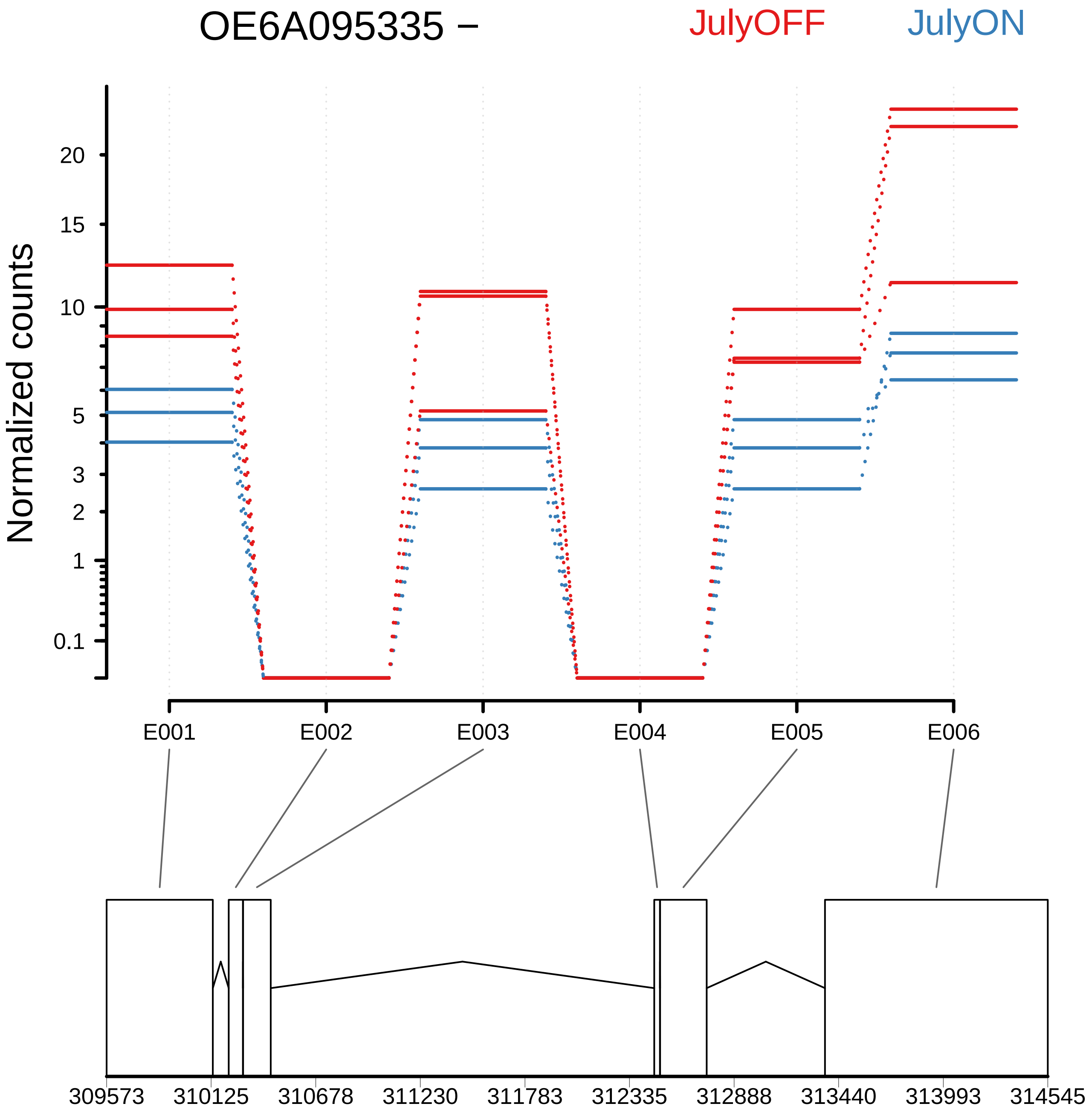
OE6A095335 −
JulyOFF
JulyON
Normalized counts
20
15
10
5
3
2
1
0.1
E001
E002
E003
E004
E005
E006
309573
310125
310678
311230
311783
312335
312888
313440
313993
314545

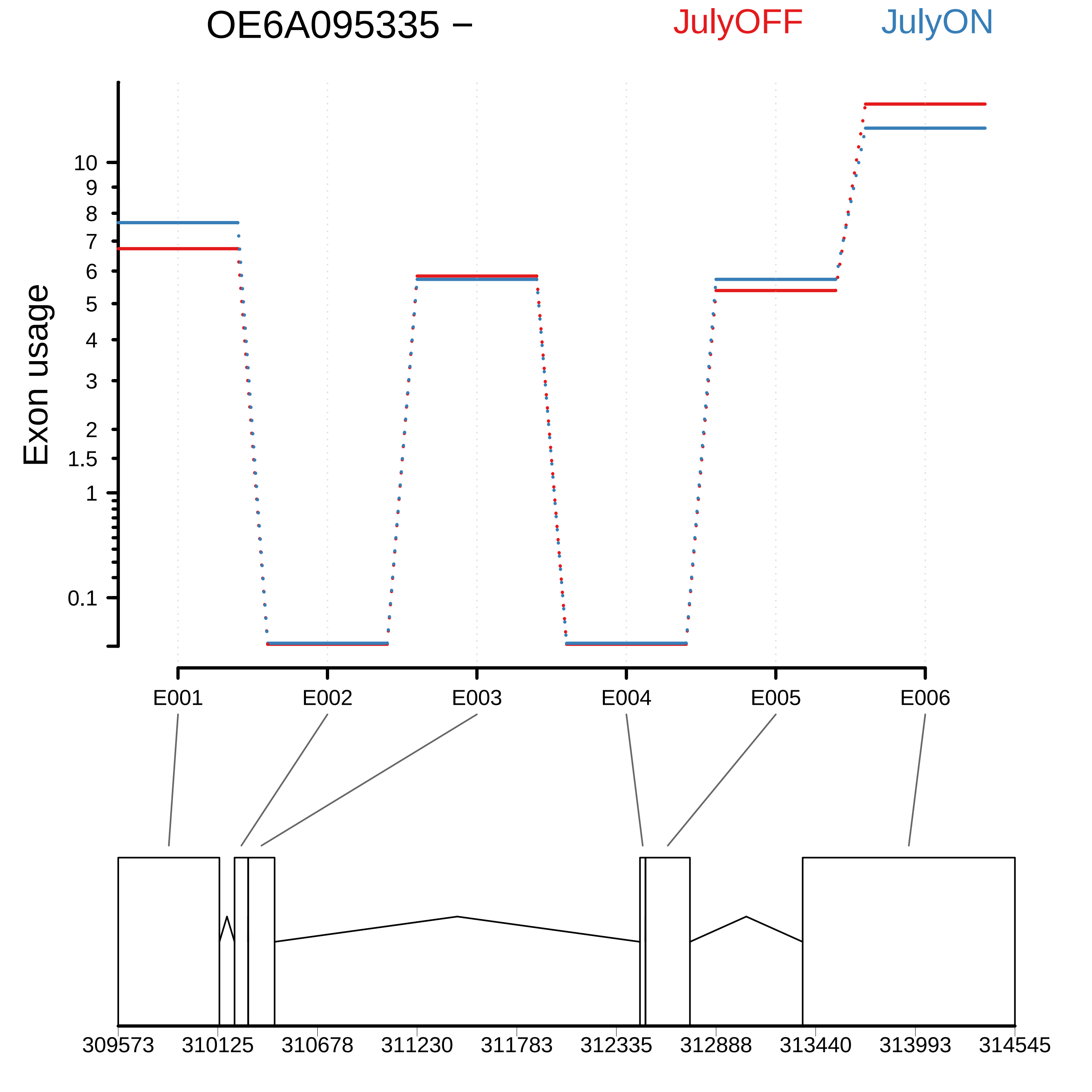

OE6A095335 −
JulyOFF
JulyON
Exon usage
10
9
8
7
6
5
4
3
2
1.5
1
0.1
E001
E002
E003
E004
E005
E006
309573
310125
310678
311230
311783
312335
312888
313440
313993
314545

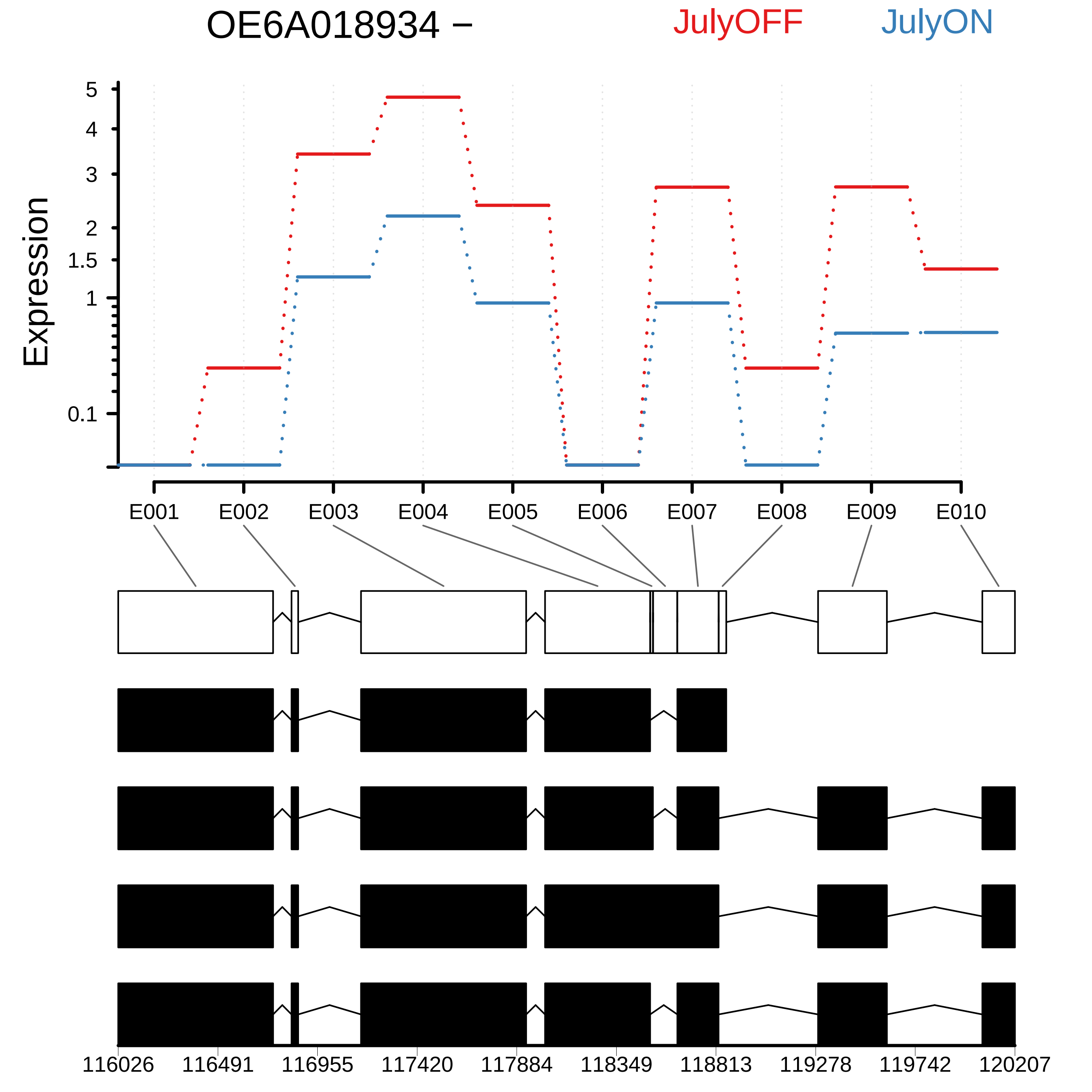

OE6A018934 –
JulyOFF
JulyON
Expression
5
4
3
2
1.5
1
0.1
E001
E002
E003
E004
E005
E006
E007
E008
E009
E010
116026
116491
116955
117420
117884
118349
118813
119278
119742
120207

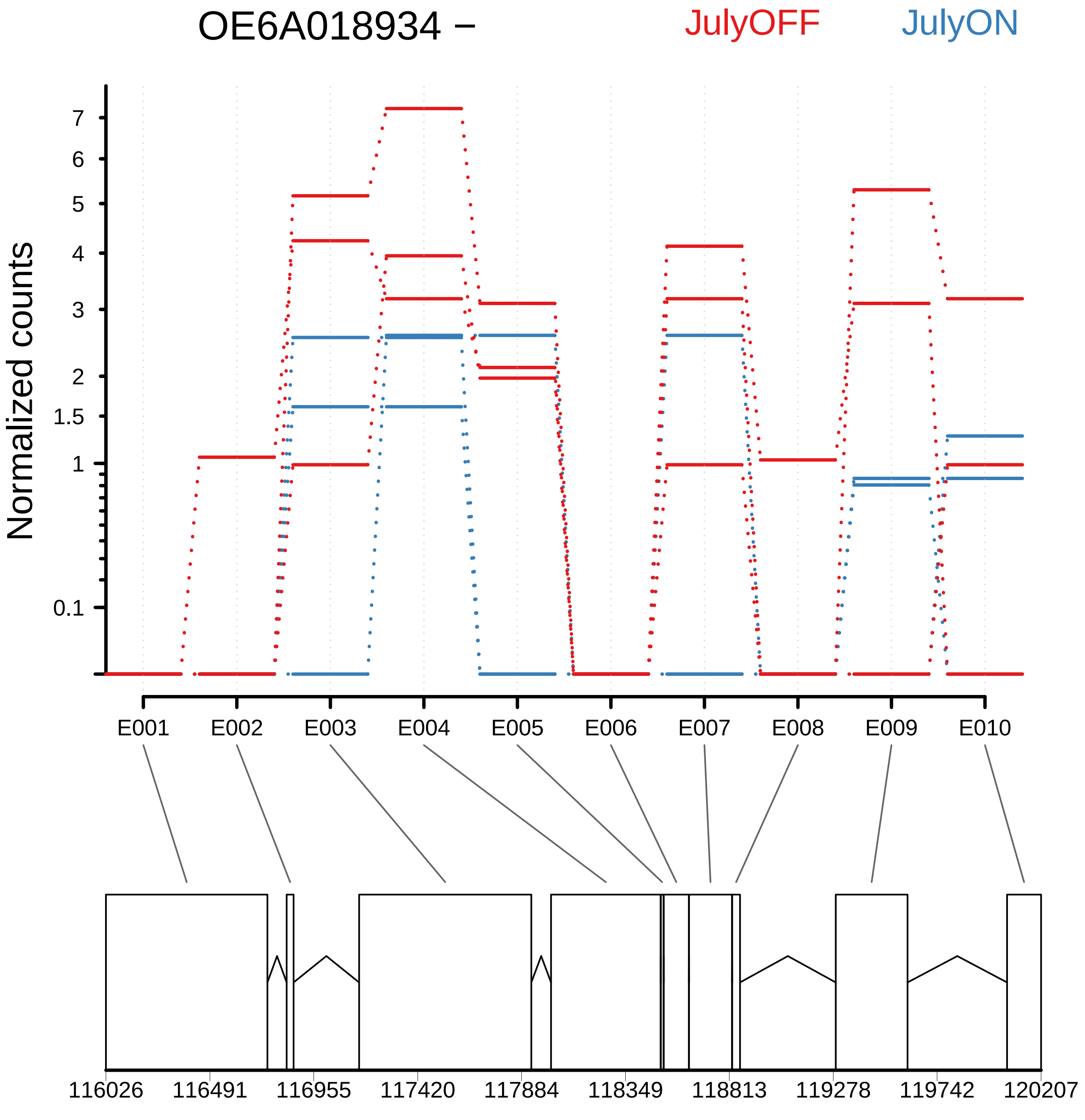

OE6A018934 −
JulyOFF
JulyON
Normalized counts
7
6
5
4
3
2
1.5
1
0.1
E001
E002
E003
E004
E005
E006
E007
E008
E009
E010
116026
116491
116955
117420
117884
118349
118813
119278
119742
120207

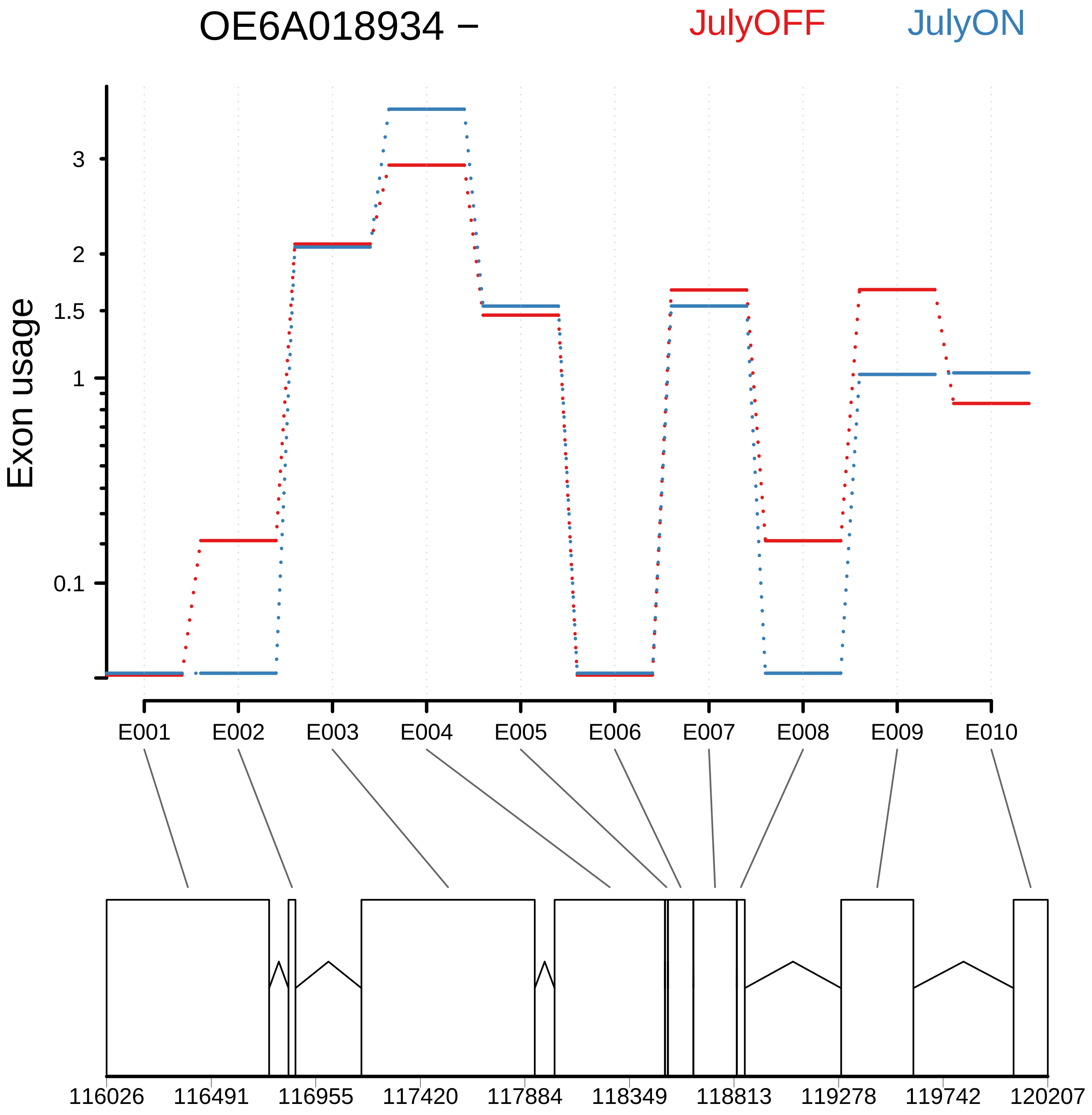
OE6A018934 −
JulyOFF
JulyON
Exon usage
3
2
1.5
1
0.1
E001
E002
E003
E004
E005
E006
E007
E008
E009
E010
116026
116491
116955
117420
117884
118349
118813
119278
119742
120207

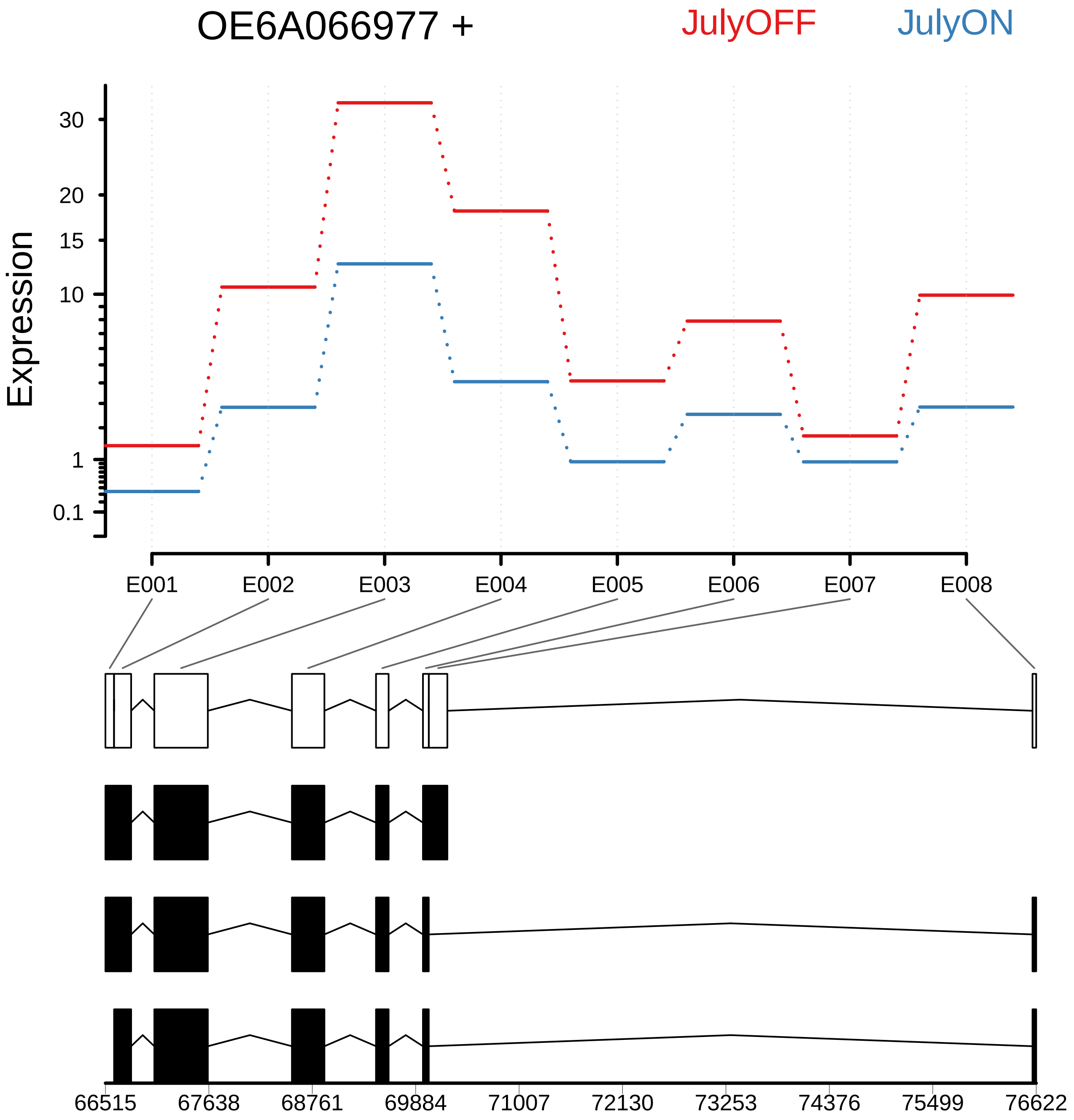
OE6A066977 +
JulyOFF
JulyON
Expression
30
20
15
10
1
0.1
E001
E002
E003
E004
E005
E006
E007
E008
66515
67638
68761
69884
71007
72130
73253
74376
75499
76622

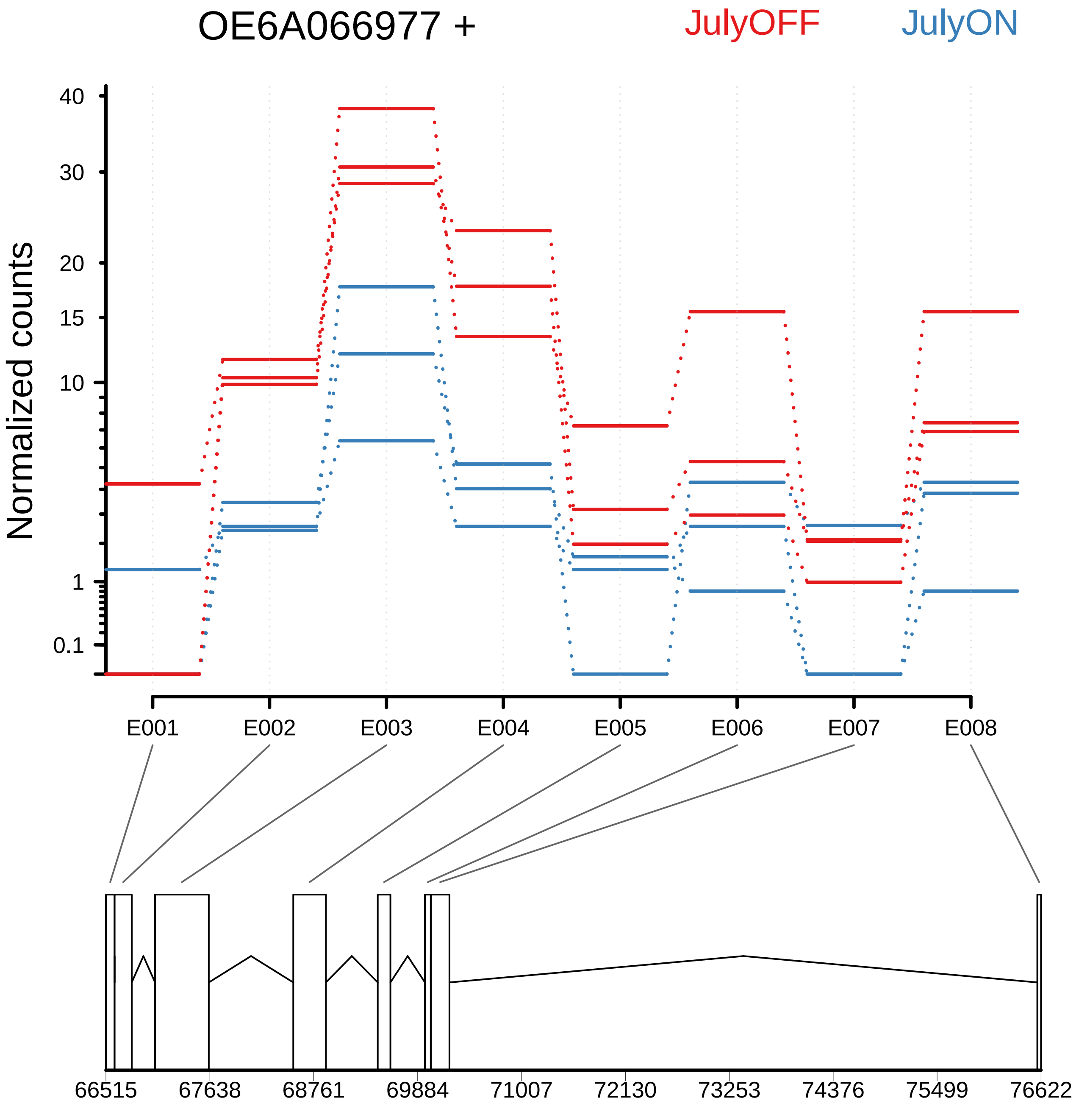

OE6A066977 +
JulyOFF
JulyON
Normalized counts
40
30
20
15
10
1
0.1
E001
E002
E003
E004
E005
E006
E007
E008
66515
67638
68761
69884
71007
72130
73253
74376
75499
76622

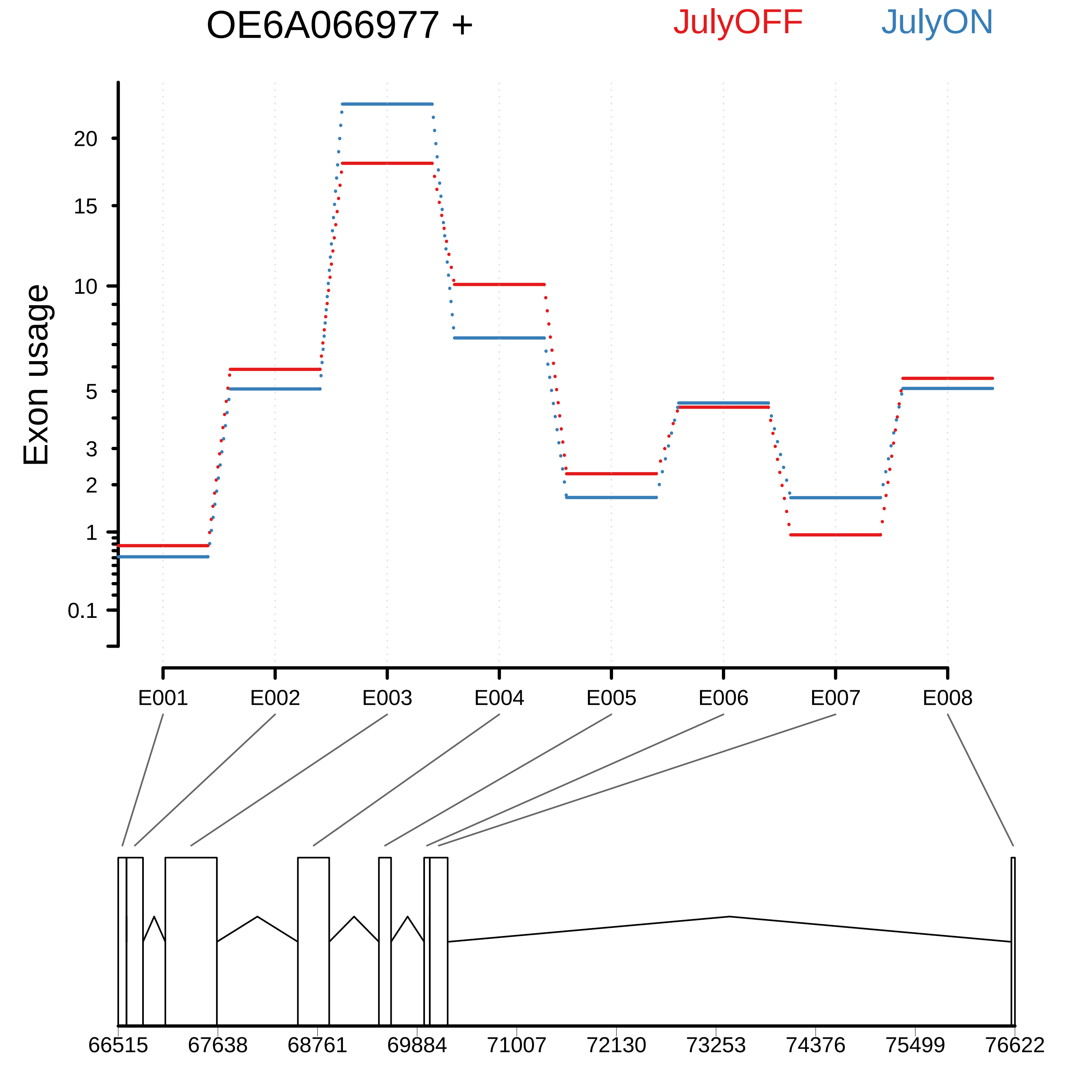

OE6A066977 +
JulyOFF
JulyON
Exon usage
20
15
10
5
3
2
1
0.1
E001
E002
E003
E004
E005
E006
E007
E008
66515
67638
68761
69884
71007
72130
73253
74376
75499
76622

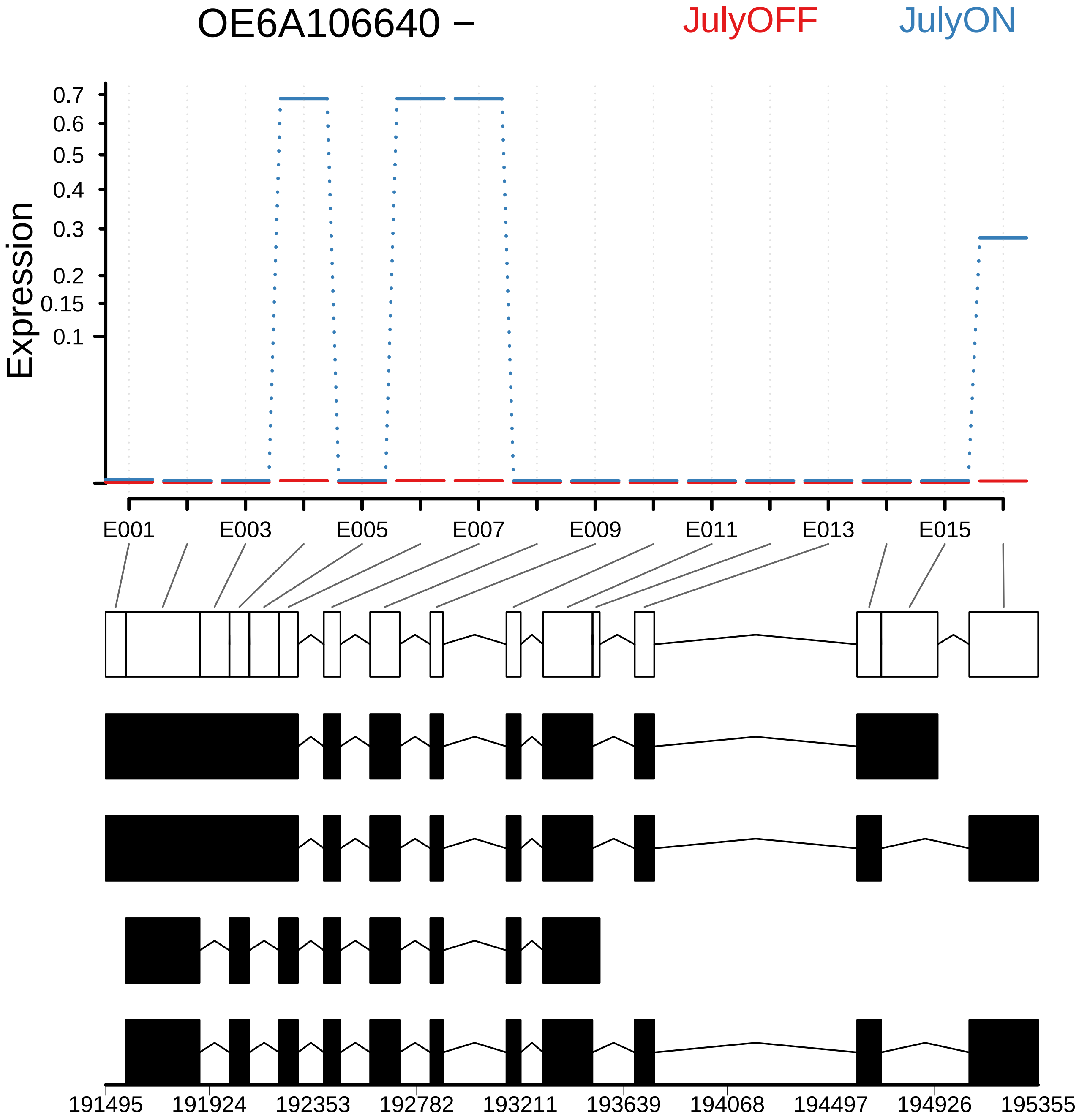
OE6A106640 –
JulyOFF
JulyON
Expression
0.7
0.6
0.5
0.4
0.3
0.2
0.15
0.1
E001
E003
E005
E007
E009
E011
E013
E015
191495
191924
192353
192782
193211
193639
194068
194497
194926
195355

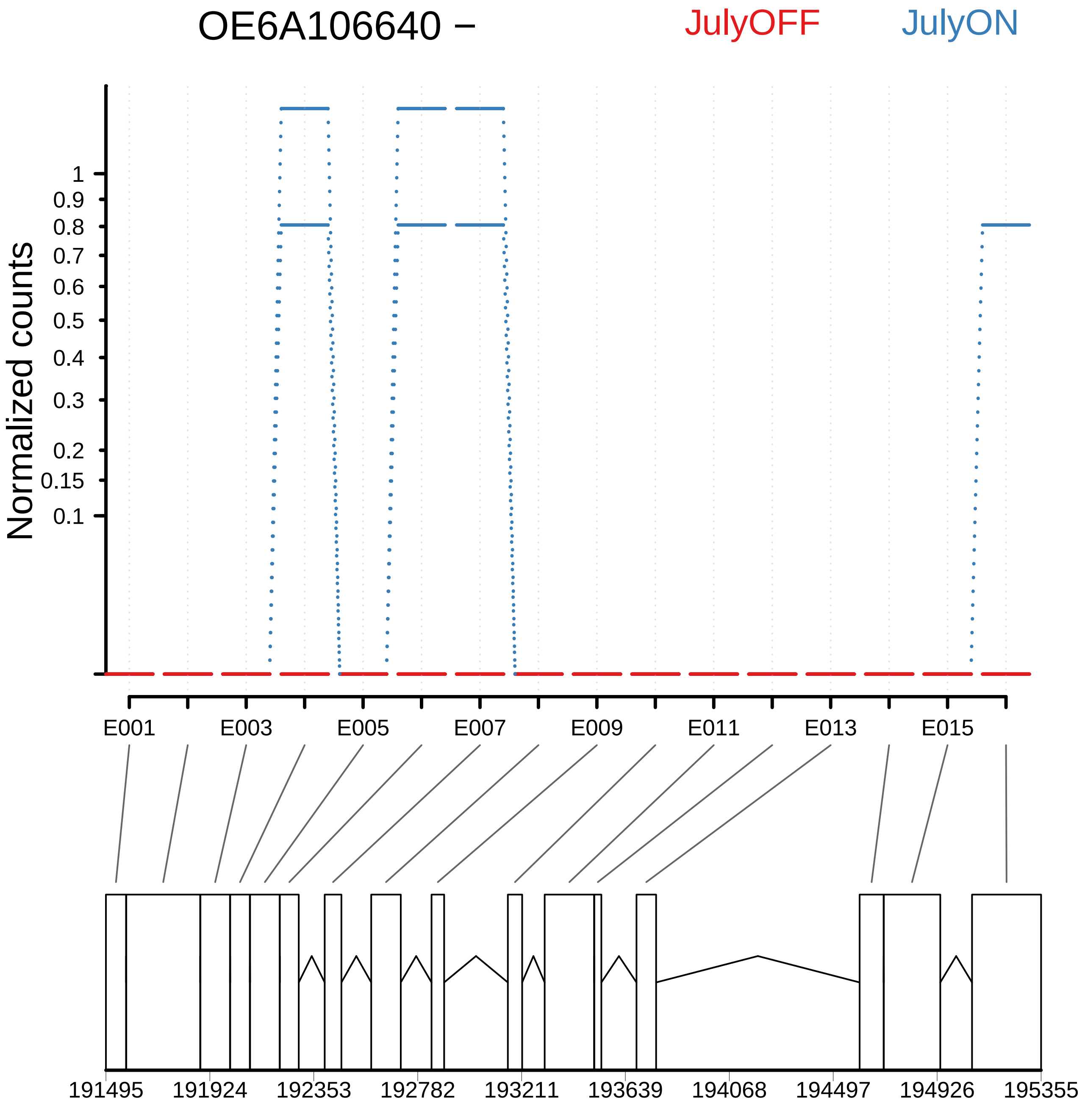
OE6A106640 −
JulyOFF
JulyON
Normalized counts
1
0.9
0.8
0.7
0.6
0.5
0.4
0.3
0.2
0.15
0.1
E001
E003
E005
E007
E009
E011
E013
E015
191495
191924
192353
192782
193211
193639
194068
194497
194926
195355

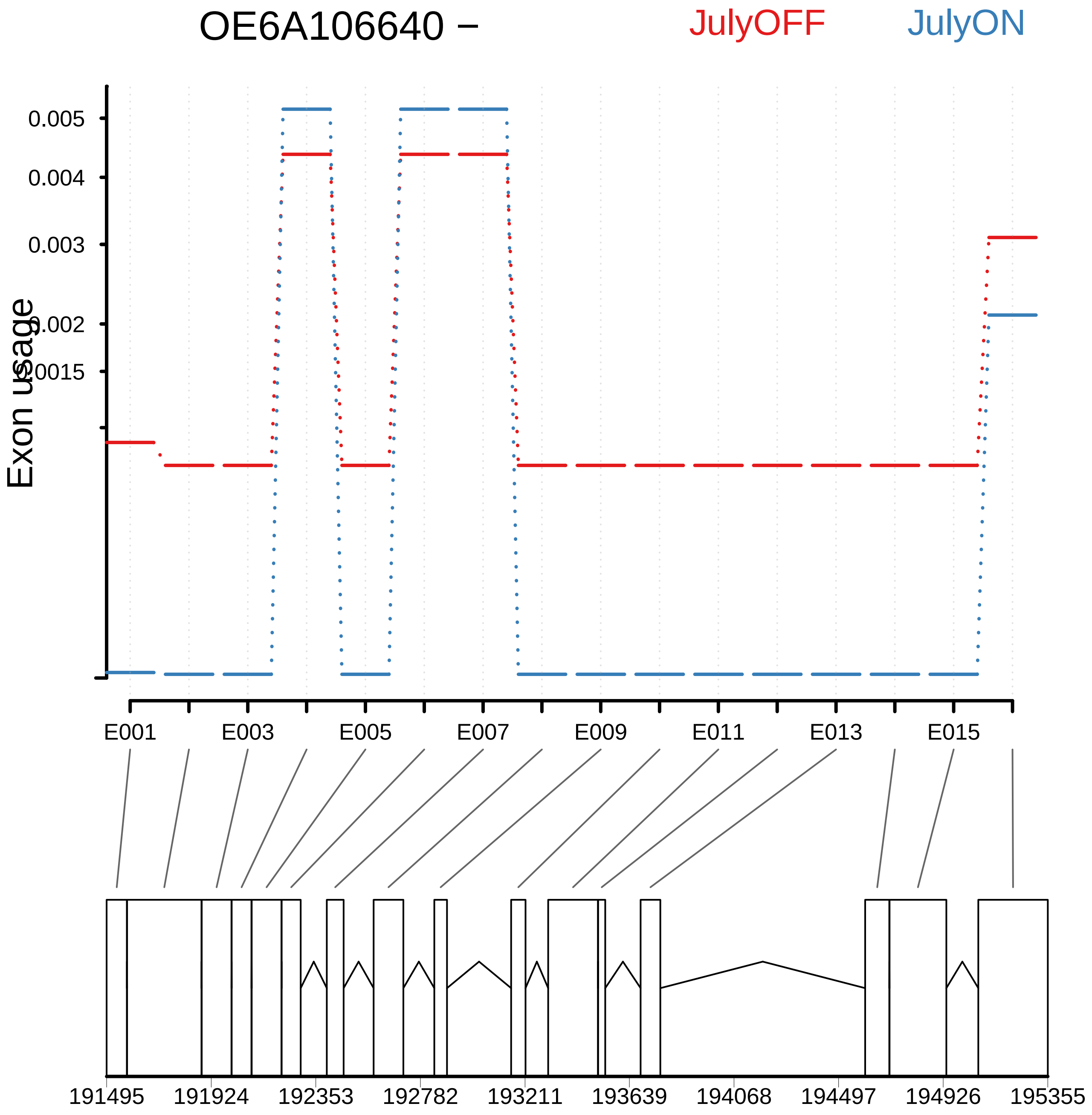
OE6A106640 −
JulyOFF
JulyON
Exon usage
0.005
0.004
0.003
0.002
0.0015
E001
E003
E005
E007
E009
E011
E013
E015
191495
191924
192353
192782
193211
193639
194068
194497
194926
195355

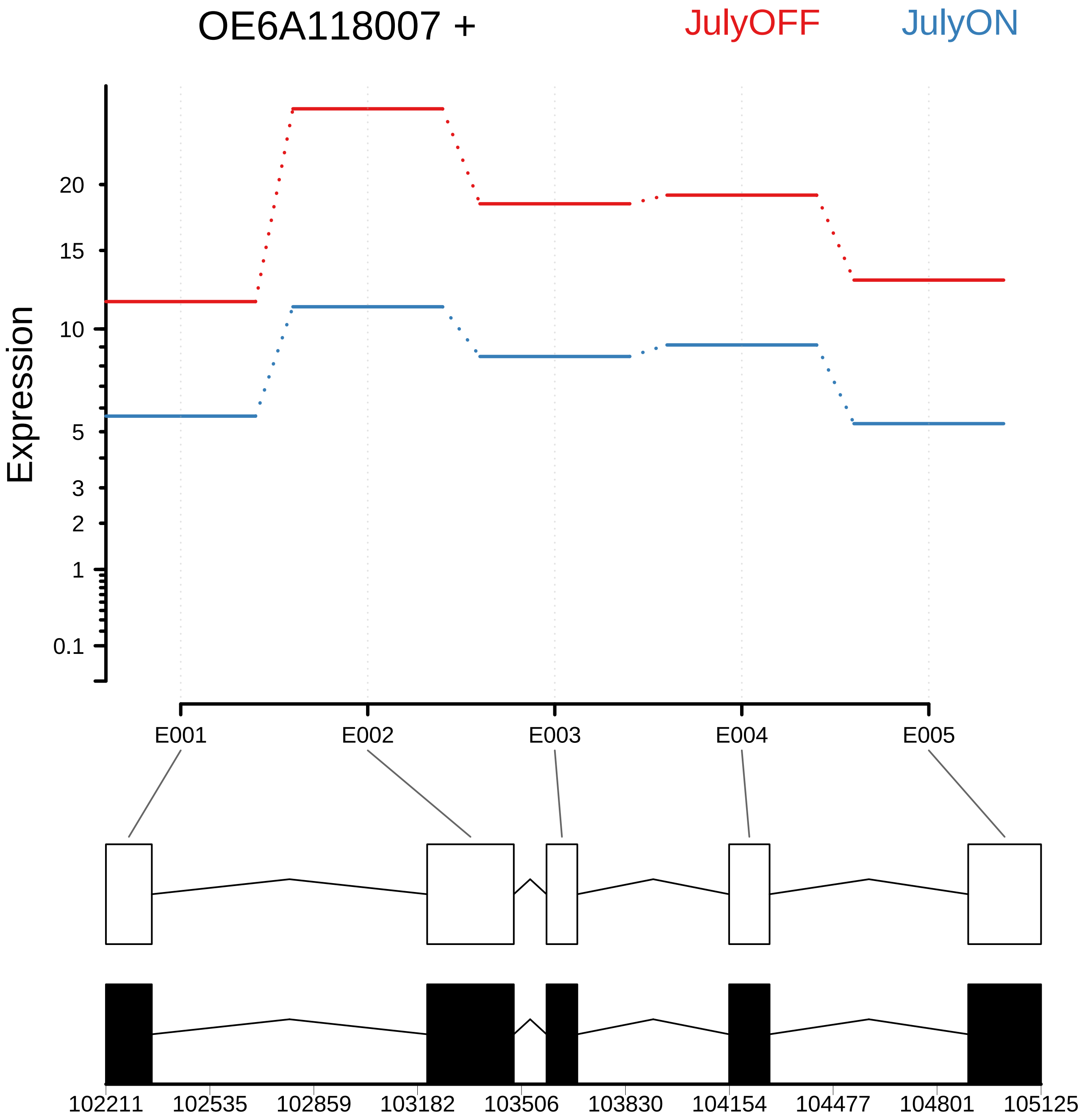

OE6A118007 +
JulyOFF
JulyON
Expression
20
15
10
5
3
2
1
0.1
E001
E002
E003
E004
E005
102211
102535
102859
103182
103506
103830
104154
104477
104801
105125

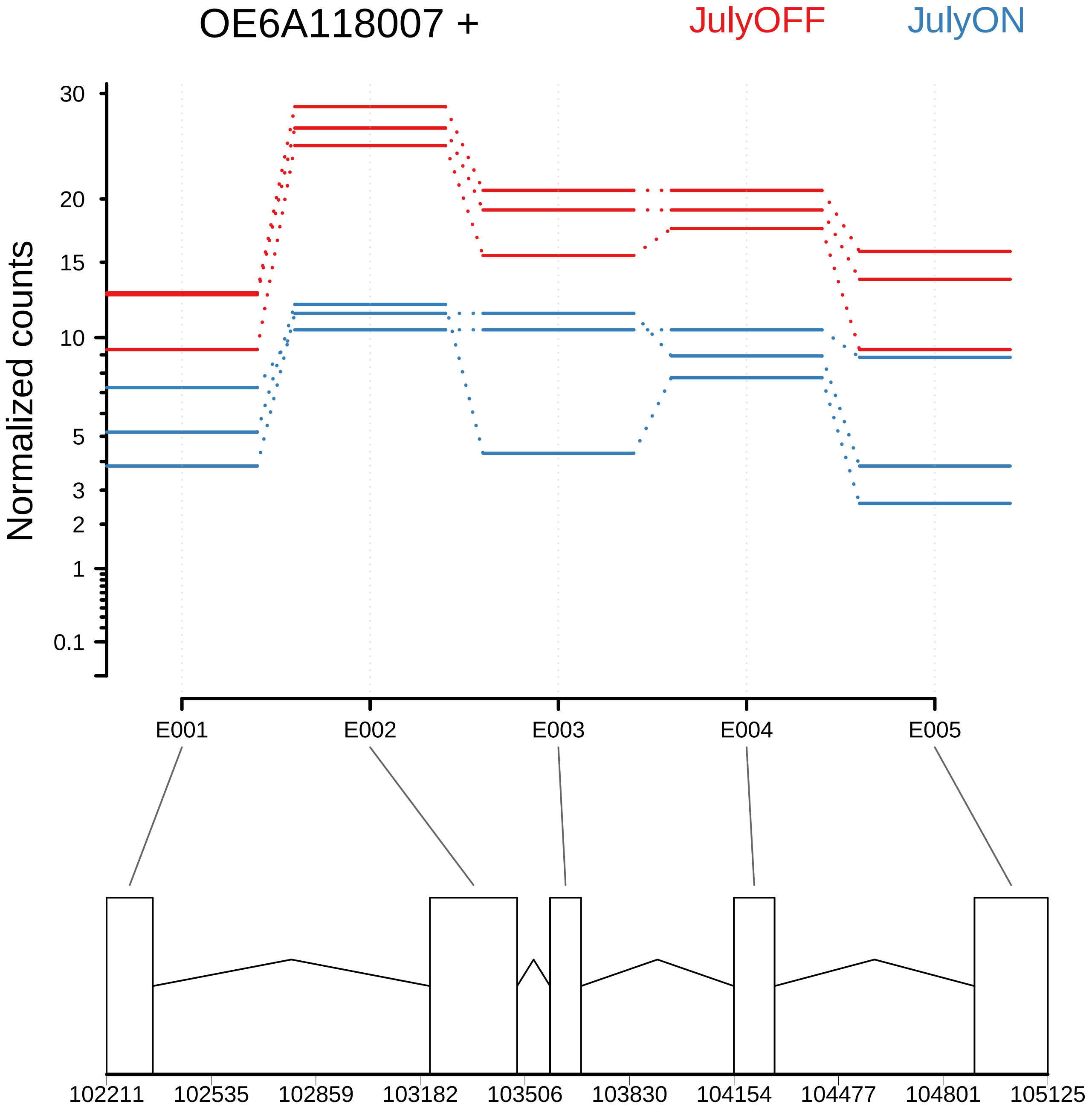

OE6A118007 +
JulyOFF
JulyON
Normalized counts
30
20
15
10
5
3
2
1
0.1
E001
E002
E003
E004
E005
102211
102535
102859
103182
103506
103830
104154
104477
104801
105125

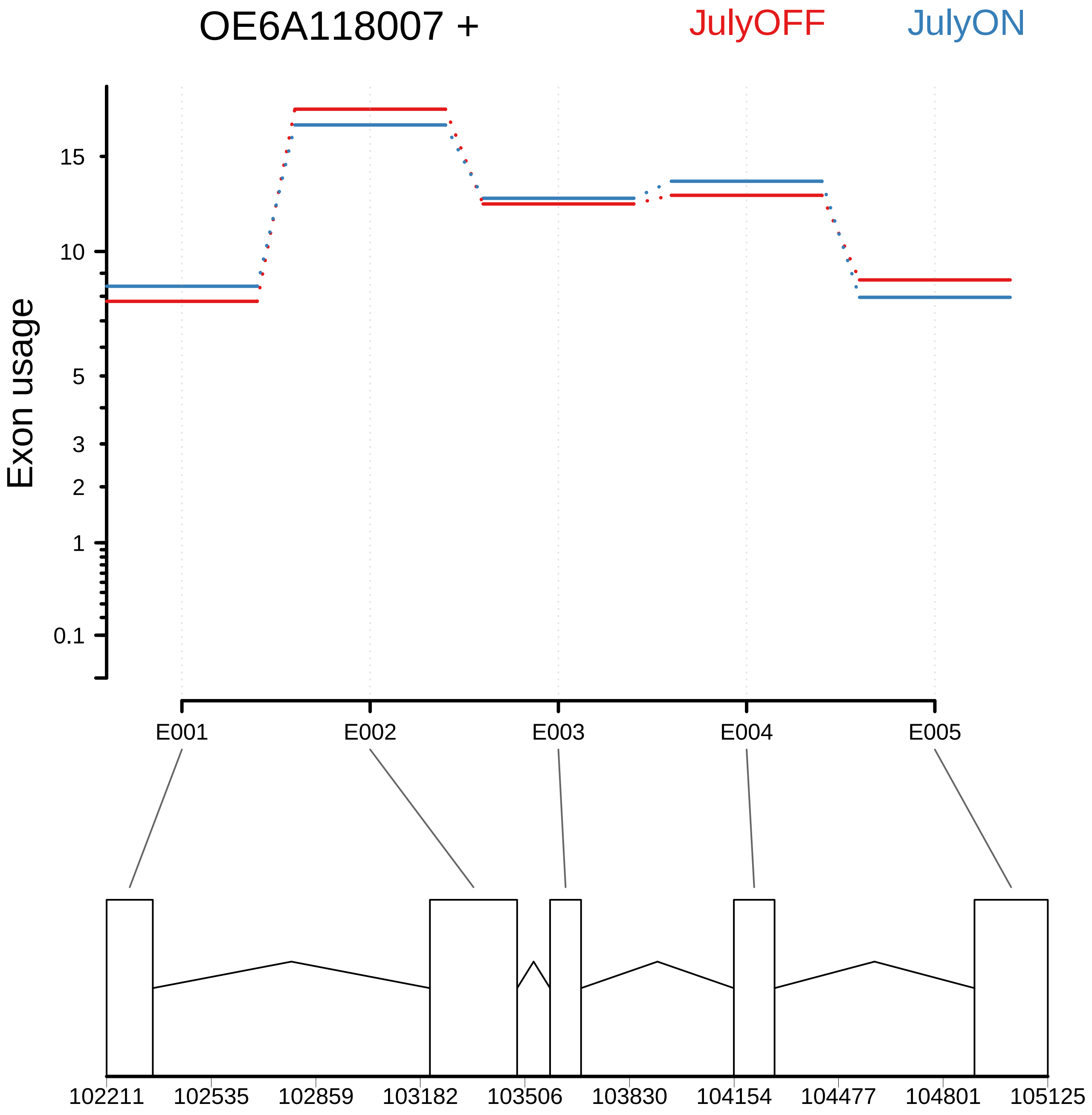

OE6A118007 +
JulyOFF
JulyON
Exon usage
15
10
5
3
2
1
0.1
E001
E002
E003
E004
E005
102211
102535
102859
103182
103506
103830
104154
104477
104801
105125

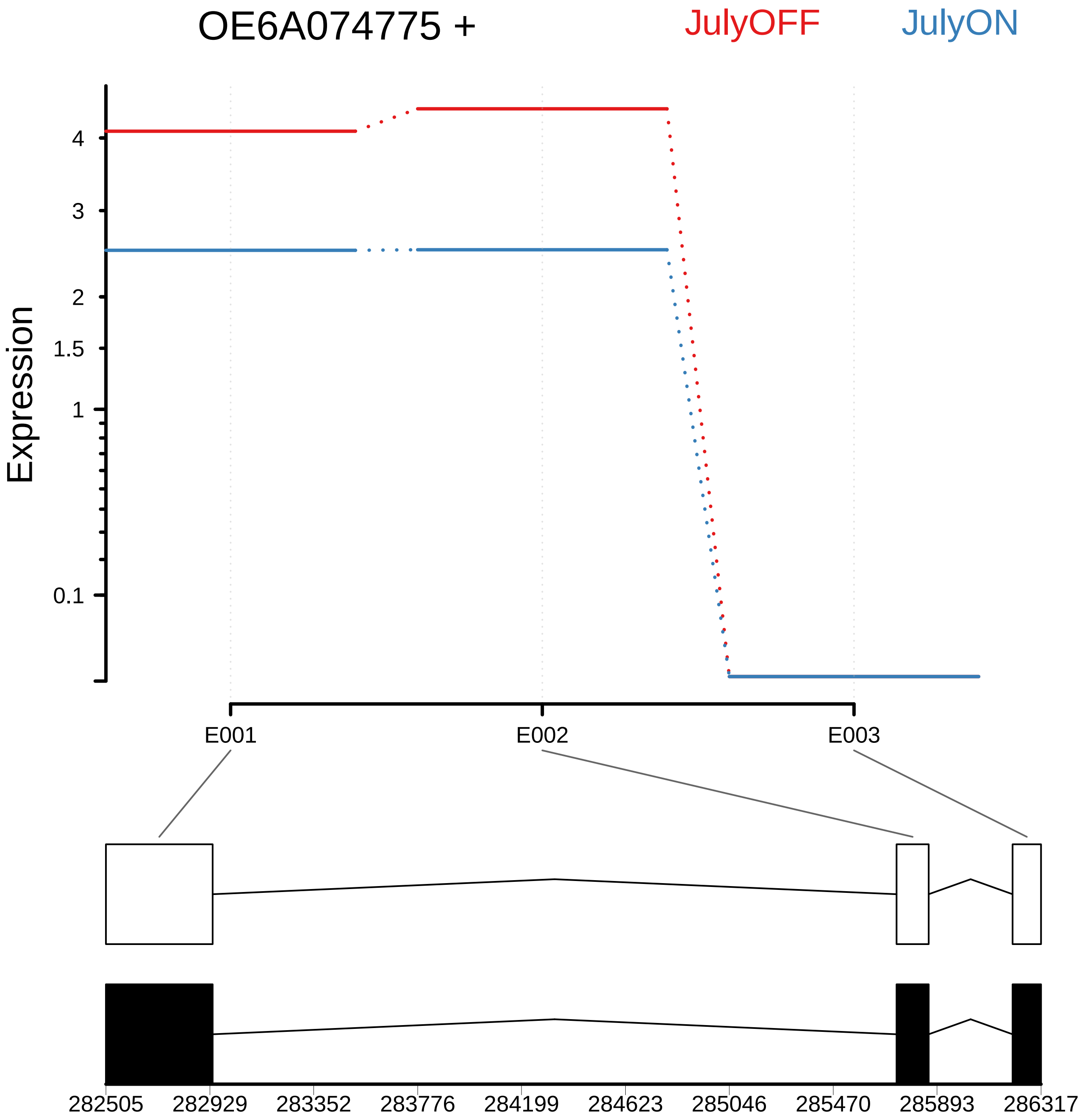

OE6A074775 +
JulyOFF
JulyON
Expression
4
3
2
1.5
1
0.1
E001
E002
E003
282505
282929
283352
283776
284199
284623
285046
285470
285893
286317

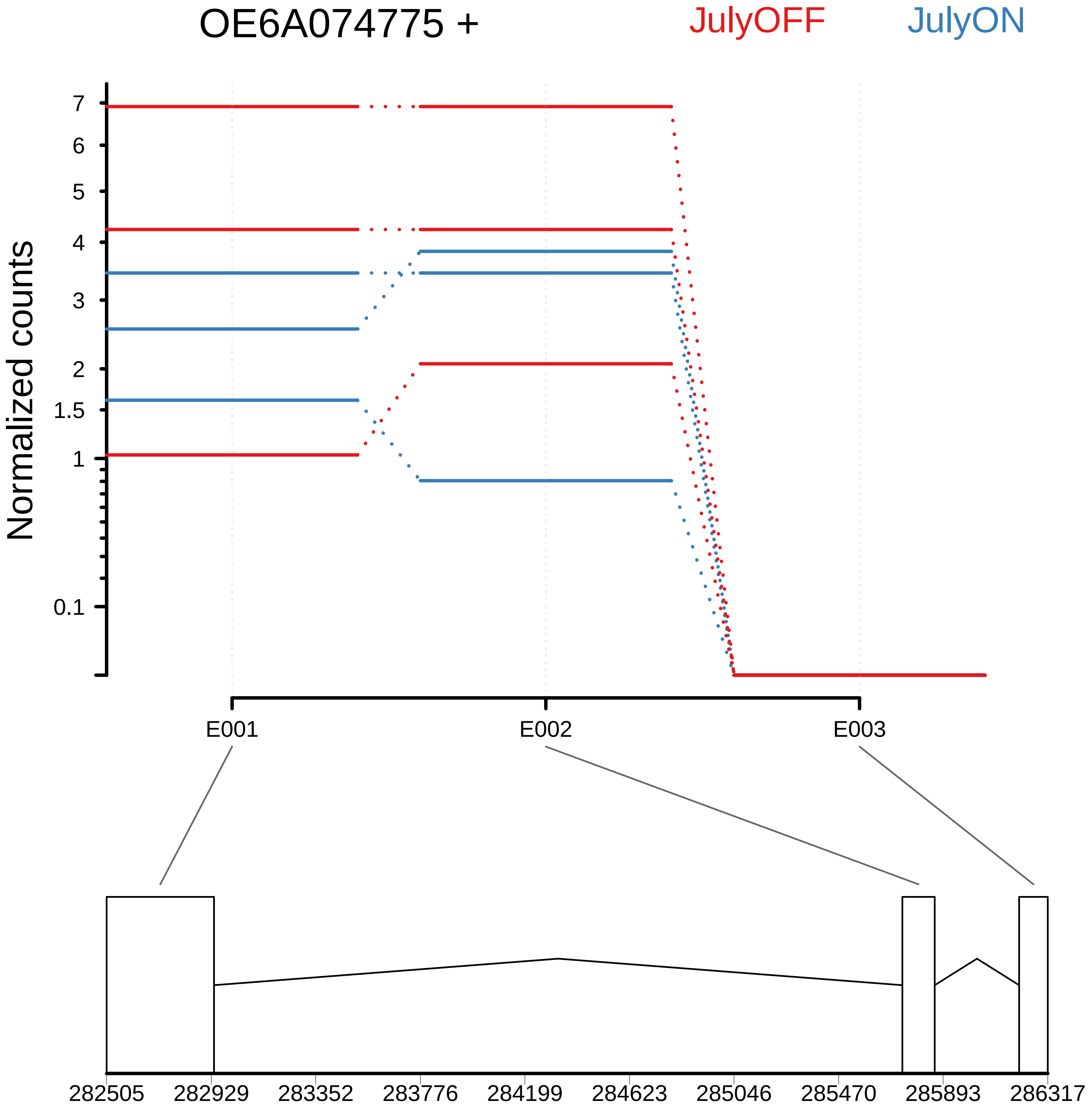
OE6A074775 +
JulyOFF
JulyON
Normalized counts
7
6
5
4
3
2
1.5
1
0.1
E001
E002
E003
282505
282929
283352
283776
284199
284623
285046
285470
285893
286317

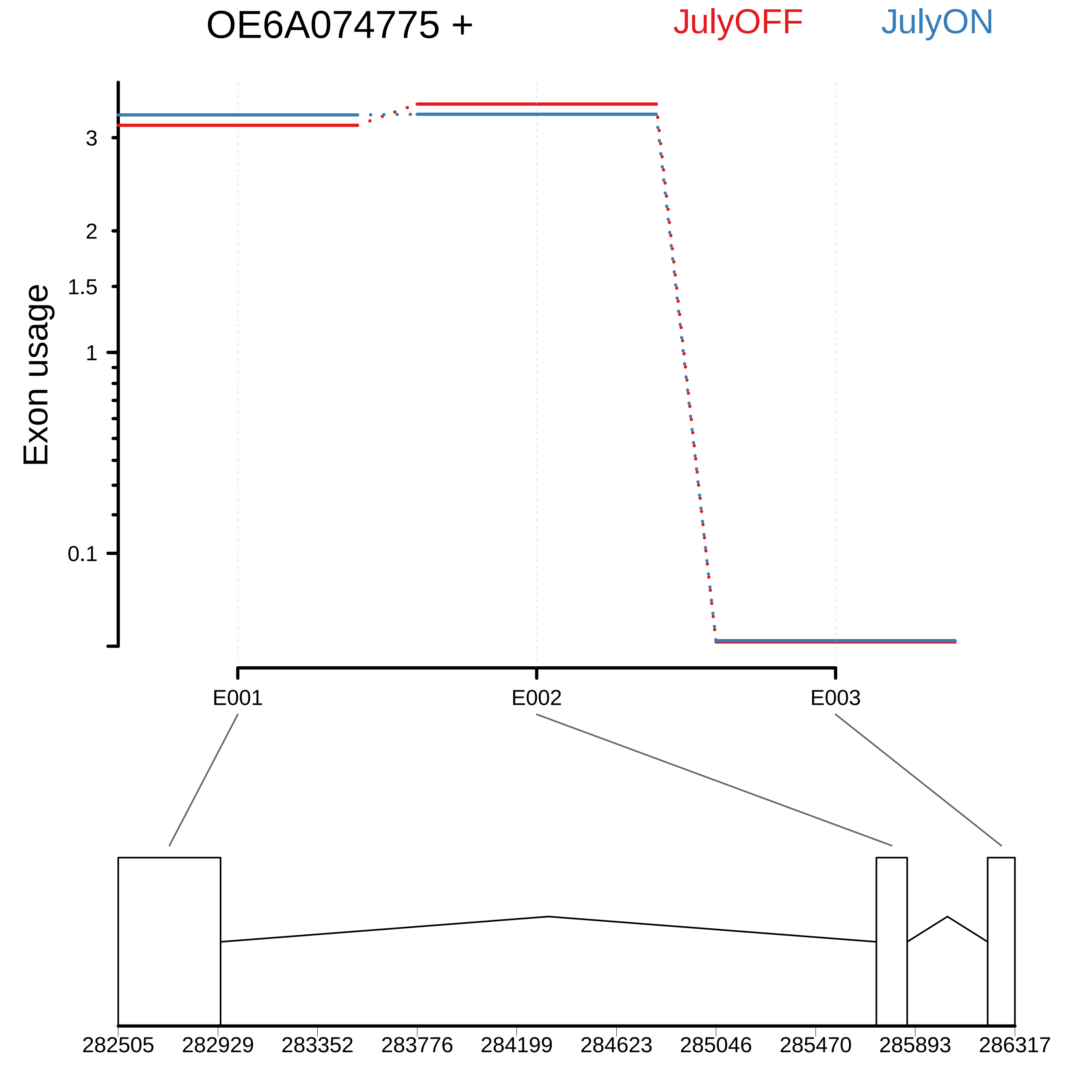

OE6A074775 +
JulyOFF
JulyON
Exon usage
3
2
1.5
1
0.1
E001
E002
E003
282505
282929
283352
283776
284199
284623
285046
285470
285893
286317

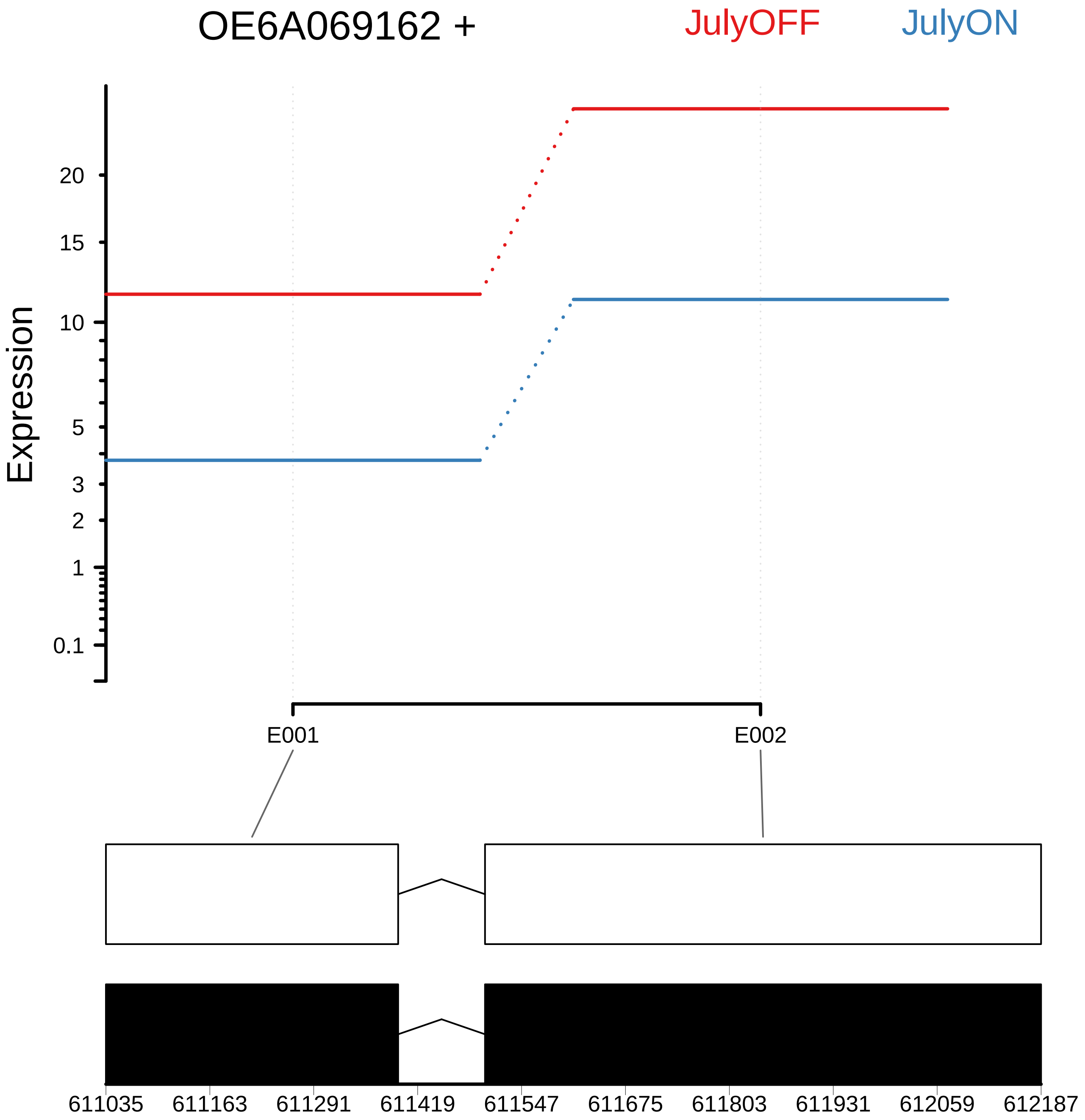

OE6A069162 +
JulyOFF
JulyON
Expression
20
15
10
5
3
2
1
0.1
E001
E002
611035
611163
611291
611419
611547
611675
611803
611931
612059
612187

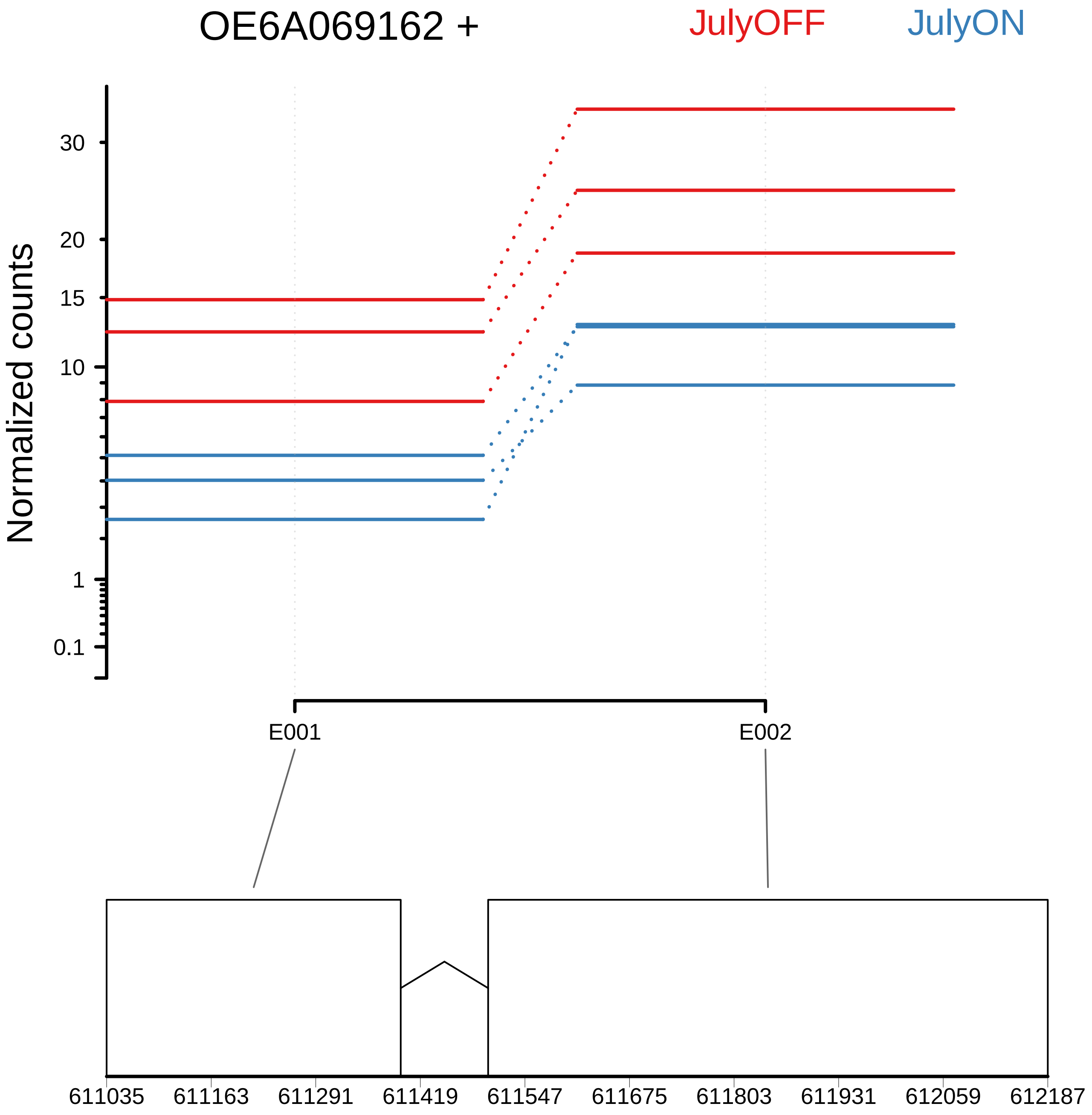
OE6A069162 +
JulyOFF
JulyON
Normalized counts
30
20
15
10
1
0.1
E001
E002
611035
611163
611291
611419
611547
611675
611803
611931
612059
612187

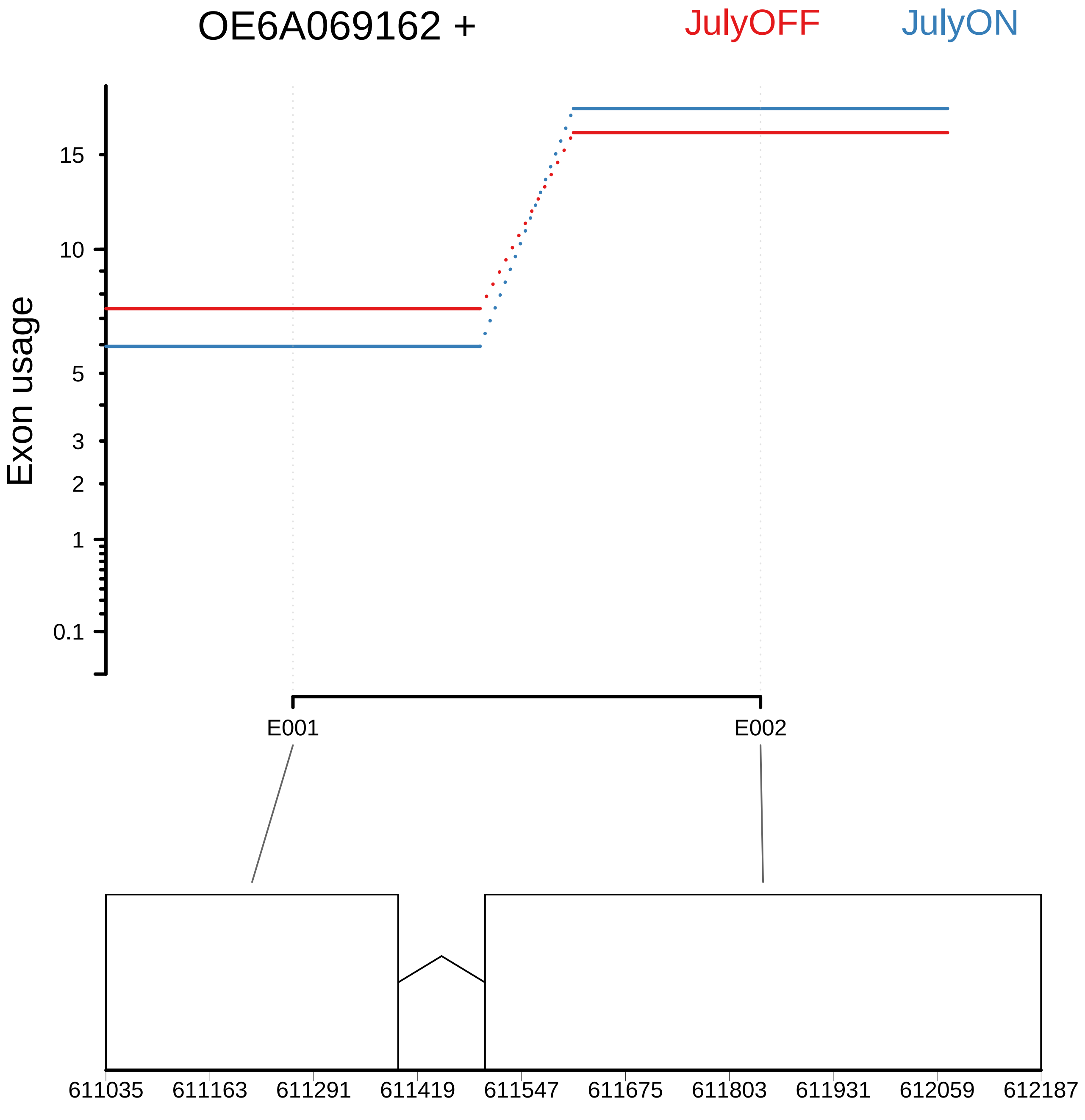
OE6A069162 +
JulyOFF
JulyON
Exon usage
15
10
5
3
2
1
0.1
E001
E002
611035
611163
611291
611419
611547
611675
611803
611931
612059
612187

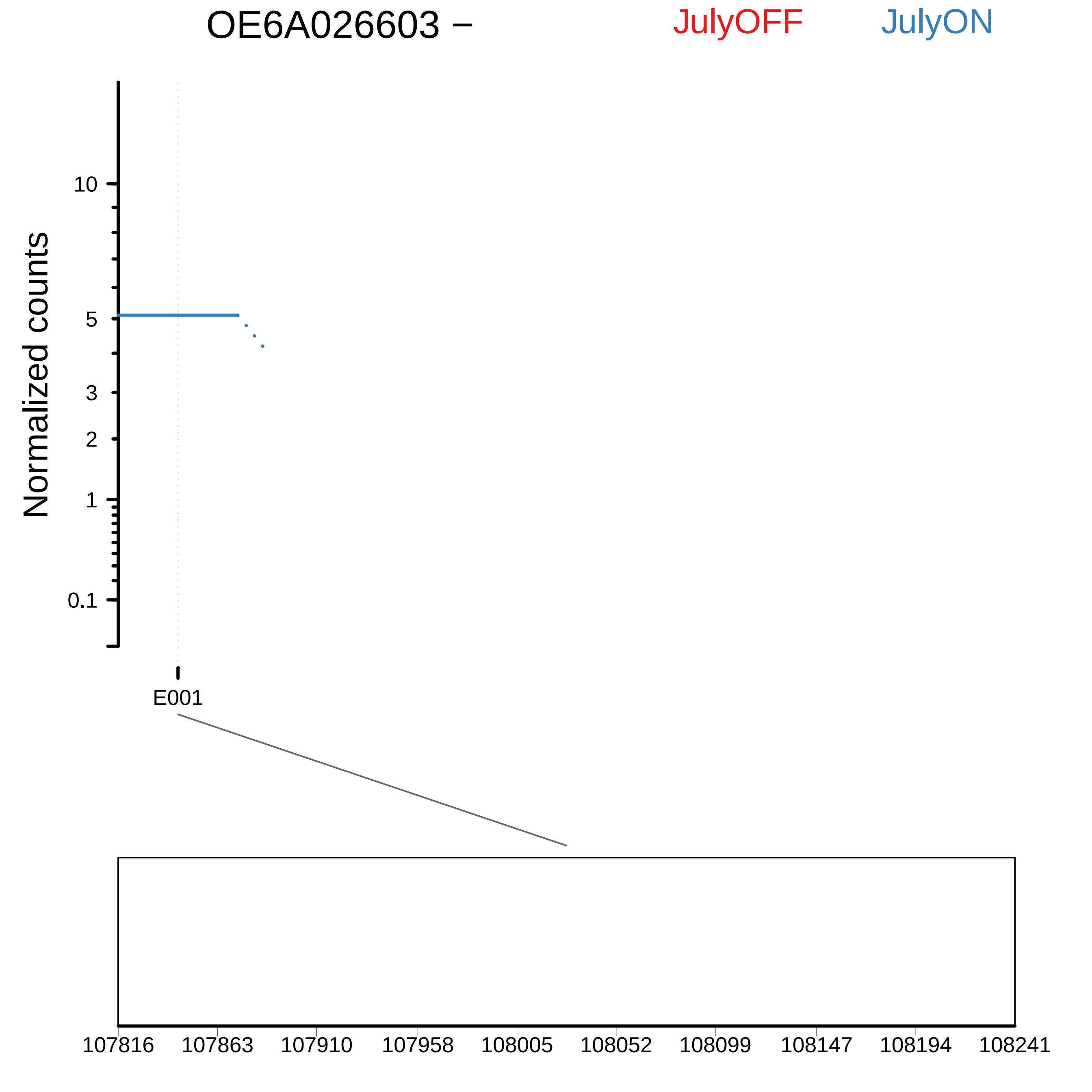
OE6A026603 −
JulyOFF
JulyON
Normalized counts
10
5
3
2
1
0.1
E001
107816
107863
107910
107958
108005
108052
108099
108147
108194
108241

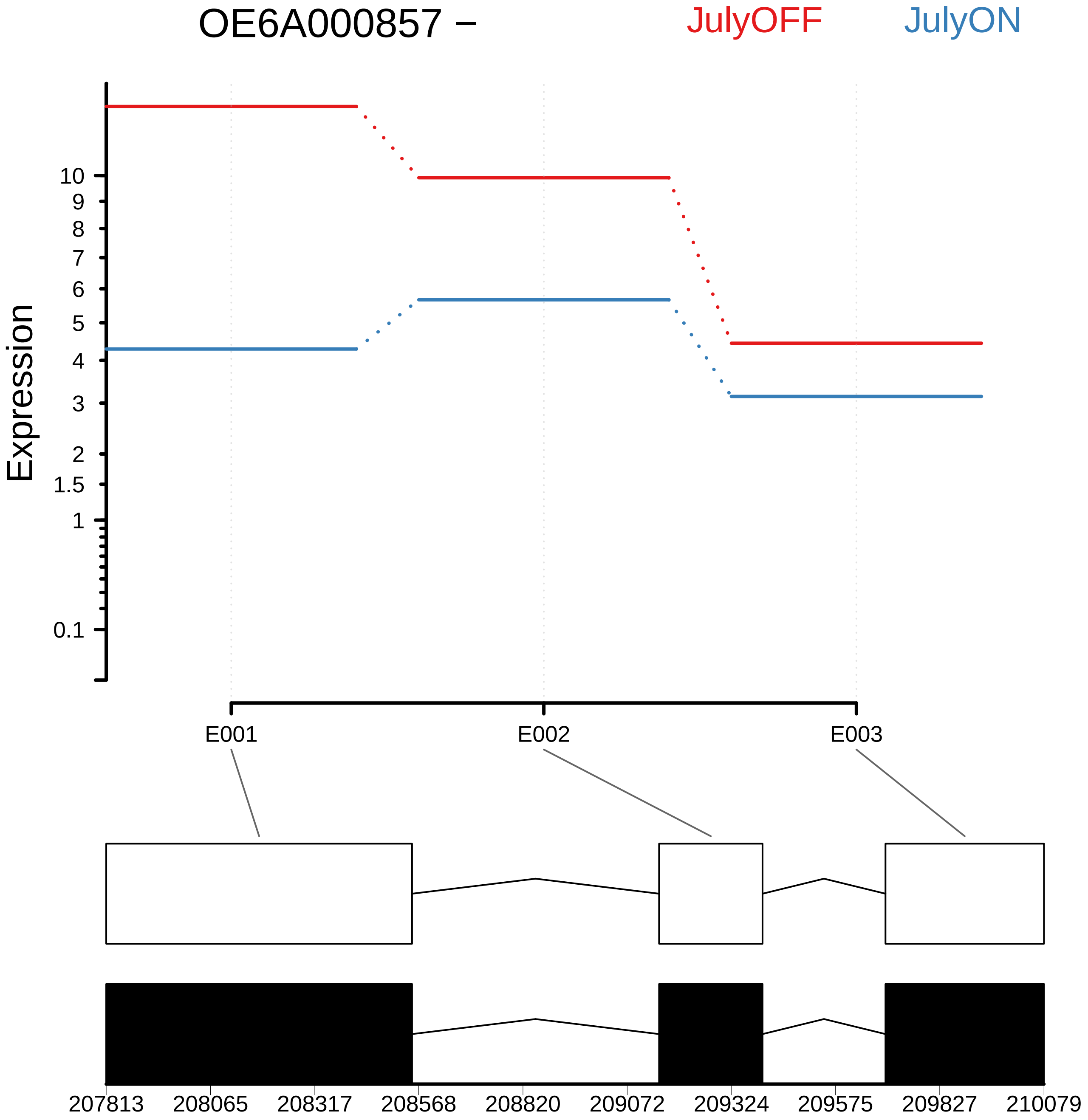

OE6A000857 −
JulyOFF
JulyON
Expression
10
9
8
7
6
5
4
3
2
1.5
1
0.1
E001
E002
E003
207813
208065
208317
208568
208820
209072
209324
209575
209827
210079

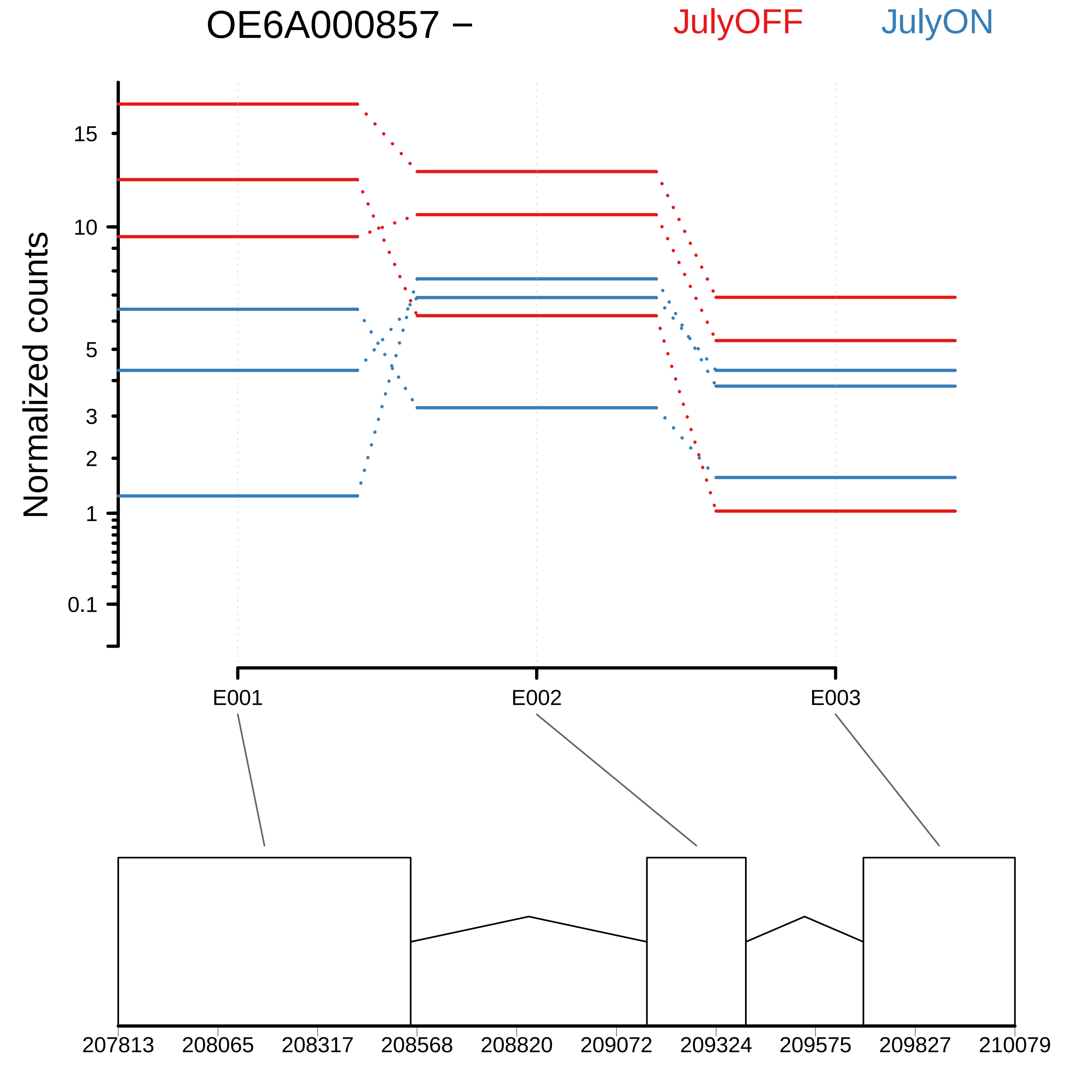
OE6A000857 −
JulyOFF
JulyON
Normalized counts
15
10
5
3
2
1
0.1
E001
E002
E003
207813
208065
208317
208568
208820
209072
209324
209575
209827
210079

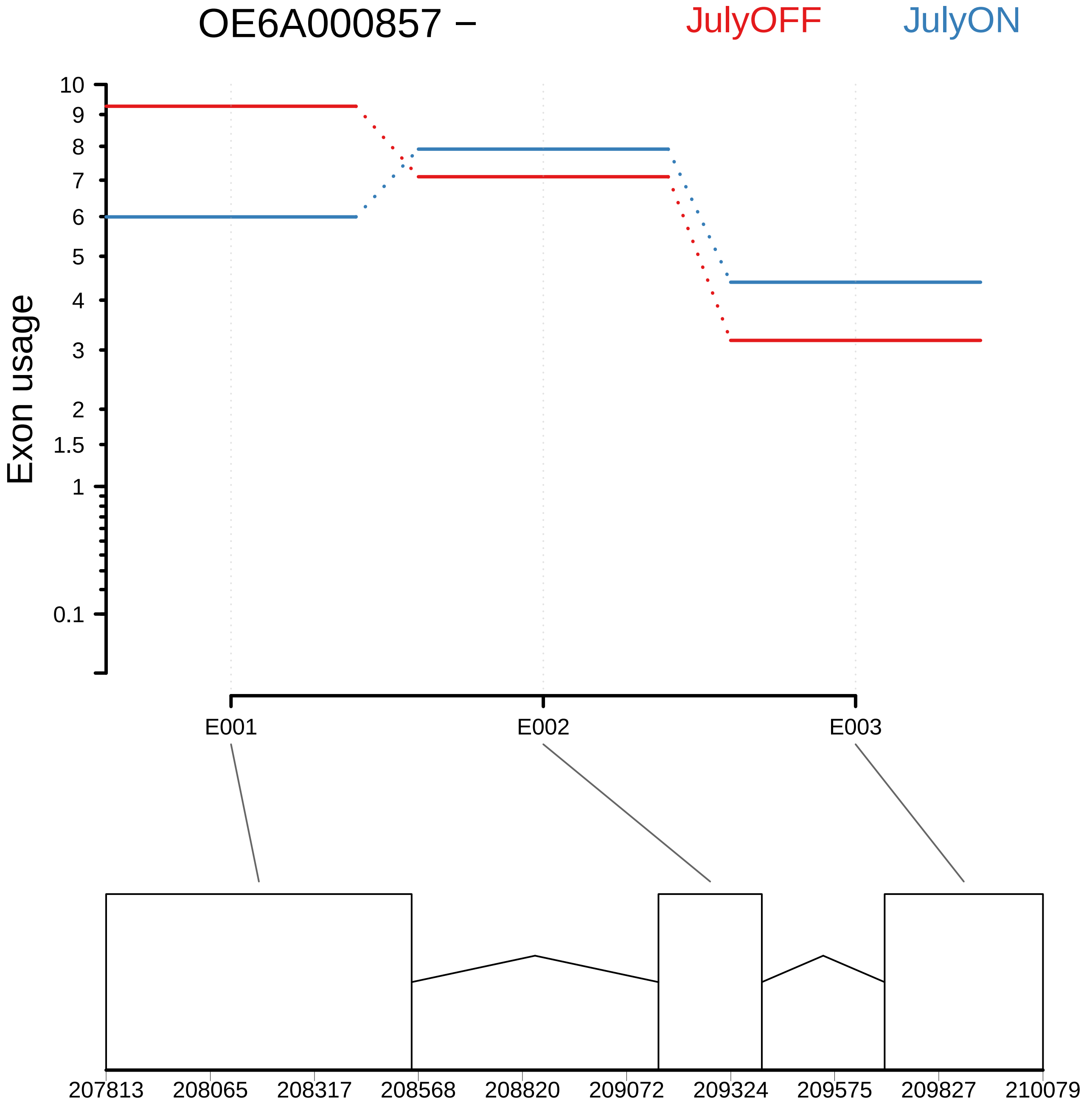
OE6A000857 −
JulyOFF
JulyON
Exon usage
10
9
8
7
6
5
4
3
2
1.5
1
0.1
E001
E002
E003
207813
208065
208317
208568
208820
209072
209324
209575
209827
210079

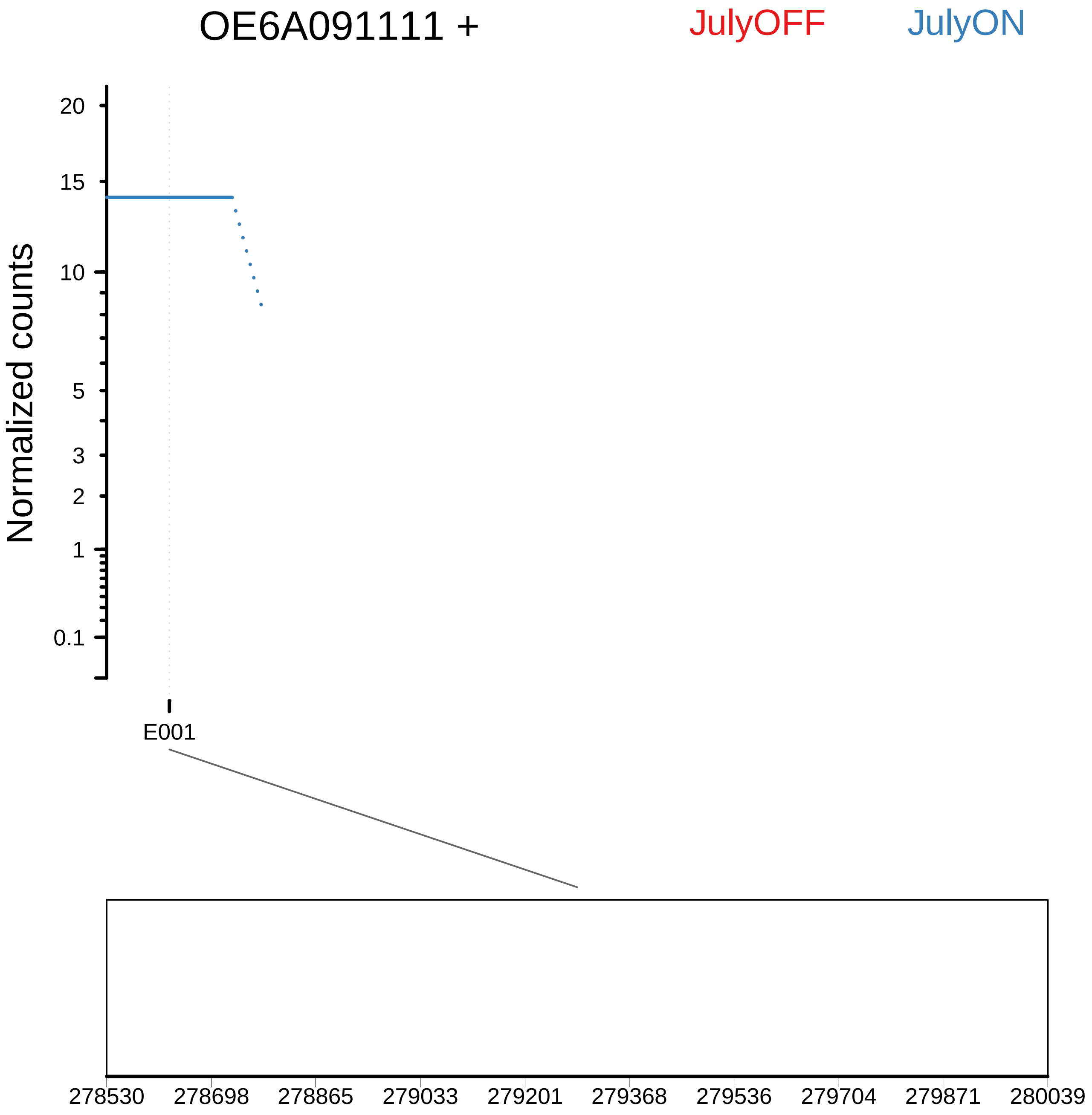

OE6A091111 +
JulyOFF
JulyON
Normalized counts
20
15
10
5
3
2
1
0.1
E001
278530
278698
278865
279033
279201
279368
279536
279704
279871
280039

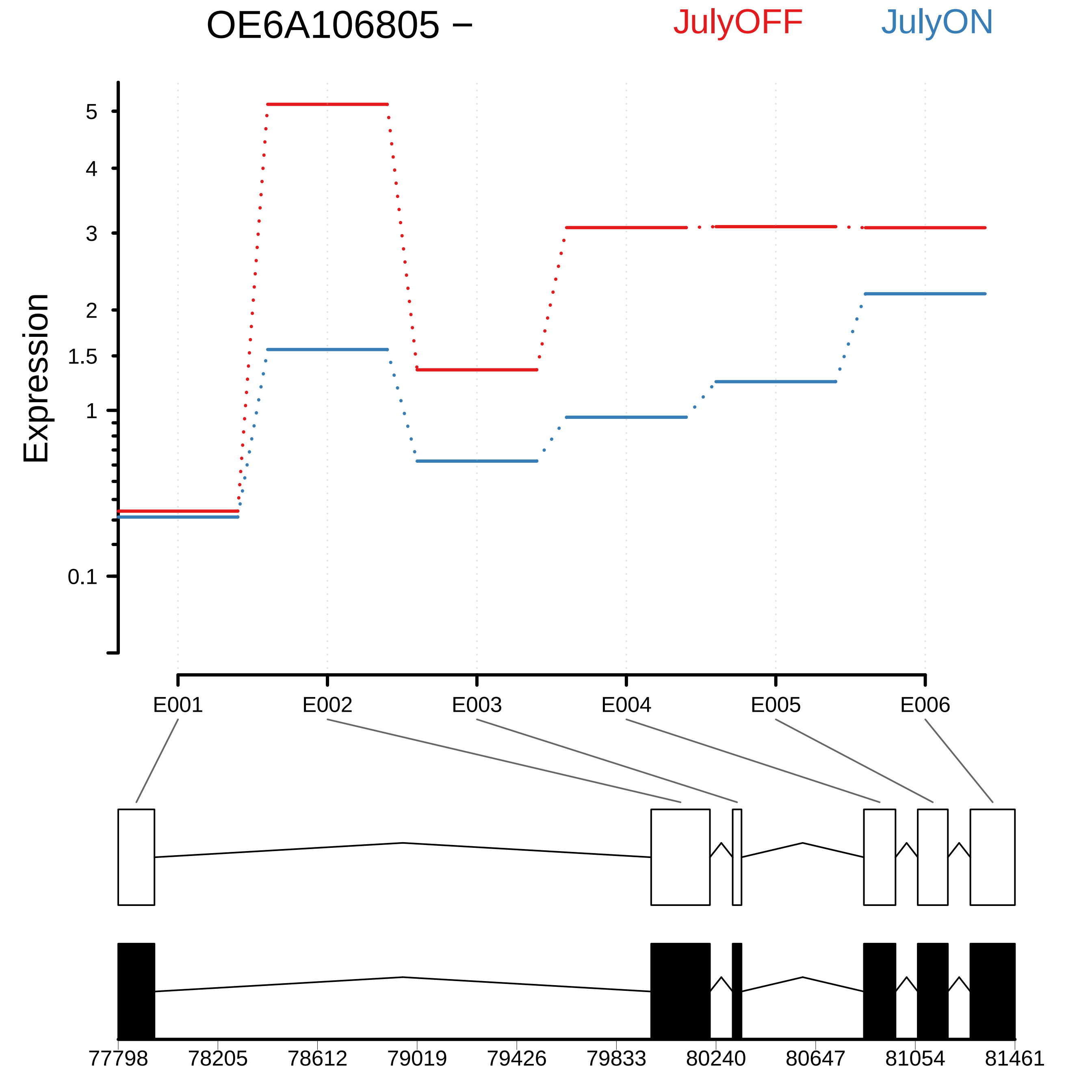

OE6A106805 −
JulyOFF
JulyON
Expression
5
4
3
2
1.5
1
0.1
E001
E002
E003
E004
E005
E006
77798
78205
78612
79019
79426
79833
80240
80647
81054
81461

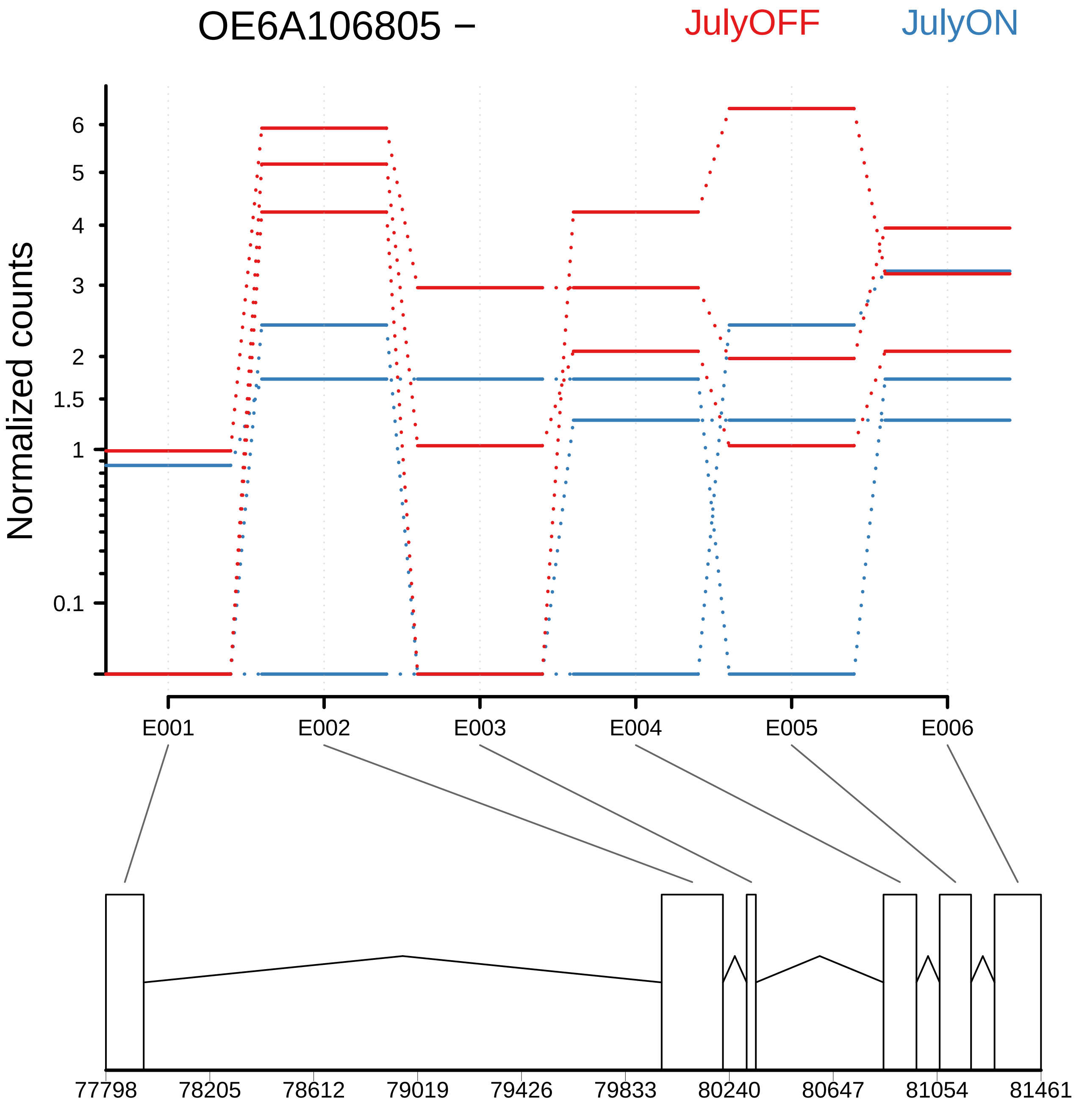
OE6A106805 −
JulyOFF
JulyON
Normalized counts
6
5
4
3
2
1.5
1
0.1
E001
E002
E003
E004
E005
E006
77798
78205
78612
79019
79426
79833
80240
80647
81054
81461

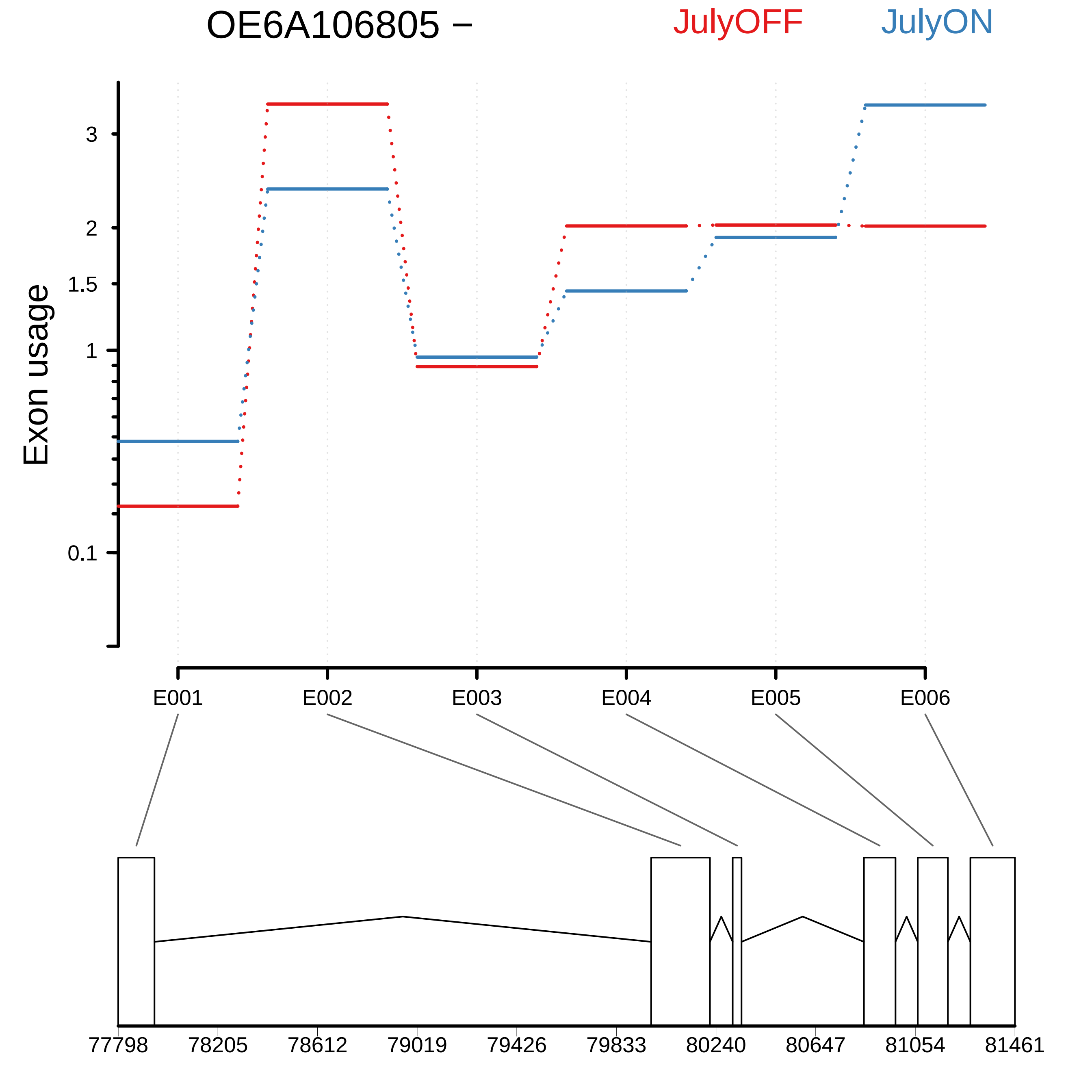

OE6A106805 −
JulyOFF
JulyON
Exon usage
3
2
1.5
1
0.1
E001
E002
E003
E004
E005
E006
77798
78205
78612
79019
79426
79833
80240
80647
81054
81461

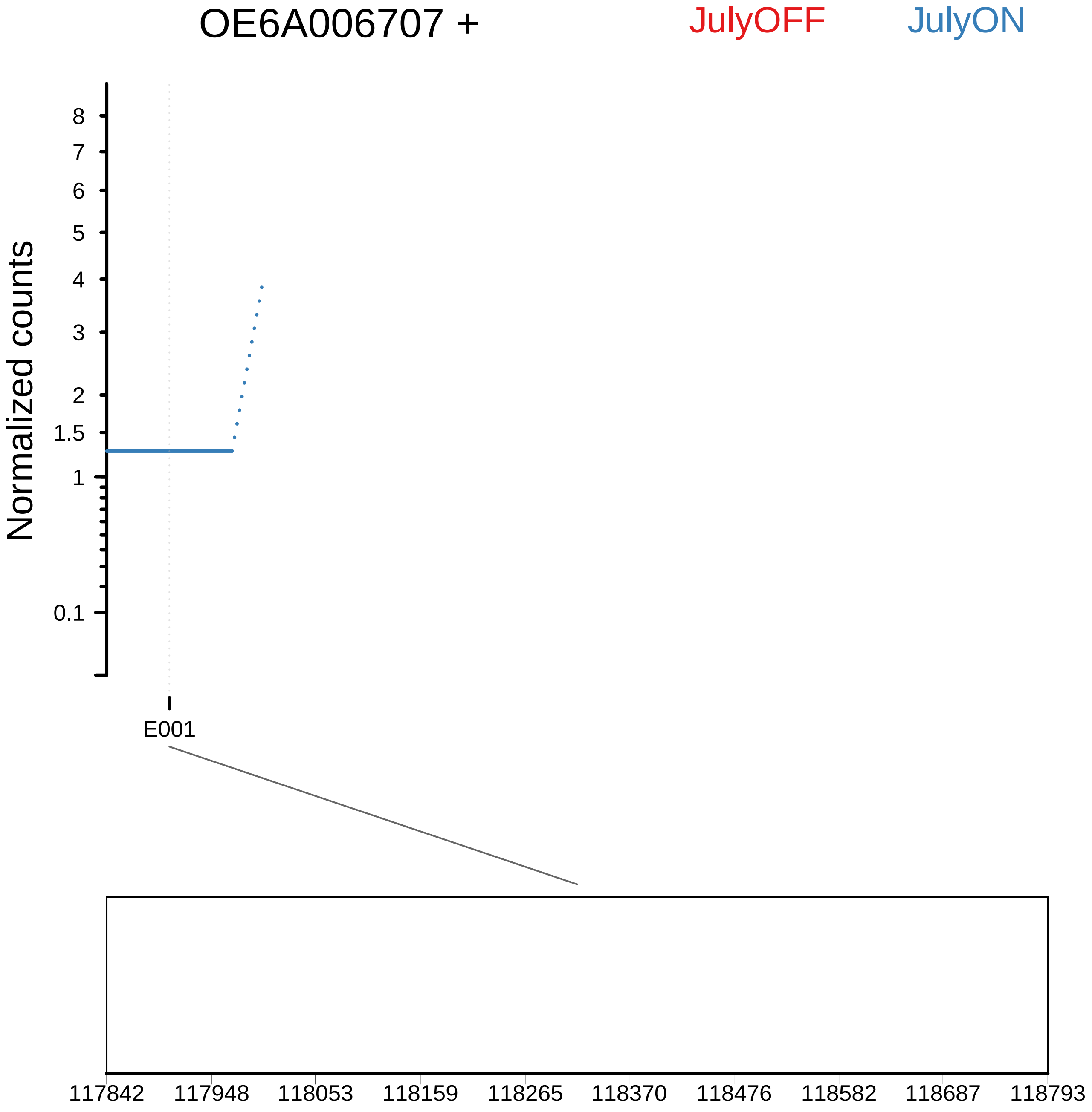
OE6A006707 +
JulyOFF
JulyON
Normalized counts
8
7
6
5
4
3
2
1.5
1
0.1
E001
117842
117948
118053
118159
118265
118370
118476
118582
118687
118793

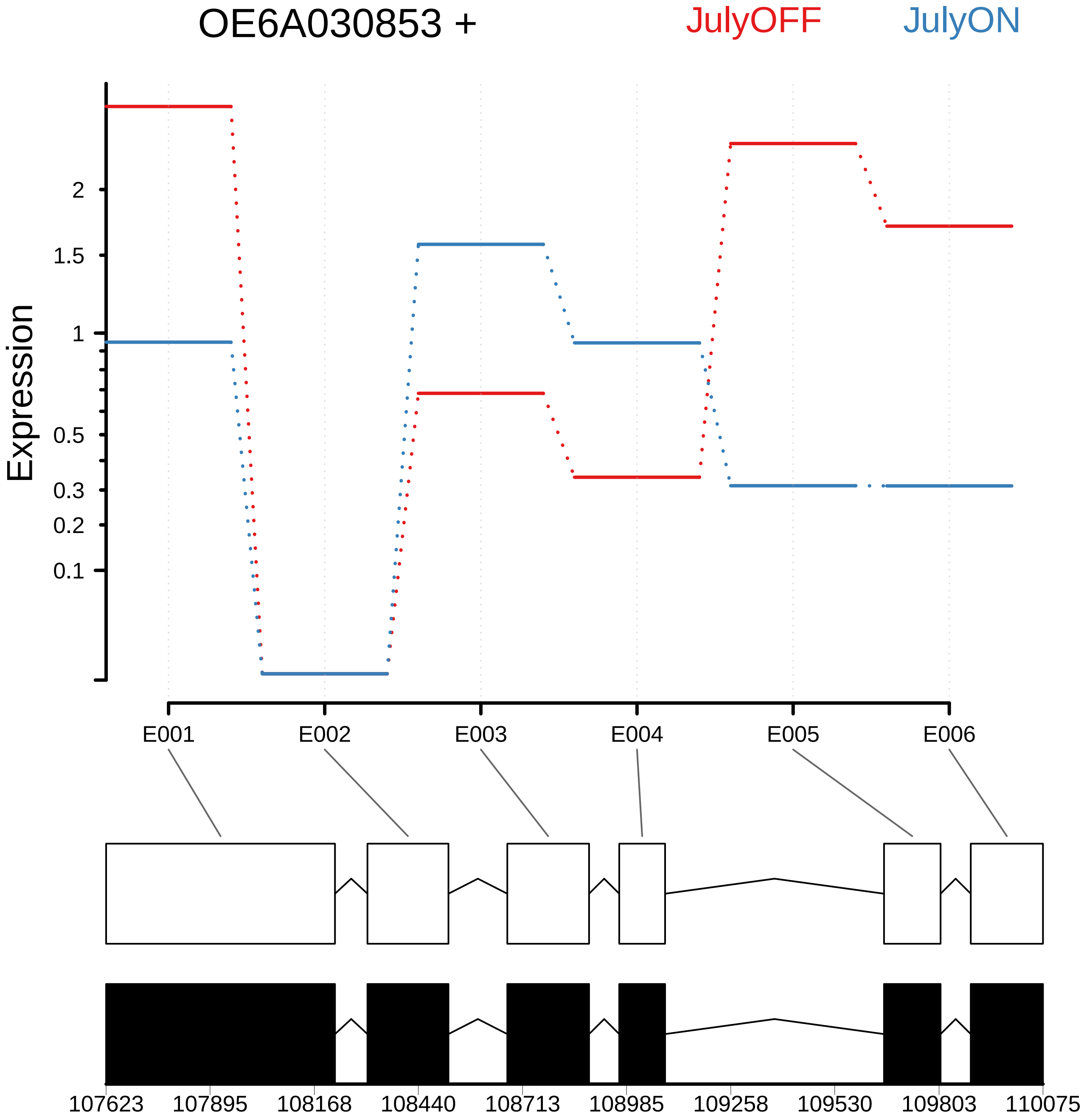
OE6A030853 +
JulyOFF
JulyON
Expression
2
1.5
1
0.5
0.3
0.2
0.1
E001
E002
E003
E004
E005
E006
107623
107895
108168
108440
108713
108985
109258
109530
109803
110075

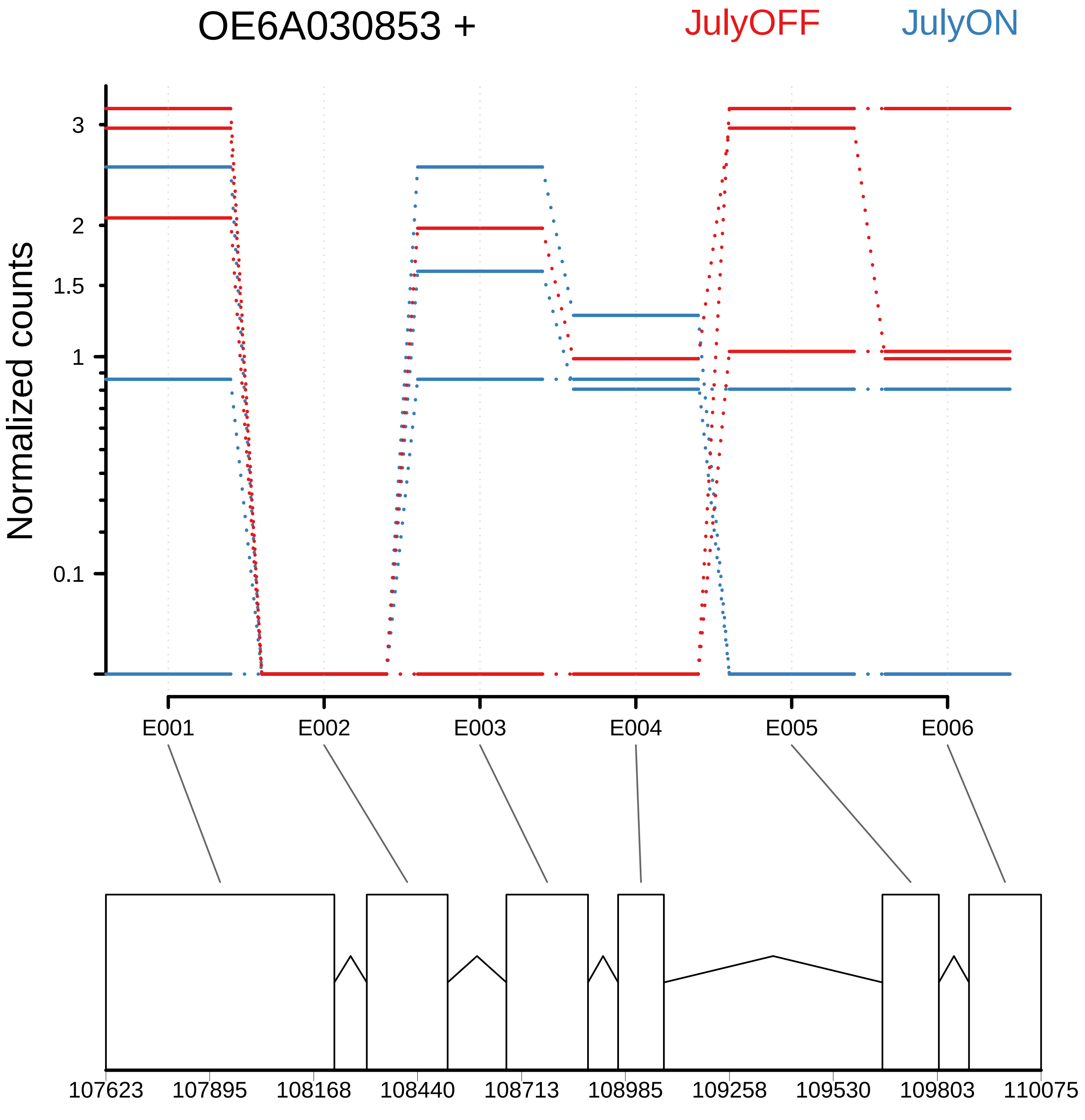
OE6A030853 +
JulyOFF
JulyON
Normalized counts
3
2
1.5
1
0.1
E001
E002
E003
E004
E005
E006
107623
107895
108168
108440
108713
108985
109258
109530
109803
110075

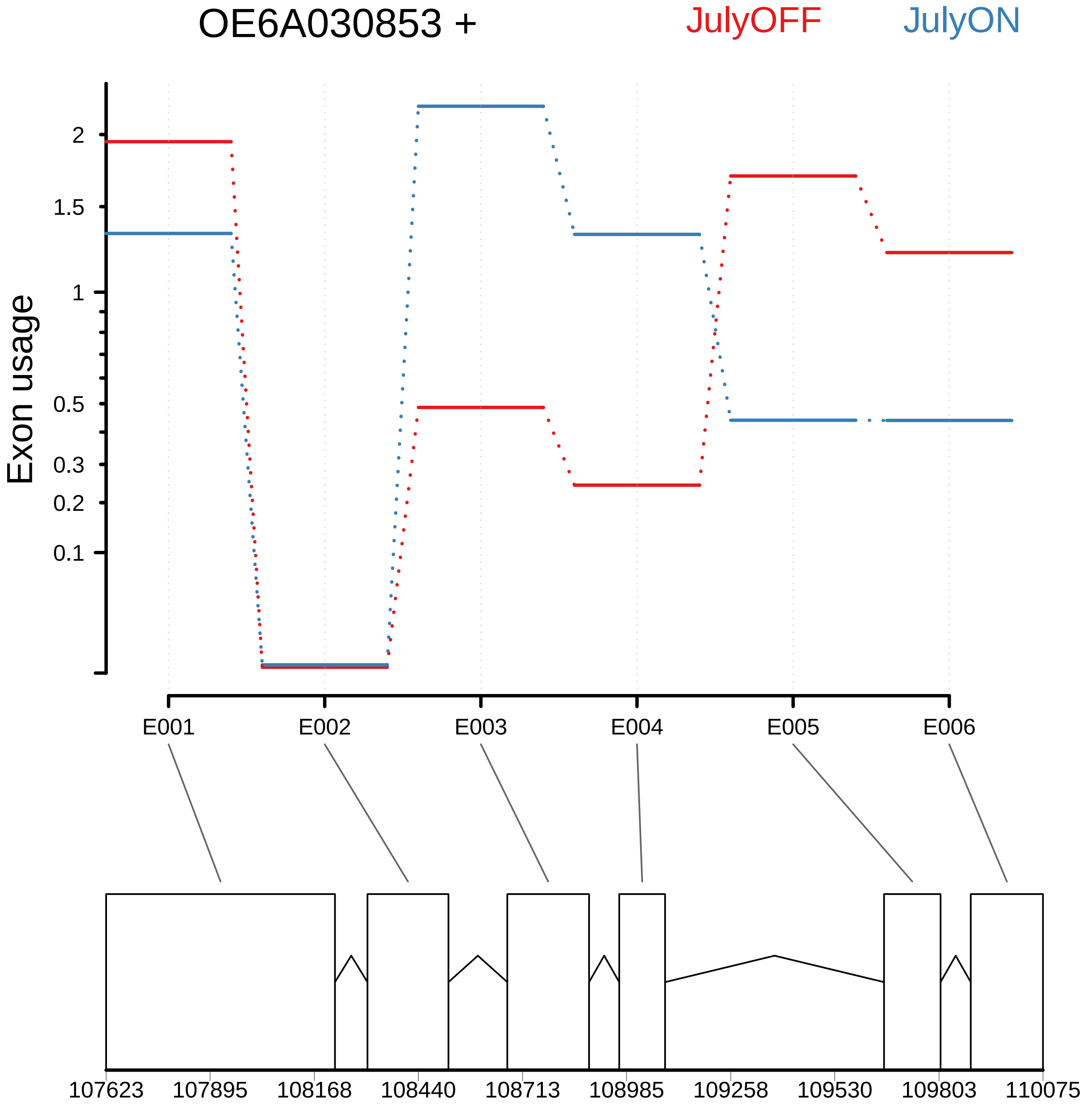

OE6A030853 +
JulyOFF
JulyON
Exon usage
2
1.5
1
0.5
0.3
0.2
0.1
E001
E002
E003
E004
E005
E006
107623
107895
108168
108440
108713
108985
109258
109530
109803
110075

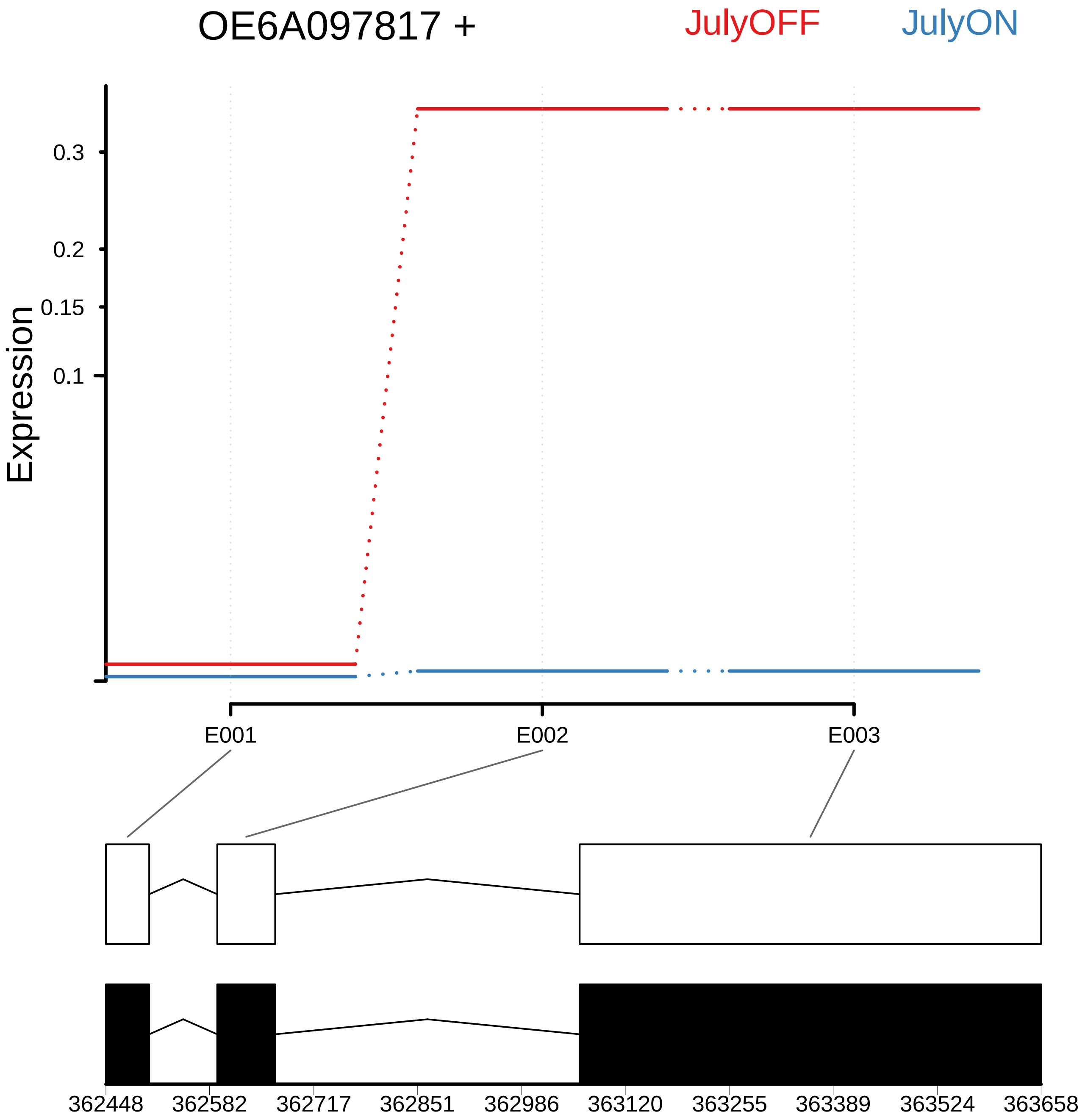
OE6A097817 +
JulyOFF
JulyON
Expression
0.3
0.2
0.15
0.1
E001
E002
E003
362448
362582
362717
362851
362986
363120
363255
363389
363524
363658

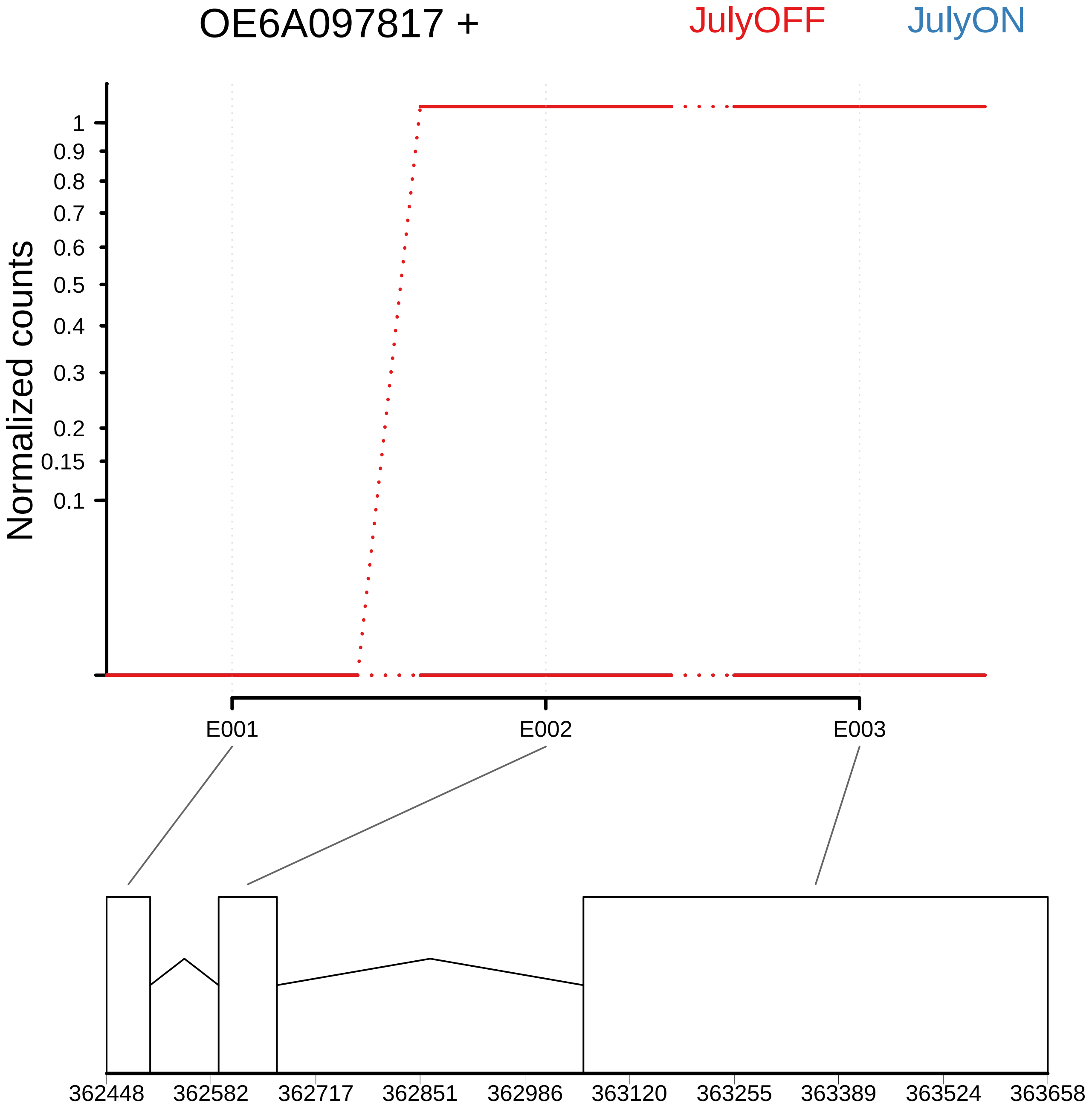

OE6A097817 +
JulyOFF
JulyON
Normalized counts
1
0.9
0.8
0.7
0.6
0.5
0.4
0.3
0.2
0.15
0.1
E001
E002
E003
362448
362582
362717
362851
362986
363120
363255
363389
363524
363658

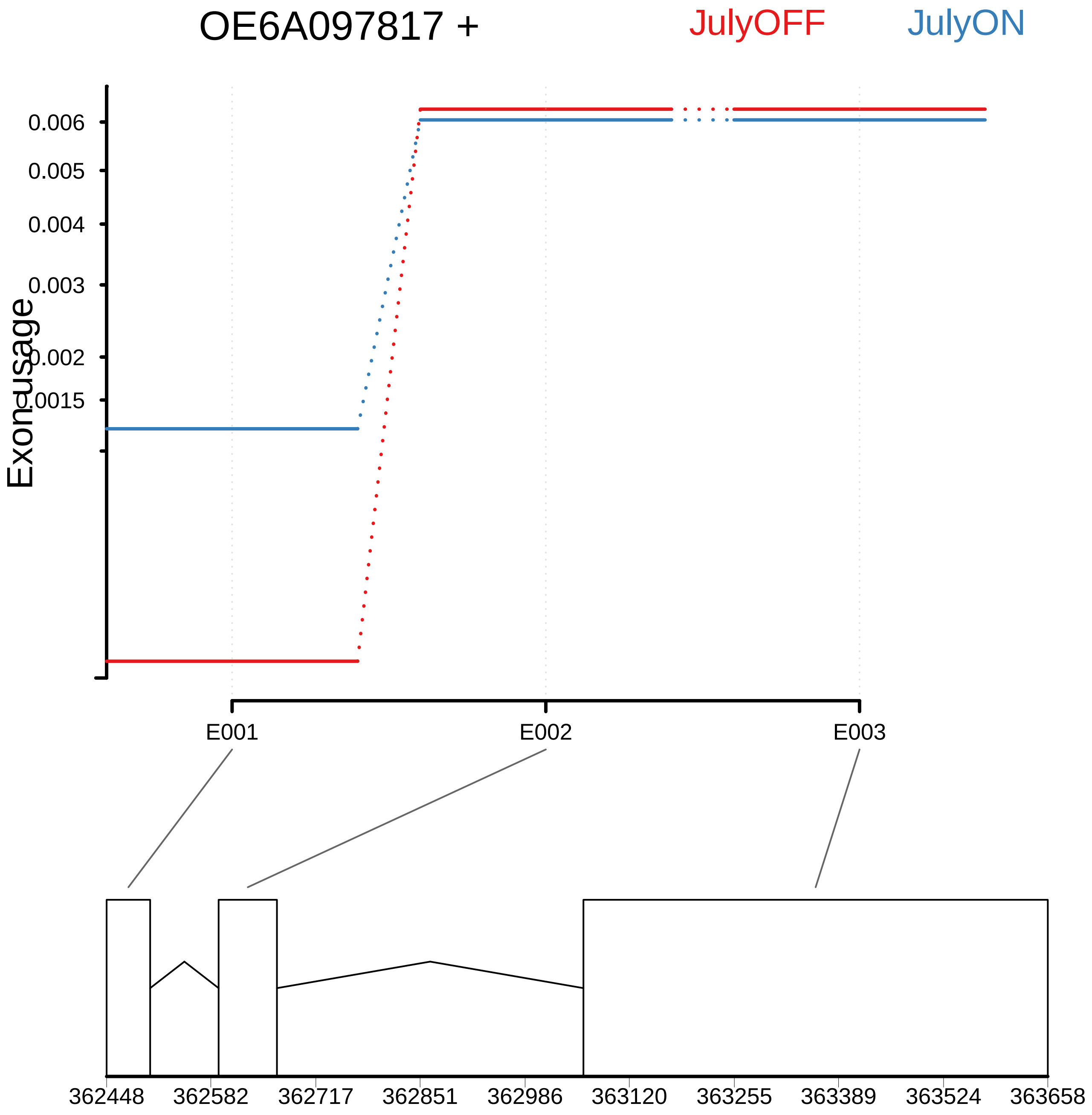
OE6A097817 +
JulyOFF
JulyON
Exon usage
0.006
0.005
0.004
0.003
0.002
0.0015
E001
E002
E003
362448
362582
362717
362851
362986
363120
363255
363389
363524
363658

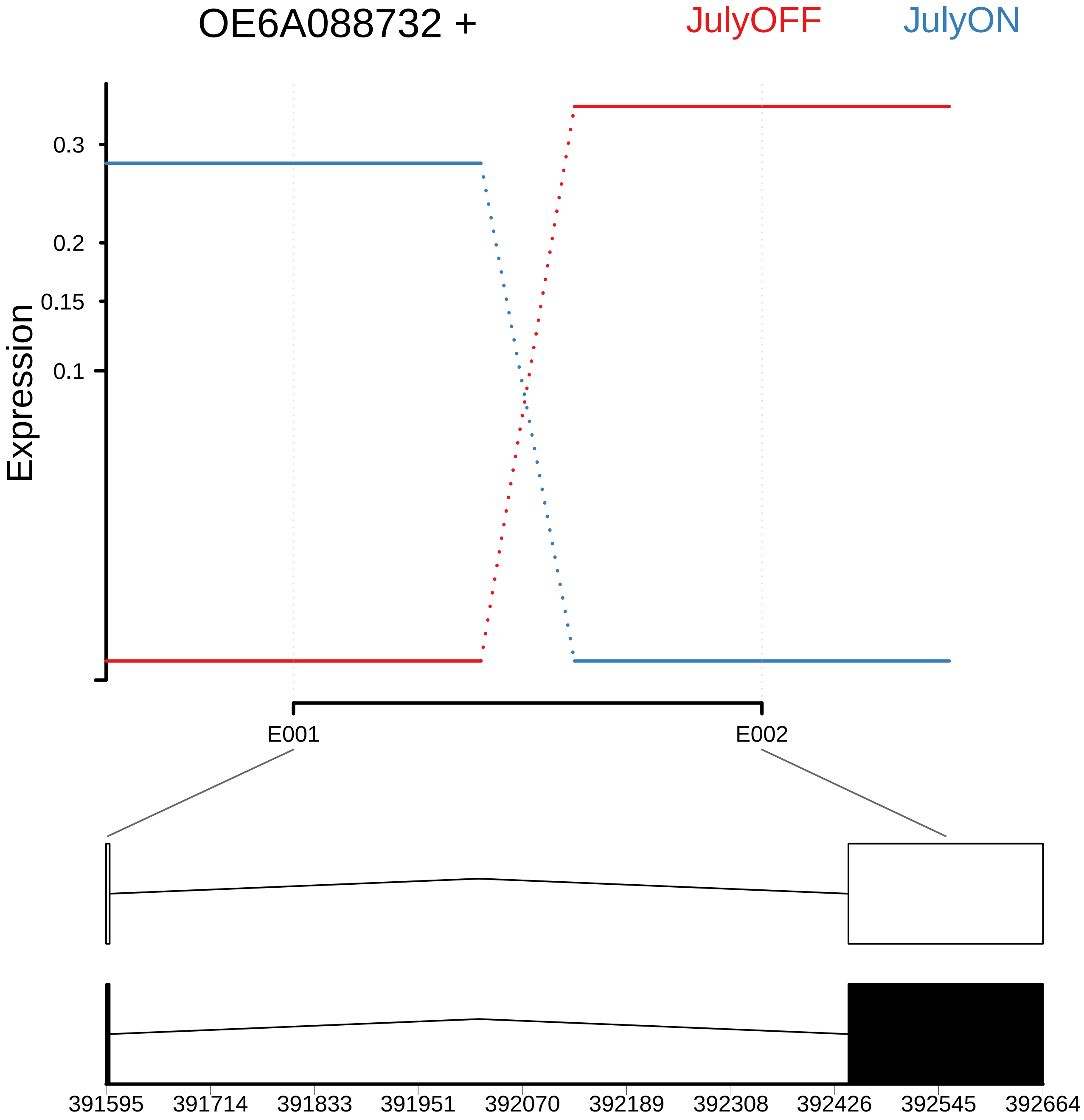

OE6A088732 +
JulyOFF
JulyON
Expression
0.3
0.2
0.15
0.1
E001
E002
391595
391714
391833
391951
392070
392189
392308
392426
392545
392664

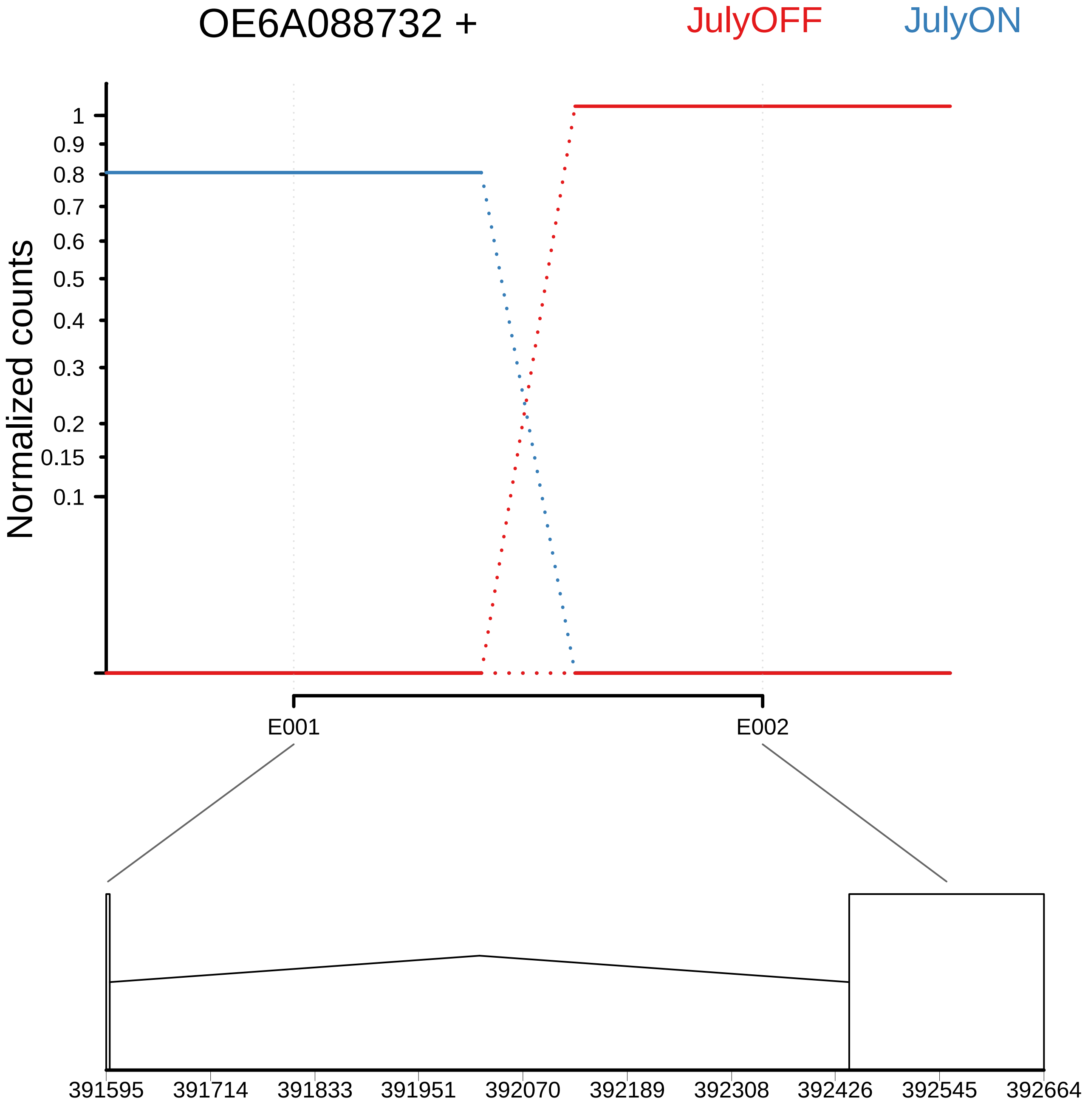

OE6A088732 +
JulyOFF
JulyON
Normalized counts
1
0.9
0.8
0.7
0.6
0.5
0.4
0.3
0.2
0.15
0.1
E001
E002
391595
391714
391833
391951
392070
392189
392308
392426
392545
392664

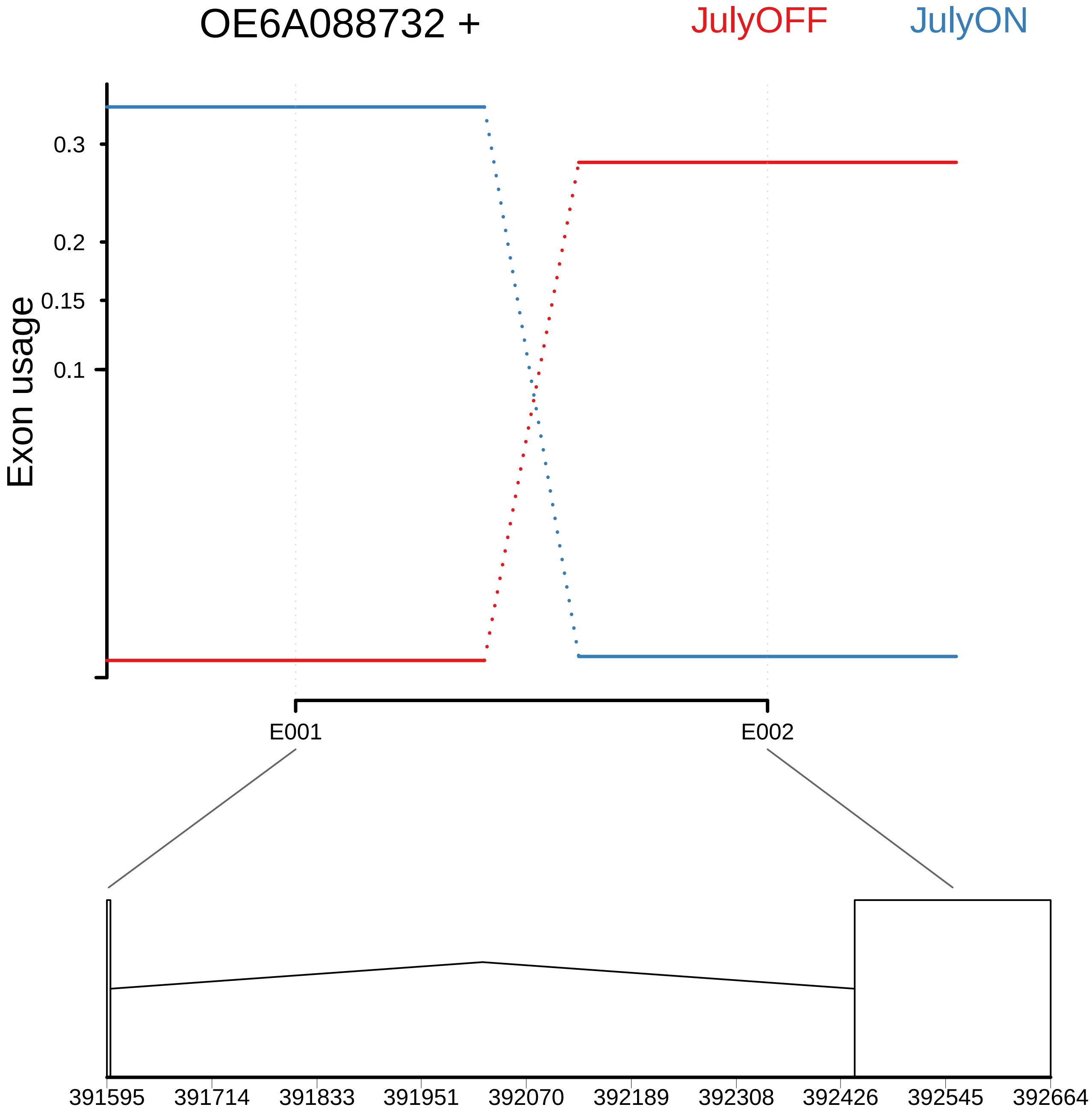

OE6A088732 +
JulyOFF
JulyON
Exon usage
0.3
0.2
0.15
0.1
E001
E002
391595
391714
391833
391951
392070
392189
392308
392426
392545
392664

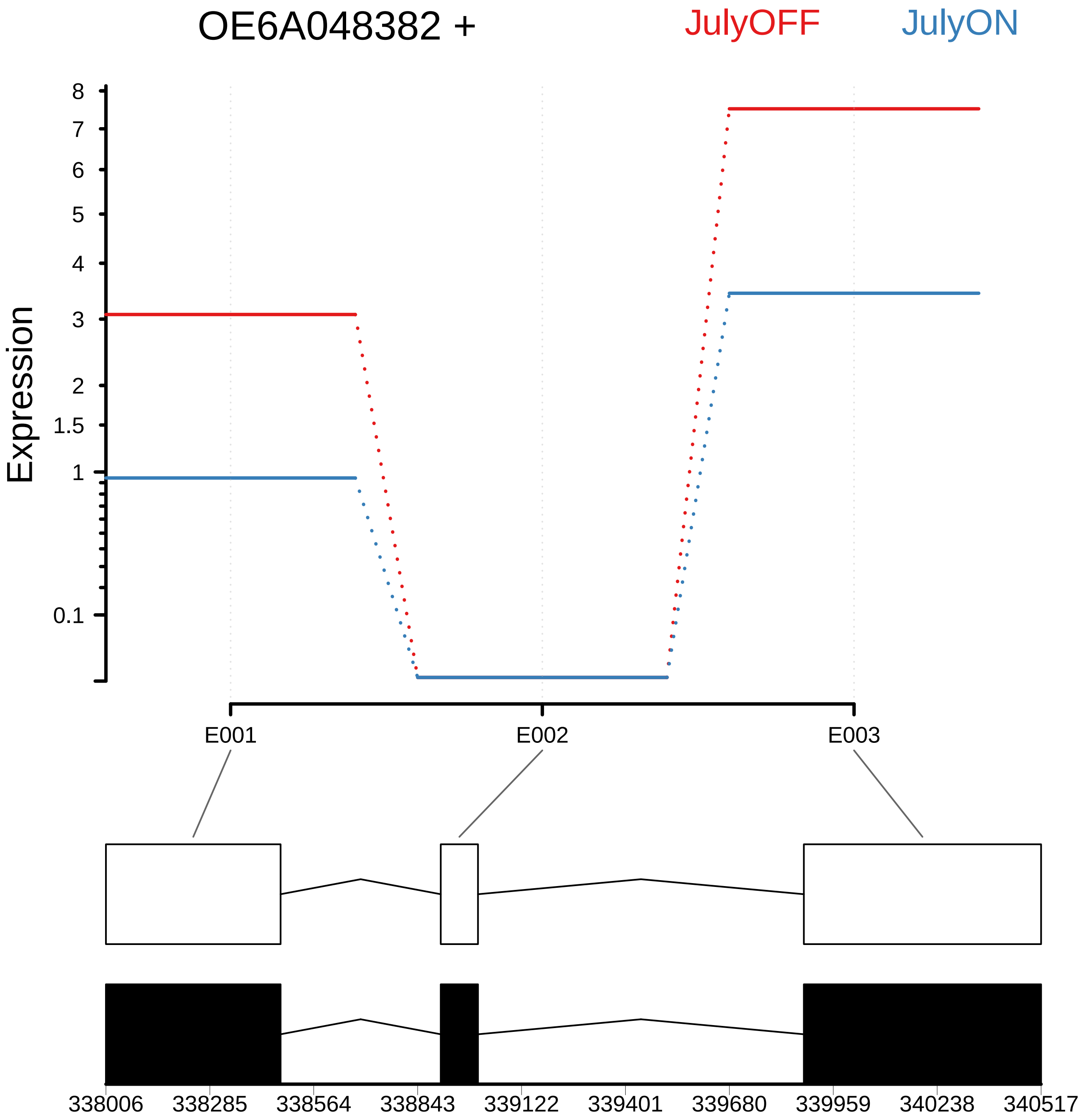

OE6A048382 +
JulyOFF
JulyON
Expression
8
7
6
5
4
3
2
1.5
1
0.1
E001
E002
E003
338006
338285
338564
338843
339122
339401
339680
339959
340238
340517

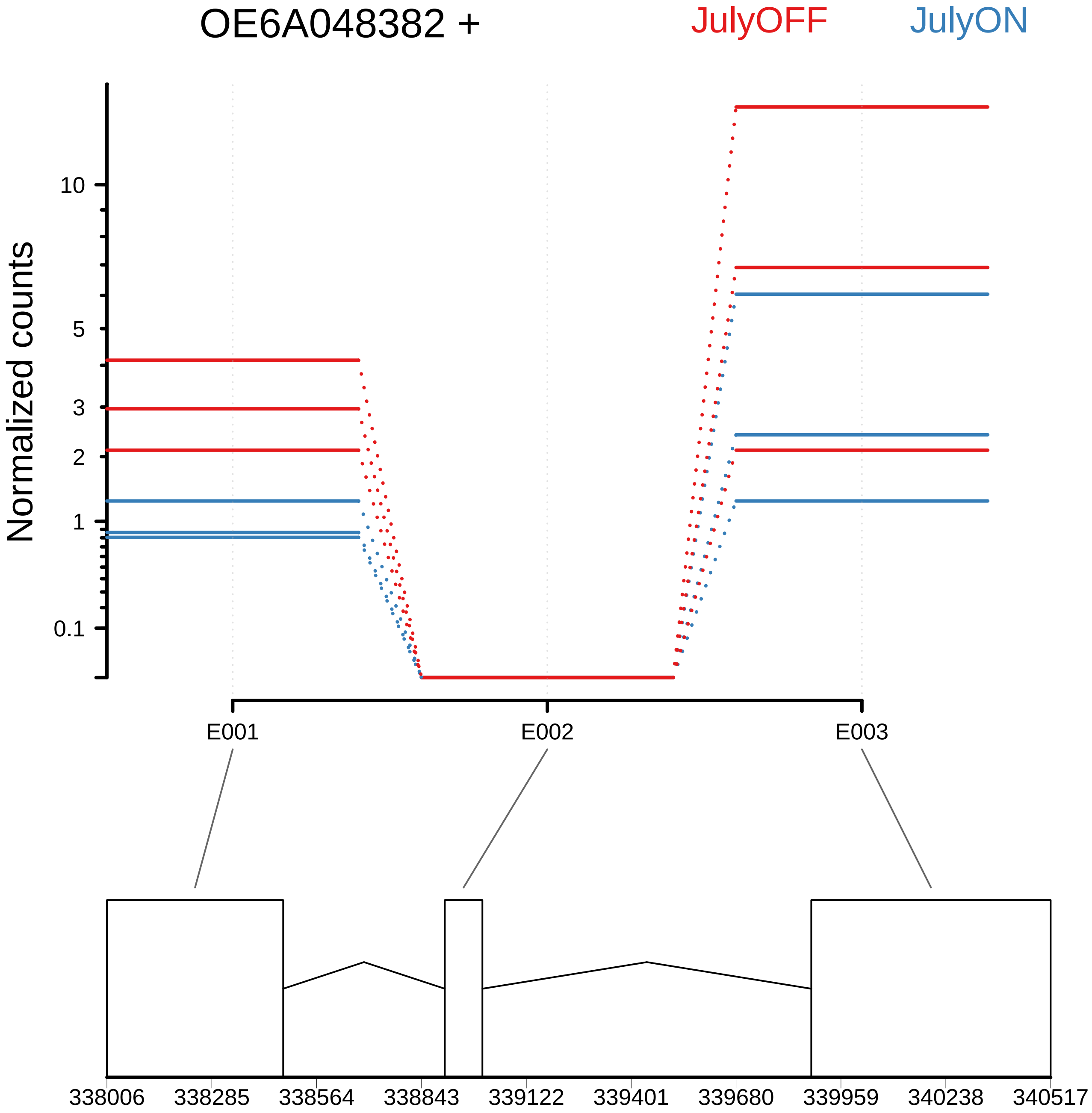
OE6A048382 +
JulyOFF
JulyON
Normalized counts
10
5
3
2
1
0.1
E001
E002
E003
338006
338285
338564
338843
339122
339401
339680
339959
340238
340517

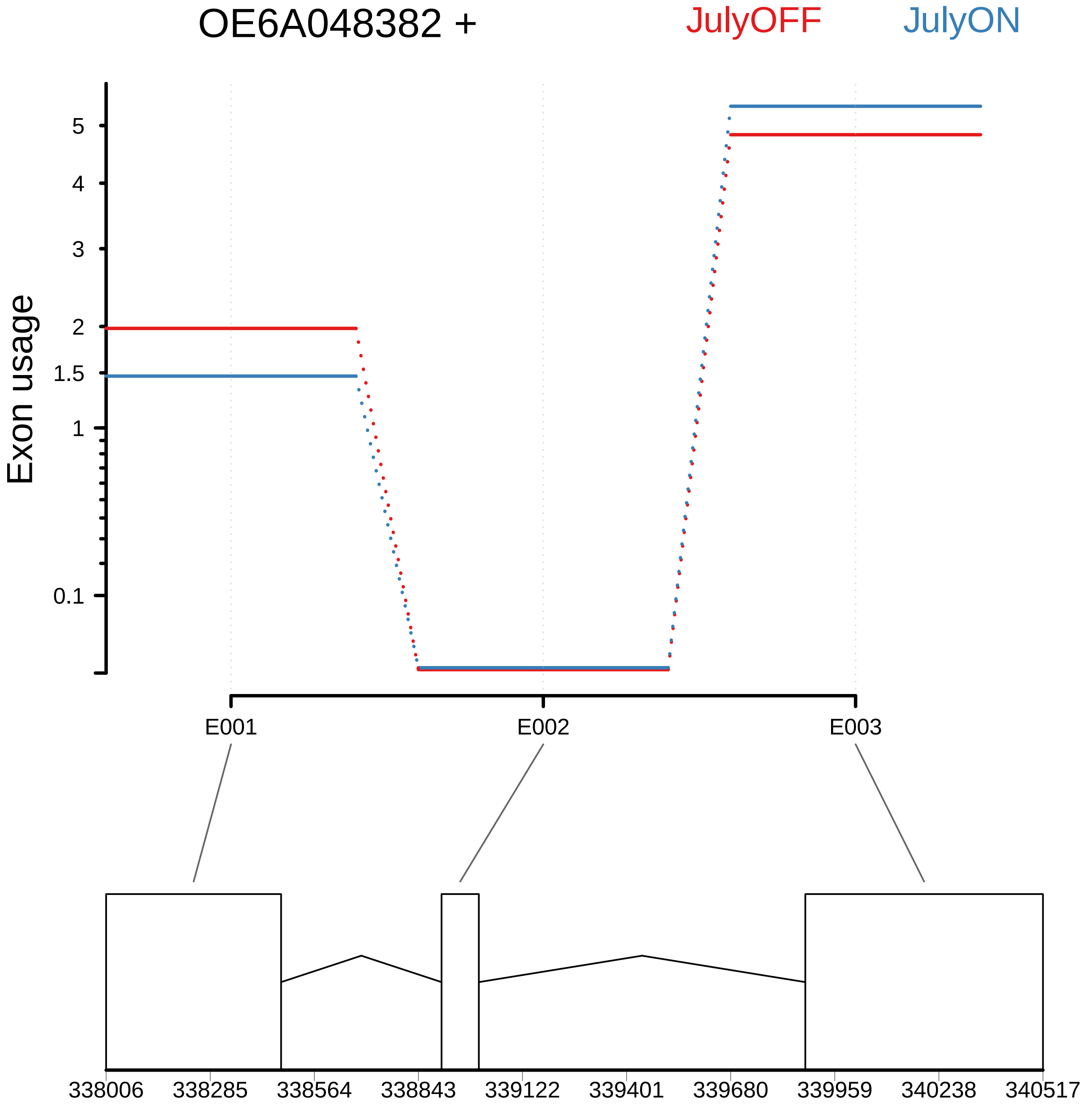

OE6A048382 +
JulyOFF
JulyON
Exon usage
5
4
3
2
1.5
1
0.1
E001
E002
E003
338006
338285
338564
338843
339122
339401
339680
339959
340238
340517

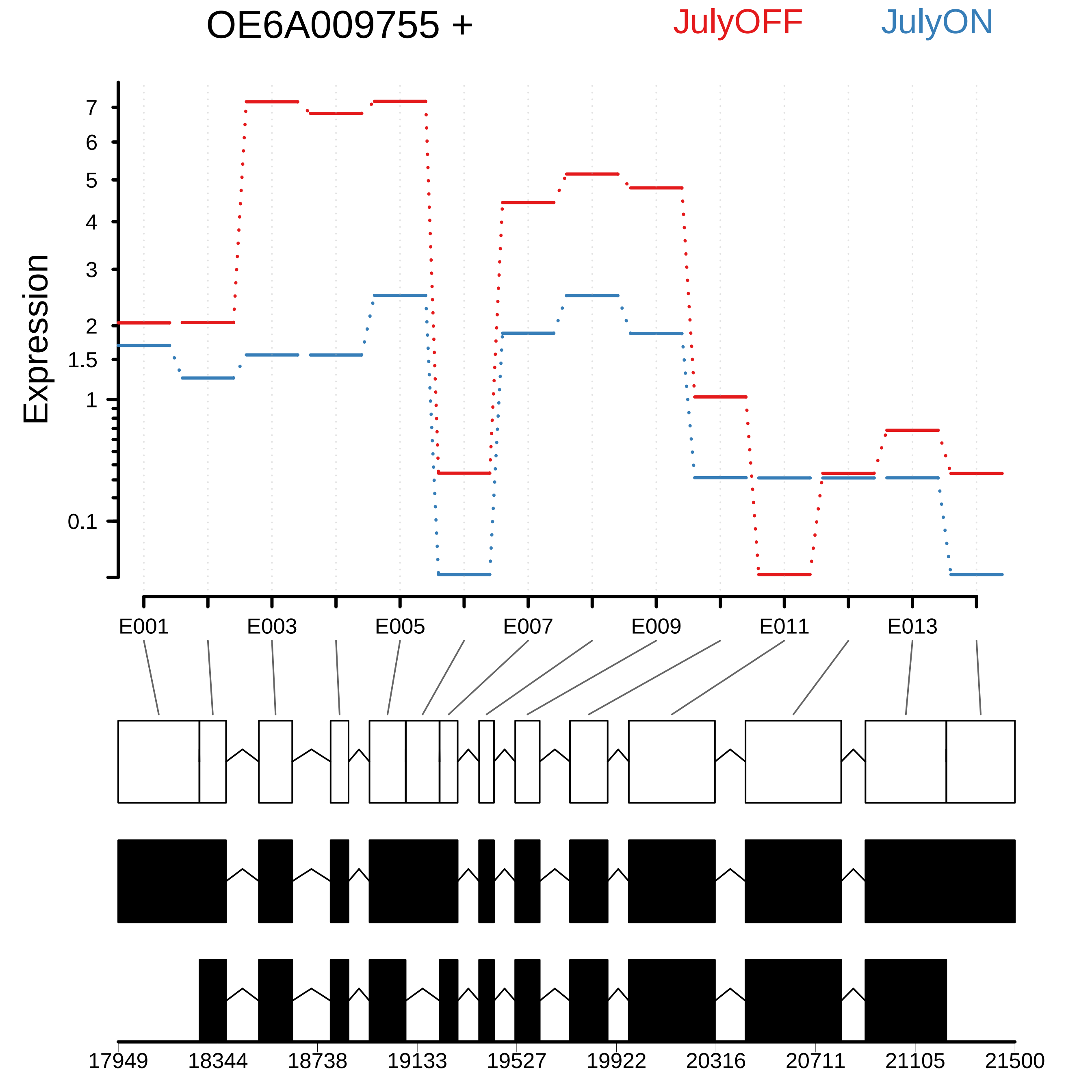

OE6A009755 +
JulyOFF
JulyON
Expression
7
6
5
4
3
2
1.5
1
0.1
E001
E003
E005
E007
E009
E011
E013
17949
18344
18738
19133
19527
19922
20316
20711
21105
21500

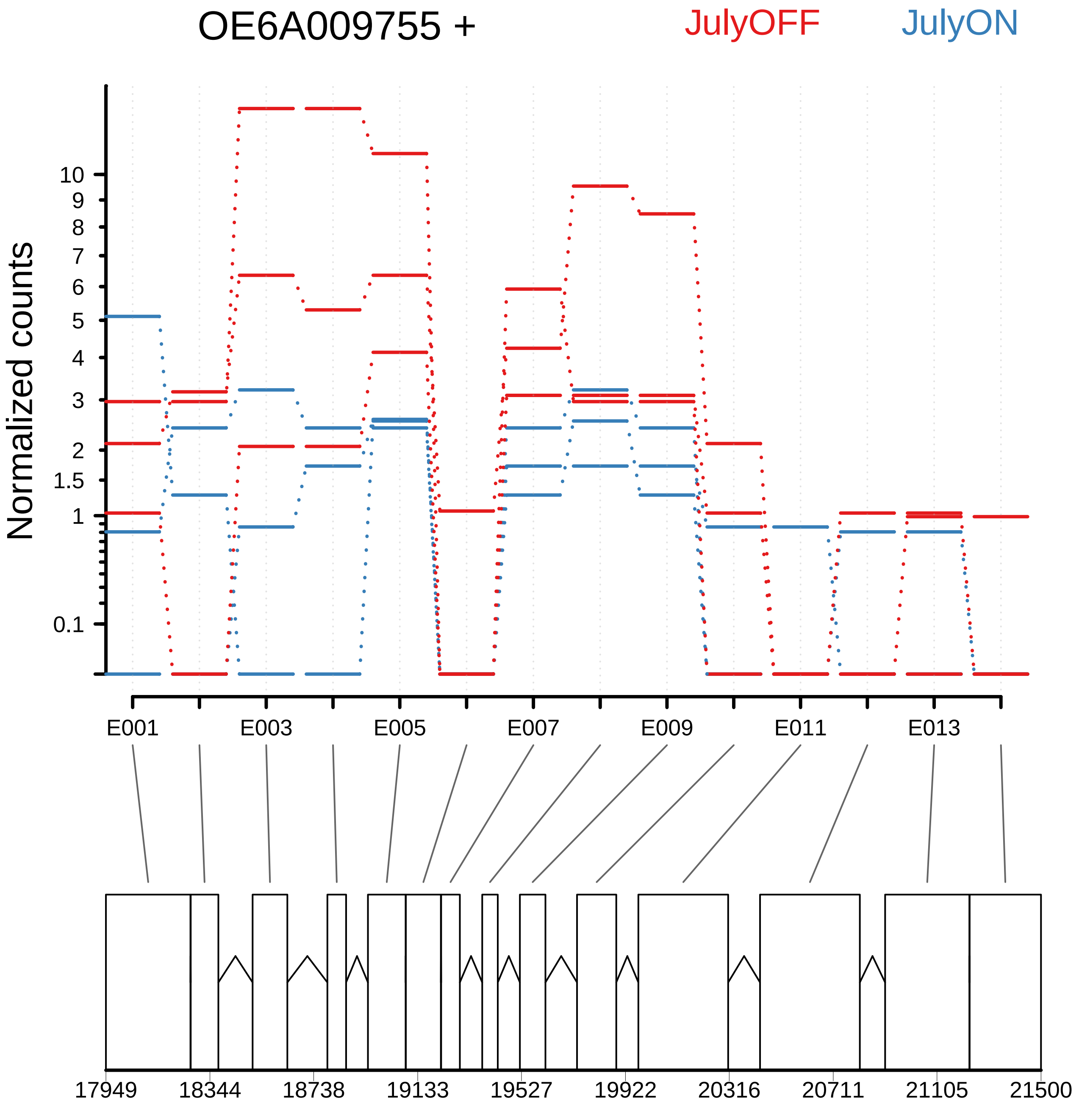
OE6A009755 +
JulyOFF
JulyON
Normalized counts
10
9
8
7
6
5
4
3
2
1.5
1
0.1
E001
E003
E005
E007
E009
E011
E013
17949
18344
18738
19133
19527
19922
20316
20711
21105
21500

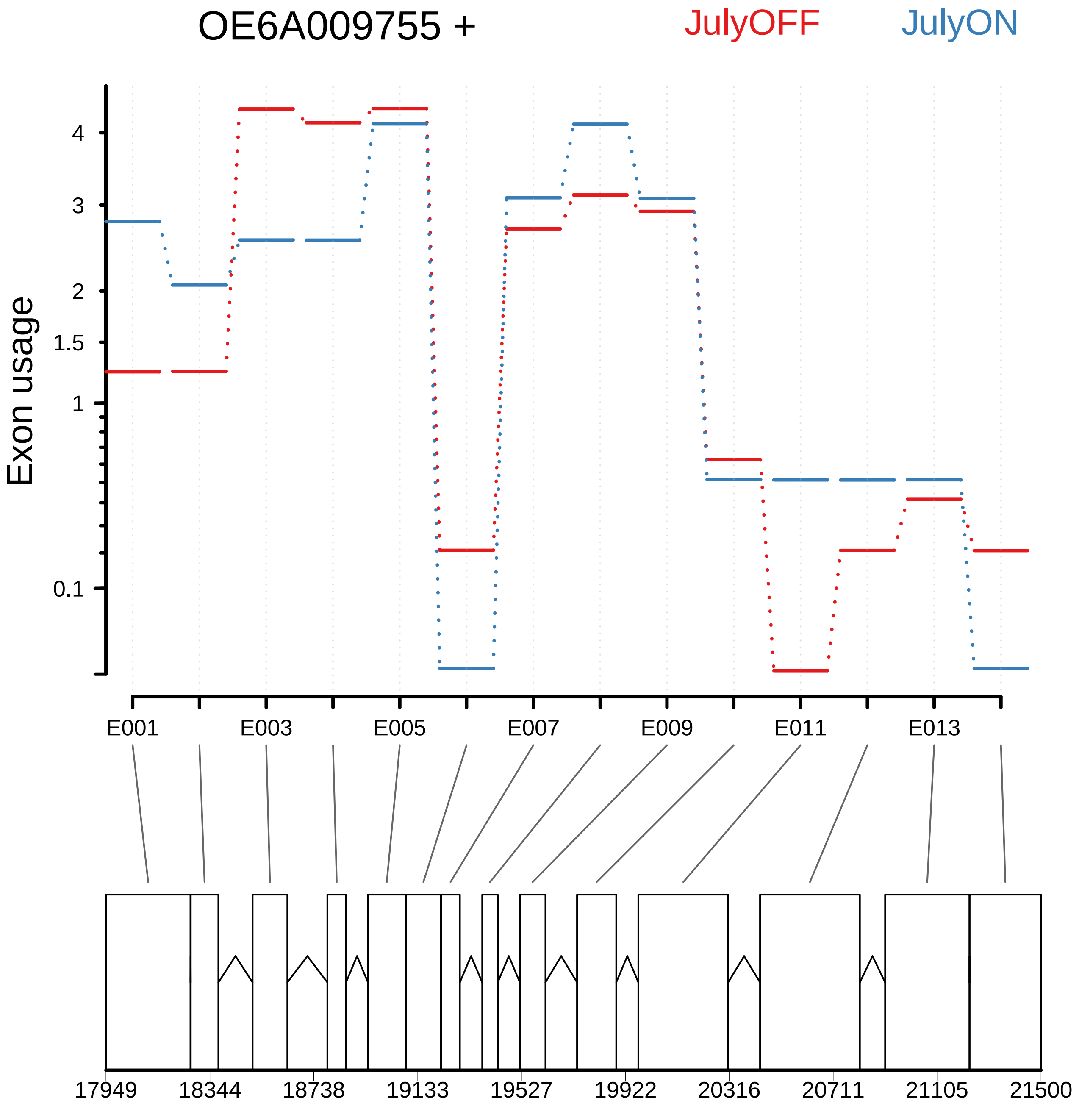
OE6A009755 +
JulyOFF
JulyON
Exon usage
4
3
2
1.5
1
0.1
E001
E003
E005
E007
E009
E011
E013
17949
18344
18738
19133
19527
19922
20316
20711
21105
21500

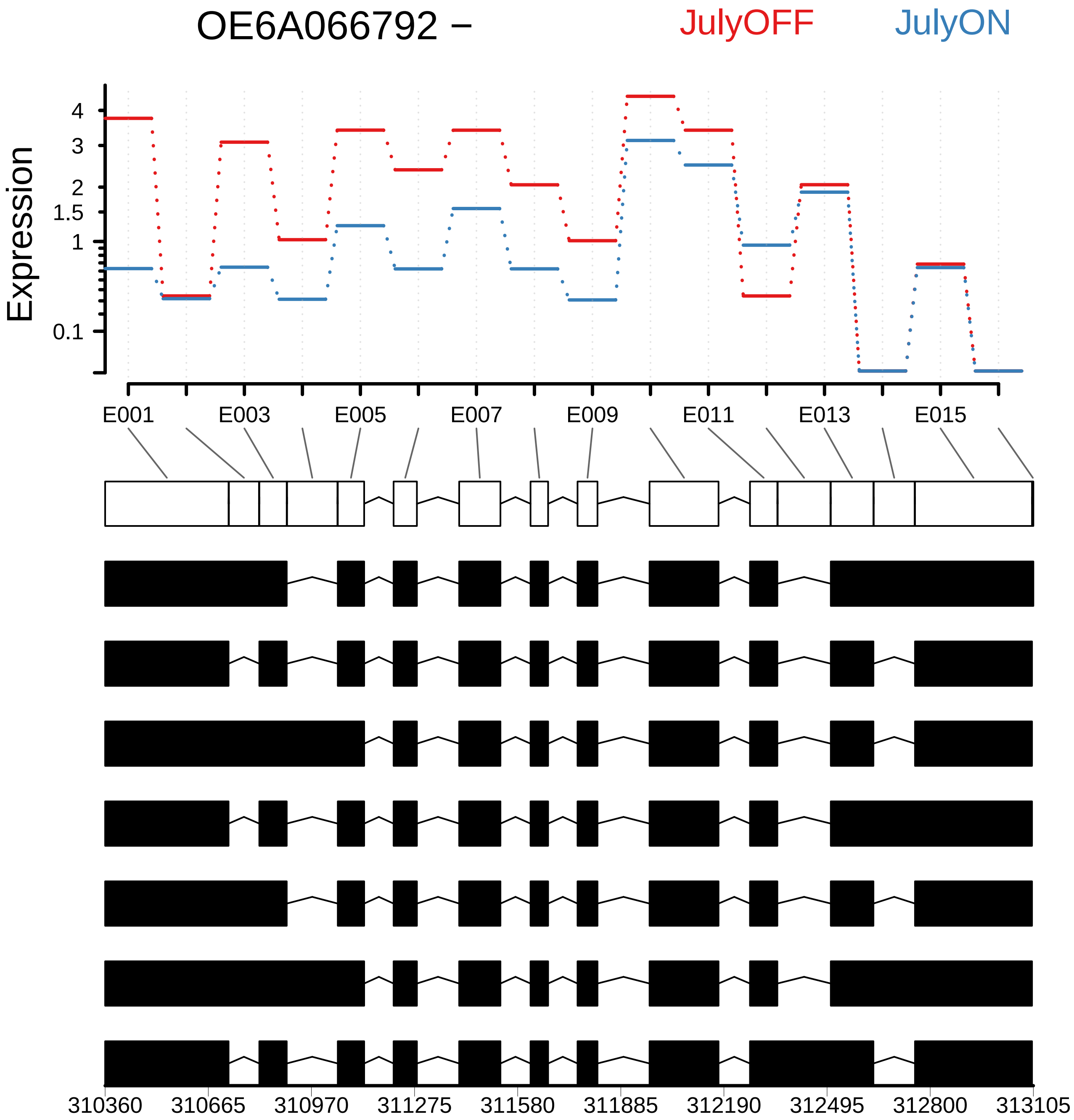

OE6A066792 −
JulyOFF
JulyON
Expression
4
3
2
1.5
1
0.1
E001
E003
E005
E007
E009
E011
E013
E015
310360
310665
310970
311275
311580
311885
312190
312495
312800
313105

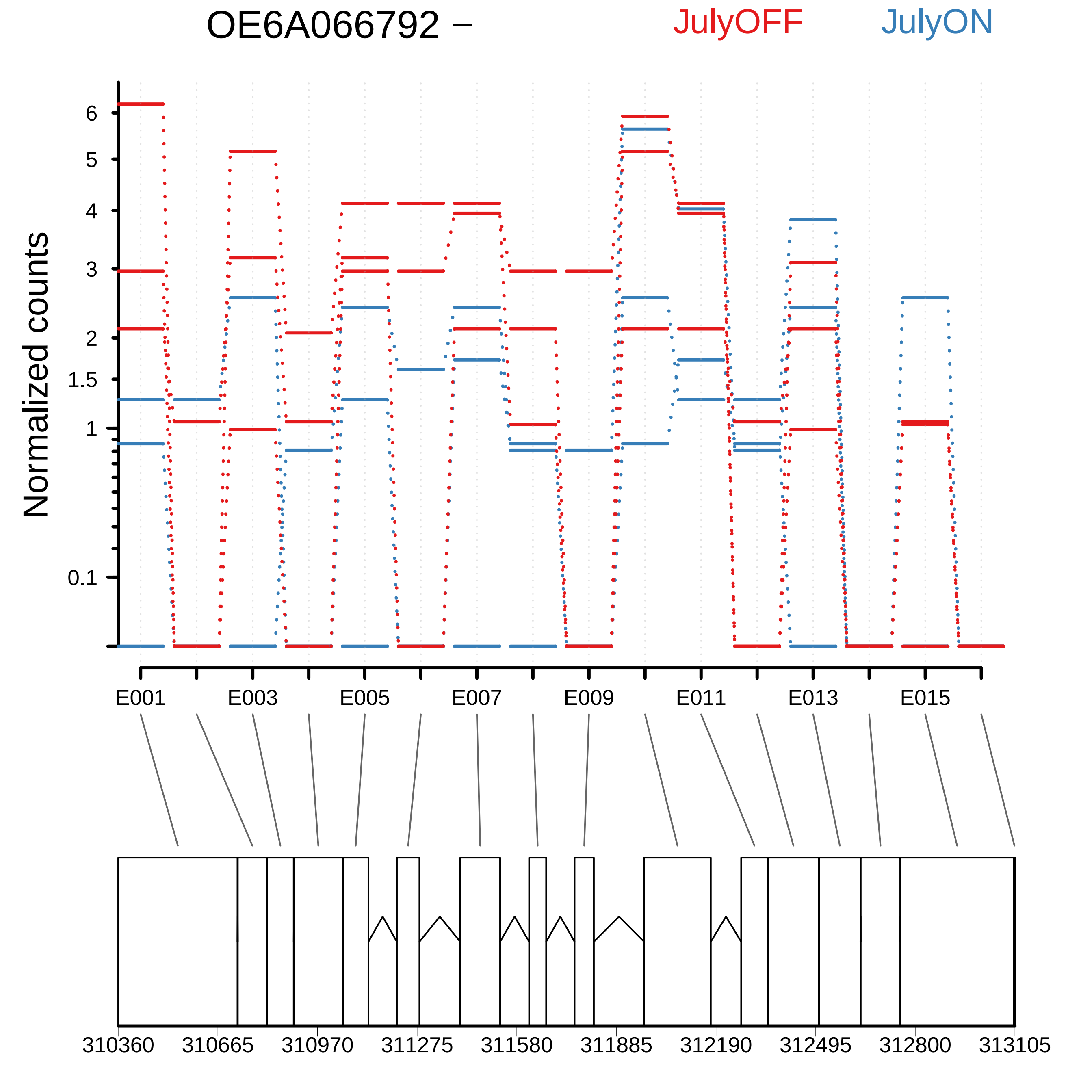
OE6A066792 −
JulyOFF
JulyON
Normalized counts
6
5
4
3
2
1.5
1
0.1
E001
E003
E005
E007
E009
E011
E013
E015
310360
310665
310970
311275
311580
311885
312190
312495
312800
313105

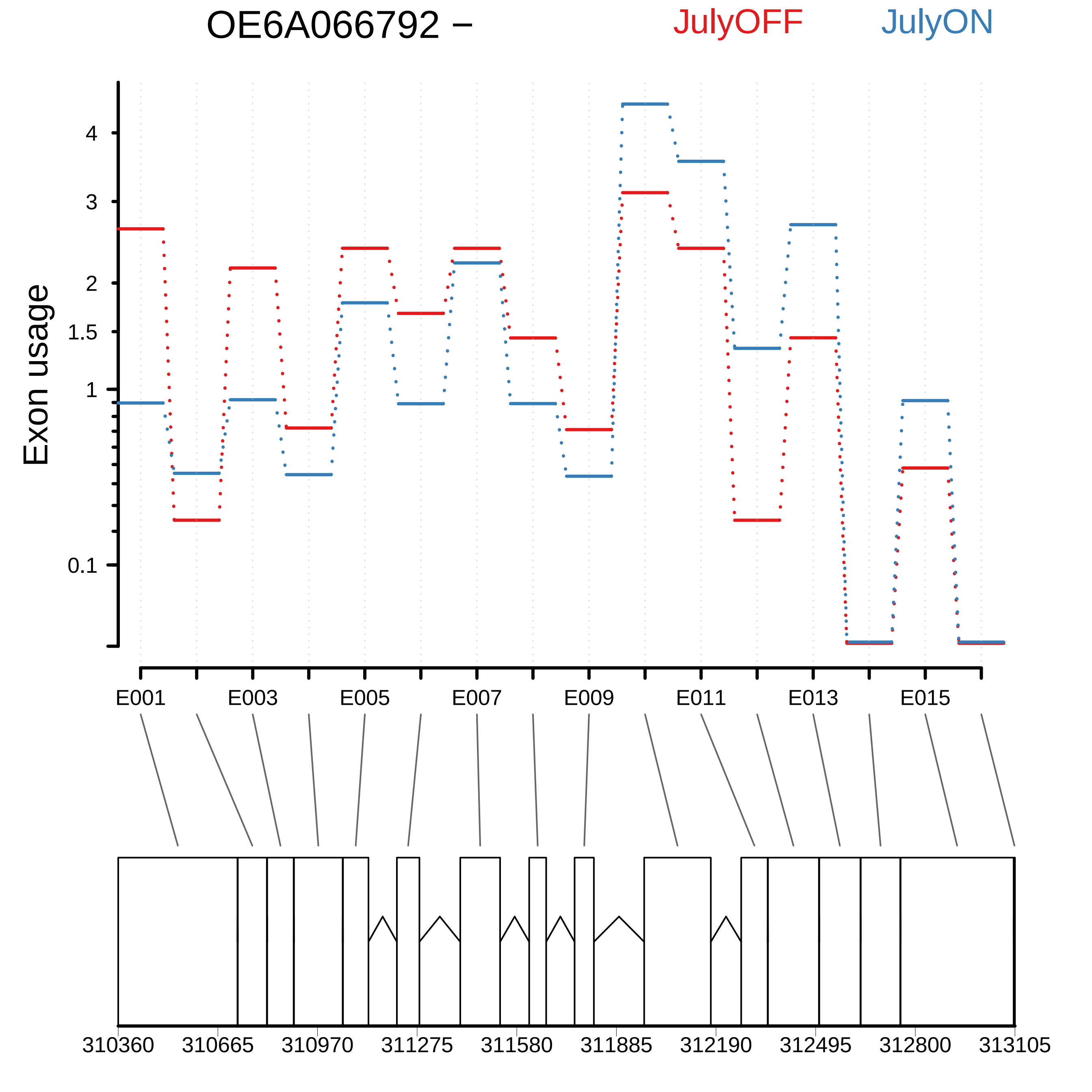

OE6A066792 −
JulyOFF
JulyON
Exon usage
4
3
2
1.5
1
0.1
E001
E003
E005
E007
E009
E011
E013
E015
310360
310665
310970
311275
311580
311885
312190
312495
312800
313105

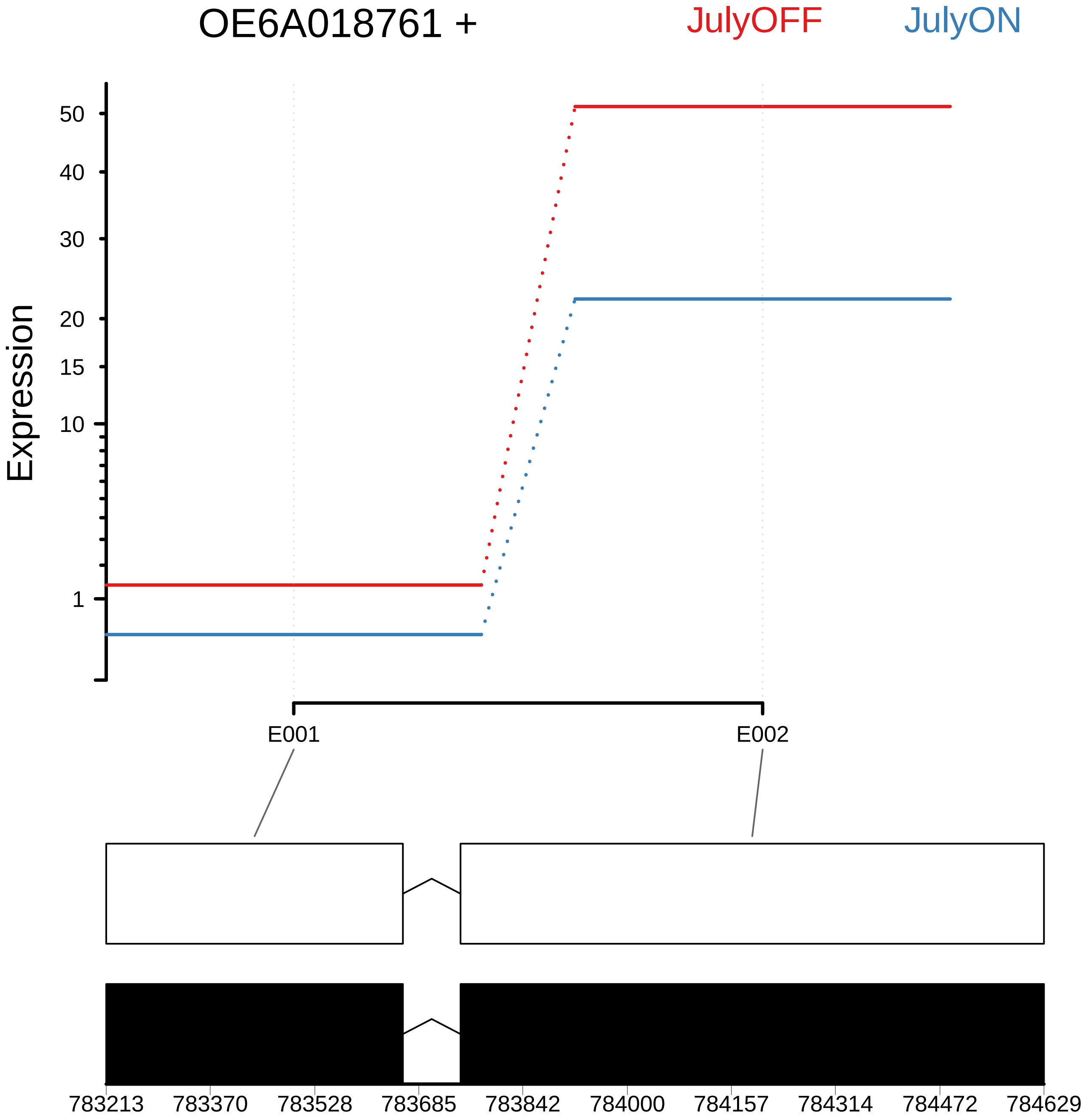

OE6A018761 +
JulyOFF
JulyON
Expression
50
40
30
20
15
10
1
E001
E002
783213
783370
783528
783685
783842
784000
784157
784314
784472
784629

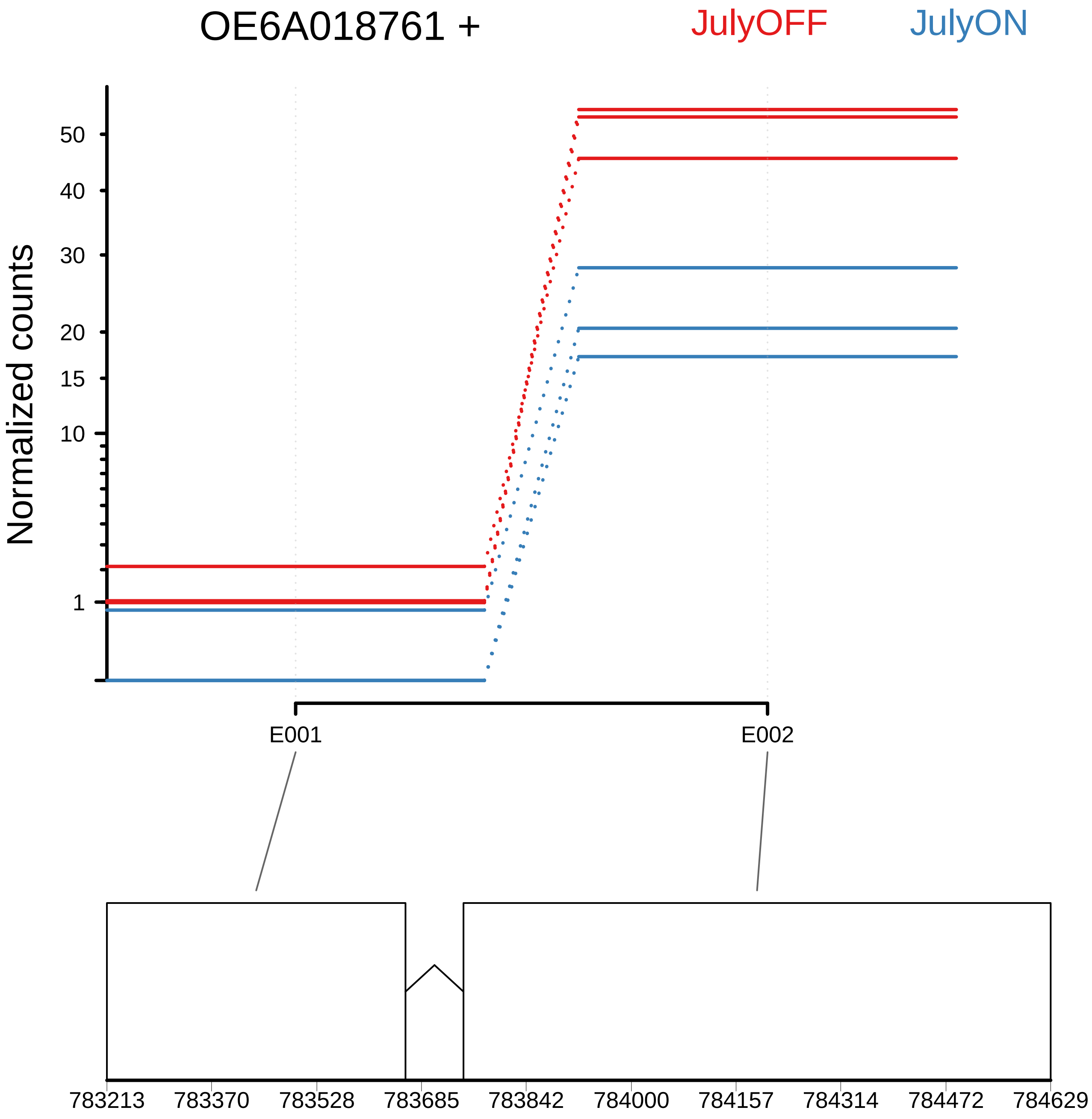
OE6A018761 +
JulyOFF
JulyON
Normalized counts
50
40
30
20
15
10
1
E001
E002
783213
783370
783528
783685
783842
784000
784157
784314
784472
784629

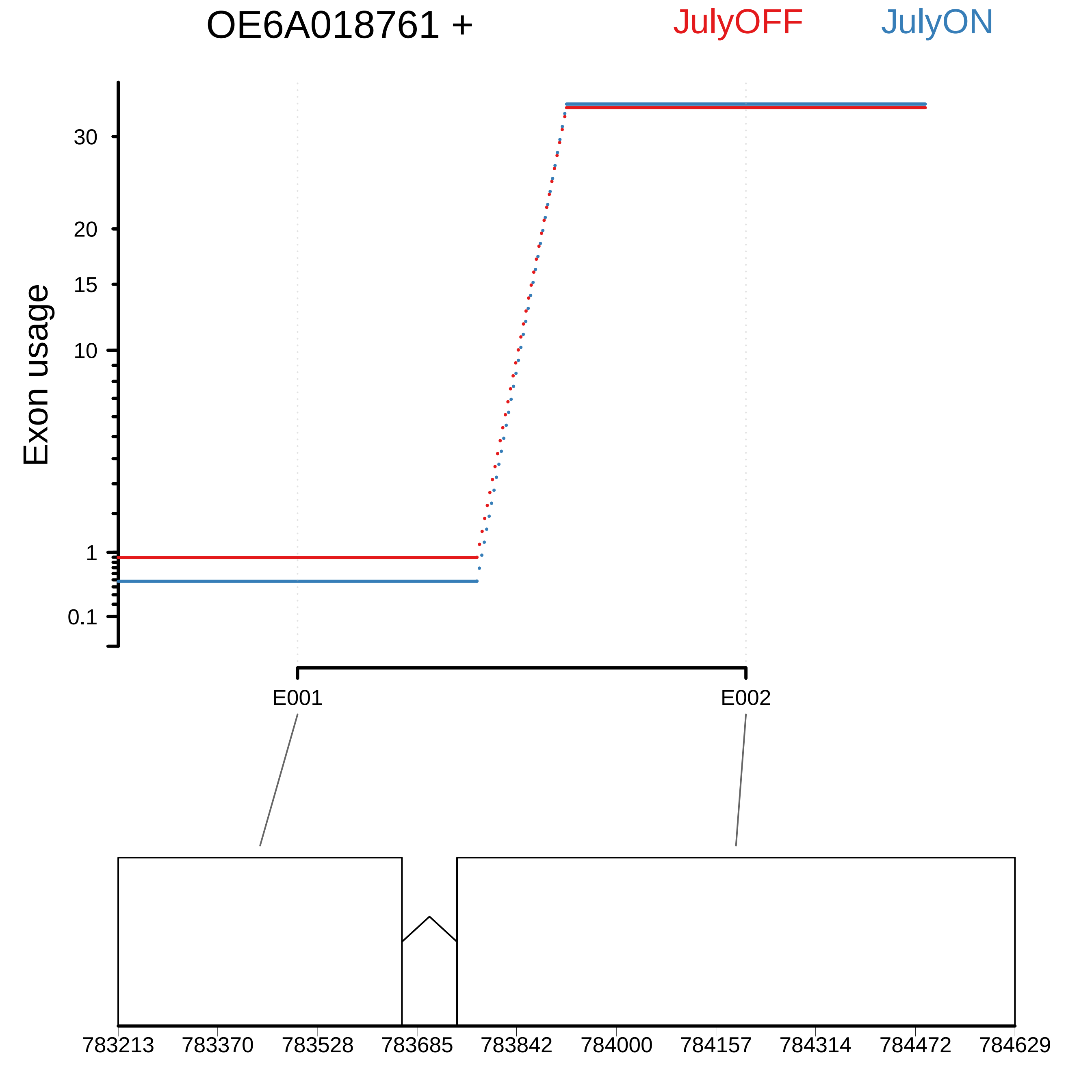

OE6A018761 +
JulyOFF
JulyON
Exon usage
30
20
15
10
1
0.1
E001
E002
783213
783370
783528
783685
783842
784000
784157
784314
784472
784629

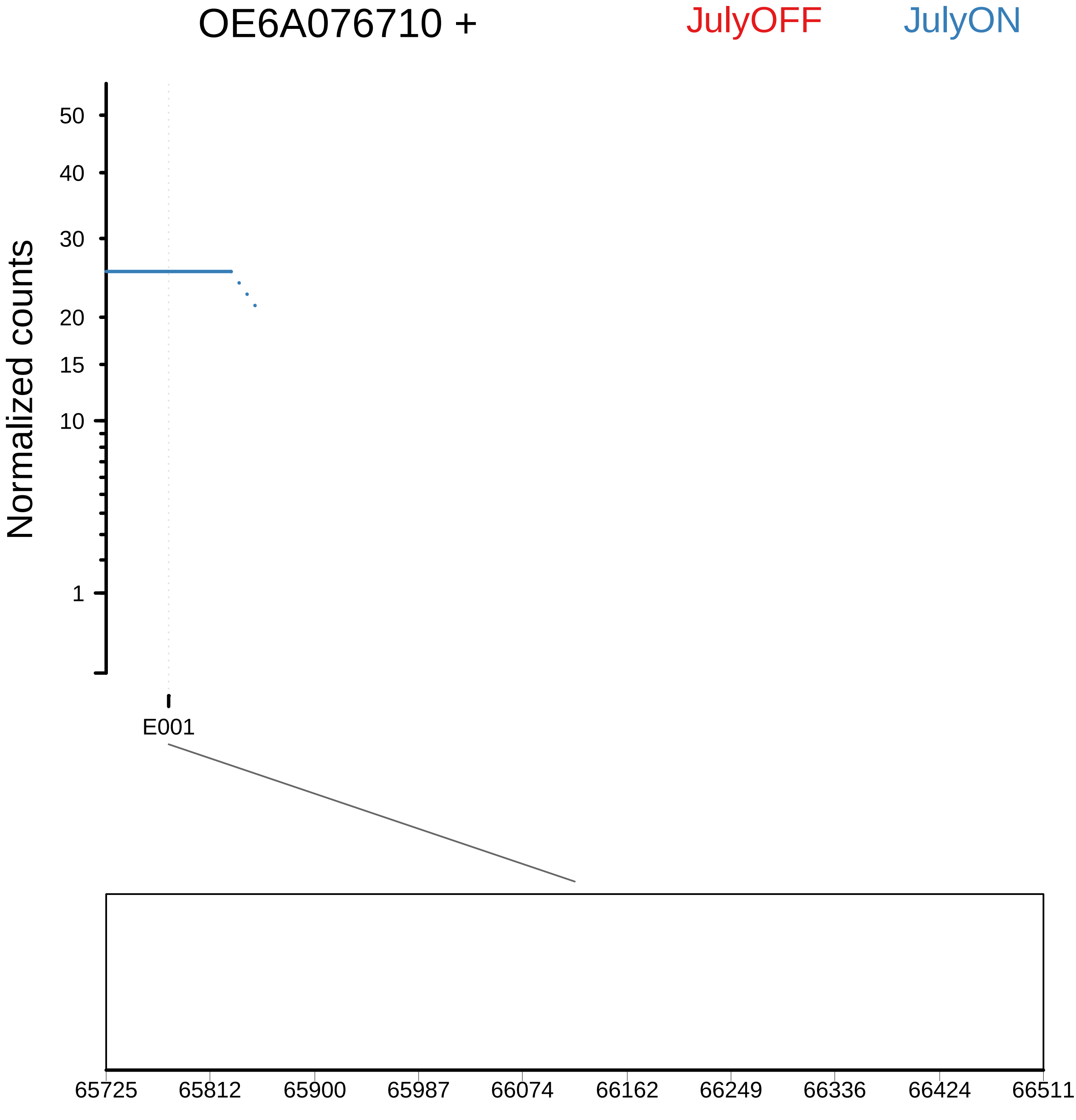

OE6A076710 +
JulyOFF
JulyON
Normalized counts
50
40
30
20
15
10
1
E001
65725
65812
65900
65987
66074
66162
66249
66336
66424
66511

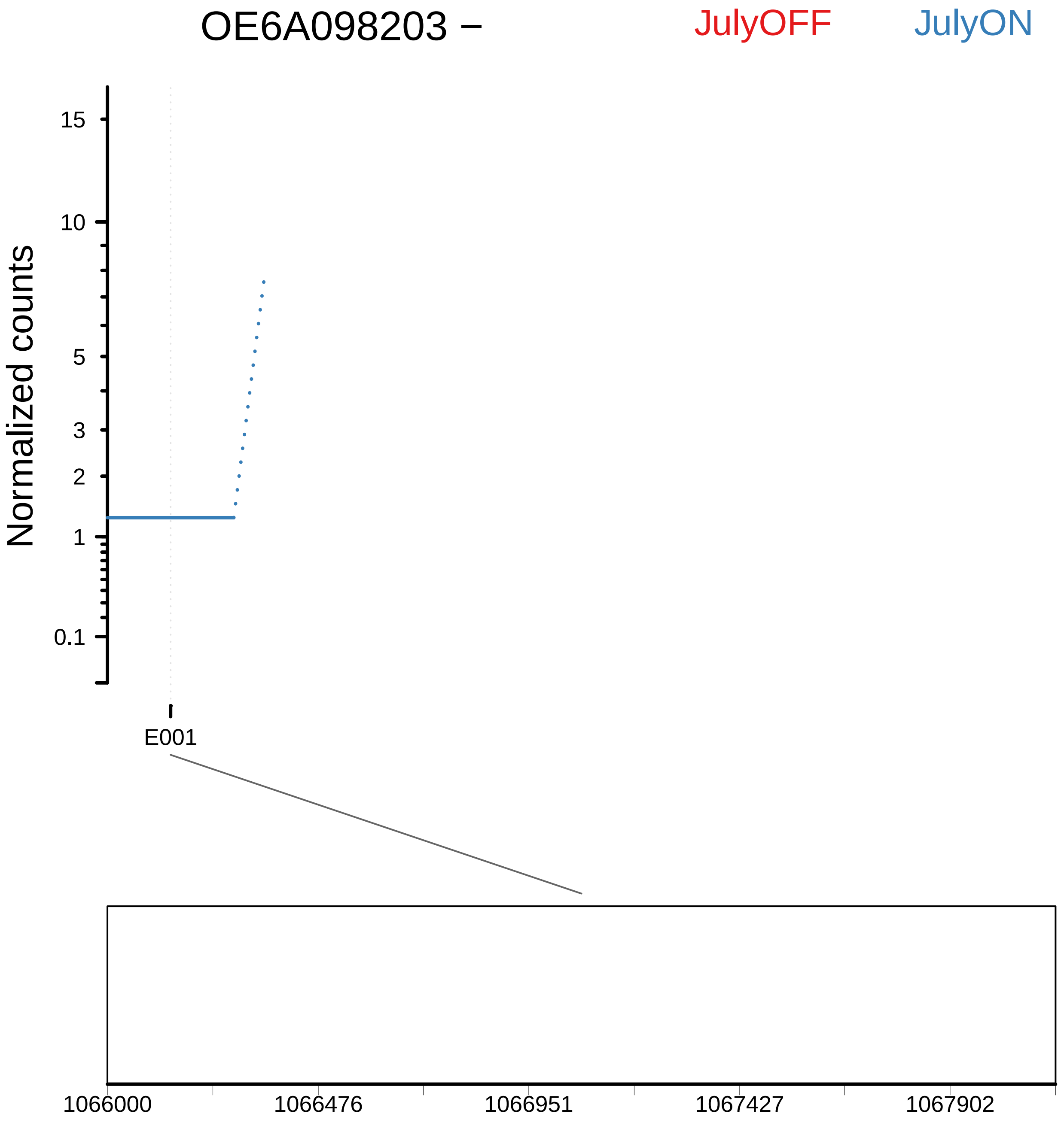
OE6A098203 −
JulyOFF
JulyON
Normalized counts
15
10
5
3
2
1
0.1
E001
1066000
1066476
1066951
1067427
1067902

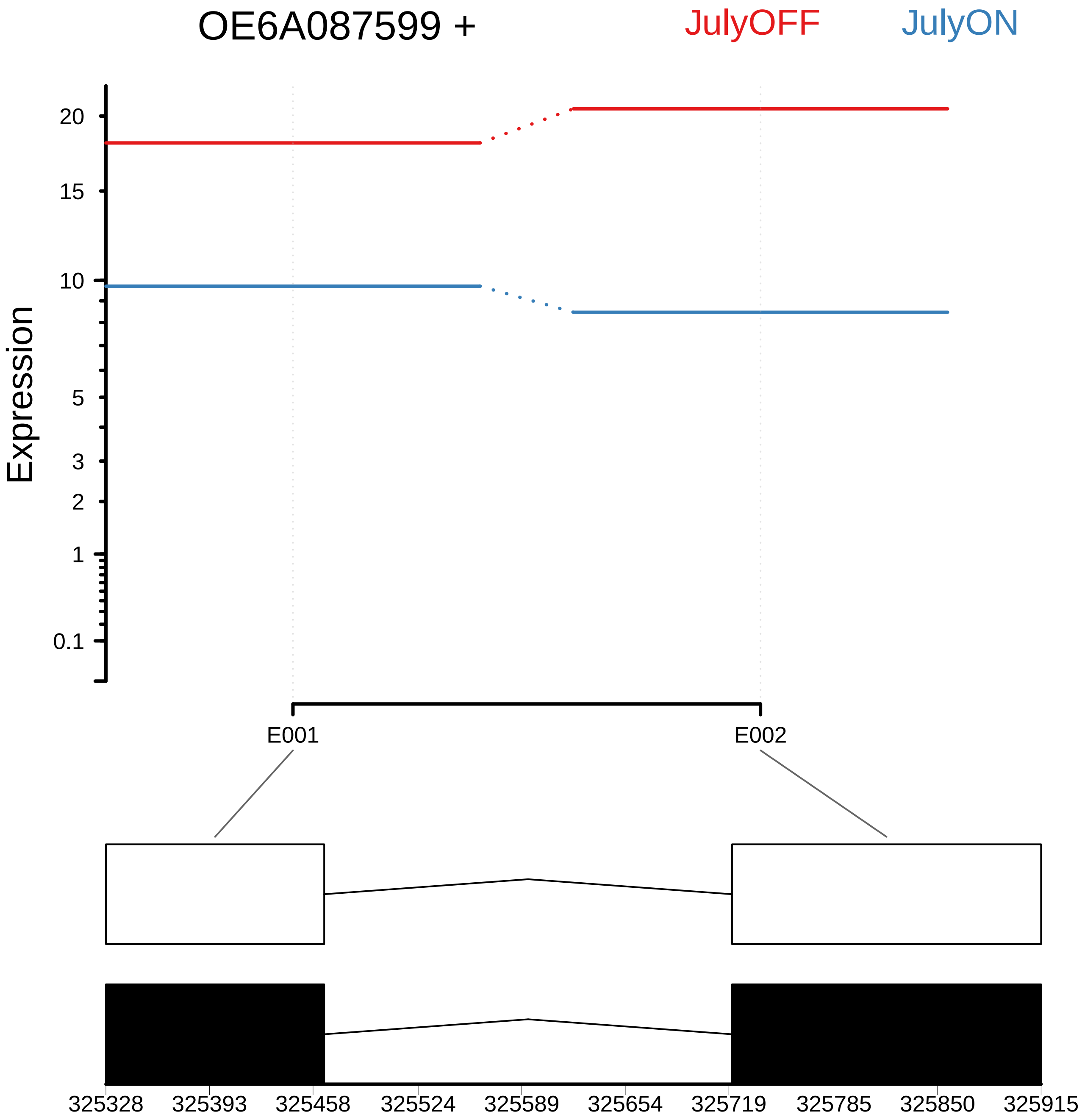
OE6A087599 +
JulyOFF
JulyON
Expression
20
15
10
5
3
2
1
0.1
E001
E002
325328
325393
325458
325524
325589
325654
325719
325785
325850
325915

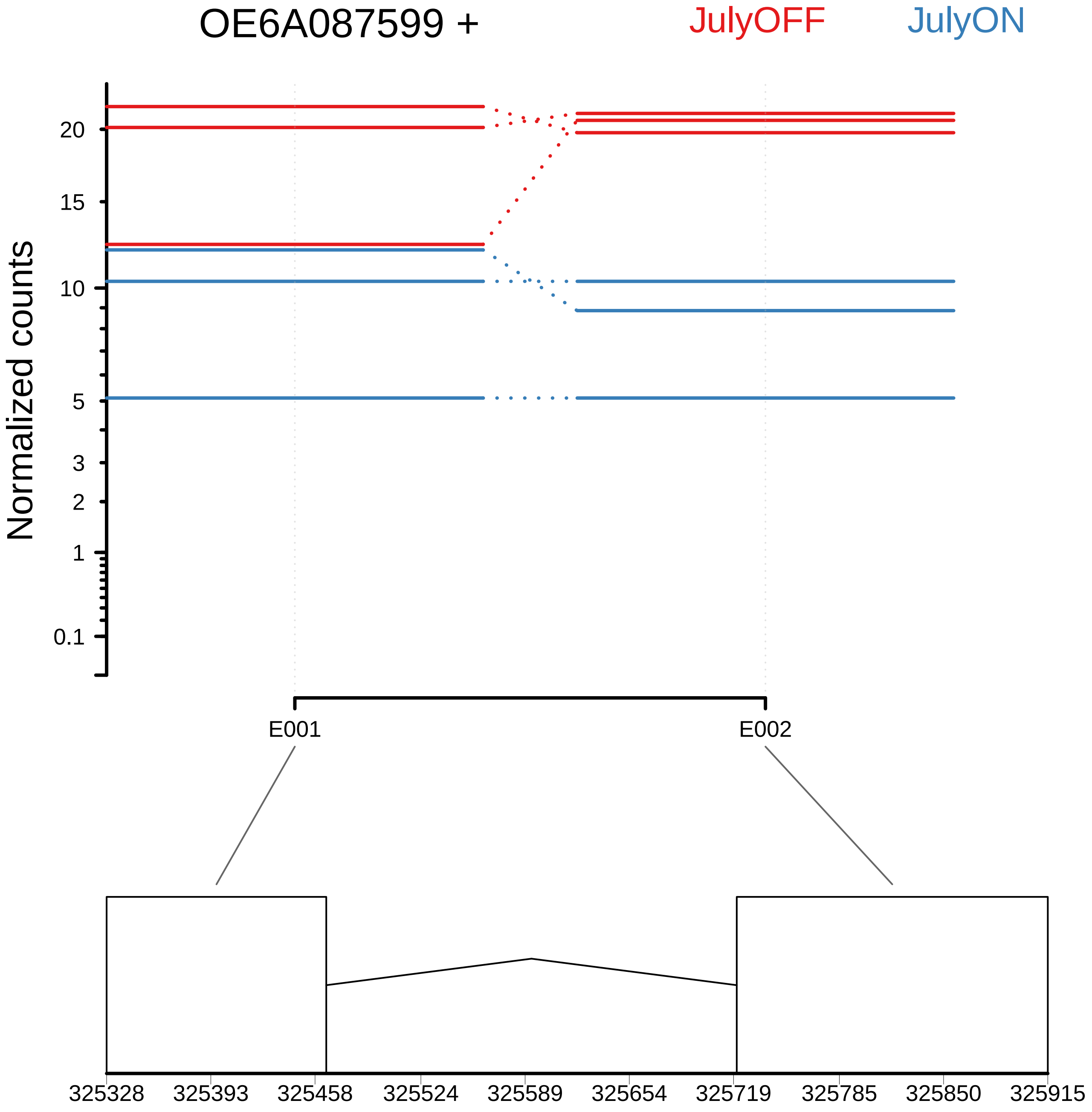

OE6A087599 +
JulyOFF
JulyON
Normalized counts
20
15
10
5
3
2
1
0.1
E001
E002
325328
325393
325458
325524
325589
325654
325719
325785
325850
325915

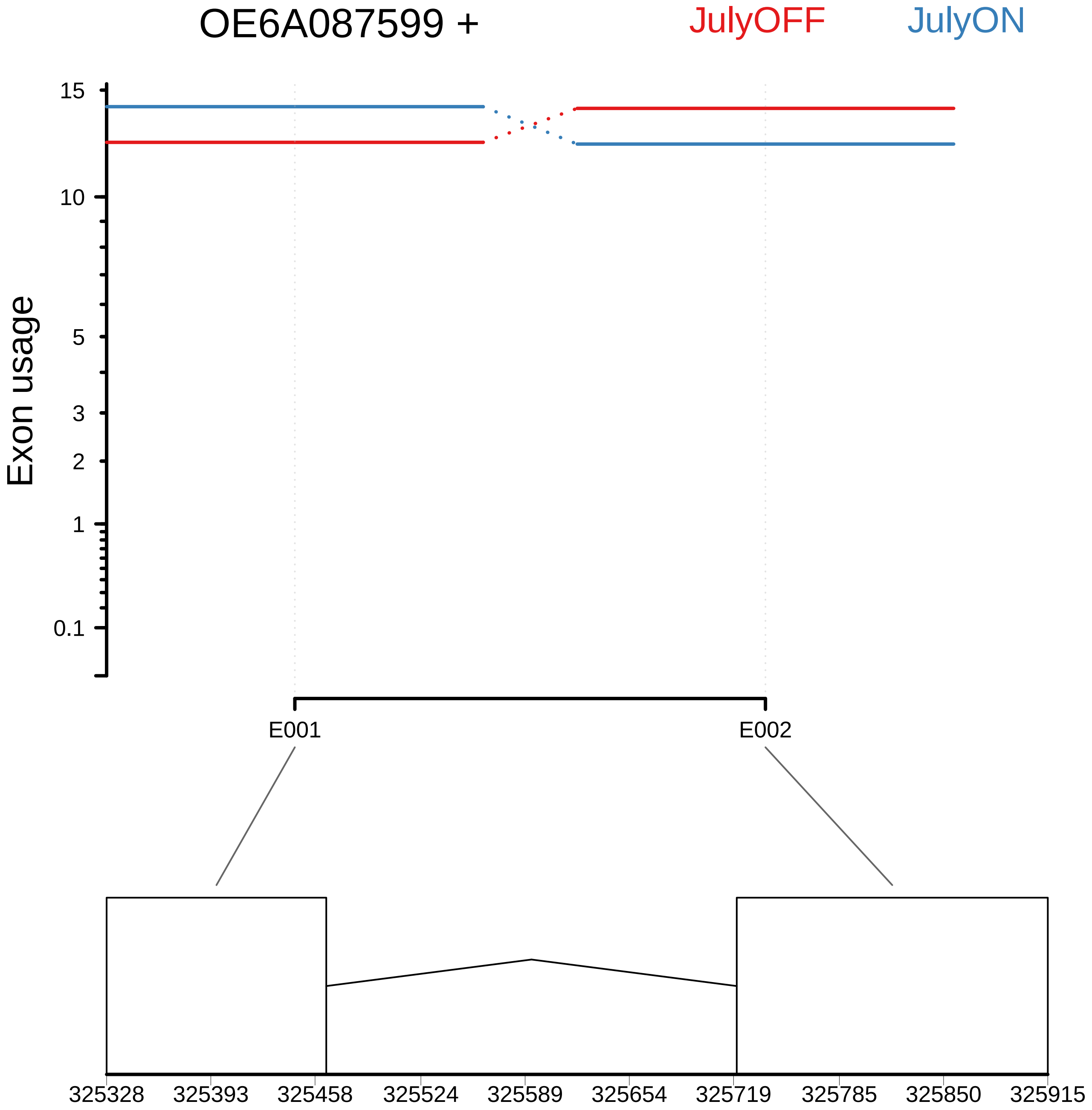

OE6A087599 +
JulyOFF
JulyON
Exon usage
15
10
5
3
2
1
0.1
E001
E002
325328
325393
325458
325524
325589
325654
325719
325785
325850
325915

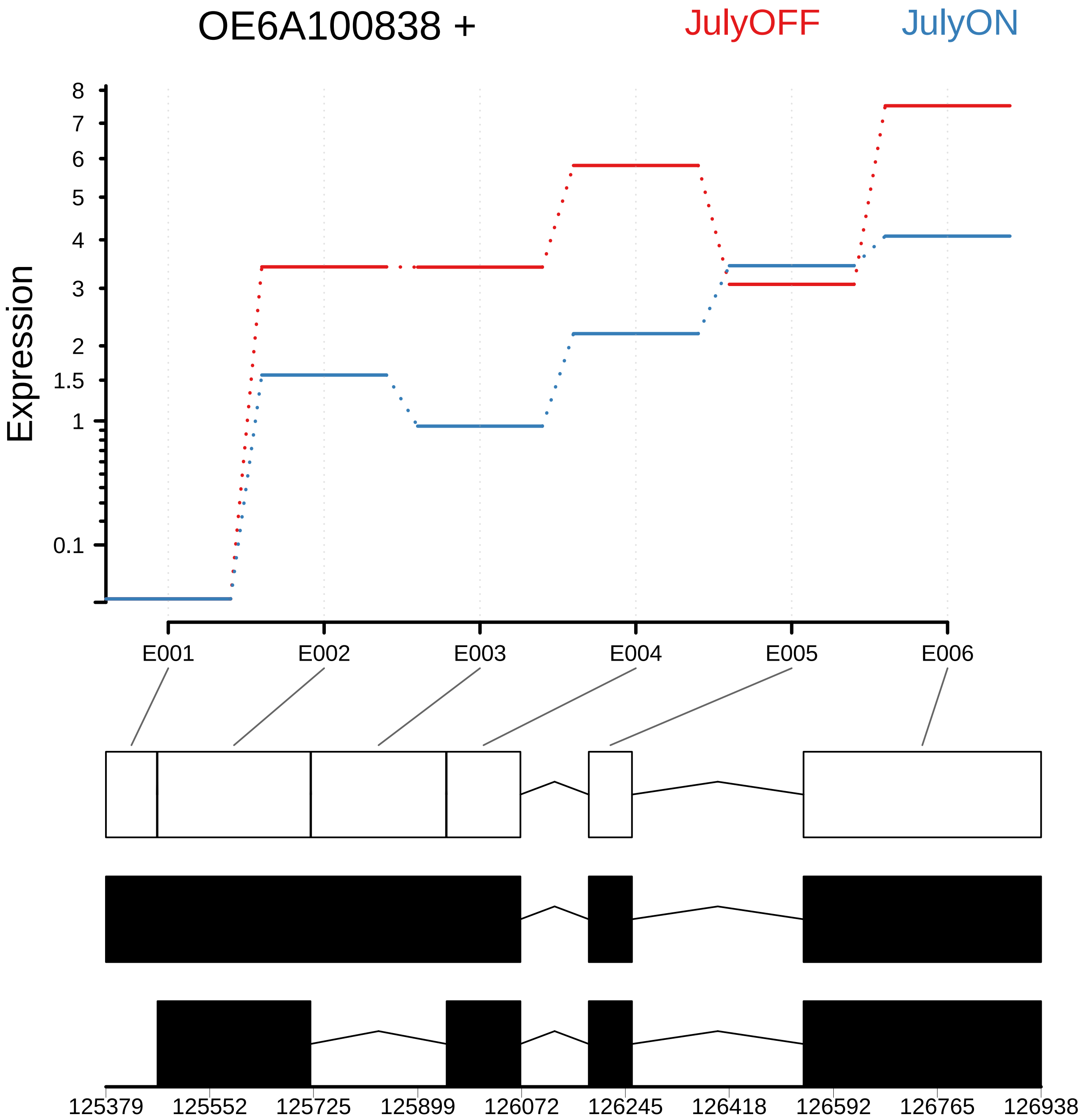

OE6A100838 +
JulyOFF
JulyON
Expression
8
7
6
5
4
3
2
1.5
1
0.1
E001
E002
E003
E004
E005
E006
125379
125552
125725
125899
126072
126245
126418
126592
126765
126938

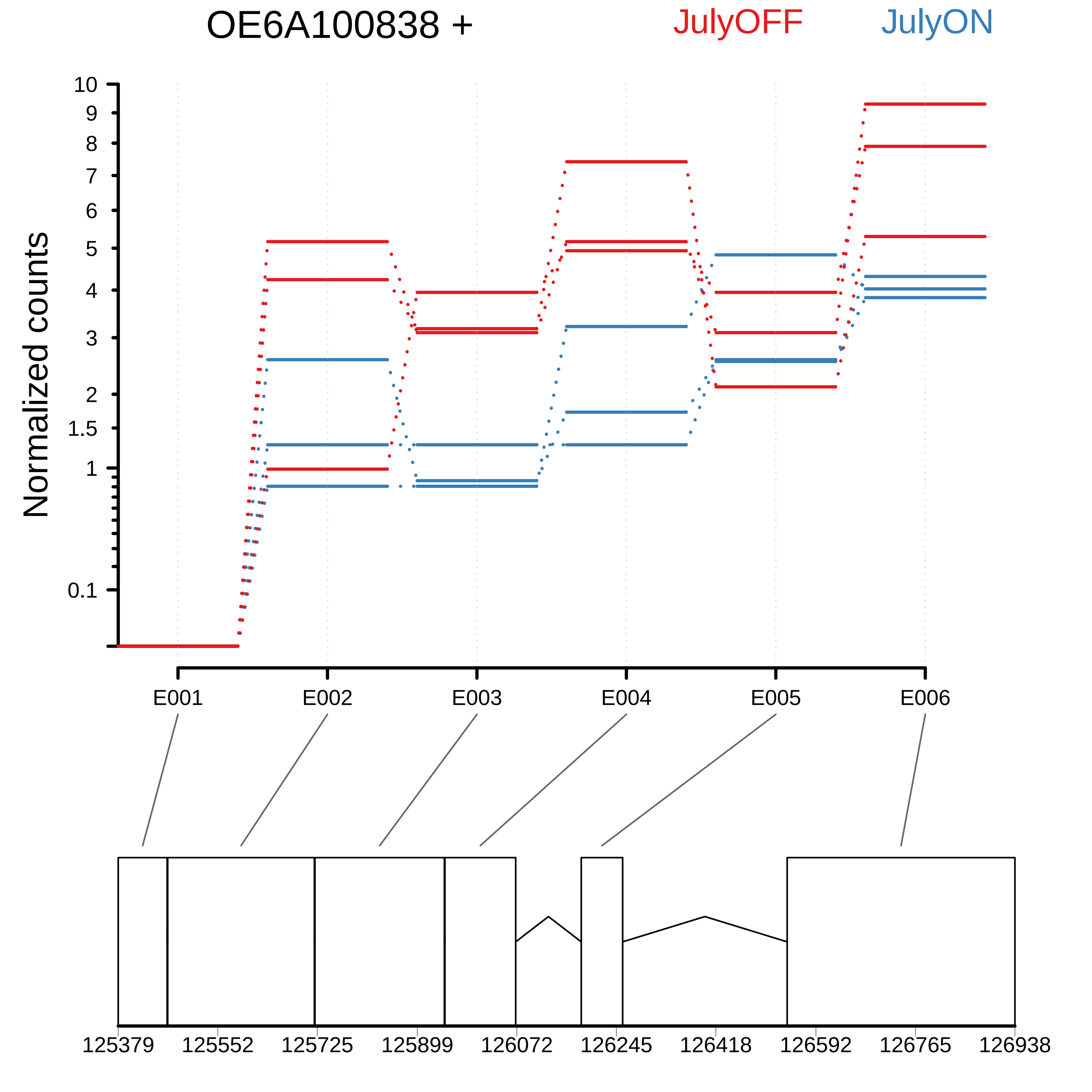
OE6A100838 +
JulyOFF
JulyON
Normalized counts
10
9
8
7
6
5
4
3
2
1.5
1
0.1
E001
E002
E003
E004
E005
E006
125379
125552
125725
125899
126072
126245
126418
126592
126765
126938

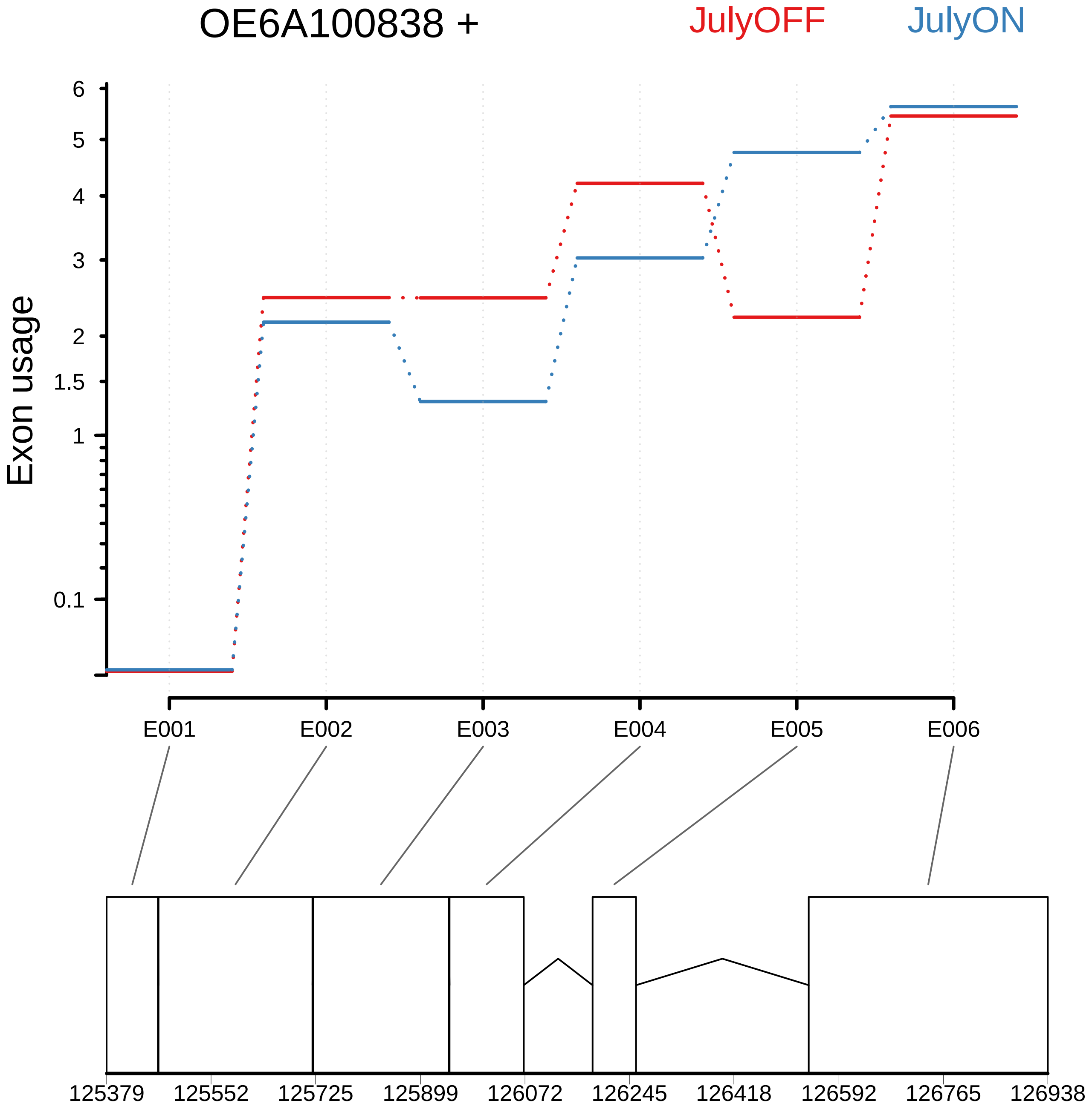
OE6A100838 +
JulyOFF
JulyON
Exon usage
6
5
4
3
2
1.5
1
0.1
E001
E002
E003
E004
E005
E006
125379
125552
125725
125899
126072
126245
126418
126592
126765
126938

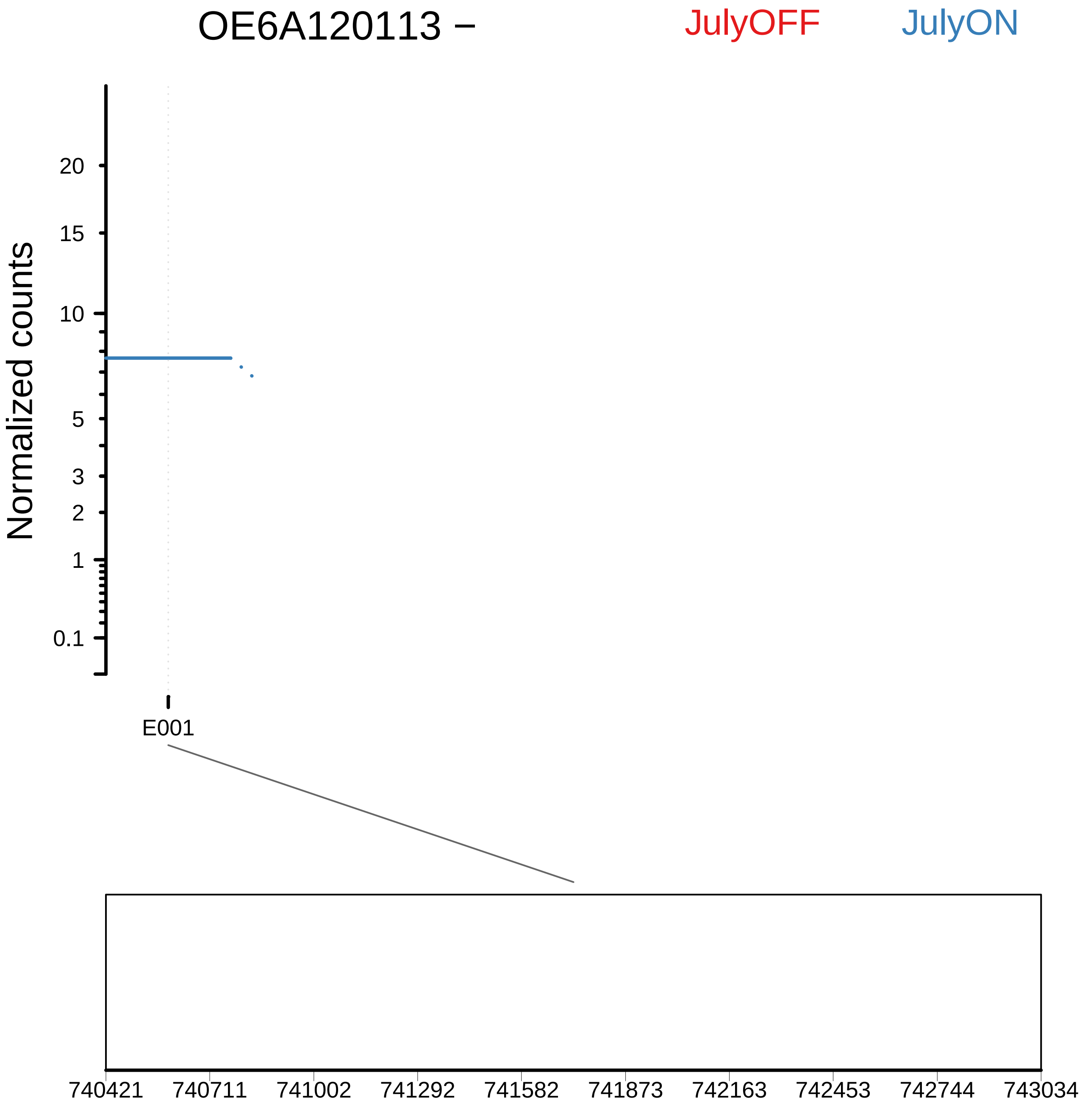

OE6A120113 −
JulyOFF
JulyON
Normalized counts
20
15
10
5
3
2
1
0.1
E001
740421
740711
741002
741292
741582
741873
742163
742453
742744
743034

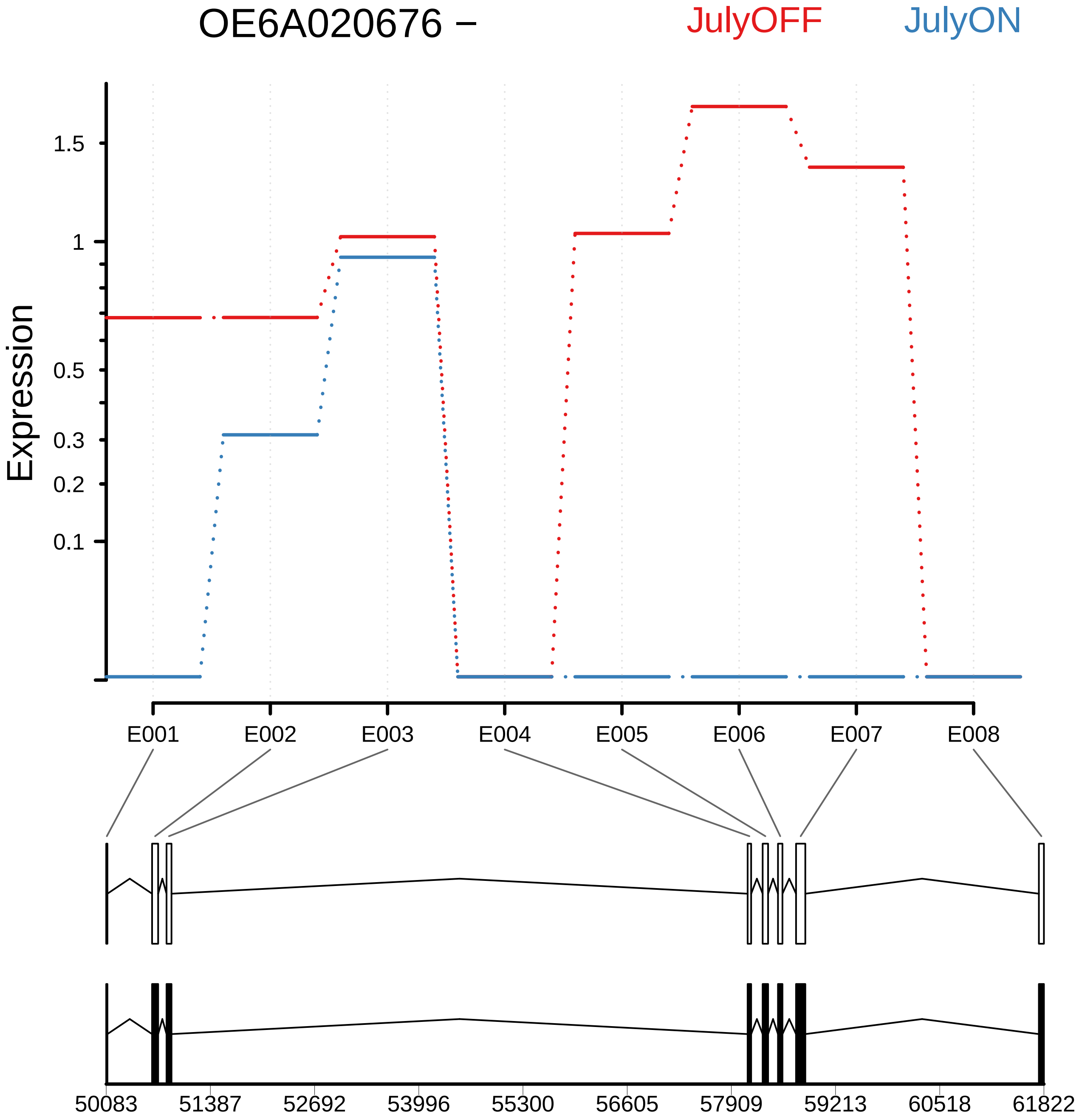

OE6A020676 −
JulyOFF
JulyON
Expression
1.5
1
0.5
0.3
0.2
0.1
E001
E002
E003
E004
E005
E006
E007
E008
50083
51387
52692
53996
55300
56605
57909
59213
60518
61822

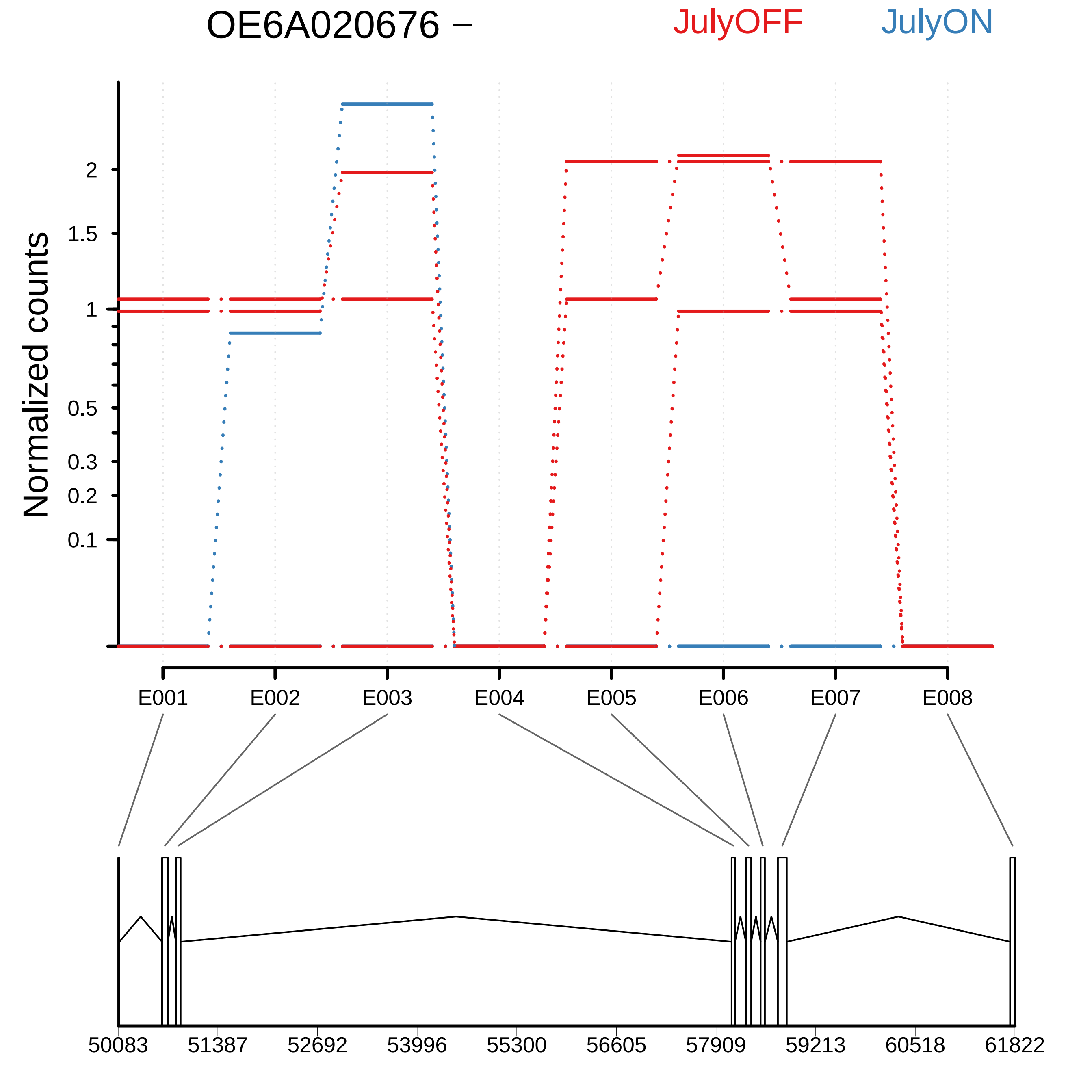

OE6A020676 −
JulyOFF
JulyON
Normalized counts
2
1.5
1
0.5
0.3
0.2
0.1
E001
E002
E003
E004
E005
E006
E007
E008
50083
51387
52692
53996
55300
56605
57909
59213
60518
61822

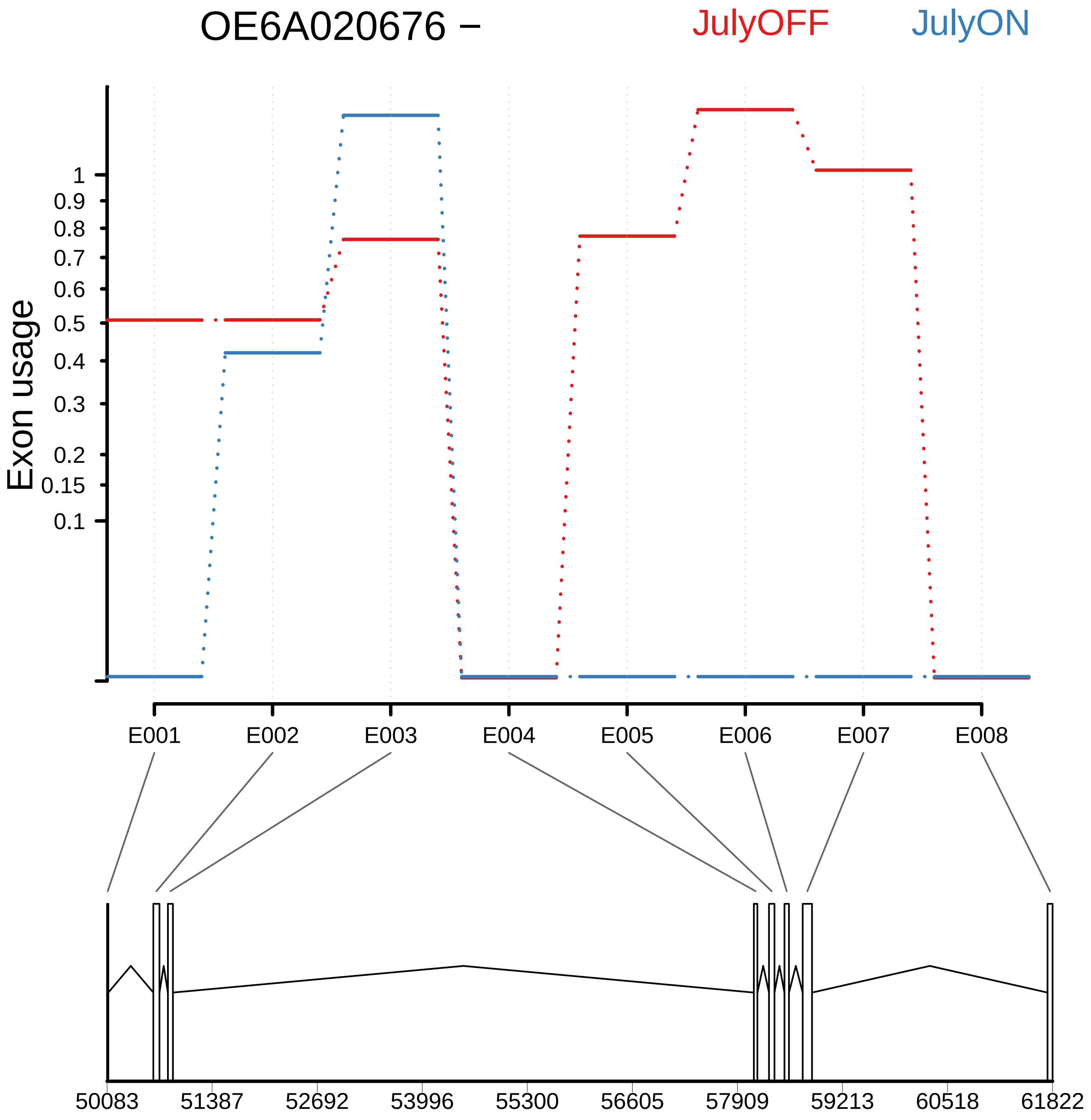

OE6A020676 −
JulyOFF
JulyON
Exon usage
1
0.9
0.8
0.7
0.6
0.5
0.4
0.3
0.2
0.15
0.1
E001
E002
E003
E004
E005
E006
E007
E008
50083
51387
52692
53996
55300
56605
57909
59213
60518
61822

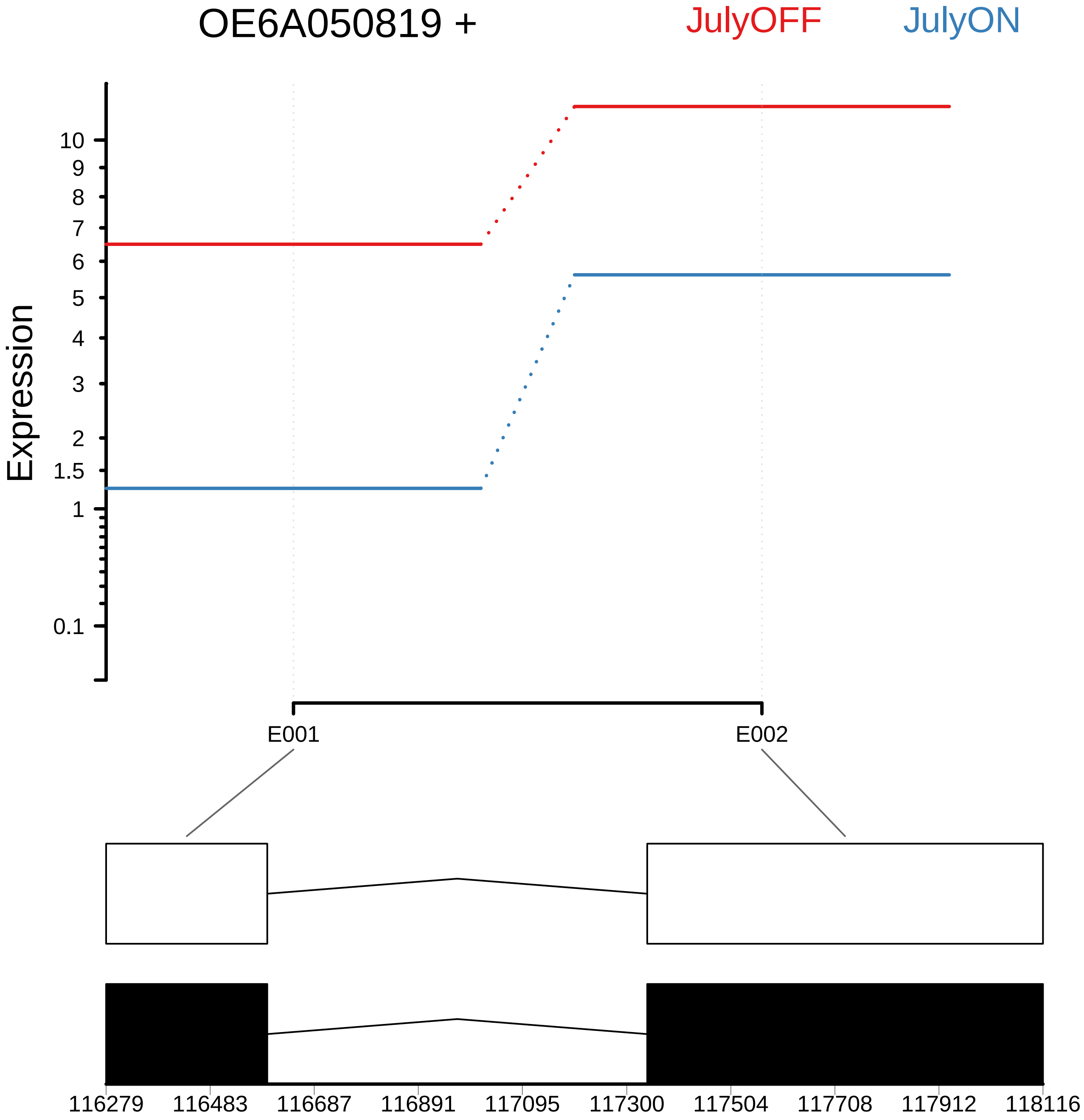
OE6A050819 +
JulyOFF
JulyON
Expression
10
9
8
7
6
5
4
3
2
1.5
1
0.1
E001
E002
116279
116483
116687
116891
117095
117300
117504
117708
117912
118116

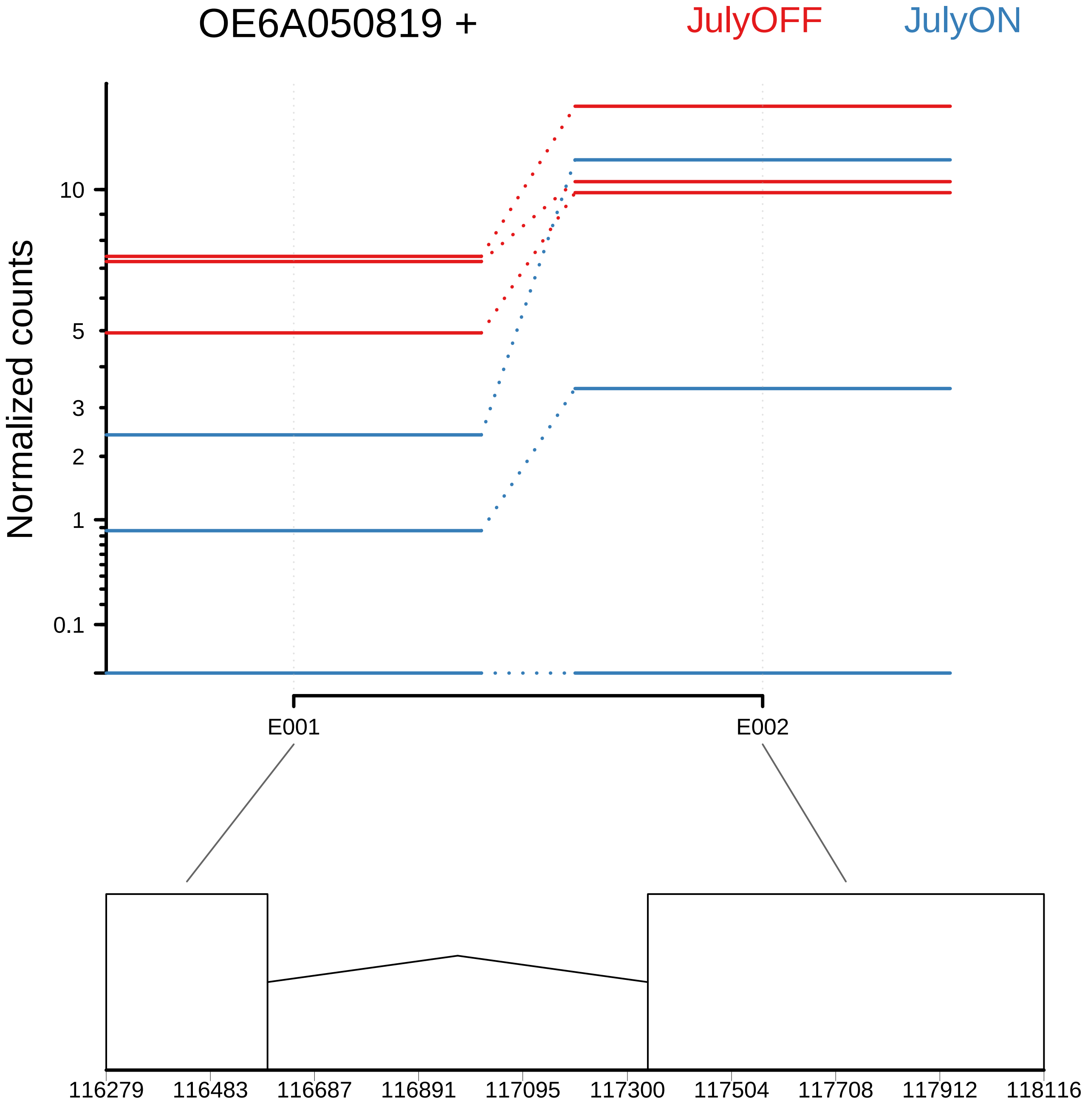
OE6A050819 +
JulyOFF
JulyON
Normalized counts
10
5
3
2
1
0.1
E001
E002
116279
116483
116687
116891
117095
117300
117504
117708
117912
118116

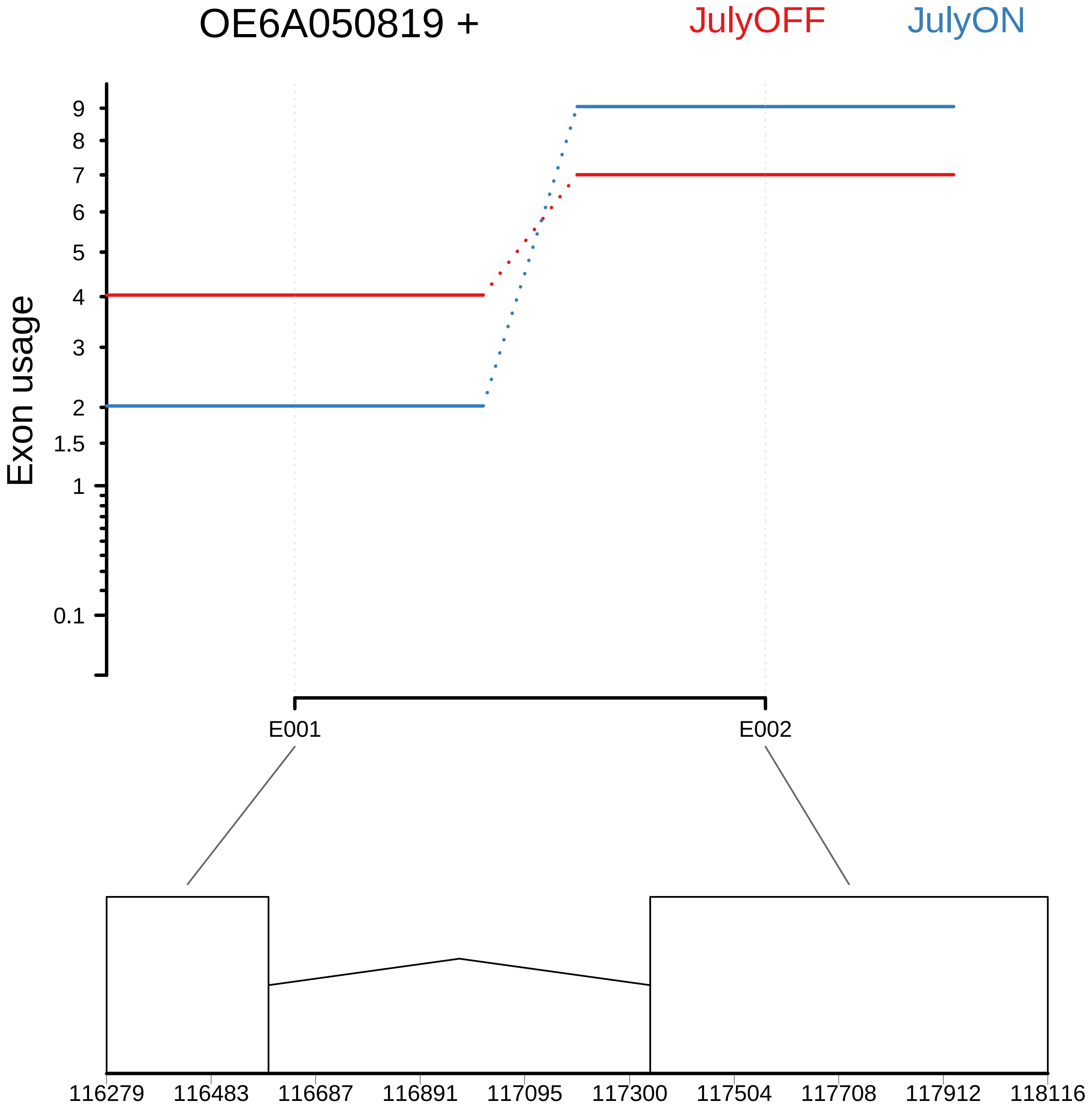

OE6A050819 +
JulyOFF
JulyON
Exon usage
9
8
7
6
5
4
3
2
1.5
1
0.1
E001
E002
116279
116483
116687
116891
117095
117300
117504
117708
117912
118116

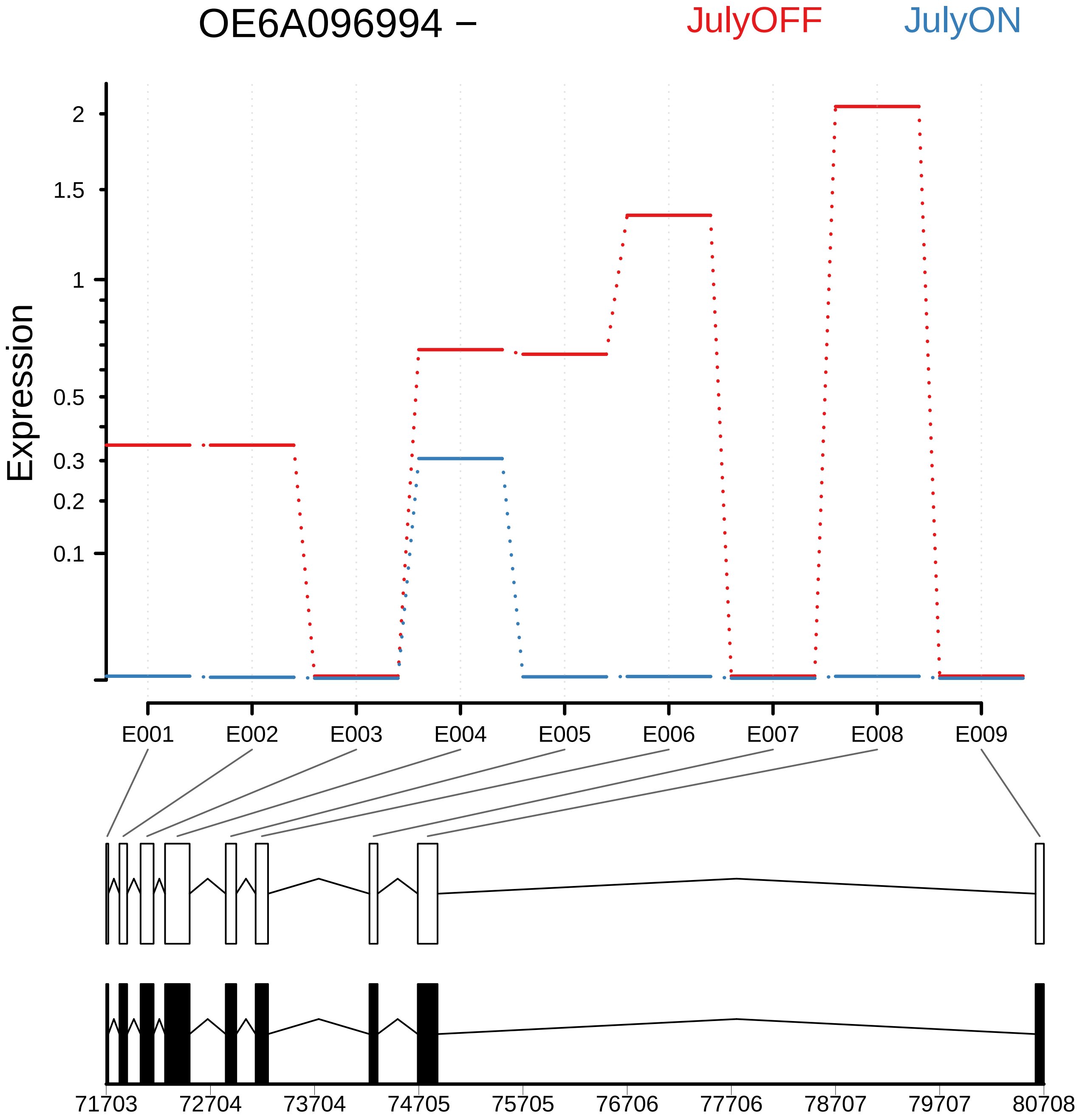
OE6A096994 –
JulyOFF
JulyON
Expression
2
1.5
1
0.5
0.3
0.2
0.1
E001
E002
E003
E004
E005
E006
E007
E008
E009
71703
72704
73704
74705
75705
76706
77706
78707
79707
80708

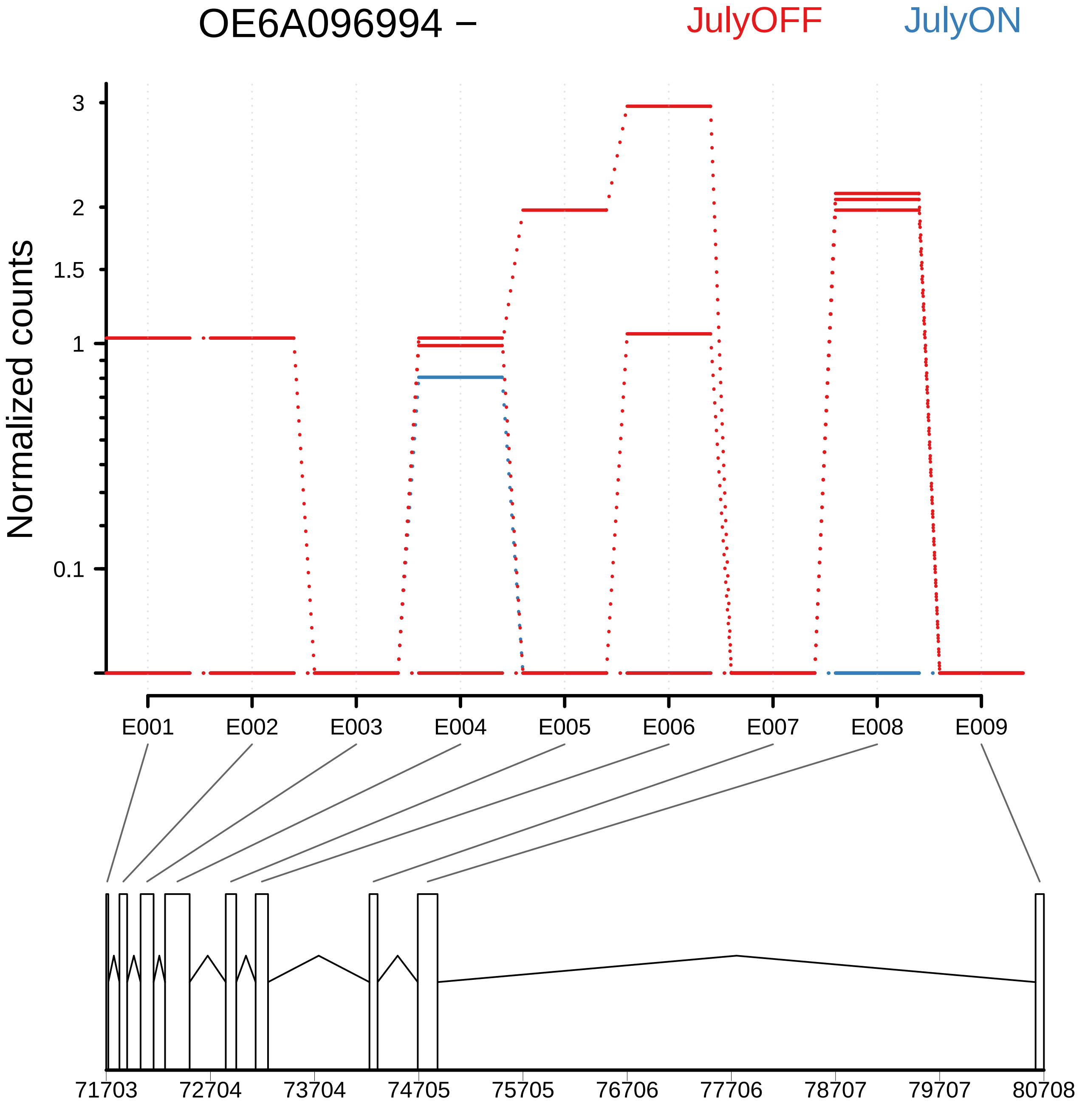

OE6A096994 −
JulyOFF
JulyON
Normalized counts
3
2
1.5
1
0.1
E001
E002
E003
E004
E005
E006
E007
E008
E009
71703
72704
73704
74705
75705
76706
77706
78707
79707
80708

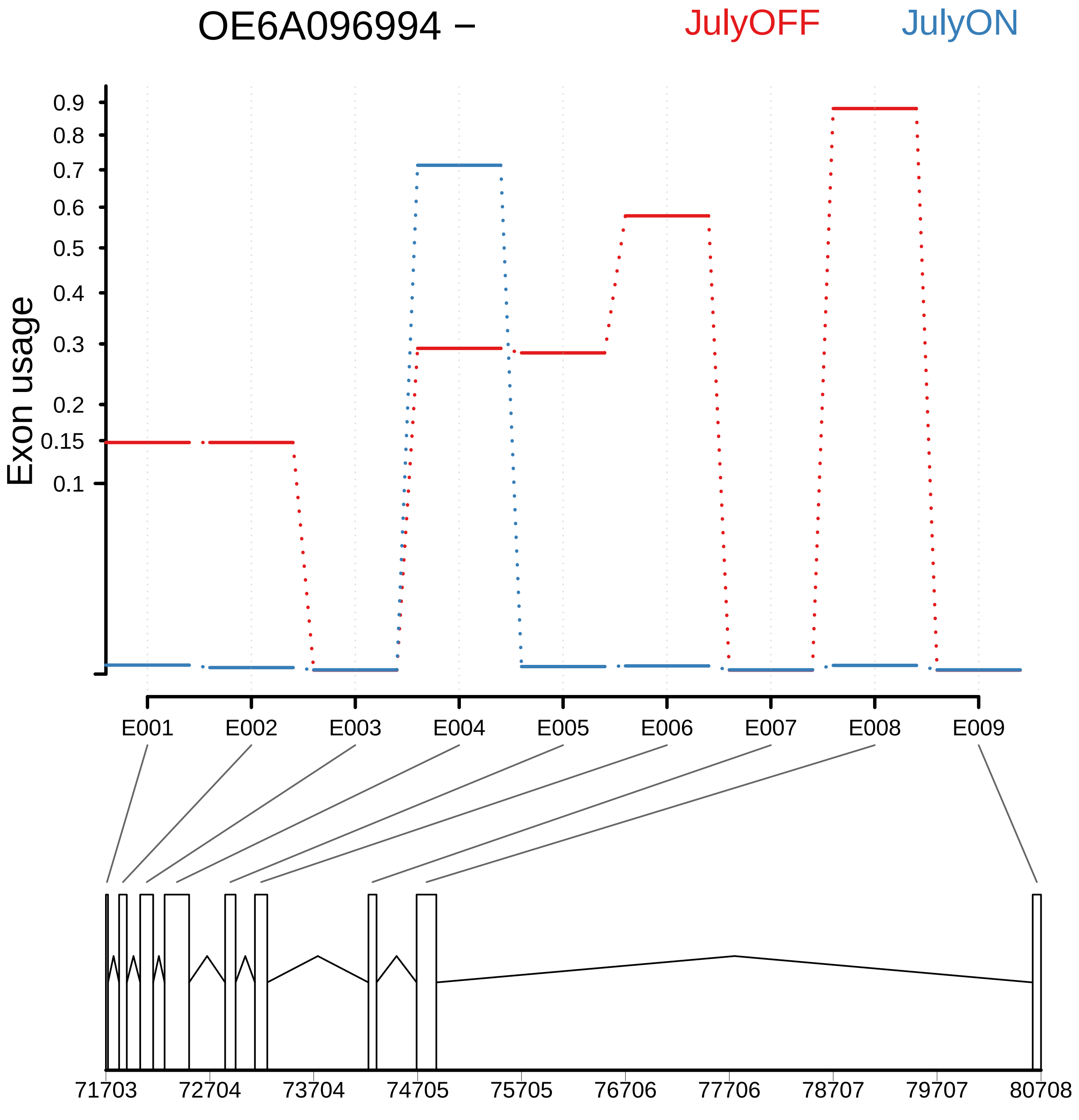
OE6A096994 −
JulyOFF
JulyON
Exon usage
0.9
0.8
0.7
0.6
0.5
0.4
0.3
0.2
0.15
0.1
E001
E002
E003
E004
E005
E006
E007
E008
E009
71703
72704
73704
74705
75705
76706
77706
78707
79707
80708

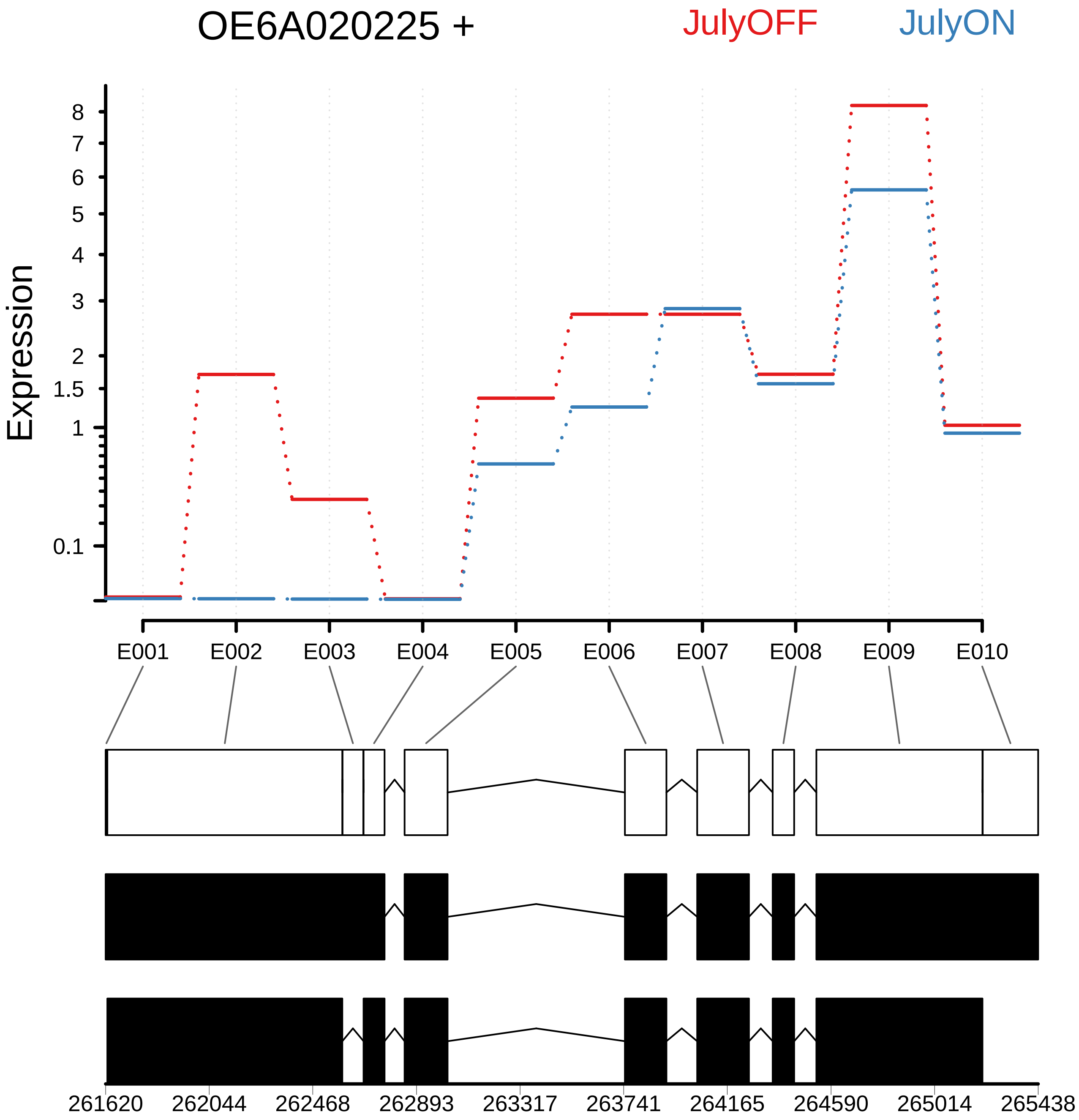
OE6A020225 +
JulyOFF
JulyON
Expression
8
7
6
5
4
3
2
1.5
1
0.1
E001
E002
E003
E004
E005
E006
E007
E008
E009
E010
261620
262044
262468
262893
263317
263741
264165
264590
265014
265438

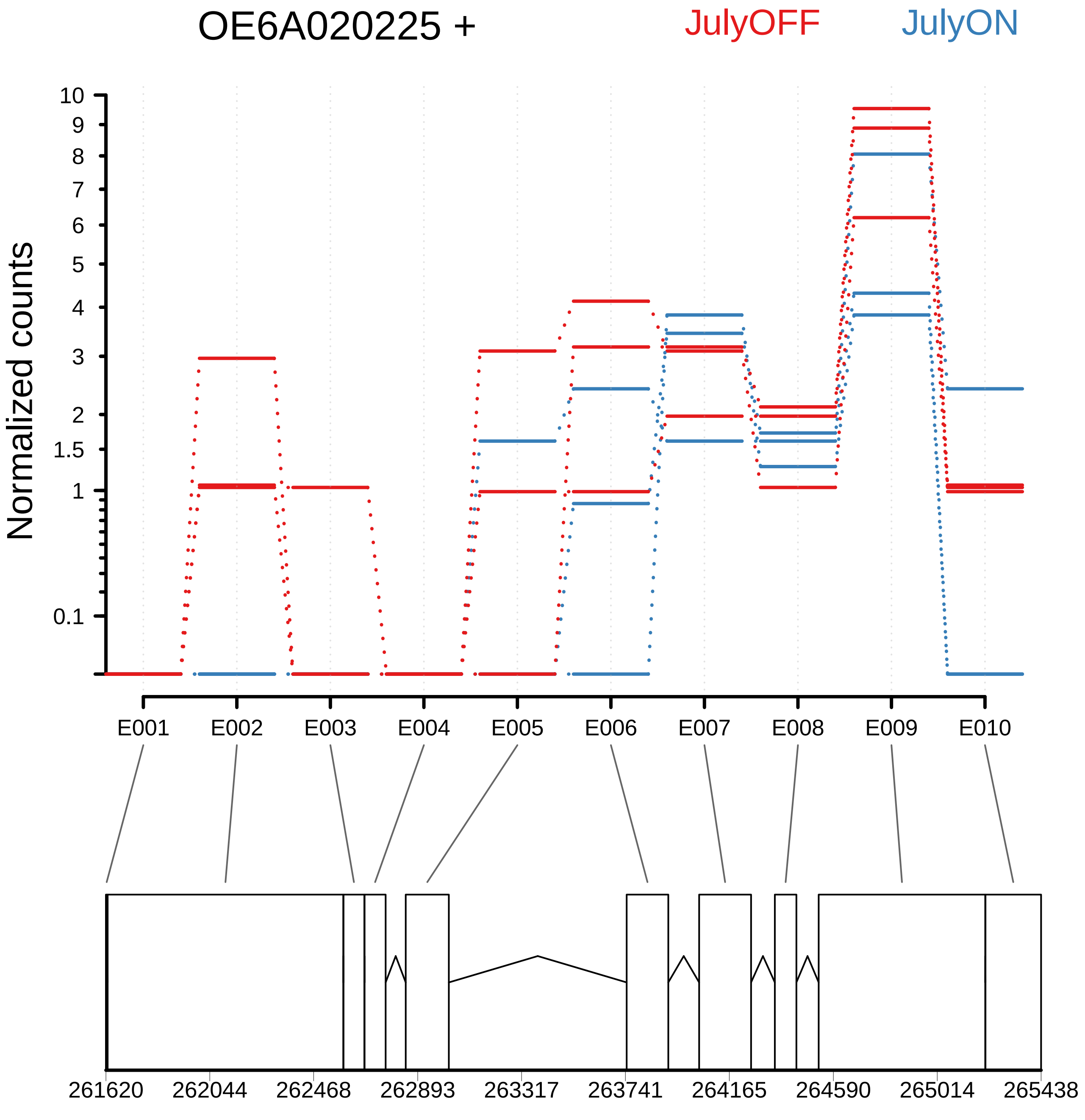
OE6A020225 +
JulyOFF
JulyON
Normalized counts
10
9
8
7
6
5
4
3
2
1.5
1
0.1
E001
E002
E003
E004
E005
E006
E007
E008
E009
E010
261620
262044
262468
262893
263317
263741
264165
264590
265014
265438

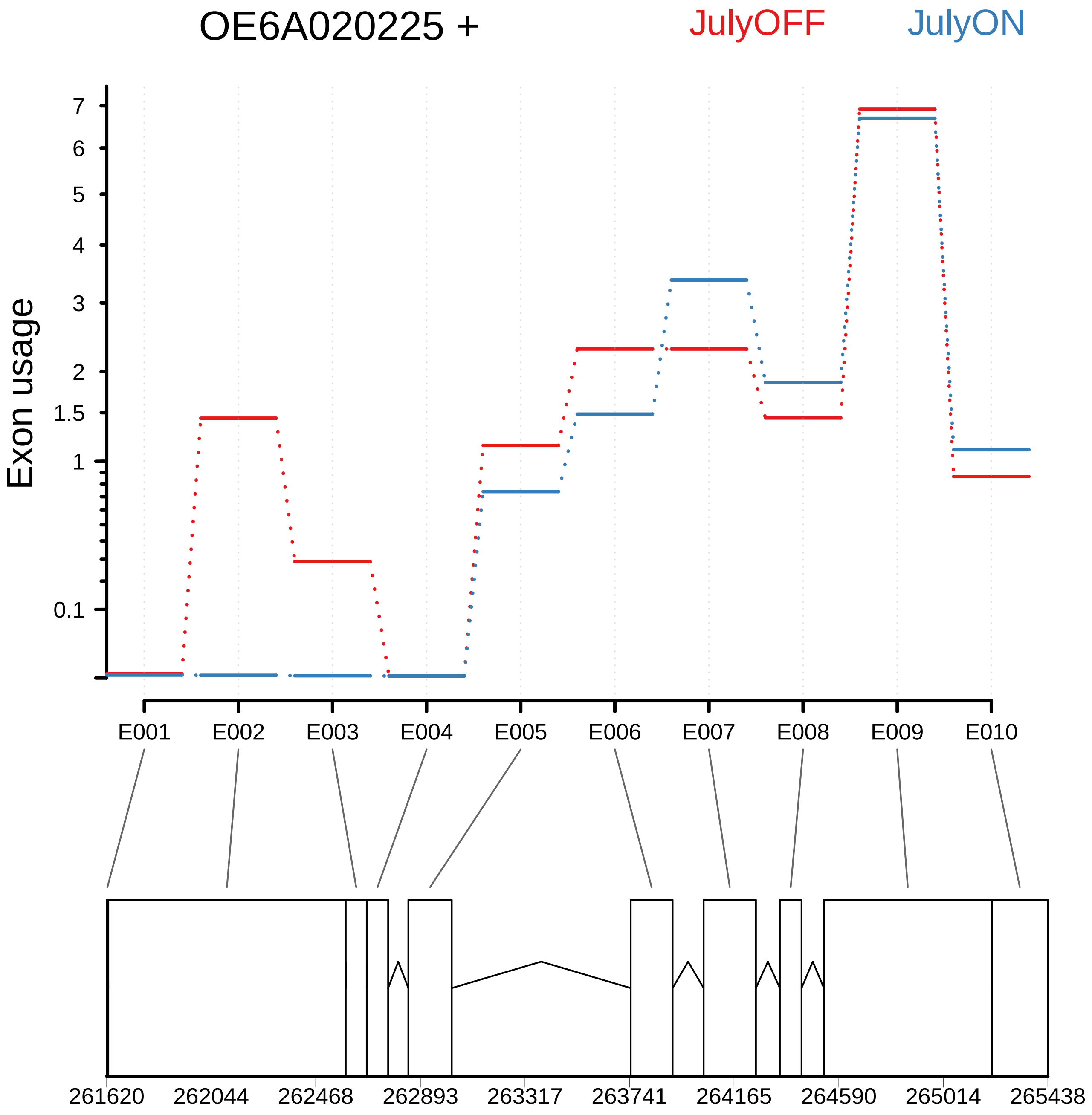

OE6A020225 +
JulyOFF
JulyON
Exon usage
7
6
5
4
3
2
1.5
1
0.1
E001
E002
E003
E004
E005
E006
E007
E008
E009
E010
261620
262044
262468
262893
263317
263741
264165
264590
265014
265438

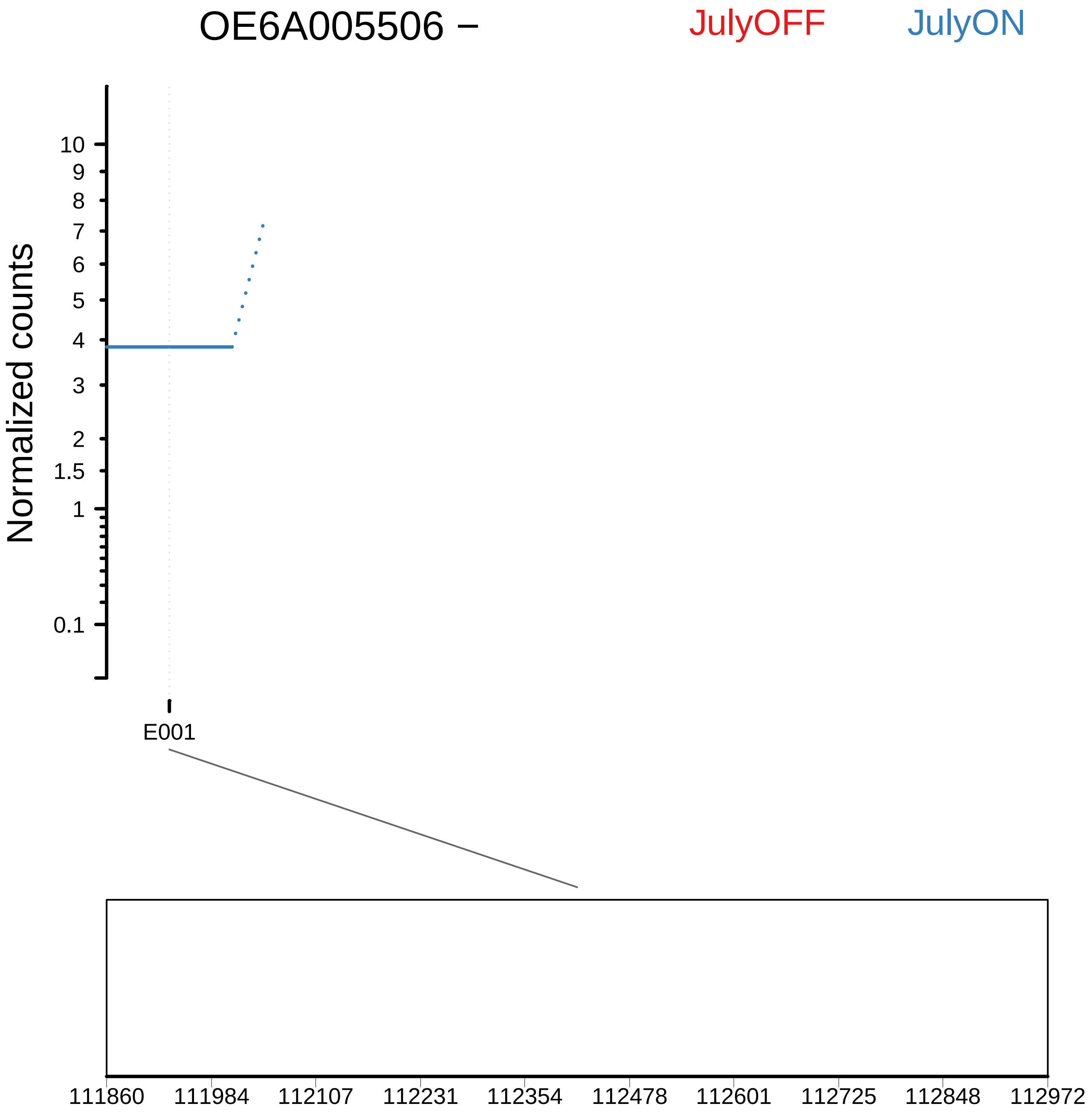

OE6A005506 −
JulyOFF
JulyON
Normalized counts
10
9
8
7
6
5
4
3
2
1.5
1
0.1
E001
111860
111984
112107
112231
112354
112478
112601
112725
112848
112972

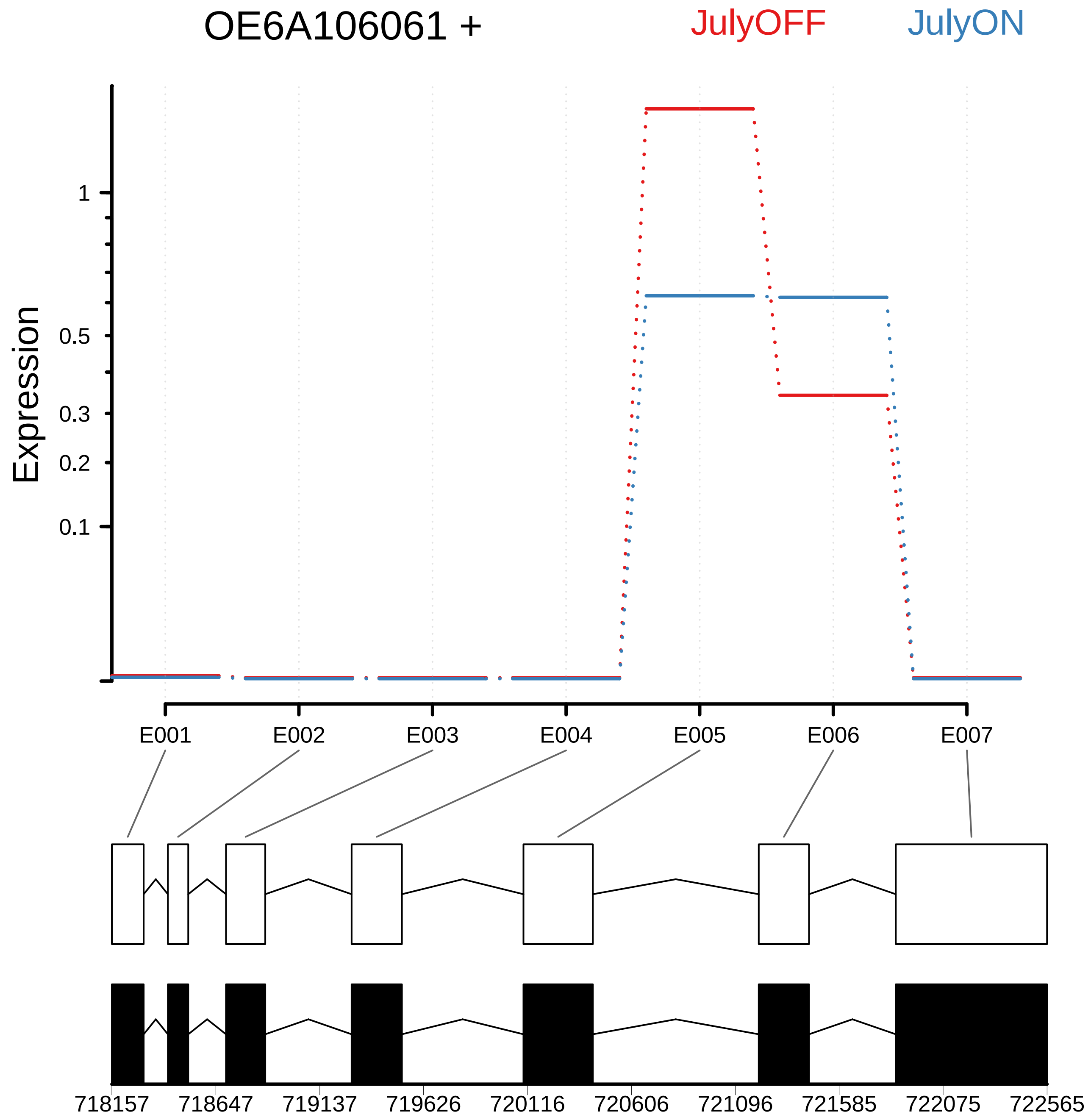

OE6A106061 +
JulyOFF
JulyON
Expression
1
0.5
0.3
0.2
0.1
E001
E002
E003
E004
E005
E006
E007
718157
718647
719137
719626
720116
720606
721096
721585
722075
722565

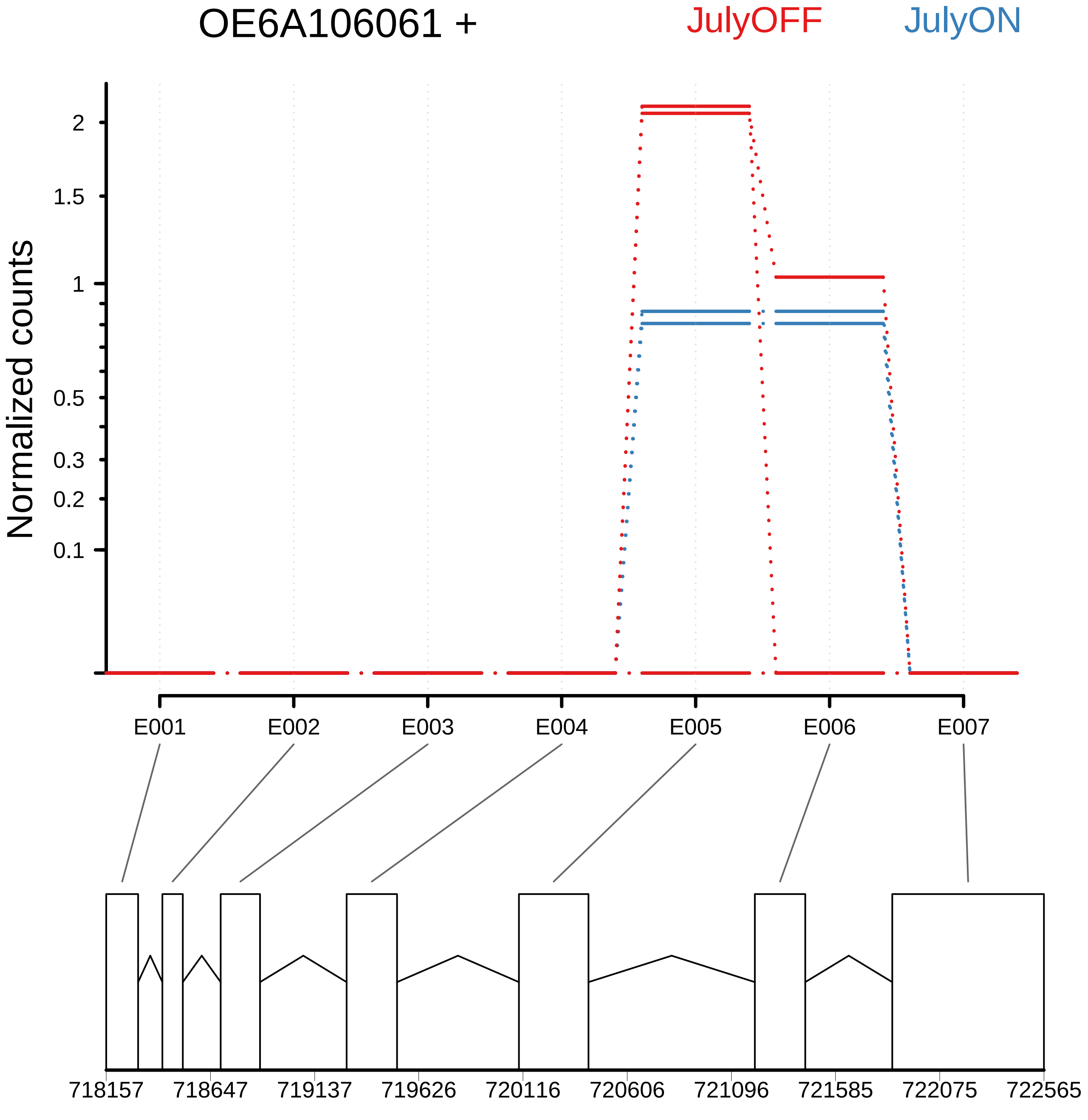

OE6A106061 +
JulyOFF
JulyON
Normalized counts
2
1.5
1
0.5
0.3
0.2
0.1
E001
E002
E003
E004
E005
E006
E007
718157
718647
719137
719626
720116
720606
721096
721585
722075
722565

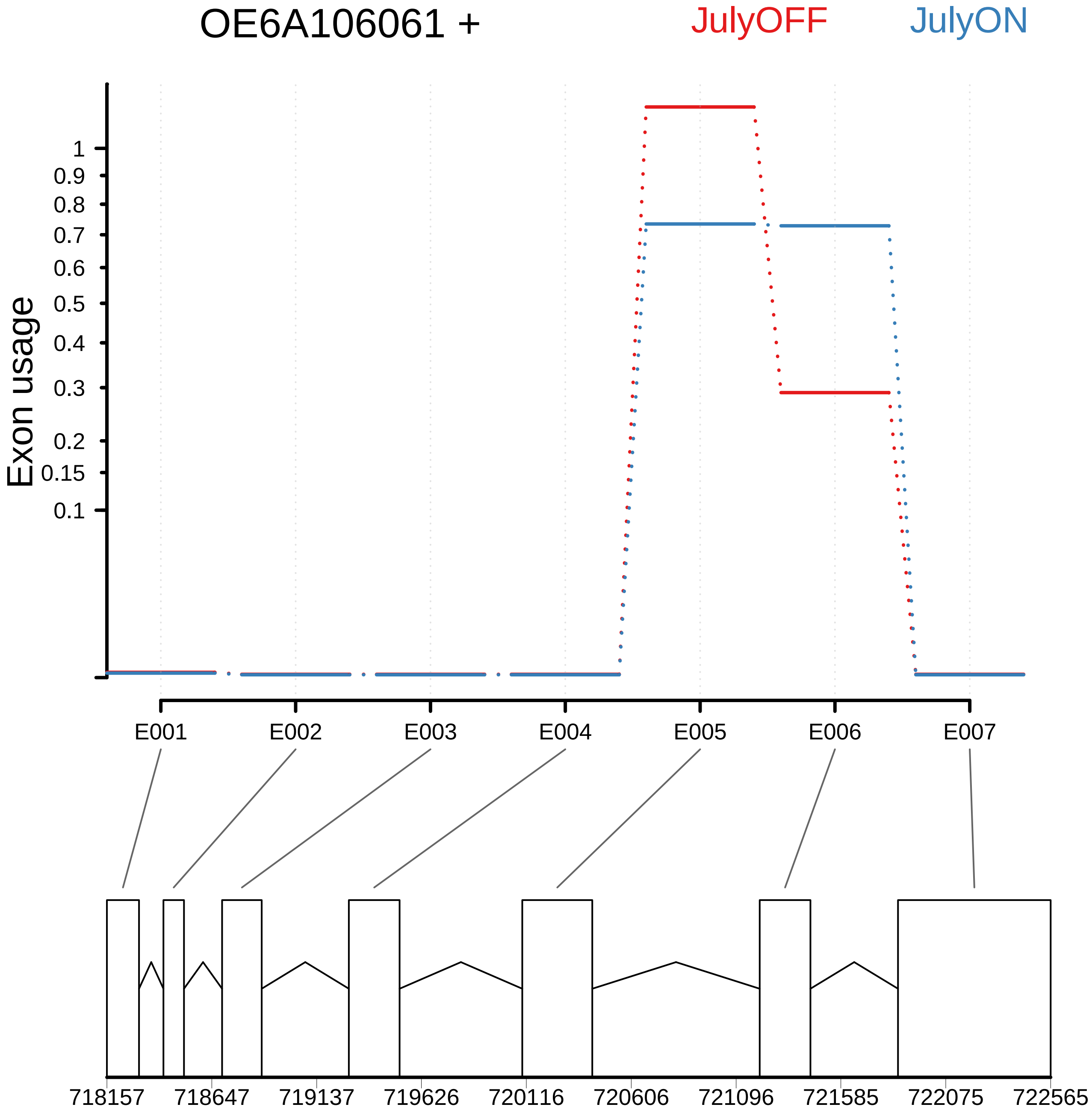

OE6A106061 +
JulyOFF
JulyON
Exon usage
1
0.9
0.8
0.7
0.6
0.5
0.4
0.3
0.2
0.15
0.1
E001
E002
E003
E004
E005
E006
E007
718157
718647
719137
719626
720116
720606
721096
721585
722075
722565

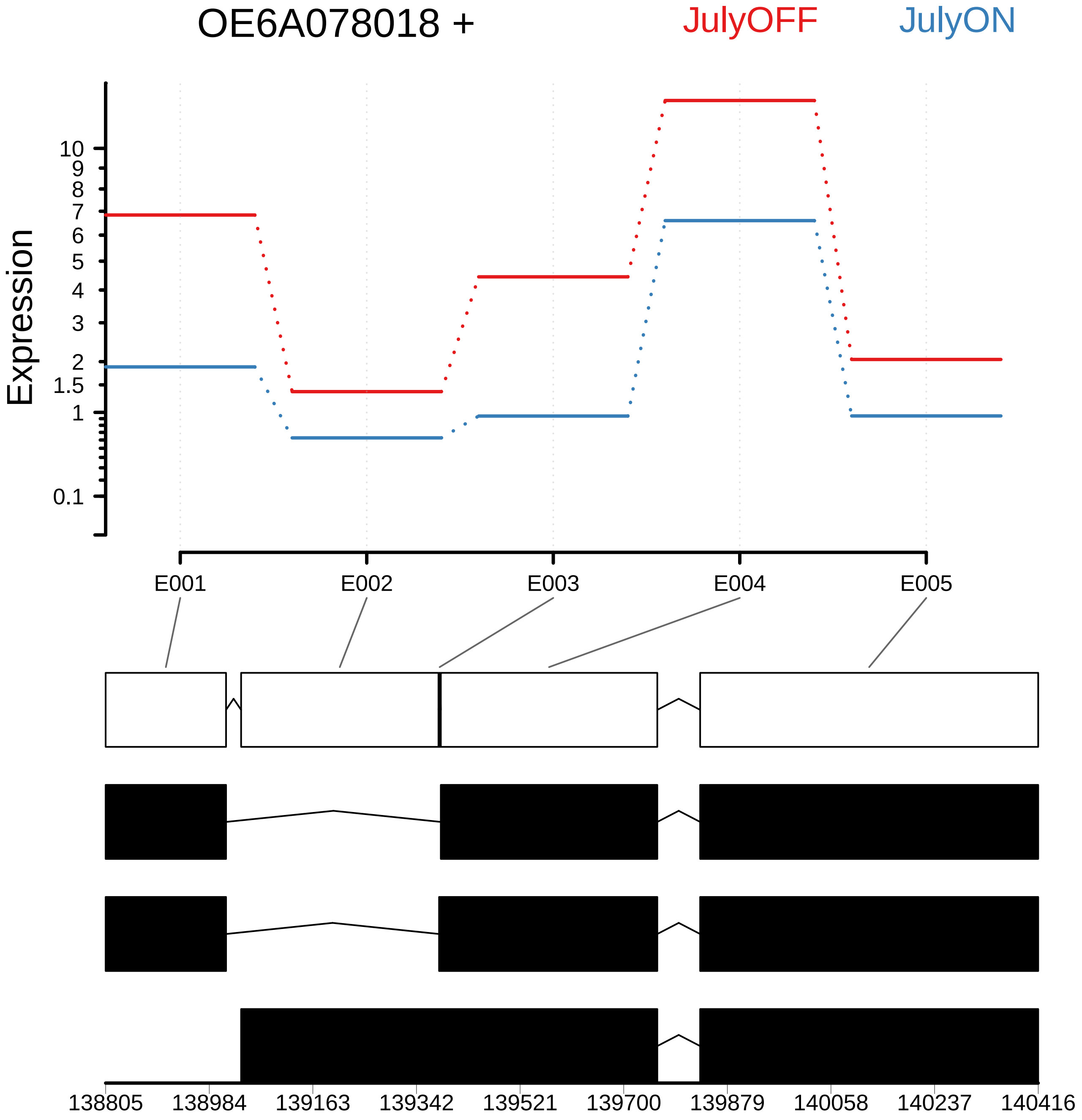

OE6A078018 +
JulyOFF
JulyON
Expression
10
9
8
7
6
5
4
3
2
1.5
1
0.1
E001
E002
E003
E004
E005
138805
138984
139163
139342
139521
139700
139879
140058
140237
140416

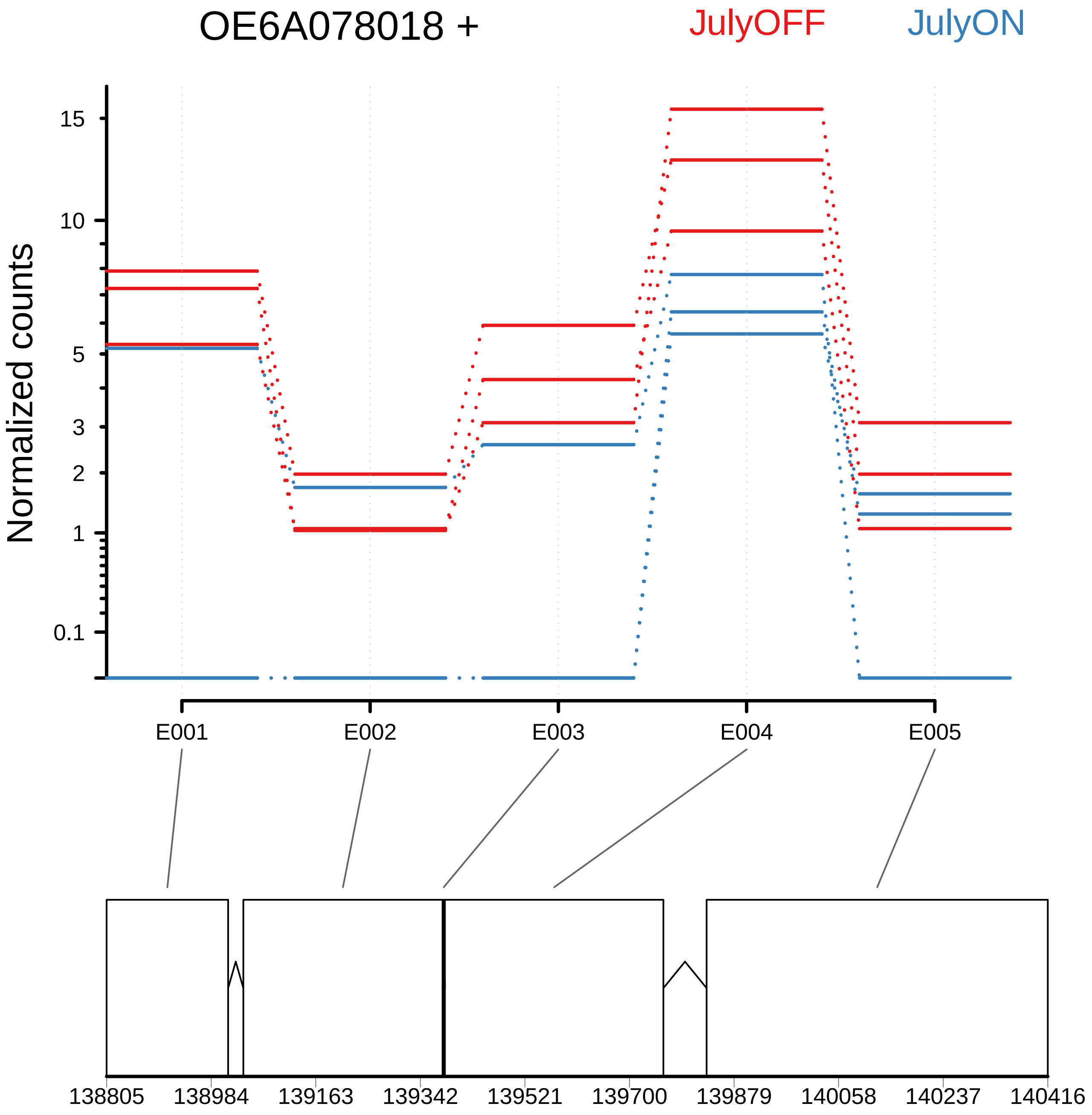
OE6A078018 +
JulyOFF
JulyON
Normalized counts
15
10
5
3
2
1
0.1
E001
E002
E003
E004
E005
138805
138984
139163
139342
139521
139700
139879
140058
140237
140416

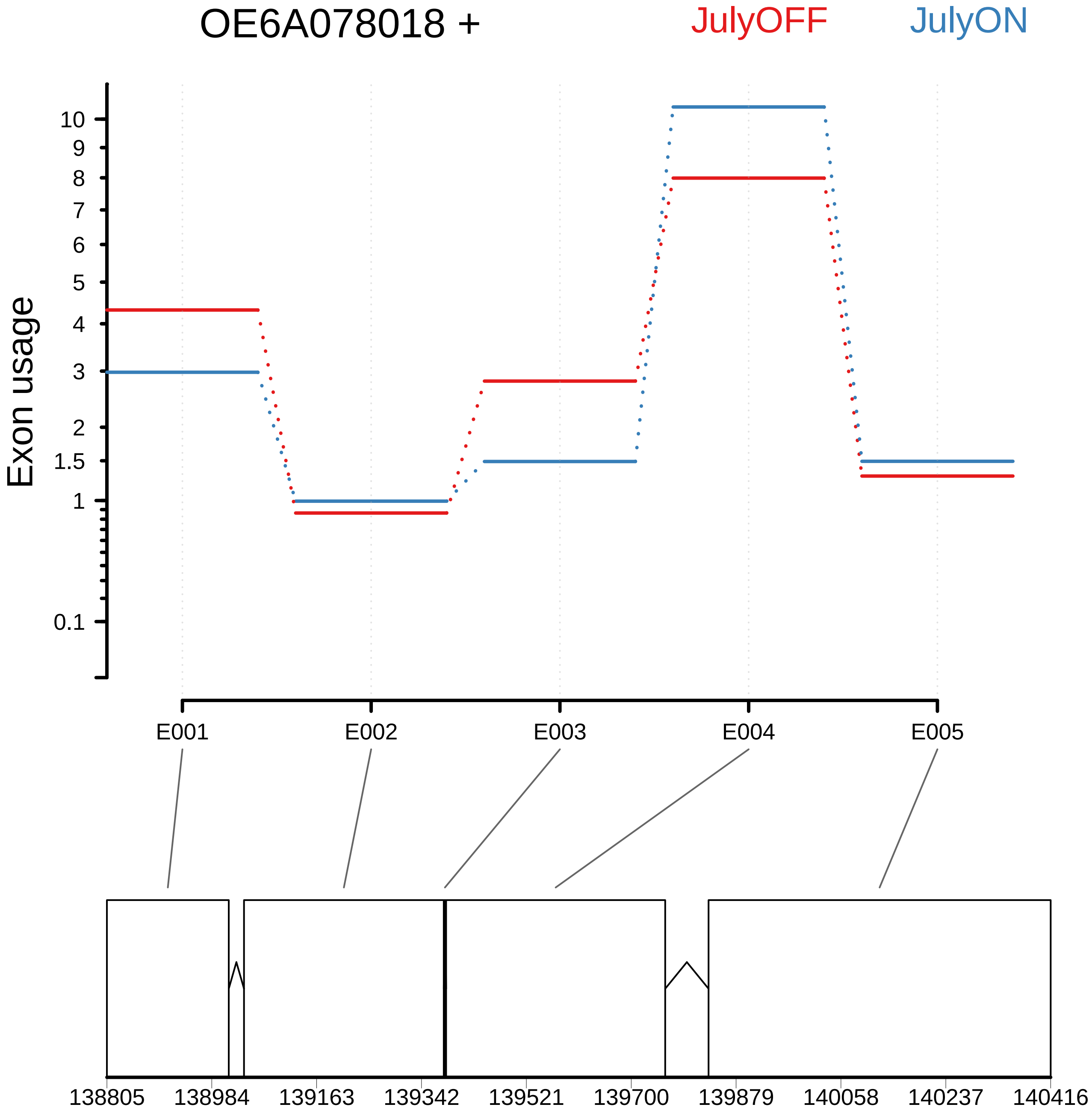
OE6A078018 +
JulyOFF
JulyON
Exon usage
10
9
8
7
6
5
4
3
2
1.5
1
0.1
E001
E002
E003
E004
E005
138805
138984
139163
139342
139521
139700
139879
140058
140237
140416

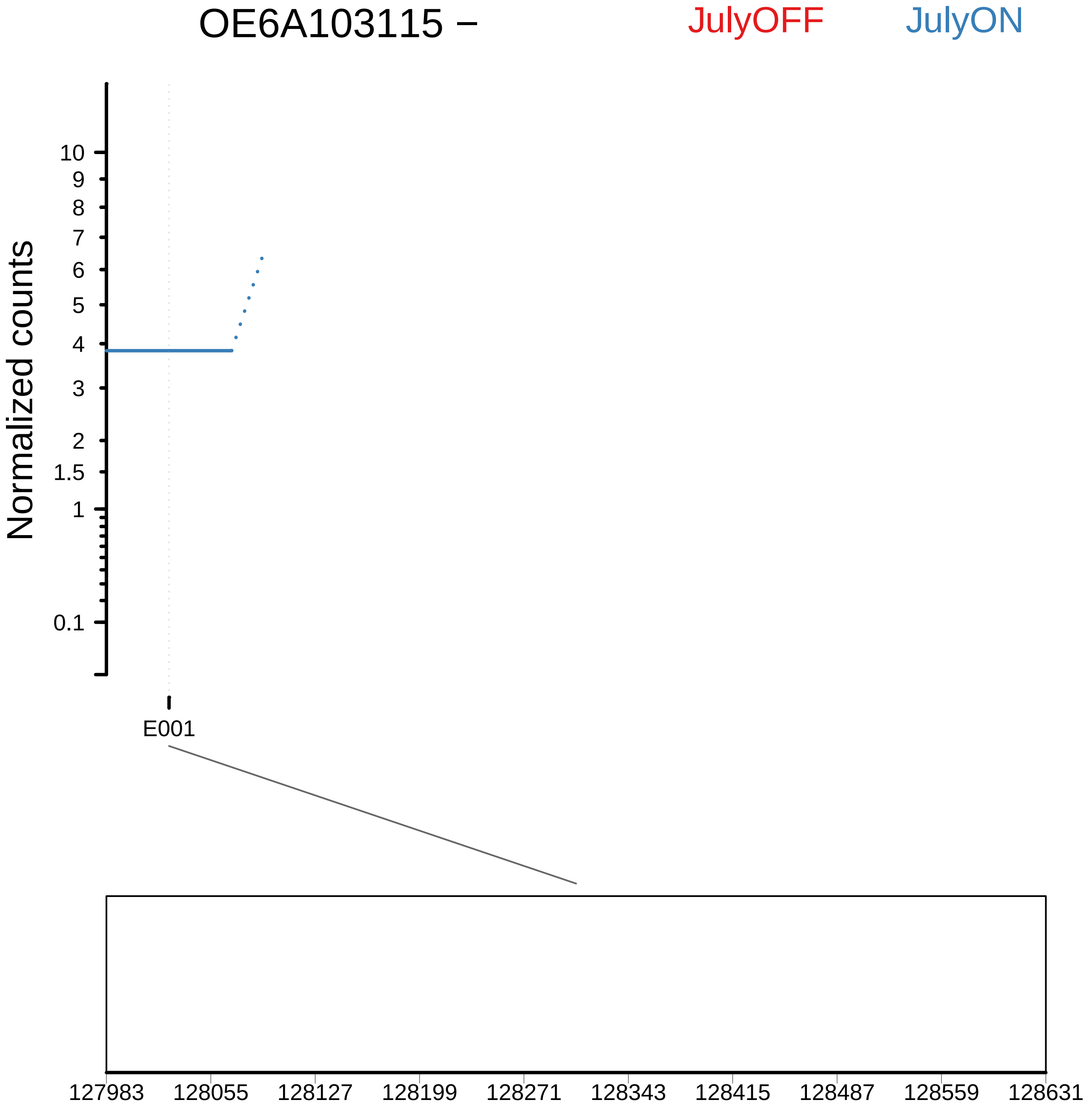
OE6A103115 −
JulyOFF
JulyON
Normalized counts
10
9
8
7
6
5
4
3
2
1.5
1
0.1
E001
127983
128055
128127
128199
128271
128343
128415
128487
128559
128631

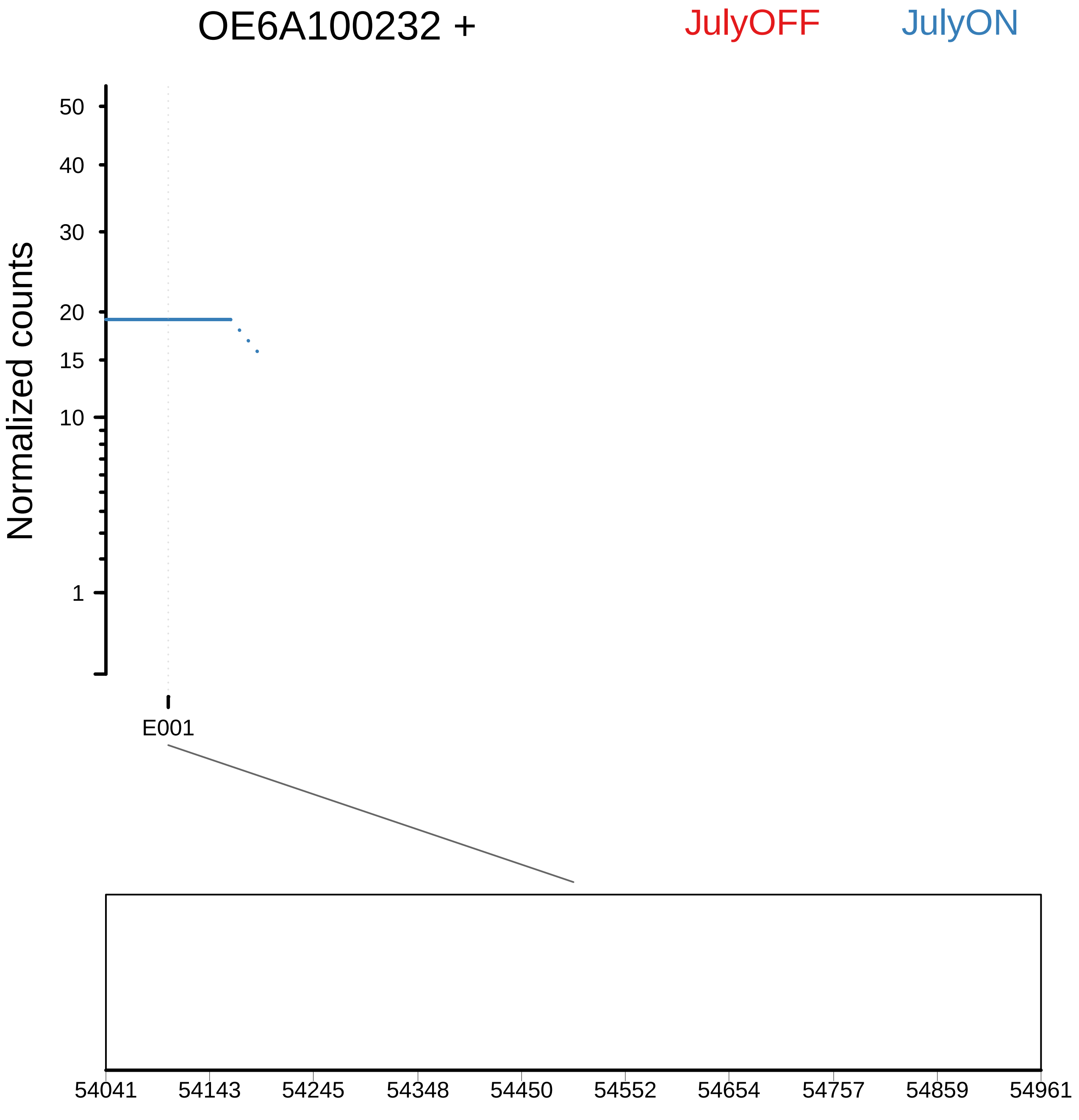
OE6A100232 +
JulyOFF
JulyON
Normalized counts
50
40
30
20
15
10
1
E001
54041
54143
54245
54348
54450
54552
54654
54757
54859
54961

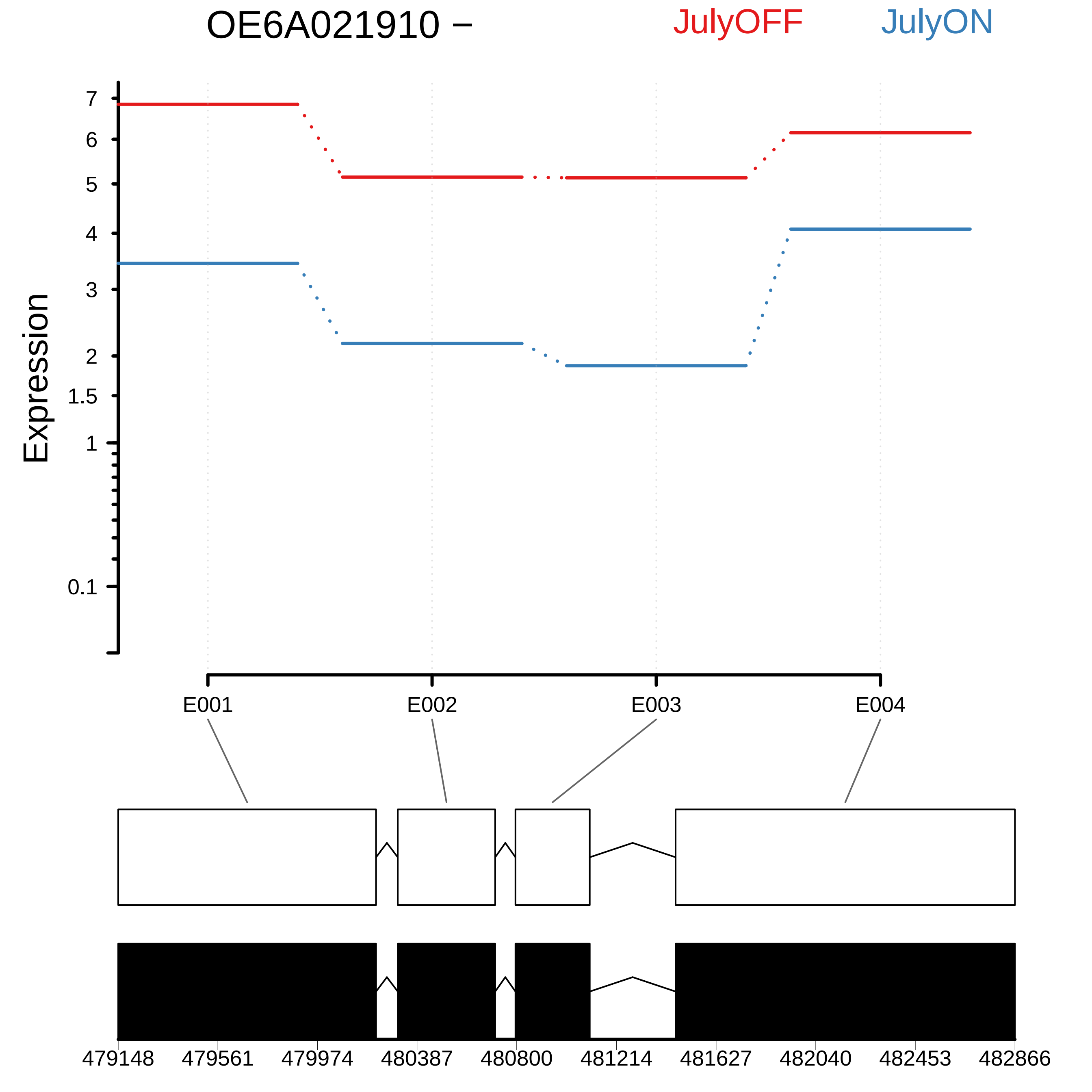

OE6A021910 −
JulyOFF
JulyON
Expression
7
6
5
4
3
2
1.5
1
0.1
E001
E002
E003
E004
479148
479561
479974
480387
480800
481214
481627
482040
482453
482866

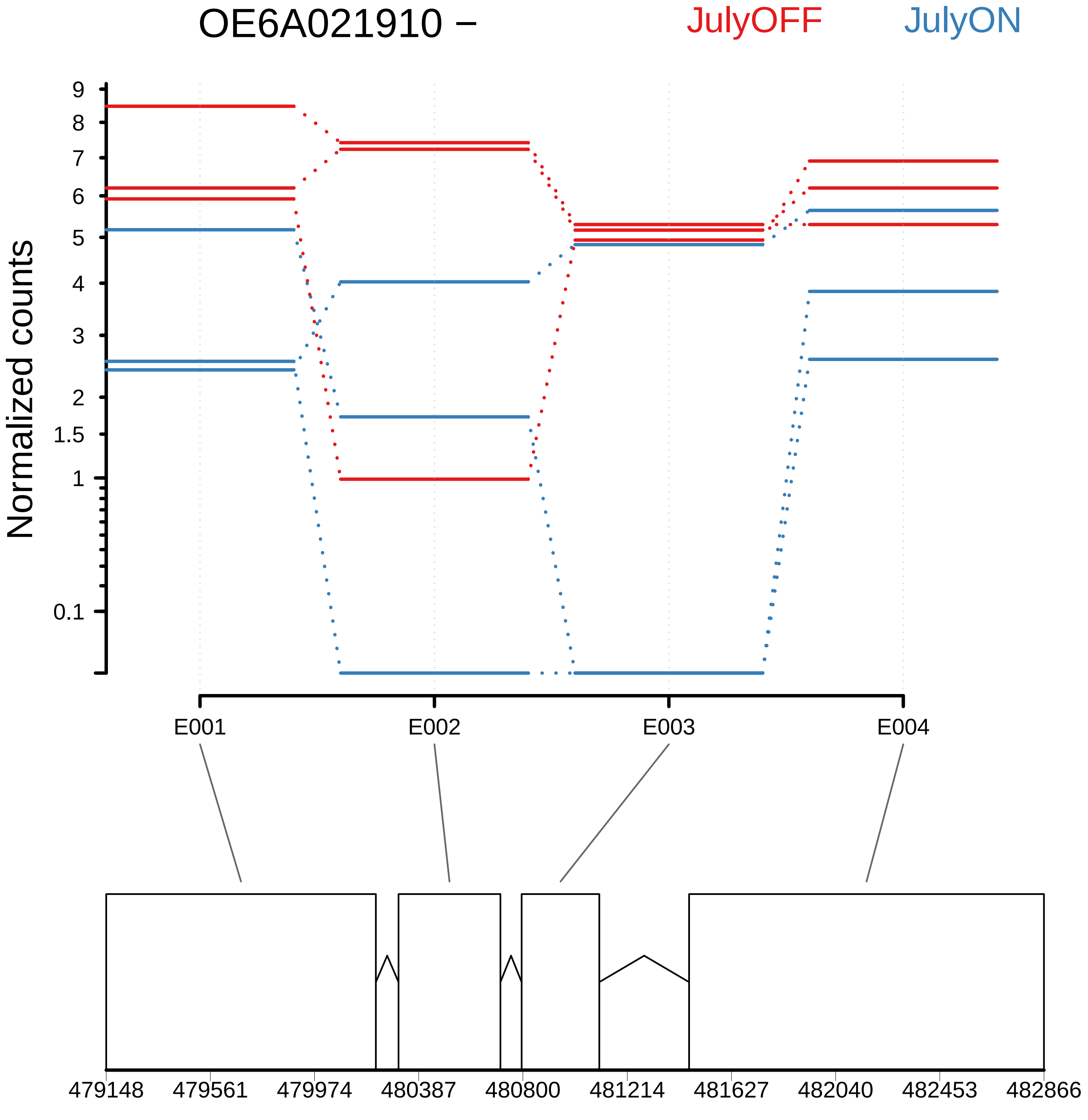
OE6A021910 −
JulyOFF
JulyON
Normalized counts
9
8
7
6
5
4
3
2
1.5
1
0.1
E001
E002
E003
E004
479148
479561
479974
480387
480800
481214
481627
482040
482453
482866

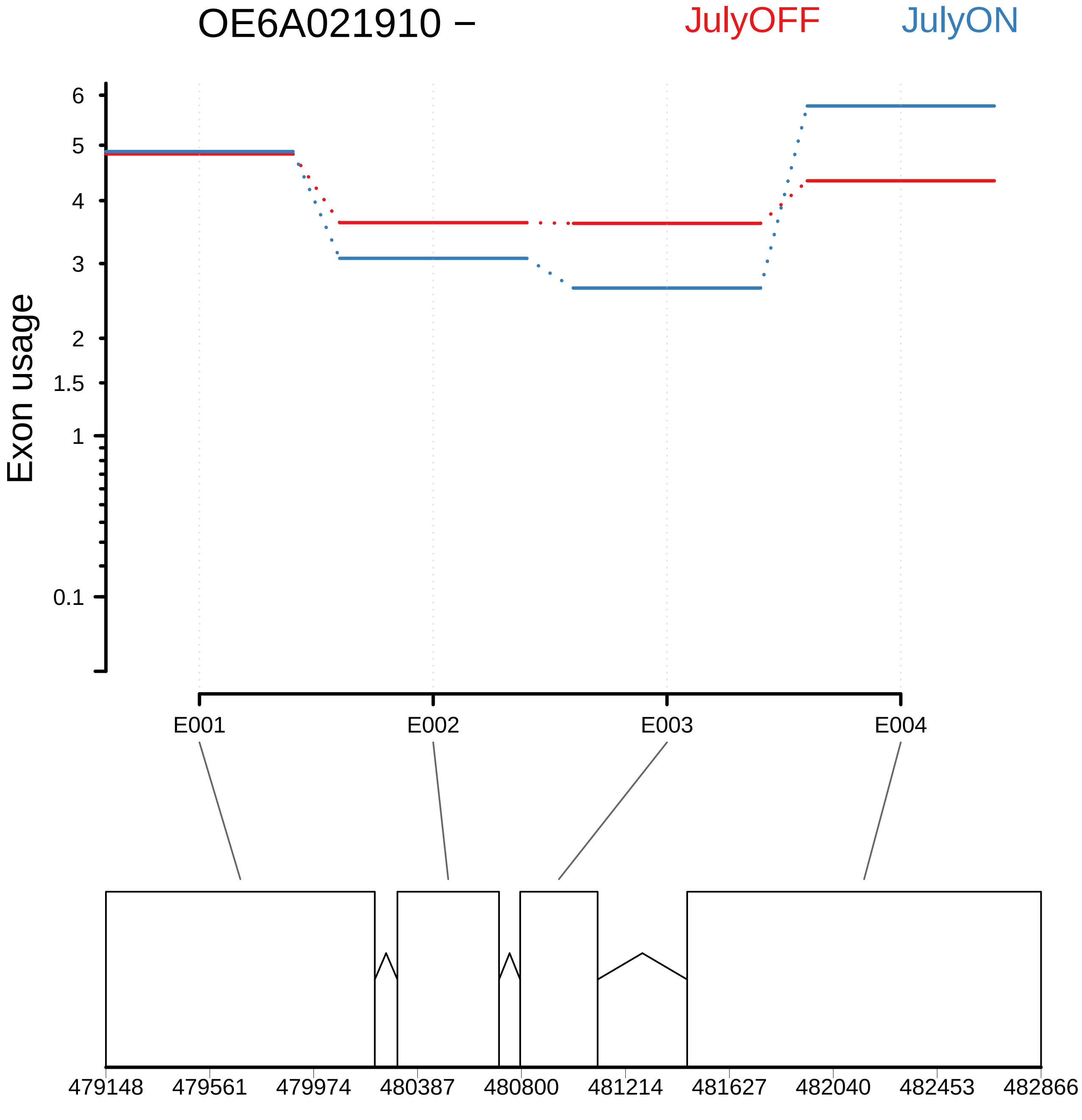

OE6A021910 −
JulyOFF
JulyON
Exon usage
6
5
4
3
2
1.5
1
0.1
E001
E002
E003
E004
479148
479561
479974
480387
480800
481214
481627
482040
482453
482866

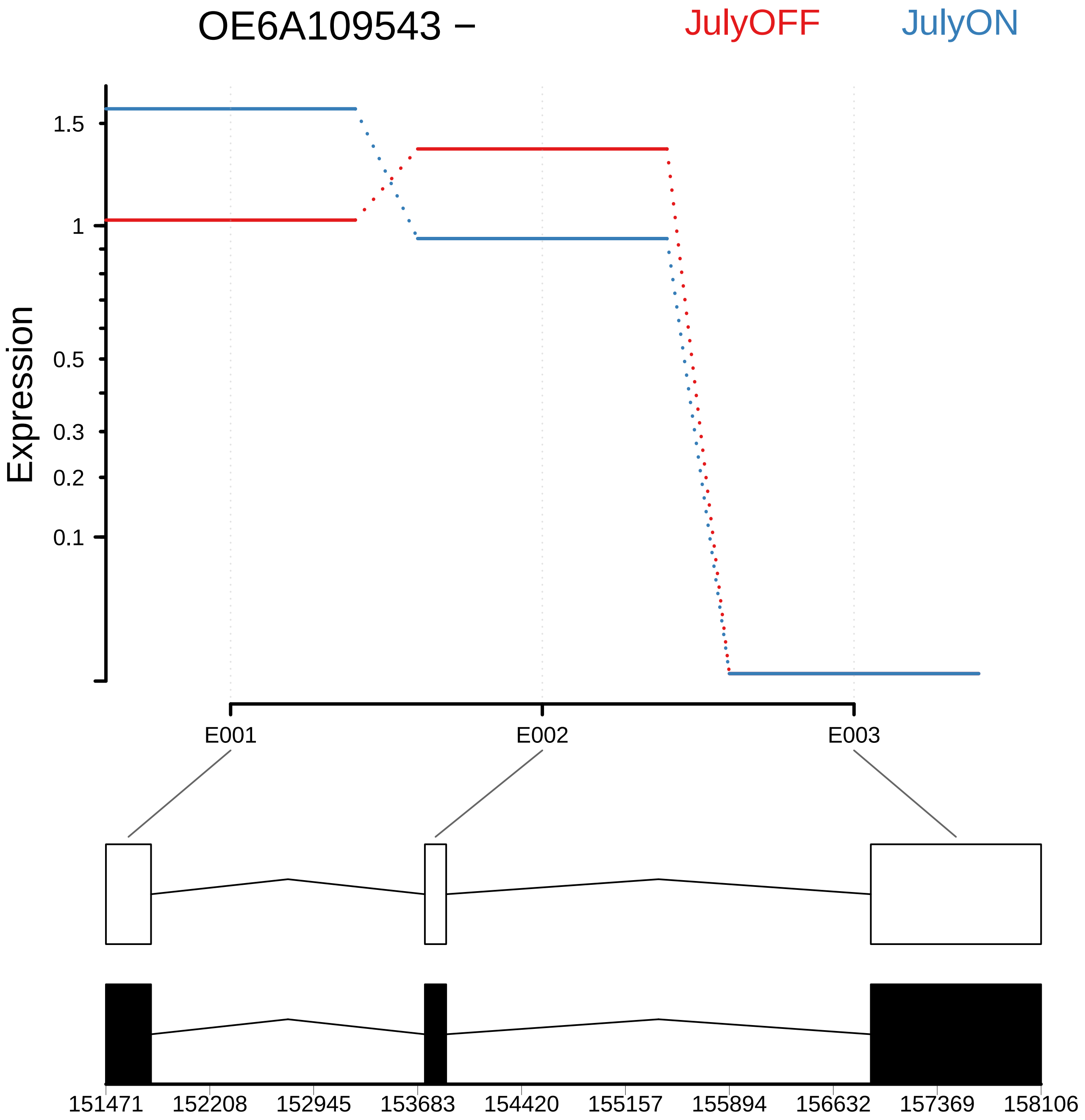

OE6A109543 −
JulyOFF
JulyON
Expression
1.5
1
0.5
0.3
0.2
0.1
E001
E002
E003
151471
152208
152945
153683
154420
155157
155894
156632
157369
158106

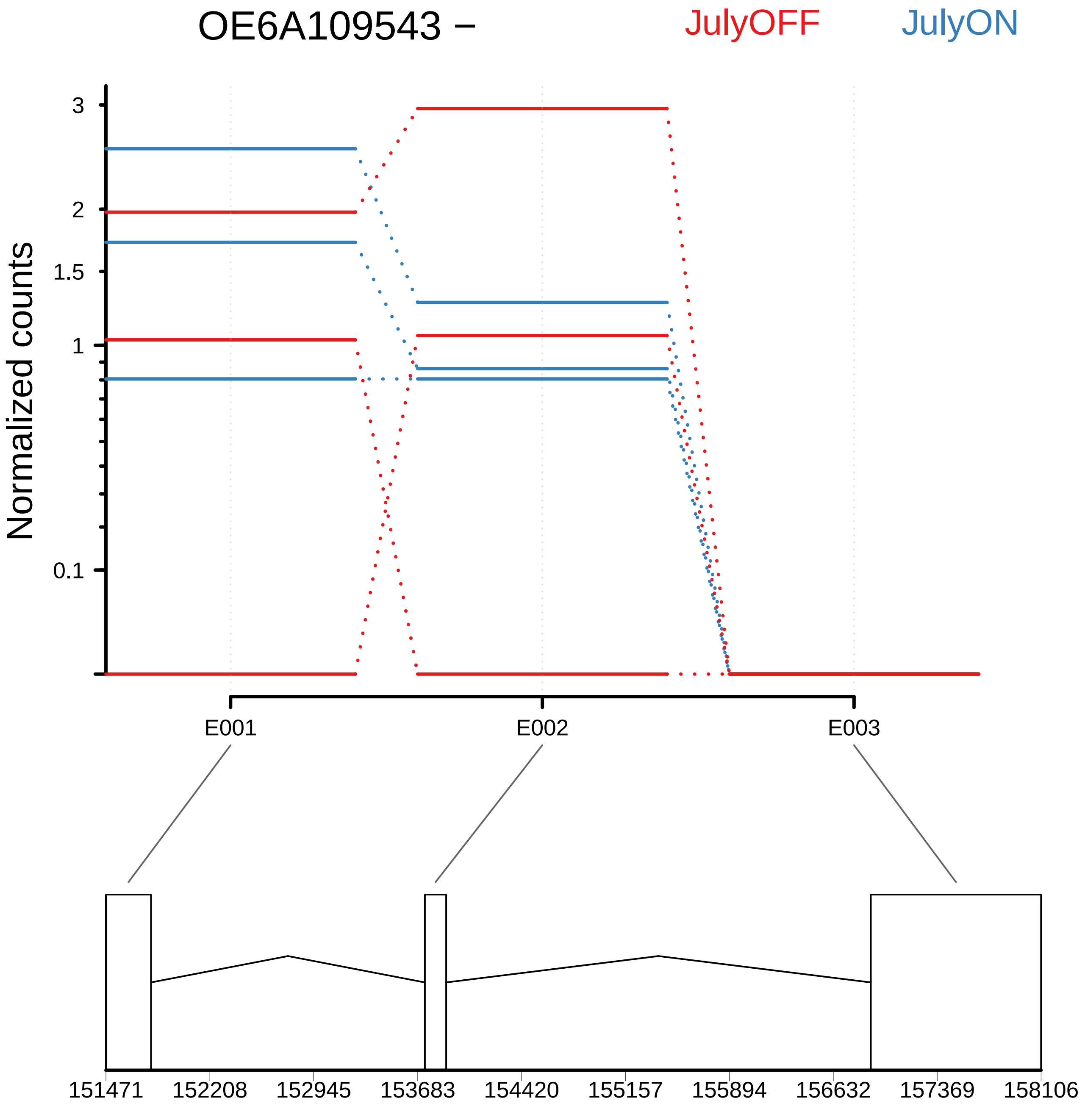
OE6A109543 −
JulyOFF
JulyON
Normalized counts
3
2
1.5
1
0.1
E001
E002
E003
151471
152208
152945
153683
154420
155157
155894
156632
157369
158106

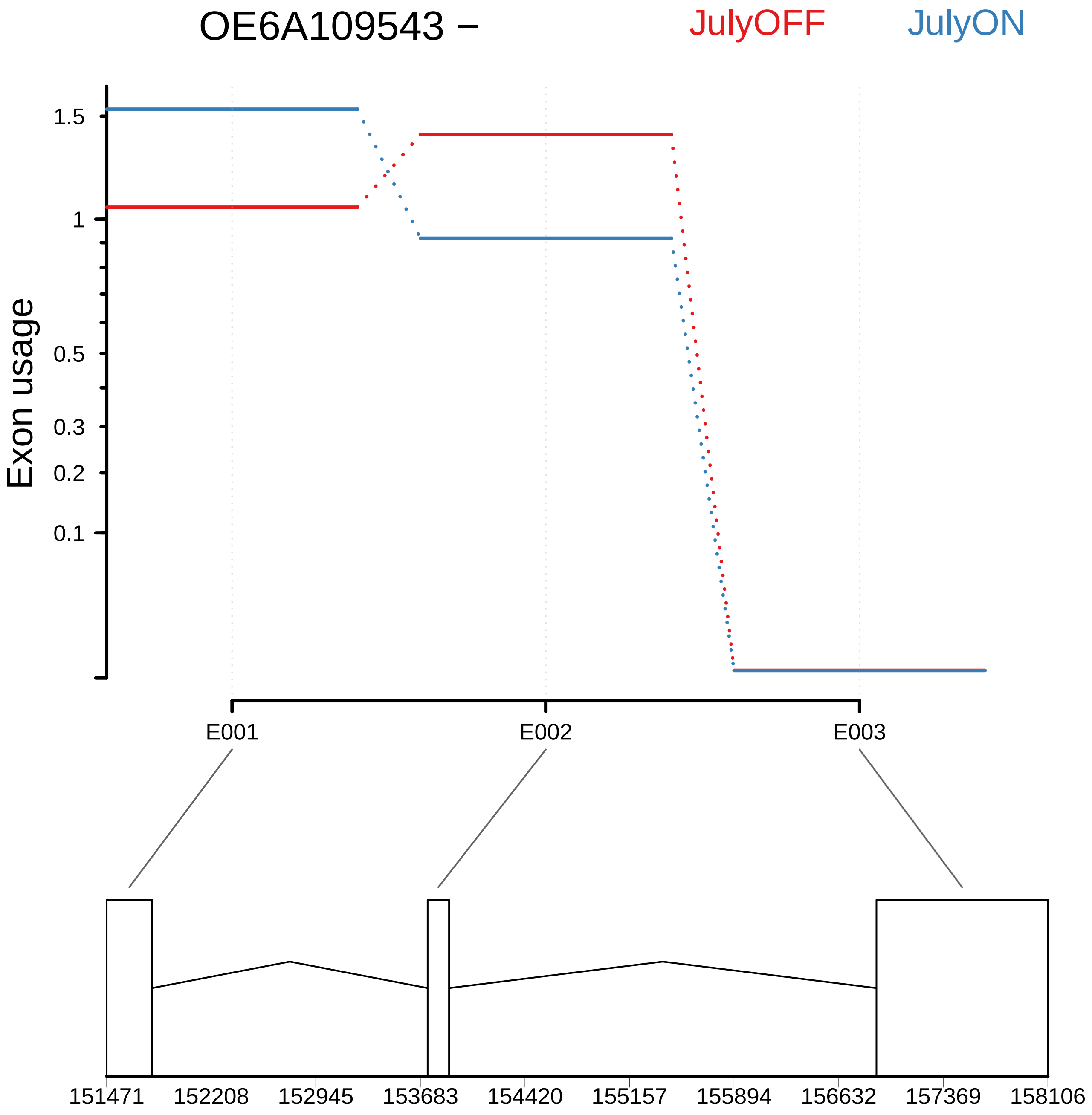

OE6A109543 −
JulyOFF
JulyON
Exon usage
1.5
1
0.5
0.3
0.2
0.1
E001
E002
E003
151471
152208
152945
153683
154420
155157
155894
156632
157369
158106

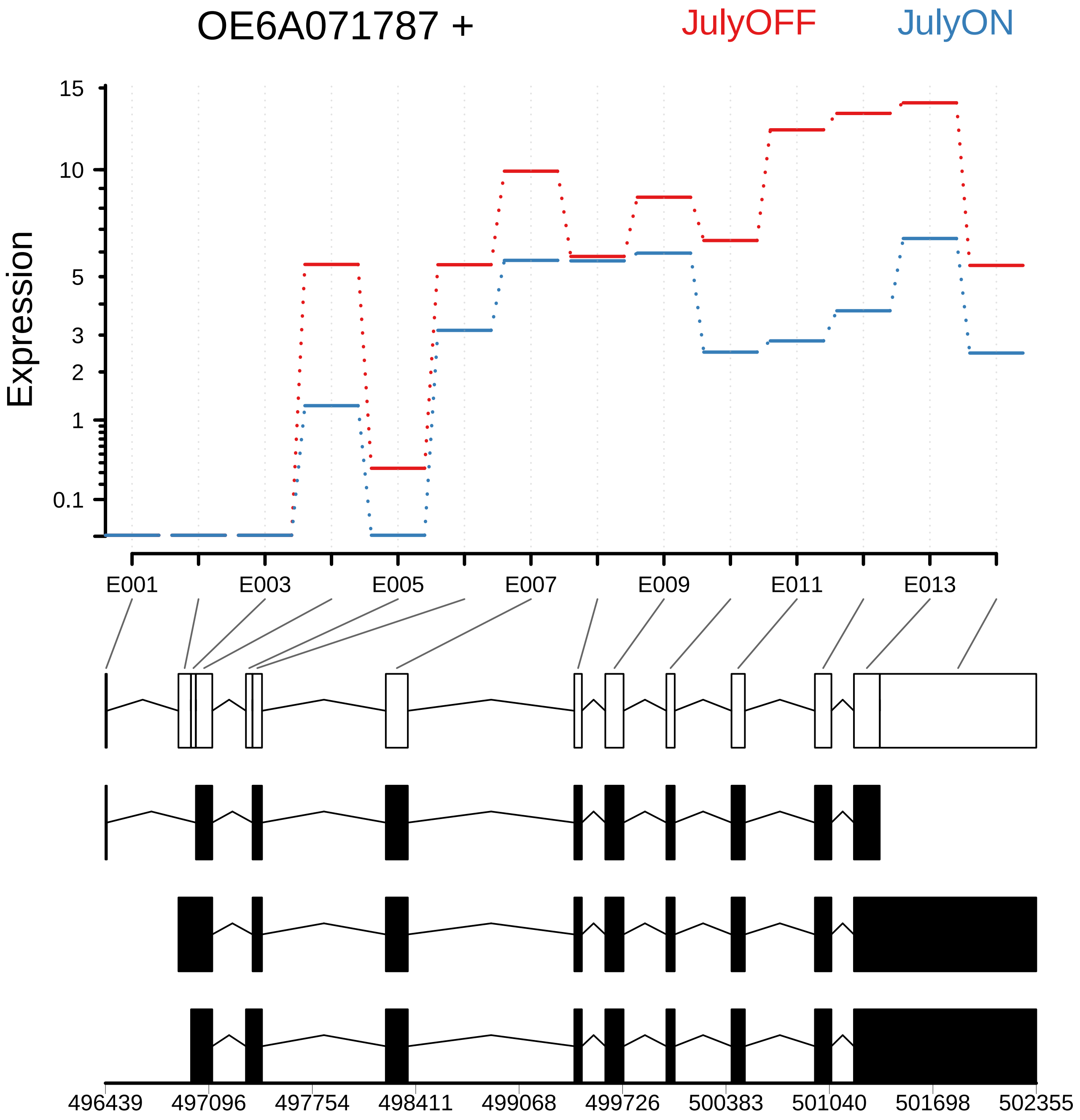
OE6A071787 +
JulyOFF
JulyON
Expression
15
10
5
3
2
1
0.1
E001
E003
E005
E007
E009
E011
E013
496439
497096
497754
498411
499068
499726
500383
501040
501698
502355

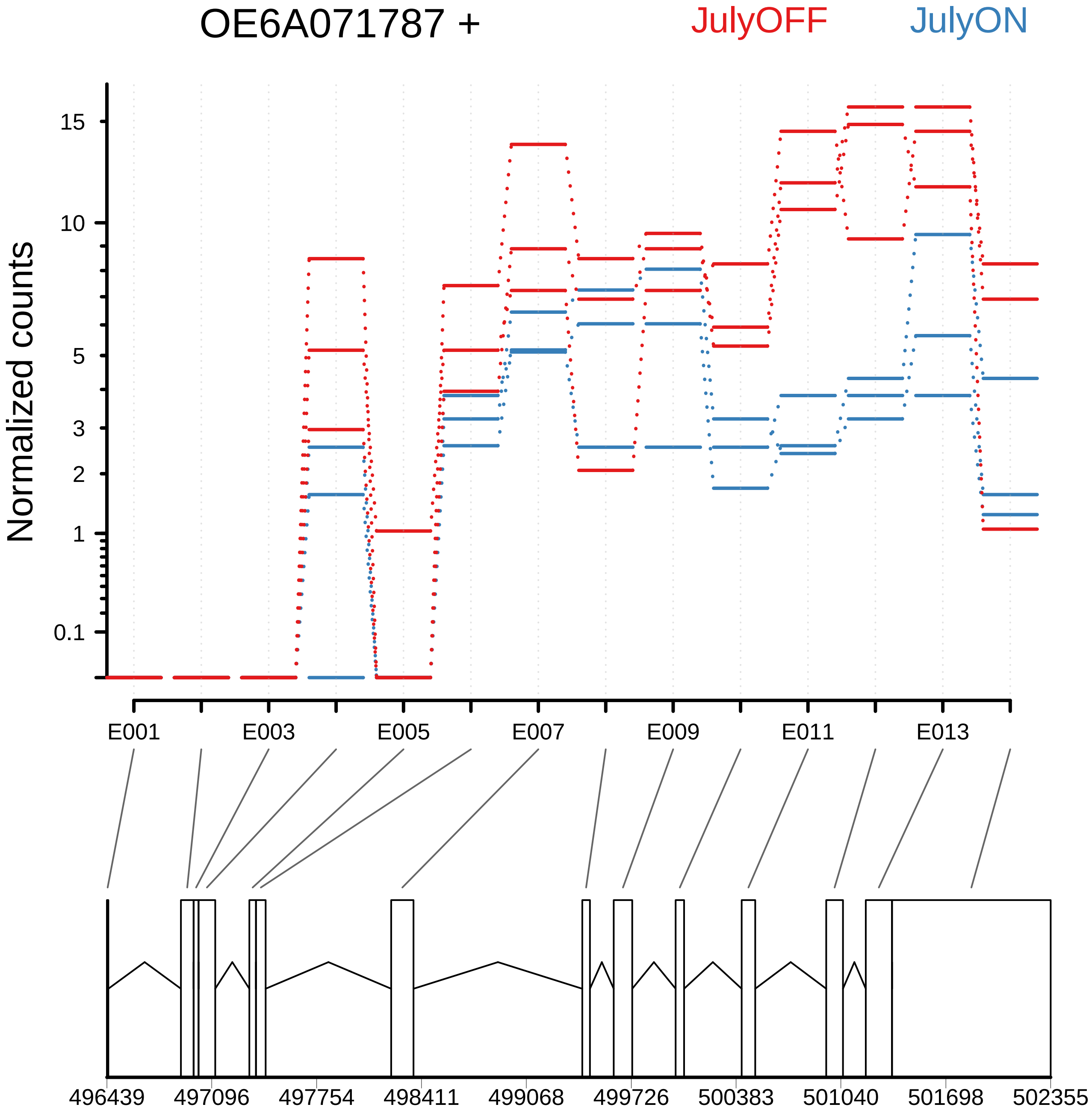

OE6A071787 +
JulyOFF
JulyON
Normalized counts
15
10
5
3
2
1
0.1
E001
E003
E005
E007
E009
E011
E013
496439
497096
497754
498411
499068
499726
500383
501040
501698
502355

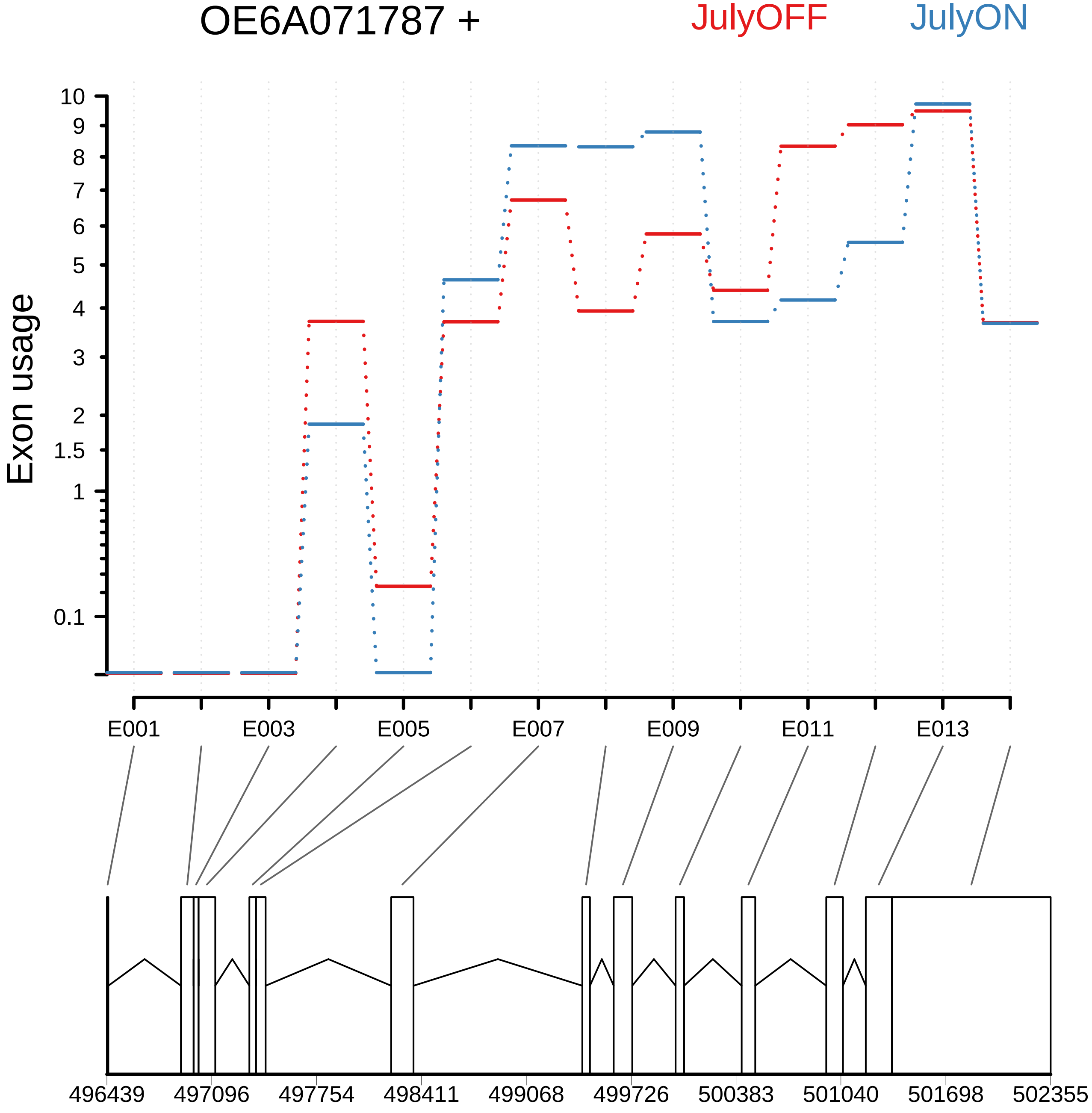
OE6A071787 +
JulyOFF
JulyON
Exon usage
10
9
8
7
6
5
4
3
2
1.5
1
0.1
E001
E003
E005
E007
E009
E011
E013
496439
497096
497754
498411
499068
499726
500383
501040
501698
502355

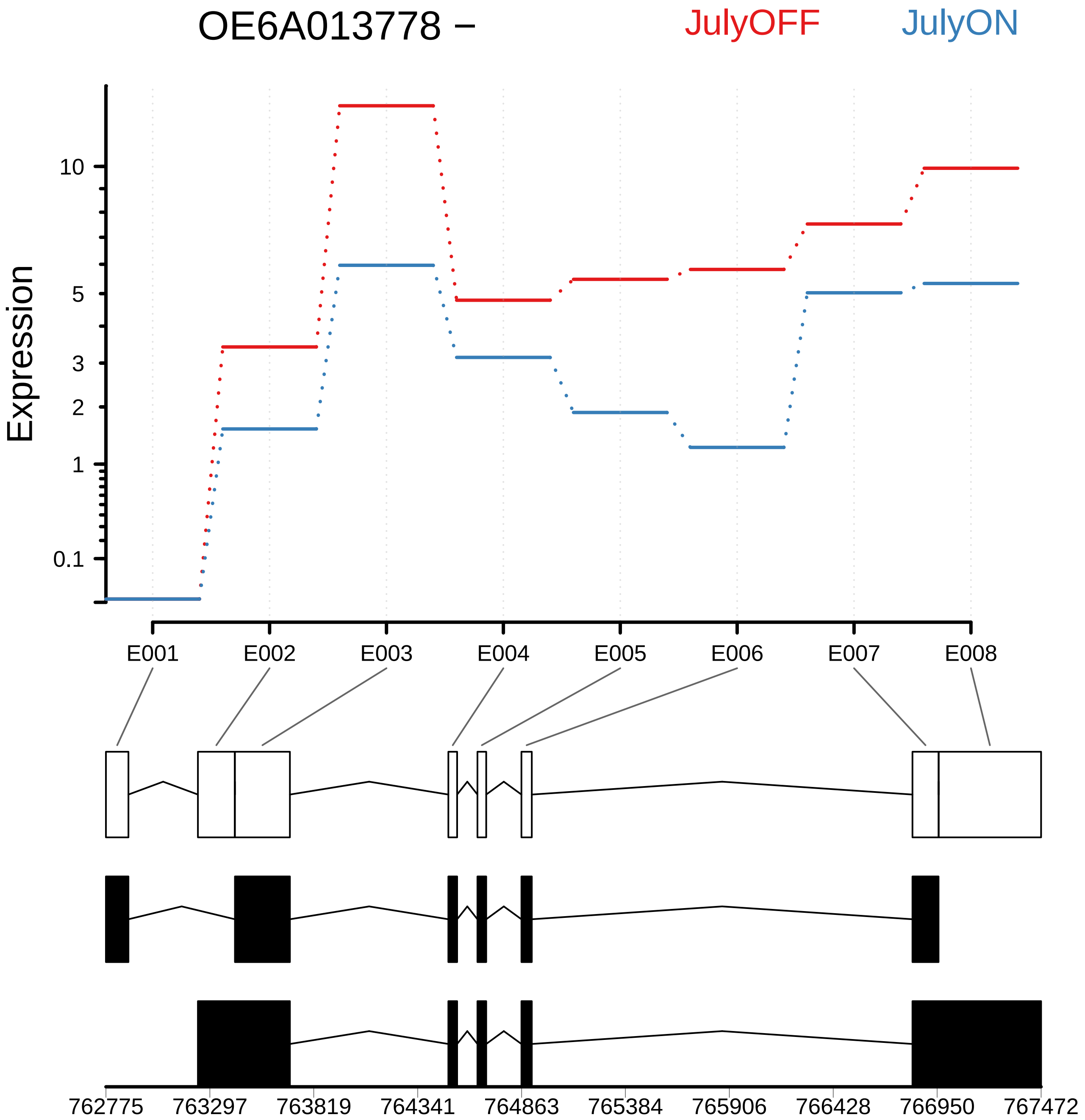

OE6A013778 –
JulyOFF
JulyON
Expression
10
5
3
2
1
0.1
E001
E002
E003
E004
E005
E006
E007
E008
762775
763297
763819
764341
764863
765384
765906
766428
766950
767472

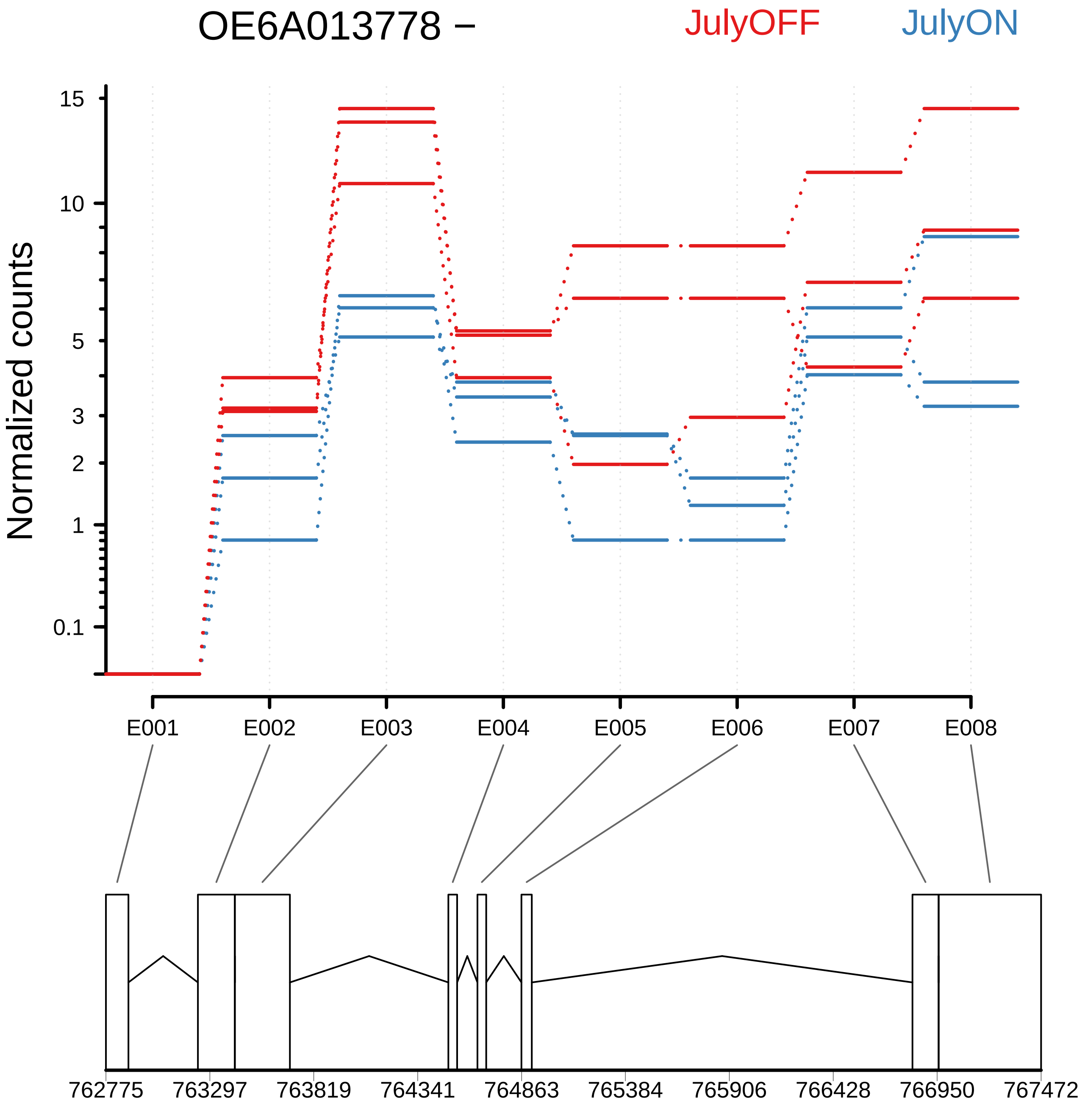

OE6A013778 −
JulyOFF
JulyON
Normalized counts
15
10
5
3
2
1
0.1
E001
E002
E003
E004
E005
E006
E007
E008
762775
763297
763819
764341
764863
765384
765906
766428
766950
767472

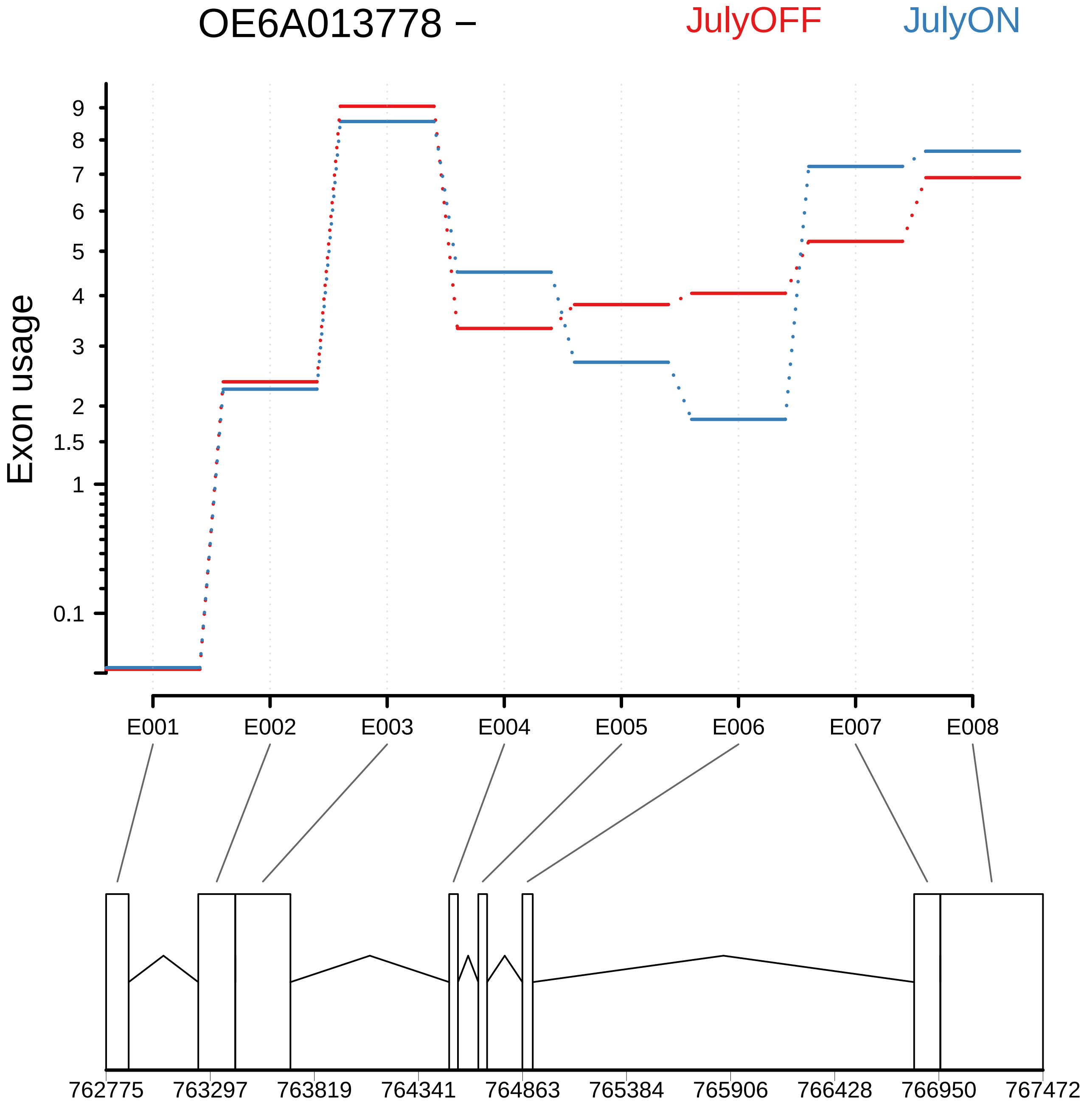
OE6A013778 −
JulyOFF
JulyON
Exon usage
9
8
7
6
5
4
3
2
1.5
1
0.1
E001
E002
E003
E004
E005
E006
E007
E008
762775
763297
763819
764341
764863
765384
765906
766428
766950
767472

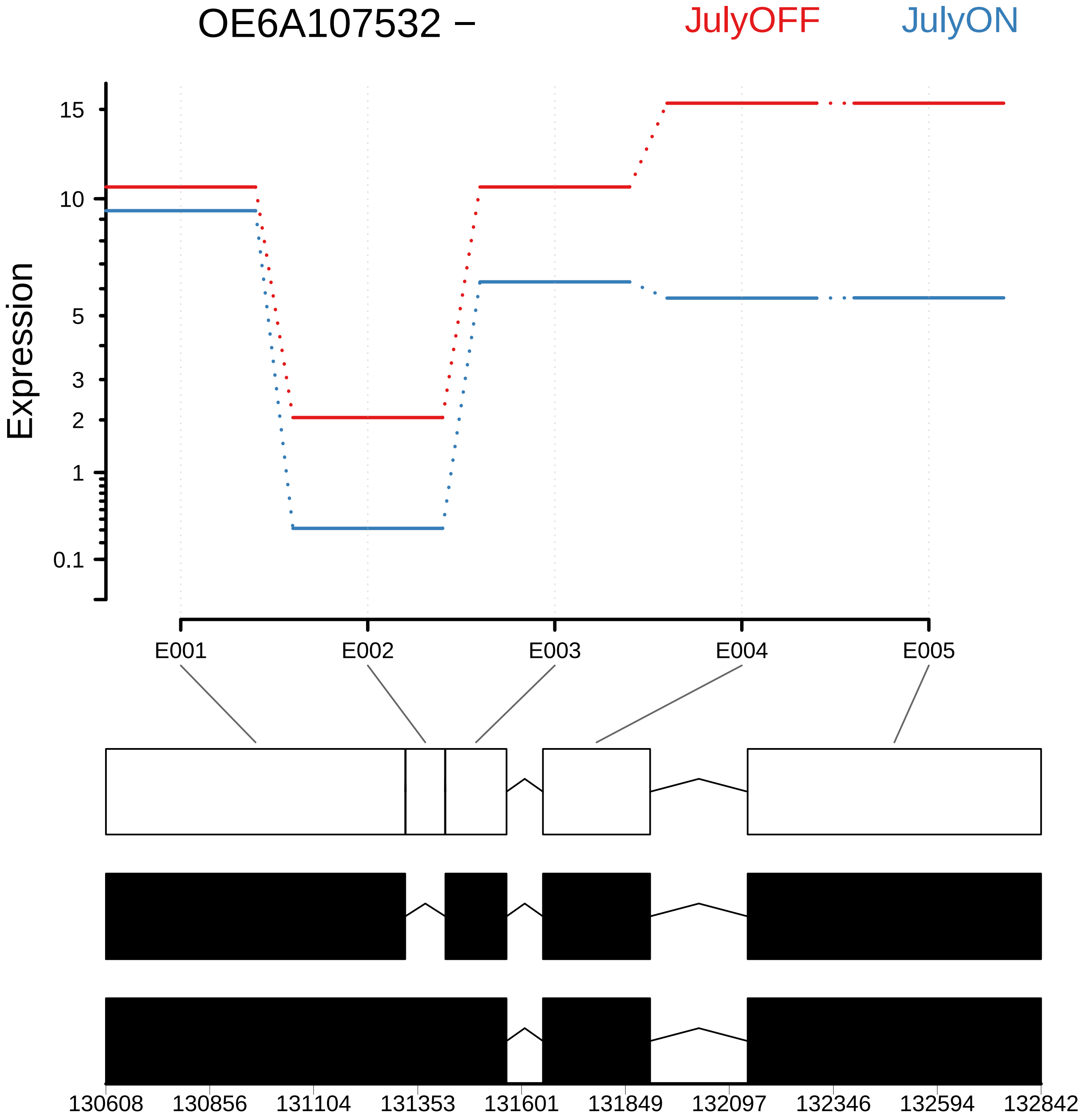

OE6A107532 −
JulyOFF
JulyON
Expression
15
10
5
3
2
1
0.1
E001
E002
E003
E004
E005
130608
130856
131104
131353
131601
131849
132097
132346
132594
132842

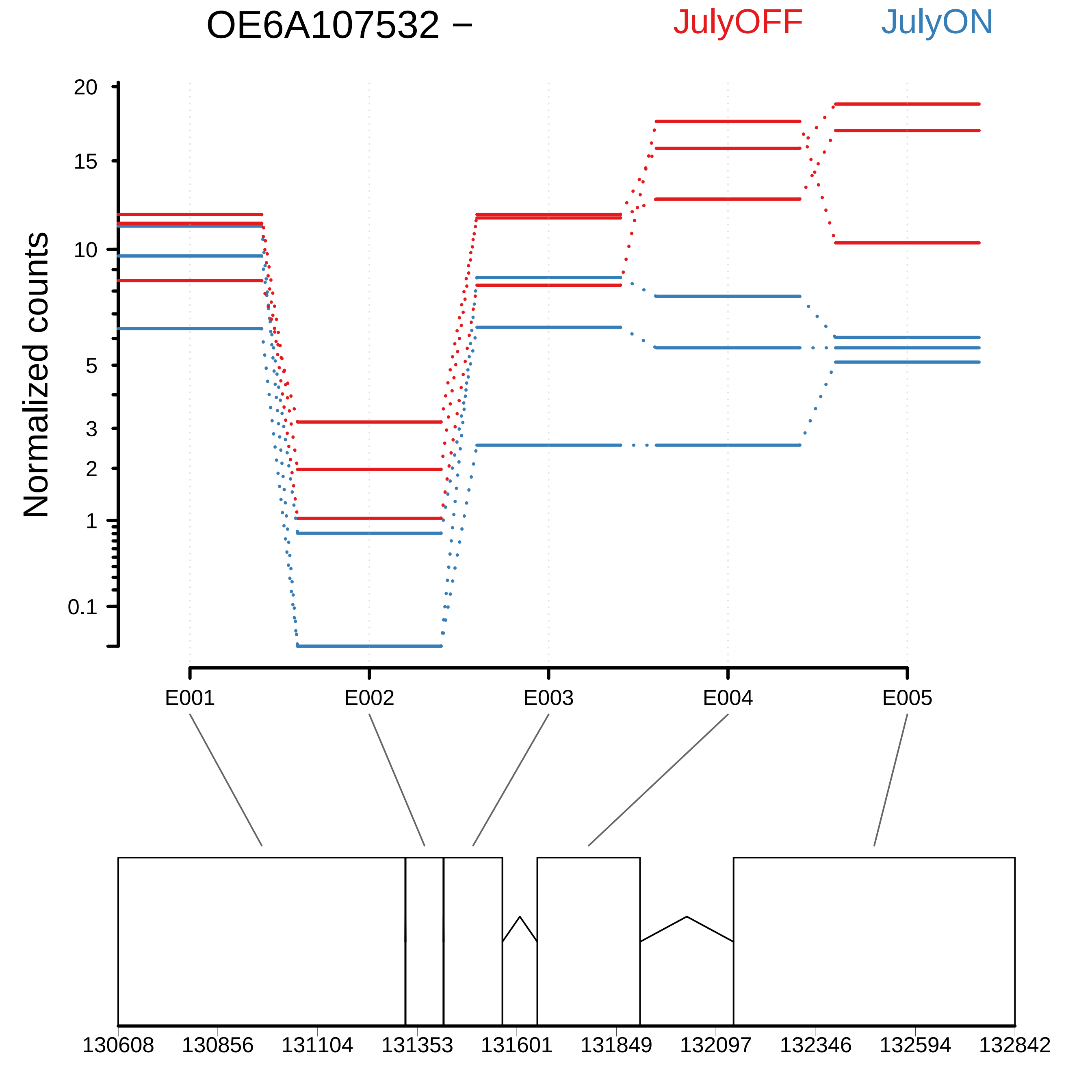
OE6A107532 −
JulyOFF
JulyON
Normalized counts
20
15
10
5
3
2
1
0.1
E001
E002
E003
E004
E005
130608
130856
131104
131353
131601
131849
132097
132346
132594
132842

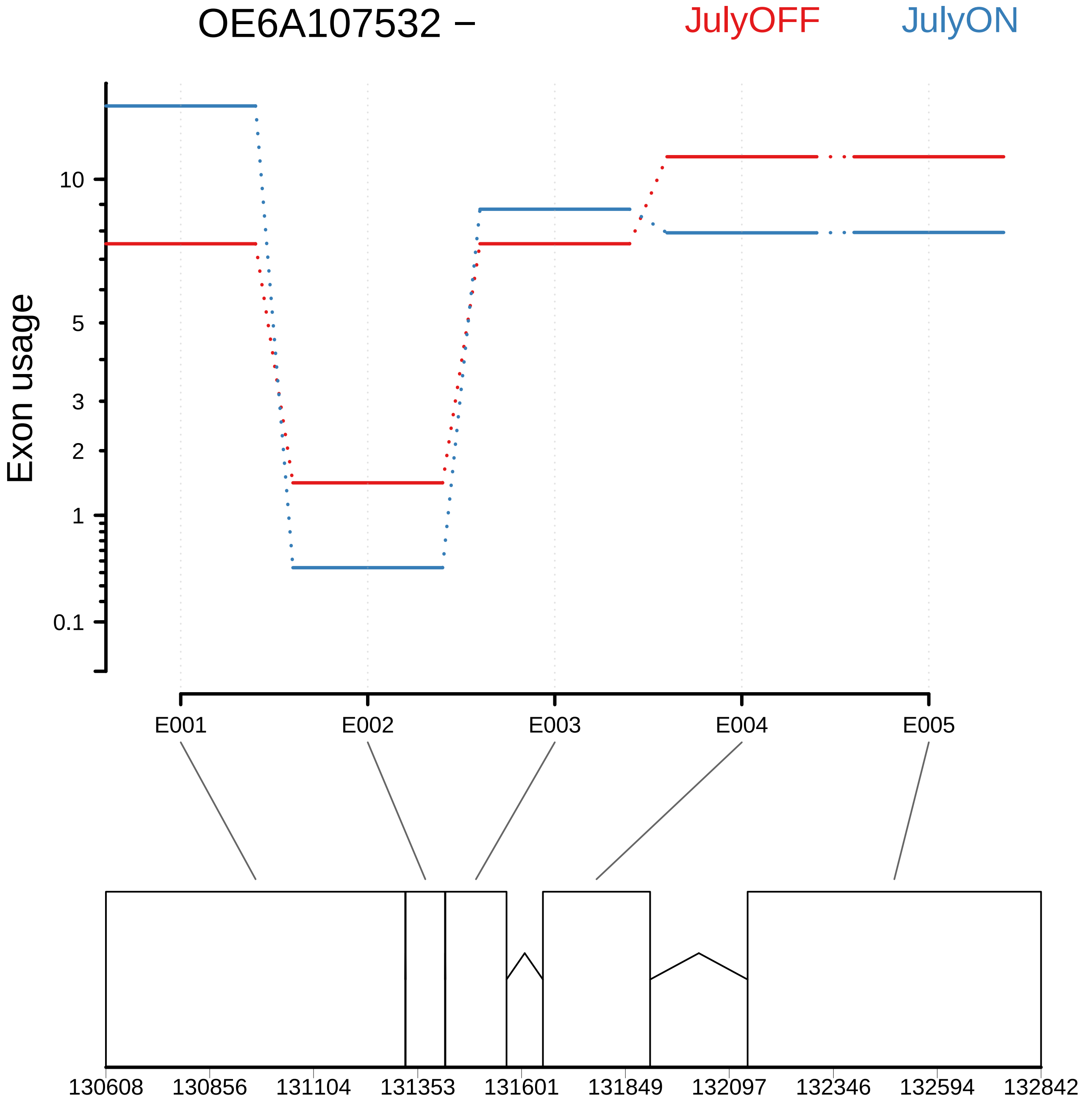

OE6A107532 −
JulyOFF
JulyON
Exon usage
10
5
3
2
1
0.1
E001
E002
E003
E004
E005
130608
130856
131104
131353
131601
131849
132097
132346
132594
132842

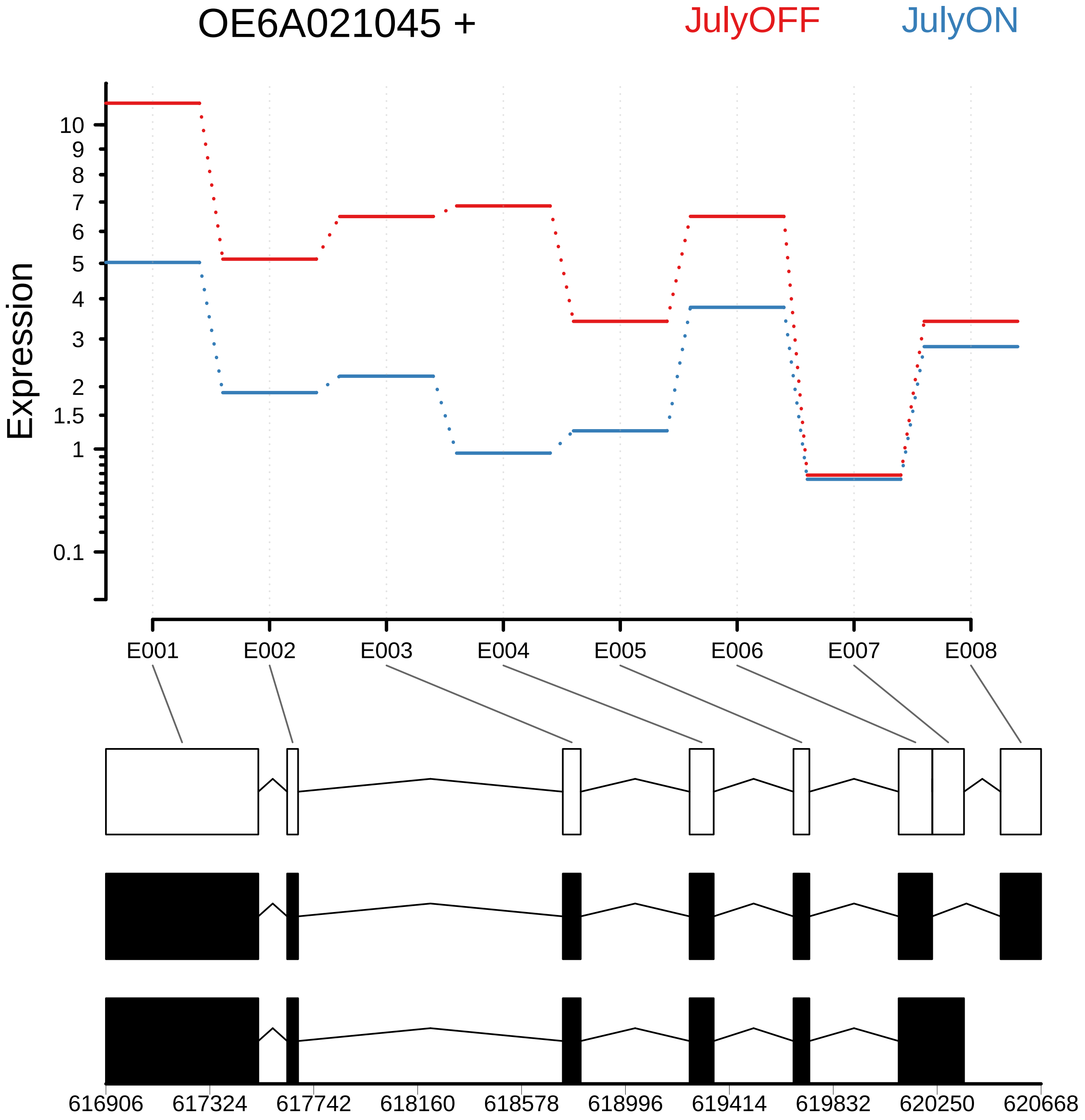
OE6A021045 +
JulyOFF
JulyON
Expression
10
9
8
7
6
5
4
3
2
1.5
1
0.1
E001
E002
E003
E004
E005
E006
E007
E008
616906
617324
617742
618160
618578
618996
619414
619832
620250
620668

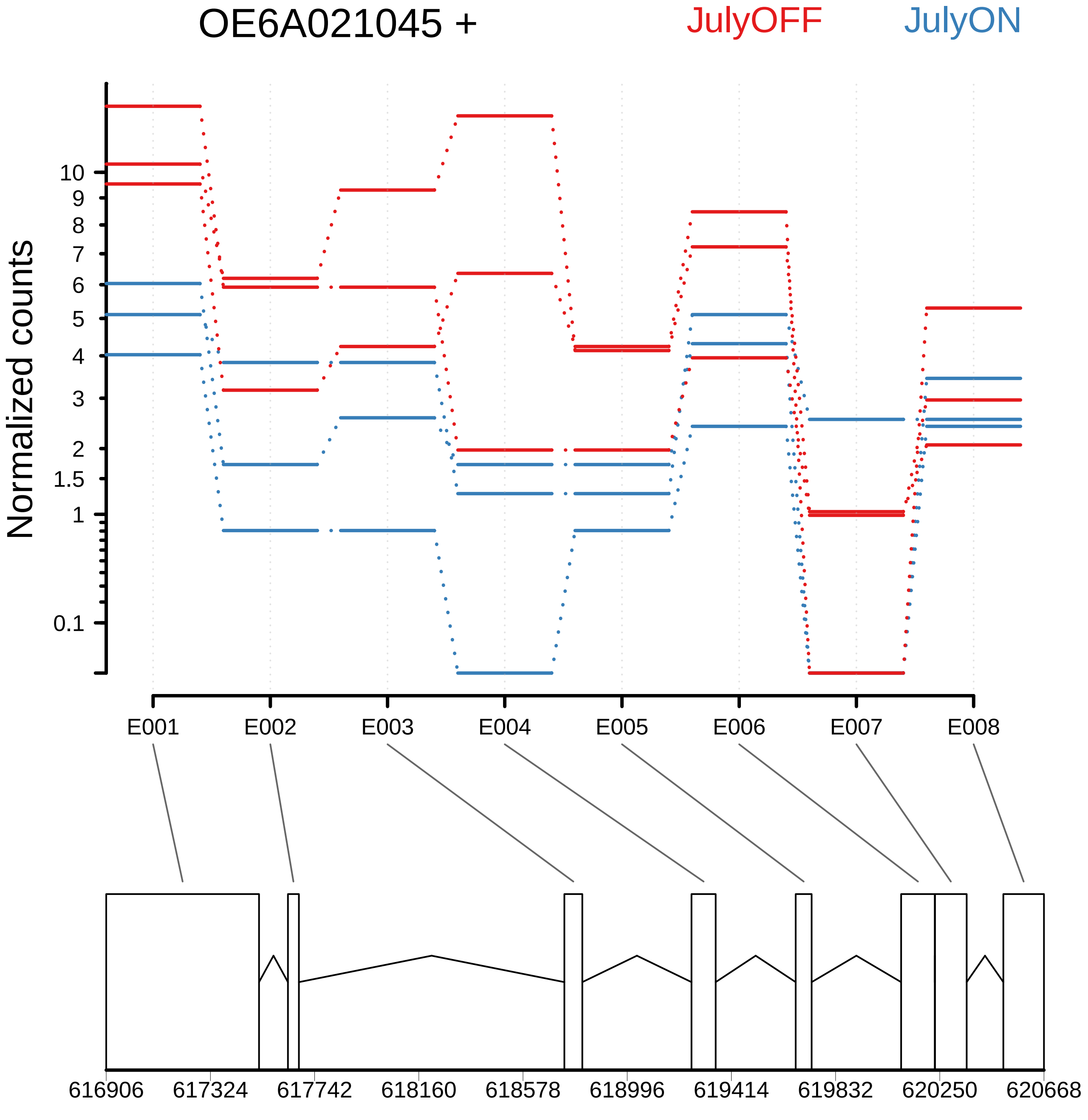

OE6A021045 +
JulyOFF
JulyON
Normalized counts
10
9
8
7
6
5
4
3
2
1.5
1
0.1
E001
E002
E003
E004
E005
E006
E007
E008
616906
617324
617742
618160
618578
618996
619414
619832
620250
620668

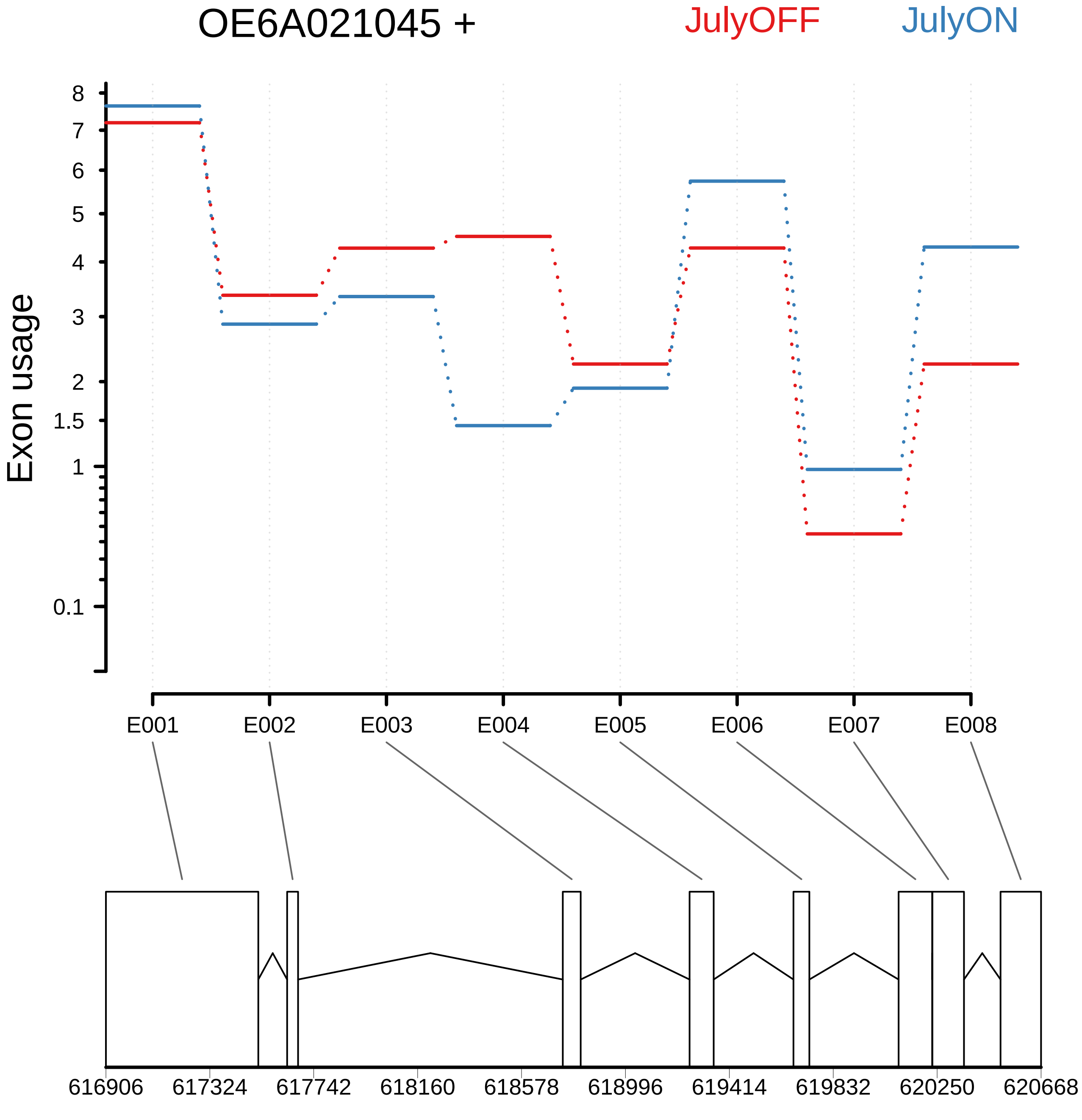
OE6A021045 +
JulyOFF
JulyON
Exon usage
8
7
6
5
4
3
2
1.5
1
0.1
E001
E002
E003
E004
E005
E006
E007
E008
616906
617324
617742
618160
618578
618996
619414
619832
620250
620668

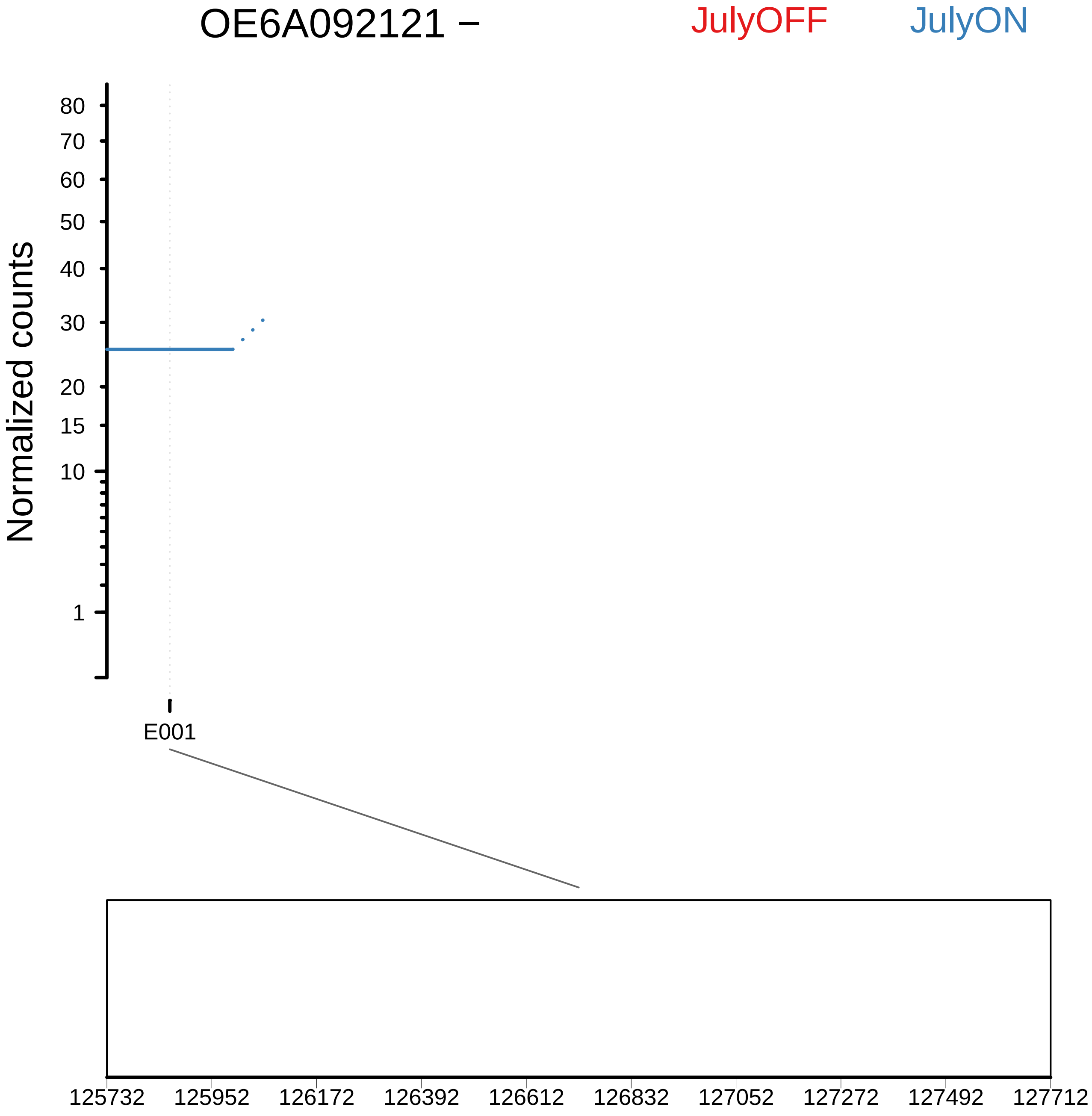
OE6A092121 −
JulyOFF
JulyON
Normalized counts
80
70
60
50
40
30
20
15
10
1
E001
125732
125952
126172
126392
126612
126832
127052
127272
127492
127712

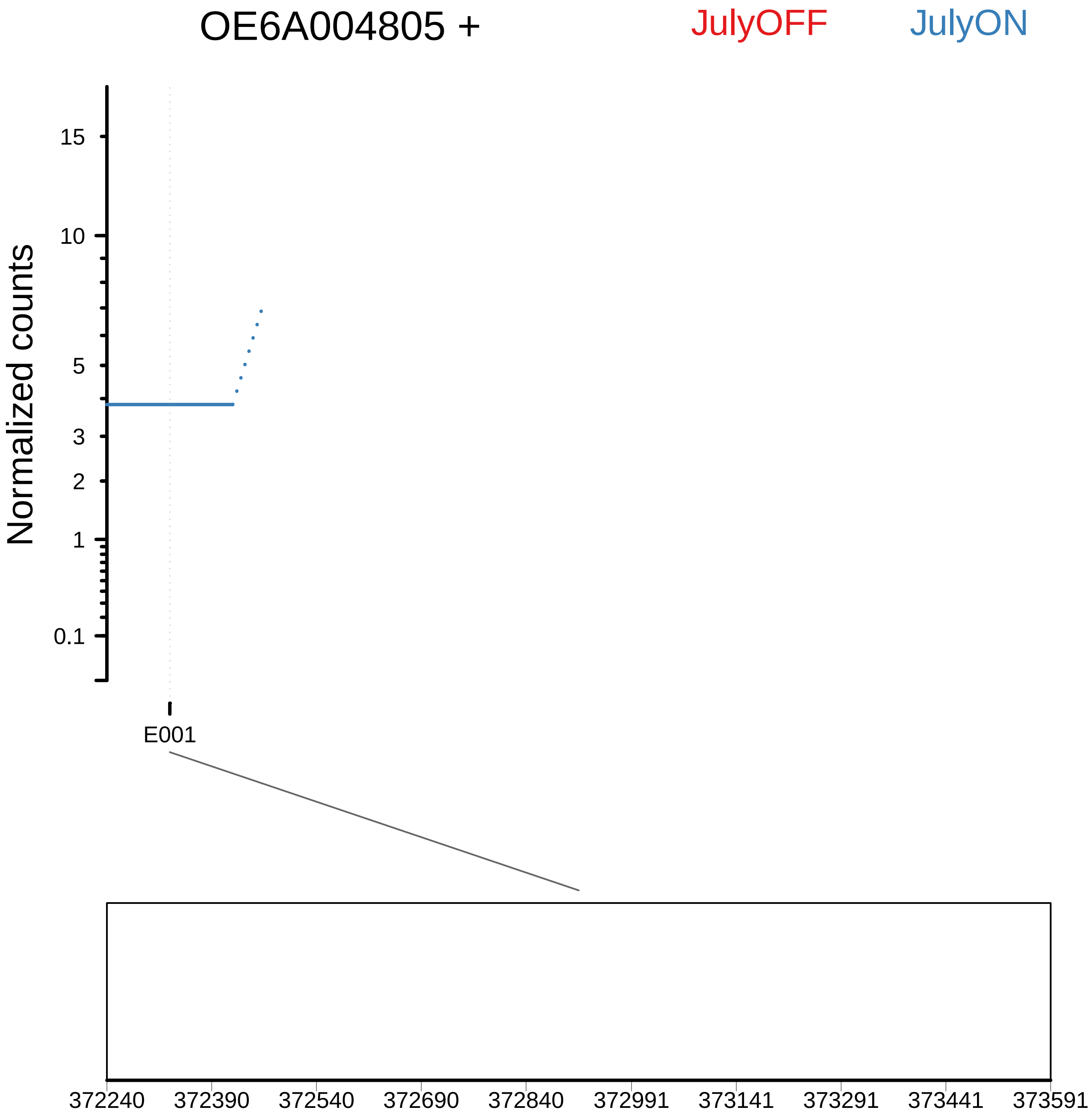
OE6A004805 +
JulyOFF
JulyON
Normalized counts
15
10
5
3
2
1
0.1
E001
372240
372390
372540
372690
372840
372991
373141
373291
373441
373591

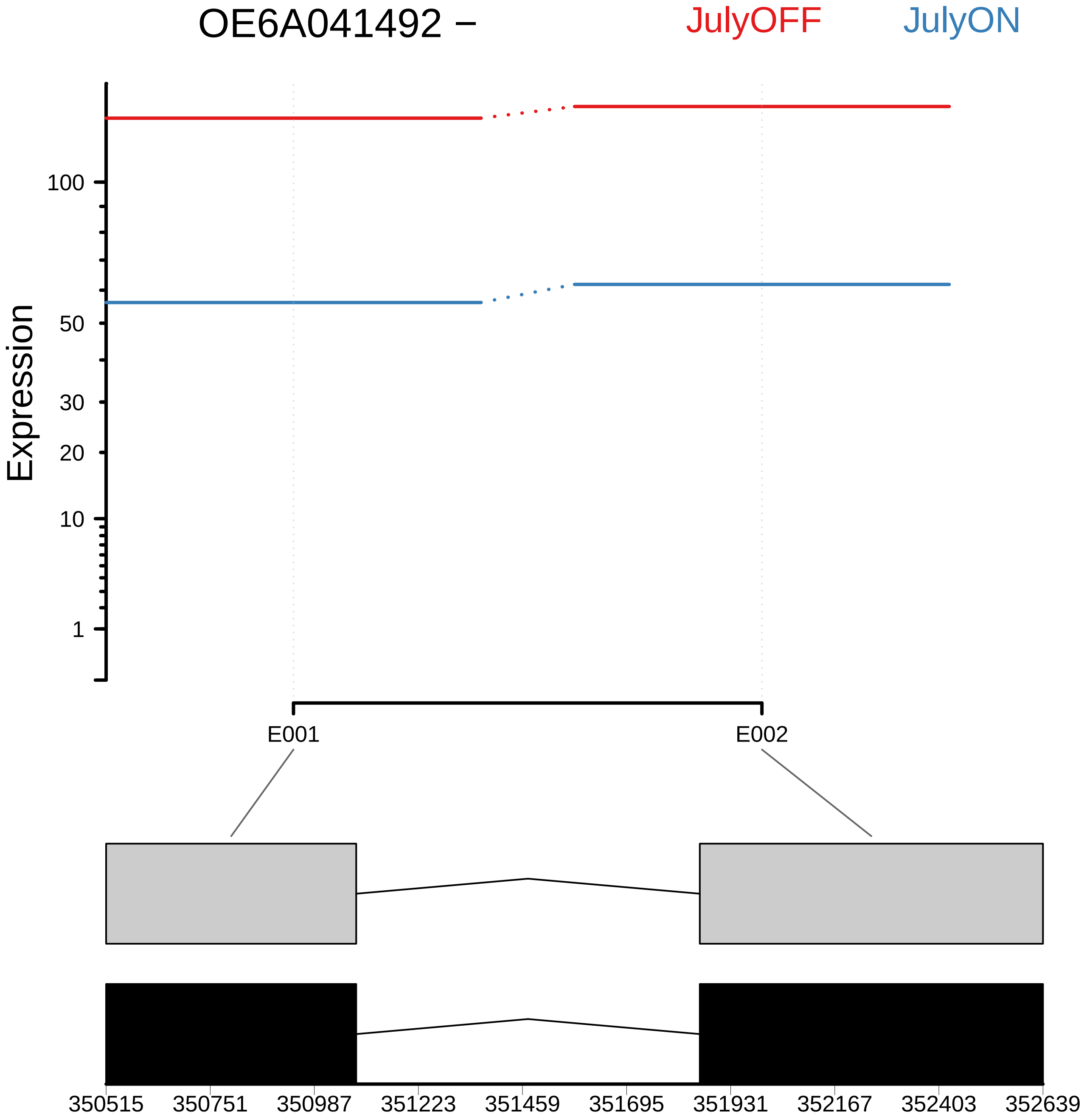

OE6A041492 −
JulyOFF
JulyON
Expression
100
50
30
20
10
1
E001
E002
350515
350751
350987
351223
351459
351695
351931
352167
352403
352639

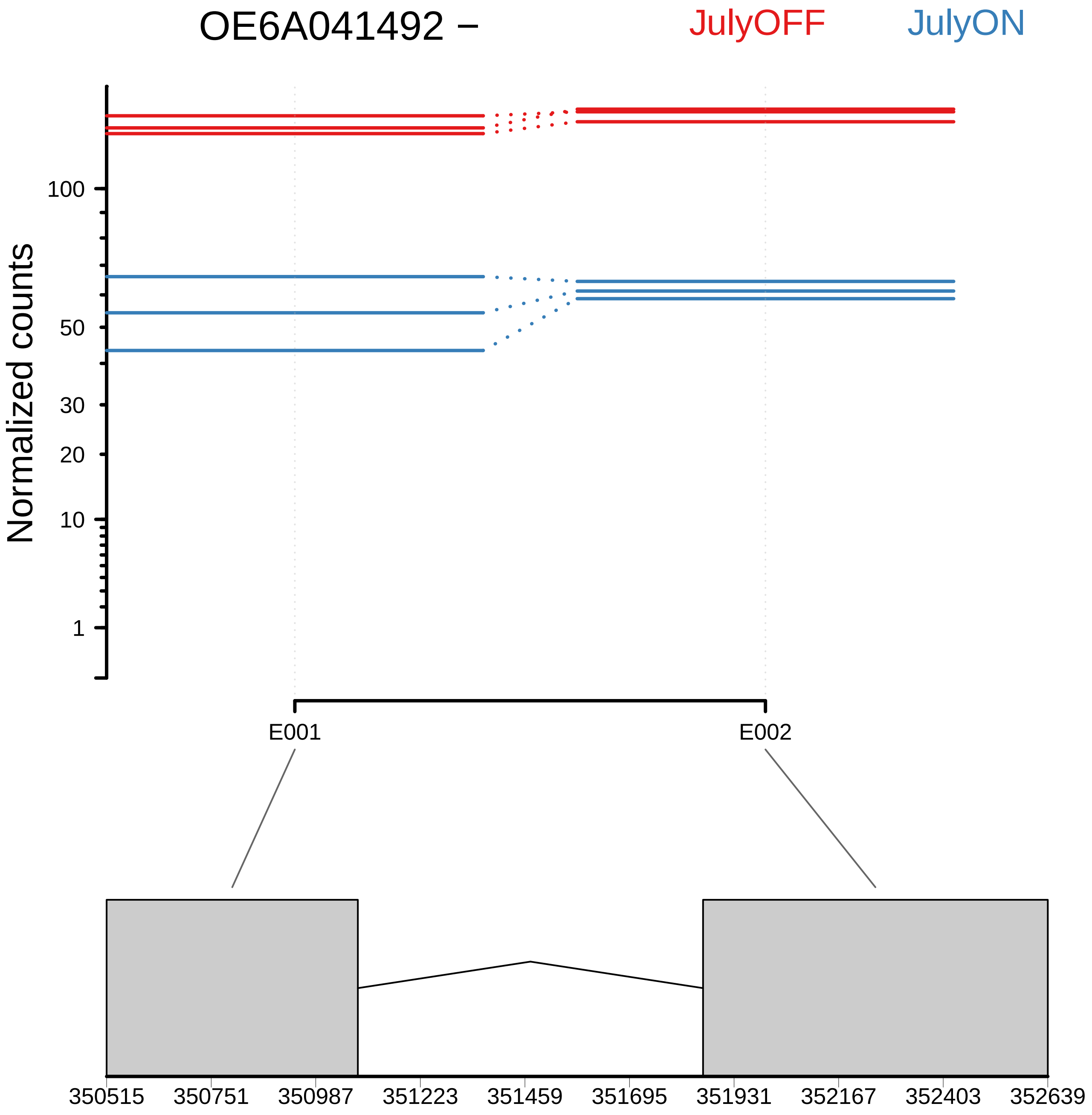
OE6A041492 −
JulyOFF
JulyON
Normalized counts
100
50
30
20
10
1
E001
E002
350515
350751
350987
351223
351459
351695
351931
352167
352403
352639

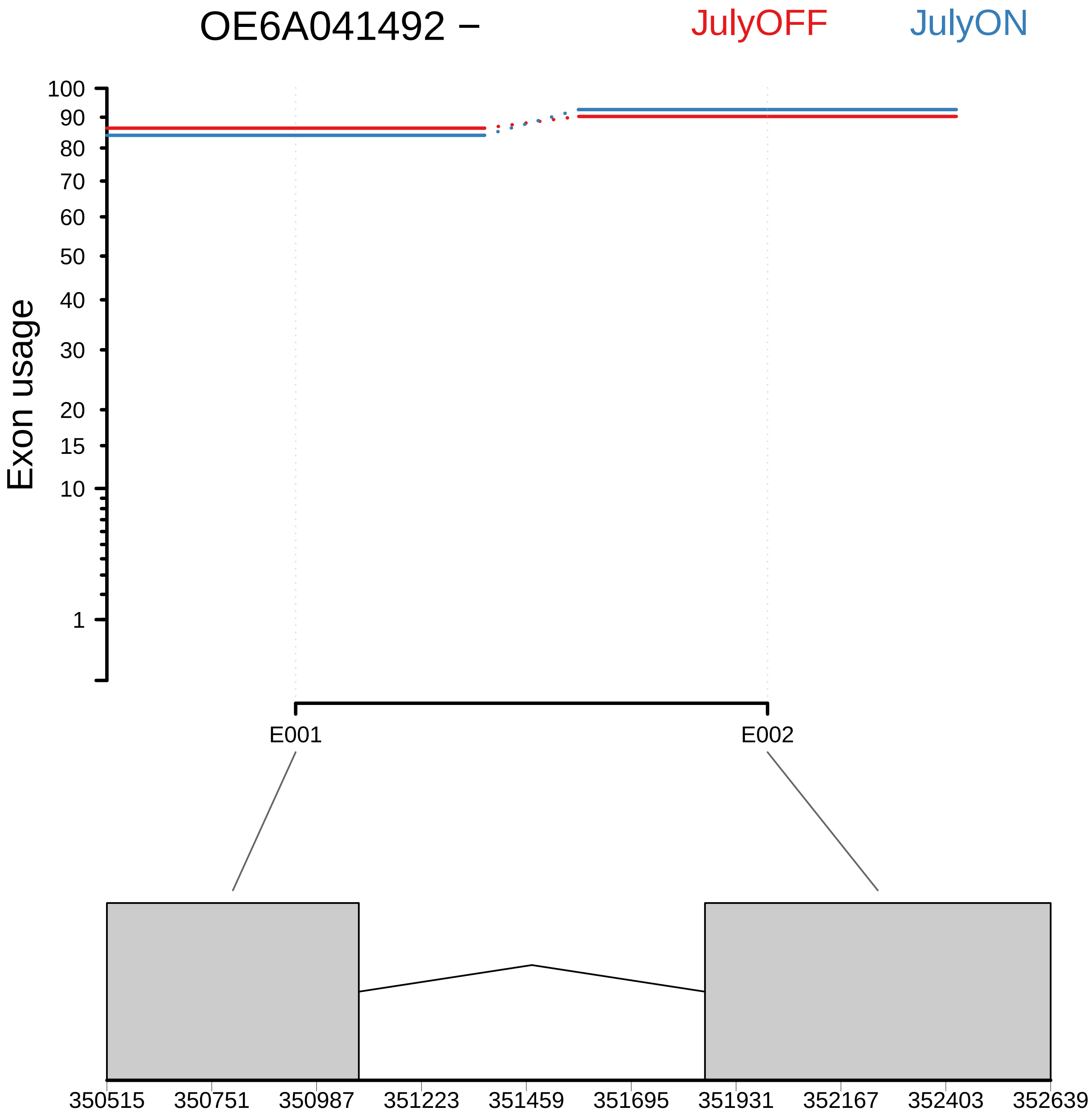

OE6A041492 −
JulyOFF
JulyON
Exon usage
100
90
80
70
60
50
40
30
20
15
10
1
E001
E002
350515
350751
350987
351223
351459
351695
351931
352167
352403
352639

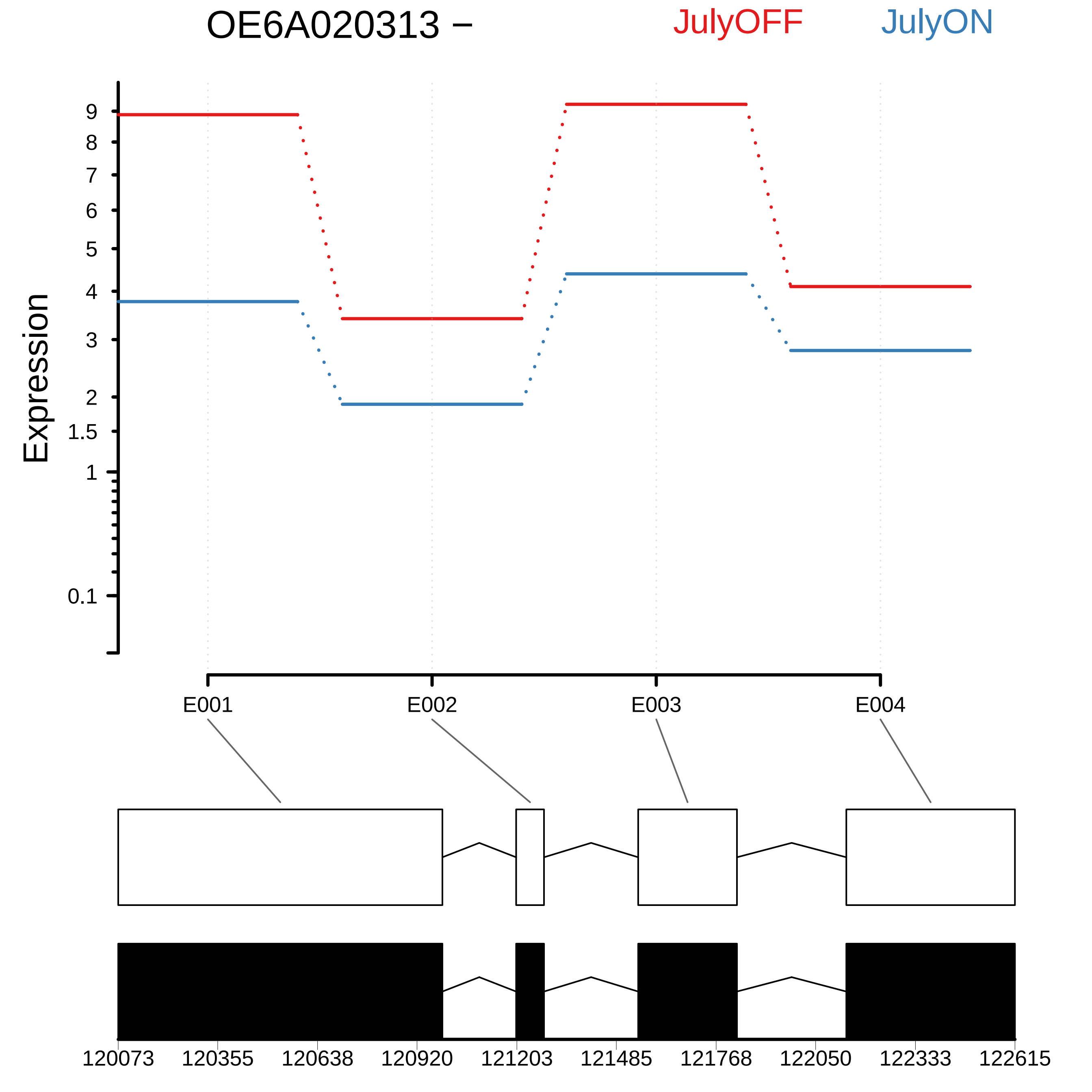

OE6A020313 −
JulyOFF
JulyON
Expression
9
8
7
6
5
4
3
2
1.5
1
0.1
E001
E002
E003
E004
120073
120355
120638
120920
121203
121485
121768
122050
122333
122615

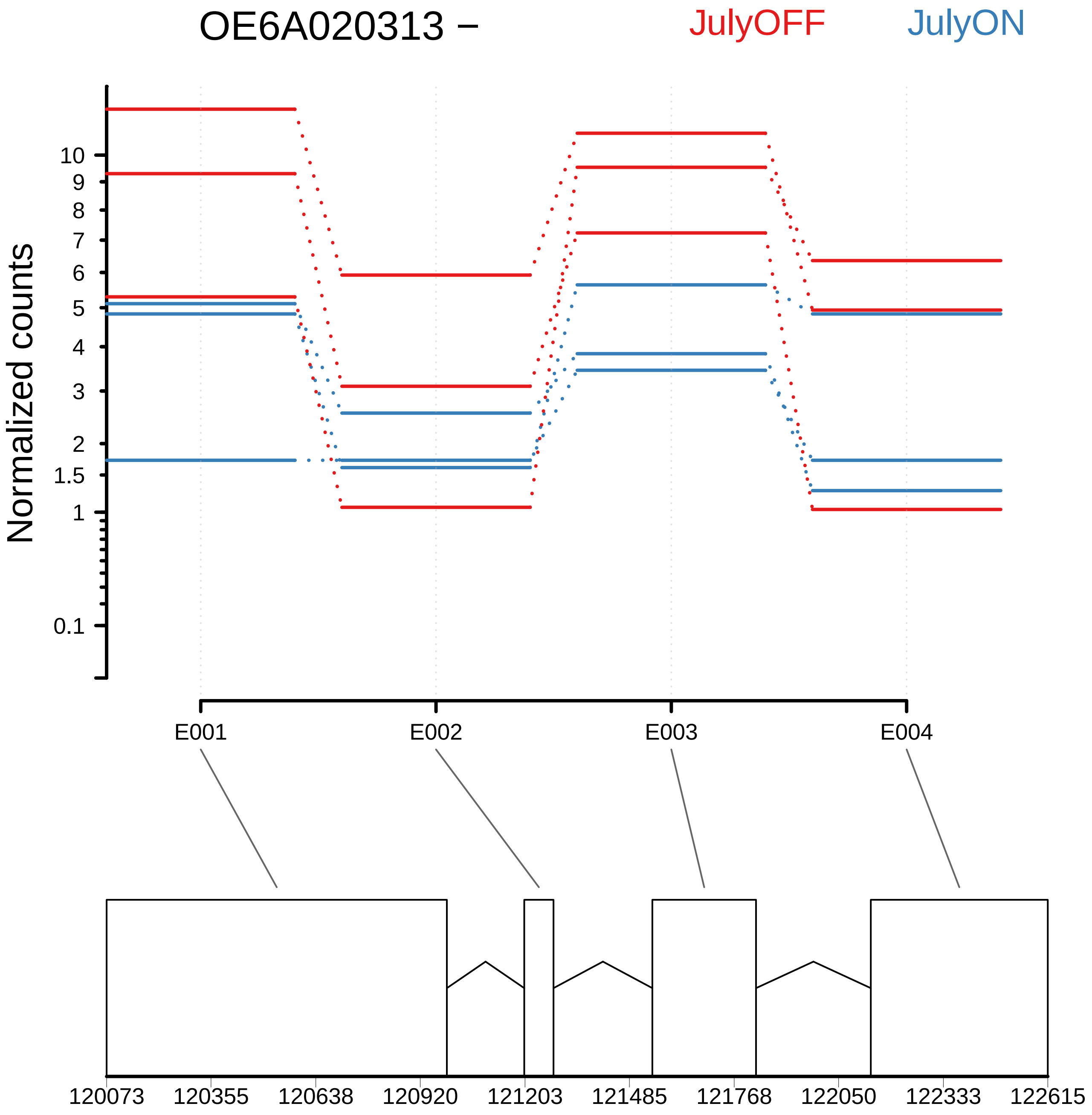

OE6A020313 −
JulyOFF
JulyON
Normalized counts
10
9
8
7
6
5
4
3
2
1.5
1
0.1
E001
E002
E003
E004
120073
120355
120638
120920
121203
121485
121768
122050
122333
122615

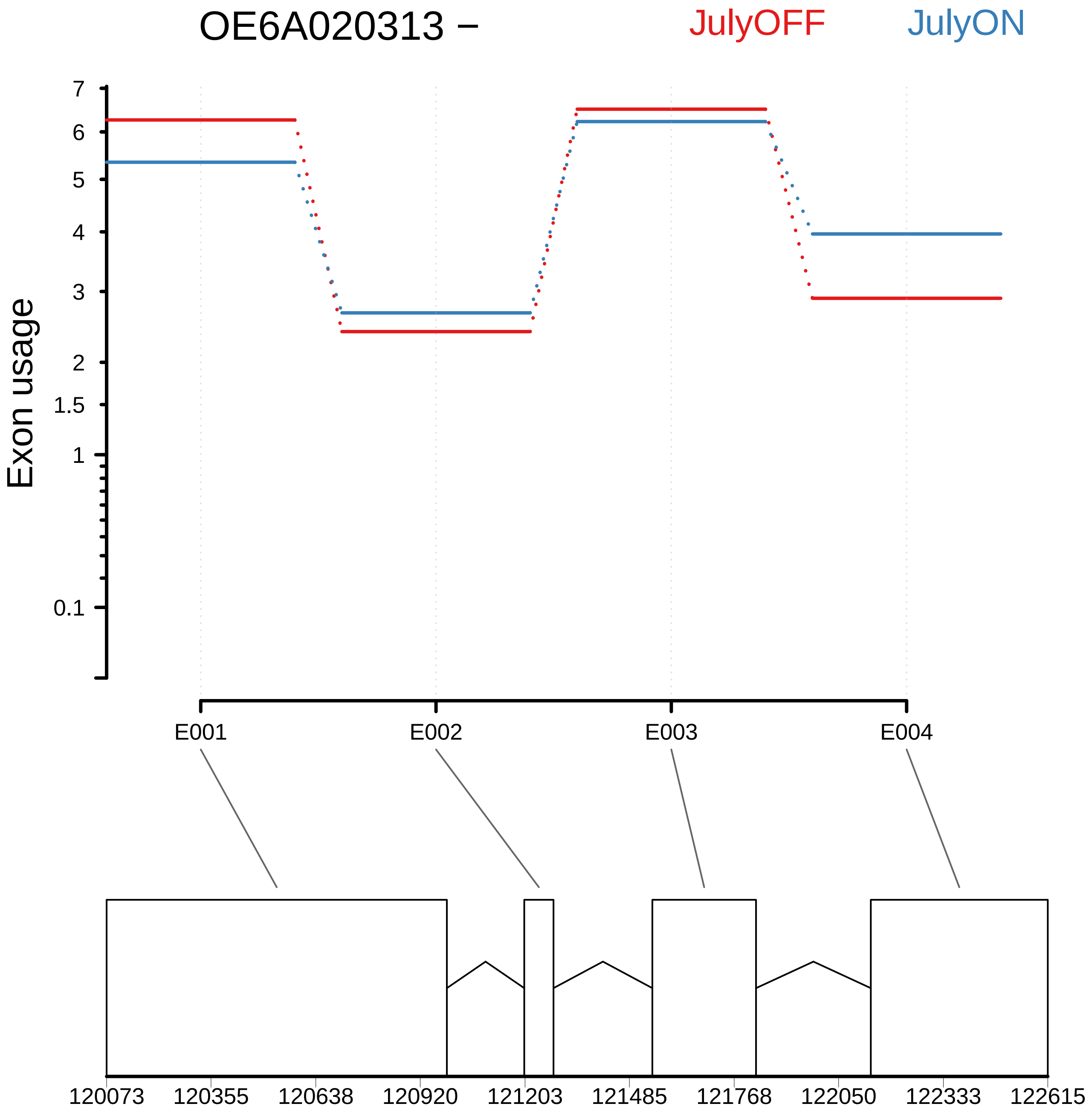

OE6A020313 −
JulyOFF
JulyON
Exon usage
7
6
5
4
3
2
1.5
1
0.1
E001
E002
E003
E004
120073
120355
120638
120920
121203
121485
121768
122050
122333
122615

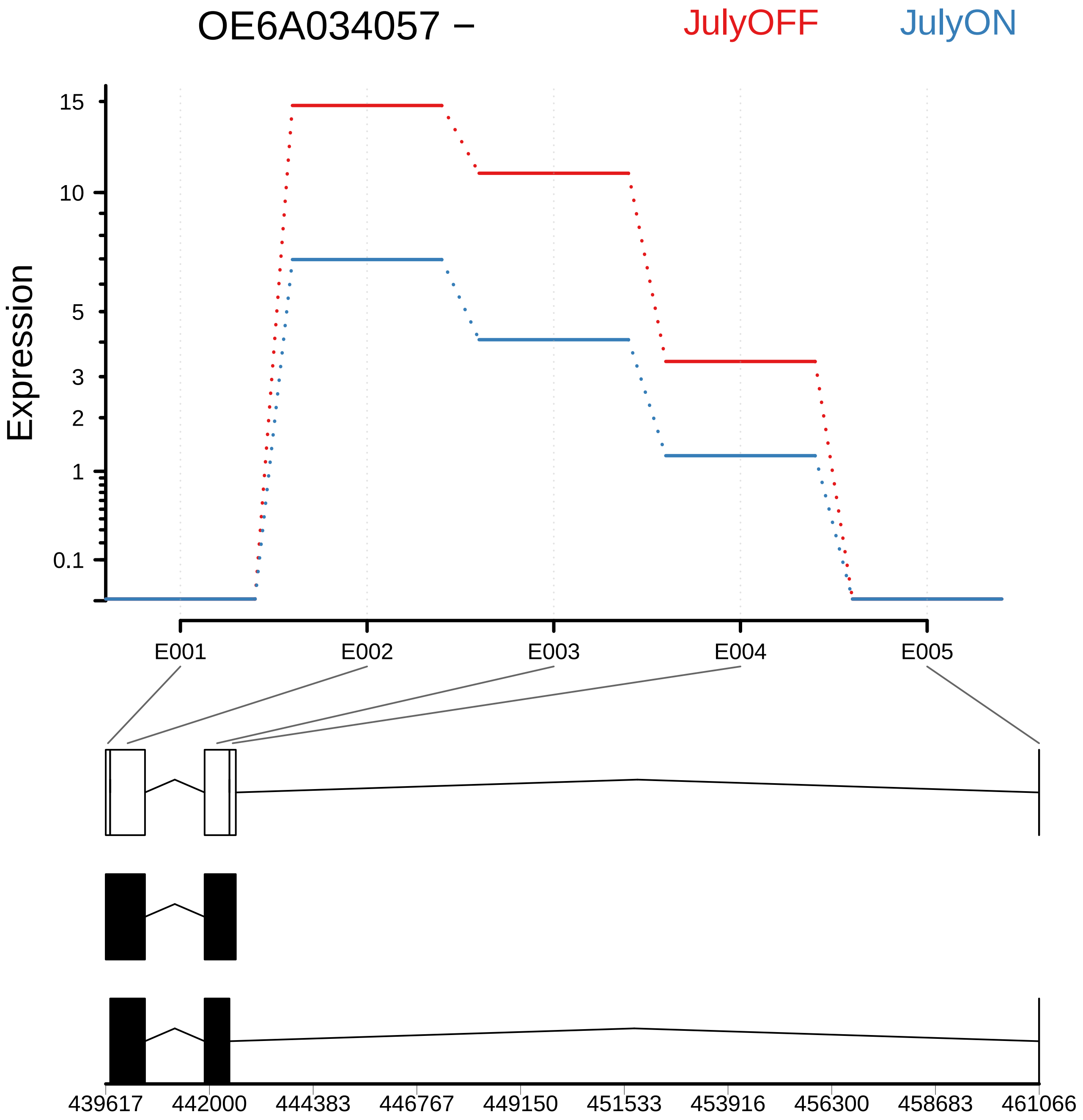

OE6A034057 −
JulyOFF
JulyON
Expression
15
10
5
3
2
1
0.1
E001
E002
E003
E004
E005
439617
442000
444383
446767
449150
451533
453916
456300
458683
461066

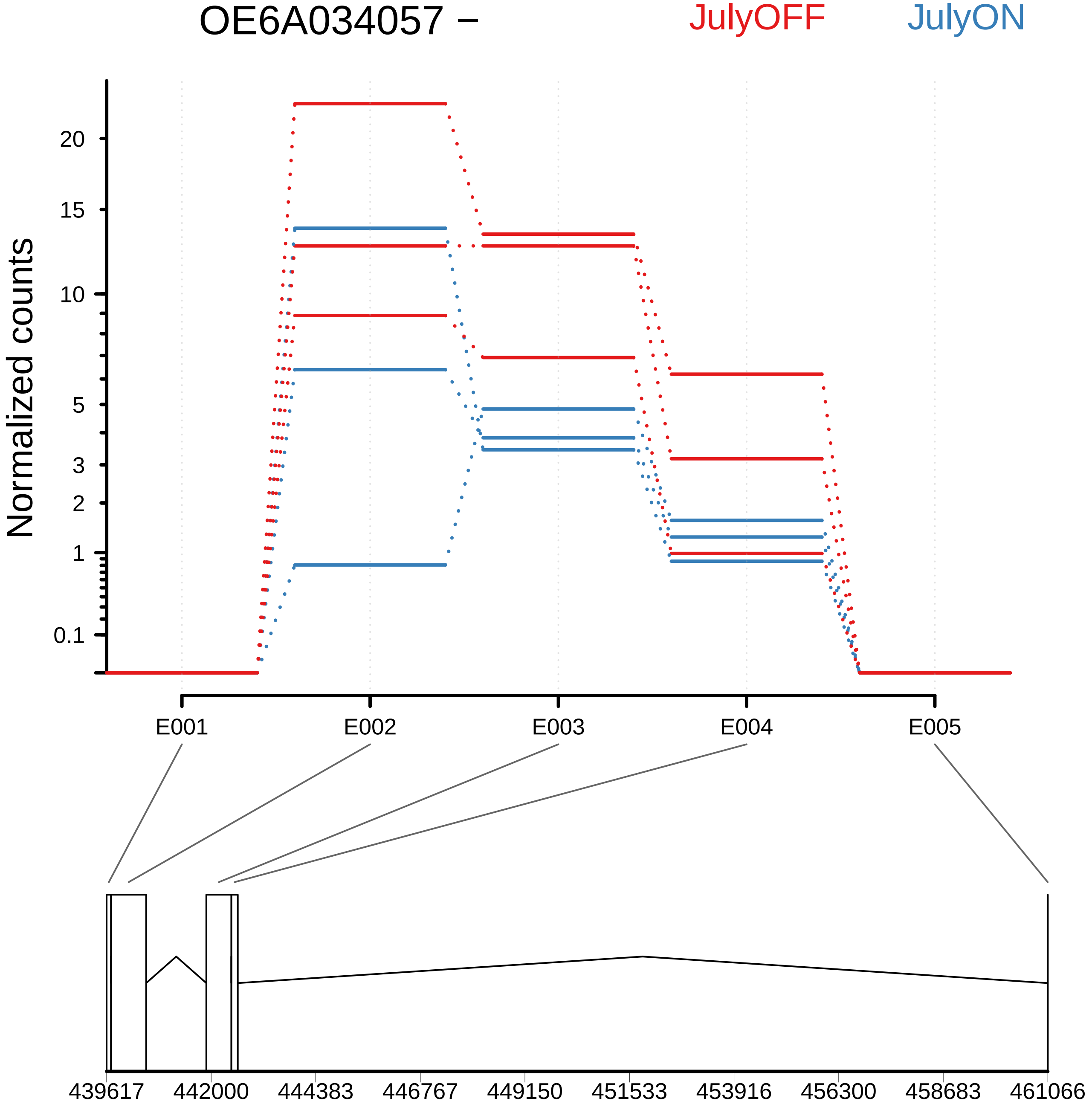

OE6A034057 −
JulyOFF
JulyON
Normalized counts
20
15
10
5
3
2
1
0.1
E001
E002
E003
E004
E005
439617
442000
444383
446767
449150
451533
453916
456300
458683
461066

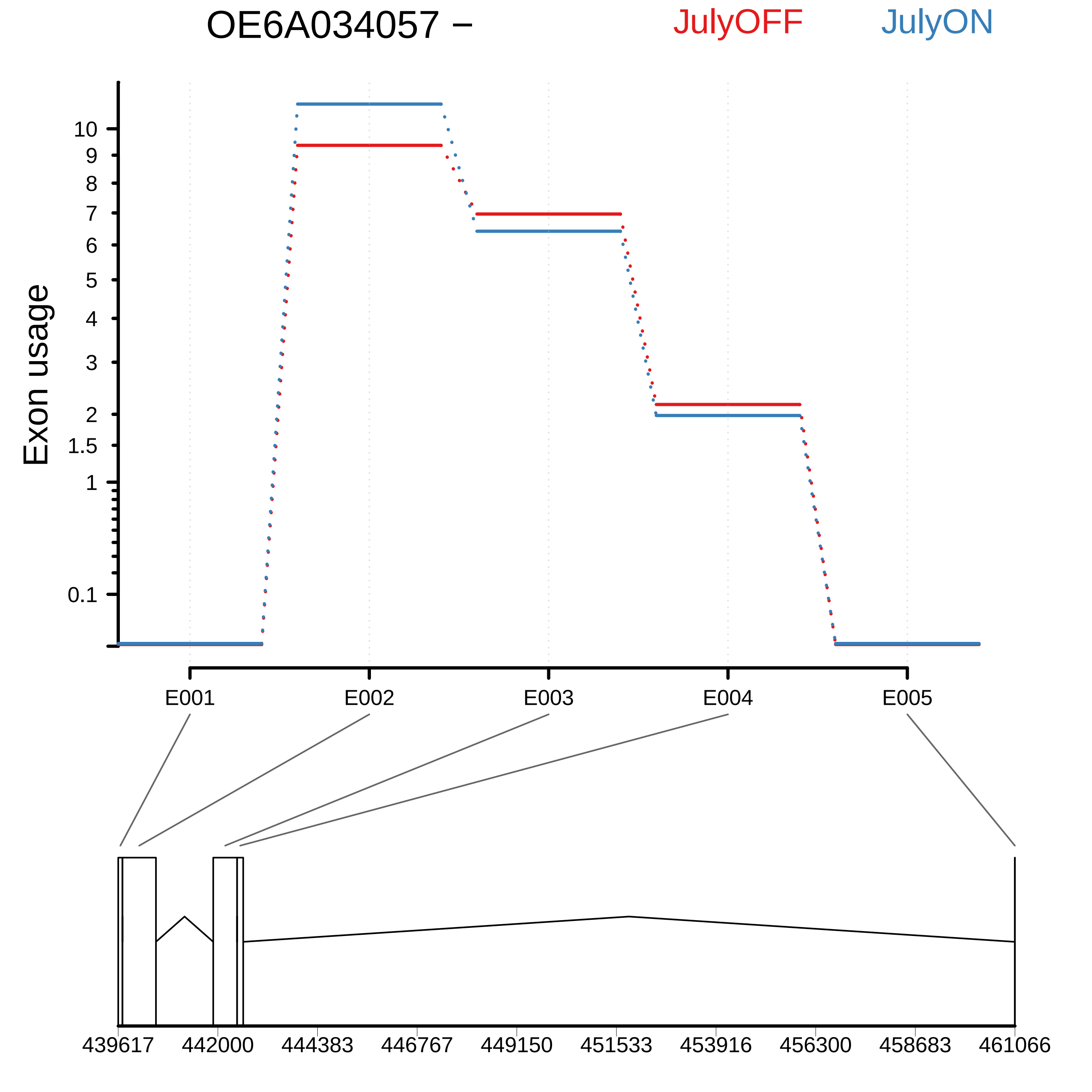

OE6A034057 −
JulyOFF
JulyON
Exon usage
10
9
8
7
6
5
4
3
2
1.5
1
0.1
E001
E002
E003
E004
E005
439617
442000
444383
446767
449150
451533
453916
456300
458683
461066

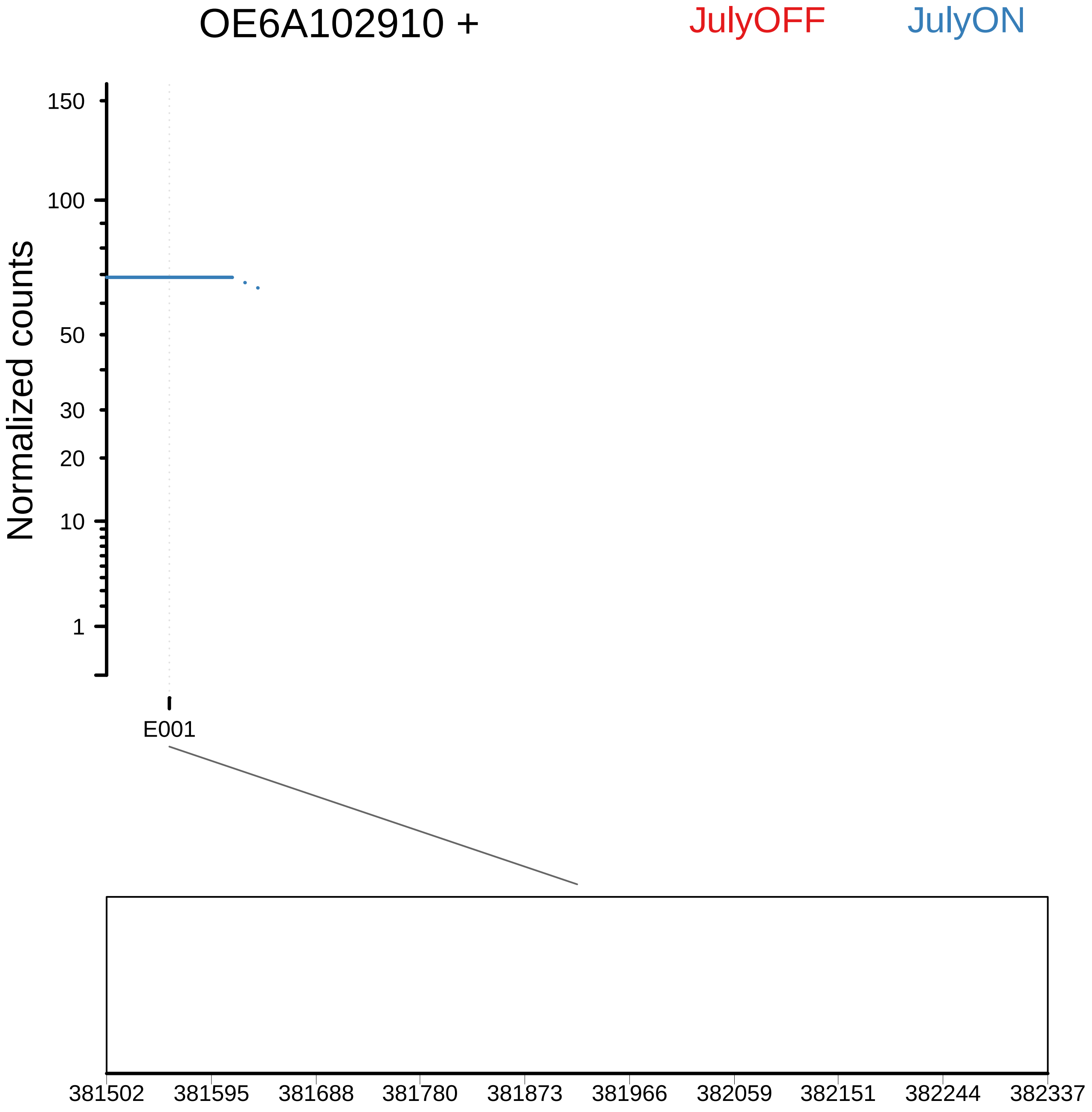

OE6A102910 +
JulyOFF
JulyON
Normalized counts
150
100
50
30
20
10
1
E001
381502
381595
381688
381780
381873
381966
382059
382151
382244
382337

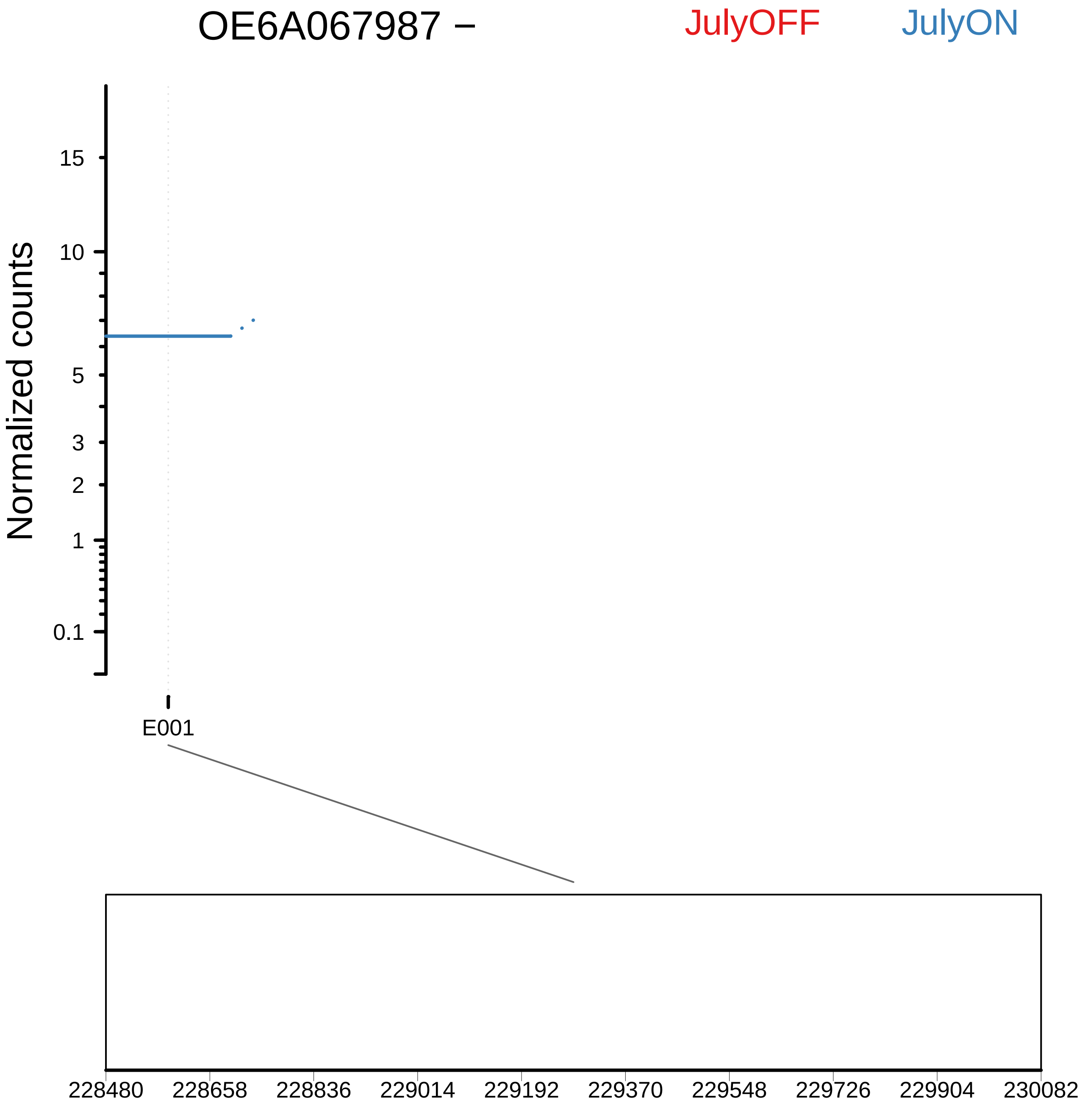

OE6A067987 −
JulyOFF
JulyON
Normalized counts
15
10
5
3
2
1
0.1
E001
228480
228658
228836
229014
229192
229370
229548
229726
229904
230082

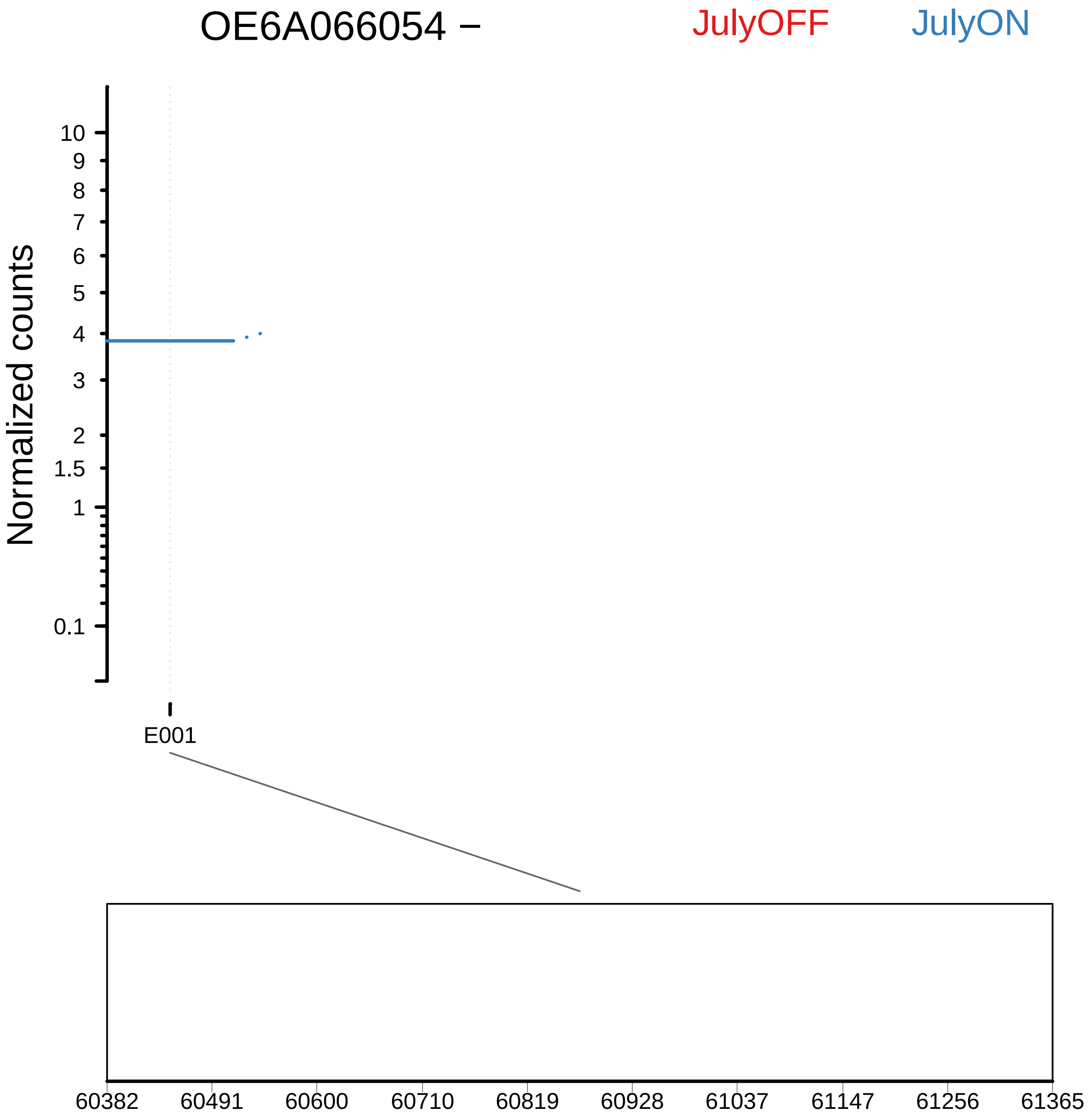

OE6A066054 −
JulyOFF
JulyON
Normalized counts
10
9
8
7
6
5
4
3
2
1.5
1
0.1
E001
60382
60491
60600
60710
60819
60928
61037
61147
61256
61365

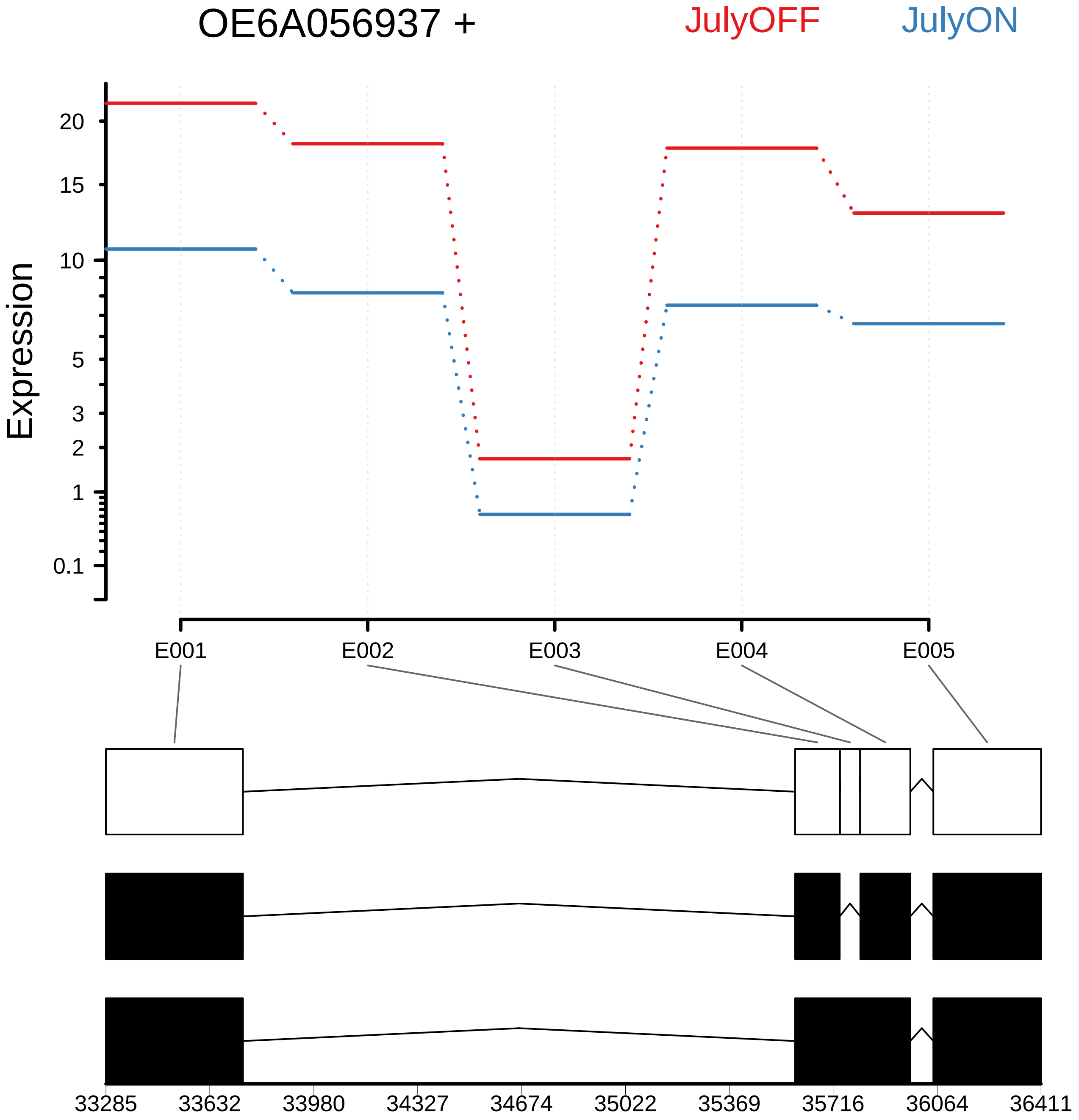

OE6A056937 +
JulyOFF
JulyON
Expression
20
15
10
5
3
2
1
0.1
E001
E002
E003
E004
E005
33285
33632
33980
34327
34674
35022
35369
35716
36064
36411

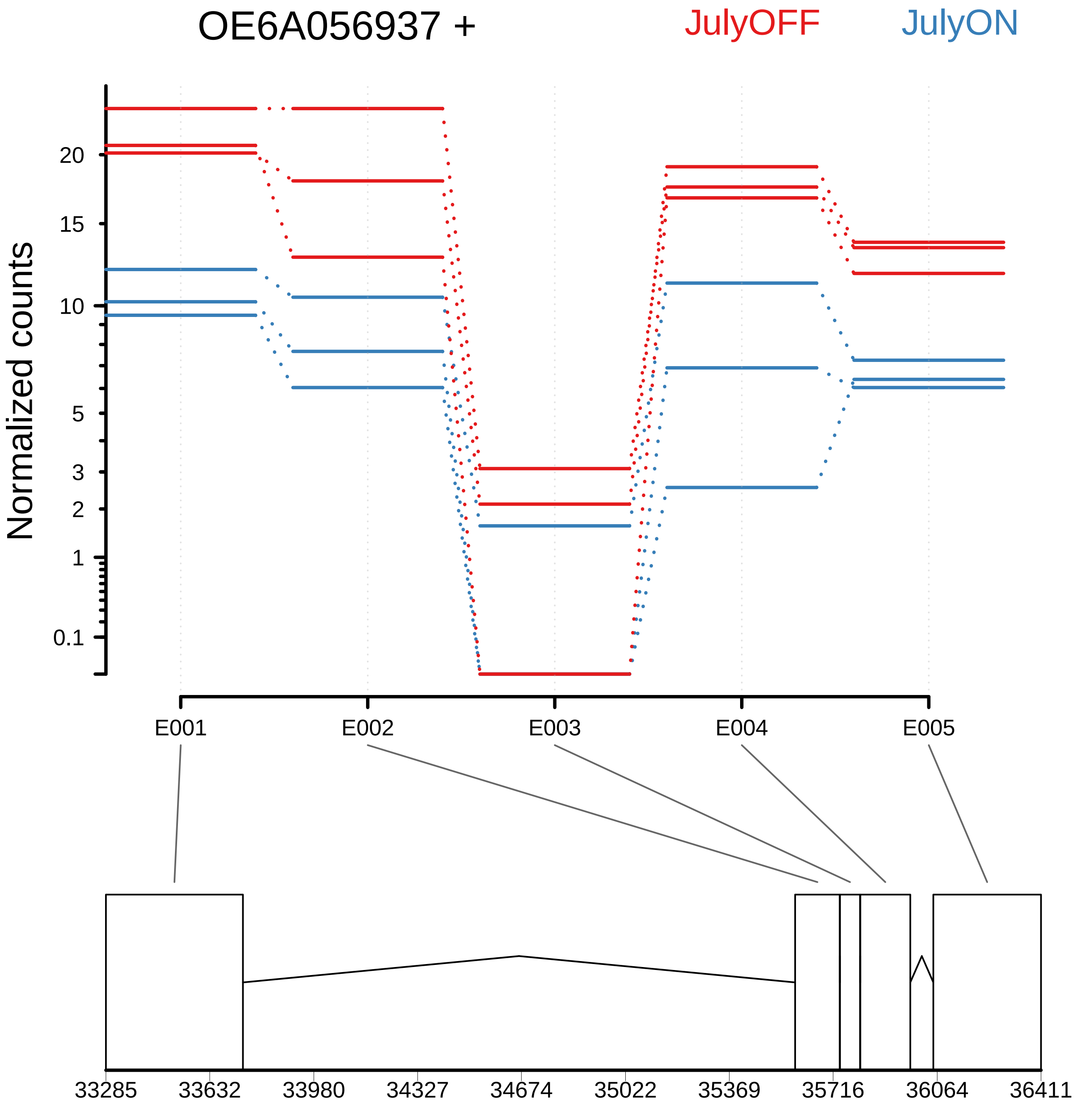

OE6A056937 +
JulyOFF
JulyON
Normalized counts
20
15
10
5
3
2
1
0.1
E001
E002
E003
E004
E005
33285
33632
33980
34327
34674
35022
35369
35716
36064
36411

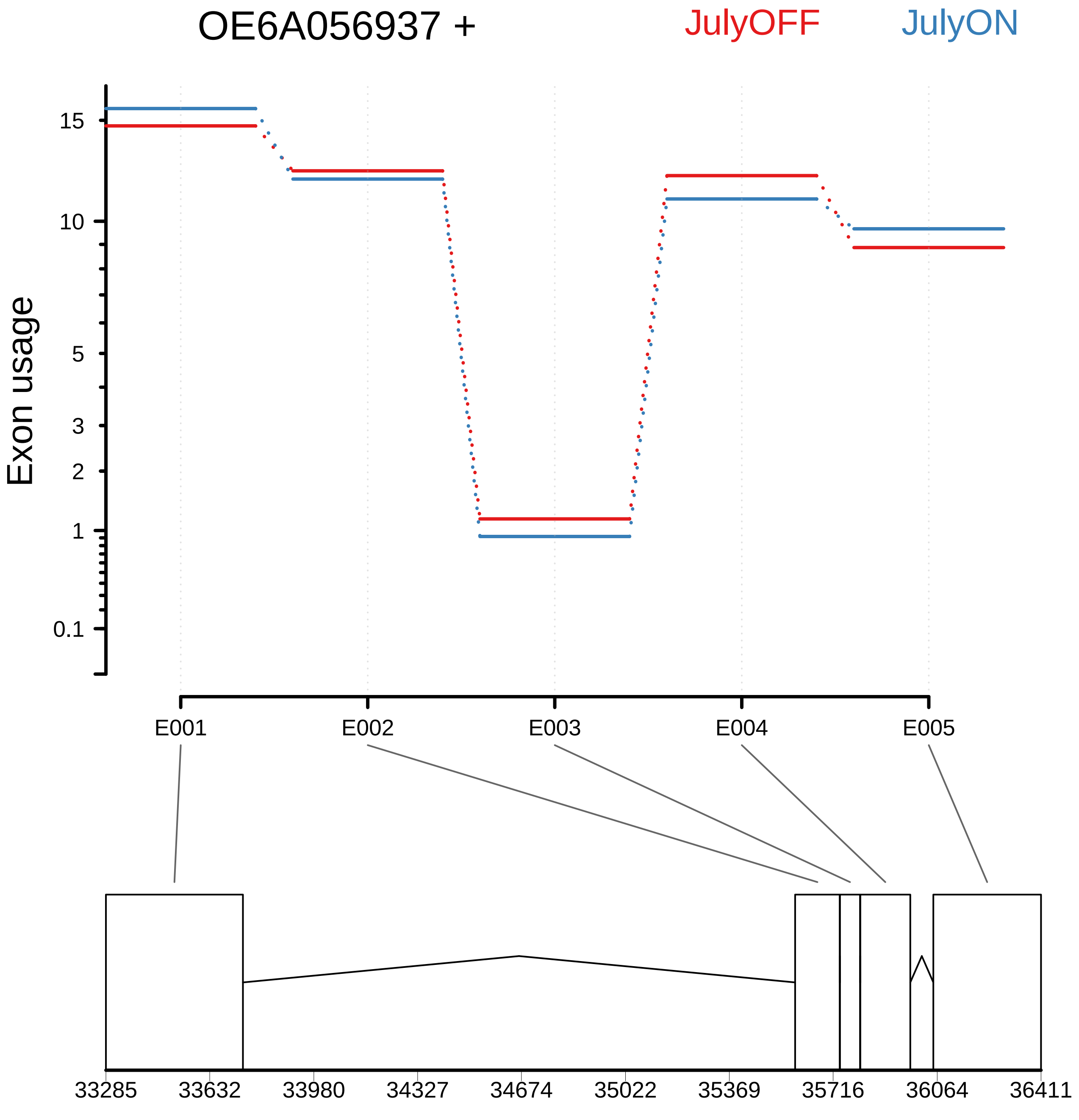
OE6A056937 +
JulyOFF
JulyON
Exon usage
15
10
5
3
2
1
0.1
E001
E002
E003
E004
E005
33285
33632
33980
34327
34674
35022
35369
35716
36064
36411

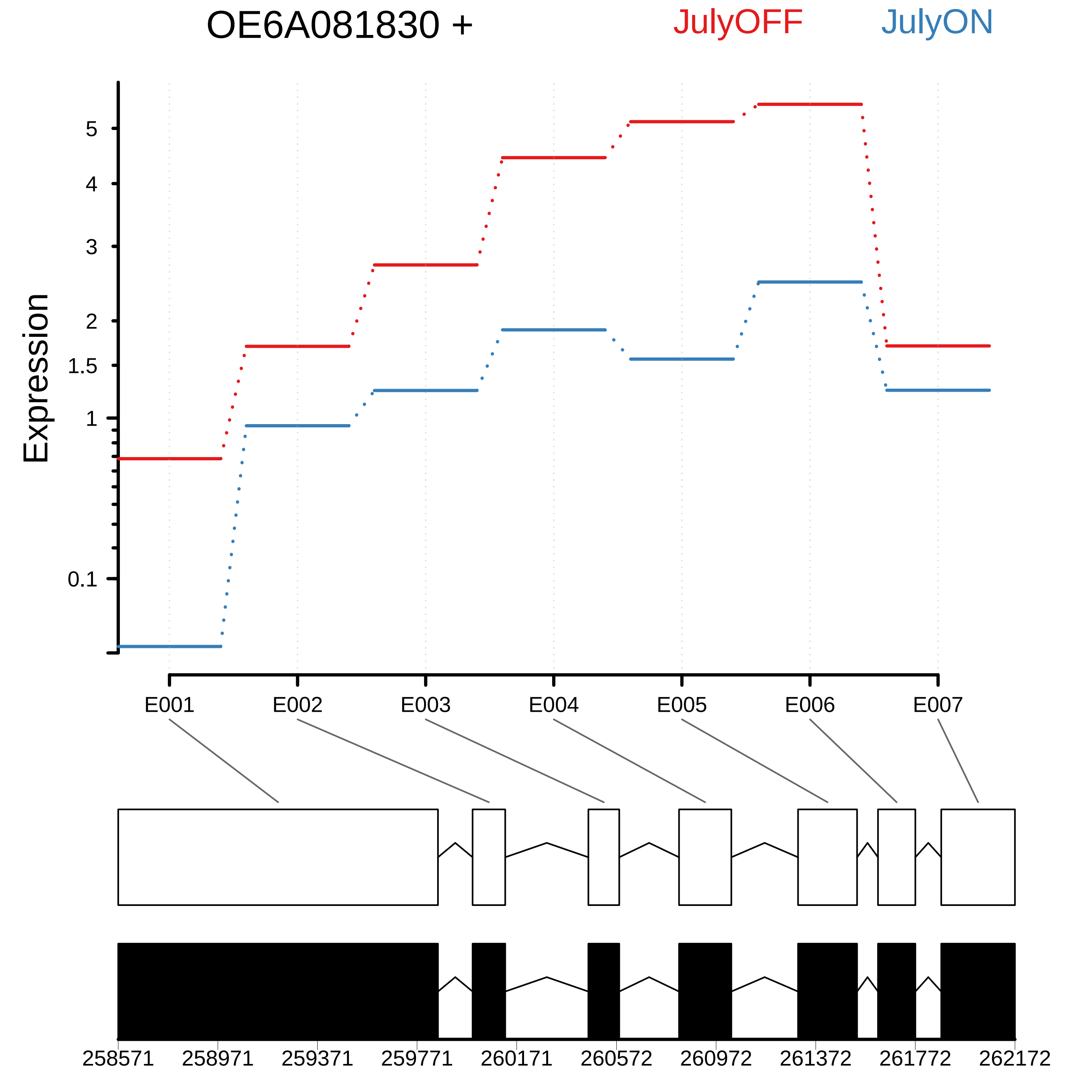
OE6A081830 +
JulyOFF
JulyON
Expression
5
4
3
2
1.5
1
0.1
E001
E002
E003
E004
E005
E006
E007
258571
258971
259371
259771
260171
260572
260972
261372
261772
262172

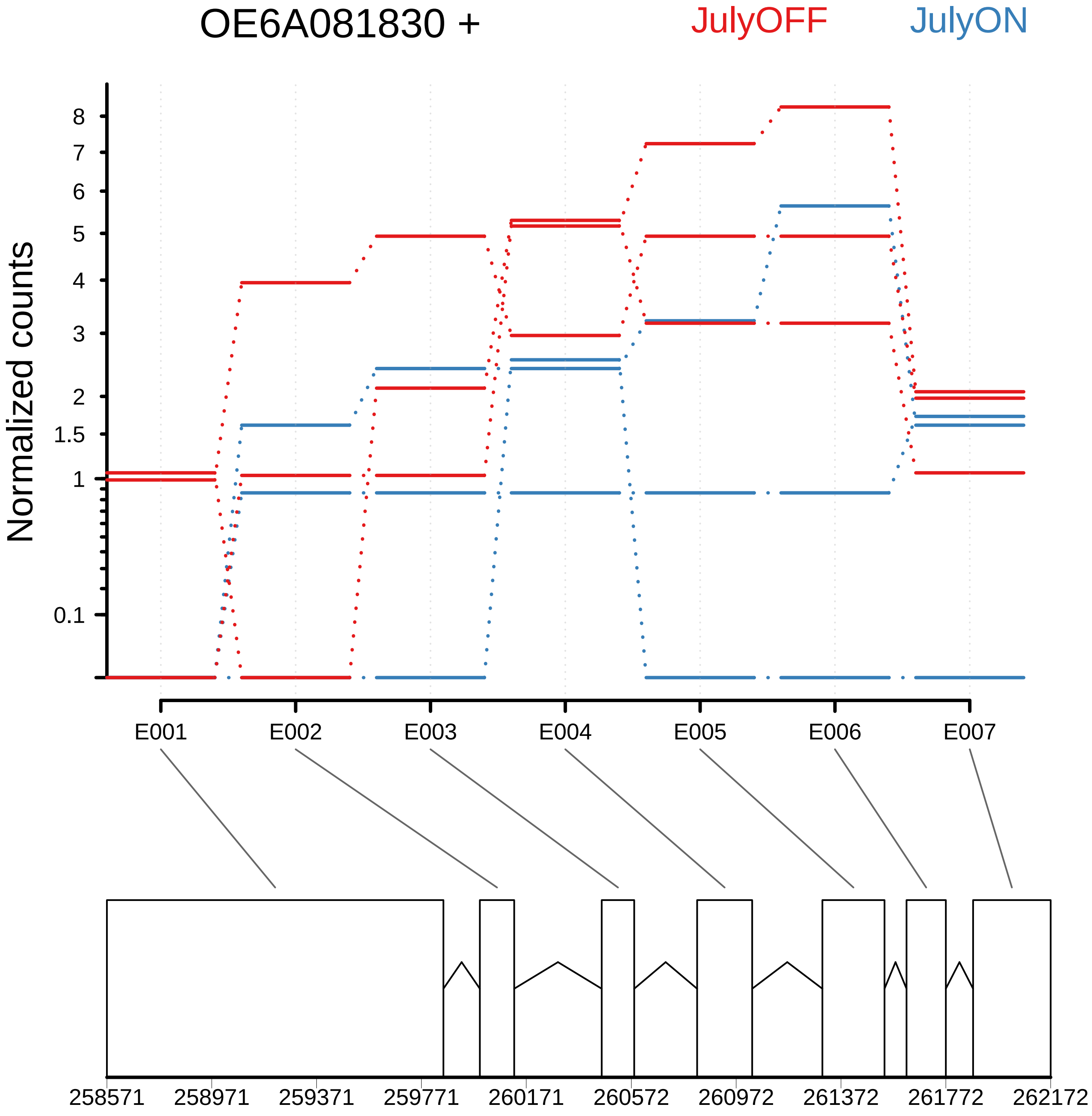

OE6A081830 +
JulyOFF
JulyON
Normalized counts
8
7
6
5
4
3
2
1.5
1
0.1
E001
E002
E003
E004
E005
E006
E007
258571
258971
259371
259771
260171
260572
260972
261372
261772
262172

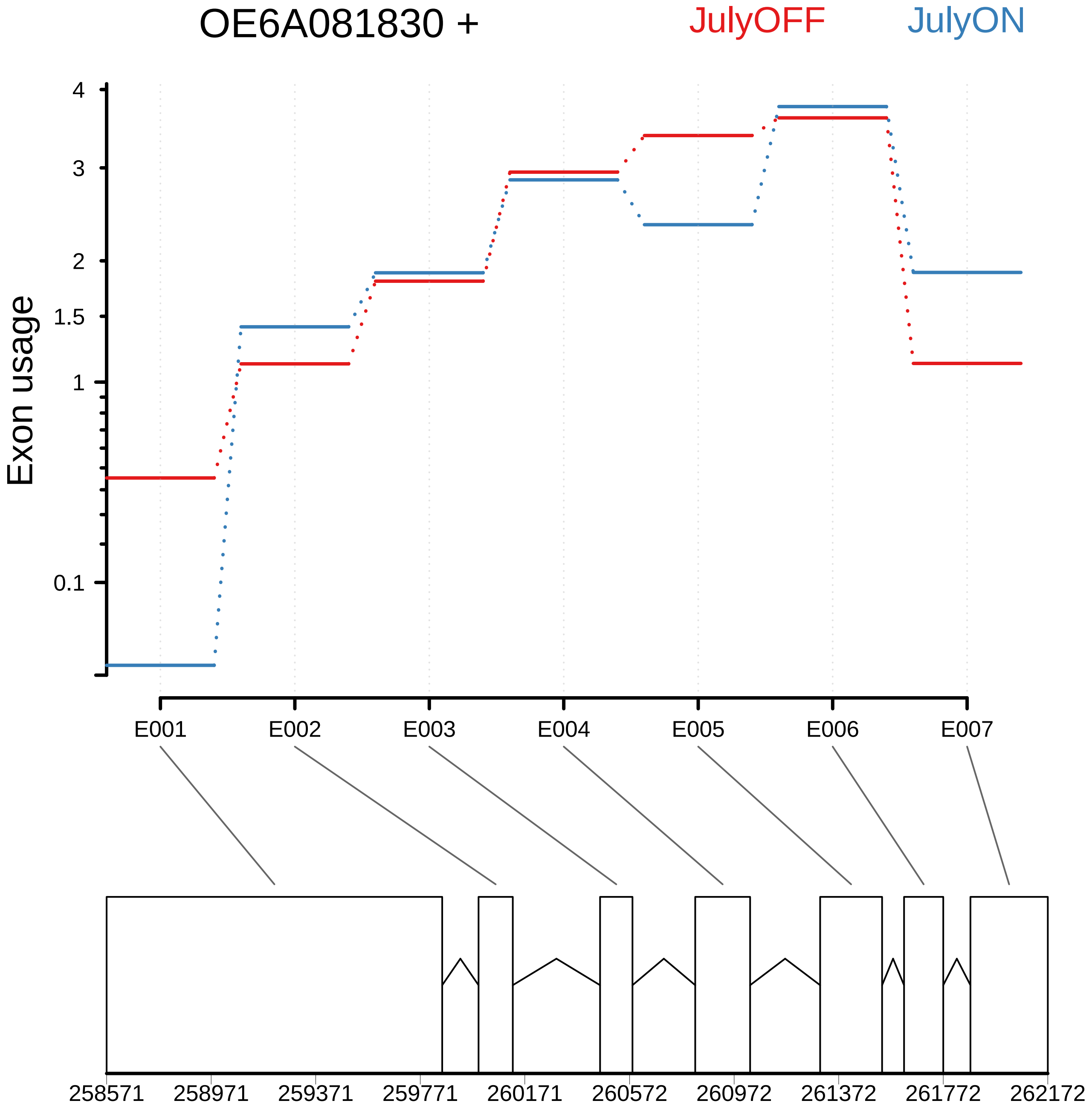
OE6A081830 +
JulyOFF
JulyON
Exon usage
4
3
2
1.5
1
0.1
E001
E002
E003
E004
E005
E006
E007
258571
258971
259371
259771
260171
260572
260972
261372
261772
262172

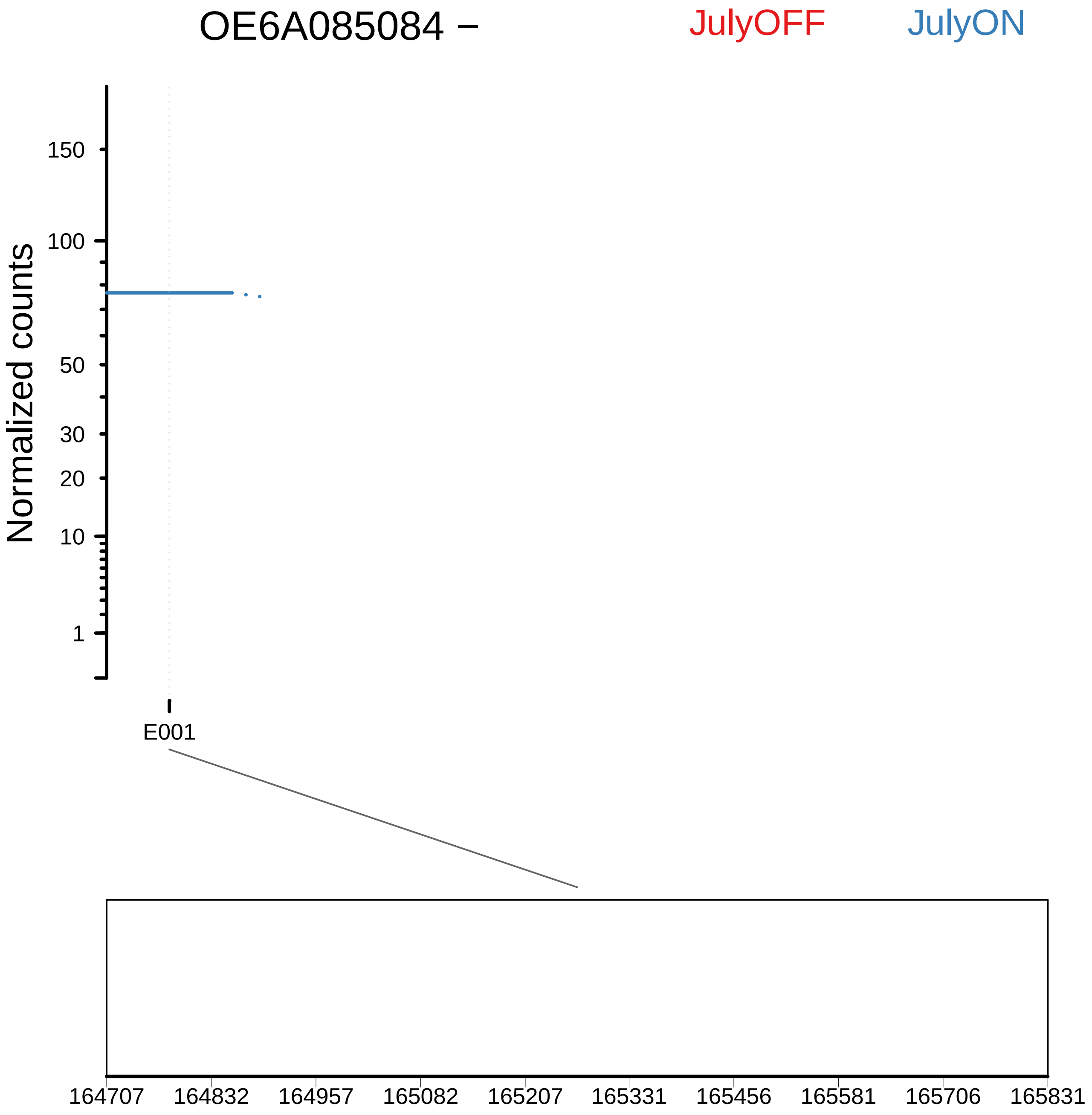

OE6A085084 −
JulyOFF
JulyON
Normalized counts
150
100
50
30
20
10
1
E001
164707
164832
164957
165082
165207
165331
165456
165581
165706
165831

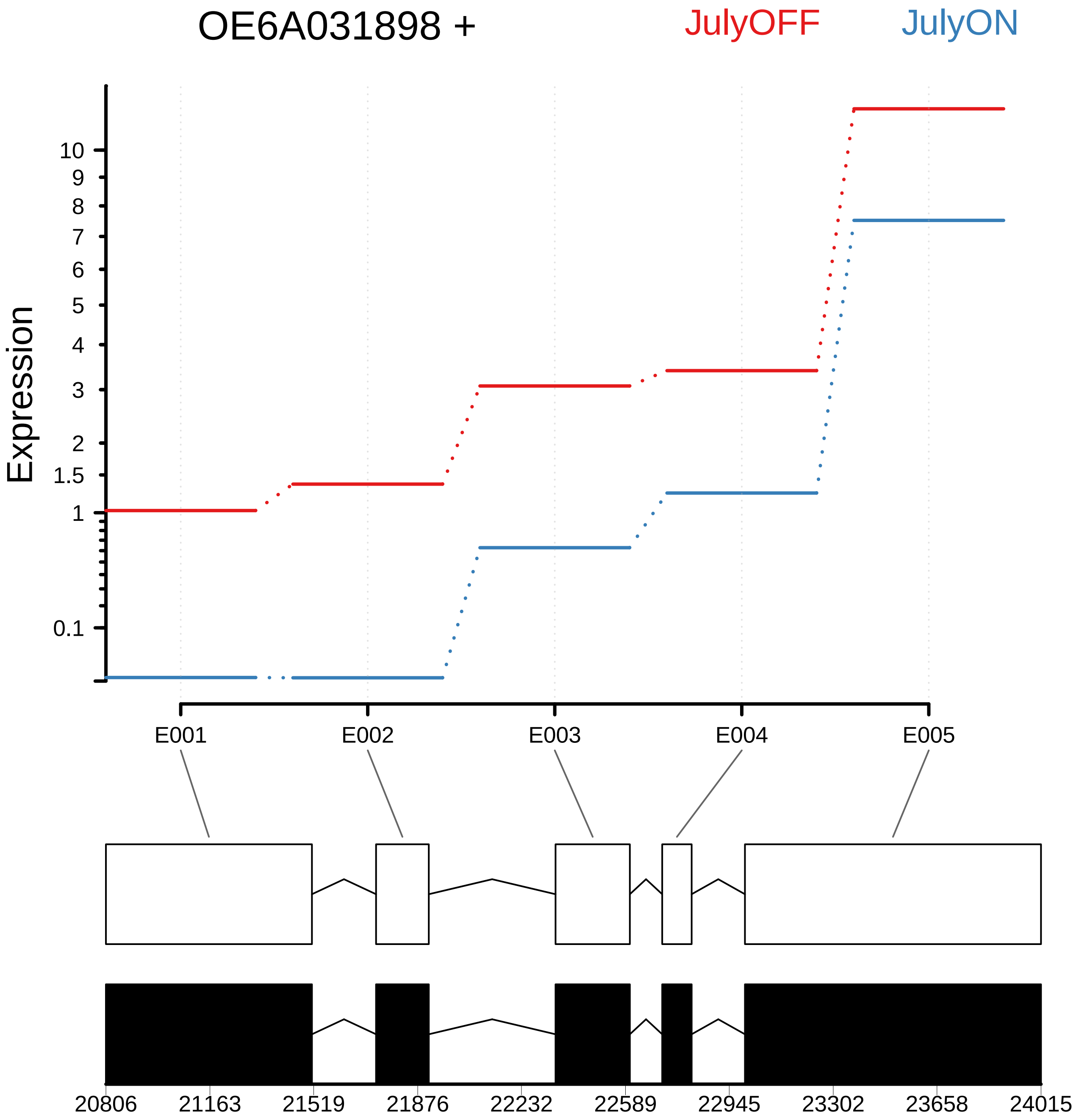

OE6A031898 +
JulyOFF
JulyON
Expression
10
9
8
7
6
5
4
3
2
1.5
1
0.1
E001
E002
E003
E004
E005
20806
21163
21519
21876
22232
22589
22945
23302
23658
24015

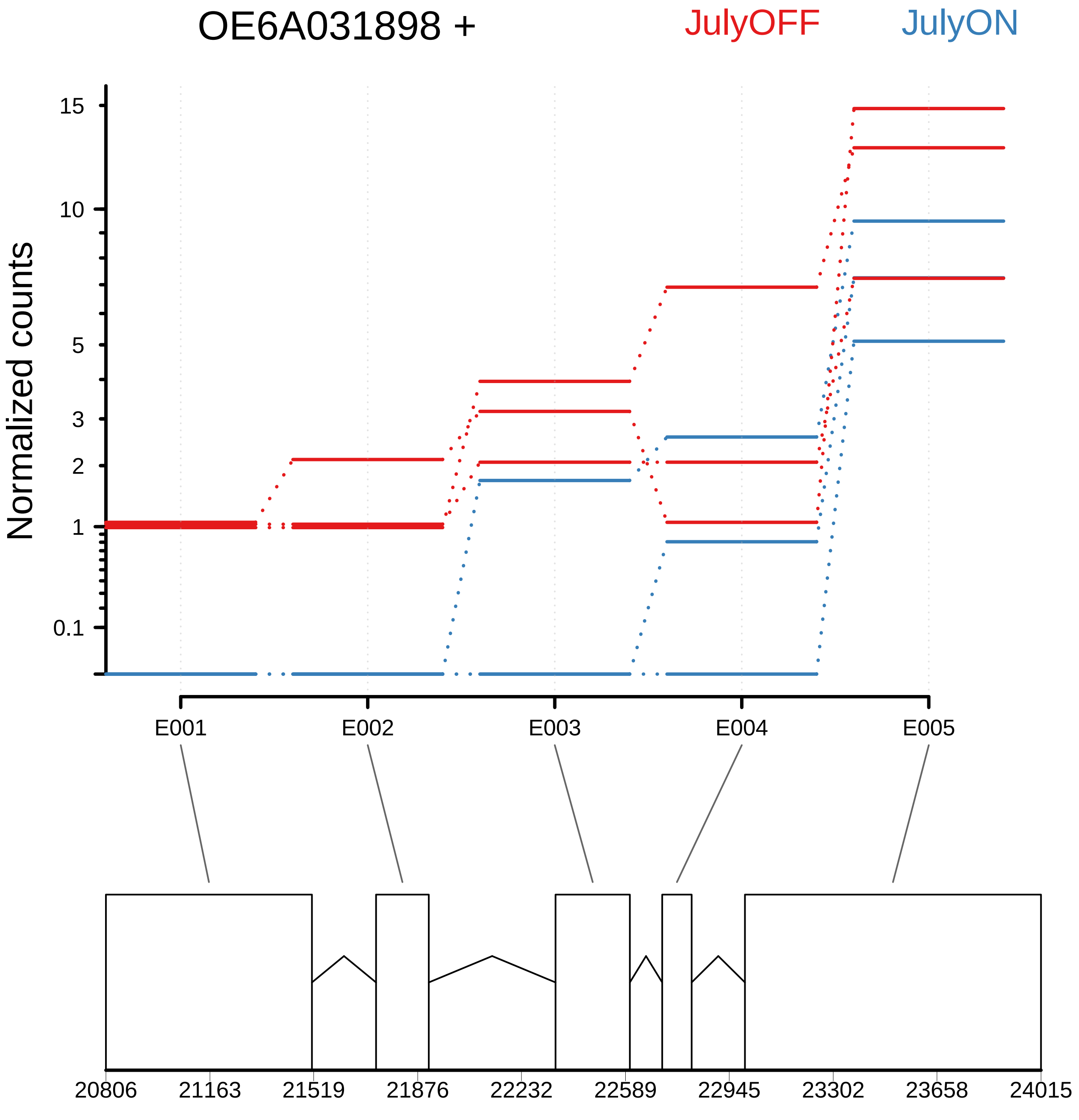

OE6A031898 +
JulyOFF
JulyON
Normalized counts
15
10
5
3
2
1
0.1
E001
E002
E003
E004
E005
20806
21163
21519
21876
22232
22589
22945
23302
23658
24015

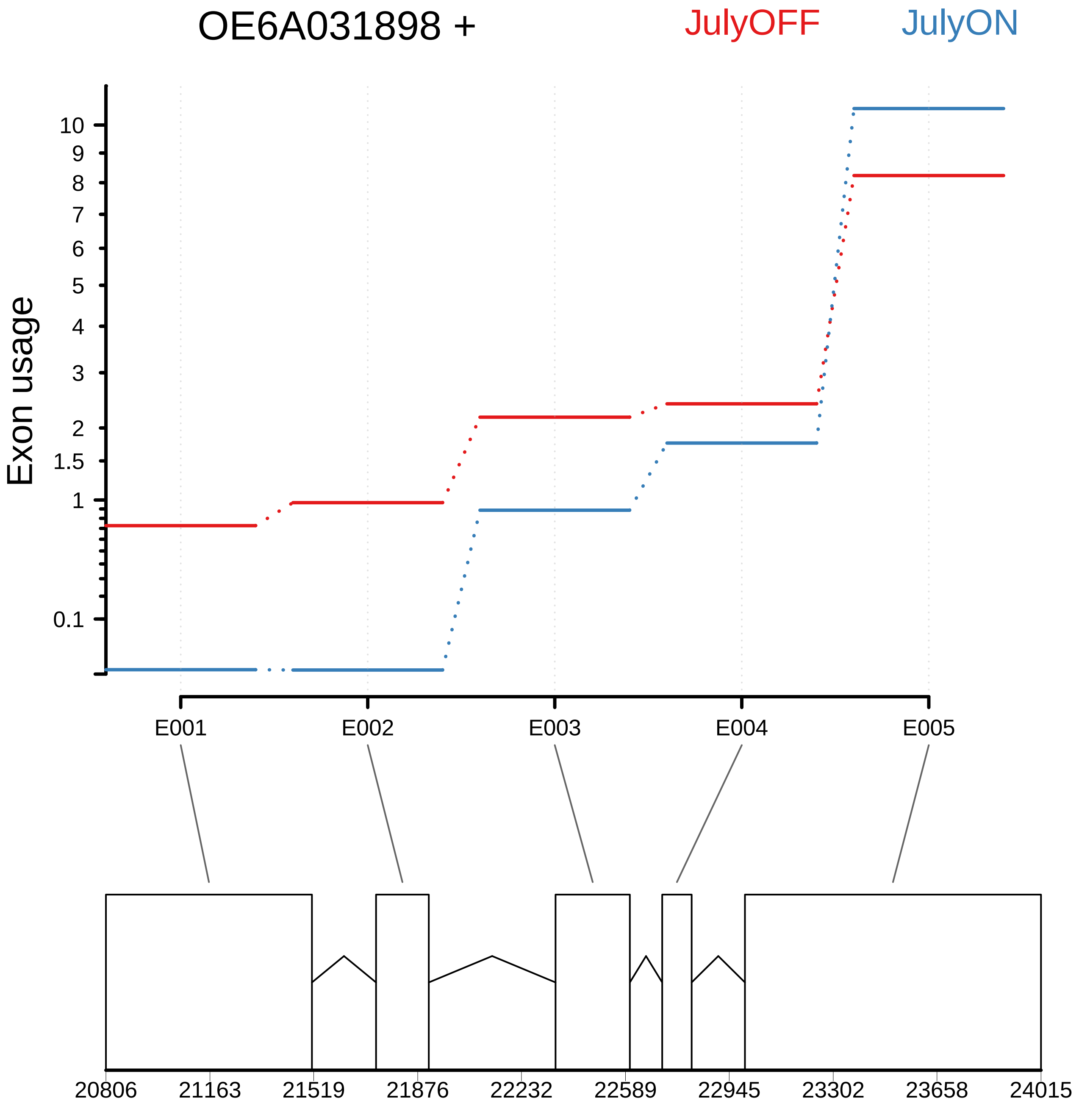
OE6A031898 +
JulyOFF
JulyON
Exon usage
10
9
8
7
6
5
4
3
2
1.5
1
0.1
E001
E002
E003
E004
E005
20806
21163
21519
21876
22232
22589
22945
23302
23658
24015

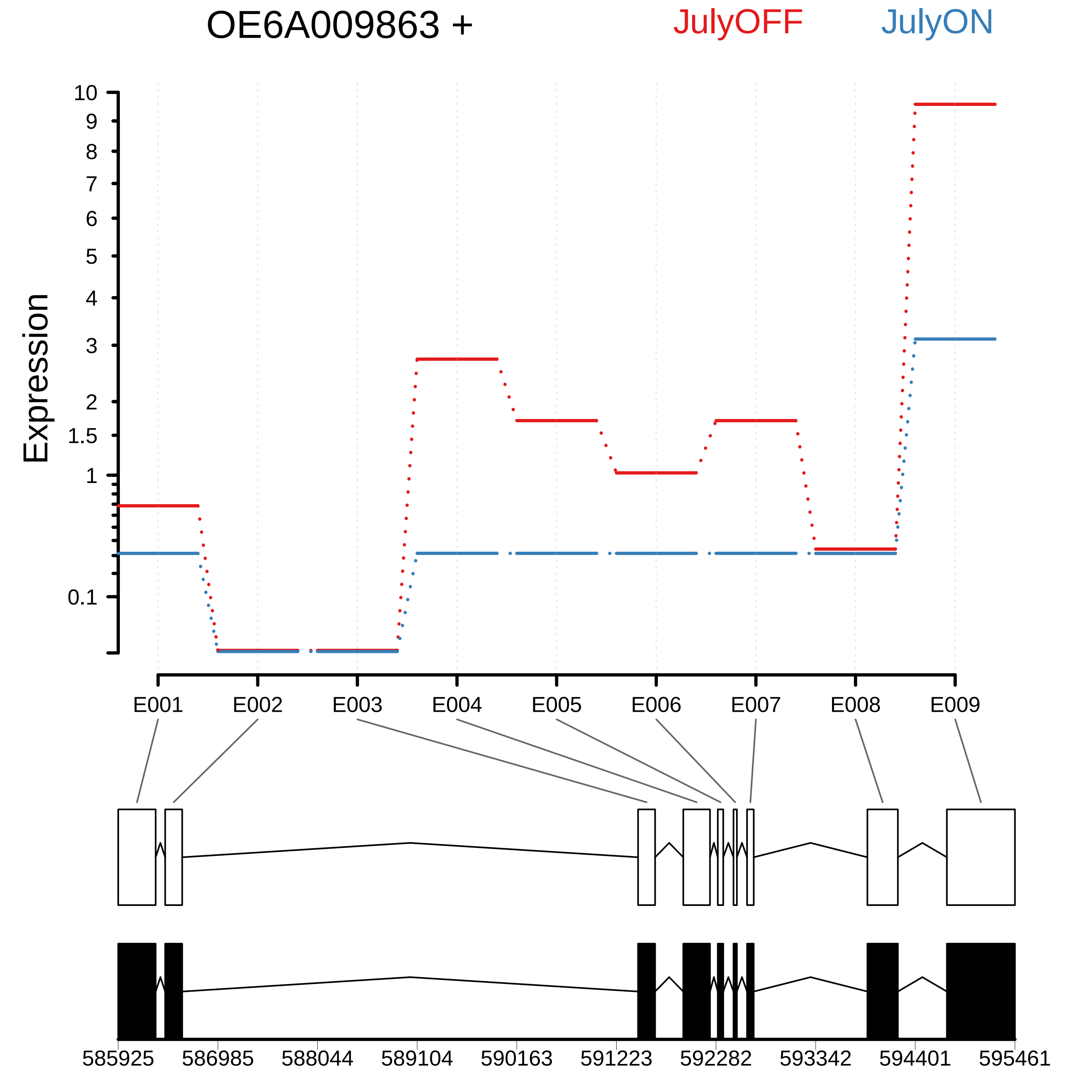

OE6A009863 +
JulyOFF
JulyON
Expression
10
9
8
7
6
5
4
3
2
1.5
1
0.1
E001
E002
E003
E004
E005
E006
E007
E008
E009
585925
586985
588044
589104
590163
591223
592282
593342
594401
595461

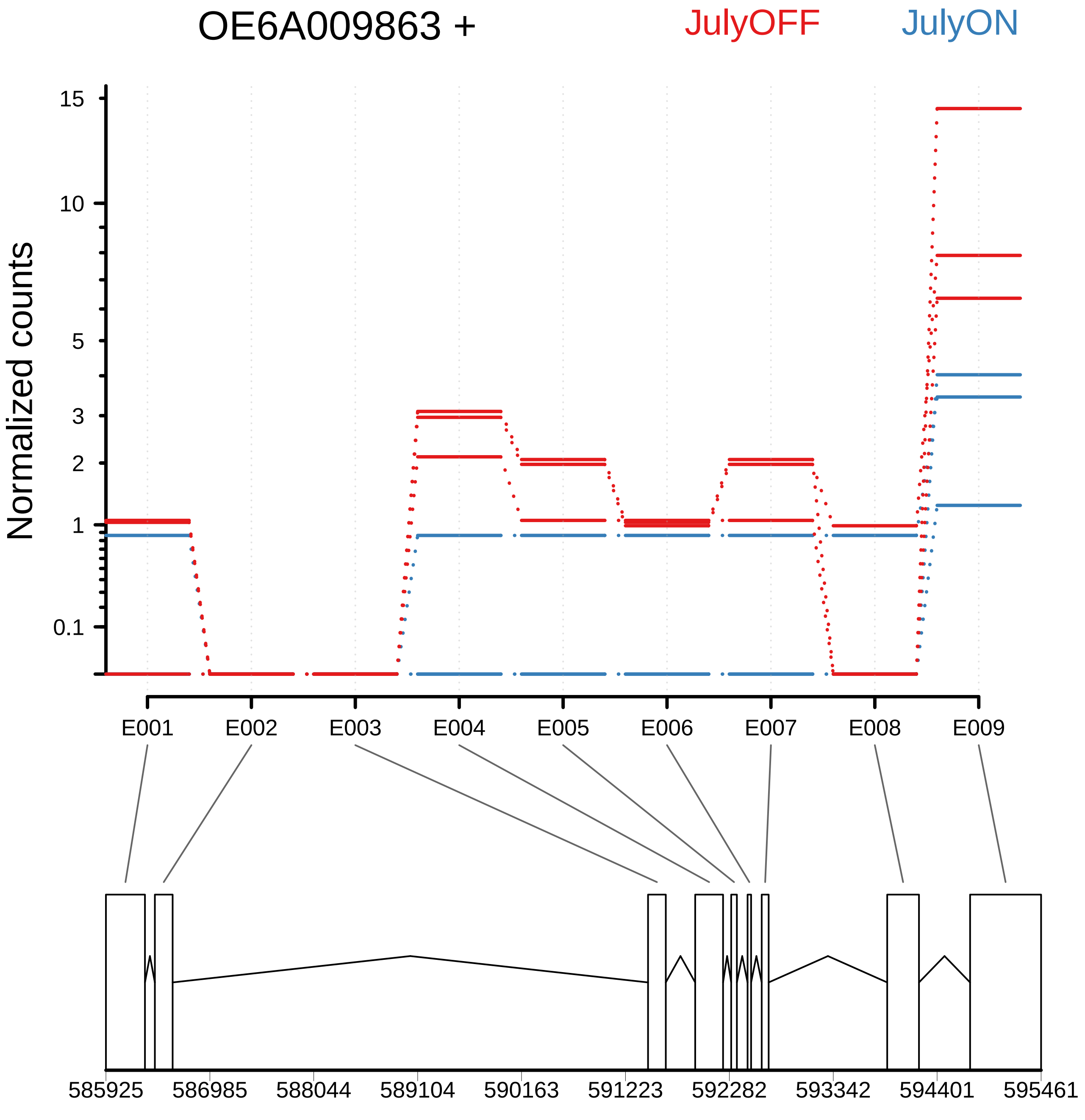
OE6A009863 +
JulyOFF
JulyON
Normalized counts
15
10
5
3
2
1
0.1
E001
E002
E003
E004
E005
E006
E007
E008
E009
585925
586985
588044
589104
590163
591223
592282
593342
594401
595461

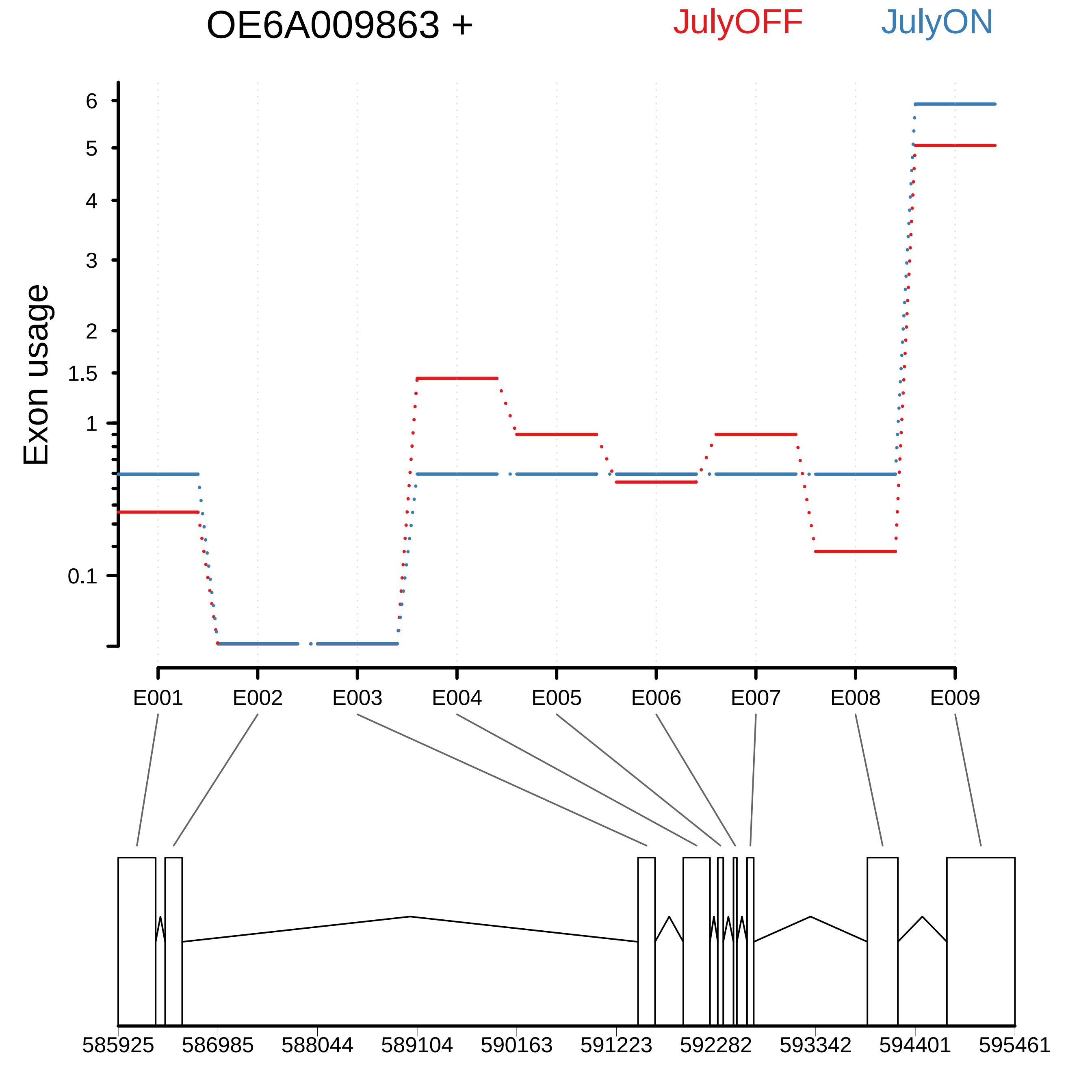
OE6A009863 +
JulyOFF
JulyON
Exon usage
6
5
4
3
2
1.5
1
0.1
E001
E002
E003
E004
E005
E006
E007
E008
E009
585925
586985
588044
589104
590163
591223
592282
593342
594401
595461

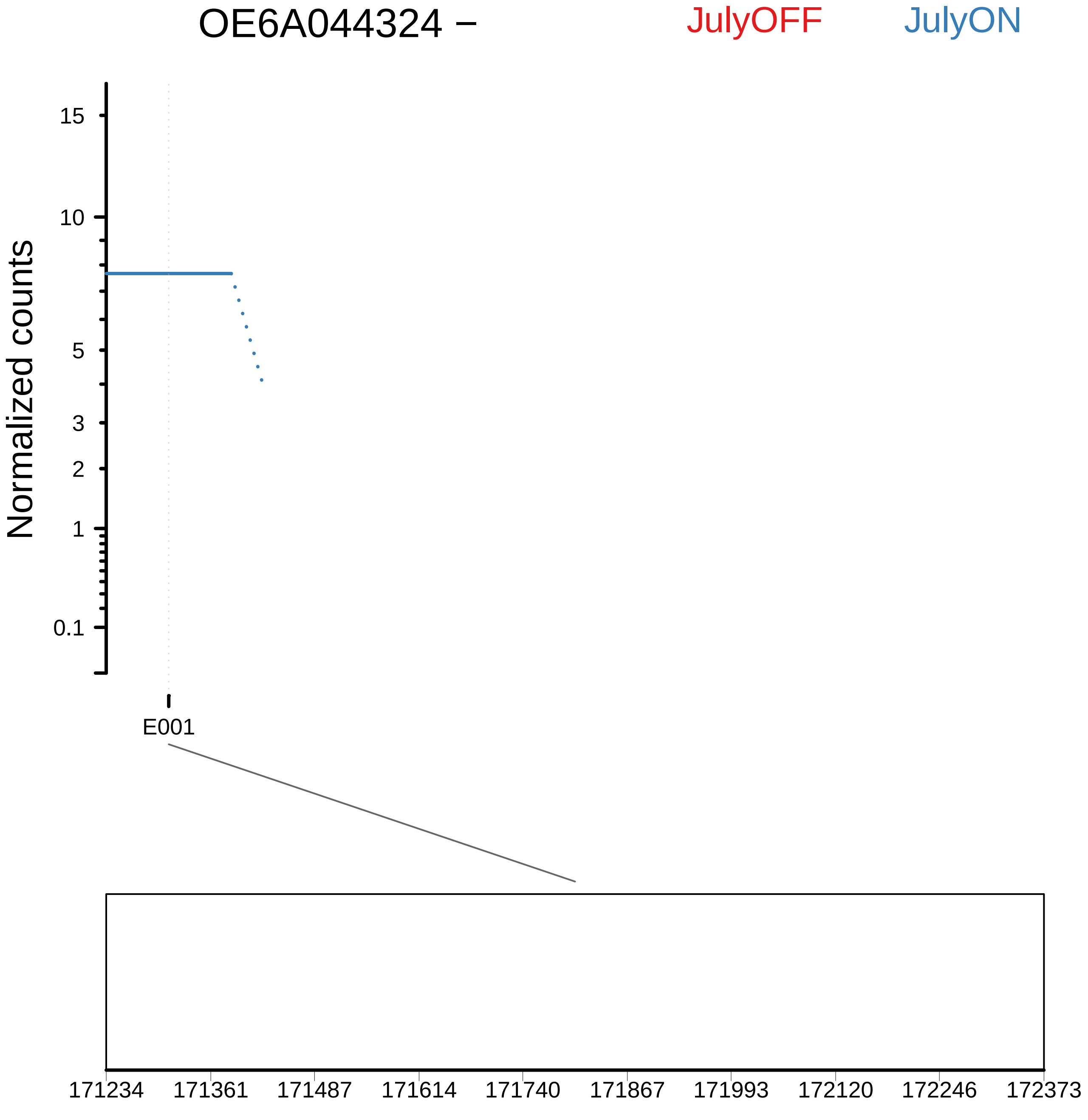
OE6A044324 −
JulyOFF
JulyON
Normalized counts
15
10
5
3
2
1
0.1
E001
171234
171361
171487
171614
171740
171867
171993
172120
172246
172373

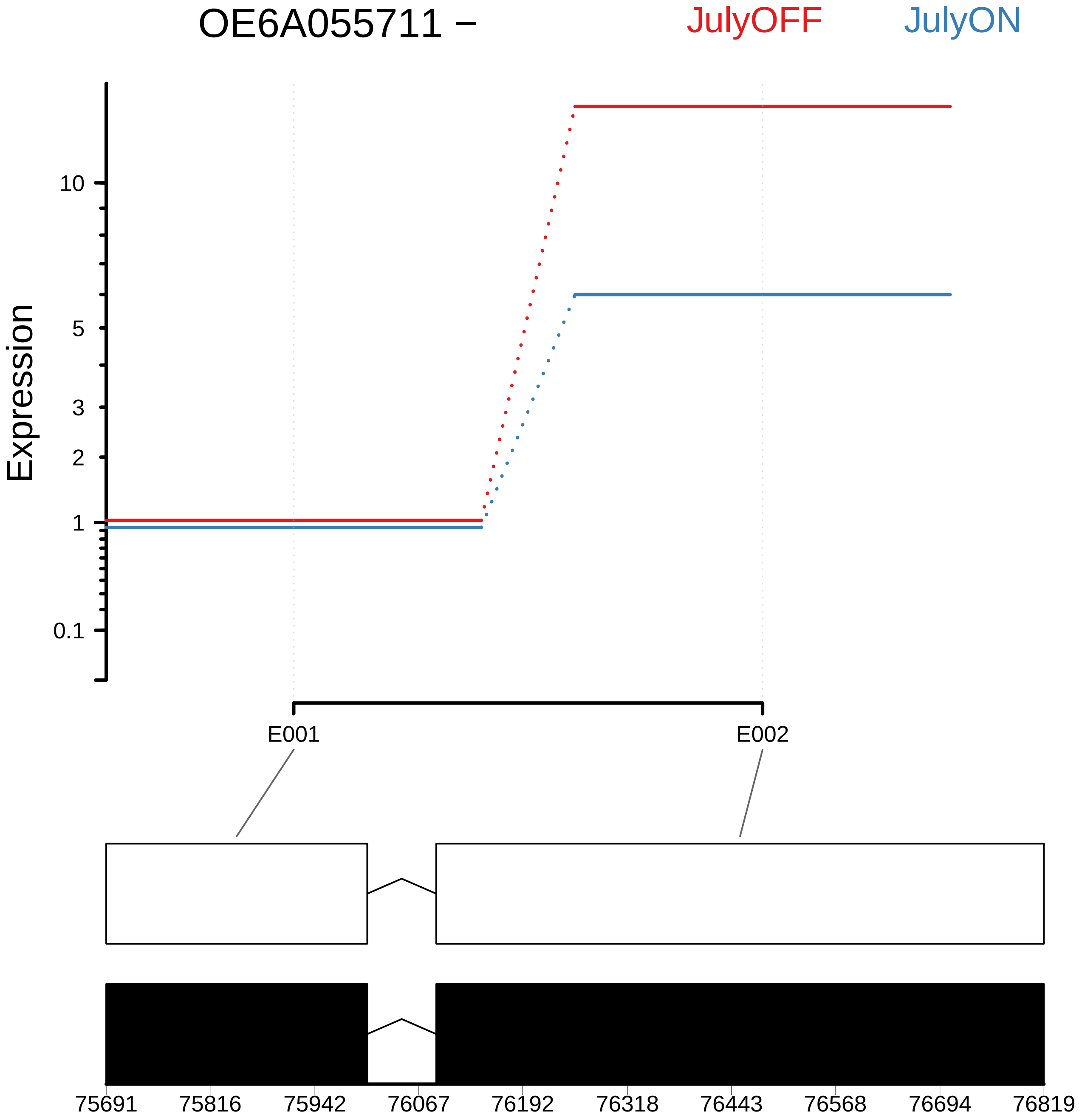
OE6A055711 −
JulyOFF
JulyON
Expression
10
5
3
2
1
0.1
E001
E002
75691
75816
75942
76067
76192
76318
76443
76568
76694
76819

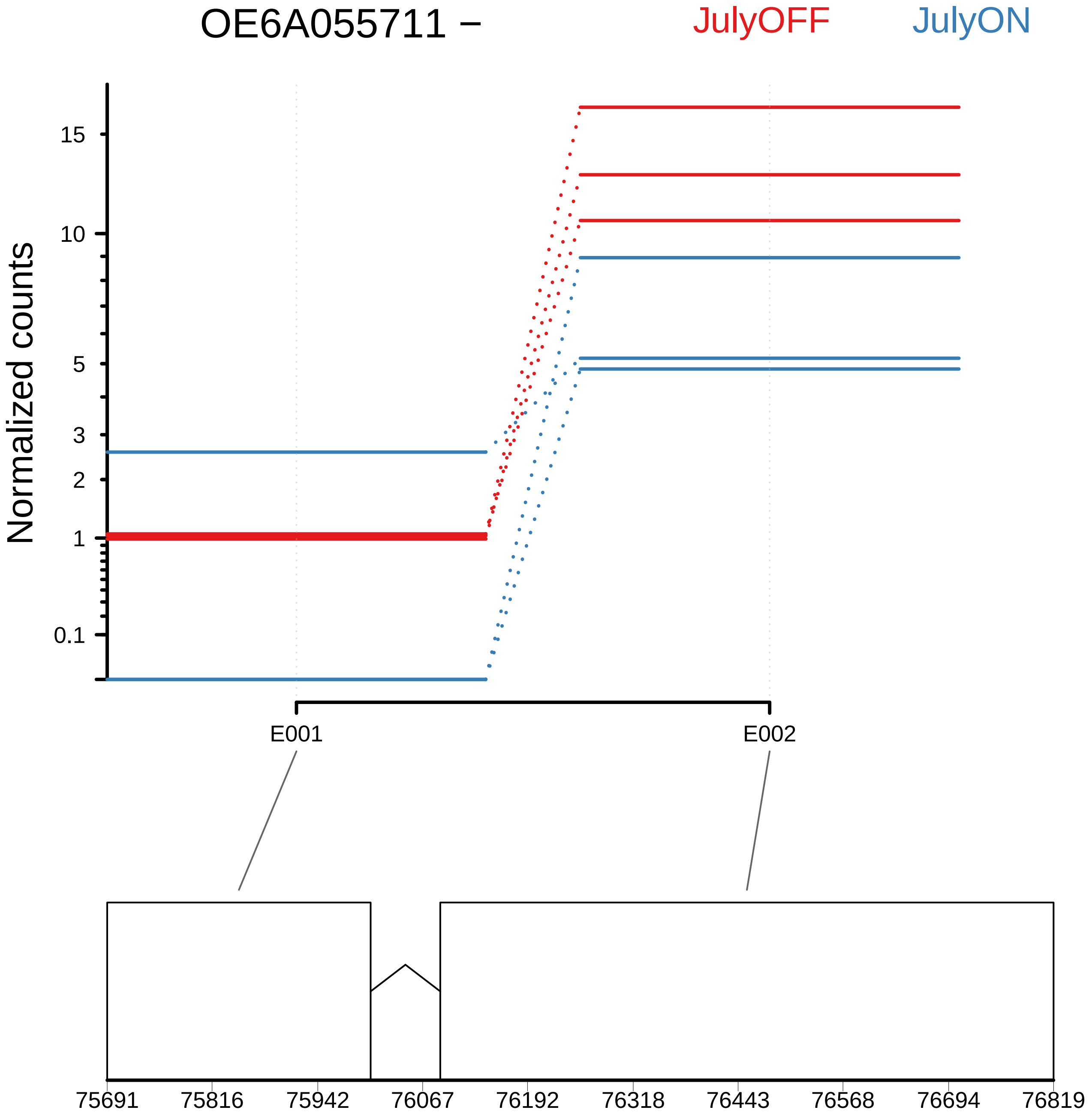
OE6A055711 −
JulyOFF
JulyON
Normalized counts
15
10
5
3
2
1
0.1
E001
E002
75691
75816
75942
76067
76192
76318
76443
76568
76694
76819

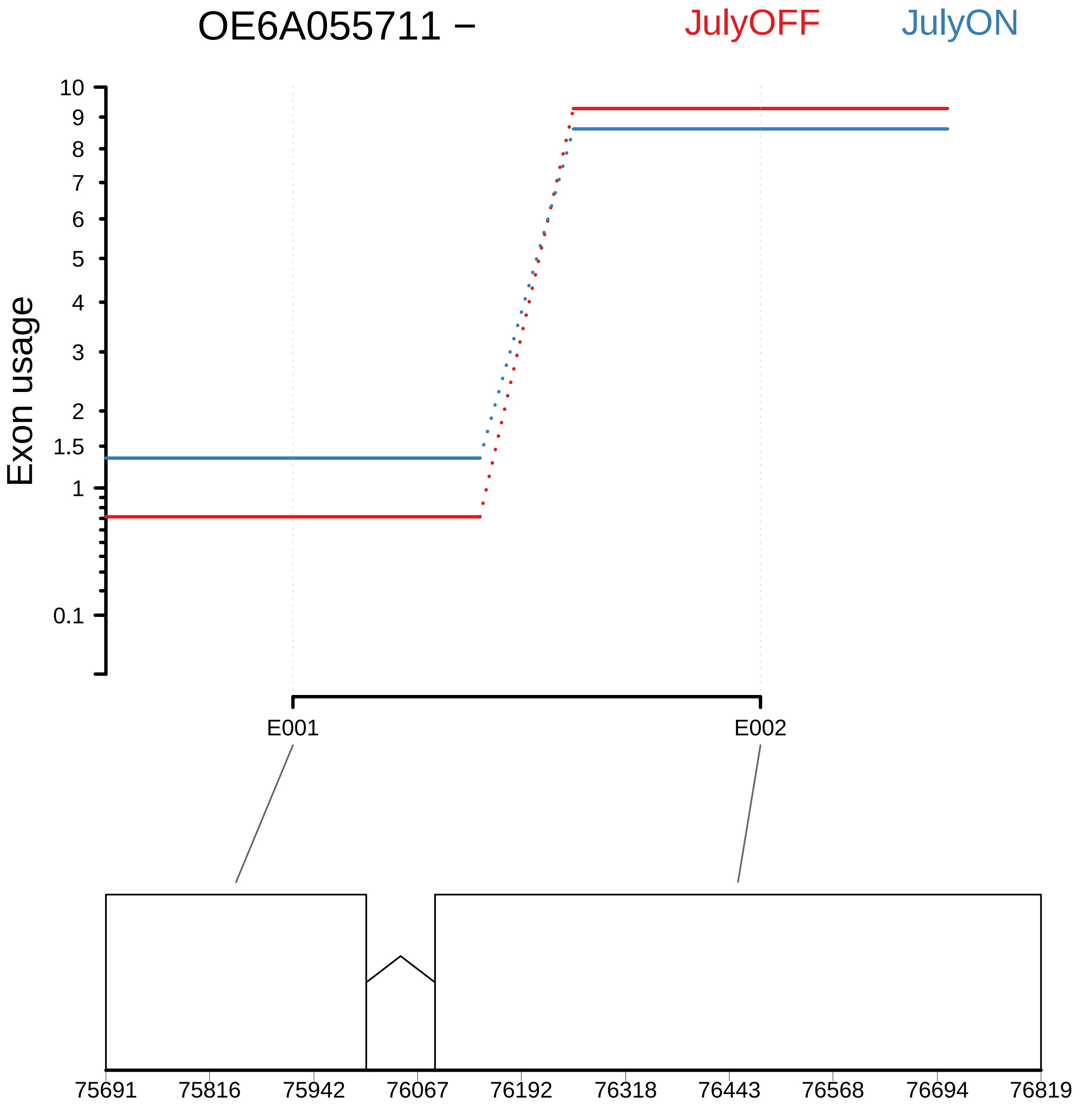
OE6A055711 −
JulyOFF
JulyON
Exon usage
10
9
8
7
6
5
4
3
2
1.5
1
0.1
E001
E002
75691
75816
75942
76067
76192
76318
76443
76568
76694
76819

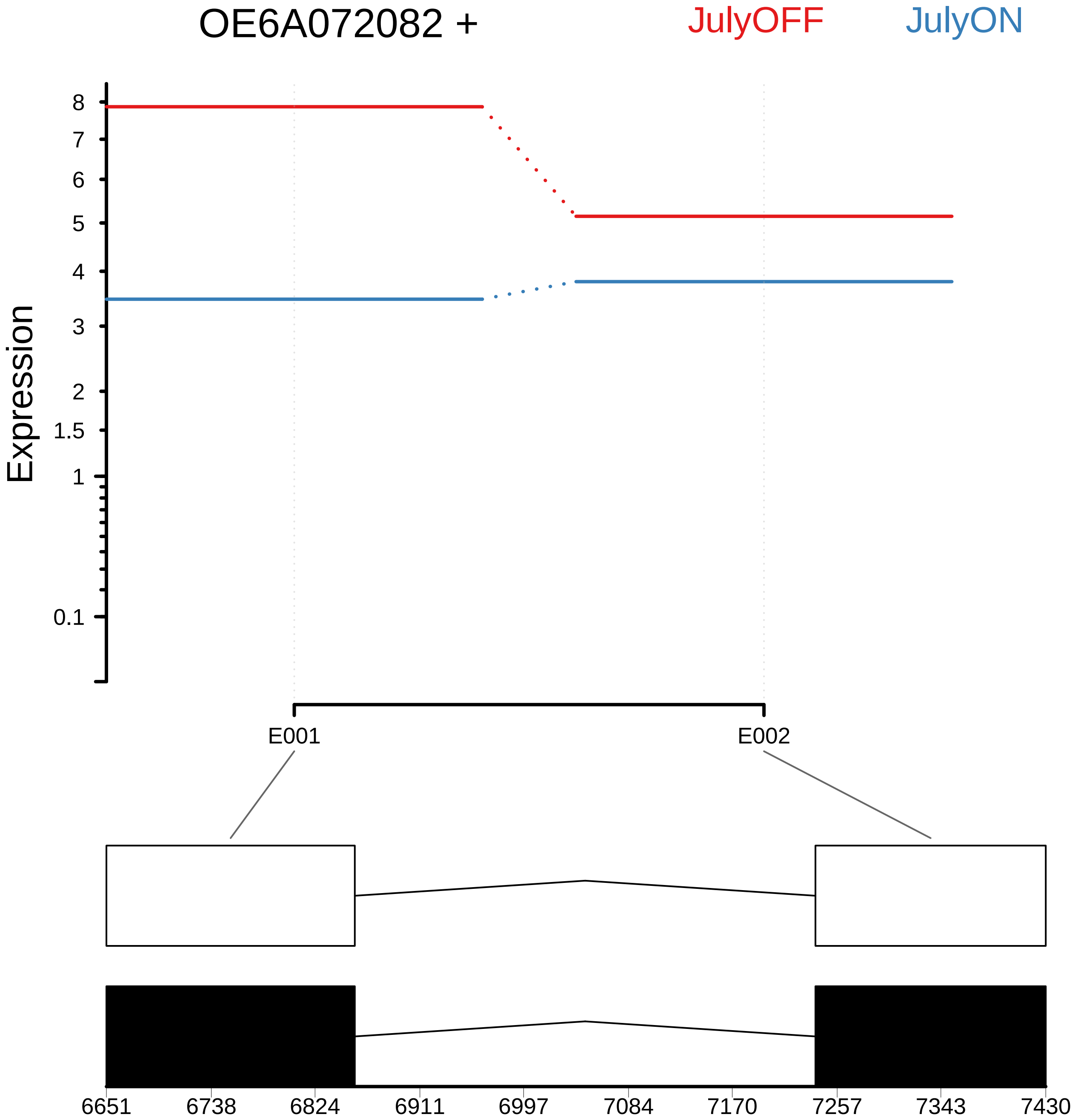
OE6A072082 +
JulyOFF
JulyON
Expression
8
7
6
5
4
3
2
1.5
1
0.1
E001
E002
6651
6738
6824
6911
6997
7084
7170
7257
7343
7430

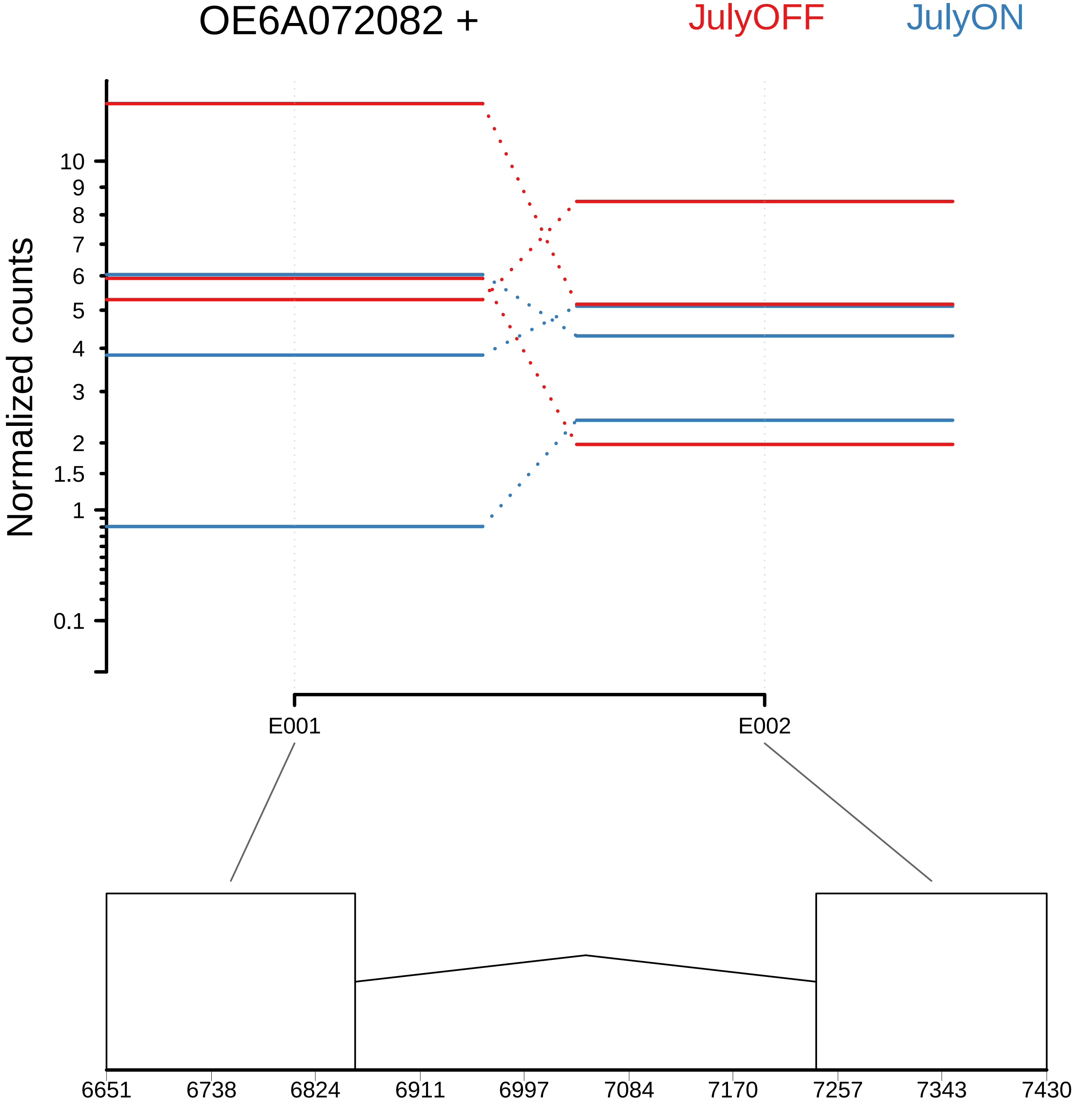

OE6A072082 +
JulyOFF
JulyON
Normalized counts
10
9
8
7
6
5
4
3
2
1.5
1
0.1
E001
E002
6651
6738
6824
6911
6997
7084
7170
7257
7343
7430

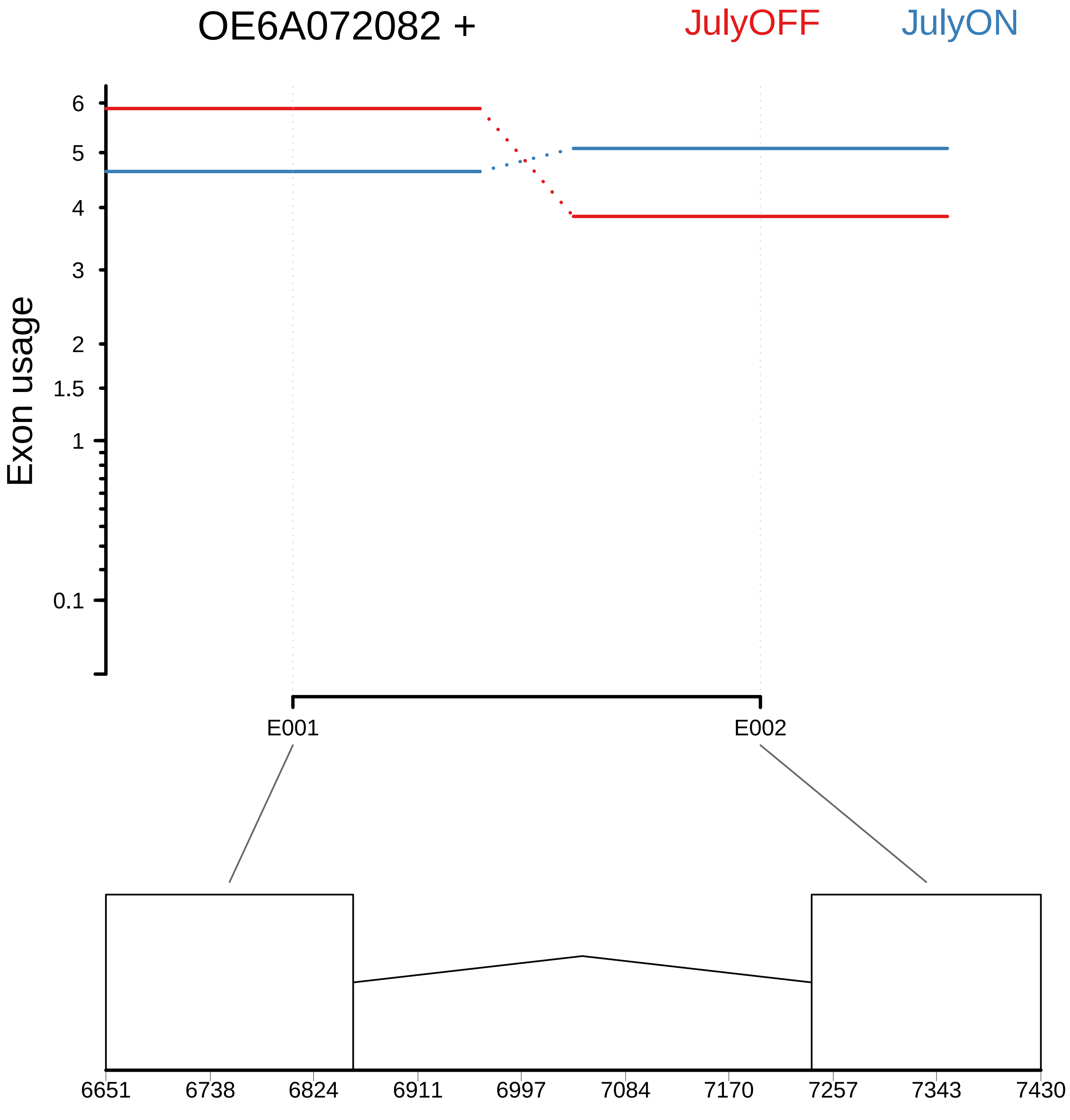
OE6A072082 +
JulyOFF
JulyON
Exon usage
6
5
4
3
2
1.5
1
0.1
E001
E002
6651
6738
6824
6911
6997
7084
7170
7257
7343
7430

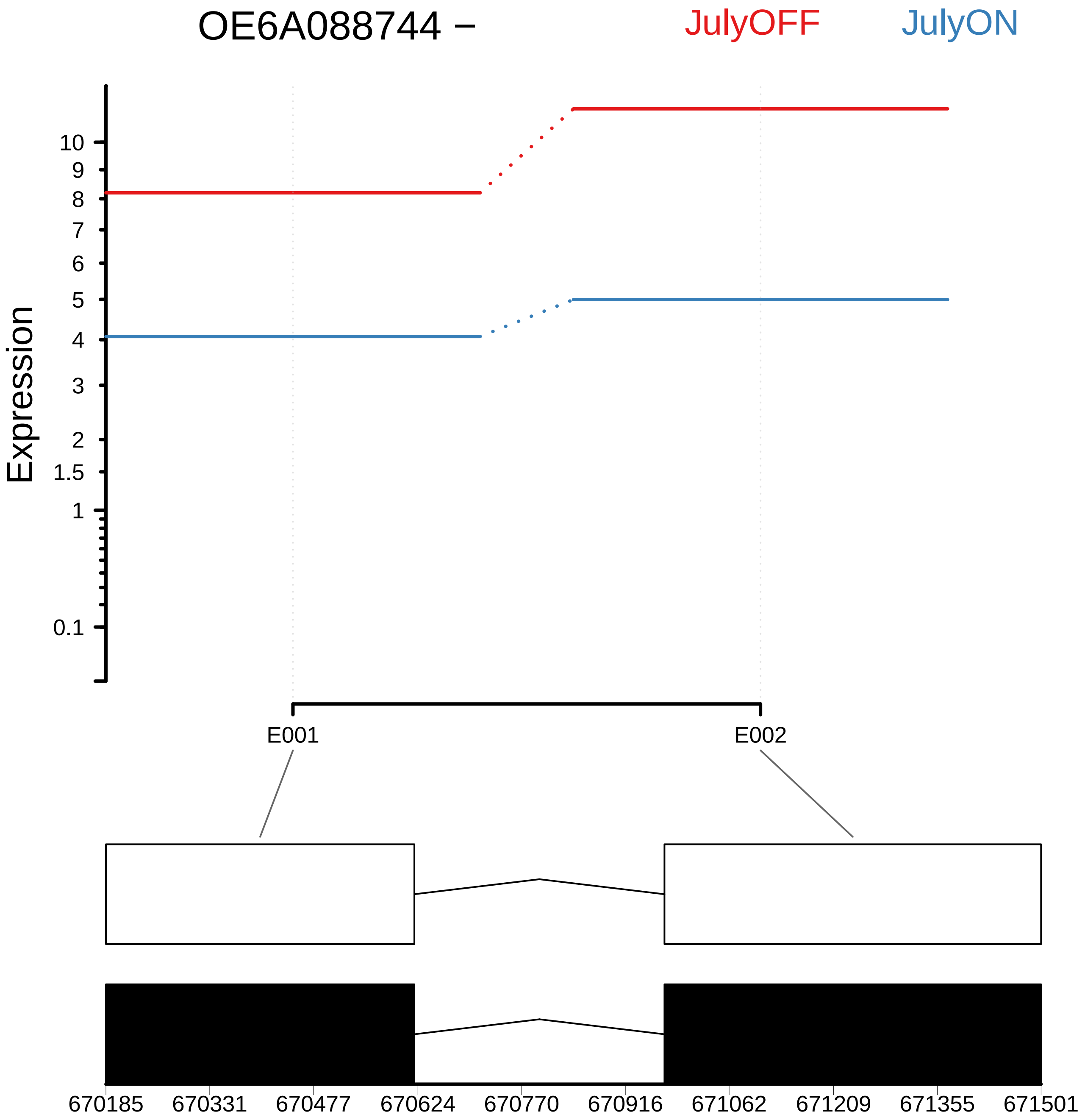
OE6A088744 −
JulyOFF
JulyON
Expression
10
9
8
7
6
5
4
3
2
1.5
1
0.1
E001
E002
670185
670331
670477
670624
670770
670916
671062
671209
671355
671501

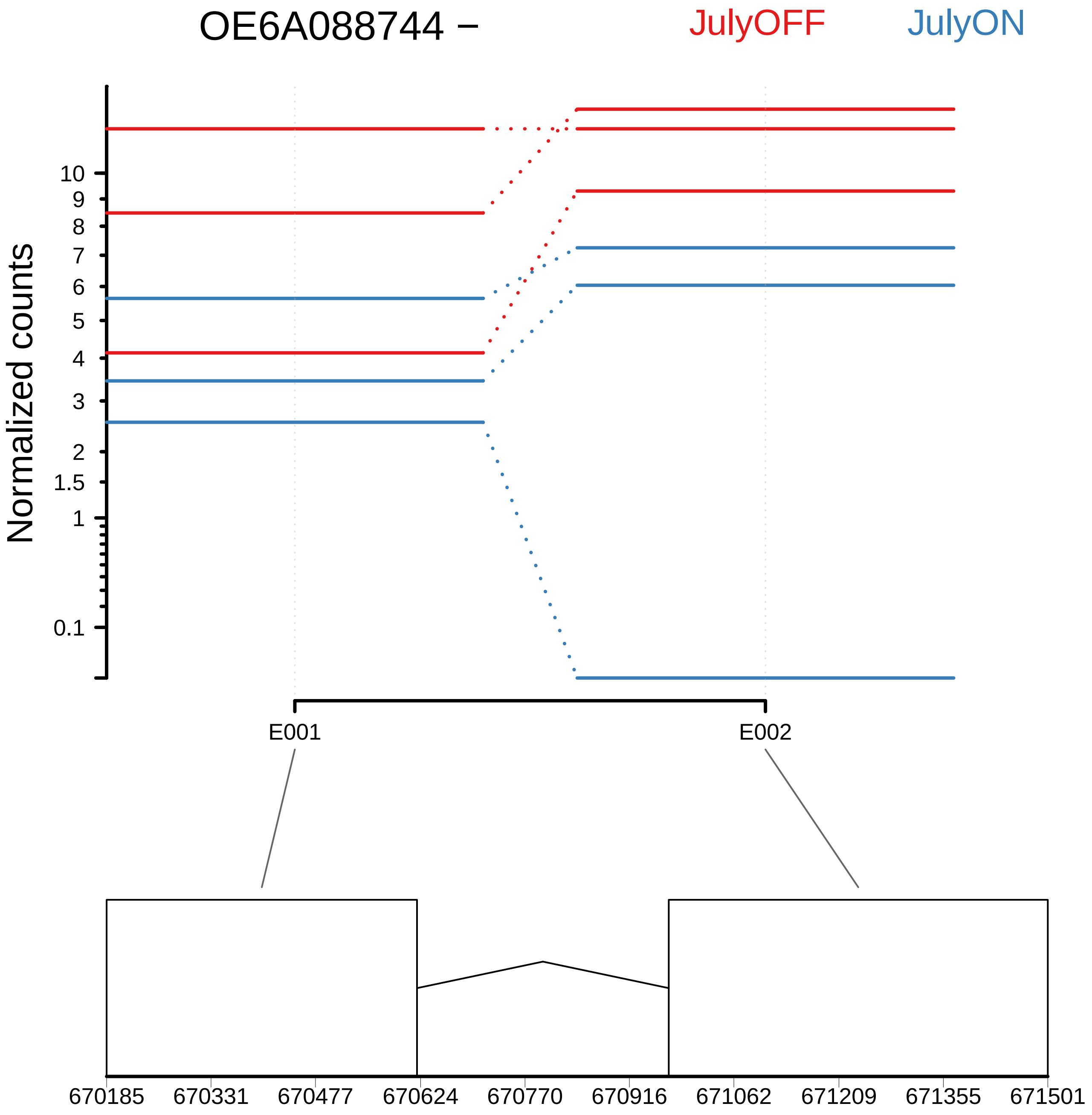

OE6A088744 −
JulyOFF
JulyON
Normalized counts
10
9
8
7
6
5
4
3
2
1.5
1
0.1
E001
E002
670185
670331
670477
670624
670770
670916
671062
671209
671355
671501

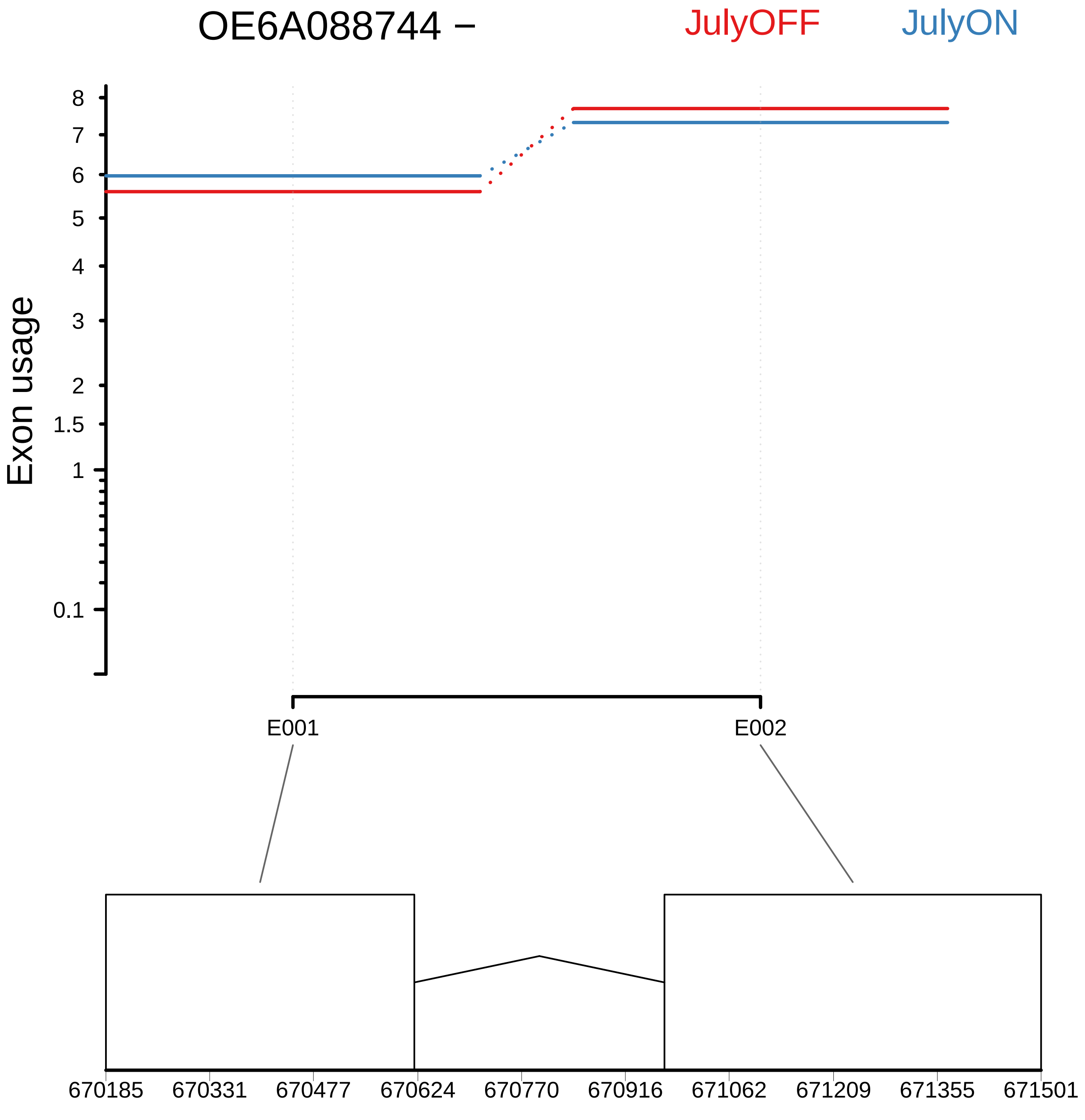

OE6A088744 −
JulyOFF
JulyON
Exon usage
8
7
6
5
4
3
2
1.5
1
0.1
E001
E002
670185
670331
670477
670624
670770
670916
671062
671209
671355
671501

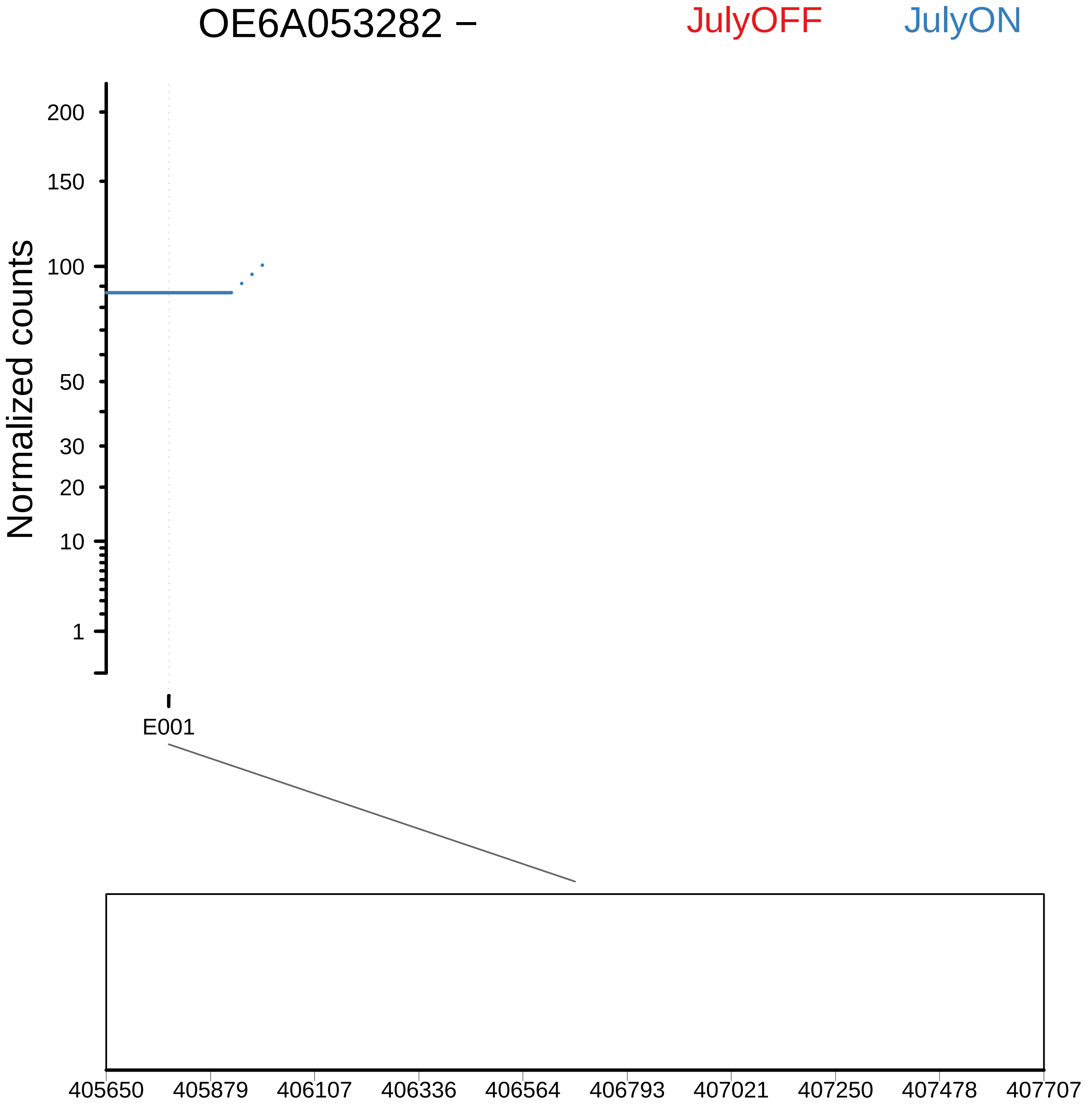
OE6A053282 −
JulyOFF
JulyON
Normalized counts
200
150
100
50
30
20
10
1
E001
405650
405879
406107
406336
406564
406793
407021
407250
407478
407707

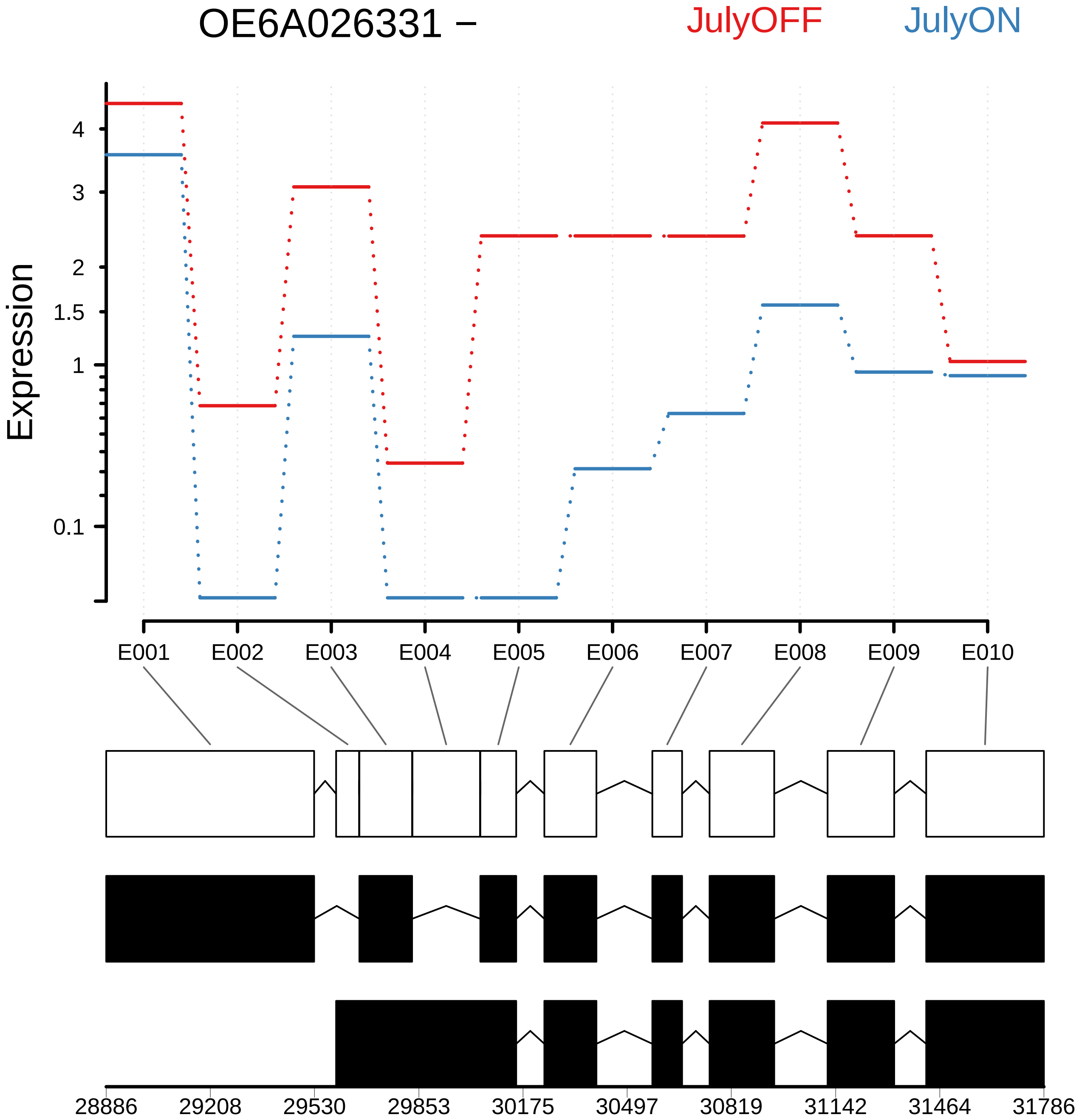

OE6A026331 –
JulyOFF
JulyON
Expression
4
3
2
1.5
1
0.1
E001
E002
E003
E004
E005
E006
E007
E008
E009
E010
28886
29208
29530
29853
30175
30497
30819
31142
31464
31786

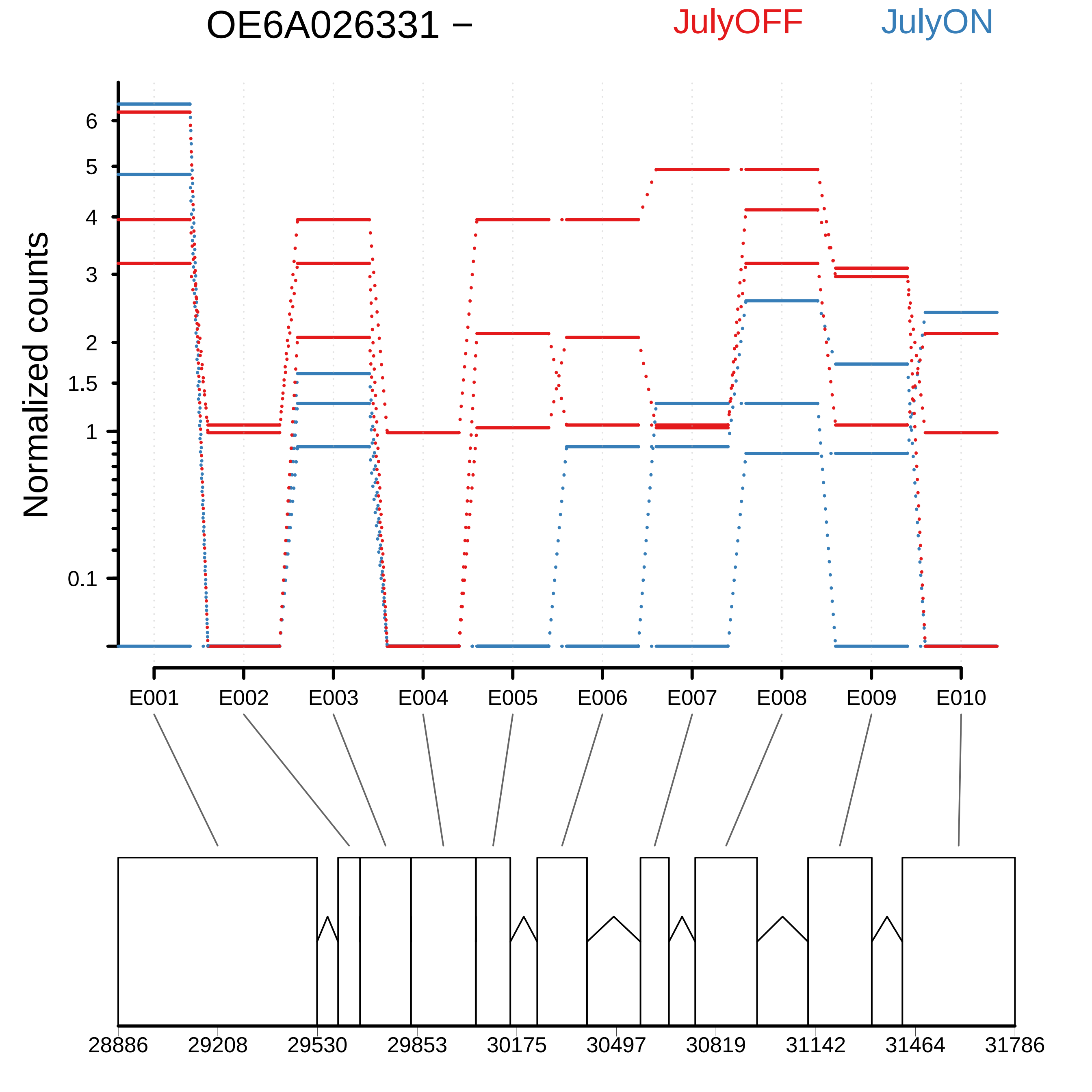

OE6A026331 −
JulyOFF
JulyON
Normalized counts
6
5
4
3
2
1.5
1
0.1
E001
E002
E003
E004
E005
E006
E007
E008
E009
E010
28886
29208
29530
29853
30175
30497
30819
31142
31464
31786

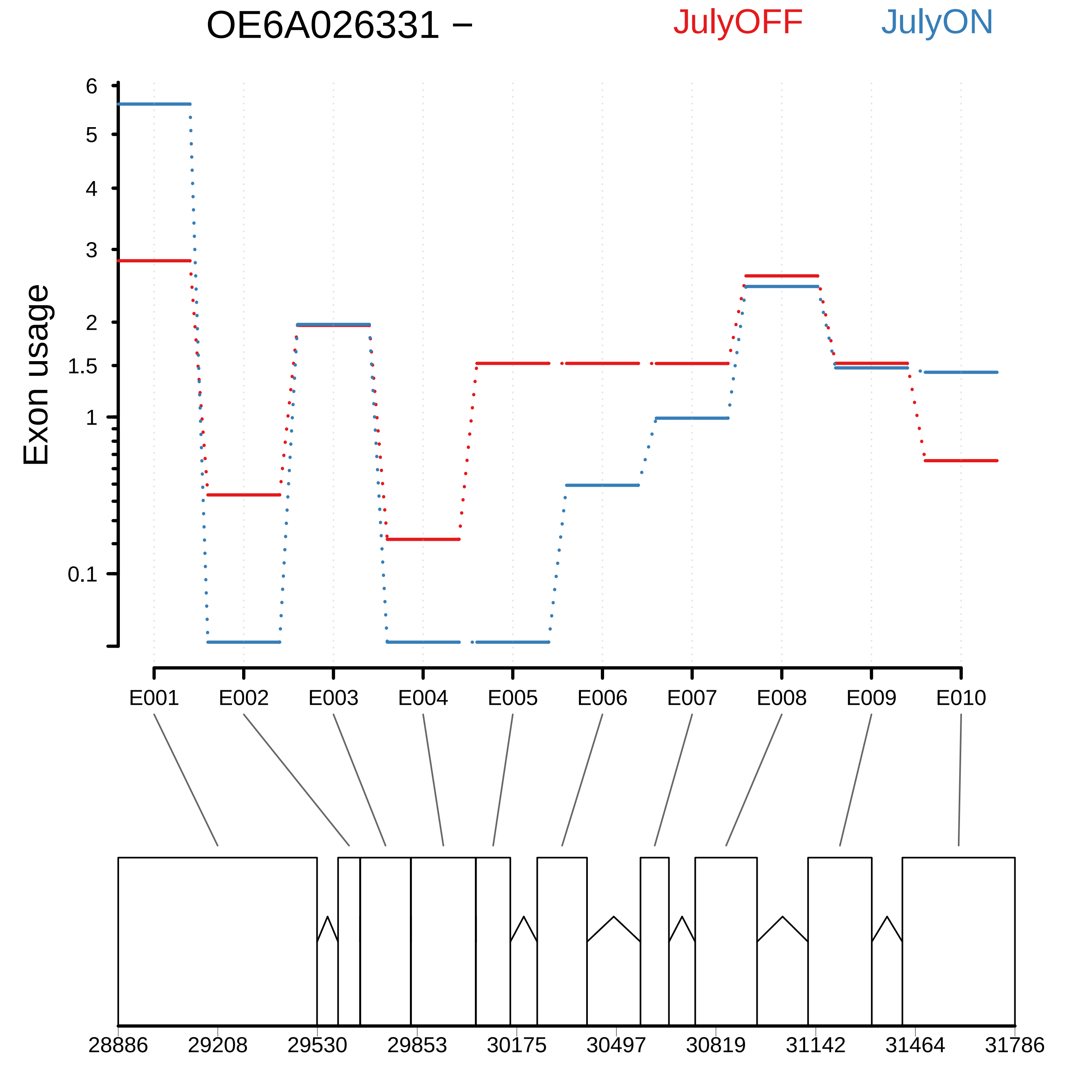
OE6A026331 −
JulyOFF
JulyON
Exon usage
6
5
4
3
2
1.5
1
0.1
E001
E002
E003
E004
E005
E006
E007
E008
E009
E010
28886
29208
29530
29853
30175
30497
30819
31142
31464
31786

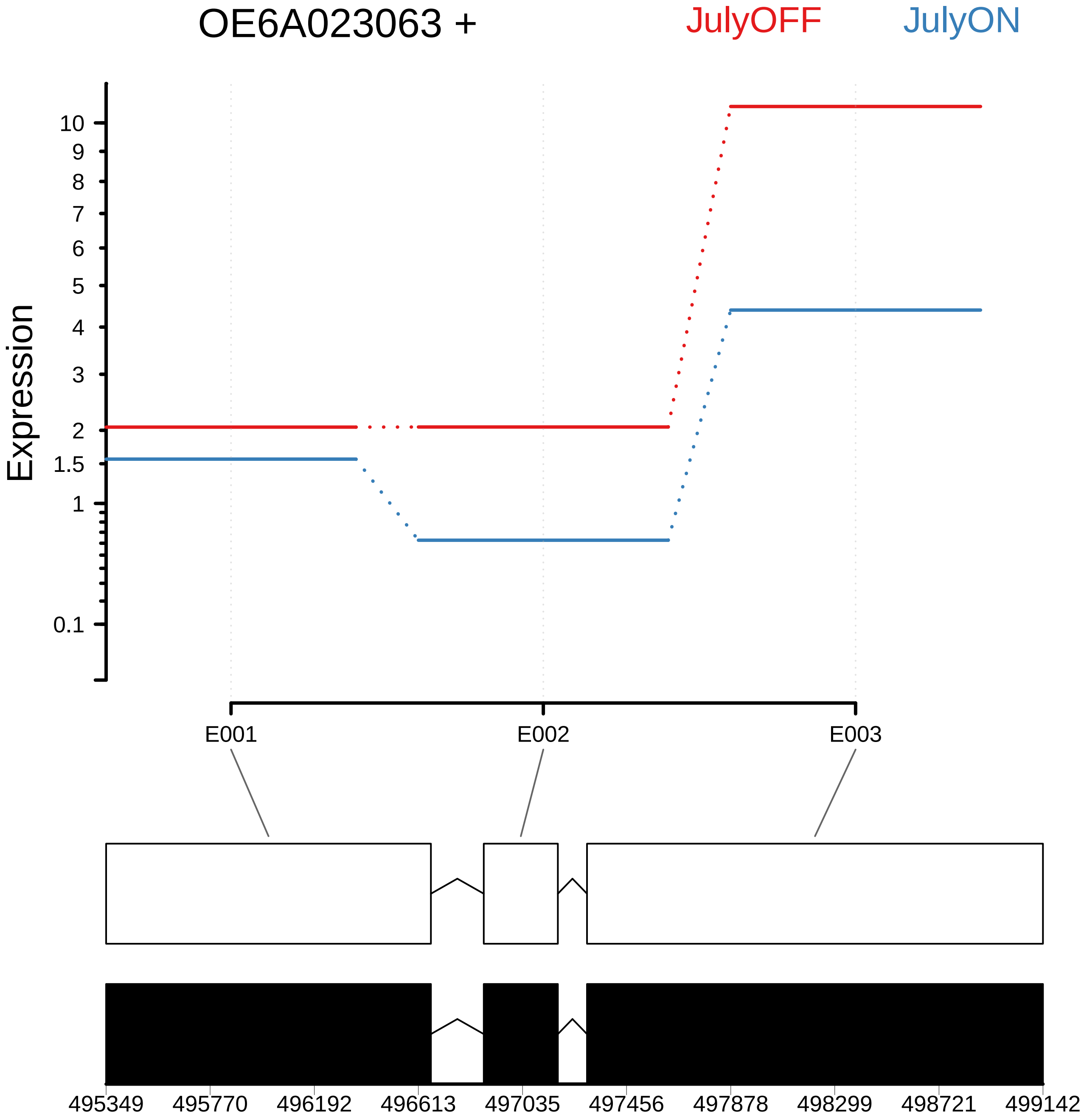
OE6A023063 +
JulyOFF
JulyON
Expression
10
9
8
7
6
5
4
3
2
1.5
1
0.1
E001
E002
E003
495349
495770
496192
496613
497035
497456
497878
498299
498721
499142

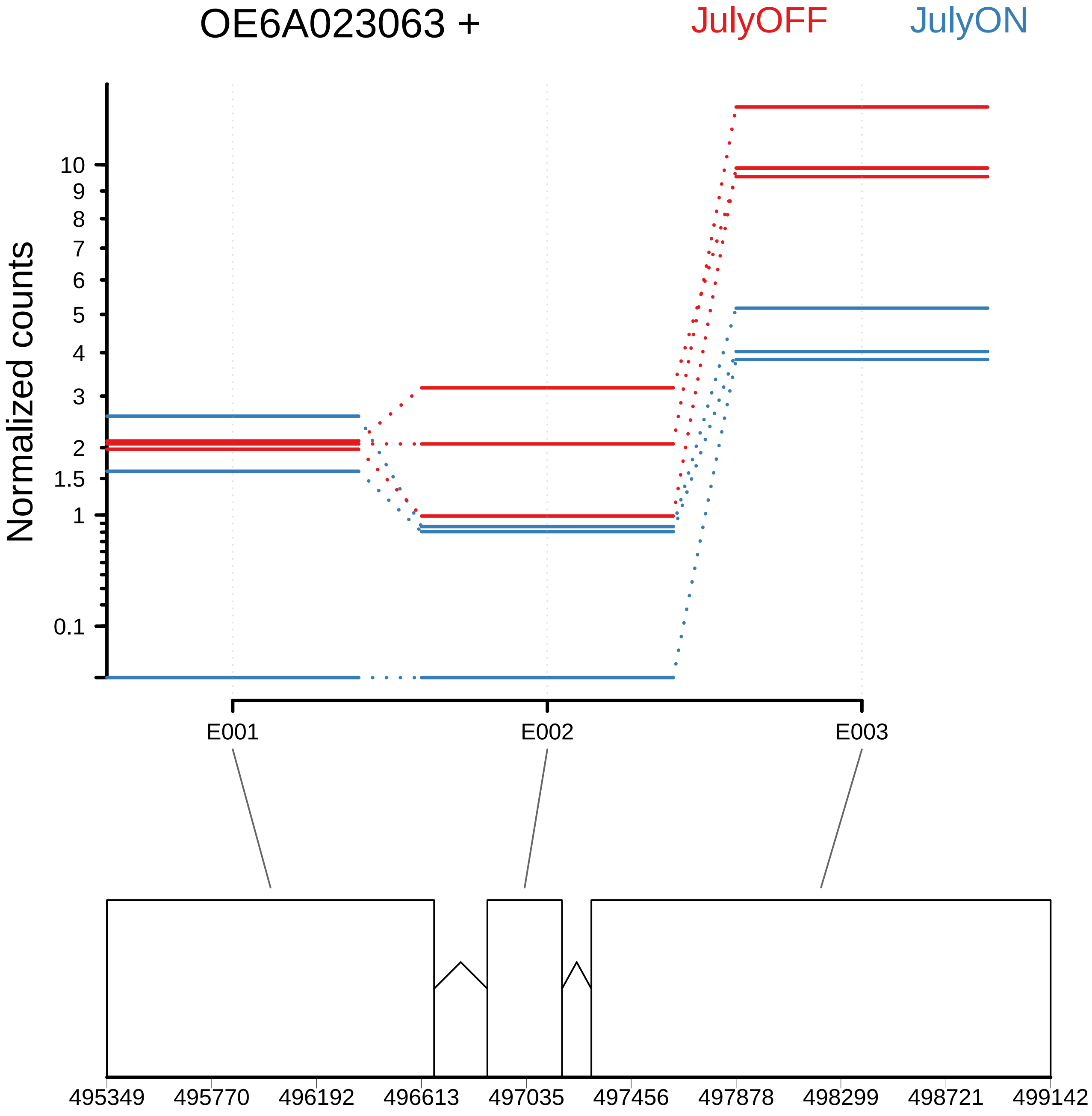
OE6A023063 +
JulyOFF
JulyON
Normalized counts
10
9
8
7
6
5
4
3
2
1.5
1
0.1
E001
E002
E003
495349
495770
496192
496613
497035
497456
497878
498299
498721
499142

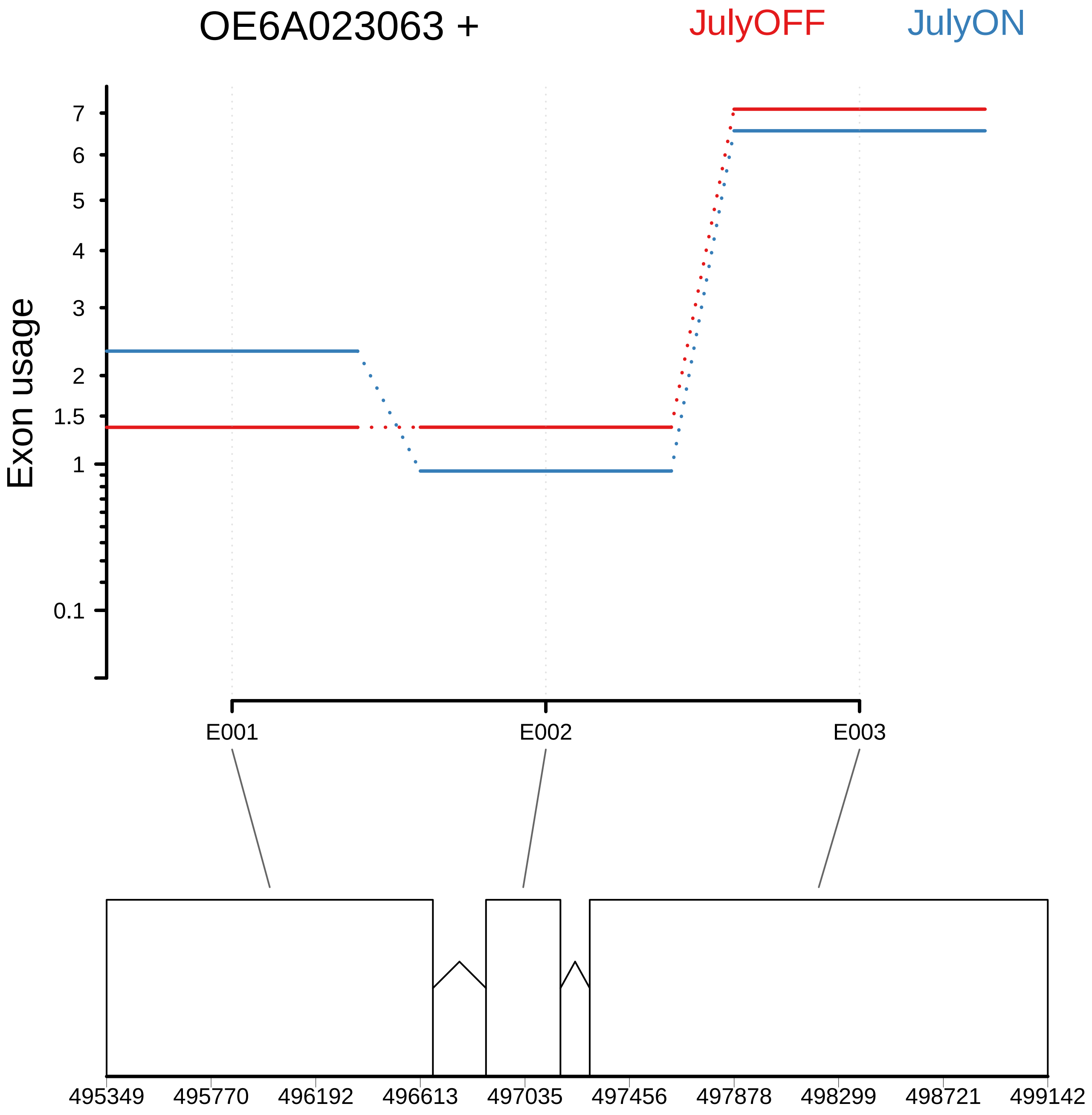
OE6A023063 +
JulyOFF
JulyON
Exon usage
7
6
5
4
3
2
1.5
1
0.1
E001
E002
E003
495349
495770
496192
496613
497035
497456
497878
498299
498721
499142

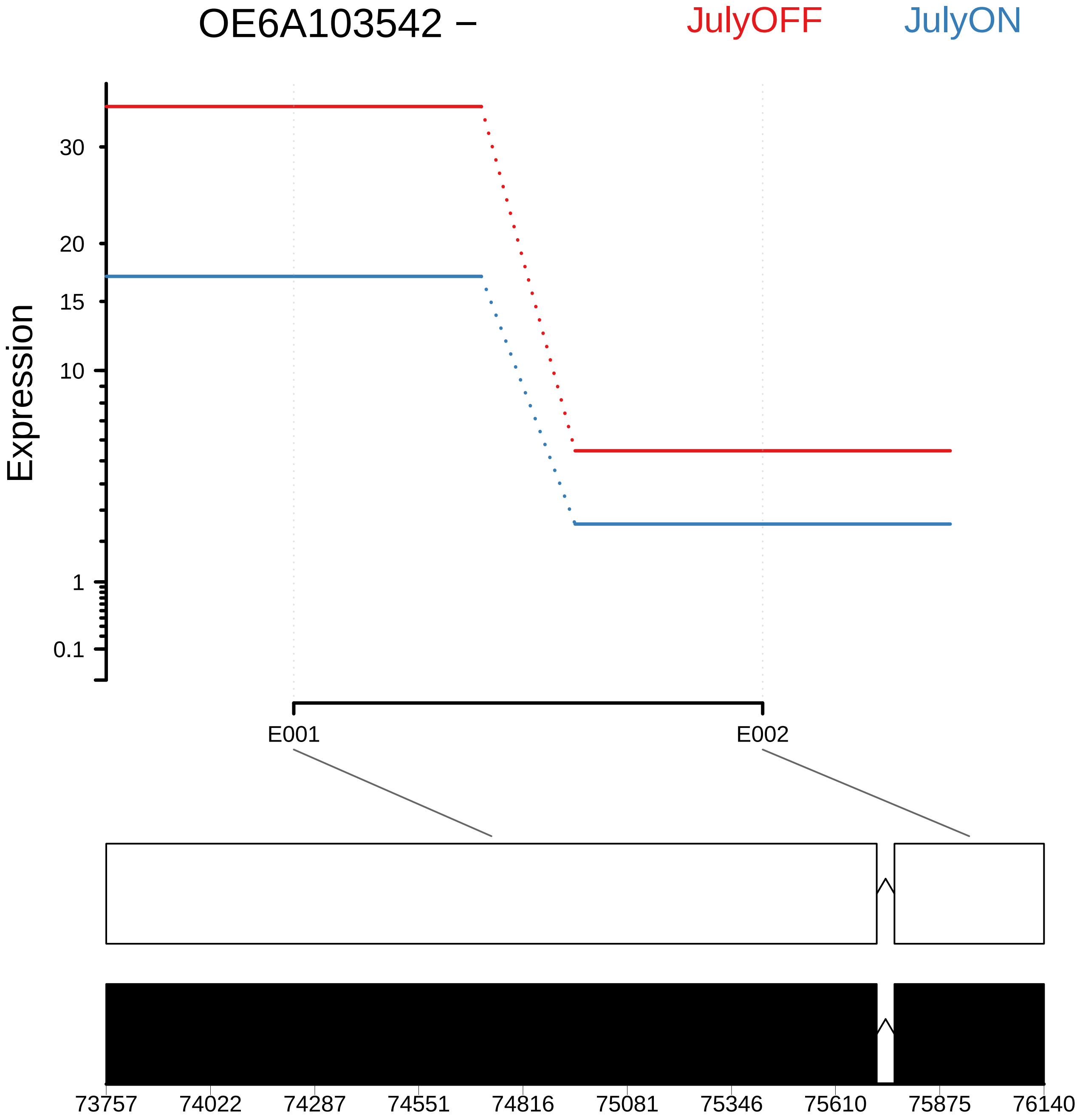
OE6A103542 −
JulyOFF
JulyON
Expression
30
20
15
10
1
0.1
E001
E002
73757
74022
74287
74551
74816
75081
75346
75610
75875
76140

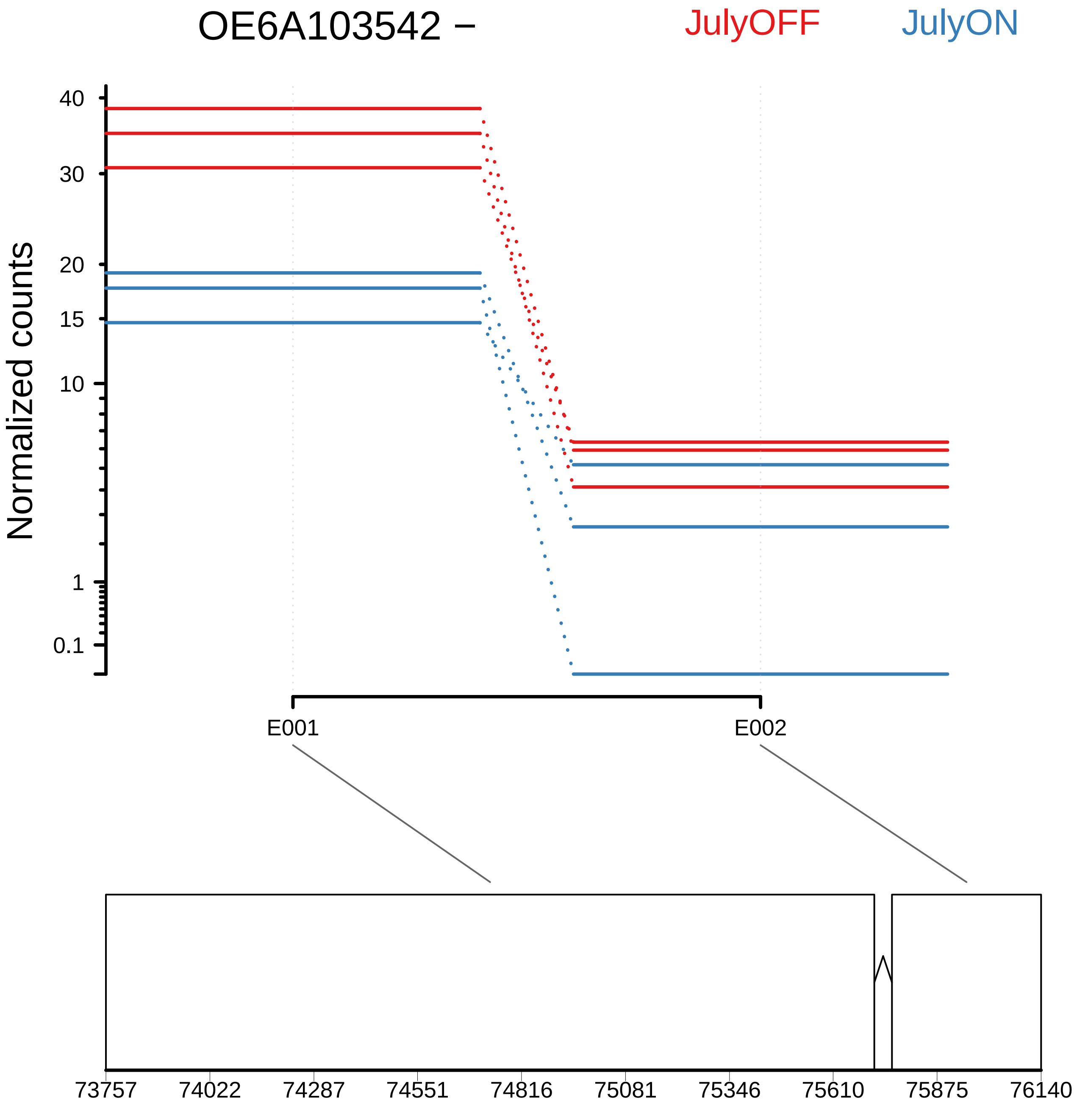
OE6A103542 −
JulyOFF
JulyON
Normalized counts
40
30
20
15
10
1
0.1
E001
E002
73757
74022
74287
74551
74816
75081
75346
75610
75875
76140

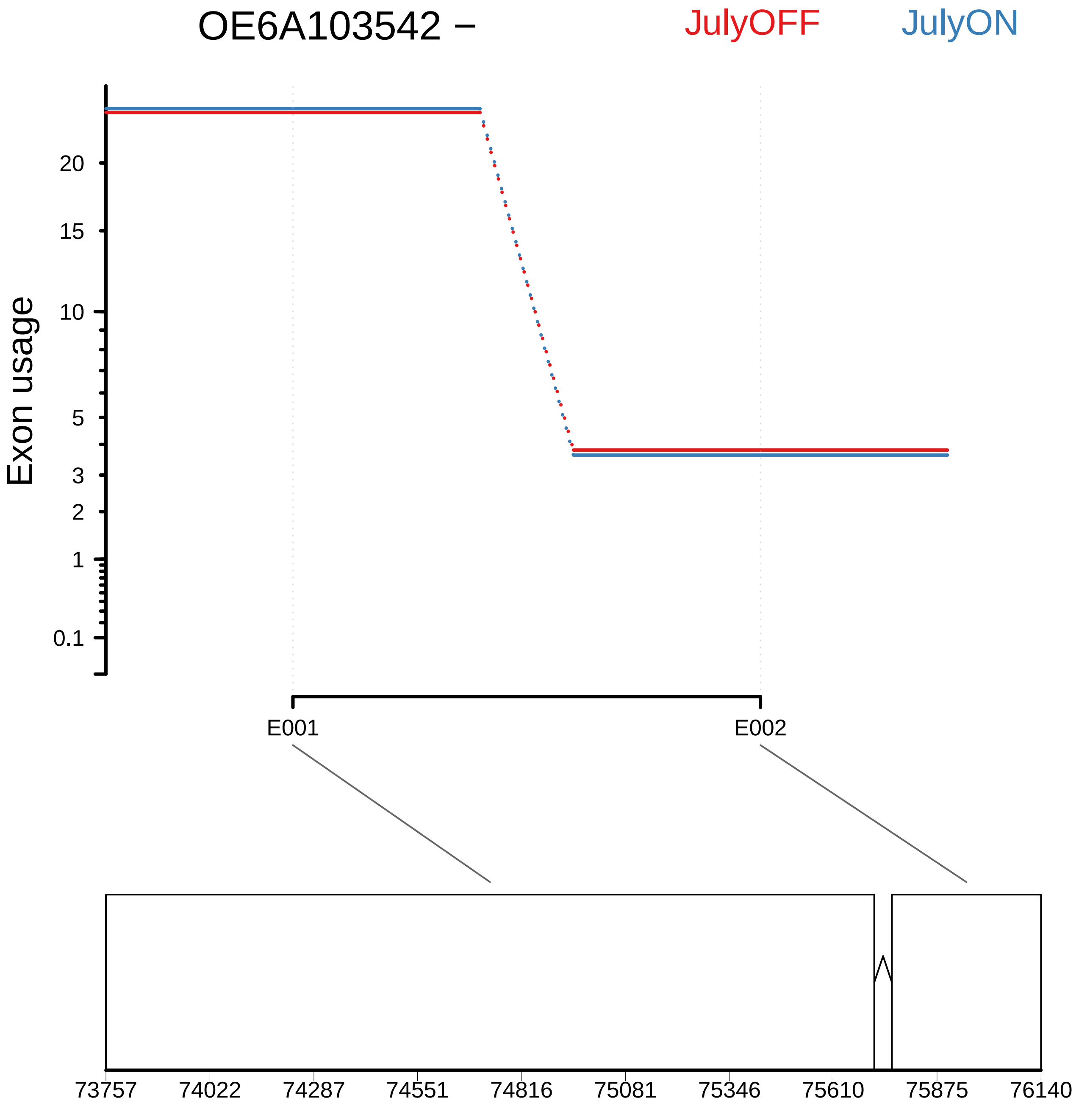
OE6A103542 −
JulyOFF
JulyON
Exon usage
20
15
10
5
3
2
1
0.1
E001
E002
73757
74022
74287
74551
74816
75081
75346
75610
75875
76140

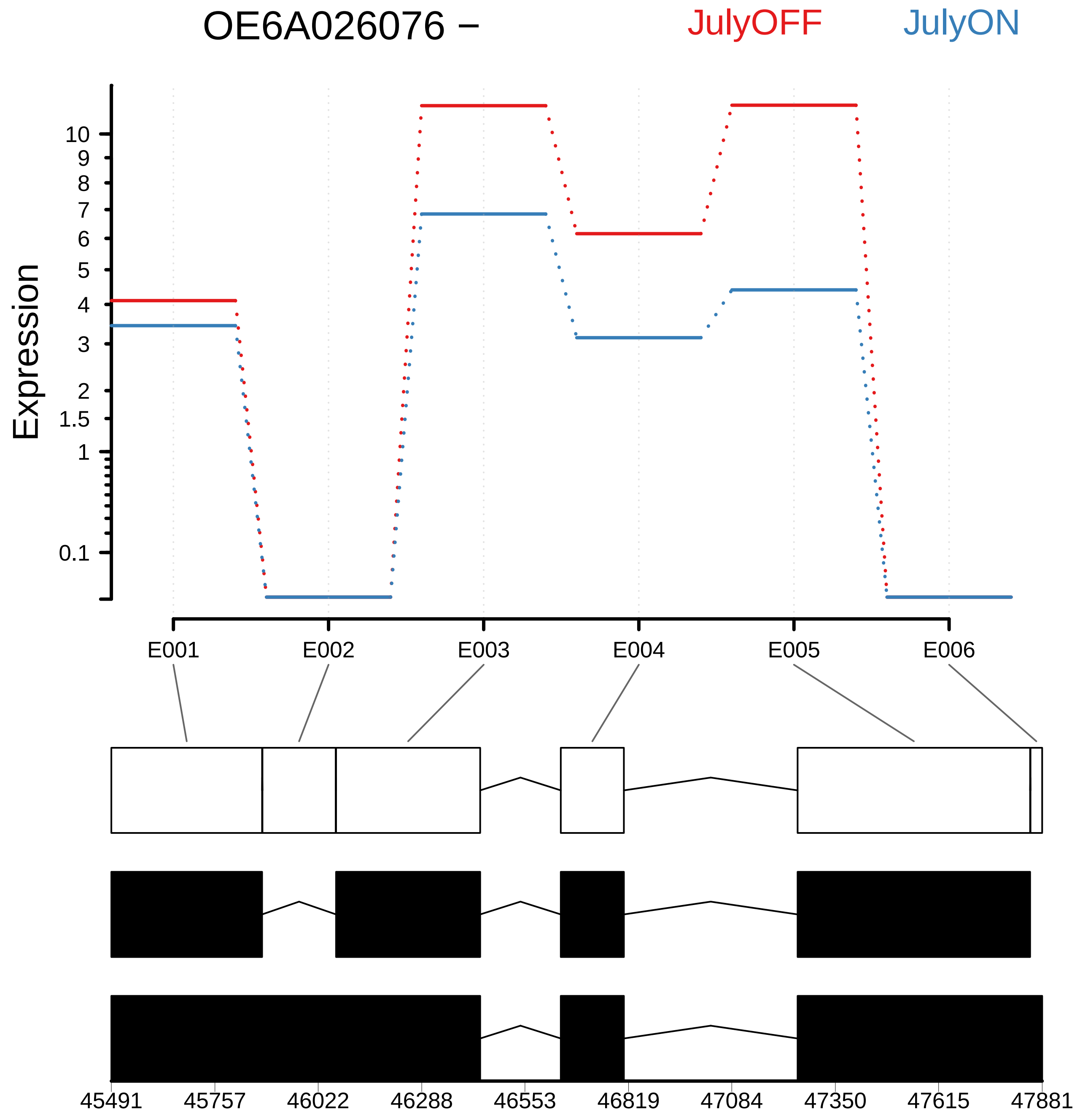
OE6A026076 −
JulyOFF
JulyON
Expression
10
9
8
7
6
5
4
3
2
1.5
1
0.1
E001
E002
E003
E004
E005
E006
45491
45757
46022
46288
46553
46819
47084
47350
47615
47881

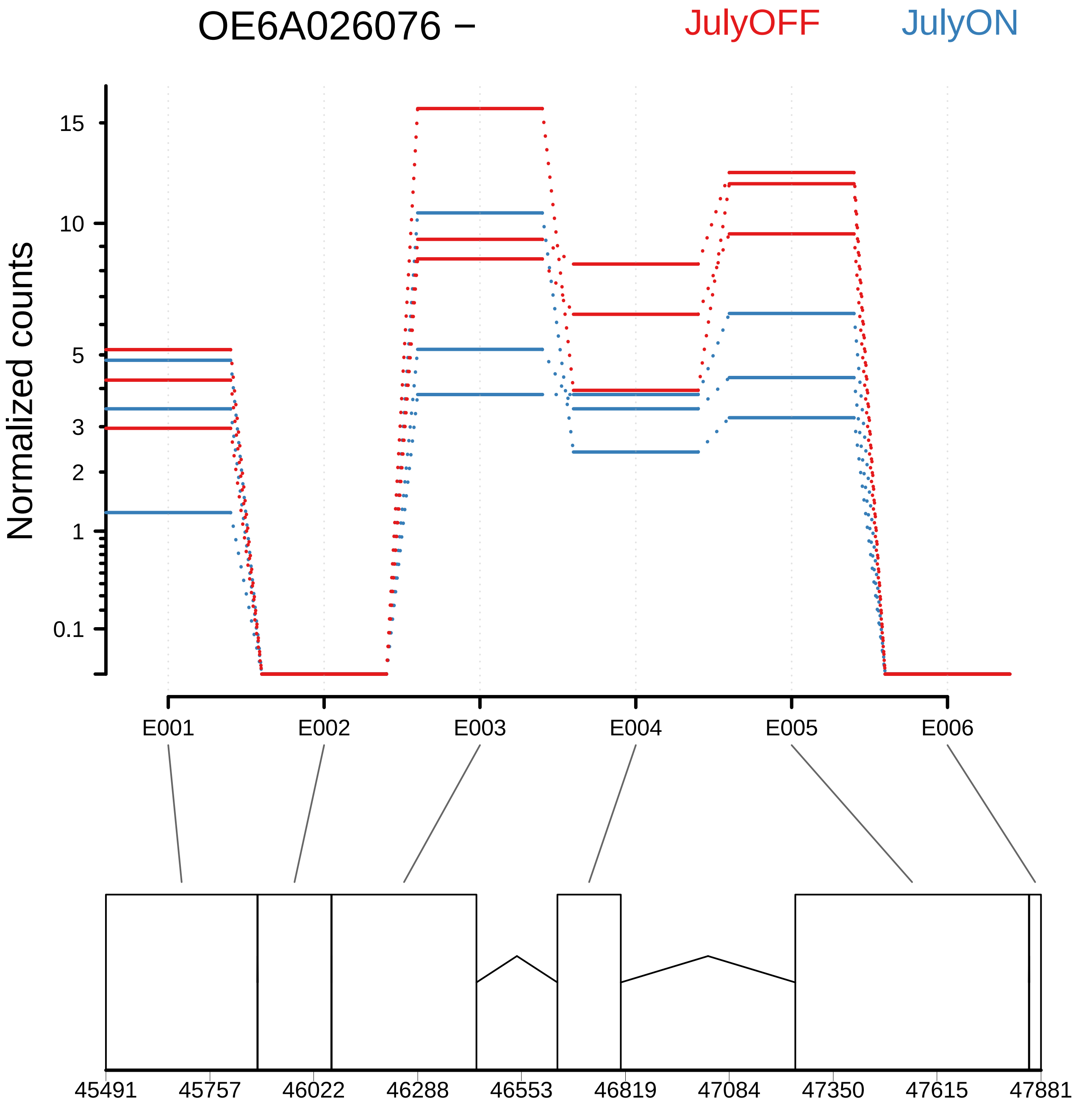

OE6A026076 −
JulyOFF
JulyON
Normalized counts
15
10
5
3
2
1
0.1
E001
E002
E003
E004
E005
E006
45491
45757
46022
46288
46553
46819
47084
47350
47615
47881

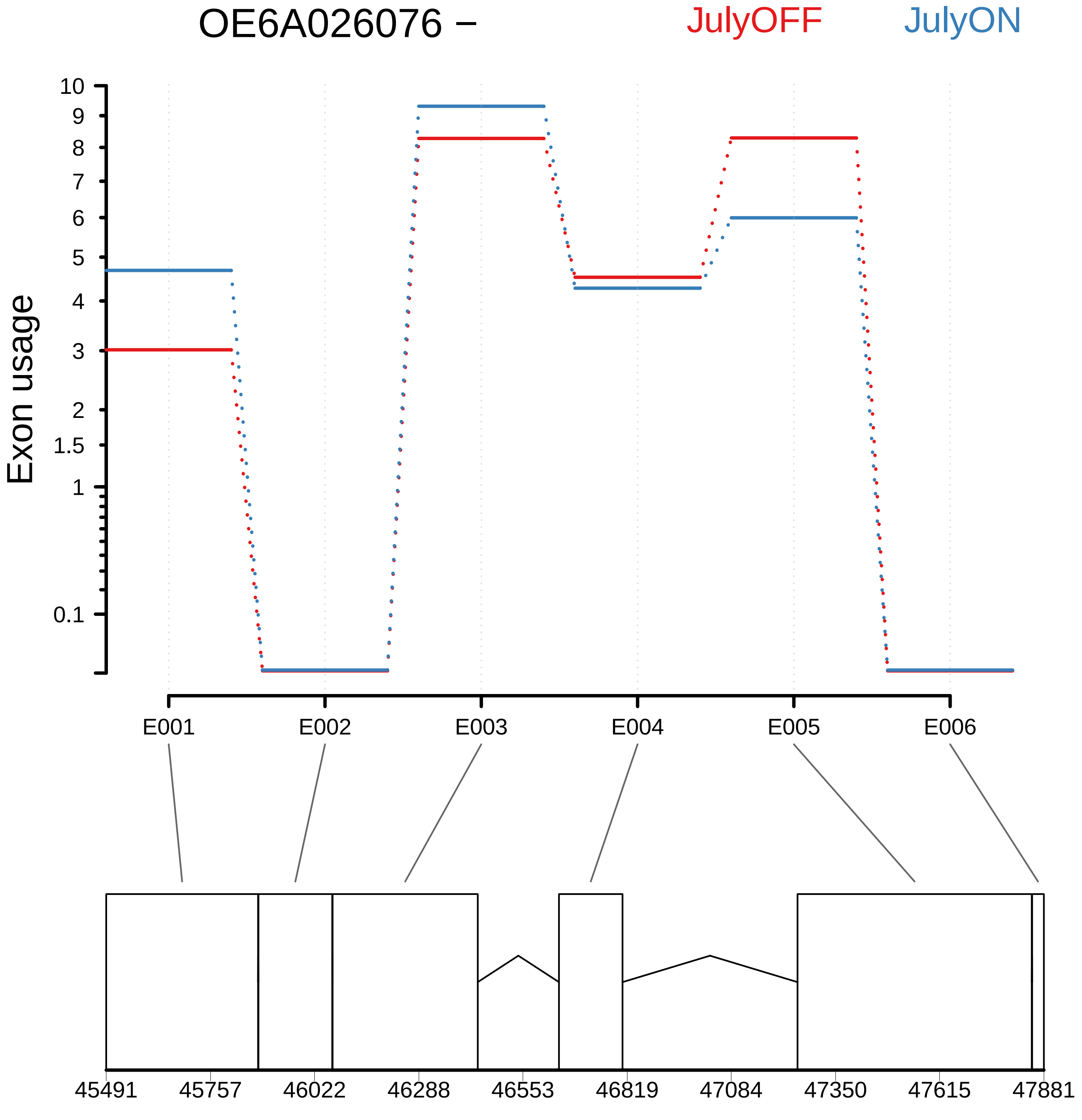
OE6A026076 −
JulyOFF
JulyON
Exon usage
10
9
8
7
6
5
4
3
2
1.5
1
0.1
E001
E002
E003
E004
E005
E006
45491
45757
46022
46288
46553
46819
47084
47350
47615
47881

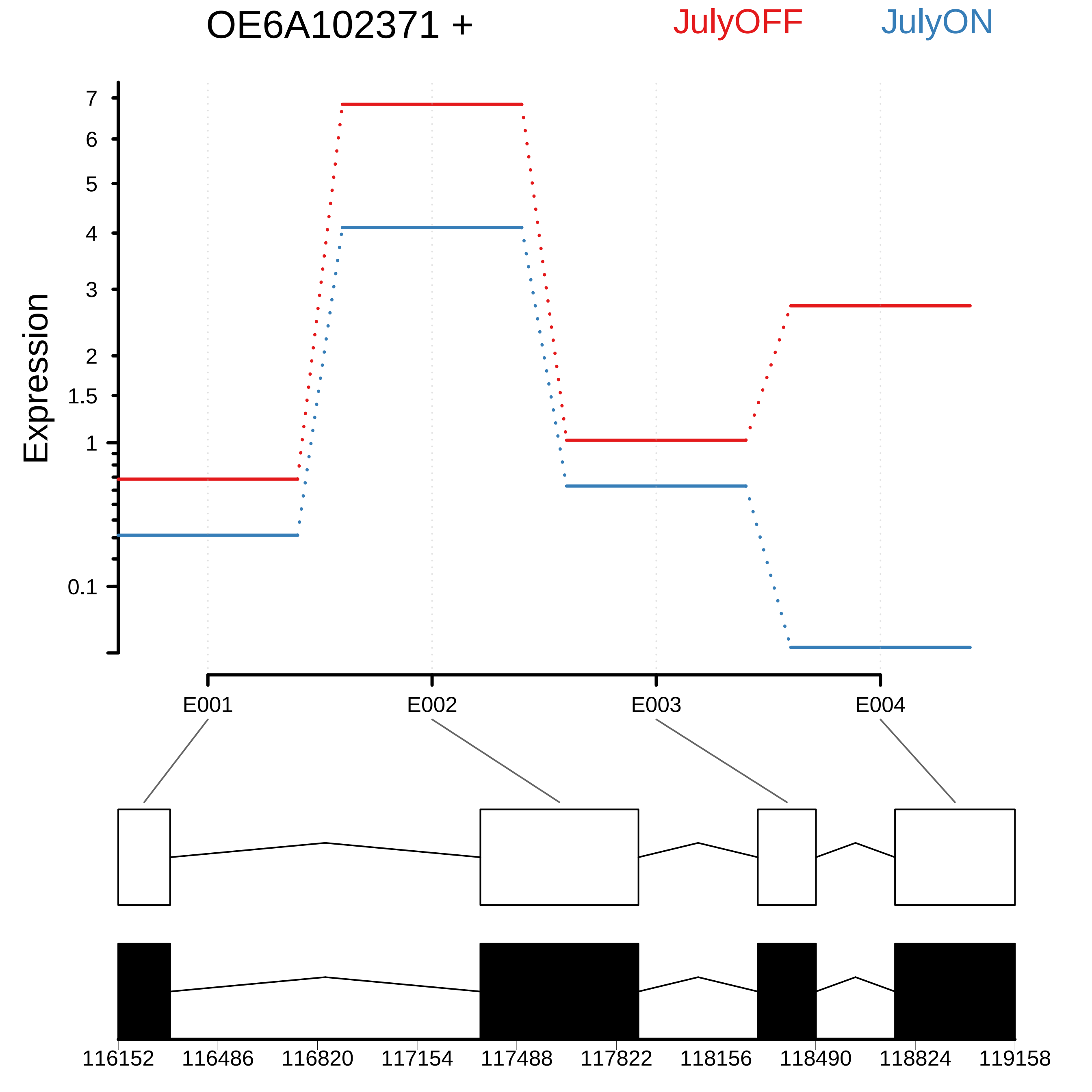
OE6A102371 +
JulyOFF
JulyON
Expression
7
6
5
4
3
2
1.5
1
0.1
E001
E002
E003
E004
116152
116486
116820
117154
117488
117822
118156
118490
118824
119158

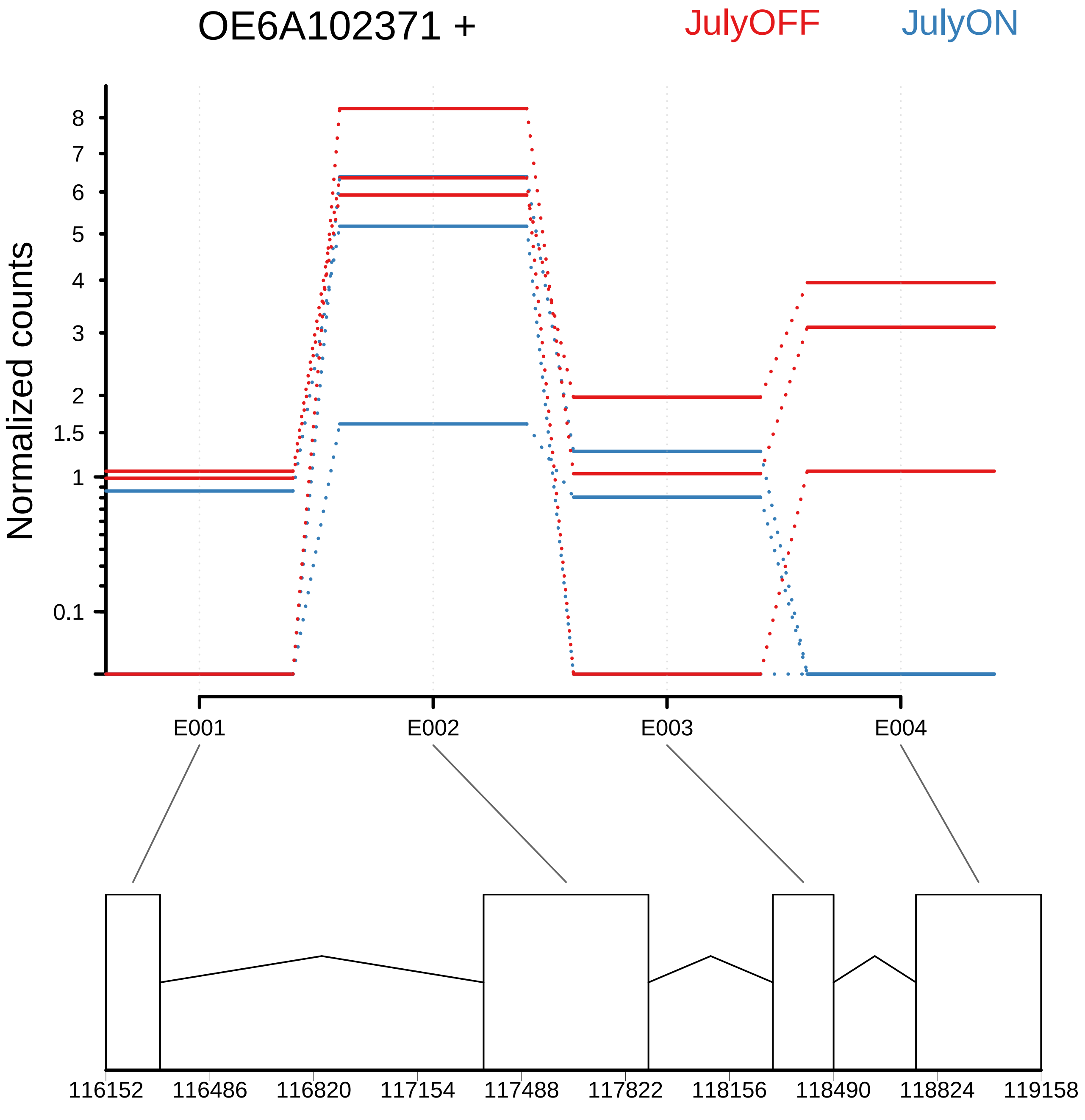

OE6A102371 +
JulyOFF
JulyON
Normalized counts
8
7
6
5
4
3
2
1.5
1
0.1
E001
E002
E003
E004
116152
116486
116820
117154
117488
117822
118156
118490
118824
119158

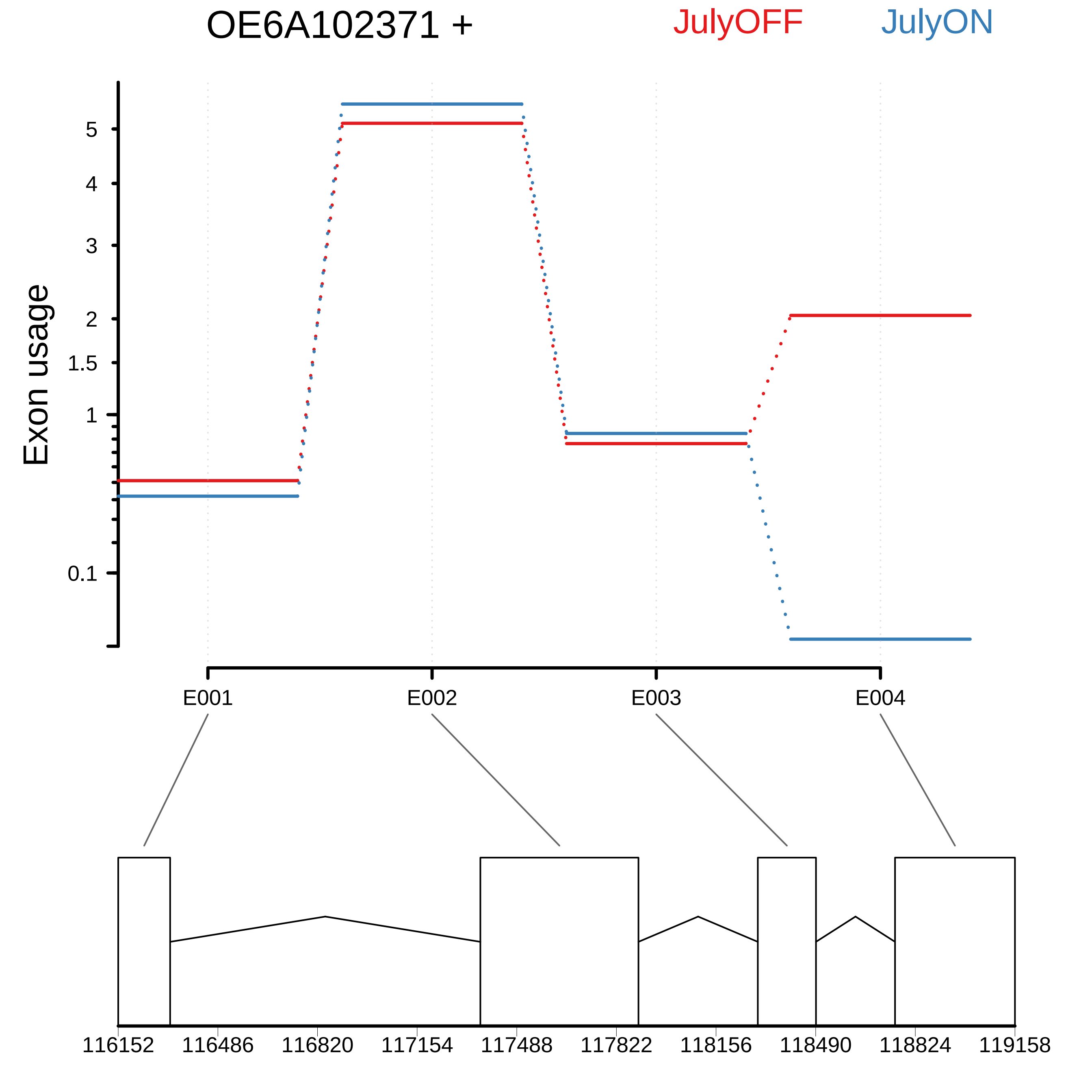
OE6A102371 +
JulyOFF
JulyON
Exon usage
5
4
3
2
1.5
1
0.1
E001
E002
E003
E004
116152
116486
116820
117154
117488
117822
118156
118490
118824
119158

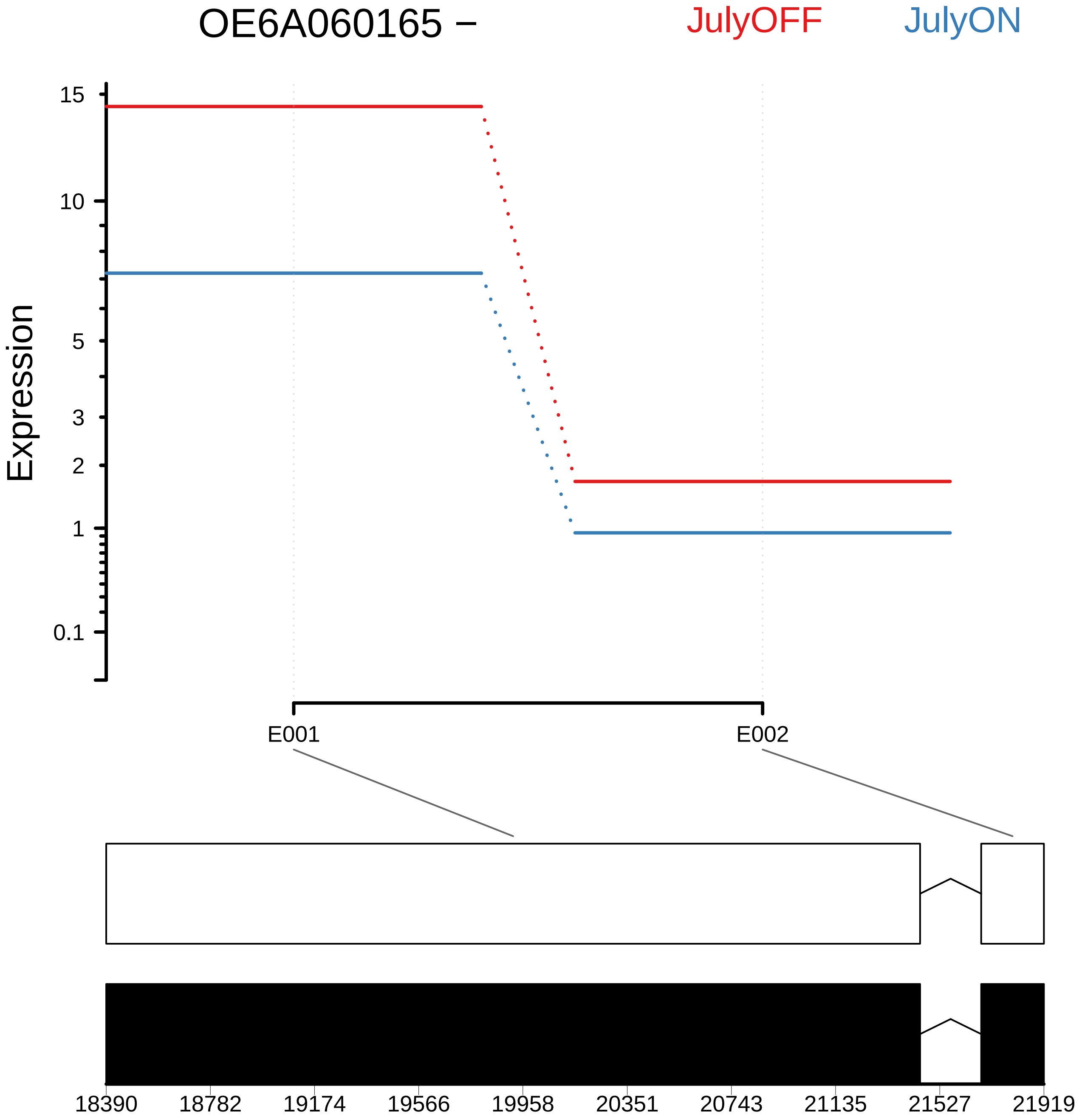

OE6A060165 −
JulyOFF
JulyON
Expression
15
10
5
3
2
1
0.1
E001
E002
18390
18782
19174
19566
19958
20351
20743
21135
21527
21919

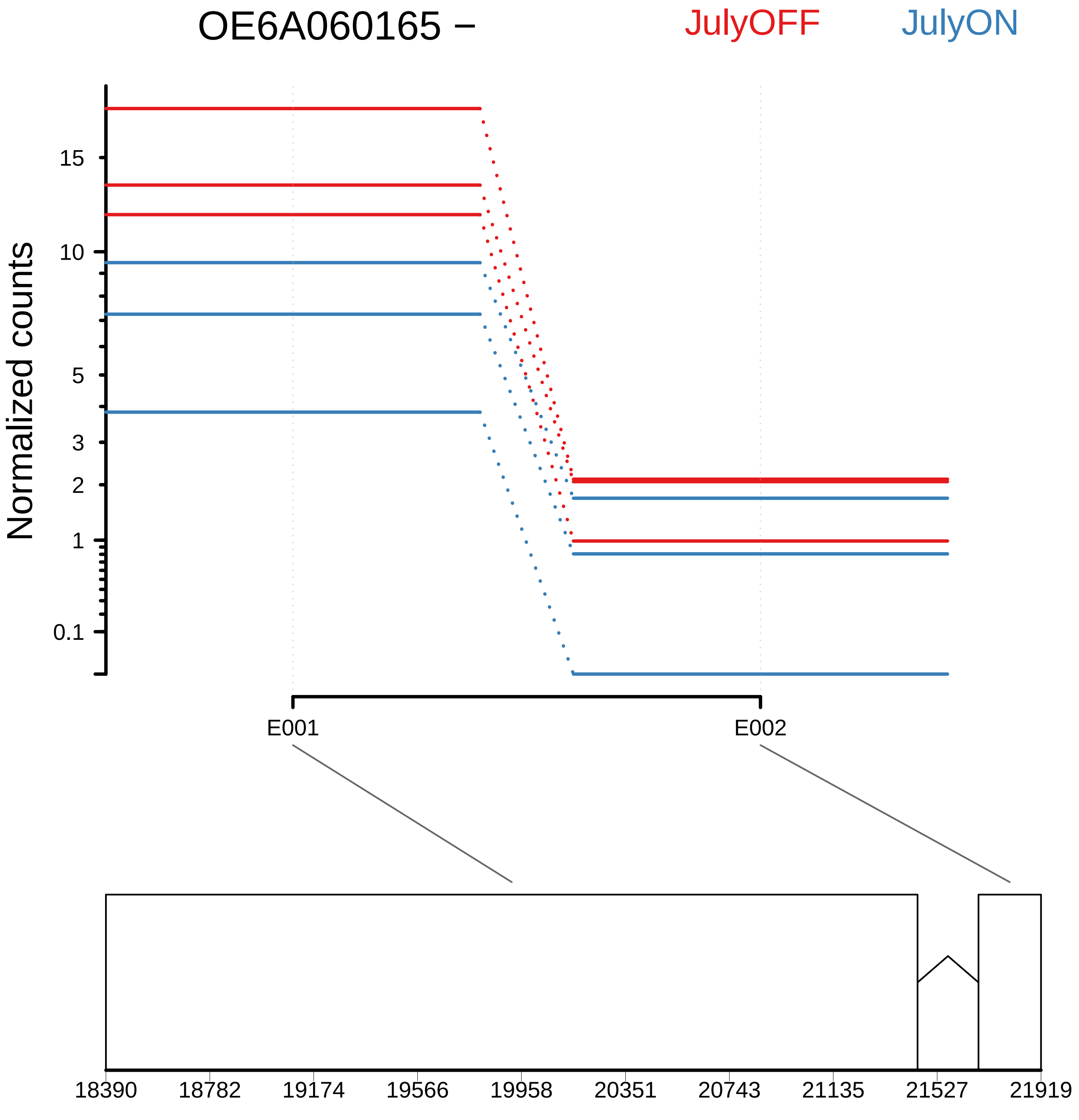
OE6A060165 −
JulyOFF
JulyON
Normalized counts
15
10
5
3
2
1
0.1
E001
E002
18390
18782
19174
19566
19958
20351
20743
21135
21527
21919

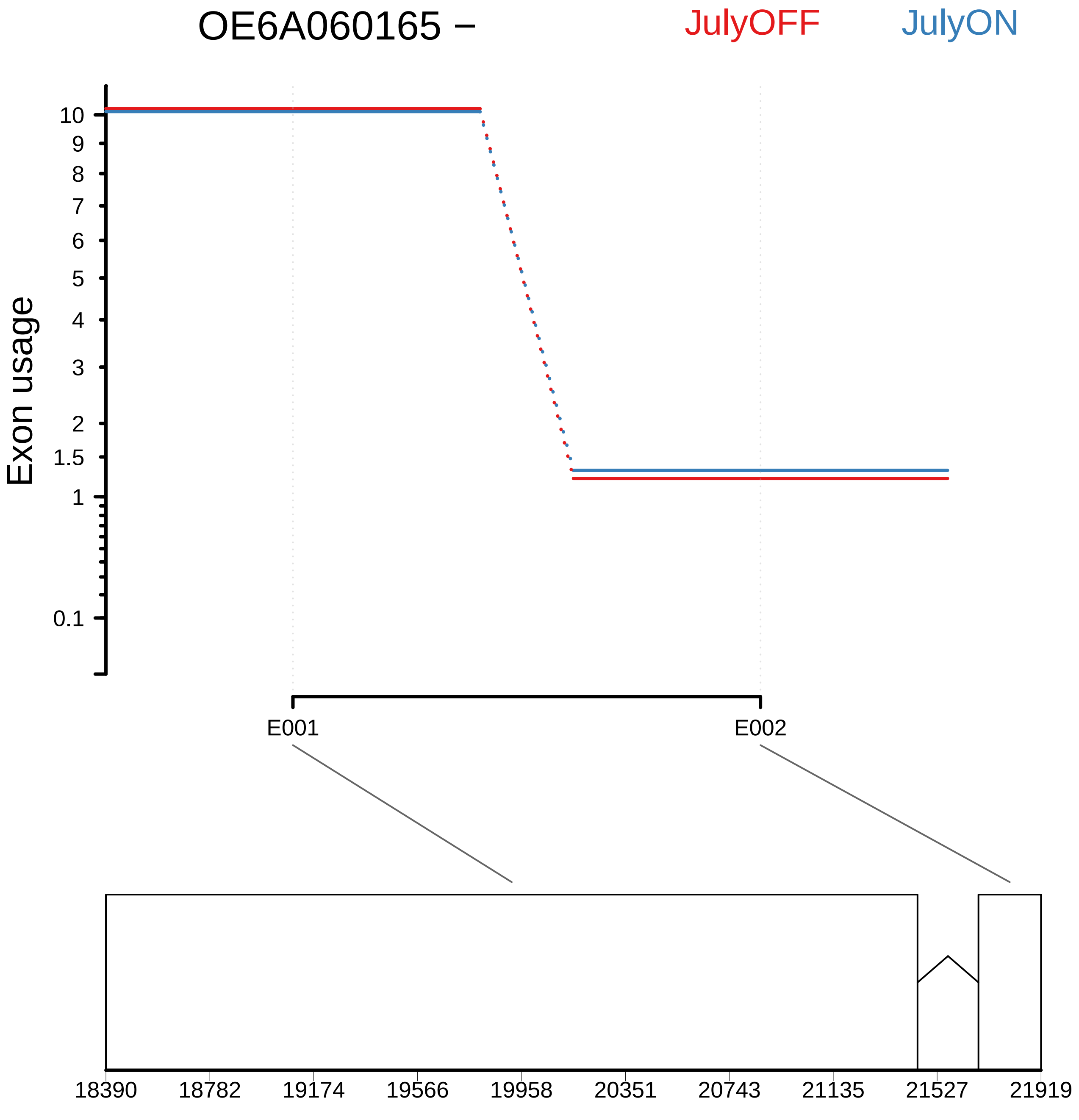

OE6A060165 −
JulyOFF
JulyON
Exon usage
10
9
8
7
6
5
4
3
2
1.5
1
0.1
E001
E002
18390
18782
19174
19566
19958
20351
20743
21135
21527
21919

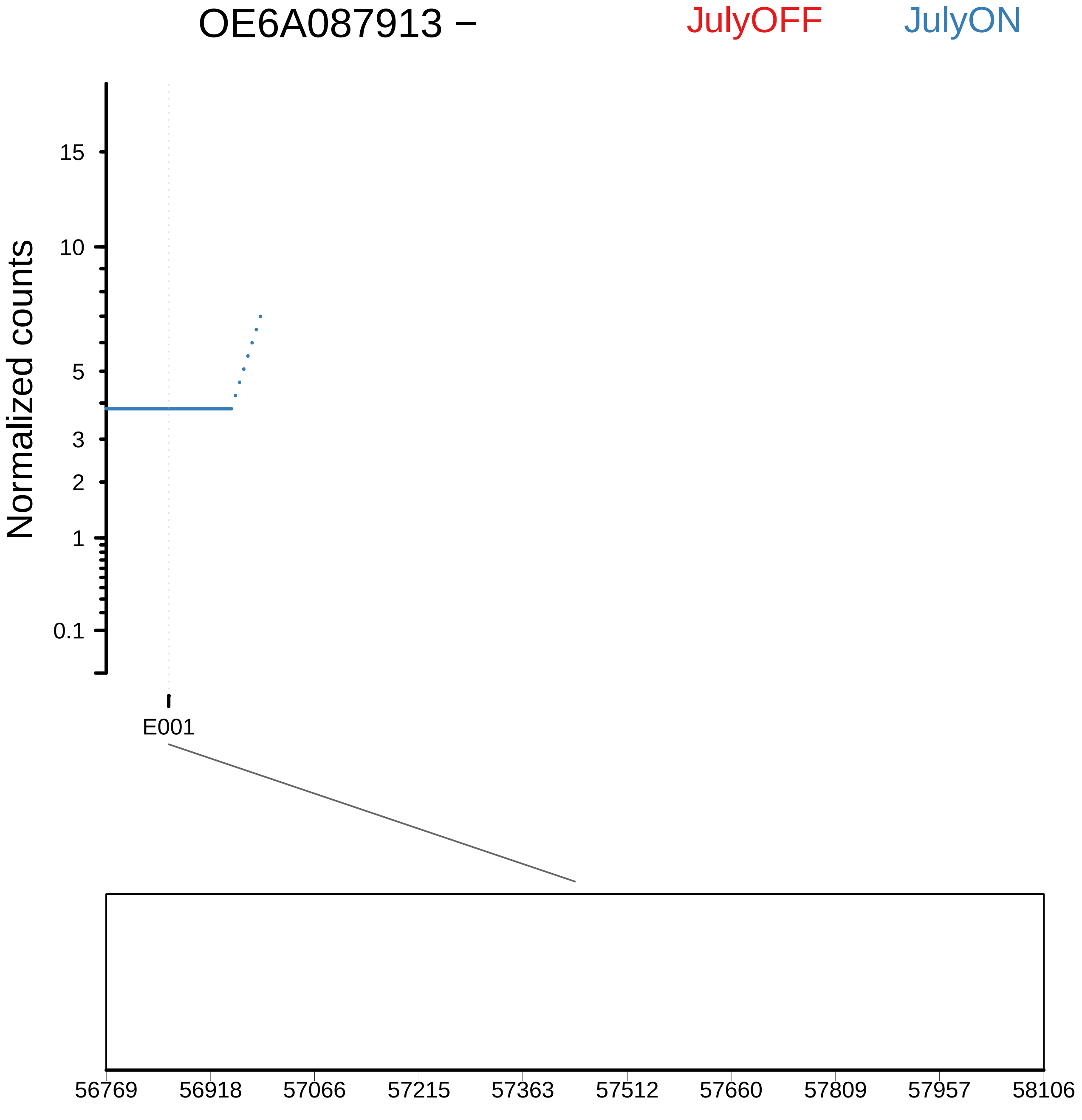
OE6A087913 −
JulyOFF
JulyON
Normalized counts
15
10
5
3
2
1
0.1
E001
56769
56918
57066
57215
57363
57512
57660
57809
57957
58106

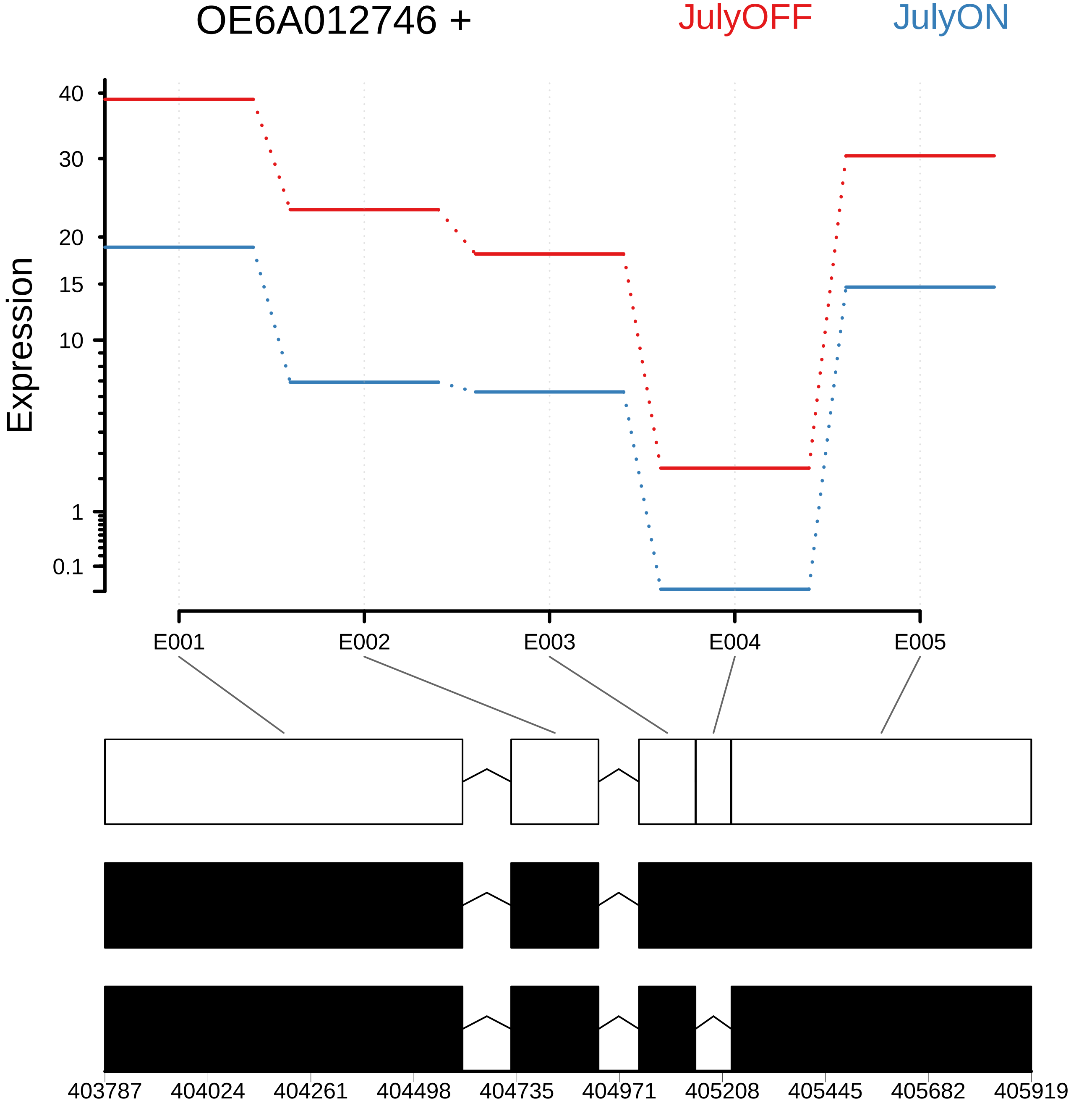

OE6A012746 +
JulyOFF
JulyON
Expression
40
30
20
15
10
1
0.1
E001
E002
E003
E004
E005
403787
404024
404261
404498
404735
404971
405208
405445
405682
405919

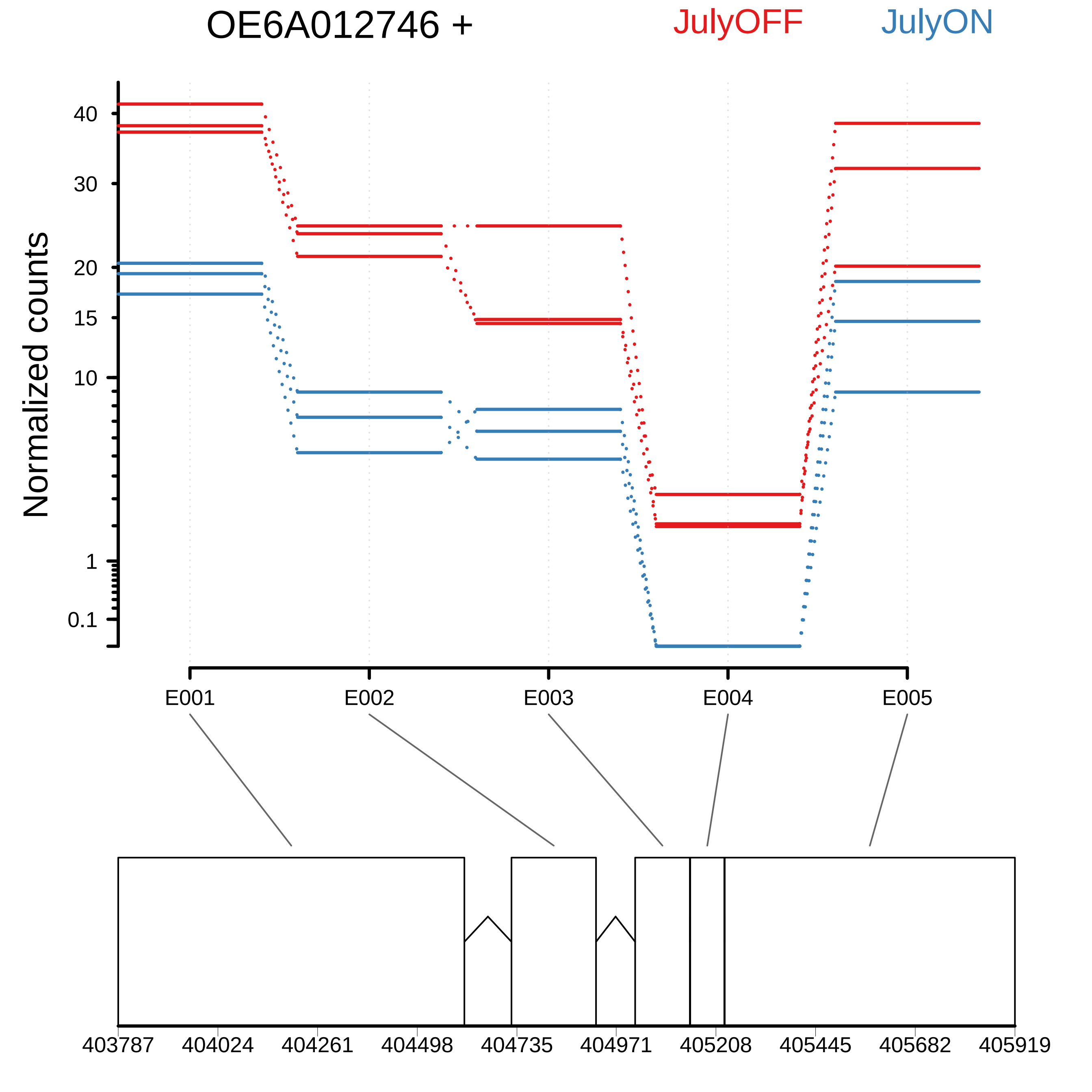

OE6A012746 +
JulyOFF
JulyON
Normalized counts
40
30
20
15
10
1
0.1
E001
E002
E003
E004
E005
403787
404024
404261
404498
404735
404971
405208
405445
405682
405919

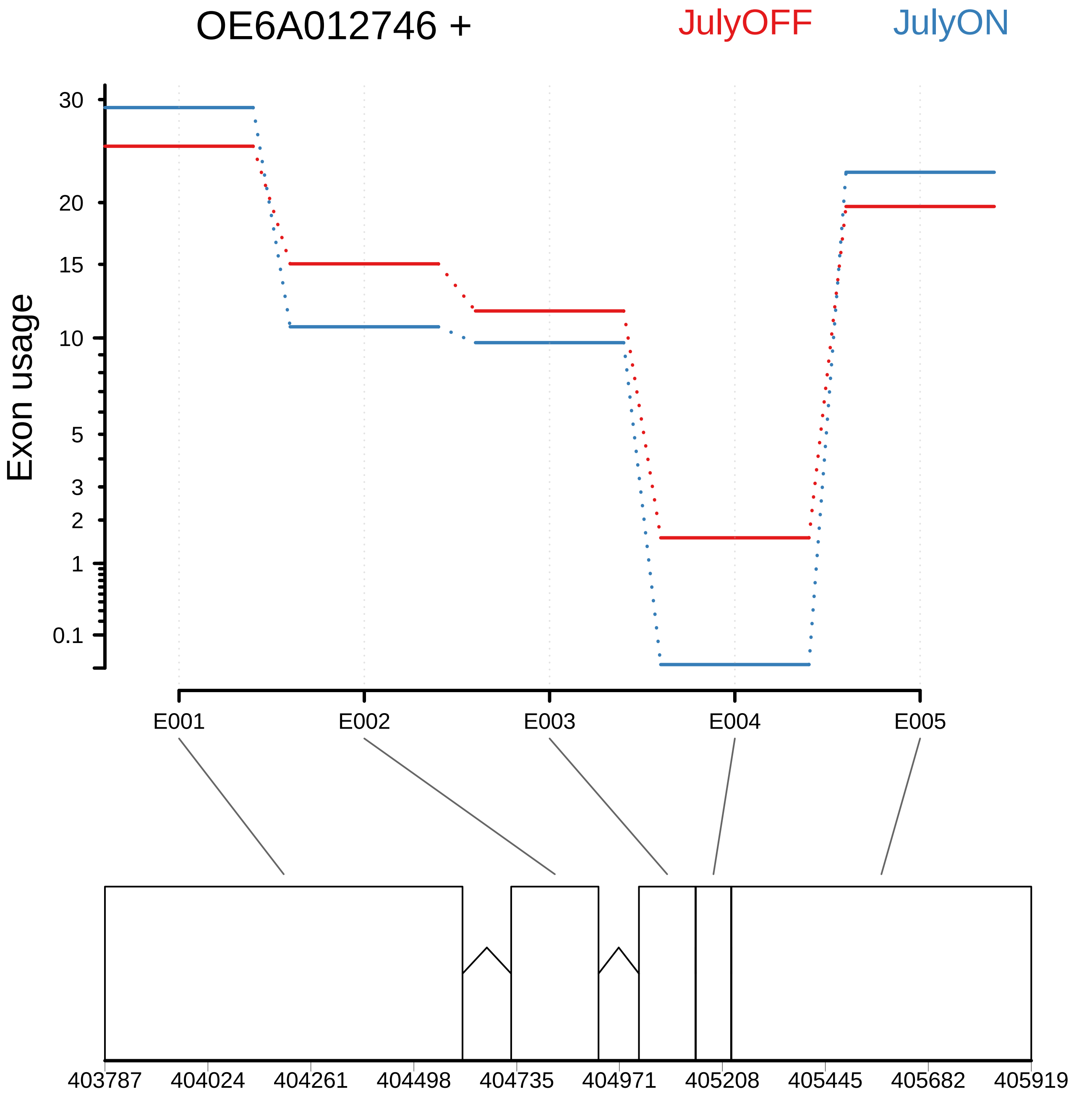

OE6A012746 +
JulyOFF
JulyON
Exon usage
30
20
15
10
5
3
2
1
0.1
E001
E002
E003
E004
E005
403787
404024
404261
404498
404735
404971
405208
405445
405682
405919

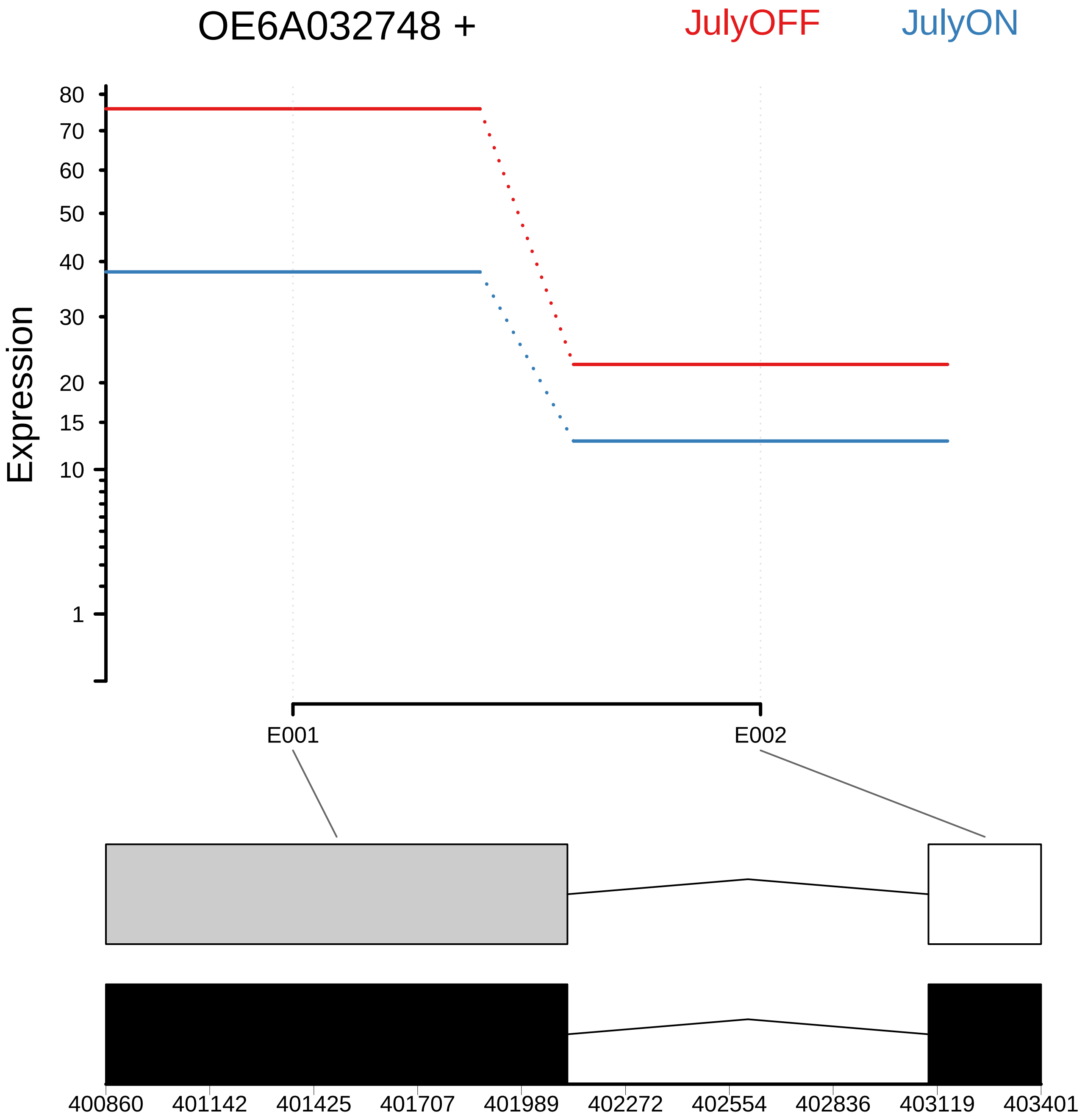
OE6A032748 +
JulyOFF
JulyON
Expression
80
70
60
50
40
30
20
15
10
1
E001
E002
400860
401142
401425
401707
401989
402272
402554
402836
403119
403401

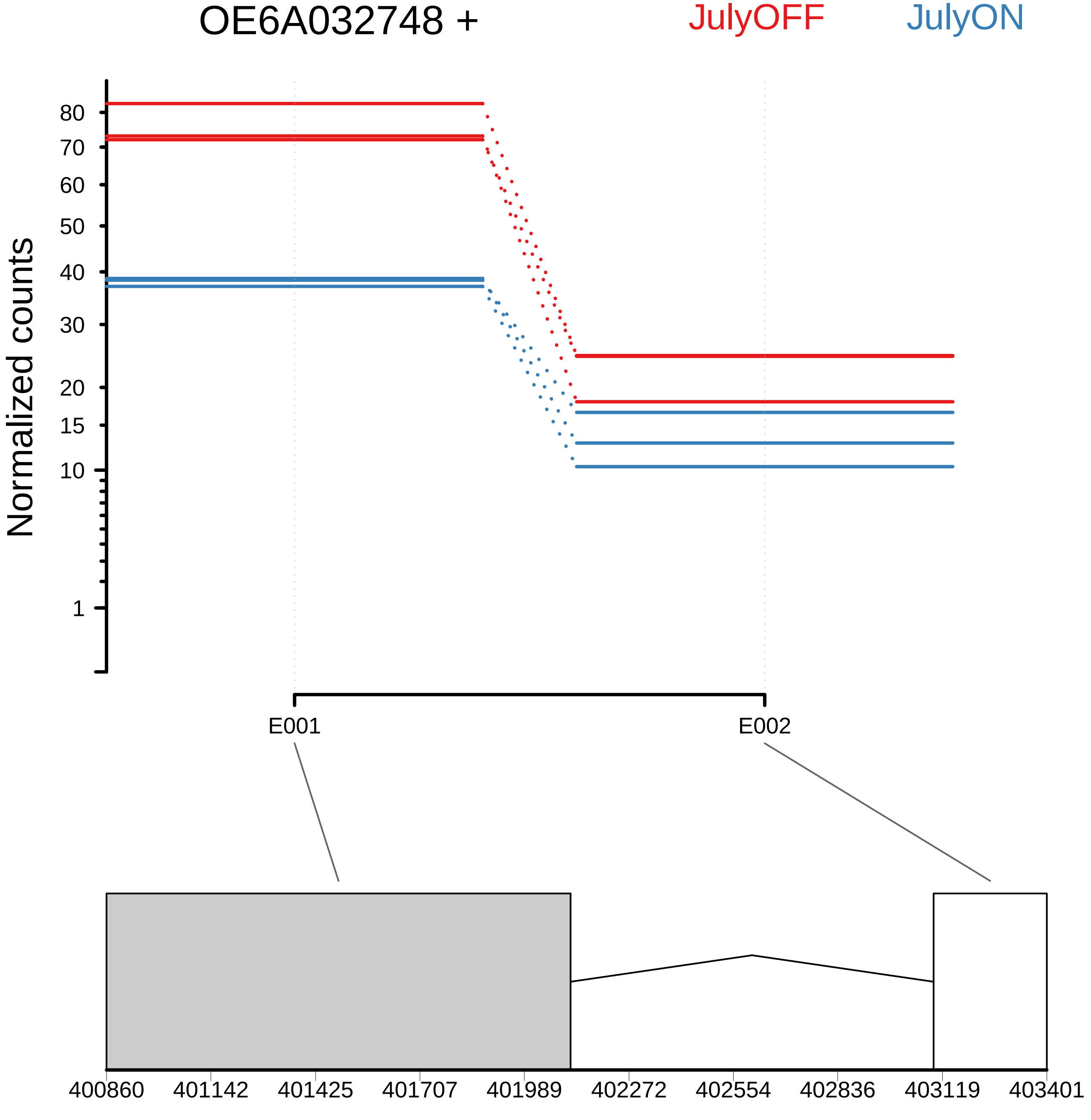

OE6A032748 +
JulyOFF
JulyON
Normalized counts
80
70
60
50
40
30
20
15
10
1
E001
E002
400860
401142
401425
401707
401989
402272
402554
402836
403119
403401

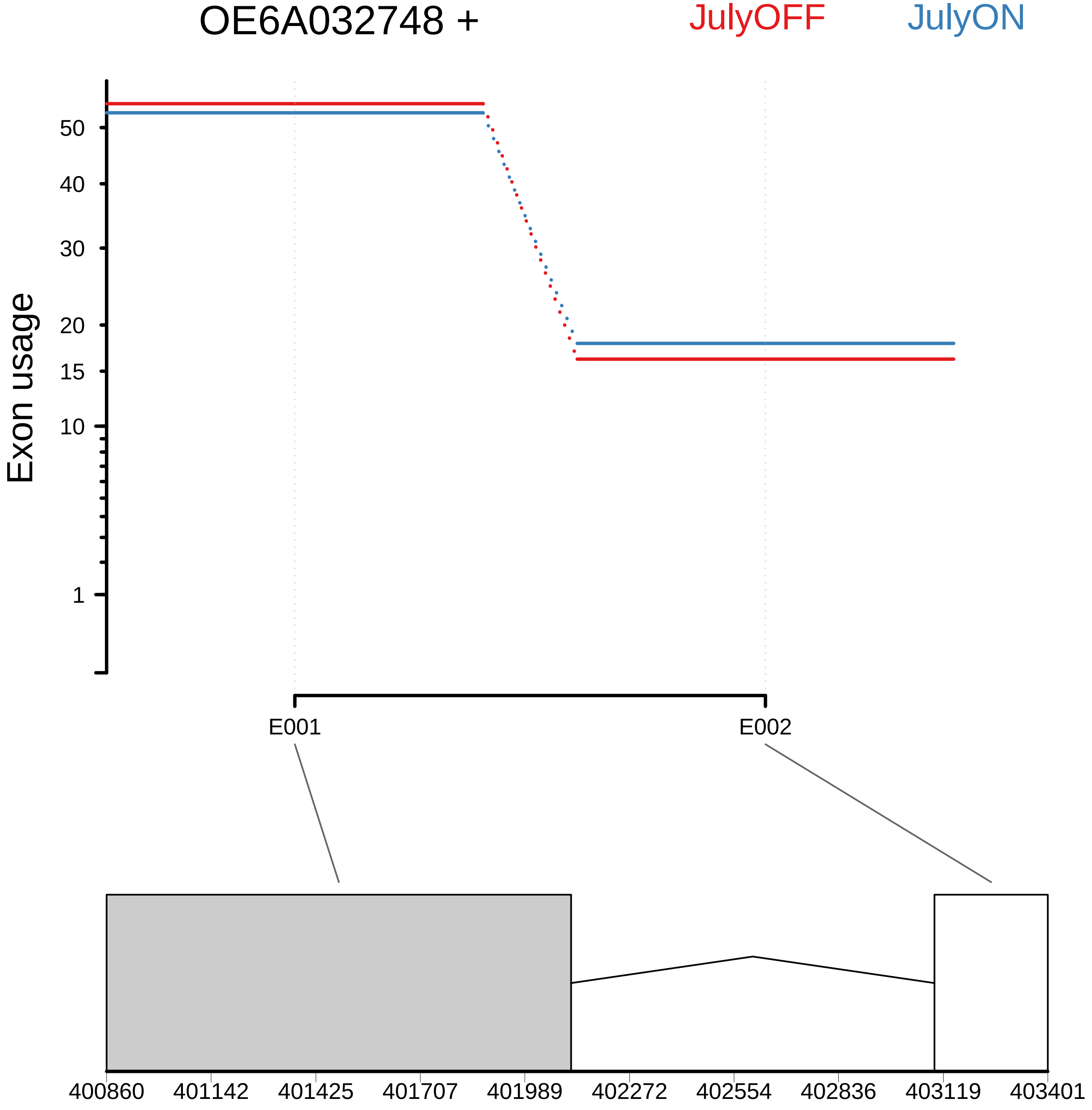
OE6A032748 +
JulyOFF
JulyON
Exon usage
50
40
30
20
15
10
1
E001
E002
400860
401142
401425
401707
401989
402272
402554
402836
403119
403401

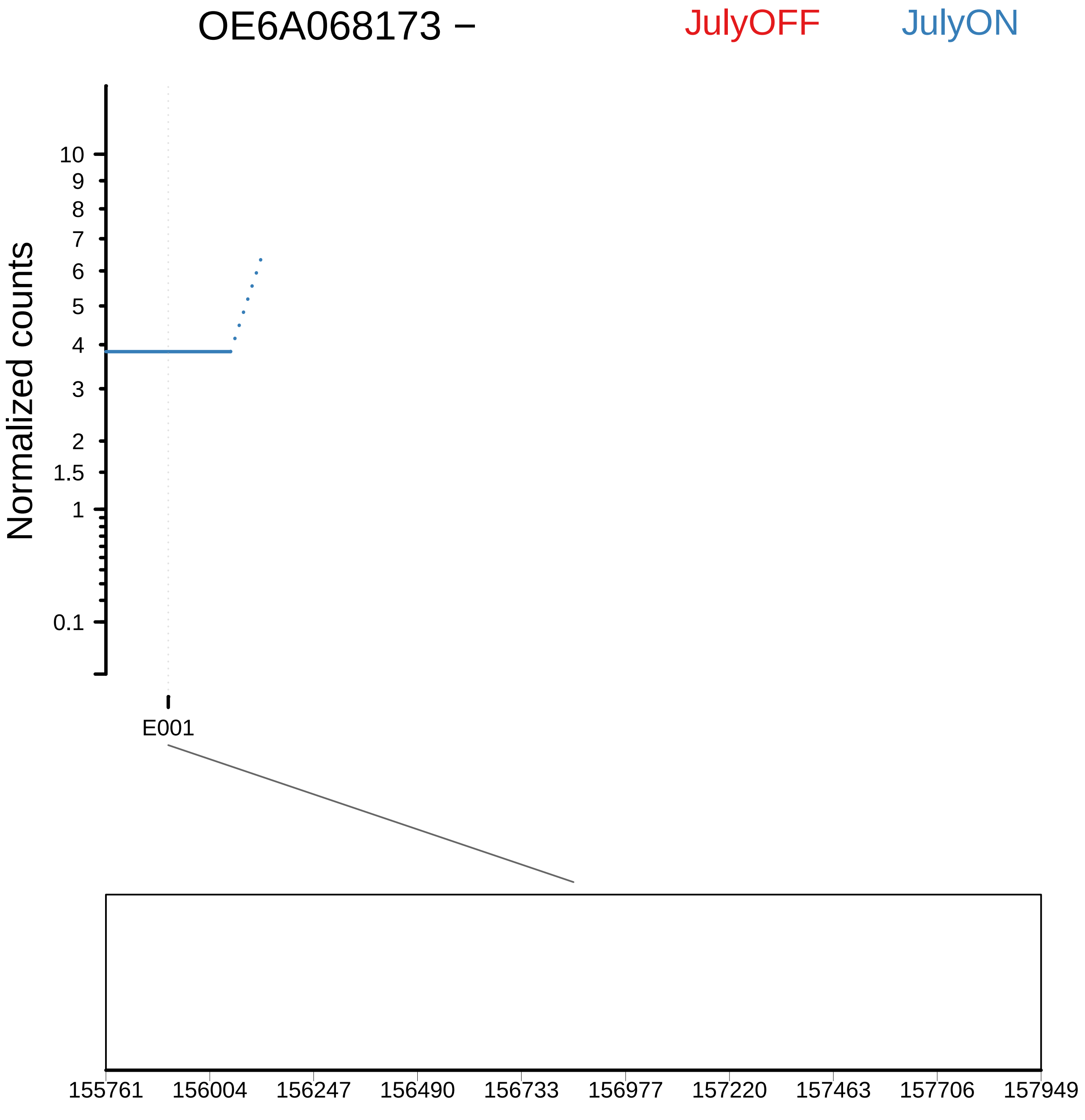
OE6A068173 −
JulyOFF
JulyON
Normalized counts
10
9
8
7
6
5
4
3
2
1.5
1
0.1
E001
155761
156004
156247
156490
156733
156977
157220
157463
157706
157949

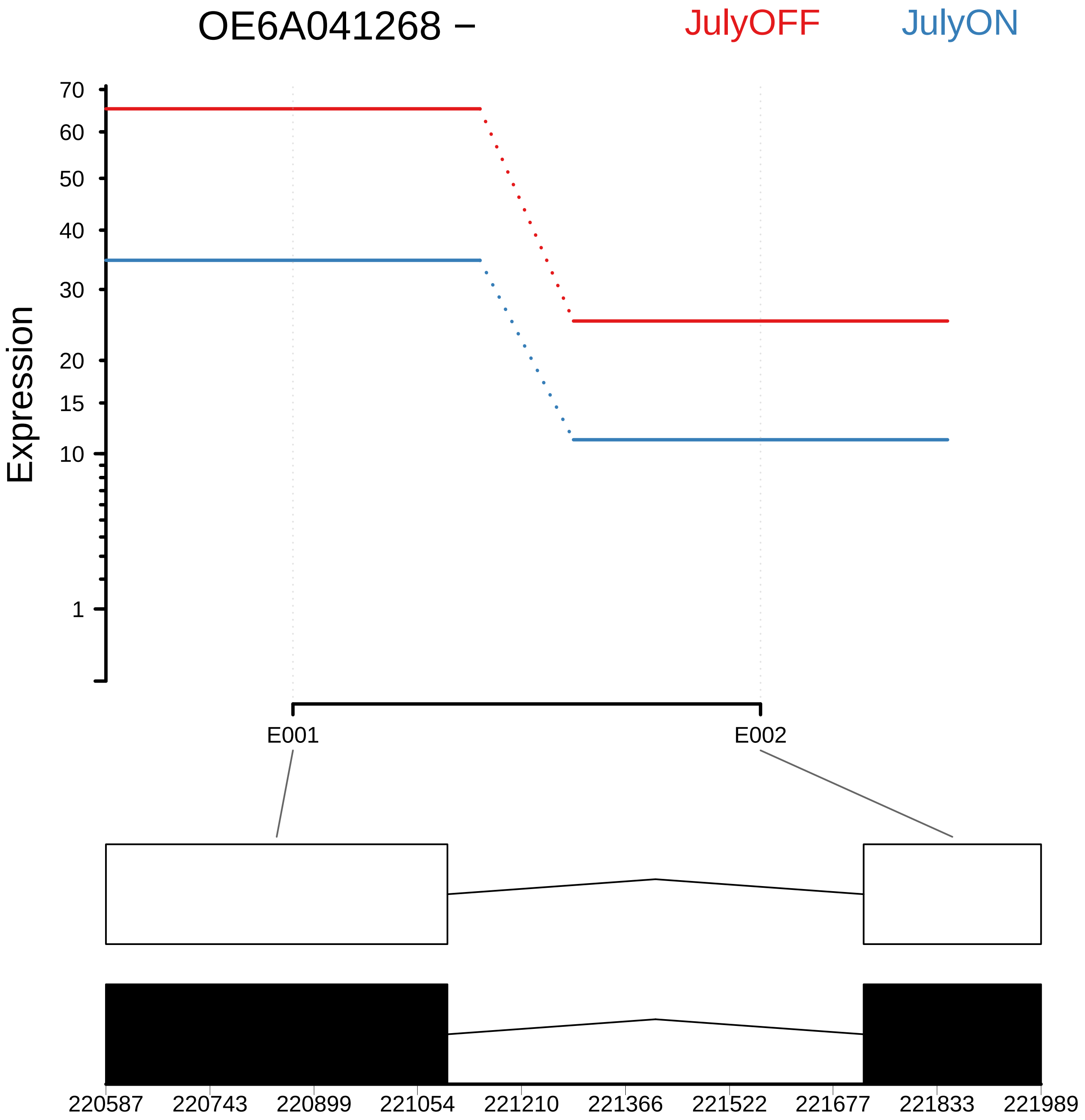

OE6A041268 −
JulyOFF
JulyON
Expression
70
60
50
40
30
20
15
10
1
E001
E002
220587
220743
220899
221054
221210
221366
221522
221677
221833
221989

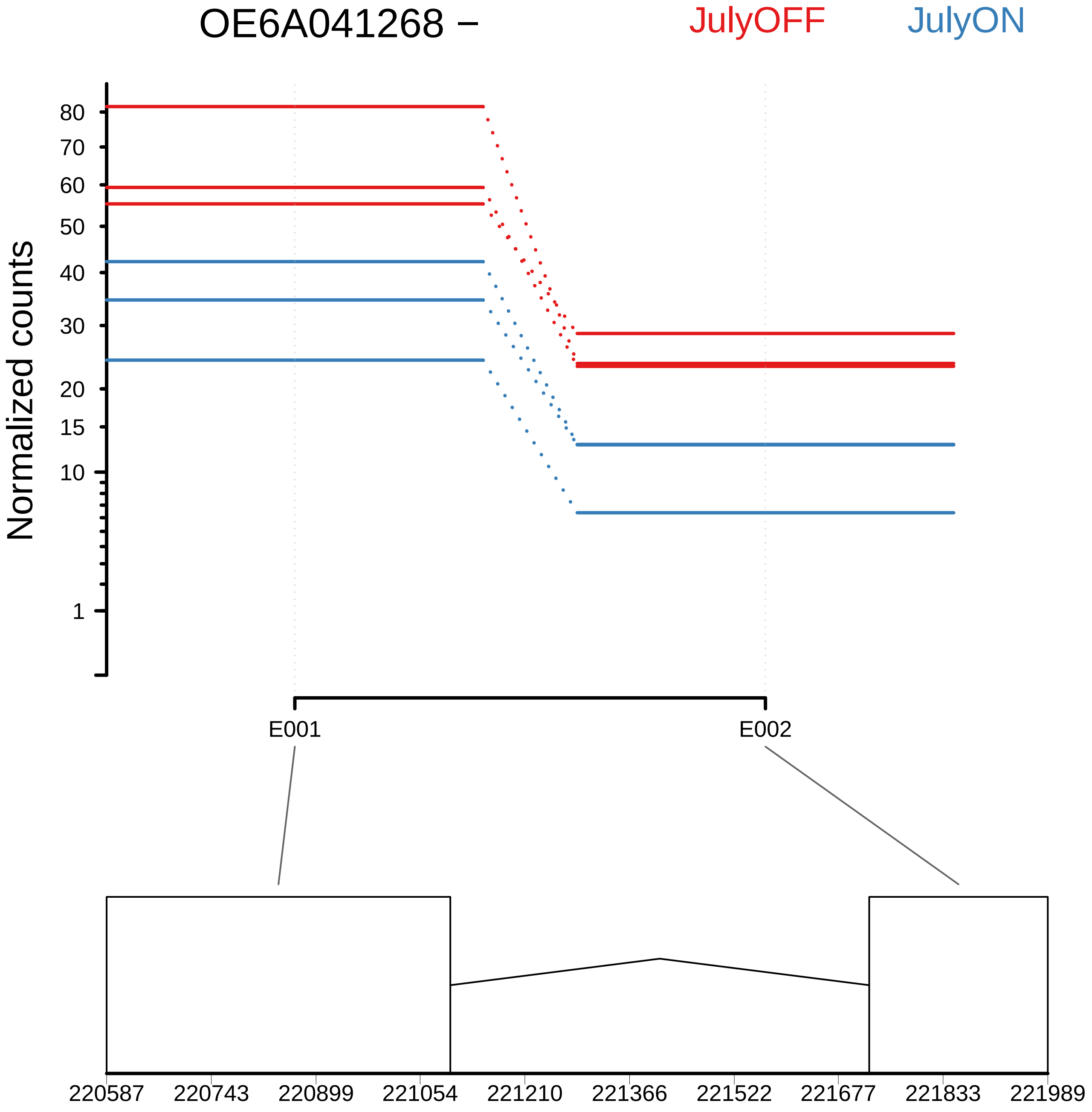
OE6A041268 −
JulyOFF
JulyON
Normalized counts
80
70
60
50
40
30
20
15
10
1
E001
E002
220587
220743
220899
221054
221210
221366
221522
221677
221833
221989

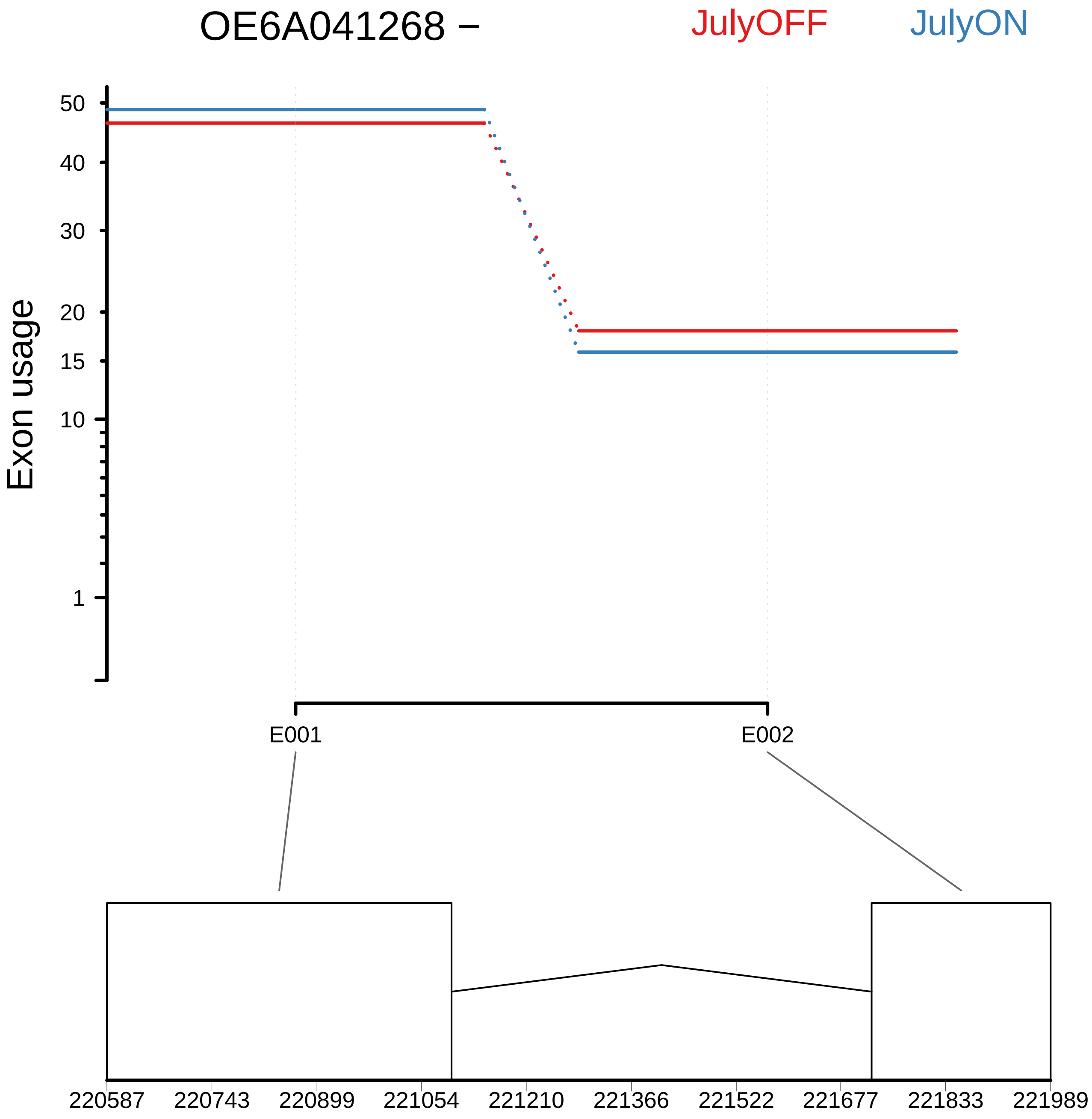

OE6A041268 −
JulyOFF
JulyON
Exon usage
50
40
30
20
15
10
1
E001
E002
220587
220743
220899
221054
221210
221366
221522
221677
221833
221989

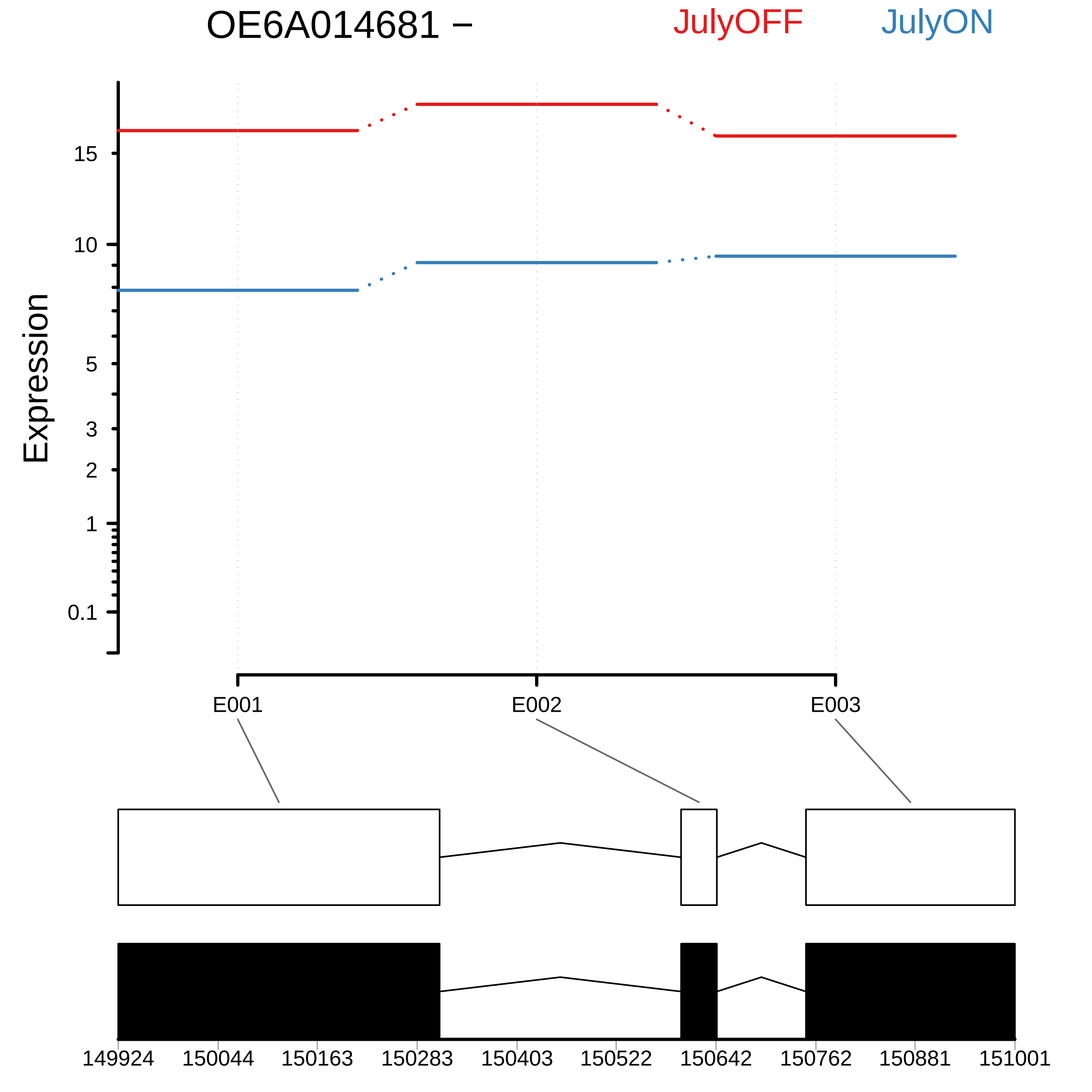

OE6A014681 −
JulyOFF
JulyON
Expression
15
10
5
3
2
1
0.1
E001
E002
E003
149924
150044
150163
150283
150403
150522
150642
150762
150881
151001

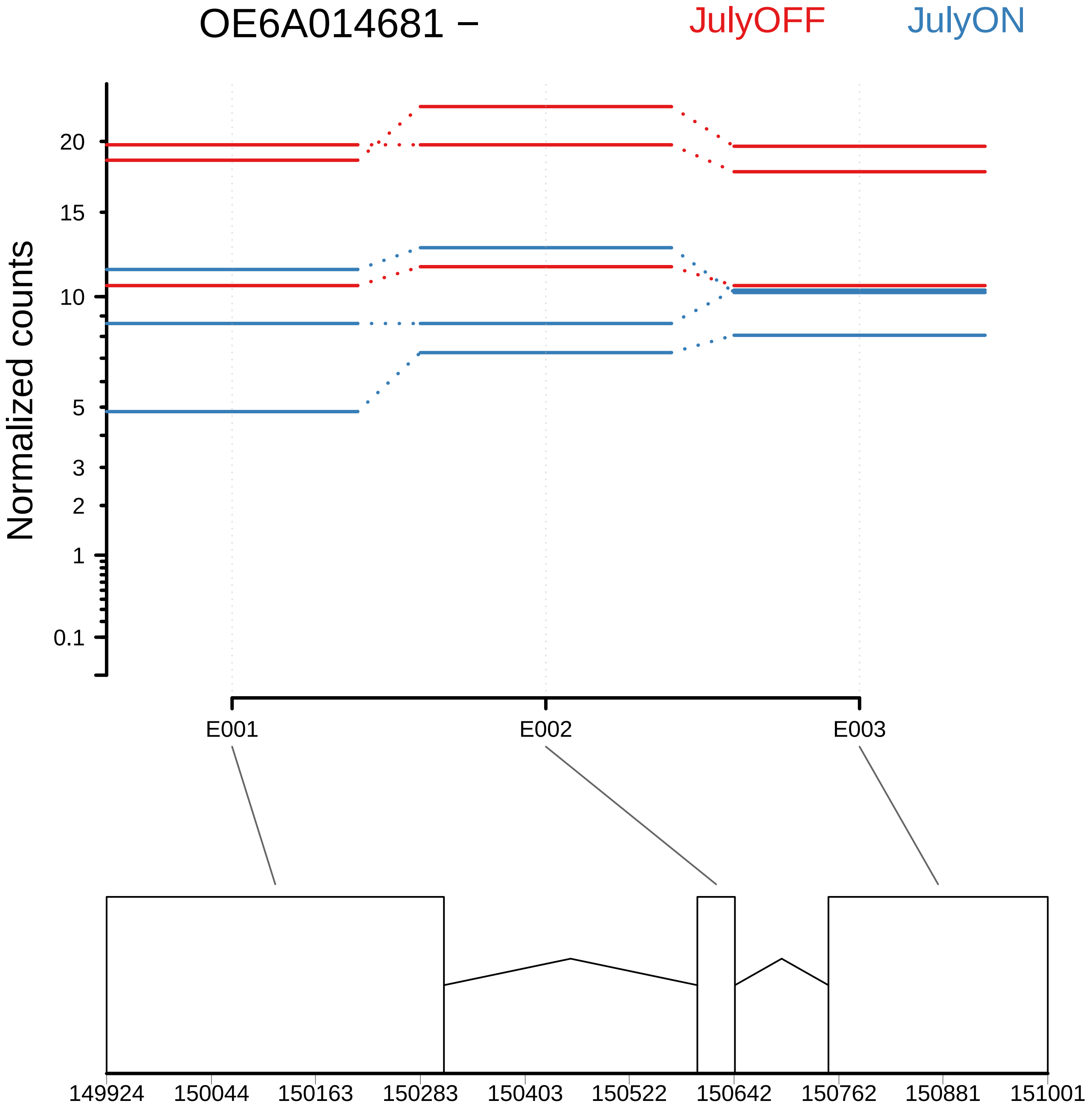

OE6A014681 −
JulyOFF
JulyON
Normalized counts
20
15
10
5
3
2
1
0.1
E001
E002
E003
149924
150044
150163
150283
150403
150522
150642
150762
150881
151001

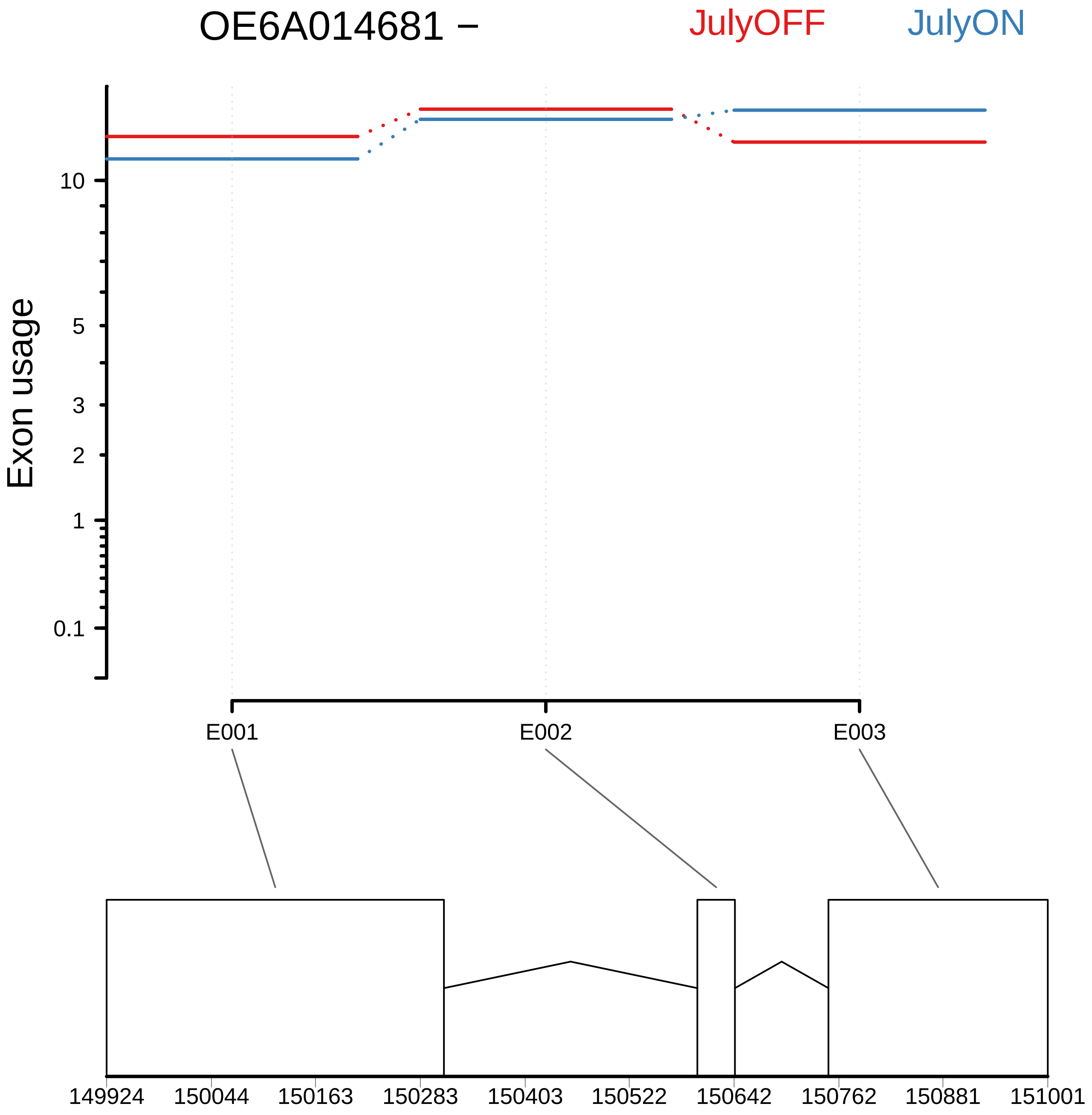
OE6A014681 −
JulyOFF
JulyON
Exon usage
10
5
3
2
1
0.1
E001
E002
E003
149924
150044
150163
150283
150403
150522
150642
150762
150881
151001

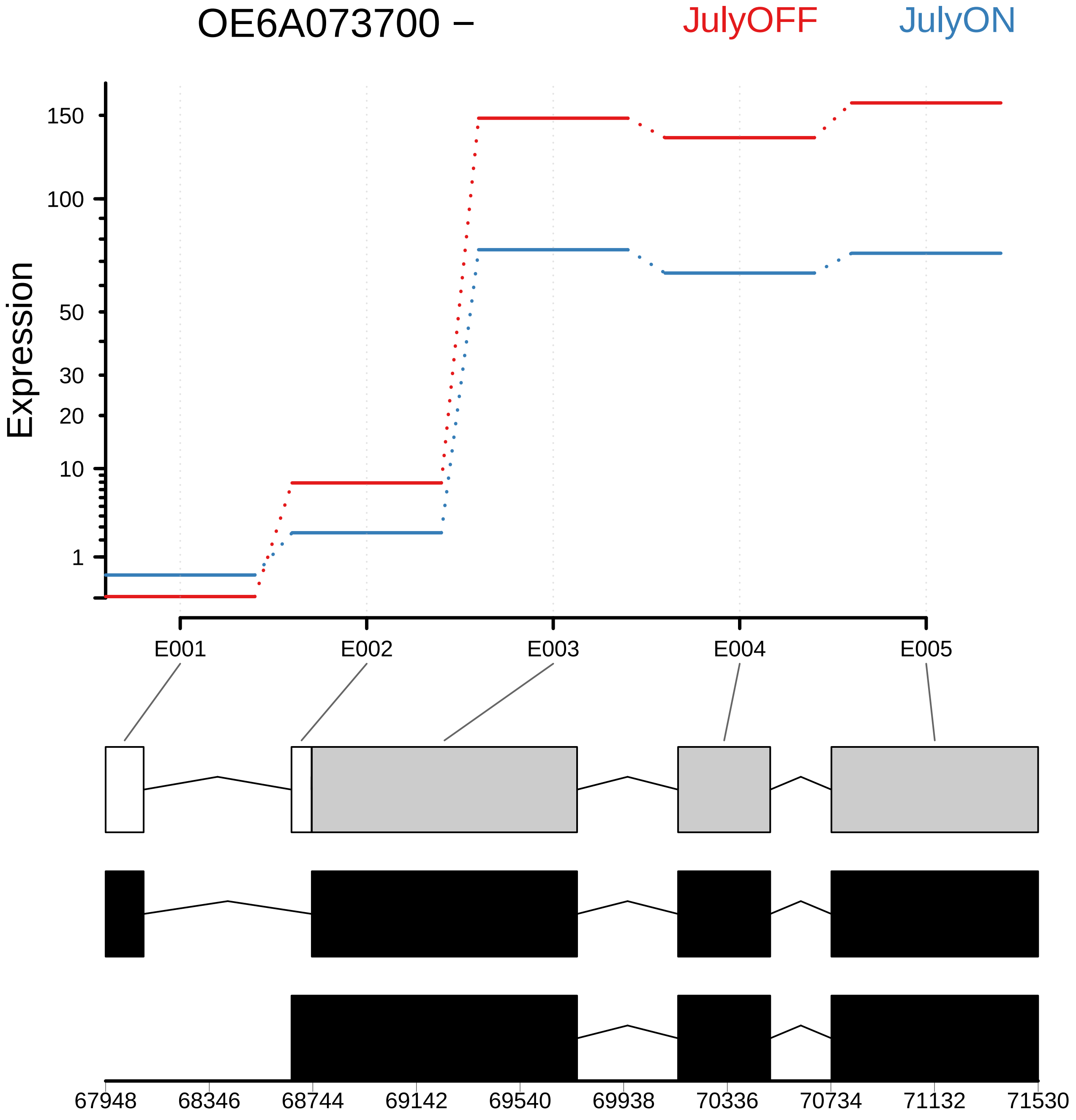

OE6A073700 –
JulyOFF
JulyON
Expression
150
100
50
30
20
10
1
E001
E002
E003
E004
E005
67948
68346
68744
69142
69540
69938
70336
70734
71132
71530

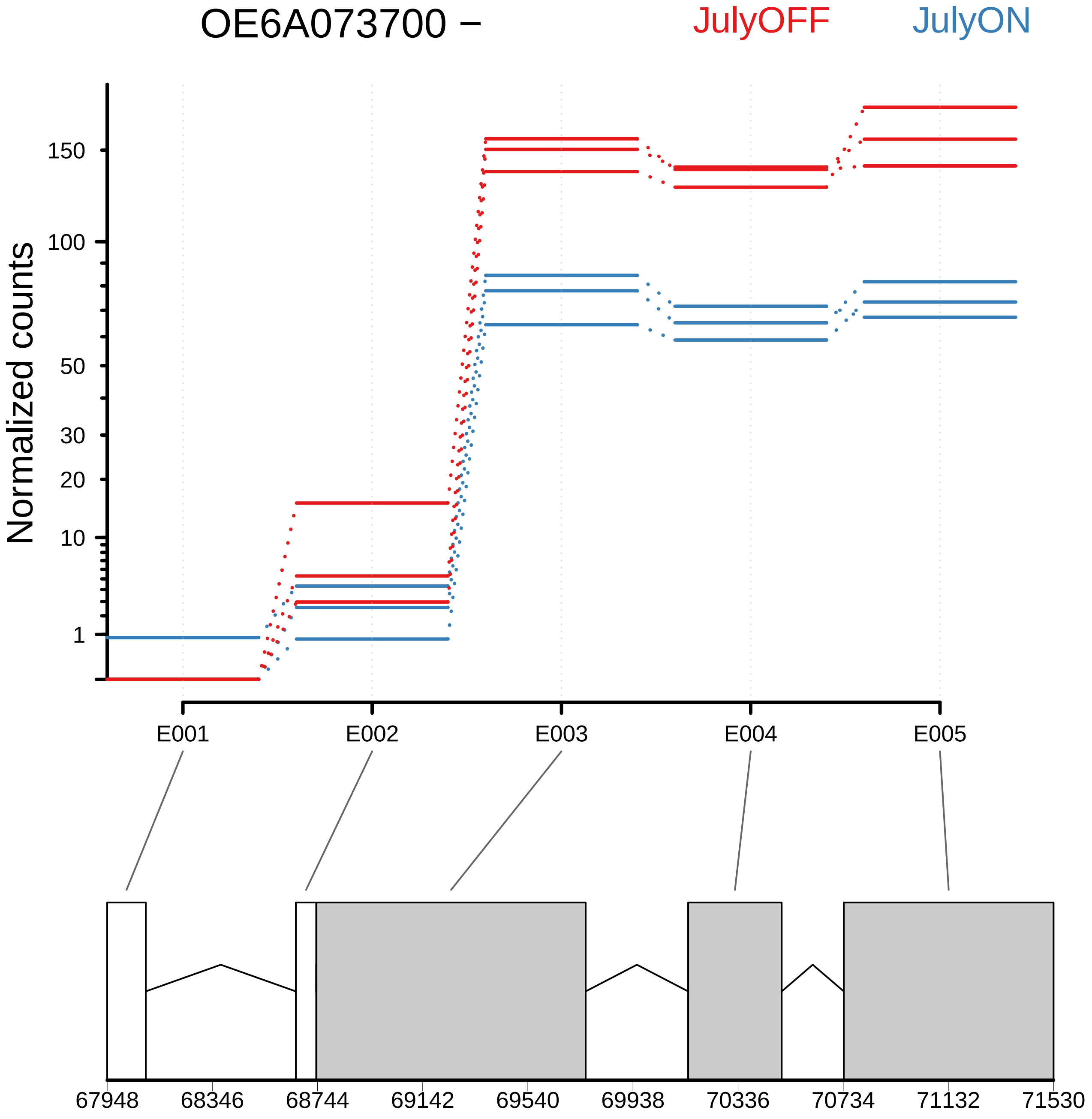
OE6A073700 −
JulyOFF
JulyON
Normalized counts
150
100
50
30
20
10
1
E001
E002
E003
E004
E005
67948
68346
68744
69142
69540
69938
70336
70734
71132
71530

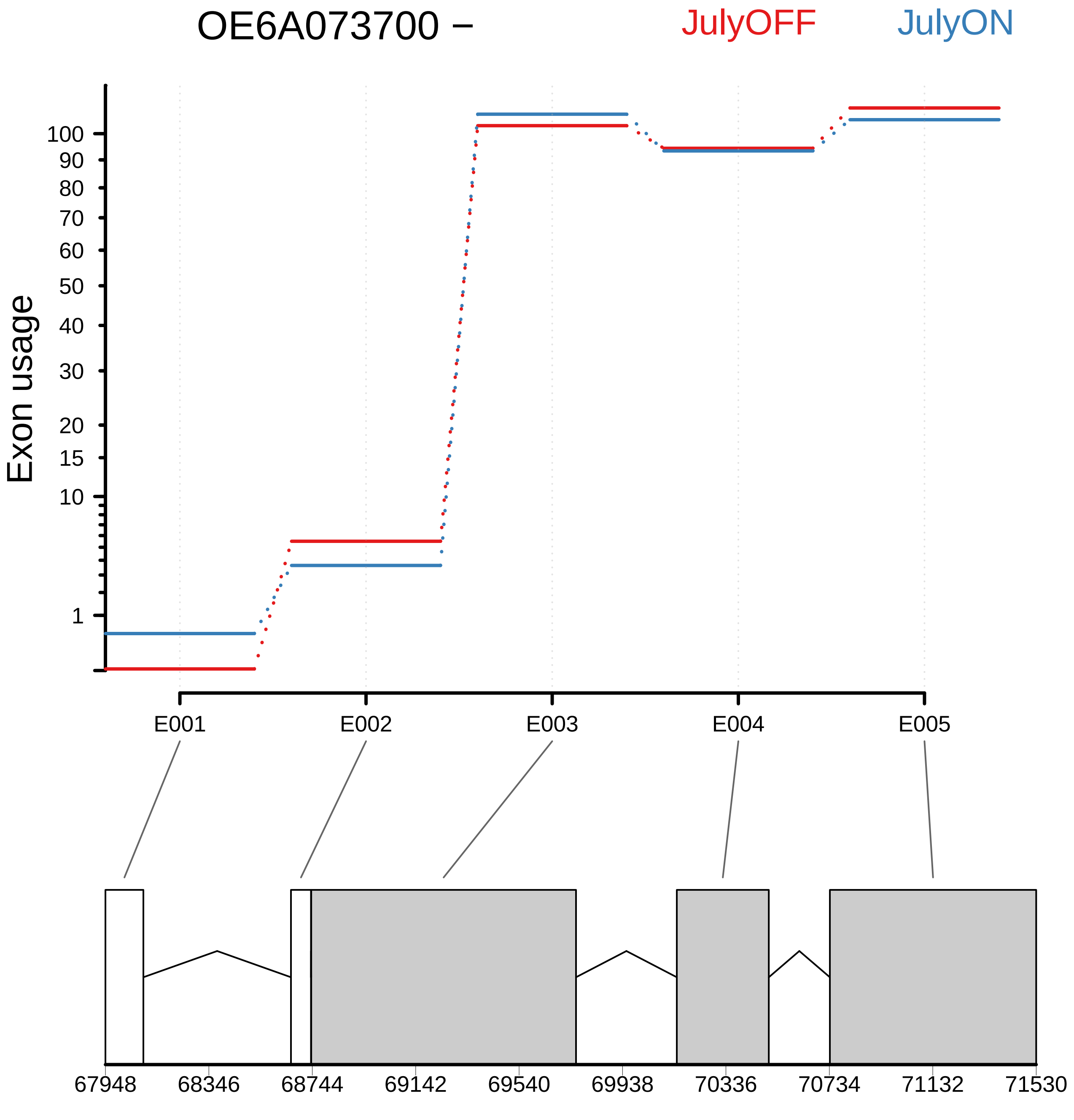

OE6A073700 −
JulyOFF
JulyON
Exon usage
100
90
80
70
60
50
40
30
20
15
10
1
E001
E002
E003
E004
E005
67948
68346
68744
69142
69540
69938
70336
70734
71132
71530

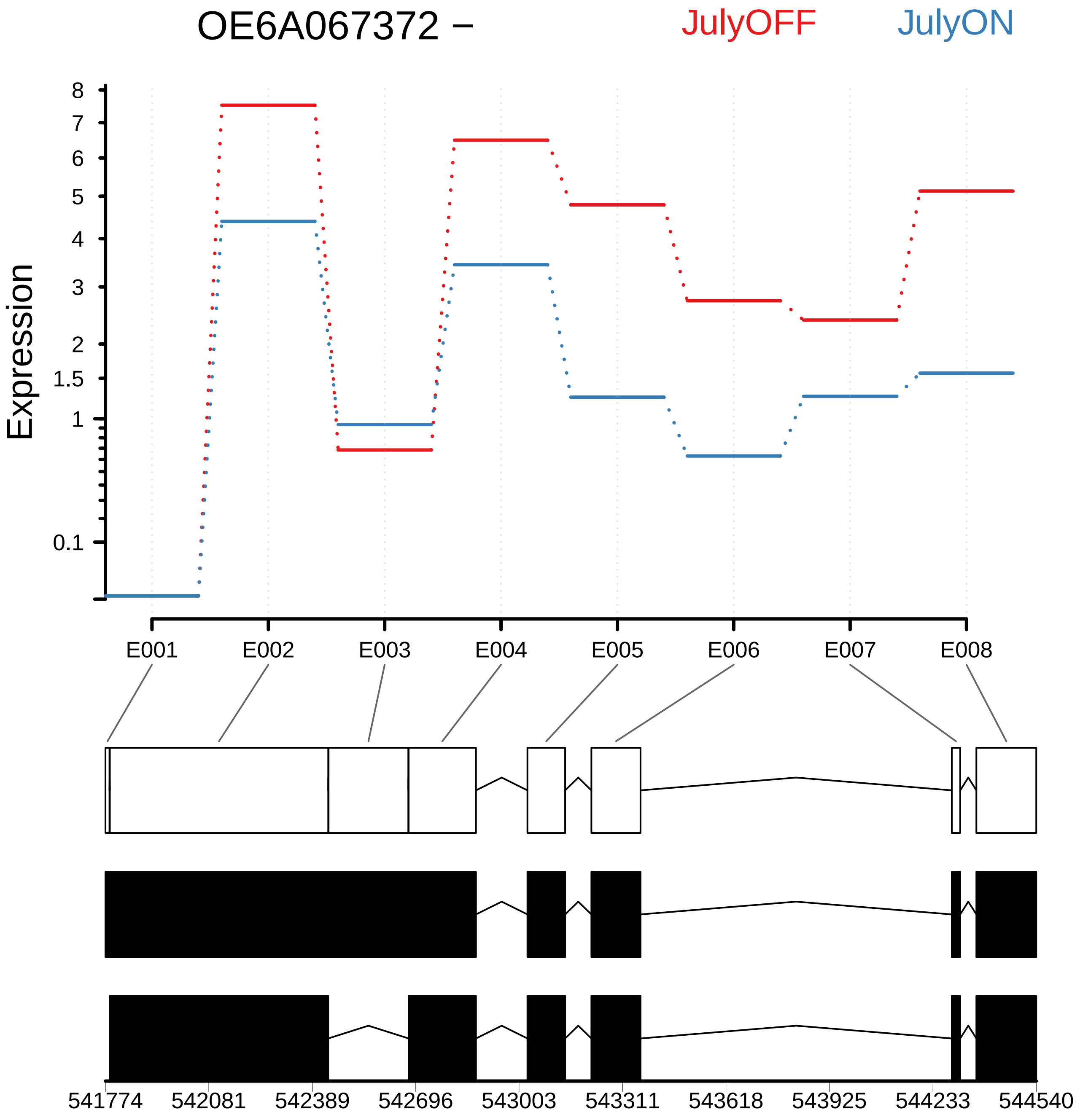

OE6A067372 −
JulyOFF
JulyON
Expression
8
7
6
5
4
3
2
1.5
1
0.1
E001
E002
E003
E004
E005
E006
E007
E008
541774
542081
542389
542696
543003
543311
543618
543925
544233
544540

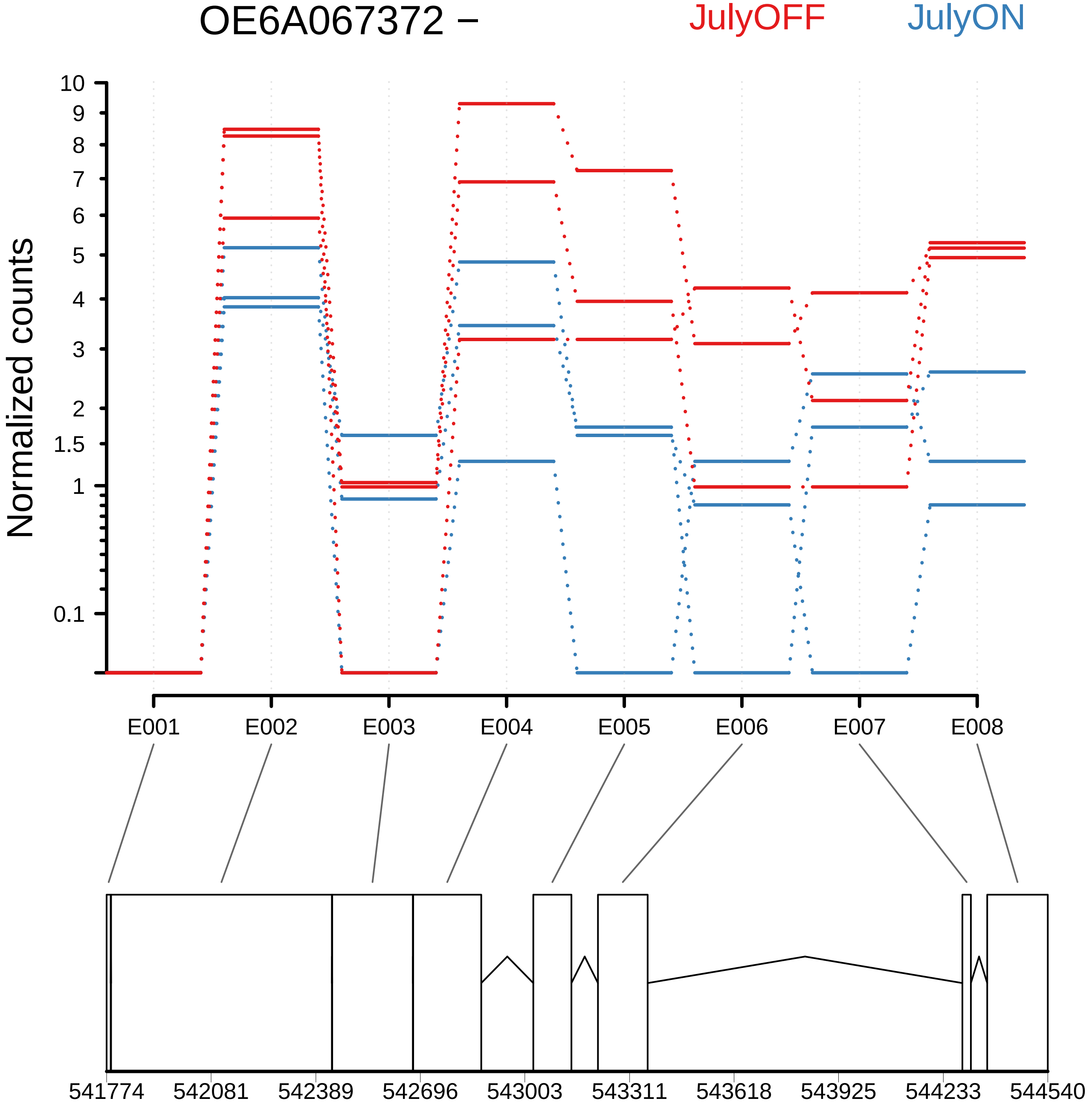
OE6A067372 −
JulyOFF
JulyON
Normalized counts
10
9
8
7
6
5
4
3
2
1.5
1
0.1
E001
E002
E003
E004
E005
E006
E007
E008
541774
542081
542389
542696
543003
543311
543618
543925
544233
544540

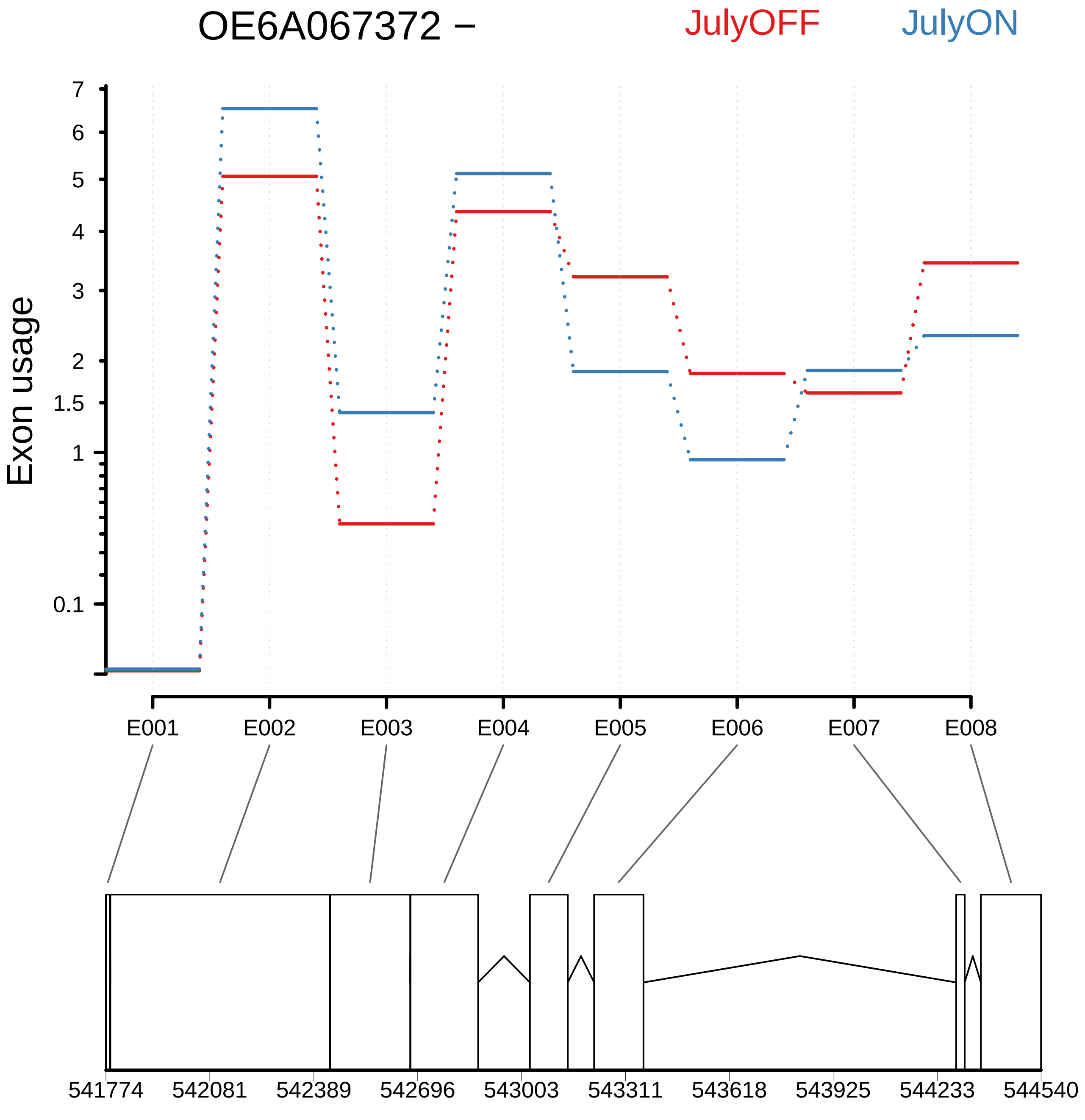

OE6A067372 −
JulyOFF
JulyON
Exon usage
7
6
5
4
3
2
1.5
1
0.1
E001
E002
E003
E004
E005
E006
E007
E008
541774
542081
542389
542696
543003
543311
543618
543925
544233
544540

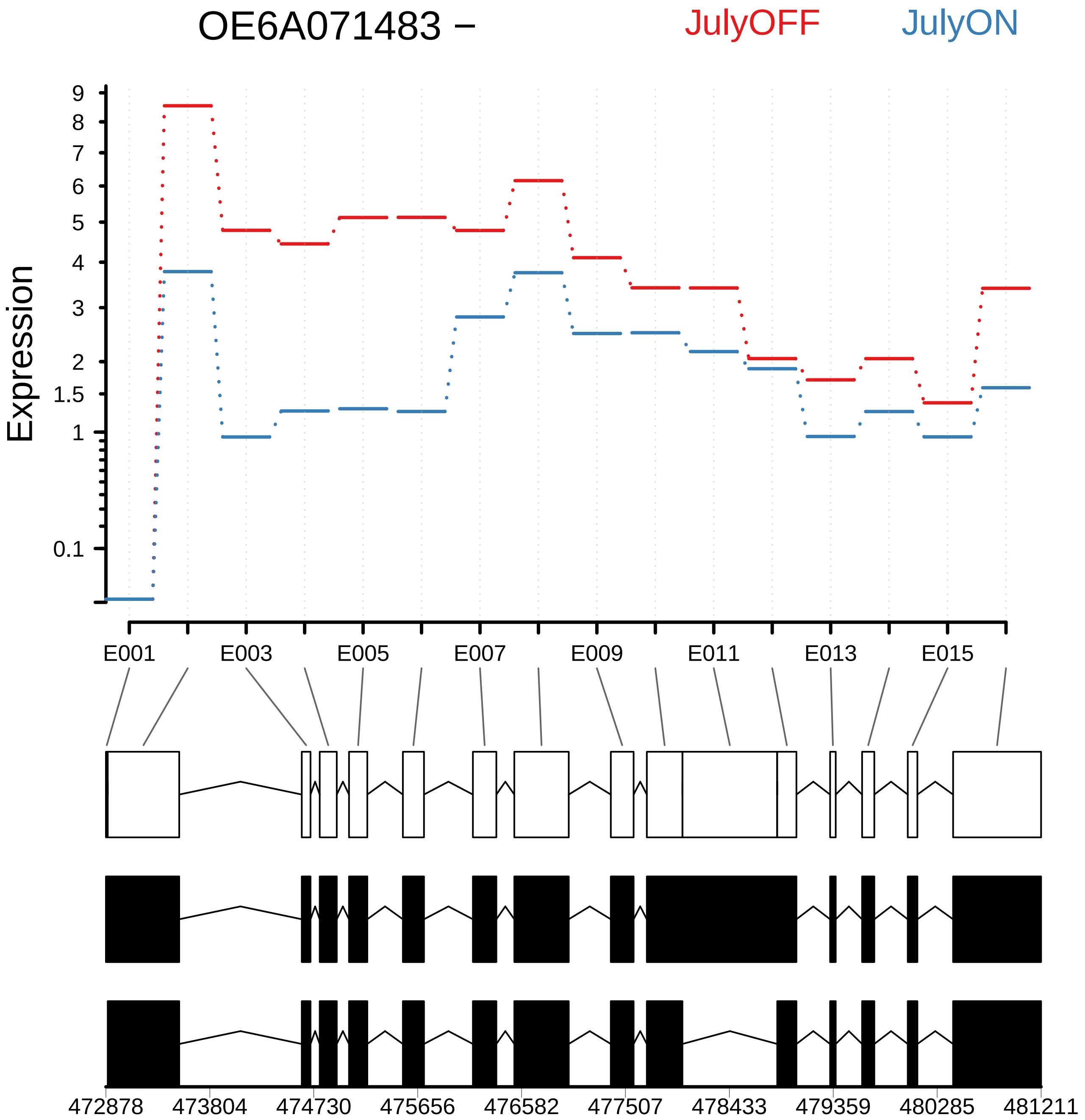
OE6A071483 −
JulyOFF
JulyON
Expression
9
8
7
6
5
4
3
2
1.5
1
0.1
E001
E003
E005
E007
E009
E011
E013
E015
472878
473804
474730
475656
476582
477507
478433
479359
480285
481211

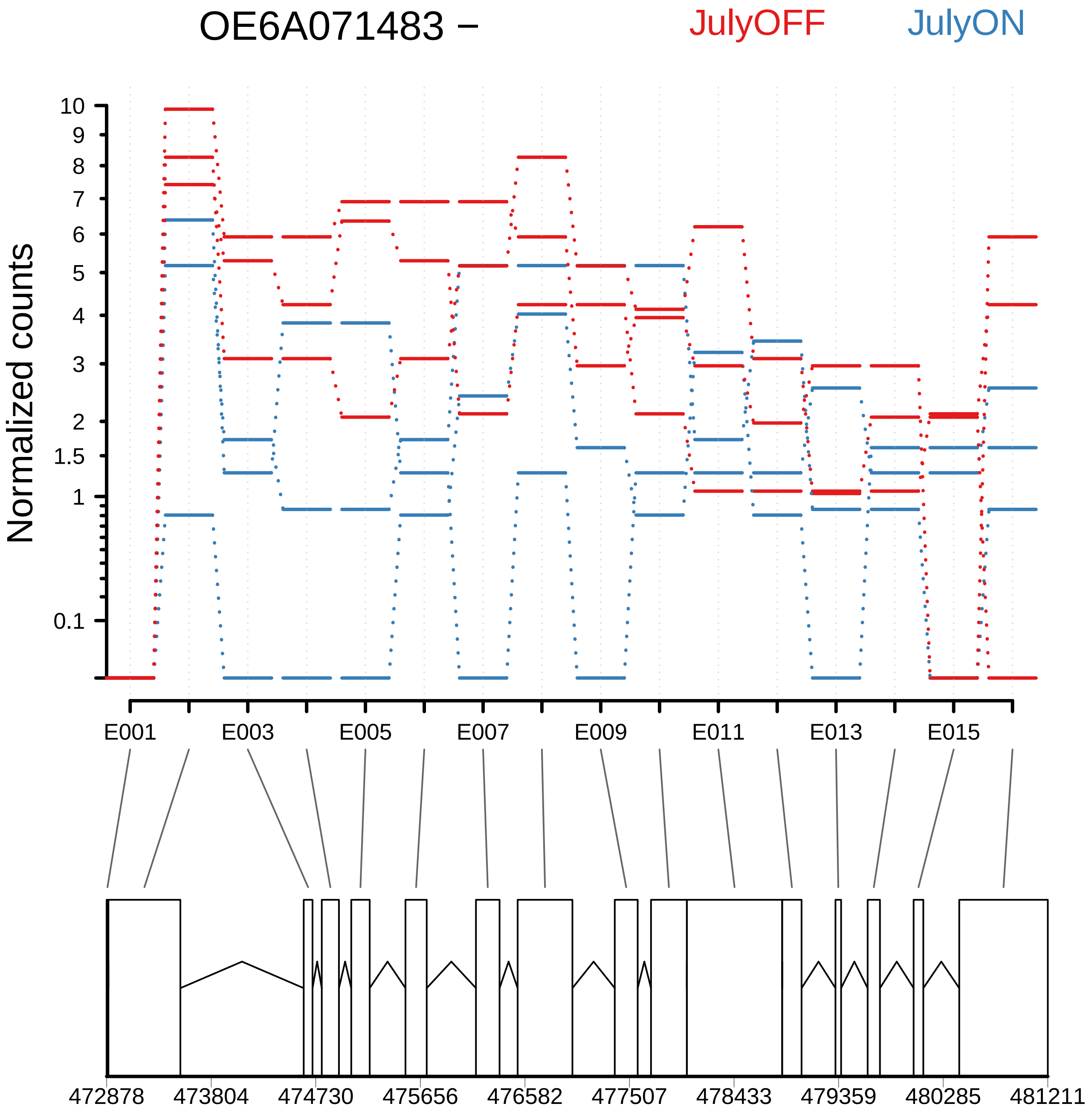

OE6A071483 −
JulyOFF
JulyON
Normalized counts
10
9
8
7
6
5
4
3
2
1.5
1
0.1
E001
E003
E005
E007
E009
E011
E013
E015
472878
473804
474730
475656
476582
477507
478433
479359
480285
481211

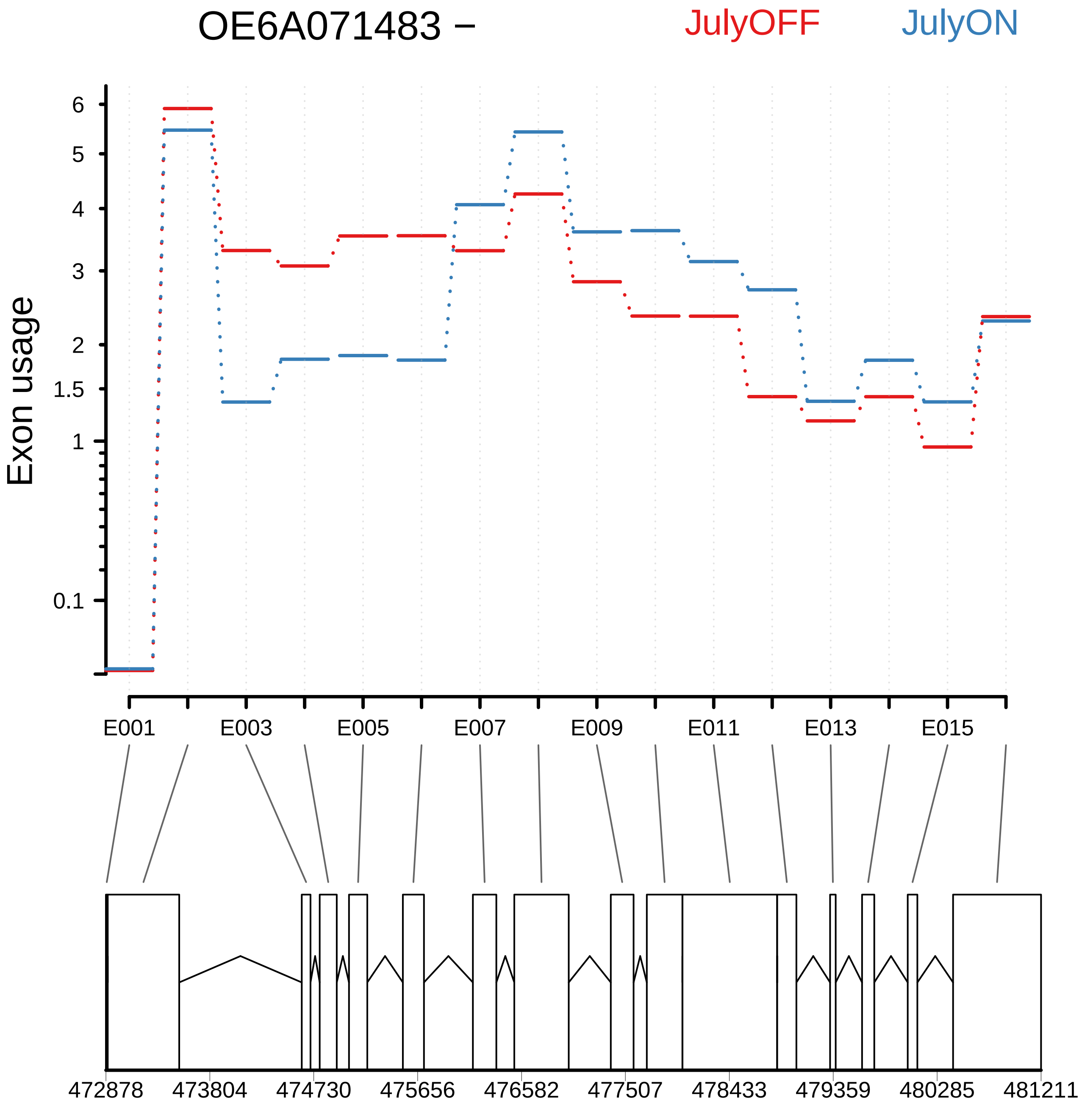

OE6A071483 −
JulyOFF
JulyON
Exon usage
6
5
4
3
2
1.5
1
0.1
E001
E003
E005
E007
E009
E011
E013
E015
472878
473804
474730
475656
476582
477507
478433
479359
480285
481211

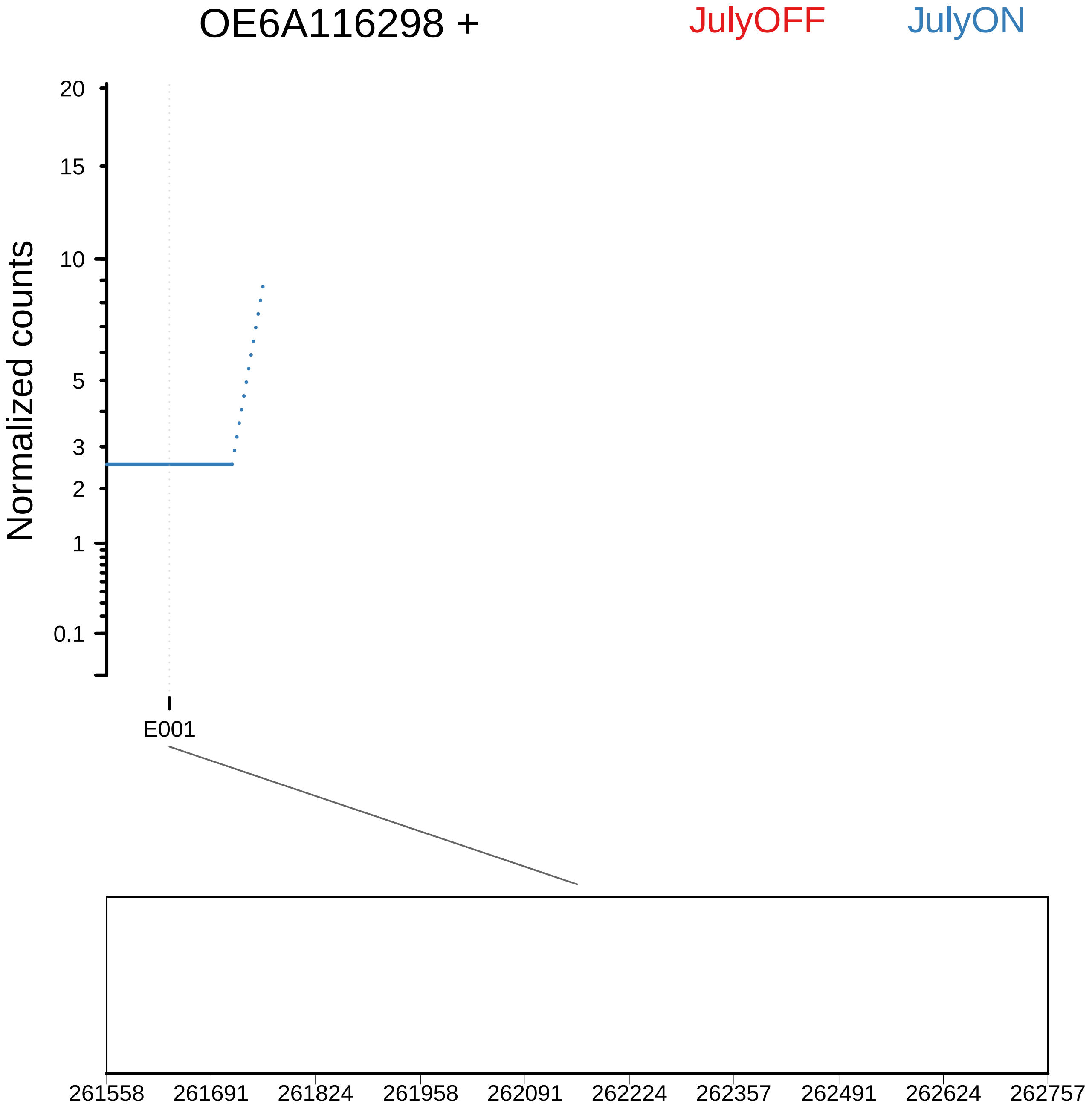

OE6A116298 +
JulyOFF
JulyON
Normalized counts
20
15
10
5
3
2
1
0.1
E001
261558
261691
261824
261958
262091
262224
262357
262491
262624
262757

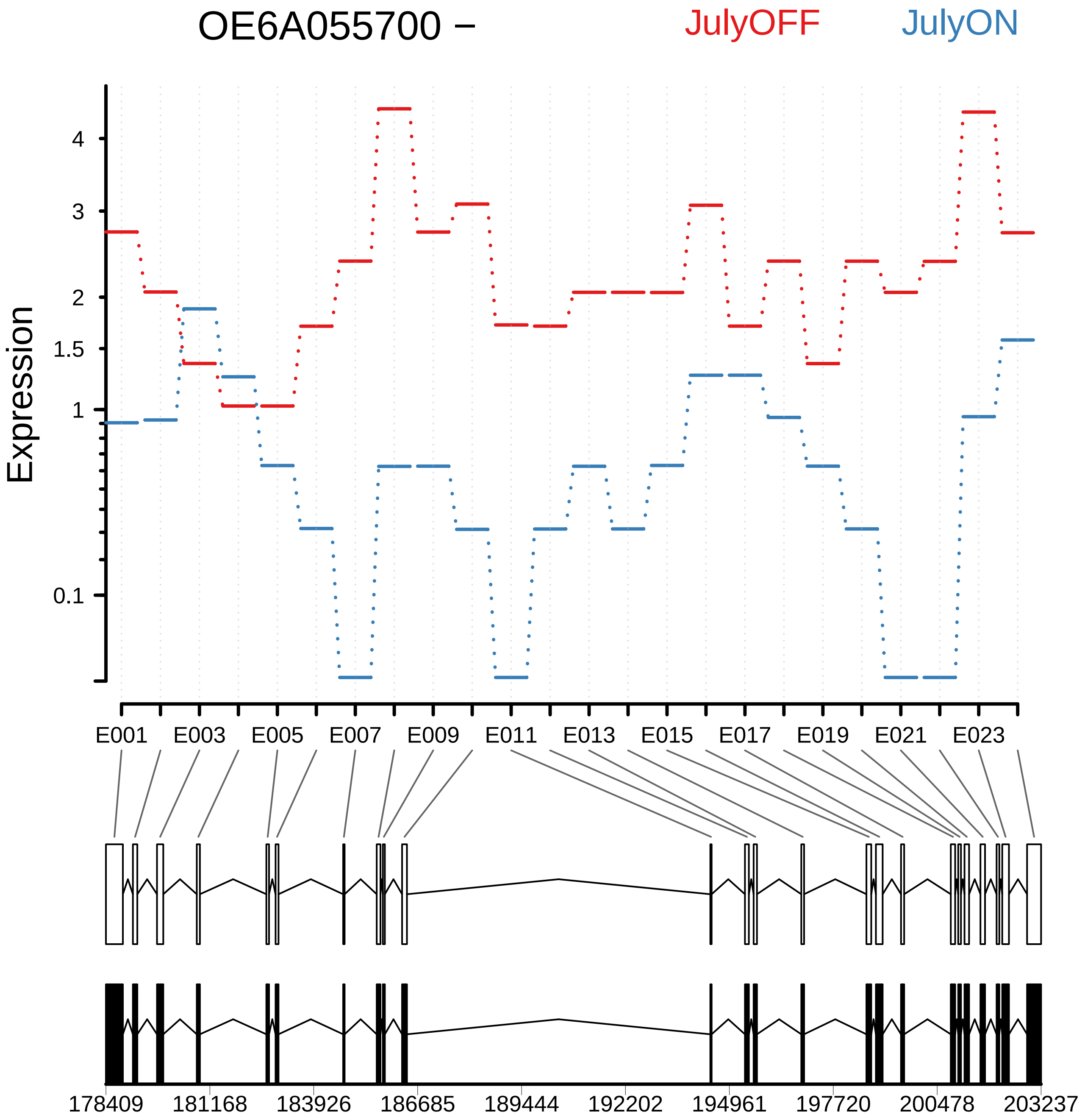
OE6A055700 −
JulyOFF
JulyON
Expression
4
3
2
1.5
1
0.1
E001
E003
E005
E007
E009
E011
E013
E015
E017
E019
E021
E023
178409
181168
183926
186685
189444
192202
194961
197720
200478
203237

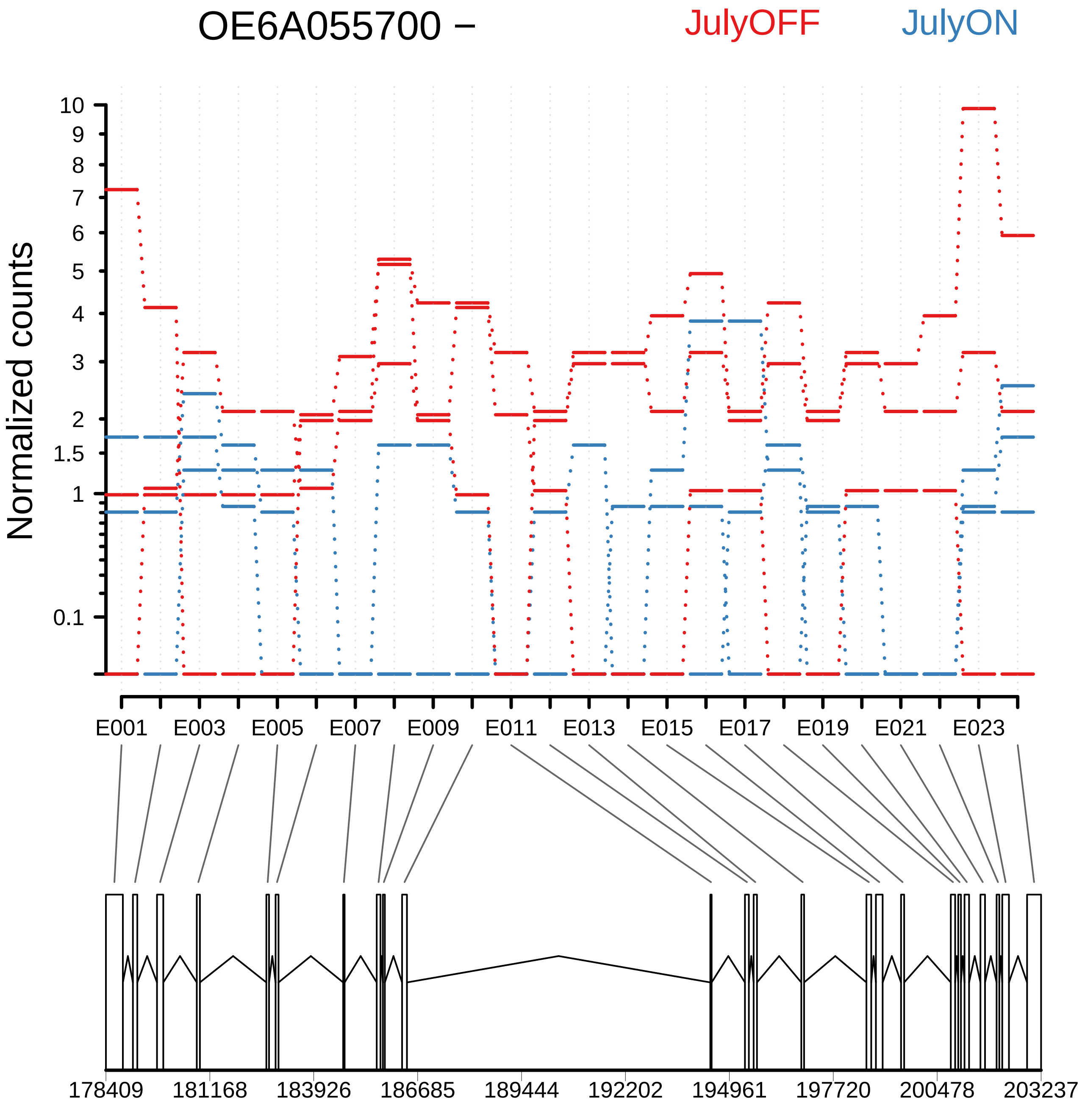

OE6A055700 −
JulyOFF
JulyON
Normalized counts
10
9
8
7
6
5
4
3
2
1.5
1
0.1
E001
E003
E005
E007
E009
E011
E013
E015
E017
E019
E021
E023
178409
181168
183926
186685
189444
192202
194961
197720
200478
203237

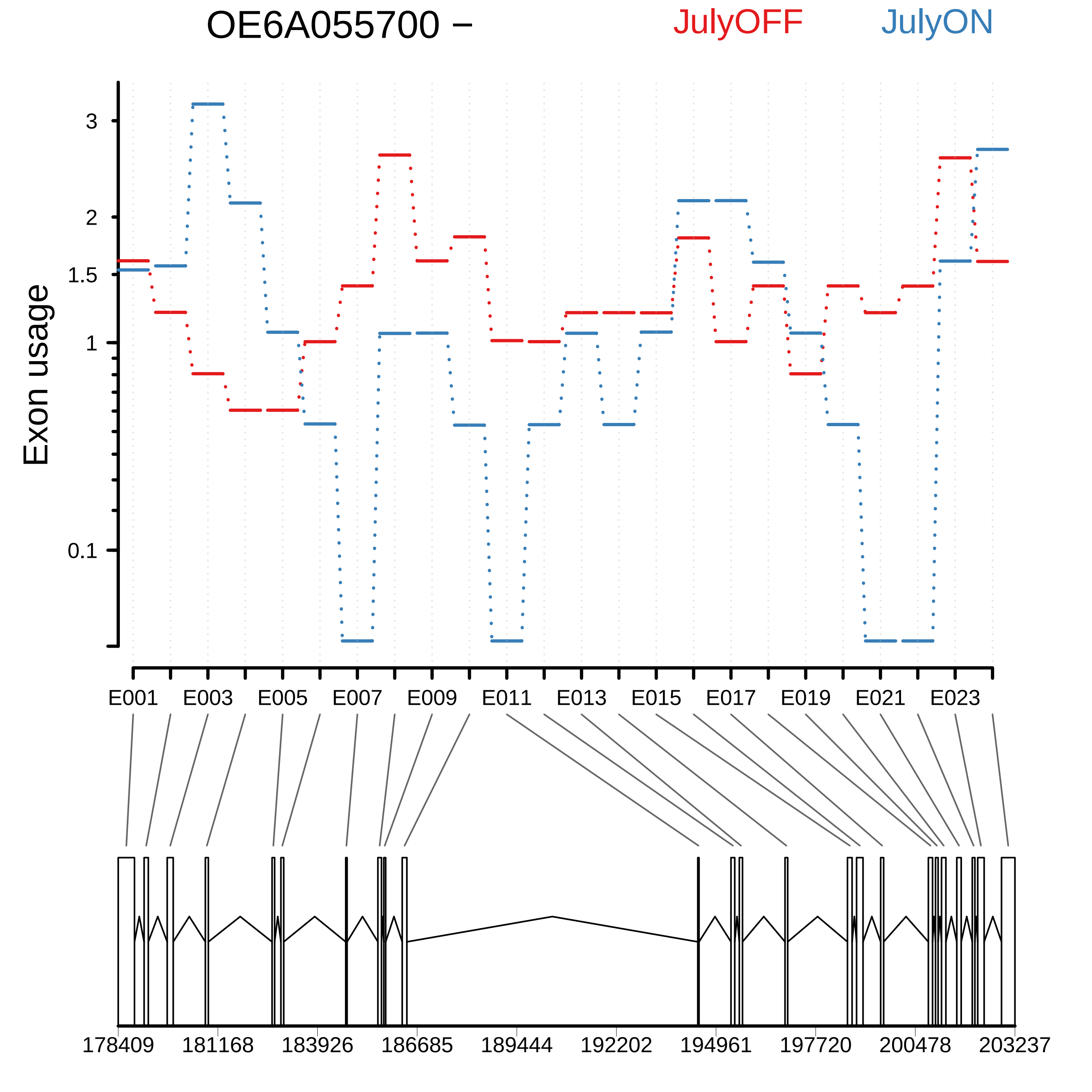

OE6A055700 −
JulyOFF
JulyON
Exon usage
3
2
1.5
1
0.1
E001
E003
E005
E007
E009
E011
E013
E015
E017
E019
E021
E023
178409
181168
183926
186685
189444
192202
194961
197720
200478
203237

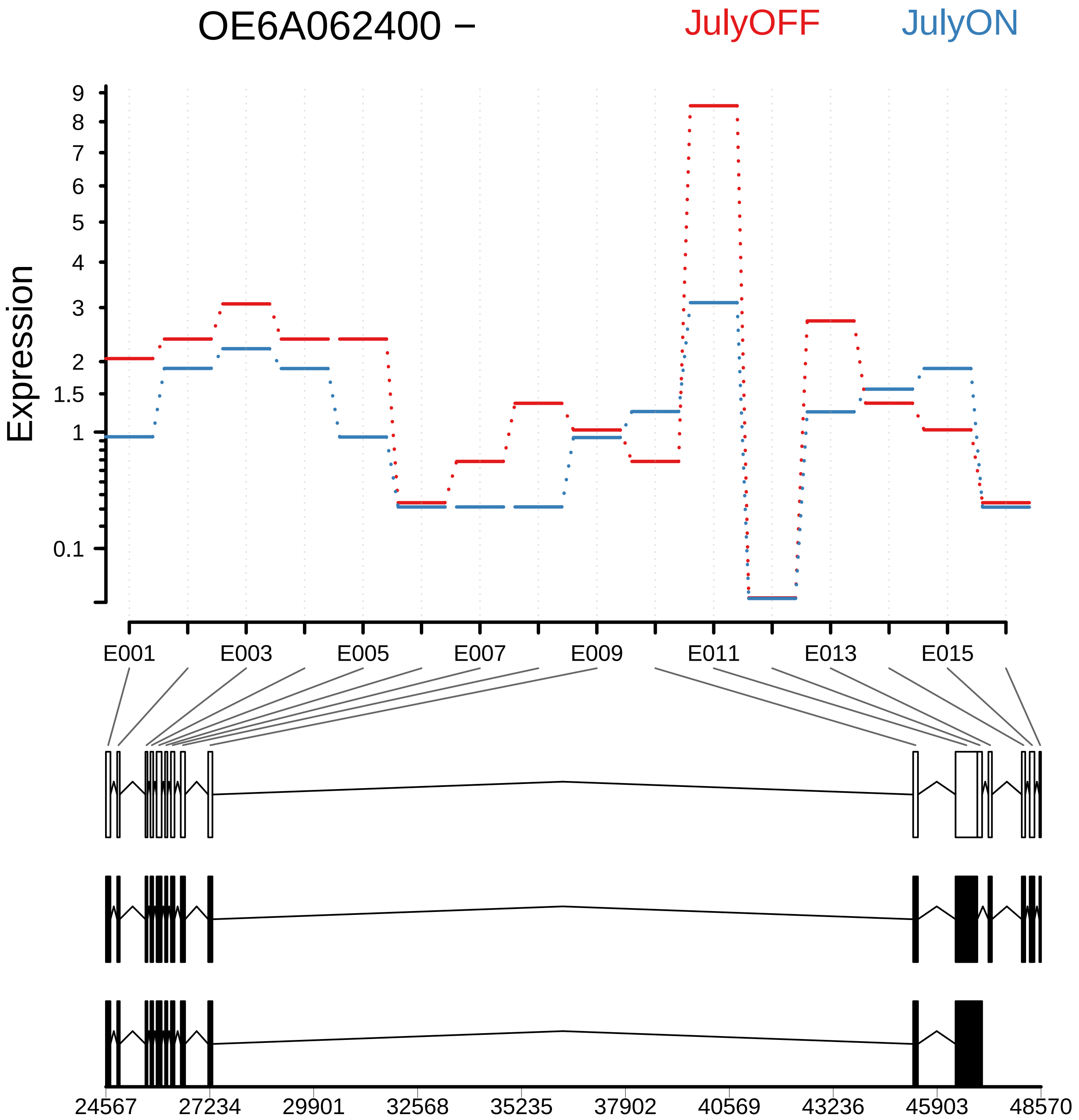

OE6A062400 –
JulyOFF
JulyON
Expression
9
8
7
6
5
4
3
2
1.5
1
0.1
E001
E003
E005
E007
E009
E011
E013
E015
24567
27234
29901
32568
35235
37902
40569
43236
45903
48570

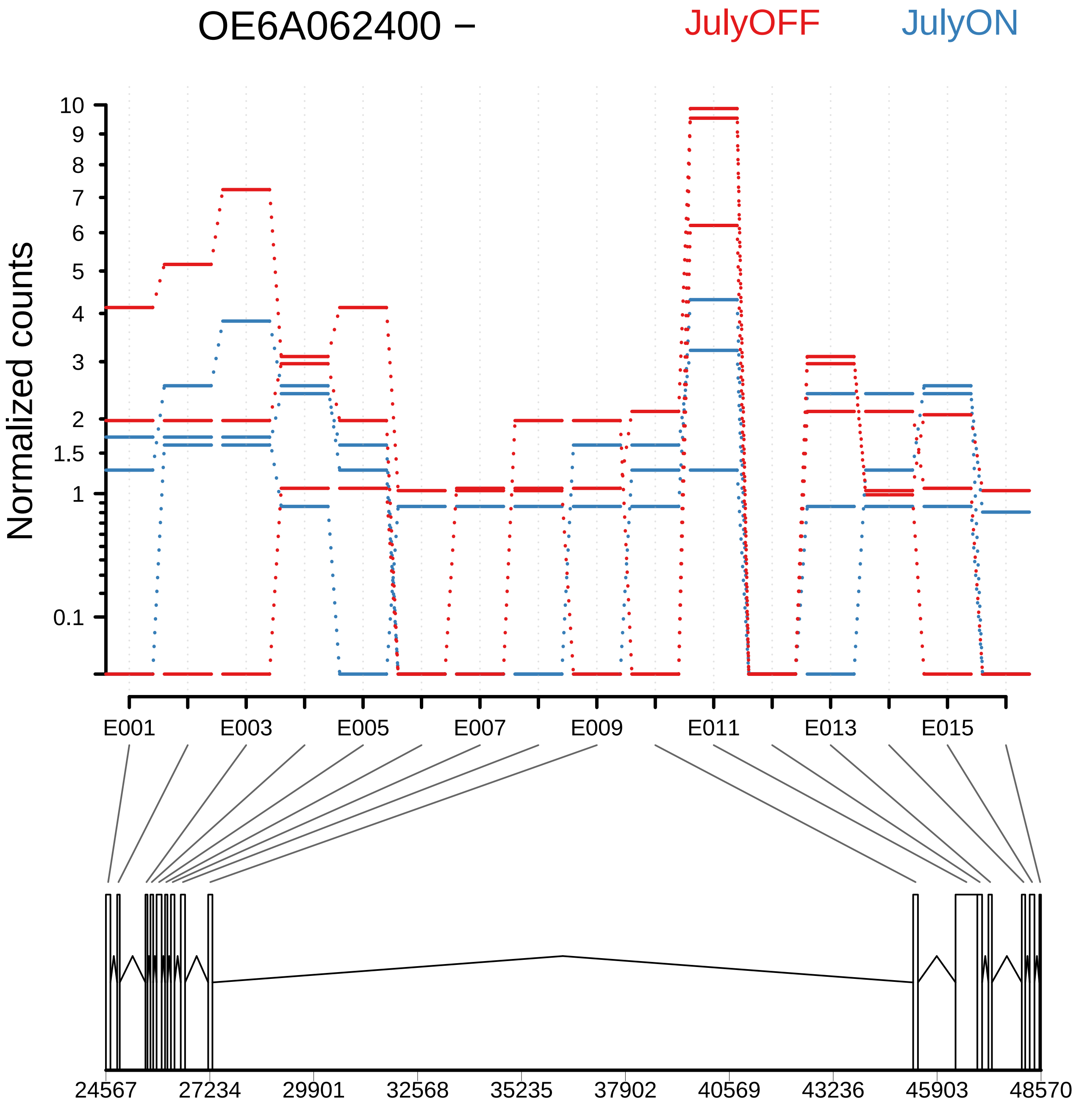

OE6A062400 –
JulyOFF
JulyON
Normalized counts
10
9
8
7
6
5
4
3
2
1.5
1
0.1
E001
E003
E005
E007
E009
E011
E013
E015
24567
27234
29901
32568
35235
37902
40569
43236
45903
48570

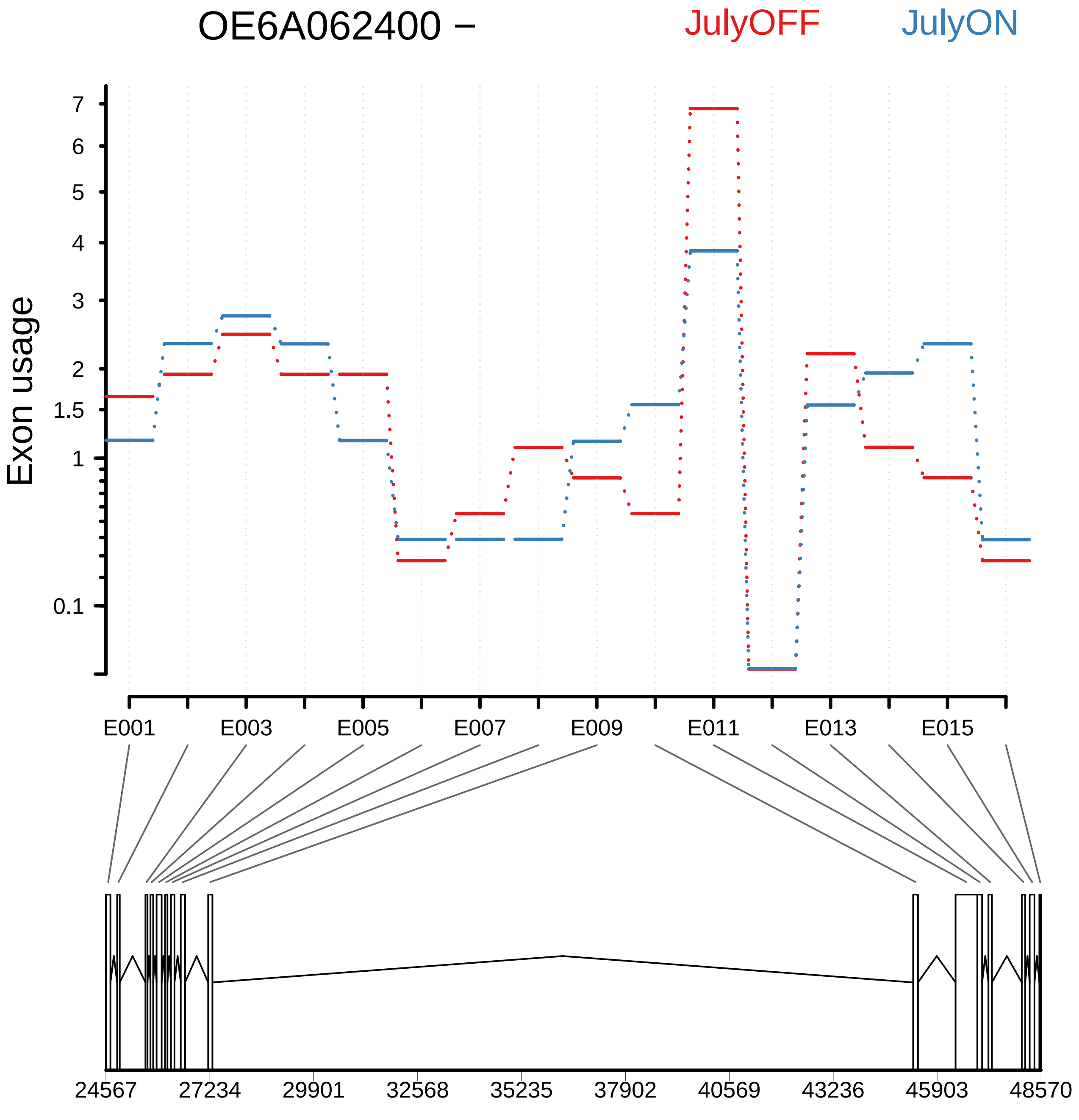

OE6A062400 −
JulyOFF
JulyON
Exon usage
7
6
5
4
3
2
1.5
1
0.1
E001
E003
E005
E007
E009
E011
E013
E015
24567
27234
29901
32568
35235
37902
40569
43236
45903
48570

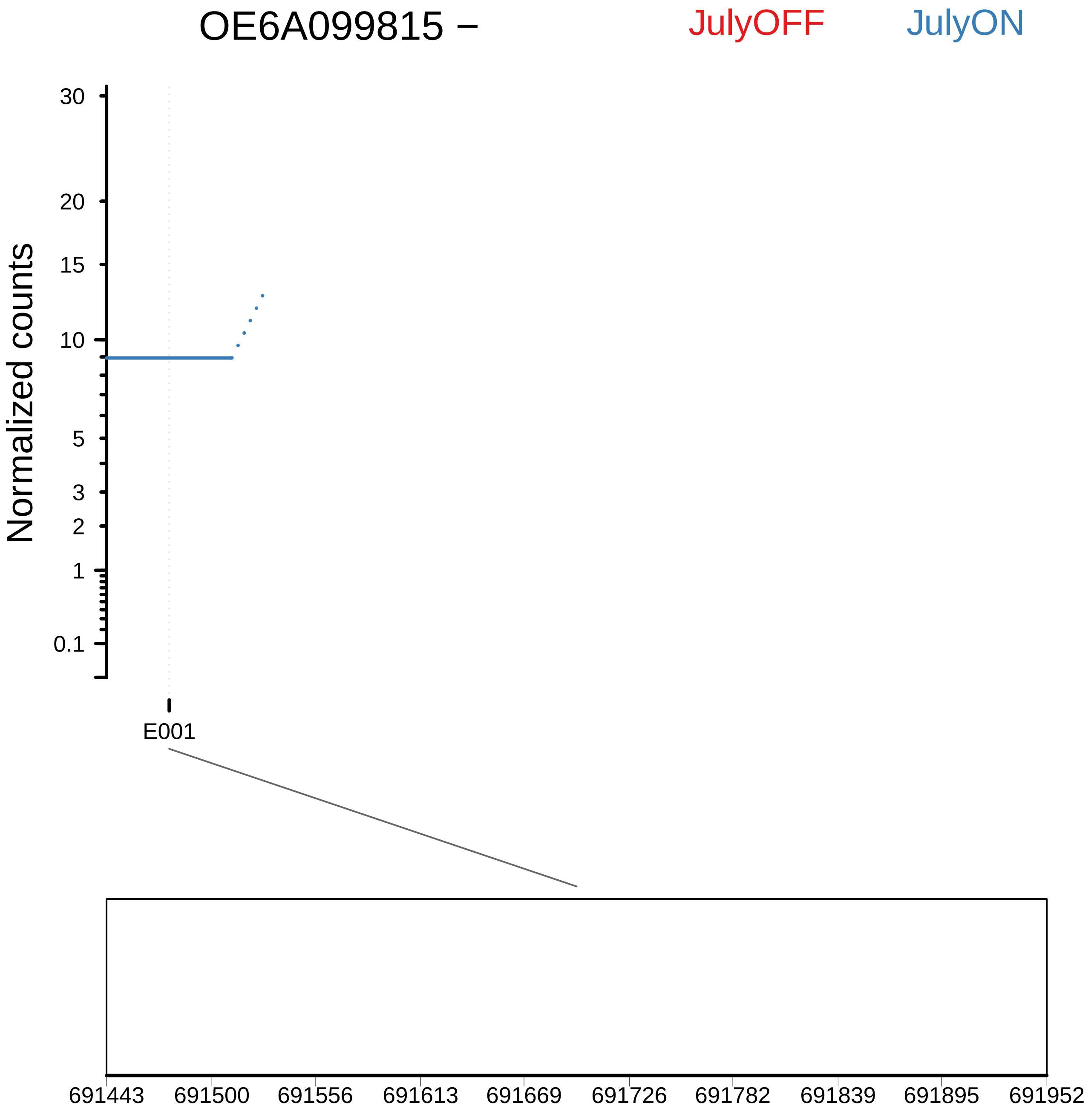
OE6A099815 −
JulyOFF
JulyON
Normalized counts
30
20
15
10
5
3
2
1
0.1
E001
691443
691500
691556
691613
691669
691726
691782
691839
691895
691952

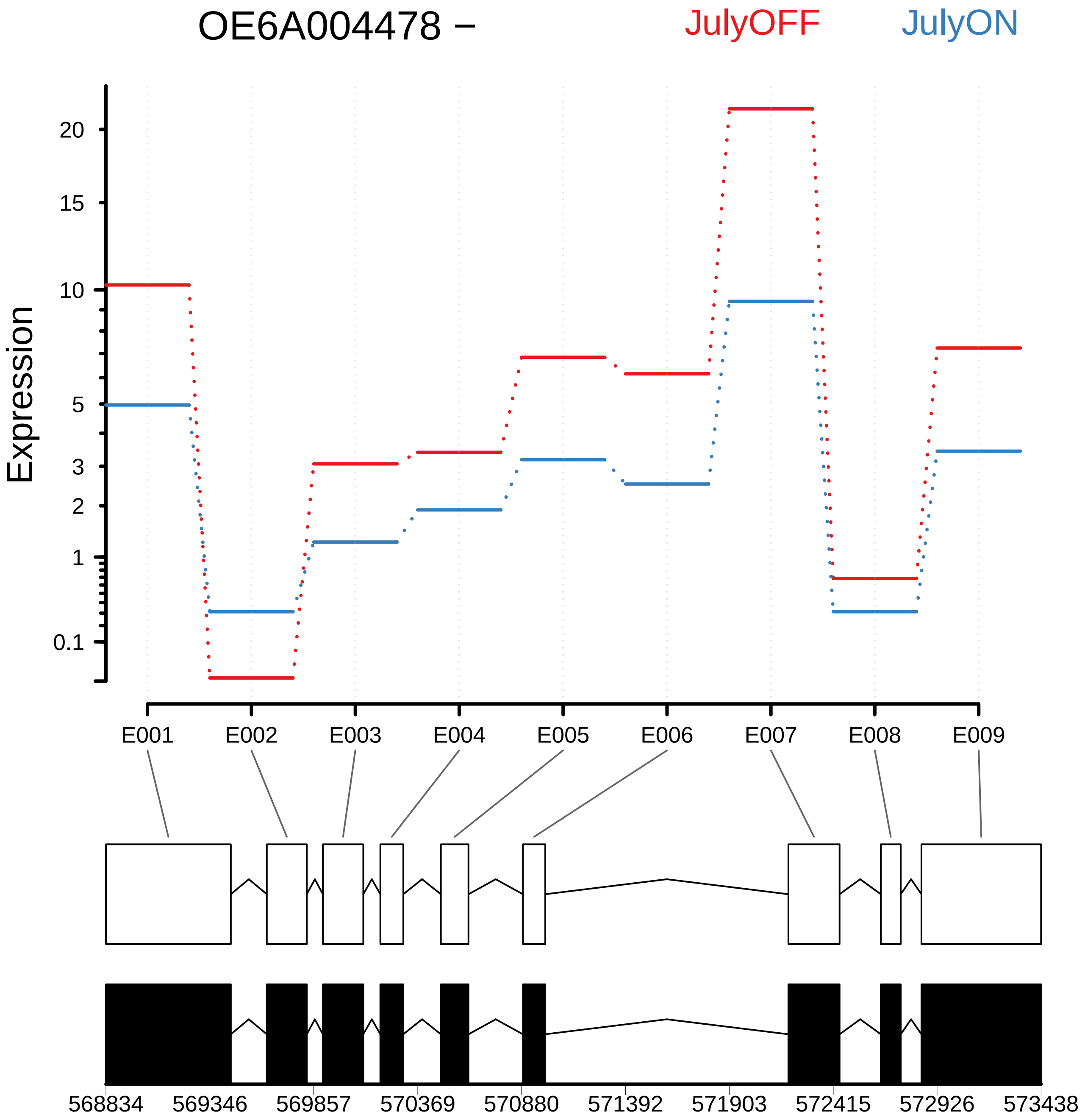

OE6A004478 –
JulyOFF
JulyON
Expression
20
15
10
5
3
2
1
0.1
E001
E002
E003
E004
E005
E006
E007
E008
E009
568834
569346
569857
570369
570880
571392
571903
572415
572926
573438

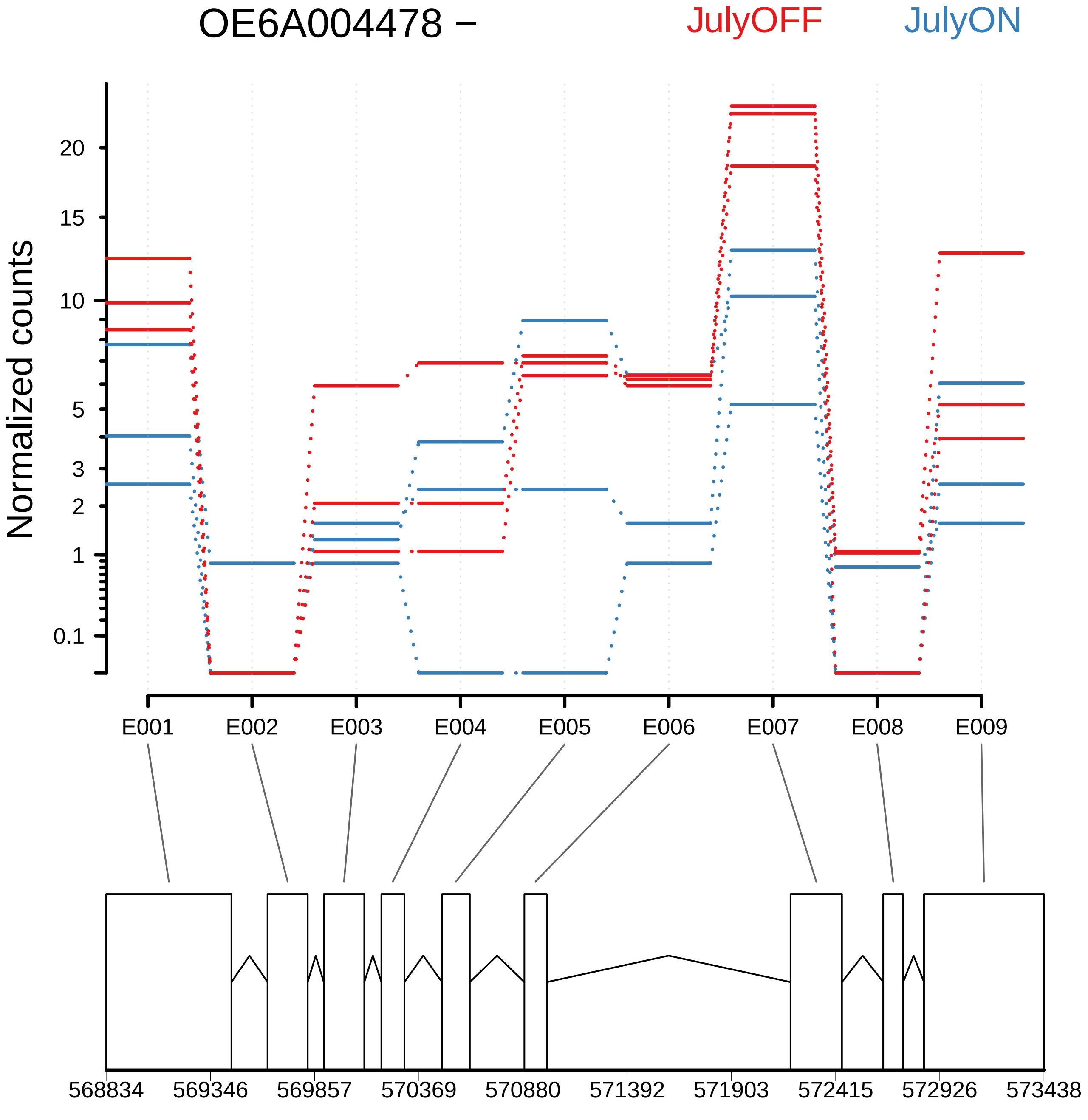

OE6A004478 −
JulyOFF
JulyON
Normalized counts
20
15
10
5
3
2
1
0.1
E001
E002
E003
E004
E005
E006
E007
E008
E009
568834
569346
569857
570369
570880
571392
571903
572415
572926
573438

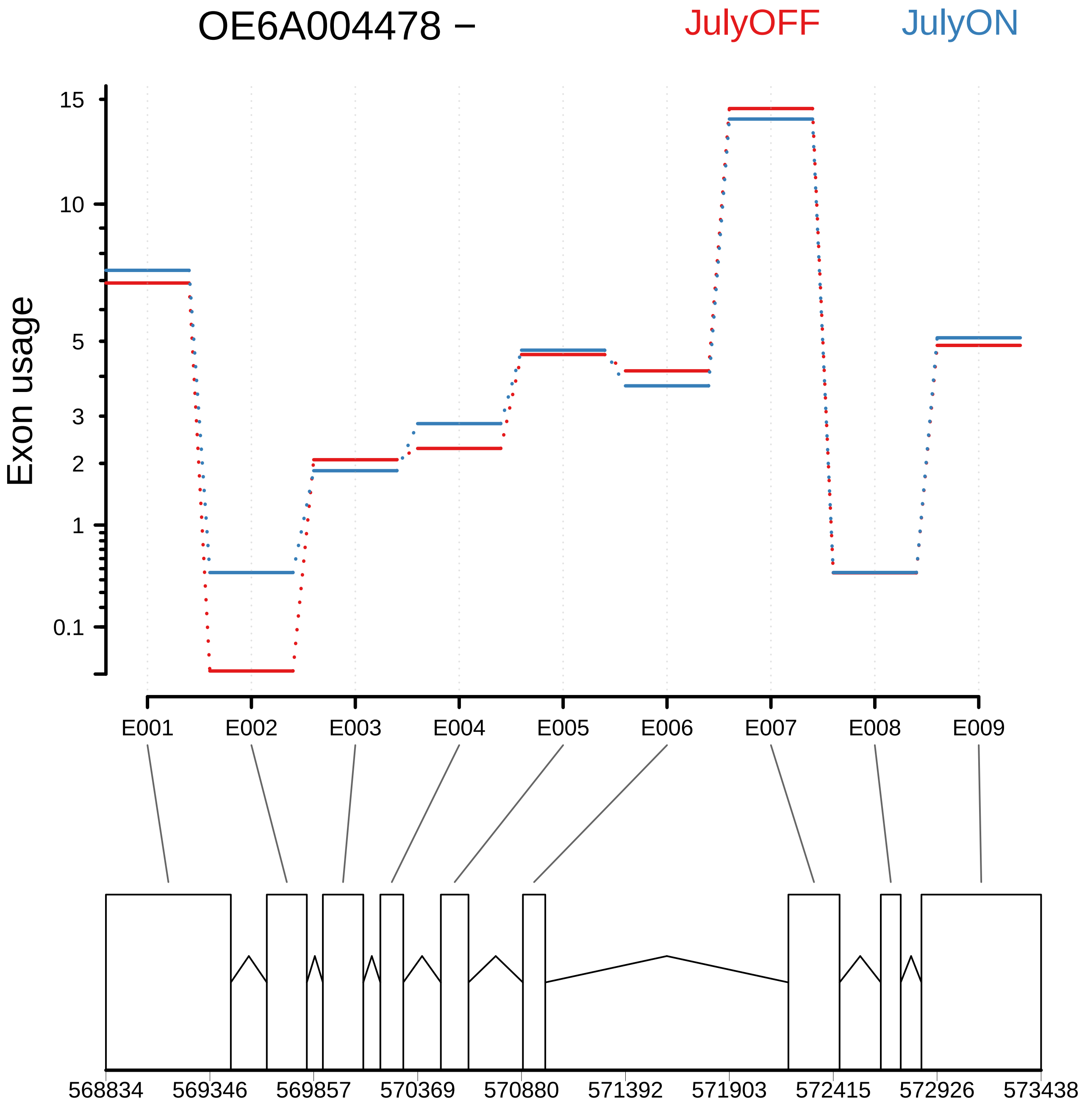

OE6A004478 −
JulyOFF
JulyON
Exon usage
15
10
5
3
2
1
0.1
E001
E002
E003
E004
E005
E006
E007
E008
E009
568834
569346
569857
570369
570880
571392
571903
572415
572926
573438

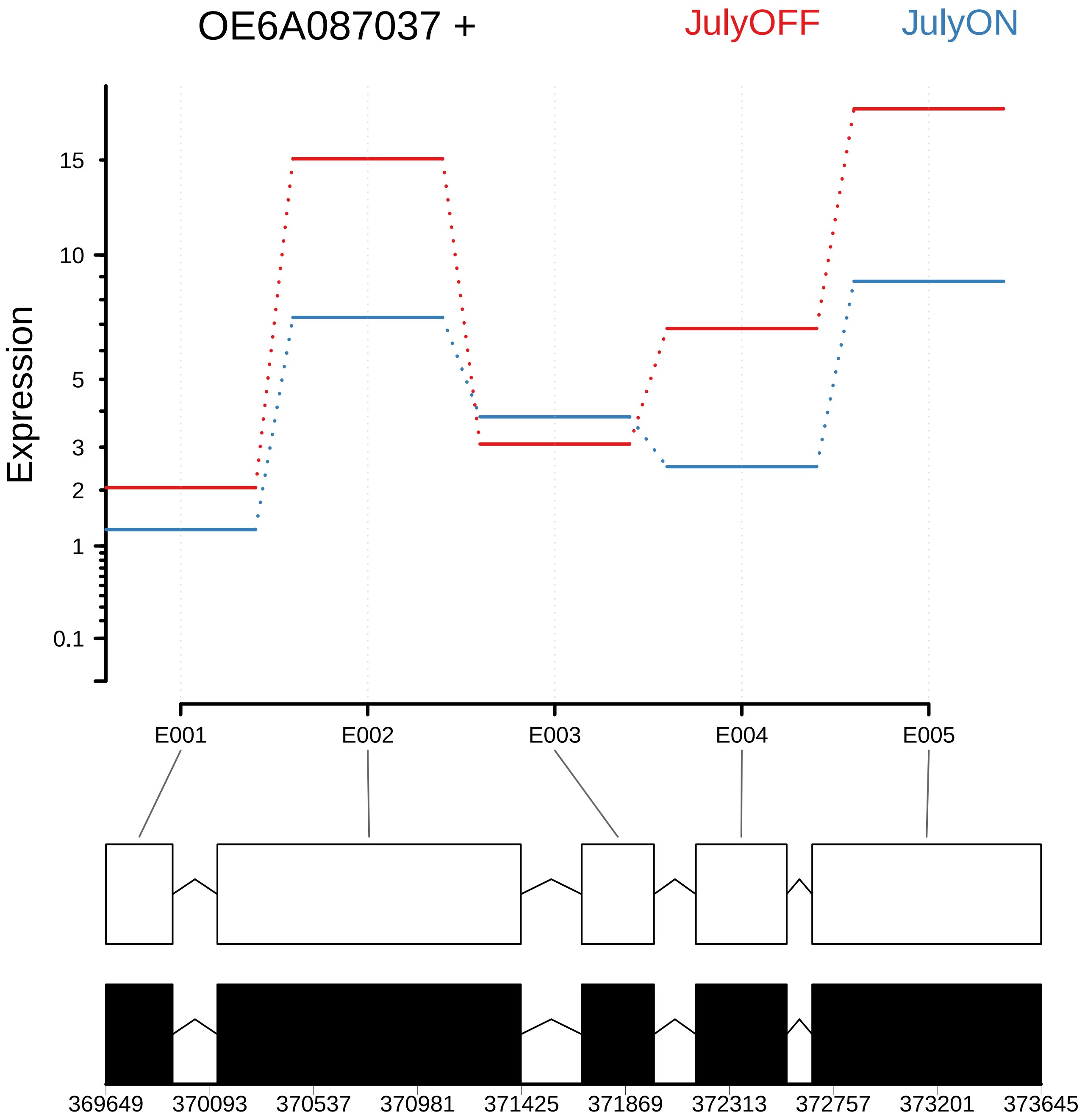
OE6A087037 +
JulyOFF
JulyON
Expression
15
10
5
3
2
1
0.1
E001
E002
E003
E004
E005
369649
370093
370537
370981
371425
371869
372313
372757
373201
373645

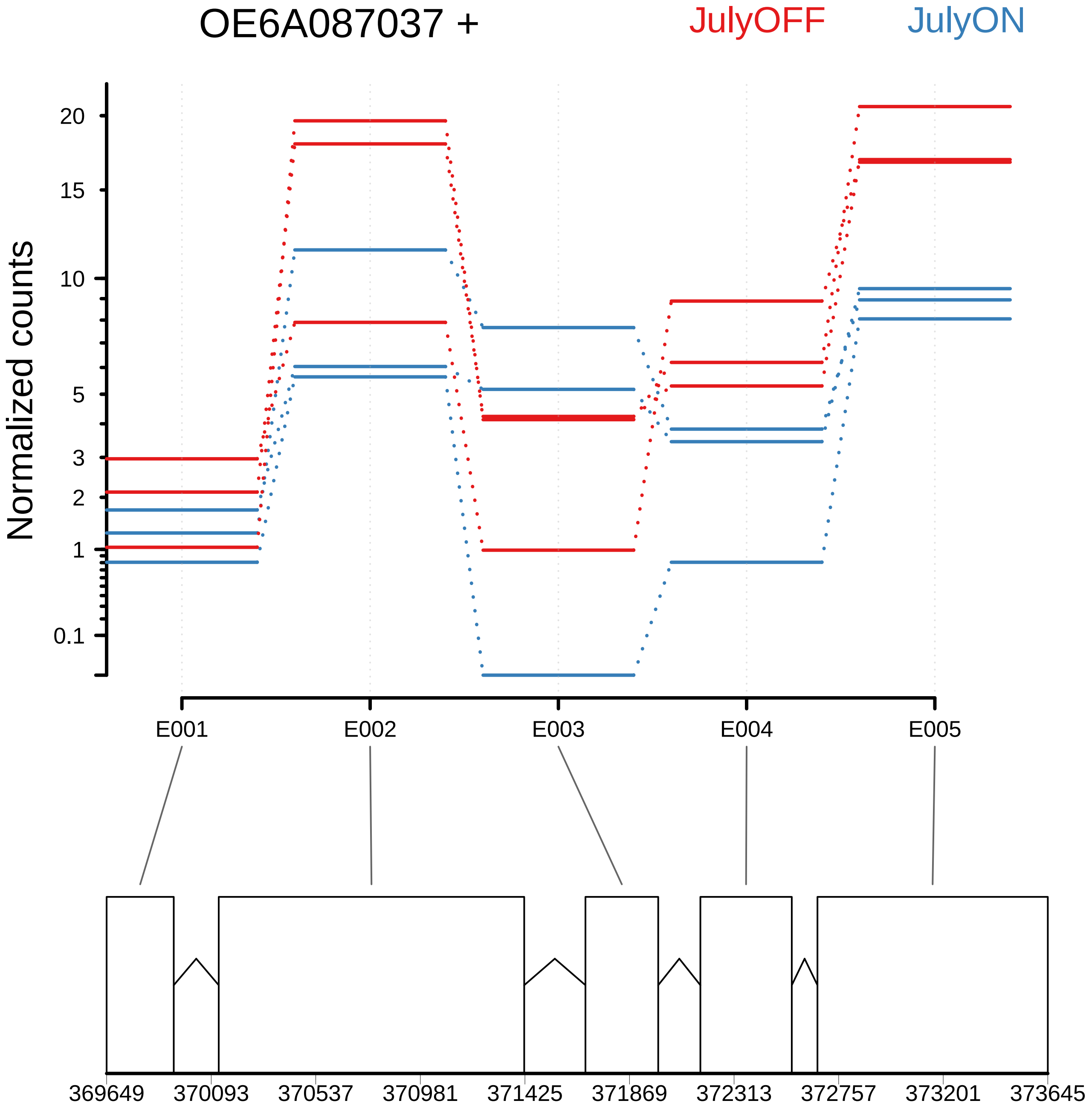
OE6A087037 +
JulyOFF
JulyON
Normalized counts
20
15
10
5
3
2
1
0.1
E001
E002
E003
E004
E005
369649
370093
370537
370981
371425
371869
372313
372757
373201
373645

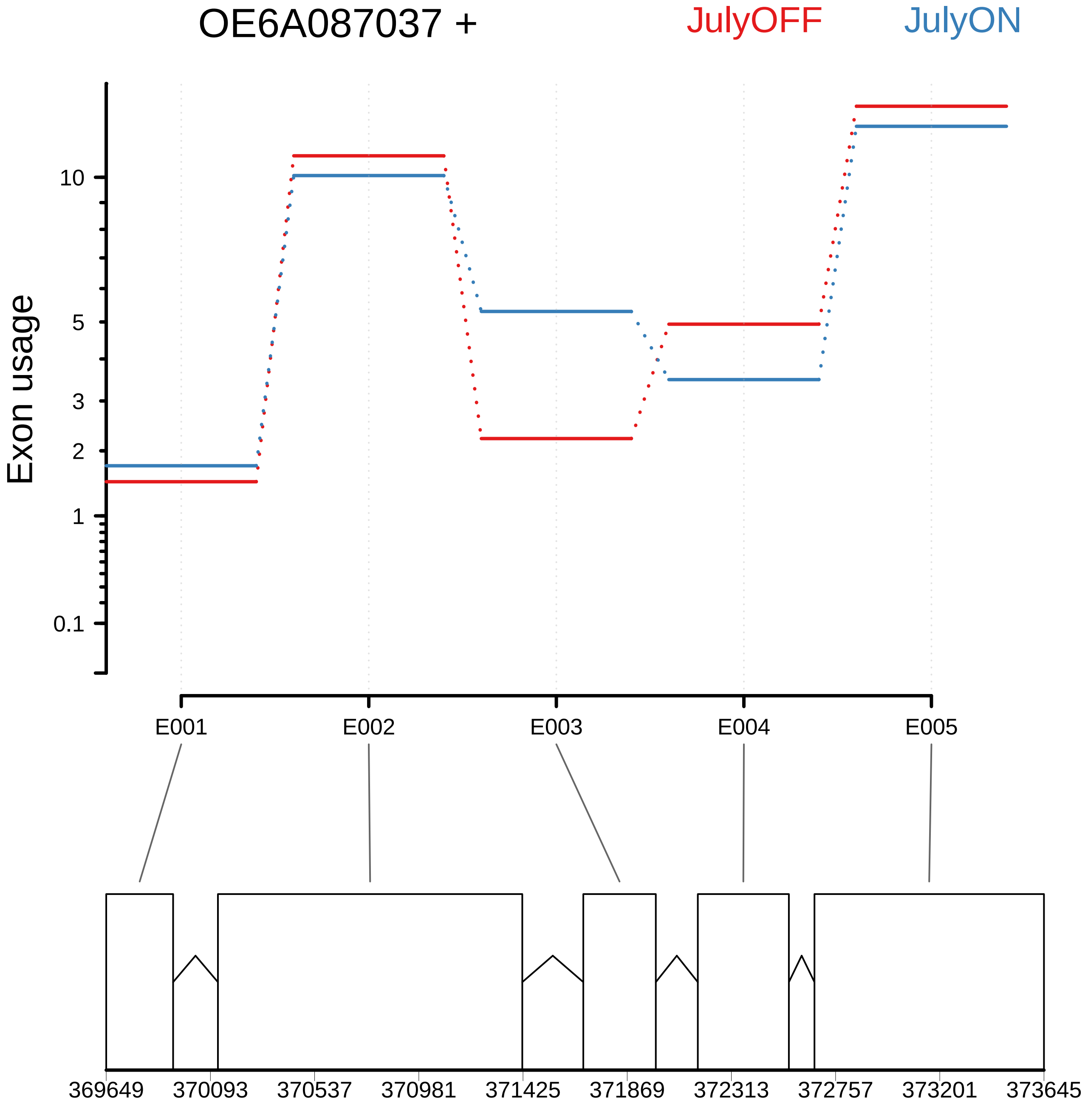

OE6A087037 +
JulyOFF
JulyON
Exon usage
10
5
3
2
1
0.1
E001
E002
E003
E004
E005
369649
370093
370537
370981
371425
371869
372313
372757
373201
373645

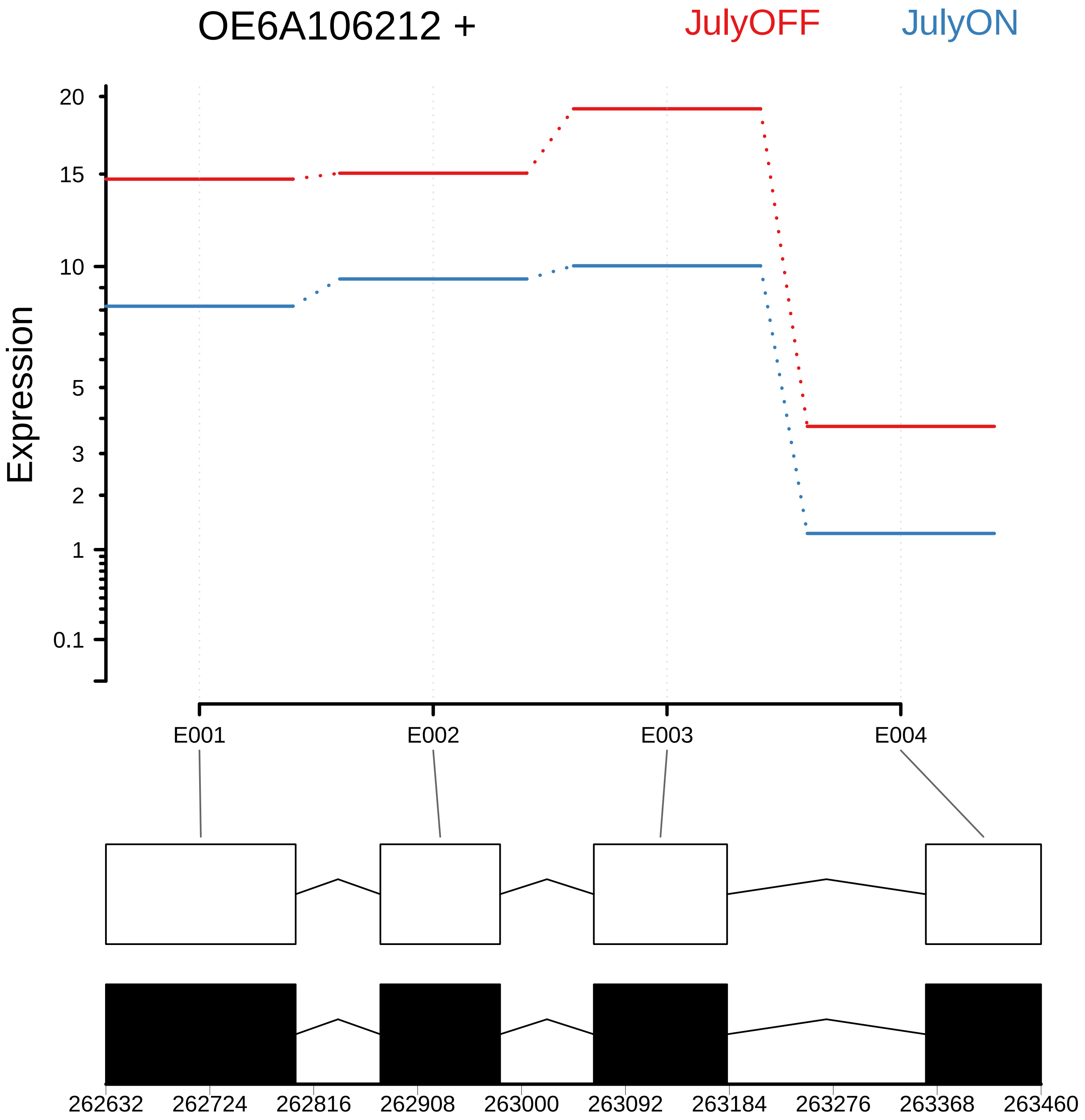

OE6A106212 +
JulyOFF
JulyON
Expression
20
15
10
5
3
2
1
0.1
E001
E002
E003
E004
262632
262724
262816
262908
263000
263092
263184
263276
263368
263460

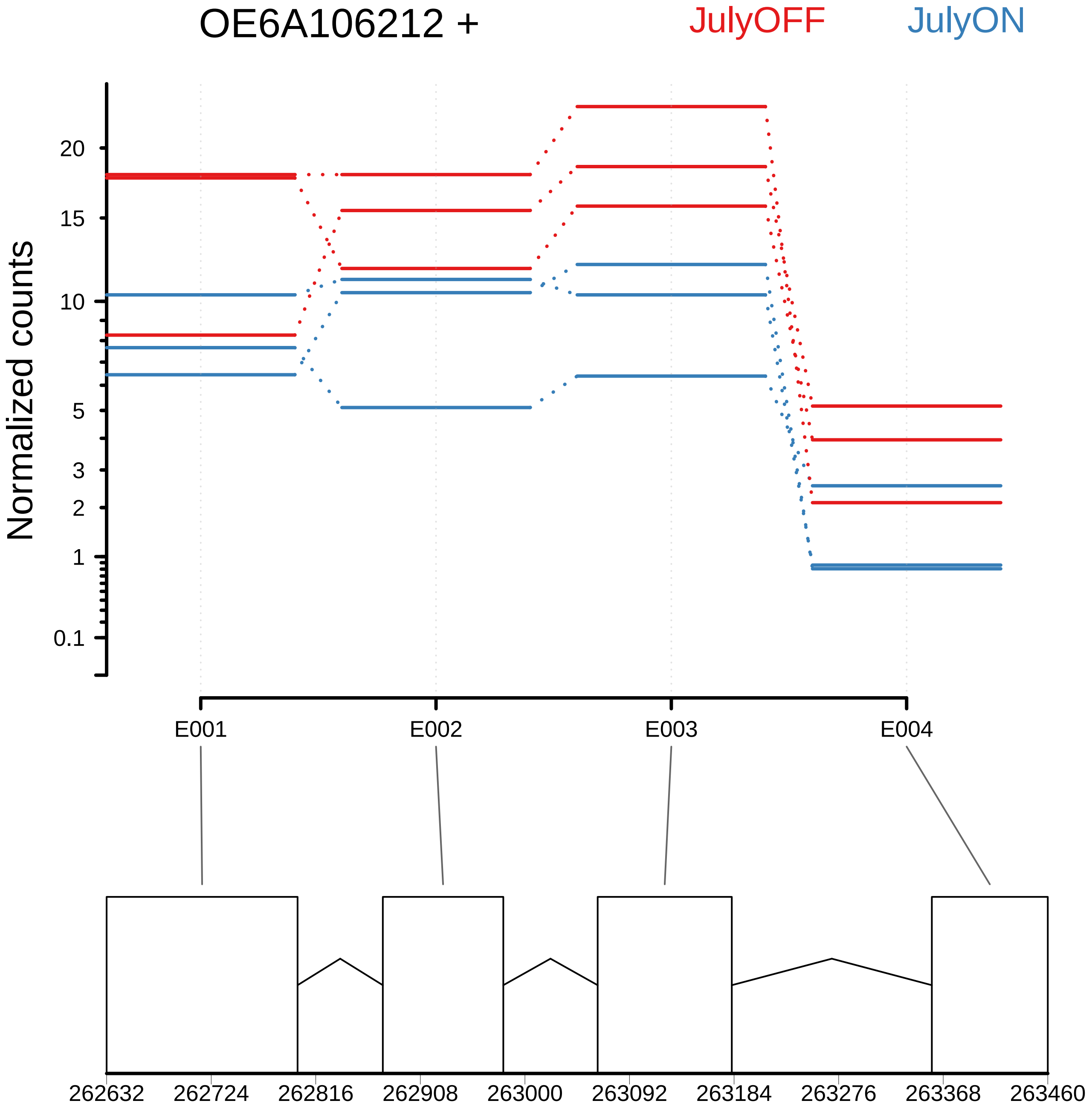
OE6A106212 +
JulyOFF
JulyON
Normalized counts
20
15
10
5
3
2
1
0.1
E001
E002
E003
E004
262632
262724
262816
262908
263000
263092
263184
263276
263368
263460

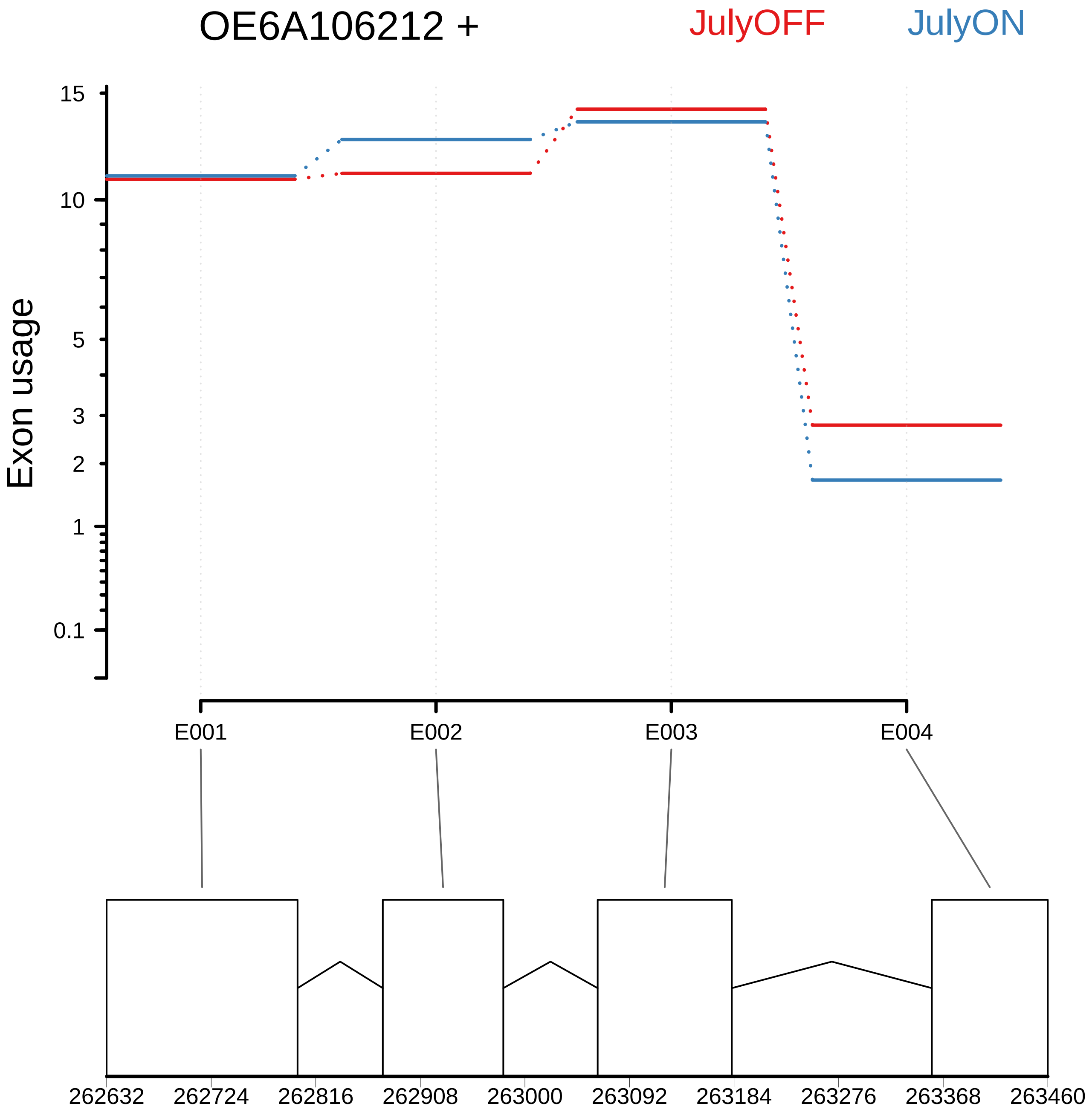

OE6A106212 +
JulyOFF
JulyON
Exon usage
15
10
5
3
2
1
0.1
E001
E002
E003
E004
262632
262724
262816
262908
263000
263092
263184
263276
263368
263460

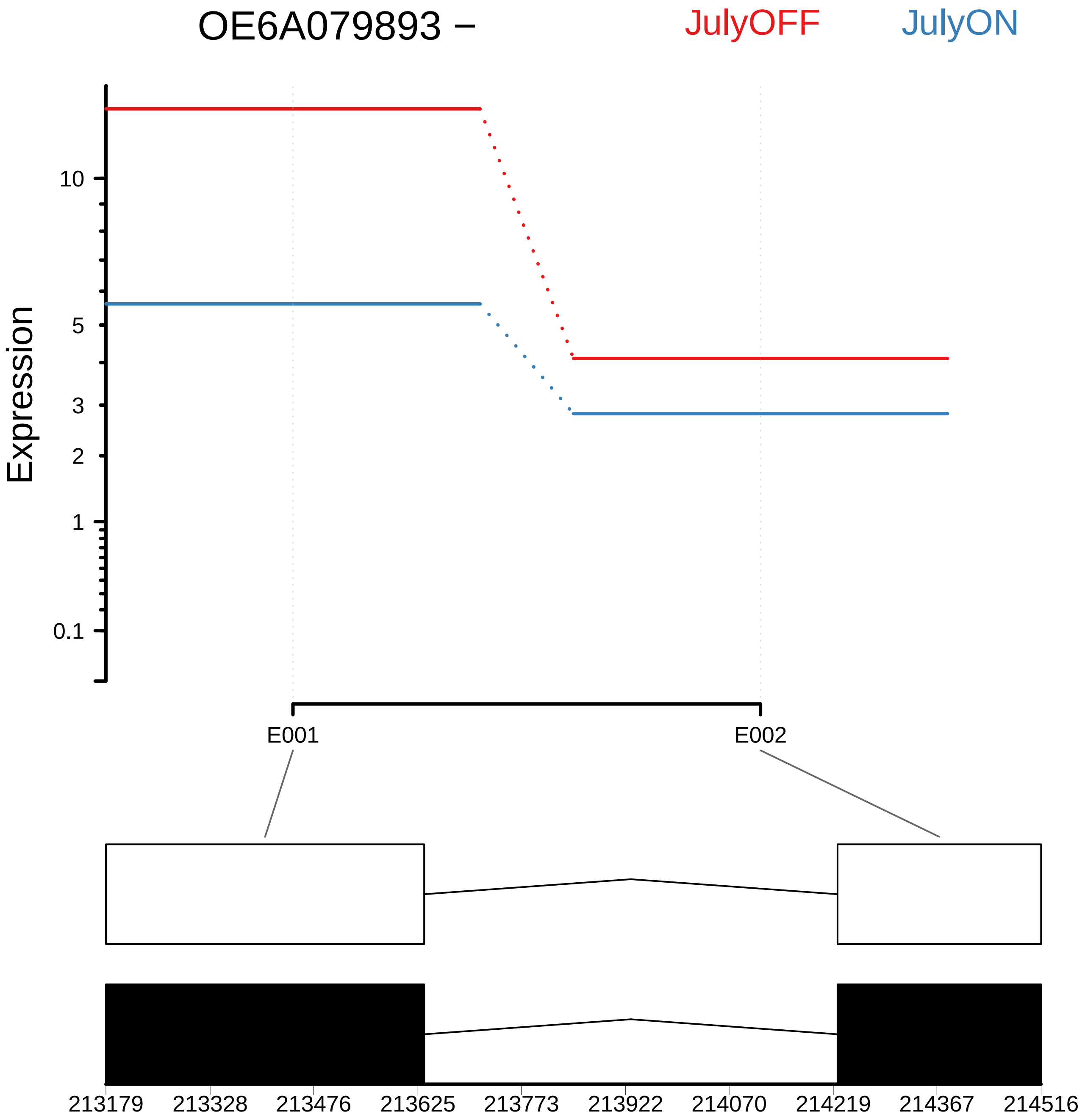

OE6A079893 −
JulyOFF
JulyON
Expression
10
5
3
2
1
0.1
E001
E002
213179
213328
213476
213625
213773
213922
214070
214219
214367
214516

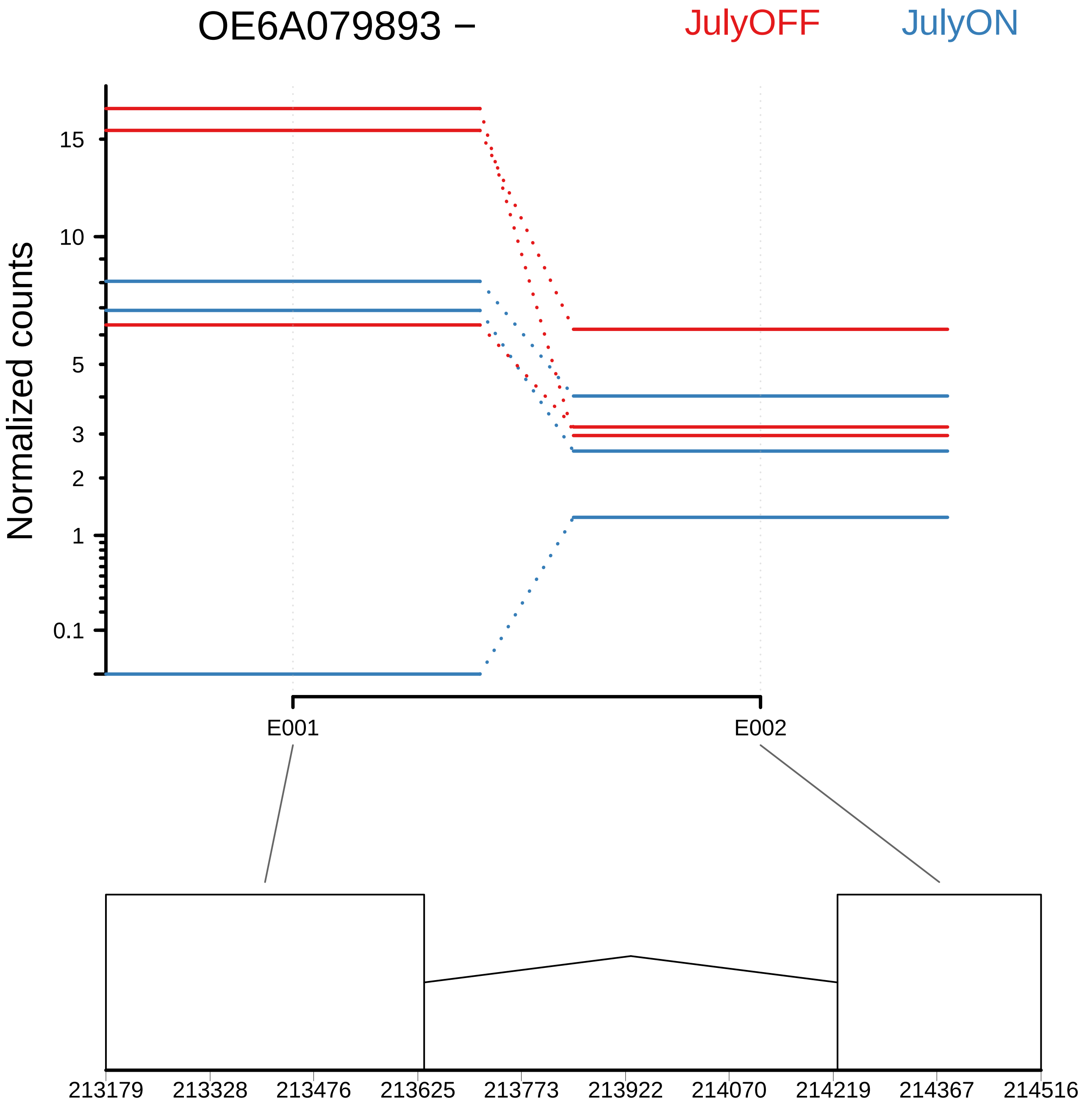

OE6A079893 −
JulyOFF
JulyON
Normalized counts
15
10
5
3
2
1
0.1
E001
E002
213179
213328
213476
213625
213773
213922
214070
214219
214367
214516

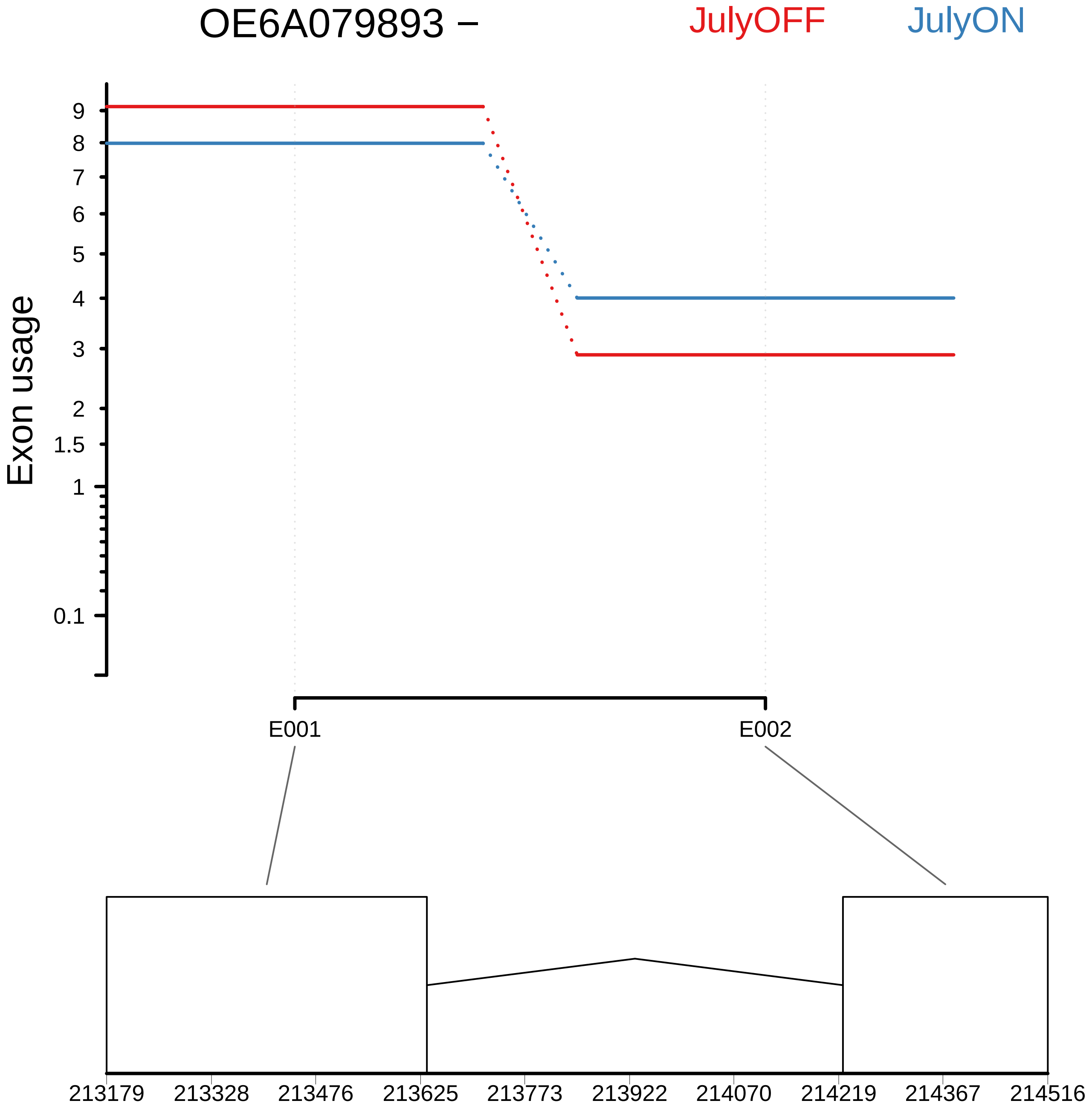

OE6A079893 −
JulyOFF
JulyON
Exon usage
9
8
7
6
5
4
3
2
1.5
1
0.1
E001
E002
213179
213328
213476
213625
213773
213922
214070
214219
214367
214516

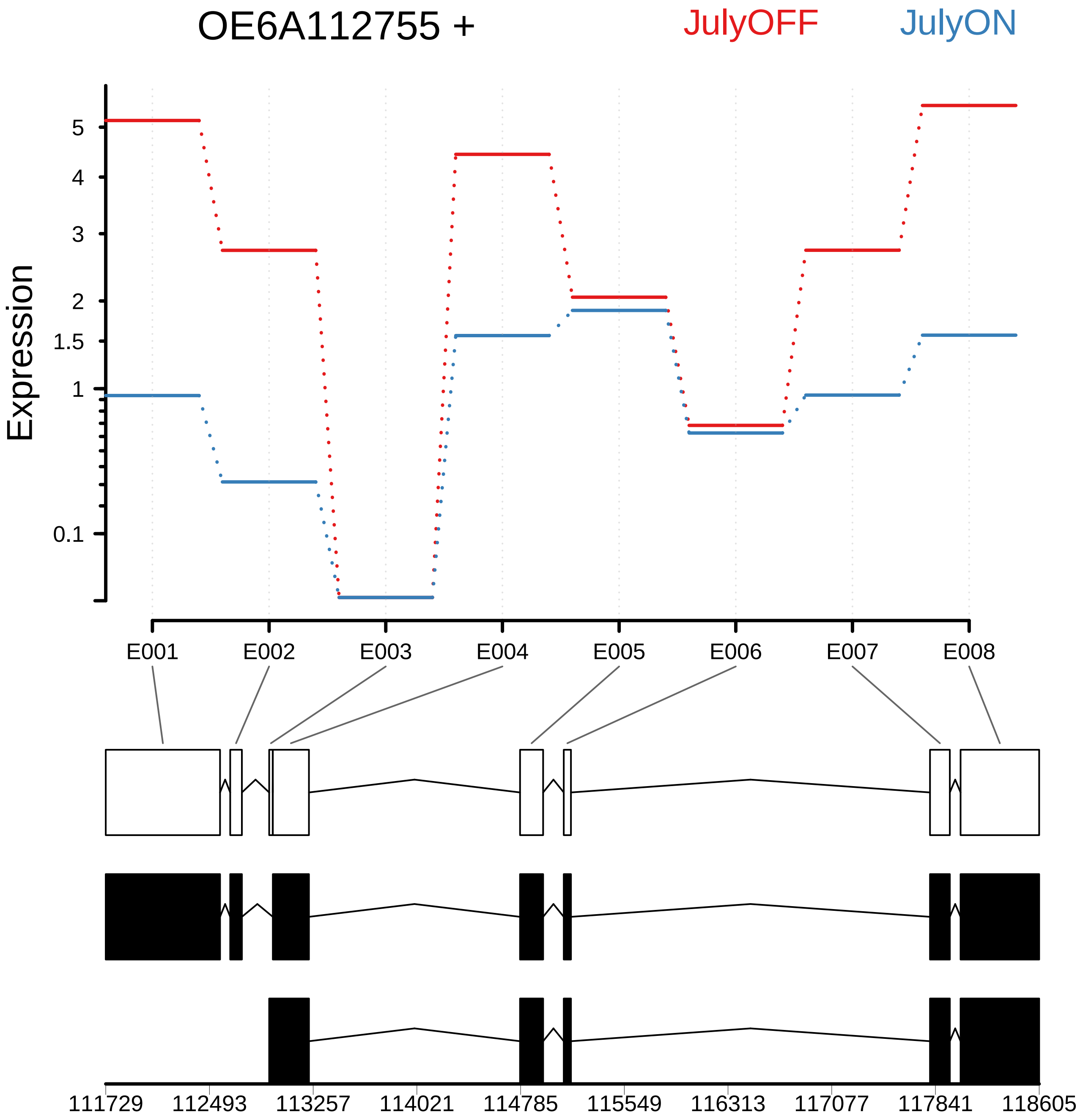

OE6A112755 +
JulyOFF
JulyON
Expression
5
4
3
2
1.5
1
0.1
E001
E002
E003
E004
E005
E006
E007
E008
111729
112493
113257
114021
114785
115549
116313
117077
117841
118605

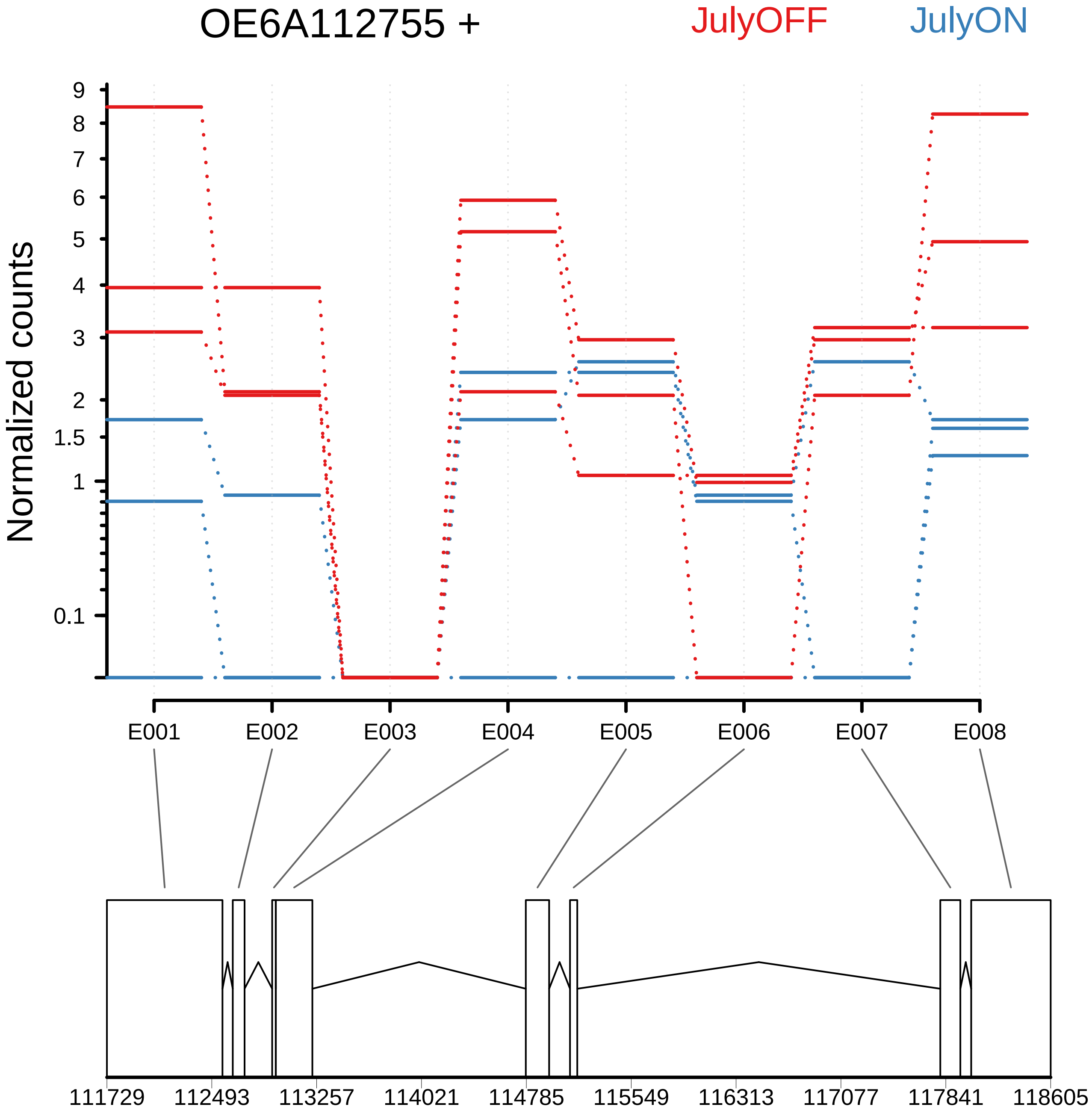
OE6A112755 +
JulyOFF
JulyON
Normalized counts
9
8
7
6
5
4
3
2
1.5
1
0.1
E001
E002
E003
E004
E005
E006
E007
E008
111729
112493
113257
114021
114785
115549
116313
117077
117841
118605

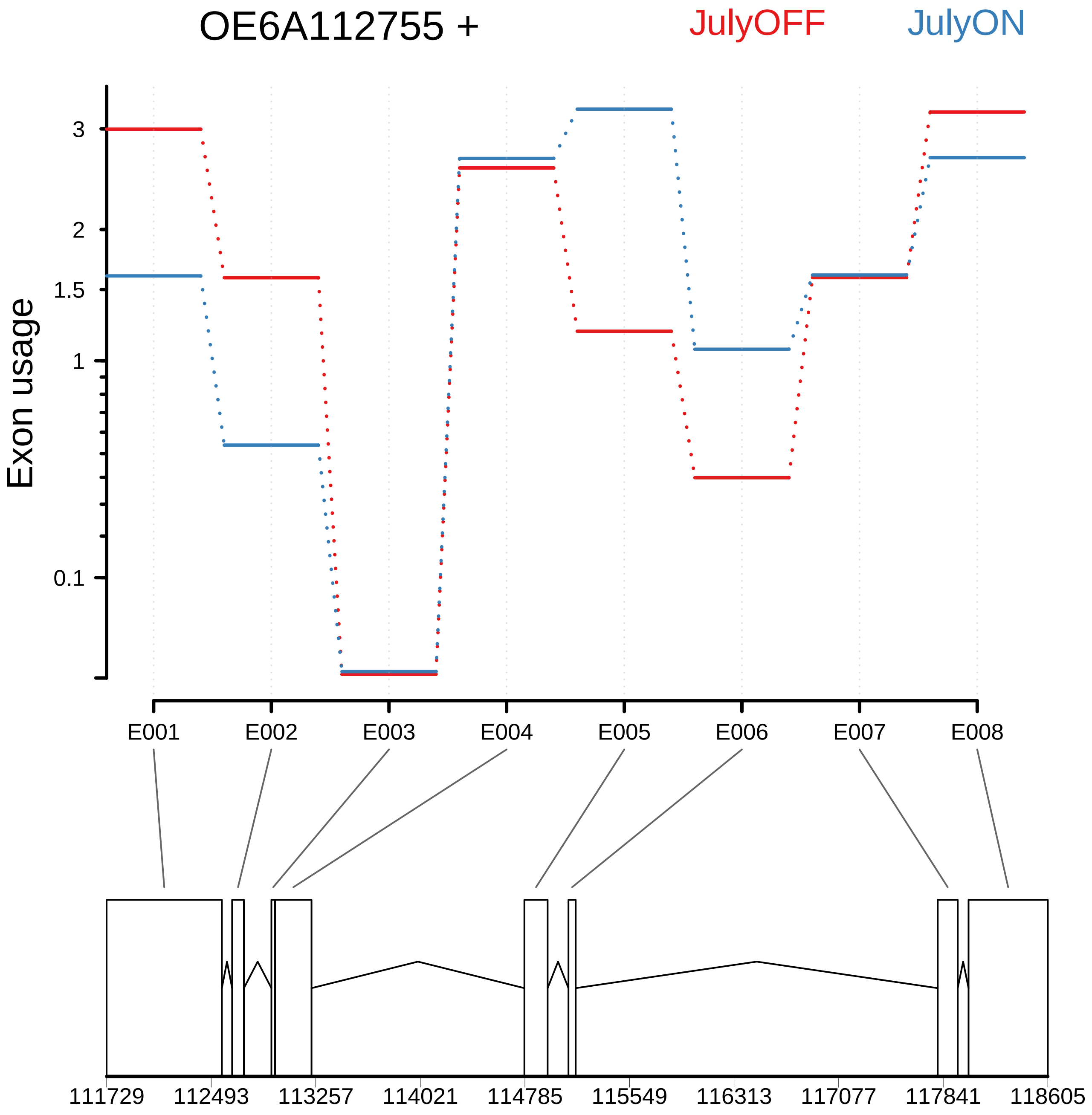

OE6A112755 +
JulyOFF
JulyON
Exon usage
3
2
1.5
1
0.1
E001
E002
E003
E004
E005
E006
E007
E008
111729
112493
113257
114021
114785
115549
116313
117077
117841
118605

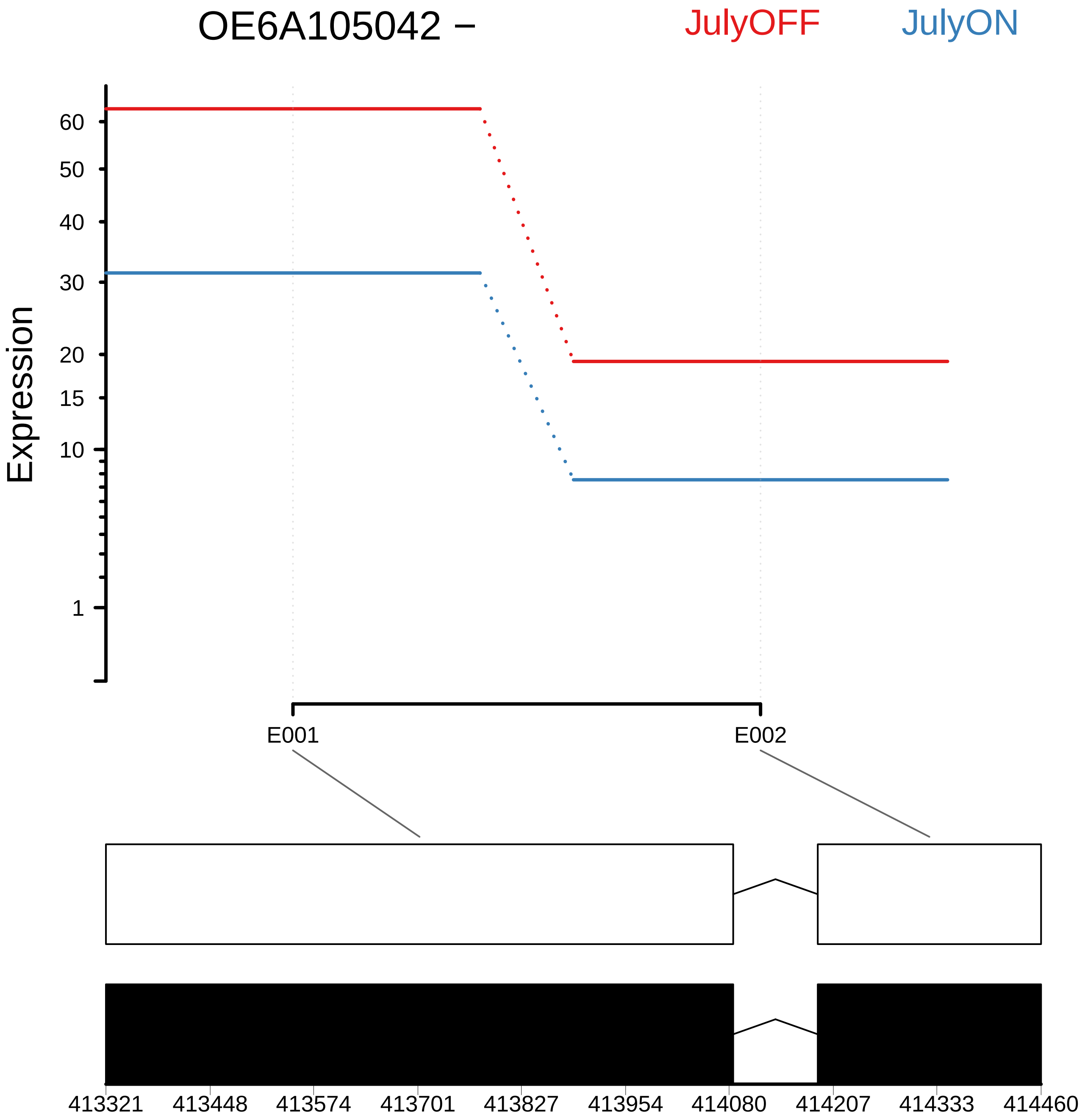

OE6A105042 −
JulyOFF
JulyON
Expression
60
50
40
30
20
15
10
1
E001
E002
413321
413448
413574
413701
413827
413954
414080
414207
414333
414460

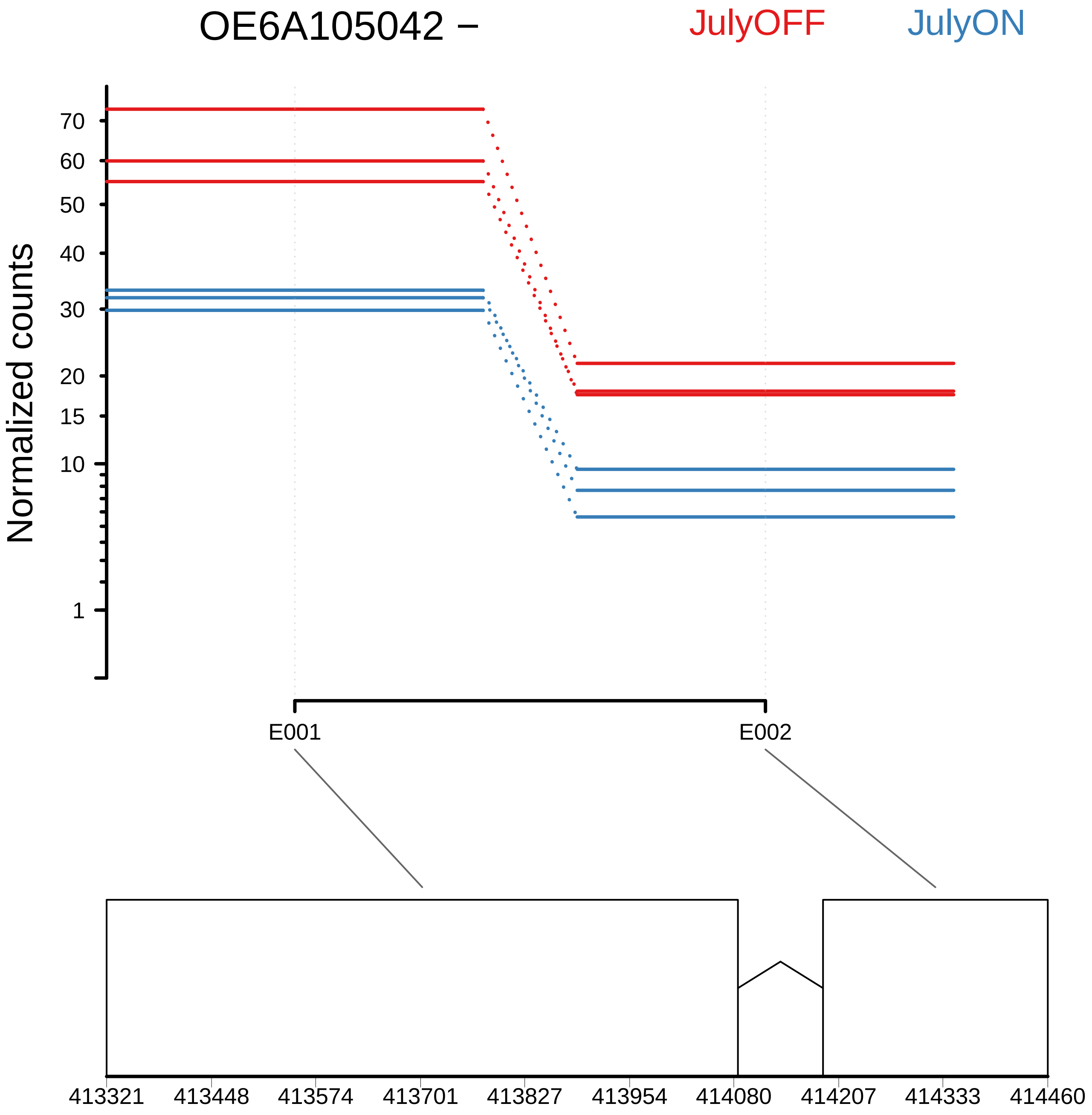

OE6A105042 −
JulyOFF
JulyON
Normalized counts
70
60
50
40
30
20
15
10
1
E001
E002
413321
413448
413574
413701
413827
413954
414080
414207
414333
414460

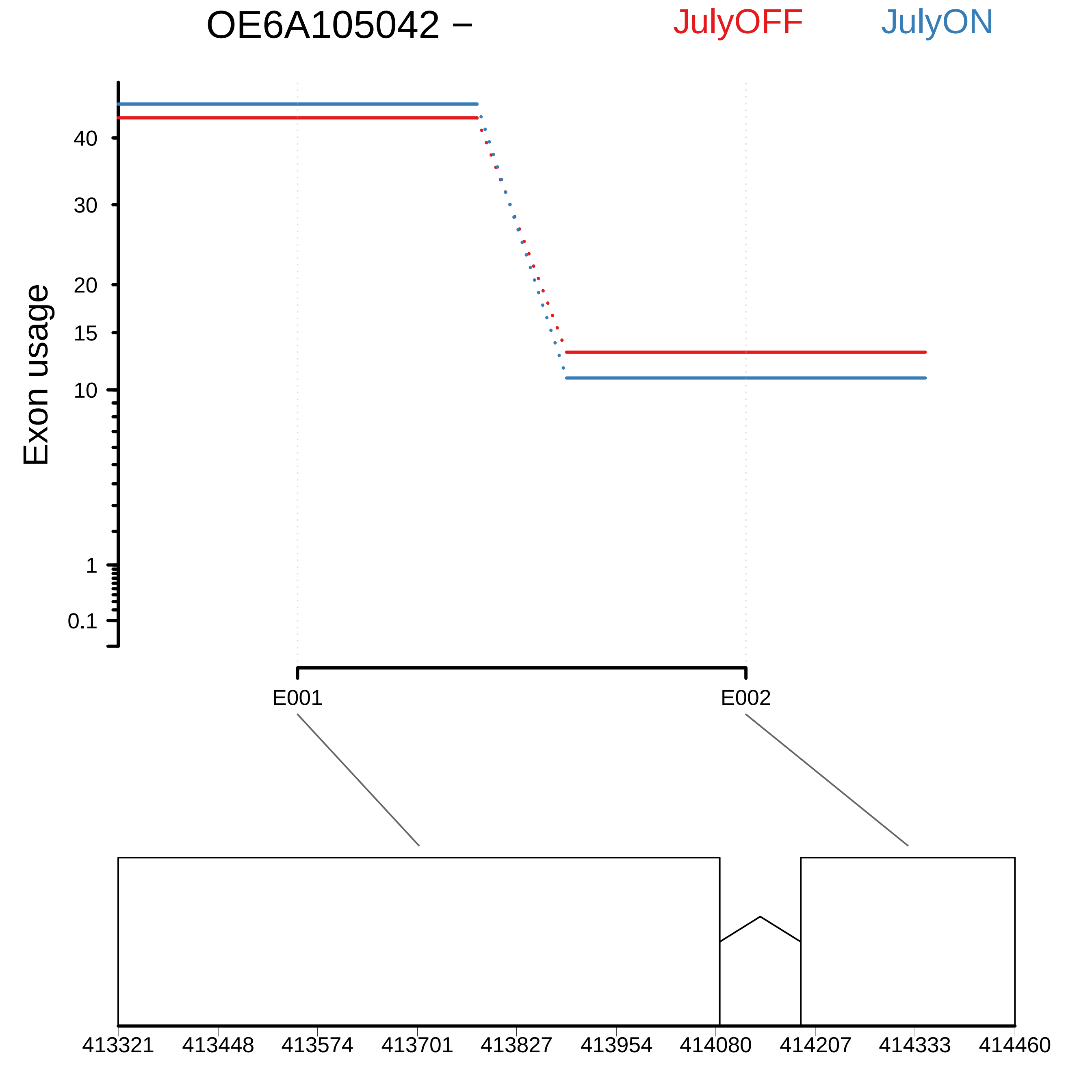

OE6A105042 −
JulyOFF
JulyON
Exon usage
40
30
20
15
10
1
0.1
E001
E002
413321
413448
413574
413701
413827
413954
414080
414207
414333
414460

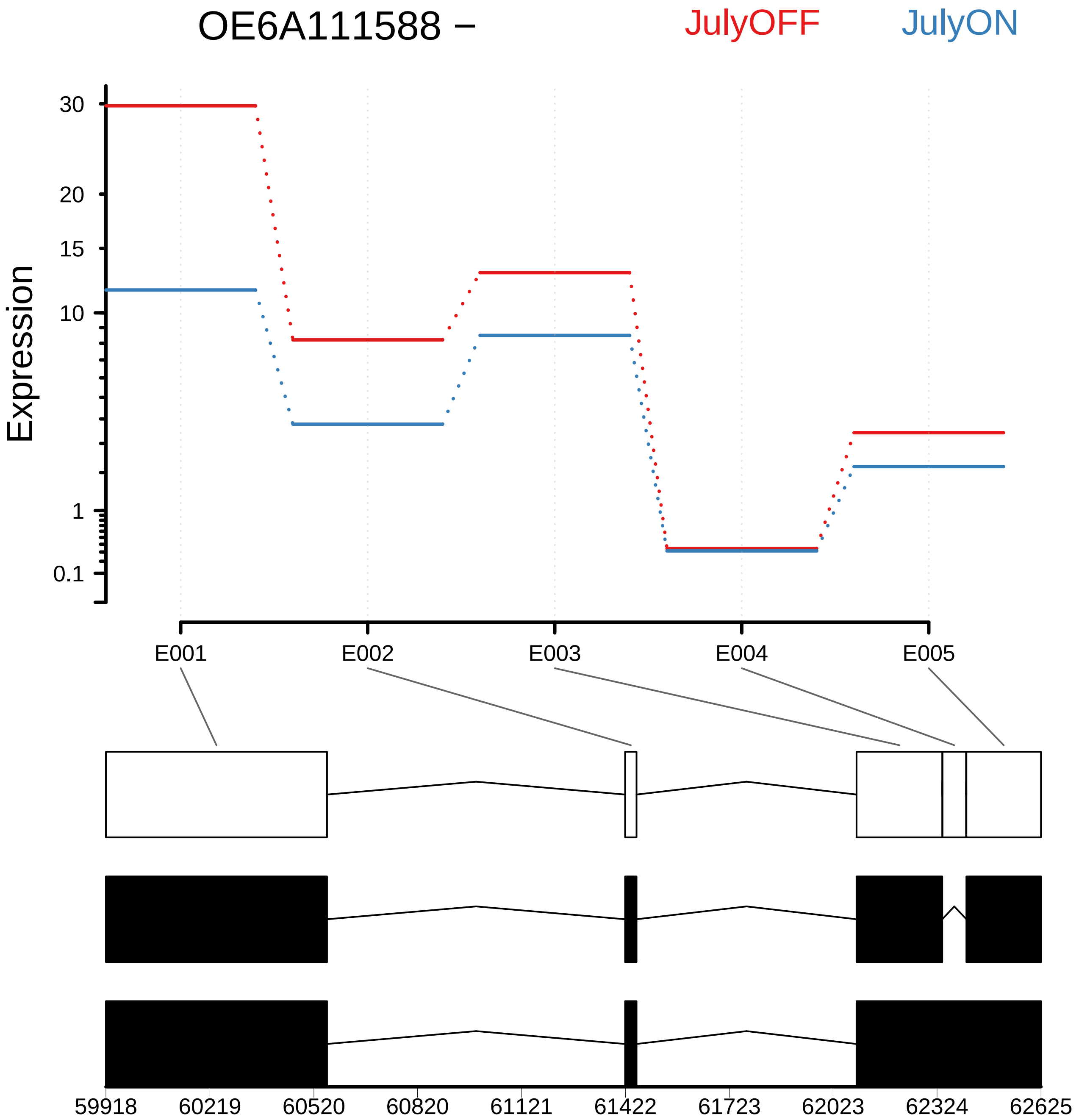
OE6A111588 –
JulyOFF
JulyON
Expression
30
20
15
10
1
0.1
E001
E002
E003
E004
E005
59918
60219
60520
60820
61121
61422
61723
62023
62324
62625

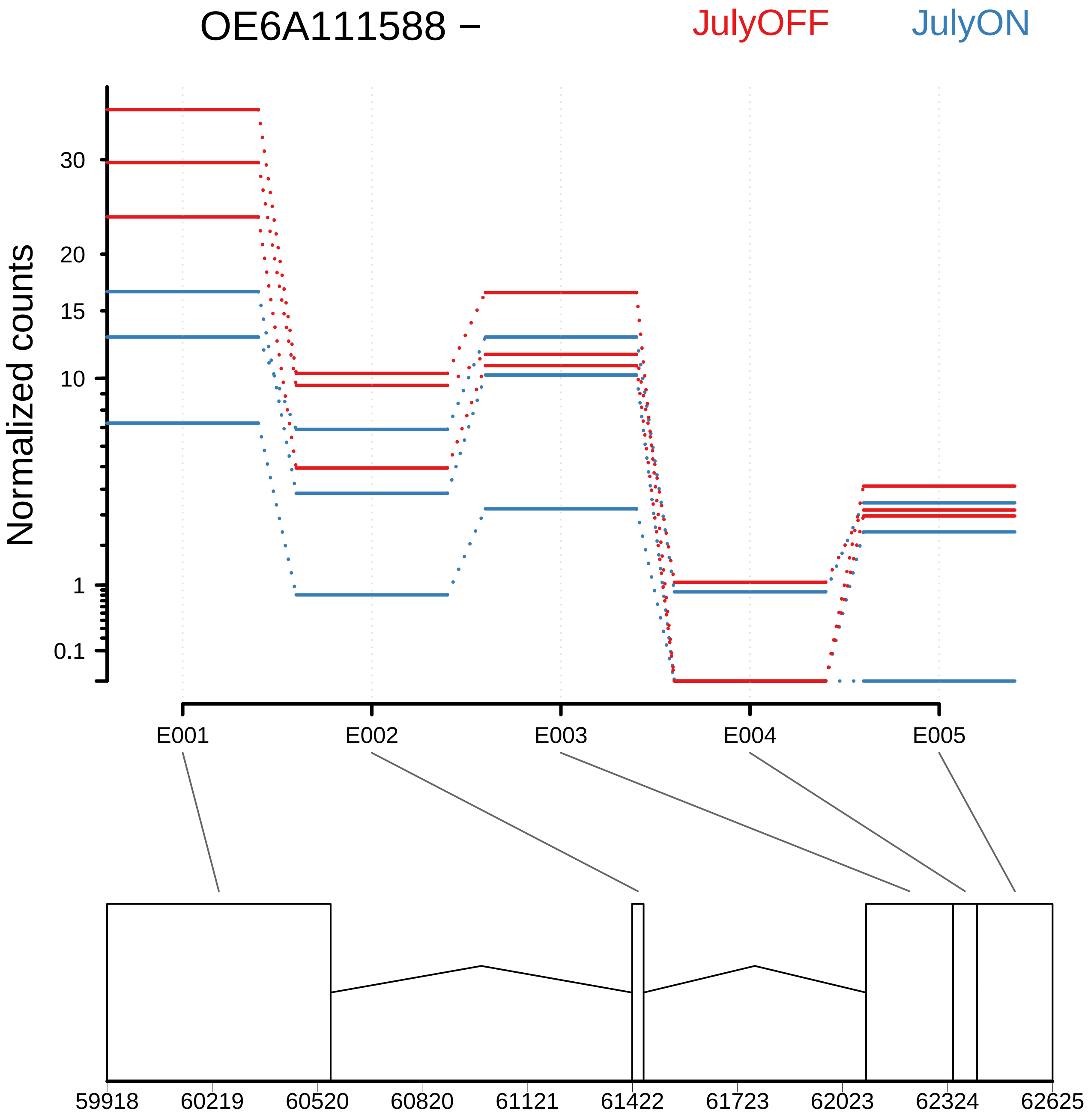
OE6A111588 –
JulyOFF
JulyON
Normalized counts
30
20
15
10
1
0.1
E001
E002
E003
E004
E005
59918
60219
60520
60820
61121
61422
61723
62023
62324
62625

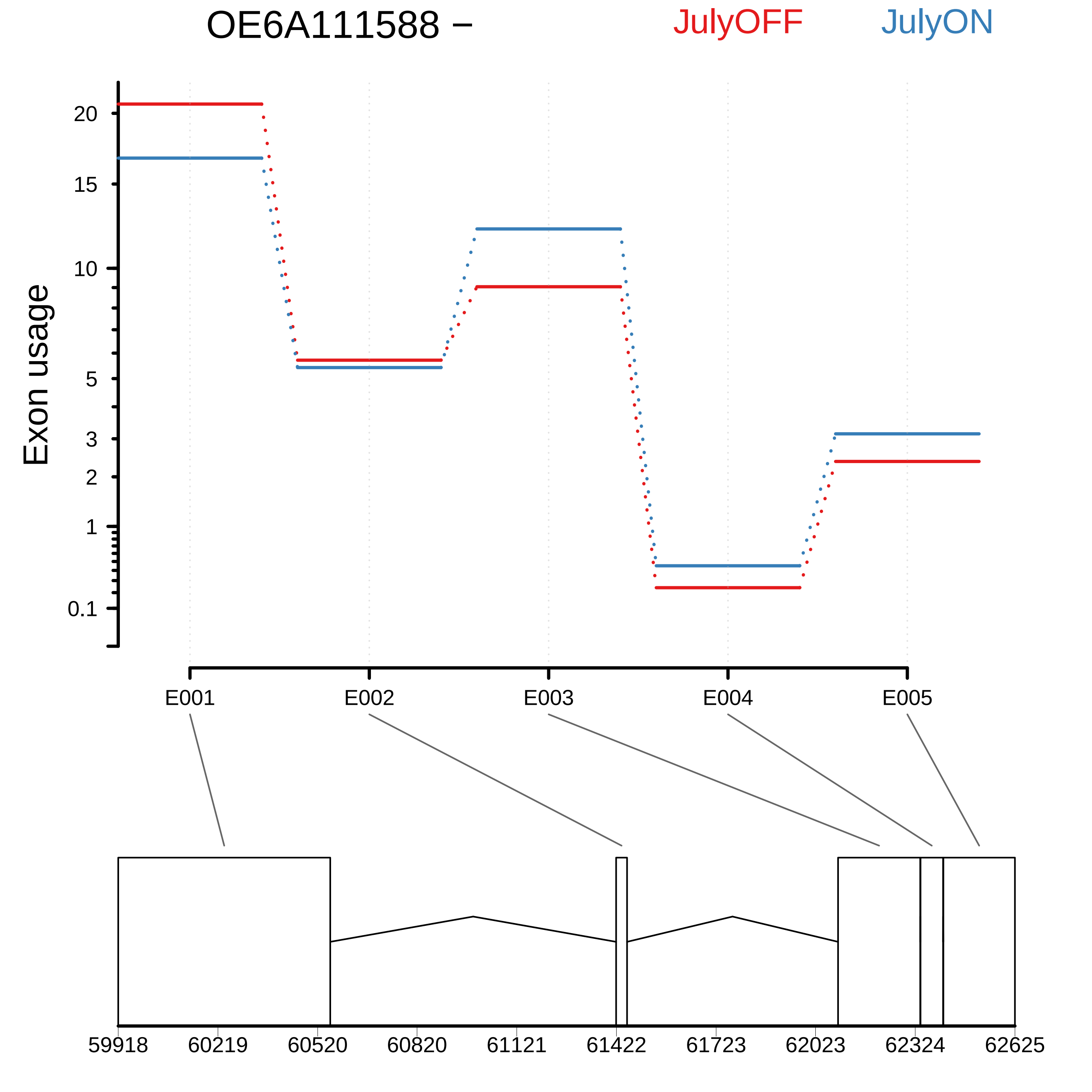
OE6A111588 –
JulyOFF
JulyON
Exon usage
20
15
10
5
3
2
1
0.1
E001
E002
E003
E004
E005
59918
60219
60520
60820
61121
61422
61723
62023
62324
62625

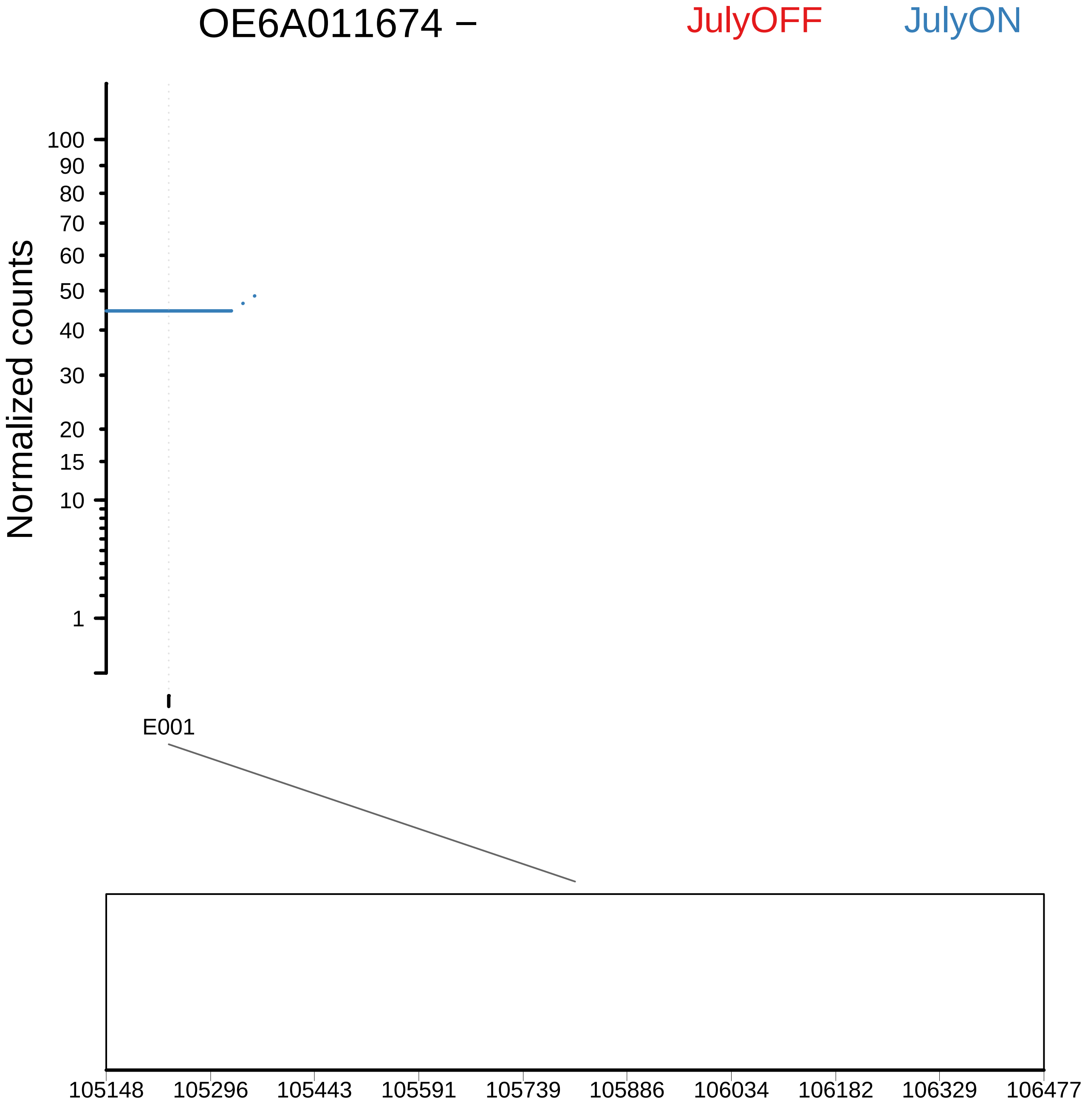

OE6A011674 −
JulyOFF
JulyON
Normalized counts
100
90
80
70
60
50
40
30
20
15
10
1
E001
105148
105296
105443
105591
105739
105886
106034
106182
106329
106477

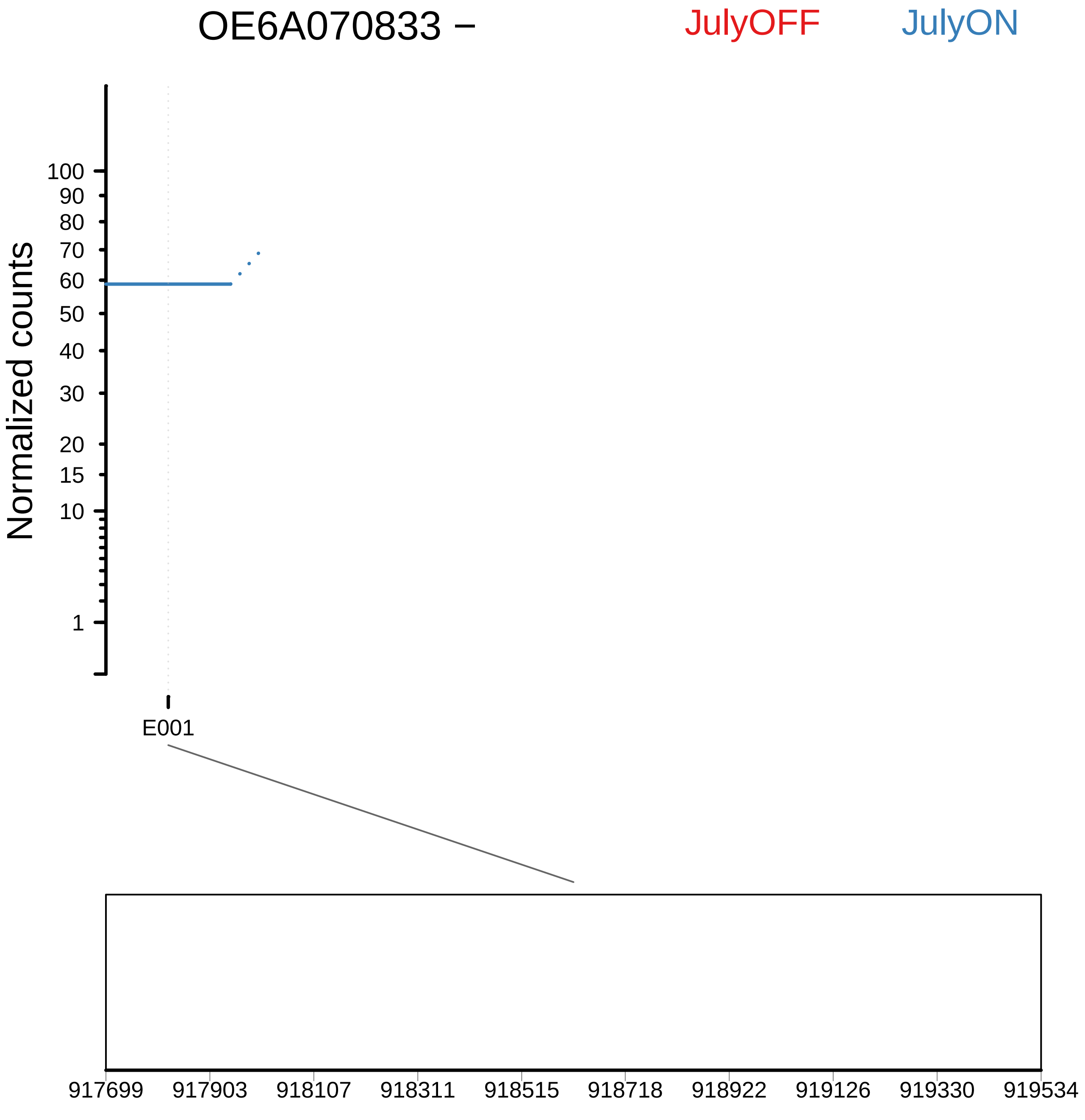

OE6A070833 −
JulyOFF
JulyON
Normalized counts
100
90
80
70
60
50
40
30
20
15
10
1
E001
917699
917903
918107
918311
918515
918718
918922
919126
919330
919534

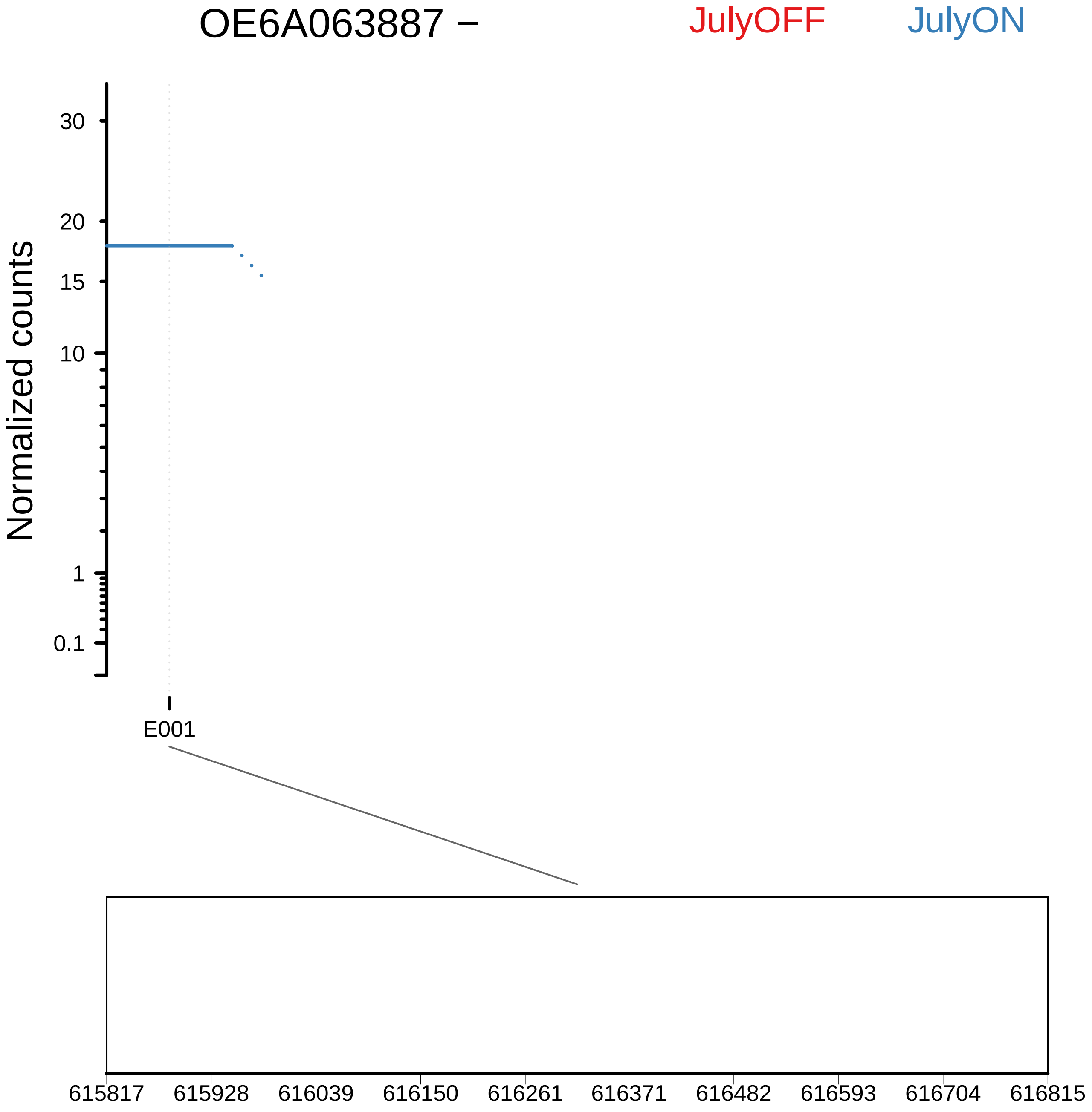
OE6A063887 −
JulyOFF
JulyON
Normalized counts
30
20
15
10
1
0.1
E001
615817
615928
616039
616150
616261
616371
616482
616593
616704
616815

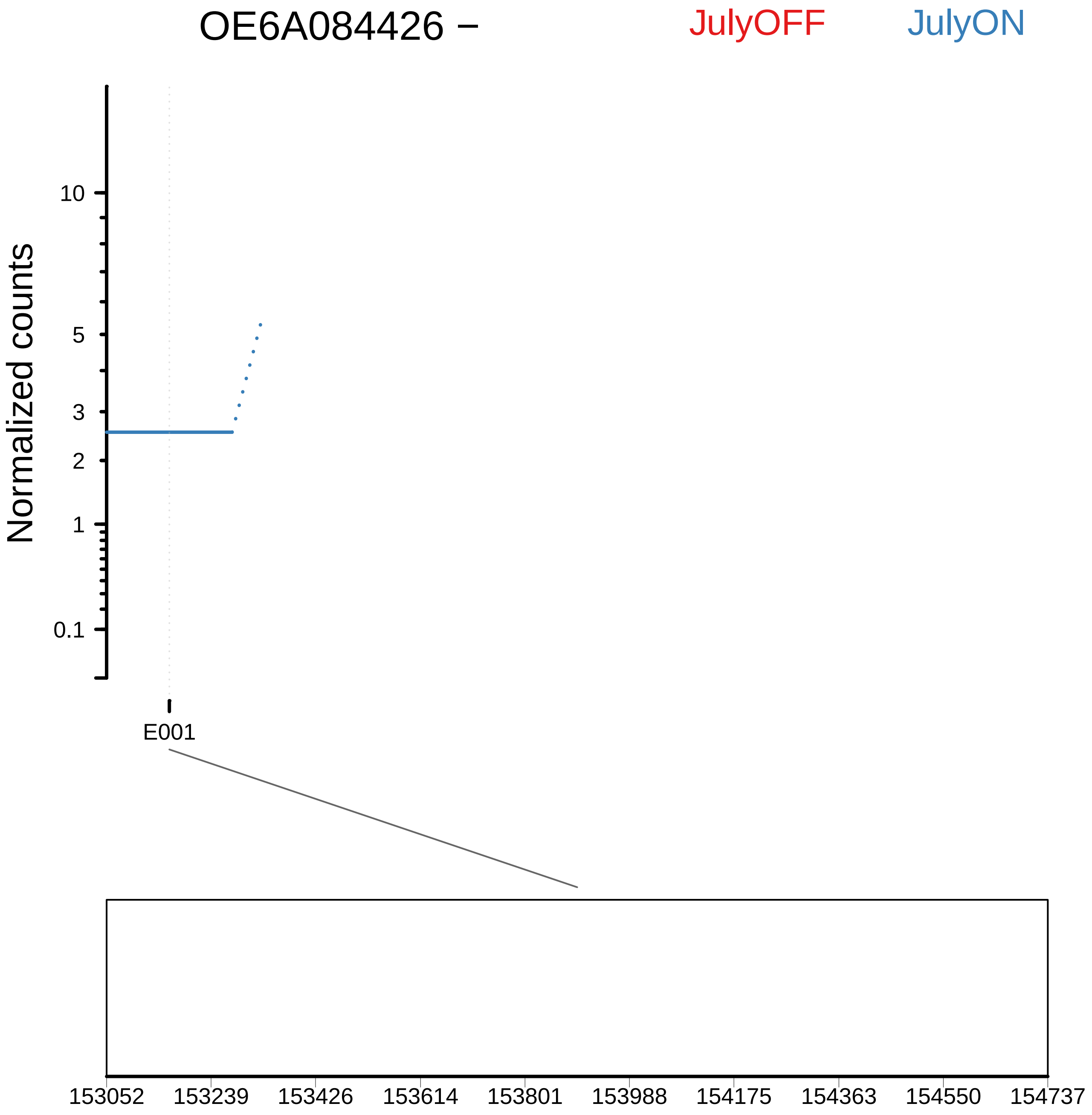

OE6A084426 −
JulyOFF
JulyON
Normalized counts
10
5
3
2
1
0.1
E001
153052
153239
153426
153614
153801
153988
154175
154363
154550
154737

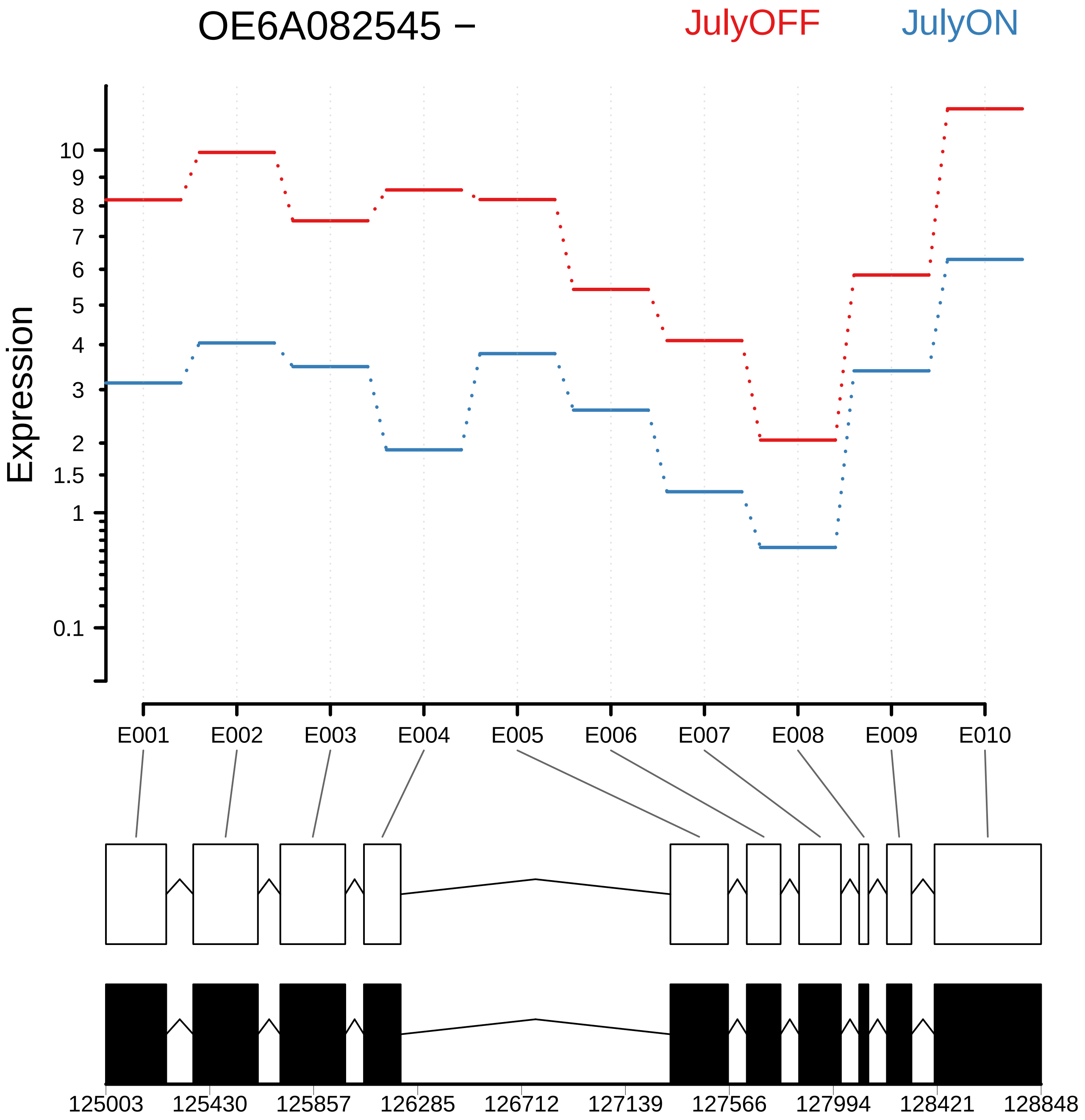

OE6A082545 –
JulyOFF
JulyON
Expression
10
9
8
7
6
5
4
3
2
1.5
1
0.1
E001
E002
E003
E004
E005
E006
E007
E008
E009
E010
125003
125430
125857
126285
126712
127139
127566
127994
128421
128848

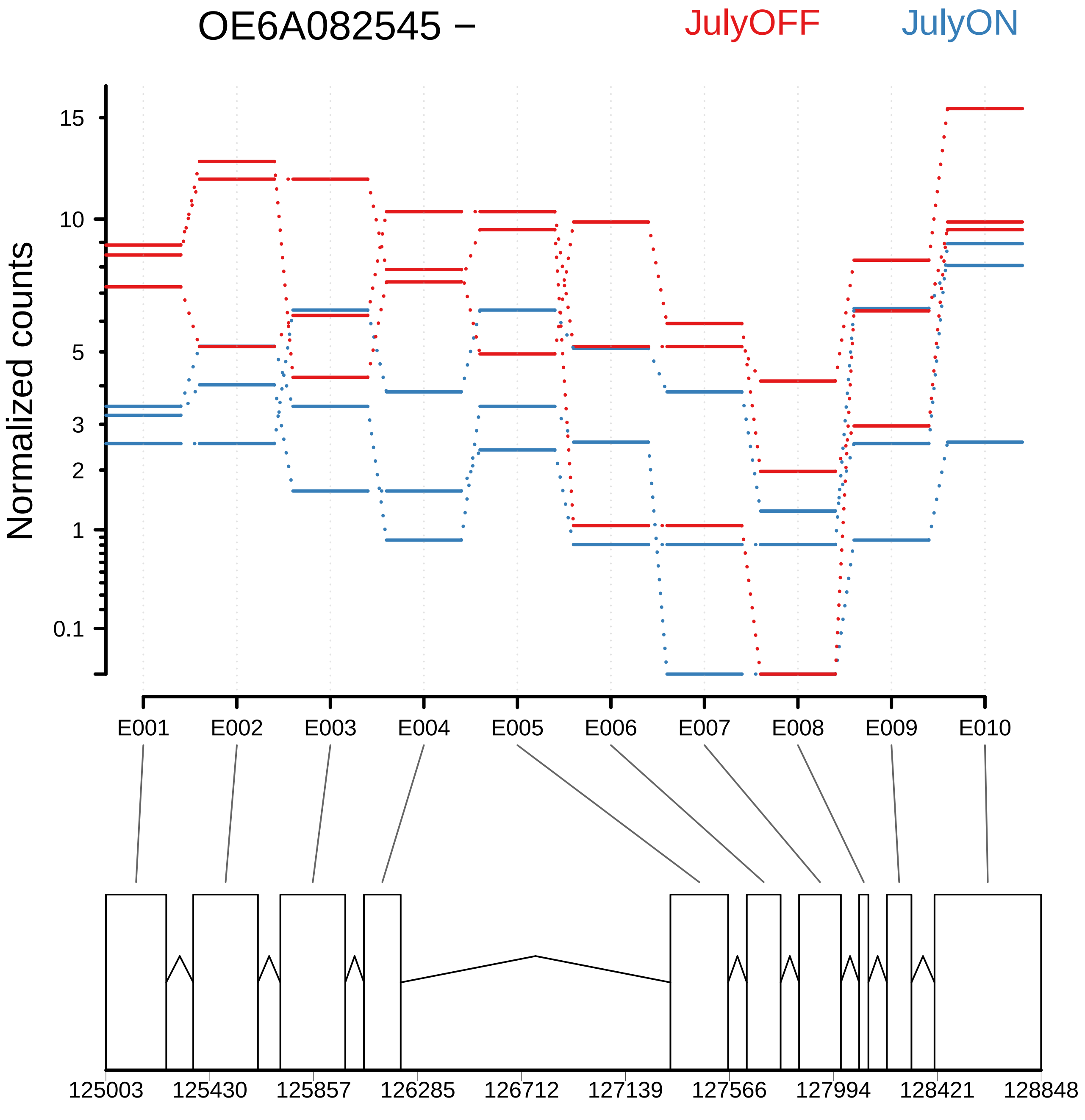
OE6A082545 −
JulyOFF
JulyON
Normalized counts
15
10
5
3
2
1
0.1
E001
E002
E003
E004
E005
E006
E007
E008
E009
E010
125003
125430
125857
126285
126712
127139
127566
127994
128421
128848

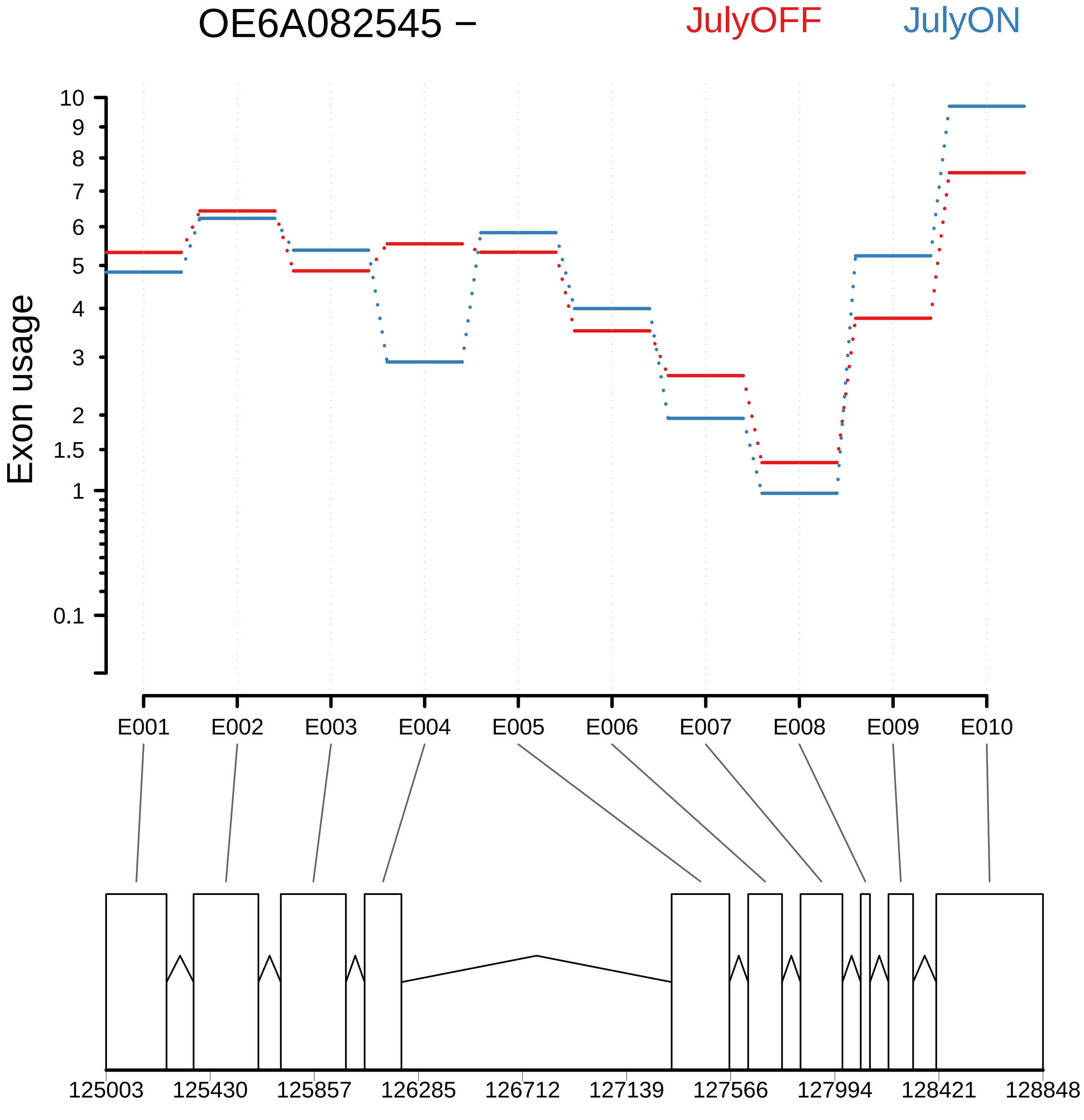
OE6A082545 –
JulyOFF
JulyON
Exon usage
10
9
8
7
6
5
4
3
2
1.5
1
0.1
E001
E002
E003
E004
E005
E006
E007
E008
E009
E010
125003
125430
125857
126285
126712
127139
127566
127994
128421
128848

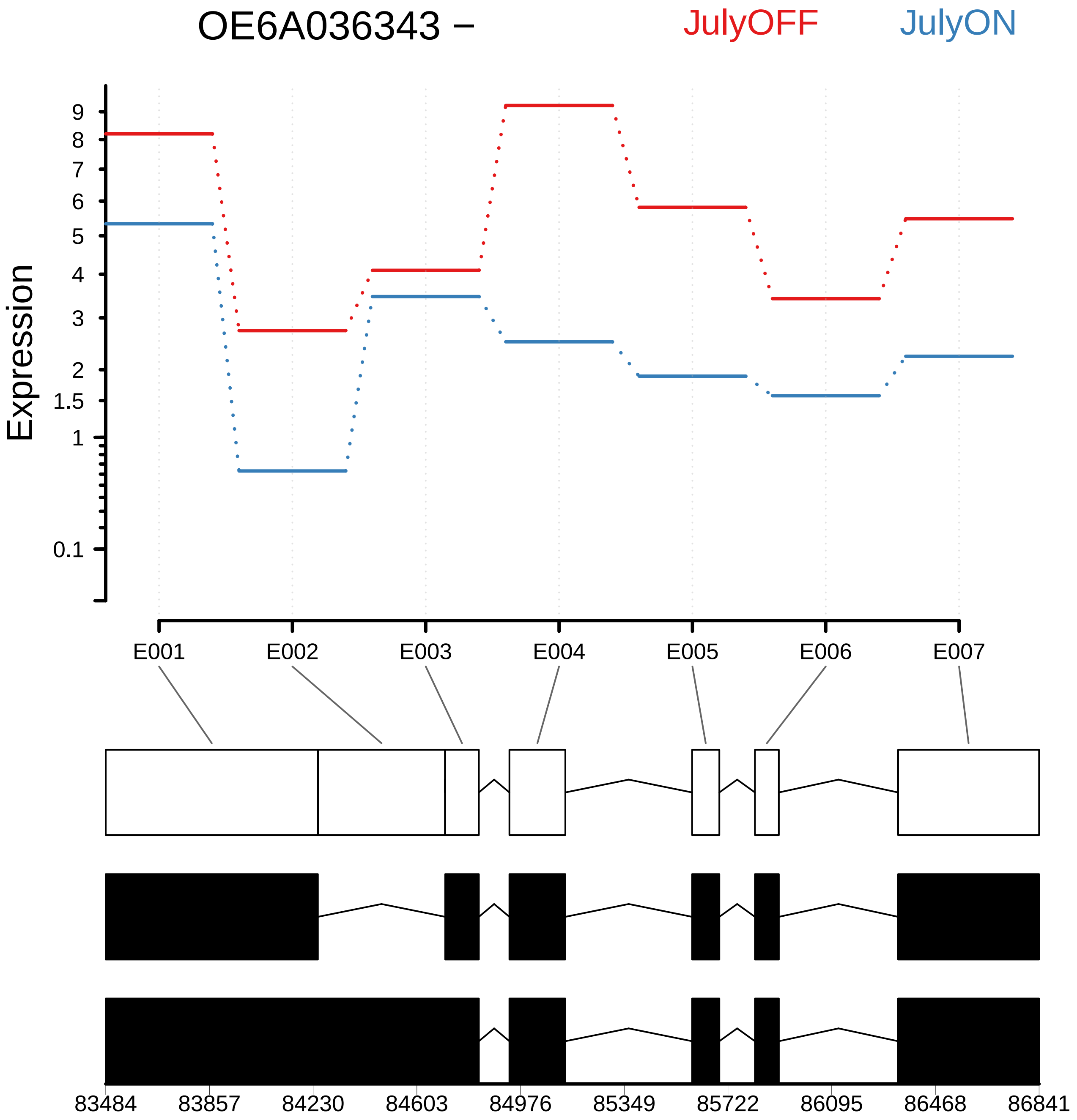

OE6A036343 −
JulyOFF
JulyON
Expression
9
8
7
6
5
4
3
2
1.5
1
0.1
E001
E002
E003
E004
E005
E006
E007
83484
83857
84230
84603
84976
85349
85722
86095
86468
86841

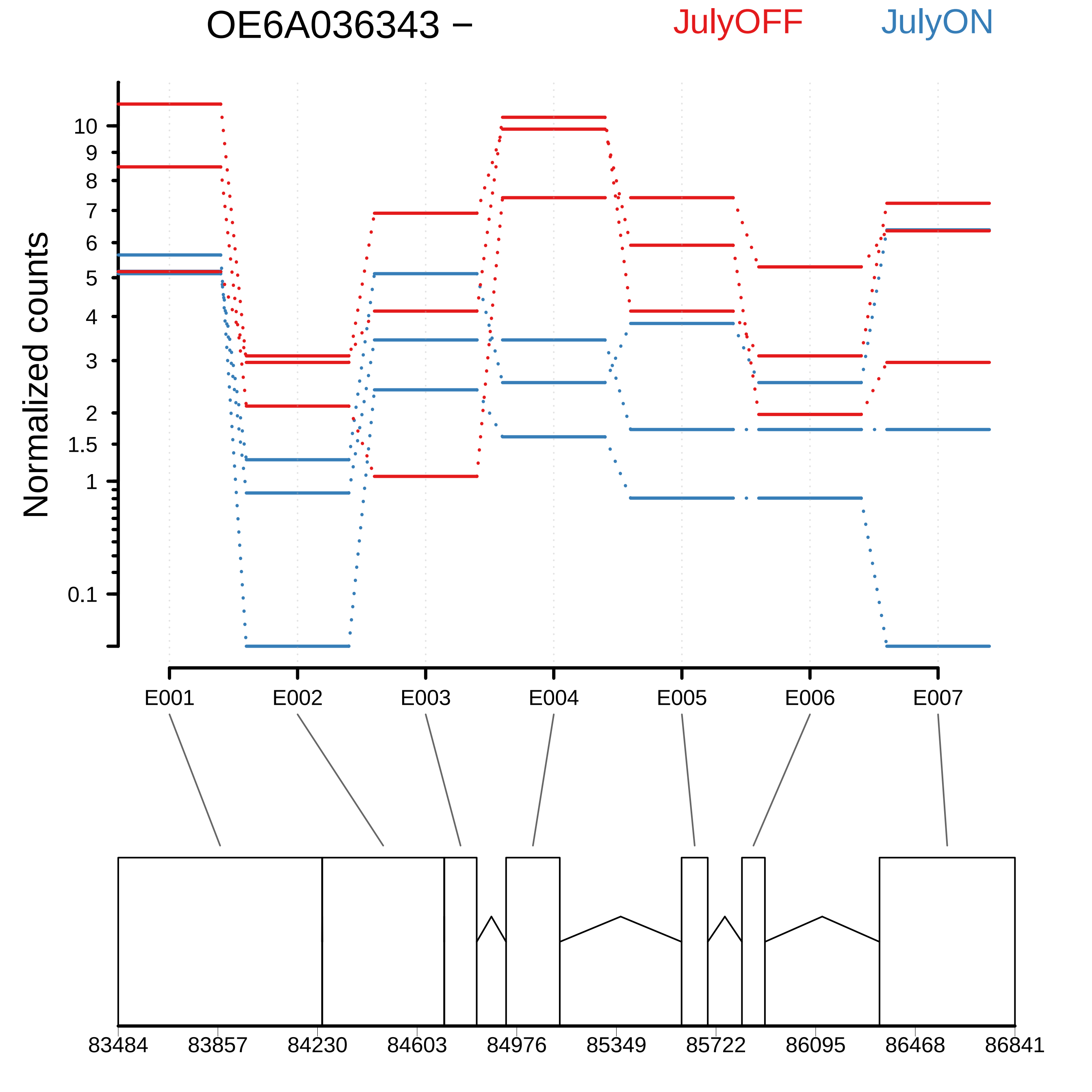

OE6A036343 −
JulyOFF
JulyON
Normalized counts
10
9
8
7
6
5
4
3
2
1.5
1
0.1
E001
E002
E003
E004
E005
E006
E007
83484
83857
84230
84603
84976
85349
85722
86095
86468
86841

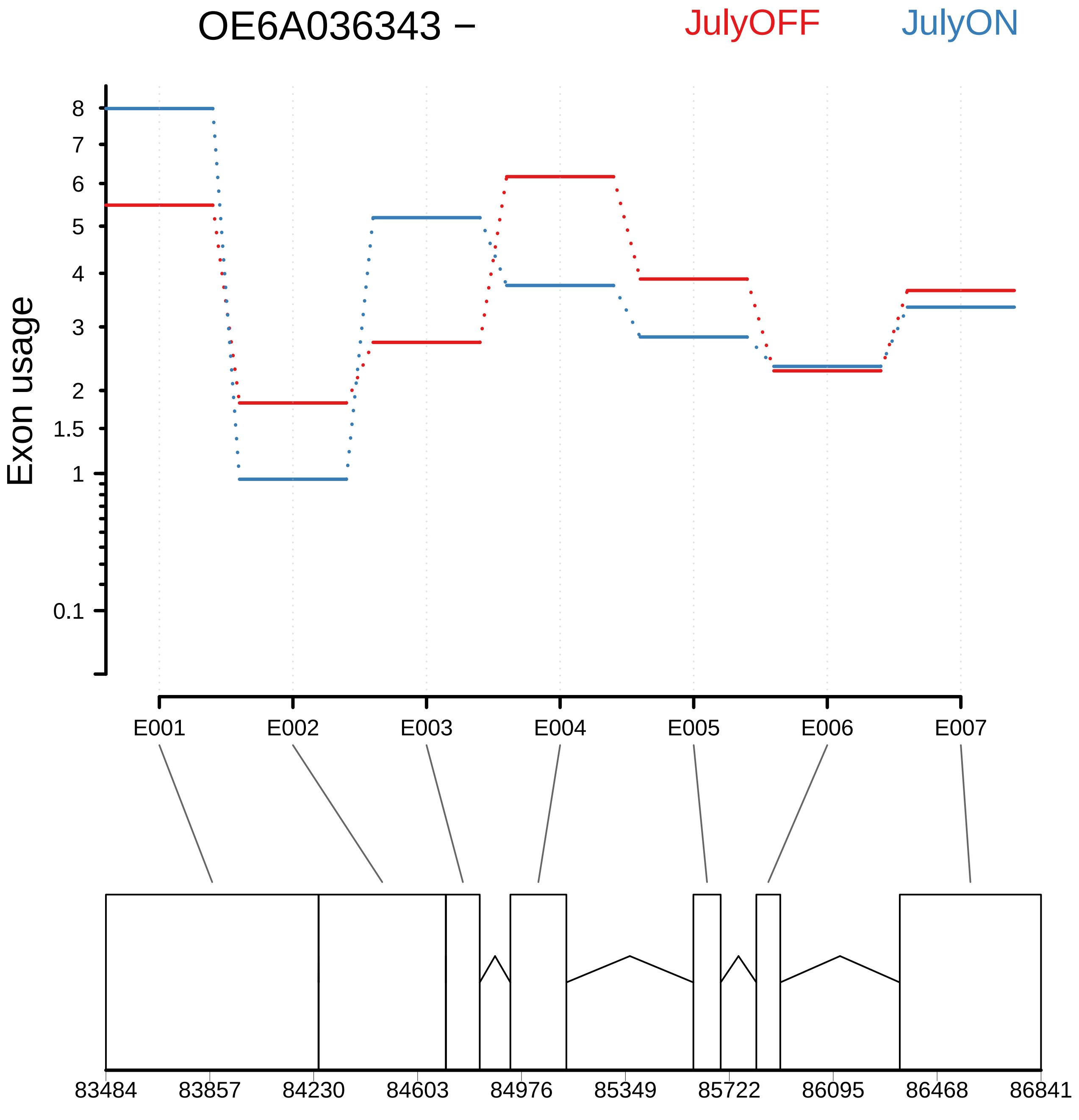
OE6A036343 −
JulyOFF
JulyON
Exon usage
8
7
6
5
4
3
2
1.5
1
0.1
E001
E002
E003
E004
E005
E006
E007
83484
83857
84230
84603
84976
85349
85722
86095
86468
86841

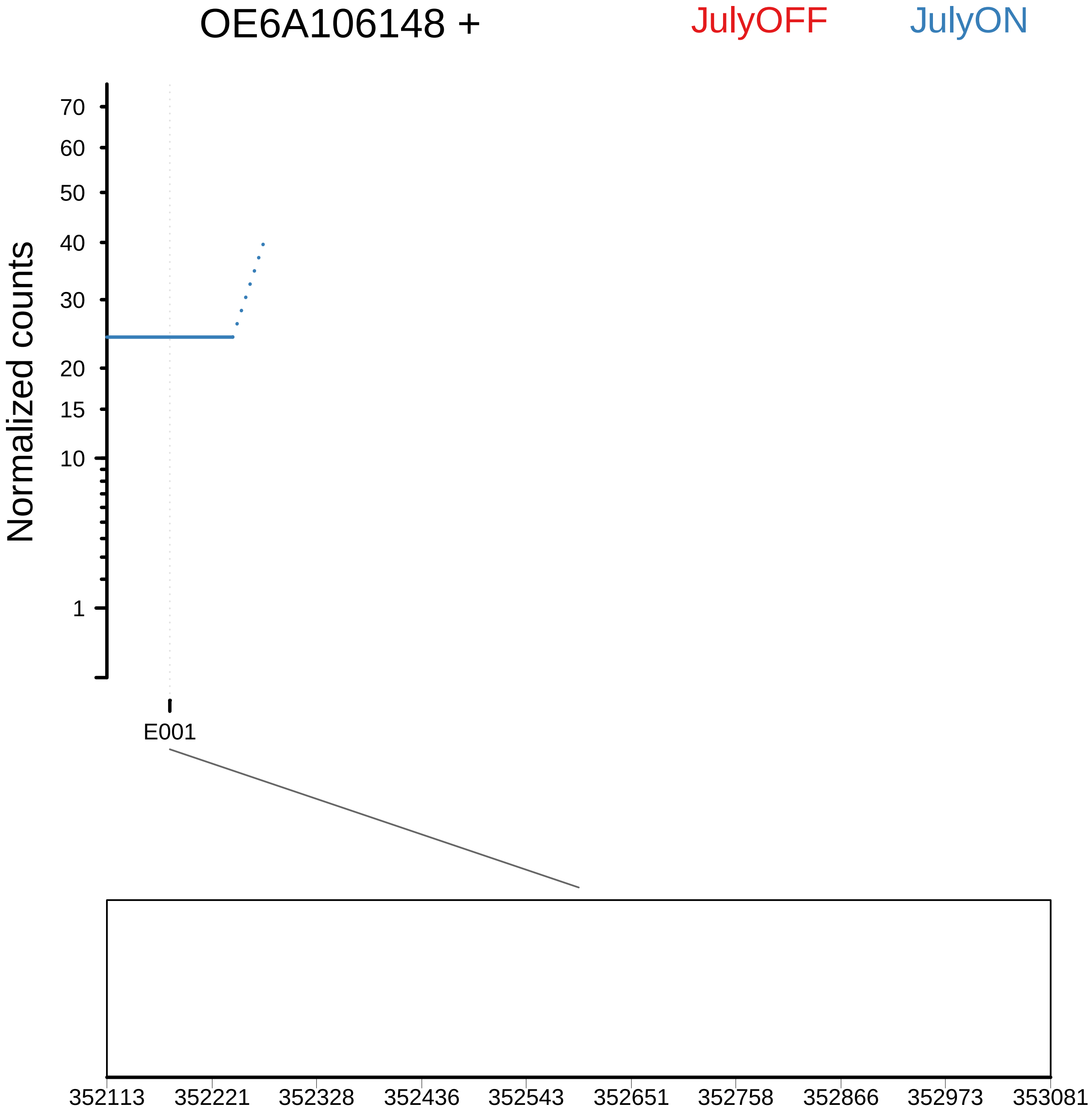
OE6A106148 +
JulyOFF
JulyON
Normalized counts
70
60
50
40
30
20
15
10
1
E001
352113
352221
352328
352436
352543
352651
352758
352866
352973
353081

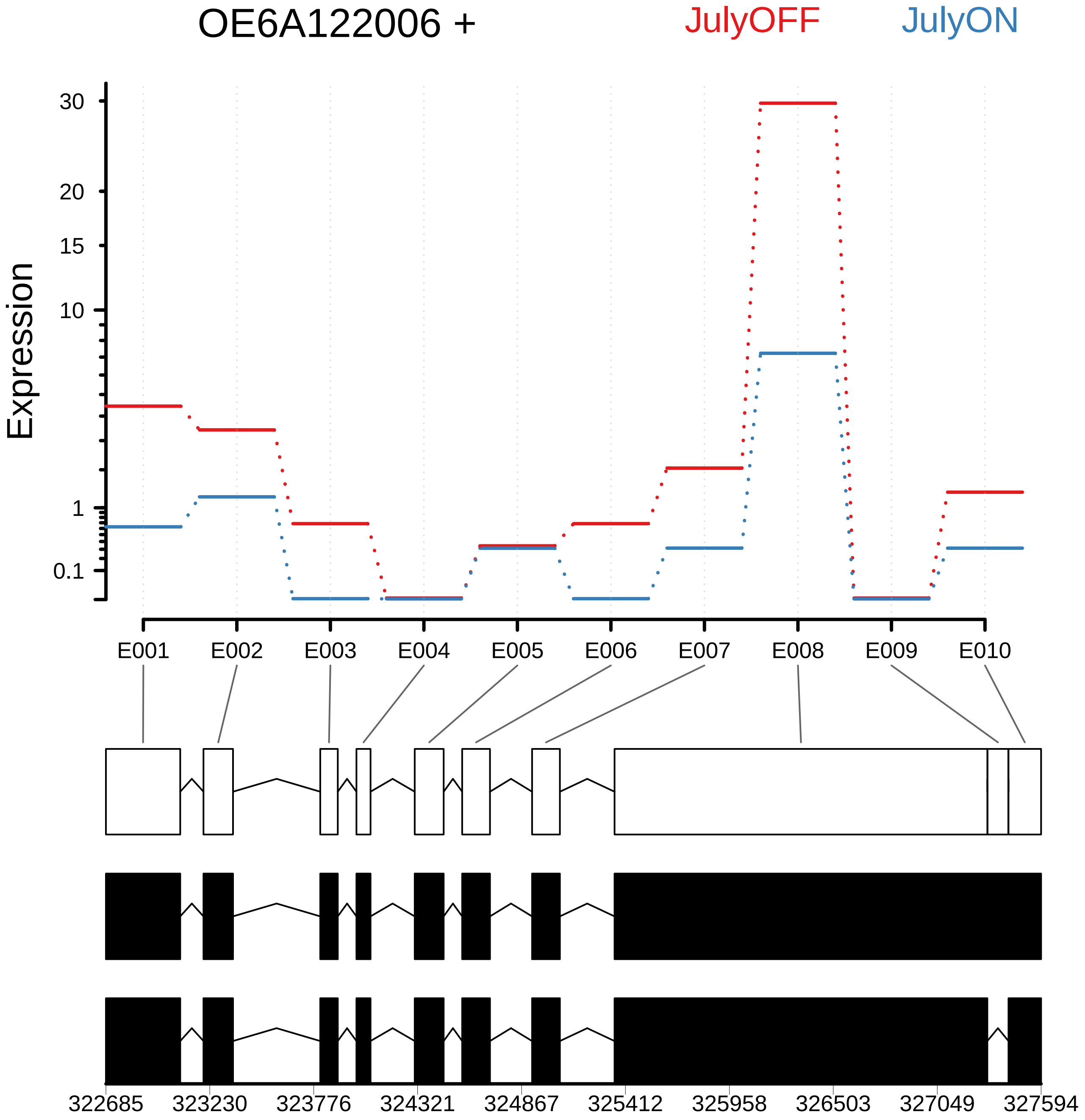
OE6A122006 +
JulyOFF
JulyON
Expression
30
20
15
10
1
0.1
E001
E002
E003
E004
E005
E006
E007
E008
E009
E010
322685
323230
323776
324321
324867
325412
325958
326503
327049
327594

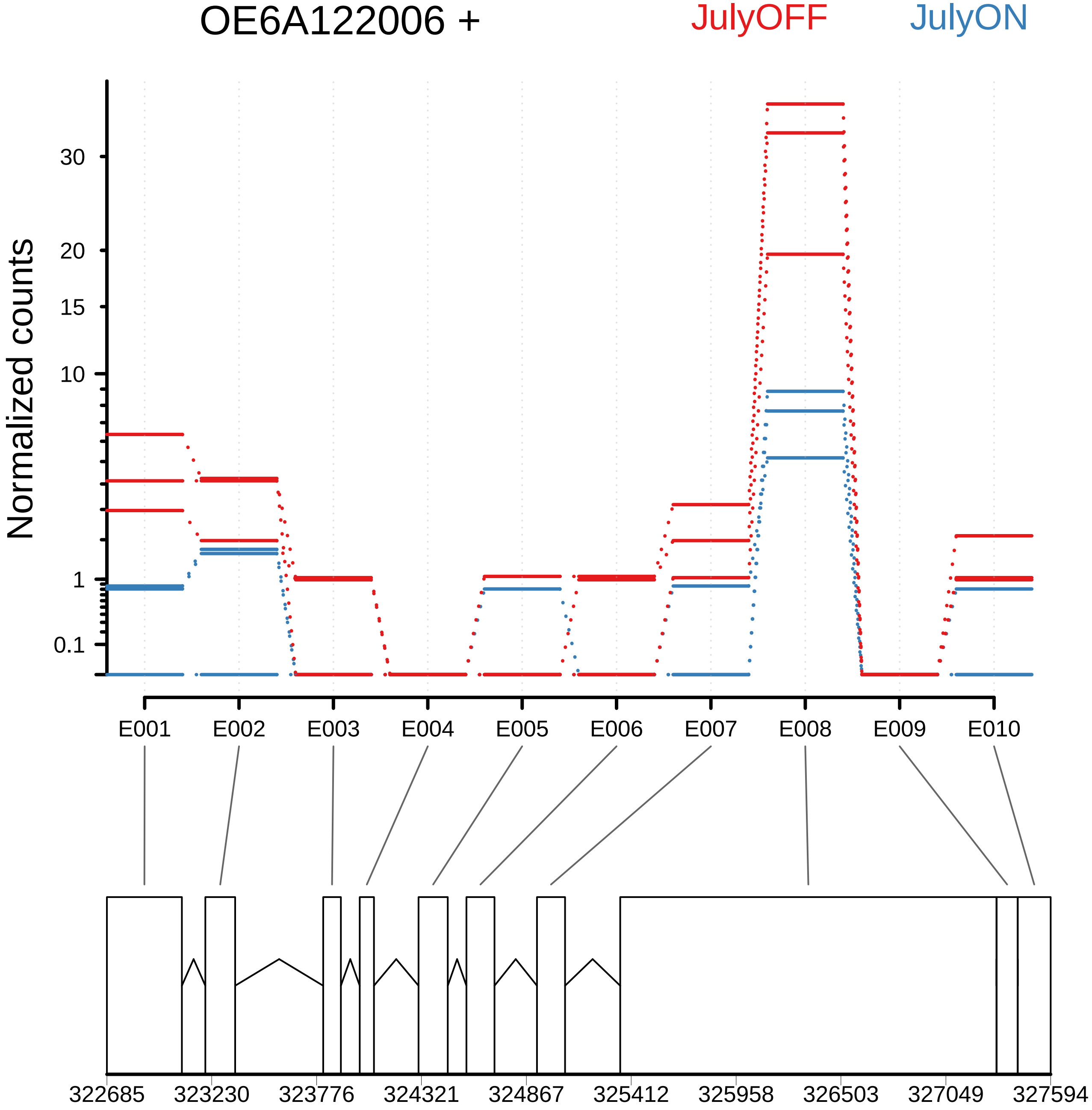

OE6A122006 +
JulyOFF
JulyON
Normalized counts
30
20
15
10
1
0.1
E001
E002
E003
E004
E005
E006
E007
E008
E009
E010
322685
323230
323776
324321
324867
325412
325958
326503
327049
327594

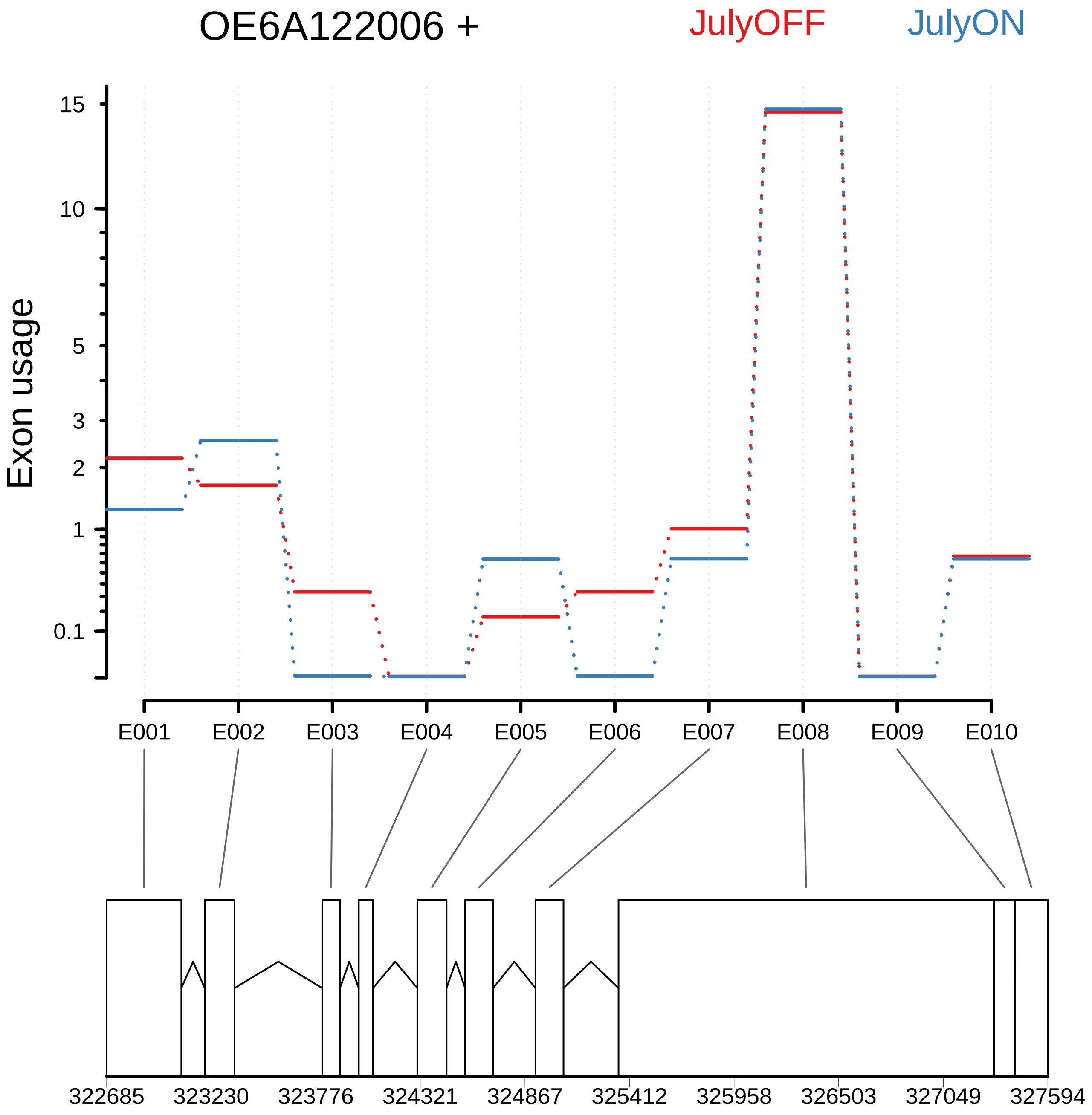

OE6A122006 +
JulyOFF
JulyON
Exon usage
15
10
5
3
2
1
0.1
E001
E002
E003
E004
E005
E006
E007
E008
E009
E010
322685
323230
323776
324321
324867
325412
325958
326503
327049
327594

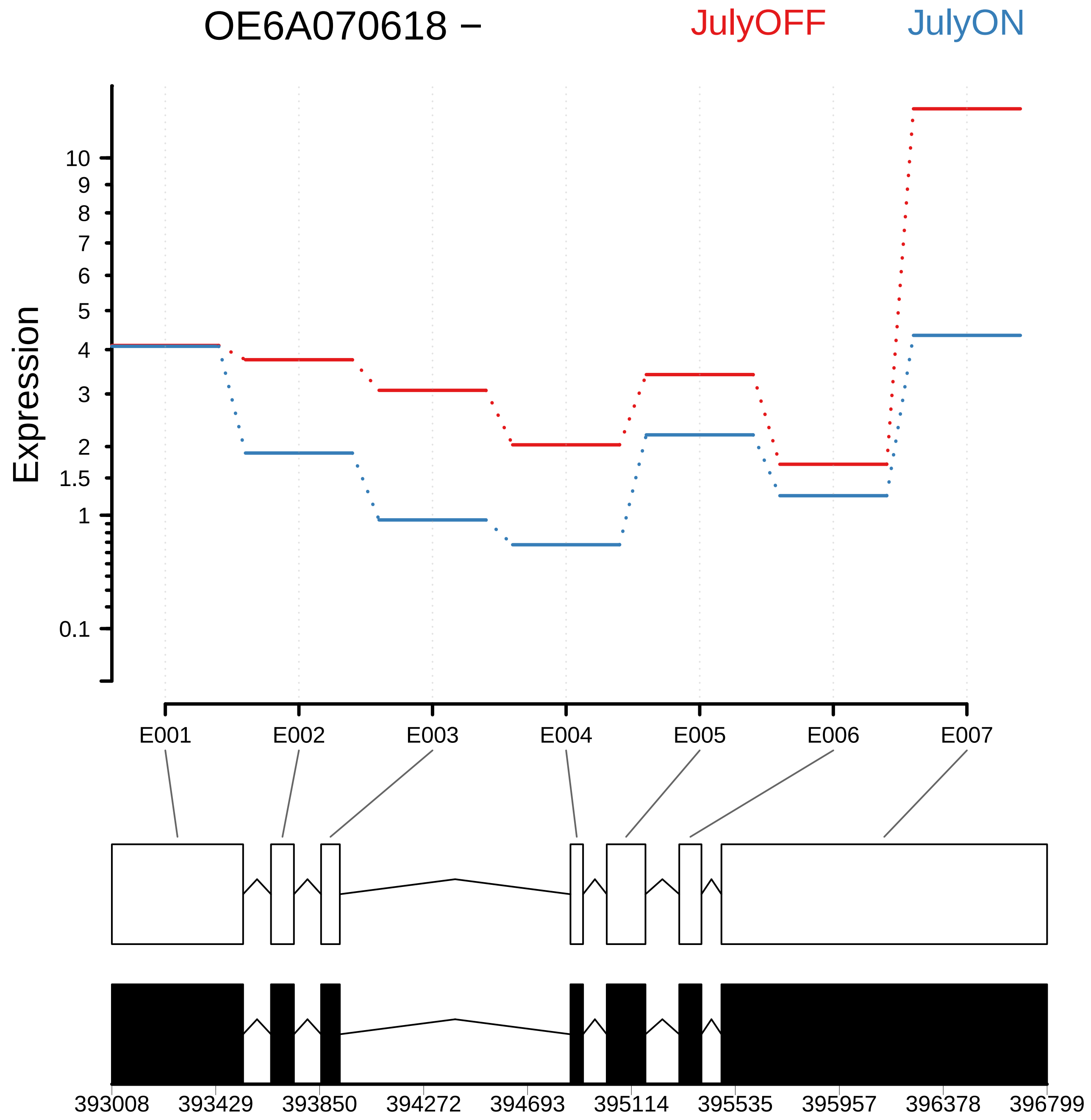

OE6A070618 –
JulyOFF
JulyON
Expression
10
9
8
7
6
5
4
3
2
1.5
1
0.1
E001
E002
E003
E004
E005
E006
E007
393008
393429
393850
394272
394693
395114
395535
395957
396378
396799

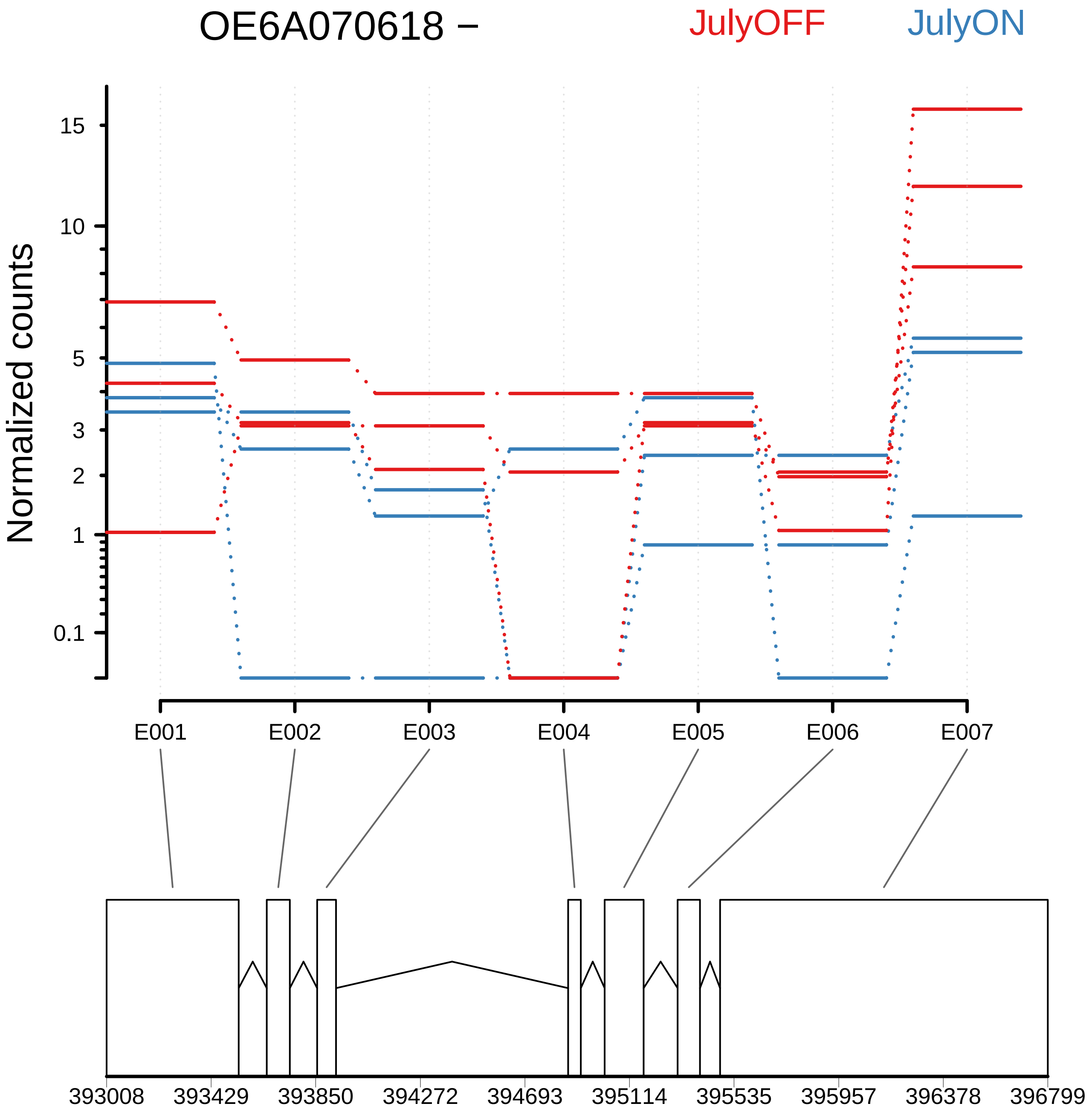

OE6A070618 −
JulyOFF
JulyON
Normalized counts
15
10
5
3
2
1
0.1
E001
E002
E003
E004
E005
E006
E007
393008
393429
393850
394272
394693
395114
395535
395957
396378
396799

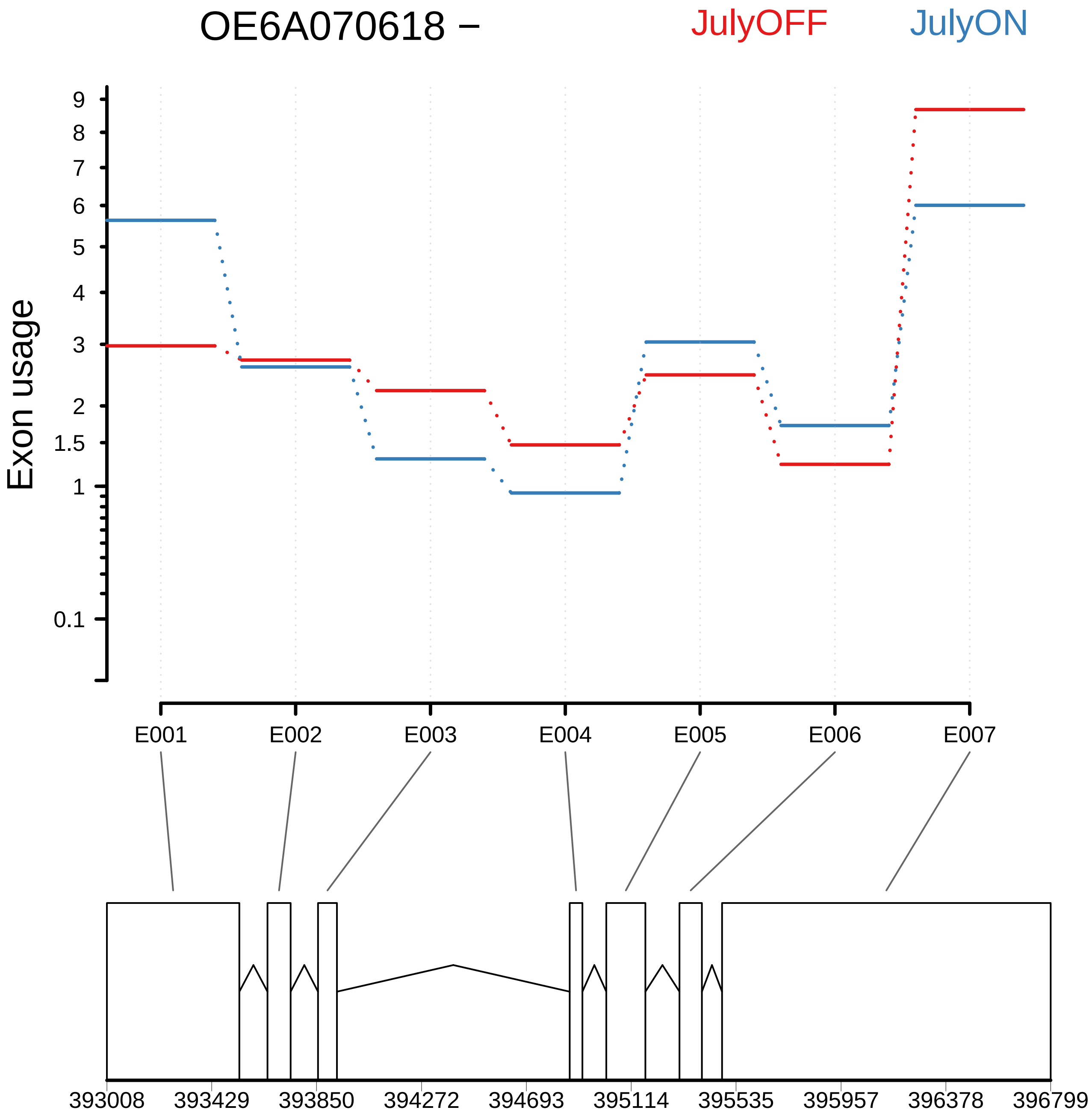
OE6A070618 −
JulyOFF
JulyON
Exon usage
9
8
7
6
5
4
3
2
1.5
1
0.1
E001
E002
E003
E004
E005
E006
E007
393008
393429
393850
394272
394693
395114
395535
395957
396378
396799

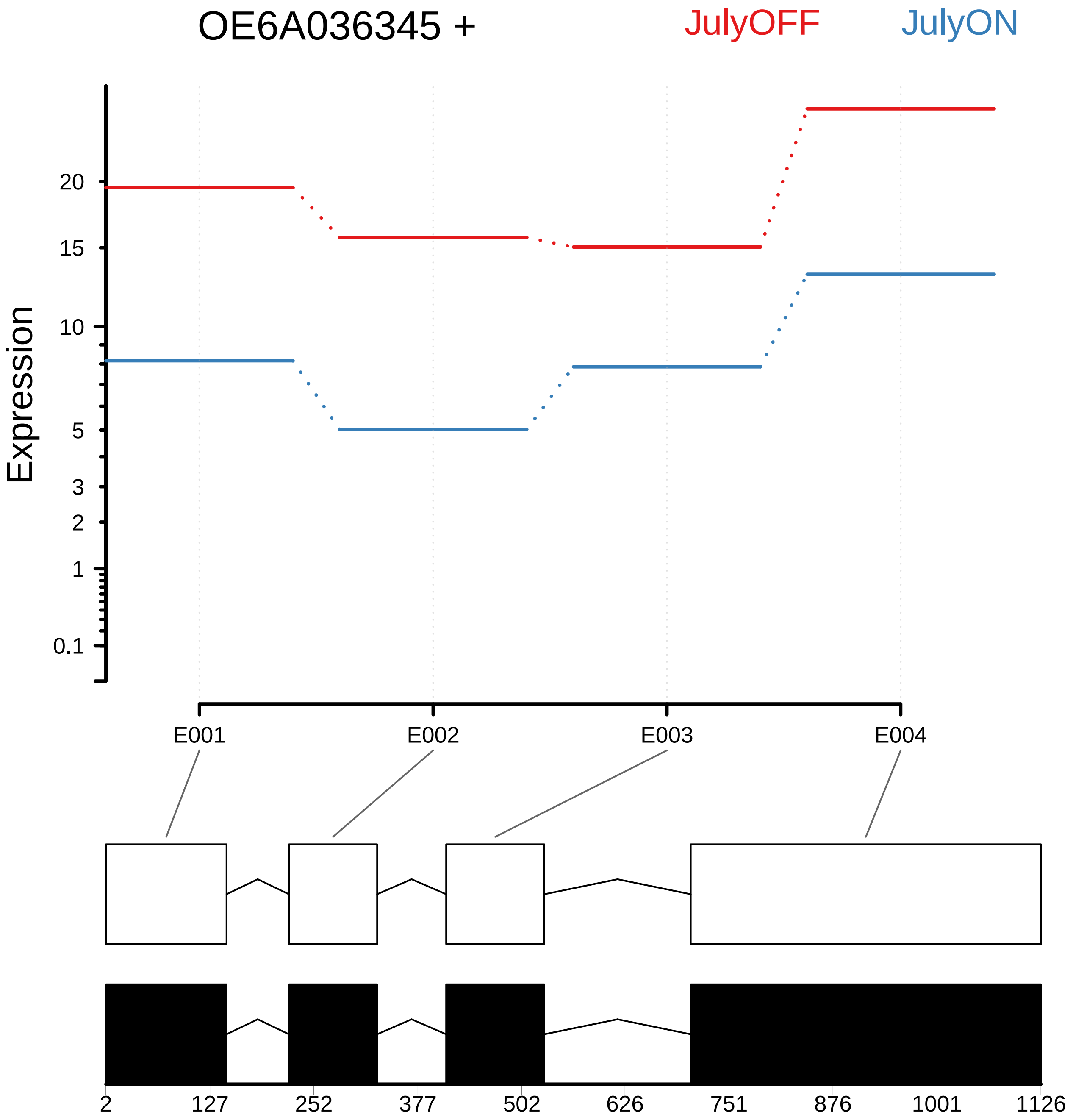

OE6A036345 +
JulyOFF
JulyON
Expression
20
15
10
5
3
2
1
0.1
E001
E002
E003
E004
2
127
252
377
502
626
751
876
1001
1126

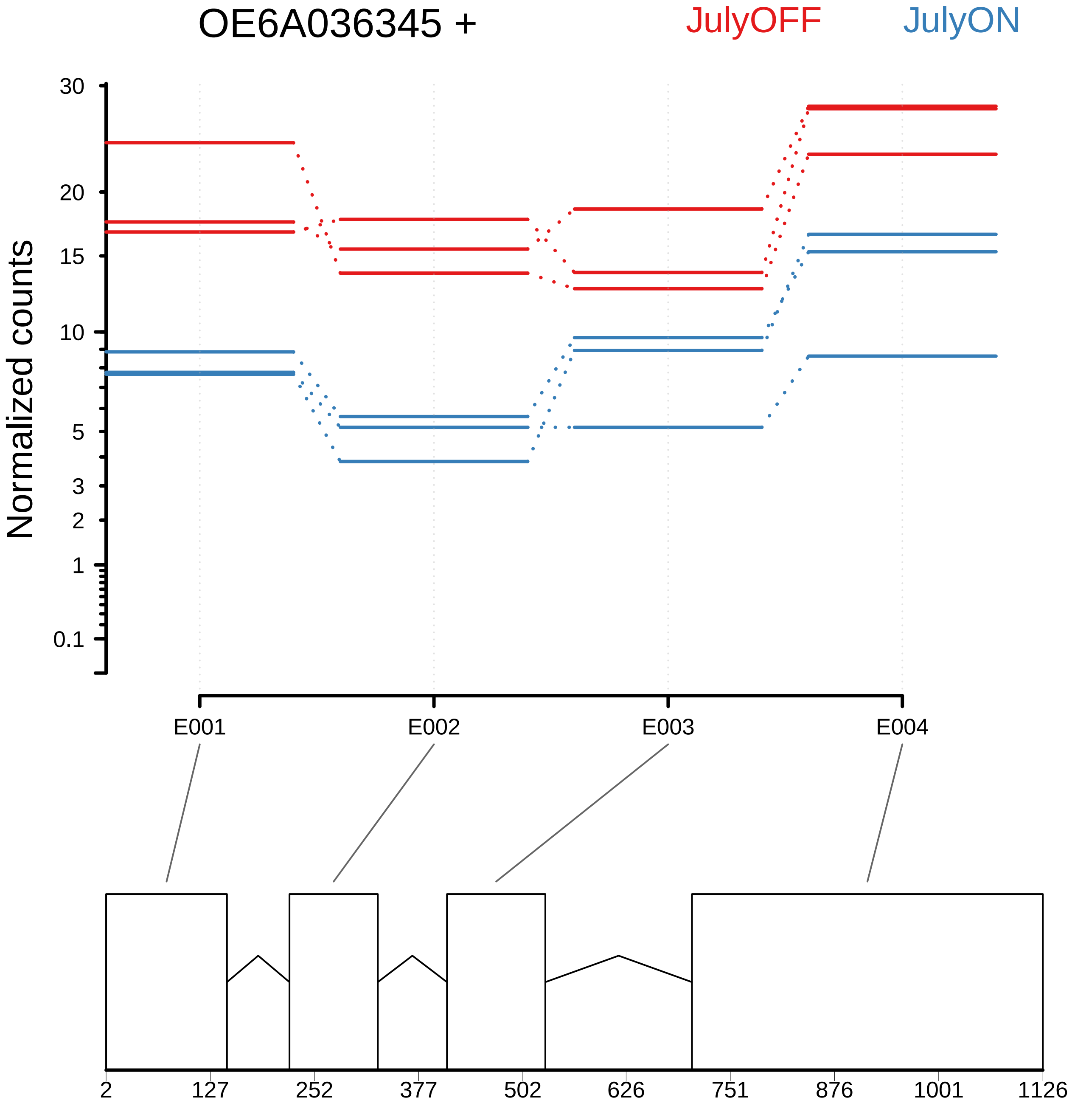

OE6A036345 +
JulyOFF
JulyON
Normalized counts
30
20
15
10
5
3
2
1
0.1
E001
E002
E003
E004
2
127
252
377
502
626
751
876
1001
1126

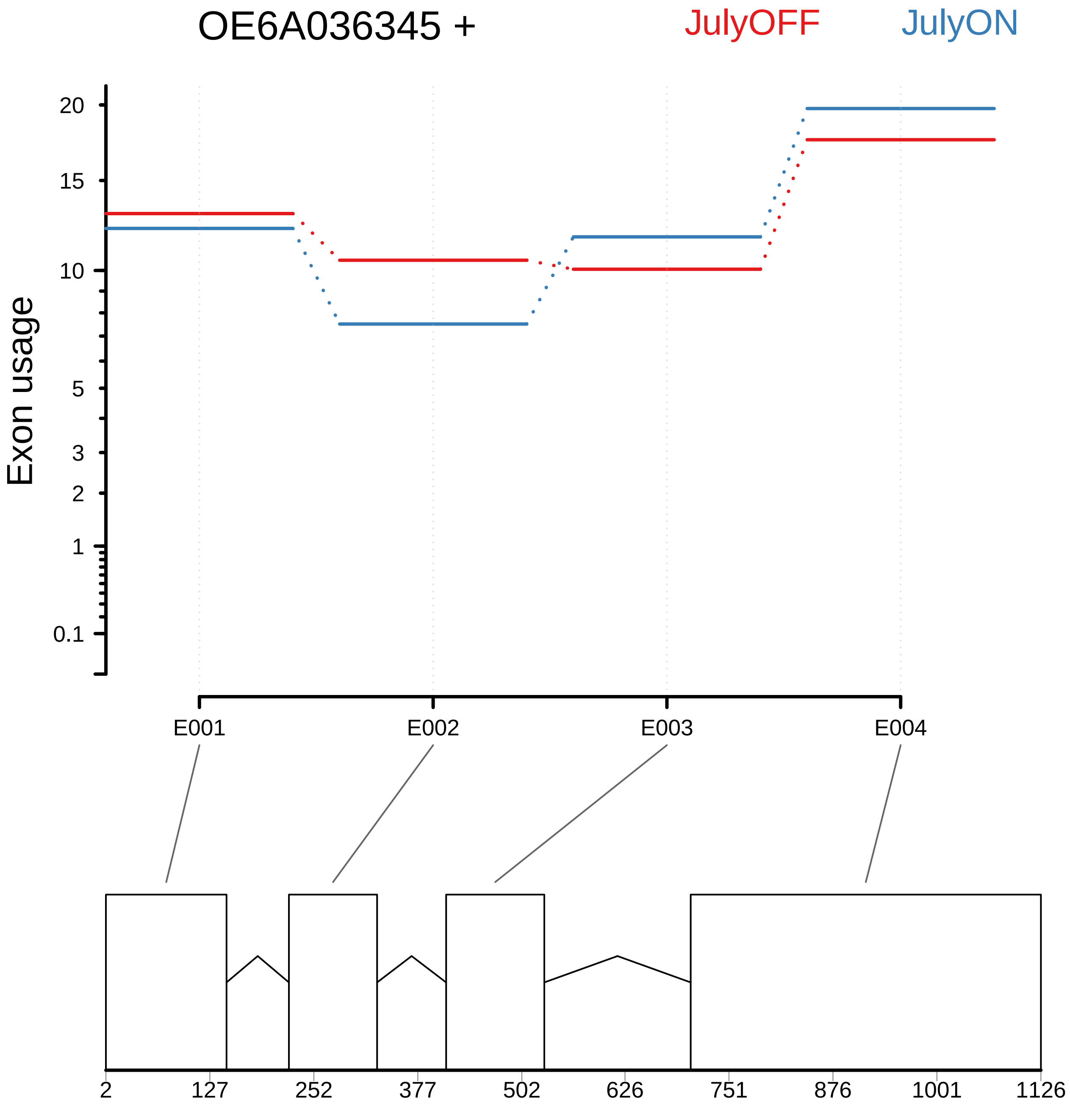

OE6A036345 +
JulyOFF
JulyON
Exon usage
20
15
10
5
3
2
1
0.1
E001
E002
E003
E004
2
127
252
377
502
626
751
876
1001
1126

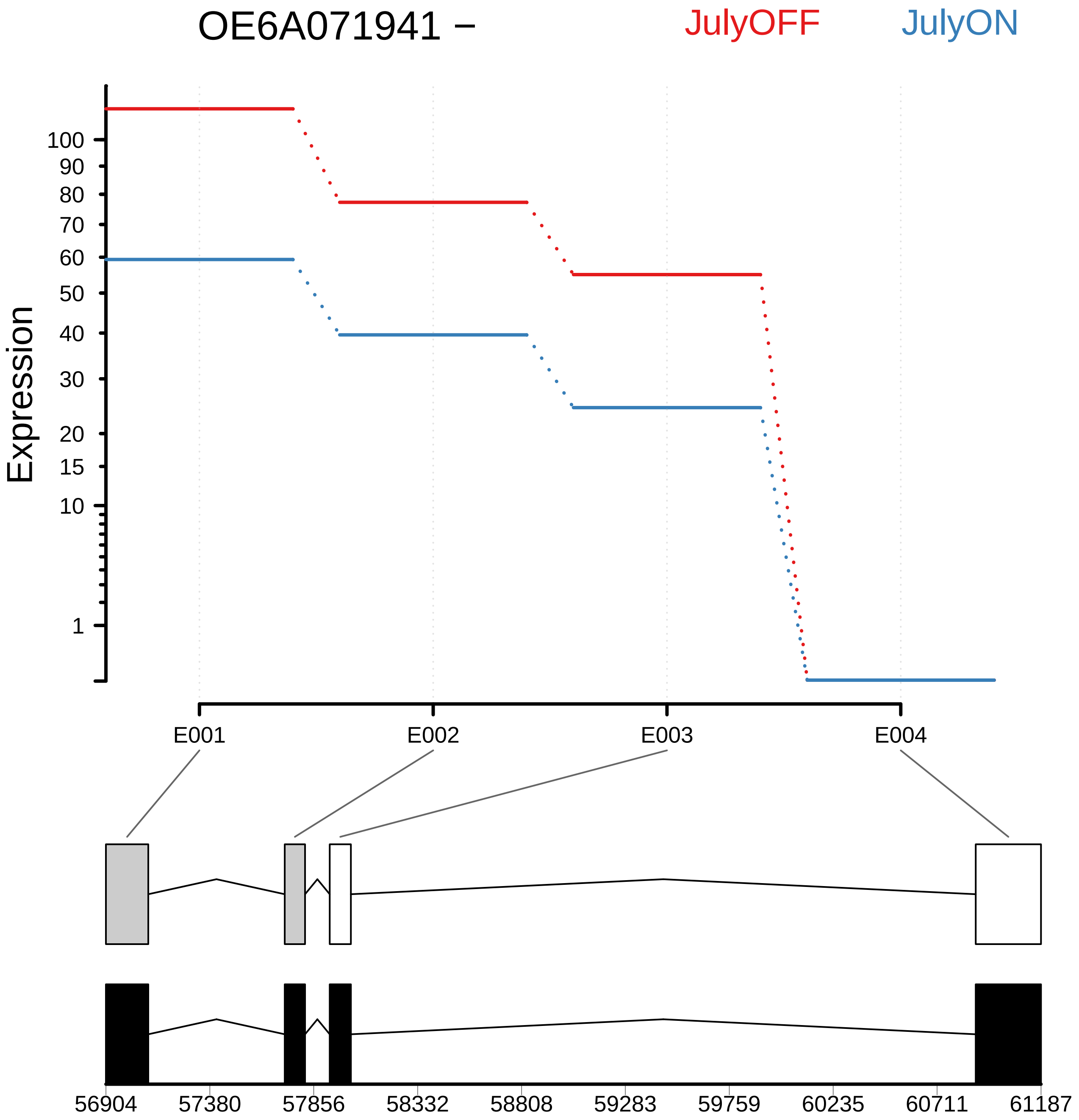

OE6A071941 −
JulyOFF
JulyON
Expression
100
90
80
70
60
50
40
30
20
15
10
1
E001
E002
E003
E004
56904
57380
57856
58332
58808
59283
59759
60235
60711
61187

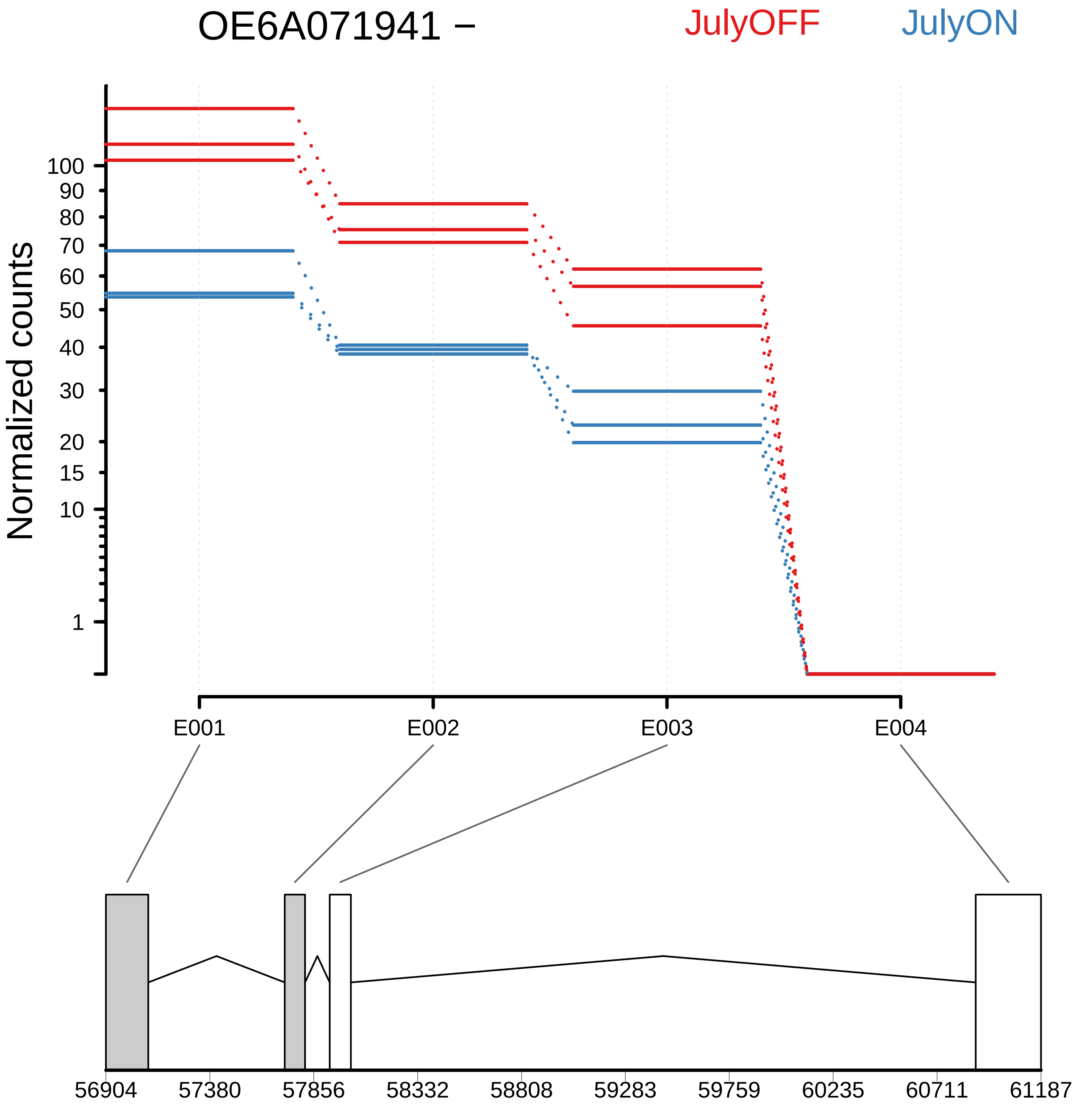

OE6A071941 −
JulyOFF
JulyON
Normalized counts
100
90
80
70
60
50
40
30
20
15
10
1
E001
E002
E003
E004
56904
57380
57856
58332
58808
59283
59759
60235
60711
61187

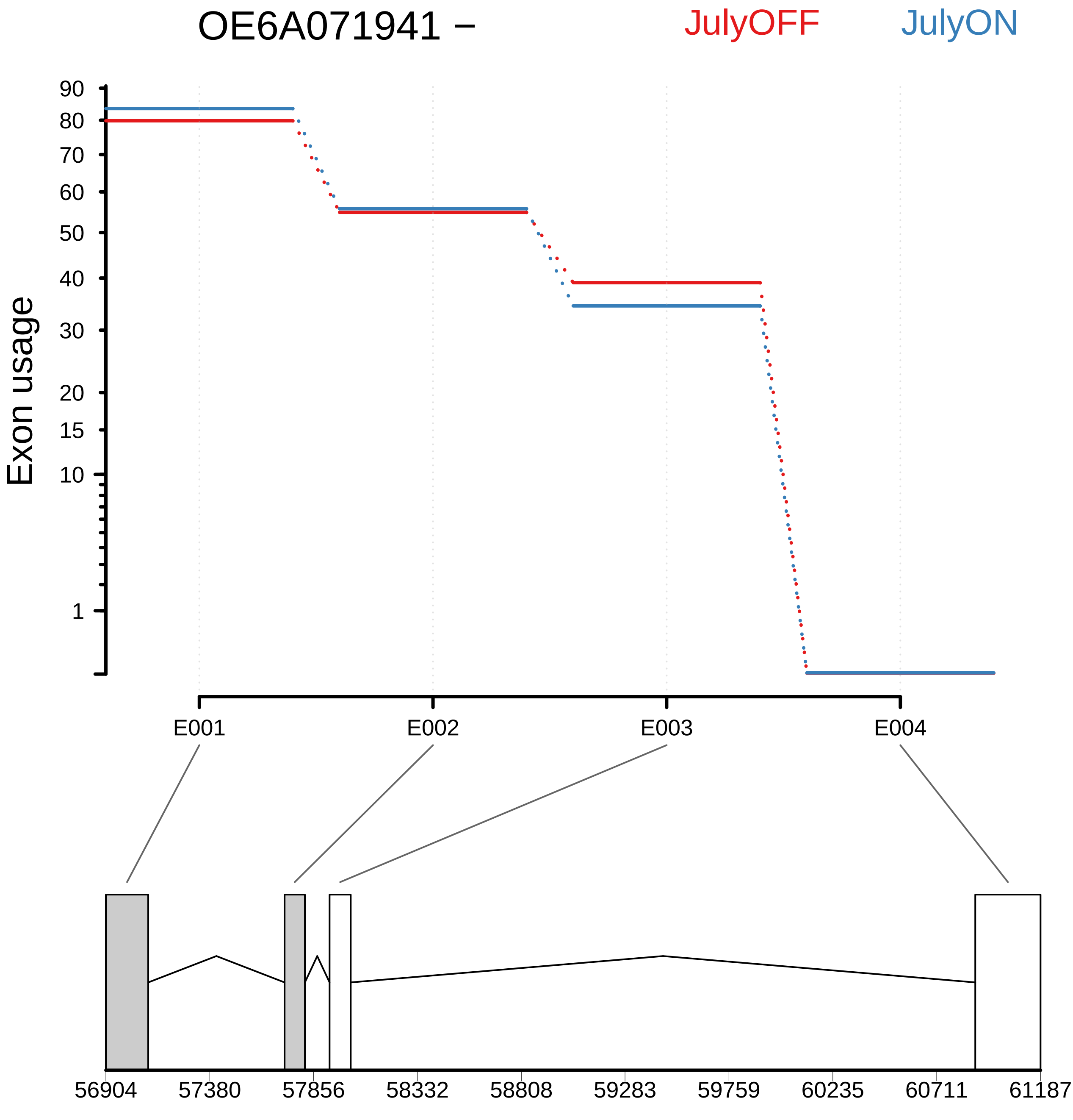

OE6A071941 −
JulyOFF
JulyON
Exon usage
90
80
70
60
50
40
30
20
15
10
1
E001
E002
E003
E004
56904
57380
57856
58332
58808
59283
59759
60235
60711
61187

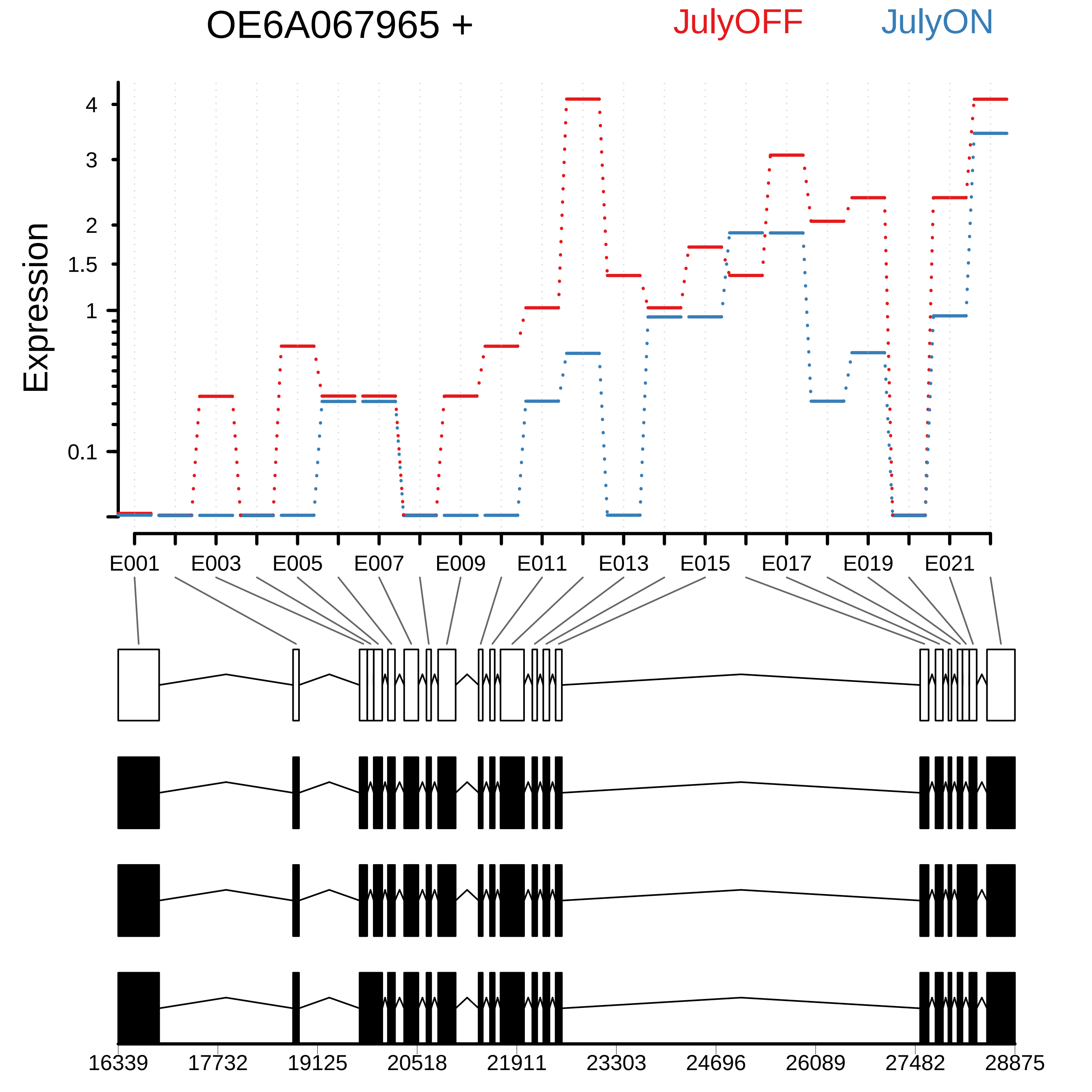
OE6A067965 +
JulyOFF
JulyON
Expression
4
3
2
1.5
1
0.1
E001
E003
E005
E007
E009
E011
E013
E015
E017
E019
E021
16339
17732
19125
20518
21911
23303
24696
26089
27482
28875

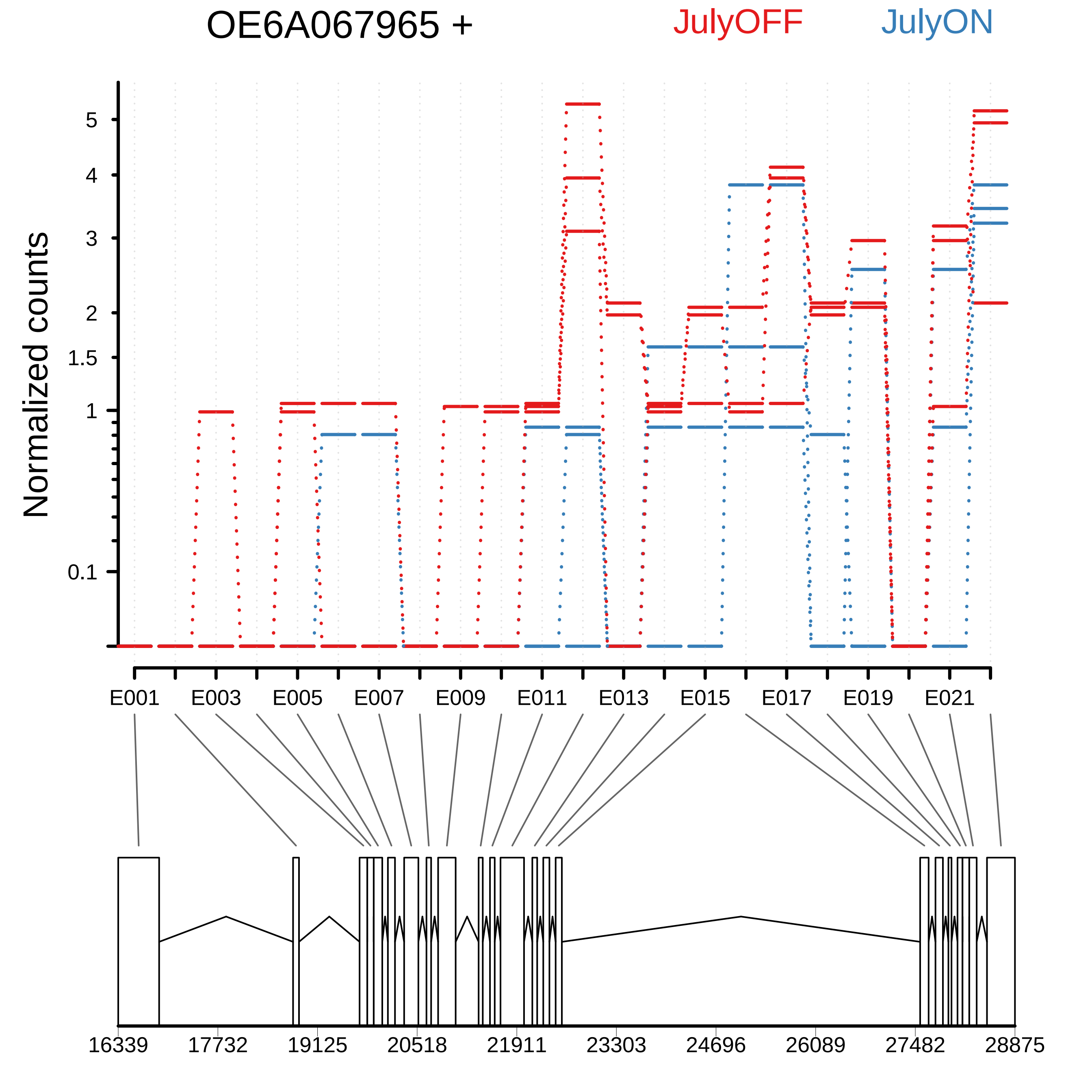
OE6A067965 +
JulyOFF
JulyON
Normalized counts
5
4
3
2
1.5
1
0.1
E001
E003
E005
E007
E009
E011
E013
E015
E017
E019
E021
16339
17732
19125
20518
21911
23303
24696
26089
27482
28875

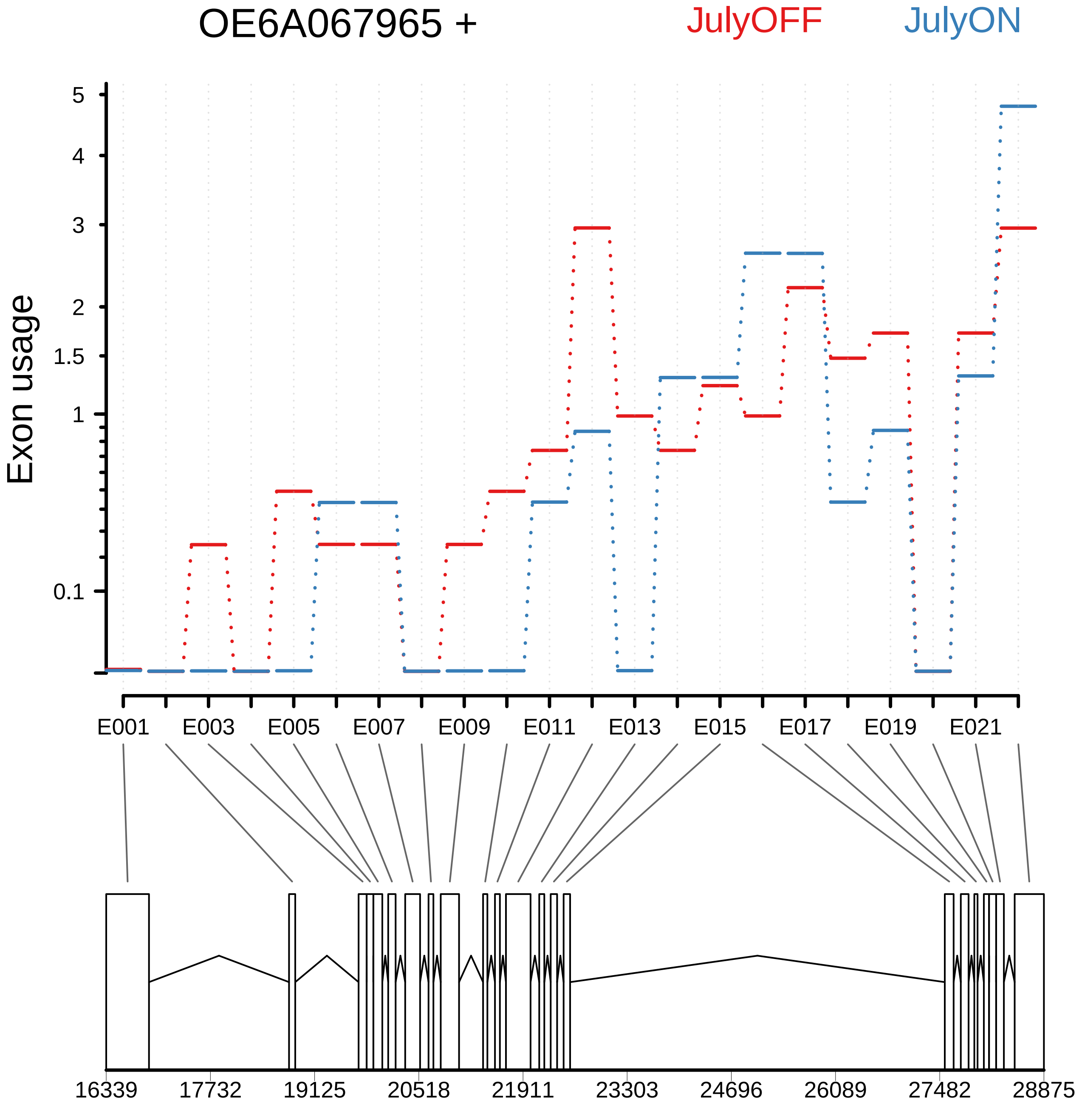

OE6A067965 +
JulyOFF
JulyON
Exon usage
5
4
3
2
1.5
1
0.1
E001
E003
E005
E007
E009
E011
E013
E015
E017
E019
E021
16339
17732
19125
20518
21911
23303
24696
26089
27482
28875

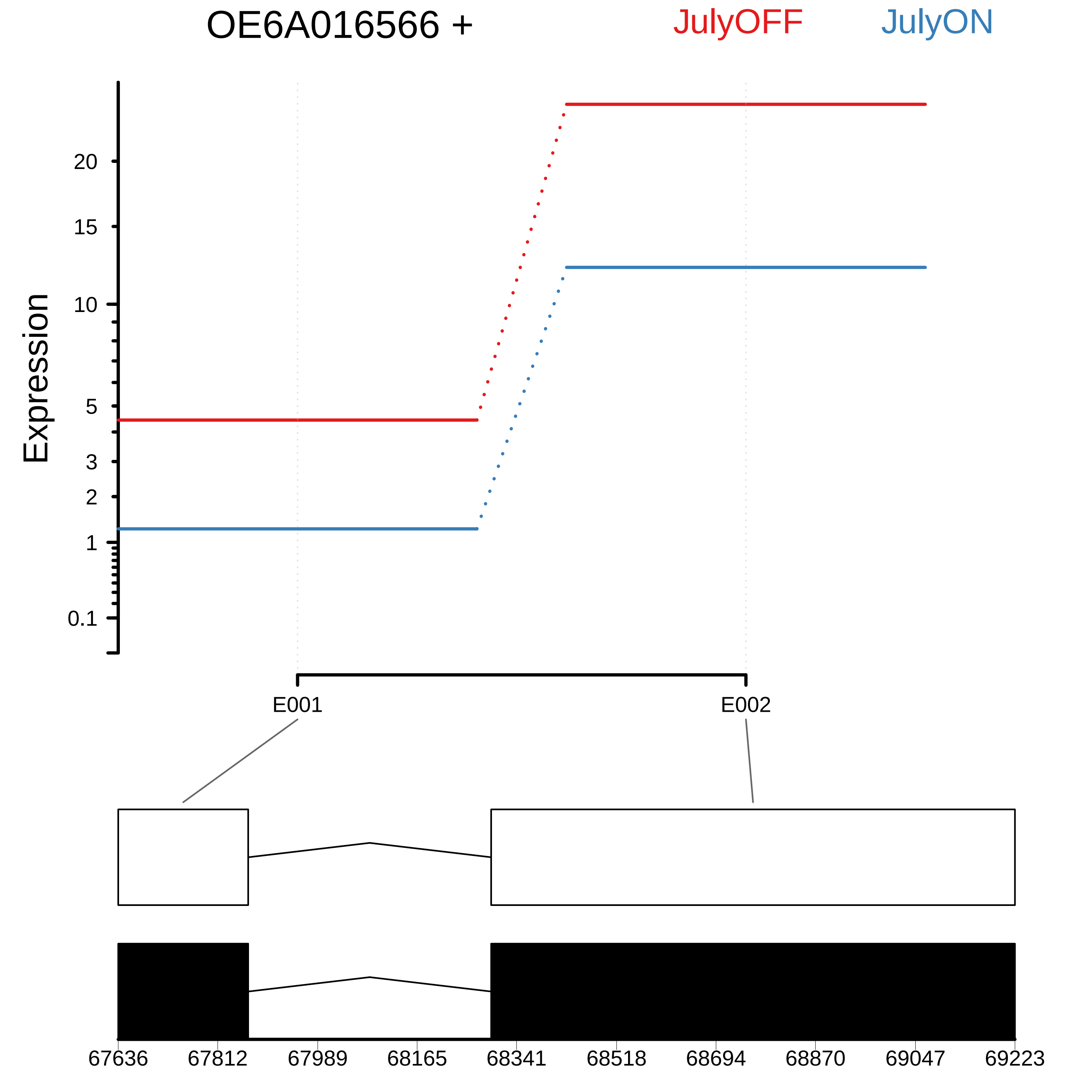

OE6A016566 +
JulyOFF
JulyON
Expression
20
15
10
5
3
2
1
0.1
E001
E002
67636
67812
67989
68165
68341
68518
68694
68870
69047
69223

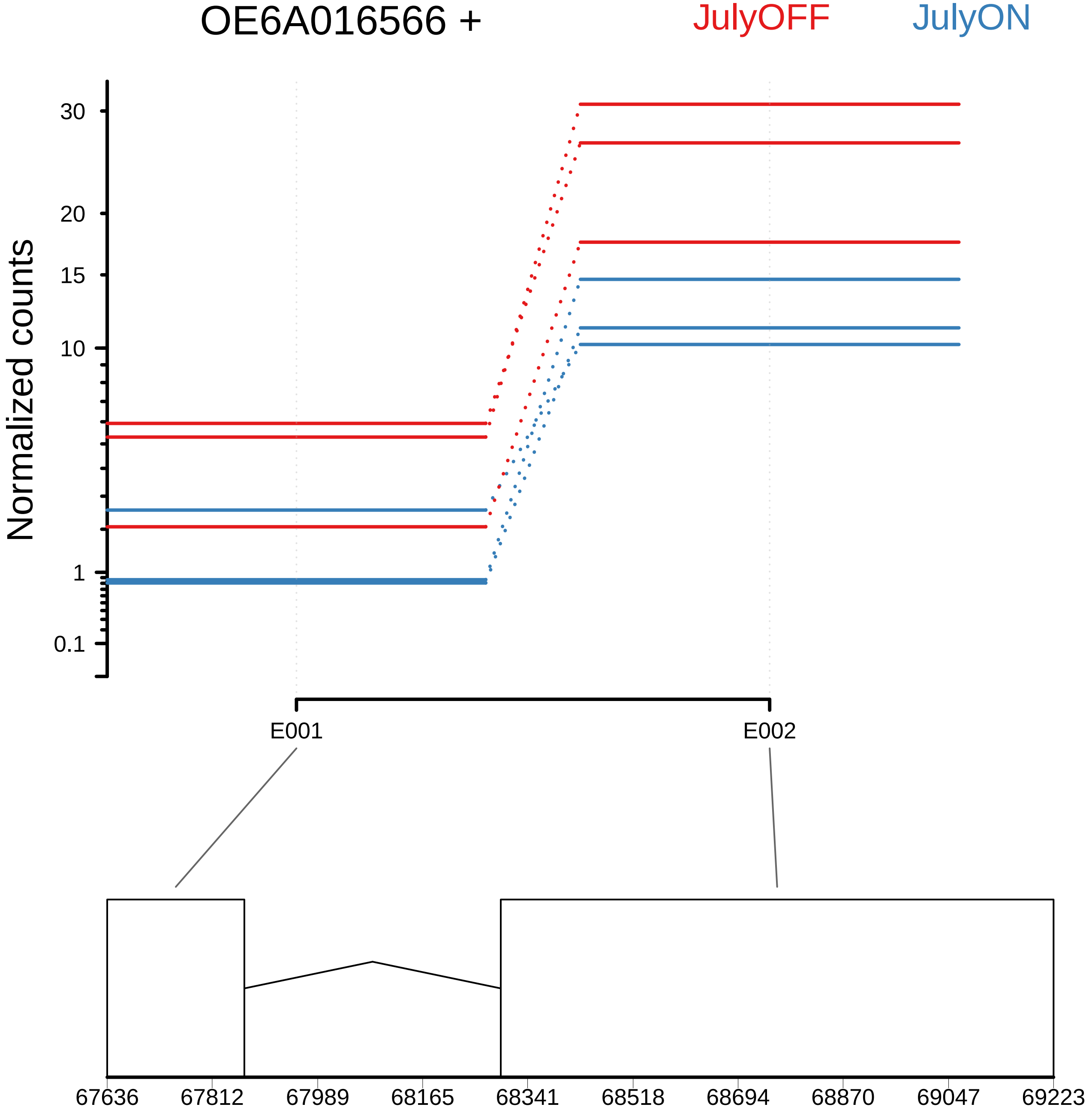

OE6A016566 +
JulyOFF
JulyON
Normalized counts
30
20
15
10
1
0.1
E001
E002
67636
67812
67989
68165
68341
68518
68694
68870
69047
69223

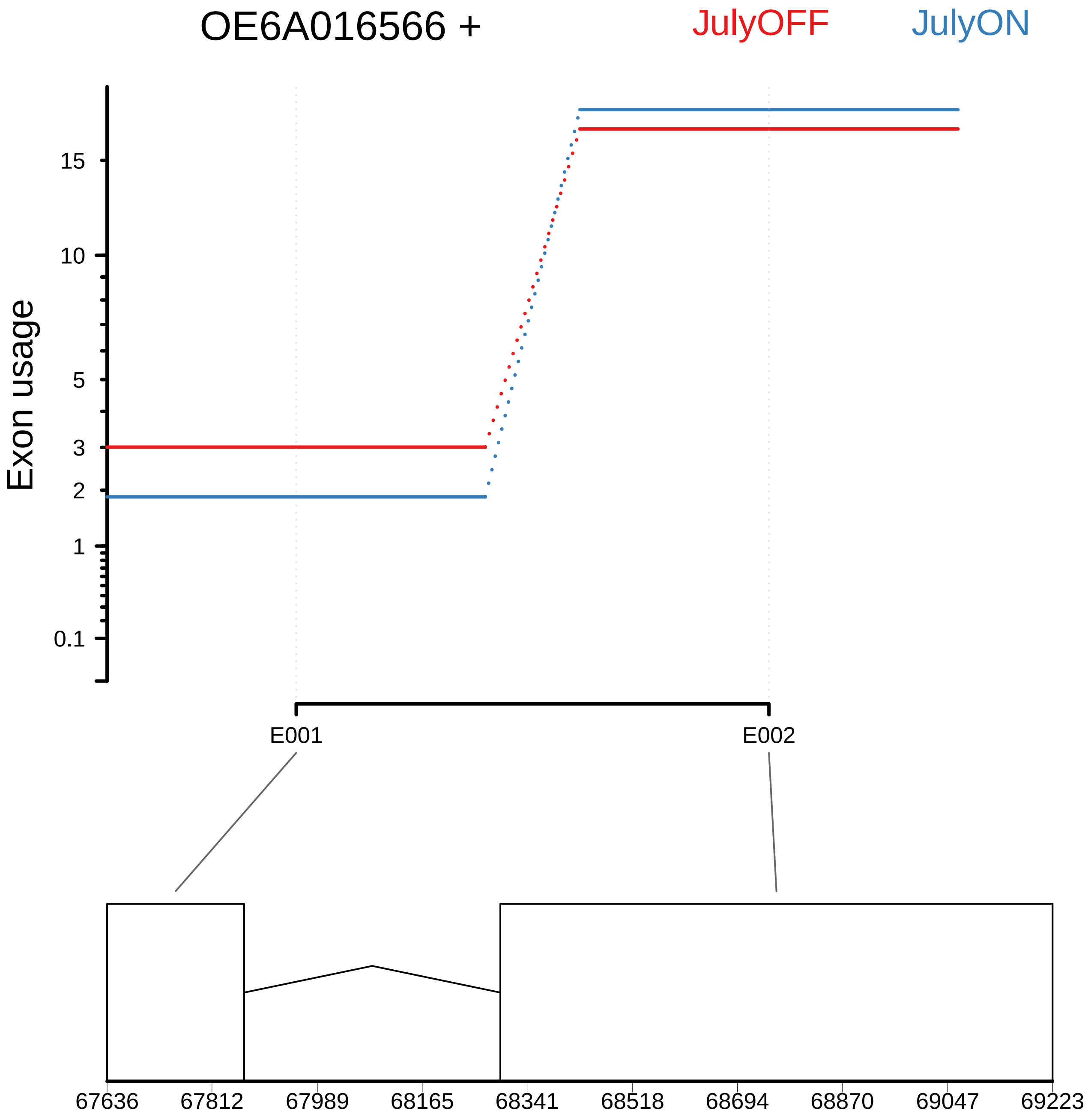

OE6A016566 +
JulyOFF
JulyON
Exon usage
15
10
5
3
2
1
0.1
E001
E002
67636
67812
67989
68165
68341
68518
68694
68870
69047
69223

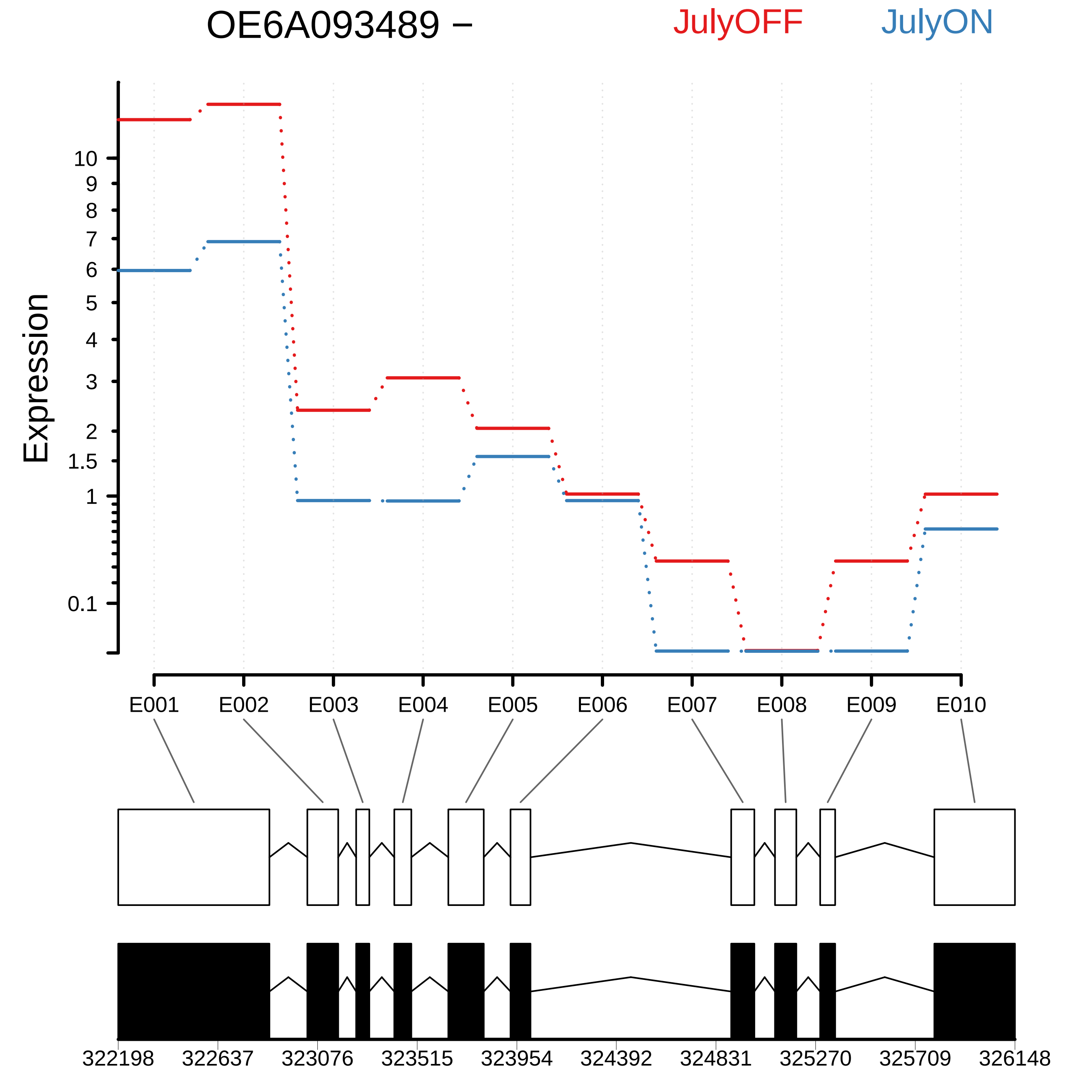

OE6A093489 –
JulyOFF
JulyON
Expression
10
9
8
7
6
5
4
3
2
1.5
1
0.1
E001
E002
E003
E004
E005
E006
E007
E008
E009
E010
322198
322637
323076
323515
323954
324392
324831
325270
325709
326148

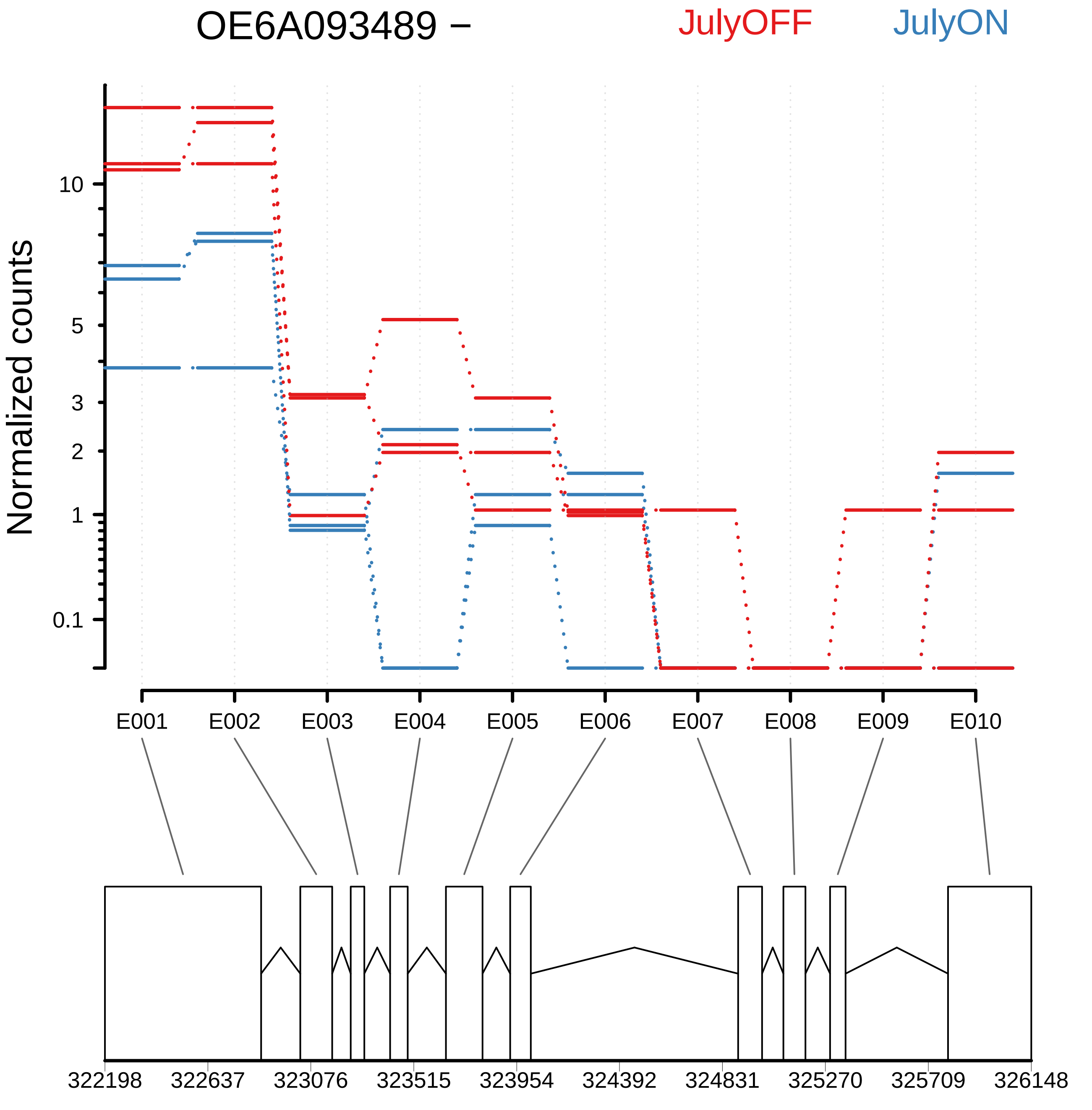
OE6A093489 –
JulyOFF
JulyON
Normalized counts
10
5
3
2
1
0.1
E001
E002
E003
E004
E005
E006
E007
E008
E009
E010
322198
322637
323076
323515
323954
324392
324831
325270
325709
326148

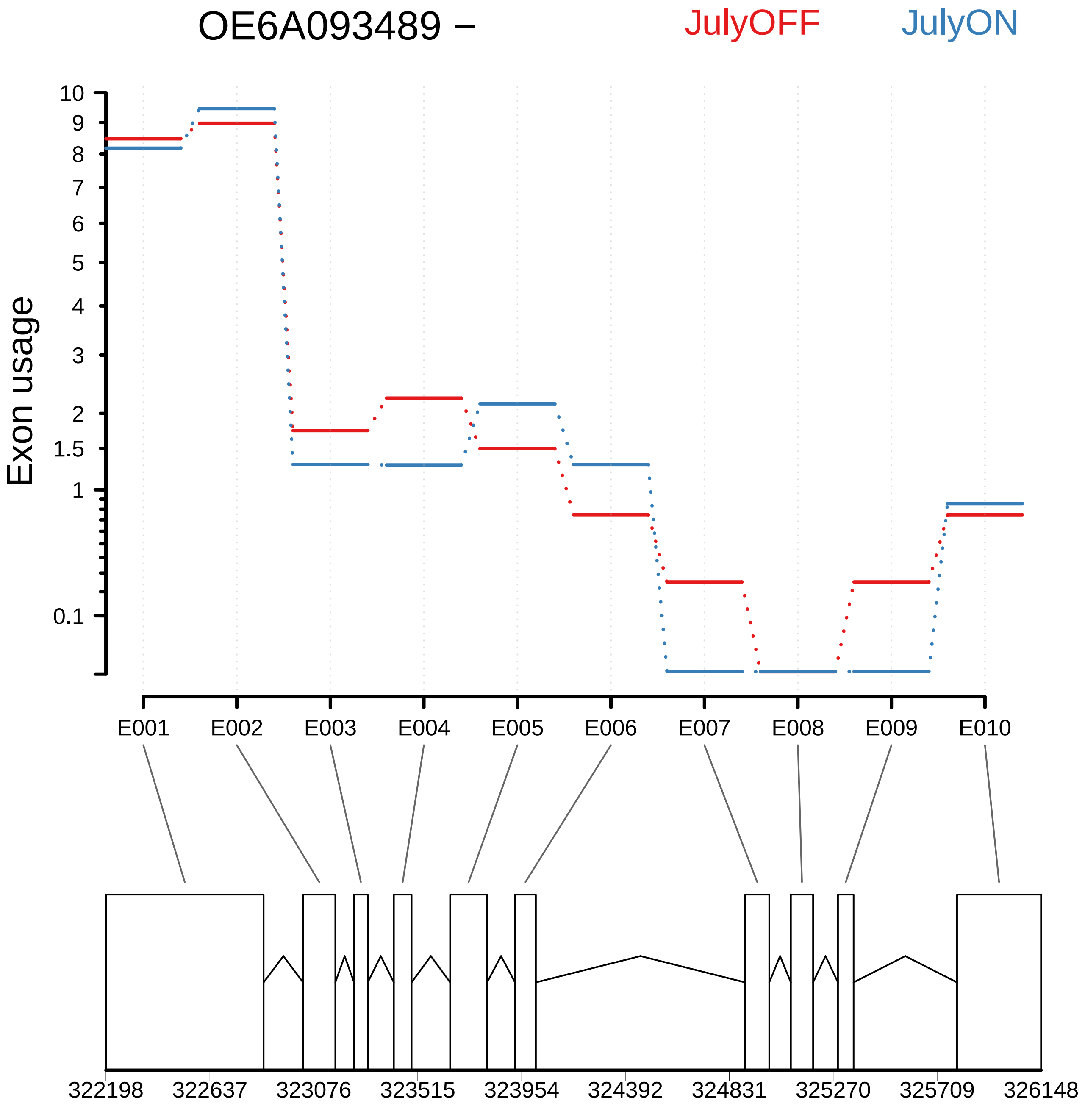

OE6A093489 –
JulyOFF
JulyON
Exon usage
10
9
8
7
6
5
4
3
2
1.5
1
0.1
E001
E002
E003
E004
E005
E006
E007
E008
E009
E010
322198
322637
323076
323515
323954
324392
324831
325270
325709
326148

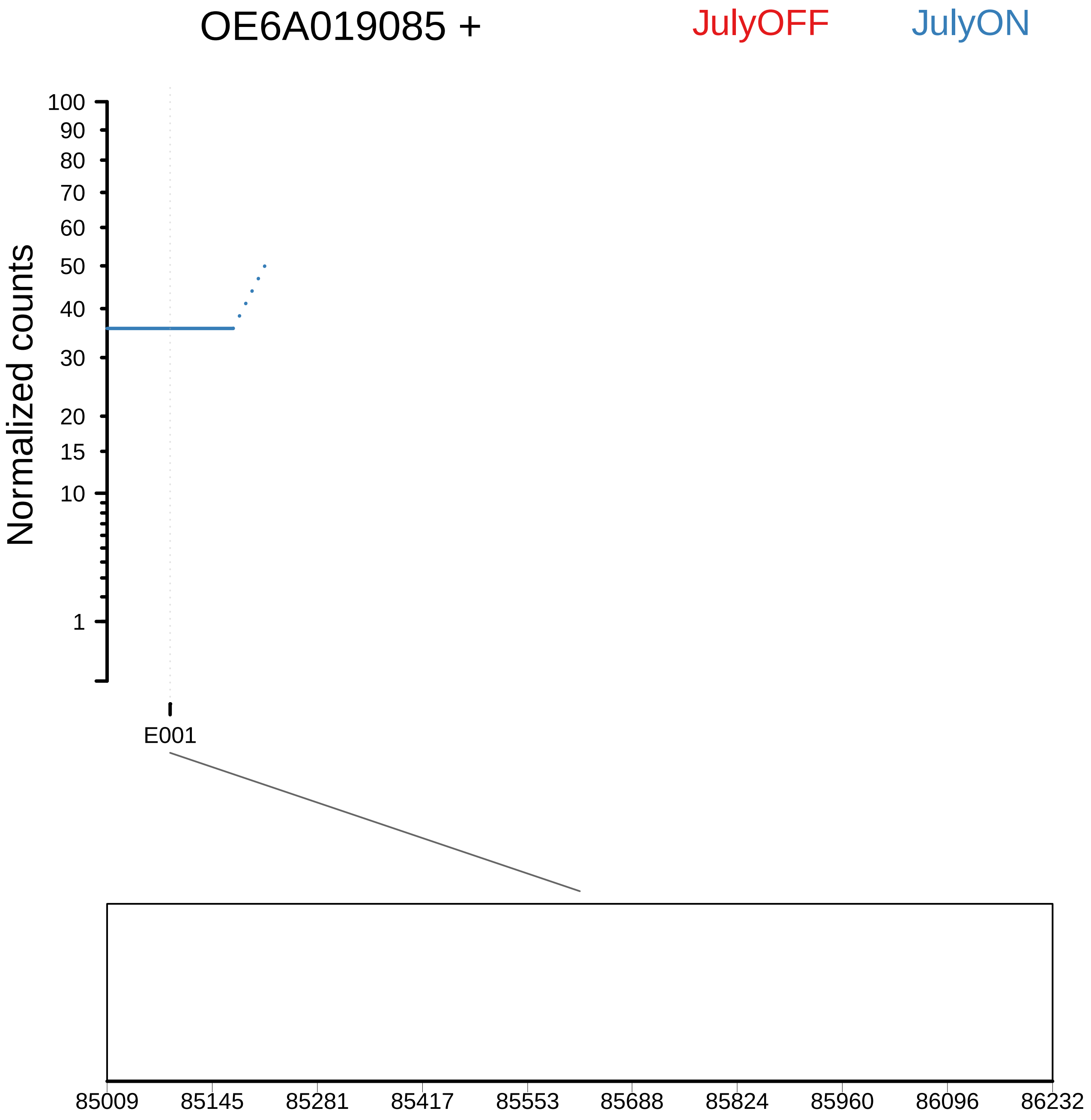
OE6A019085 +
JulyOFF
JulyON
Normalized counts
100
90
80
70
60
50
40
30
20
15
10
1
E001
85009
85145
85281
85417
85553
85688
85824
85960
86096
86232

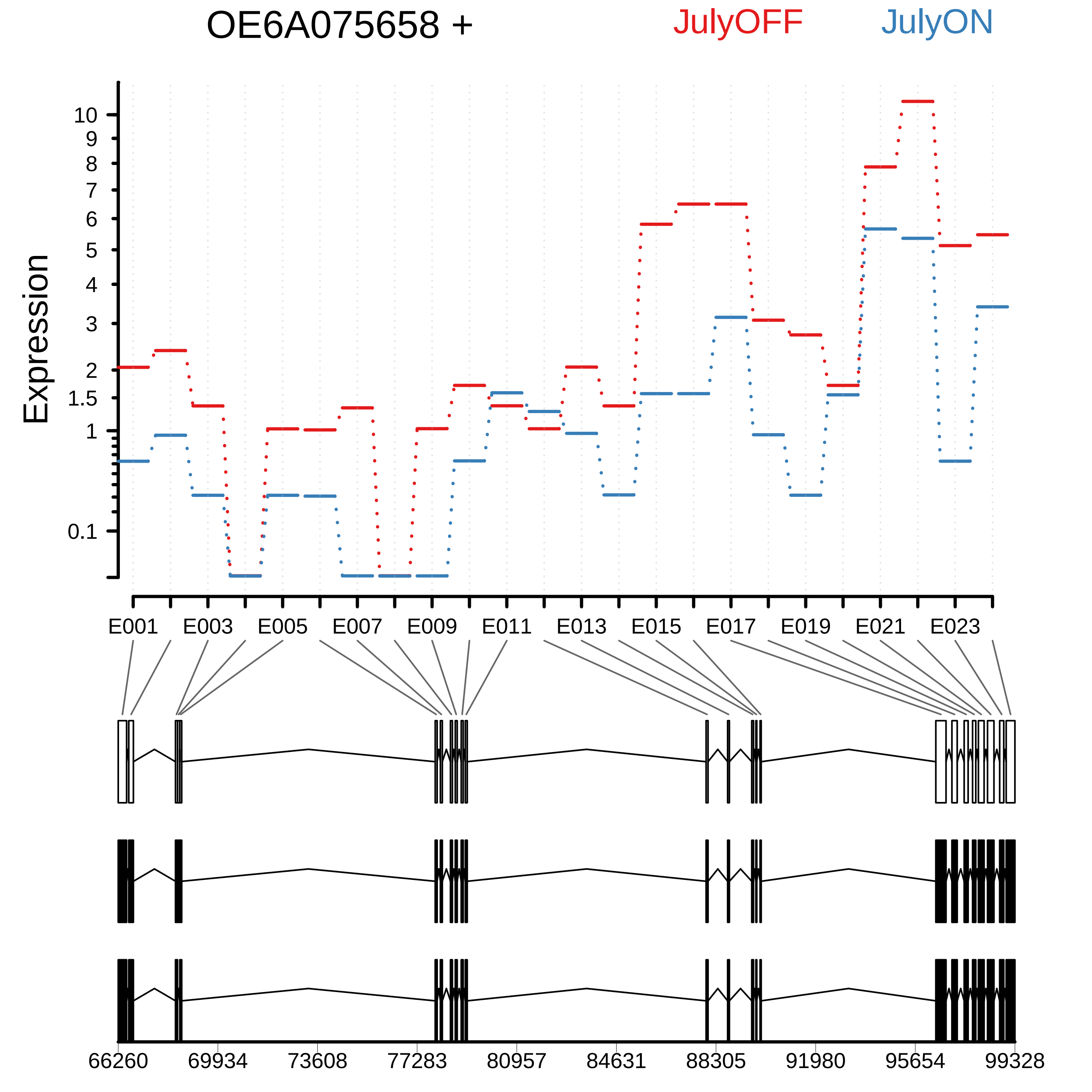
OE6A075658 +
JulyOFF
JulyON
Expression
10
9
8
7
6
5
4
3
2
1.5
1
0.1
E001
E003
E005
E007
E009
E011
E013
E015
E017
E019
E021
E023
66260
69934
73608
77283
80957
84631
88305
91980
95654
99328

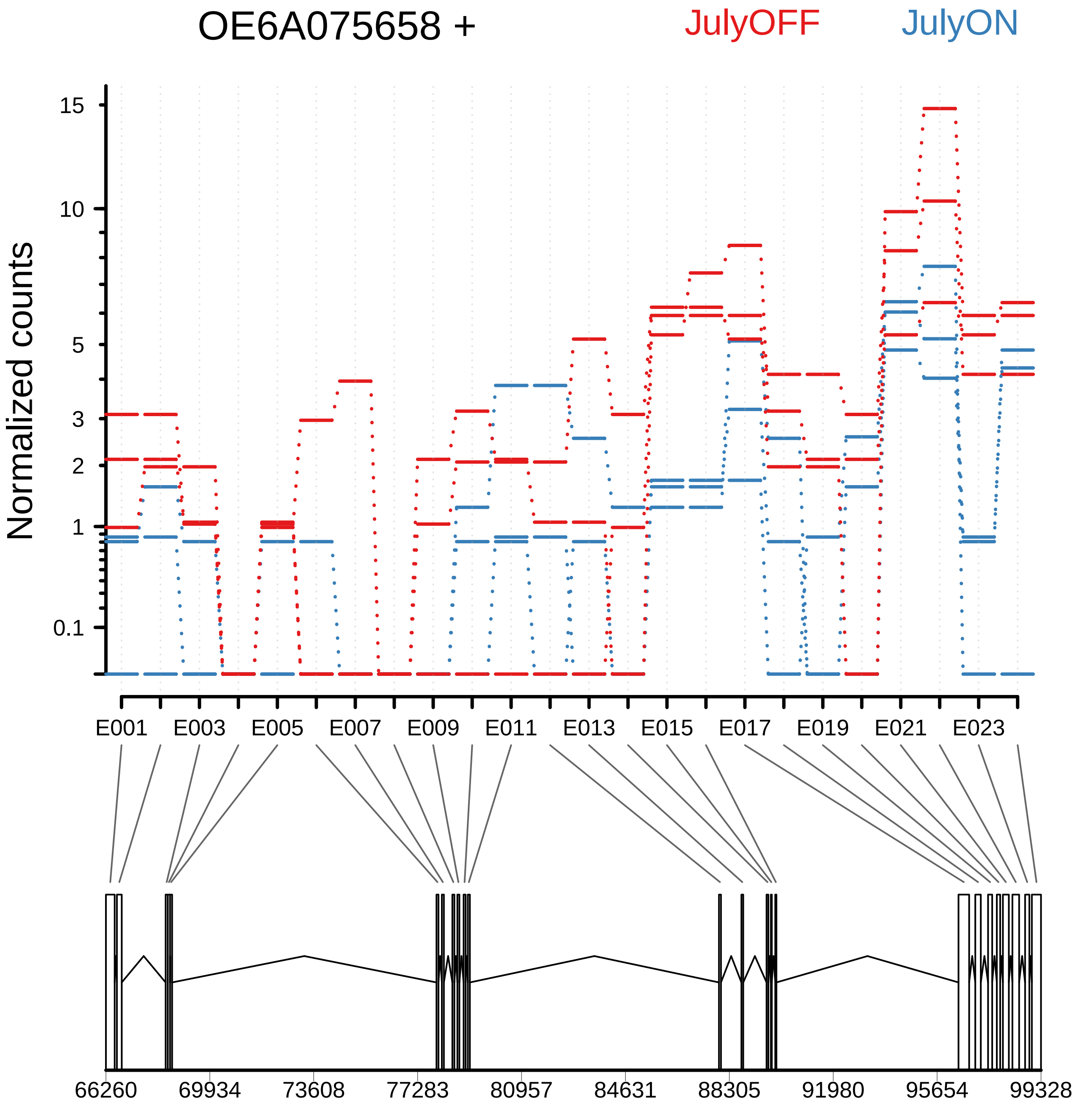

OE6A075658 +
JulyOFF
JulyON
Normalized counts
15
10
5
3
2
1
0.1
E001
E003
E005
E007
E009
E011
E013
E015
E017
E019
E021
E023
66260
69934
73608
77283
80957
84631
88305
91980
95654
99328

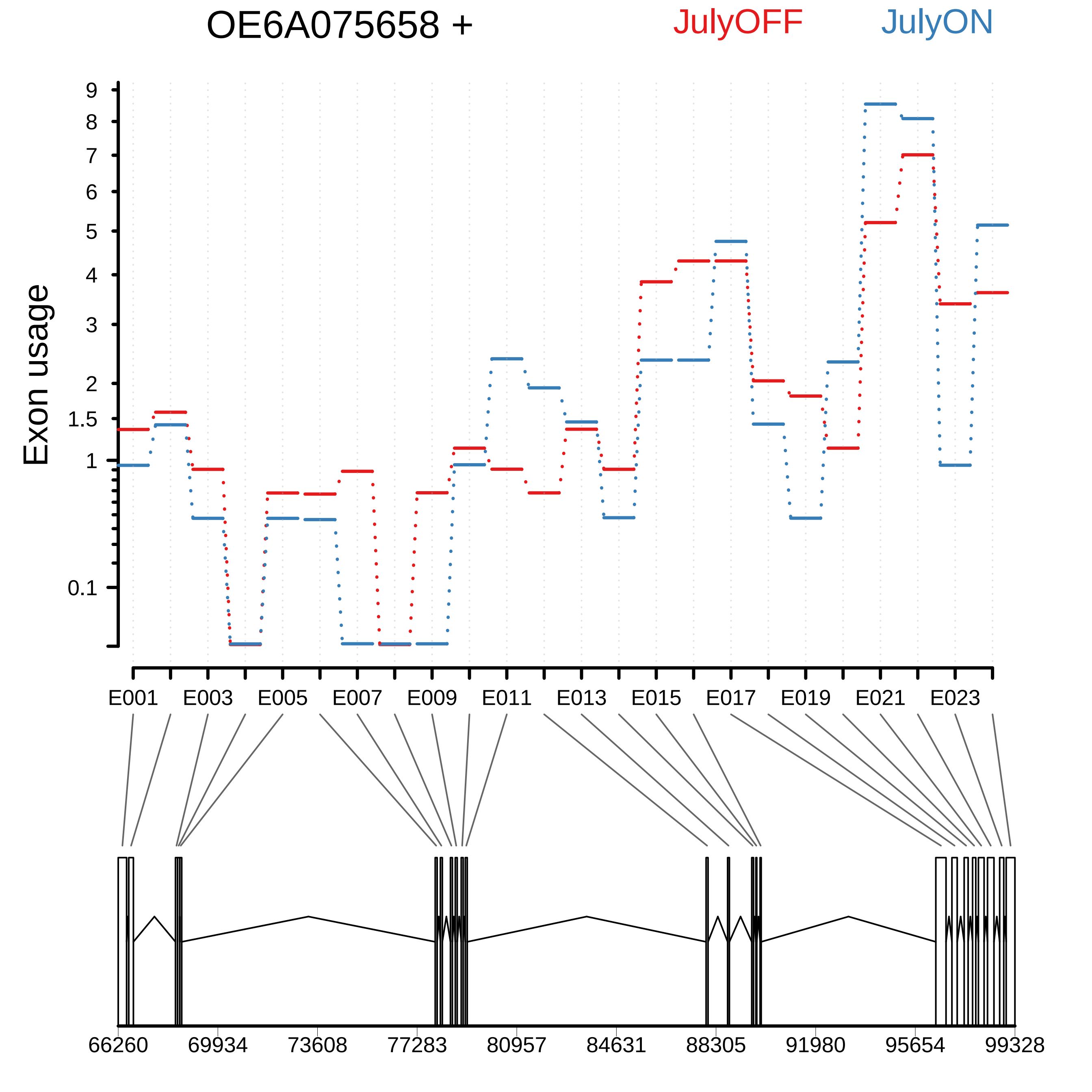

OE6A075658 +
JulyOFF
JulyON
Exon usage
9
8
7
6
5
4
3
2
1.5
1
0.1
E001
E003
E005
E007
E009
E011
E013
E015
E017
E019
E021
E023
66260
69934
73608
77283
80957
84631
88305
91980
95654
99328

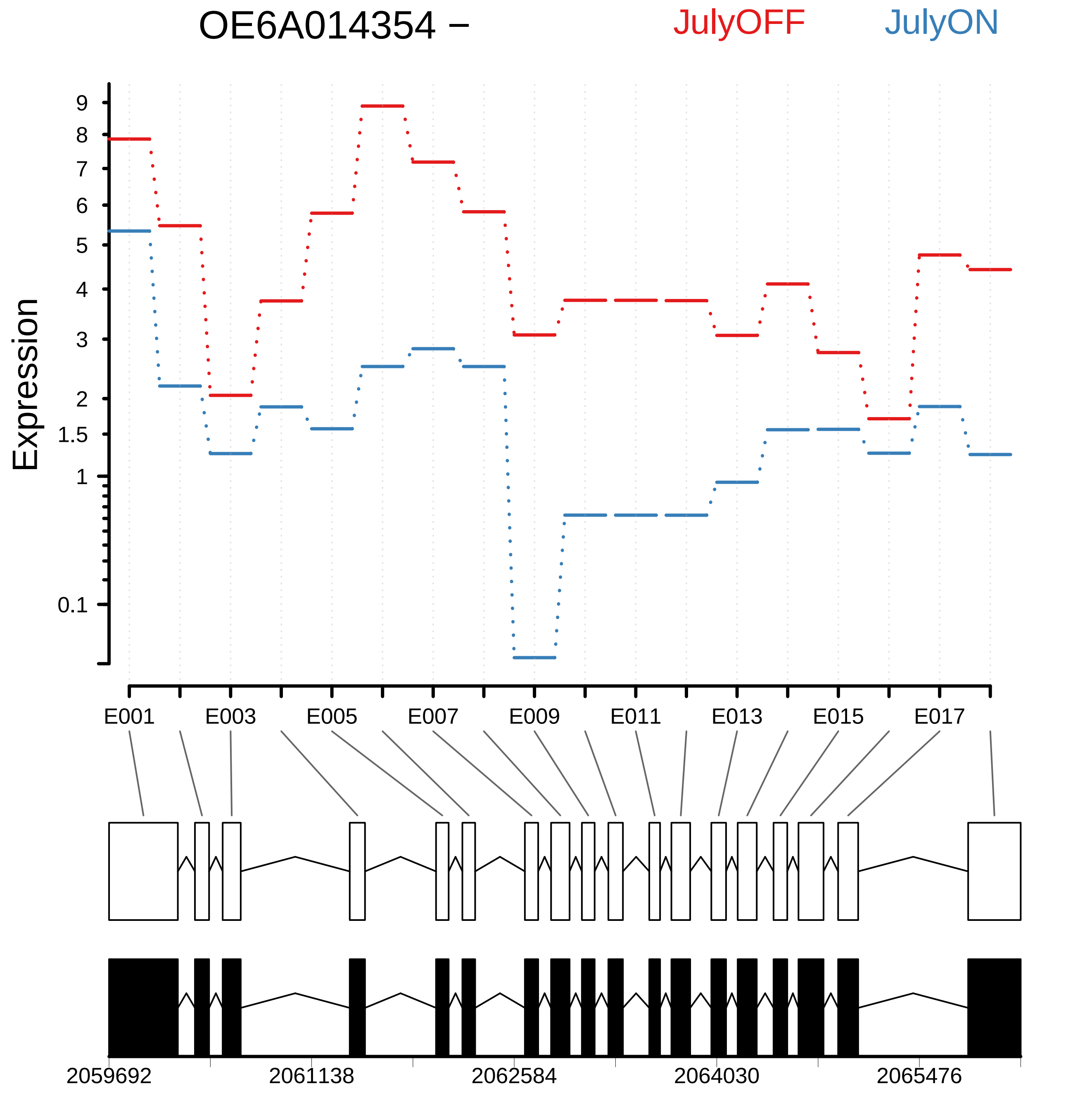
OE6A014354 −
JulyOFF
JulyON
Expression
9
8
7
6
5
4
3
2
1.5
1
0.1
E001
E003
E005
E007
E009
E011
E013
E015
E017
2059692
2061138
2062584
2064030
2065476

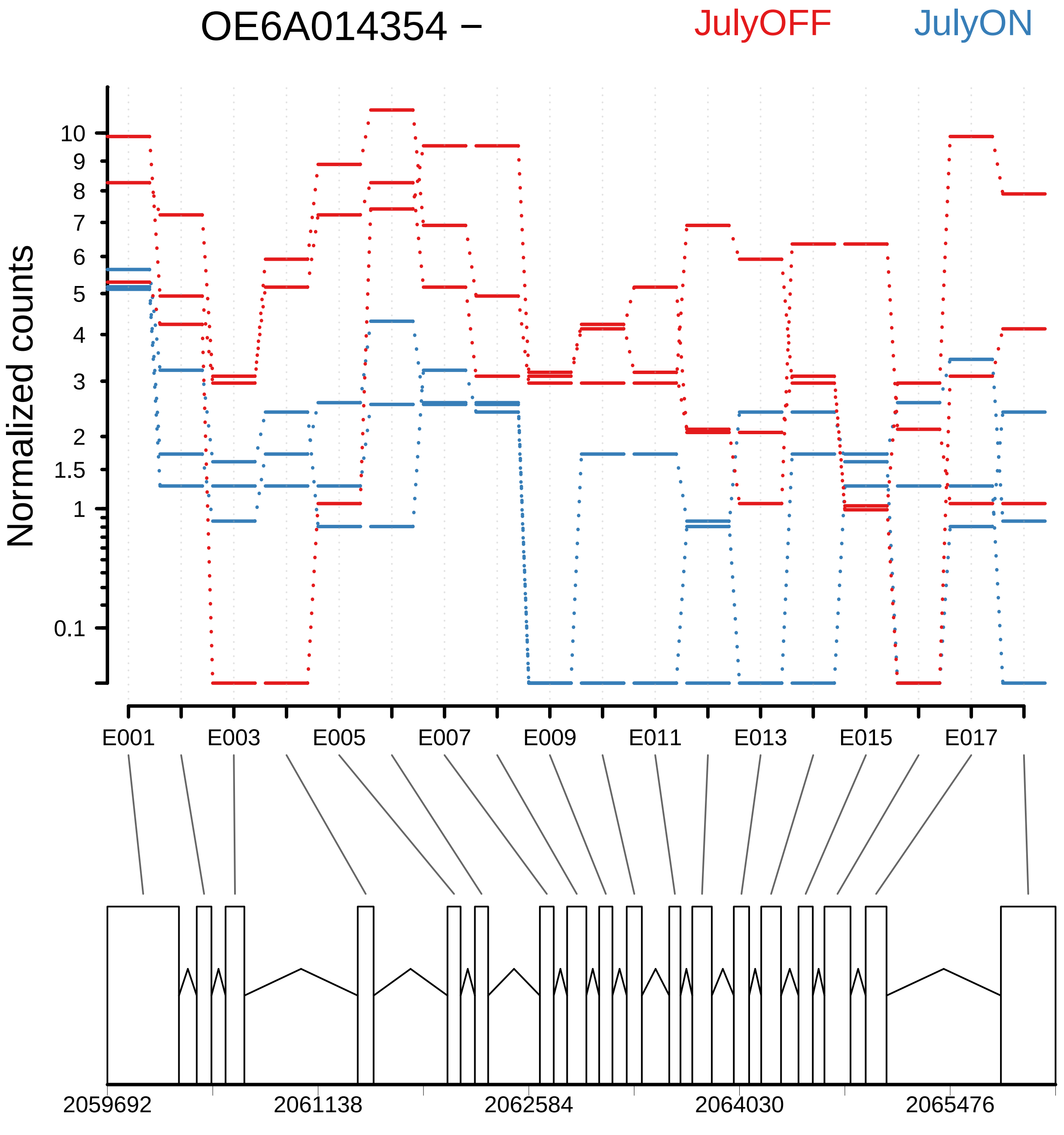
OE6A014354 −
JulyOFF
JulyON
Normalized counts
10
9
8
7
6
5
4
3
2
1.5
1
0.1
E001
E003
E005
E007
E009
E011
E013
E015
E017
2059692
2061138
2062584
2064030
2065476

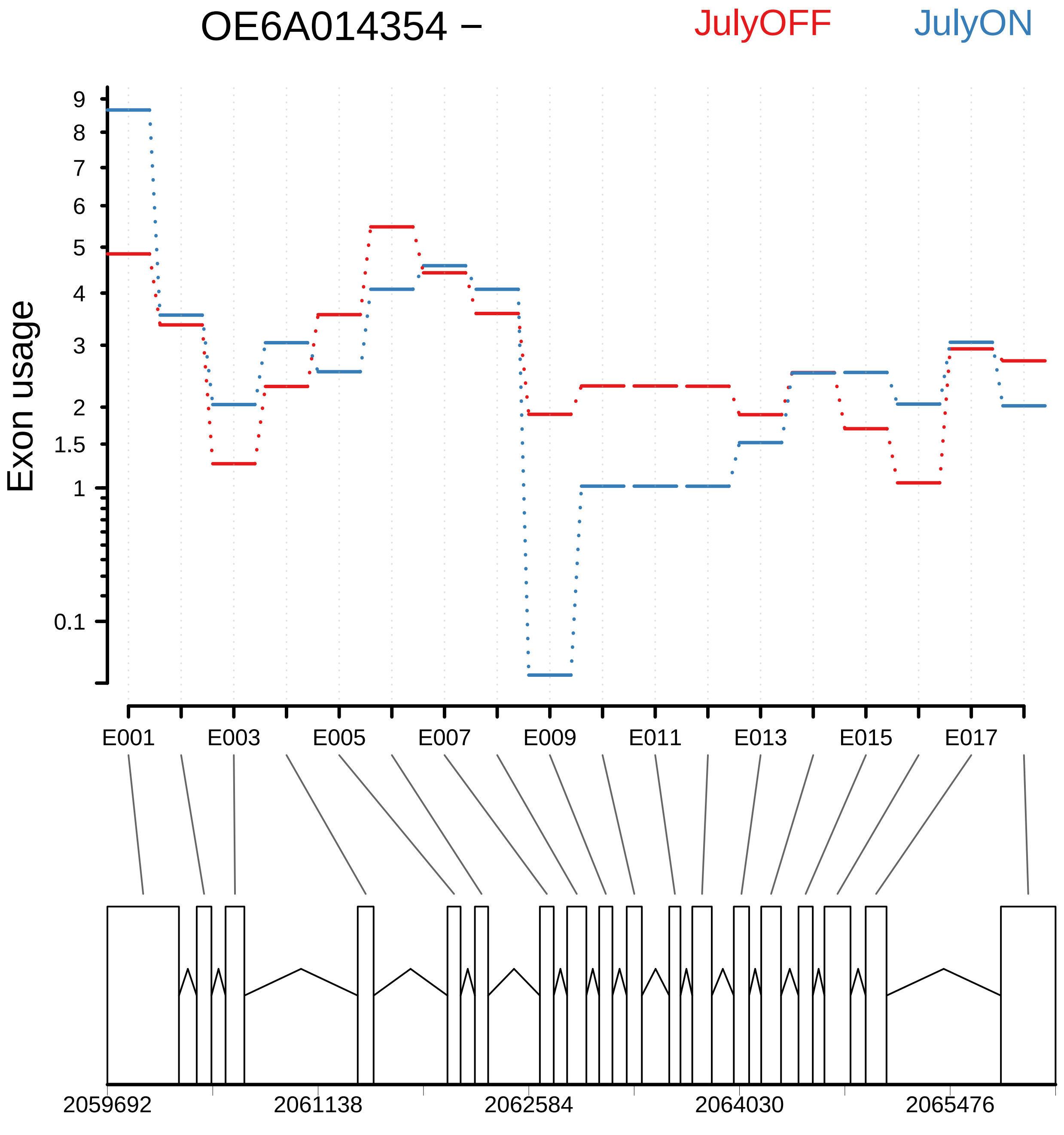

OE6A014354 −
JulyOFF
JulyON
Exon usage
9
8
7
6
5
4
3
2
1.5
1
0.1
E001
E003
E005
E007
E009
E011
E013
E015
E017
2059692
2061138
2062584
2064030
2065476

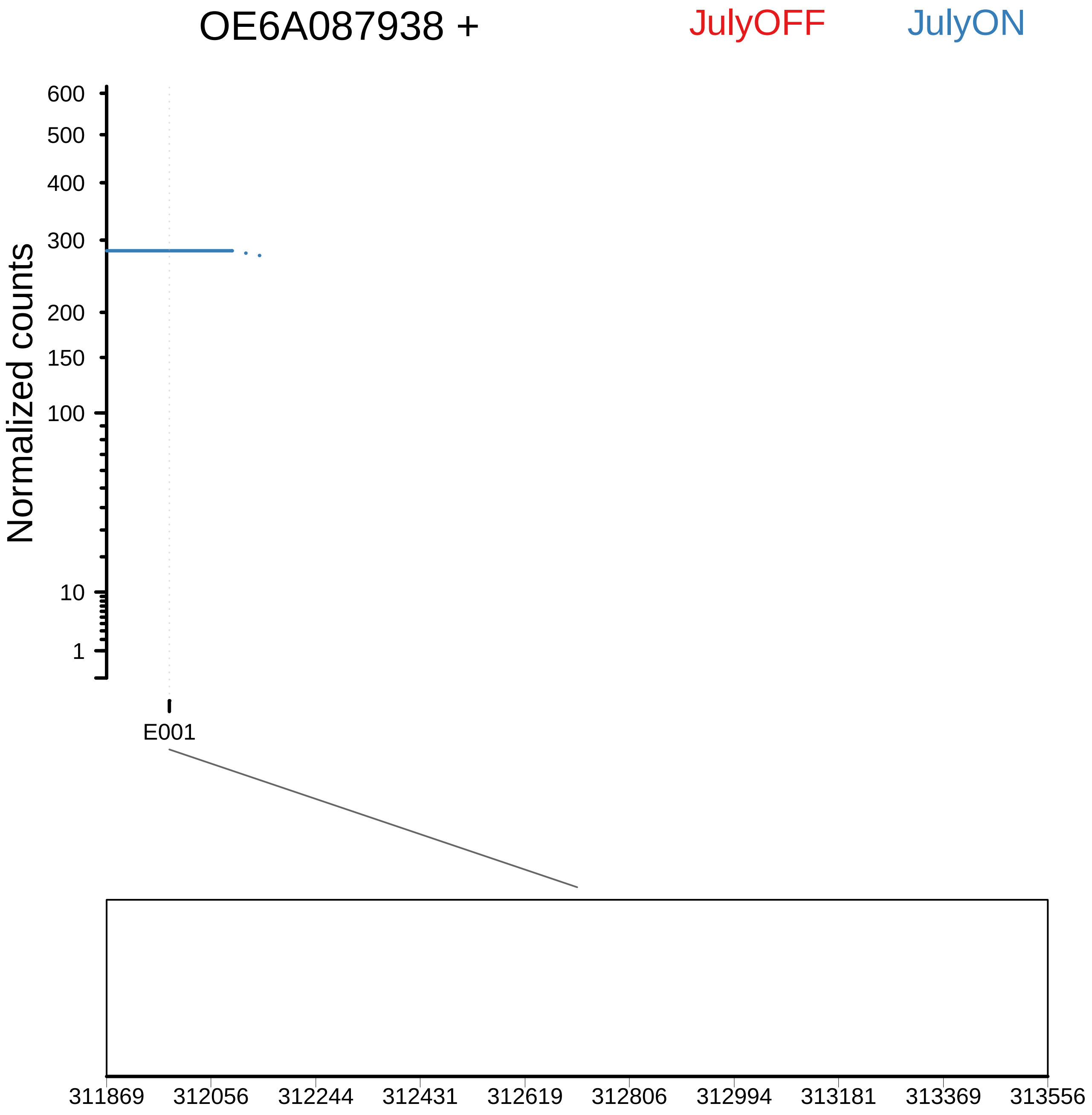
OE6A087938 +
JulyOFF
JulyON
Normalized counts
600
500
400
300
200
150
100
10
1
E001
311869
312056
312244
312431
312619
312806
312994
313181
313369
313556

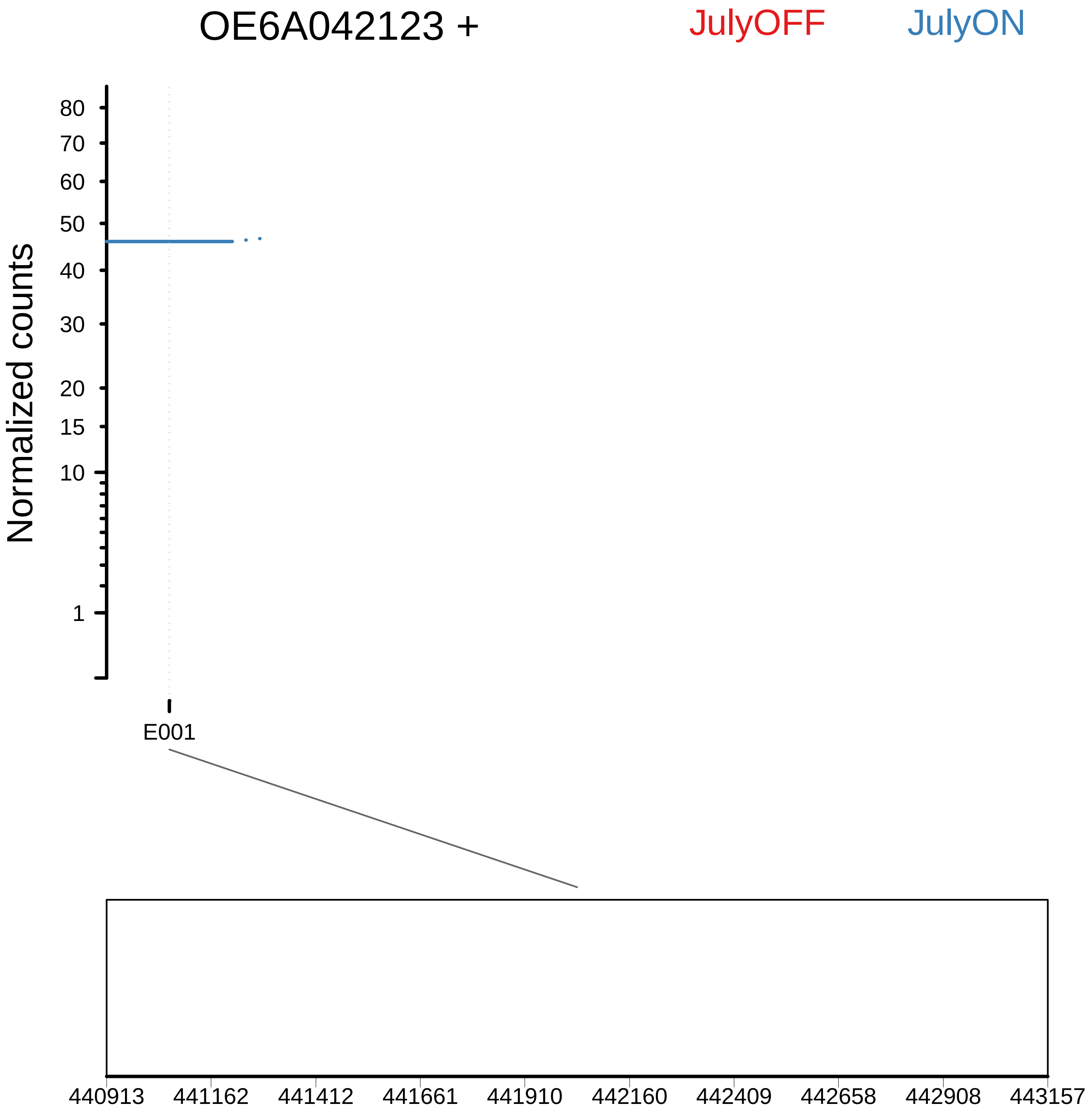

OE6A042123 +
JulyOFF
JulyON
Normalized counts
80
70
60
50
40
30
20
15
10
1
E001
440913
441162
441412
441661
441910
442160
442409
442658
442908
443157

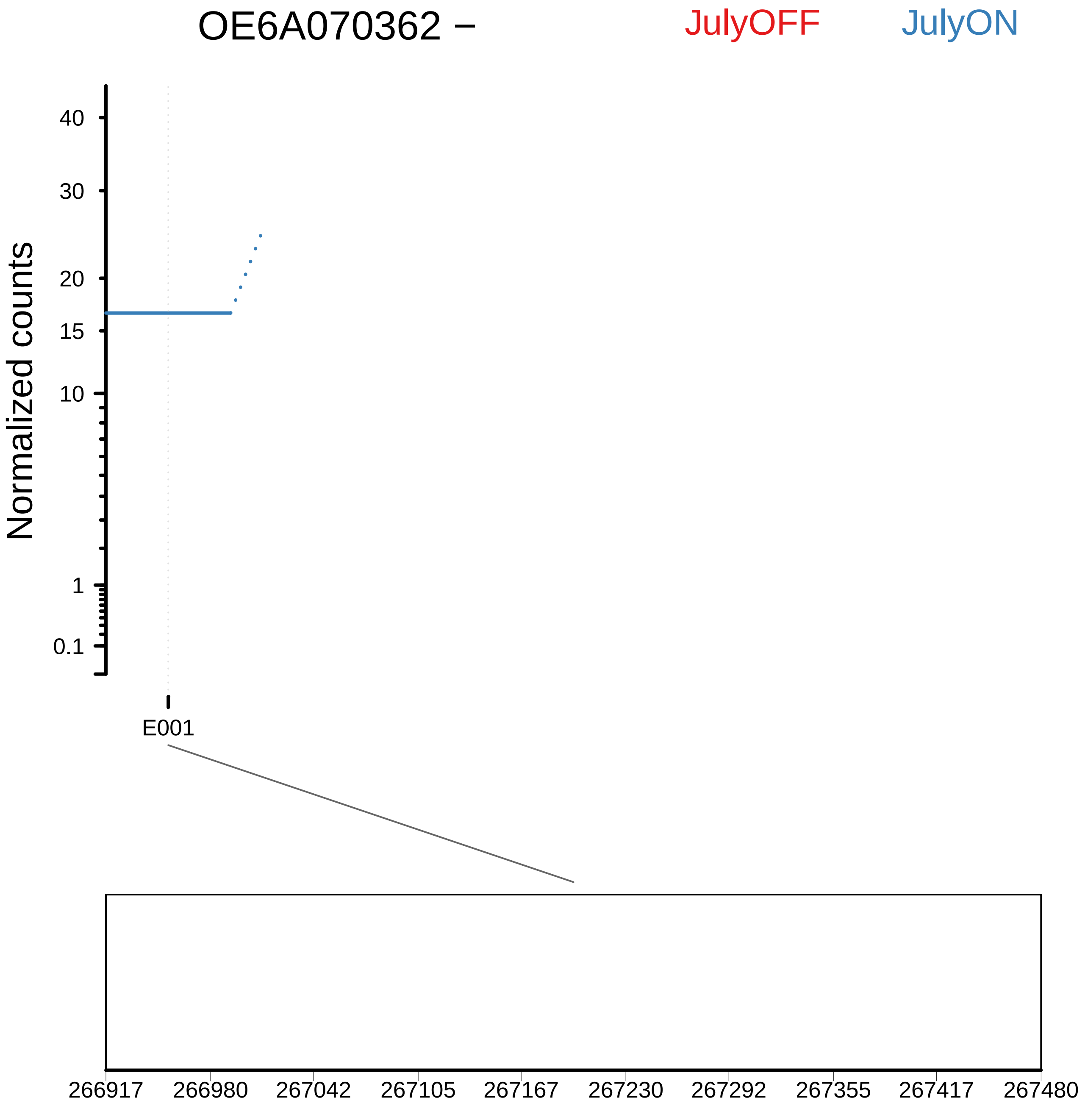
OE6A070362 −
JulyOFF
JulyON
Normalized counts
40
30
20
15
10
1
0.1
E001
266917
266980
267042
267105
267167
267230
267292
267355
267417
267480

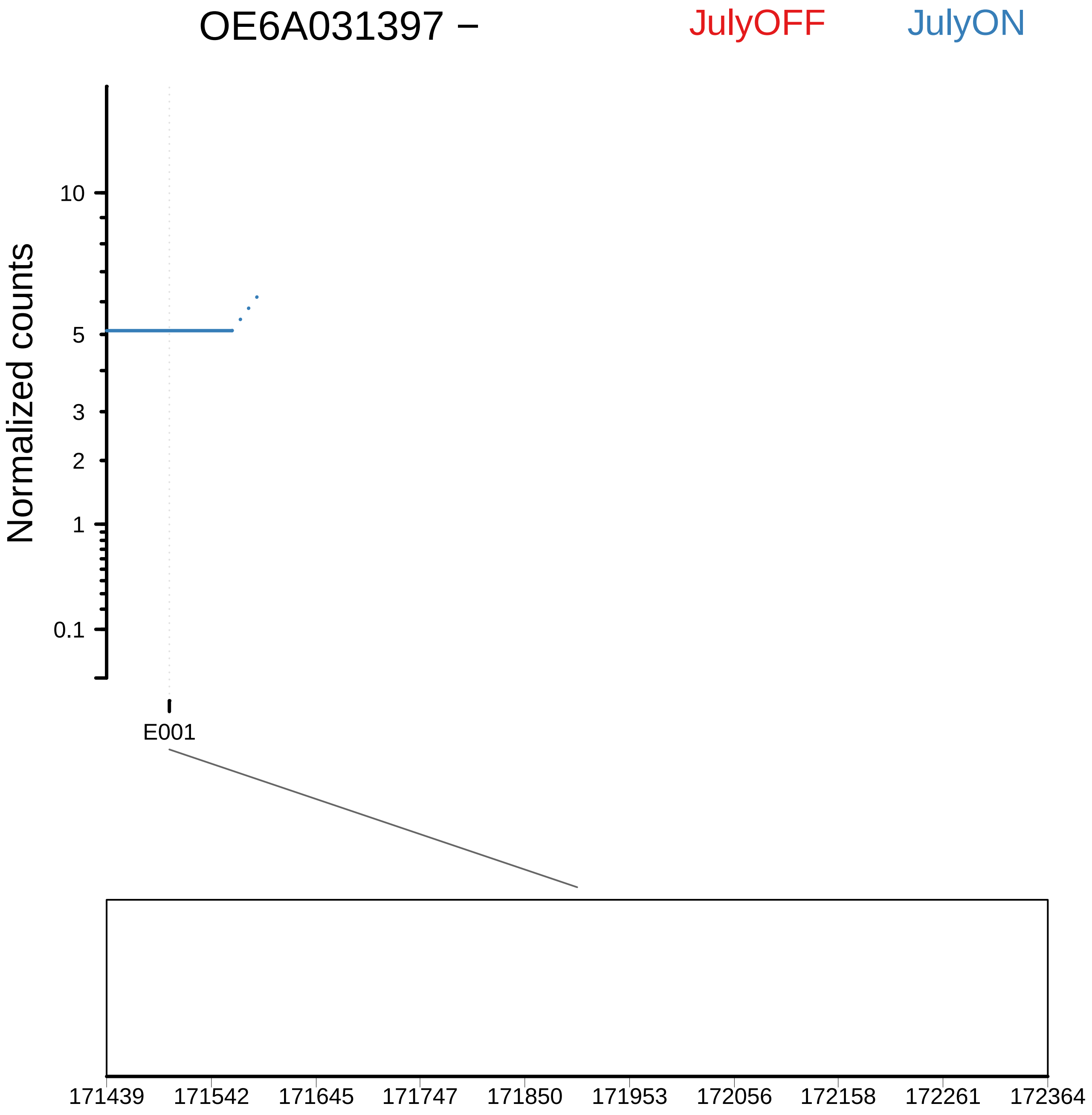

OE6A031397 −
JulyOFF
JulyON
Normalized counts
10
5
3
2
1
0.1
E001
171439
171542
171645
171747
171850
171953
172056
172158
172261
172364

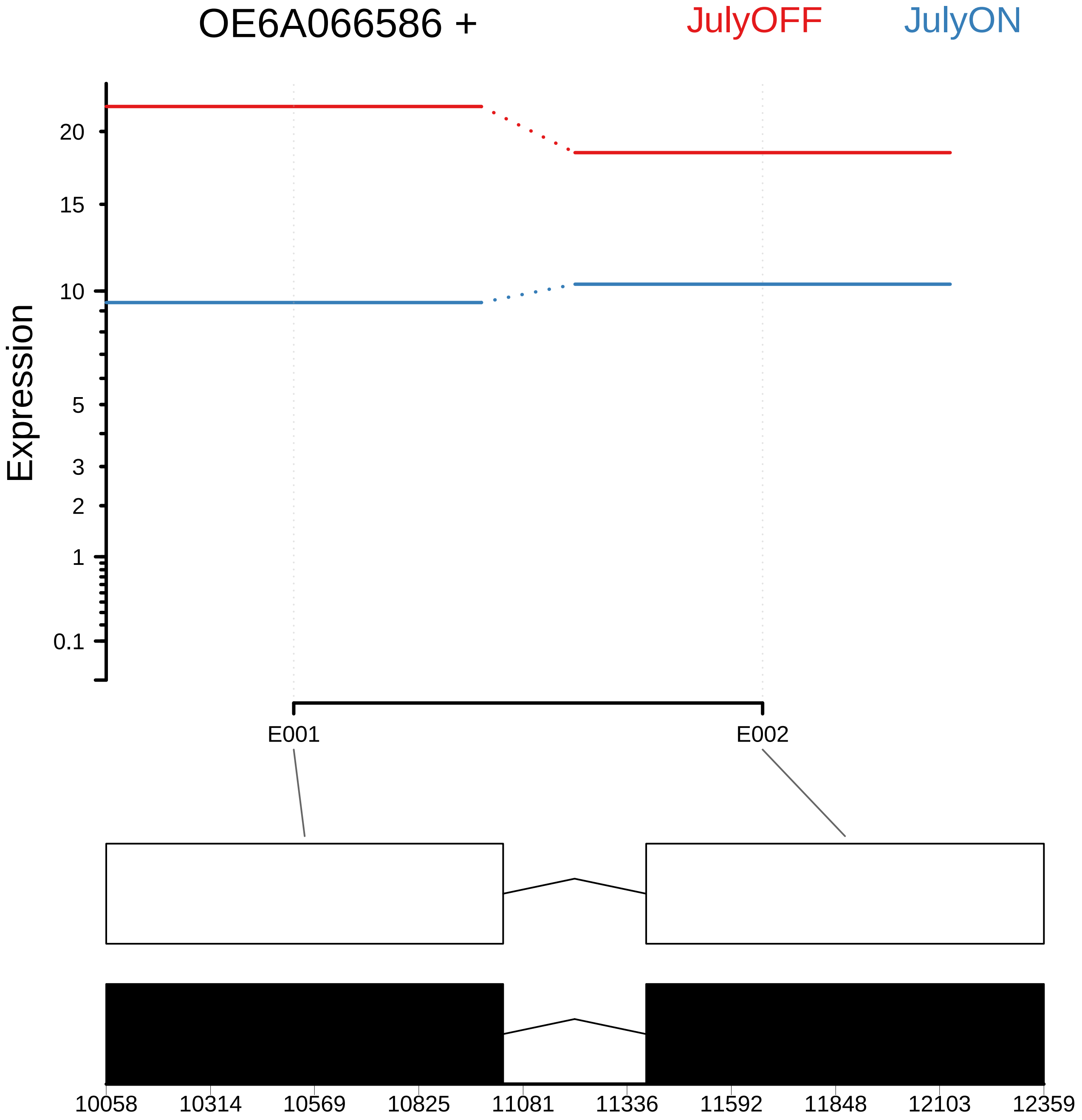

OE6A066586 +
JulyOFF
JulyON
Expression
20
15
10
5
3
2
1
0.1
E001
E002
10058
10314
10569
10825
11081
11336
11592
11848
12103
12359

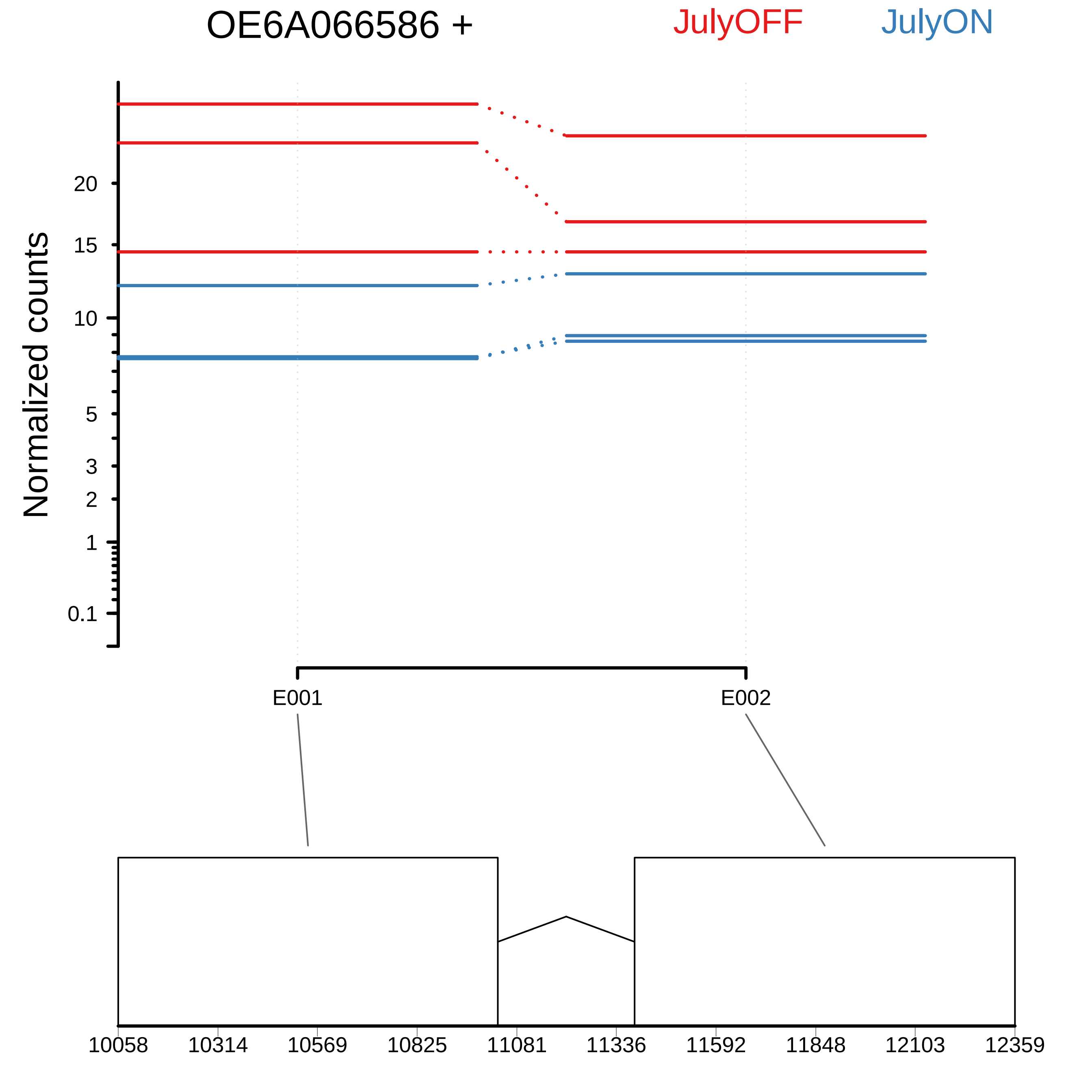

OE6A066586 +
JulyOFF
JulyON
Normalized counts
20
15
10
5
3
2
1
0.1
E001
E002
10058
10314
10569
10825
11081
11336
11592
11848
12103
12359

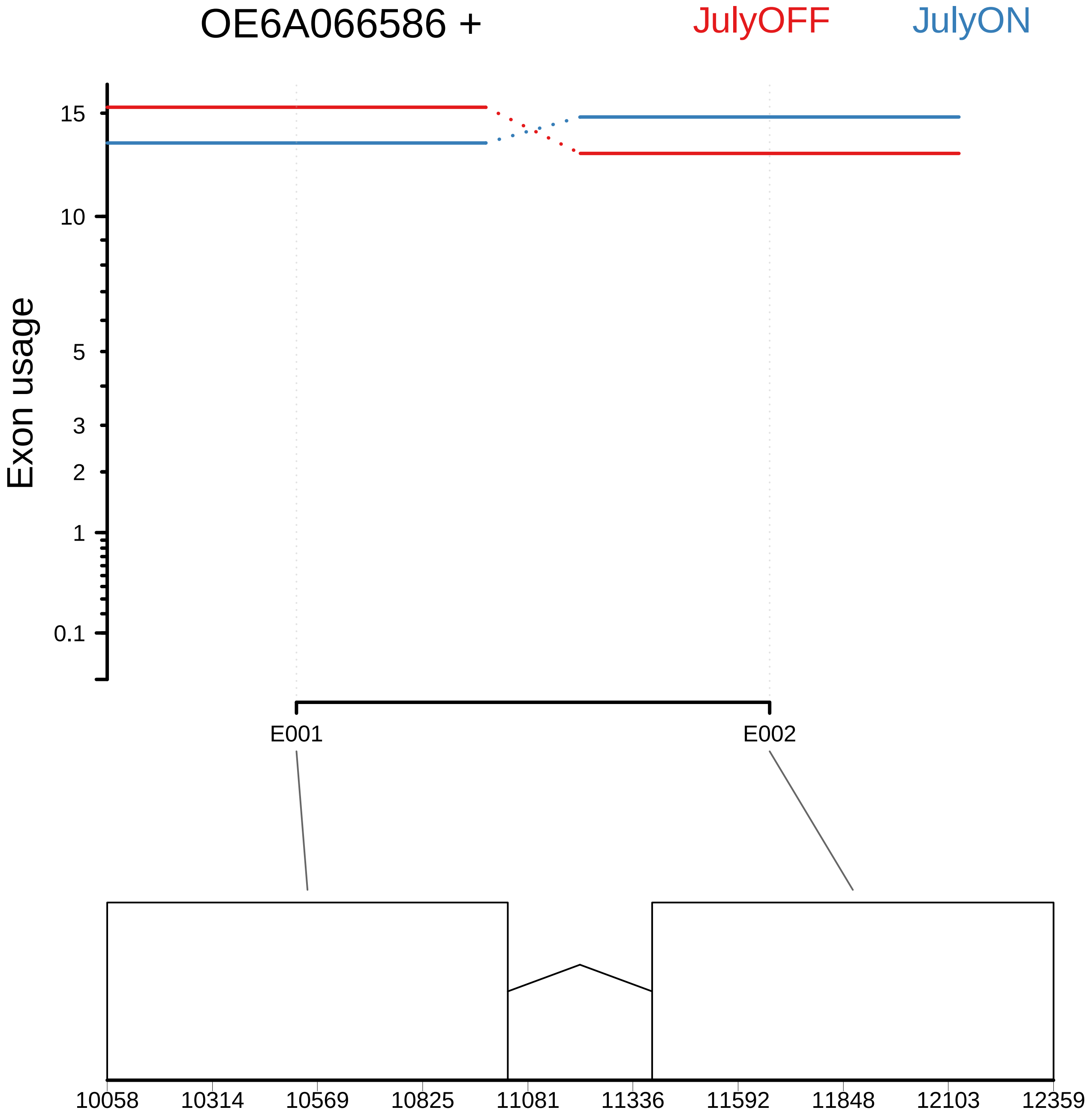

OE6A066586 +
JulyOFF
JulyON
Exon usage
15
10
5
3
2
1
0.1
E001
E002
10058
10314
10569
10825
11081
11336
11592
11848
12103
12359

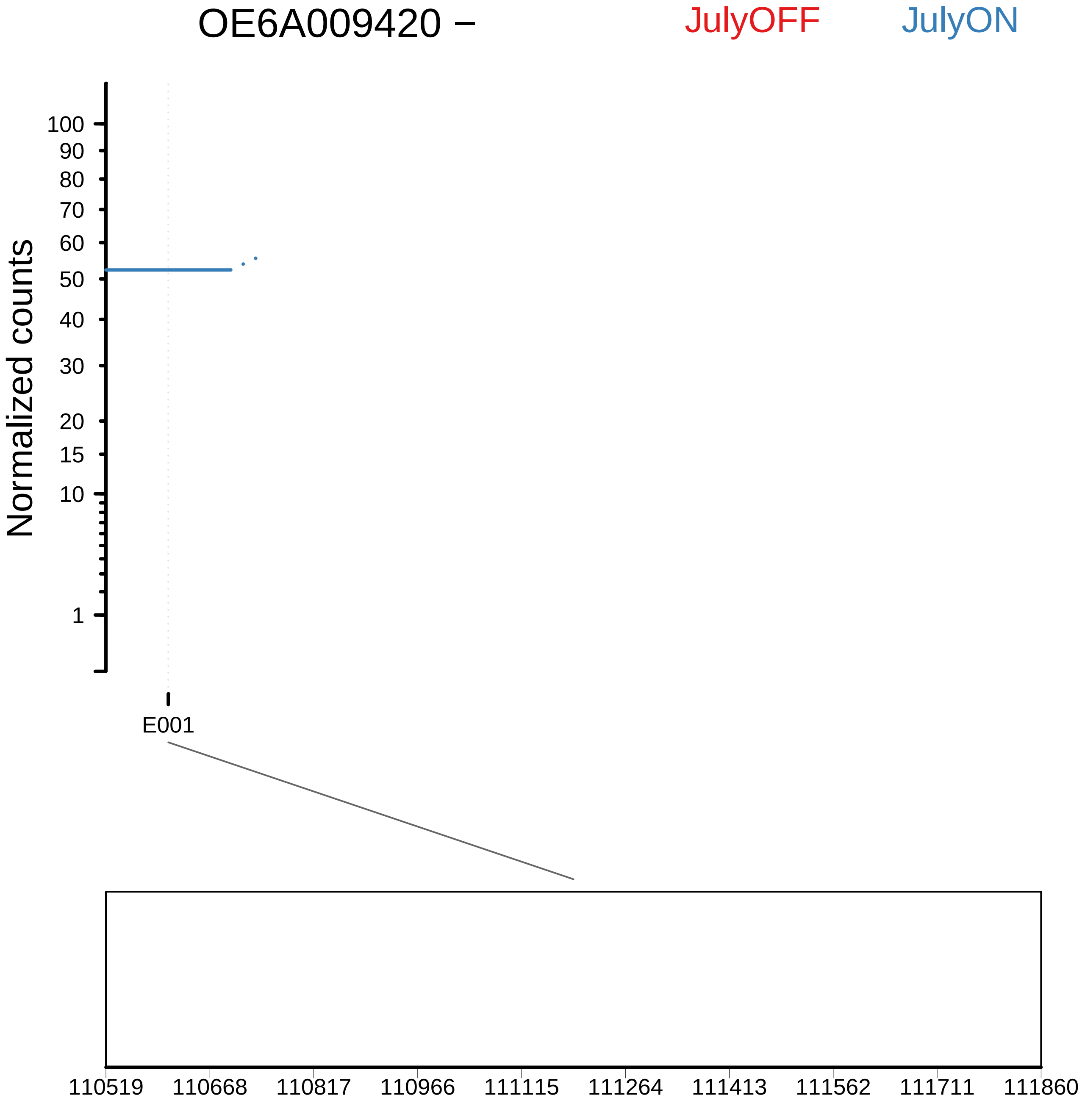

OE6A009420 −
JulyOFF
JulyON
Normalized counts
100
90
80
70
60
50
40
30
20
15
10
1
E001
110519
110668
110817
110966
111115
111264
111413
111562
111711
111860

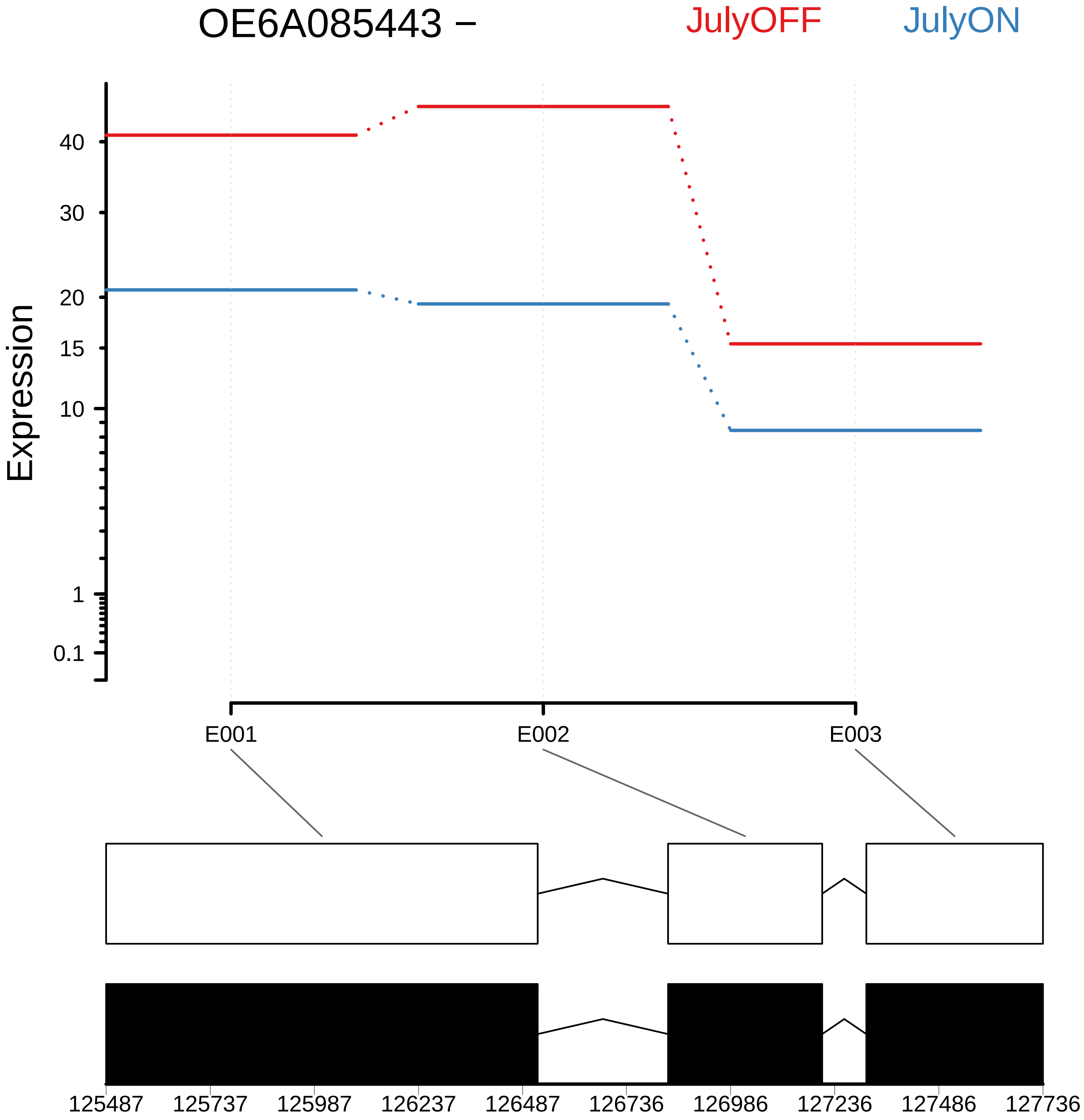
OE6A085443 −
JulyOFF
JulyON
Expression
40
30
20
15
10
1
0.1
E001
E002
E003
125487
125737
125987
126237
126487
126736
126986
127236
127486
127736

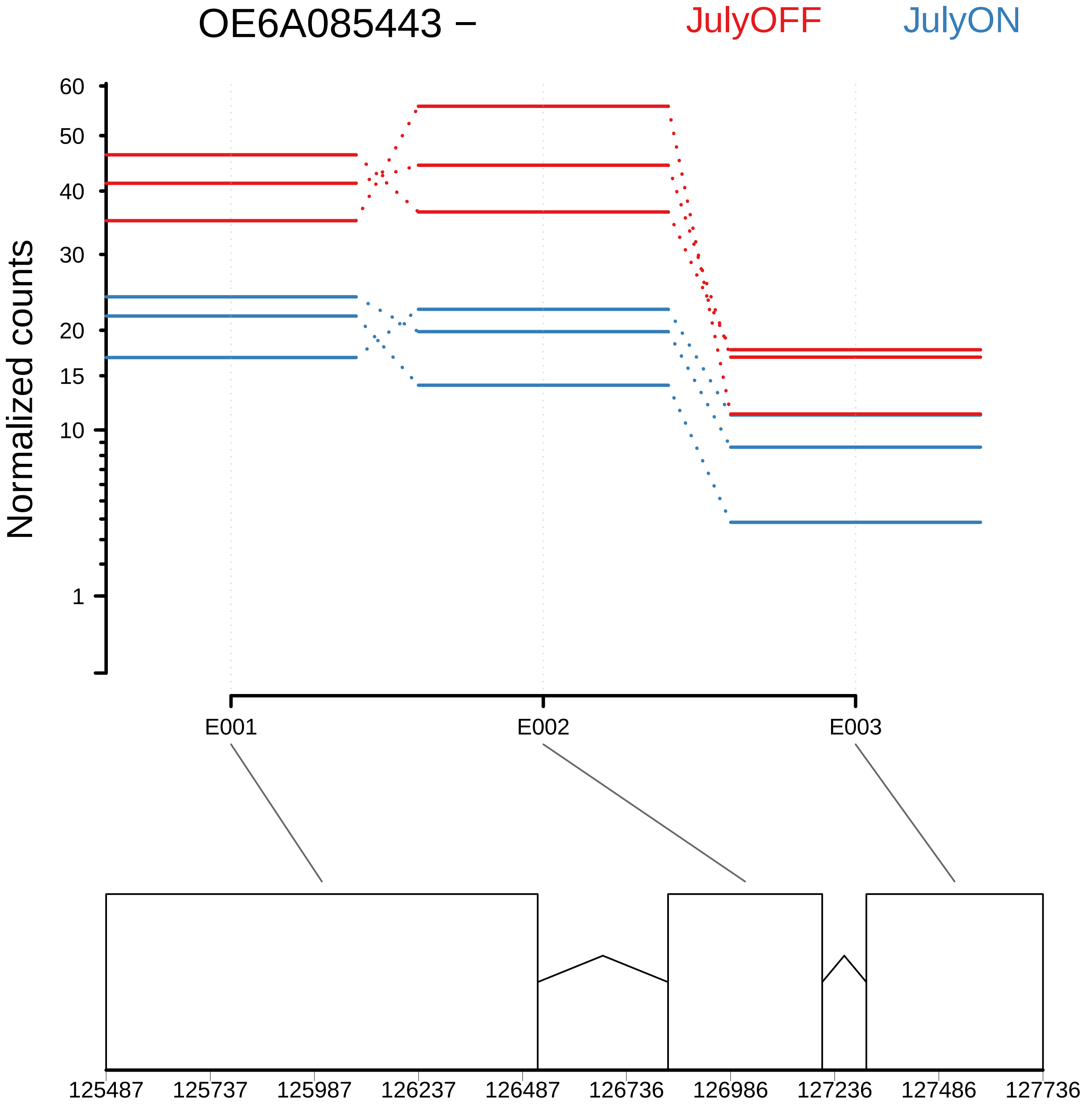
OE6A085443 −
JulyOFF
JulyON
Normalized counts
60
50
40
30
20
15
10
1
E001
E002
E003
125487
125737
125987
126237
126487
126736
126986
127236
127486
127736

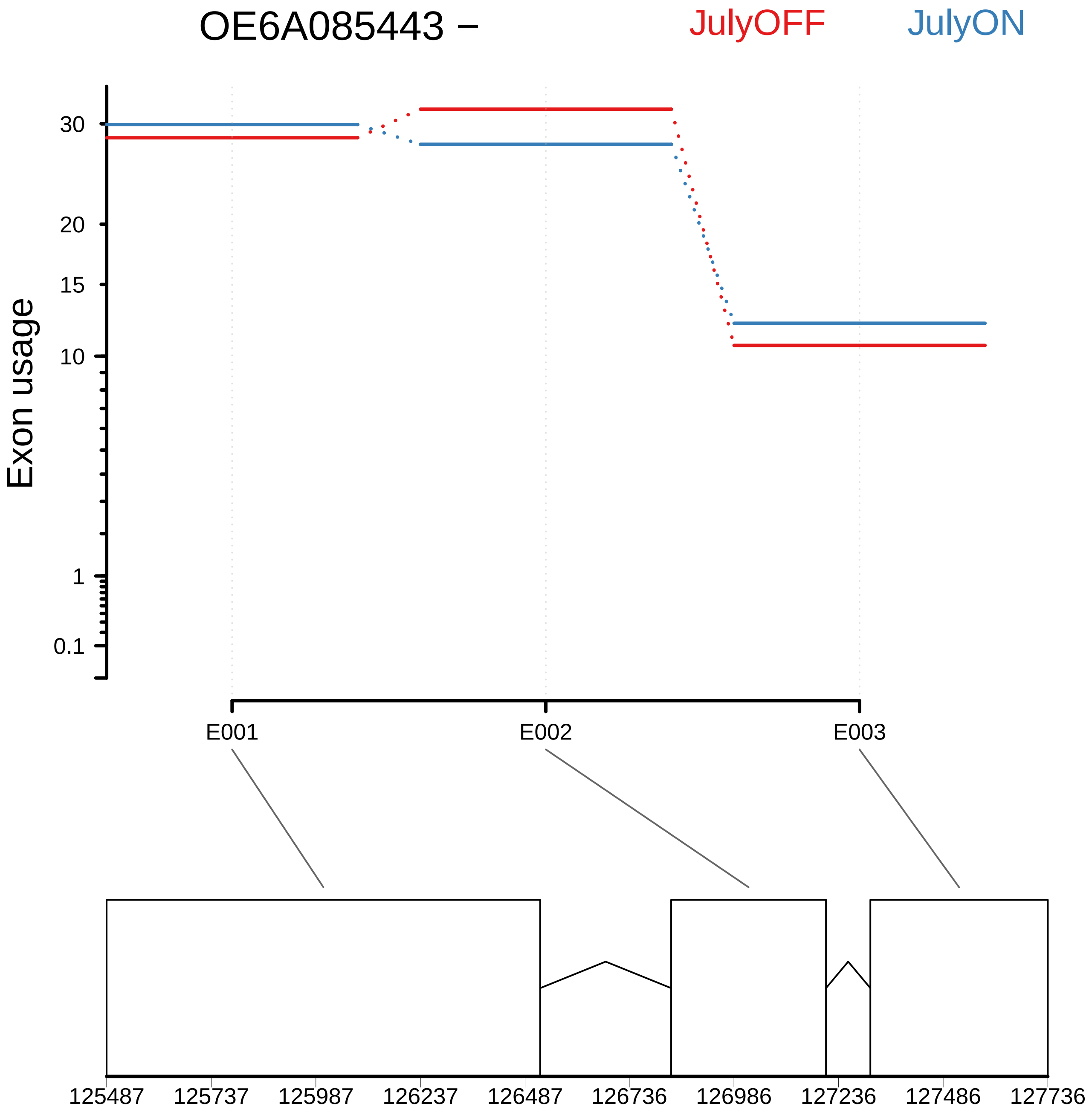
OE6A085443 −
JulyOFF
JulyON
Exon usage
30
20
15
10
1
0.1
E001
E002
E003
125487
125737
125987
126237
126487
126736
126986
127236
127486
127736

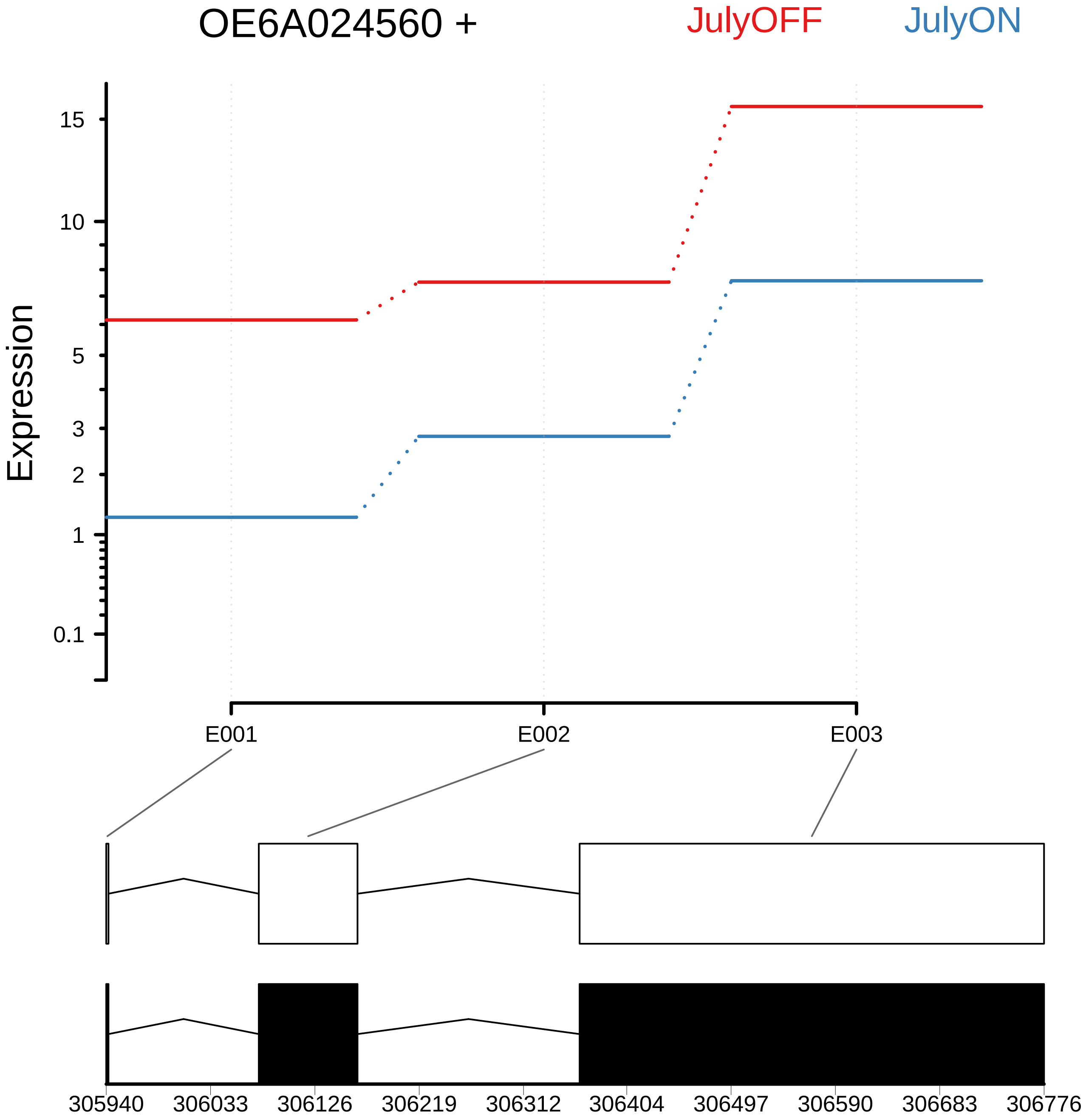

OE6A024560 +
JulyOFF
JulyON
Expression
15
10
5
3
2
1
0.1
E001
E002
E003
305940
306033
306126
306219
306312
306404
306497
306590
306683
306776

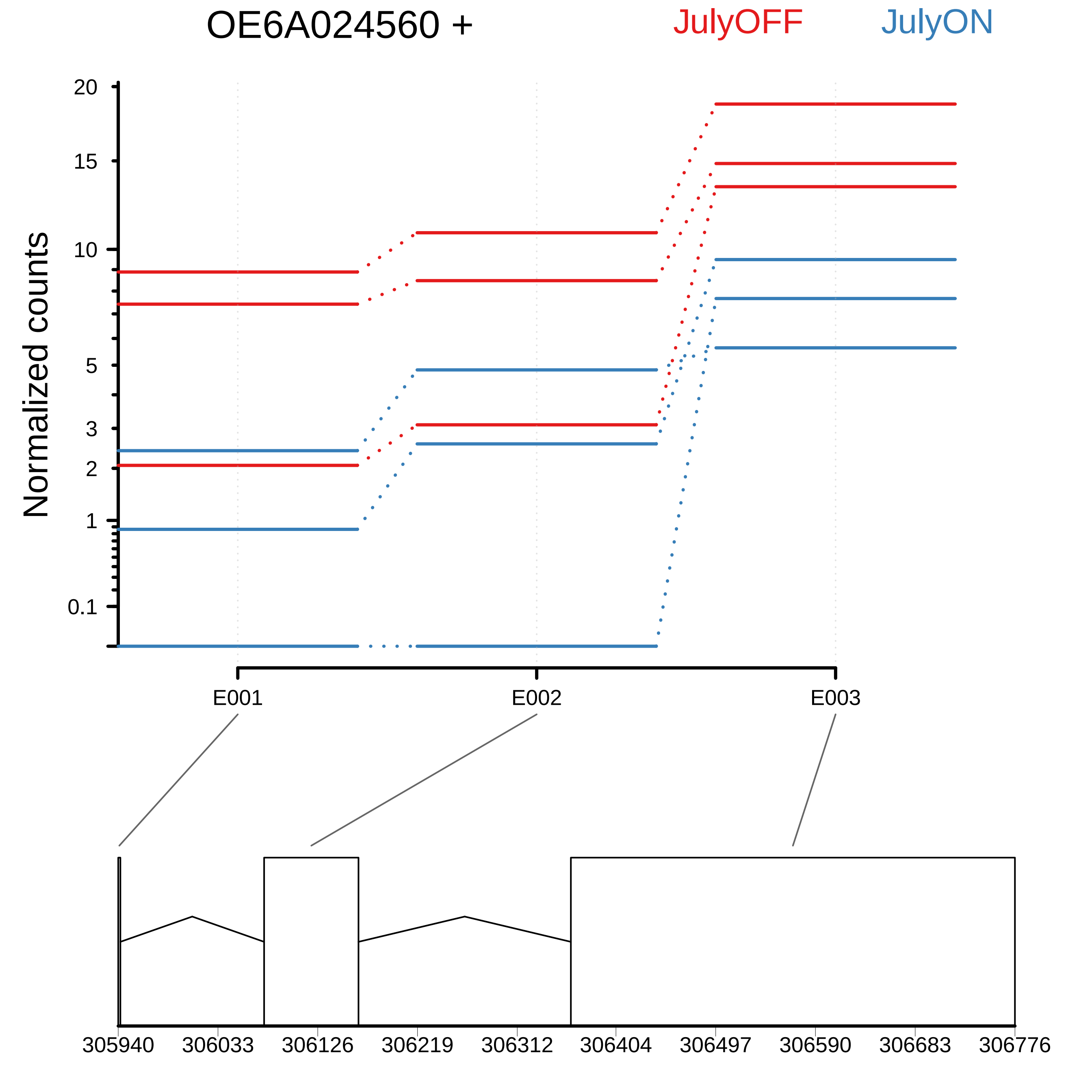

OE6A024560 +
JulyOFF
JulyON
Normalized counts
20
15
10
5
3
2
1
0.1
E001
E002
E003
305940
306033
306126
306219
306312
306404
306497
306590
306683
306776

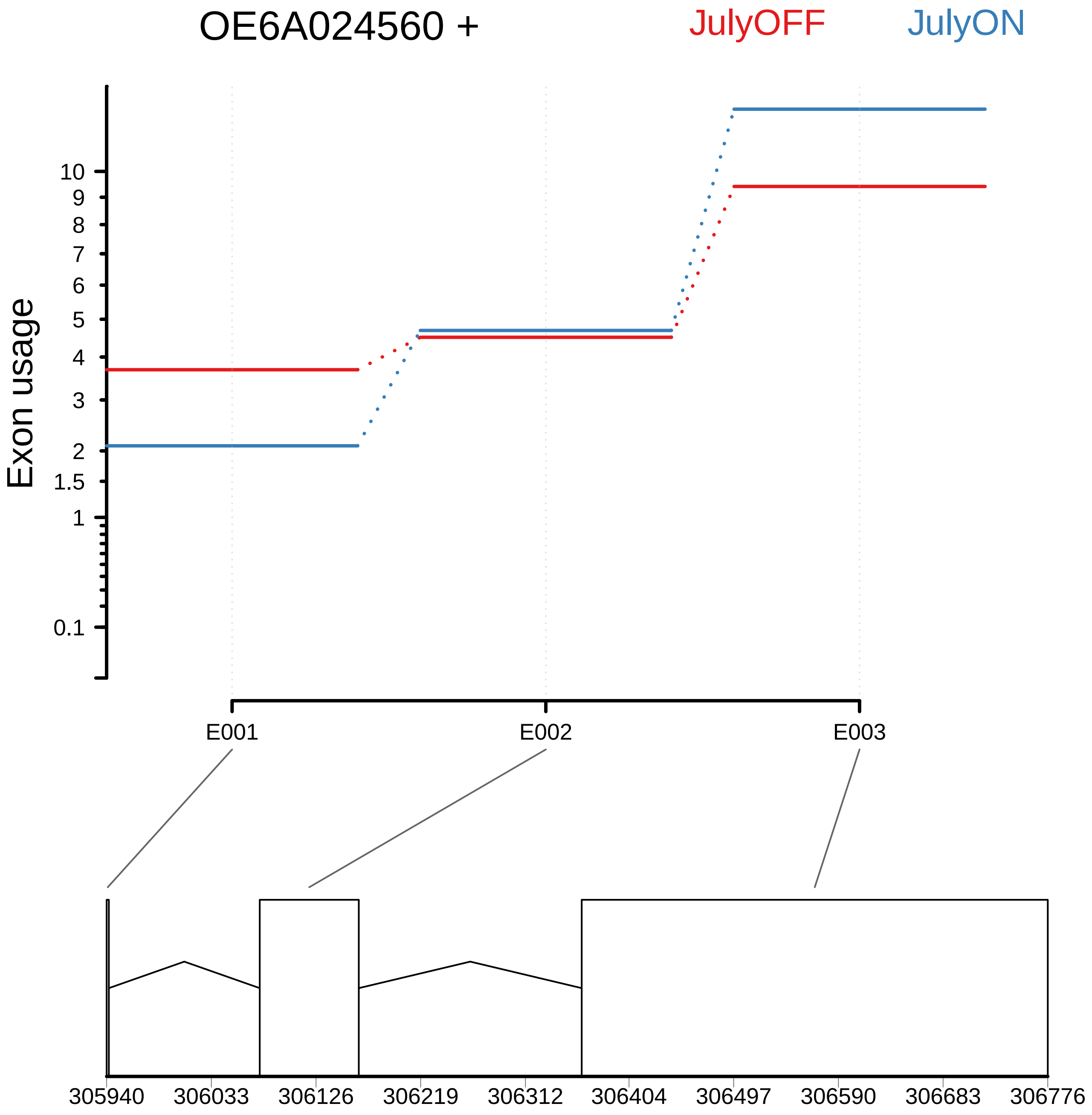
OE6A024560 +
JulyOFF
JulyON
Exon usage
10
9
8
7
6
5
4
3
2
1.5
1
0.1
E001
E002
E003
305940
306033
306126
306219
306312
306404
306497
306590
306683
306776

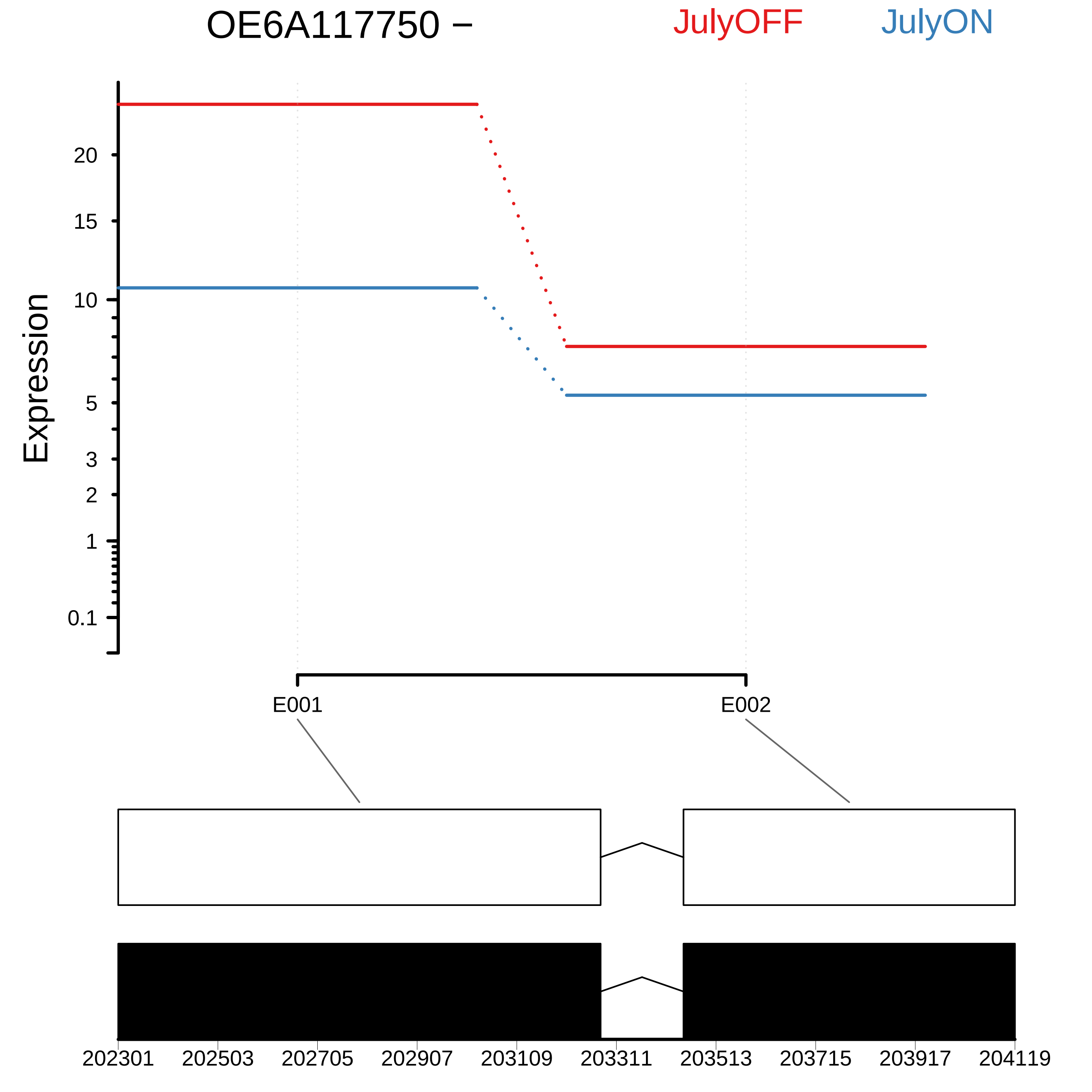
OE6A117750 −
JulyOFF
JulyON
Expression
20
15
10
5
3
2
1
0.1
E001
E002
202301
202503
202705
202907
203109
203311
203513
203715
203917
204119

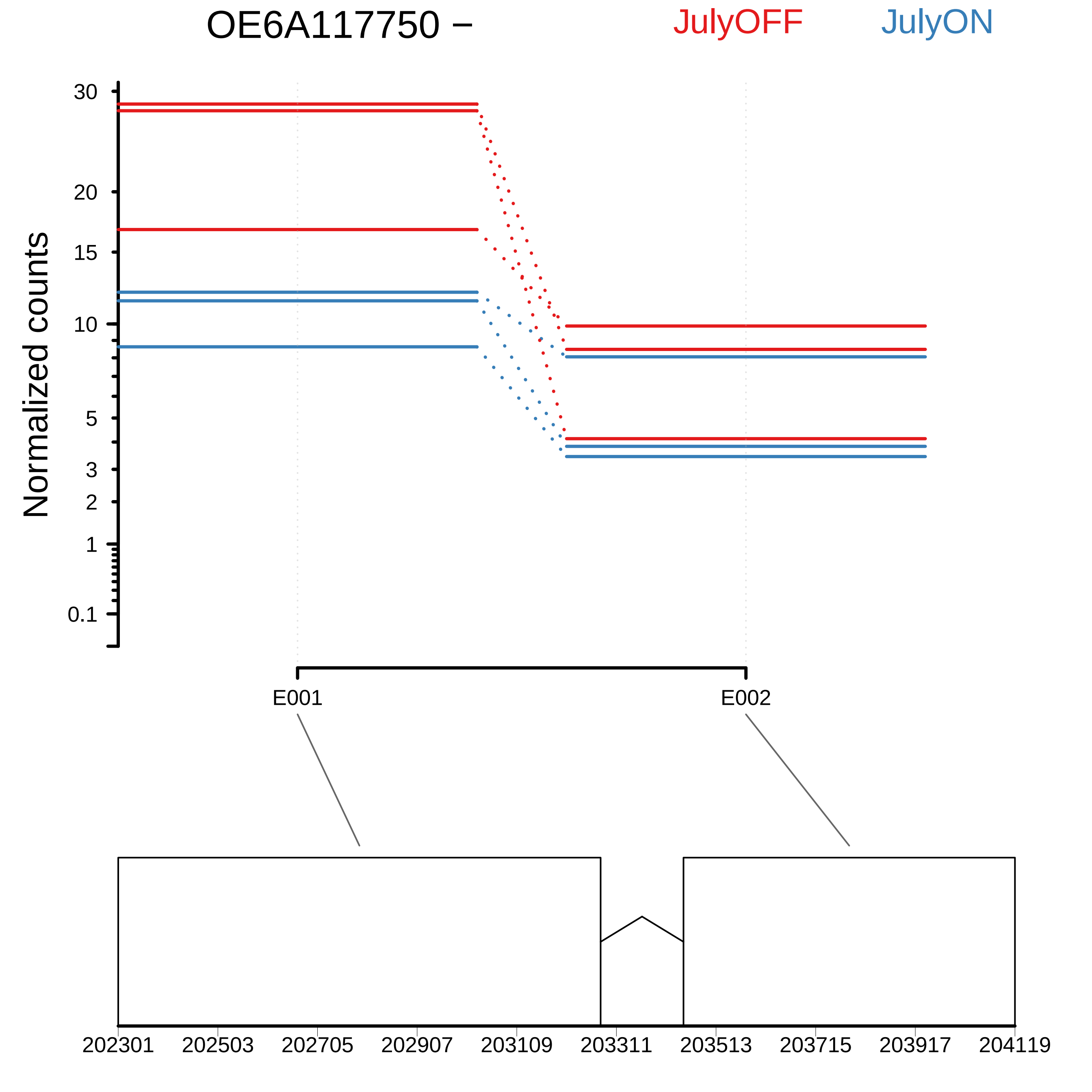

OE6A117750 −
JulyOFF
JulyON
Normalized counts
30
20
15
10
5
3
2
1
0.1
E001
E002
202301
202503
202705
202907
203109
203311
203513
203715
203917
204119

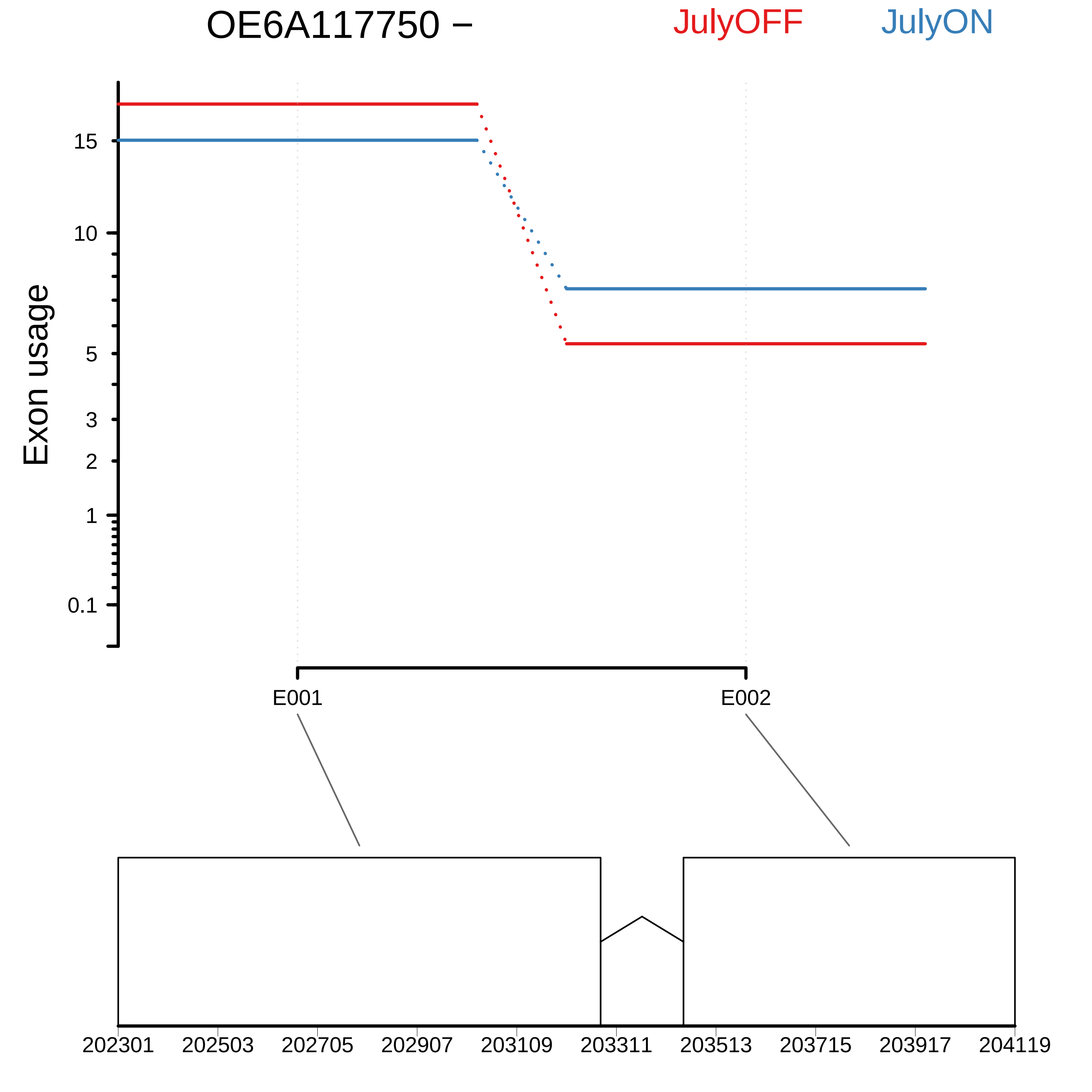
OE6A117750 −
JulyOFF
JulyON
Exon usage
15
10
5
3
2
1
0.1
E001
E002
202301
202503
202705
202907
203109
203311
203513
203715
203917
204119

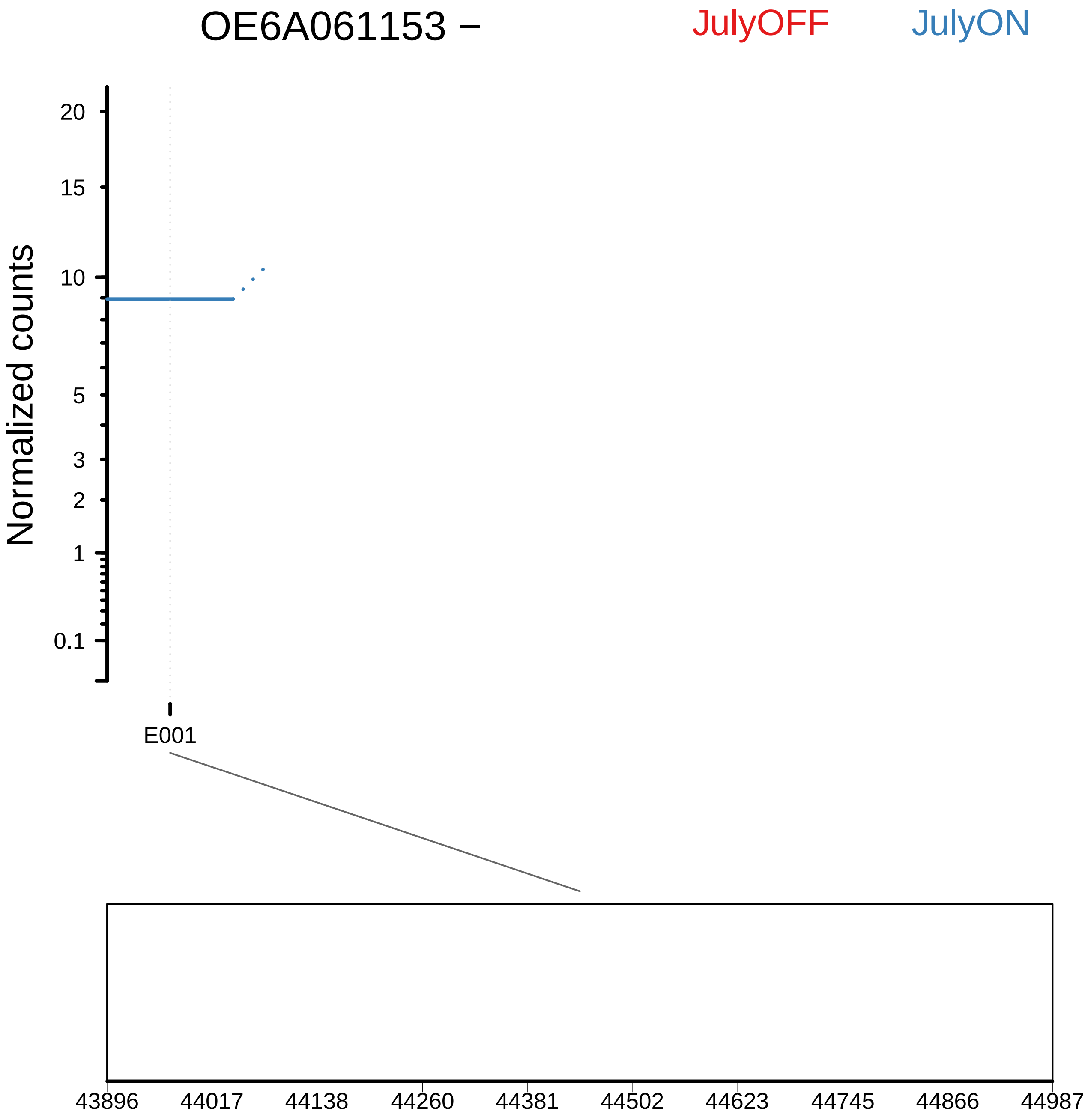
OE6A061153 −
JulyOFF
JulyON
Normalized counts
20
15
10
5
3
2
1
0.1
E001
43896
44017
44138
44260
44381
44502
44623
44745
44866
44987

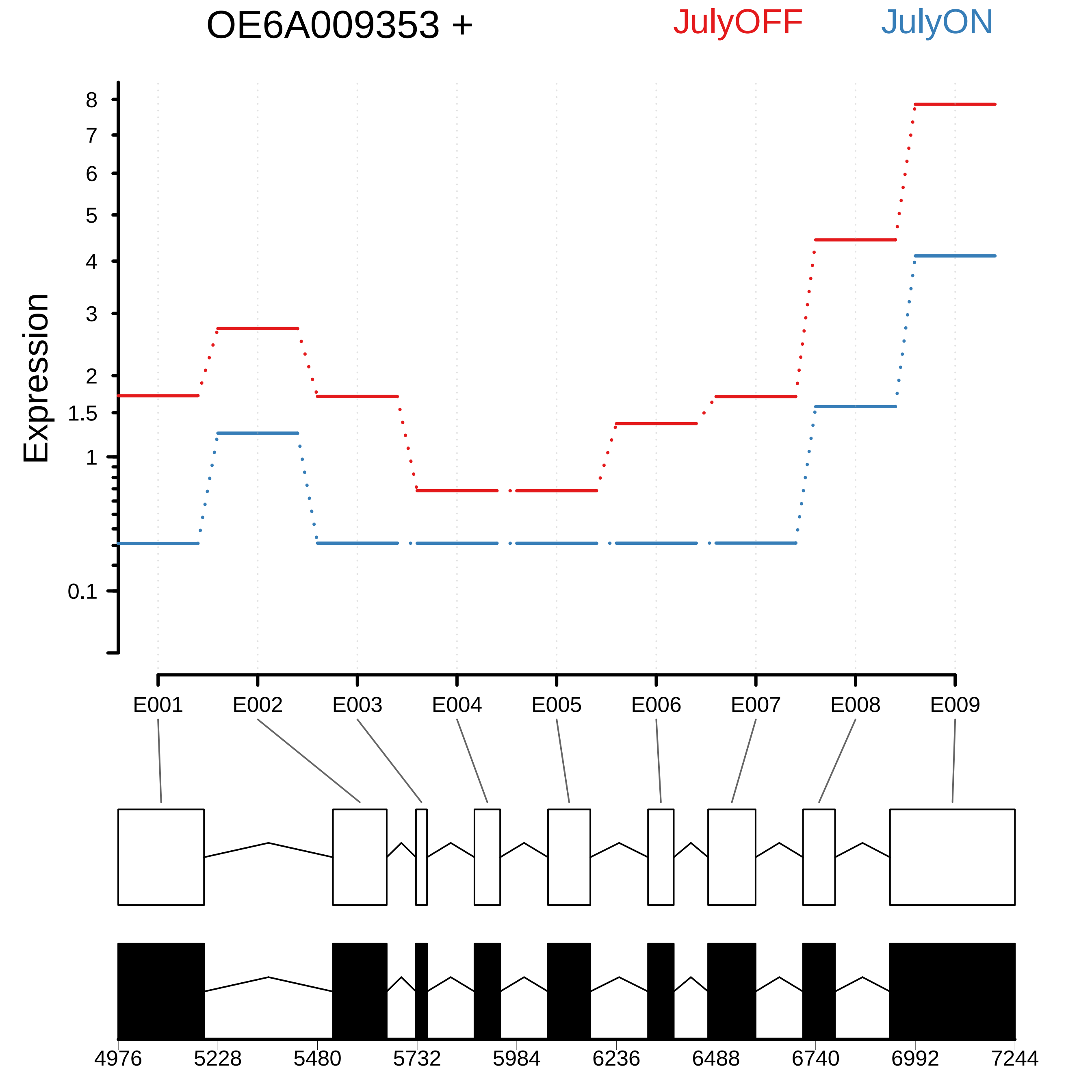

OE6A009353 +
JulyOFF
JulyON
Expression
8
7
6
5
4
3
2
1.5
1
0.1
E001
E002
E003
E004
E005
E006
E007
E008
E009
4976
5228
5480
5732
5984
6236
6488
6740
6992
7244

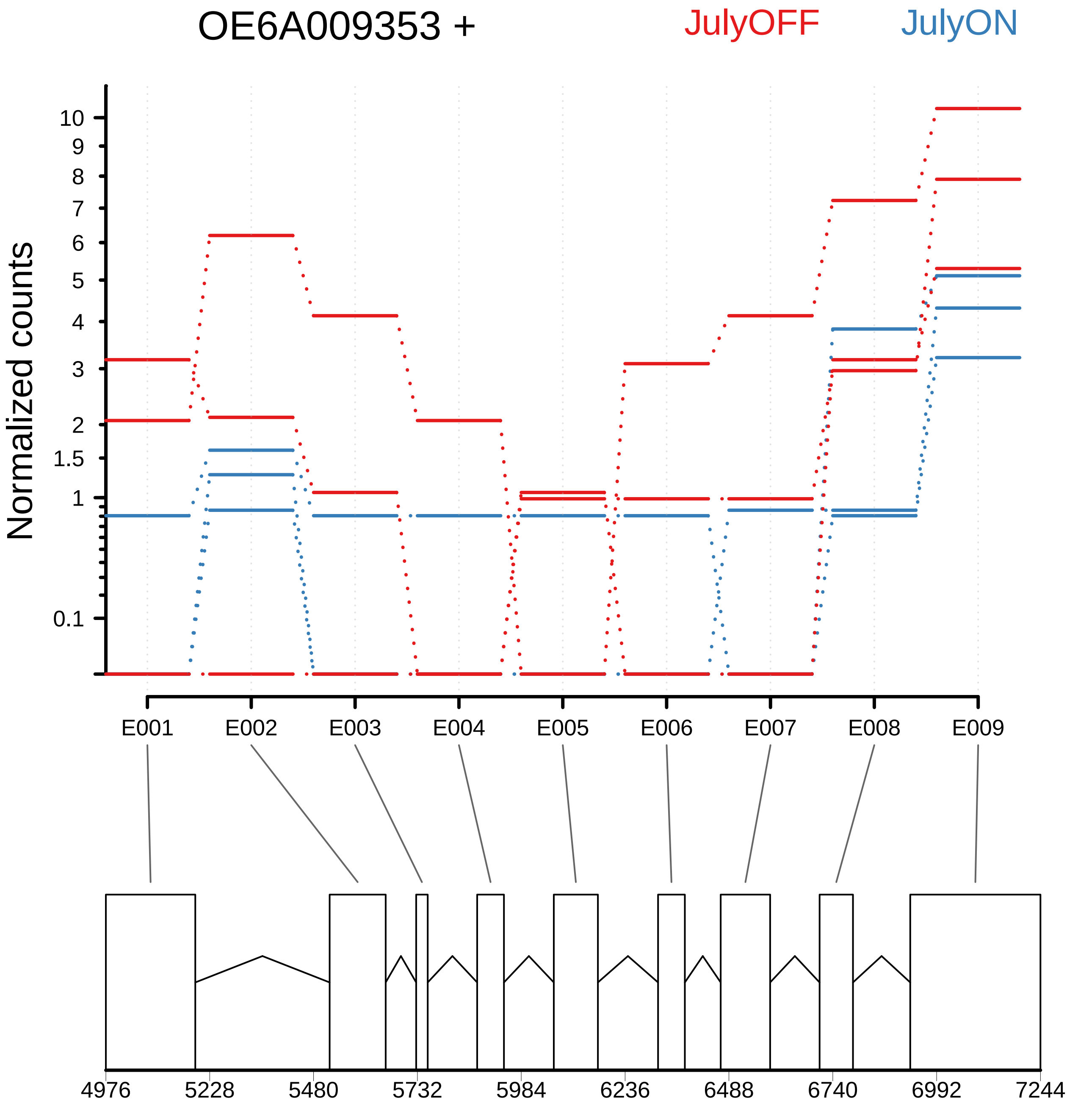

OE6A009353 +
JulyOFF
JulyON
Normalized counts
10
9
8
7
6
5
4
3
2
1.5
1
0.1
E001
E002
E003
E004
E005
E006
E007
E008
E009
4976
5228
5480
5732
5984
6236
6488
6740
6992
7244

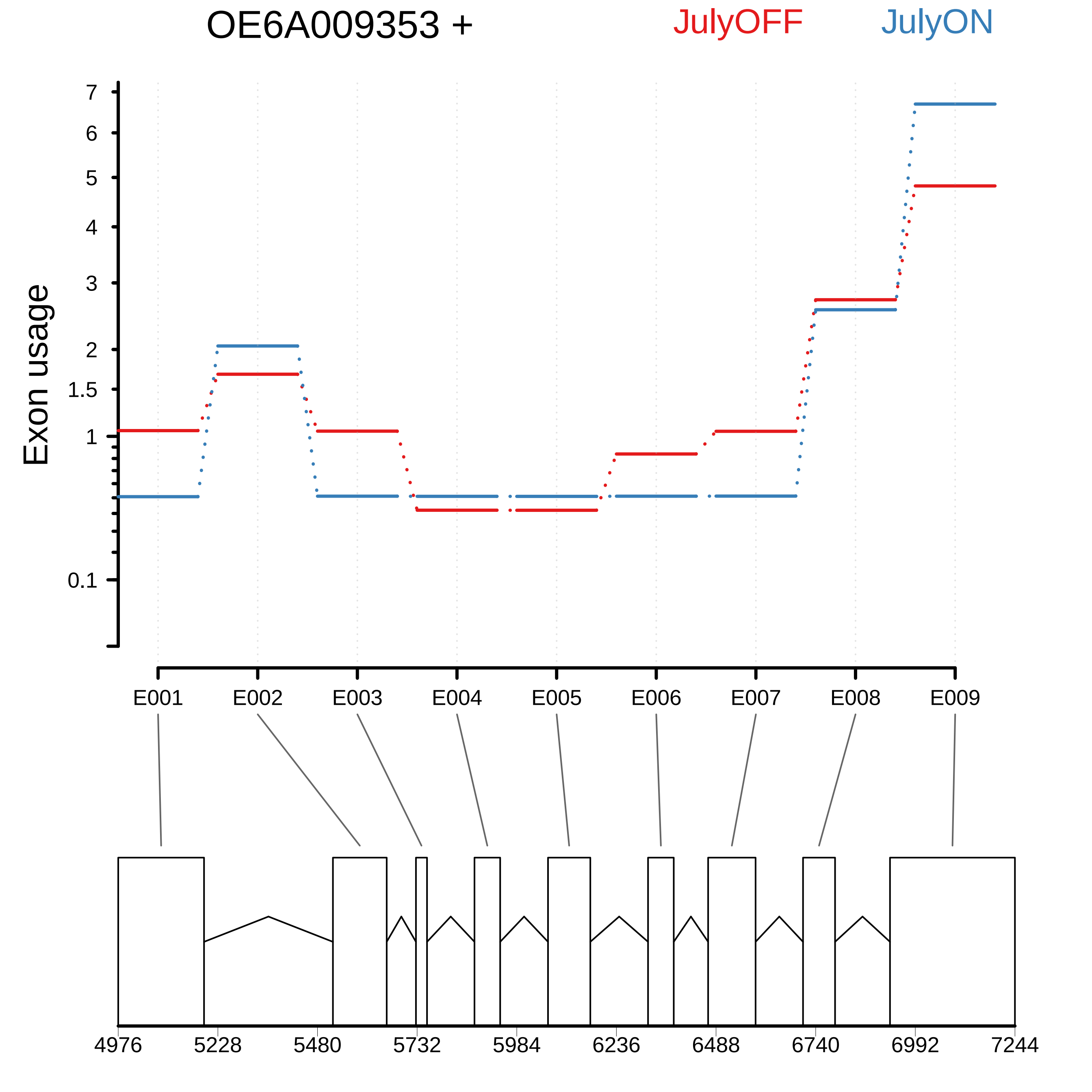
OE6A009353 +
JulyOFF
JulyON
Exon usage
7
6
5
4
3
2
1.5
1
0.1
E001
E002
E003
E004
E005
E006
E007
E008
E009
4976
5228
5480
5732
5984
6236
6488
6740
6992
7244

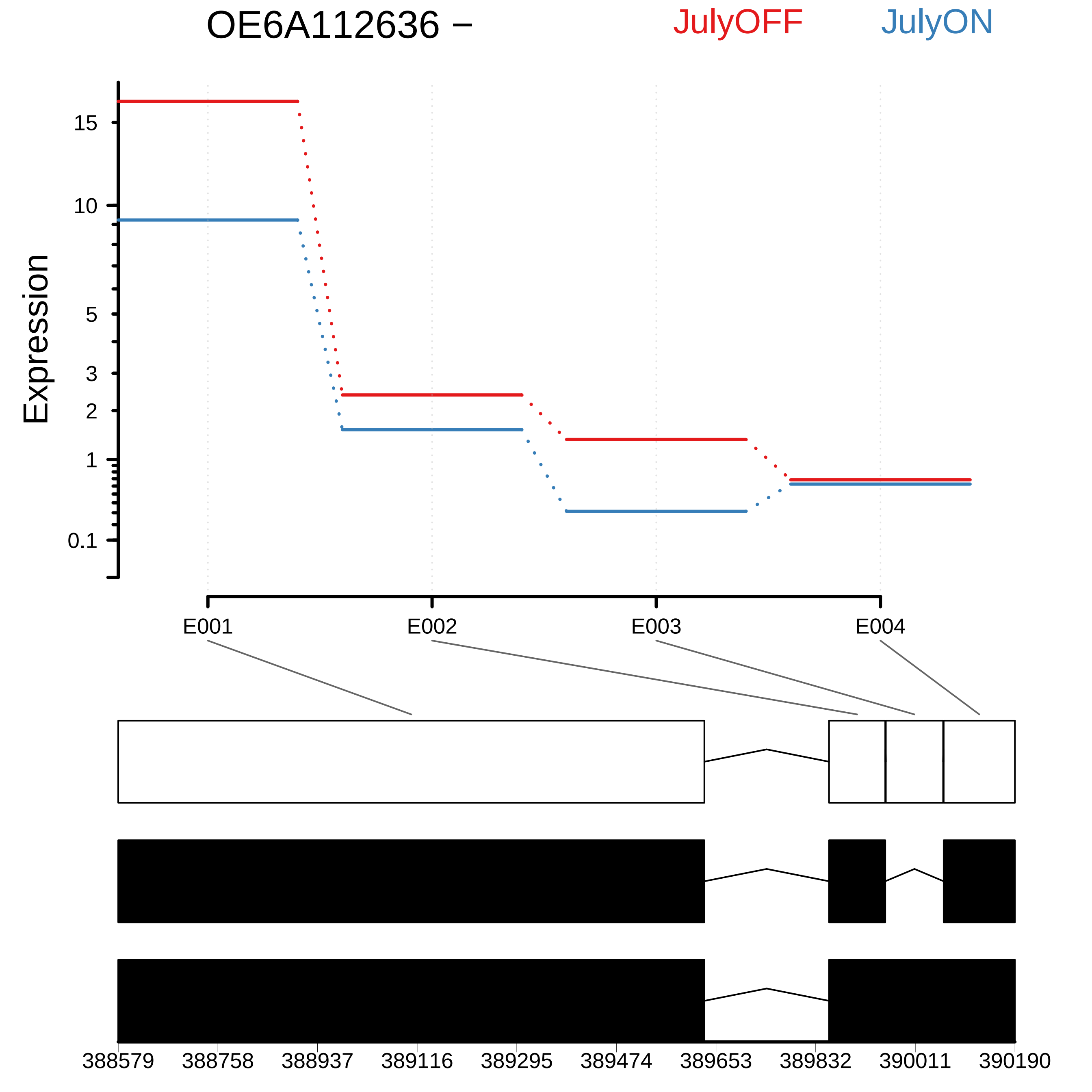
OE6A112636 −
JulyOFF
JulyON
Expression
15
10
5
3
2
1
0.1
E001
E002
E003
E004
388579
388758
388937
389116
389295
389474
389653
389832
390011
390190

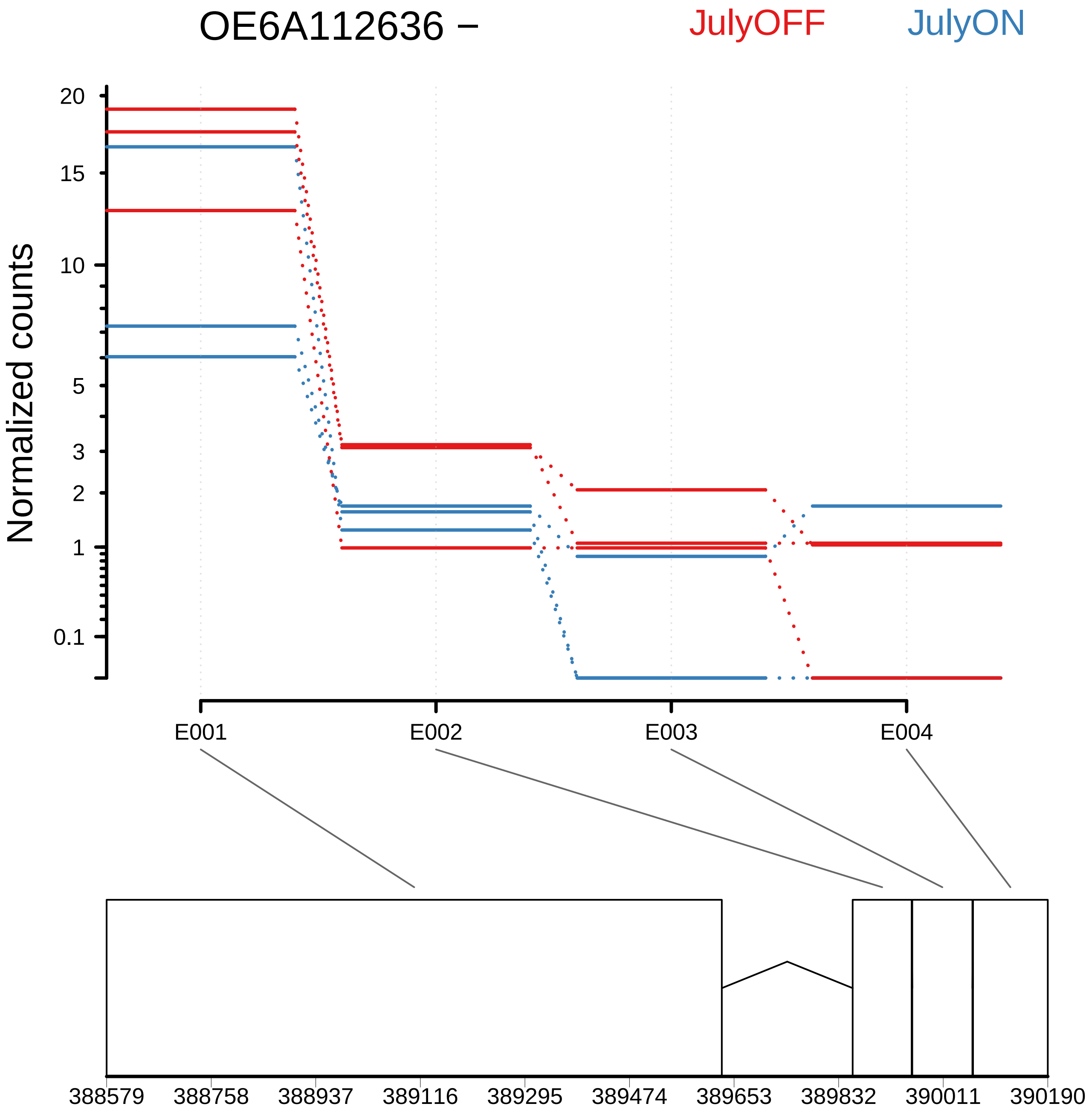
OE6A112636 −
JulyOFF
JulyON
Normalized counts
20
15
10
5
3
2
1
0.1
E001
E002
E003
E004
388579
388758
388937
389116
389295
389474
389653
389832
390011
390190

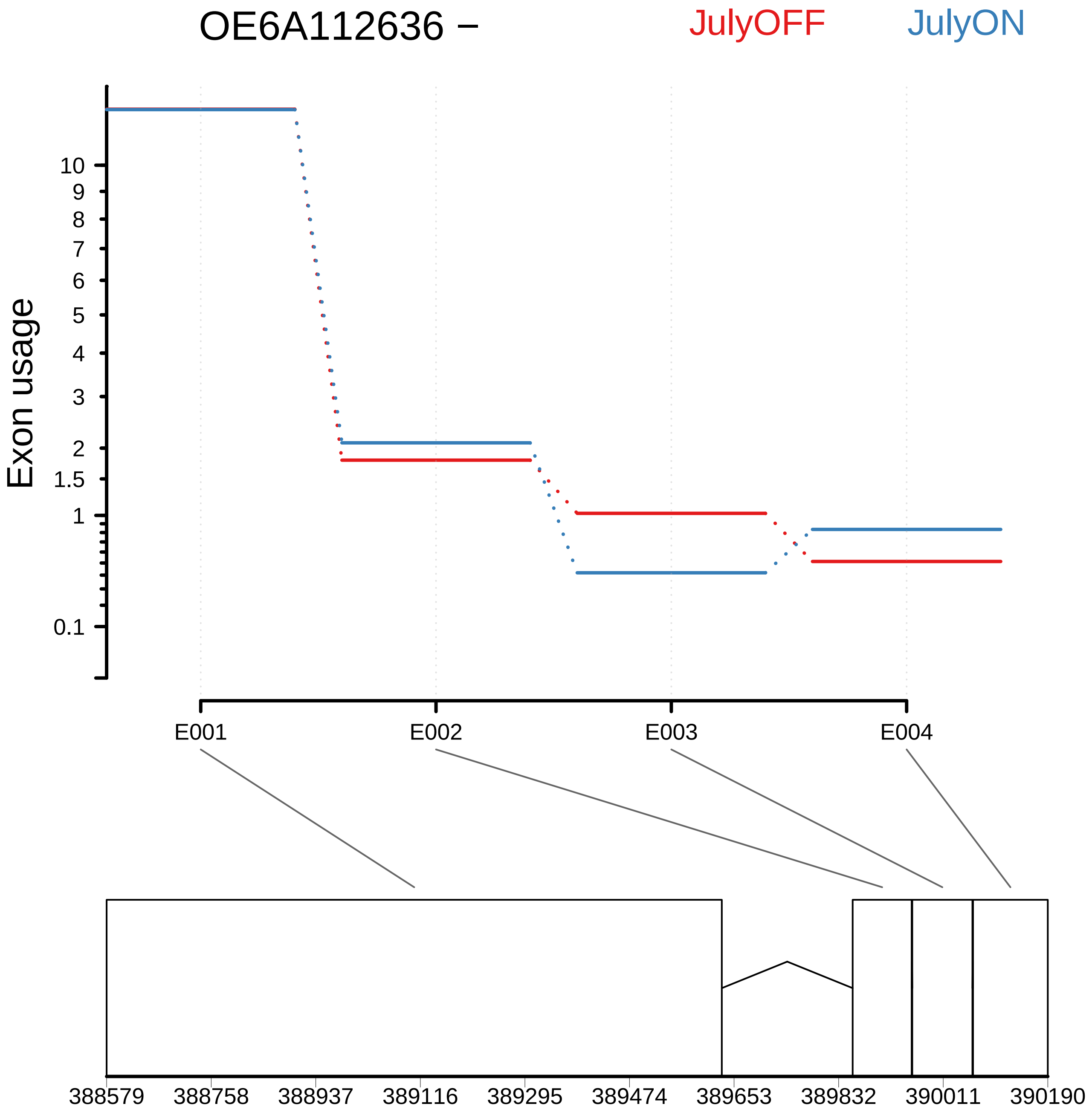
OE6A112636 −
JulyOFF
JulyON
Exon usage
10
9
8
7
6
5
4
3
2
1.5
1
0.1
E001
E002
E003
E004
388579
388758
388937
389116
389295
389474
389653
389832
390011
390190

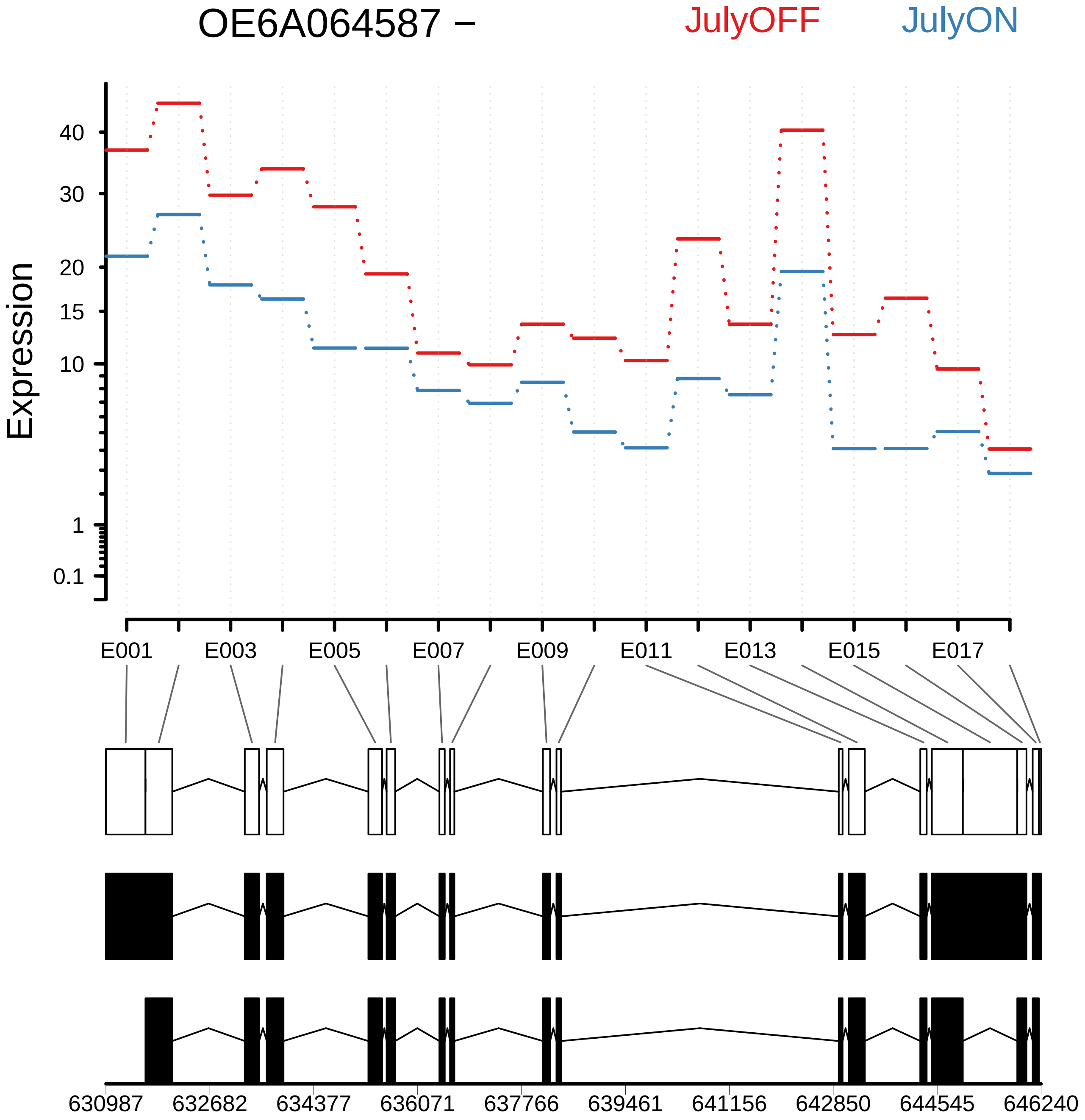

OE6A064587 −
JulyOFF
JulyON
Expression
40
30
20
15
10
1
0.1
E001
E003
E005
E007
E009
E011
E013
E015
E017
630987
632682
634377
636071
637766
639461
641156
642850
644545
646240

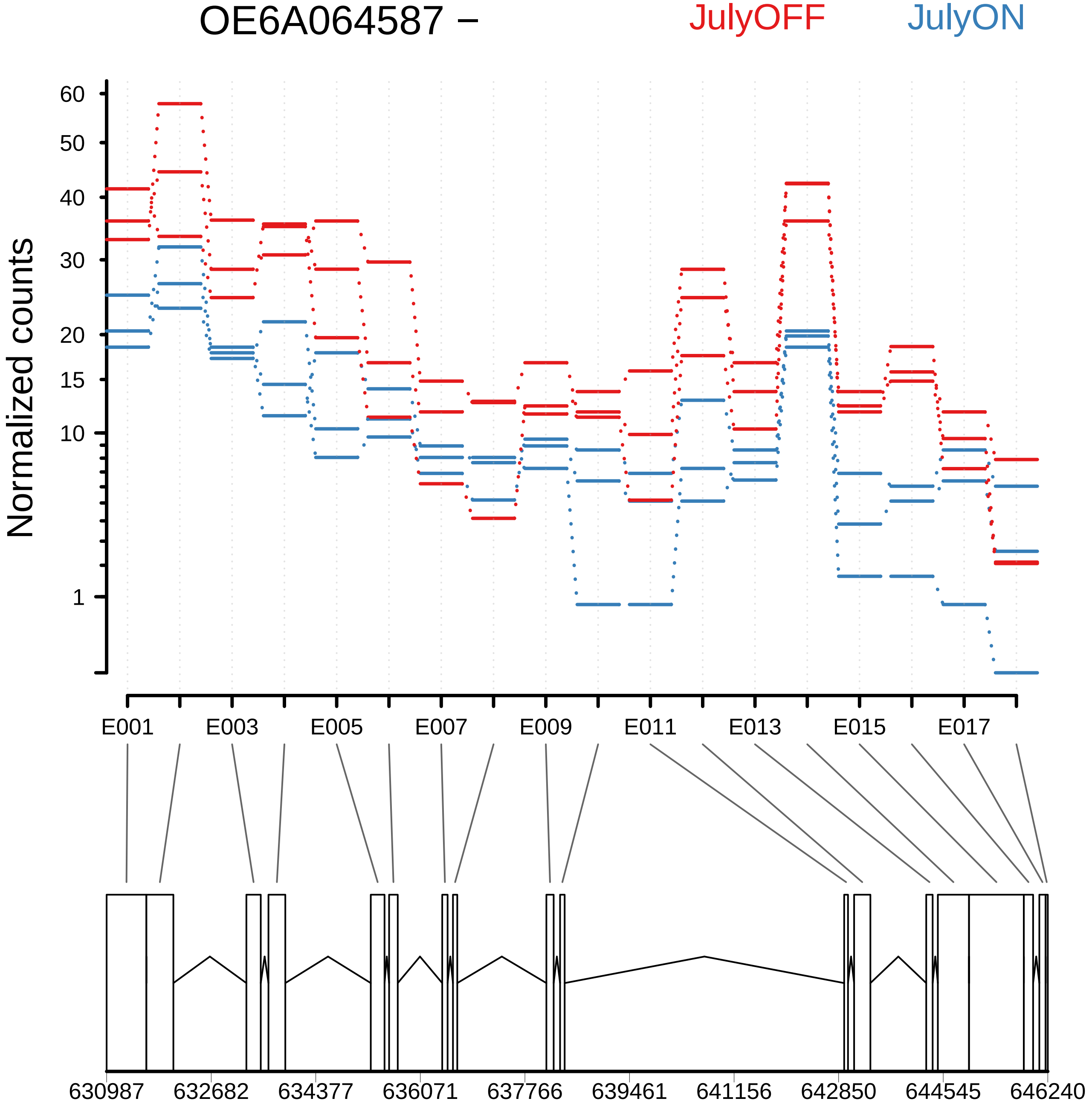
OE6A064587 –
JulyOFF
JulyON
Normalized counts
60
50
40
30
20
15
10
1
E001
E003
E005
E007
E009
E011
E013
E015
E017
630987
632682
634377
636071
637766
639461
641156
642850
644545
646240

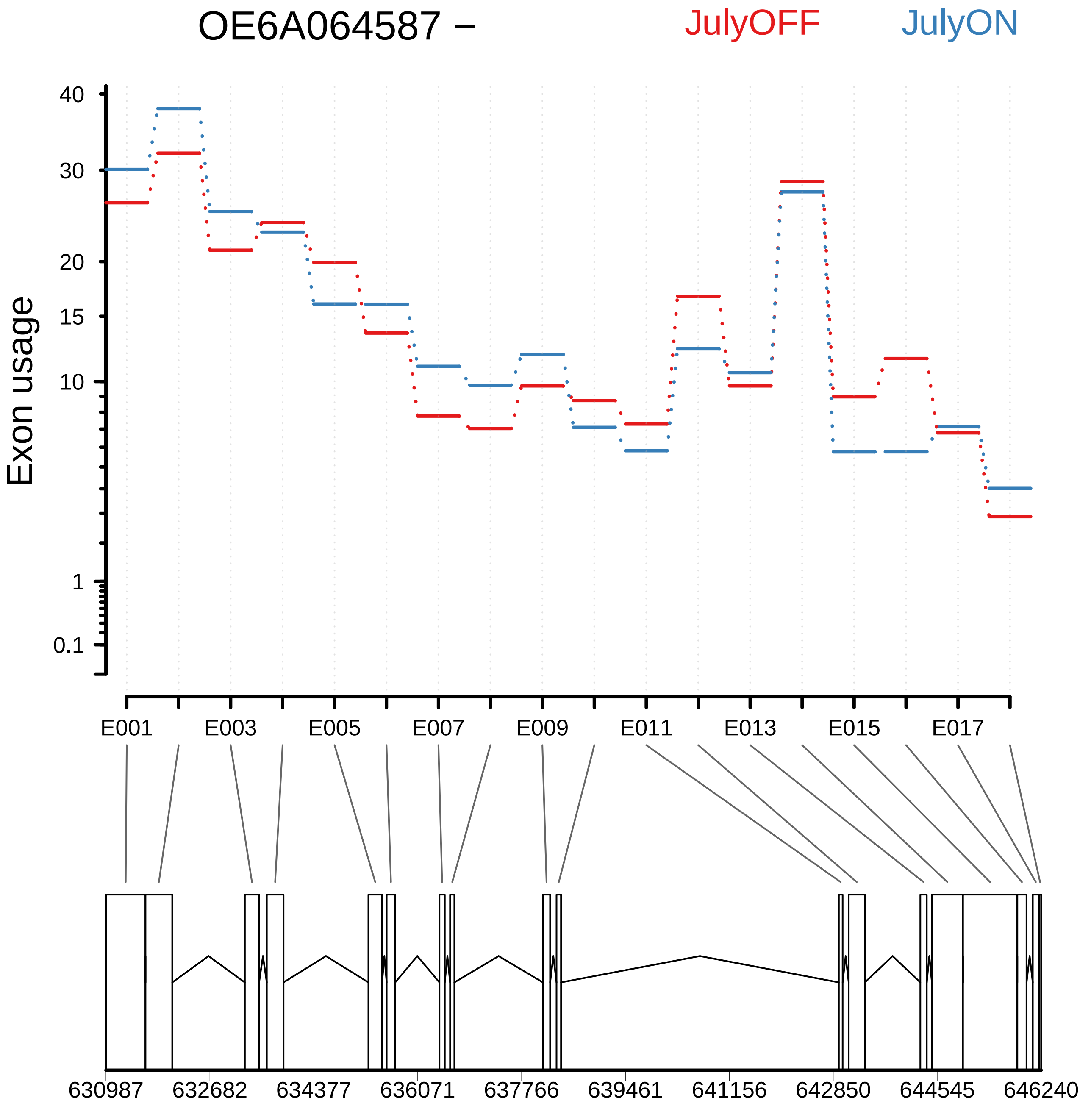

OE6A064587 −
JulyOFF
JulyON
Exon usage
40
30
20
15
10
1
0.1
E001
E003
E005
E007
E009
E011
E013
E015
E017
630987
632682
634377
636071
637766
639461
641156
642850
644545
646240

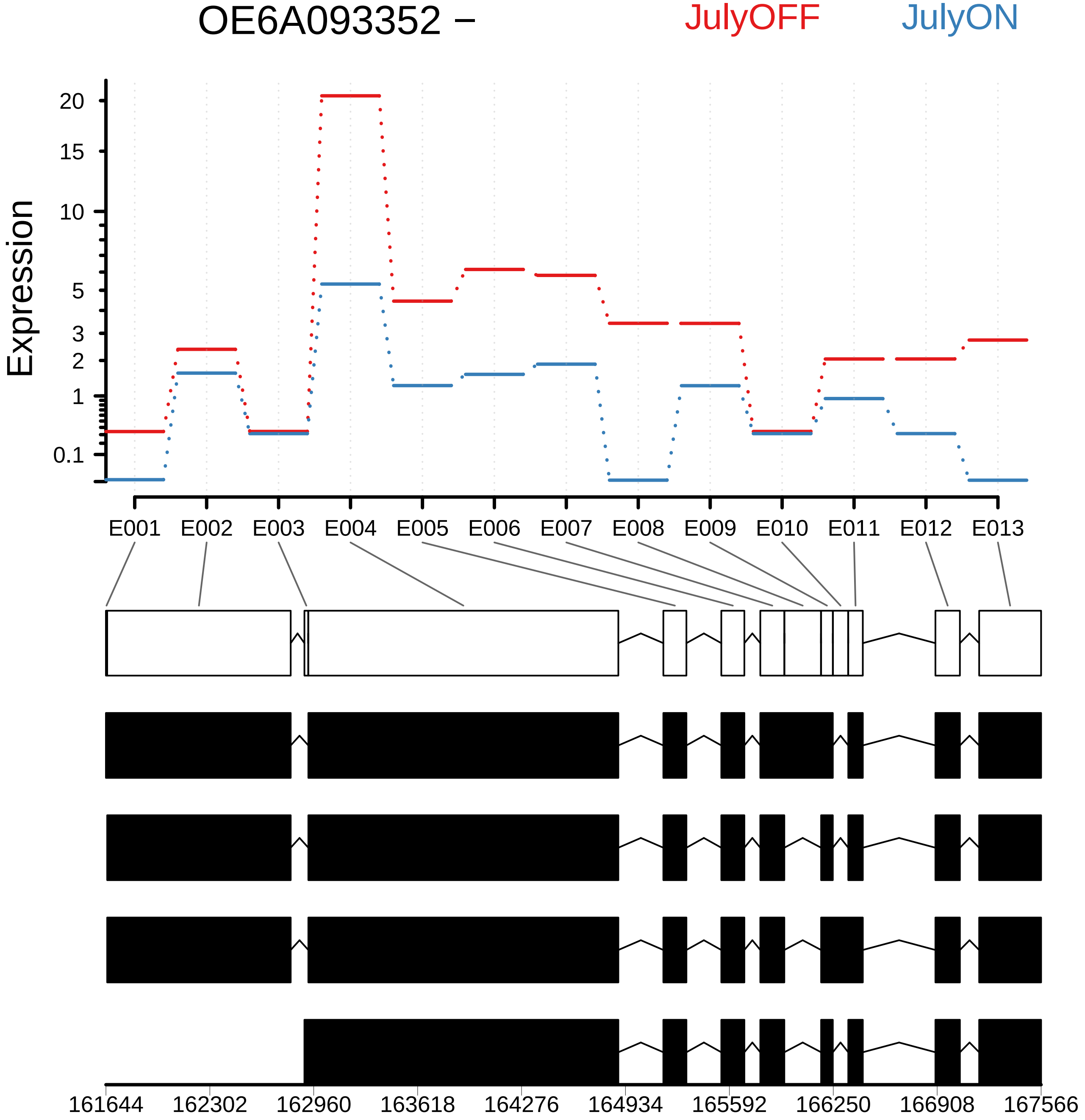
OE6A093352 –
JulyOFF
JulyON
Expression
20
15
10
5
3
2
1
0.1
E001
E002
E003
E004
E005
E006
E007
E008
E009
E010
E011
E012
E013
161644
162302
162960
163618
164276
164934
165592
166250
166908
167566

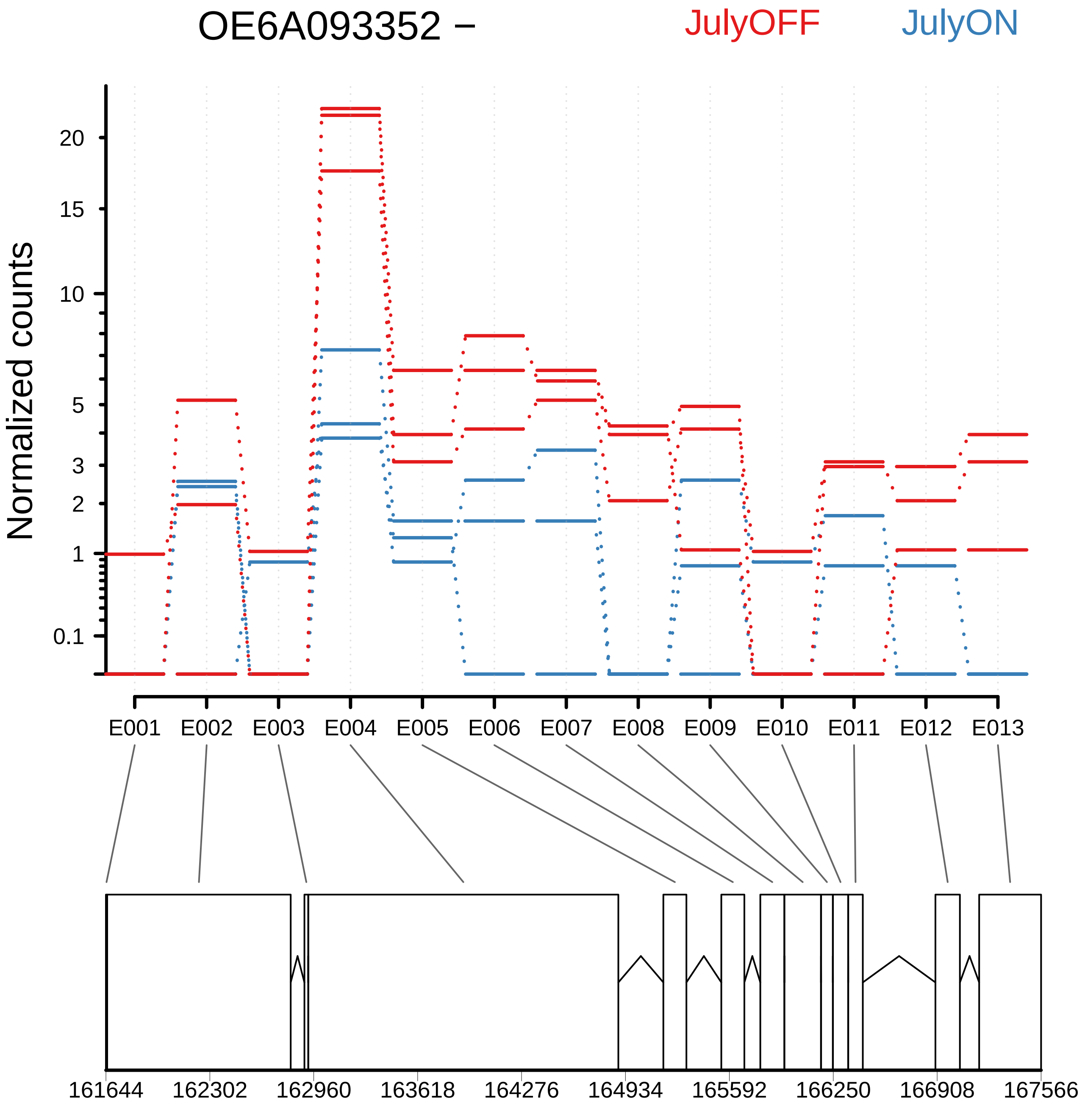
OE6A093352 −
JulyOFF
JulyON
Normalized counts
20
15
10
5
3
2
1
0.1
E001
E002
E003
E004
E005
E006
E007
E008
E009
E010
E011
E012
E013
161644
162302
162960
163618
164276
164934
165592
166250
166908
167566

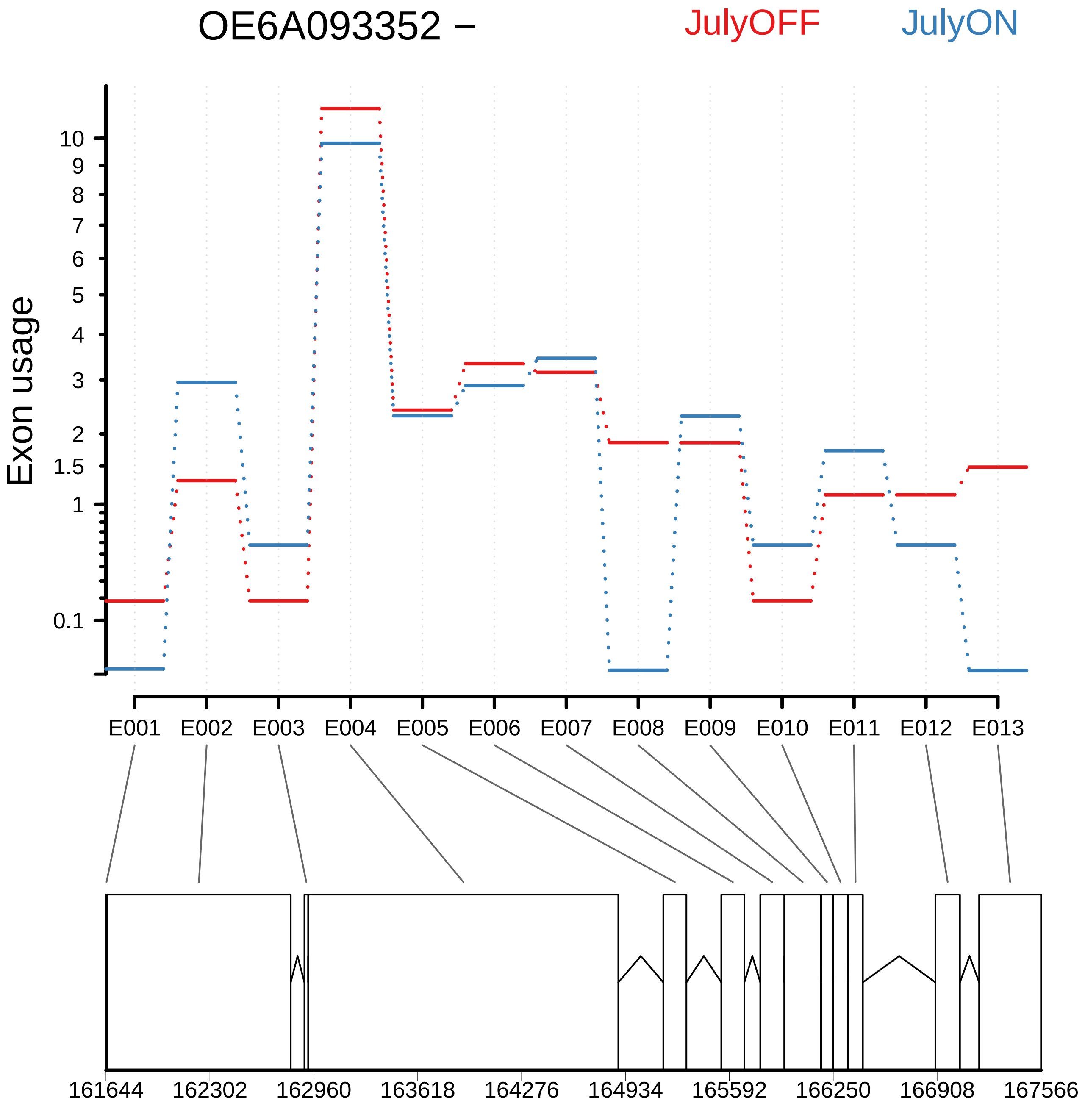
OE6A093352 −
JulyOFF
JulyON
Exon usage
10
9
8
7
6
5
4
3
2
1.5
1
0.1
E001
E002
E003
E004
E005
E006
E007
E008
E009
E010
E011
E012
E013
161644
162302
162960
163618
164276
164934
165592
166250
166908
167566

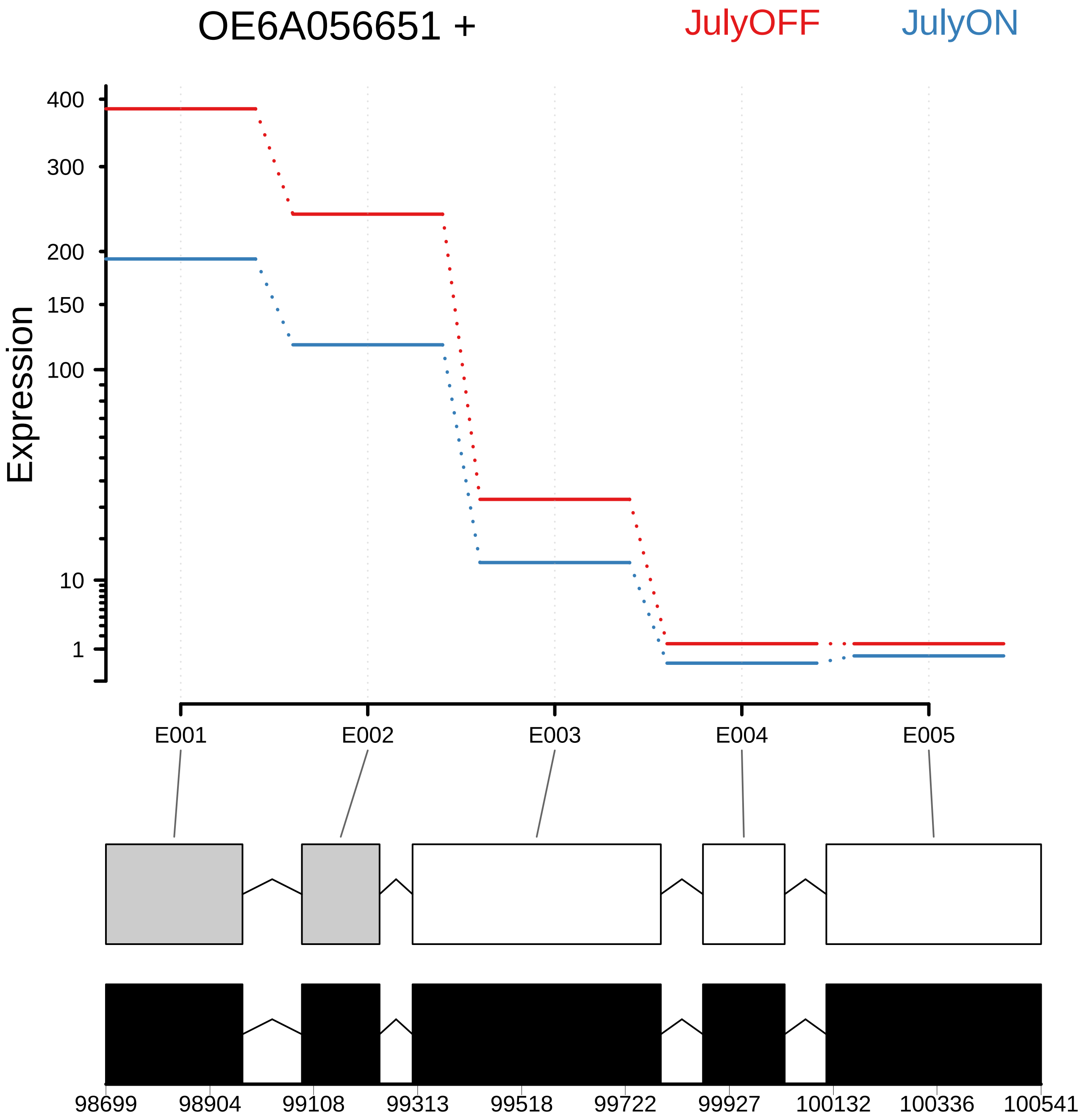
OE6A056651 +
JulyOFF
JulyON
Expression
400
300
200
150
100
10
1
E001
E002
E003
E004
E005
98699
98904
99108
99313
99518
99722
99927
100132
100336
100541

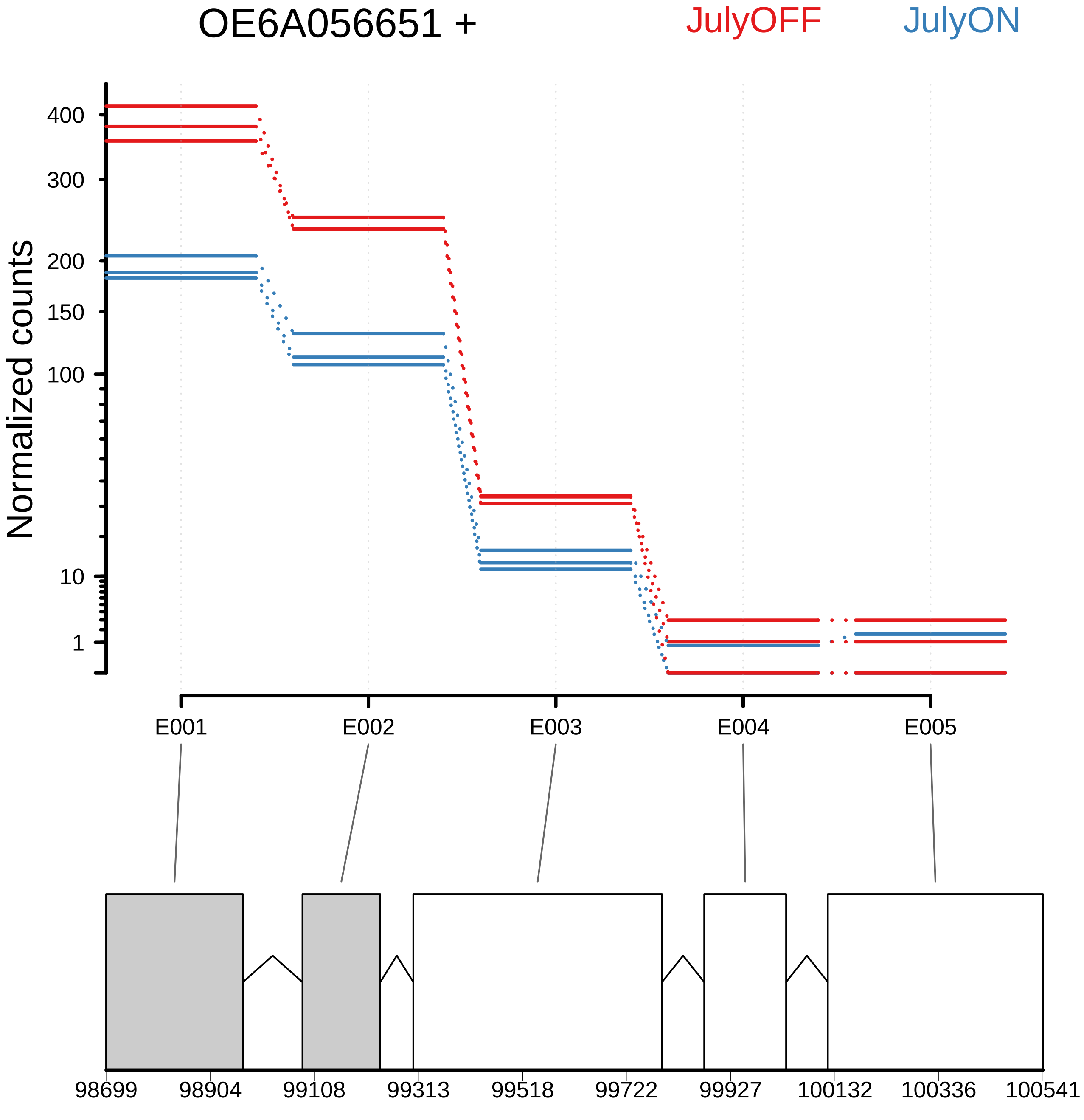
OE6A056651 +
JulyOFF
JulyON
Normalized counts
400
300
200
150
100
10
1
E001
E002
E003
E004
E005
98699
98904
99108
99313
99518
99722
99927
100132
100336
100541

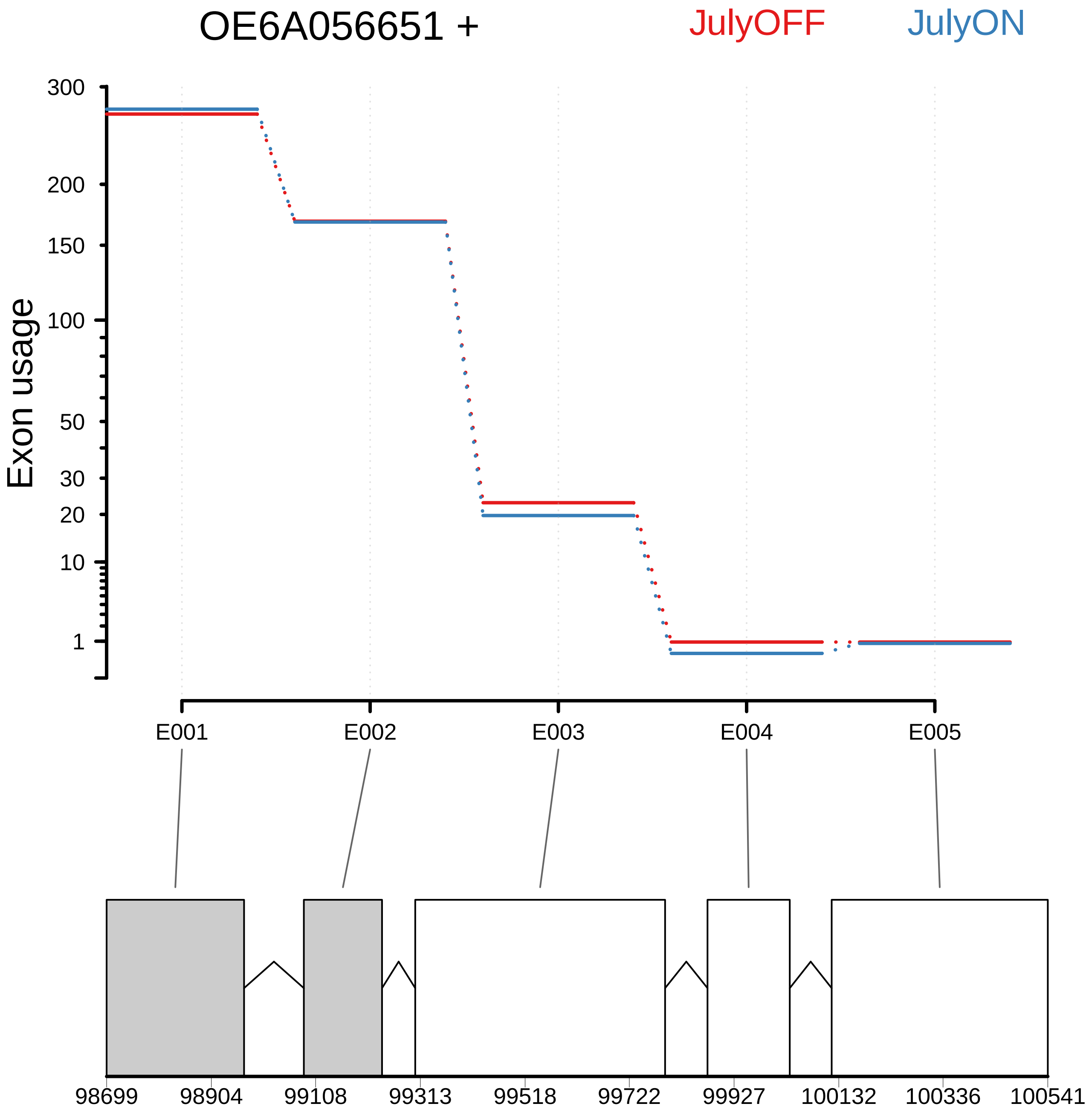

OE6A056651 +
JulyOFF
JulyON
Exon usage
300
200
150
100
50
30
20
10
1
E001
E002
E003
E004
E005
98699
98904
99108
99313
99518
99722
99927
100132
100336
100541

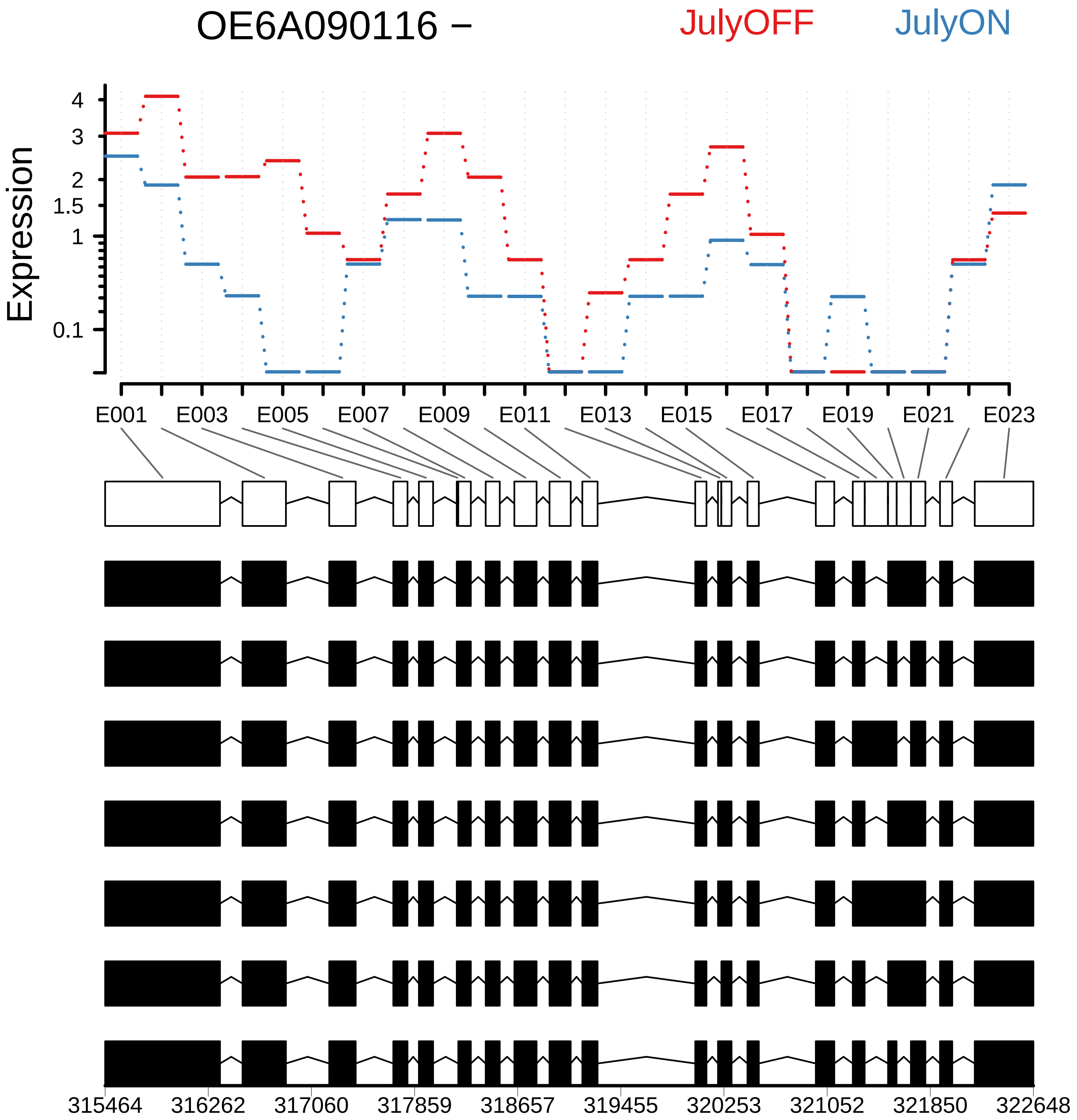
OE6A090116 −
JulyOFF
JulyON
Expression
4
3
2
1.5
1
0.1
E001
E003
E005
E007
E009
E011
E013
E015
E017
E019
E021
E023
315464
316262
317060
317859
318657
319455
320253
321052
321850
322648

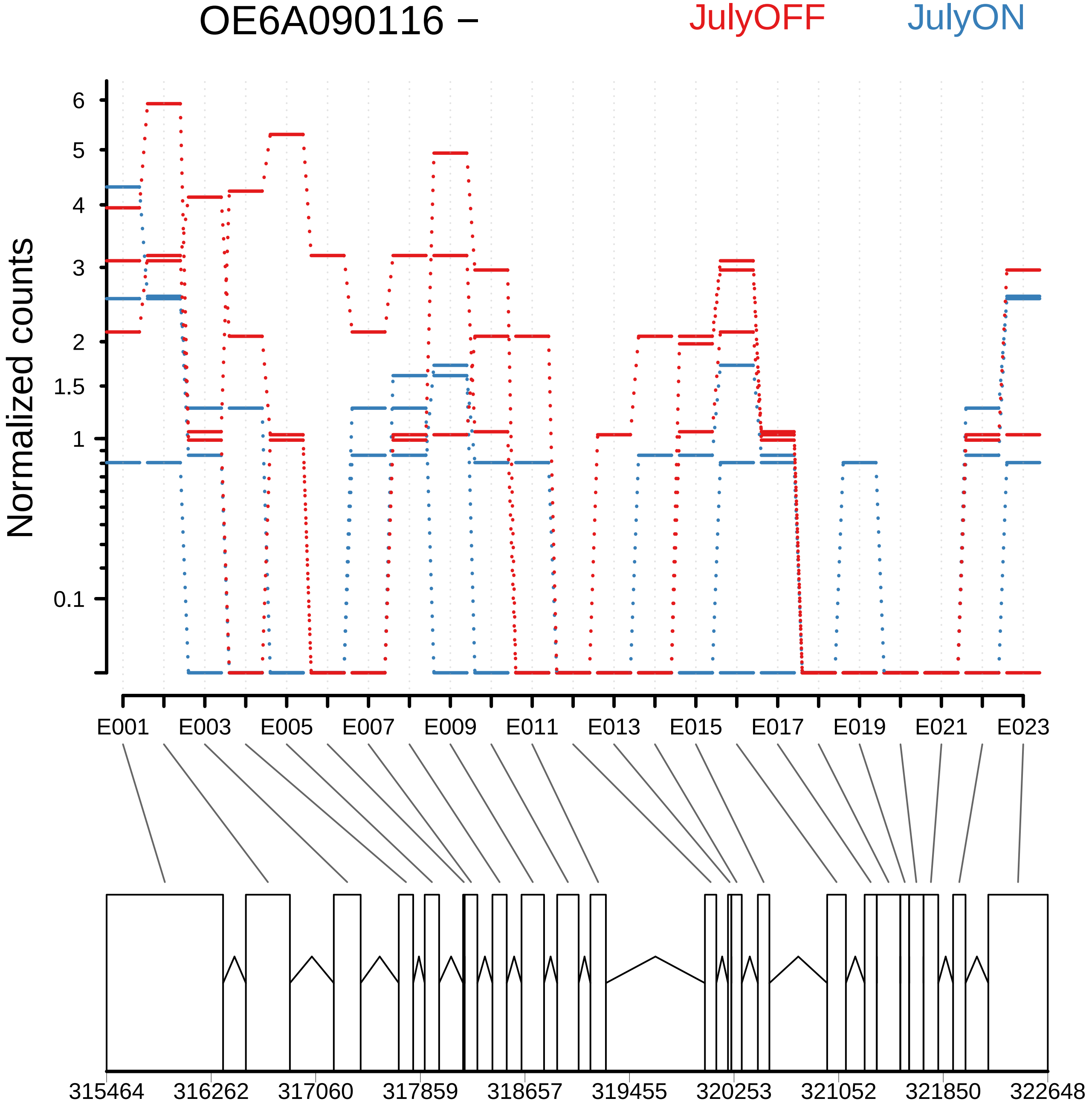
OE6A090116 −
JulyOFF
JulyON
Normalized counts
6
5
4
3
2
1.5
1
0.1
E001
E003
E005
E007
E009
E011
E013
E015
E017
E019
E021
E023
315464
316262
317060
317859
318657
319455
320253
321052
321850
322648

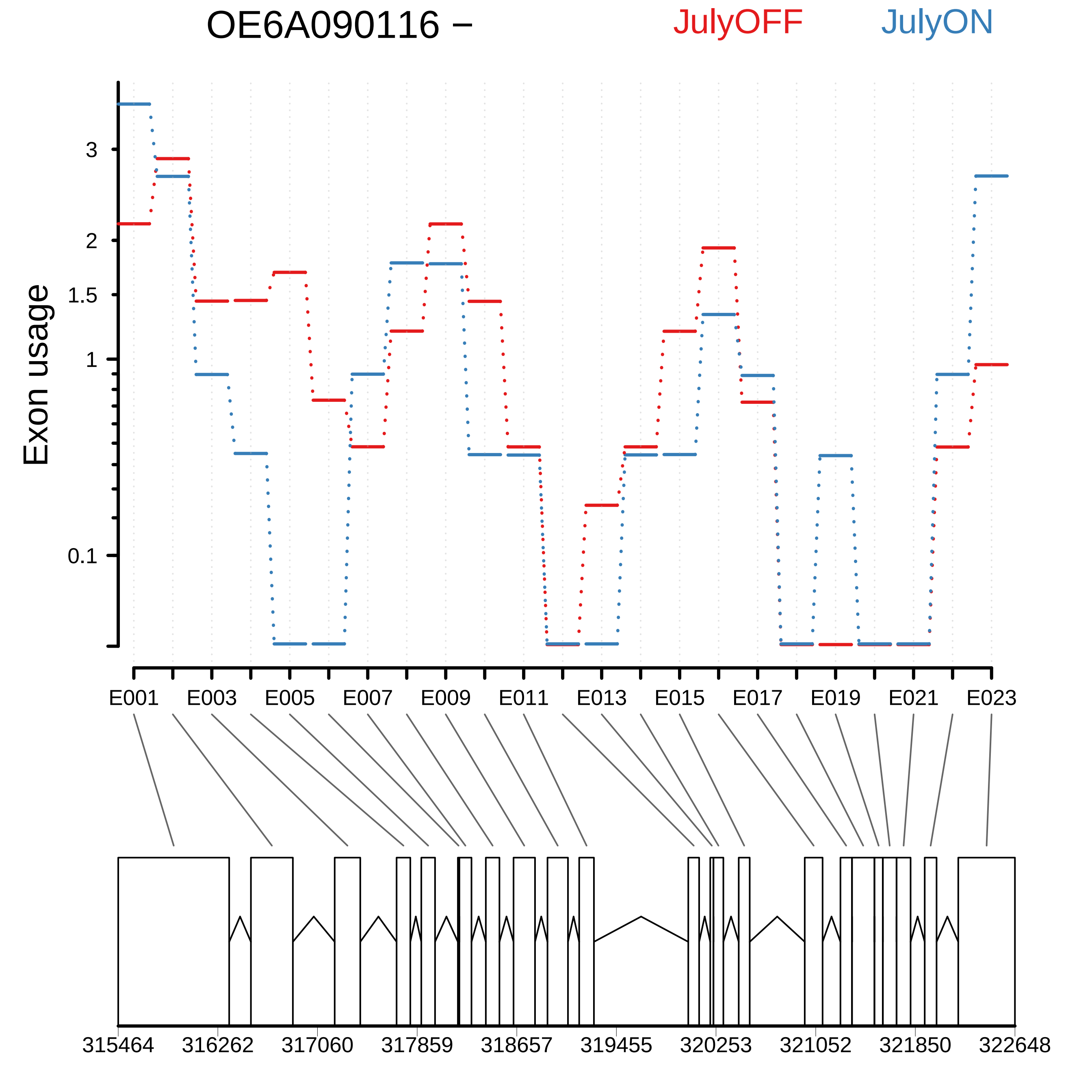
OE6A090116 −
JulyOFF
JulyON
Exon usage
3
2
1.5
1
0.1
E001
E003
E005
E007
E009
E011
E013
E015
E017
E019
E021
E023
315464
316262
317060
317859
318657
319455
320253
321052
321850
322648

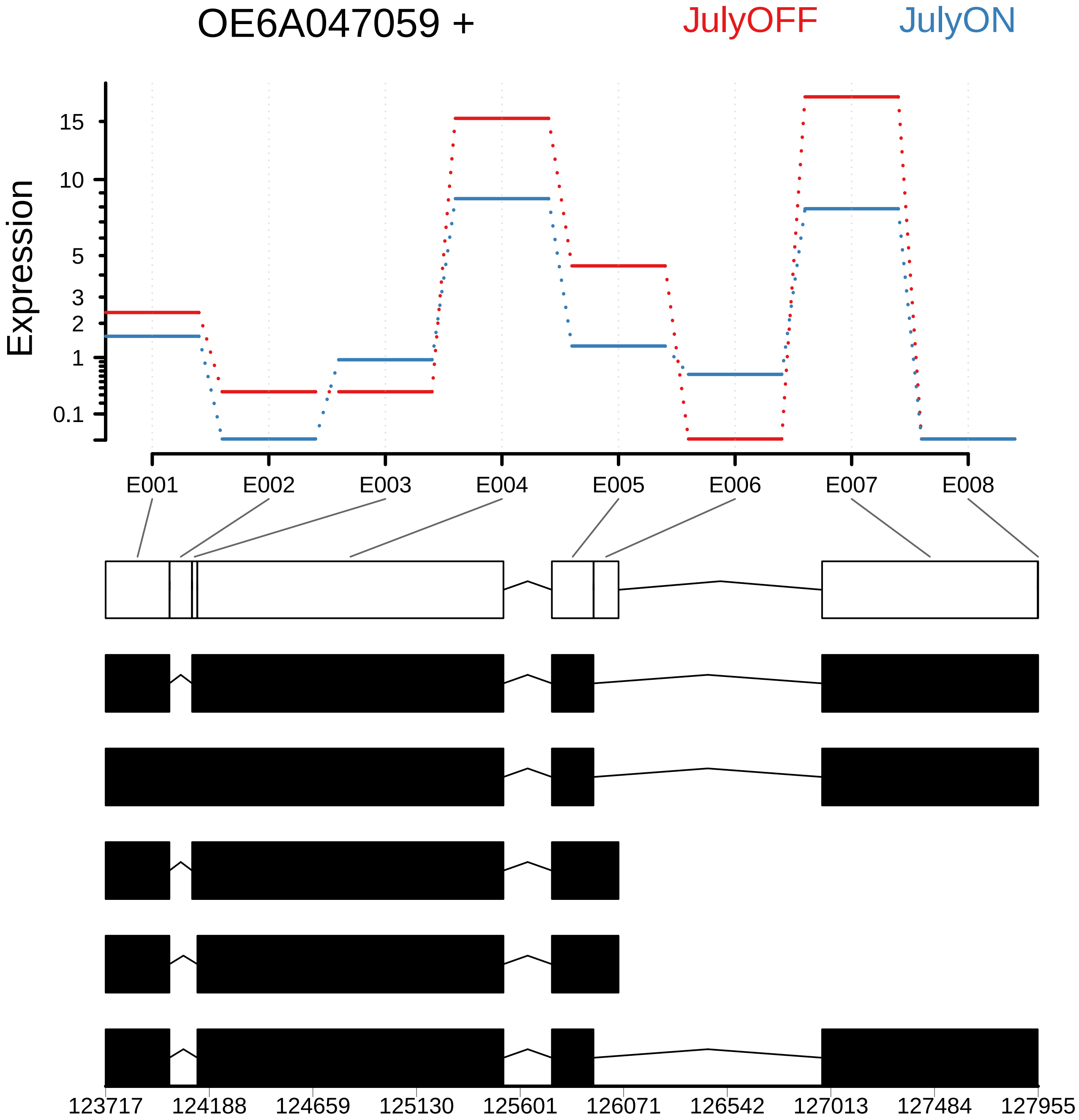

OE6A047059 +
JulyOFF
JulyON
Expression
15
10
5
3
2
1
0.1
E001
E002
E003
E004
E005
E006
E007
E008
123717
124188
124659
125130
125601
126071
126542
127013
127484
127955

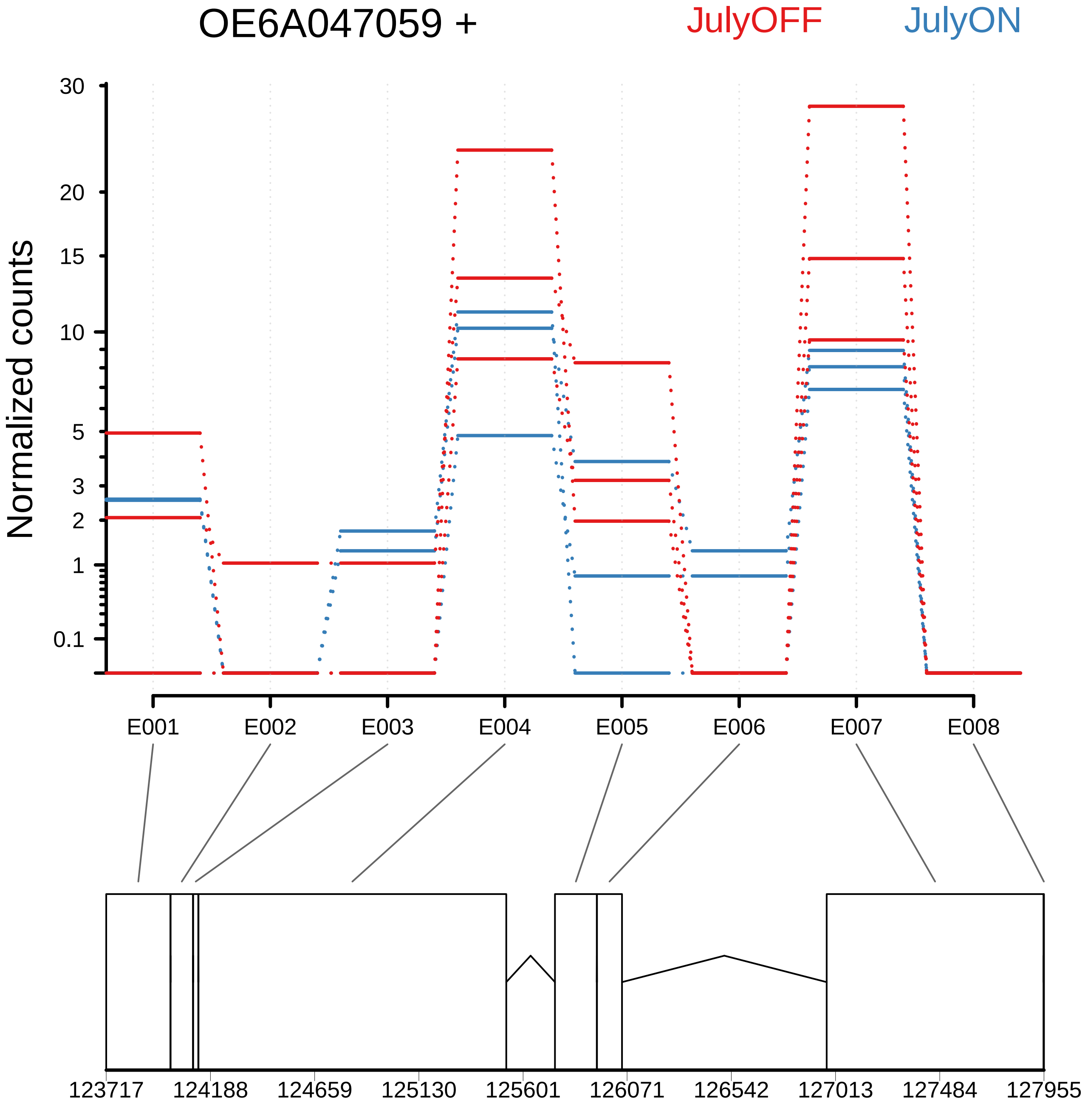
OE6A047059 +
JulyOFF
JulyON
Normalized counts
30
20
15
10
5
3
2
1
0.1
E001
E002
E003
E004
E005
E006
E007
E008
123717
124188
124659
125130
125601
126071
126542
127013
127484
127955

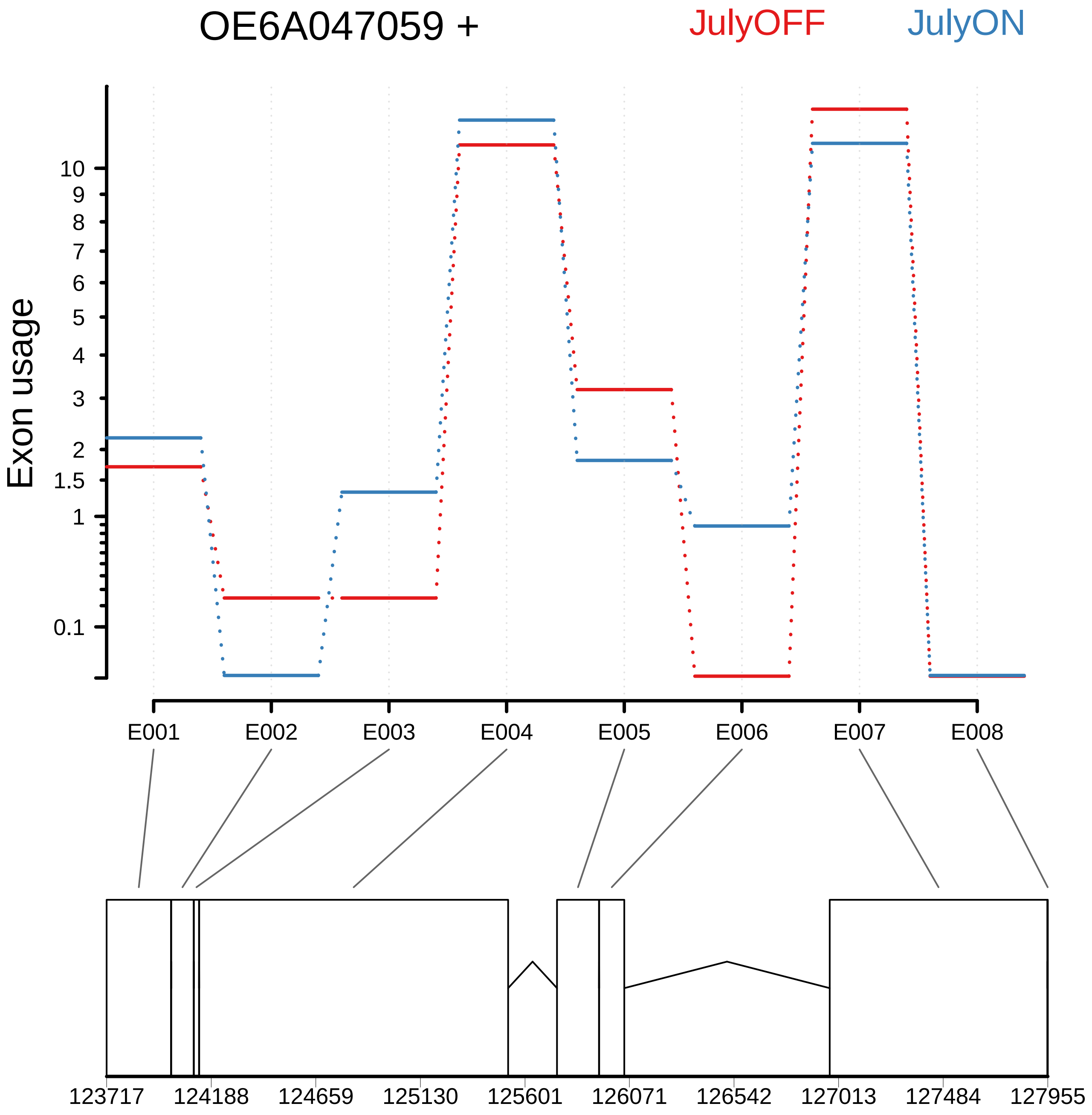
OE6A047059 +
JulyOFF
JulyON
Exon usage
10
9
8
7
6
5
4
3
2
1.5
1
0.1
E001
E002
E003
E004
E005
E006
E007
E008
123717
124188
124659
125130
125601
126071
126542
127013
127484
127955

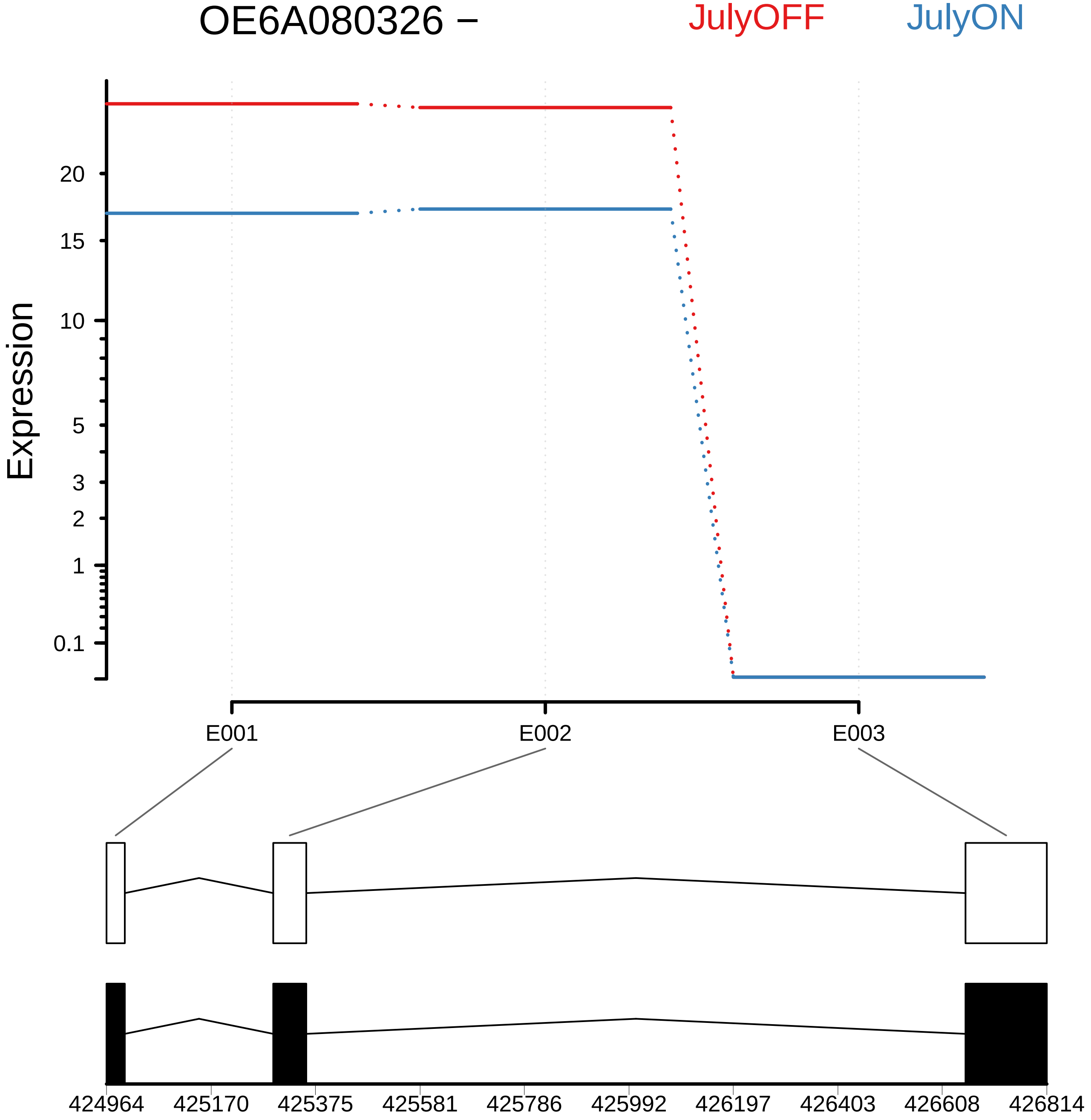

OE6A080326 −
JulyOFF
JulyON
Expression
20
15
10
5
3
2
1
0.1
E001
E002
E003
424964
425170
425375
425581
425786
425992
426197
426403
426608
426814

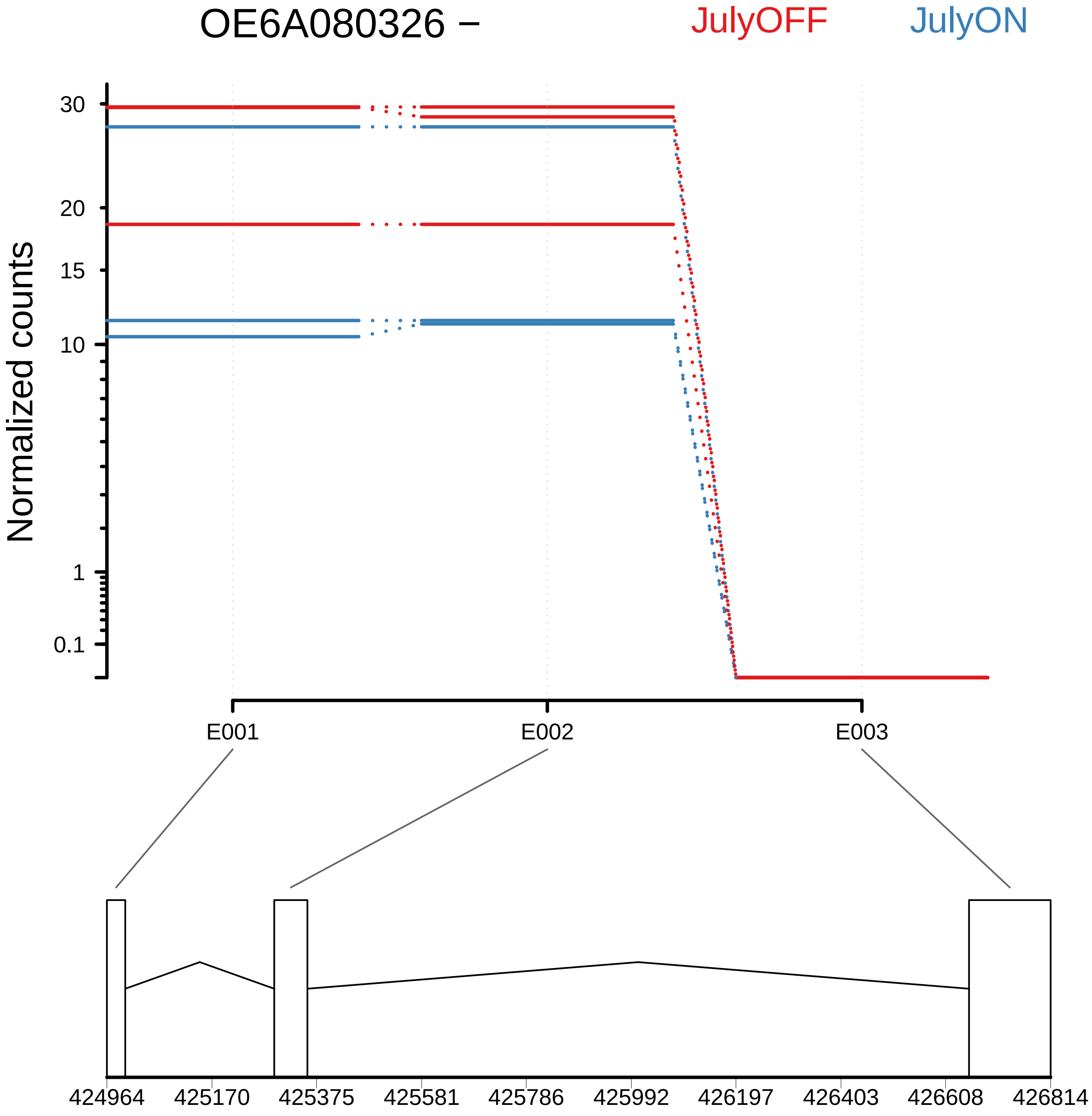

OE6A080326 −
JulyOFF
JulyON
Normalized counts
30
20
15
10
1
0.1
E001
E002
E003
424964
425170
425375
425581
425786
425992
426197
426403
426608
426814

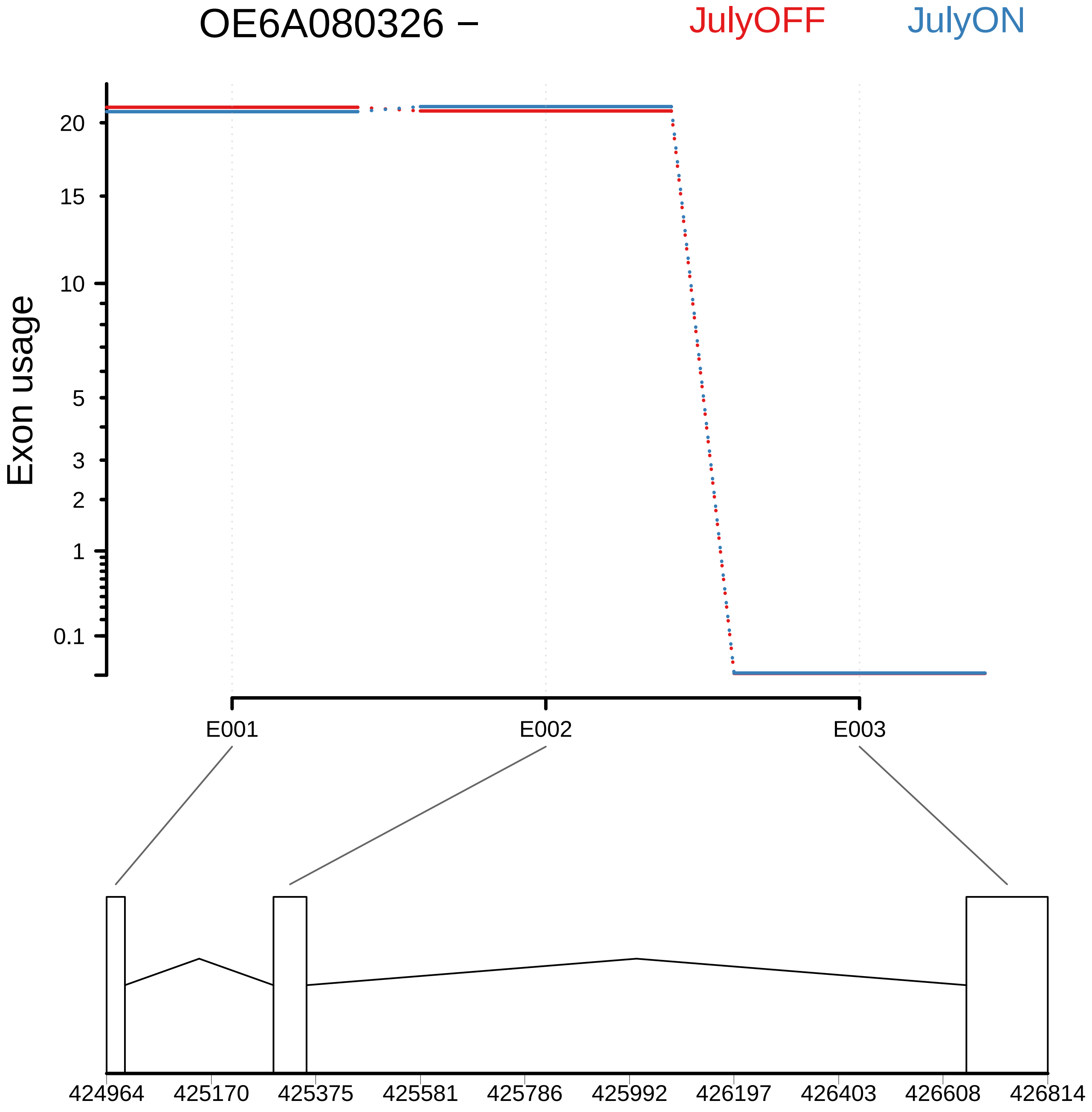
OE6A080326 −
JulyOFF
JulyON
Exon usage
20
15
10
5
3
2
1
0.1
E001
E002
E003
424964
425170
425375
425581
425786
425992
426197
426403
426608
426814

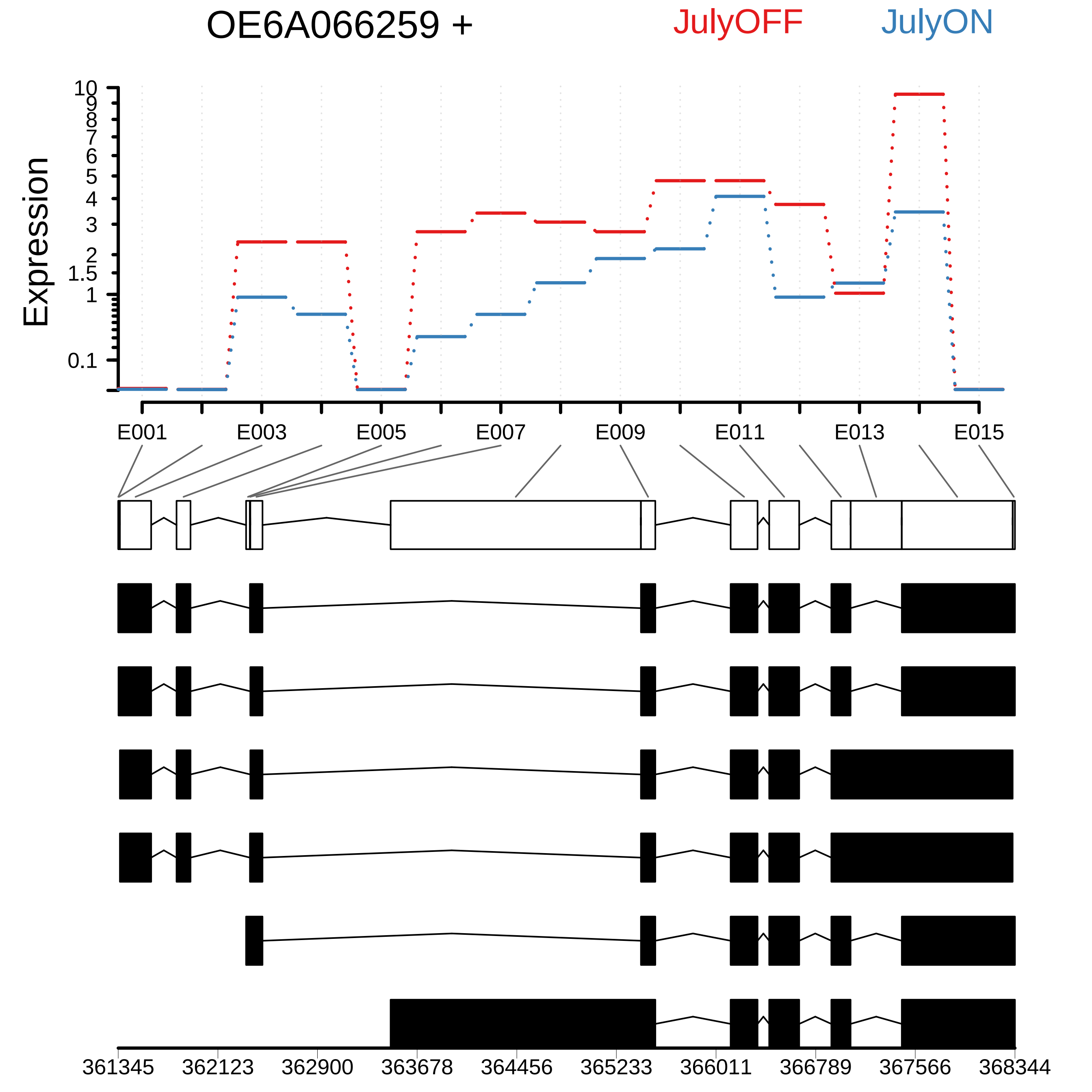
OE6A066259 +
JulyOFF
JulyON
Expression
10
9
8
7
6
5
4
3
2
1.5
1
0.1
E001
E003
E005
E007
E009
E011
E013
E015
361345
362123
362900
363678
364456
365233
366011
366789
367566
368344

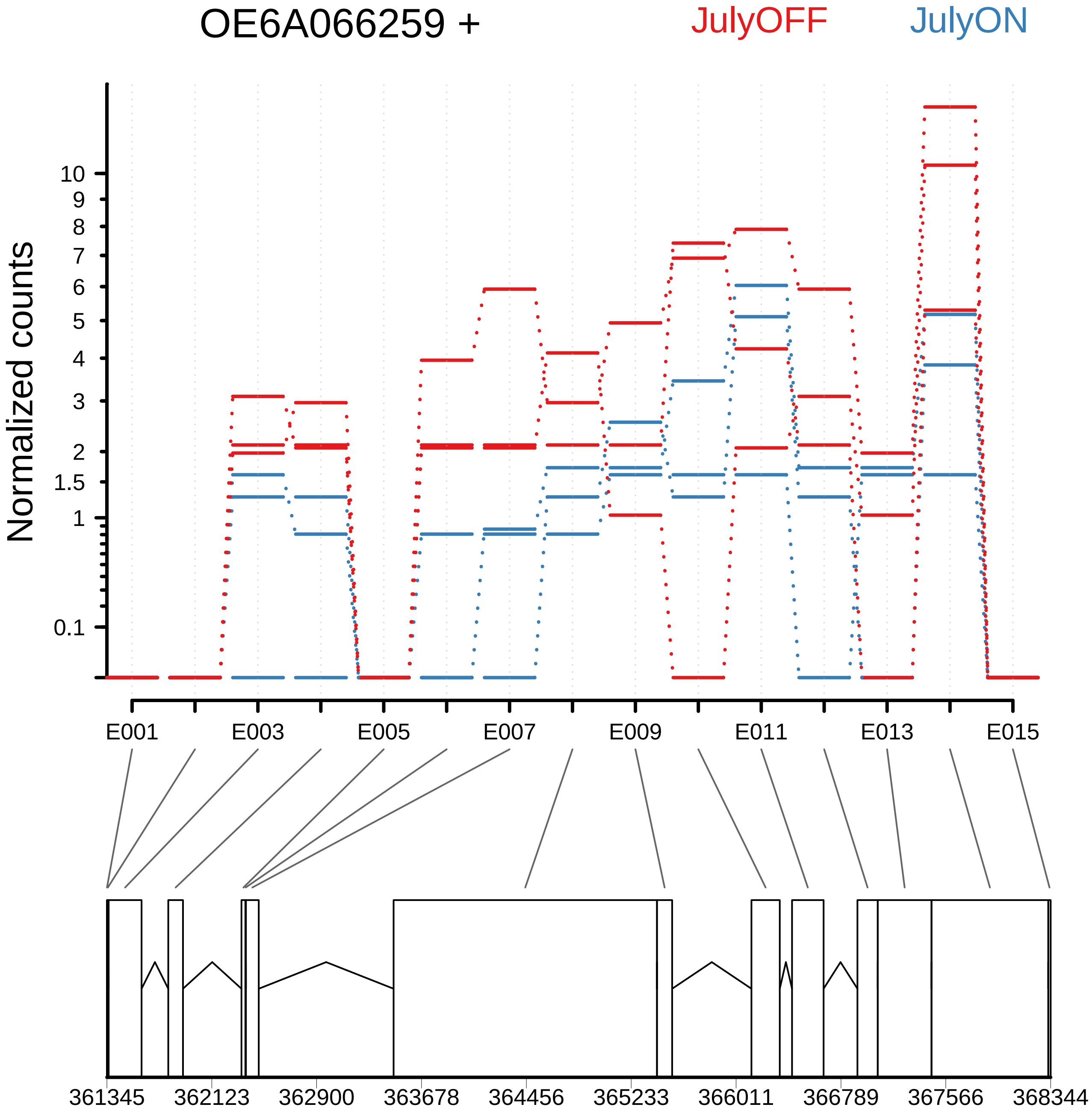

OE6A066259 +
JulyOFF
JulyON
Normalized counts
10
9
8
7
6
5
4
3
2
1.5
1
0.1
E001
E003
E005
E007
E009
E011
E013
E015
361345
362123
362900
363678
364456
365233
366011
366789
367566
368344

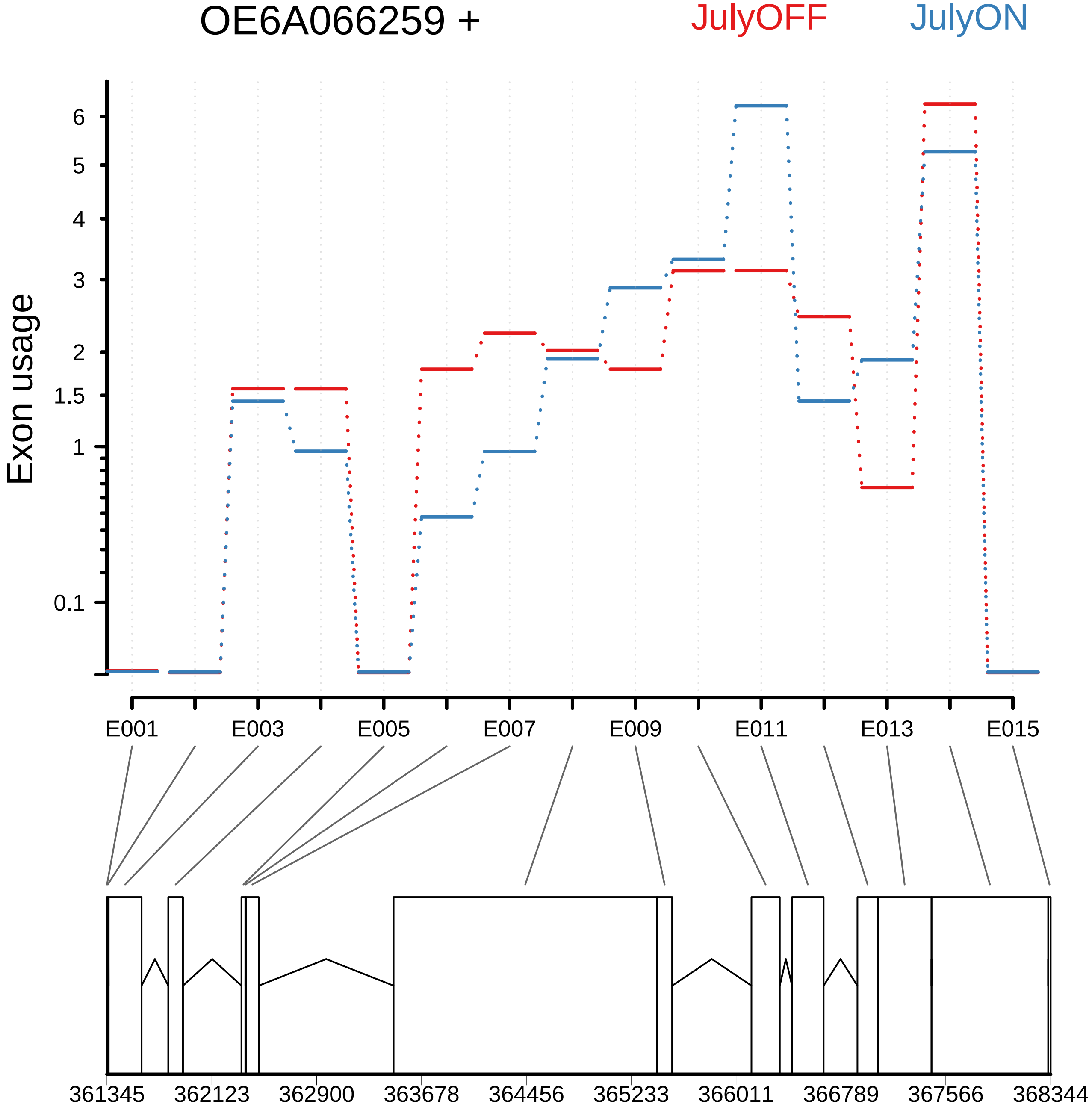

OE6A066259 +
JulyOFF
JulyON
Exon usage
6
5
4
3
2
1.5
1
0.1
E001
E003
E005
E007
E009
E011
E013
E015
361345
362123
362900
363678
364456
365233
366011
366789
367566
368344

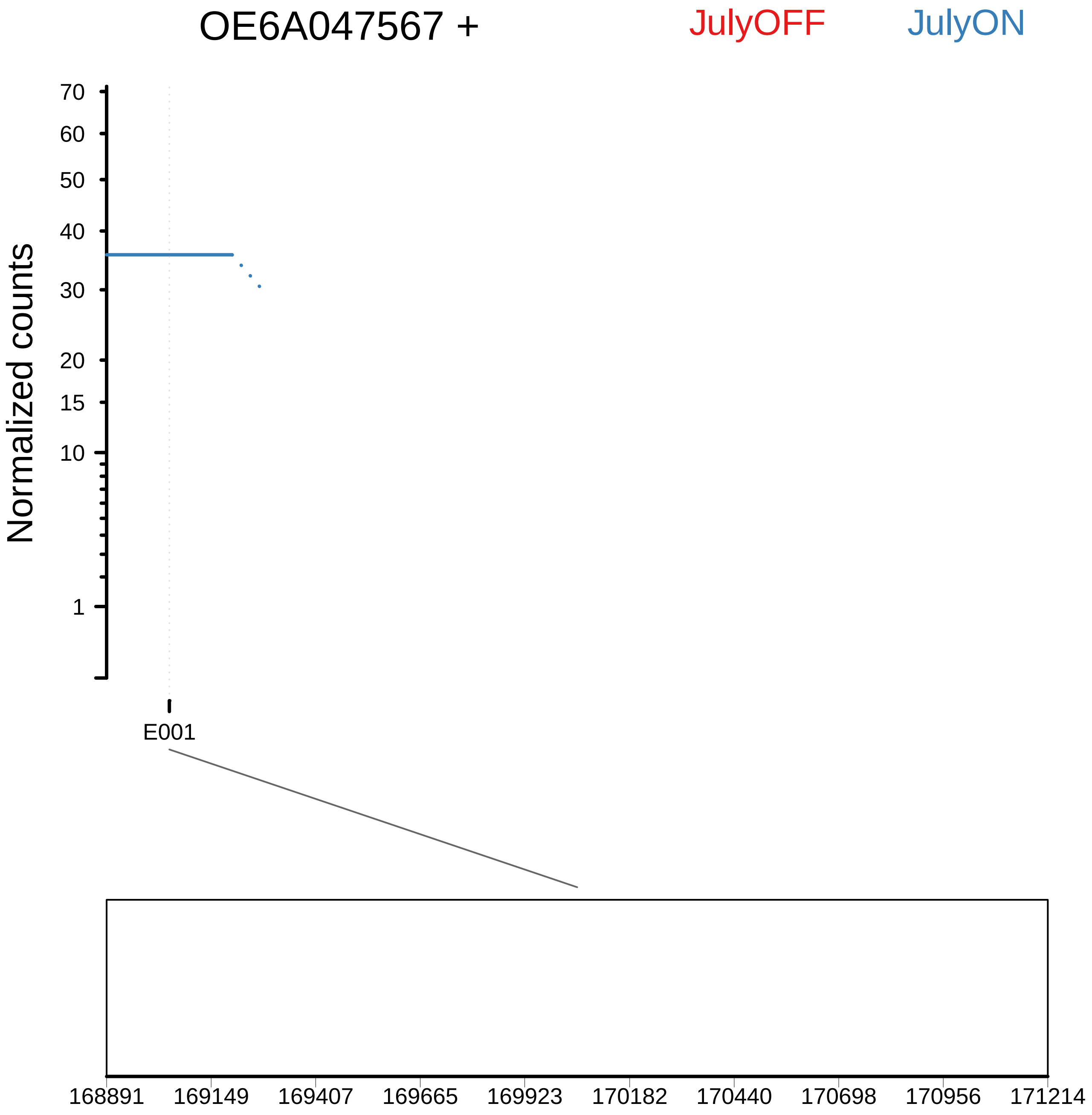
OE6A047567 +
JulyOFF
JulyON
Normalized counts
70
60
50
40
30
20
15
10
1
E001
168891
169149
169407
169665
169923
170182
170440
170698
170956
171214

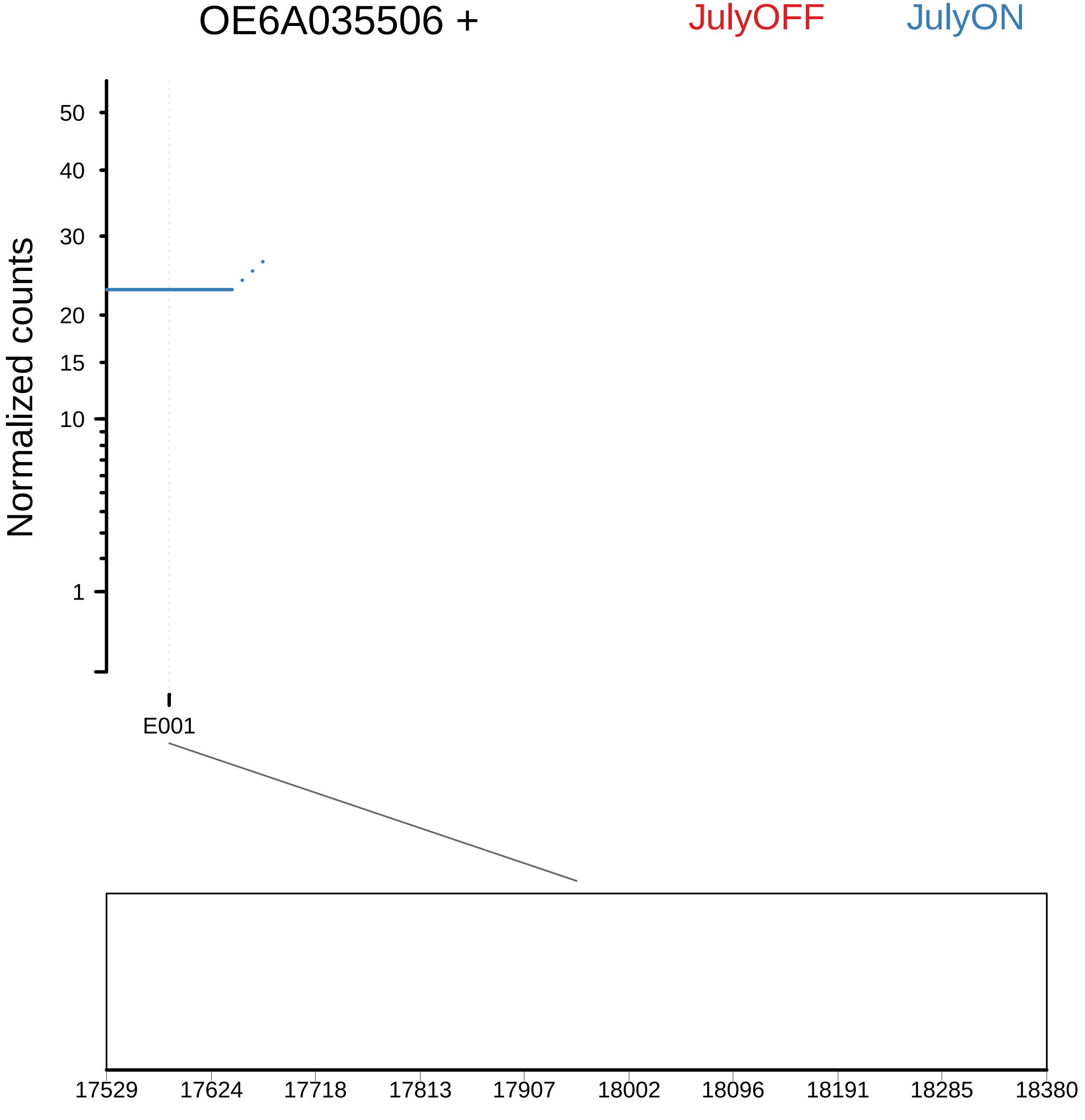
OE6A035506 +
JulyOFF
JulyON
Normalized counts
50
40
30
20
15
10
1
E001
17529
17624
17718
17813
17907
18002
18096
18191
18285
18380
