# Supplementary material for: A Complex Gene Network Mediated by Ethylene Signal Transduction TFs Defines the Flower Induction and Differentiation in Olea europaea L
Source: Genes (Basel). 2021 Apr 9;12(4):545. doi: 10.3390/genes12040545 (PMC8070190; doi:10.3390/genes12040545)
Supplement: Supplementary file 1 [file FigureS5.pdf › SupplementaryData/FigureS5.pdf]

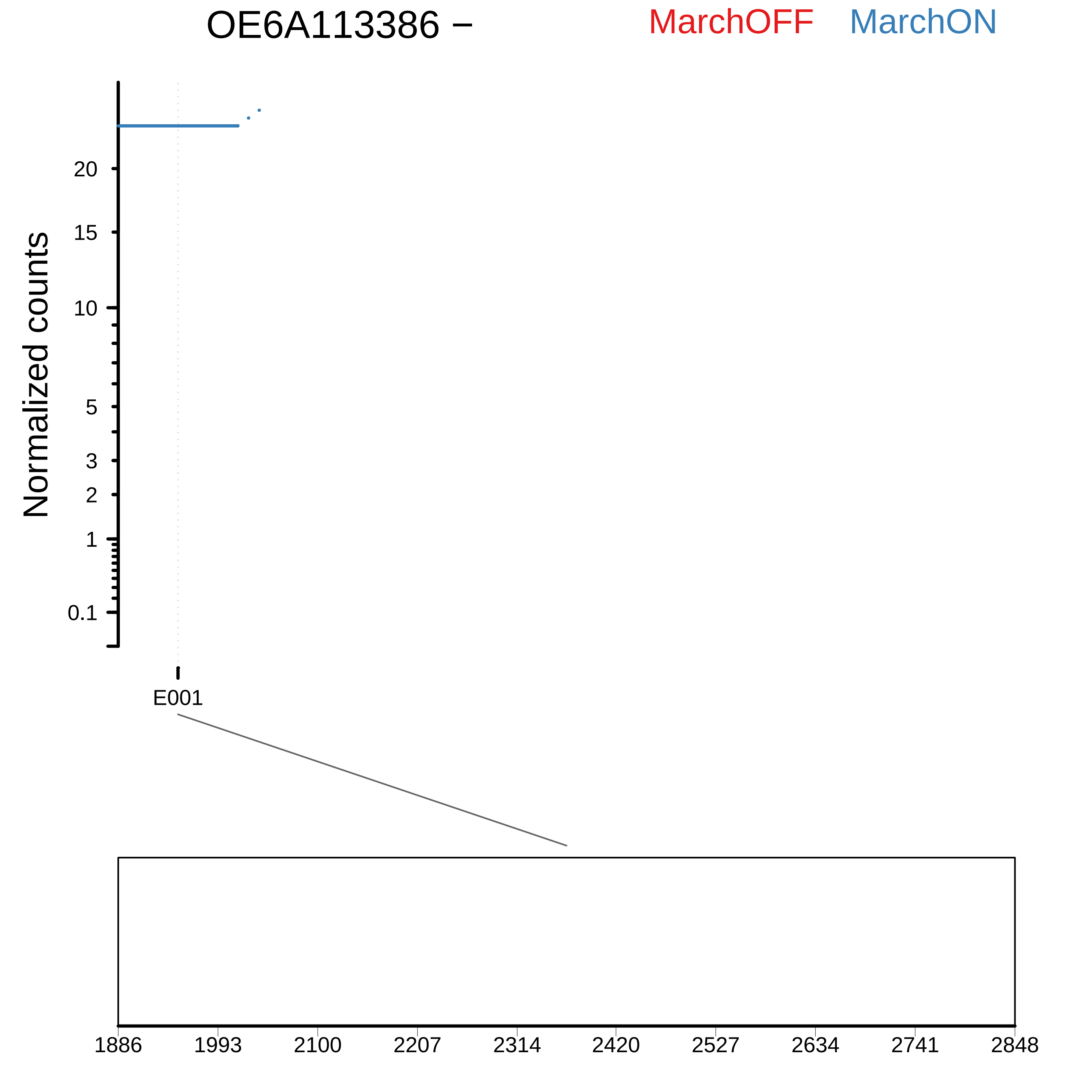

OE6A113386 −
MarchOFF
MarchON
Normalized counts
20
15
10
5
3
2
1
0.1
E001
1886
1993
2100
2207
2314
2420
2527
2634
2741
2848

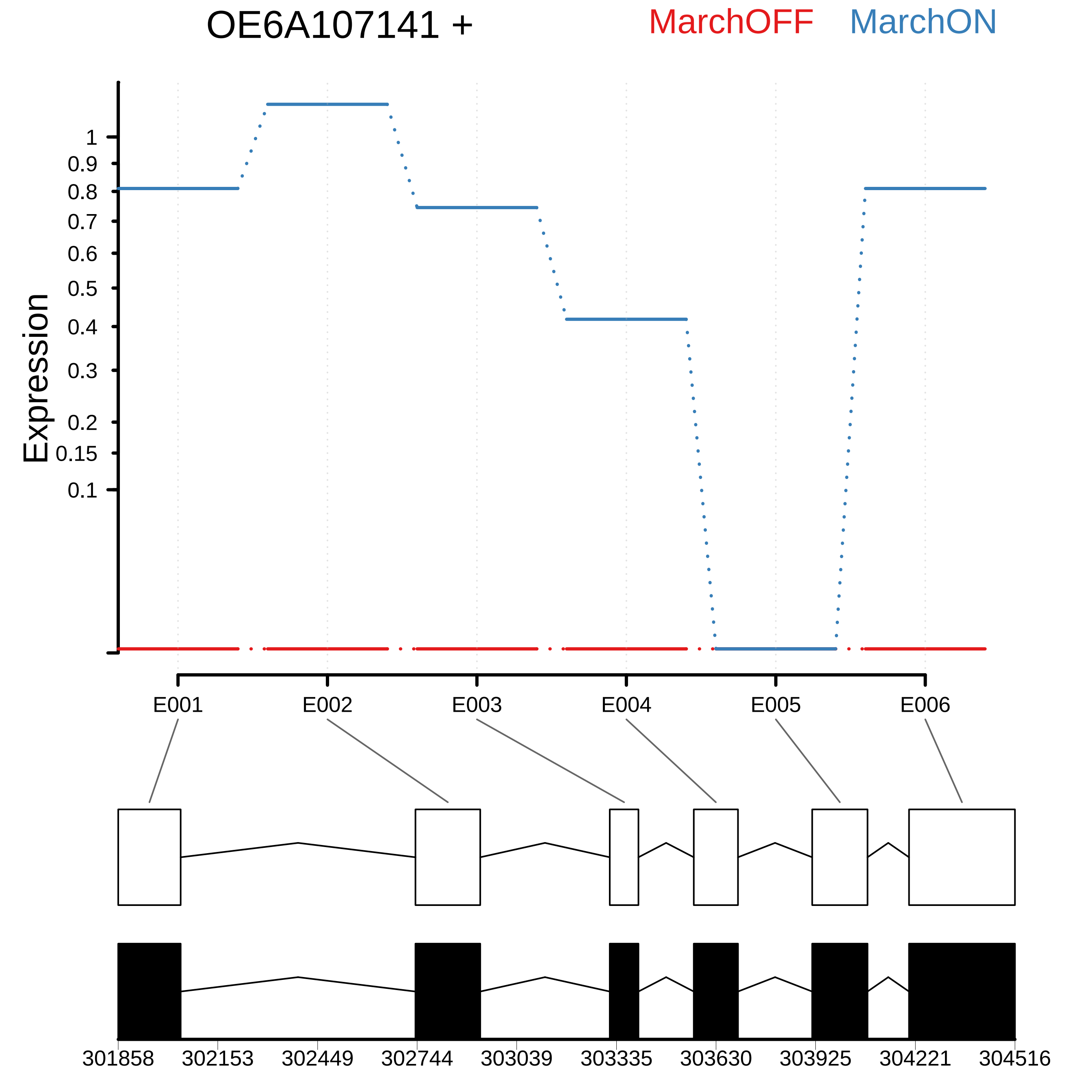

OE6A107141 +
MarchOFF
MarchON
Expression
1
0.9
0.8
0.7
0.6
0.5
0.4
0.3
0.2
0.15
0.1
E001
E002
E003
E004
E005
E006
301858
302153
302449
302744
303039
303335
303630
303925
304221
304516

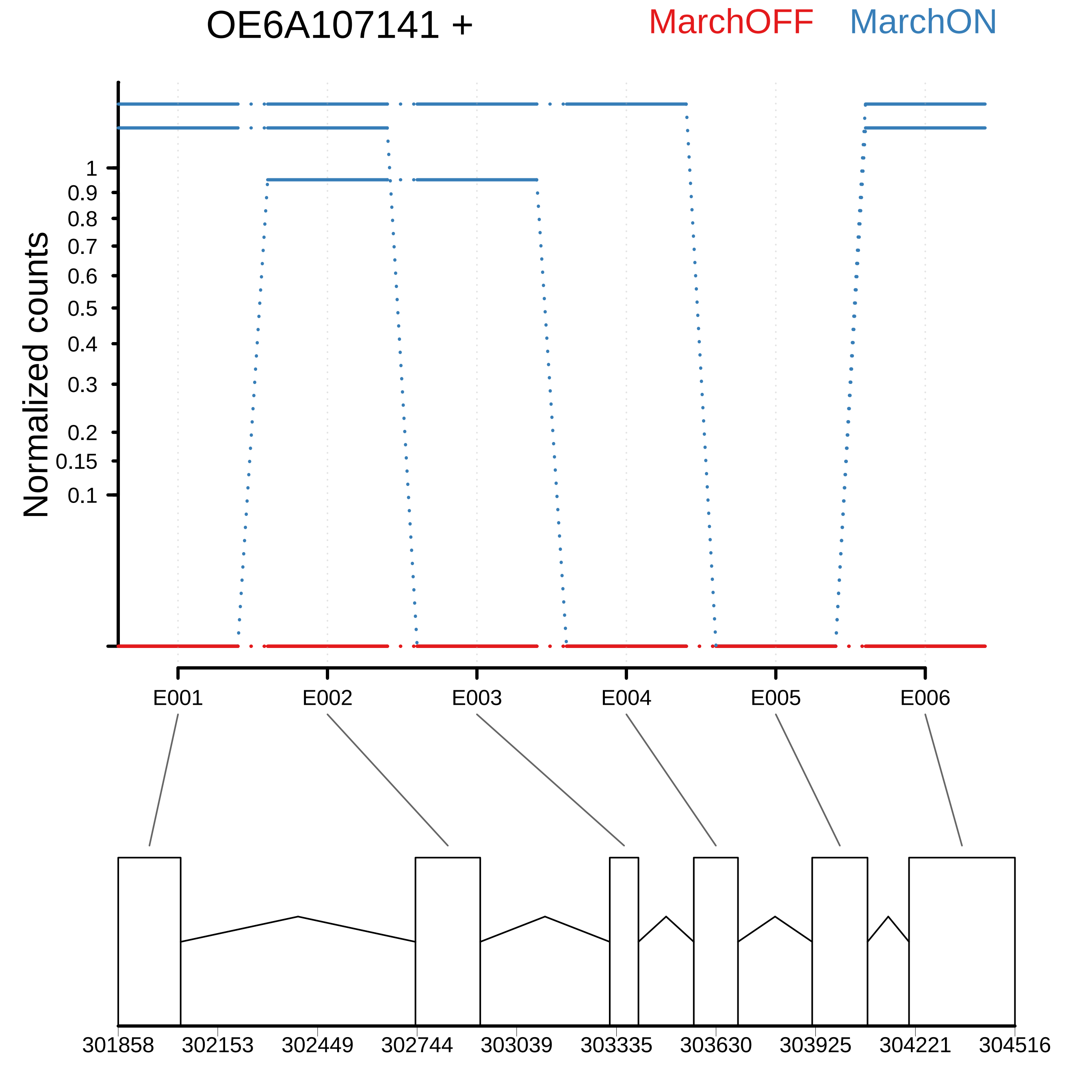
OE6A107141 +
MarchOFF
MarchON
Normalized counts
1
0.9
0.8
0.7
0.6
0.5
0.4
0.3
0.2
0.15
0.1
E001
E002
E003
E004
E005
E006
301858
302153
302449
302744
303039
303335
303630
303925
304221
304516

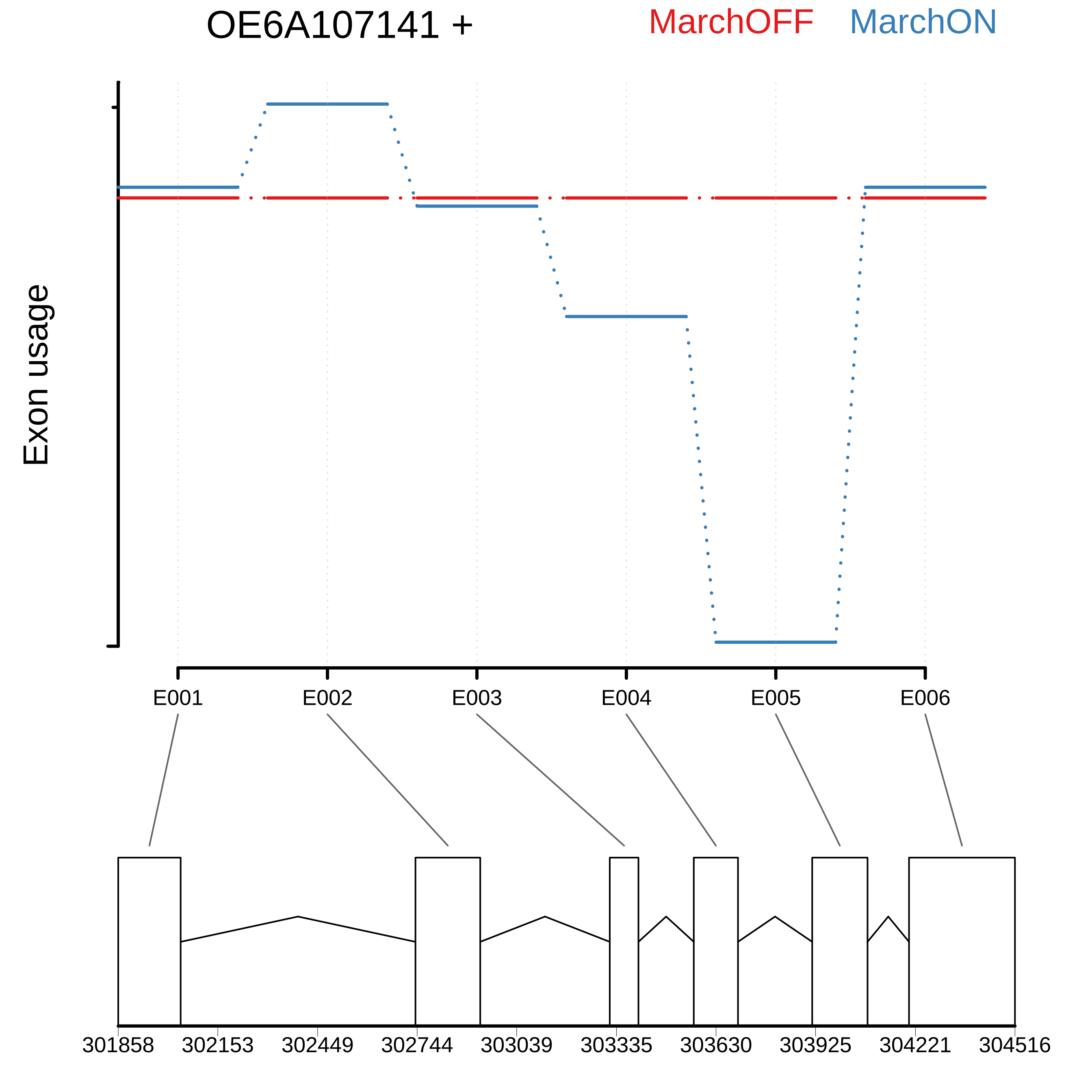

OE6A107141 +
MarchOFF
MarchON
Exon usage
E001
E002
E003
E004
E005
E006
301858
302153
302449
302744
303039
303335
303630
303925
304221
304516

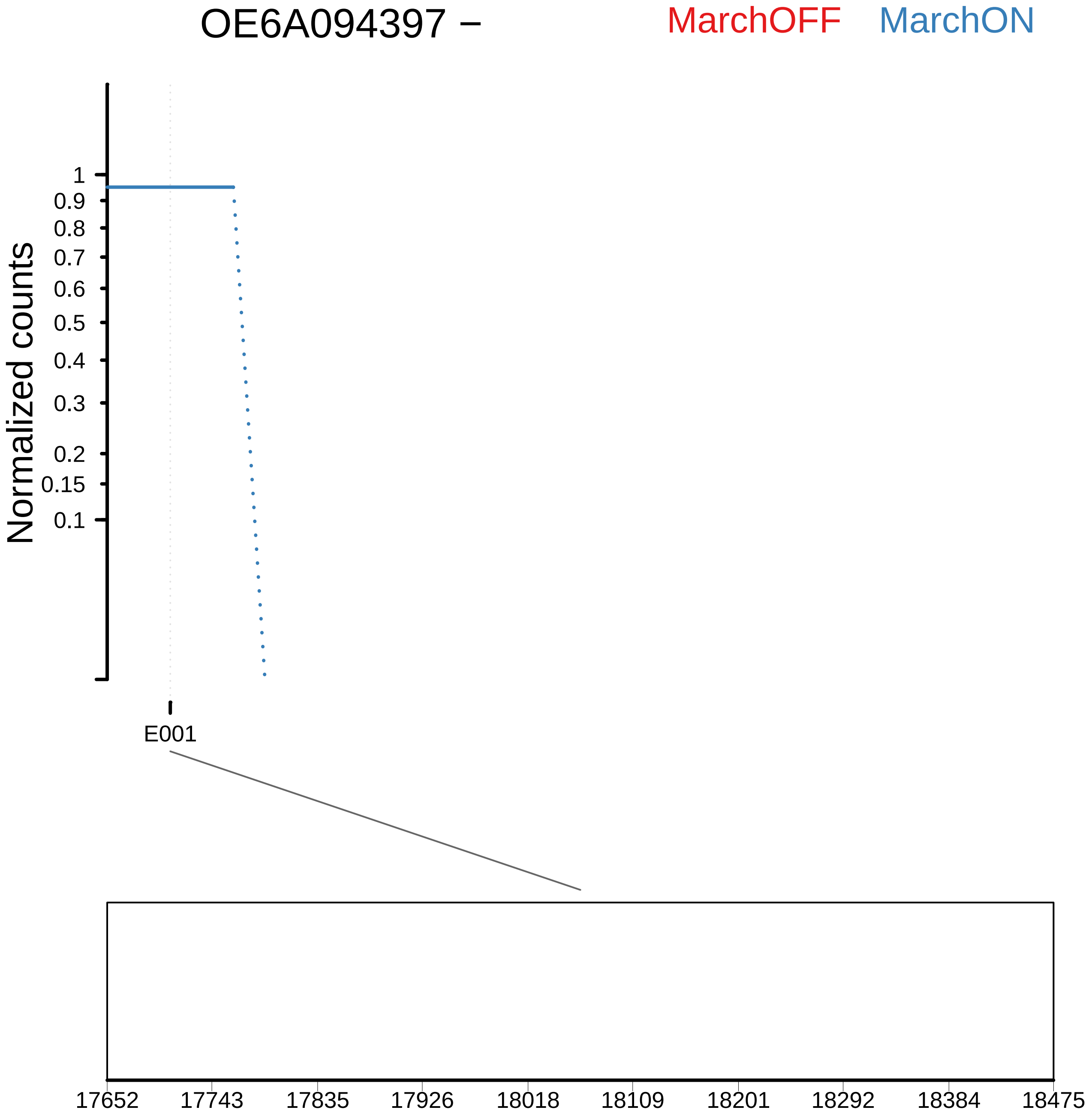

OE6A094397 −
MarchOFF
MarchON
Normalized counts
1
0.9
0.8
0.7
0.6
0.5
0.4
0.3
0.2
0.15
0.1
E001
17652
17743
17835
17926
18018
18109
18201
18292
18384
18475

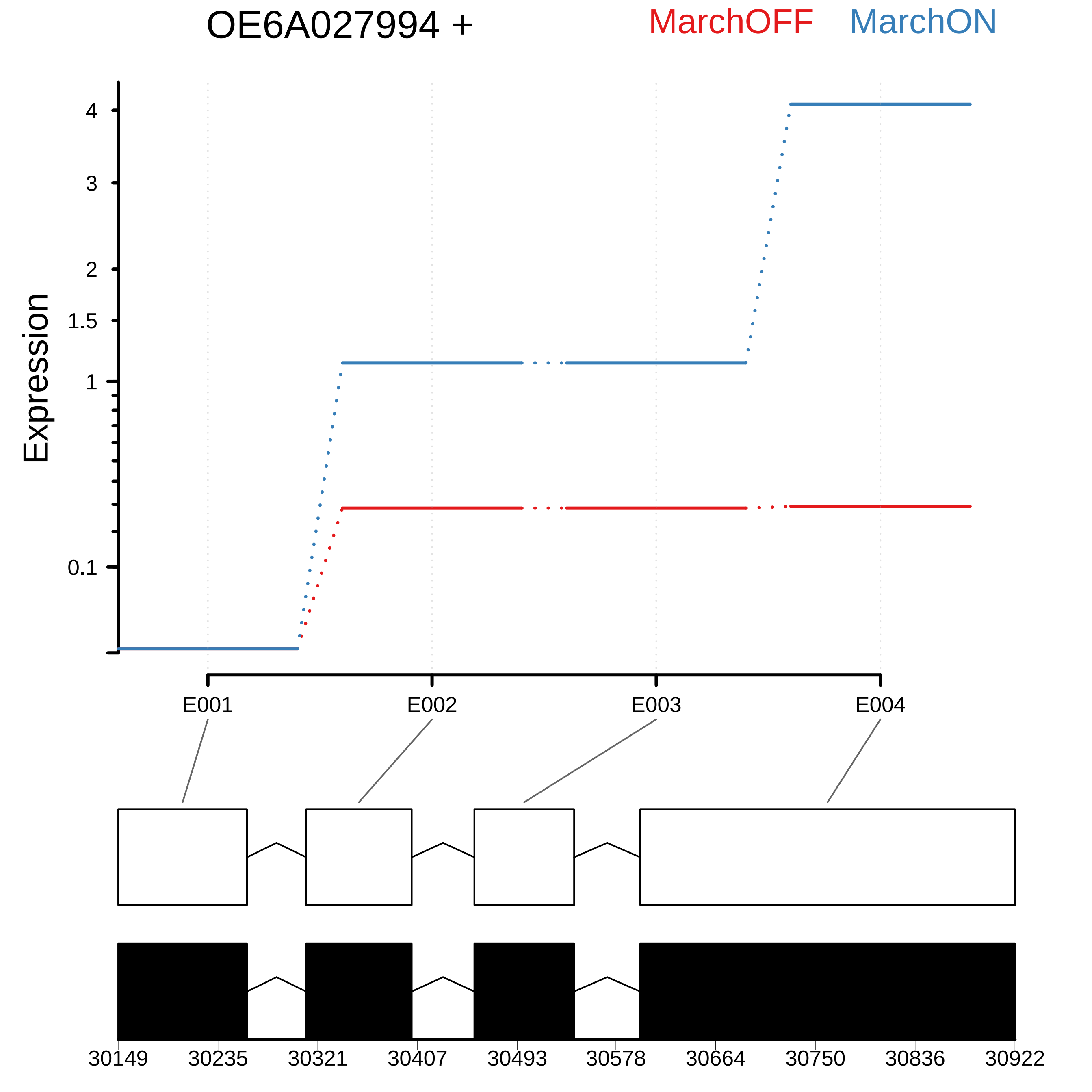
OE6A027994 +
MarchOFF
MarchON
Expression
4
3
2
1.5
1
0.1
E001
E002
E003
E004
30149
30235
30321
30407
30493
30578
30664
30750
30836
30922

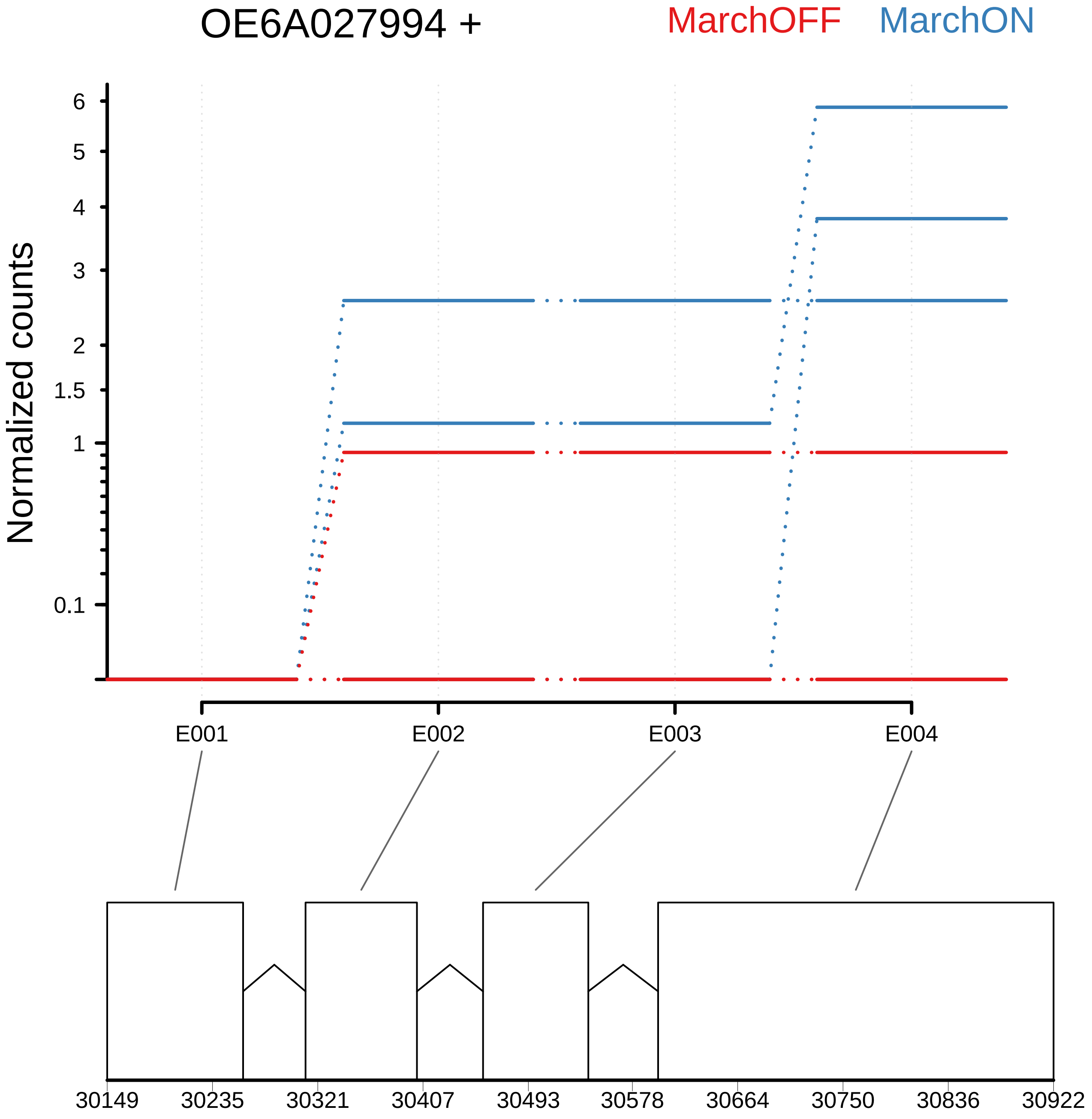
OE6A027994 +
MarchOFF
MarchON
Normalized counts
6
5
4
3
2
1.5
1
0.1
E001
E002
E003
E004
30149
30235
30321
30407
30493
30578
30664
30750
30836
30922

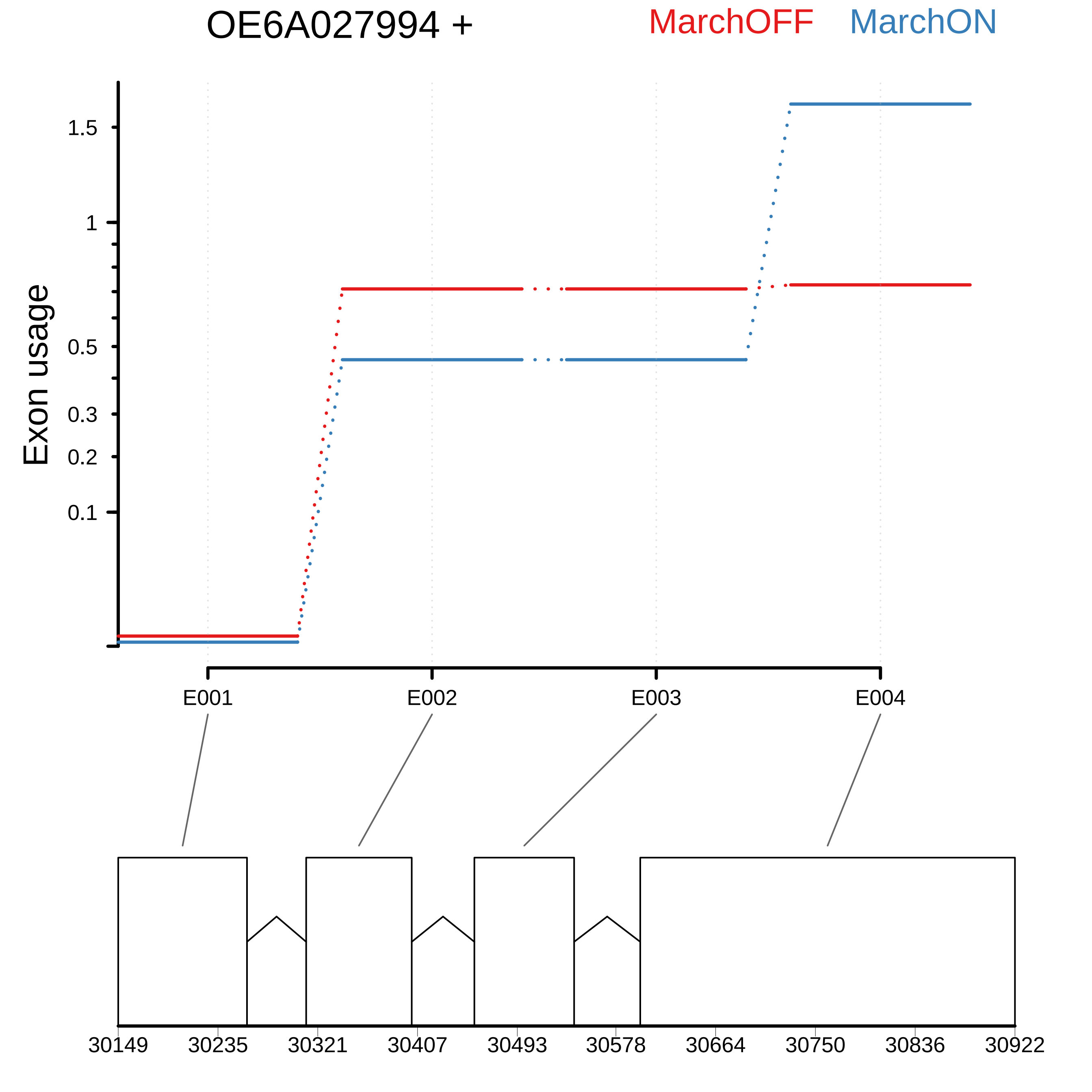
OE6A027994 +
MarchOFF
MarchON
Exon usage
1.5
1
0.5
0.3
0.2
0.1
E001
E002
E003
E004
30149
30235
30321
30407
30493
30578
30664
30750
30836
30922

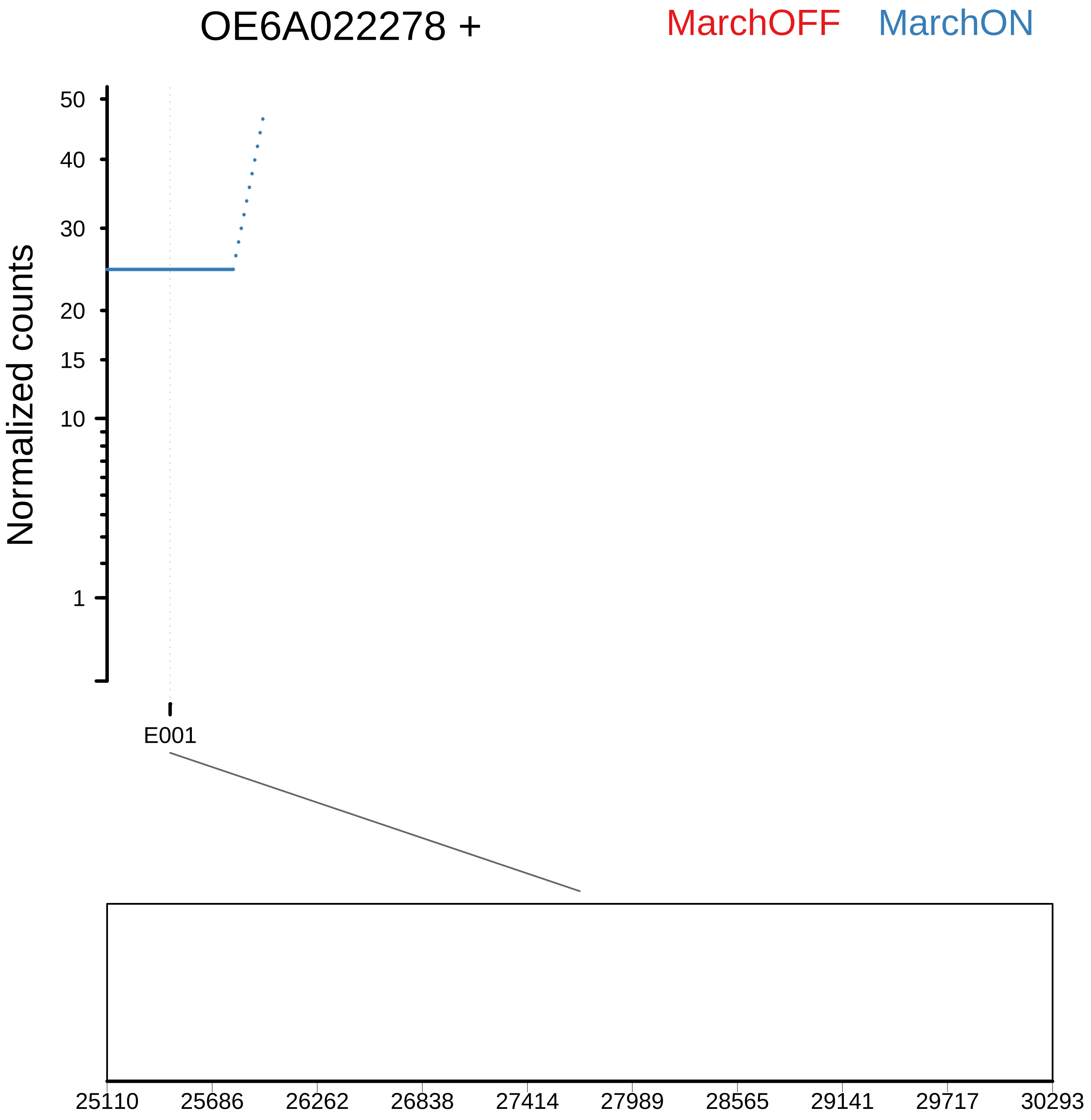
OE6A022278 +
MarchOFF
MarchON
Normalized counts
50
40
30
20
15
10
1
E001
25110
25686
26262
26838
27414
27989
28565
29141
29717
30293

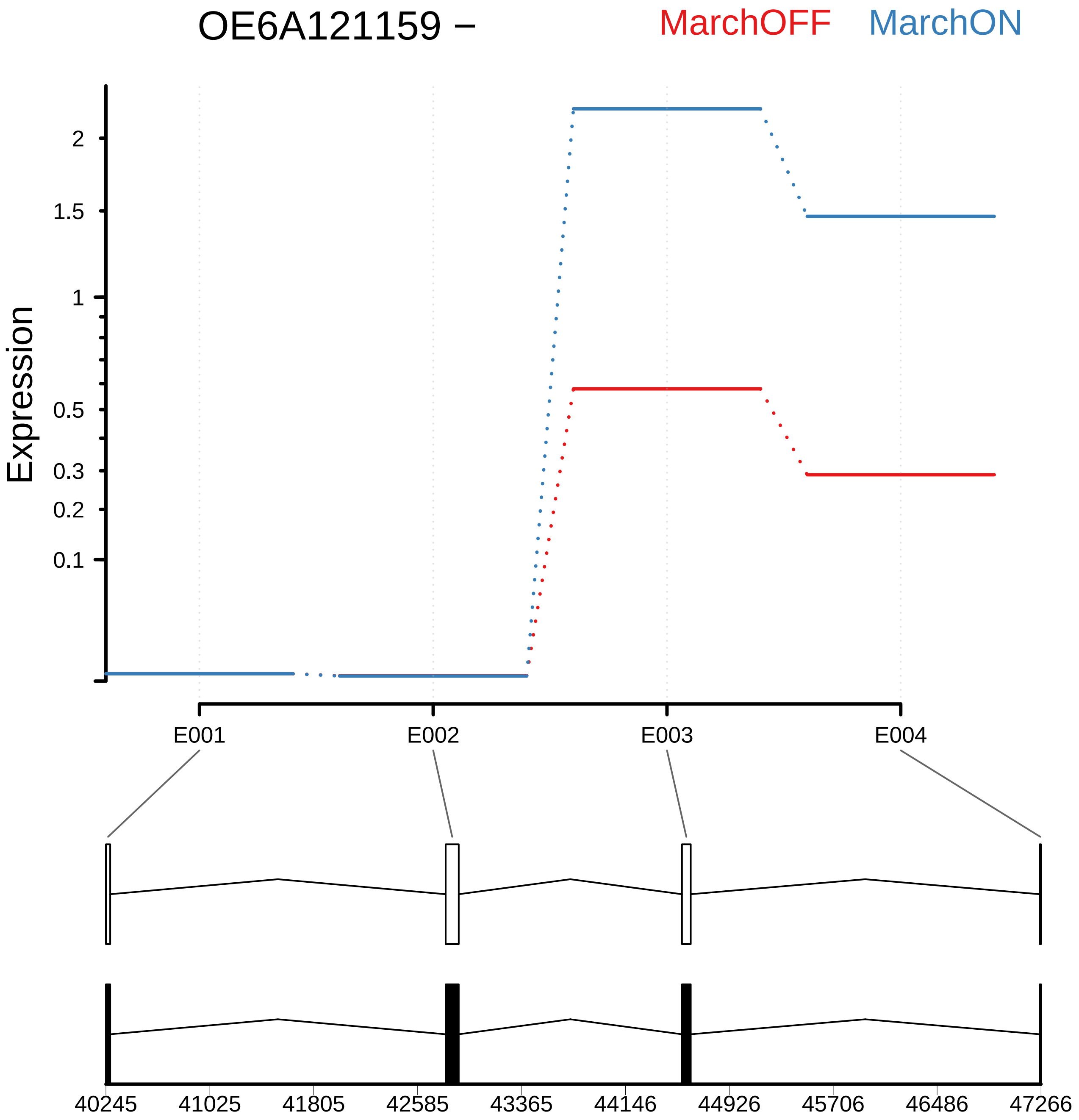

OE6A121159 −
MarchOFF
MarchON
Expression
2
1.5
1
0.5
0.3
0.2
0.1
E001
E002
E003
E004
40245
41025
41805
42585
43365
44146
44926
45706
46486
47266

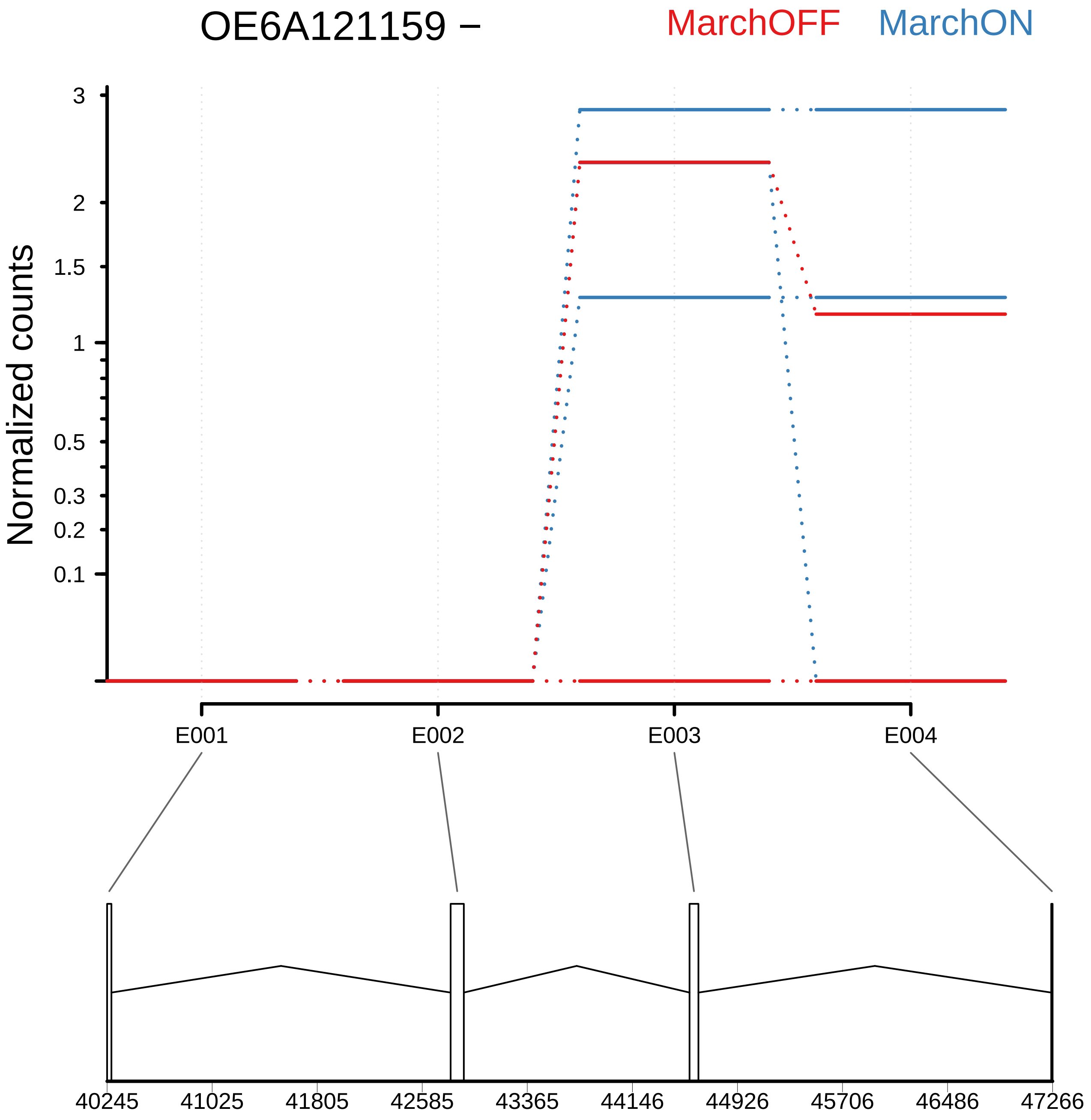

OE6A121159 −
MarchOFF
MarchON
Normalized counts
3
2
1.5
1
0.5
0.3
0.2
0.1
E001
E002
E003
E004
40245
41025
41805
42585
43365
44146
44926
45706
46486
47266

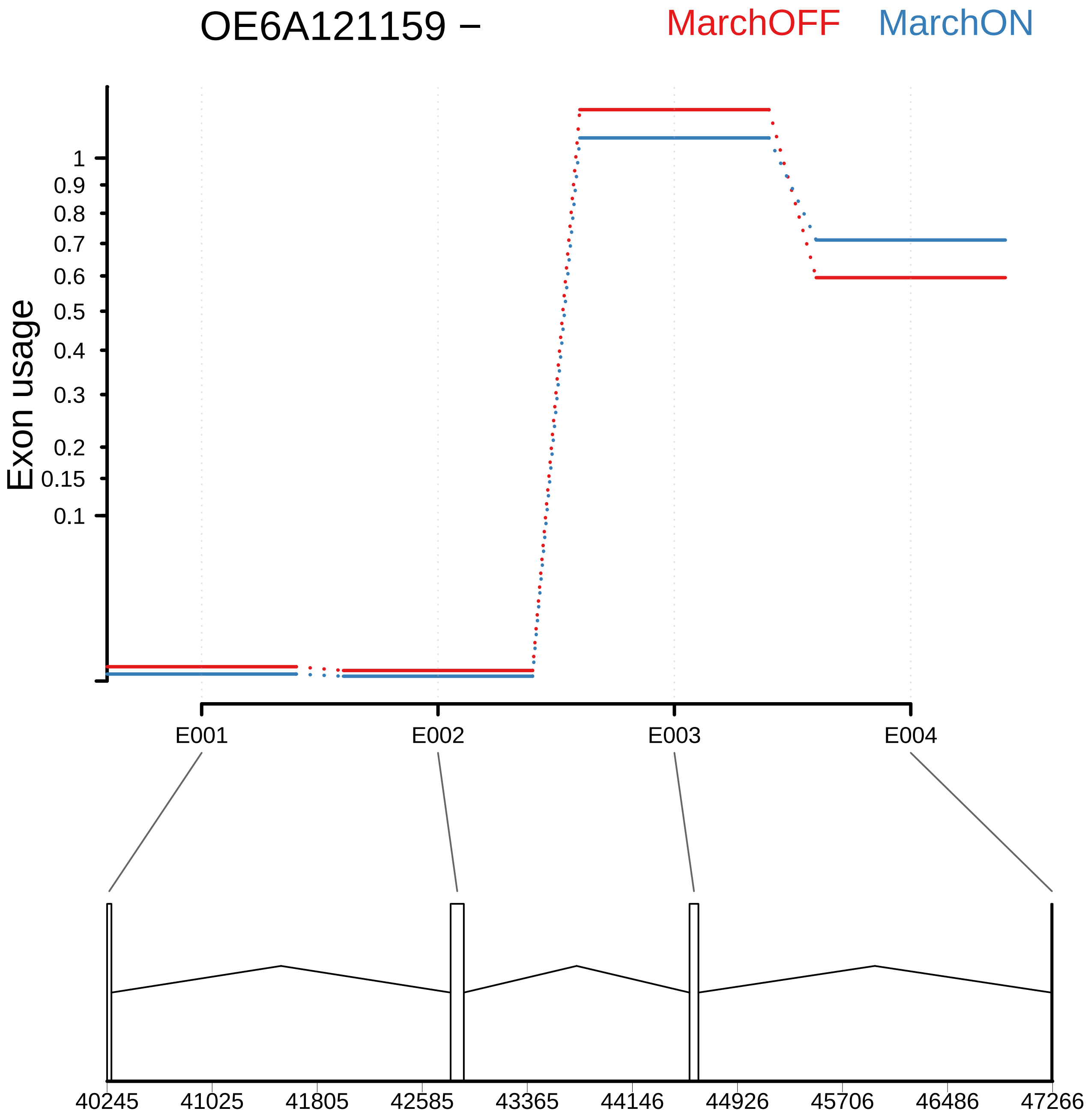
OE6A121159 −
MarchOFF
MarchON
Exon usage
1
0.9
0.8
0.7
0.6
0.5
0.4
0.3
0.2
0.15
0.1
E001
E002
E003
E004
40245
41025
41805
42585
43365
44146
44926
45706
46486
47266

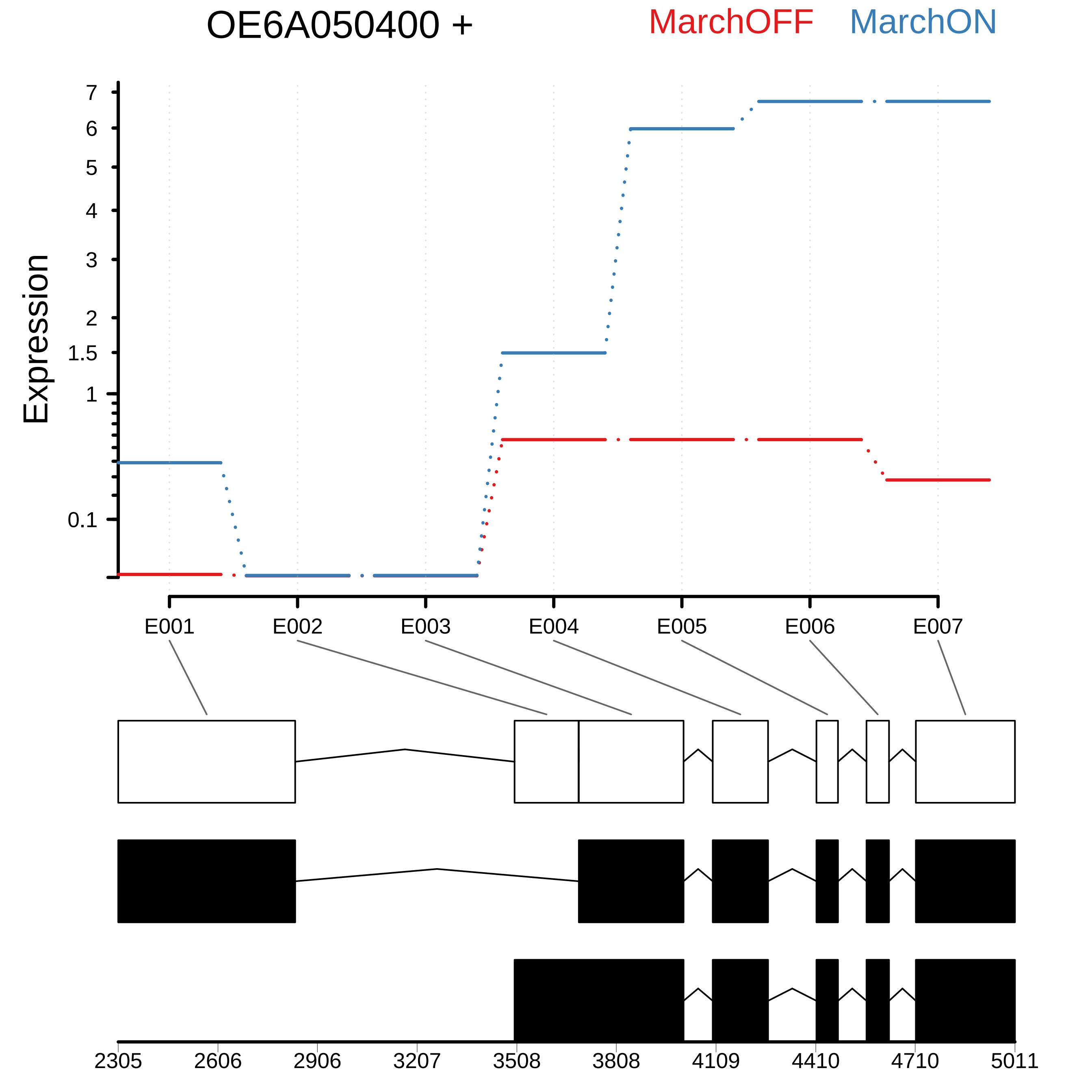

OE6A050400 +
MarchOFF
MarchON
Expression
7
6
5
4
3
2
1.5
1
0.1
E001
E002
E003
E004
E005
E006
E007
2305
2606
2906
3207
3508
3808
4109
4410
4710
5011

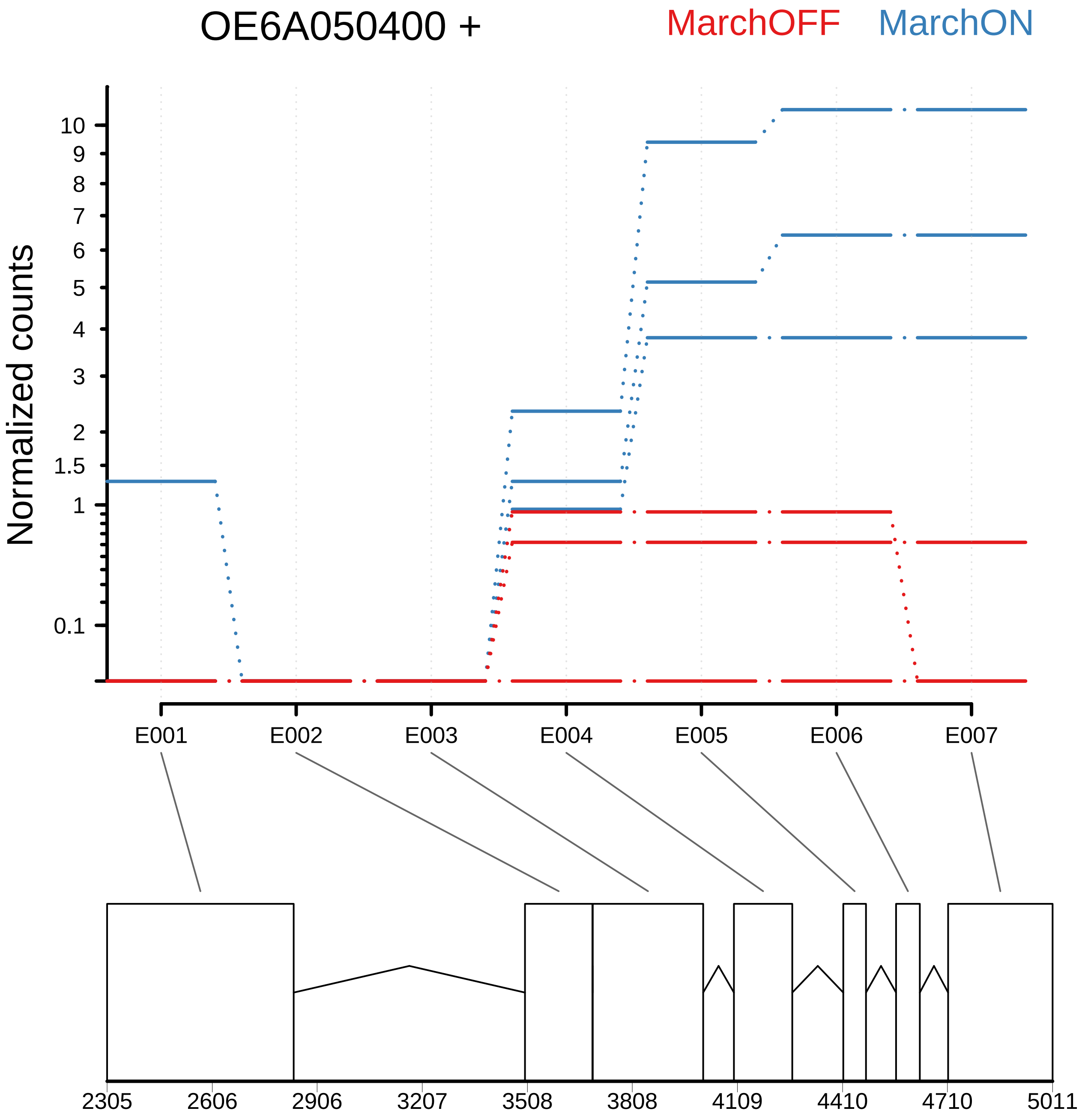
OE6A050400 +
MarchOFF
MarchON
Normalized counts
10
9
8
7
6
5
4
3
2
1.5
1
0.1
E001
E002
E003
E004
E005
E006
E007
2305
2606
2906
3207
3508
3808
4109
4410
4710
5011

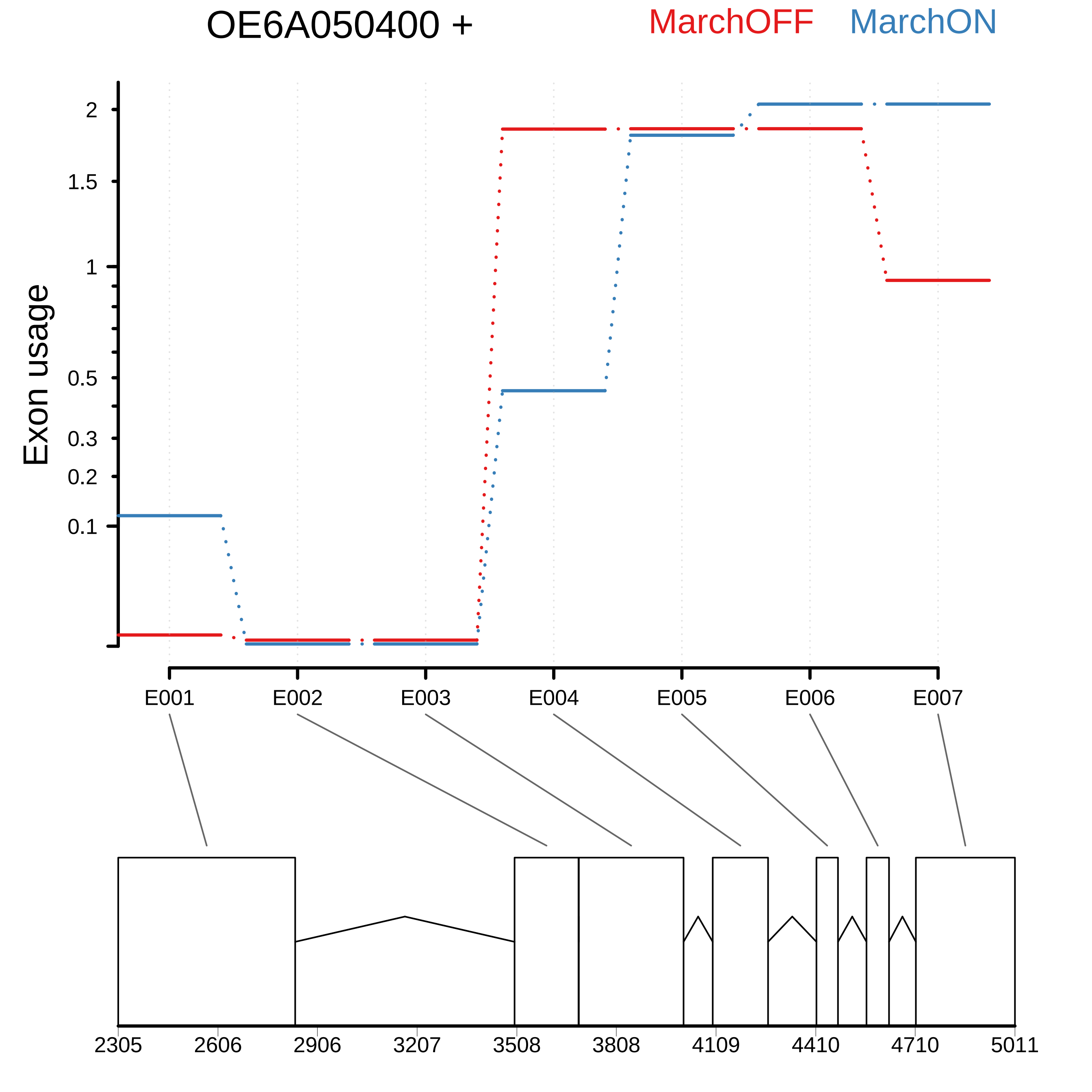

OE6A050400 +
MarchOFF
MarchON
Exon usage
2
1.5
1
0.5
0.3
0.2
0.1
E001
E002
E003
E004
E005
E006
E007
2305
2606
2906
3207
3508
3808
4109
4410
4710
5011

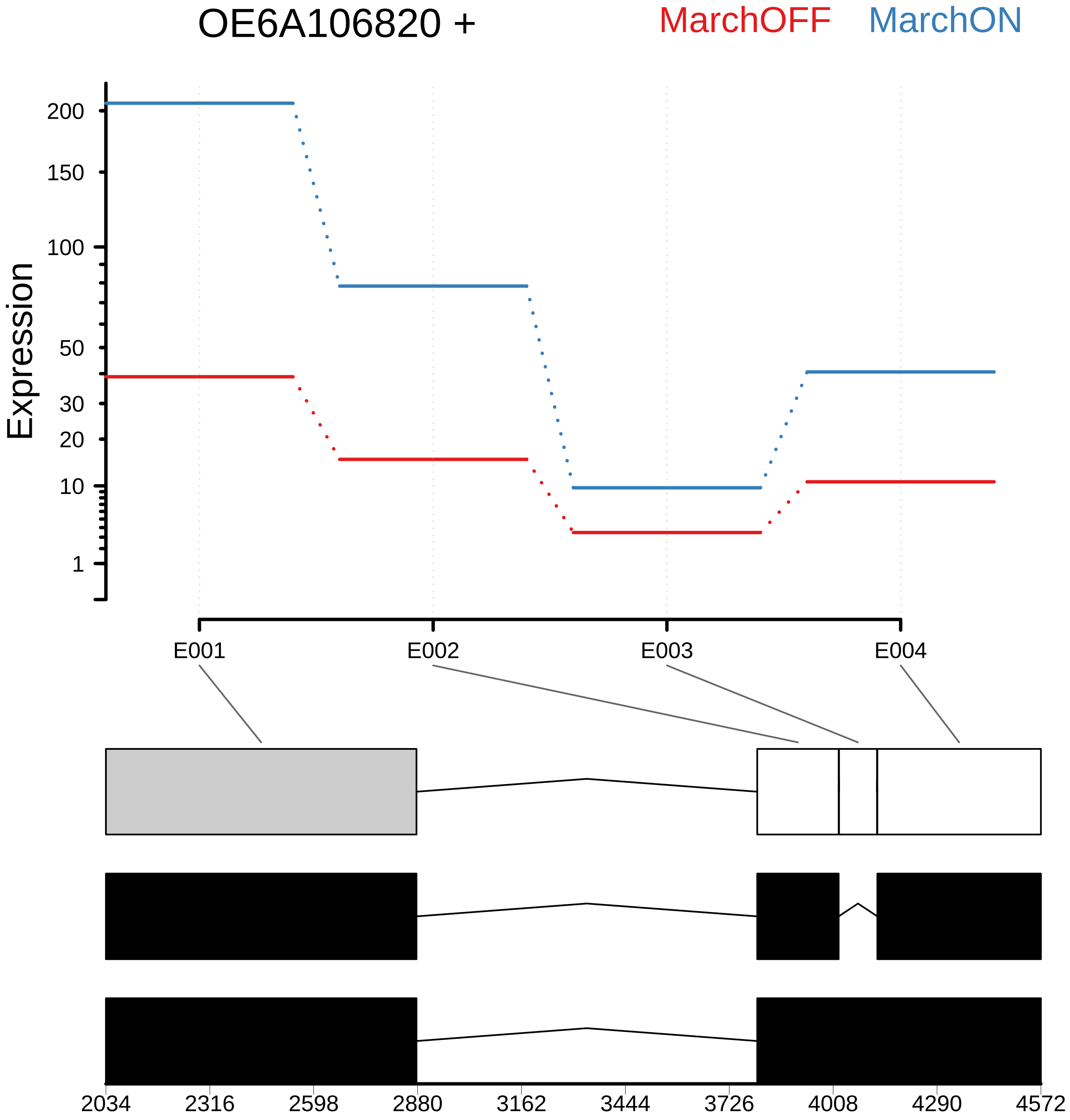

OE6A106820 +
MarchOFF
MarchON
Expression
200
150
100
50
30
20
10
1
E001
E002
E003
E004
2034
2316
2598
2880
3162
3444
3726
4008
4290
4572

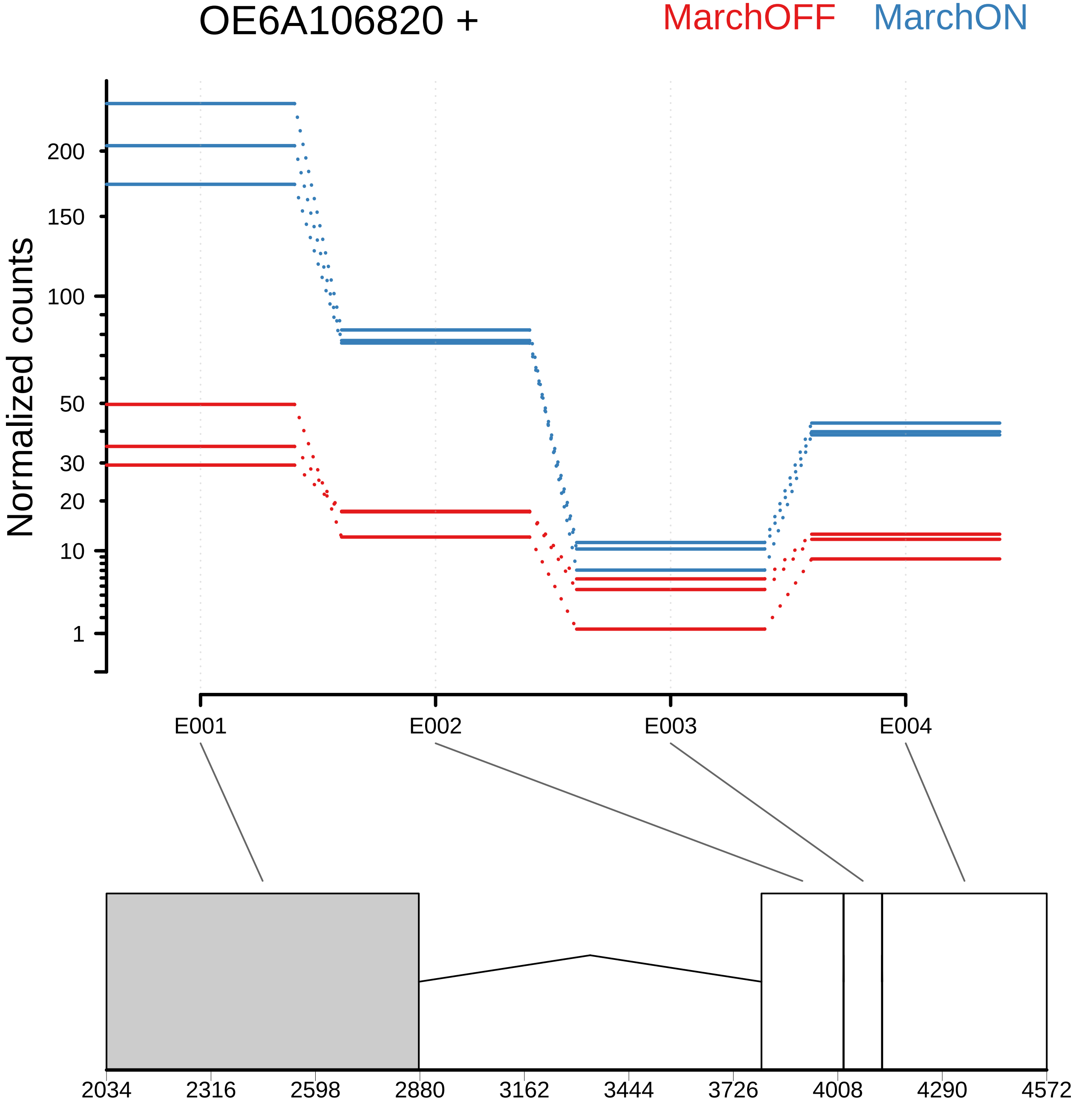
OE6A106820 +
MarchOFF
MarchON
Normalized counts
200
150
100
50
30
20
10
1
E001
E002
E003
E004
2034
2316
2598
2880
3162
3444
3726
4008
4290
4572

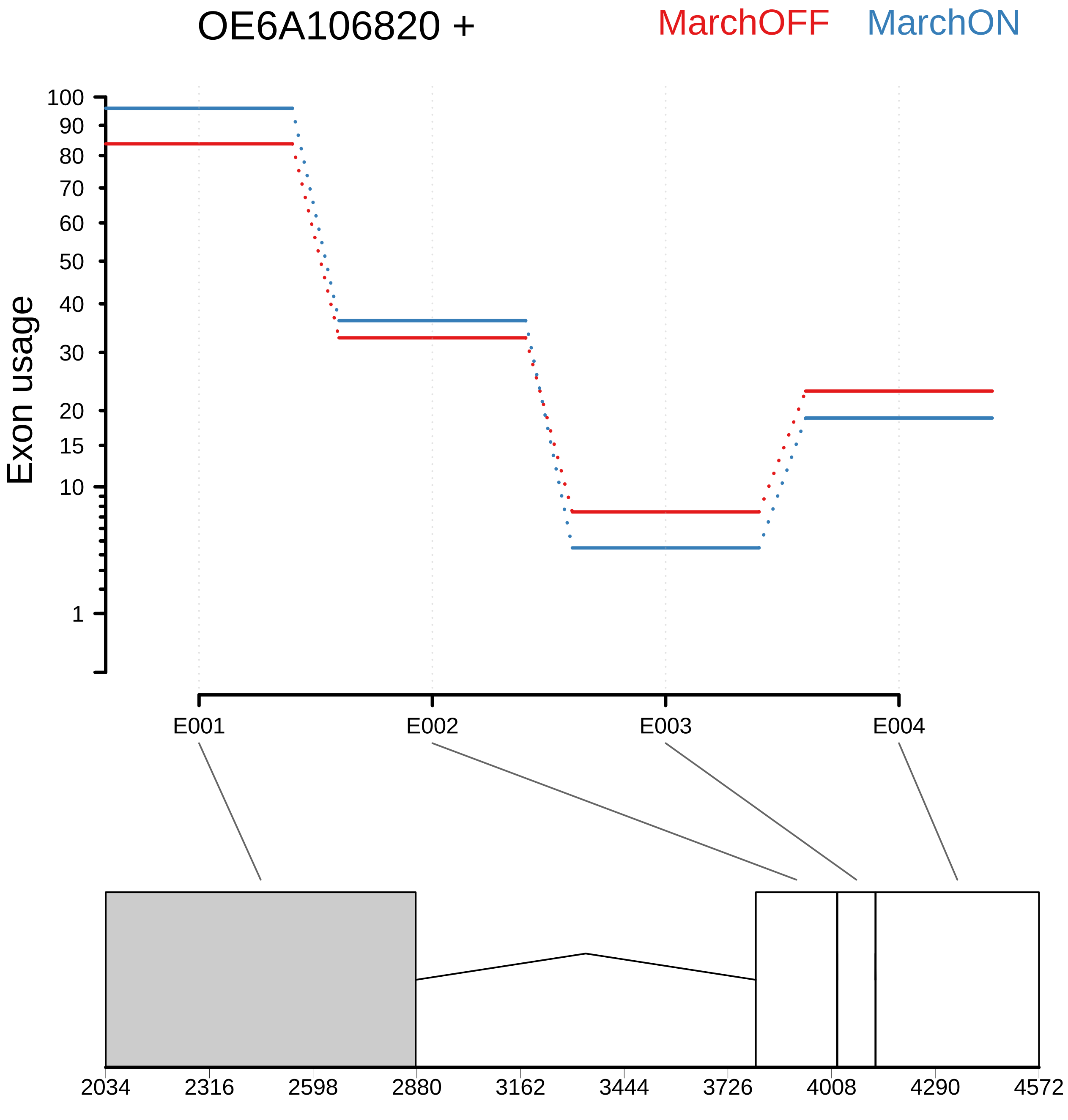

OE6A106820 +
MarchOFF
MarchON
Exon usage
100
90
80
70
60
50
40
30
20
15
10
1
E001
E002
E003
E004
2034
2316
2598
2880
3162
3444
3726
4008
4290
4572

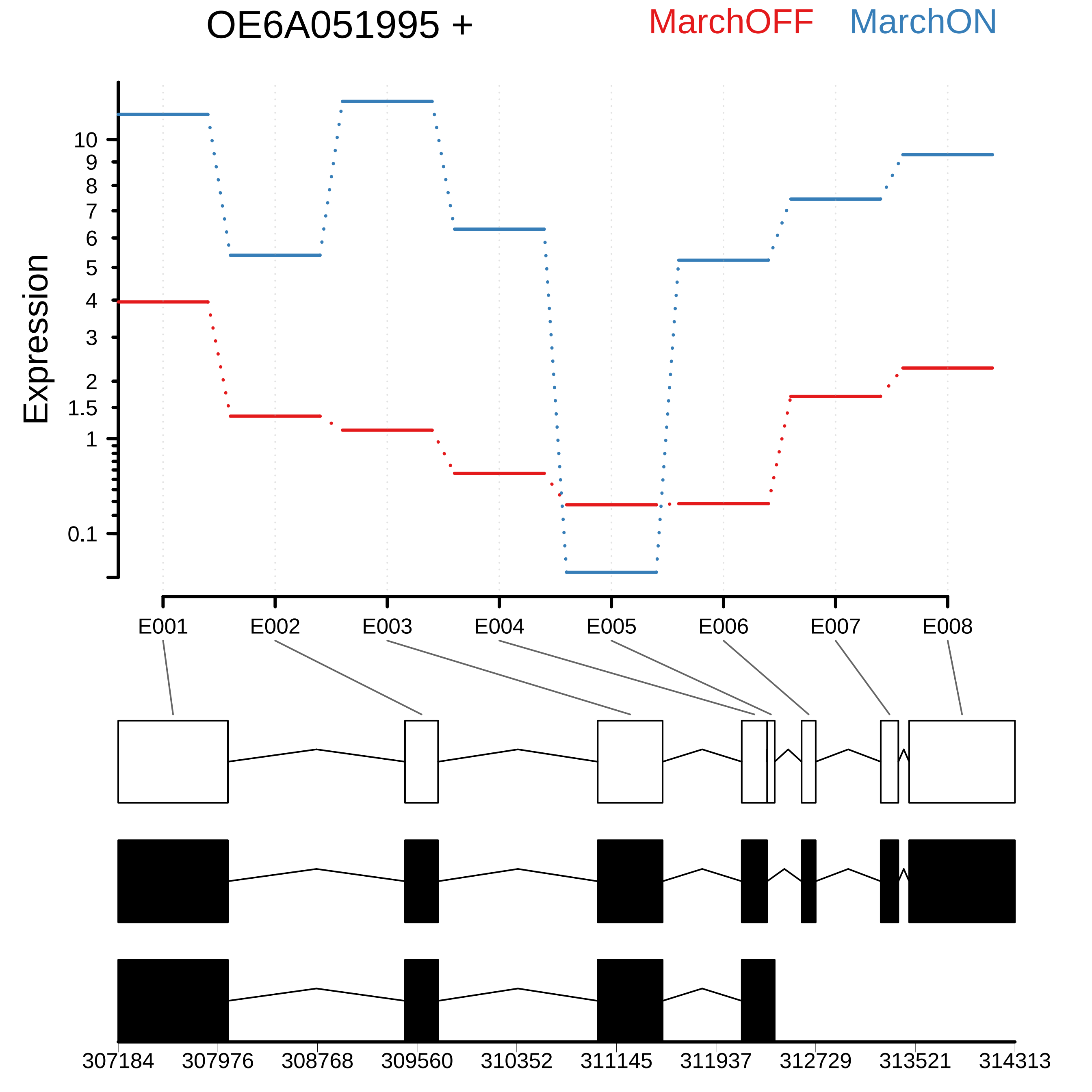
OE6A051995 +
MarchOFF
MarchON
Expression
10
9
8
7
6
5
4
3
2
1.5
1
0.1
E001
E002
E003
E004
E005
E006
E007
E008
307184
307976
308768
309560
310352
311145
311937
312729
313521
314313

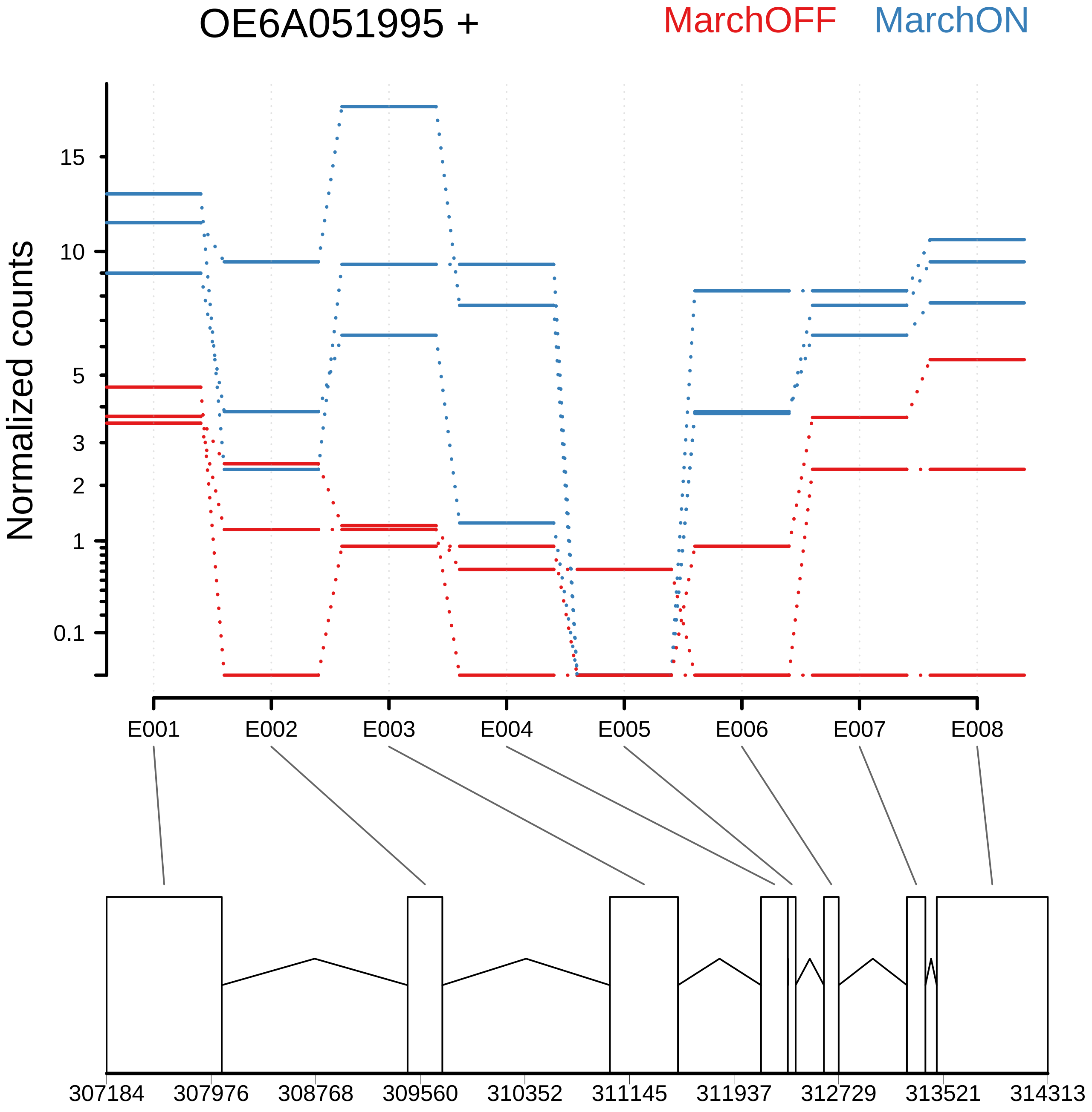
OE6A051995 +
MarchOFF
MarchON
Normalized counts
15
10
5
3
2
1
0.1
E001
E002
E003
E004
E005
E006
E007
E008
307184
307976
308768
309560
310352
311145
311937
312729
313521
314313

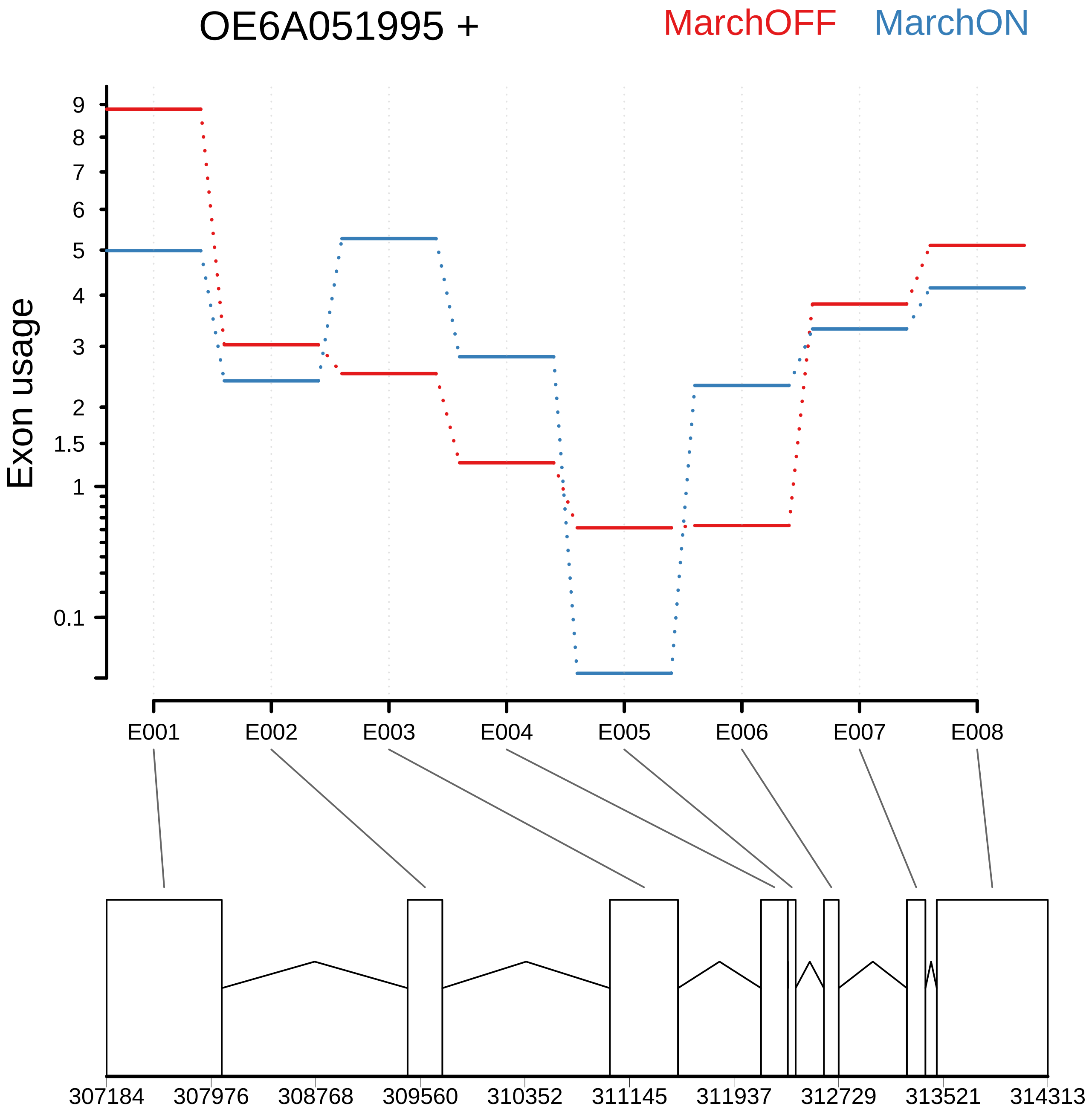

OE6A051995 +
MarchOFF
MarchON
Exon usage
9
8
7
6
5
4
3
2
1.5
1
0.1
E001
E002
E003
E004
E005
E006
E007
E008
307184
307976
308768
309560
310352
311145
311937
312729
313521
314313

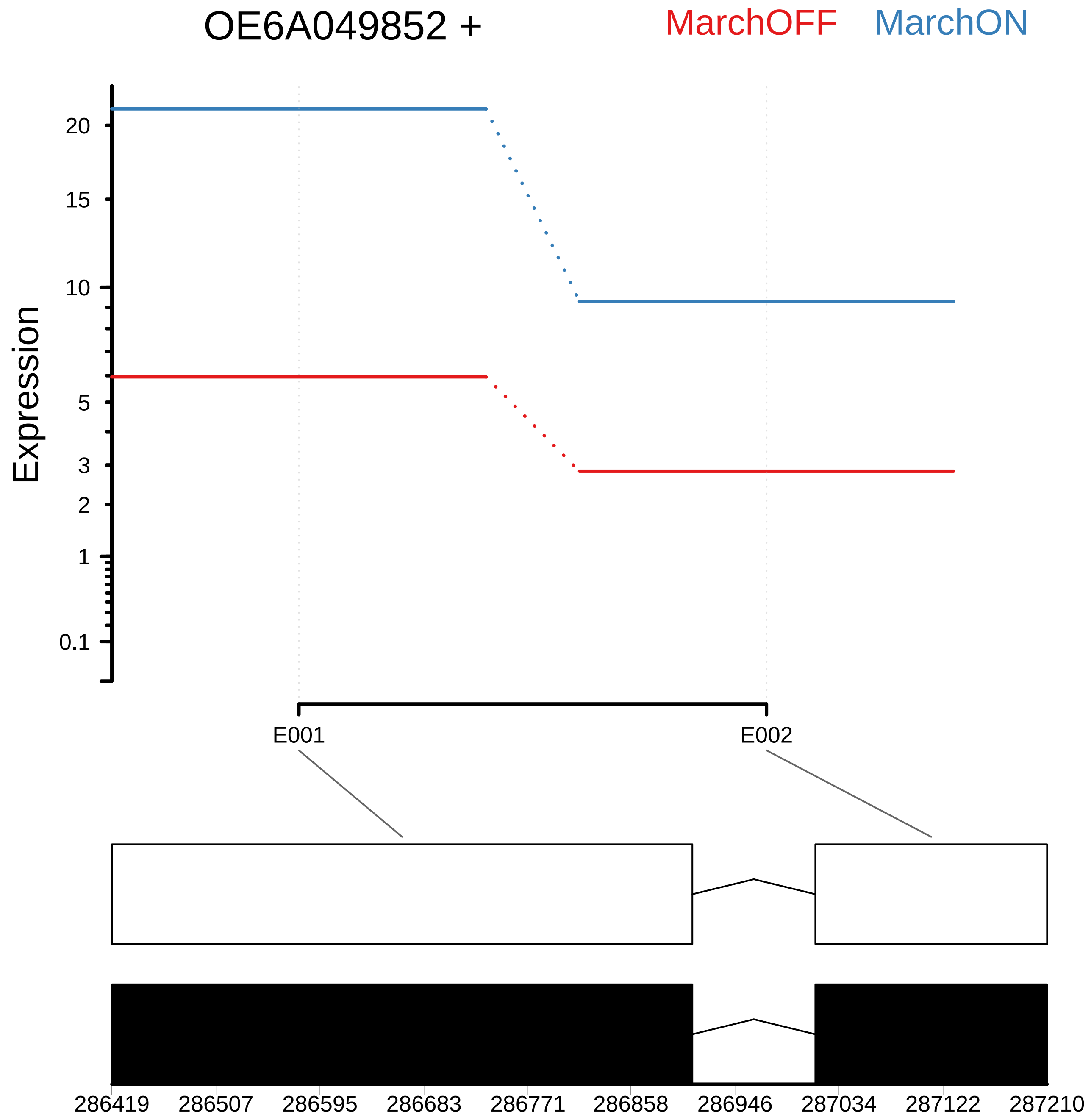

OE6A049852 +
MarchOFF
MarchON
Expression
20
15
10
5
3
2
1
0.1
E001
E002
286419
286507
286595
286683
286771
286858
286946
287034
287122
287210

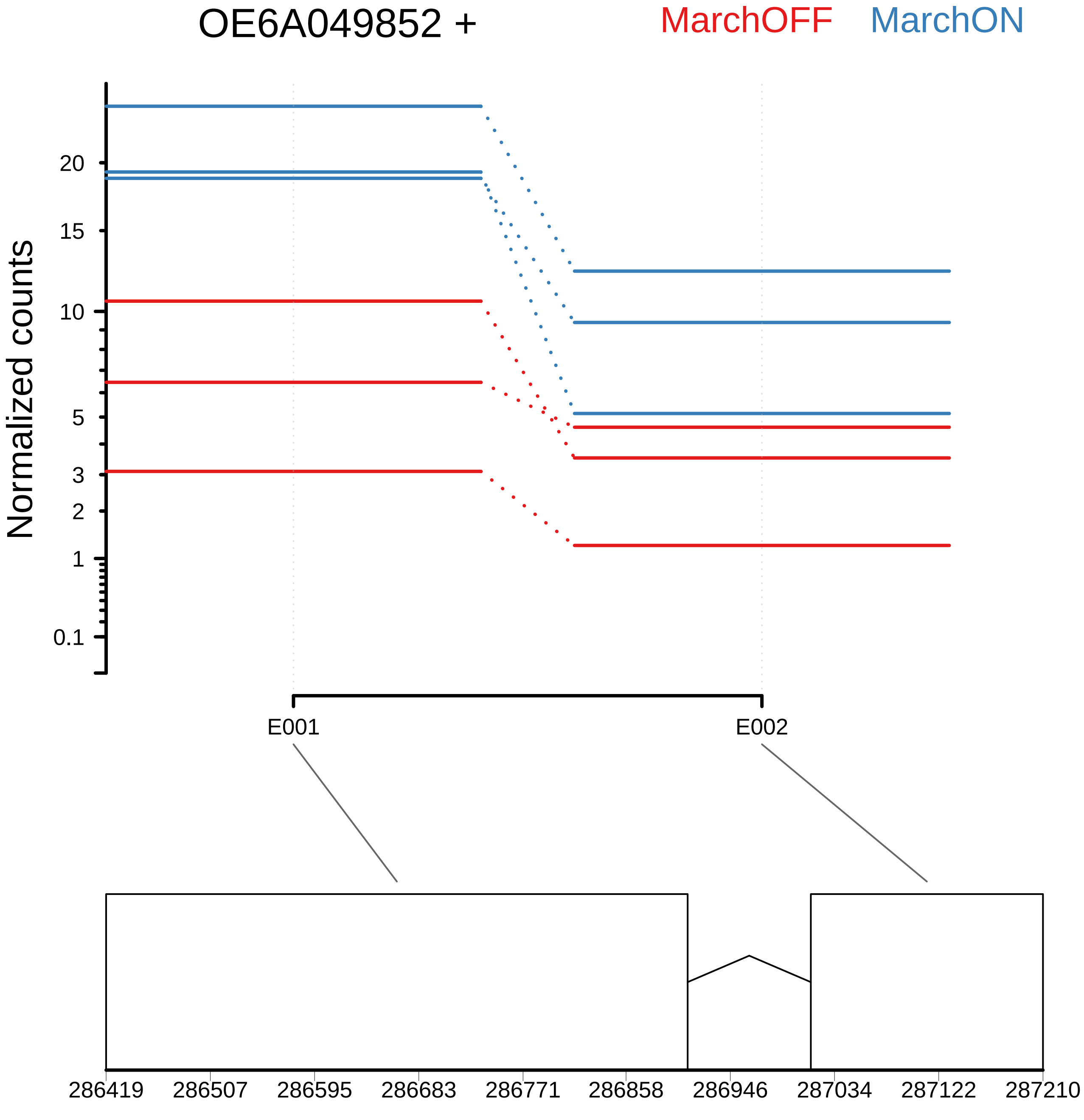

OE6A049852 +
MarchOFF
MarchON
Normalized counts
20
15
10
5
3
2
1
0.1
E001
E002
286419
286507
286595
286683
286771
286858
286946
287034
287122
287210

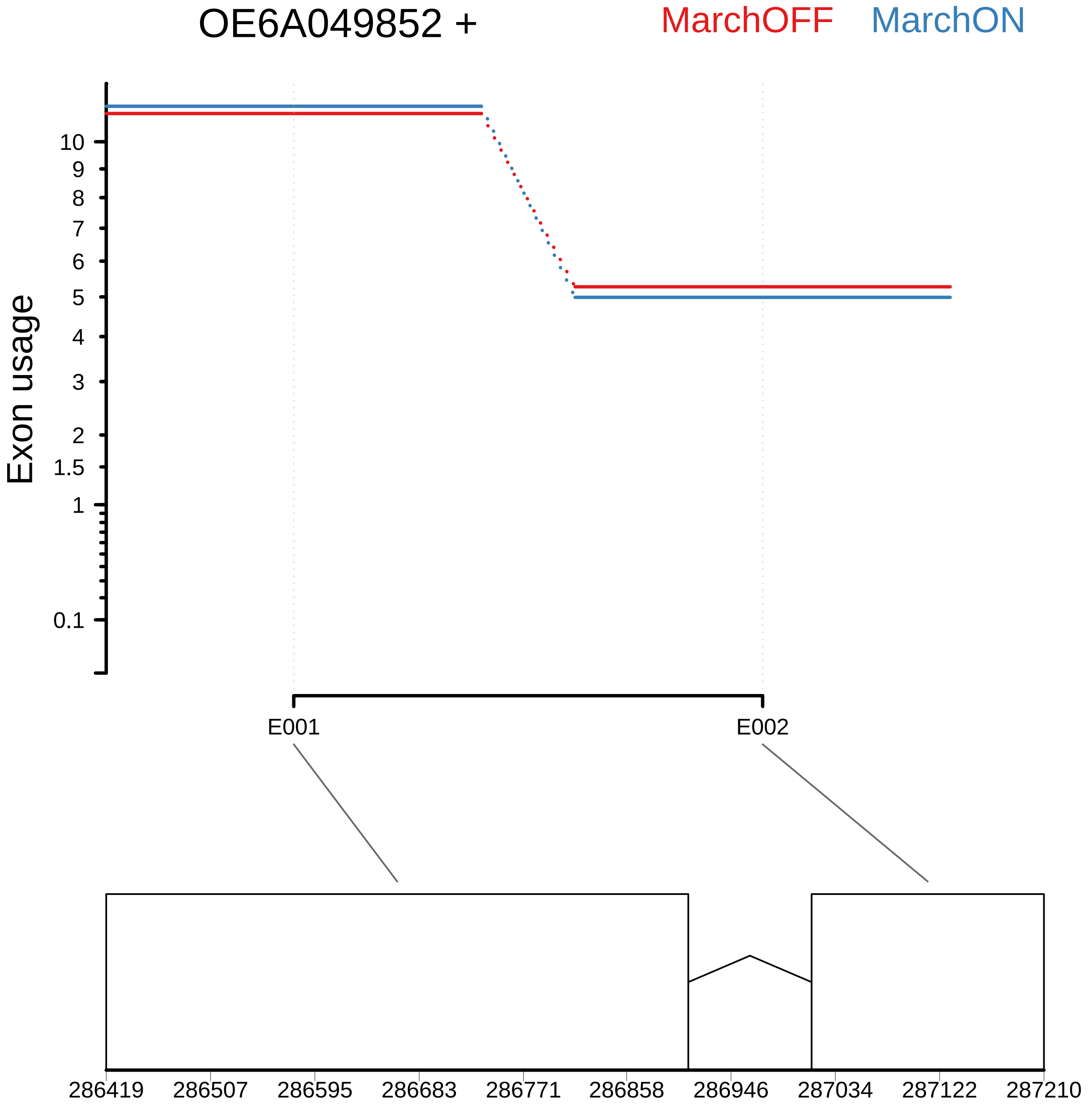
OE6A049852 +
MarchOFF
MarchON
Exon usage
10
9
8
7
6
5
4
3
2
1.5
1
0.1
E001
E002
286419
286507
286595
286683
286771
286858
286946
287034
287122
287210

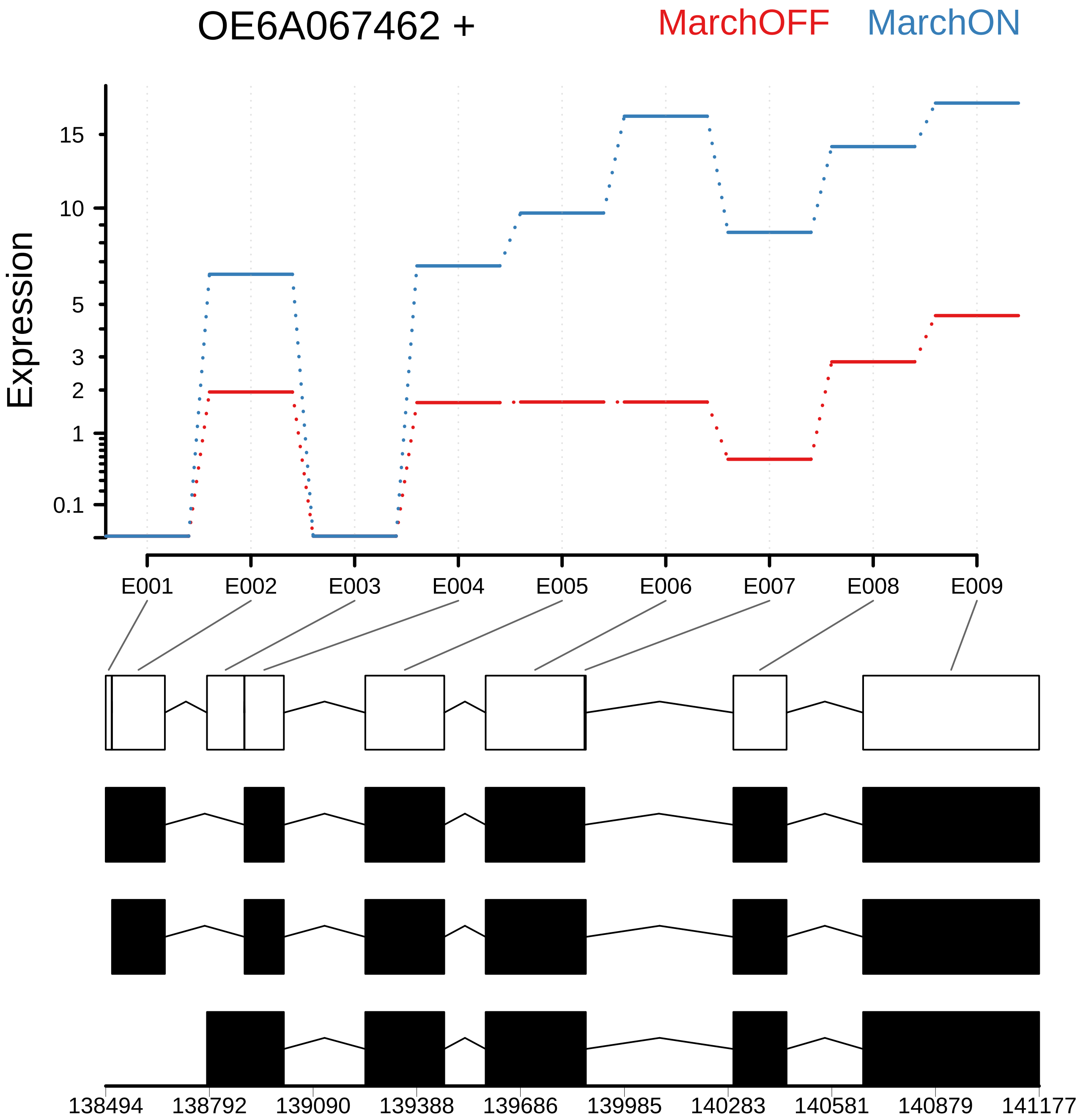
OE6A067462 +
MarchOFF
MarchON
Expression
15
10
5
3
2
1
0.1
E001
E002
E003
E004
E005
E006
E007
E008
E009
138494
138792
139090
139388
139686
139985
140283
140581
140879
141177

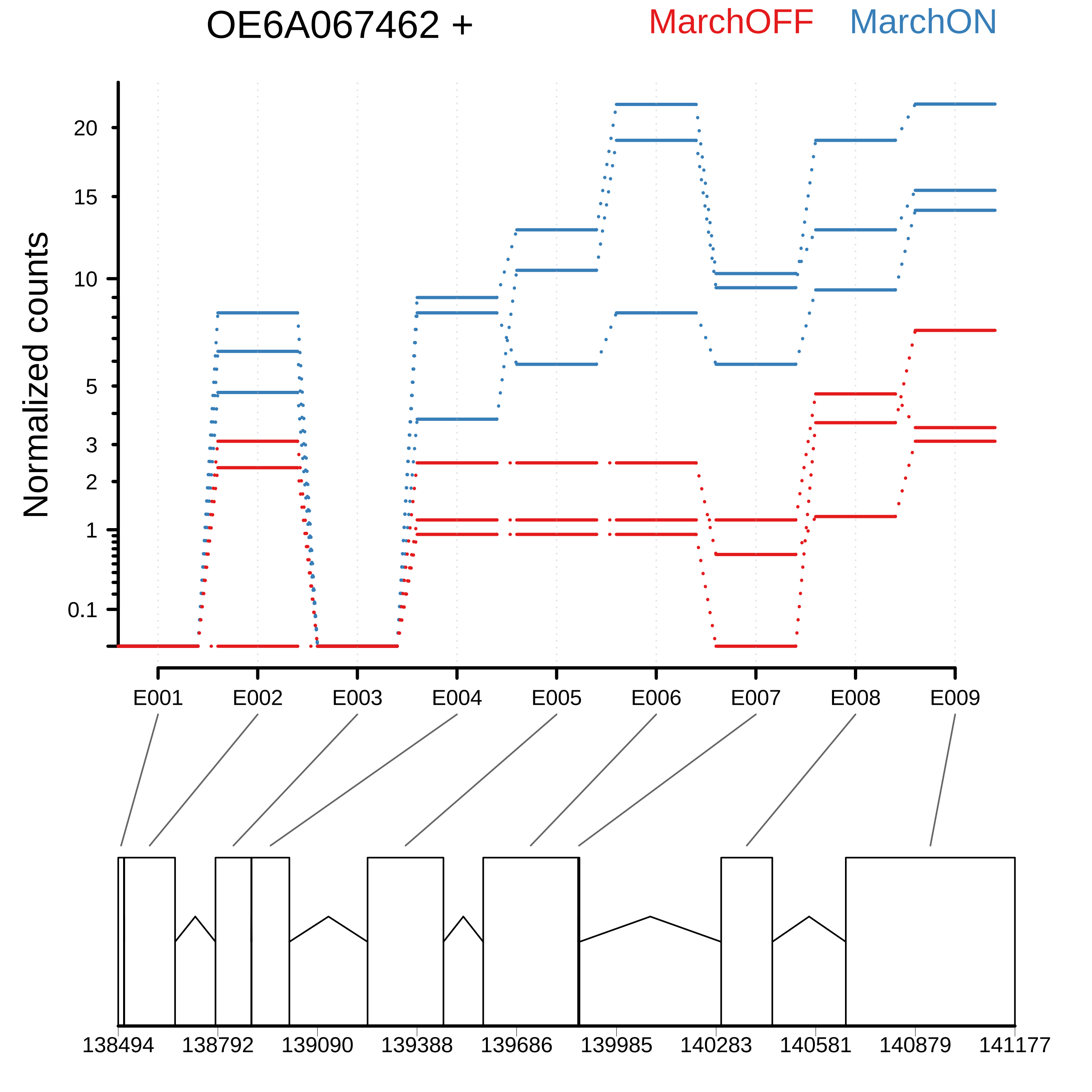

OE6A067462 +
MarchOFF
MarchON
Normalized counts
20
15
10
5
3
2
1
0.1
E001
E002
E003
E004
E005
E006
E007
E008
E009
138494
138792
139090
139388
139686
139985
140283
140581
140879
141177

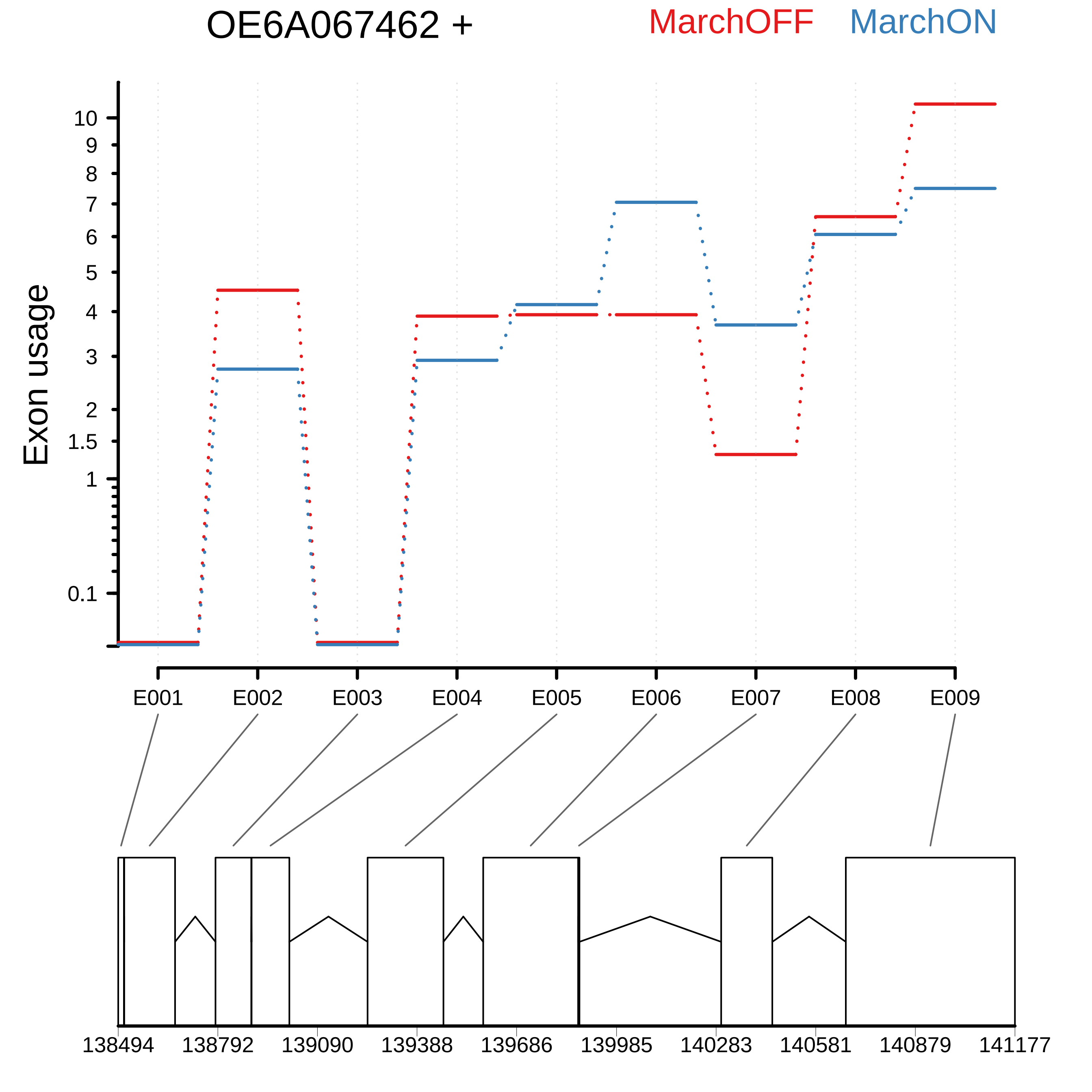

OE6A067462 +
MarchOFF
MarchON
Exon usage
10
9
8
7
6
5
4
3
2
1.5
1
0.1
E001
E002
E003
E004
E005
E006
E007
E008
E009
138494
138792
139090
139388
139686
139985
140283
140581
140879
141177

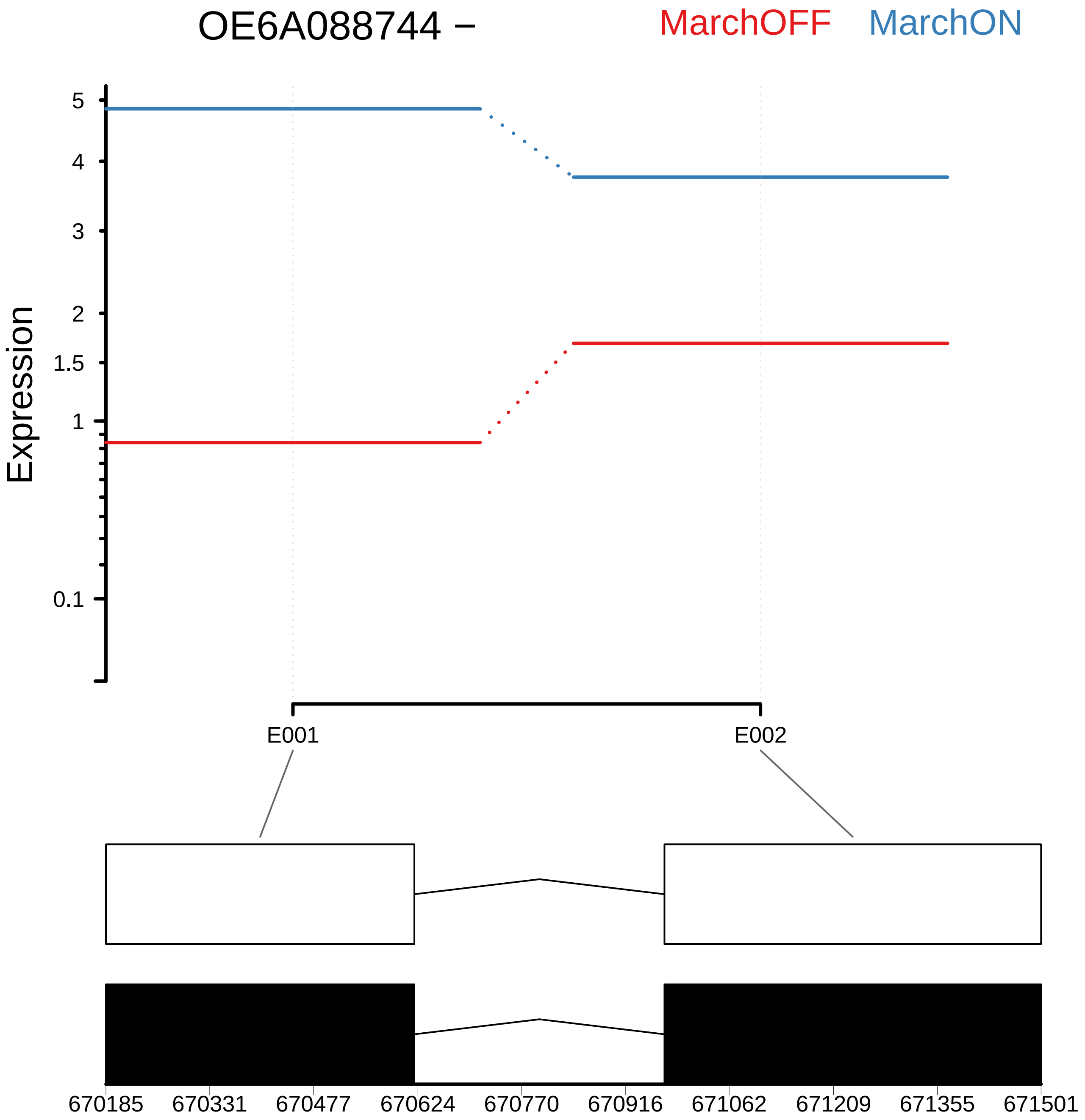

OE6A088744 −
MarchOFF
MarchON
Expression
5
4
3
2
1.5
1
0.1
E001
E002
670185
670331
670477
670624
670770
670916
671062
671209
671355
671501

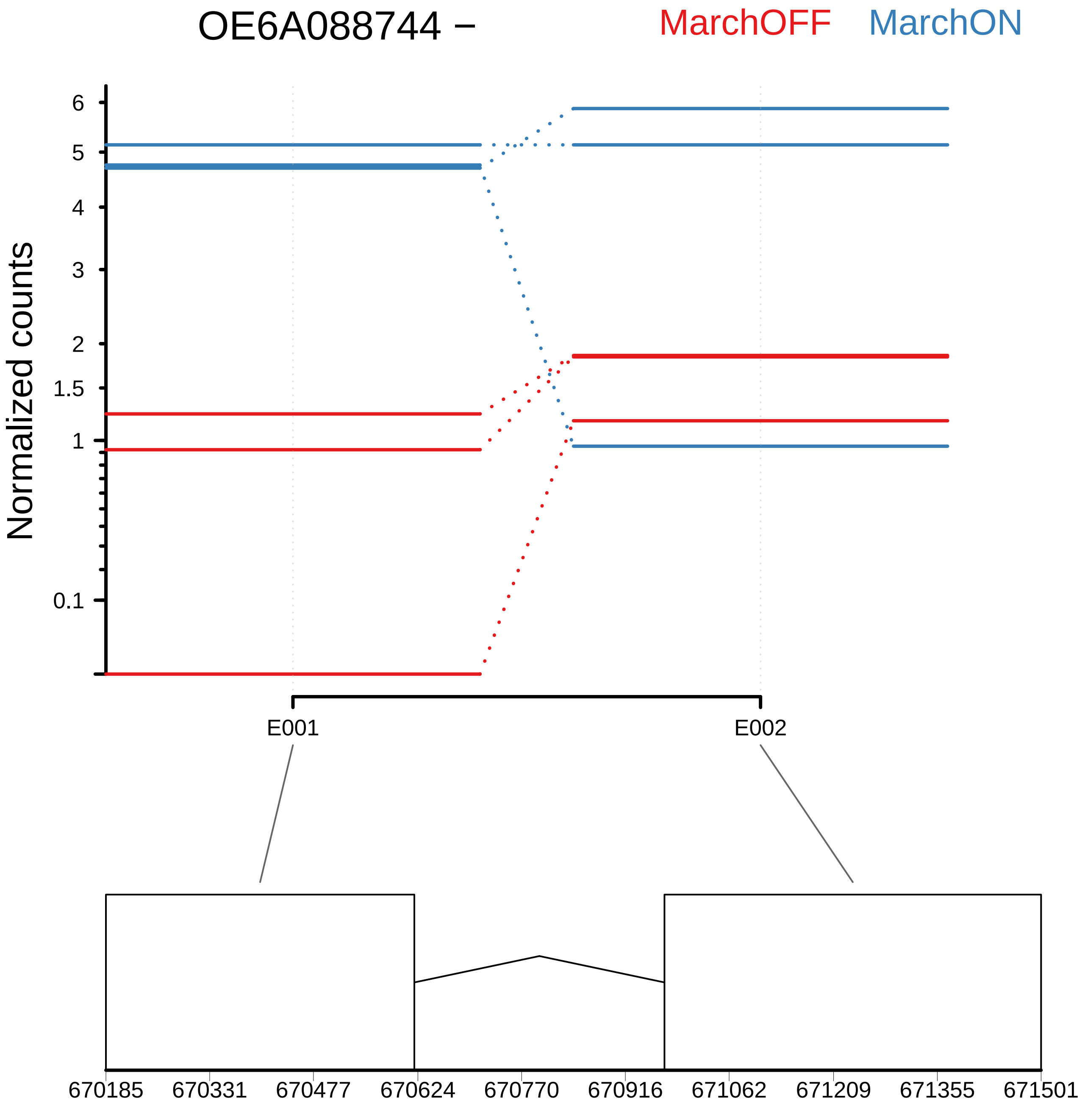

OE6A088744 −
MarchOFF
MarchON
Normalized counts
6
5
4
3
2
1.5
1
0.1
E001
E002
670185
670331
670477
670624
670770
670916
671062
671209
671355
671501

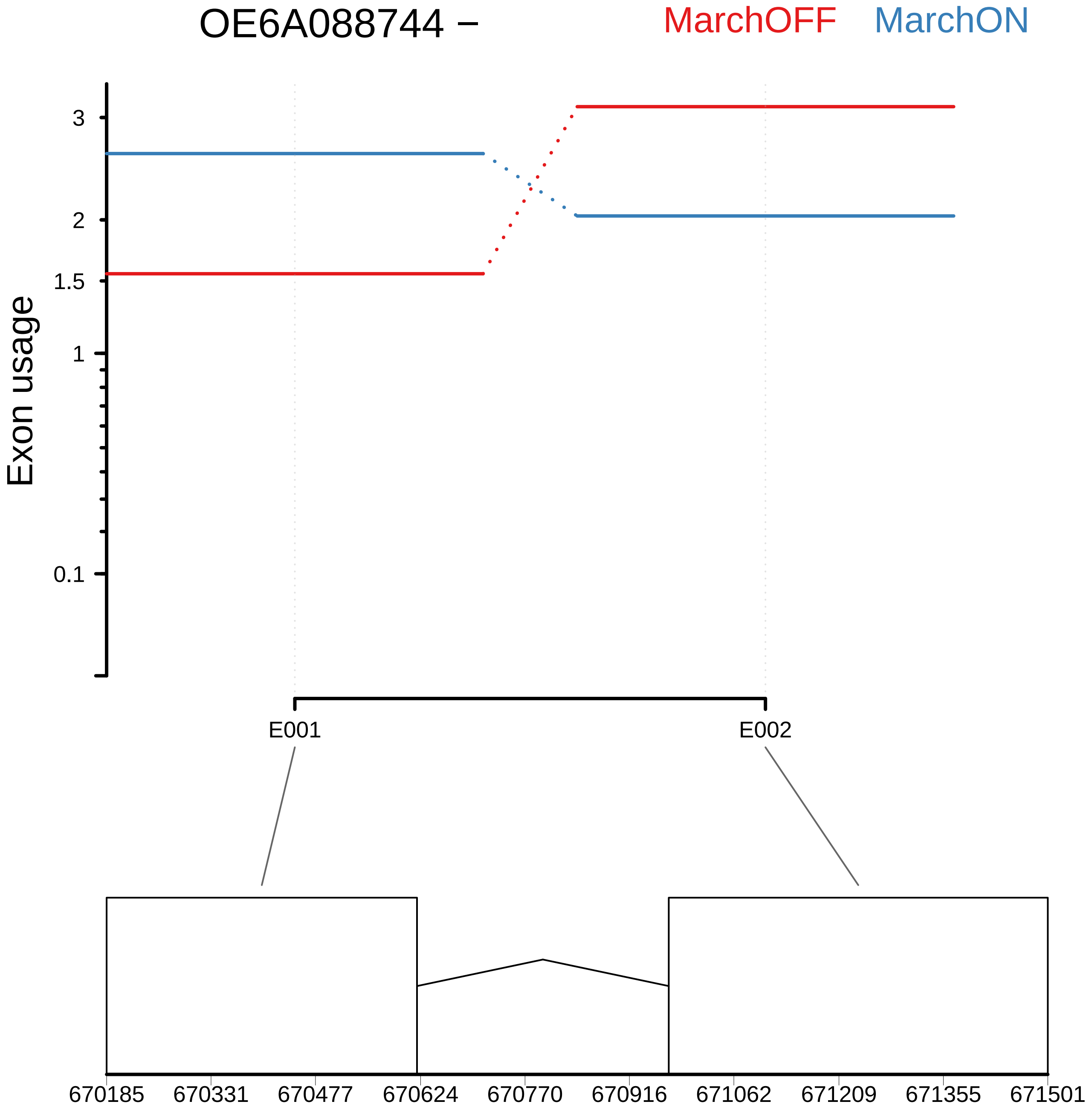
OE6A088744 −
MarchOFF
MarchON
Exon usage
3
2
1.5
1
0.1
E001
E002
670185
670331
670477
670624
670770
670916
671062
671209
671355
671501

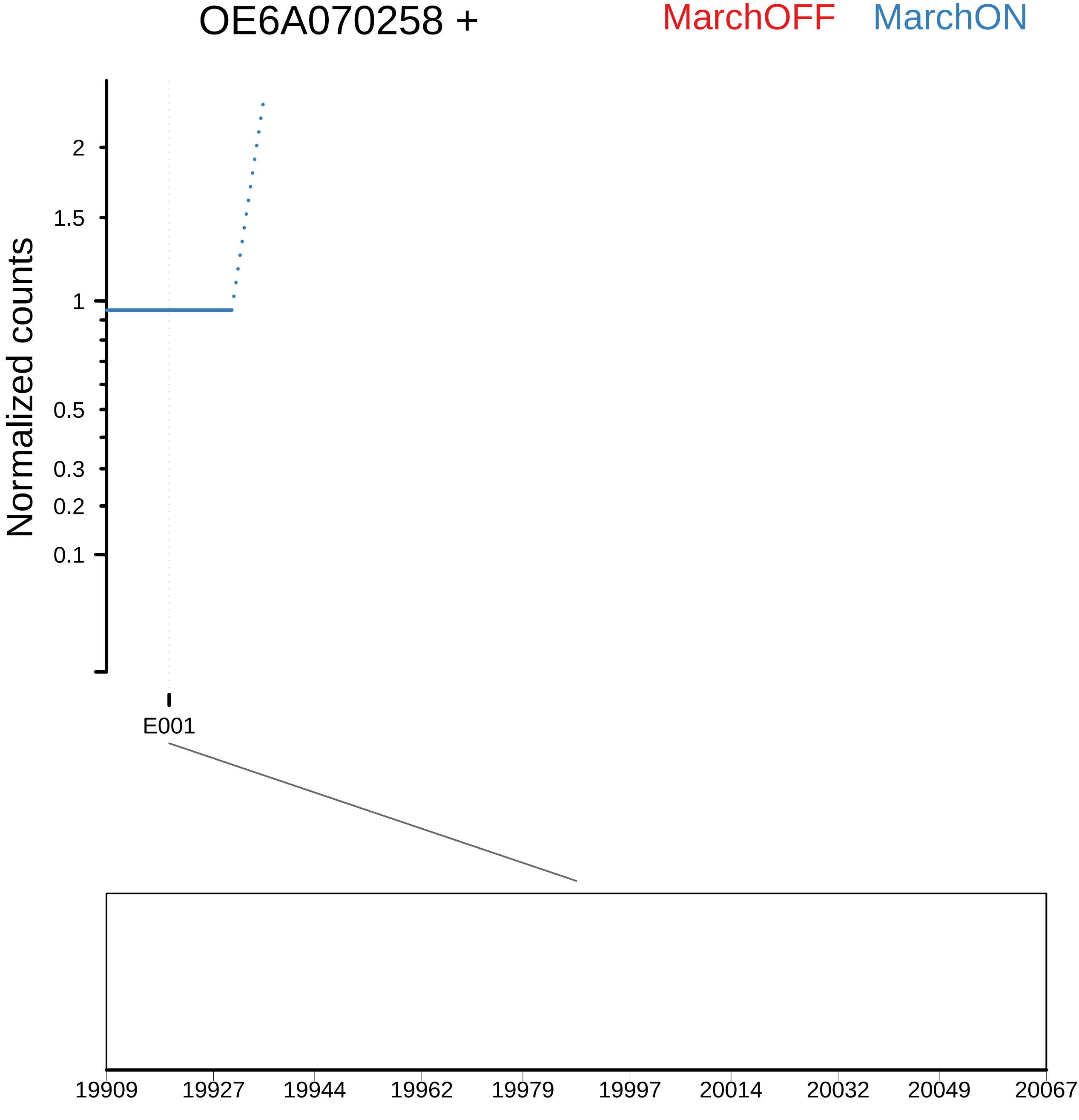

OE6A070258 +
MarchOFF
MarchON
Normalized counts
2
1.5
1
0.5
0.3
0.2
0.1
E001
19909
19927
19944
19962
19979
19997
20014
20032
20049
20067

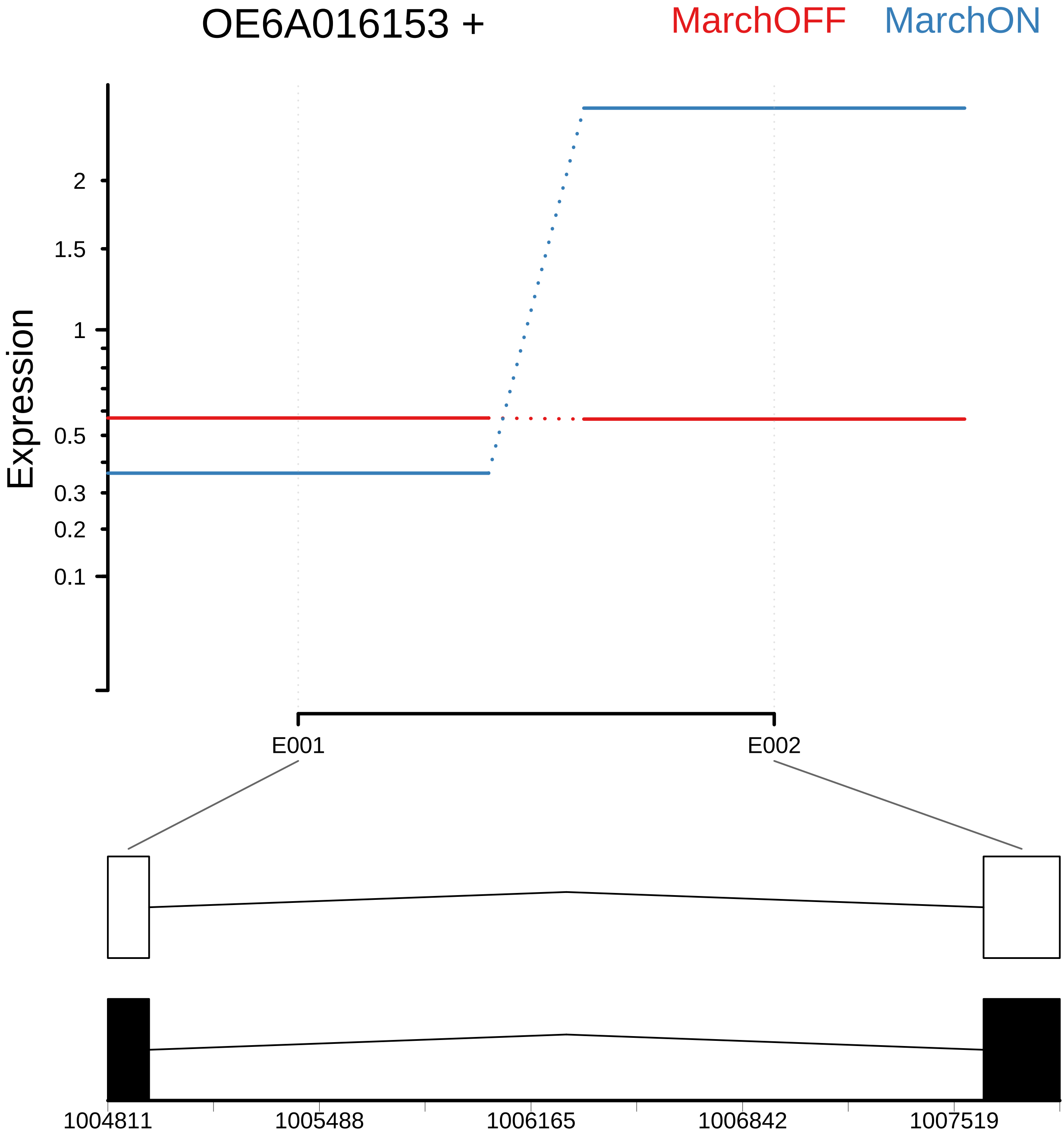

OE6A016153 +
MarchOFF
MarchON
Expression
2
1.5
1
0.5
0.3
0.2
0.1
E001
E002
1004811
1005488
1006165
1006842
1007519

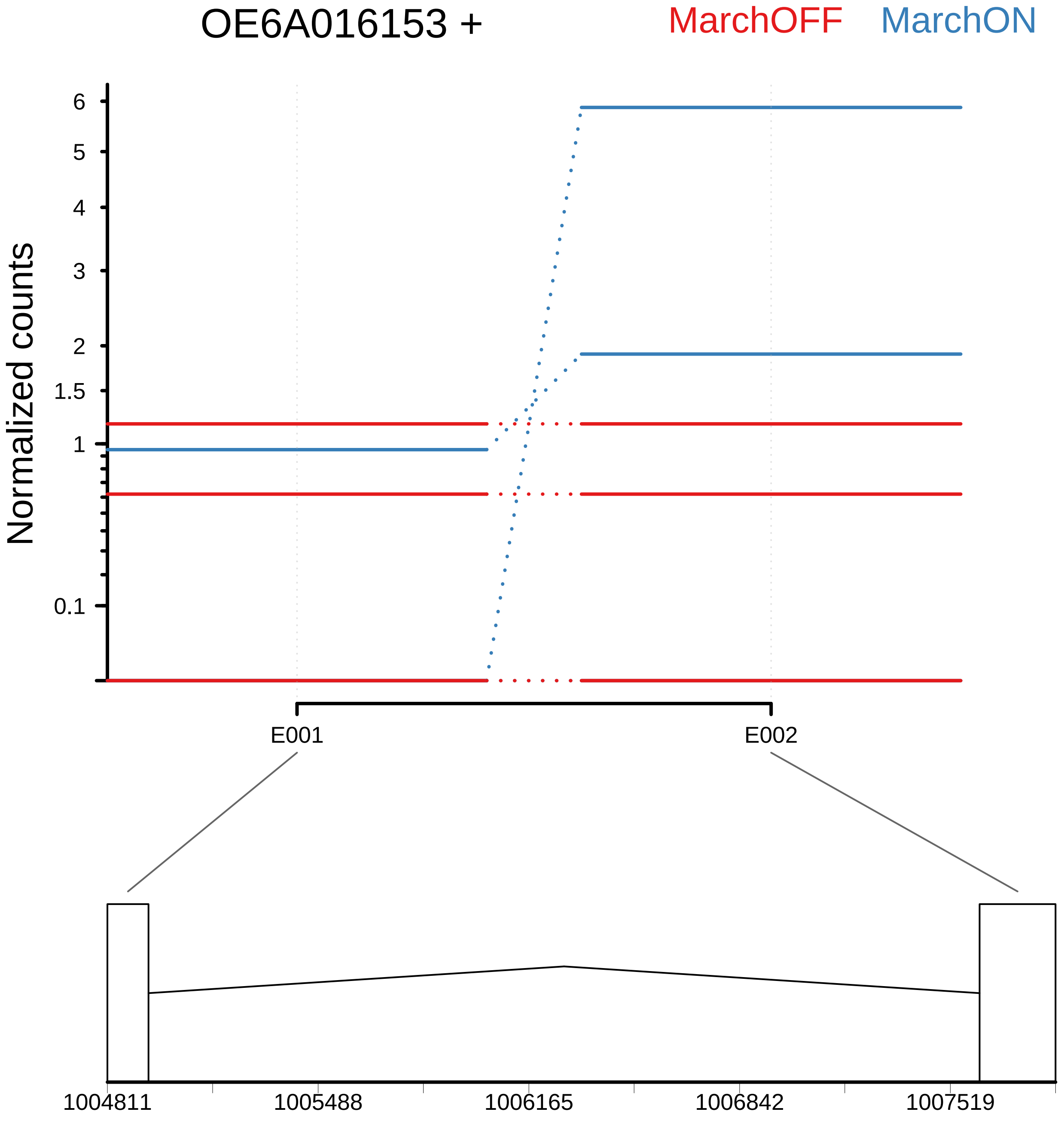
OE6A016153 +
MarchOFF
MarchON
Normalized counts
6
5
4
3
2
1.5
1
0.1
E001
E002
1004811
1005488
1006165
1006842
1007519

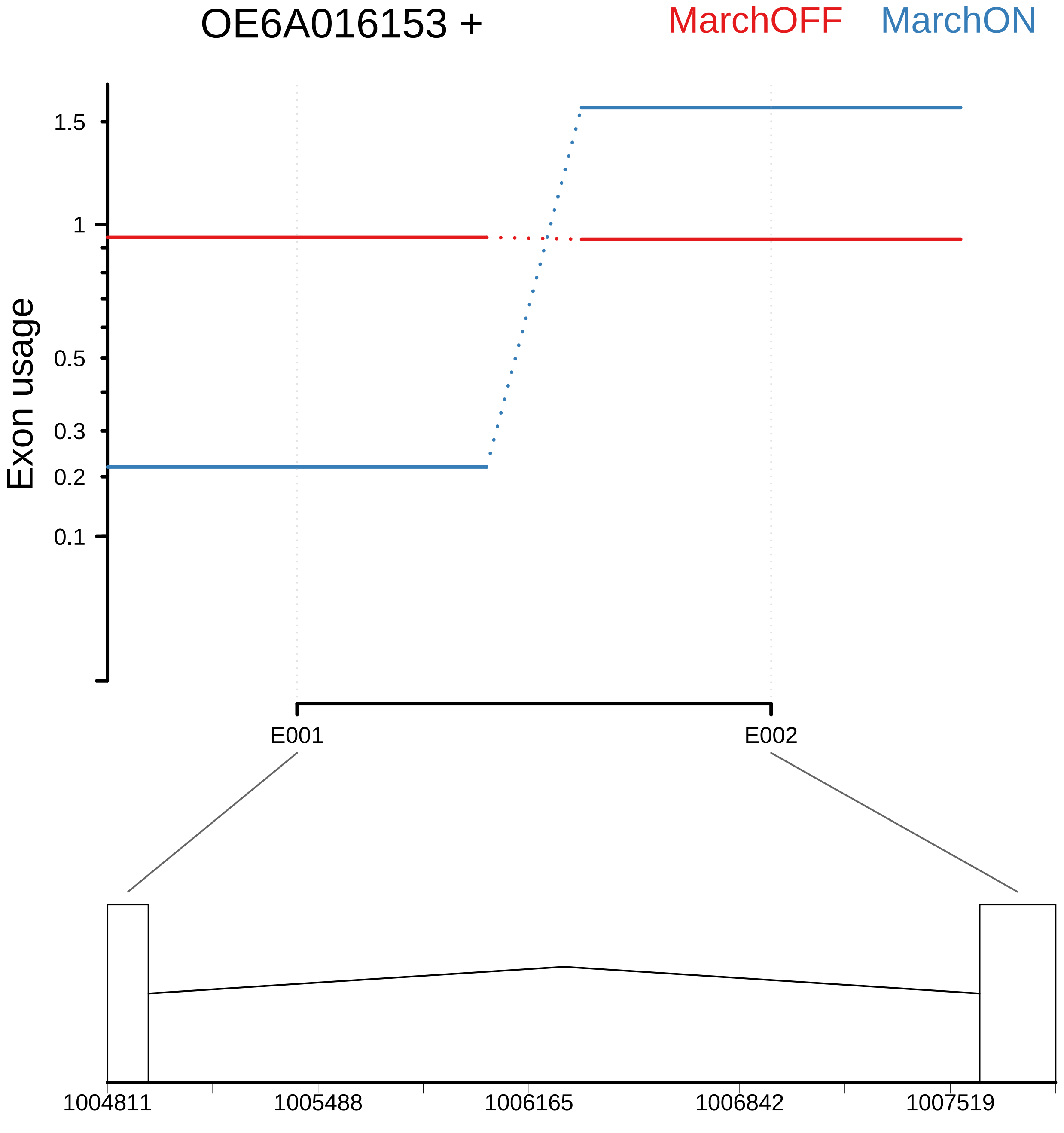
OE6A016153 +
MarchOFF
MarchON
Exon usage
1.5
1
0.5
0.3
0.2
0.1
E001
E002
1004811
1005488
1006165
1006842
1007519

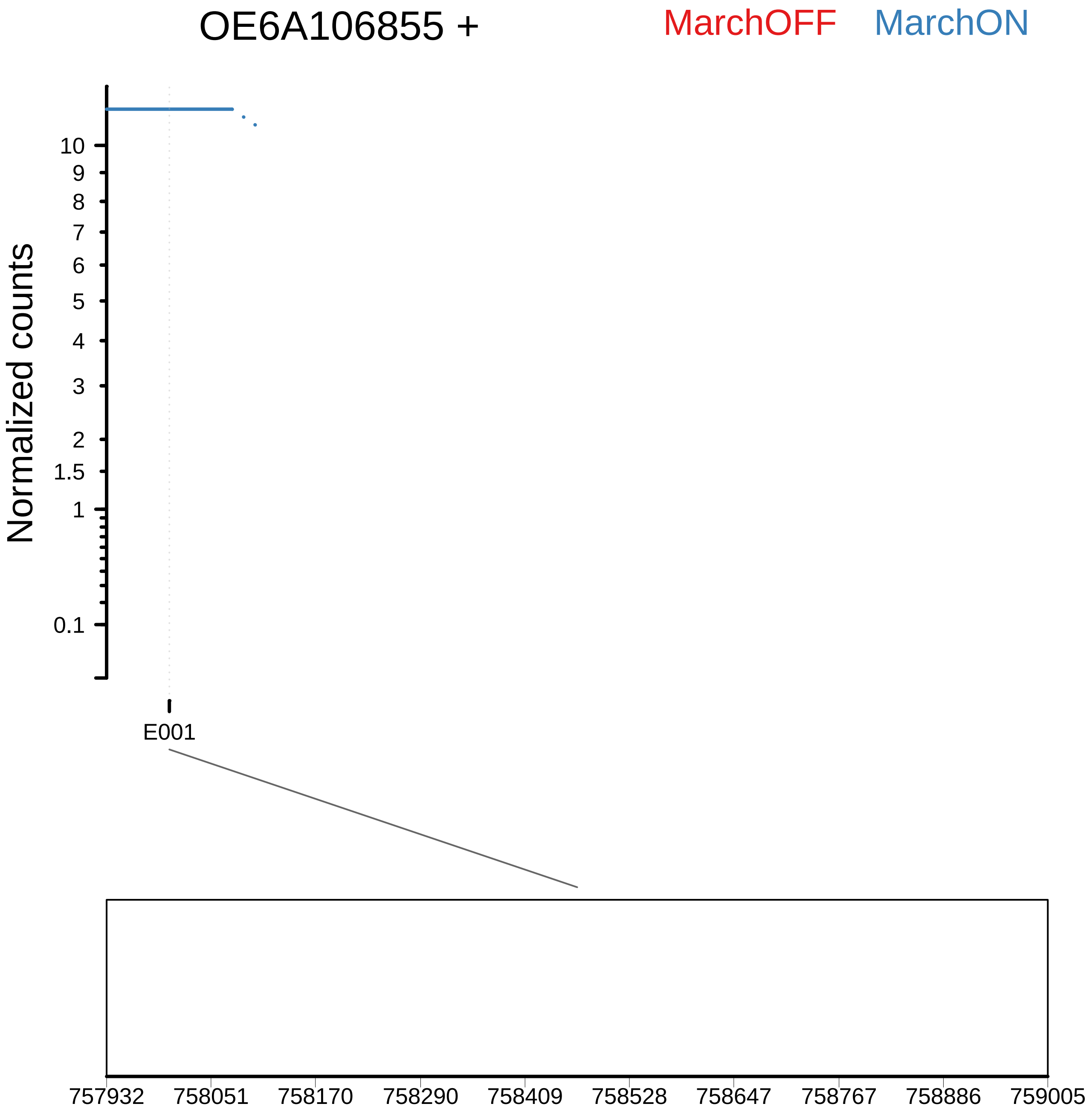
OE6A106855 +
MarchOFF
MarchON
Normalized counts
10
9
8
7
6
5
4
3
2
1.5
1
0.1
E001
757932
758051
758170
758290
758409
758528
758647
758767
758886
759005

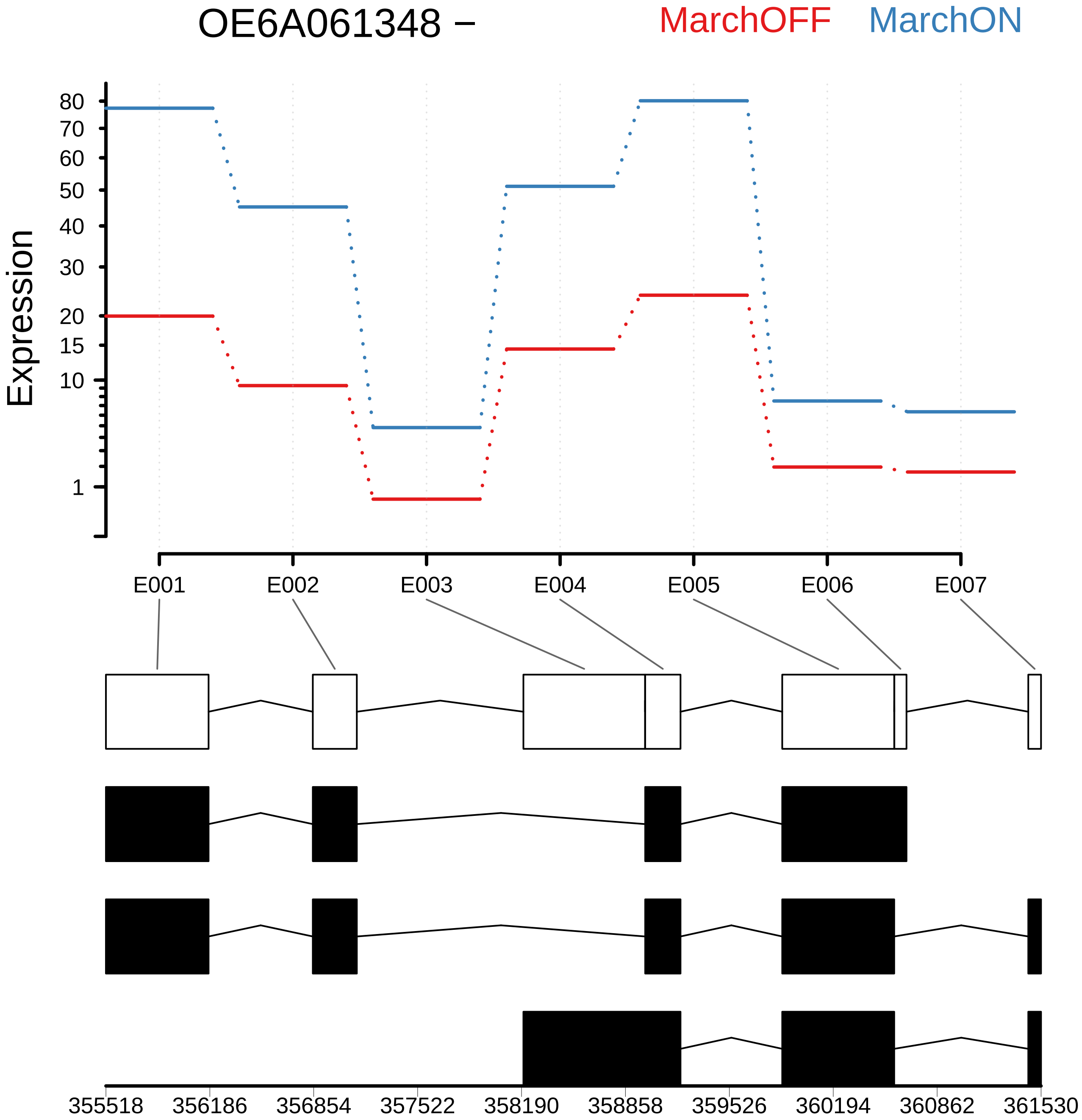

OE6A061348 −
MarchOFF
MarchON
Expression
80
70
60
50
40
30
20
15
10
1
E001
E002
E003
E004
E005
E006
E007
355518
356186
356854
357522
358190
358858
359526
360194
360862
361530

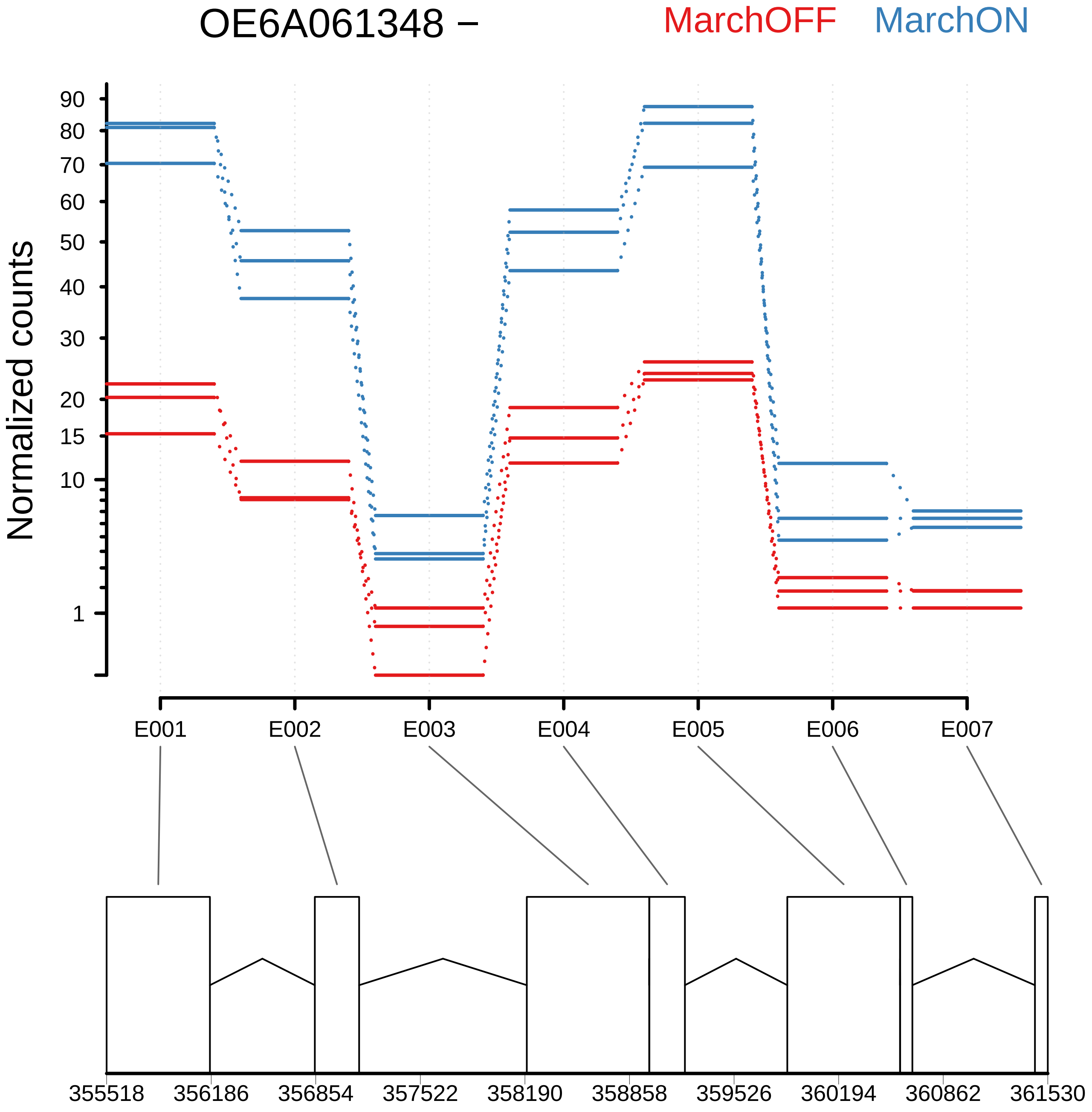

OE6A061348 −
MarchOFF
MarchON
Normalized counts
90
80
70
60
50
40
30
20
15
10
1
E001
E002
E003
E004
E005
E006
E007
355518
356186
356854
357522
358190
358858
359526
360194
360862
361530

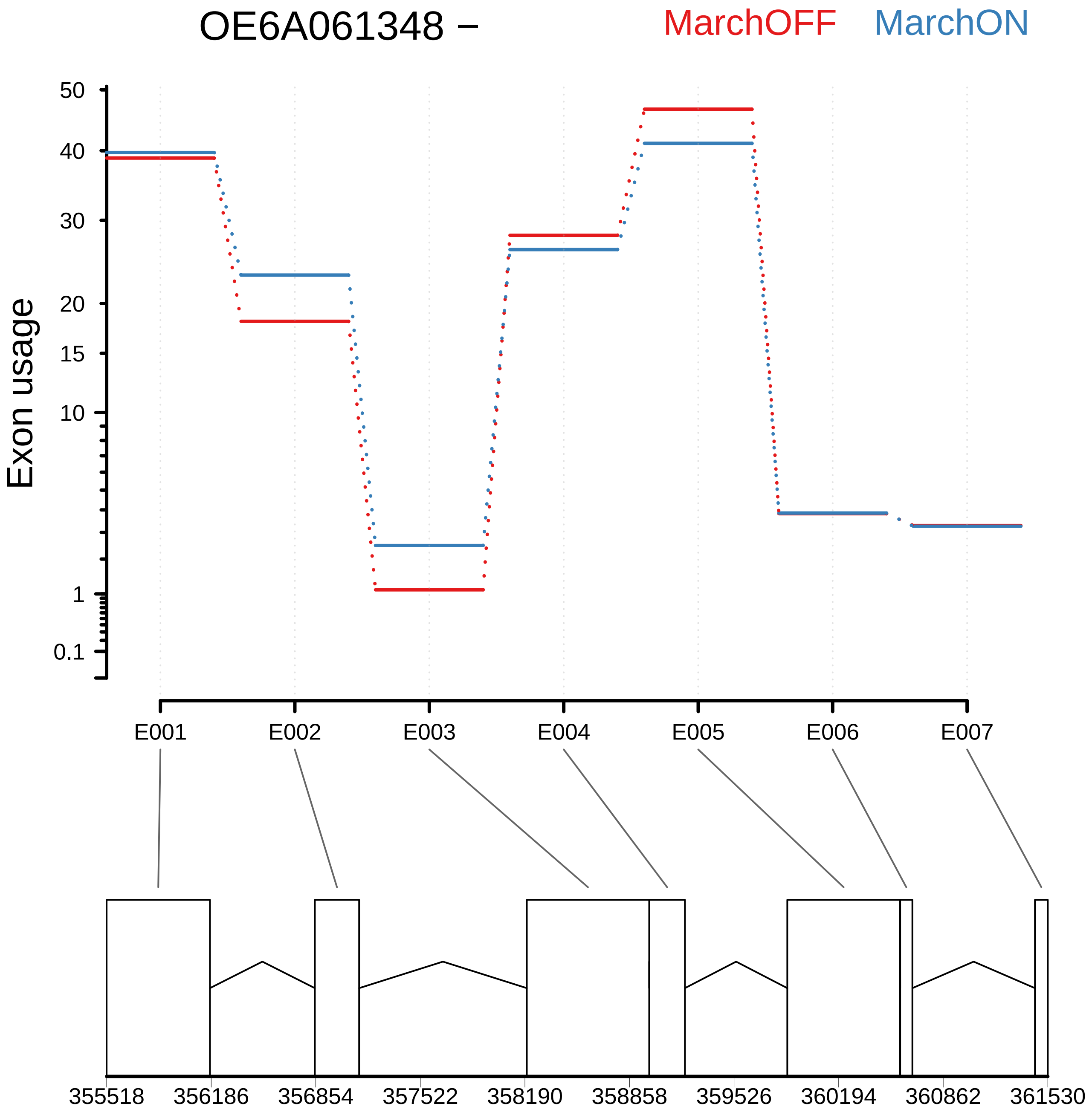
OE6A061348 −
MarchOFF
MarchON
Exon usage
50
40
30
20
15
10
1
0.1
E001
E002
E003
E004
E005
E006
E007
355518
356186
356854
357522
358190
358858
359526
360194
360862
361530

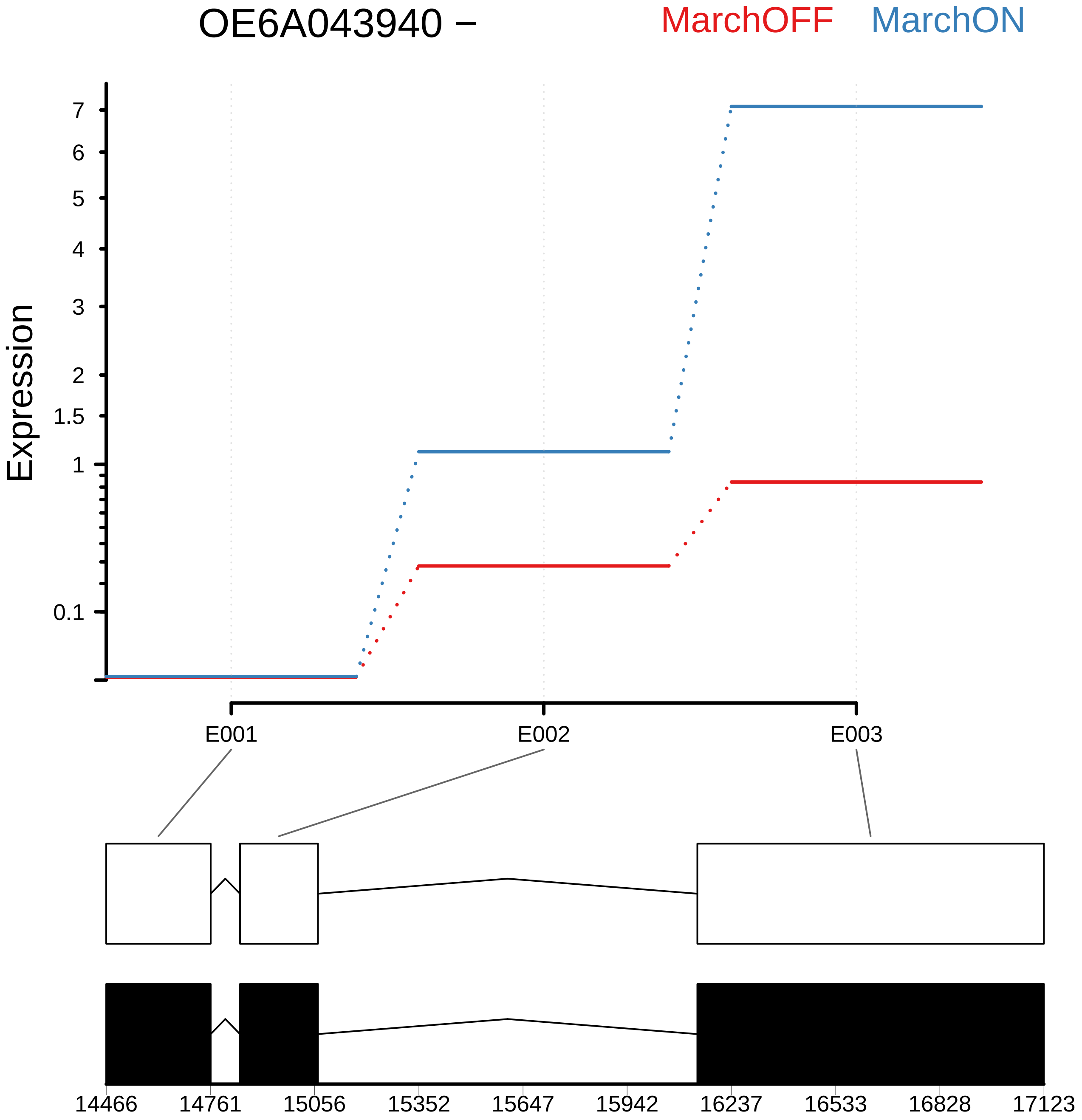

OE6A043940 −
MarchOFF
MarchON
Expression
7
6
5
4
3
2
1.5
1
0.1
E001
E002
E003
14466
14761
15056
15352
15647
15942
16237
16533
16828
17123

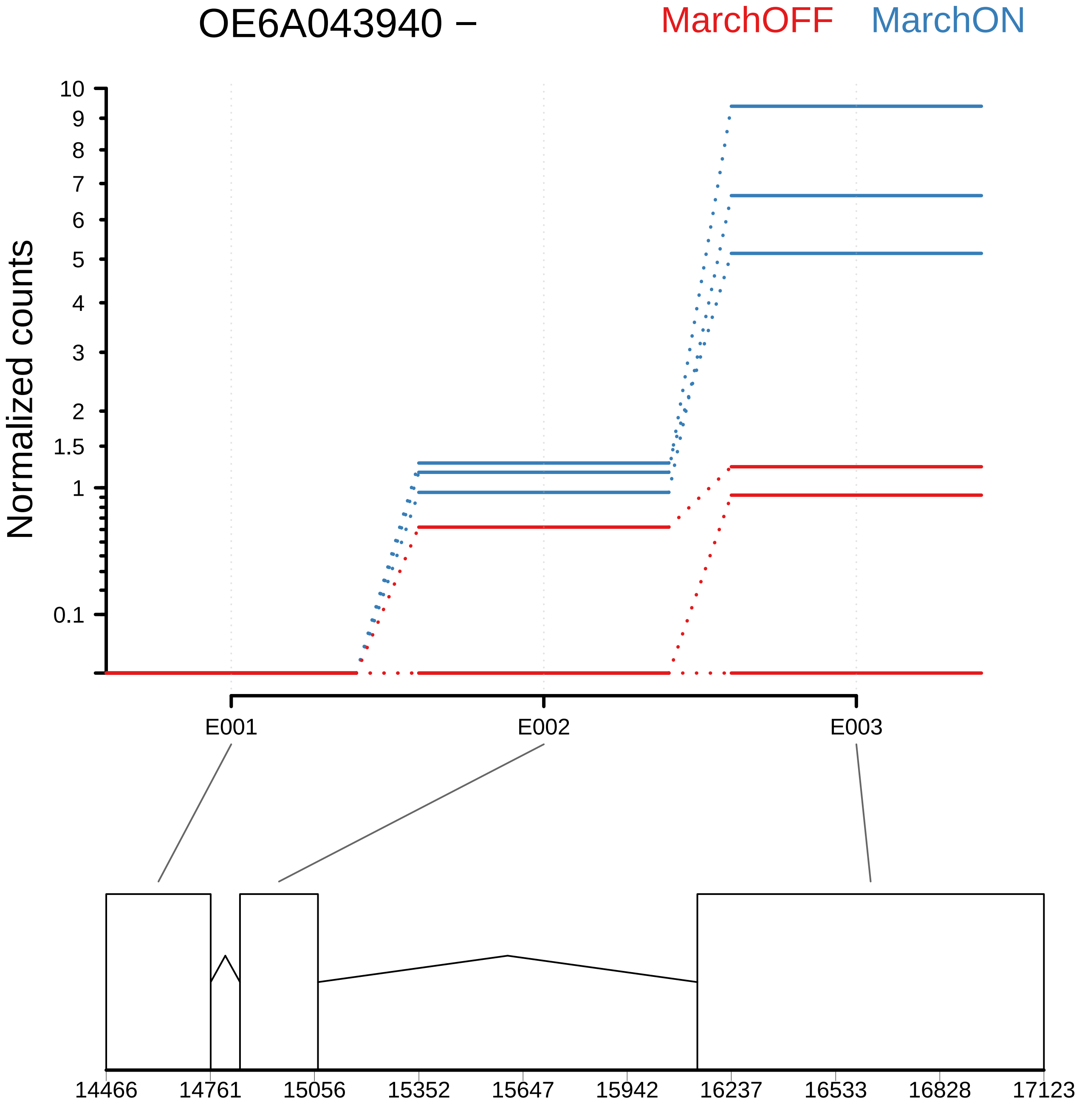

OE6A043940 −
MarchOFF
MarchON
Normalized counts
10
9
8
7
6
5
4
3
2
1.5
1
0.1
E001
E002
E003
14466
14761
15056
15352
15647
15942
16237
16533
16828
17123

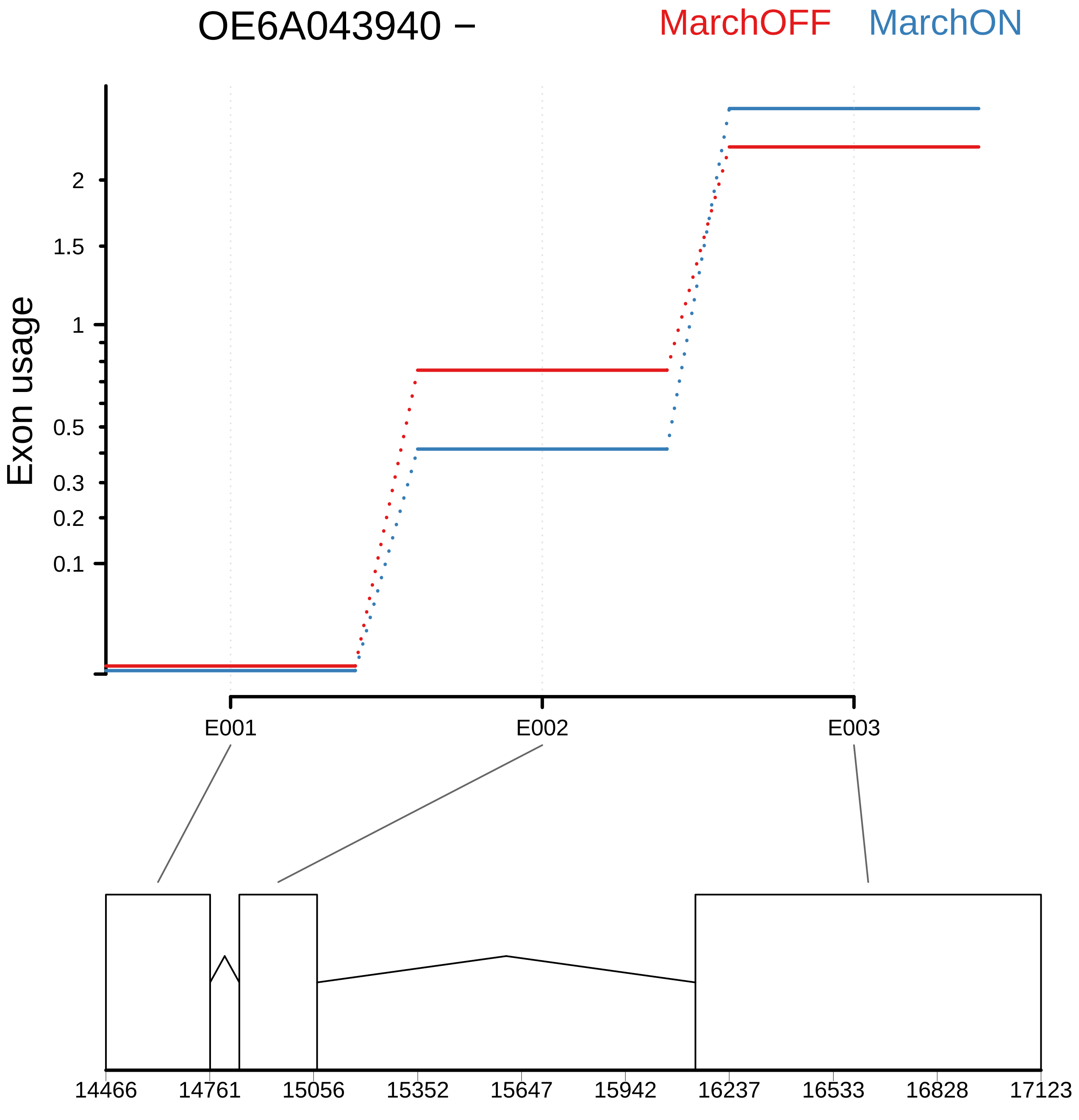

OE6A043940 −
MarchOFF
MarchON
Exon usage
2
1.5
1
0.5
0.3
0.2
0.1
E001
E002
E003
14466
14761
15056
15352
15647
15942
16237
16533
16828
17123

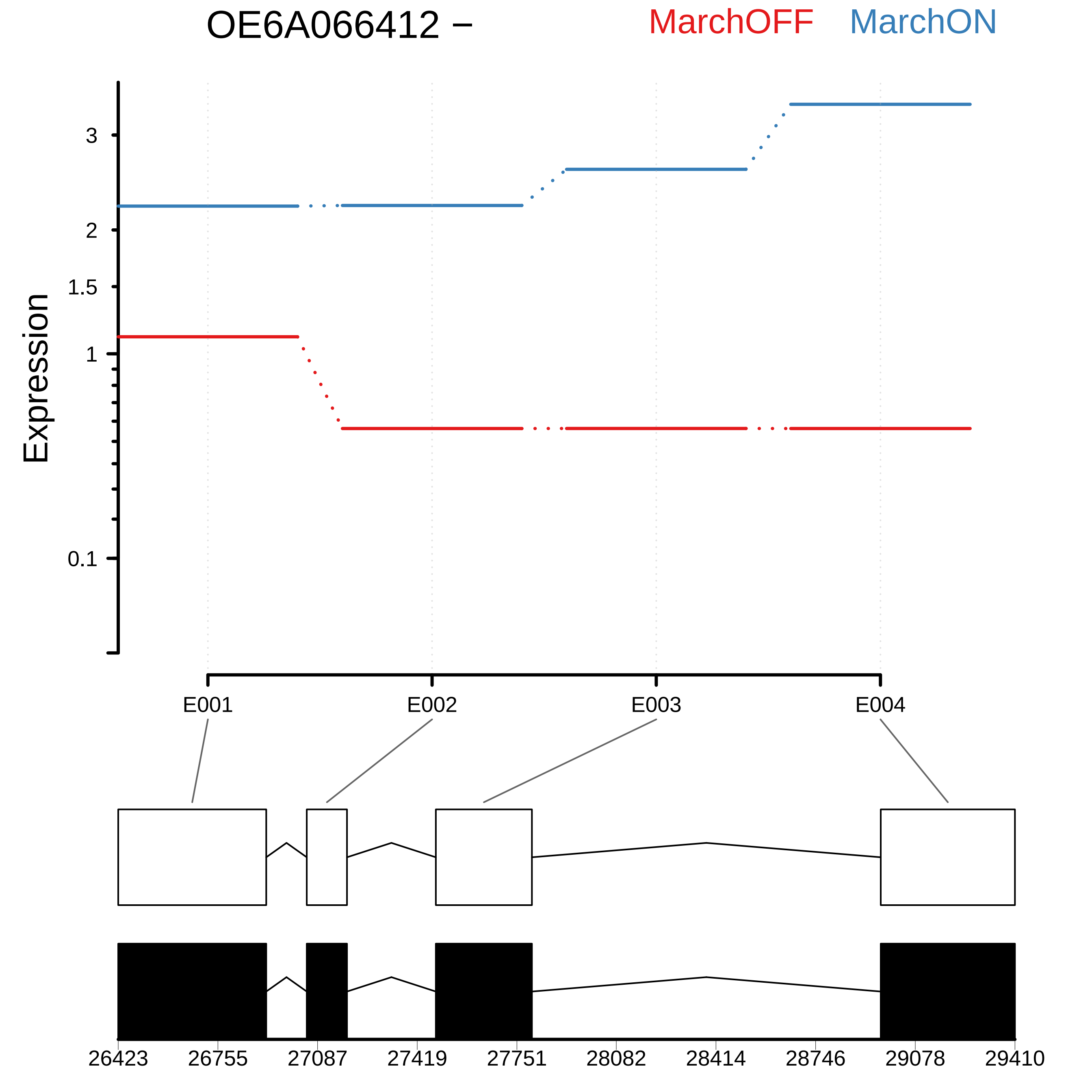

OE6A066412 −
MarchOFF
MarchON
Expression
3
2
1.5
1
0.1
E001
E002
E003
E004
26423
26755
27087
27419
27751
28082
28414
28746
29078
29410

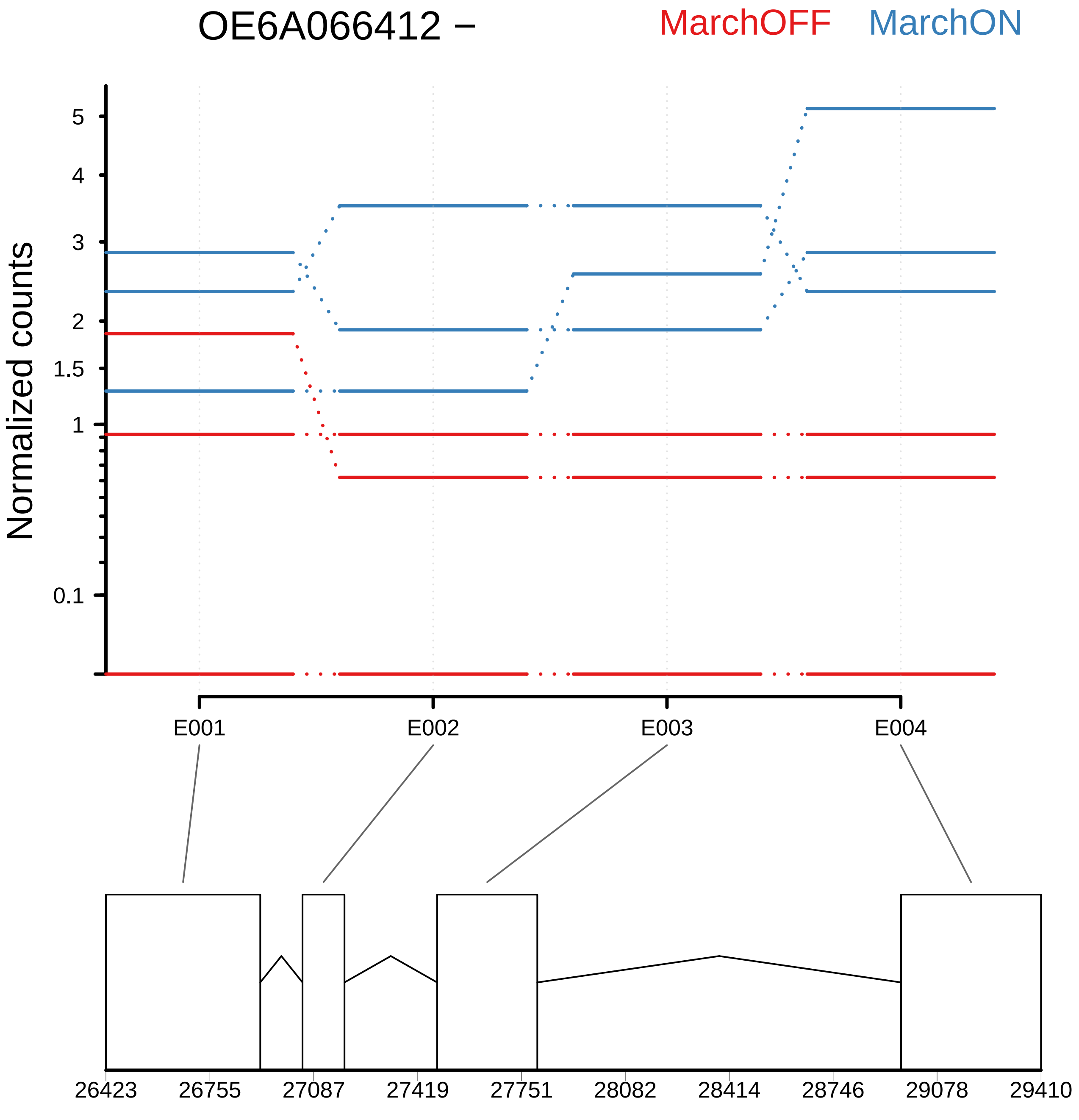

OE6A066412 −
MarchOFF
MarchON
Normalized counts
5
4
3
2
1.5
1
0.1
E001
E002
E003
E004
26423
26755
27087
27419
27751
28082
28414
28746
29078
29410

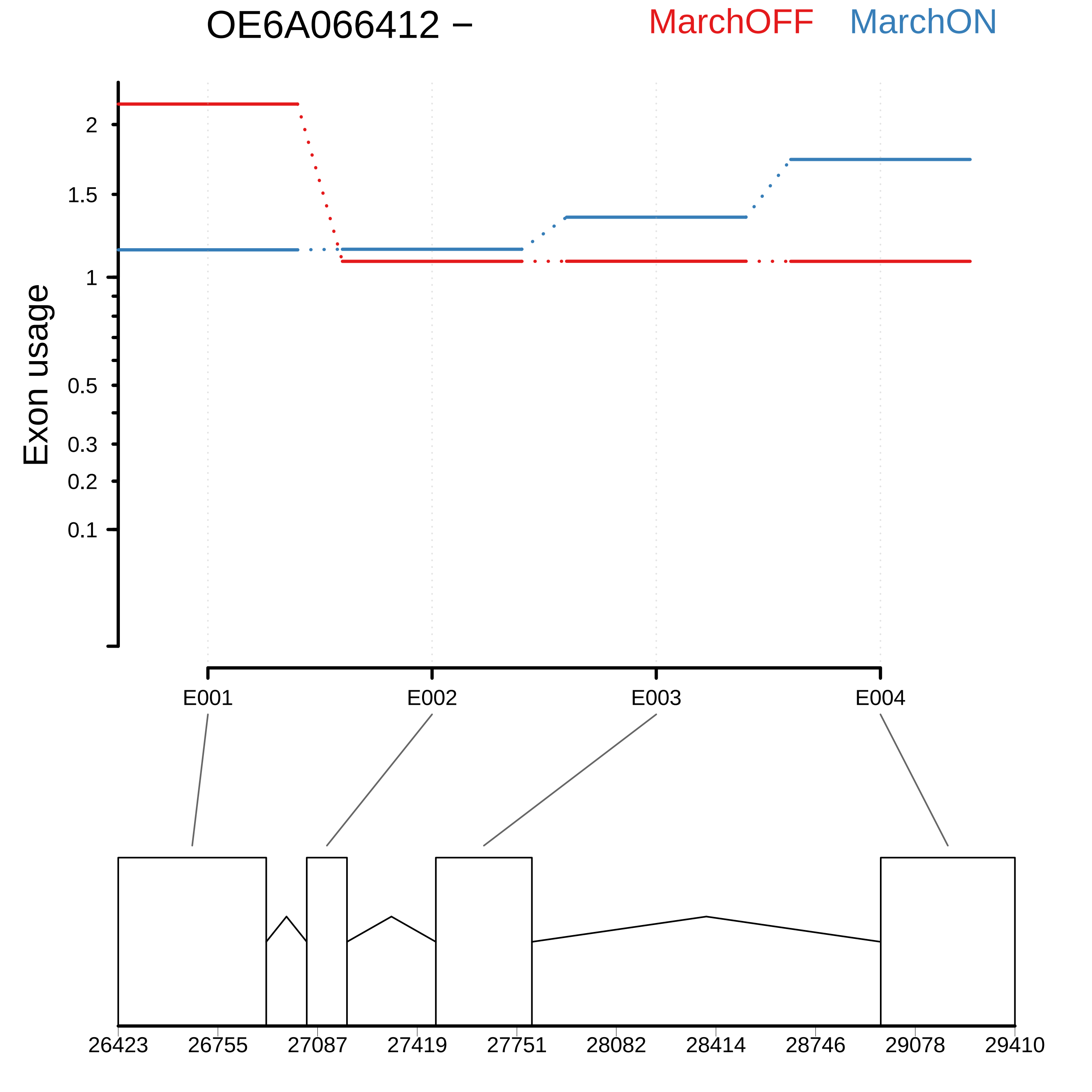

OE6A066412 −
MarchOFF
MarchON
Exon usage
2
1.5
1
0.5
0.3
0.2
0.1
E001
E002
E003
E004
26423
26755
27087
27419
27751
28082
28414
28746
29078
29410

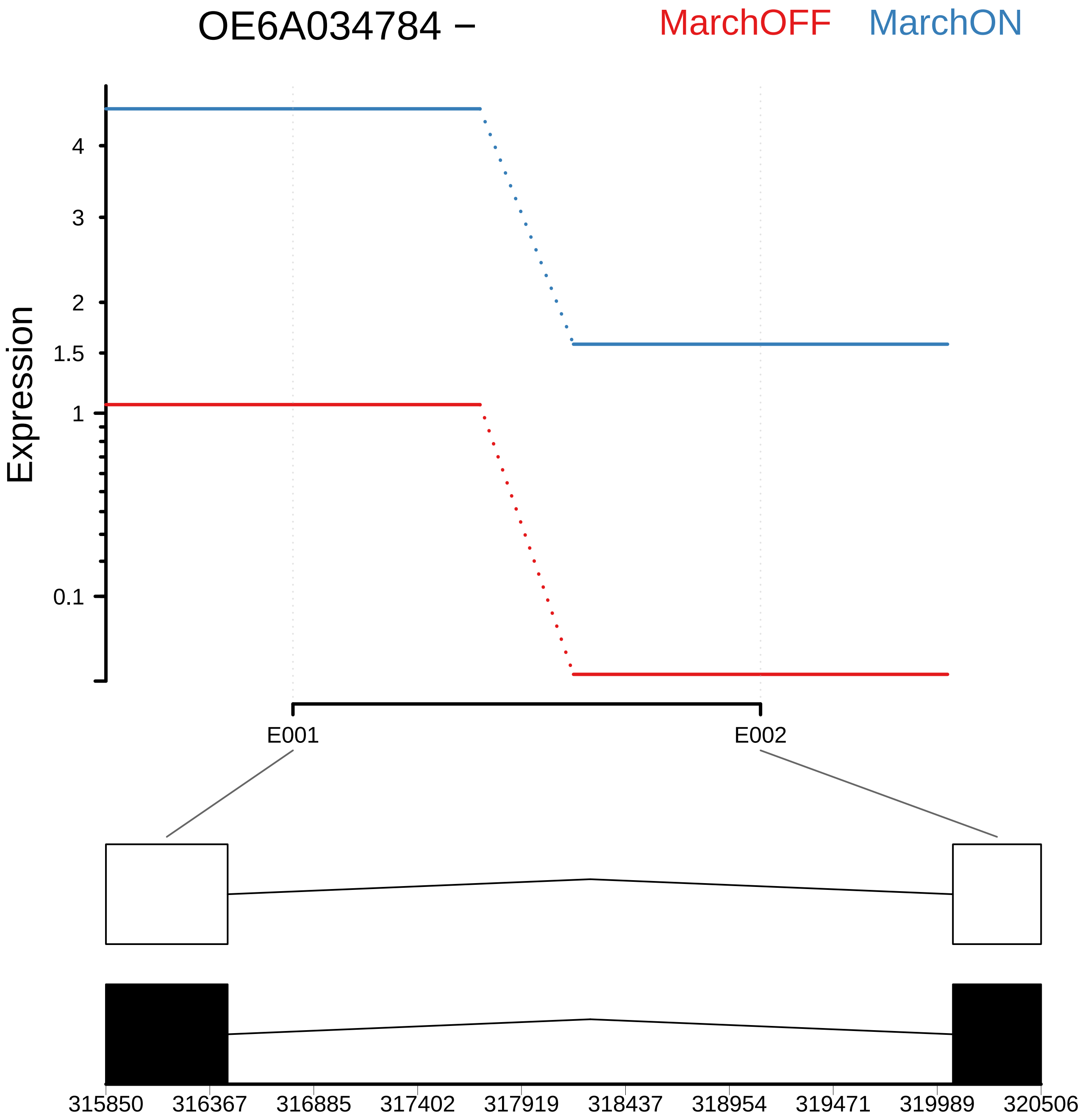

OE6A034784 −
MarchOFF
MarchON
Expression
4
3
2
1.5
1
0.1
E001
E002
315850
316367
316885
317402
317919
318437
318954
319471
319989
320506

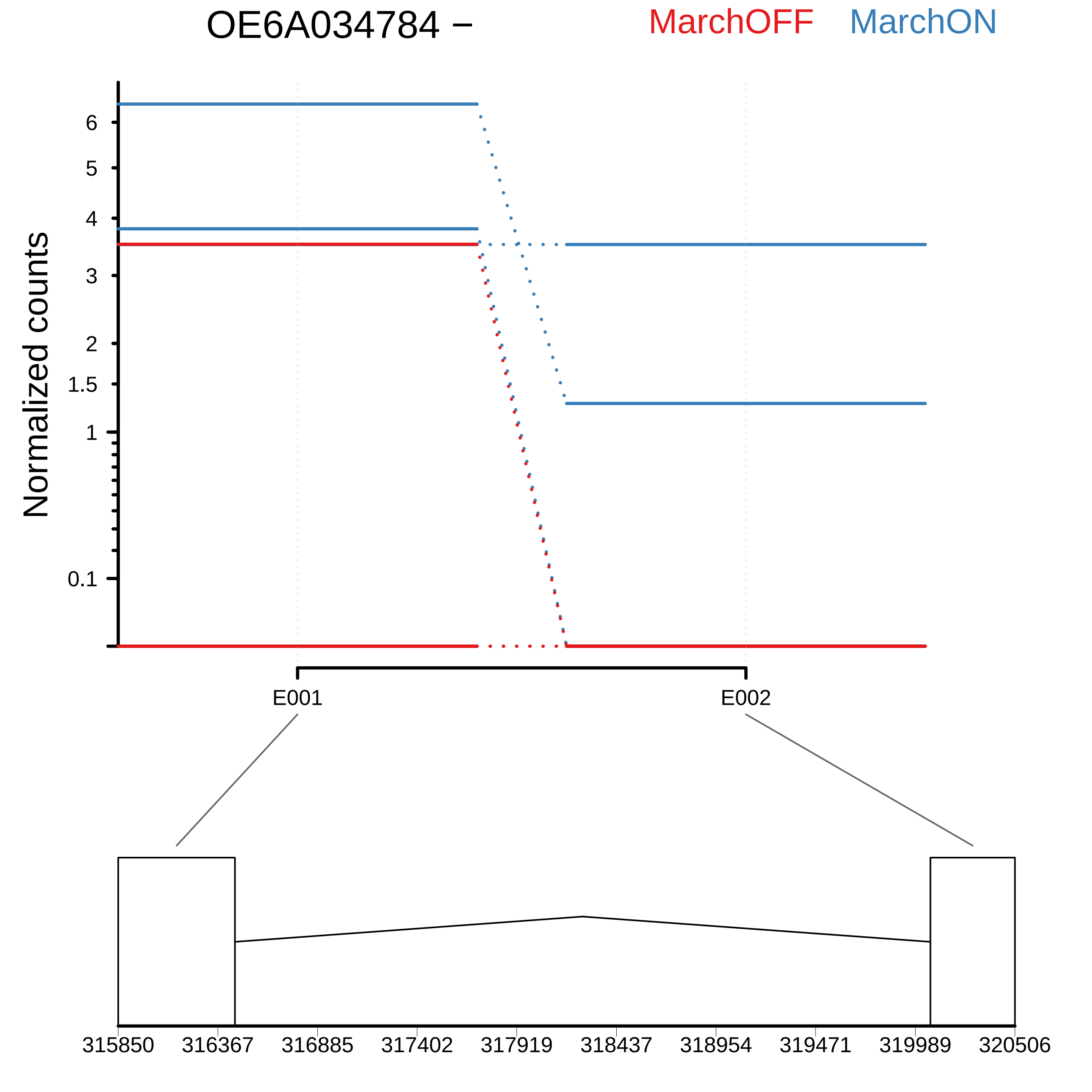
OE6A034784 −
MarchOFF
MarchON
Normalized counts
6
5
4
3
2
1.5
1
0.1
E001
E002
315850
316367
316885
317402
317919
318437
318954
319471
319989
320506

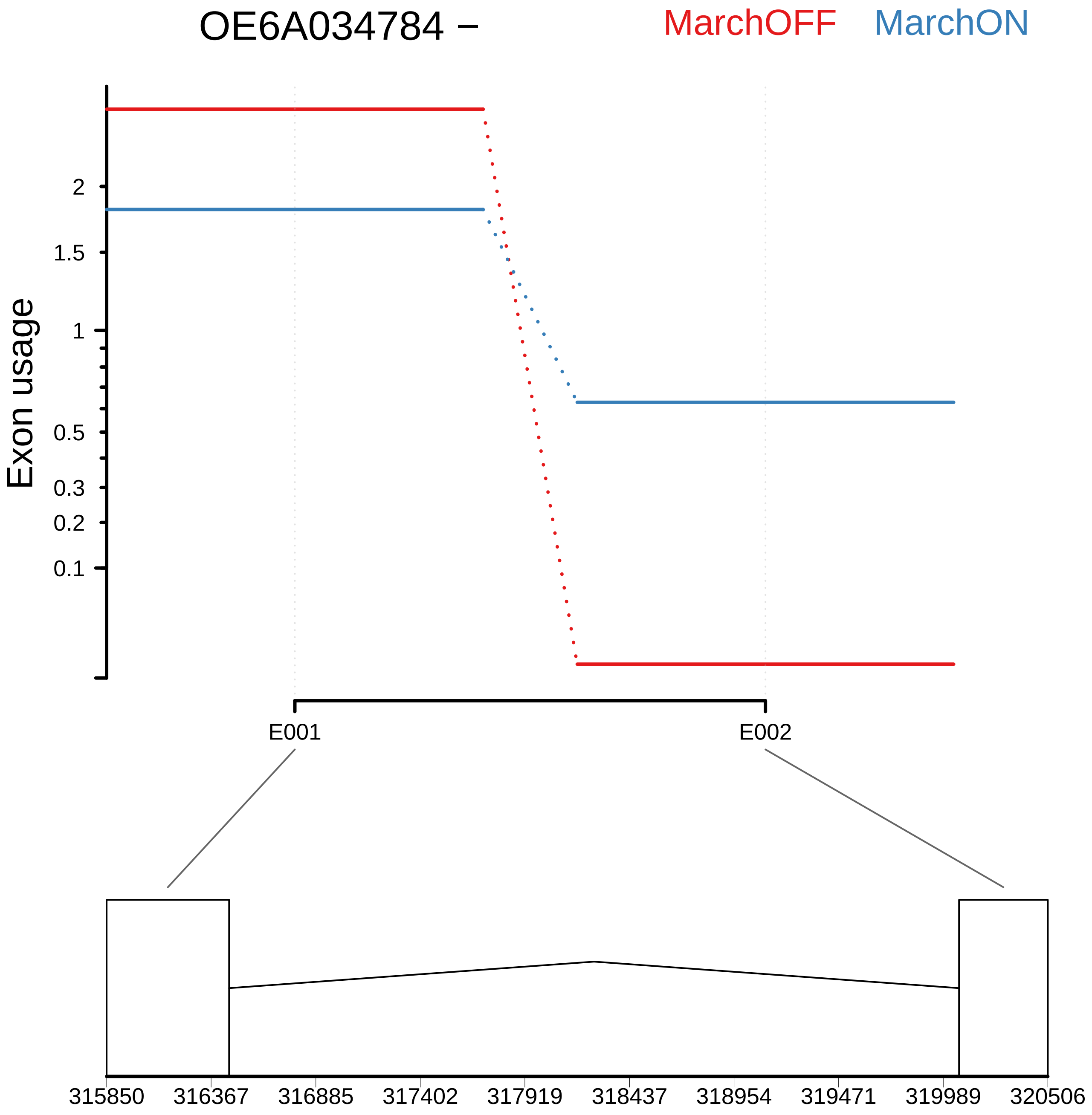

OE6A034784 −
MarchOFF
MarchON
Exon usage
2
1.5
1
0.5
0.3
0.2
0.1
E001
E002
315850
316367
316885
317402
317919
318437
318954
319471
319989
320506

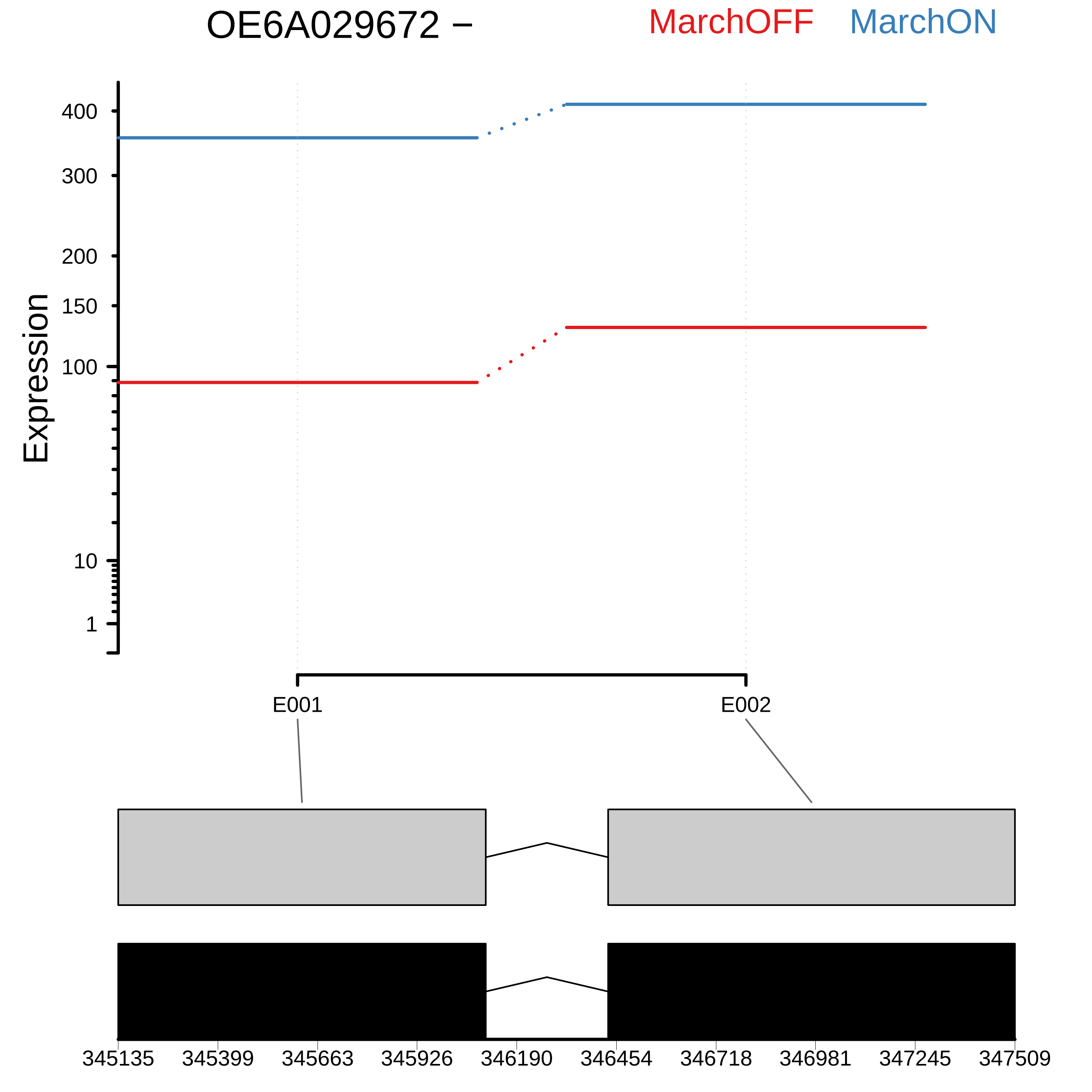

OE6A029672 −
MarchOFF
MarchON
Expression
400
300
200
150
100
10
1
E001
E002
345135
345399
345663
345926
346190
346454
346718
346981
347245
347509

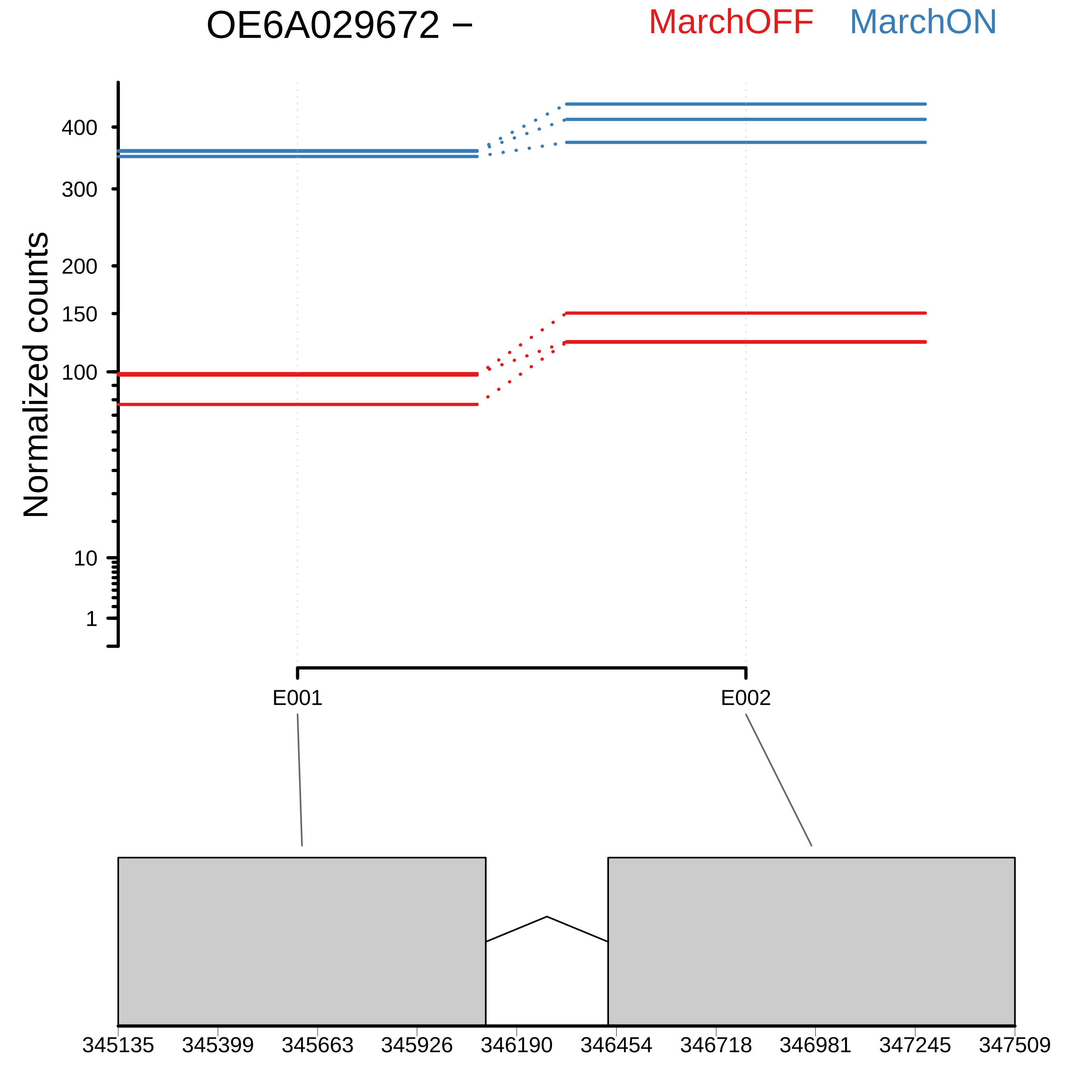

OE6A029672 −
MarchOFF
MarchON
Normalized counts
400
300
200
150
100
10
1
E001
E002
345135
345399
345663
345926
346190
346454
346718
346981
347245
347509

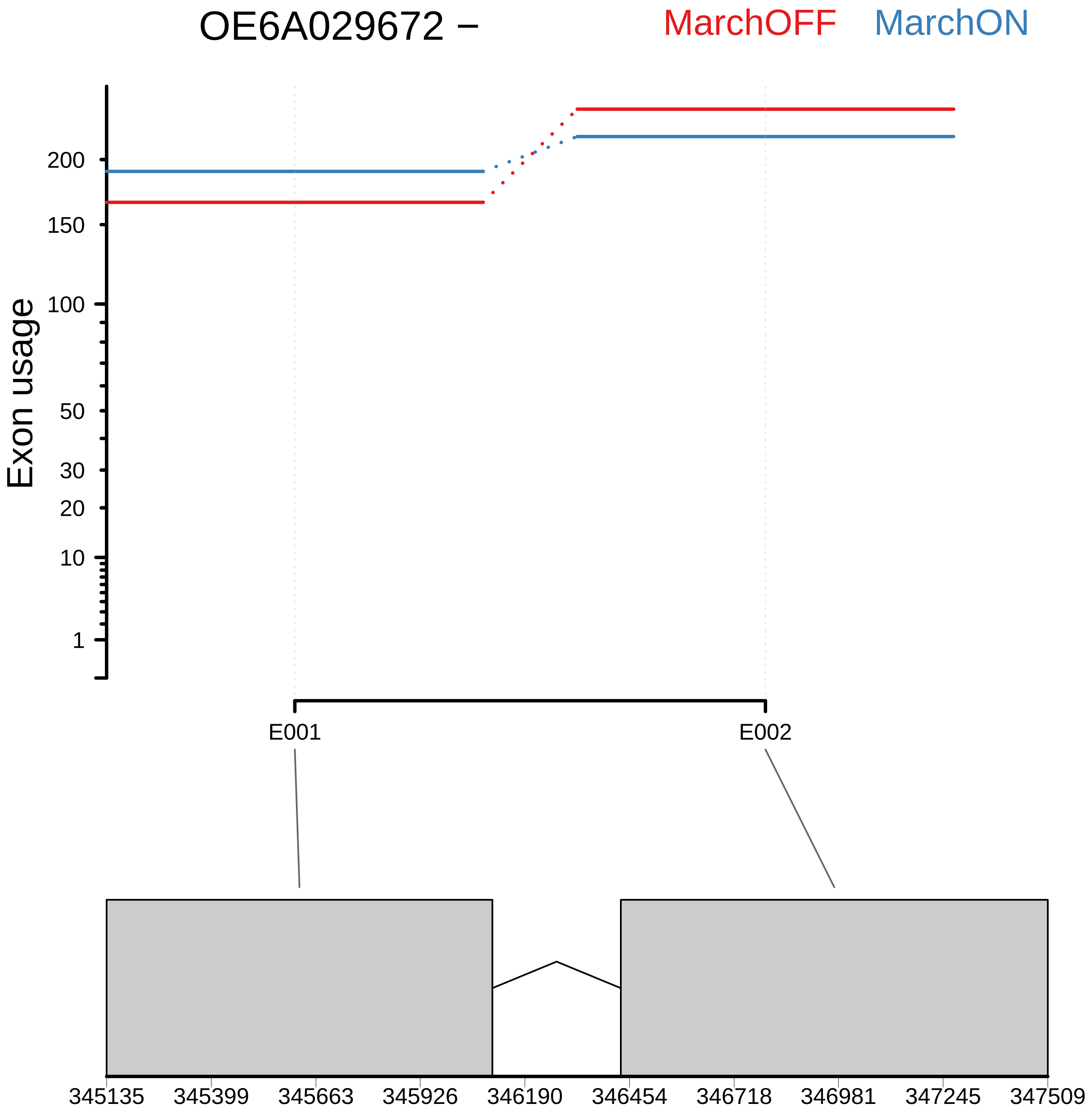
OE6A029672 −
MarchOFF
MarchON
Exon usage
200
150
100
50
30
20
10
1
E001
E002
345135
345399
345663
345926
346190
346454
346718
346981
347245
347509

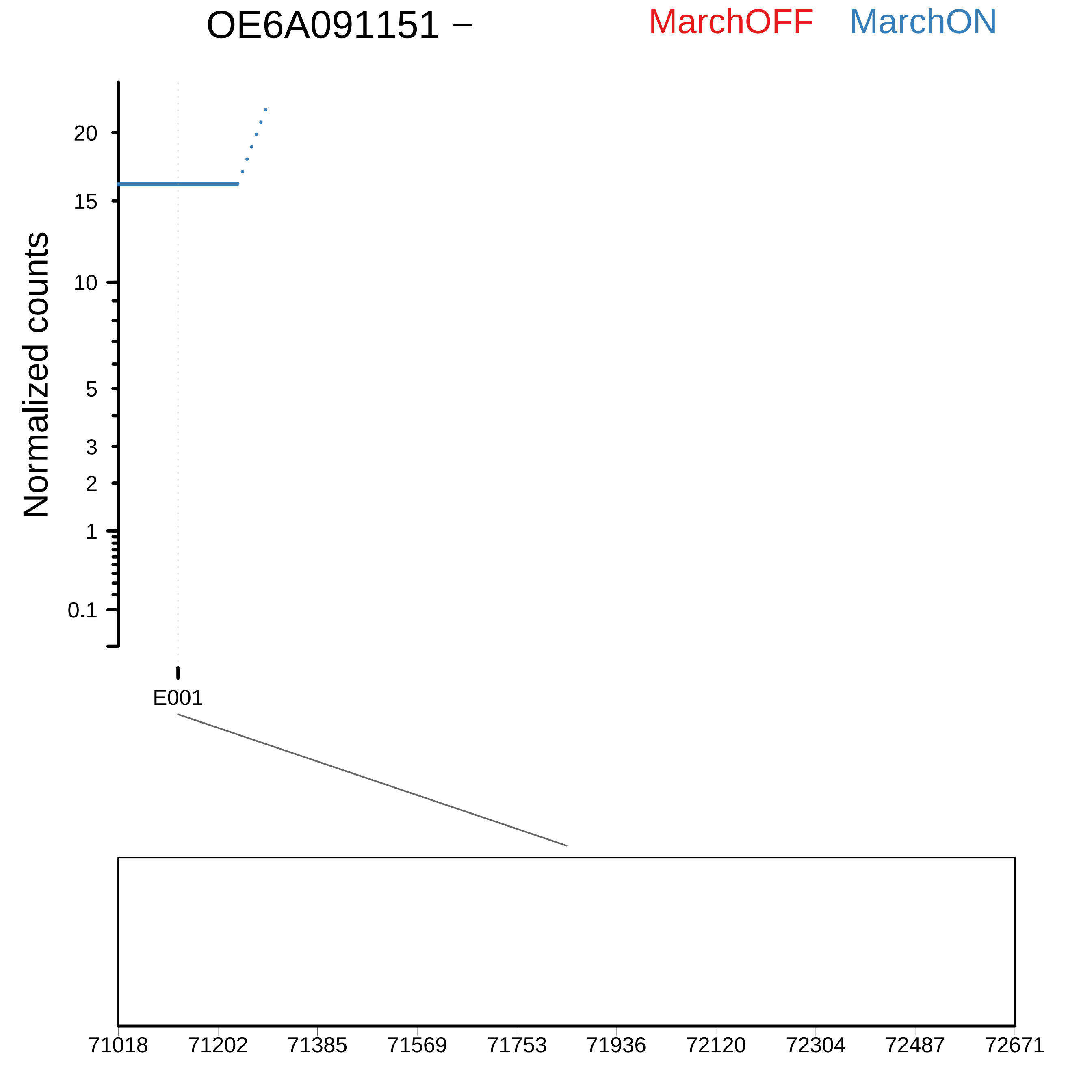
OE6A091151 −
MarchOFF
MarchON
Normalized counts
20
15
10
5
3
2
1
0.1
E001
71018
71202
71385
71569
71753
71936
72120
72304
72487
72671

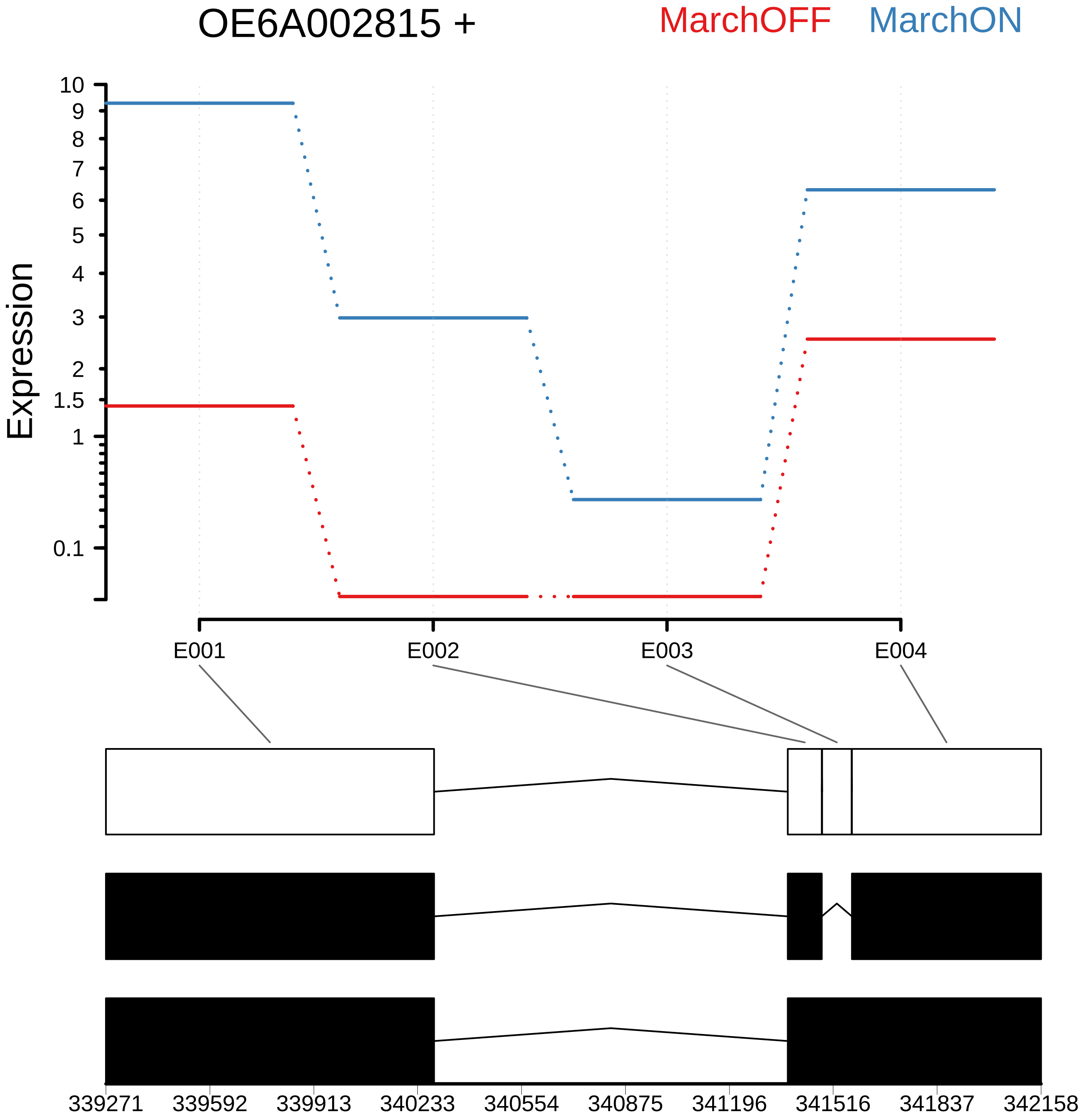
OE6A002815 +
MarchOFF
MarchON
Expression
10
9
8
7
6
5
4
3
2
1.5
1
0.1
E001
E002
E003
E004
339271
339592
339913
340233
340554
340875
341196
341516
341837
342158

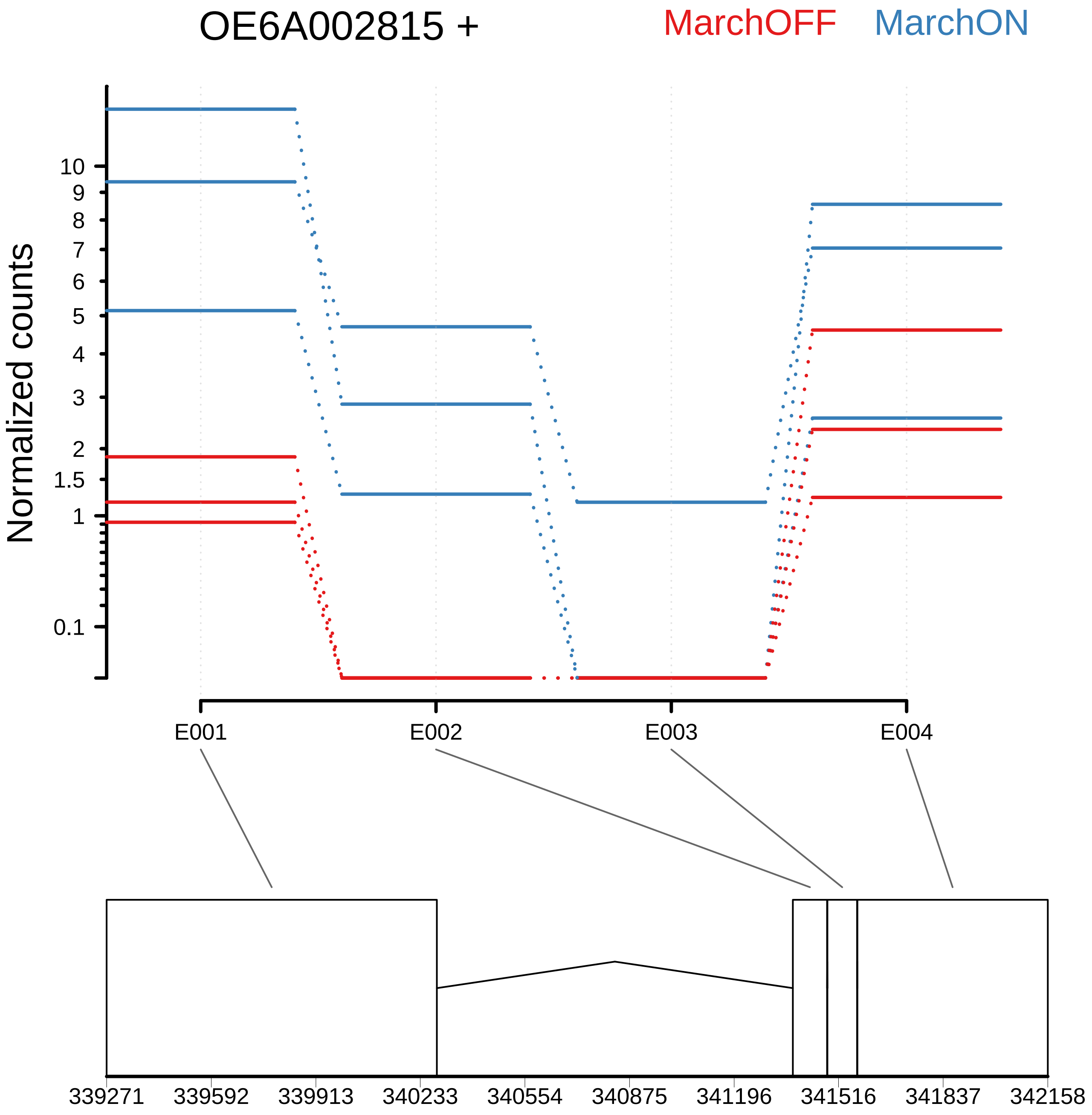

OE6A002815 +
MarchOFF
MarchON
Normalized counts
10
9
8
7
6
5
4
3
2
1.5
1
0.1
E001
E002
E003
E004
339271
339592
339913
340233
340554
340875
341196
341516
341837
342158

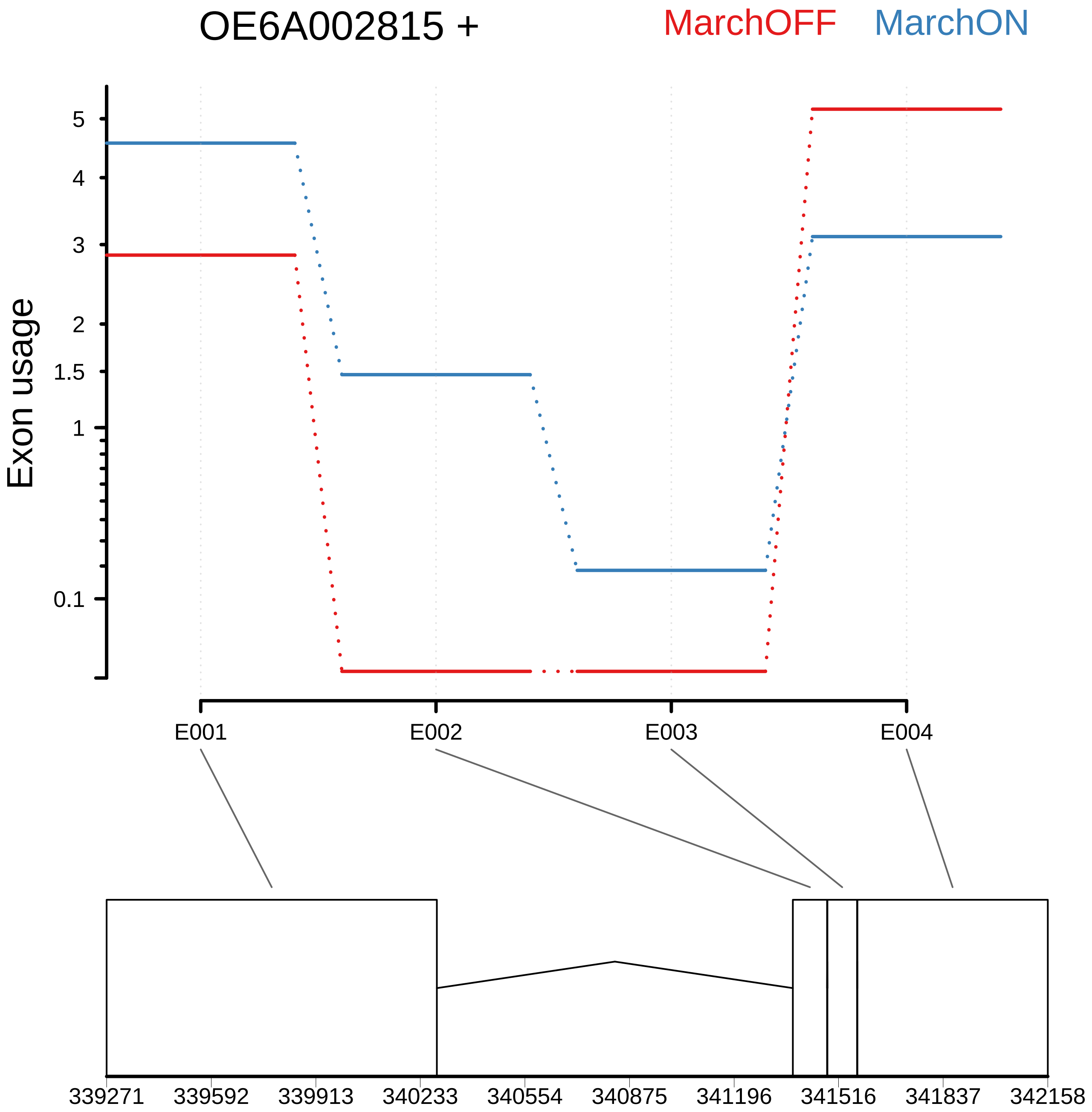

OE6A002815 +
MarchOFF
MarchON
Exon usage
5
4
3
2
1.5
1
0.1
E001
E002
E003
E004
339271
339592
339913
340233
340554
340875
341196
341516
341837
342158

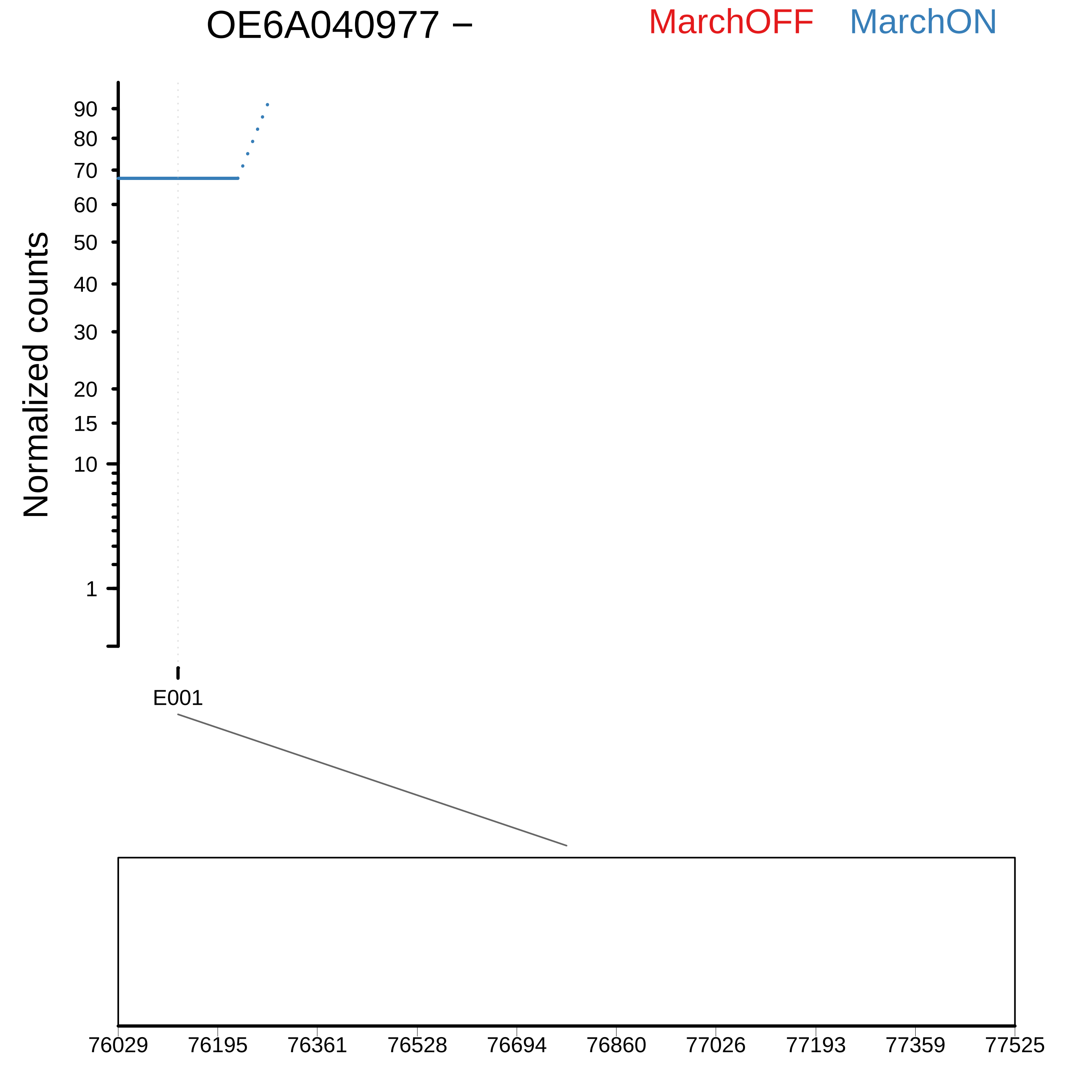
OE6A040977 −
MarchOFF
MarchON
Normalized counts
90
80
70
60
50
40
30
20
15
10
1
E001
76029
76195
76361
76528
76694
76860
77026
77193
77359
77525

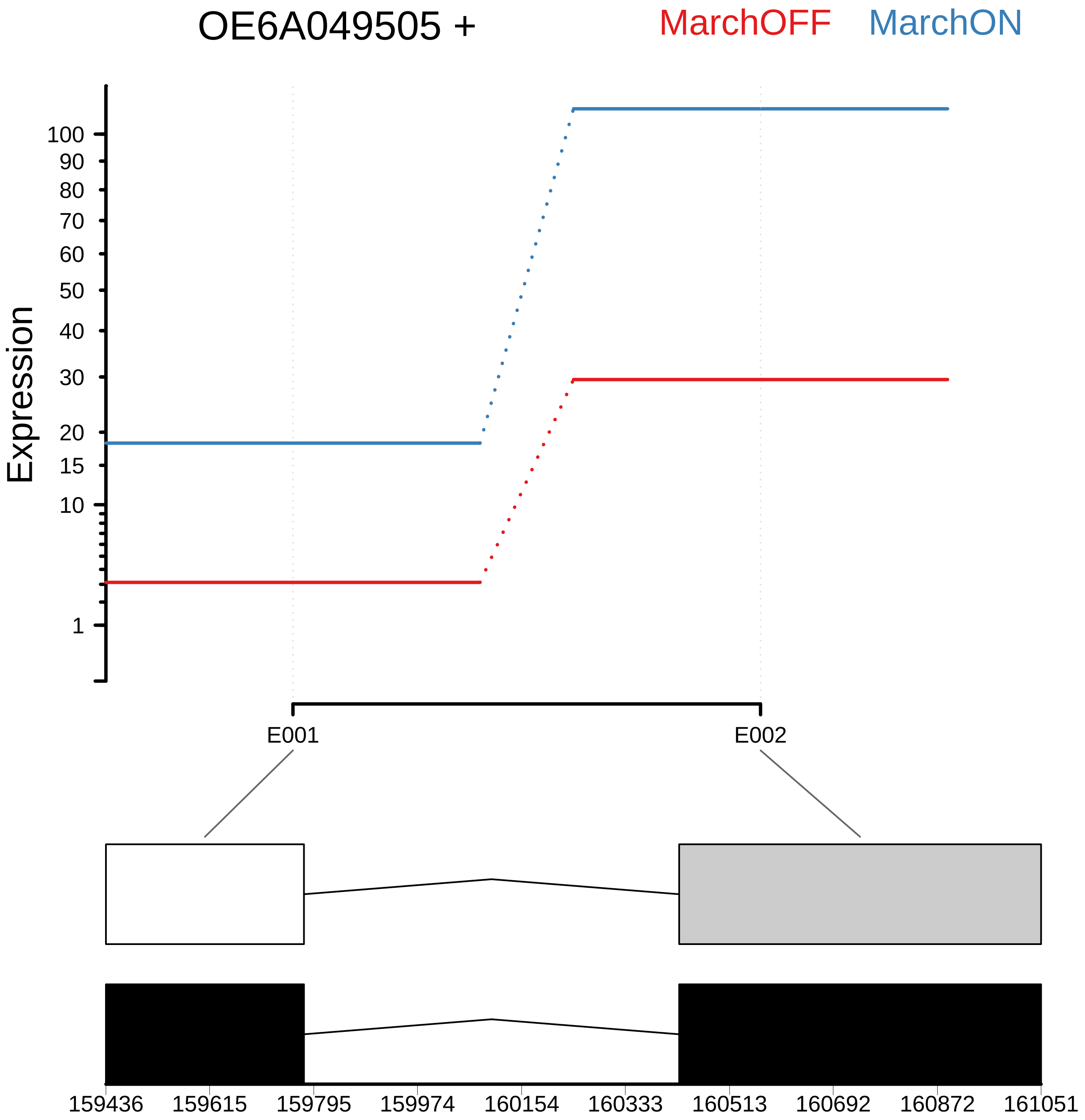

OE6A049505 +
MarchOFF
MarchON
Expression
100
90
80
70
60
50
40
30
20
15
10
1
E001
E002
159436
159615
159795
159974
160154
160333
160513
160692
160872
161051

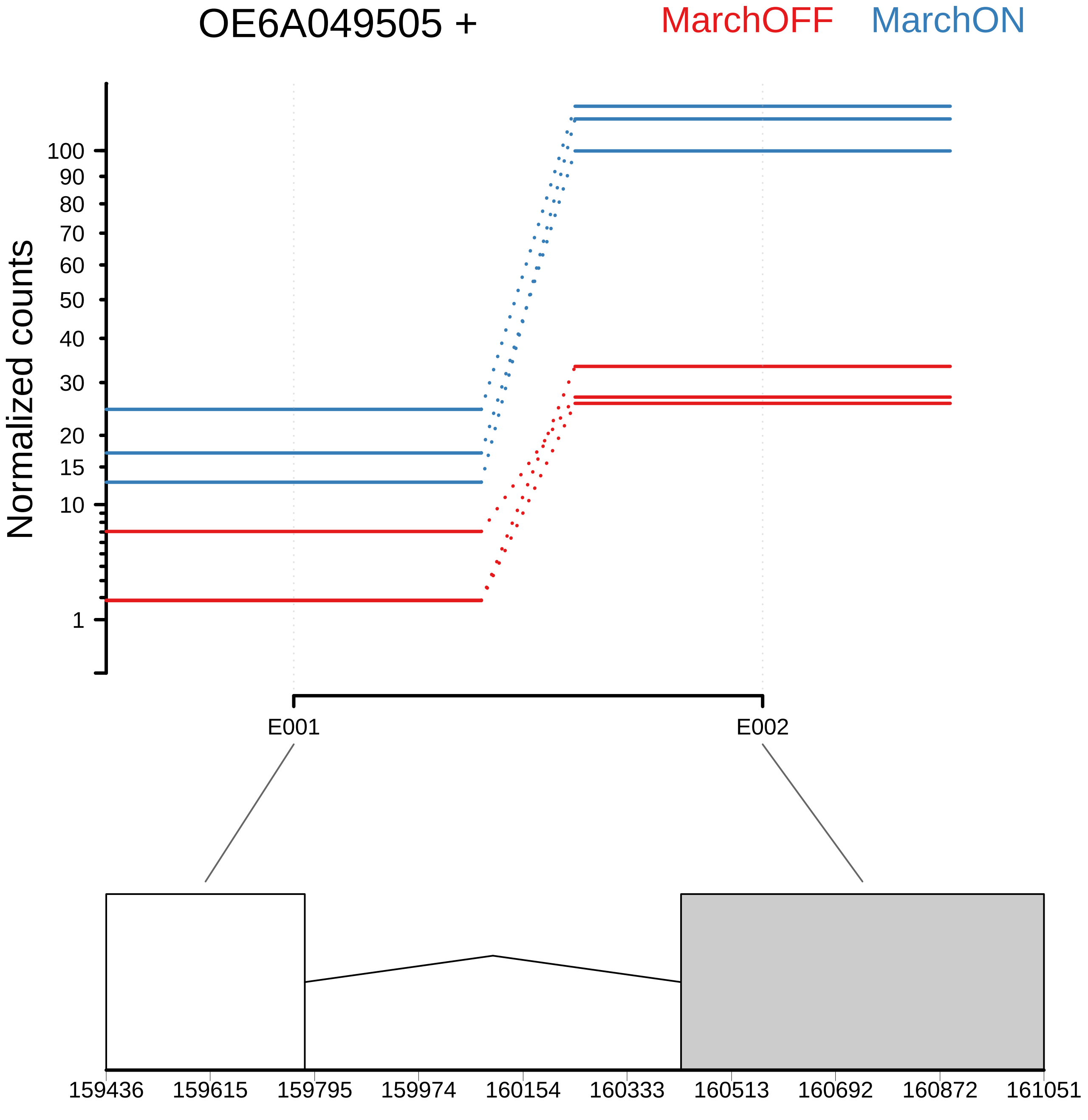

OE6A049505 +
MarchOFF
MarchON
Normalized counts
100
90
80
70
60
50
40
30
20
15
10
1
E001
E002
159436
159615
159795
159974
160154
160333
160513
160692
160872
161051

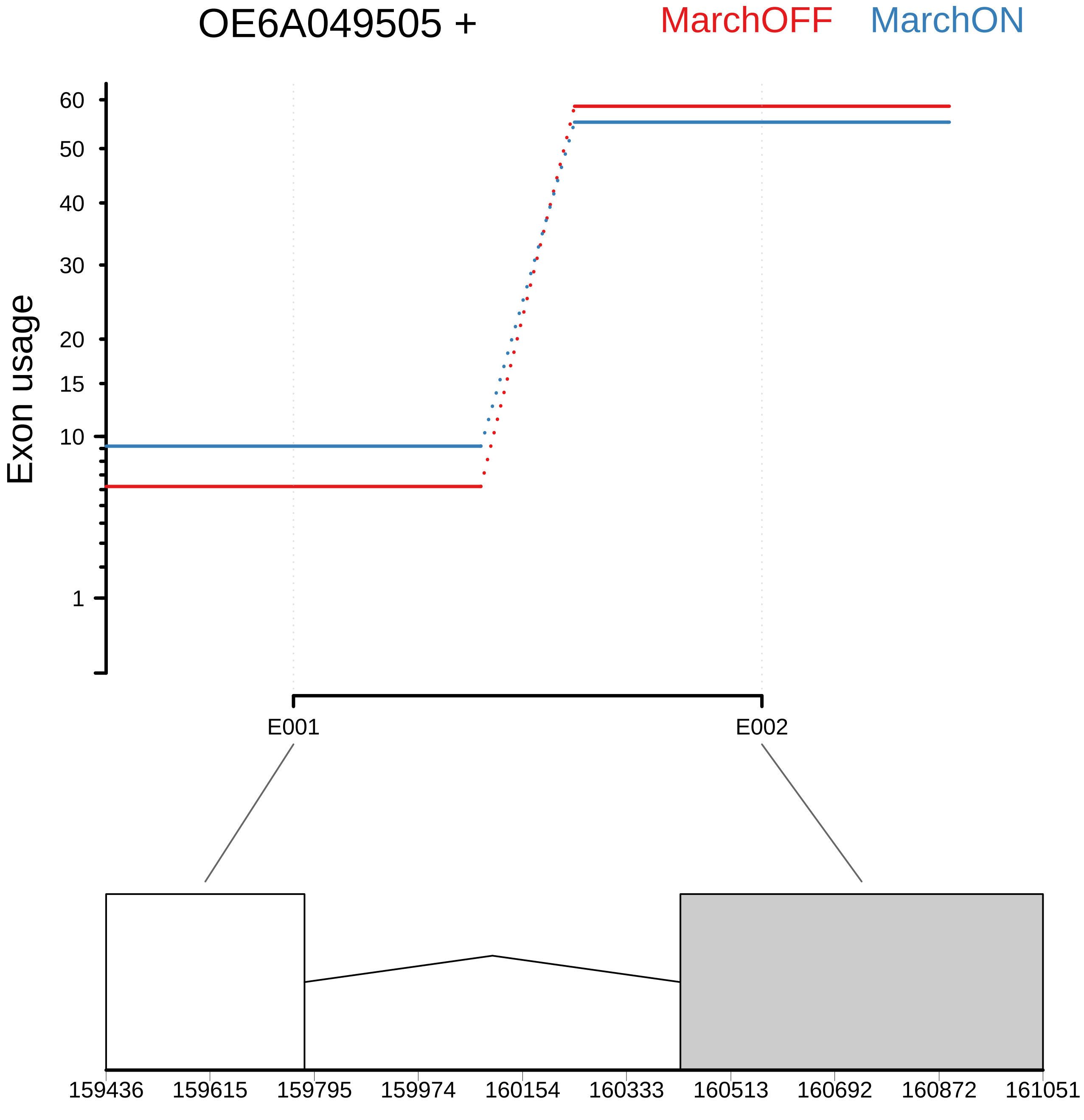

OE6A049505 +
MarchOFF
MarchON
Exon usage
60
50
40
30
20
15
10
1
E001
E002
159436
159615
159795
159974
160154
160333
160513
160692
160872
161051

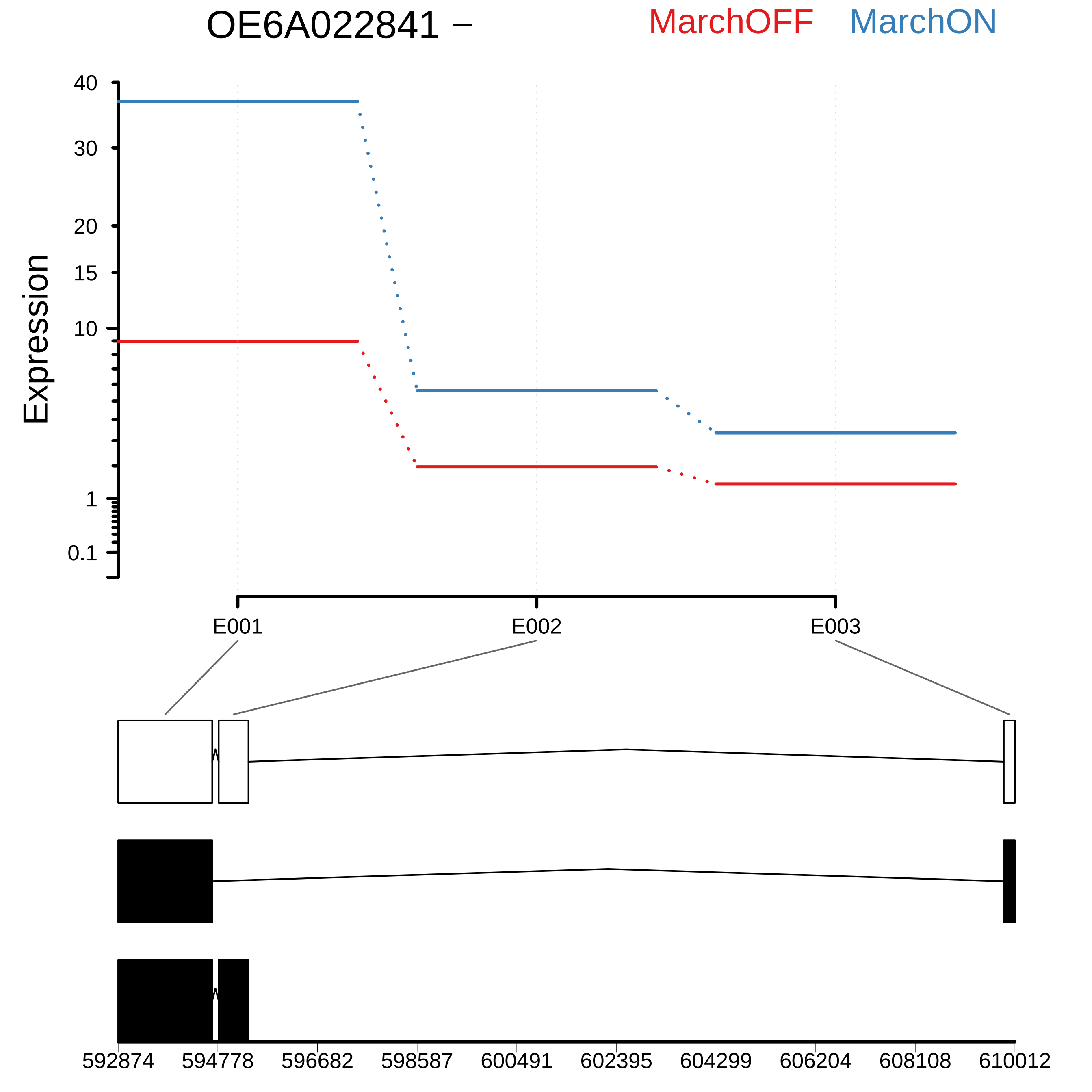

OE6A022841 −
MarchOFF
MarchON
Expression
40
30
20
15
10
1
0.1
E001
E002
E003
592874
594778
596682
598587
600491
602395
604299
606204
608108
610012

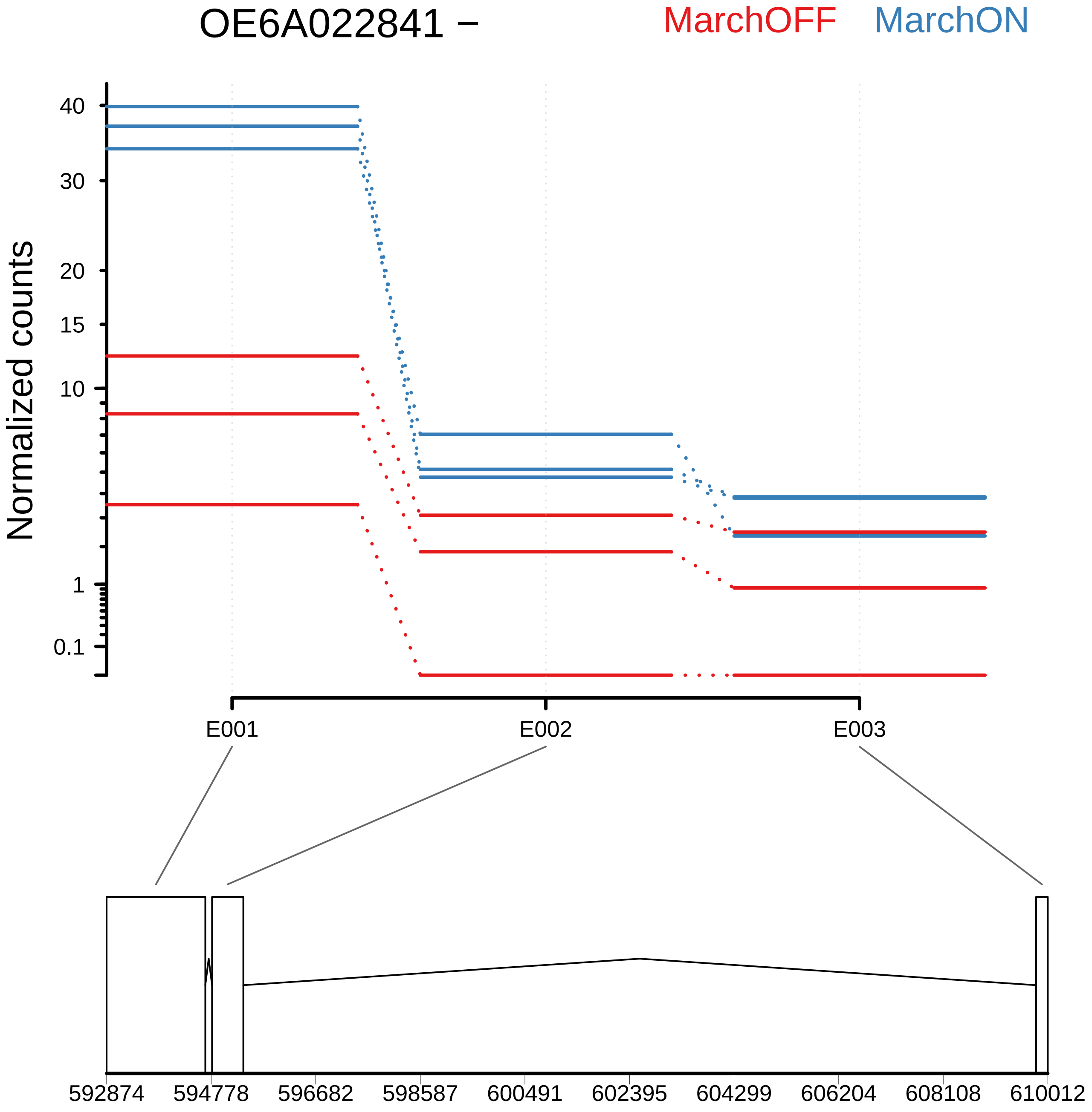

OE6A022841 −
MarchOFF
MarchON
Normalized counts
40
30
20
15
10
1
0.1
E001
E002
E003
592874
594778
596682
598587
600491
602395
604299
606204
608108
610012

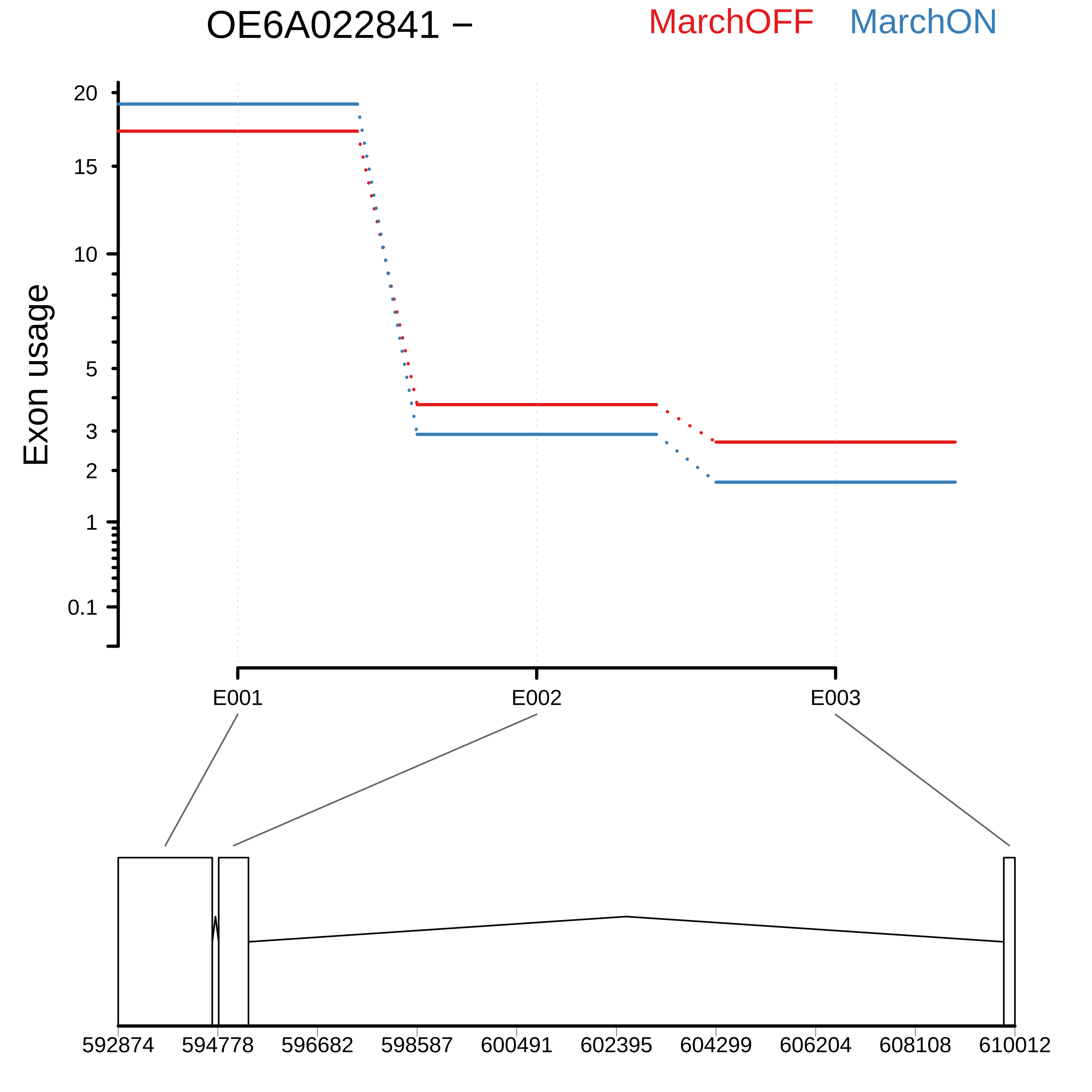
OE6A022841 −
MarchOFF
MarchON
Exon usage
20
15
10
5
3
2
1
0.1
E001
E002
E003
592874
594778
596682
598587
600491
602395
604299
606204
608108
610012

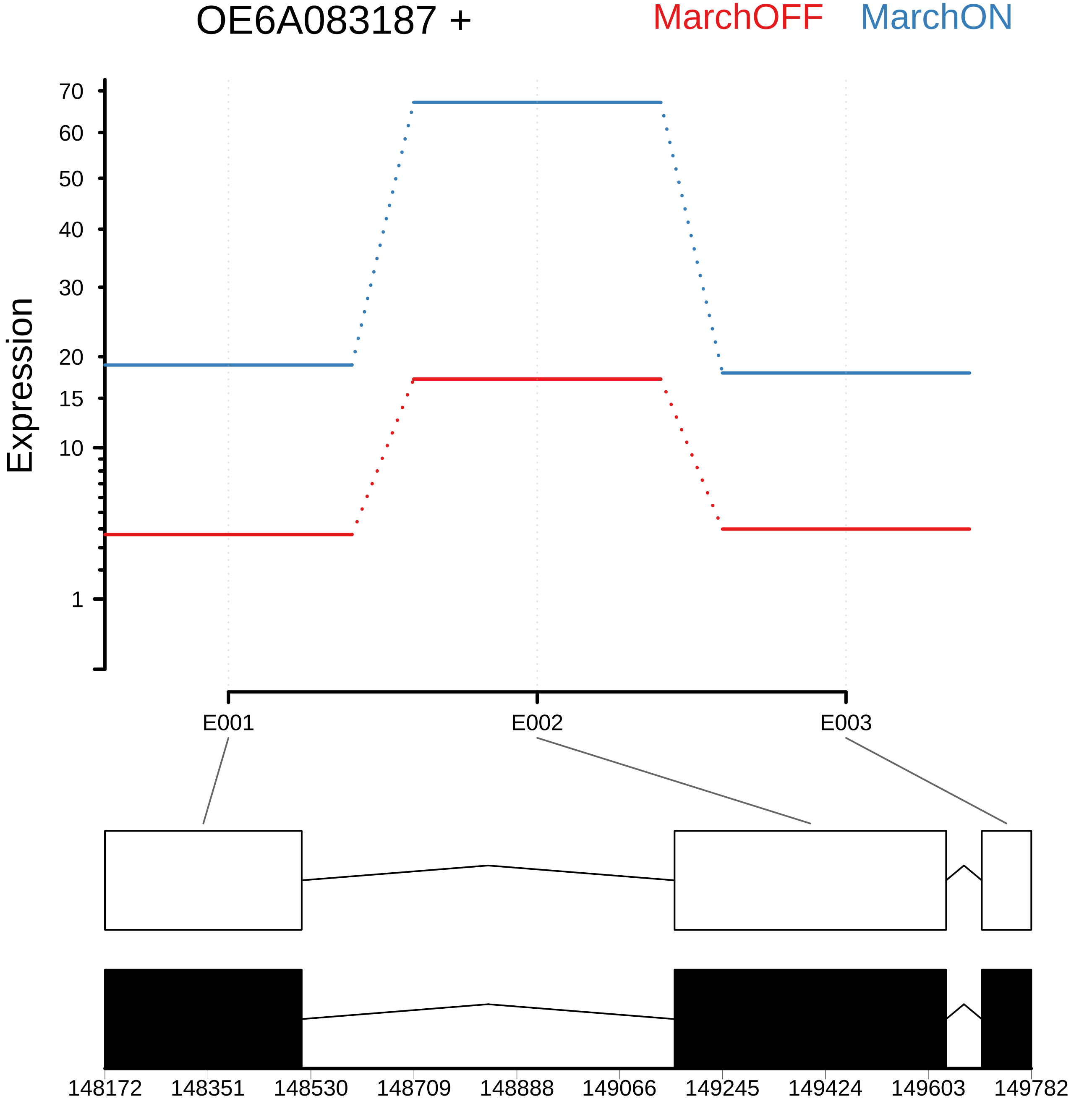

OE6A083187 +
MarchOFF
MarchON
Expression
70
60
50
40
30
20
15
10
1
E001
E002
E003
148172
148351
148530
148709
148888
149066
149245
149424
149603
149782

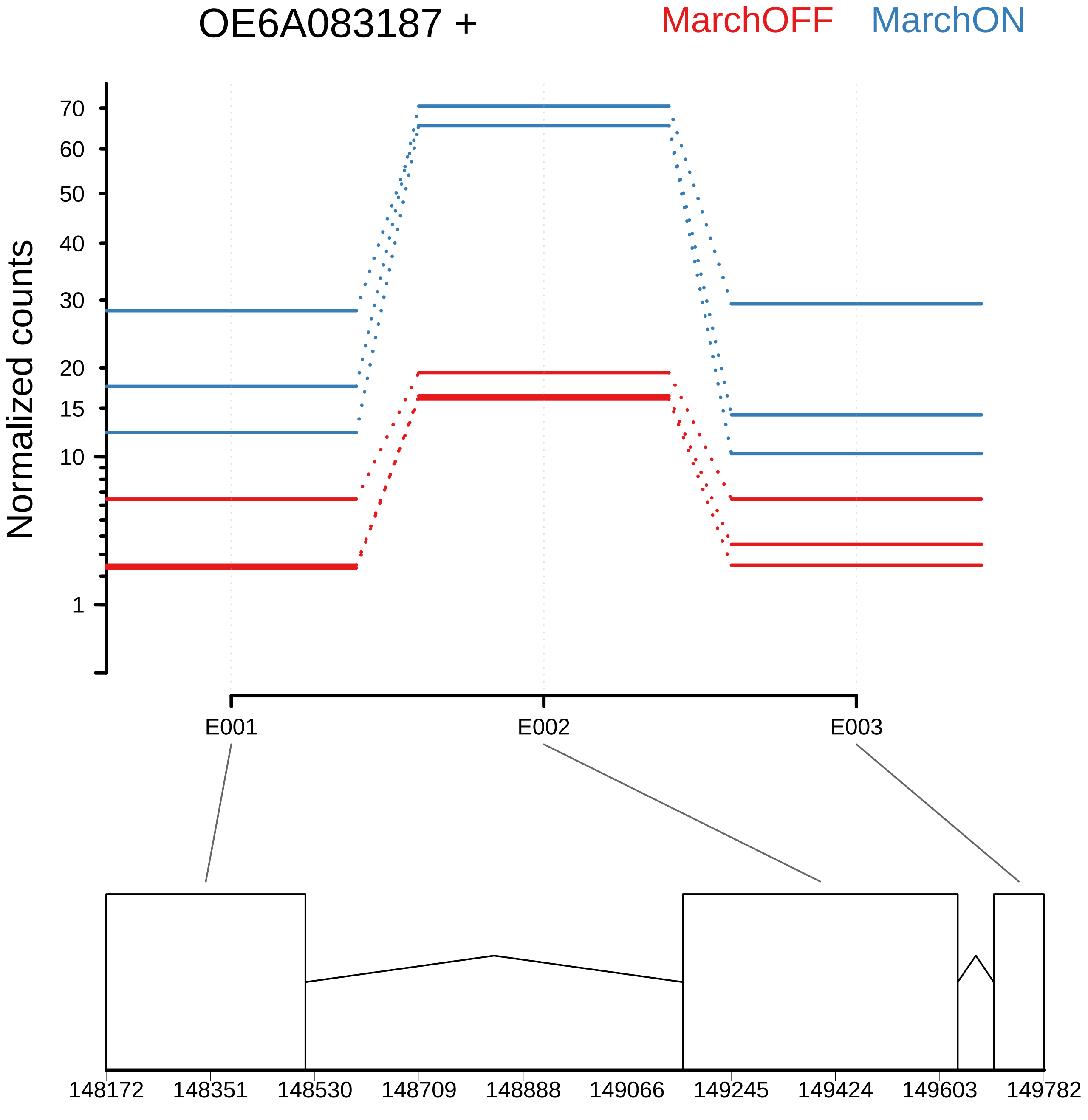
OE6A083187 +
MarchOFF
MarchON
Normalized counts
70
60
50
40
30
20
15
10
1
E001
E002
E003
148172
148351
148530
148709
148888
149066
149245
149424
149603
149782

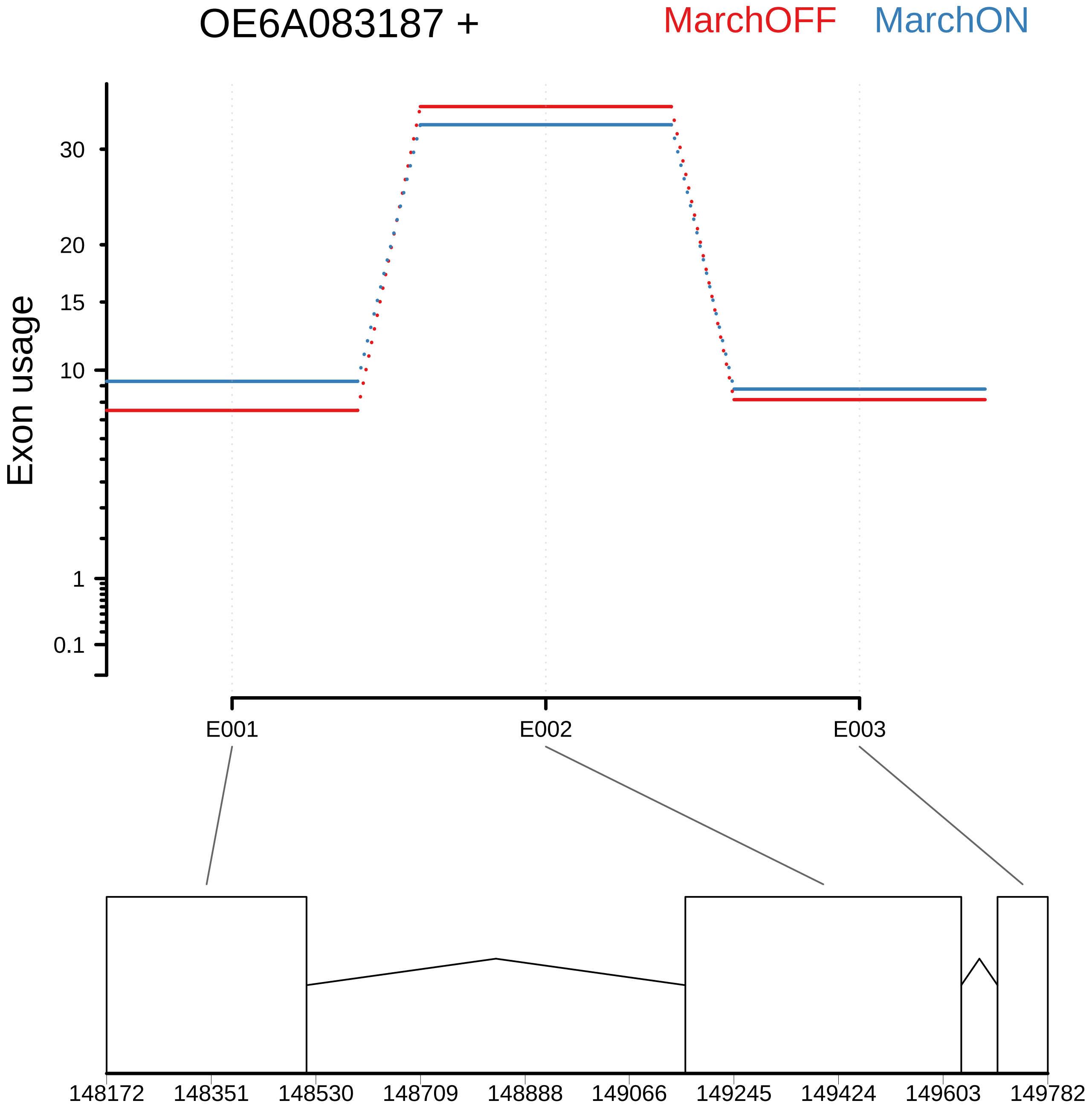
OE6A083187 +
MarchOFF
MarchON
Exon usage
30
20
15
10
1
0.1
E001
E002
E003
148172
148351
148530
148709
148888
149066
149245
149424
149603
149782

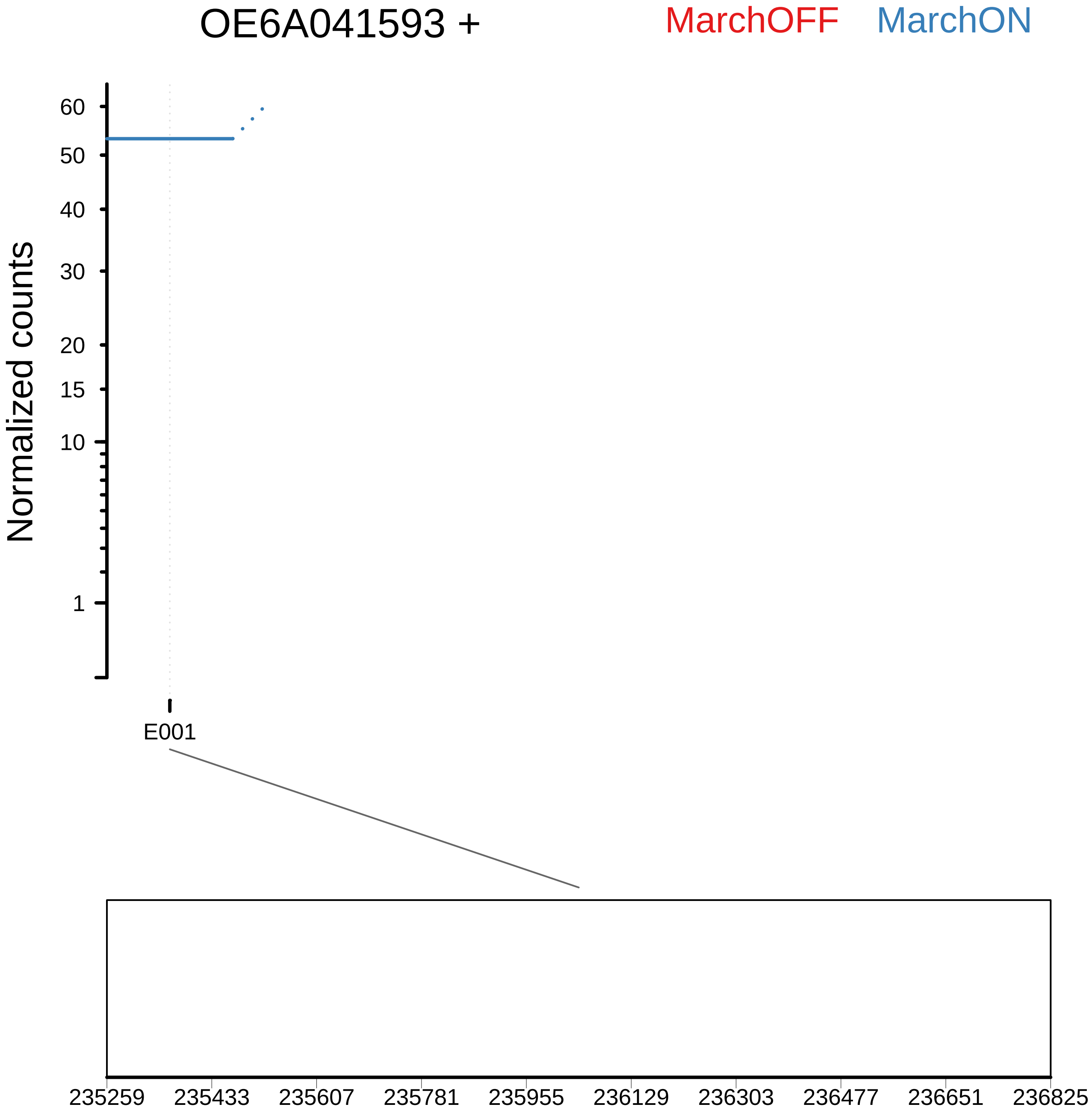
OE6A041593 +
MarchOFF
MarchON
Normalized counts
60
50
40
30
20
15
10
1
E001
235259
235433
235607
235781
235955
236129
236303
236477
236651
236825

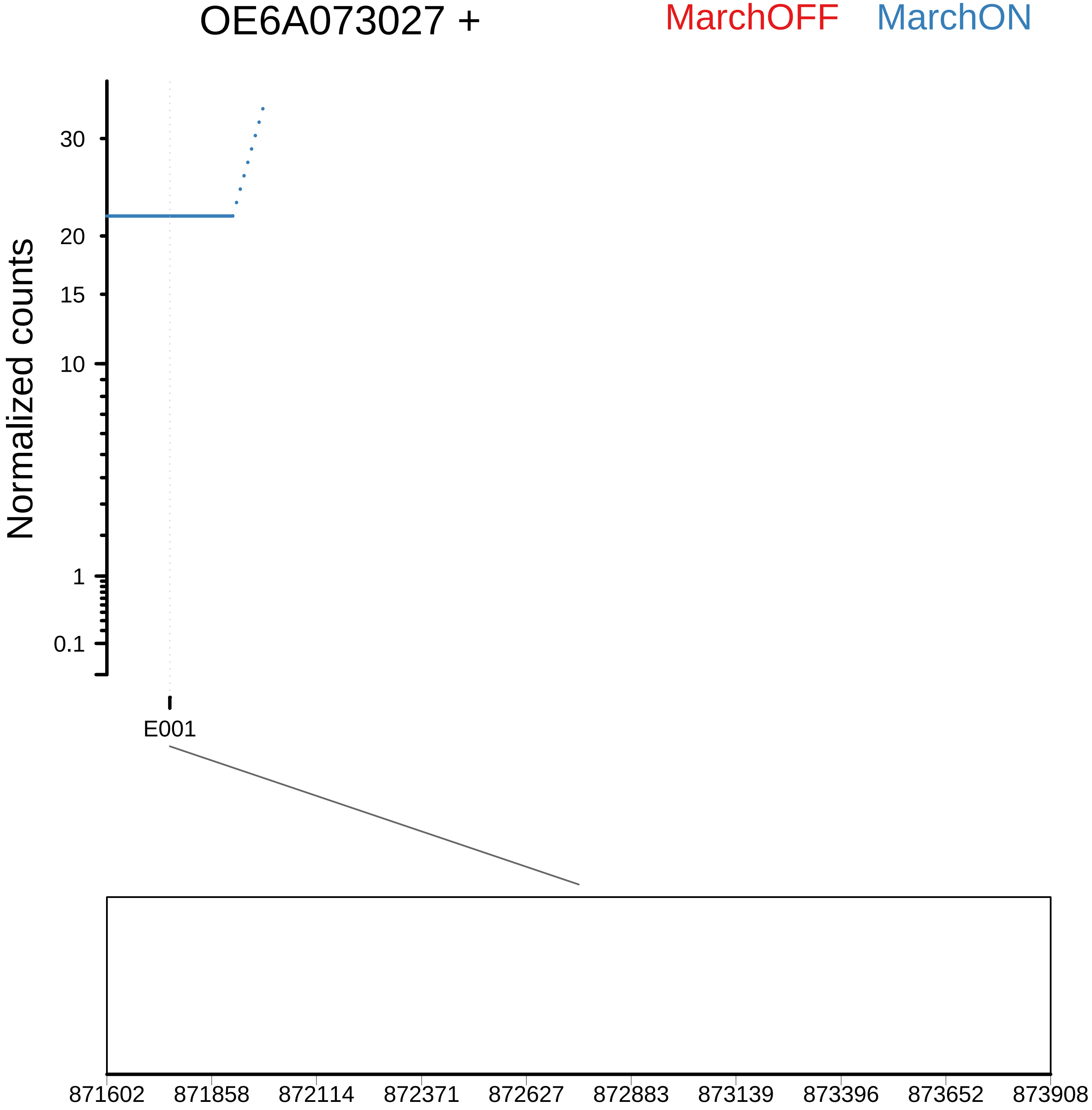
OE6A073027 +
MarchOFF
MarchON
Normalized counts
30
20
15
10
1
0.1
E001
871602
871858
872114
872371
872627
872883
873139
873396
873652
873908

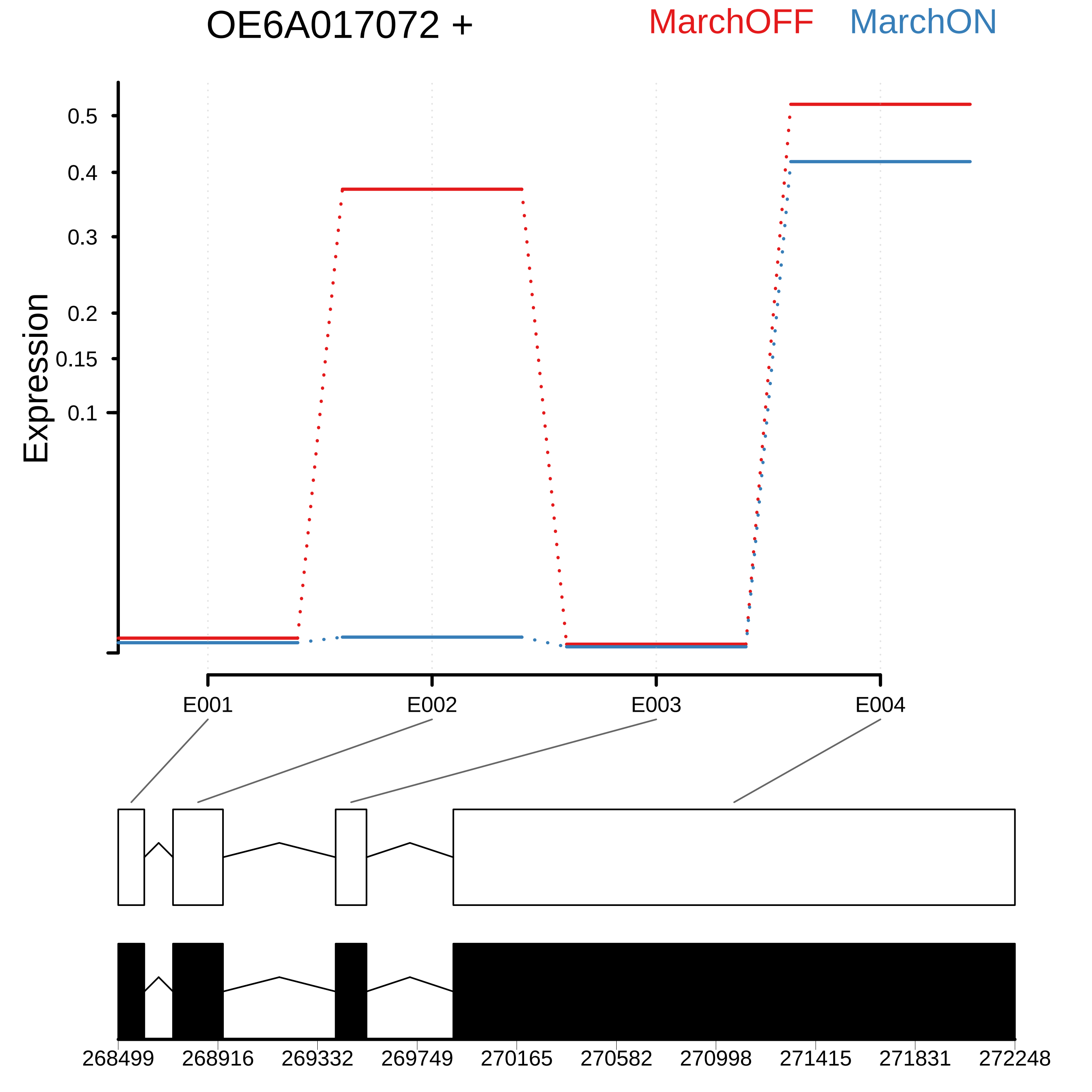

OE6A017072 +
MarchOFF
MarchON
Expression
0.5
0.4
0.3
0.2
0.15
0.1
E001
E002
E003
E004
268499
268916
269332
269749
270165
270582
270998
271415
271831
272248

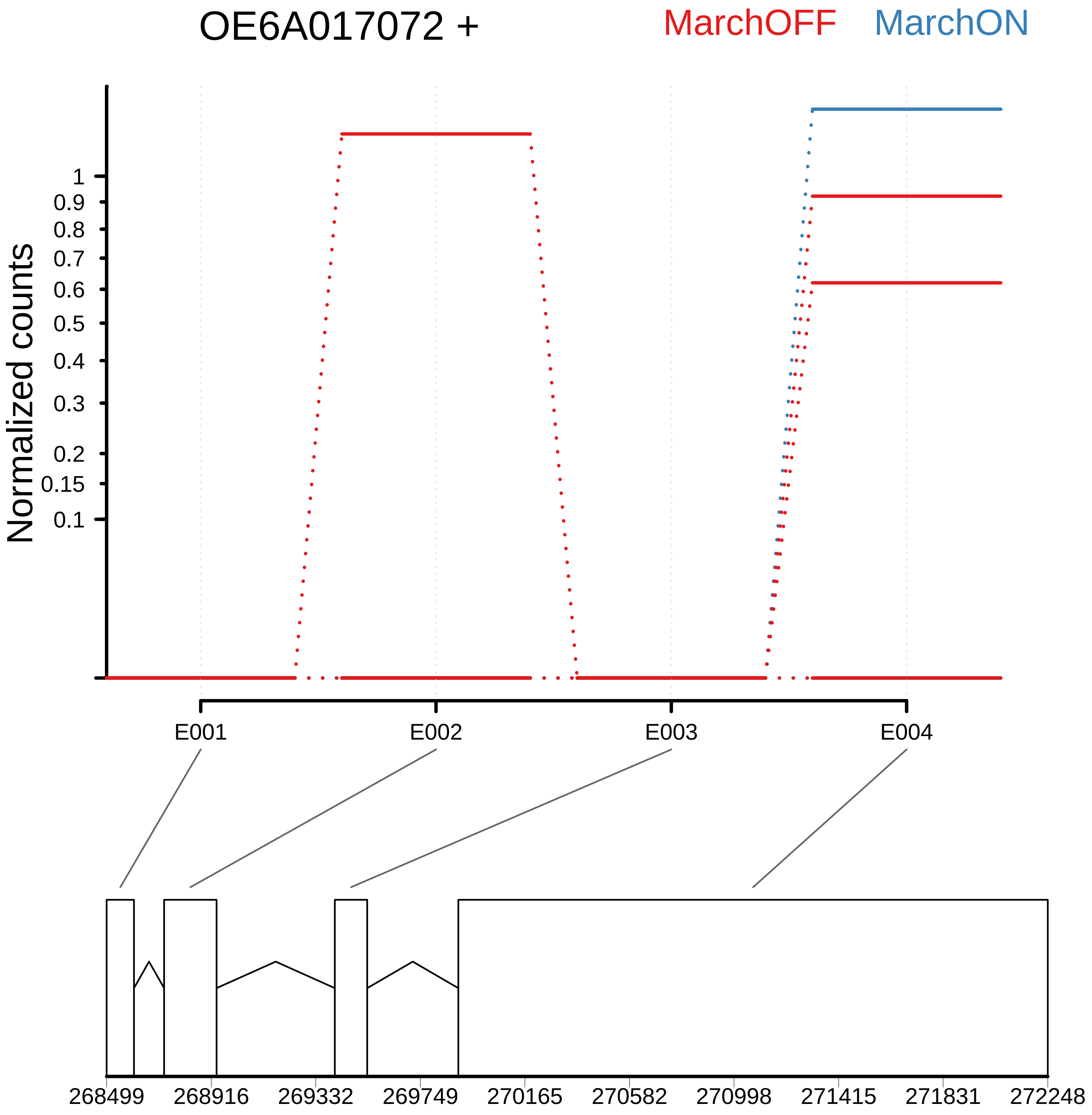

OE6A017072 +
MarchOFF
MarchON
Normalized counts
1
0.9
0.8
0.7
0.6
0.5
0.4
0.3
0.2
0.15
0.1
E001
E002
E003
E004
268499
268916
269332
269749
270165
270582
270998
271415
271831
272248

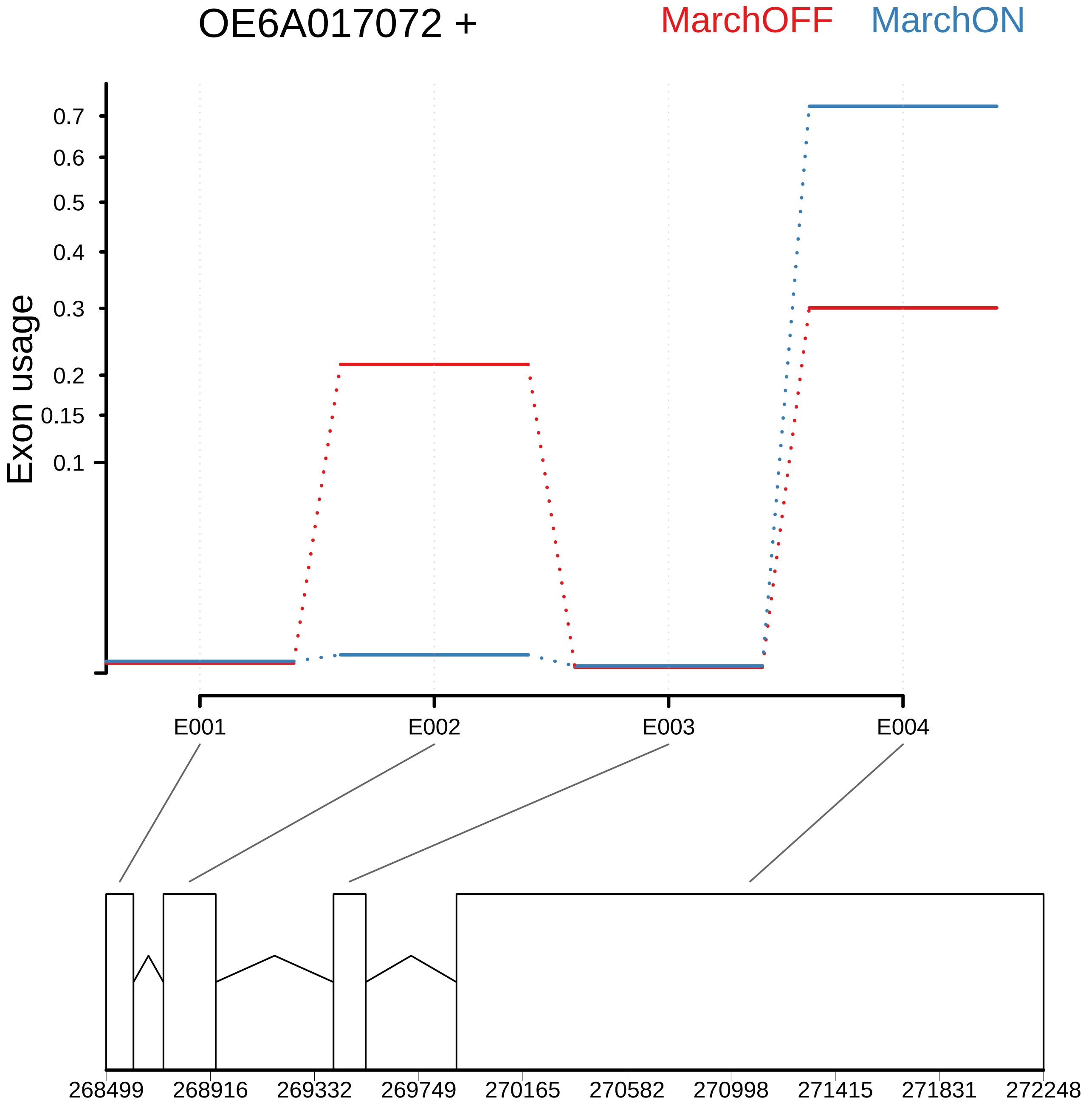
OE6A017072 +
MarchOFF
MarchON
Exon usage
0.7
0.6
0.5
0.4
0.3
0.2
0.15
0.1
E001
E002
E003
E004
268499
268916
269332
269749
270165
270582
270998
271415
271831
272248

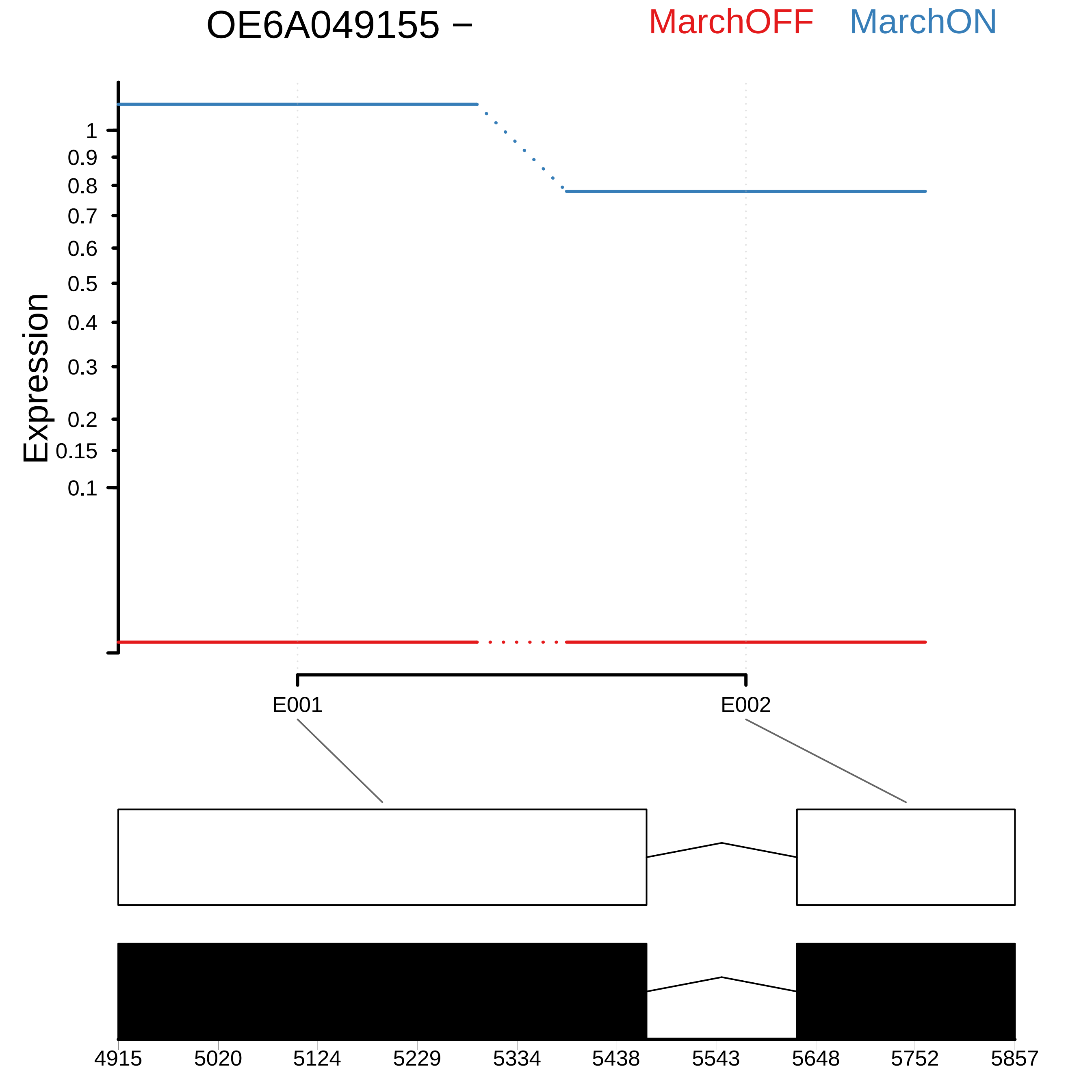

OE6A049155 −
MarchOFF
MarchON
Expression
1
0.9
0.8
0.7
0.6
0.5
0.4
0.3
0.2
0.15
0.1
E001
E002
4915
5020
5124
5229
5334
5438
5543
5648
5752
5857

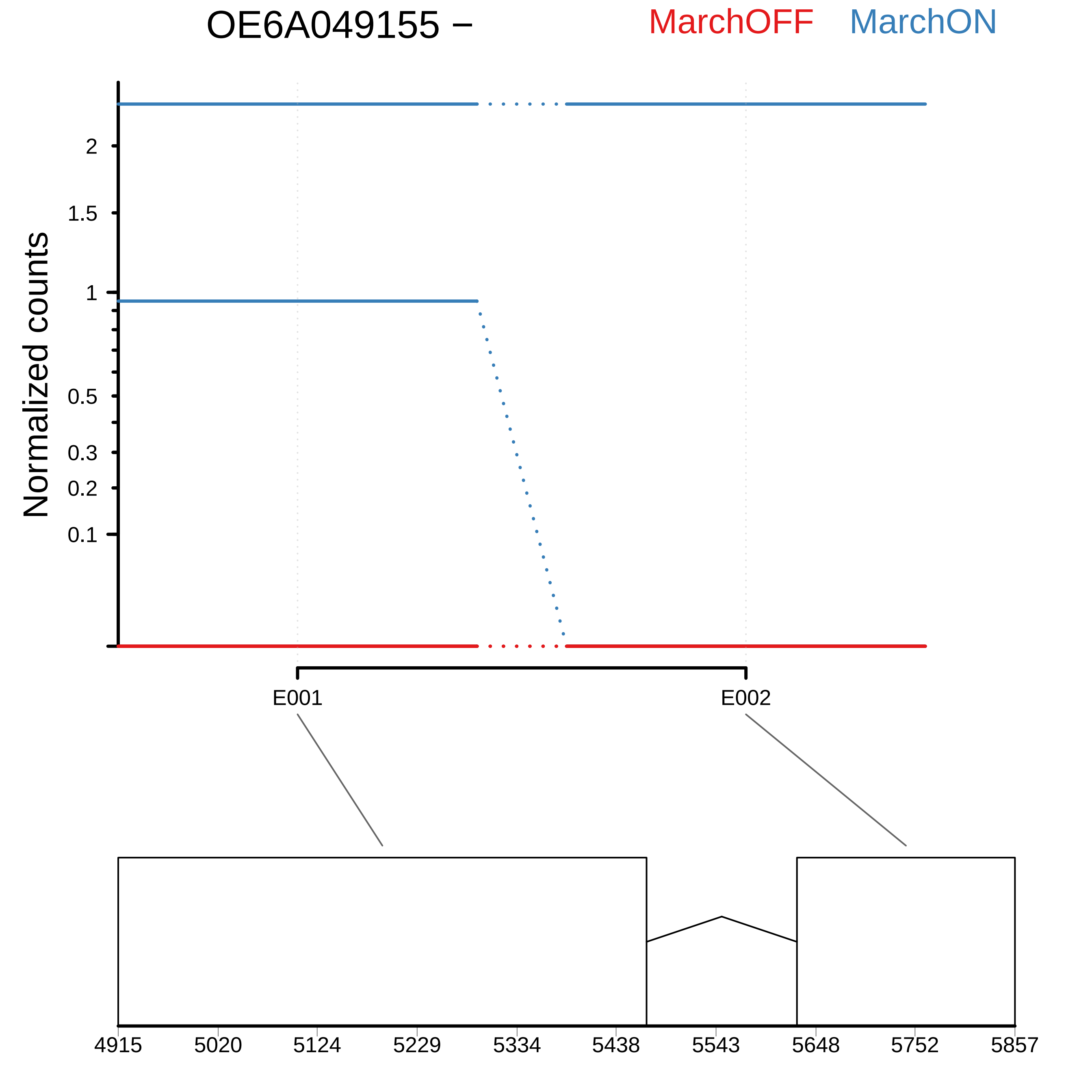

OE6A049155 −
MarchOFF
MarchON
Normalized counts
2
1.5
1
0.5
0.3
0.2
0.1
E001
E002
4915
5020
5124
5229
5334
5438
5543
5648
5752
5857

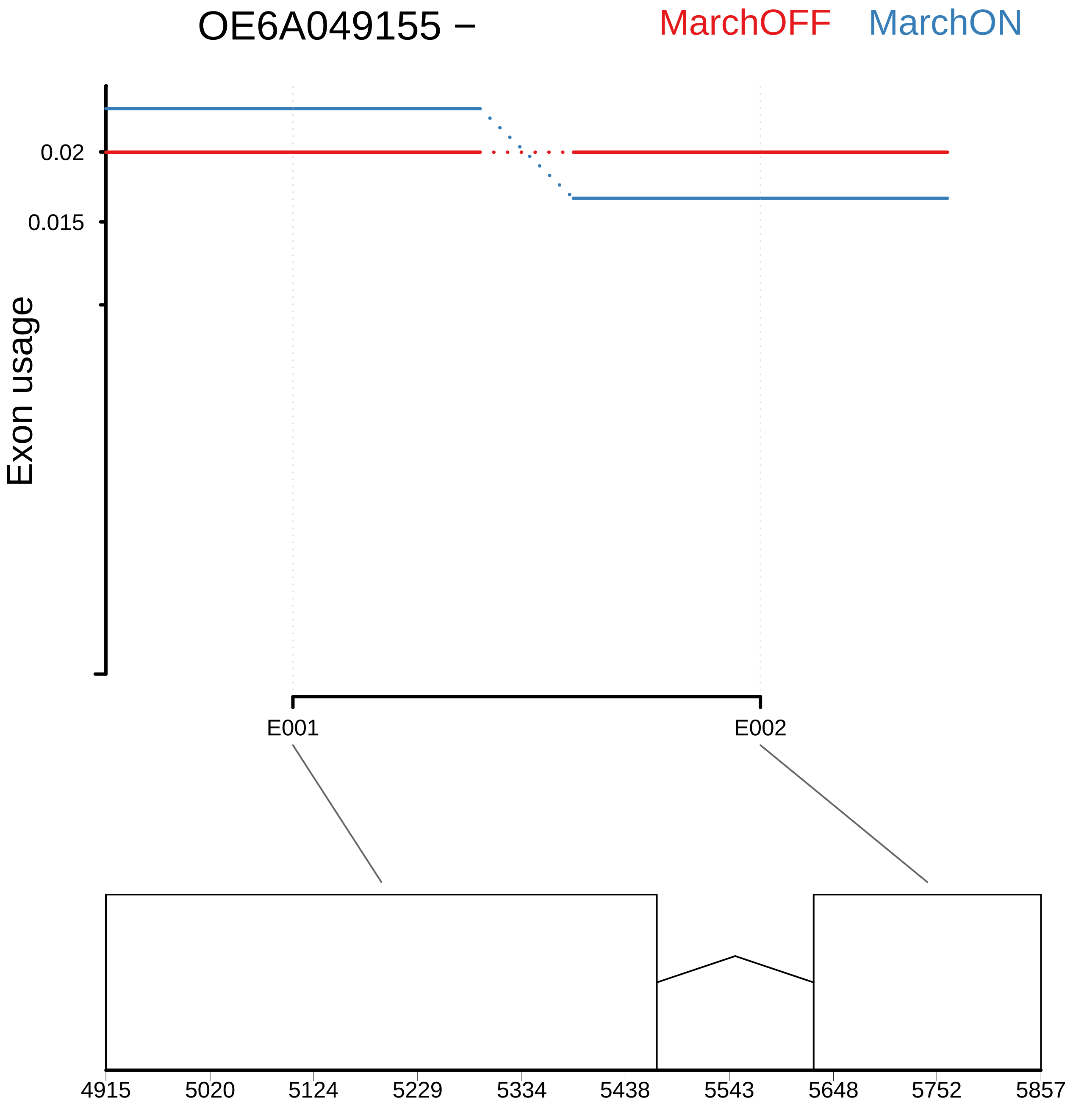

OE6A049155 −
MarchOFF
MarchON
Exon usage
0.02
0.015
E001
E002
4915
5020
5124
5229
5334
5438
5543
5648
5752
5857

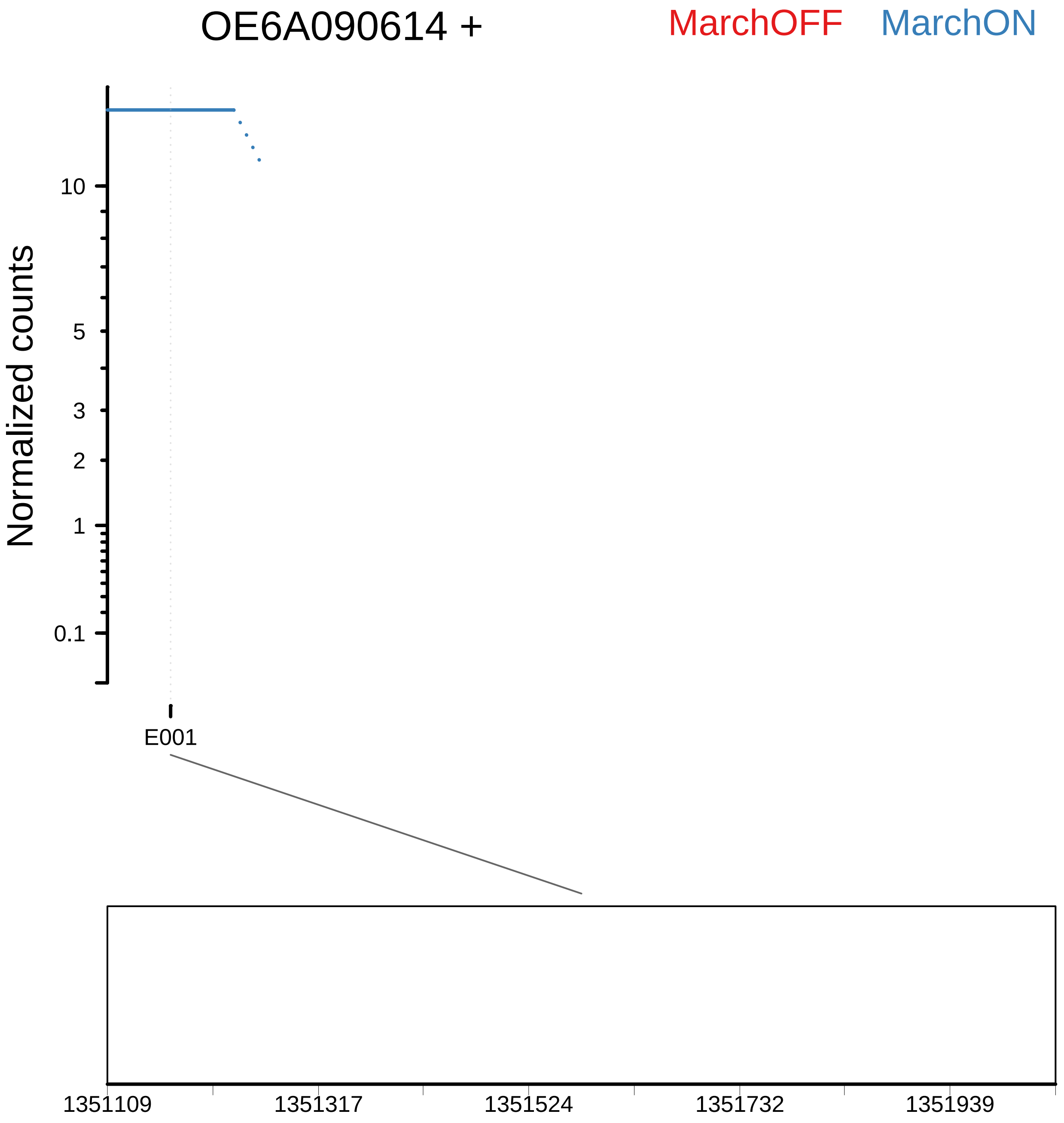
OE6A090614 +
MarchOFF
MarchON
Normalized counts
10
5
3
2
1
0.1
E001
1351109
1351317
1351524
1351732
1351939

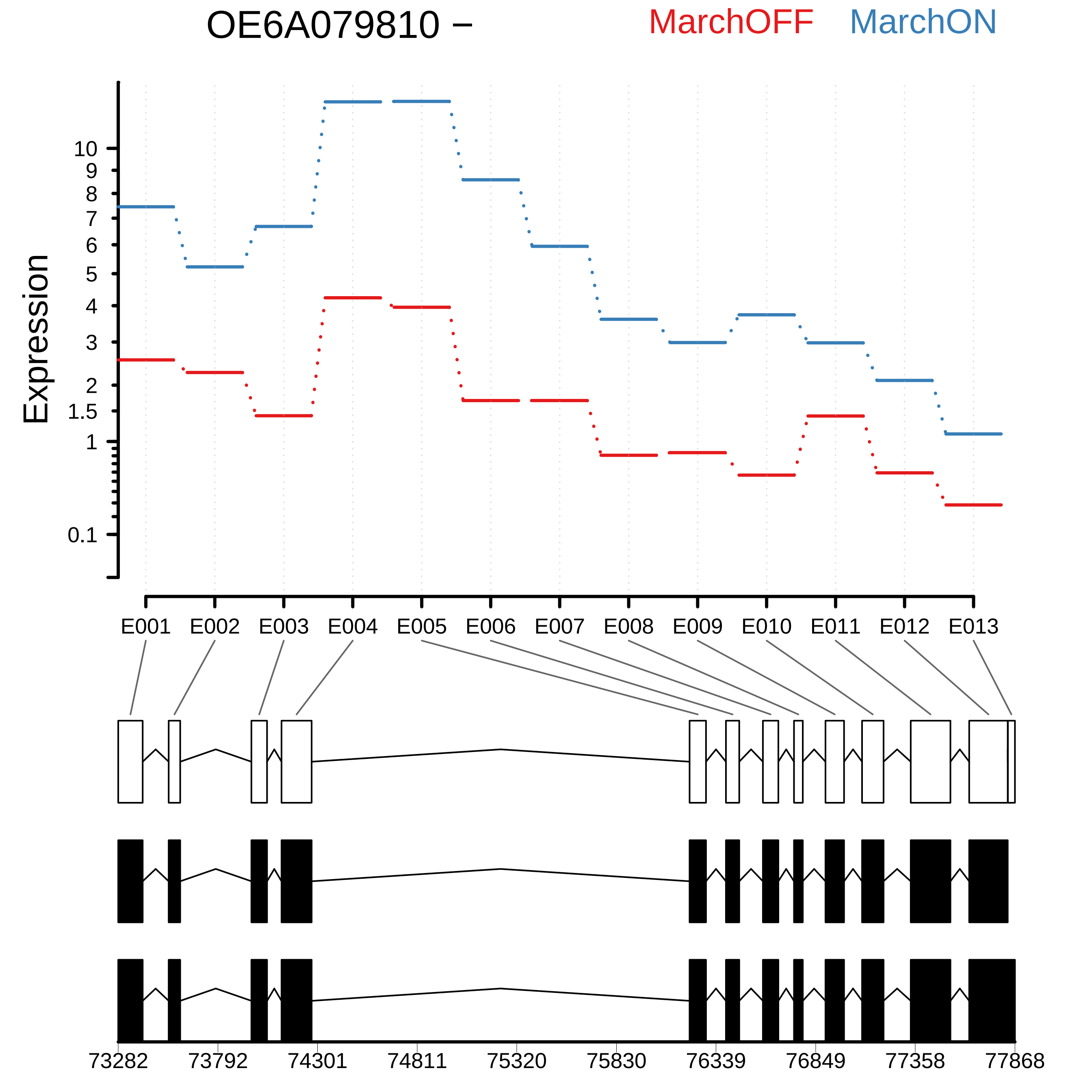
OE6A079810 −
MarchOFF
MarchON
Expression
10
9
8
7
6
5
4
3
2
1.5
1
0.1
E001
E002
E003
E004
E005
E006
E007
E008
E009
E010
E011
E012
E013
73282
73792
74301
74811
75320
75830
76339
76849
77358
77868

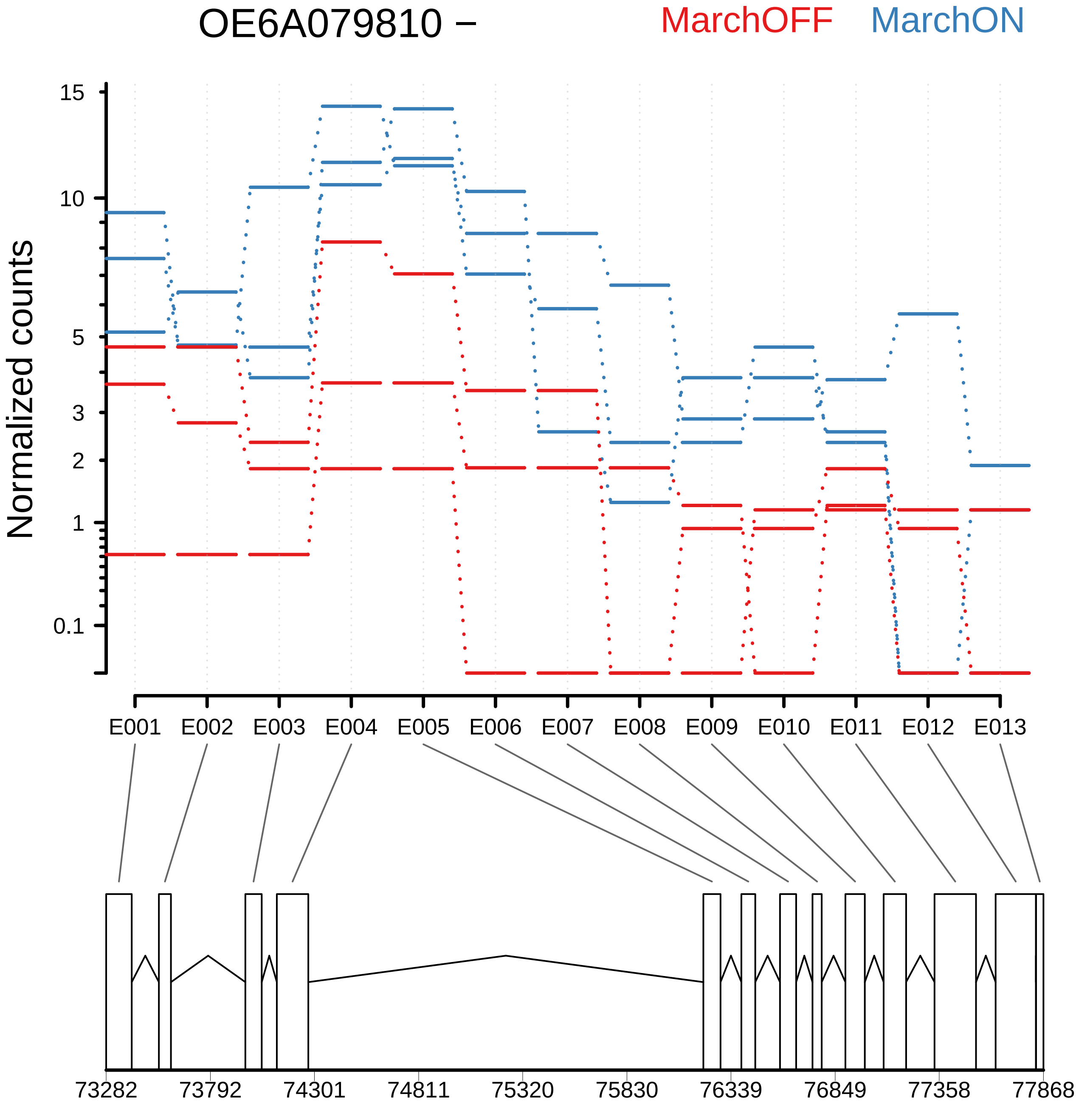

OE6A079810 −
MarchOFF
MarchON
Normalized counts
15
10
5
3
2
1
0.1
E001
E002
E003
E004
E005
E006
E007
E008
E009
E010
E011
E012
E013
73282
73792
74301
74811
75320
75830
76339
76849
77358
77868

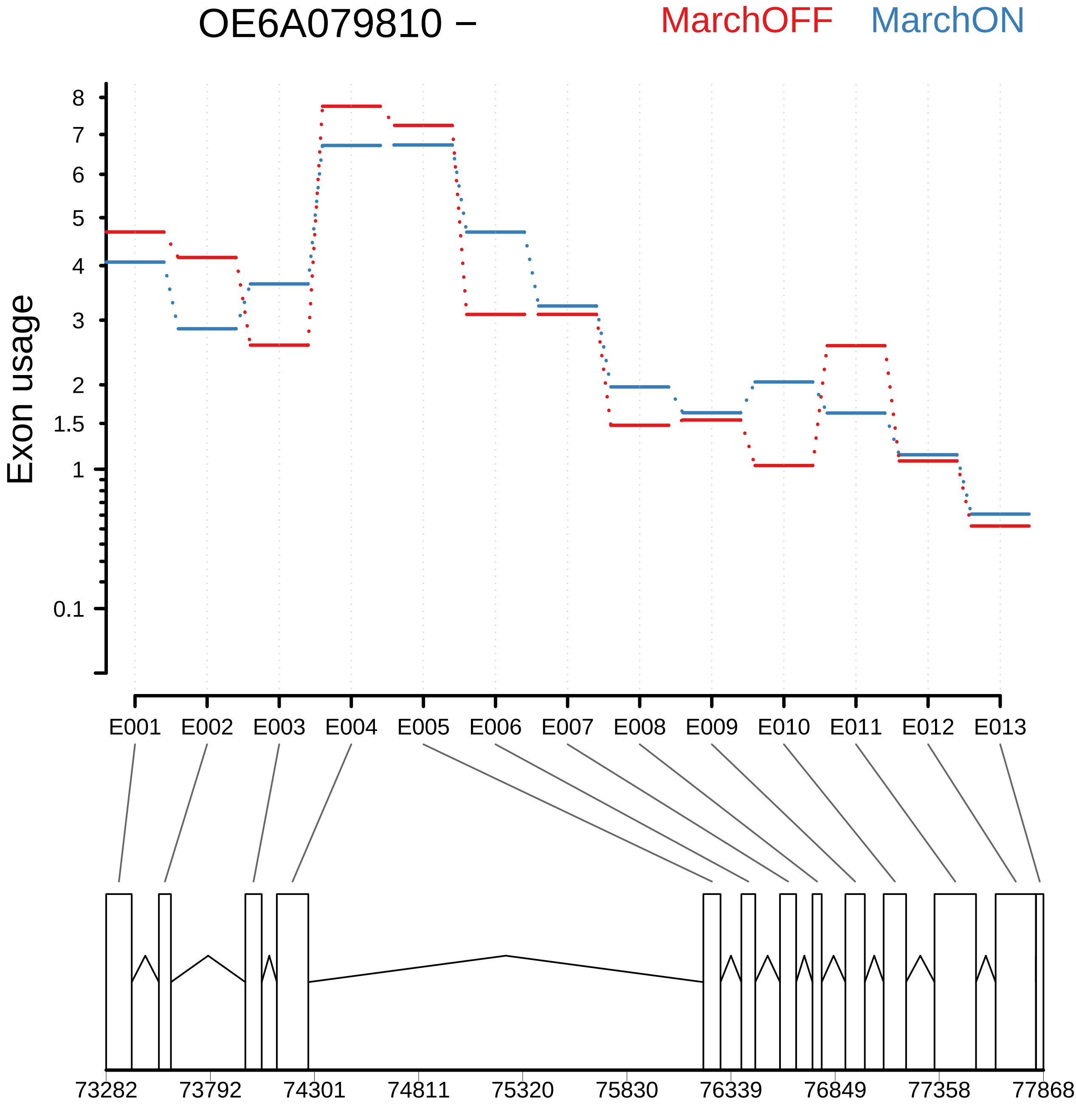

OE6A079810 −
MarchOFF
MarchON
Exon usage
8
7
6
5
4
3
2
1.5
1
0.1
E001
E002
E003
E004
E005
E006
E007
E008
E009
E010
E011
E012
E013
73282
73792
74301
74811
75320
75830
76339
76849
77358
77868

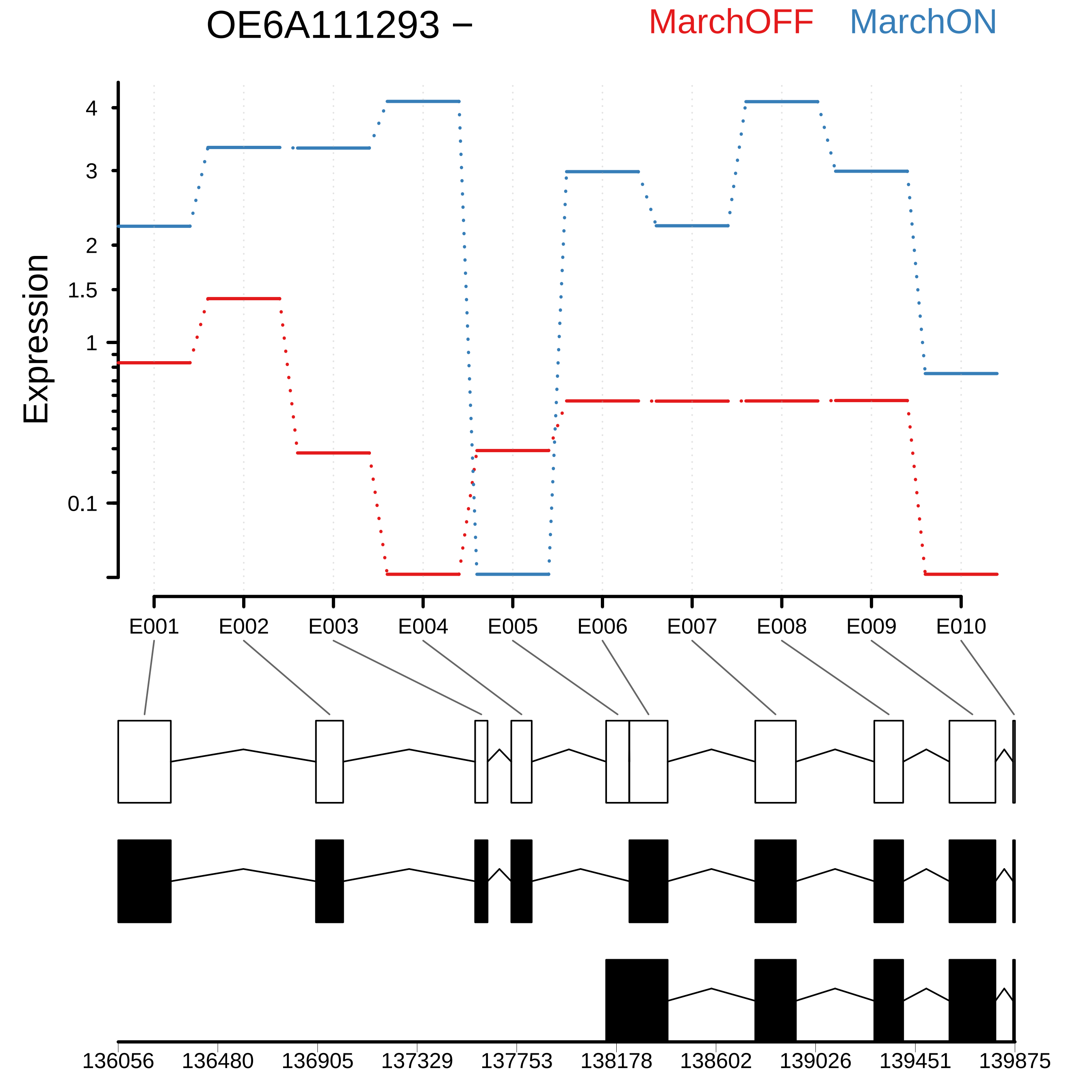

OE6A111293 −
MarchOFF
MarchON
Expression
4
3
2
1.5
1
0.1
E001
E002
E003
E004
E005
E006
E007
E008
E009
E010
136056
136480
136905
137329
137753
138178
138602
139026
139451
139875

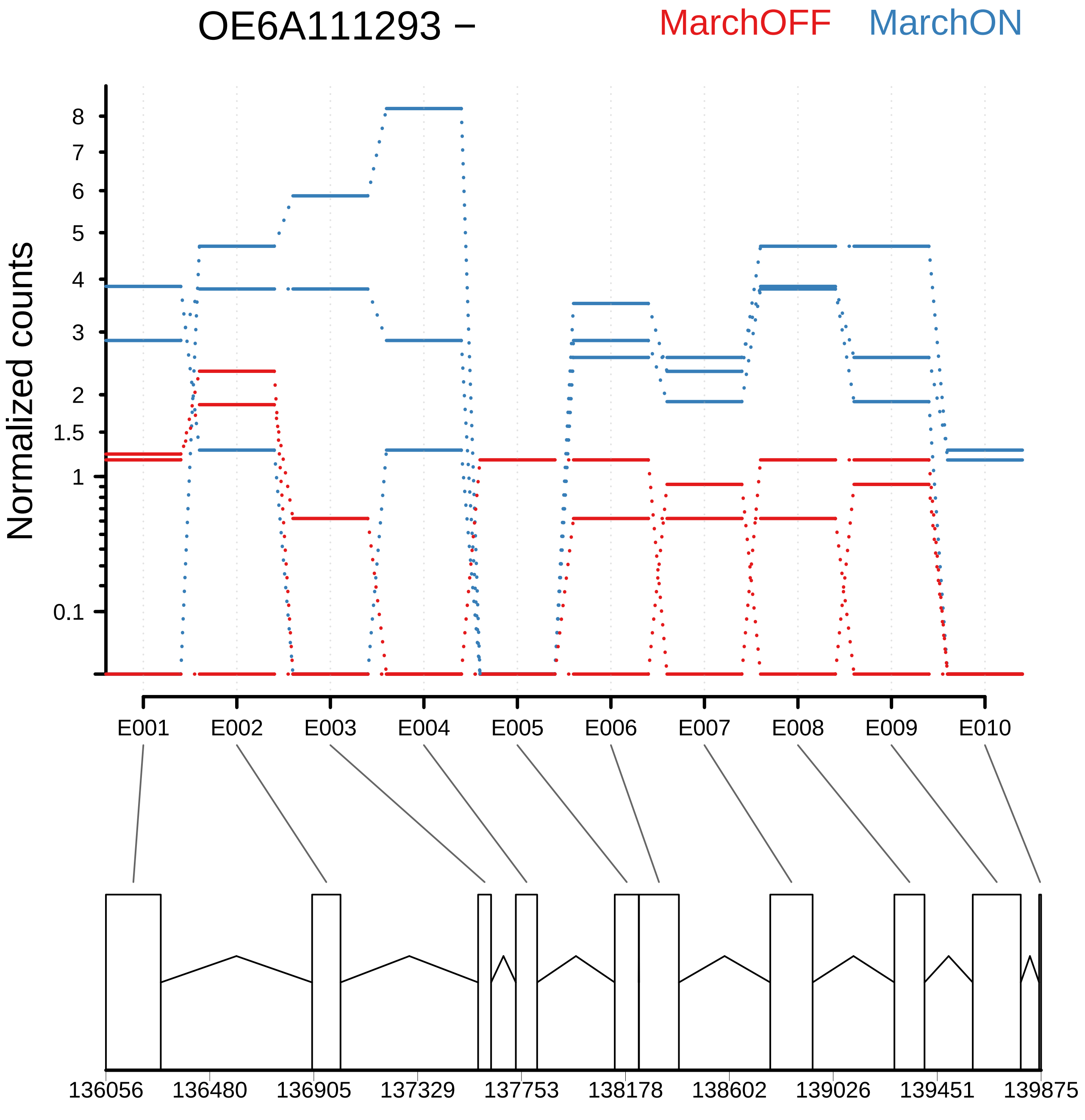
OE6A111293 −
MarchOFF
MarchON
Normalized counts
8
7
6
5
4
3
2
1.5
1
0.1
E001
E002
E003
E004
E005
E006
E007
E008
E009
E010
136056
136480
136905
137329
137753
138178
138602
139026
139451
139875

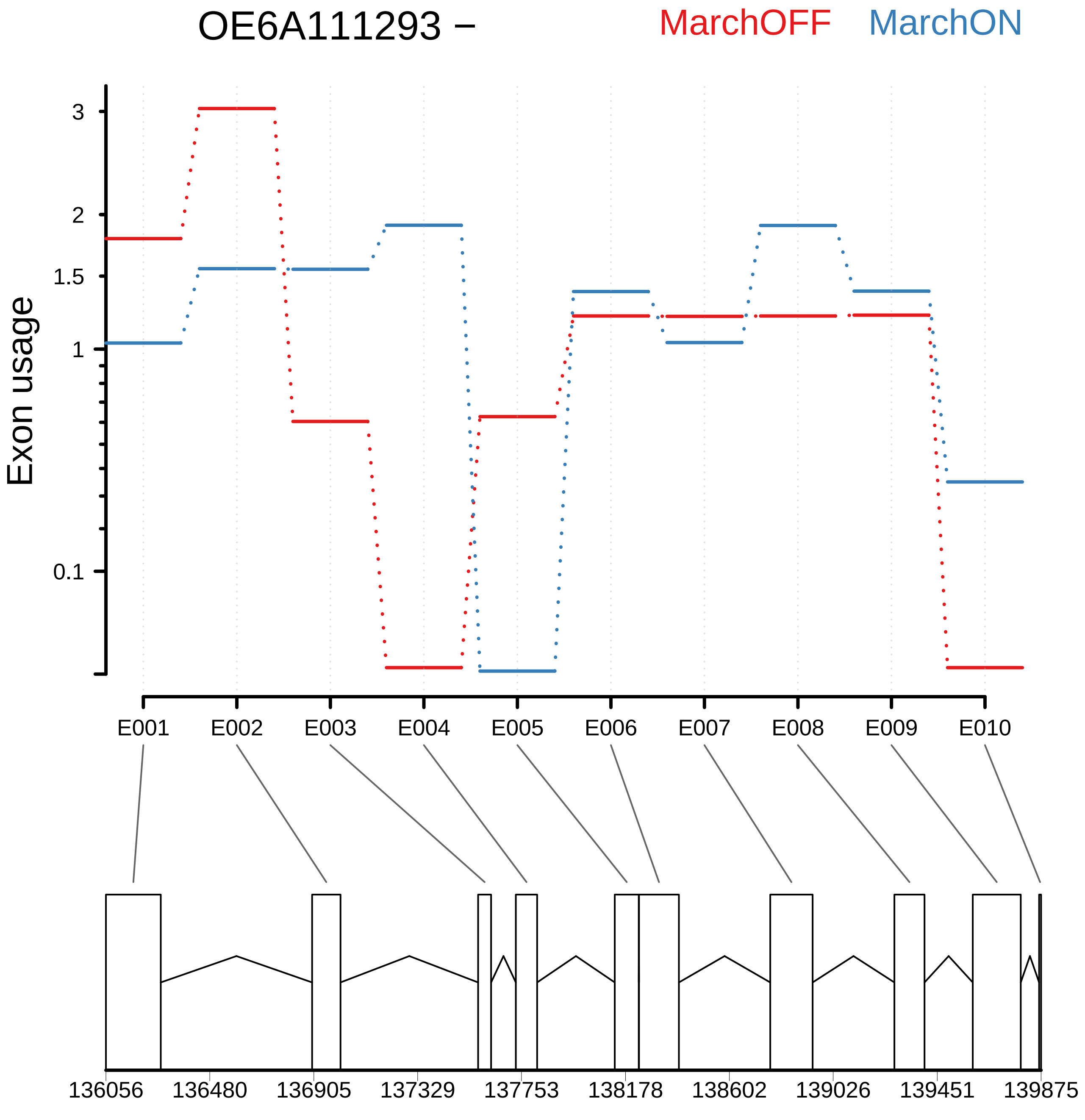
OE6A111293 −
MarchOFF
MarchON
Exon usage
3
2
1.5
1
0.1
E001
E002
E003
E004
E005
E006
E007
E008
E009
E010
136056
136480
136905
137329
137753
138178
138602
139026
139451
139875

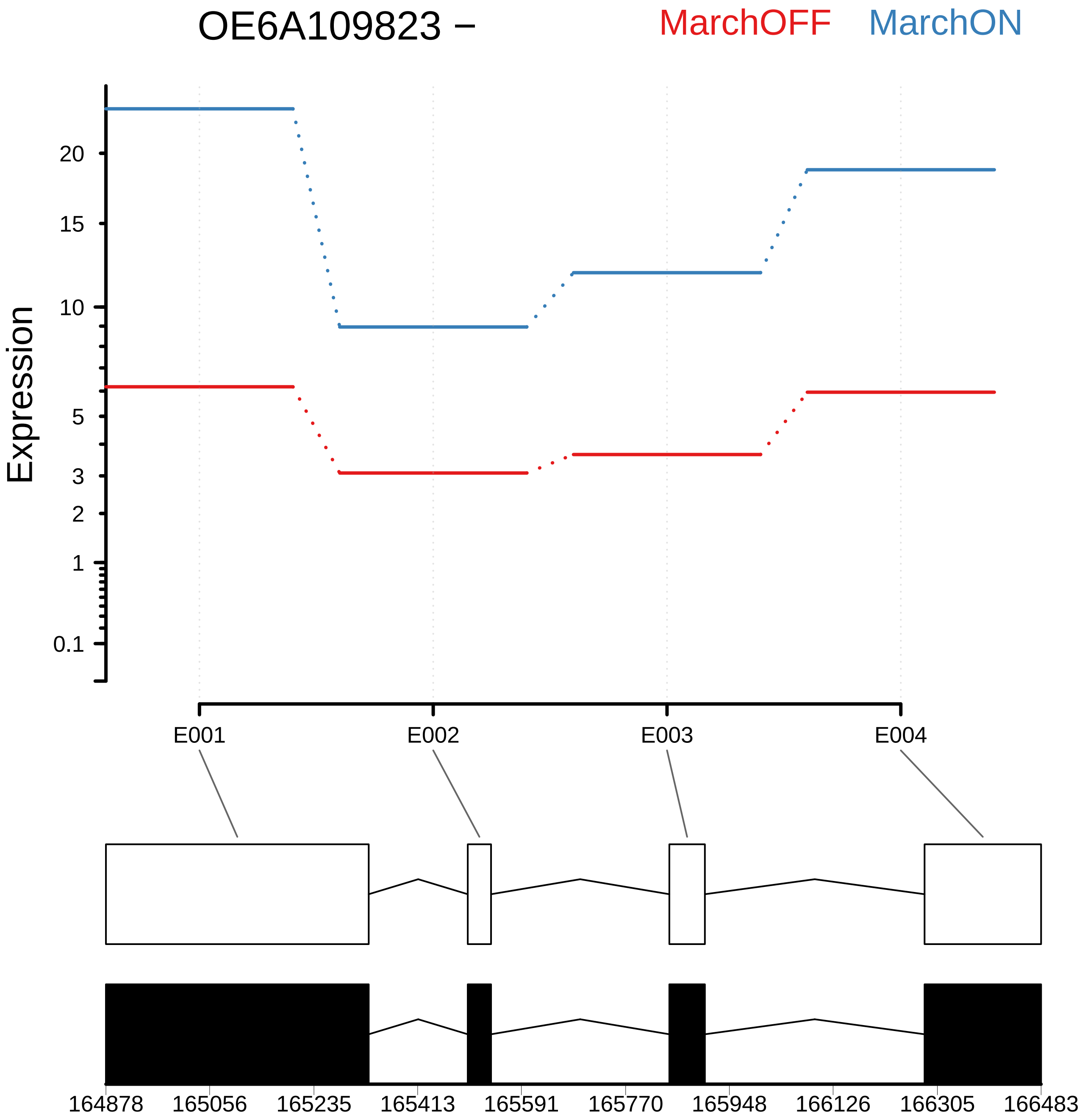

OE6A109823 −
MarchOFF
MarchON
Expression
20
15
10
5
3
2
1
0.1
E001
E002
E003
E004
164878
165056
165235
165413
165591
165770
165948
166126
166305
166483

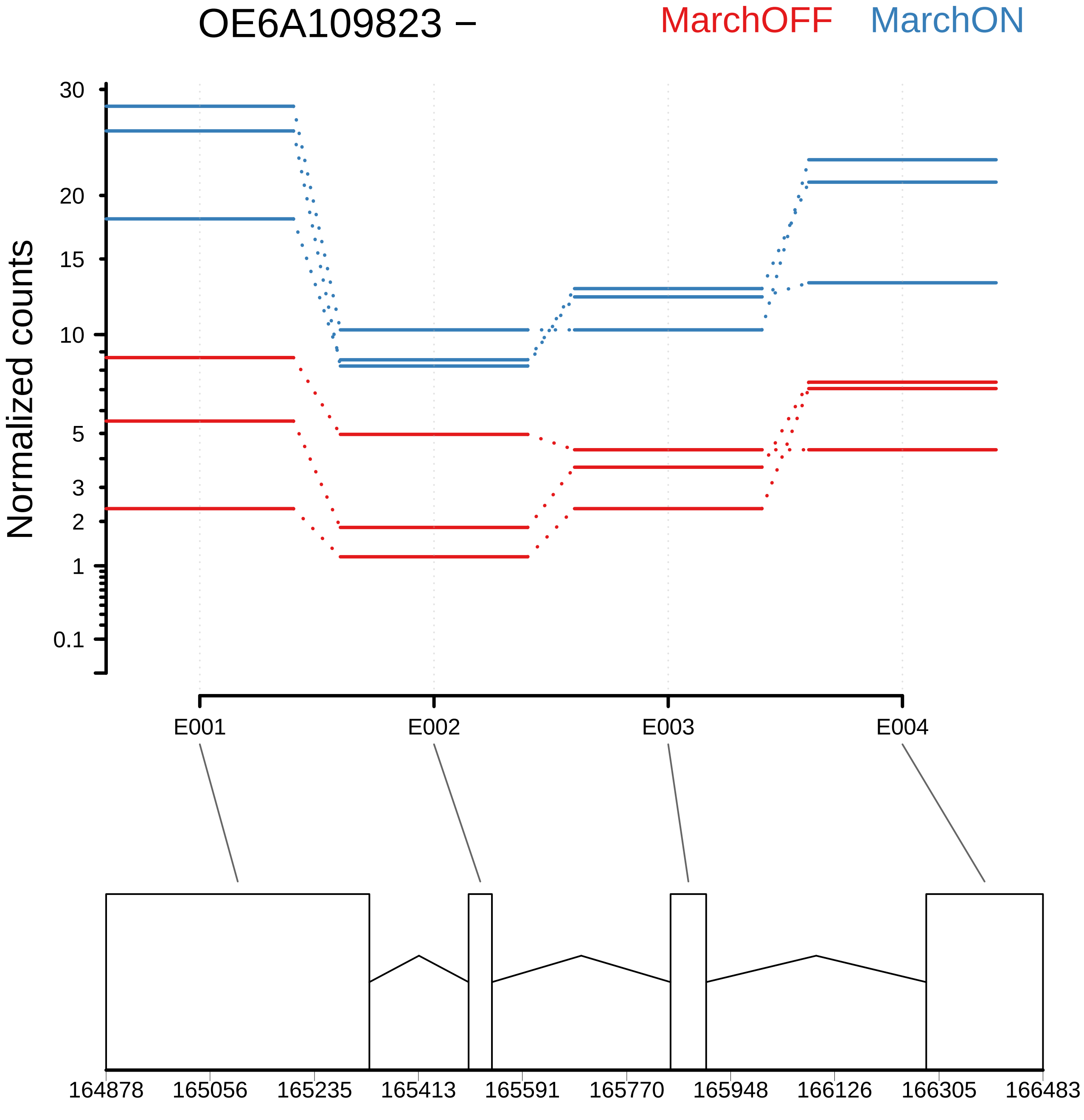

OE6A109823 −
MarchOFF
MarchON
Normalized counts
30
20
15
10
5
3
2
1
0.1
E001
E002
E003
E004
164878
165056
165235
165413
165591
165770
165948
166126
166305
166483

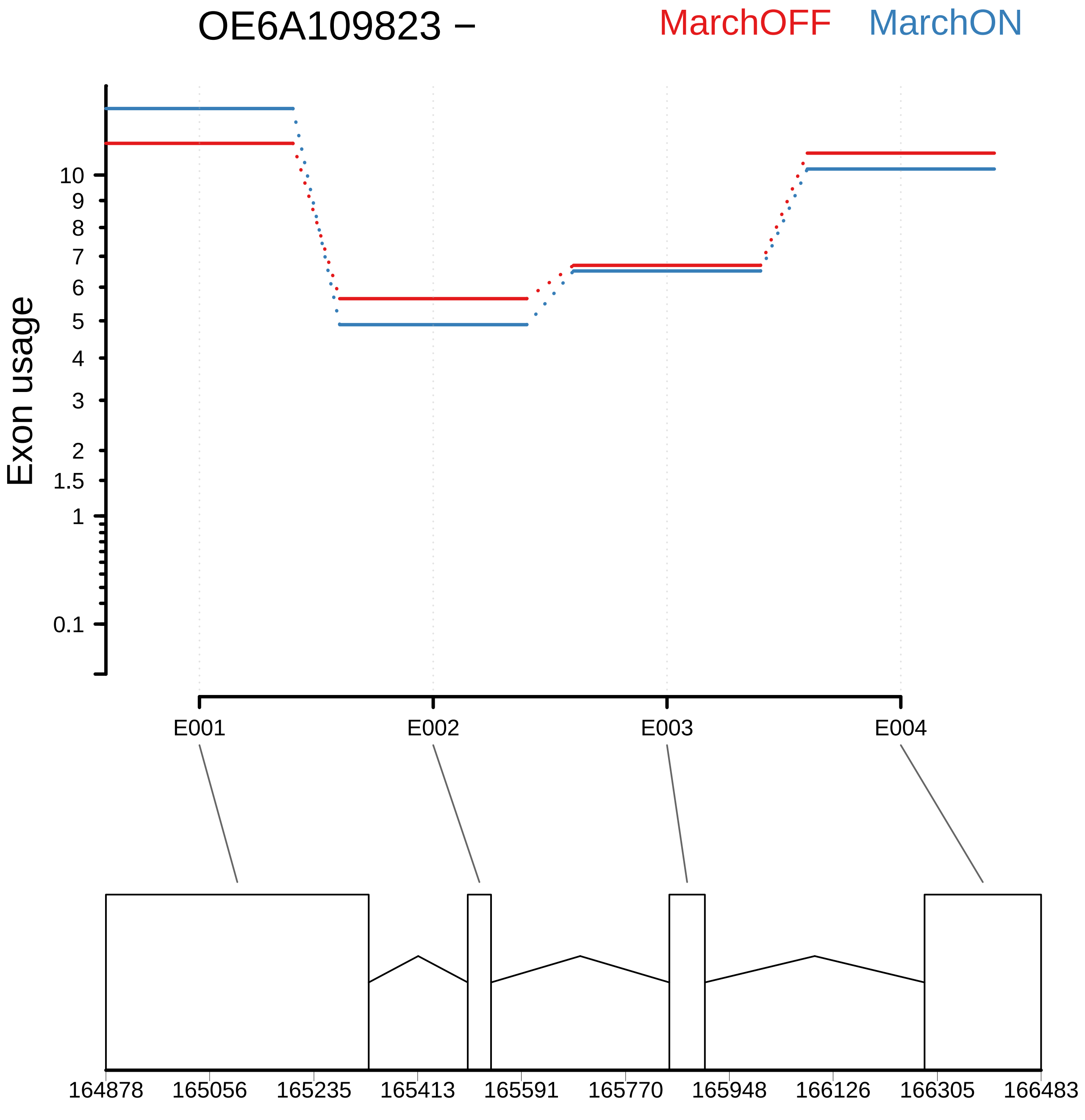

OE6A109823 −
MarchOFF
MarchON
Exon usage
10
9
8
7
6
5
4
3
2
1.5
1
0.1
E001
E002
E003
E004
164878
165056
165235
165413
165591
165770
165948
166126
166305
166483

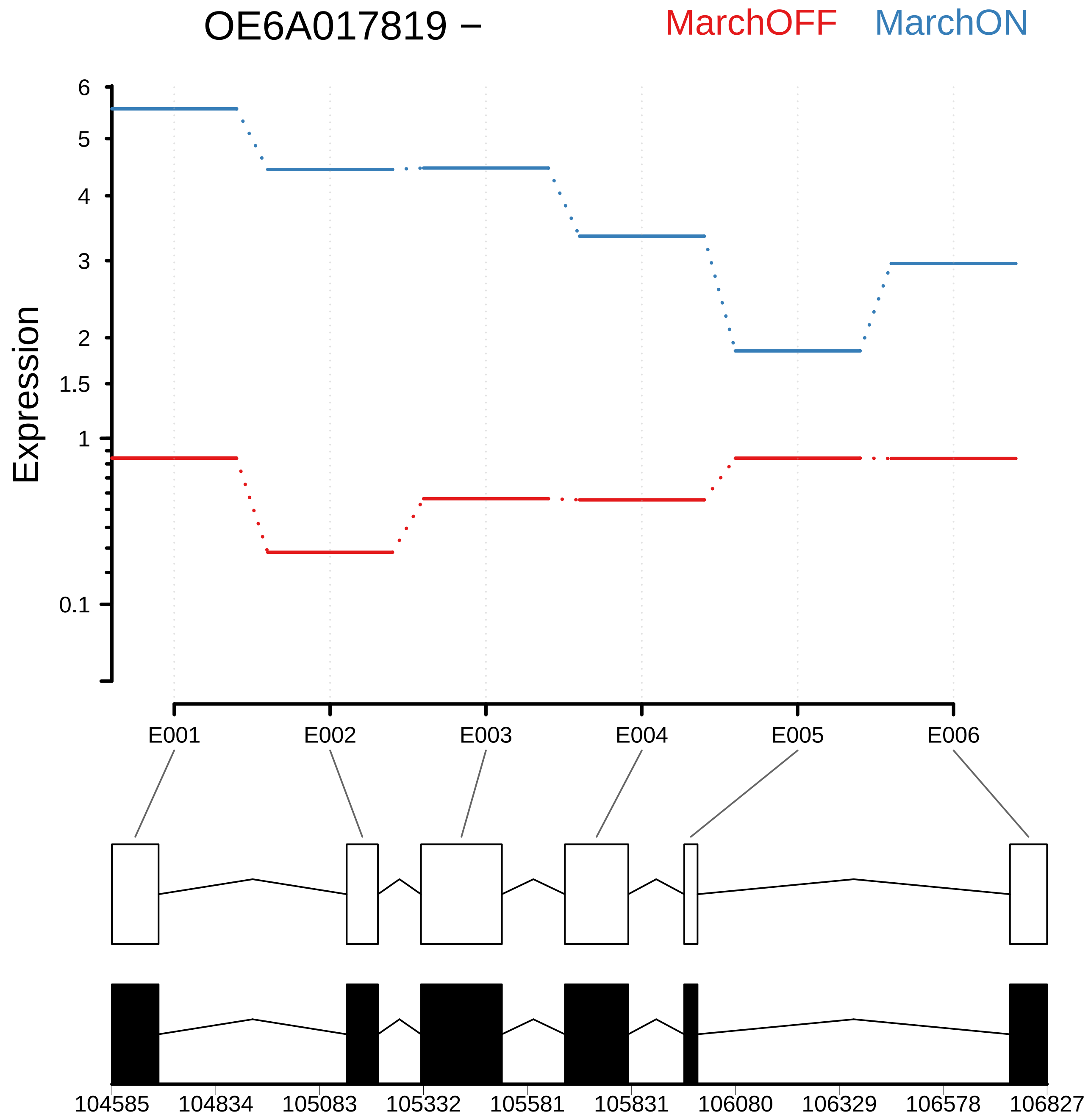

OE6A017819 –
MarchOFF
MarchON
Expression
6
5
4
3
2
1.5
1
0.1
E001
E002
E003
E004
E005
E006
104585
104834
105083
105332
105581
105831
106080
106329
106578
106827

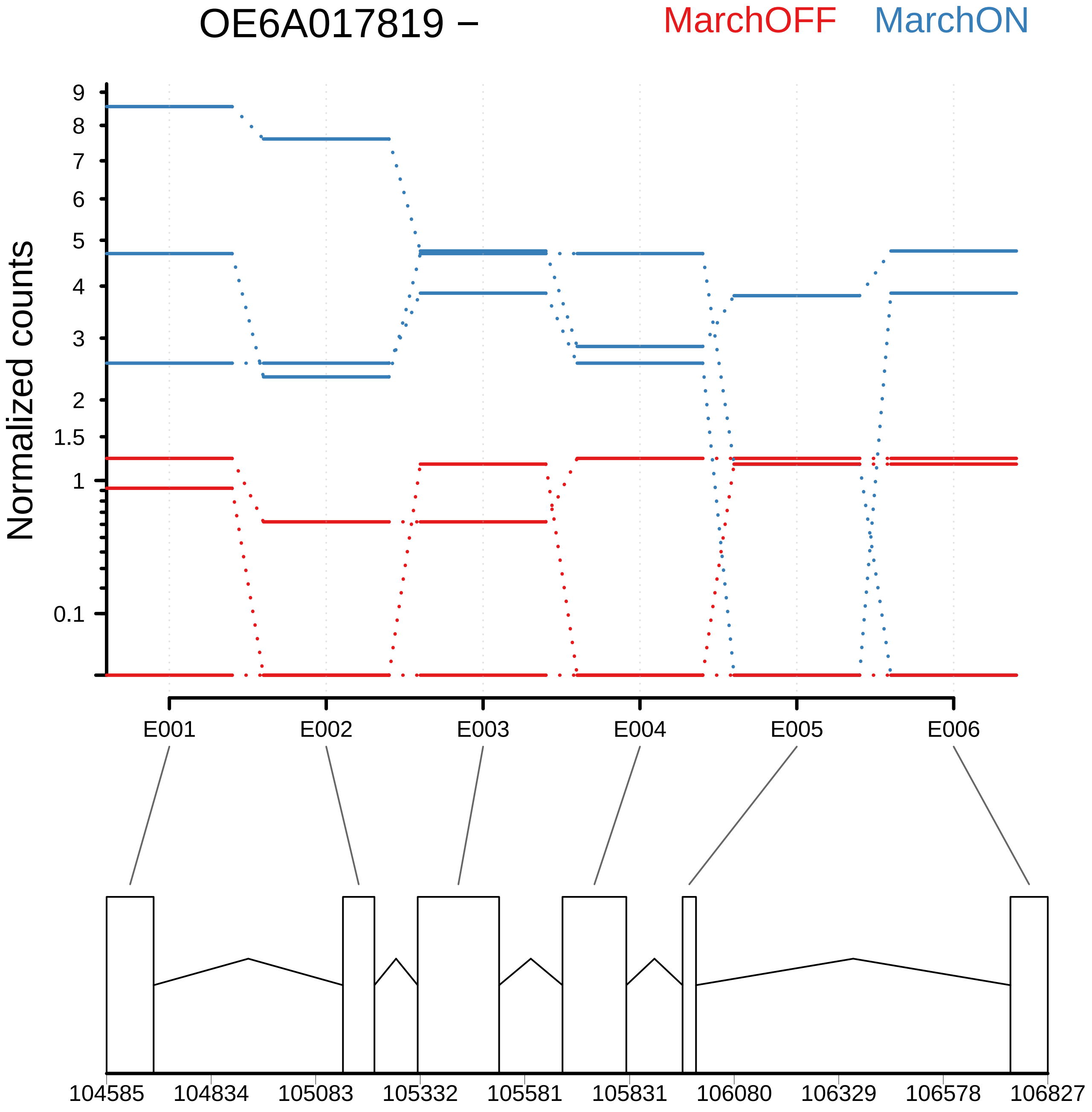
OE6A017819 −
MarchOFF
MarchON
Normalized counts
9
8
7
6
5
4
3
2
1.5
1
0.1
E001
E002
E003
E004
E005
E006
104585
104834
105083
105332
105581
105831
106080
106329
106578
106827

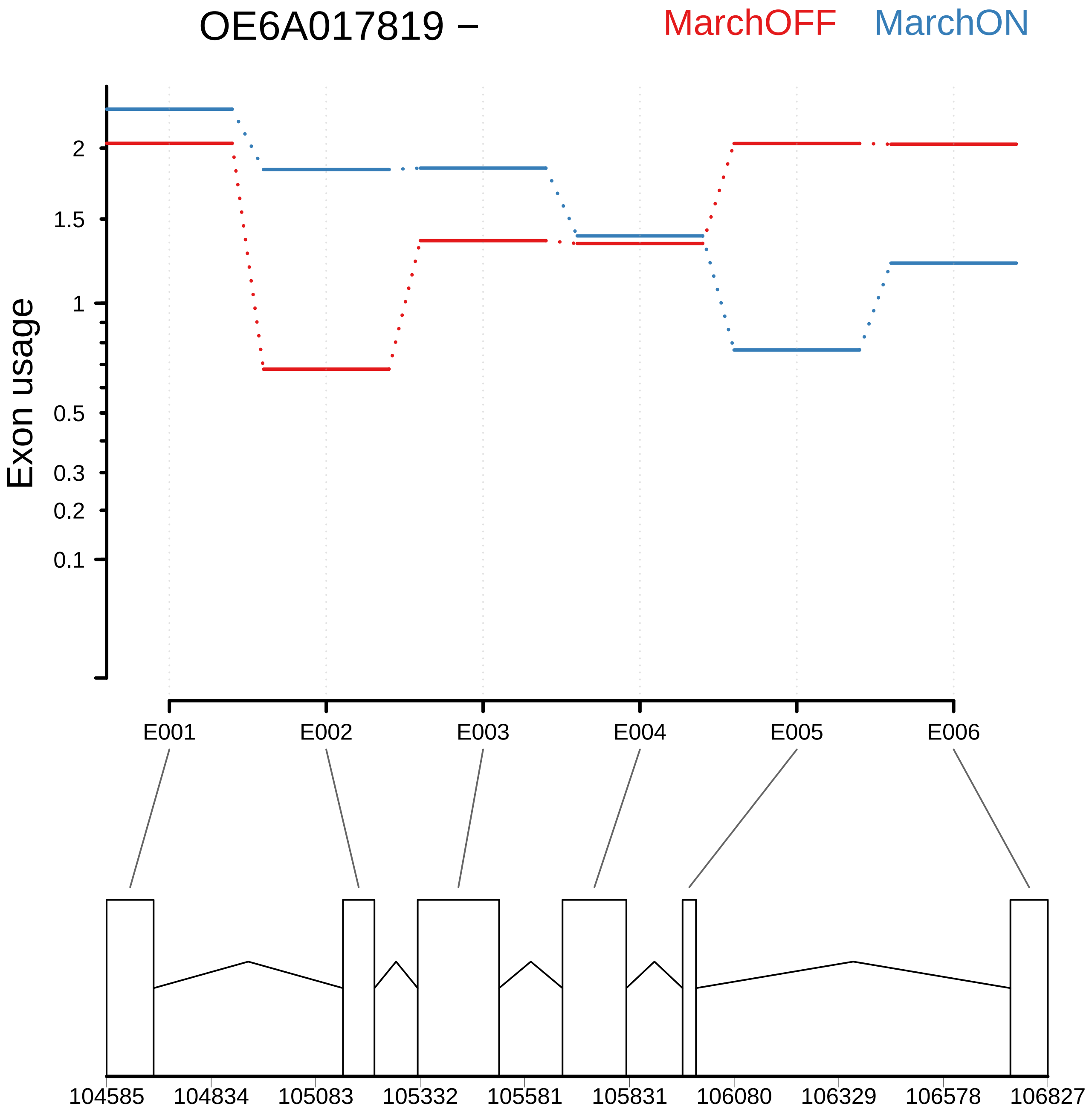

OE6A017819 −
MarchOFF
MarchON
Exon usage
2
1.5
1
0.5
0.3
0.2
0.1
E001
E002
E003
E004
E005
E006
104585
104834
105083
105332
105581
105831
106080
106329
106578
106827

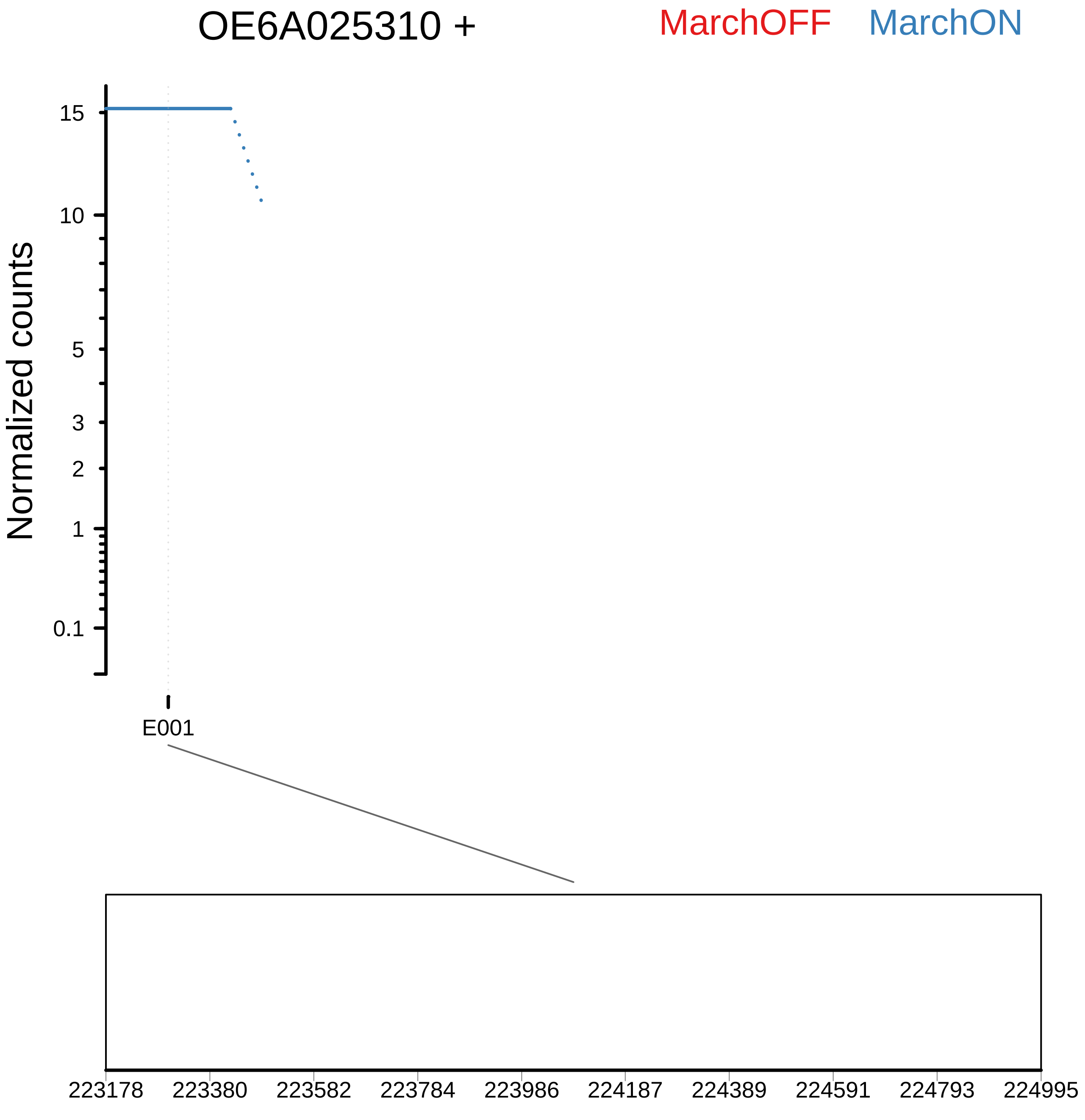
OE6A025310 +
MarchOFF
MarchON
Normalized counts
15
10
5
3
2
1
0.1
E001
223178
223380
223582
223784
223986
224187
224389
224591
224793
224995

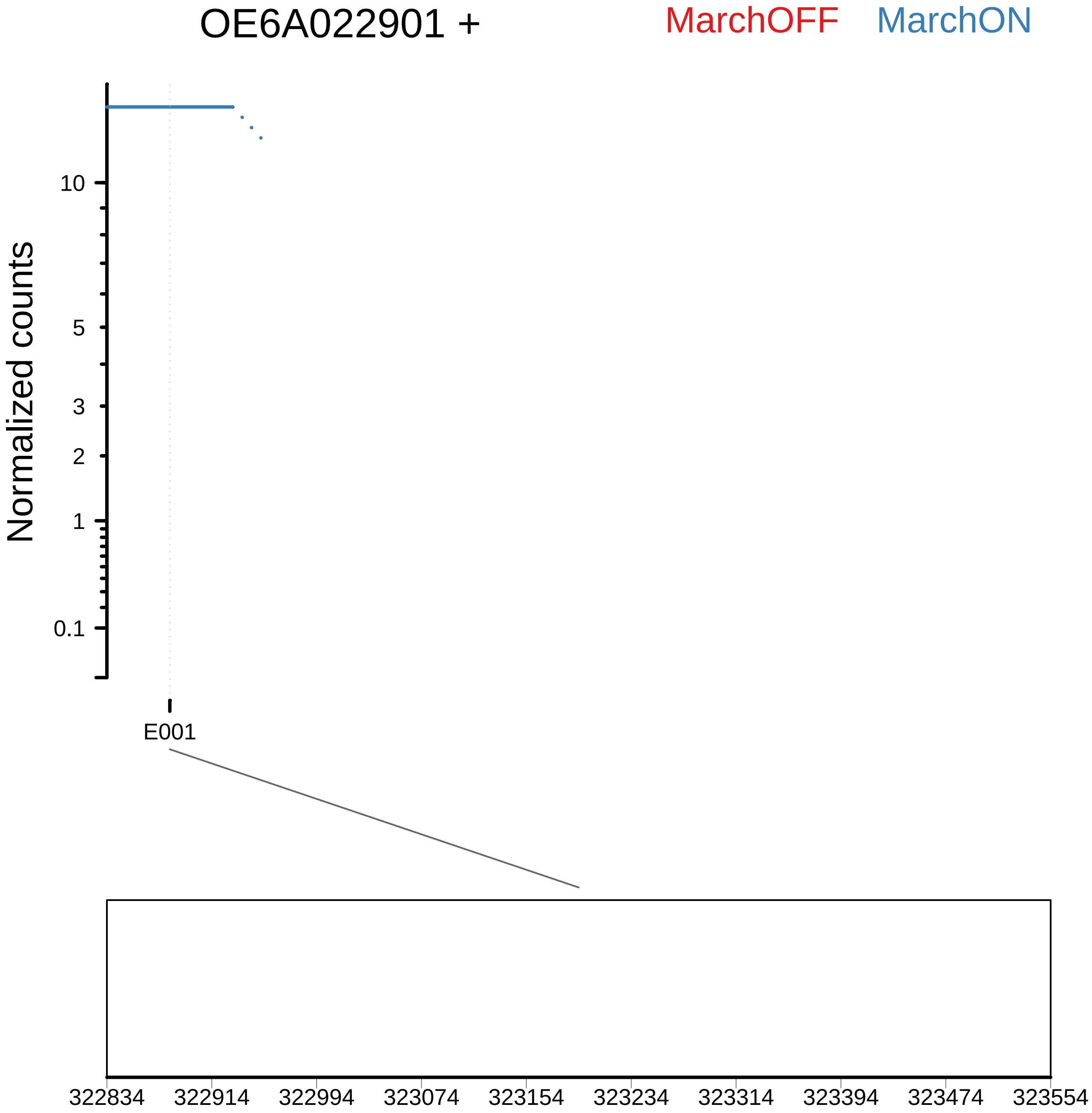
OE6A022901 +
MarchOFF
MarchON
Normalized counts
10
5
3
2
1
0.1
E001
322834
322914
322994
323074
323154
323234
323314
323394
323474
323554

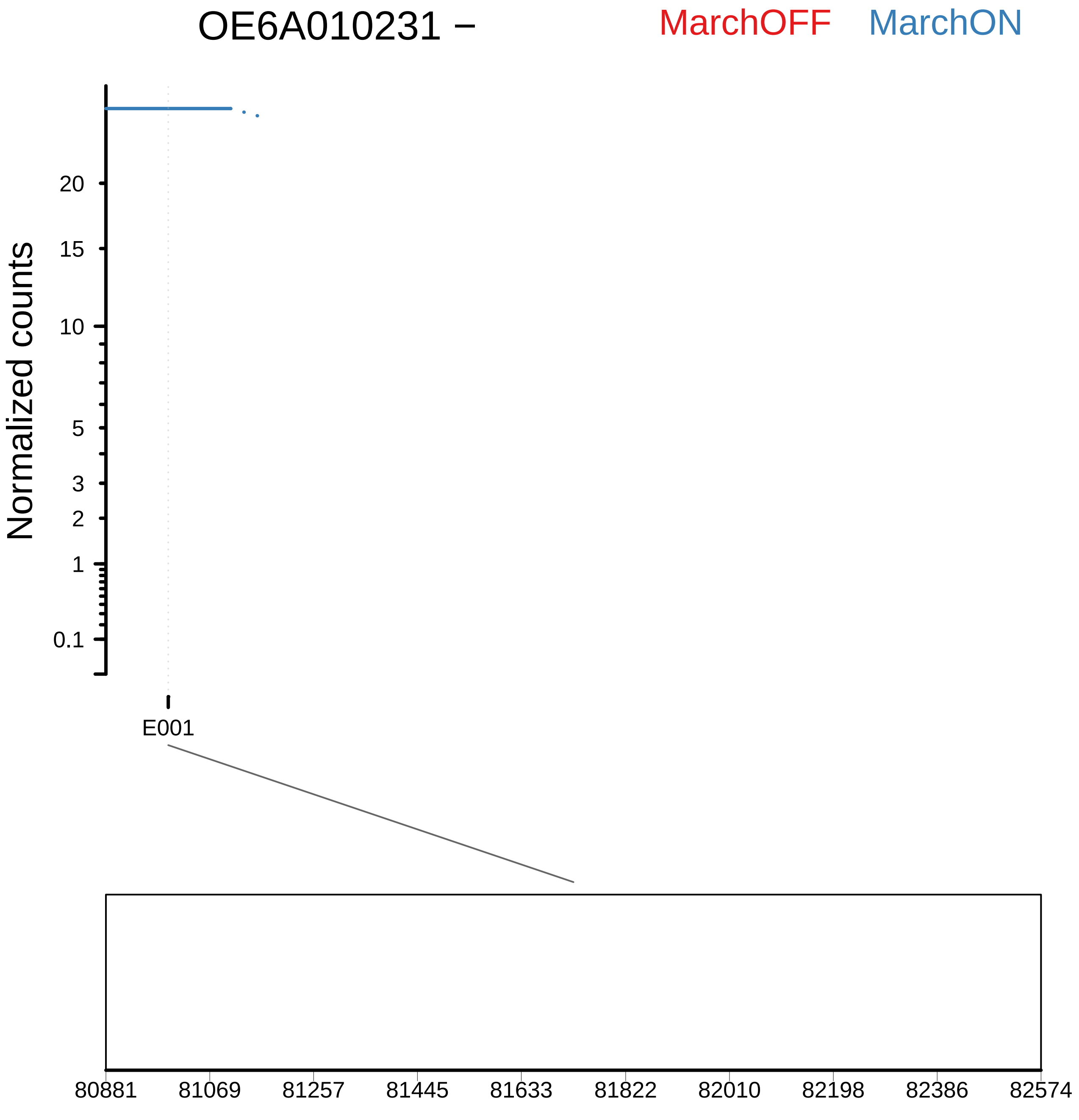
OE6A010231 −
MarchOFF
MarchON
Normalized counts
20
15
10
5
3
2
1
0.1
E001
80881
81069
81257
81445
81633
81822
82010
82198
82386
82574

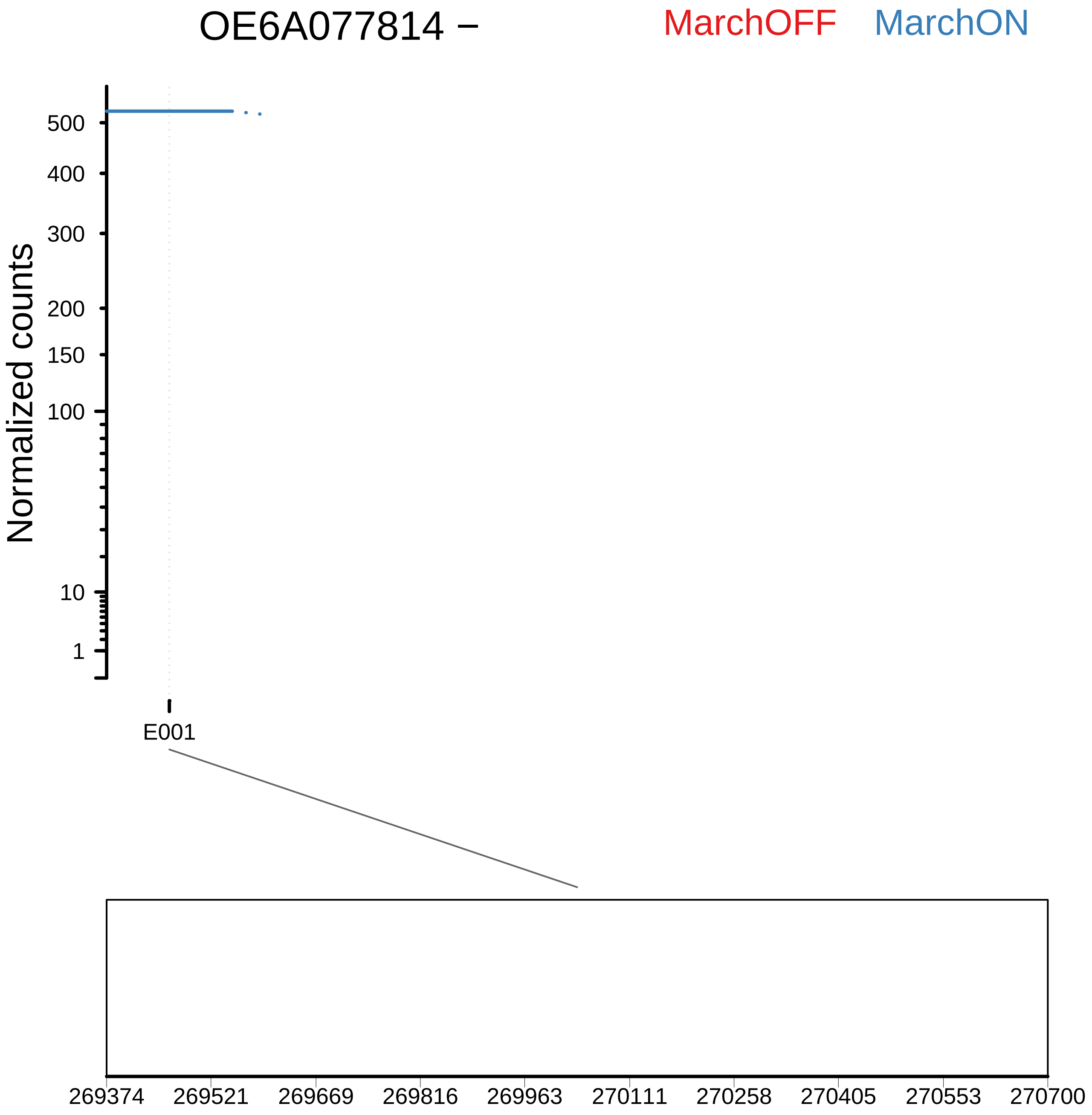

OE6A077814 −
MarchOFF
MarchON
Normalized counts
500
400
300
200
150
100
10
1
E001
269374
269521
269669
269816
269963
270111
270258
270405
270553
270700

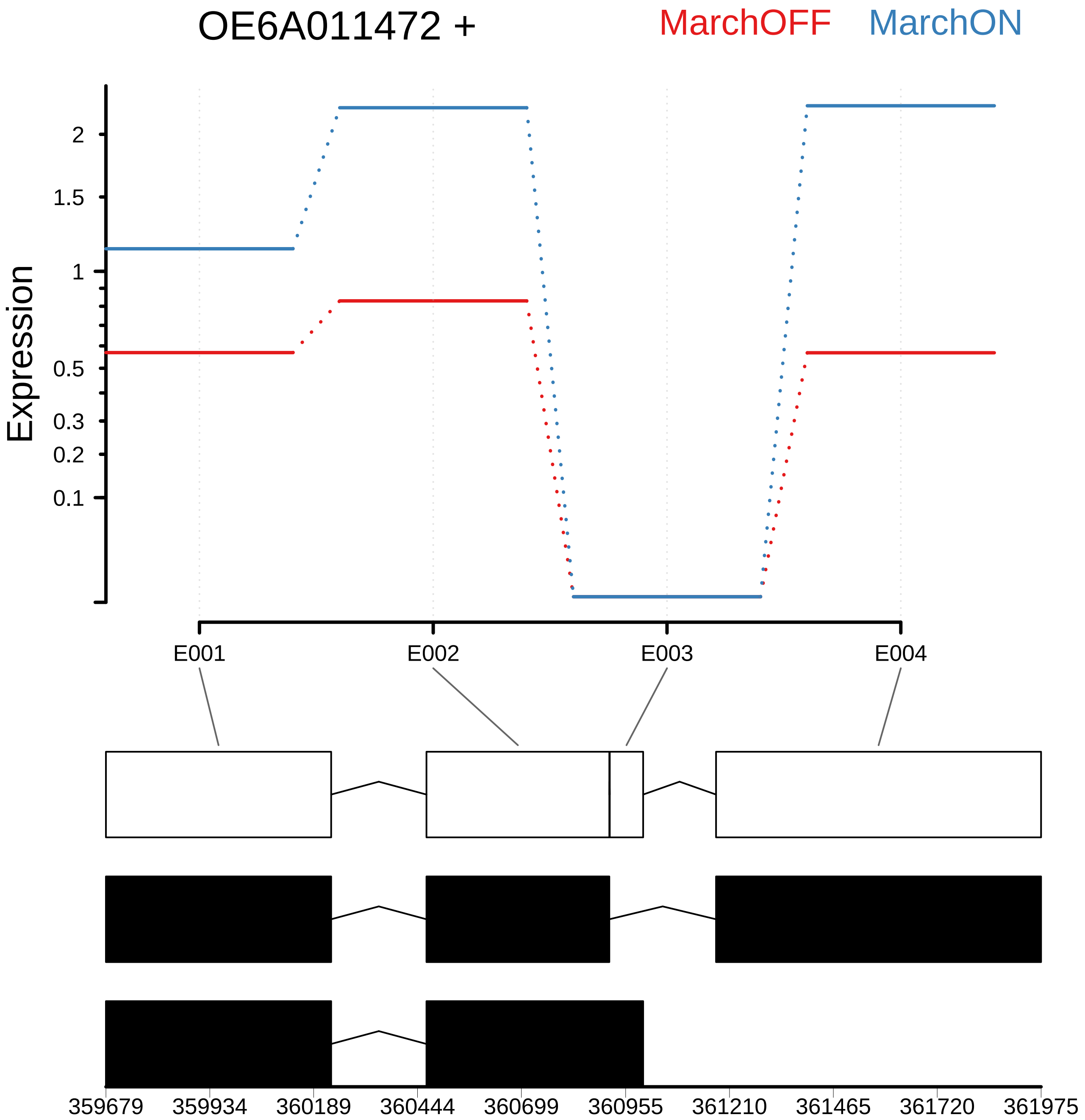
OE6A011472 +
MarchOFF
MarchON
Expression
2
1.5
1
0.5
0.3
0.2
0.1
E001
E002
E003
E004
359679
359934
360189
360444
360699
360955
361210
361465
361720
361975

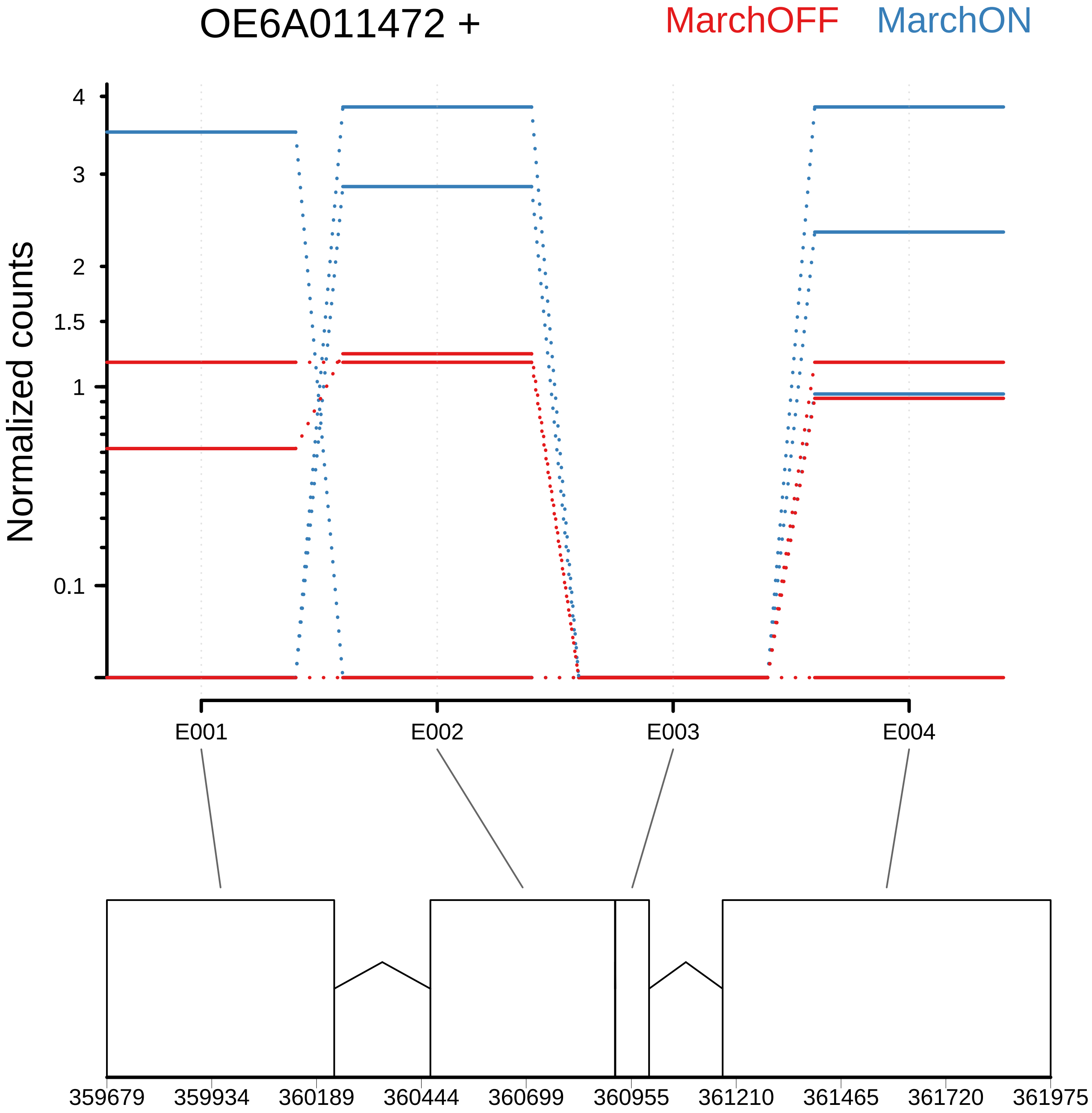

OE6A011472 +
MarchOFF
MarchON
Normalized counts
4
3
2
1.5
1
0.1
E001
E002
E003
E004
359679
359934
360189
360444
360699
360955
361210
361465
361720
361975

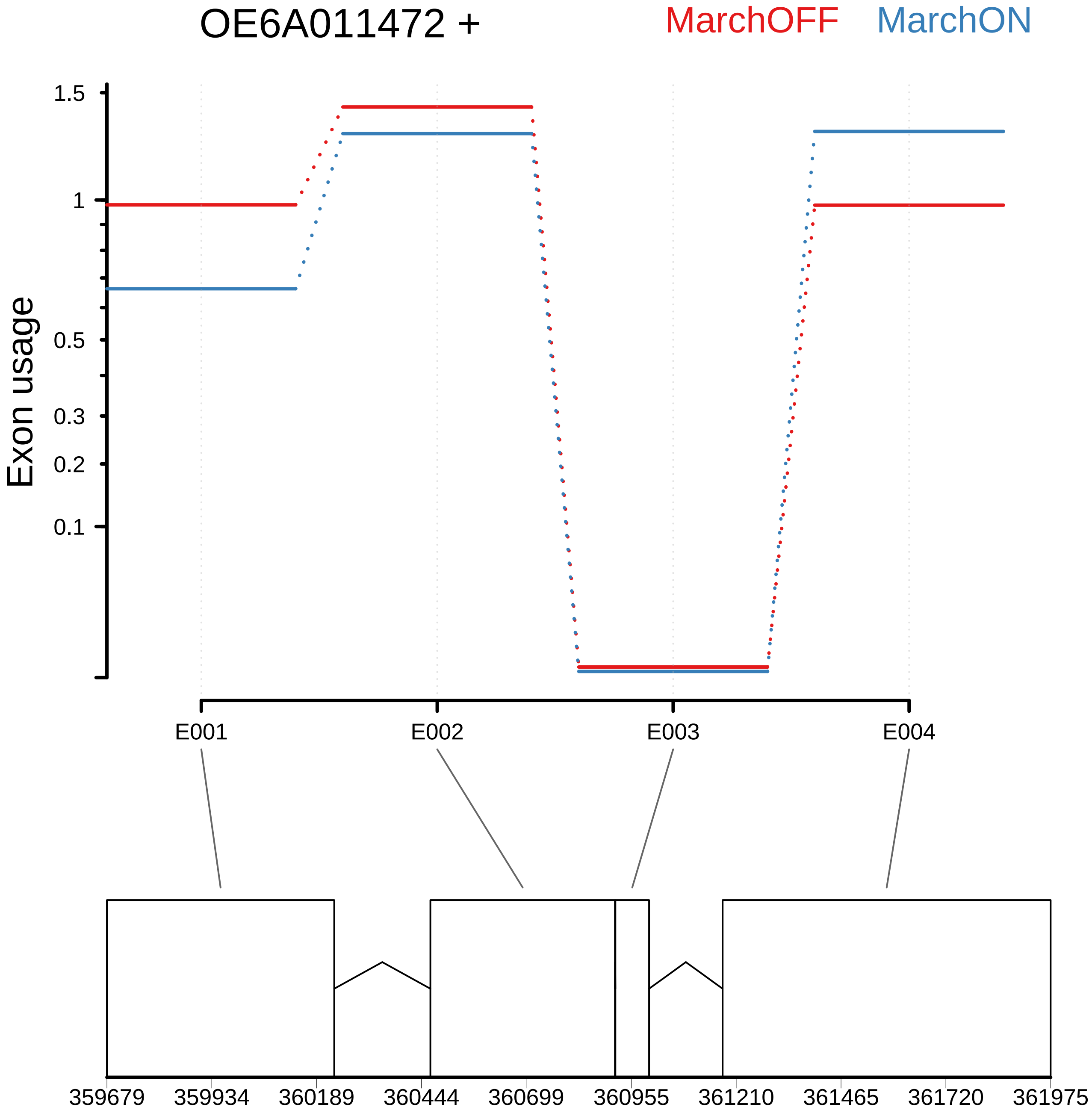

OE6A011472 +
MarchOFF
MarchON
Exon usage
1.5
1
0.5
0.3
0.2
0.1
E001
E002
E003
E004
359679
359934
360189
360444
360699
360955
361210
361465
361720
361975

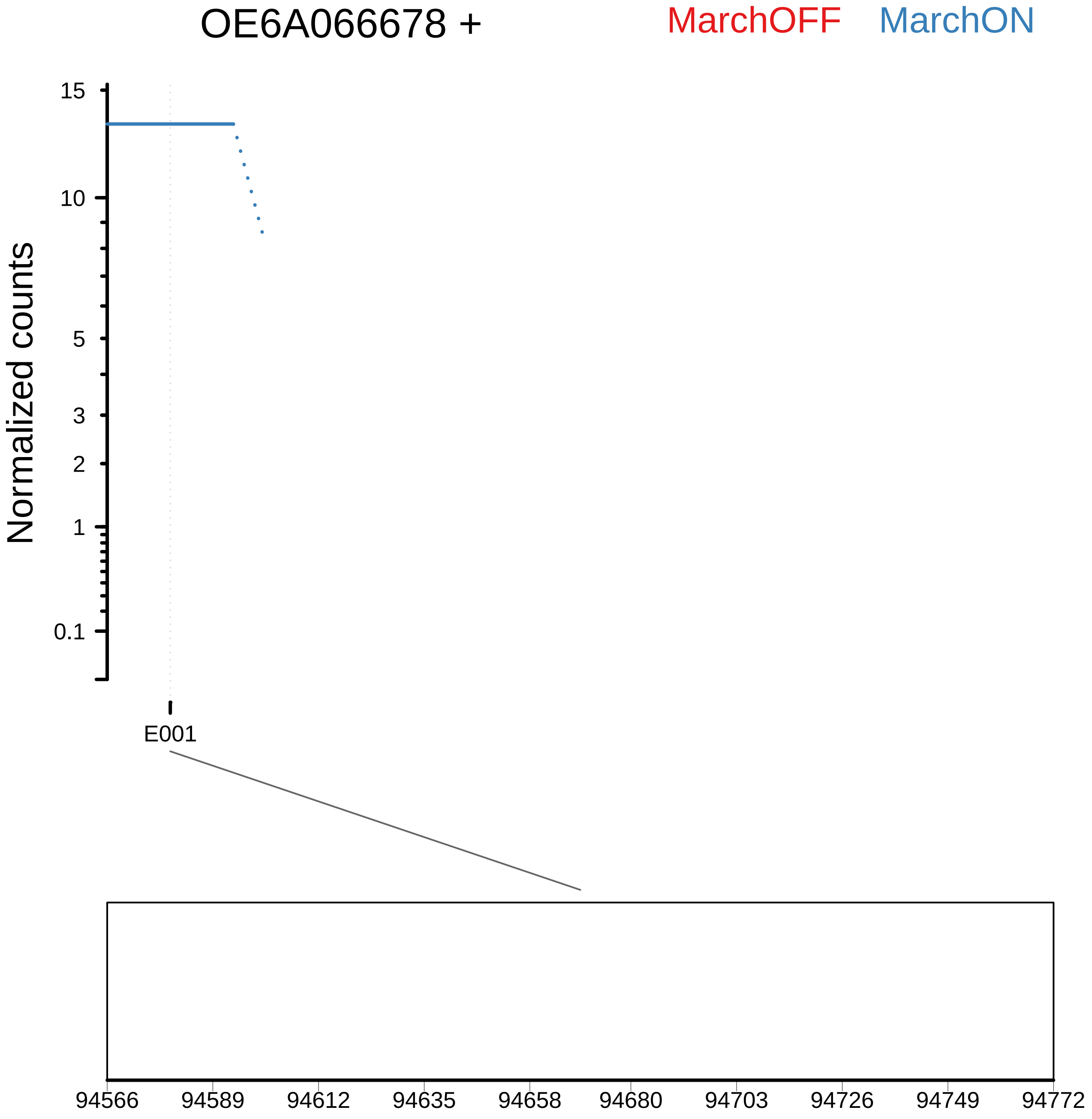

OE6A066678 +
MarchOFF
MarchON
Normalized counts
15
10
5
3
2
1
0.1
E001
94566
94589
94612
94635
94658
94680
94703
94726
94749
94772

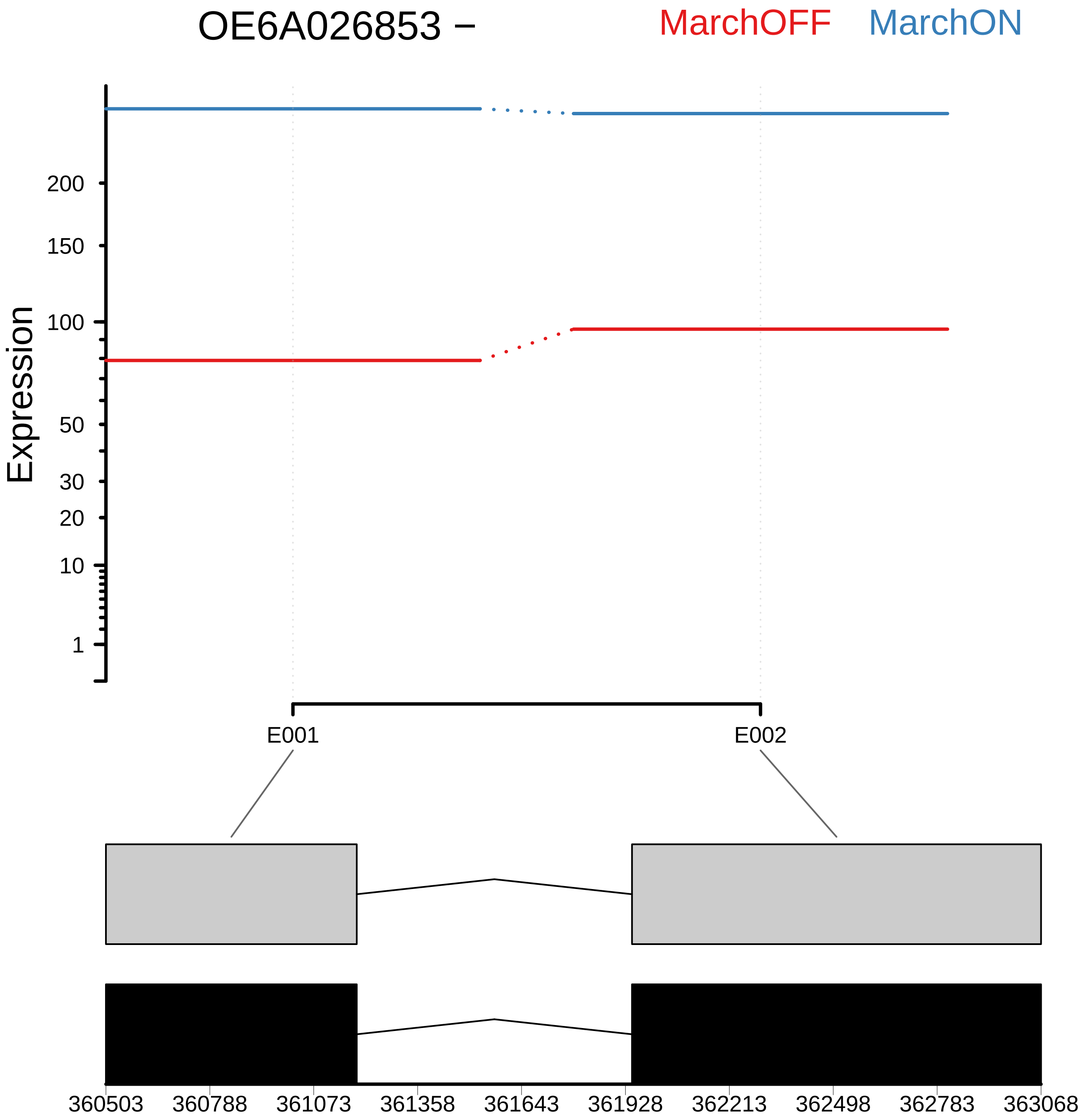
OE6A026853 −
MarchOFF
MarchON
Expression
200
150
100
50
30
20
10
1
E001
E002
360503
360788
361073
361358
361643
361928
362213
362498
362783
363068

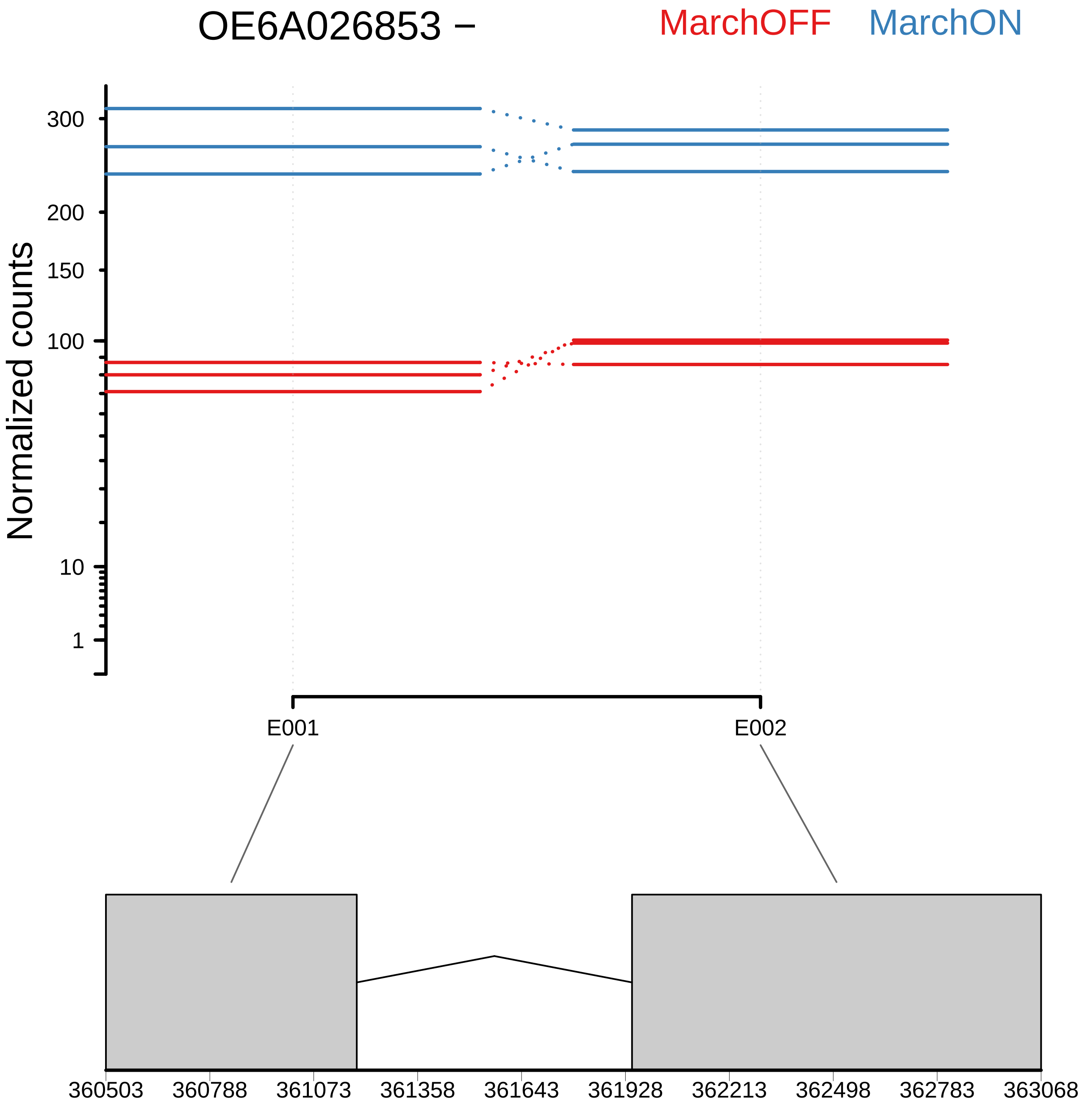

OE6A026853 −
MarchOFF
MarchON
Normalized counts
300
200
150
100
10
1
E001
E002
360503
360788
361073
361358
361643
361928
362213
362498
362783
363068

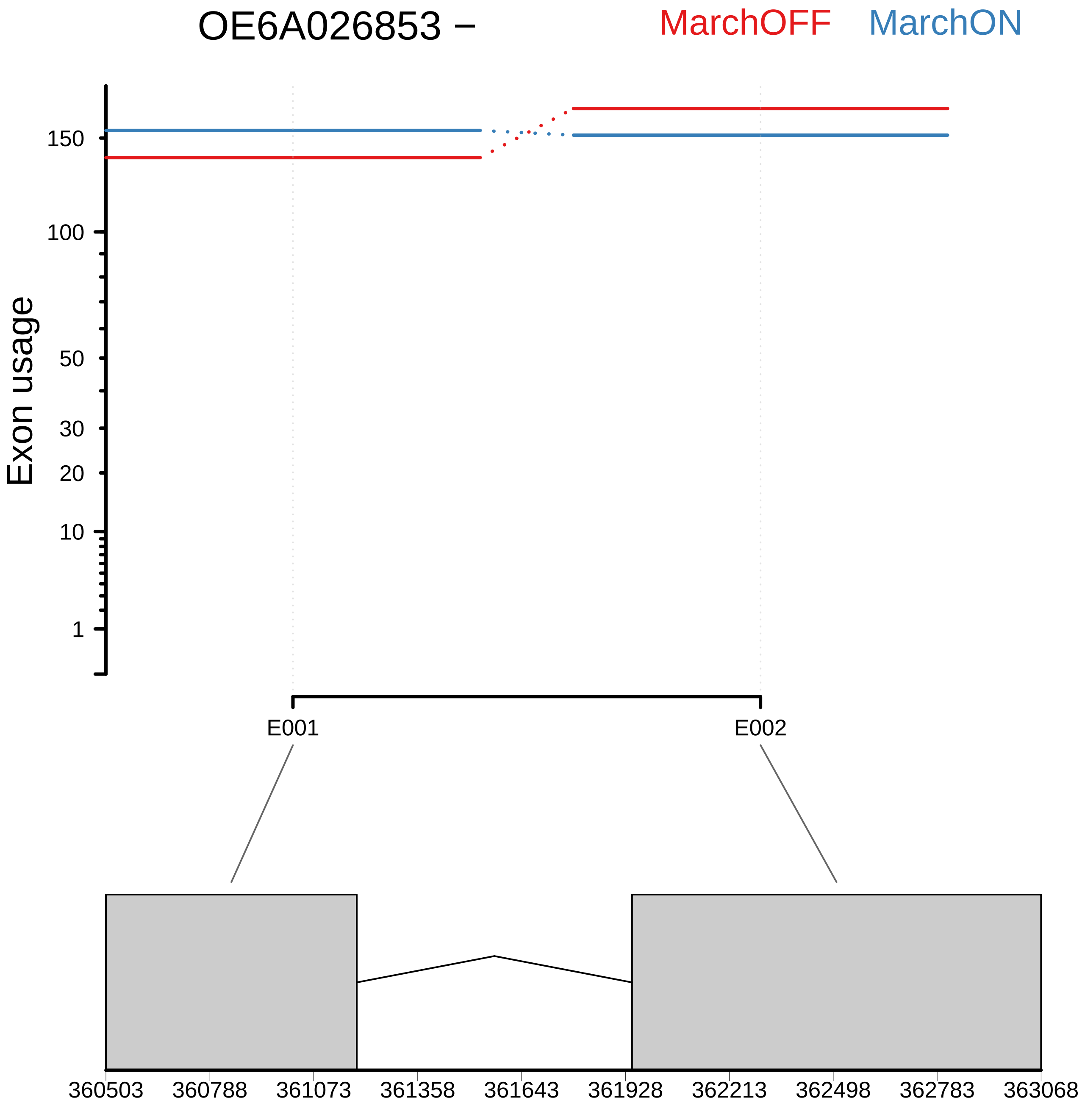

OE6A026853 −
MarchOFF
MarchON
Exon usage
150
100
50
30
20
10
1
E001
E002
360503
360788
361073
361358
361643
361928
362213
362498
362783
363068

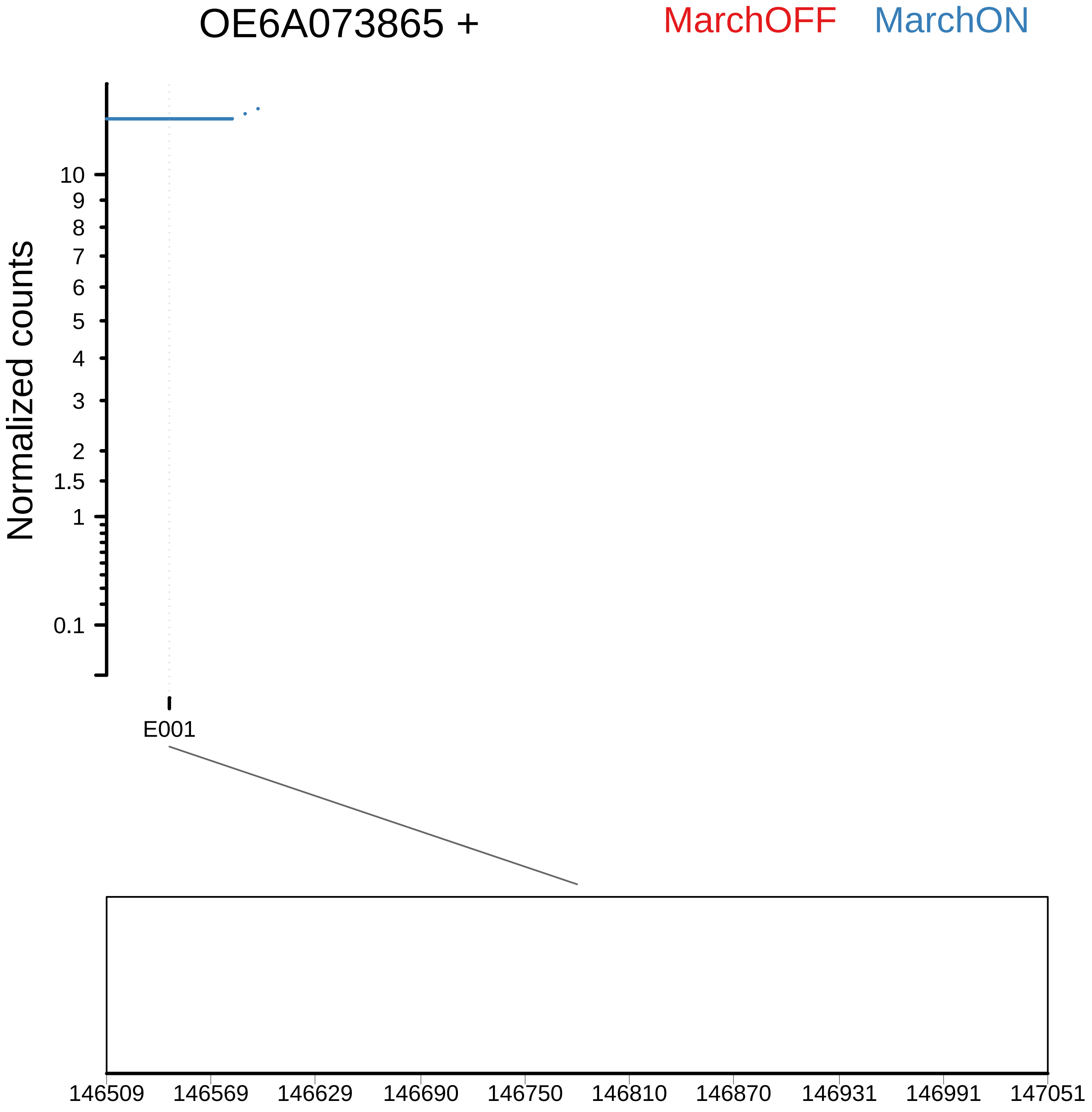

OE6A073865 +
MarchOFF
MarchON
Normalized counts
10
9
8
7
6
5
4
3
2
1.5
1
0.1
E001
146509
146569
146629
146690
146750
146810
146870
146931
146991
147051

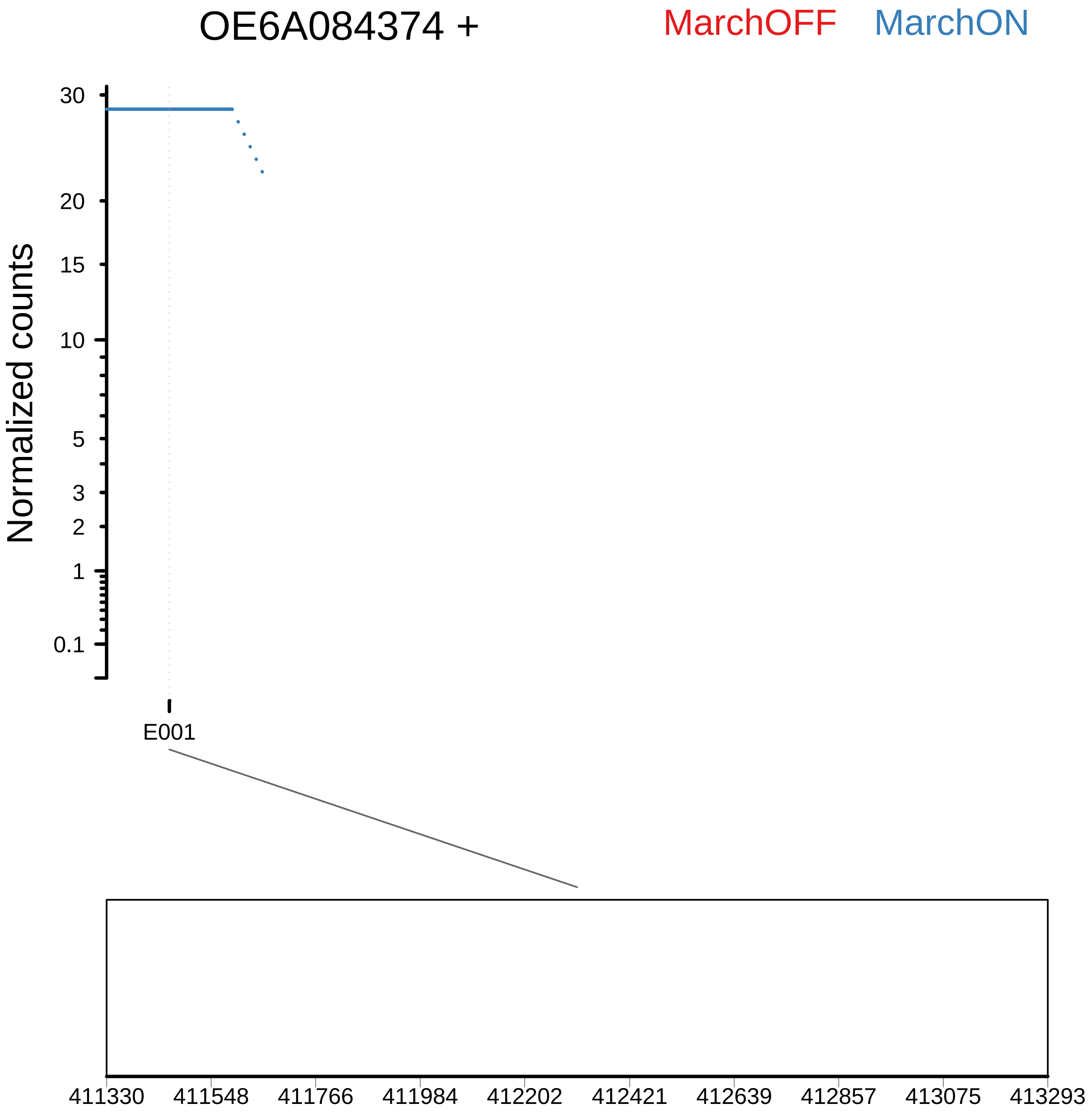

OE6A084374 +
MarchOFF
MarchON
Normalized counts
30
20
15
10
5
3
2
1
0.1
E001
411330
411548
411766
411984
412202
412421
412639
412857
413075
413293

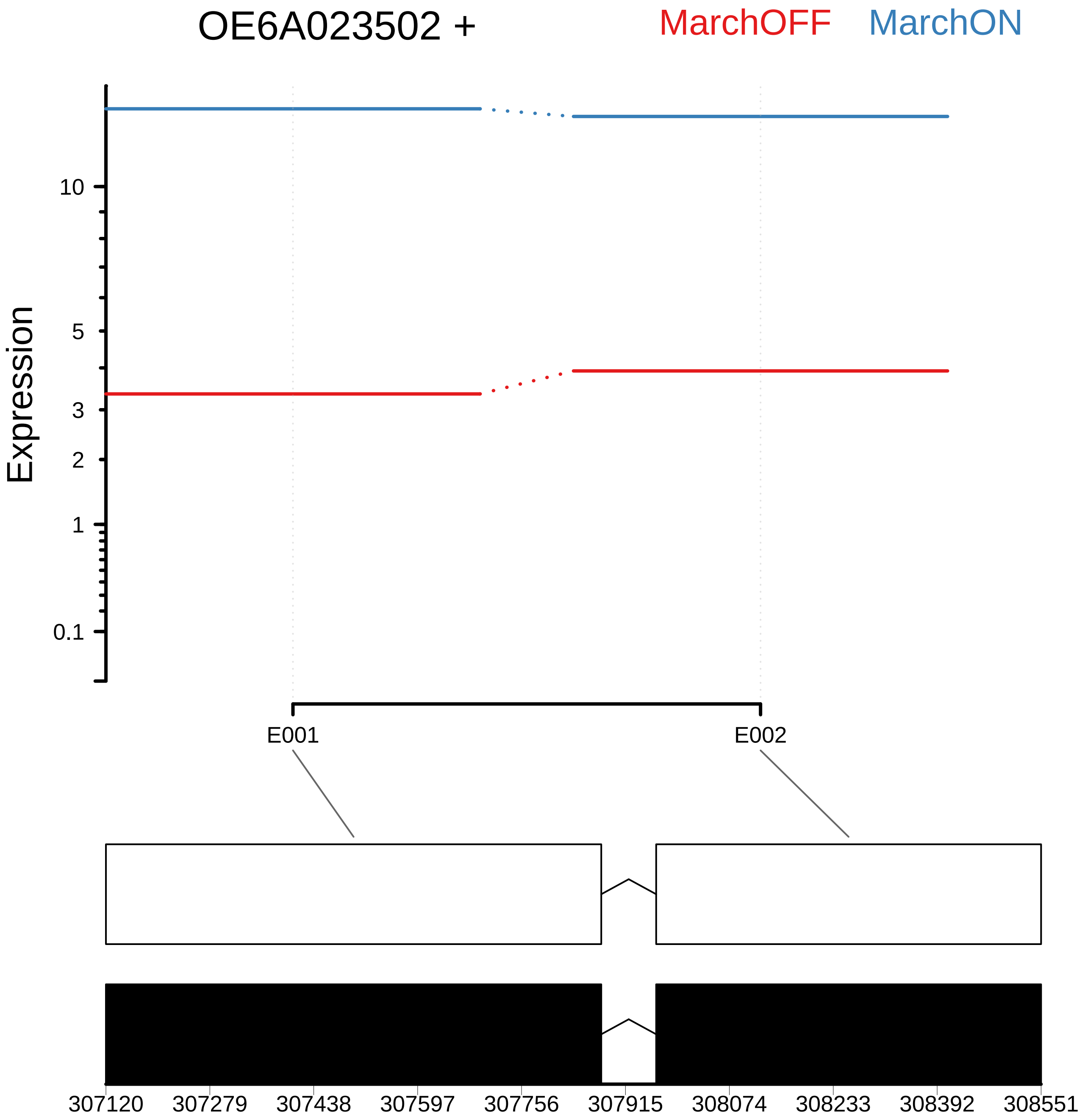

OE6A023502 +
MarchOFF
MarchON
Expression
10
5
3
2
1
0.1
E001
E002
307120
307279
307438
307597
307756
307915
308074
308233
308392
308551

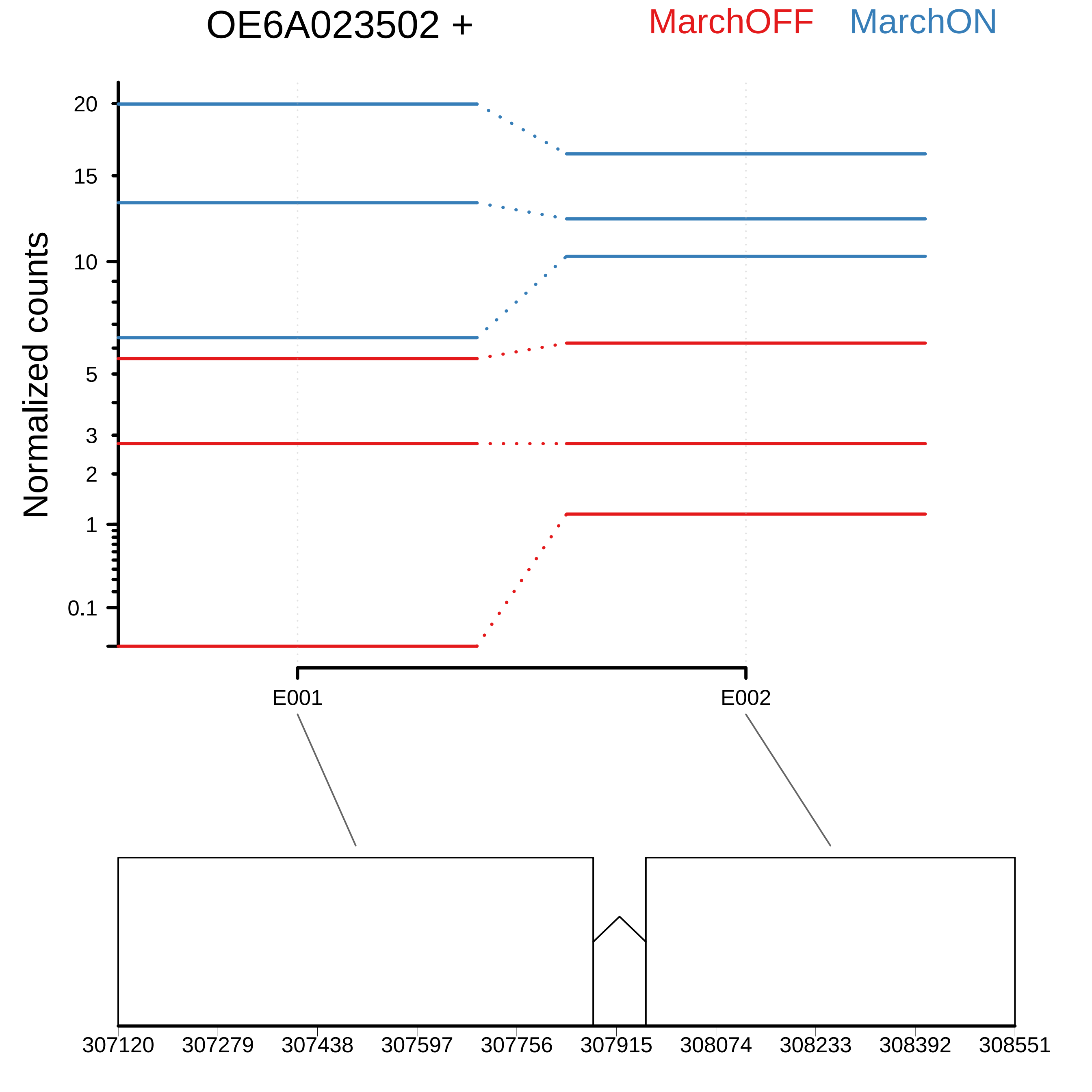
OE6A023502 +
MarchOFF
MarchON
Normalized counts
20
15
10
5
3
2
1
0.1
E001
E002
307120
307279
307438
307597
307756
307915
308074
308233
308392
308551

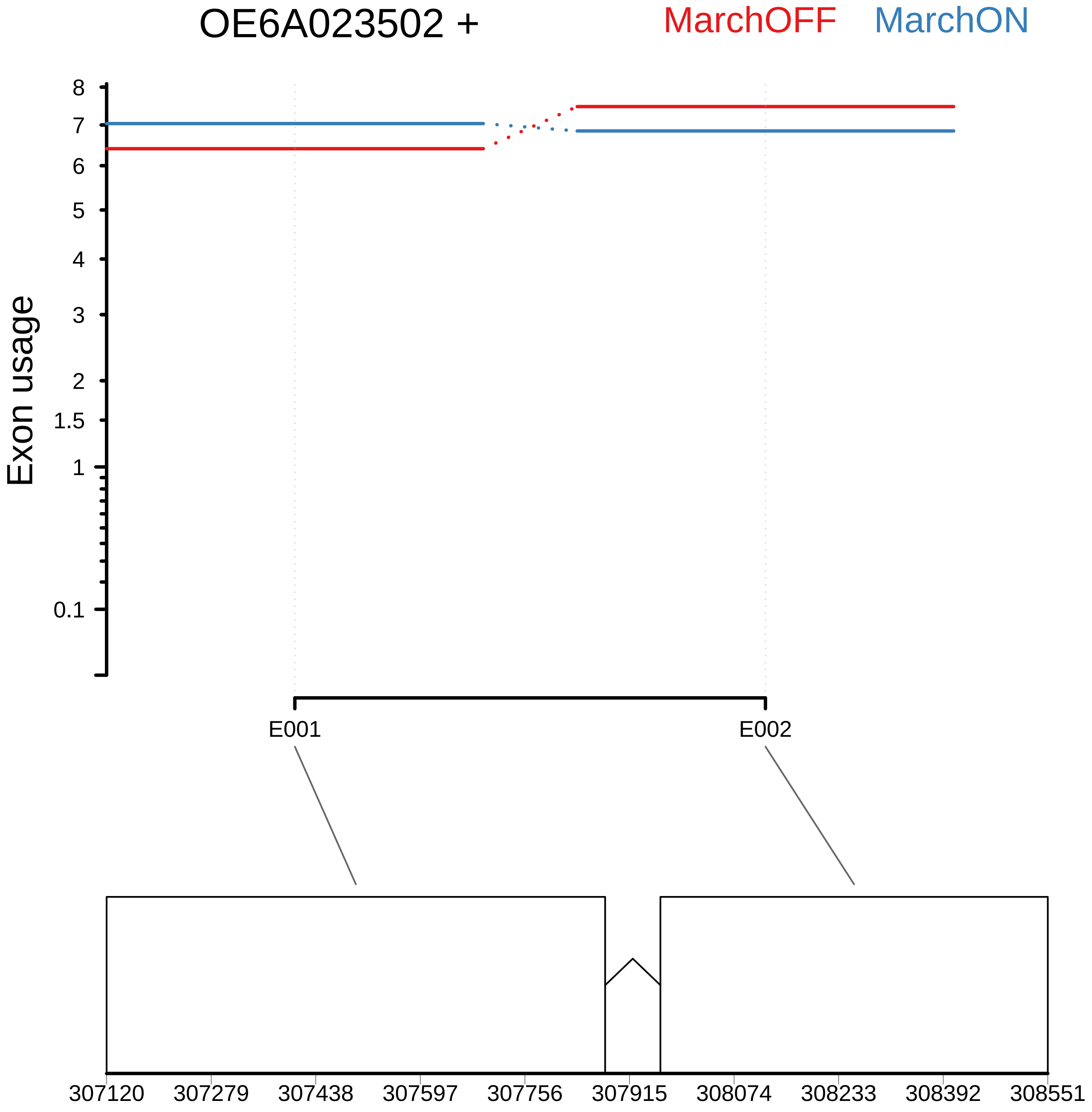
OE6A023502 +
MarchOFF
MarchON
Exon usage
8
7
6
5
4
3
2
1.5
1
0.1
E001
E002
307120
307279
307438
307597
307756
307915
308074
308233
308392
308551

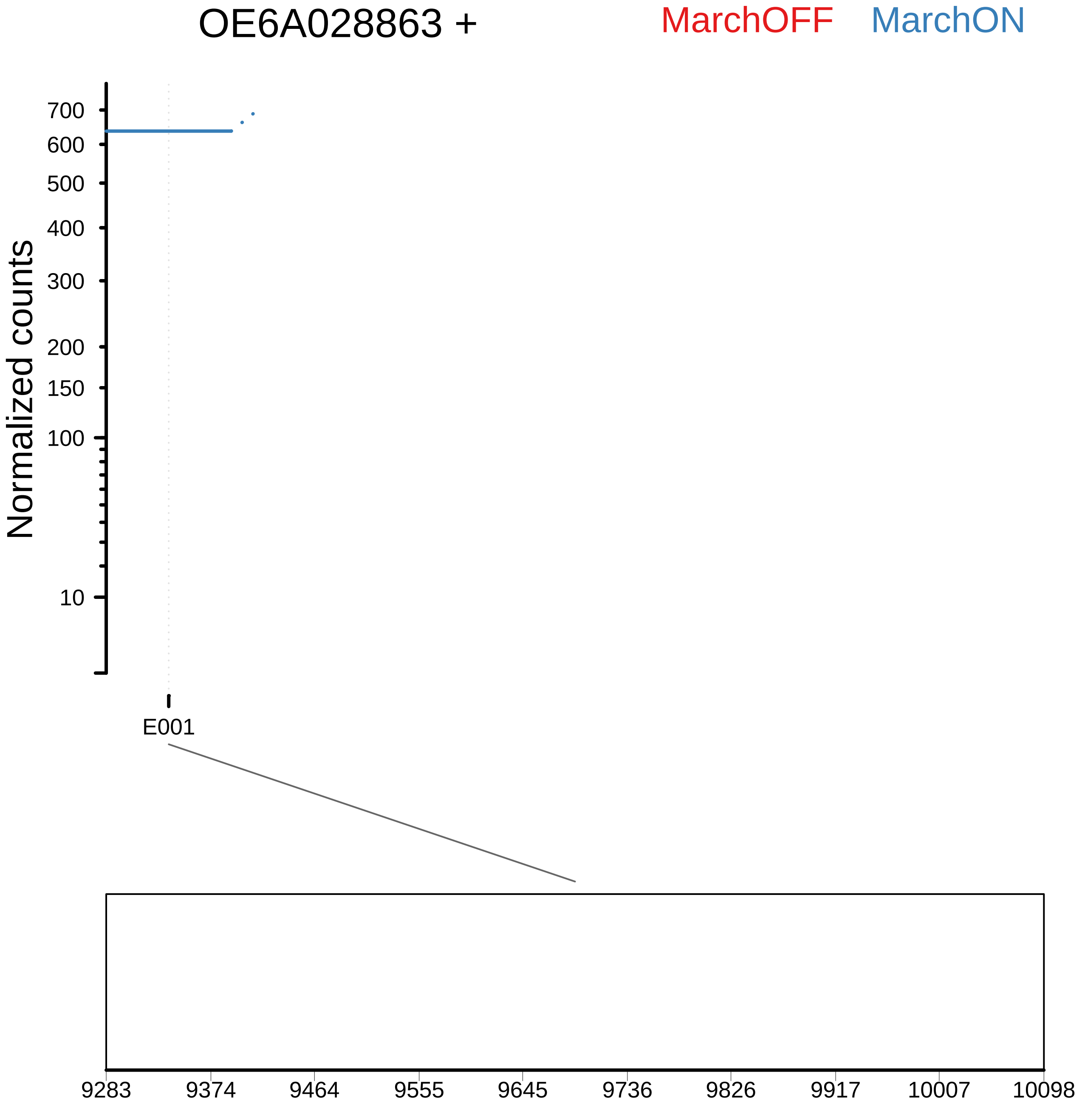
OE6A028863 +
MarchOFF
MarchON
Normalized counts
700
600
500
400
300
200
150
100
10
E001
9283
9374
9464
9555
9645
9736
9826
9917
10007
10098

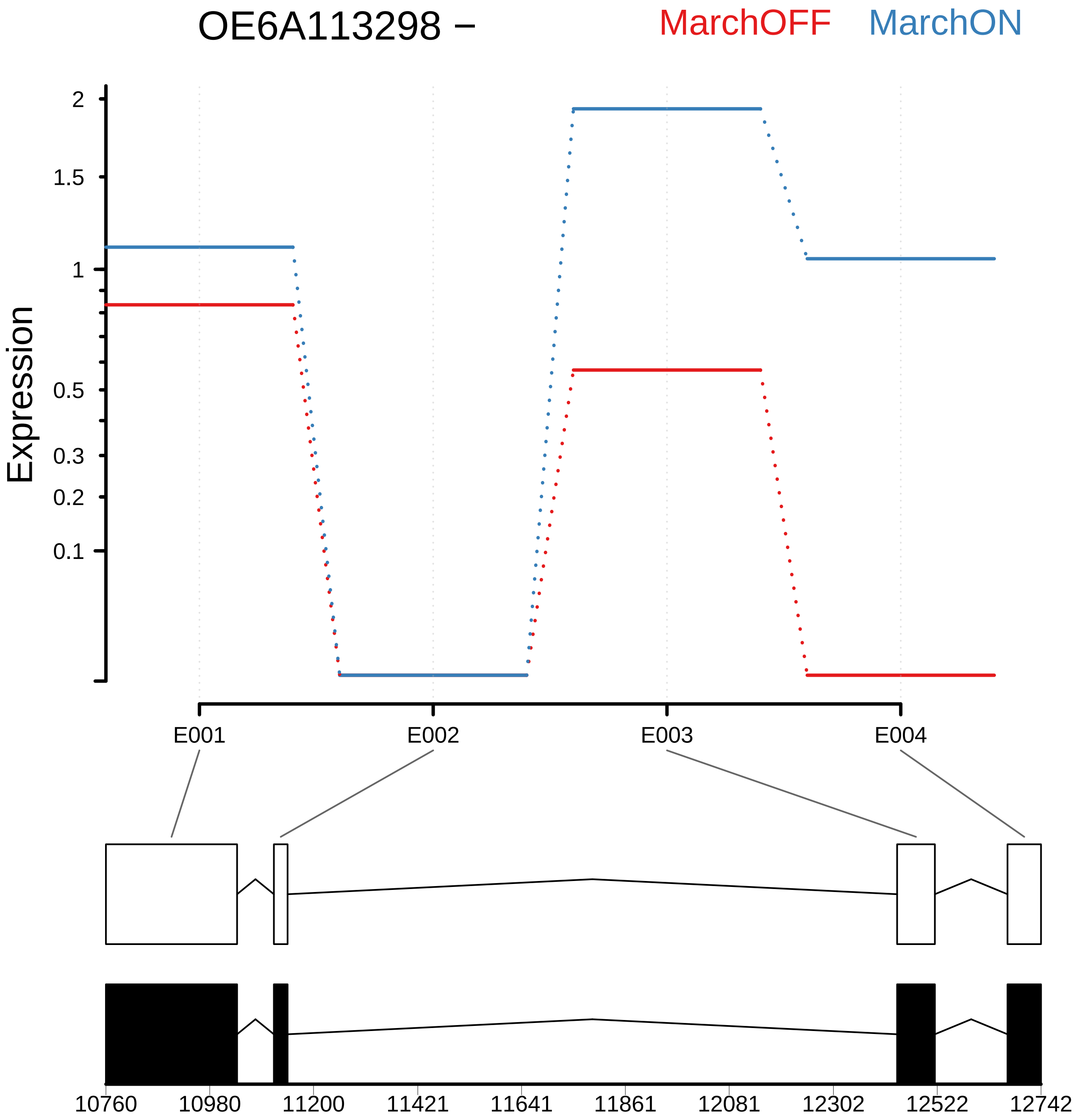

OE6A113298 −
MarchOFF
MarchON
Expression
2
1.5
1
0.5
0.3
0.2
0.1
E001
E002
E003
E004
10760
10980
11200
11421
11641
11861
12081
12302
12522
12742

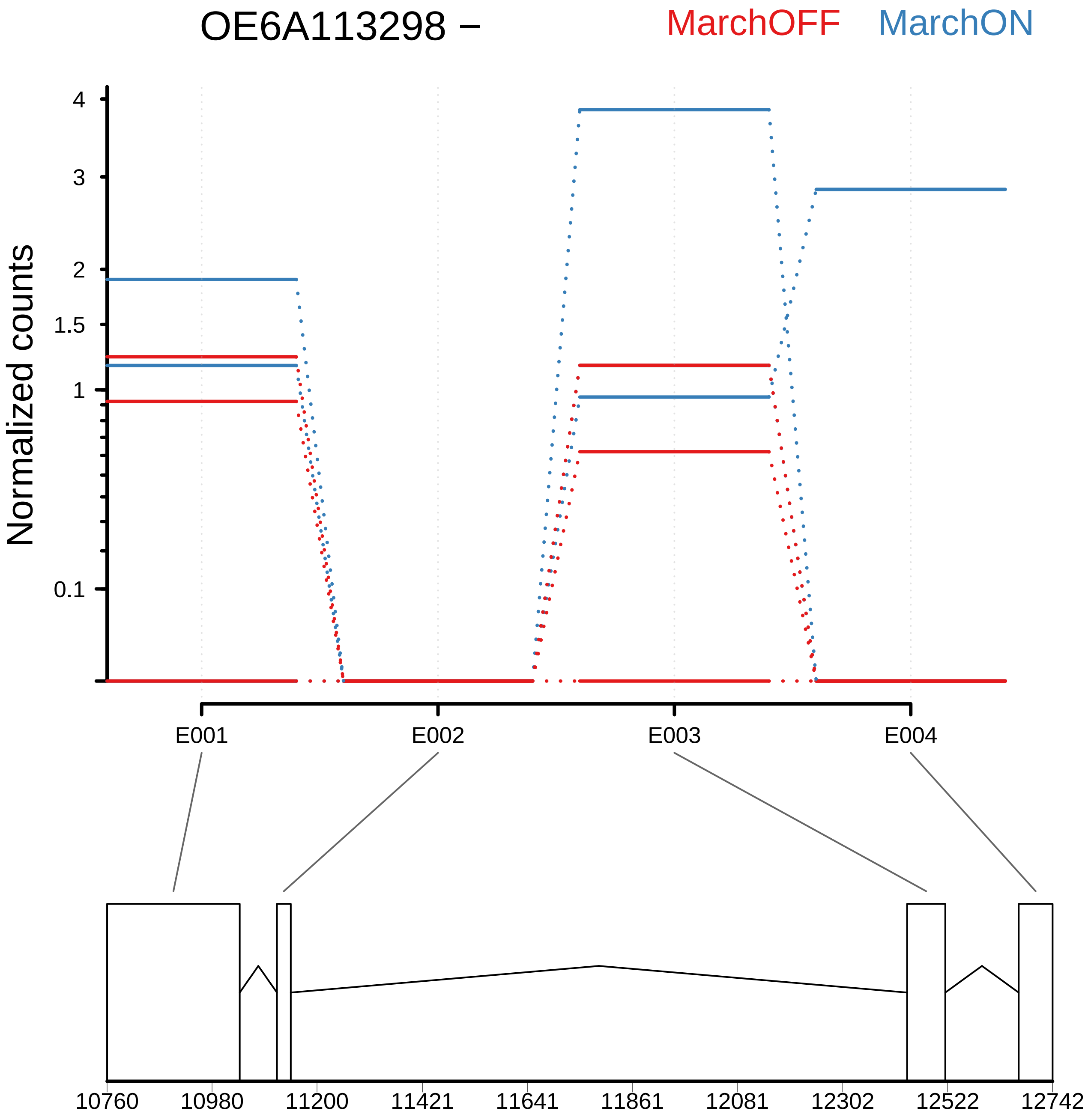
OE6A113298 −
MarchOFF
MarchON
Normalized counts
4
3
2
1.5
1
0.1
E001
E002
E003
E004
10760
10980
11200
11421
11641
11861
12081
12302
12522
12742

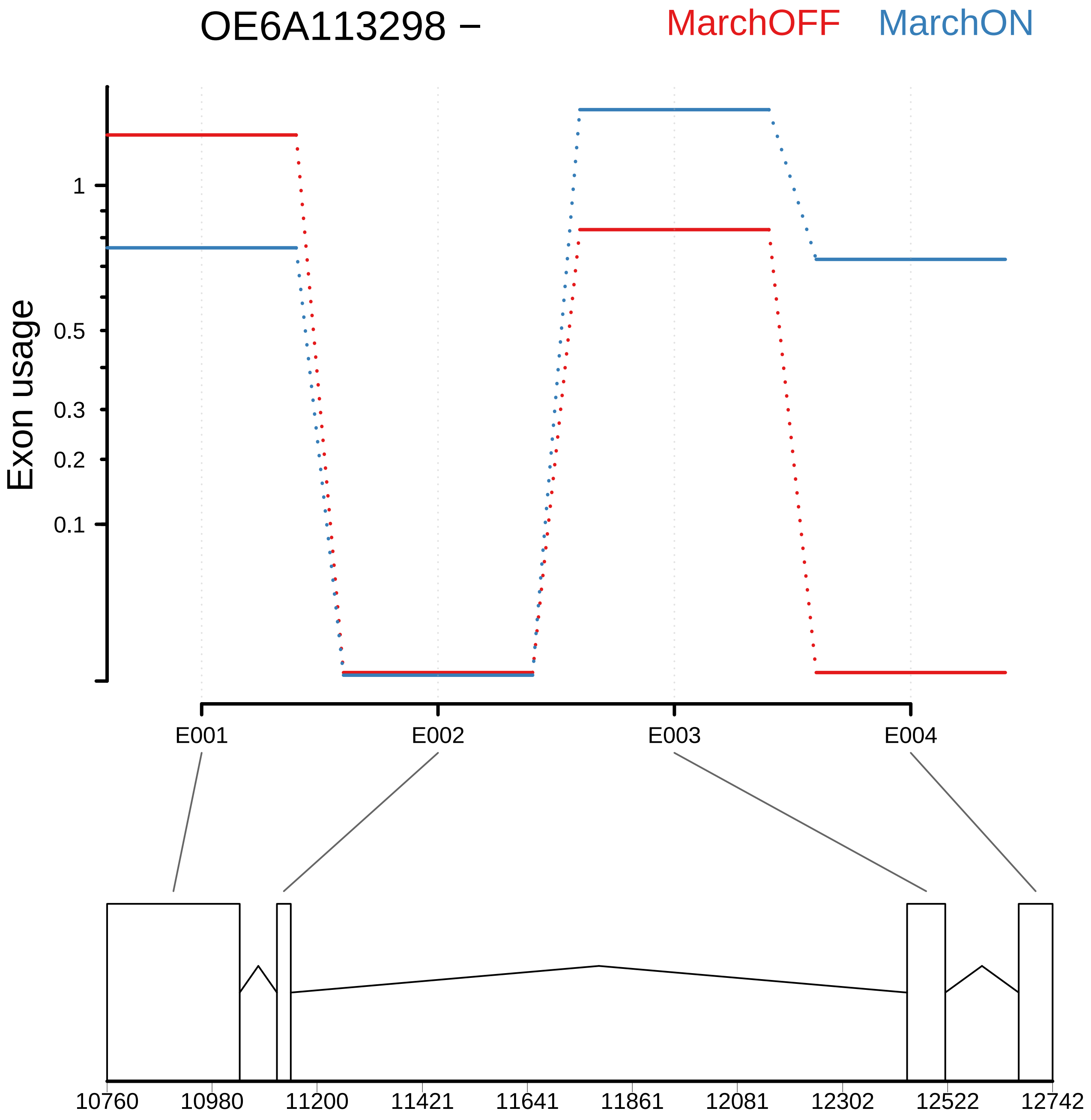
OE6A113298 −
MarchOFF
MarchON
Exon usage
1
0.5
0.3
0.2
0.1
E001
E002
E003
E004
10760
10980
11200
11421
11641
11861
12081
12302
12522
12742

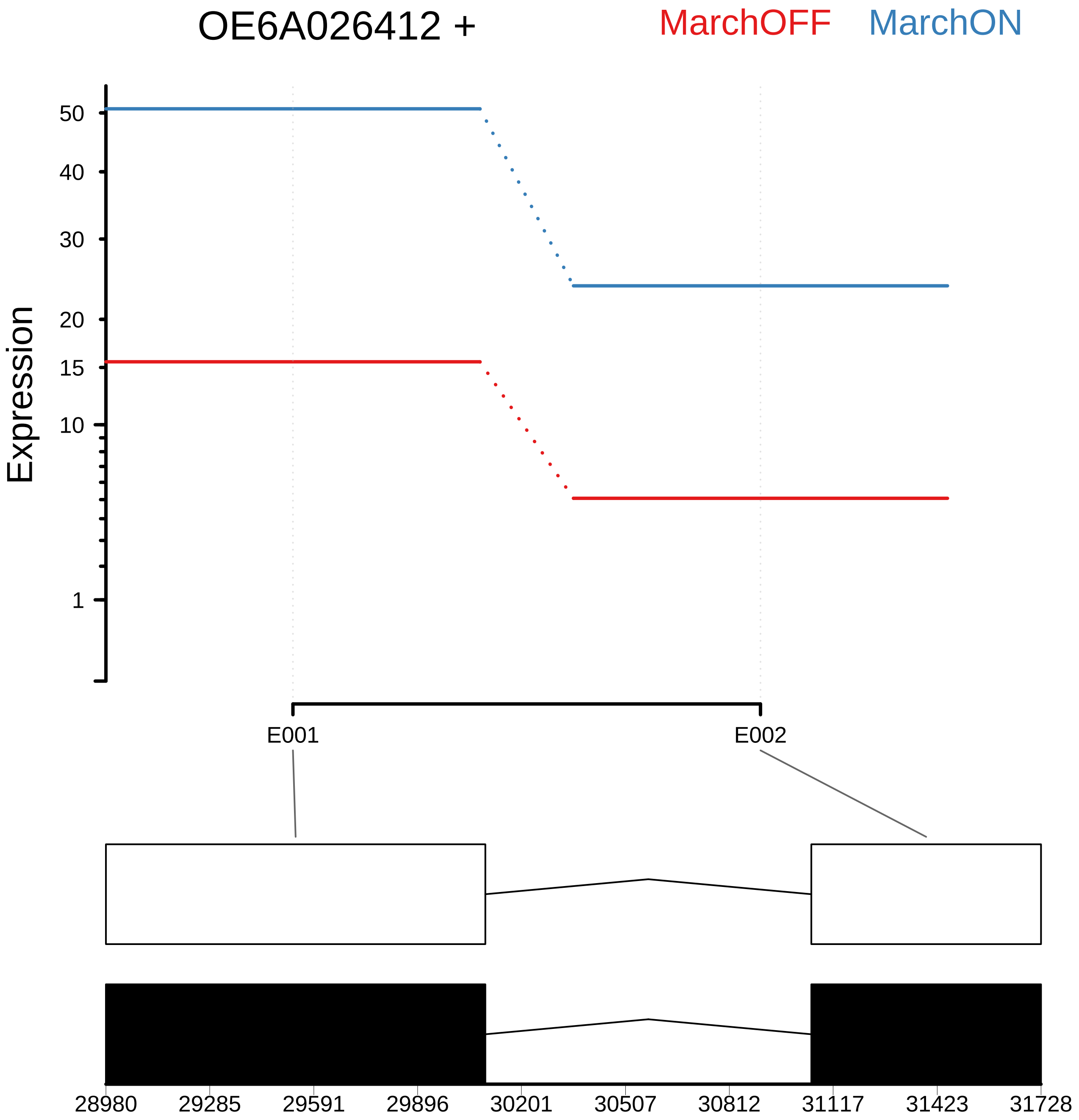
OE6A026412 +
MarchOFF
MarchON
Expression
50
40
30
20
15
10
1
E001
E002
28980
29285
29591
29896
30201
30507
30812
31117
31423
31728

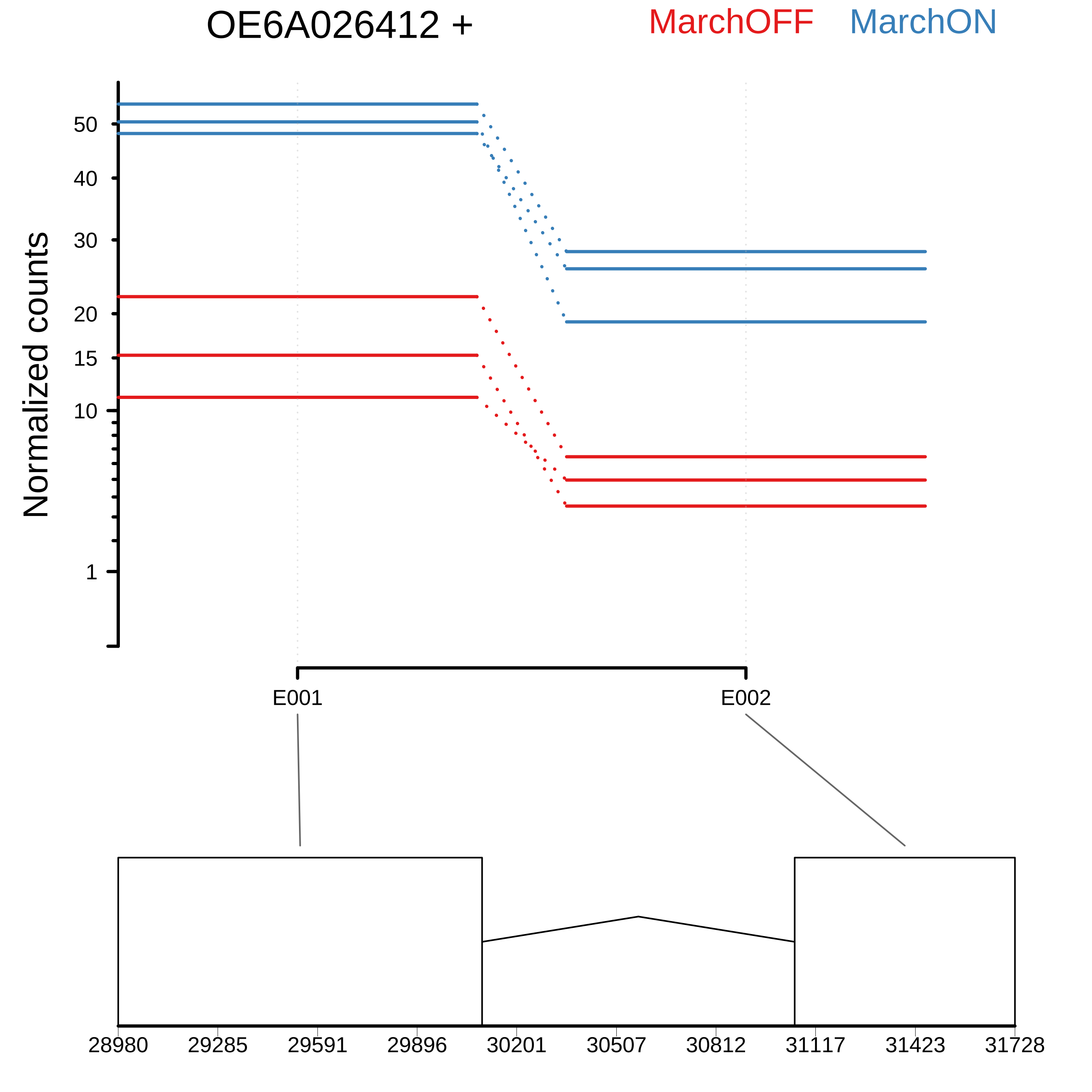
OE6A026412 +
MarchOFF
MarchON
Normalized counts
50
40
30
20
15
10
1
E001
E002
28980
29285
29591
29896
30201
30507
30812
31117
31423
31728

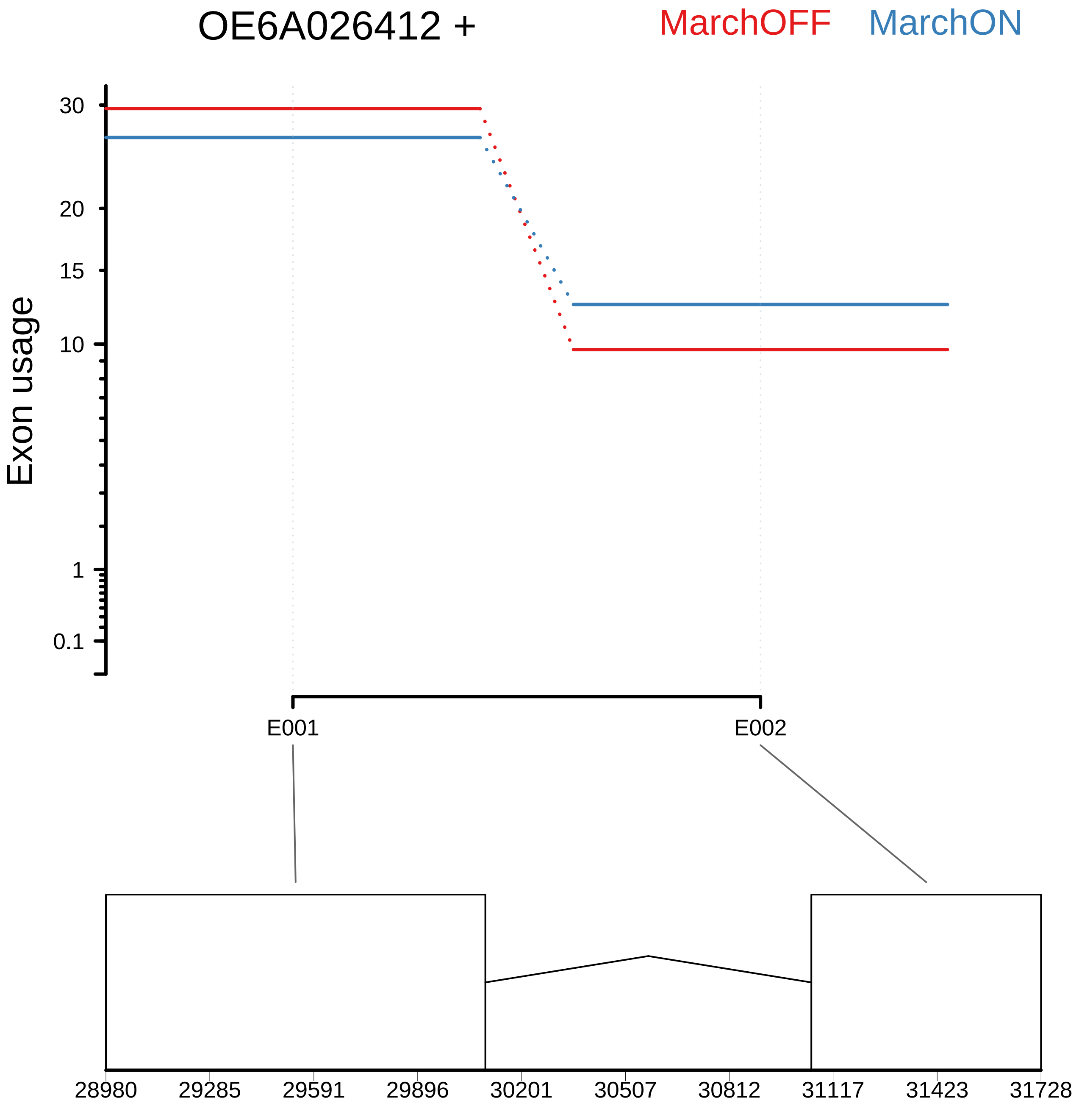
OE6A026412 +
MarchOFF
MarchON
Exon usage
30
20
15
10
1
0.1
E001
E002
28980
29285
29591
29896
30201
30507
30812
31117
31423
31728

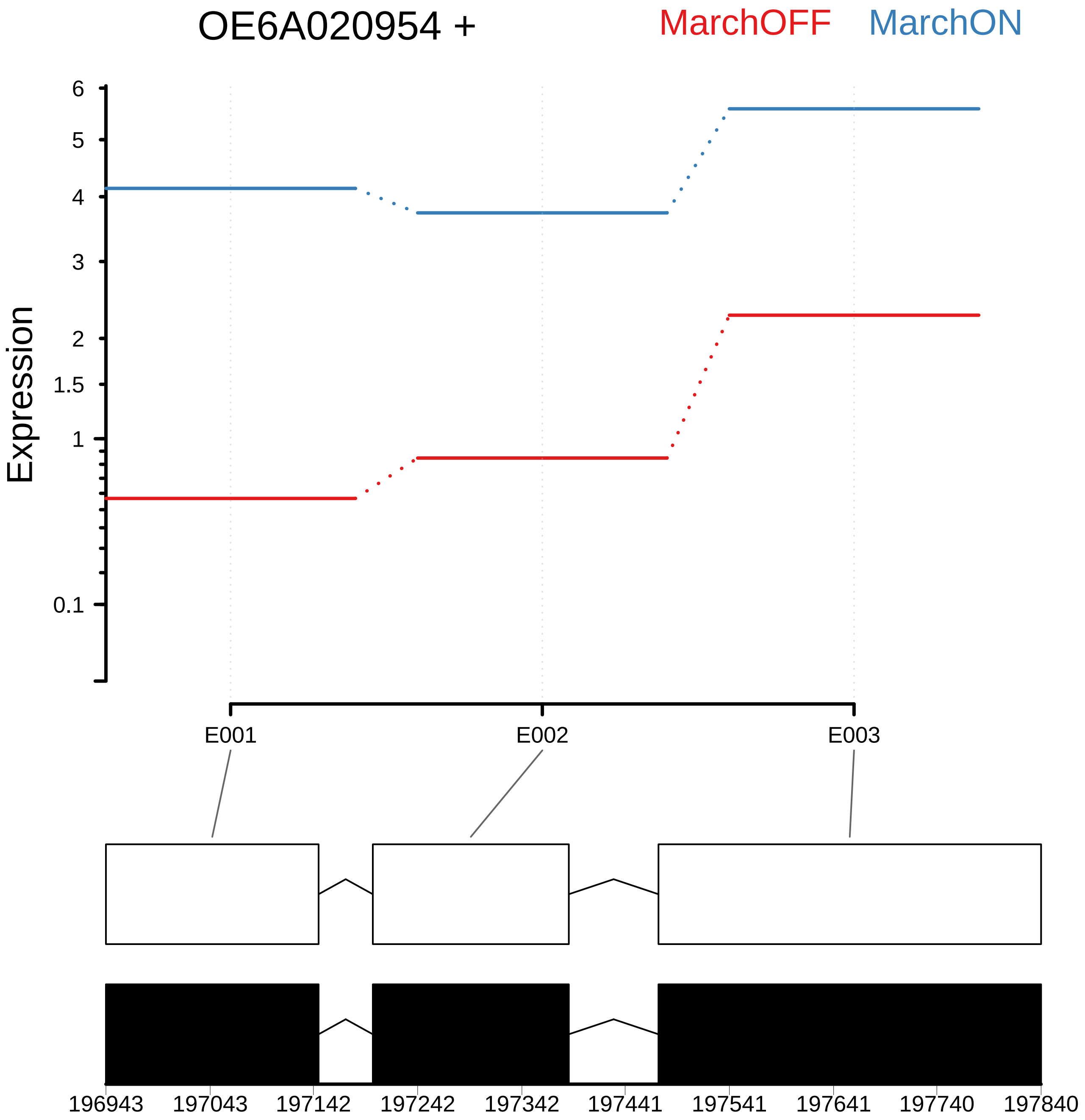

OE6A020954 +
MarchOFF
MarchON
Expression
6
5
4
3
2
1.5
1
0.1
E001
E002
E003
196943
197043
197142
197242
197342
197441
197541
197641
197740
197840

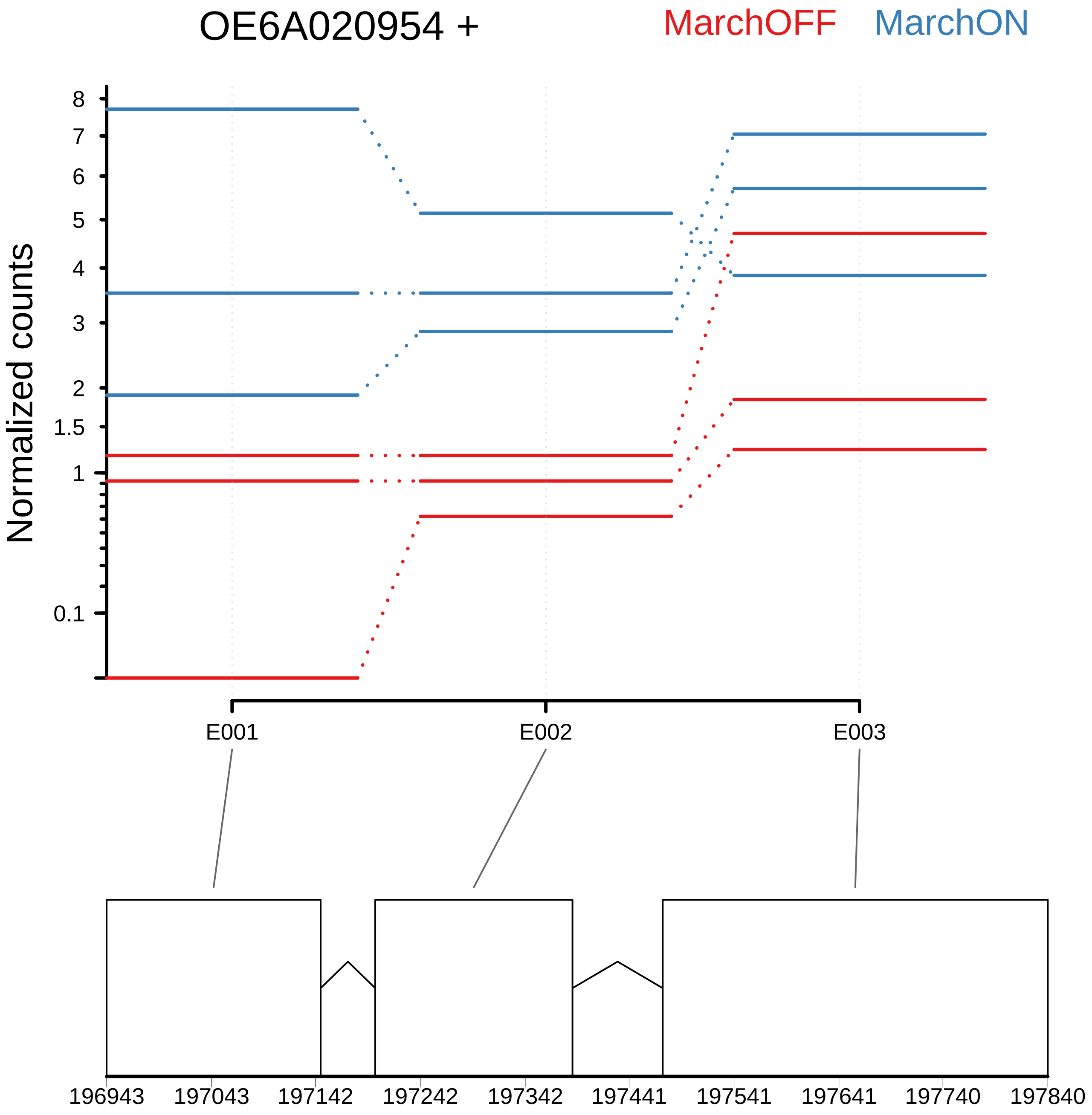

OE6A020954 +
MarchOFF
MarchON
Normalized counts
8
7
6
5
4
3
2
1.5
1
0.1
E001
E002
E003
196943
197043
197142
197242
197342
197441
197541
197641
197740
197840

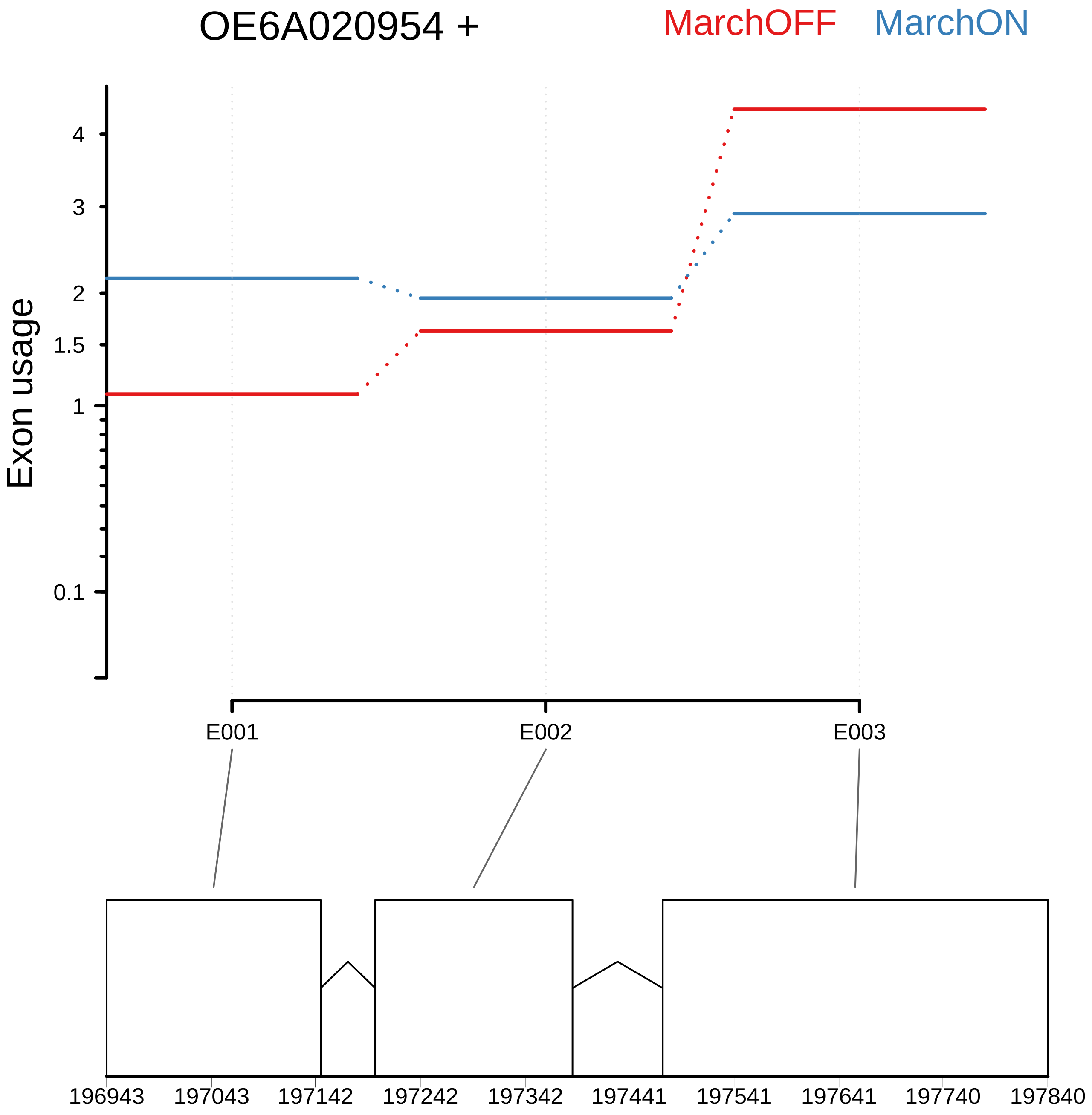
OE6A020954 +
MarchOFF
MarchON
Exon usage
4
3
2
1.5
1
0.1
E001
E002
E003
196943
197043
197142
197242
197342
197441
197541
197641
197740
197840

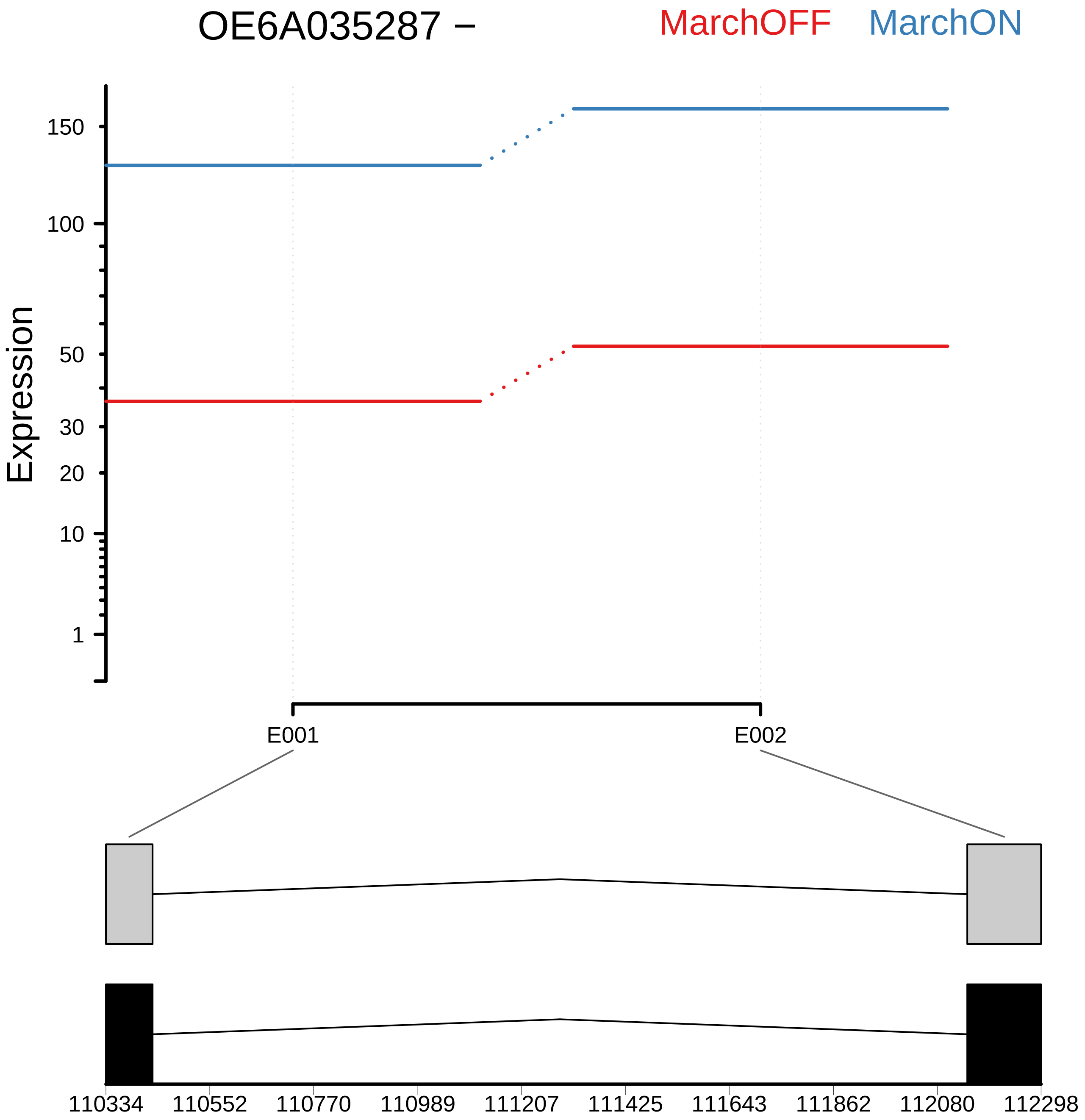

OE6A035287 −
MarchOFF
MarchON
Expression
150
100
50
30
20
10
1
E001
E002
110334
110552
110770
110989
111207
111425
111643
111862
112080
112298

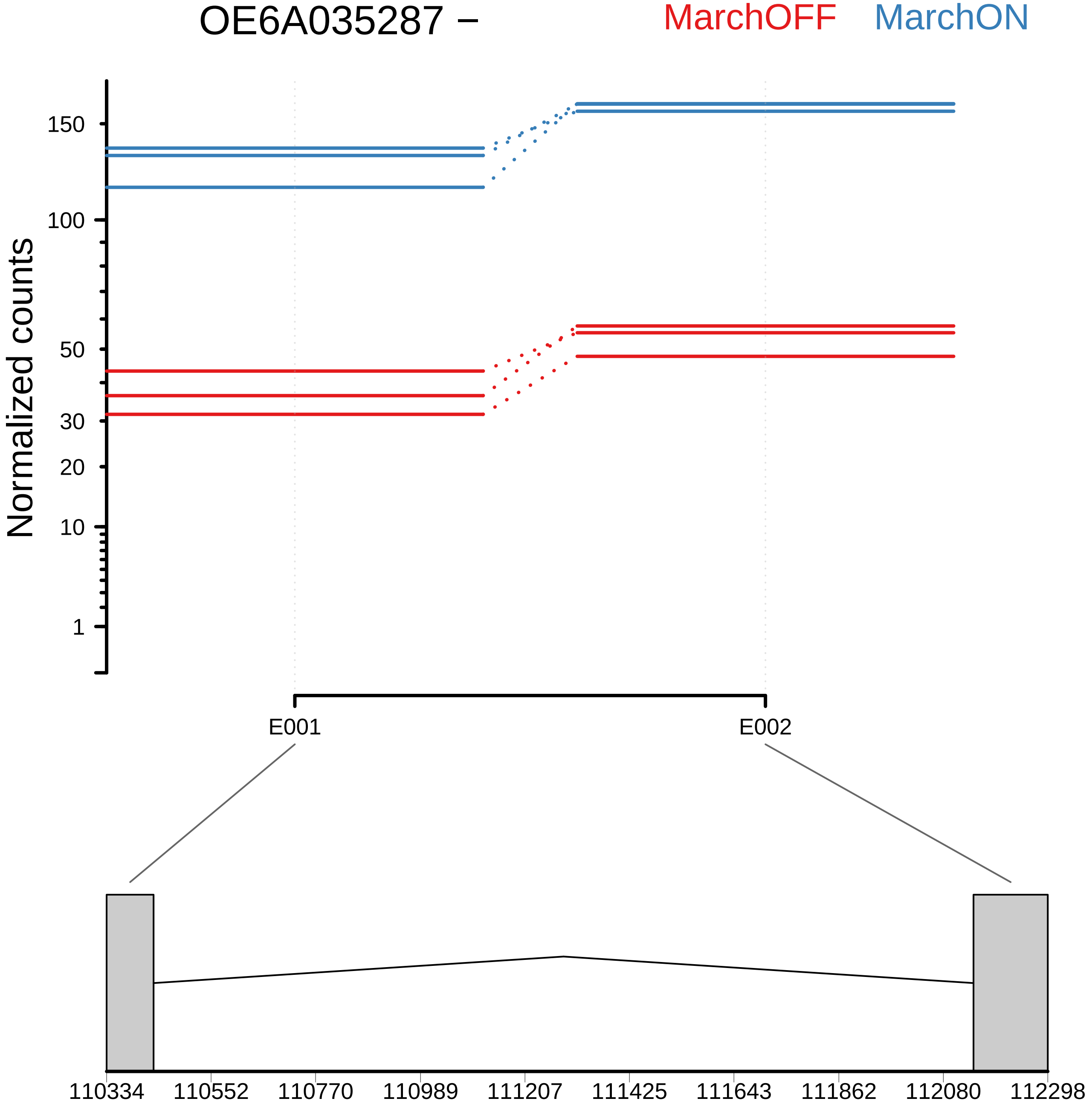
OE6A035287 −
MarchOFF
MarchON
Normalized counts
150
100
50
30
20
10
1
E001
E002
110334
110552
110770
110989
111207
111425
111643
111862
112080
112298

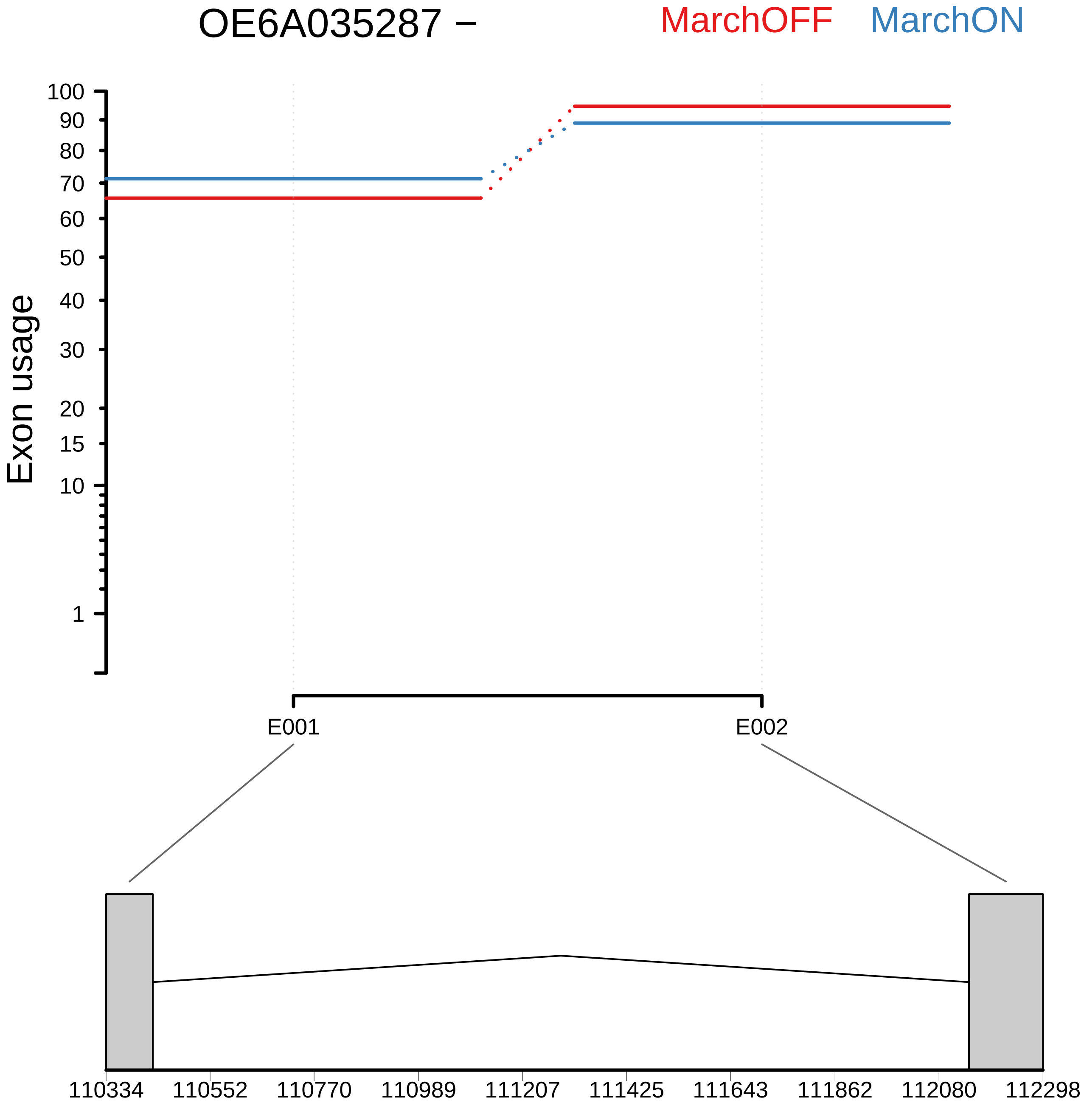

OE6A035287 −
MarchOFF
MarchON
Exon usage
100
90
80
70
60
50
40
30
20
15
10
1
E001
E002
110334
110552
110770
110989
111207
111425
111643
111862
112080
112298

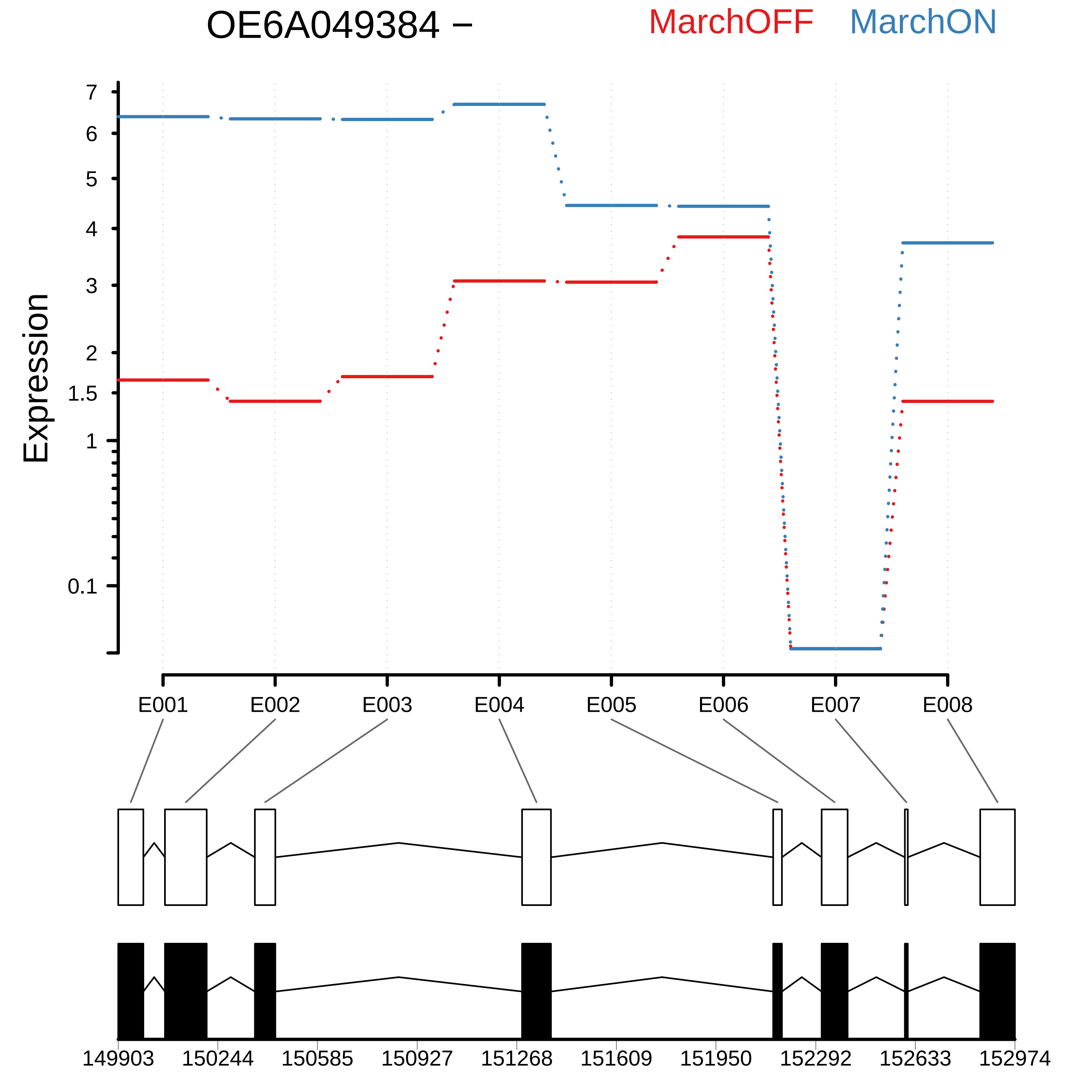

OE6A049384 −
MarchOFF
MarchON
Expression
7
6
5
4
3
2
1.5
1
0.1
E001
E002
E003
E004
E005
E006
E007
E008
149903
150244
150585
150927
151268
151609
151950
152292
152633
152974

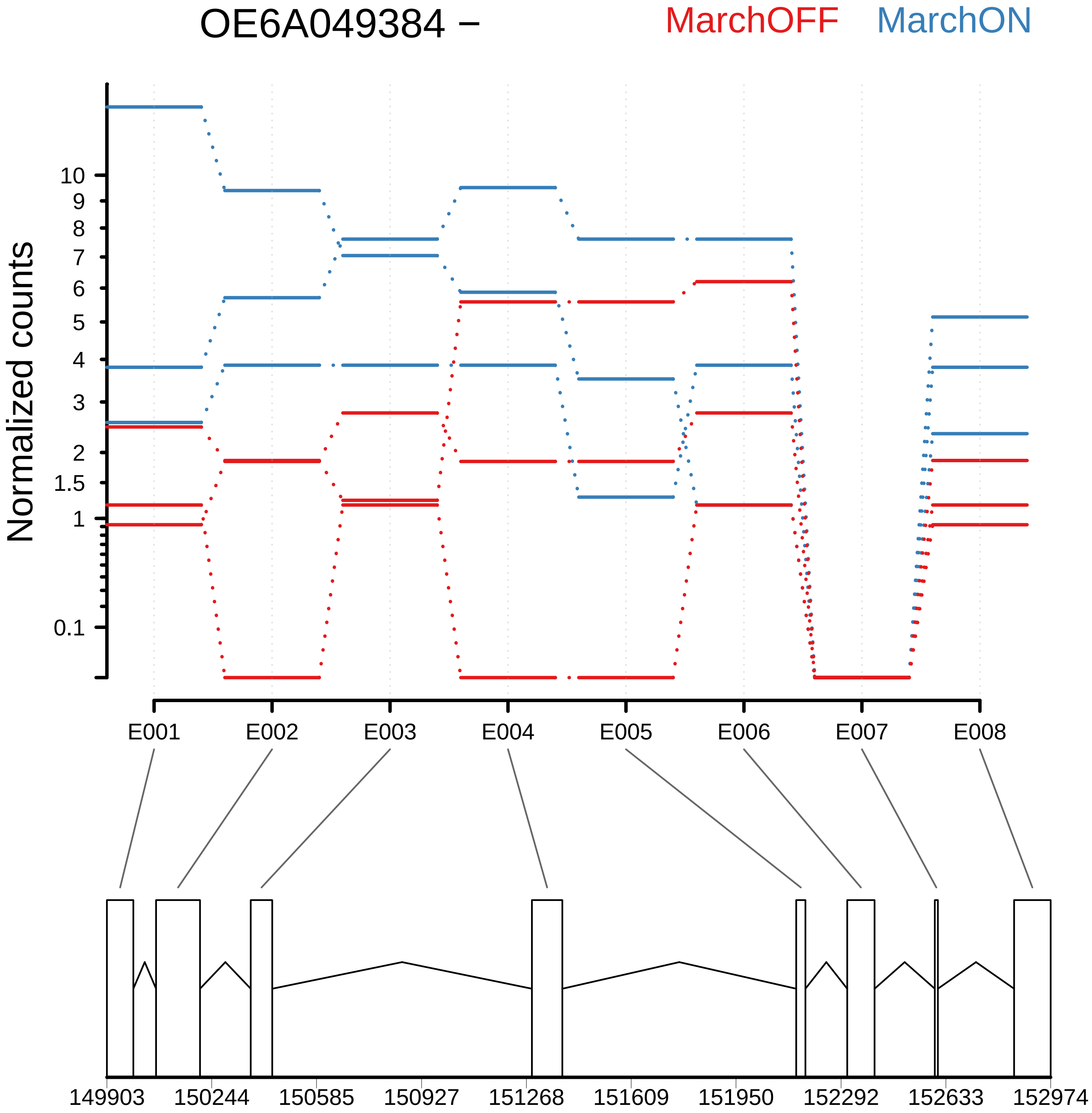

OE6A049384 −
MarchOFF
MarchON
Normalized counts
10
9
8
7
6
5
4
3
2
1.5
1
0.1
E001
E002
E003
E004
E005
E006
E007
E008
149903
150244
150585
150927
151268
151609
151950
152292
152633
152974

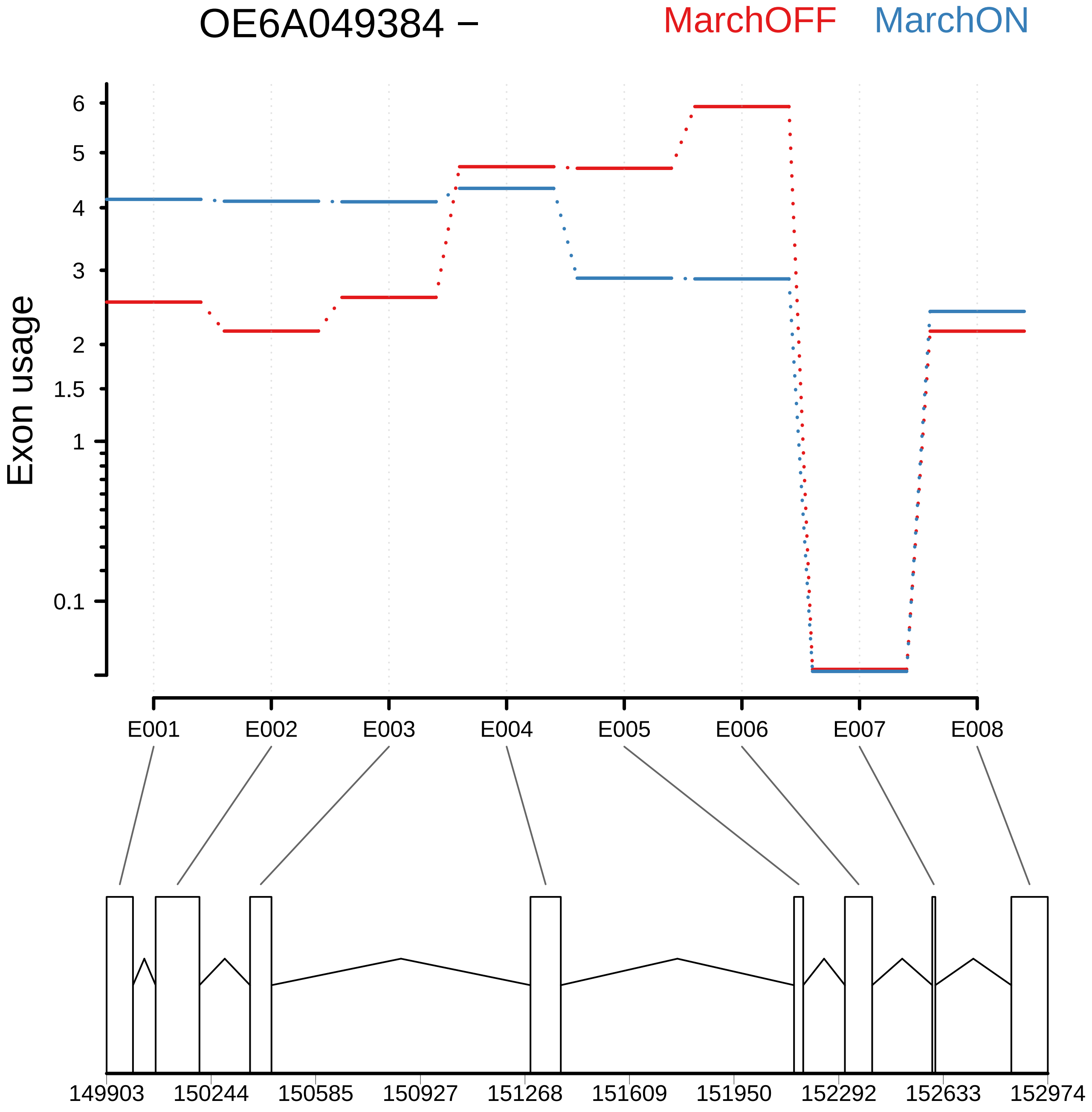
OE6A049384 –
MarchOFF
MarchON
Exon usage
6
5
4
3
2
1.5
1
0.1
E001
E002
E003
E004
E005
E006
E007
E008
149903
150244
150585
150927
151268
151609
151950
152292
152633
152974

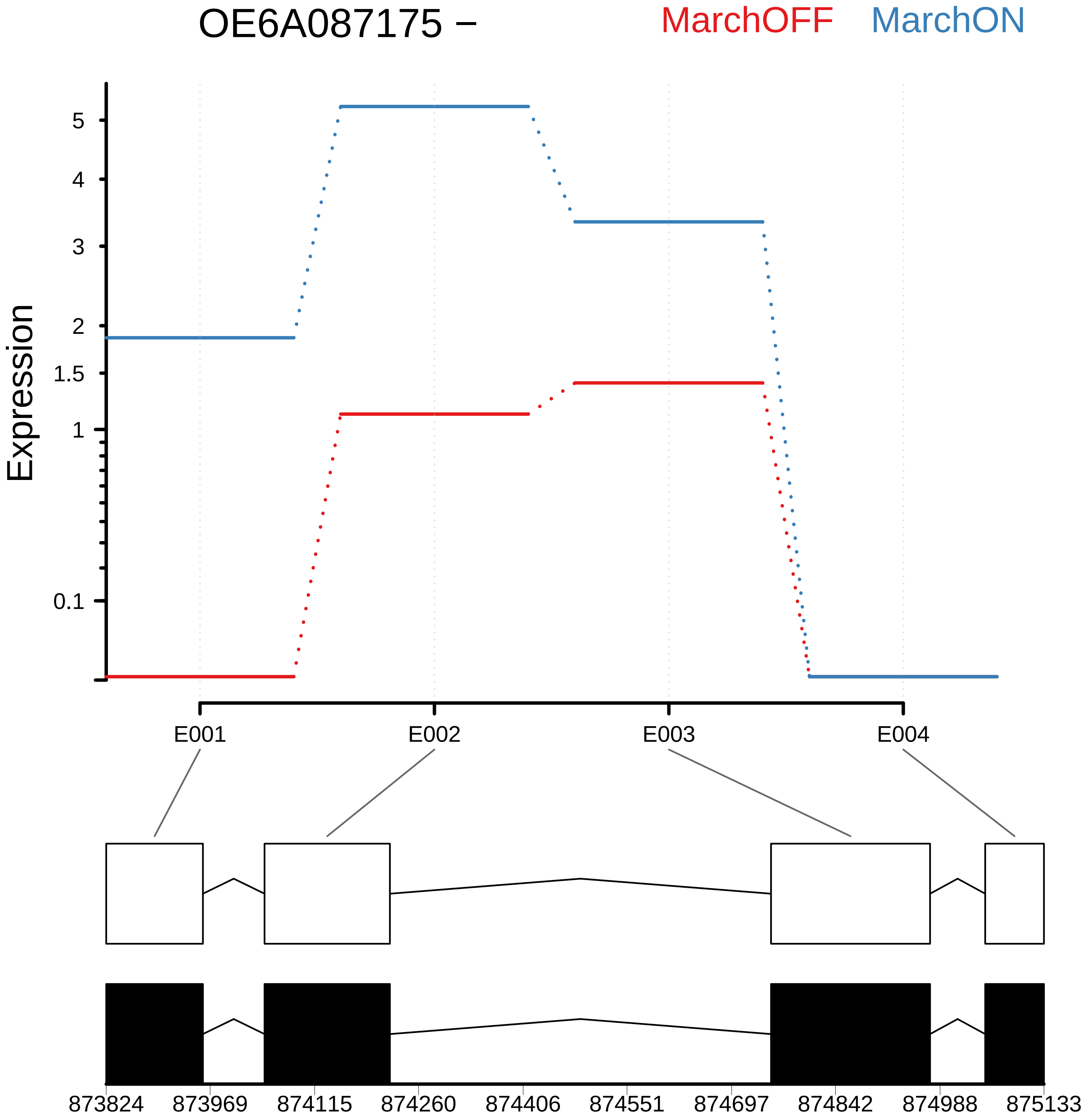

OE6A087175 −
MarchOFF
MarchON
Expression
5
4
3
2
1.5
1
0.1
E001
E002
E003
E004
873824
873969
874115
874260
874406
874551
874697
874842
874988
875133

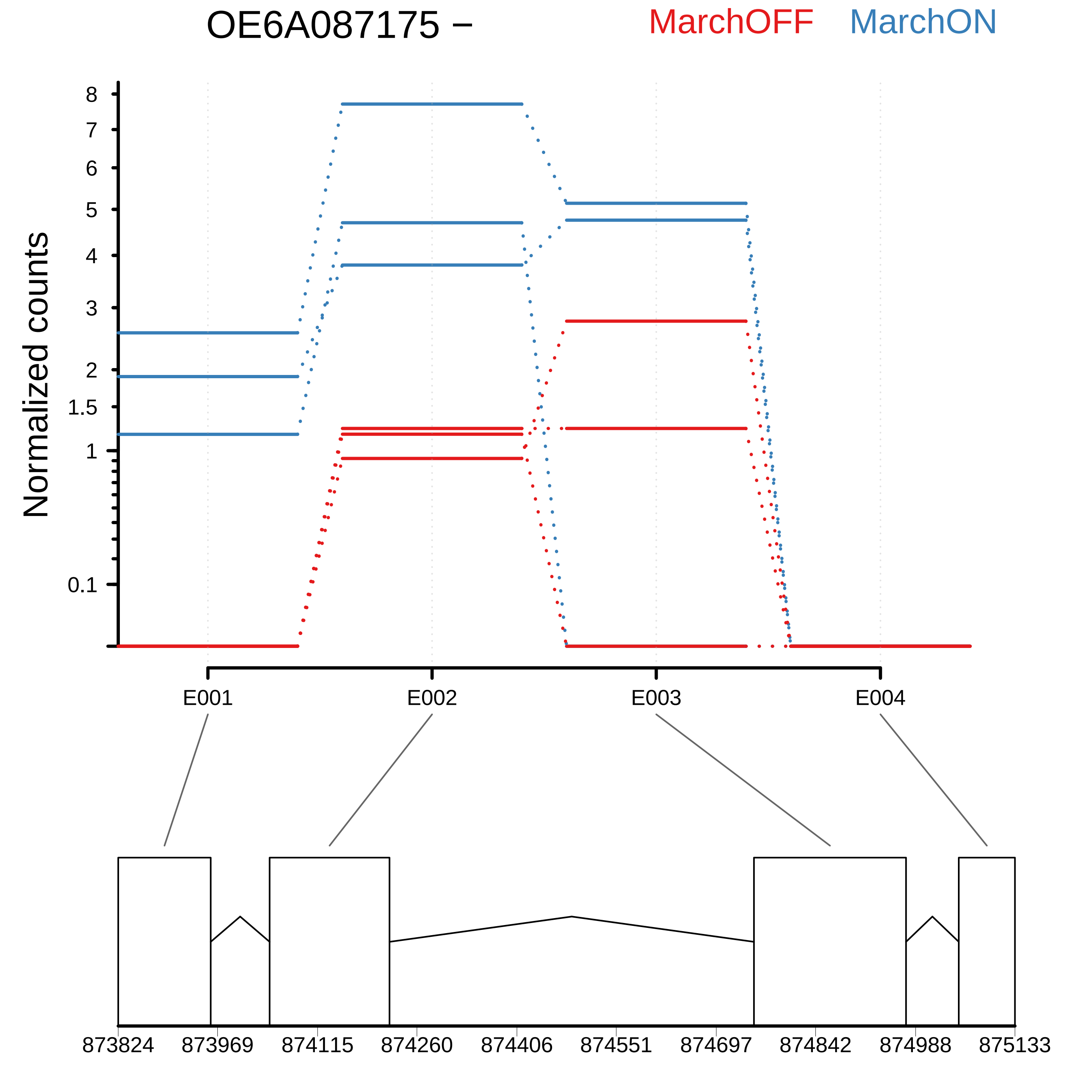

OE6A087175 −
MarchOFF
MarchON
Normalized counts
8
7
6
5
4
3
2
1.5
1
0.1
E001
E002
E003
E004
873824
873969
874115
874260
874406
874551
874697
874842
874988
875133

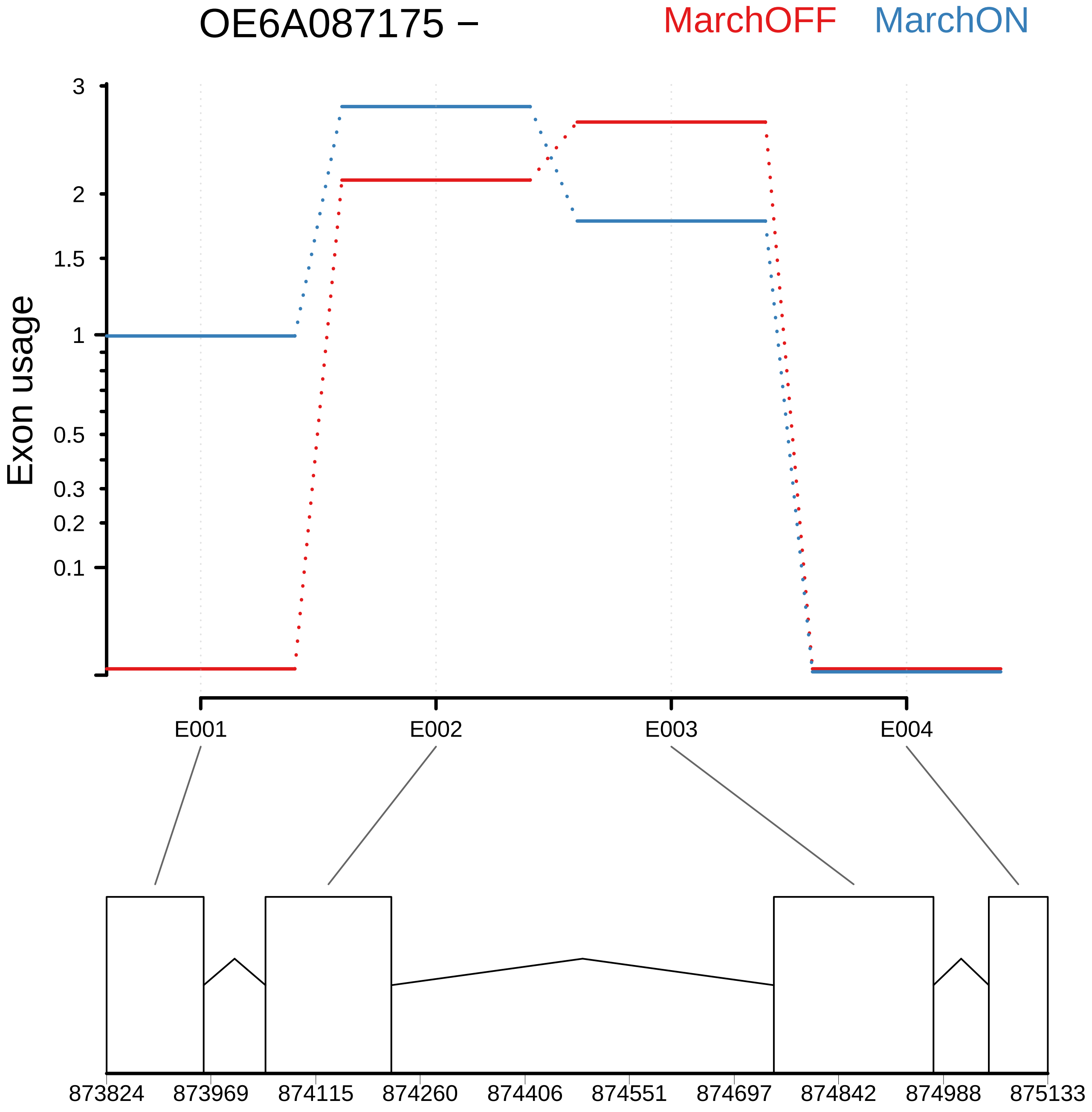
OE6A087175 −
MarchOFF
MarchON
Exon usage
3
2
1.5
1
0.5
0.3
0.2
0.1
E001
E002
E003
E004
873824
873969
874115
874260
874406
874551
874697
874842
874988
875133

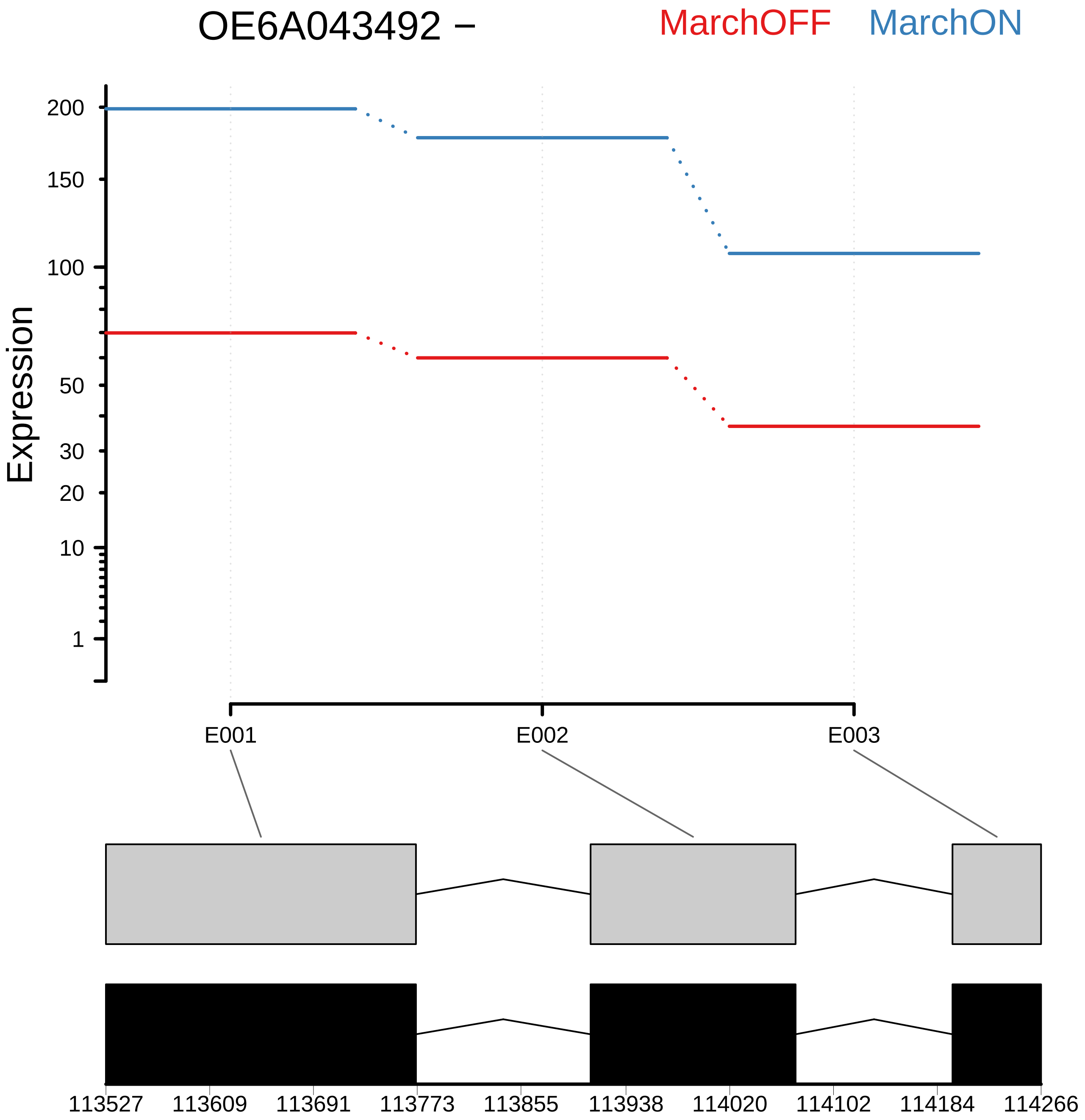
OE6A043492 −
MarchOFF
MarchON
Expression
200
150
100
50
30
20
10
1
E001
E002
E003
113527
113609
113691
113773
113855
113938
114020
114102
114184
114266

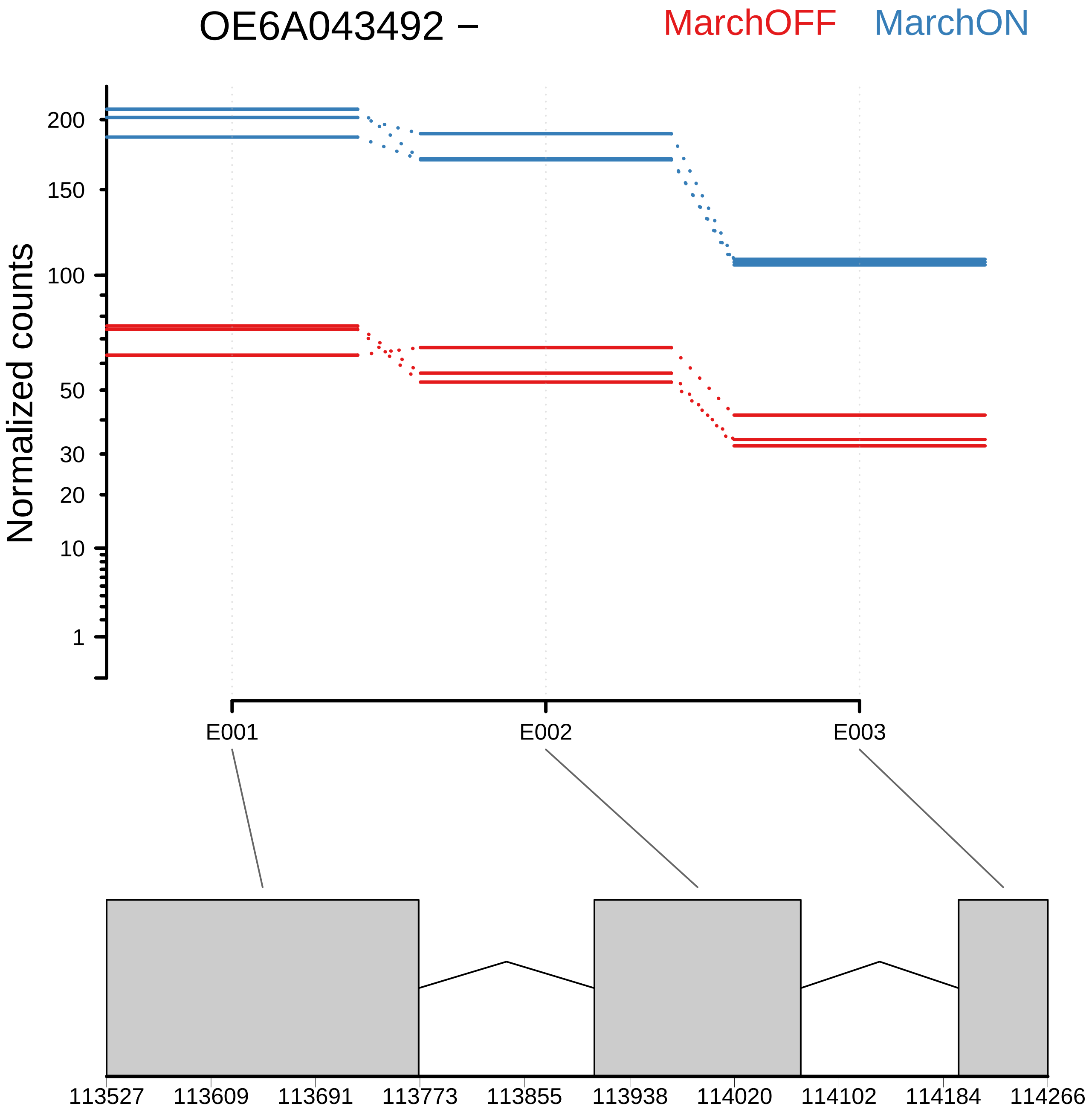
OE6A043492 −
MarchOFF
MarchON
Normalized counts
200
150
100
50
30
20
10
1
E001
E002
E003
113527
113609
113691
113773
113855
113938
114020
114102
114184
114266

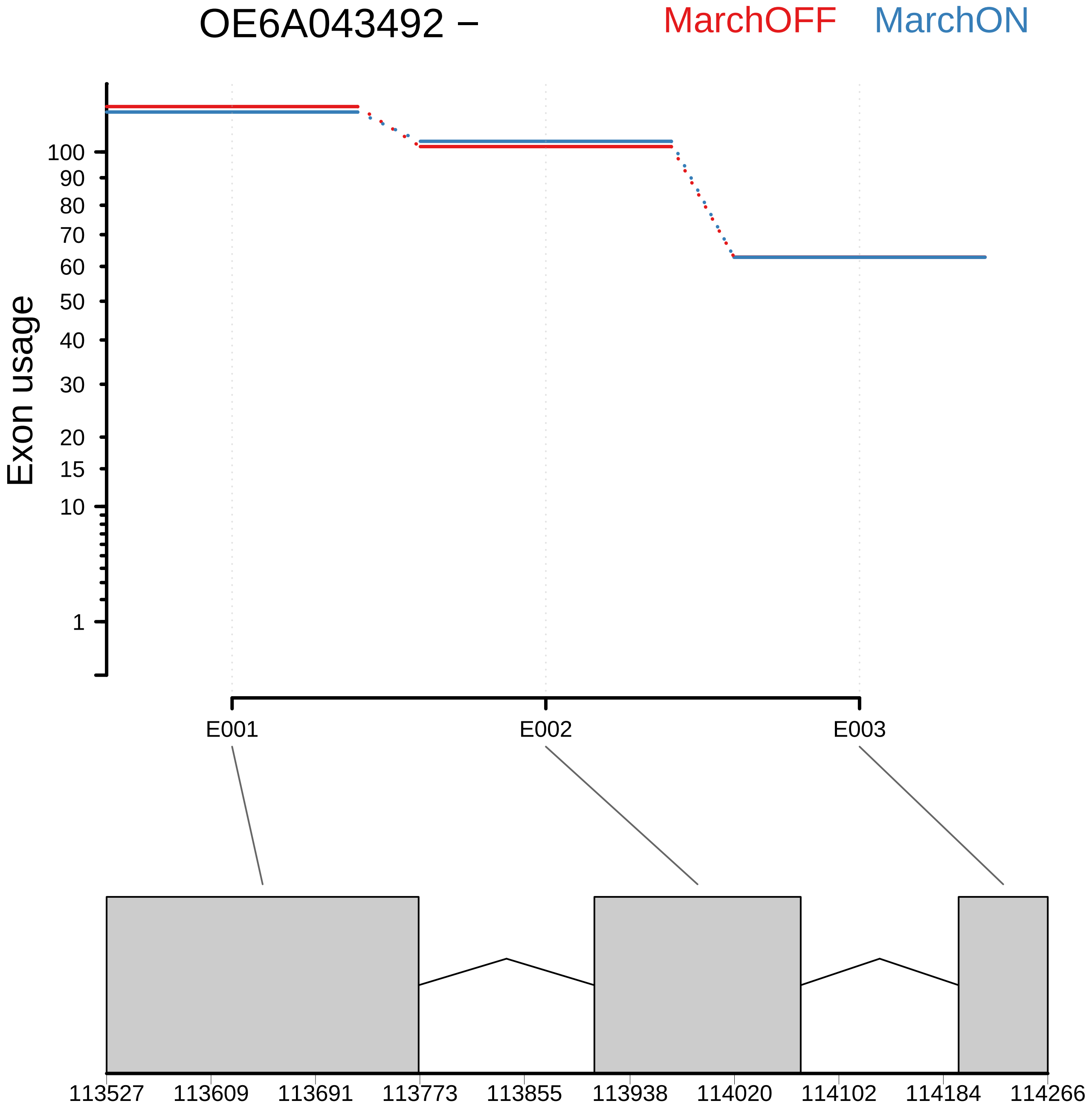
OE6A043492 −
MarchOFF
MarchON
Exon usage
100
90
80
70
60
50
40
30
20
15
10
1
E001
E002
E003
113527
113609
113691
113773
113855
113938
114020
114102
114184
114266

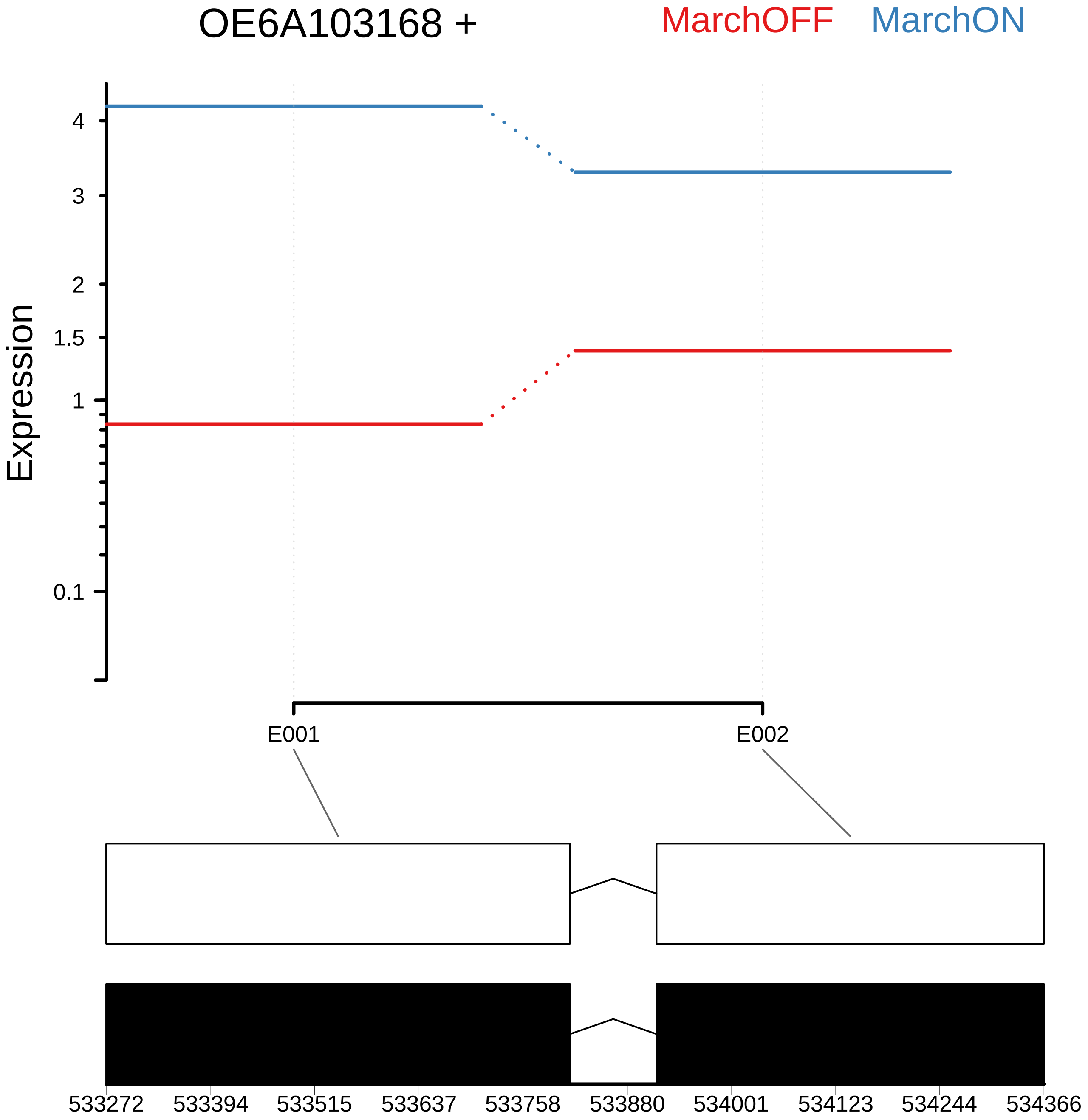
OE6A103168 +
MarchOFF
MarchON
Expression
4
3
2
1.5
1
0.1
E001
E002
533272
533394
533515
533637
533758
533880
534001
534123
534244
534366

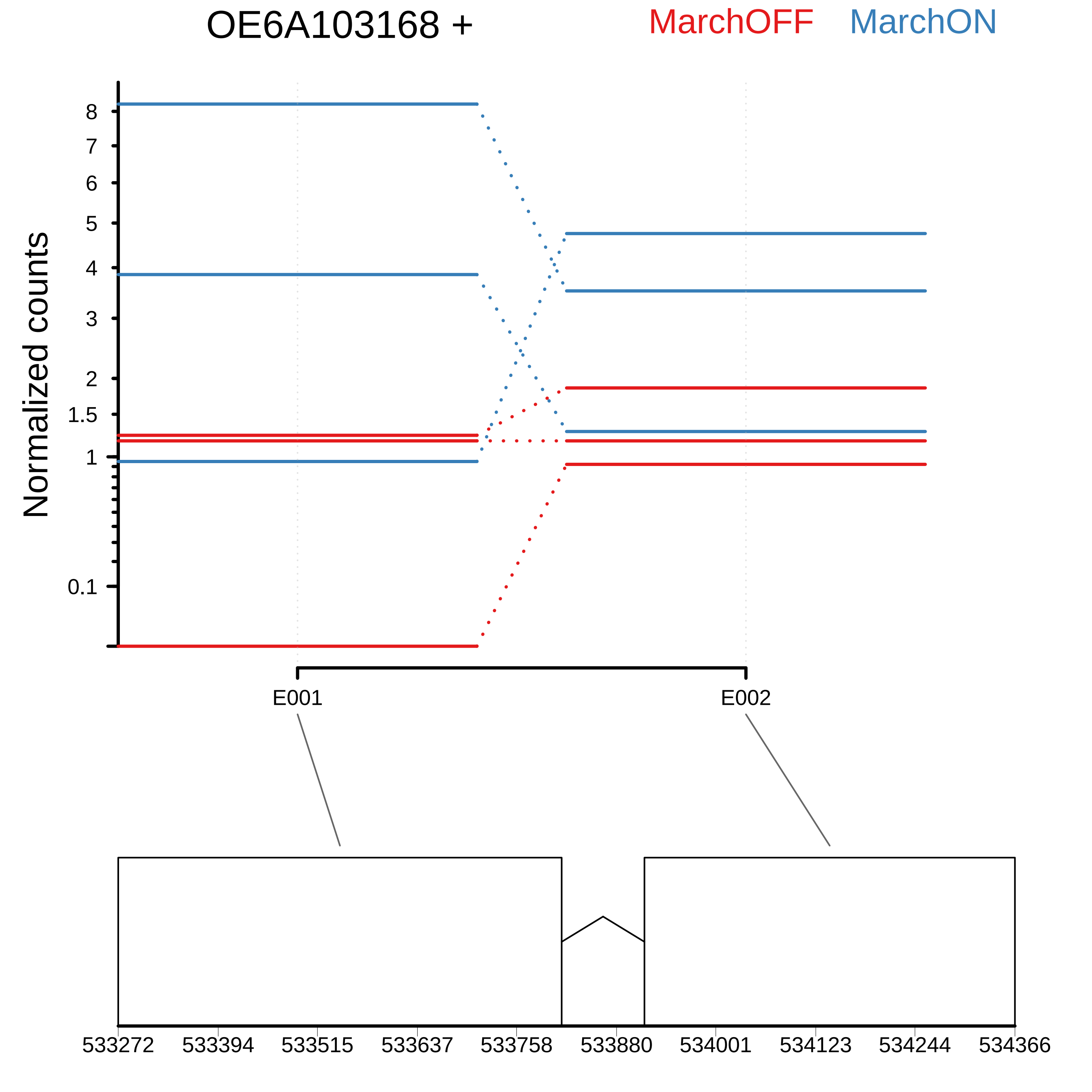

OE6A103168 +
MarchOFF
MarchON
Normalized counts
8
7
6
5
4
3
2
1.5
1
0.1
E001
E002
533272
533394
533515
533637
533758
533880
534001
534123
534244
534366

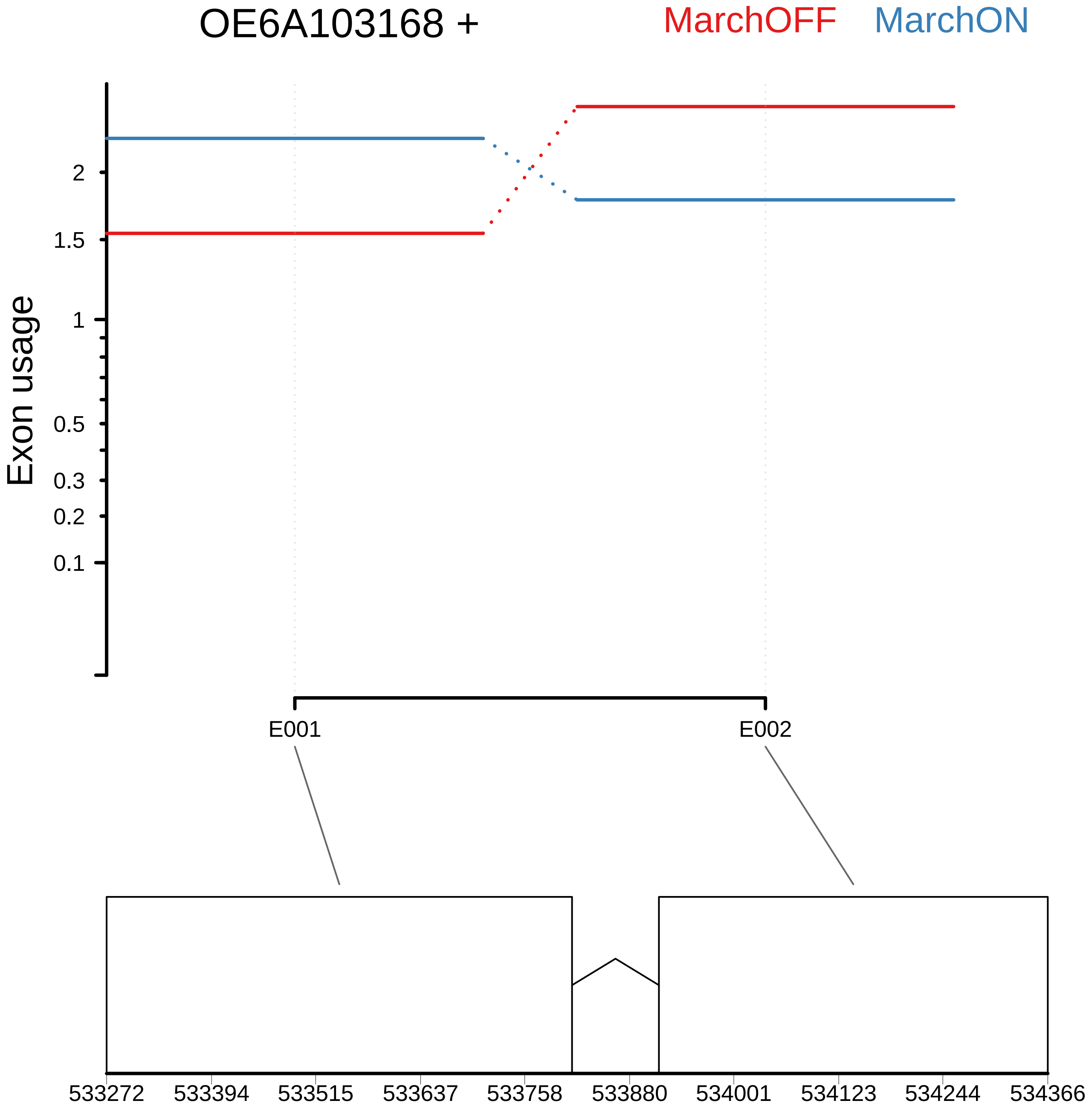
OE6A103168 +
MarchOFF
MarchON
Exon usage
2
1.5
1
0.5
0.3
0.2
0.1
E001
E002
533272
533394
533515
533637
533758
533880
534001
534123
534244
534366

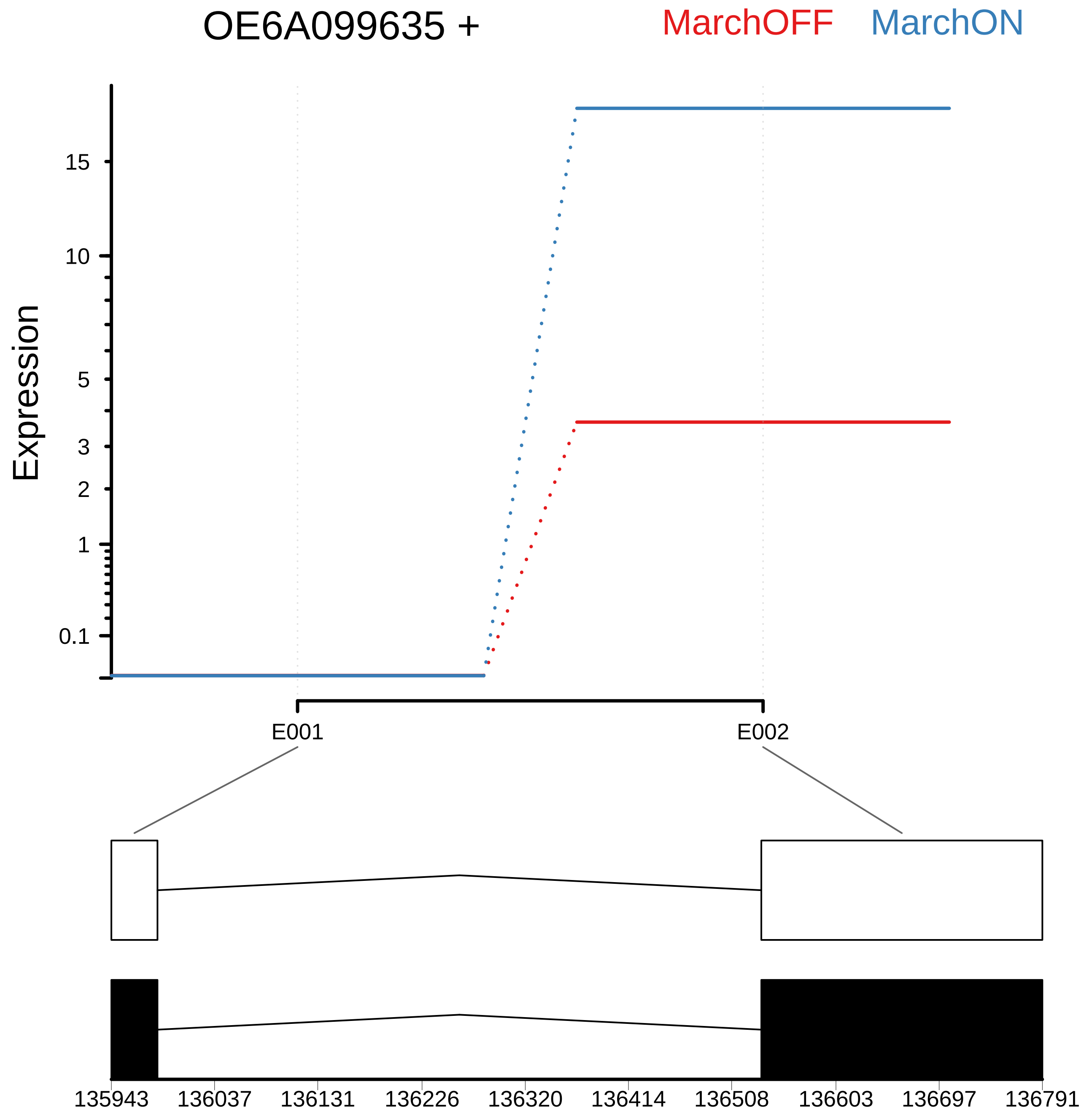
OE6A099635 +
MarchOFF
MarchON
Expression
15
10
5
3
2
1
0.1
E001
E002
135943
136037
136131
136226
136320
136414
136508
136603
136697
136791

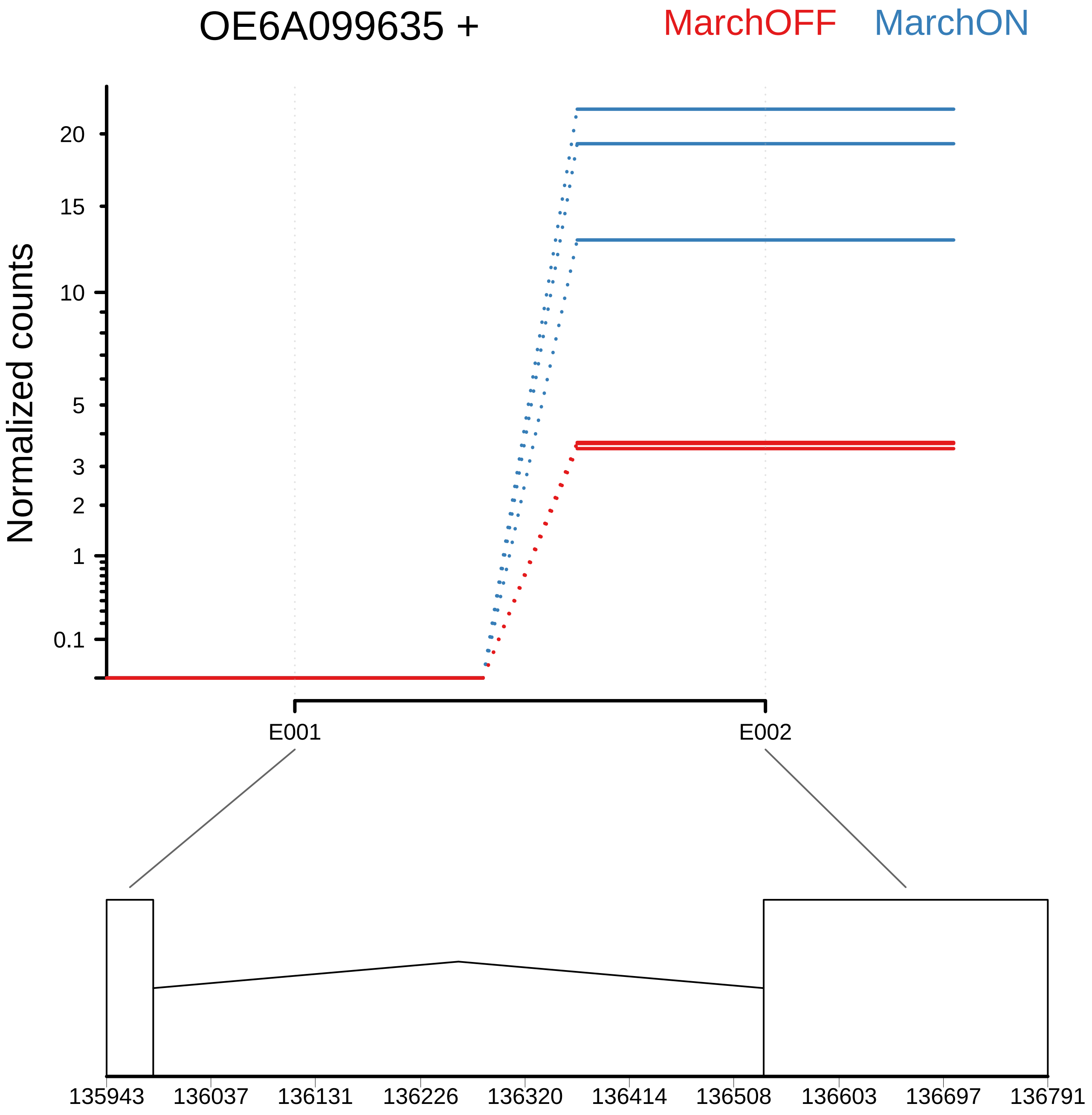
OE6A099635 +
MarchOFF
MarchON
Normalized counts
20
15
10
5
3
2
1
0.1
E001
E002
135943
136037
136131
136226
136320
136414
136508
136603
136697
136791

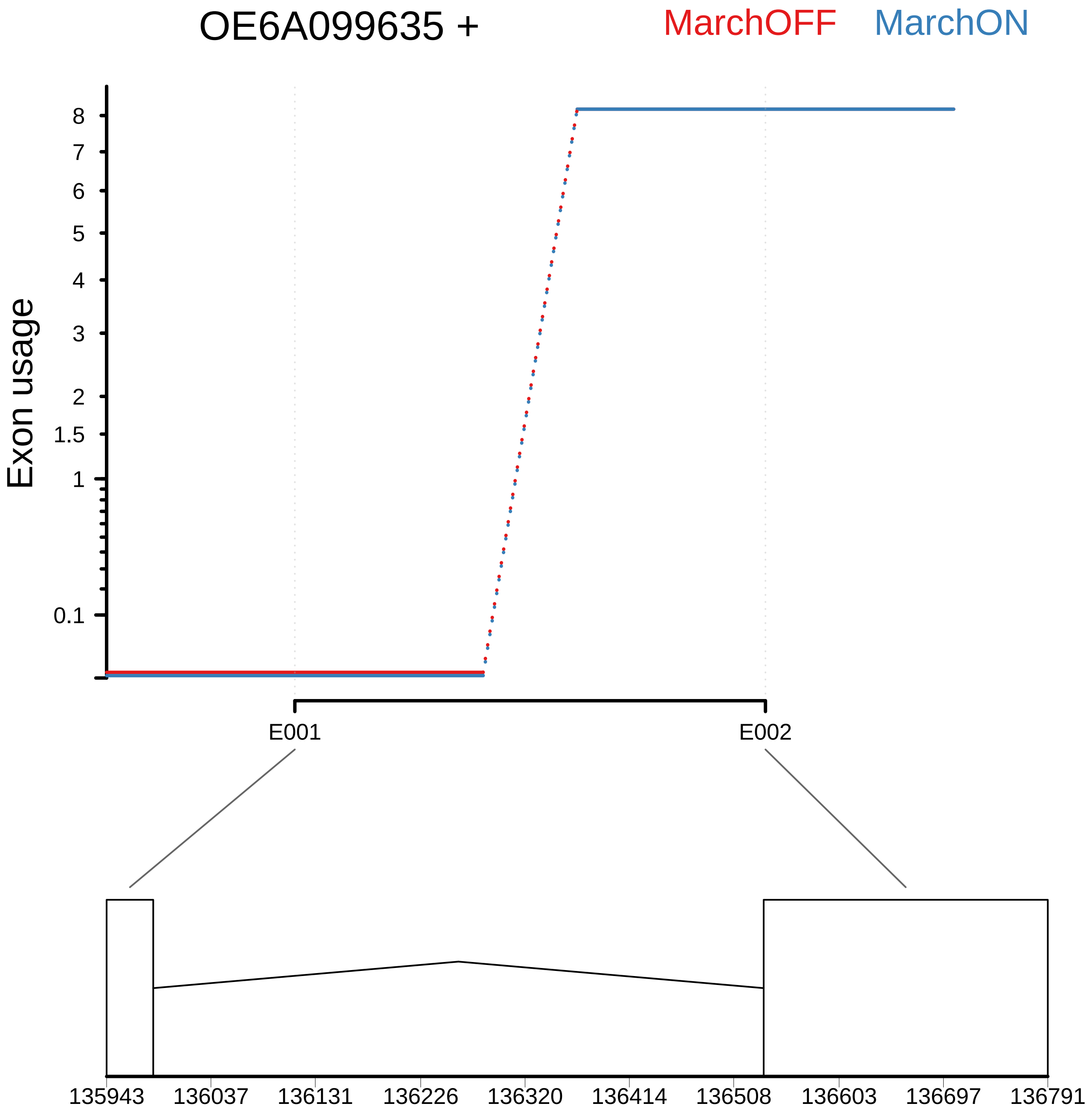
OE6A099635 +
MarchOFF
MarchON
Exon usage
8
7
6
5
4
3
2
1.5
1
0.1
E001
E002
135943
136037
136131
136226
136320
136414
136508
136603
136697
136791

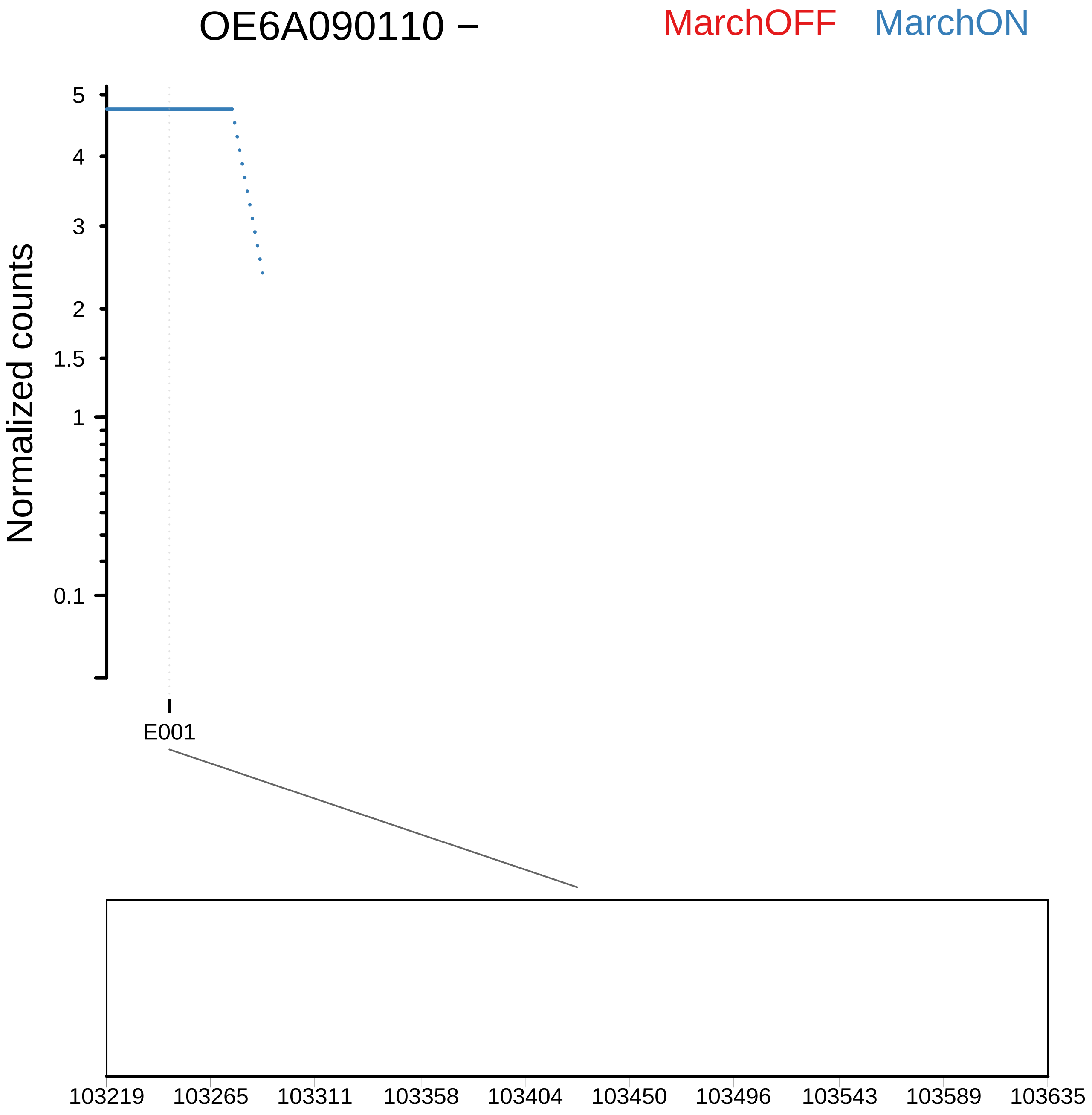
OE6A090110 −
MarchOFF
MarchON
Normalized counts
5
4
3
2
1.5
1
0.1
E001
103219
103265
103311
103358
103404
103450
103496
103543
103589
103635

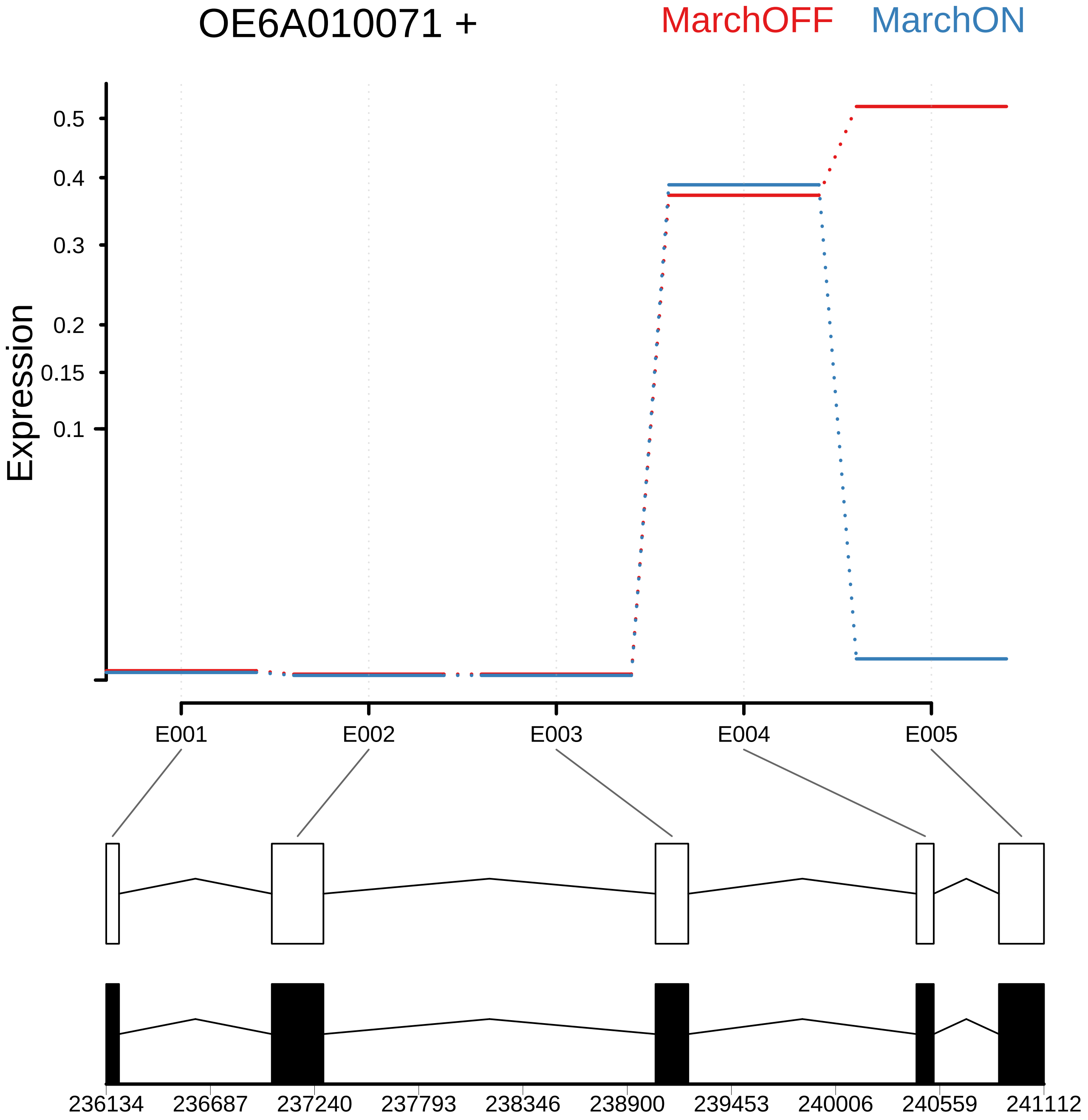
OE6A010071 +
MarchOFF
MarchON
Expression
0.5
0.4
0.3
0.2
0.15
0.1
E001
E002
E003
E004
E005
236134
236687
237240
237793
238346
238900
239453
240006
240559
241112

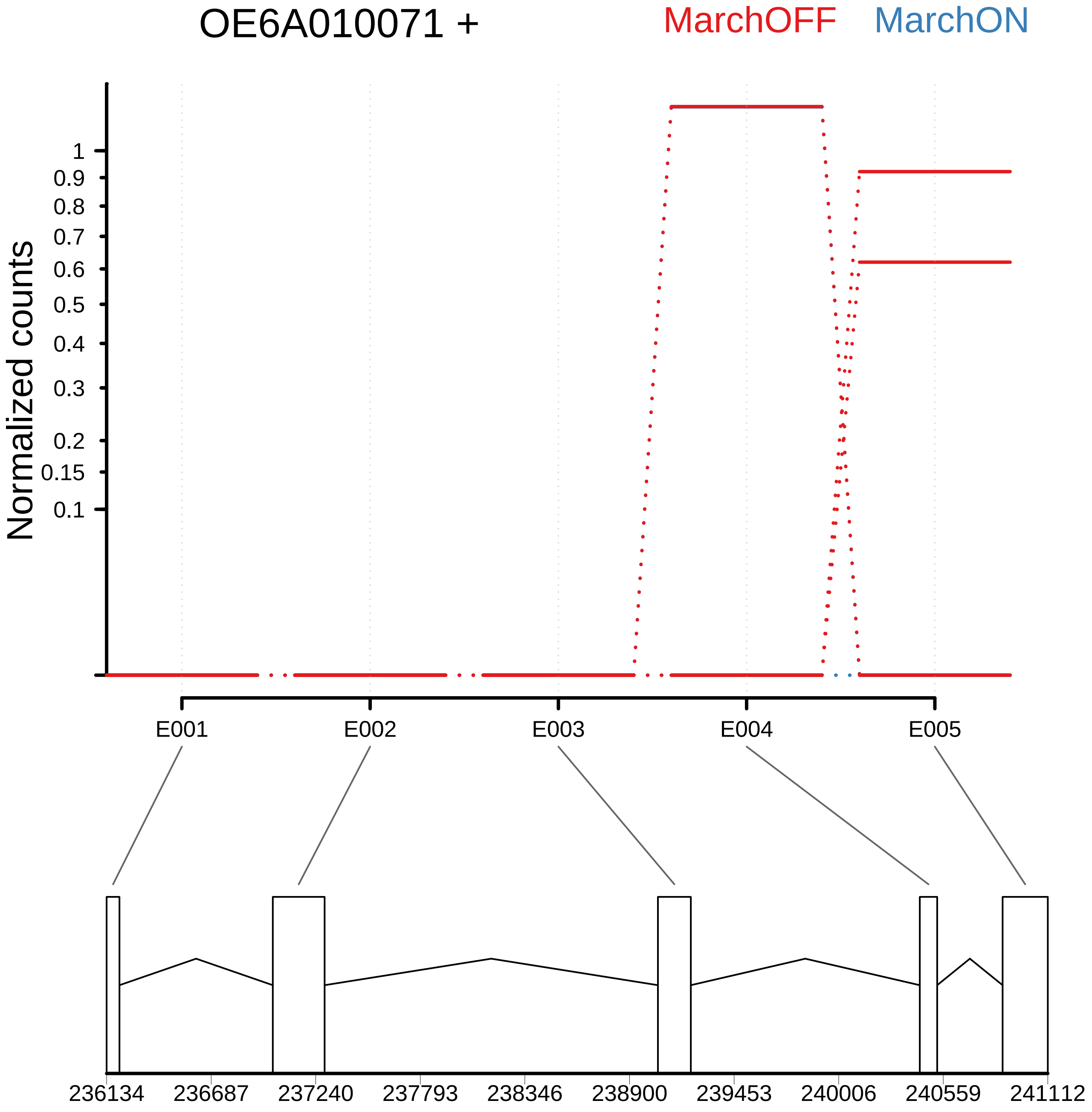

OE6A010071 +
MarchOFF
MarchON
Normalized counts
1
0.9
0.8
0.7
0.6
0.5
0.4
0.3
0.2
0.15
0.1
E001
E002
E003
E004
E005
236134
236687
237240
237793
238346
238900
239453
240006
240559
241112

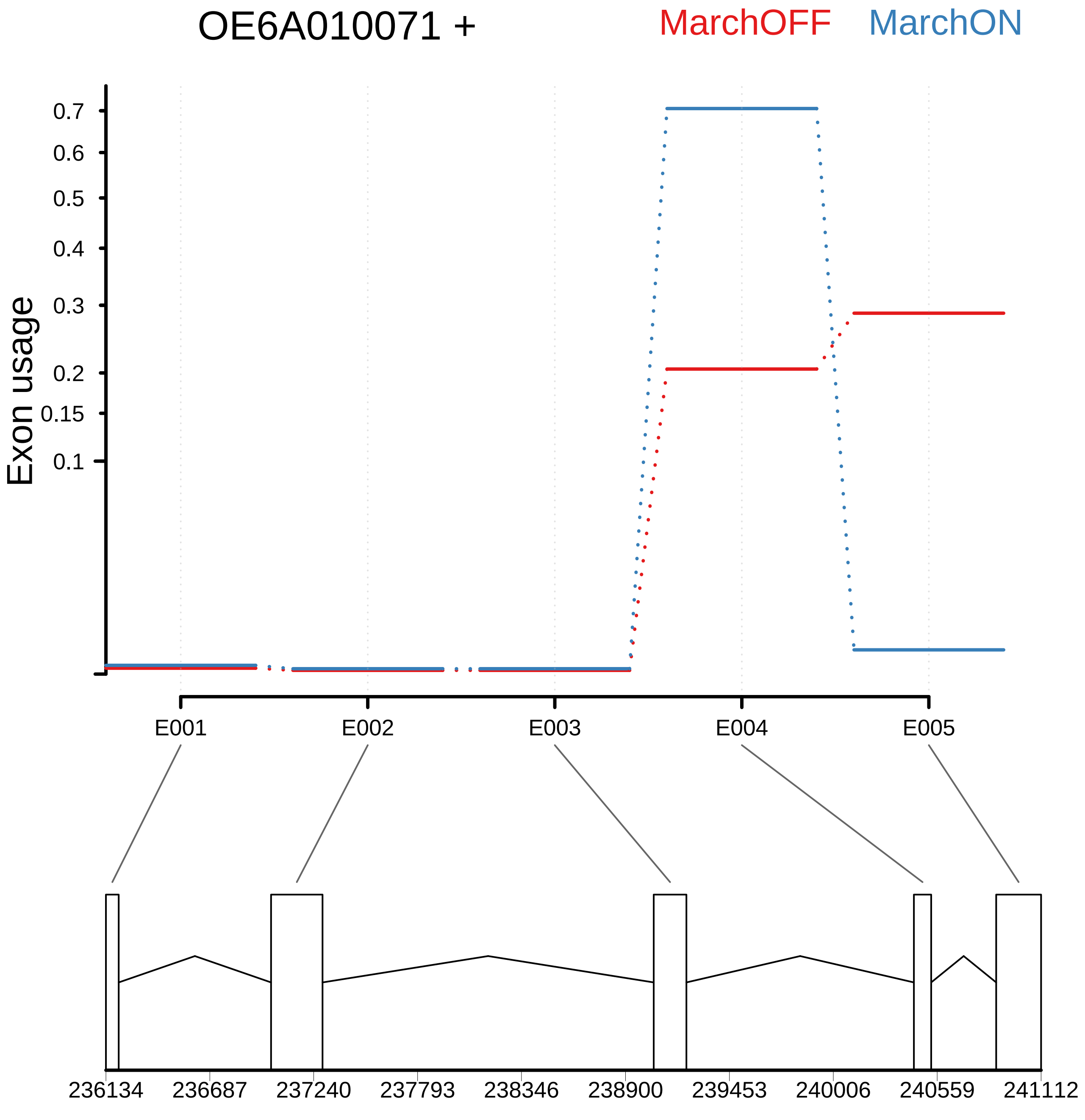

OE6A010071 +
MarchOFF
MarchON
Exon usage
0.7
0.6
0.5
0.4
0.3
0.2
0.15
0.1
E001
E002
E003
E004
E005
236134
236687
237240
237793
238346
238900
239453
240006
240559
241112

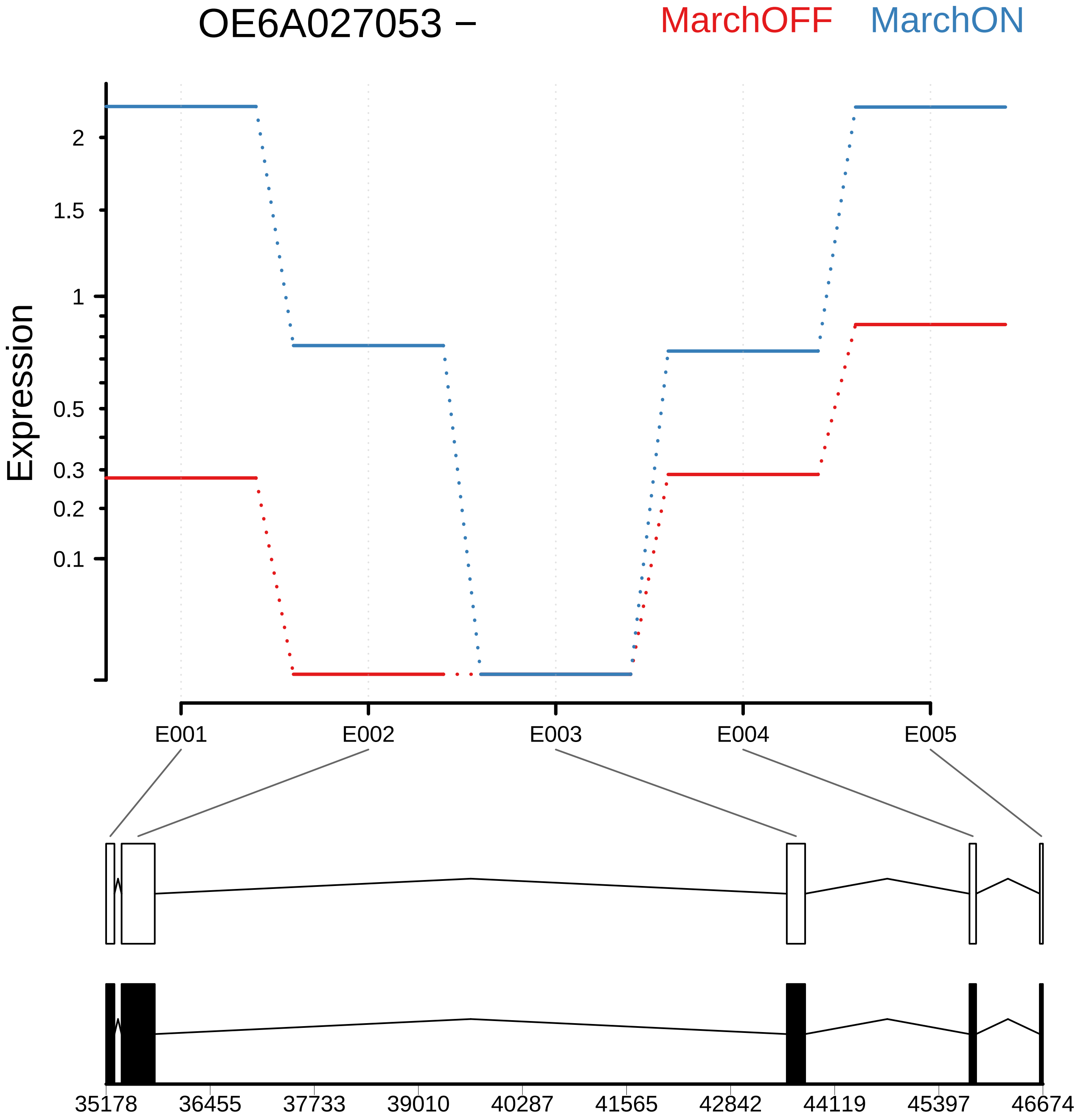

OE6A027053 −
MarchOFF
MarchON
Expression
2
1.5
1
0.5
0.3
0.2
0.1
E001
E002
E003
E004
E005
35178
36455
37733
39010
40287
41565
42842
44119
45397
46674

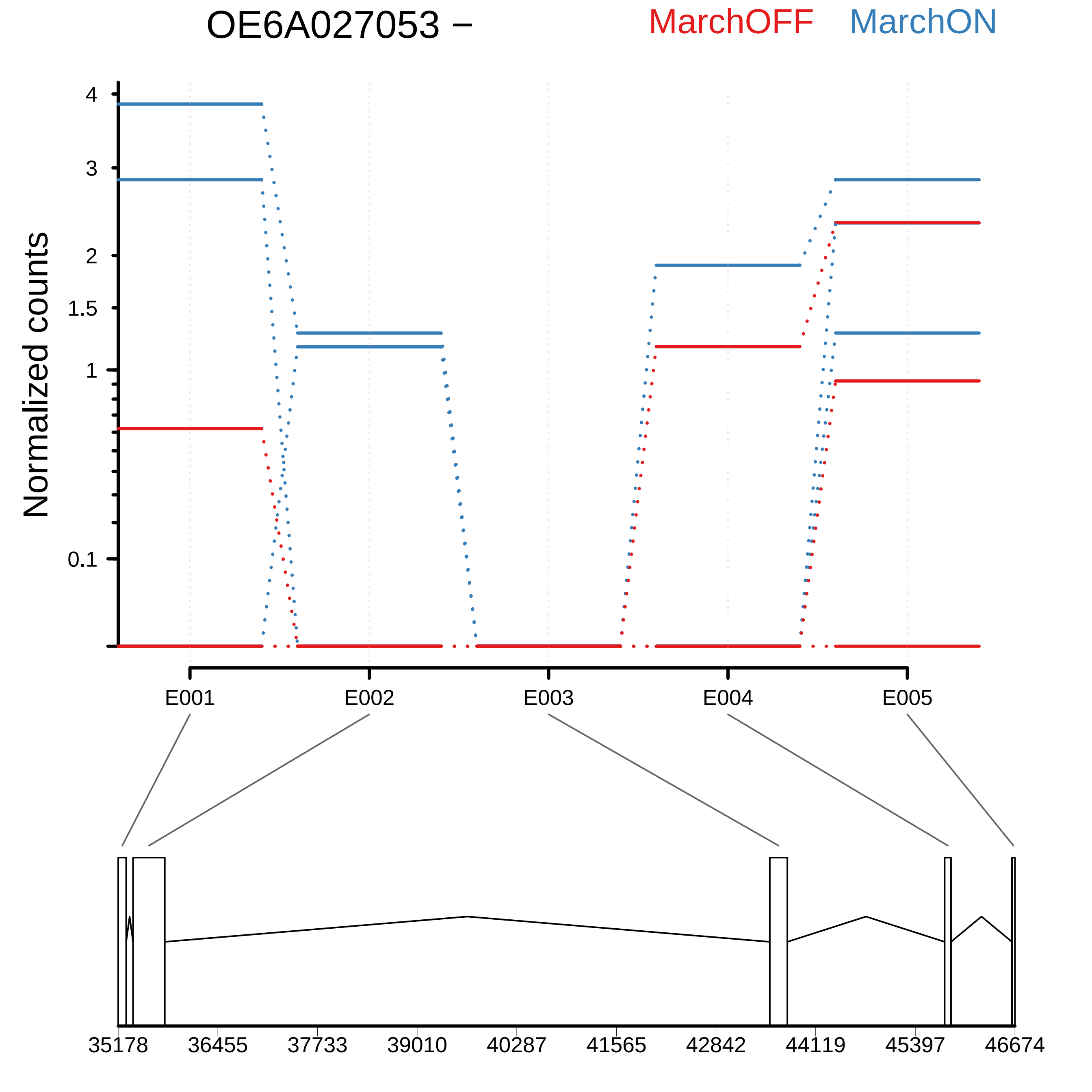
OE6A027053 −
MarchOFF
MarchON
Normalized counts
4
3
2
1.5
1
0.1
E001
E002
E003
E004
E005
35178
36455
37733
39010
40287
41565
42842
44119
45397
46674

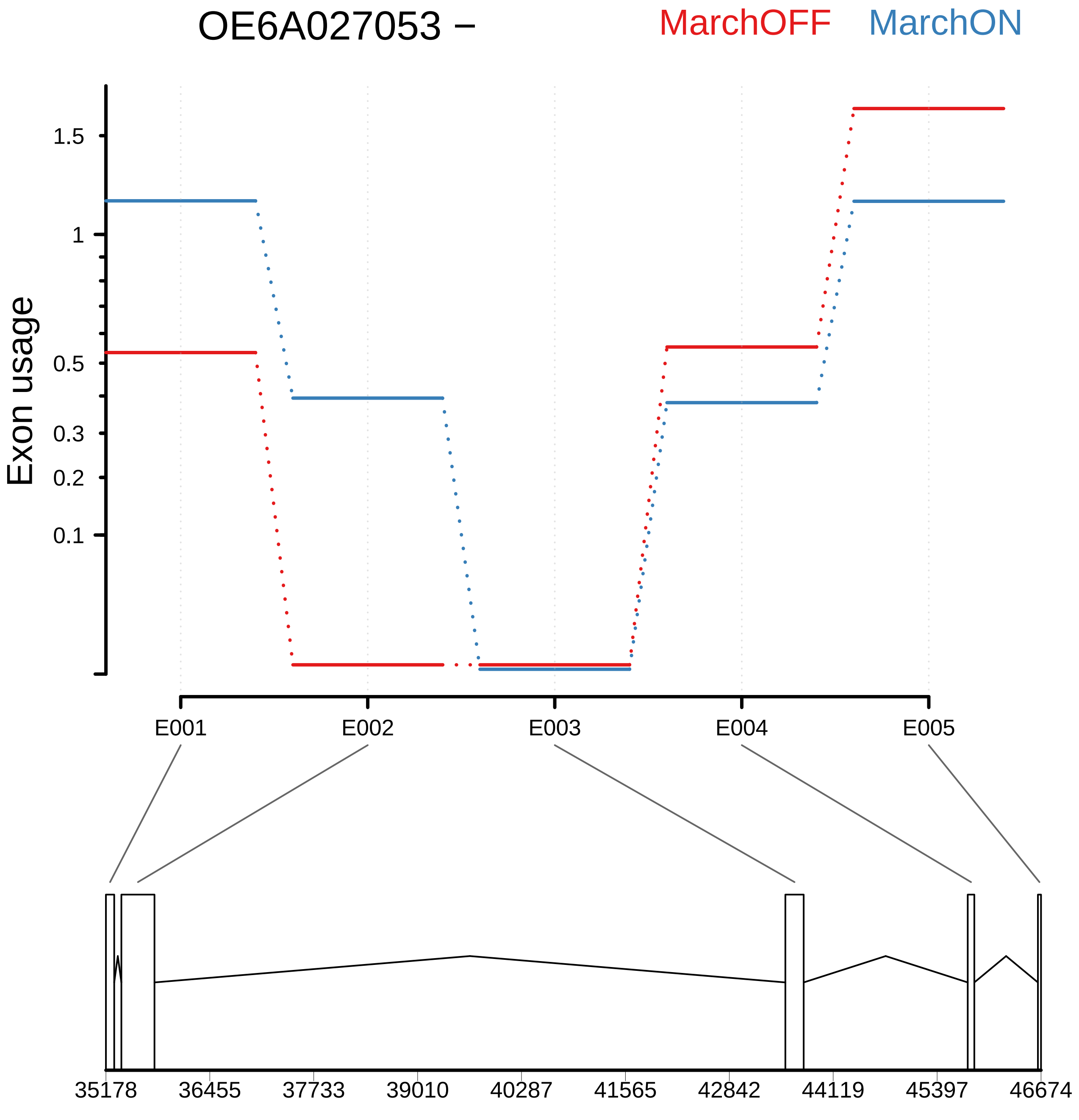

OE6A027053 −
MarchOFF
MarchON
Exon usage
1.5
1
0.5
0.3
0.2
0.1
E001
E002
E003
E004
E005
35178
36455
37733
39010
40287
41565
42842
44119
45397
46674

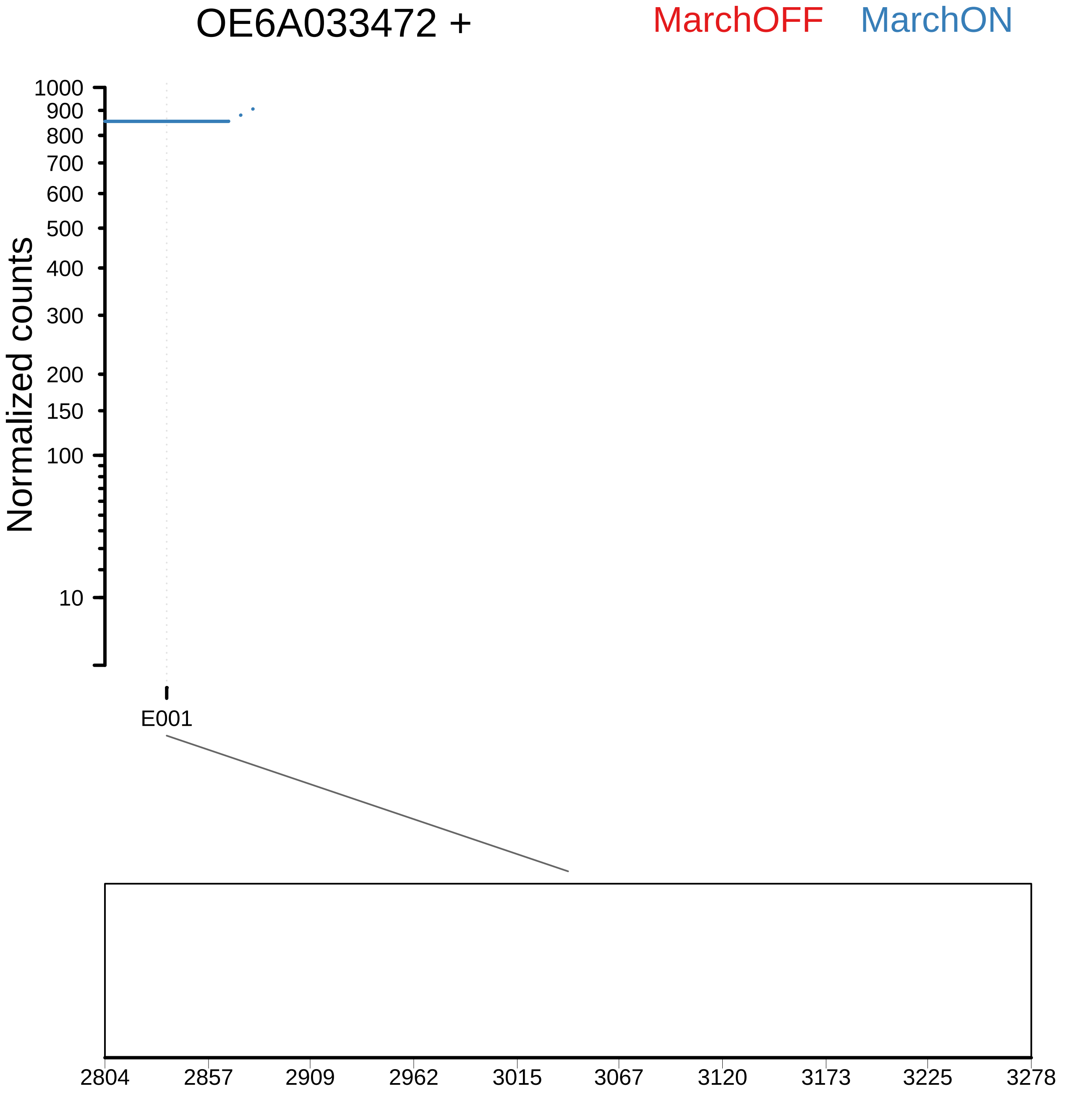
OE6A033472 +
MarchOFF
MarchON
Normalized counts
1000
900
800
700
600
500
400
300
200
150
100
10
E001
2804
2857
2909
2962
3015
3067
3120
3173
3225
3278

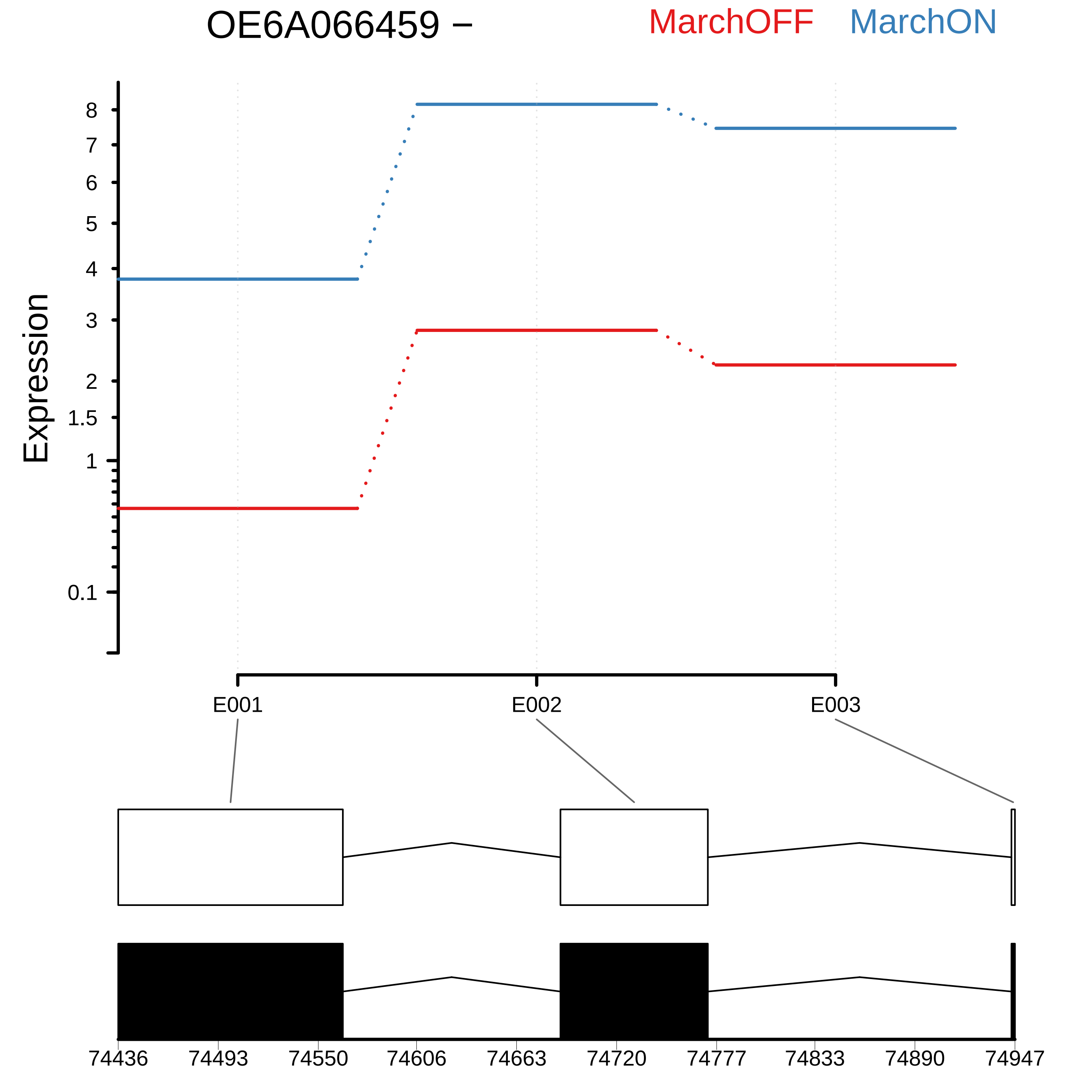

OE6A066459 −
MarchOFF
MarchON
Expression
8
7
6
5
4
3
2
1.5
1
0.1
E001
E002
E003
74436
74493
74550
74606
74663
74720
74777
74833
74890
74947

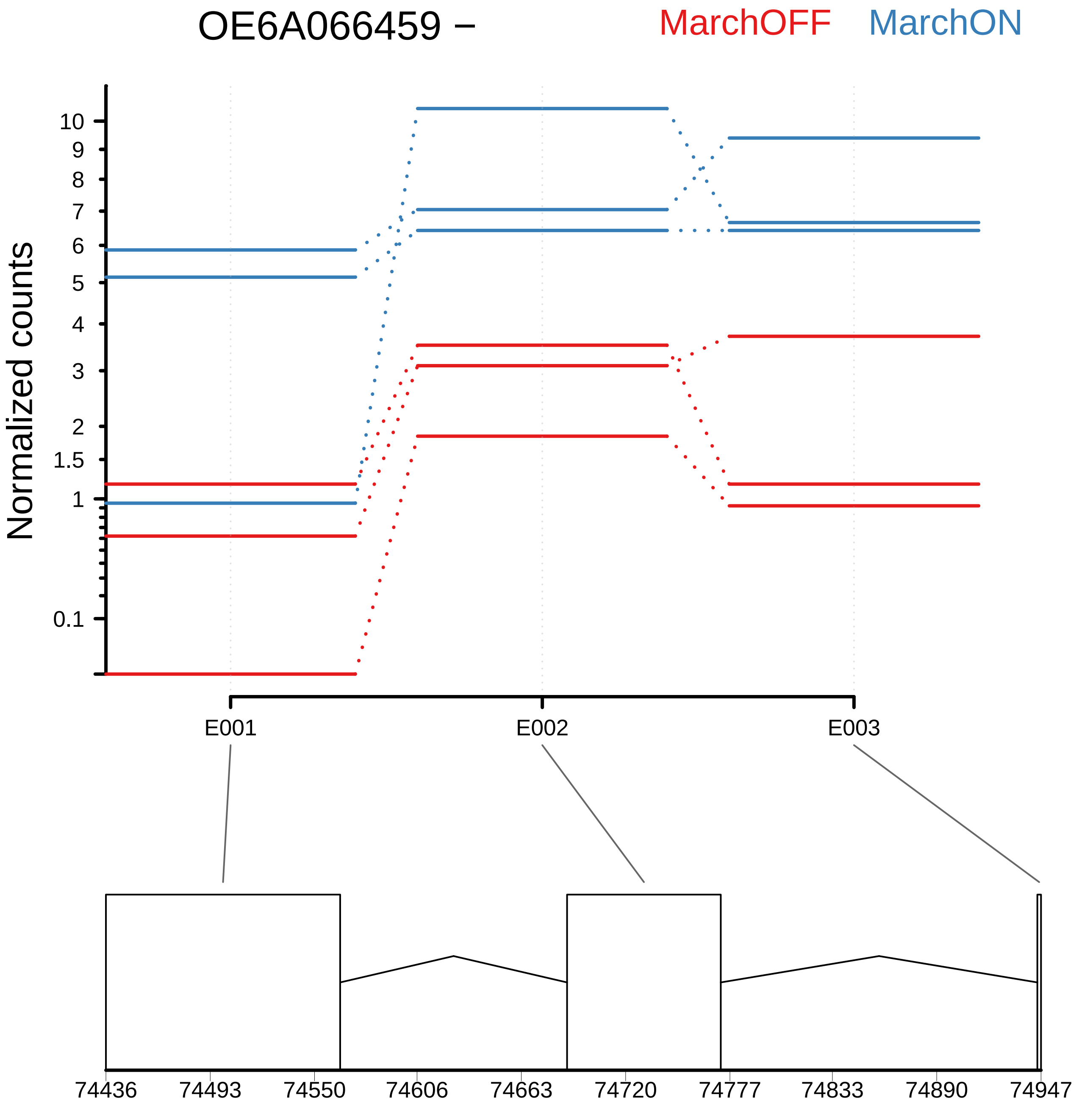

OE6A066459 −
MarchOFF
MarchON
Normalized counts
10
9
8
7
6
5
4
3
2
1.5
1
0.1
E001
E002
E003
74436
74493
74550
74606
74663
74720
74777
74833
74890
74947

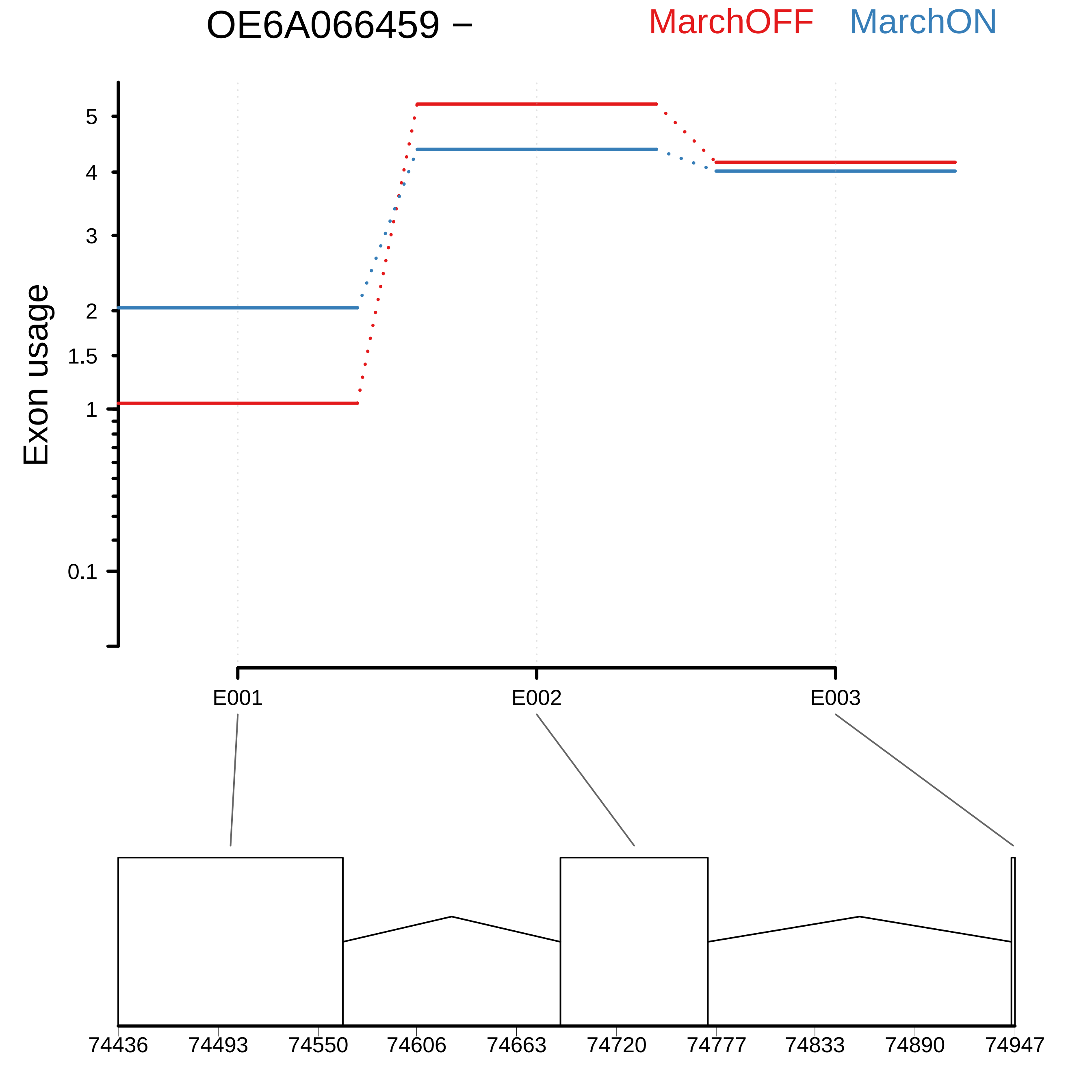
OE6A066459 −
MarchOFF
MarchON
Exon usage
5
4
3
2
1.5
1
0.1
E001
E002
E003
74436
74493
74550
74606
74663
74720
74777
74833
74890
74947

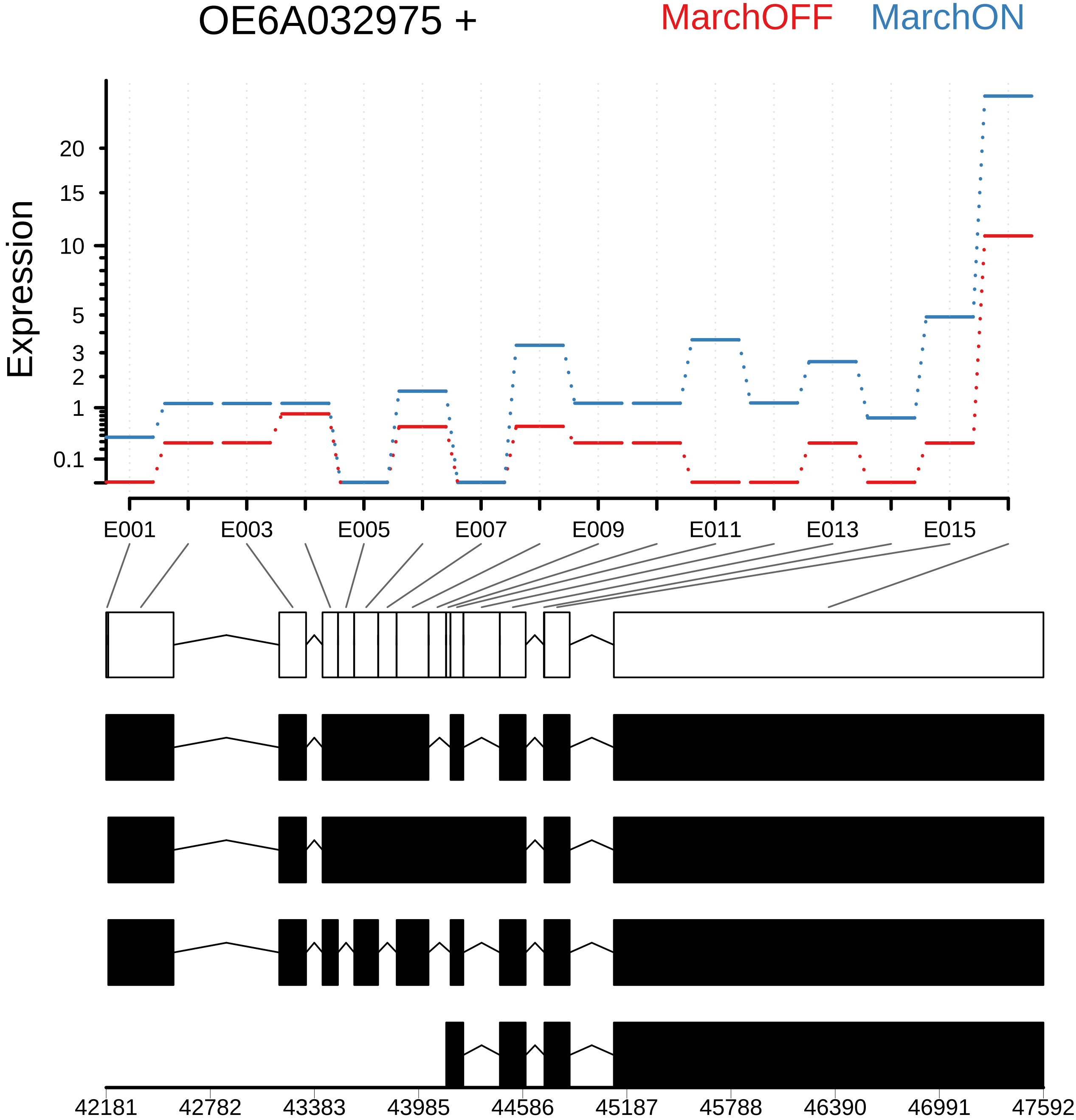
OE6A032975 +
MarchOFF
MarchON
Expression
20
15
10
5
3
2
1
0.1
E001
E003
E005
E007
E009
E011
E013
E015
42181
42782
43383
43985
44586
45187
45788
46390
46991
47592

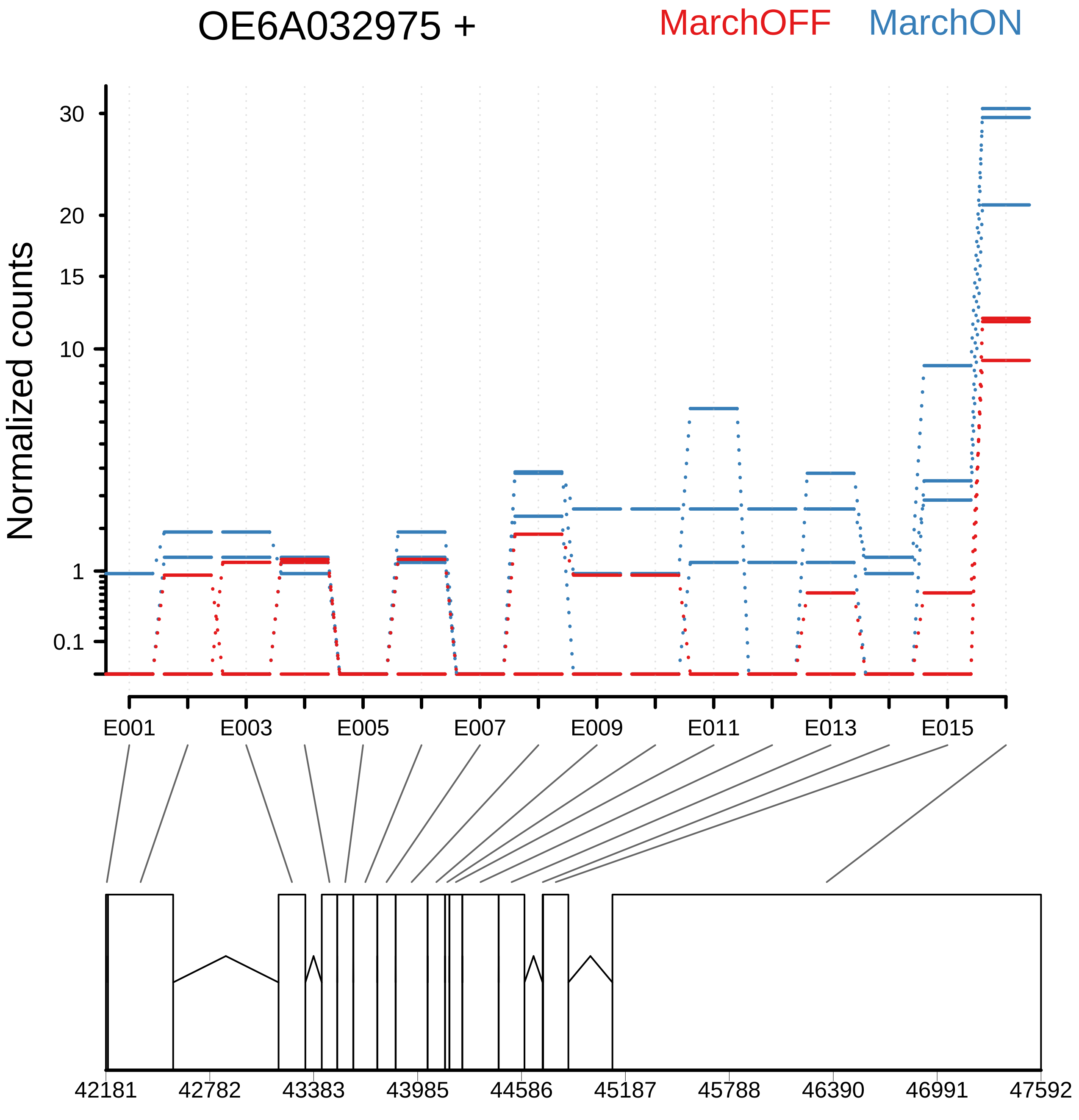

OE6A032975 +
MarchOFF
MarchON
Normalized counts
30
20
15
10
1
0.1
E001
E003
E005
E007
E009
E011
E013
E015
42181
42782
43383
43985
44586
45187
45788
46390
46991
47592

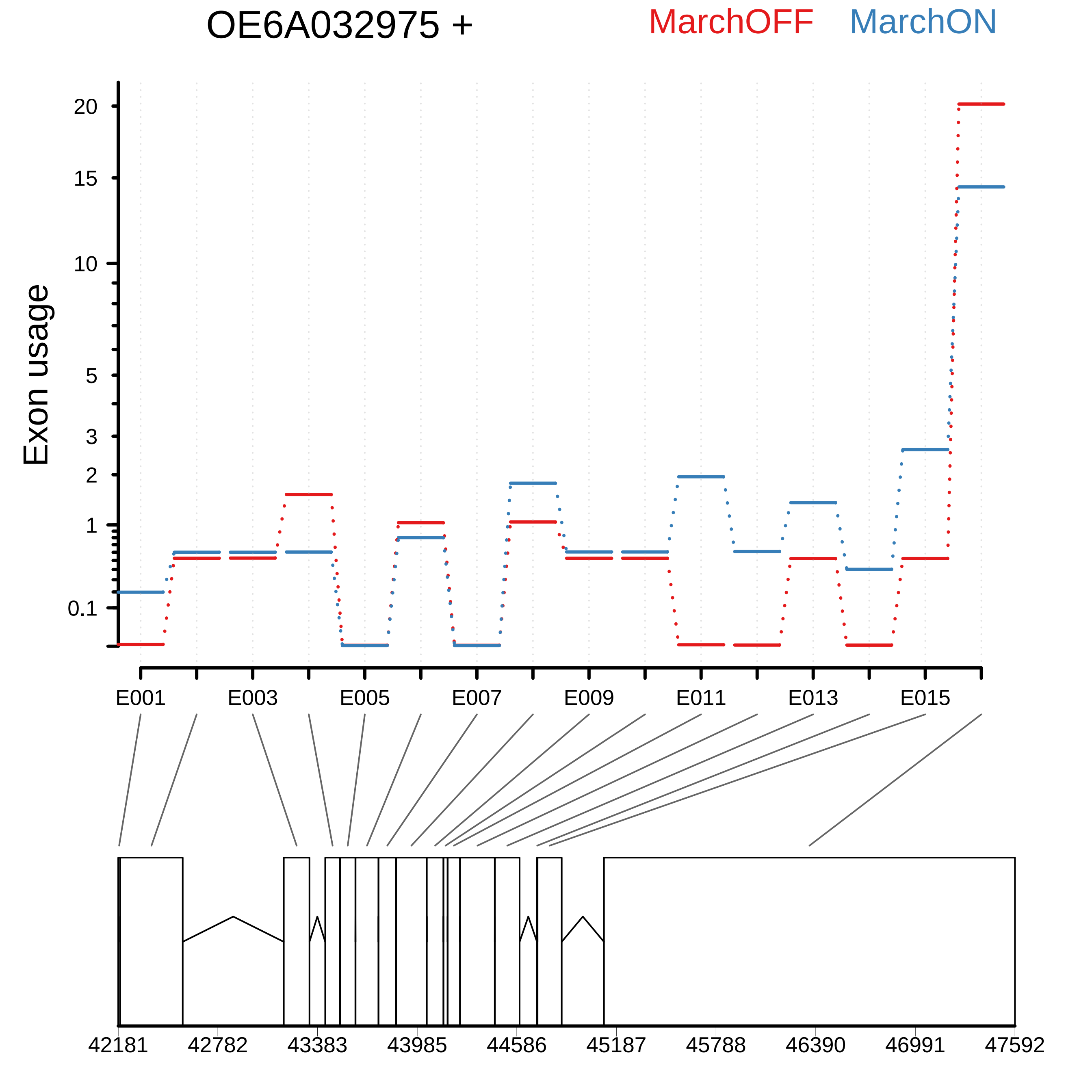

OE6A032975 +
MarchOFF
MarchON
Exon usage
20
15
10
5
3
2
1
0.1
E001
E003
E005
E007
E009
E011
E013
E015
42181
42782
43383
43985
44586
45187
45788
46390
46991
47592

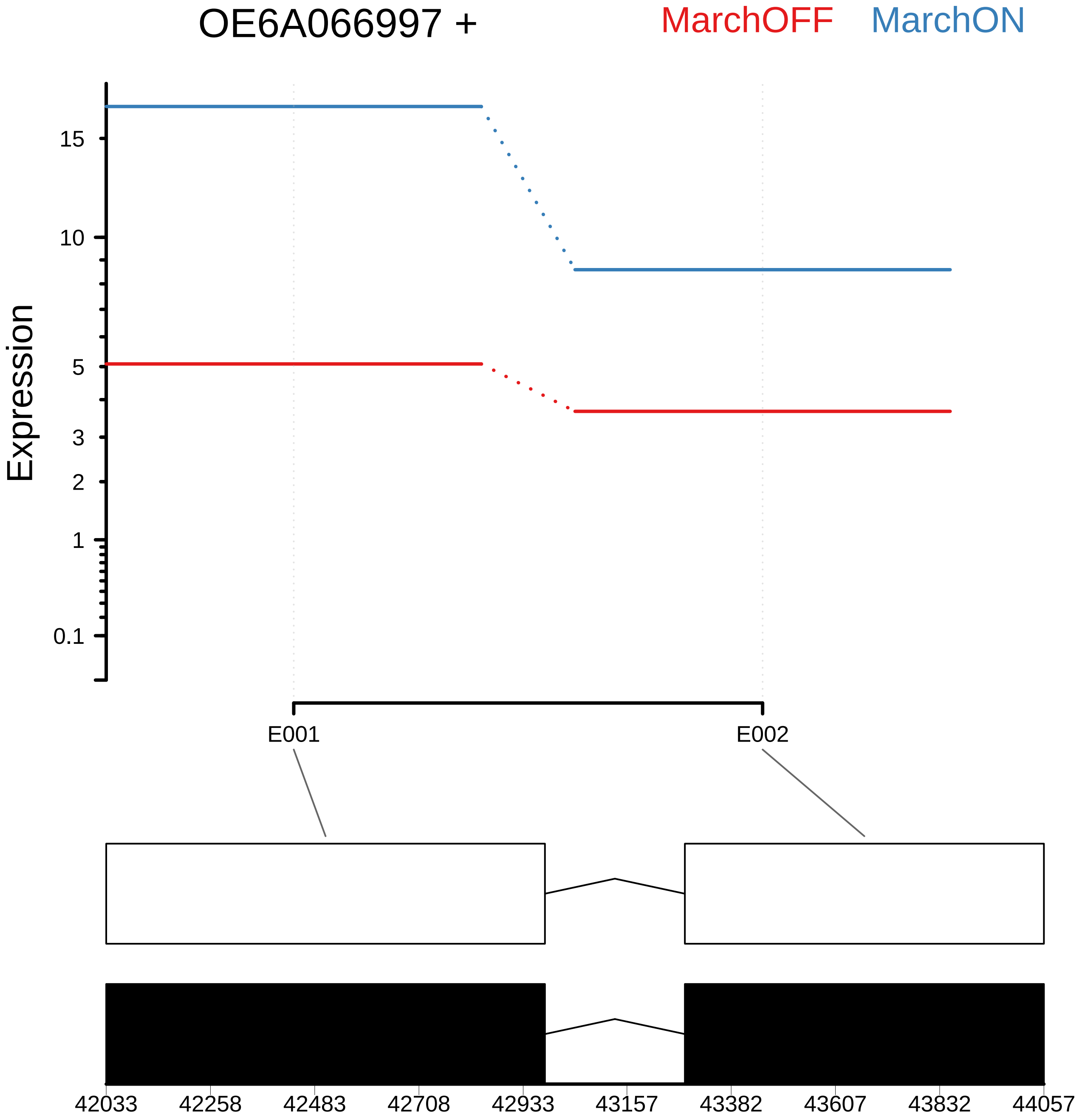

OE6A066997 +
MarchOFF
MarchON
Expression
15
10
5
3
2
1
0.1
E001
E002
42033
42258
42483
42708
42933
43157
43382
43607
43832
44057

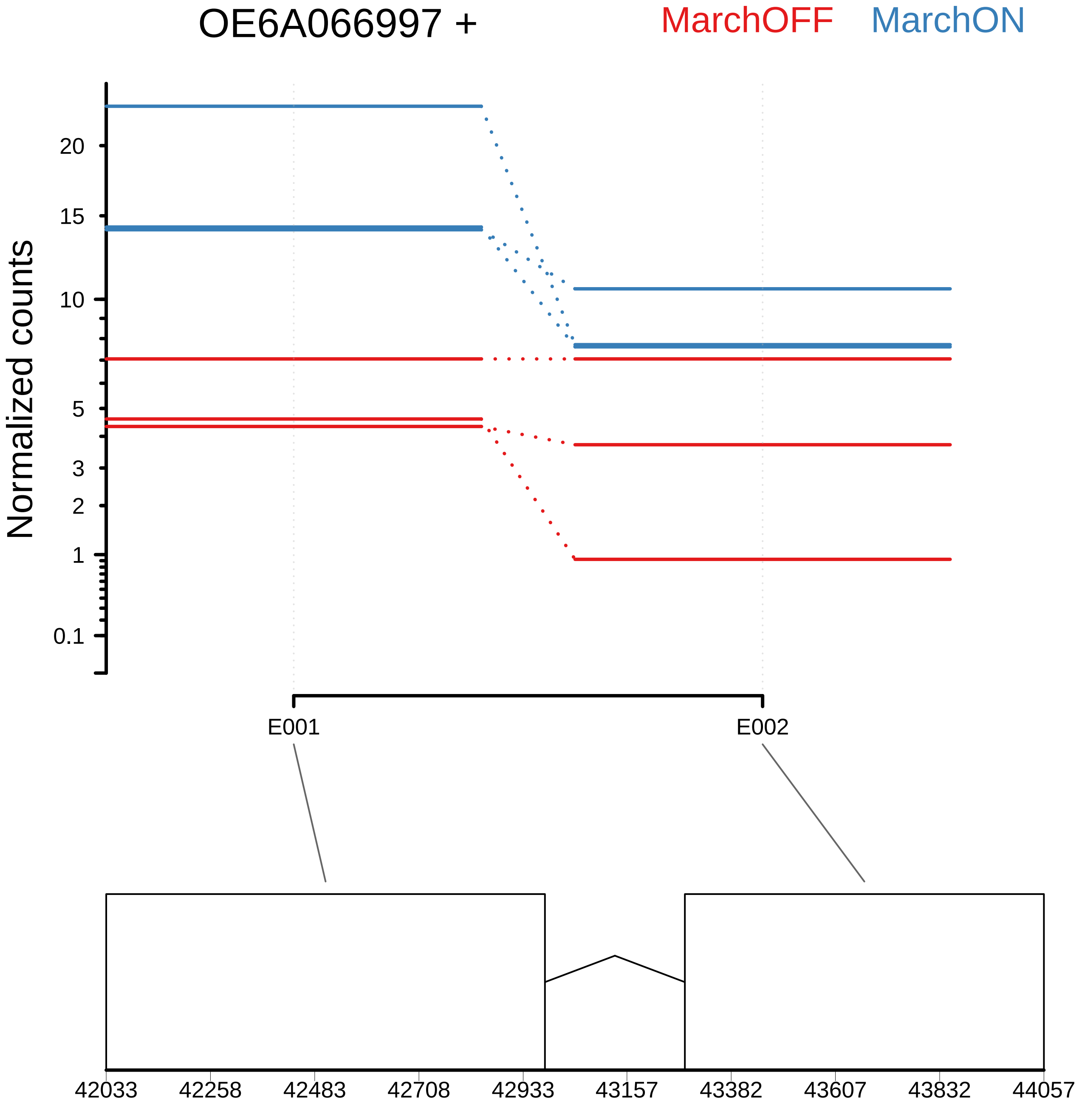

OE6A066997 +
MarchOFF
MarchON
Normalized counts
20
15
10
5
3
2
1
0.1
E001
E002
42033
42258
42483
42708
42933
43157
43382
43607
43832
44057

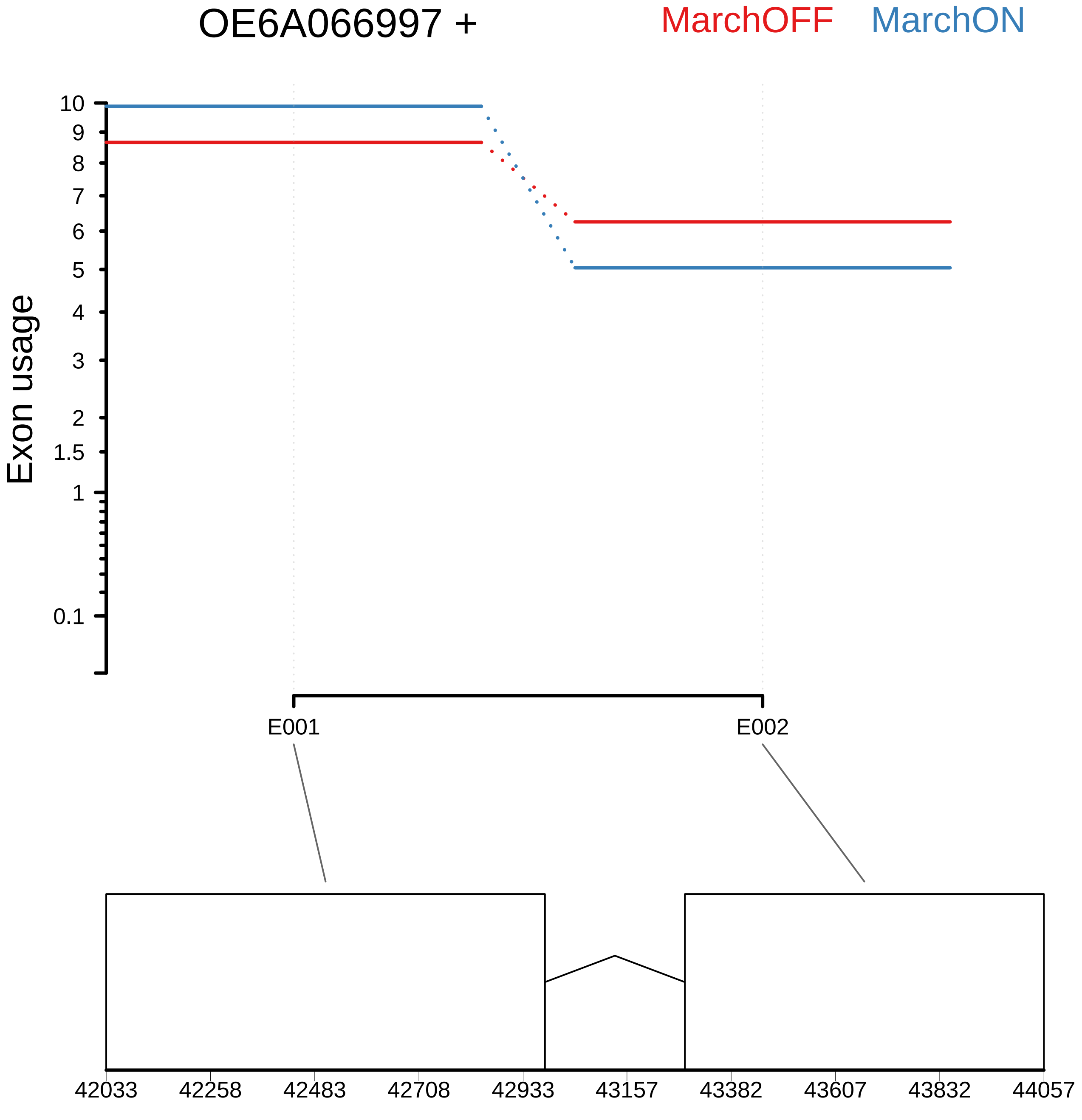

OE6A066997 +
MarchOFF
MarchON
Exon usage
10
9
8
7
6
5
4
3
2
1.5
1
0.1
E001
E002
42033
42258
42483
42708
42933
43157
43382
43607
43832
44057

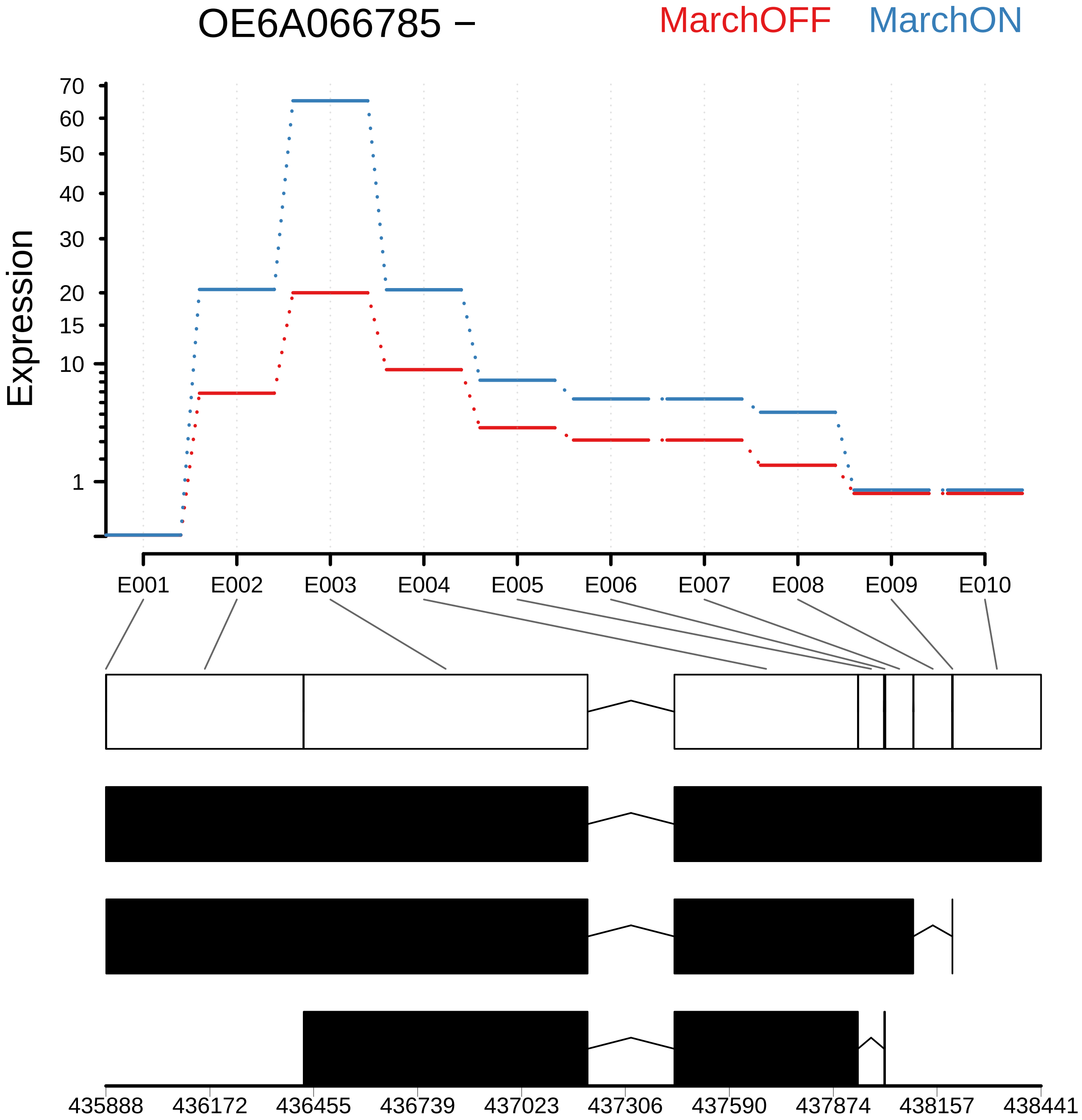

OE6A066785 –
MarchOFF
MarchON
Expression
70
60
50
40
30
20
15
10
1
E001
E002
E003
E004
E005
E006
E007
E008
E009
E010
435888
436172
436455
436739
437023
437306
437590
437874
438157
438441

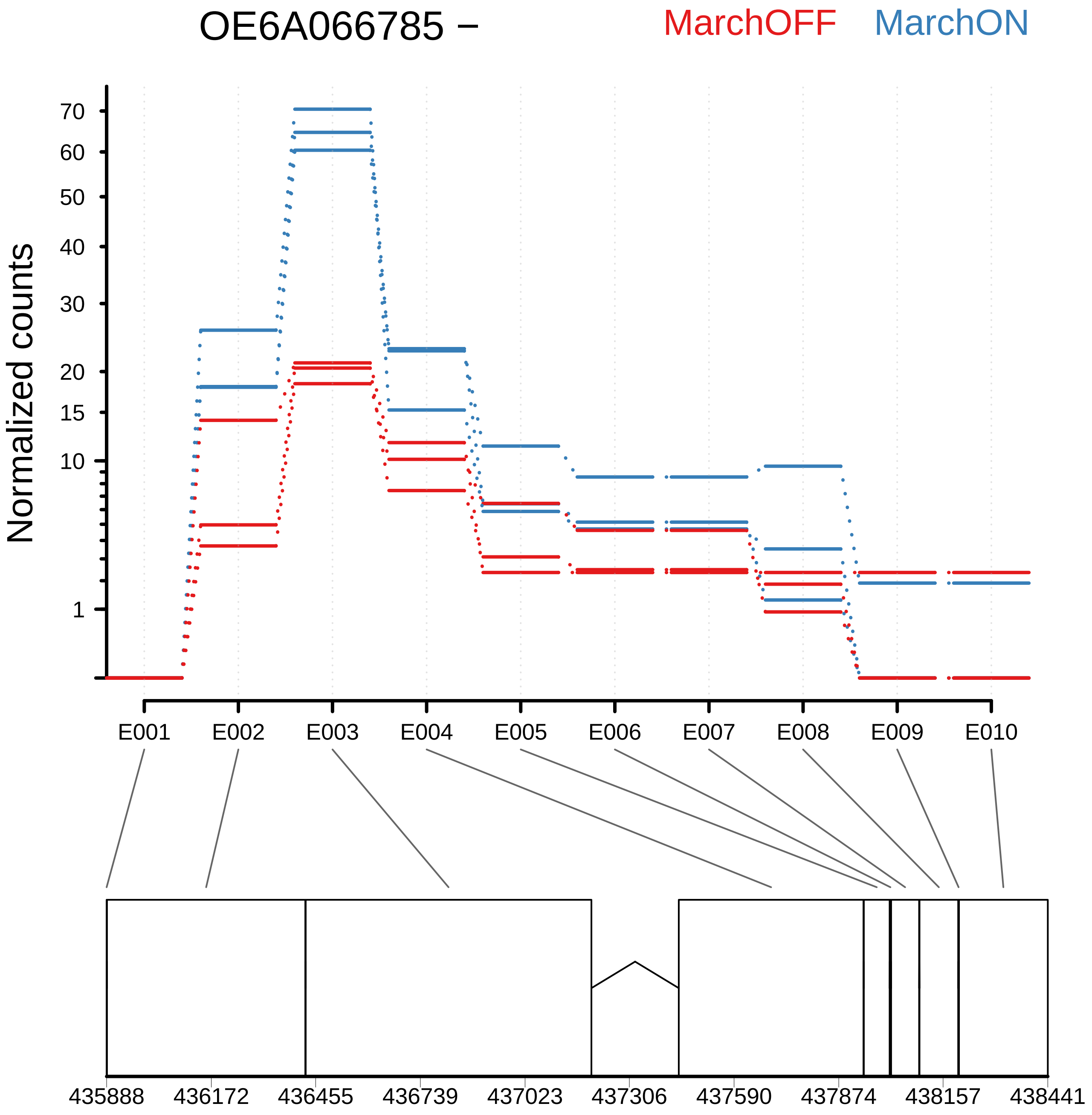

OE6A066785 −
MarchOFF
MarchON
Normalized counts
70
60
50
40
30
20
15
10
1
E001
E002
E003
E004
E005
E006
E007
E008
E009
E010
435888
436172
436455
436739
437023
437306
437590
437874
438157
438441

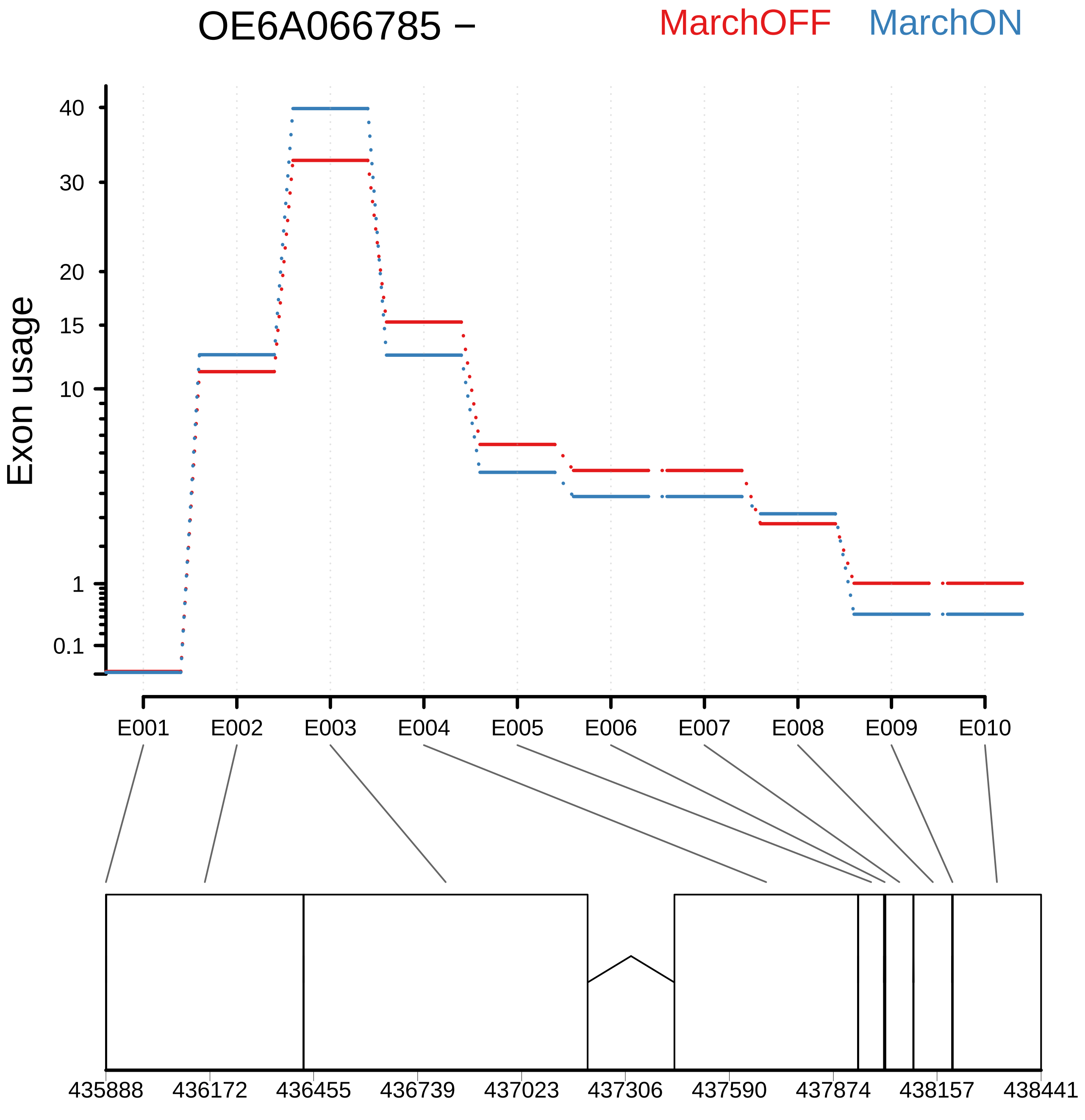

OE6A066785 −
MarchOFF
MarchON
Exon usage
40
30
20
15
10
1
0.1
E001
E002
E003
E004
E005
E006
E007
E008
E009
E010
435888
436172
436455
436739
437023
437306
437590
437874
438157
438441

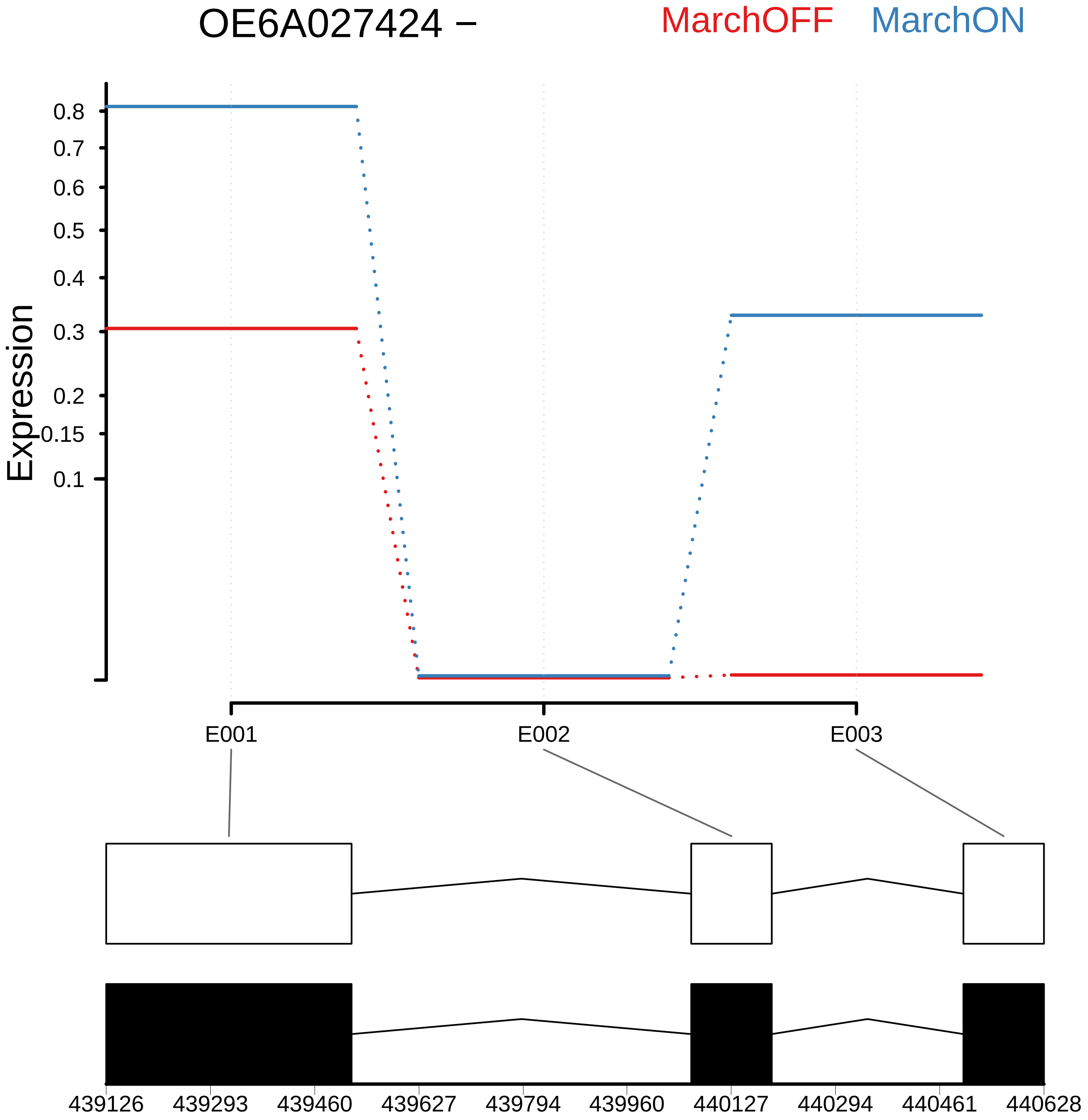

OE6A027424 −
MarchOFF
MarchON
Expression
0.8
0.7
0.6
0.5
0.4
0.3
0.2
0.15
0.1
E001
E002
E003
439126
439293
439460
439627
439794
439960
440127
440294
440461
440628

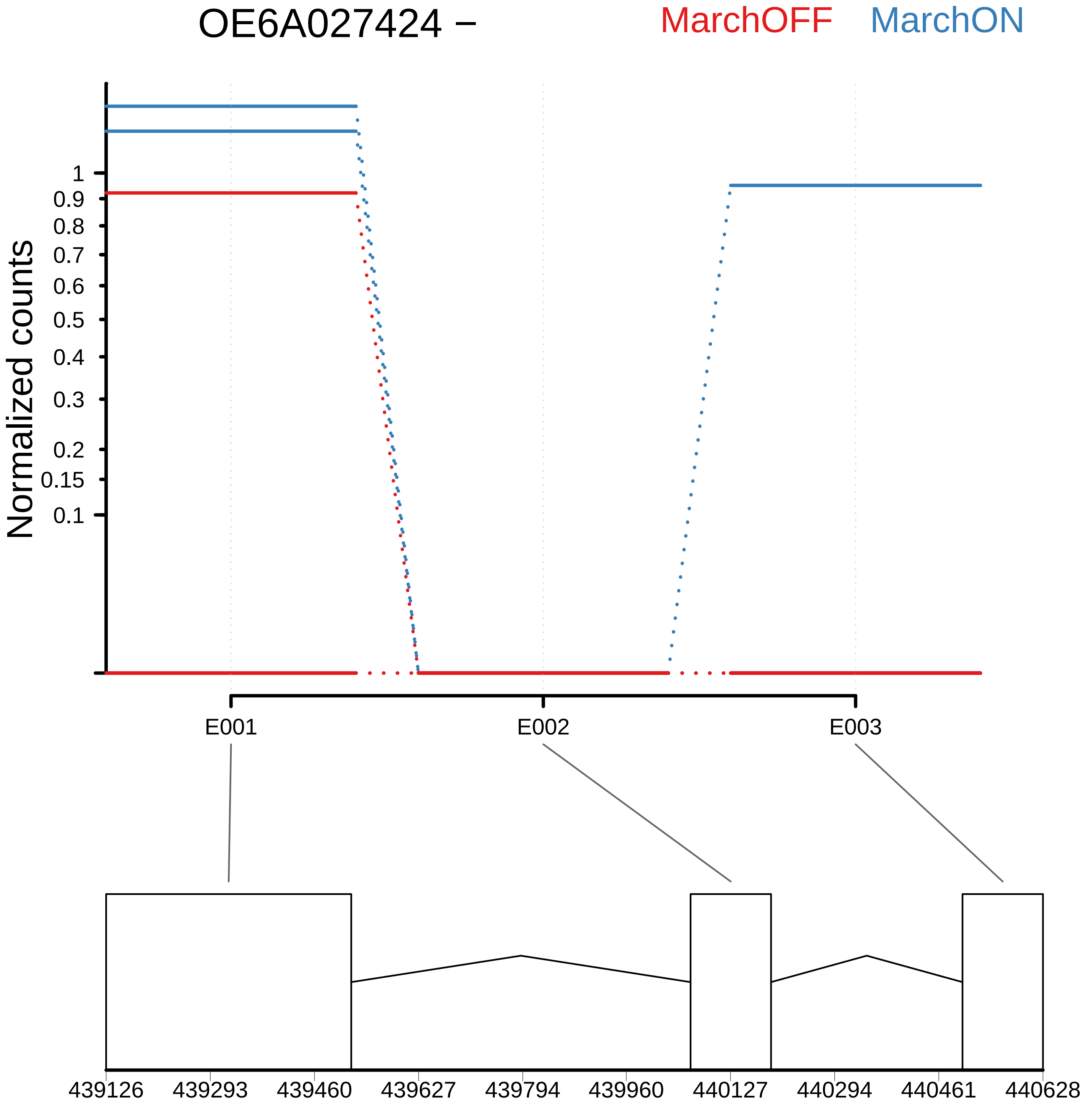
OE6A027424 −
MarchOFF
MarchON
Normalized counts
1
0.9
0.8
0.7
0.6
0.5
0.4
0.3
0.2
0.15
0.1
E001
E002
E003
439126
439293
439460
439627
439794
439960
440127
440294
440461
440628

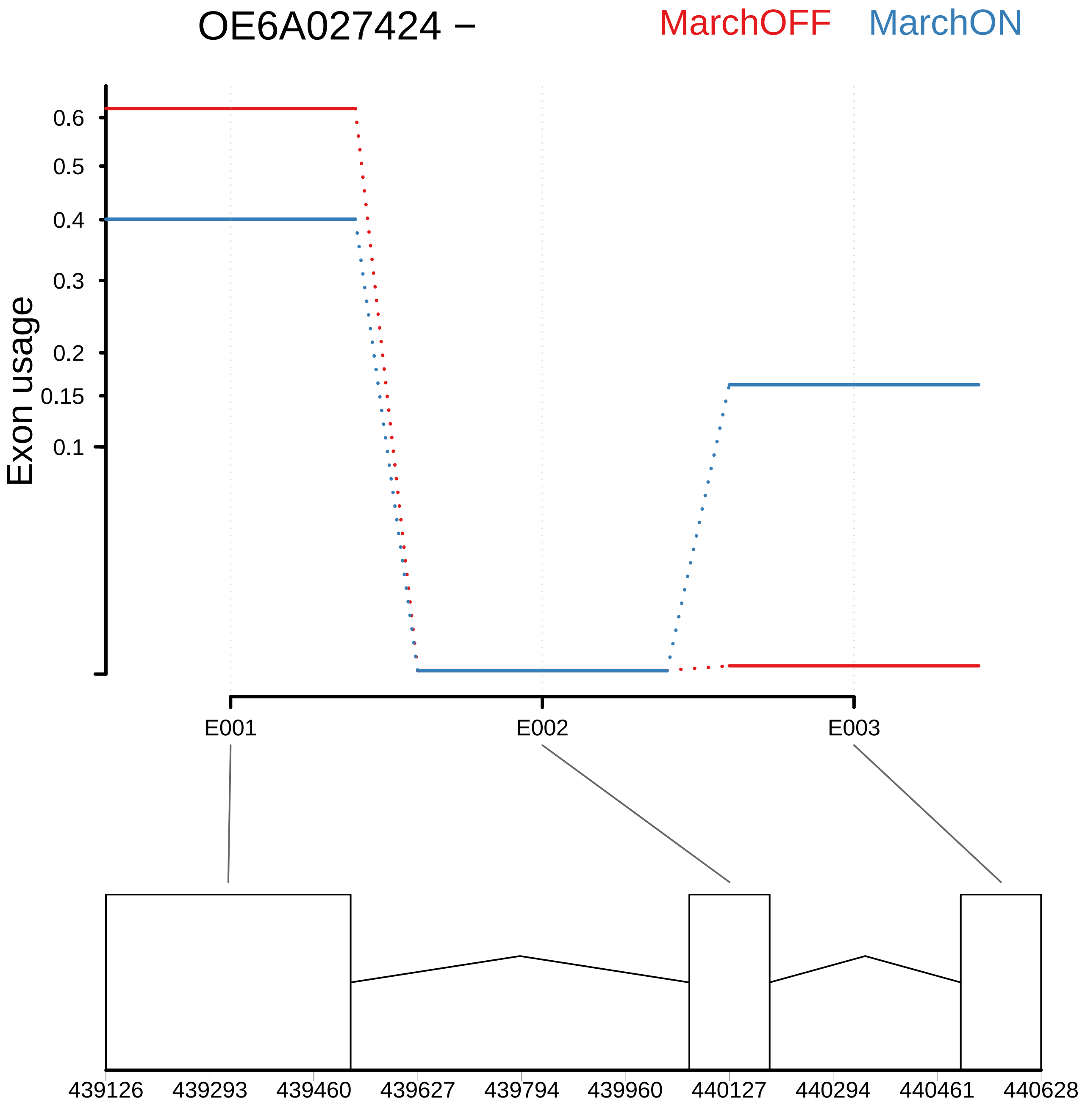
OE6A027424 −
MarchOFF
MarchON
Exon usage
0.6
0.5
0.4
0.3
0.2
0.15
0.1
E001
E002
E003
439126
439293
439460
439627
439794
439960
440127
440294
440461
440628

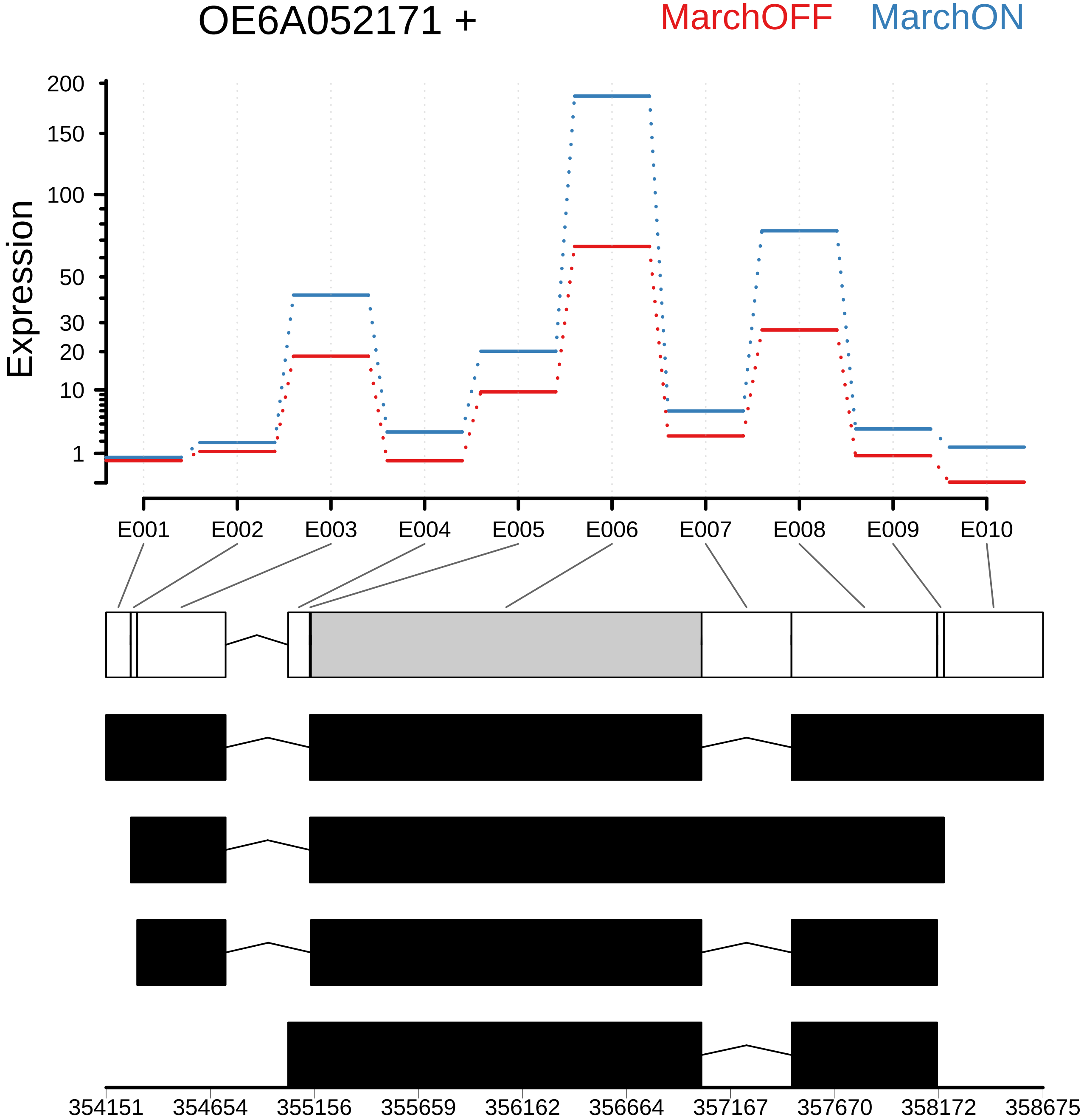
OE6A052171 +
MarchOFF
MarchON
Expression
200
150
100
50
30
20
10
1
E001
E002
E003
E004
E005
E006
E007
E008
E009
E010
354151
354654
355156
355659
356162
356664
357167
357670
358172
358675

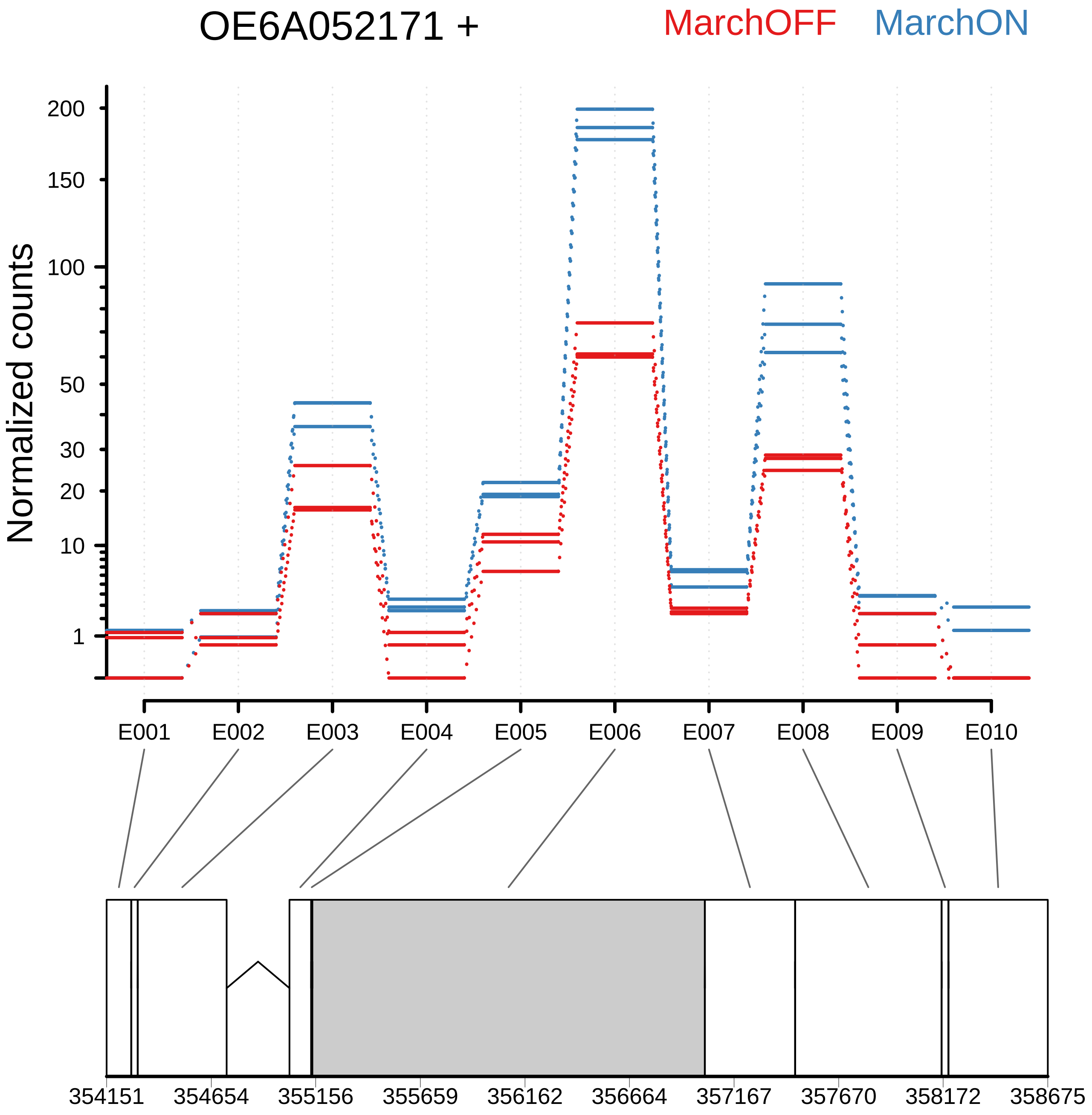

OE6A052171 +
MarchOFF
MarchON
Normalized counts
200
150
100
50
30
20
10
1
E001
E002
E003
E004
E005
E006
E007
E008
E009
E010
354151
354654
355156
355659
356162
356664
357167
357670
358172
358675

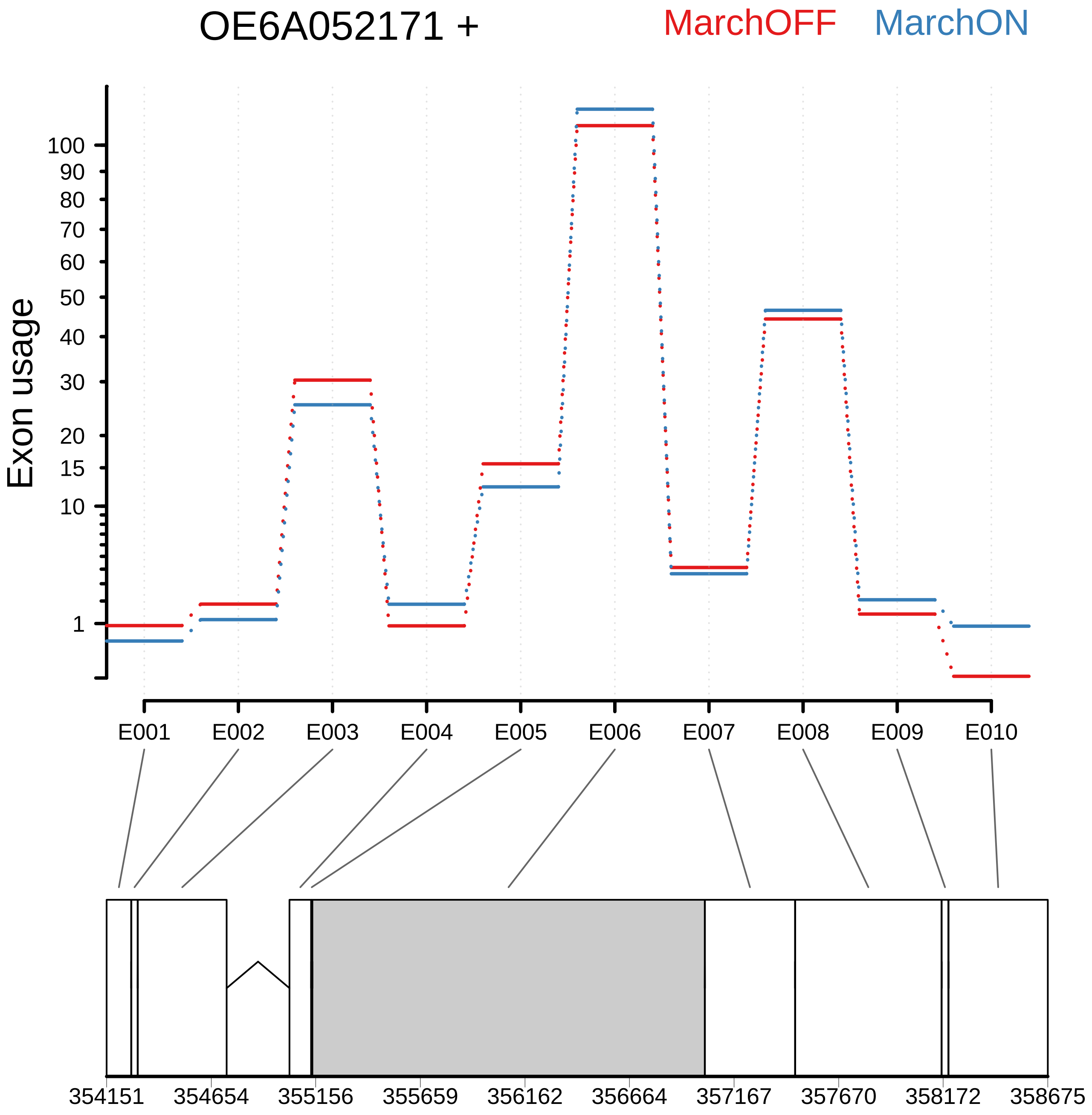

OE6A052171 +
MarchOFF
MarchON
Exon usage
100
90
80
70
60
50
40
30
20
15
10
1
E001
E002
E003
E004
E005
E006
E007
E008
E009
E010
354151
354654
355156
355659
356162
356664
357167
357670
358172
358675

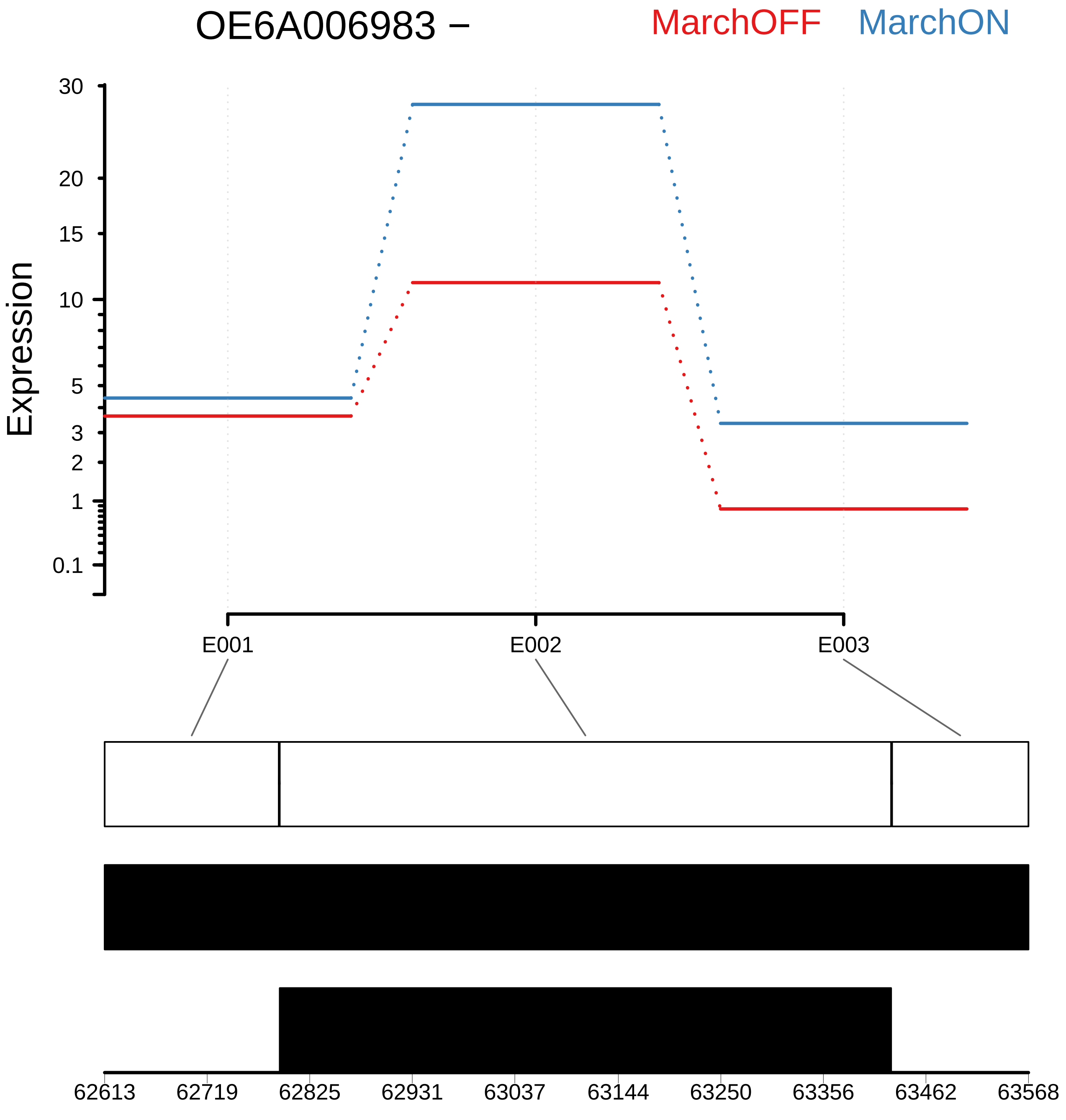

OE6A006983 −
MarchOFF
MarchON
Expression
30
20
15
10
5
3
2
1
0.1
E001
E002
E003
62613
62719
62825
62931
63037
63144
63250
63356
63462
63568

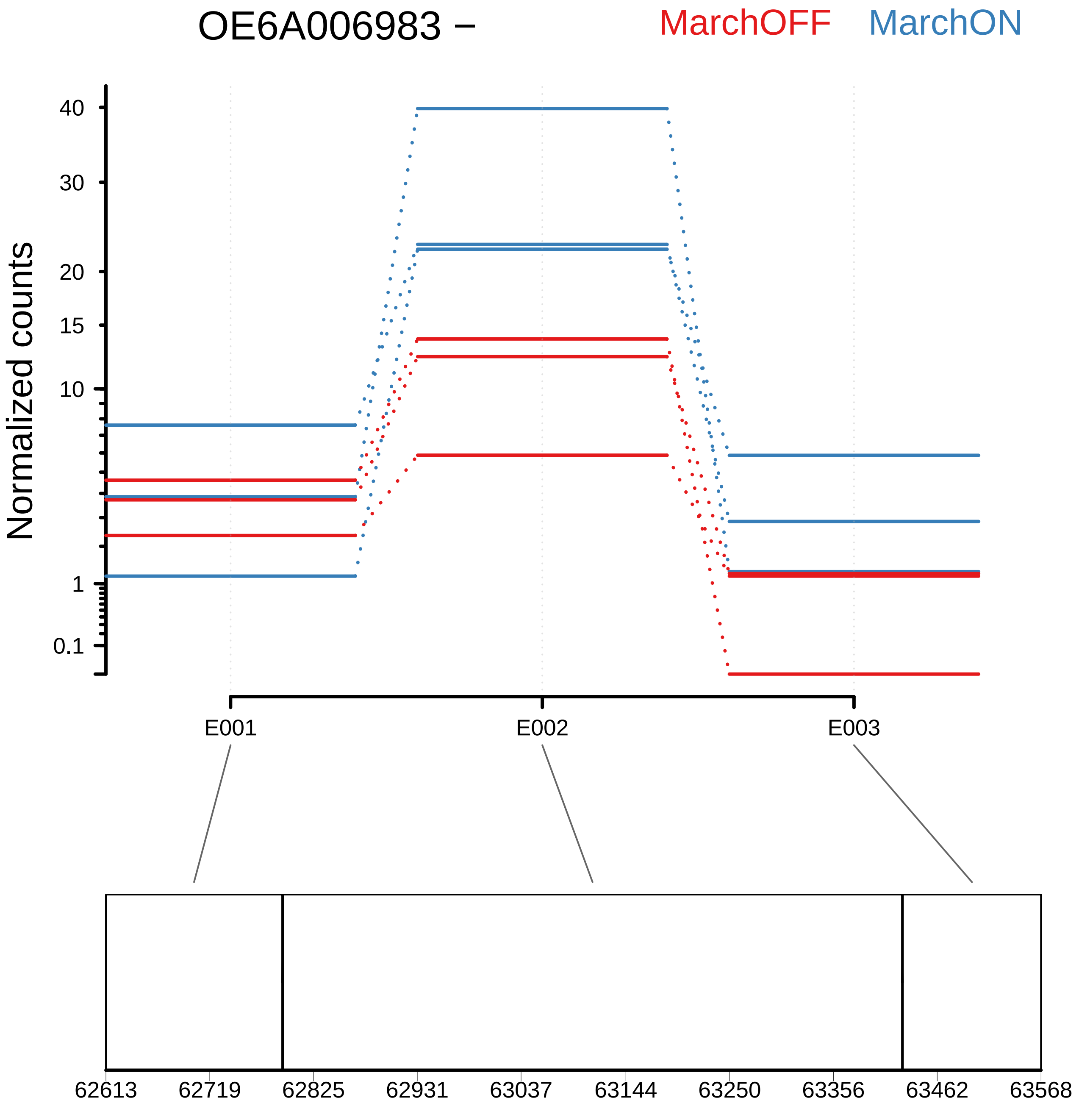

OE6A006983 −
MarchOFF
MarchON
Normalized counts
40
30
20
15
10
1
0.1
E001
E002
E003
62613
62719
62825
62931
63037
63144
63250
63356
63462
63568

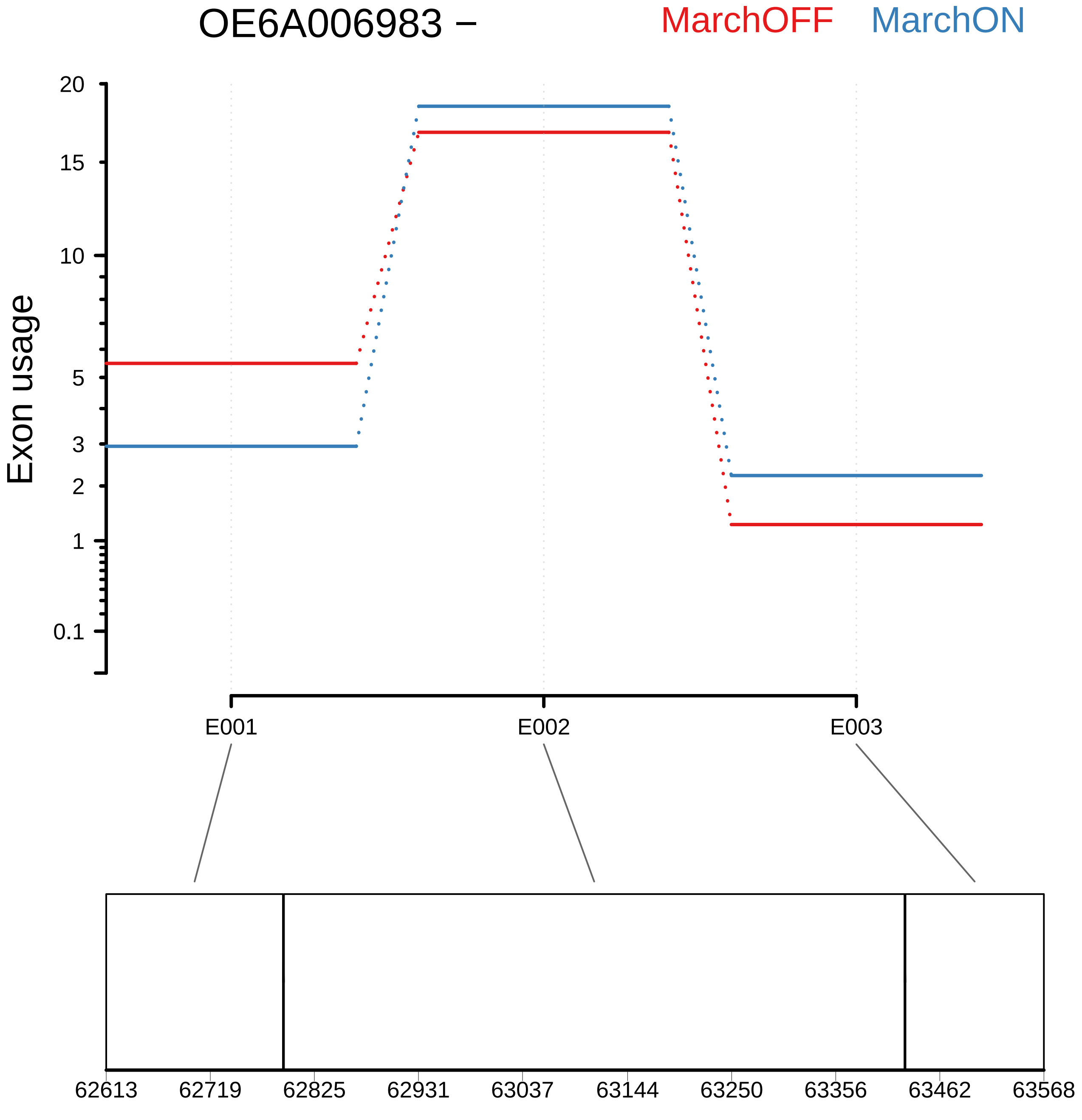
OE6A006983 −
MarchOFF
MarchON
Exon usage
20
15
10
5
3
2
1
0.1
E001
E002
E003
62613
62719
62825
62931
63037
63144
63250
63356
63462
63568

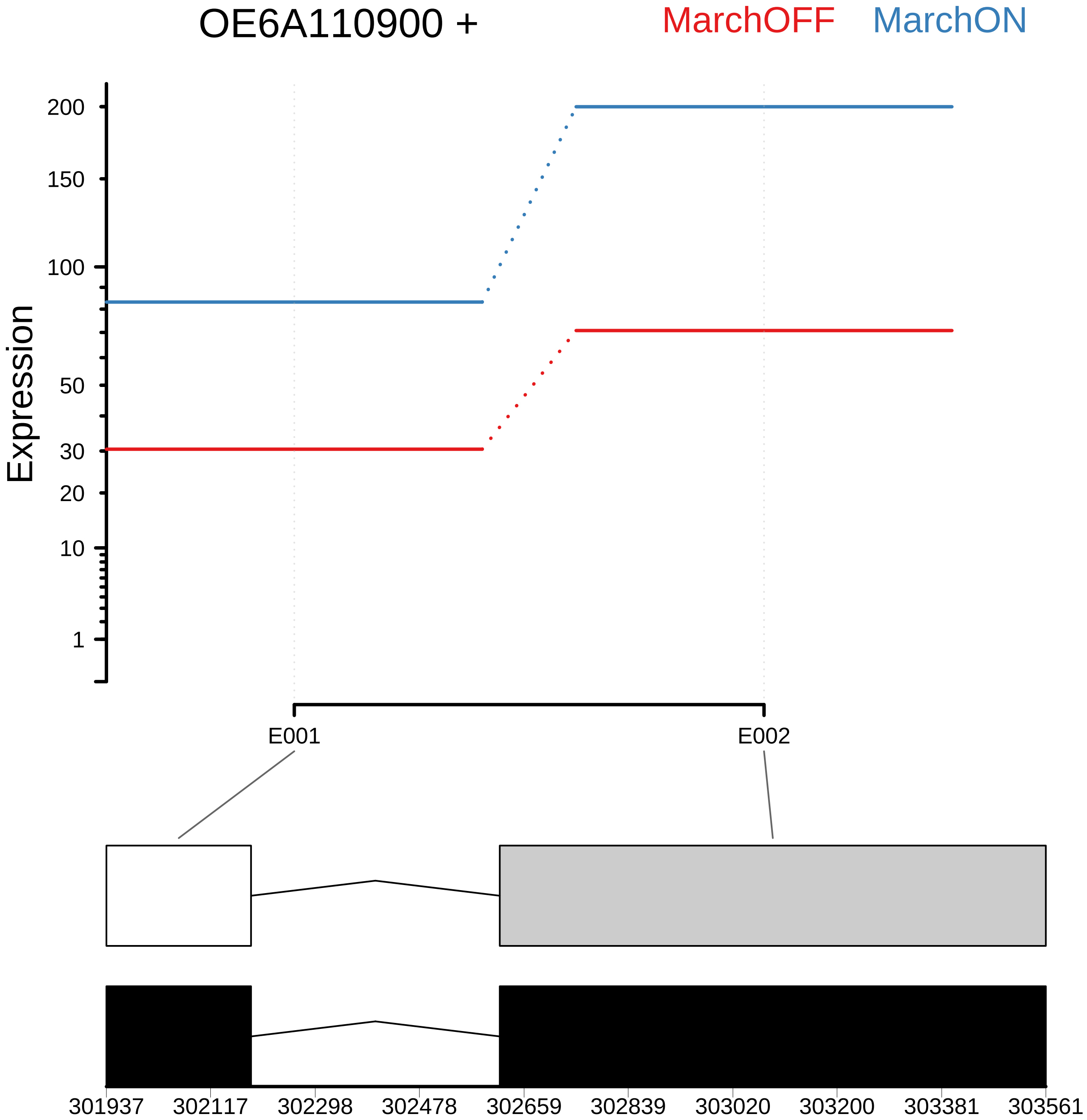
OE6A110900 +
MarchOFF
MarchON
Expression
200
150
100
50
30
20
10
1
E001
E002
301937
302117
302298
302478
302659
302839
303020
303200
303381
303561

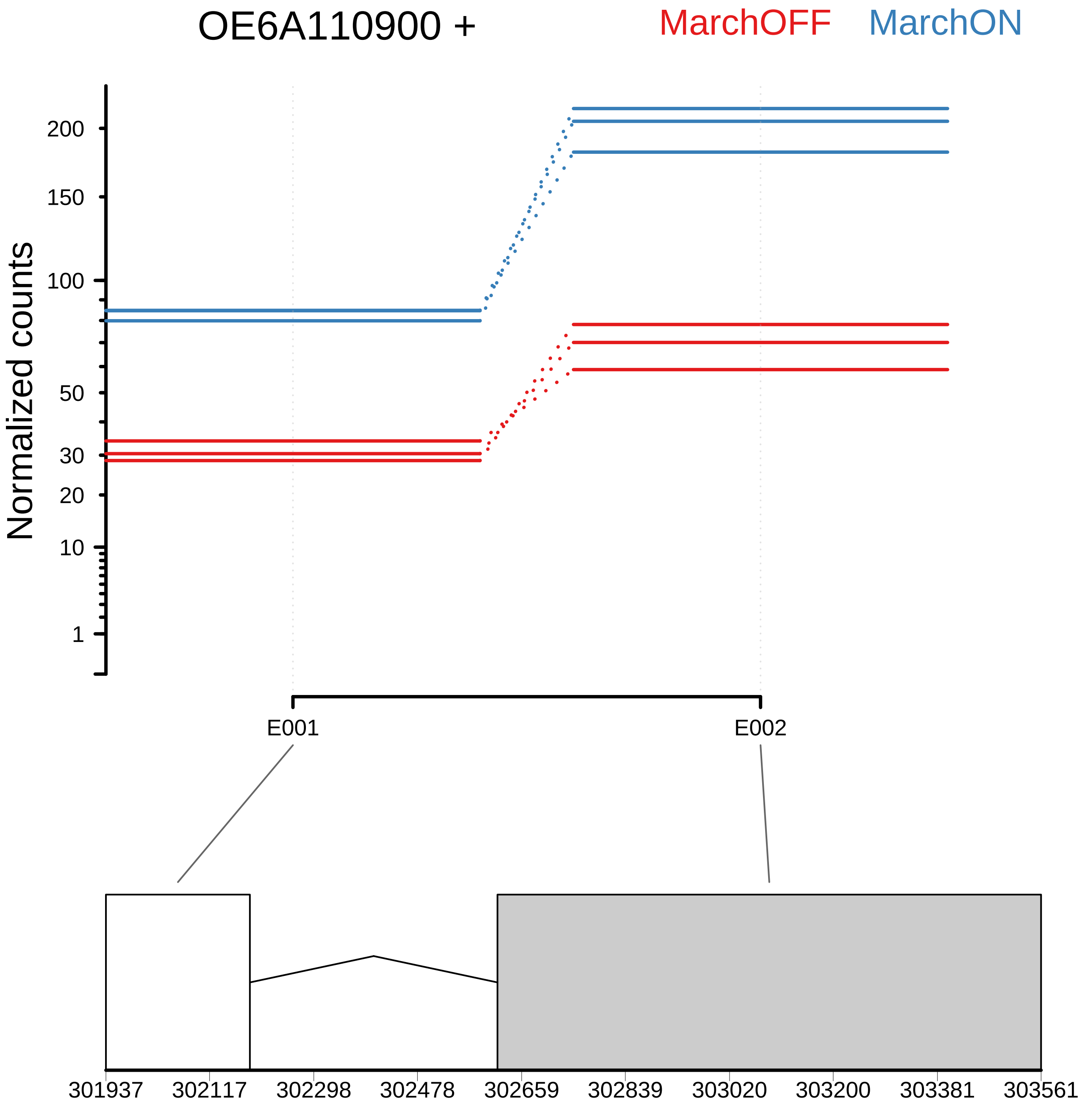
OE6A110900 +
MarchOFF
MarchON
Normalized counts
200
150
100
50
30
20
10
1
E001
E002
301937
302117
302298
302478
302659
302839
303020
303200
303381
303561

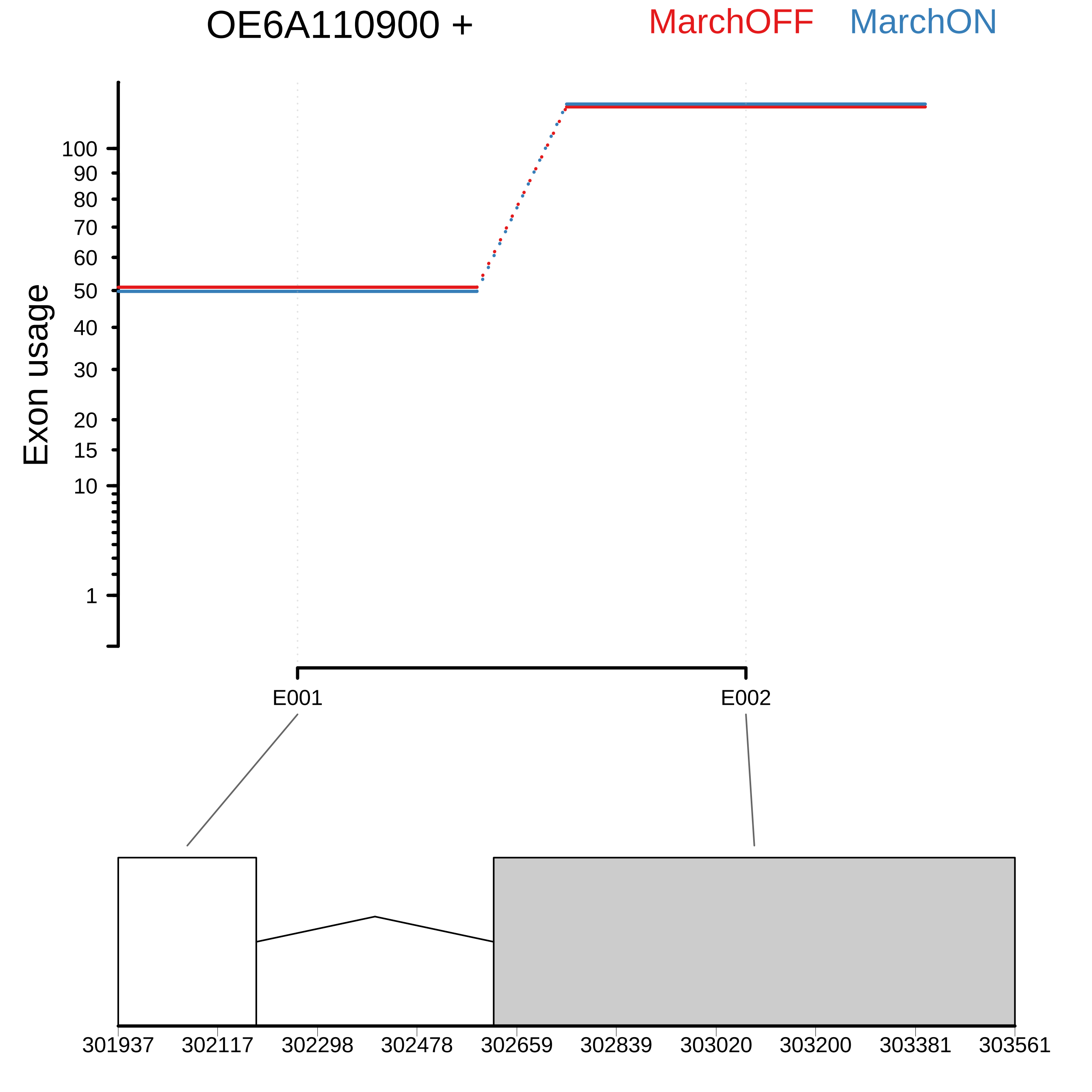

OE6A110900 +
MarchOFF
MarchON
Exon usage
100
90
80
70
60
50
40
30
20
15
10
1
E001
E002
301937
302117
302298
302478
302659
302839
303020
303200
303381
303561

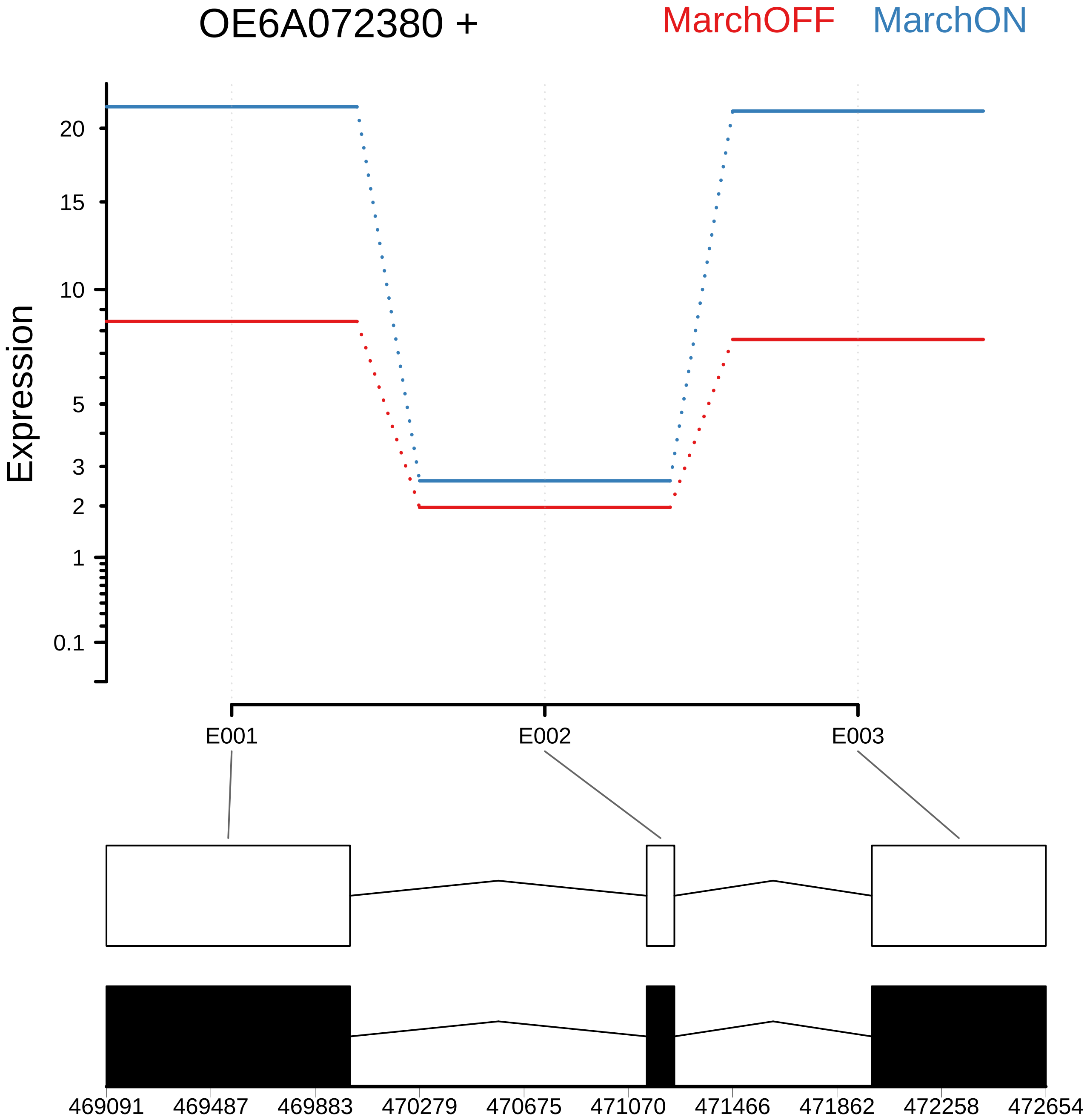
OE6A072380 +
MarchOFF
MarchON
Expression
20
15
10
5
3
2
1
0.1
E001
E002
E003
469091
469487
469883
470279
470675
471070
471466
471862
472258
472654

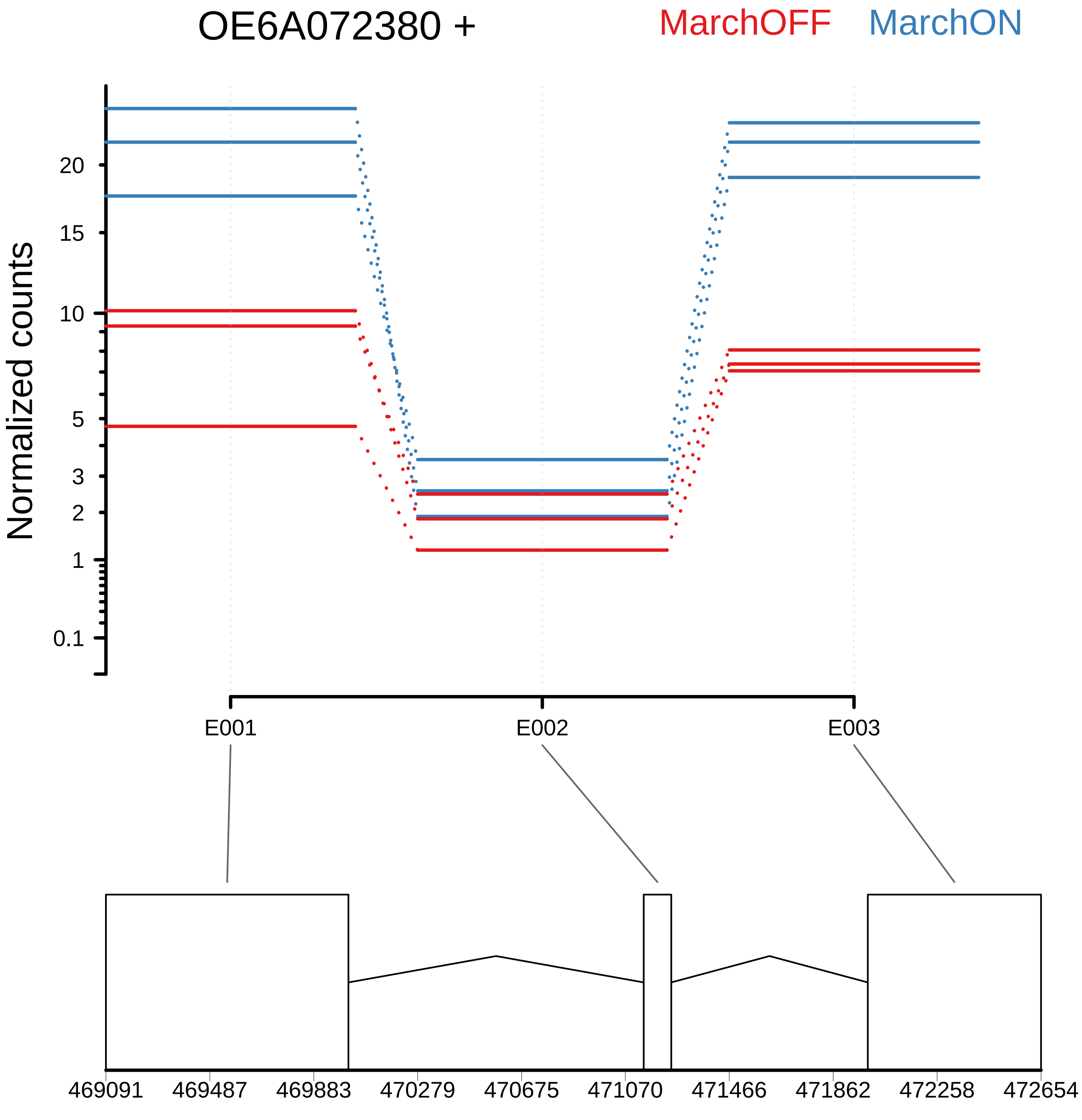
OE6A072380 +
MarchOFF
MarchON
Normalized counts
20
15
10
5
3
2
1
0.1
E001
E002
E003
469091
469487
469883
470279
470675
471070
471466
471862
472258
472654

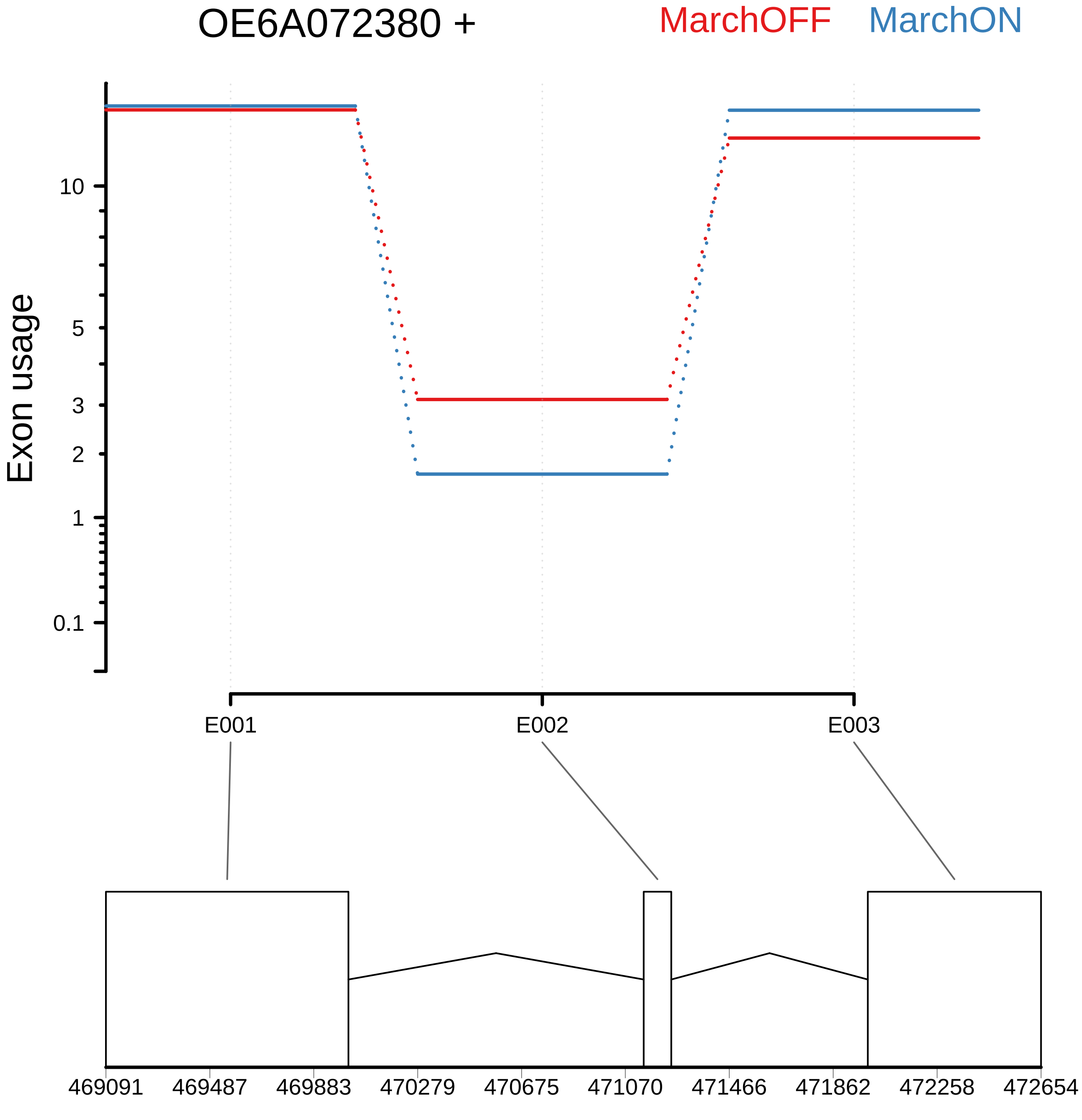

OE6A072380 +
MarchOFF
MarchON
Exon usage
10
5
3
2
1
0.1
E001
E002
E003
469091
469487
469883
470279
470675
471070
471466
471862
472258
472654

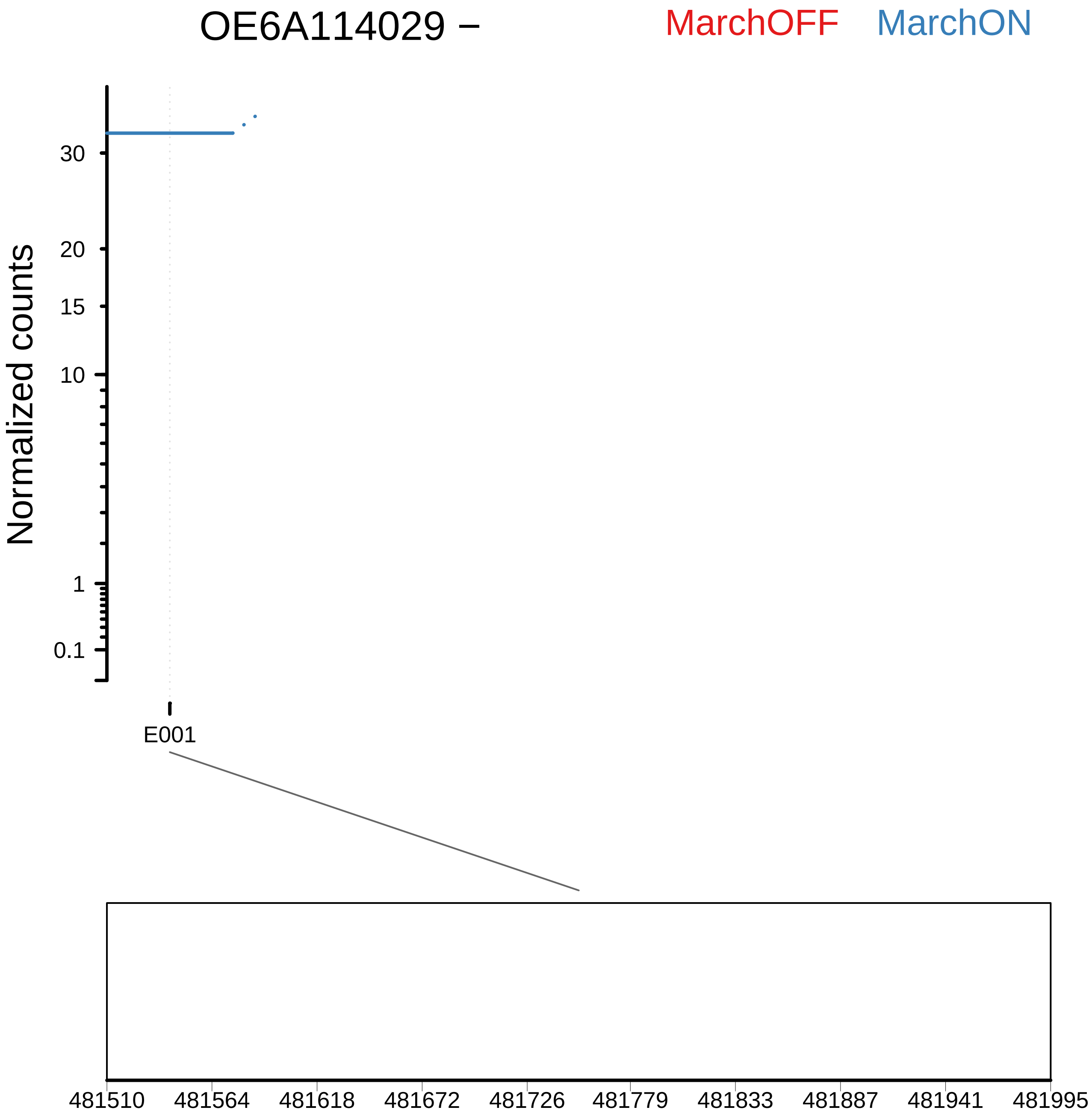

OE6A114029 −
MarchOFF
MarchON
Normalized counts
30
20
15
10
1
0.1
E001
481510
481564
481618
481672
481726
481779
481833
481887
481941
481995

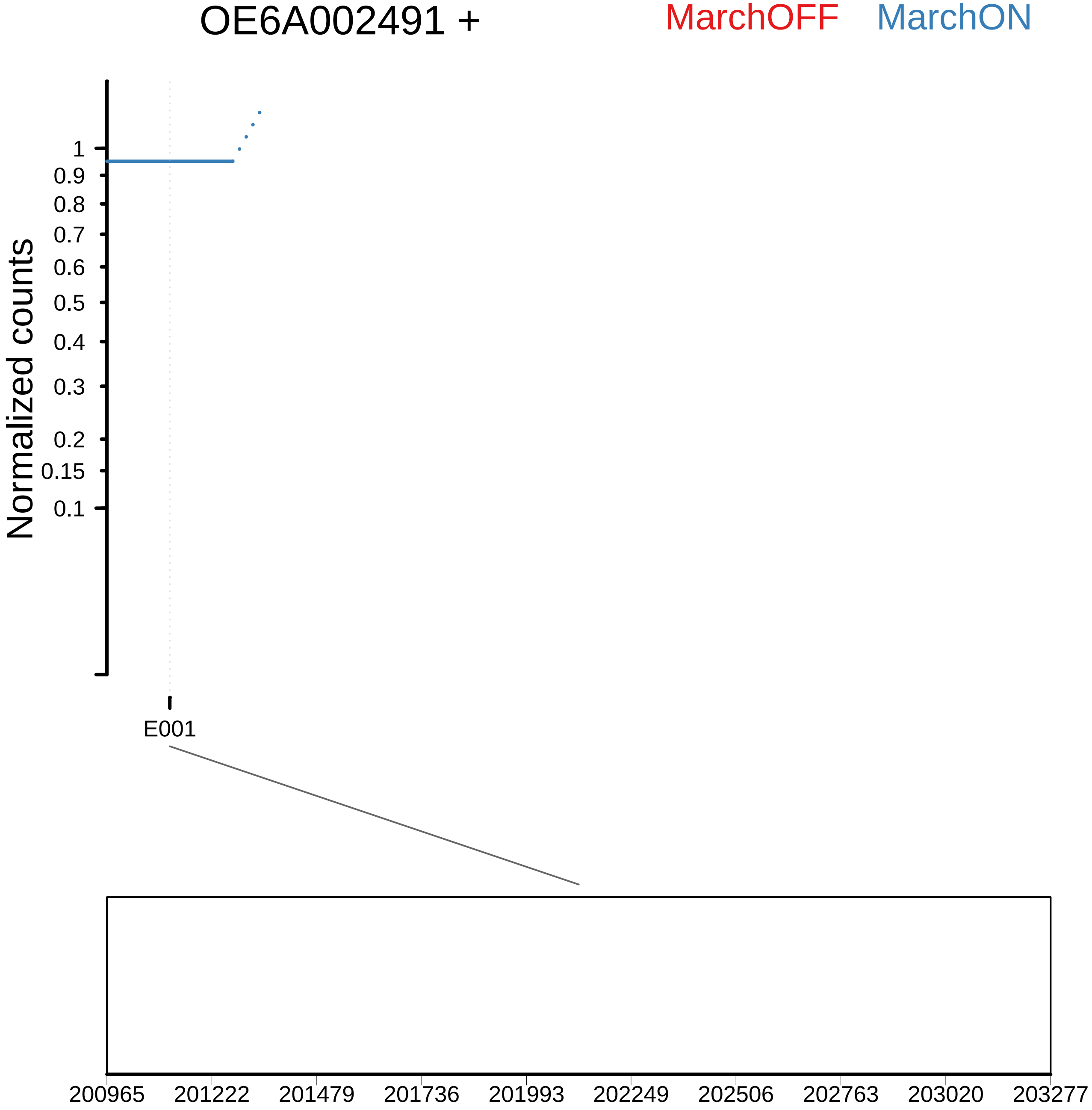
OE6A002491 +
MarchOFF
MarchON
Normalized counts
1
0.9
0.8
0.7
0.6
0.5
0.4
0.3
0.2
0.15
0.1
E001
200965
201222
201479
201736
201993
202249
202506
202763
203020
203277

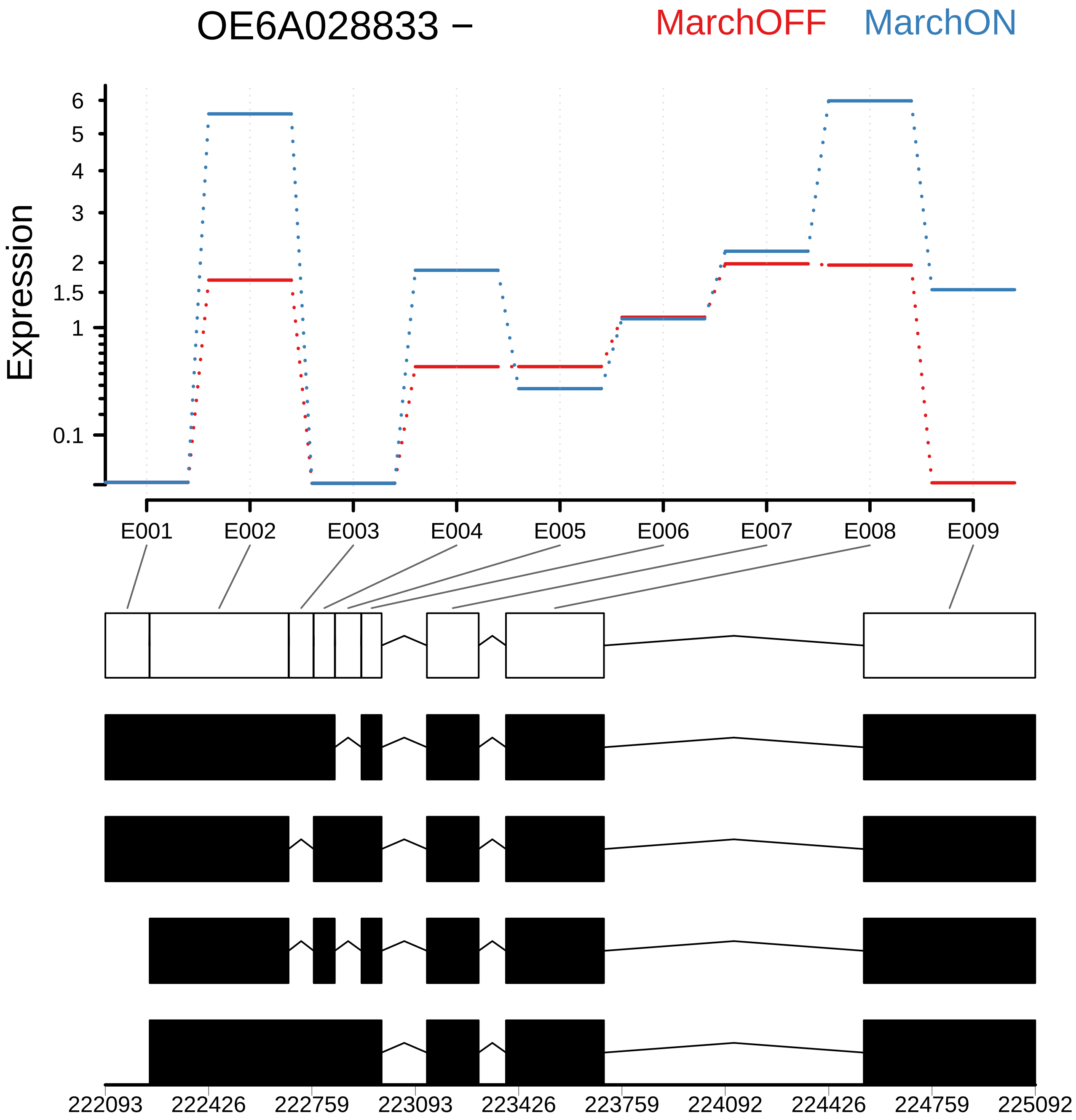
OE6A028833 –
MarchOFF
MarchON
Expression
6
5
4
3
2
1.5
1
0.1
E001
E002
E003
E004
E005
E006
E007
E008
E009
222093
222426
222759
223093
223426
223759
224092
224426
224759
225092

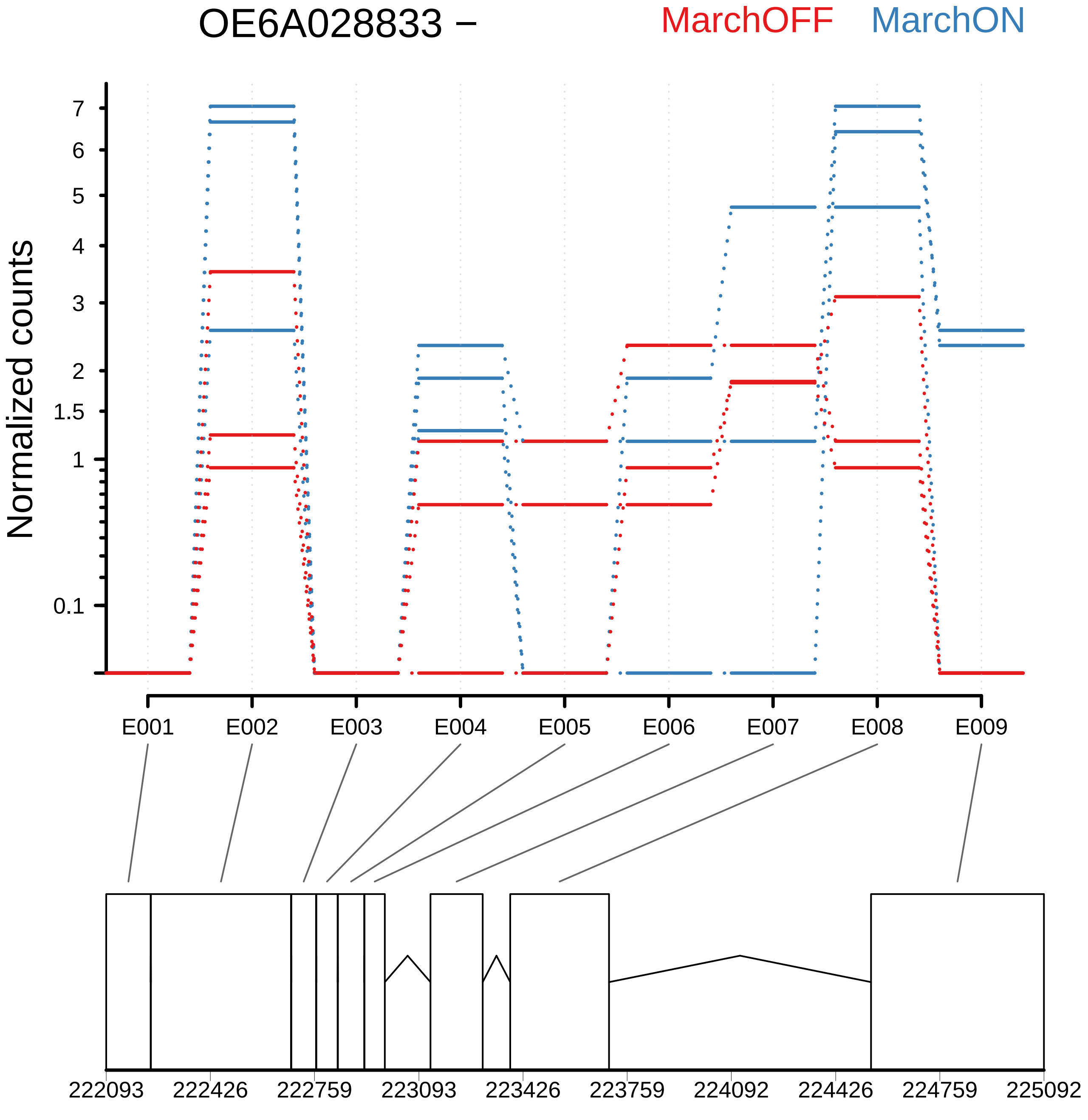

OE6A028833 −
MarchOFF
MarchON
Normalized counts
7
6
5
4
3
2
1.5
1
0.1
E001
E002
E003
E004
E005
E006
E007
E008
E009
222093
222426
222759
223093
223426
223759
224092
224426
224759
225092

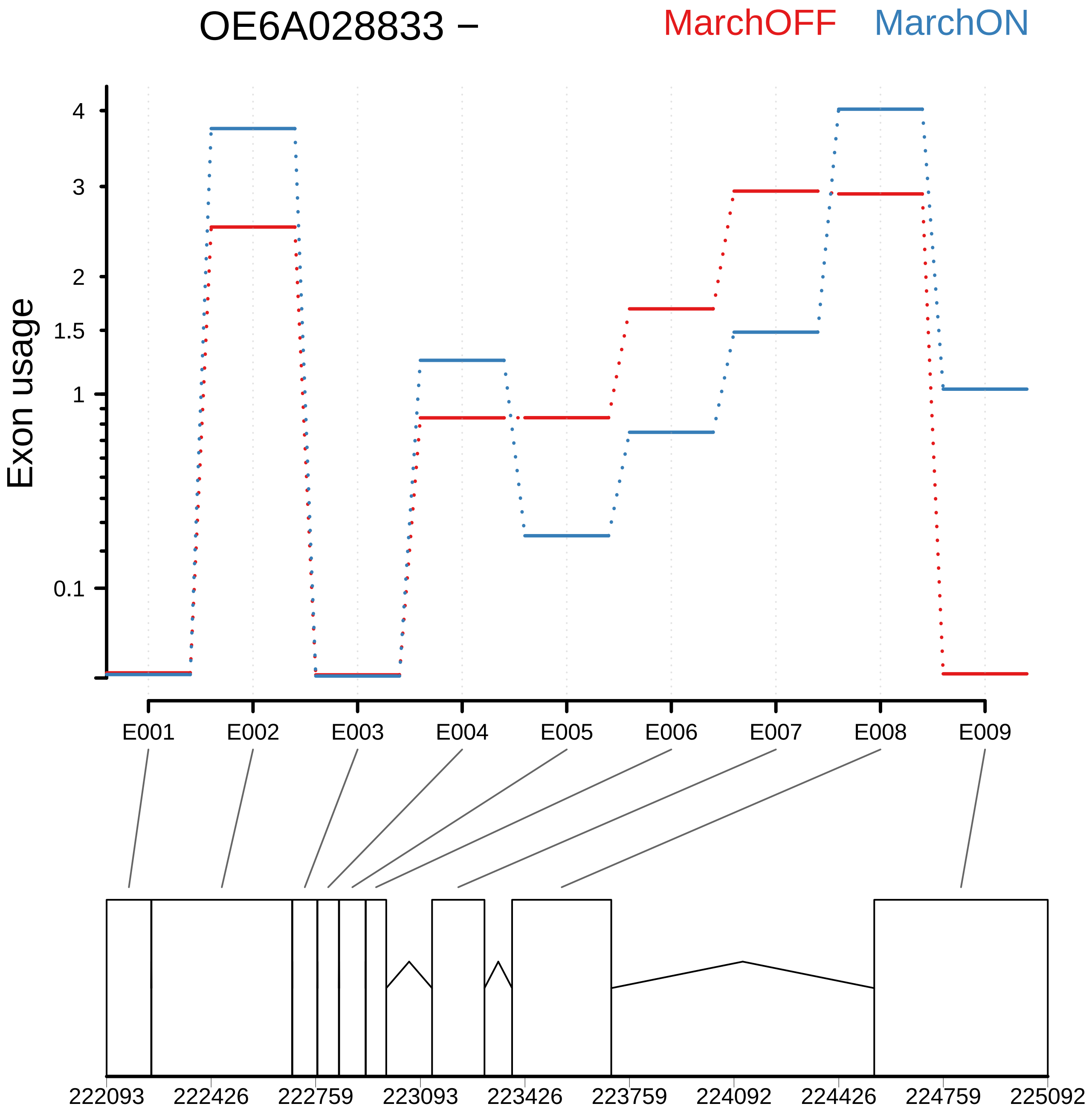
OE6A028833 −
MarchOFF
MarchON
Exon usage
4
3
2
1.5
1
0.1
E001
E002
E003
E004
E005
E006
E007
E008
E009
222093
222426
222759
223093
223426
223759
224092
224426
224759
225092

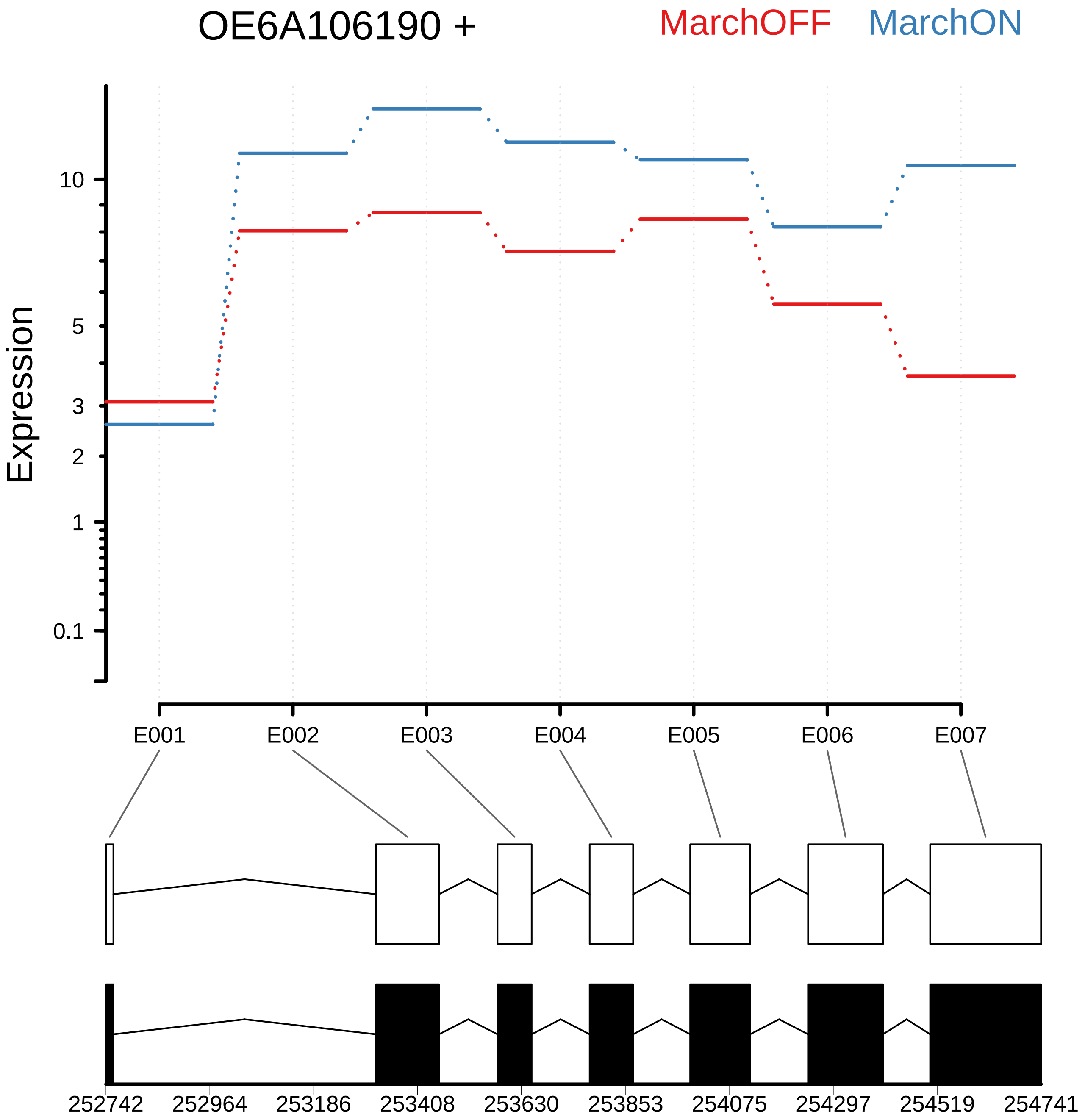
OE6A106190 +
MarchOFF
MarchON
Expression
10
5
3
2
1
0.1
E001
E002
E003
E004
E005
E006
E007
252742
252964
253186
253408
253630
253853
254075
254297
254519
254741

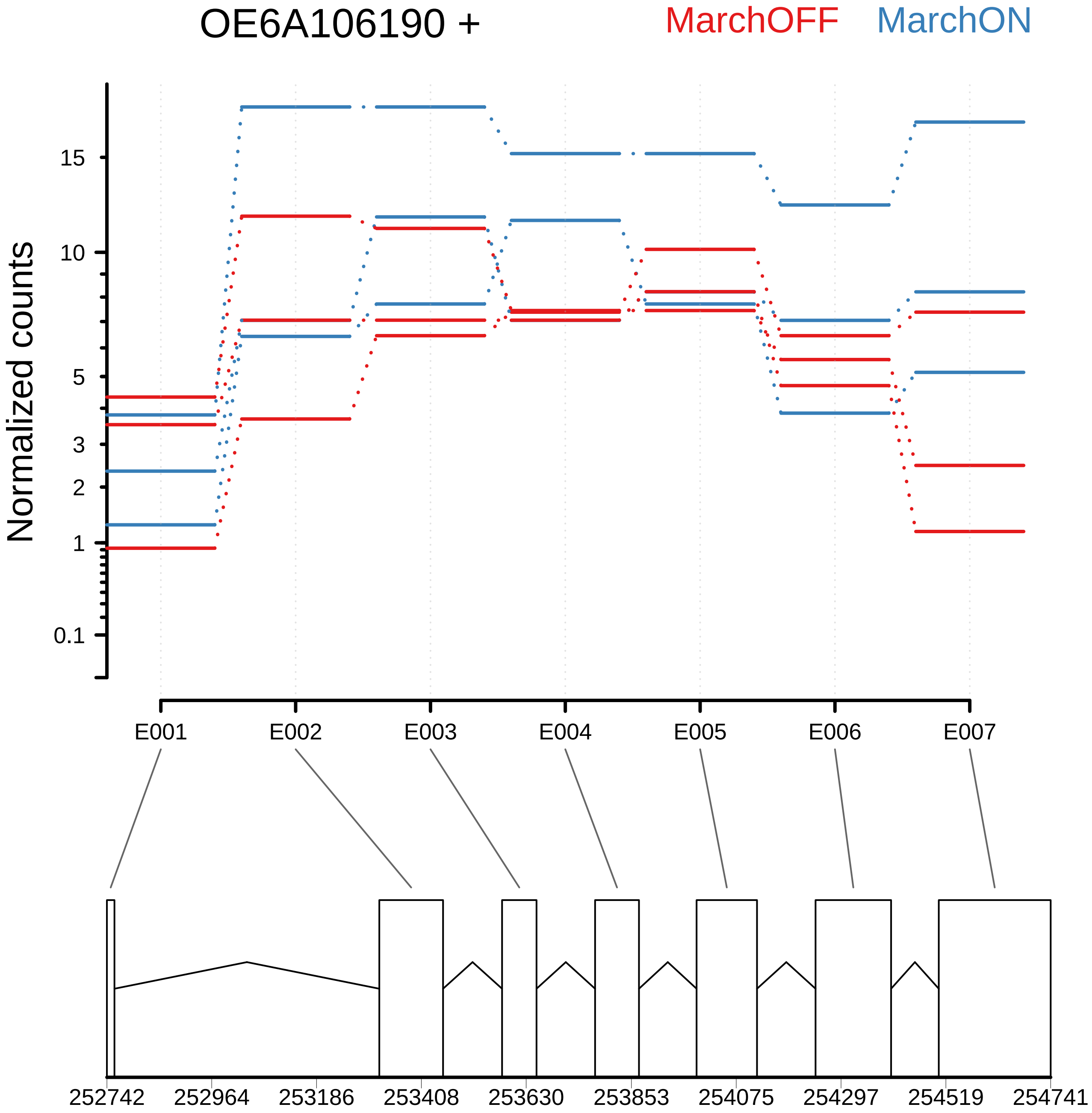

OE6A106190 +
MarchOFF
MarchON
Normalized counts
15
10
5
3
2
1
0.1
E001
E002
E003
E004
E005
E006
E007
252742
252964
253186
253408
253630
253853
254075
254297
254519
254741

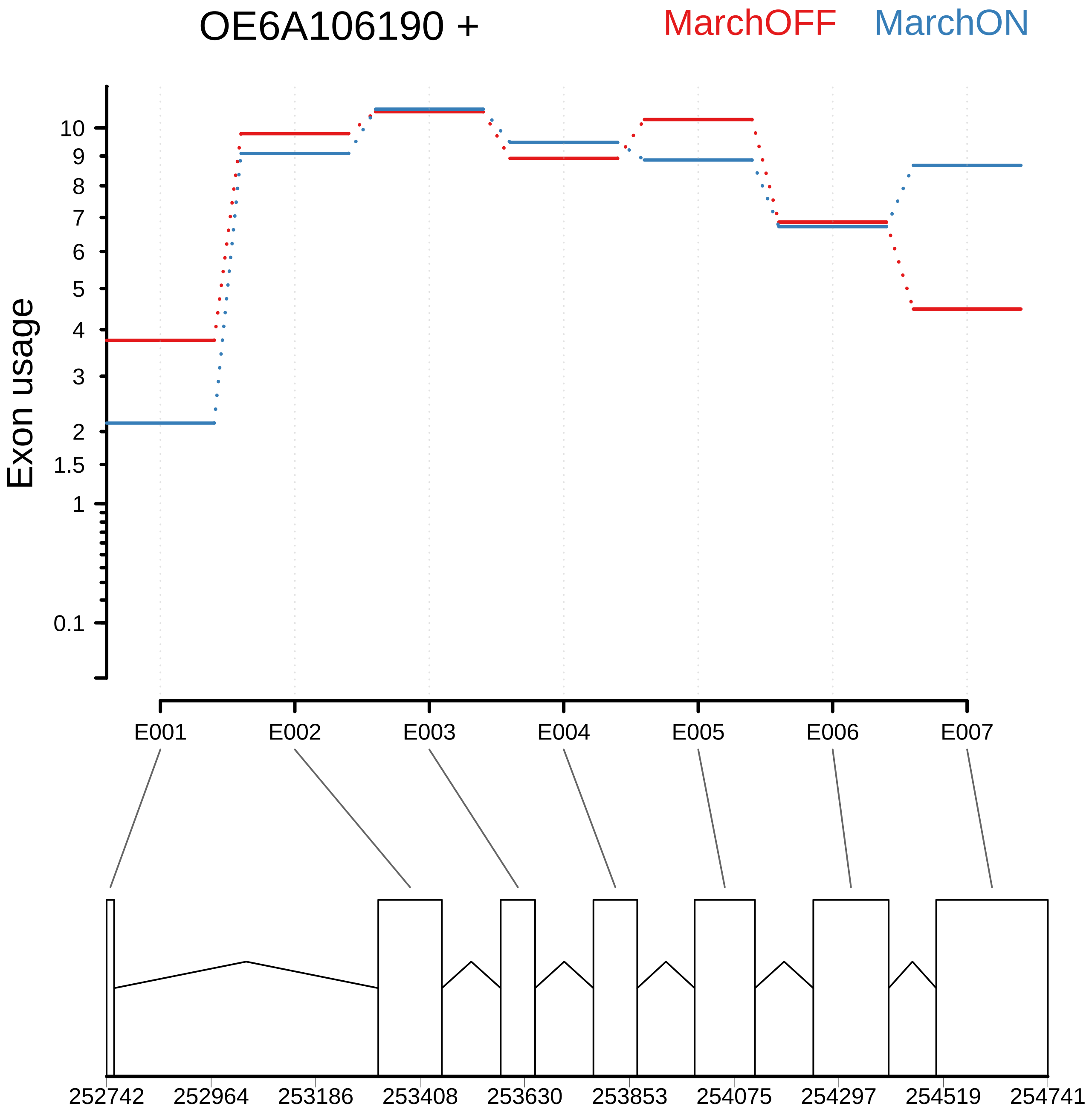
OE6A106190 +
MarchOFF
MarchON
Exon usage
10
9
8
7
6
5
4
3
2
1.5
1
0.1
E001
E002
E003
E004
E005
E006
E007
252742
252964
253186
253408
253630
253853
254075
254297
254519
254741

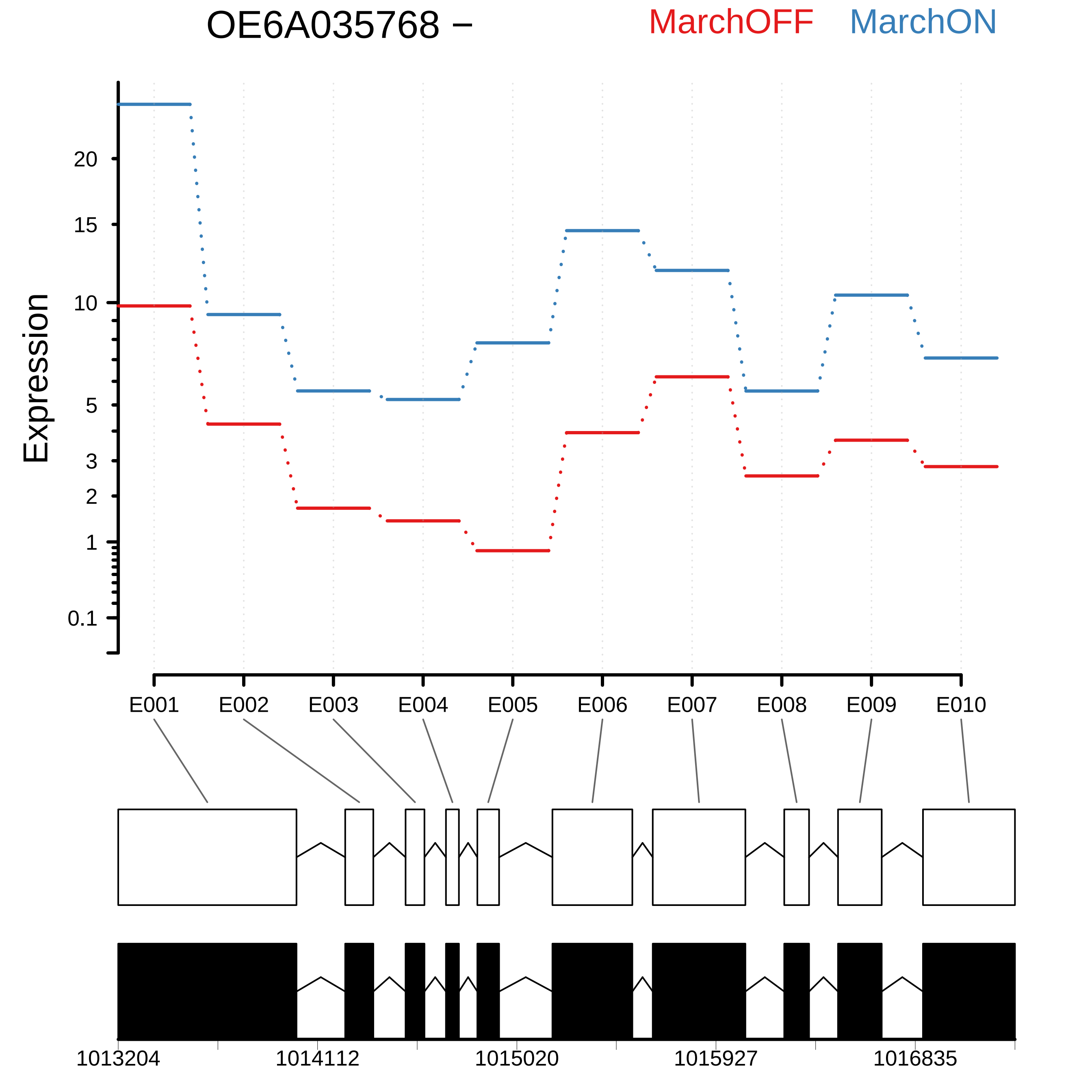
OE6A035768 –
MarchOFF
MarchON
Expression
20
15
10
5
3
2
1
0.1
E001
E002
E003
E004
E005
E006
E007
E008
E009
E010
1013204
1014112
1015020
1015927
1016835

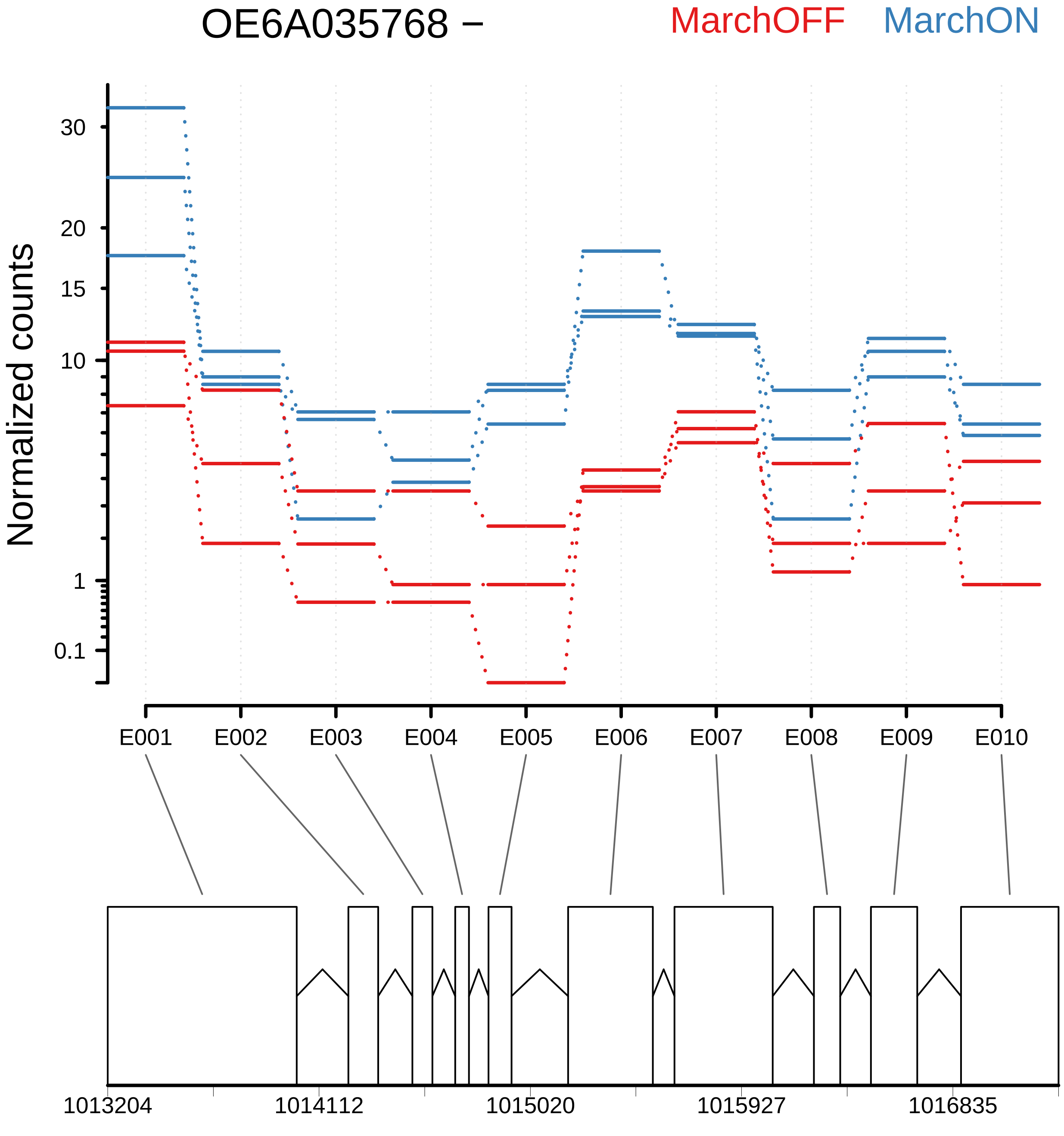

OE6A035768 −
MarchOFF
MarchON
Normalized counts
30
20
15
10
1
0.1
E001
E002
E003
E004
E005
E006
E007
E008
E009
E010
1013204
1014112
1015020
1015927
1016835

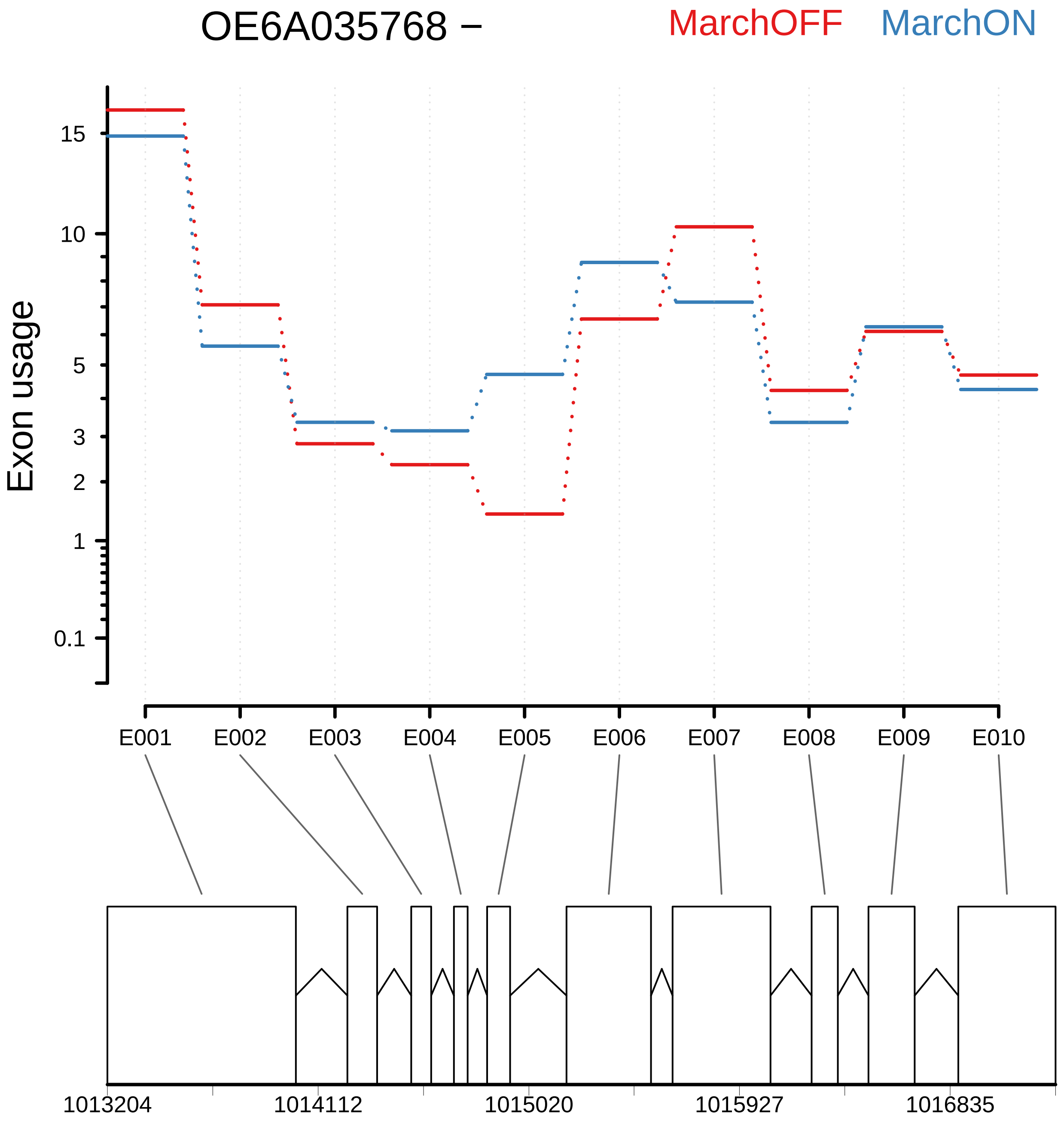

OE6A035768 −
MarchOFF
MarchON
Exon usage
15
10
5
3
2
1
0.1
E001
E002
E003
E004
E005
E006
E007
E008
E009
E010
1013204
1014112
1015020
1015927
1016835

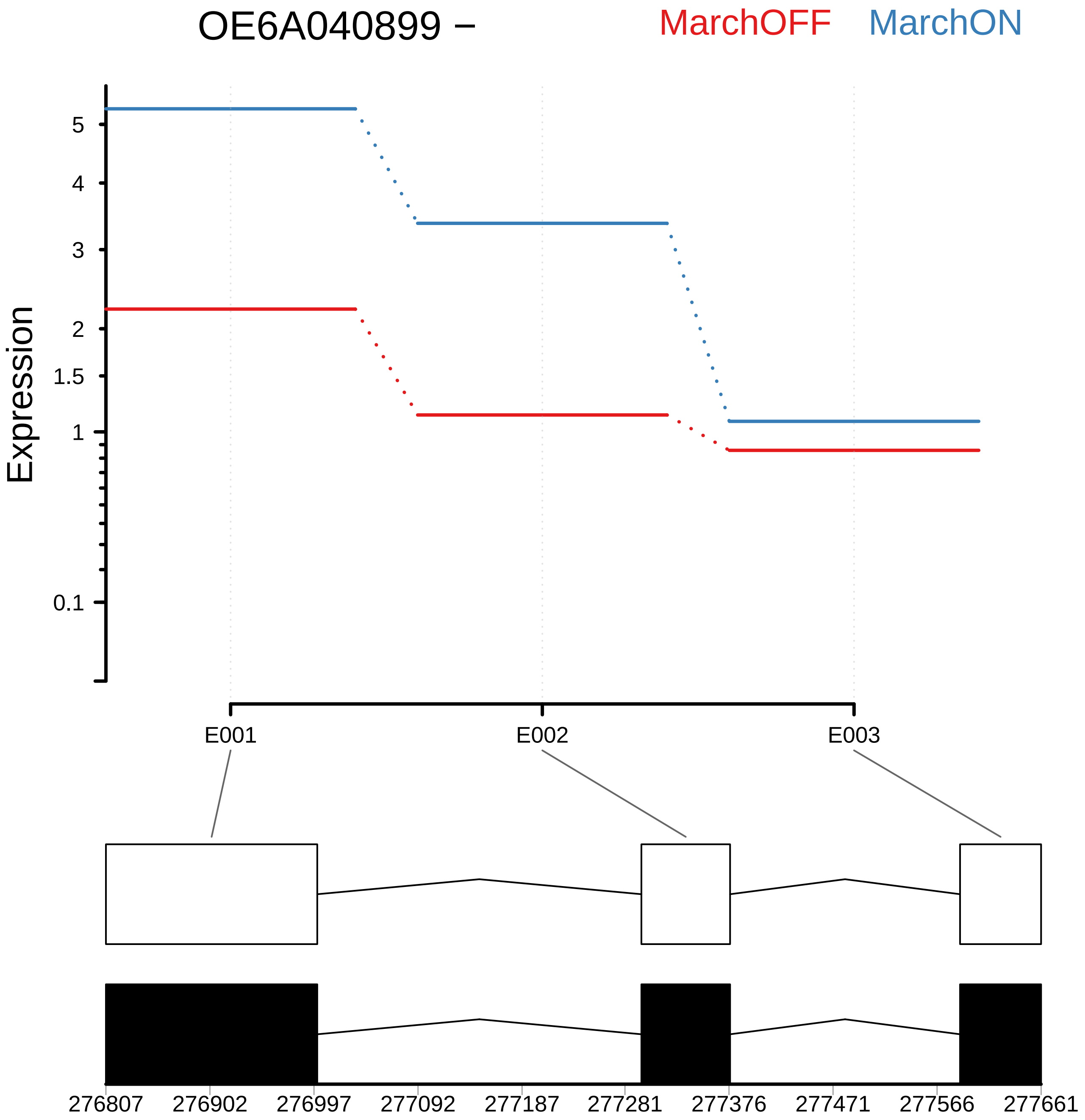

OE6A040899 −
MarchOFF
MarchON
Expression
5
4
3
2
1.5
1
0.1
E001
E002
E003
276807
276902
276997
277092
277187
277281
277376
277471
277566
277661

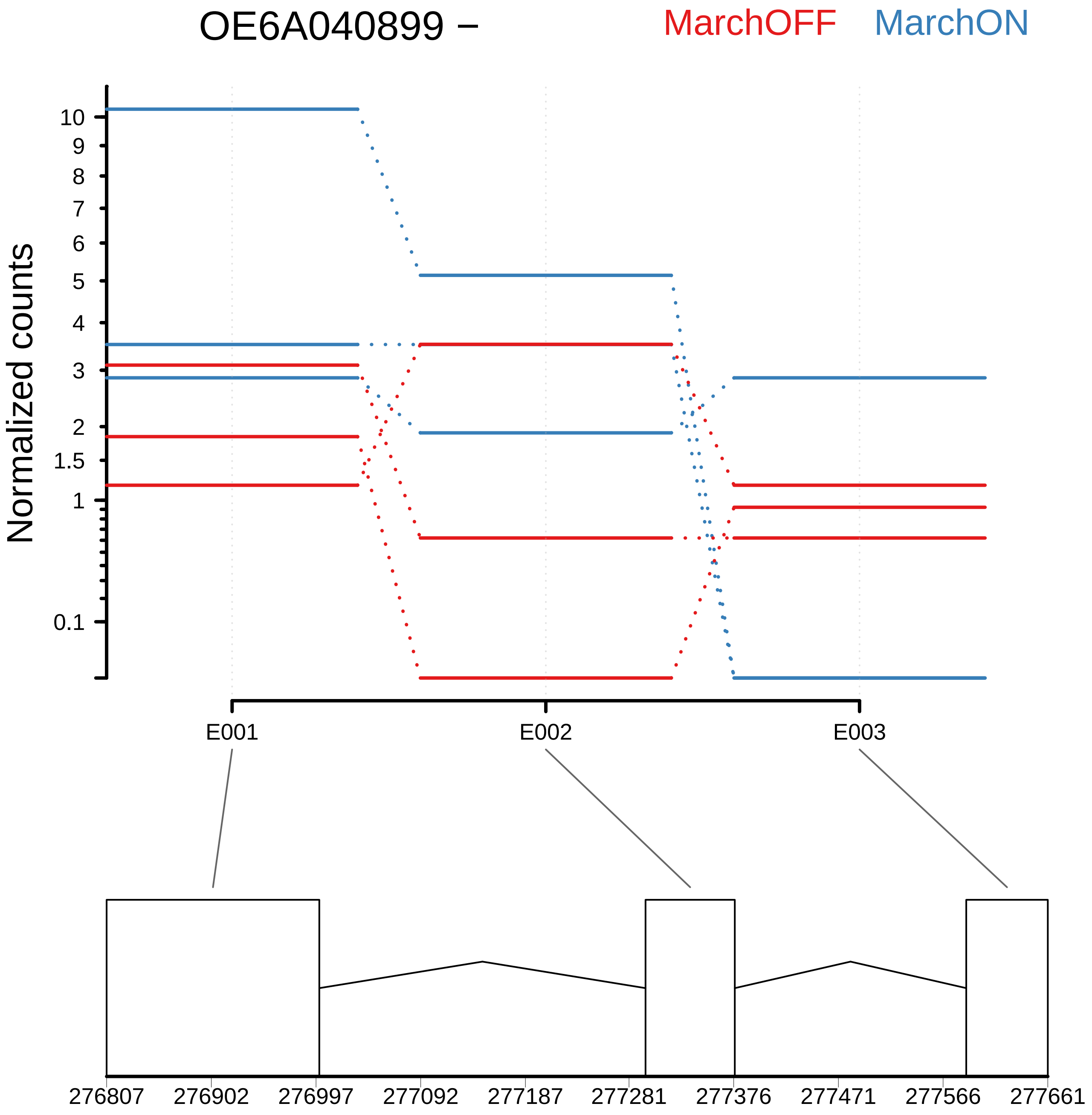
OE6A040899 −
MarchOFF
MarchON
Normalized counts
10
9
8
7
6
5
4
3
2
1.5
1
0.1
E001
E002
E003
276807
276902
276997
277092
277187
277281
277376
277471
277566
277661

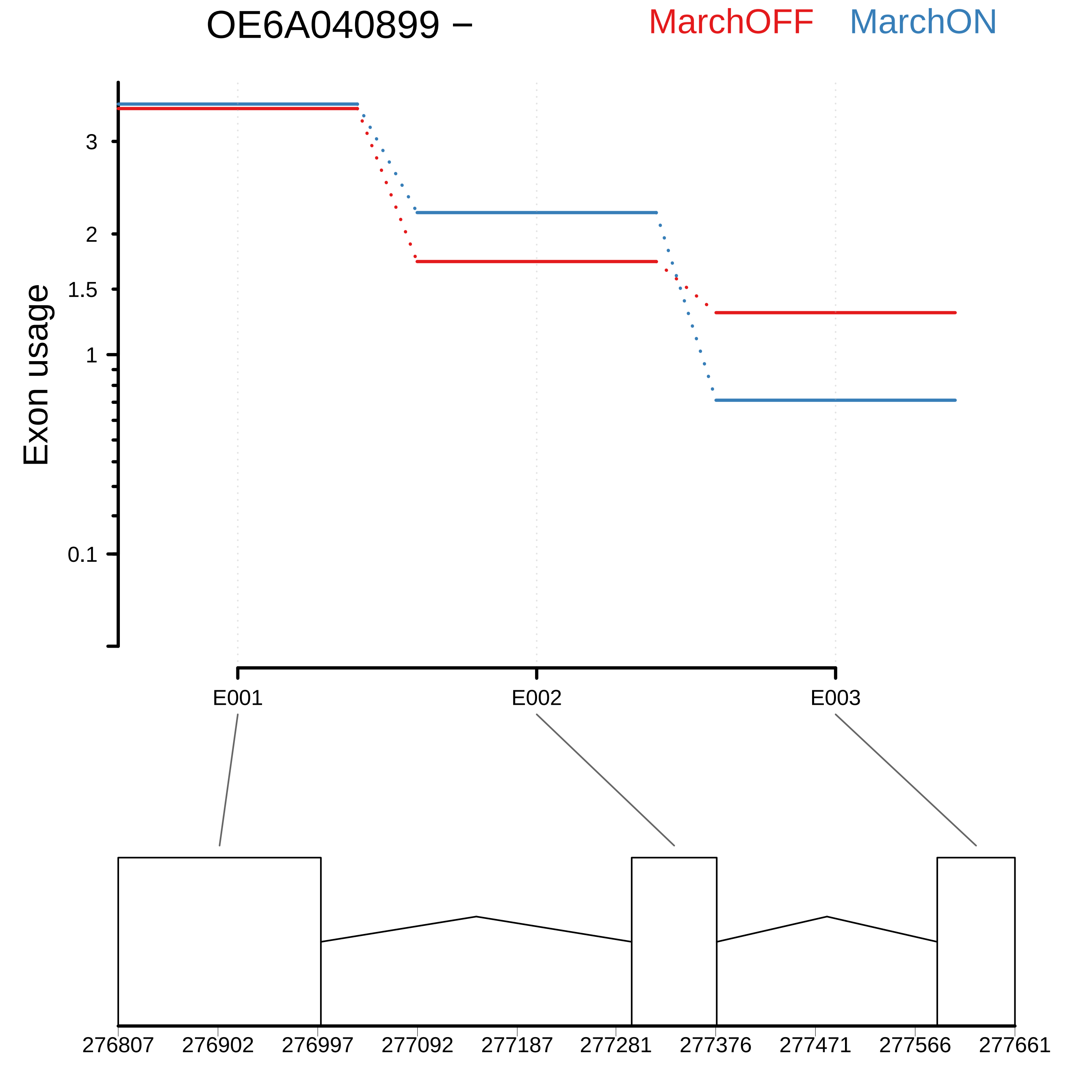

OE6A040899 −
MarchOFF
MarchON
Exon usage
3
2
1.5
1
0.1
E001
E002
E003
276807
276902
276997
277092
277187
277281
277376
277471
277566
277661

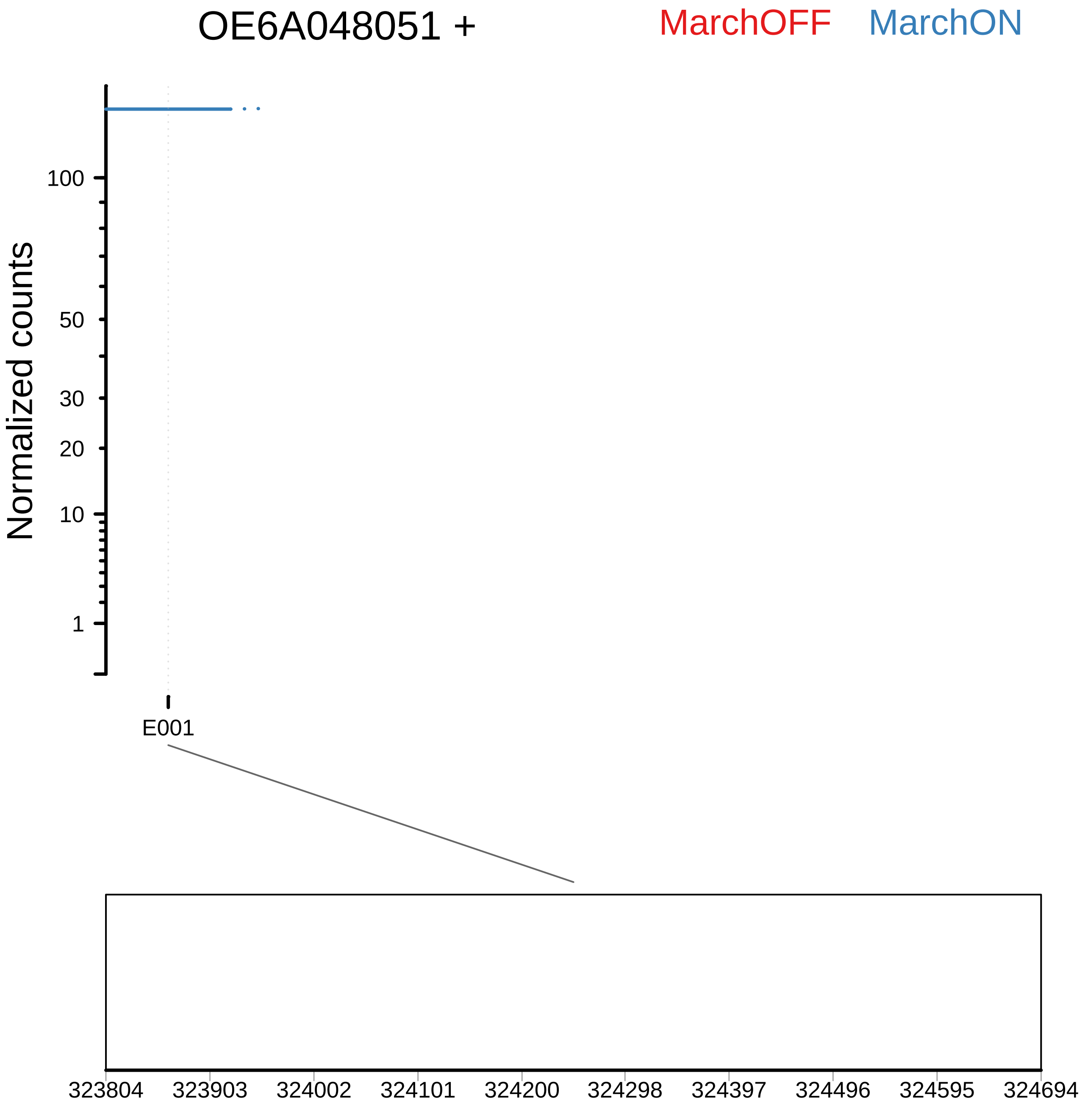

OE6A048051 +
MarchOFF
MarchON
Normalized counts
100
50
30
20
10
1
E001
323804
323903
324002
324101
324200
324298
324397
324496
324595
324694

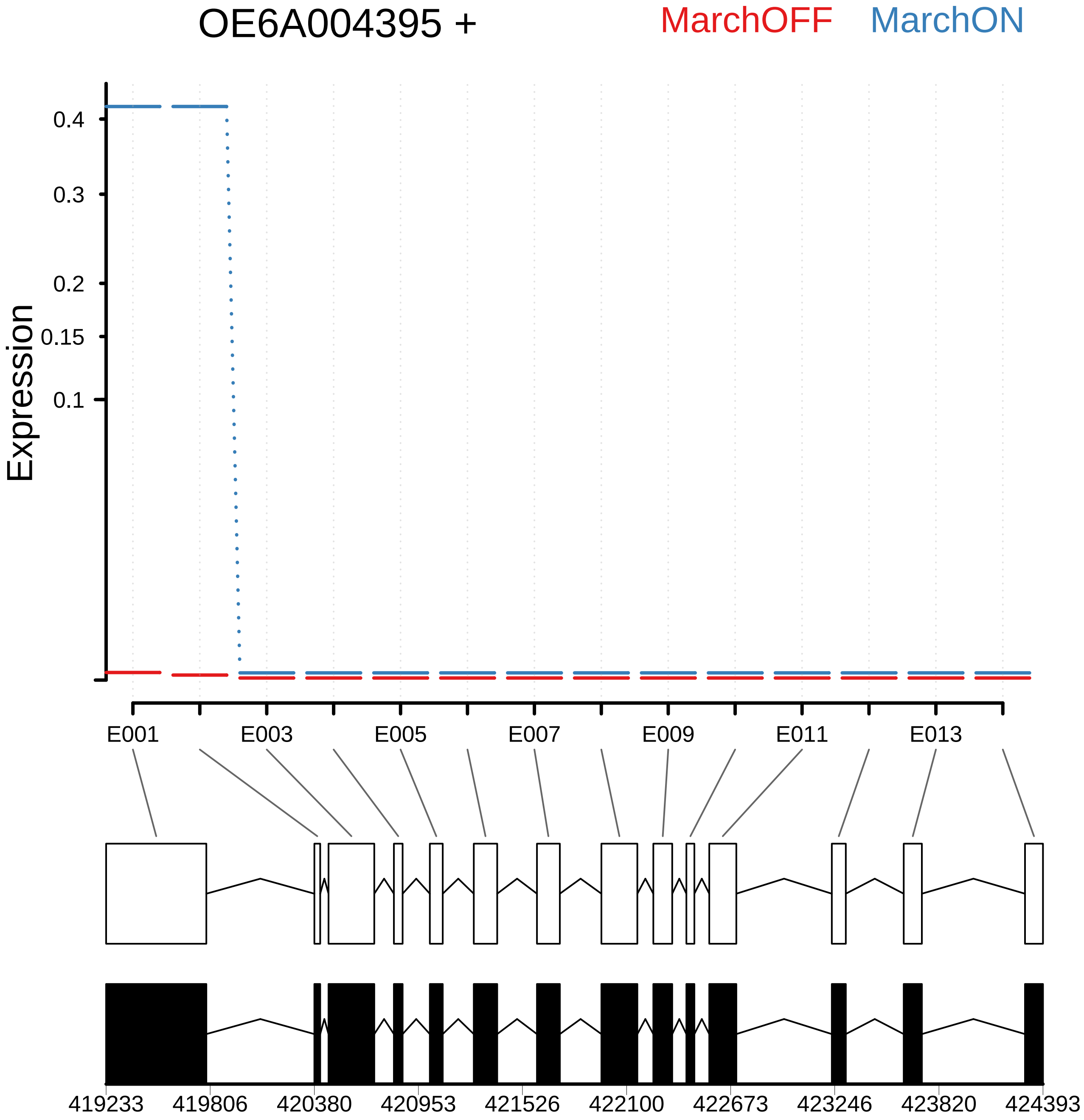
OE6A004395 +
MarchOFF
MarchON
Expression
0.4
0.3
0.2
0.15
0.1
E001
E003
E005
E007
E009
E011
E013
419233
419806
420380
420953
421526
422100
422673
423246
423820
424393

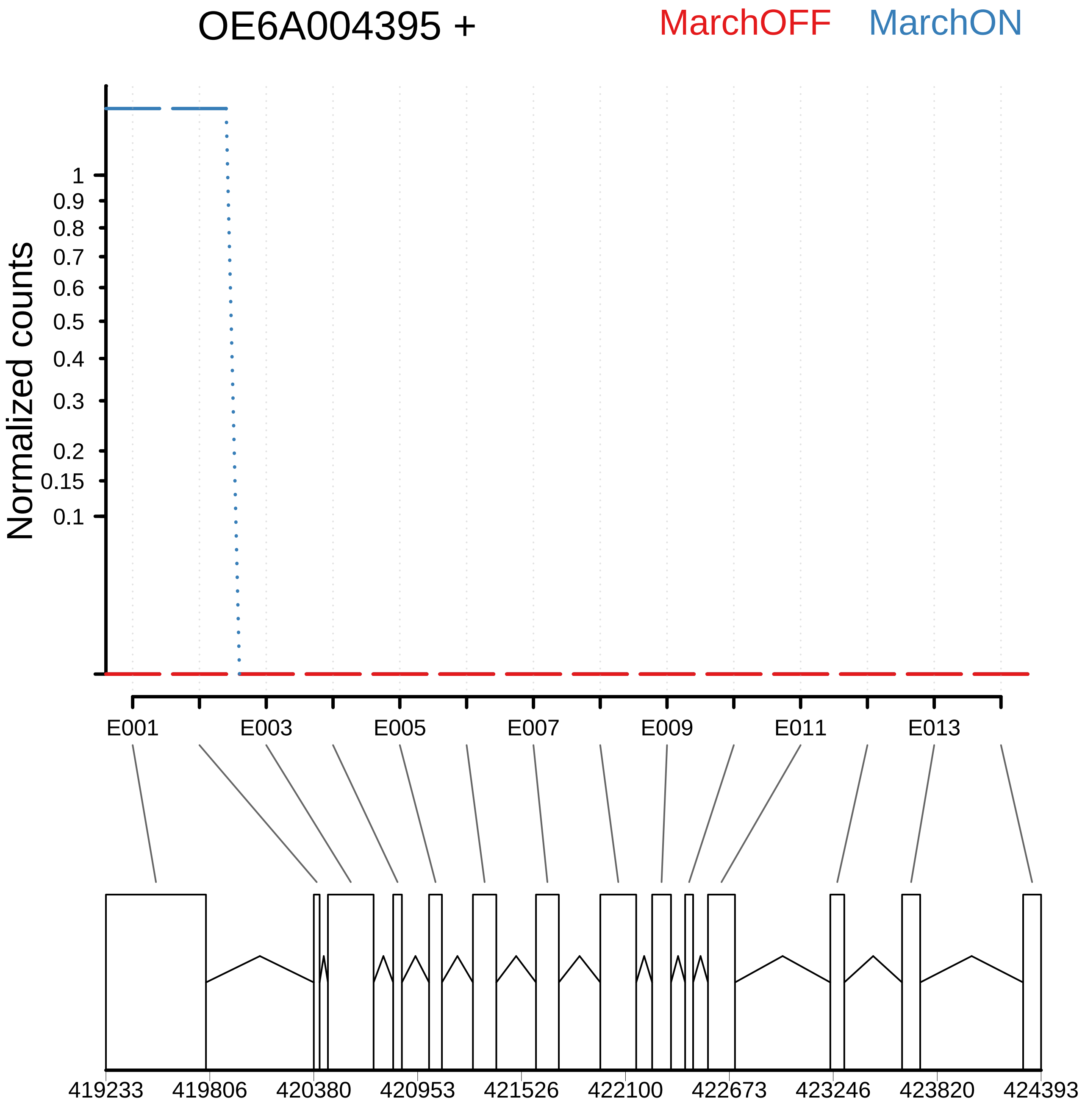
OE6A004395 +
MarchOFF
MarchON
Normalized counts
1
0.9
0.8
0.7
0.6
0.5
0.4
0.3
0.2
0.15
0.1
E001
E003
E005
E007
E009
E011
E013
419233
419806
420380
420953
421526
422100
422673
423246
423820
424393

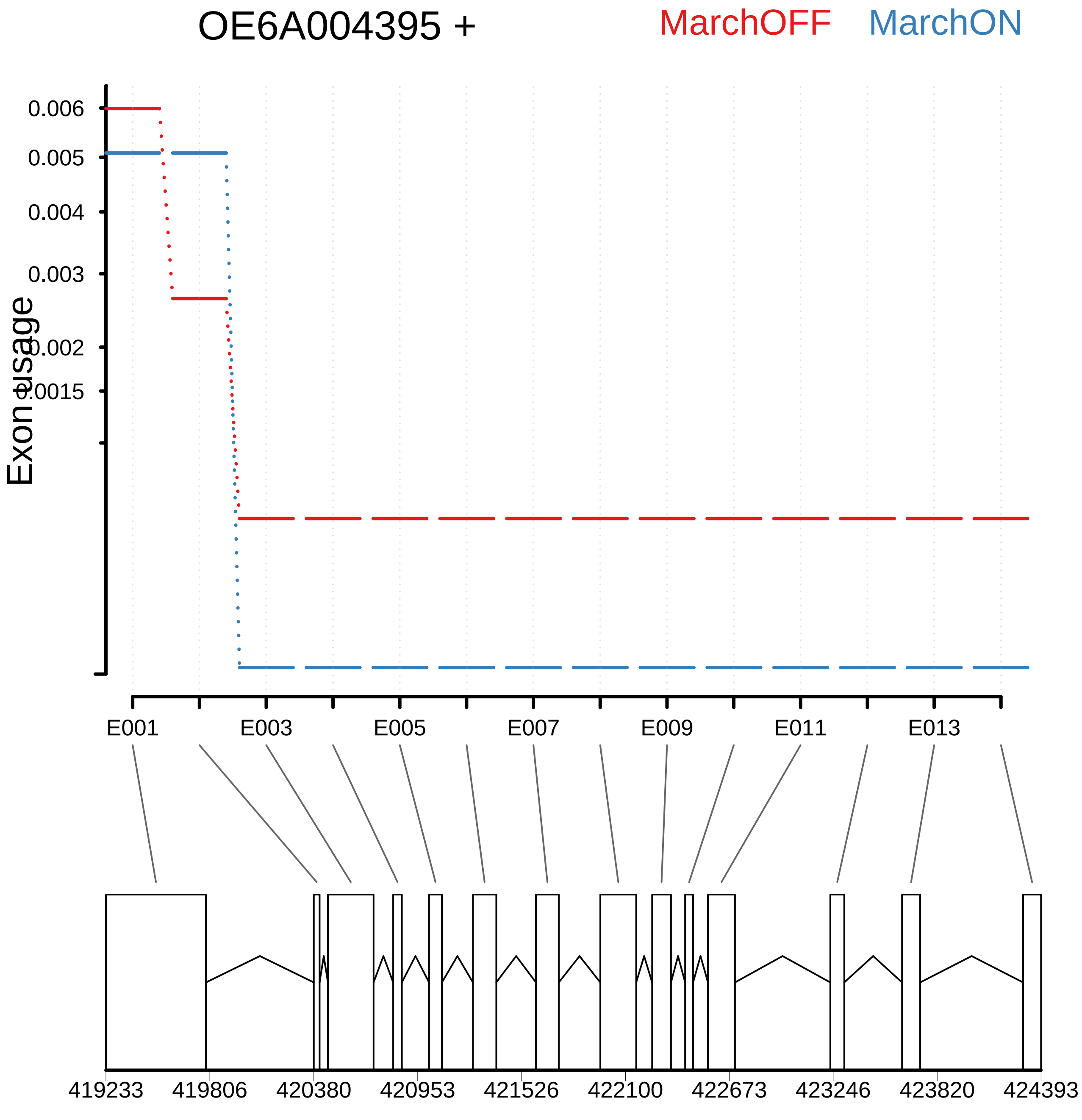

OE6A004395 +
MarchOFF
MarchON
Exon usage
0.006
0.005
0.004
0.003
0.002
0.0015
E001
E003
E005
E007
E009
E011
E013
419233
419806
420380
420953
421526
422100
422673
423246
423820
424393

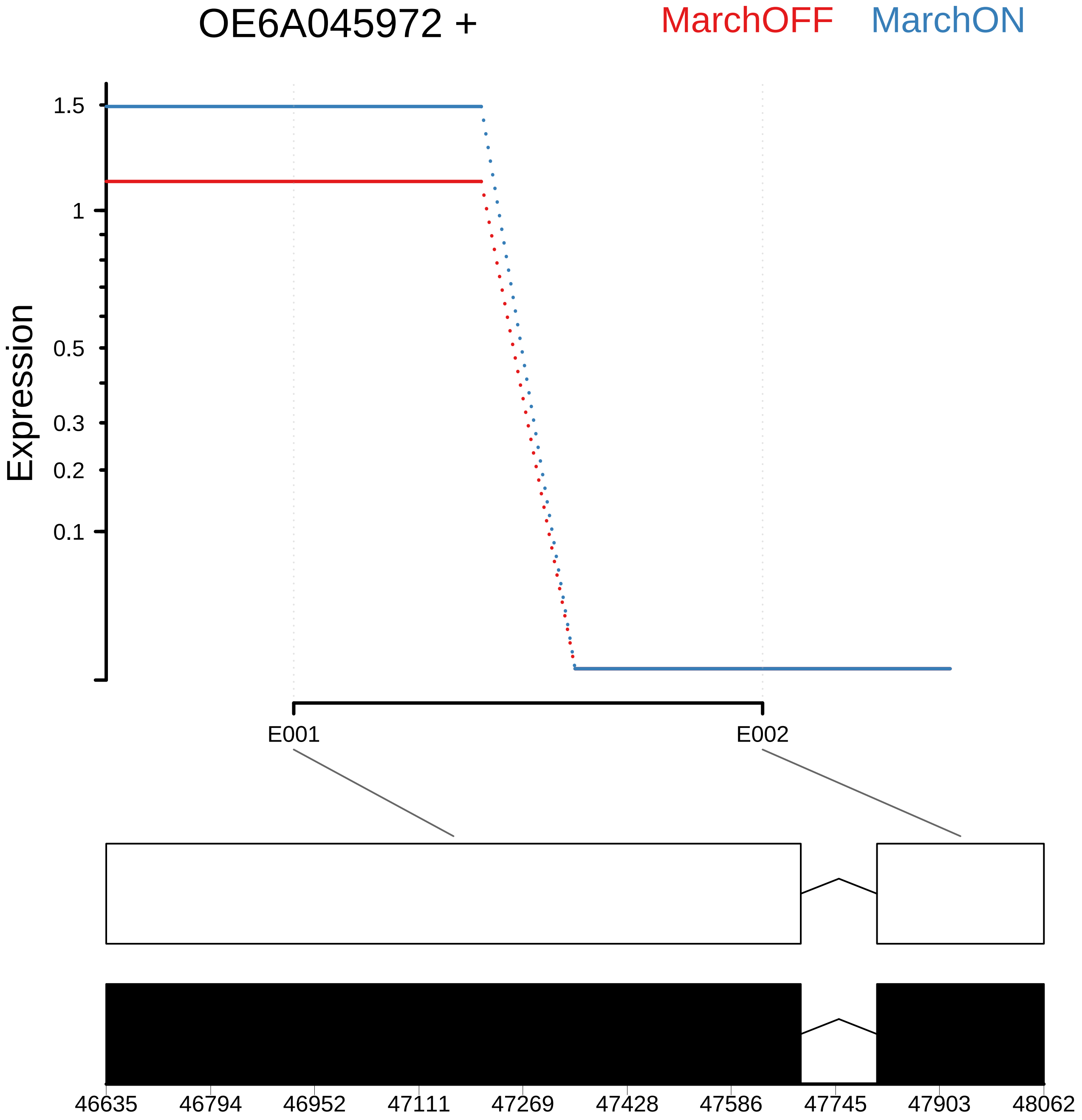
OE6A045972 +
MarchOFF
MarchON
Expression
1.5
1
0.5
0.3
0.2
0.1
E001
E002
46635
46794
46952
47111
47269
47428
47586
47745
47903
48062

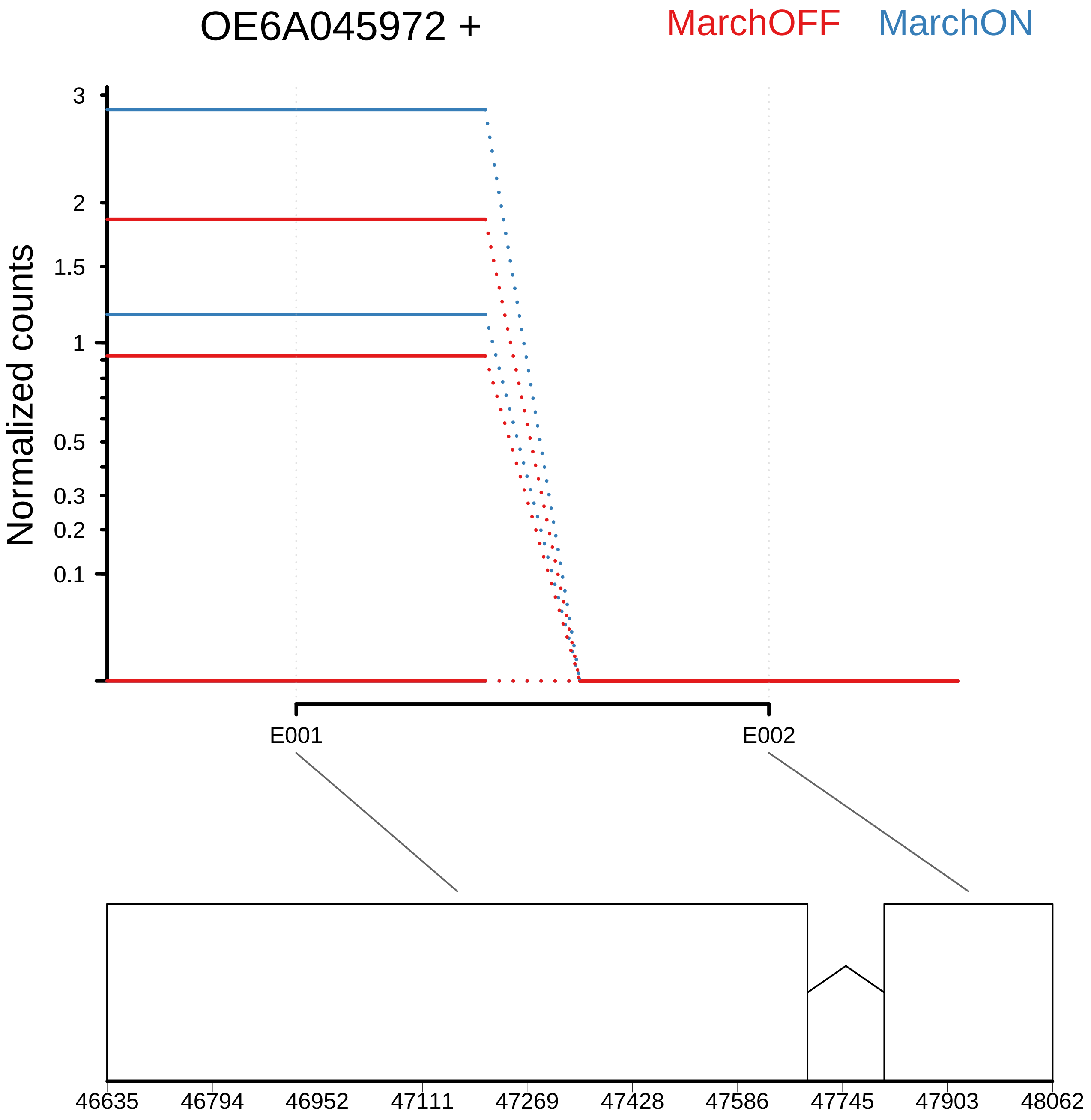

OE6A045972 +
MarchOFF
MarchON
Normalized counts
3
2
1.5
1
0.5
0.3
0.2
0.1
E001
E002
46635
46794
46952
47111
47269
47428
47586
47745
47903
48062

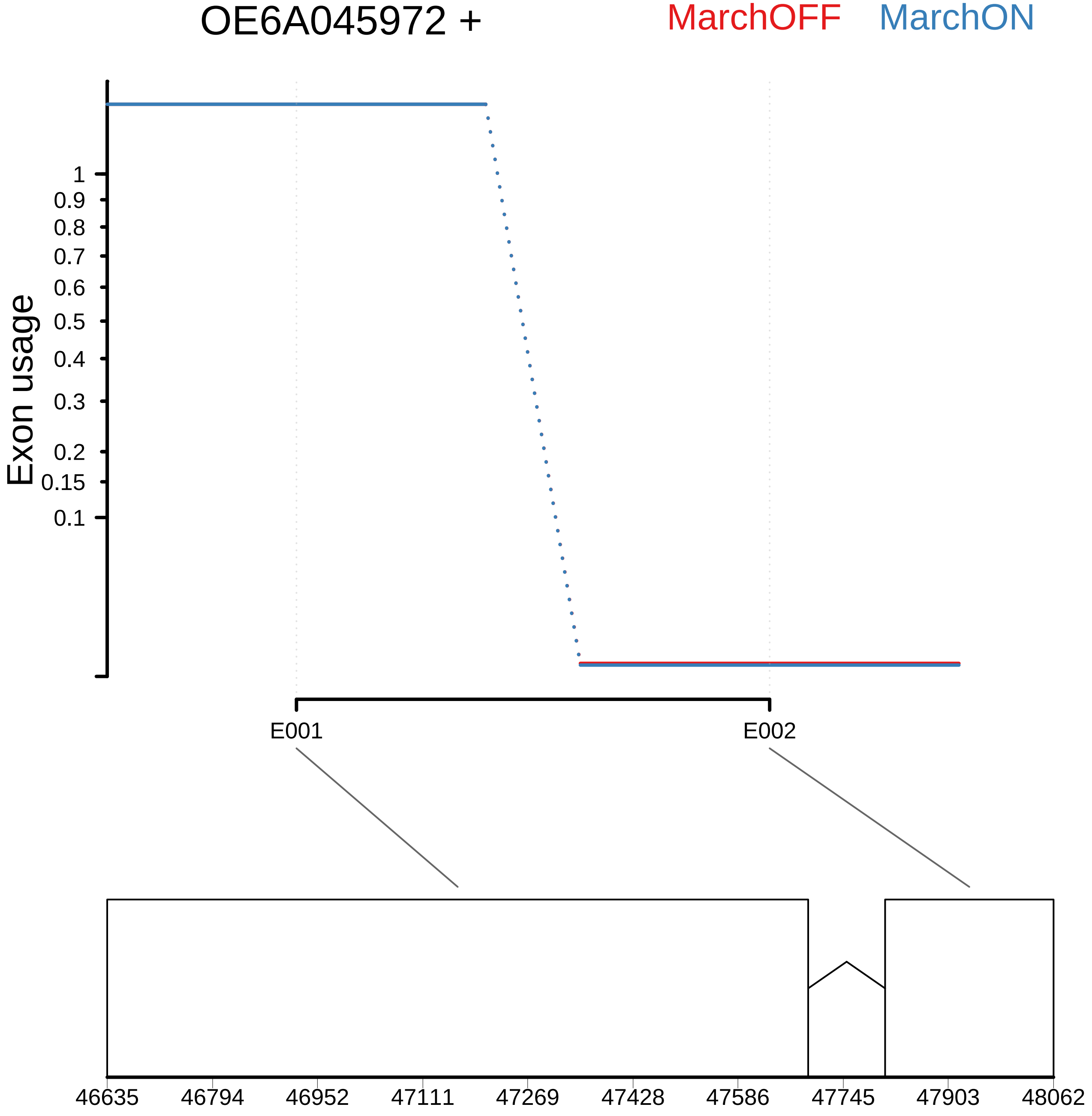

OE6A045972 +
MarchOFF
MarchON
Exon usage
1
0.9
0.8
0.7
0.6
0.5
0.4
0.3
0.2
0.15
0.1
E001
E002
46635
46794
46952
47111
47269
47428
47586
47745
47903
48062

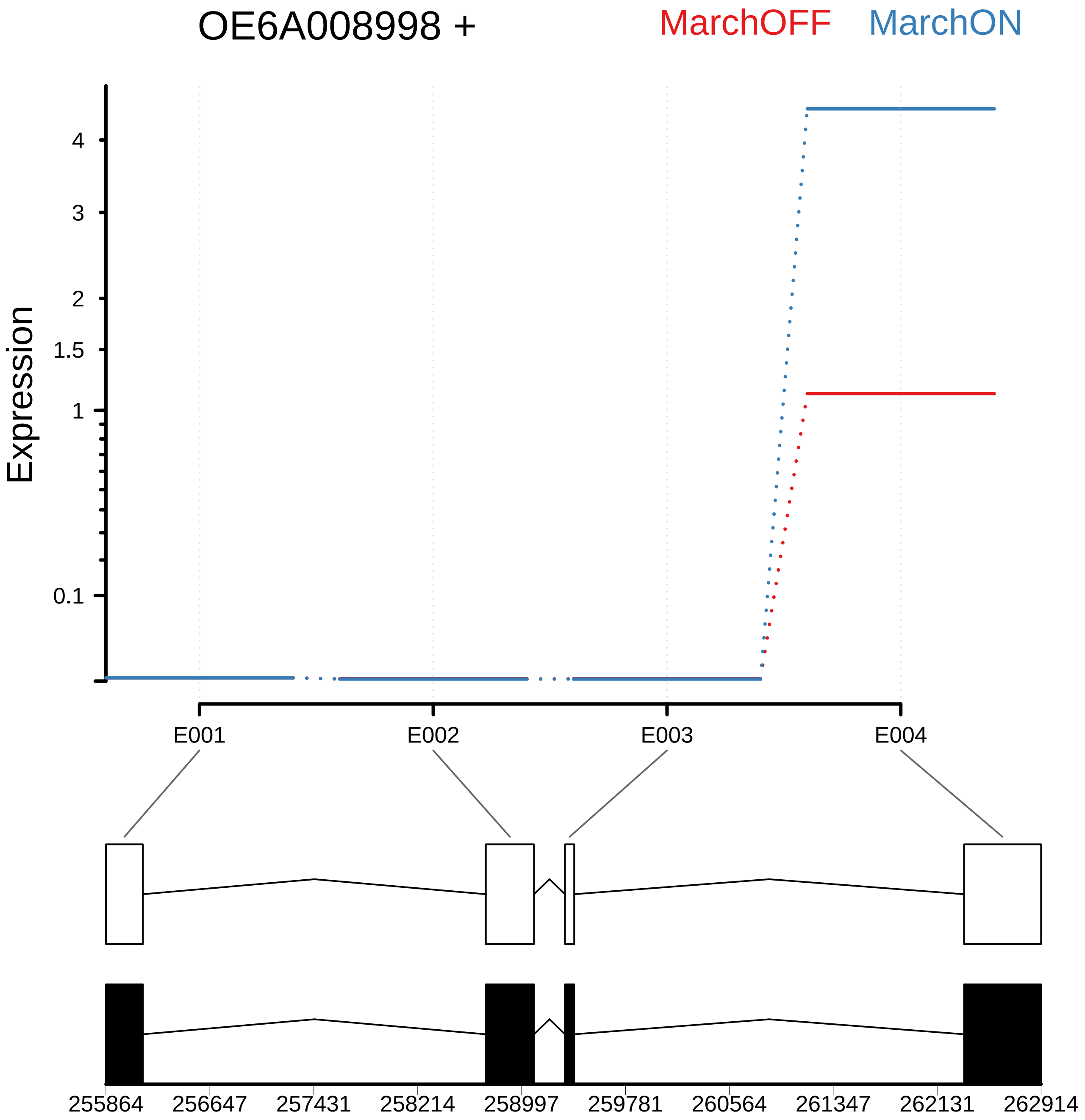

OE6A008998 +
MarchOFF
MarchON
Expression
4
3
2
1.5
1
0.1
E001
E002
E003
E004
255864
256647
257431
258214
258997
259781
260564
261347
262131
262914

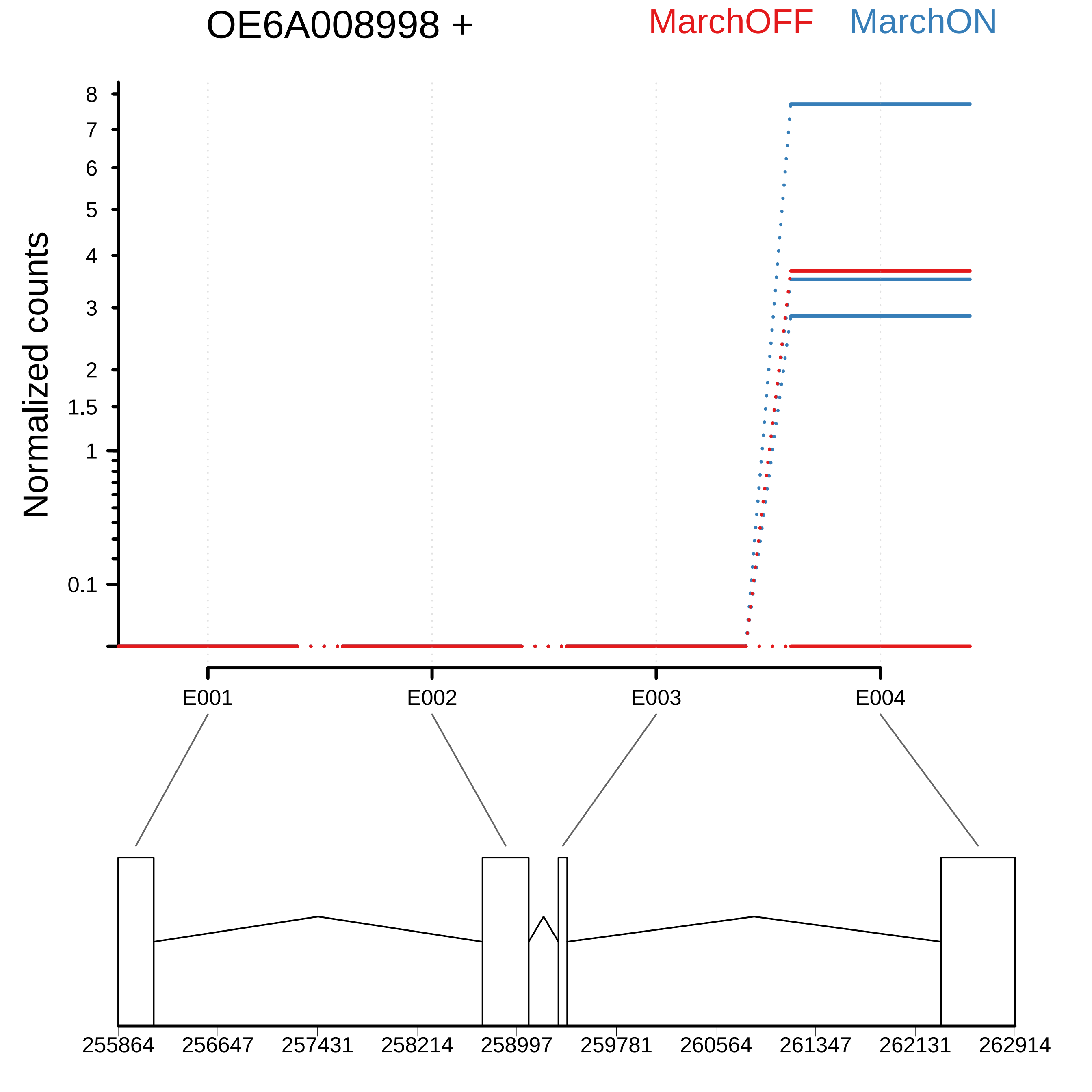

OE6A008998 +
MarchOFF
MarchON
Normalized counts
8
7
6
5
4
3
2
1.5
1
0.1
E001
E002
E003
E004
255864
256647
257431
258214
258997
259781
260564
261347
262131
262914

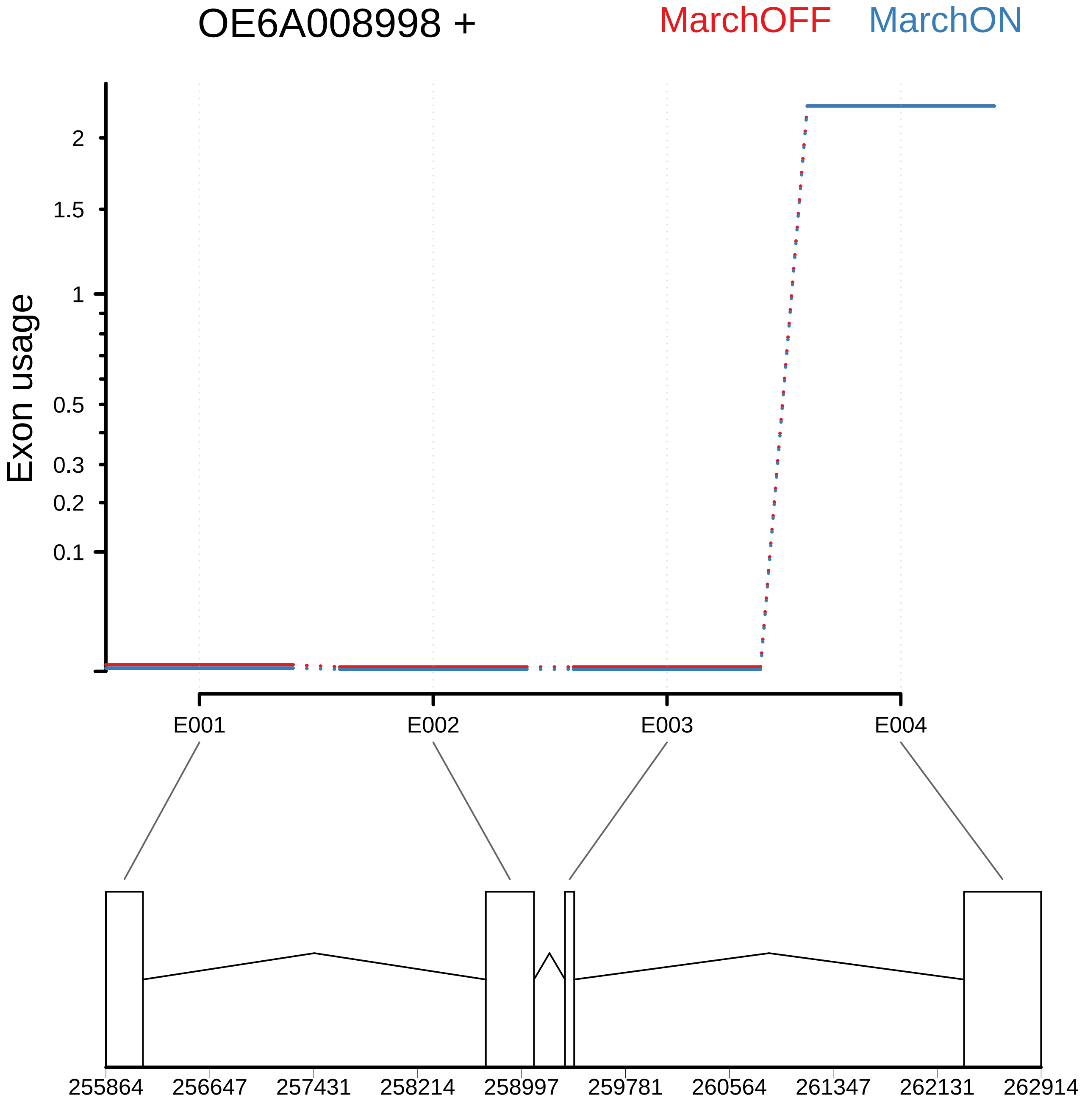

OE6A008998 +
MarchOFF
MarchON
Exon usage
2
1.5
1
0.5
0.3
0.2
0.1
E001
E002
E003
E004
255864
256647
257431
258214
258997
259781
260564
261347
262131
262914

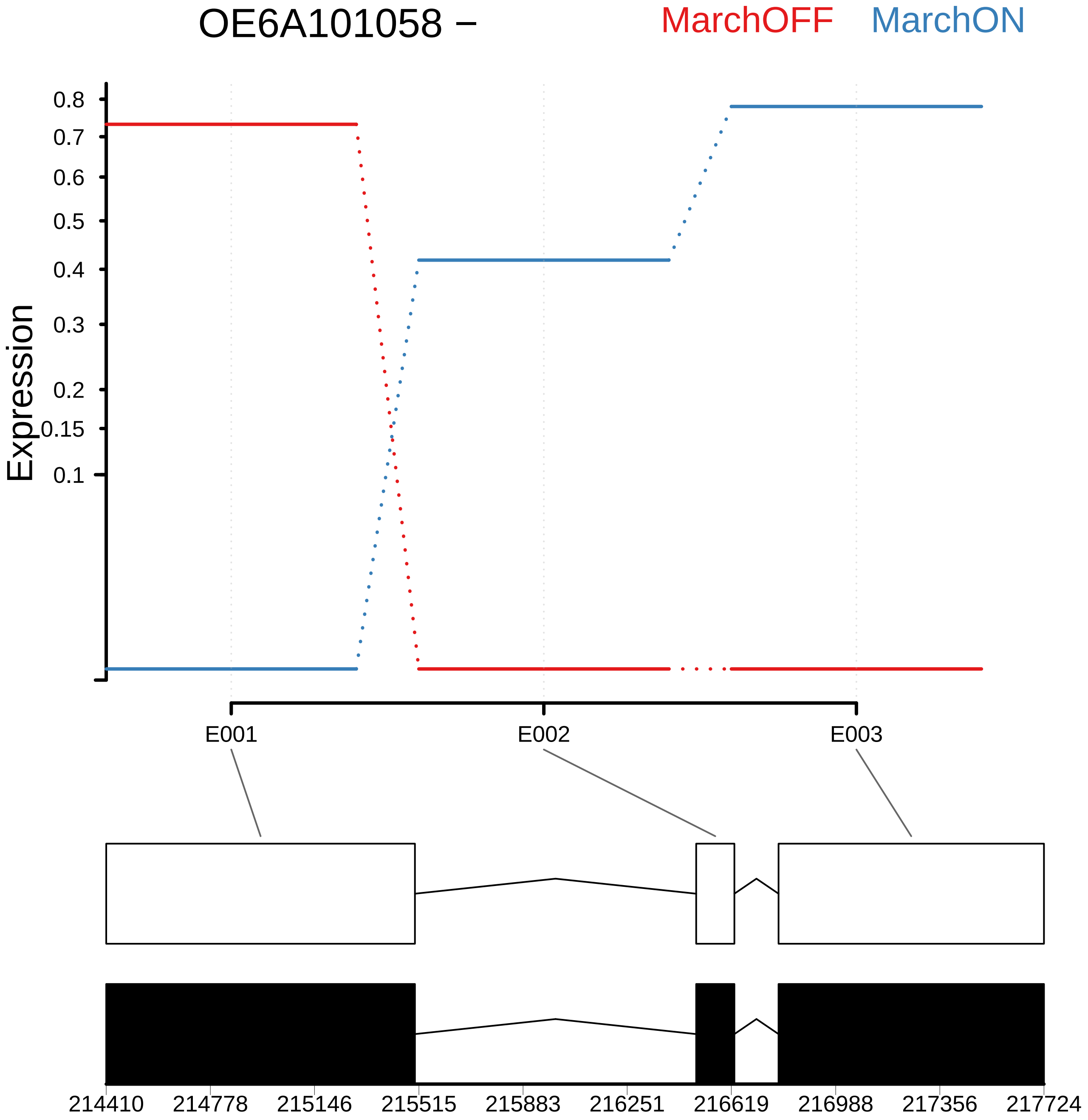

OE6A101058 −
MarchOFF
MarchON
Expression
0.8
0.7
0.6
0.5
0.4
0.3
0.2
0.15
0.1
E001
E002
E003
214410
214778
215146
215515
215883
216251
216619
216988
217356
217724

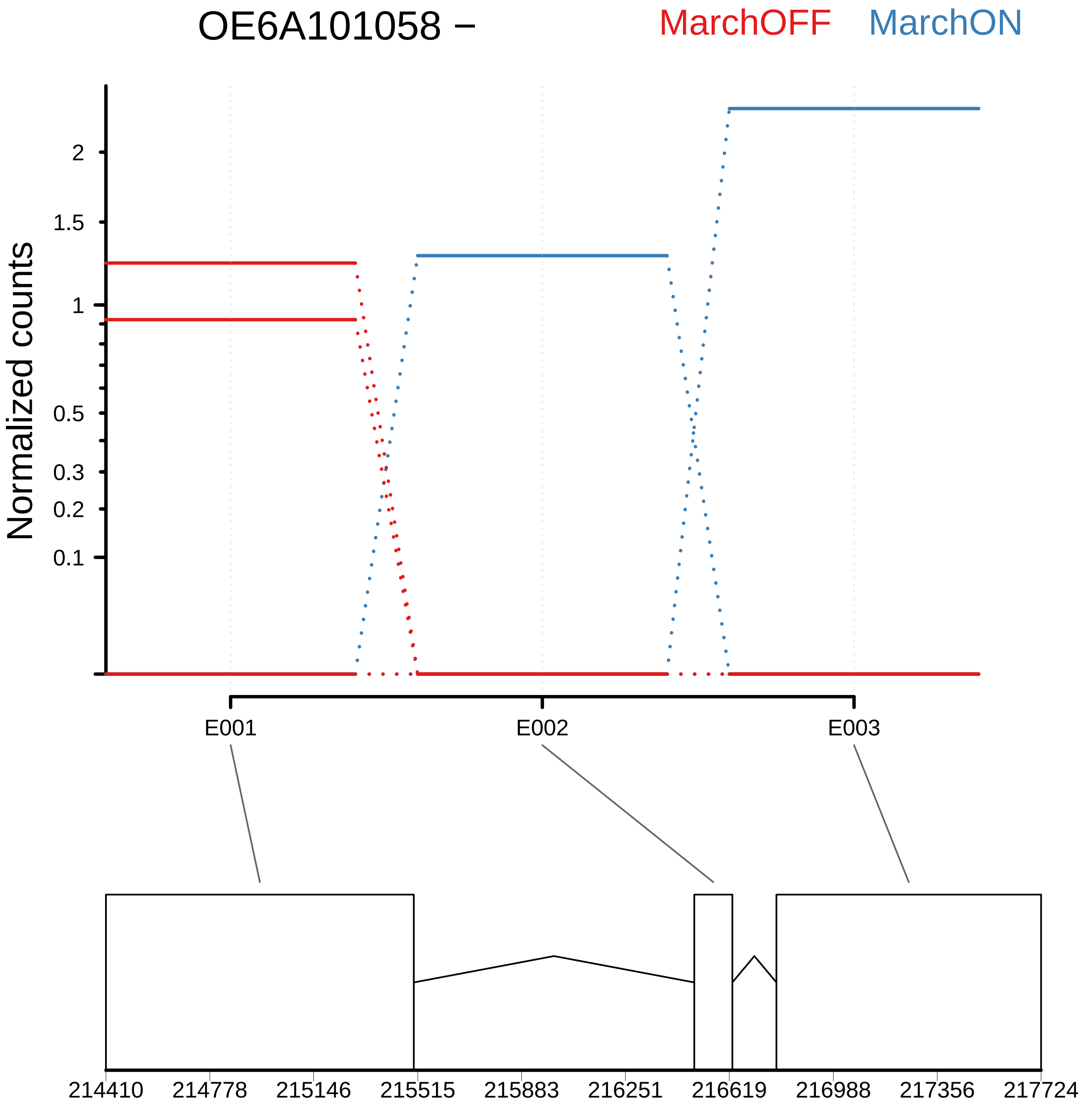
OE6A101058 −
MarchOFF
MarchON
Normalized counts
2
1.5
1
0.5
0.3
0.2
0.1
E001
E002
E003
214410
214778
215146
215515
215883
216251
216619
216988
217356
217724

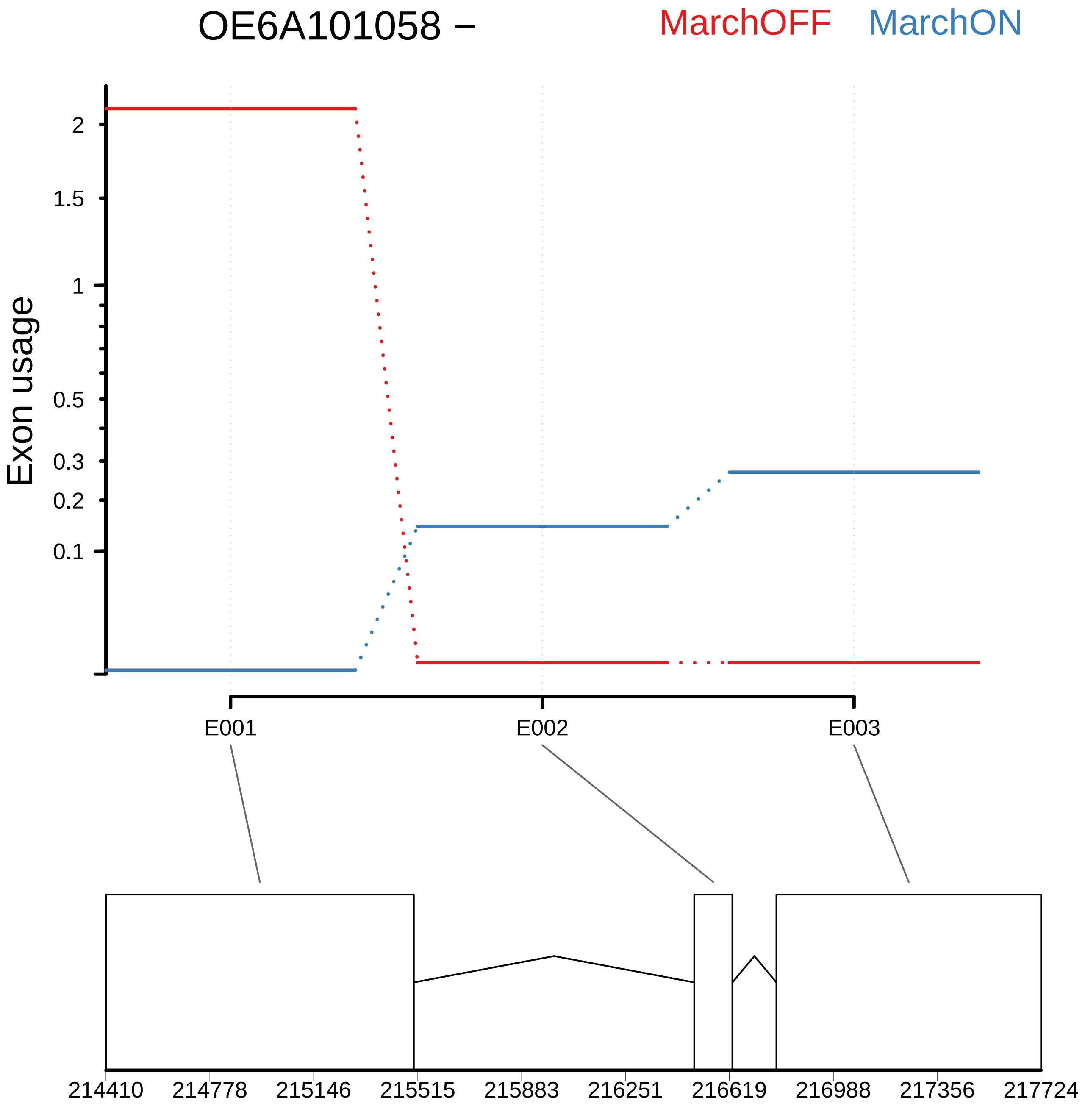

OE6A101058 −
MarchOFF
MarchON
Exon usage
2
1.5
1
0.5
0.3
0.2
0.1
E001
E002
E003
214410
214778
215146
215515
215883
216251
216619
216988
217356
217724

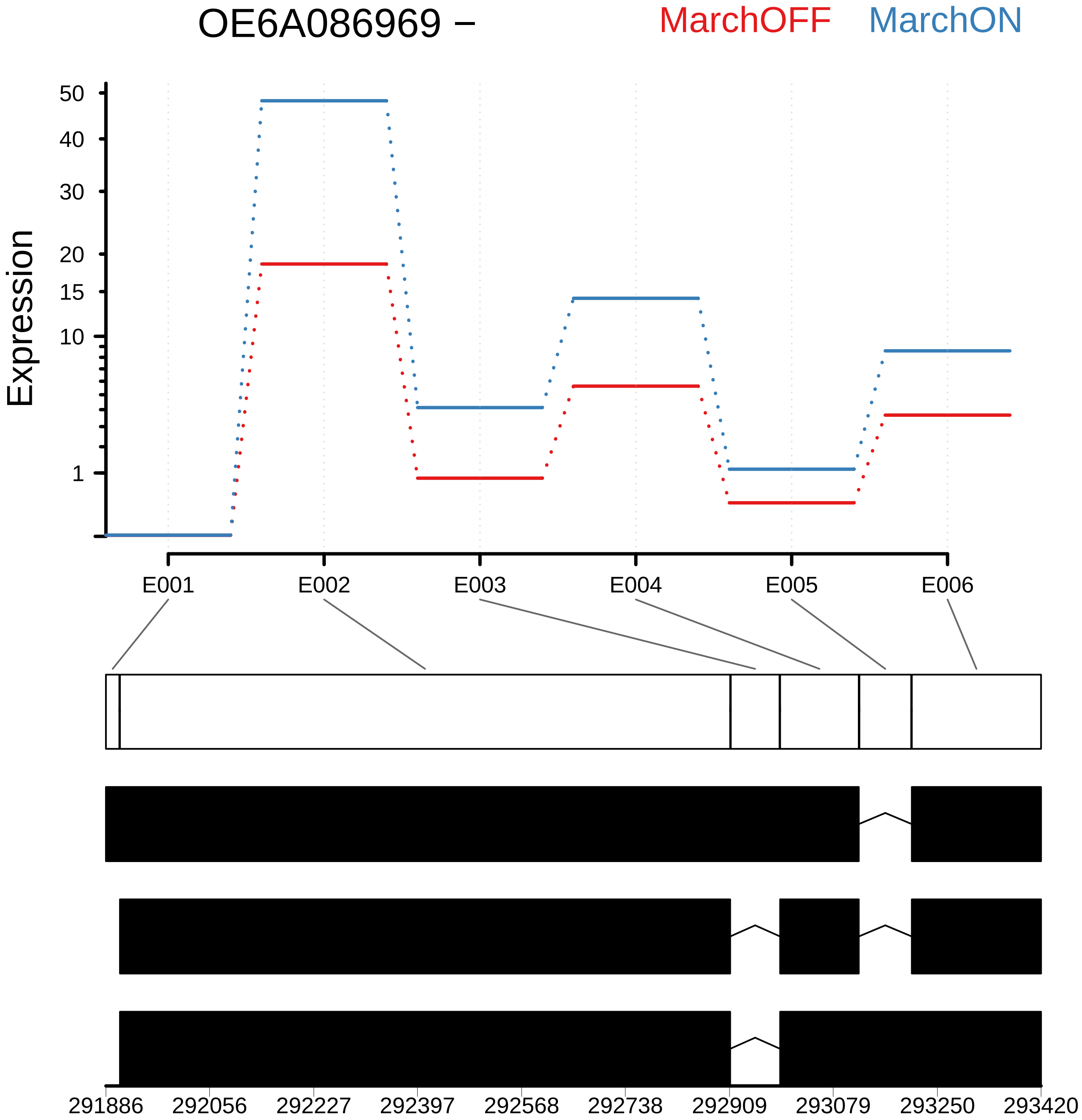

OE6A086969 –
MarchOFF
MarchON
Expression
50
40
30
20
15
10
1
E001
E002
E003
E004
E005
E006
291886
292056
292227
292397
292568
292738
292909
293079
293250
293420

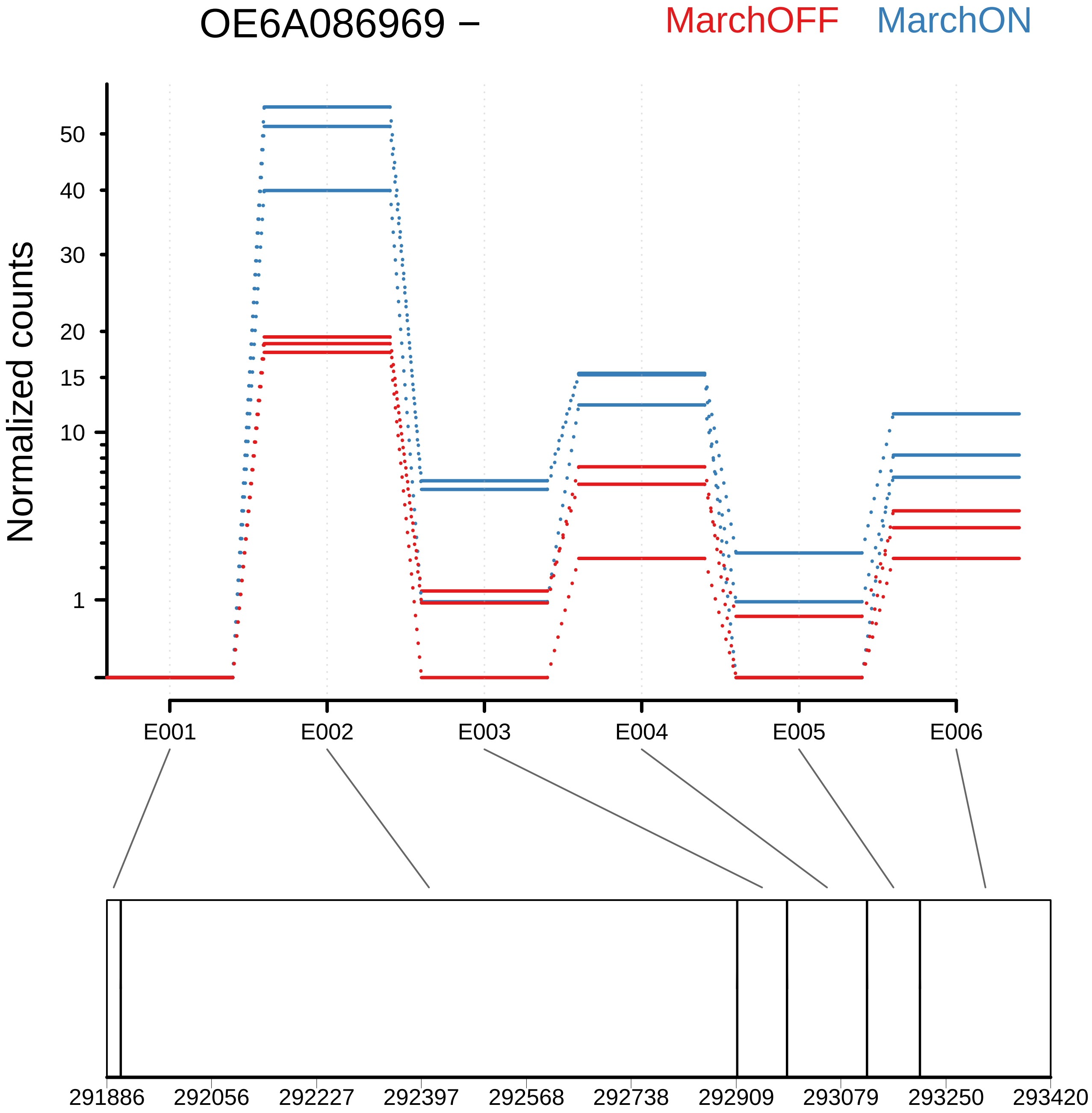
OE6A086969 −
MarchOFF
MarchON
Normalized counts
50
40
30
20
15
10
1
E001
E002
E003
E004
E005
E006
291886
292056
292227
292397
292568
292738
292909
293079
293250
293420

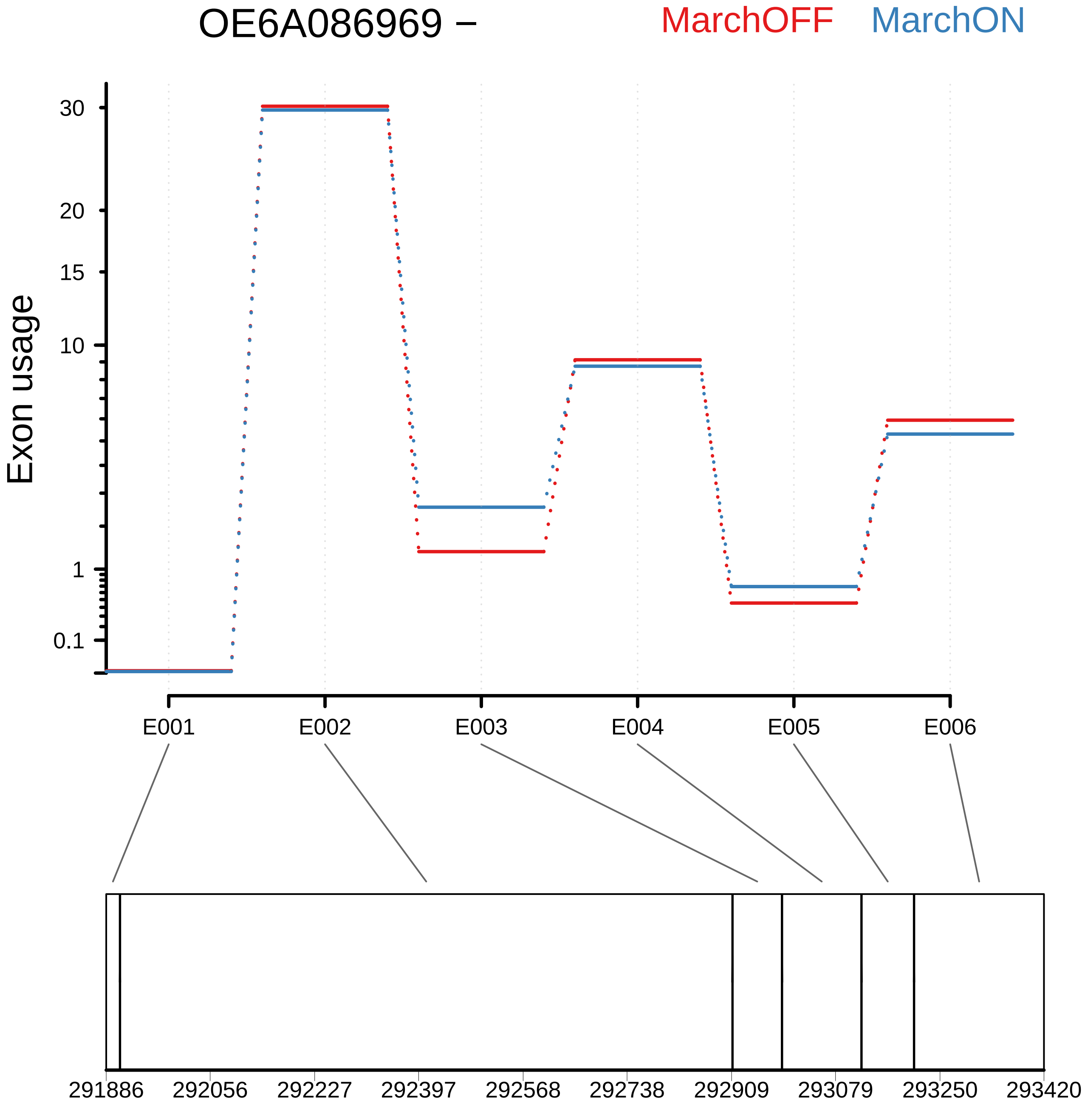

OE6A086969 −
MarchOFF
MarchON
Exon usage
30
20
15
10
1
0.1
E001
E002
E003
E004
E005
E006
291886
292056
292227
292397
292568
292738
292909
293079
293250
293420

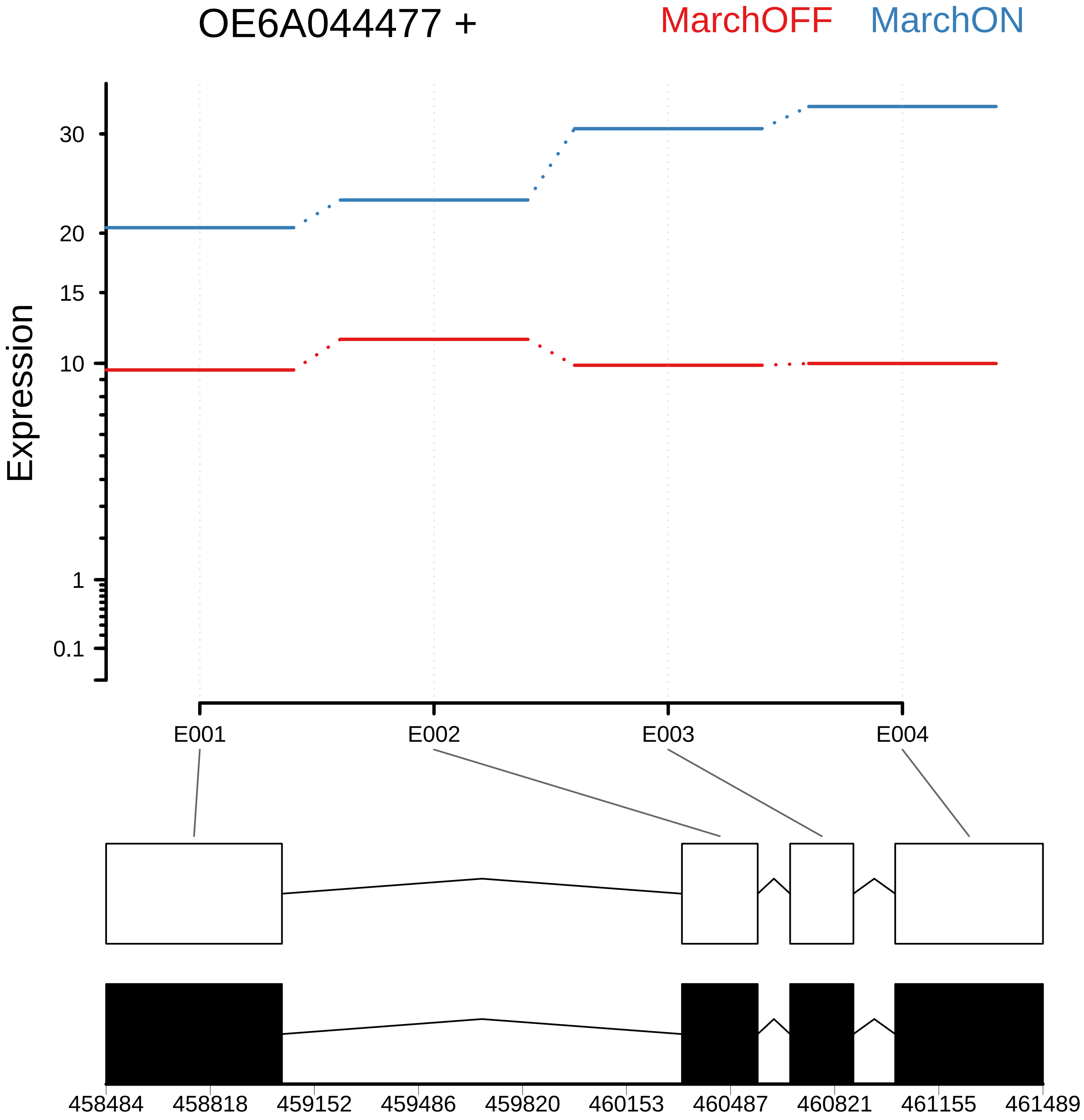

OE6A044477 +
MarchOFF
MarchON
Expression
30
20
15
10
1
0.1
E001
E002
E003
E004
458484
458818
459152
459486
459820
460153
460487
460821
461155
461489

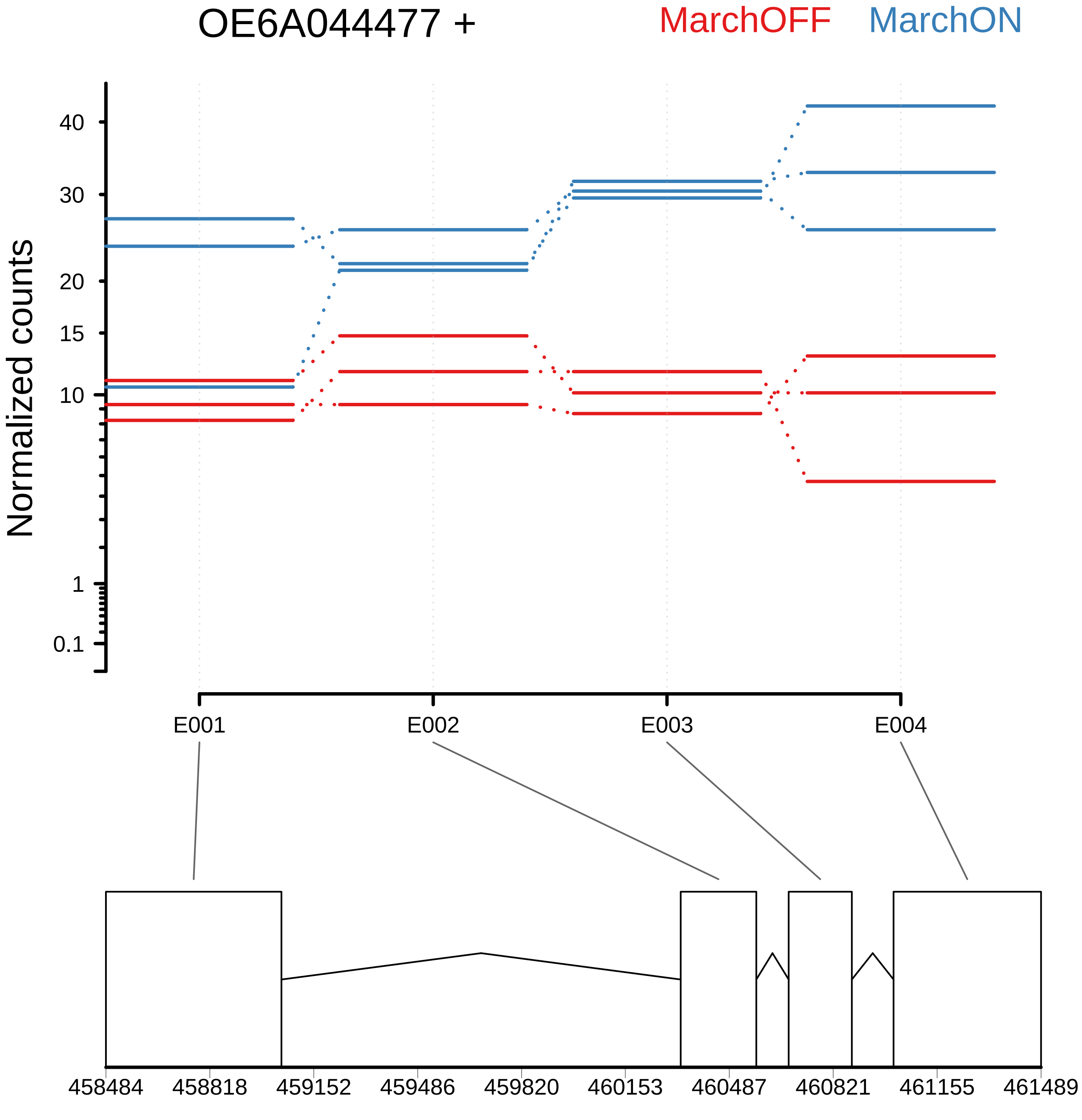

OE6A044477 +
MarchOFF
MarchON
Normalized counts
40
30
20
15
10
1
0.1
E001
E002
E003
E004
458484
458818
459152
459486
459820
460153
460487
460821
461155
461489

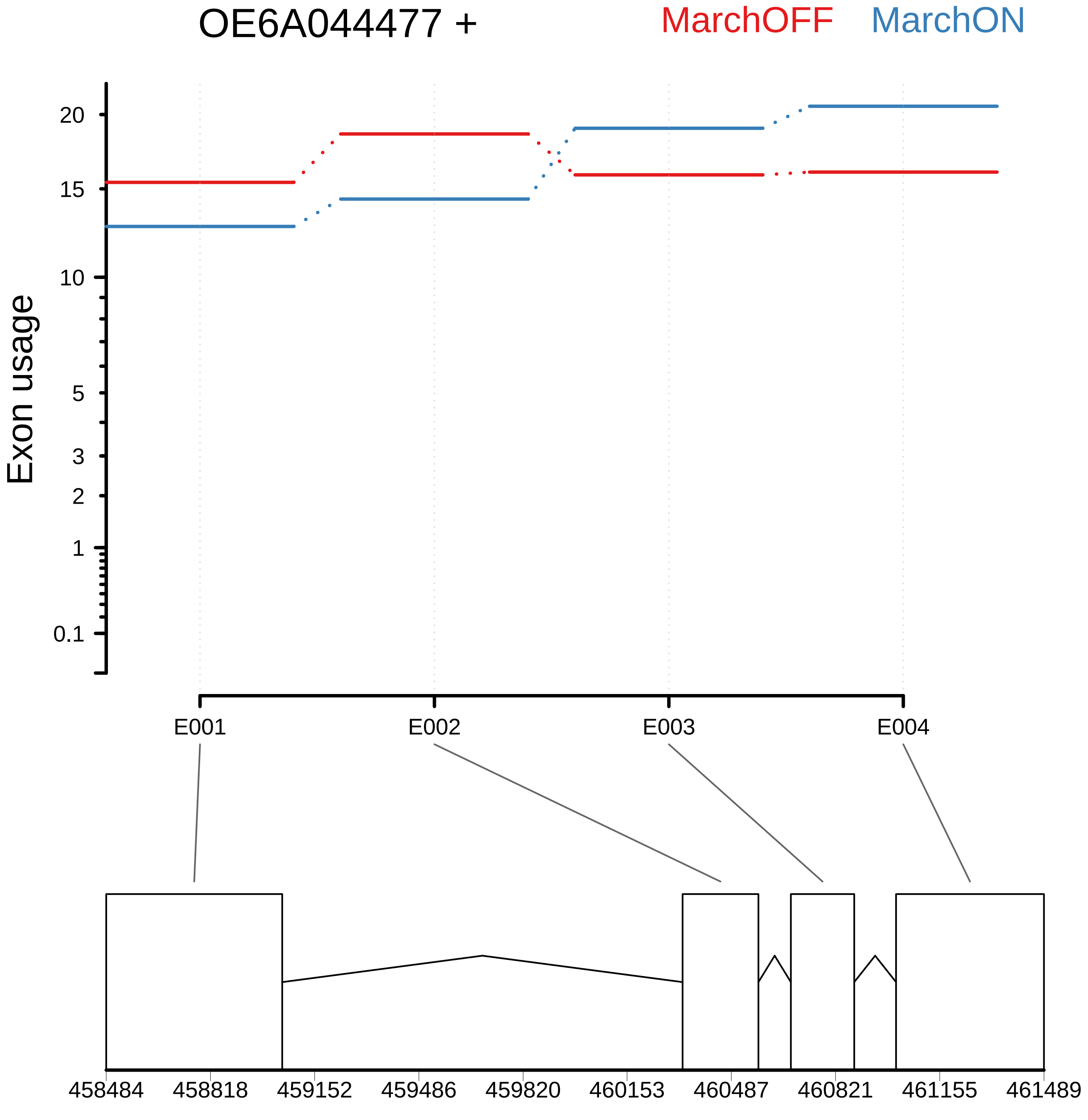
OE6A044477 +
MarchOFF
MarchON
Exon usage
20
15
10
5
3
2
1
0.1
E001
E002
E003
E004
458484
458818
459152
459486
459820
460153
460487
460821
461155
461489

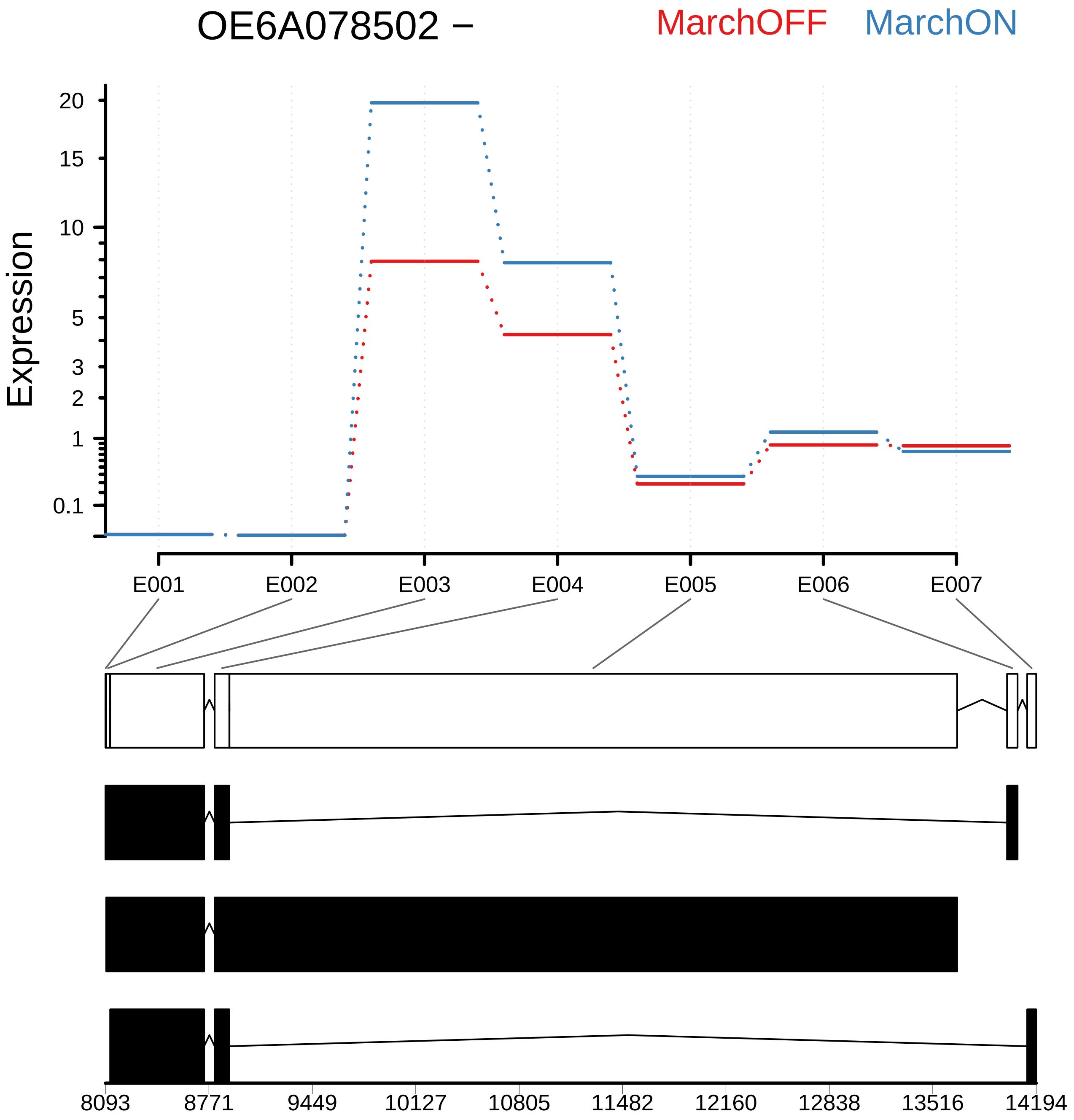

OE6A078502 −
MarchOFF
MarchON
Expression
20
15
10
5
3
2
1
0.1
E001
E002
E003
E004
E005
E006
E007
8093
8771
9449
10127
10805
11482
12160
12838
13516
14194

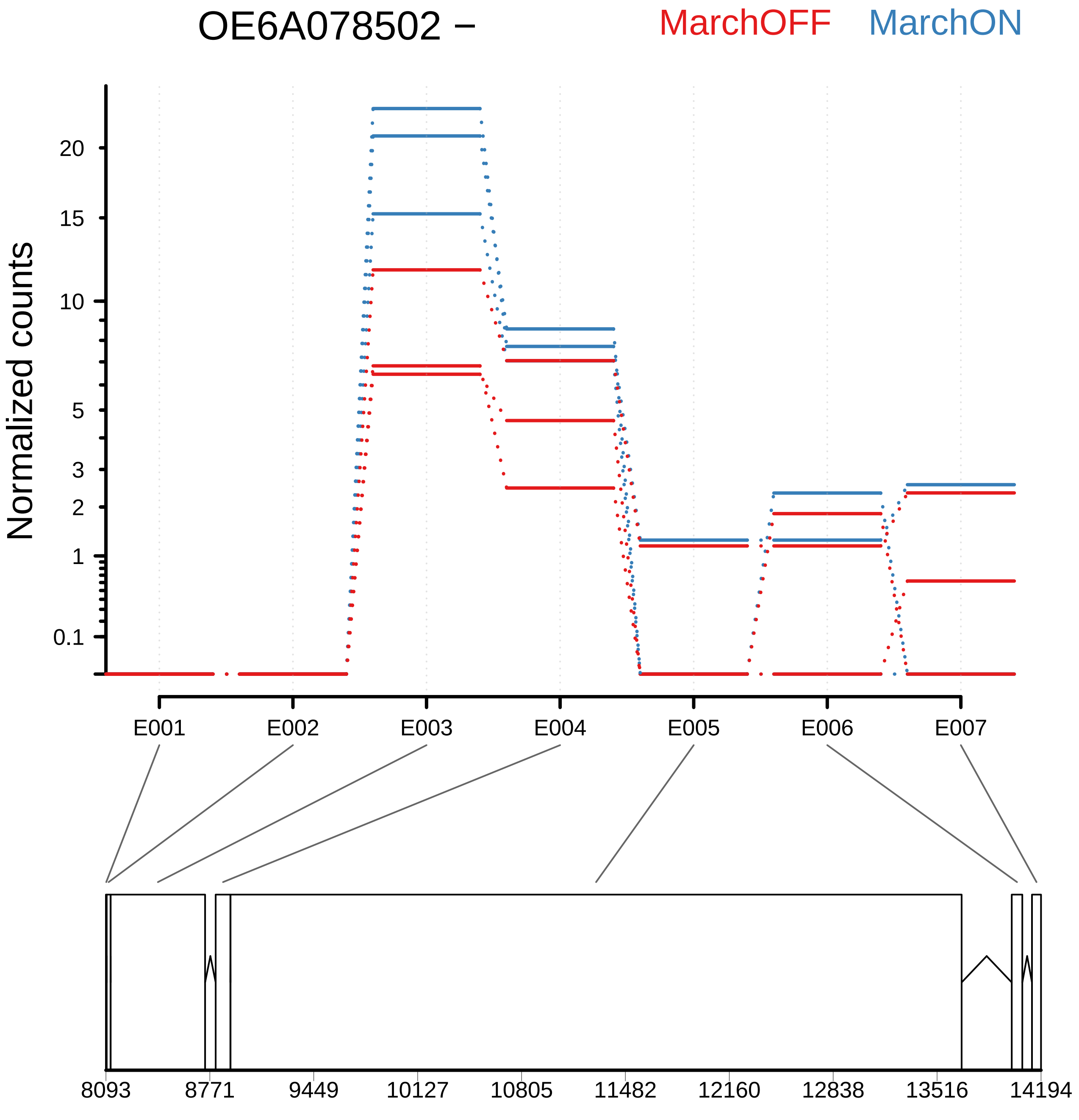

OE6A078502 −
MarchOFF
MarchON
Normalized counts
20
15
10
5
3
2
1
0.1
E001
E002
E003
E004
E005
E006
E007
8093
8771
9449
10127
10805
11482
12160
12838
13516
14194

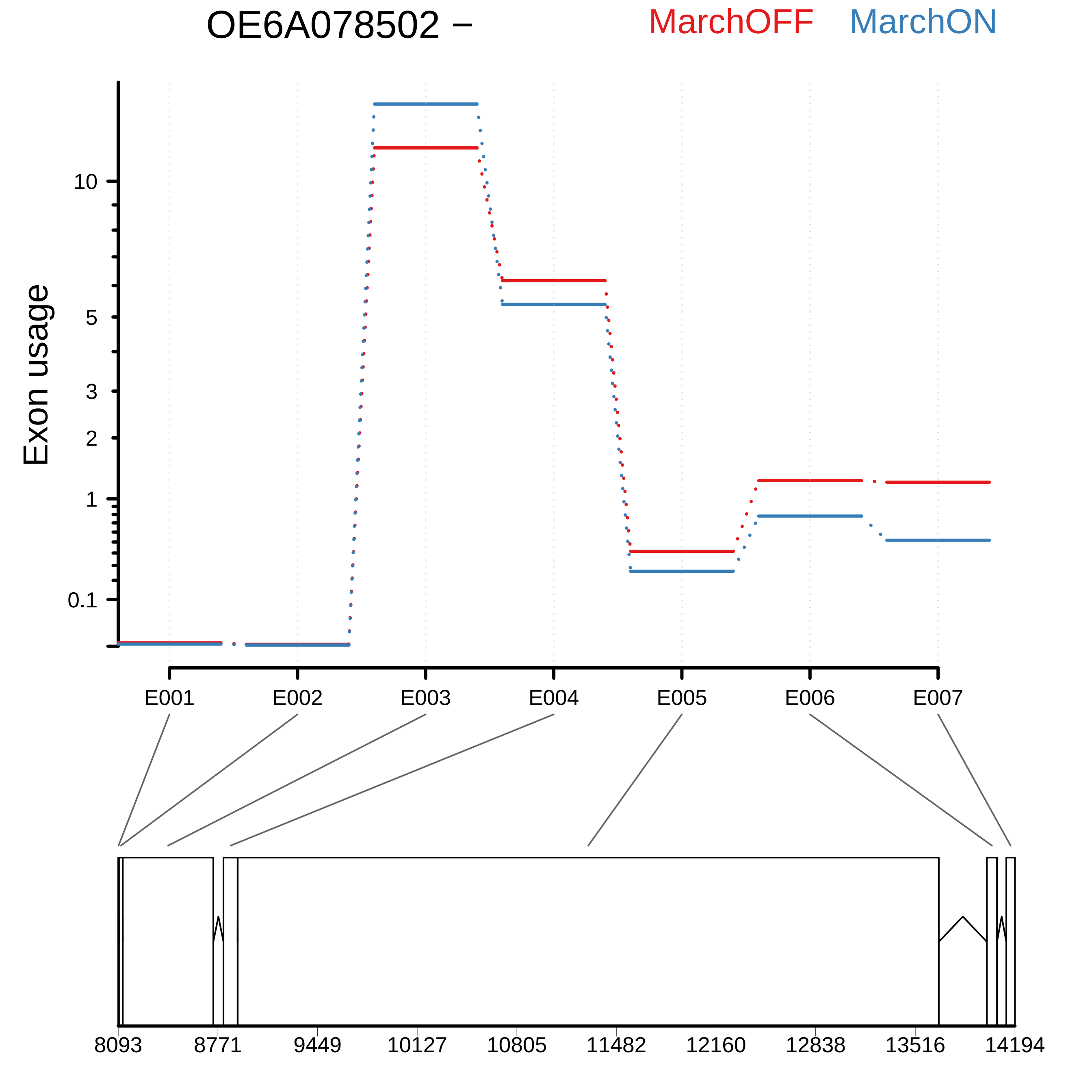

OE6A078502 −
MarchOFF
MarchON
Exon usage
10
5
3
2
1
0.1
E001
E002
E003
E004
E005
E006
E007
8093
8771
9449
10127
10805
11482
12160
12838
13516
14194

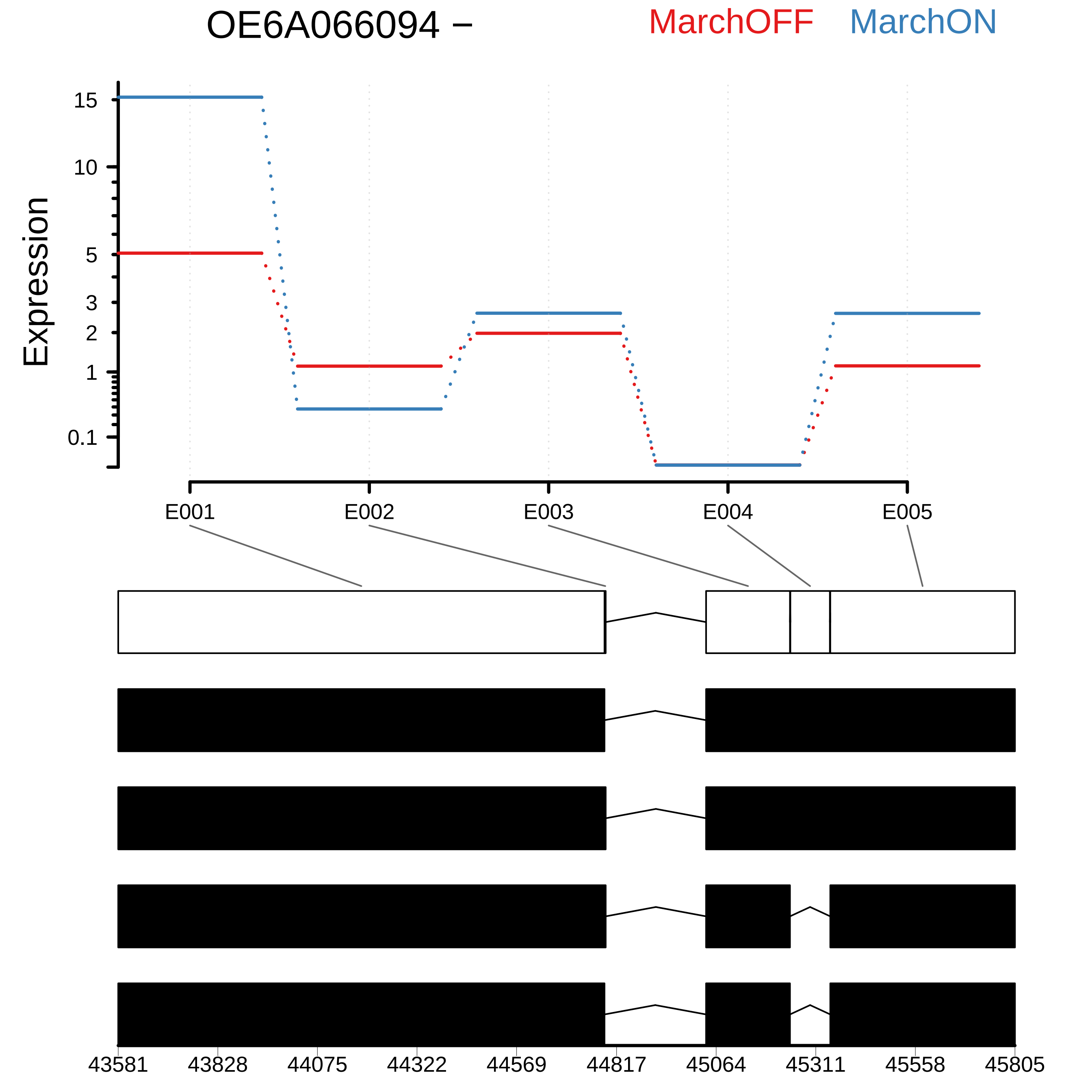

OE6A066094 –
MarchOFF
MarchON
Expression
15
10
5
3
2
1
0.1
E001
E002
E003
E004
E005
43581
43828
44075
44322
44569
44817
45064
45311
45558
45805

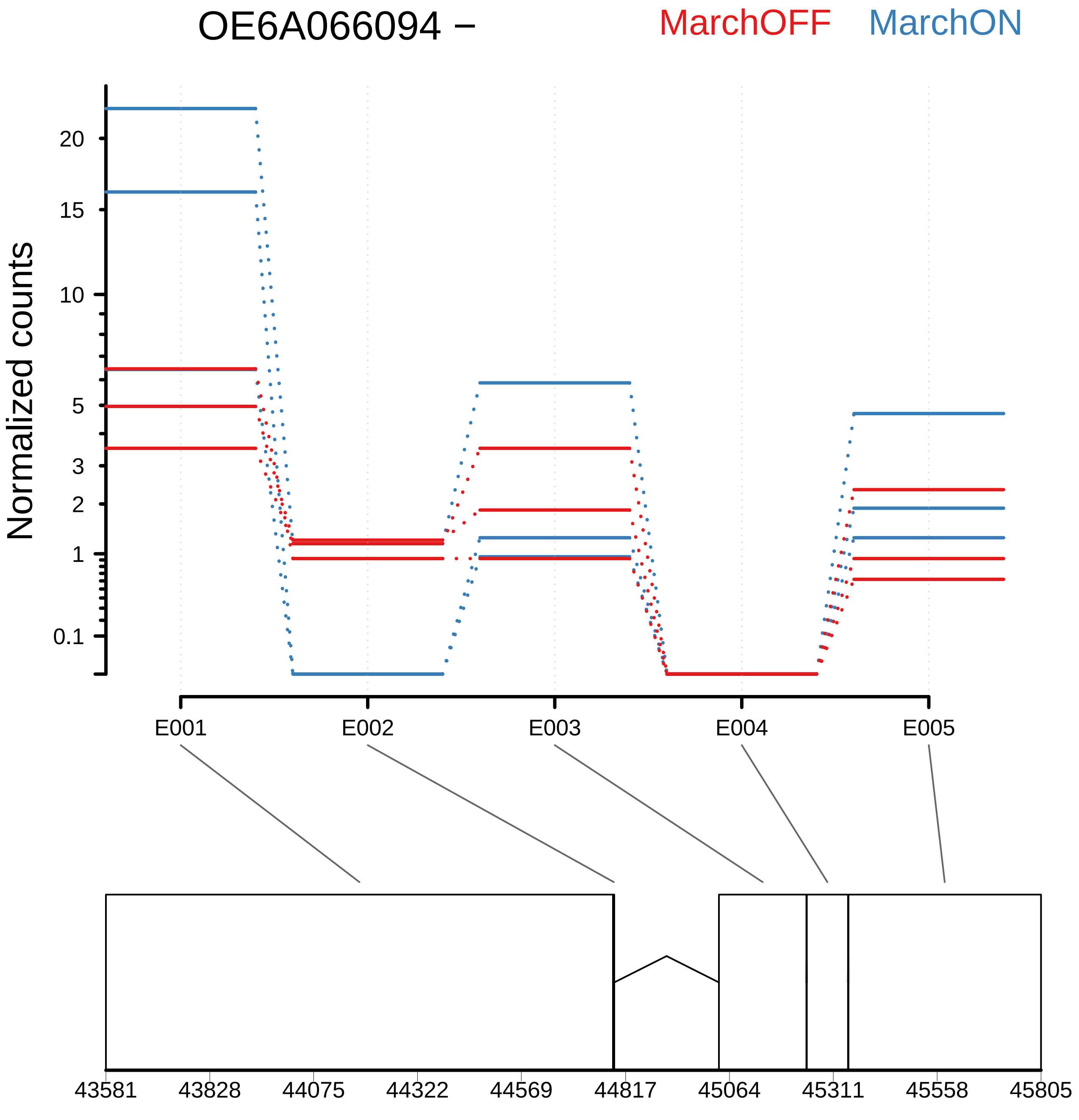
OE6A066094 −
MarchOFF
MarchON
Normalized counts
20
15
10
5
3
2
1
0.1
E001
E002
E003
E004
E005
43581
43828
44075
44322
44569
44817
45064
45311
45558
45805

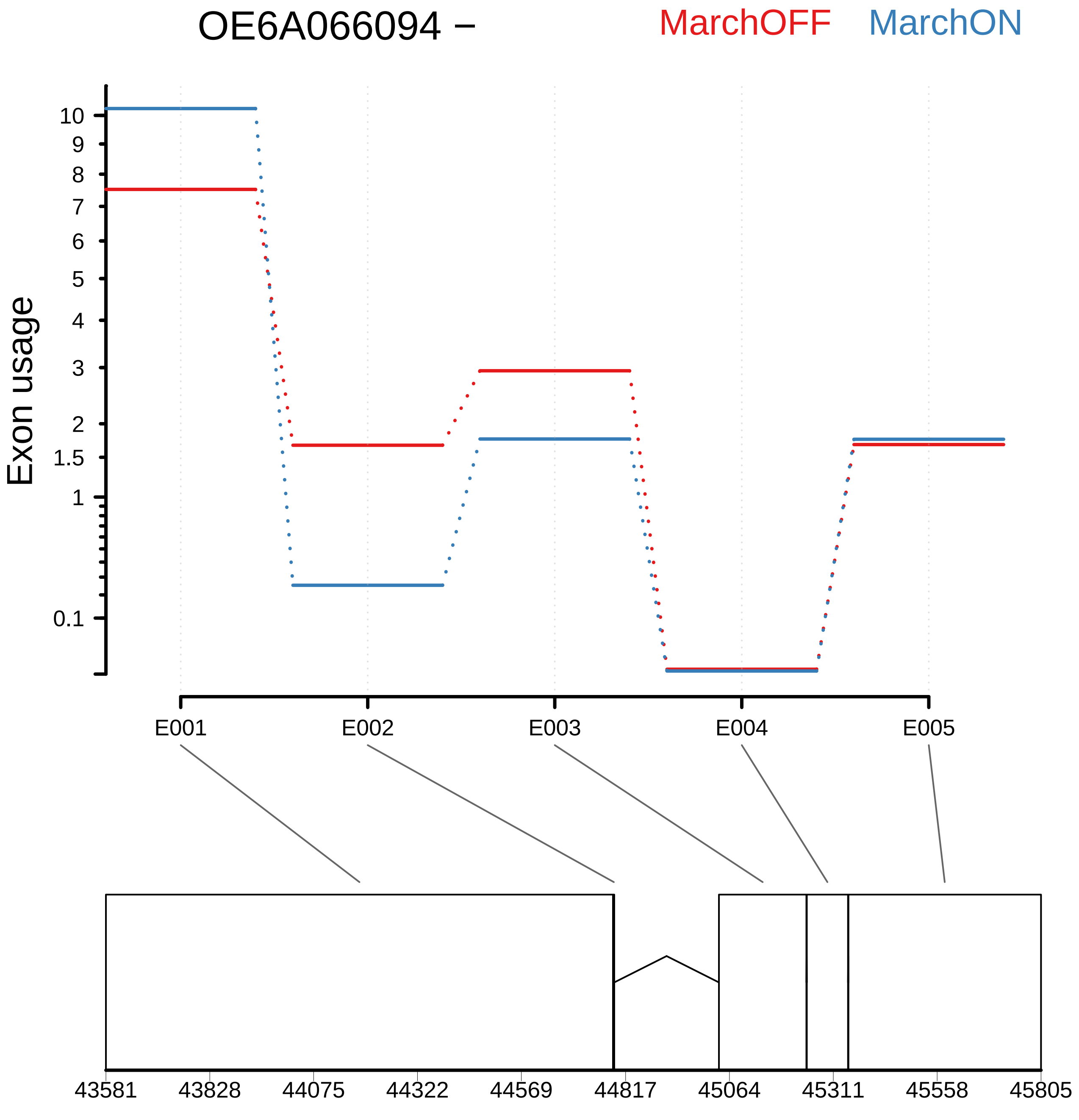

OE6A066094 −
MarchOFF
MarchON
Exon usage
10
9
8
7
6
5
4
3
2
1.5
1
0.1
E001
E002
E003
E004
E005
43581
43828
44075
44322
44569
44817
45064
45311
45558
45805

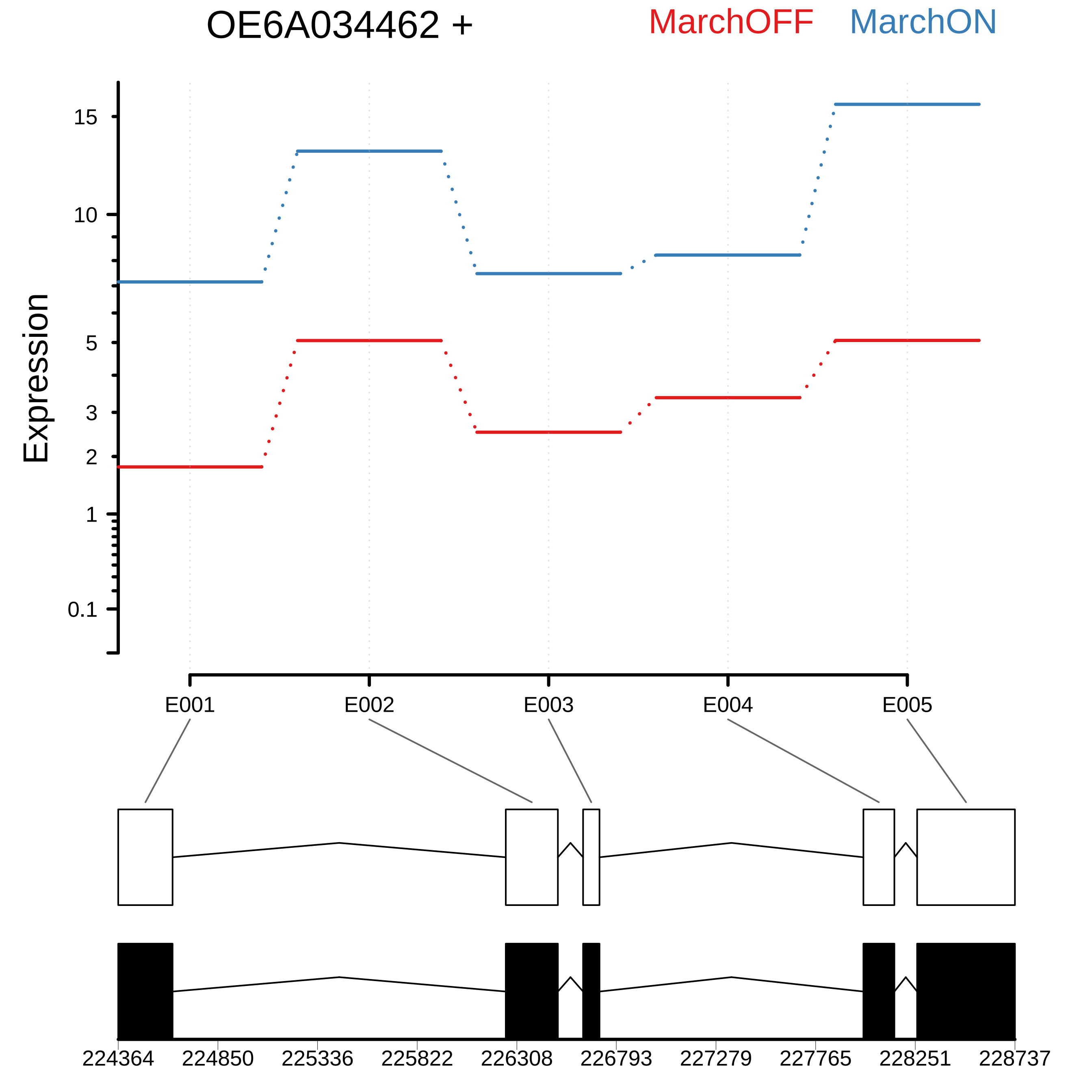
OE6A034462 +
MarchOFF
MarchON
Expression
15
10
5
3
2
1
0.1
E001
E002
E003
E004
E005
224364
224850
225336
225822
226308
226793
227279
227765
228251
228737

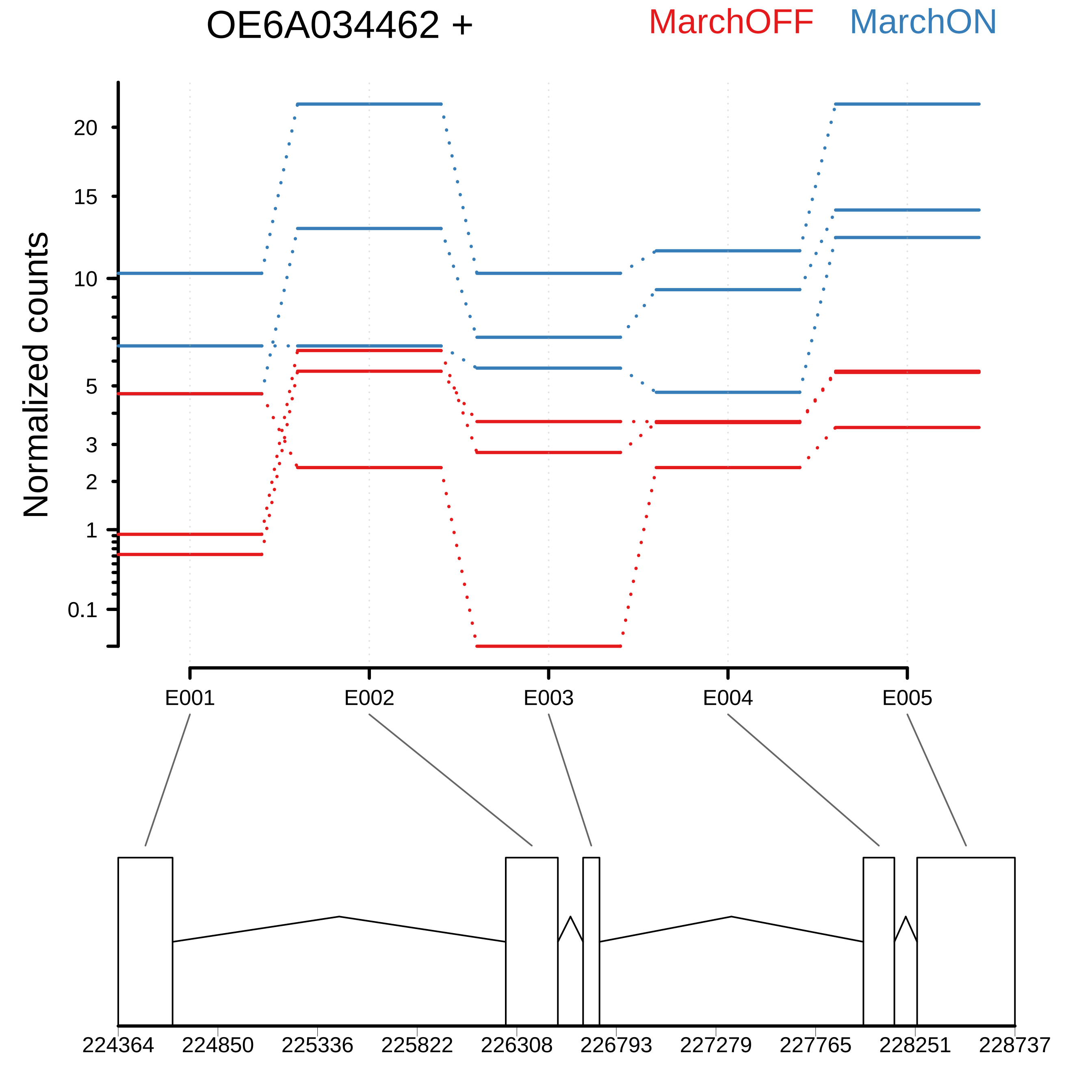

OE6A034462 +
MarchOFF
MarchON
Normalized counts
20
15
10
5
3
2
1
0.1
E001
E002
E003
E004
E005
224364
224850
225336
225822
226308
226793
227279
227765
228251
228737

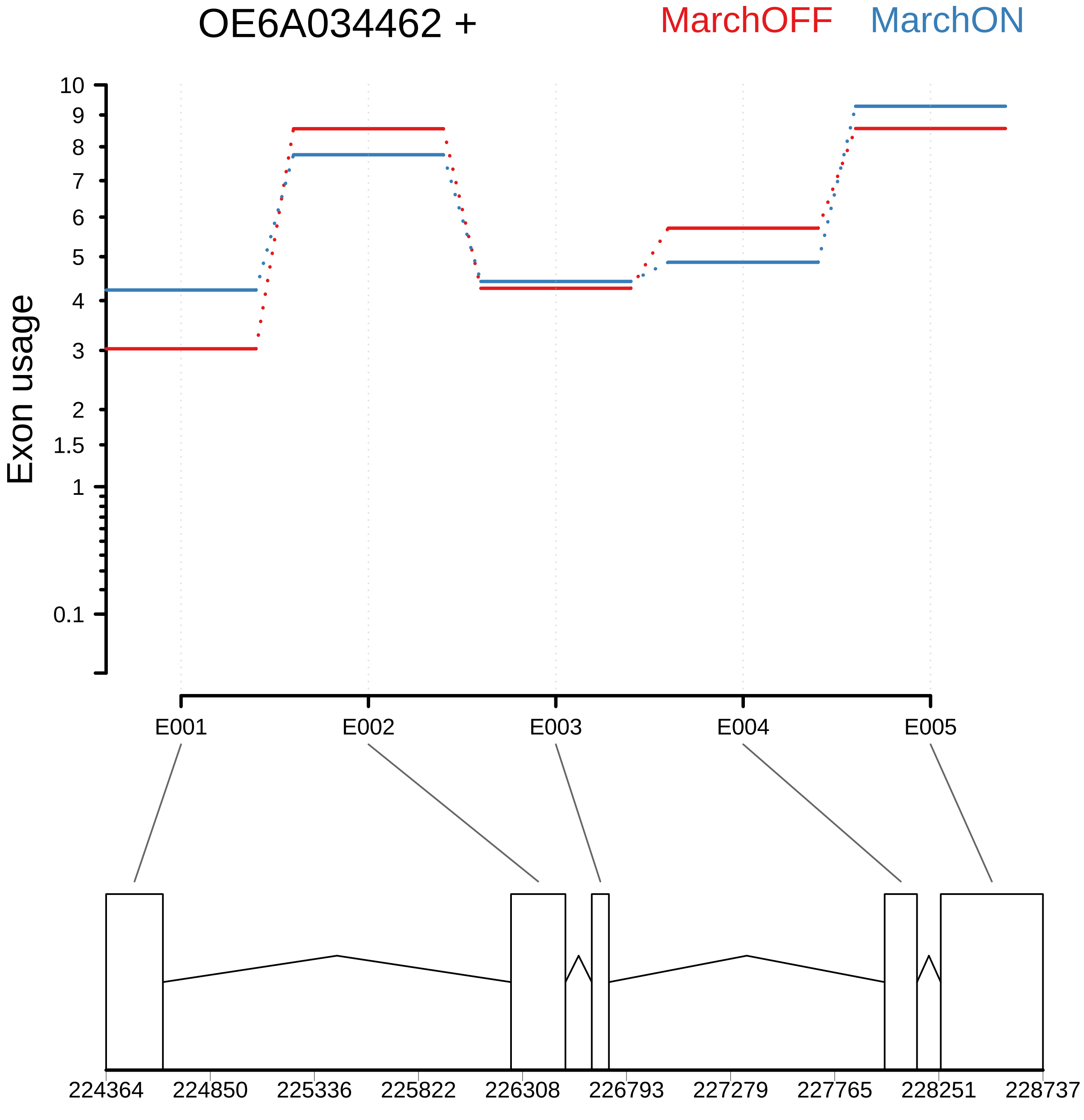

OE6A034462 +
MarchOFF
MarchON
Exon usage
10
9
8
7
6
5
4
3
2
1.5
1
0.1
E001
E002
E003
E004
E005
224364
224850
225336
225822
226308
226793
227279
227765
228251
228737

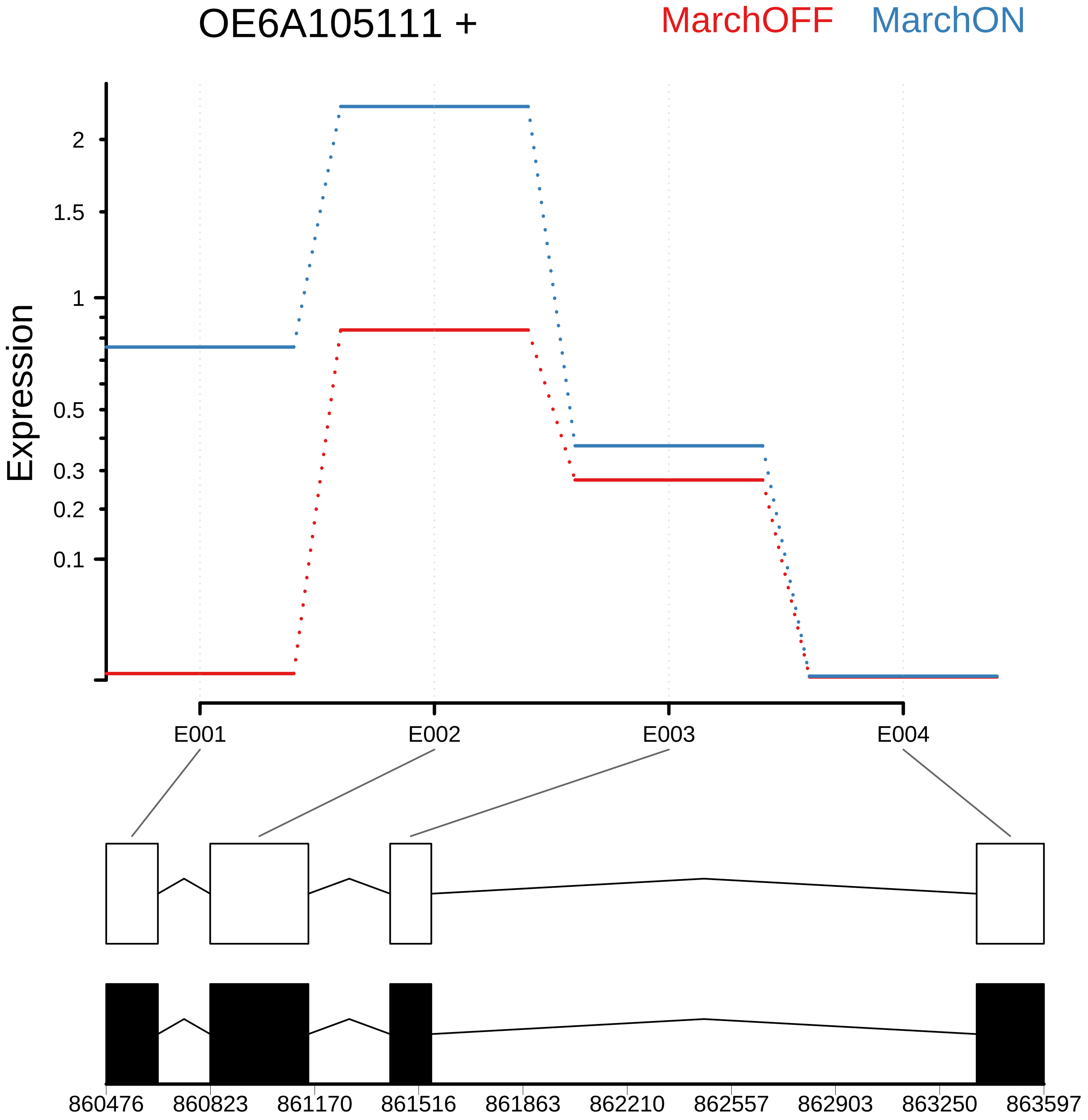
OE6A105111 +
MarchOFF
MarchON
Expression
2
1.5
1
0.5
0.3
0.2
0.1
E001
E002
E003
E004
860476
860823
861170
861516
861863
862210
862557
862903
863250
863597

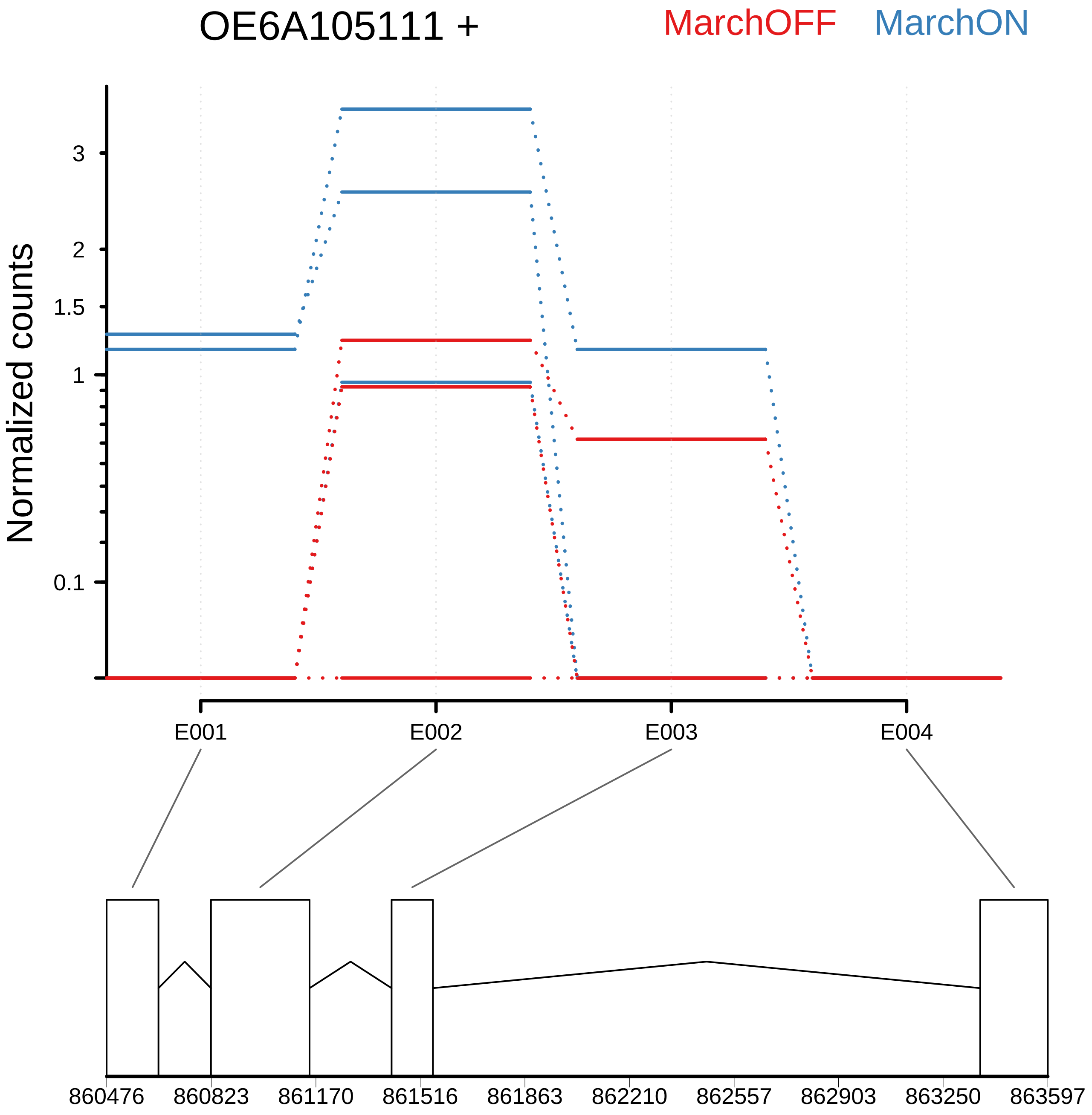

OE6A105111 +
MarchOFF
MarchON
Normalized counts
3
2
1.5
1
0.1
E001
E002
E003
E004
860476
860823
861170
861516
861863
862210
862557
862903
863250
863597

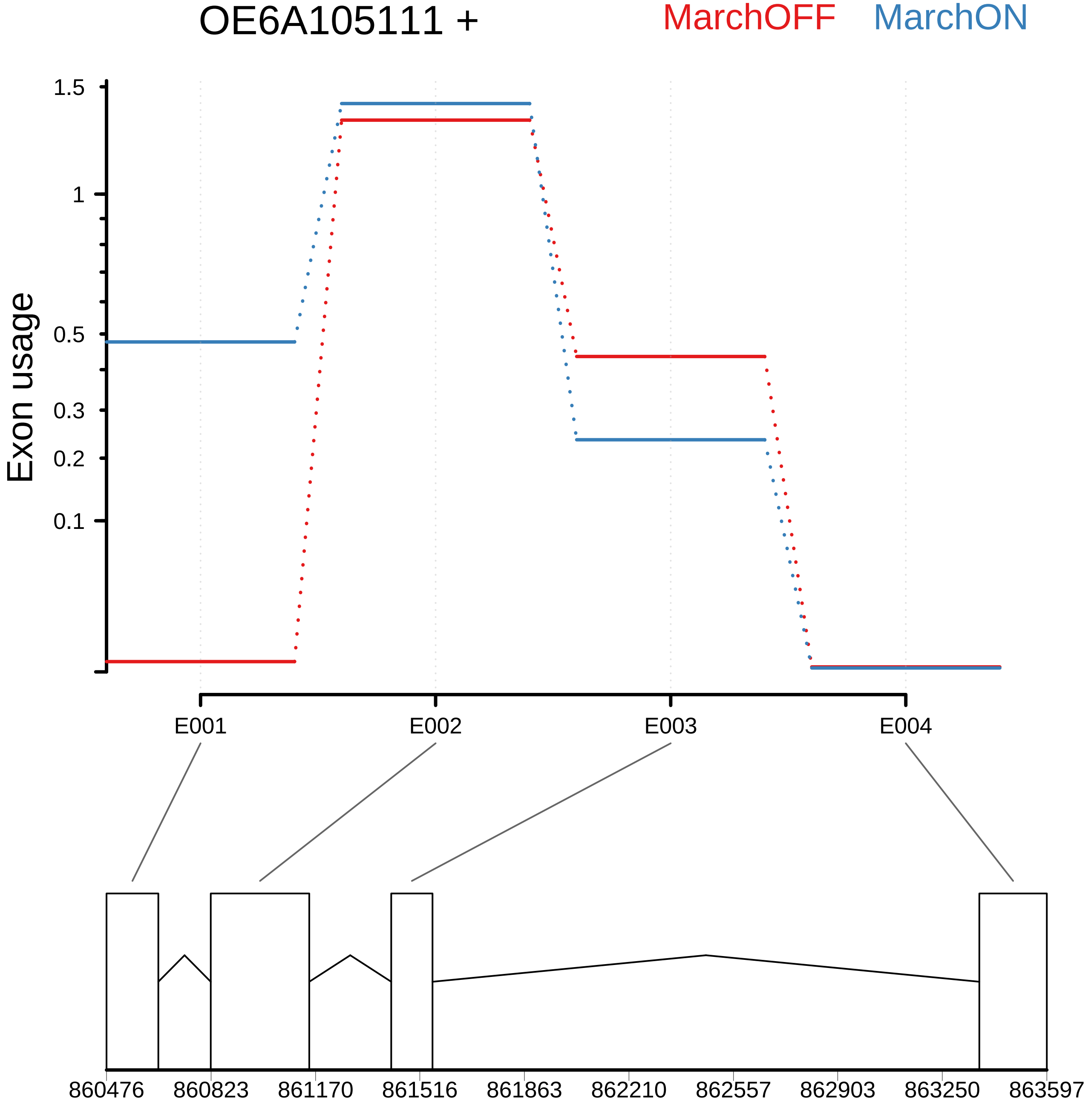

OE6A105111 +
MarchOFF
MarchON
Exon usage
1.5
1
0.5
0.3
0.2
0.1
E001
E002
E003
E004
860476
860823
861170
861516
861863
862210
862557
862903
863250
863597

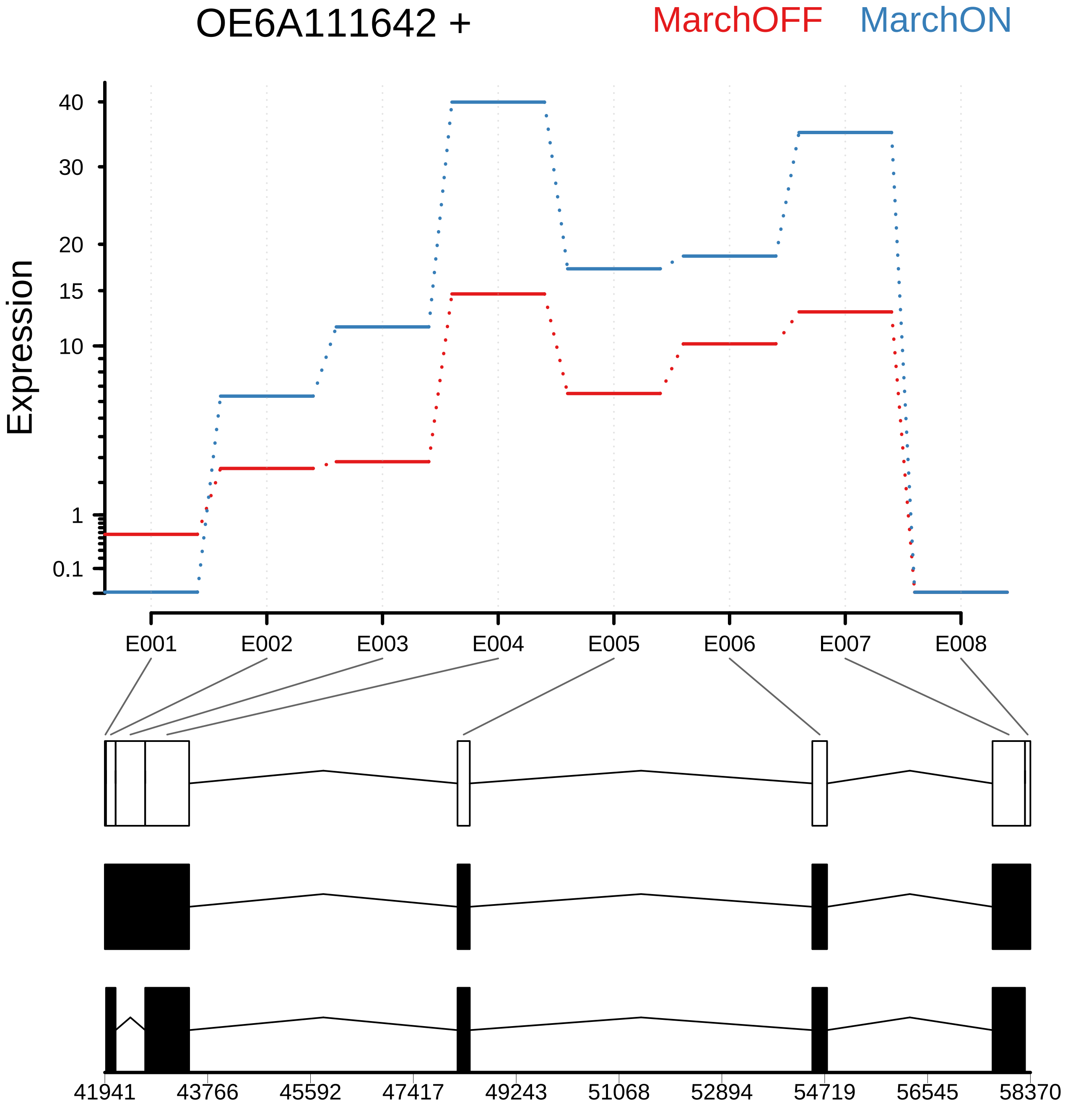

OE6A111642 +
MarchOFF
MarchON
Expression
40
30
20
15
10
1
0.1
E001
E002
E003
E004
E005
E006
E007
E008
41941
43766
45592
47417
49243
51068
52894
54719
56545
58370

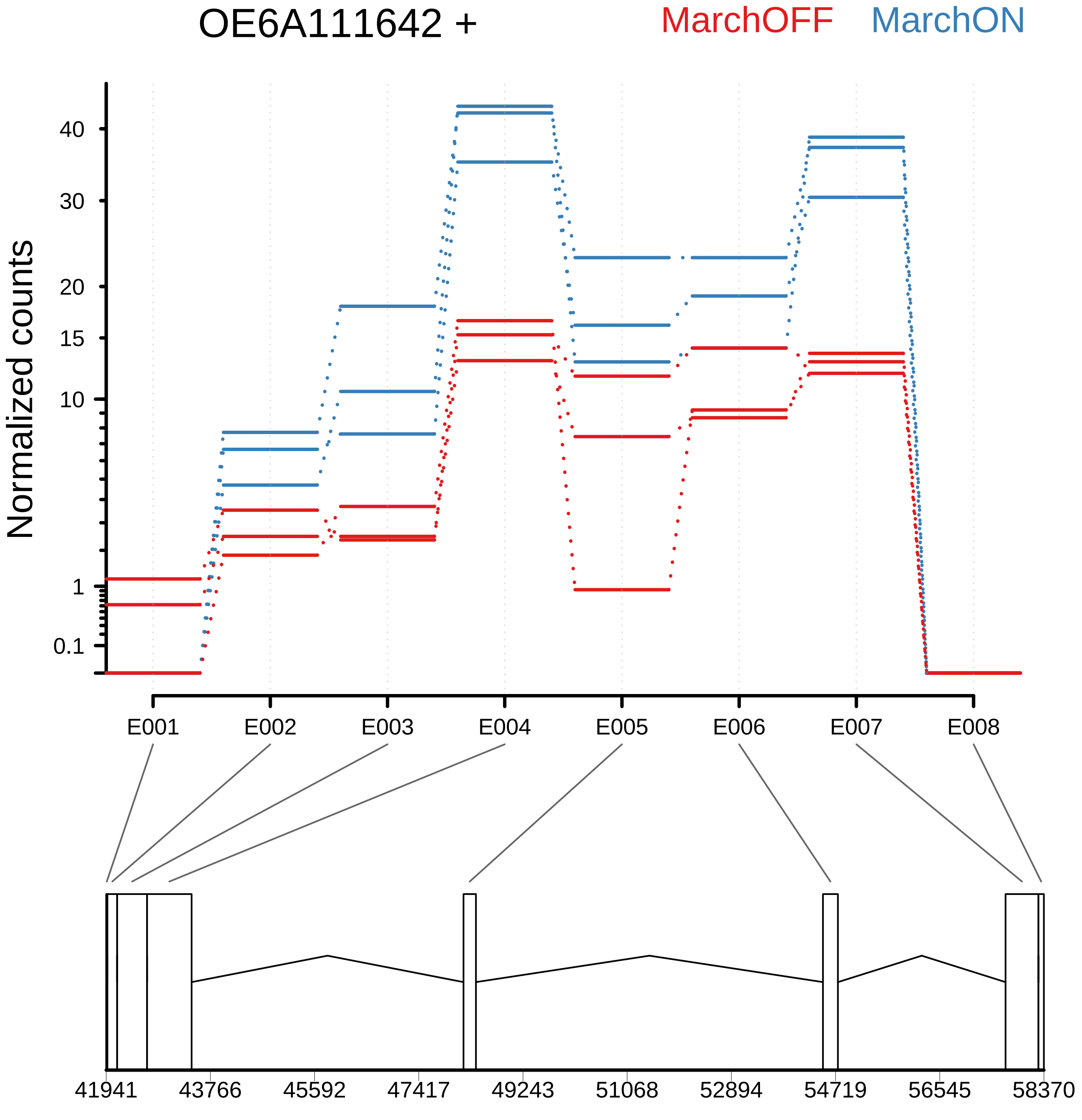

OE6A111642 +
MarchOFF
MarchON
Normalized counts
40
30
20
15
10
1
0.1
E001
E002
E003
E004
E005
E006
E007
E008
41941
43766
45592
47417
49243
51068
52894
54719
56545
58370

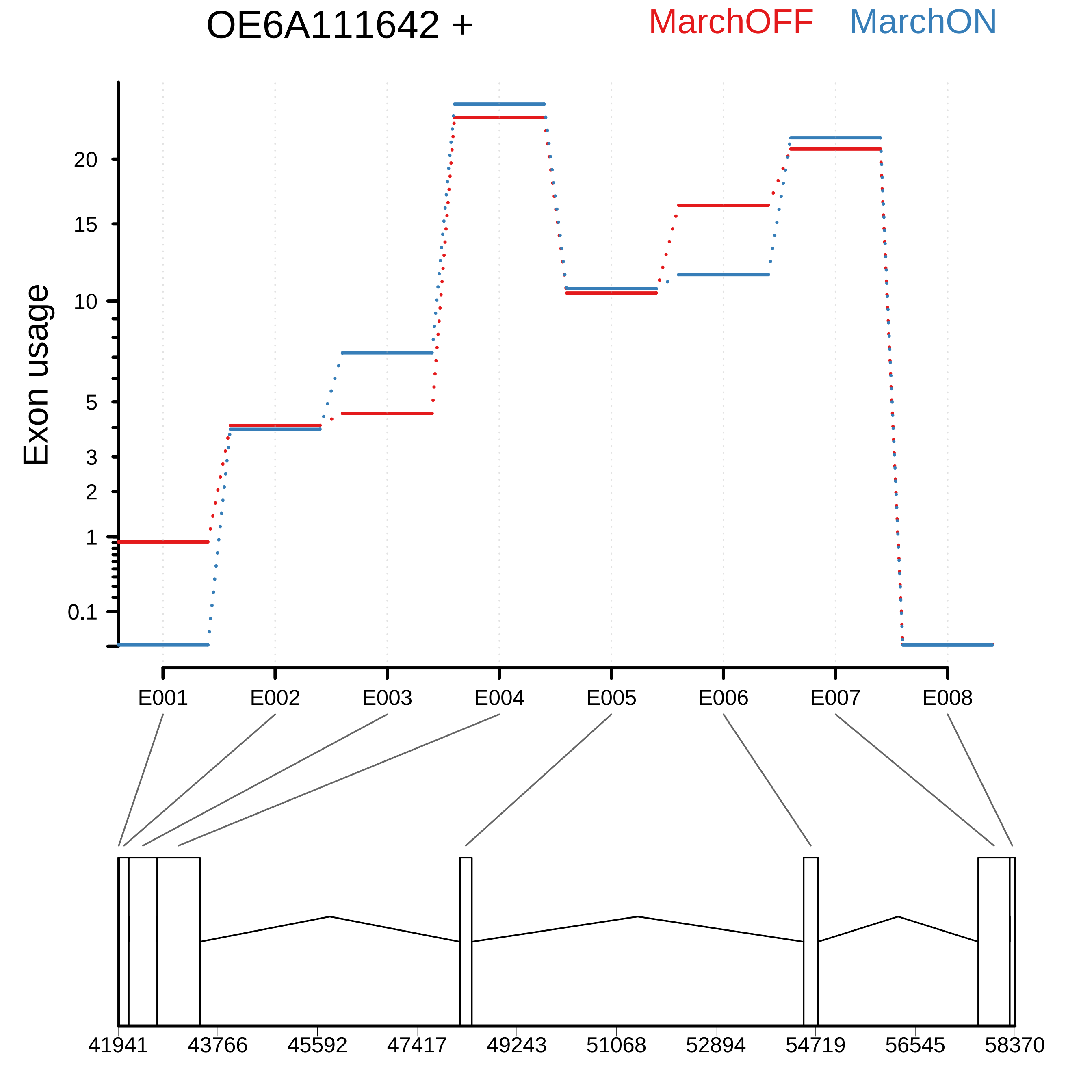

OE6A111642 +
MarchOFF
MarchON
Exon usage
20
15
10
5
3
2
1
0.1
E001
E002
E003
E004
E005
E006
E007
E008
41941
43766
45592
47417
49243
51068
52894
54719
56545
58370

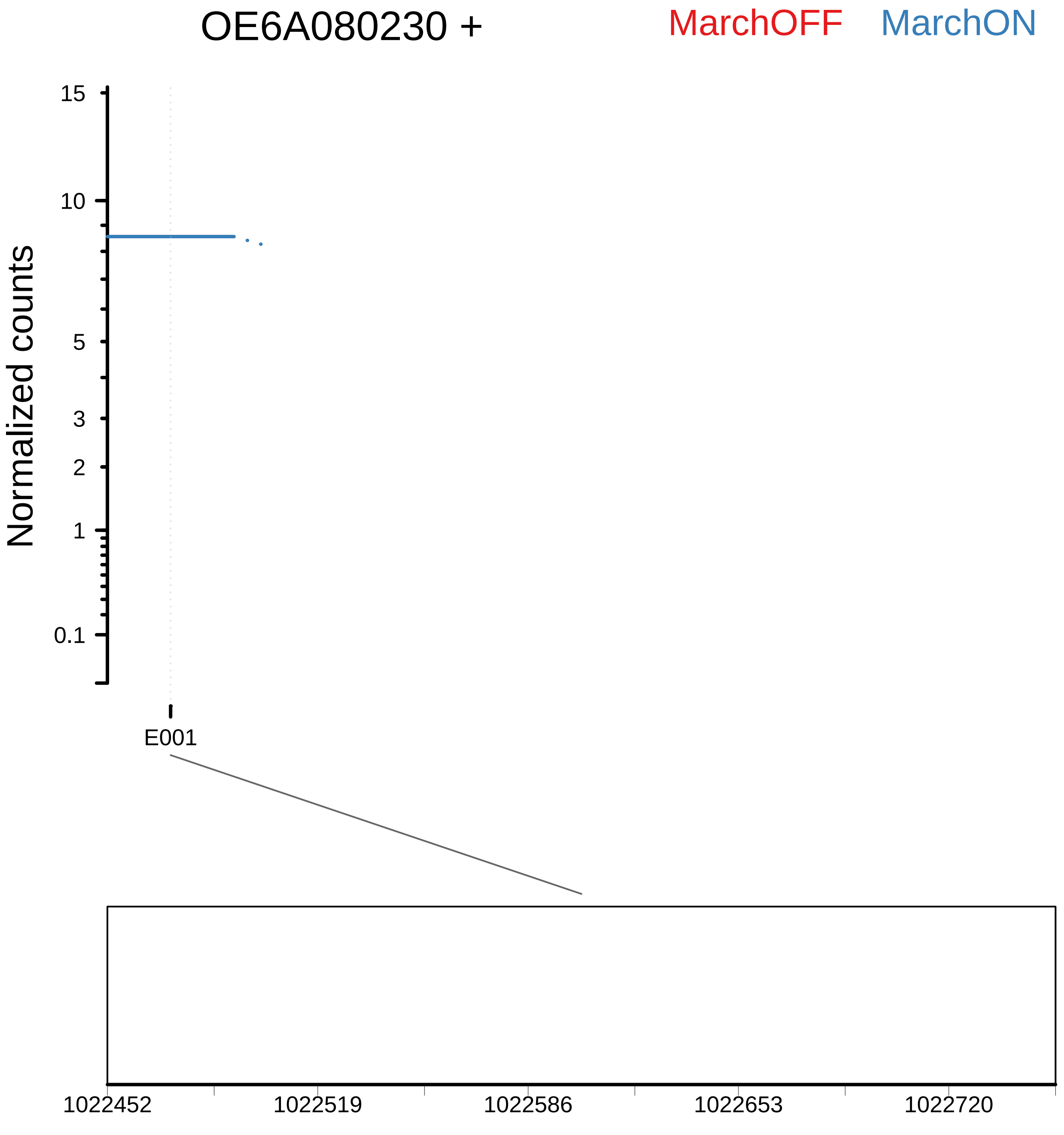

OE6A080230 +
MarchOFF
MarchON
Normalized counts
15
10
5
3
2
1
0.1
E001
1022452
1022519
1022586
1022653
1022720

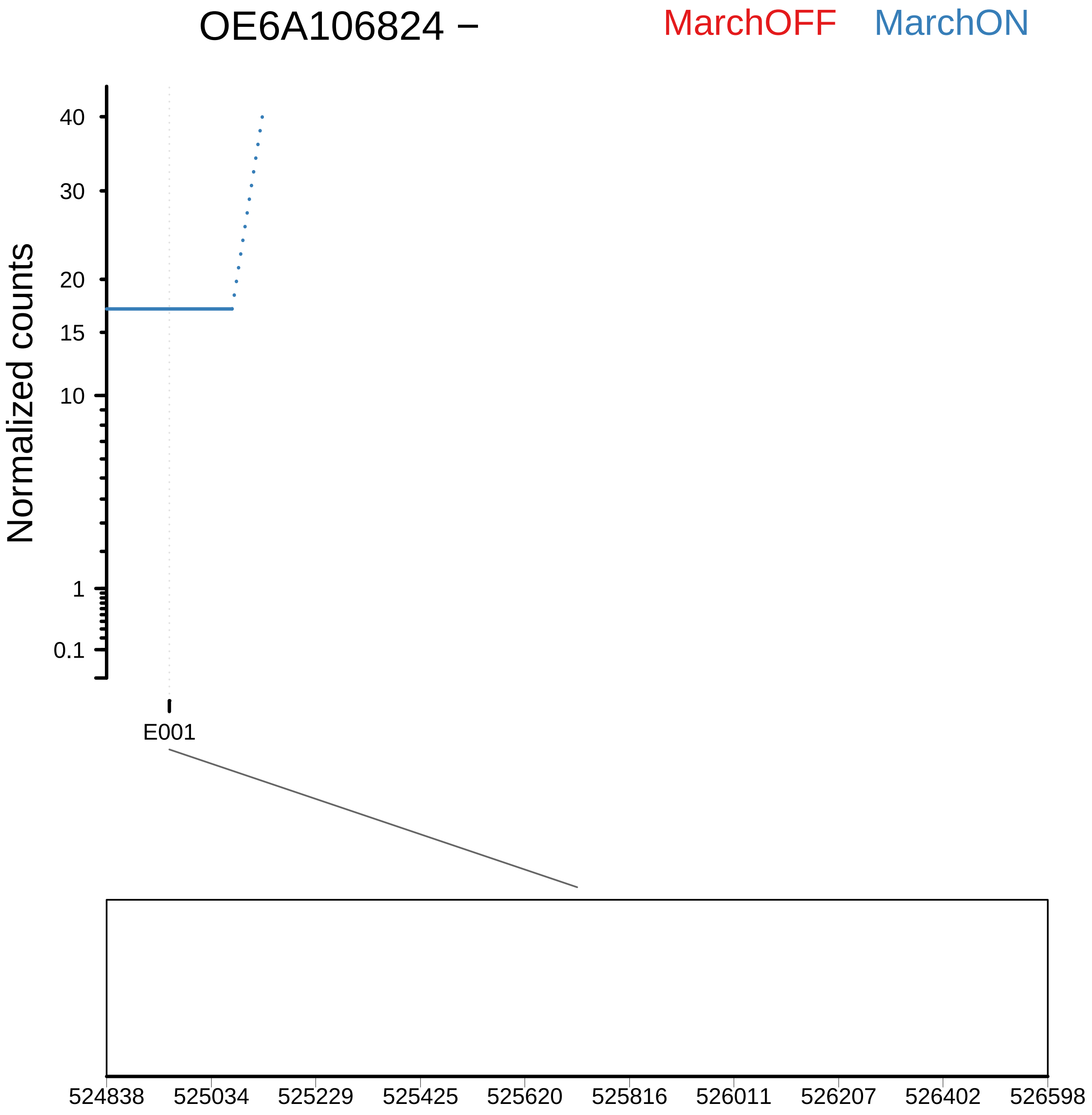

OE6A106824 −
MarchOFF
MarchON
Normalized counts
40
30
20
15
10
1
0.1
E001
524838
525034
525229
525425
525620
525816
526011
526207
526402
526598

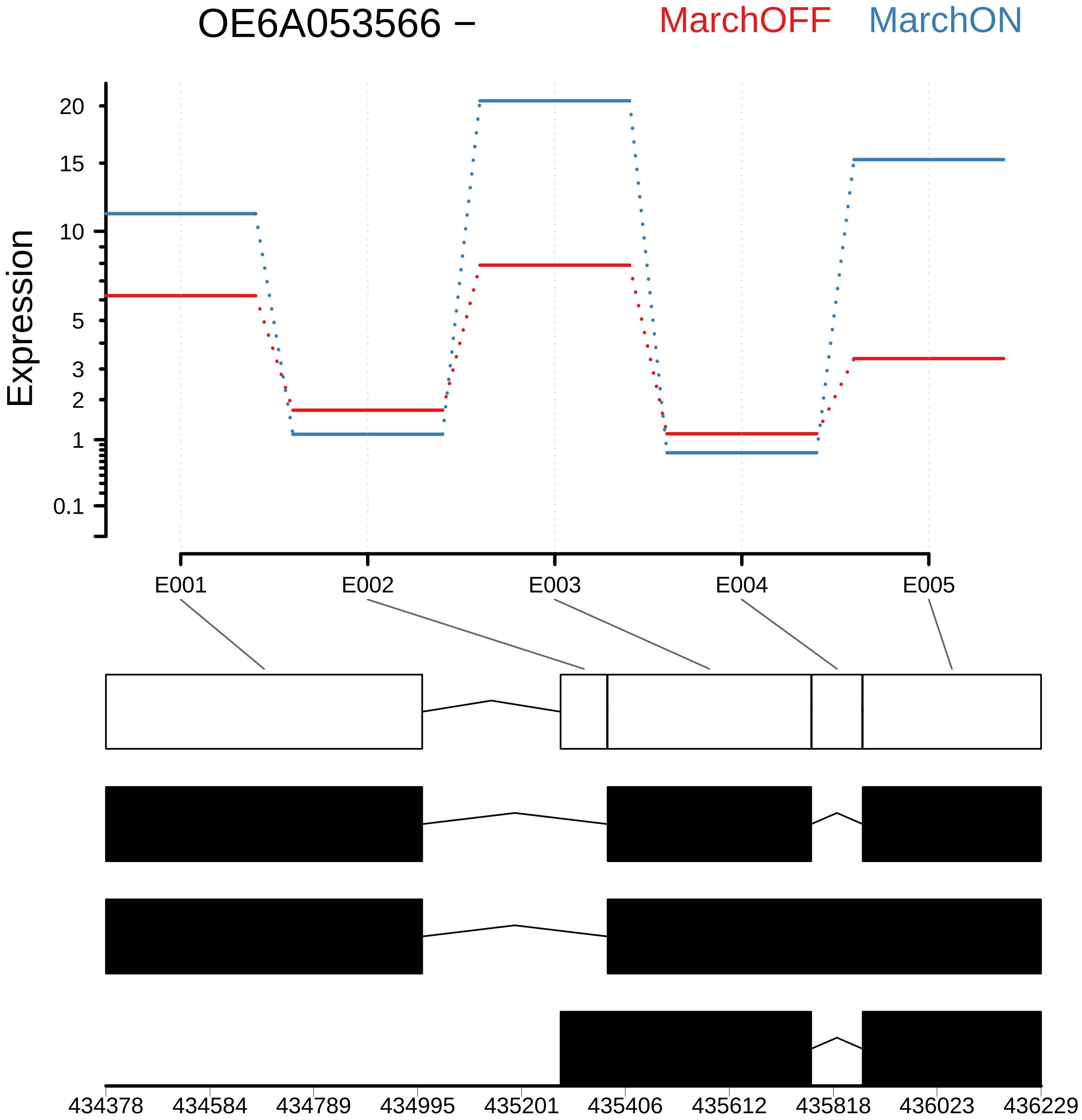

OE6A053566 −
MarchOFF
MarchON
Expression
20
15
10
5
3
2
1
0.1
E001
E002
E003
E004
E005
434378
434584
434789
434995
435201
435406
435612
435818
436023
436229

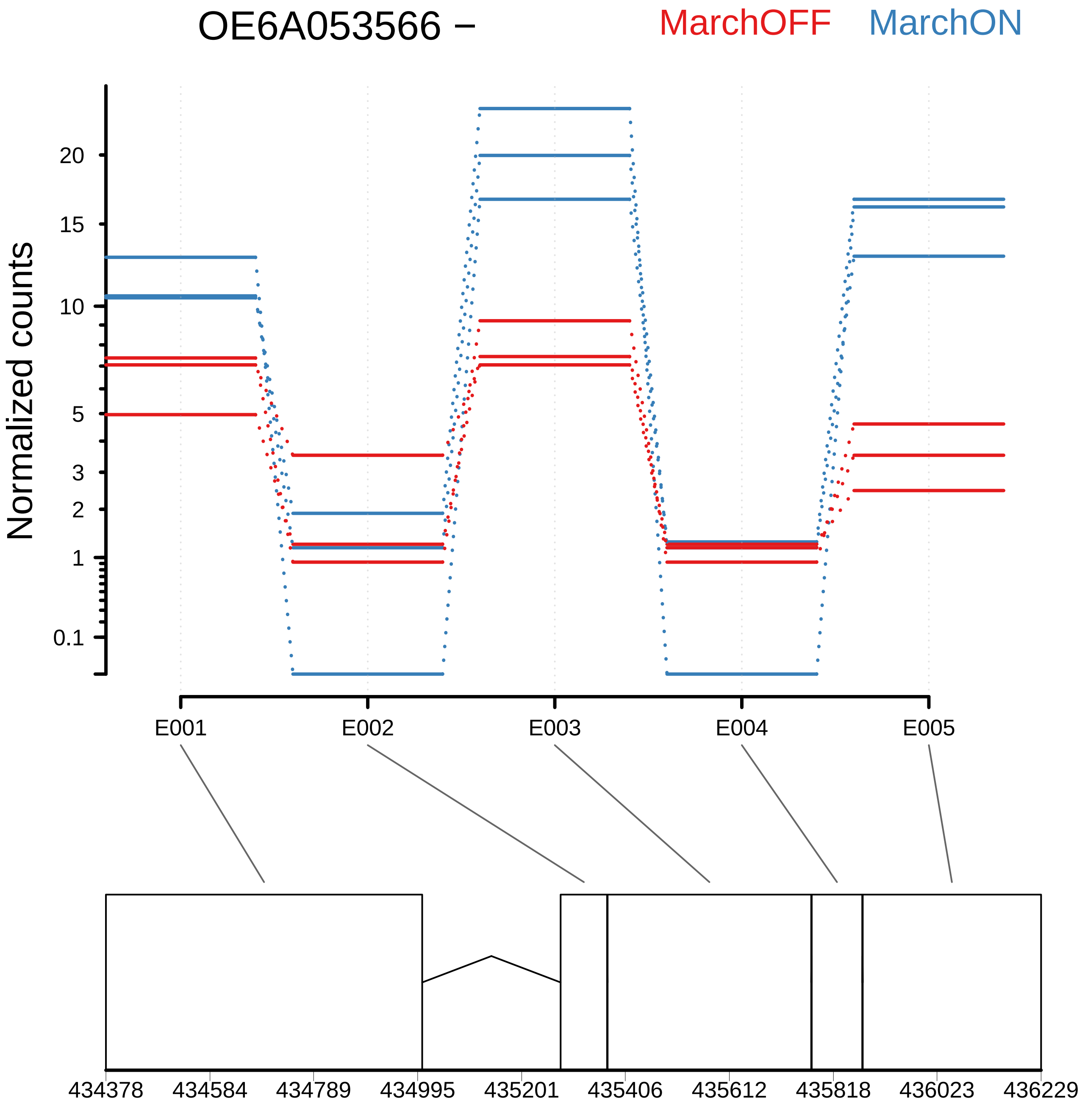

OE6A053566 −
MarchOFF
MarchON
Normalized counts
20
15
10
5
3
2
1
0.1
E001
E002
E003
E004
E005
434378
434584
434789
434995
435201
435406
435612
435818
436023
436229

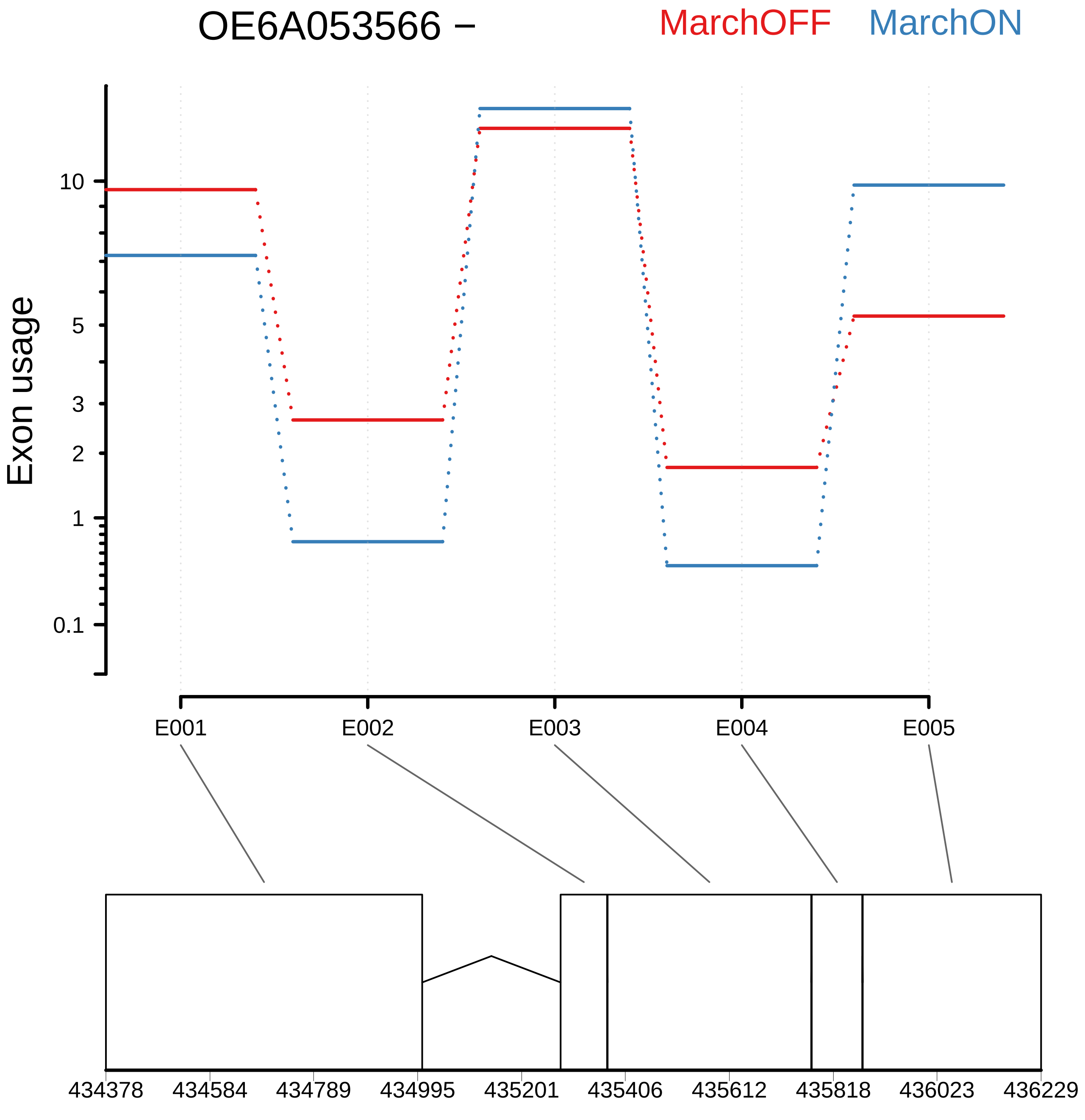
OE6A053566 −
MarchOFF
MarchON
Exon usage
10
5
3
2
1
0.1
E001
E002
E003
E004
E005
434378
434584
434789
434995
435201
435406
435612
435818
436023
436229

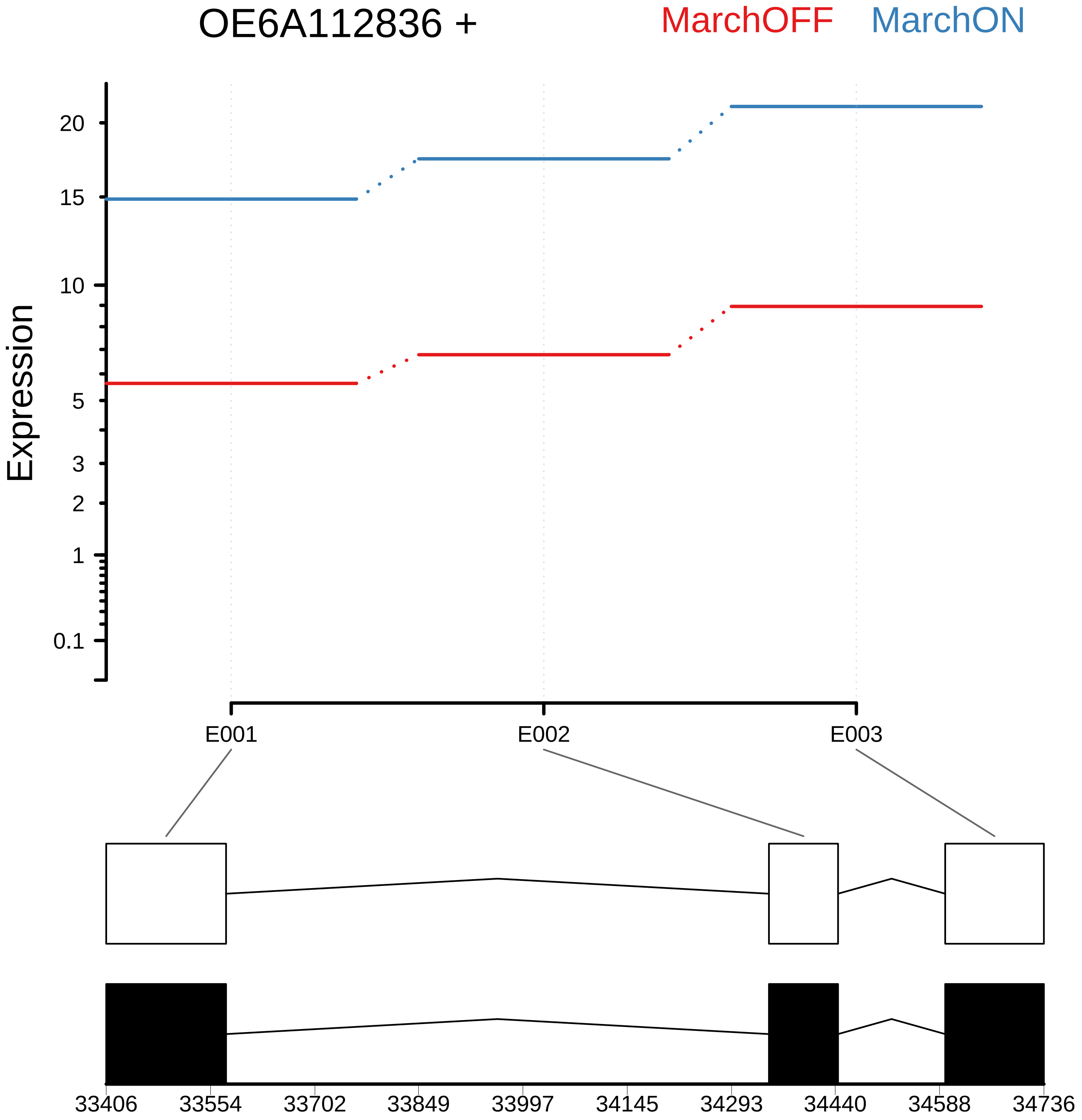

OE6A112836 +
MarchOFF
MarchON
Expression
20
15
10
5
3
2
1
0.1
E001
E002
E003
33406
33554
33702
33849
33997
34145
34293
34440
34588
34736

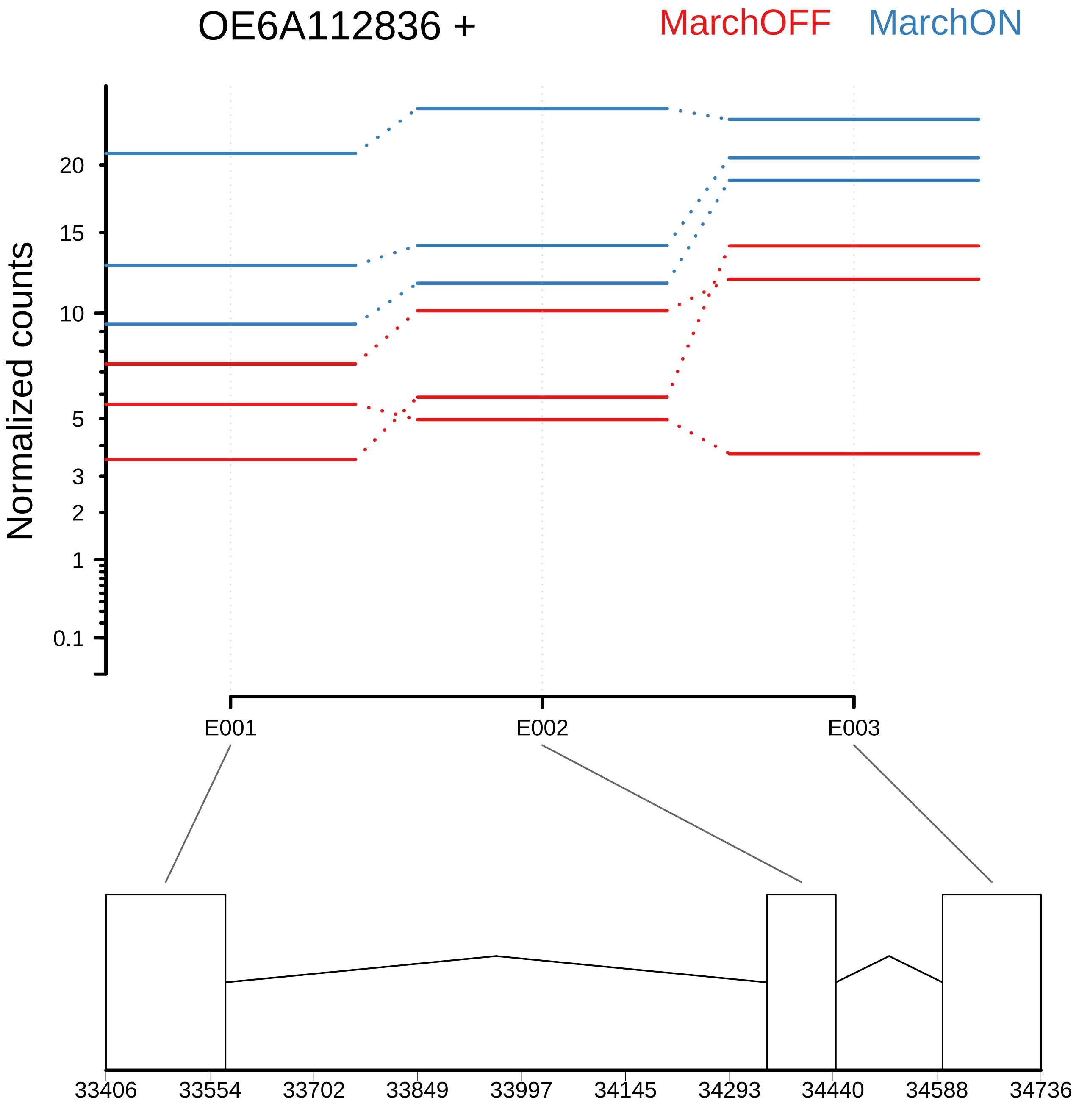

OE6A112836 +
MarchOFF
MarchON
Normalized counts
20
15
10
5
3
2
1
0.1
E001
E002
E003
33406
33554
33702
33849
33997
34145
34293
34440
34588
34736

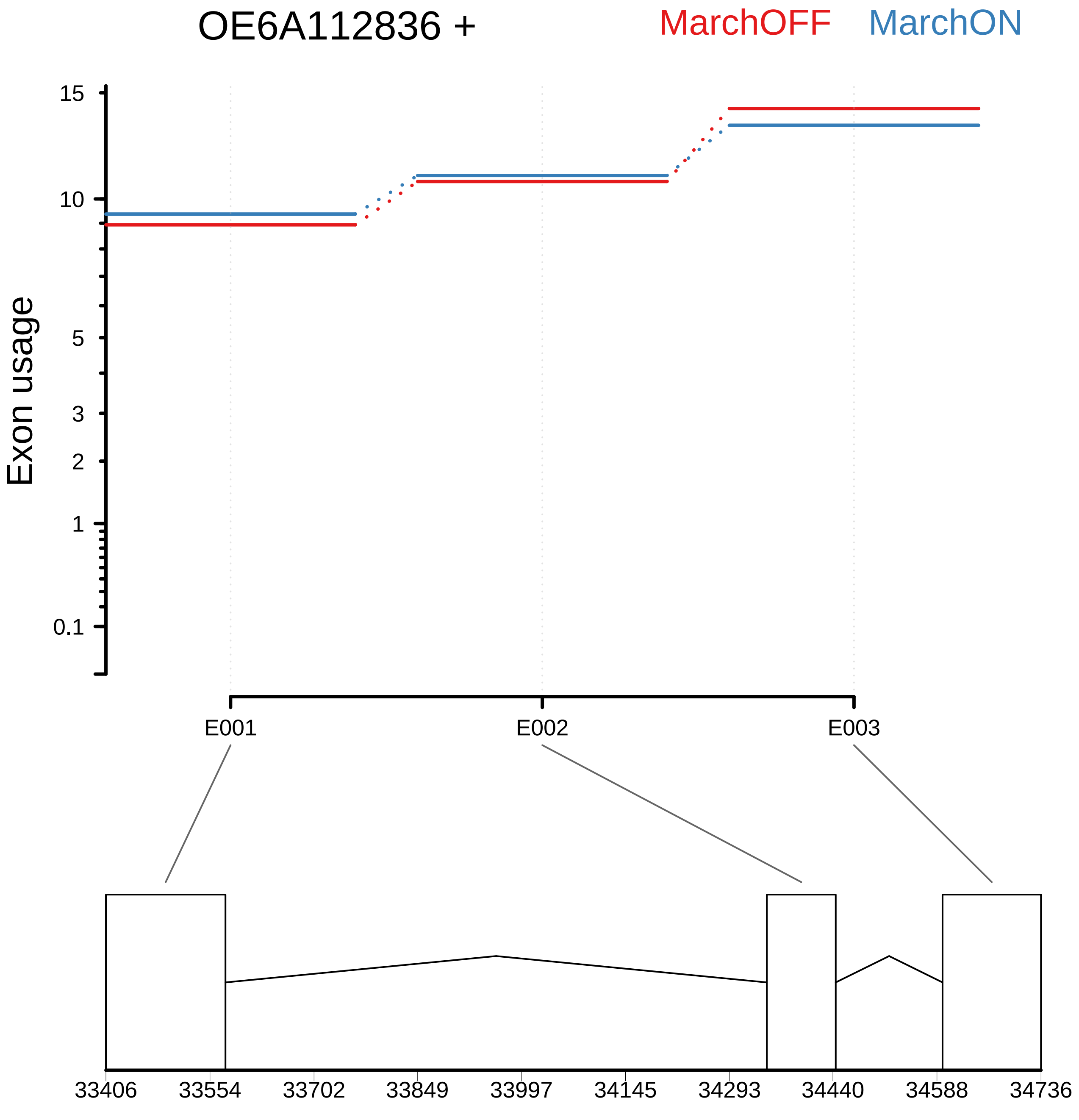
OE6A112836 +
MarchOFF
MarchON
Exon usage
15
10
5
3
2
1
0.1
E001
E002
E003
33406
33554
33702
33849
33997
34145
34293
34440
34588
34736

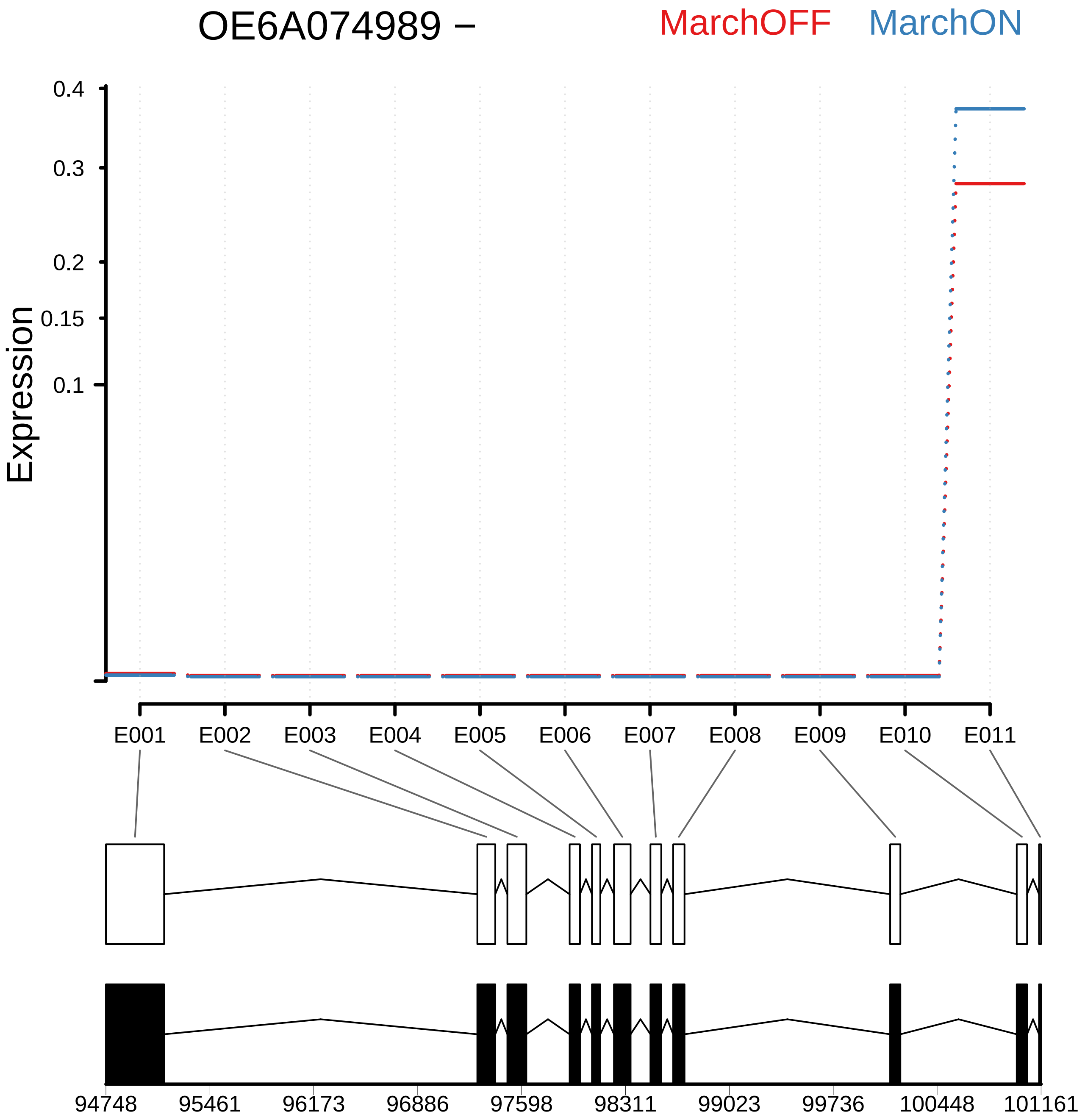

OE6A074989 −
MarchOFF
MarchON
Expression
0.4
0.3
0.2
0.15
0.1
E001
E002
E003
E004
E005
E006
E007
E008
E009
E010
E011
94748
95461
96173
96886
97598
98311
99023
99736
100448
101161

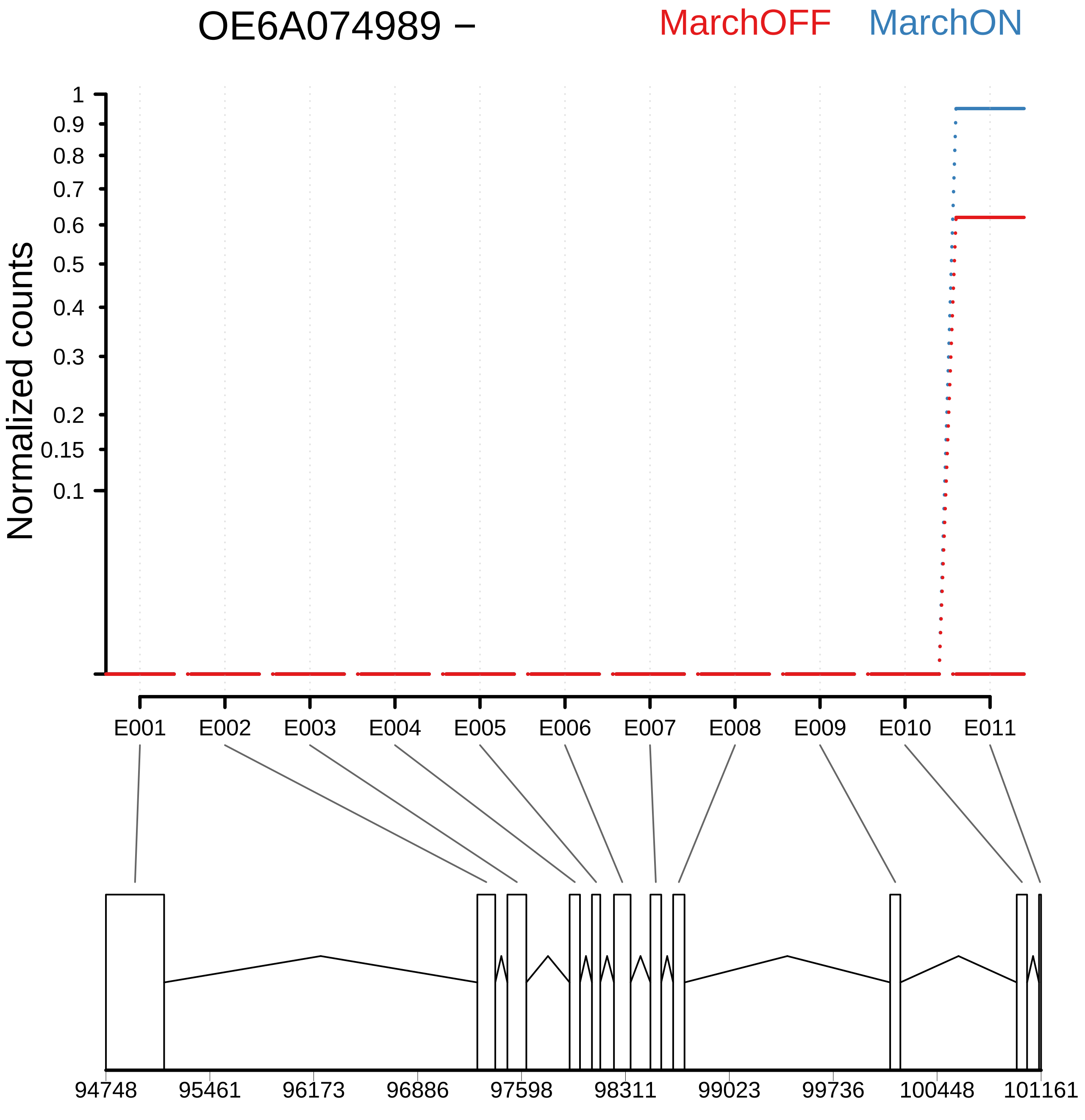
OE6A074989 –
MarchOFF
MarchON
Normalized counts
1
0.9
0.8
0.7
0.6
0.5
0.4
0.3
0.2
0.15
0.1
E001
E002
E003
E004
E005
E006
E007
E008
E009
E010
E011
94748
95461
96173
96886
97598
98311
99023
99736
100448
101161

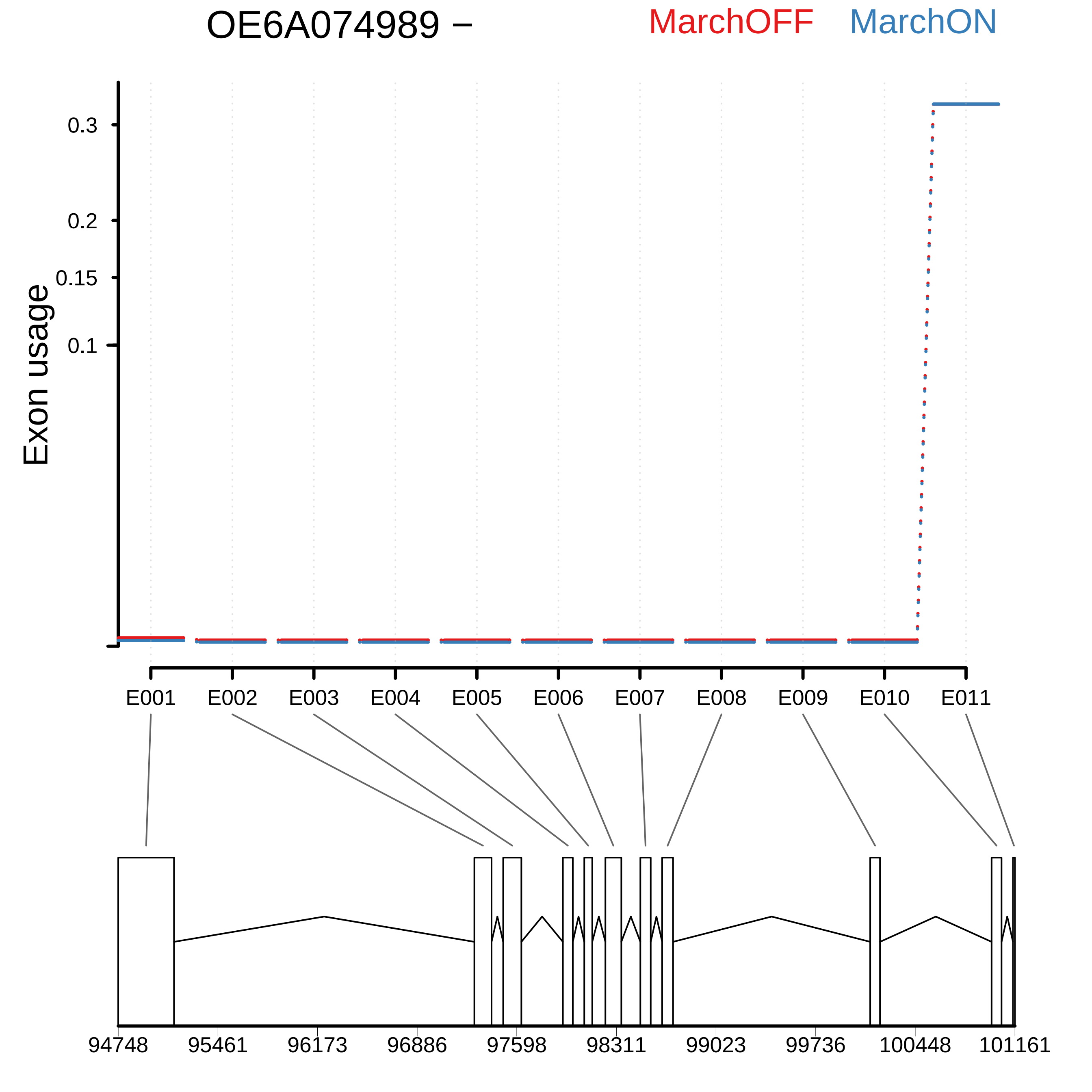
OE6A074989 −
MarchOFF
MarchON
Exon usage
0.3
0.2
0.15
0.1
E001
E002
E003
E004
E005
E006
E007
E008
E009
E010
E011
94748
95461
96173
96886
97598
98311
99023
99736
100448
101161

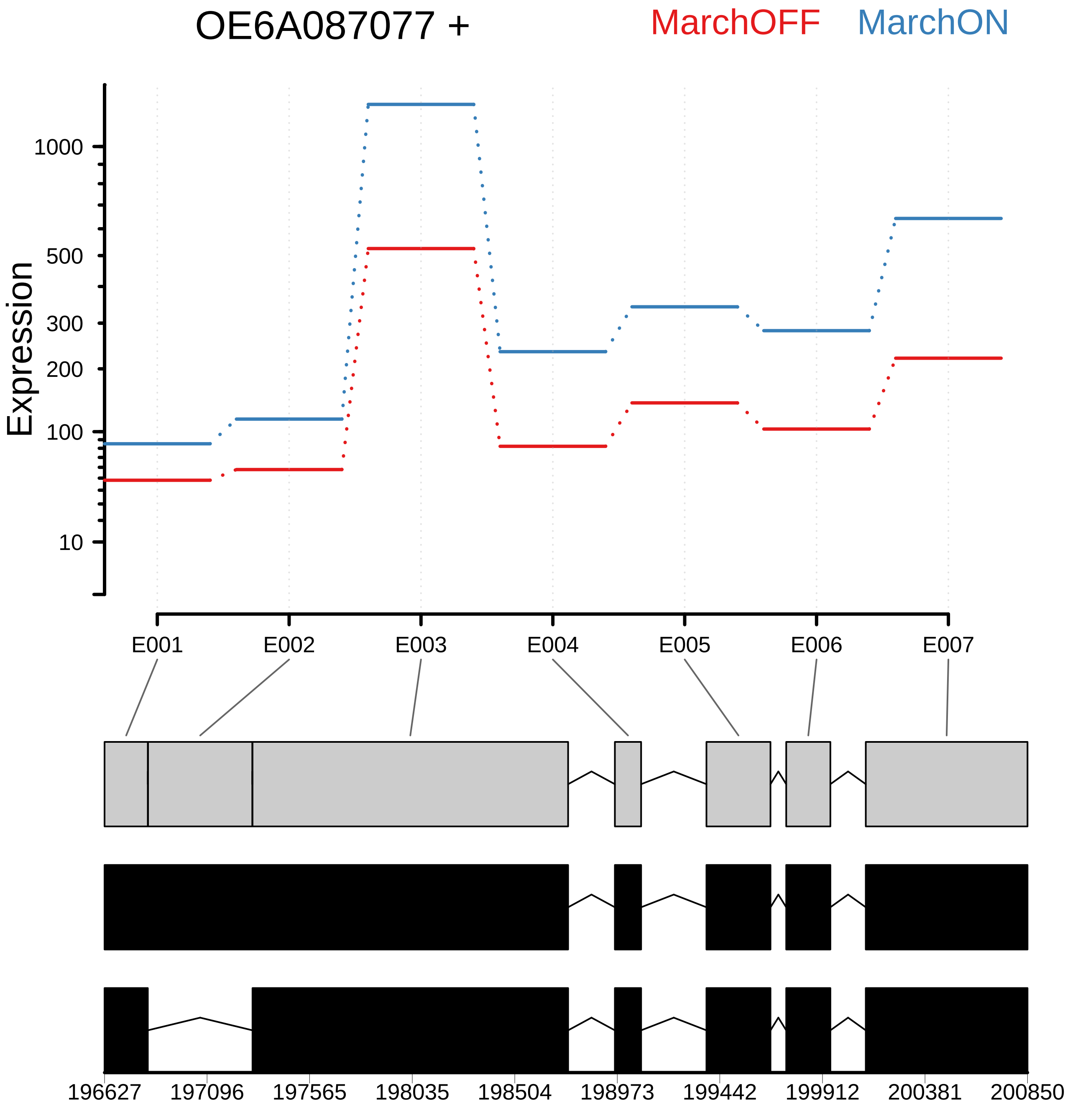
OE6A087077 +
MarchOFF
MarchON
Expression
1000
500
300
200
100
10
E001
E002
E003
E004
E005
E006
E007
196627
197096
197565
198035
198504
198973
199442
199912
200381
200850

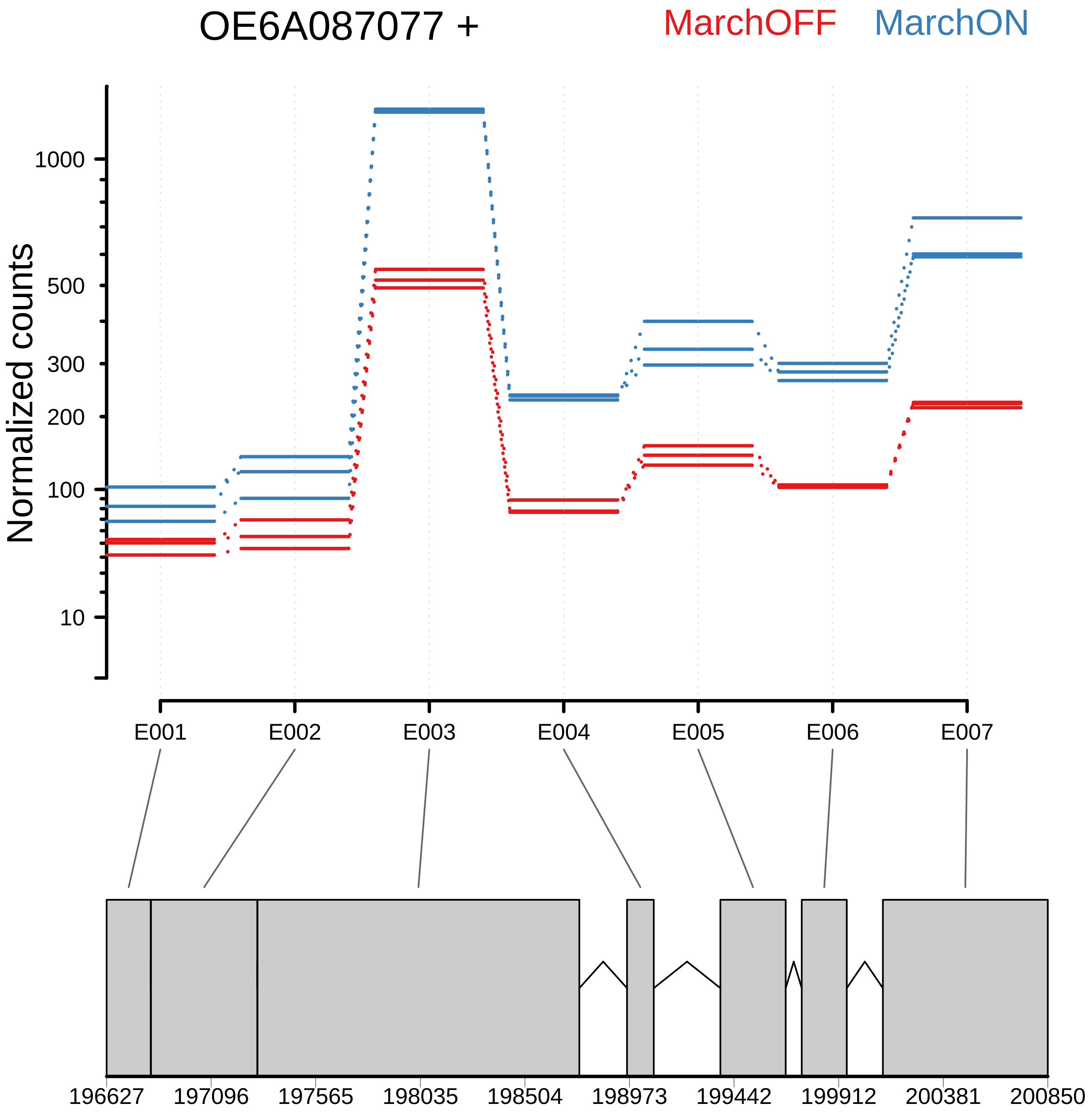

OE6A087077 +
MarchOFF
MarchON
Normalized counts
1000
500
300
200
100
10
E001
E002
E003
E004
E005
E006
E007
196627
197096
197565
198035
198504
198973
199442
199912
200381
200850

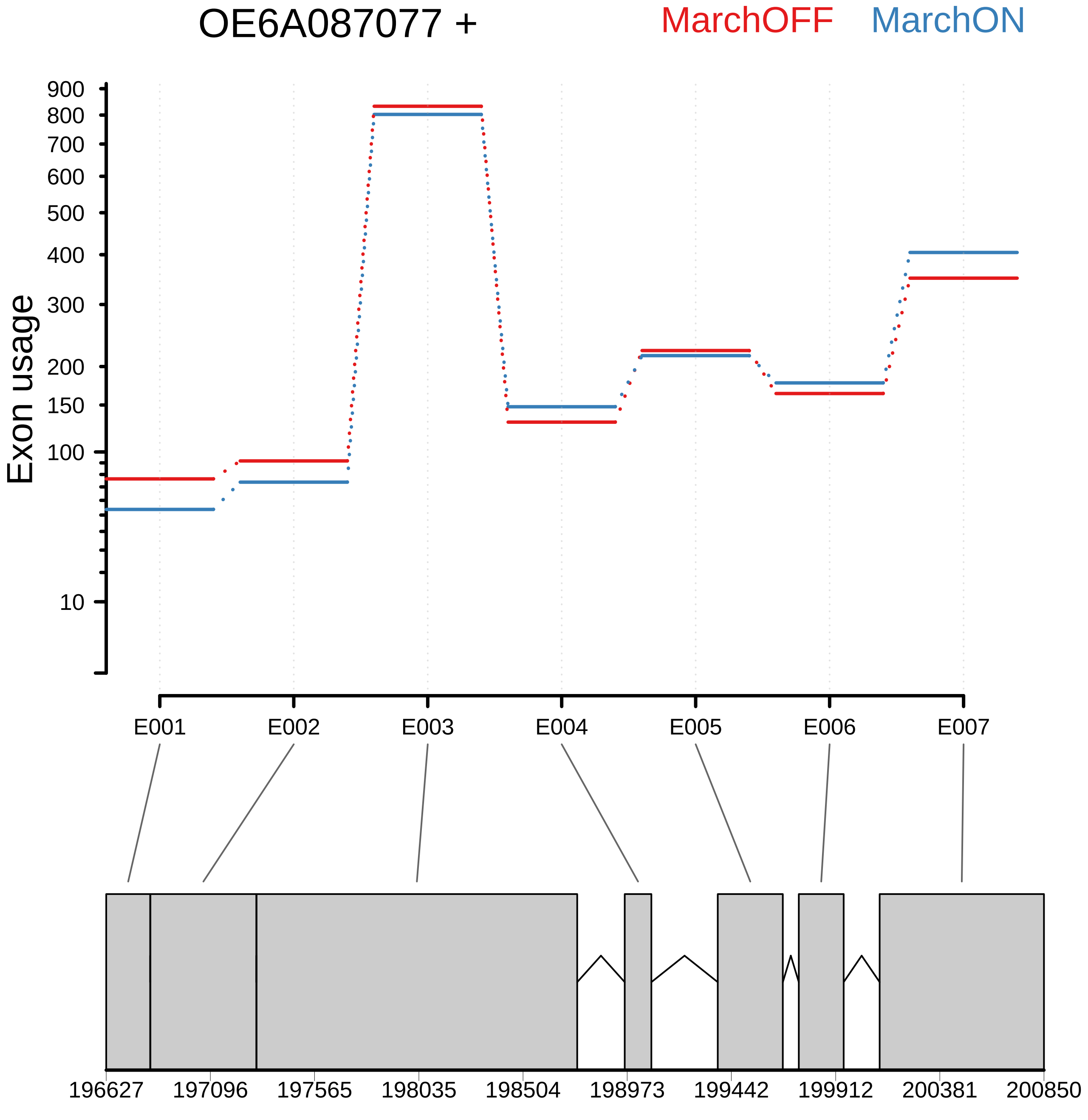
OE6A087077 +
MarchOFF
MarchON
Exon usage
900
800
700
600
500
400
300
200
150
100
10
E001
E002
E003
E004
E005
E006
E007
196627
197096
197565
198035
198504
198973
199442
199912
200381
200850

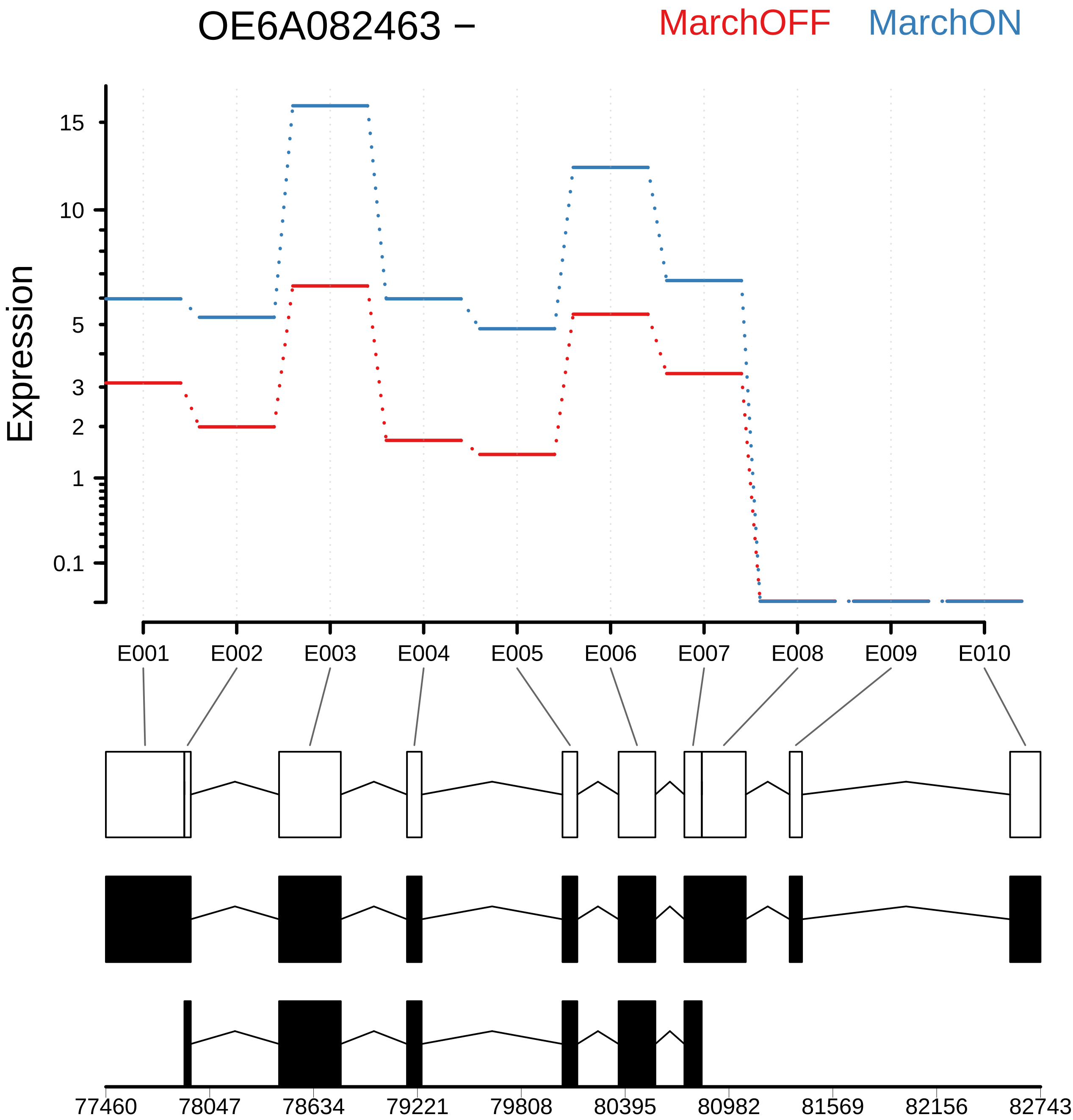

OE6A082463 −
MarchOFF
MarchON
Expression
15
10
5
3
2
1
0.1
E001
E002
E003
E004
E005
E006
E007
E008
E009
E010
77460
78047
78634
79221
79808
80395
80982
81569
82156
82743

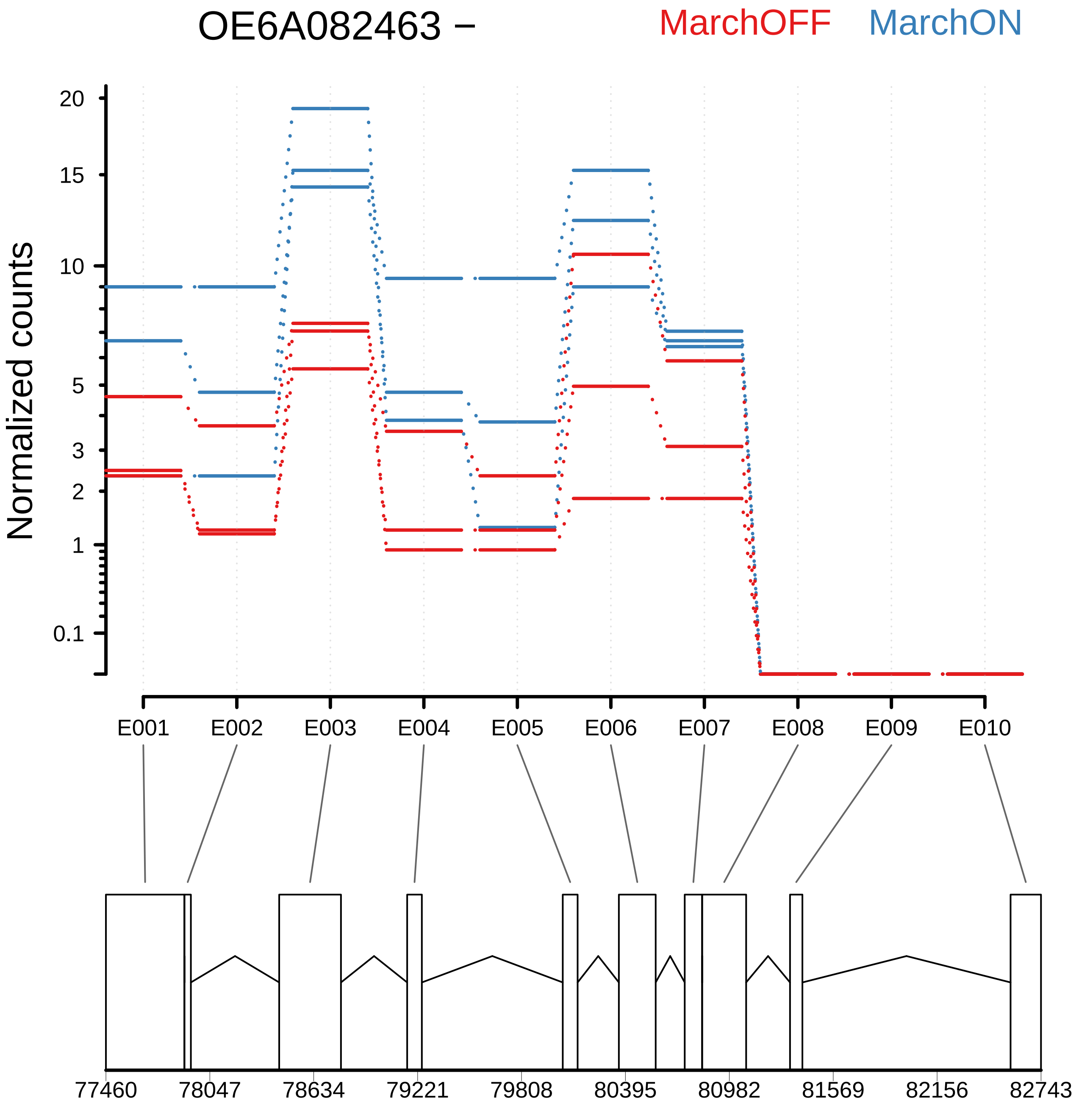
OE6A082463 −
MarchOFF
MarchON
Normalized counts
20
15
10
5
3
2
1
0.1
E001
E002
E003
E004
E005
E006
E007
E008
E009
E010
77460
78047
78634
79221
79808
80395
80982
81569
82156
82743

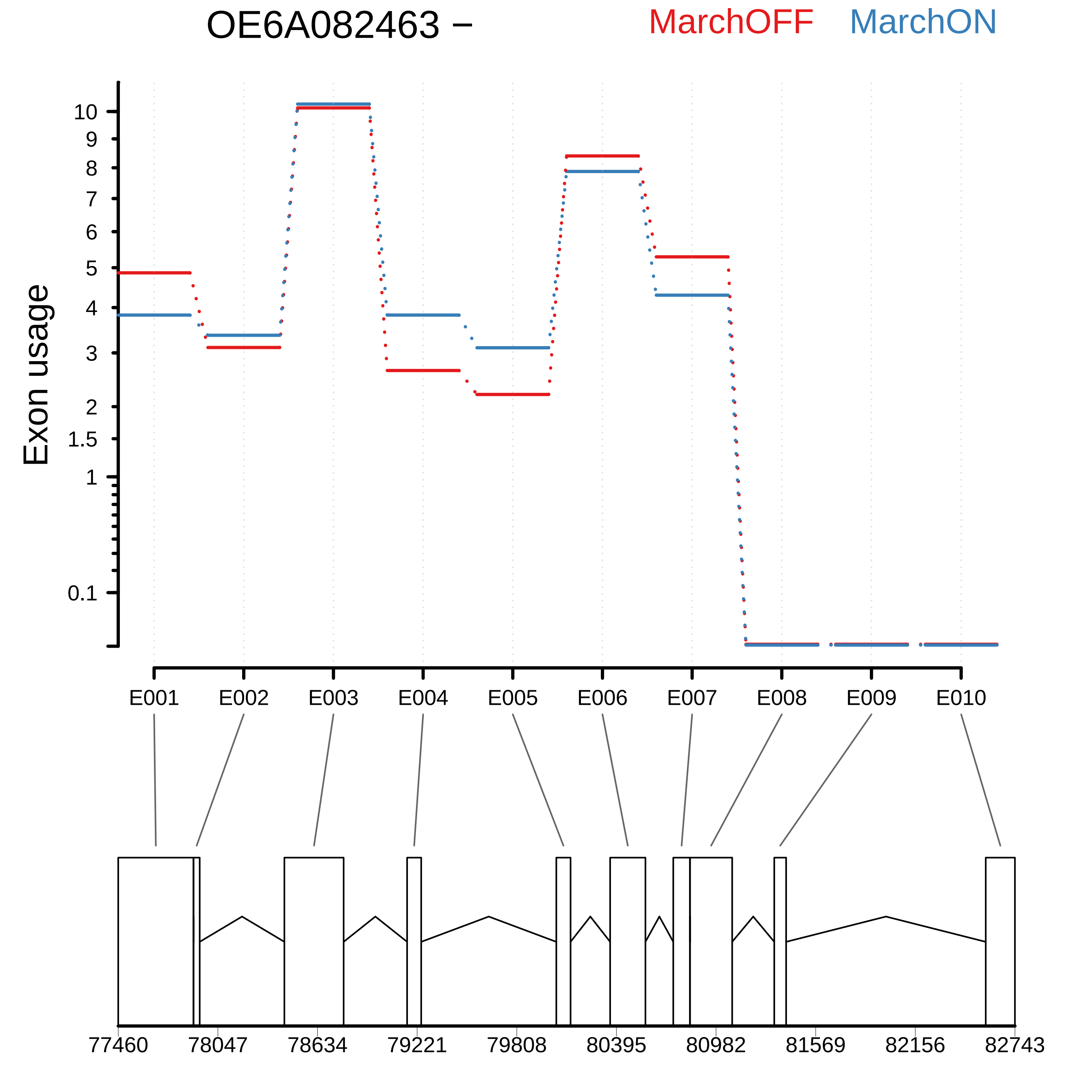

OE6A082463 −
MarchOFF
MarchON
Exon usage
10
9
8
7
6
5
4
3
2
1.5
1
0.1
E001
E002
E003
E004
E005
E006
E007
E008
E009
E010
77460
78047
78634
79221
79808
80395
80982
81569
82156
82743

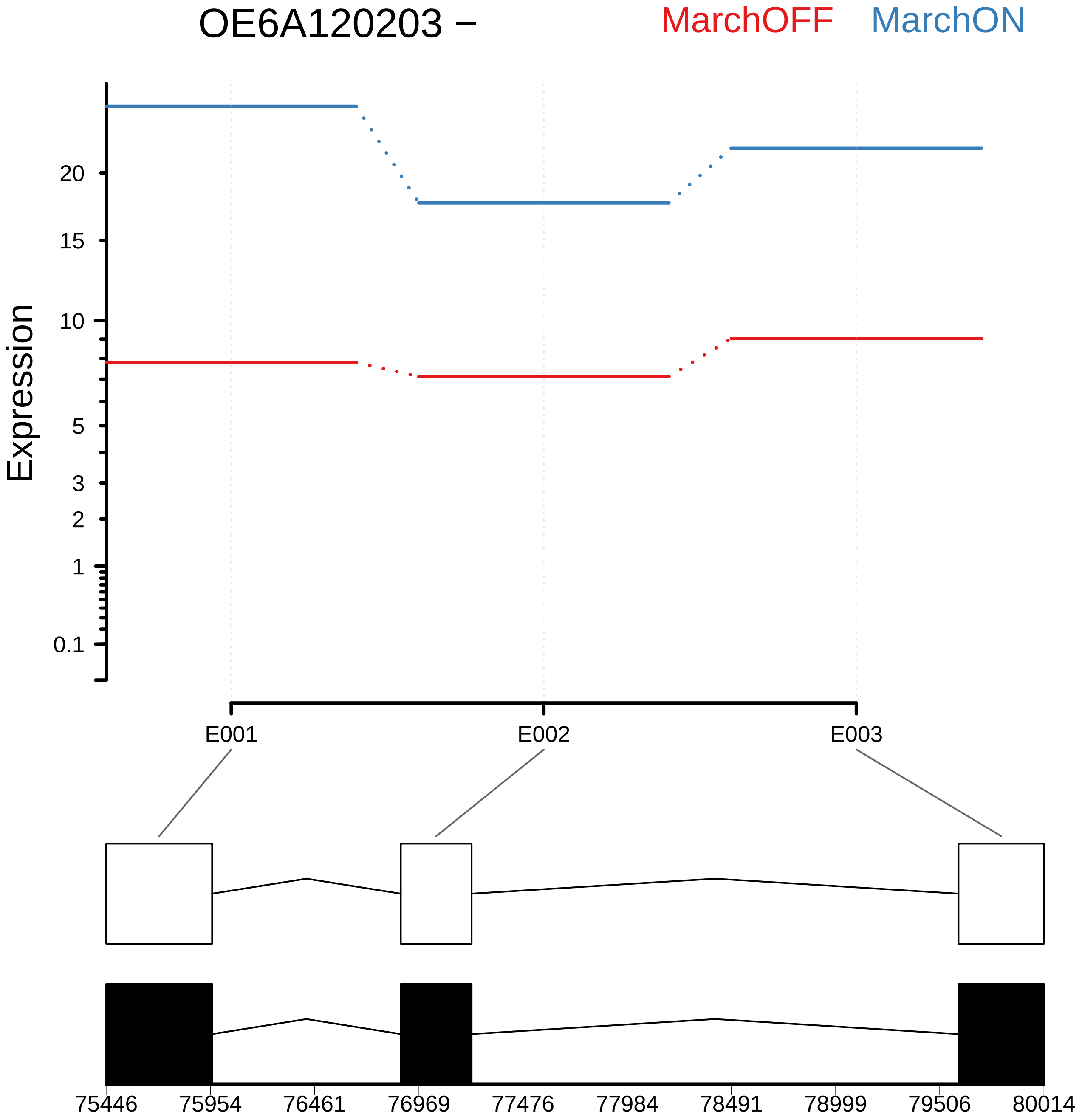

OE6A120203 −
MarchOFF
MarchON
Expression
20
15
10
5
3
2
1
0.1
E001
E002
E003
75446
75954
76461
76969
77476
77984
78491
78999
79506
80014

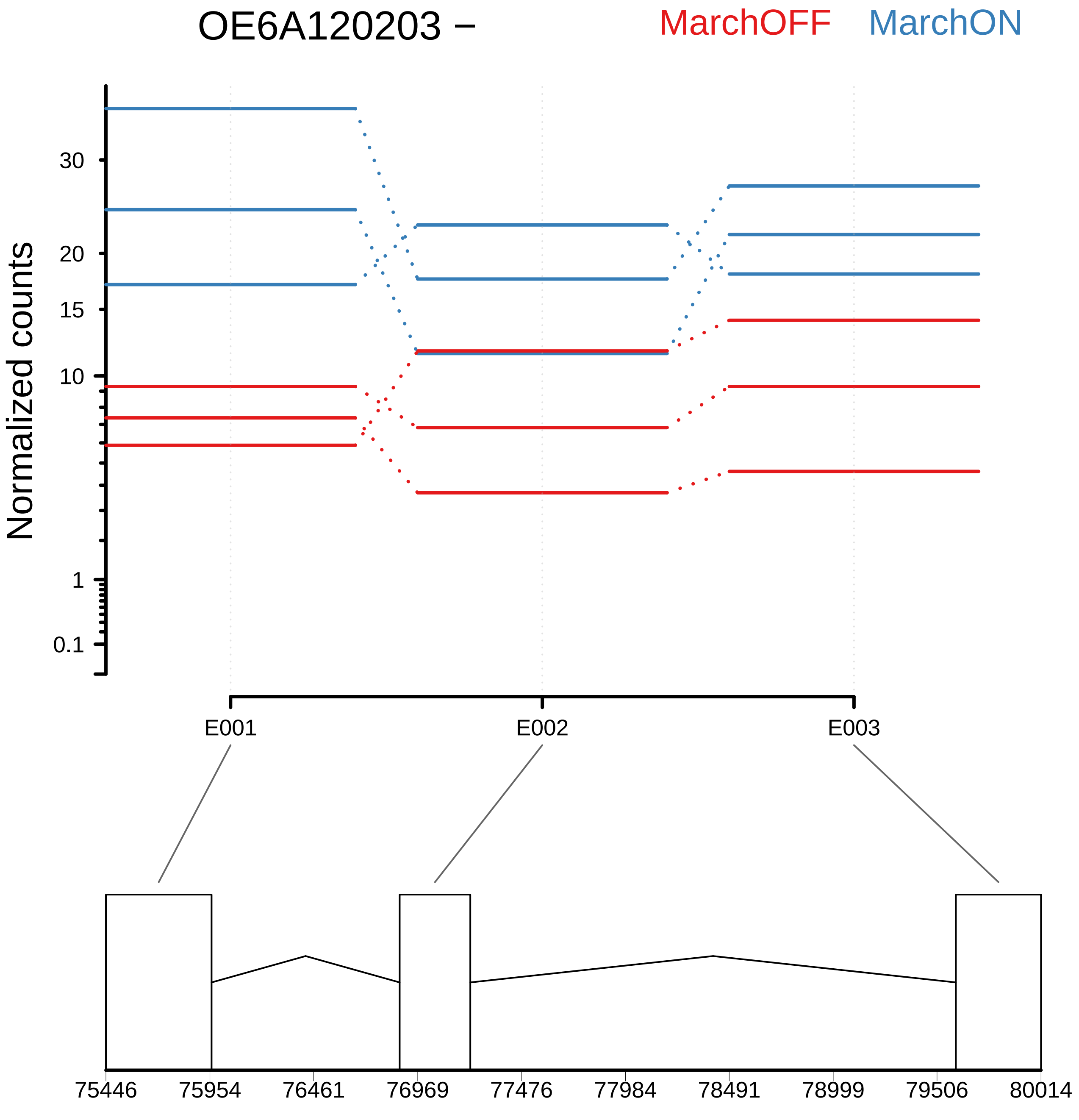
OE6A120203 −
MarchOFF
MarchON
Normalized counts
30
20
15
10
1
0.1
E001
E002
E003
75446
75954
76461
76969
77476
77984
78491
78999
79506
80014

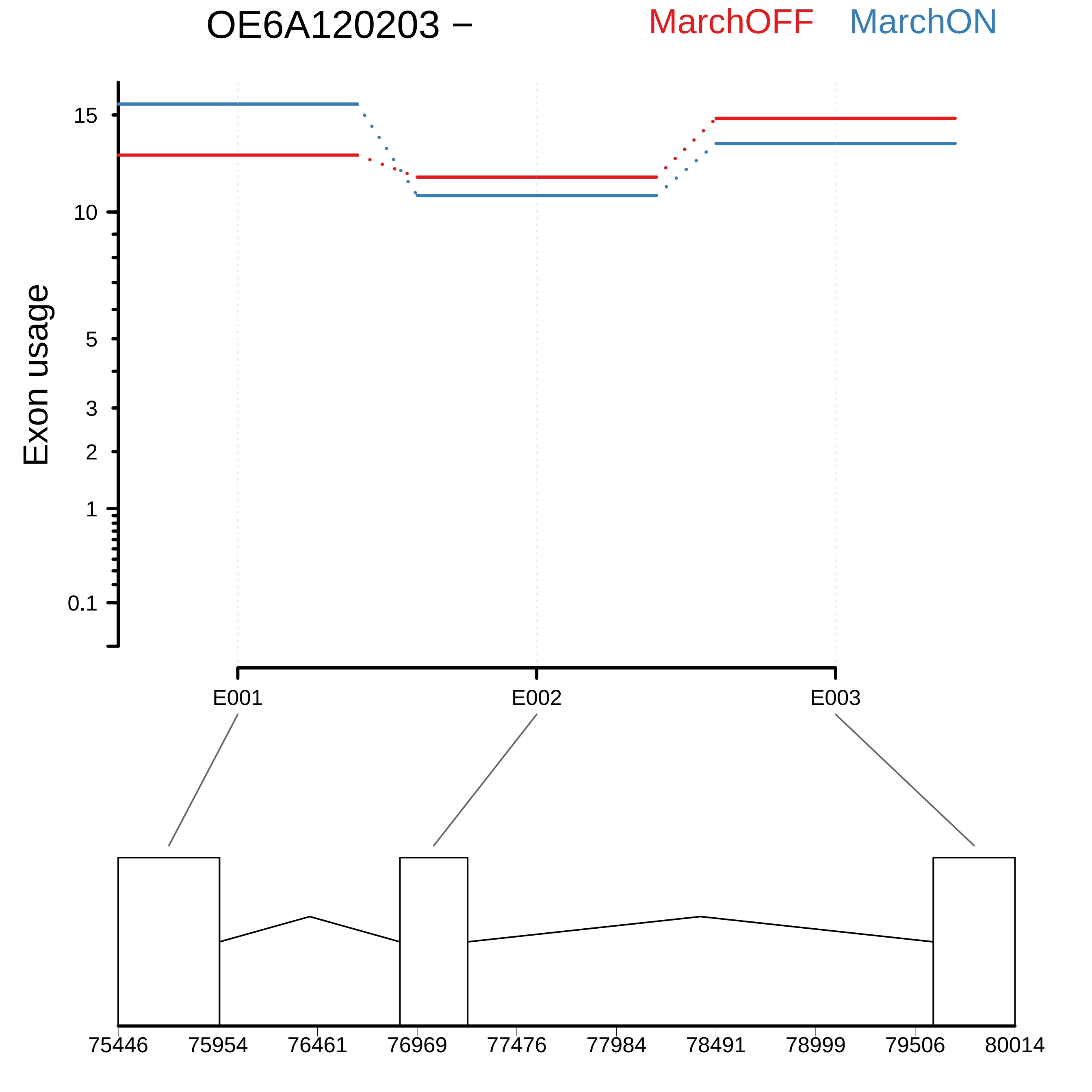

OE6A120203 −
MarchOFF
MarchON
Exon usage
15
10
5
3
2
1
0.1
E001
E002
E003
75446
75954
76461
76969
77476
77984
78491
78999
79506
80014

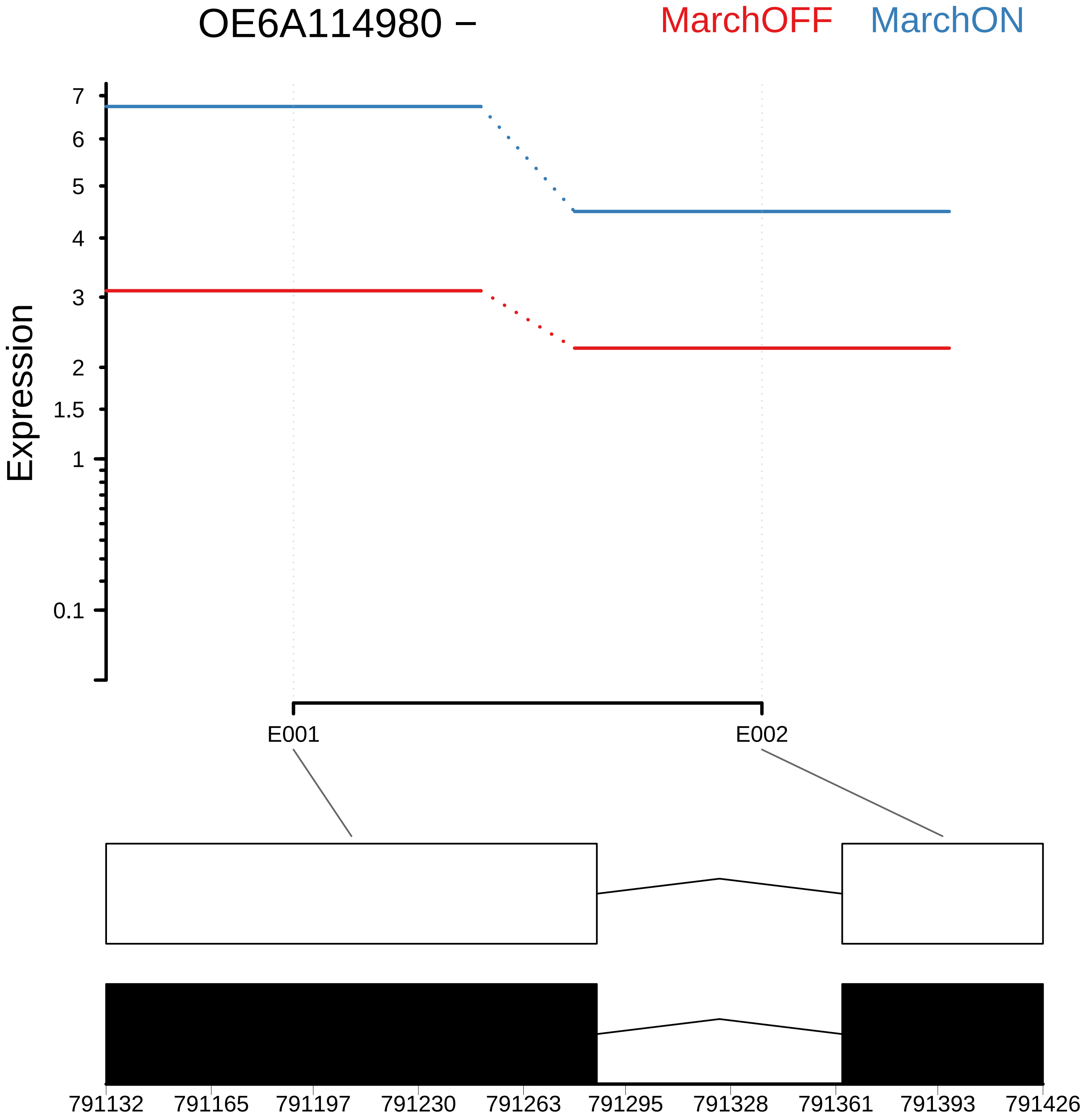
OE6A114980 −
MarchOFF
MarchON
Expression
7
6
5
4
3
2
1.5
1
0.1
E001
E002
791132
791165
791197
791230
791263
791295
791328
791361
791393
791426

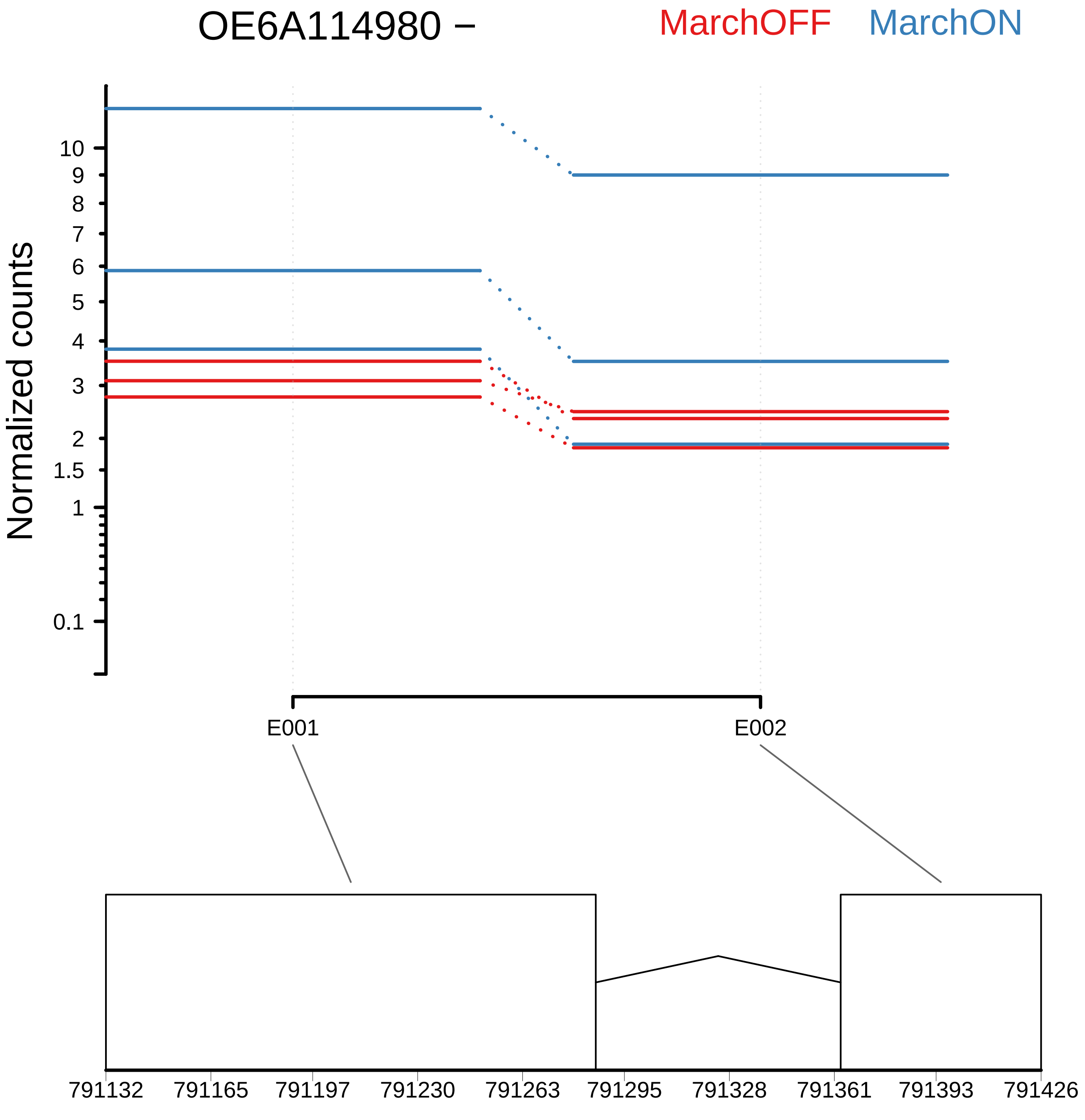
OE6A114980 −
MarchOFF
MarchON
Normalized counts
10
9
8
7
6
5
4
3
2
1.5
1
0.1
E001
E002
791132
791165
791197
791230
791263
791295
791328
791361
791393
791426

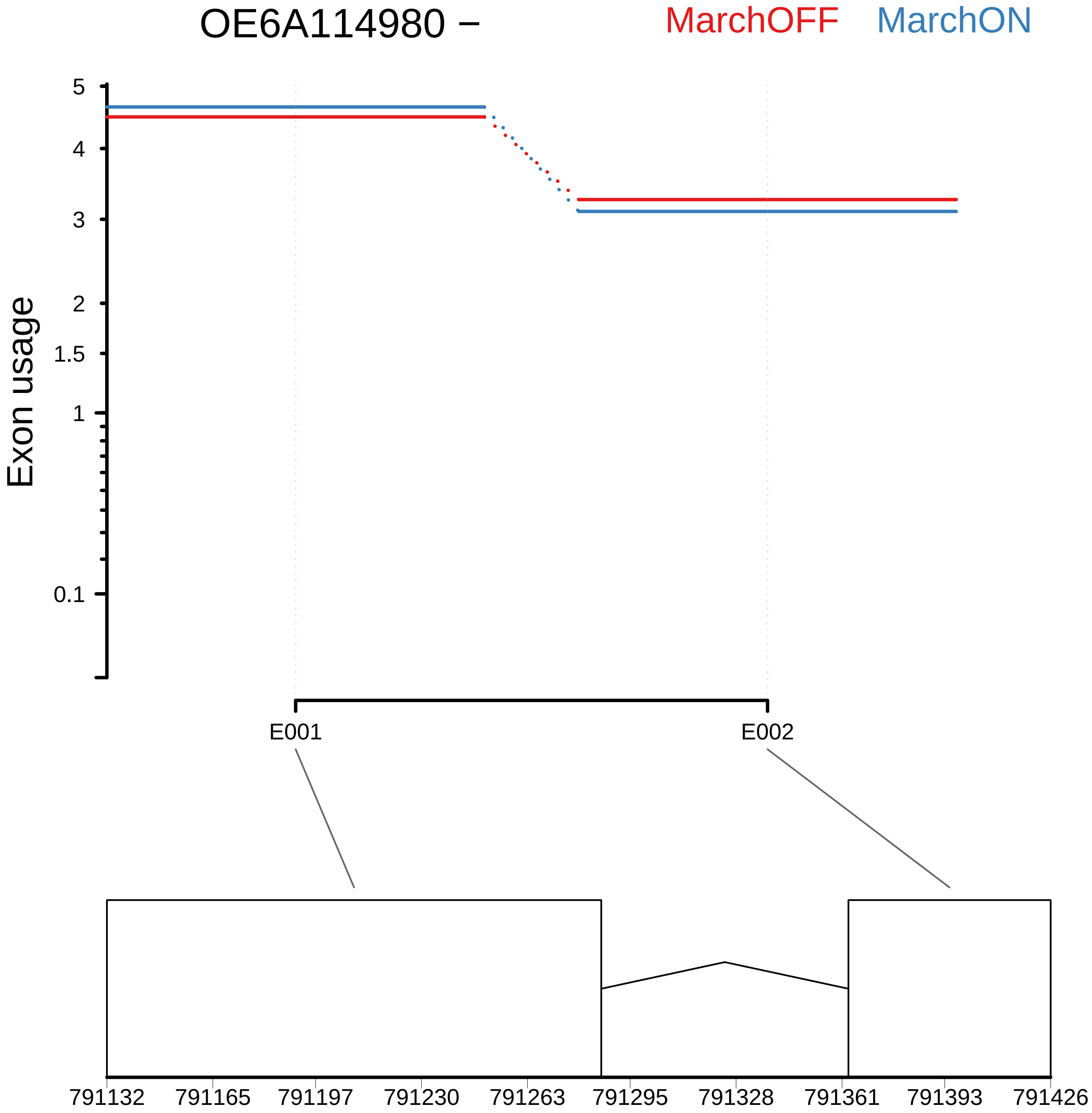

OE6A114980 −
MarchOFF
MarchON
Exon usage
5
4
3
2
1.5
1
0.1
E001
E002
791132
791165
791197
791230
791263
791295
791328
791361
791393
791426

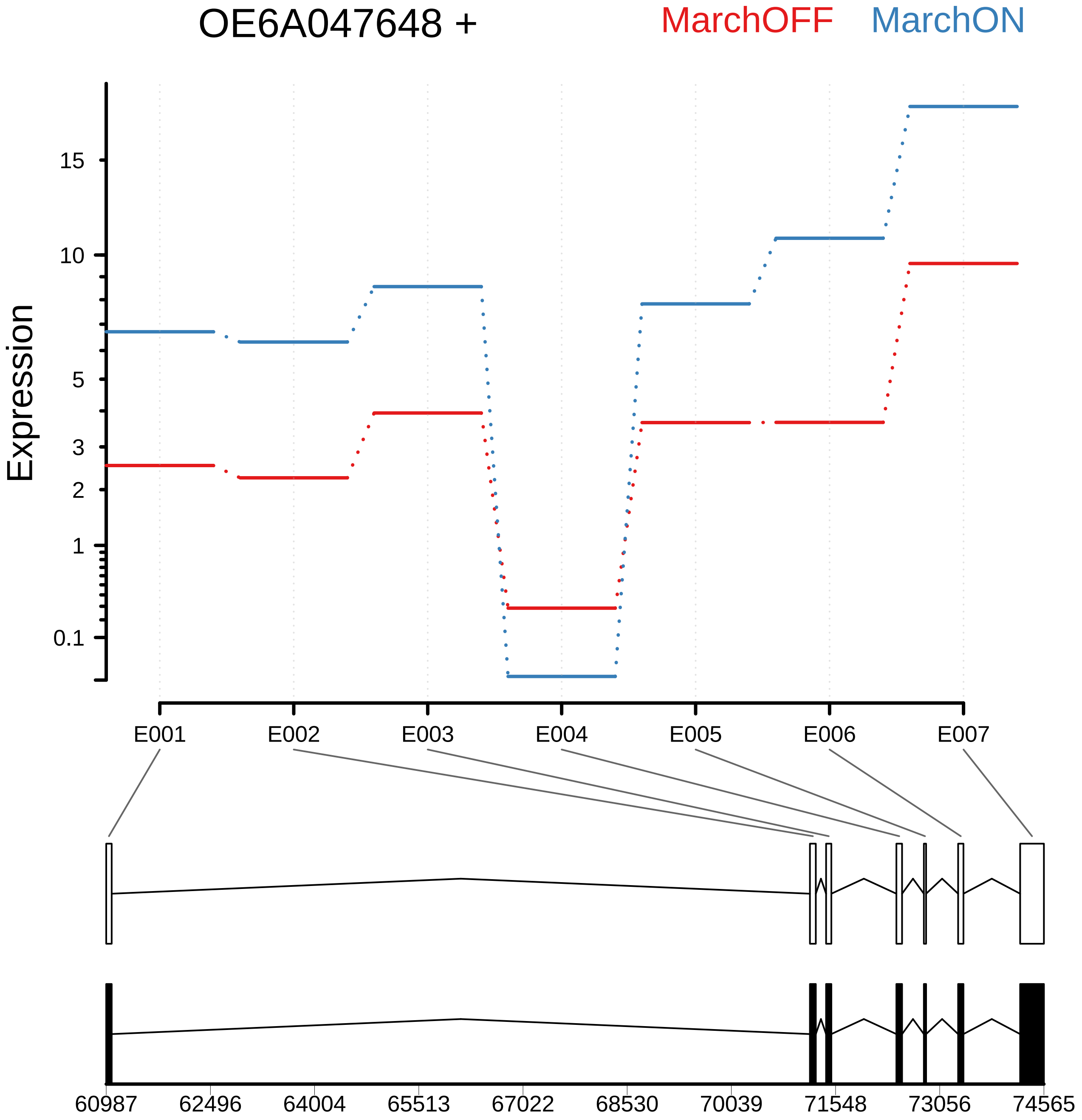

OE6A047648 +
MarchOFF
MarchON
Expression
15
10
5
3
2
1
0.1
E001
E002
E003
E004
E005
E006
E007
60987
62496
64004
65513
67022
68530
70039
71548
73056
74565

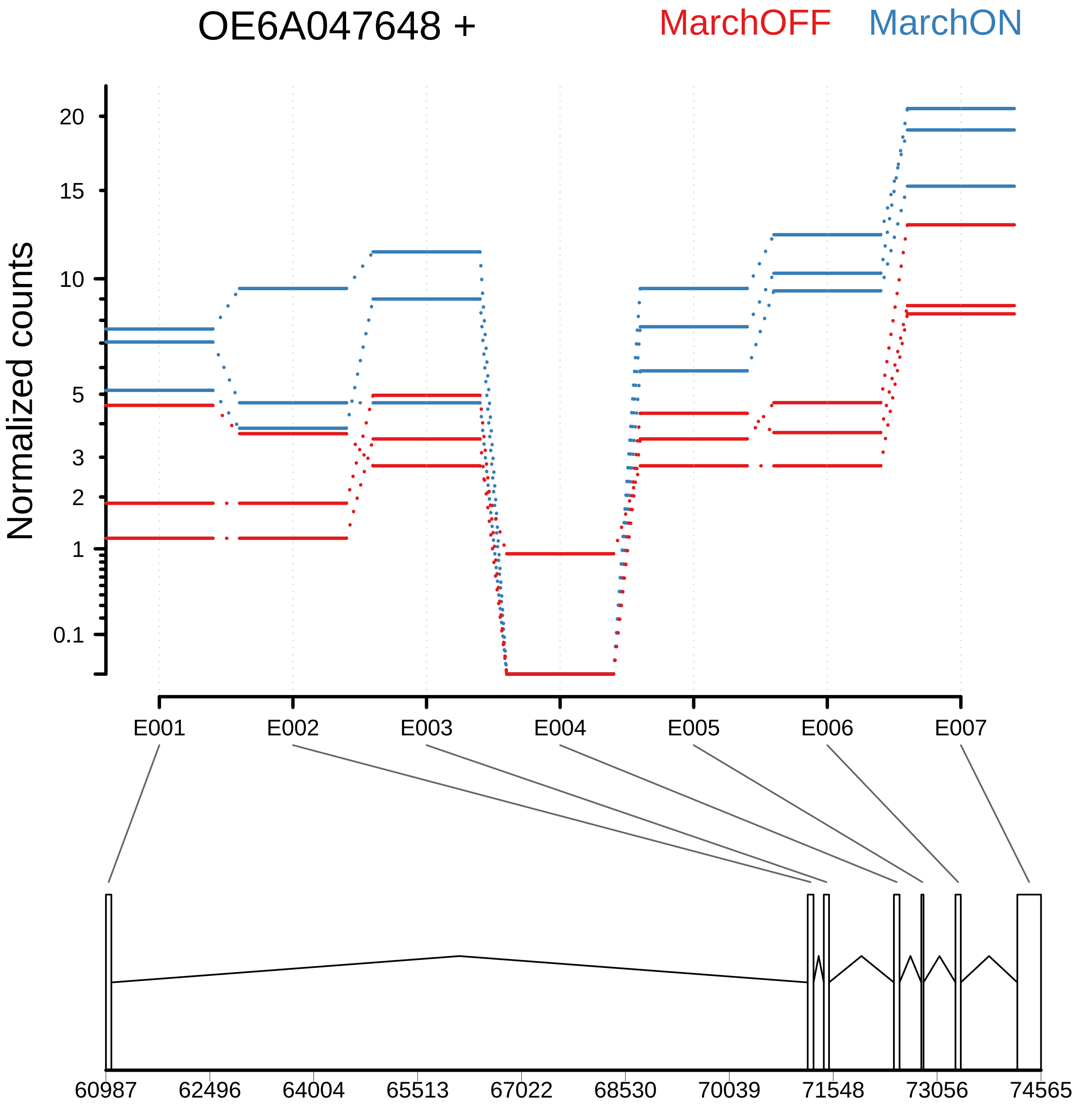

OE6A047648 +
MarchOFF
MarchON
Normalized counts
20
15
10
5
3
2
1
0.1
E001
E002
E003
E004
E005
E006
E007
60987
62496
64004
65513
67022
68530
70039
71548
73056
74565

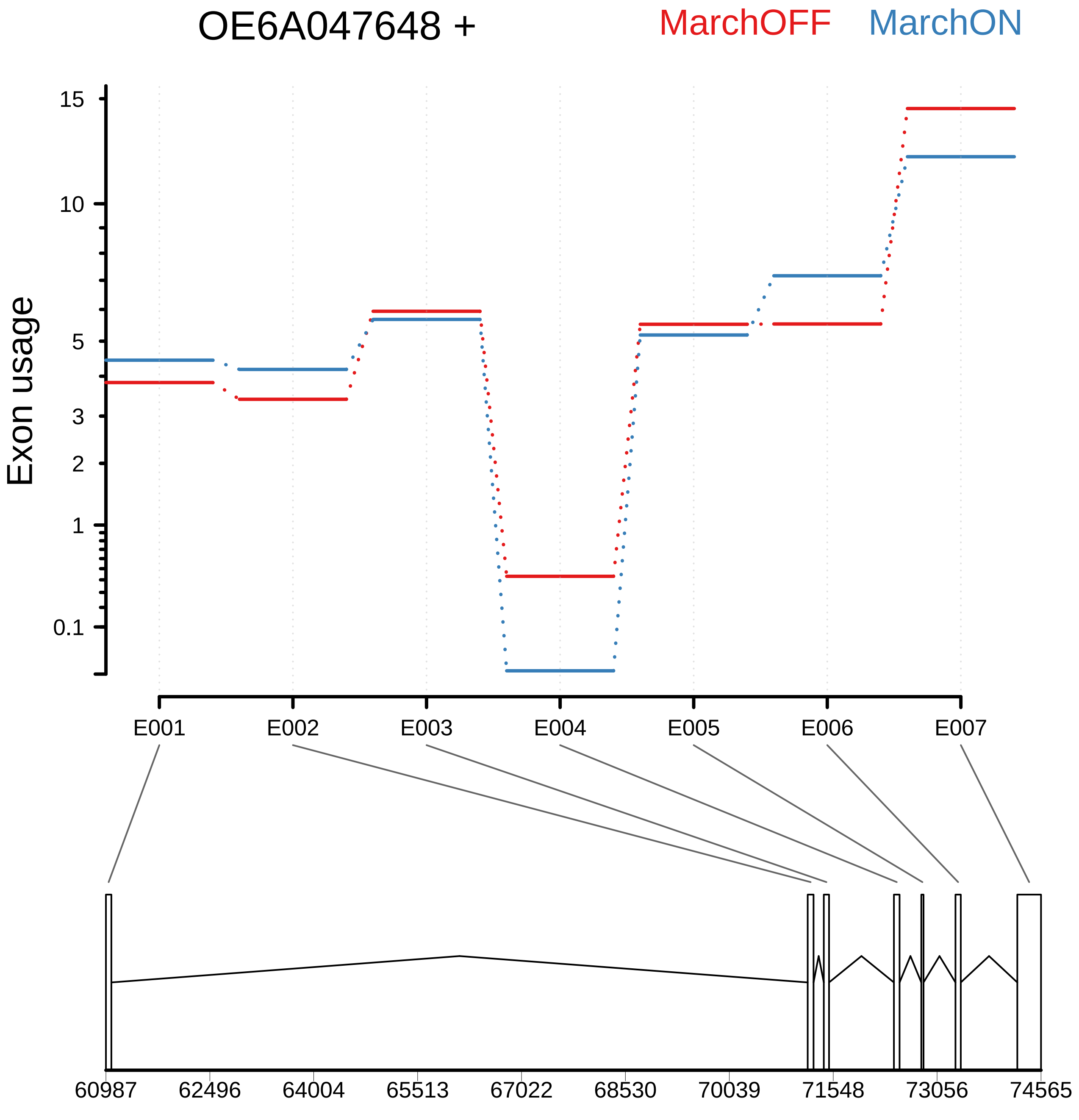

OE6A047648 +
MarchOFF
MarchON
Exon usage
15
10
5
3
2
1
0.1
E001
E002
E003
E004
E005
E006
E007
60987
62496
64004
65513
67022
68530
70039
71548
73056
74565

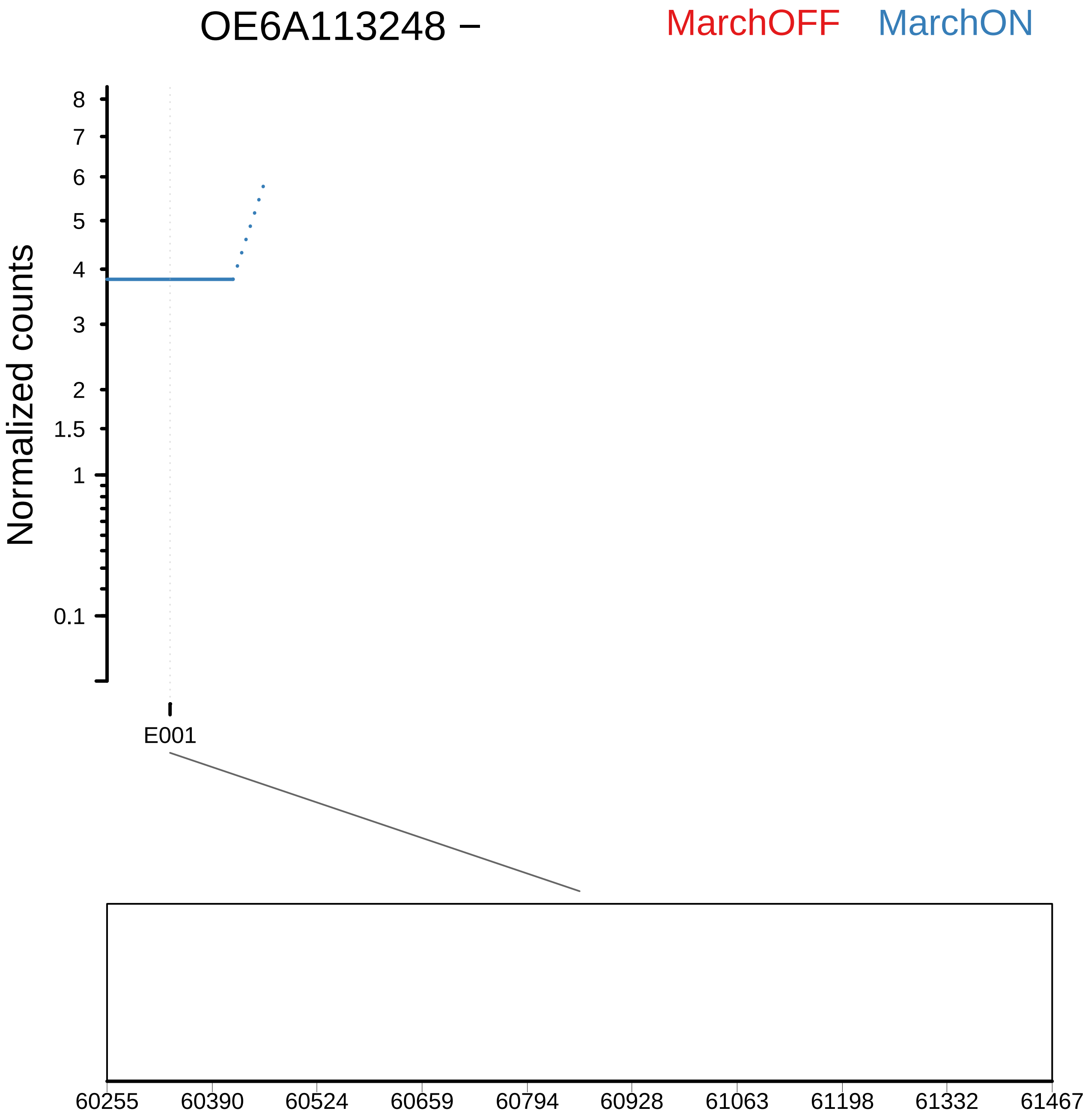

OE6A113248 −
MarchOFF
MarchON
Normalized counts
8
7
6
5
4
3
2
1.5
1
0.1
E001
60255
60390
60524
60659
60794
60928
61063
61198
61332
61467

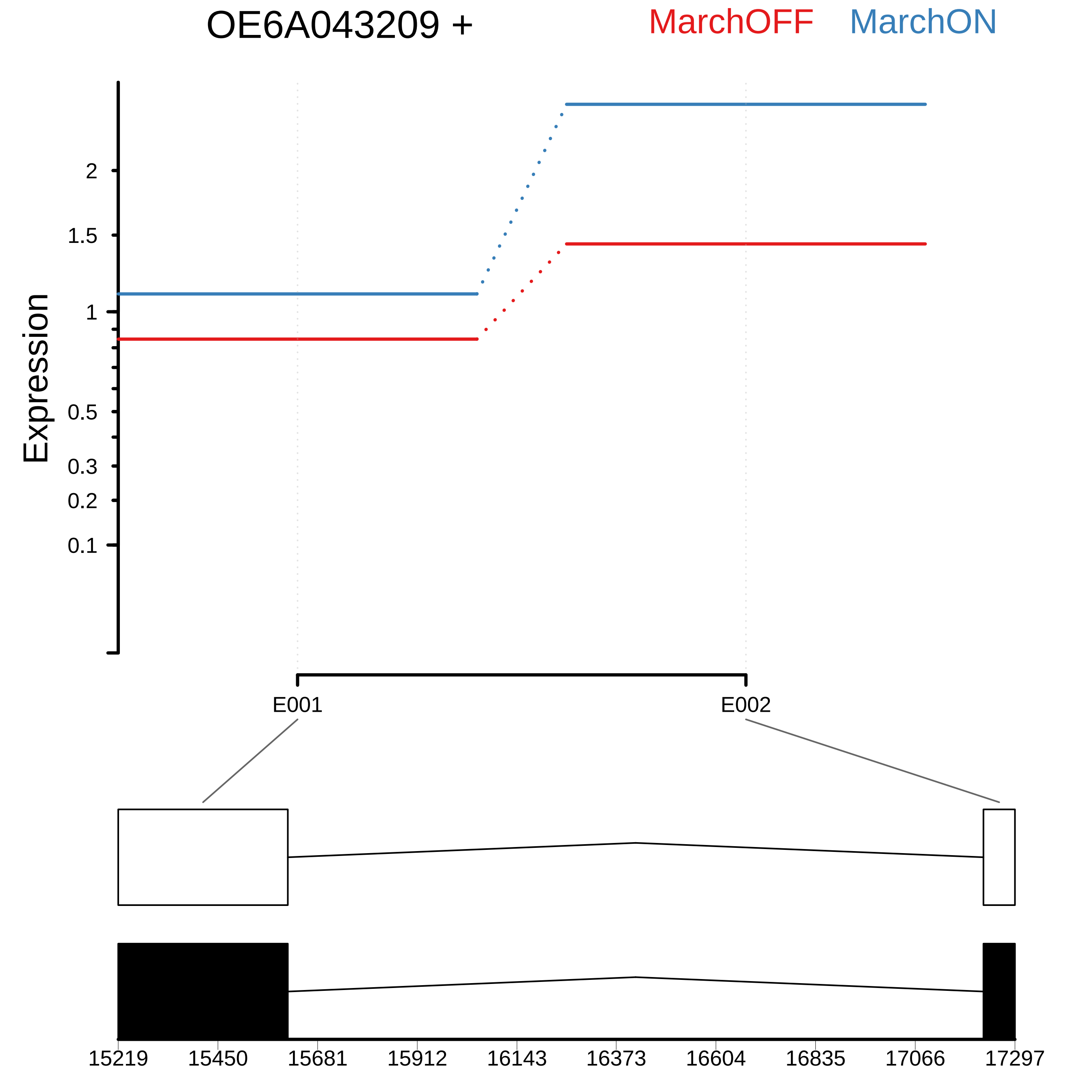
OE6A043209 +
MarchOFF
MarchON
Expression
2
1.5
1
0.5
0.3
0.2
0.1
E001
E002
15219
15450
15681
15912
16143
16373
16604
16835
17066
17297

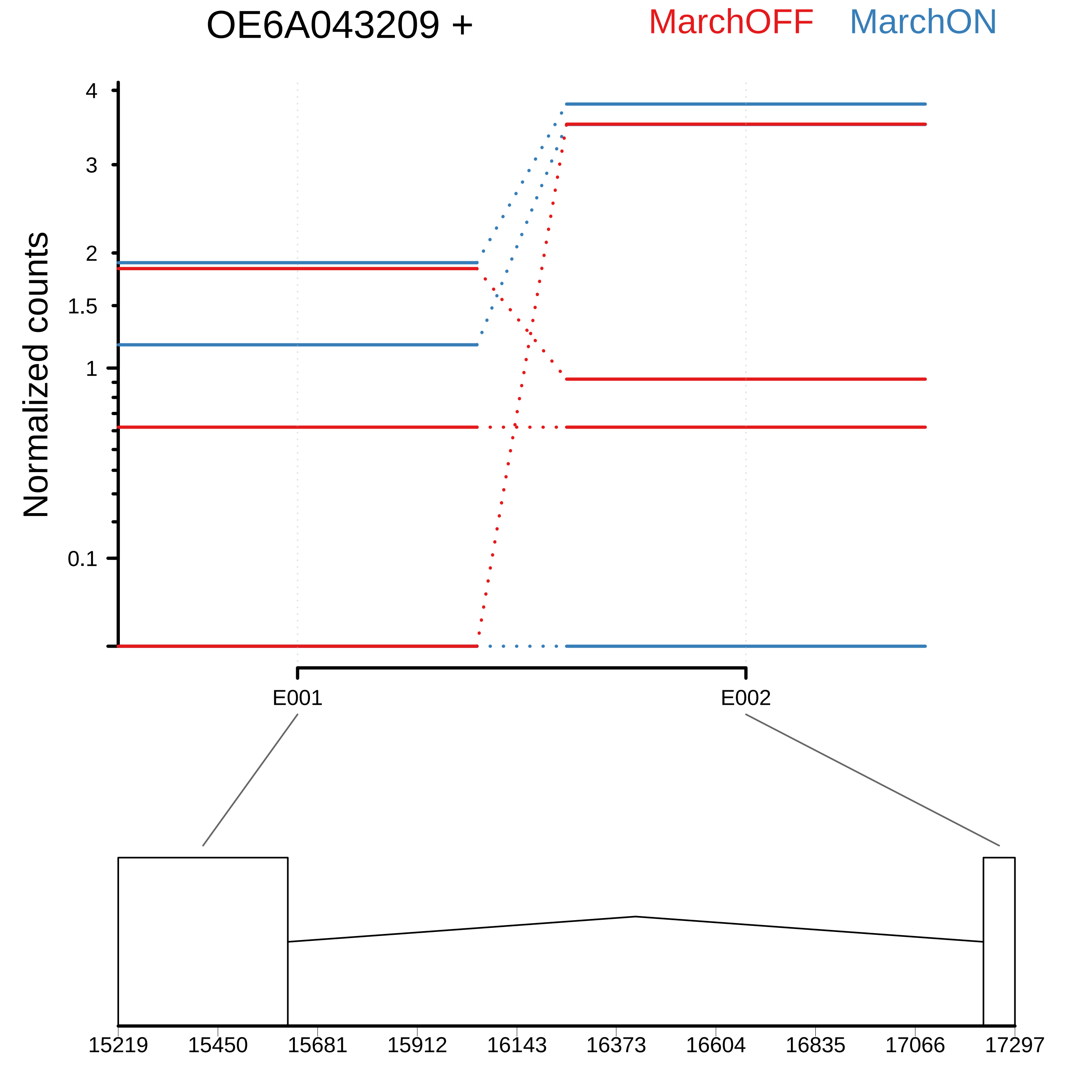

OE6A043209 +
MarchOFF
MarchON
Normalized counts
4
3
2
1.5
1
0.1
E001
E002
15219
15450
15681
15912
16143
16373
16604
16835
17066
17297

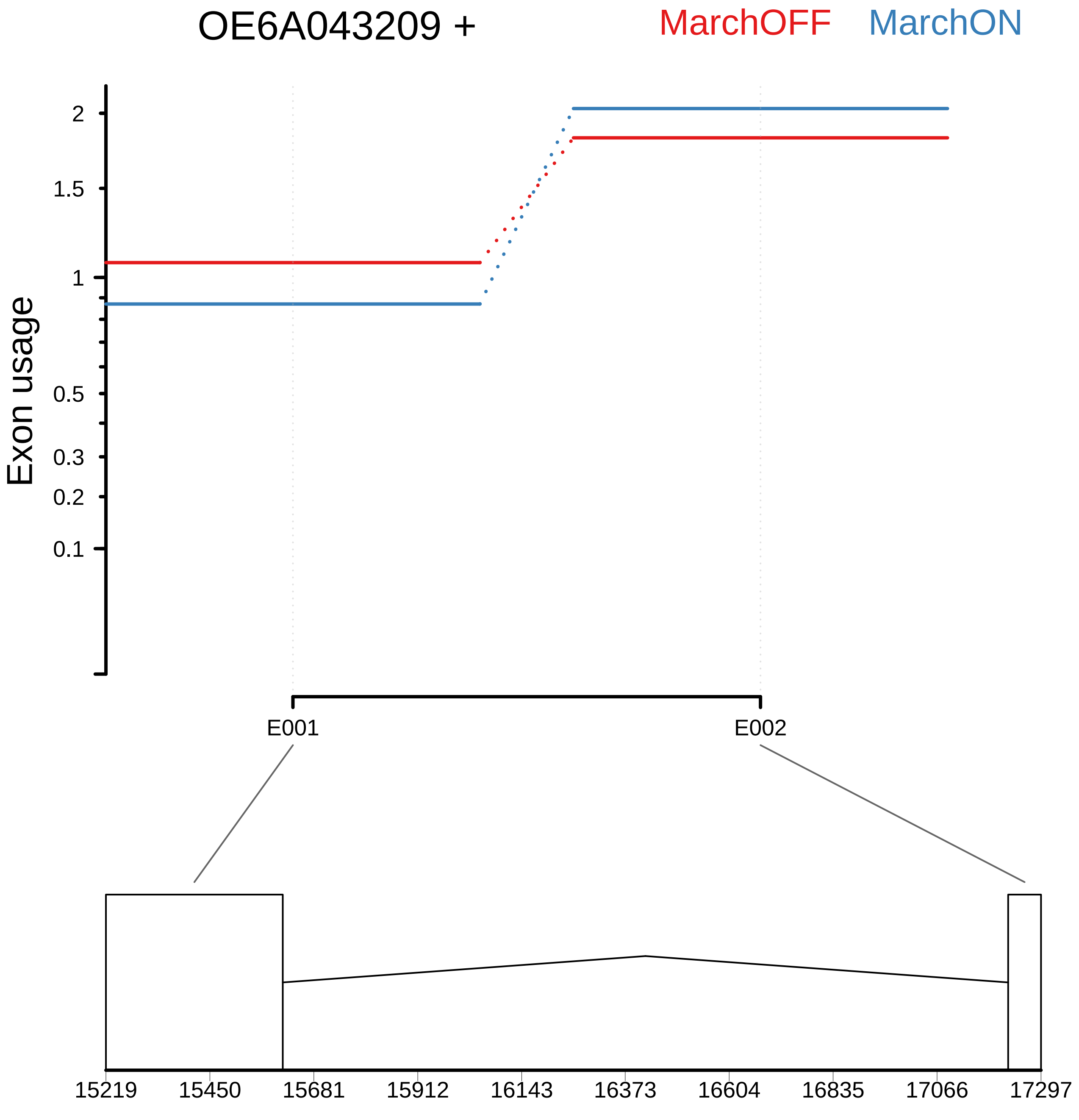

OE6A043209 +
MarchOFF
MarchON
Exon usage
2
1.5
1
0.5
0.3
0.2
0.1
E001
E002
15219
15450
15681
15912
16143
16373
16604
16835
17066
17297

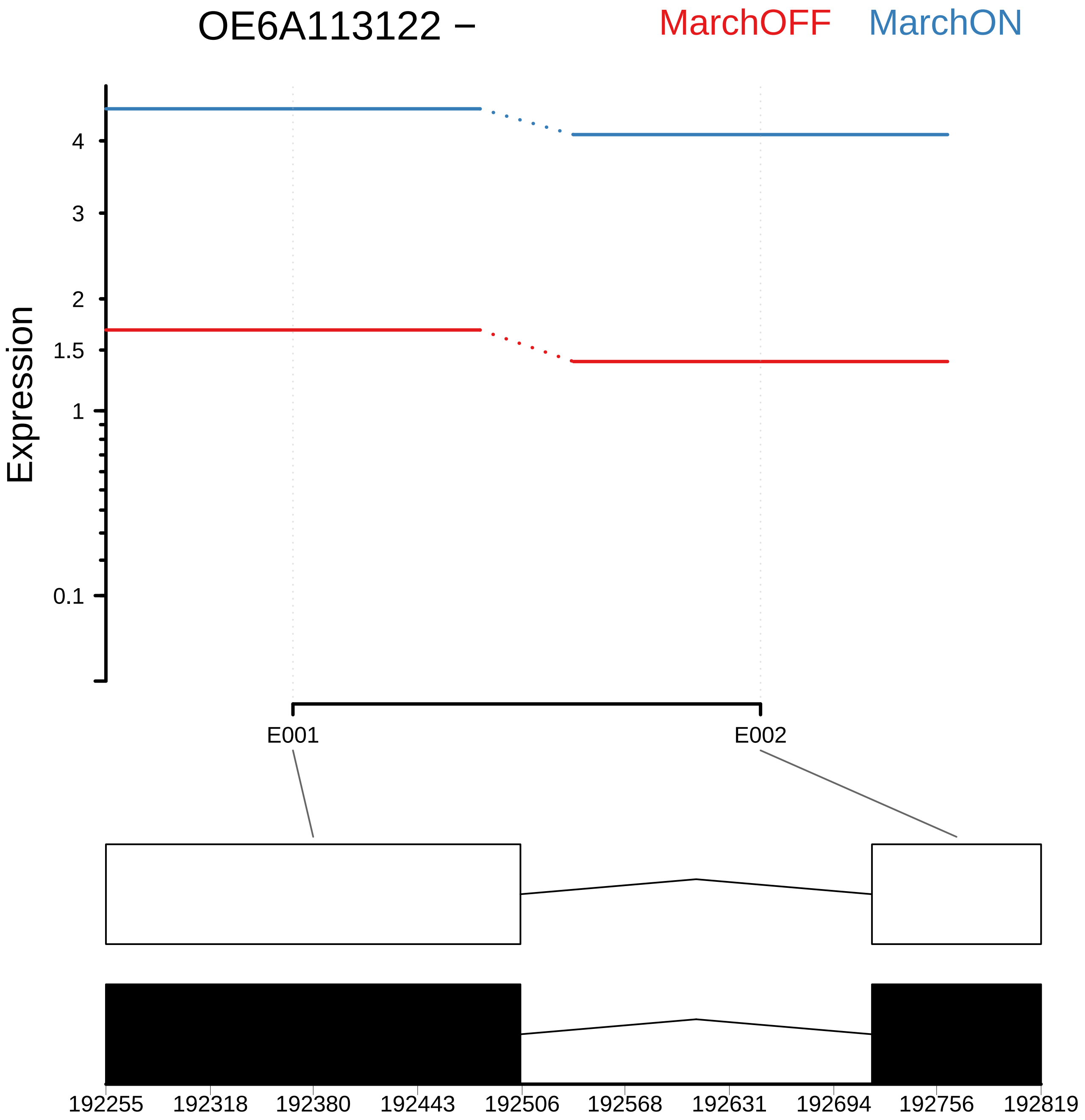
OE6A113122 −
MarchOFF
MarchON
Expression
4
3
2
1.5
1
0.1
E001
E002
192255
192318
192380
192443
192506
192568
192631
192694
192756
192819

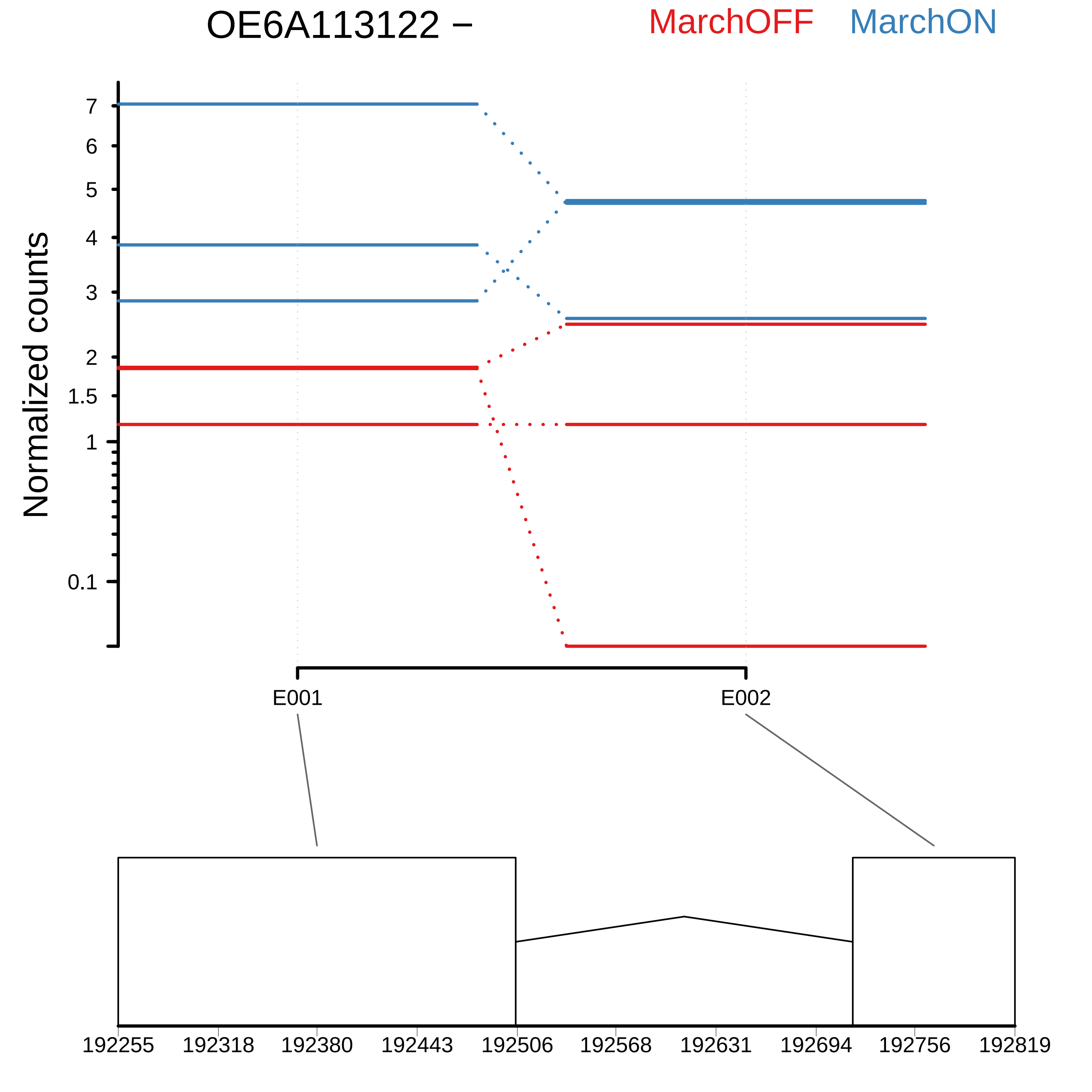

OE6A113122 −
MarchOFF
MarchON
Normalized counts
7
6
5
4
3
2
1.5
1
0.1
E001
E002
192255
192318
192380
192443
192506
192568
192631
192694
192756
192819

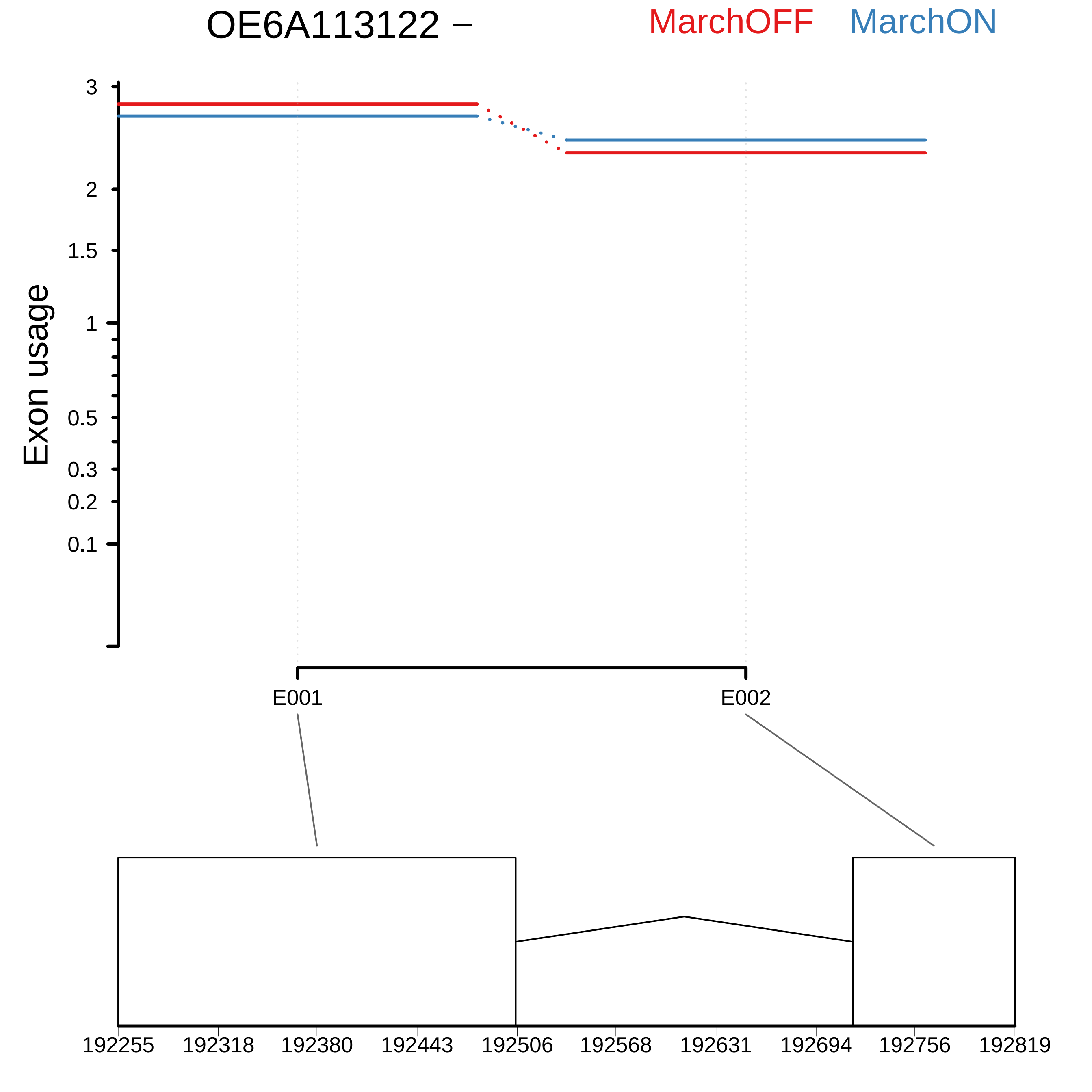

OE6A113122 −
MarchOFF
MarchON
Exon usage
3
2
1.5
1
0.5
0.3
0.2
0.1
E001
E002
192255
192318
192380
192443
192506
192568
192631
192694
192756
192819

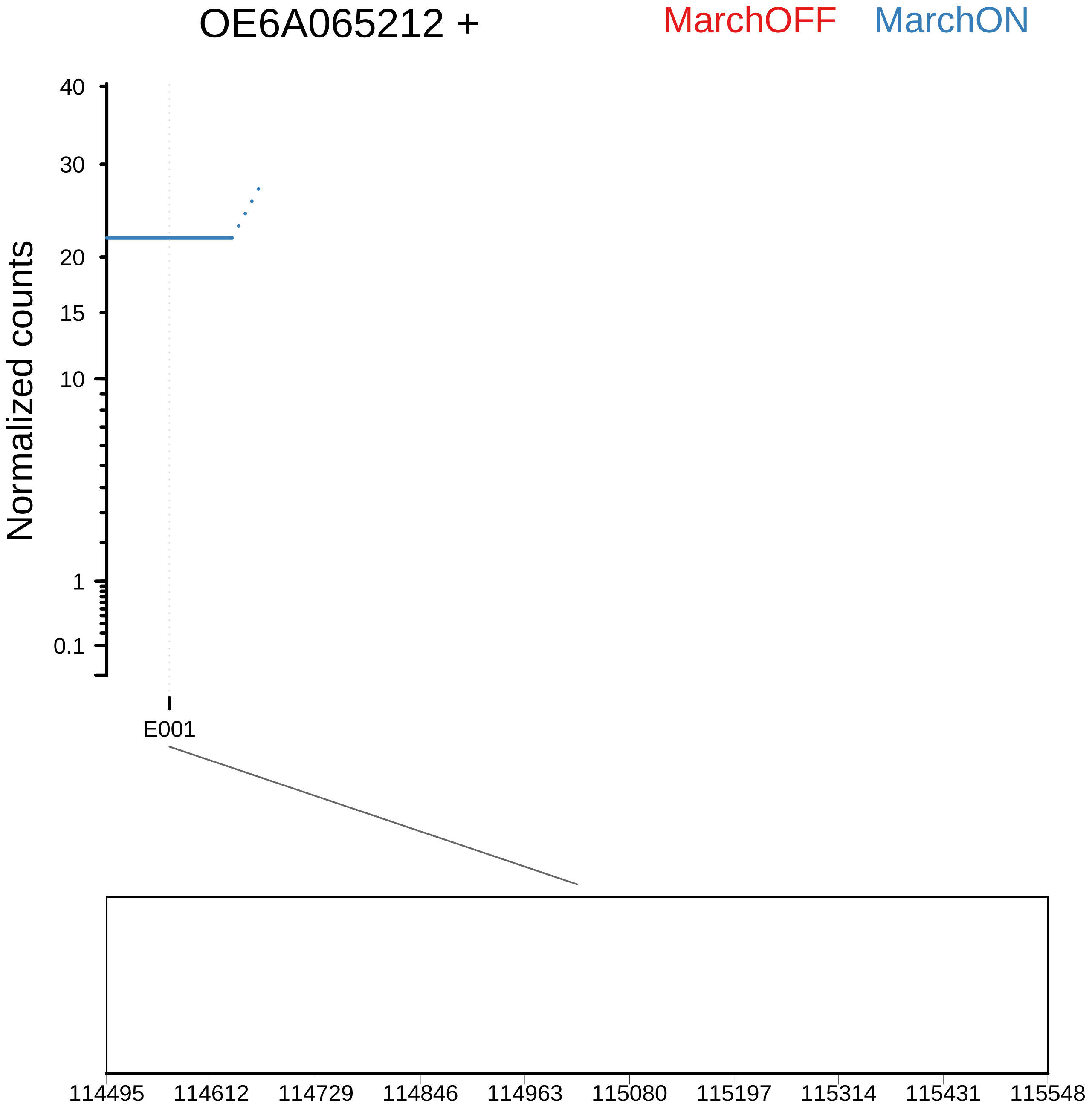

OE6A065212 +
MarchOFF
MarchON
Normalized counts
40
30
20
15
10
1
0.1
E001
114495
114612
114729
114846
114963
115080
115197
115314
115431
115548

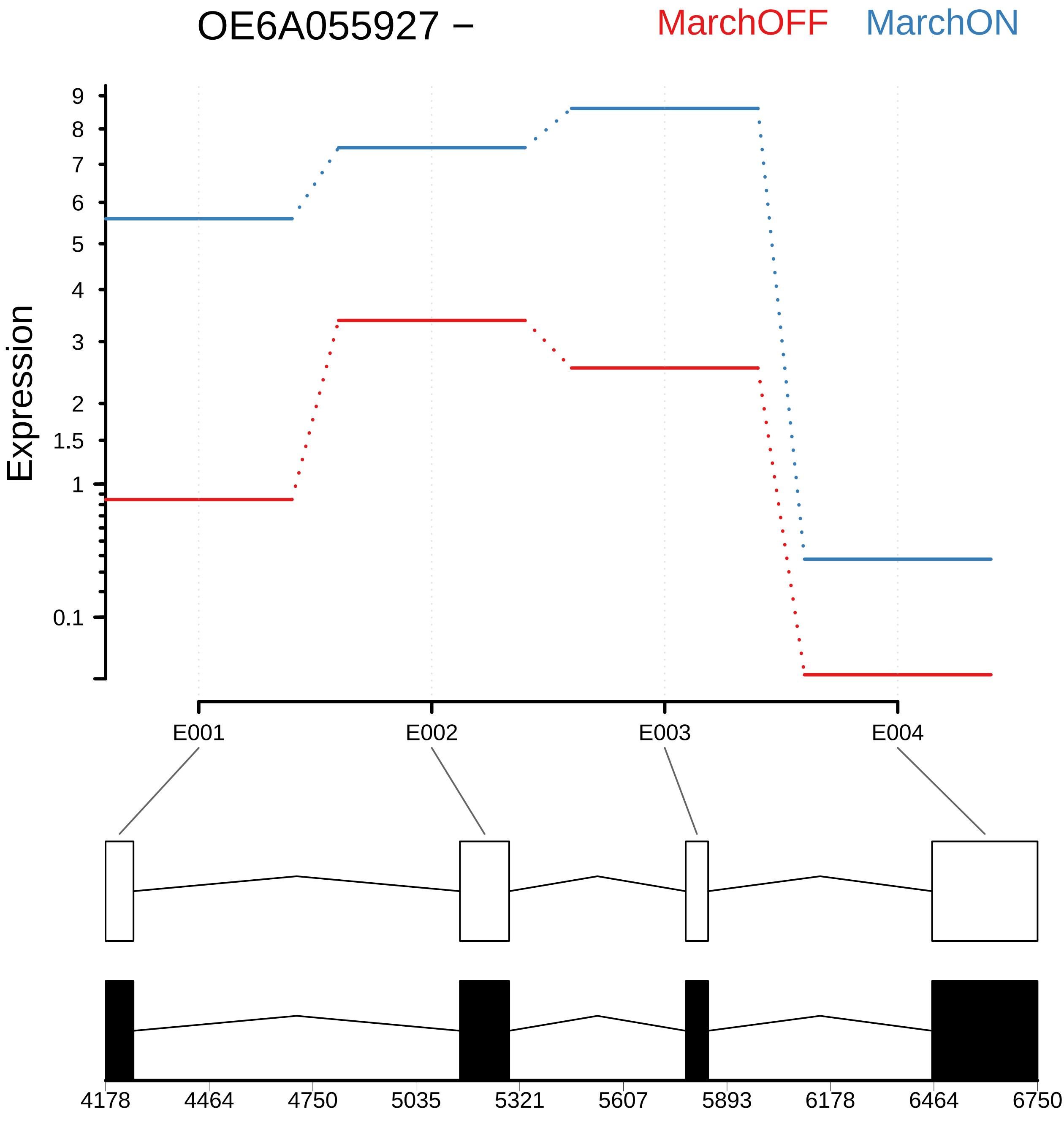
OE6A055927 −
MarchOFF
MarchON
Expression
9
8
7
6
5
4
3
2
1.5
1
0.1
E001
E002
E003
E004
4178
4464
4750
5035
5321
5607
5893
6178
6464
6750

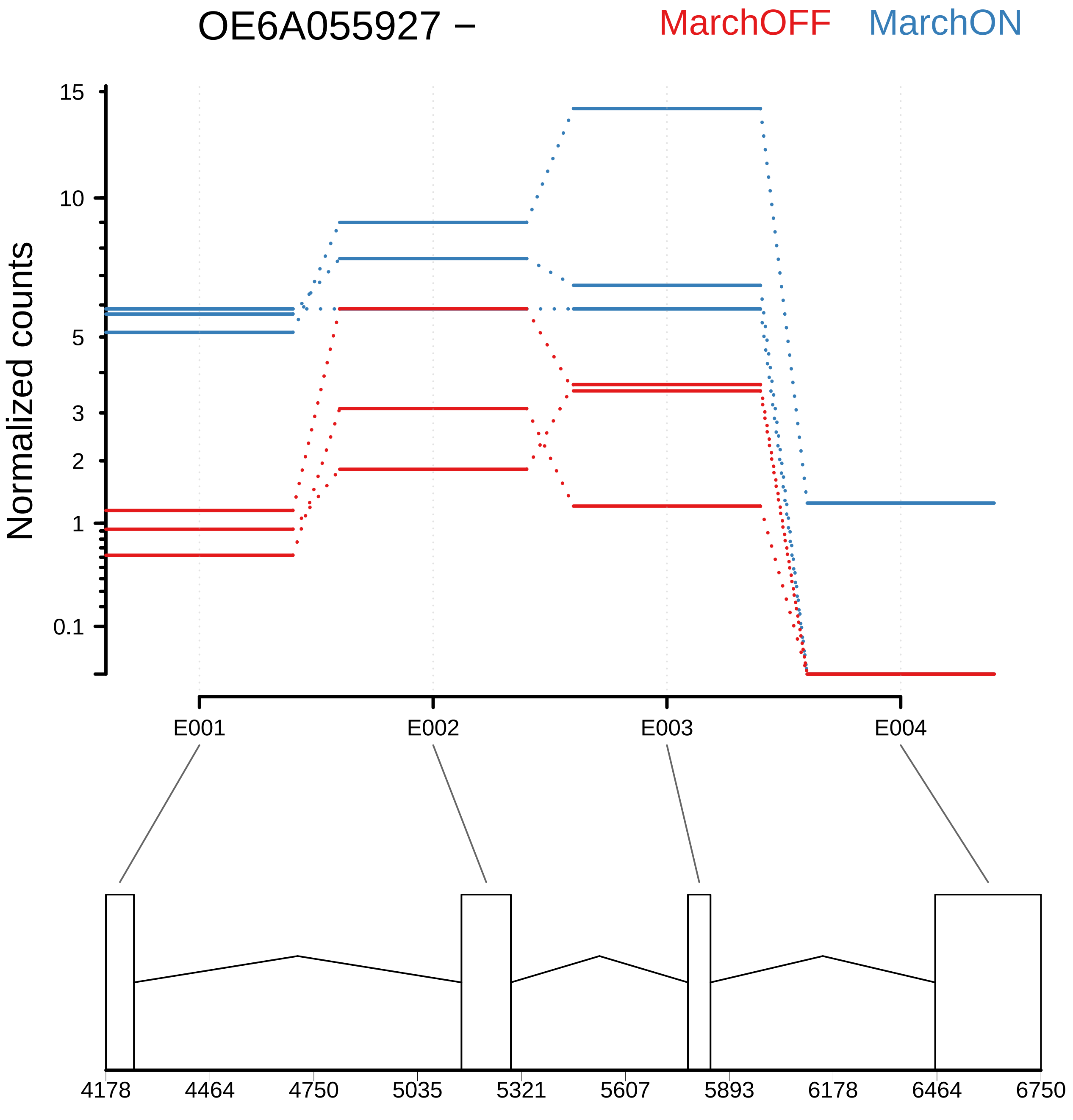

OE6A055927 −
MarchOFF
MarchON
Normalized counts
15
10
5
3
2
1
0.1
E001
E002
E003
E004
4178
4464
4750
5035
5321
5607
5893
6178
6464
6750

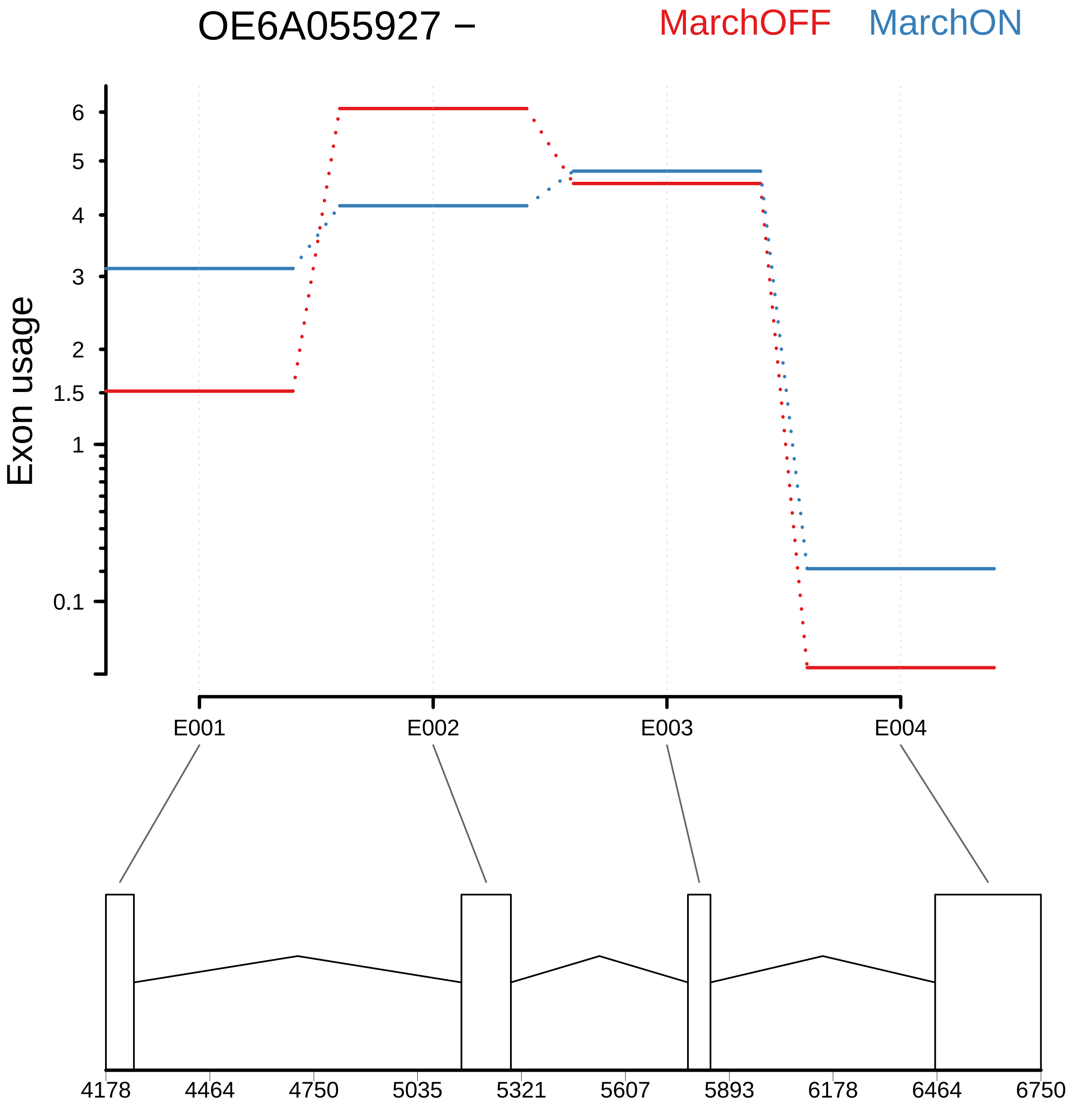

OE6A055927 −
MarchOFF
MarchON
Exon usage
6
5
4
3
2
1.5
1
0.1
E001
E002
E003
E004
4178
4464
4750
5035
5321
5607
5893
6178
6464
6750

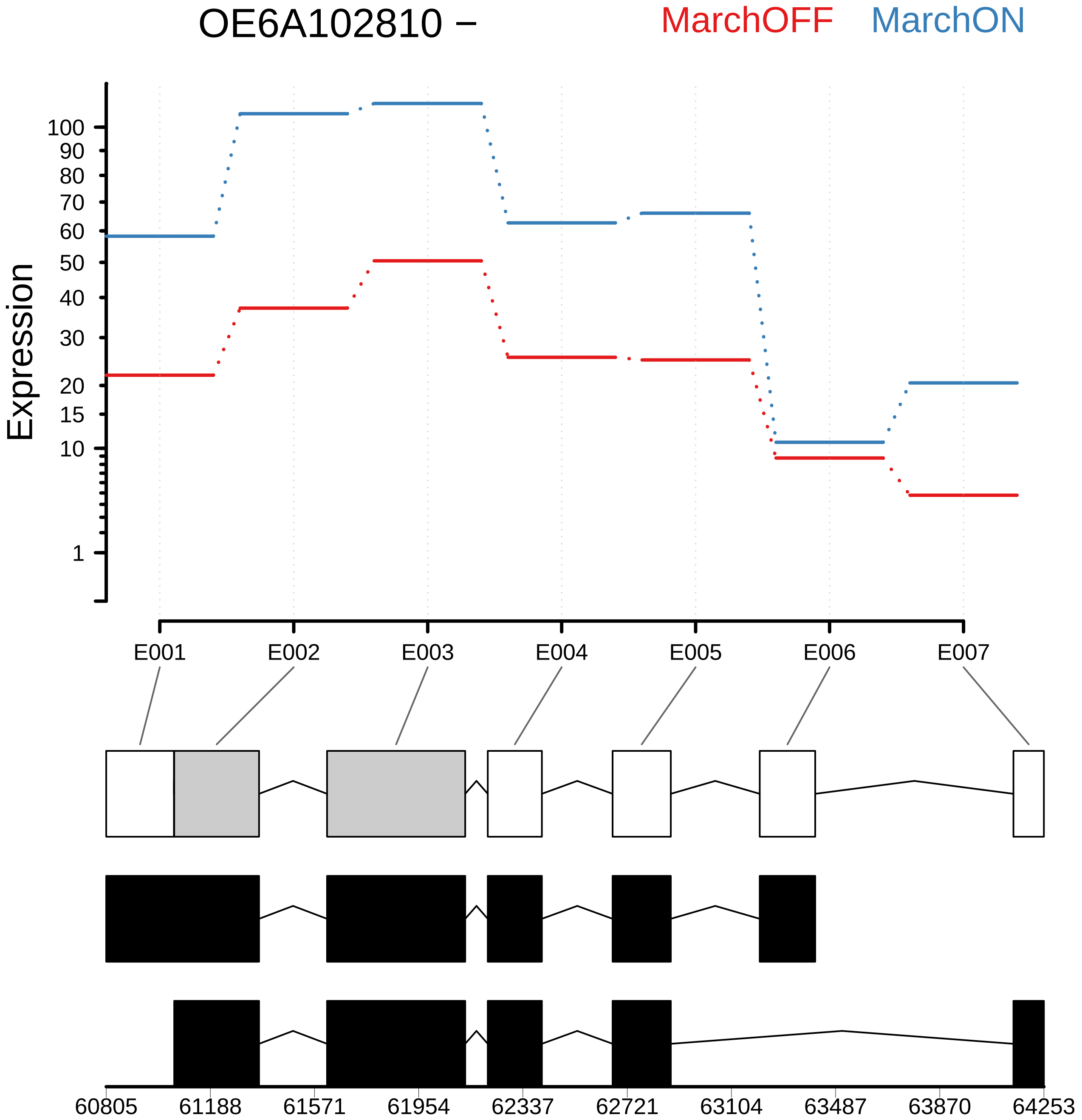

OE6A102810 −
MarchOFF
MarchON
Expression
100
90
80
70
60
50
40
30
20
15
10
1
E001
E002
E003
E004
E005
E006
E007
60805
61188
61571
61954
62337
62721
63104
63487
63870
64253

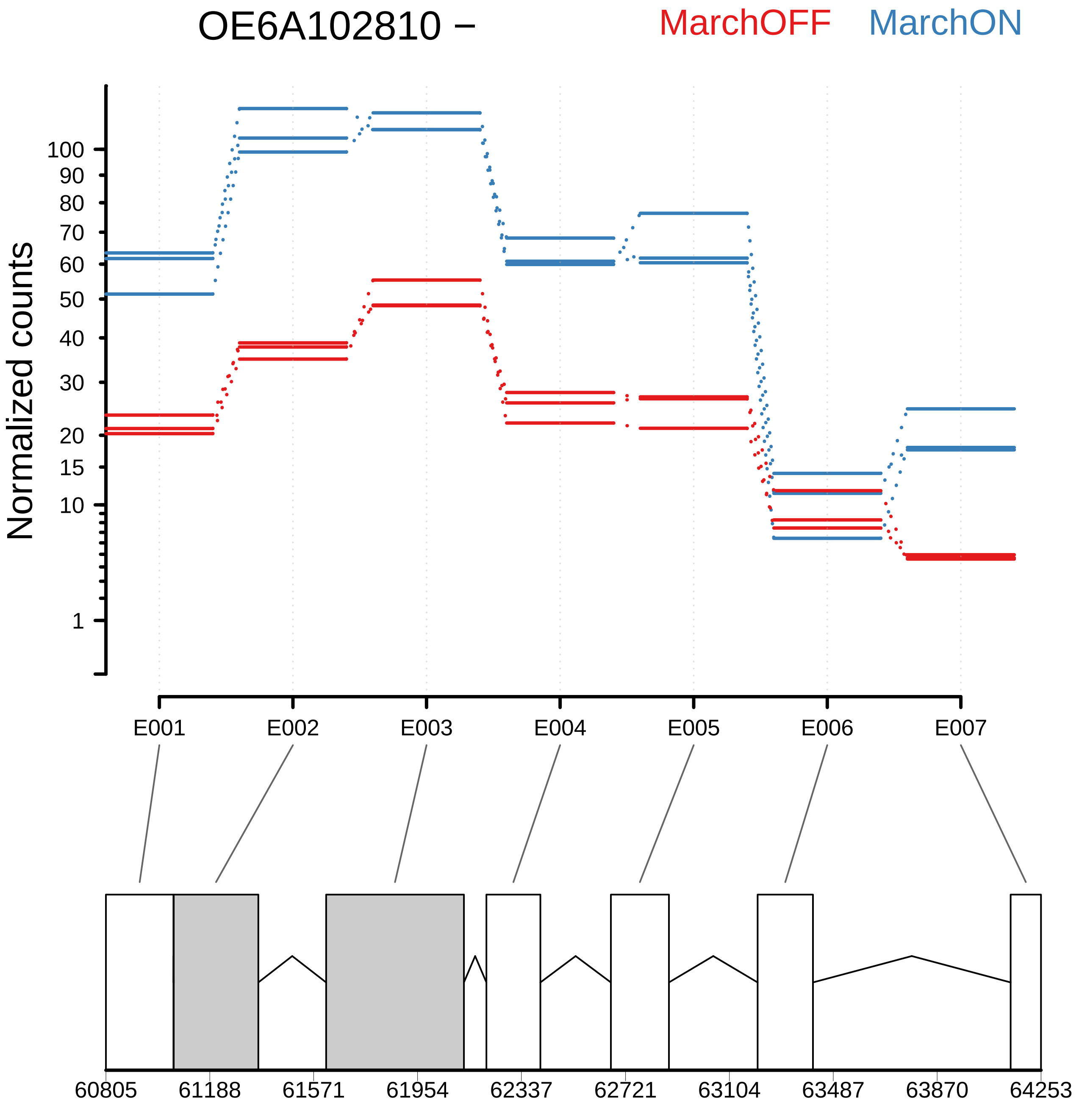

OE6A102810 −
MarchOFF
MarchON
Normalized counts
100
90
80
70
60
50
40
30
20
15
10
1
E001
E002
E003
E004
E005
E006
E007
60805
61188
61571
61954
62337
62721
63104
63487
63870
64253

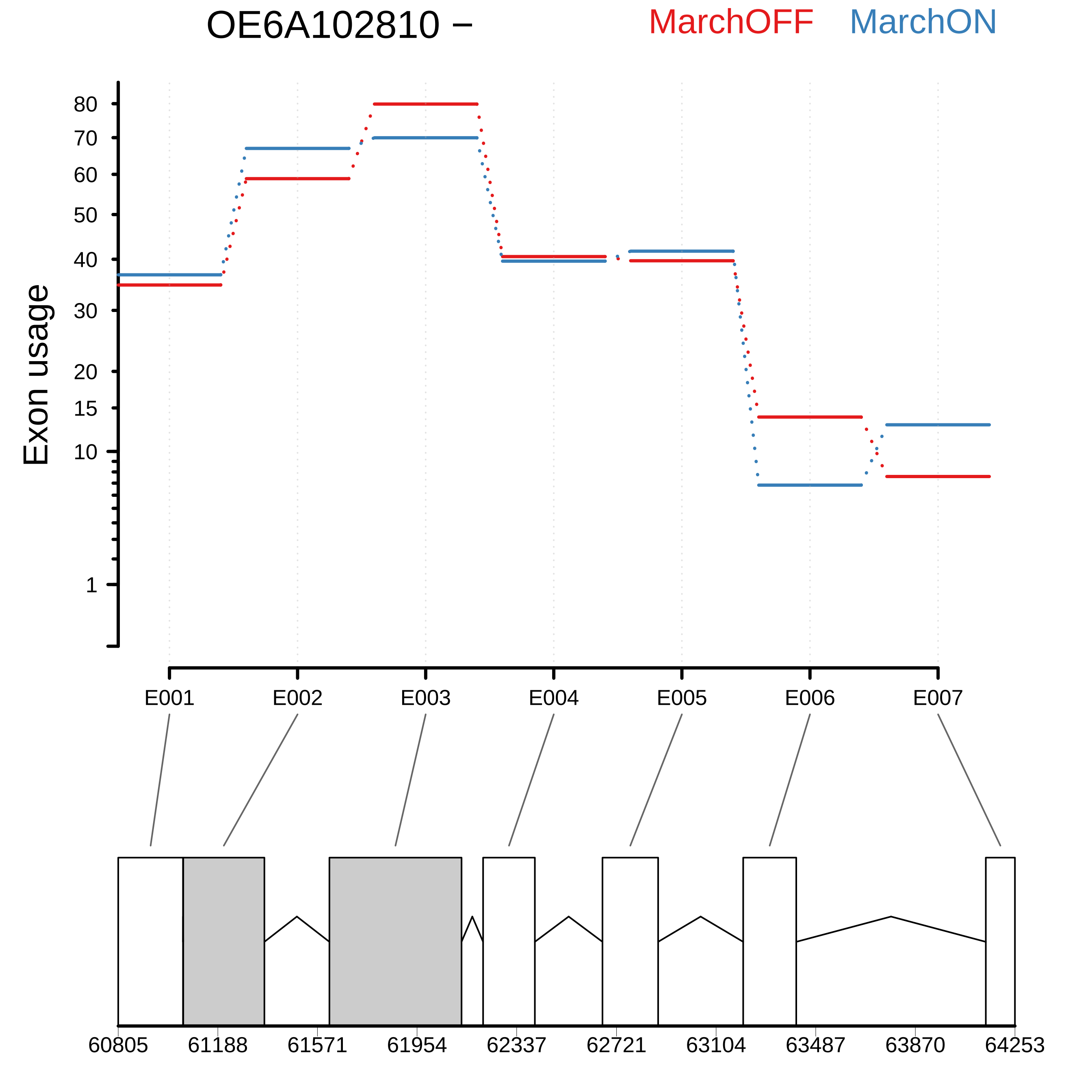

OE6A102810 −
MarchOFF
MarchON
Exon usage
80
70
60
50
40
30
20
15
10
1
E001
E002
E003
E004
E005
E006
E007
60805
61188
61571
61954
62337
62721
63104
63487
63870
64253

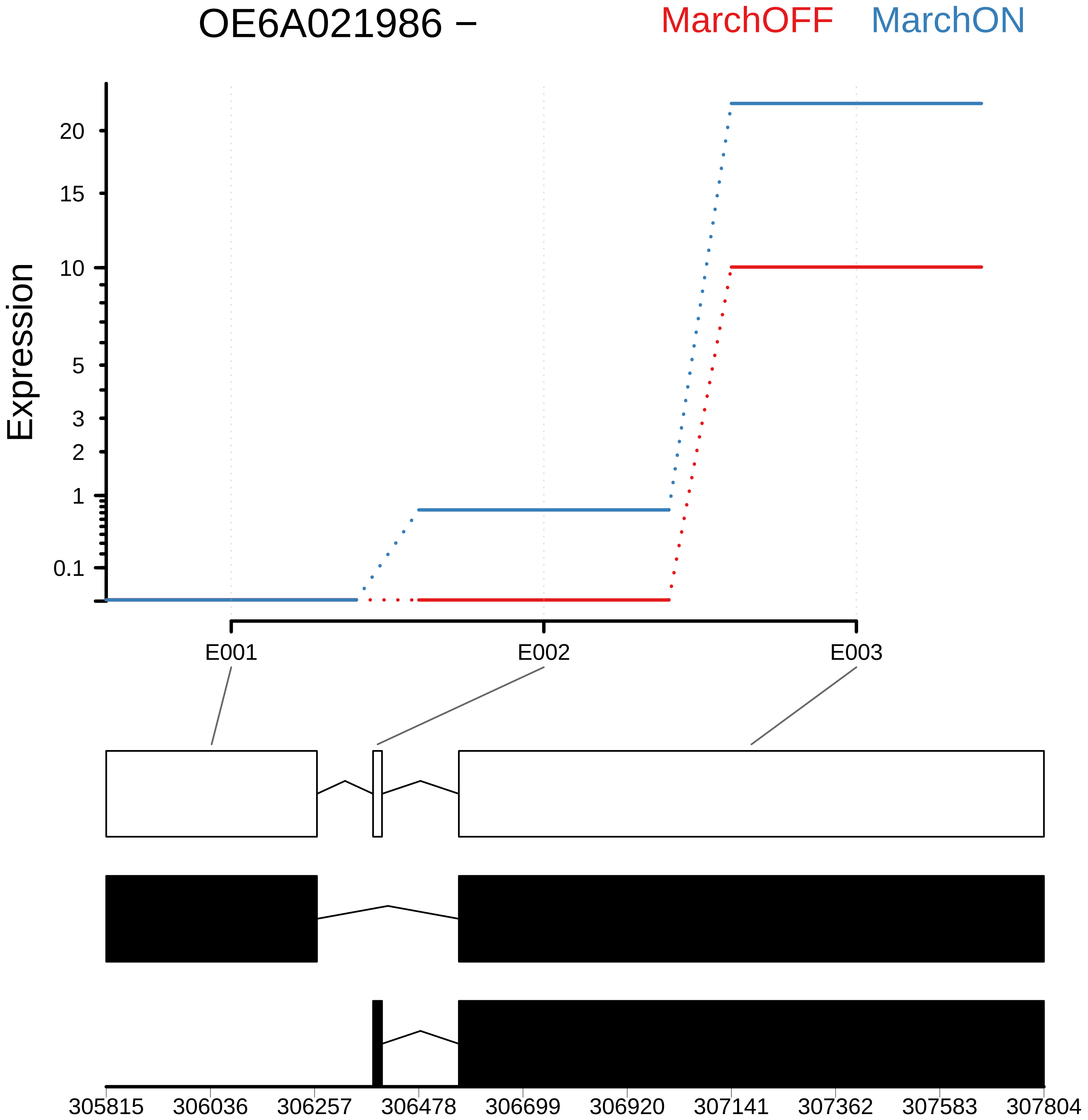
OE6A021986 –
MarchOFF
MarchON
Expression
20
15
10
5
3
2
1
0.1
E001
E002
E003
305815
306036
306257
306478
306699
306920
307141
307362
307583
307804

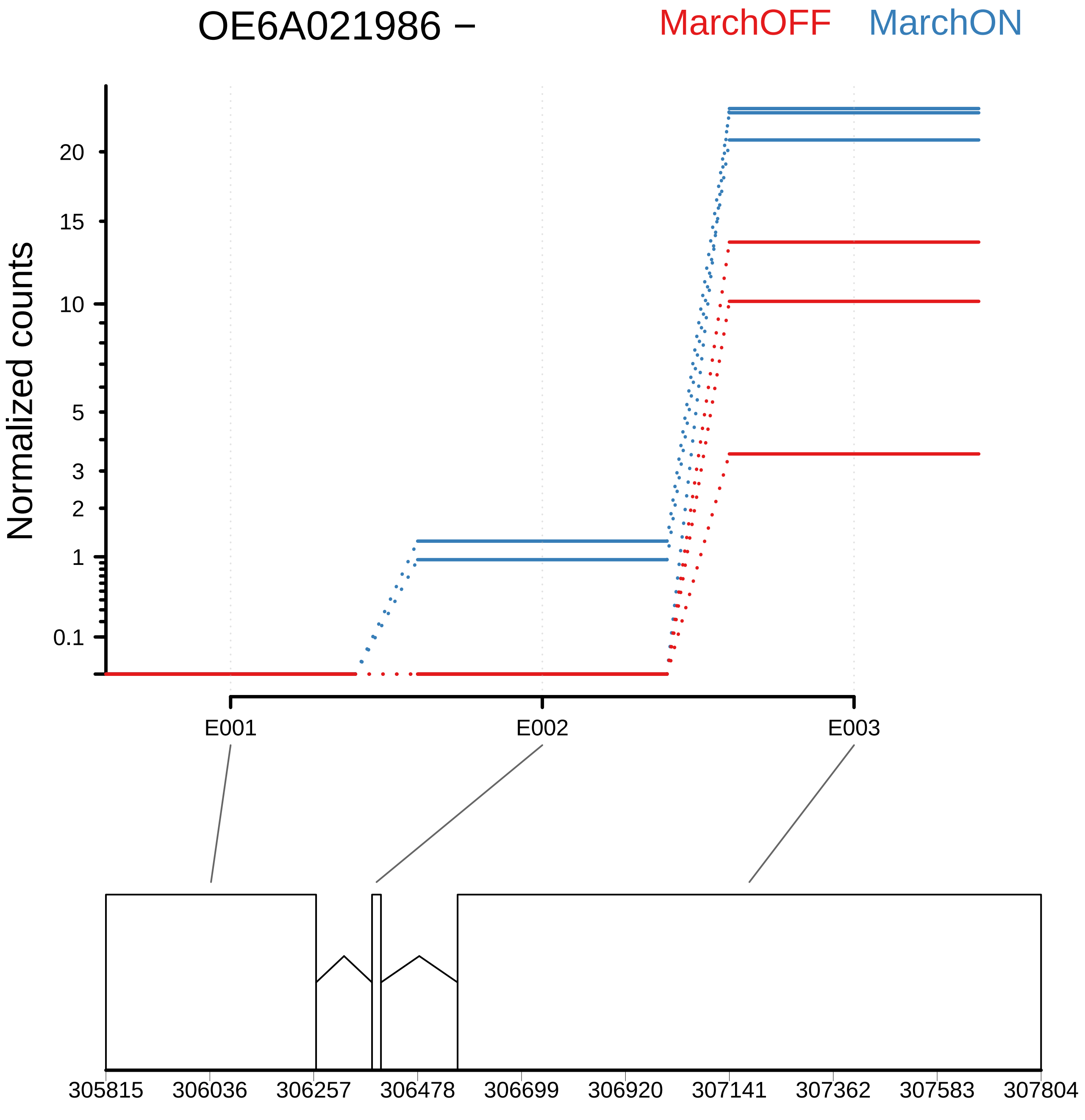

OE6A021986 −
MarchOFF
MarchON
Normalized counts
20
15
10
5
3
2
1
0.1
E001
E002
E003
305815
306036
306257
306478
306699
306920
307141
307362
307583
307804

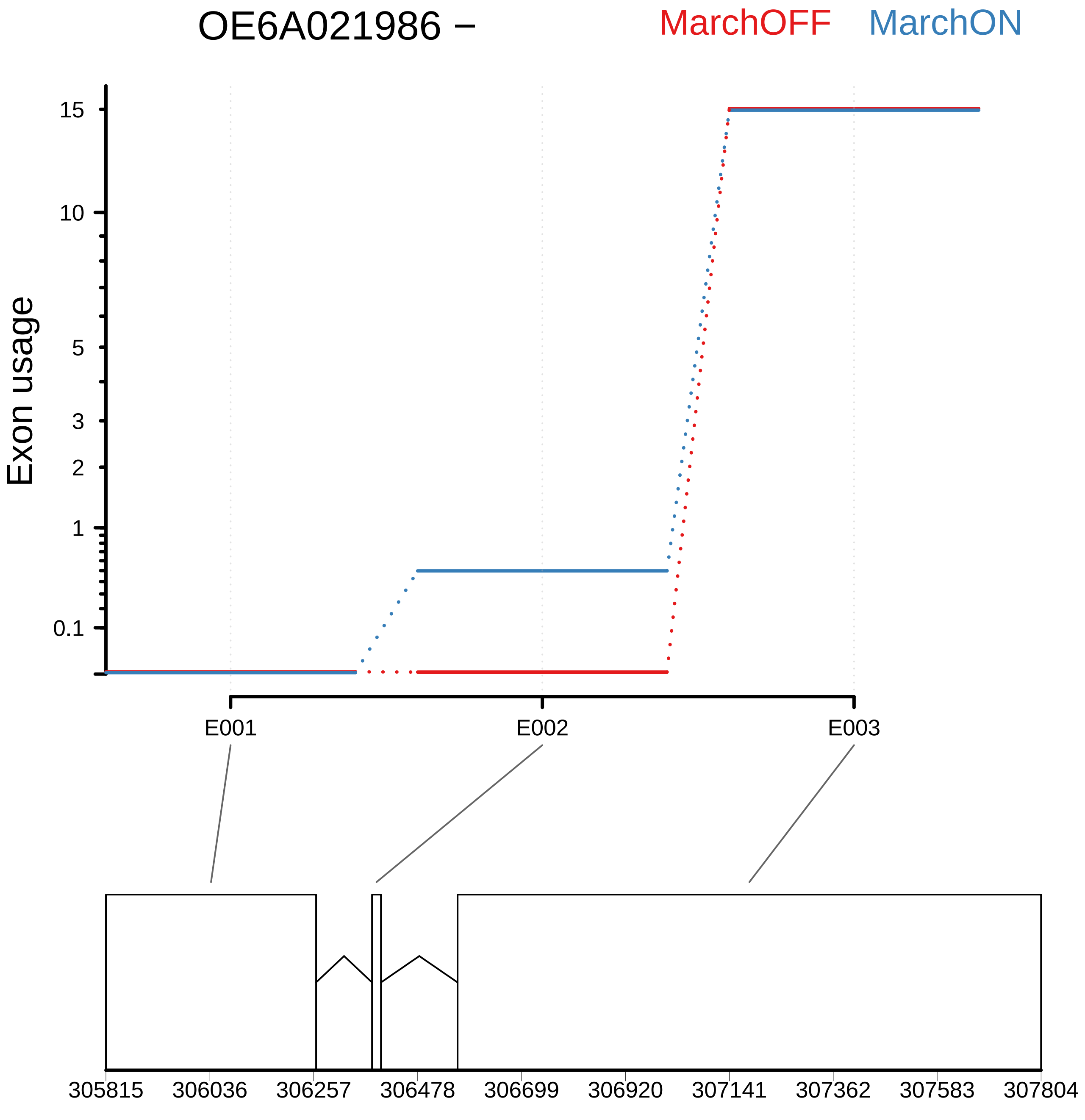
OE6A021986 −
MarchOFF
MarchON
Exon usage
15
10
5
3
2
1
0.1
E001
E002
E003
305815
306036
306257
306478
306699
306920
307141
307362
307583
307804

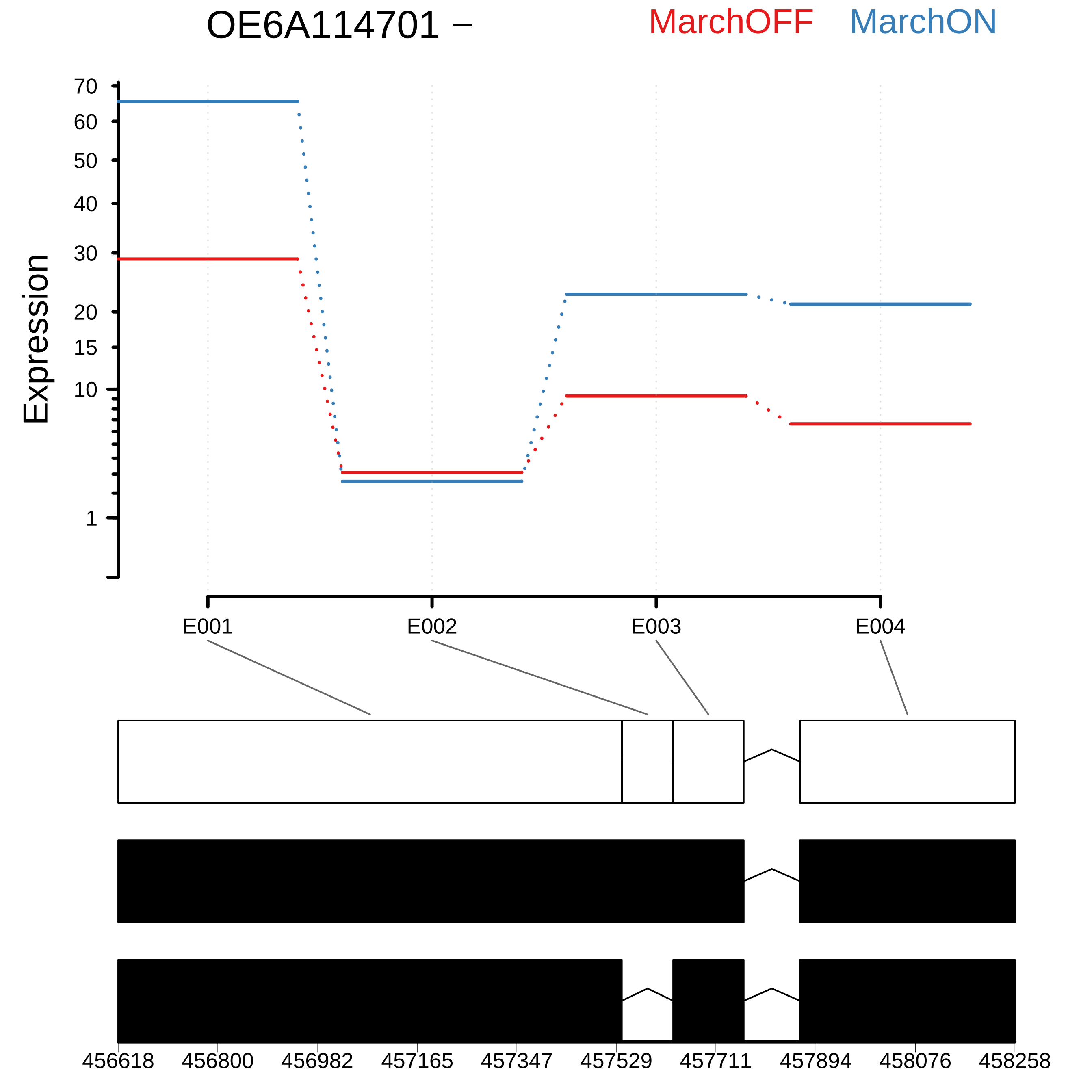
OE6A114701 −
MarchOFF
MarchON
Expression
70
60
50
40
30
20
15
10
1
E001
E002
E003
E004
456618
456800
456982
457165
457347
457529
457711
457894
458076
458258

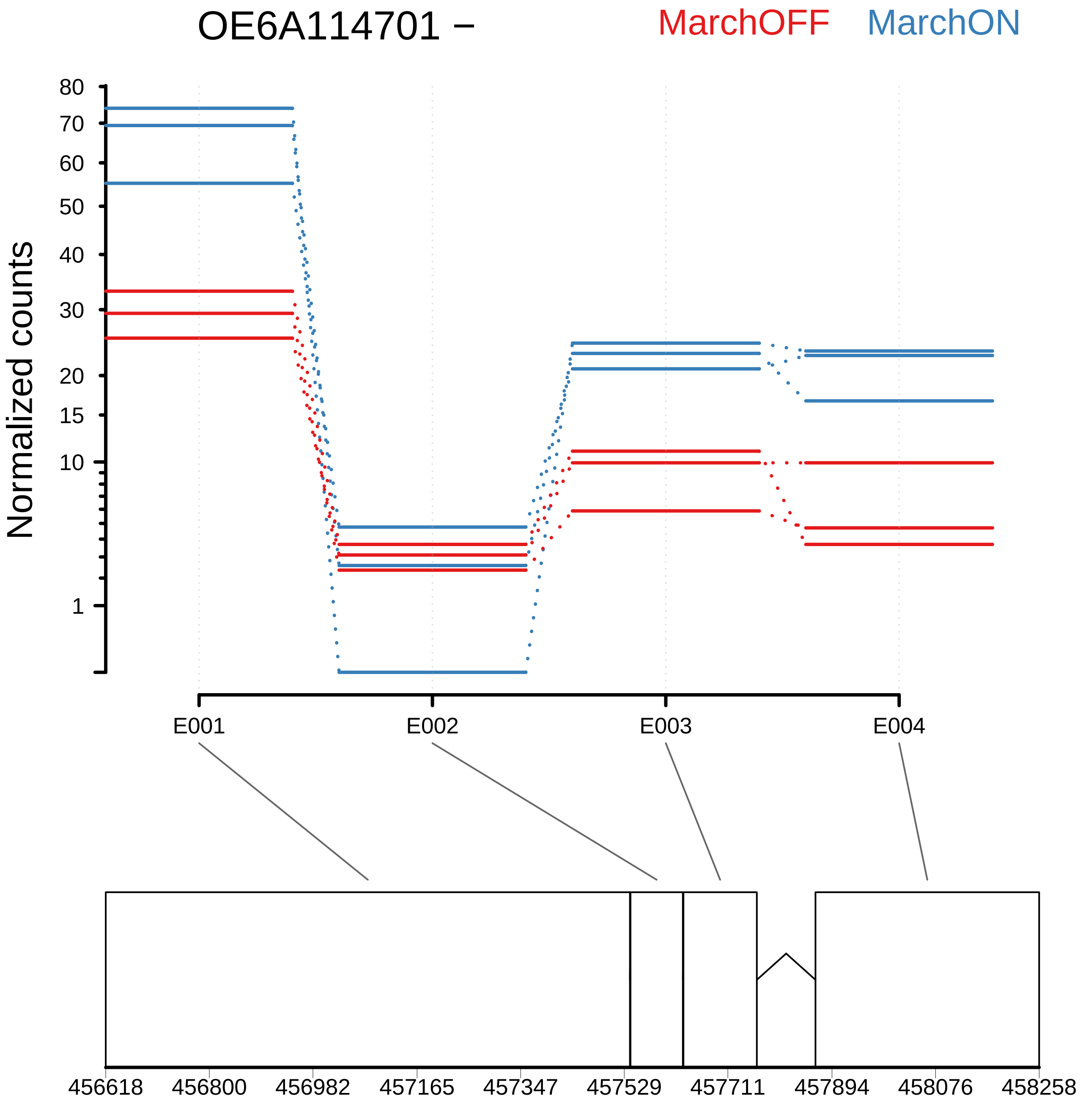

OE6A114701 −
MarchOFF
MarchON
Normalized counts
80
70
60
50
40
30
20
15
10
1
E001
E002
E003
E004
456618
456800
456982
457165
457347
457529
457711
457894
458076
458258

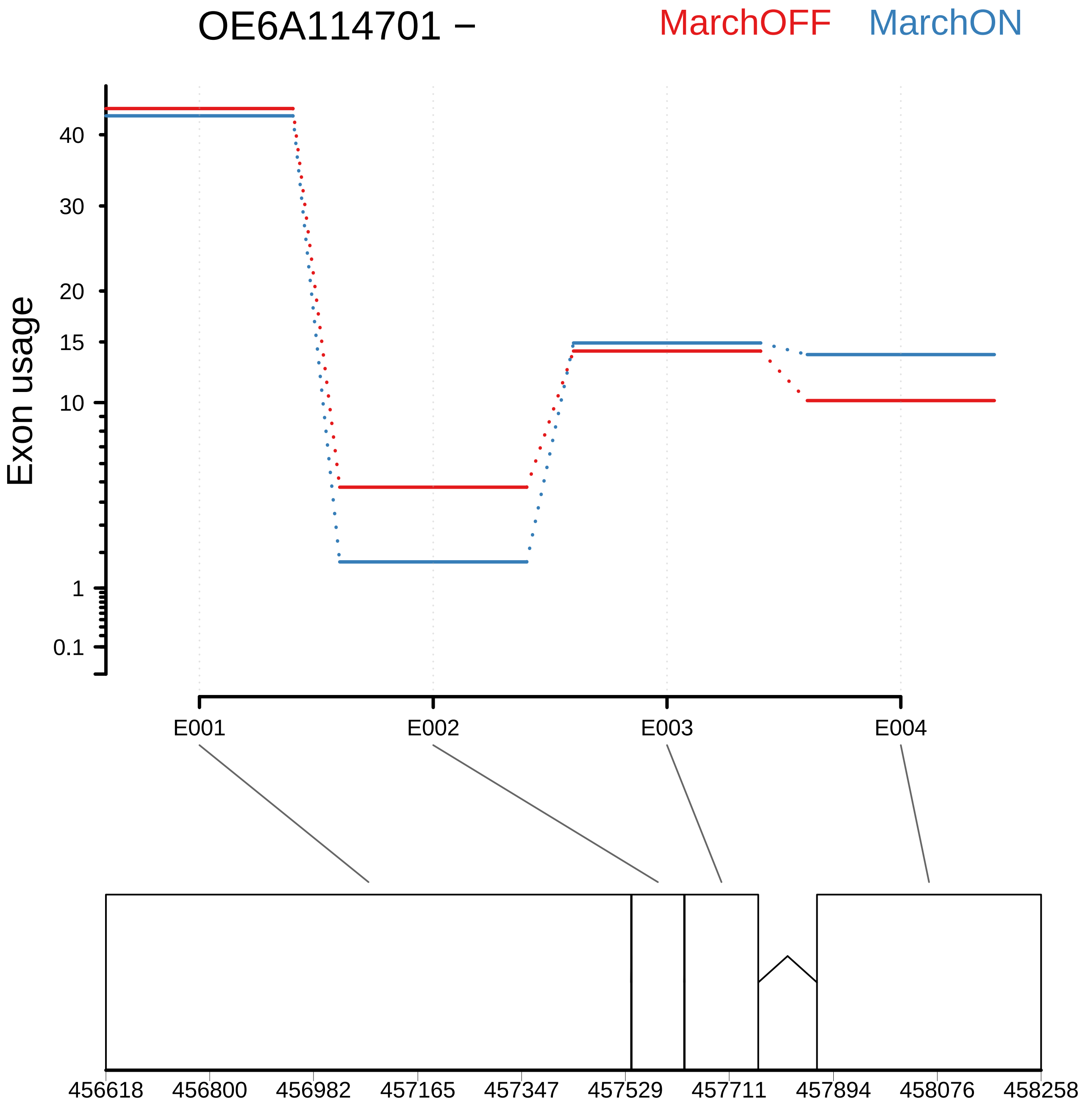
OE6A114701 −
MarchOFF
MarchON
Exon usage
40
30
20
15
10
1
0.1
E001
E002
E003
E004
456618
456800
456982
457165
457347
457529
457711
457894
458076
458258

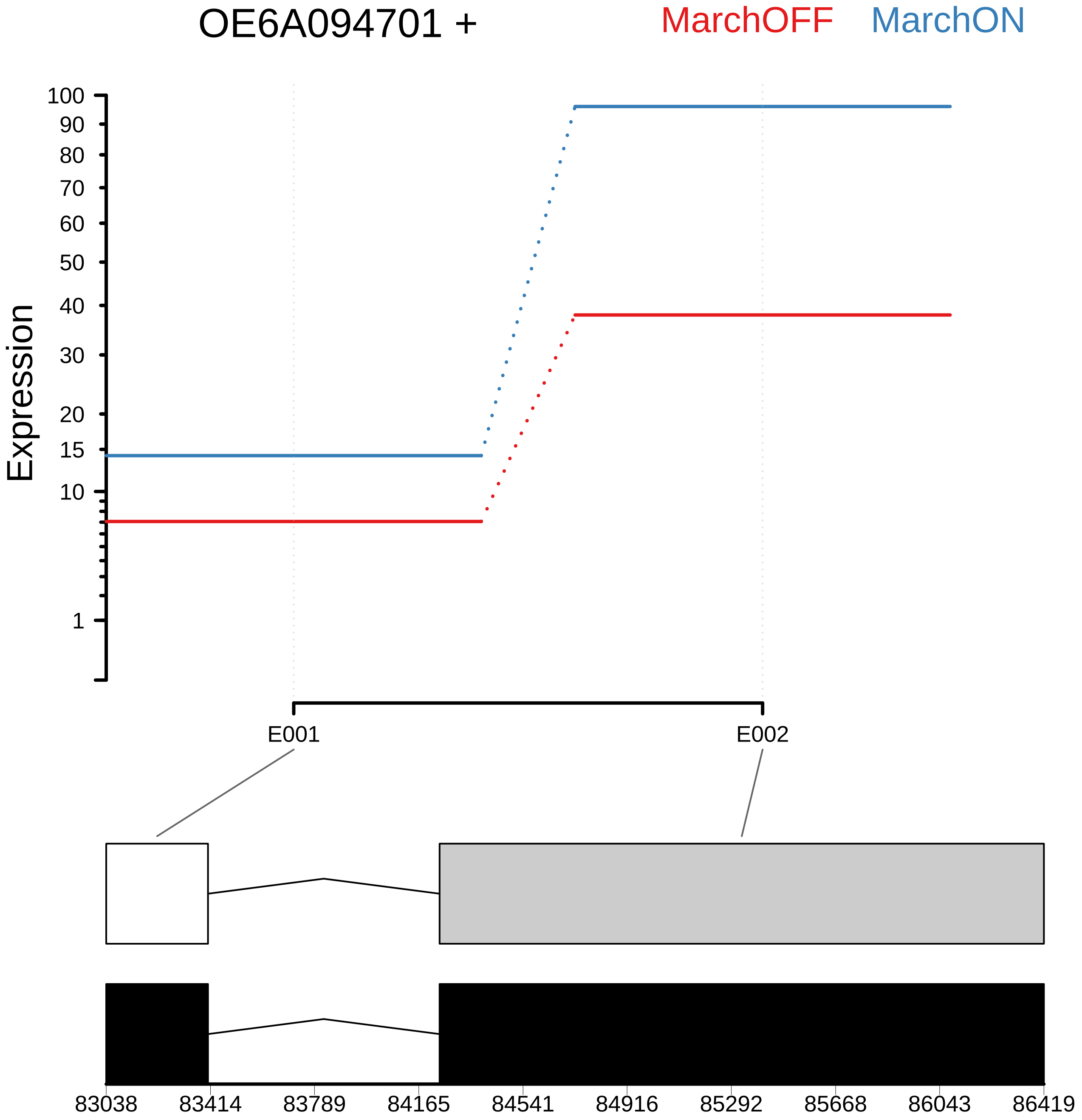

OE6A094701 +
MarchOFF
MarchON
Expression
100
90
80
70
60
50
40
30
20
15
10
1
E001
E002
83038
83414
83789
84165
84541
84916
85292
85668
86043
86419

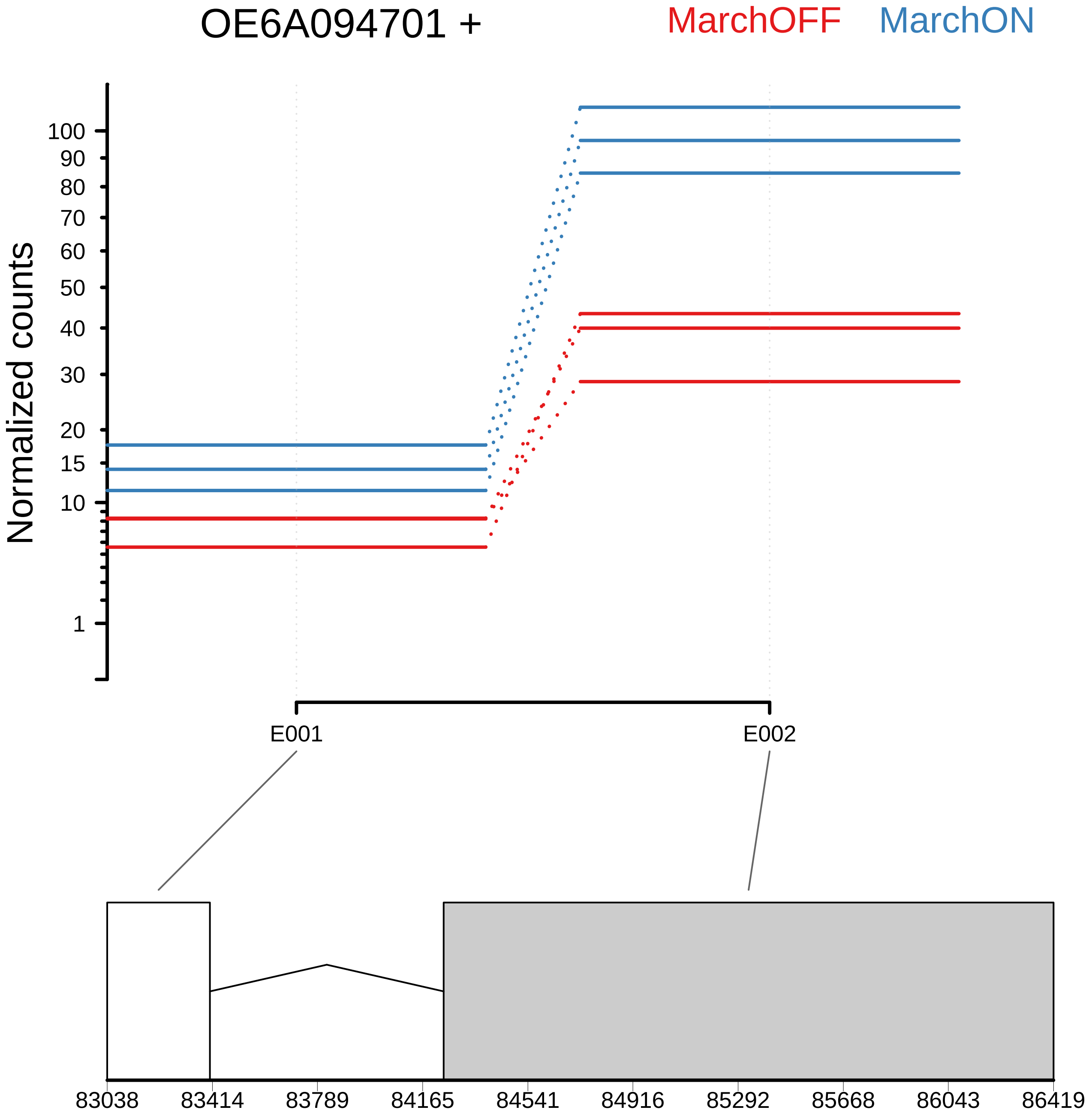

OE6A094701 +
MarchOFF
MarchON
Normalized counts
100
90
80
70
60
50
40
30
20
15
10
1
E001
E002
83038
83414
83789
84165
84541
84916
85292
85668
86043
86419

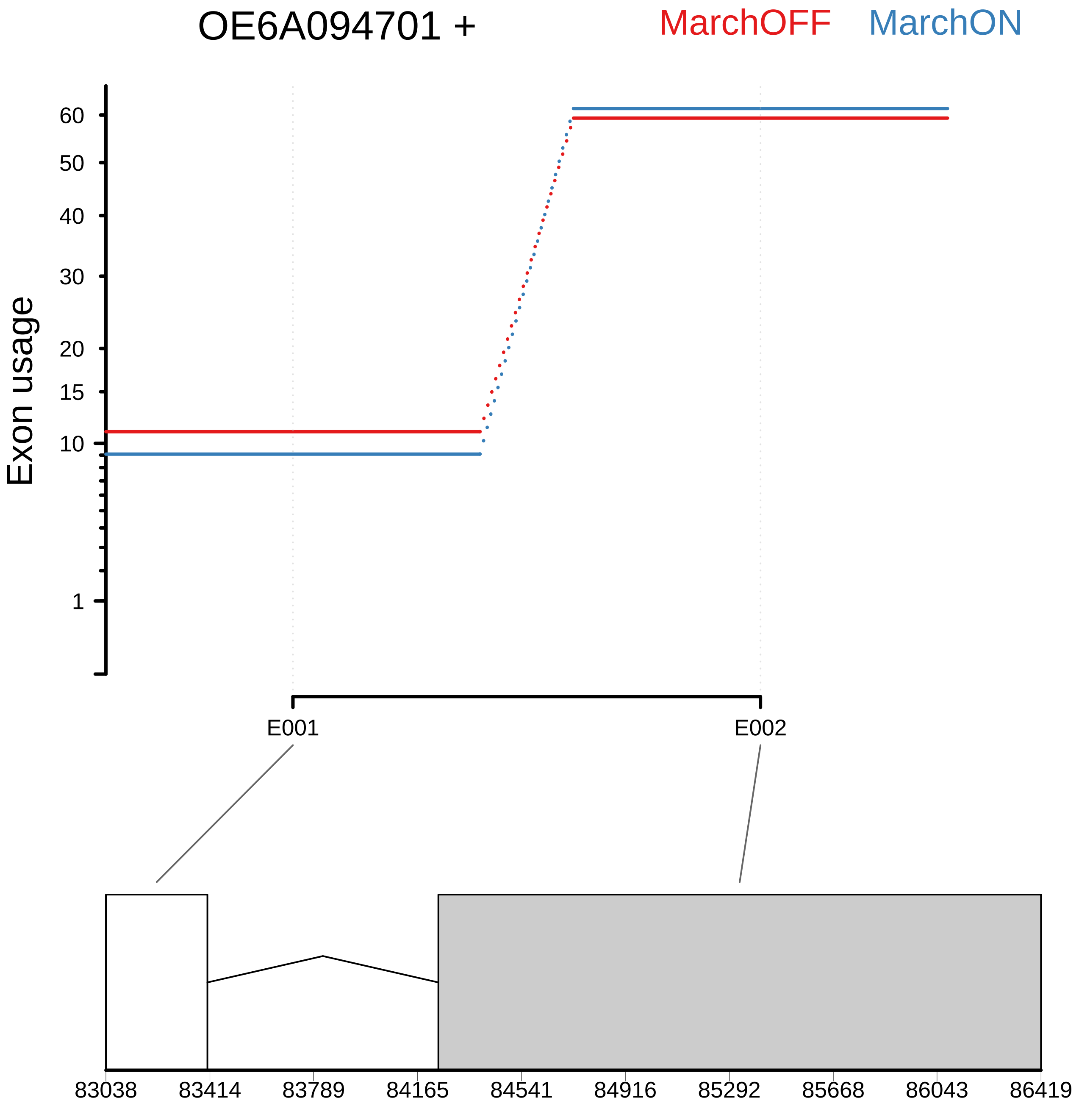

OE6A094701 +
MarchOFF
MarchON
Exon usage
60
50
40
30
20
15
10
1
E001
E002
83038
83414
83789
84165
84541
84916
85292
85668
86043
86419

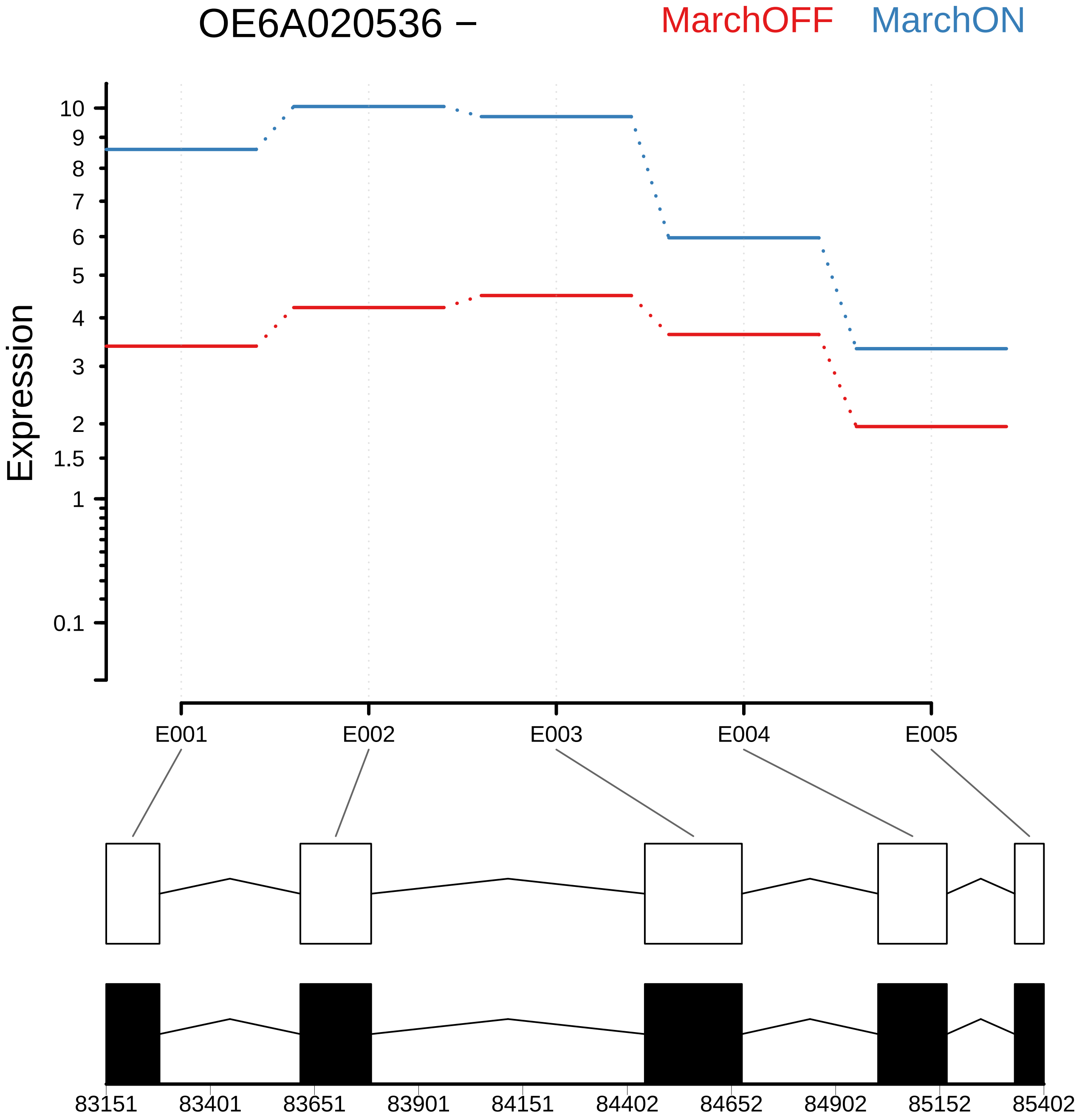

OE6A020536 −
MarchOFF
MarchON
Expression
10
9
8
7
6
5
4
3
2
1.5
1
0.1
E001
E002
E003
E004
E005
83151
83401
83651
83901
84151
84402
84652
84902
85152
85402

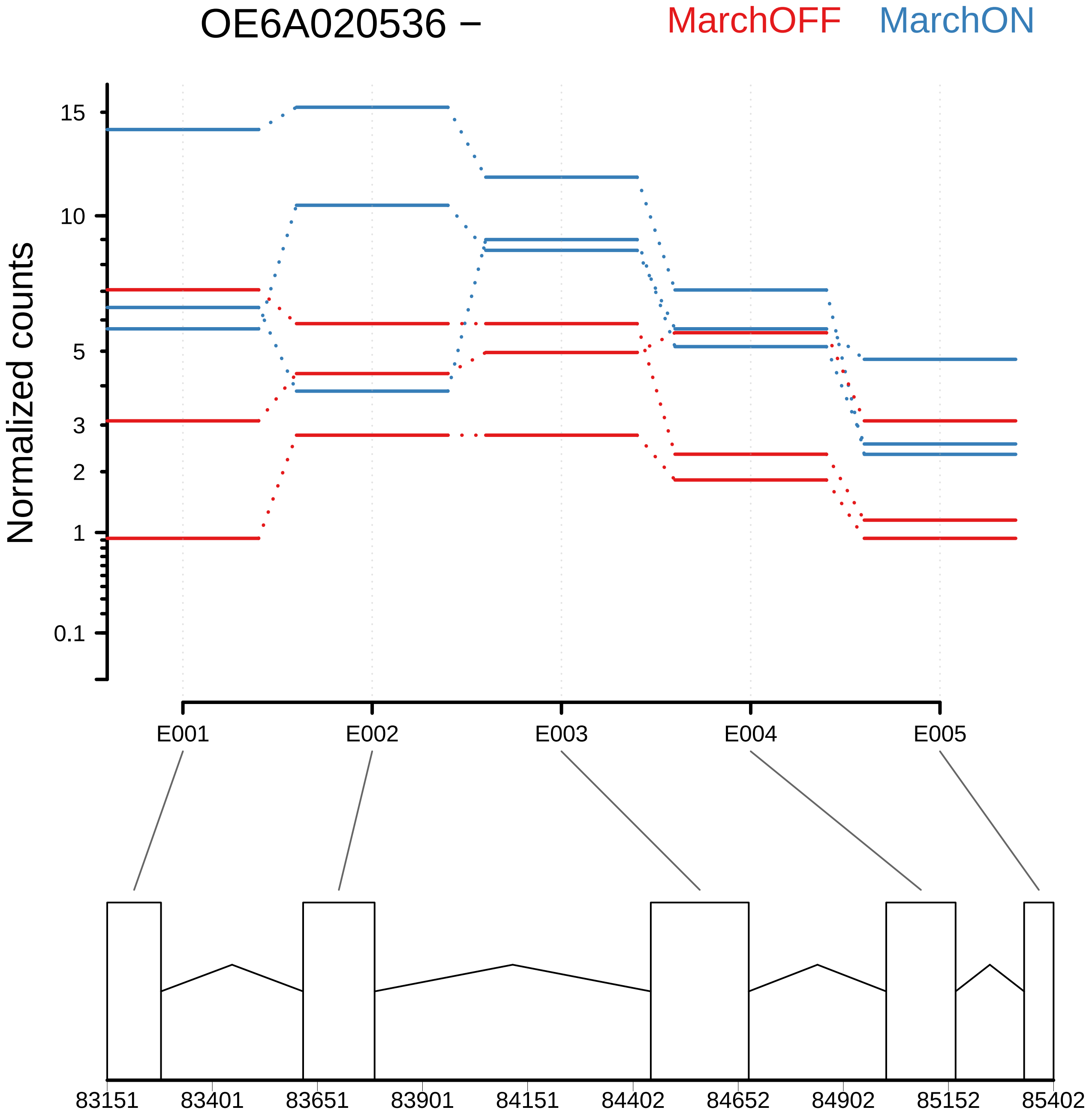
OE6A020536 −
MarchOFF
MarchON
Normalized counts
15
10
5
3
2
1
0.1
E001
E002
E003
E004
E005
83151
83401
83651
83901
84151
84402
84652
84902
85152
85402

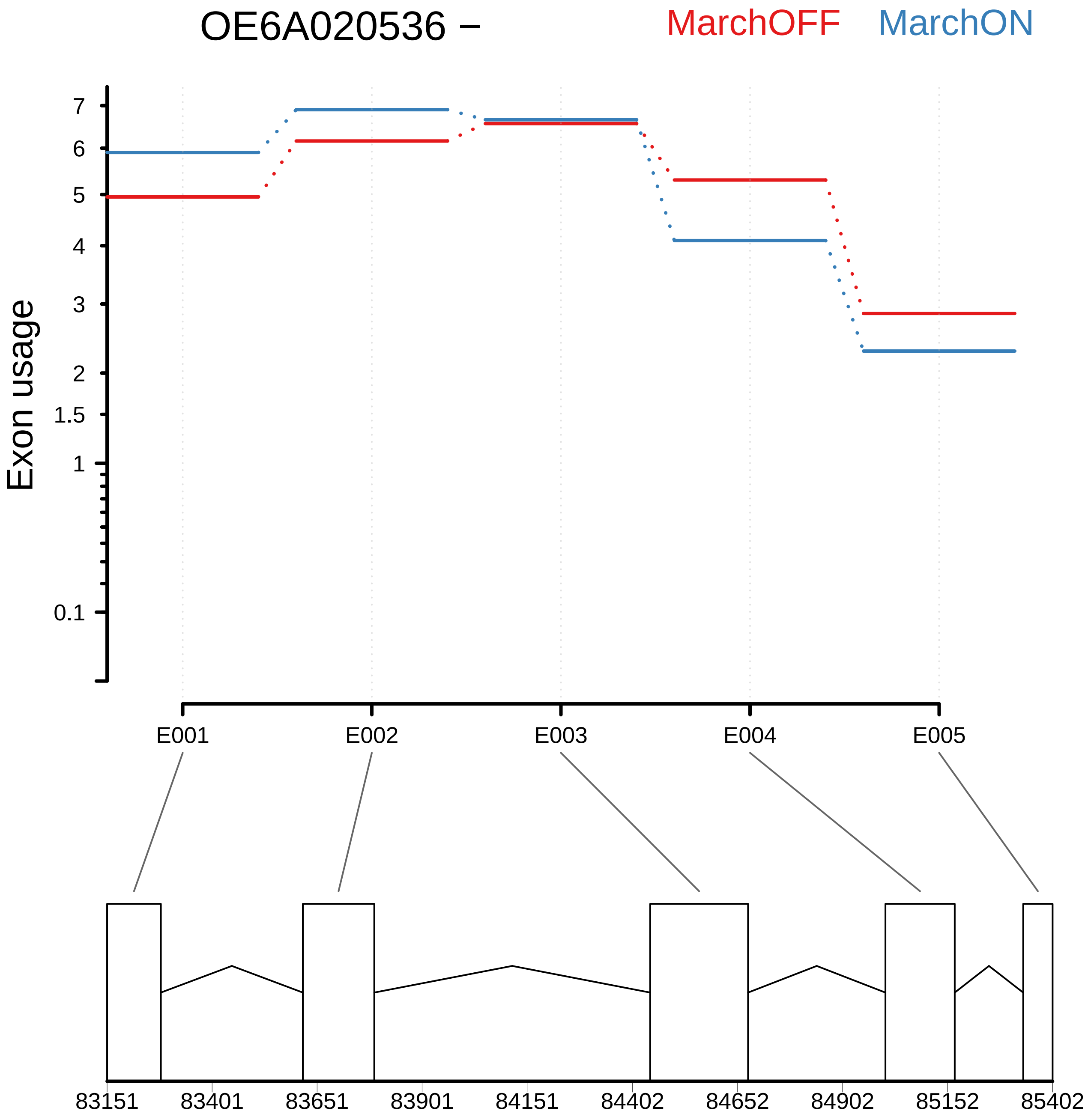

OE6A020536 −
MarchOFF
MarchON
Exon usage
7
6
5
4
3
2
1.5
1
0.1
E001
E002
E003
E004
E005
83151
83401
83651
83901
84151
84402
84652
84902
85152
85402

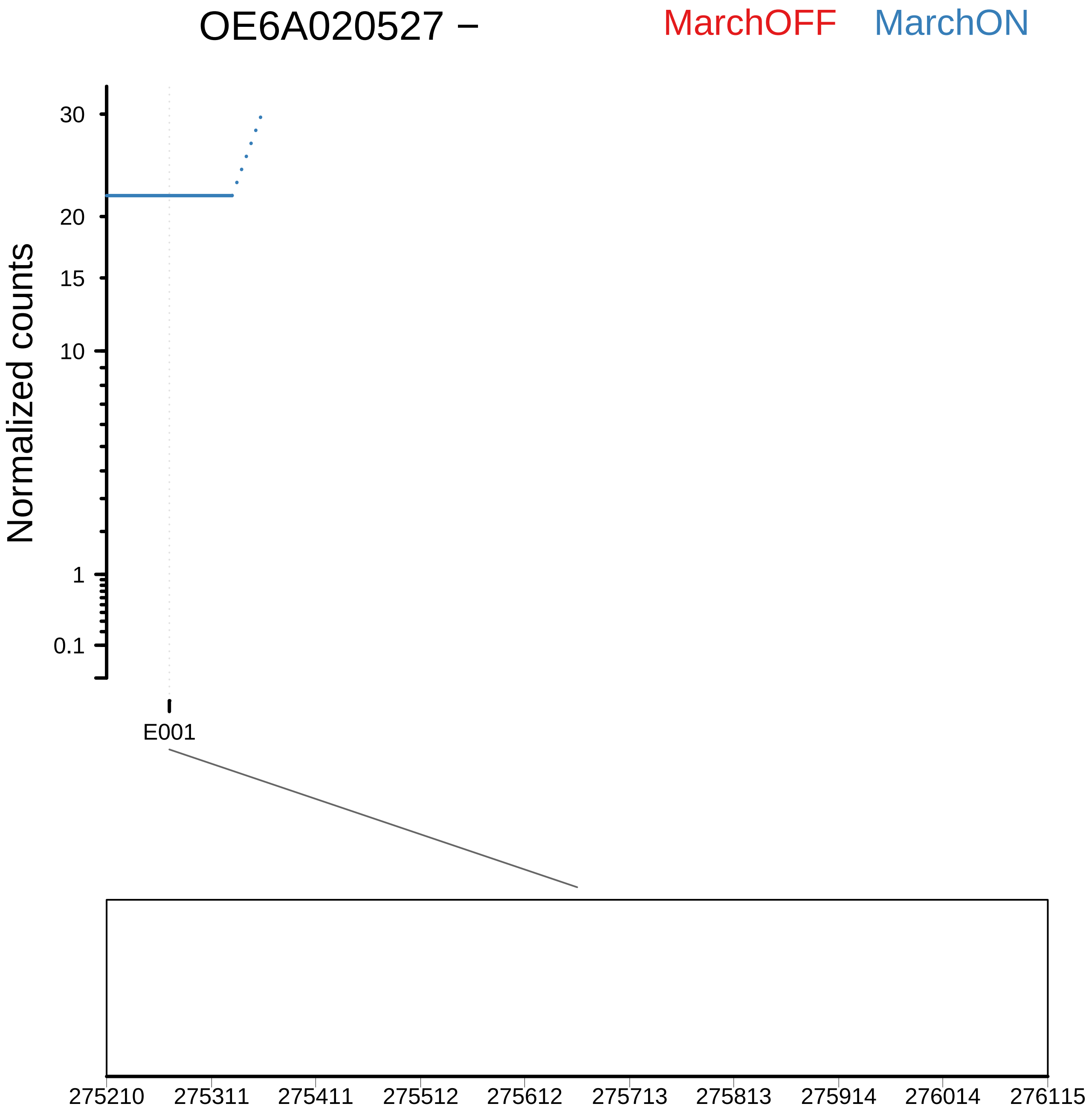
OE6A020527 −
MarchOFF
MarchON
Normalized counts
30
20
15
10
1
0.1
E001
275210
275311
275411
275512
275612
275713
275813
275914
276014
276115

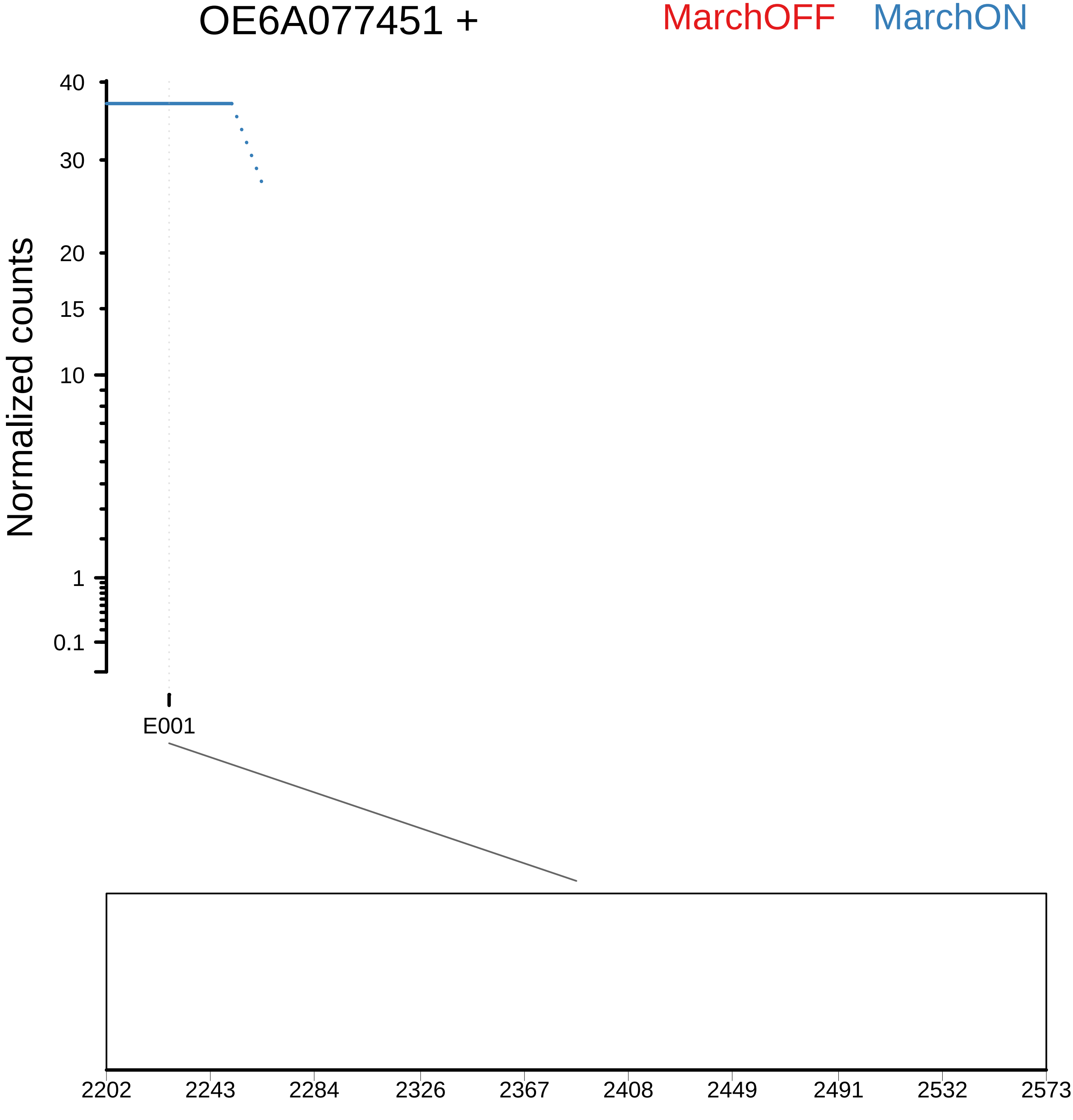

OE6A077451 +
MarchOFF
MarchON
Normalized counts
40
30
20
15
10
1
0.1
E001
2202
2243
2284
2326
2367
2408
2449
2491
2532
2573

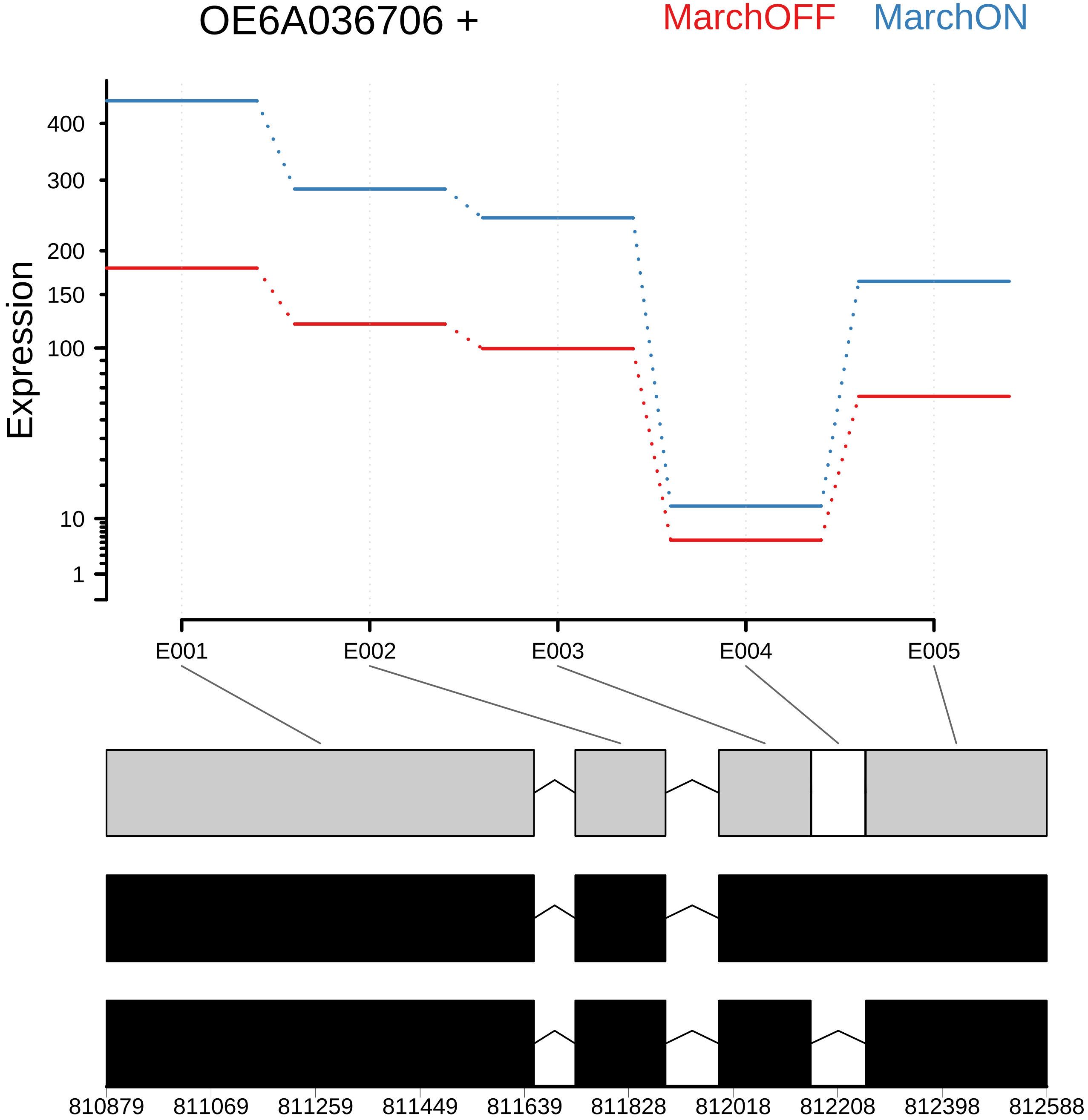

OE6A036706 +
MarchOFF
MarchON
Expression
400
300
200
150
100
10
1
E001
E002
E003
E004
E005
810879
811069
811259
811449
811639
811828
812018
812208
812398
812588

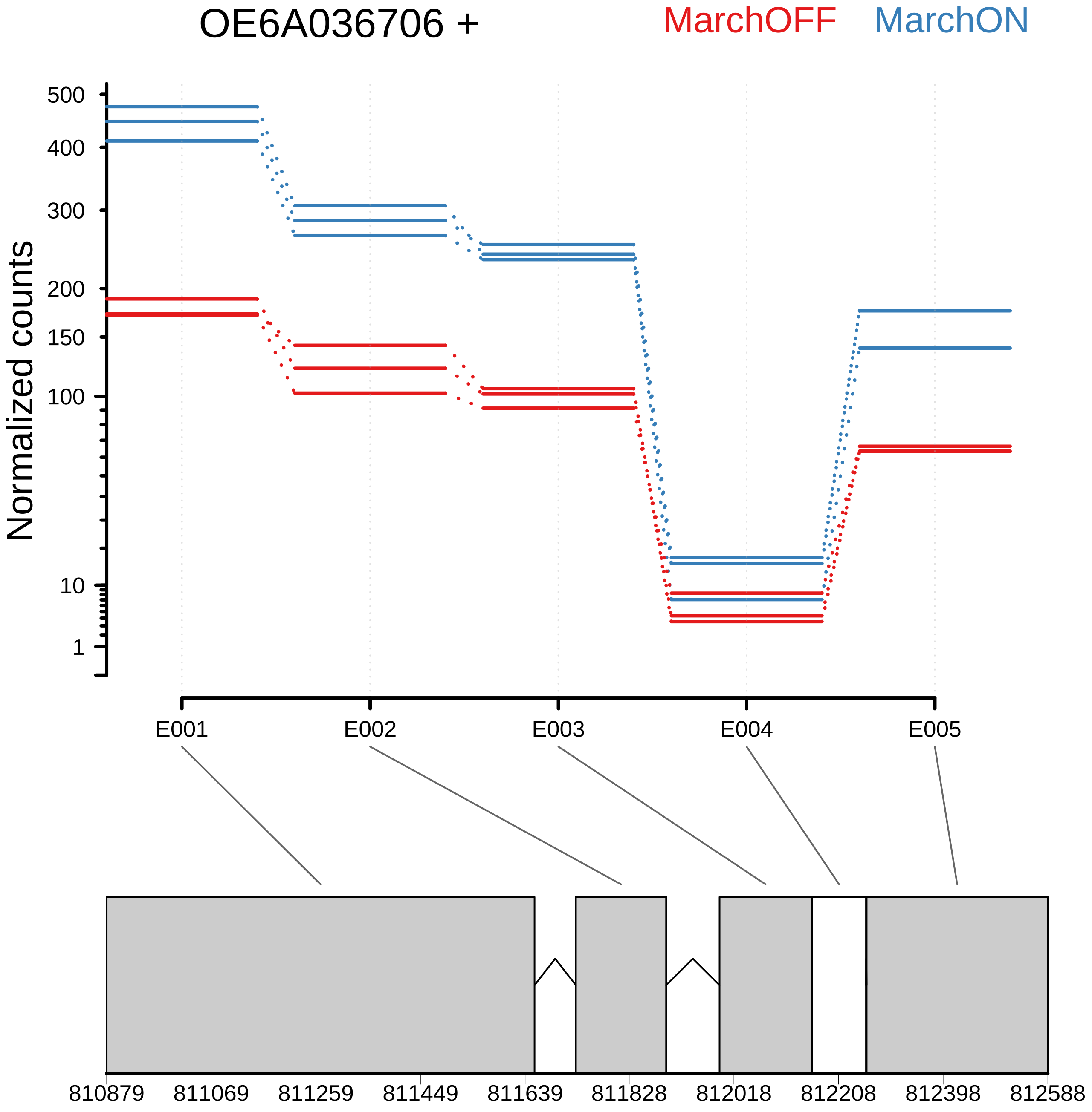
OE6A036706 +
MarchOFF
MarchON
Normalized counts
500
400
300
200
150
100
10
1
E001
E002
E003
E004
E005
810879
811069
811259
811449
811639
811828
812018
812208
812398
812588

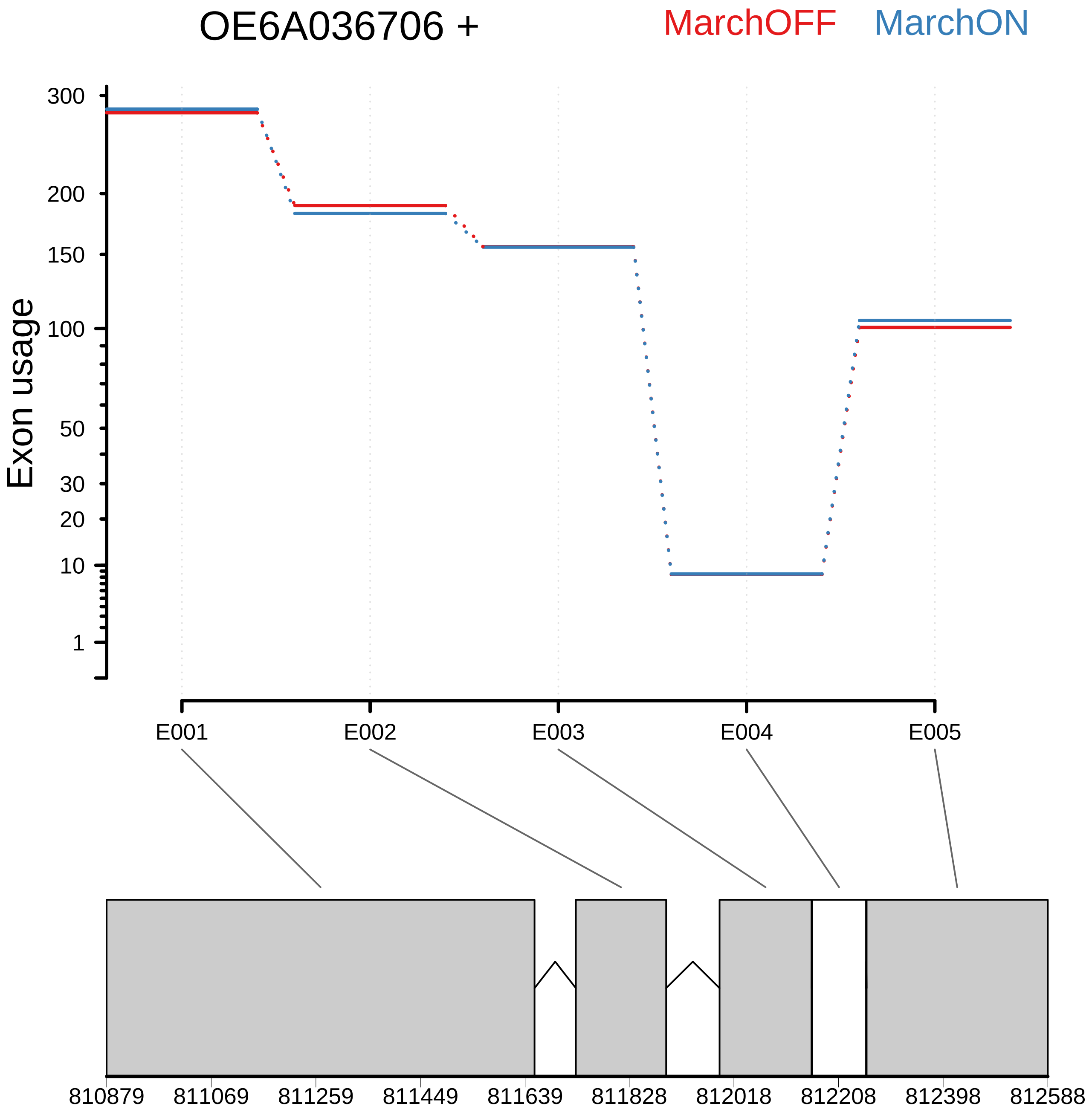

OE6A036706 +
MarchOFF
MarchON
Exon usage
300
200
150
100
50
30
20
10
1
E001
E002
E003
E004
E005
810879
811069
811259
811449
811639
811828
812018
812208
812398
812588

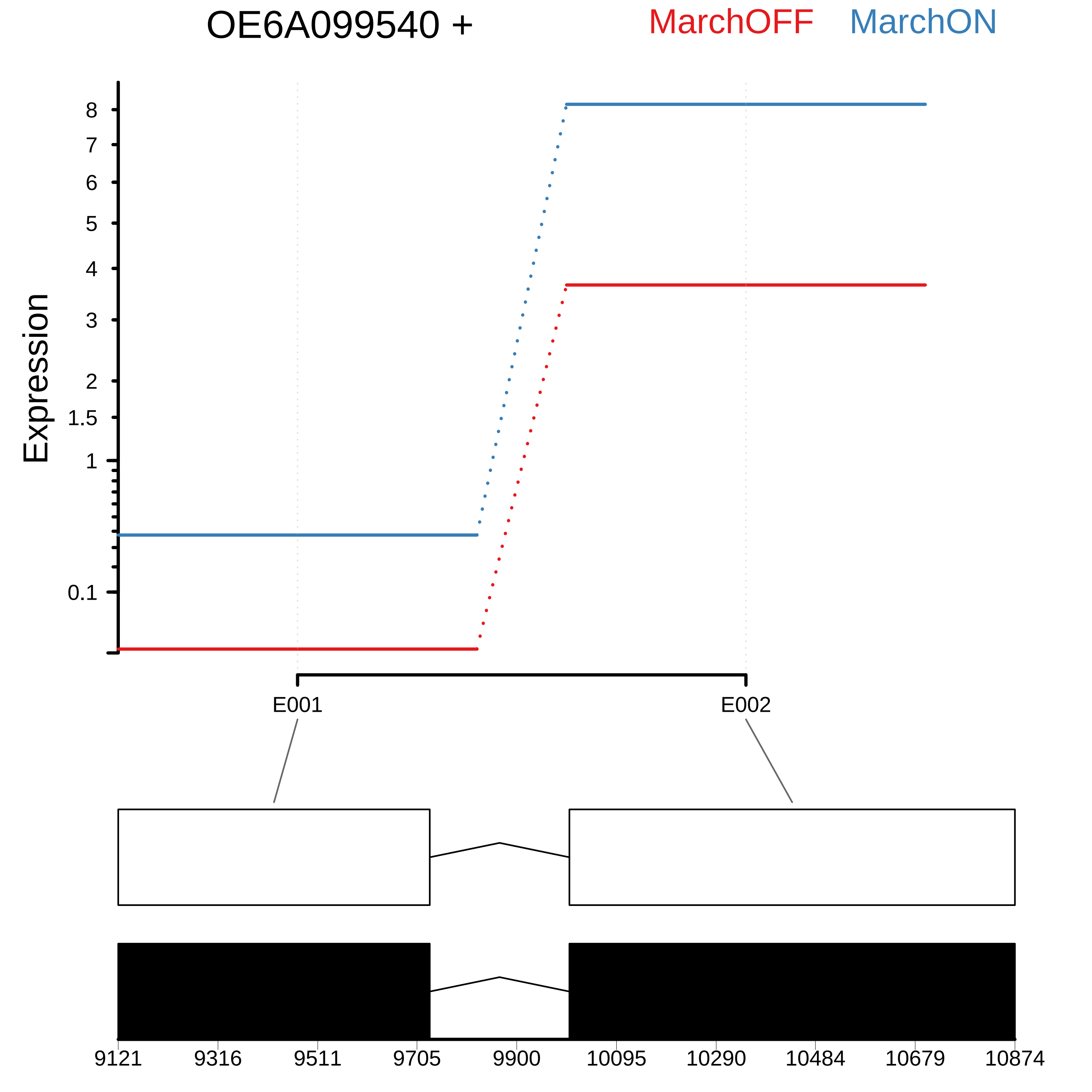

OE6A099540 +
MarchOFF
MarchON
Expression
8
7
6
5
4
3
2
1.5
1
0.1
E001
E002
9121
9316
9511
9705
9900
10095
10290
10484
10679
10874

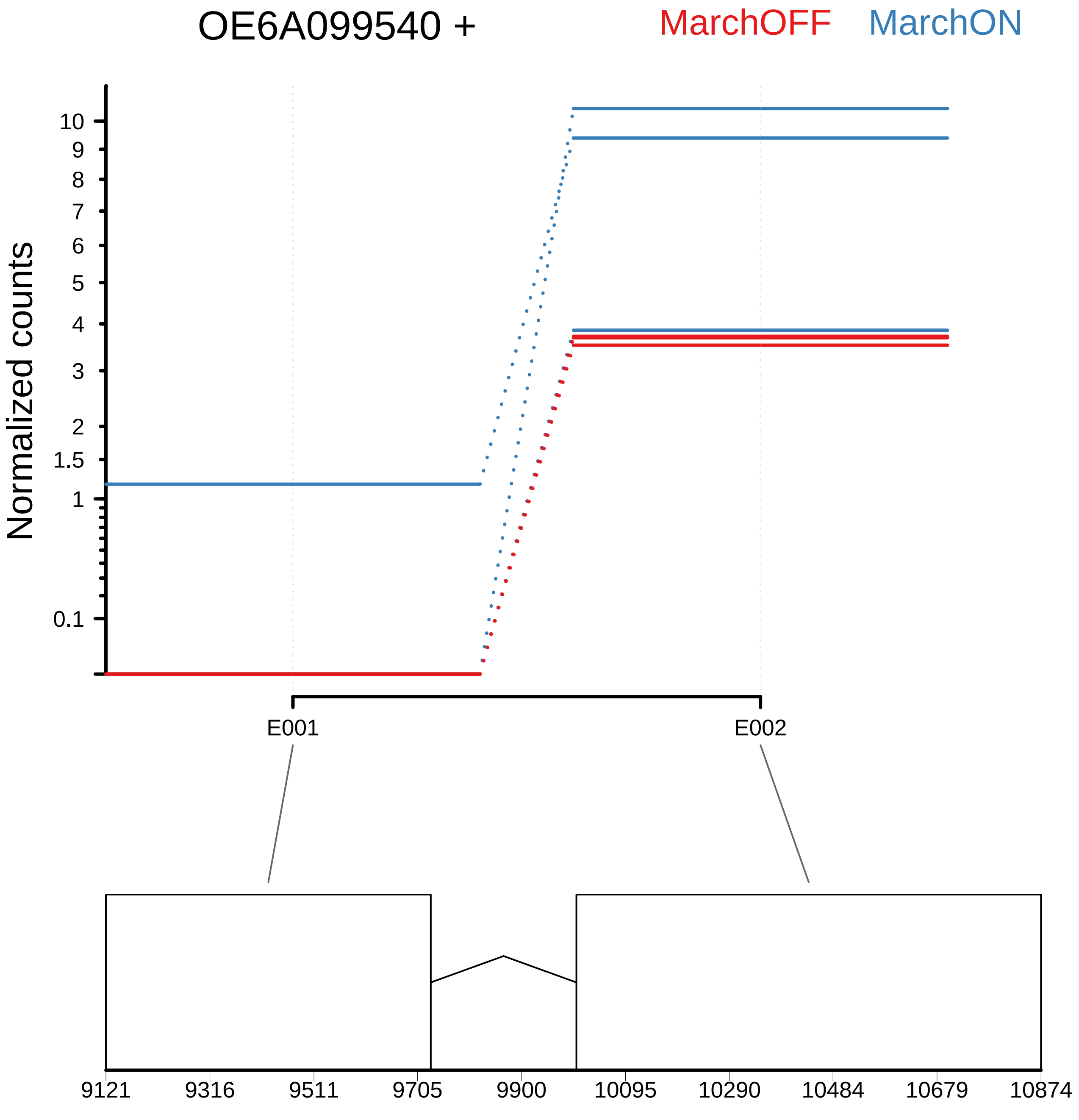
OE6A099540 +
MarchOFF
MarchON
Normalized counts
10
9
8
7
6
5
4
3
2
1.5
1
0.1
E001
E002
9121
9316
9511
9705
9900
10095
10290
10484
10679
10874

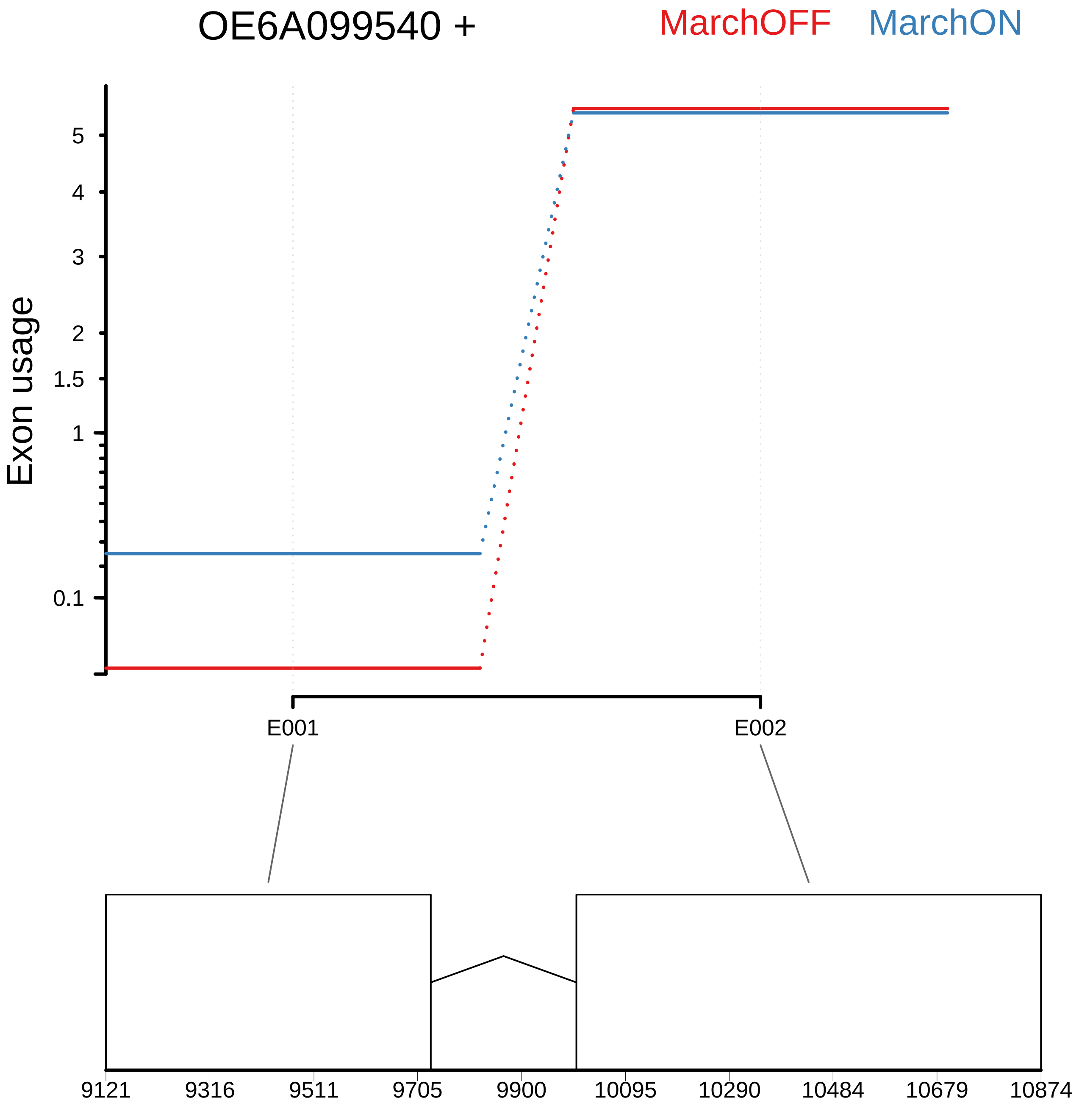
OE6A099540 +
MarchOFF
MarchON
Exon usage
5
4
3
2
1.5
1
0.1
E001
E002
9121
9316
9511
9705
9900
10095
10290
10484
10679
10874

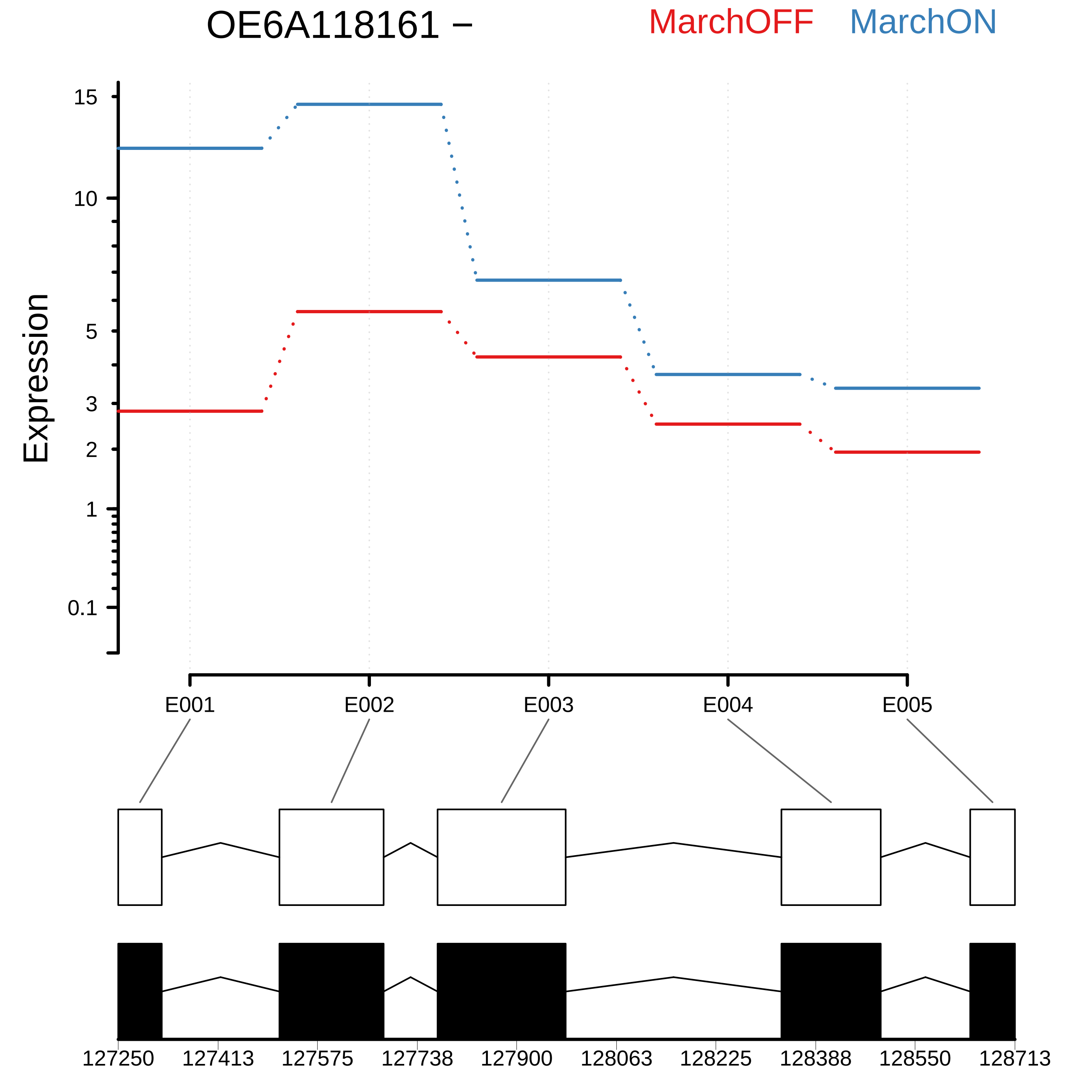

OE6A118161 −
MarchOFF
MarchON
Expression
15
10
5
3
2
1
0.1
E001
E002
E003
E004
E005
127250
127413
127575
127738
127900
128063
128225
128388
128550
128713

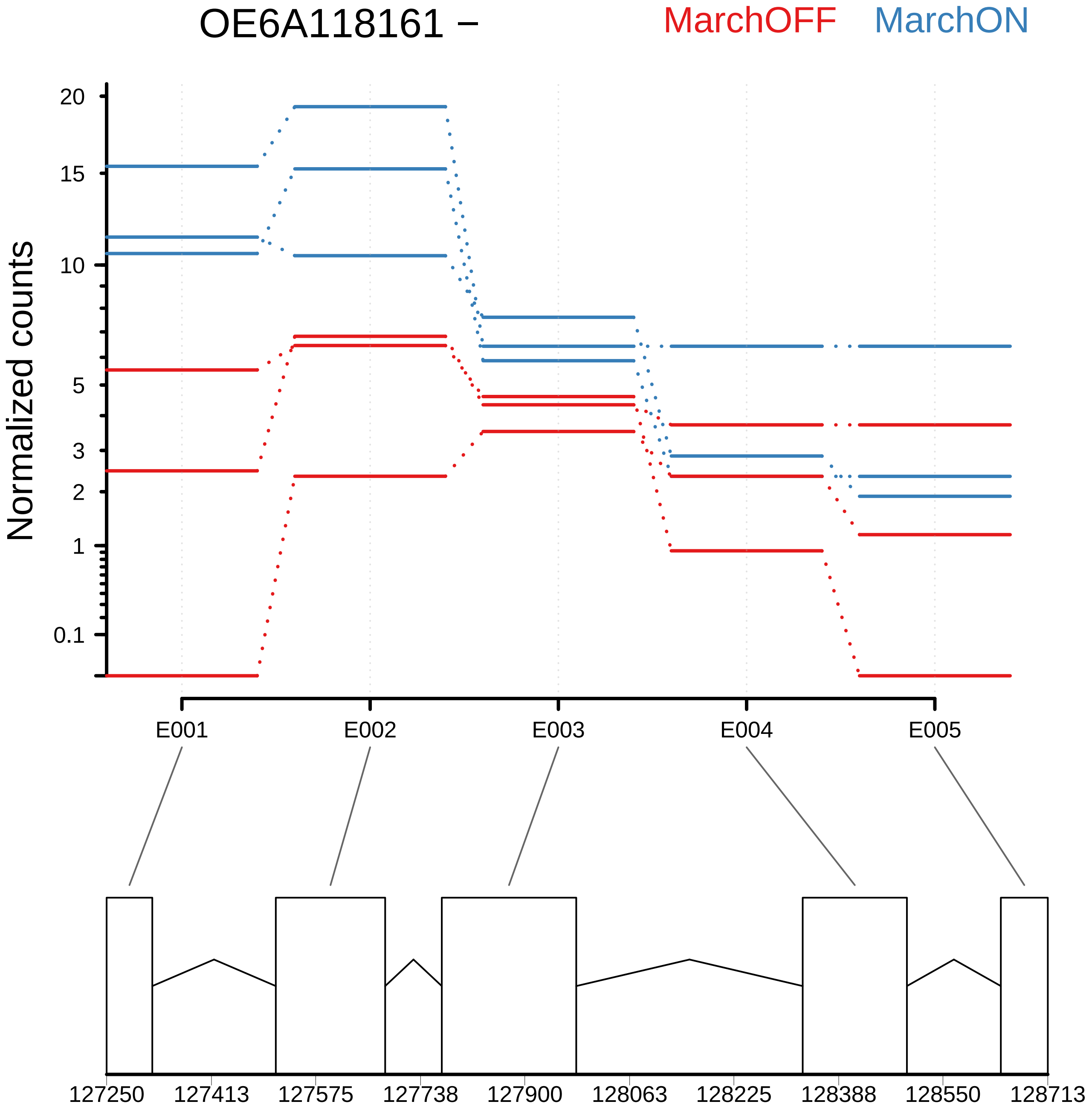
OE6A118161 −
MarchOFF
MarchON
Normalized counts
20
15
10
5
3
2
1
0.1
E001
E002
E003
E004
E005
127250
127413
127575
127738
127900
128063
128225
128388
128550
128713

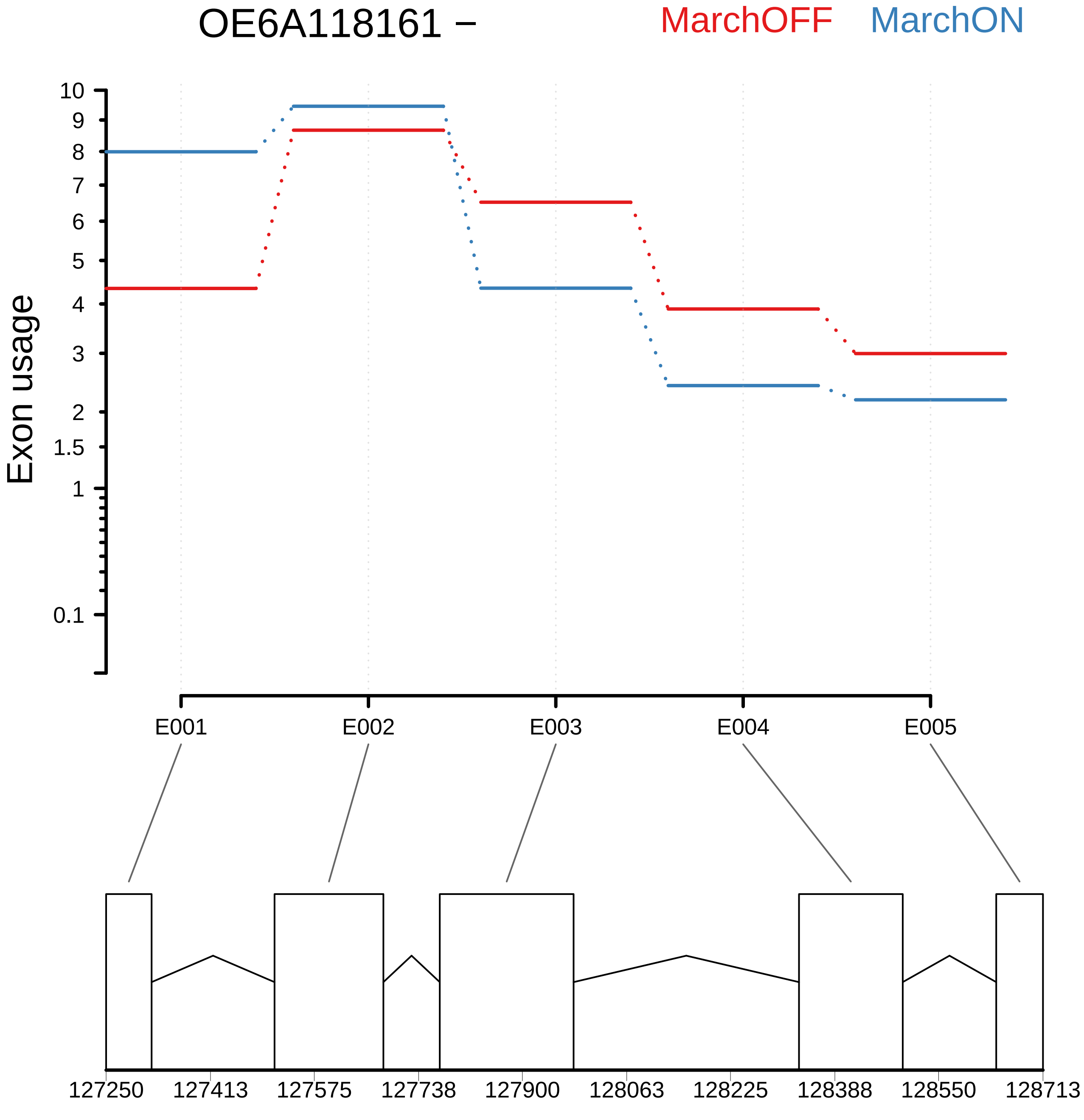
OE6A118161 −
MarchOFF
MarchON
Exon usage
10
9
8
7
6
5
4
3
2
1.5
1
0.1
E001
E002
E003
E004
E005
127250
127413
127575
127738
127900
128063
128225
128388
128550
128713

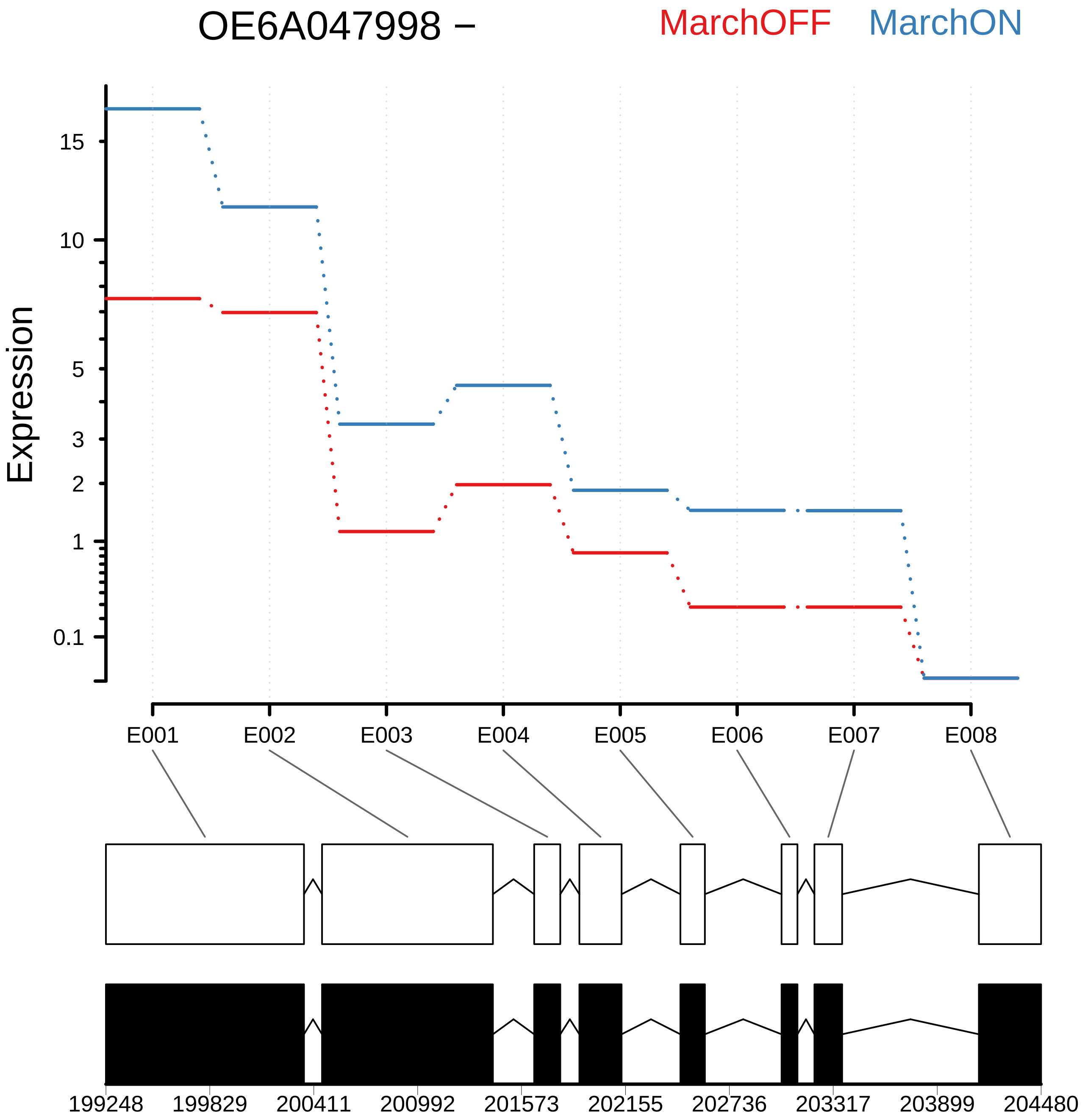

OE6A047998 −
MarchOFF
MarchON
Expression
15
10
5
3
2
1
0.1
E001
E002
E003
E004
E005
E006
E007
E008
199248
199829
200411
200992
201573
202155
202736
203317
203899
204480

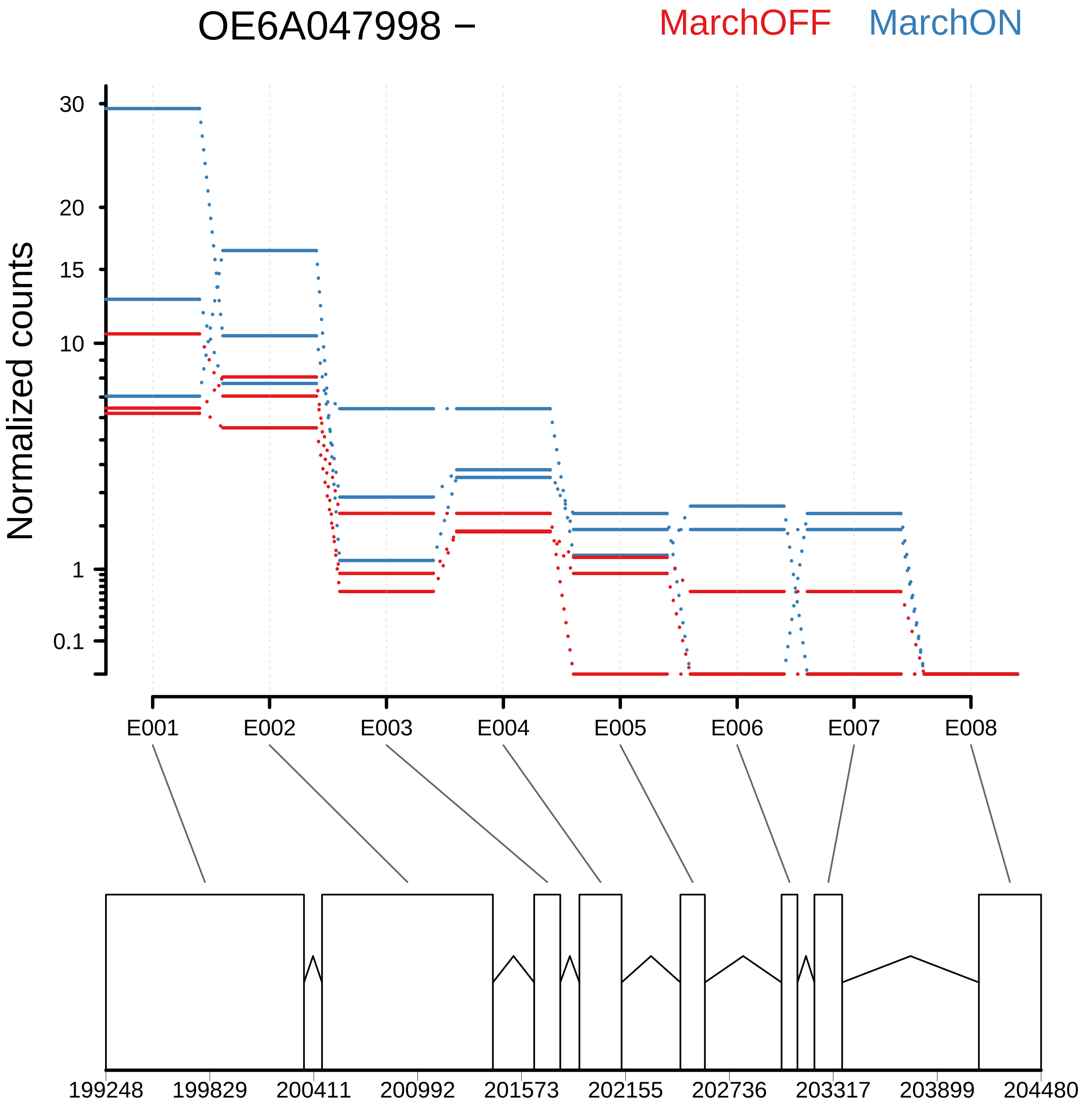

OE6A047998 –
MarchOFF
MarchON
Normalized counts
30
20
15
10
1
0.1
E001
E002
E003
E004
E005
E006
E007
E008
199248
199829
200411
200992
201573
202155
202736
203317
203899
204480

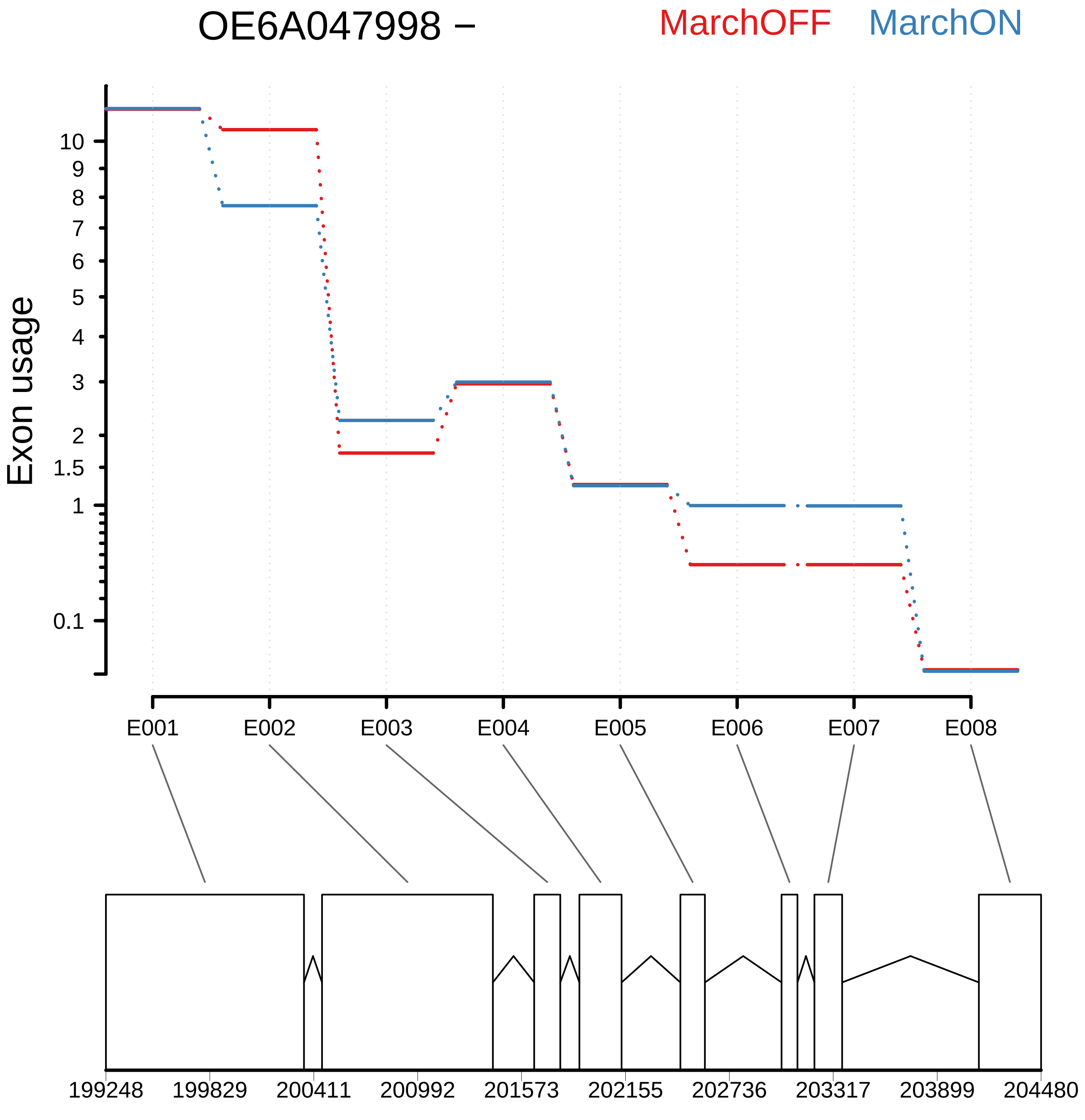

OE6A047998 −
MarchOFF
MarchON
Exon usage
10
9
8
7
6
5
4
3
2
1.5
1
0.1
E001
E002
E003
E004
E005
E006
E007
E008
199248
199829
200411
200992
201573
202155
202736
203317
203899
204480

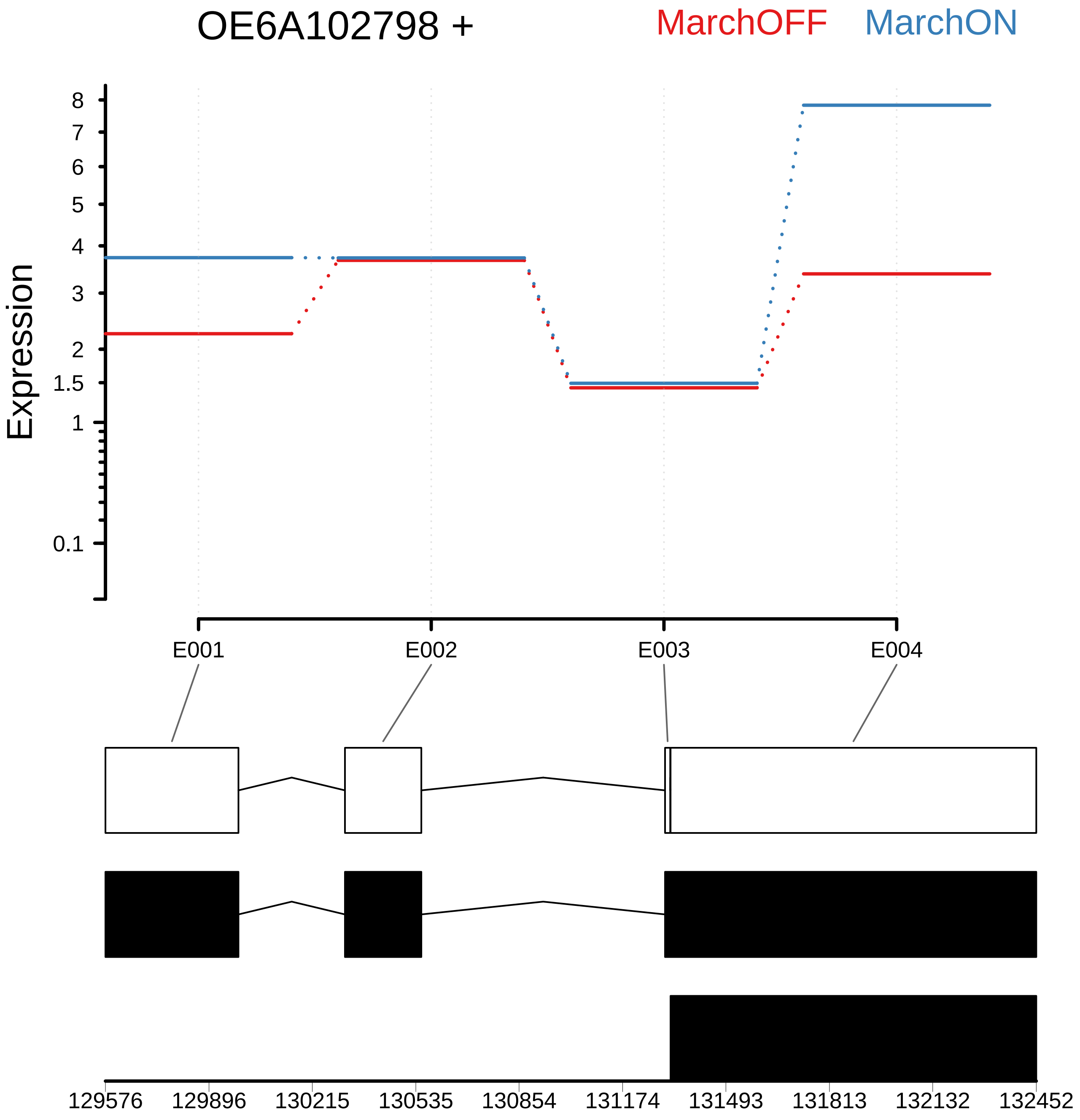
OE6A102798 +
MarchOFF
MarchON
Expression
8
7
6
5
4
3
2
1.5
1
0.1
E001
E002
E003
E004
129576
129896
130215
130535
130854
131174
131493
131813
132132
132452

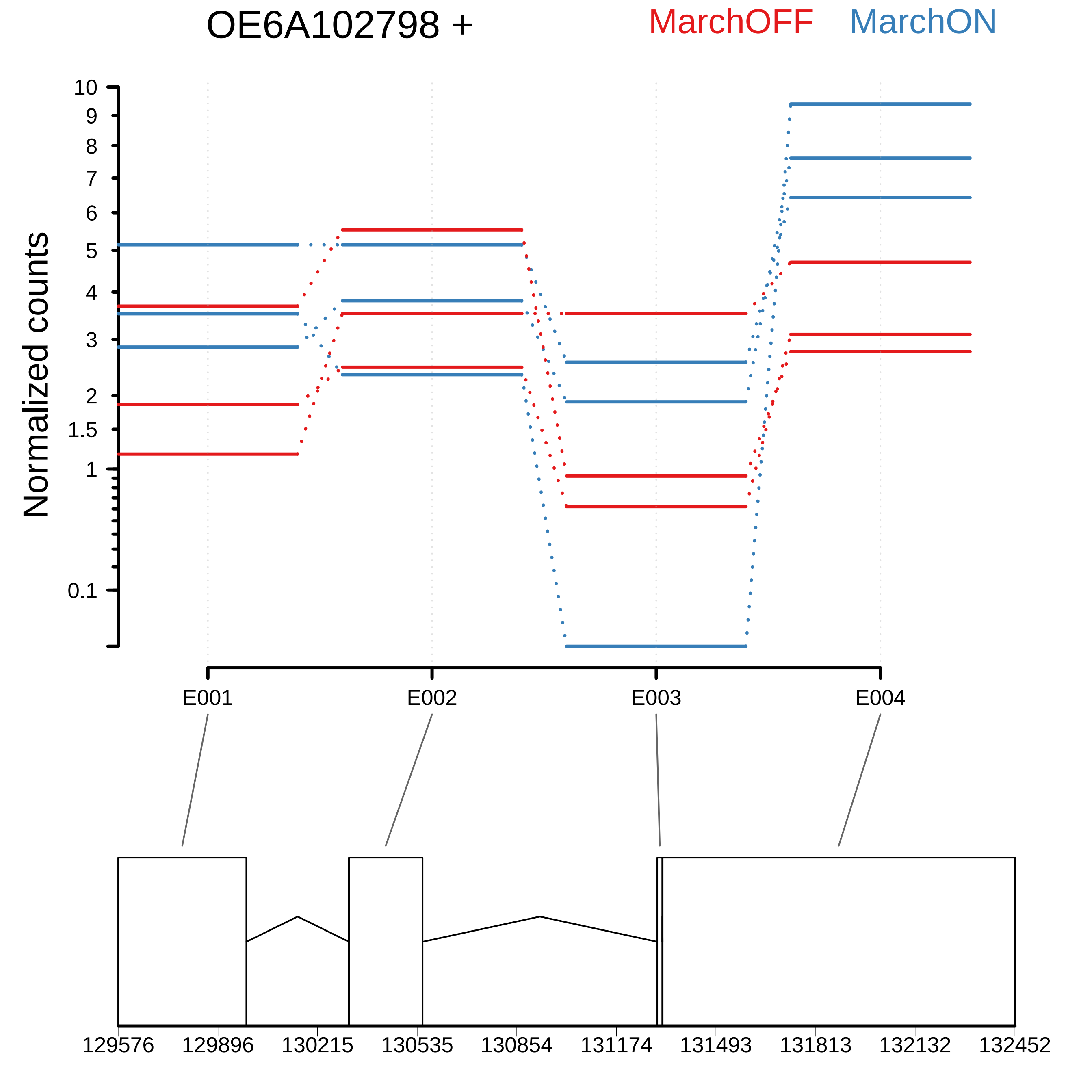

OE6A102798 +
MarchOFF
MarchON
Normalized counts
10
9
8
7
6
5
4
3
2
1.5
1
0.1
E001
E002
E003
E004
129576
129896
130215
130535
130854
131174
131493
131813
132132
132452

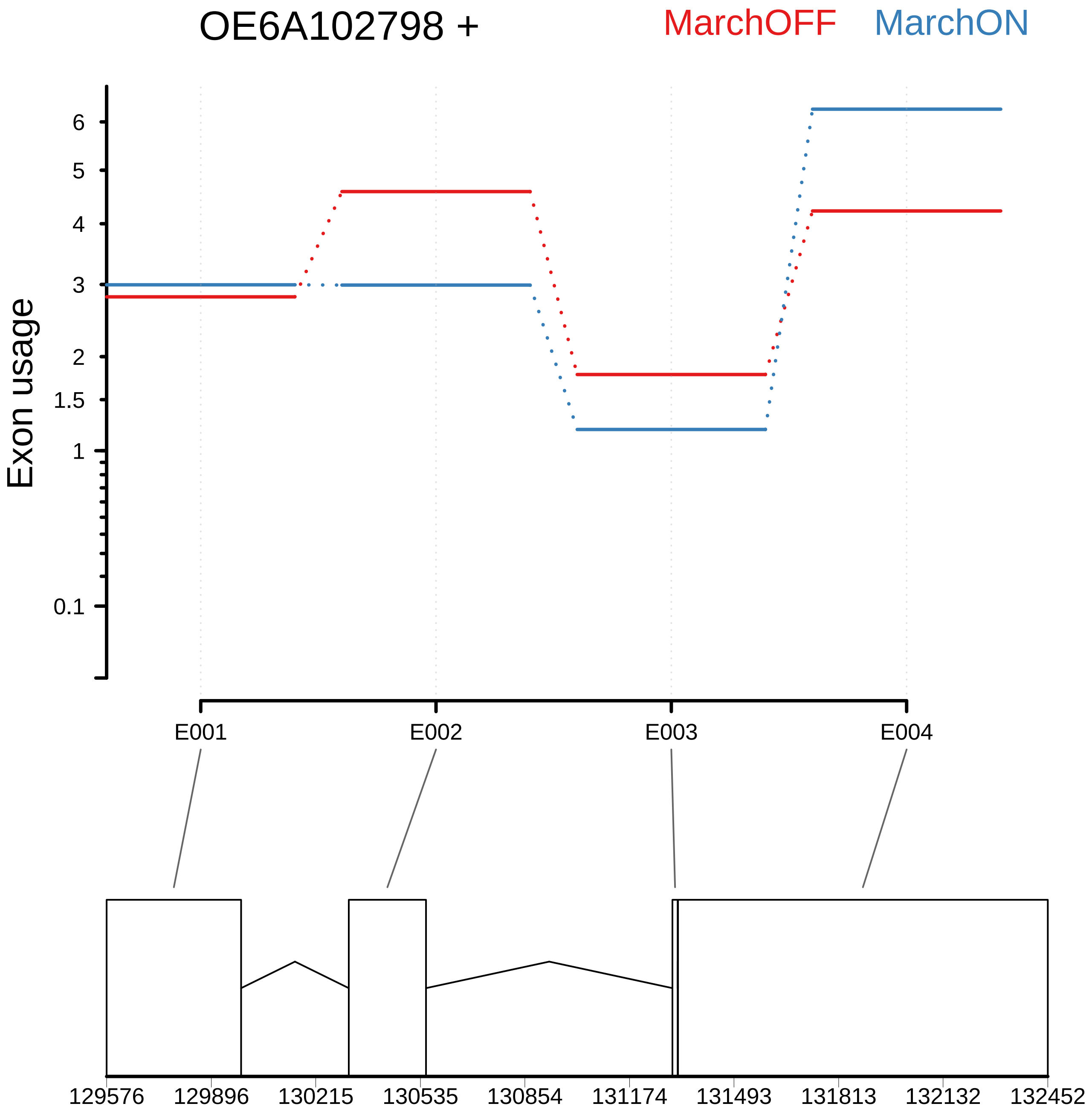

OE6A102798 +
MarchOFF
MarchON
Exon usage
6
5
4
3
2
1.5
1
0.1
E001
E002
E003
E004
129576
129896
130215
130535
130854
131174
131493
131813
132132
132452

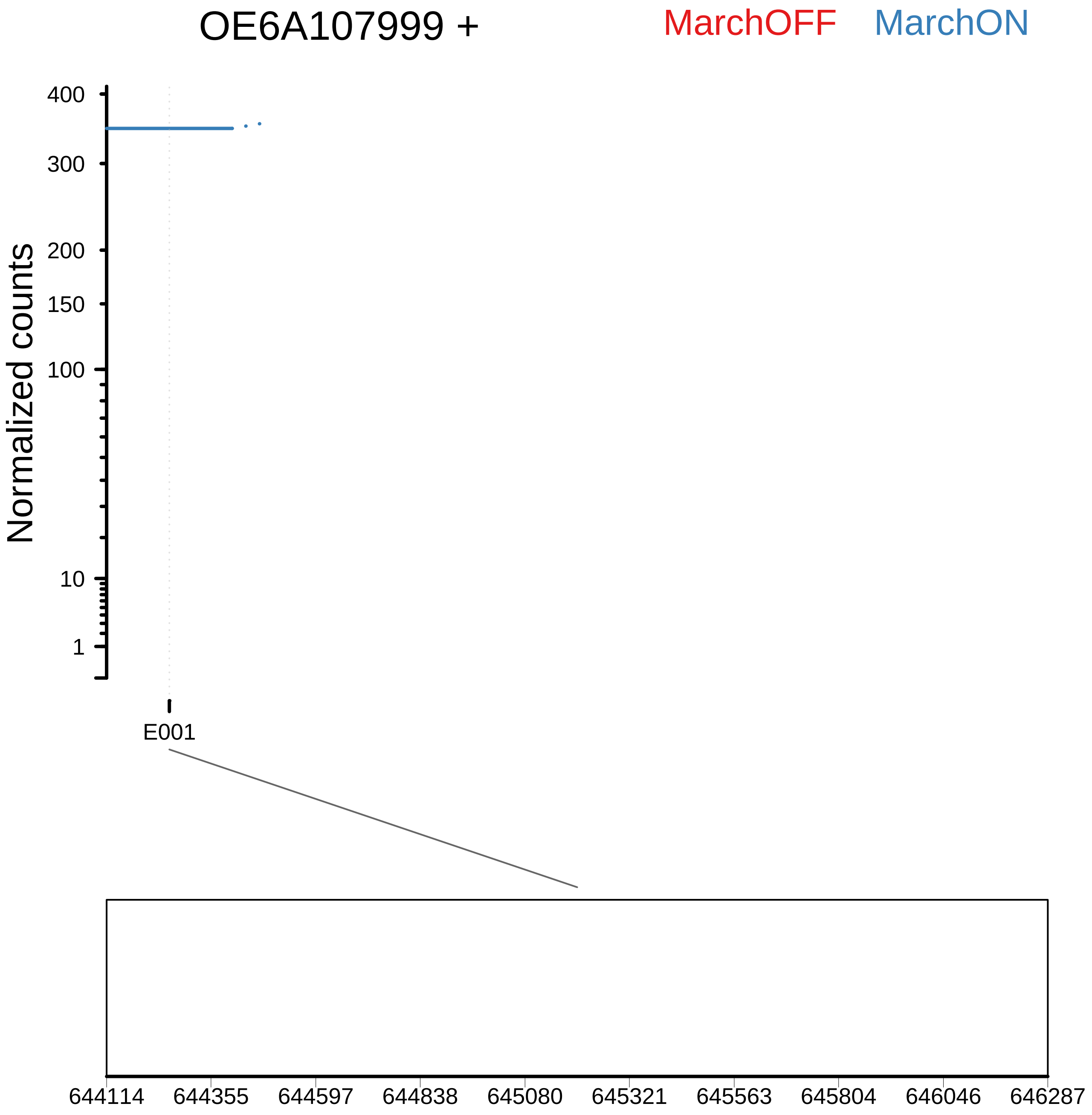

OE6A107999 +
MarchOFF
MarchON
Normalized counts
400
300
200
150
100
10
1
E001
644114
644355
644597
644838
645080
645321
645563
645804
646046
646287

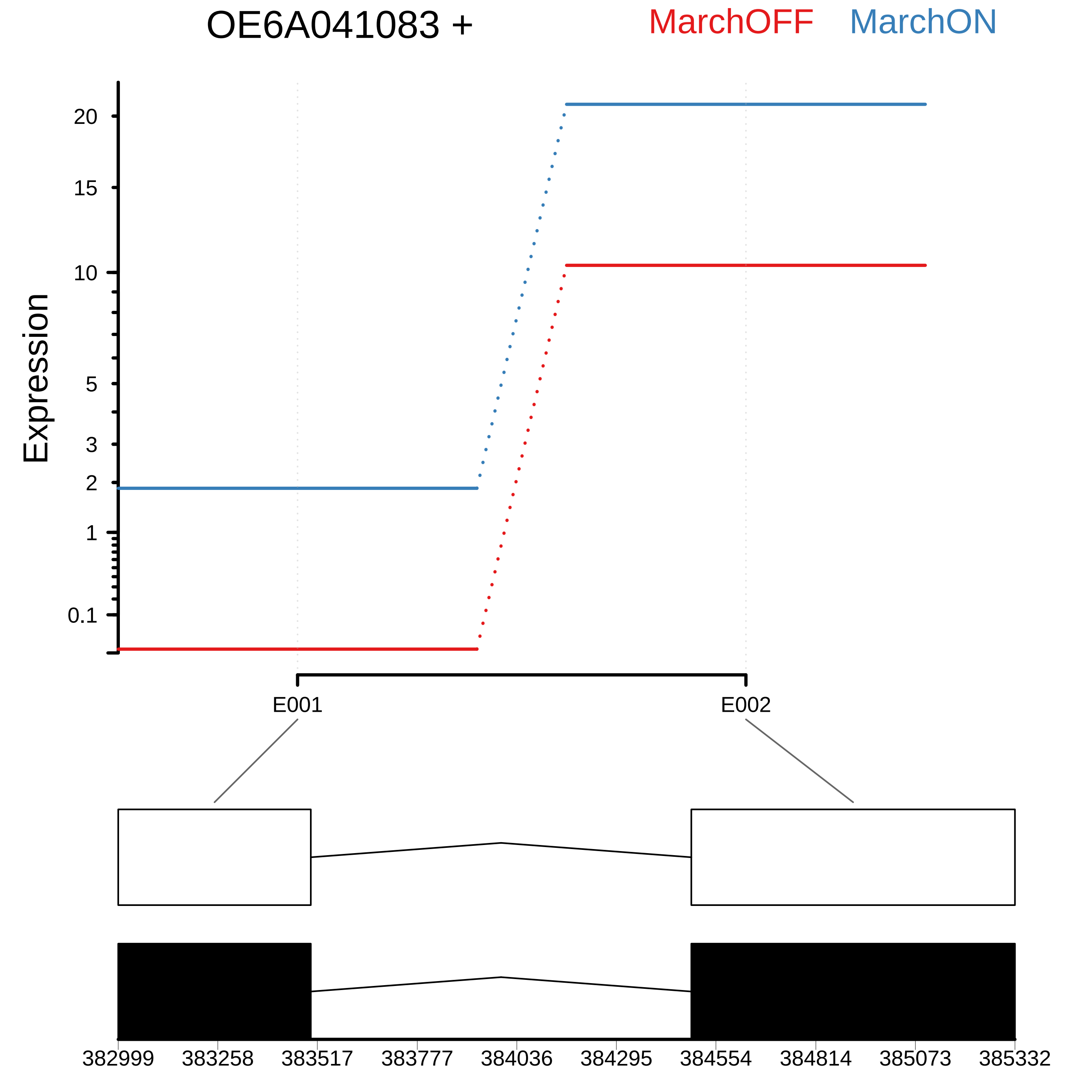
OE6A041083 +
MarchOFF
MarchON
Expression
20
15
10
5
3
2
1
0.1
E001
E002
382999
383258
383517
383777
384036
384295
384554
384814
385073
385332

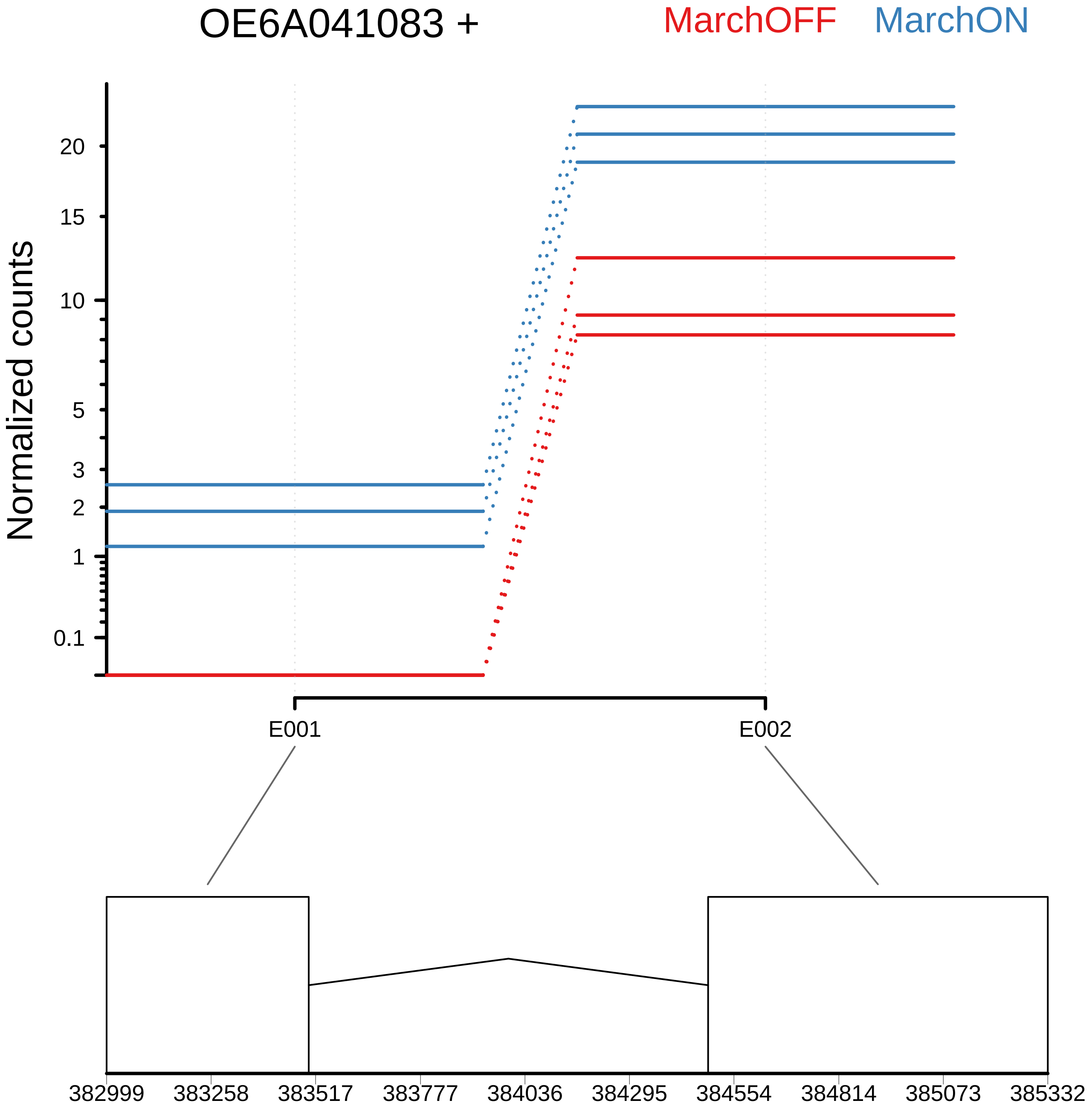
OE6A041083 +
MarchOFF
MarchON
Normalized counts
20
15
10
5
3
2
1
0.1
E001
E002
382999
383258
383517
383777
384036
384295
384554
384814
385073
385332

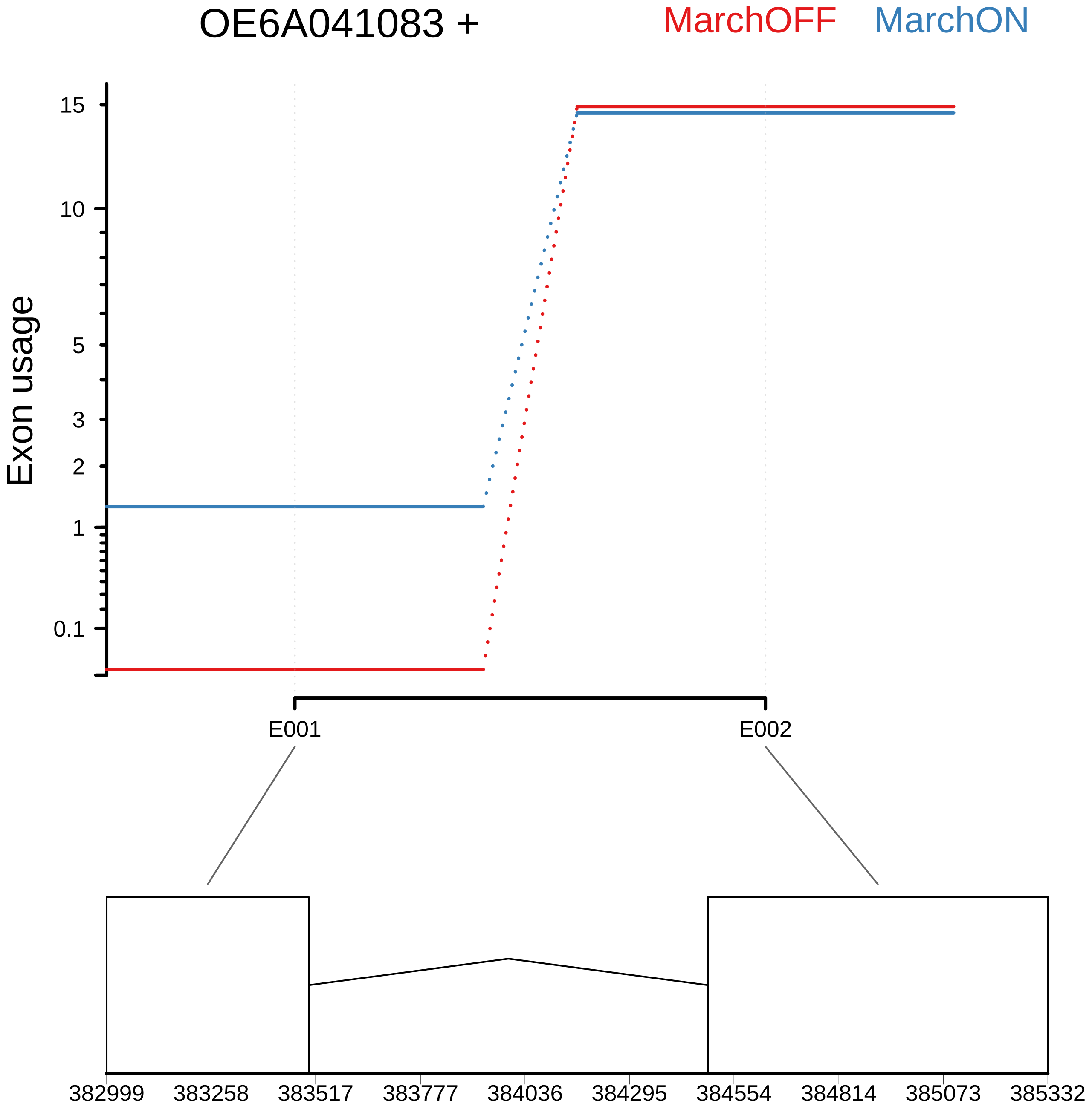

OE6A041083 +
MarchOFF
MarchON
Exon usage
15
10
5
3
2
1
0.1
E001
E002
382999
383258
383517
383777
384036
384295
384554
384814
385073
385332

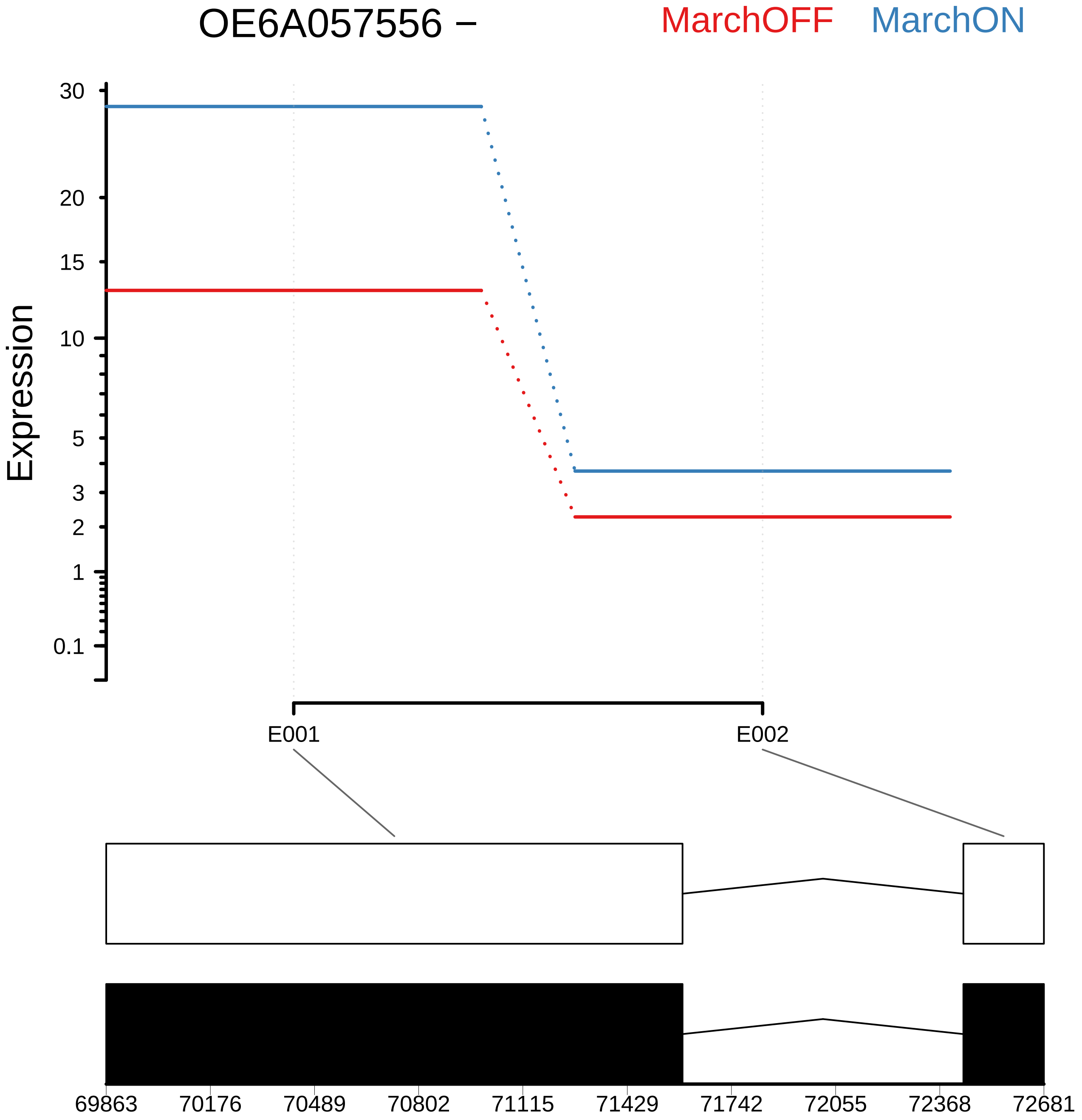

OE6A057556 −
MarchOFF
MarchON
Expression
30
20
15
10
5
3
2
1
0.1
E001
E002
69863
70176
70489
70802
71115
71429
71742
72055
72368
72681

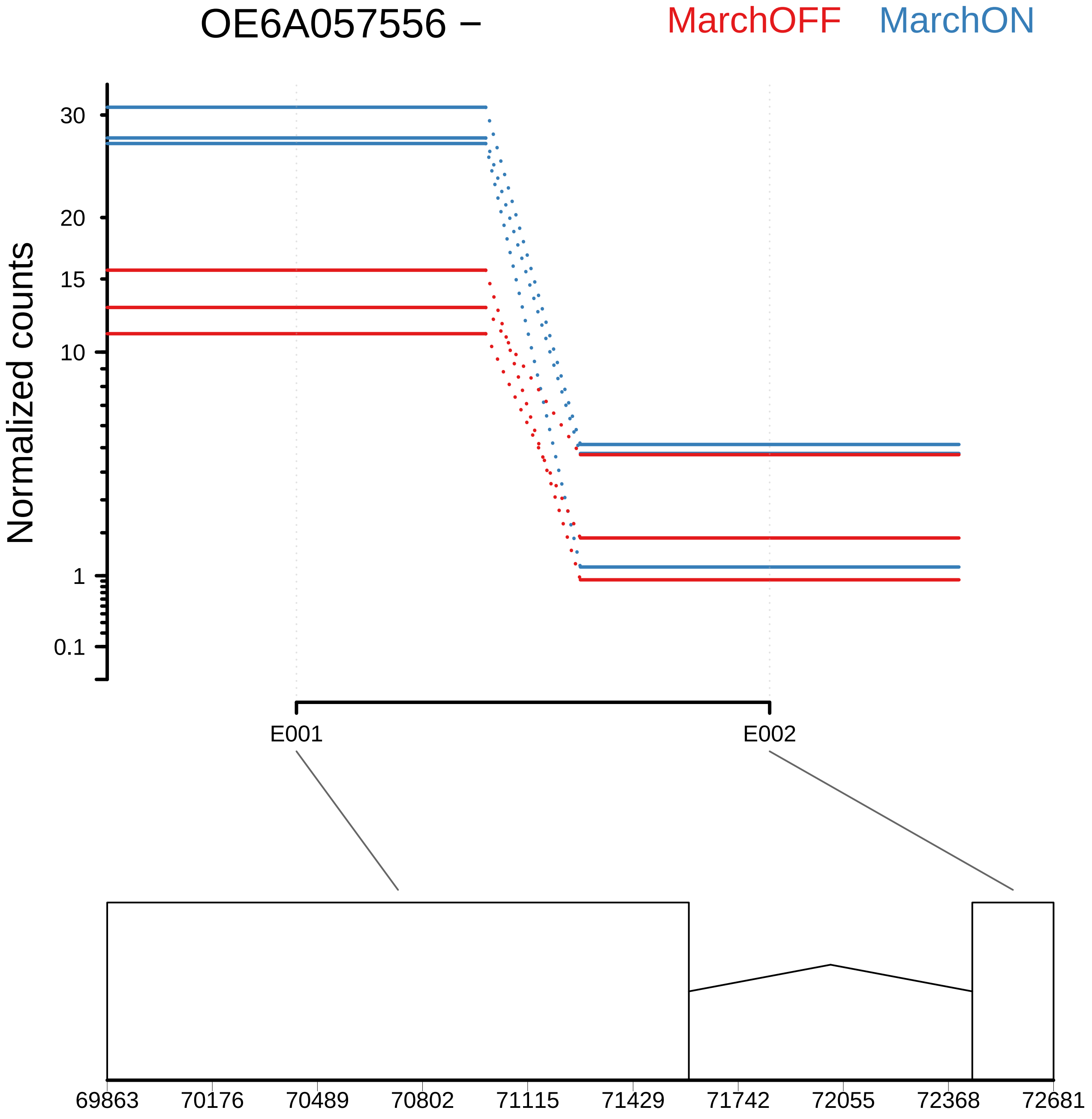

OE6A057556 −
MarchOFF
MarchON
Normalized counts
30
20
15
10
1
0.1
E001
E002
69863
70176
70489
70802
71115
71429
71742
72055
72368
72681

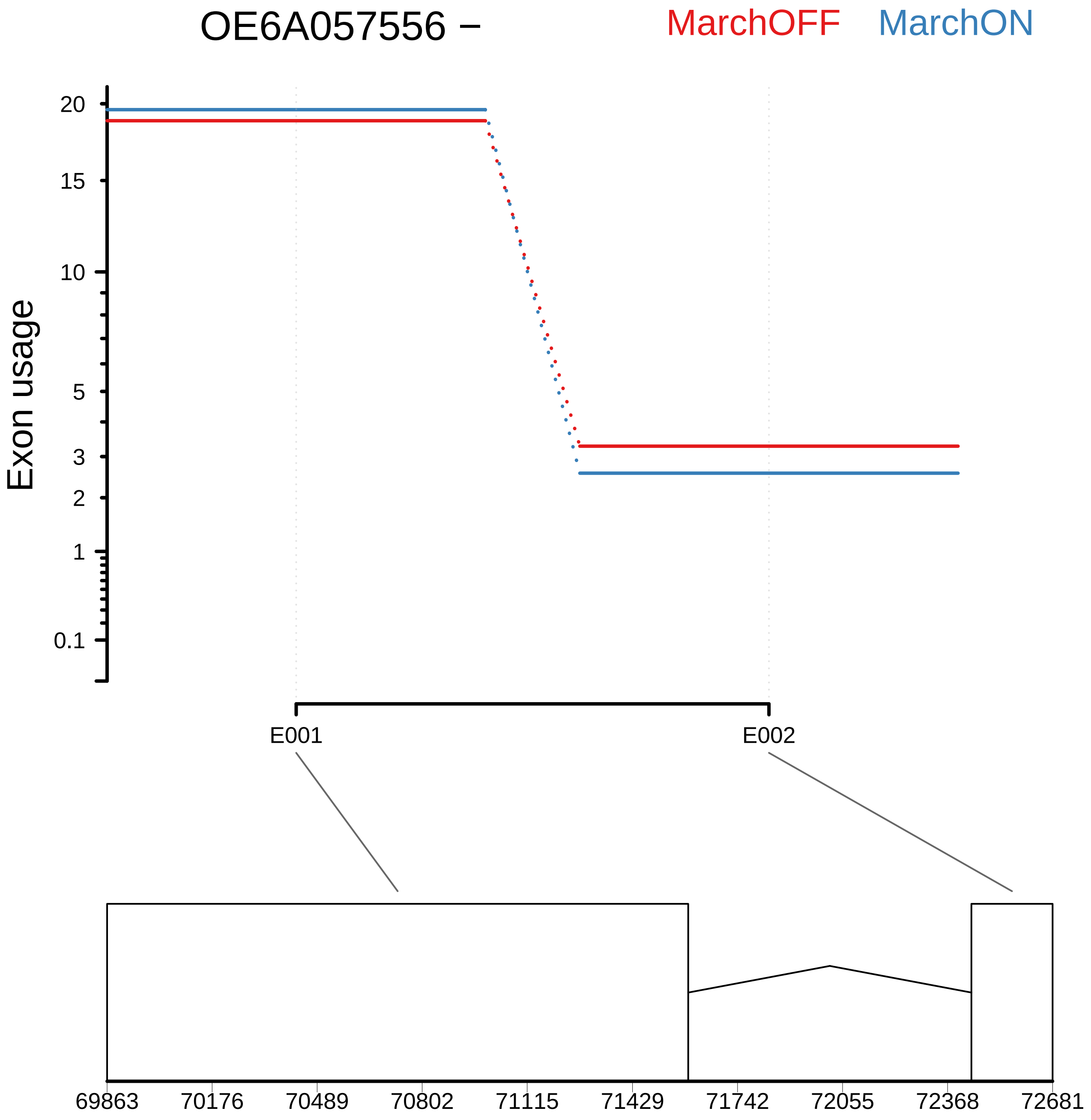
OE6A057556 −
MarchOFF
MarchON
Exon usage
20
15
10
5
3
2
1
0.1
E001
E002
69863
70176
70489
70802
71115
71429
71742
72055
72368
72681

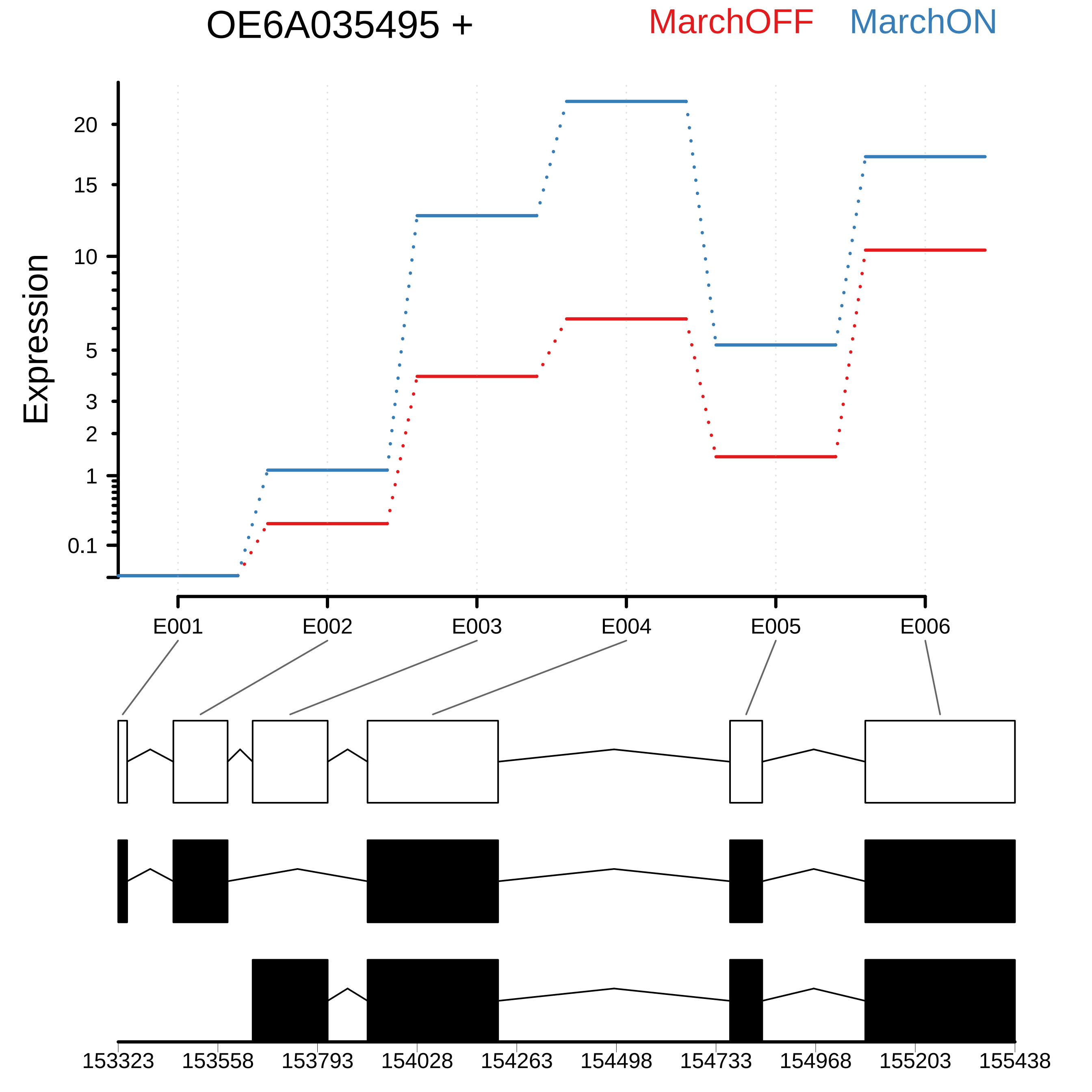

OE6A035495 +
MarchOFF
MarchON
Expression
20
15
10
5
3
2
1
0.1
E001
E002
E003
E004
E005
E006
153323
153558
153793
154028
154263
154498
154733
154968
155203
155438

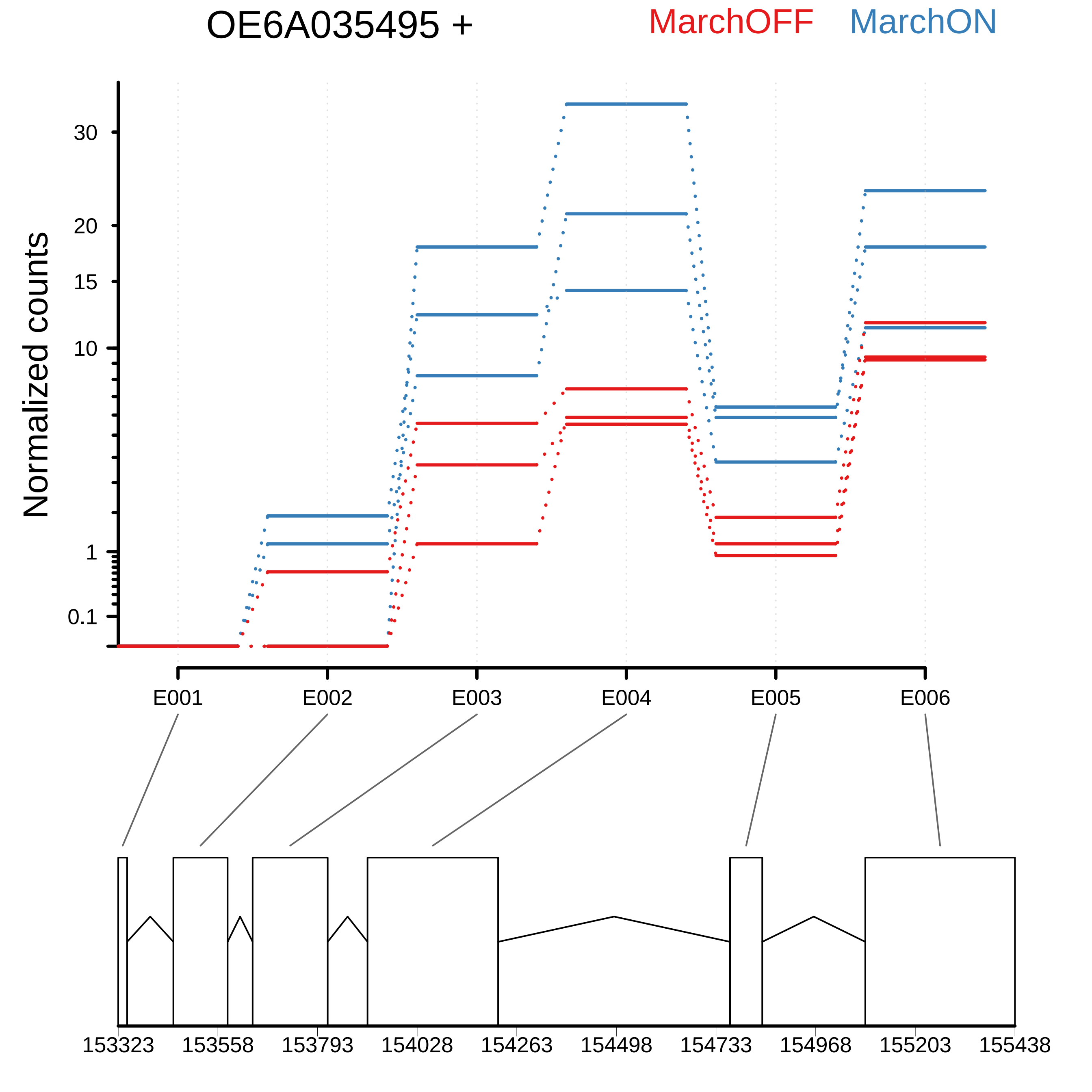
OE6A035495 +
MarchOFF
MarchON
Normalized counts
30
20
15
10
1
0.1
E001
E002
E003
E004
E005
E006
153323
153558
153793
154028
154263
154498
154733
154968
155203
155438

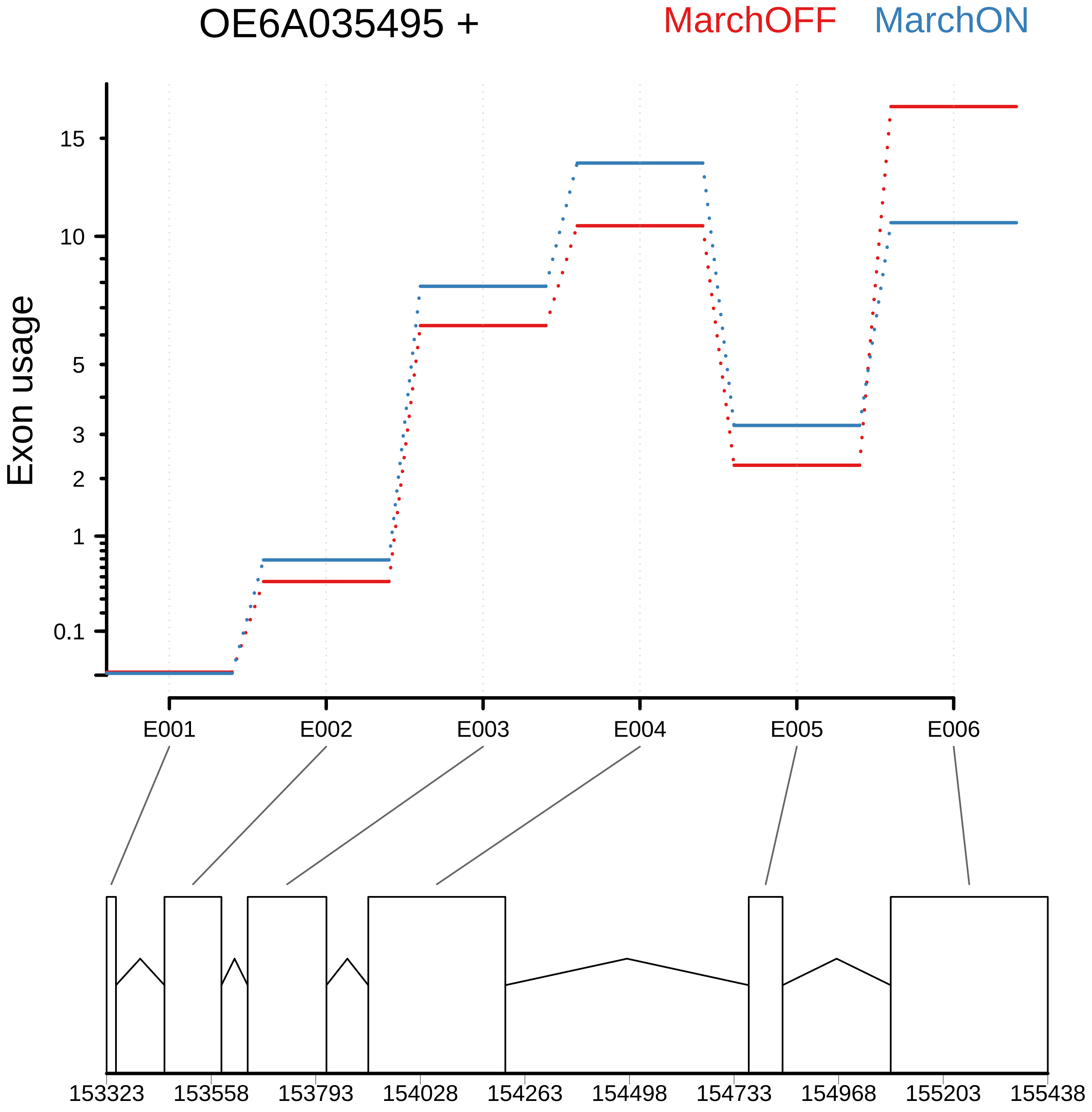

OE6A035495 +
MarchOFF
MarchON
Exon usage
15
10
5
3
2
1
0.1
E001
E002
E003
E004
E005
E006
153323
153558
153793
154028
154263
154498
154733
154968
155203
155438

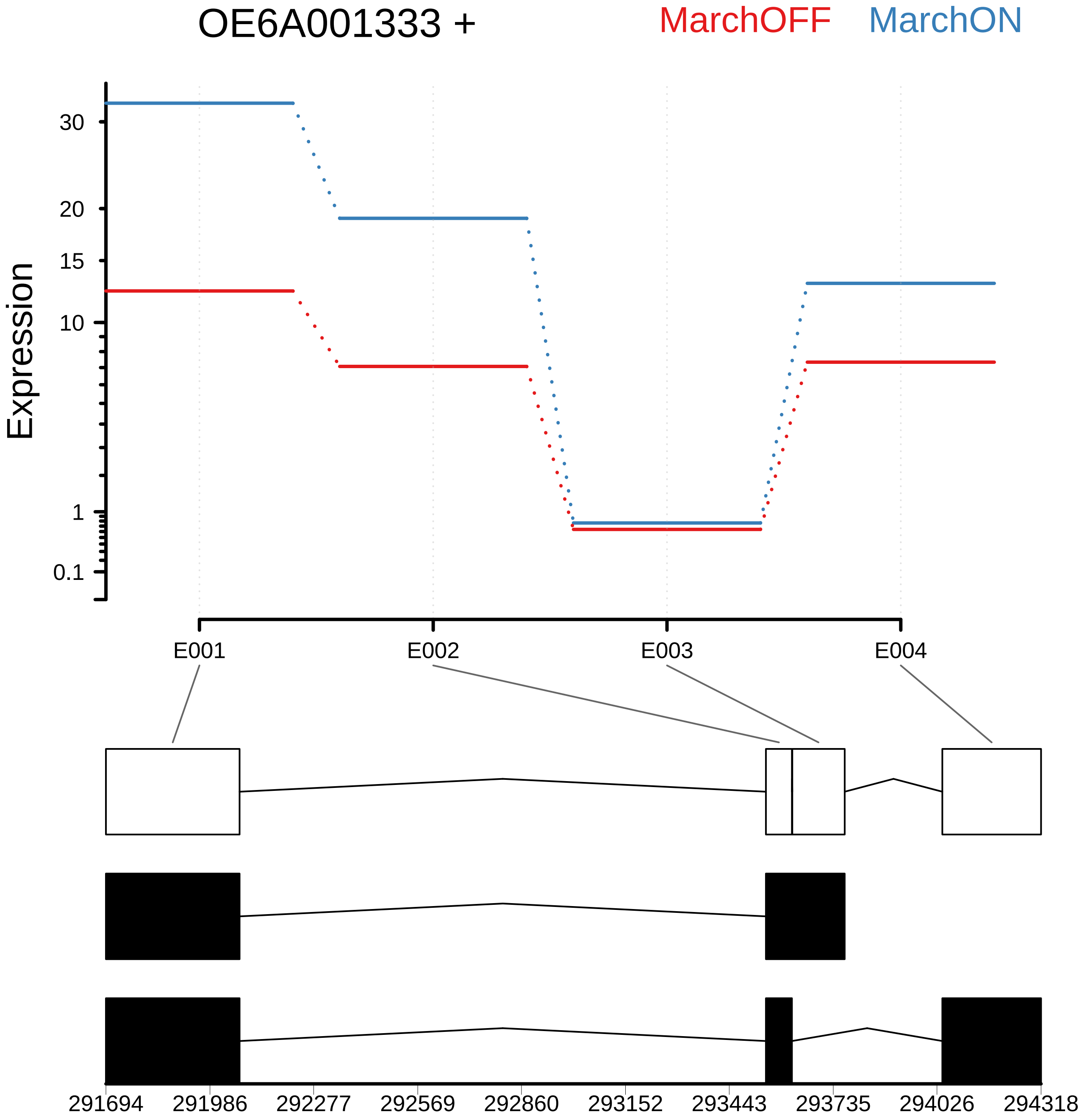
OE6A001333 +
MarchOFF
MarchON
Expression
30
20
15
10
1
0.1
E001
E002
E003
E004
291694
291986
292277
292569
292860
293152
293443
293735
294026
294318

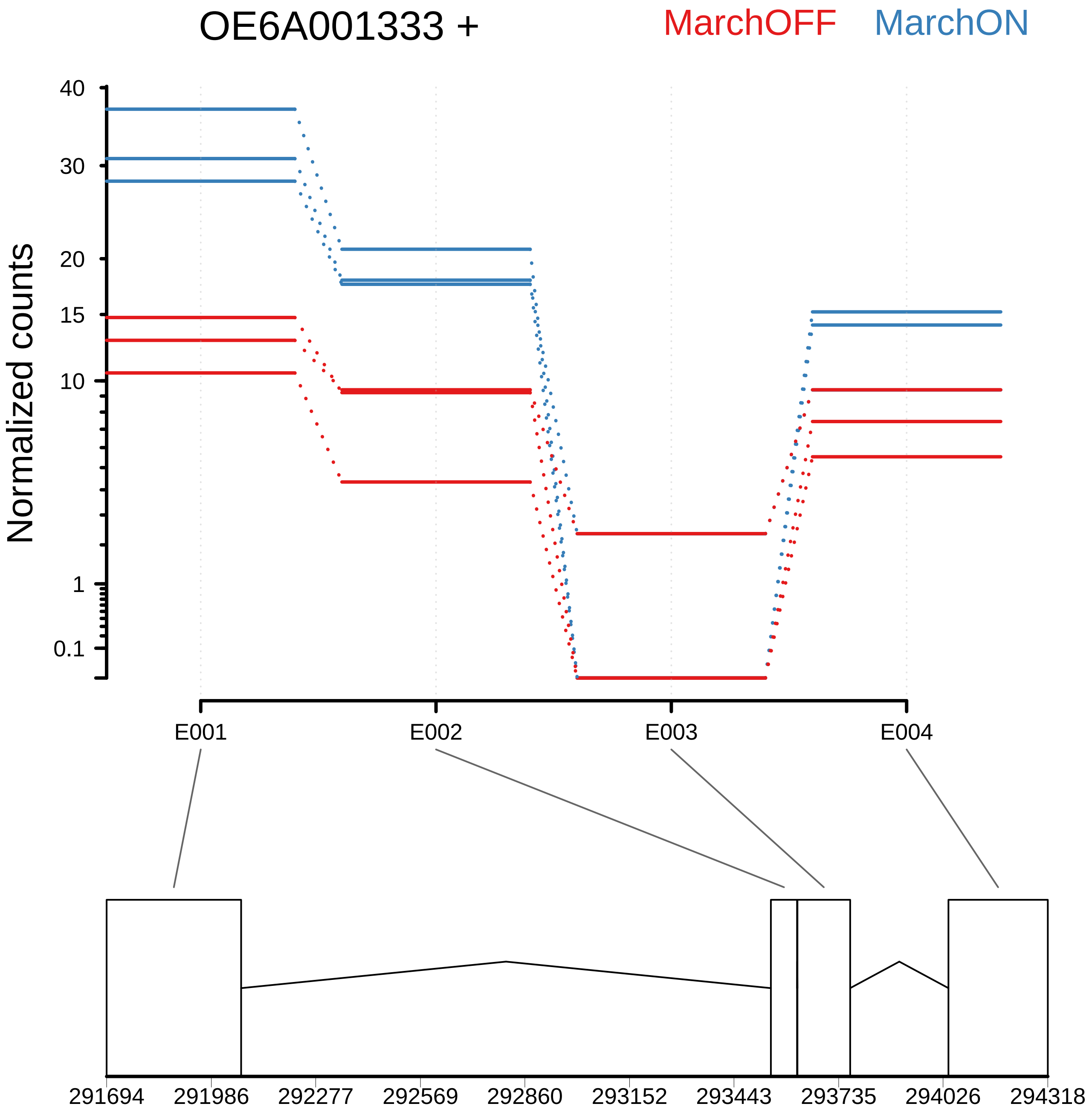

OE6A001333 +
MarchOFF
MarchON
Normalized counts
40
30
20
15
10
1
0.1
E001
E002
E003
E004
291694
291986
292277
292569
292860
293152
293443
293735
294026
294318

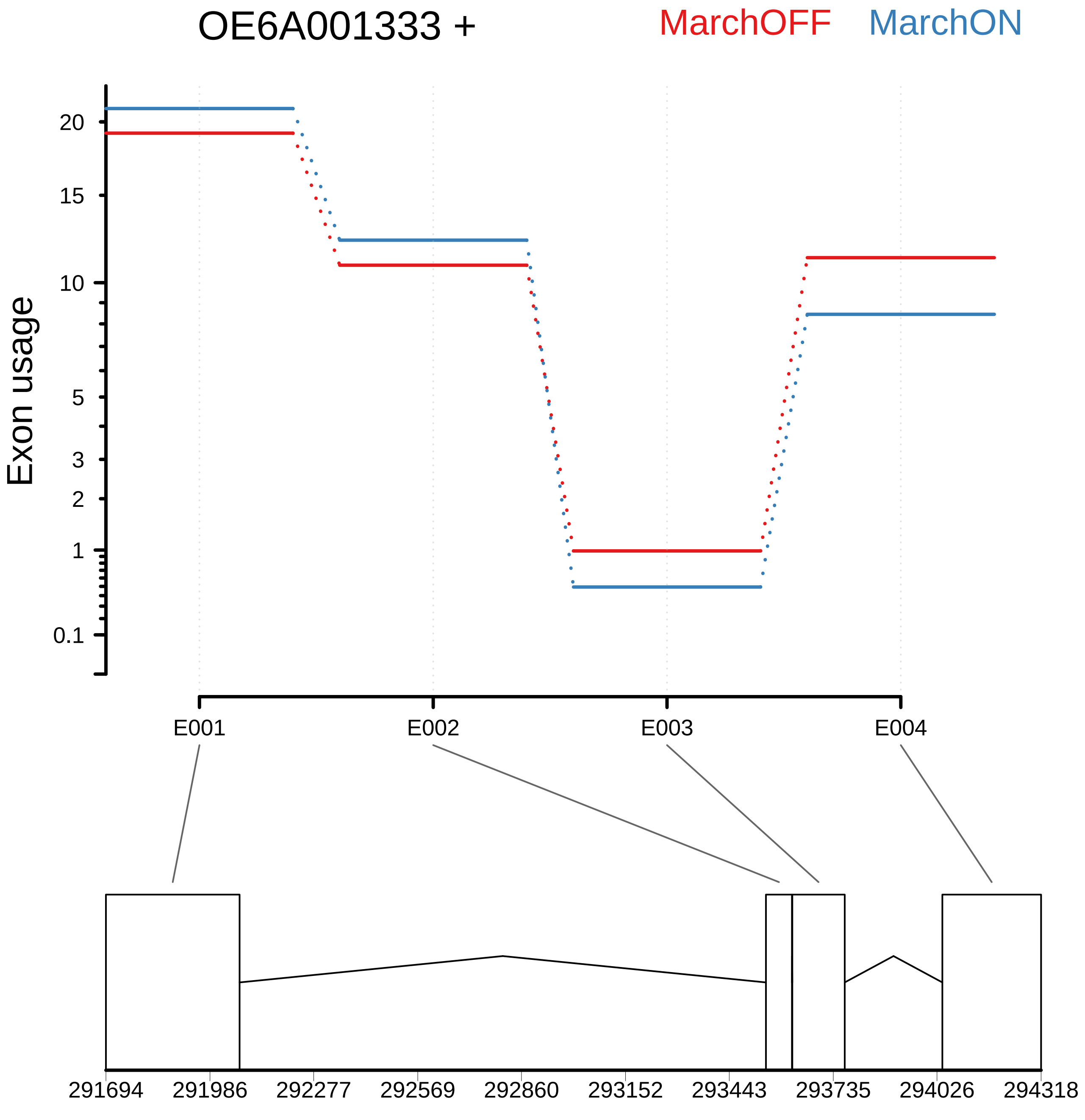

OE6A001333 +
MarchOFF
MarchON
Exon usage
20
15
10
5
3
2
1
0.1
E001
E002
E003
E004
291694
291986
292277
292569
292860
293152
293443
293735
294026
294318

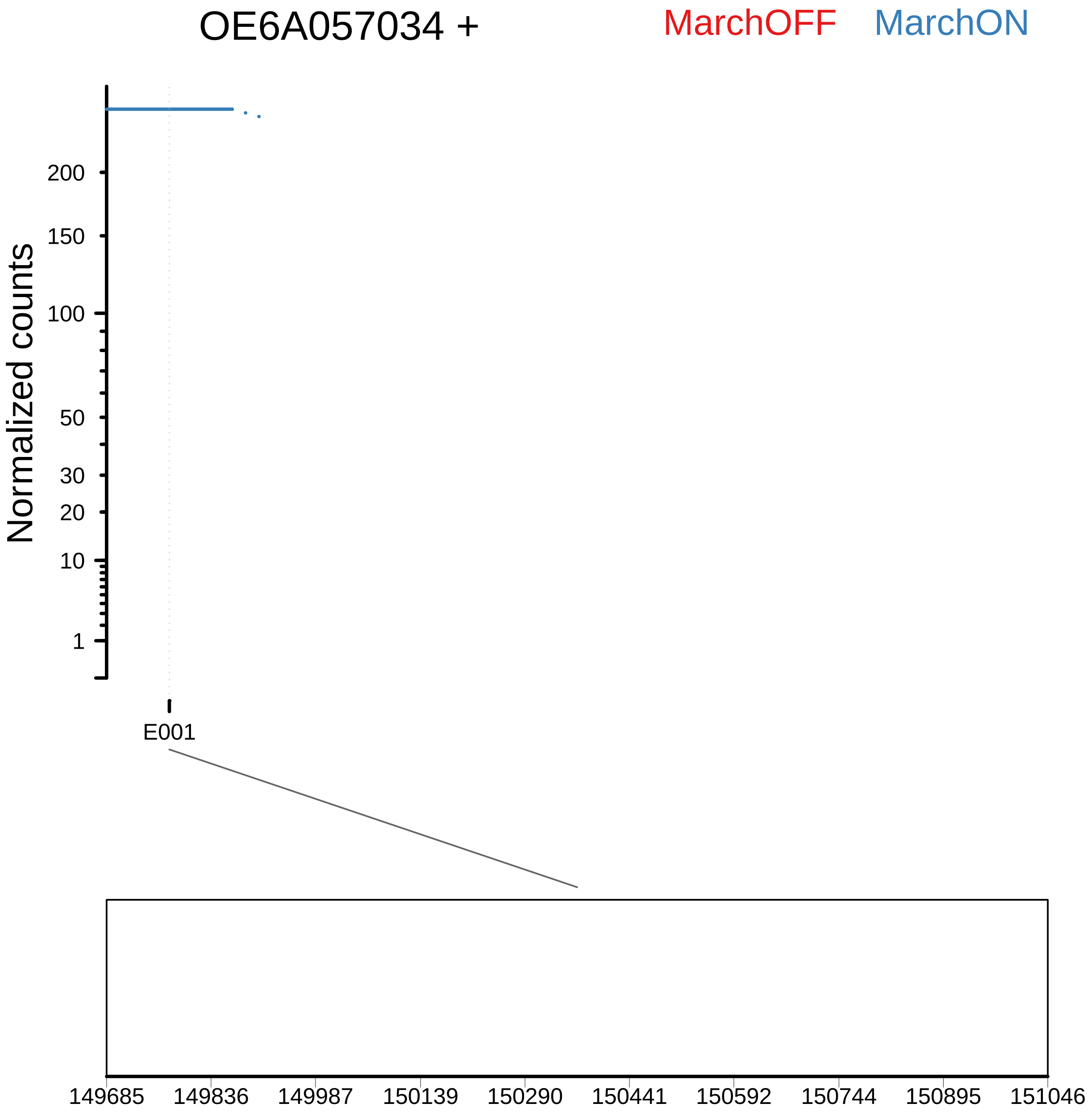

OE6A057034 +
MarchOFF
MarchON
Normalized counts
200
150
100
50
30
20
10
1
E001
149685
149836
149987
150139
150290
150441
150592
150744
150895
151046

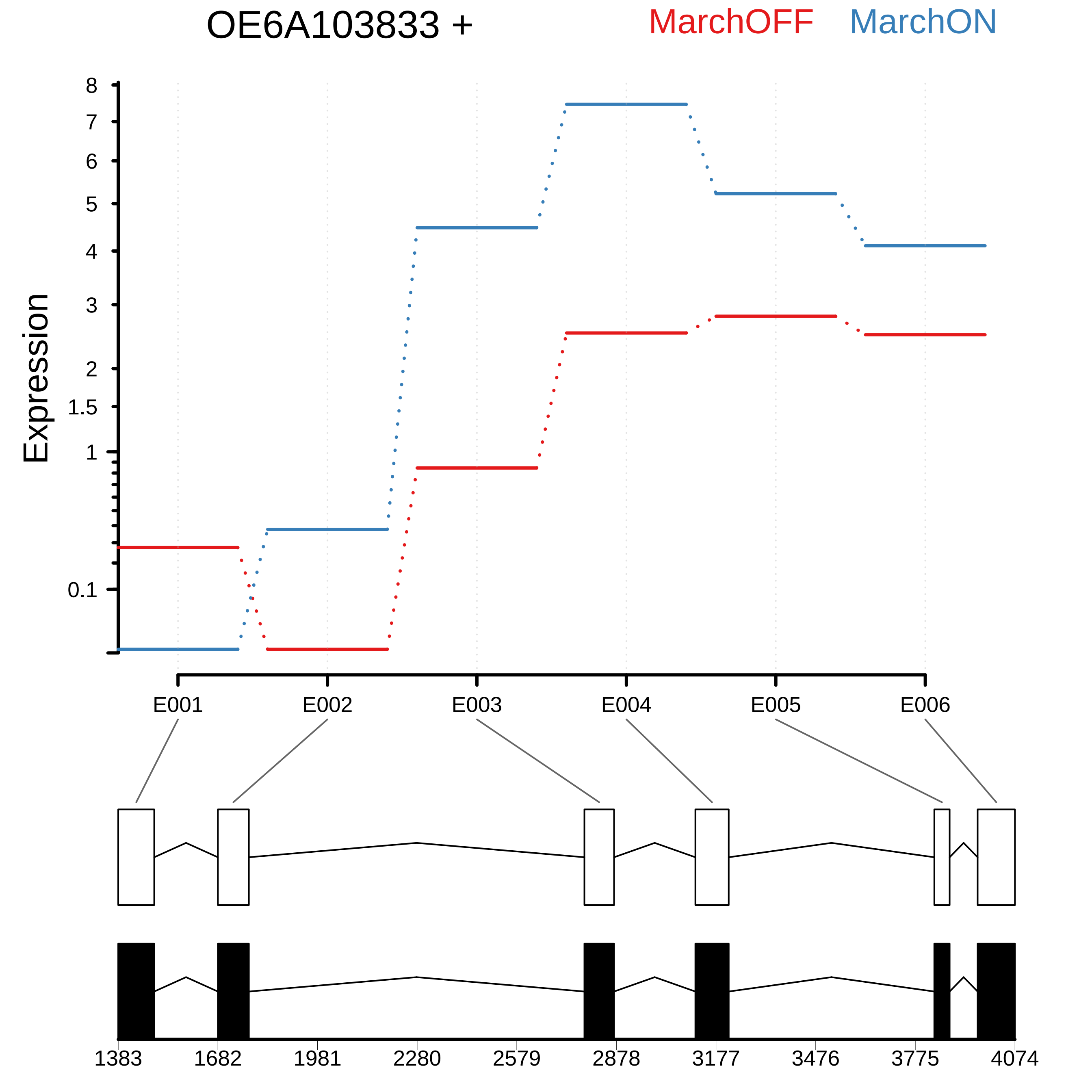

OE6A103833 +
MarchOFF
MarchON
Expression
8
7
6
5
4
3
2
1.5
1
0.1
E001
E002
E003
E004
E005
E006
1383
1682
1981
2280
2579
2878
3177
3476
3775
4074

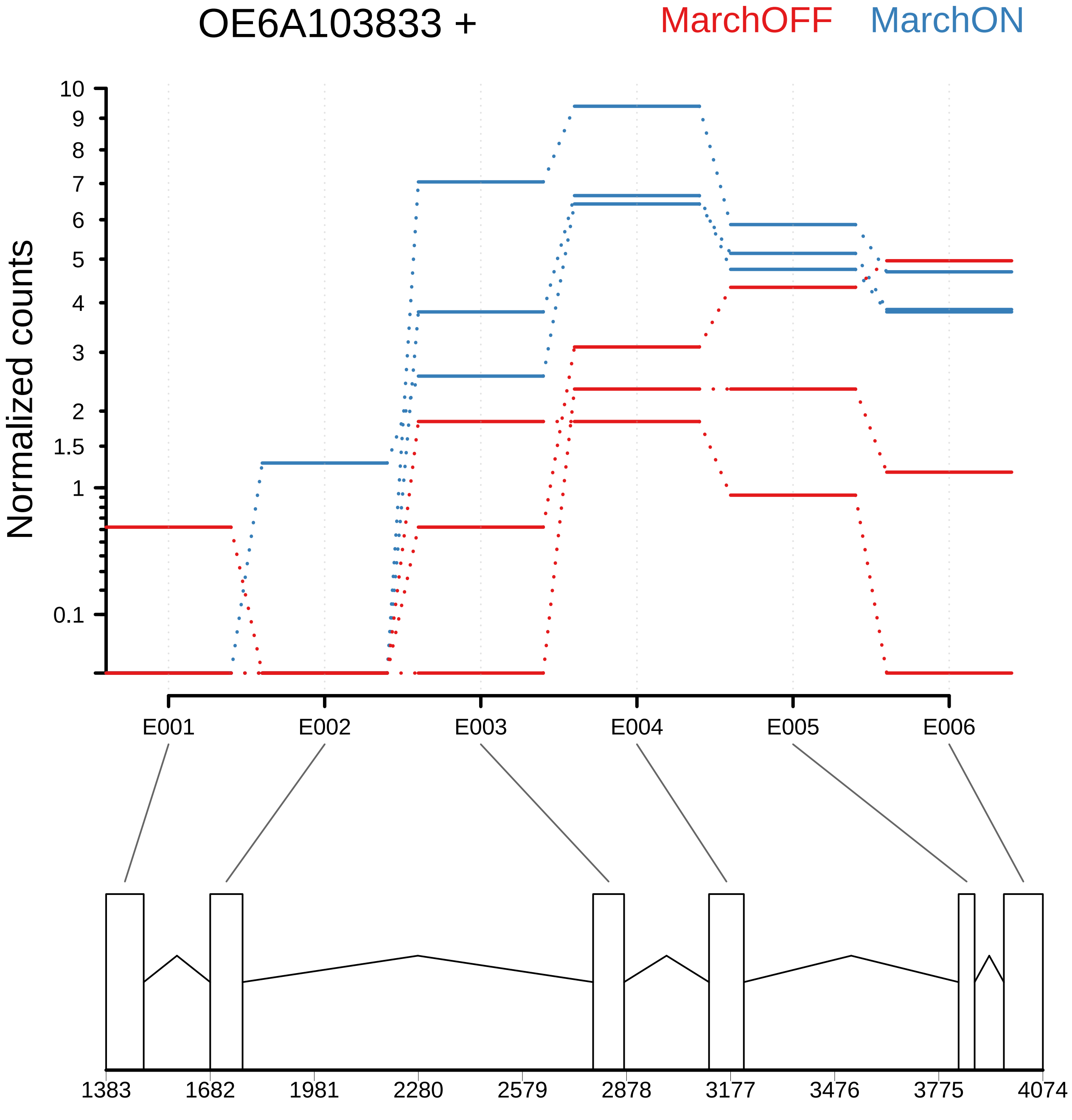

OE6A103833 +
MarchOFF
MarchON
Normalized counts
10
9
8
7
6
5
4
3
2
1.5
1
0.1
E001
E002
E003
E004
E005
E006
1383
1682
1981
2280
2579
2878
3177
3476
3775
4074

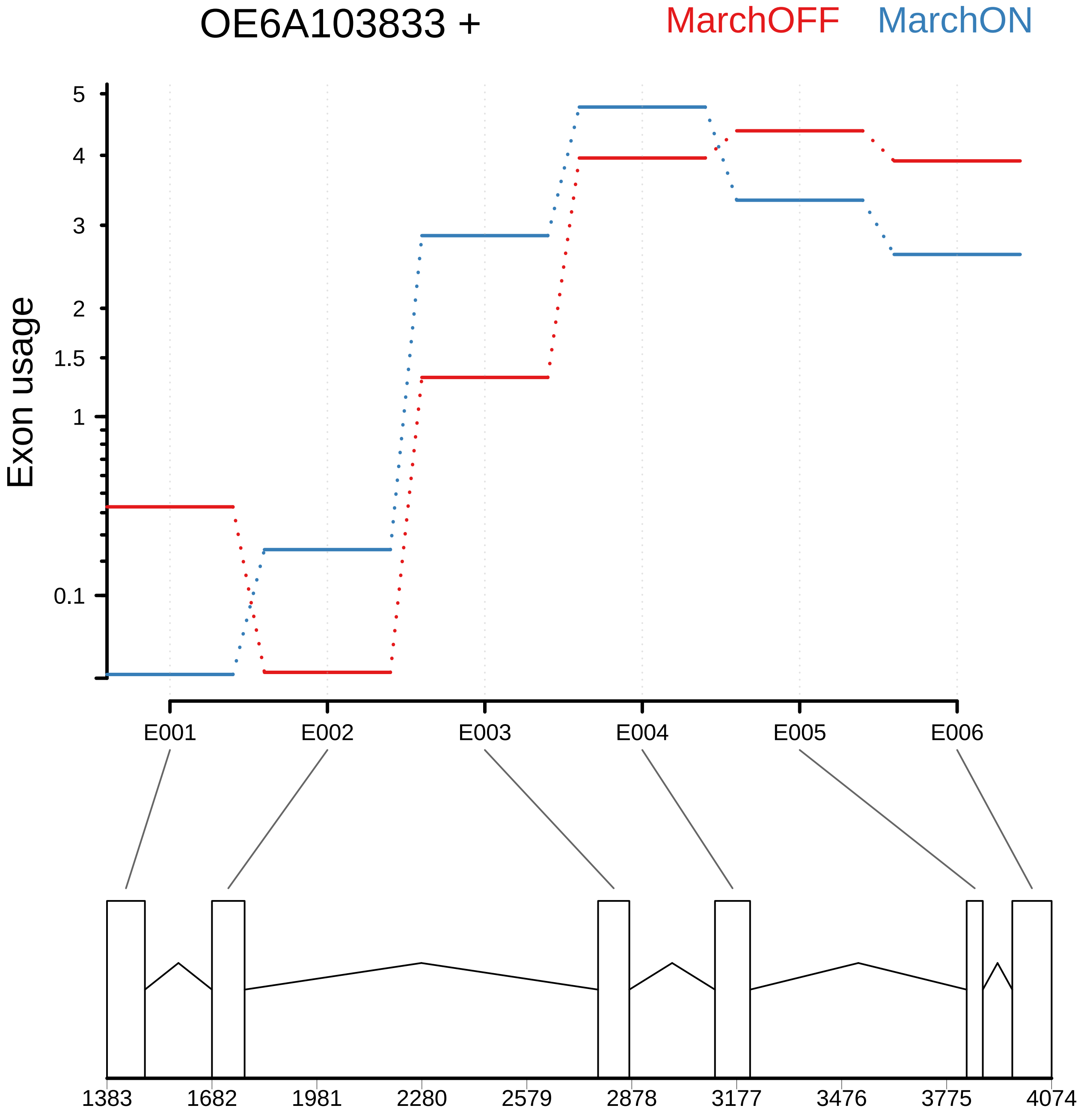
OE6A103833 +
MarchOFF
MarchON
Exon usage
5
4
3
2
1.5
1
0.1
E001
E002
E003
E004
E005
E006
1383
1682
1981
2280
2579
2878
3177
3476
3775
4074

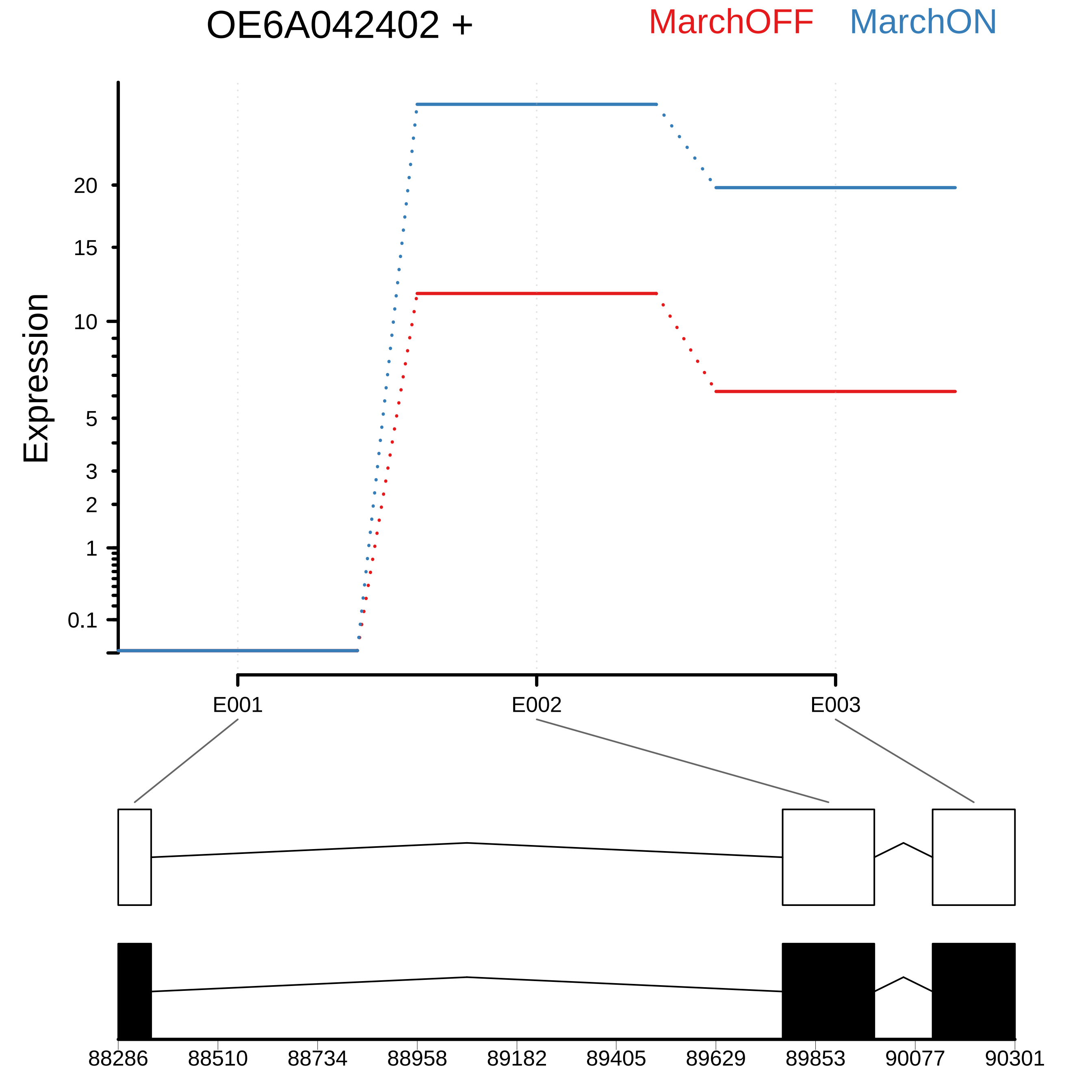

OE6A042402 +
MarchOFF
MarchON
Expression
20
15
10
5
3
2
1
0.1
E001
E002
E003
88286
88510
88734
88958
89182
89405
89629
89853
90077
90301

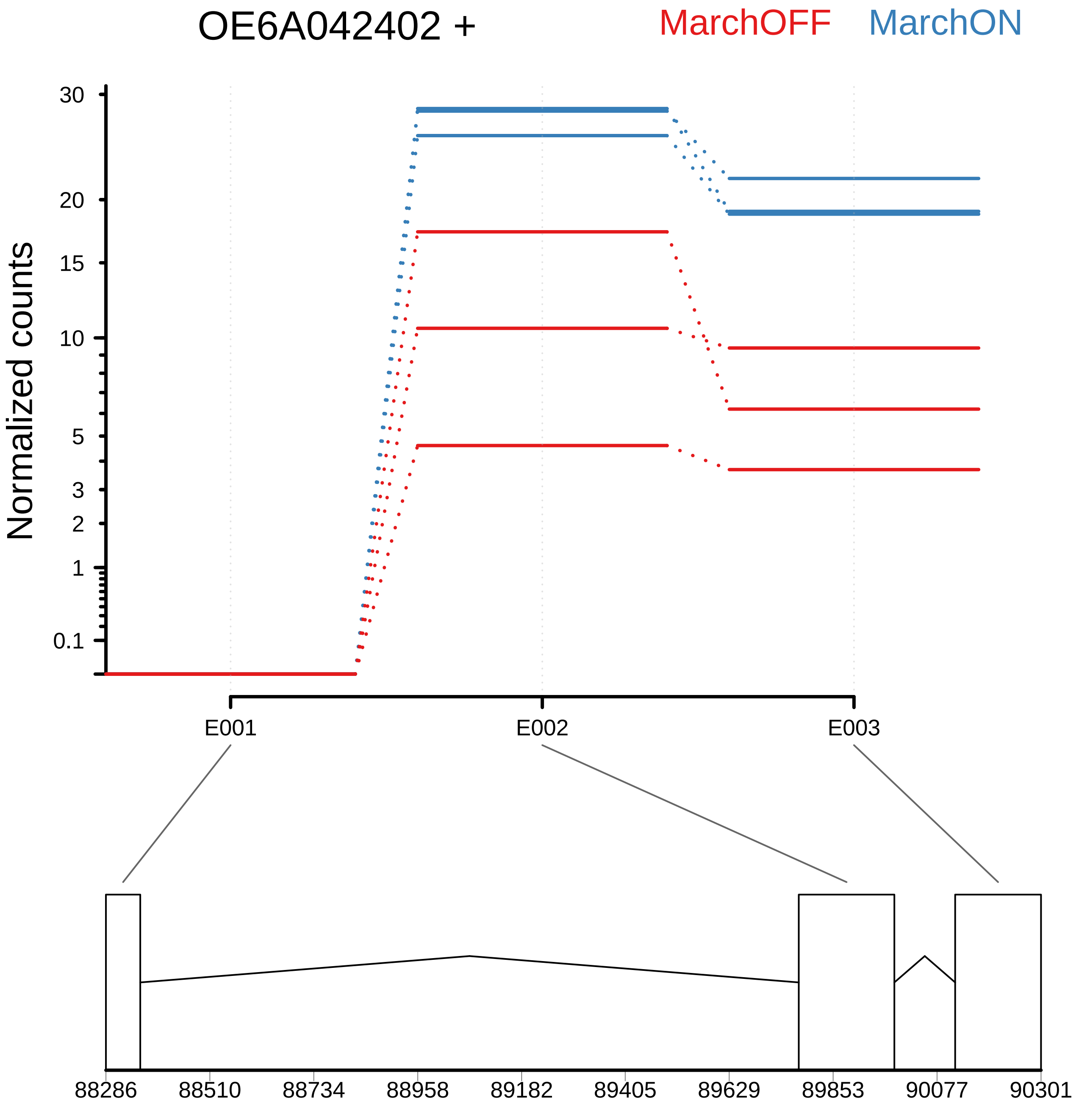

OE6A042402 +
MarchOFF
MarchON
Normalized counts
30
20
15
10
5
3
2
1
0.1
E001
E002
E003
88286
88510
88734
88958
89182
89405
89629
89853
90077
90301

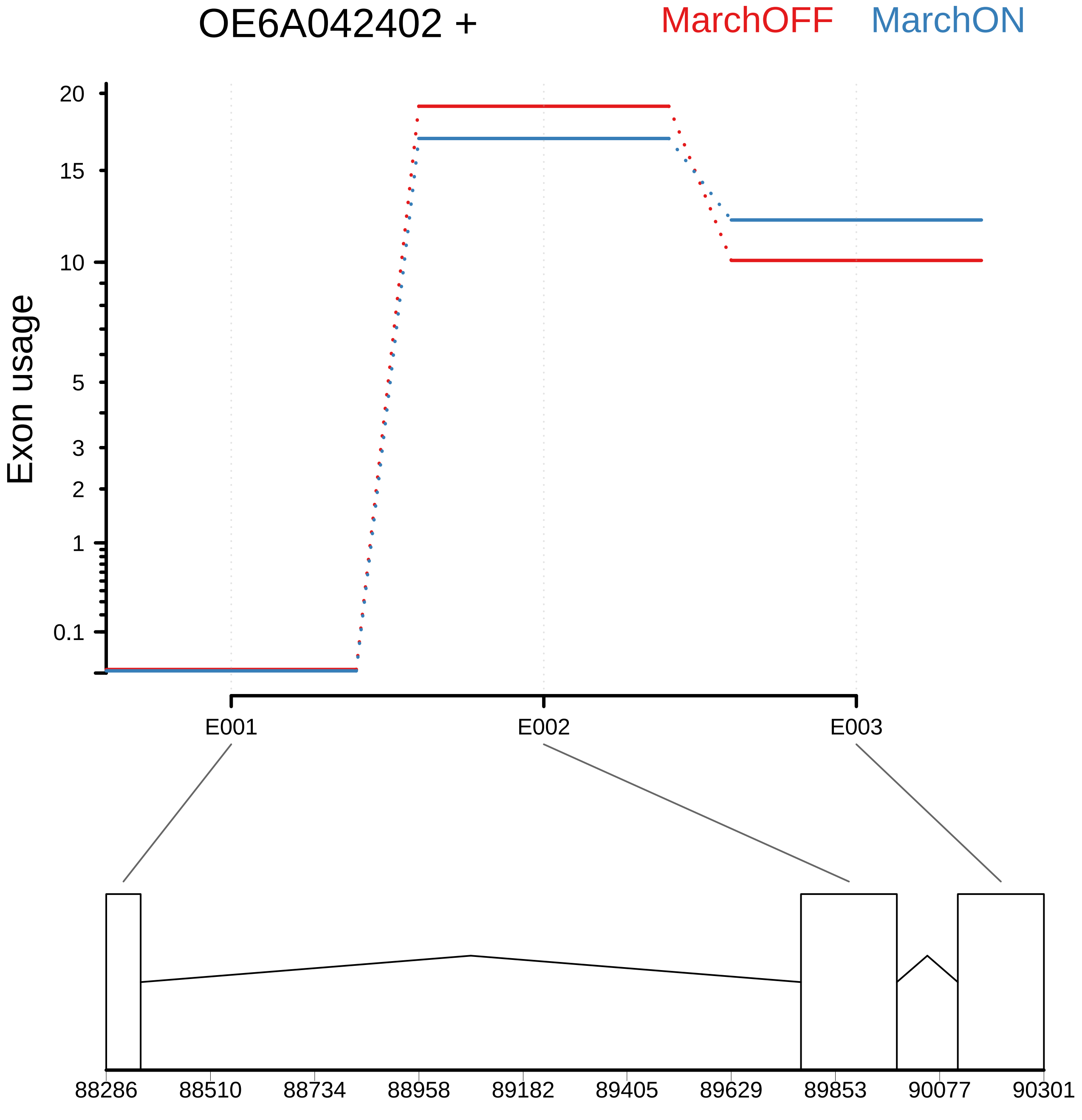

OE6A042402 +
MarchOFF
MarchON
Exon usage
20
15
10
5
3
2
1
0.1
E001
E002
E003
88286
88510
88734
88958
89182
89405
89629
89853
90077
90301

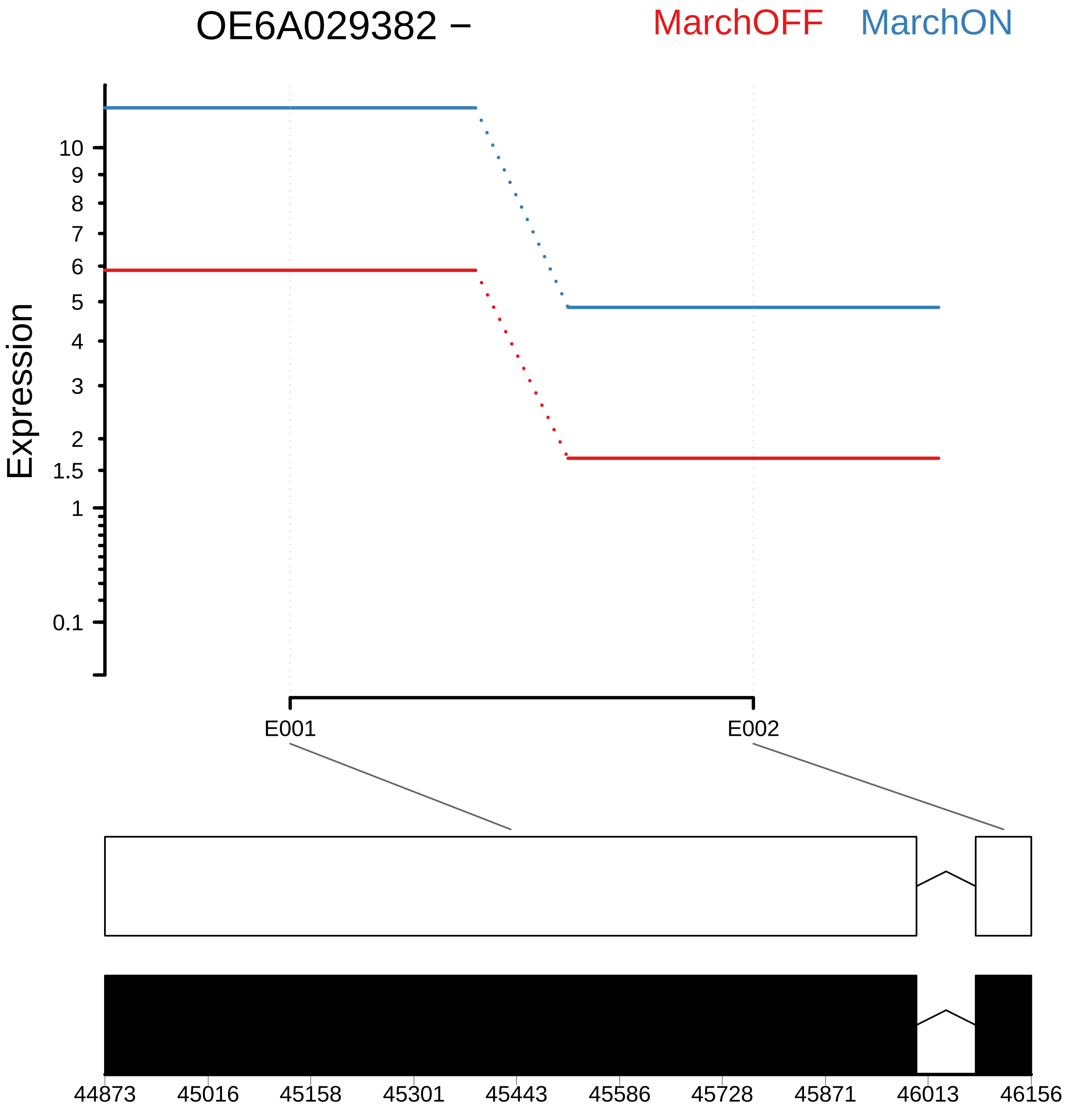

OE6A029382 −
MarchOFF
MarchON
Expression
10
9
8
7
6
5
4
3
2
1.5
1
0.1
E001
E002
44873
45016
45158
45301
45443
45586
45728
45871
46013
46156

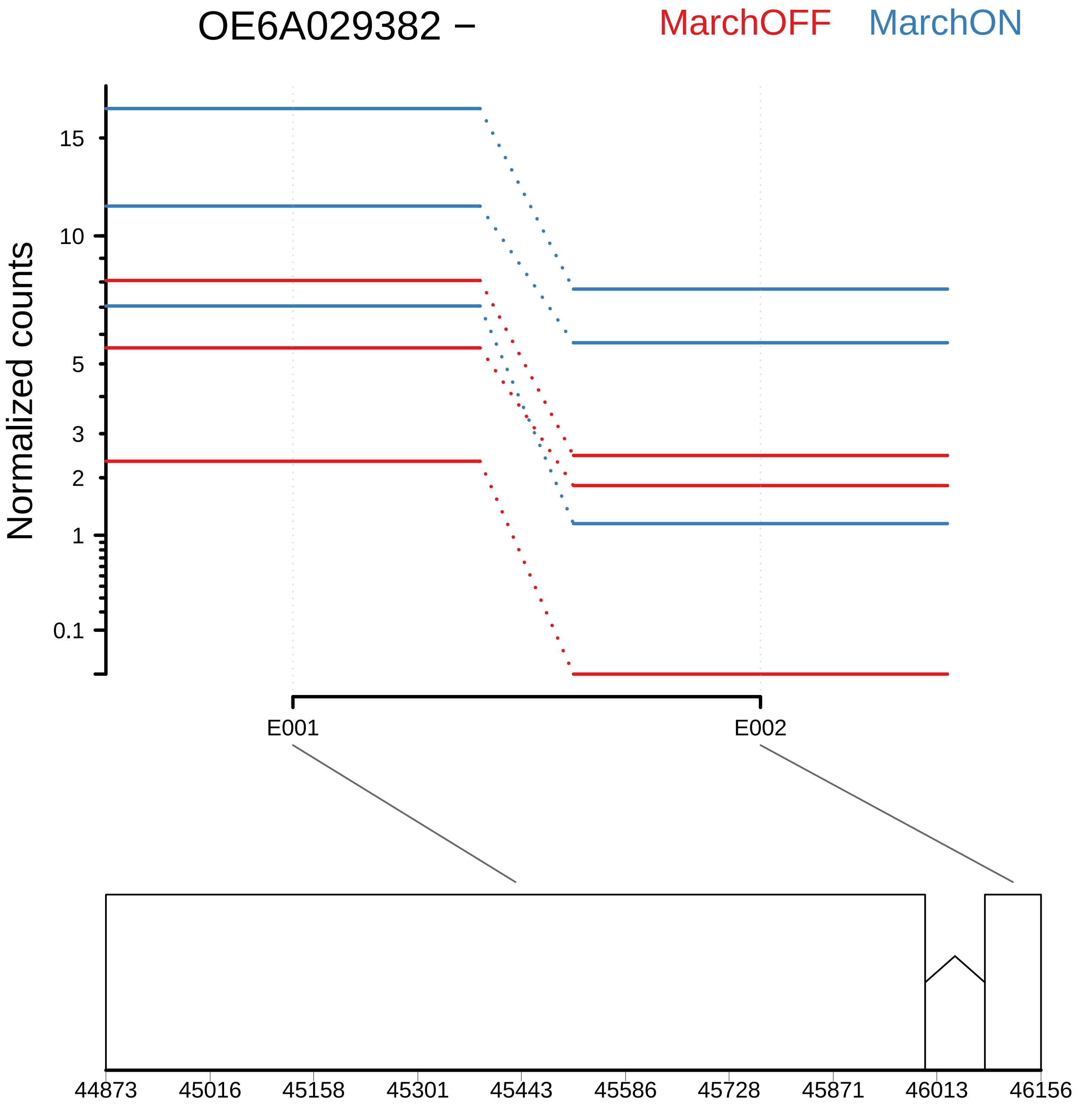

OE6A029382 −
MarchOFF
MarchON
Normalized counts
15
10
5
3
2
1
0.1
E001
E002
44873
45016
45158
45301
45443
45586
45728
45871
46013
46156

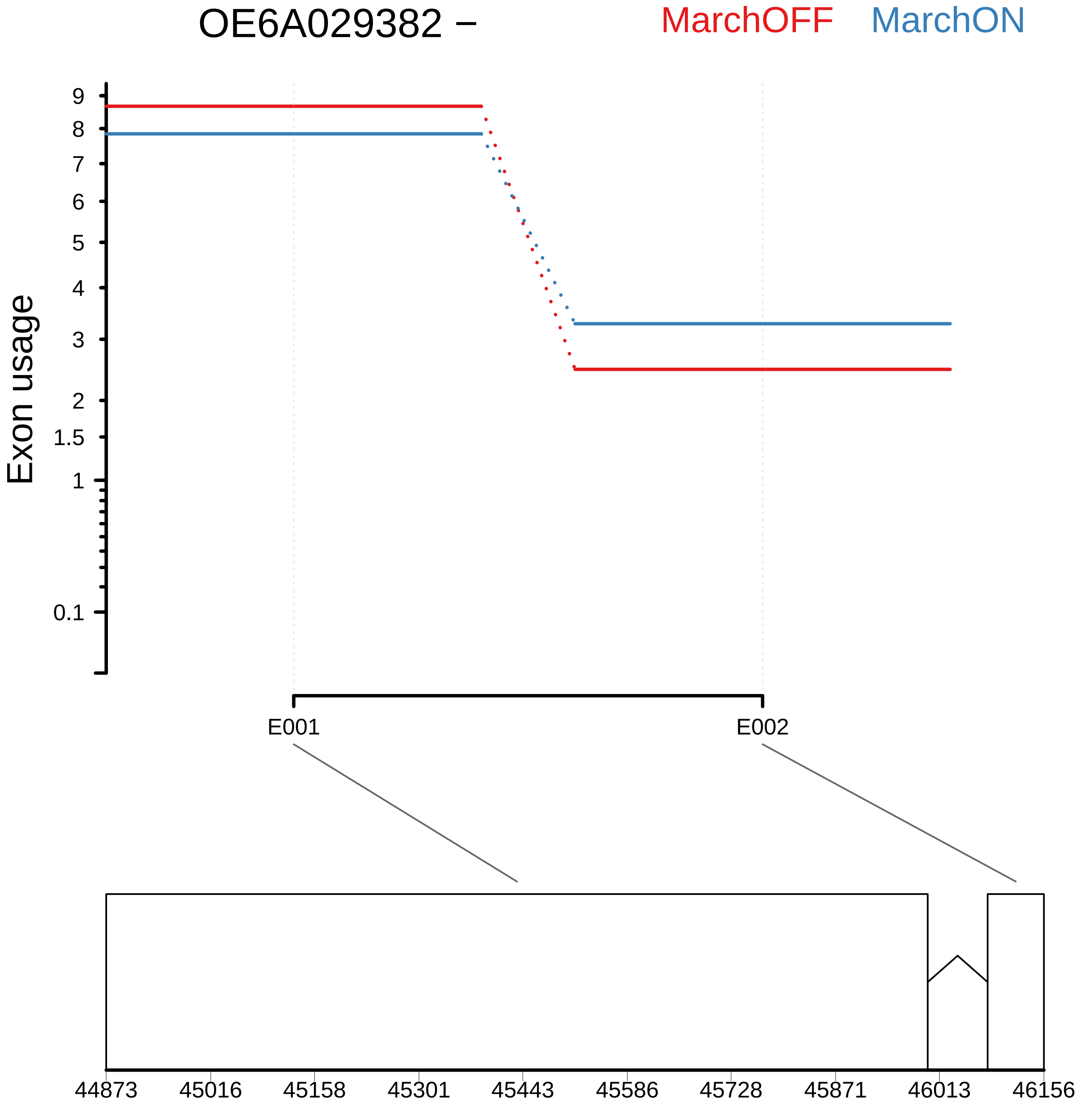
OE6A029382 −
MarchOFF
MarchON
Exon usage
9
8
7
6
5
4
3
2
1.5
1
0.1
E001
E002
44873
45016
45158
45301
45443
45586
45728
45871
46013
46156

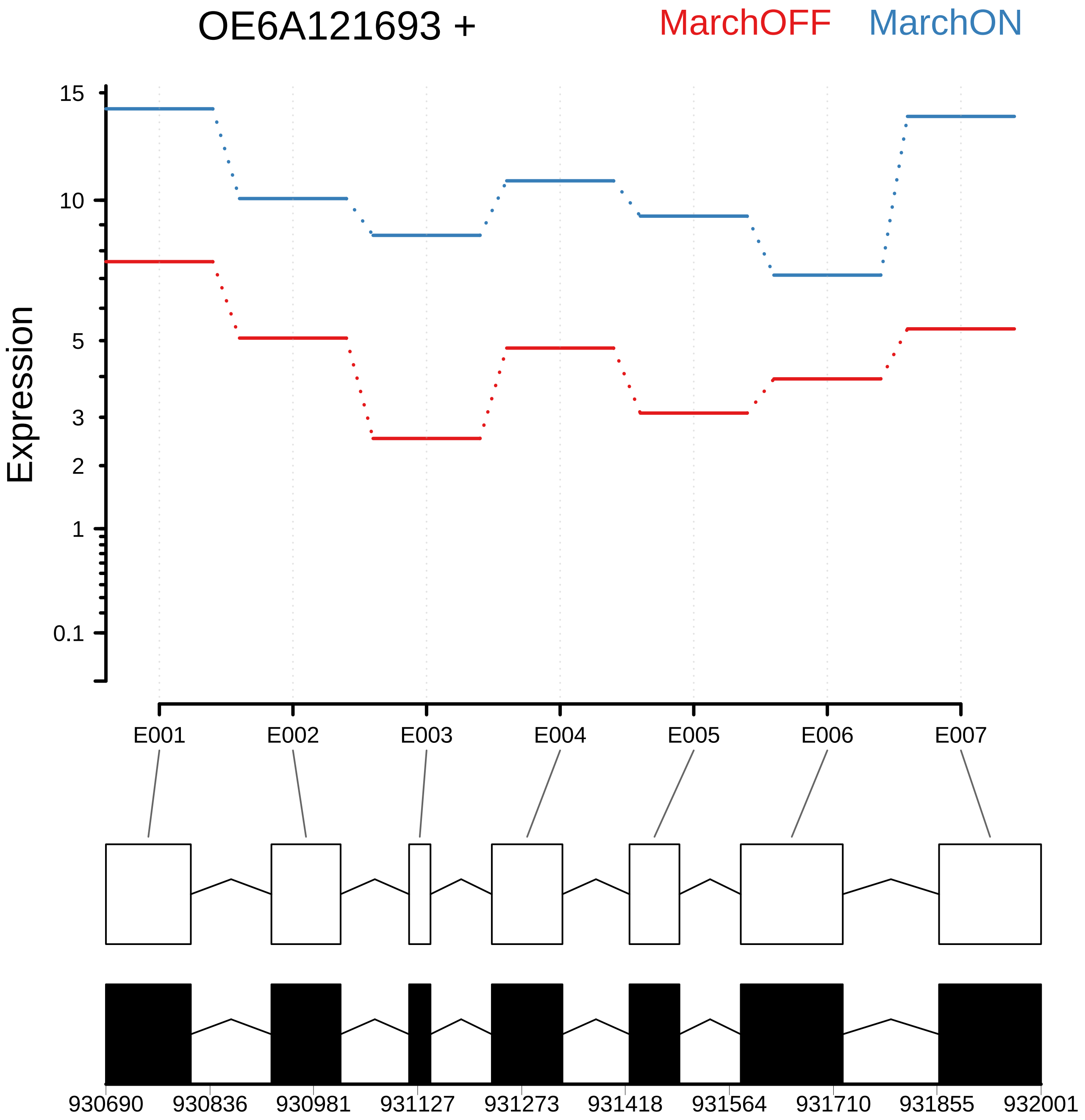
OE6A121693 +
MarchOFF
MarchON
Expression
15
10
5
3
2
1
0.1
E001
E002
E003
E004
E005
E006
E007
930690
930836
930981
931127
931273
931418
931564
931710
931855
932001

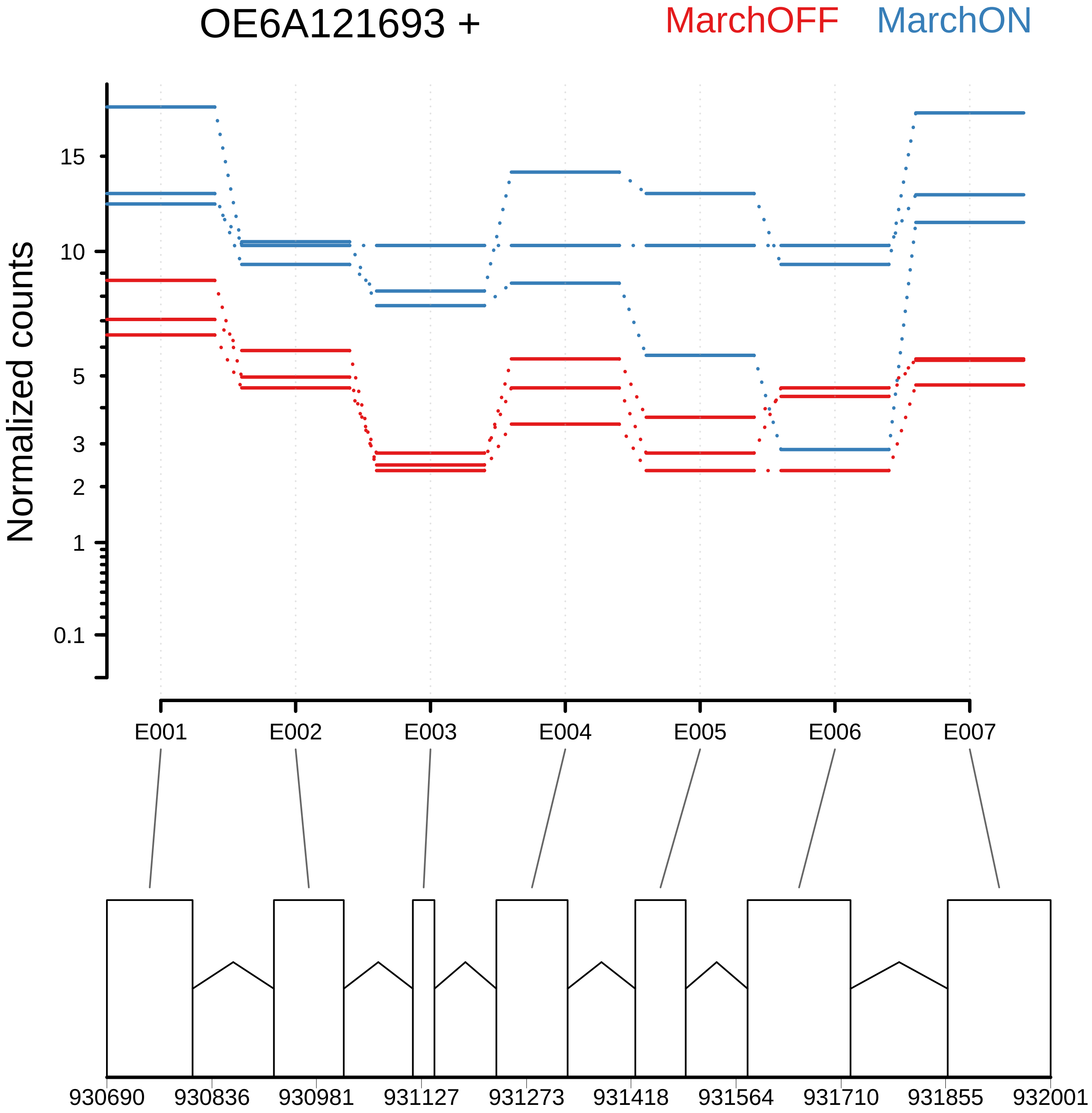

OE6A121693 +
MarchOFF
MarchON
Normalized counts
15
10
5
3
2
1
0.1
E001
E002
E003
E004
E005
E006
E007
930690
930836
930981
931127
931273
931418
931564
931710
931855
932001

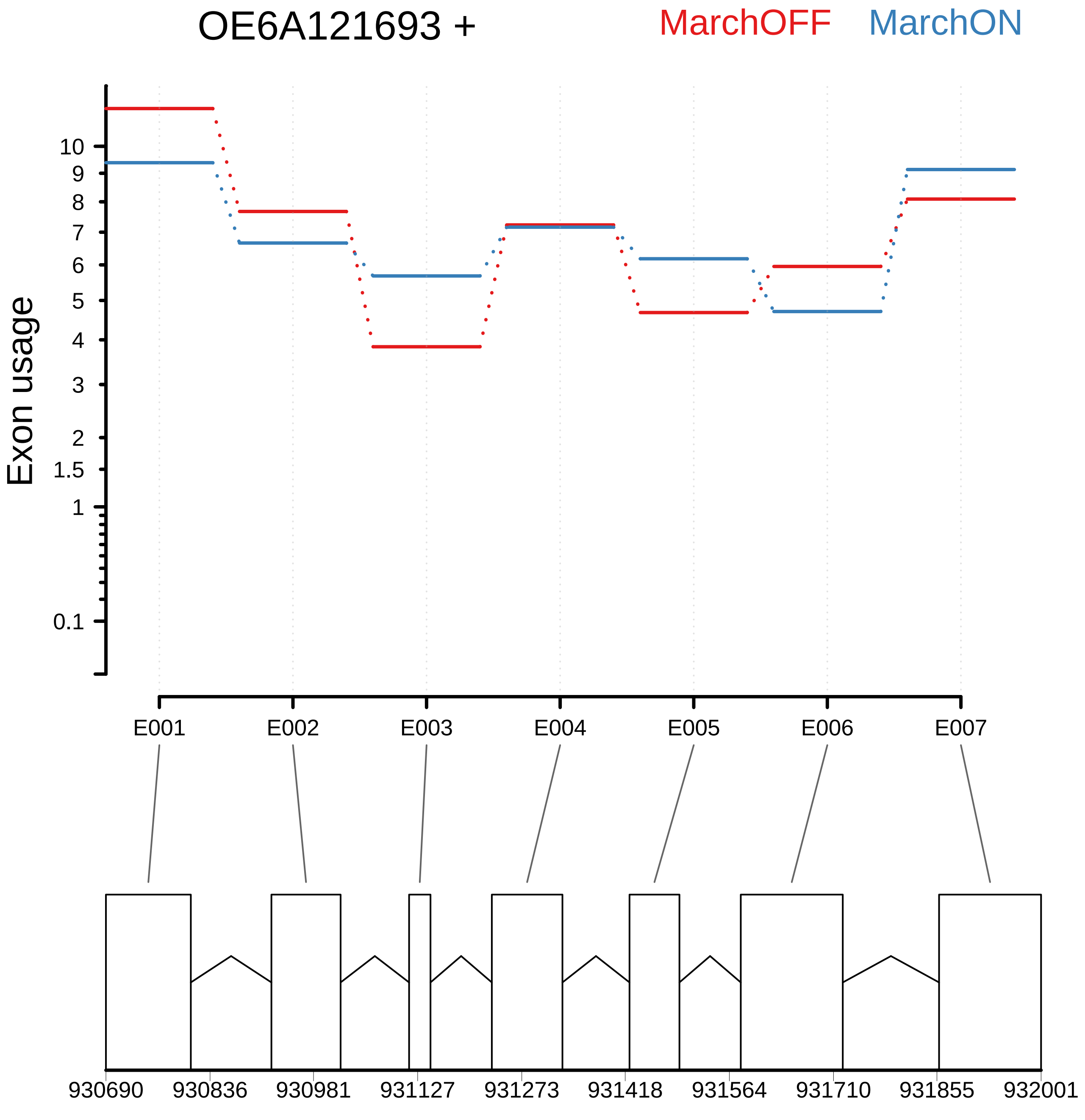
OE6A121693 +
MarchOFF
MarchON
Exon usage
10
9
8
7
6
5
4
3
2
1.5
1
0.1
E001
E002
E003
E004
E005
E006
E007
930690
930836
930981
931127
931273
931418
931564
931710
931855
932001

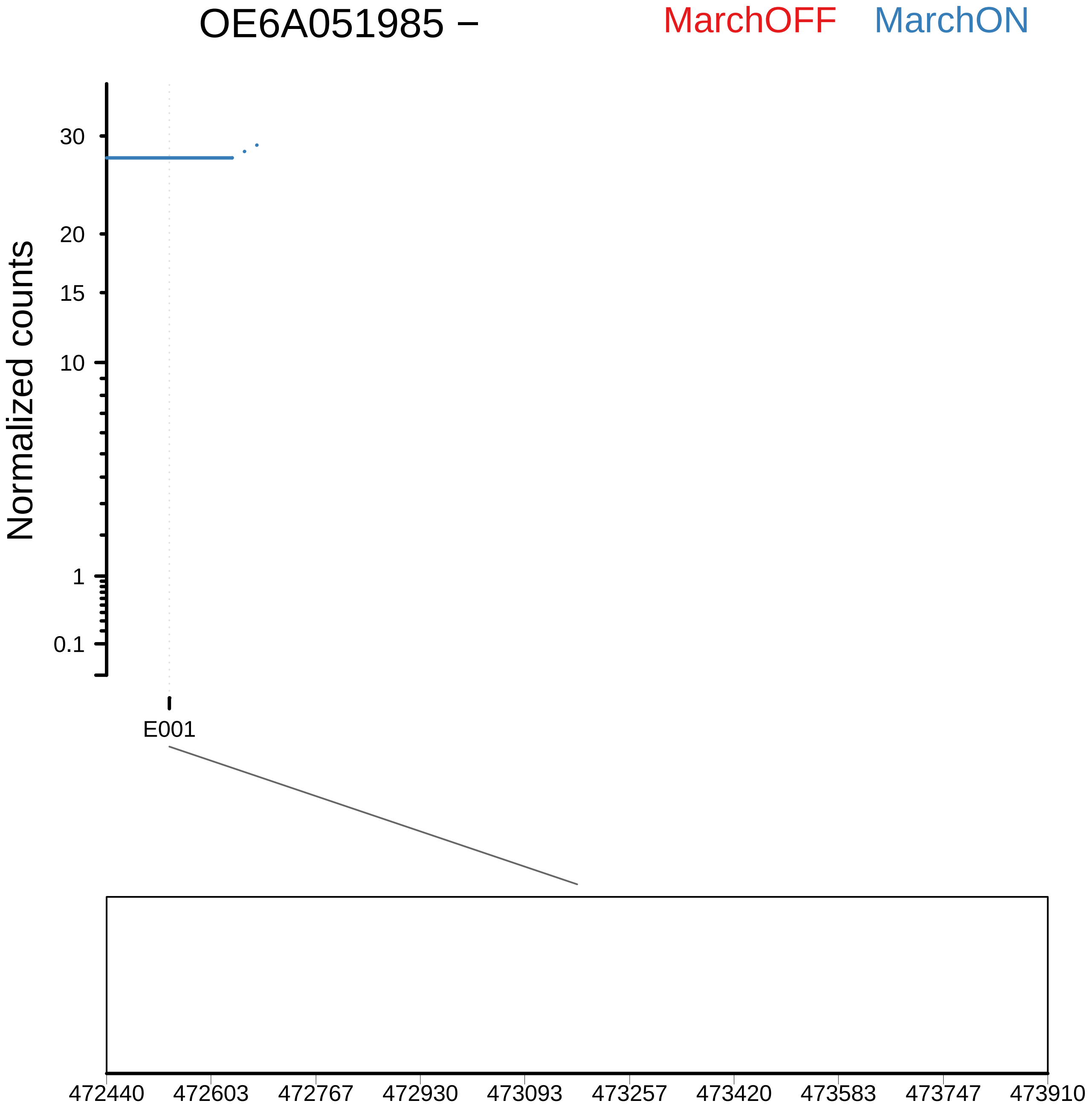

OE6A051985 −
MarchOFF
MarchON
Normalized counts
30
20
15
10
1
0.1
E001
472440
472603
472767
472930
473093
473257
473420
473583
473747
473910

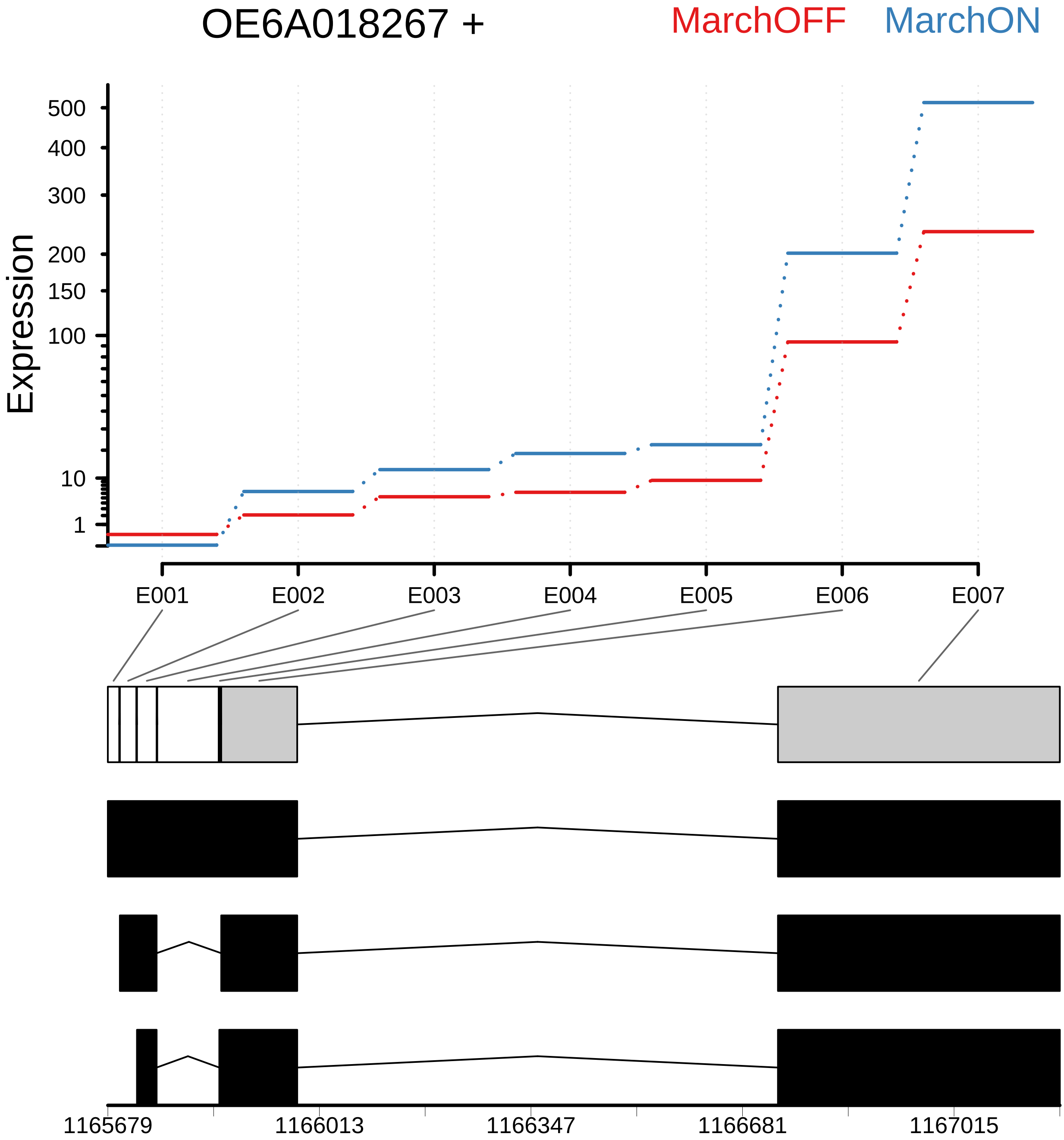

OE6A018267 +
MarchOFF
MarchON
Expression
500
400
300
200
150
100
10
1
E001
E002
E003
E004
E005
E006
E007
1165679
1166013
1166347
1166681
1167015

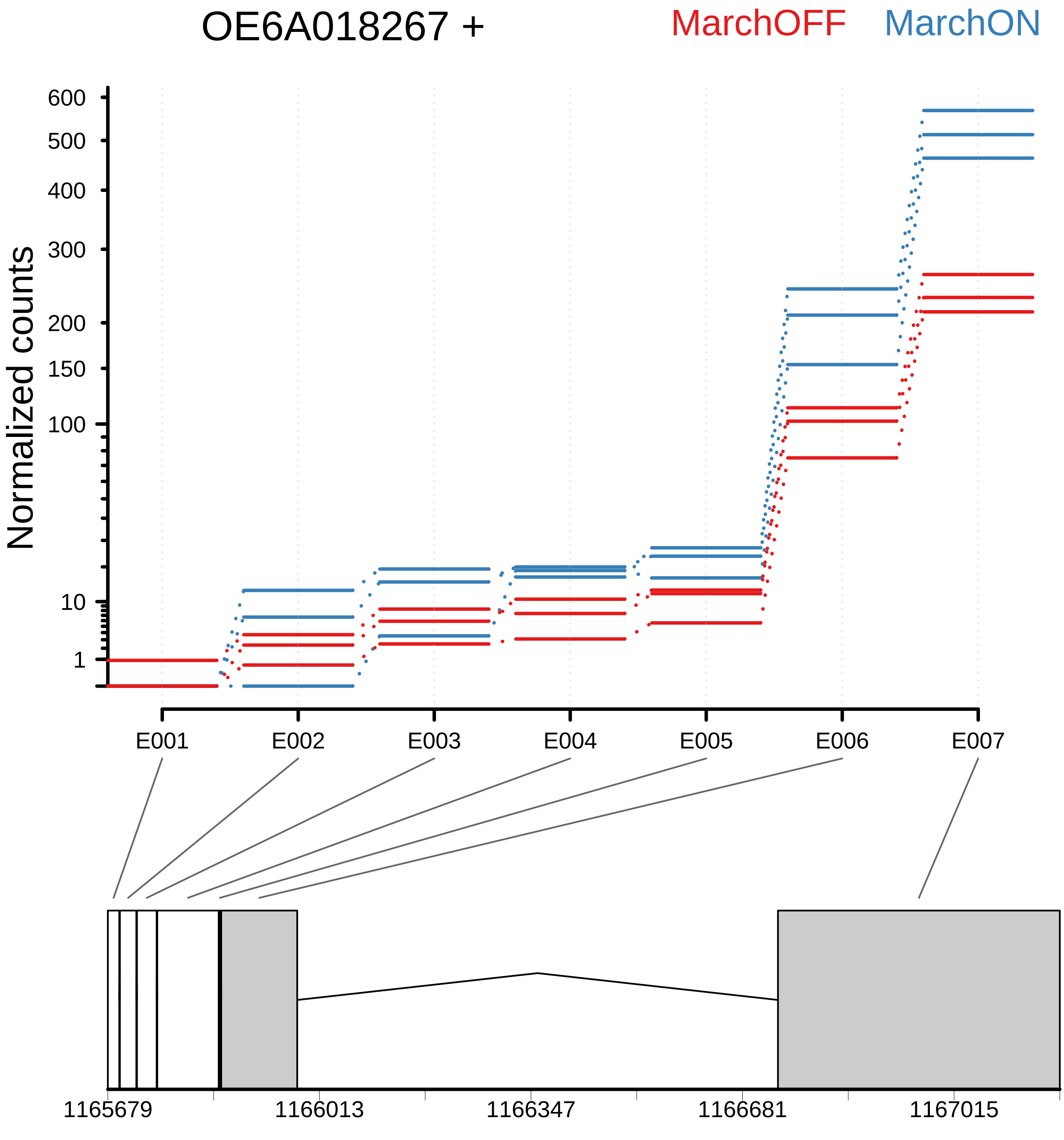

OE6A018267 +
MarchOFF
MarchON
Normalized counts
600
500
400
300
200
150
100
10
1
E001
E002
E003
E004
E005
E006
E007
1165679
1166013
1166347
1166681
1167015

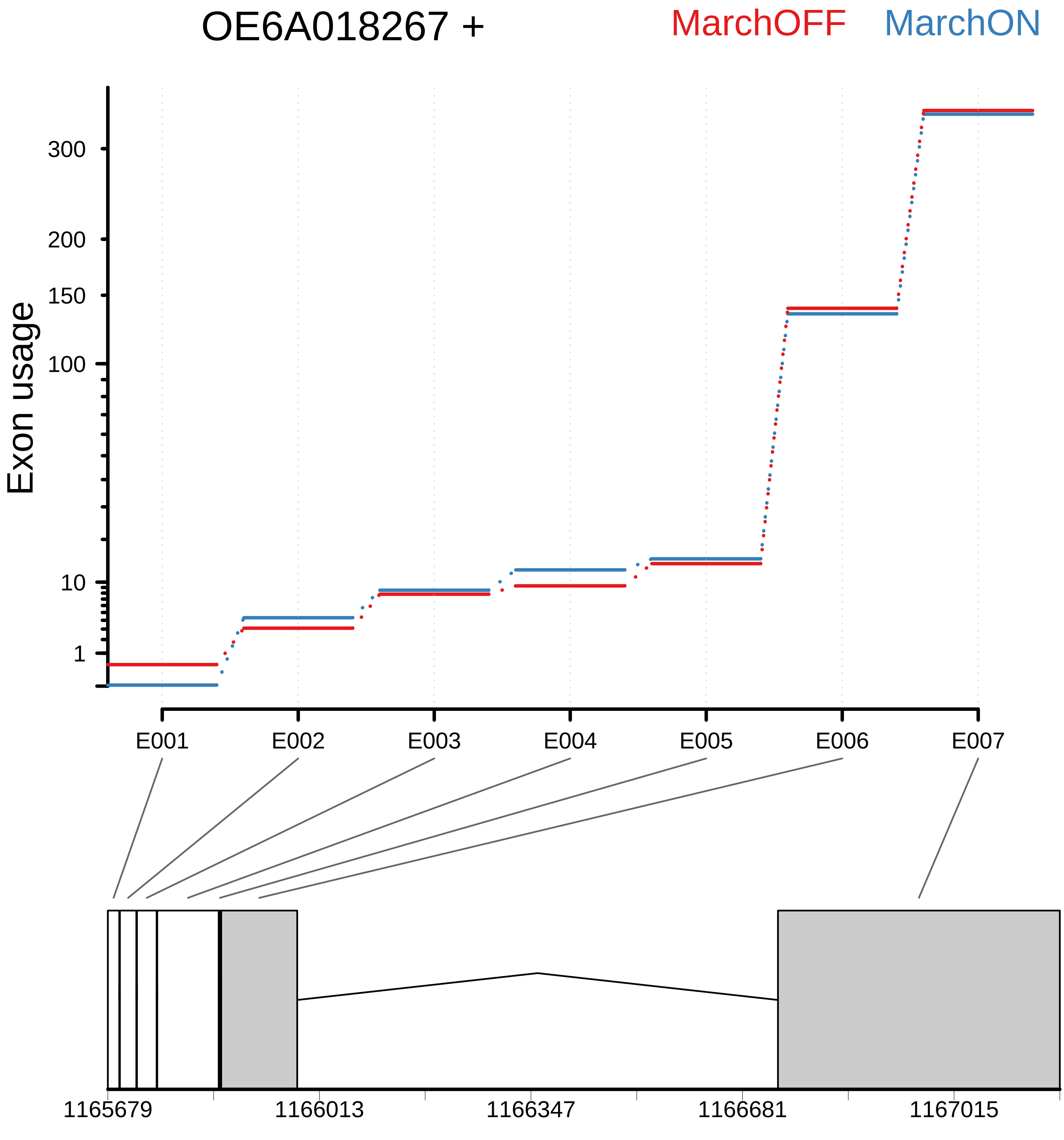

OE6A018267 +
MarchOFF
MarchON
Exon usage
300
200
150
100
10
1
E001
E002
E003
E004
E005
E006
E007
1165679
1166013
1166347
1166681
1167015

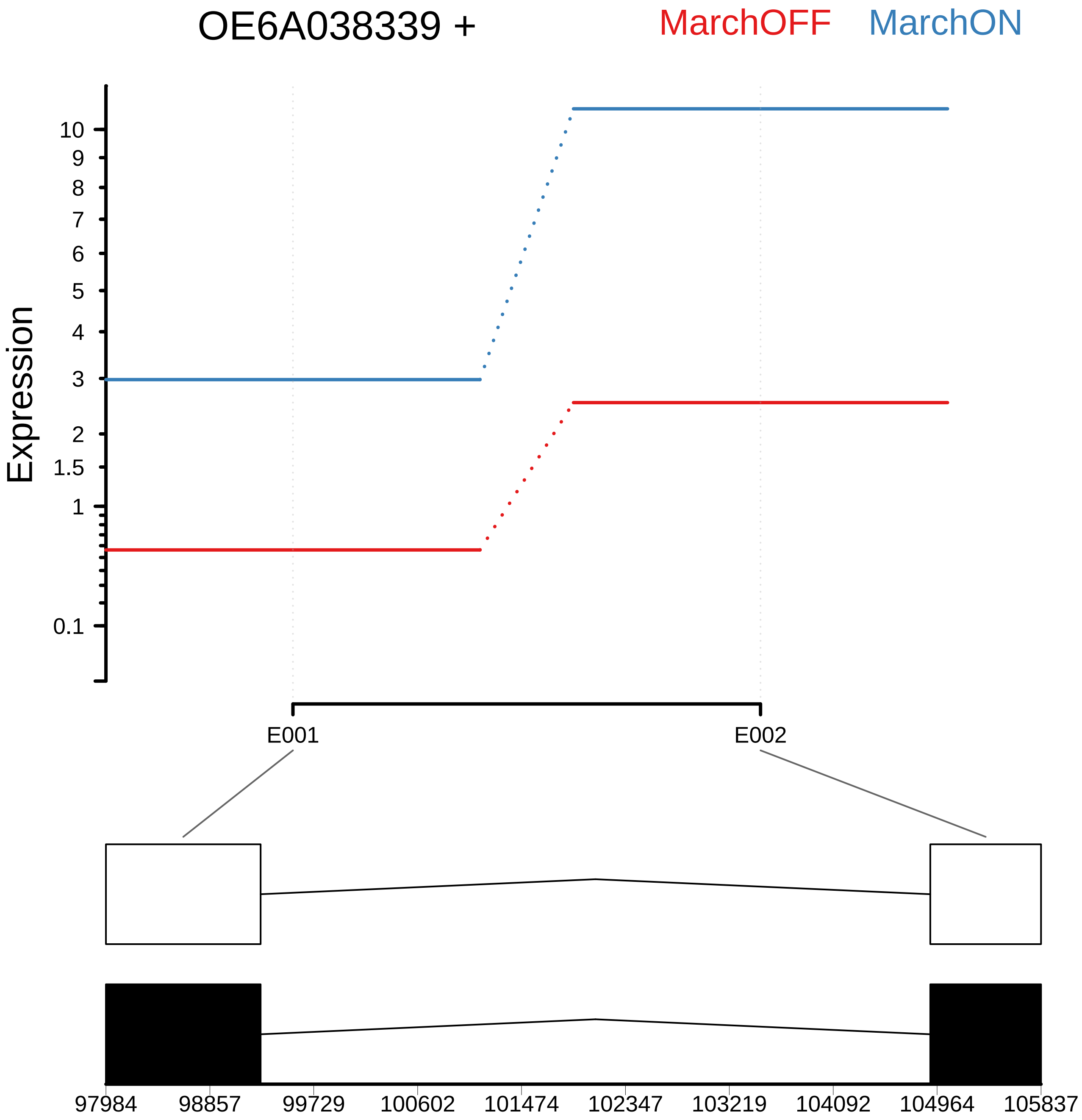

OE6A038339 +
MarchOFF
MarchON
Expression
10
9
8
7
6
5
4
3
2
1.5
1
0.1
E001
E002
97984
98857
99729
100602
101474
102347
103219
104092
104964
105837

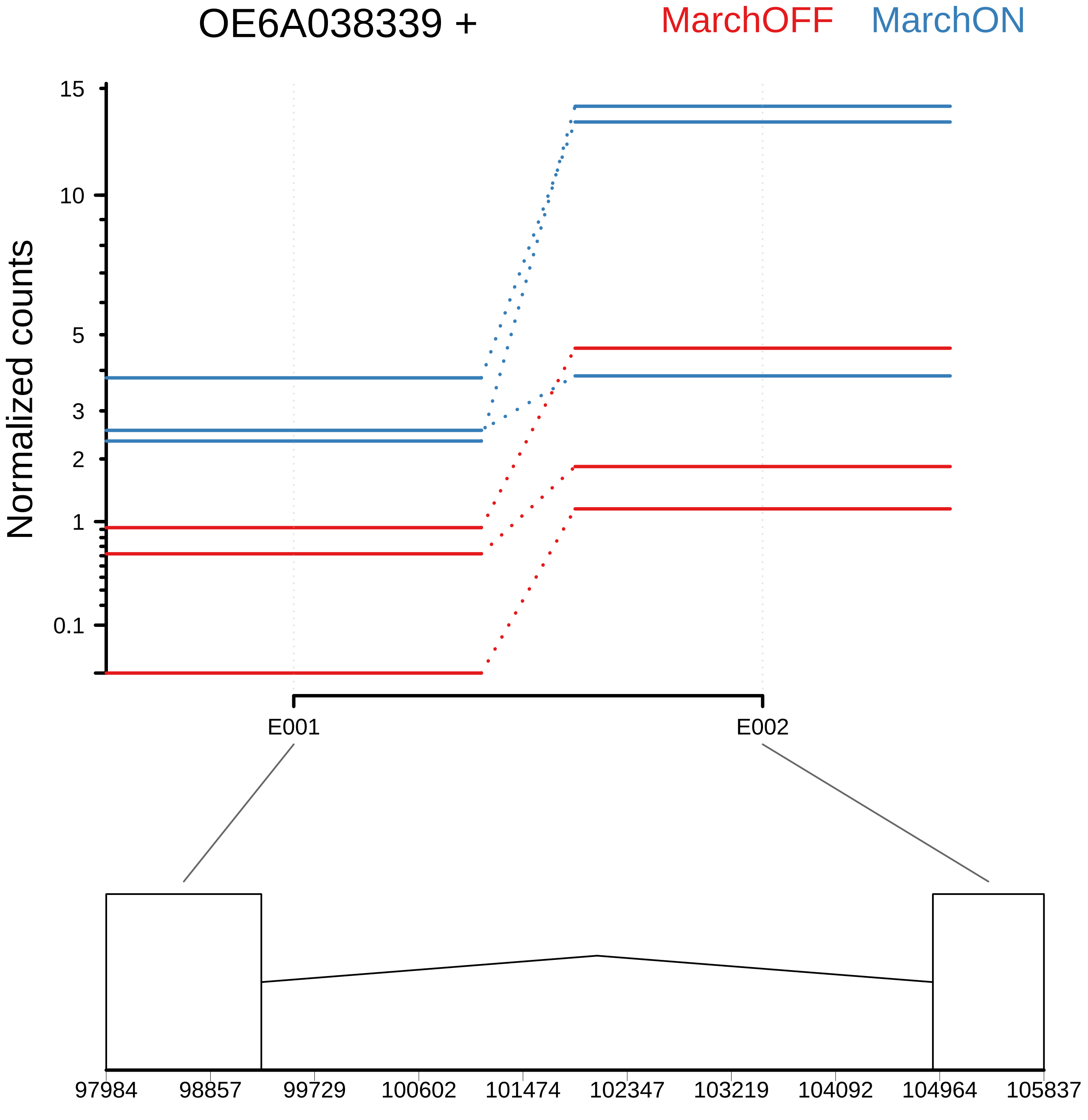
OE6A038339 +
MarchOFF
MarchON
Normalized counts
15
10
5
3
2
1
0.1
E001
E002
97984
98857
99729
100602
101474
102347
103219
104092
104964
105837

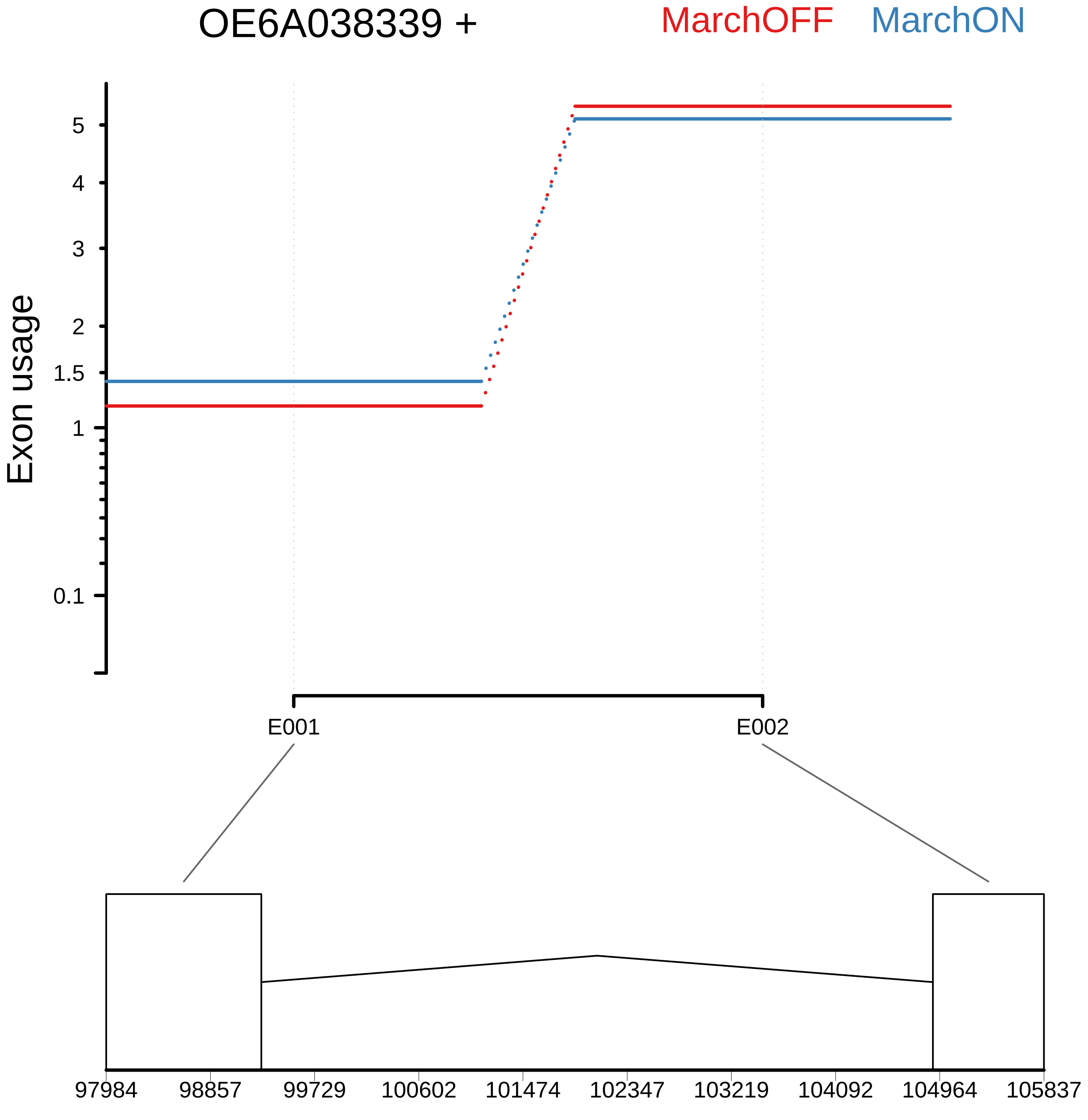

OE6A038339 +
MarchOFF
MarchON
Exon usage
5
4
3
2
1.5
1
0.1
E001
E002
97984
98857
99729
100602
101474
102347
103219
104092
104964
105837

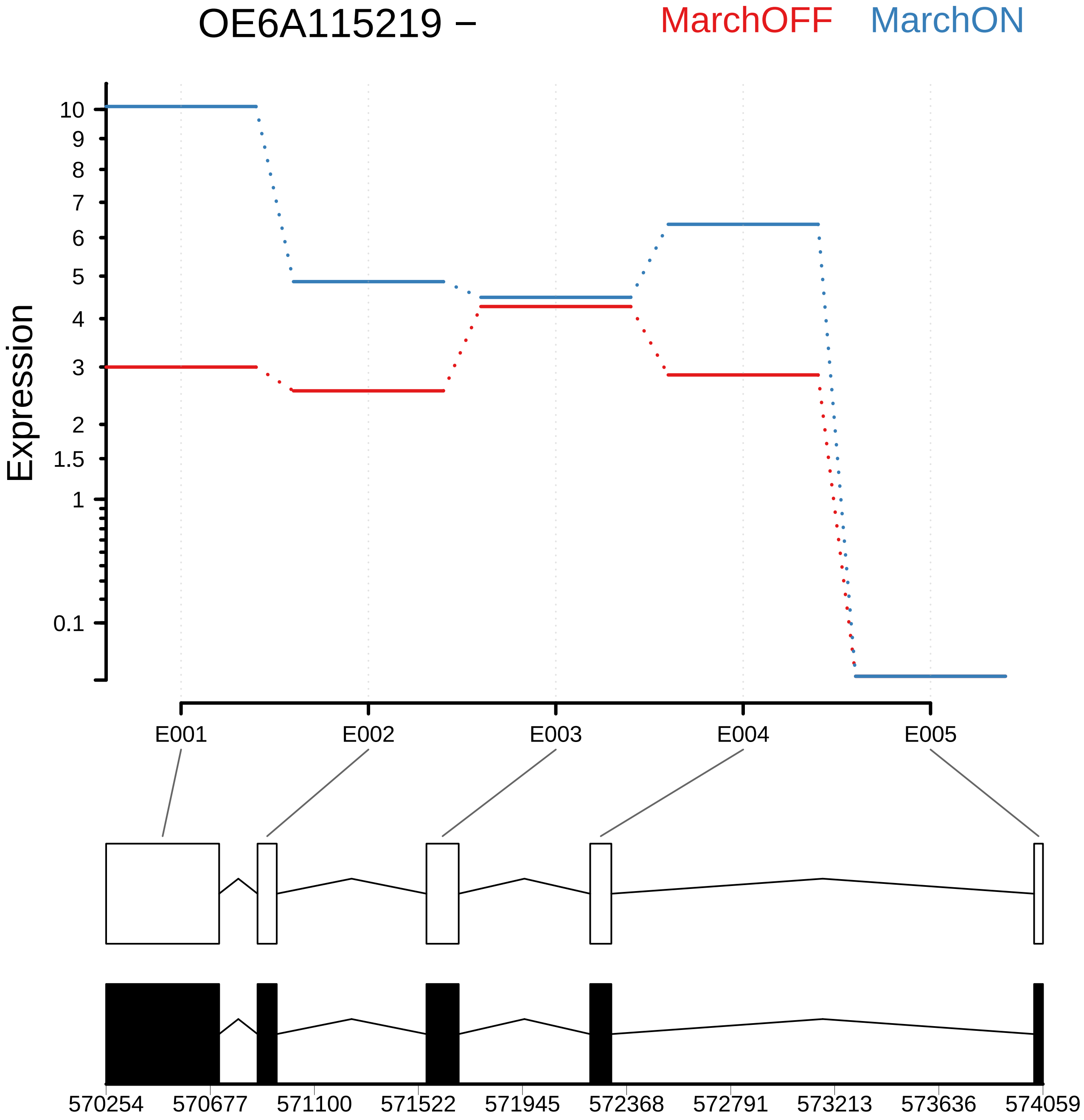

OE6A115219 –
MarchOFF
MarchON
Expression
10
9
8
7
6
5
4
3
2
1.5
1
0.1
E001
E002
E003
E004
E005
570254
570677
571100
571522
571945
572368
572791
573213
573636
574059

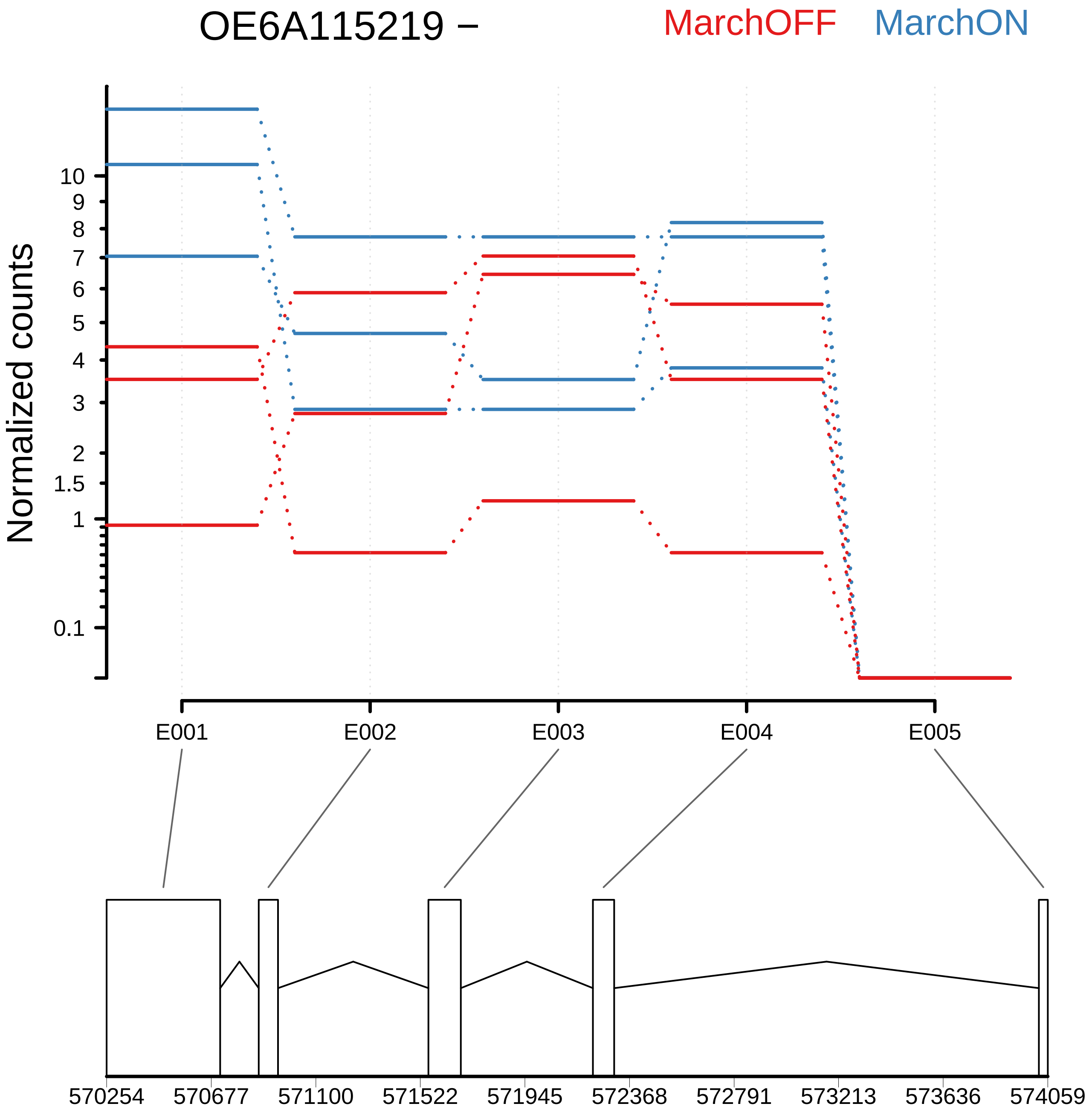

OE6A115219 −
MarchOFF
MarchON
Normalized counts
10
9
8
7
6
5
4
3
2
1.5
1
0.1
E001
E002
E003
E004
E005
570254
570677
571100
571522
571945
572368
572791
573213
573636
574059

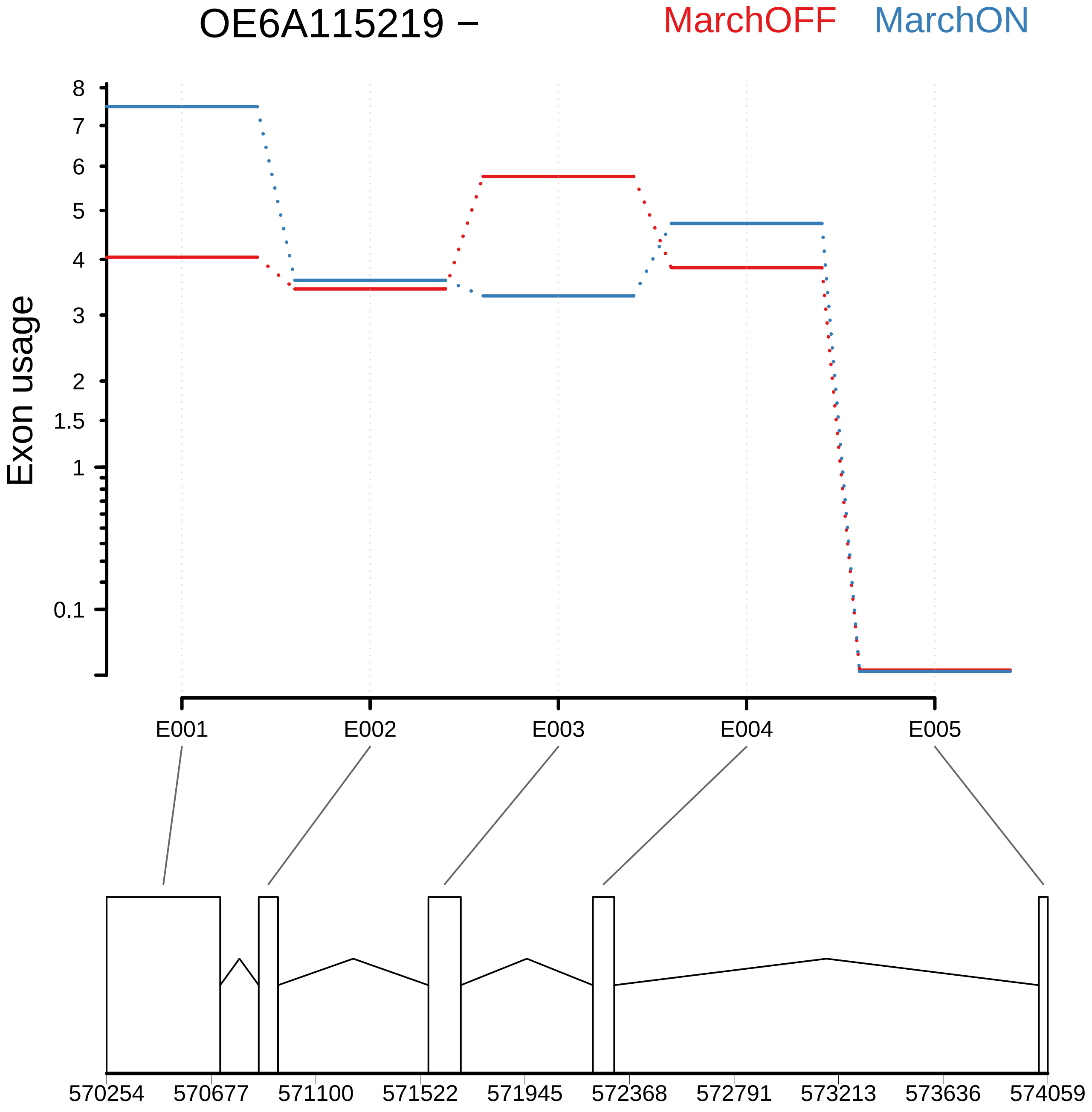

OE6A115219 −
MarchOFF
MarchON
Exon usage
8
7
6
5
4
3
2
1.5
1
0.1
E001
E002
E003
E004
E005
570254
570677
571100
571522
571945
572368
572791
573213
573636
574059

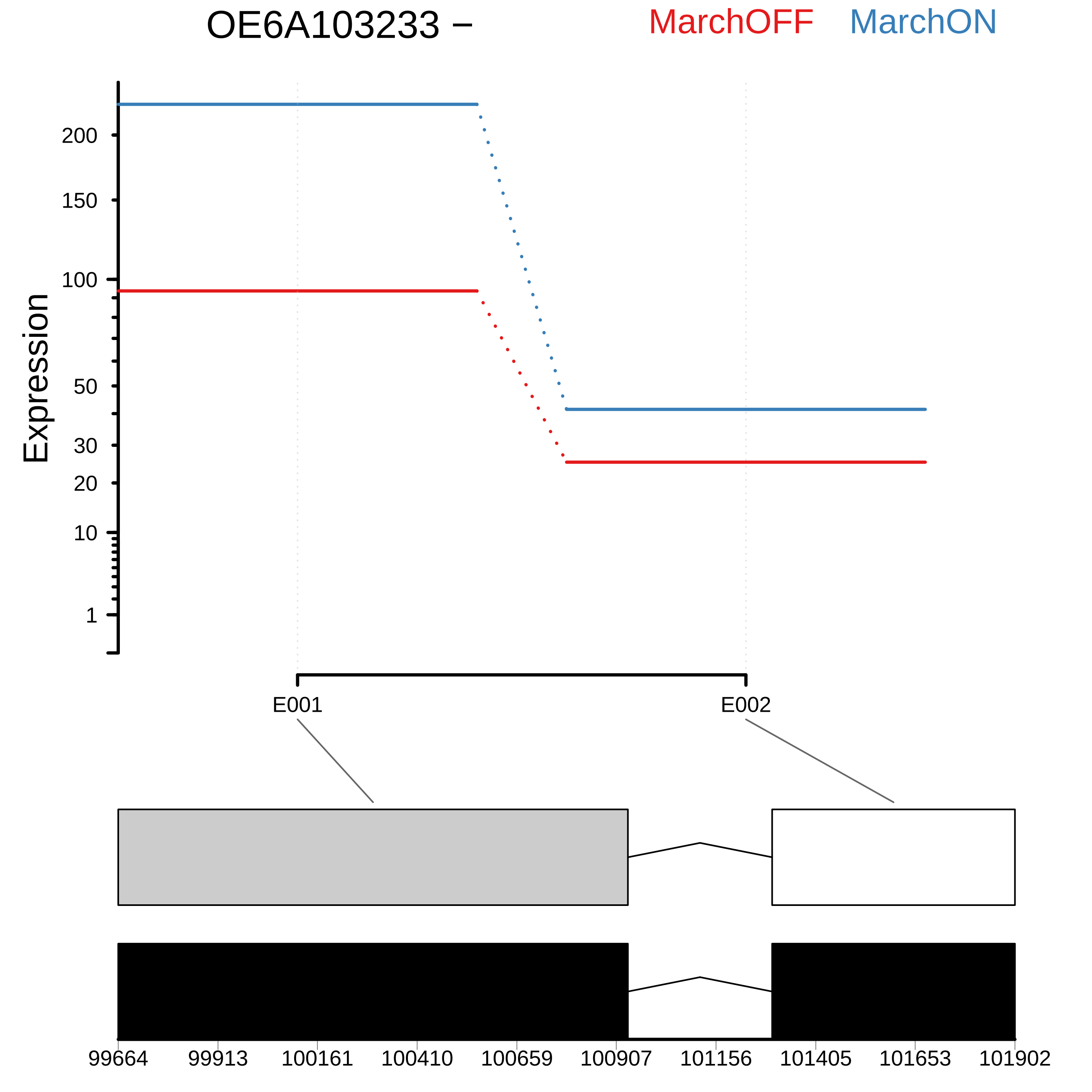
OE6A103233 −
MarchOFF
MarchON
Expression
200
150
100
50
30
20
10
1
E001
E002
99664
99913
100161
100410
100659
100907
101156
101405
101653
101902

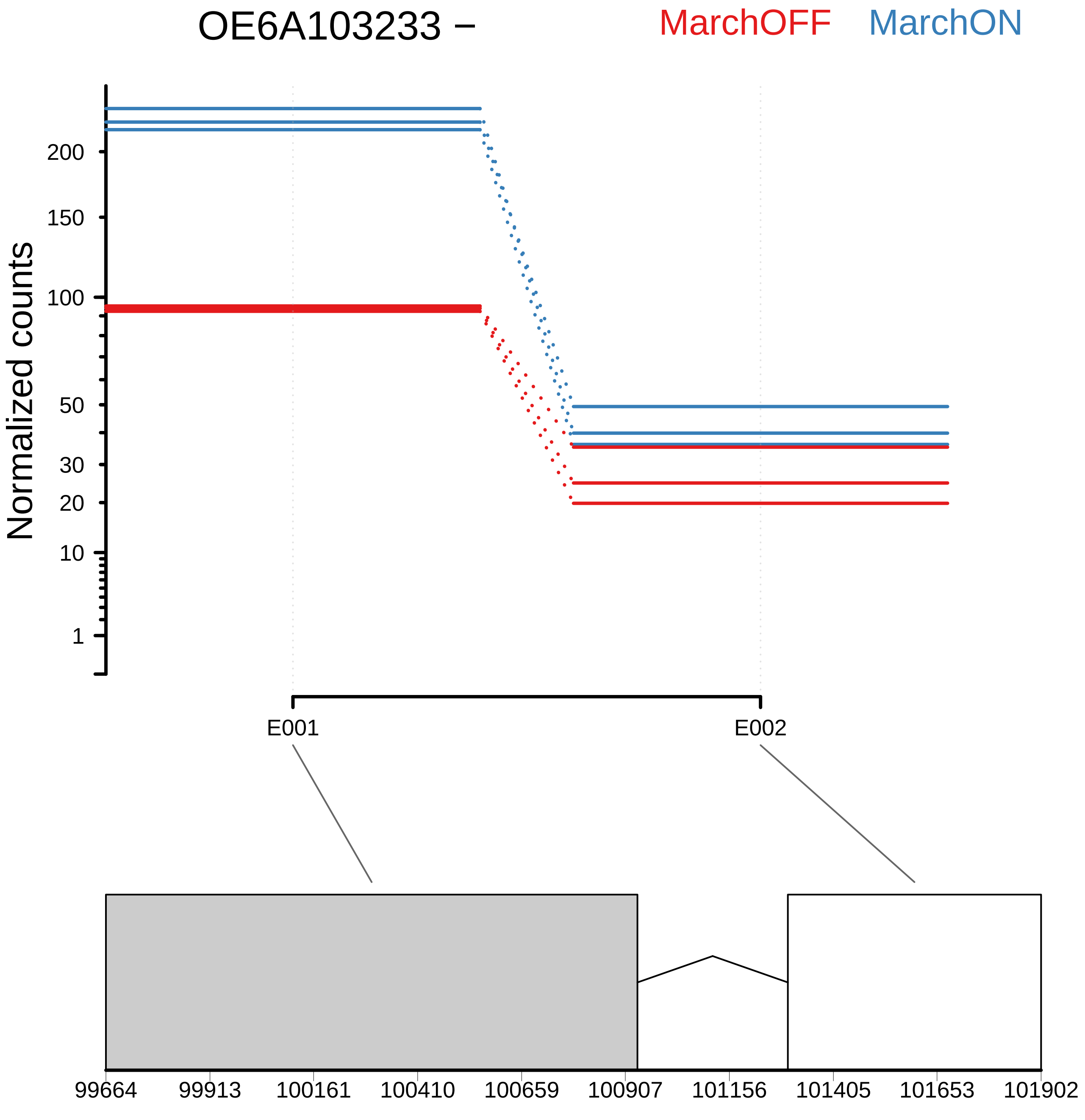
OE6A103233 −
MarchOFF
MarchON
Normalized counts
200
150
100
50
30
20
10
1
E001
E002
99664
99913
100161
100410
100659
100907
101156
101405
101653
101902

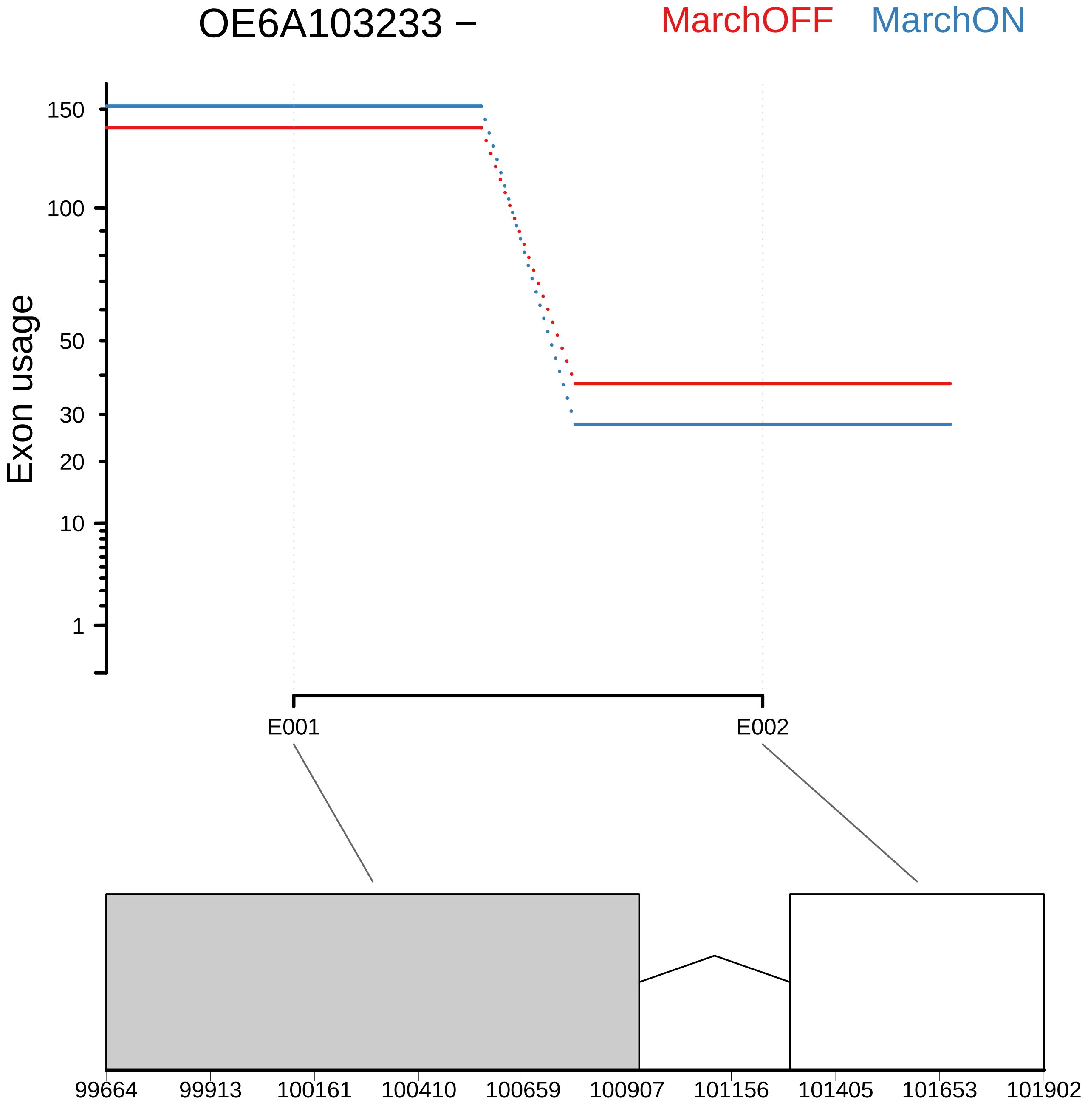

OE6A103233 −
MarchOFF
MarchON
Exon usage
150
100
50
30
20
10
1
E001
E002
99664
99913
100161
100410
100659
100907
101156
101405
101653
101902

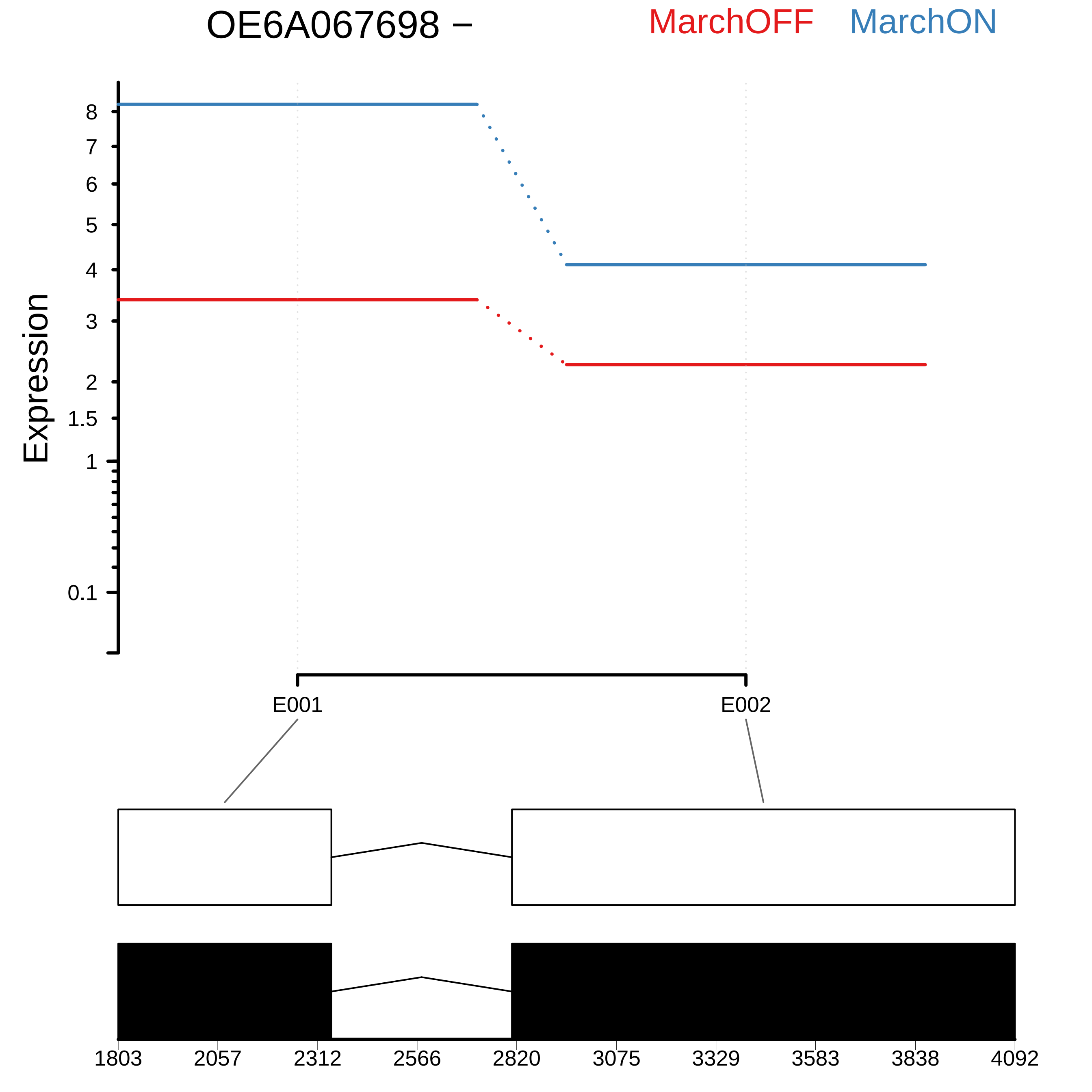
OE6A067698 −
MarchOFF
MarchON
Expression
8
7
6
5
4
3
2
1.5
1
0.1
E001
E002
1803
2057
2312
2566
2820
3075
3329
3583
3838
4092

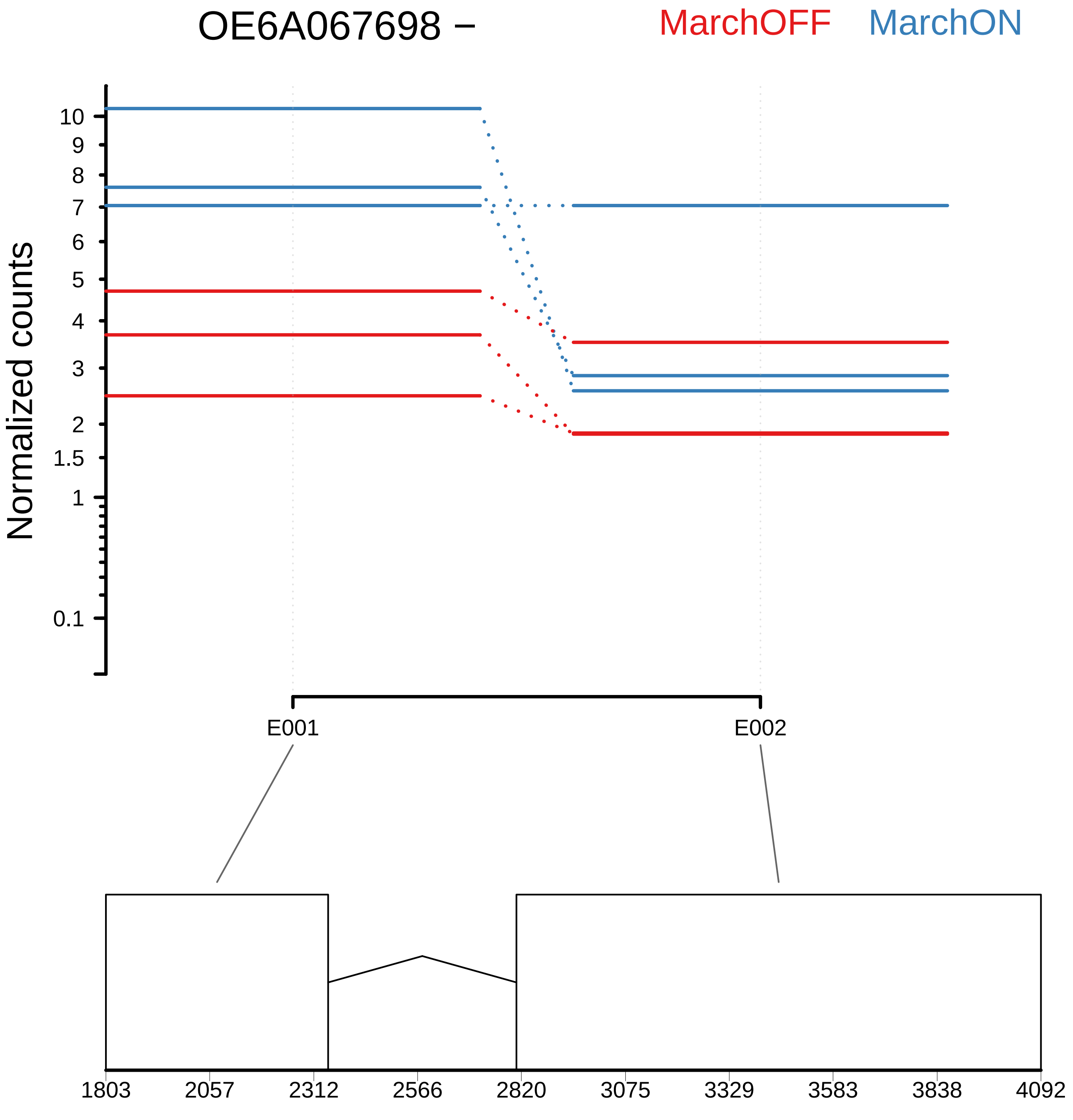
OE6A067698 −
MarchOFF
MarchON
Normalized counts
10
9
8
7
6
5
4
3
2
1.5
1
0.1
E001
E002
1803
2057
2312
2566
2820
3075
3329
3583
3838
4092

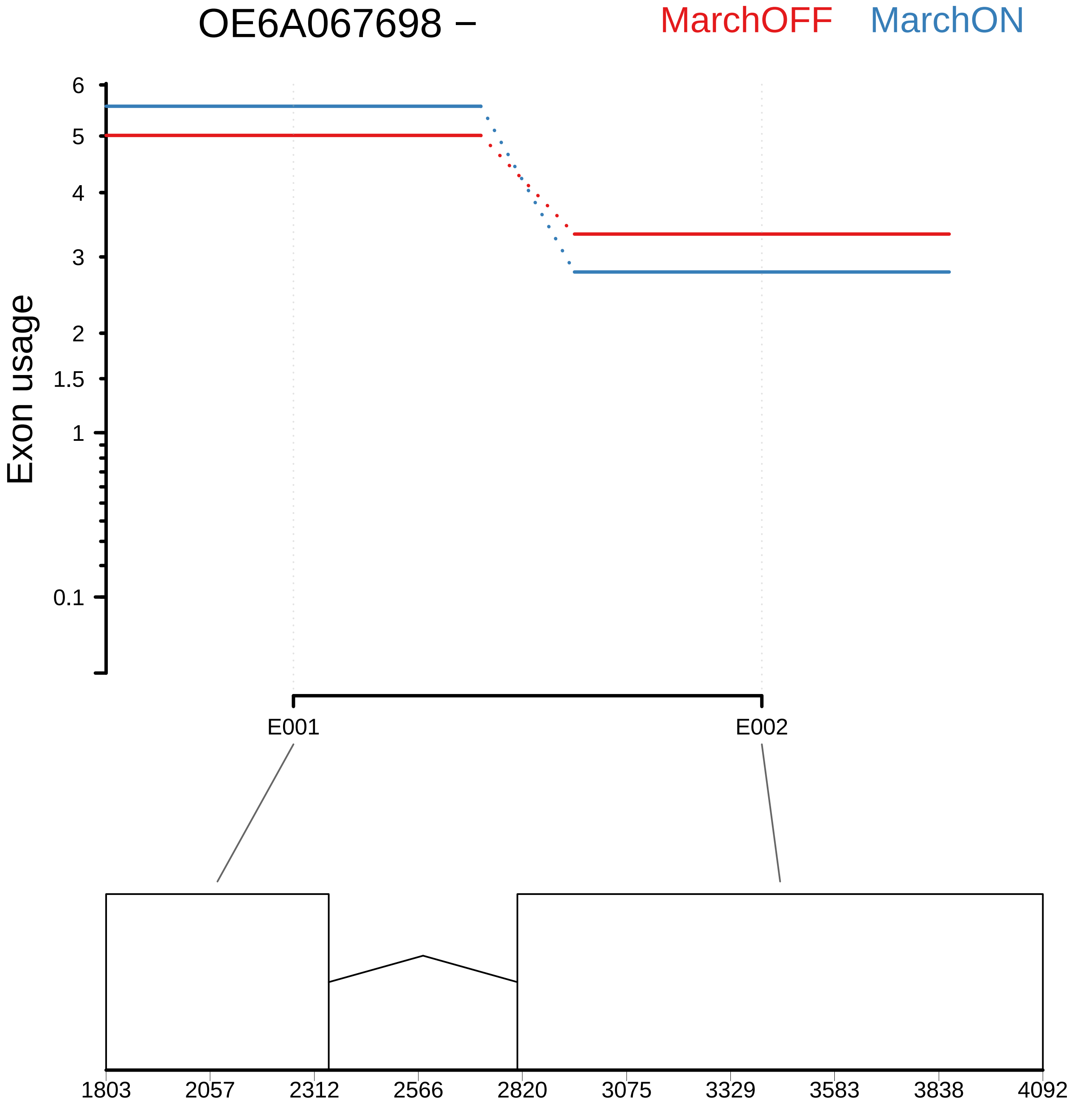
OE6A067698 −
MarchOFF
MarchON
Exon usage
6
5
4
3
2
1.5
1
0.1
E001
E002
1803
2057
2312
2566
2820
3075
3329
3583
3838
4092

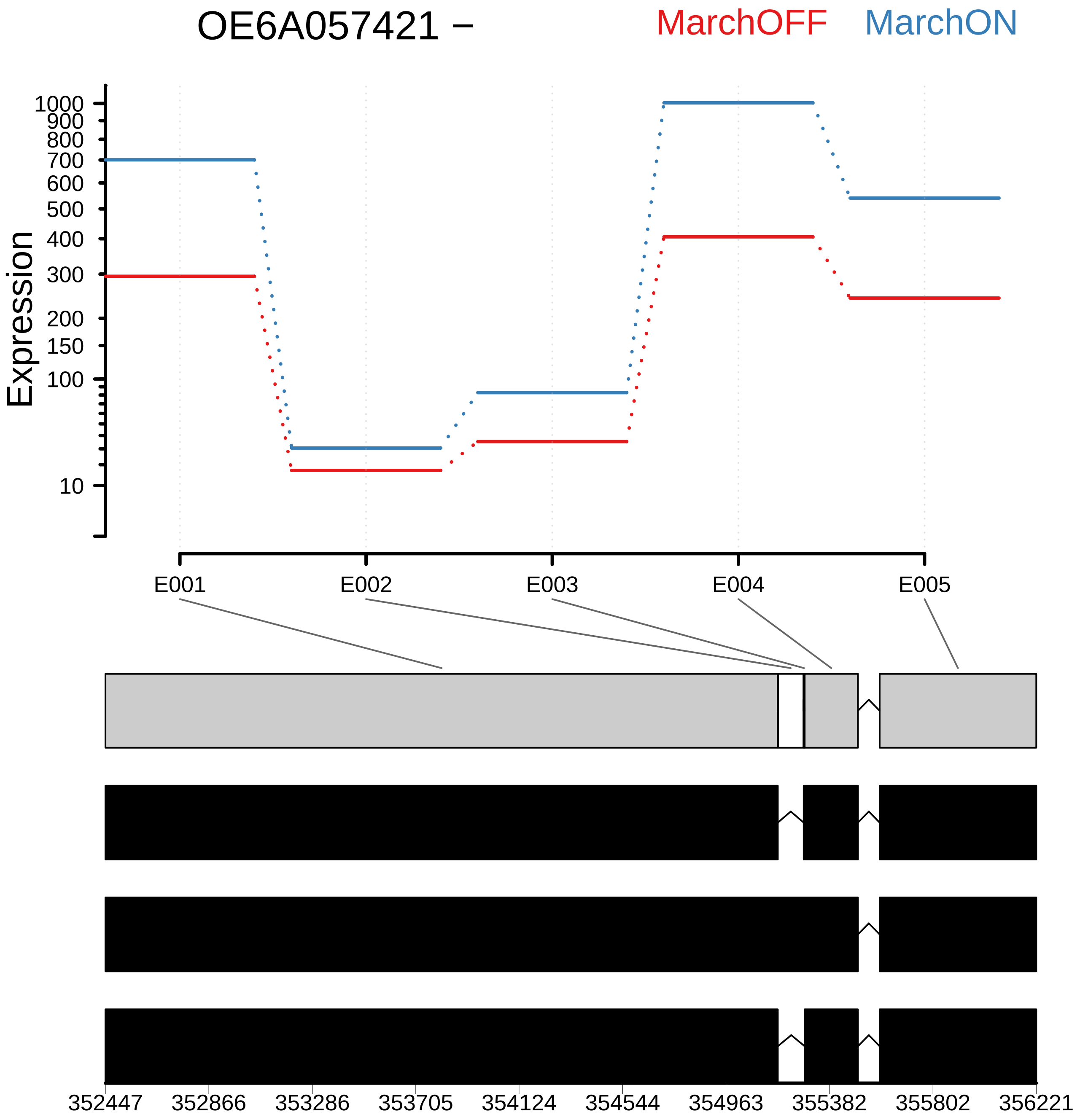
OE6A057421 −
MarchOFF
MarchON
Expression
1000
900
800
700
600
500
400
300
200
150
100
10
E001
E002
E003
E004
E005
352447
352866
353286
353705
354124
354544
354963
355382
355802
356221

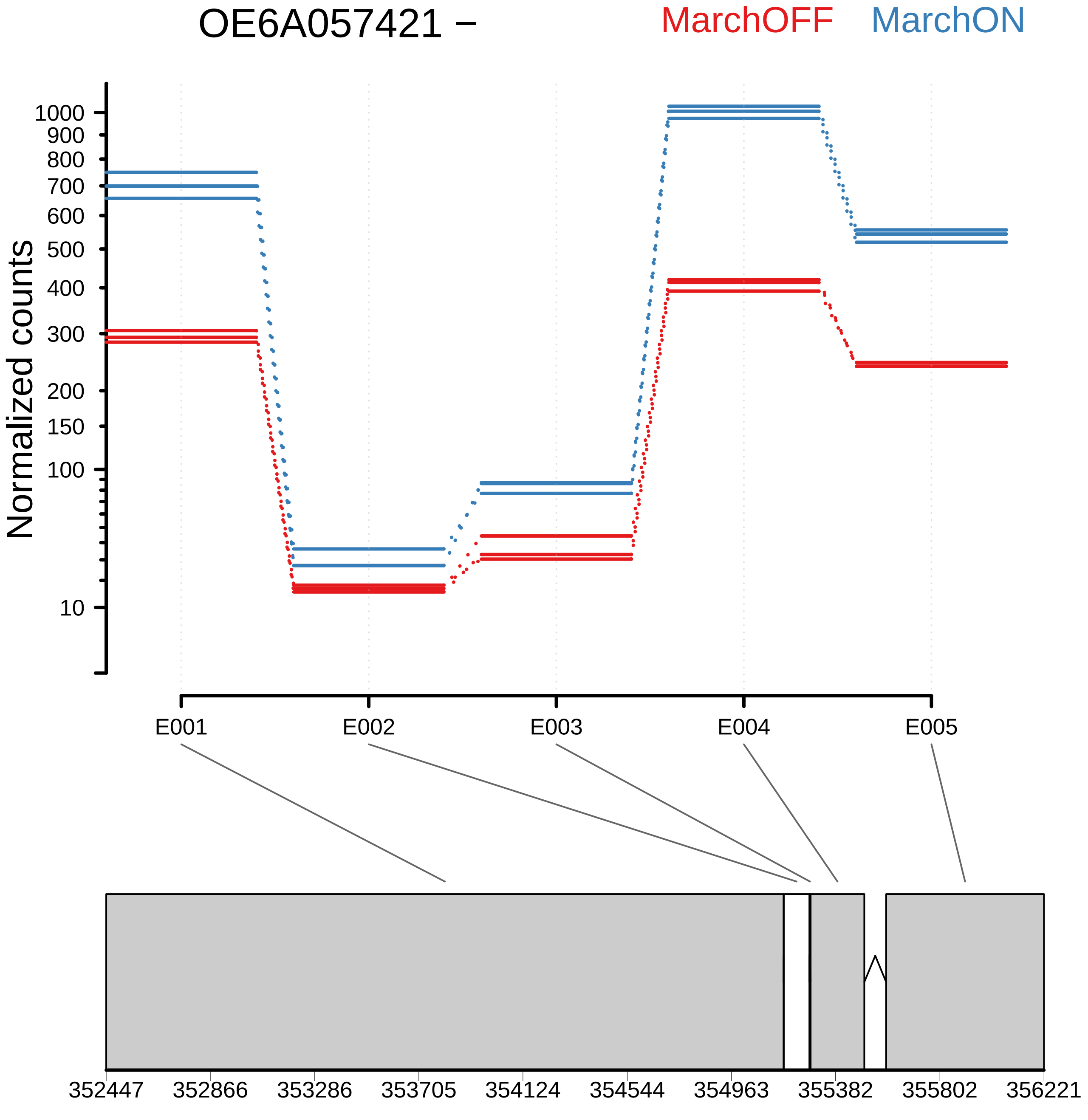

OE6A057421 −
MarchOFF
MarchON
Normalized counts
1000
900
800
700
600
500
400
300
200
150
100
10
E001
E002
E003
E004
E005
352447
352866
353286
353705
354124
354544
354963
355382
355802
356221

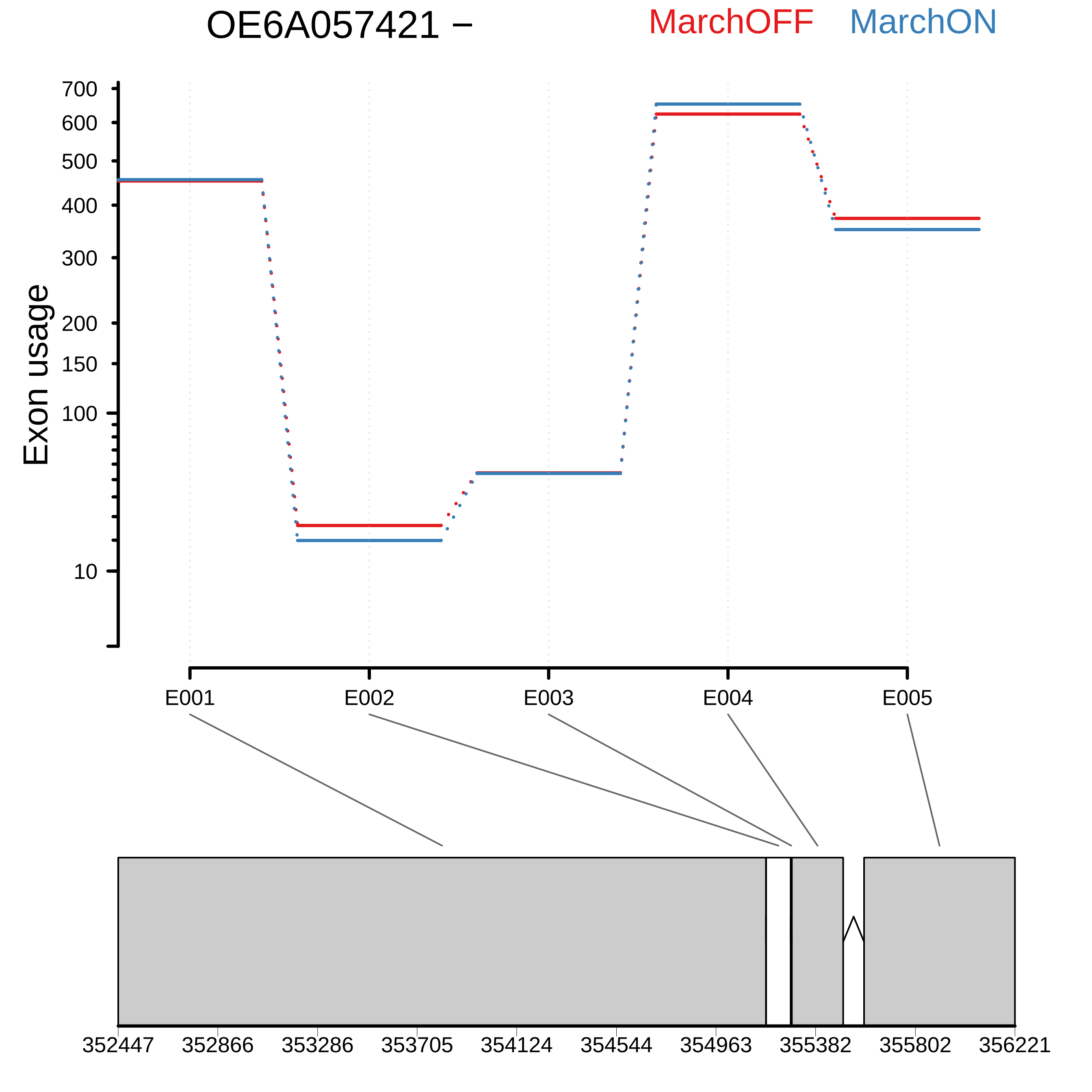

OE6A057421 −
MarchOFF
MarchON
Exon usage
700
600
500
400
300
200
150
100
10
E001
E002
E003
E004
E005
352447
352866
353286
353705
354124
354544
354963
355382
355802
356221

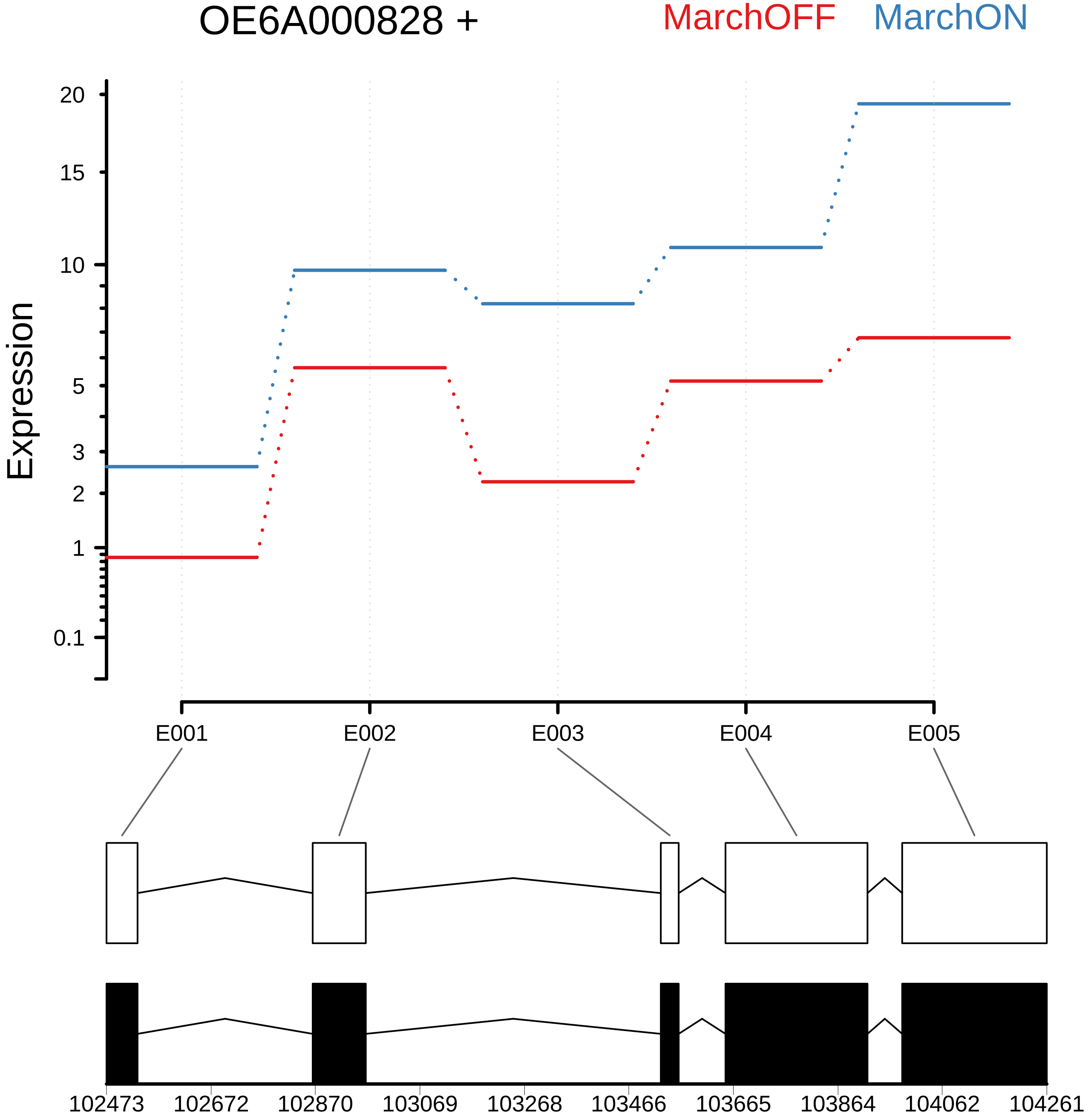
OE6A000828 +
MarchOFF
MarchON
Expression
20
15
10
5
3
2
1
0.1
E001
E002
E003
E004
E005
102473
102672
102870
103069
103268
103466
103665
103864
104062
104261

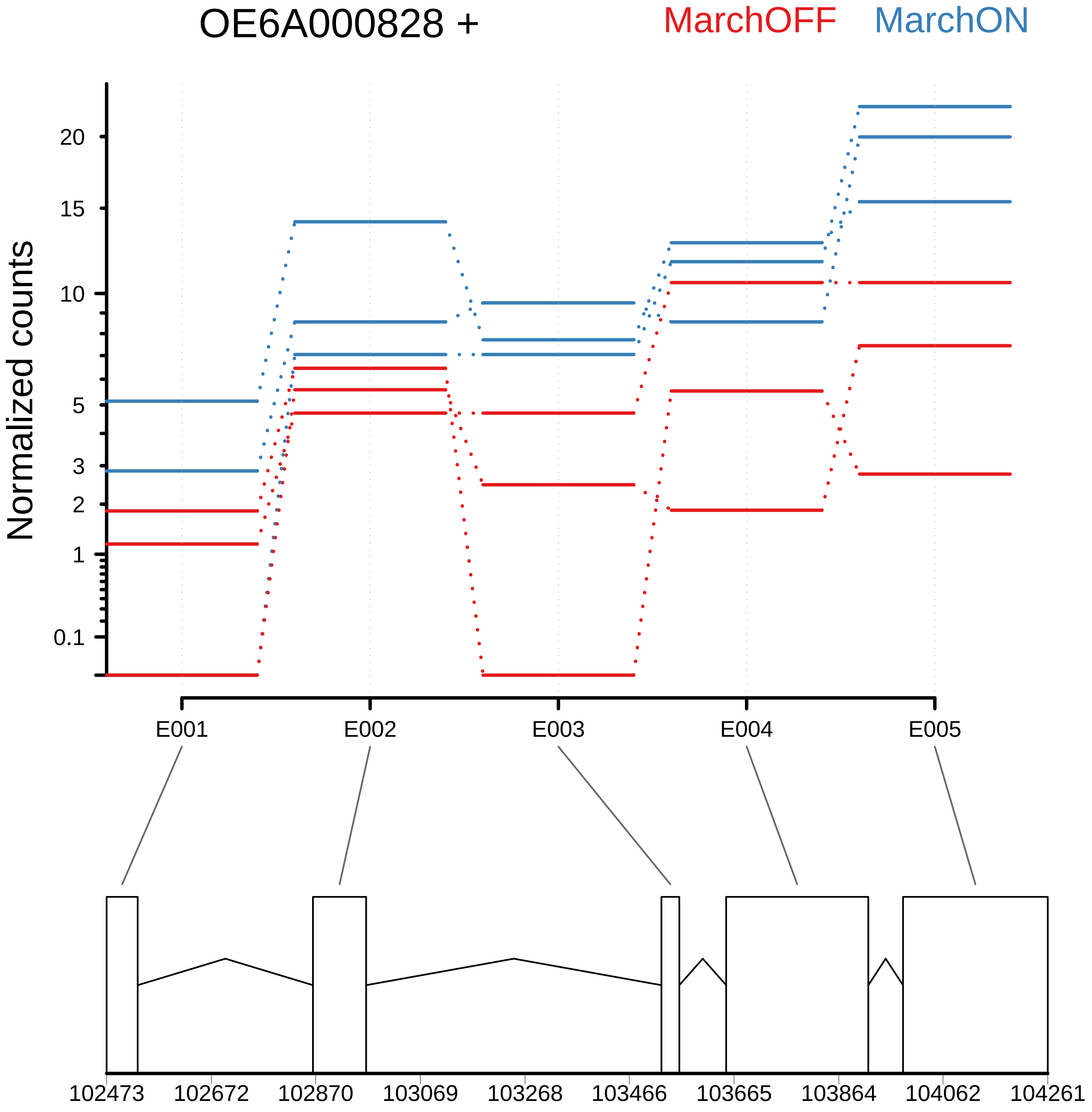

OE6A000828 +
MarchOFF
MarchON
Normalized counts
20
15
10
5
3
2
1
0.1
E001
E002
E003
E004
E005
102473
102672
102870
103069
103268
103466
103665
103864
104062
104261

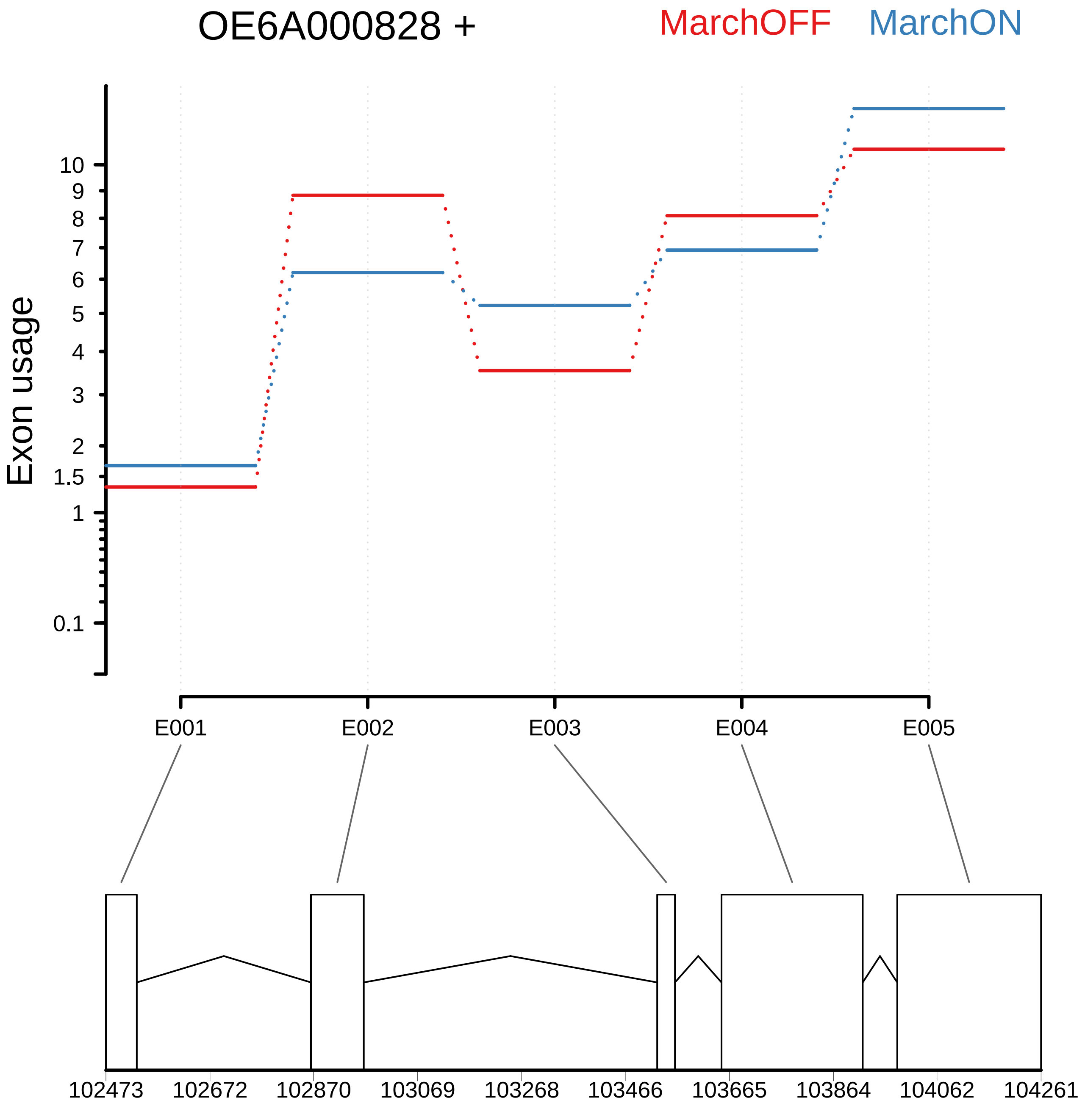

OE6A000828 +
MarchOFF
MarchON
Exon usage
10
9
8
7
6
5
4
3
2
1.5
1
0.1
E001
E002
E003
E004
E005
102473
102672
102870
103069
103268
103466
103665
103864
104062
104261

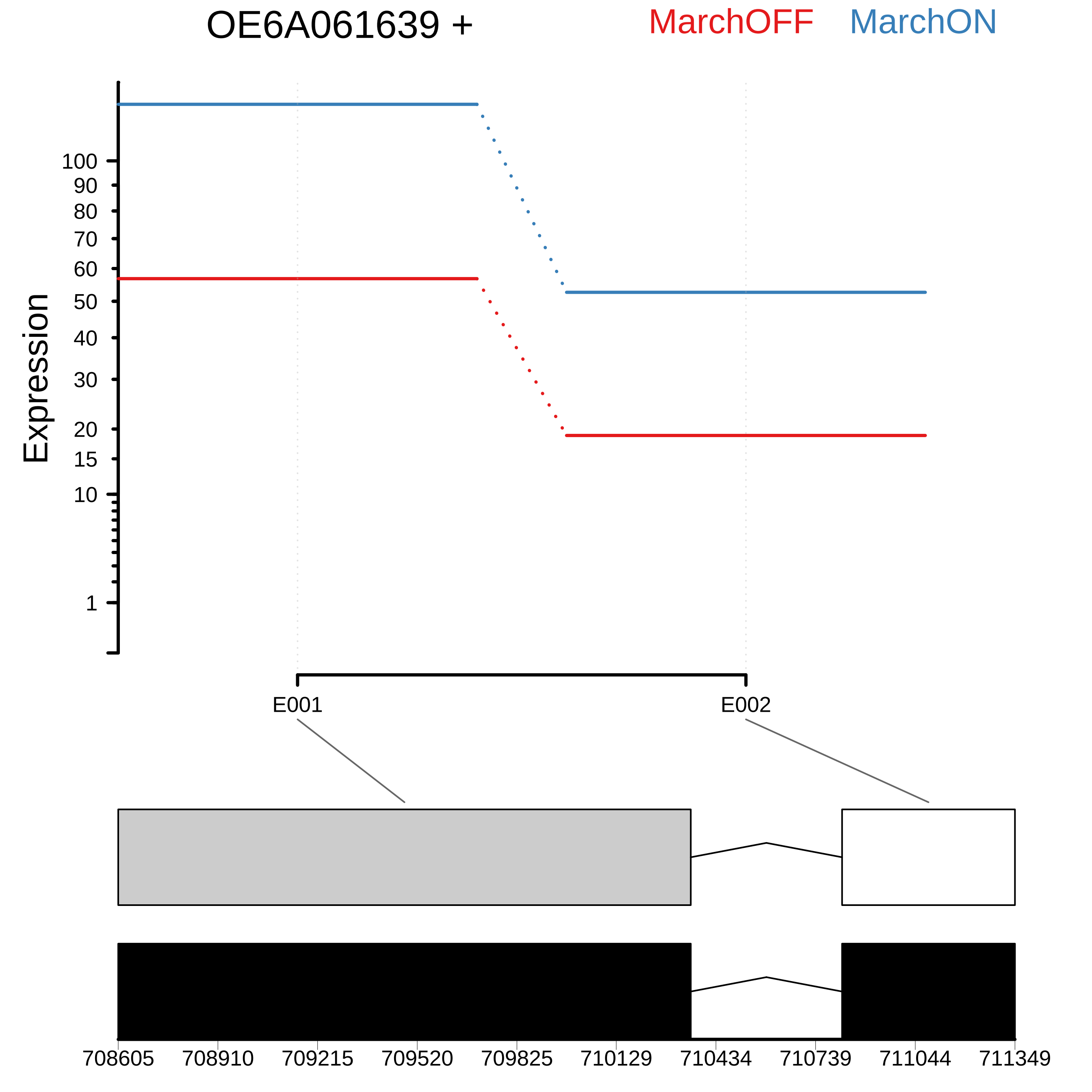
OE6A061639 +
MarchOFF
MarchON
Expression
100
90
80
70
60
50
40
30
20
15
10
1
E001
E002
708605
708910
709215
709520
709825
710129
710434
710739
711044
711349

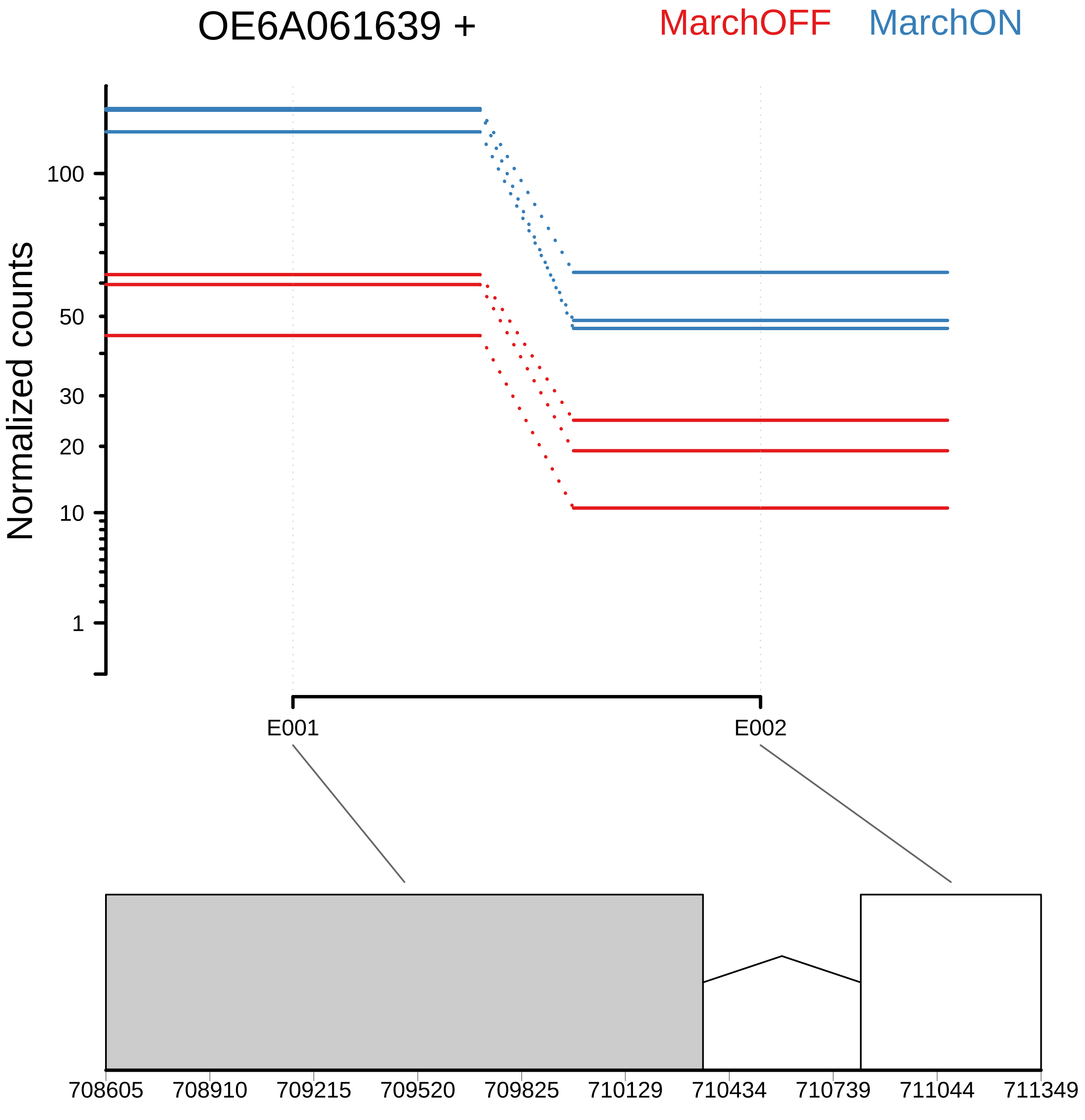
OE6A061639 +
MarchOFF
MarchON
Normalized counts
100
50
30
20
10
1
E001
E002
708605
708910
709215
709520
709825
710129
710434
710739
711044
711349

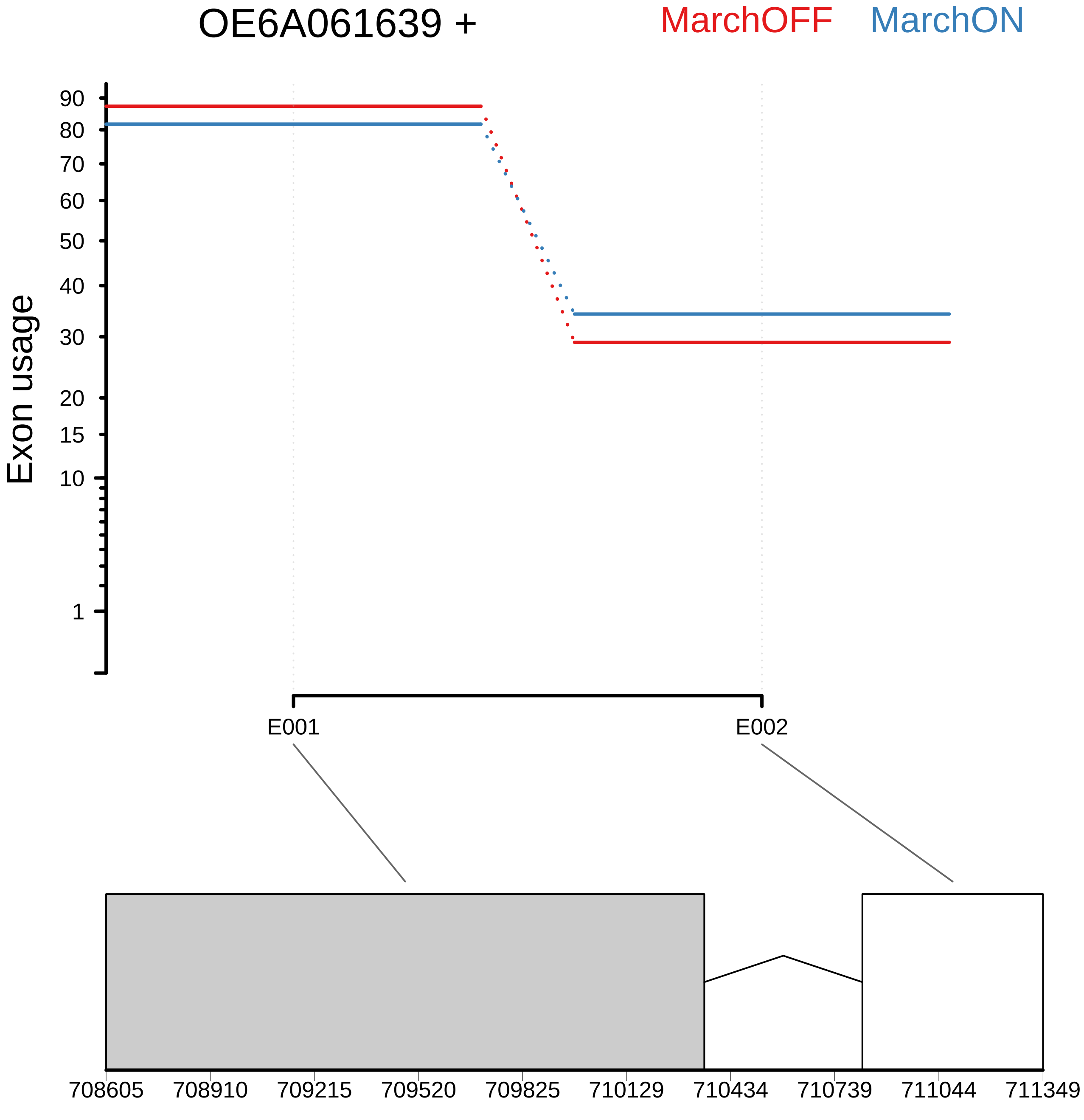

OE6A061639 +
MarchOFF
MarchON
Exon usage
90
80
70
60
50
40
30
20
15
10
1
E001
E002
708605
708910
709215
709520
709825
710129
710434
710739
711044
711349

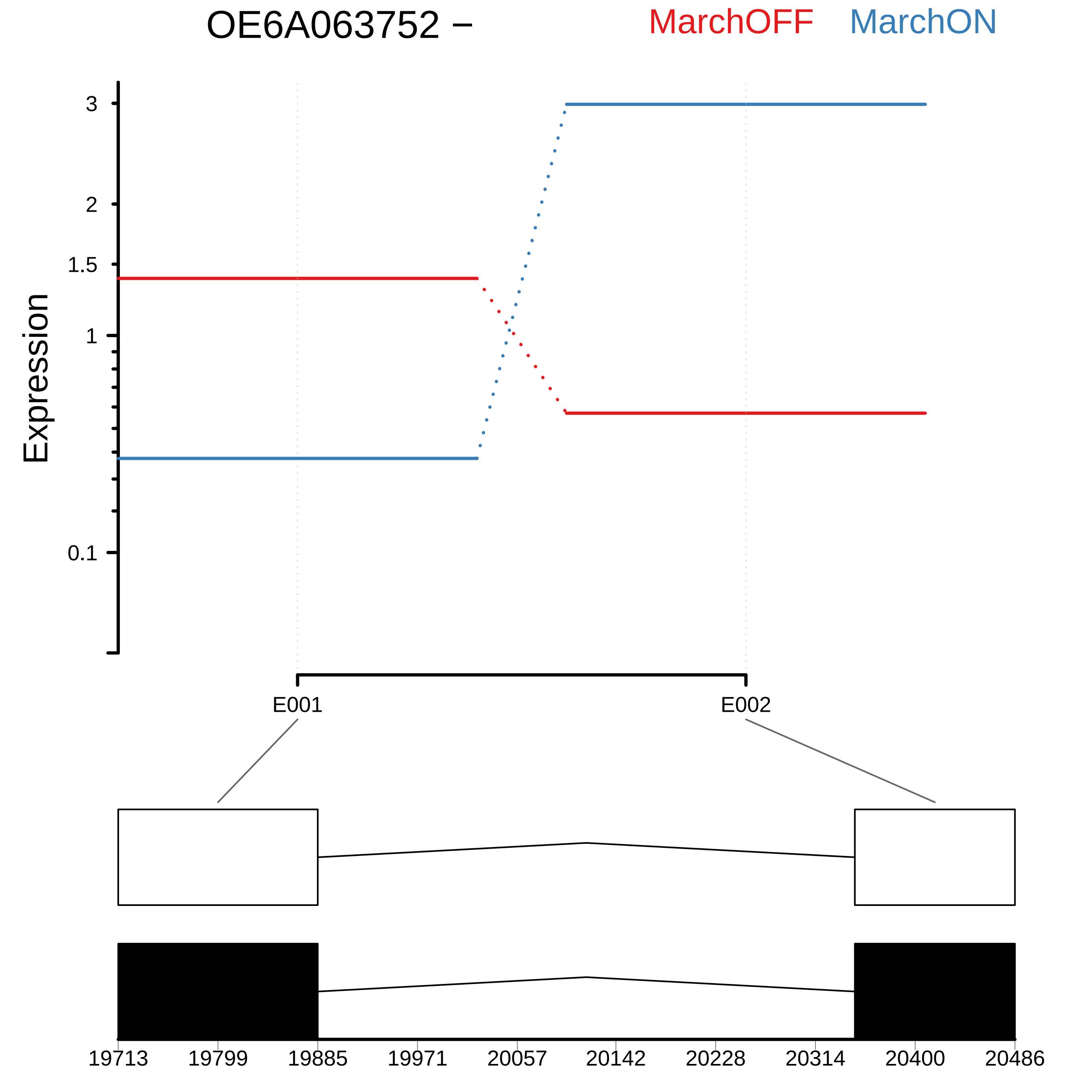

OE6A063752 −
MarchOFF
MarchON
Expression
3
2
1.5
1
0.1
E001
E002
19713
19799
19885
19971
20057
20142
20228
20314
20400
20486

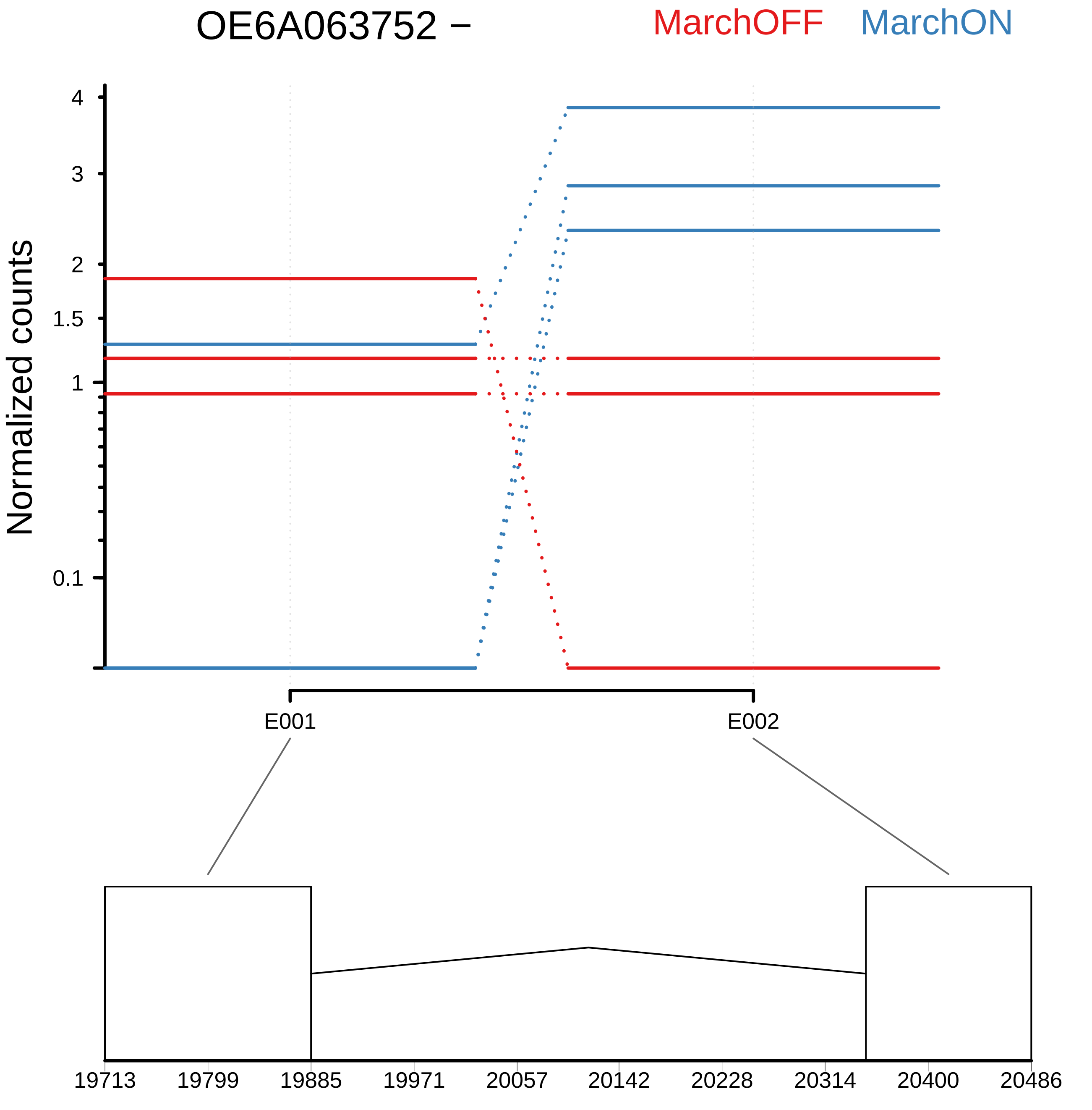
OE6A063752 −
MarchOFF
MarchON
Normalized counts
4
3
2
1.5
1
0.1
E001
E002
19713
19799
19885
19971
20057
20142
20228
20314
20400
20486

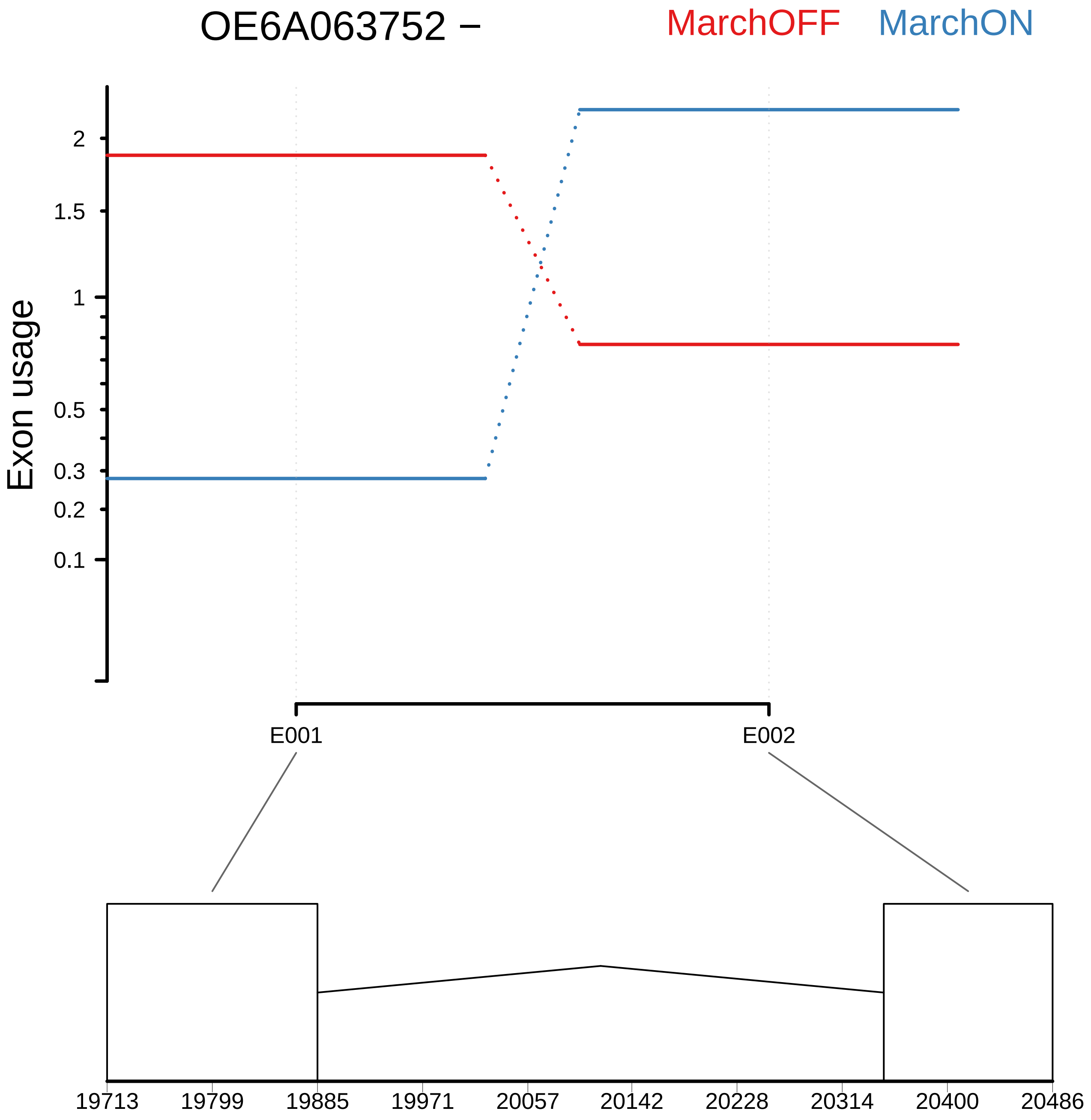

OE6A063752 −
MarchOFF
MarchON
Exon usage
2
1.5
1
0.5
0.3
0.2
0.1
E001
E002
19713
19799
19885
19971
20057
20142
20228
20314
20400
20486

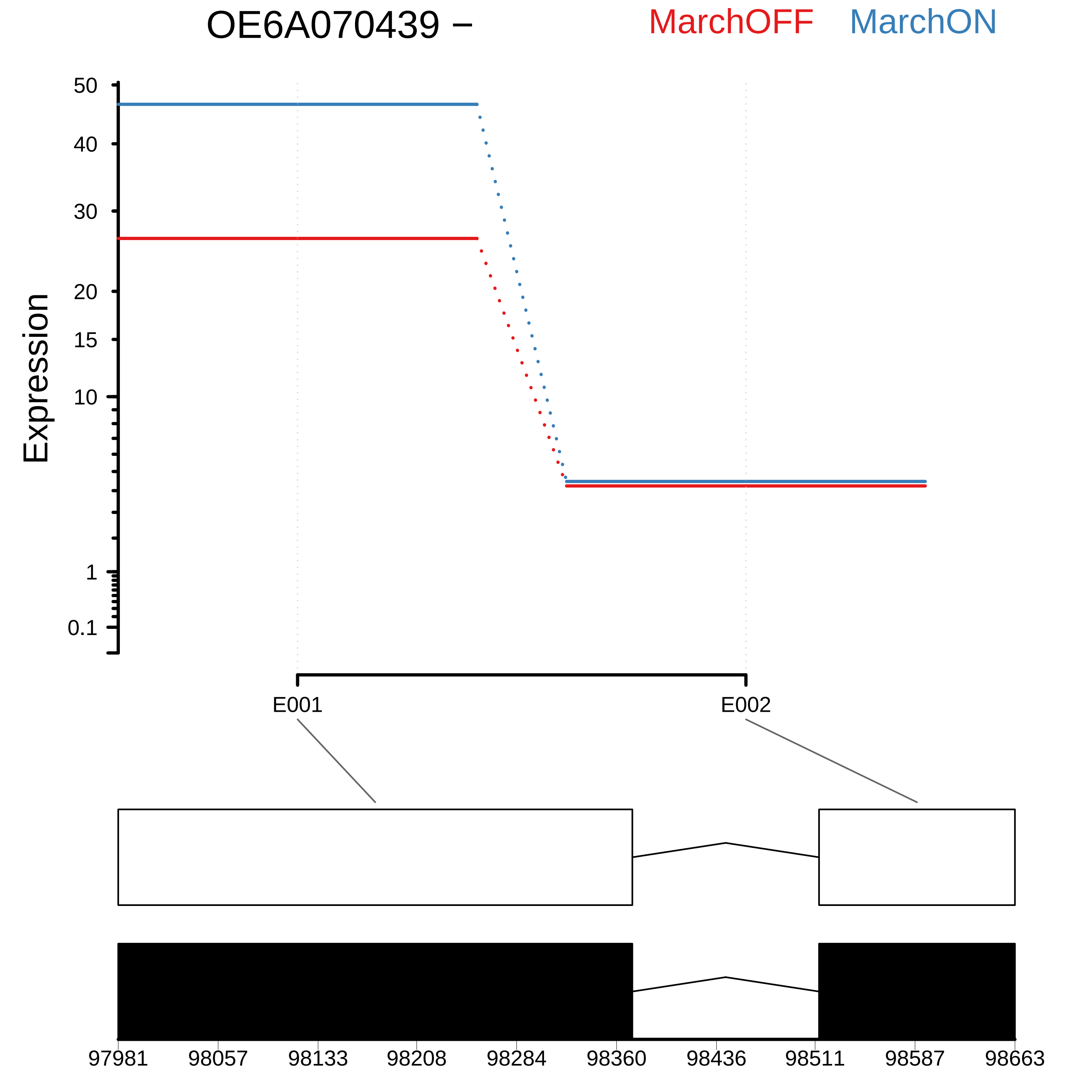

OE6A070439 −
MarchOFF
MarchON
Expression
50
40
30
20
15
10
1
0.1
E001
E002
97981
98057
98133
98208
98284
98360
98436
98511
98587
98663

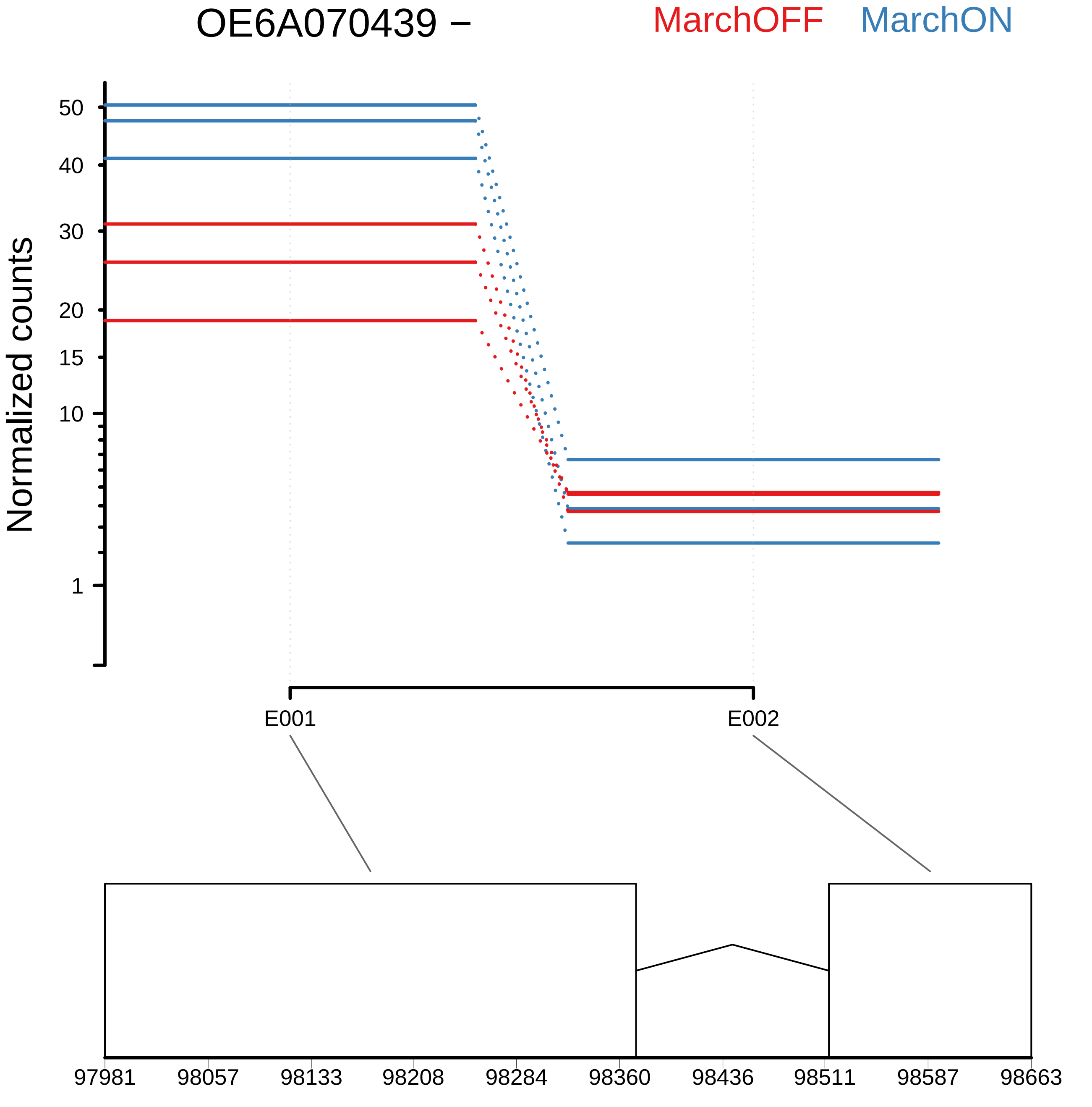
OE6A070439 −
MarchOFF
MarchON
Normalized counts
50
40
30
20
15
10
1
E001
E002
97981
98057
98133
98208
98284
98360
98436
98511
98587
98663

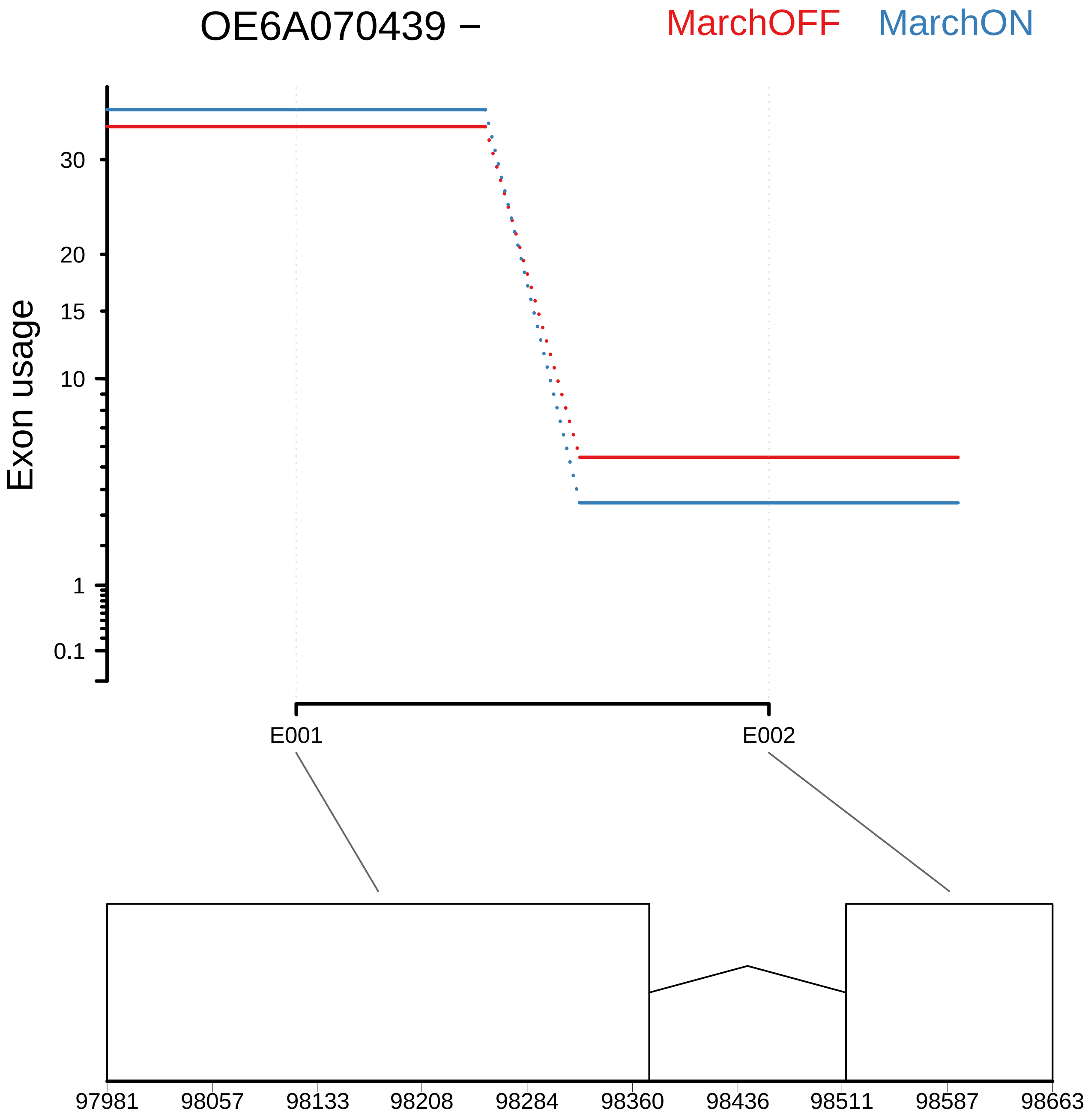

OE6A070439 −
MarchOFF
MarchON
Exon usage
30
20
15
10
1
0.1
E001
E002
97981
98057
98133
98208
98284
98360
98436
98511
98587
98663

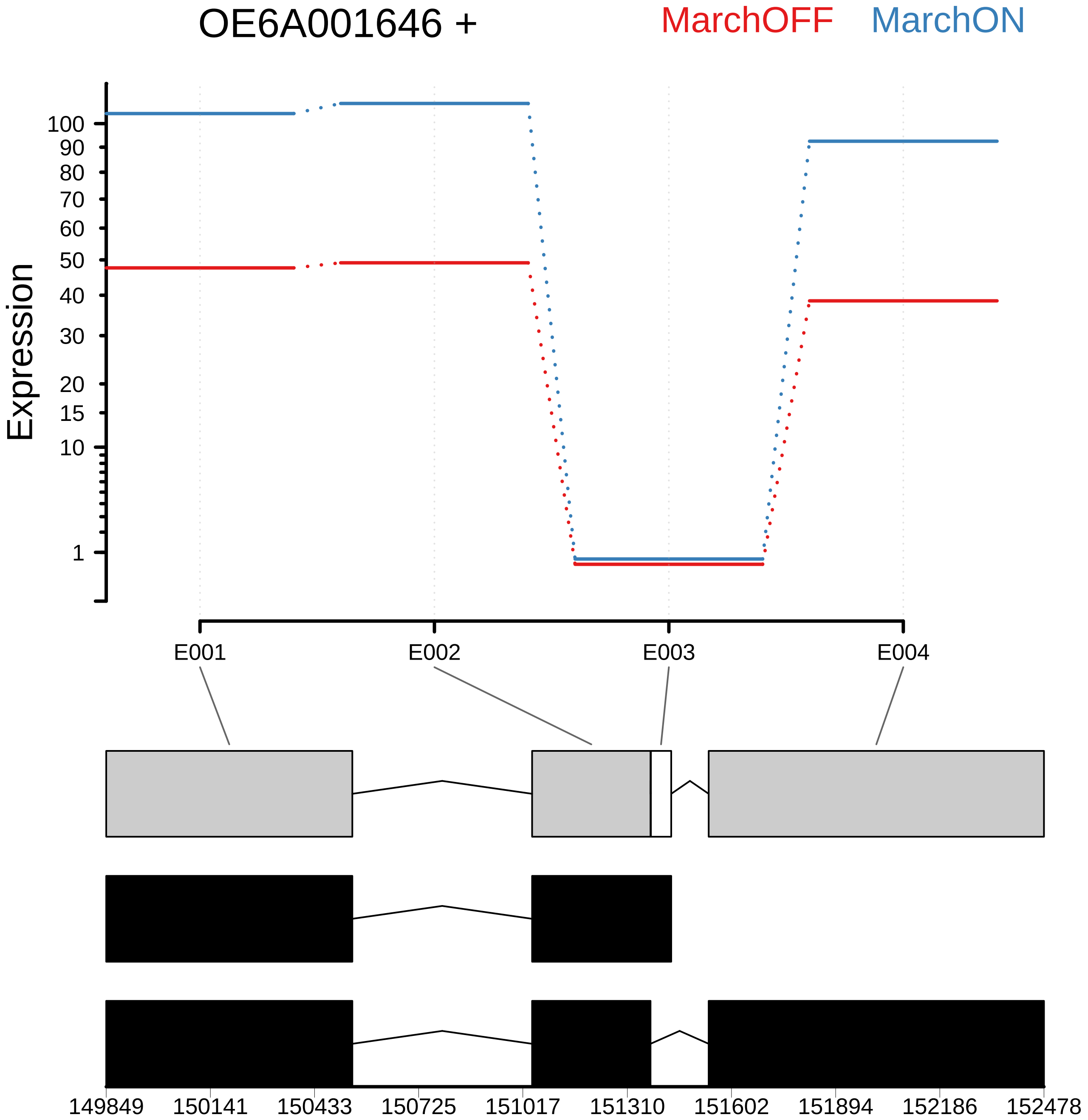
OE6A001646 +
MarchOFF
MarchON
Expression
100
90
80
70
60
50
40
30
20
15
10
1
E001
E002
E003
E004
149849
150141
150433
150725
151017
151310
151602
151894
152186
152478

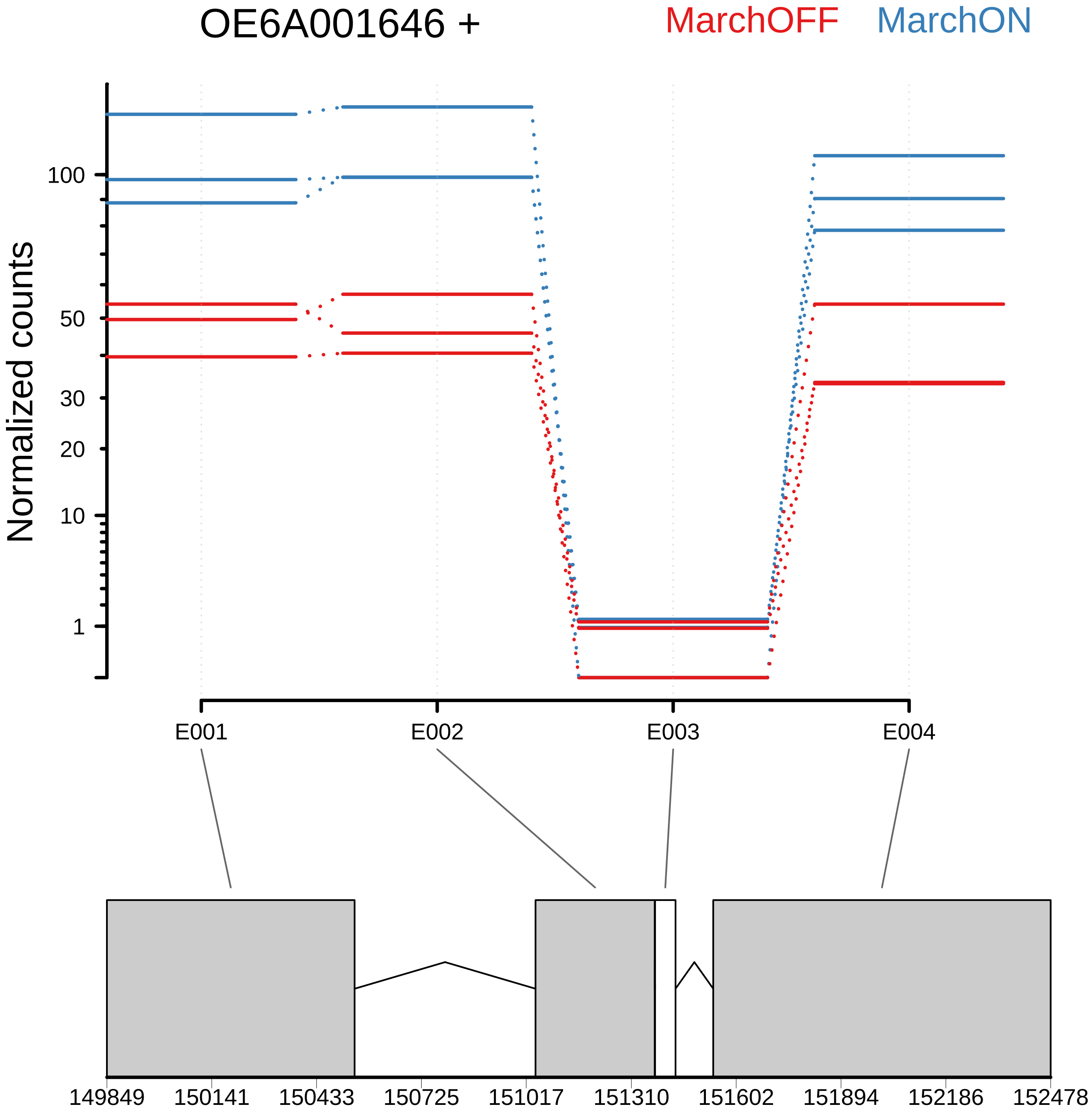
OE6A001646 +
MarchOFF
MarchON
Normalized counts
100
50
30
20
10
1
E001
E002
E003
E004
149849
150141
150433
150725
151017
151310
151602
151894
152186
152478

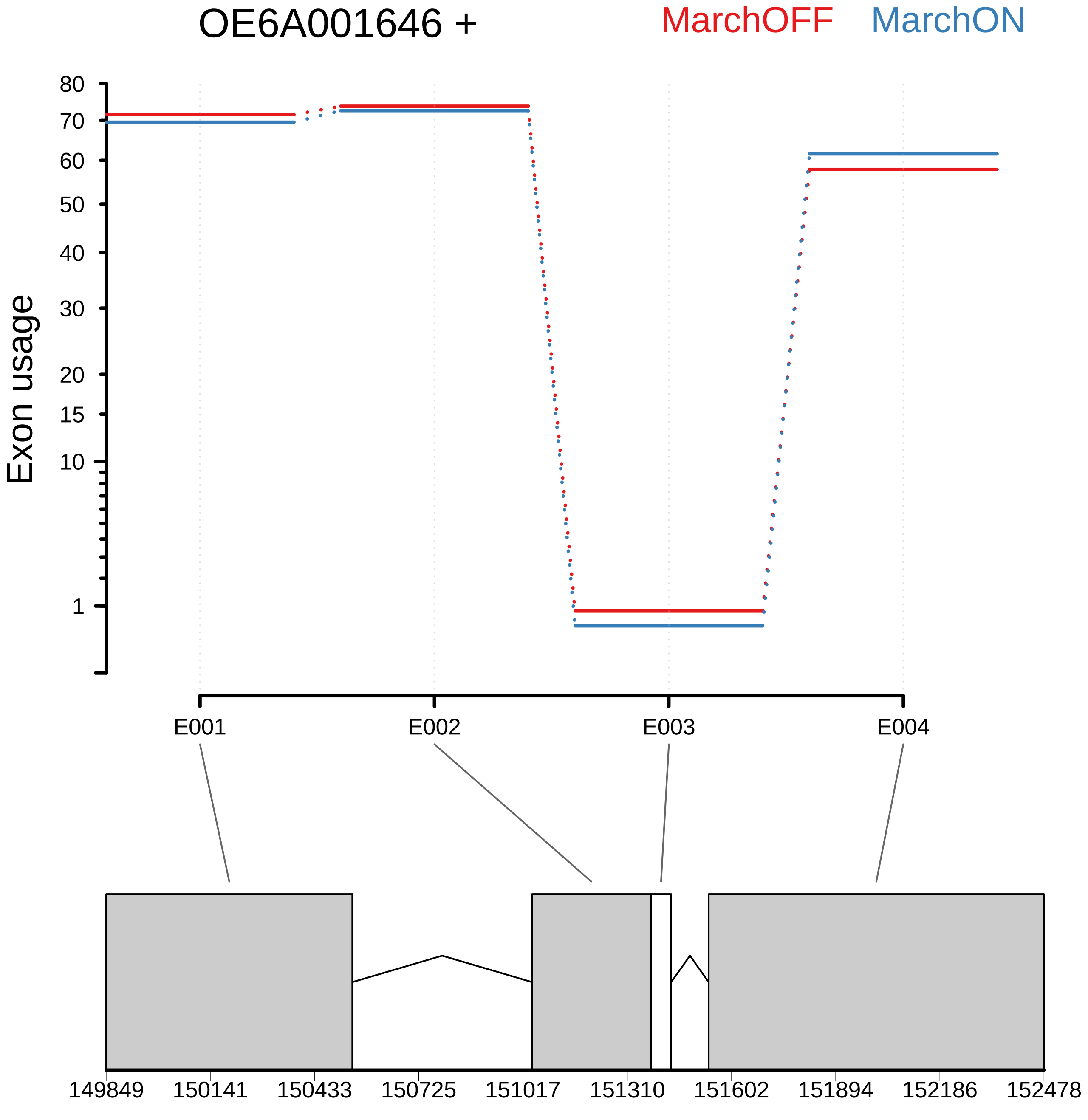

OE6A001646 +
MarchOFF
MarchON
Exon usage
80
70
60
50
40
30
20
15
10
1
E001
E002
E003
E004
149849
150141
150433
150725
151017
151310
151602
151894
152186
152478

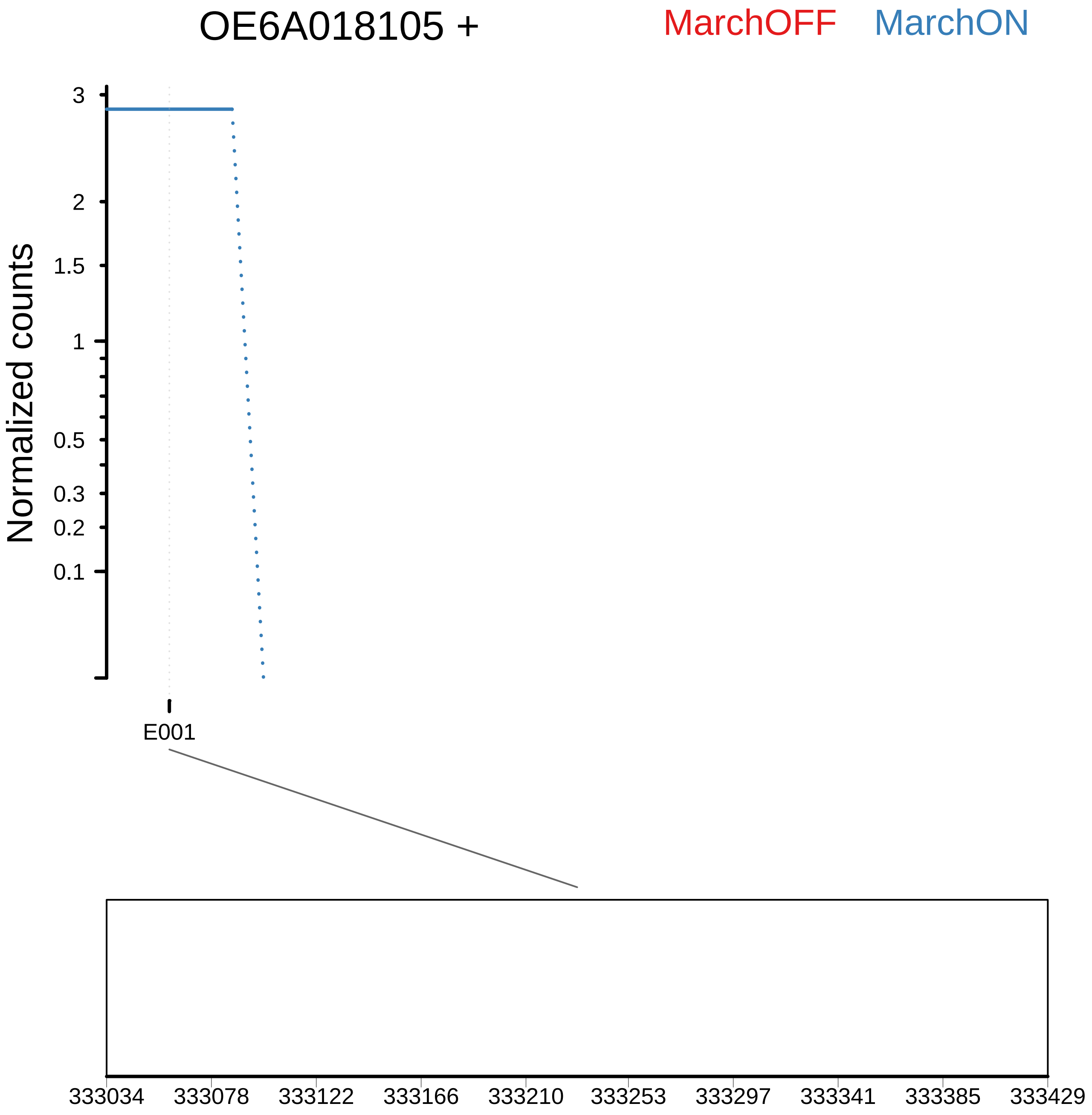

OE6A018105 +
MarchOFF
MarchON
Normalized counts
3
2
1.5
1
0.5
0.3
0.2
0.1
E001
333034
333078
333122
333166
333210
333253
333297
333341
333385
333429

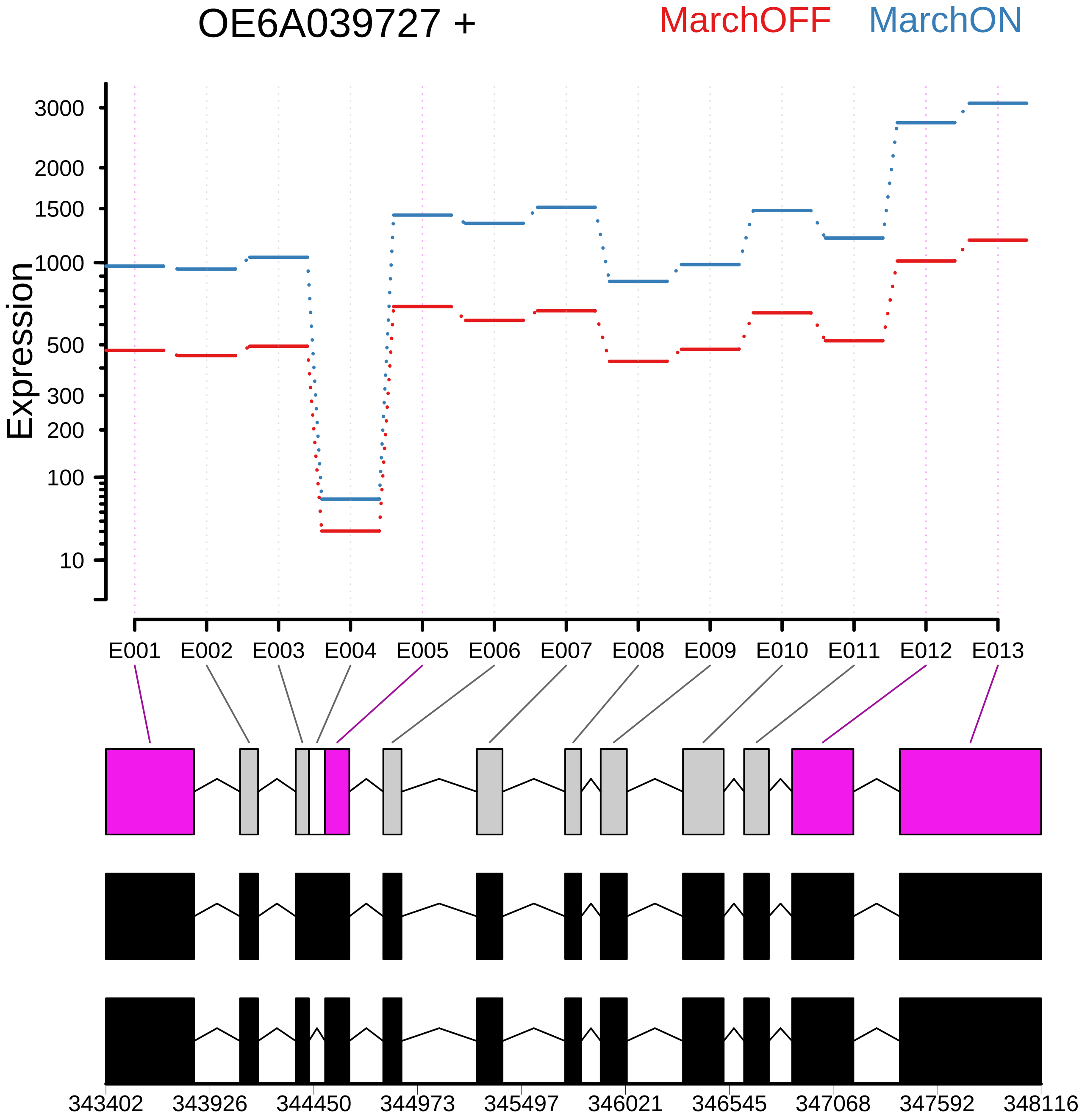

OE6A039727 +
MarchOFF
MarchON
Expression
3000
2000
1500
1000
500
300
200
100
10
E001
E002
E003
E004
E005
E006
E007
E008
E009
E010
E011
E012
E013
343402
343926
344450
344973
345497
346021
346545
347068
347592
348116

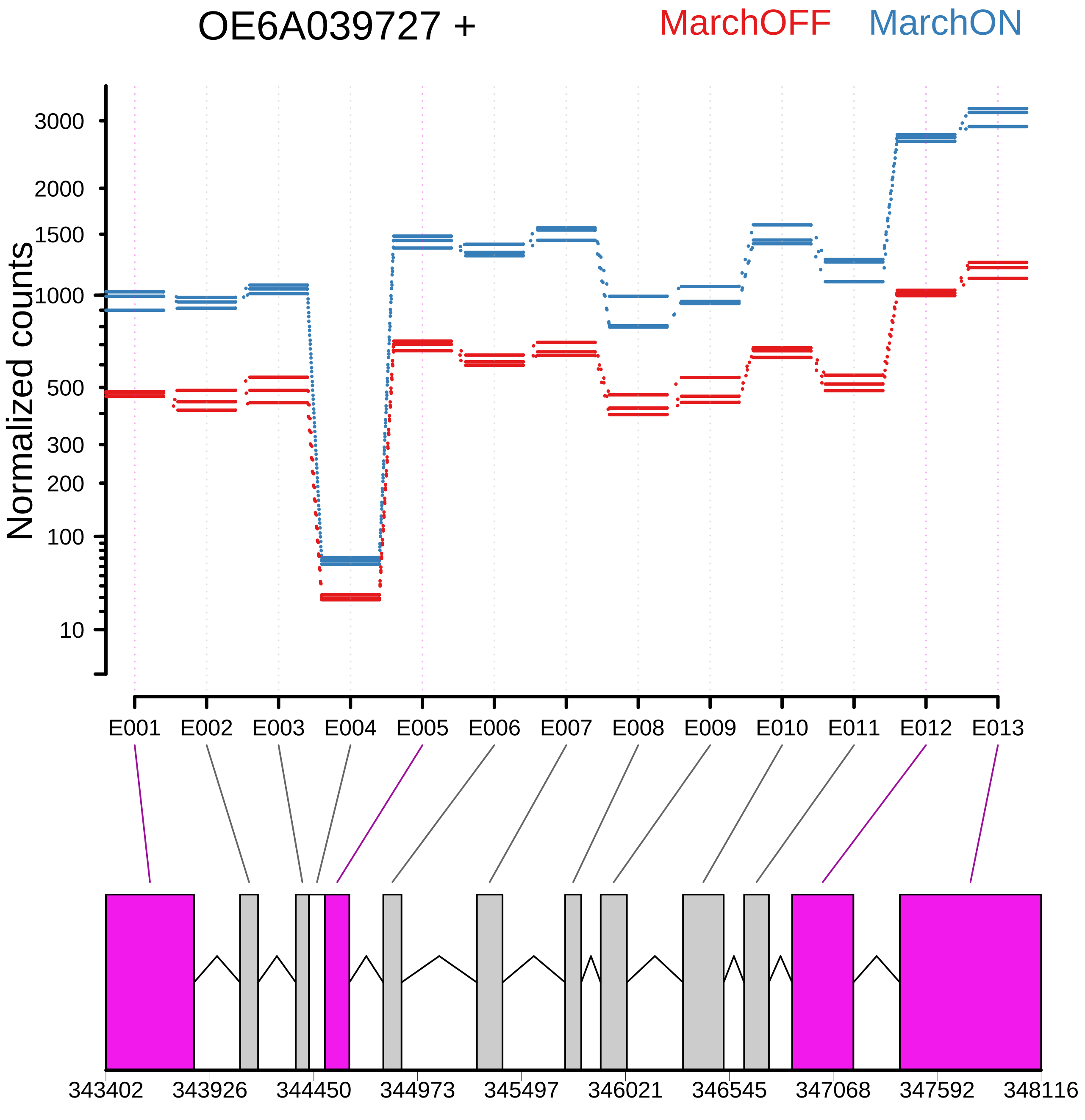

OE6A039727 +
MarchOFF
MarchON
Normalized counts
3000
2000
1500
1000
500
300
200
100
10
E001
E002
E003
E004
E005
E006
E007
E008
E009
E010
E011
E012
E013
343402
343926
344450
344973
345497
346021
346545
347068
347592
348116

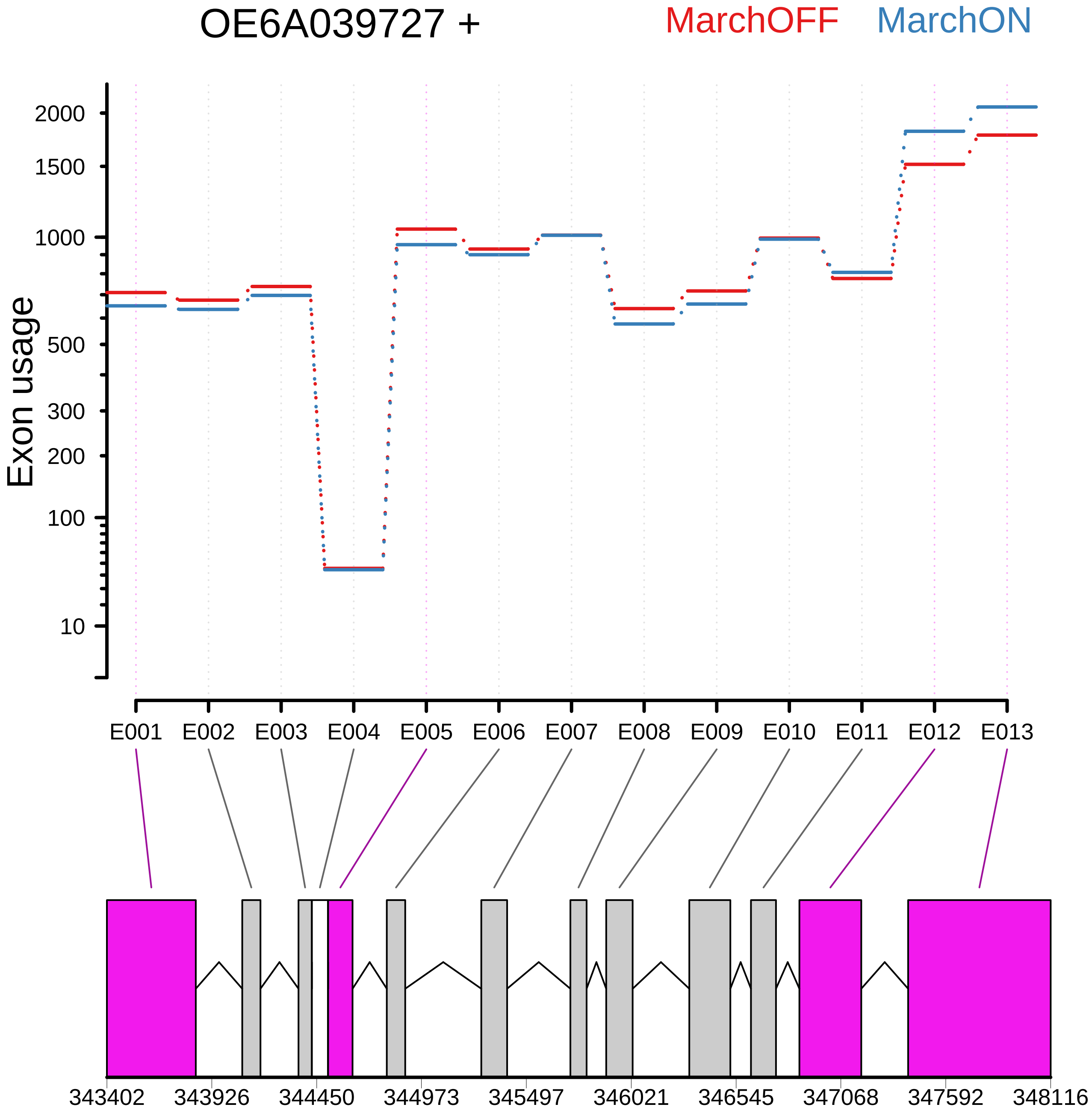

OE6A039727 +
MarchOFF
MarchON
Exon usage
2000
1500
1000
500
300
200
100
10
E001
E002
E003
E004
E005
E006
E007
E008
E009
E010
E011
E012
E013
343402
343926
344450
344973
345497
346021
346545
347068
347592
348116

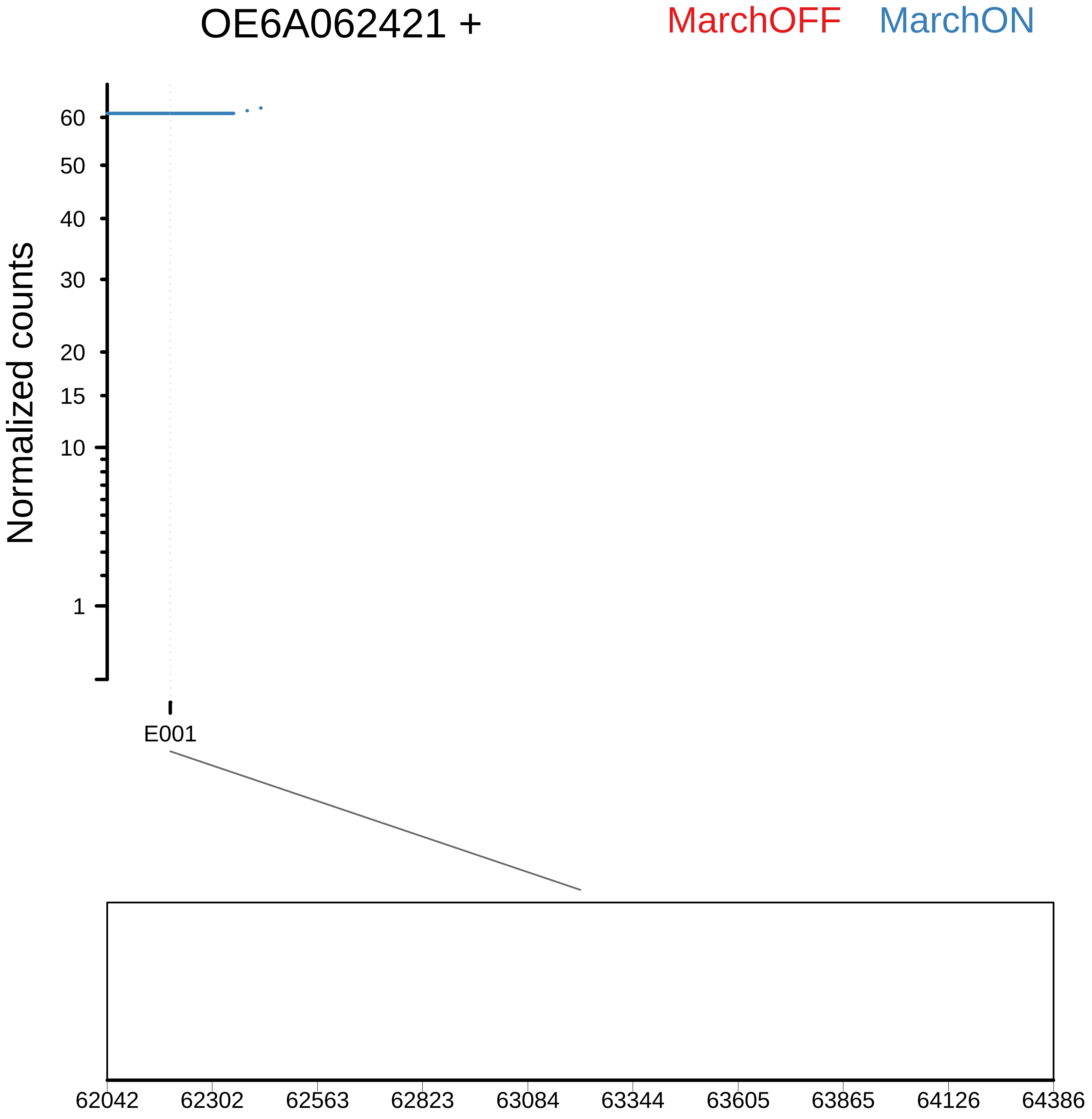

OE6A062421 +
MarchOFF
MarchON
Normalized counts
60
50
40
30
20
15
10
1
E001
62042
62302
62563
62823
63084
63344
63605
63865
64126
64386

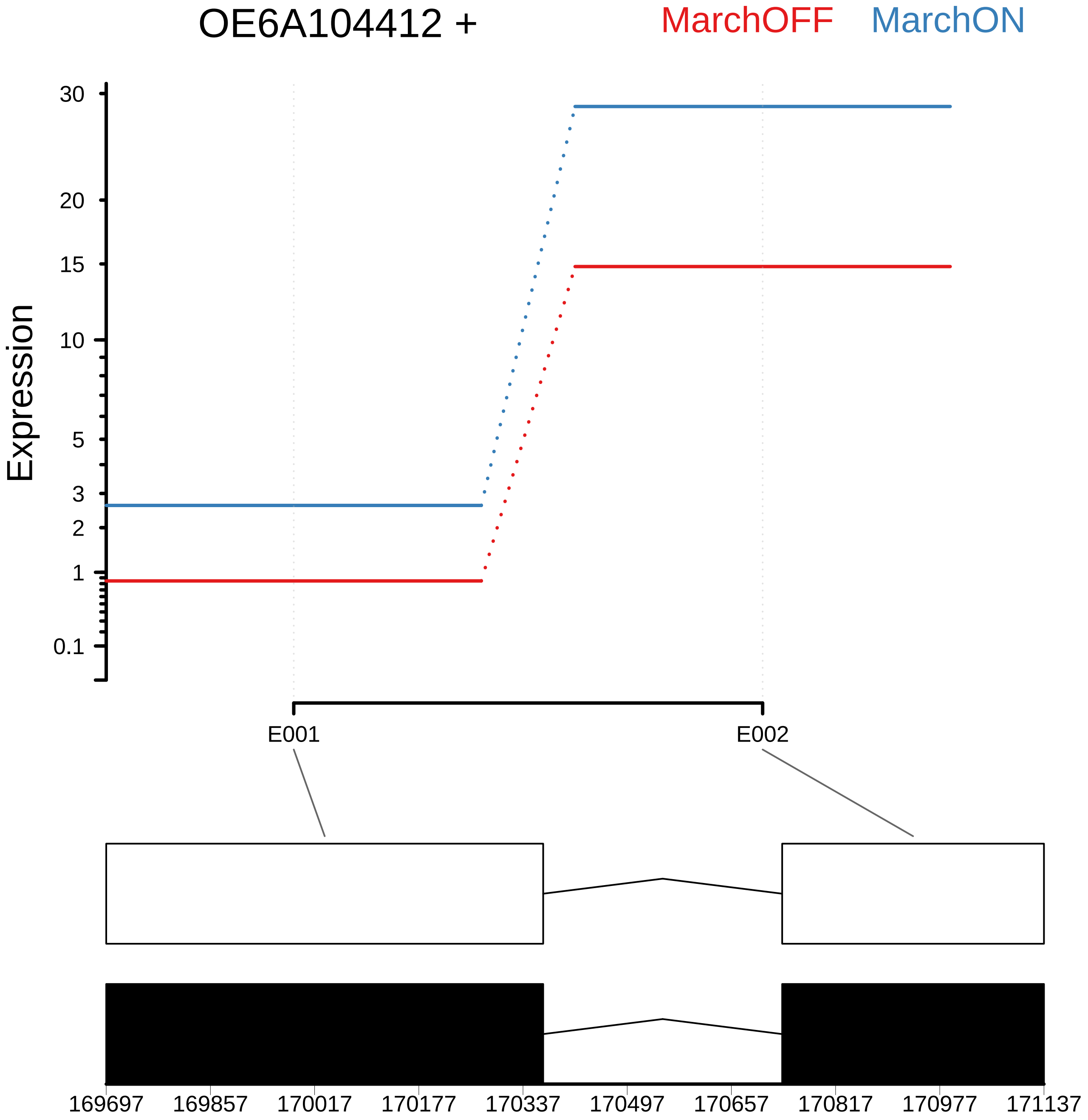

OE6A104412 +
MarchOFF
MarchON
Expression
30
20
15
10
5
3
2
1
0.1
E001
E002
169697
169857
170017
170177
170337
170497
170657
170817
170977
171137

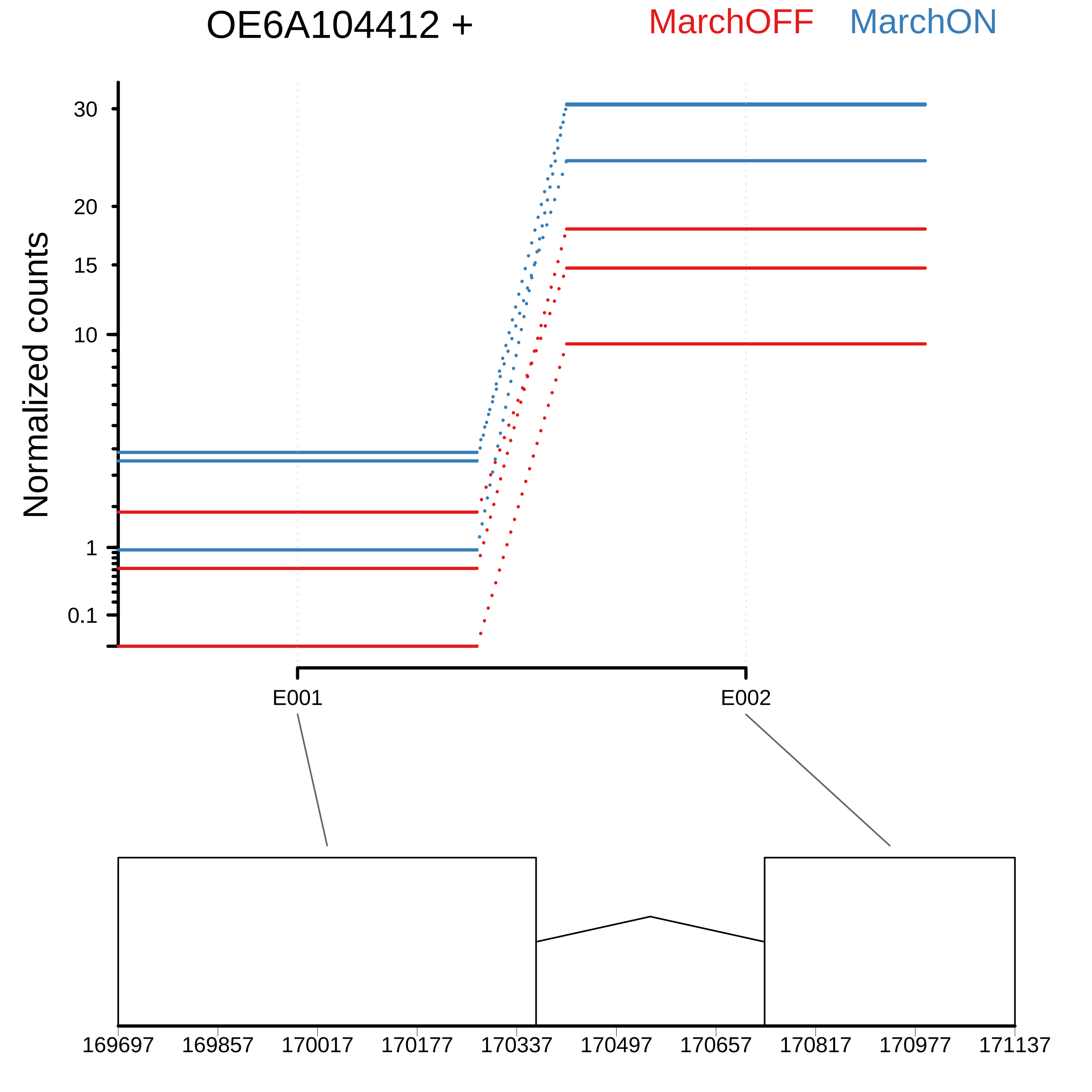
OE6A104412 +
MarchOFF
MarchON
Normalized counts
30
20
15
10
1
0.1
E001
E002
169697
169857
170017
170177
170337
170497
170657
170817
170977
171137

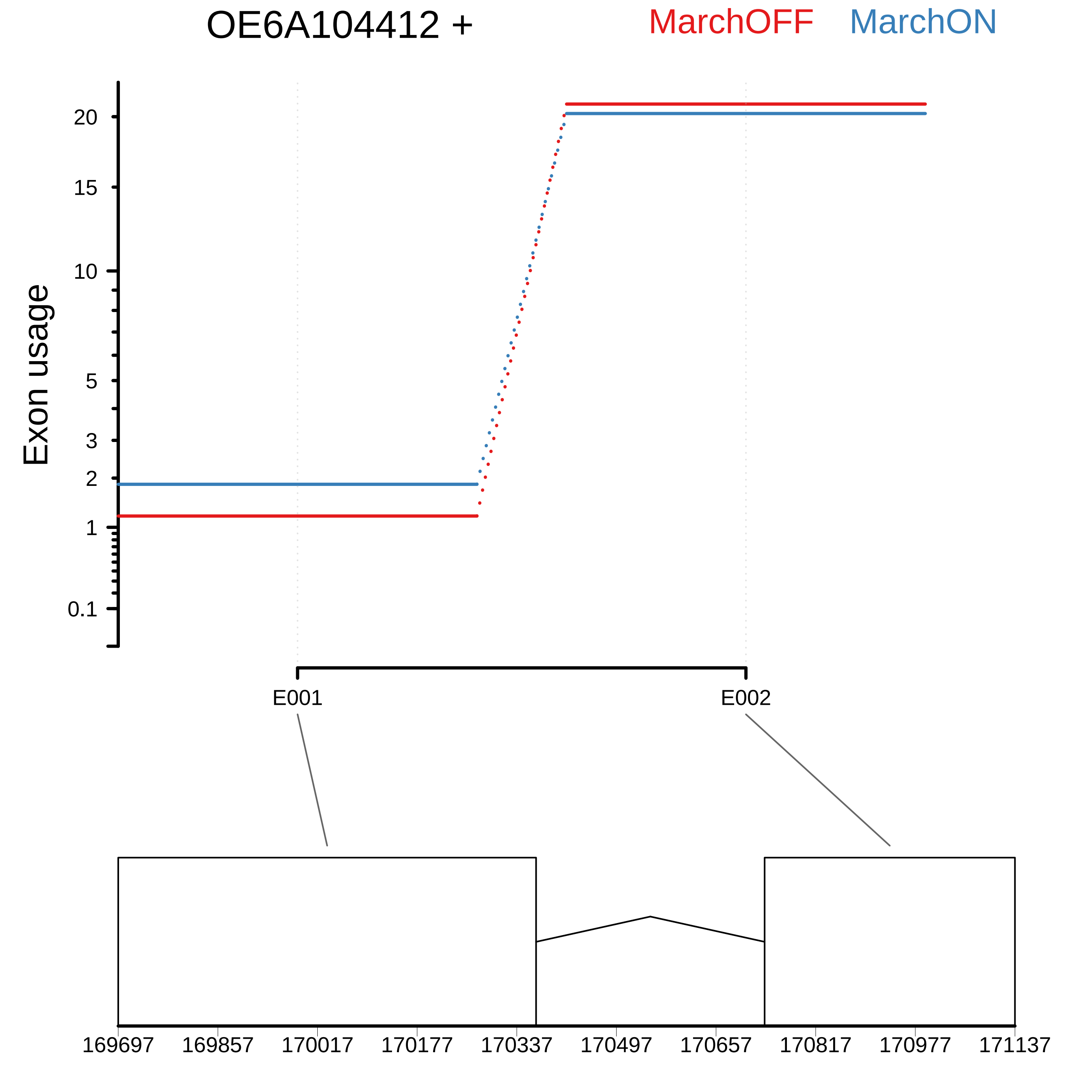
OE6A104412 +
MarchOFF
MarchON
Exon usage
20
15
10
5
3
2
1
0.1
E001
E002
169697
169857
170017
170177
170337
170497
170657
170817
170977
171137

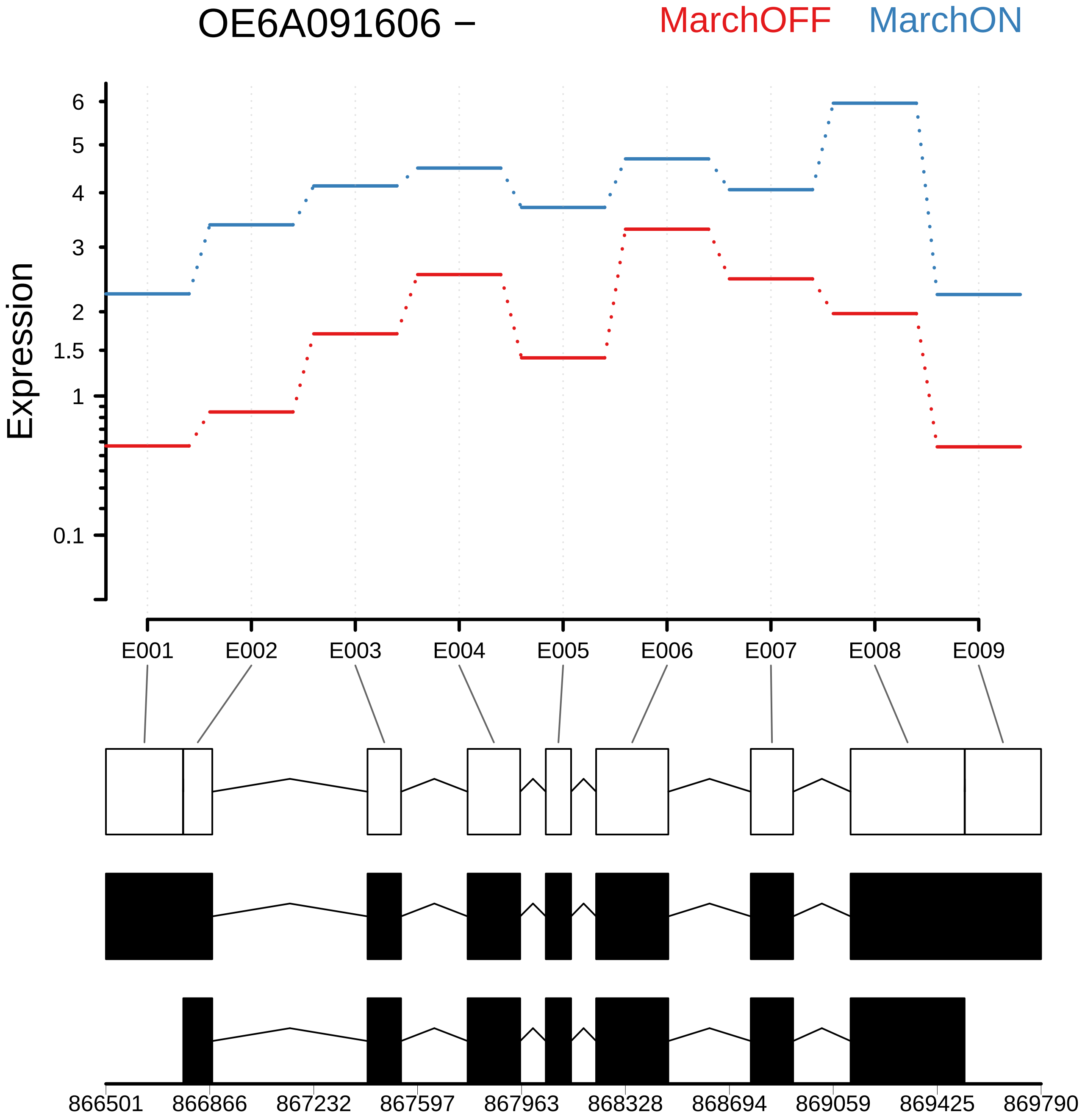

OE6A091606 –
MarchOFF
MarchON
Expression
6
5
4
3
2
1.5
1
0.1
E001
E002
E003
E004
E005
E006
E007
E008
E009
866501
866866
867232
867597
867963
868328
868694
869059
869425
869790

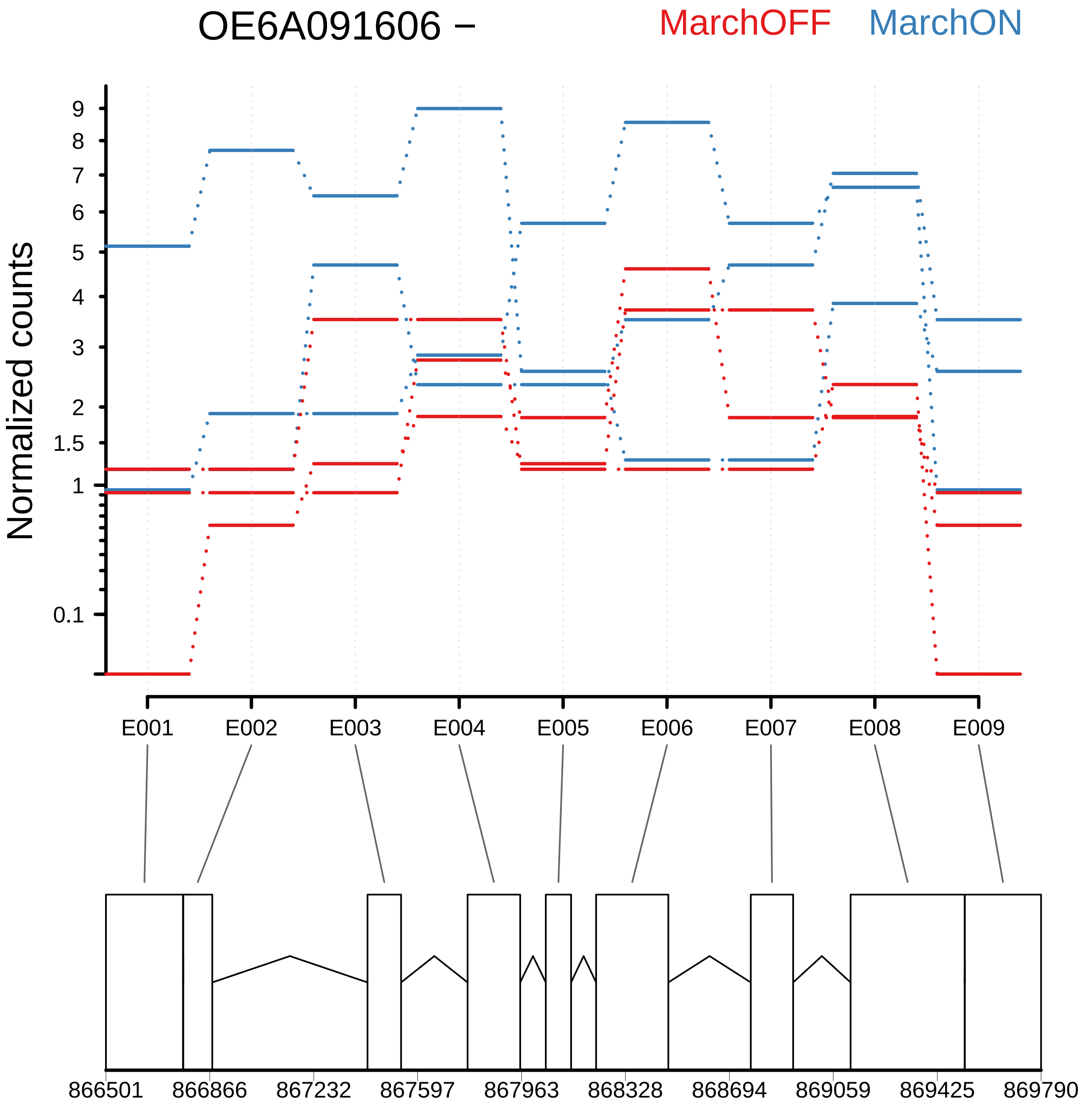

OE6A091606 −
MarchOFF
MarchON
Normalized counts
9
8
7
6
5
4
3
2
1.5
1
0.1
E001
E002
E003
E004
E005
E006
E007
E008
E009
866501
866866
867232
867597
867963
868328
868694
869059
869425
869790

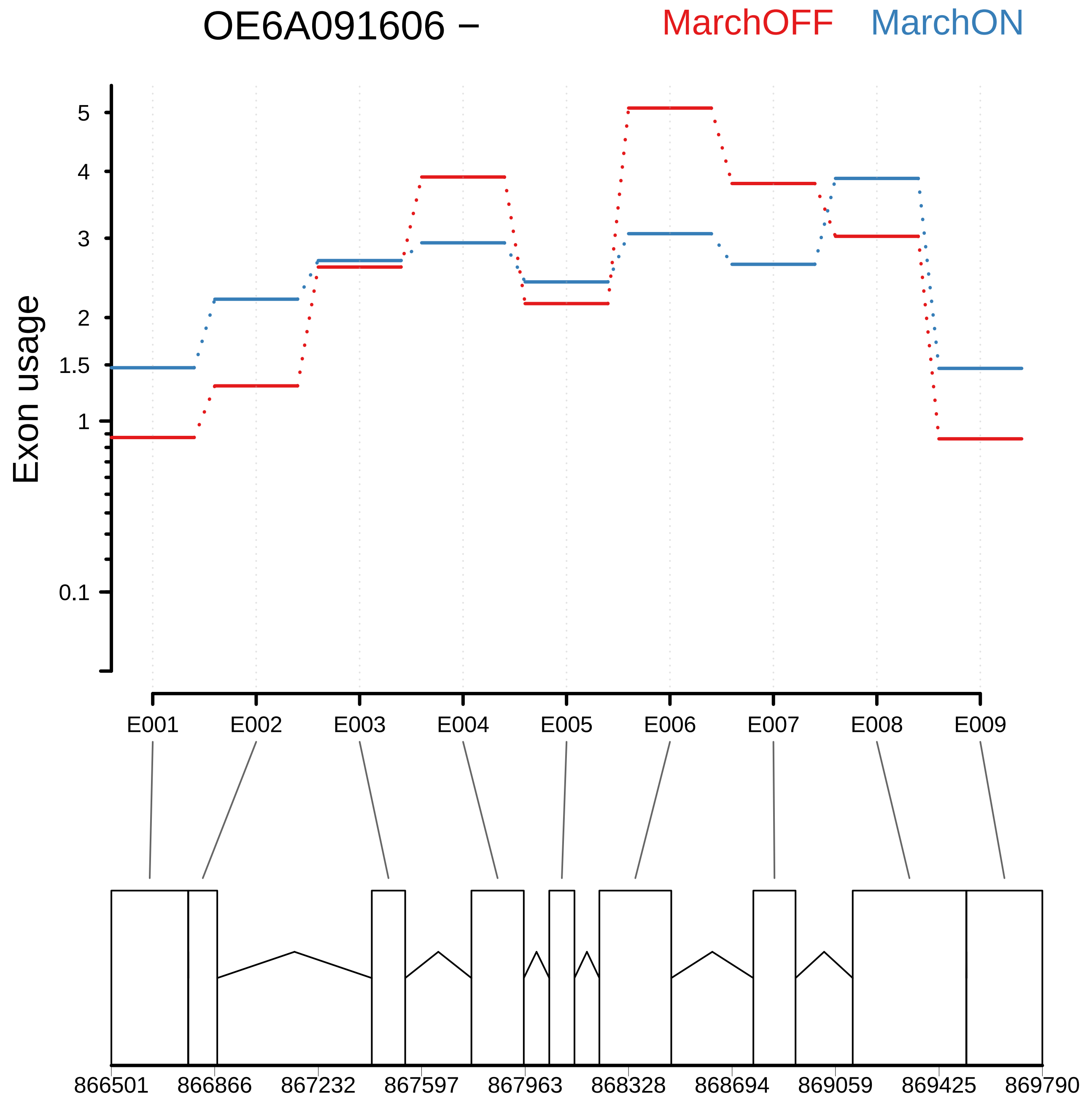
OE6A091606 −
MarchOFF
MarchON
Exon usage
5
4
3
2
1.5
1
0.1
E001
E002
E003
E004
E005
E006
E007
E008
E009
866501
866866
867232
867597
867963
868328
868694
869059
869425
869790

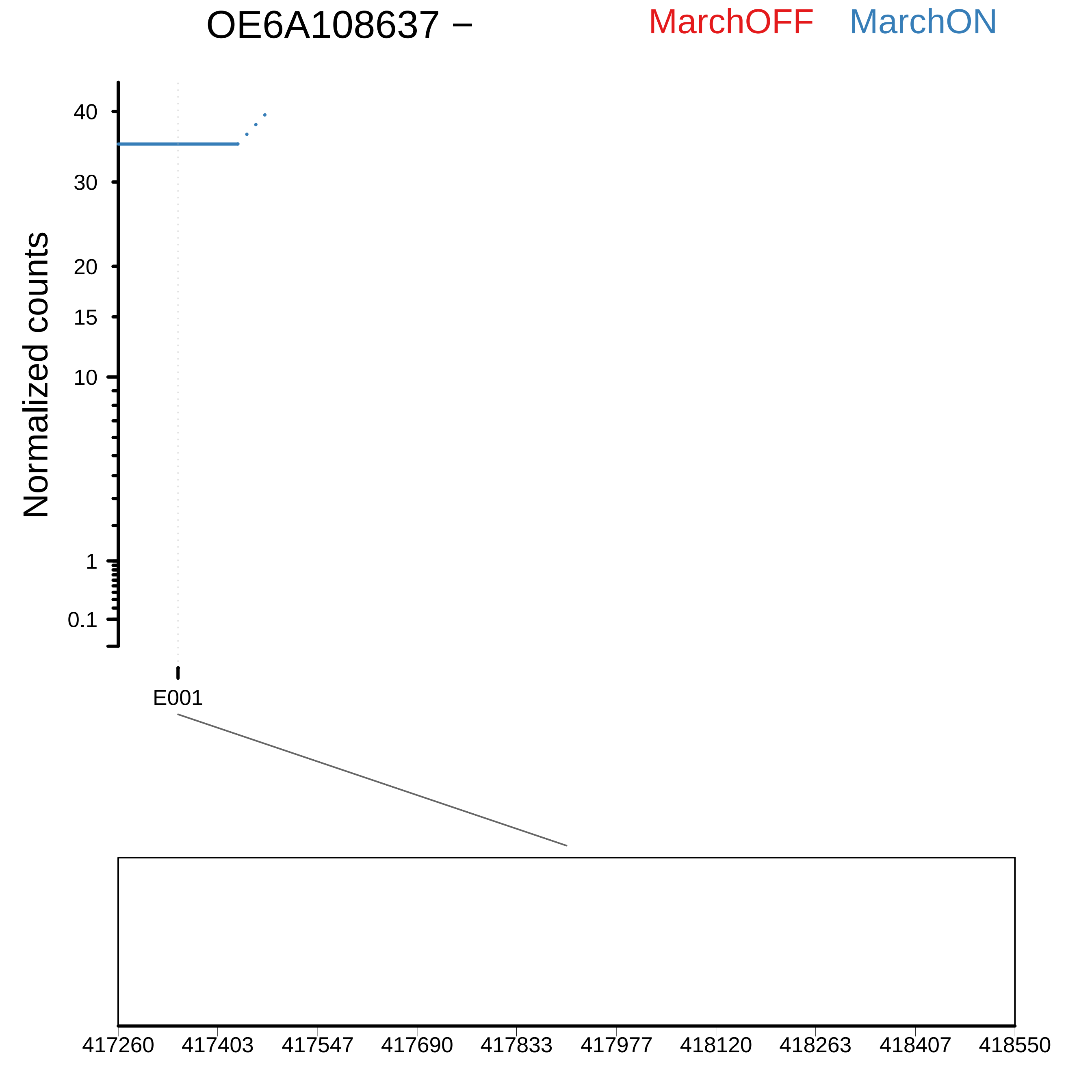

OE6A108637 −
MarchOFF
MarchON
Normalized counts
40
30
20
15
10
1
0.1
E001
417260
417403
417547
417690
417833
417977
418120
418263
418407
418550

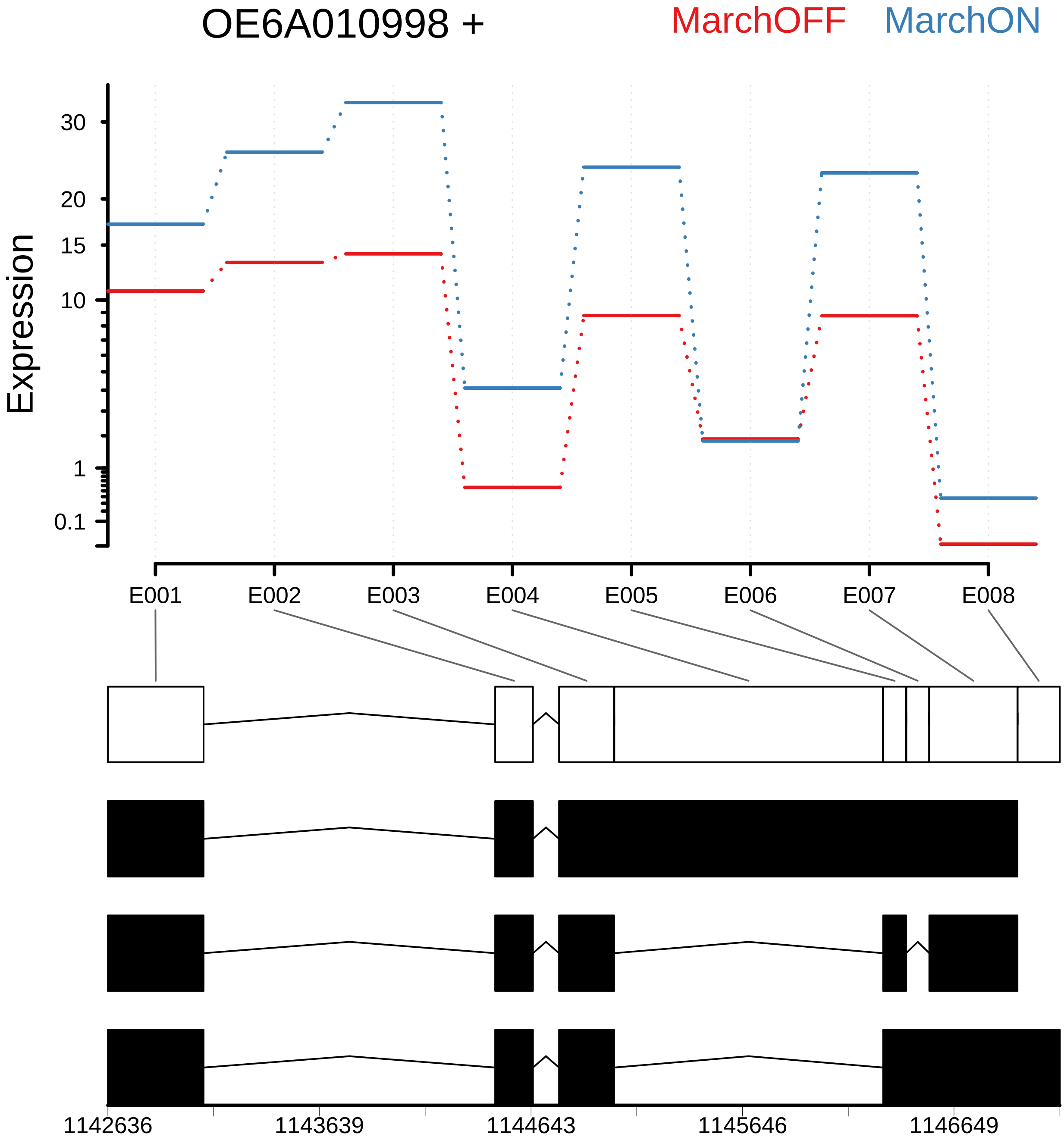

OE6A010998 +
MarchOFF
MarchON
Expression
30
20
15
10
1
0.1
E001
E002
E003
E004
E005
E006
E007
E008
1142636
1143639
1144643
1145646
1146649

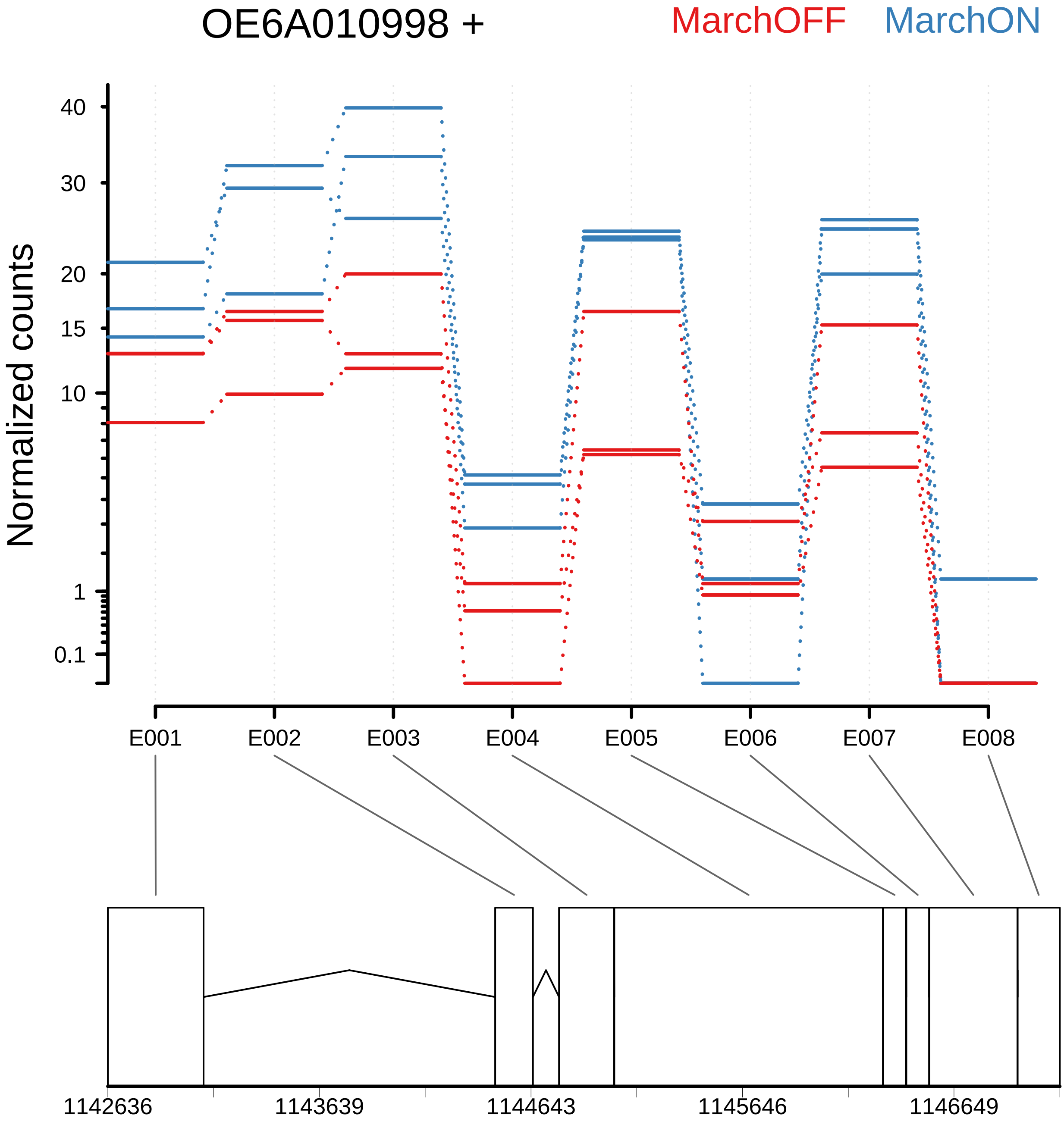

OE6A010998 +
MarchOFF
MarchON
Normalized counts
40
30
20
15
10
1
0.1
E001
E002
E003
E004
E005
E006
E007
E008
1142636
1143639
1144643
1145646
1146649

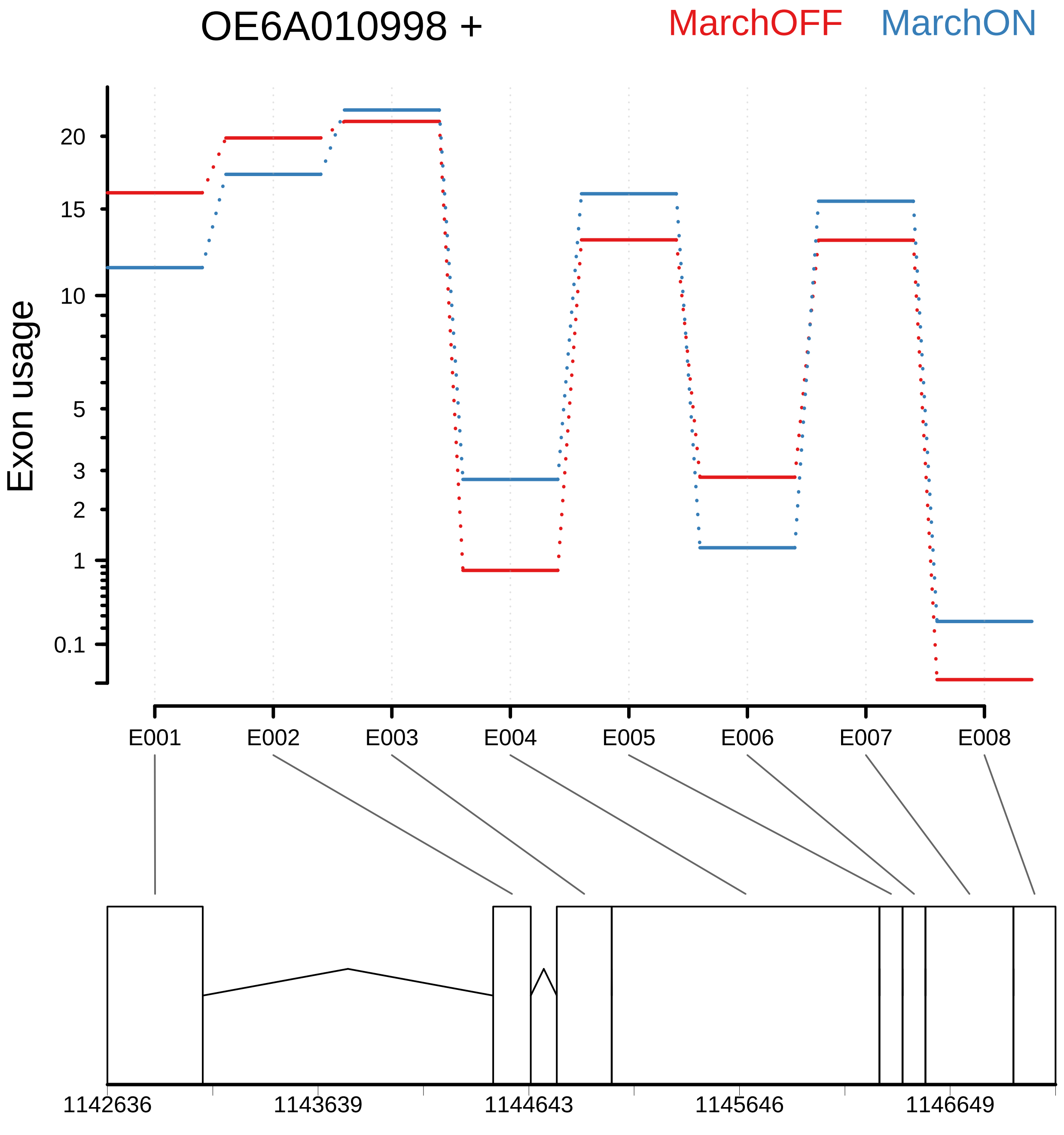
OE6A010998 +
MarchOFF
MarchON
Exon usage
20
15
10
5
3
2
1
0.1
E001
E002
E003
E004
E005
E006
E007
E008
1142636
1143639
1144643
1145646
1146649

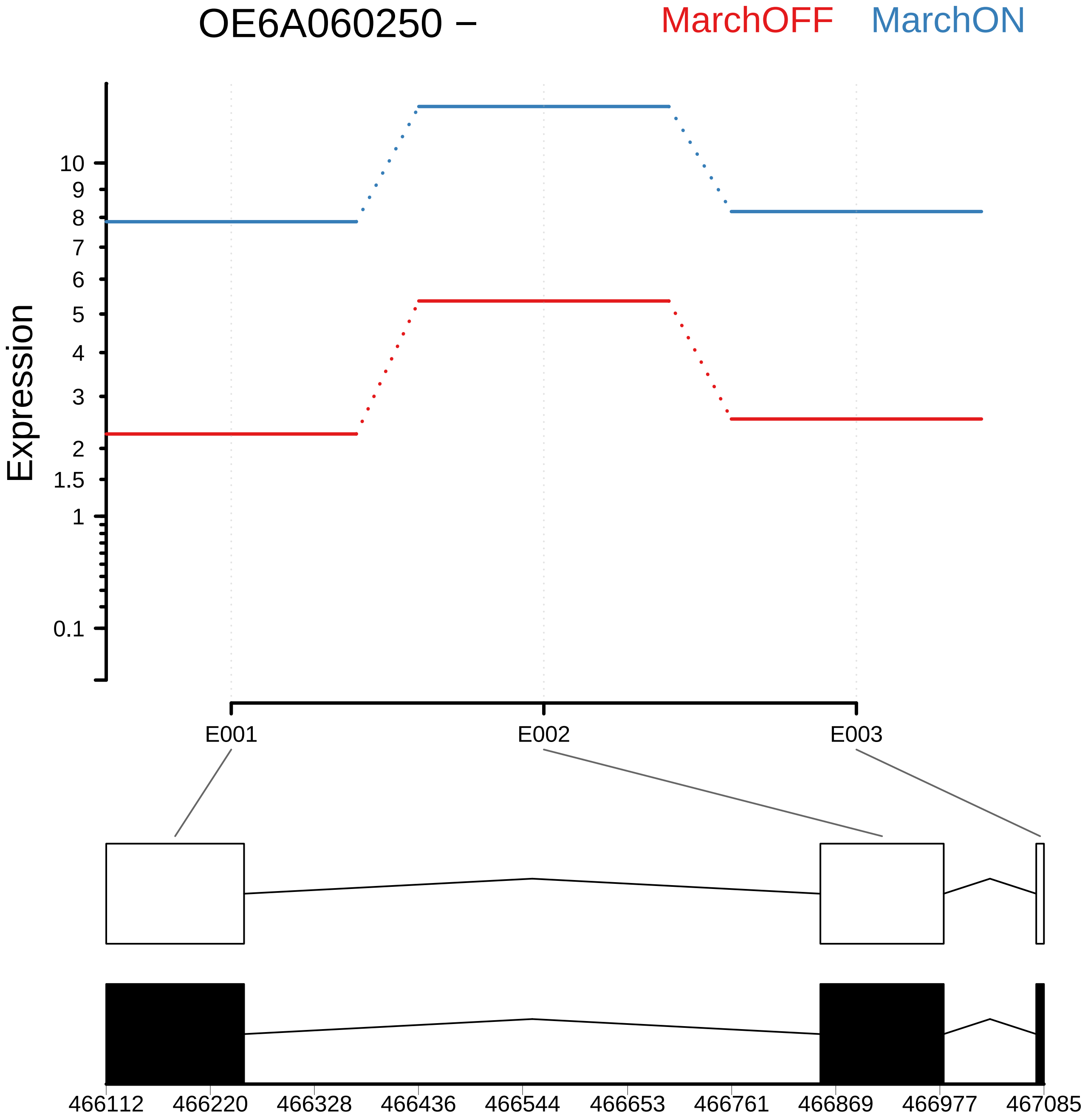

OE6A060250 −
MarchOFF
MarchON
Expression
10
9
8
7
6
5
4
3
2
1.5
1
0.1
E001
E002
E003
466112
466220
466328
466436
466544
466653
466761
466869
466977
467085

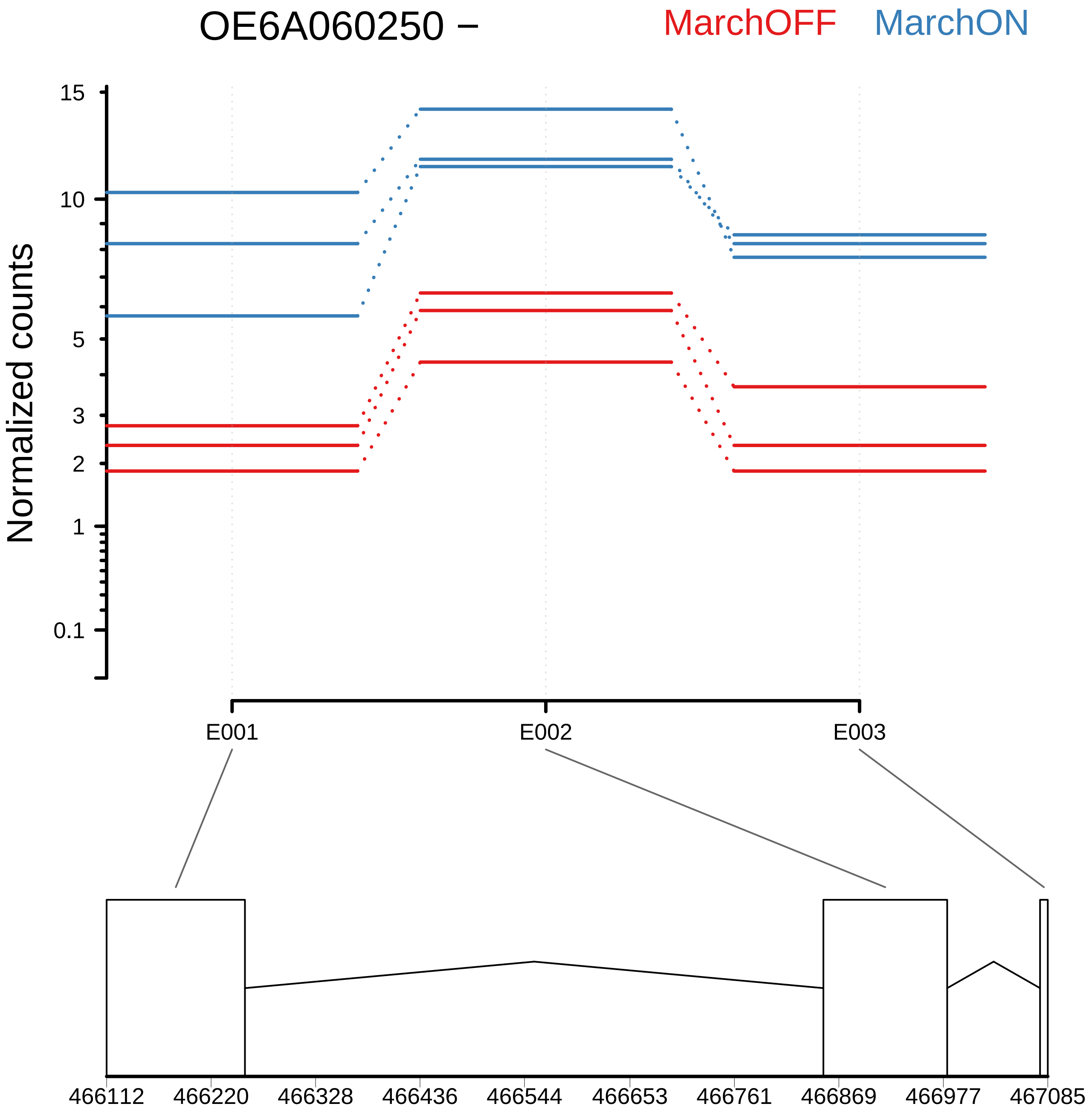
OE6A060250 −
MarchOFF
MarchON
Normalized counts
15
10
5
3
2
1
0.1
E001
E002
E003
466112
466220
466328
466436
466544
466653
466761
466869
466977
467085

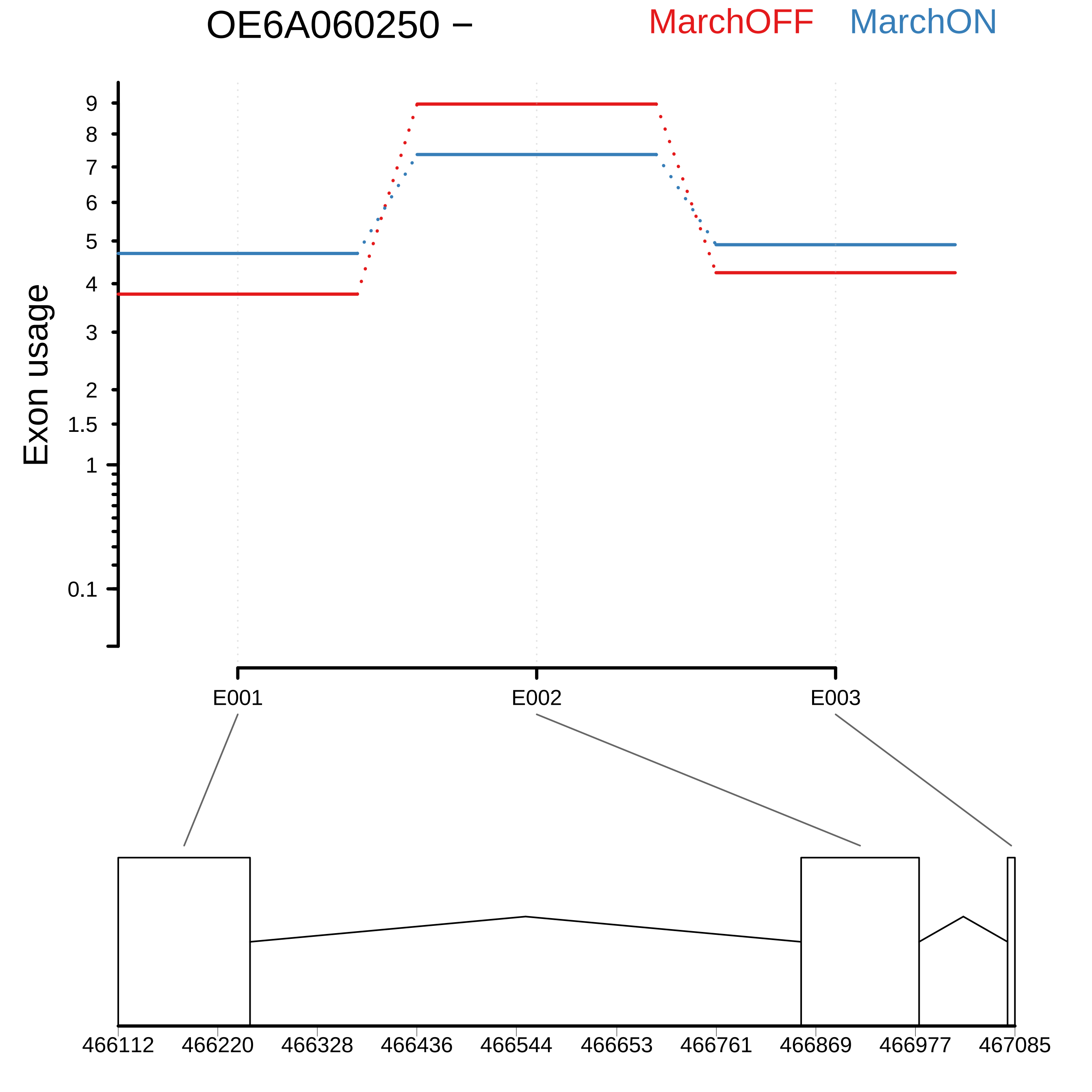

OE6A060250 −
MarchOFF
MarchON
Exon usage
9
8
7
6
5
4
3
2
1.5
1
0.1
E001
E002
E003
466112
466220
466328
466436
466544
466653
466761
466869
466977
467085

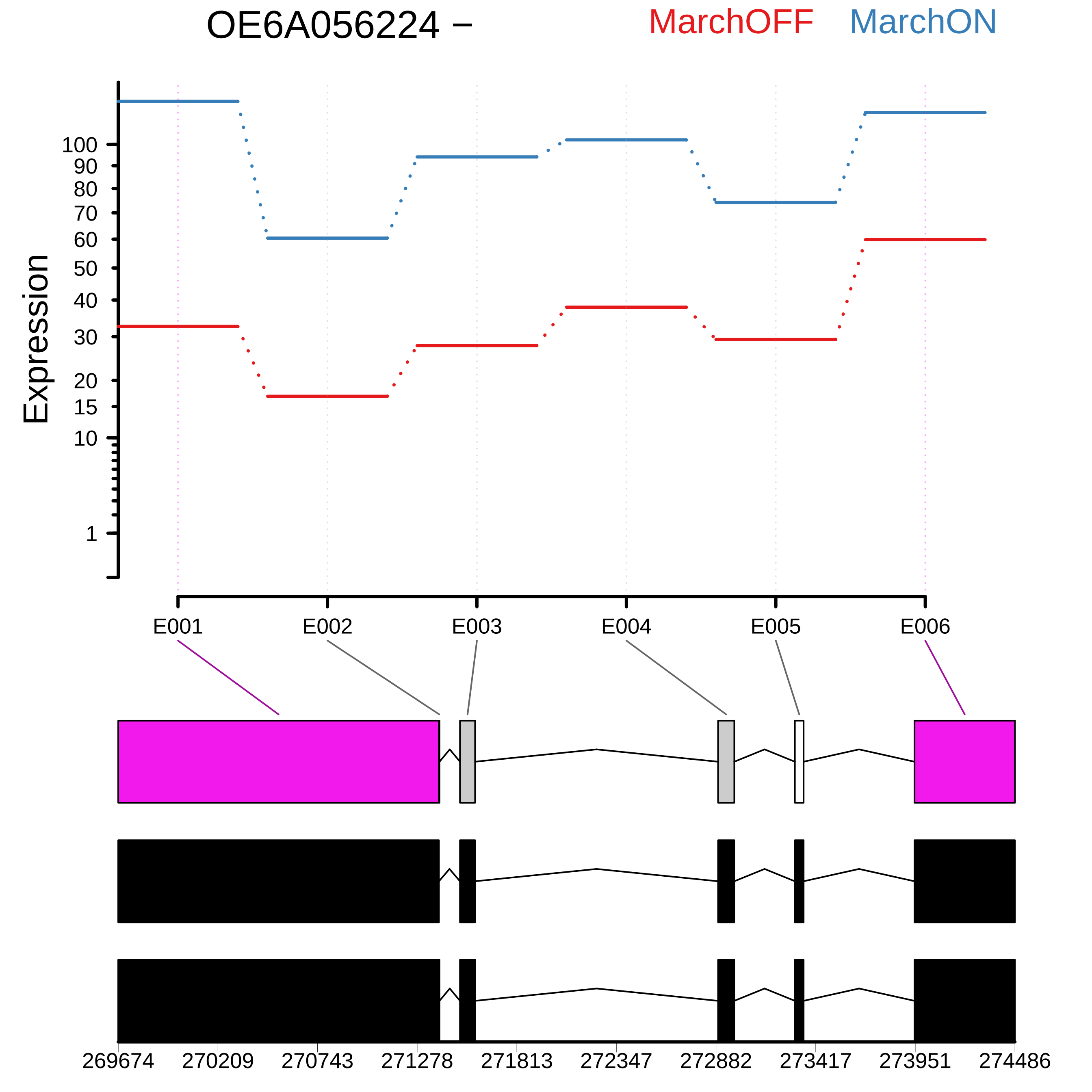

OE6A056224 –
MarchOFF
MarchON
Expression
100
90
80
70
60
50
40
30
20
15
10
1
E001
E002
E003
E004
E005
E006
269674
270209
270743
271278
271813
272347
272882
273417
273951
274486

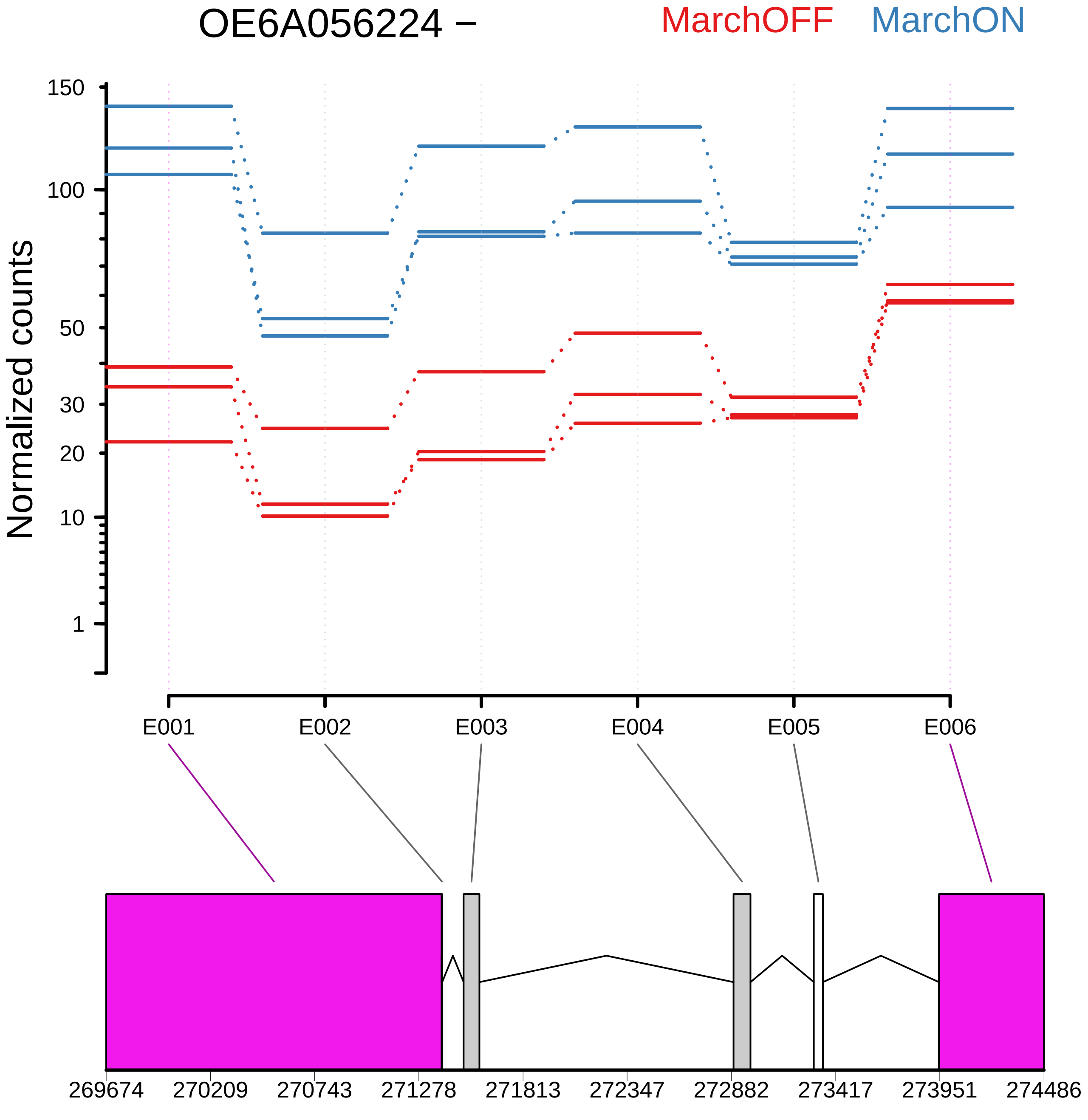
OE6A056224 −
MarchOFF
MarchON
Normalized counts
150
100
50
30
20
10
1
E001
E002
E003
E004
E005
E006
269674
270209
270743
271278
271813
272347
272882
273417
273951
274486

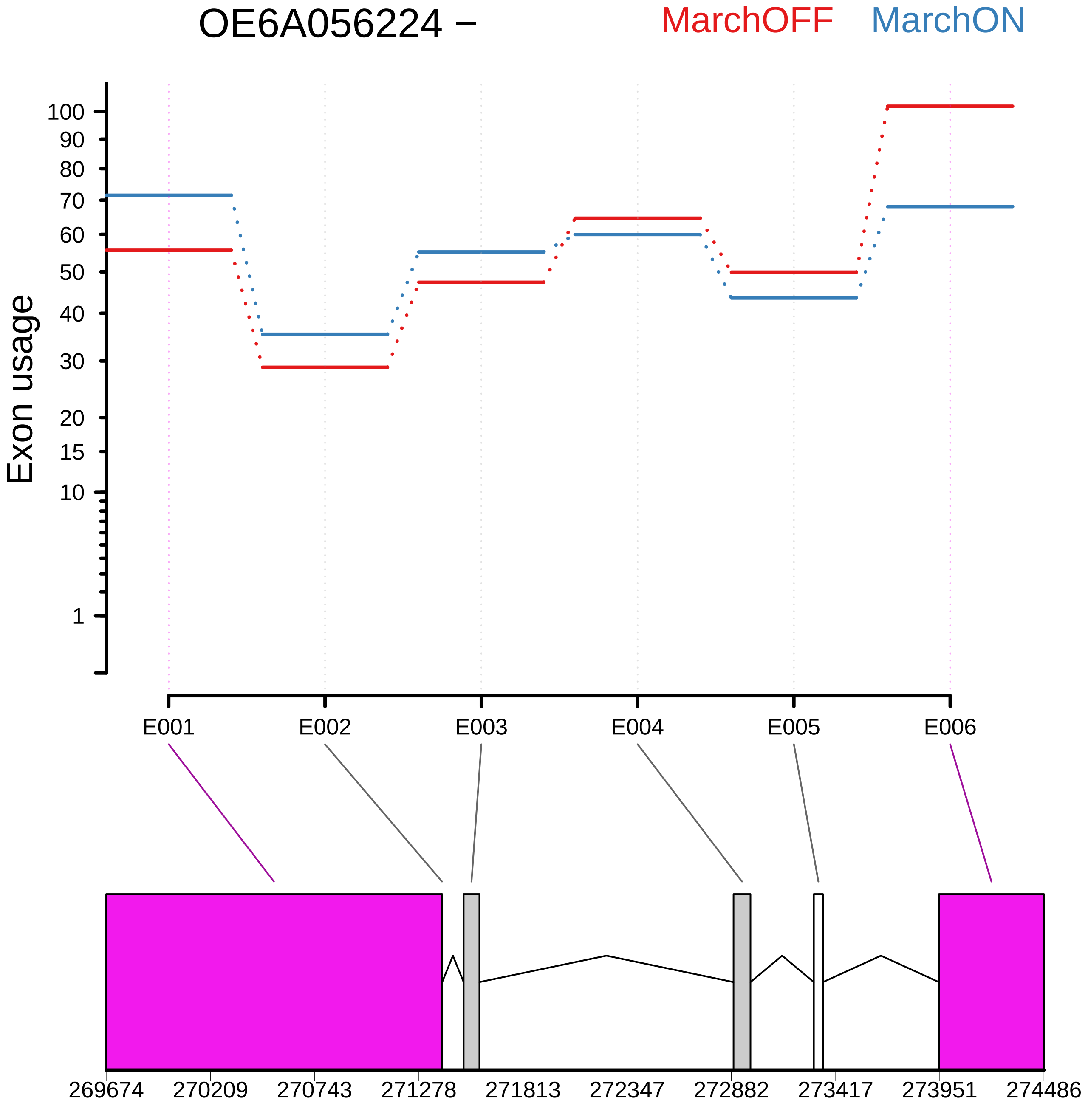

OE6A056224 −
MarchOFF
MarchON
Exon usage
100
90
80
70
60
50
40
30
20
15
10
1
E001
E002
E003
E004
E005
E006
269674
270209
270743
271278
271813
272347
272882
273417
273951
274486

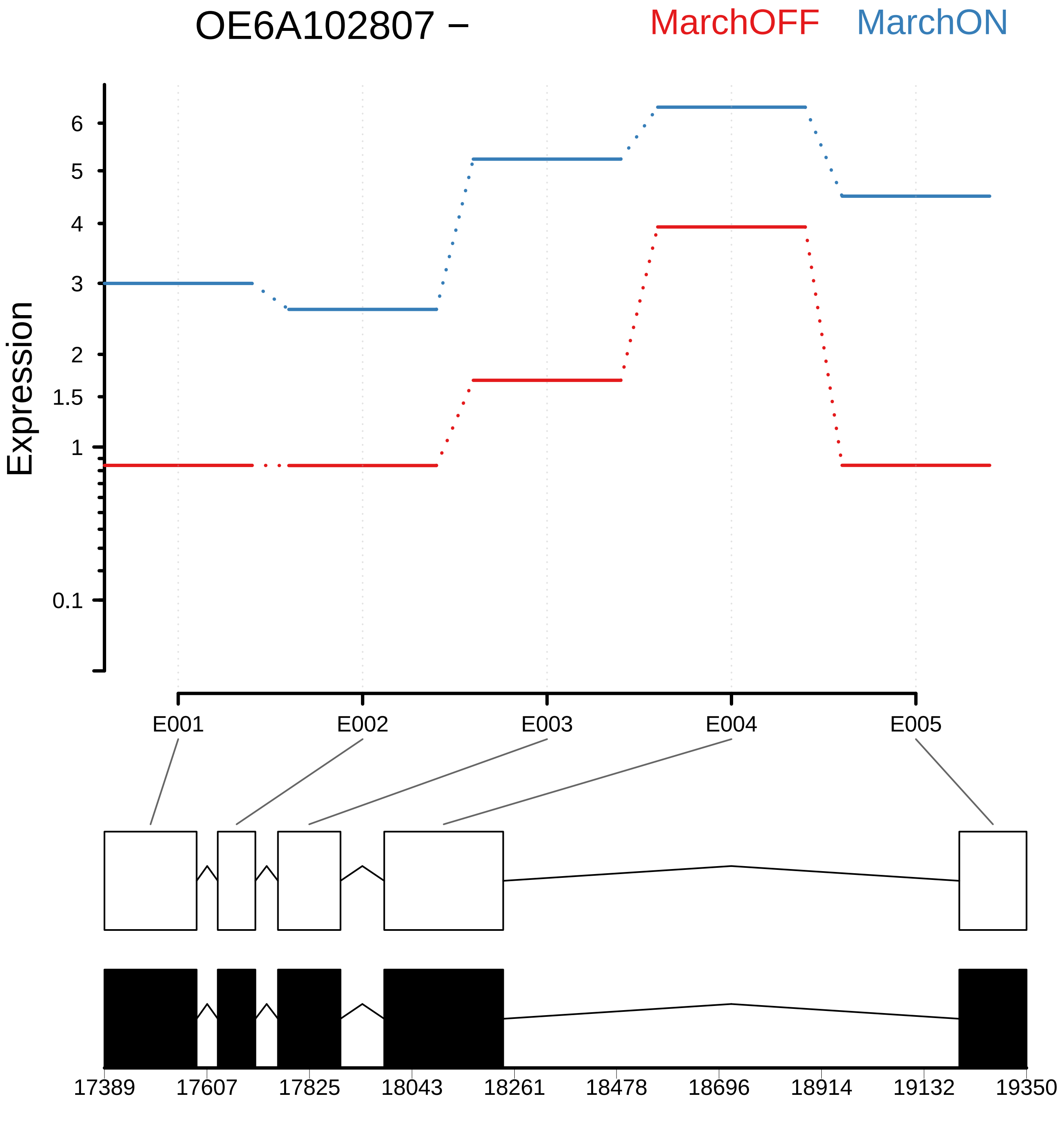
OE6A102807 −
MarchOFF
MarchON
Expression
6
5
4
3
2
1.5
1
0.1
E001
E002
E003
E004
E005
17389
17607
17825
18043
18261
18478
18696
18914
19132
19350

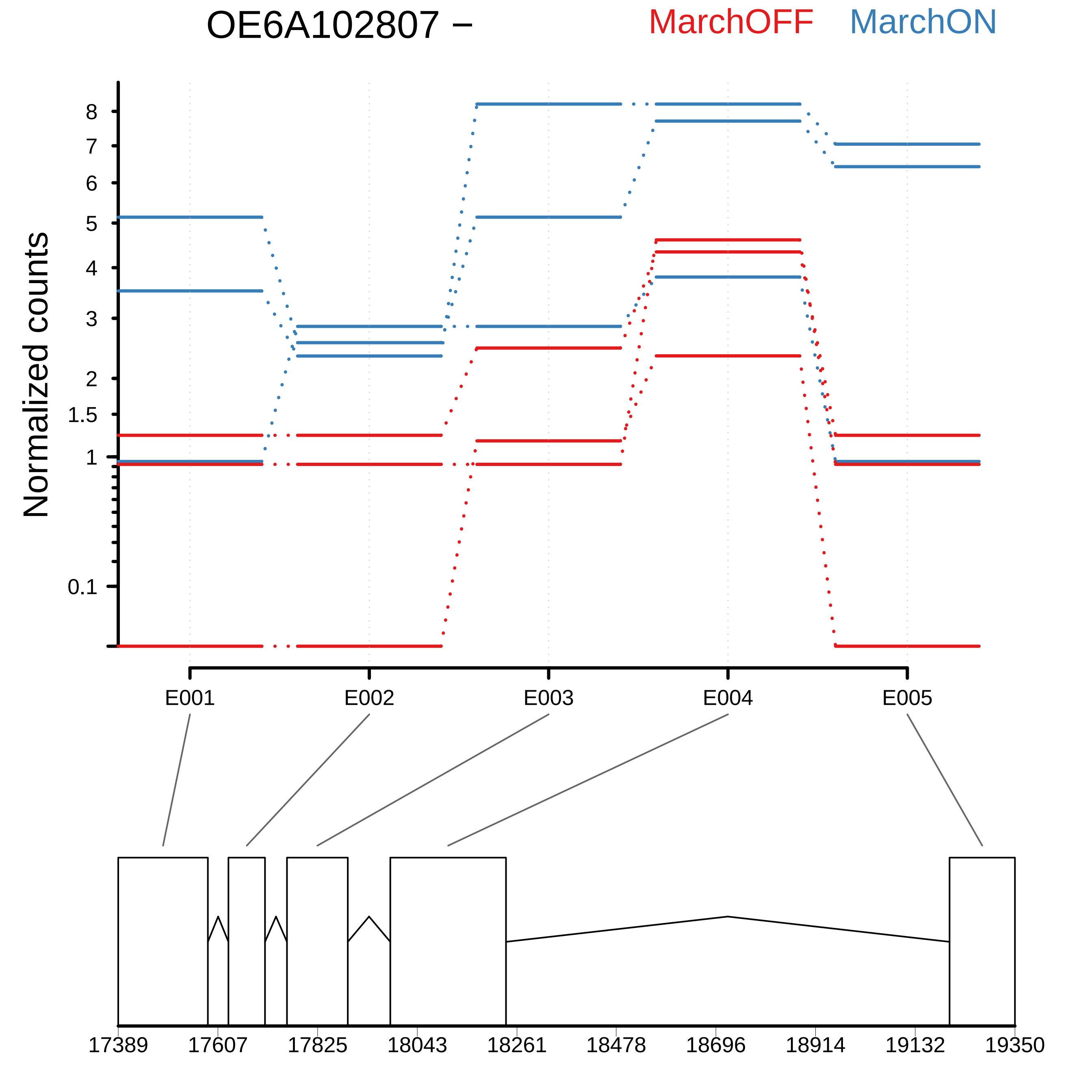
OE6A102807 −
MarchOFF
MarchON
Normalized counts
8
7
6
5
4
3
2
1.5
1
0.1
E001
E002
E003
E004
E005
17389
17607
17825
18043
18261
18478
18696
18914
19132
19350

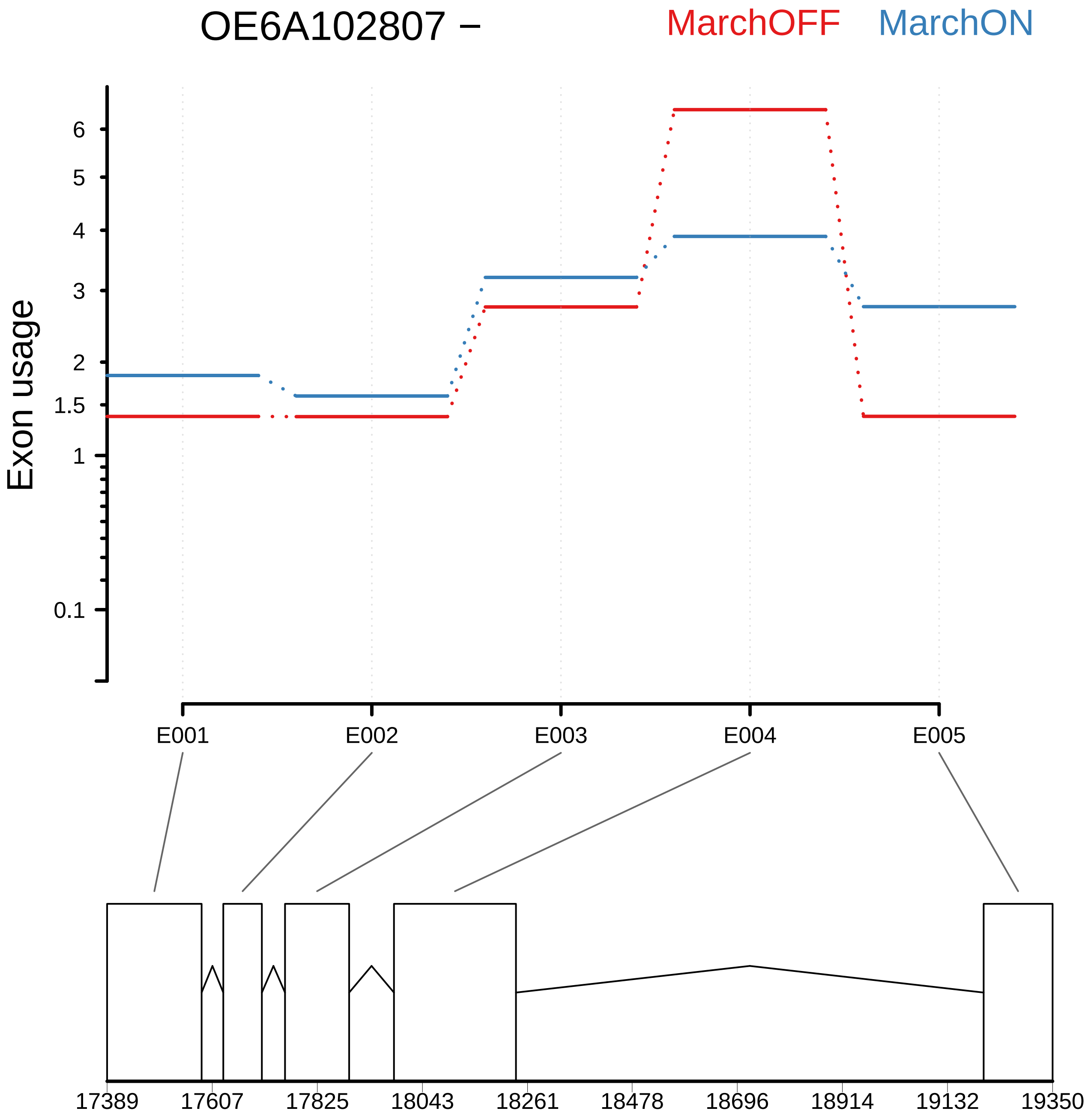

OE6A102807 −
MarchOFF
MarchON
Exon usage
6
5
4
3
2
1.5
1
0.1
E001
E002
E003
E004
E005
17389
17607
17825
18043
18261
18478
18696
18914
19132
19350

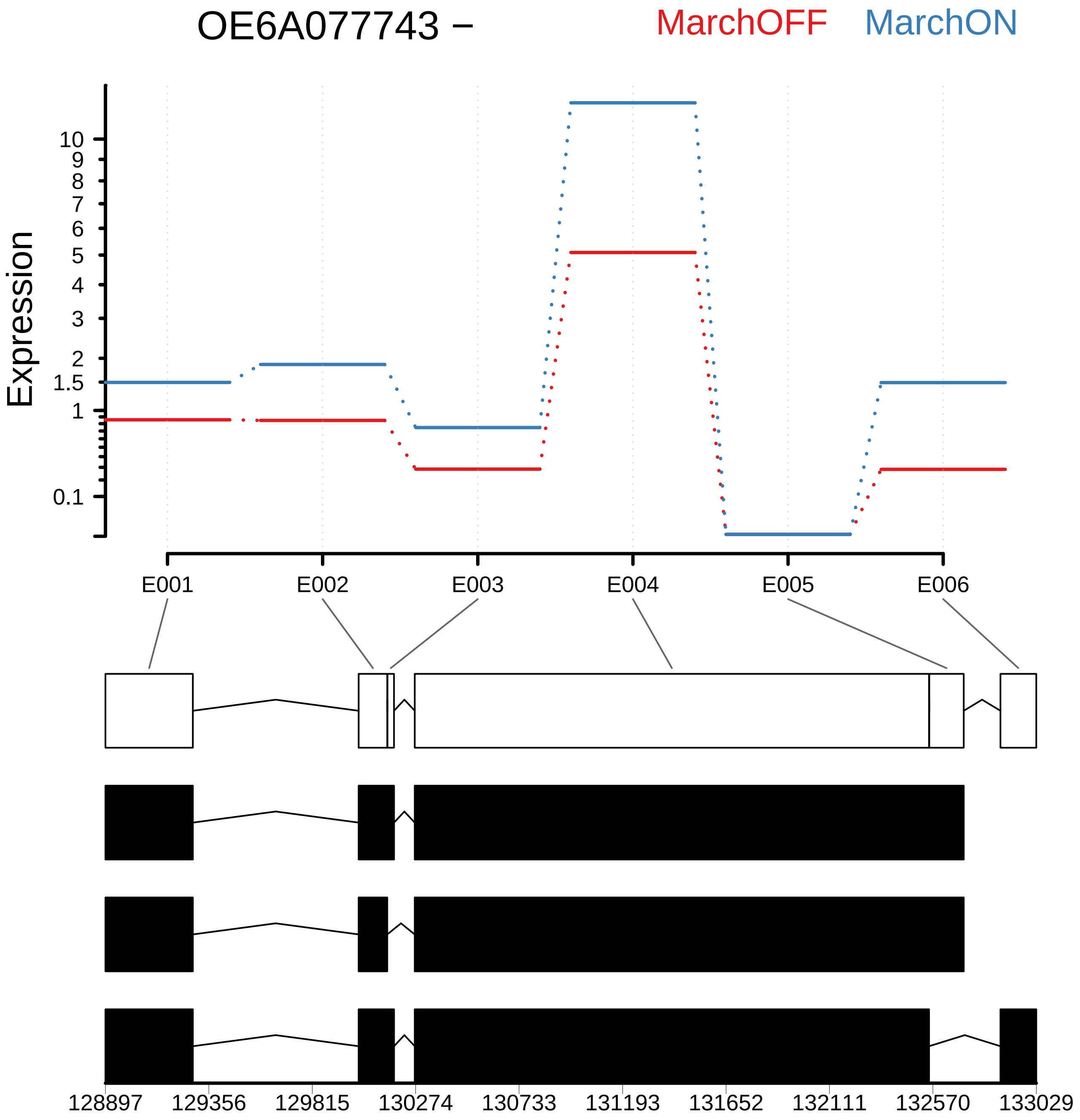

OE6A077743 −
MarchOFF
MarchON
Expression
10
9
8
7
6
5
4
3
2
1.5
1
0.1
E001
E002
E003
E004
E005
E006
128897
129356
129815
130274
130733
131193
131652
132111
132570
133029

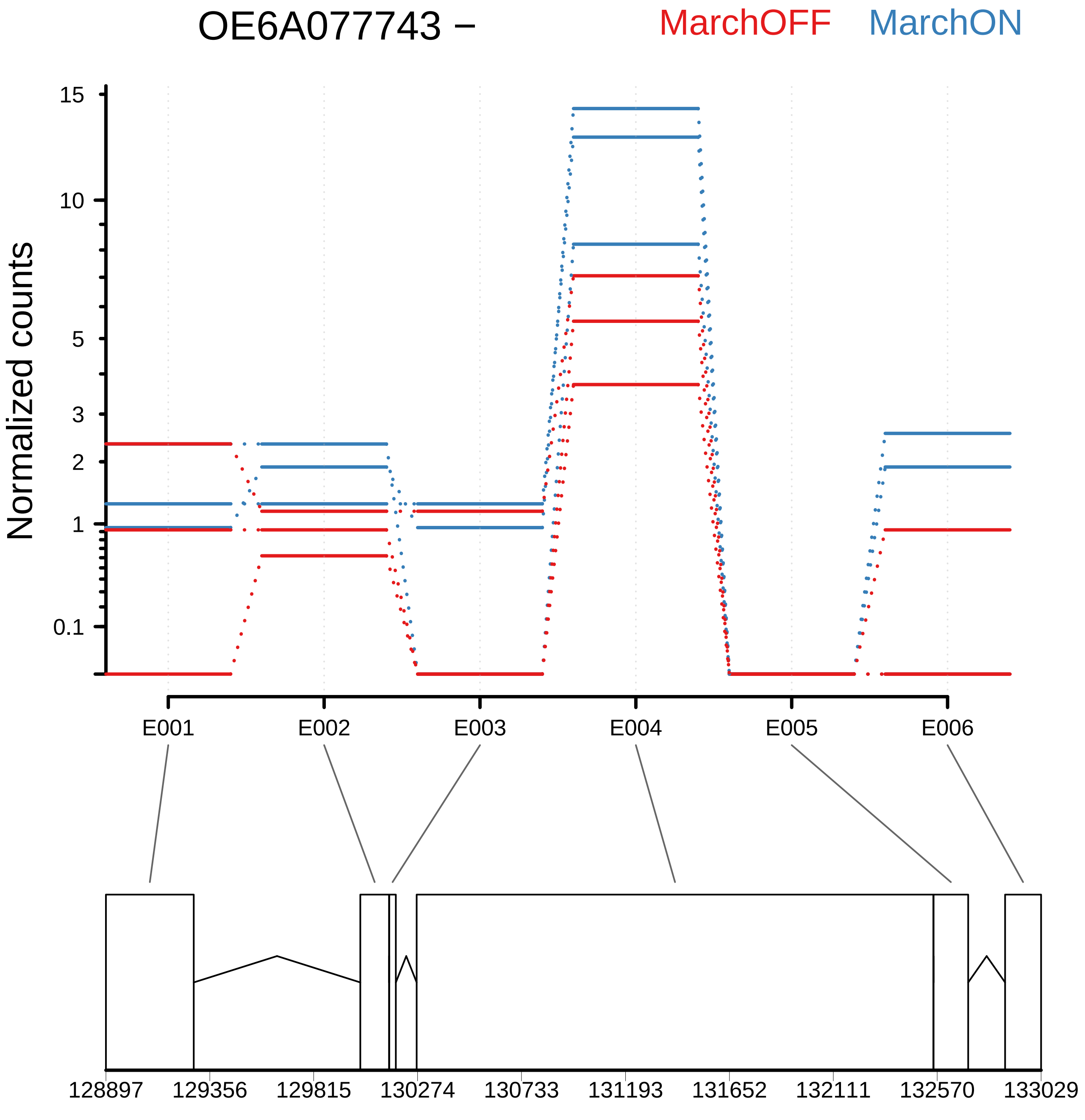

OE6A077743 −
MarchOFF
MarchON
Normalized counts
15
10
5
3
2
1
0.1
E001
E002
E003
E004
E005
E006
128897
129356
129815
130274
130733
131193
131652
132111
132570
133029

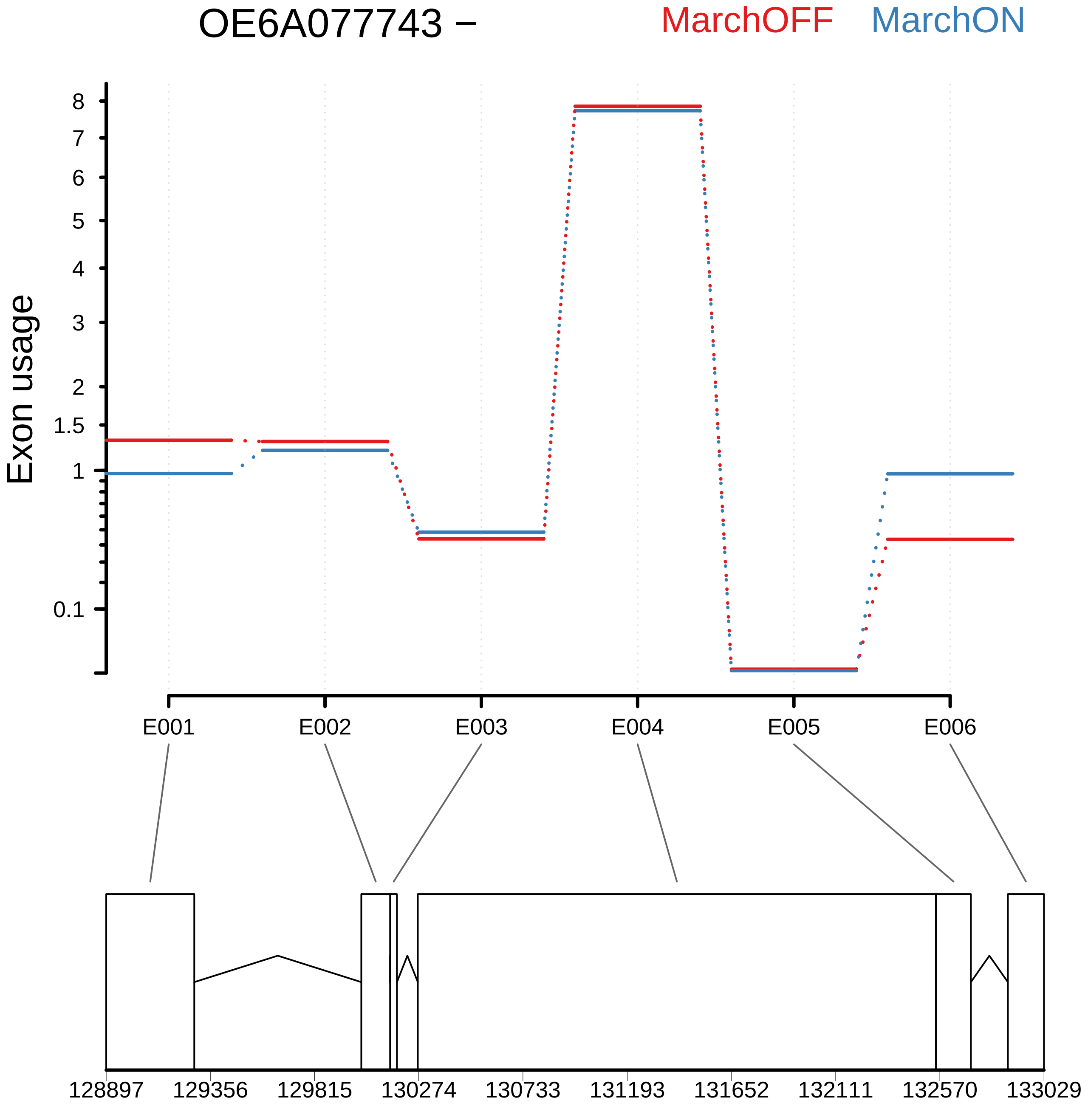

OE6A077743 −
MarchOFF
MarchON
Exon usage
8
7
6
5
4
3
2
1.5
1
0.1
E001
E002
E003
E004
E005
E006
128897
129356
129815
130274
130733
131193
131652
132111
132570
133029

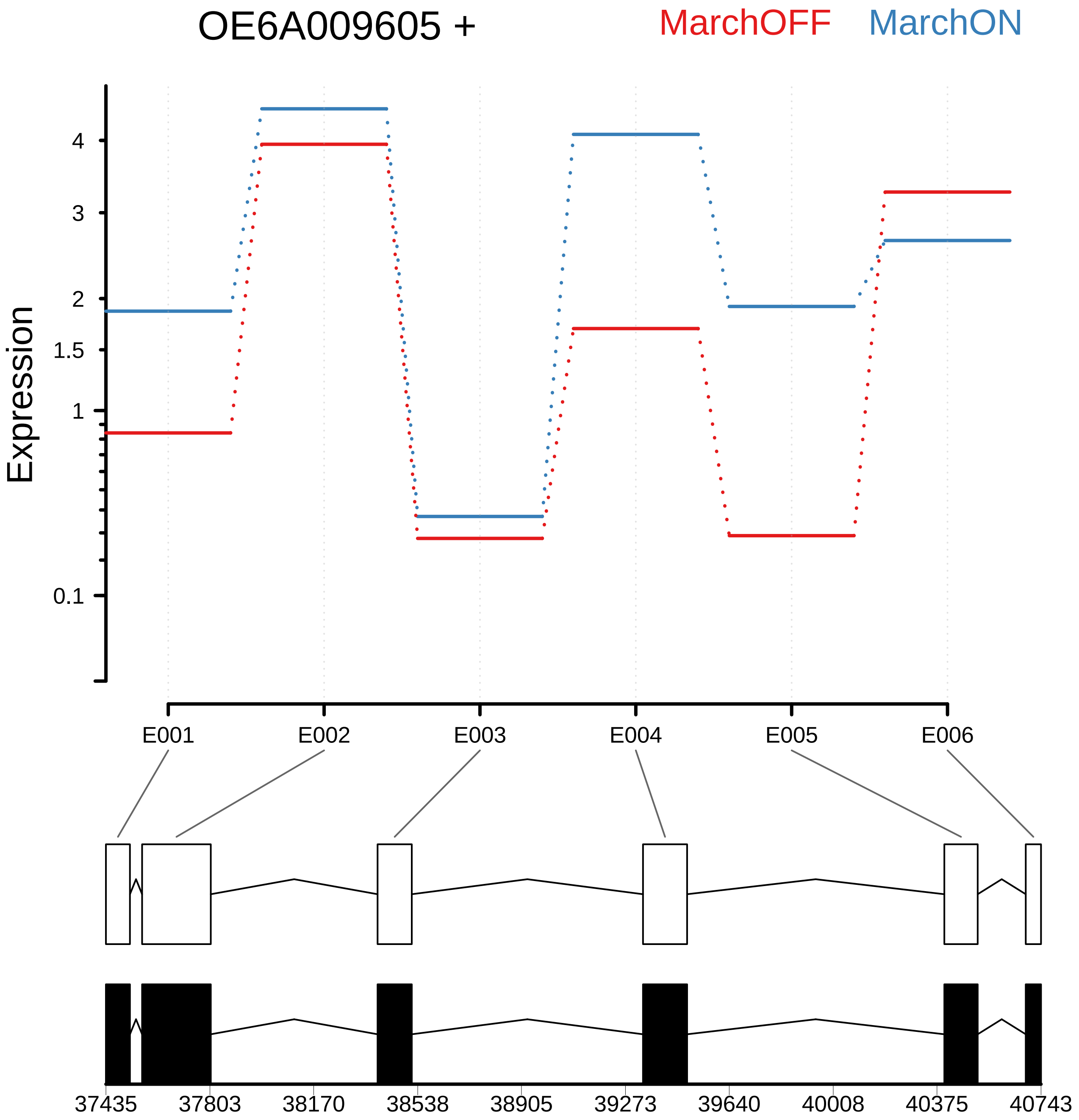

OE6A009605 +
MarchOFF
MarchON
Expression
4
3
2
1.5
1
0.1
E001
E002
E003
E004
E005
E006
37435
37803
38170
38538
38905
39273
39640
40008
40375
40743

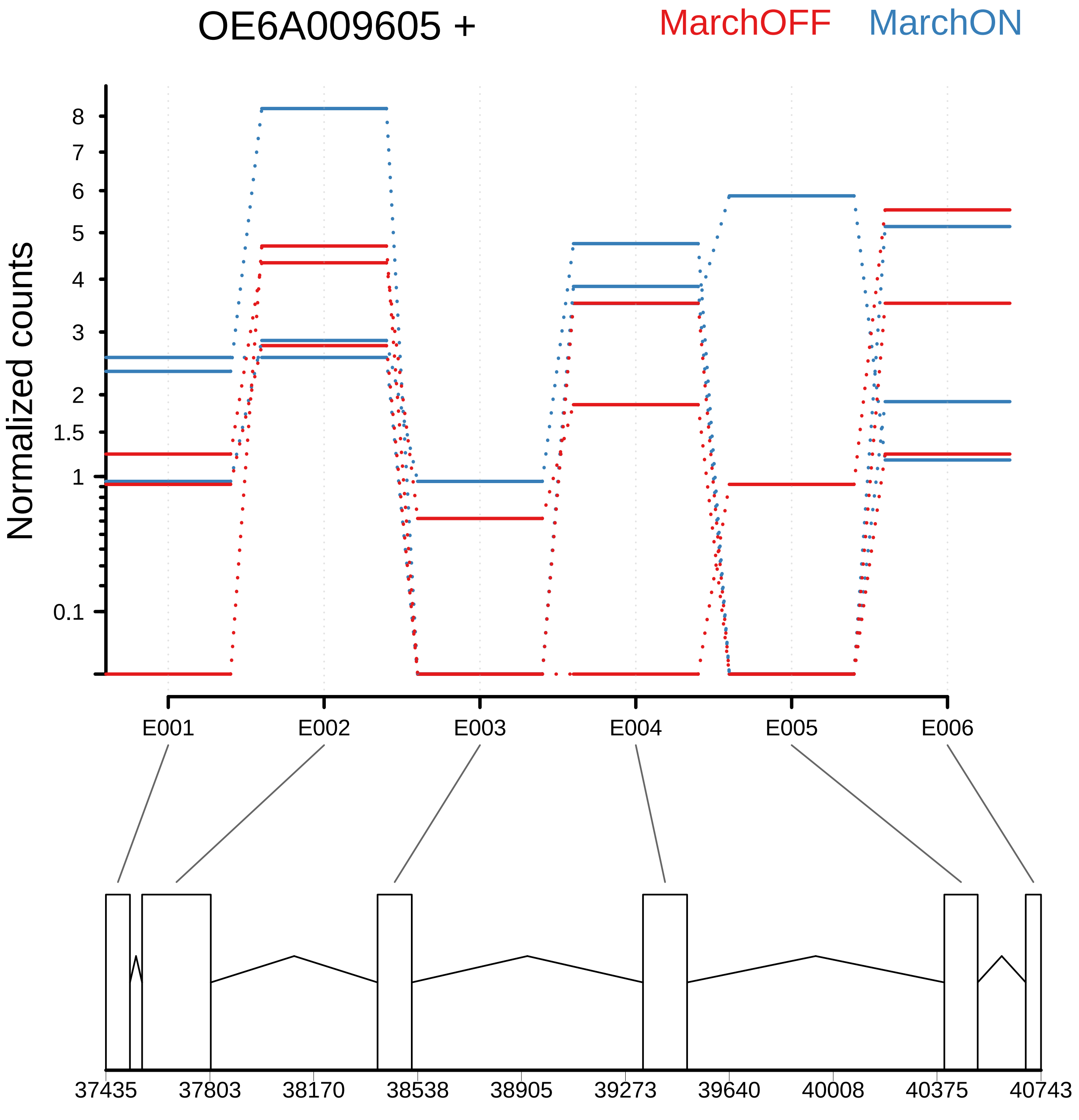
OE6A009605 +
MarchOFF
MarchON
Normalized counts
8
7
6
5
4
3
2
1.5
1
0.1
E001
E002
E003
E004
E005
E006
37435
37803
38170
38538
38905
39273
39640
40008
40375
40743

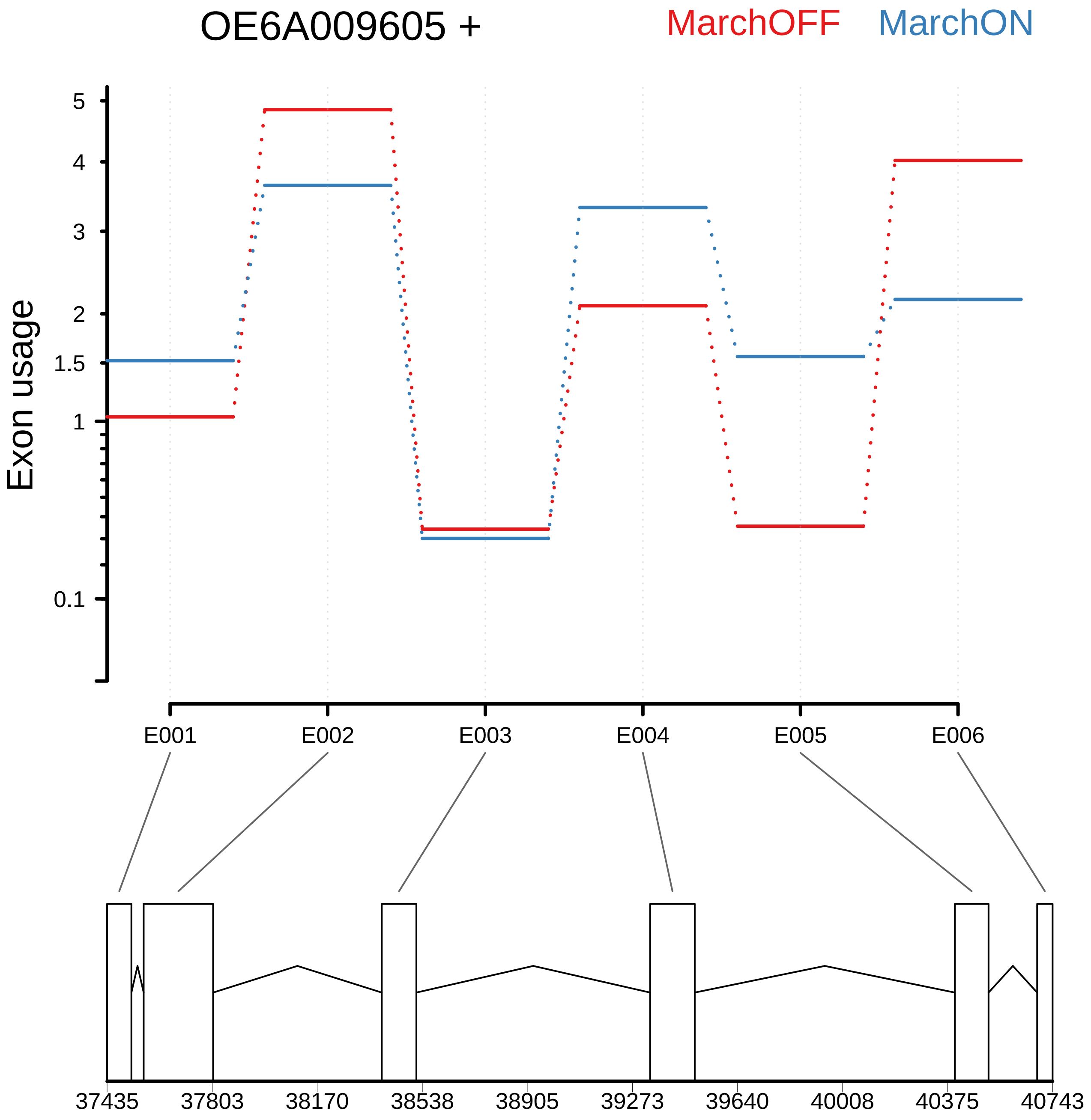

OE6A009605 +
MarchOFF
MarchON
Exon usage
5
4
3
2
1.5
1
0.1
E001
E002
E003
E004
E005
E006
37435
37803
38170
38538
38905
39273
39640
40008
40375
40743

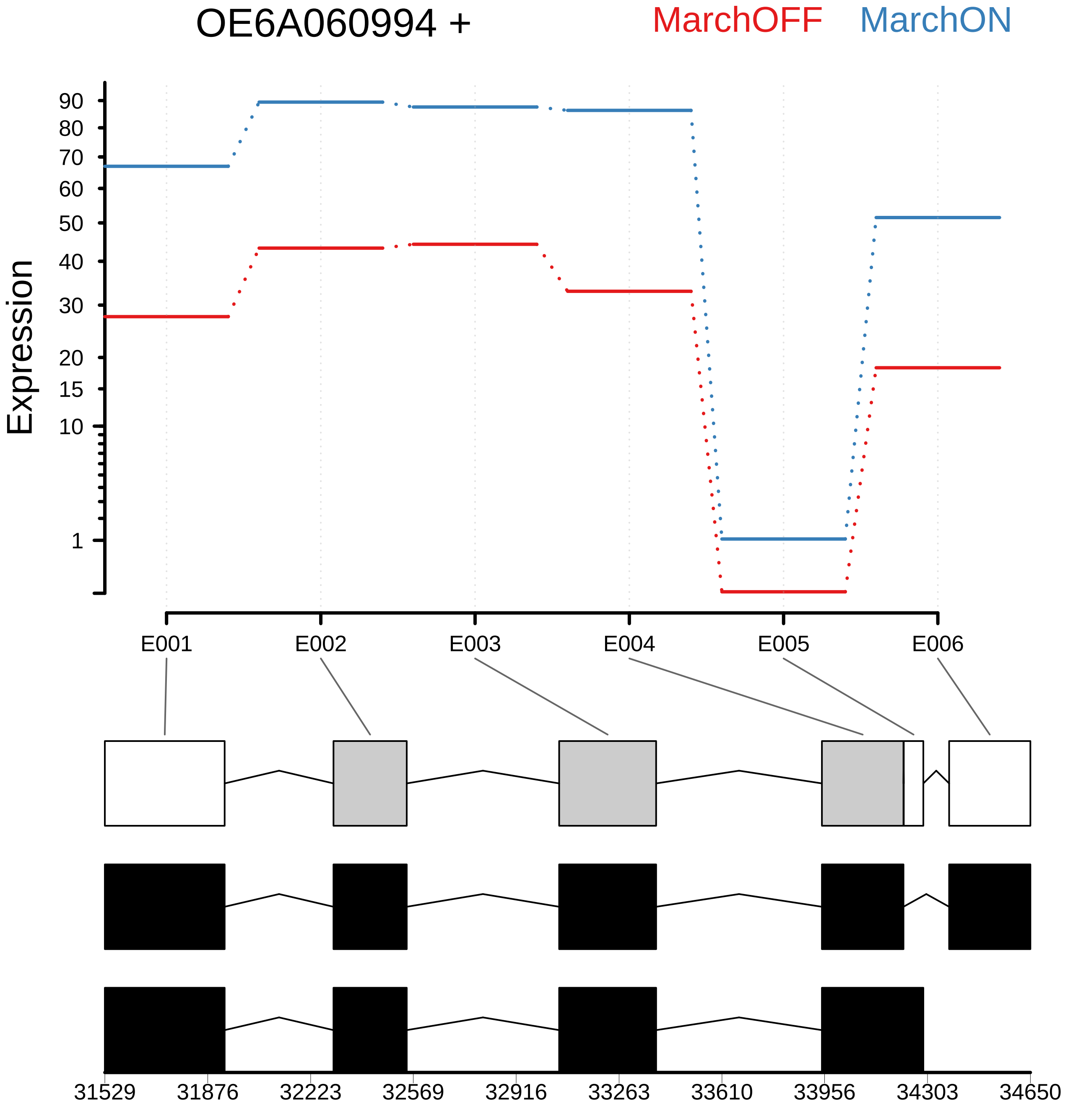
OE6A060994 +
MarchOFF
MarchON
Expression
90
80
70
60
50
40
30
20
15
10
1
E001
E002
E003
E004
E005
E006
31529
31876
32223
32569
32916
33263
33610
33956
34303
34650

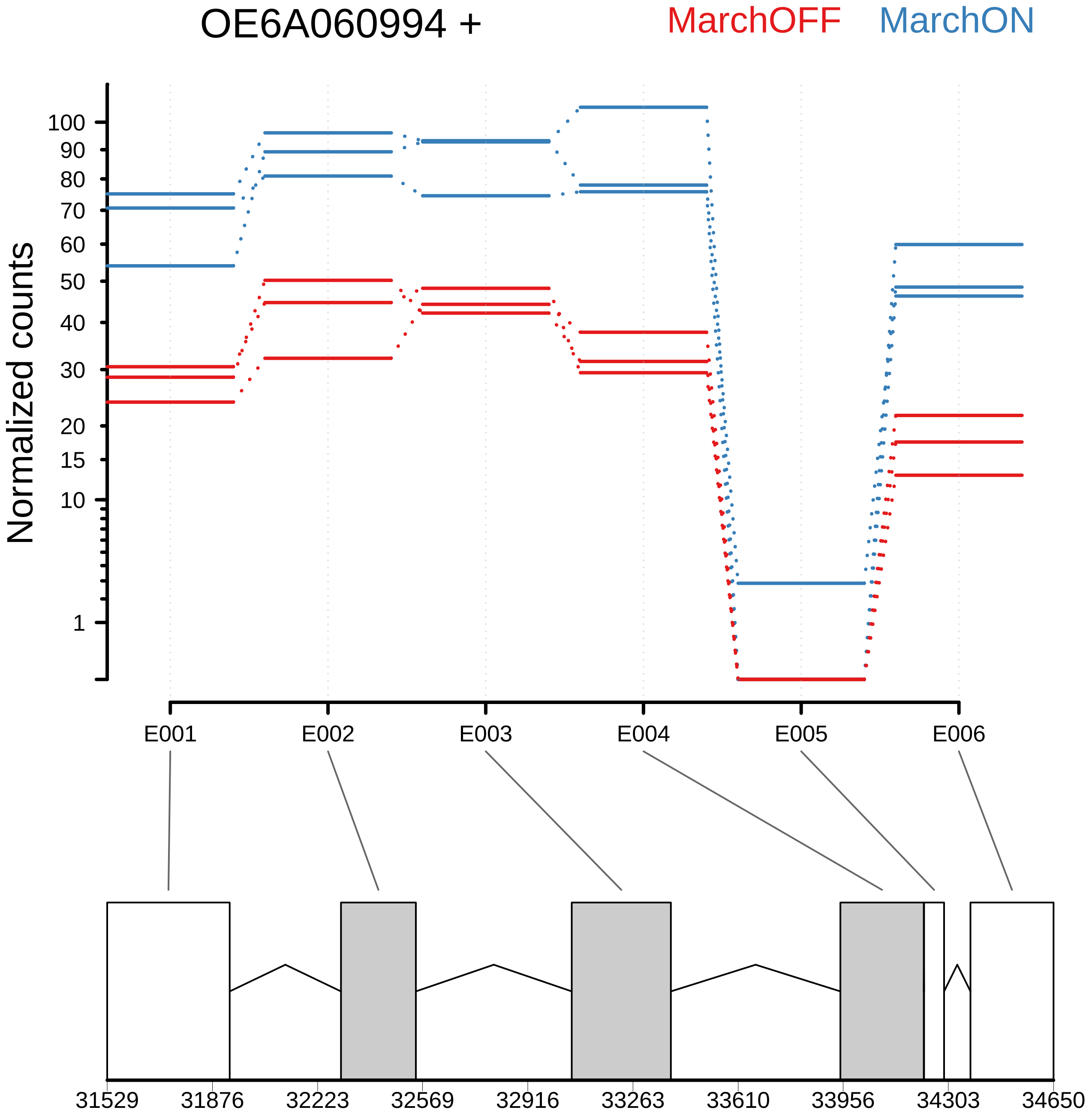

OE6A060994 +
MarchOFF
MarchON
Normalized counts
100
90
80
70
60
50
40
30
20
15
10
1
E001
E002
E003
E004
E005
E006
31529
31876
32223
32569
32916
33263
33610
33956
34303
34650

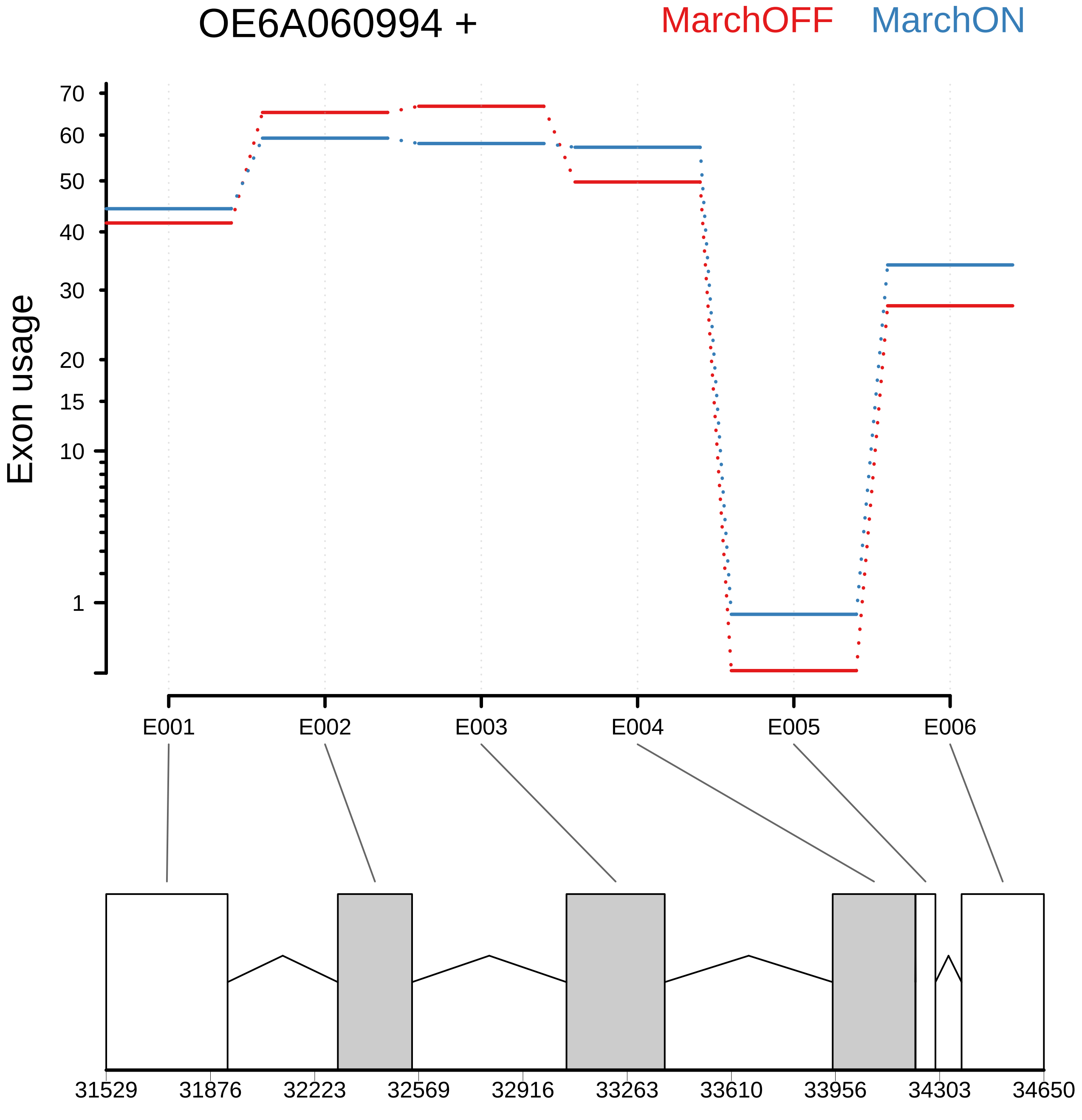
OE6A060994 +
MarchOFF
MarchON
Exon usage
70
60
50
40
30
20
15
10
1
E001
E002
E003
E004
E005
E006
31529
31876
32223
32569
32916
33263
33610
33956
34303
34650

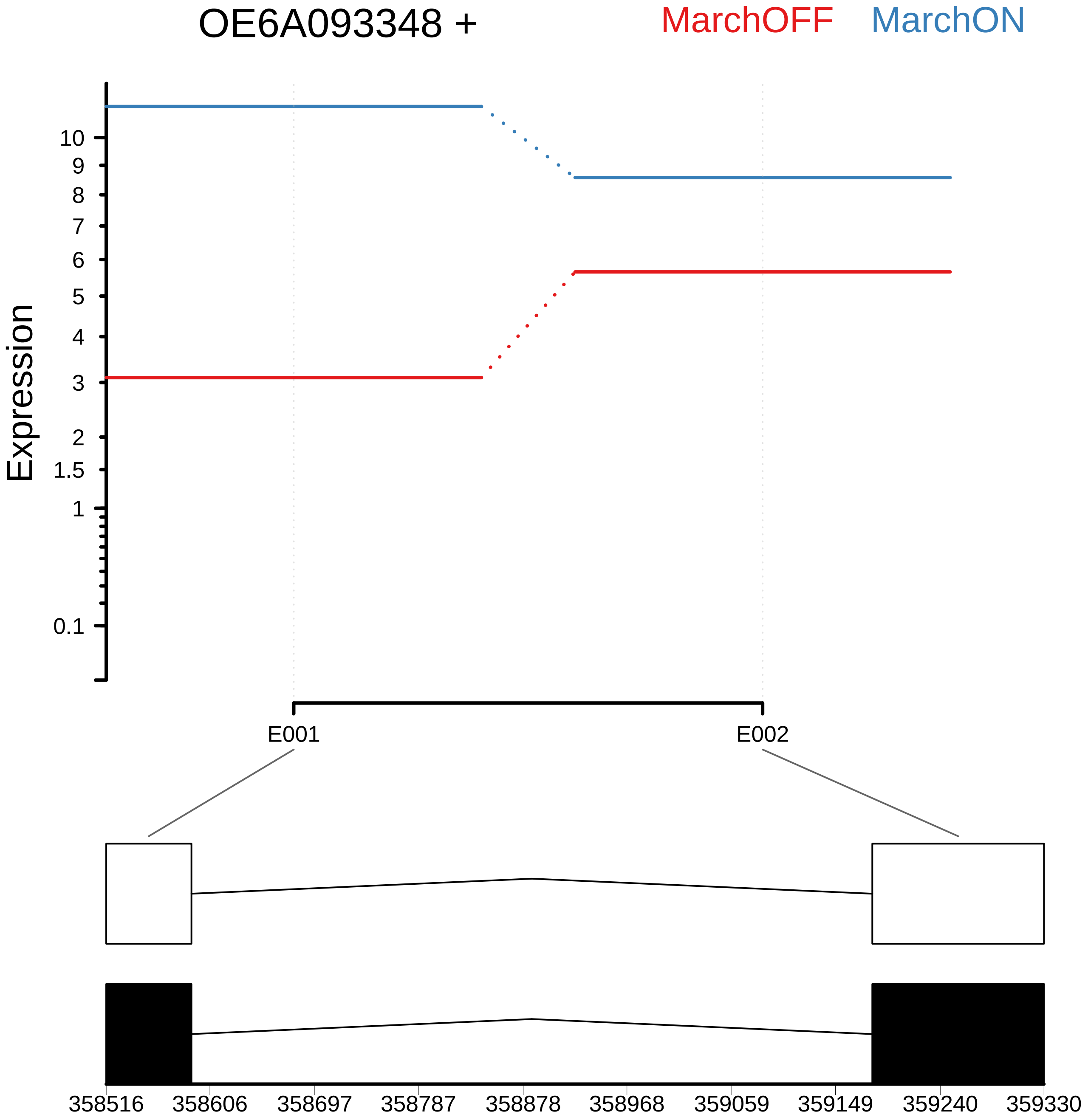

OE6A093348 +
MarchOFF
MarchON
Expression
10
9
8
7
6
5
4
3
2
1.5
1
0.1
E001
E002
358516
358606
358697
358787
358878
358968
359059
359149
359240
359330

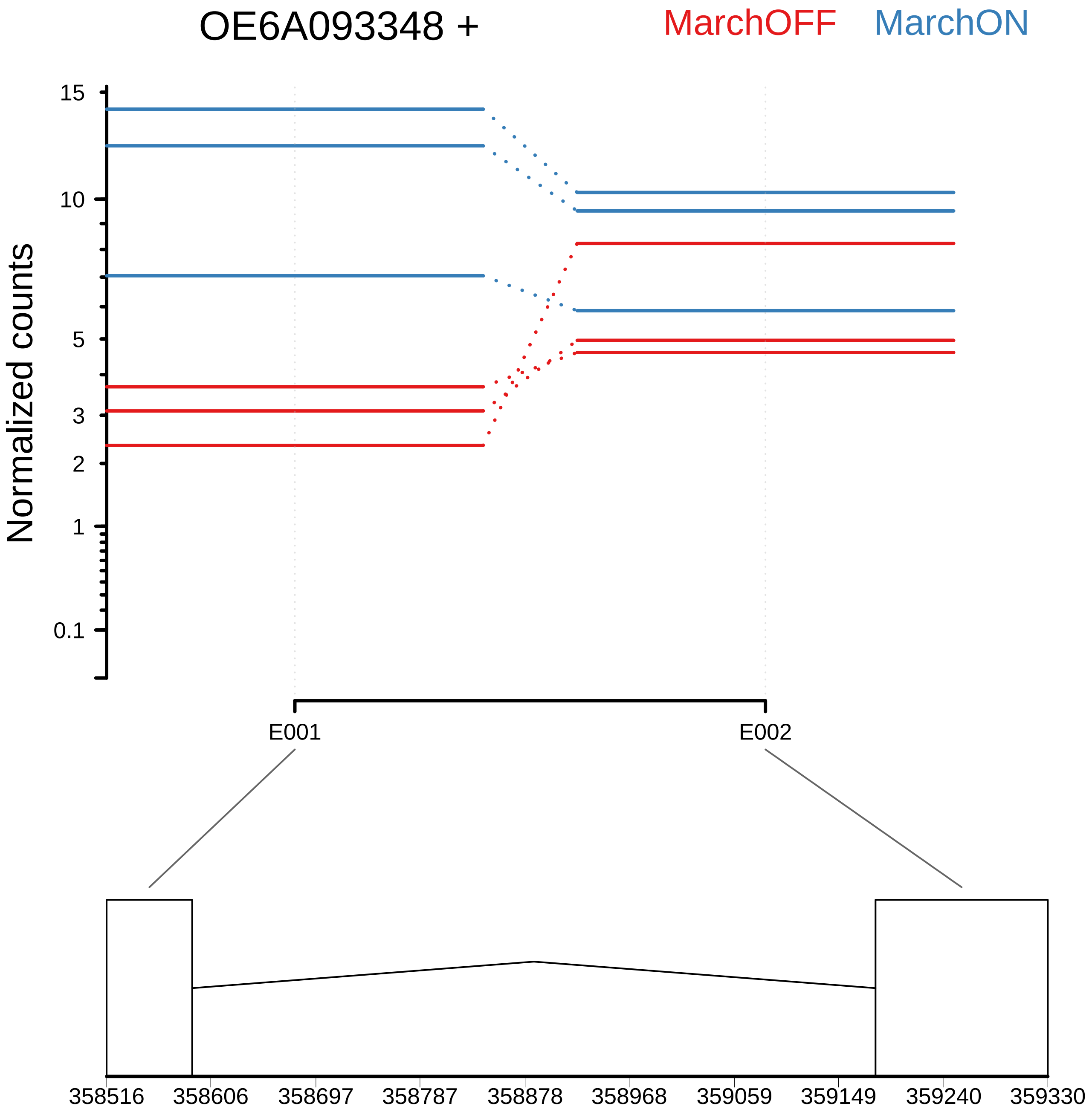
OE6A093348 +
MarchOFF
MarchON
Normalized counts
15
10
5
3
2
1
0.1
E001
E002
358516
358606
358697
358787
358878
358968
359059
359149
359240
359330

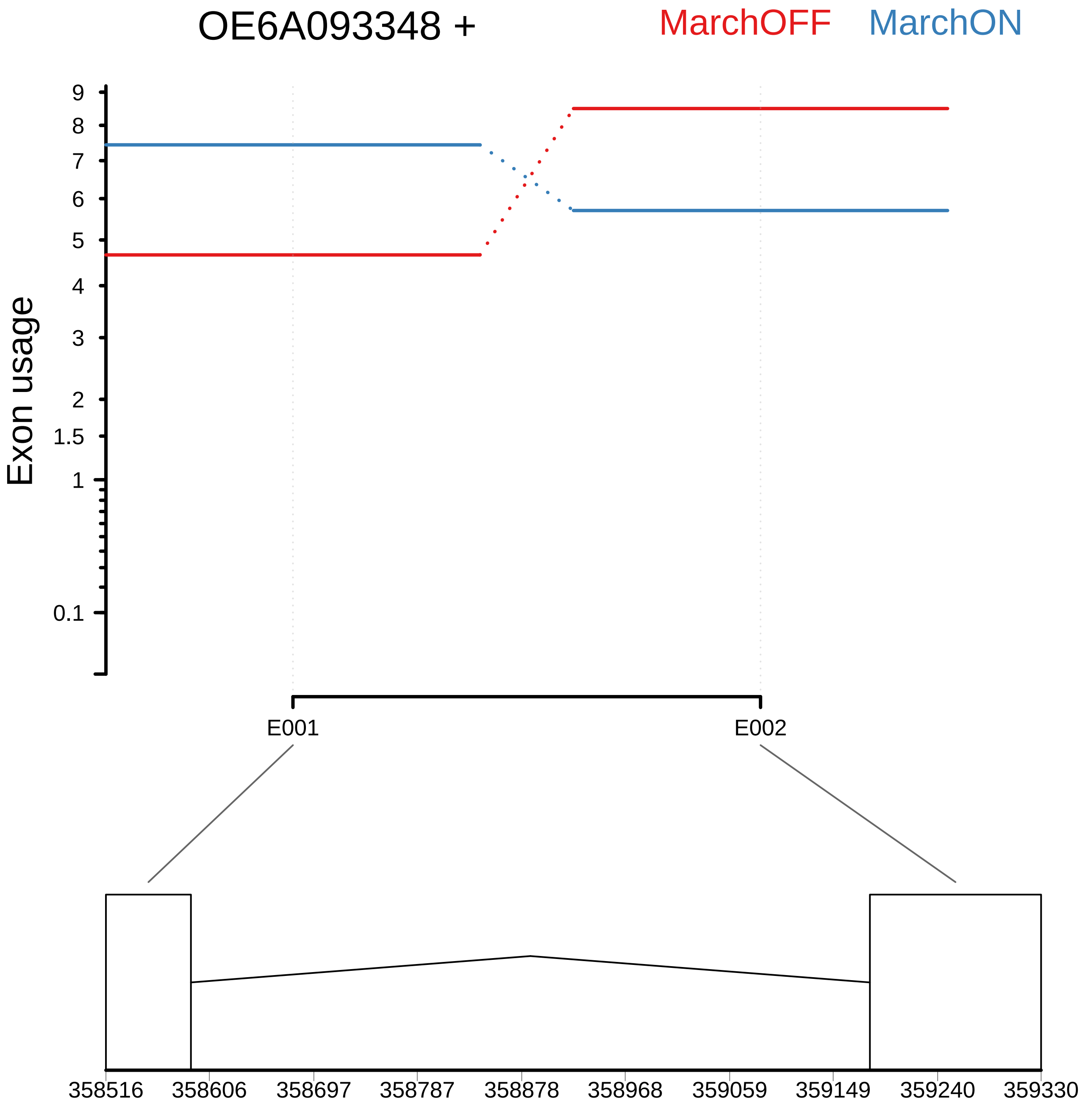

OE6A093348 +
MarchOFF
MarchON
Exon usage
9
8
7
6
5
4
3
2
1.5
1
0.1
E001
E002
358516
358606
358697
358787
358878
358968
359059
359149
359240
359330

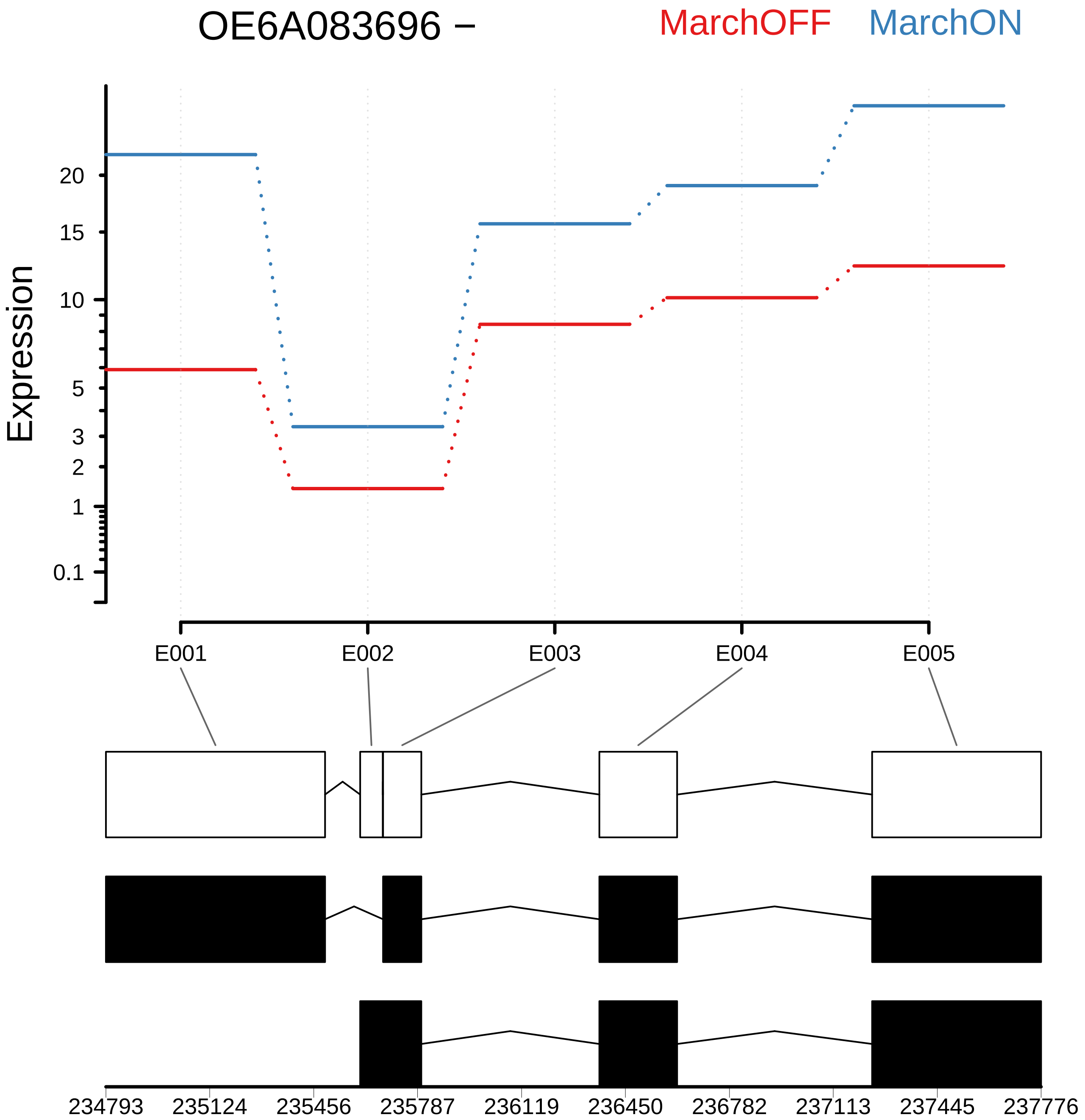

OE6A083696 −
MarchOFF
MarchON
Expression
20
15
10
5
3
2
1
0.1
E001
E002
E003
E004
E005
234793
235124
235456
235787
236119
236450
236782
237113
237445
237776

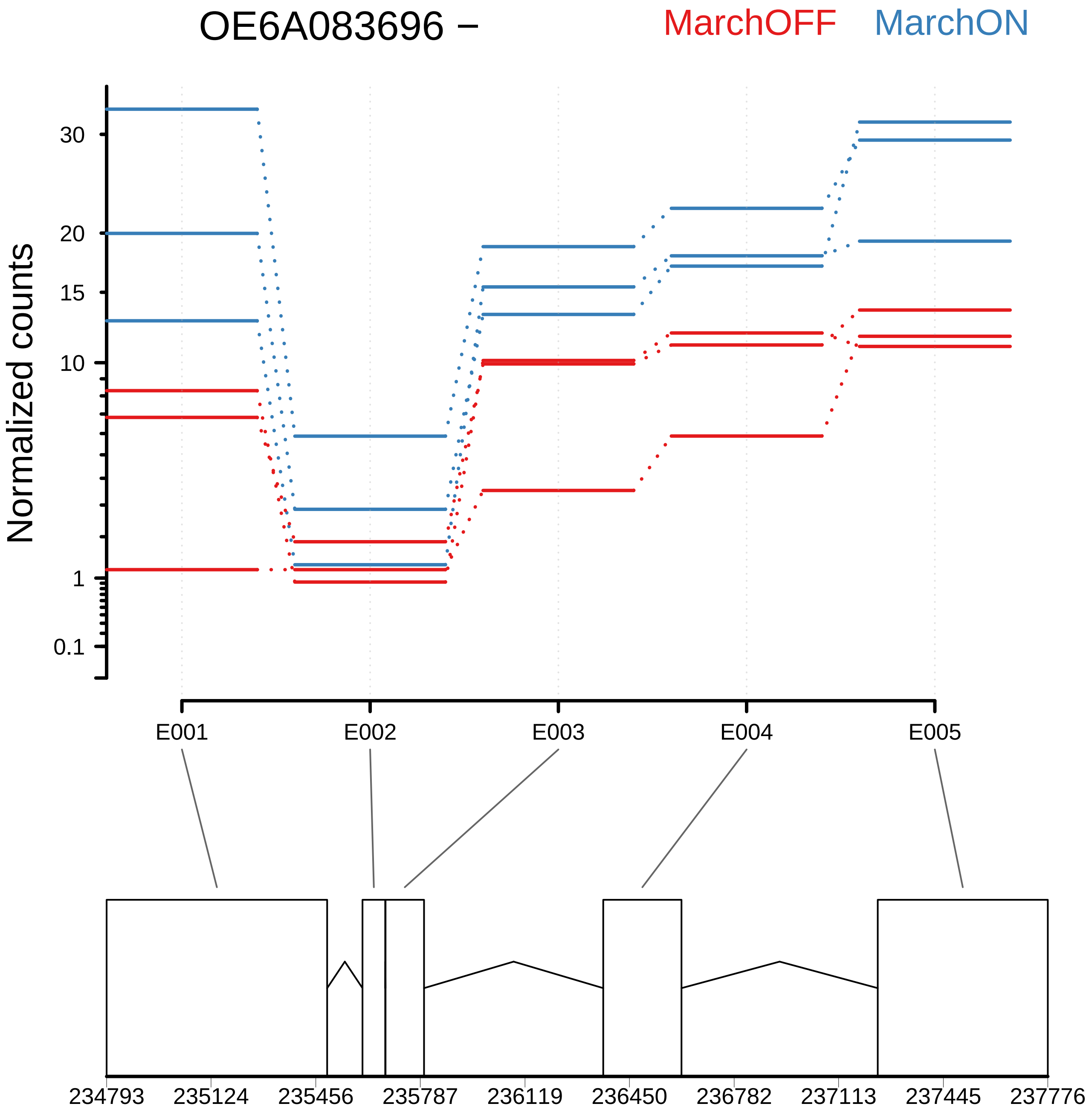
OE6A083696 −
MarchOFF
MarchON
Normalized counts
30
20
15
10
1
0.1
E001
E002
E003
E004
E005
234793
235124
235456
235787
236119
236450
236782
237113
237445
237776

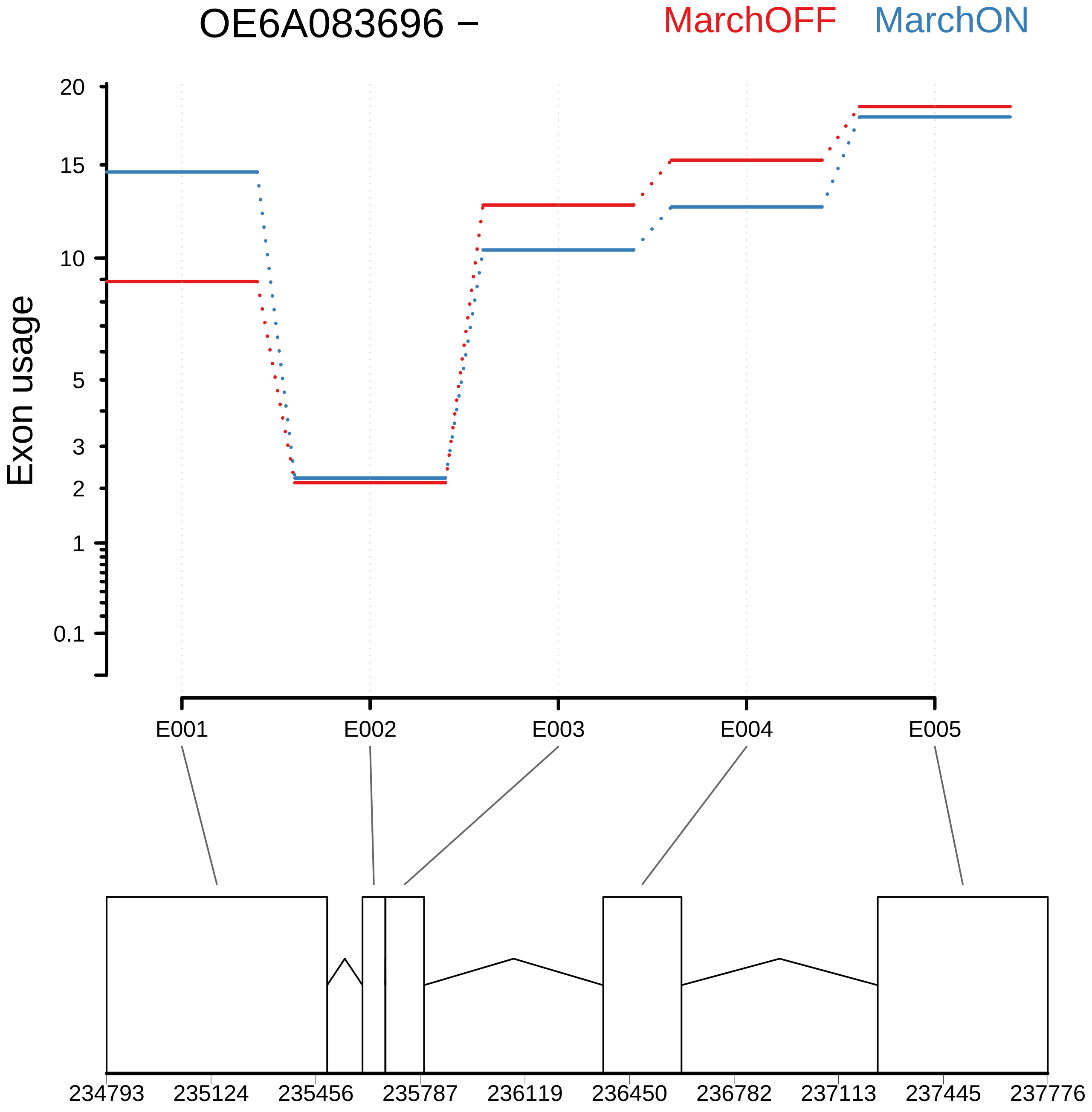
OE6A083696 −
MarchOFF
MarchON
Exon usage
20
15
10
5
3
2
1
0.1
E001
E002
E003
E004
E005
234793
235124
235456
235787
236119
236450
236782
237113
237445
237776

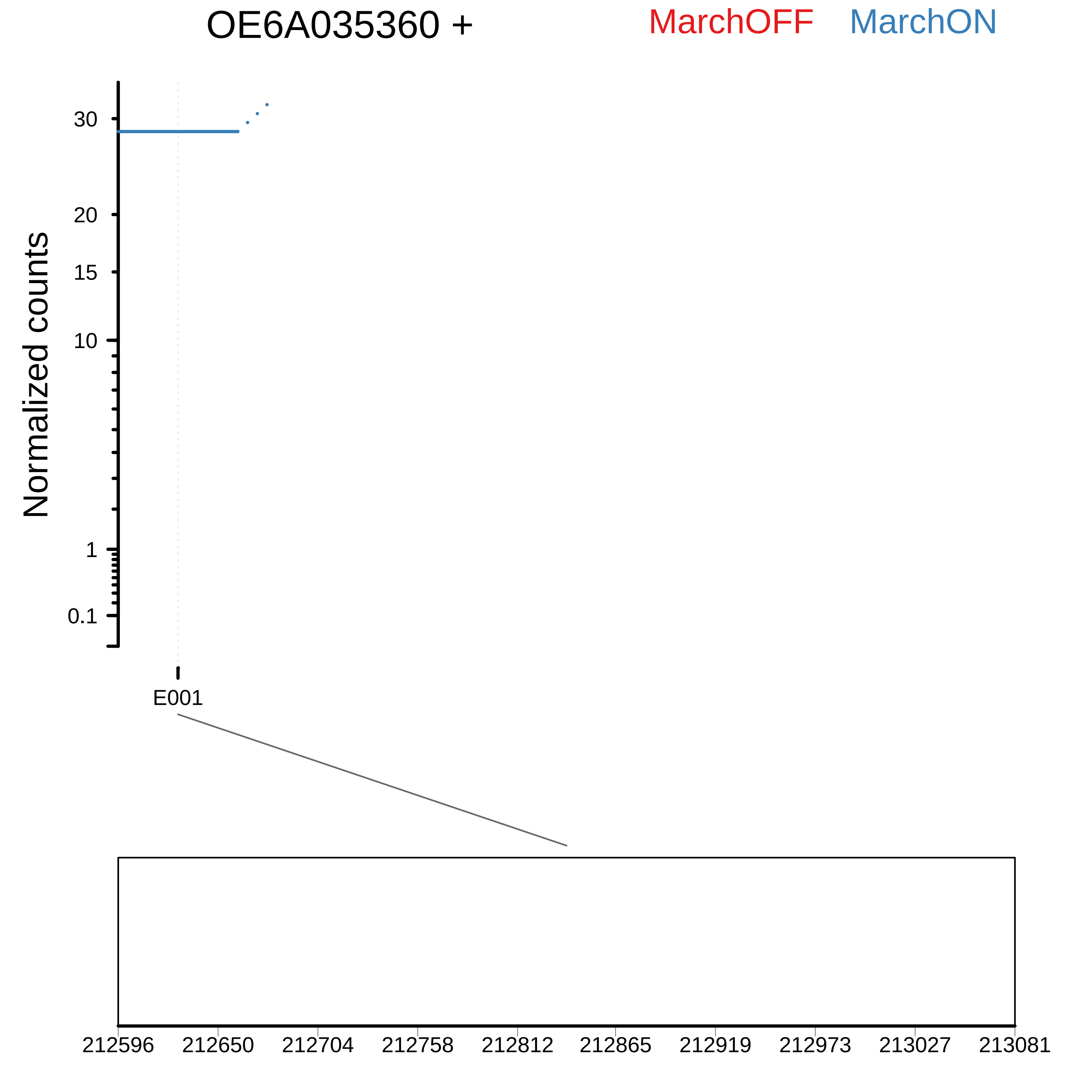

OE6A035360 +
MarchOFF
MarchON
Normalized counts
30
20
15
10
1
0.1
E001
212596
212650
212704
212758
212812
212865
212919
212973
213027
213081

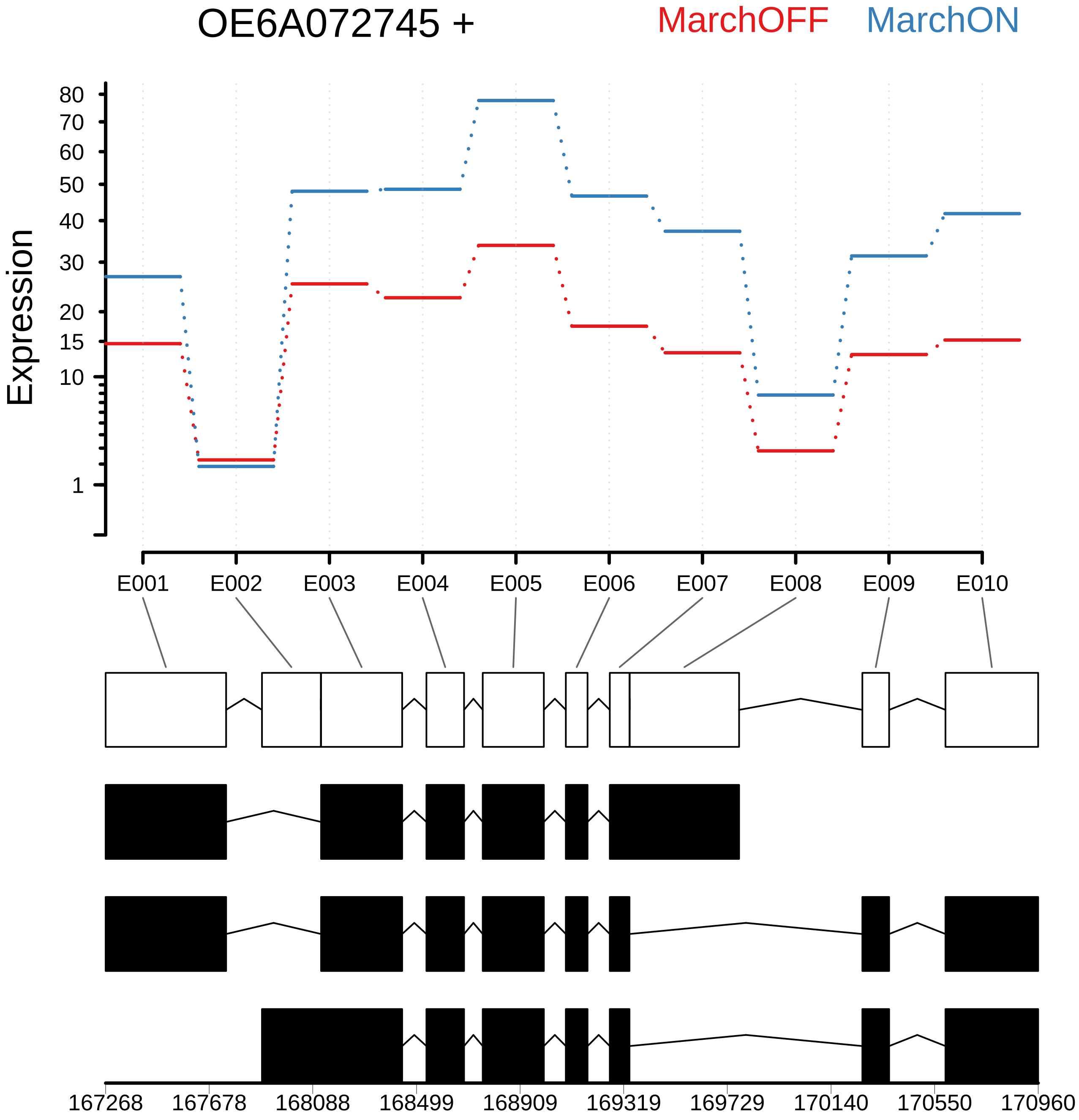

OE6A072745 +
MarchOFF
MarchON
Expression
80
70
60
50
40
30
20
15
10
1
E001
E002
E003
E004
E005
E006
E007
E008
E009
E010
167268
167678
168088
168499
168909
169319
169729
170140
170550
170960

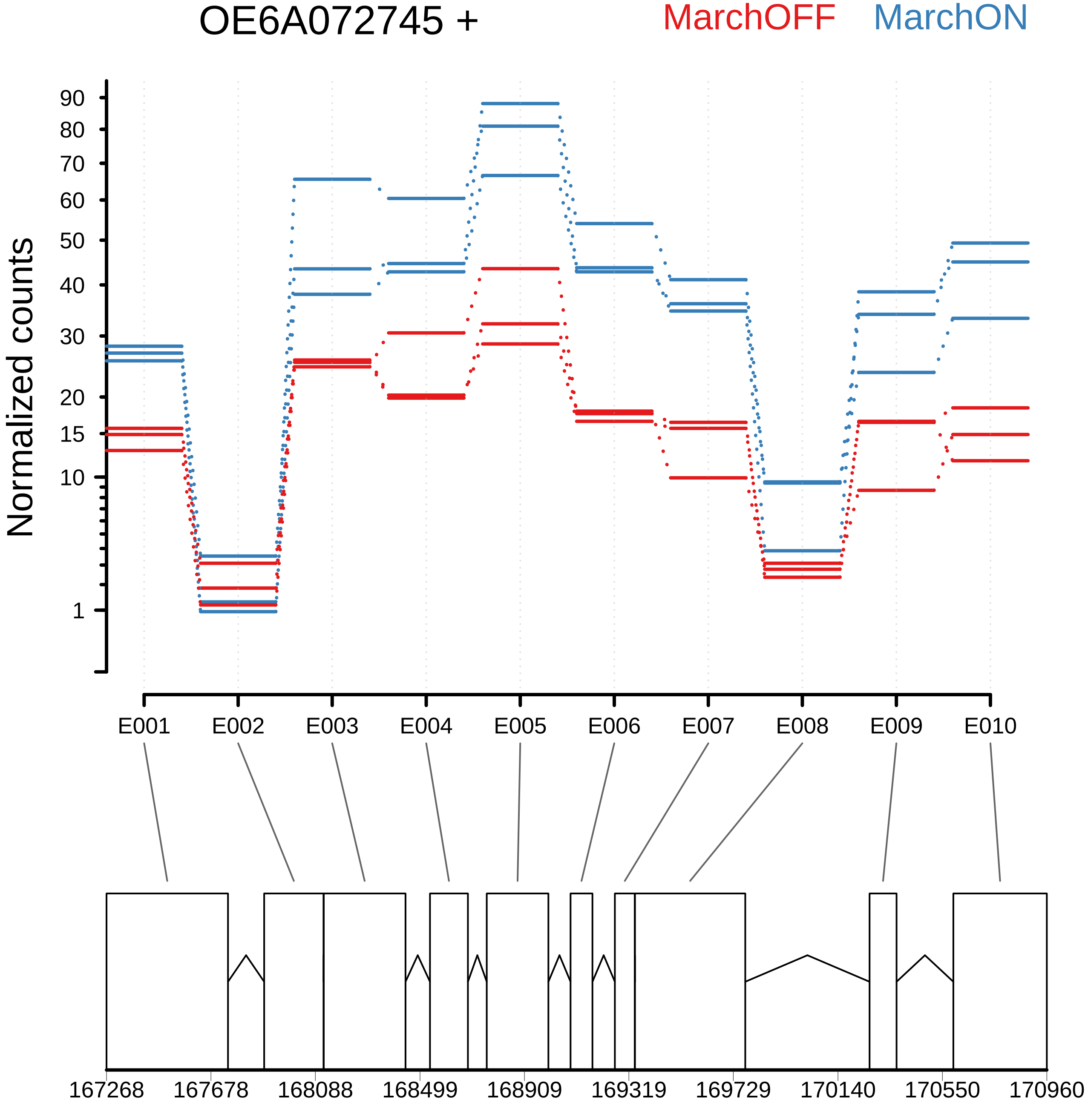

OE6A072745 +
MarchOFF
MarchON
Normalized counts
90
80
70
60
50
40
30
20
15
10
1
E001
E002
E003
E004
E005
E006
E007
E008
E009
E010
167268
167678
168088
168499
168909
169319
169729
170140
170550
170960

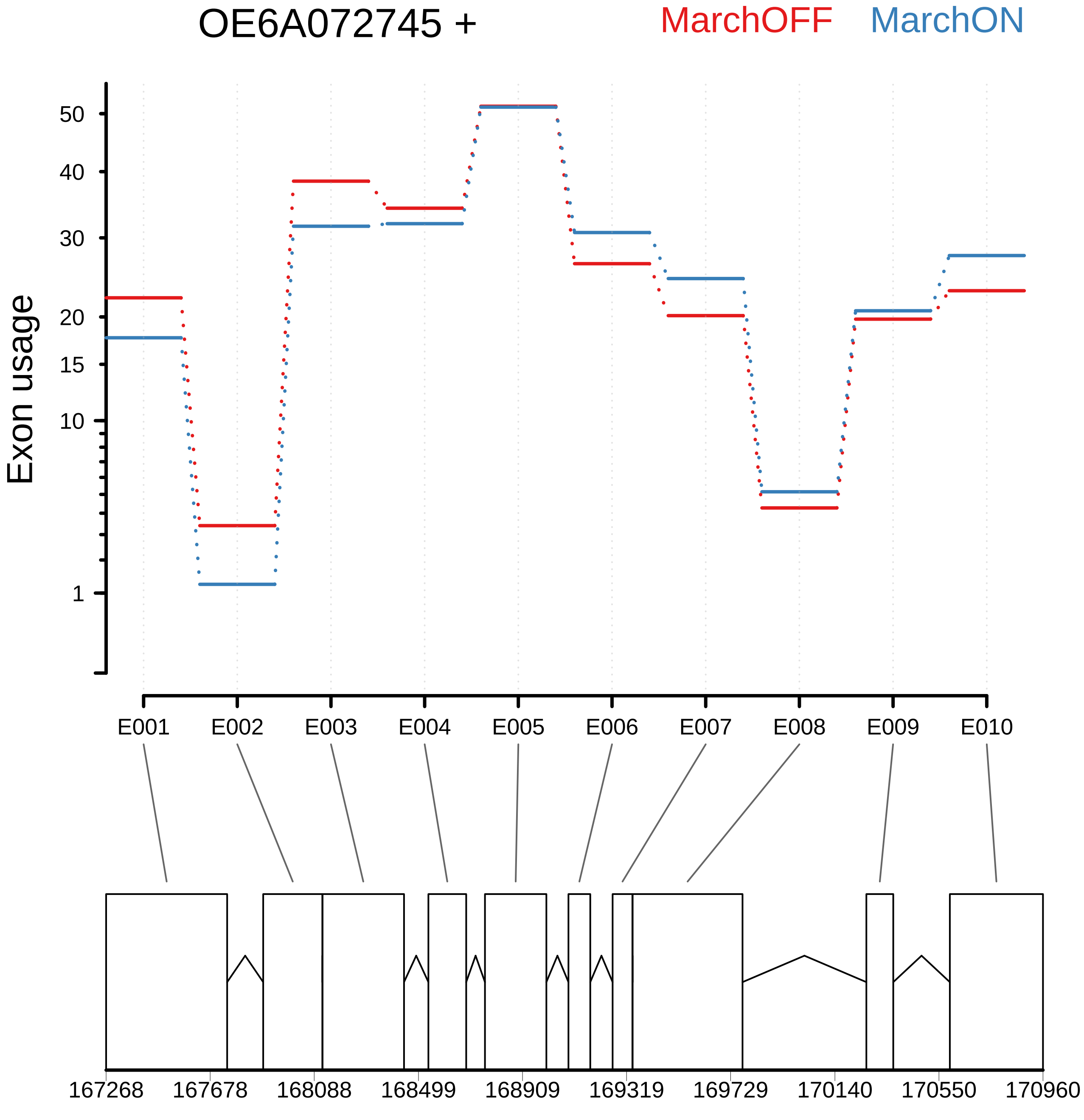

OE6A072745 +
MarchOFF
MarchON
Exon usage
50
40
30
20
15
10
1
E001
E002
E003
E004
E005
E006
E007
E008
E009
E010
167268
167678
168088
168499
168909
169319
169729
170140
170550
170960

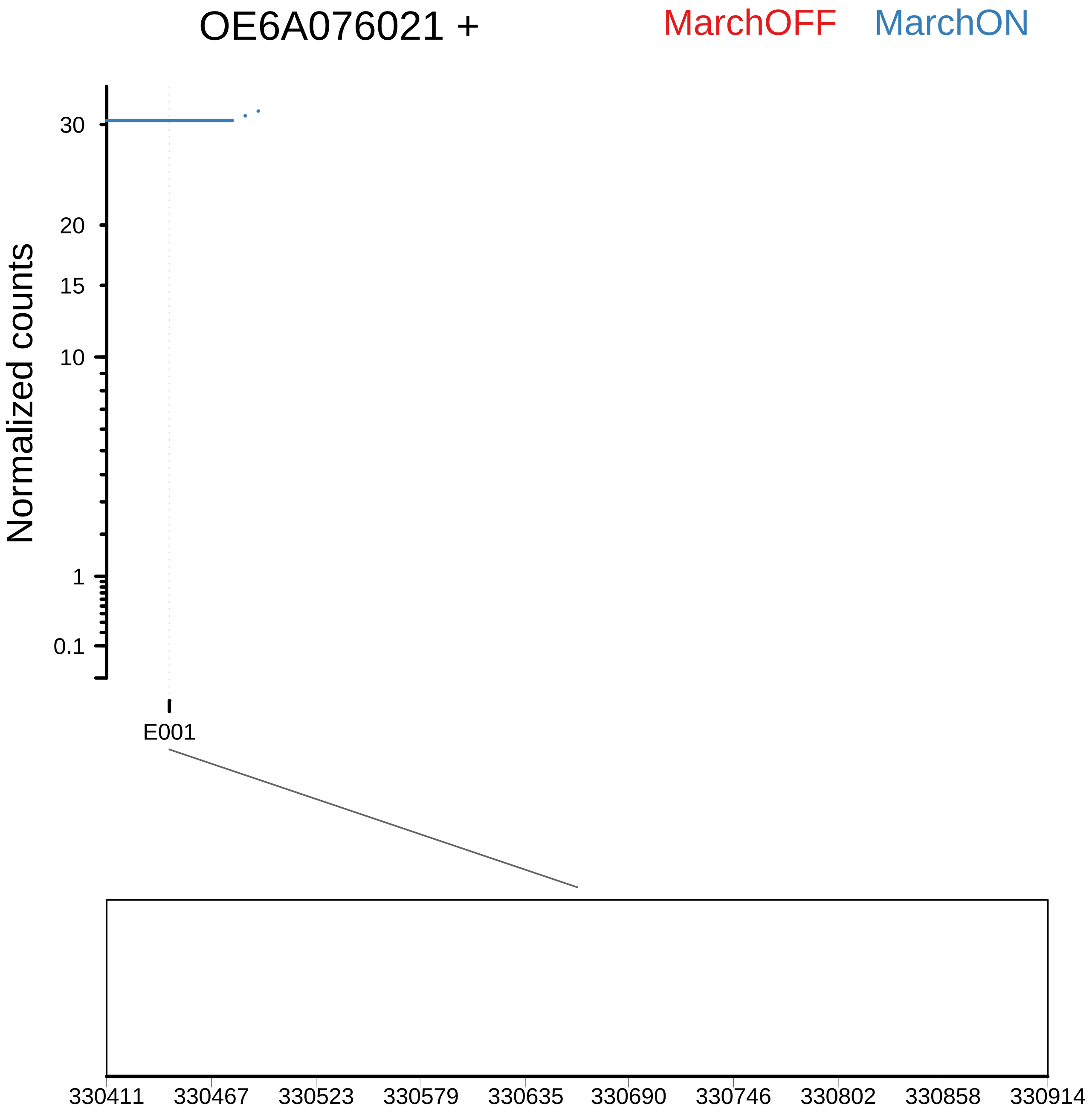

OE6A076021 +
MarchOFF
MarchON
Normalized counts
30
20
15
10
1
0.1
E001
330411
330467
330523
330579
330635
330690
330746
330802
330858
330914

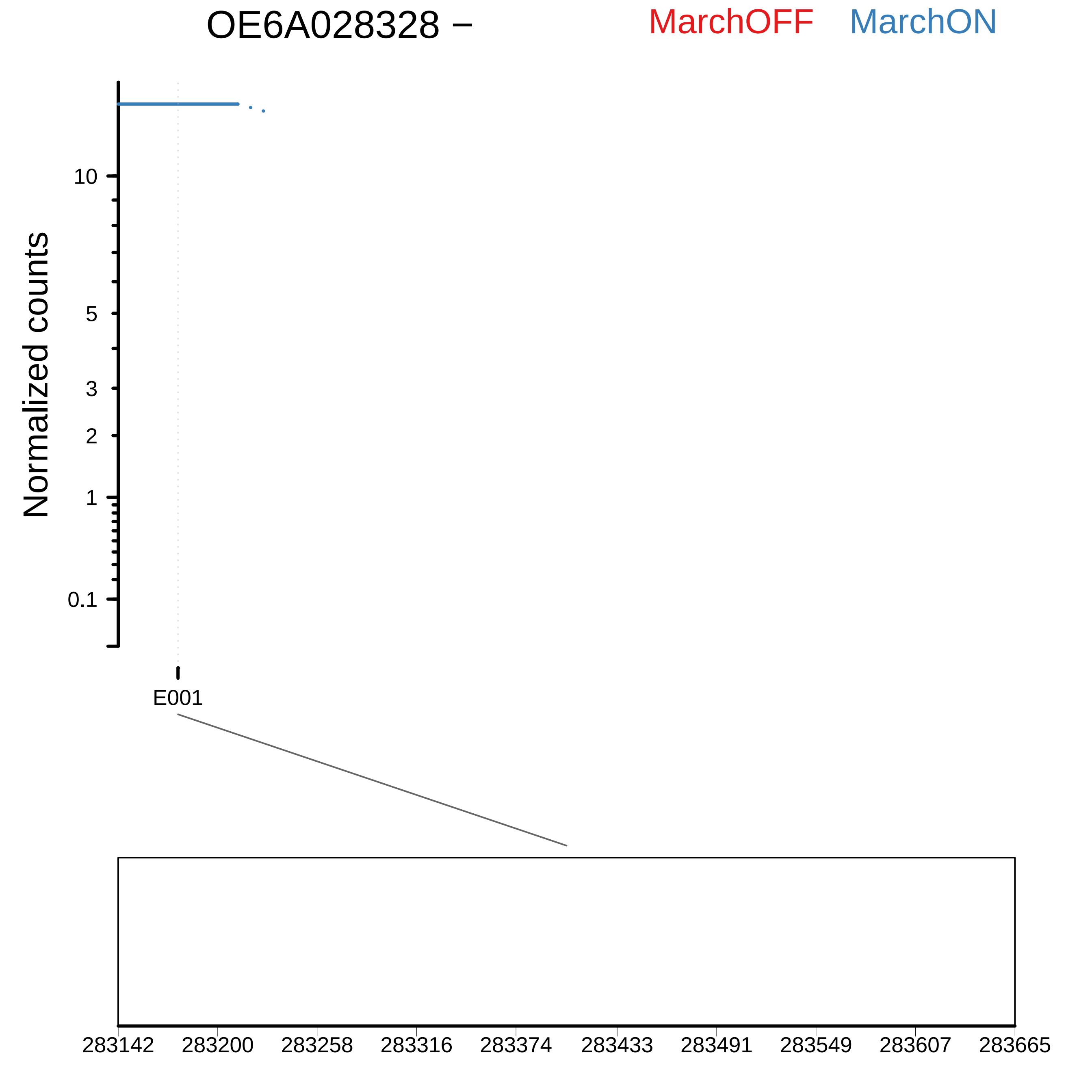
OE6A028328 −
MarchOFF
MarchON
Normalized counts
10
5
3
2
1
0.1
E001
283142
283200
283258
283316
283374
283433
283491
283549
283607
283665

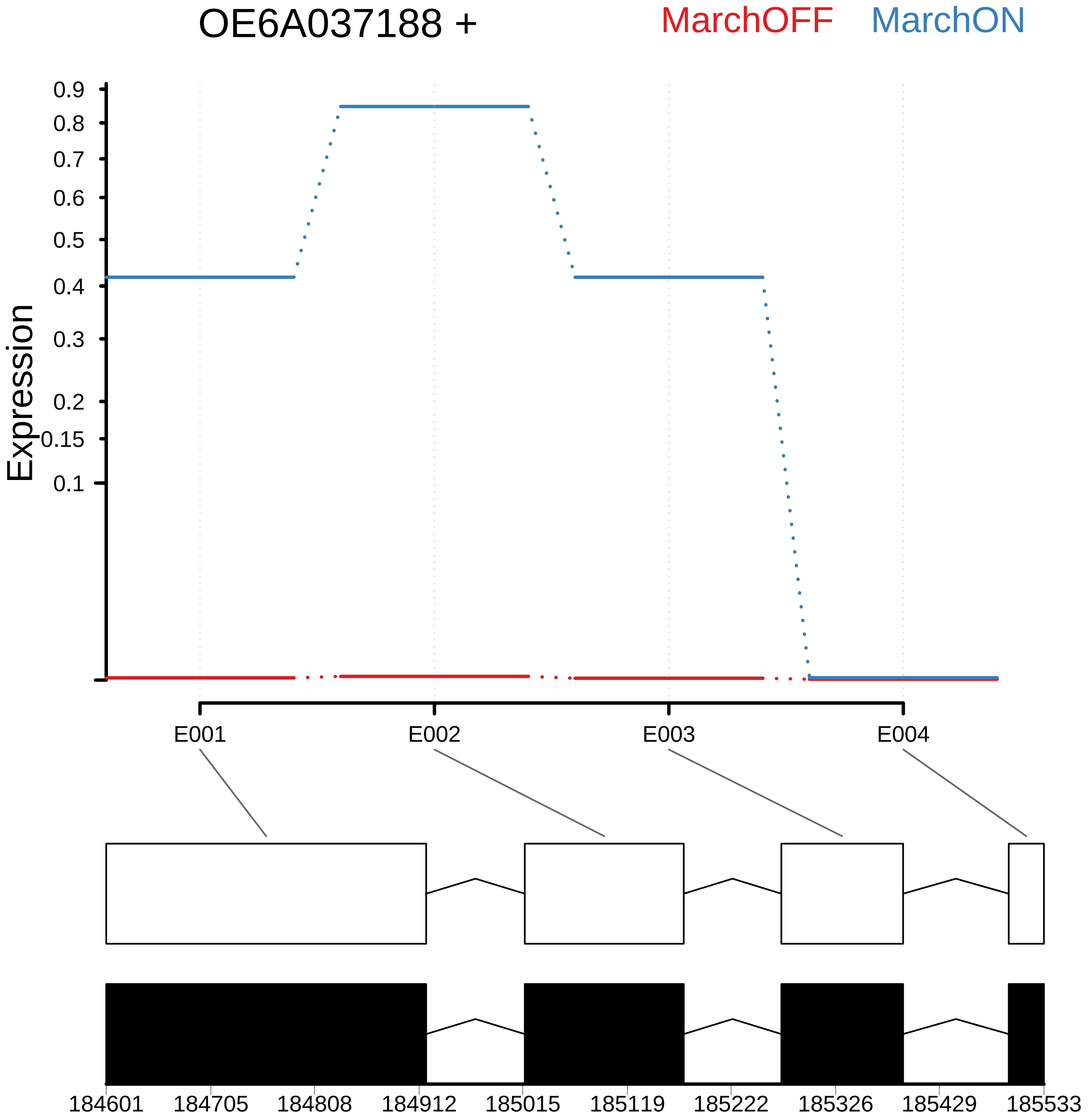

OE6A037188 +
MarchOFF
MarchON
Expression
0.9
0.8
0.7
0.6
0.5
0.4
0.3
0.2
0.15
0.1
E001
E002
E003
E004
184601
184705
184808
184912
185015
185119
185222
185326
185429
185533

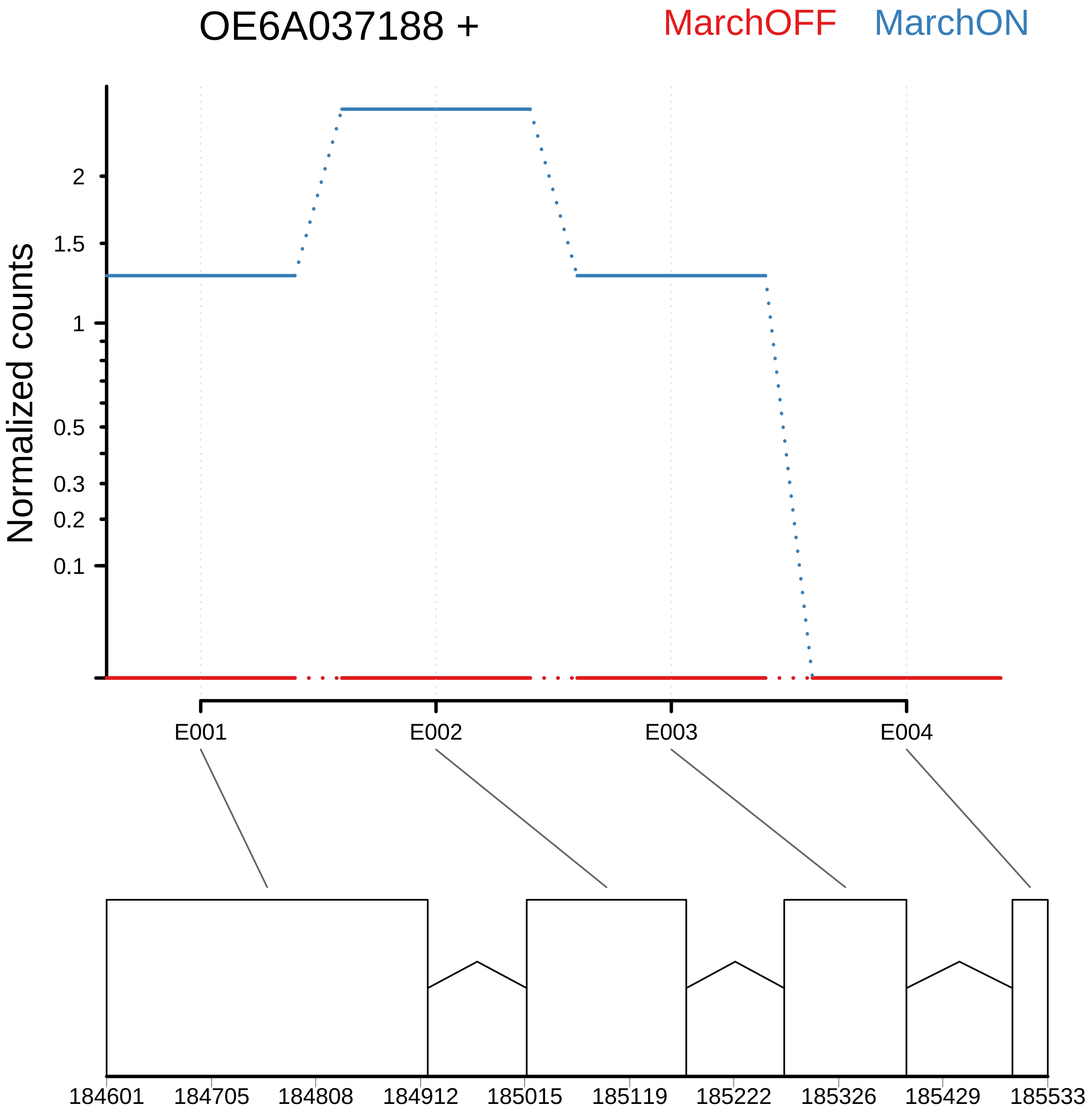
OE6A037188 +
MarchOFF
MarchON
Normalized counts
2
1.5
1
0.5
0.3
0.2
0.1
E001
E002
E003
E004
184601
184705
184808
184912
185015
185119
185222
185326
185429
185533

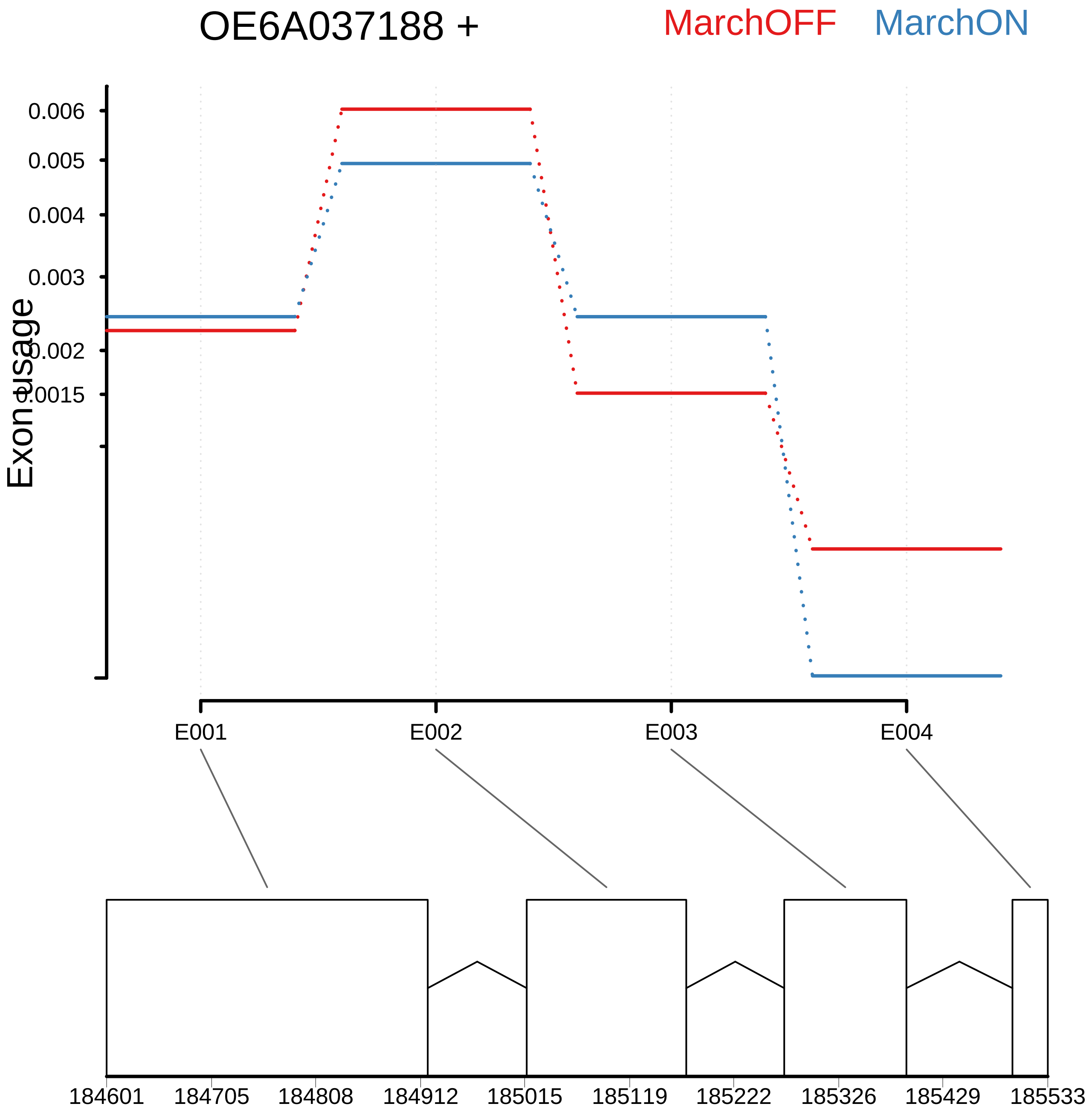

OE6A037188 +
MarchOFF
MarchON
Exon usage
0.006
0.005
0.004
0.003
0.002
0.0015
E001
E002
E003
E004
184601
184705
184808
184912
185015
185119
185222
185326
185429
185533

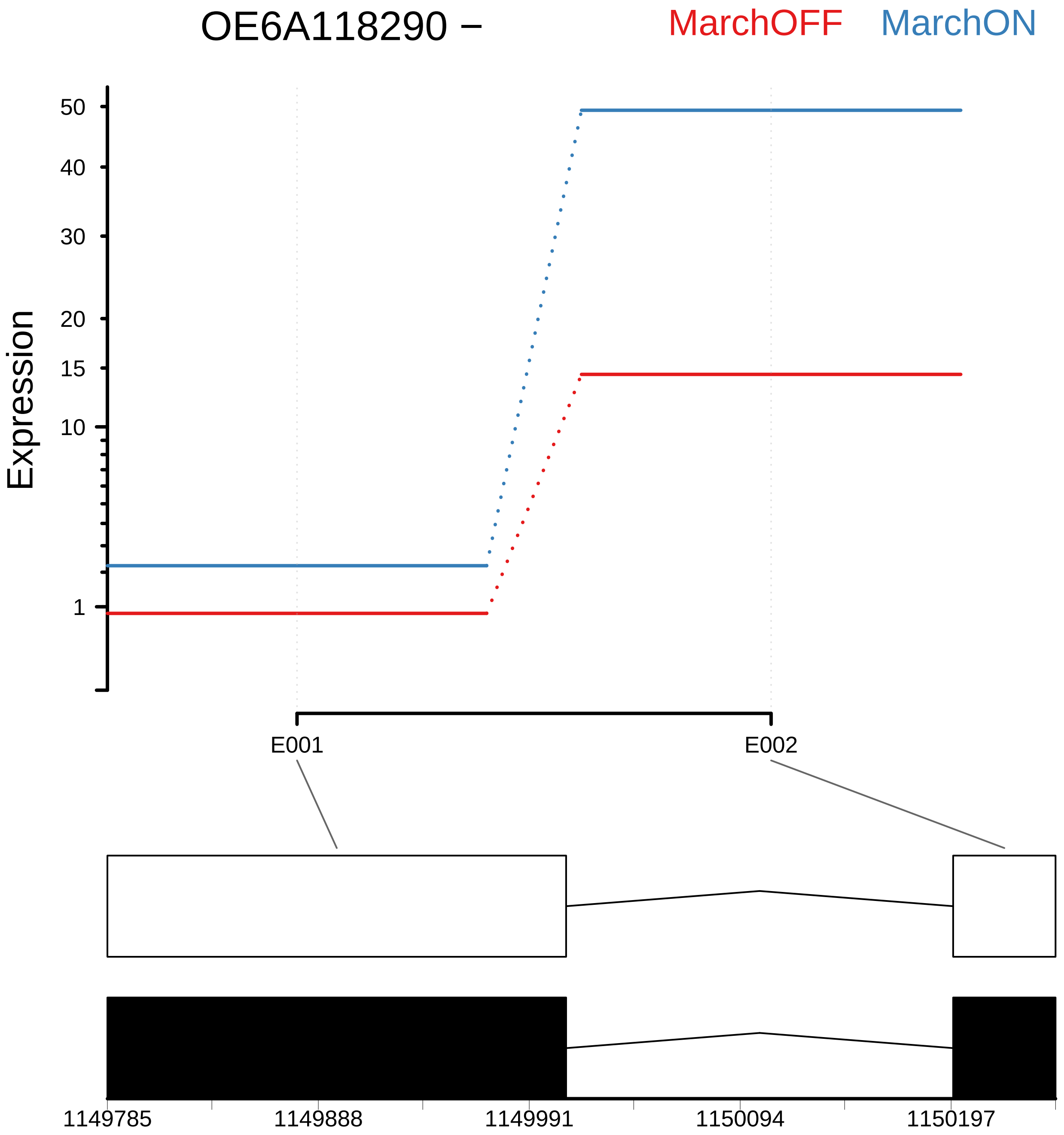

OE6A118290 −
MarchOFF
MarchON
Expression
50
40
30
20
15
10
1
E001
E002
1149785
1149888
1149991
1150094
1150197

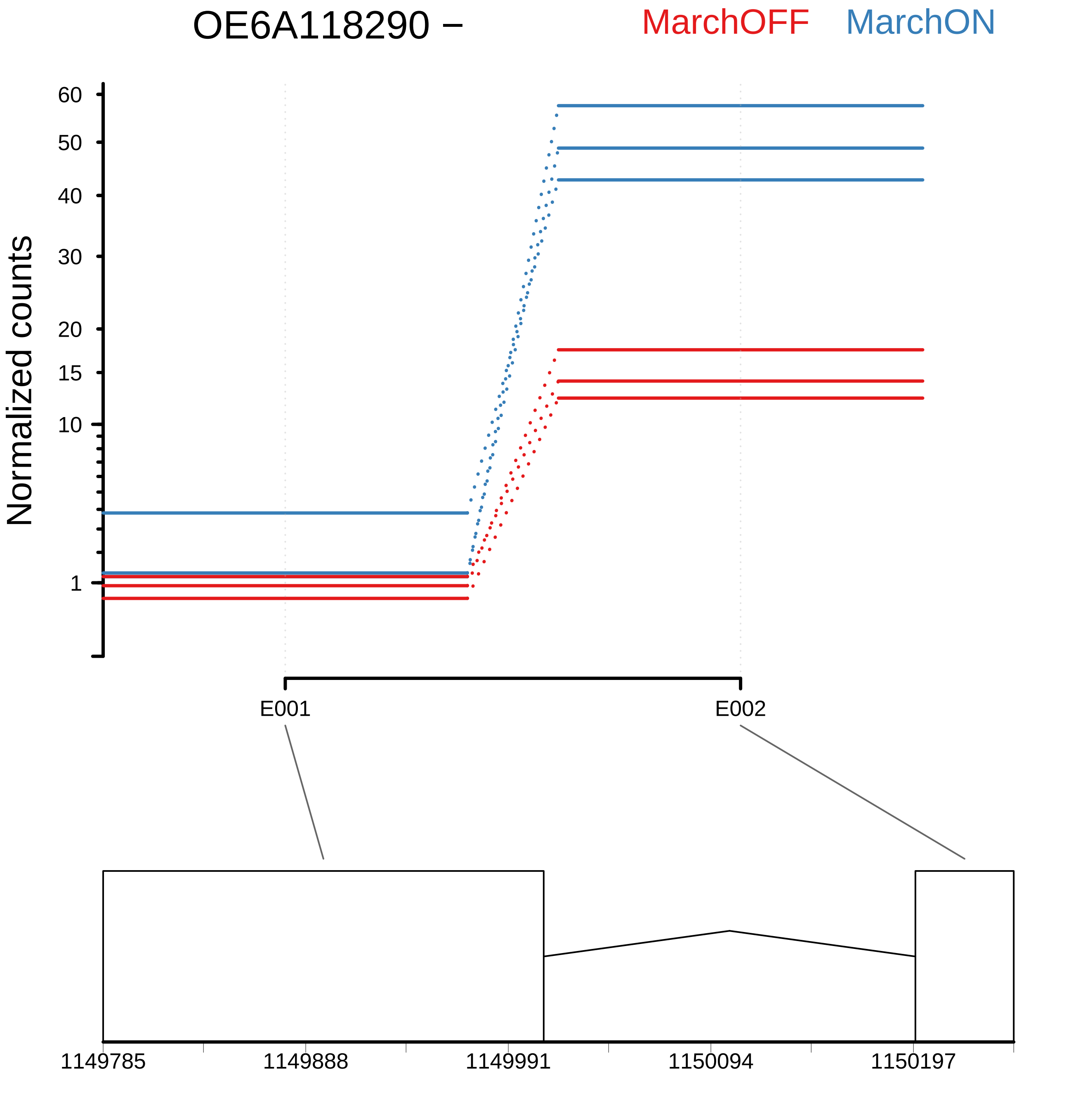
OE6A118290 −
MarchOFF
MarchON
Normalized counts
60
50
40
30
20
15
10
1
E001
E002
1149785
1149888
1149991
1150094
1150197

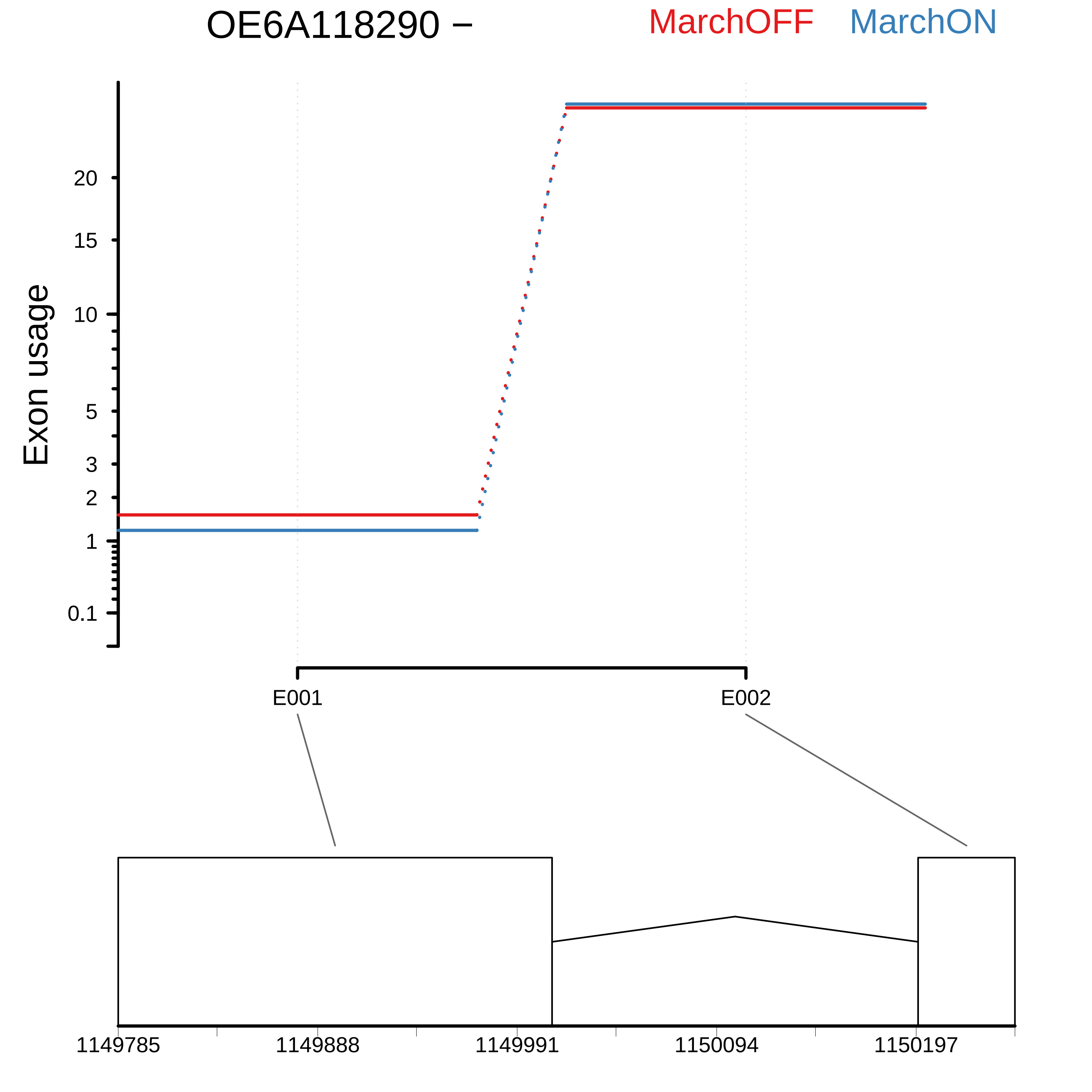

OE6A118290 −
MarchOFF
MarchON
Exon usage
20
15
10
5
3
2
1
0.1
E001
E002
1149785
1149888
1149991
1150094
1150197

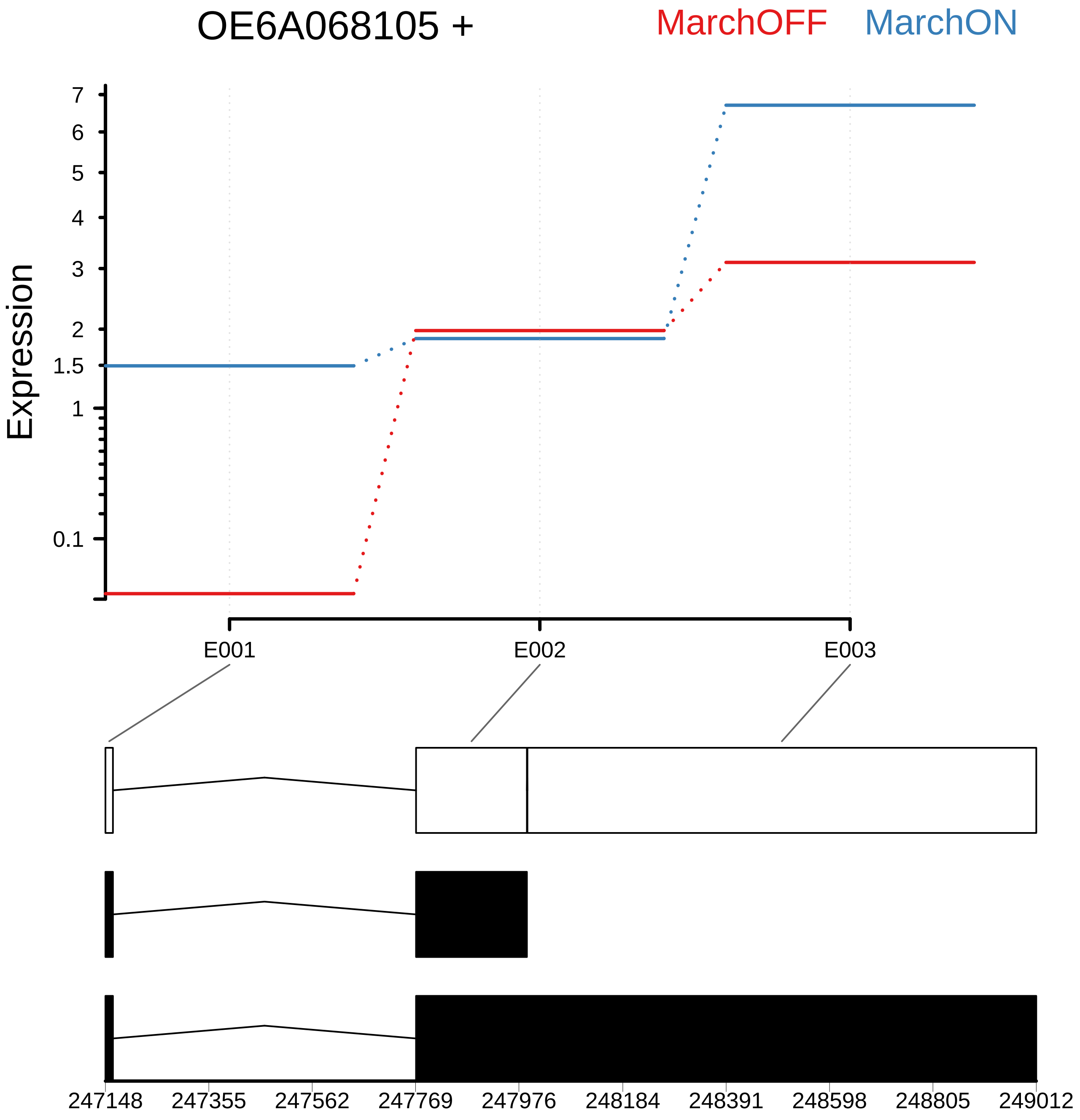

OE6A068105 +
MarchOFF
MarchON
Expression
7
6
5
4
3
2
1.5
1
0.1
E001
E002
E003
247148
247355
247562
247769
247976
248184
248391
248598
248805
249012

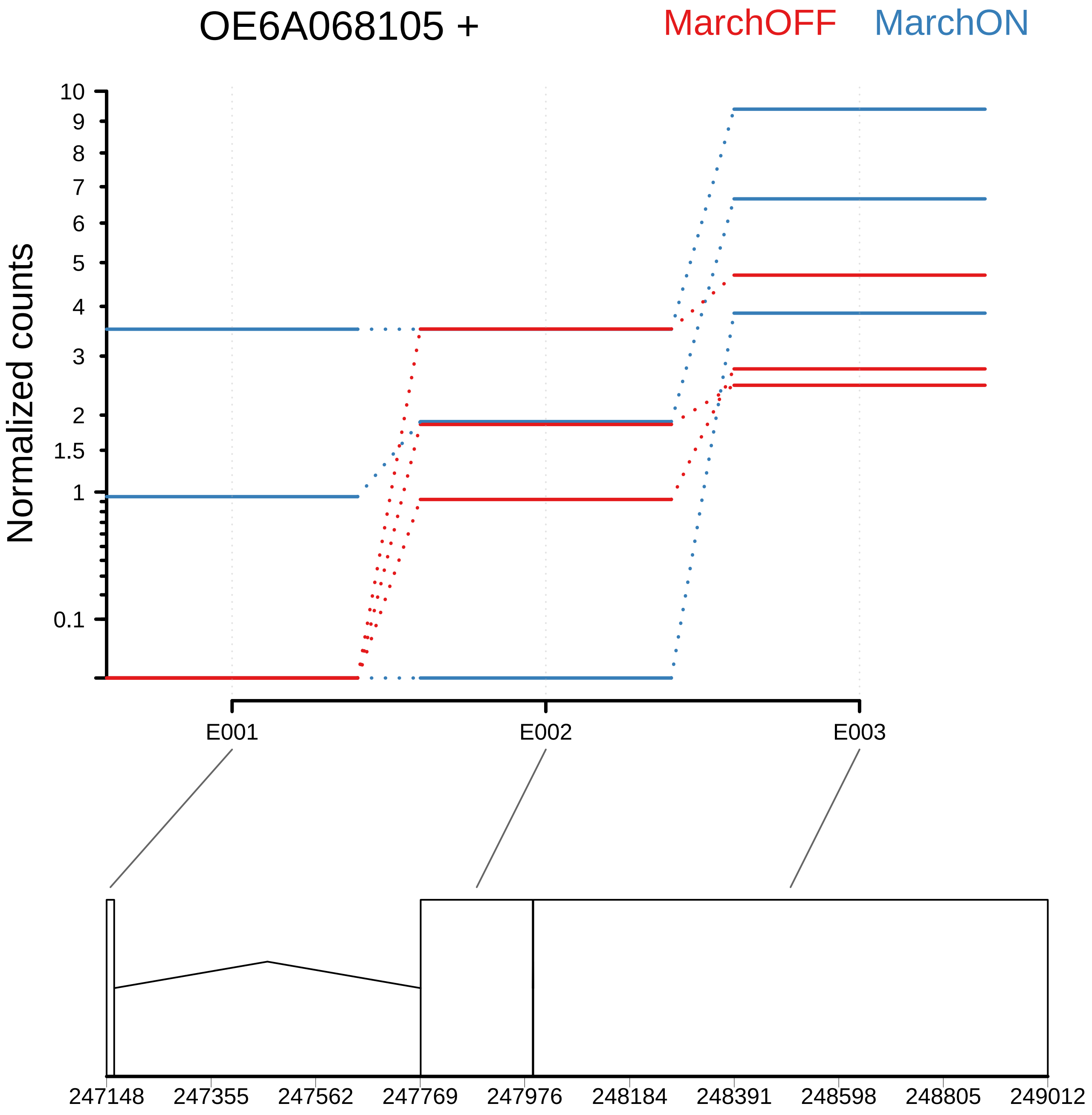

OE6A068105 +
MarchOFF
MarchON
Normalized counts
10
9
8
7
6
5
4
3
2
1.5
1
0.1
E001
E002
E003
247148
247355
247562
247769
247976
248184
248391
248598
248805
249012

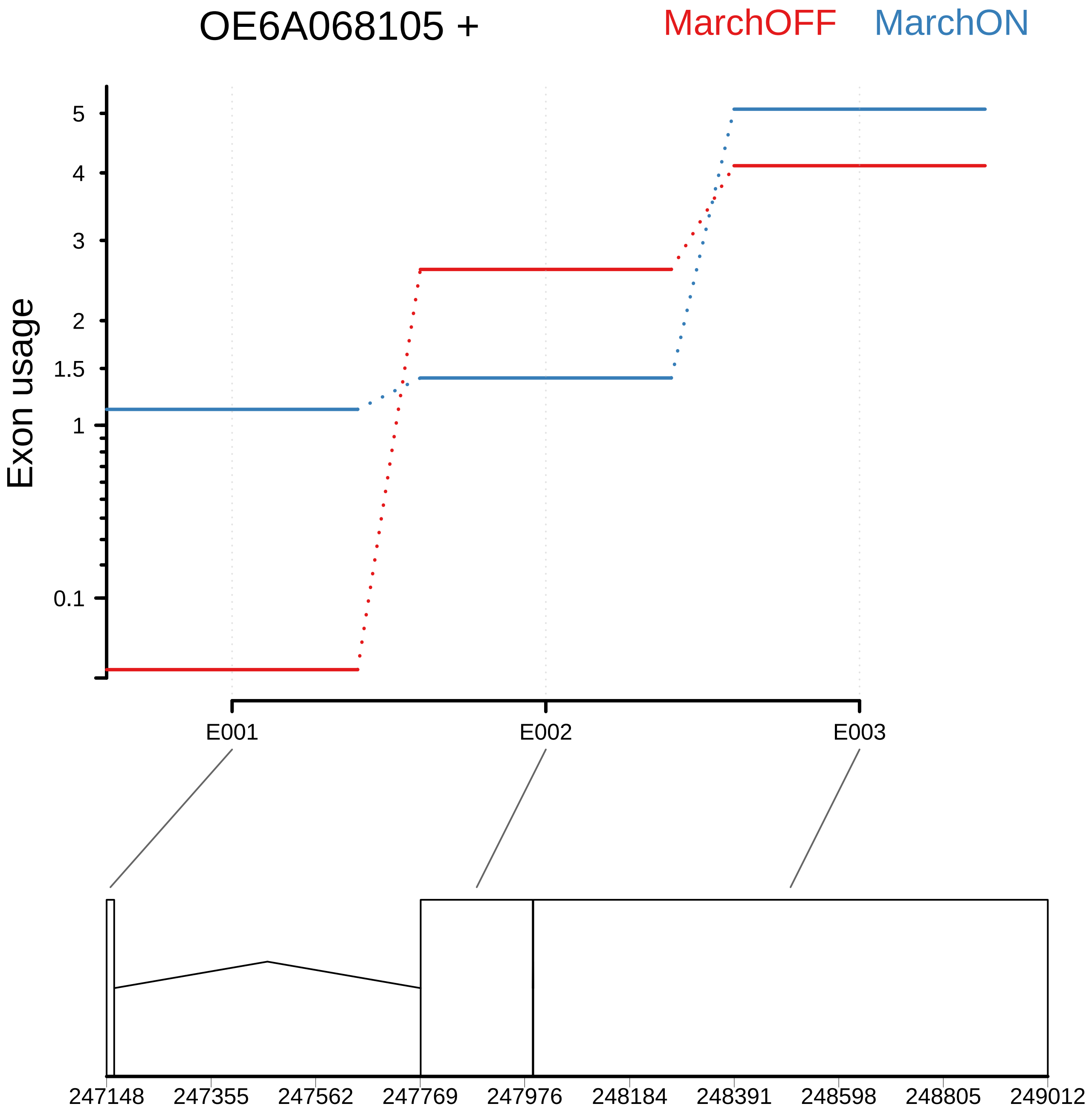

OE6A068105 +
MarchOFF
MarchON
Exon usage
5
4
3
2
1.5
1
0.1
E001
E002
E003
247148
247355
247562
247769
247976
248184
248391
248598
248805
249012

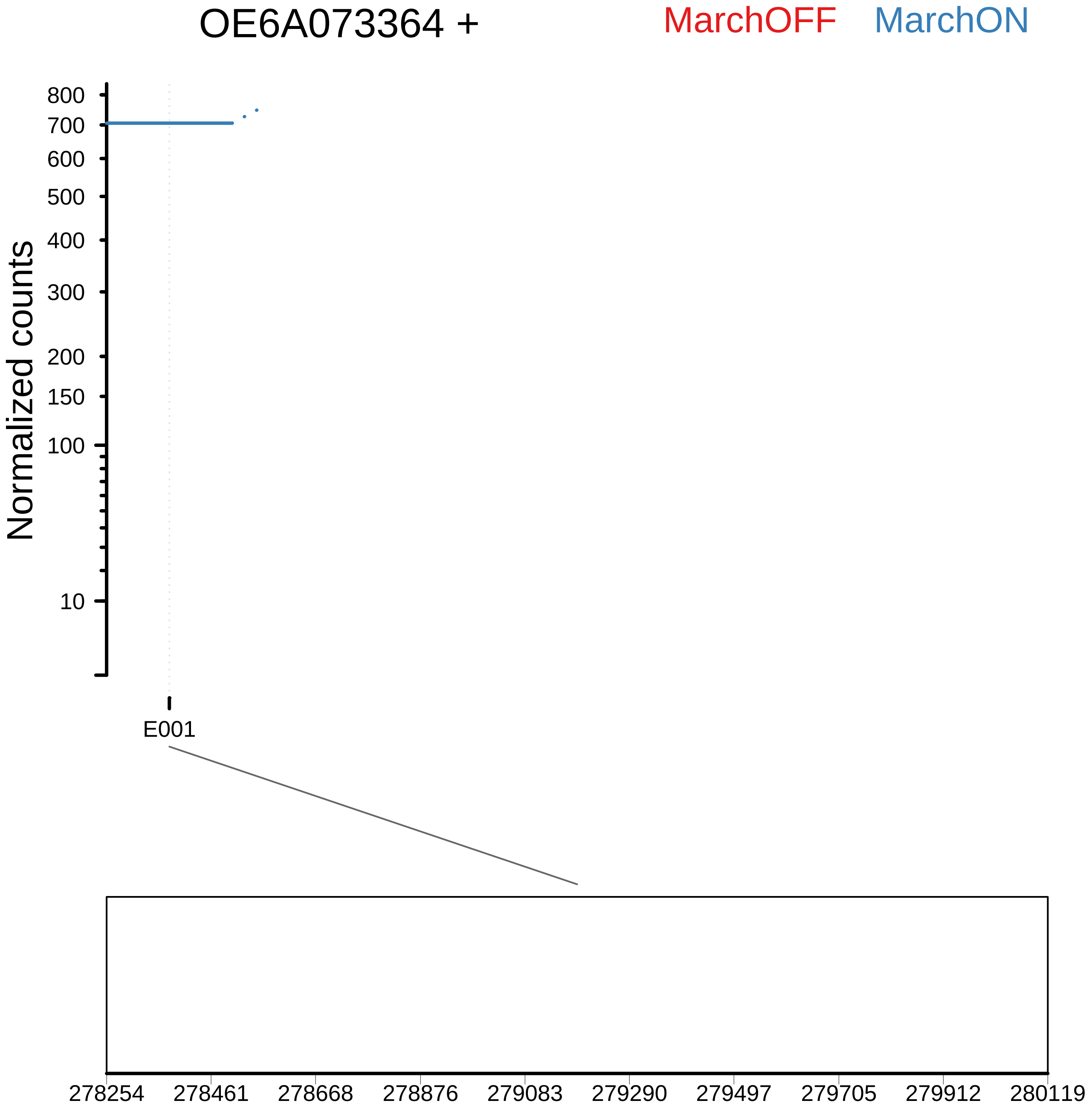

OE6A073364 +
MarchOFF
MarchON
Normalized counts
800
700
600
500
400
300
200
150
100
10
E001
278254
278461
278668
278876
279083
279290
279497
279705
279912
280119

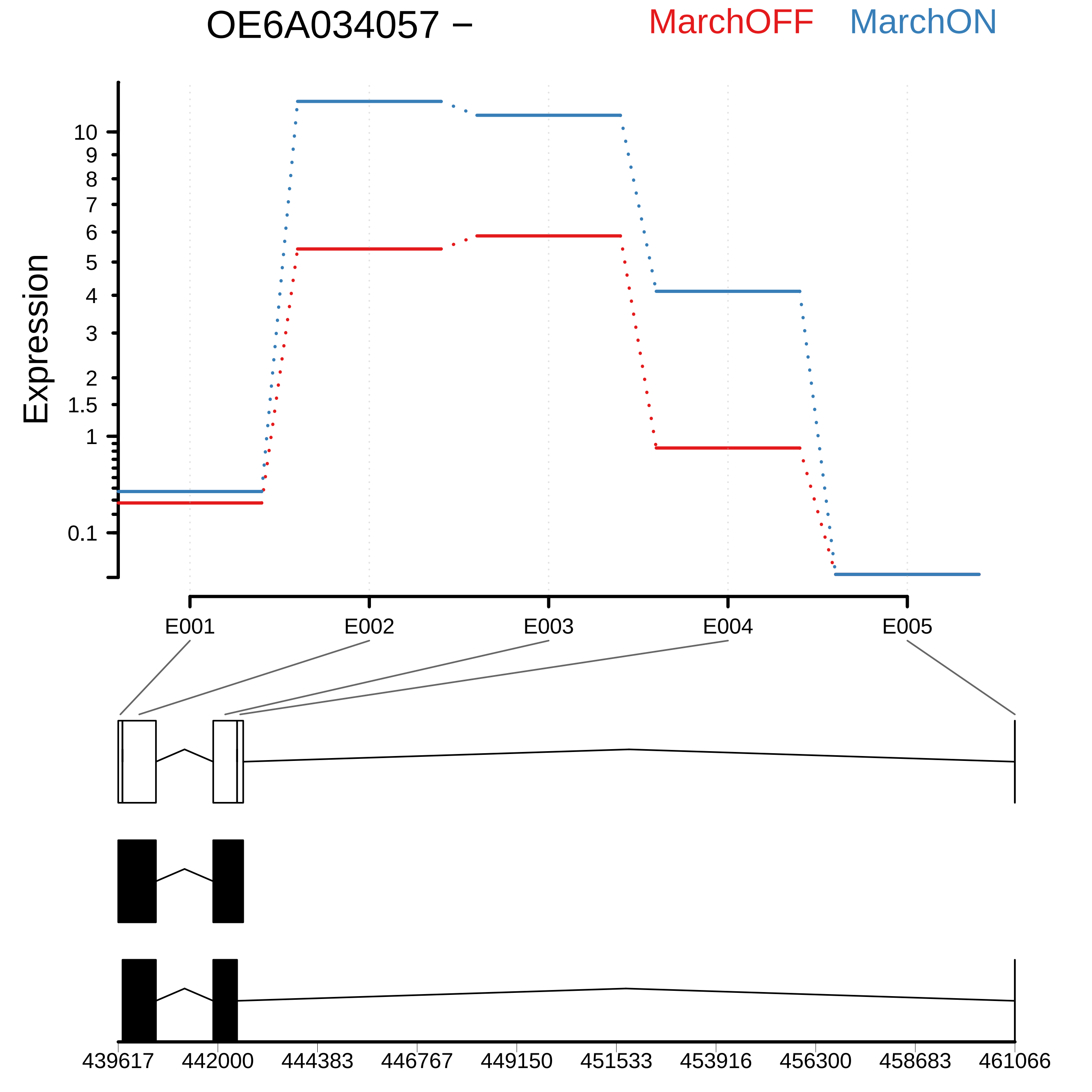

OE6A034057 −
MarchOFF
MarchON
Expression
10
9
8
7
6
5
4
3
2
1.5
1
0.1
E001
E002
E003
E004
E005
439617
442000
444383
446767
449150
451533
453916
456300
458683
461066

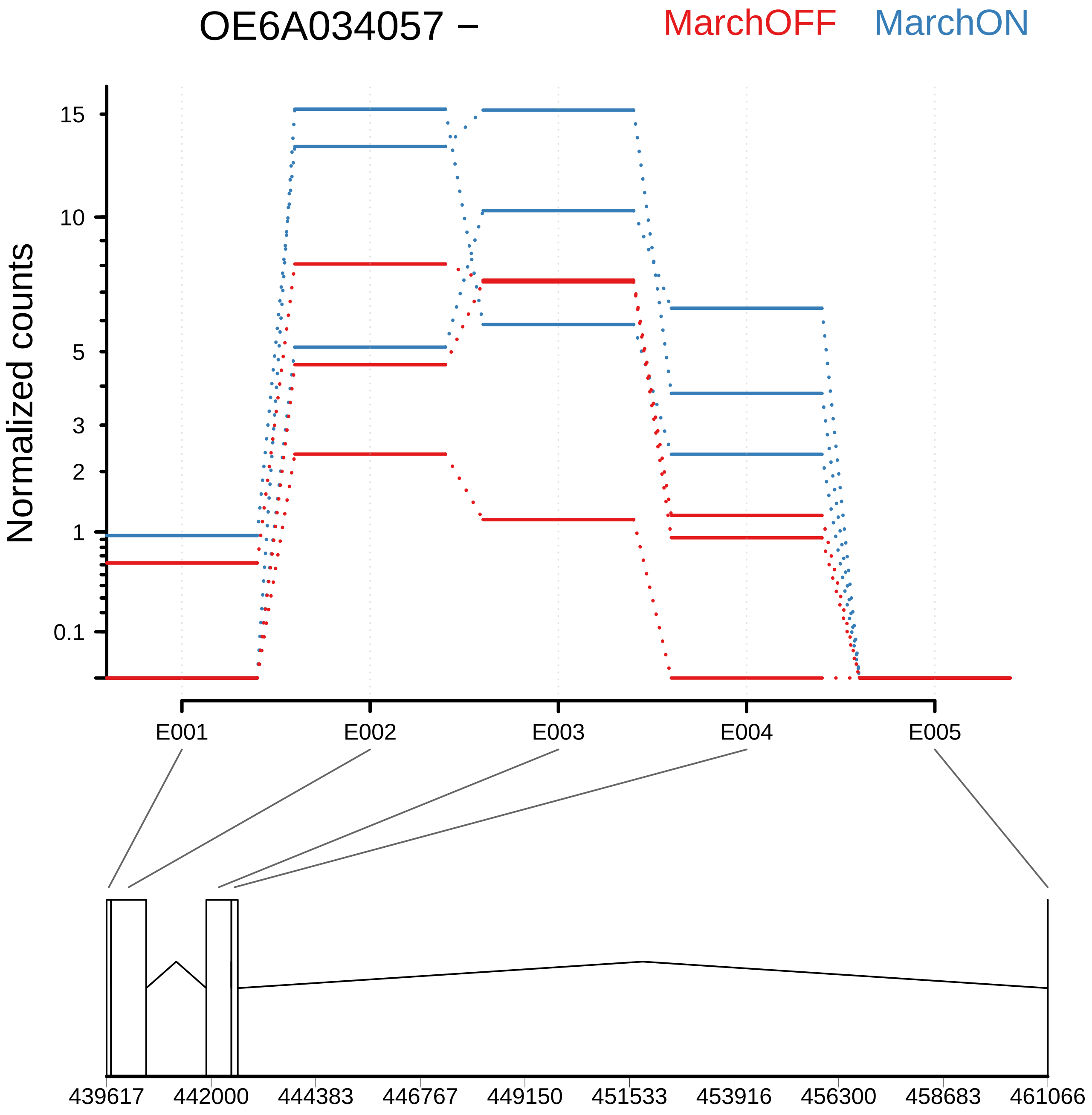

OE6A034057 −
MarchOFF
MarchON
Normalized counts
15
10
5
3
2
1
0.1
E001
E002
E003
E004
E005
439617
442000
444383
446767
449150
451533
453916
456300
458683
461066

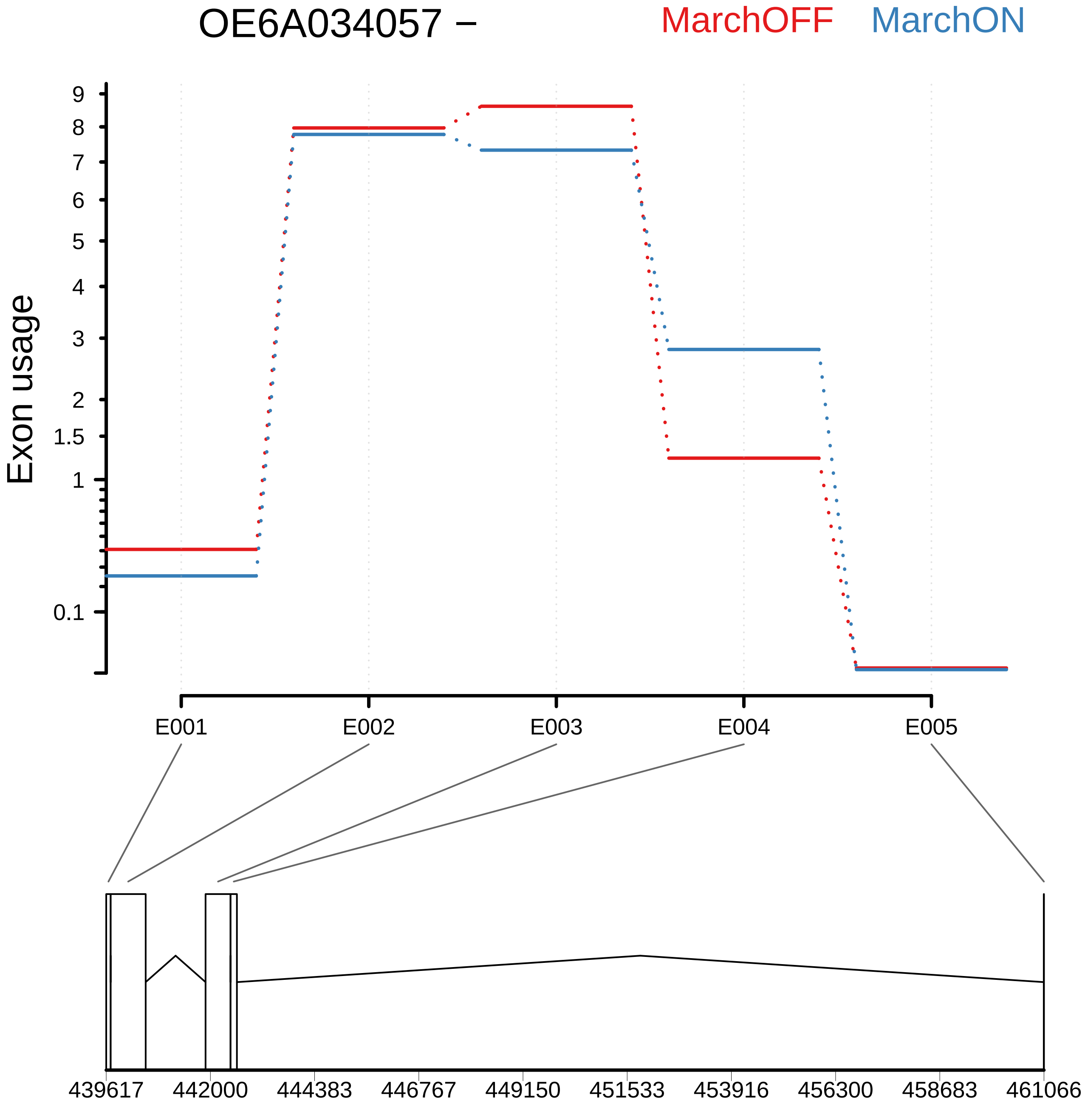

OE6A034057 −
MarchOFF
MarchON
Exon usage
9
8
7
6
5
4
3
2
1.5
1
0.1
E001
E002
E003
E004
E005
439617
442000
444383
446767
449150
451533
453916
456300
458683
461066

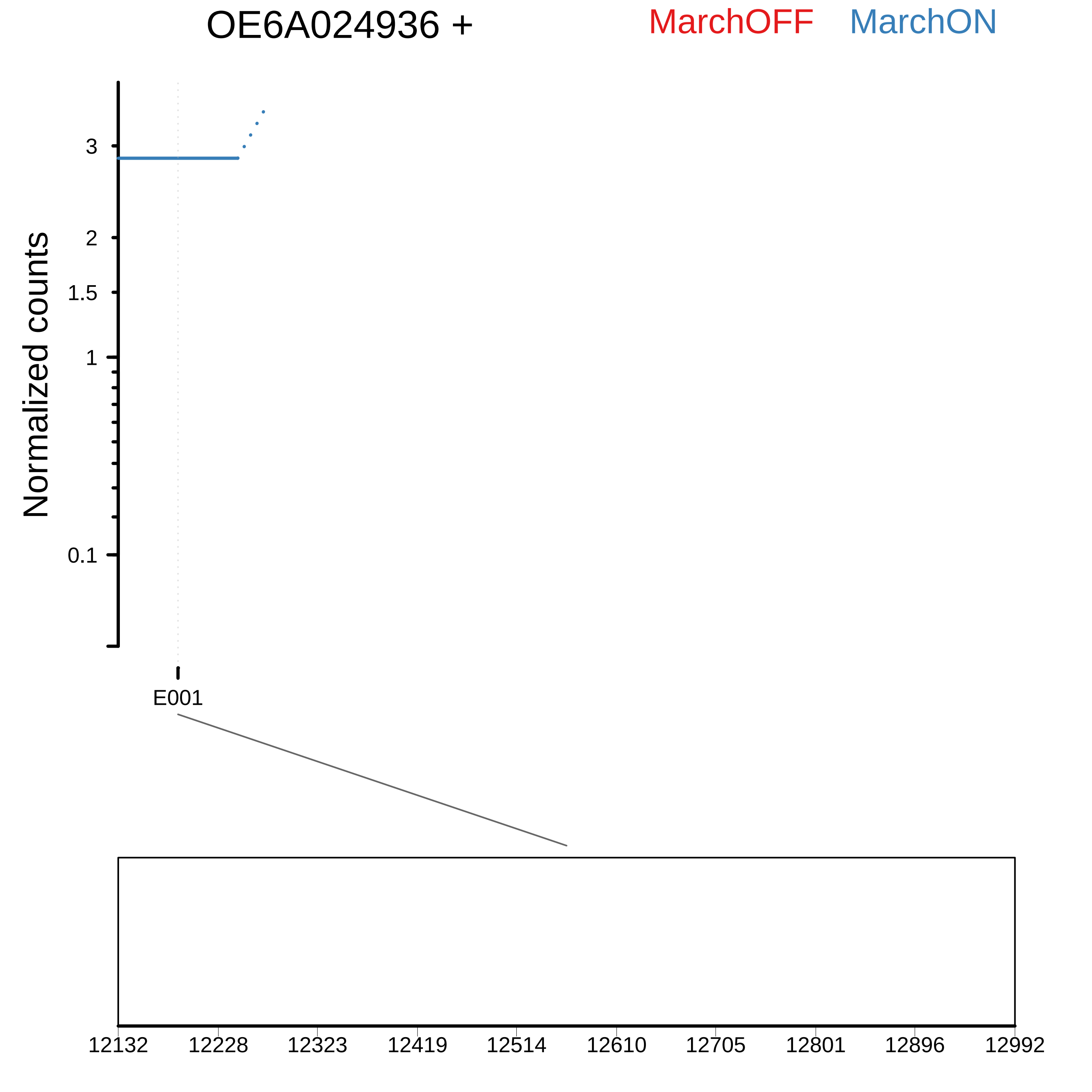

OE6A024936 +
MarchOFF
MarchON
Normalized counts
3
2
1.5
1
0.1
E001
12132
12228
12323
12419
12514
12610
12705
12801
12896
12992

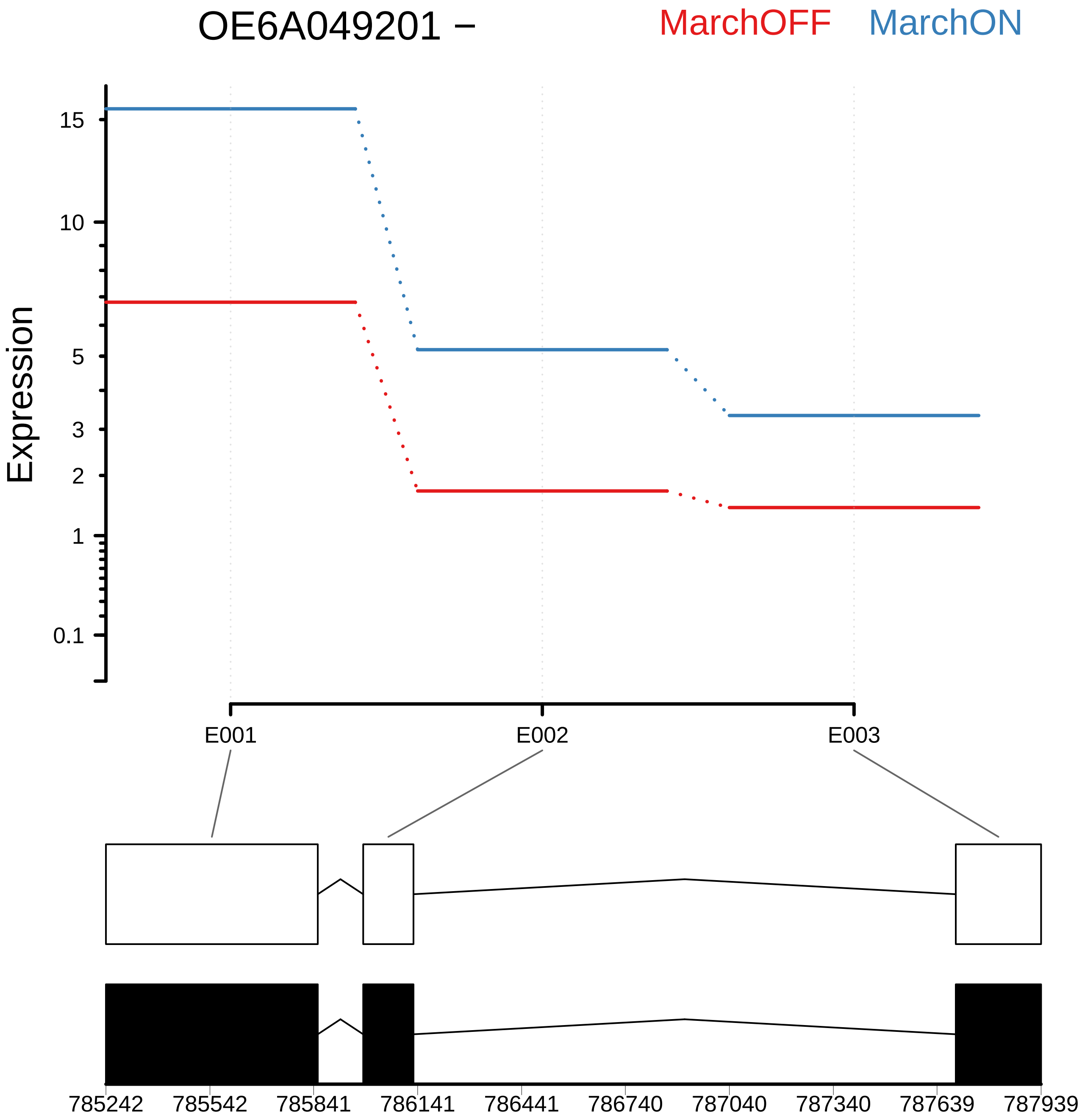

OE6A049201 −
MarchOFF
MarchON
Expression
15
10
5
3
2
1
0.1
E001
E002
E003
785242
785542
785841
786141
786441
786740
787040
787340
787639
787939

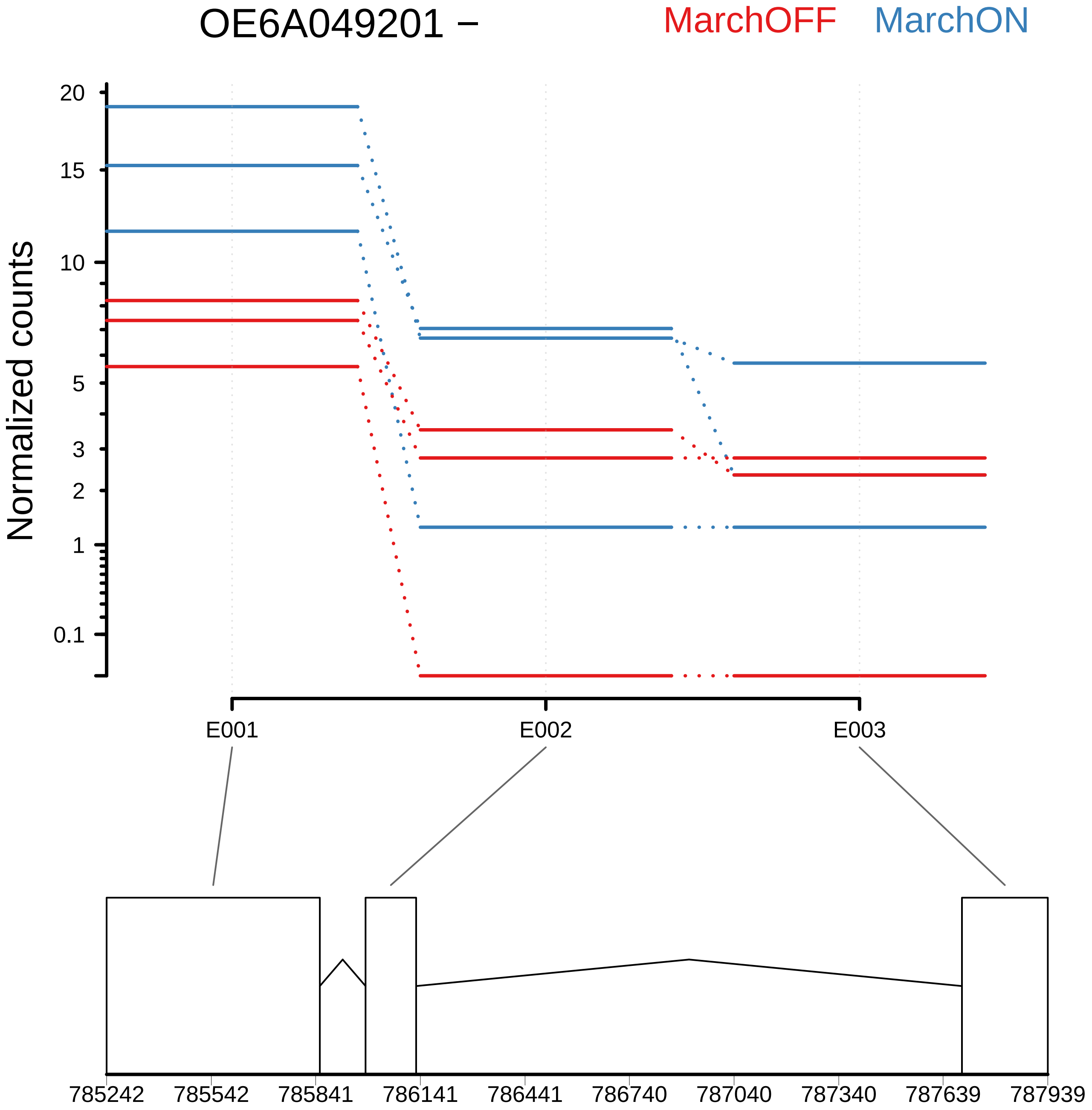

OE6A049201 −
MarchOFF
MarchON
Normalized counts
20
15
10
5
3
2
1
0.1
E001
E002
E003
785242
785542
785841
786141
786441
786740
787040
787340
787639
787939

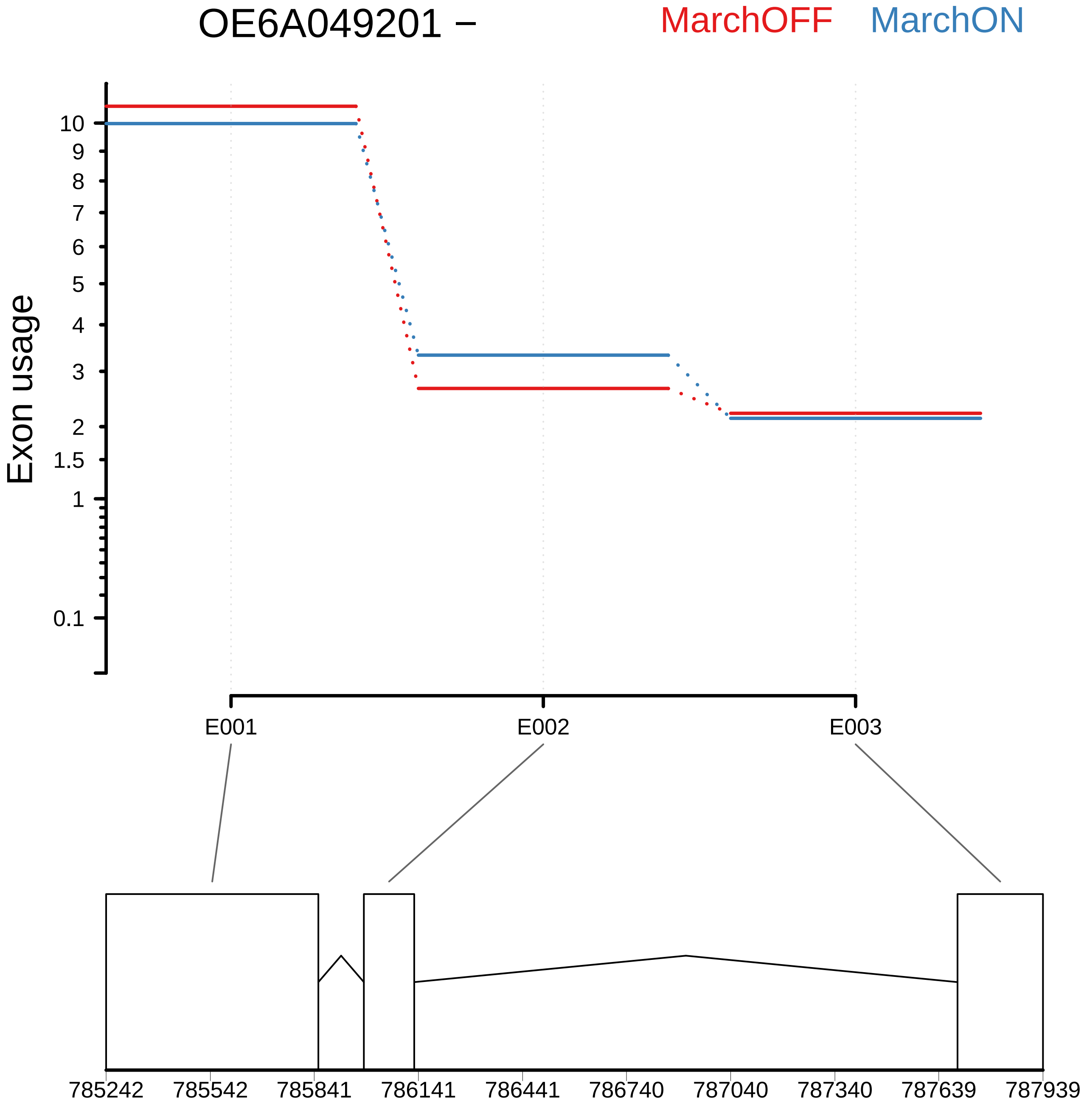
OE6A049201 −
MarchOFF
MarchON
Exon usage
10
9
8
7
6
5
4
3
2
1.5
1
0.1
E001
E002
E003
785242
785542
785841
786141
786441
786740
787040
787340
787639
787939

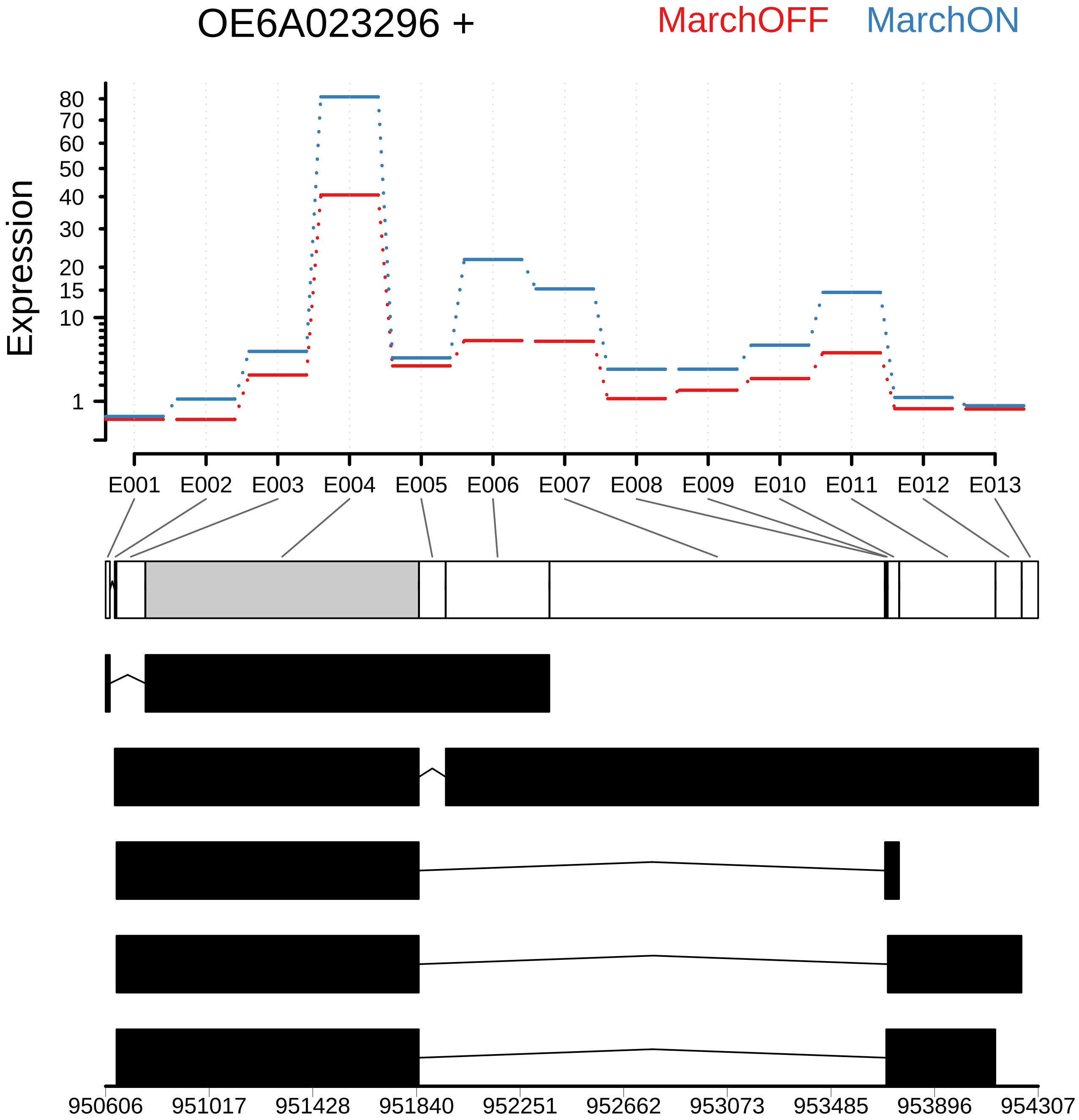

OE6A023296 +
MarchOFF
MarchON
Expression
80
70
60
50
40
30
20
15
10
1
E001
E002
E003
E004
E005
E006
E007
E008
E009
E010
E011
E012
E013
950606
951017
951428
951840
952251
952662
953073
953485
953896
954307

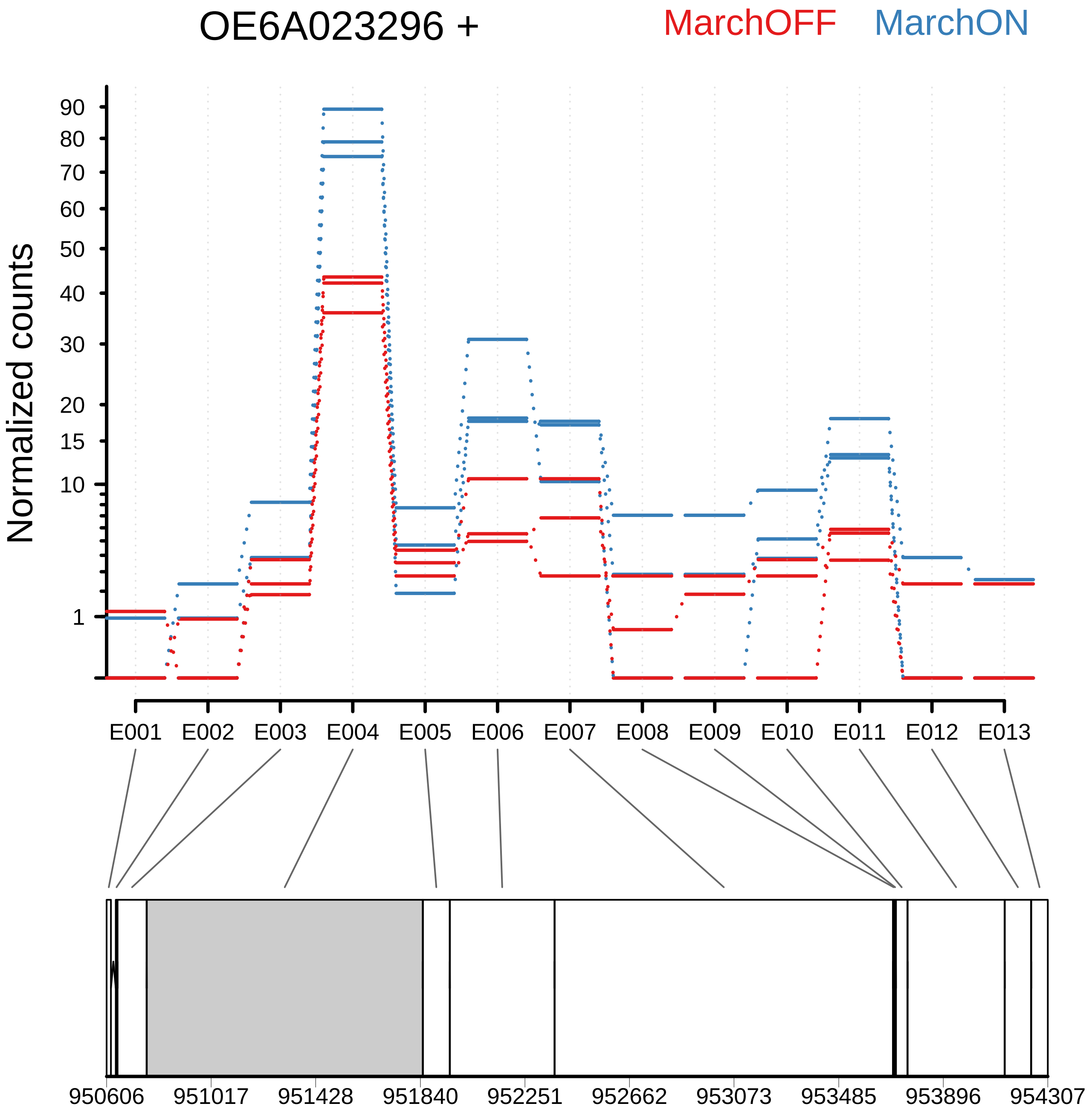
OE6A023296 +
MarchOFF
MarchON
Normalized counts
90
80
70
60
50
40
30
20
15
10
1
E001
E002
E003
E004
E005
E006
E007
E008
E009
E010
E011
E012
E013
950606
951017
951428
951840
952251
952662
953073
953485
953896
954307

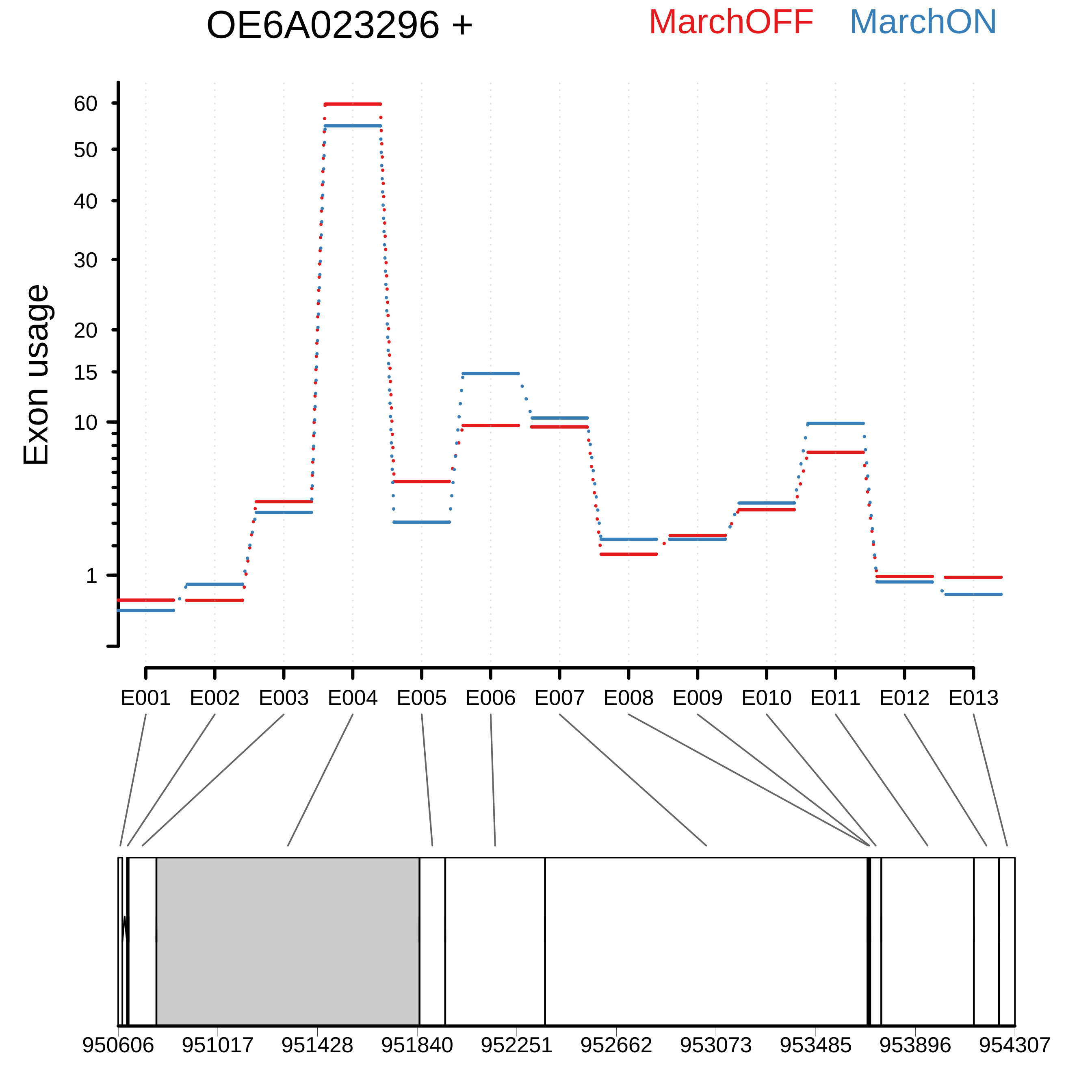

OE6A023296 +
MarchOFF
MarchON
Exon usage
60
50
40
30
20
15
10
1
E001
E002
E003
E004
E005
E006
E007
E008
E009
E010
E011
E012
E013
950606
951017
951428
951840
952251
952662
953073
953485
953896
954307

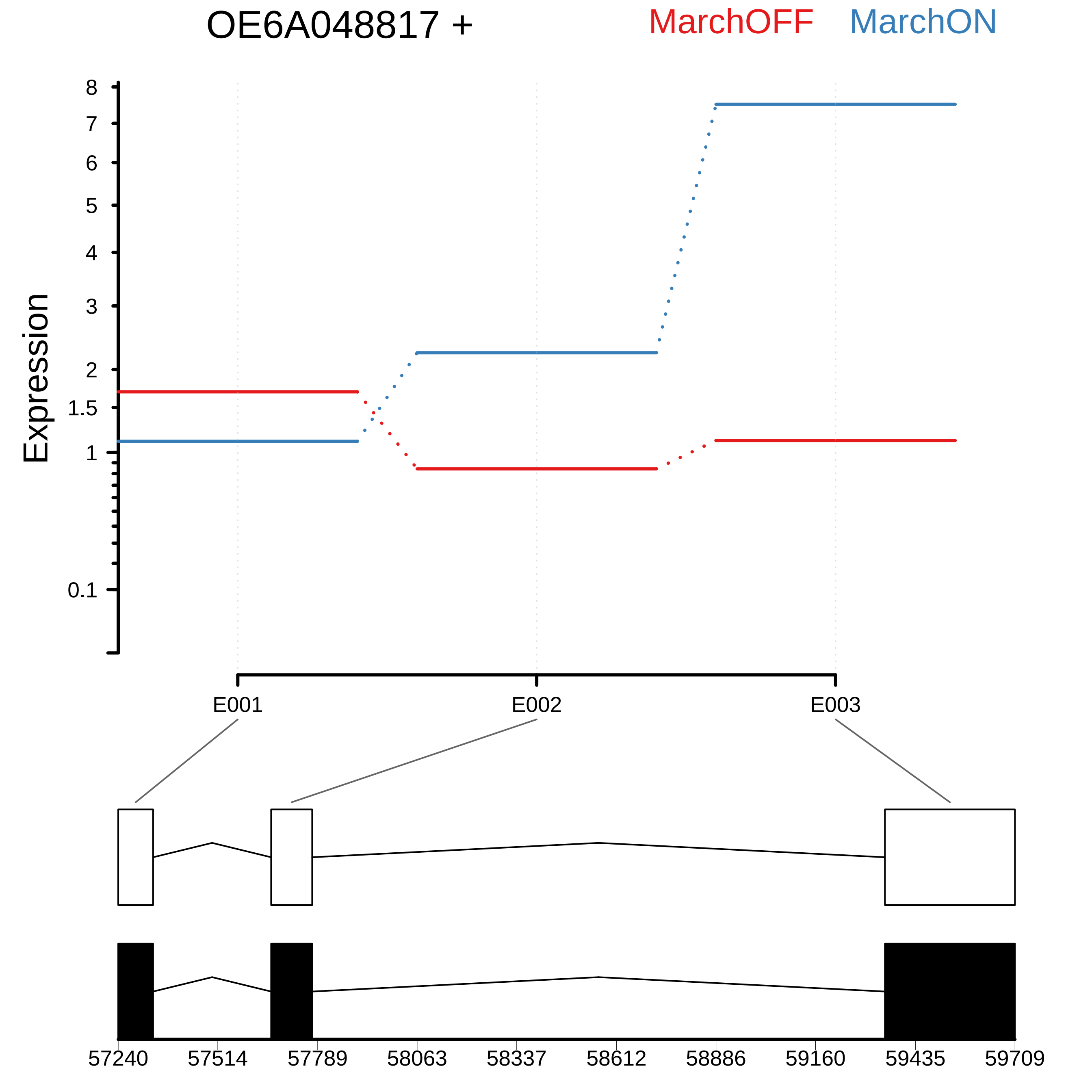

OE6A048817 +
MarchOFF
MarchON
Expression
8
7
6
5
4
3
2
1.5
1
0.1
E001
E002
E003
57240
57514
57789
58063
58337
58612
58886
59160
59435
59709

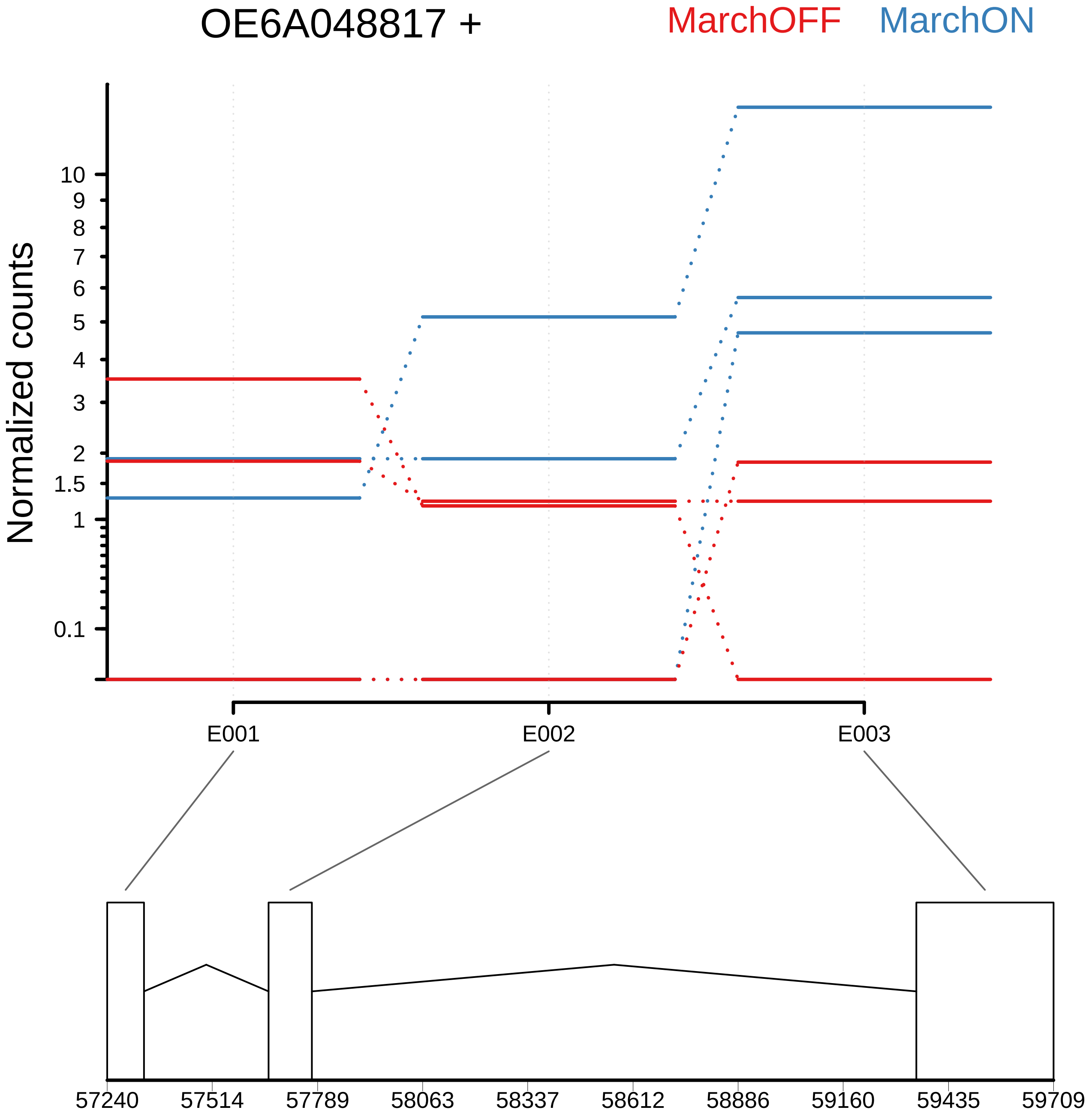

OE6A048817 +
MarchOFF
MarchON
Normalized counts
10
9
8
7
6
5
4
3
2
1.5
1
0.1
E001
E002
E003
57240
57514
57789
58063
58337
58612
58886
59160
59435
59709

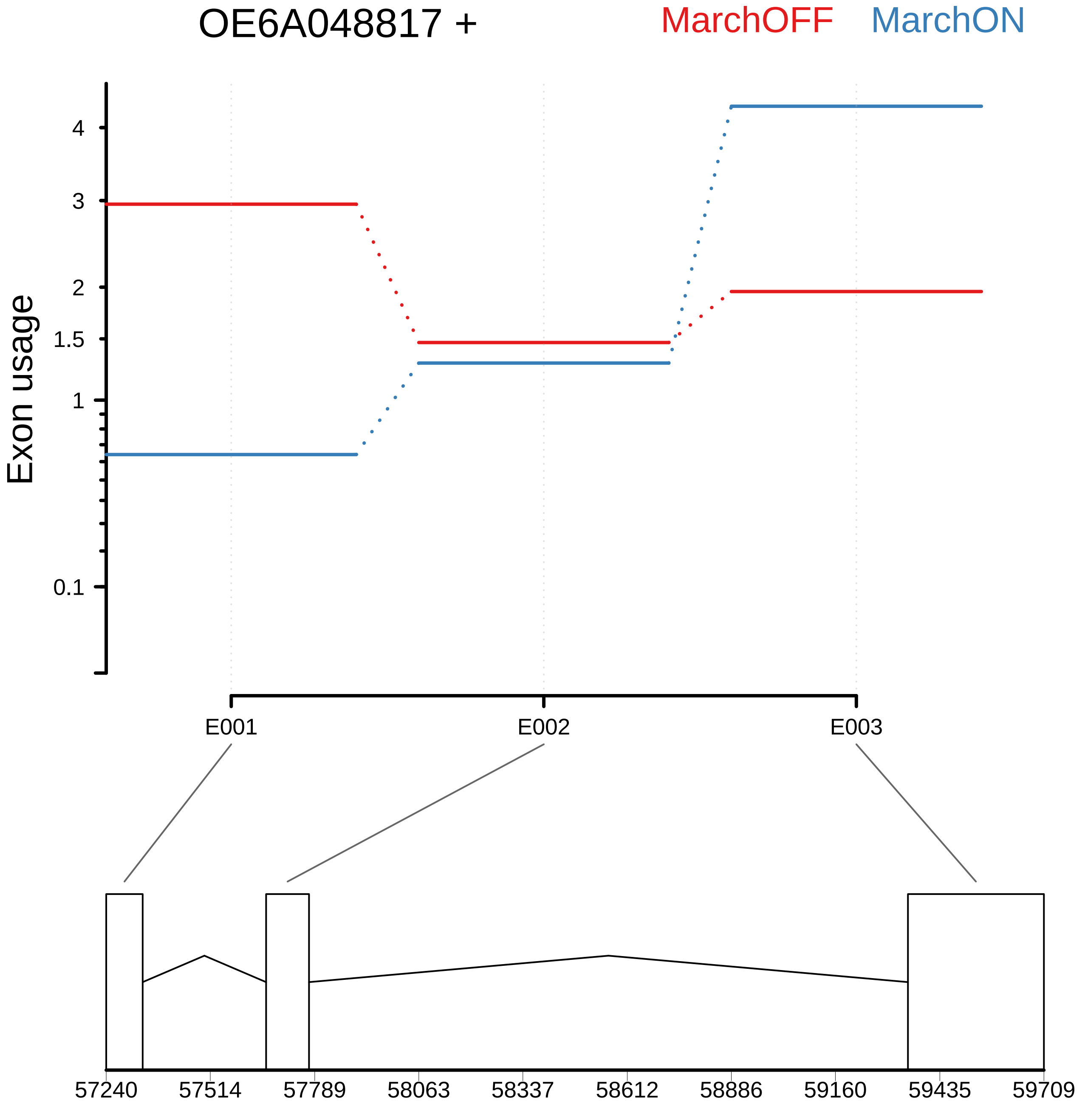
OE6A048817 +
MarchOFF
MarchON
Exon usage
4
3
2
1.5
1
0.1
E001
E002
E003
57240
57514
57789
58063
58337
58612
58886
59160
59435
59709

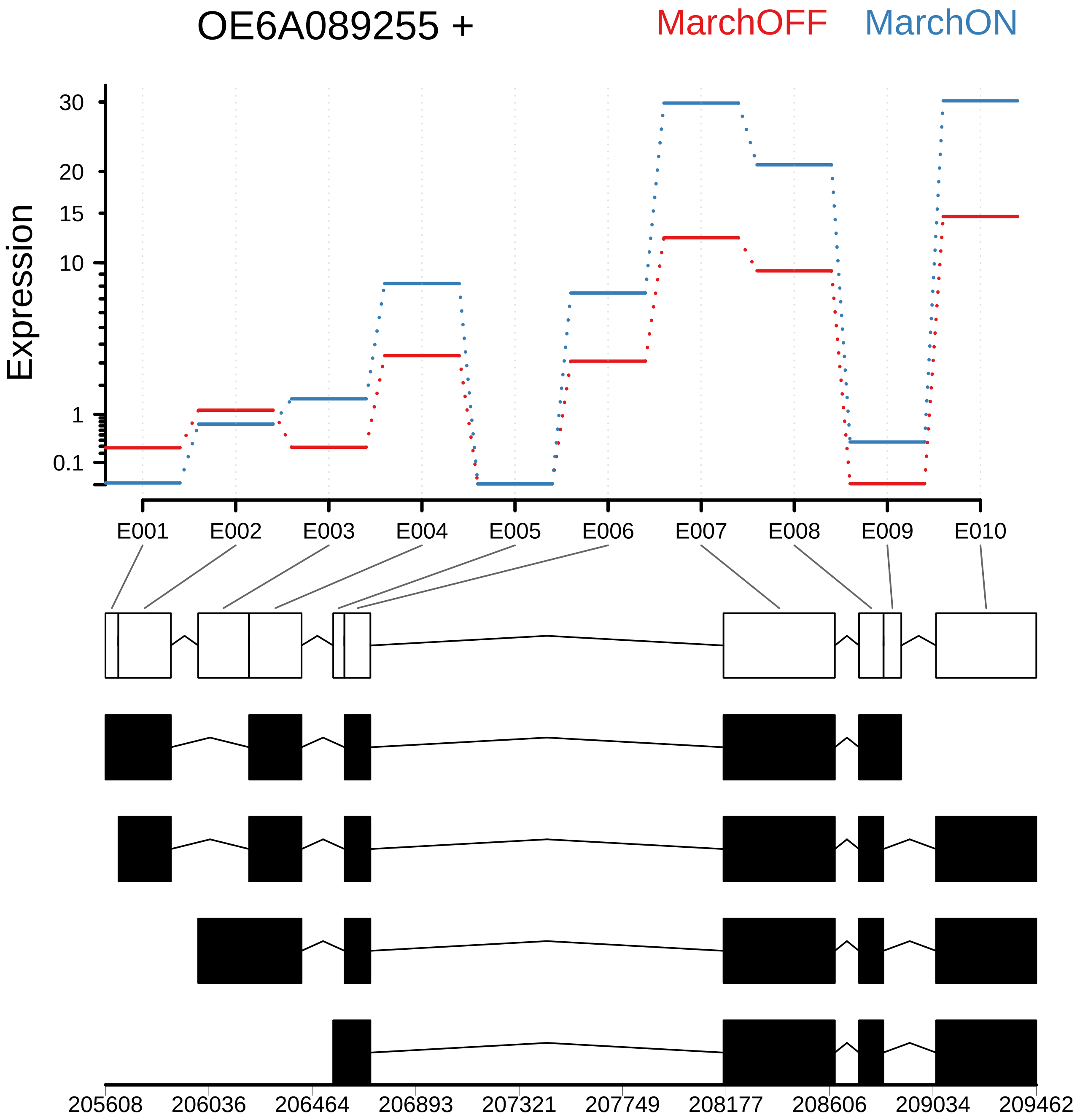

OE6A089255 +
MarchOFF
MarchON
Expression
30
20
15
10
1
0.1
E001
E002
E003
E004
E005
E006
E007
E008
E009
E010
205608
206036
206464
206893
207321
207749
208177
208606
209034
209462

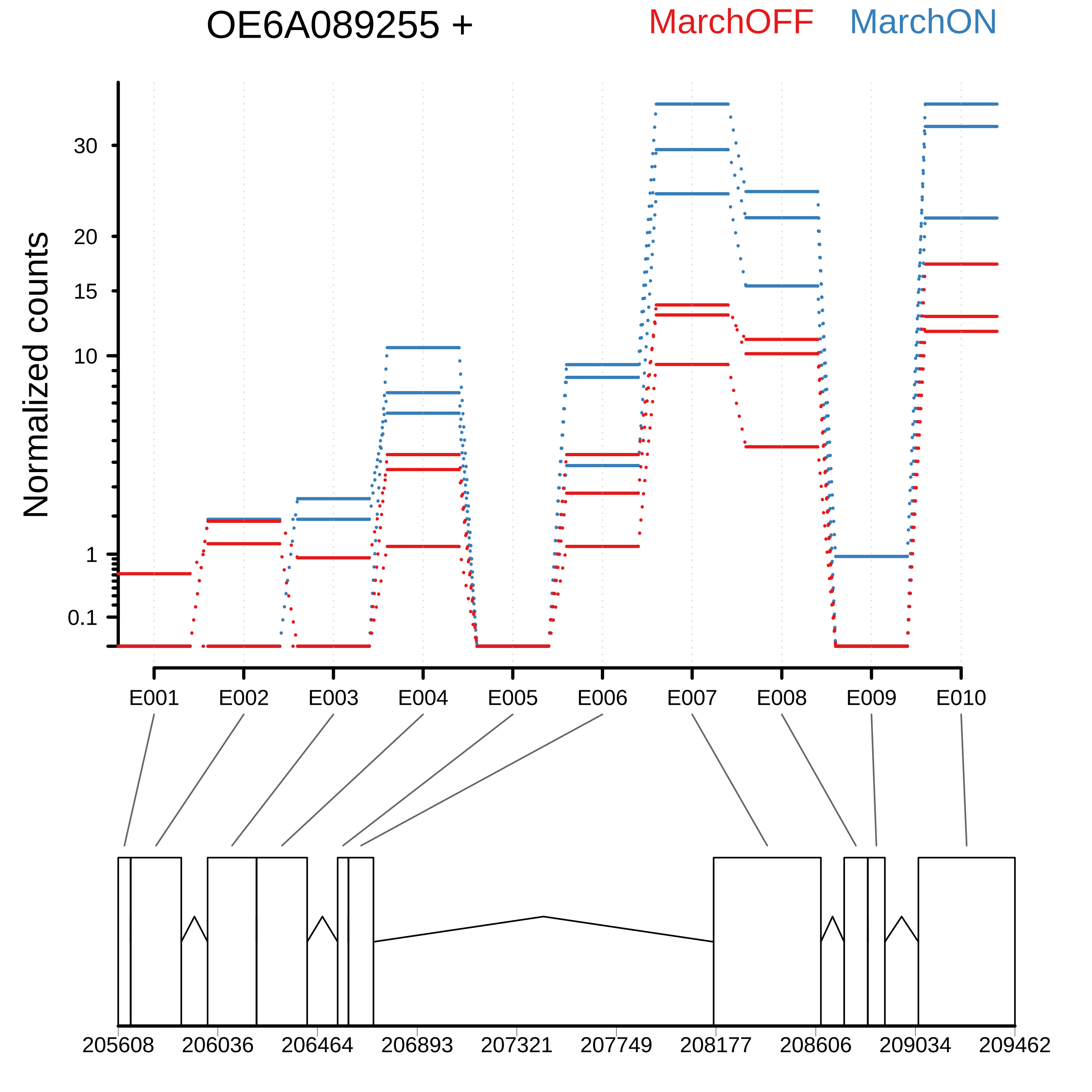

OE6A089255 +
MarchOFF
MarchON
Normalized counts
30
20
15
10
1
0.1
E001
E002
E003
E004
E005
E006
E007
E008
E009
E010
205608
206036
206464
206893
207321
207749
208177
208606
209034
209462

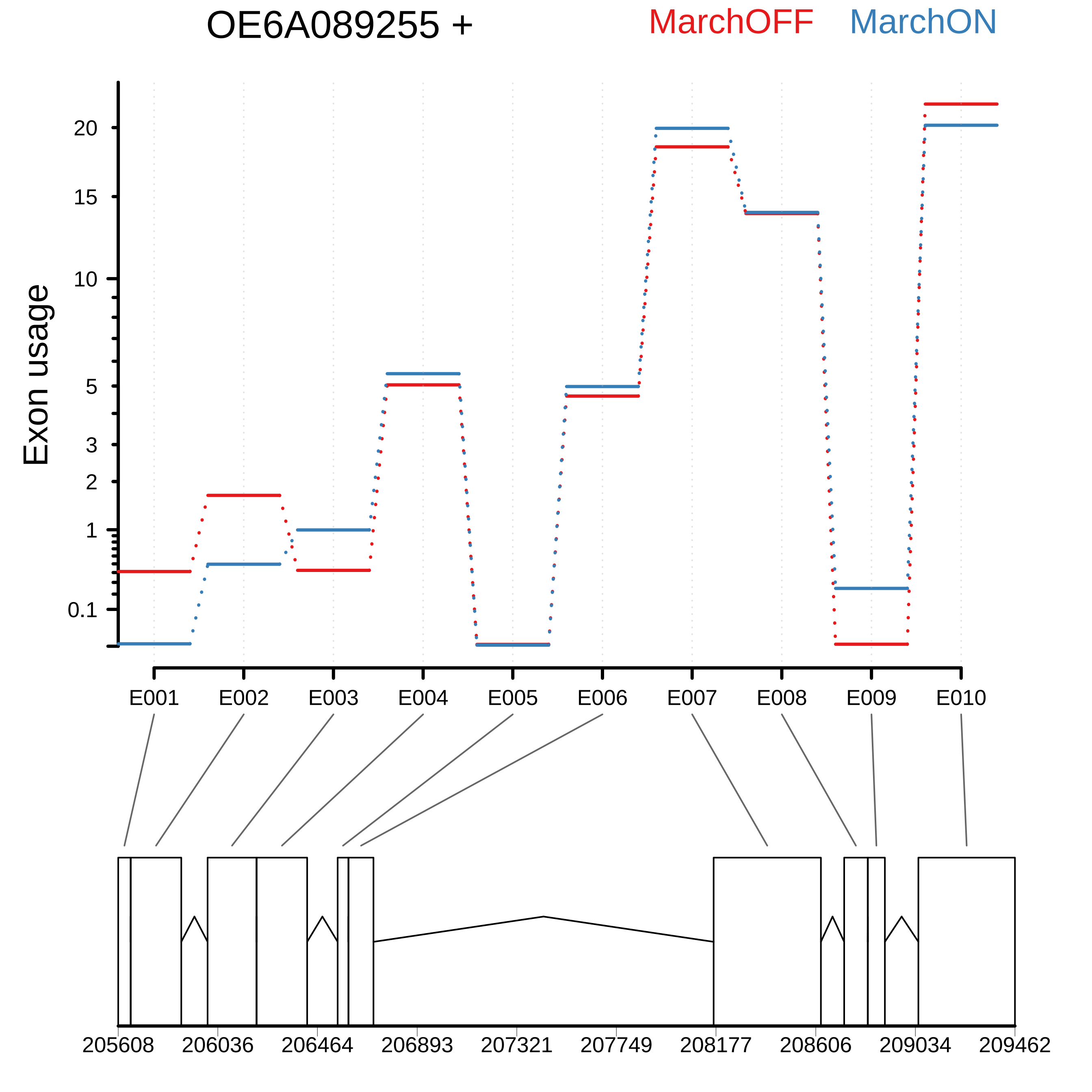

OE6A089255 +
MarchOFF
MarchON
Exon usage
20
15
10
5
3
2
1
0.1
E001
E002
E003
E004
E005
E006
E007
E008
E009
E010
205608
206036
206464
206893
207321
207749
208177
208606
209034
209462

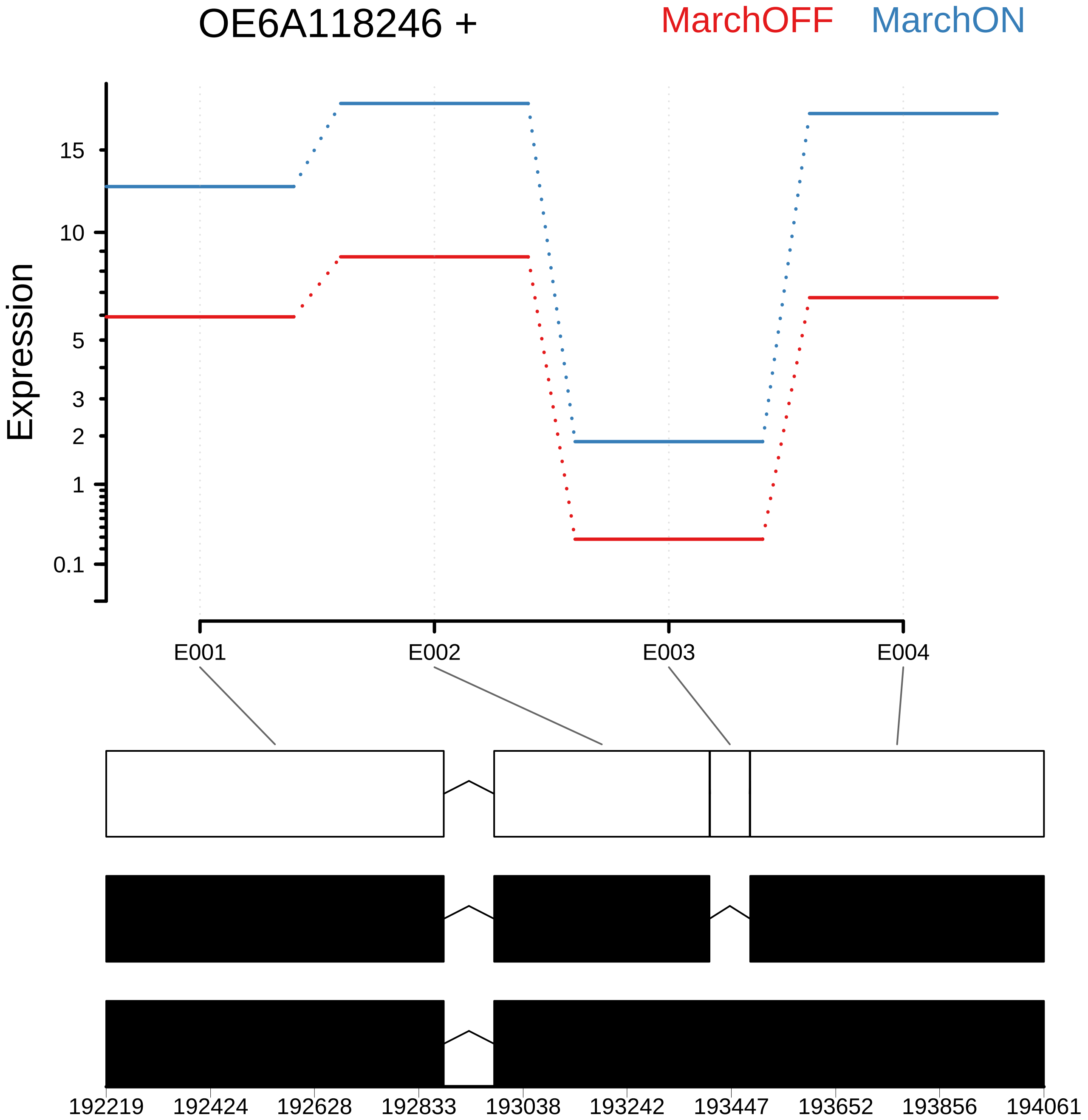
OE6A118246 +
MarchOFF
MarchON
Expression
15
10
5
3
2
1
0.1
E001
E002
E003
E004
192219
192424
192628
192833
193038
193242
193447
193652
193856
194061

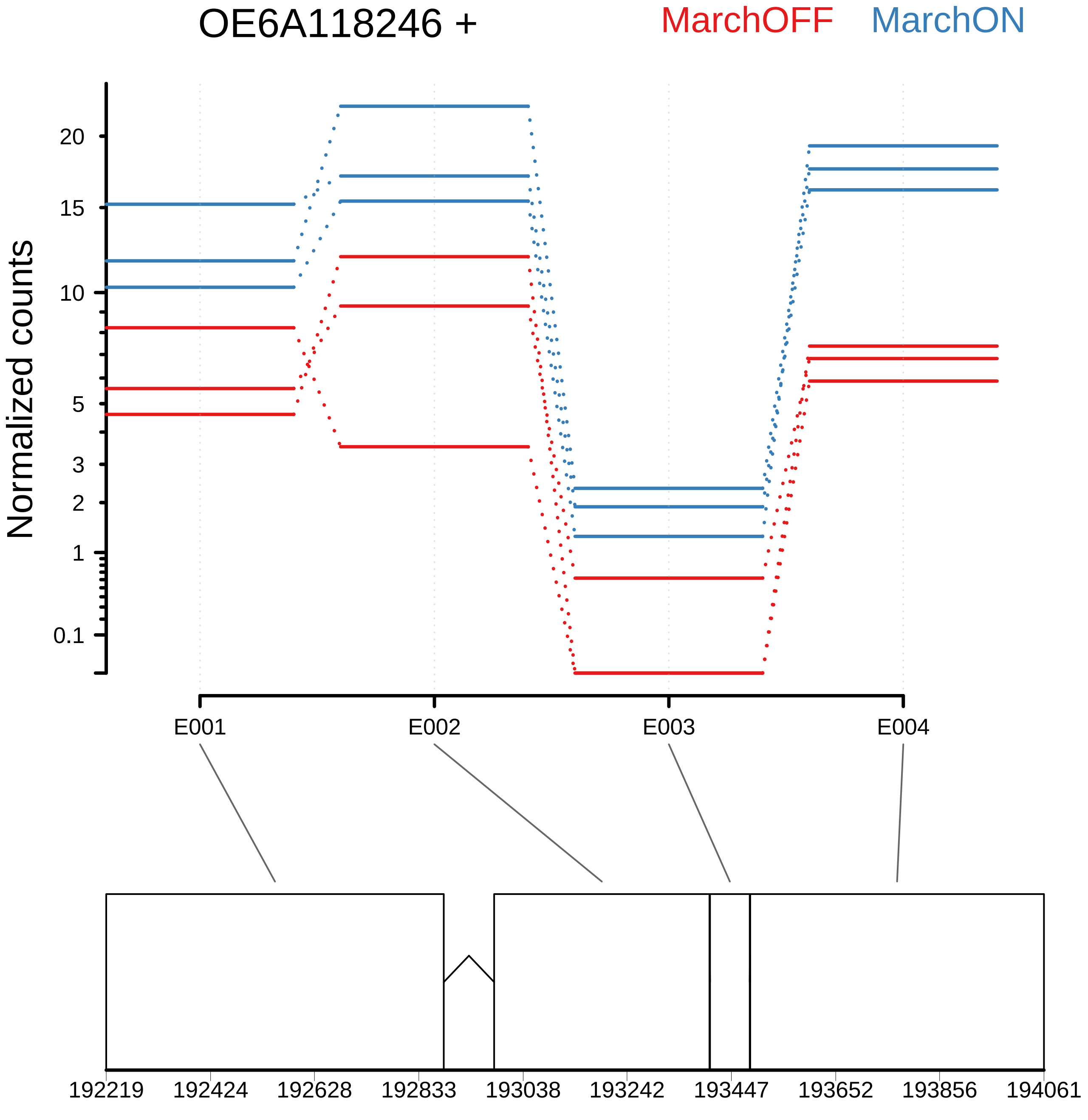
OE6A118246 +
MarchOFF
MarchON
Normalized counts
20
15
10
5
3
2
1
0.1
E001
E002
E003
E004
192219
192424
192628
192833
193038
193242
193447
193652
193856
194061

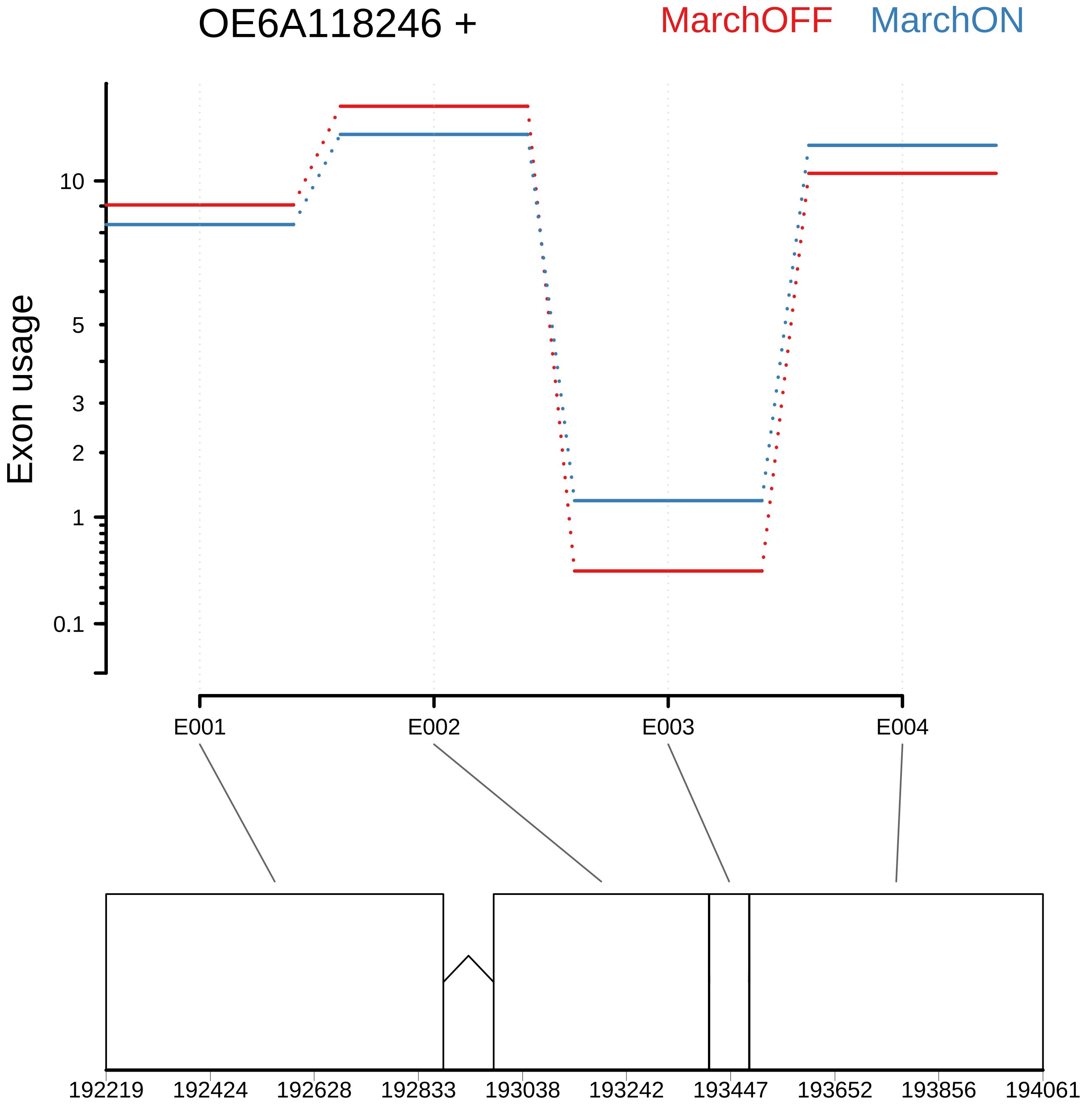
OE6A118246 +
MarchOFF
MarchON
Exon usage
10
5
3
2
1
0.1
E001
E002
E003
E004
192219
192424
192628
192833
193038
193242
193447
193652
193856
194061

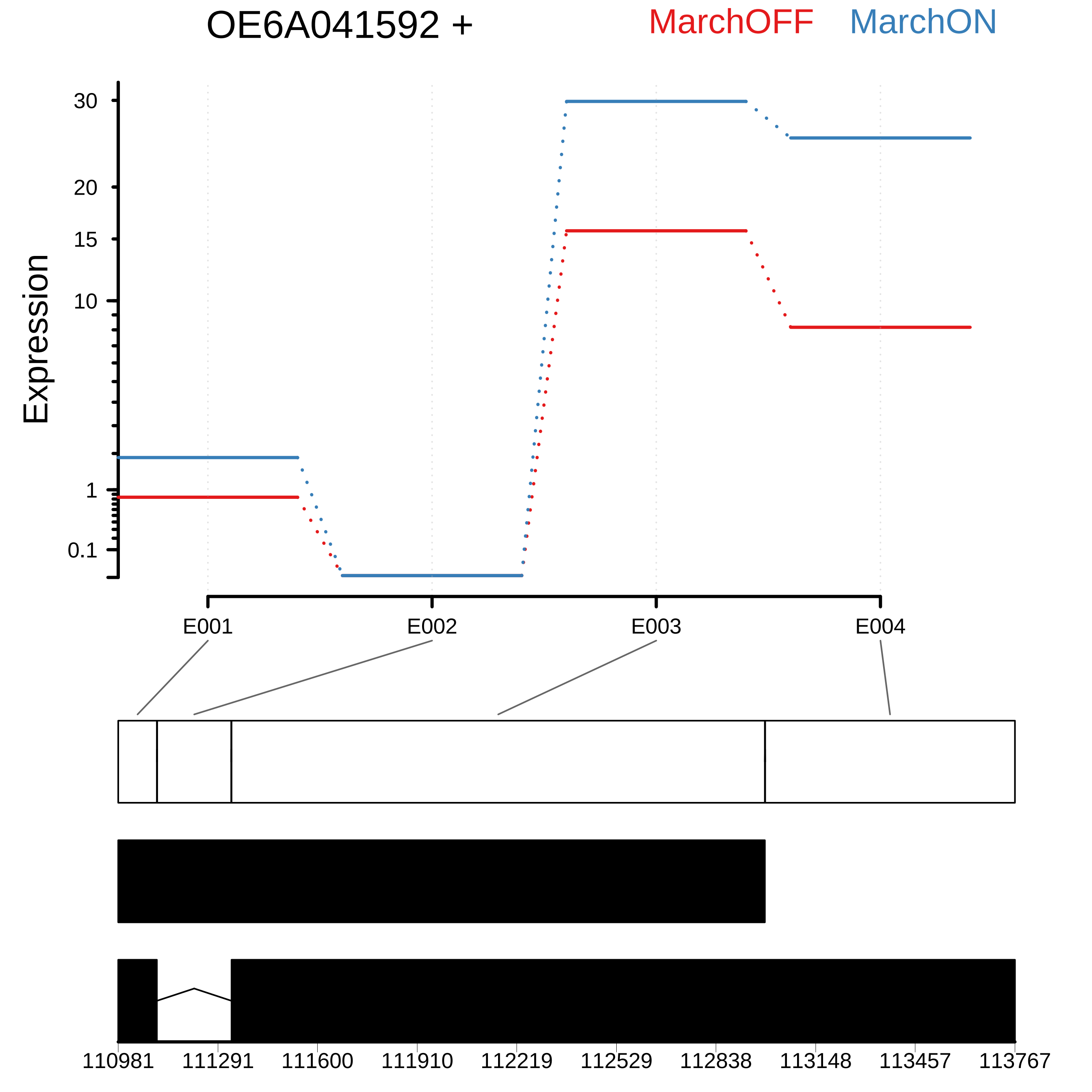
OE6A041592 +
MarchOFF
MarchON
Expression
30
20
15
10
1
0.1
E001
E002
E003
E004
110981
111291
111600
111910
112219
112529
112838
113148
113457
113767

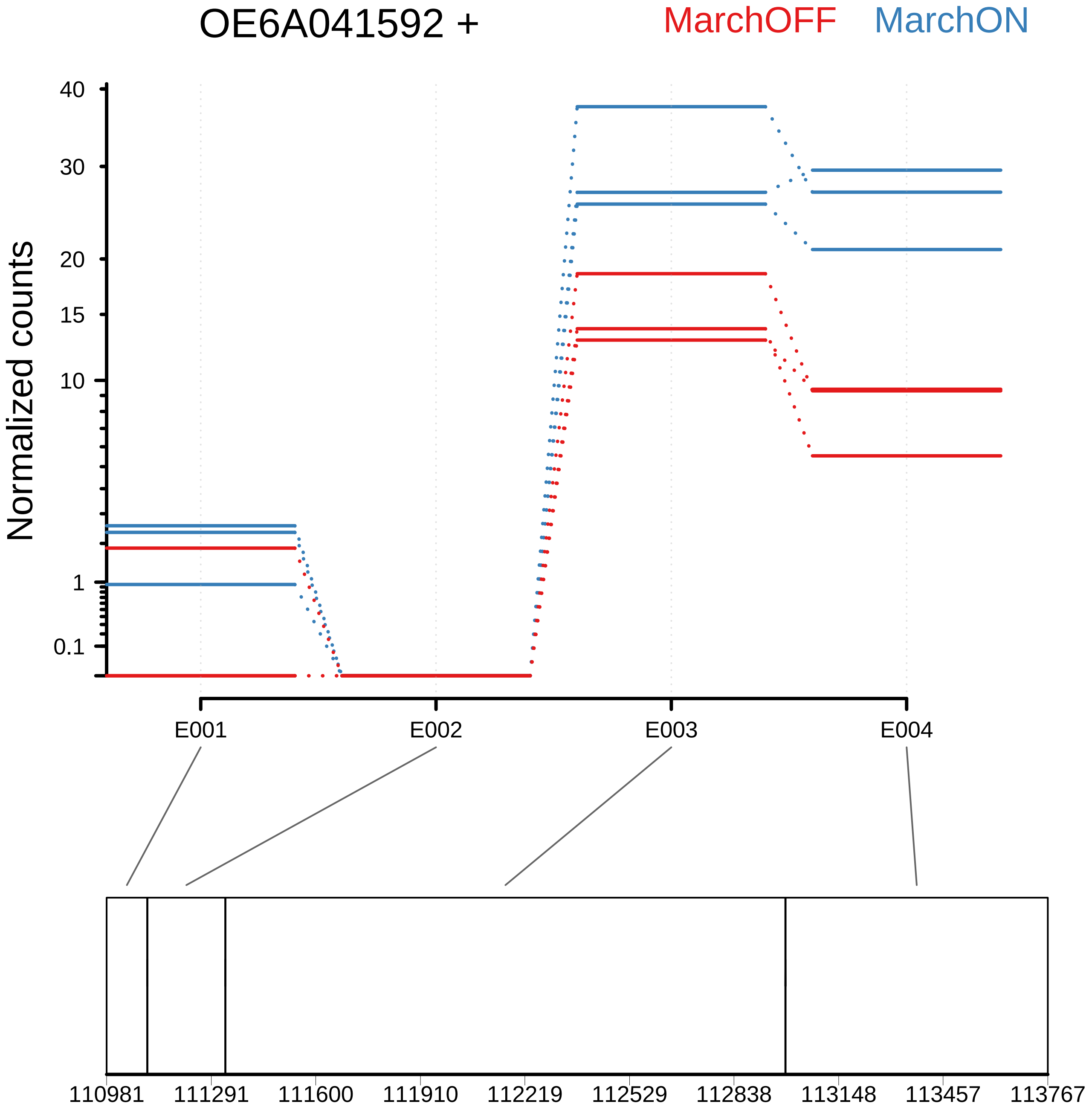

OE6A041592 +
MarchOFF
MarchON
Normalized counts
40
30
20
15
10
1
0.1
E001
E002
E003
E004
110981
111291
111600
111910
112219
112529
112838
113148
113457
113767

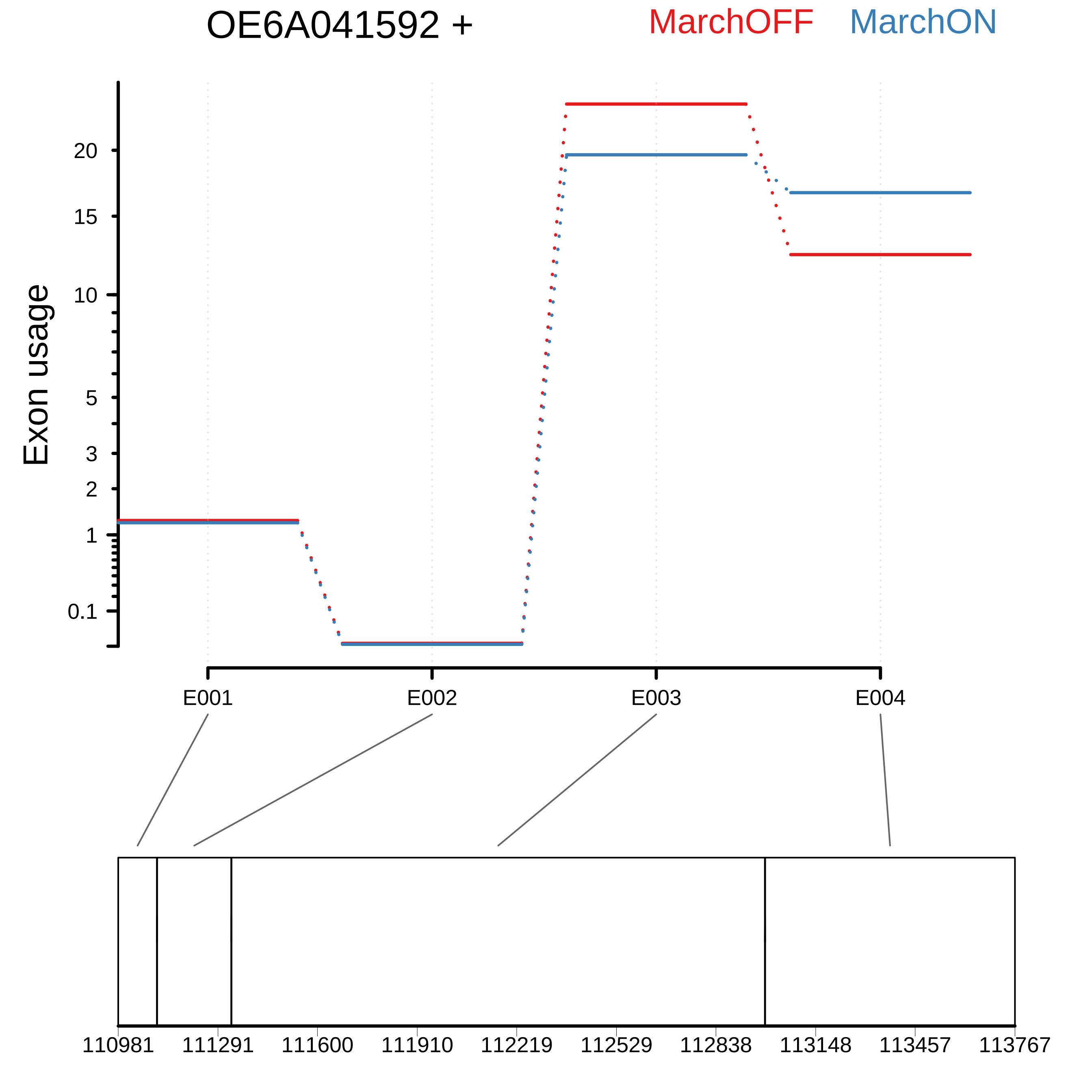

OE6A041592 +
MarchOFF
MarchON
Exon usage
20
15
10
5
3
2
1
0.1
E001
E002
E003
E004
110981
111291
111600
111910
112219
112529
112838
113148
113457
113767

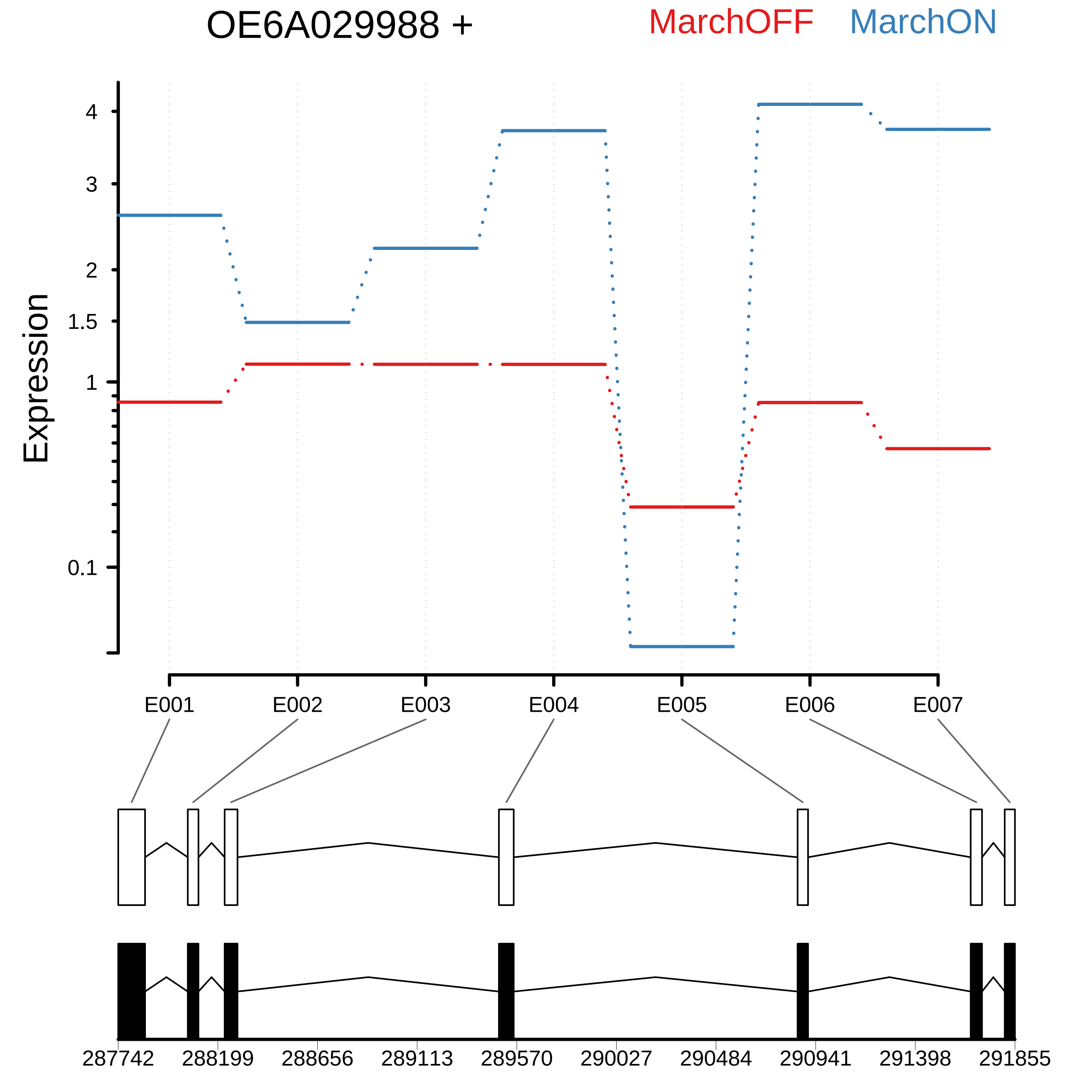
OE6A029988 +
MarchOFF
MarchON
Expression
4
3
2
1.5
1
0.1
E001
E002
E003
E004
E005
E006
E007
287742
288199
288656
289113
289570
290027
290484
290941
291398
291855

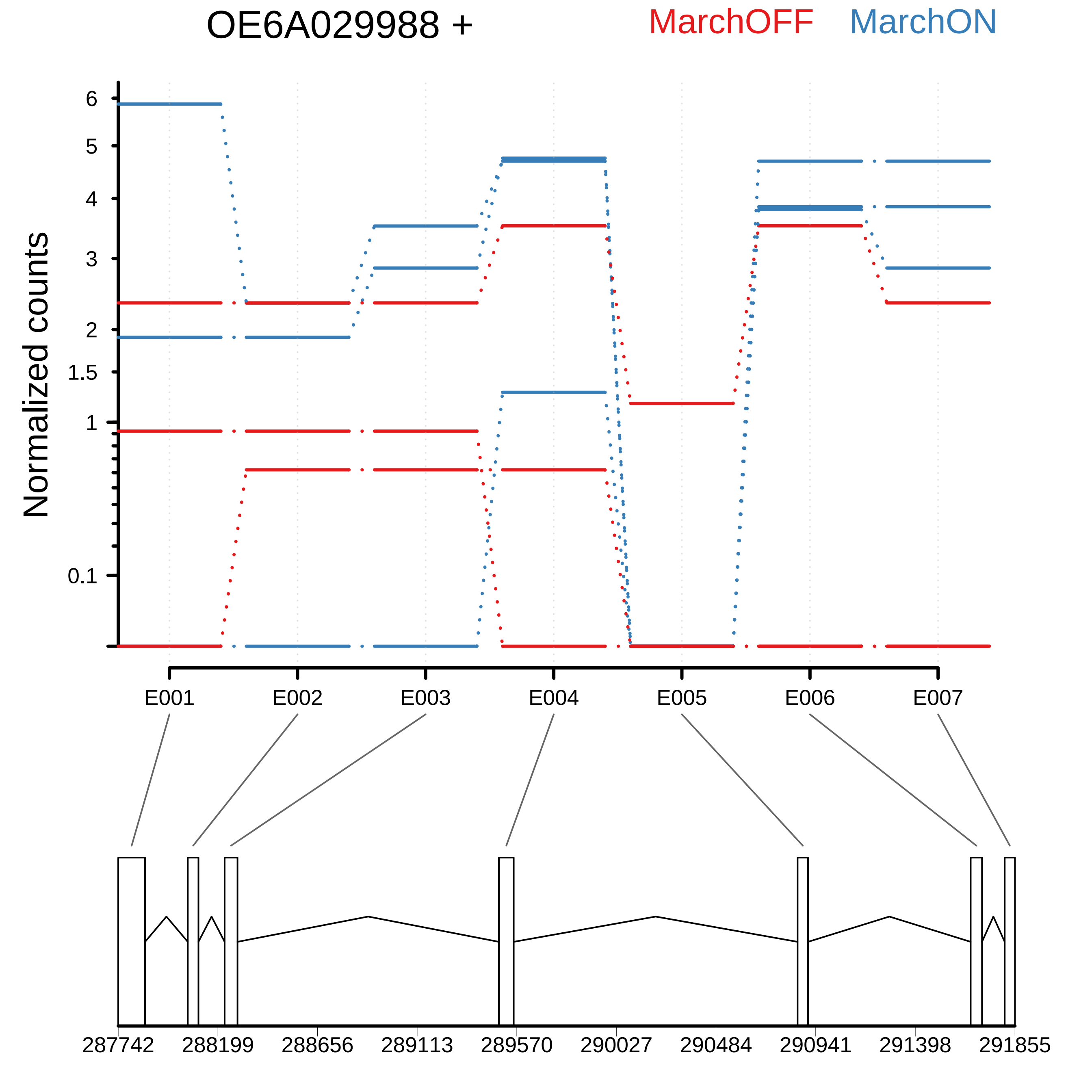
OE6A029988 +
MarchOFF
MarchON
Normalized counts
6
5
4
3
2
1.5
1
0.1
E001
E002
E003
E004
E005
E006
E007
287742
288199
288656
289113
289570
290027
290484
290941
291398
291855

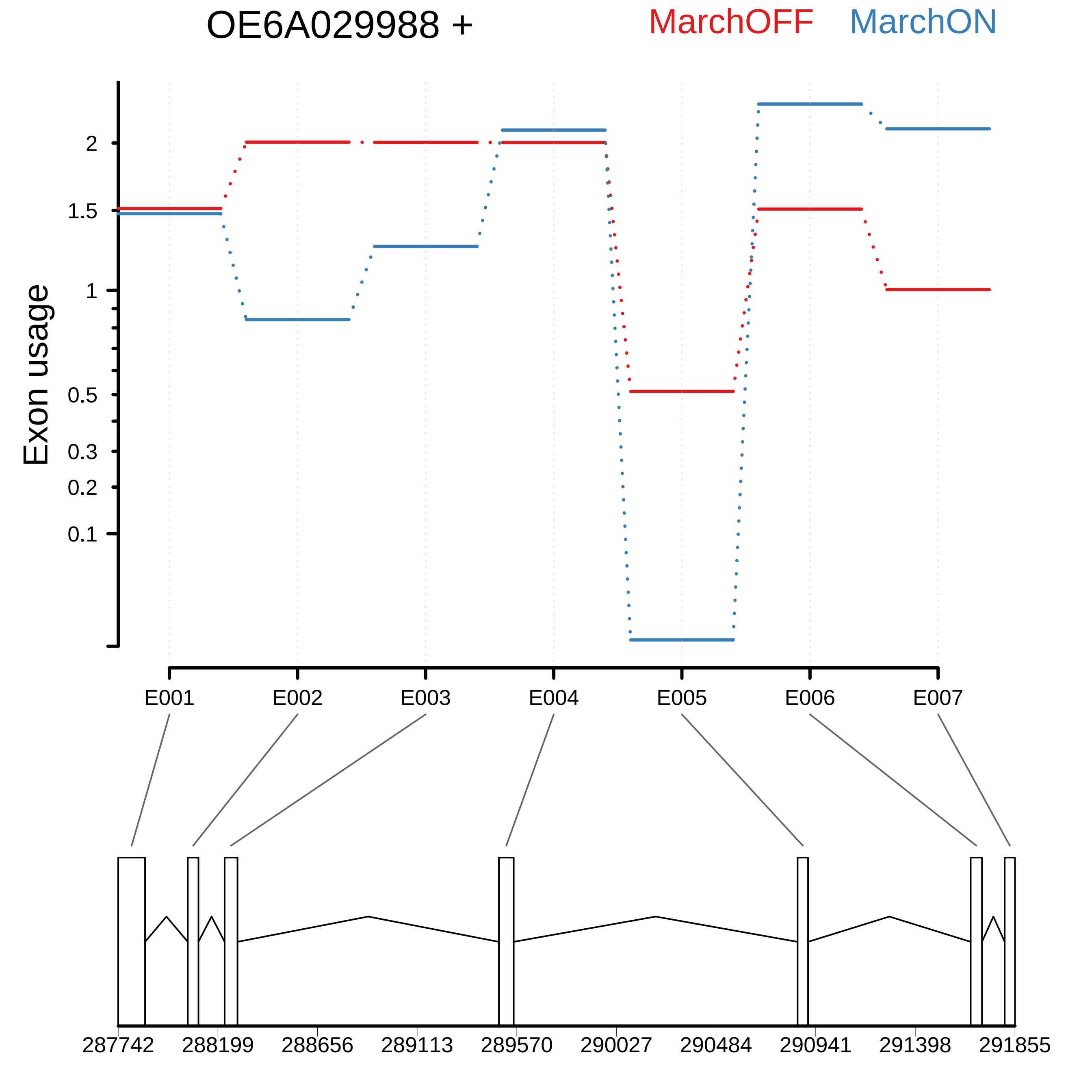

OE6A029988 +
MarchOFF
MarchON
Exon usage
2
1.5
1
0.5
0.3
0.2
0.1
E001
E002
E003
E004
E005
E006
E007
287742
288199
288656
289113
289570
290027
290484
290941
291398
291855

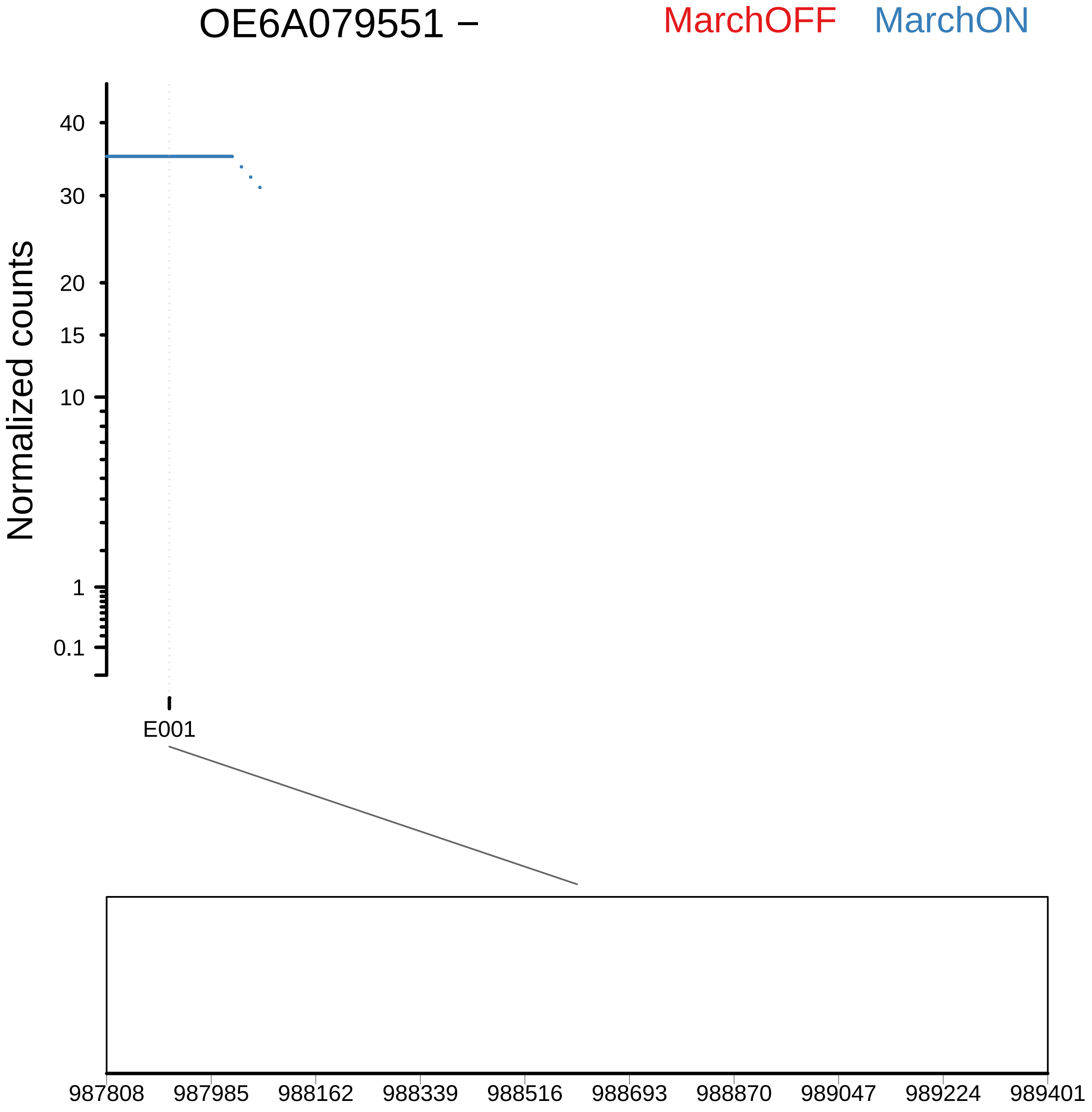

OE6A079551 −
MarchOFF
MarchON
Normalized counts
40
30
20
15
10
1
0.1
E001
987808
987985
988162
988339
988516
988693
988870
989047
989224
989401

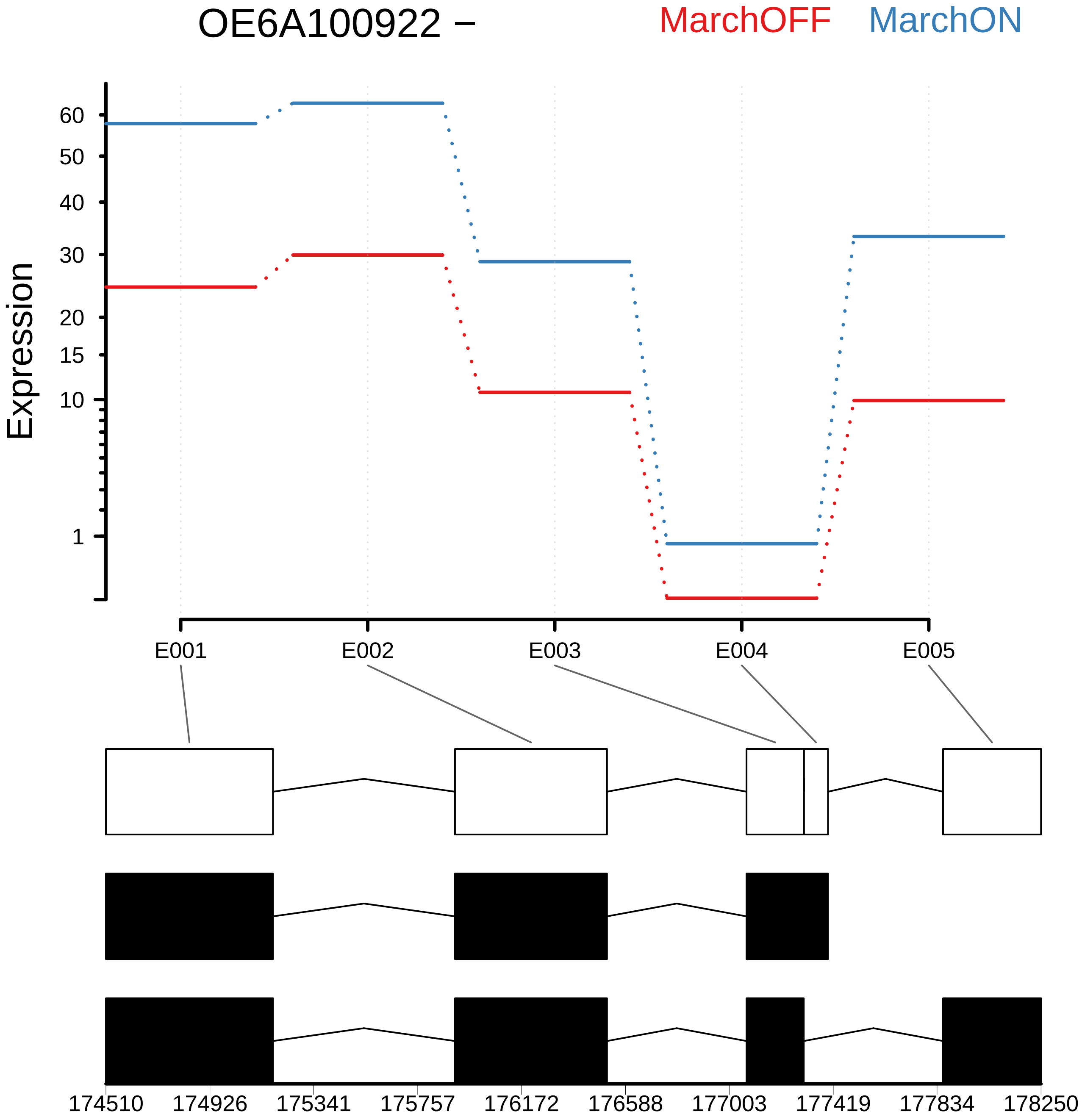
OE6A100922 –
MarchOFF
MarchON
Expression
60
50
40
30
20
15
10
1
E001
E002
E003
E004
E005
174510
174926
175341
175757
176172
176588
177003
177419
177834
178250

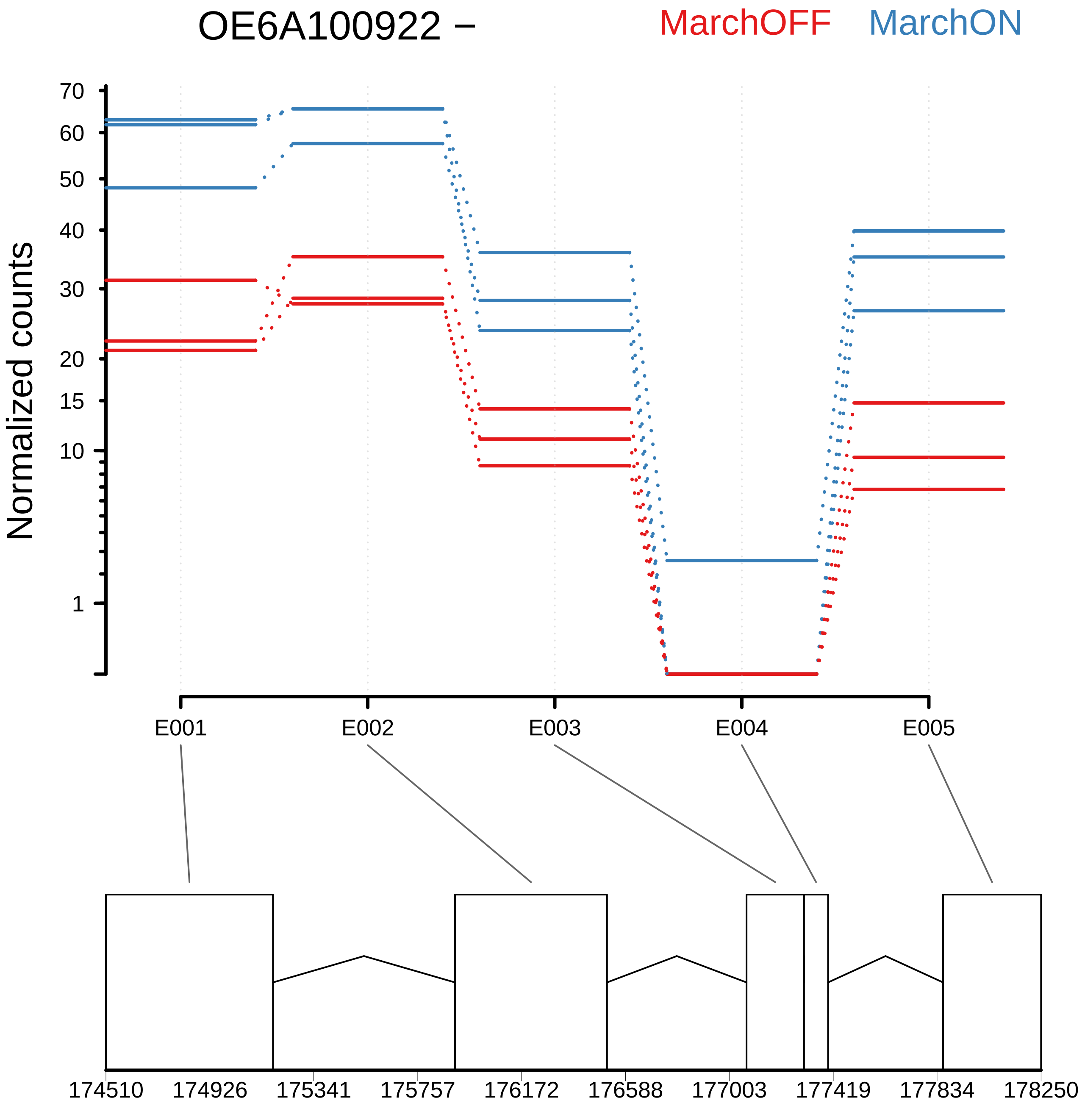

OE6A100922 –
MarchOFF
MarchON
Normalized counts
70
60
50
40
30
20
15
10
1
E001
E002
E003
E004
E005
174510
174926
175341
175757
176172
176588
177003
177419
177834
178250

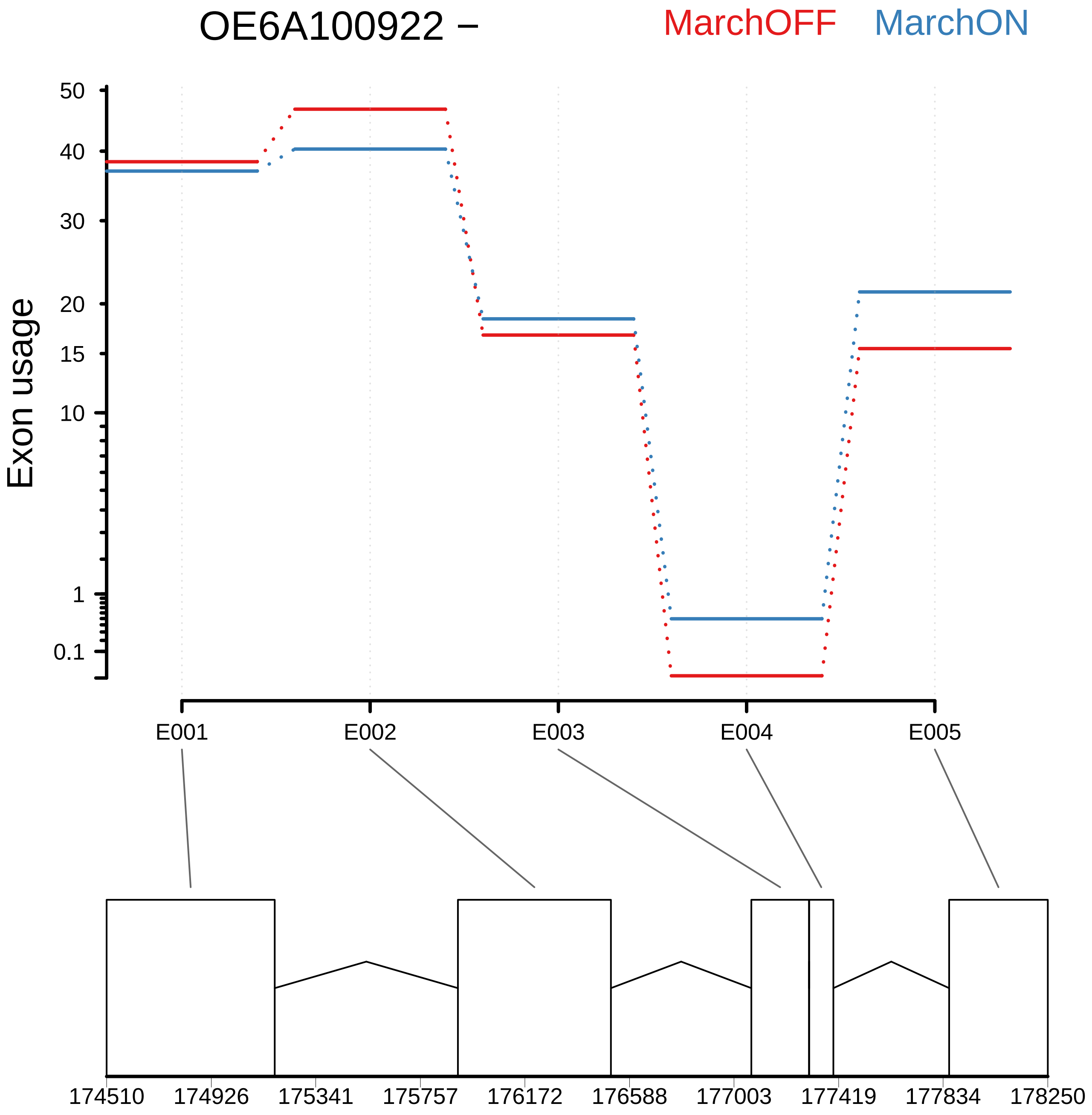

OE6A100922 −
MarchOFF
MarchON
Exon usage
50
40
30
20
15
10
1
0.1
E001
E002
E003
E004
E005
174510
174926
175341
175757
176172
176588
177003
177419
177834
178250

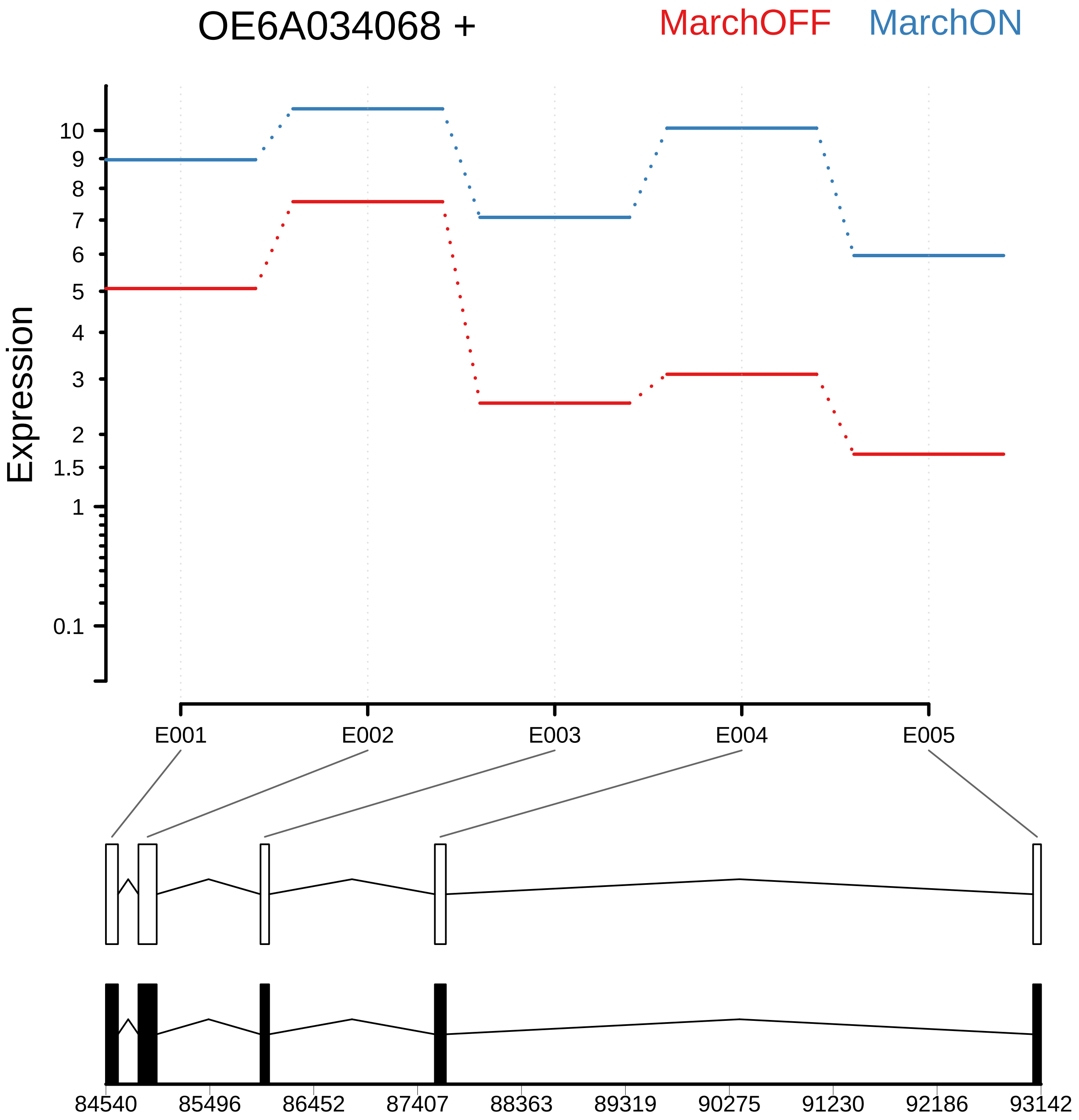

OE6A034068 +
MarchOFF
MarchON
Expression
10
9
8
7
6
5
4
3
2
1.5
1
0.1
E001
E002
E003
E004
E005
84540
85496
86452
87407
88363
89319
90275
91230
92186
93142

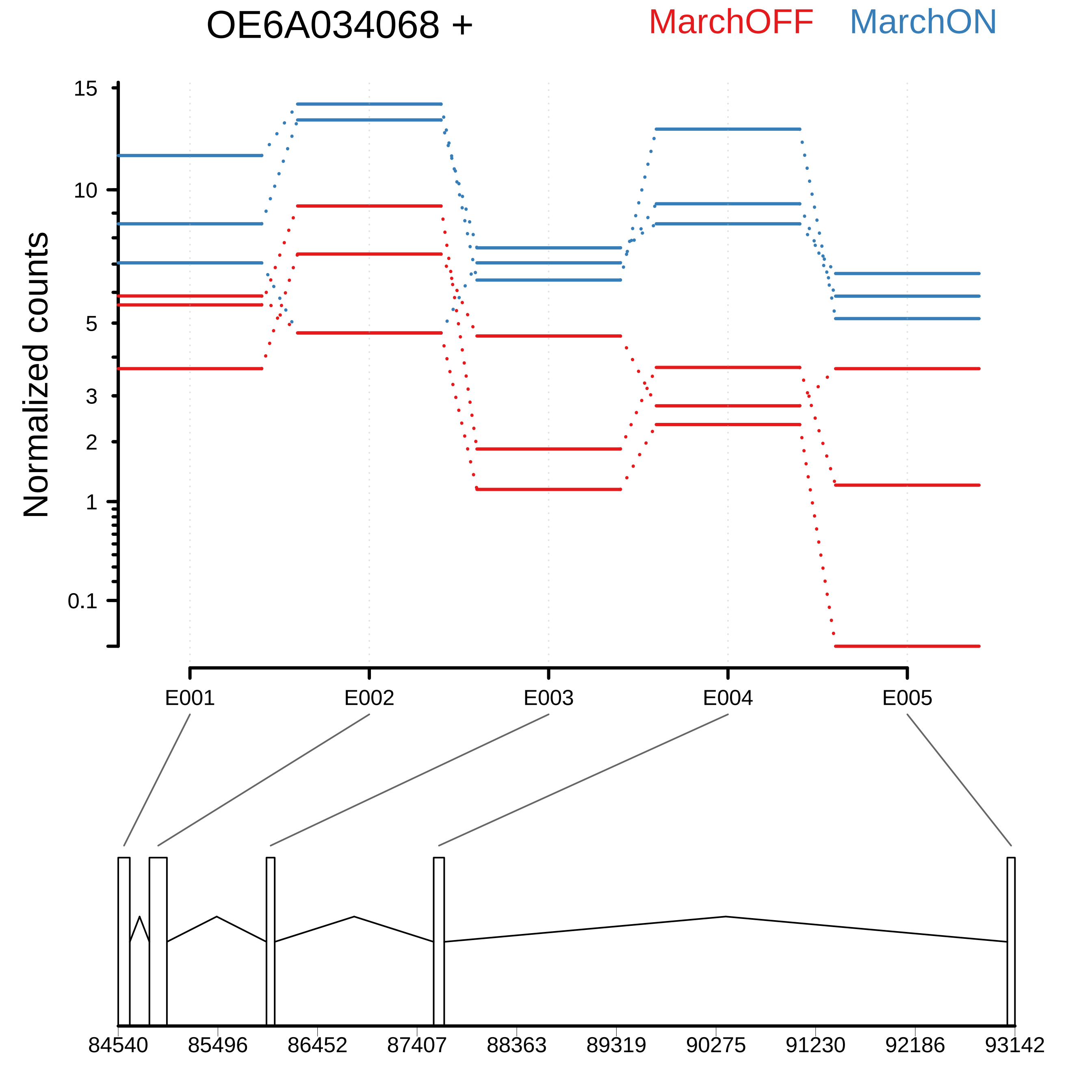
OE6A034068 +
MarchOFF
MarchON
Normalized counts
15
10
5
3
2
1
0.1
E001
E002
E003
E004
E005
84540
85496
86452
87407
88363
89319
90275
91230
92186
93142

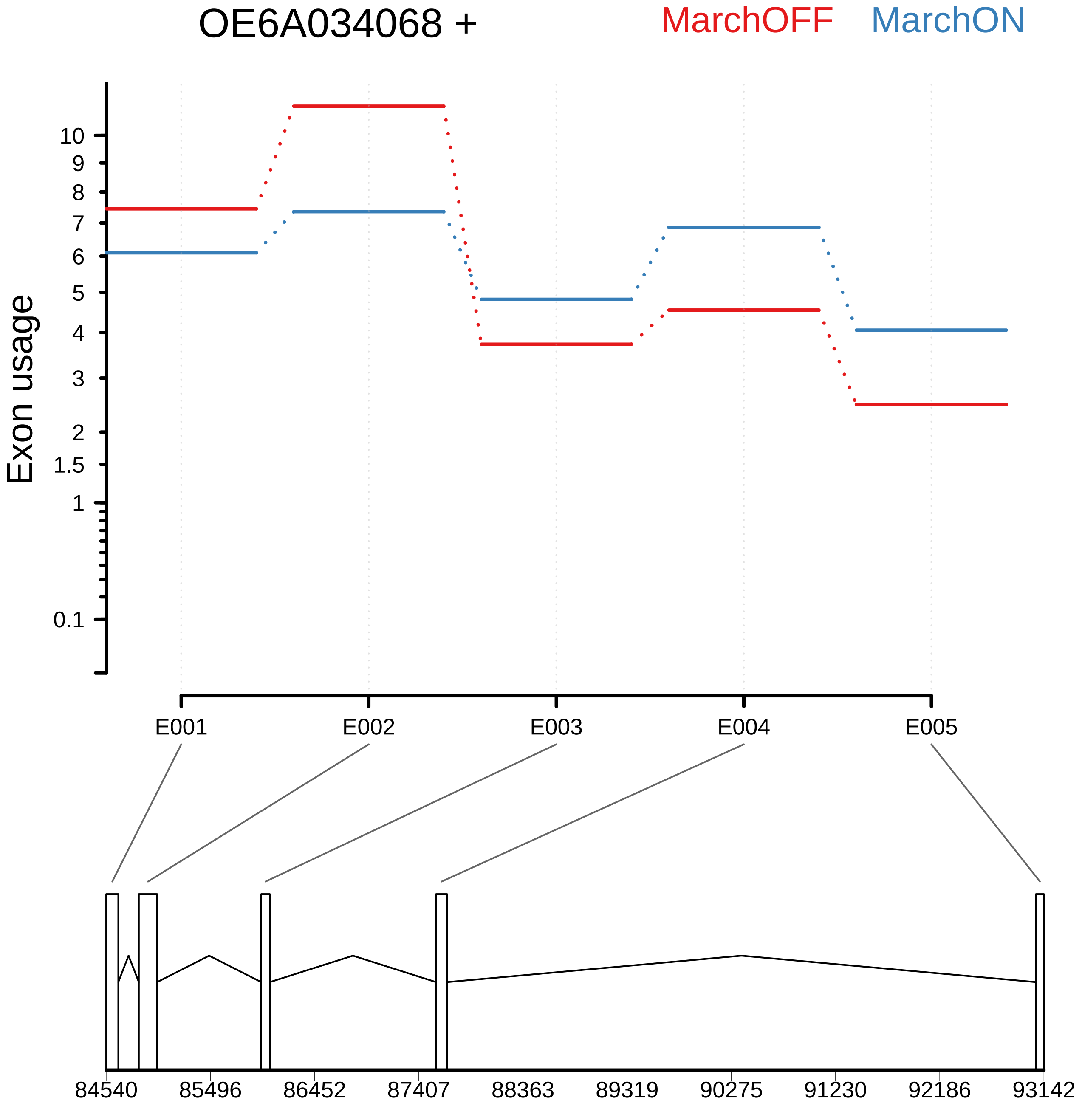
OE6A034068 +
MarchOFF
MarchON
Exon usage
10
9
8
7
6
5
4
3
2
1.5
1
0.1
E001
E002
E003
E004
E005
84540
85496
86452
87407
88363
89319
90275
91230
92186
93142

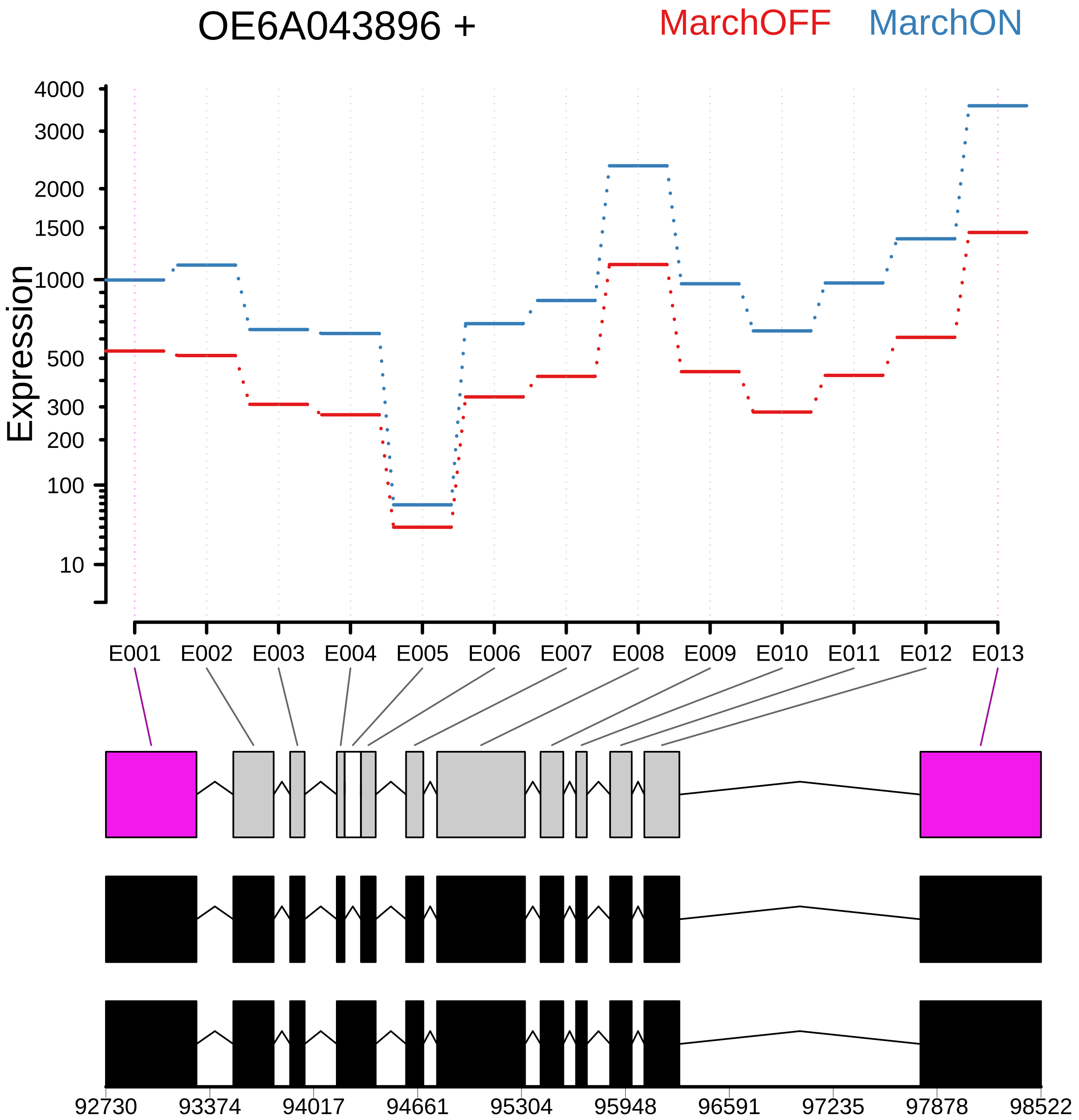
OE6A043896 +
MarchOFF
MarchON
Expression
4000
3000
2000
1500
1000
500
300
200
100
10
E001
E002
E003
E004
E005
E006
E007
E008
E009
E010
E011
E012
E013
92730
93374
94017
94661
95304
95948
96591
97235
97878
98522

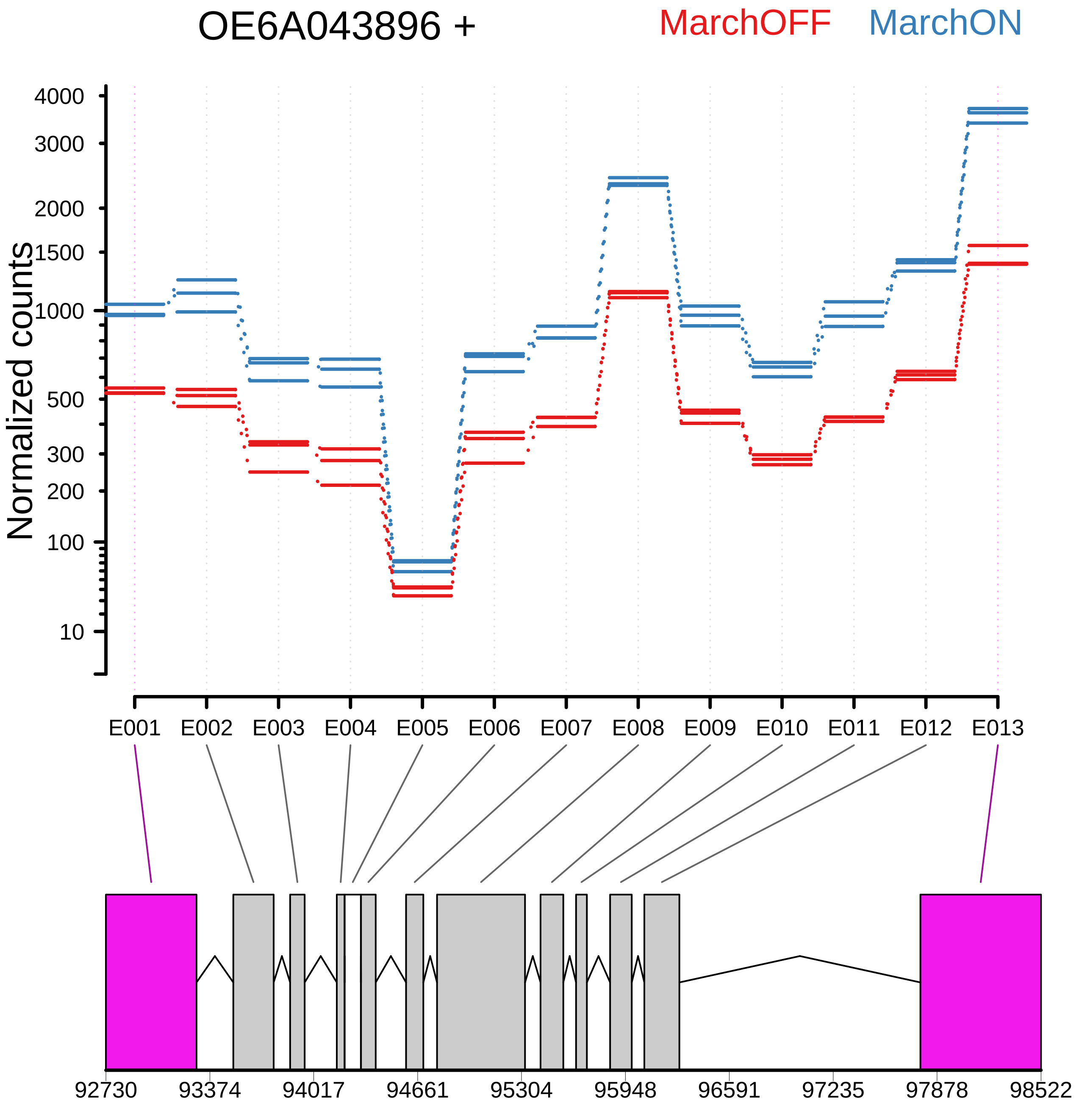

OE6A043896 +
MarchOFF
MarchON
Normalized counts
4000
3000
2000
1500
1000
500
300
200
100
10
E001
E002
E003
E004
E005
E006
E007
E008
E009
E010
E011
E012
E013
92730
93374
94017
94661
95304
95948
96591
97235
97878
98522

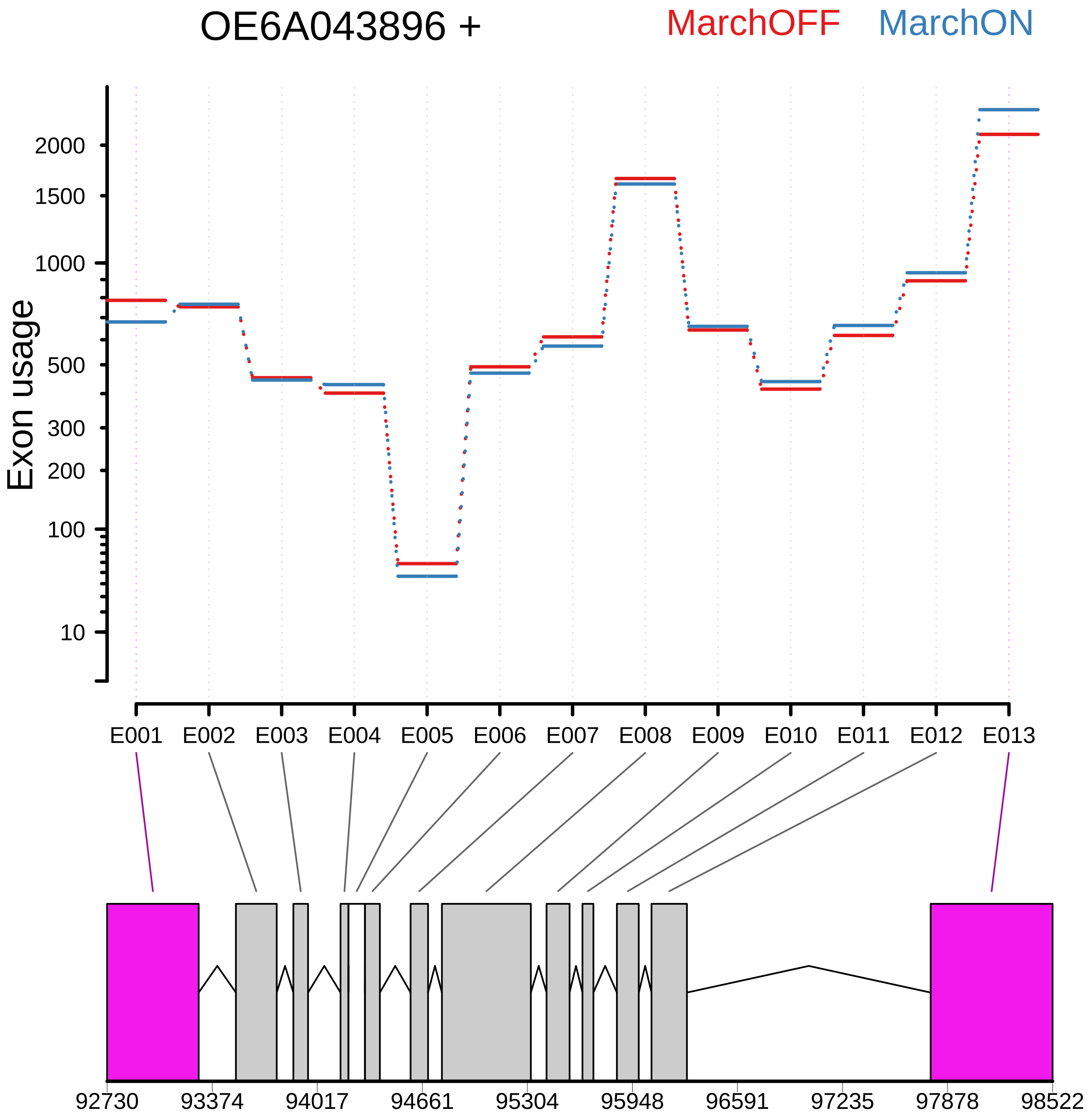

OE6A043896 +
MarchOFF
MarchON
Exon usage
2000
1500
1000
500
300
200
100
10
E001
E002
E003
E004
E005
E006
E007
E008
E009
E010
E011
E012
E013
92730
93374
94017
94661
95304
95948
96591
97235
97878
98522

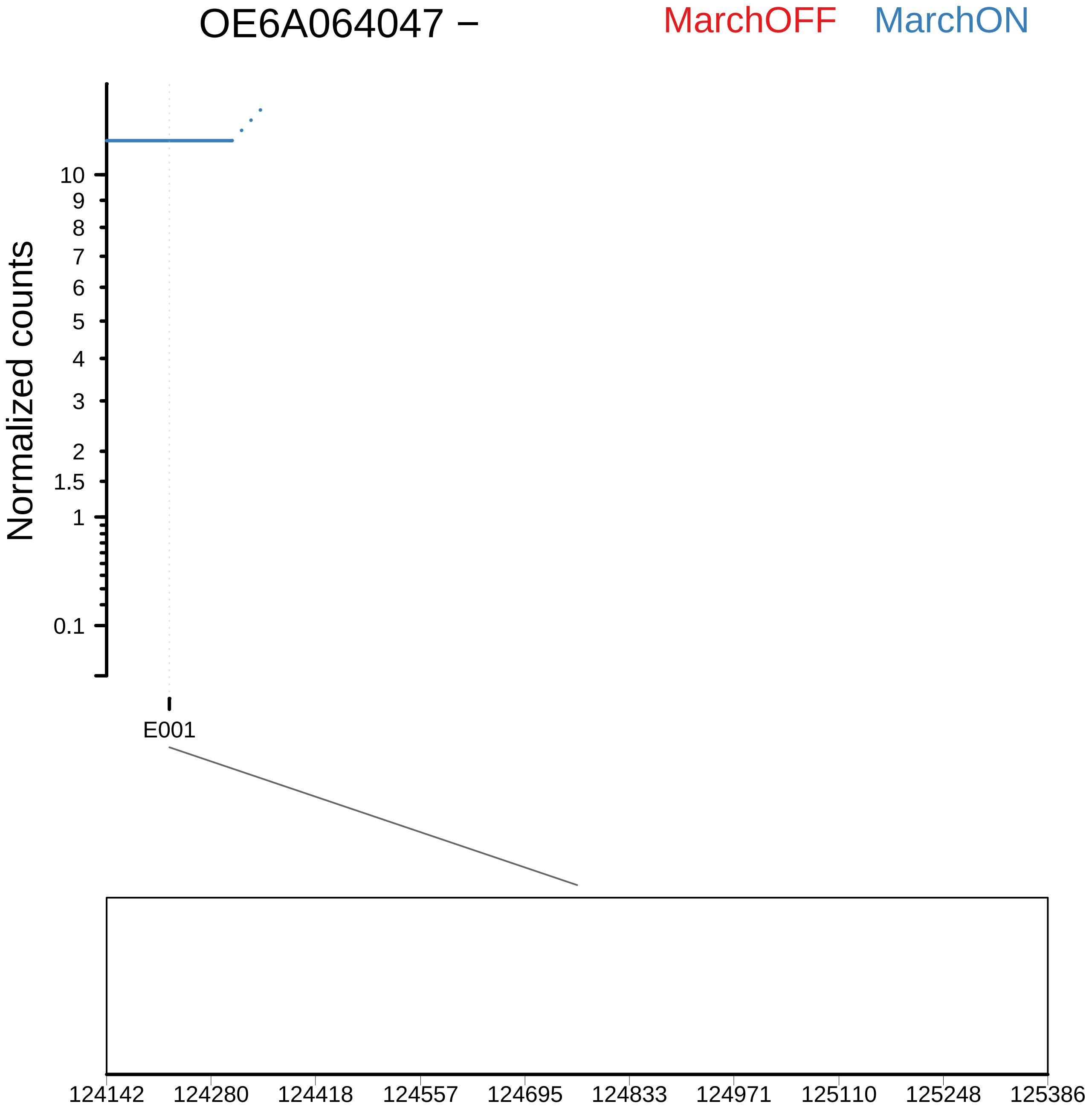
OE6A064047 −
MarchOFF
MarchON
Normalized counts
10
9
8
7
6
5
4
3
2
1.5
1
0.1
E001
124142
124280
124418
124557
124695
124833
124971
125110
125248
125386

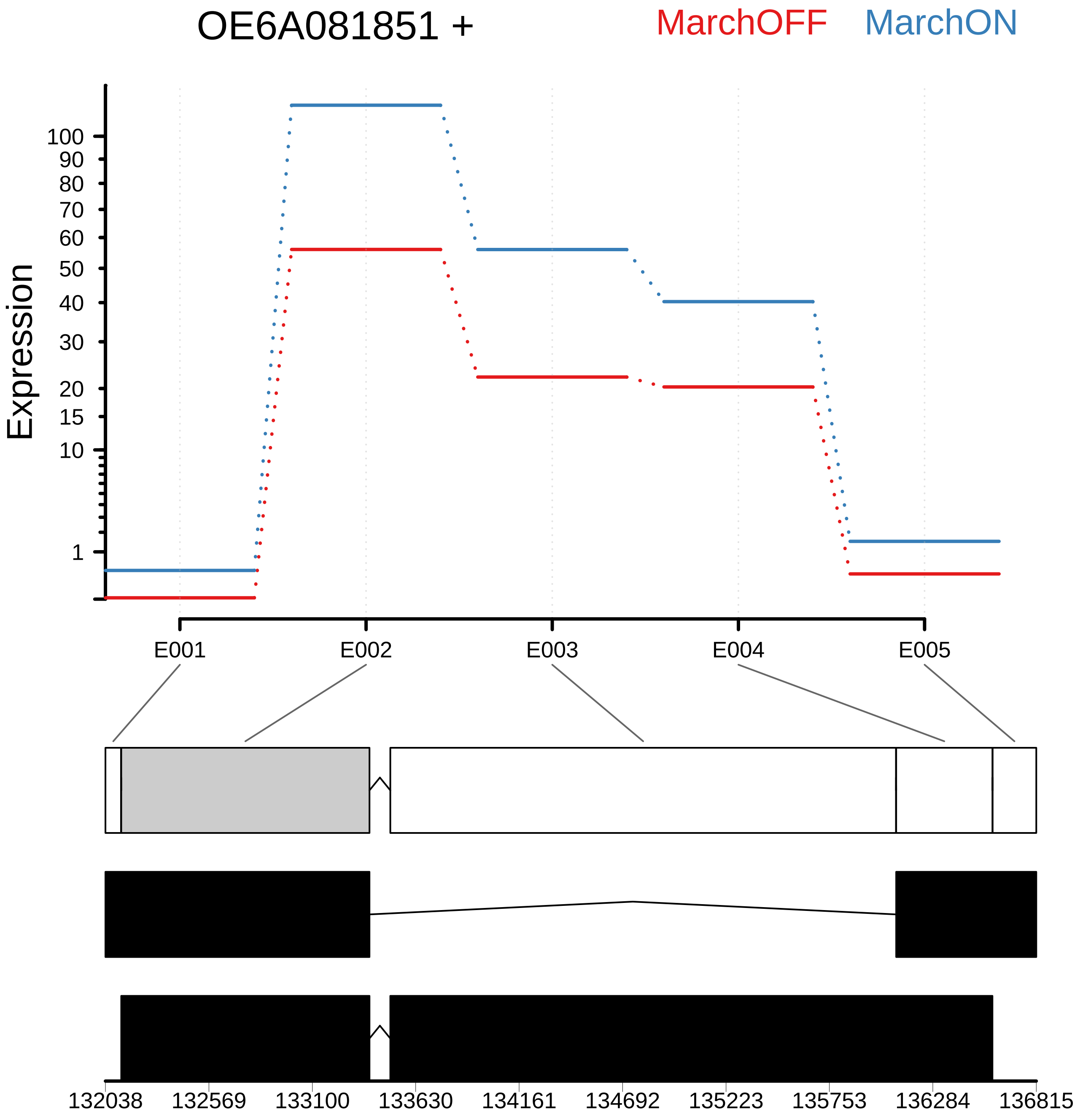
OE6A081851 +
MarchOFF
MarchON
Expression
100
90
80
70
60
50
40
30
20
15
10
1
E001
E002
E003
E004
E005
132038
132569
133100
133630
134161
134692
135223
135753
136284
136815

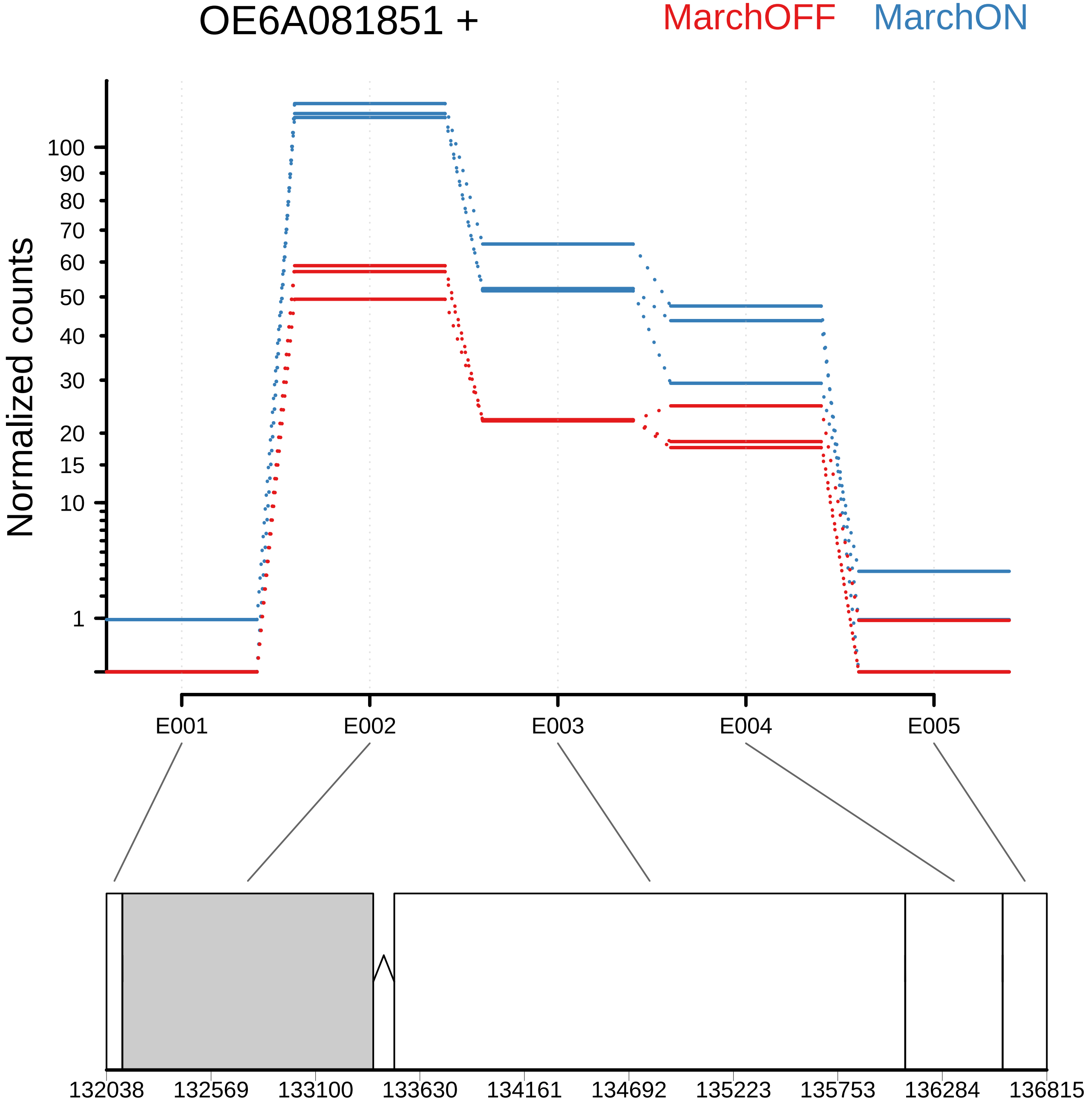

OE6A081851 +
MarchOFF
MarchON
Normalized counts
100
90
80
70
60
50
40
30
20
15
10
1
E001
E002
E003
E004
E005
132038
132569
133100
133630
134161
134692
135223
135753
136284
136815

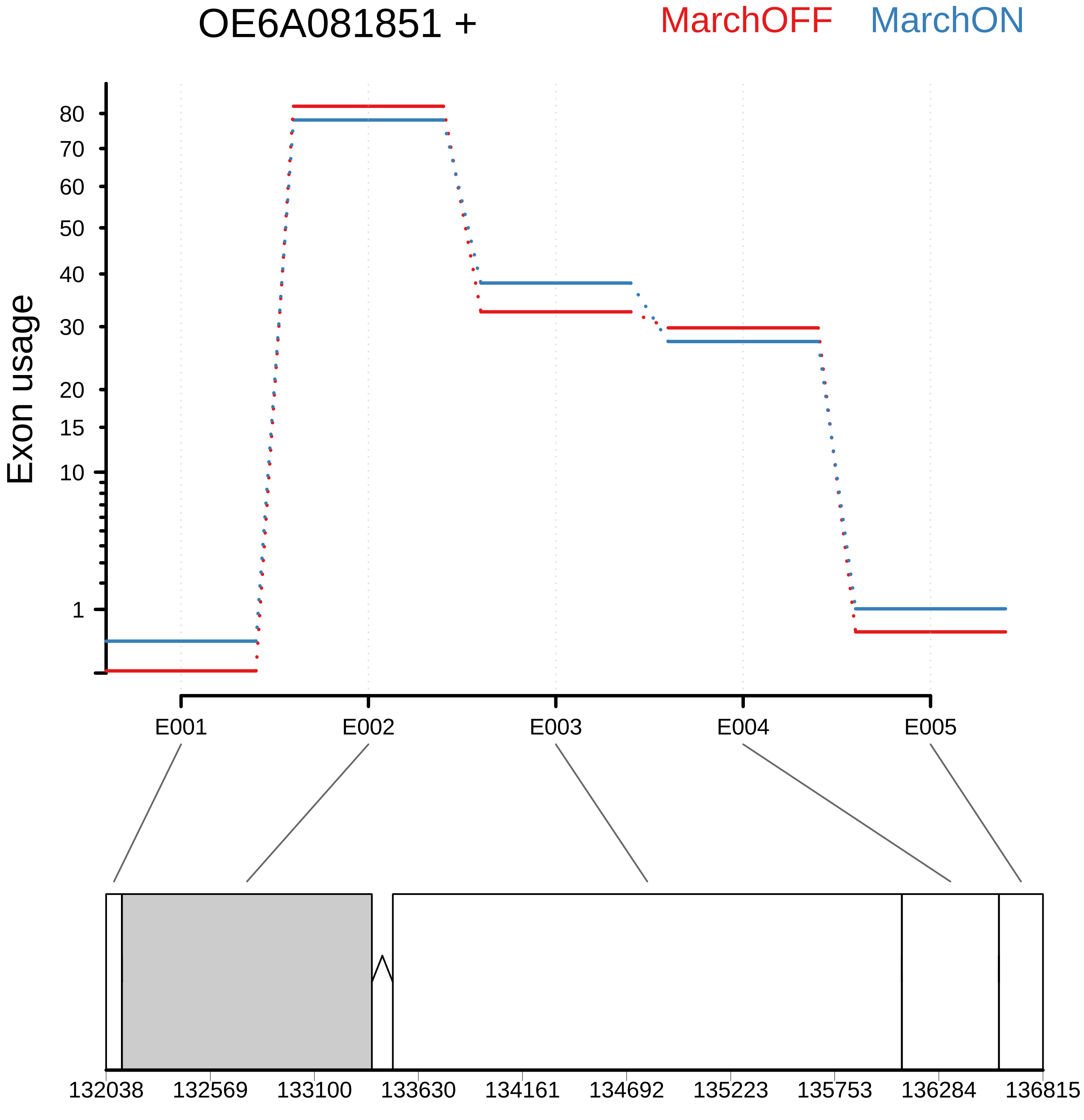

OE6A081851 +
MarchOFF
MarchON
Exon usage
80
70
60
50
40
30
20
15
10
1
E001
E002
E003
E004
E005
132038
132569
133100
133630
134161
134692
135223
135753
136284
136815

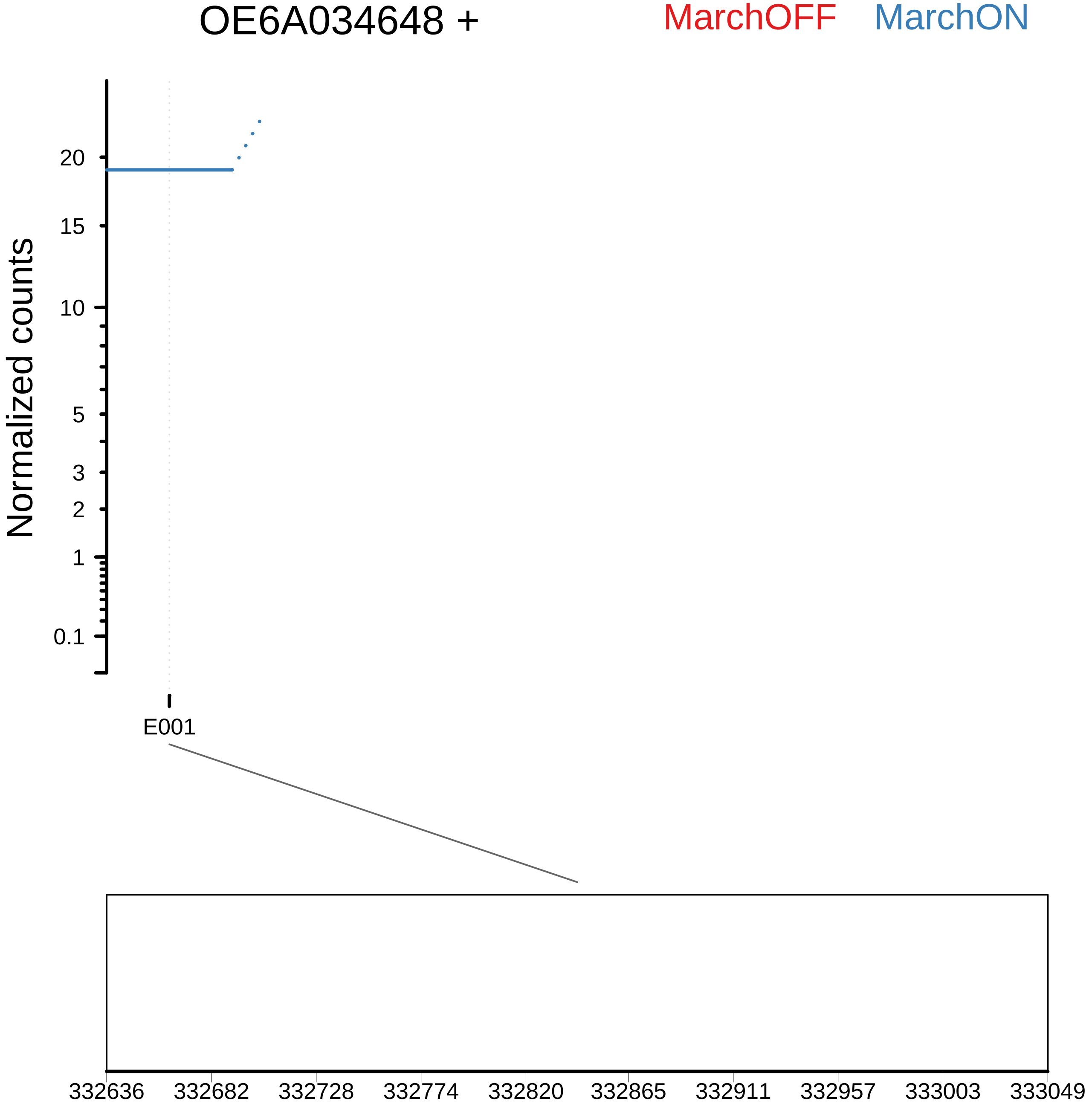

OE6A034648 +
MarchOFF
MarchON
Normalized counts
20
15
10
5
3
2
1
0.1
E001
332636
332682
332728
332774
332820
332865
332911
332957
333003
333049

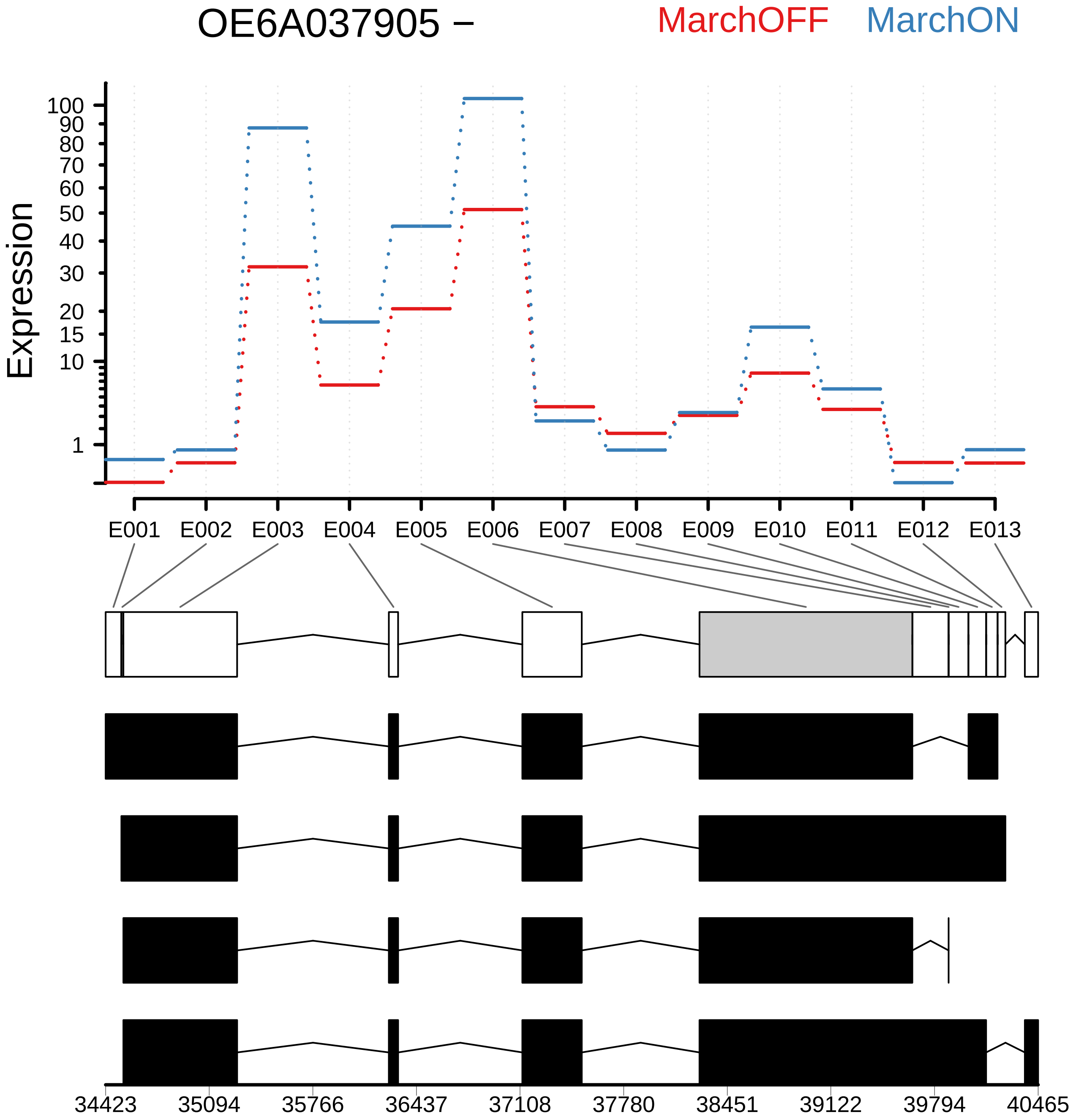
OE6A037905 –
MarchOFF
MarchON
Expression
100
90
80
70
60
50
40
30
20
15
10
1
E001
E002
E003
E004
E005
E006
E007
E008
E009
E010
E011
E012
E013
34423
35094
35766
36437
37108
37780
38451
39122
39794
40465

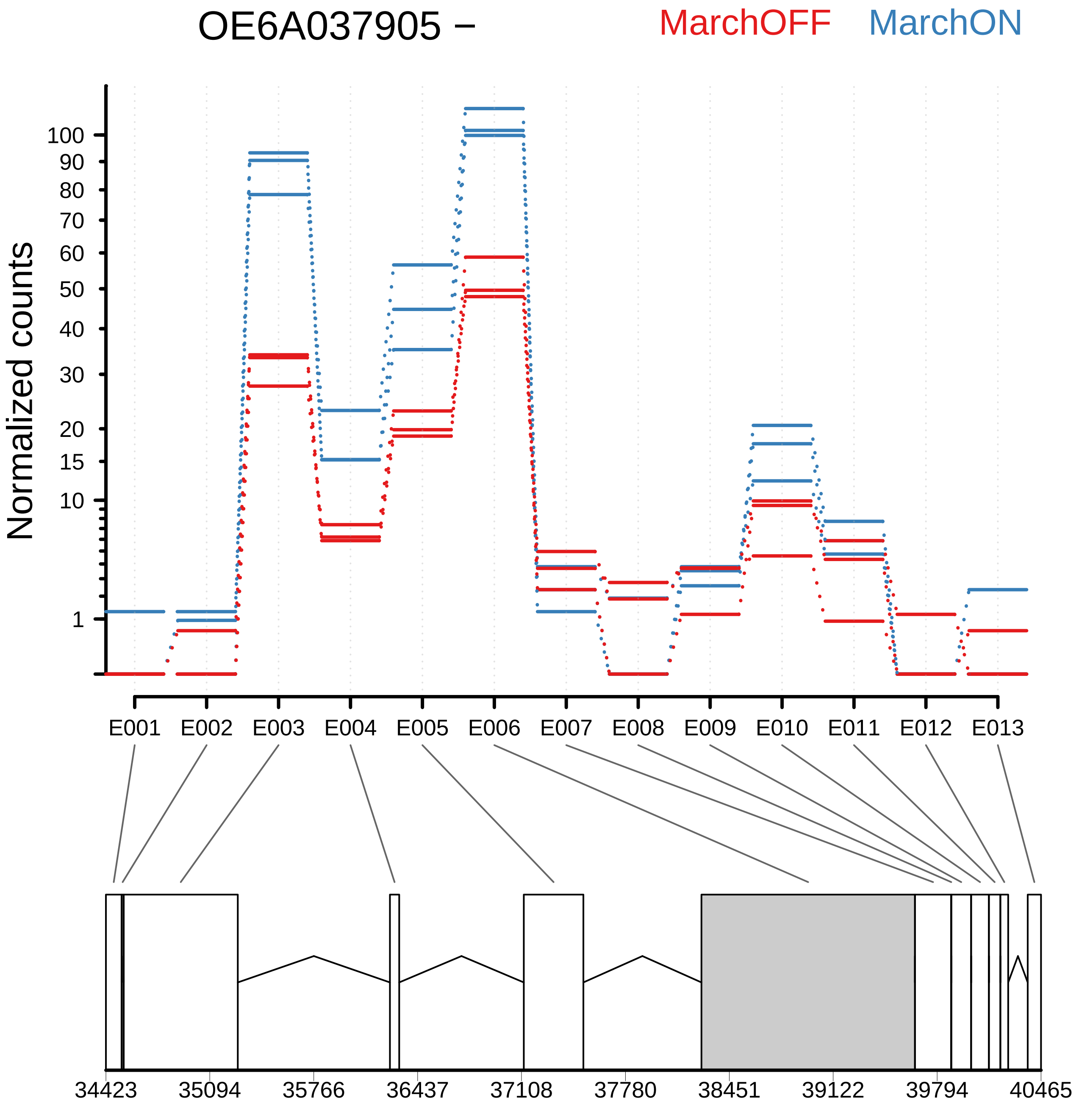
OE6A037905 −
MarchOFF
MarchON
Normalized counts
100
90
80
70
60
50
40
30
20
15
10
1
E001
E002
E003
E004
E005
E006
E007
E008
E009
E010
E011
E012
E013
34423
35094
35766
36437
37108
37780
38451
39122
39794
40465

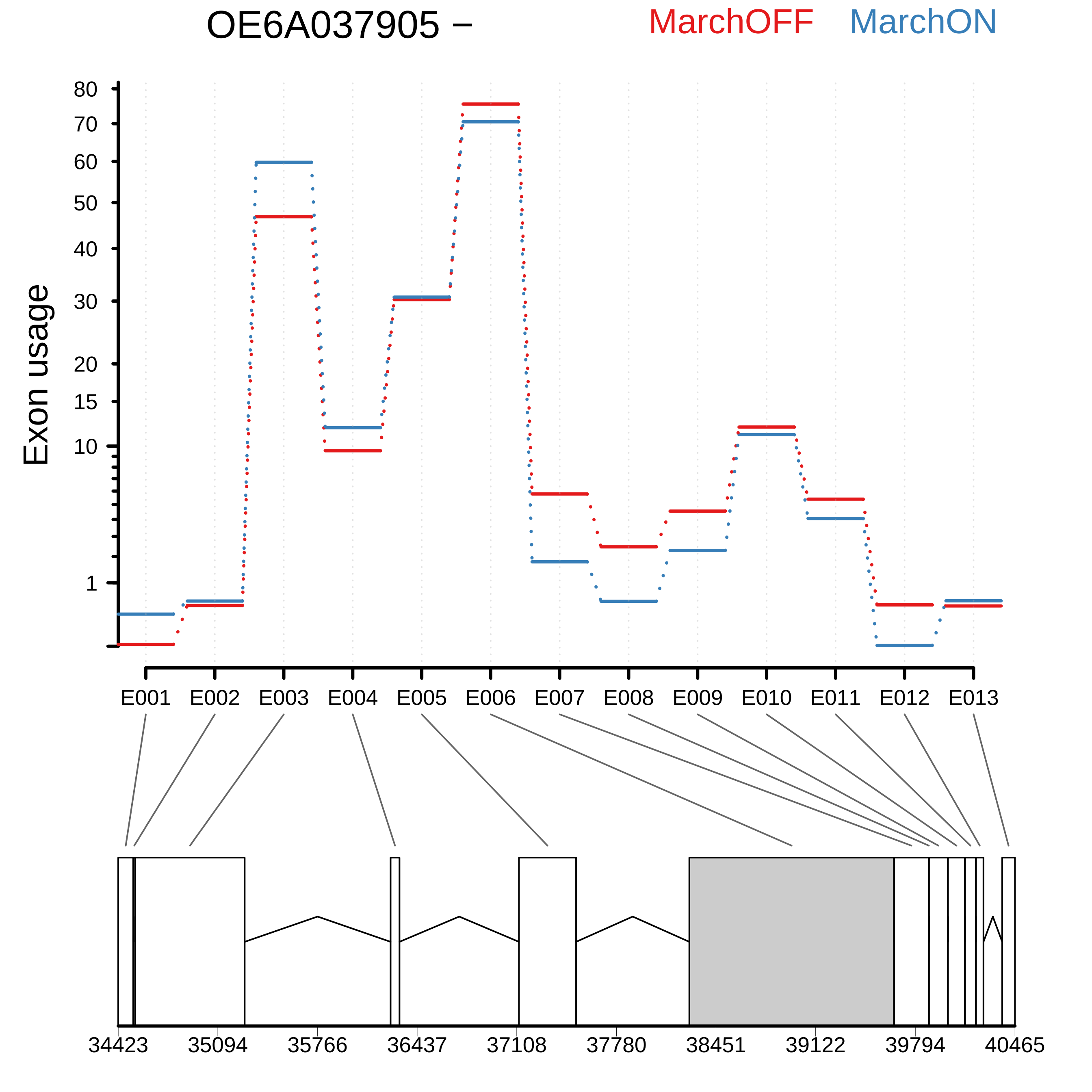
OE6A037905 −
MarchOFF
MarchON
Exon usage
80
70
60
50
40
30
20
15
10
1
E001
E002
E003
E004
E005
E006
E007
E008
E009
E010
E011
E012
E013
34423
35094
35766
36437
37108
37780
38451
39122
39794
40465

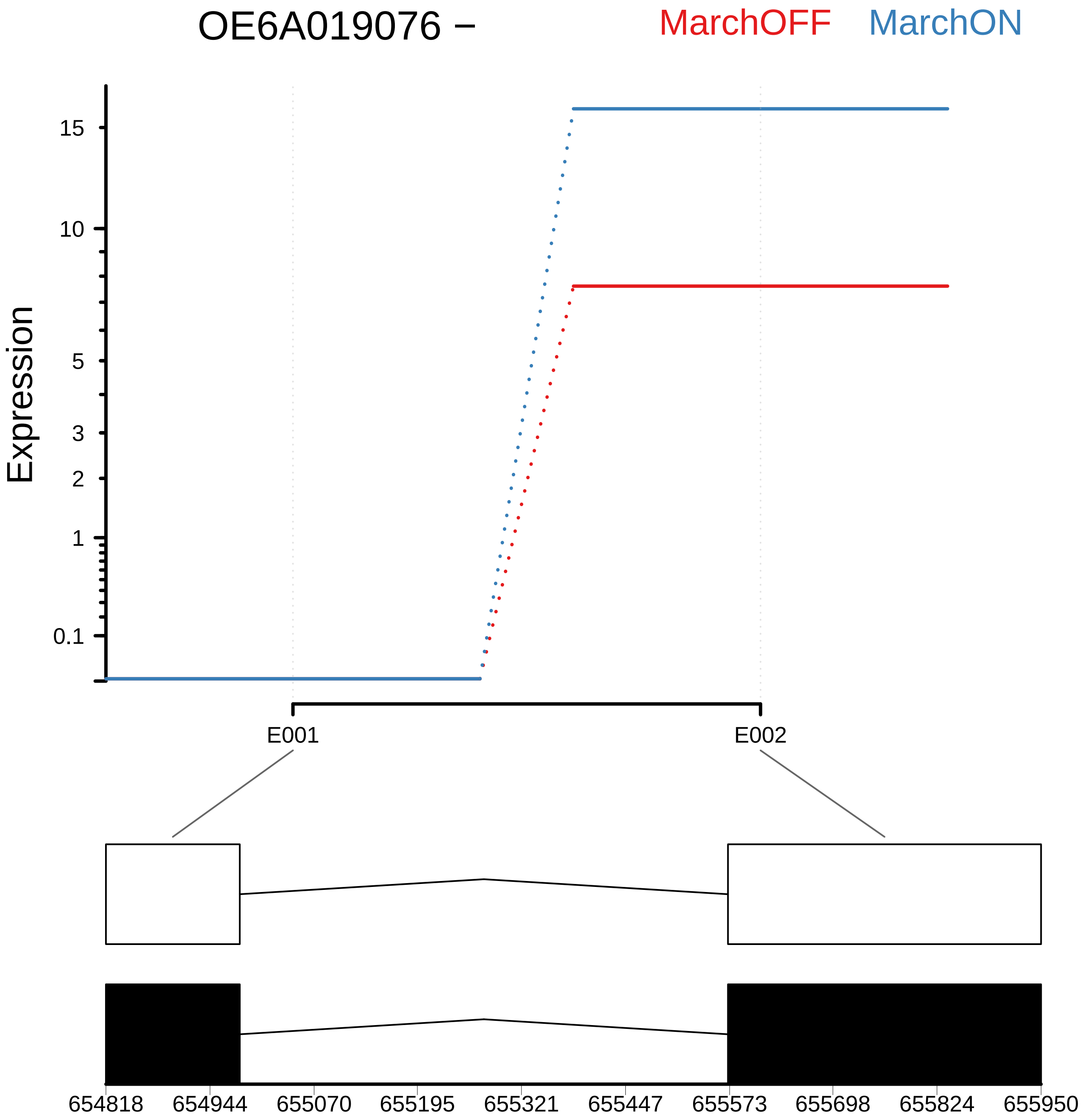
OE6A019076 −
MarchOFF
MarchON
Expression
15
10
5
3
2
1
0.1
E001
E002
654818
654944
655070
655195
655321
655447
655573
655698
655824
655950

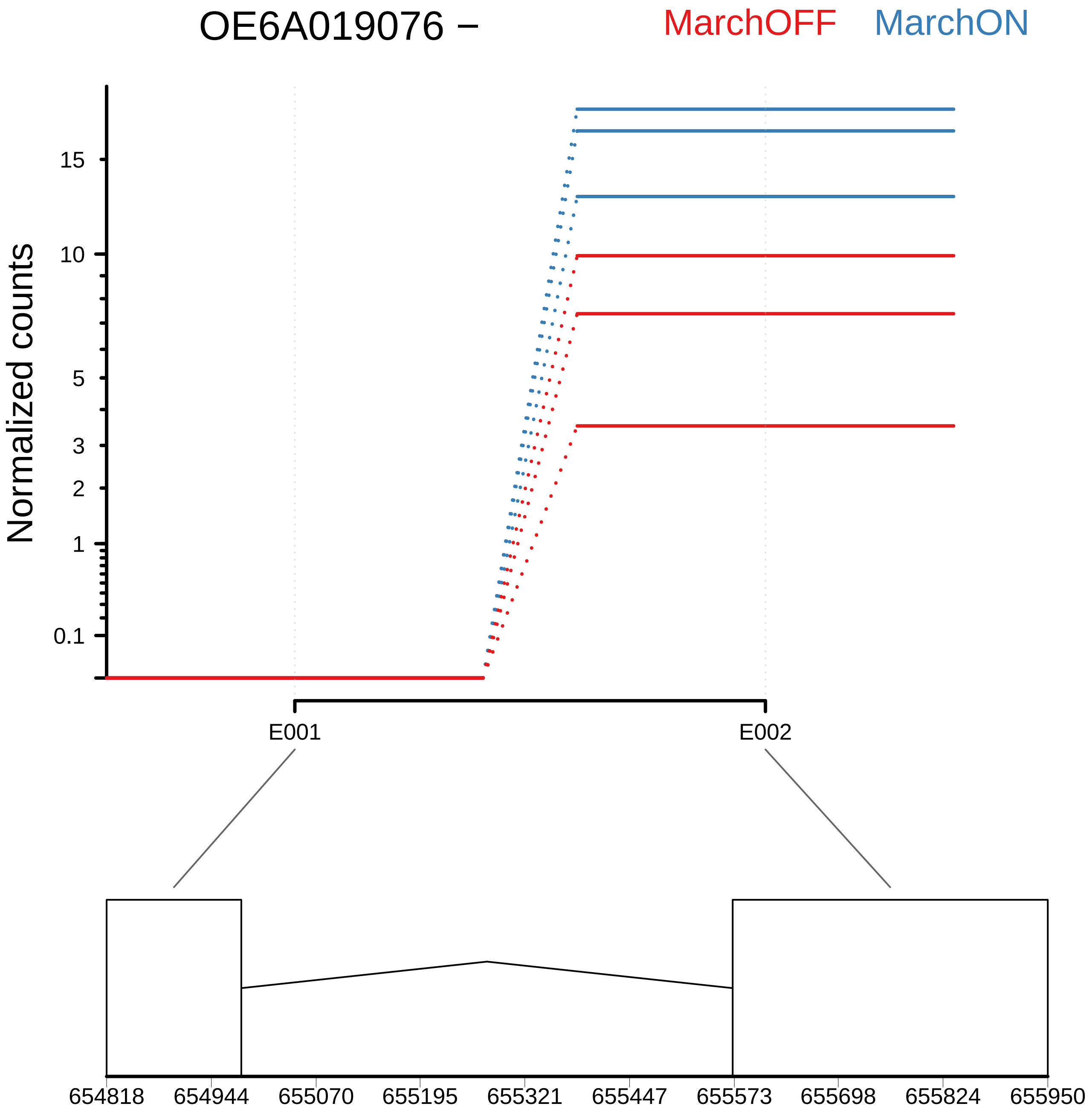

OE6A019076 −
MarchOFF
MarchON
Normalized counts
15
10
5
3
2
1
0.1
E001
E002
654818
654944
655070
655195
655321
655447
655573
655698
655824
655950

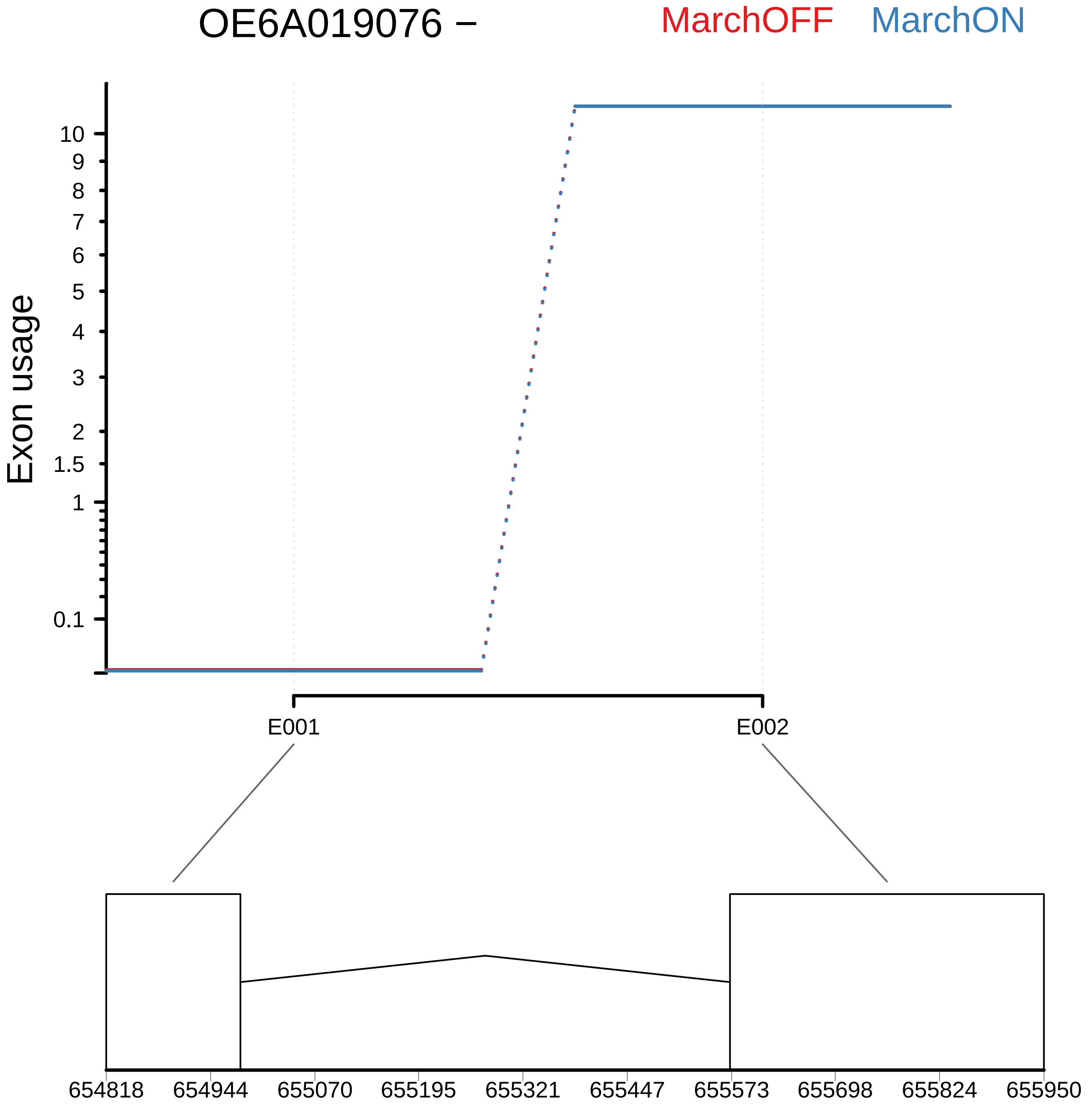

OE6A019076 −
MarchOFF
MarchON
Exon usage
10
9
8
7
6
5
4
3
2
1.5
1
0.1
E001
E002
654818
654944
655070
655195
655321
655447
655573
655698
655824
655950

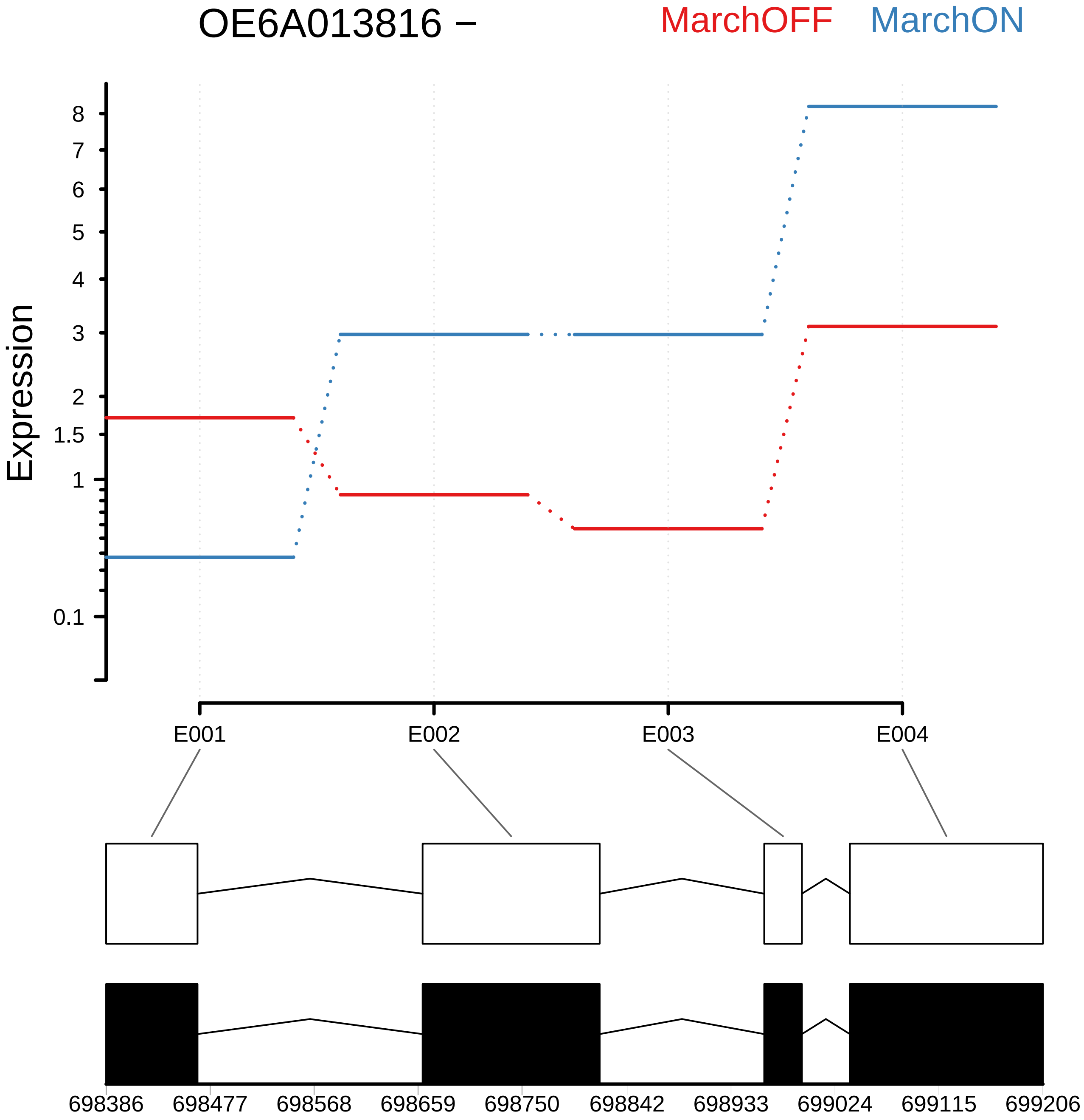

OE6A013816 −
MarchOFF
MarchON
Expression
8
7
6
5
4
3
2
1.5
1
0.1
E001
E002
E003
E004
698386
698477
698568
698659
698750
698842
698933
699024
699115
699206

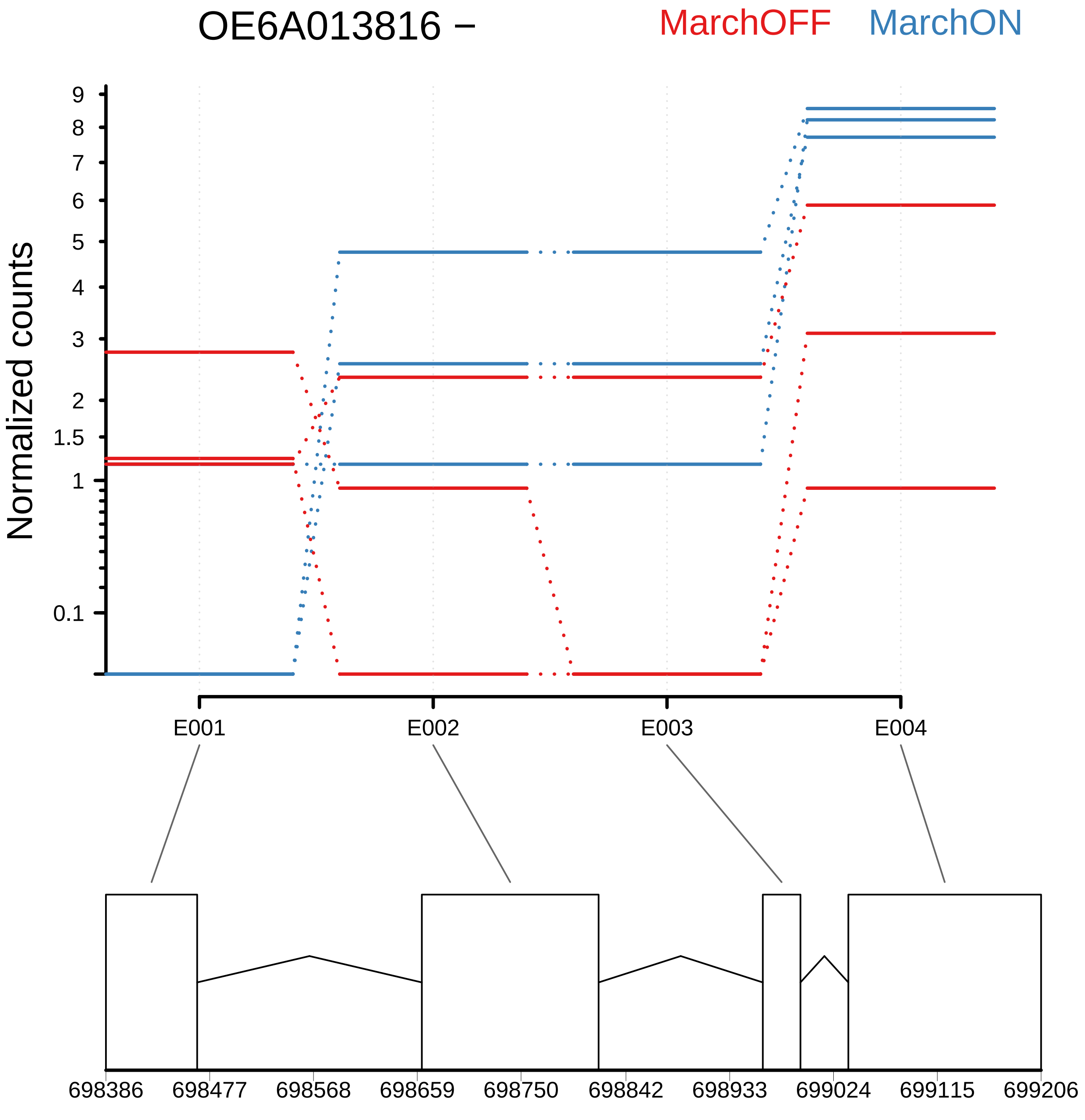

OE6A013816 −
MarchOFF
MarchON
Normalized counts
9
8
7
6
5
4
3
2
1.5
1
0.1
E001
E002
E003
E004
698386
698477
698568
698659
698750
698842
698933
699024
699115
699206

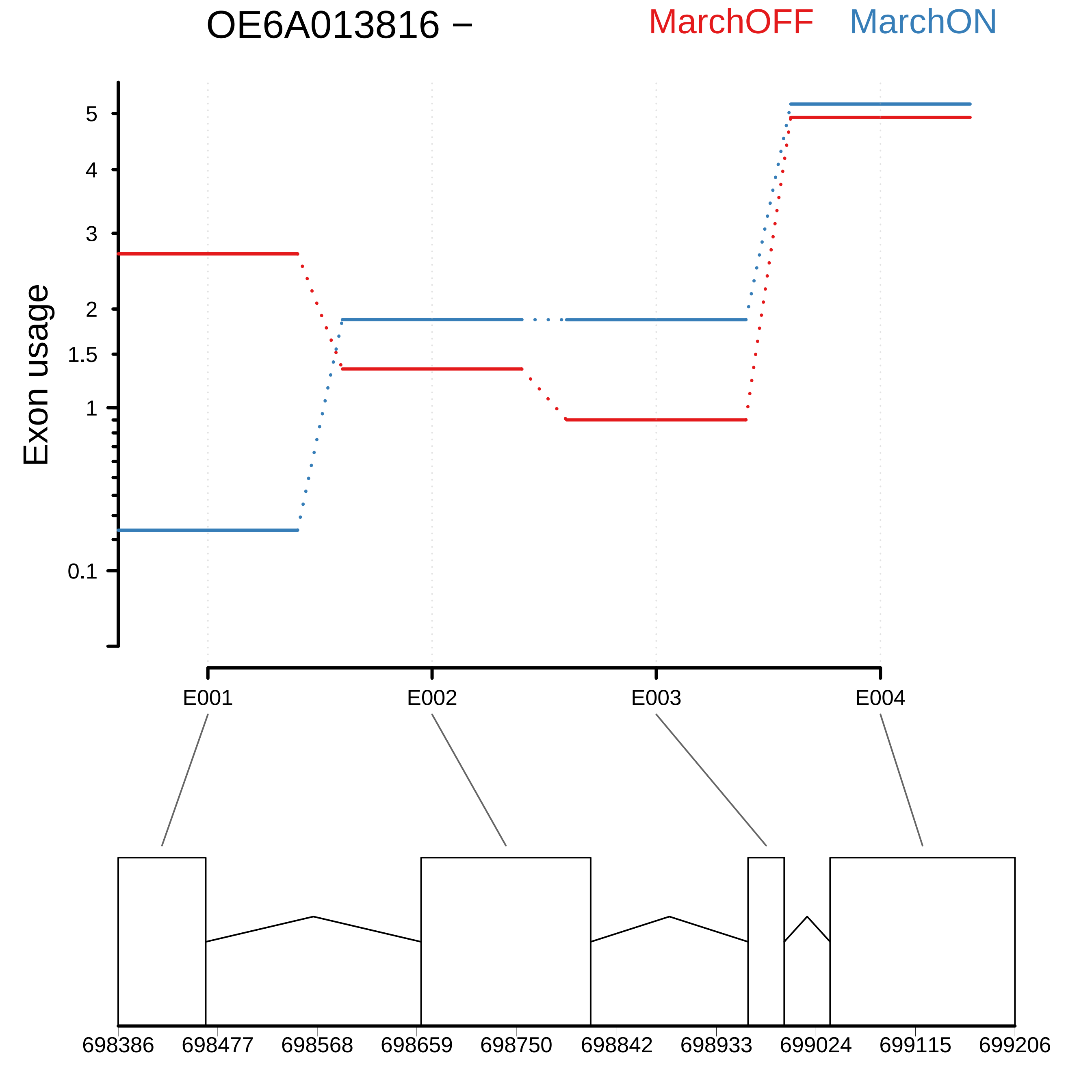

OE6A013816 −
MarchOFF
MarchON
Exon usage
5
4
3
2
1.5
1
0.1
E001
E002
E003
E004
698386
698477
698568
698659
698750
698842
698933
699024
699115
699206

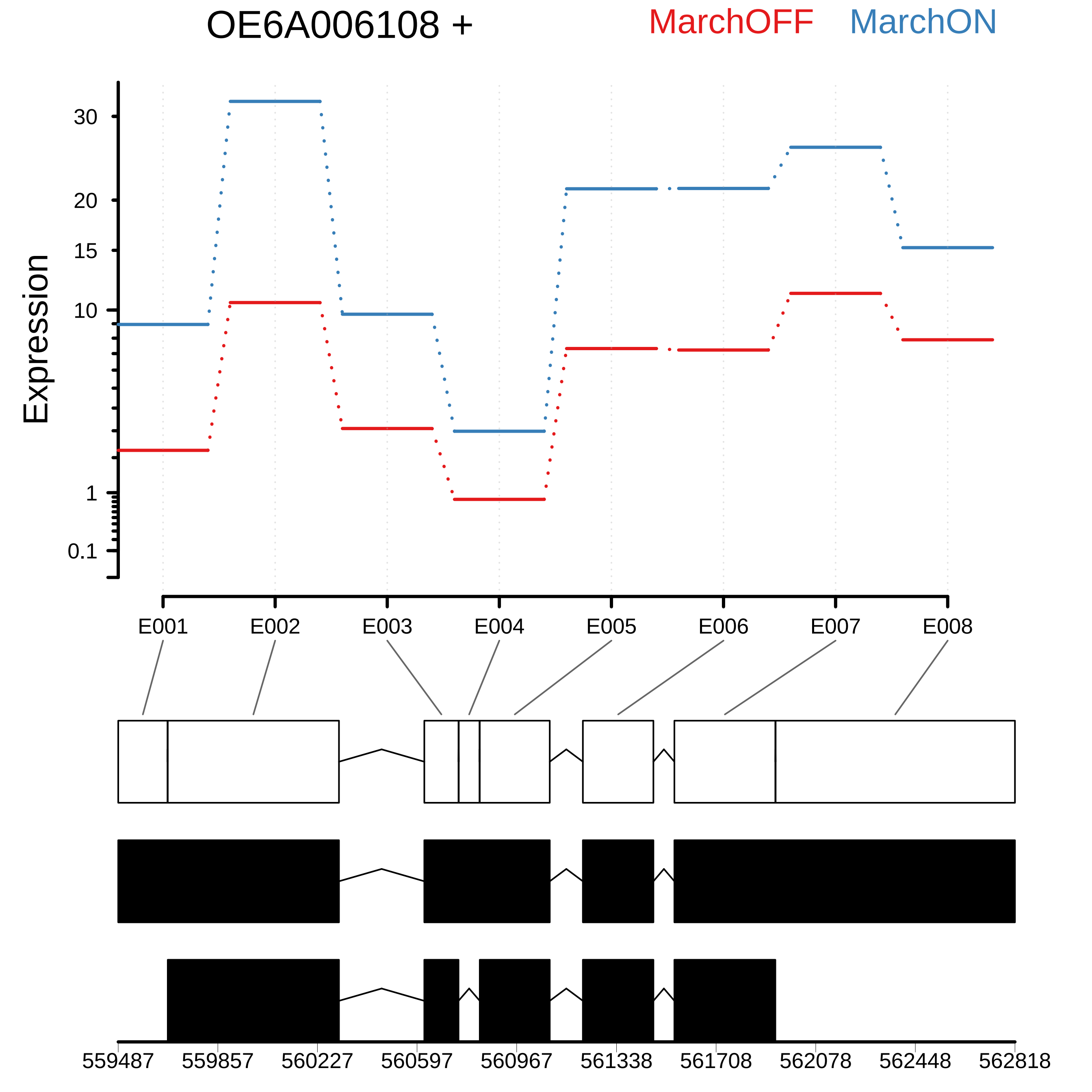

OE6A006108 +
MarchOFF
MarchON
Expression
30
20
15
10
1
0.1
E001
E002
E003
E004
E005
E006
E007
E008
559487
559857
560227
560597
560967
561338
561708
562078
562448
562818

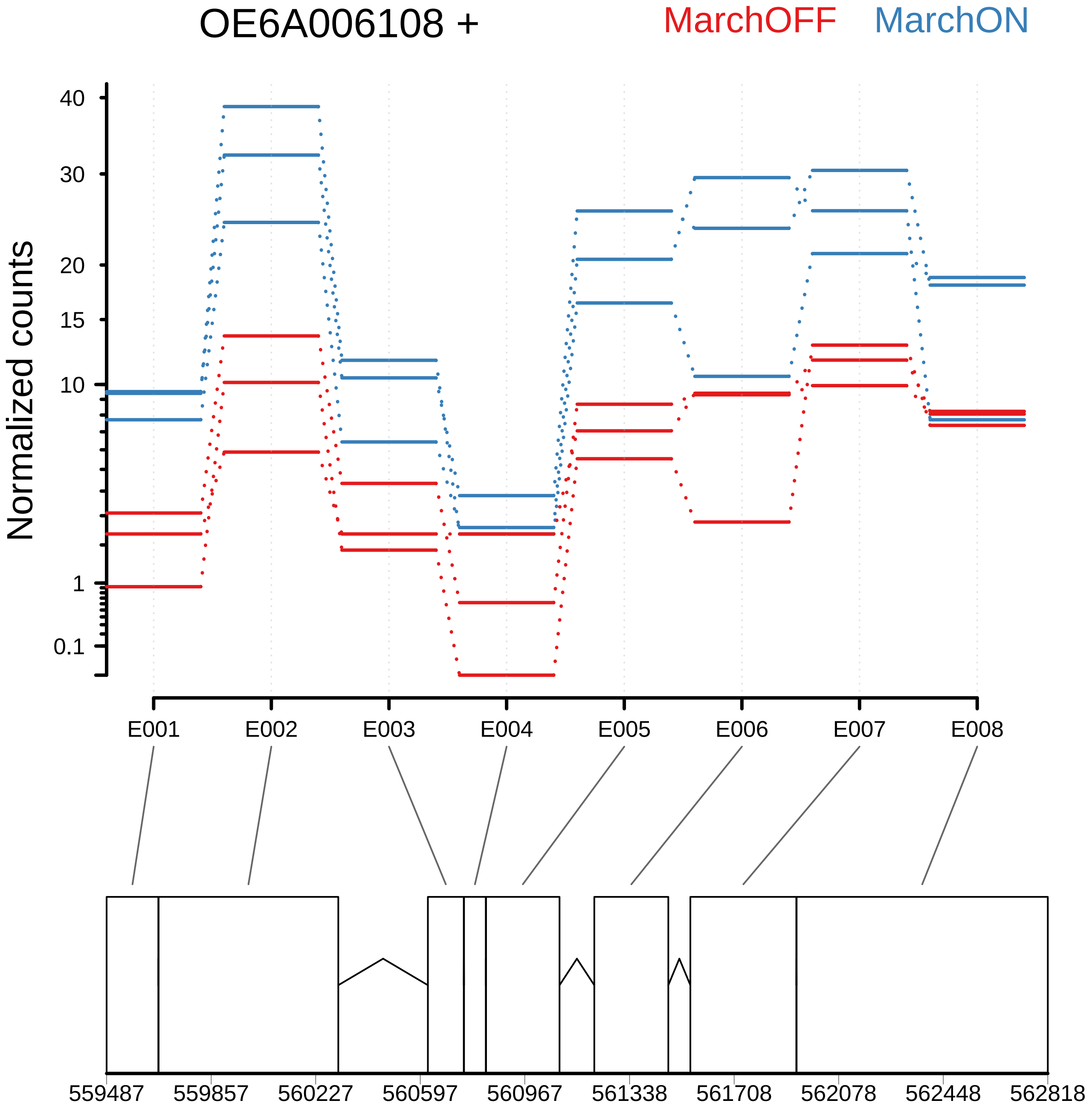

OE6A006108 +
MarchOFF
MarchON
Normalized counts
40
30
20
15
10
1
0.1
E001
E002
E003
E004
E005
E006
E007
E008
559487
559857
560227
560597
560967
561338
561708
562078
562448
562818

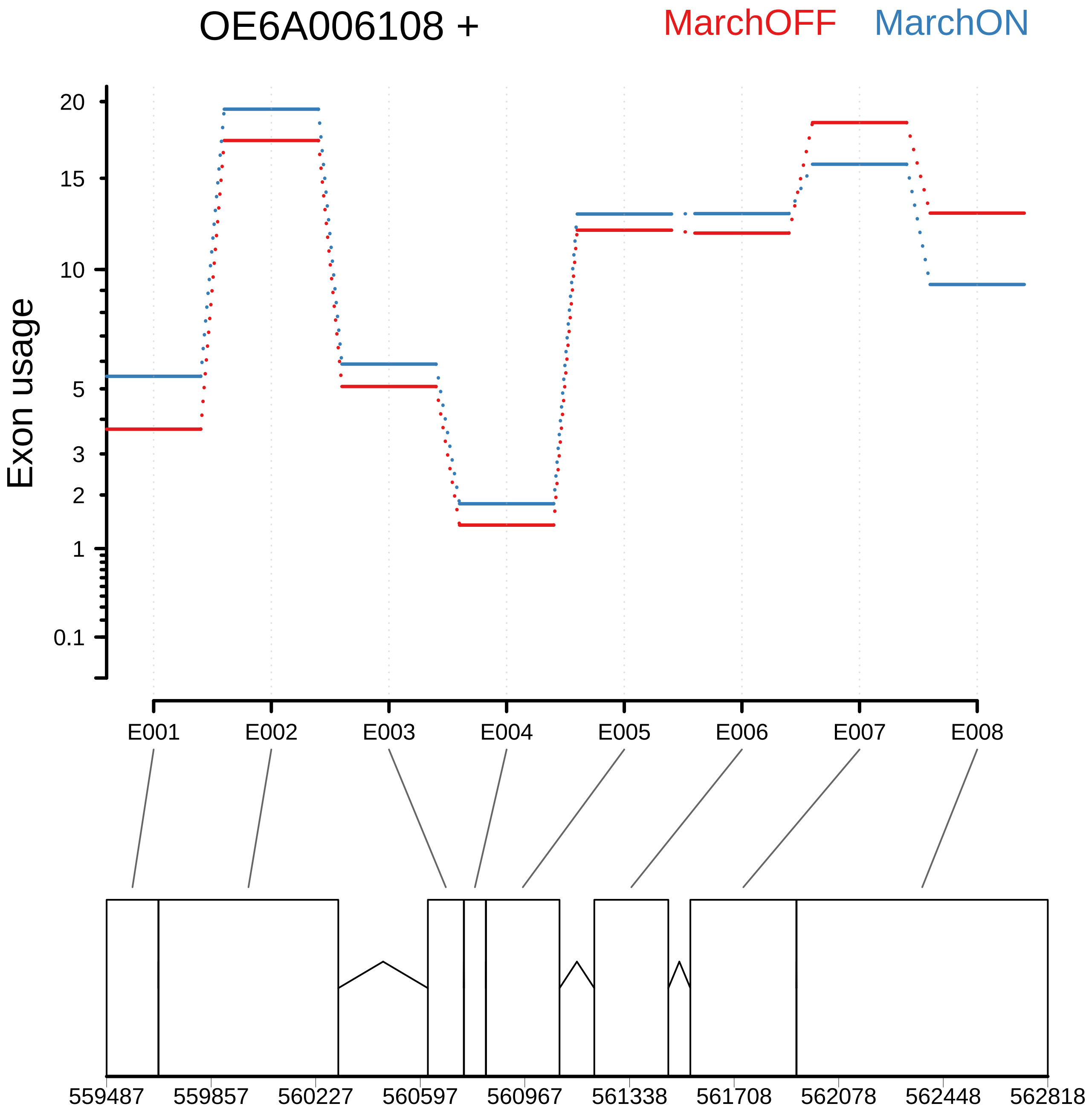
OE6A006108 +
MarchOFF
MarchON
Exon usage
20
15
10
5
3
2
1
0.1
E001
E002
E003
E004
E005
E006
E007
E008
559487
559857
560227
560597
560967
561338
561708
562078
562448
562818

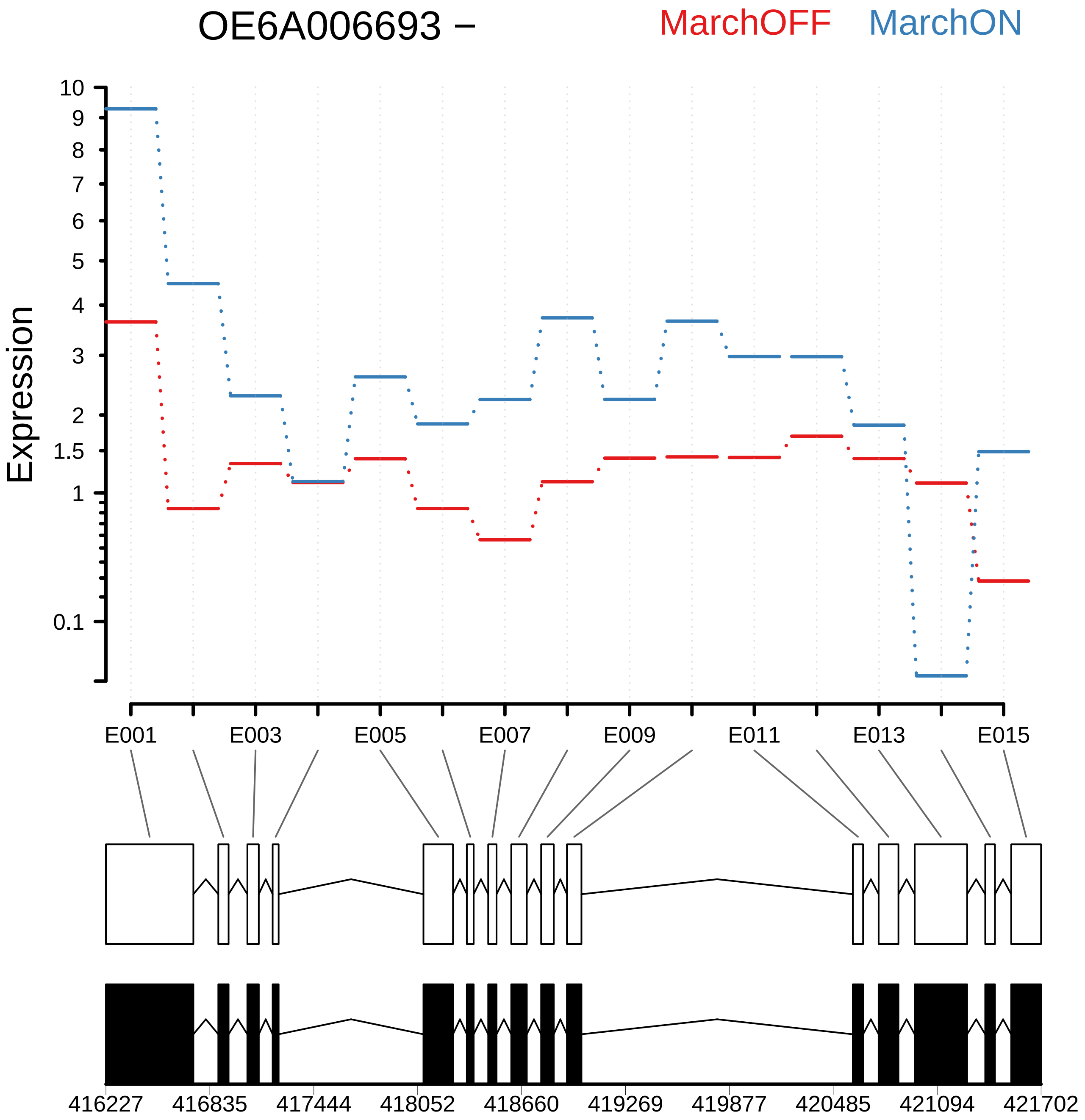

OE6A006693 −
MarchOFF
MarchON
Expression
10
9
8
7
6
5
4
3
2
1.5
1
0.1
E001
E003
E005
E007
E009
E011
E013
E015
416227
416835
417444
418052
418660
419269
419877
420485
421094
421702

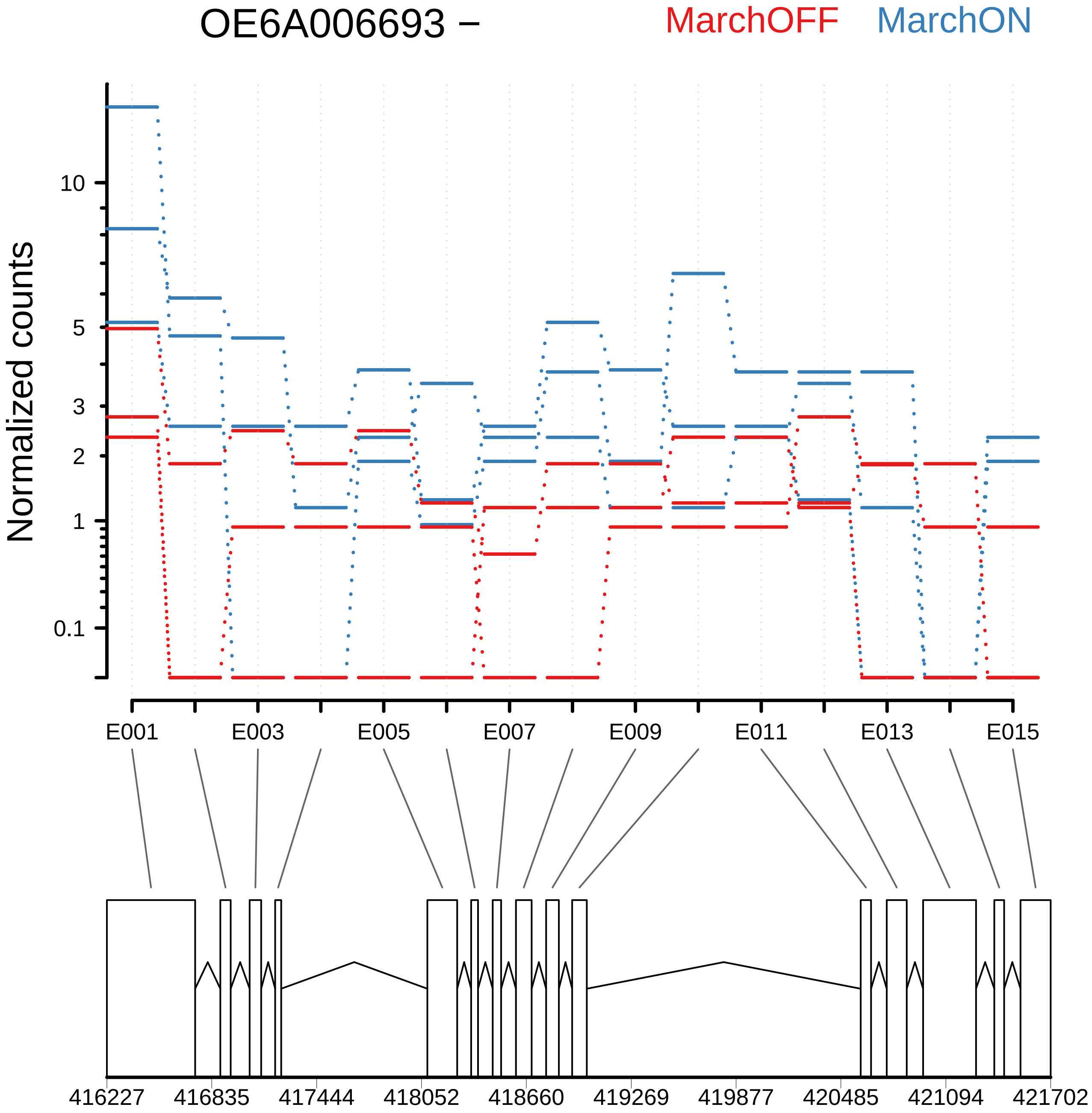

OE6A006693 −
MarchOFF
MarchON
Normalized counts
10
5
3
2
1
0.1
E001
E003
E005
E007
E009
E011
E013
E015
416227
416835
417444
418052
418660
419269
419877
420485
421094
421702

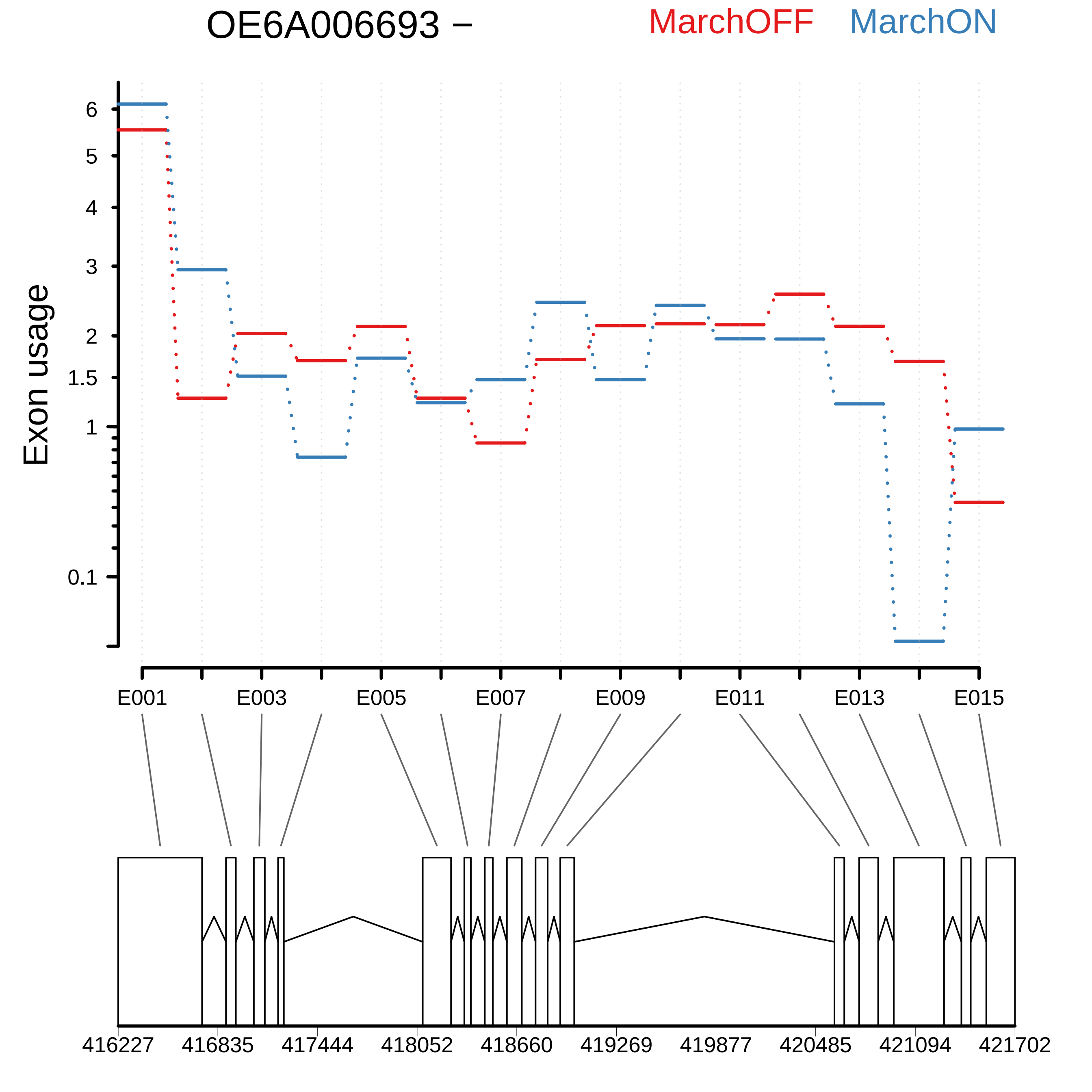

OE6A006693 −
MarchOFF
MarchON
Exon usage
6
5
4
3
2
1.5
1
0.1
E001
E003
E005
E007
E009
E011
E013
E015
416227
416835
417444
418052
418660
419269
419877
420485
421094
421702

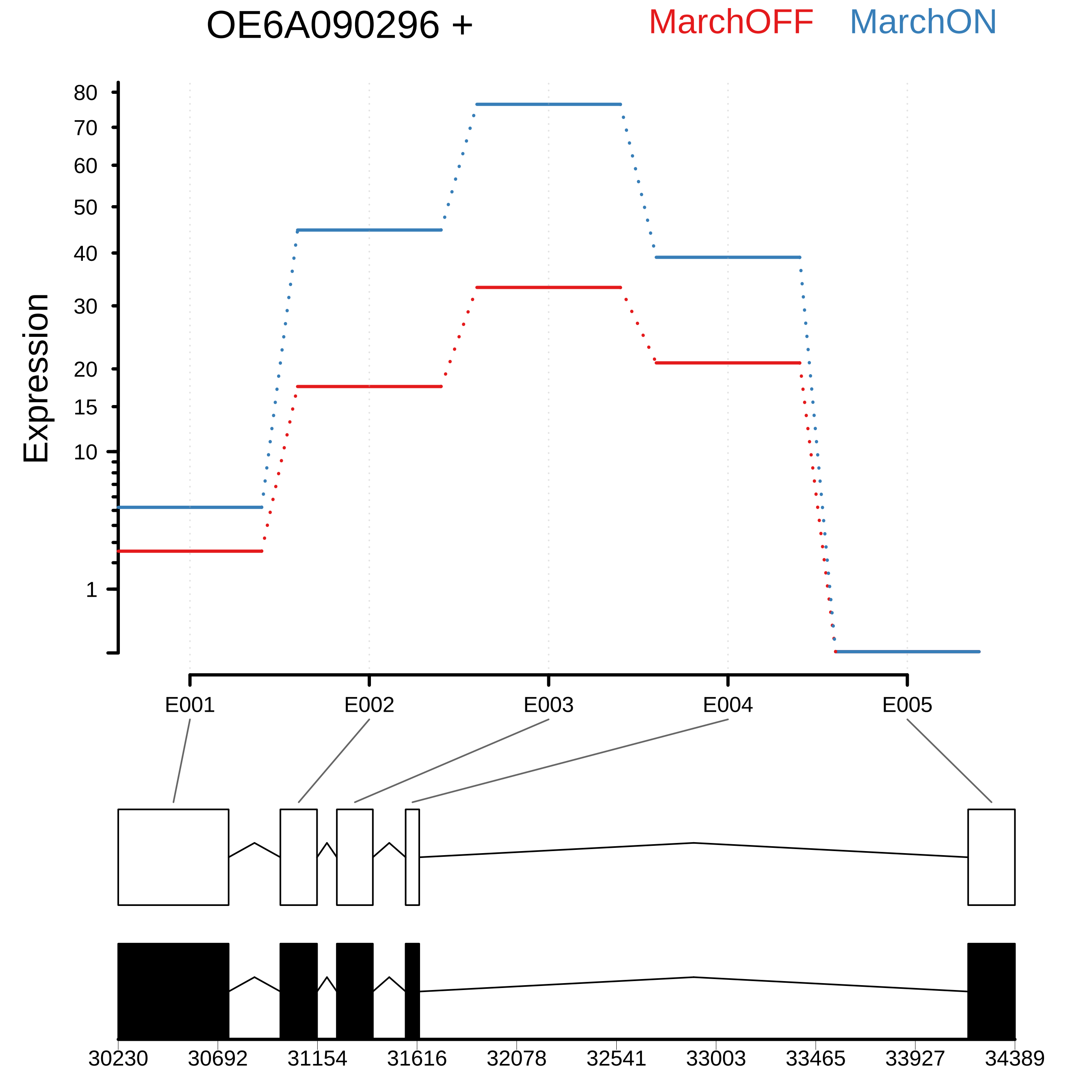

OE6A090296 +
MarchOFF
MarchON
Expression
80
70
60
50
40
30
20
15
10
1
E001
E002
E003
E004
E005
30230
30692
31154
31616
32078
32541
33003
33465
33927
34389

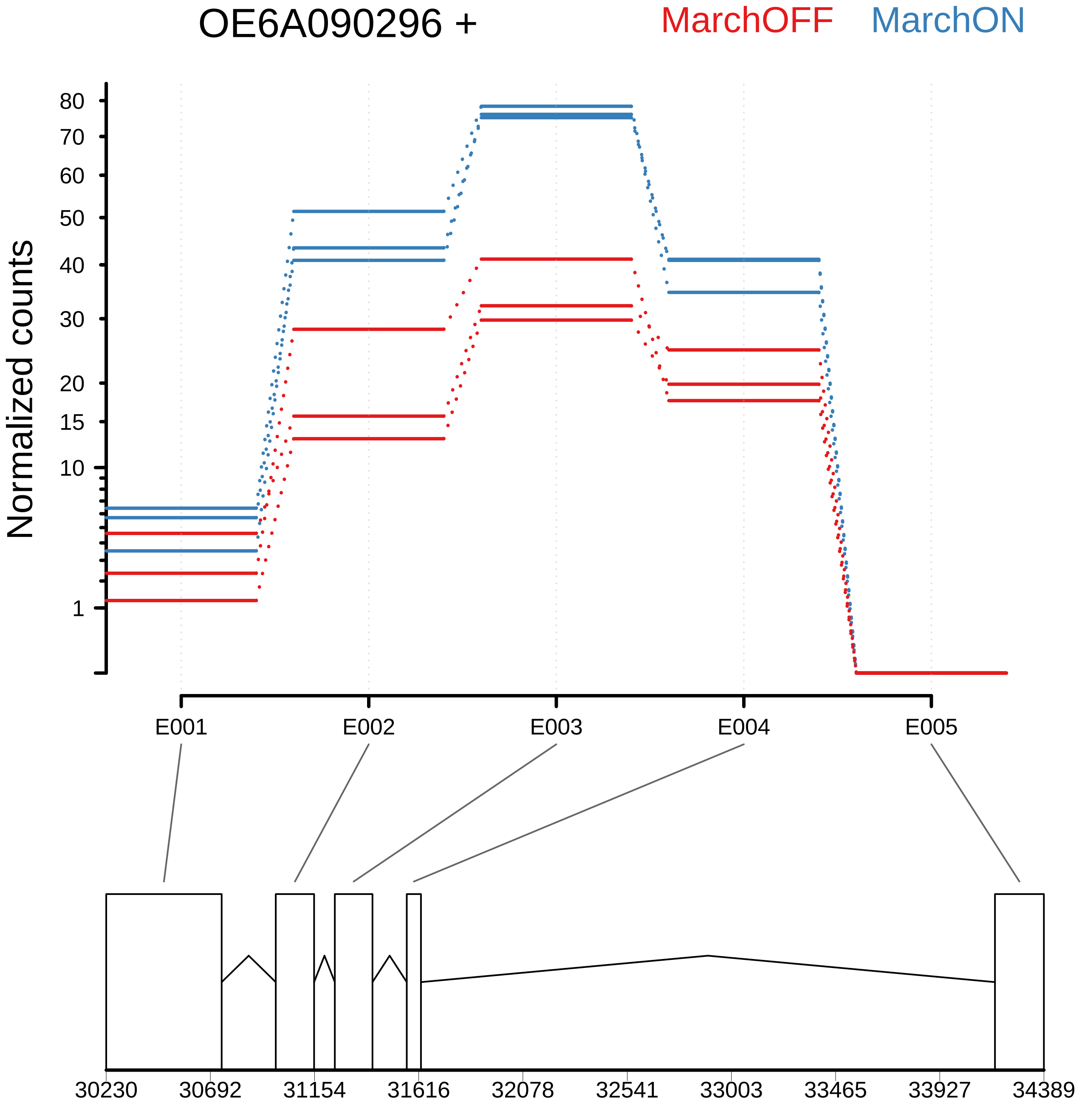

OE6A090296 +
MarchOFF
MarchON
Normalized counts
80
70
60
50
40
30
20
15
10
1
E001
E002
E003
E004
E005
30230
30692
31154
31616
32078
32541
33003
33465
33927
34389

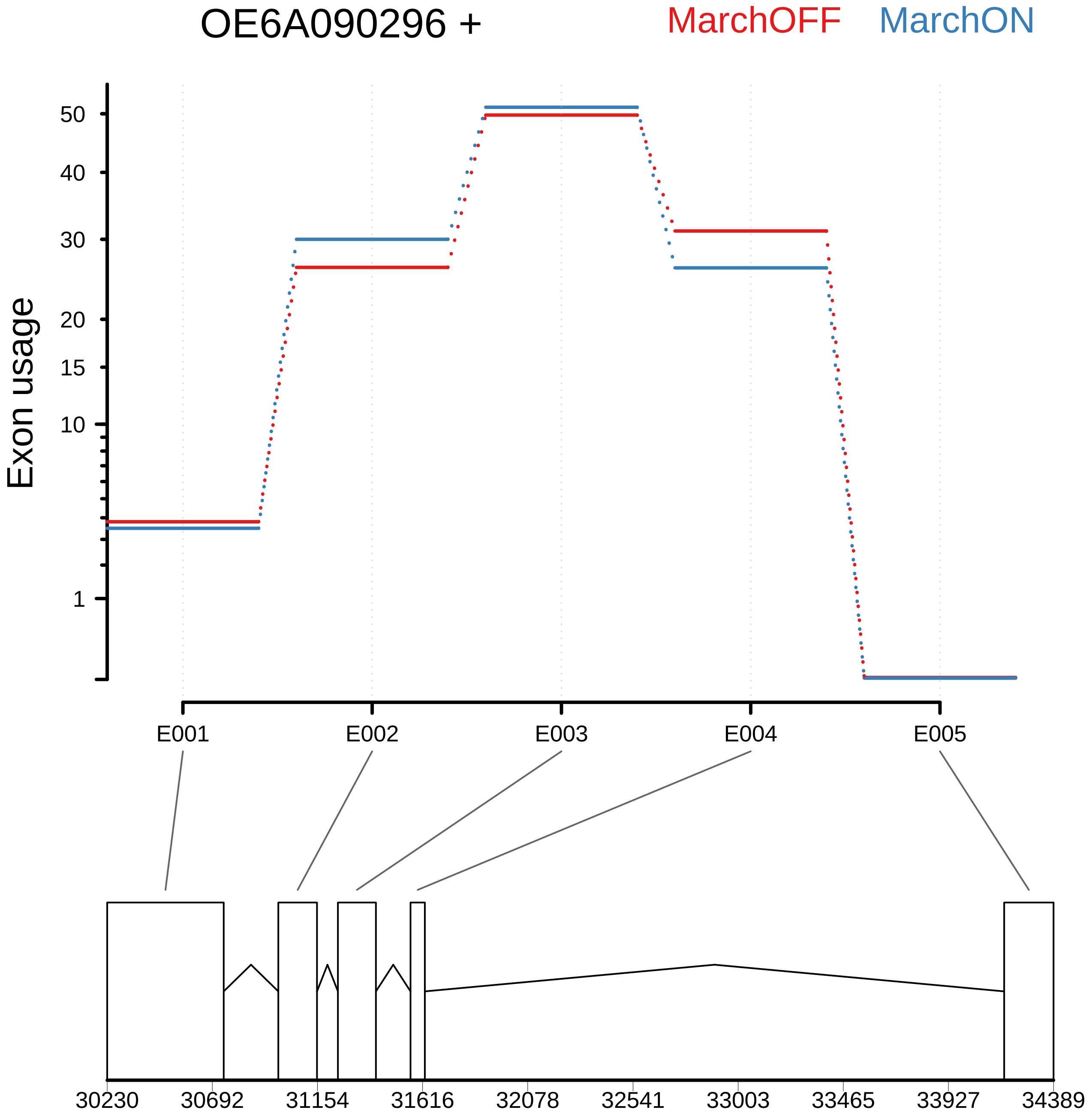
OE6A090296 +
MarchOFF
MarchON
Exon usage
50
40
30
20
15
10
1
E001
E002
E003
E004
E005
30230
30692
31154
31616
32078
32541
33003
33465
33927
34389

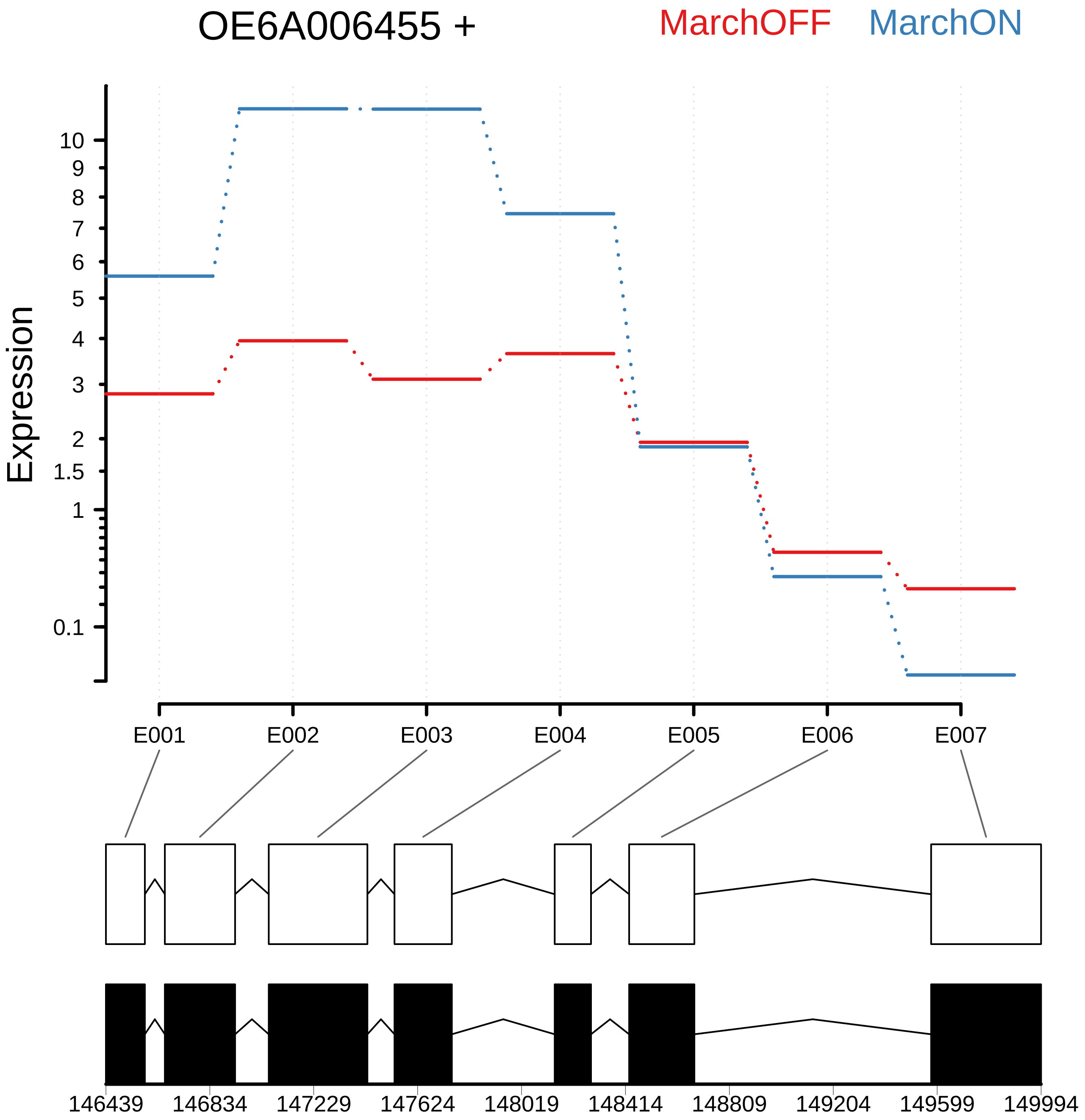

OE6A006455 +
MarchOFF
MarchON
Expression
10
9
8
7
6
5
4
3
2
1.5
1
0.1
E001
E002
E003
E004
E005
E006
E007
146439
146834
147229
147624
148019
148414
148809
149204
149599
149994

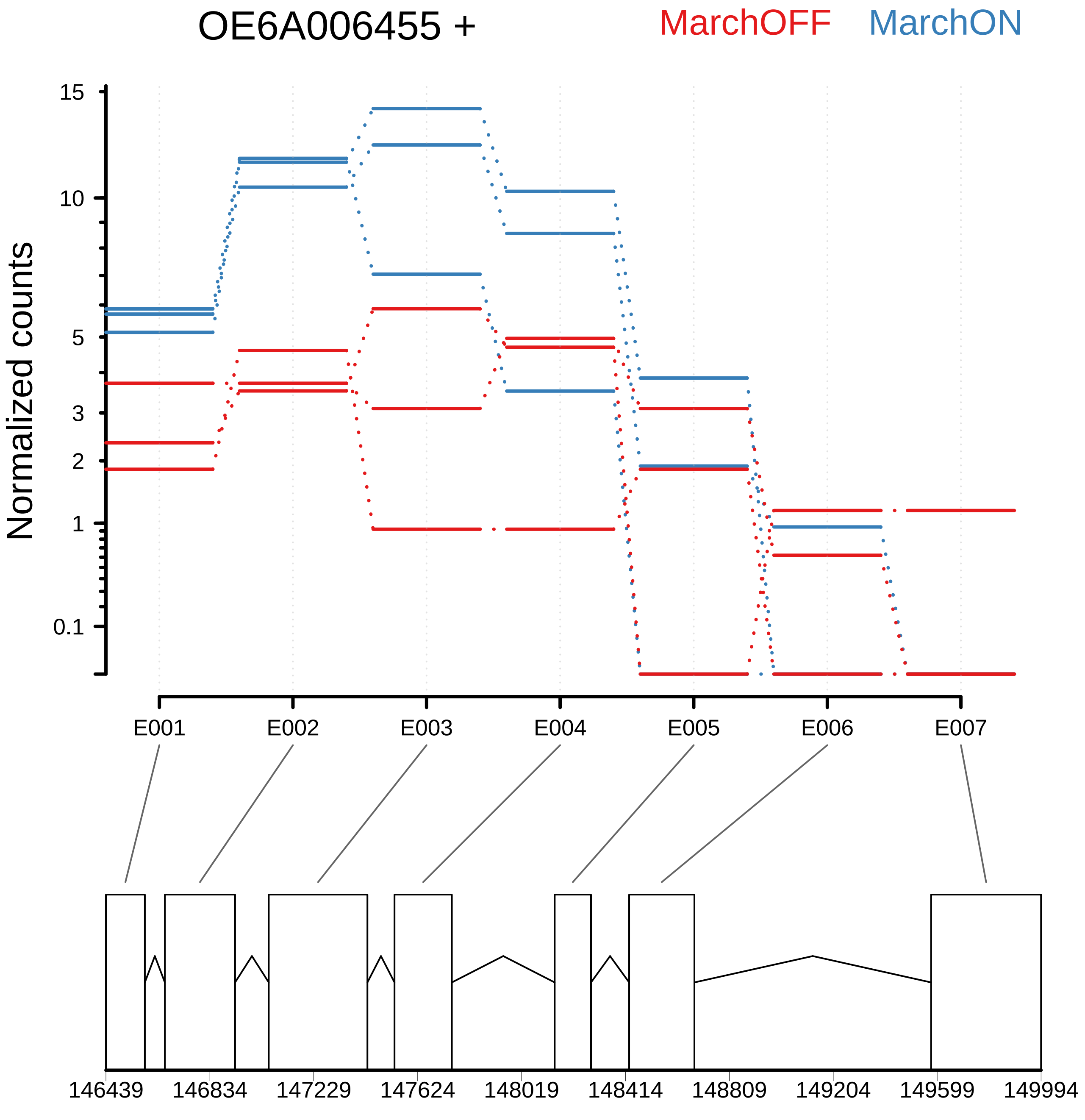

OE6A006455 +
MarchOFF
MarchON
Normalized counts
15
10
5
3
2
1
0.1
E001
E002
E003
E004
E005
E006
E007
146439
146834
147229
147624
148019
148414
148809
149204
149599
149994

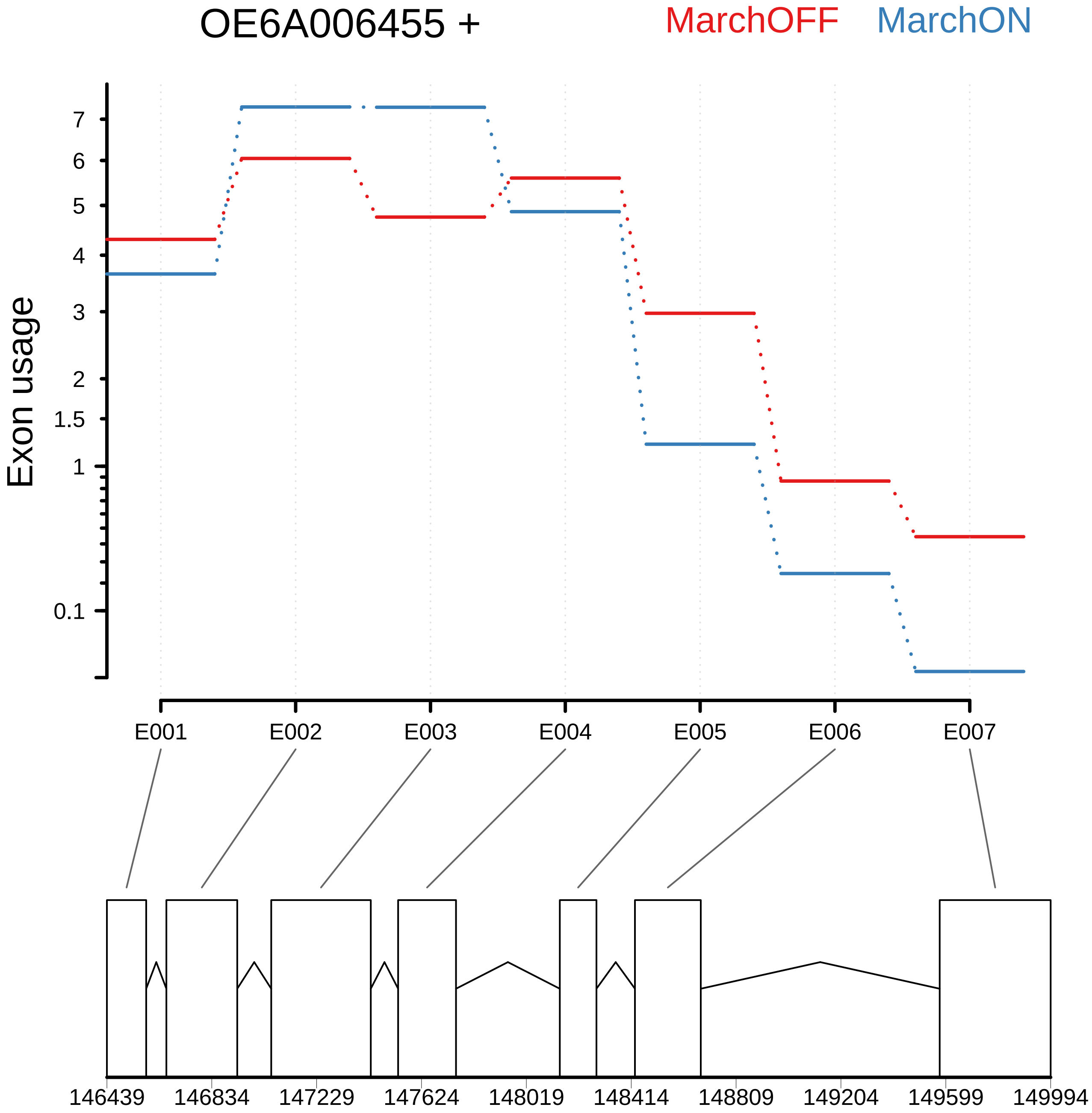

OE6A006455 +
MarchOFF
MarchON
Exon usage
7
6
5
4
3
2
1.5
1
0.1
E001
E002
E003
E004
E005
E006
E007
146439
146834
147229
147624
148019
148414
148809
149204
149599
149994

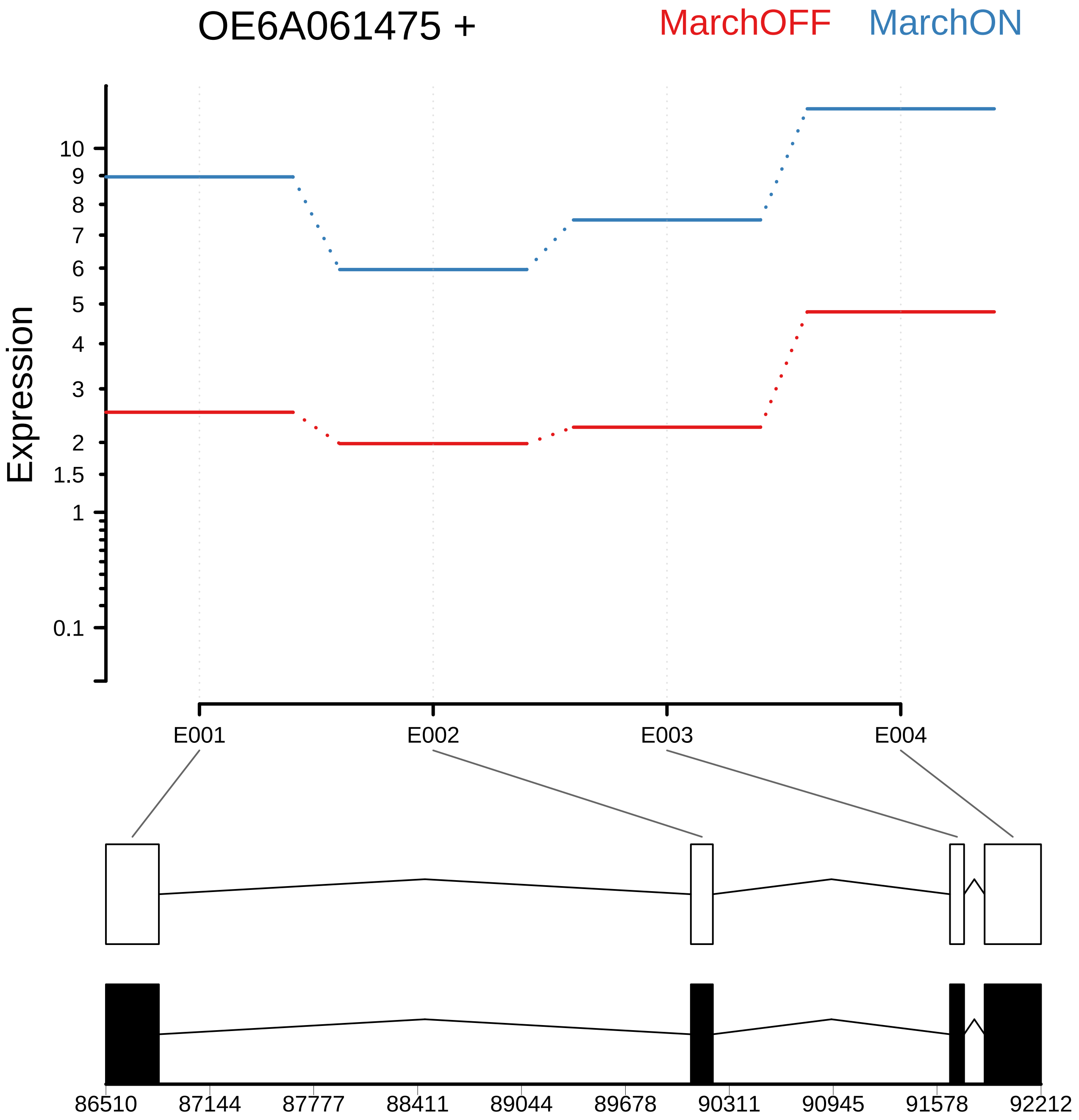
OE6A061475 +
MarchOFF
MarchON
Expression
10
9
8
7
6
5
4
3
2
1.5
1
0.1
E001
E002
E003
E004
86510
87144
87777
88411
89044
89678
90311
90945
91578
92212

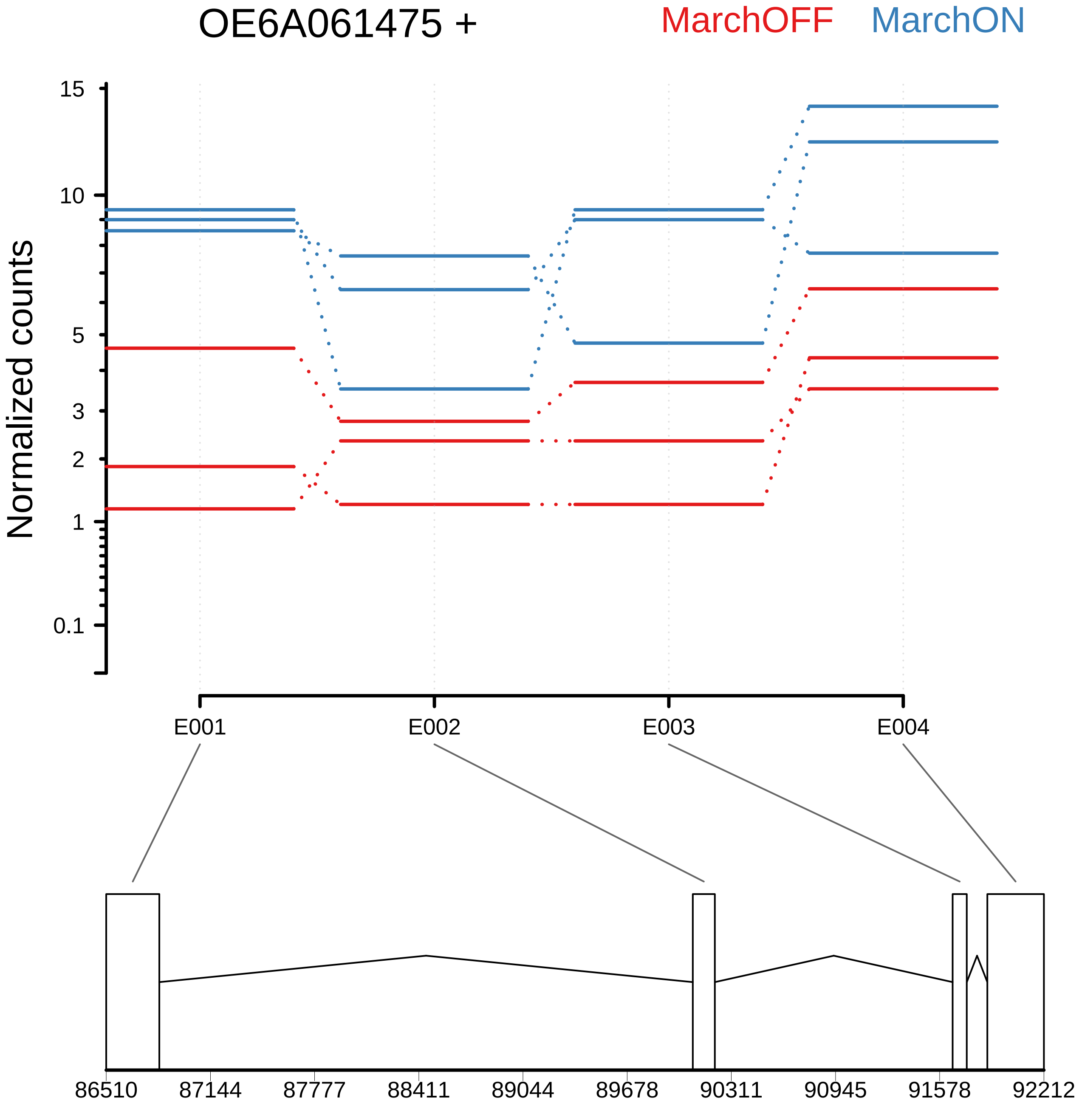

OE6A061475 +
MarchOFF
MarchON
Normalized counts
15
10
5
3
2
1
0.1
E001
E002
E003
E004
86510
87144
87777
88411
89044
89678
90311
90945
91578
92212

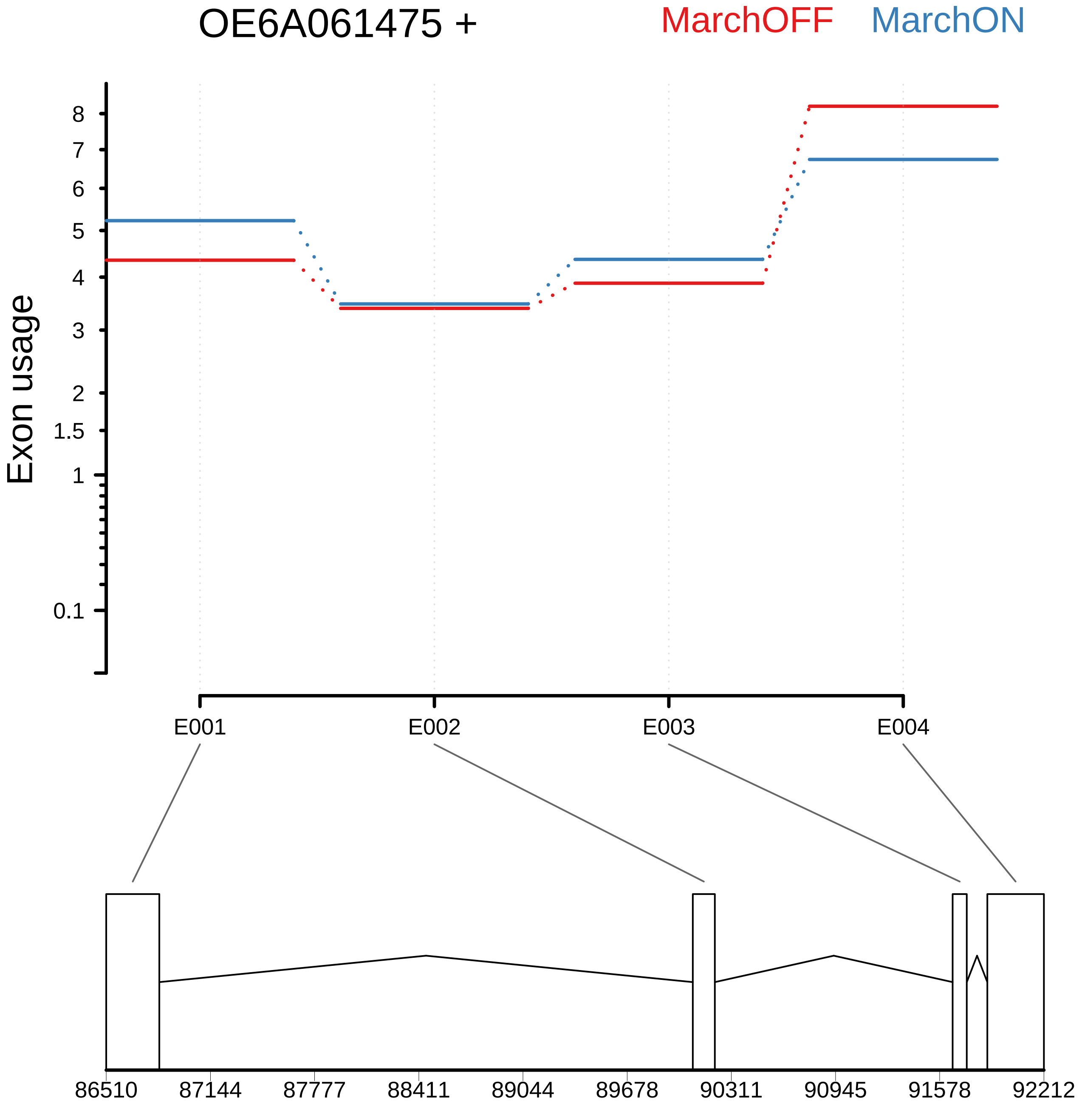

OE6A061475 +
MarchOFF
MarchON
Exon usage
8
7
6
5
4
3
2
1.5
1
0.1
E001
E002
E003
E004
86510
87144
87777
88411
89044
89678
90311
90945
91578
92212

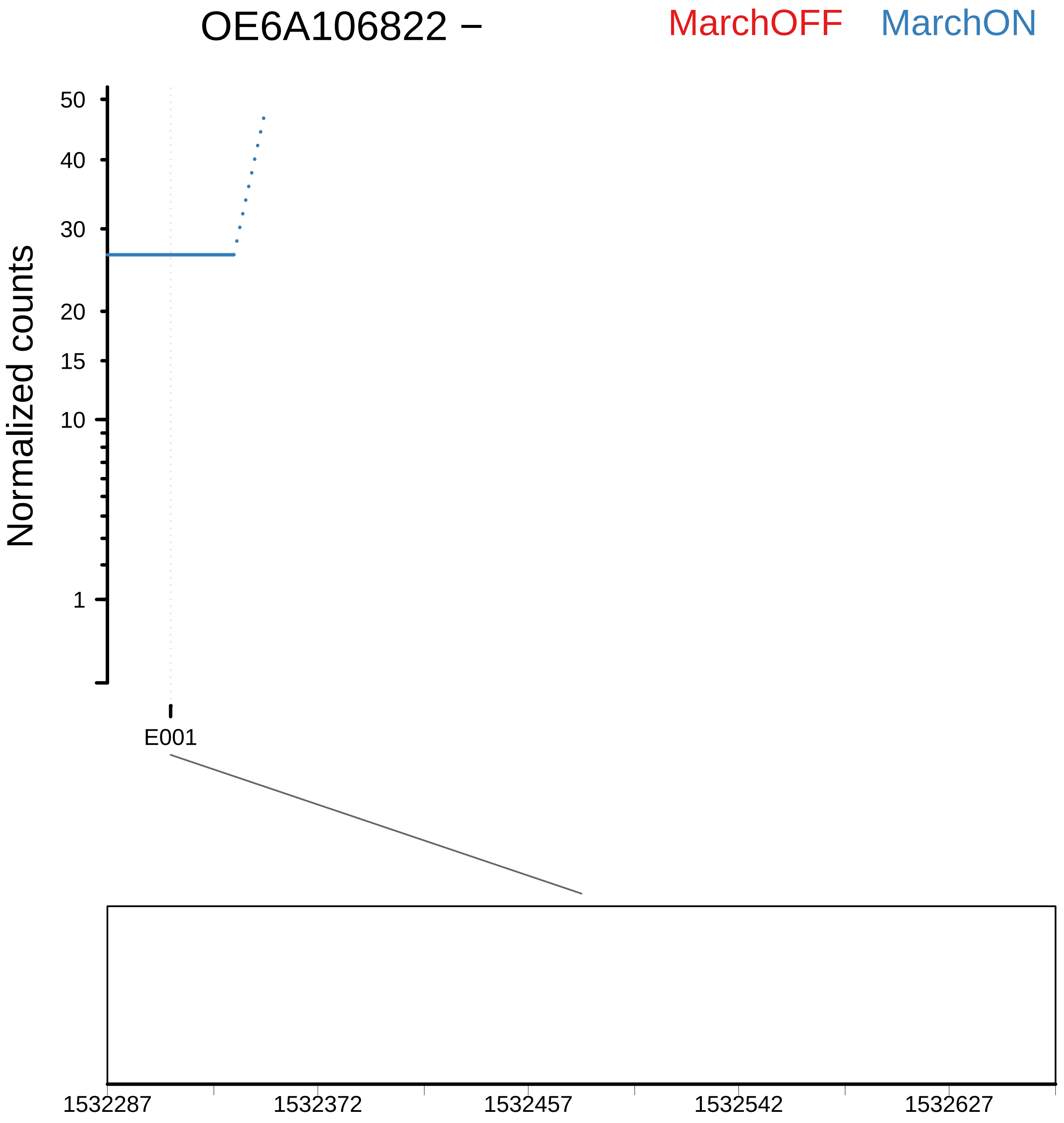
OE6A106822 −
MarchOFF
MarchON
Normalized counts
50
40
30
20
15
10
1
E001
1532287
1532372
1532457
1532542
1532627

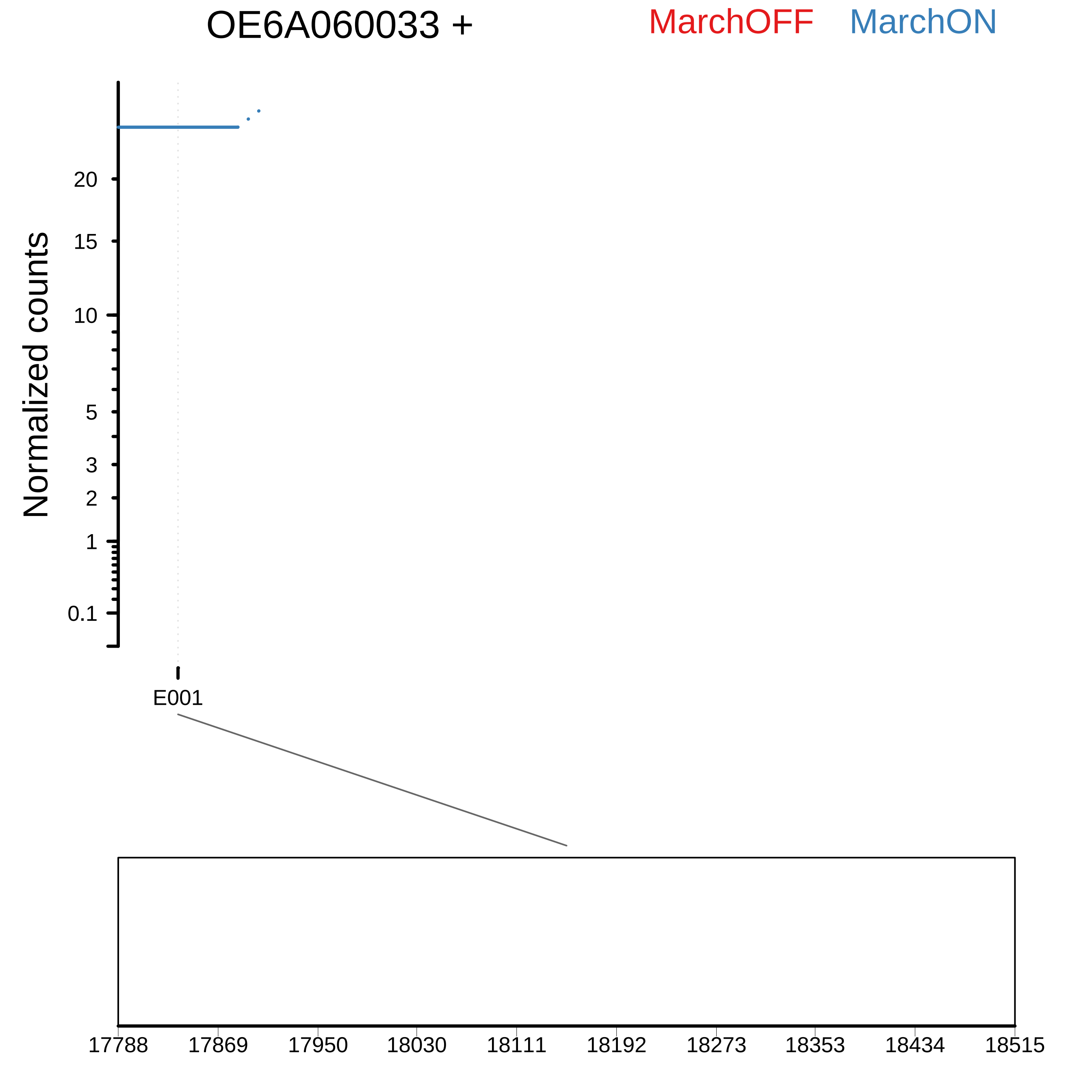
OE6A060033 +
MarchOFF
MarchON
Normalized counts
20
15
10
5
3
2
1
0.1
E001
17788
17869
17950
18030
18111
18192
18273
18353
18434
18515

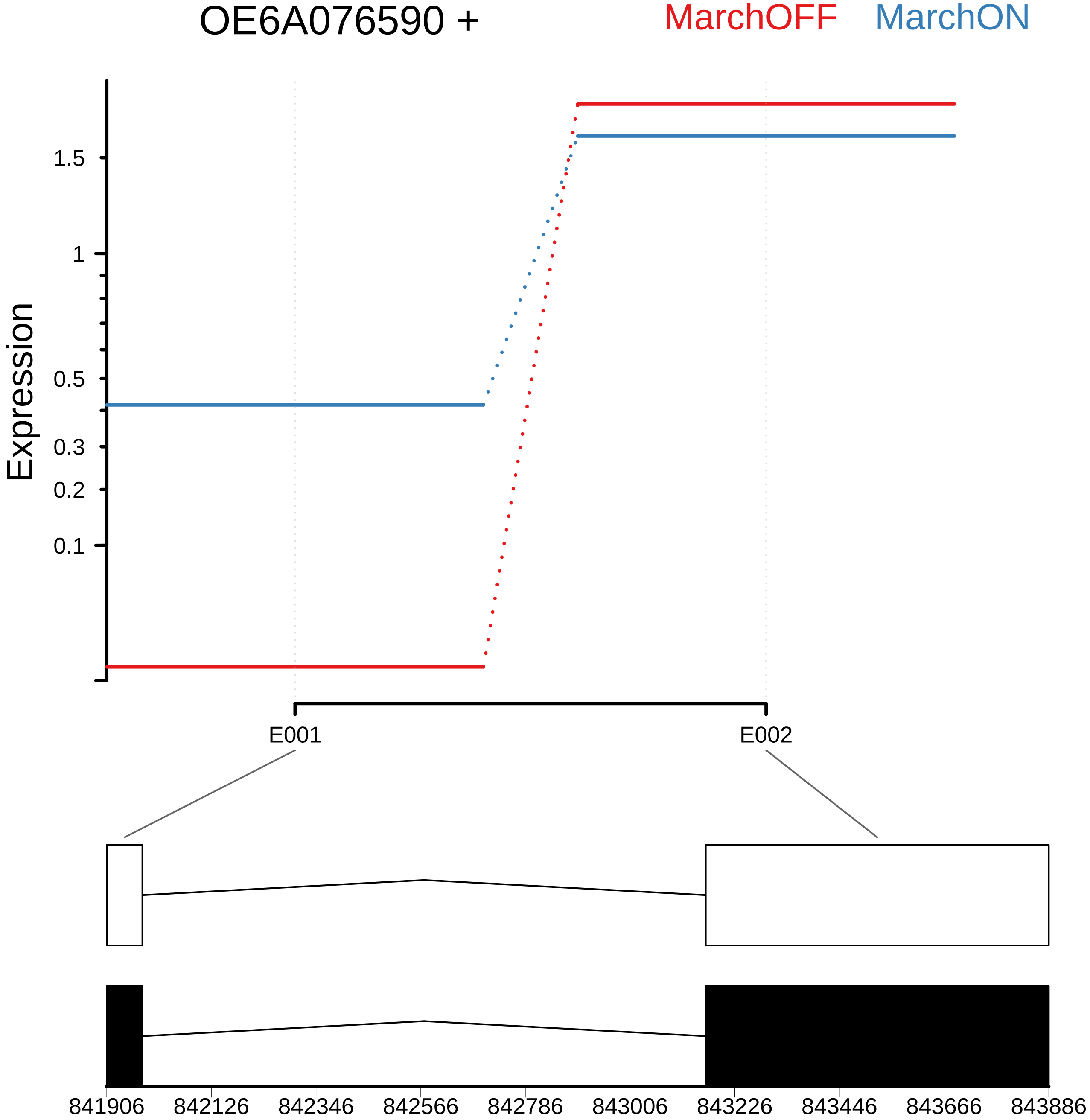

OE6A076590 +
MarchOFF
MarchON
Expression
1.5
1
0.5
0.3
0.2
0.1
E001
E002
841906
842126
842346
842566
842786
843006
843226
843446
843666
843886

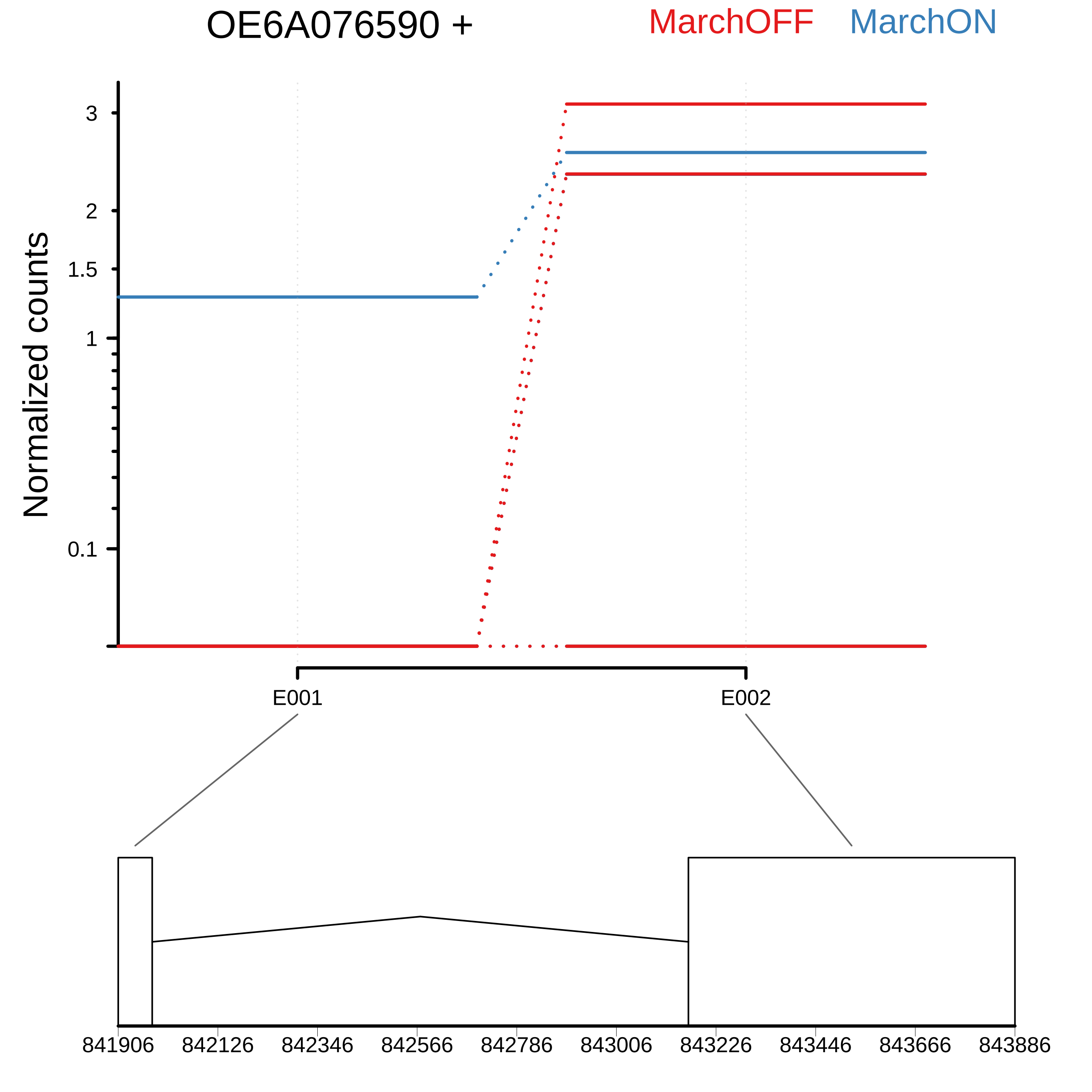
OE6A076590 +
MarchOFF
MarchON
Normalized counts
3
2
1.5
1
0.1
E001
E002
841906
842126
842346
842566
842786
843006
843226
843446
843666
843886

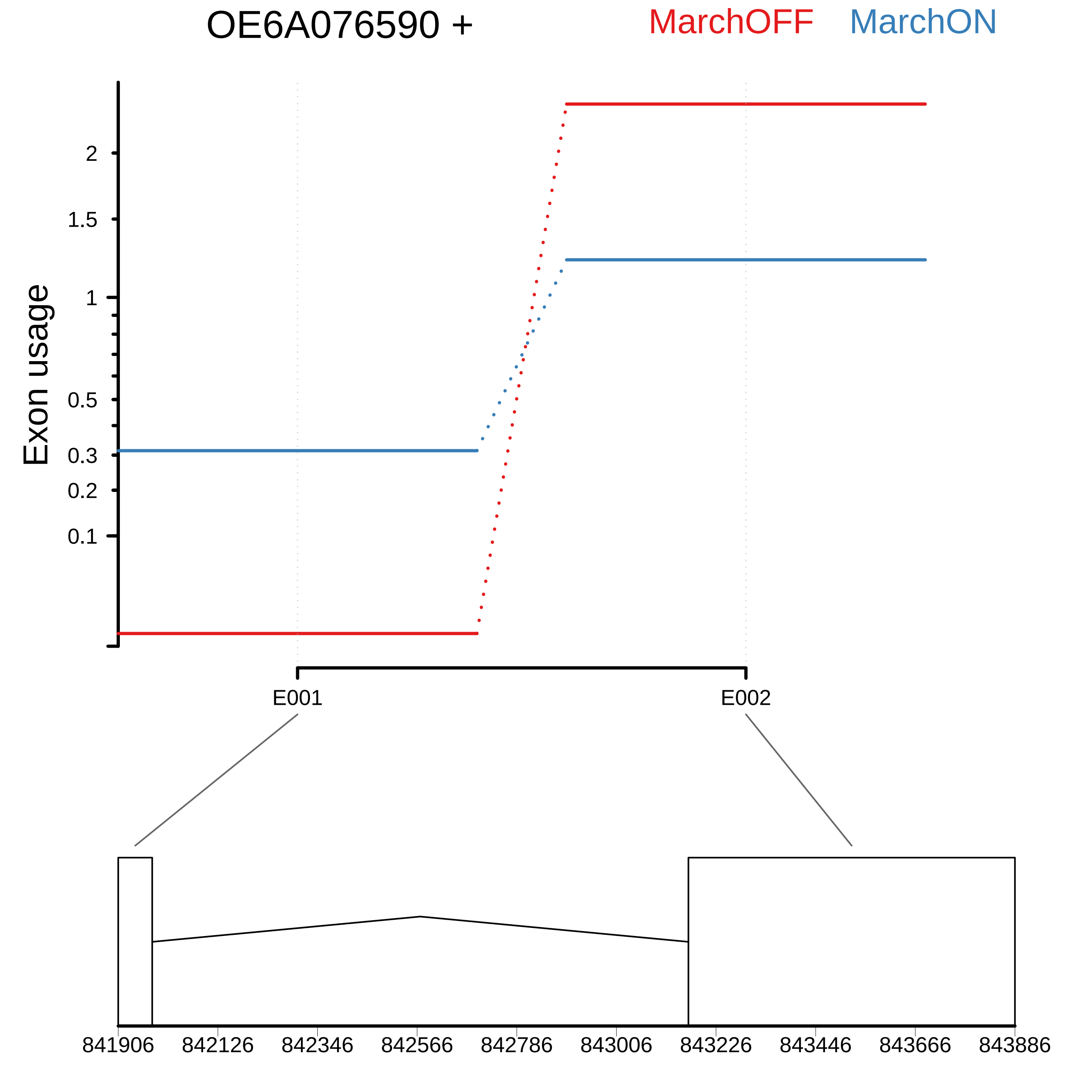

OE6A076590 +
MarchOFF
MarchON
Exon usage
2
1.5
1
0.5
0.3
0.2
0.1
E001
E002
841906
842126
842346
842566
842786
843006
843226
843446
843666
843886

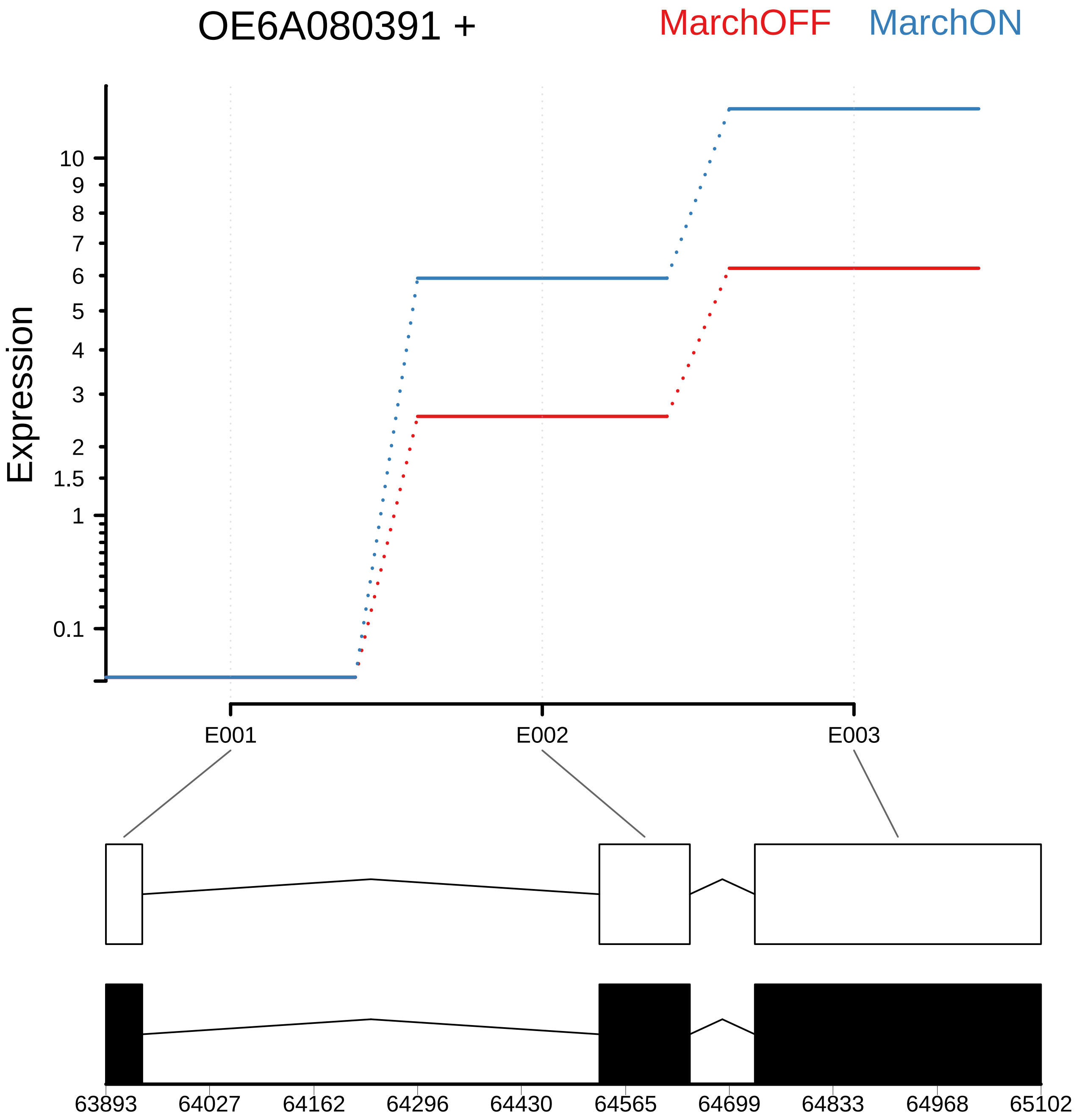
OE6A080391 +
MarchOFF
MarchON
Expression
10
9
8
7
6
5
4
3
2
1.5
1
0.1
E001
E002
E003
63893
64027
64162
64296
64430
64565
64699
64833
64968
65102

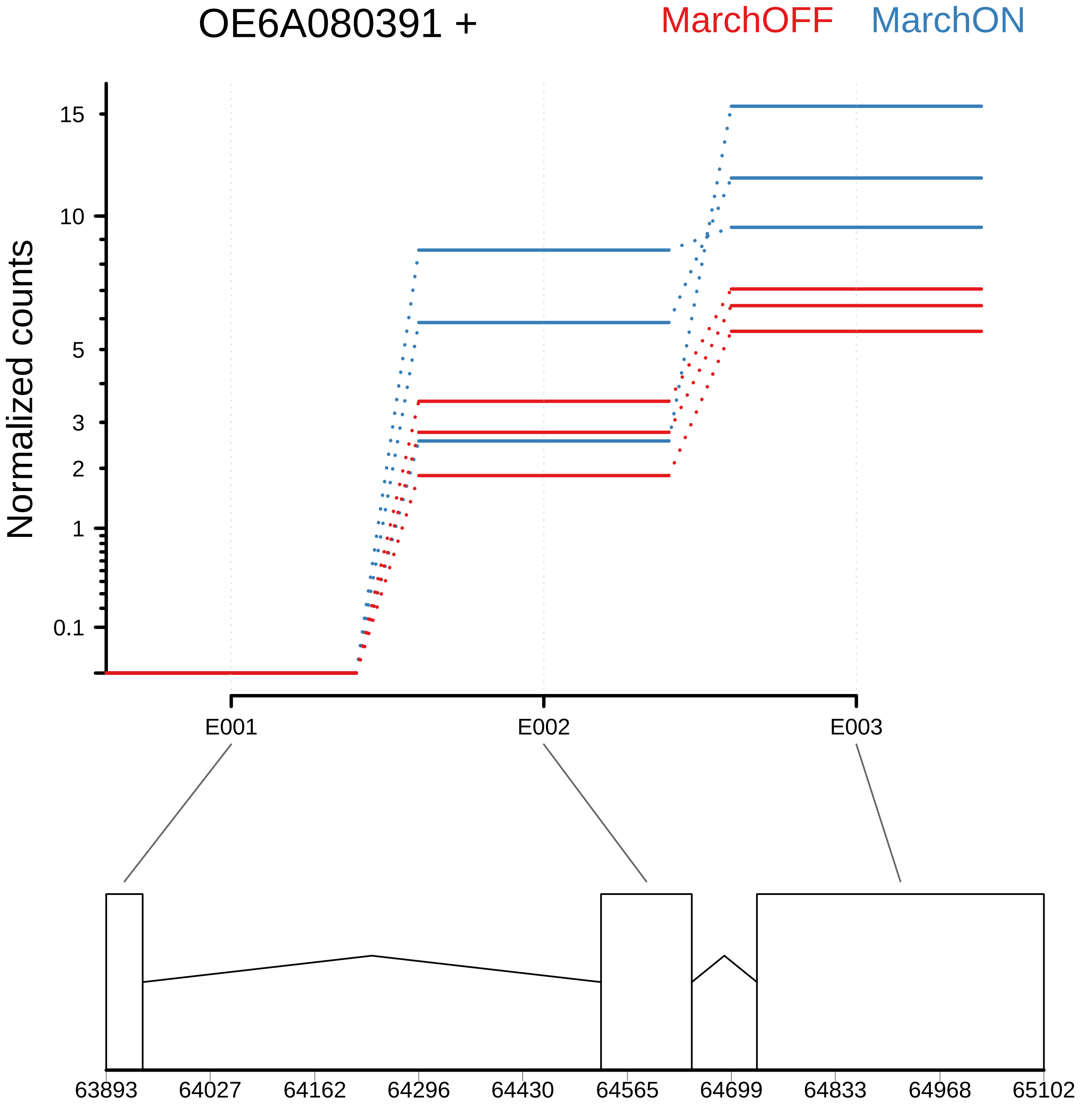

OE6A080391 +
MarchOFF
MarchON
Normalized counts
15
10
5
3
2
1
0.1
E001
E002
E003
63893
64027
64162
64296
64430
64565
64699
64833
64968
65102

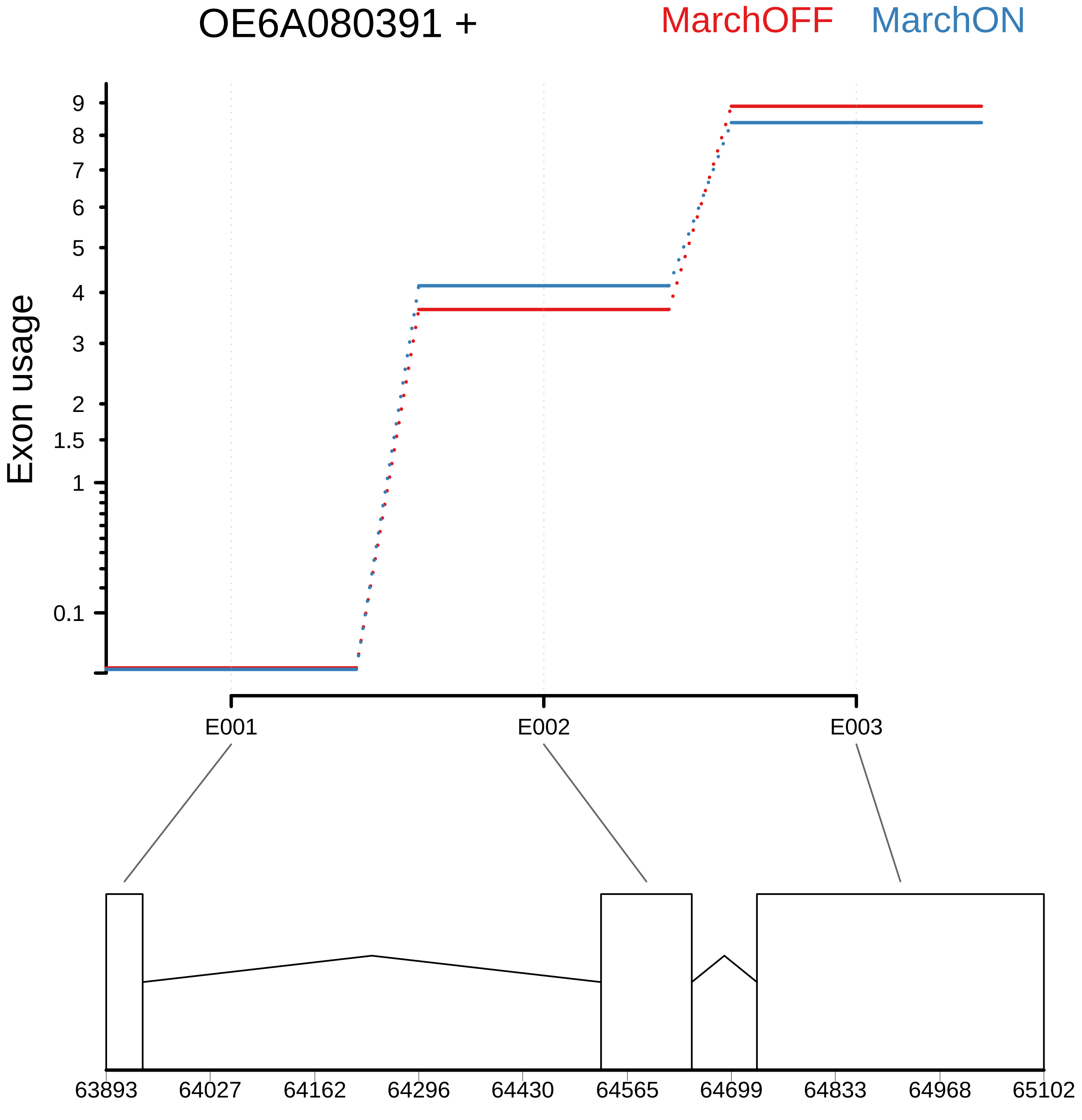

OE6A080391 +
MarchOFF
MarchON
Exon usage
9
8
7
6
5
4
3
2
1.5
1
0.1
E001
E002
E003
63893
64027
64162
64296
64430
64565
64699
64833
64968
65102

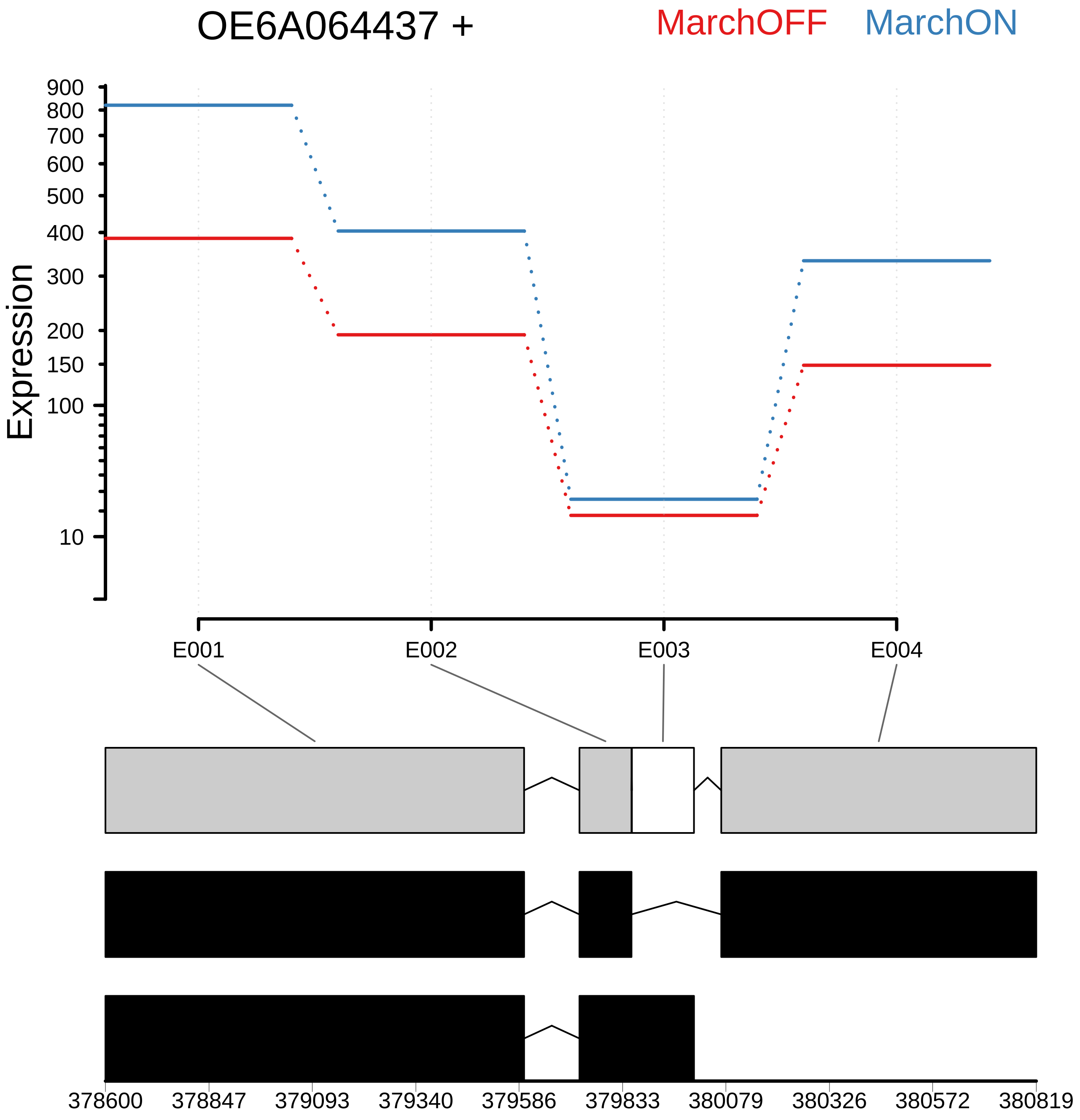
OE6A064437 +
MarchOFF
MarchON
Expression
900
800
700
600
500
400
300
200
150
100
10
E001
E002
E003
E004
378600
378847
379093
379340
379586
379833
380079
380326
380572
380819

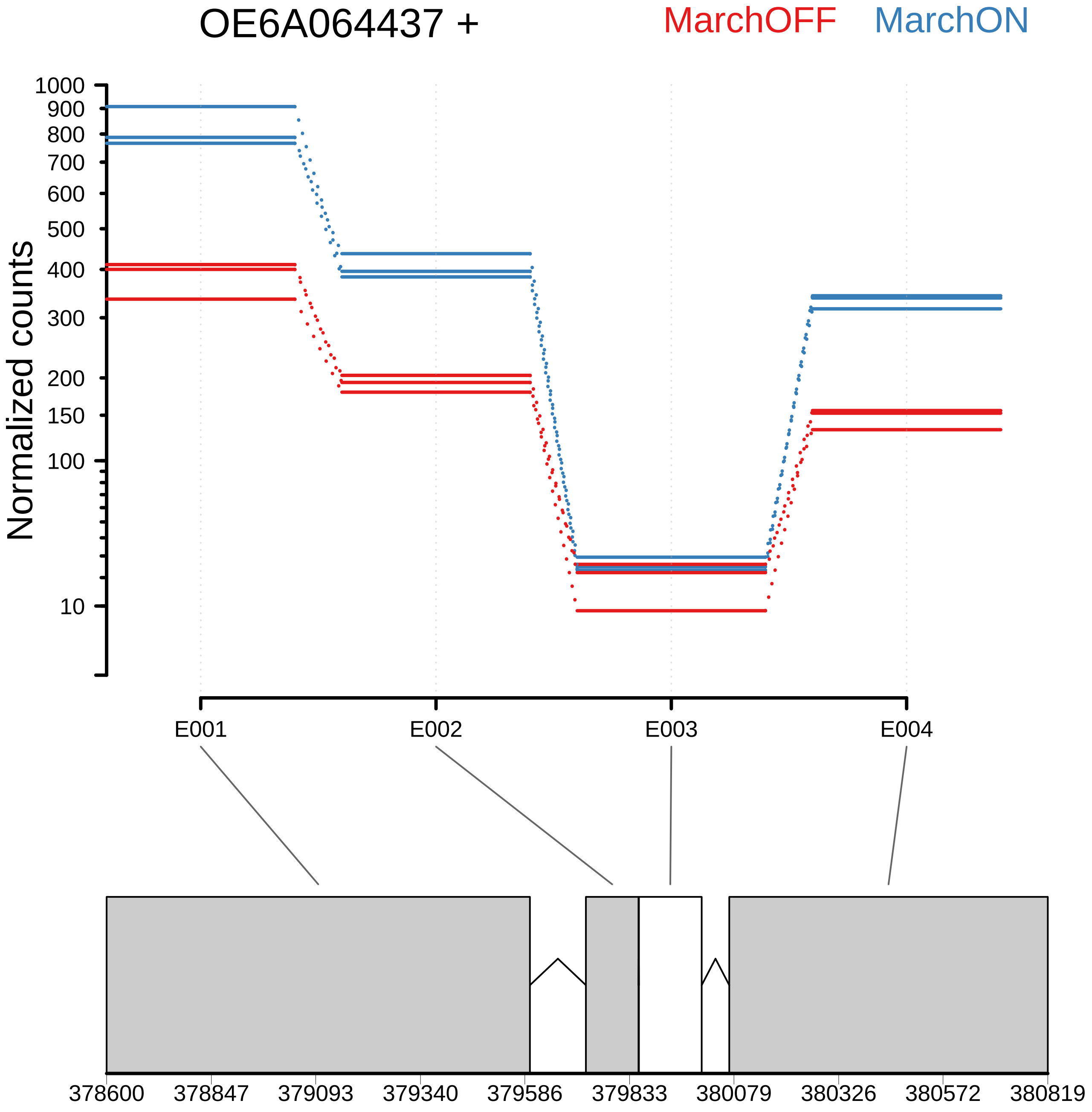

OE6A064437 +
MarchOFF
MarchON
Normalized counts
1000
900
800
700
600
500
400
300
200
150
100
10
E001
E002
E003
E004
378600
378847
379093
379340
379586
379833
380079
380326
380572
380819

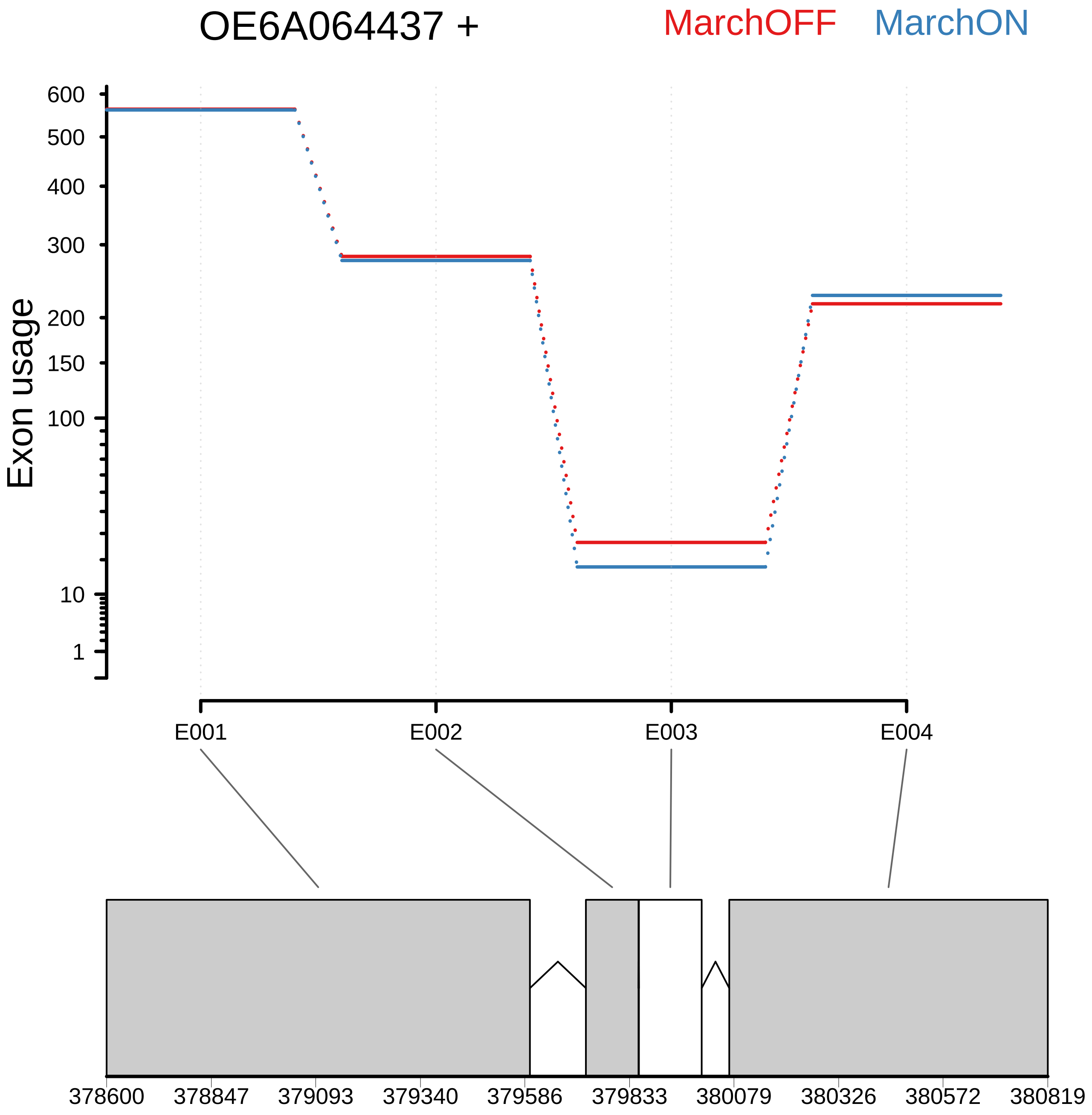

OE6A064437 +
MarchOFF
MarchON
Exon usage
600
500
400
300
200
150
100
10
1
E001
E002
E003
E004
378600
378847
379093
379340
379586
379833
380079
380326
380572
380819

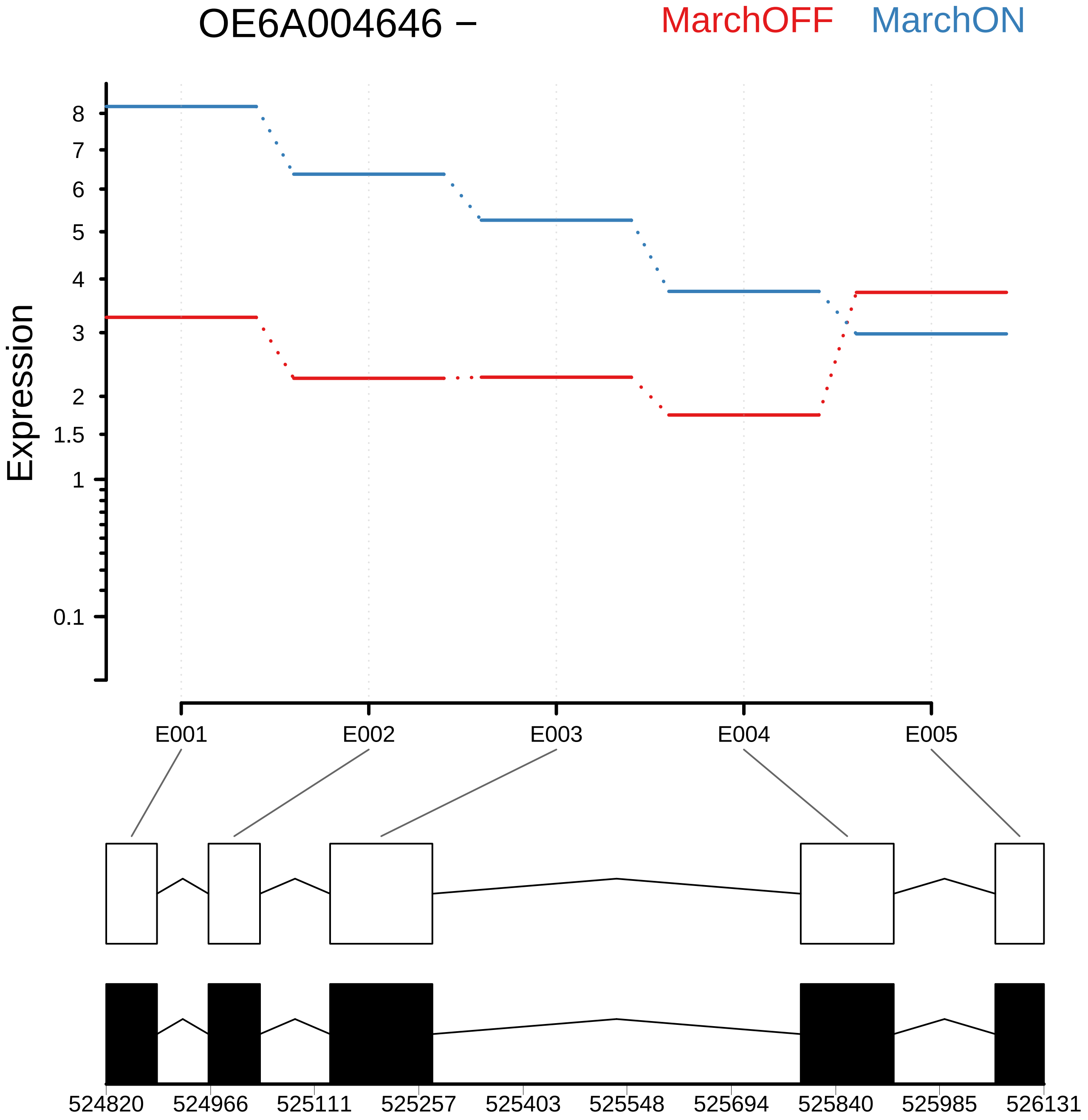
OE6A004646 −
MarchOFF
MarchON
Expression
8
7
6
5
4
3
2
1.5
1
0.1
E001
E002
E003
E004
E005
524820
524966
525111
525257
525403
525548
525694
525840
525985
526131

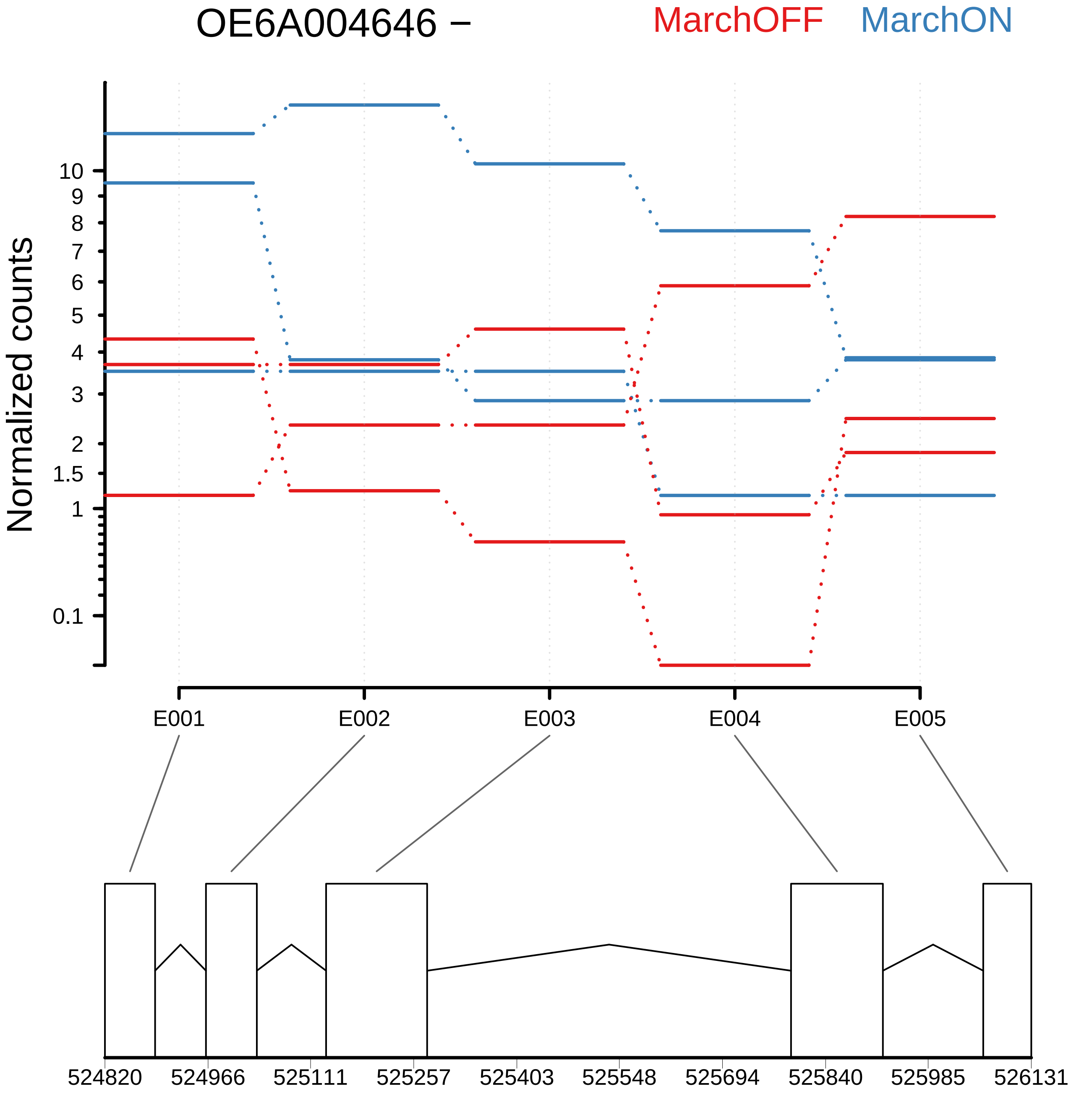

OE6A004646 −
MarchOFF
MarchON
Normalized counts
10
9
8
7
6
5
4
3
2
1.5
1
0.1
E001
E002
E003
E004
E005
524820
524966
525111
525257
525403
525548
525694
525840
525985
526131

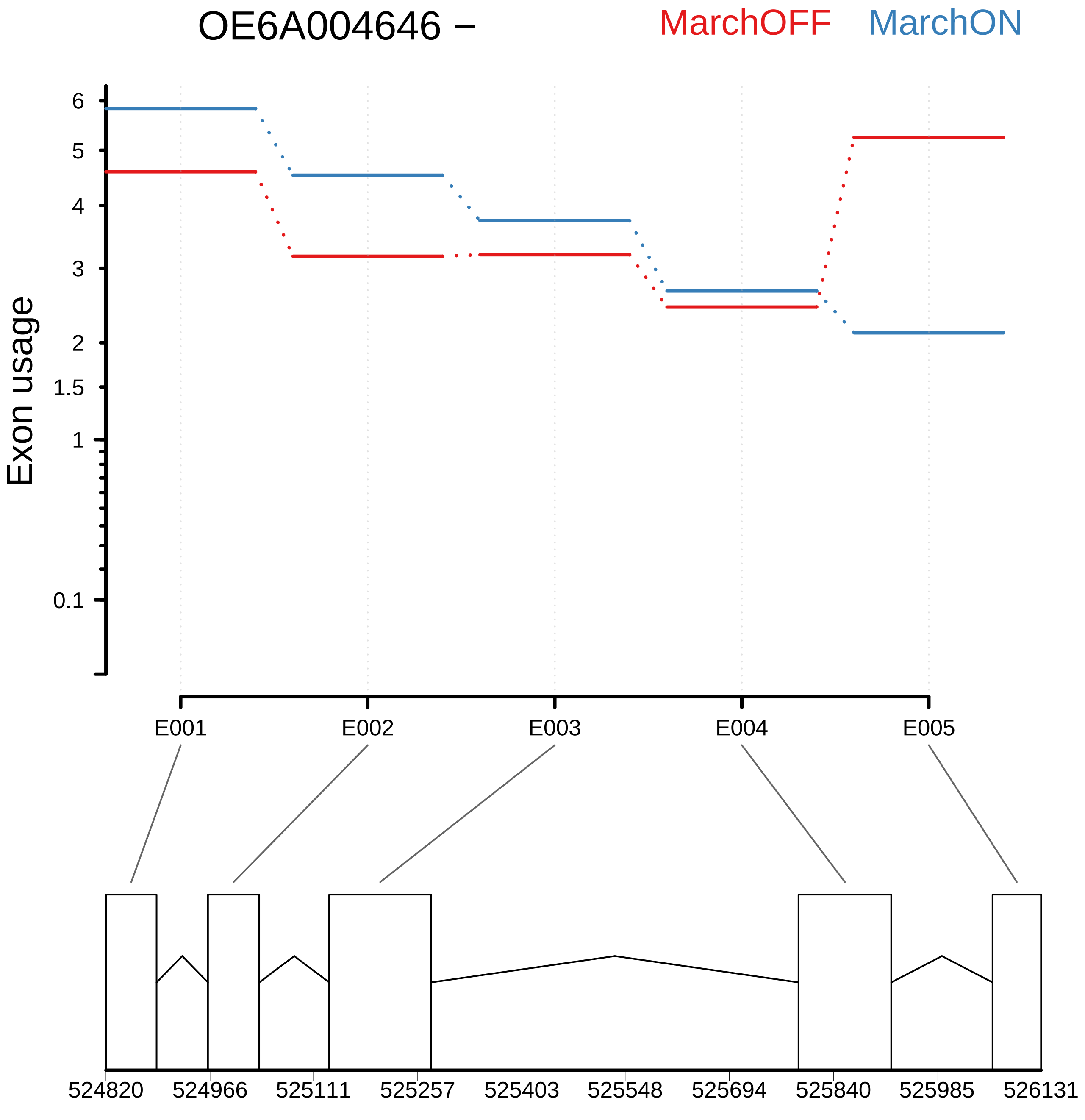

OE6A004646 −
MarchOFF
MarchON
Exon usage
6
5
4
3
2
1.5
1
0.1
E001
E002
E003
E004
E005
524820
524966
525111
525257
525403
525548
525694
525840
525985
526131

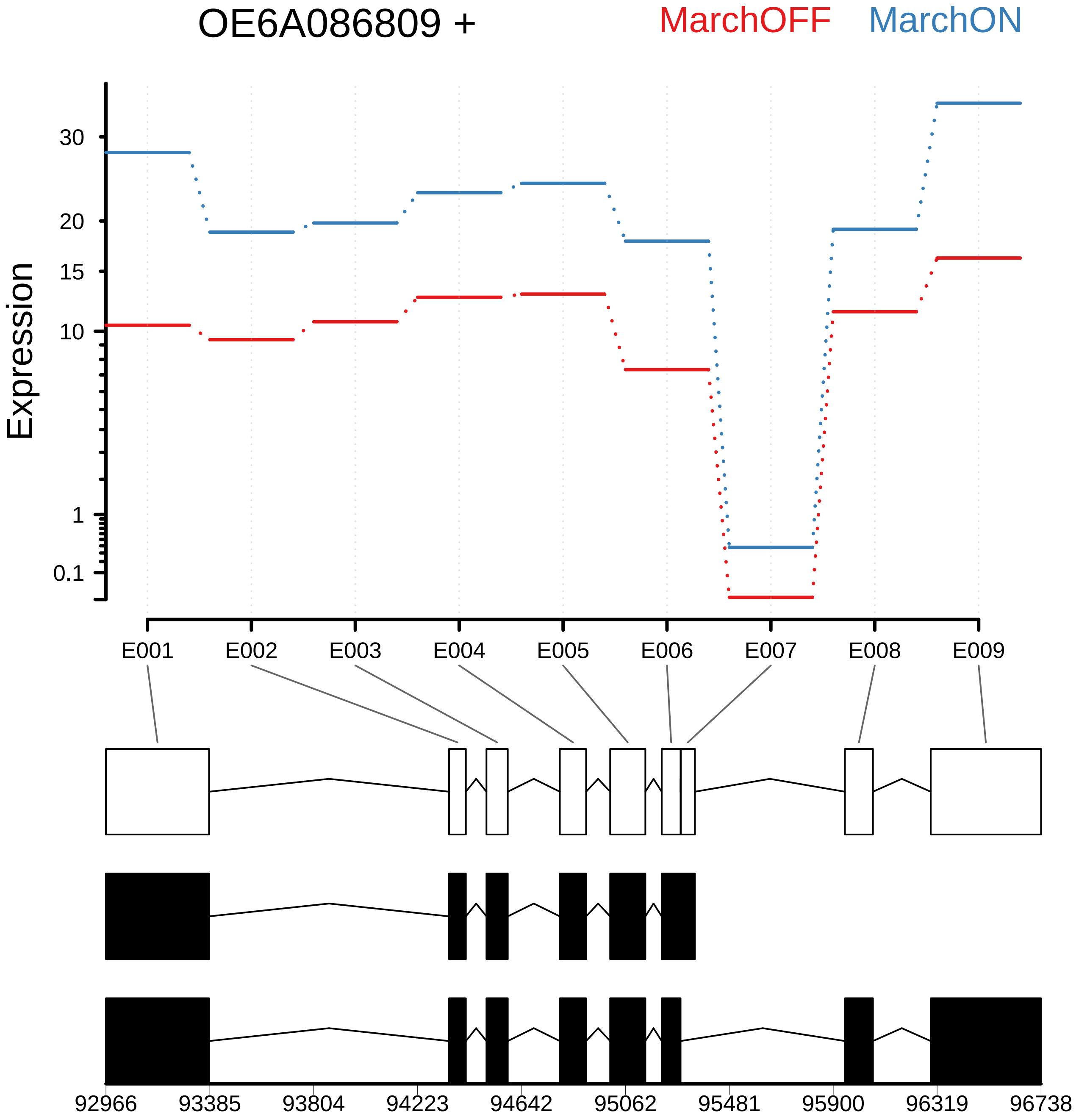

OE6A086809 +
MarchOFF
MarchON
Expression
30
20
15
10
1
0.1
E001
E002
E003
E004
E005
E006
E007
E008
E009
92966
93385
93804
94223
94642
95062
95481
95900
96319
96738

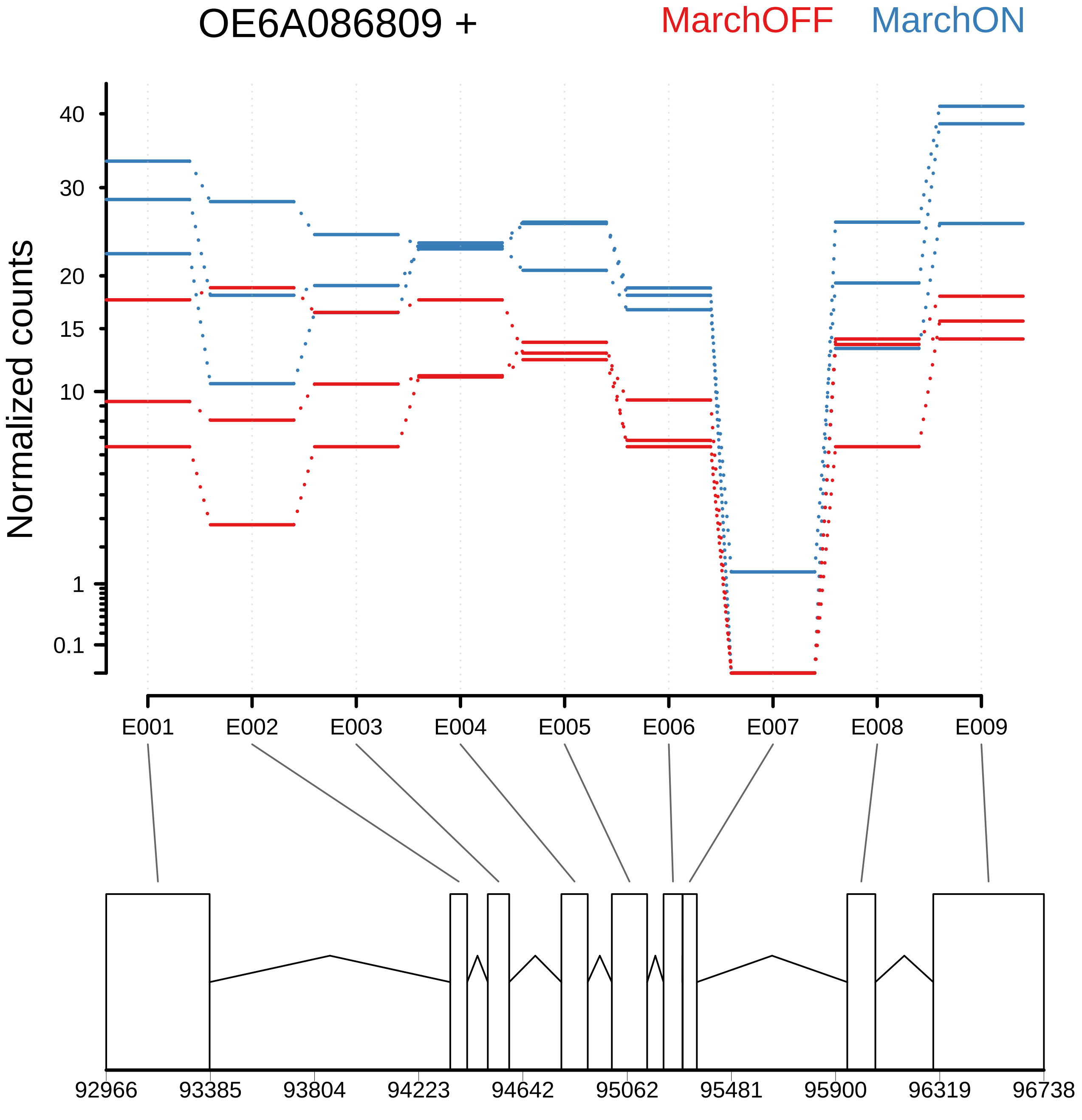
OE6A086809 +
MarchOFF
MarchON
Normalized counts
40
30
20
15
10
1
0.1
E001
E002
E003
E004
E005
E006
E007
E008
E009
92966
93385
93804
94223
94642
95062
95481
95900
96319
96738

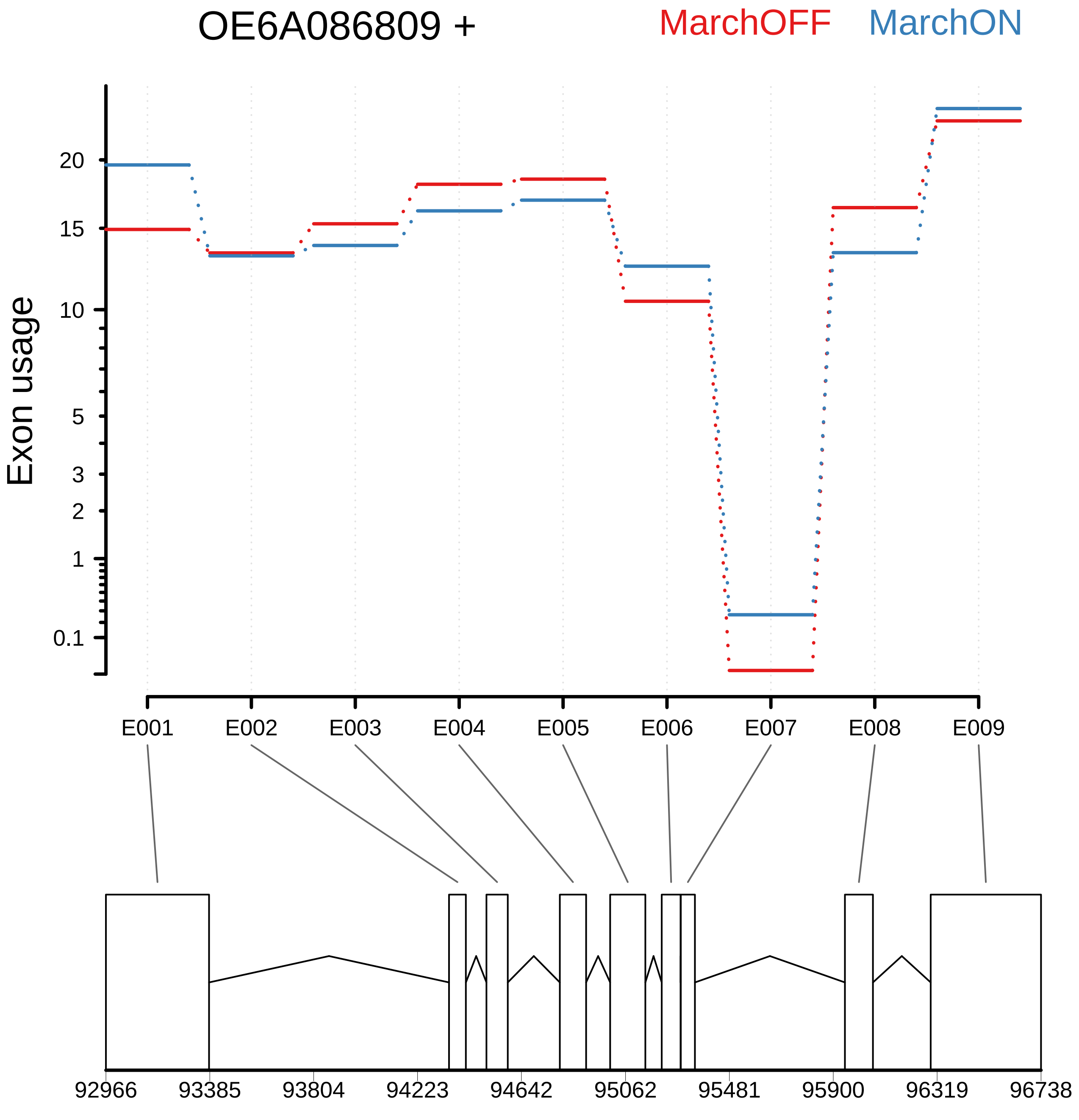

OE6A086809 +
MarchOFF
MarchON
Exon usage
20
15
10
5
3
2
1
0.1
E001
E002
E003
E004
E005
E006
E007
E008
E009
92966
93385
93804
94223
94642
95062
95481
95900
96319
96738

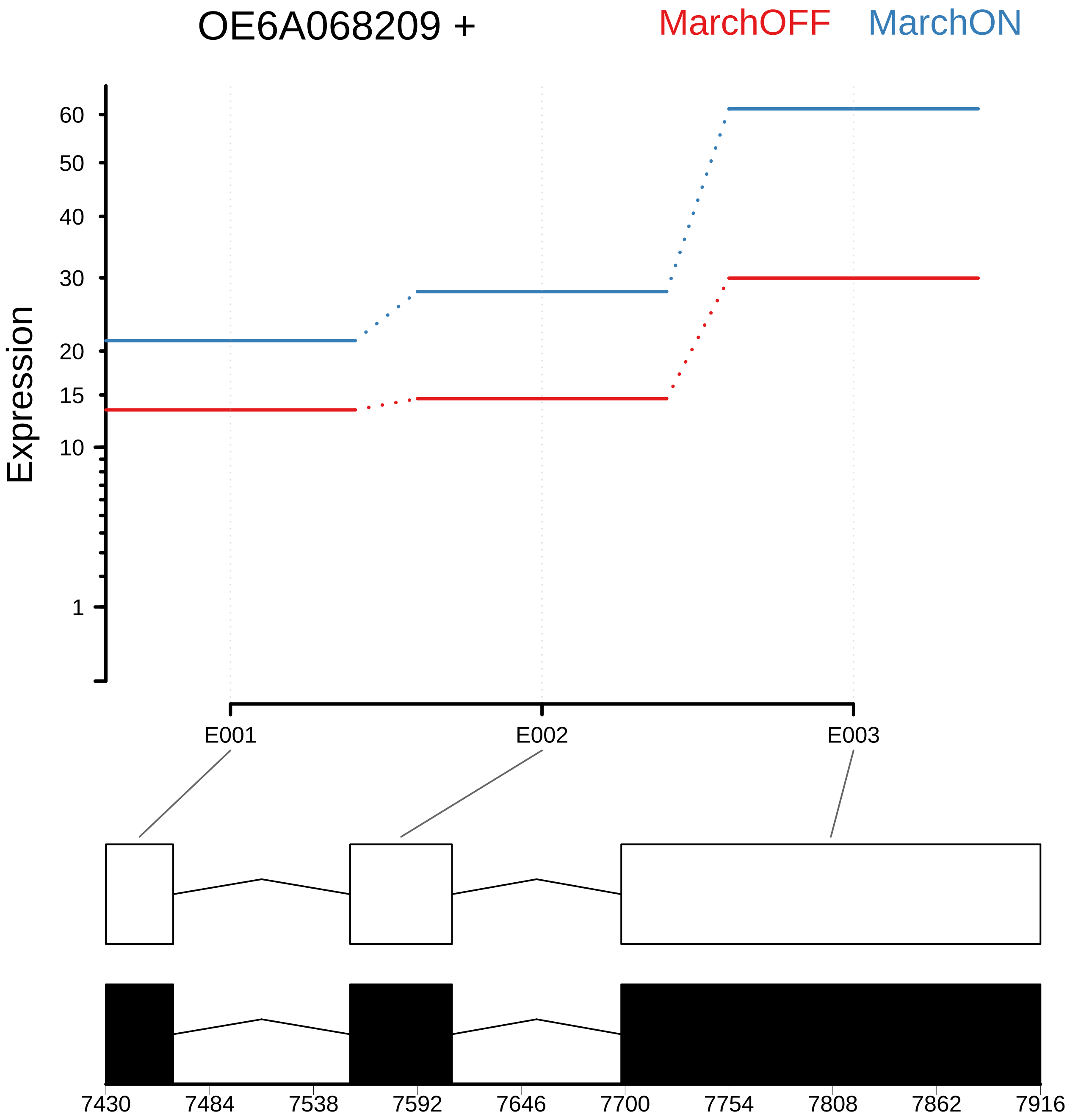

OE6A068209 +
MarchOFF
MarchON
Expression
60
50
40
30
20
15
10
1
E001
E002
E003
7430
7484
7538
7592
7646
7700
7754
7808
7862
7916

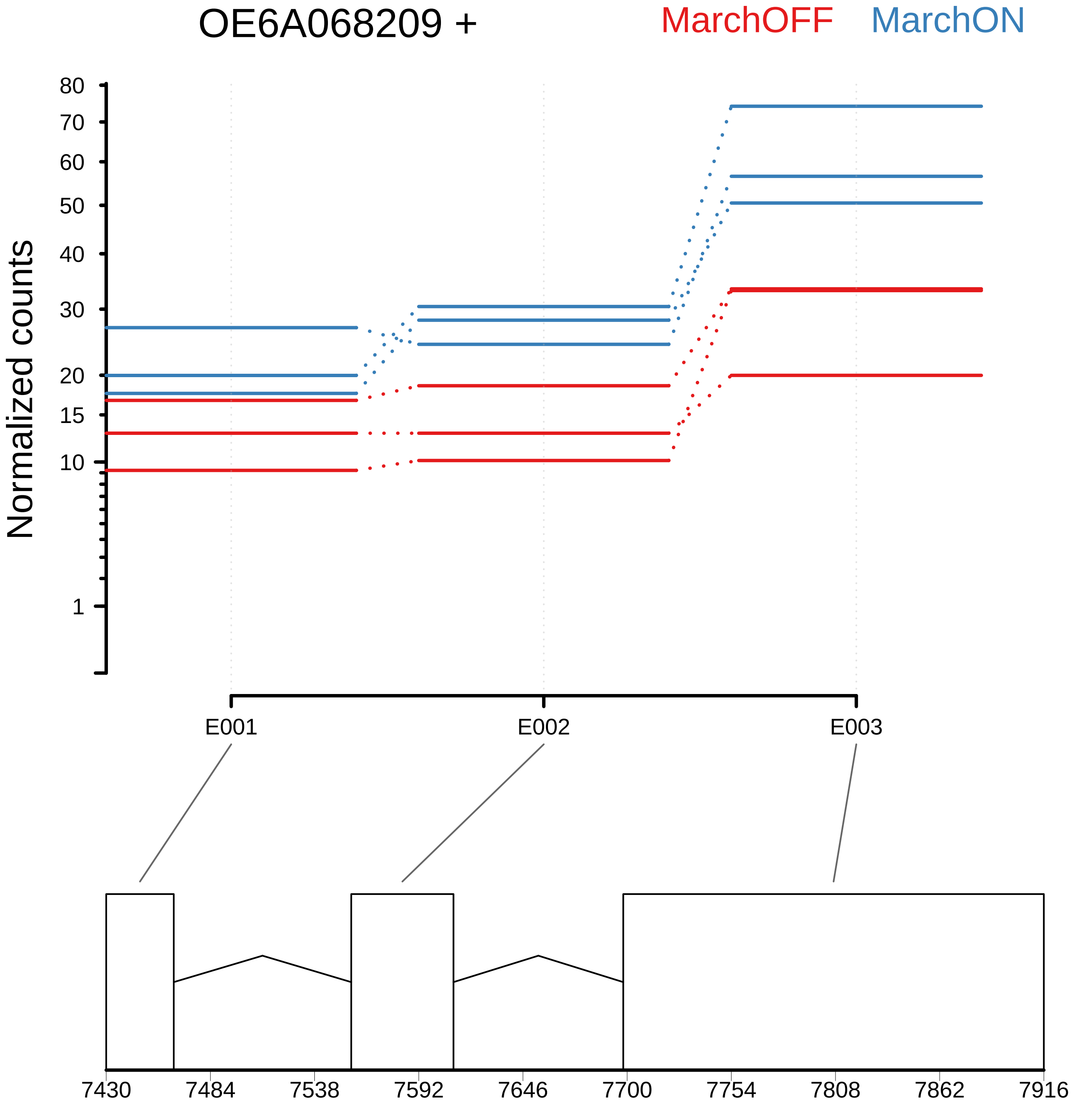

OE6A068209 +
MarchOFF
MarchON
Normalized counts
80
70
60
50
40
30
20
15
10
1
E001
E002
E003
7430
7484
7538
7592
7646
7700
7754
7808
7862
7916

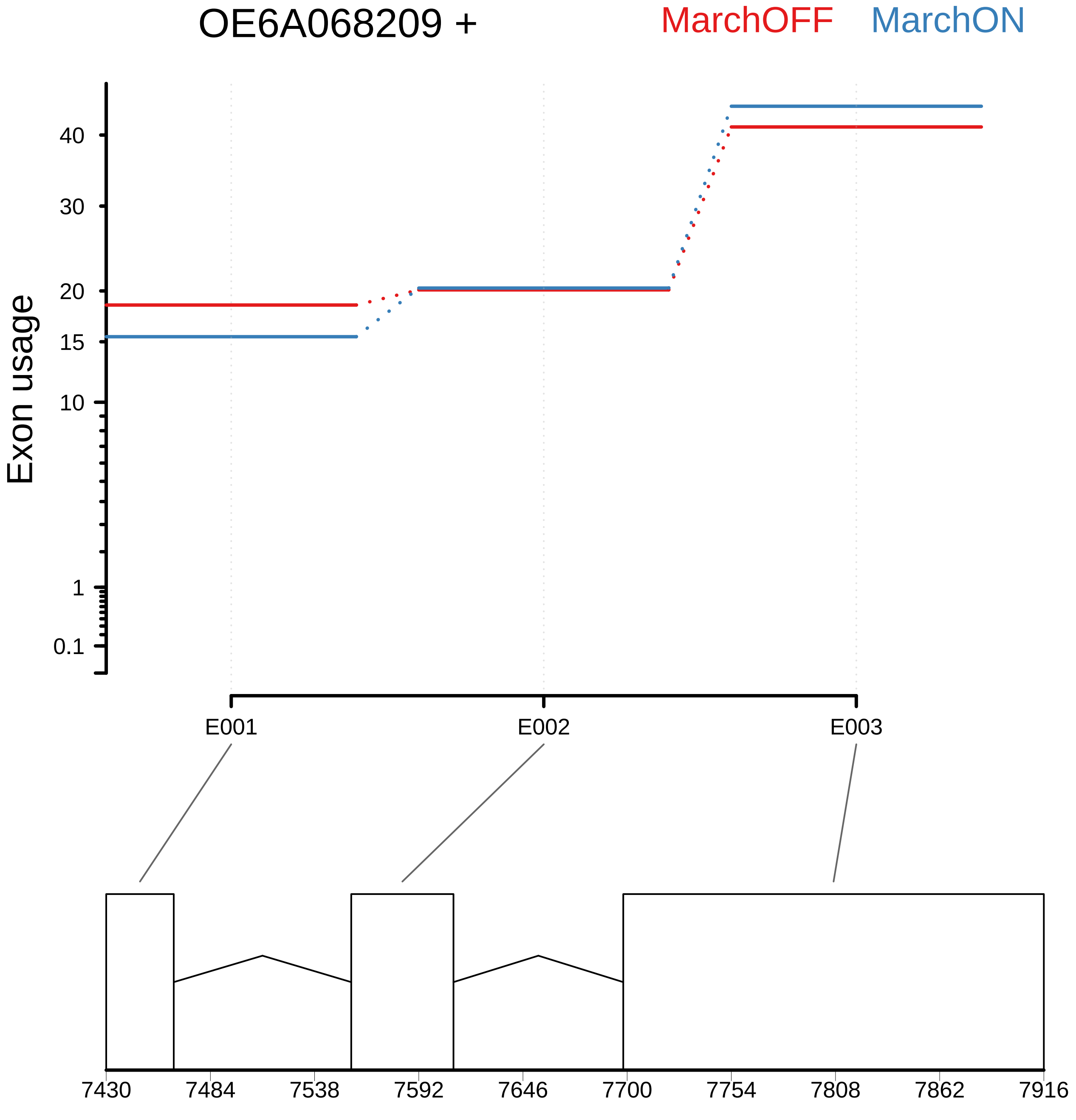

OE6A068209 +
MarchOFF
MarchON
Exon usage
40
30
20
15
10
1
0.1
E001
E002
E003
7430
7484
7538
7592
7646
7700
7754
7808
7862
7916

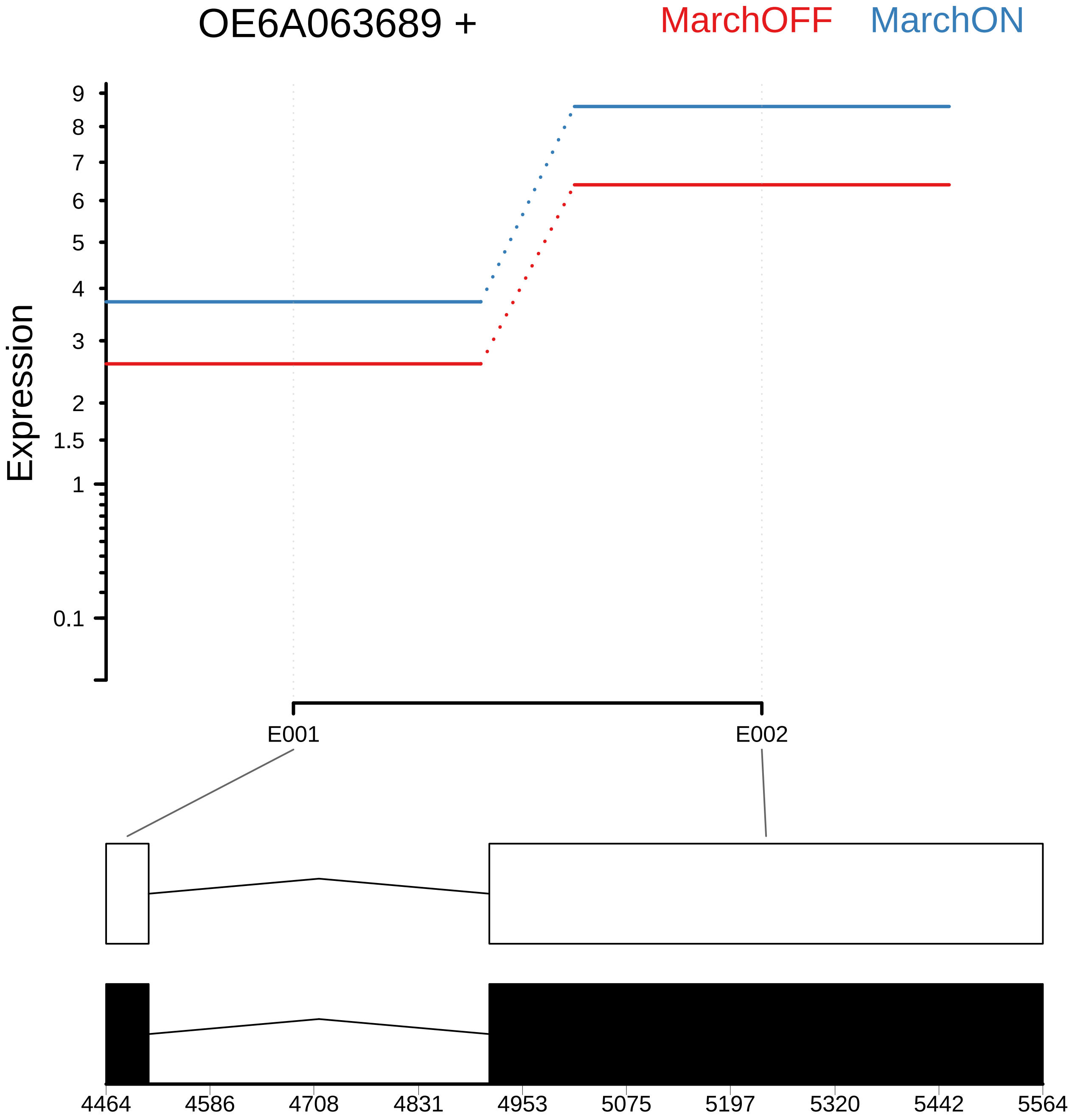

OE6A063689 +
MarchOFF
MarchON
Expression
9
8
7
6
5
4
3
2
1.5
1
0.1
E001
E002
4464
4586
4708
4831
4953
5075
5197
5320
5442
5564

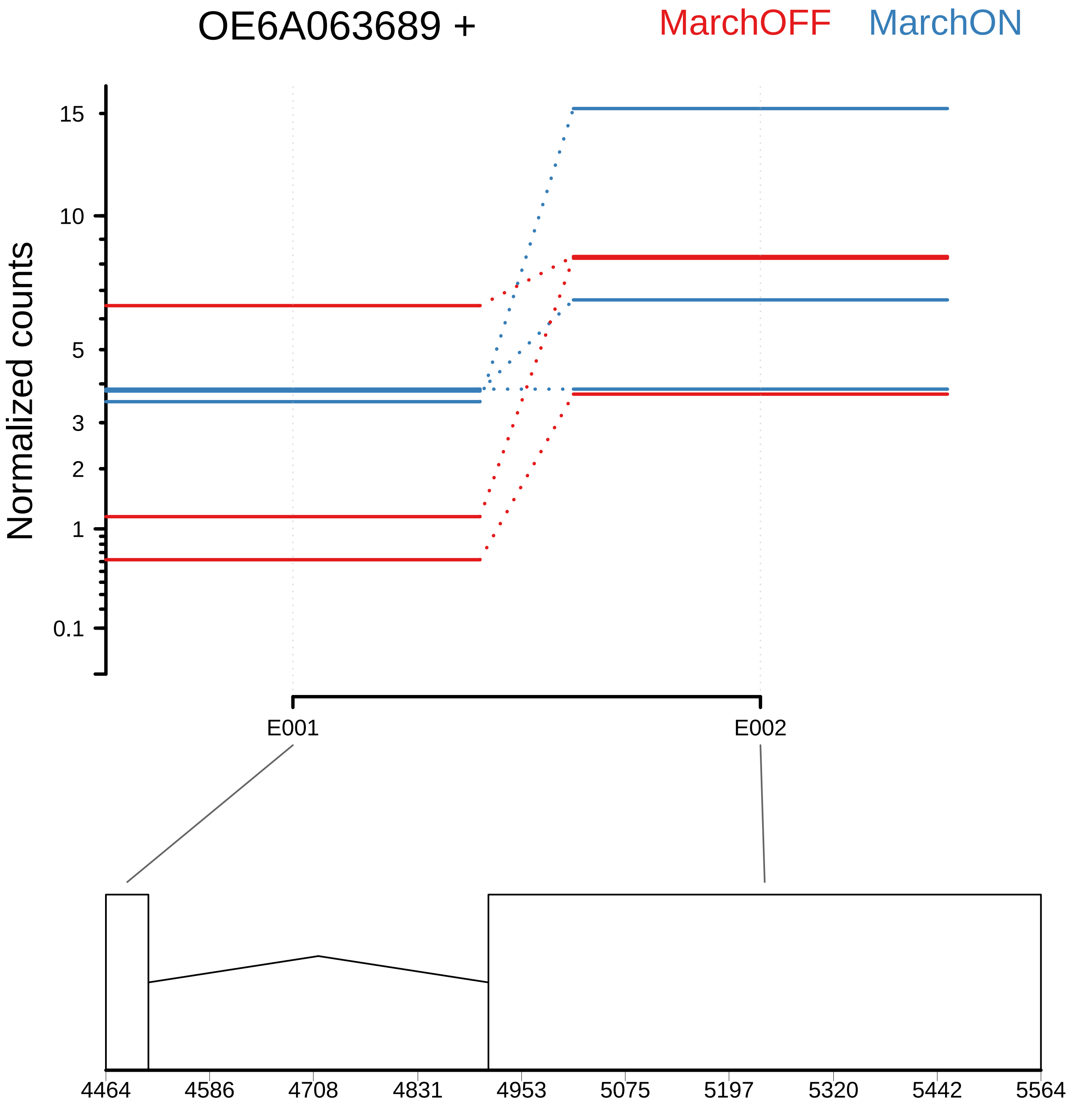

OE6A063689 +
MarchOFF
MarchON
Normalized counts
15
10
5
3
2
1
0.1
E001
E002
4464
4586
4708
4831
4953
5075
5197
5320
5442
5564

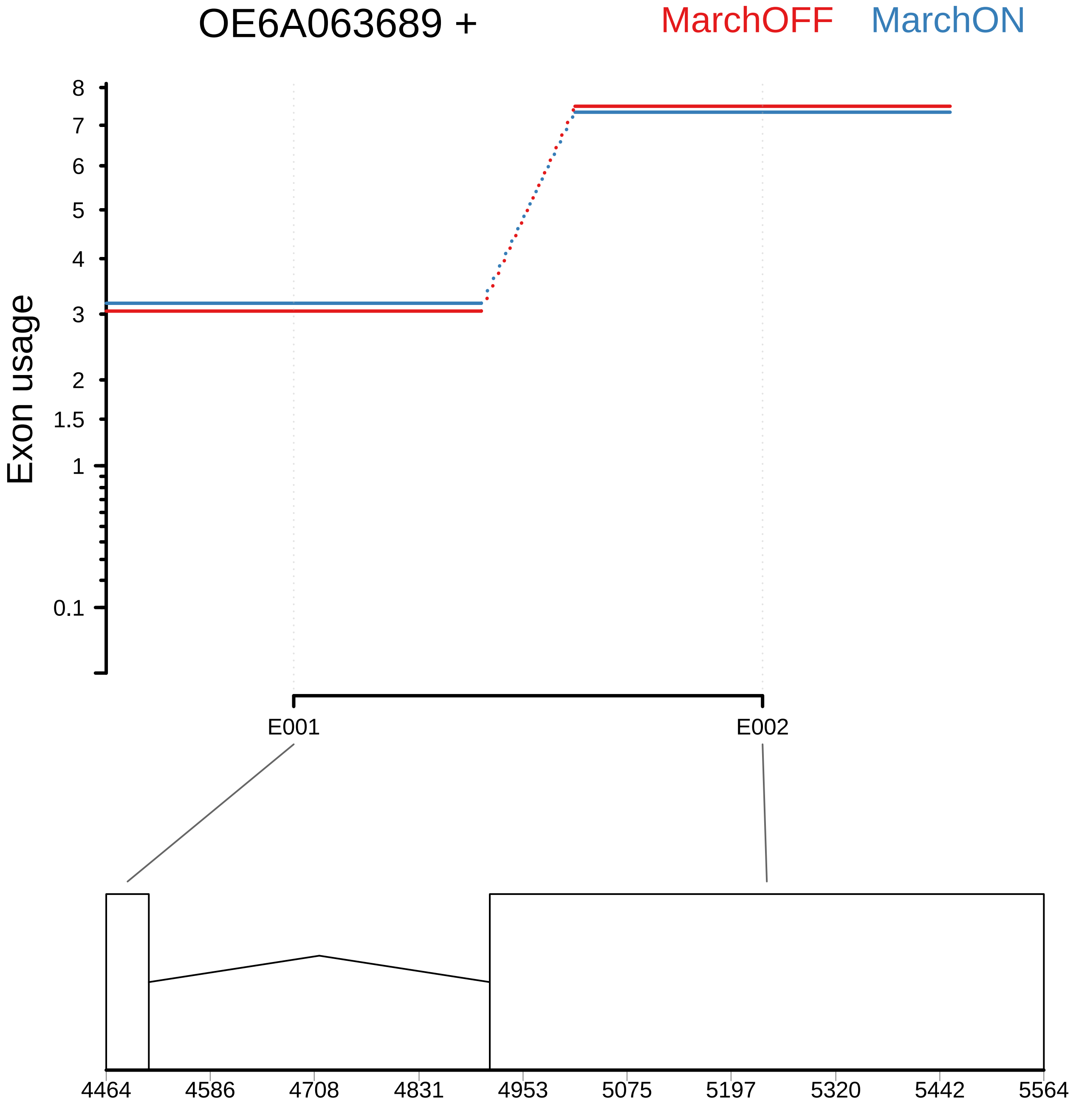

OE6A063689 +
MarchOFF
MarchON
Exon usage
8
7
6
5
4
3
2
1.5
1
0.1
E001
E002
4464
4586
4708
4831
4953
5075
5197
5320
5442
5564

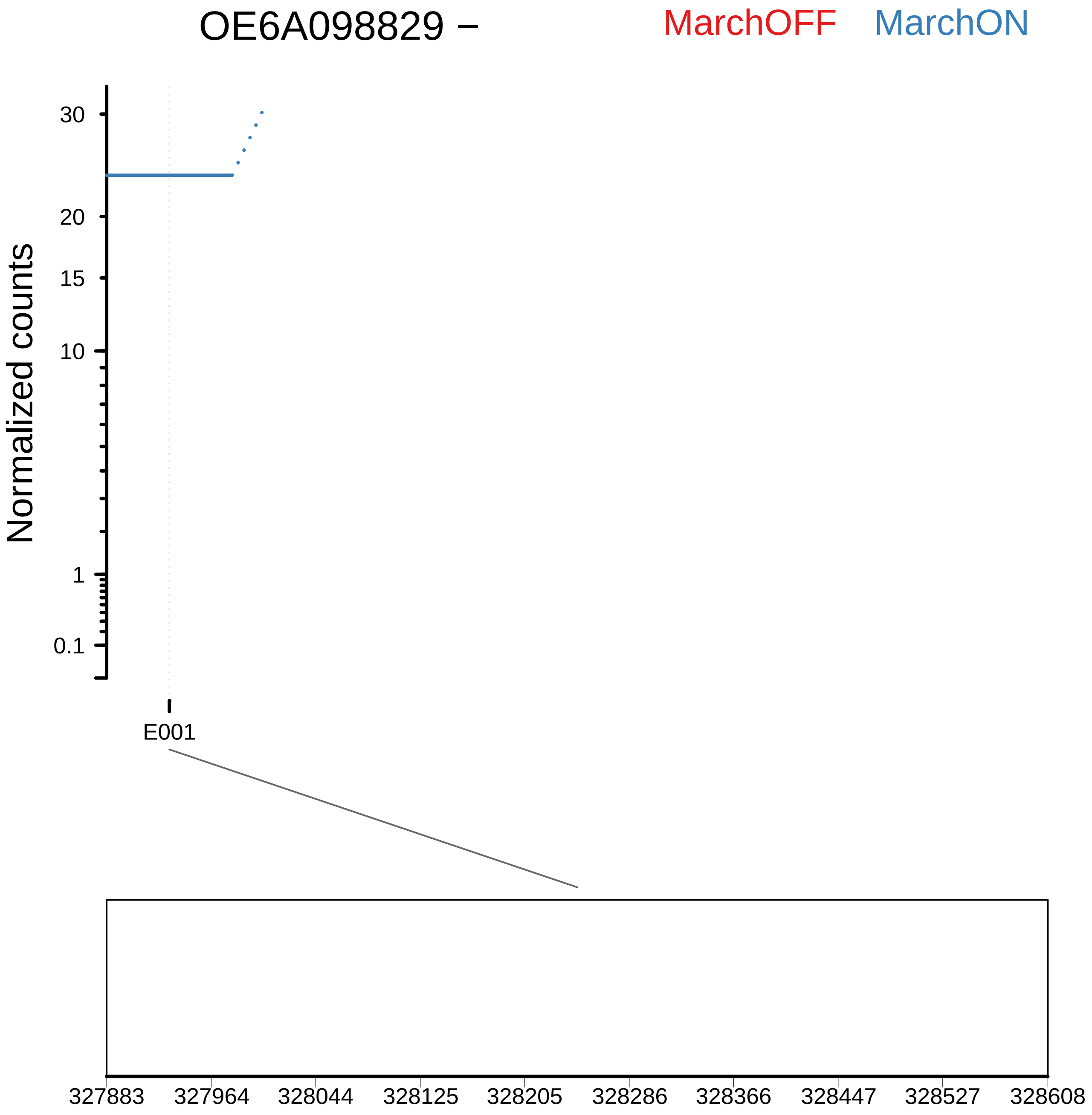

OE6A098829 −
MarchOFF
MarchON
Normalized counts
30
20
15
10
1
0.1
E001
327883
327964
328044
328125
328205
328286
328366
328447
328527
328608

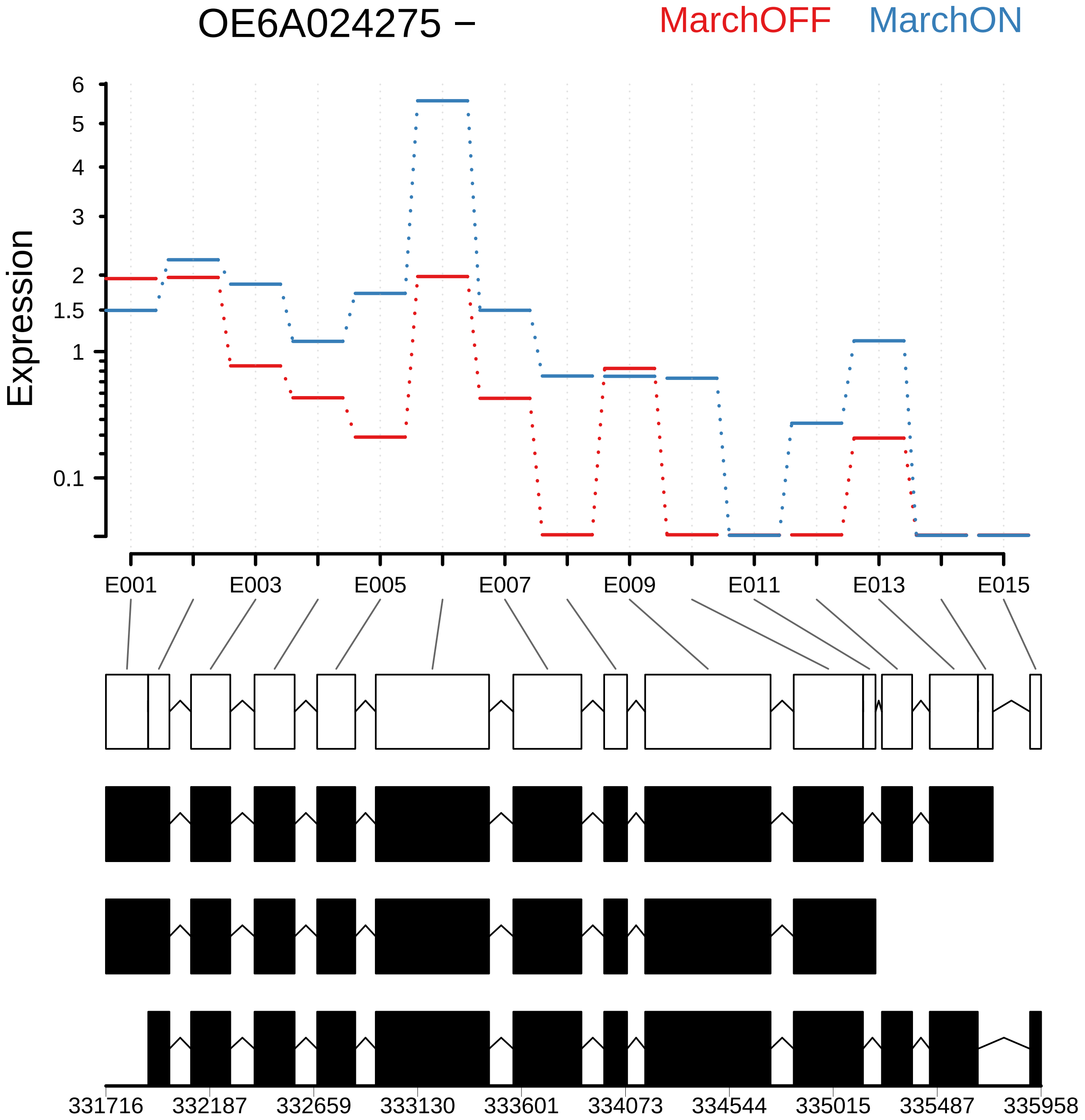

OE6A024275 −
MarchOFF
MarchON
Expression
6
5
4
3
2
1.5
1
0.1
E001
E003
E005
E007
E009
E011
E013
E015
331716
332187
332659
333130
333601
334073
334544
335015
335487
335958

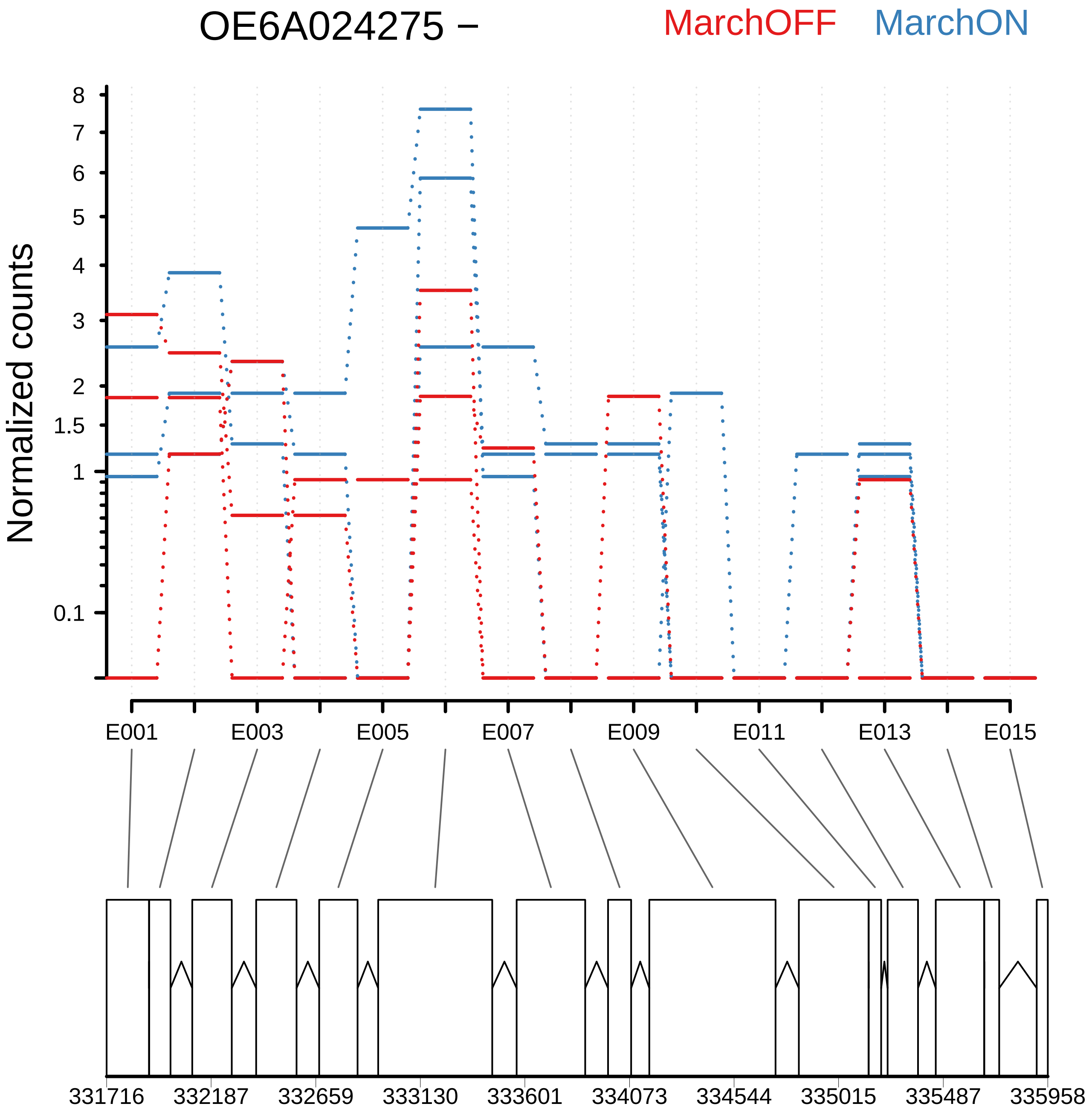
OE6A024275 −
MarchOFF
MarchON
Normalized counts
8
7
6
5
4
3
2
1.5
1
0.1
E001
E003
E005
E007
E009
E011
E013
E015
331716
332187
332659
333130
333601
334073
334544
335015
335487
335958

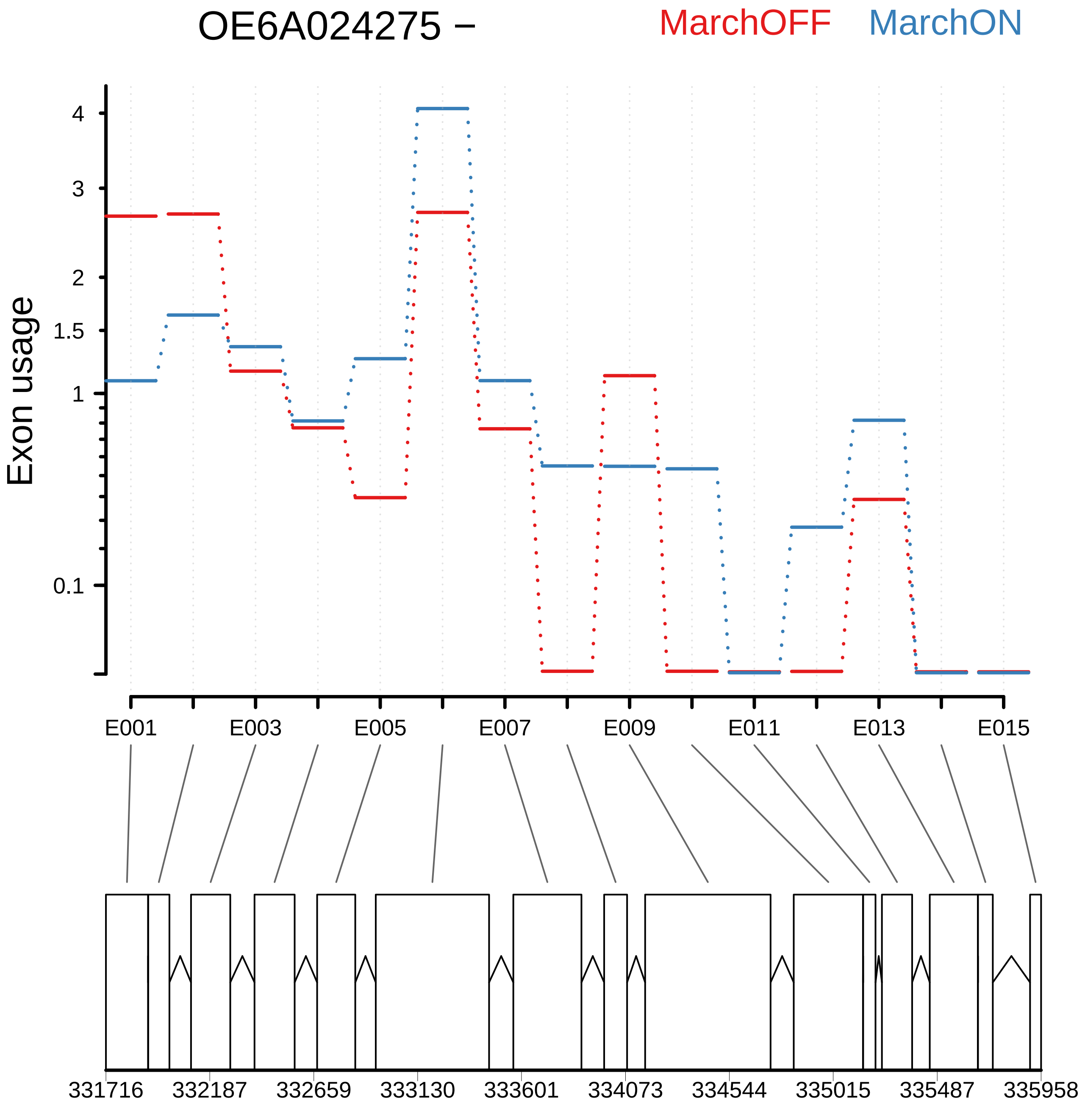
OE6A024275 −
MarchOFF
MarchON
Exon usage
4
3
2
1.5
1
0.1
E001
E003
E005
E007
E009
E011
E013
E015
331716
332187
332659
333130
333601
334073
334544
335015
335487
335958

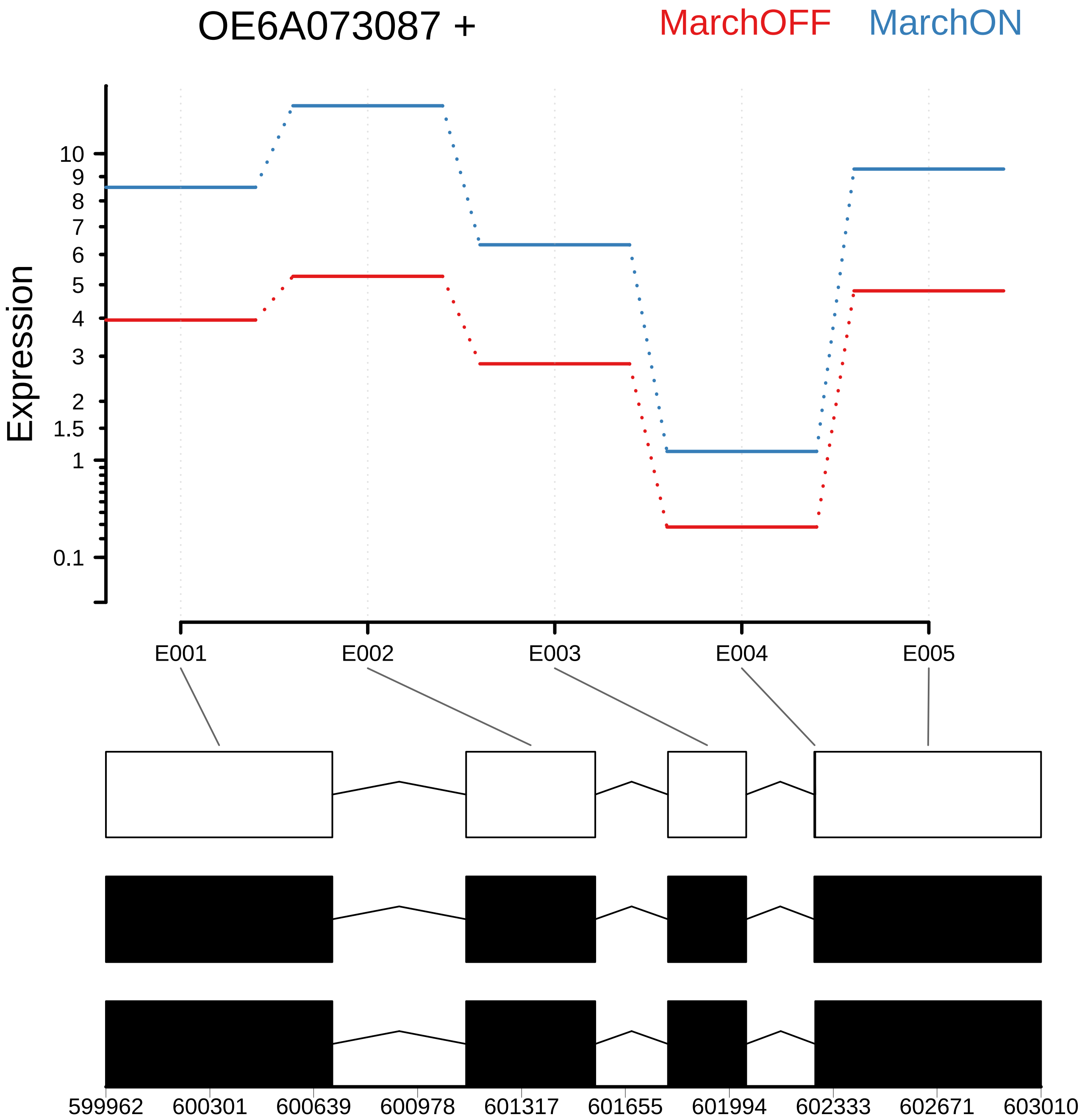

OE6A073087 +
MarchOFF
MarchON
Expression
10
9
8
7
6
5
4
3
2
1.5
1
0.1
E001
E002
E003
E004
E005
599962
600301
600639
600978
601317
601655
601994
602333
602671
603010

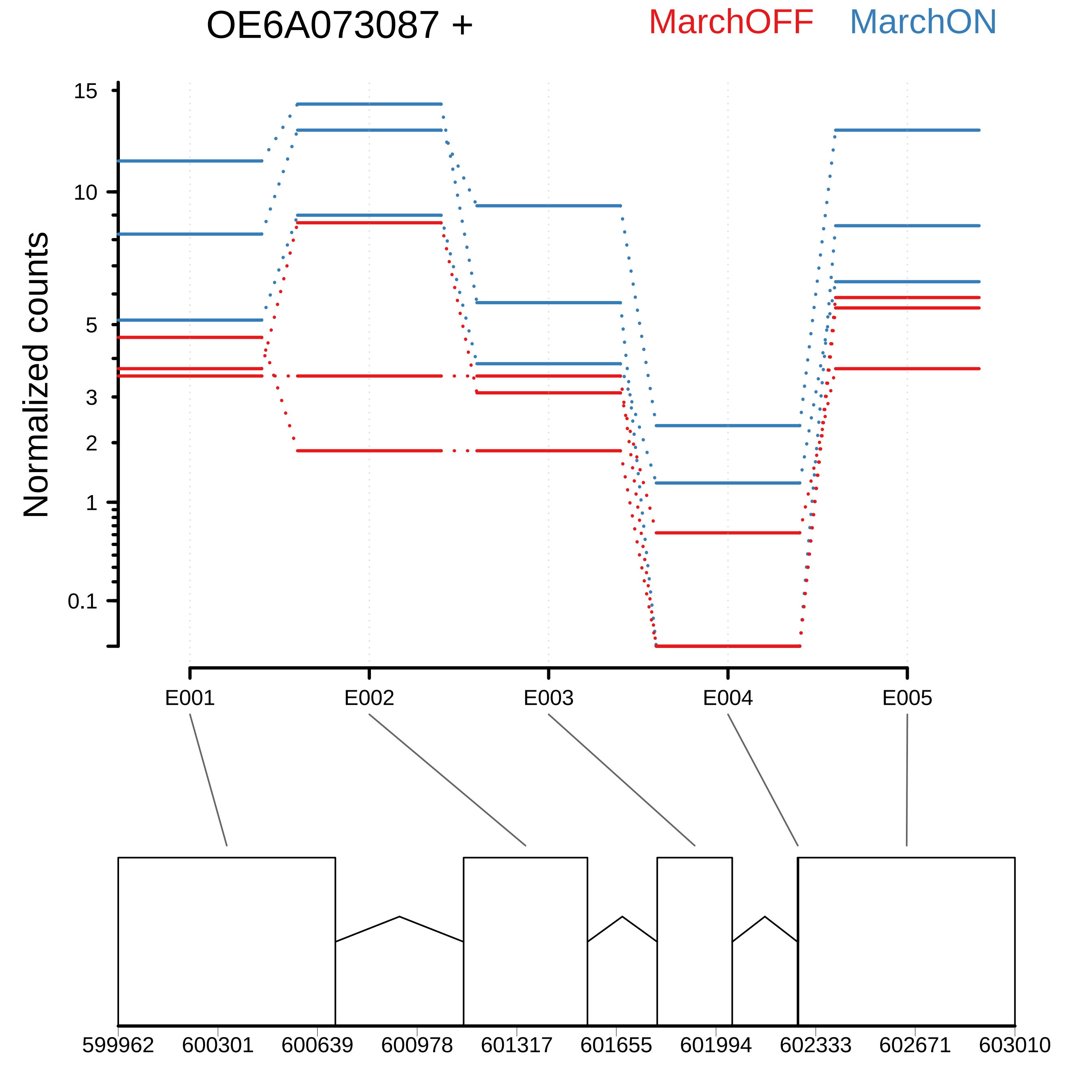

OE6A073087 +
MarchOFF
MarchON
Normalized counts
15
10
5
3
2
1
0.1
E001
E002
E003
E004
E005
599962
600301
600639
600978
601317
601655
601994
602333
602671
603010

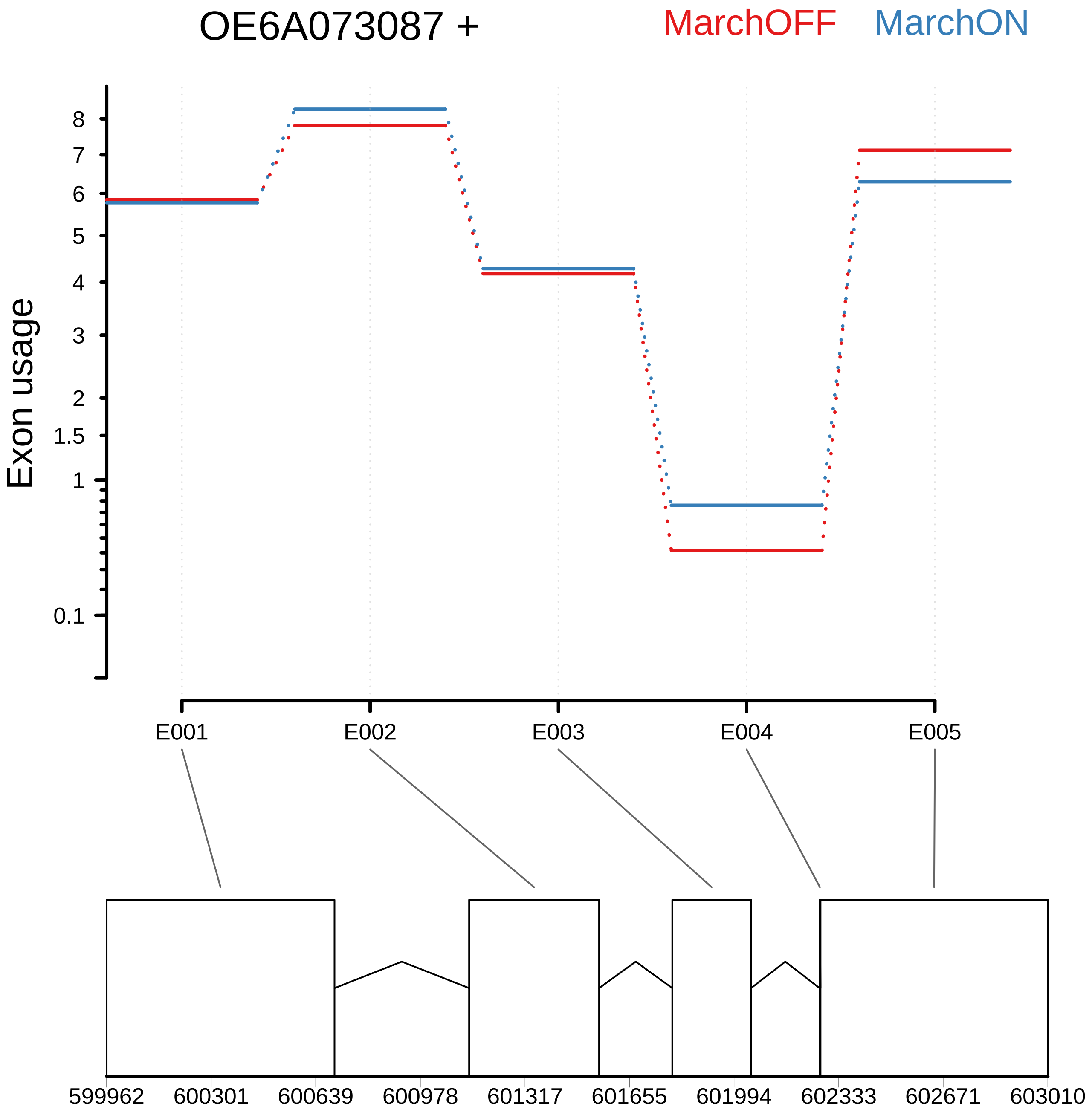
OE6A073087 +
MarchOFF
MarchON
Exon usage
8
7
6
5
4
3
2
1.5
1
0.1
E001
E002
E003
E004
E005
599962
600301
600639
600978
601317
601655
601994
602333
602671
603010

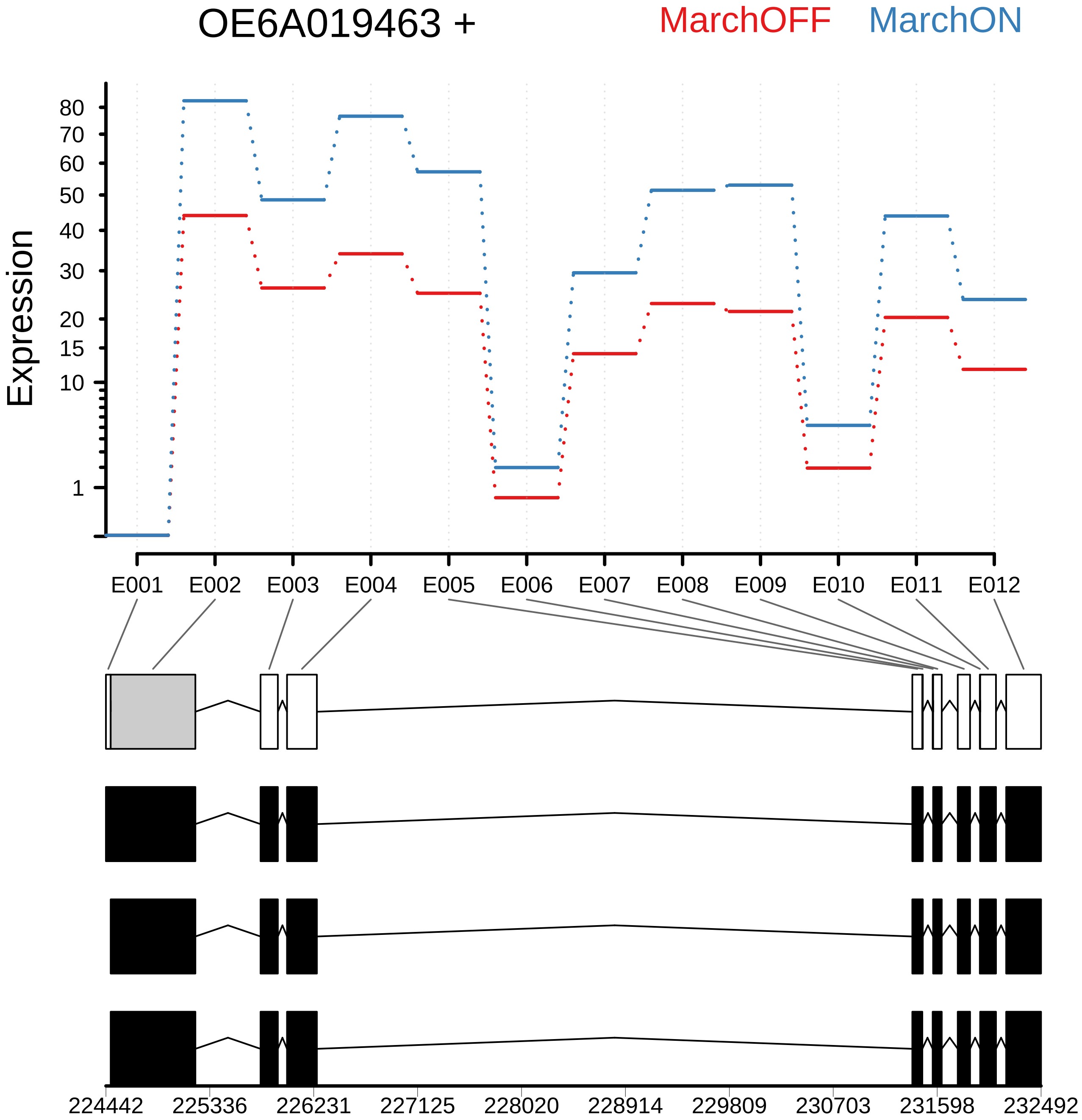

OE6A019463 +
MarchOFF
MarchON
Expression
80
70
60
50
40
30
20
15
10
1
E001
E002
E003
E004
E005
E006
E007
E008
E009
E010
E011
E012
224442
225336
226231
227125
228020
228914
229809
230703
231598
232492

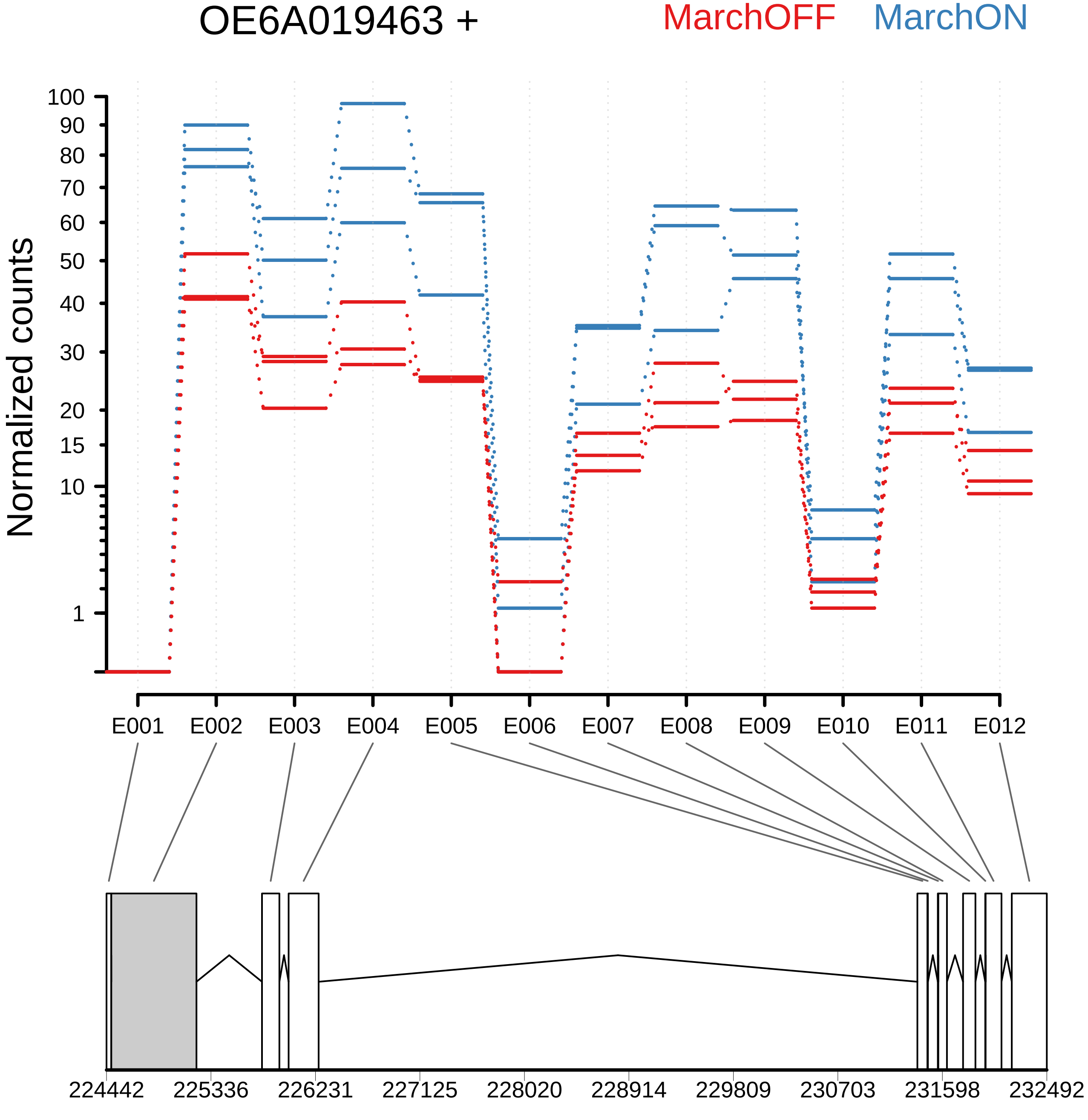
OE6A019463 +
MarchOFF
MarchON
Normalized counts
100
90
80
70
60
50
40
30
20
15
10
1
E001
E002
E003
E004
E005
E006
E007
E008
E009
E010
E011
E012
224442
225336
226231
227125
228020
228914
229809
230703
231598
232492

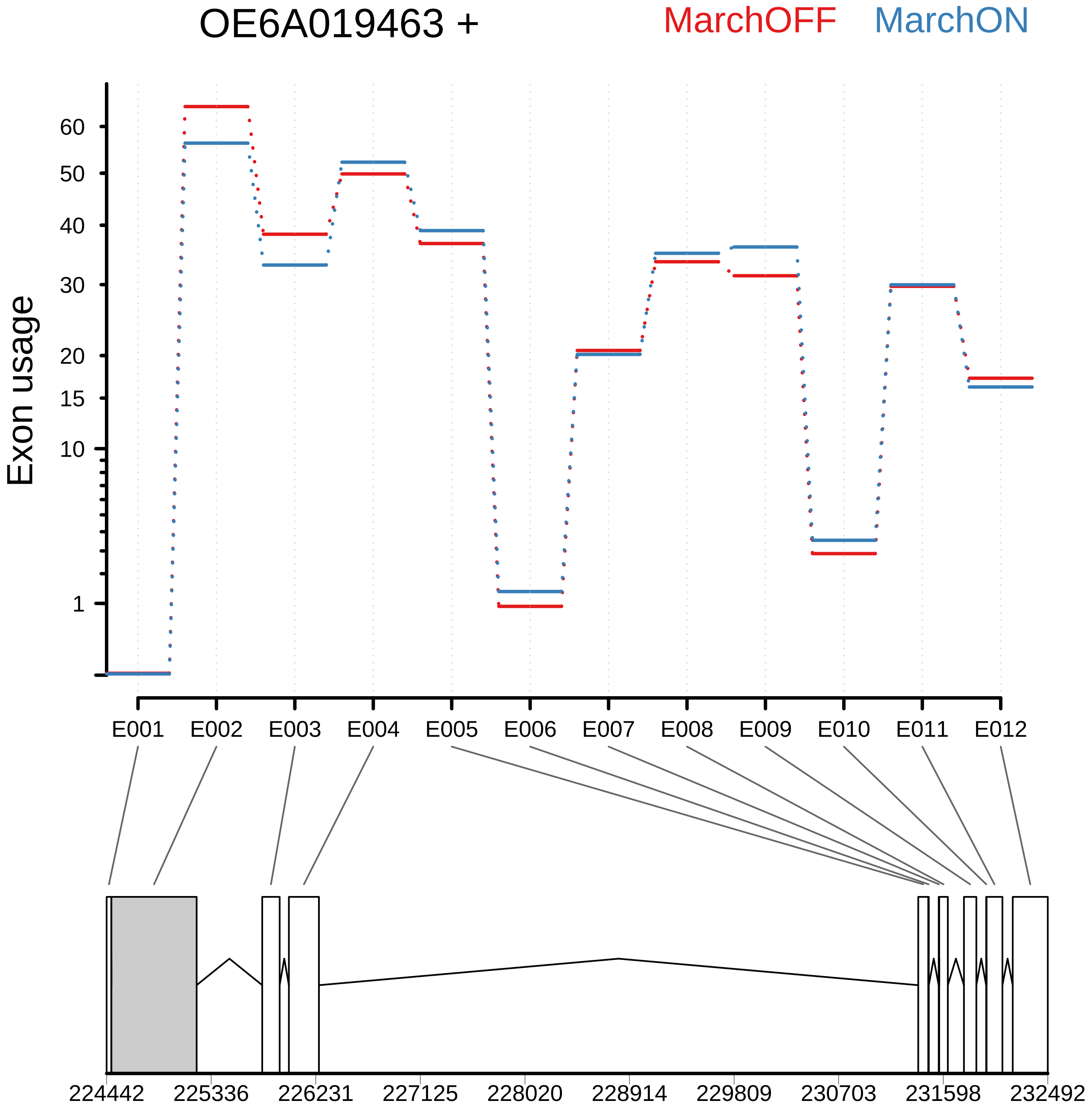

OE6A019463 +
MarchOFF
MarchON
Exon usage
60
50
40
30
20
15
10
1
E001
E002
E003
E004
E005
E006
E007
E008
E009
E010
E011
E012
224442
225336
226231
227125
228020
228914
229809
230703
231598
232492

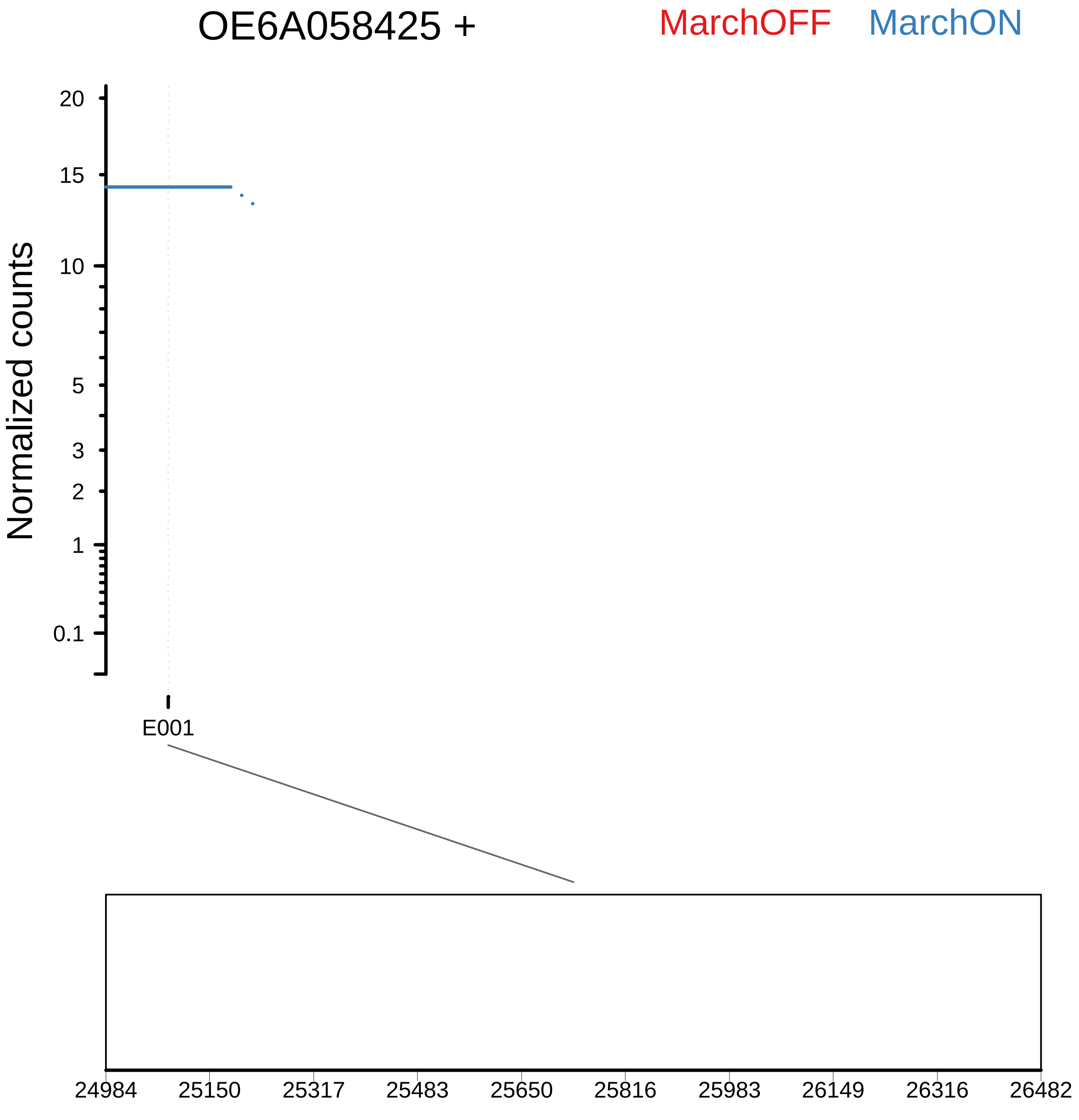

OE6A058425 +
MarchOFF
MarchON
Normalized counts
20
15
10
5
3
2
1
0.1
E001
24984
25150
25317
25483
25650
25816
25983
26149
26316
26482

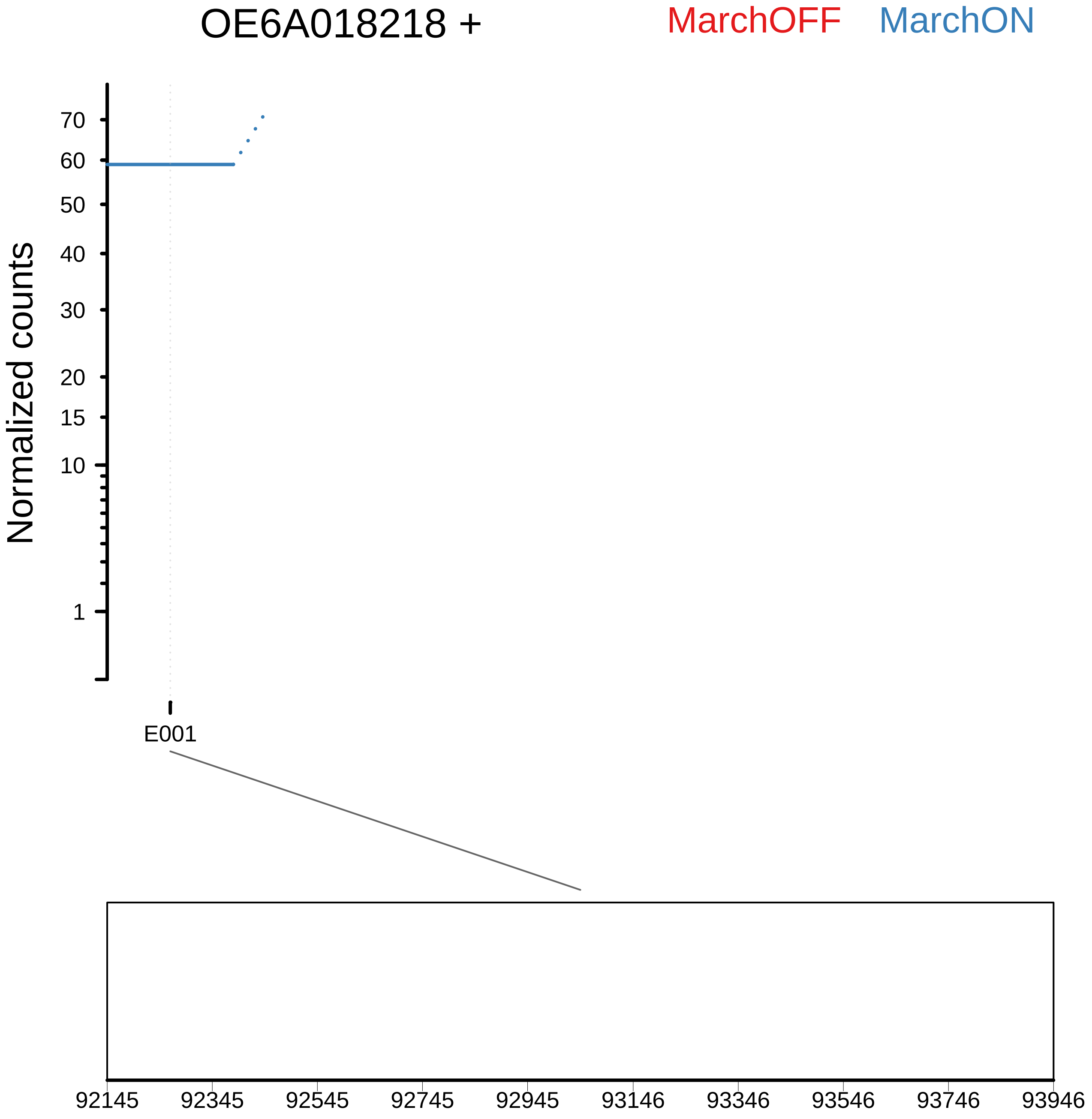
OE6A018218 +
MarchOFF
MarchON
Normalized counts
70
60
50
40
30
20
15
10
1
E001
92145
92345
92545
92745
92945
93146
93346
93546
93746
93946

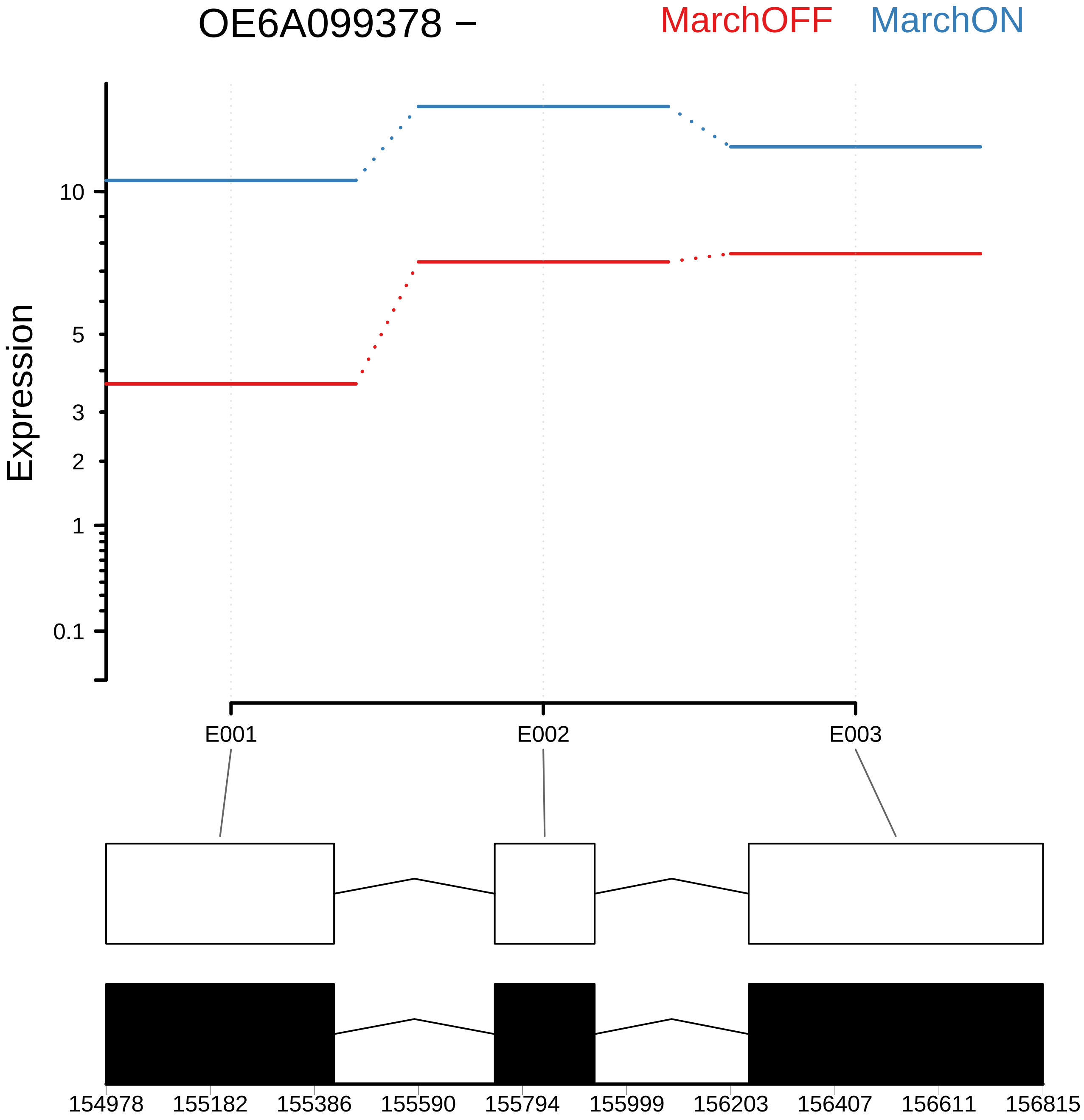
OE6A099378 −
MarchOFF
MarchON
Expression
10
5
3
2
1
0.1
E001
E002
E003
154978
155182
155386
155590
155794
155999
156203
156407
156611
156815

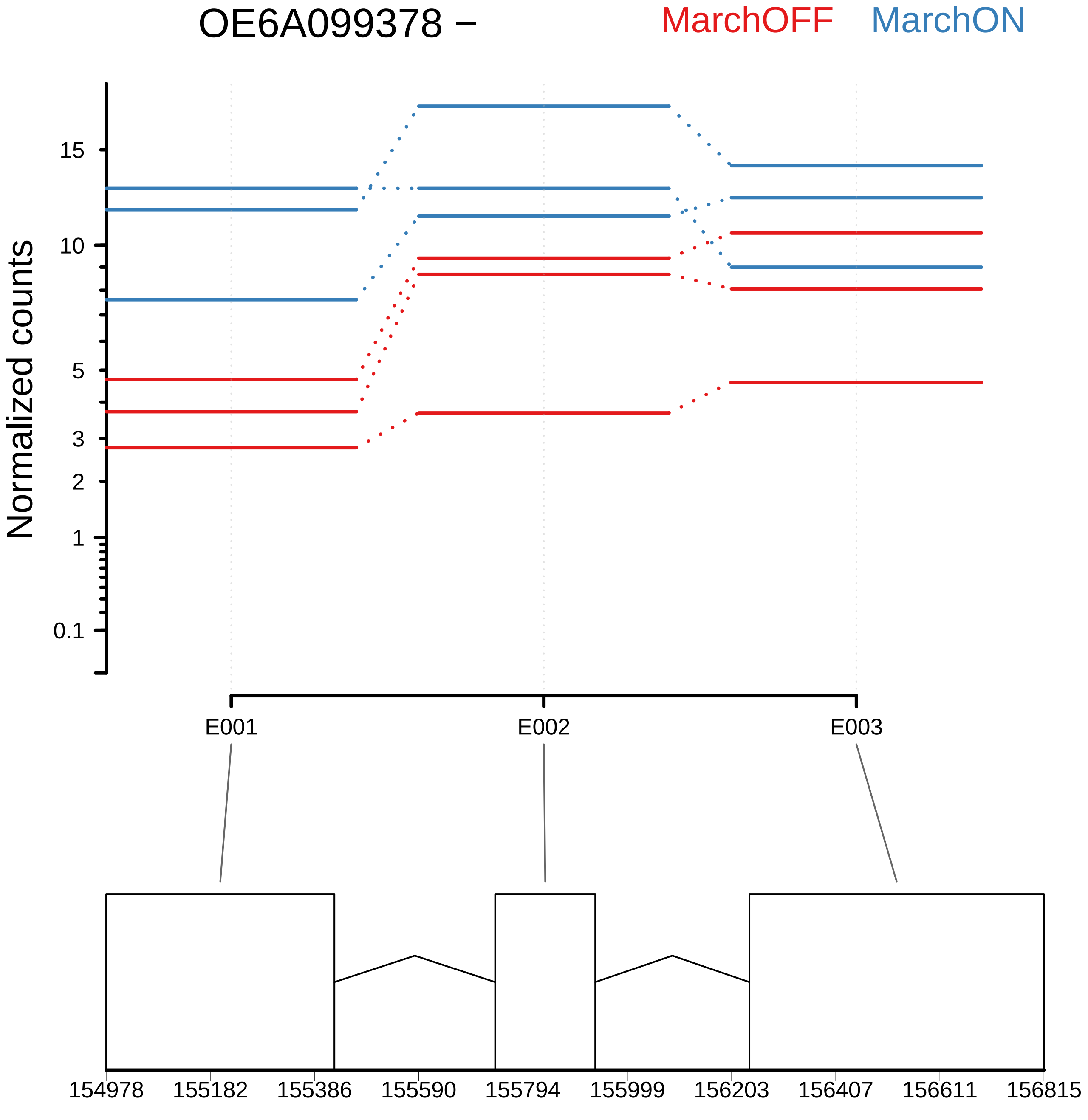

OE6A099378 −
MarchOFF
MarchON
Normalized counts
15
10
5
3
2
1
0.1
E001
E002
E003
154978
155182
155386
155590
155794
155999
156203
156407
156611
156815

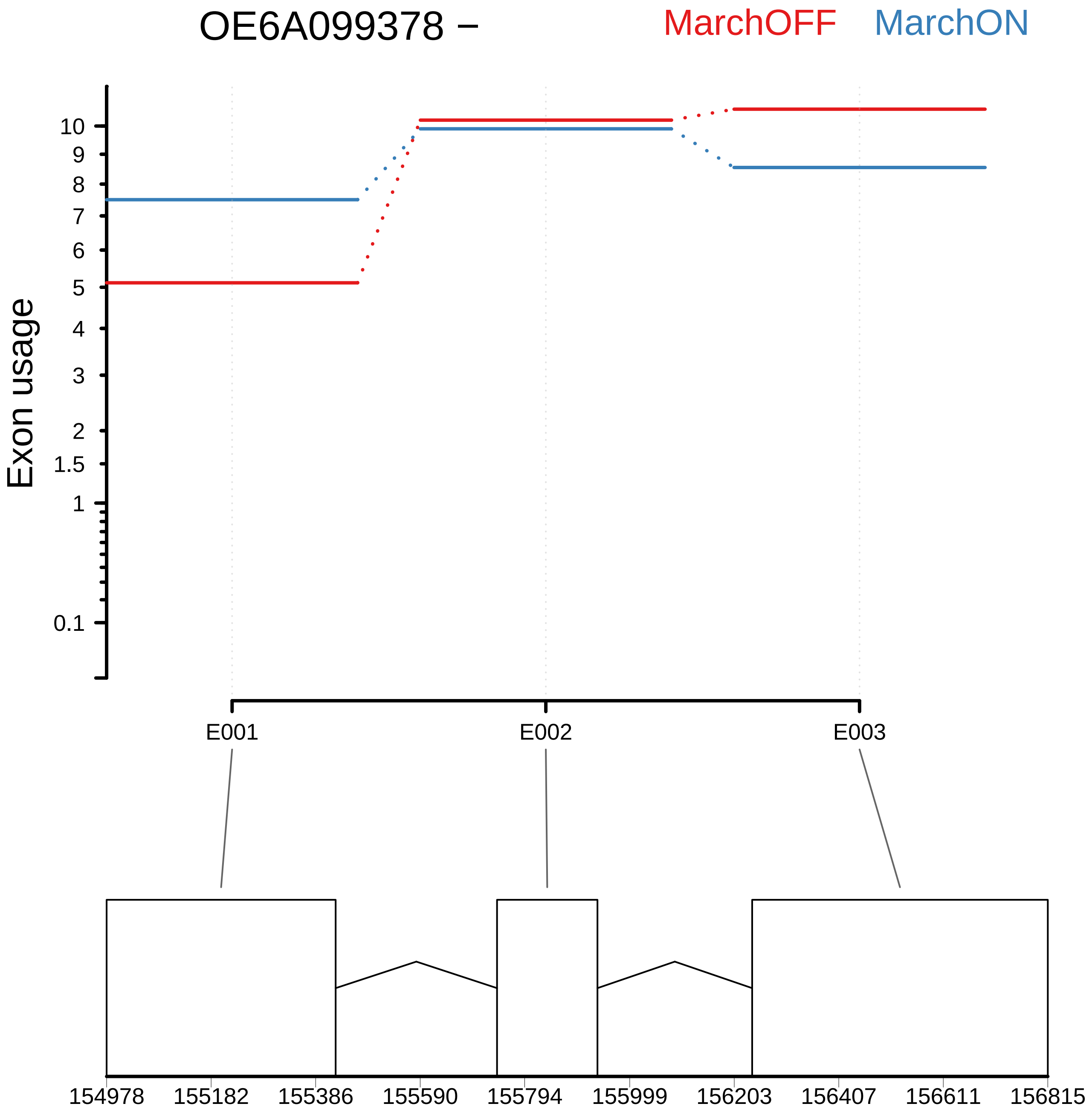

OE6A099378 −
MarchOFF
MarchON
Exon usage
10
9
8
7
6
5
4
3
2
1.5
1
0.1
E001
E002
E003
154978
155182
155386
155590
155794
155999
156203
156407
156611
156815

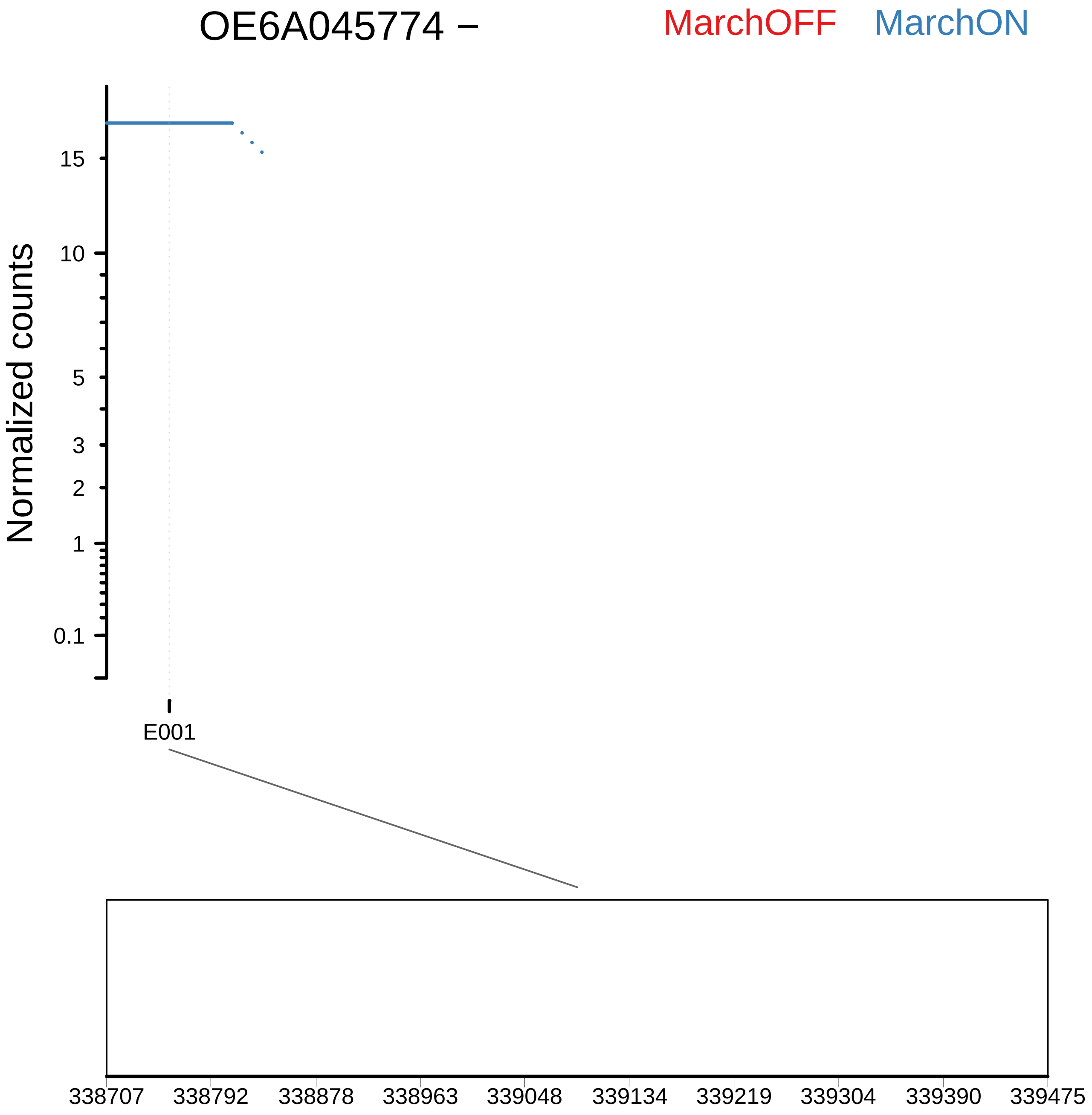

OE6A045774 −
MarchOFF
MarchON
Normalized counts
15
10
5
3
2
1
0.1
E001
338707
338792
338878
338963
339048
339134
339219
339304
339390
339475

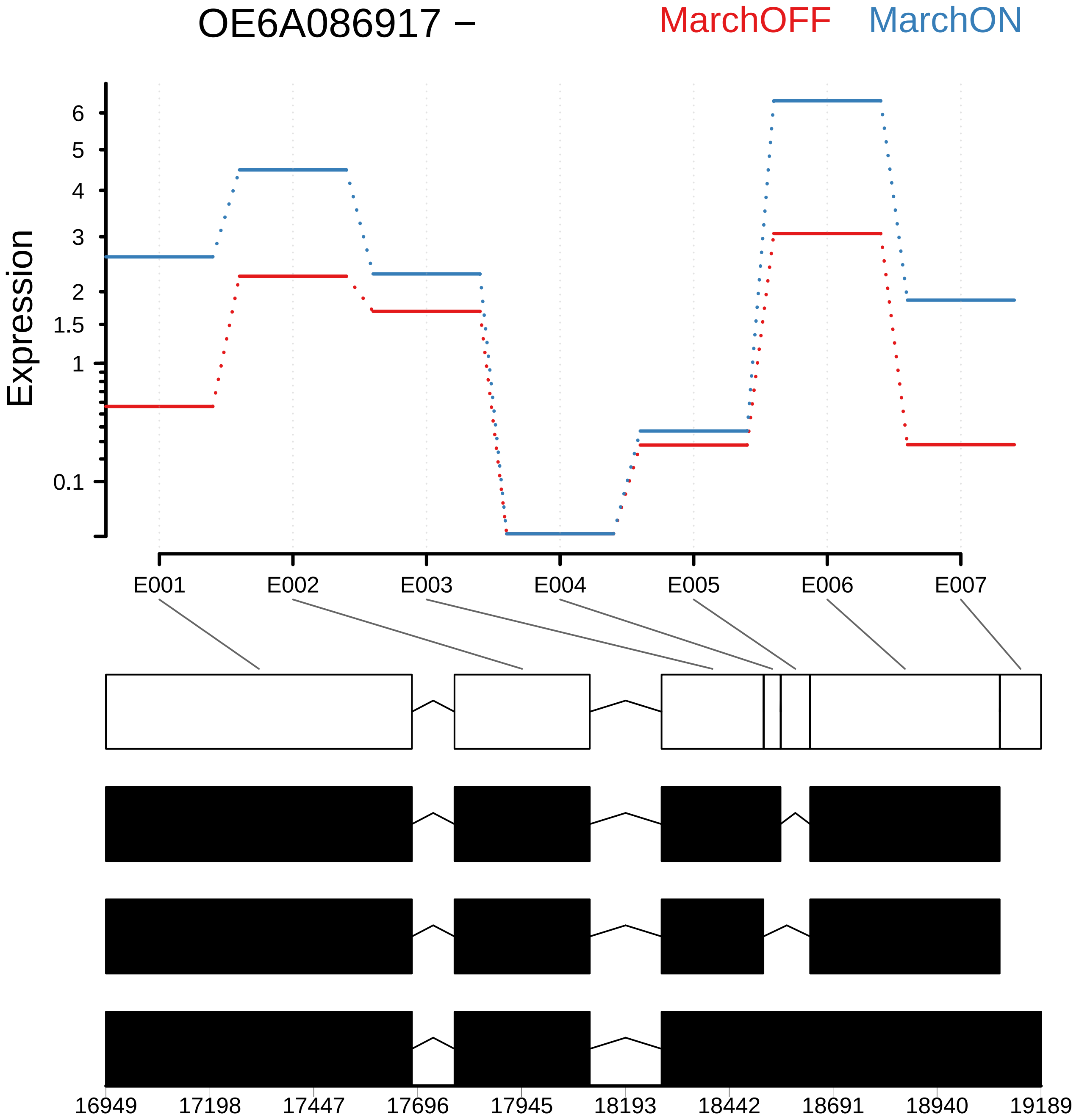

OE6A086917 –
MarchOFF
MarchON
Expression
6
5
4
3
2
1.5
1
0.1
E001
E002
E003
E004
E005
E006
E007
16949
17198
17447
17696
17945
18193
18442
18691
18940
19189

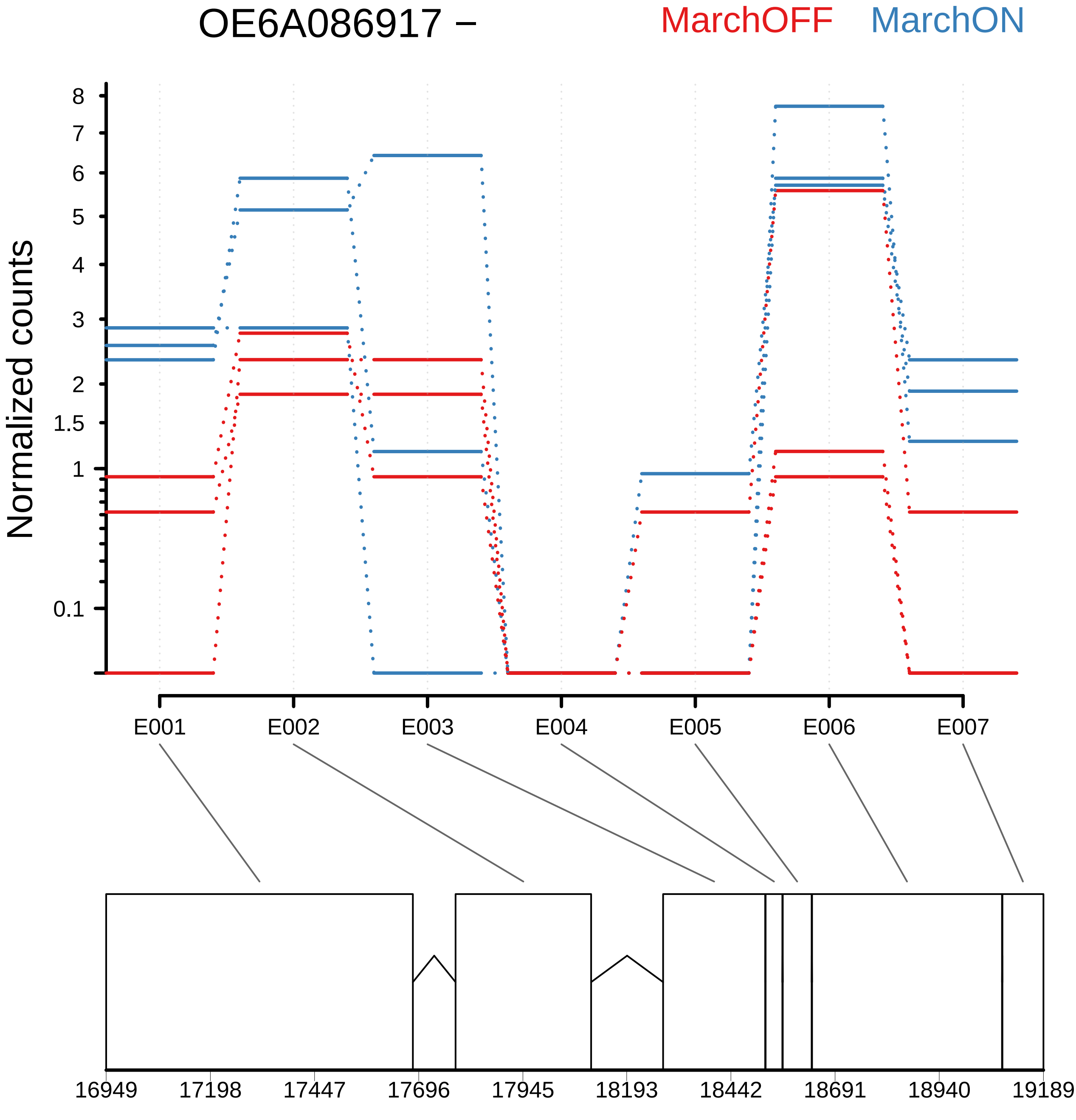

OE6A086917 −
MarchOFF
MarchON
Normalized counts
8
7
6
5
4
3
2
1.5
1
0.1
E001
E002
E003
E004
E005
E006
E007
16949
17198
17447
17696
17945
18193
18442
18691
18940
19189

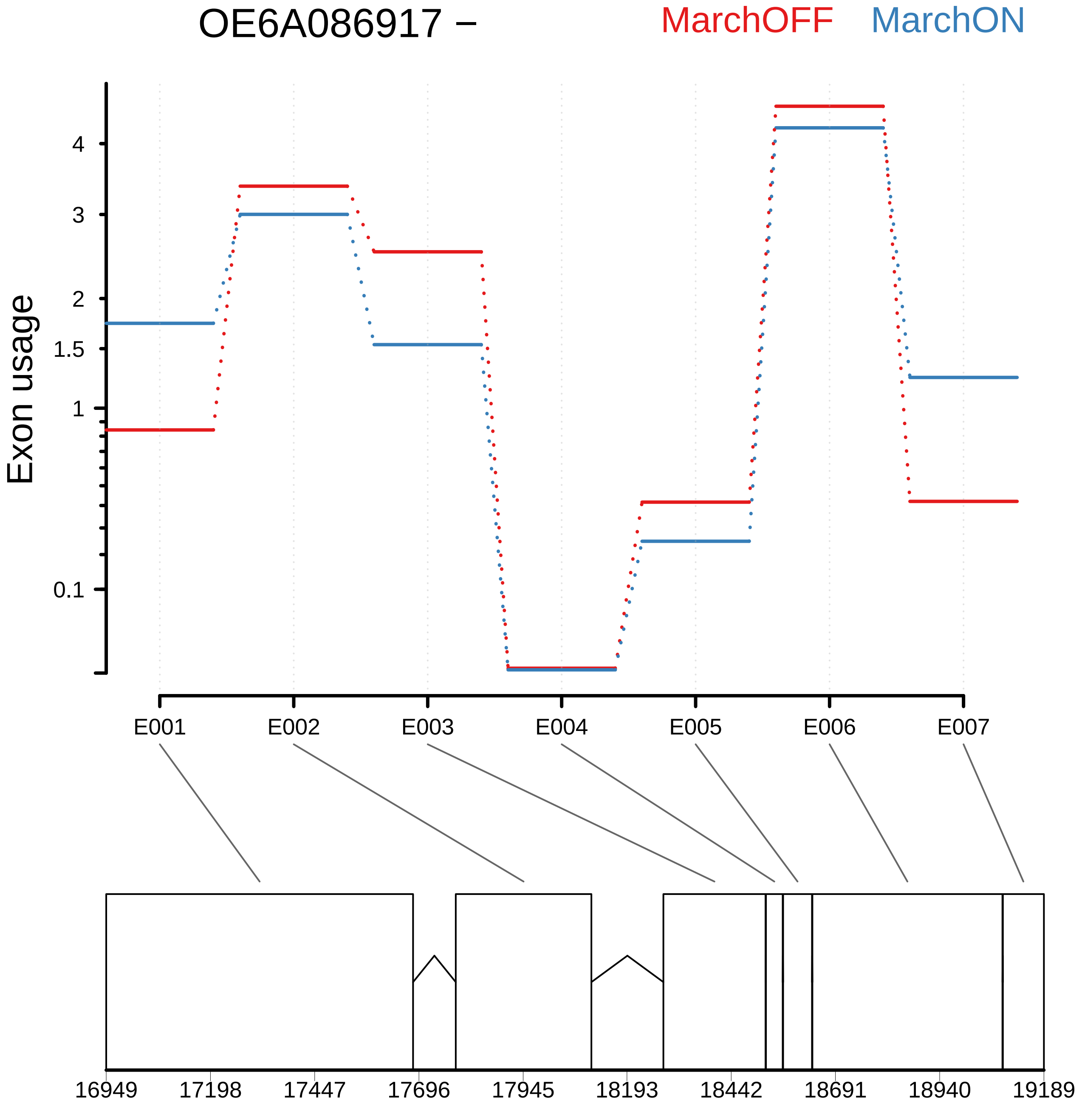

OE6A086917 −
MarchOFF
MarchON
Exon usage
4
3
2
1.5
1
0.1
E001
E002
E003
E004
E005
E006
E007
16949
17198
17447
17696
17945
18193
18442
18691
18940
19189

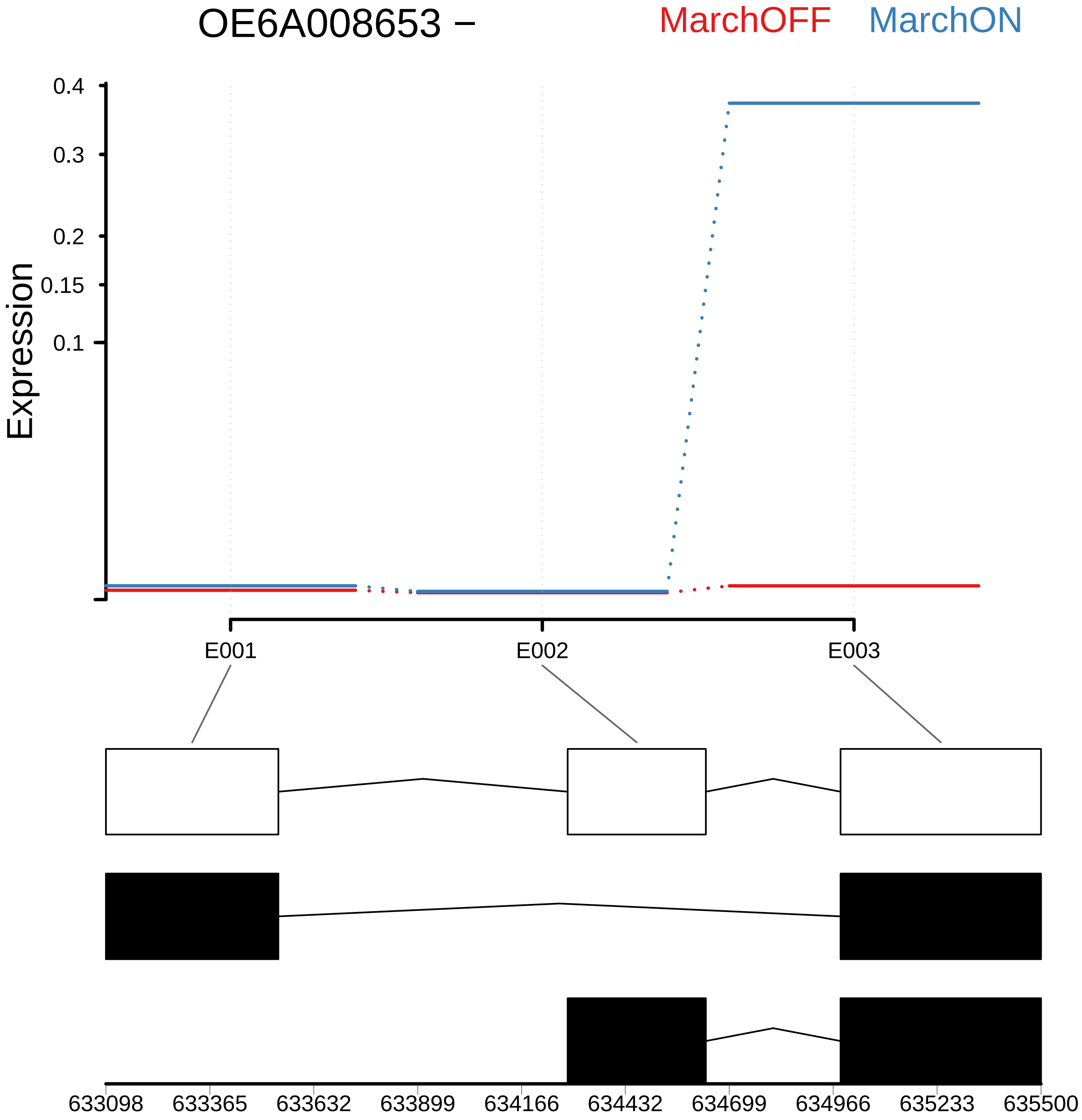
OE6A008653 −
MarchOFF
MarchON
Expression
0.4
0.3
0.2
0.15
0.1
E001
E002
E003
633098
633365
633632
633899
634166
634432
634699
634966
635233
635500

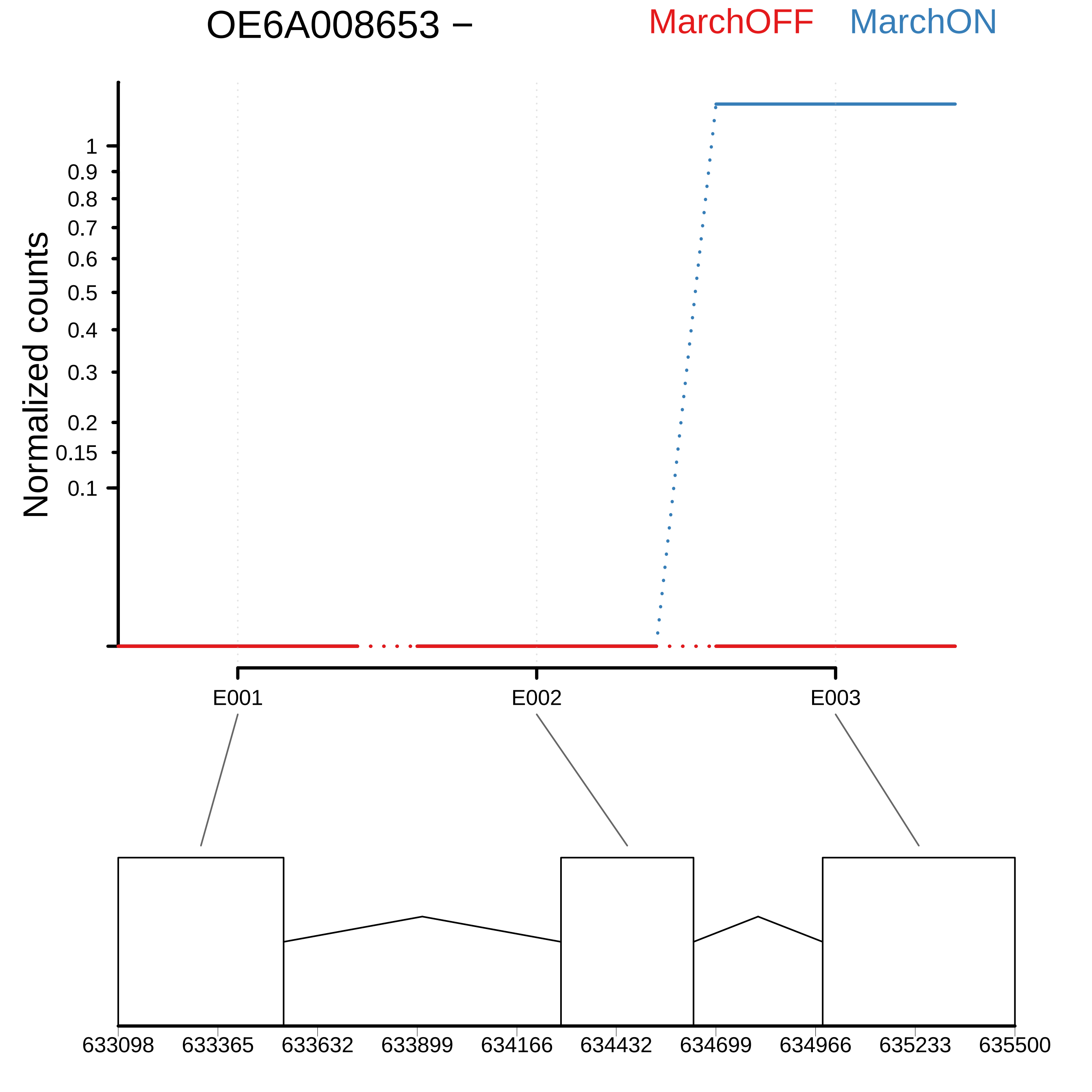
OE6A008653 −
MarchOFF
MarchON
Normalized counts
1
0.9
0.8
0.7
0.6
0.5
0.4
0.3
0.2
0.15
0.1
E001
E002
E003
633098
633365
633632
633899
634166
634432
634699
634966
635233
635500

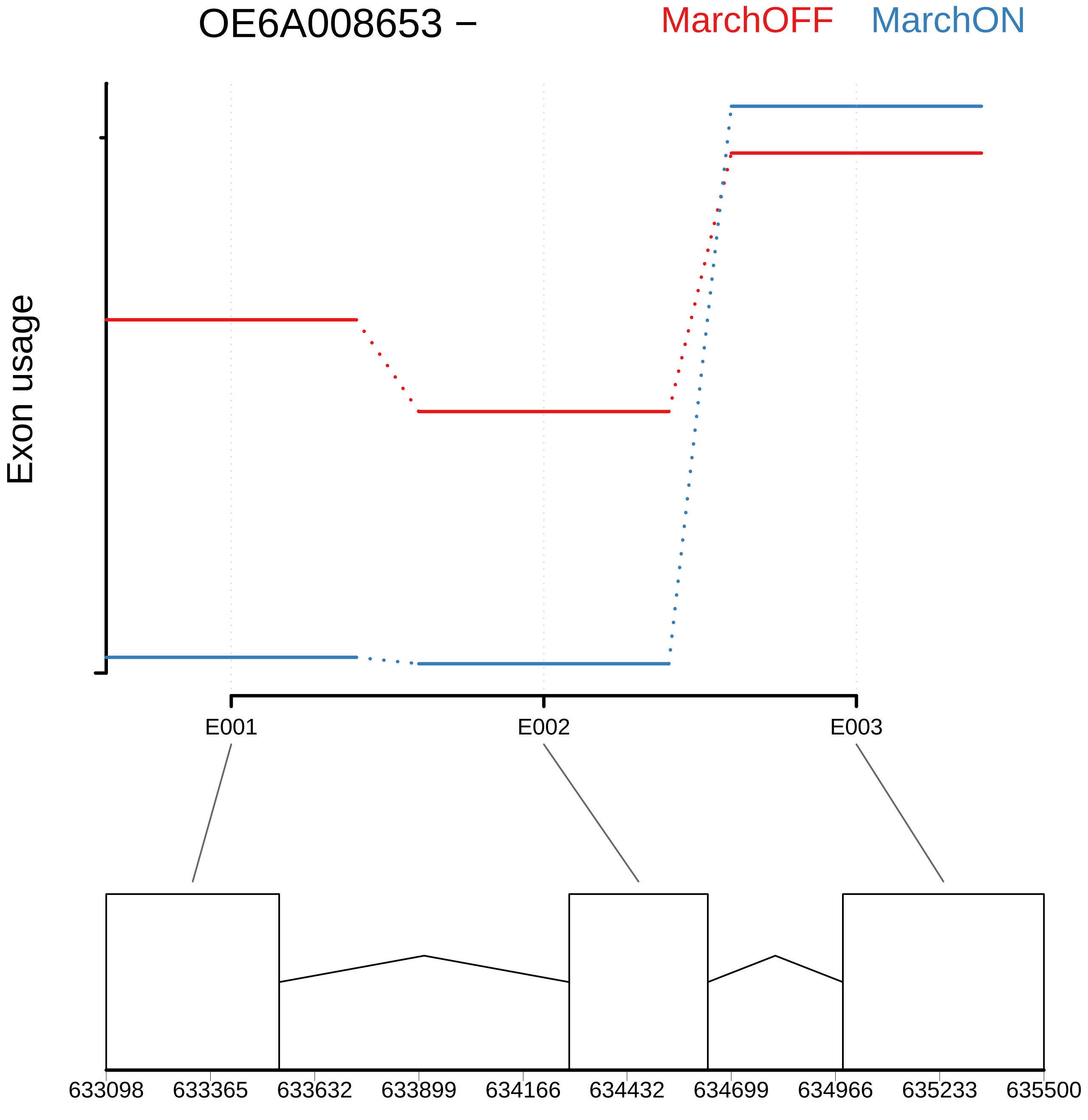

OE6A008653 −
MarchOFF
MarchON
Exon usage
E001
E002
E003
633098
633365
633632
633899
634166
634432
634699
634966
635233
635500

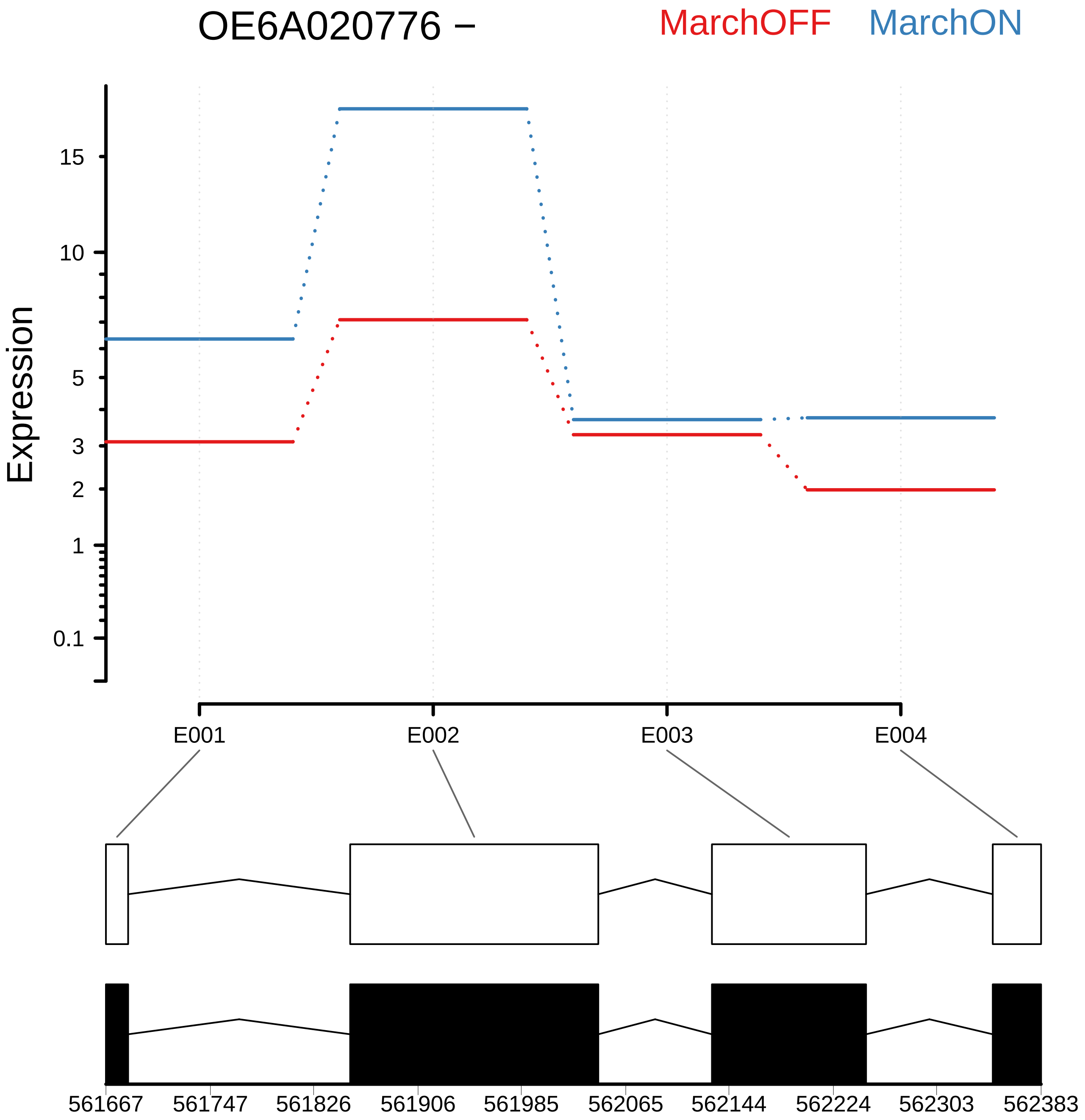
OE6A020776 −
MarchOFF
MarchON
Expression
15
10
5
3
2
1
0.1
E001
E002
E003
E004
561667
561747
561826
561906
561985
562065
562144
562224
562303
562383

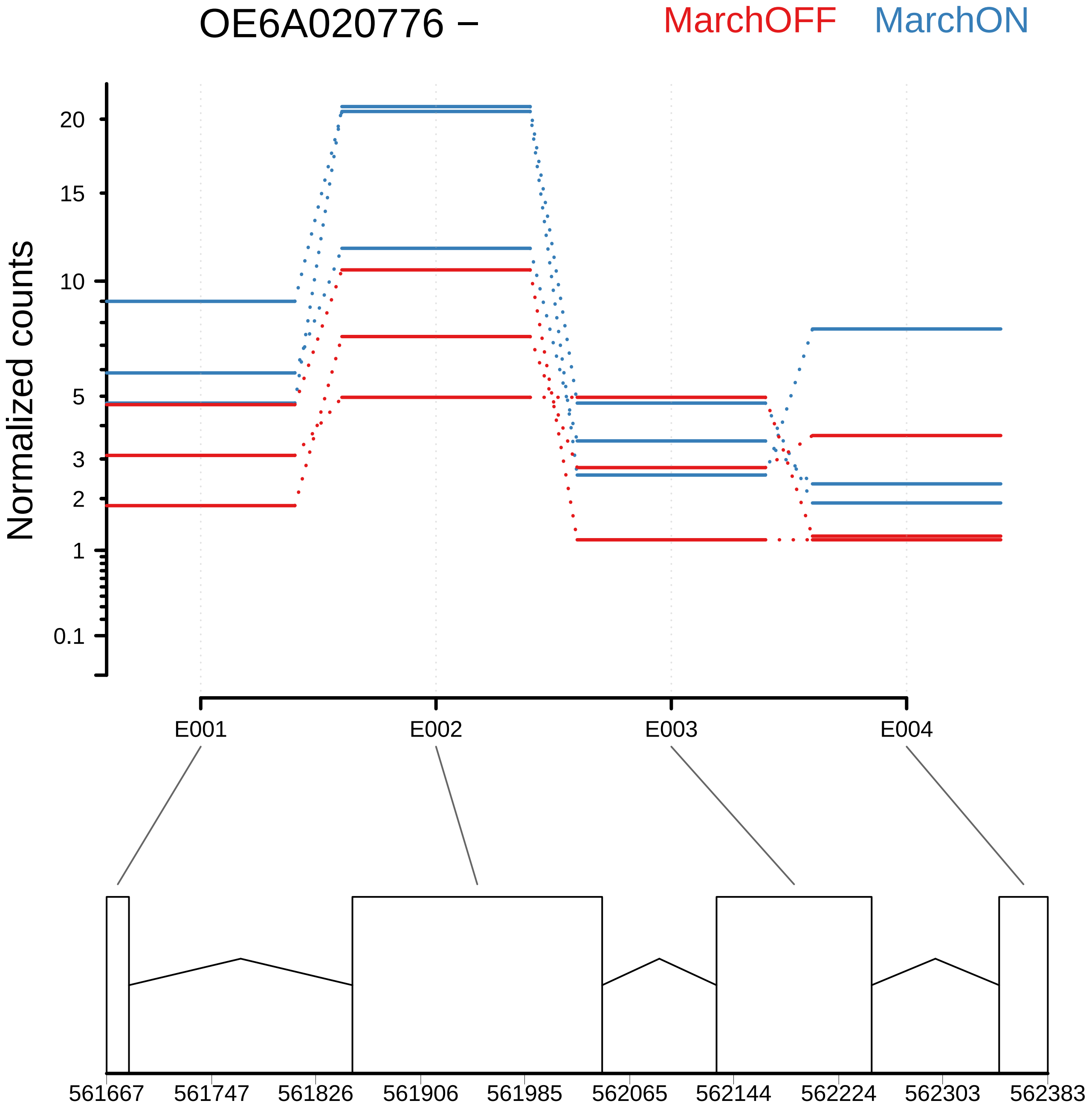

OE6A020776 −
MarchOFF
MarchON
Normalized counts
20
15
10
5
3
2
1
0.1
E001
E002
E003
E004
561667
561747
561826
561906
561985
562065
562144
562224
562303
562383

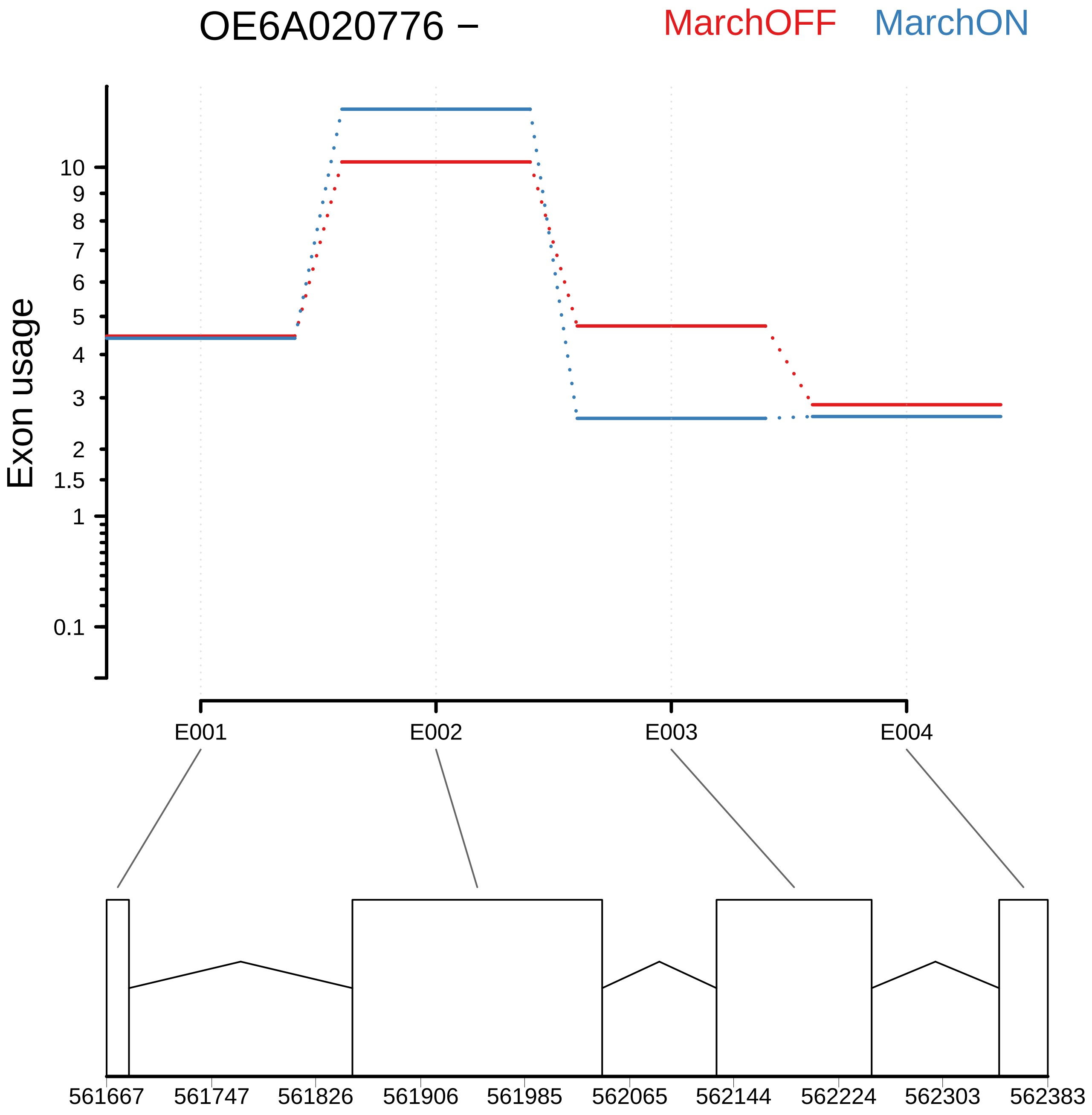
OE6A020776 −
MarchOFF
MarchON
Exon usage
10
9
8
7
6
5
4
3
2
1.5
1
0.1
E001
E002
E003
E004
561667
561747
561826
561906
561985
562065
562144
562224
562303
562383

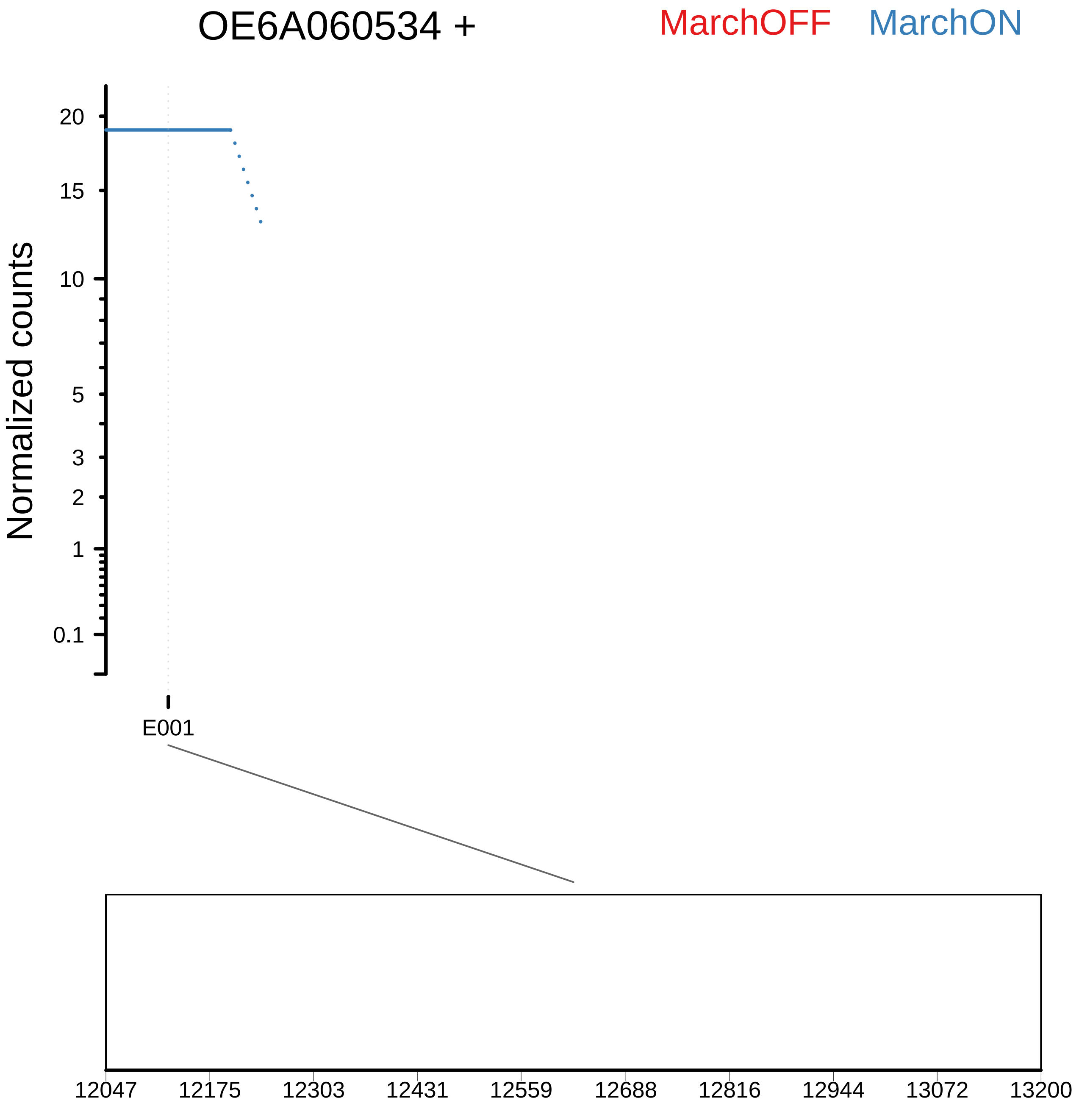
OE6A060534 +
MarchOFF
MarchON
Normalized counts
20
15
10
5
3
2
1
0.1
E001
12047
12175
12303
12431
12559
12688
12816
12944
13072
13200

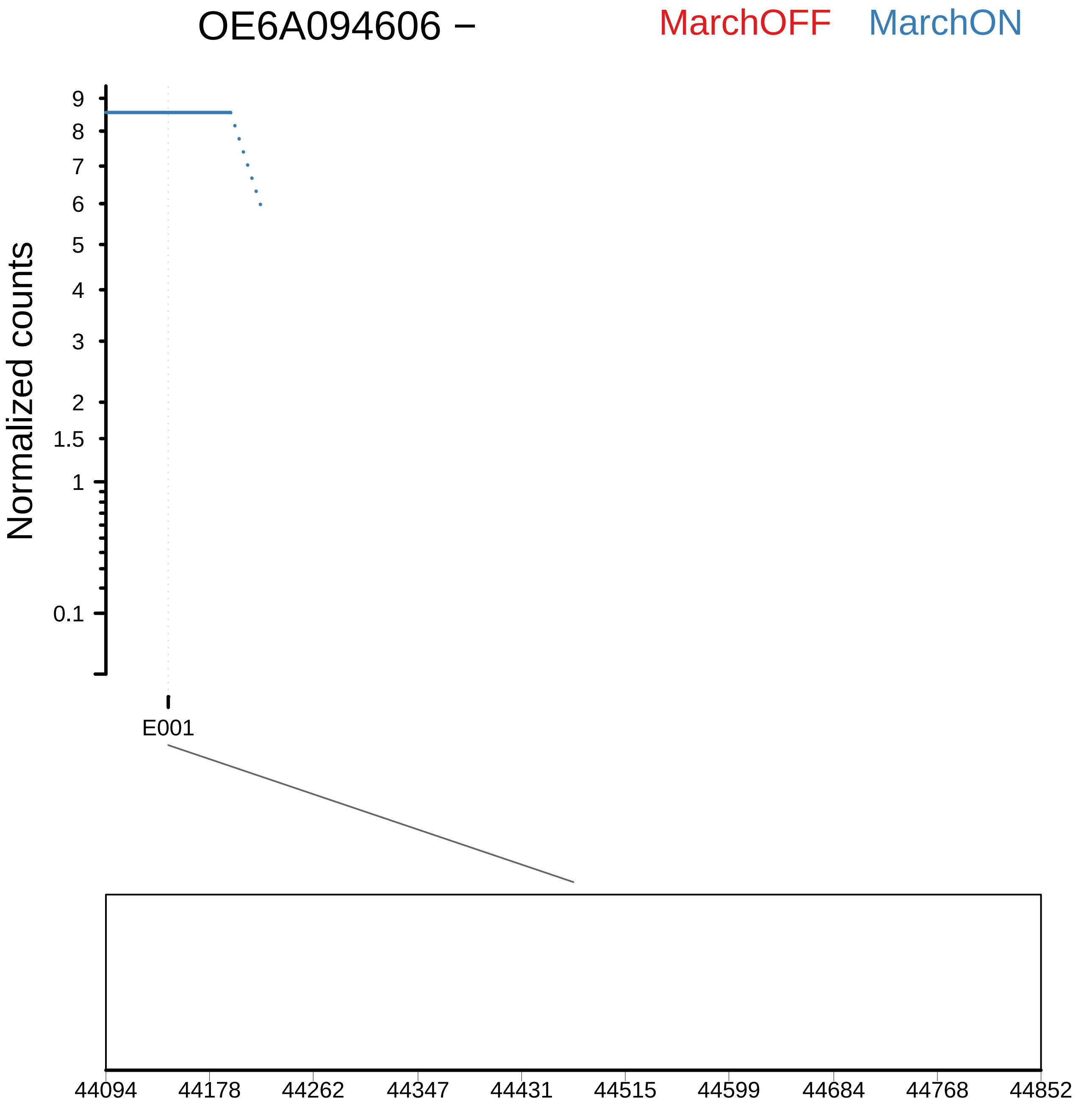

OE6A094606 −
MarchOFF
MarchON
Normalized counts
9
8
7
6
5
4
3
2
1.5
1
0.1
E001
44094
44178
44262
44347
44431
44515
44599
44684
44768
44852

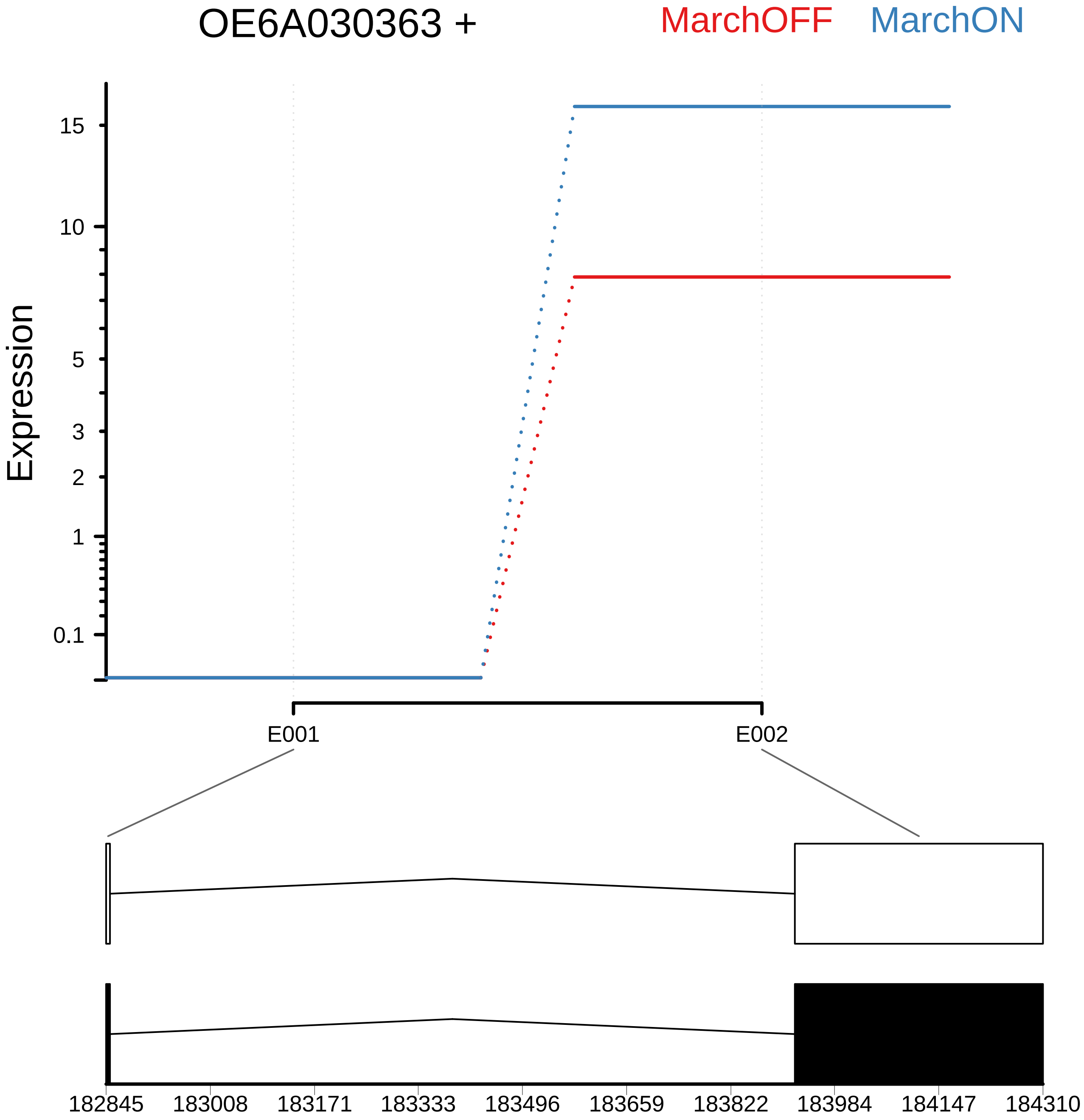

OE6A030363 +
MarchOFF
MarchON
Expression
15
10
5
3
2
1
0.1
E001
E002
182845
183008
183171
183333
183496
183659
183822
183984
184147
184310

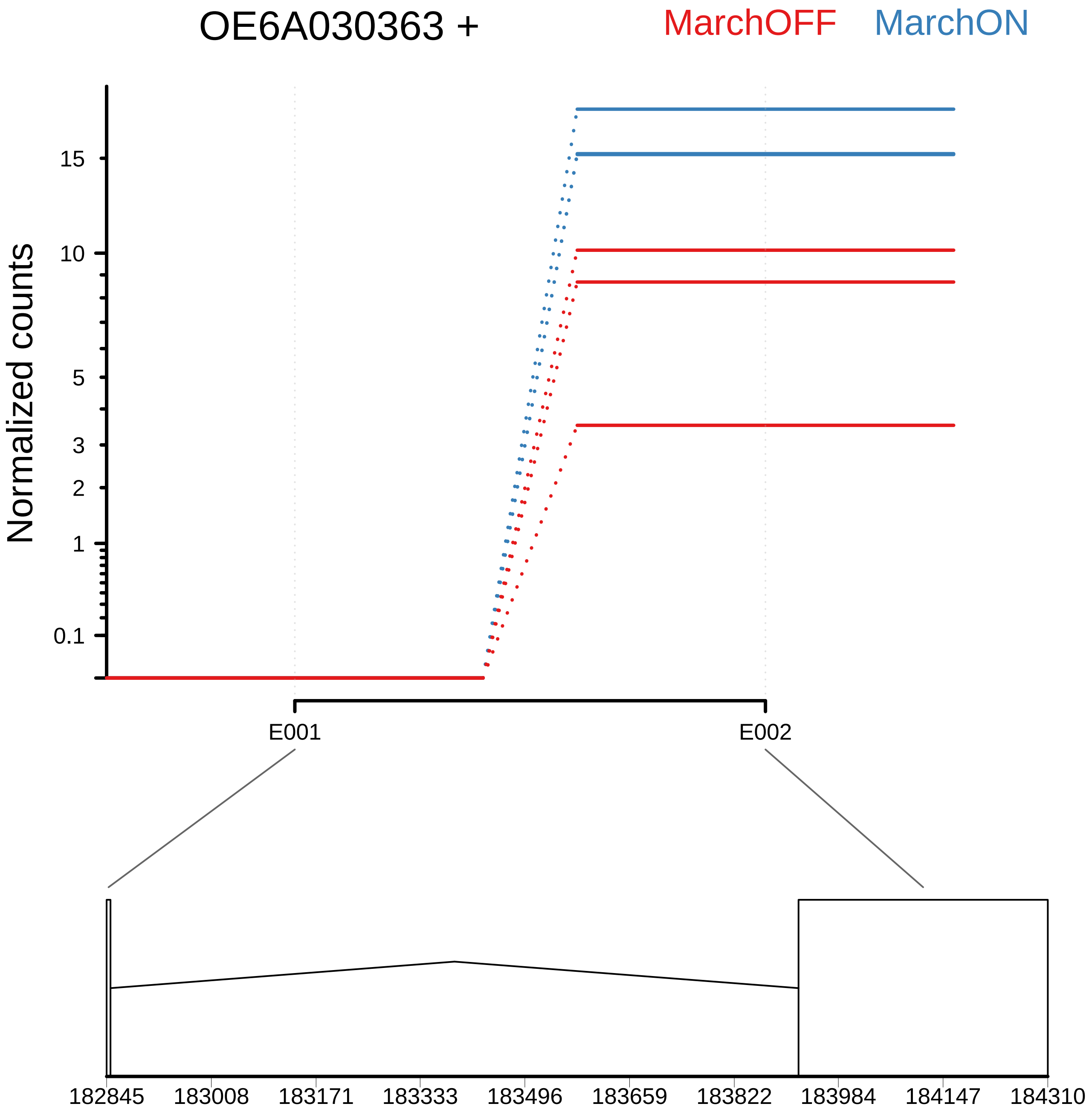

OE6A030363 +
MarchOFF
MarchON
Normalized counts
15
10
5
3
2
1
0.1
E001
E002
182845
183008
183171
183333
183496
183659
183822
183984
184147
184310

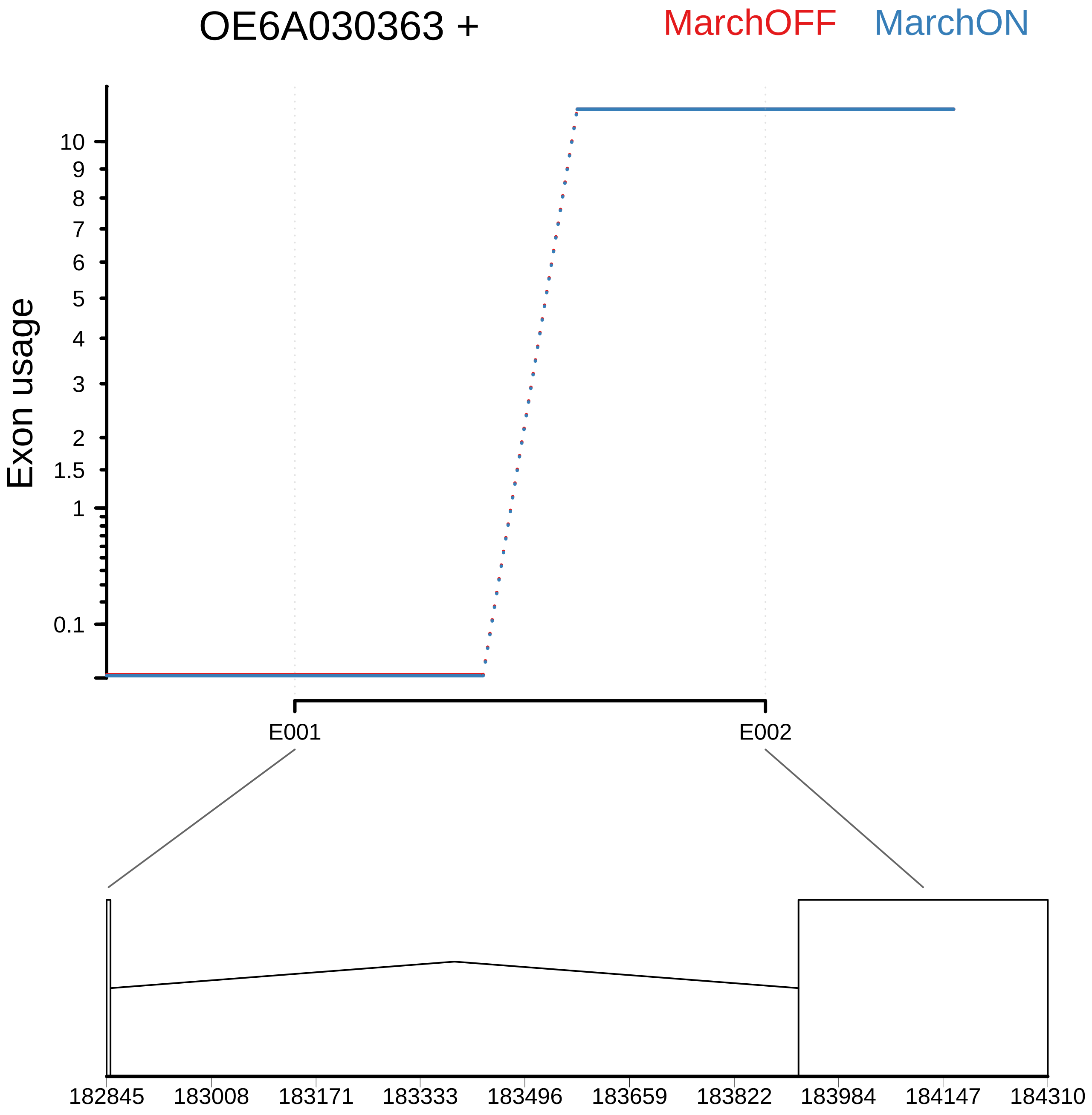
OE6A030363 +
MarchOFF
MarchON
Exon usage
10
9
8
7
6
5
4
3
2
1.5
1
0.1
E001
E002
182845
183008
183171
183333
183496
183659
183822
183984
184147
184310

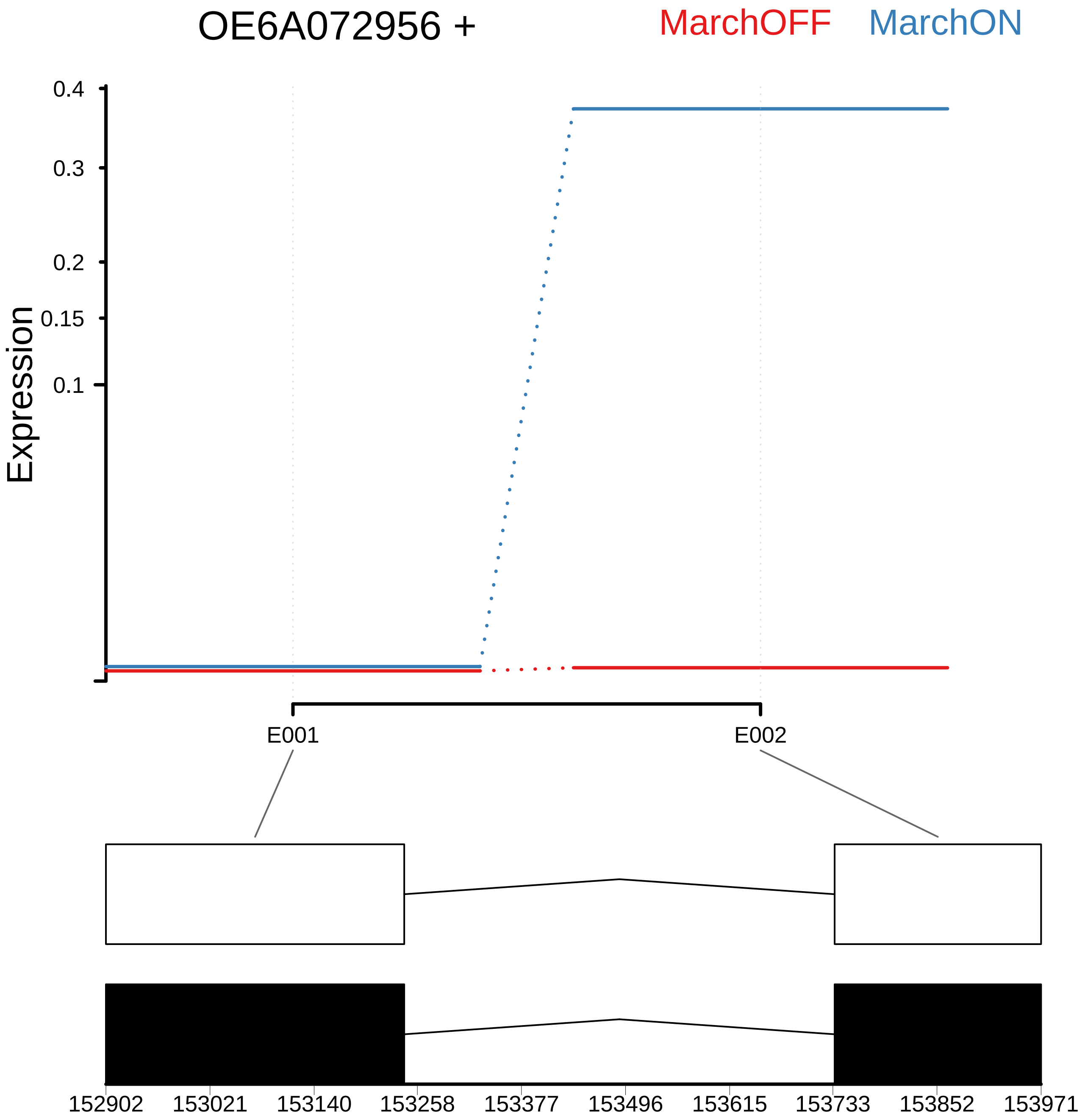

OE6A072956 +
MarchOFF
MarchON
Expression
0.4
0.3
0.2
0.15
0.1
E001
E002
152902
153021
153140
153258
153377
153496
153615
153733
153852
153971

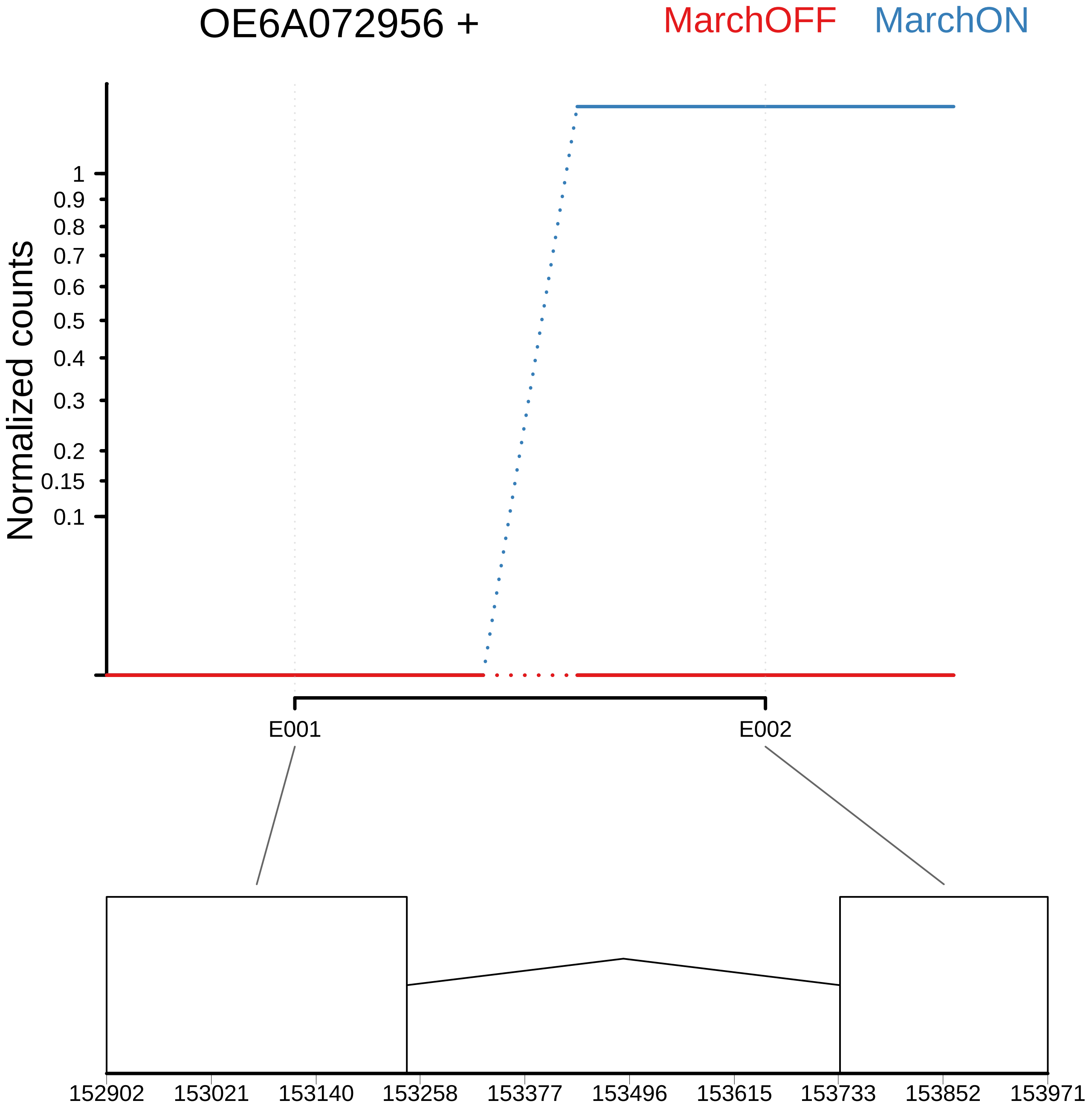
OE6A072956 +
MarchOFF
MarchON
Normalized counts
1
0.9
0.8
0.7
0.6
0.5
0.4
0.3
0.2
0.15
0.1
E001
E002
152902
153021
153140
153258
153377
153496
153615
153733
153852
153971

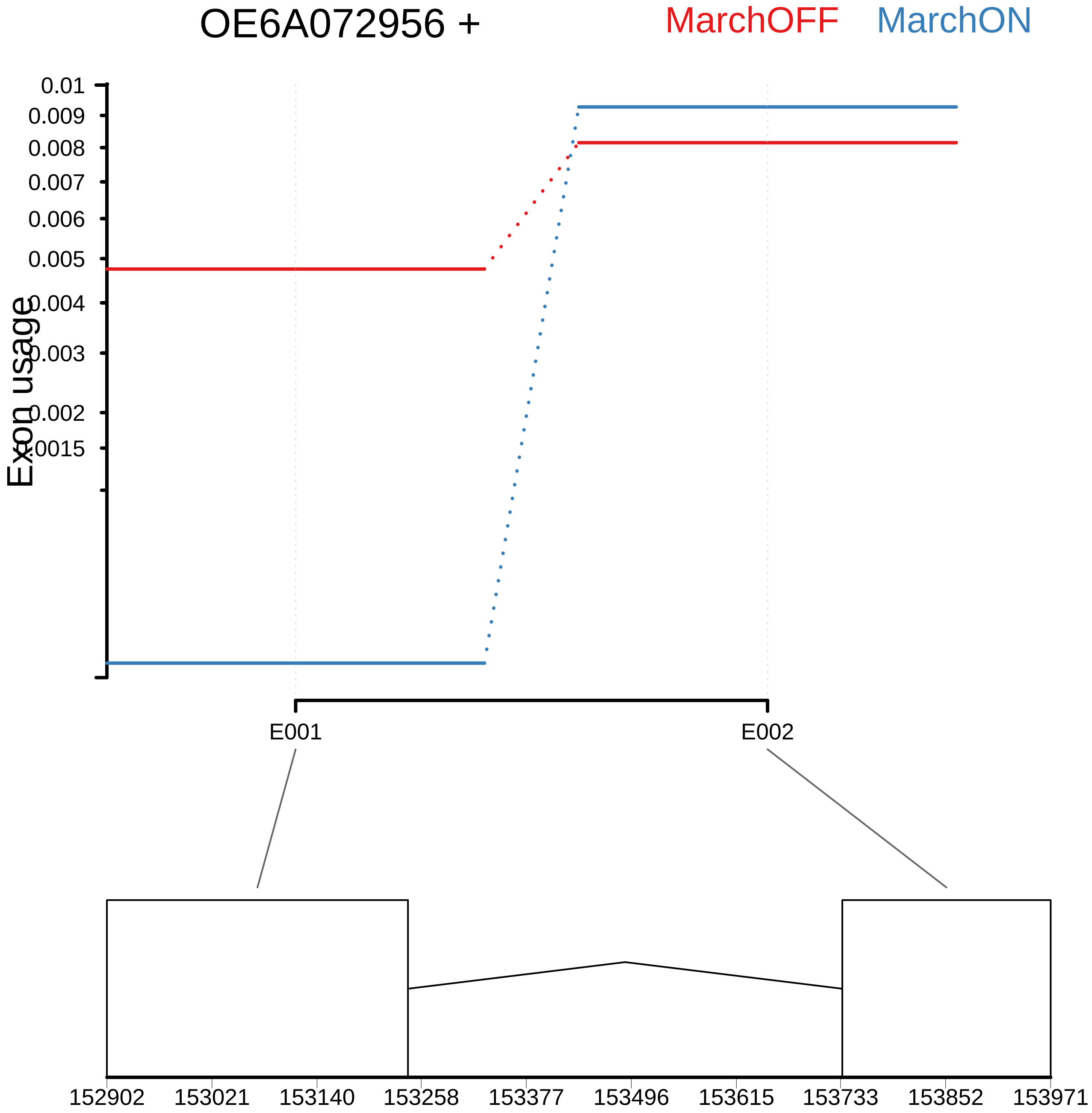

OE6A072956 +
MarchOFF
MarchON
Exon usage
0.01
0.009
0.008
0.007
0.006
0.005
0.004
0.003
0.002
0.0015
E001
E002
152902
153021
153140
153258
153377
153496
153615
153733
153852
153971

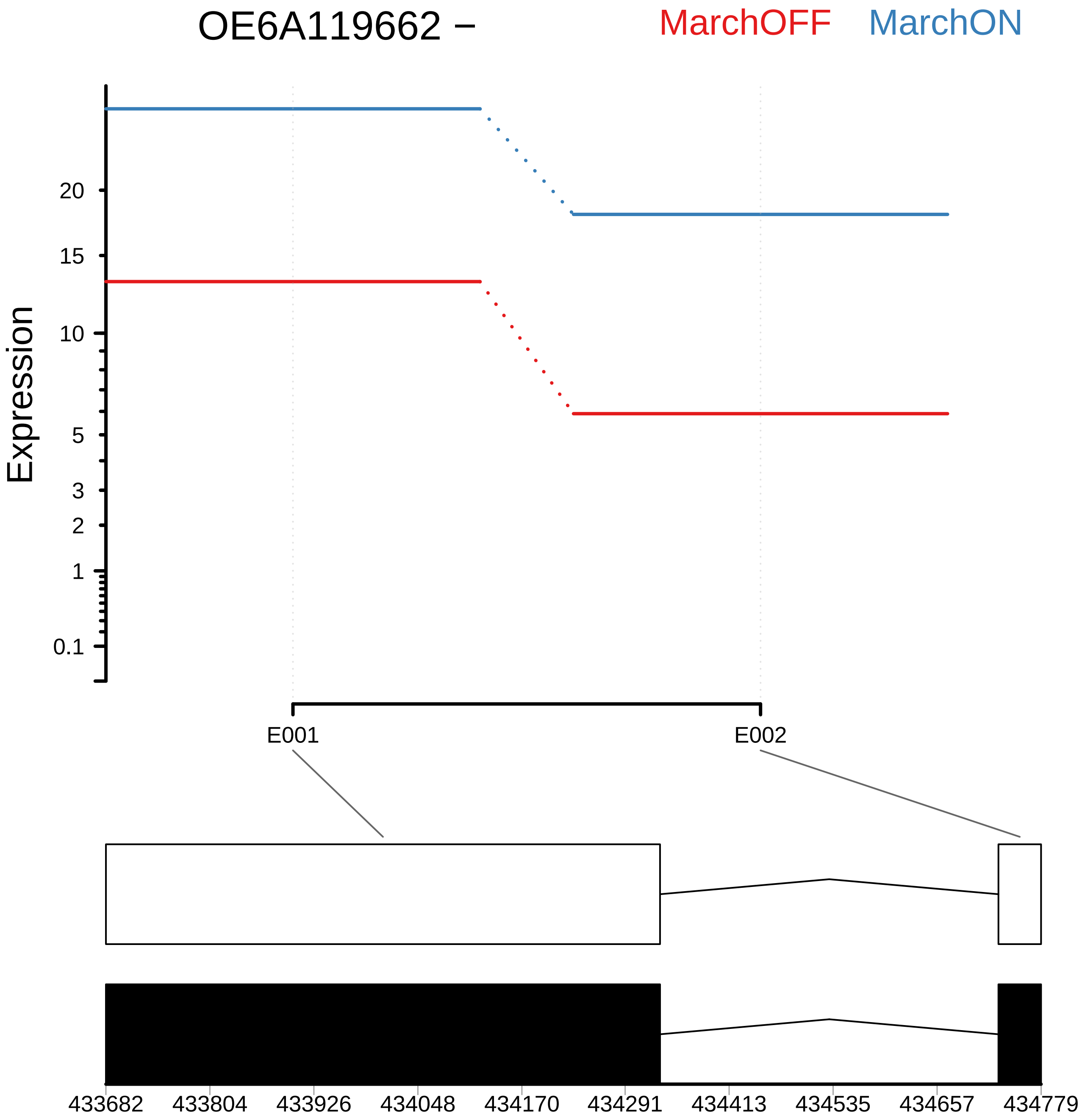

OE6A119662 −
MarchOFF
MarchON
Expression
20
15
10
5
3
2
1
0.1
E001
E002
433682
433804
433926
434048
434170
434291
434413
434535
434657
434779

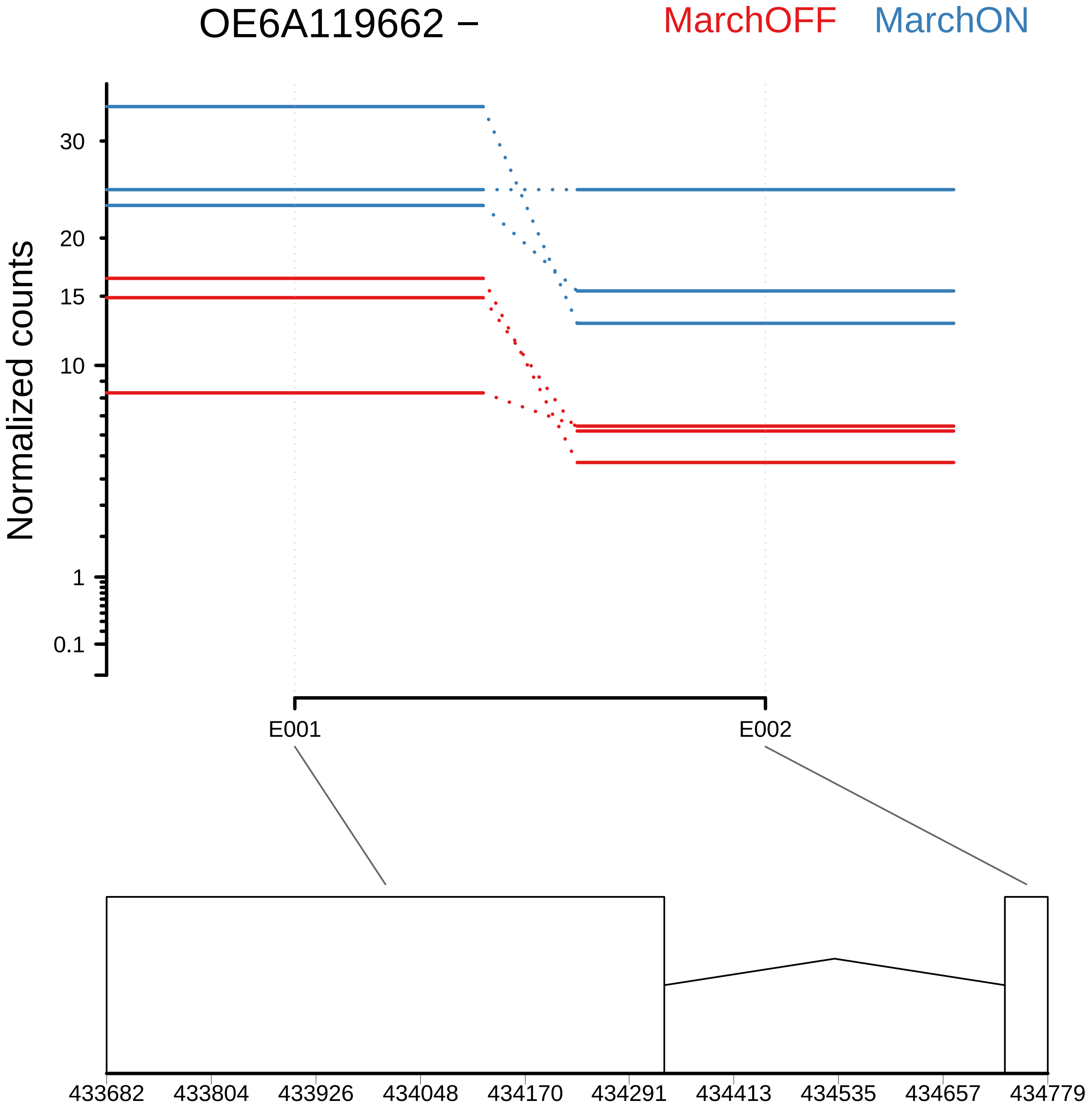
OE6A119662 −
MarchOFF
MarchON
Normalized counts
30
20
15
10
1
0.1
E001
E002
433682
433804
433926
434048
434170
434291
434413
434535
434657
434779

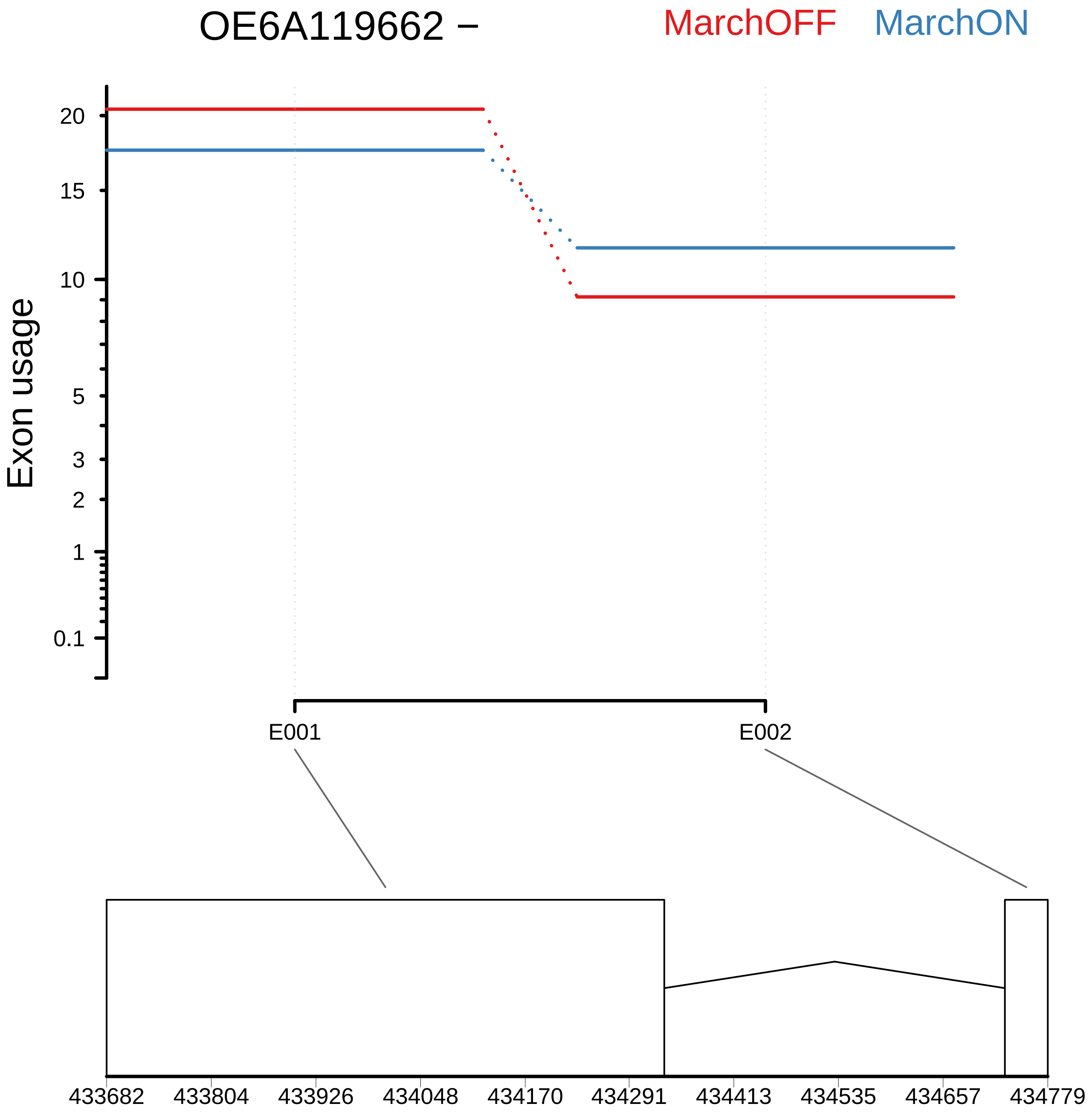

OE6A119662 −
MarchOFF
MarchON
Exon usage
20
15
10
5
3
2
1
0.1
E001
E002
433682
433804
433926
434048
434170
434291
434413
434535
434657
434779

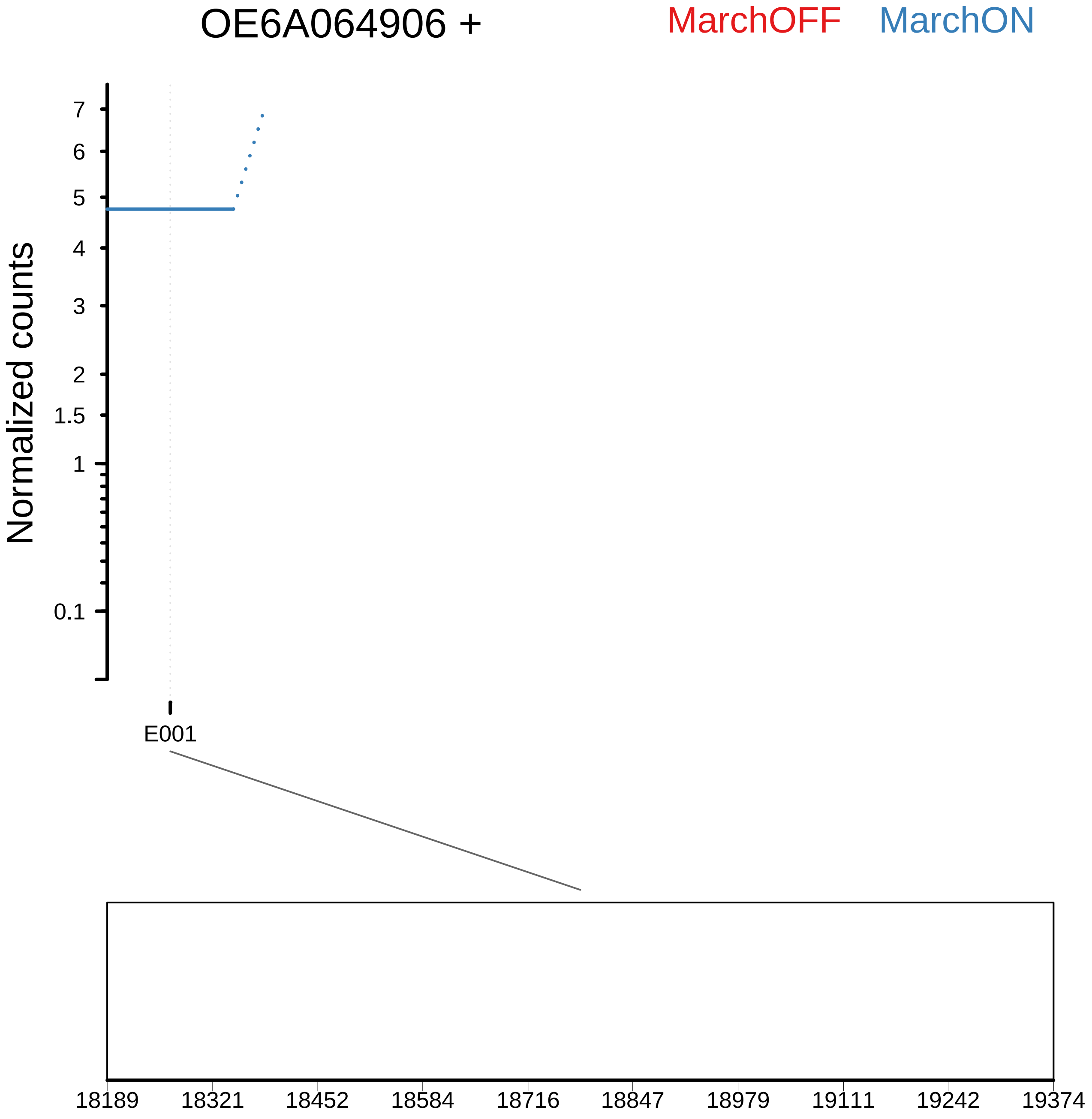
OE6A064906 +
MarchOFF
MarchON
Normalized counts
7
6
5
4
3
2
1.5
1
0.1
E001
18189
18321
18452
18584
18716
18847
18979
19111
19242
19374

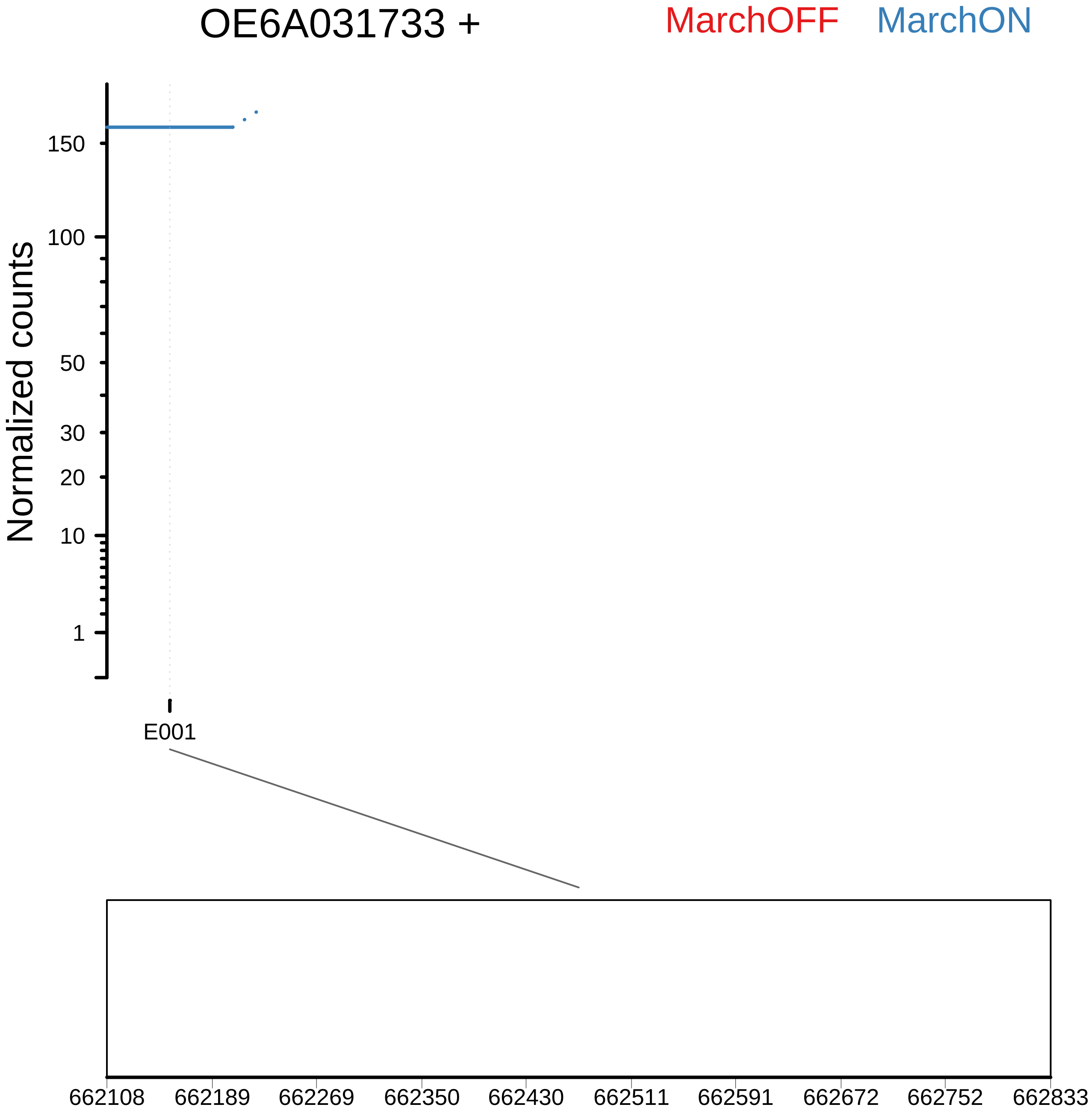

OE6A031733 +
MarchOFF
MarchON
Normalized counts
150
100
50
30
20
10
1
E001
662108
662189
662269
662350
662430
662511
662591
662672
662752
662833

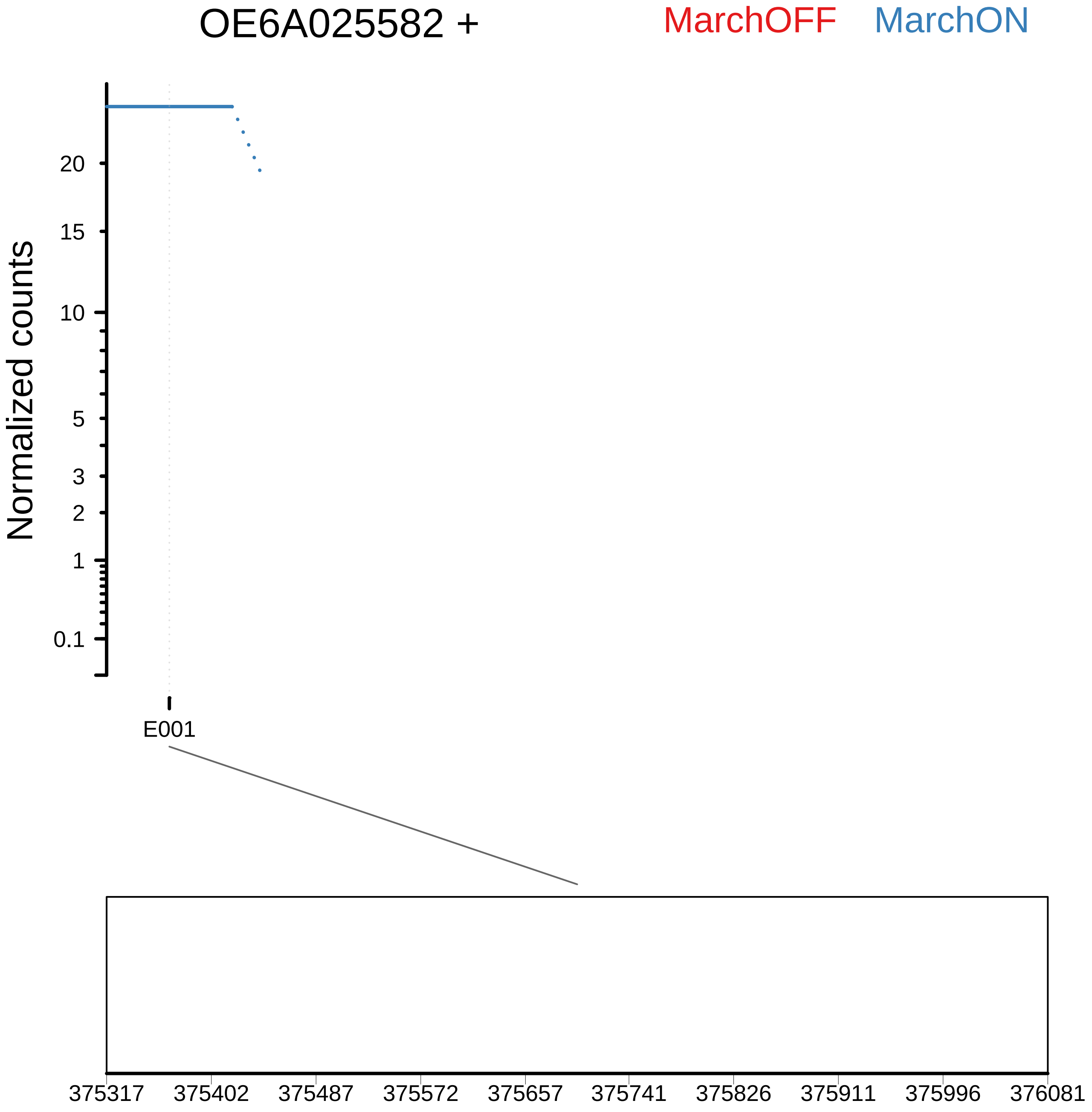
OE6A025582 +
MarchOFF
MarchON
Normalized counts
20
15
10
5
3
2
1
0.1
E001
375317
375402
375487
375572
375657
375741
375826
375911
375996
376081

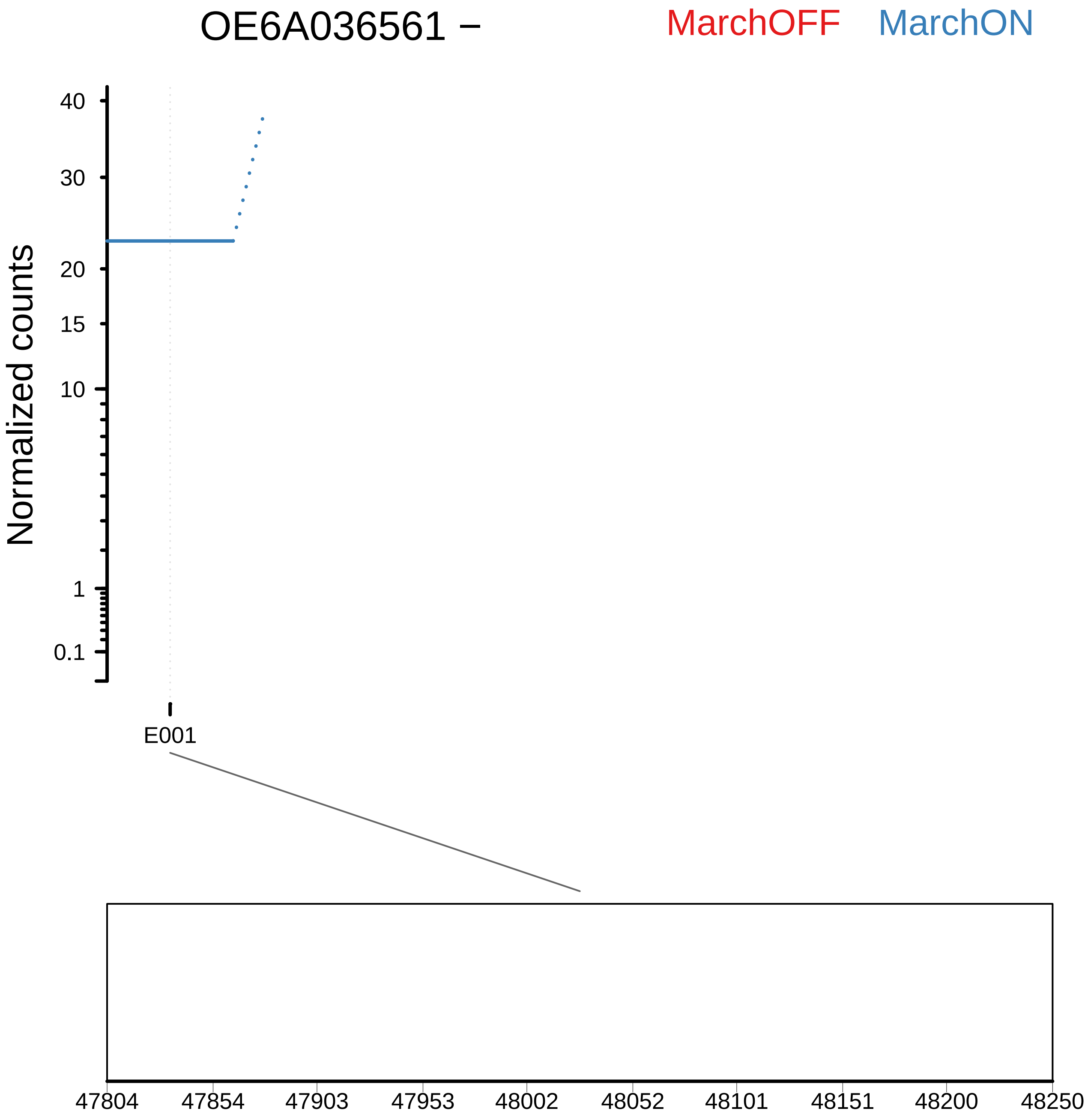

OE6A036561 −
MarchOFF
MarchON
Normalized counts
40
30
20
15
10
1
0.1
E001
47804
47854
47903
47953
48002
48052
48101
48151
48200
48250

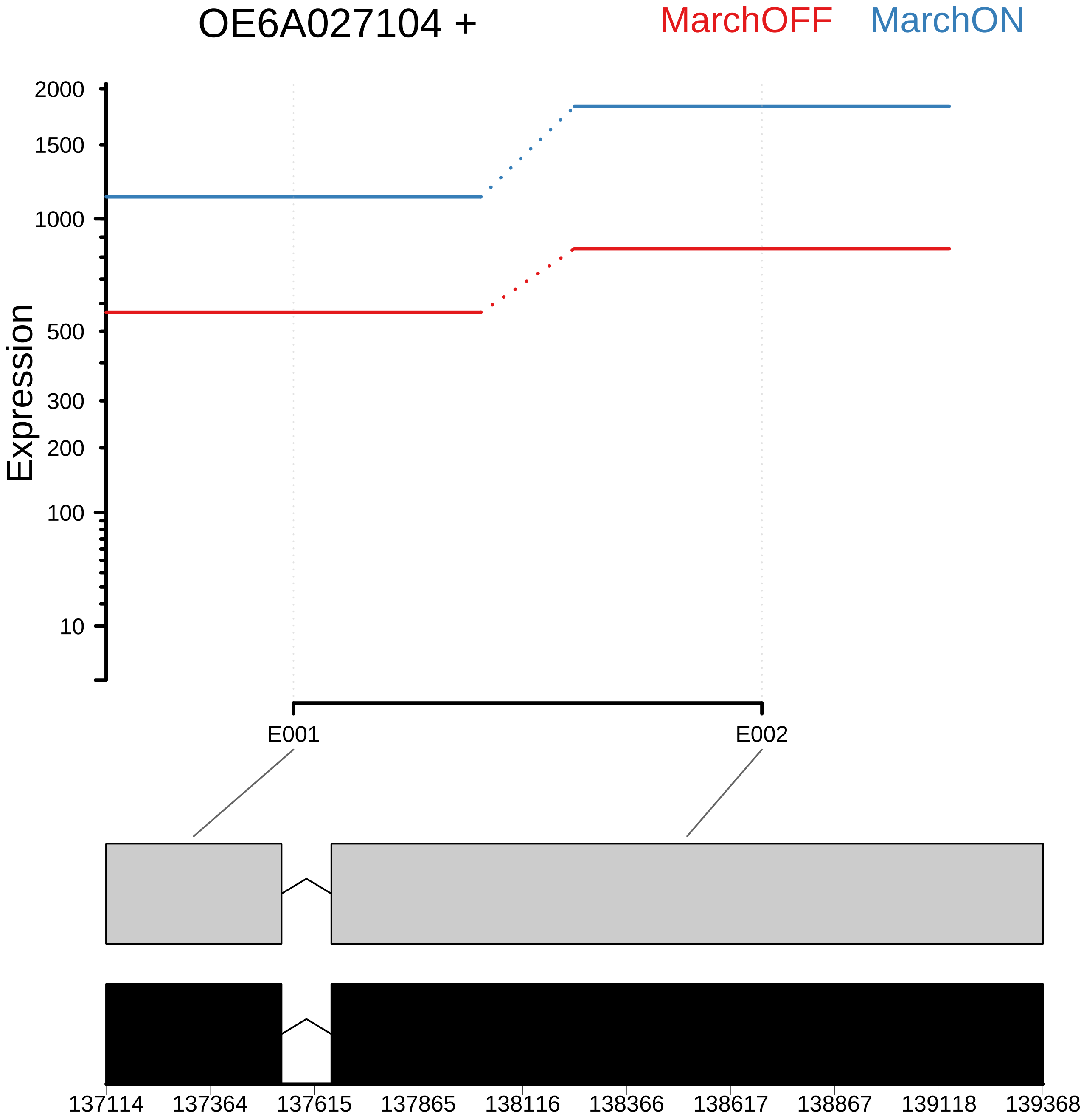

OE6A027104 +
MarchOFF
MarchON
Expression
2000
1500
1000
500
300
200
100
10
E001
E002
137114
137364
137615
137865
138116
138366
138617
138867
139118
139368

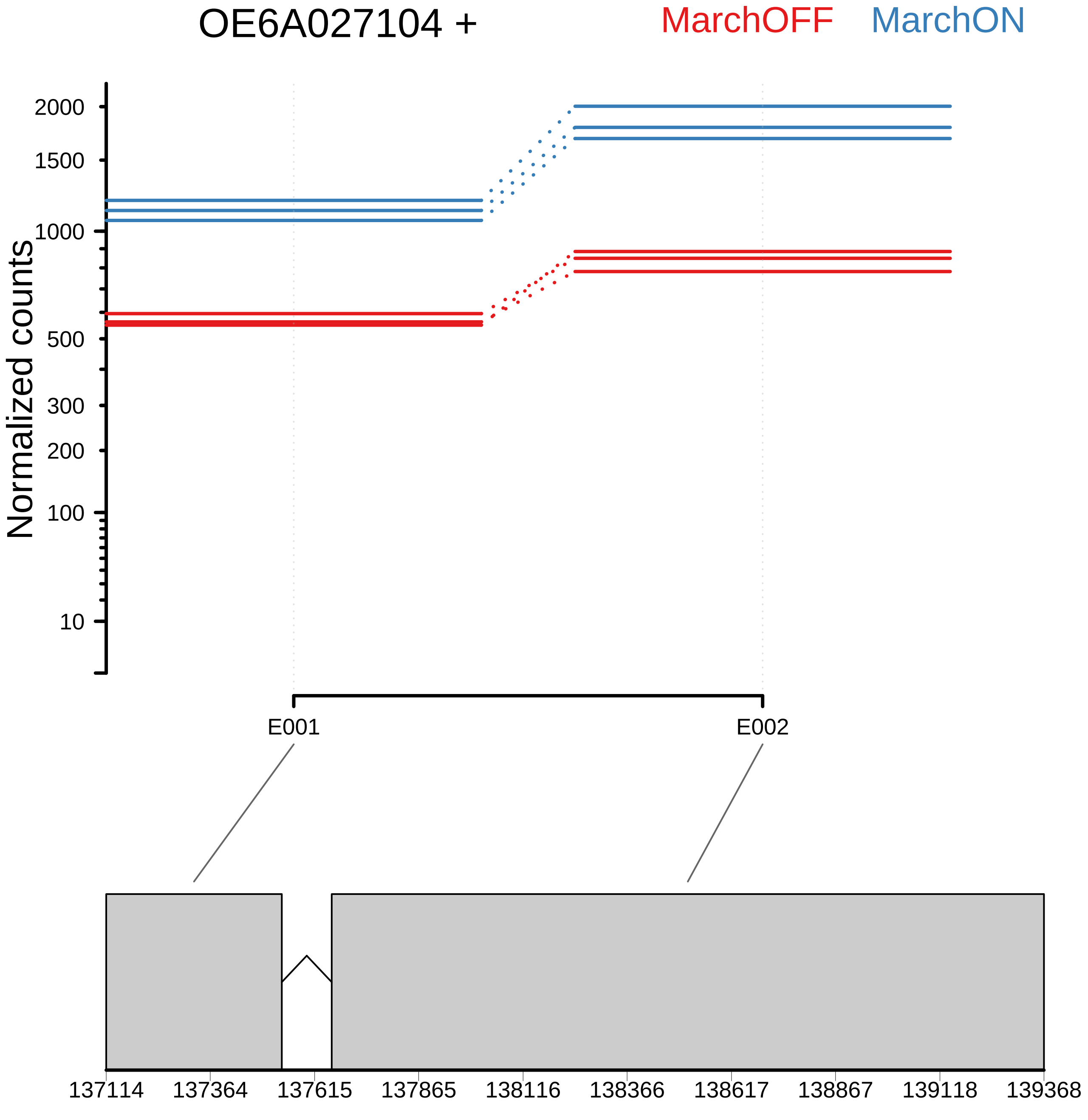

OE6A027104 +
MarchOFF
MarchON
Normalized counts
2000
1500
1000
500
300
200
100
10
E001
E002
137114
137364
137615
137865
138116
138366
138617
138867
139118
139368

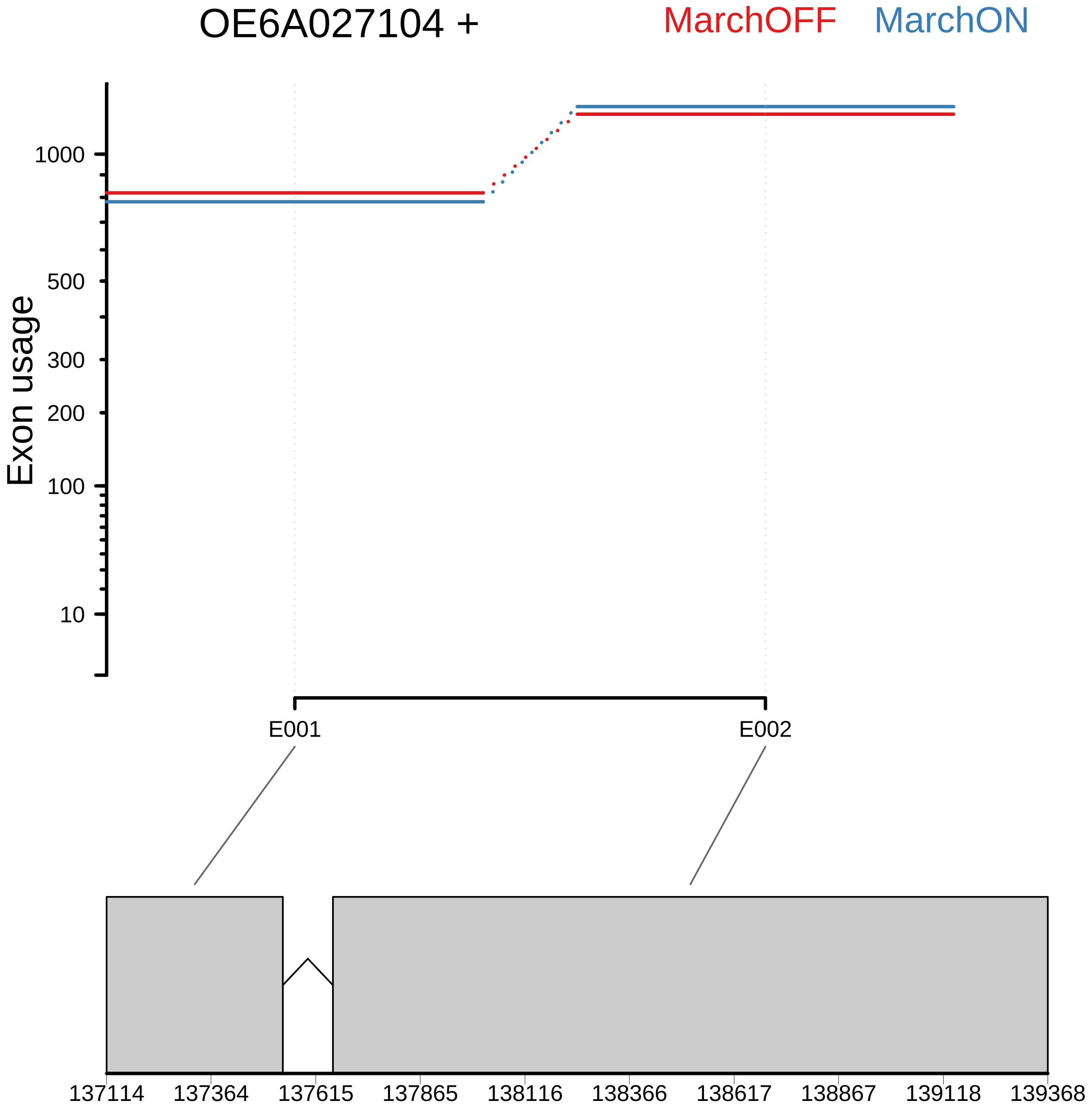

OE6A027104 +
MarchOFF
MarchON
Exon usage
1000
500
300
200
100
10
E001
E002
137114
137364
137615
137865
138116
138366
138617
138867
139118
139368

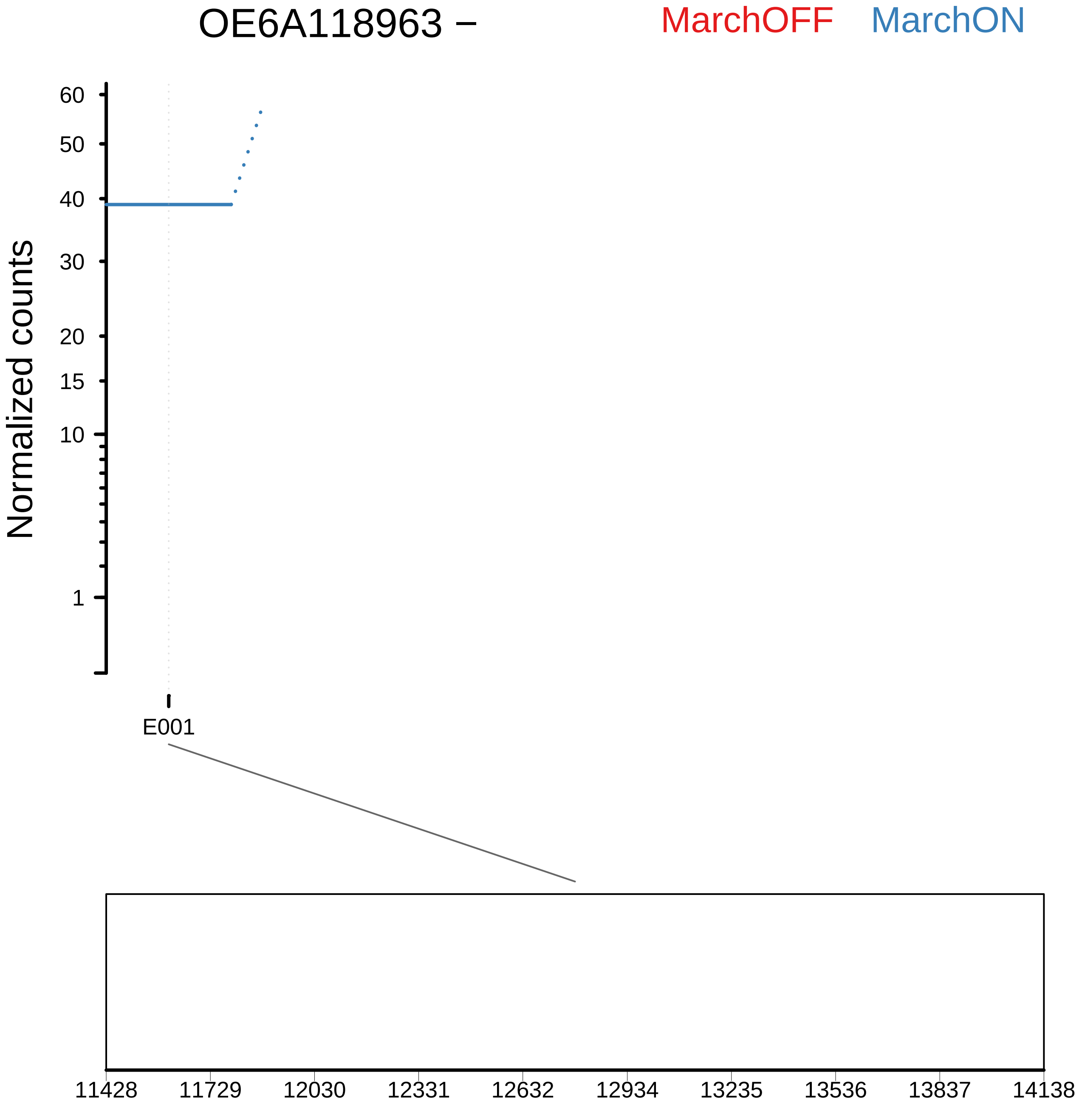

OE6A118963 −
MarchOFF
MarchON
Normalized counts
60
50
40
30
20
15
10
1
E001
11428
11729
12030
12331
12632
12934
13235
13536
13837
14138

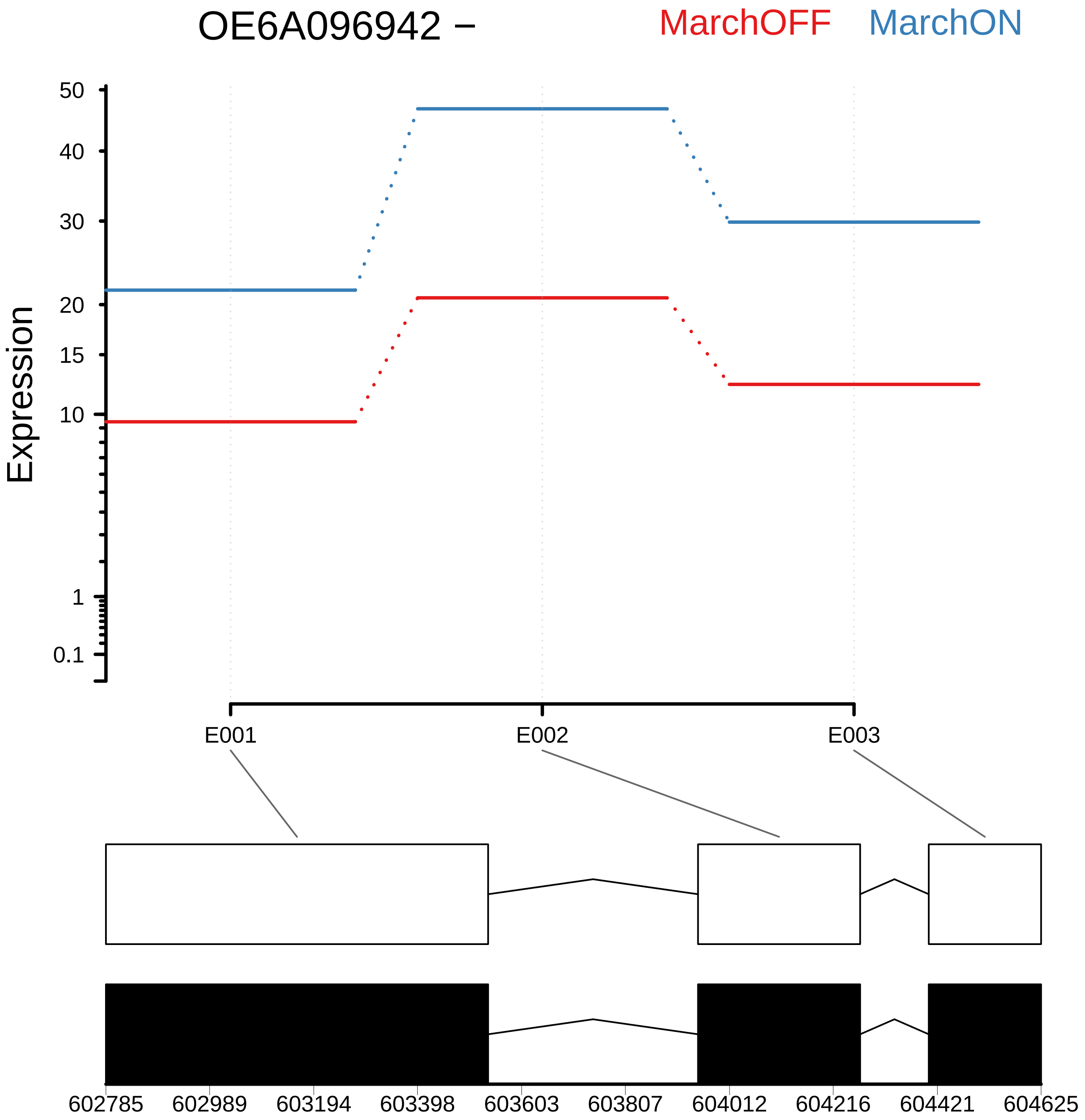

OE6A096942 −
MarchOFF
MarchON
Expression
50
40
30
20
15
10
1
0.1
E001
E002
E003
602785
602989
603194
603398
603603
603807
604012
604216
604421
604625

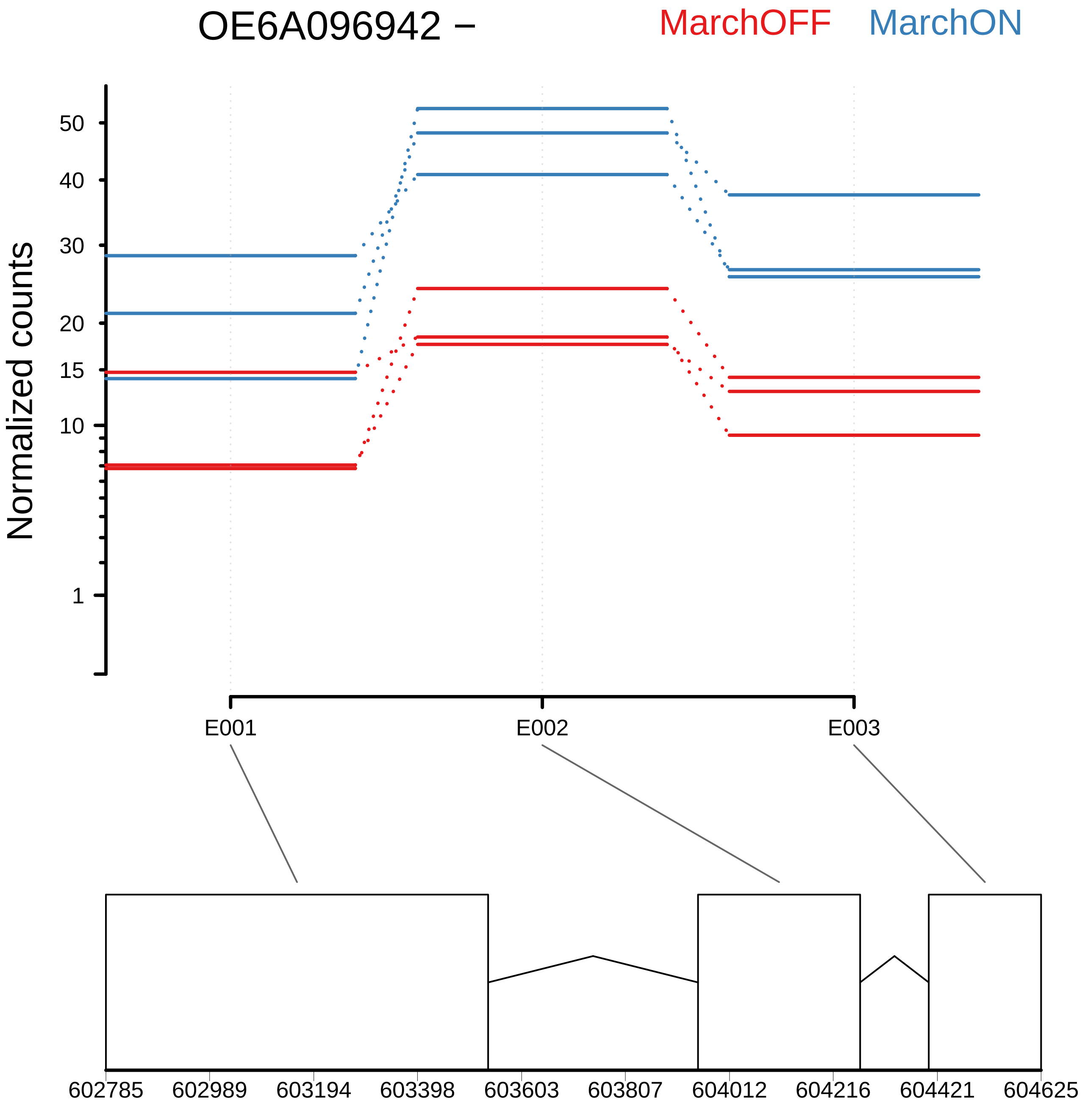
OE6A096942 −
MarchOFF
MarchON
Normalized counts
50
40
30
20
15
10
1
E001
E002
E003
602785
602989
603194
603398
603603
603807
604012
604216
604421
604625

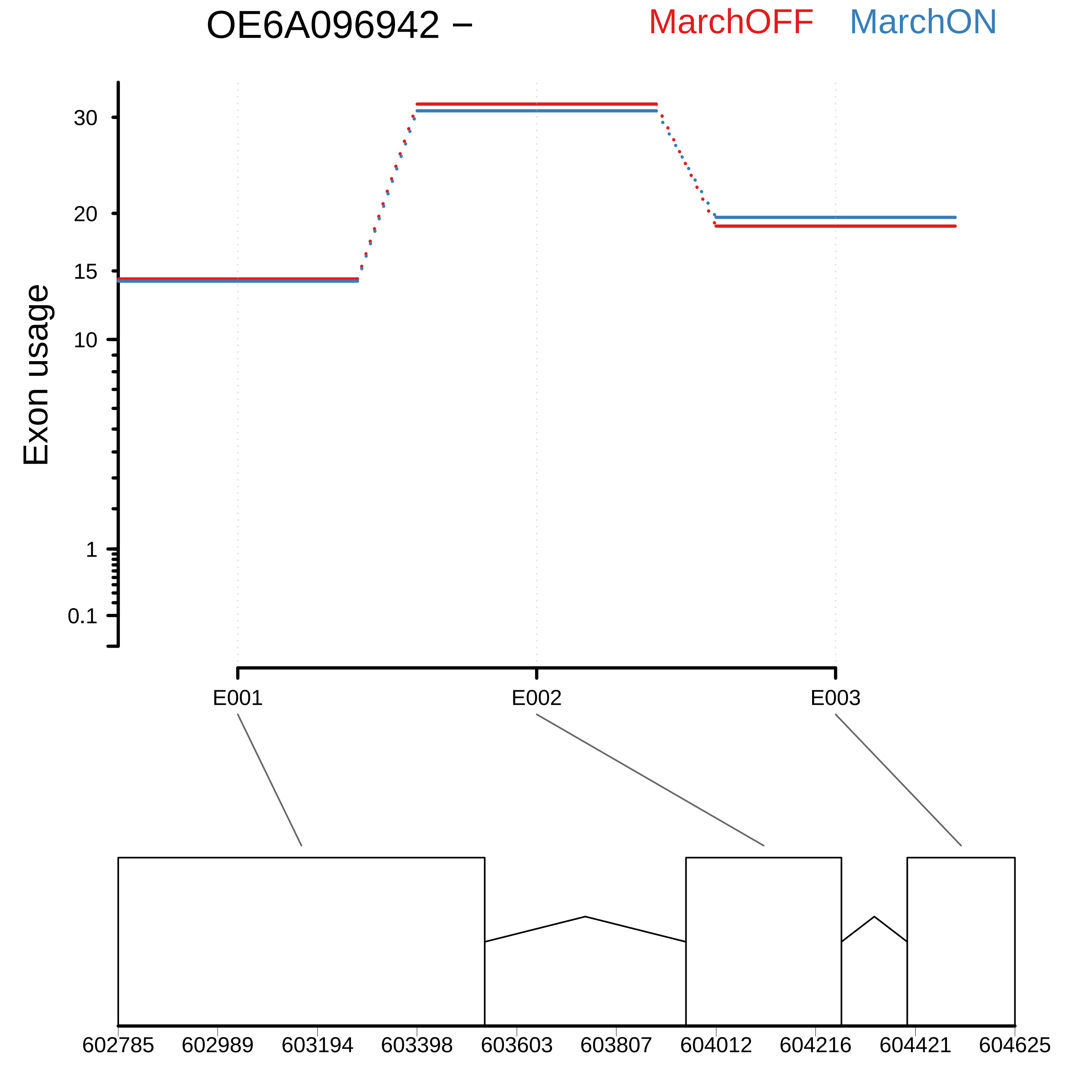

OE6A096942 −
MarchOFF
MarchON
Exon usage
30
20
15
10
1
0.1
E001
E002
E003
602785
602989
603194
603398
603603
603807
604012
604216
604421
604625

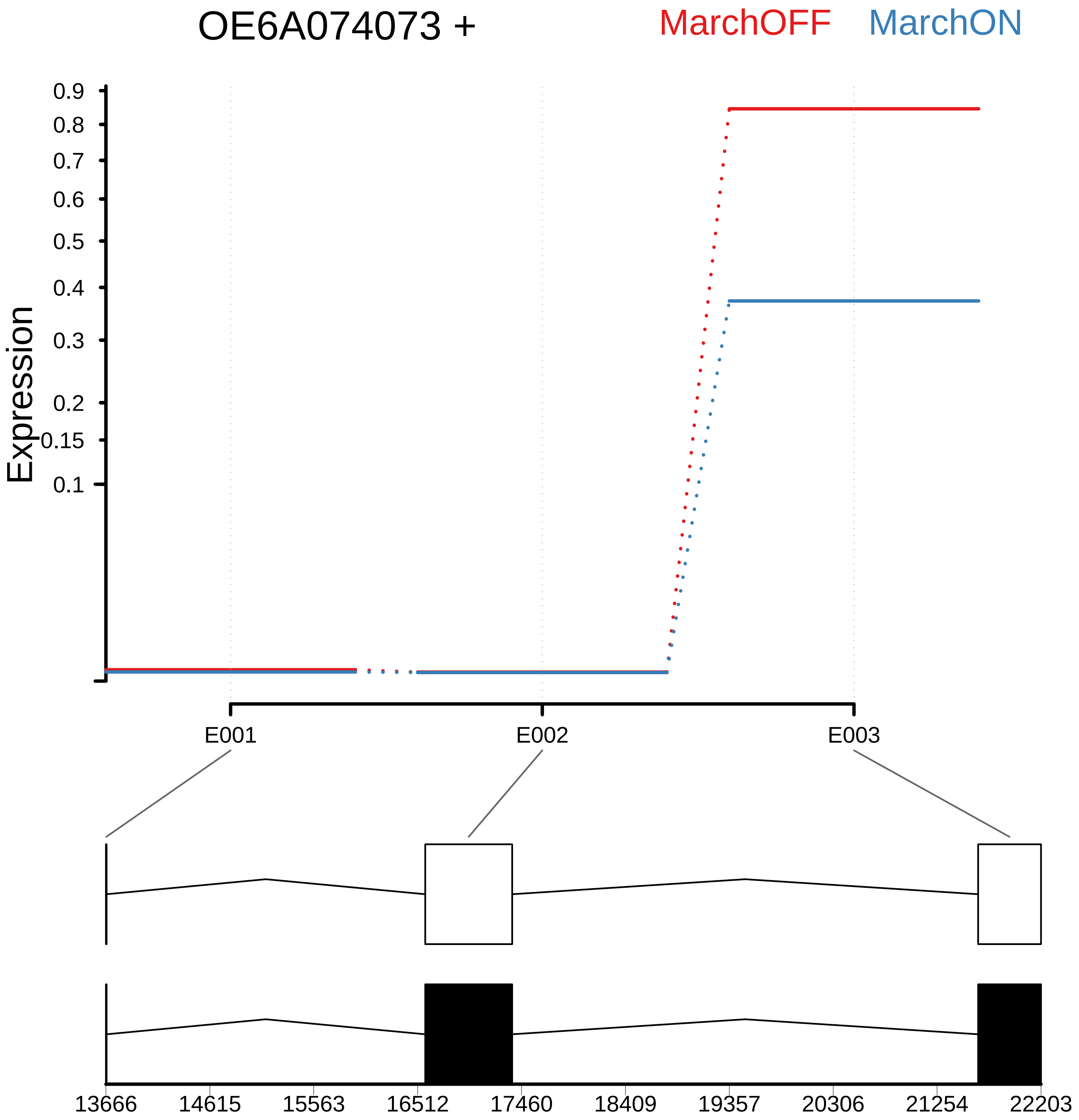

OE6A074073 +
MarchOFF
MarchON
Expression
0.9
0.8
0.7
0.6
0.5
0.4
0.3
0.2
0.15
0.1
E001
E002
E003
13666
14615
15563
16512
17460
18409
19357
20306
21254
22203

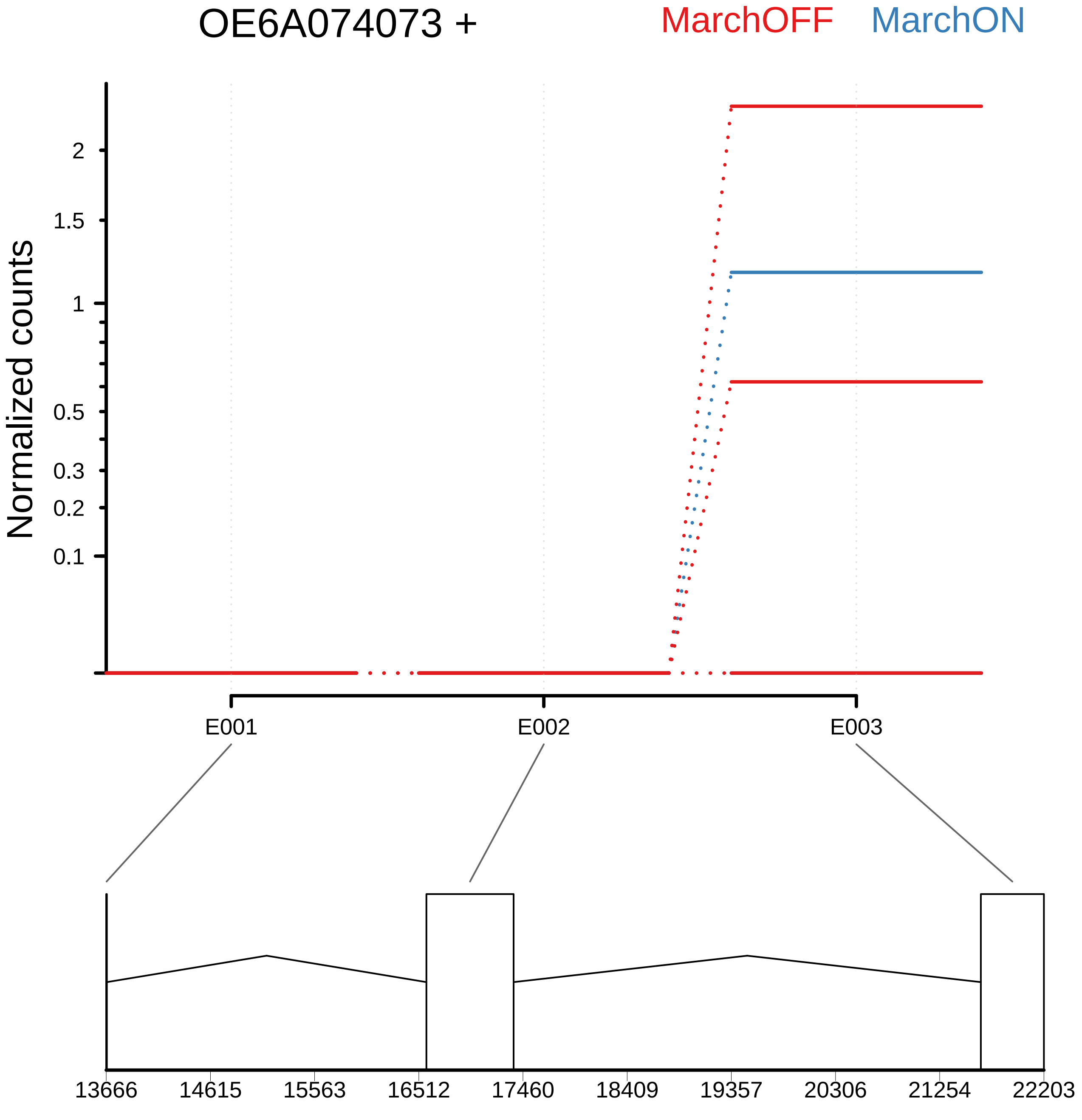

OE6A074073 +
MarchOFF
MarchON
Normalized counts
2
1.5
1
0.5
0.3
0.2
0.1
E001
E002
E003
13666
14615
15563
16512
17460
18409
19357
20306
21254
22203

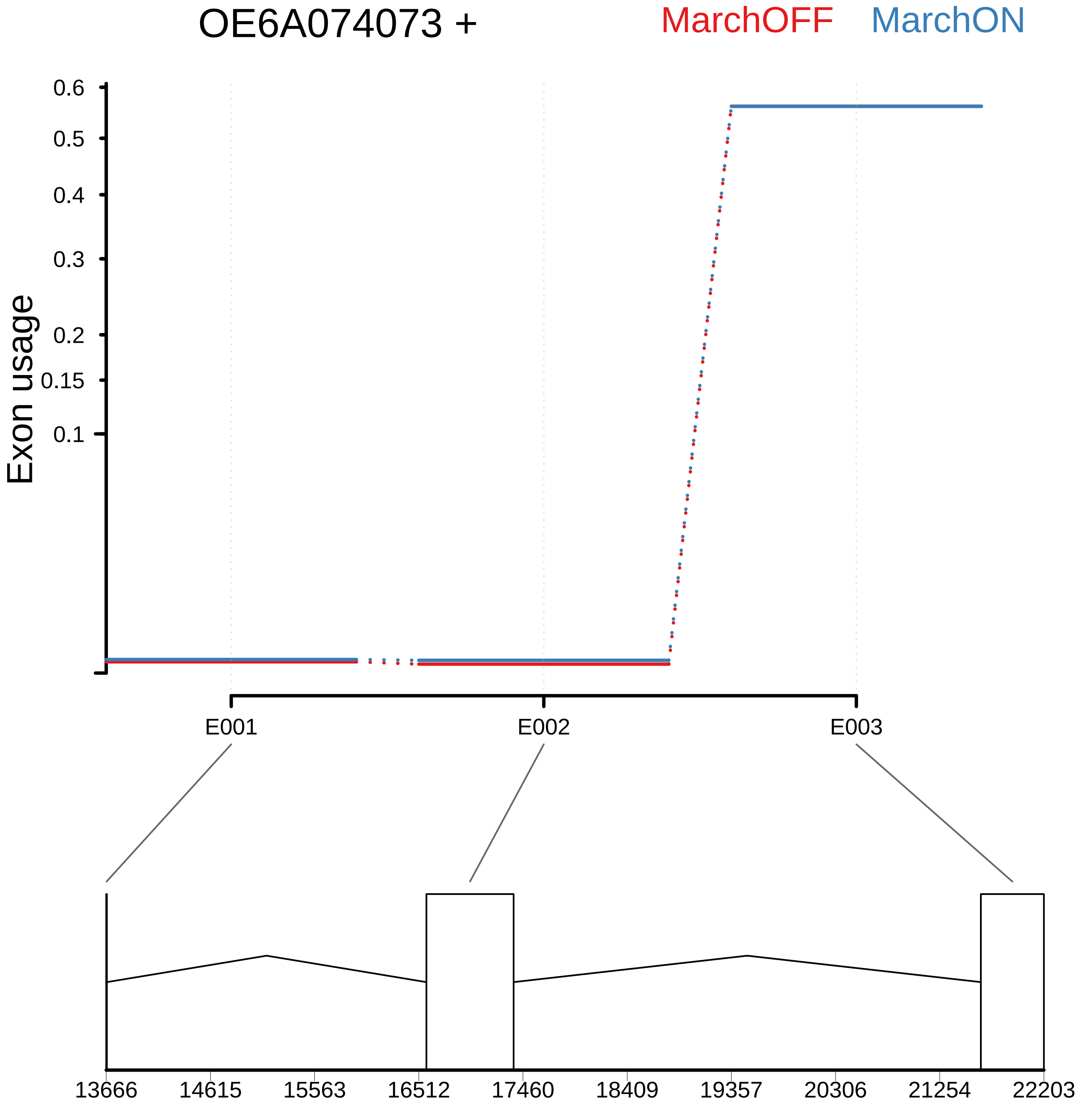
OE6A074073 +
MarchOFF
MarchON
Exon usage
0.6
0.5
0.4
0.3
0.2
0.15
0.1
E001
E002
E003
13666
14615
15563
16512
17460
18409
19357
20306
21254
22203

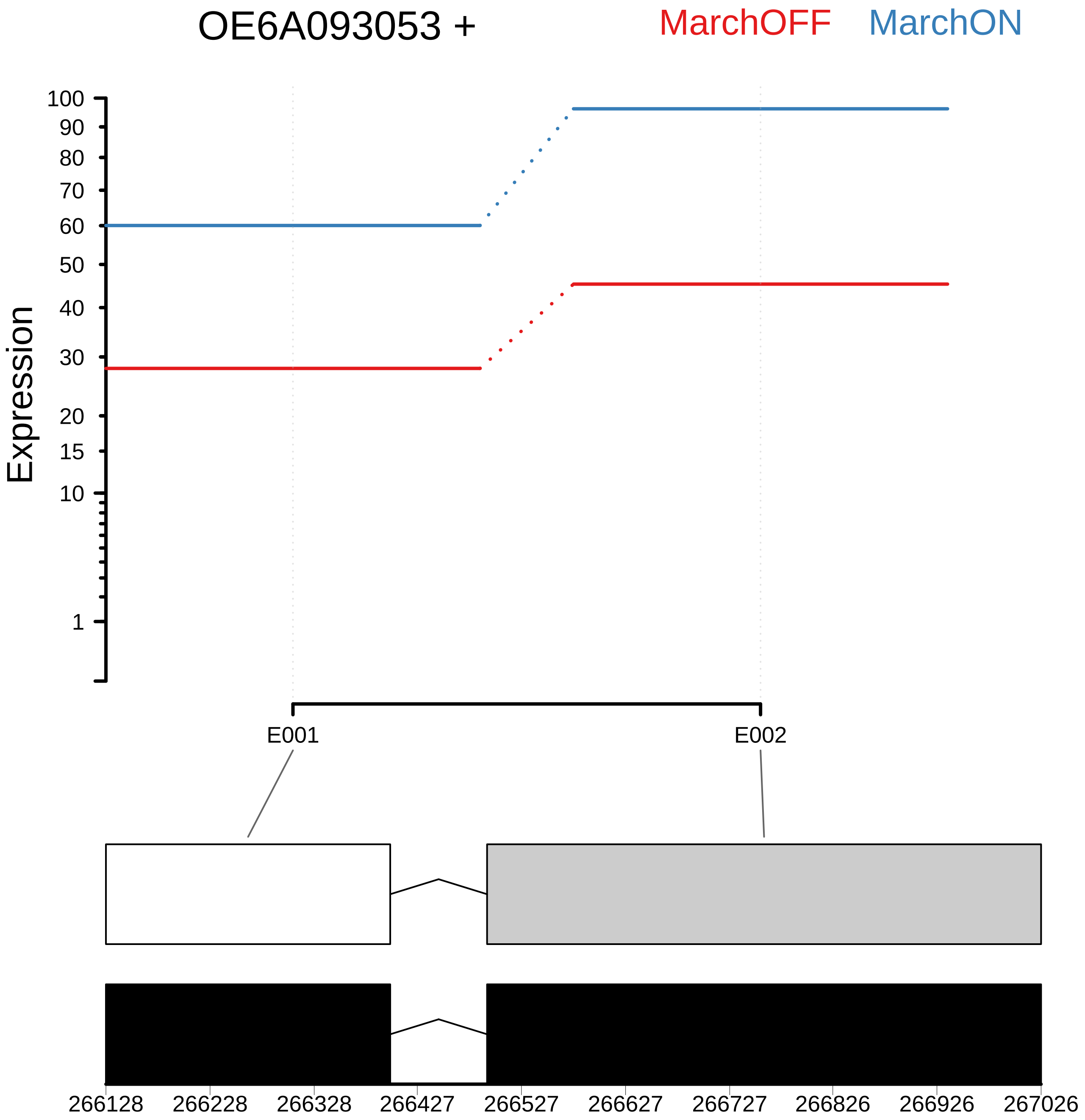
OE6A093053 +
MarchOFF
MarchON
Expression
100
90
80
70
60
50
40
30
20
15
10
1
E001
E002
266128
266228
266328
266427
266527
266627
266727
266826
266926
267026

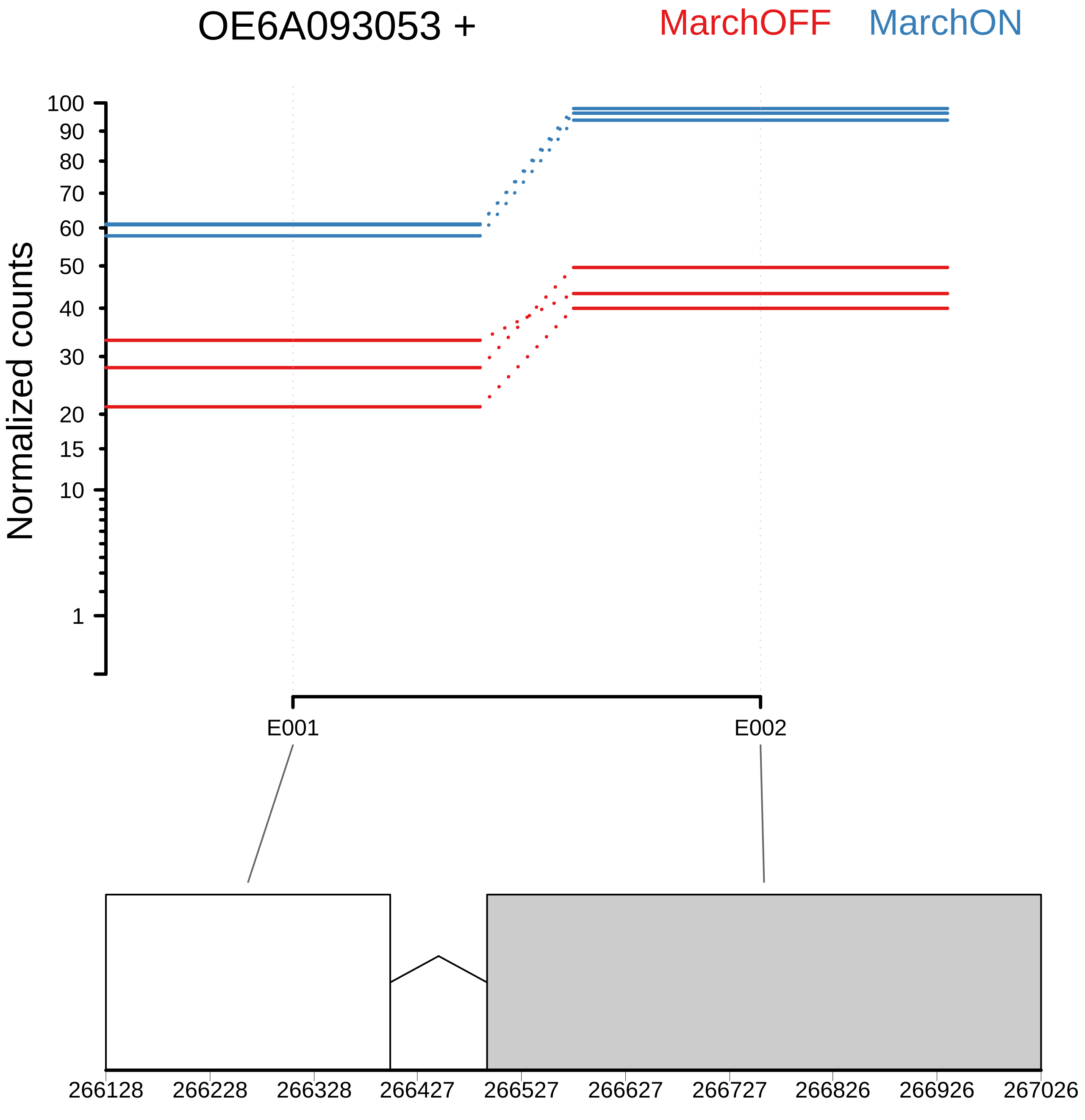

OE6A093053 +
MarchOFF
MarchON
Normalized counts
100
90
80
70
60
50
40
30
20
15
10
1
E001
E002
266128
266228
266328
266427
266527
266627
266727
266826
266926
267026

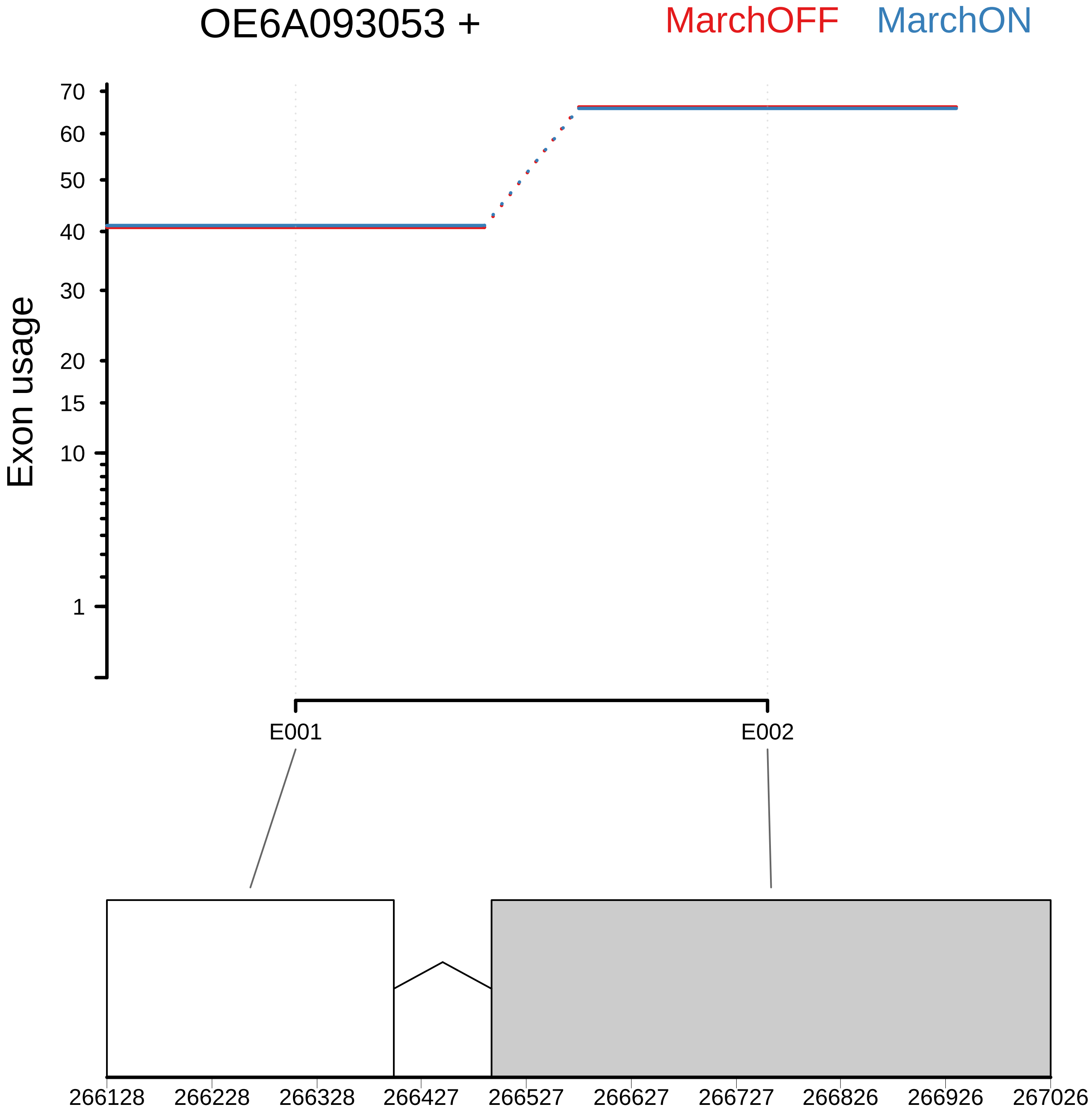

OE6A093053 +
MarchOFF
MarchON
Exon usage
70
60
50
40
30
20
15
10
1
E001
E002
266128
266228
266328
266427
266527
266627
266727
266826
266926
267026

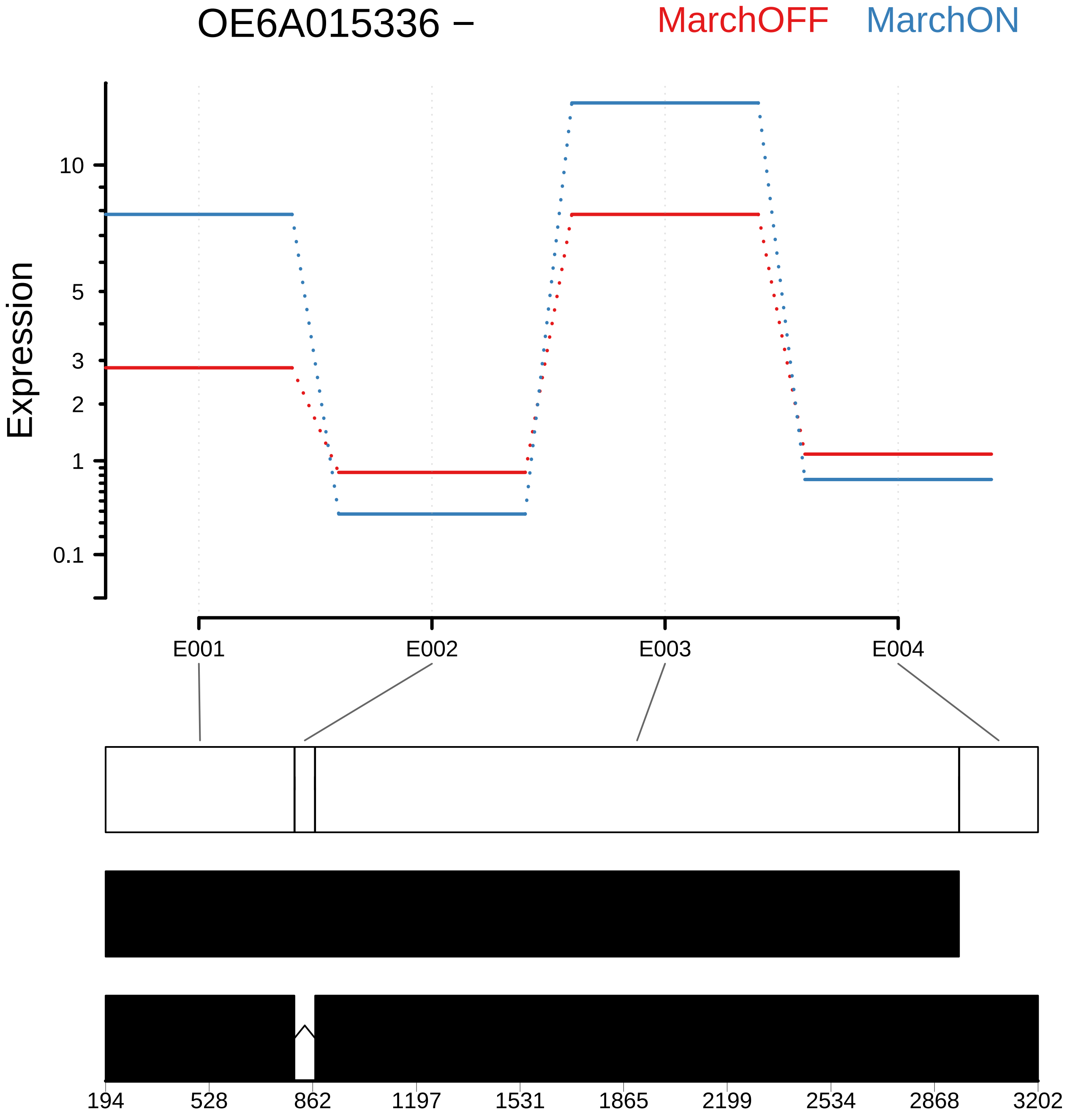

OE6A015336 −
MarchOFF
MarchON
Expression
10
5
3
2
1
0.1
E001
E002
E003
E004
194
528
862
1197
1531
1865
2199
2534
2868
3202

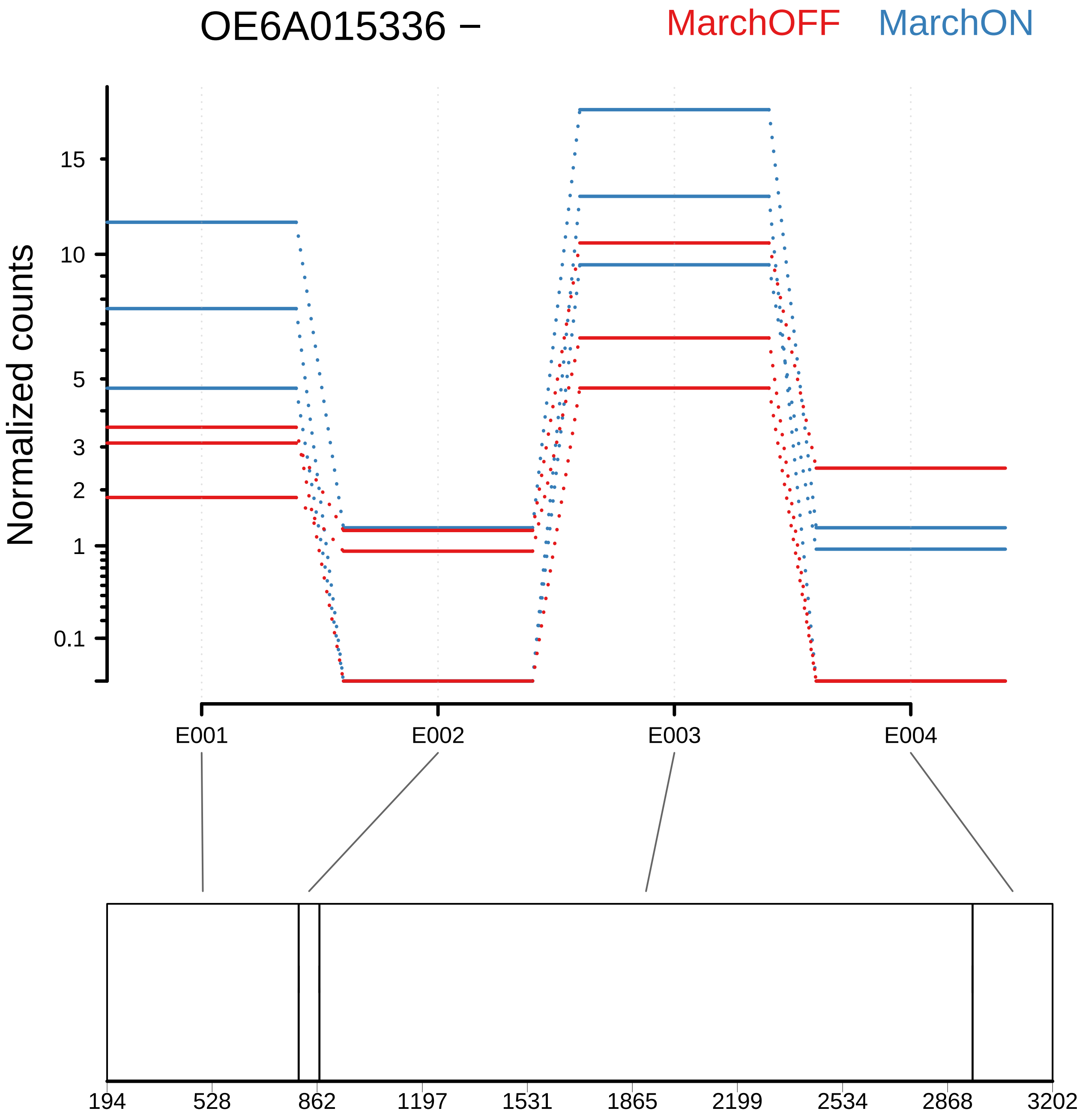

OE6A015336 −
MarchOFF
MarchON
Normalized counts
15
10
5
3
2
1
0.1
E001
E002
E003
E004
194
528
862
1197
1531
1865
2199
2534
2868
3202

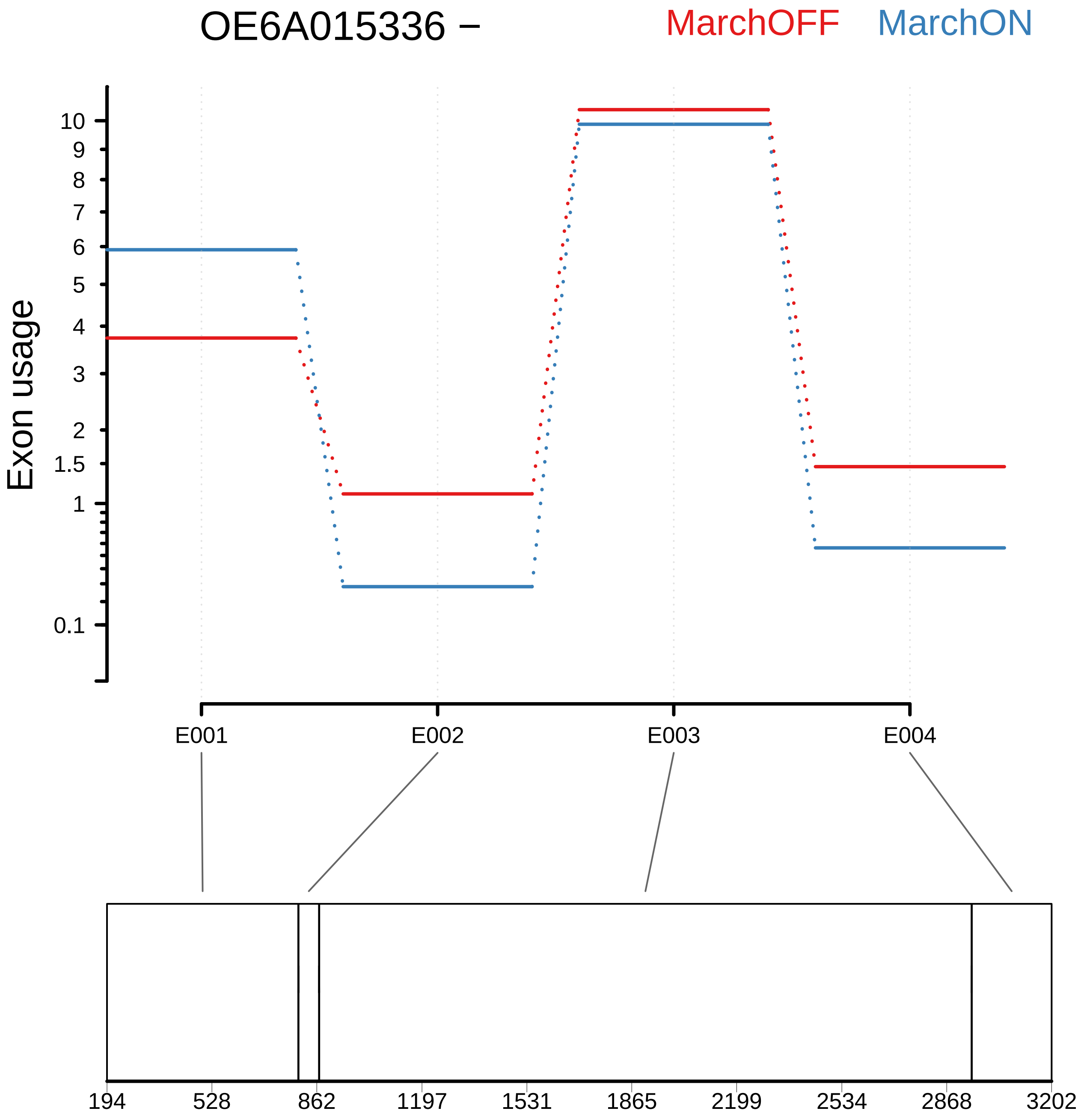

OE6A015336 −
MarchOFF
MarchON
Exon usage
10
9
8
7
6
5
4
3
2
1.5
1
0.1
E001
E002
E003
E004
194
528
862
1197
1531
1865
2199
2534
2868
3202

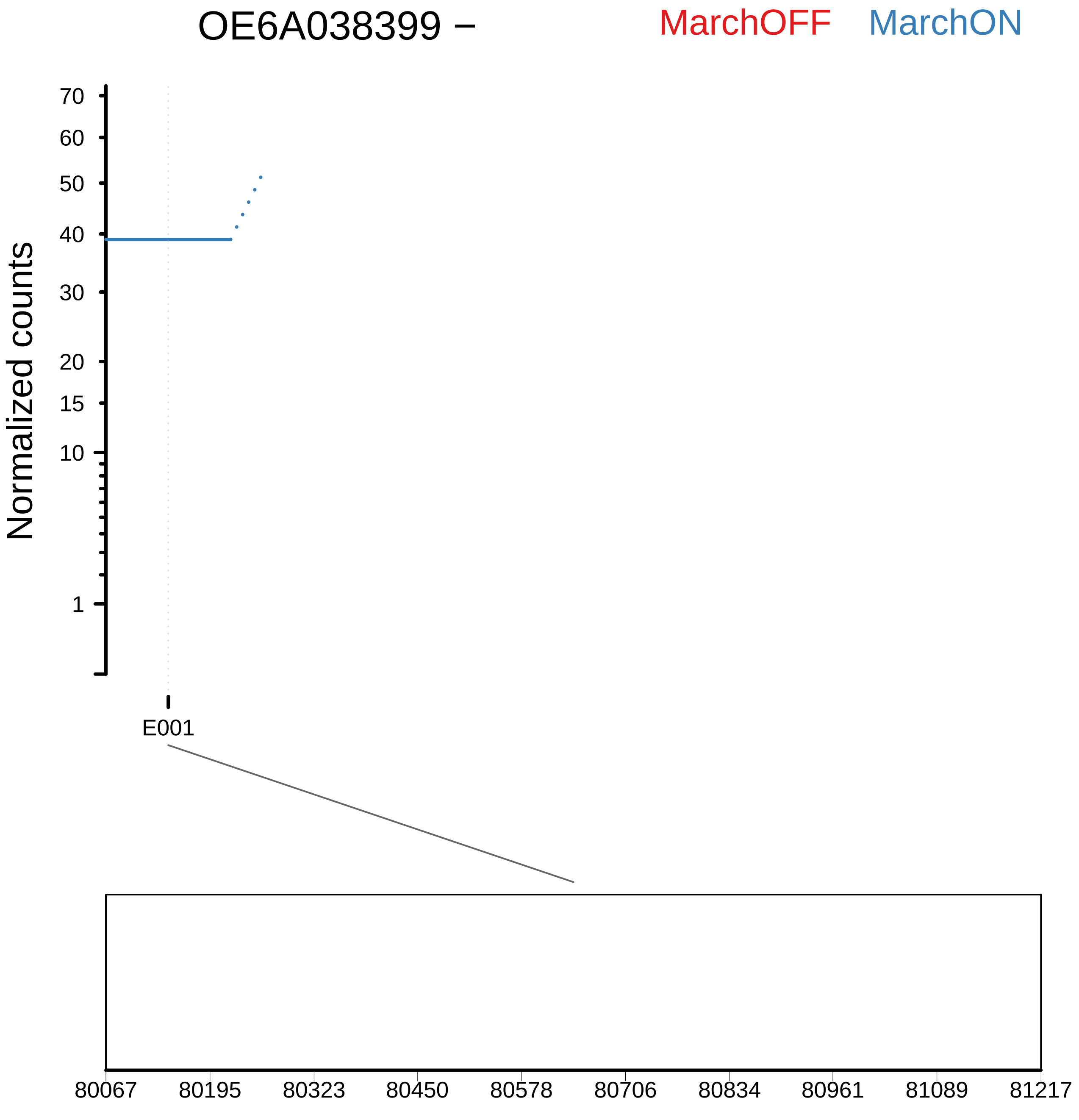

OE6A038399 −
MarchOFF
MarchON
Normalized counts
70
60
50
40
30
20
15
10
1
E001
80067
80195
80323
80450
80578
80706
80834
80961
81089
81217

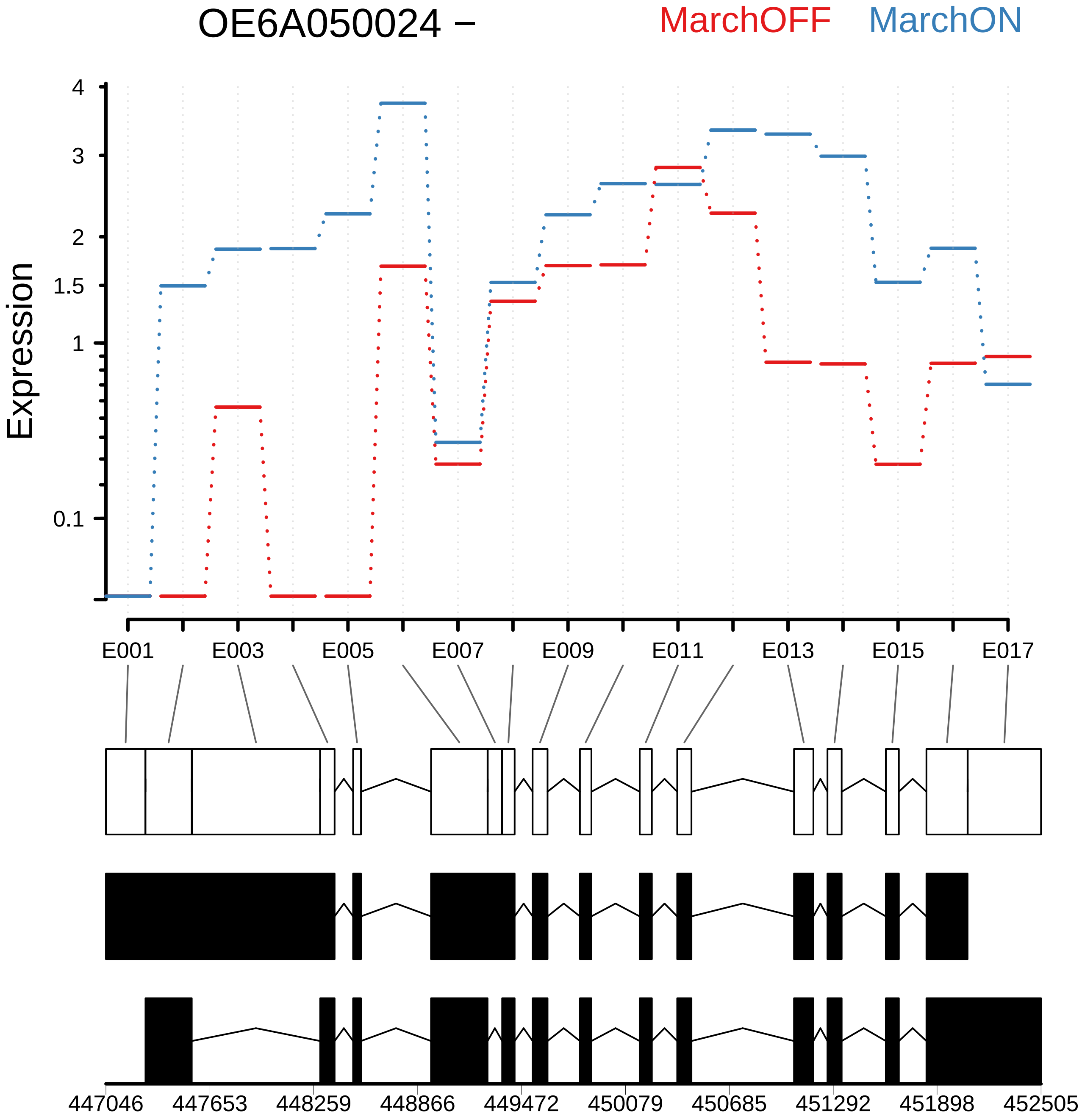

OE6A050024 −
MarchOFF
MarchON
Expression
4
3
2
1.5
1
0.1
E001
E003
E005
E007
E009
E011
E013
E015
E017
447046
447653
448259
448866
449472
450079
450685
451292
451898
452505

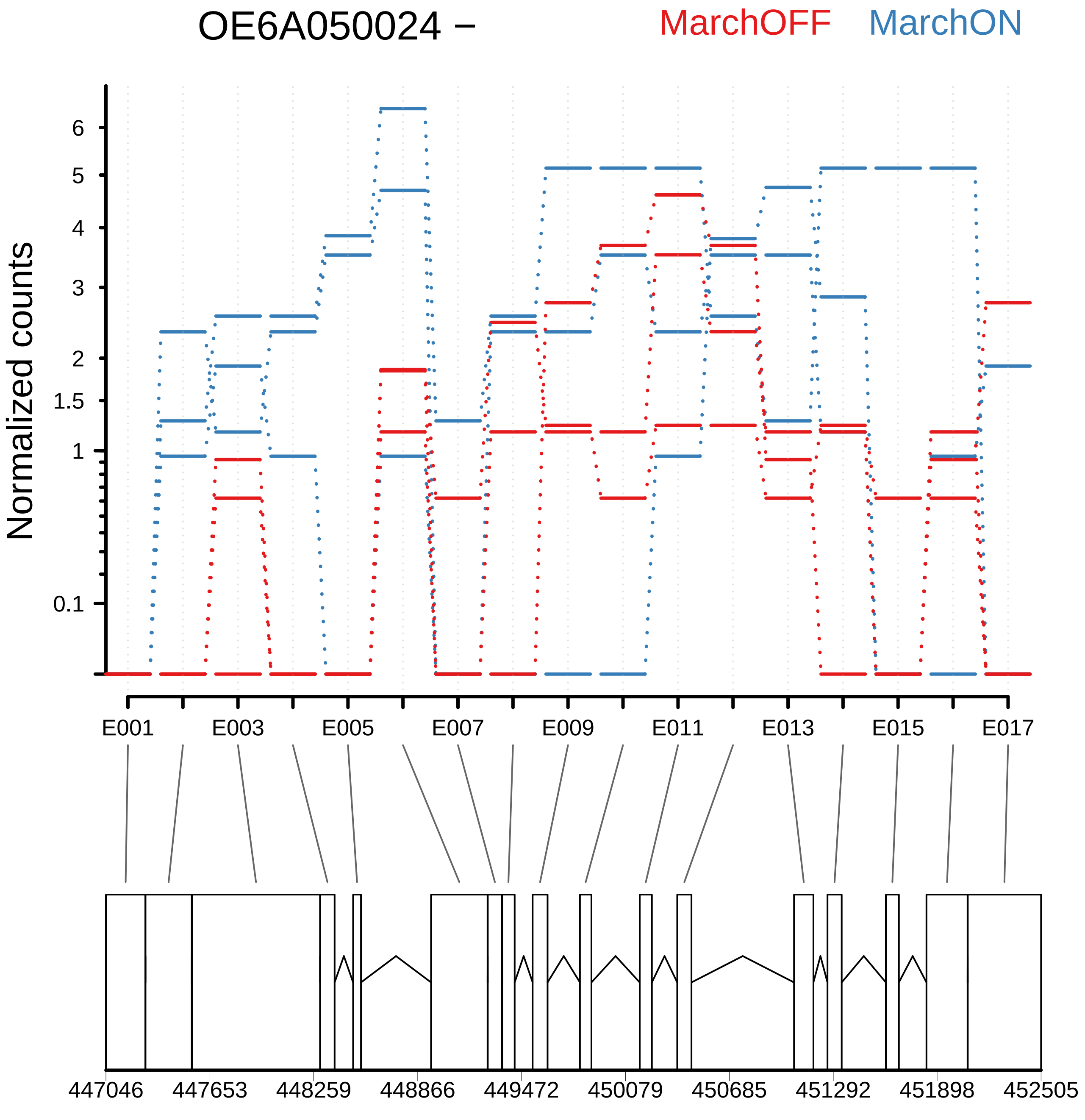

OE6A050024 −
MarchOFF
MarchON
Normalized counts
6
5
4
3
2
1.5
1
0.1
E001
E003
E005
E007
E009
E011
E013
E015
E017
447046
447653
448259
448866
449472
450079
450685
451292
451898
452505

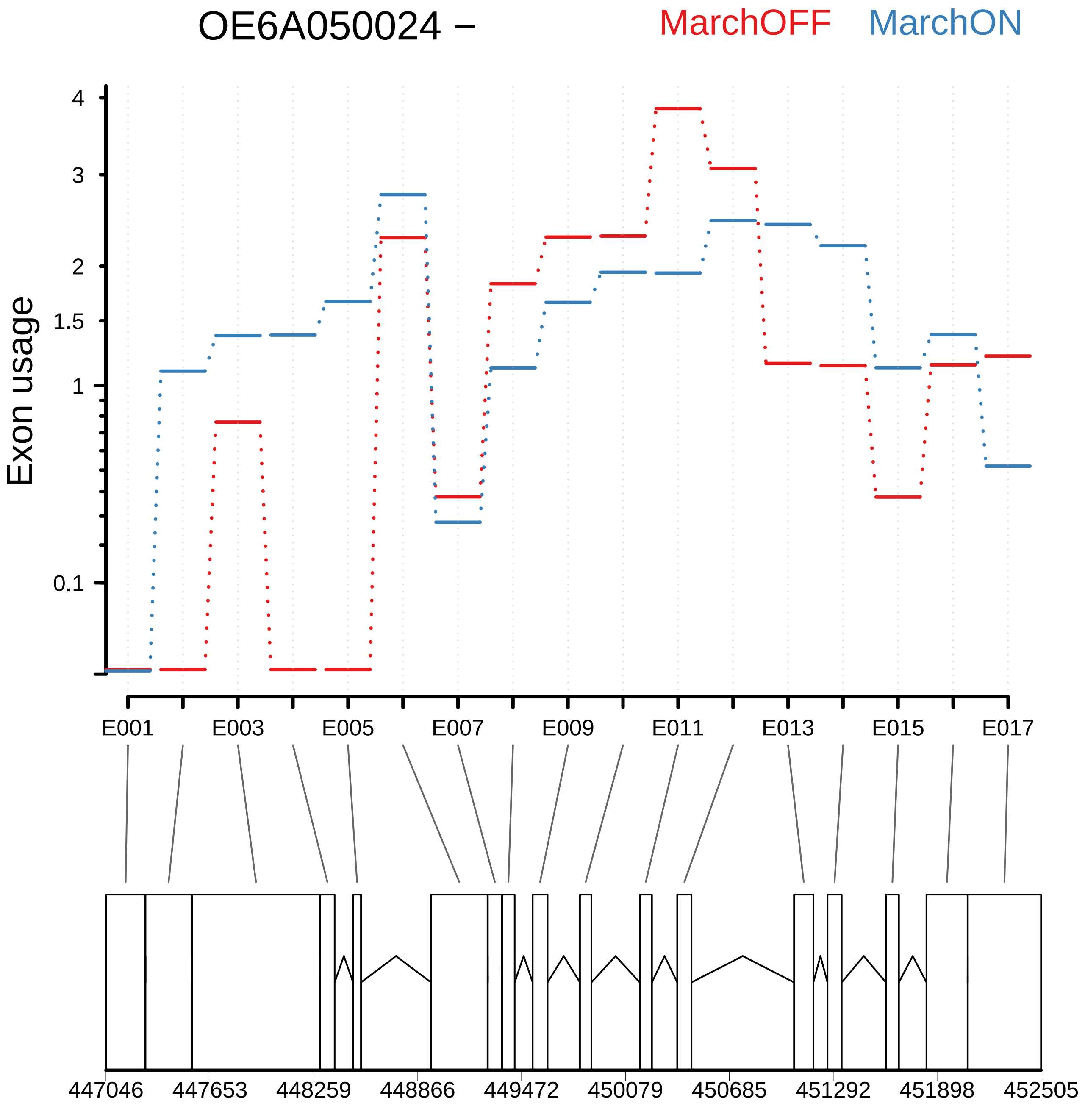
OE6A050024 −
MarchOFF
MarchON
Exon usage
4
3
2
1.5
1
0.1
E001
E003
E005
E007
E009
E011
E013
E015
E017
447046
447653
448259
448866
449472
450079
450685
451292
451898
452505

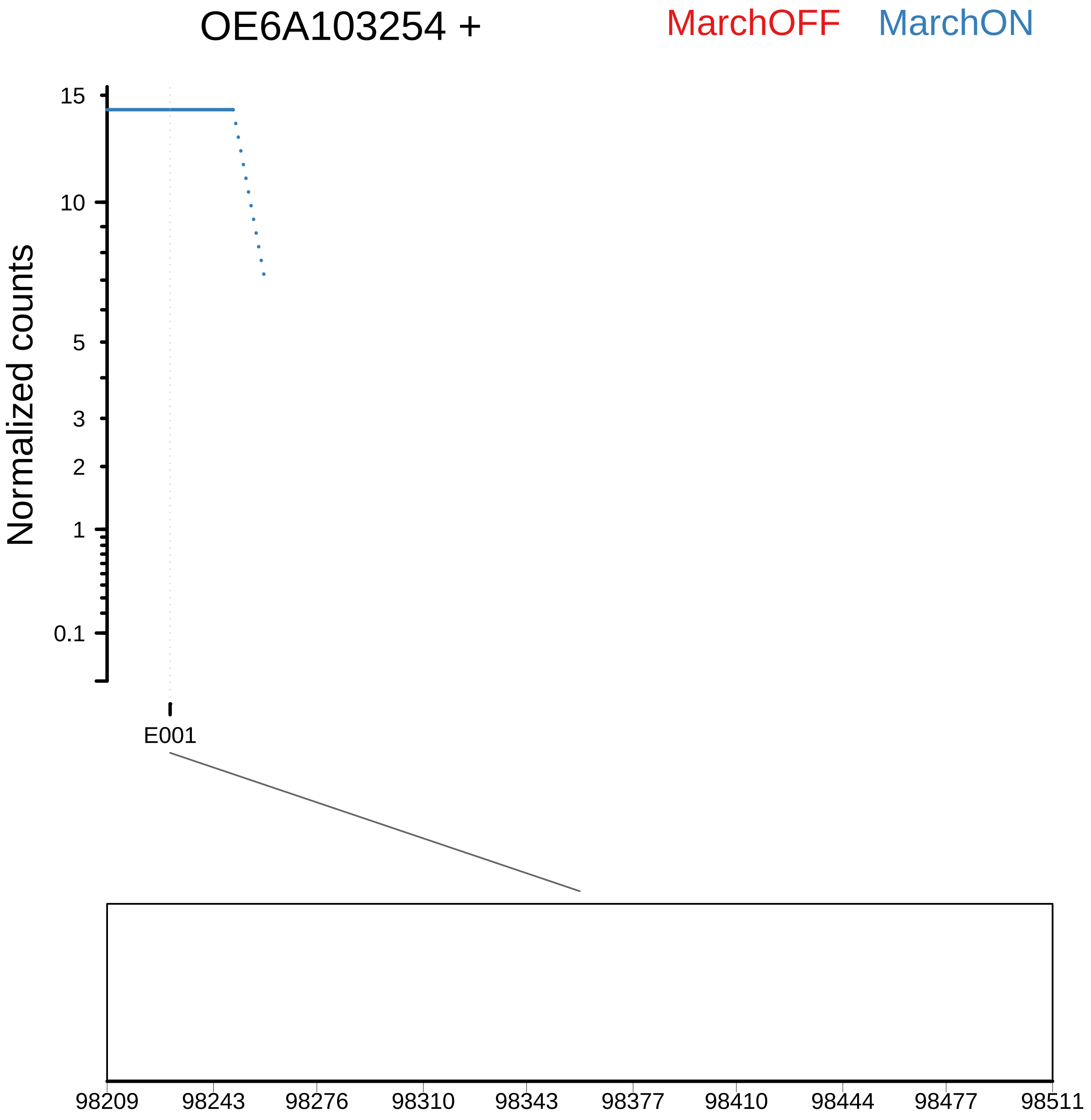
OE6A103254 +
MarchOFF
MarchON
Normalized counts
15
10
5
3
2
1
0.1
E001
98209
98243
98276
98310
98343
98377
98410
98444
98477
98511

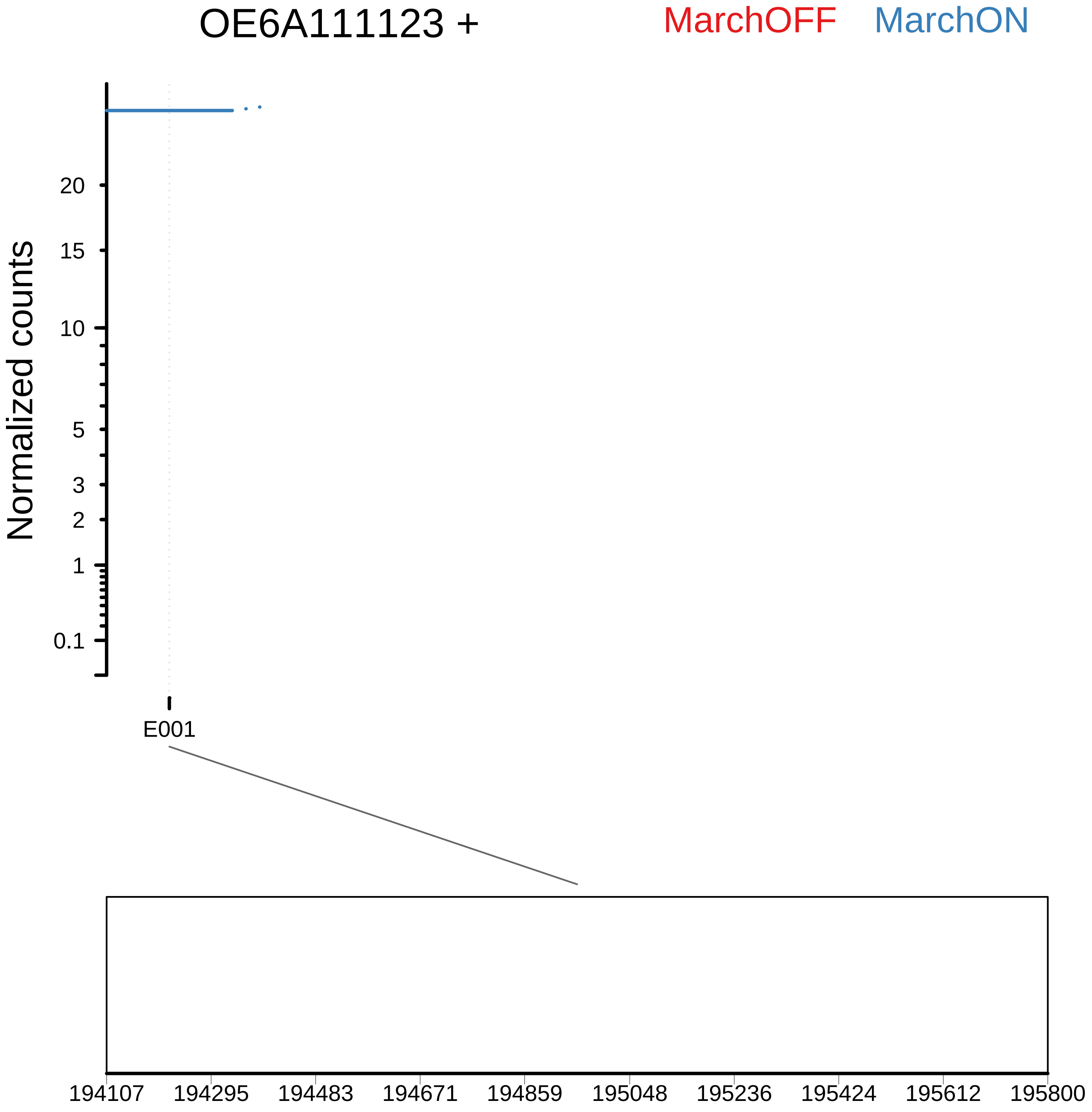
OE6A111123 +
MarchOFF
MarchON
Normalized counts
20
15
10
5
3
2
1
0.1
E001
194107
194295
194483
194671
194859
195048
195236
195424
195612
195800

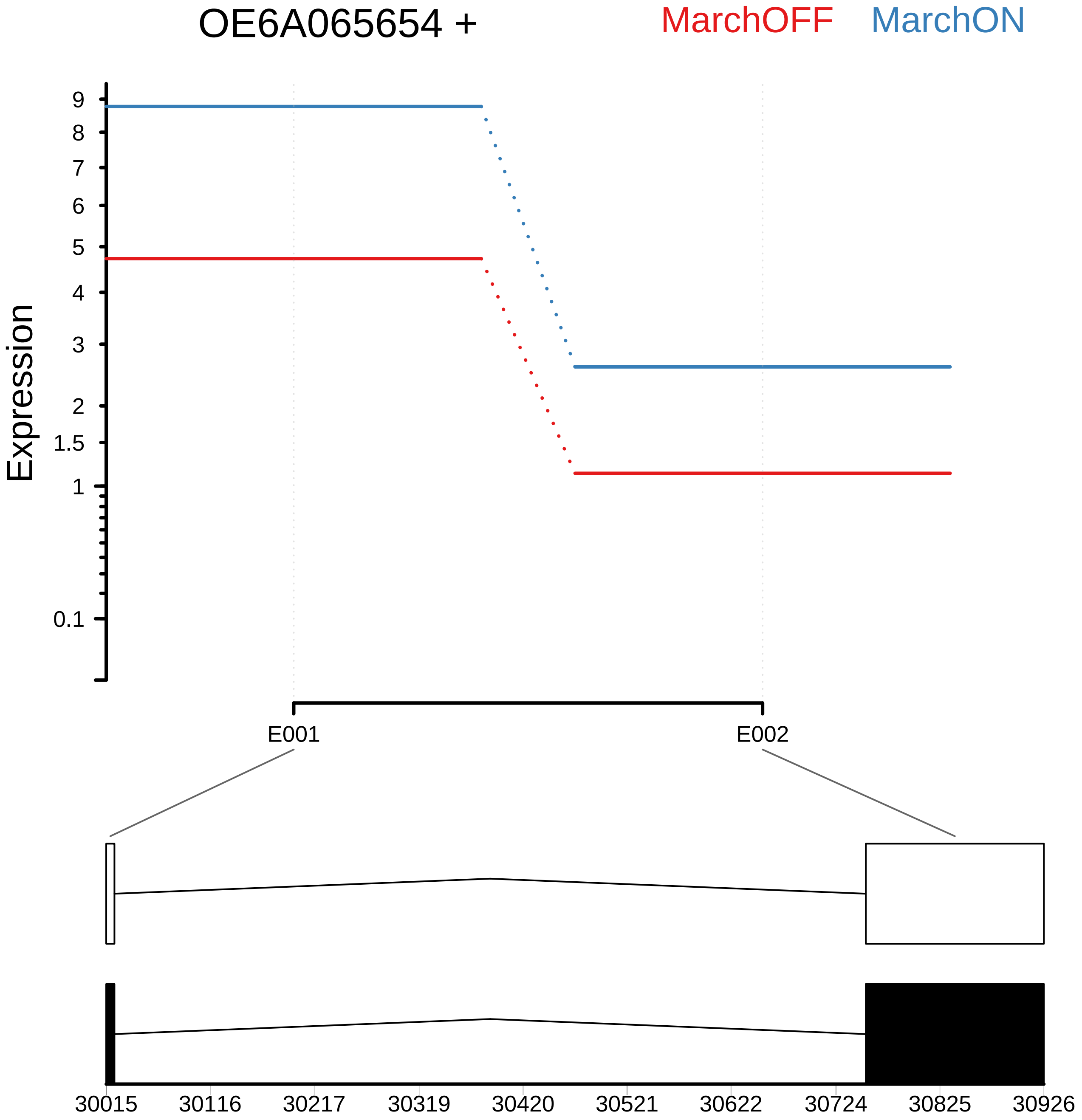
OE6A065654 +
MarchOFF
MarchON
Expression
9
8
7
6
5
4
3
2
1.5
1
0.1
E001
E002
30015
30116
30217
30319
30420
30521
30622
30724
30825
30926

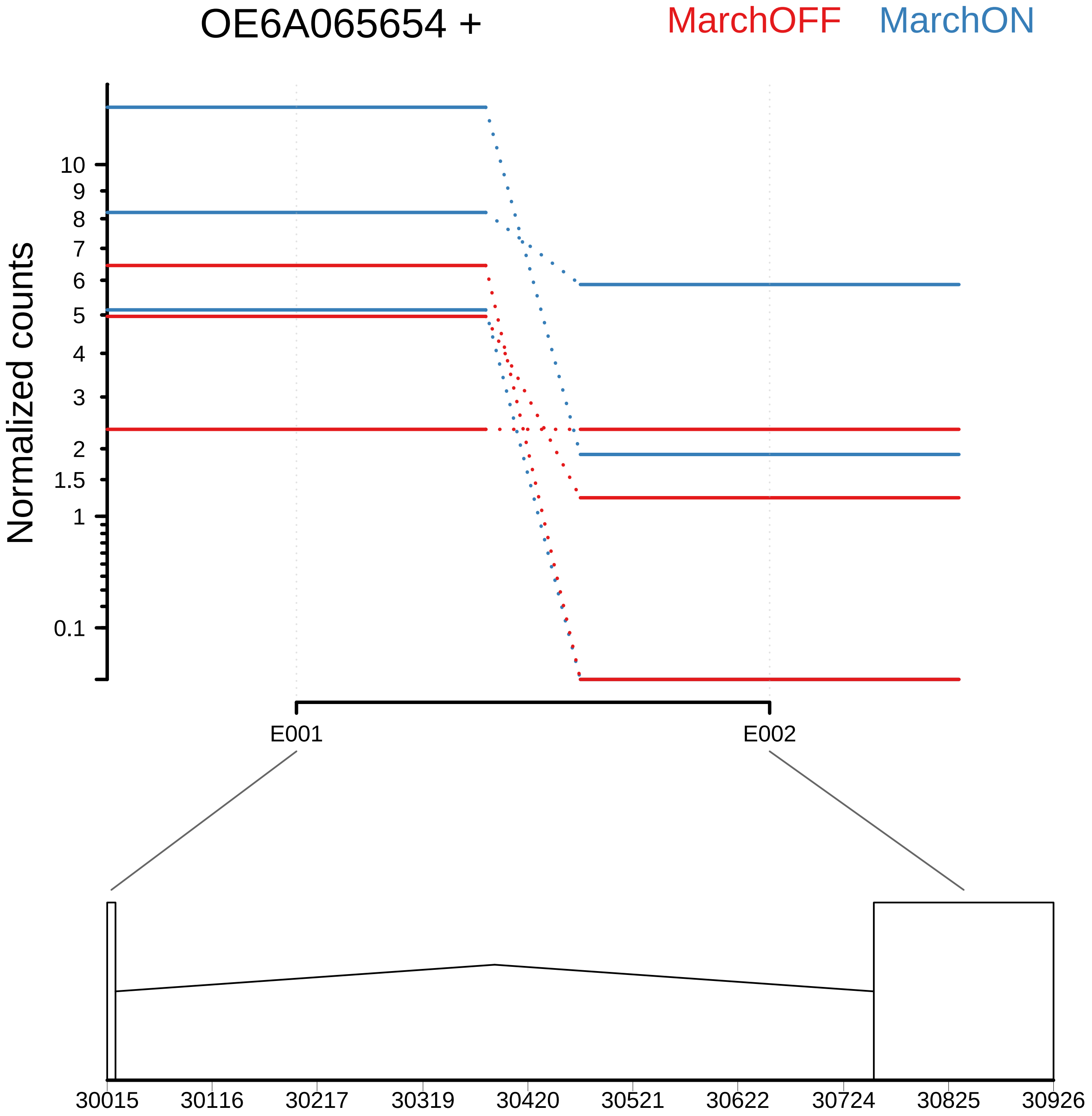

OE6A065654 +
MarchOFF
MarchON
Normalized counts
10
9
8
7
6
5
4
3
2
1.5
1
0.1
E001
E002
30015
30116
30217
30319
30420
30521
30622
30724
30825
30926

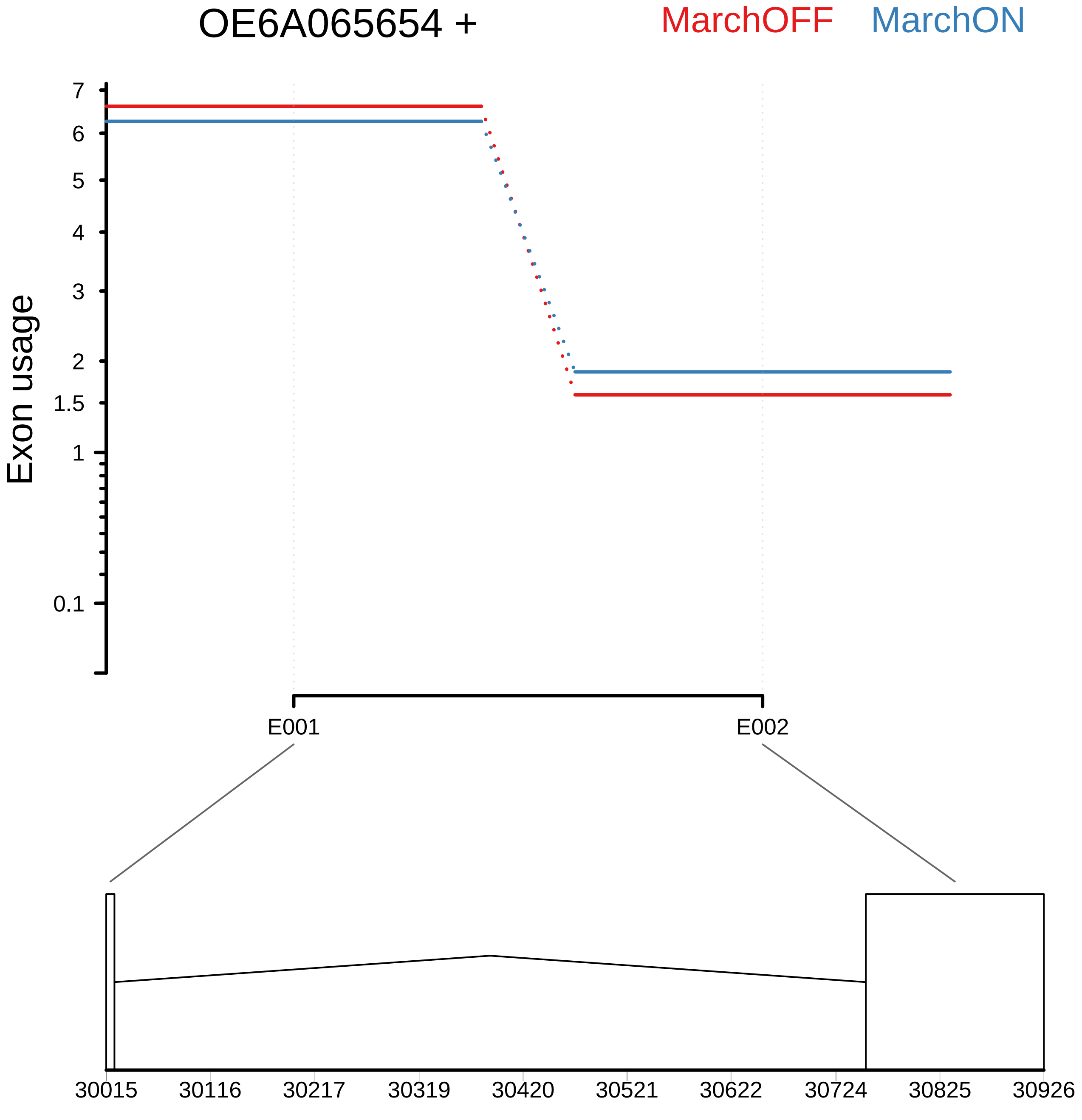
OE6A065654 +
MarchOFF
MarchON
Exon usage
7
6
5
4
3
2
1.5
1
0.1
E001
E002
30015
30116
30217
30319
30420
30521
30622
30724
30825
30926

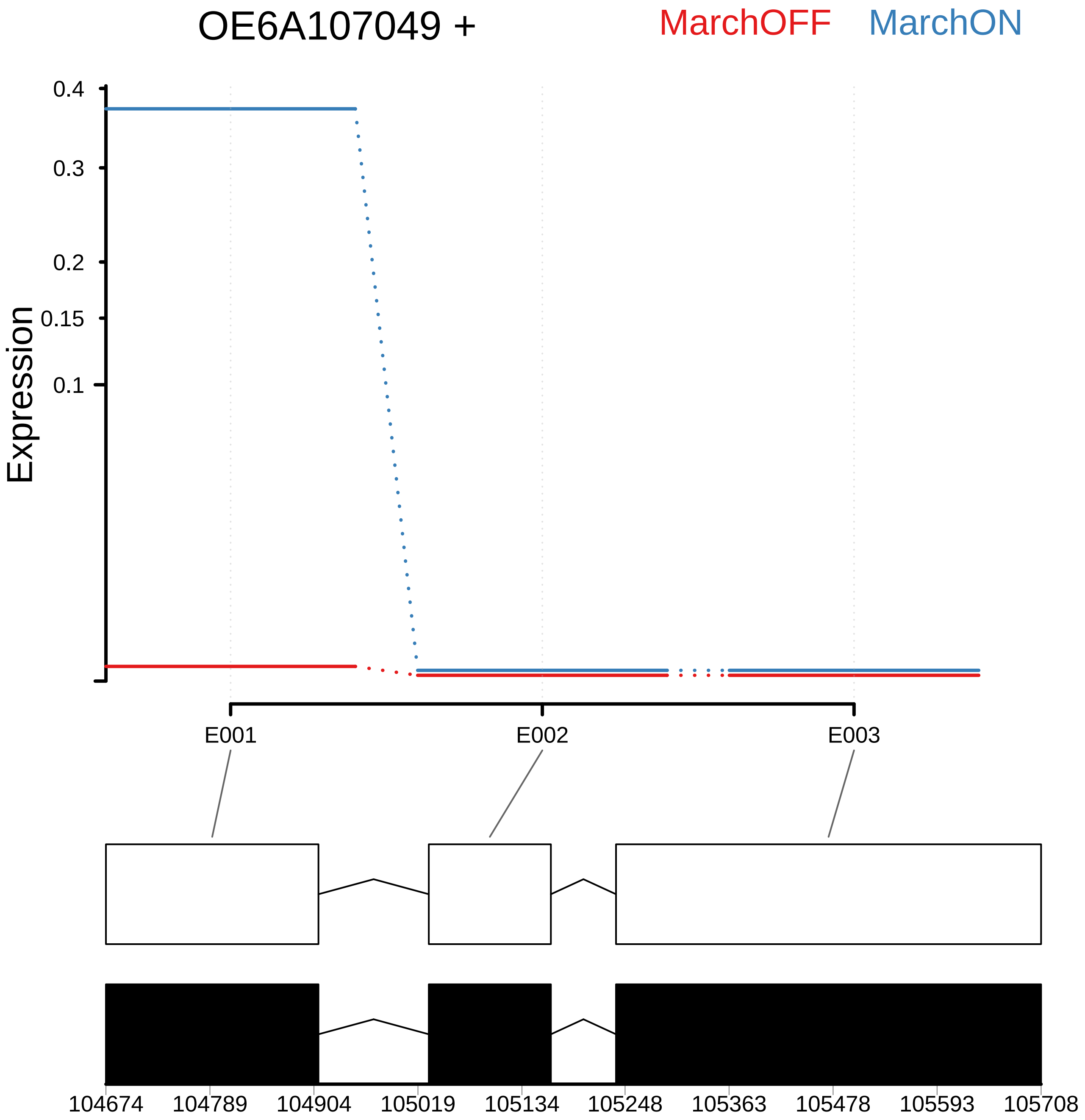
OE6A107049 +
MarchOFF
MarchON
Expression
0.4
0.3
0.2
0.15
0.1
E001
E002
E003
104674
104789
104904
105019
105134
105248
105363
105478
105593
105708

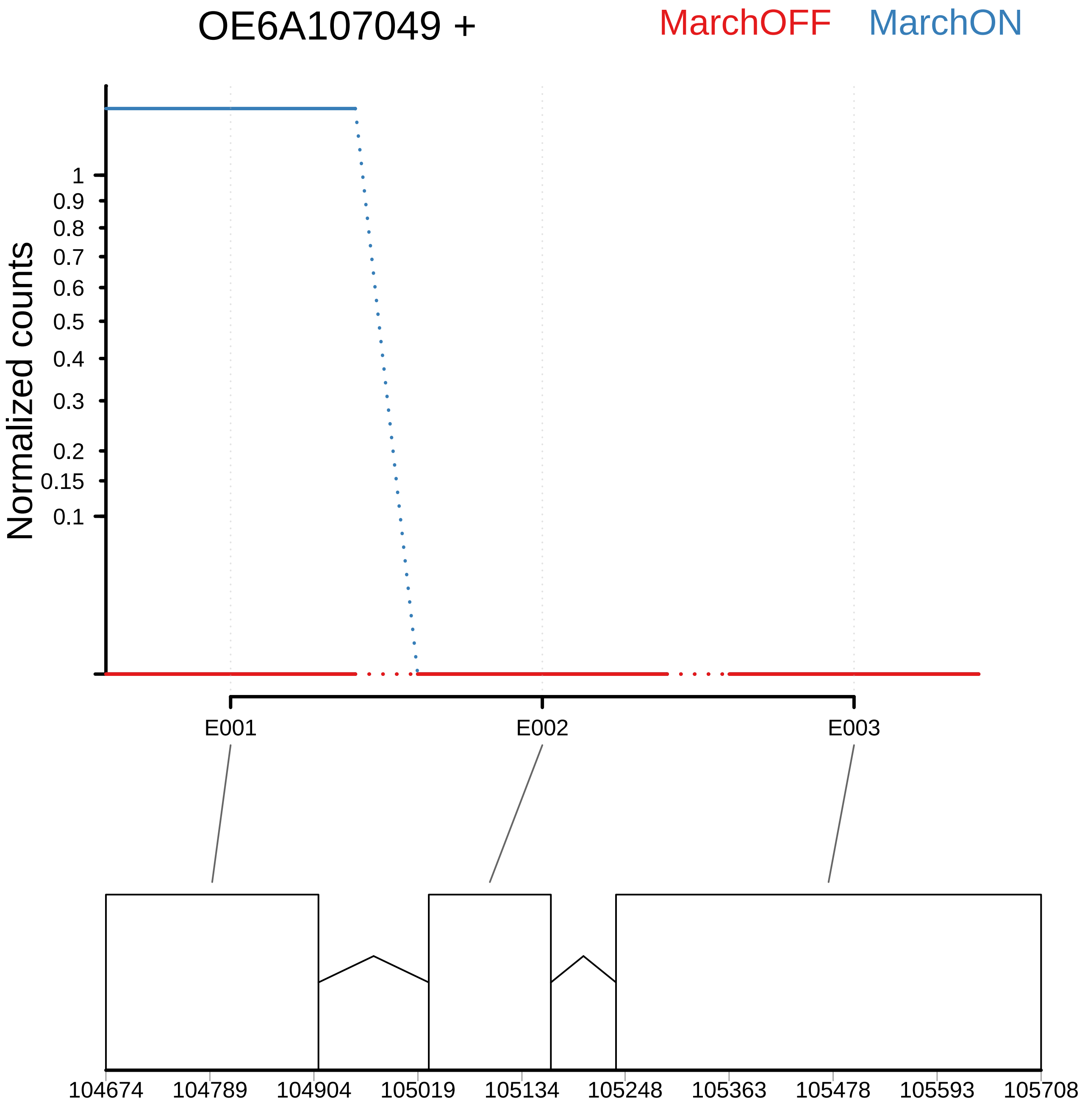

OE6A107049 +
MarchOFF
MarchON
Normalized counts
1
0.9
0.8
0.7
0.6
0.5
0.4
0.3
0.2
0.15
0.1
E001
E002
E003
104674
104789
104904
105019
105134
105248
105363
105478
105593
105708

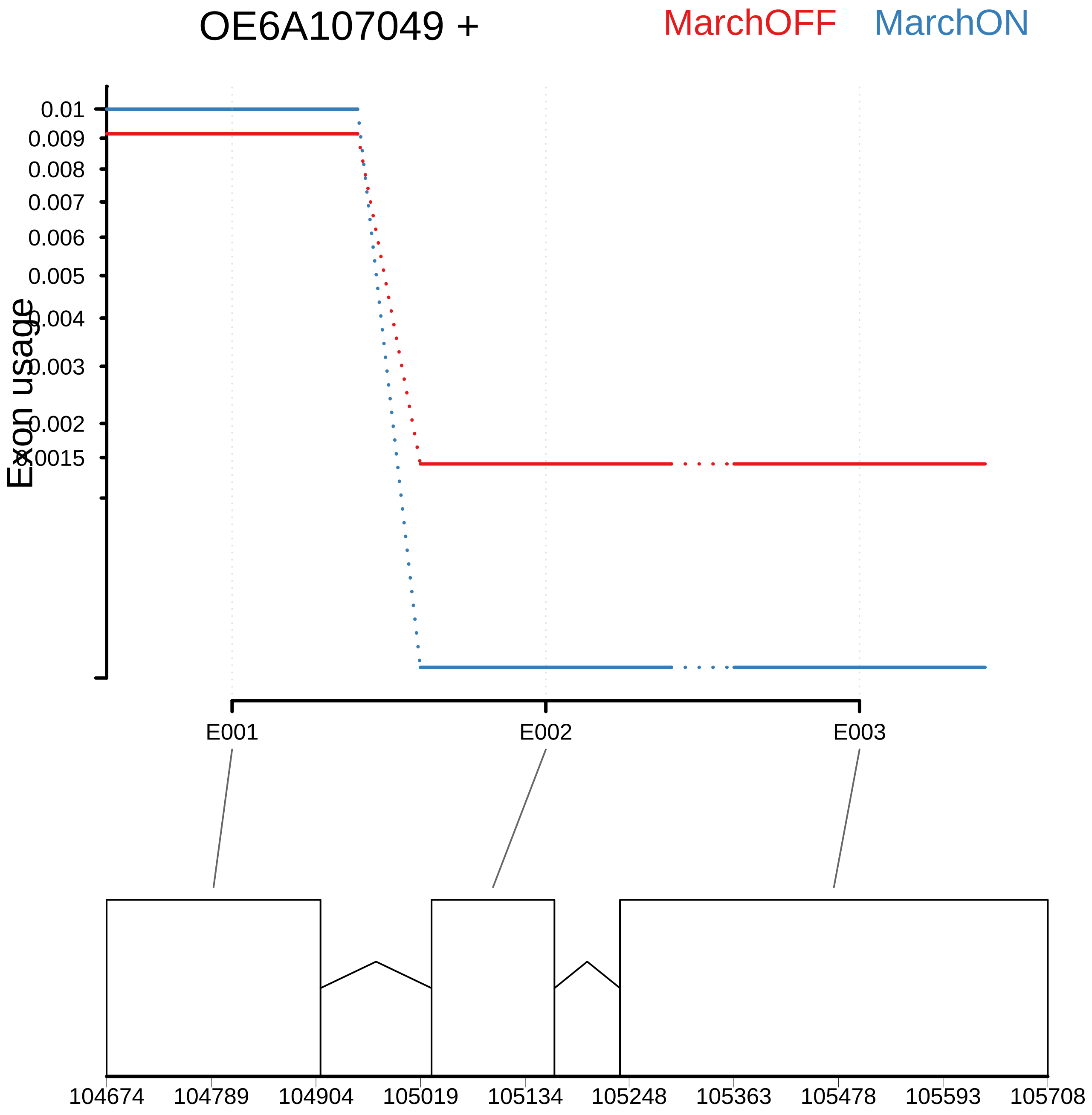

OE6A107049 +
MarchOFF
MarchON
Exon usage
0.01
0.009
0.008
0.007
0.006
0.005
0.004
0.003
0.002
0.0015
E001
E002
E003
104674
104789
104904
105019
105134
105248
105363
105478
105593
105708

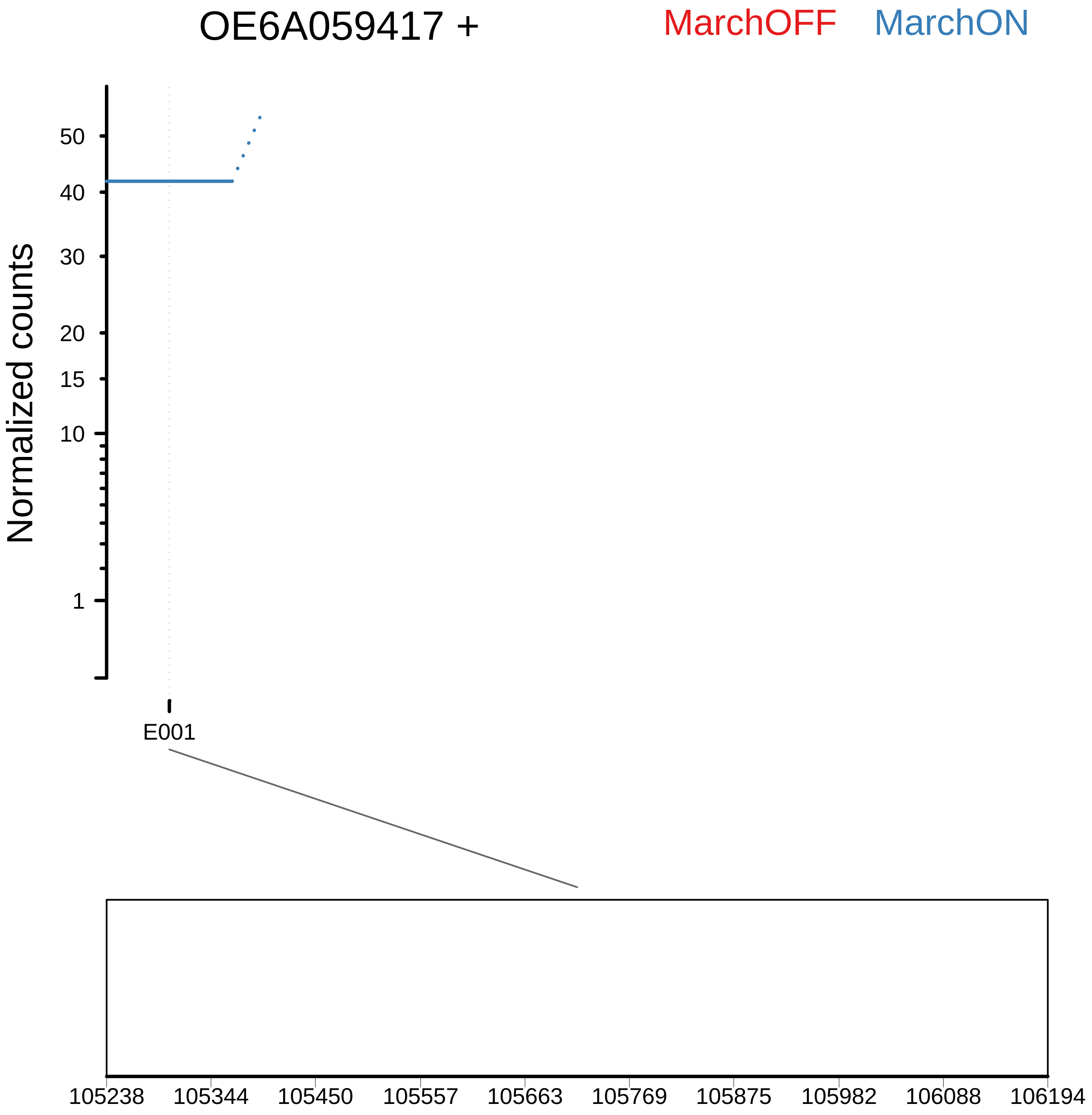

OE6A059417 +
MarchOFF
MarchON
Normalized counts
50
40
30
20
15
10
1
E001
105238
105344
105450
105557
105663
105769
105875
105982
106088
106194

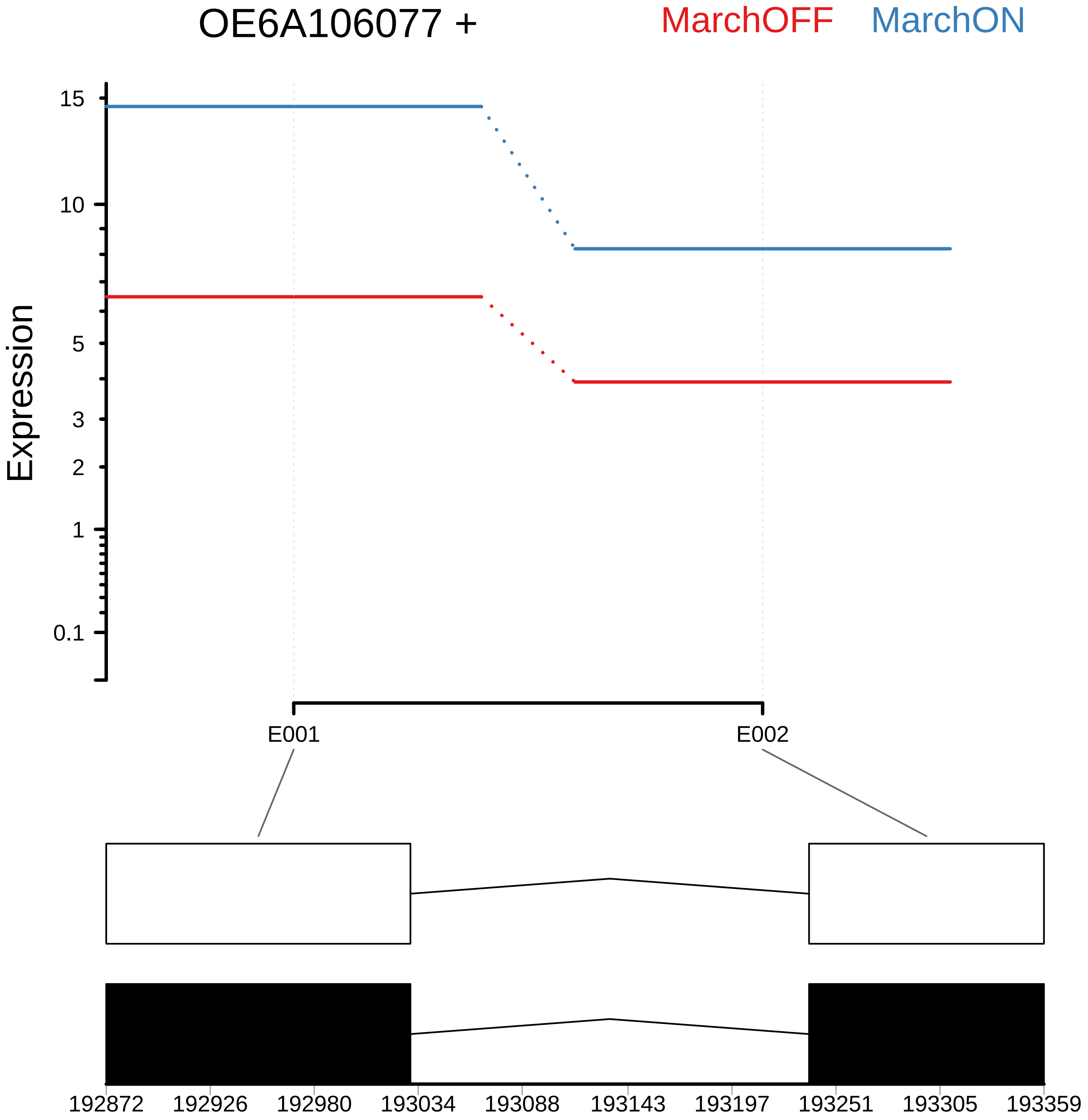
OE6A106077 +
MarchOFF
MarchON
Expression
15
10
5
3
2
1
0.1
E001
E002
192872
192926
192980
193034
193088
193143
193197
193251
193305
193359

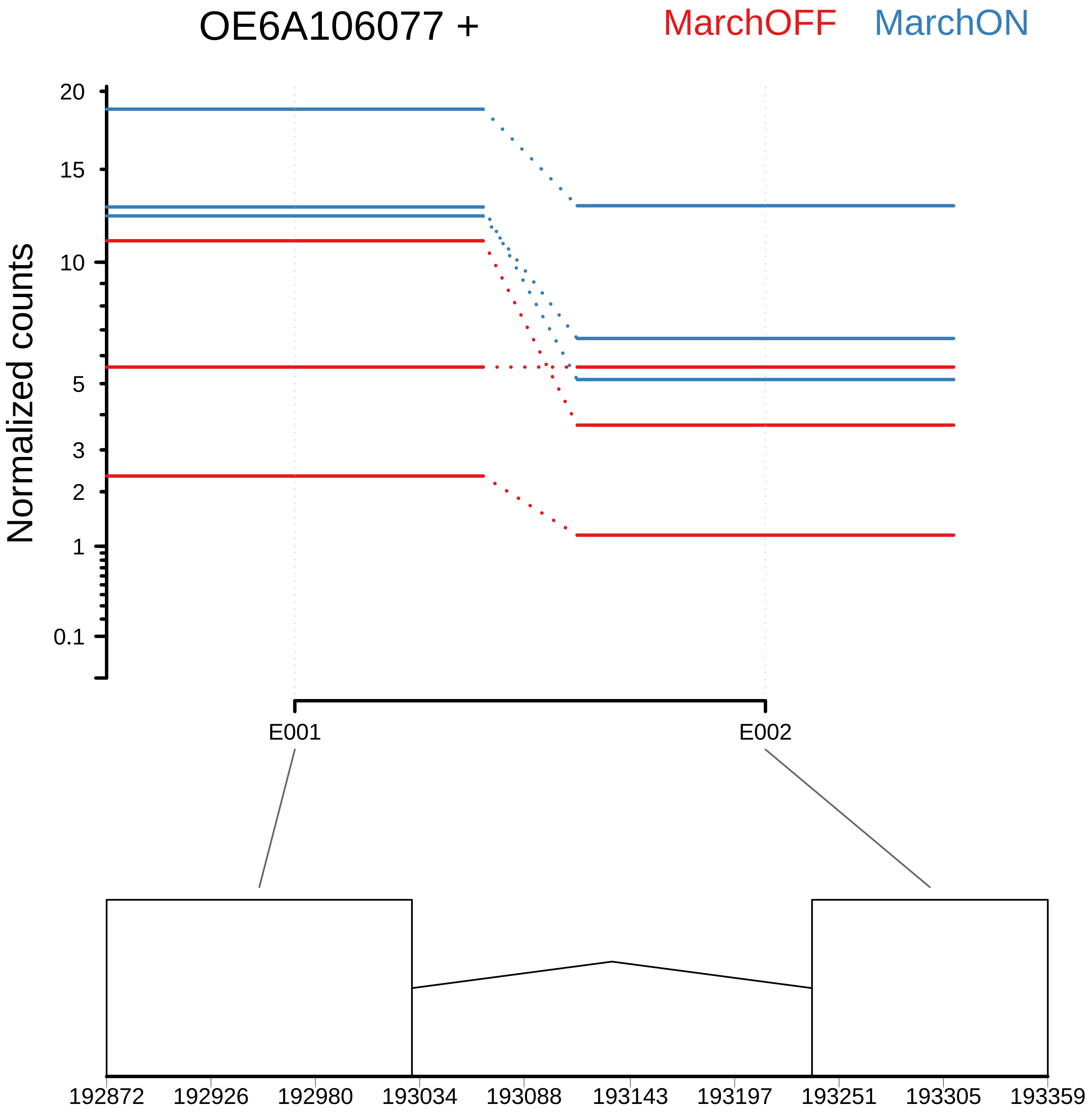
OE6A106077 +
MarchOFF
MarchON
Normalized counts
20
15
10
5
3
2
1
0.1
E001
E002
192872
192926
192980
193034
193088
193143
193197
193251
193305
193359

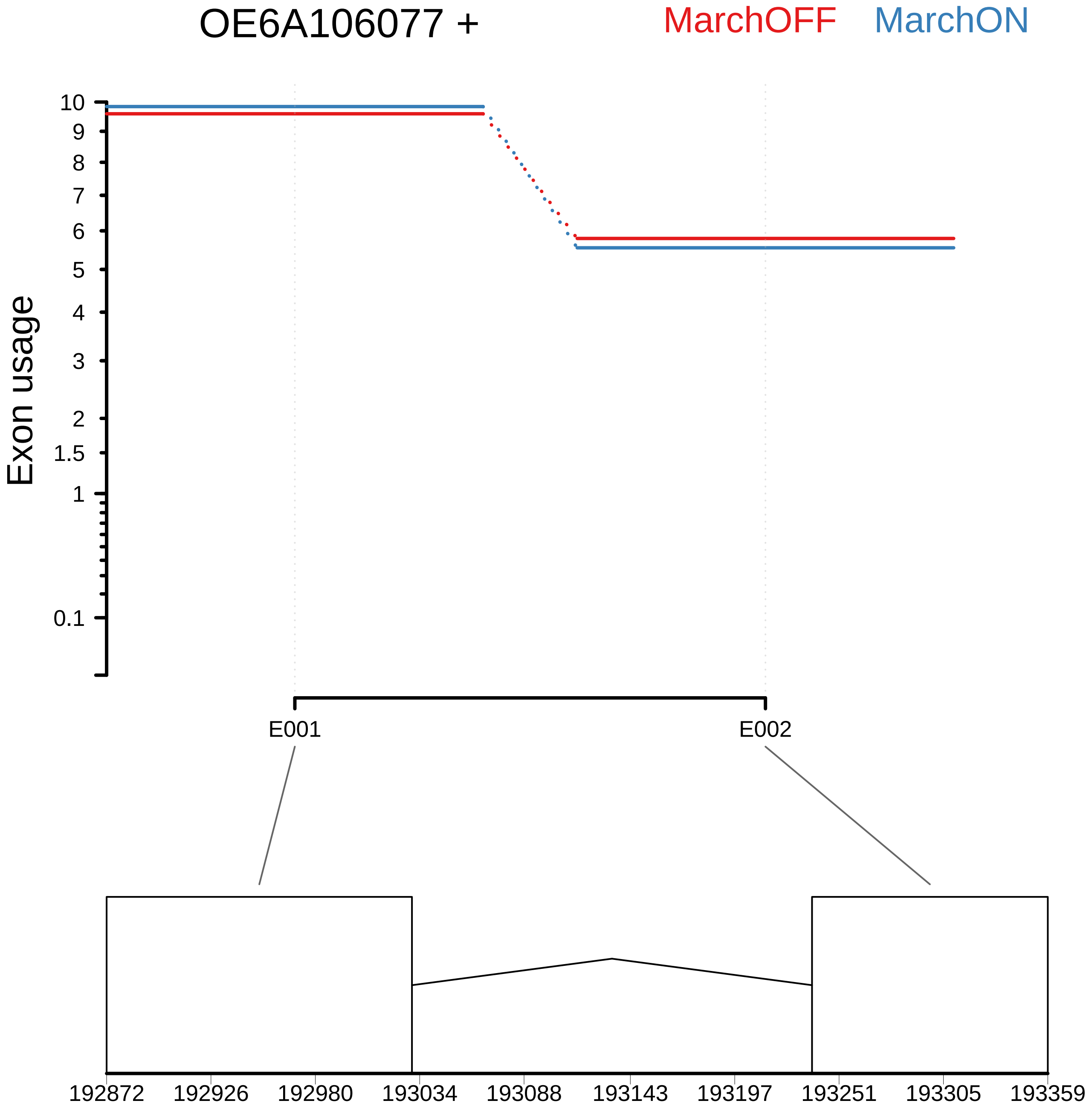

OE6A106077 +
MarchOFF
MarchON
Exon usage
10
9
8
7
6
5
4
3
2
1.5
1
0.1
E001
E002
192872
192926
192980
193034
193088
193143
193197
193251
193305
193359

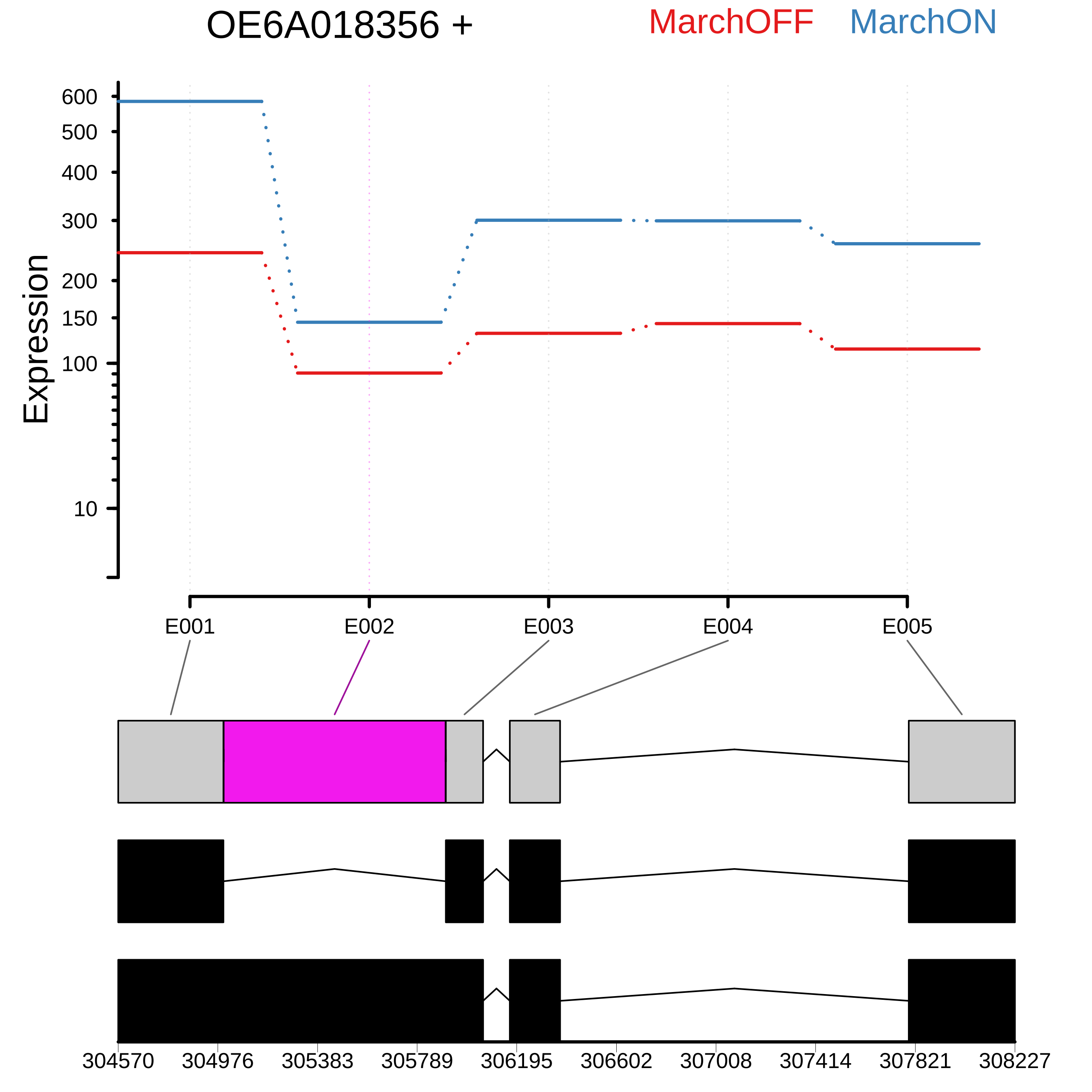
OE6A018356 +
MarchOFF
MarchON
Expression
600
500
400
300
200
150
100
10
E001
E002
E003
E004
E005
304570
304976
305383
305789
306195
306602
307008
307414
307821
308227

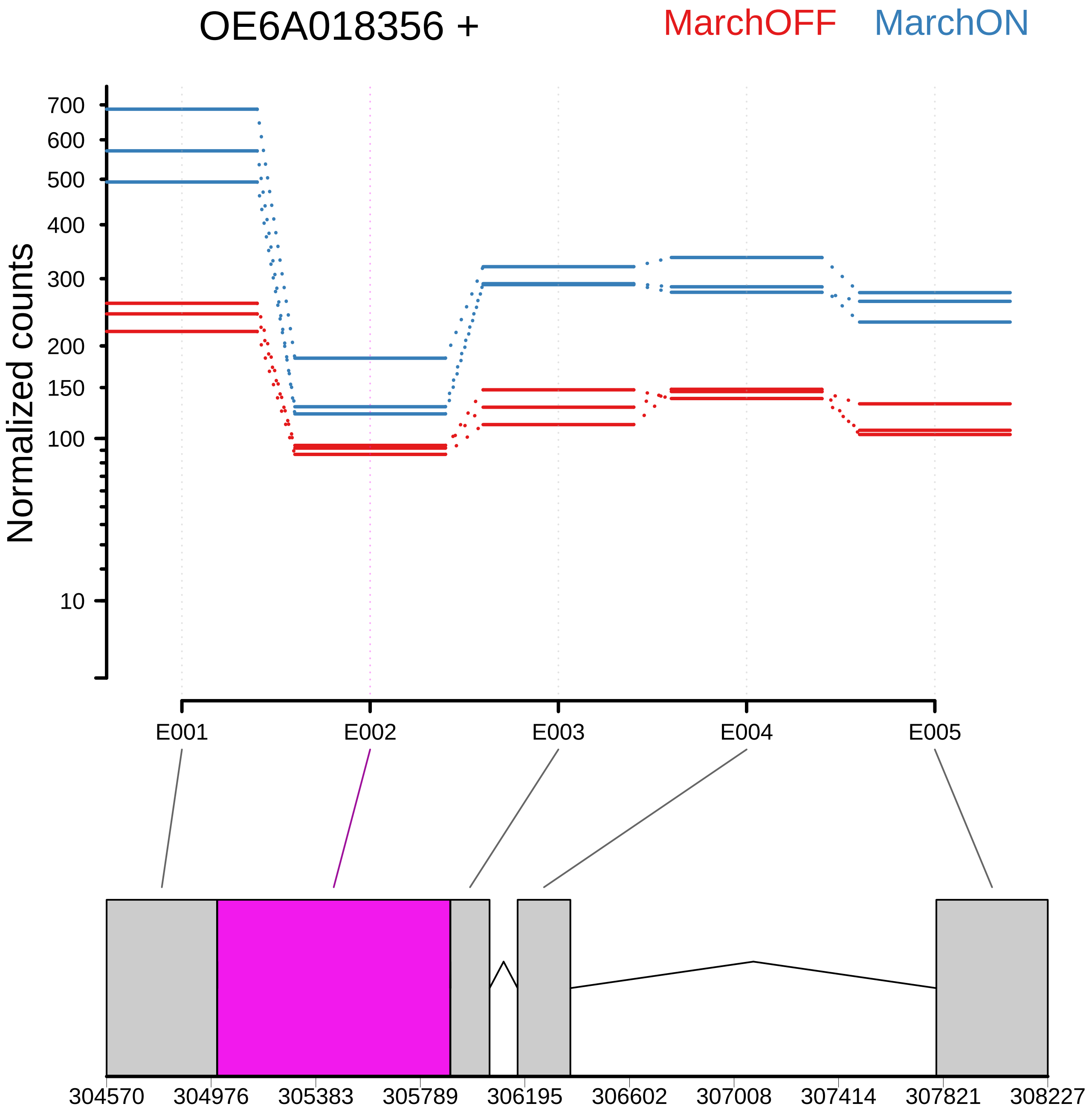

OE6A018356 +
MarchOFF
MarchON
Normalized counts
700
600
500
400
300
200
150
100
10
E001
E002
E003
E004
E005
304570
304976
305383
305789
306195
306602
307008
307414
307821
308227

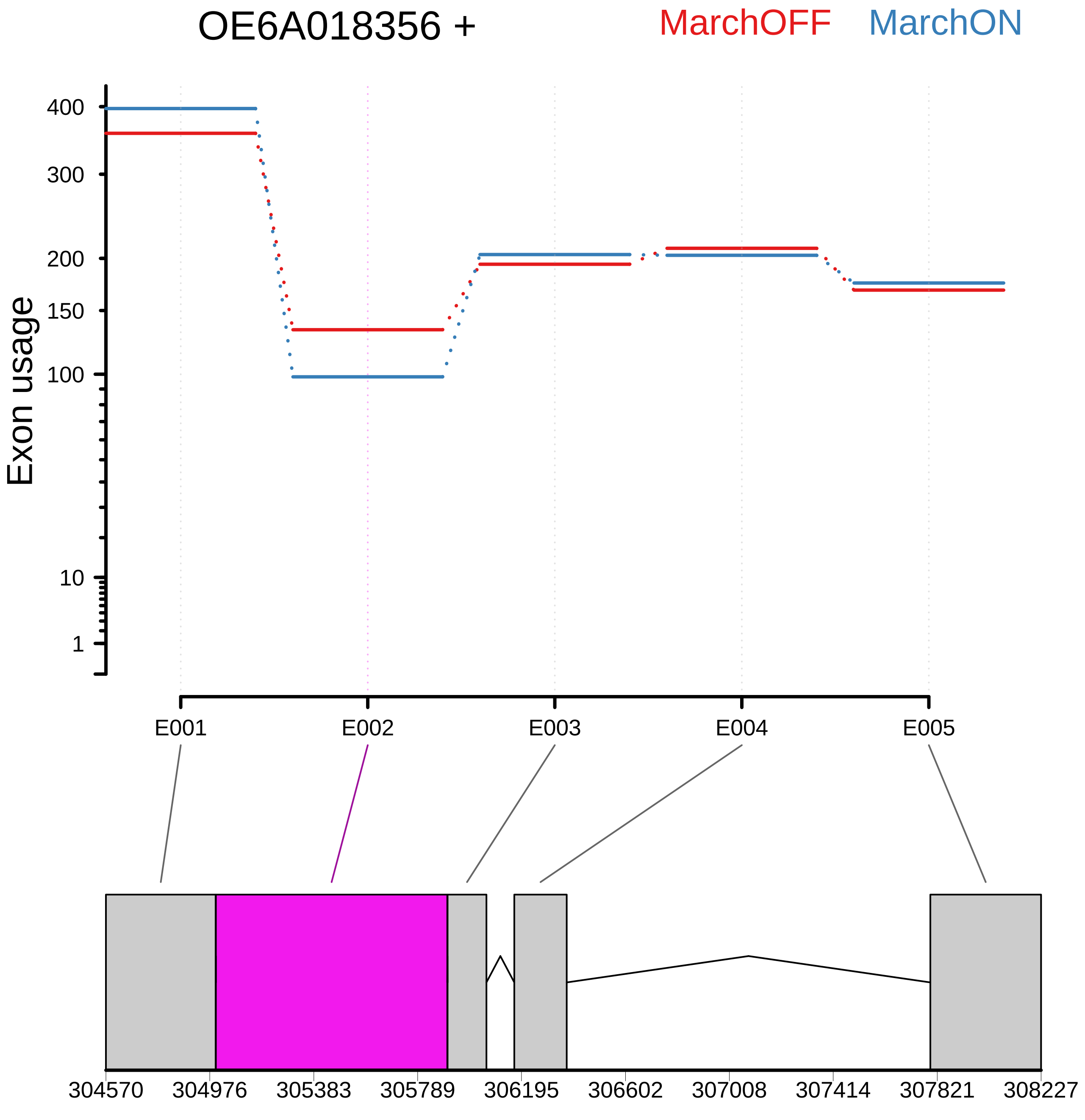
OE6A018356 +
MarchOFF
MarchON
Exon usage
400
300
200
150
100
10
1
E001
E002
E003
E004
E005
304570
304976
305383
305789
306195
306602
307008
307414
307821
308227

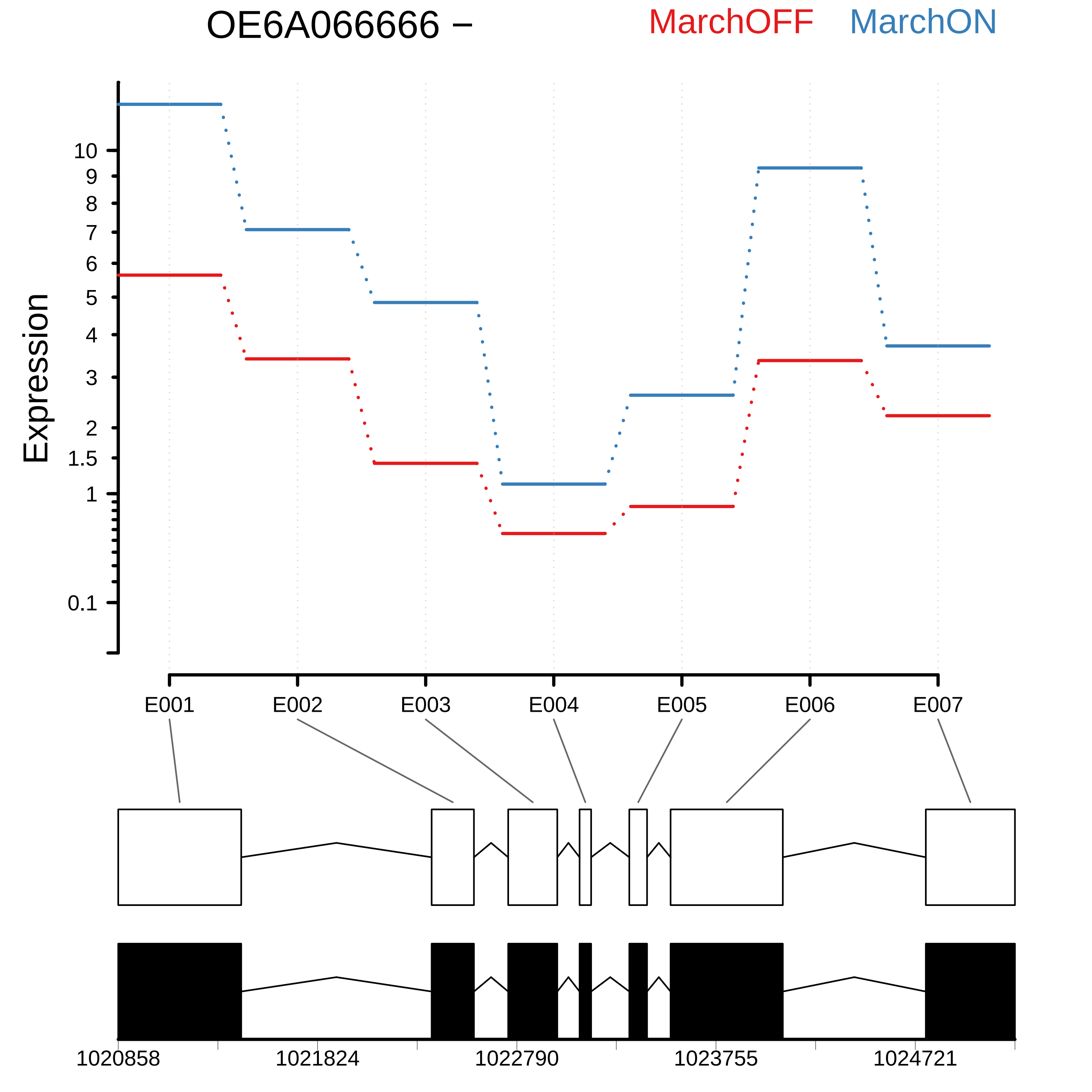
OE6A066666 −
MarchOFF
MarchON
Expression
10
9
8
7
6
5
4
3
2
1.5
1
0.1
E001
E002
E003
E004
E005
E006
E007
1020858
1021824
1022790
1023755
1024721

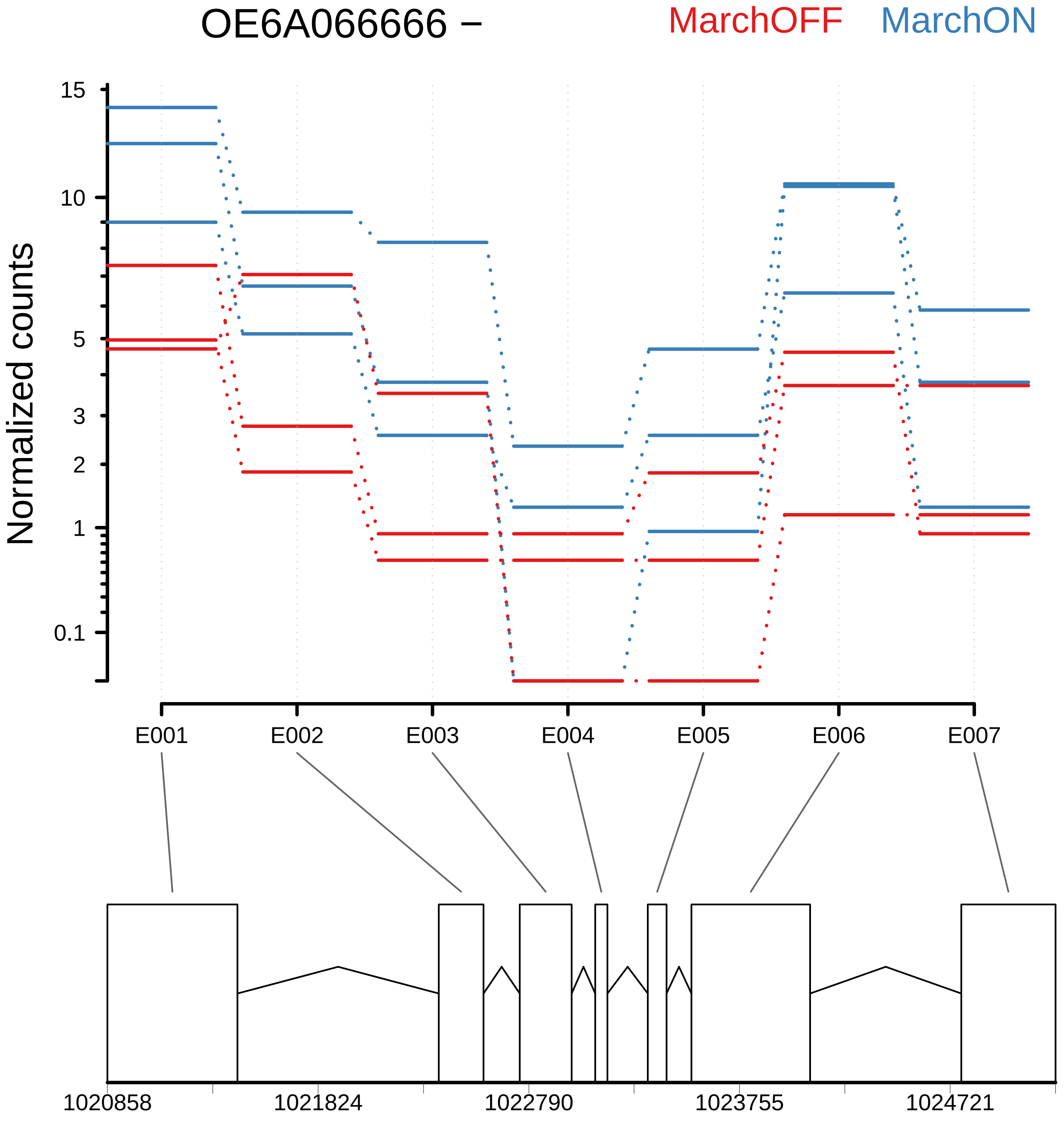

OE6A066666 −
MarchOFF
MarchON
Normalized counts
15
10
5
3
2
1
0.1
E001
E002
E003
E004
E005
E006
E007
1020858
1021824
1022790
1023755
1024721

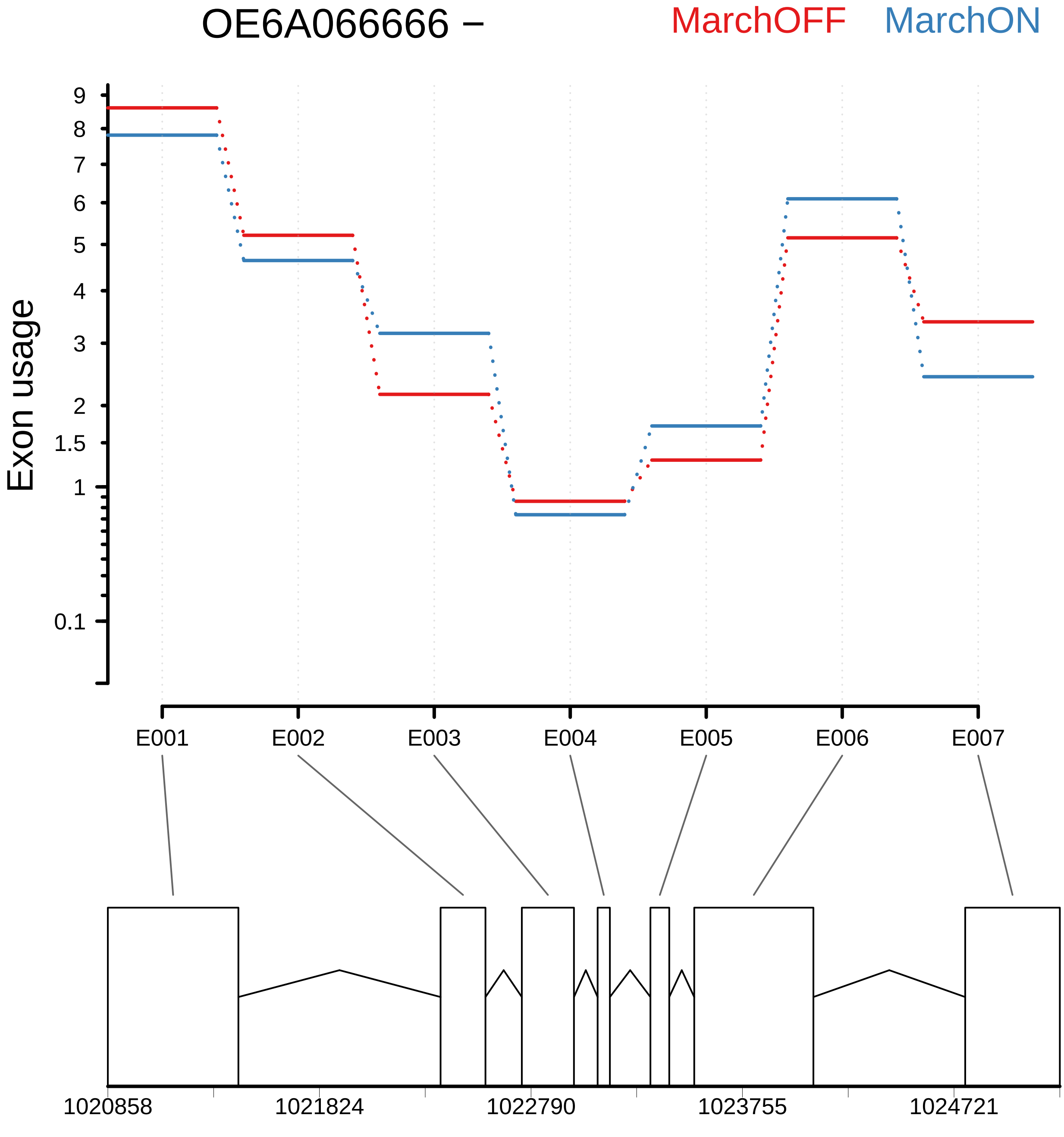

OE6A066666 −
MarchOFF
MarchON
Exon usage
9
8
7
6
5
4
3
2
1.5
1
0.1
E001
E002
E003
E004
E005
E006
E007
1020858
1021824
1022790
1023755
1024721

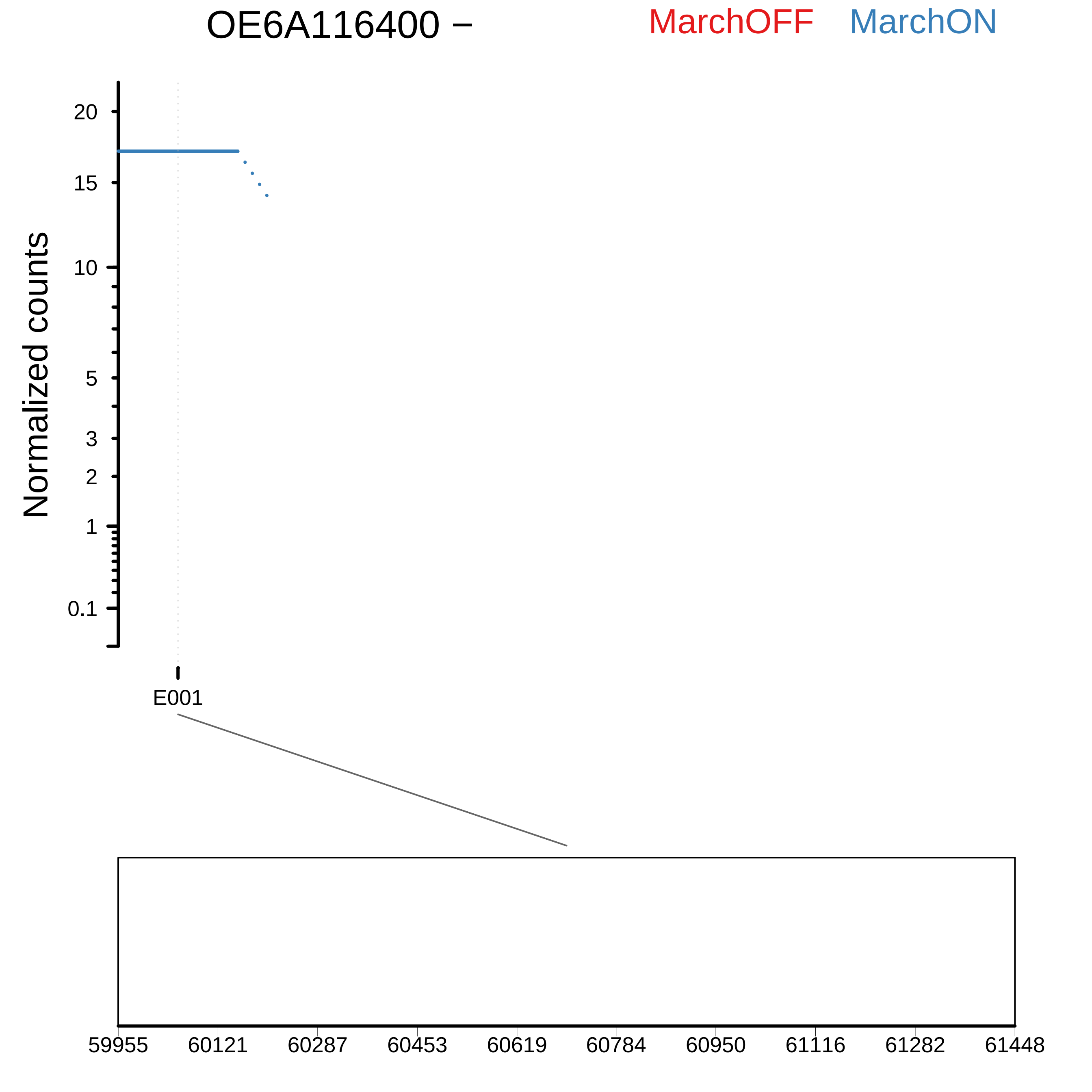

OE6A116400 −
MarchOFF
MarchON
Normalized counts
20
15
10
5
3
2
1
0.1
E001
59955
60121
60287
60453
60619
60784
60950
61116
61282
61448

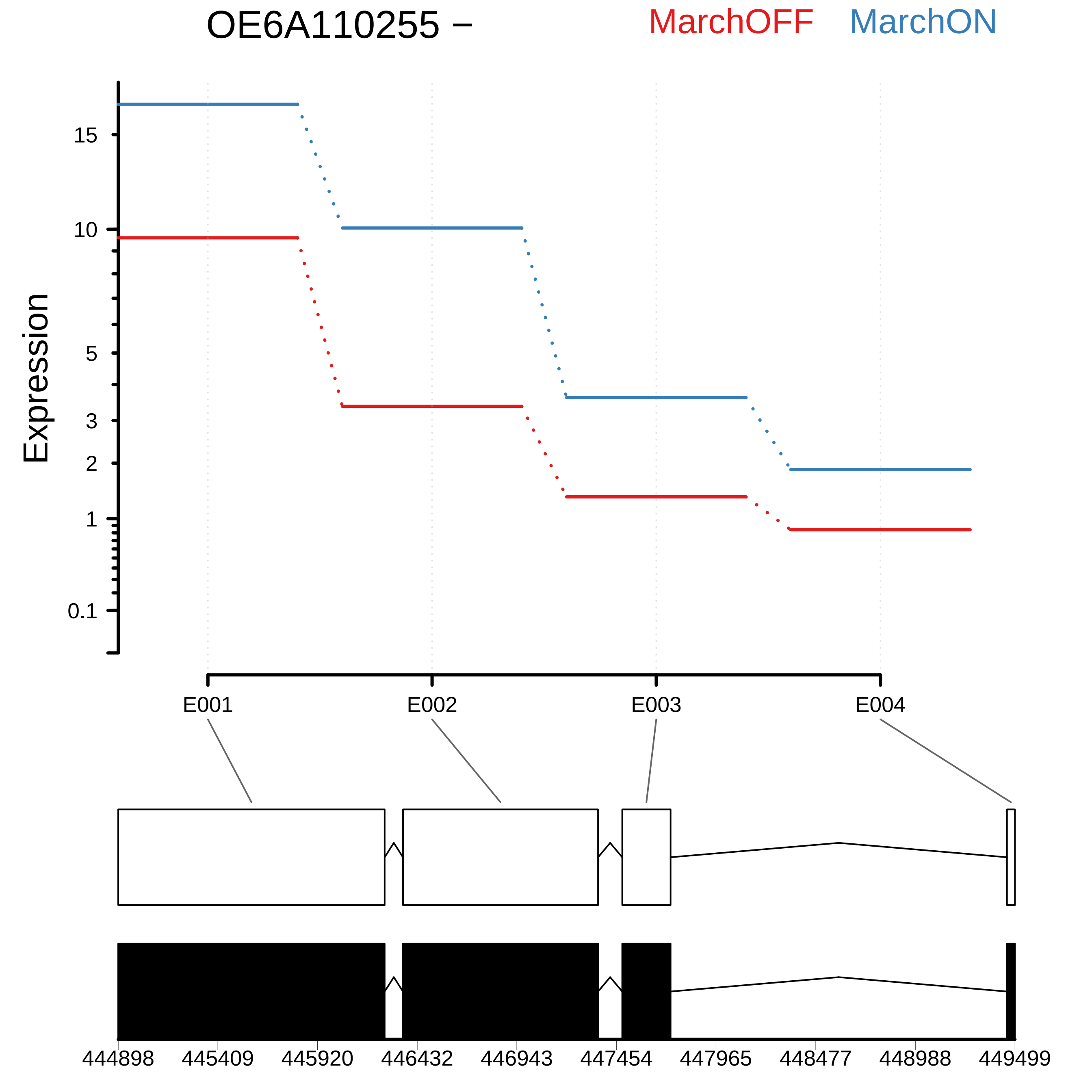

OE6A110255 −
MarchOFF
MarchON
Expression
15
10
5
3
2
1
0.1
E001
E002
E003
E004
444898
445409
445920
446432
446943
447454
447965
448477
448988
449499

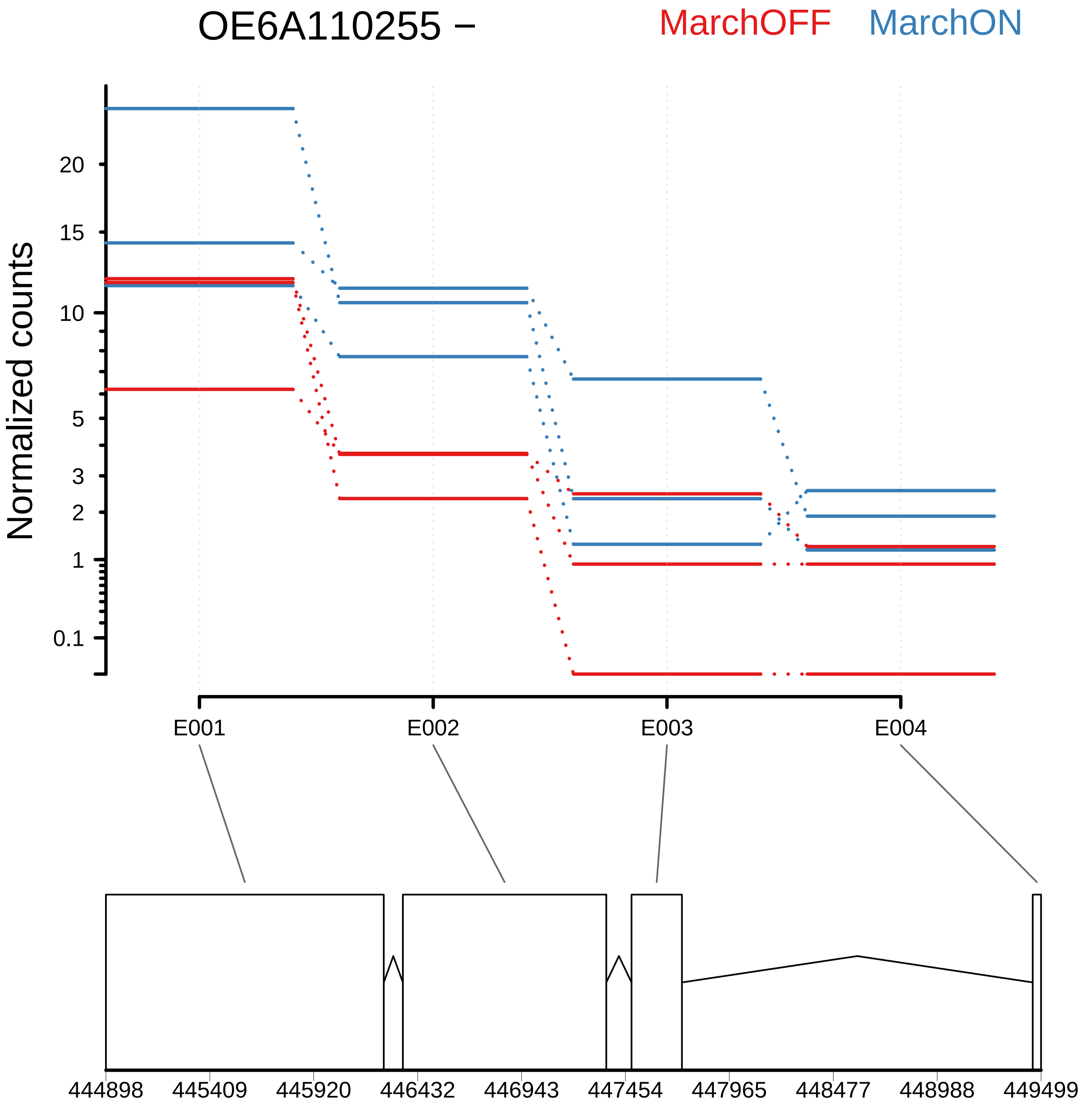

OE6A110255 −
MarchOFF
MarchON
Normalized counts
20
15
10
5
3
2
1
0.1
E001
E002
E003
E004
444898
445409
445920
446432
446943
447454
447965
448477
448988
449499

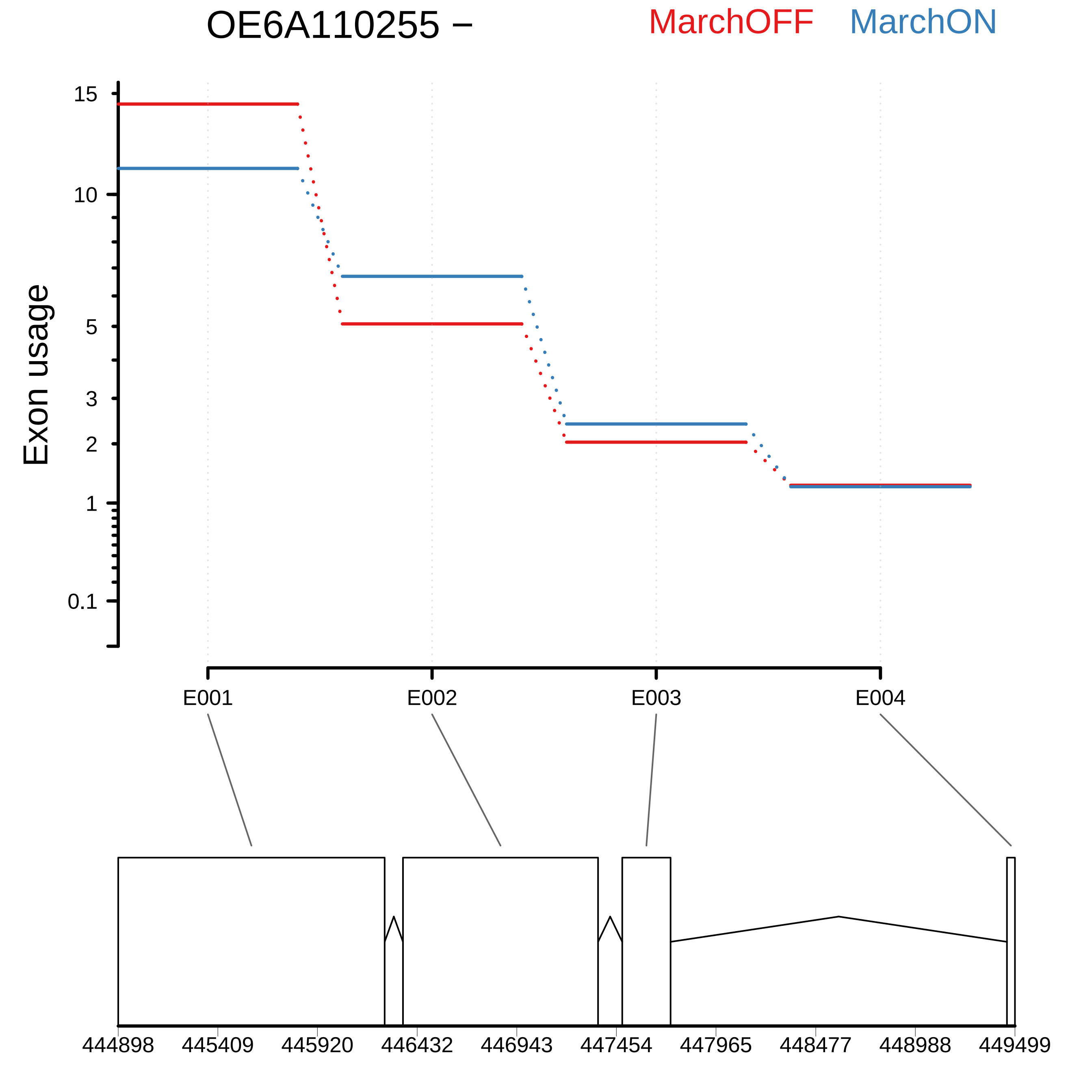
OE6A110255 −
MarchOFF
MarchON
Exon usage
15
10
5
3
2
1
0.1
E001
E002
E003
E004
444898
445409
445920
446432
446943
447454
447965
448477
448988
449499

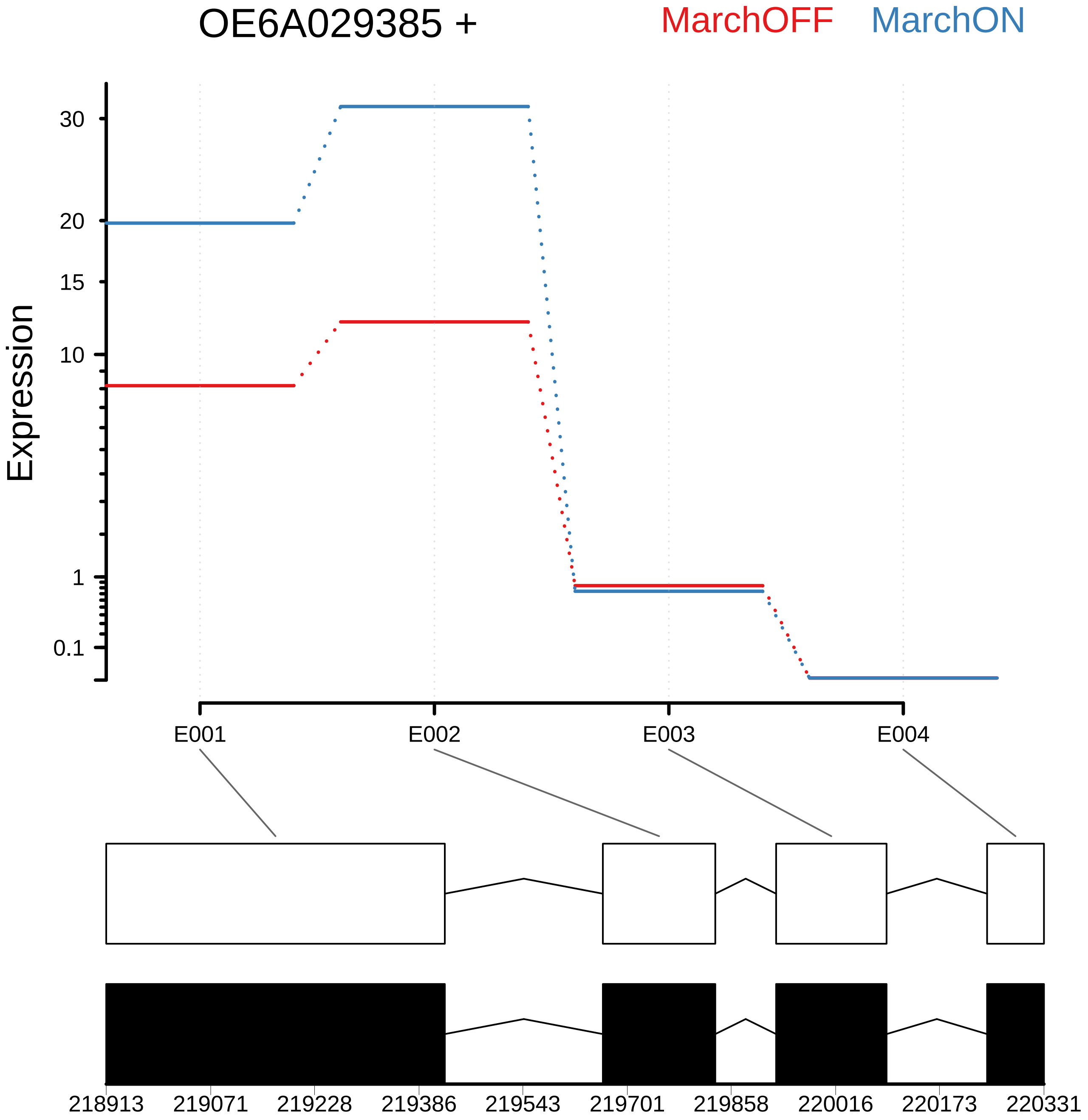

OE6A029385 +
MarchOFF
MarchON
Expression
30
20
15
10
1
0.1
E001
E002
E003
E004
218913
219071
219228
219386
219543
219701
219858
220016
220173
220331

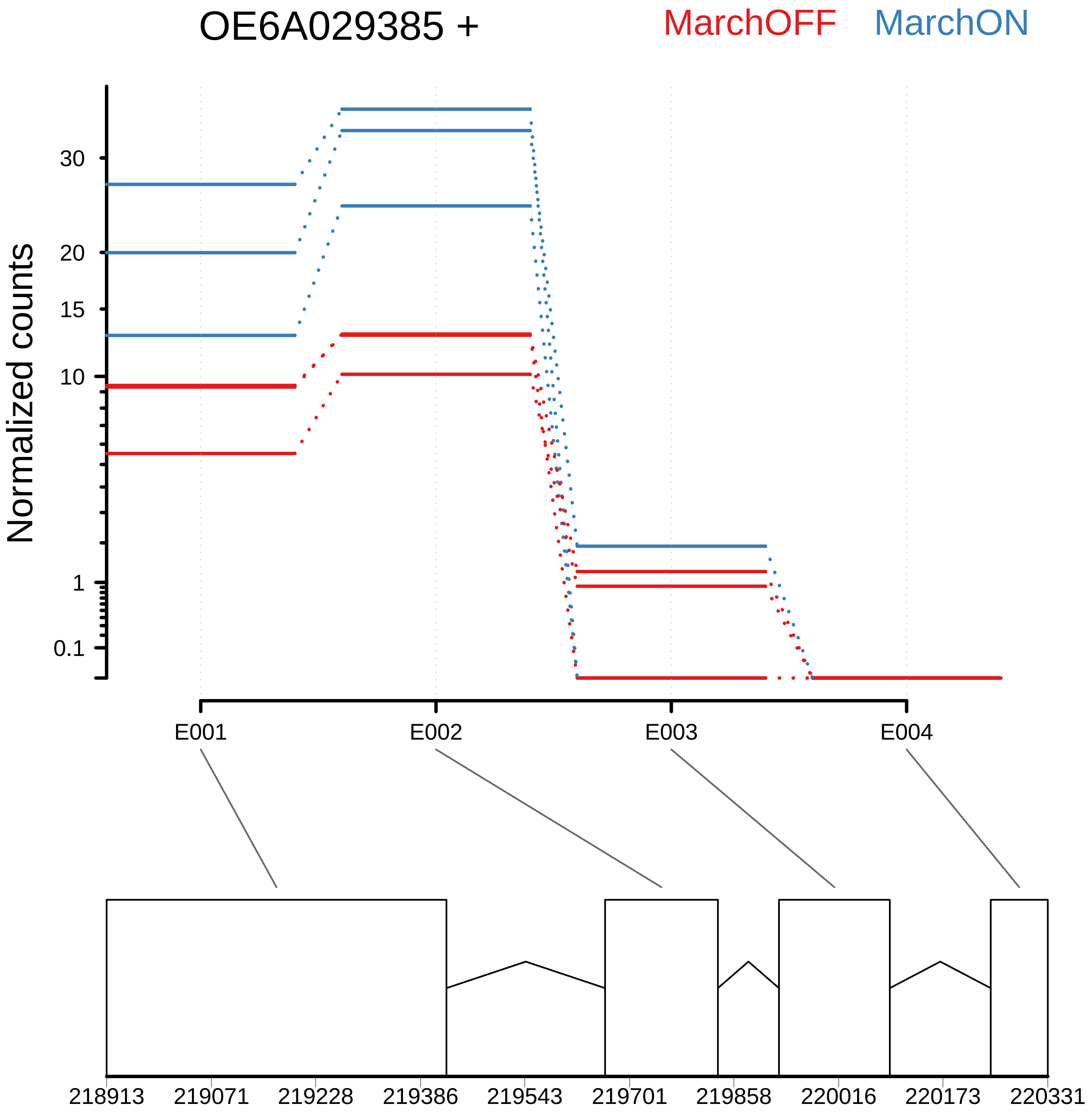
OE6A029385 +
MarchOFF
MarchON
Normalized counts
30
20
15
10
1
0.1
E001
E002
E003
E004
218913
219071
219228
219386
219543
219701
219858
220016
220173
220331

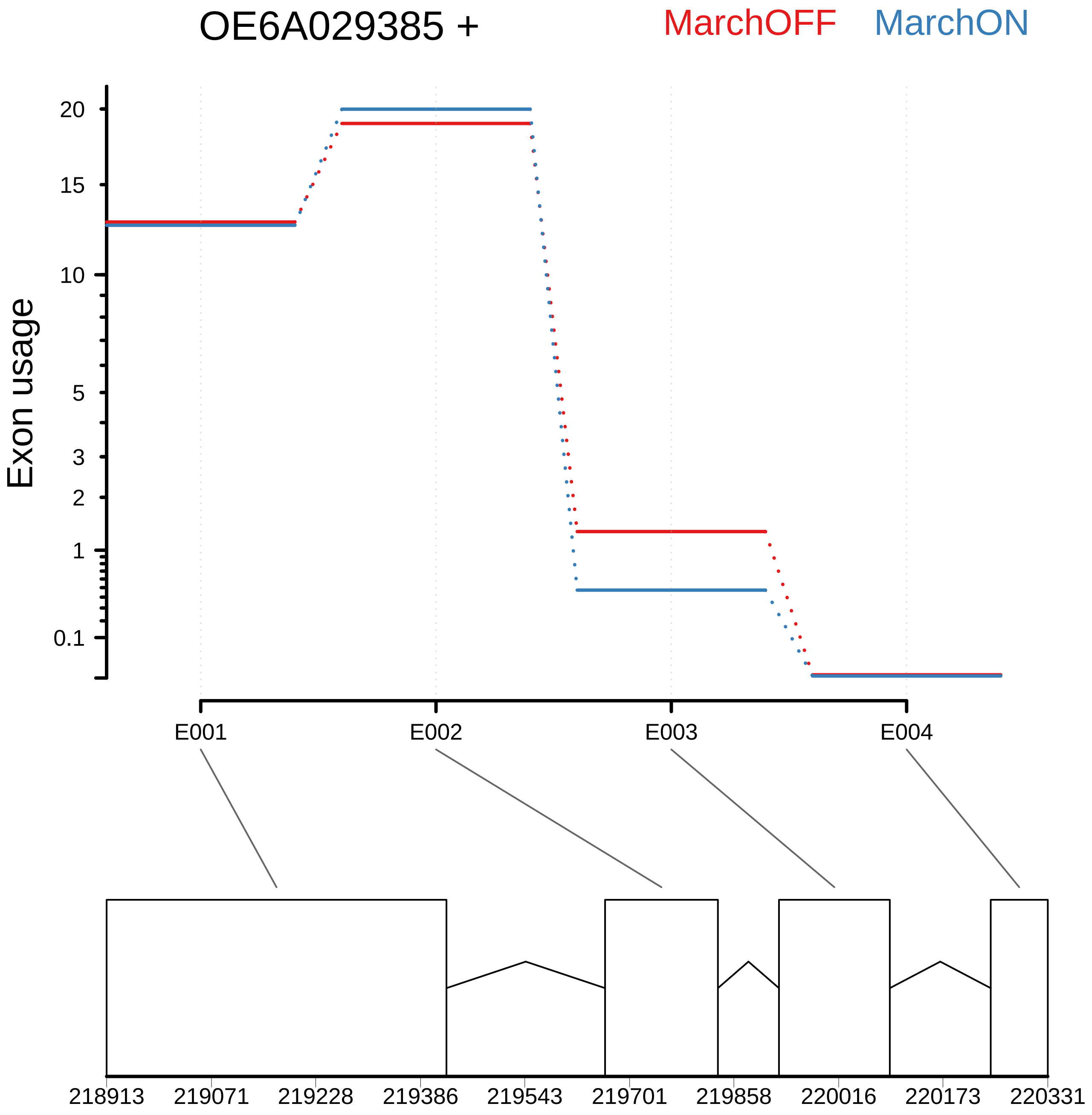

OE6A029385 +
MarchOFF
MarchON
Exon usage
20
15
10
5
3
2
1
0.1
E001
E002
E003
E004
218913
219071
219228
219386
219543
219701
219858
220016
220173
220331

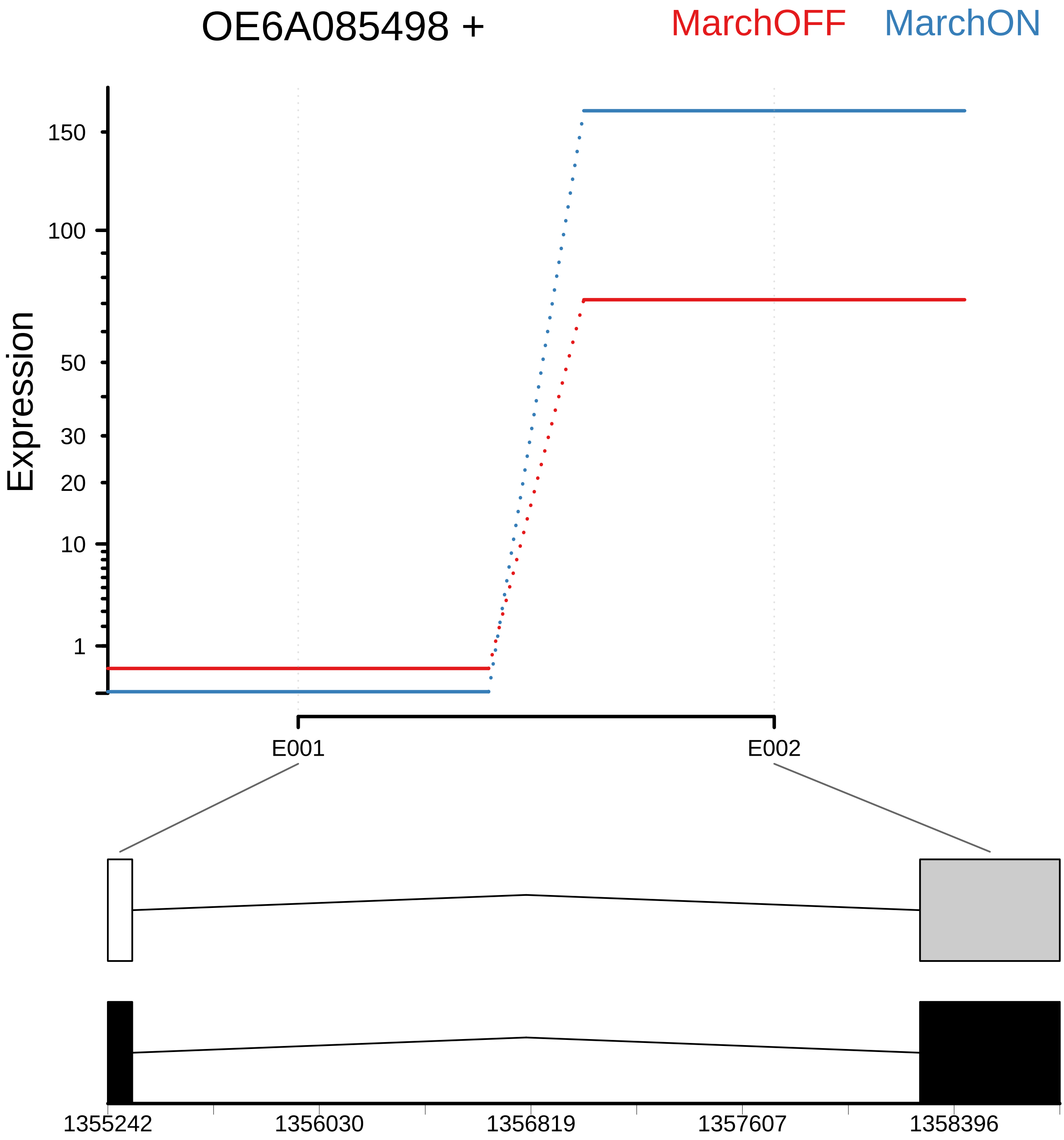
OE6A085498 +
MarchOFF
MarchON
Expression
150
100
50
30
20
10
1
E001
E002
1355242
1356030
1356819
1357607
1358396

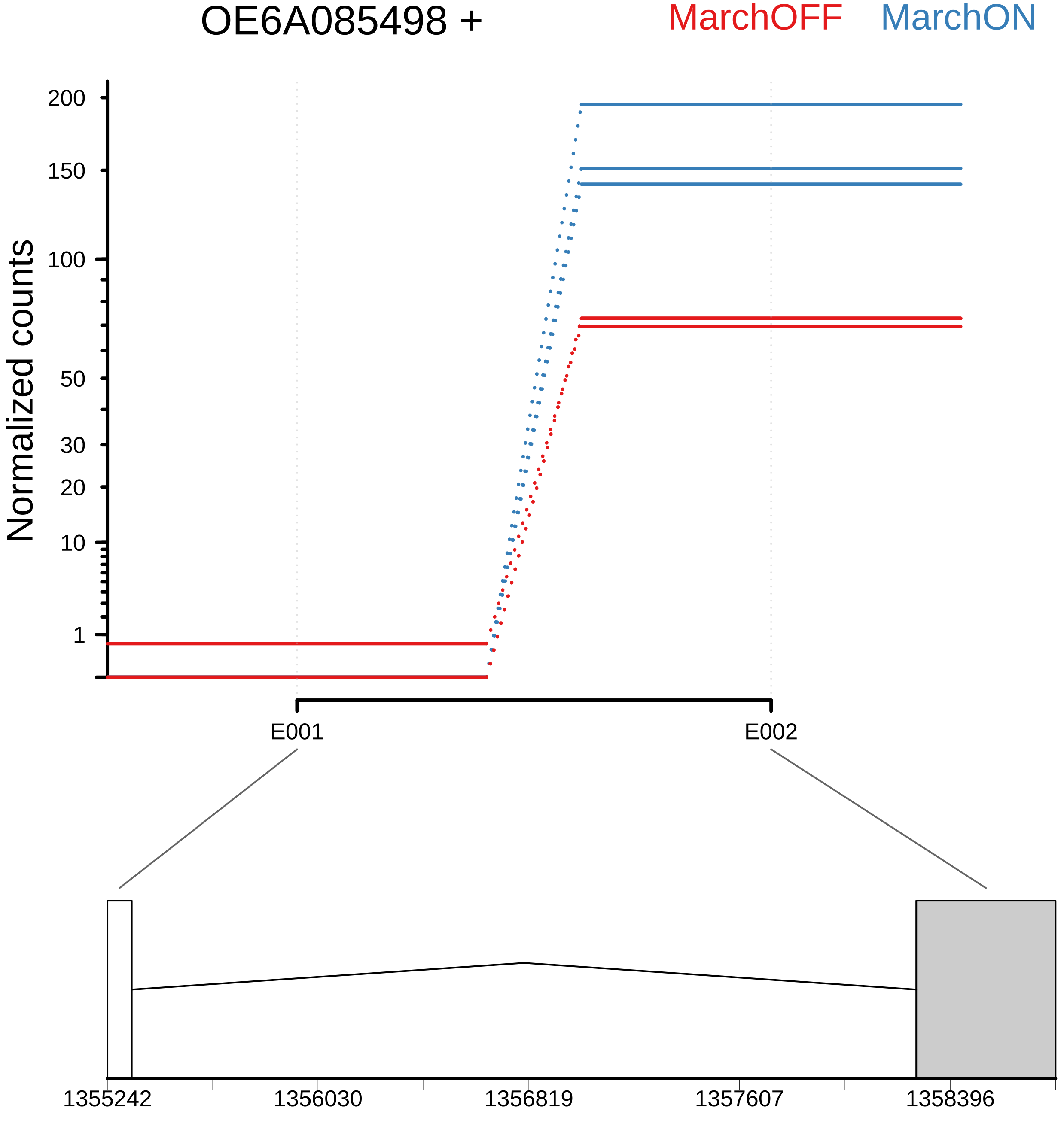
OE6A085498 +
MarchOFF
MarchON
Normalized counts
200
150
100
50
30
20
10
1
E001
E002
1355242
1356030
1356819
1357607
1358396

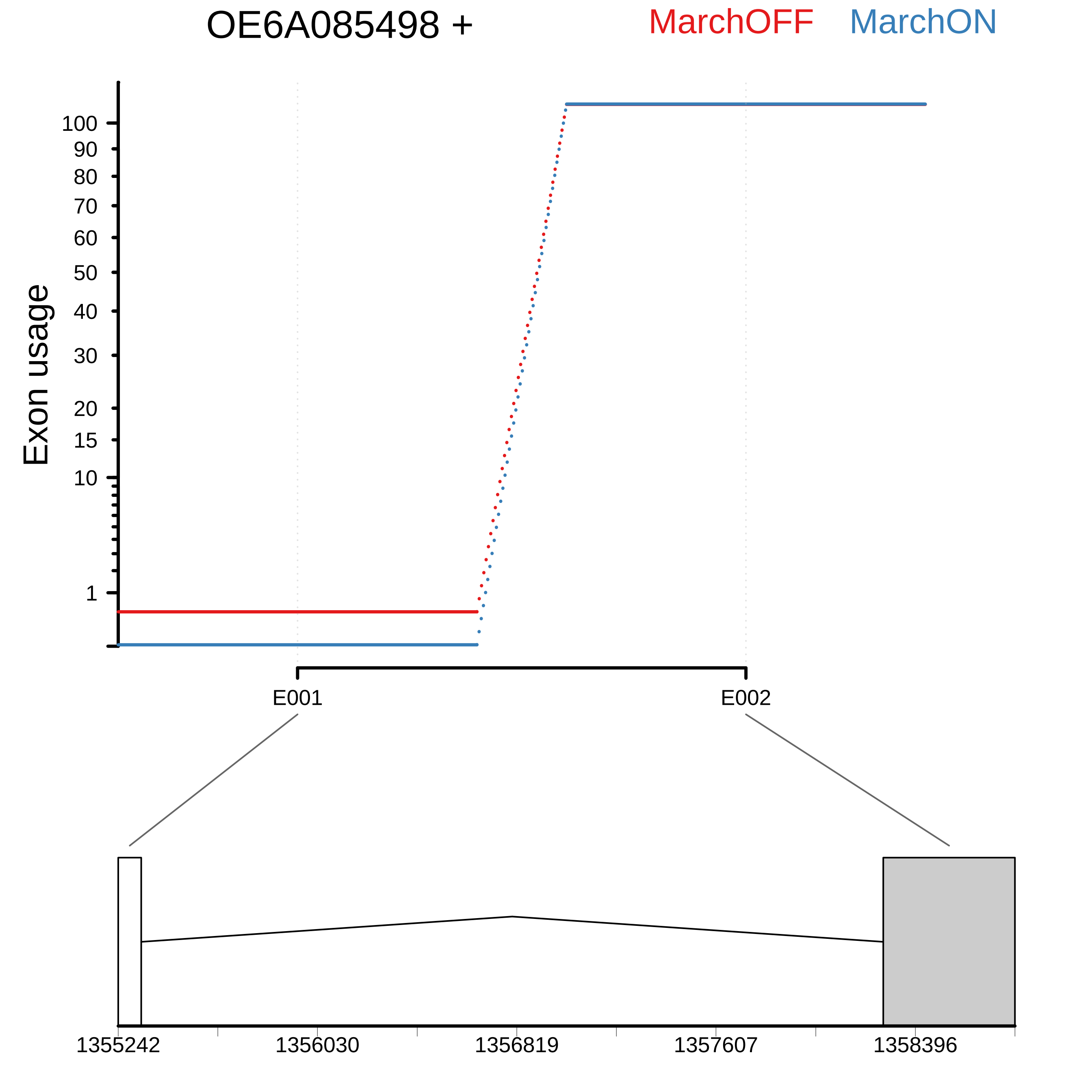

OE6A085498 +
MarchOFF
MarchON
Exon usage
100
90
80
70
60
50
40
30
20
15
10
1
E001
E002
1355242
1356030
1356819
1357607
1358396

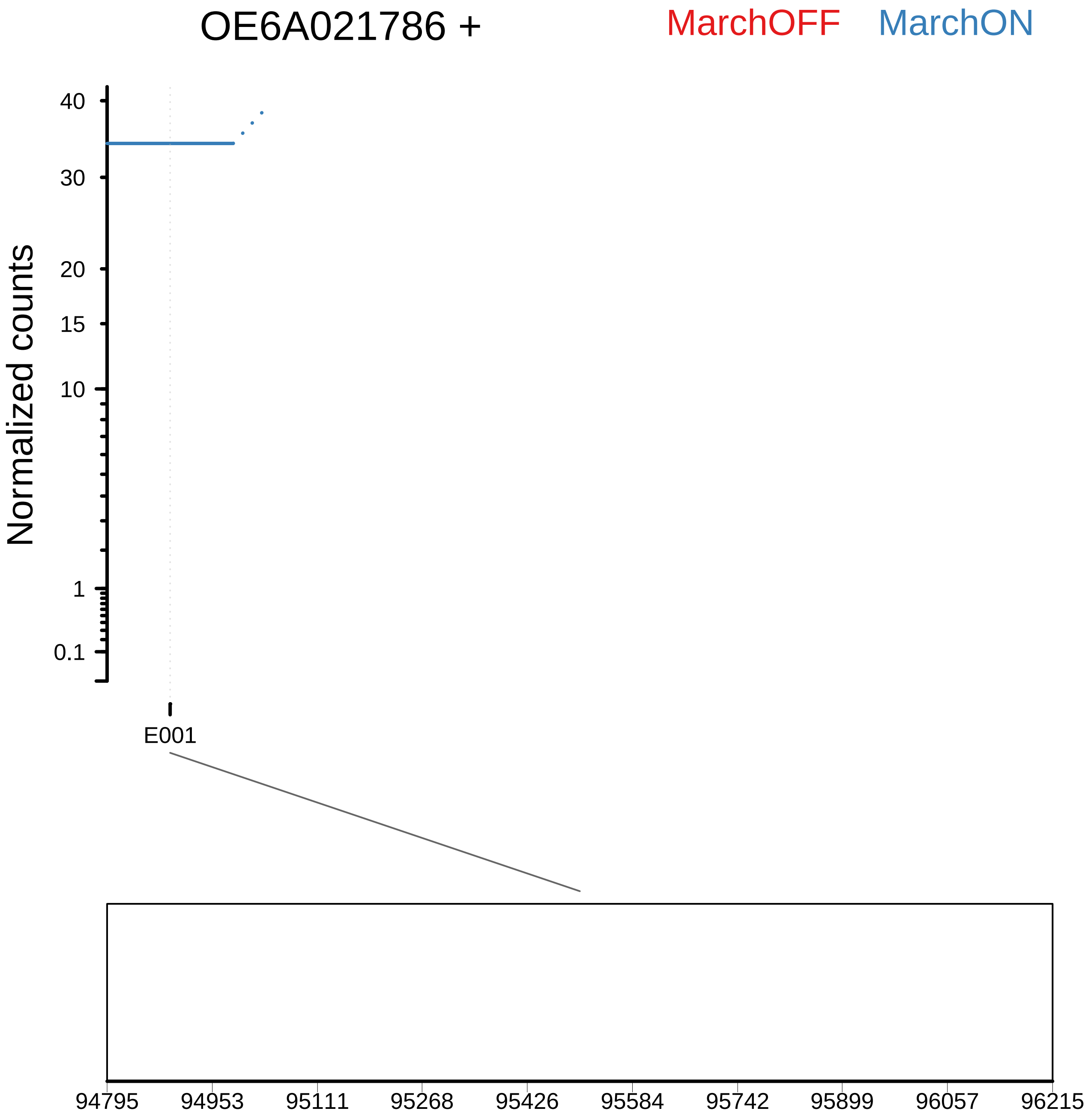
OE6A021786 +
MarchOFF
MarchON
Normalized counts
40
30
20
15
10
1
0.1
E001
94795
94953
95111
95268
95426
95584
95742
95899
96057
96215

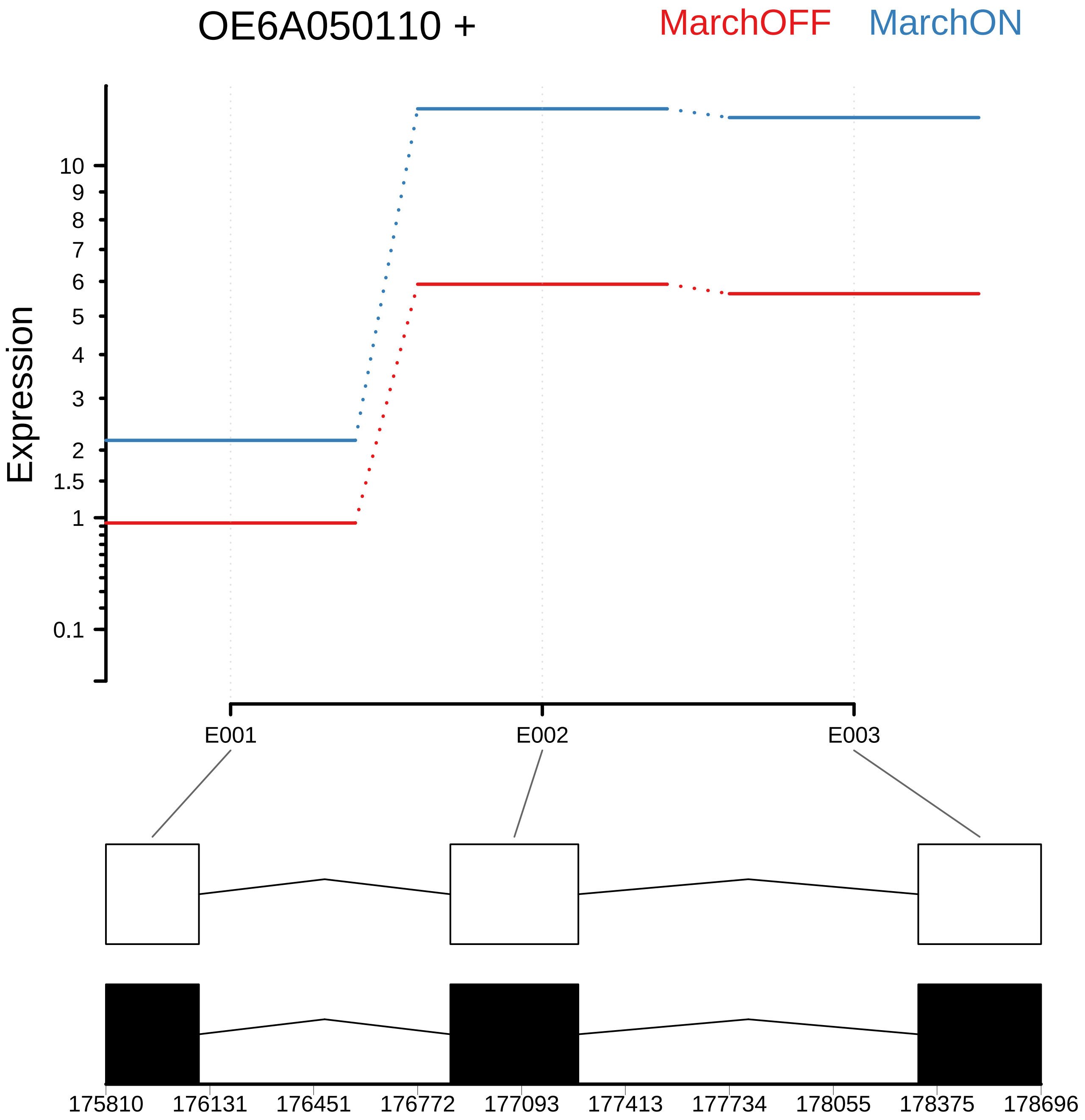
OE6A050110 +
MarchOFF
MarchON
Expression
10
9
8
7
6
5
4
3
2
1.5
1
0.1
E001
E002
E003
175810
176131
176451
176772
177093
177413
177734
178055
178375
178696

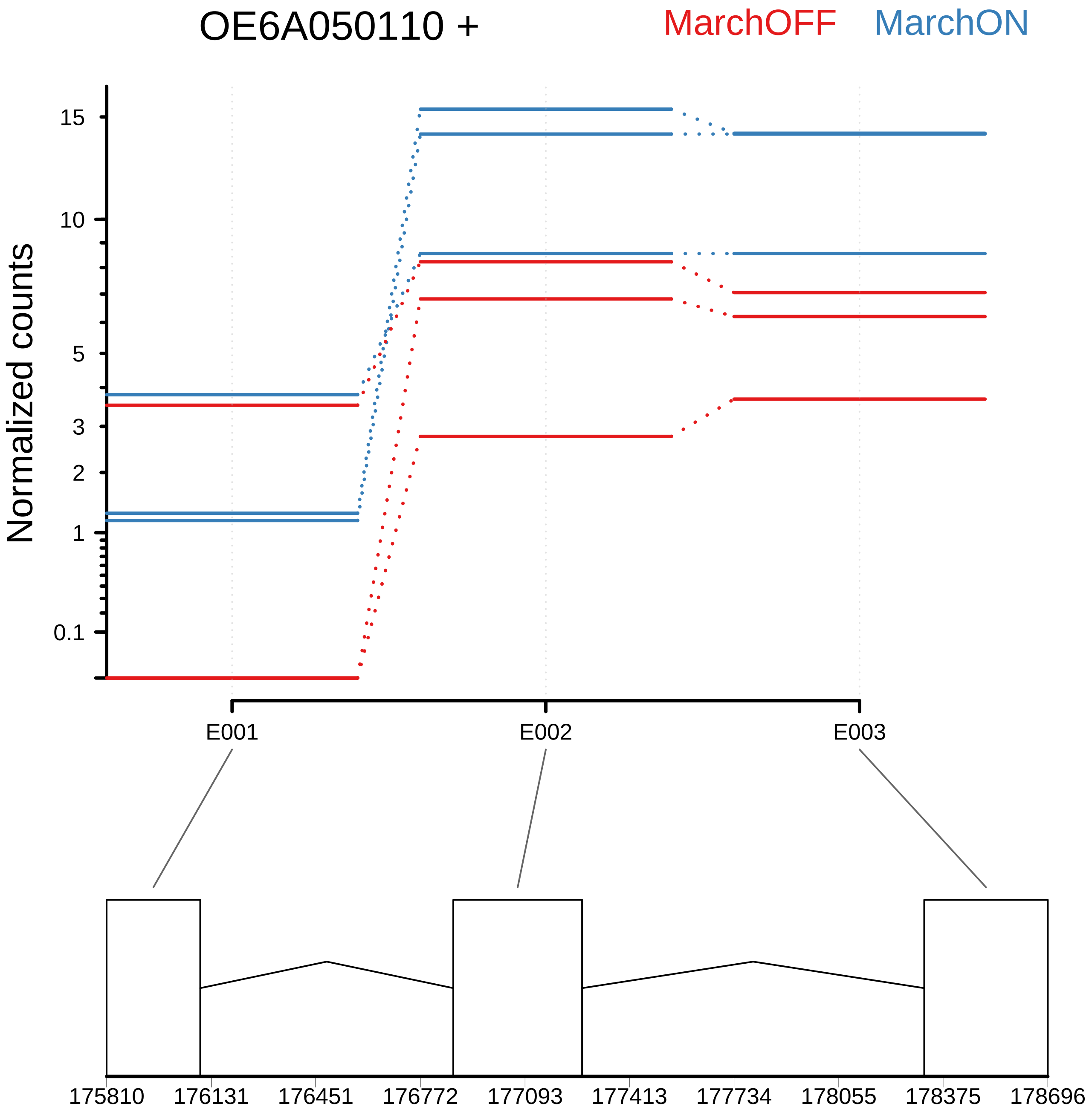

OE6A050110 +
MarchOFF
MarchON
Normalized counts
15
10
5
3
2
1
0.1
E001
E002
E003
175810
176131
176451
176772
177093
177413
177734
178055
178375
178696

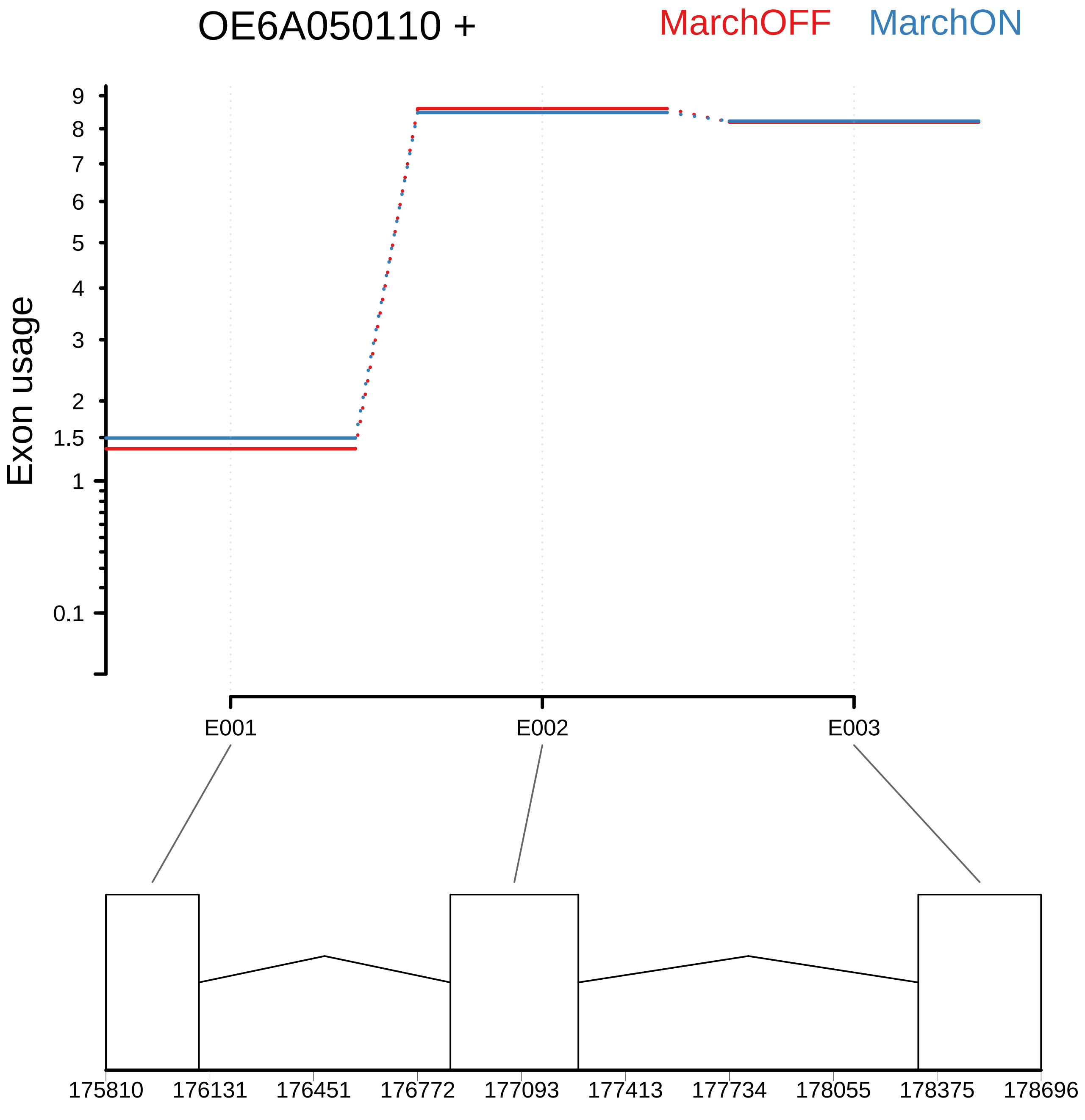
OE6A050110 +
MarchOFF
MarchON
Exon usage
9
8
7
6
5
4
3
2
1.5
1
0.1
E001
E002
E003
175810
176131
176451
176772
177093
177413
177734
178055
178375
178696

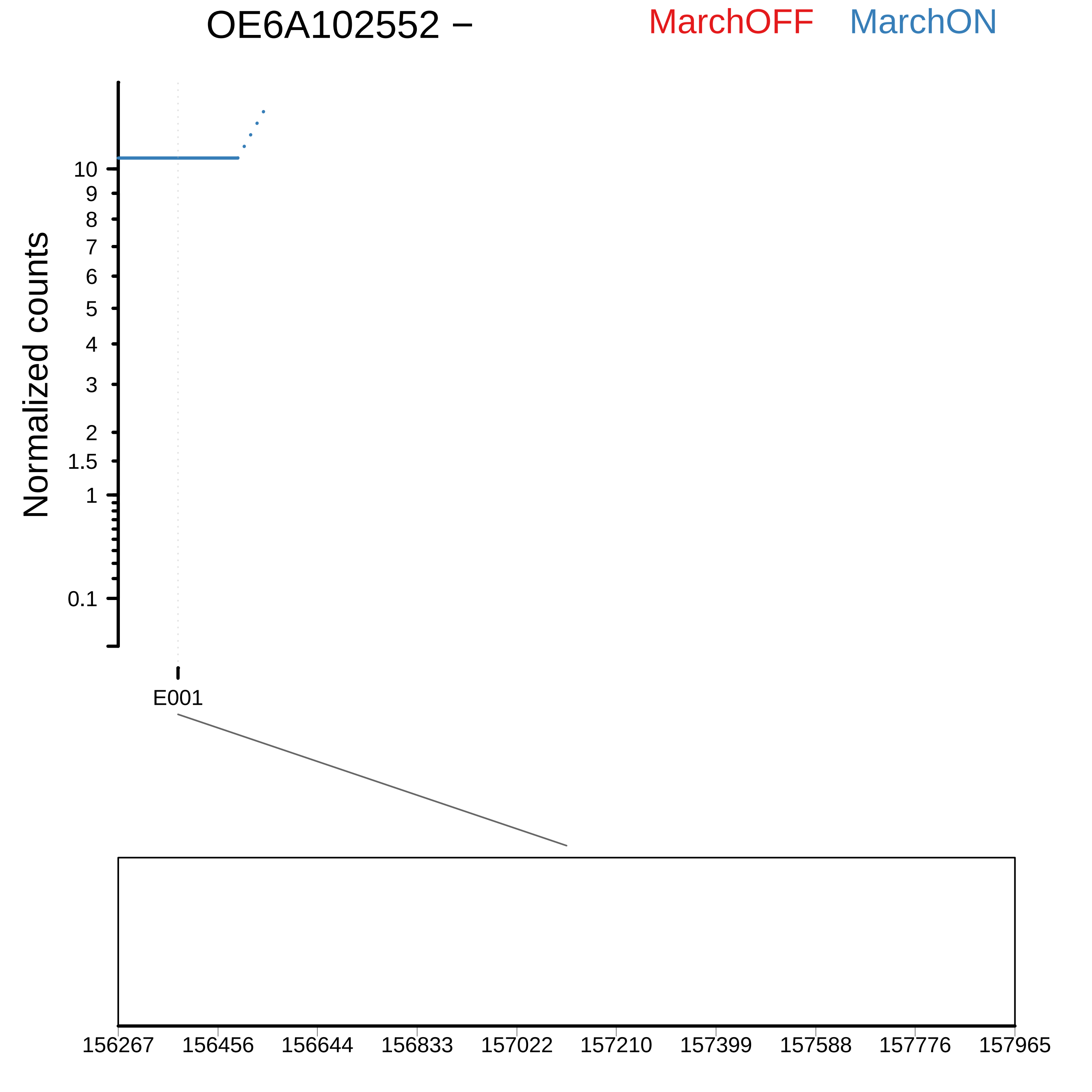
OE6A102552 −
MarchOFF
MarchON
Normalized counts
10
9
8
7
6
5
4
3
2
1.5
1
0.1
E001
156267
156456
156644
156833
157022
157210
157399
157588
157776
157965

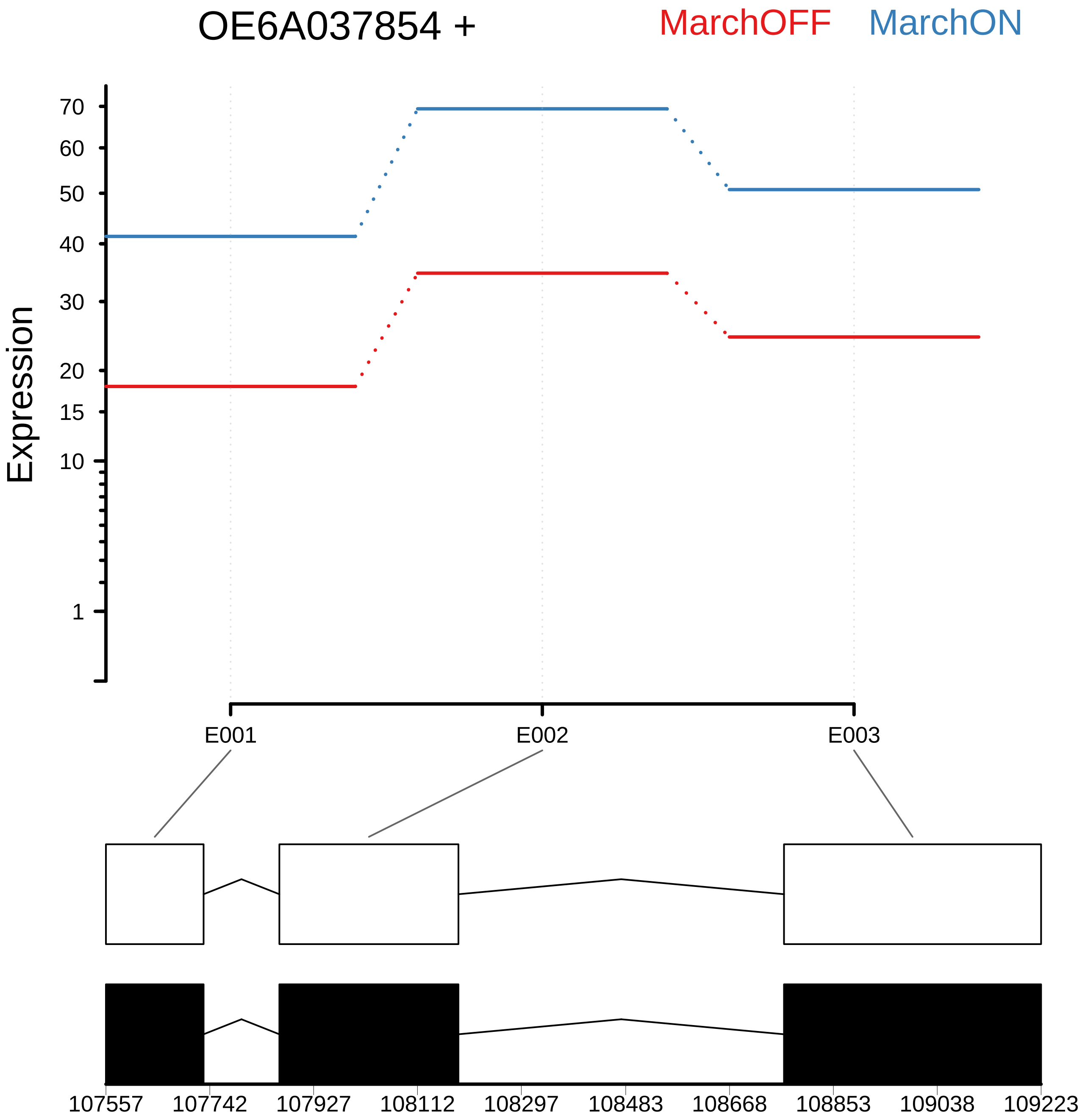
OE6A037854 +
MarchOFF
MarchON
Expression
70
60
50
40
30
20
15
10
1
E001
E002
E003
107557
107742
107927
108112
108297
108483
108668
108853
109038
109223

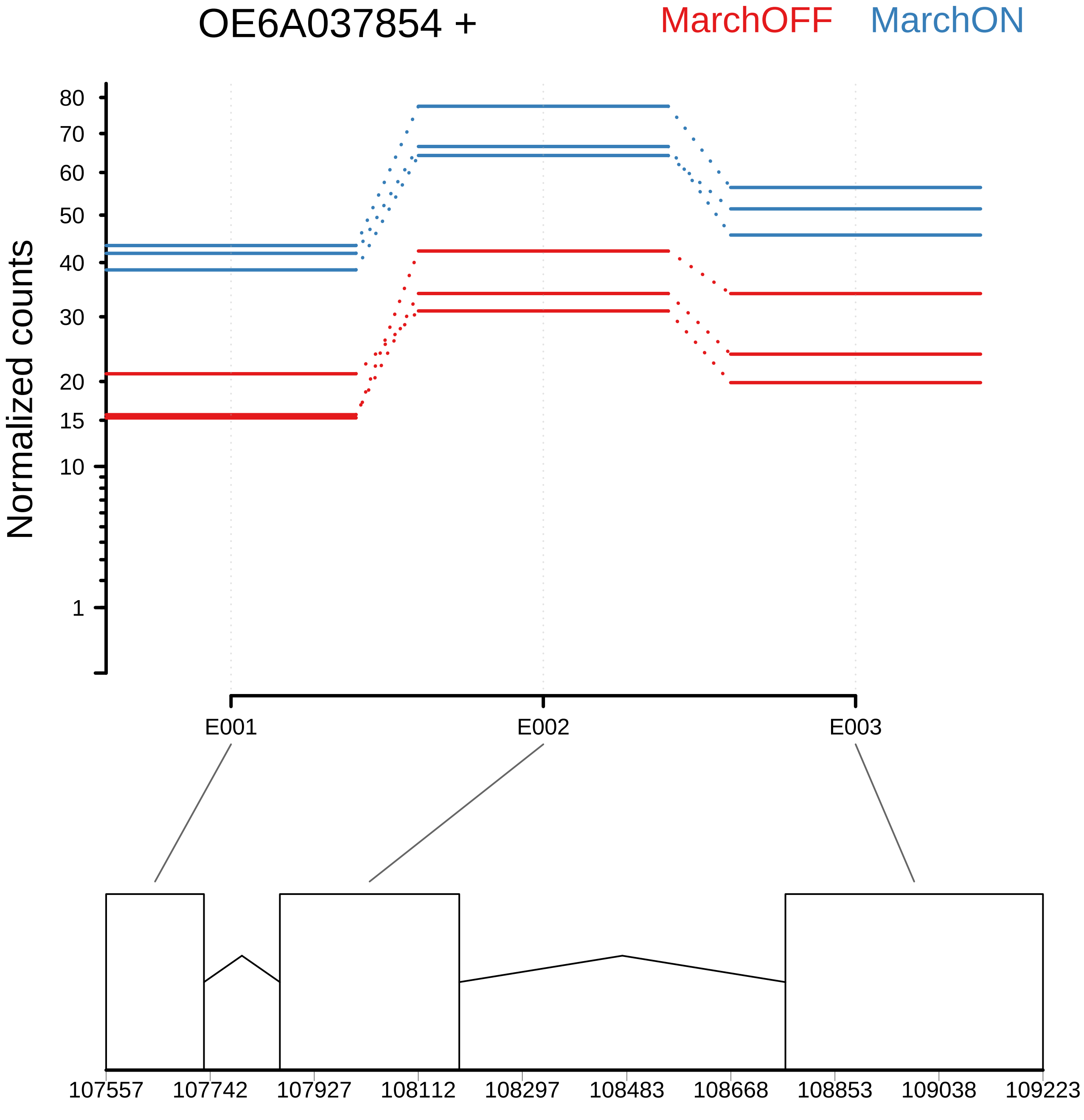

OE6A037854 +
MarchOFF
MarchON
Normalized counts
80
70
60
50
40
30
20
15
10
1
E001
E002
E003
107557
107742
107927
108112
108297
108483
108668
108853
109038
109223

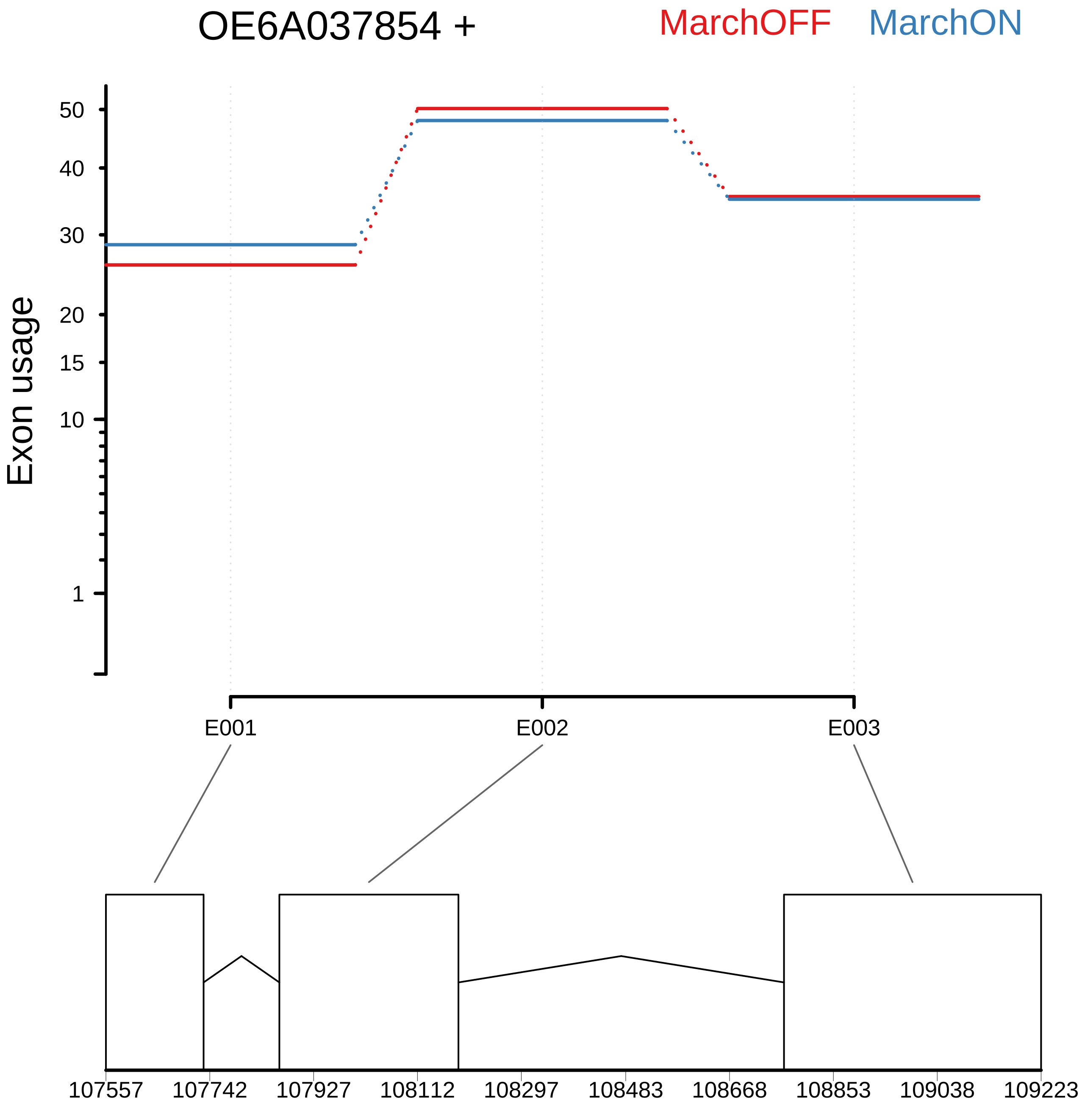
OE6A037854 +
MarchOFF
MarchON
Exon usage
50
40
30
20
15
10
1
E001
E002
E003
107557
107742
107927
108112
108297
108483
108668
108853
109038
109223

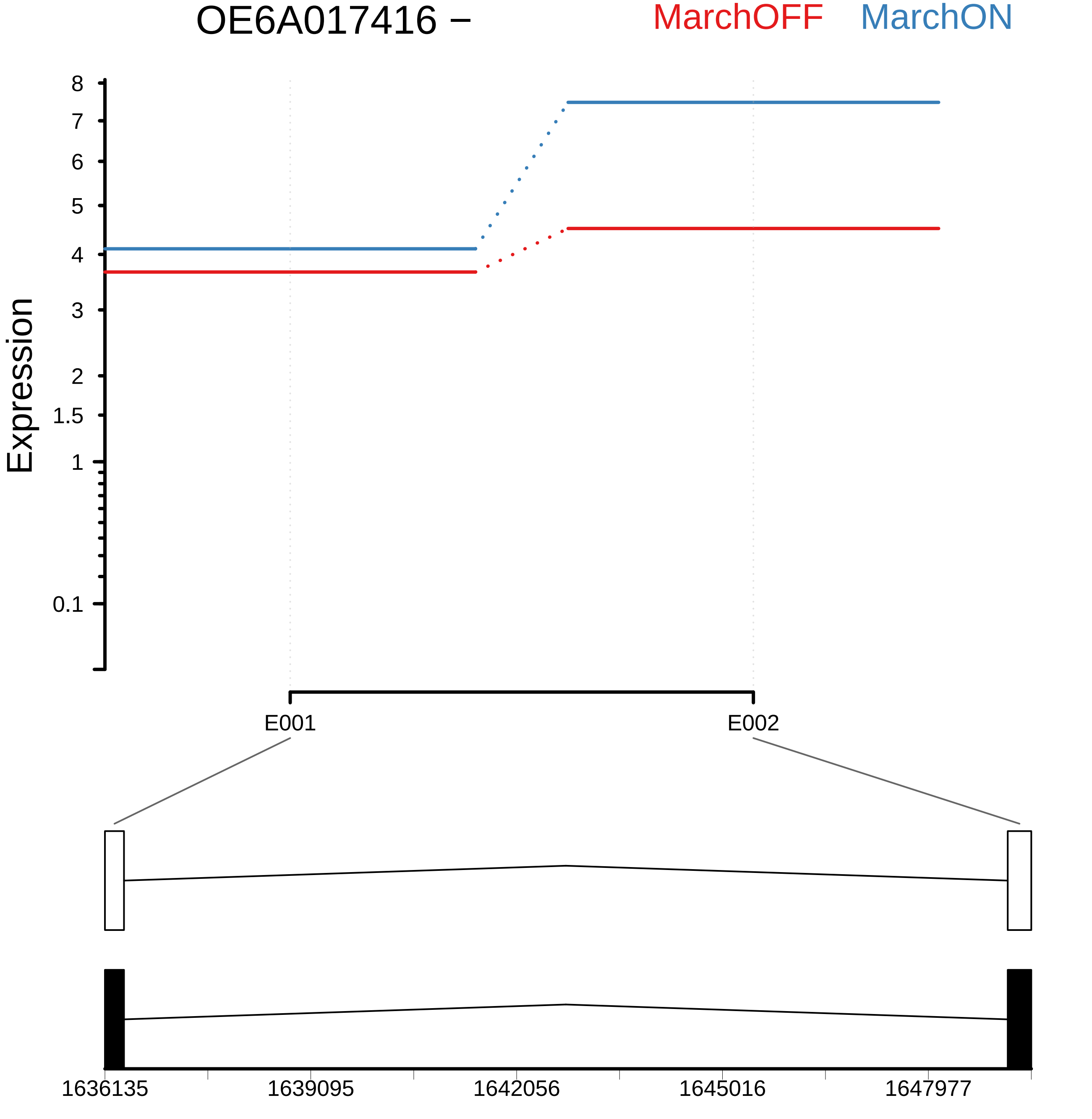

OE6A017416 −
MarchOFF
MarchON
Expression
8
7
6
5
4
3
2
1.5
1
0.1
E001
E002
1636135
1639095
1642056
1645016
1647977

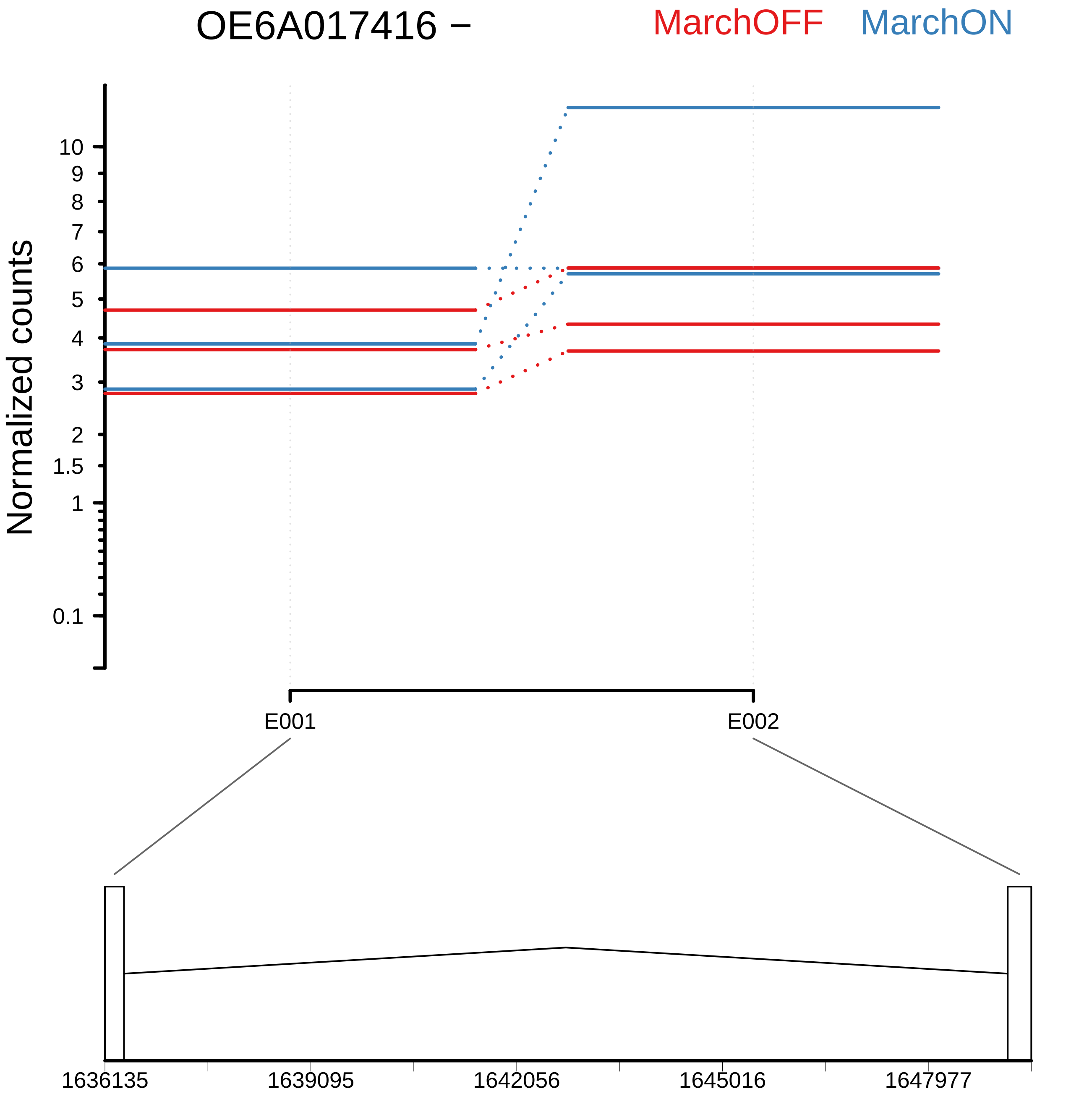

OE6A017416 −
MarchOFF
MarchON
Normalized counts
10
9
8
7
6
5
4
3
2
1.5
1
0.1
E001
E002
1636135
1639095
1642056
1645016
1647977

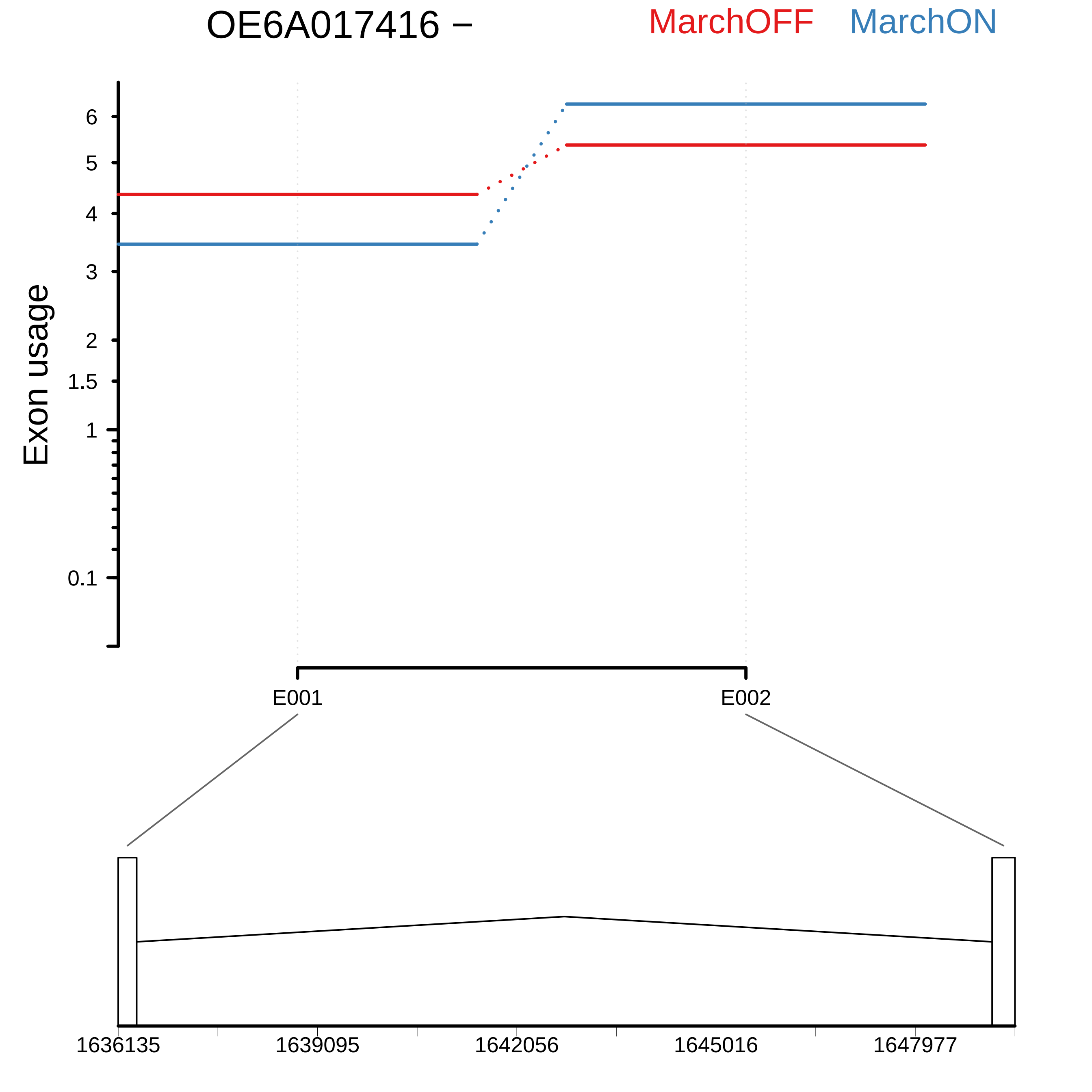
OE6A017416 −
MarchOFF
MarchON
Exon usage
6
5
4
3
2
1.5
1
0.1
E001
E002
1636135
1639095
1642056
1645016
1647977

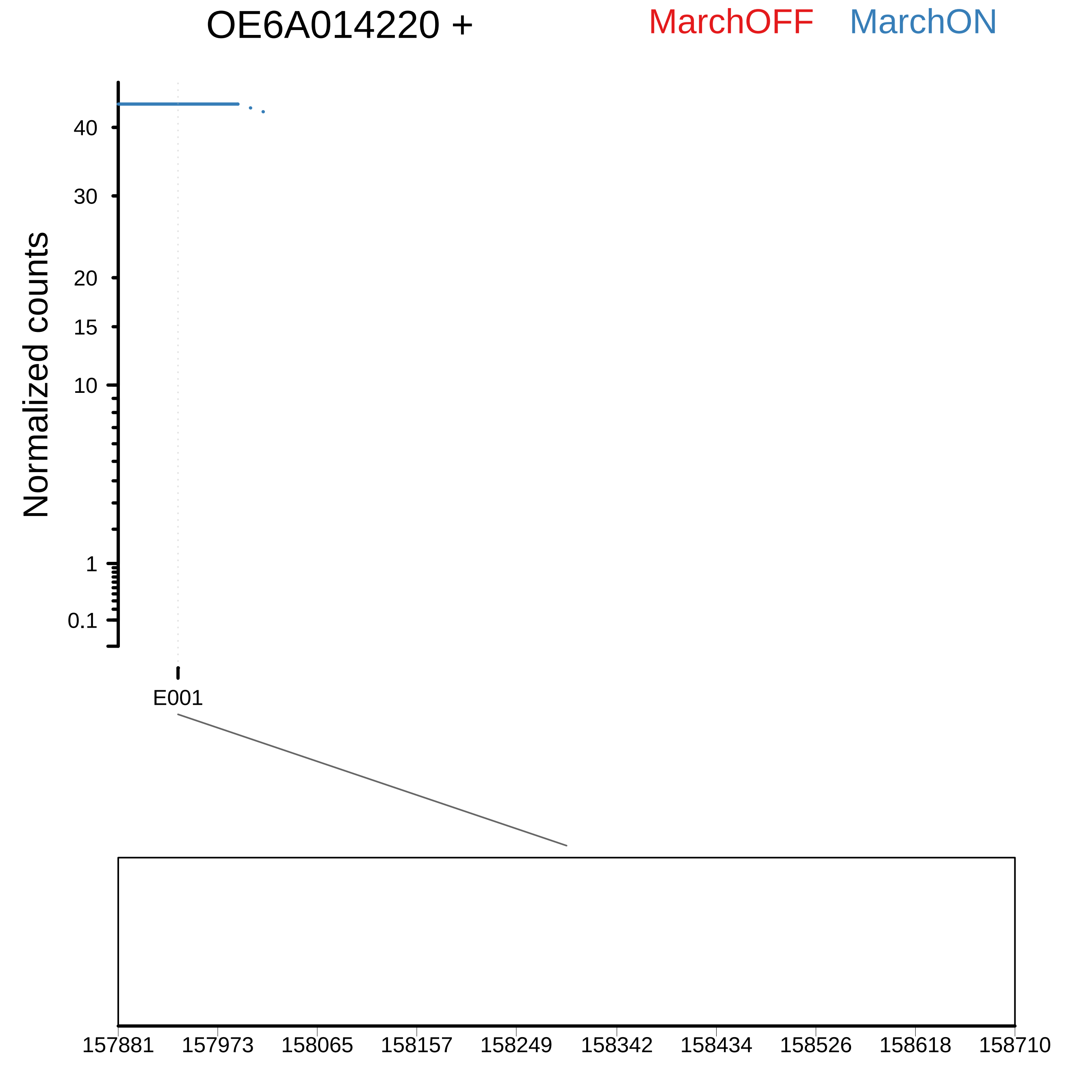

OE6A014220 +
MarchOFF
MarchON
Normalized counts
40
30
20
15
10
1
0.1
E001
157881
157973
158065
158157
158249
158342
158434
158526
158618
158710

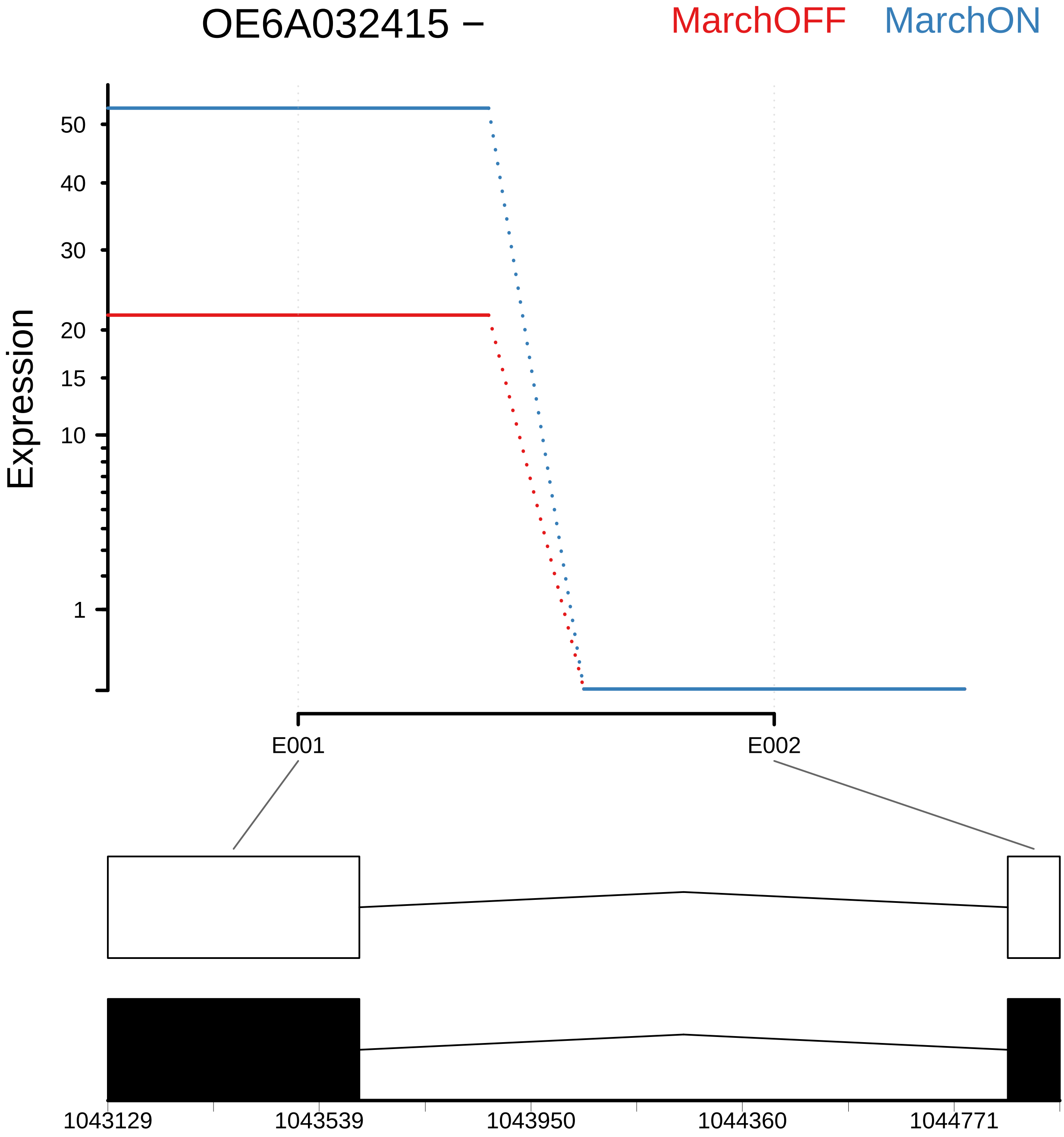
OE6A032415 −
MarchOFF
MarchON
Expression
50
40
30
20
15
10
1
E001
E002
1043129
1043539
1043950
1044360
1044771

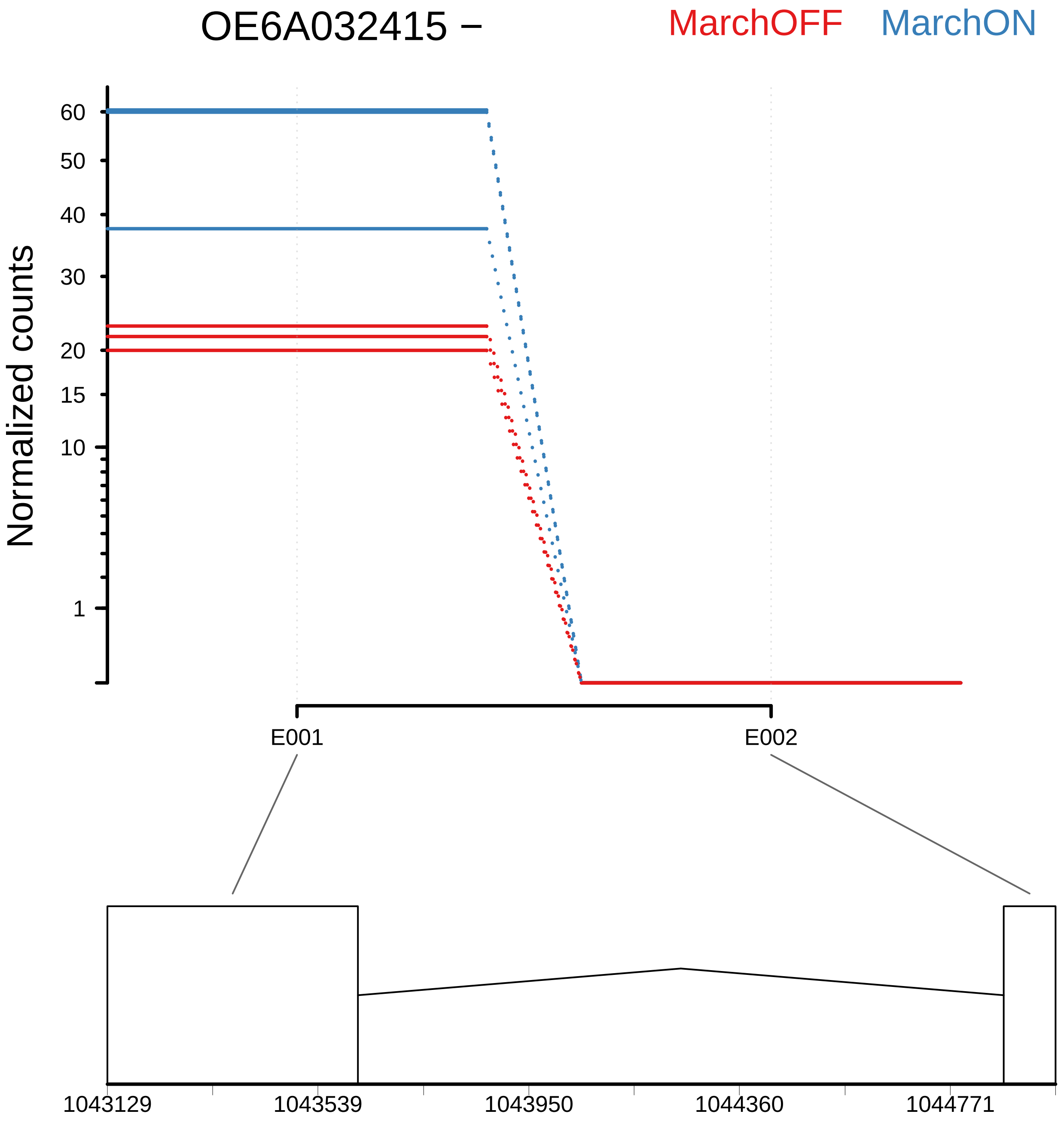
OE6A032415 −
MarchOFF
MarchON
Normalized counts
60
50
40
30
20
15
10
1
E001
E002
1043129
1043539
1043950
1044360
1044771

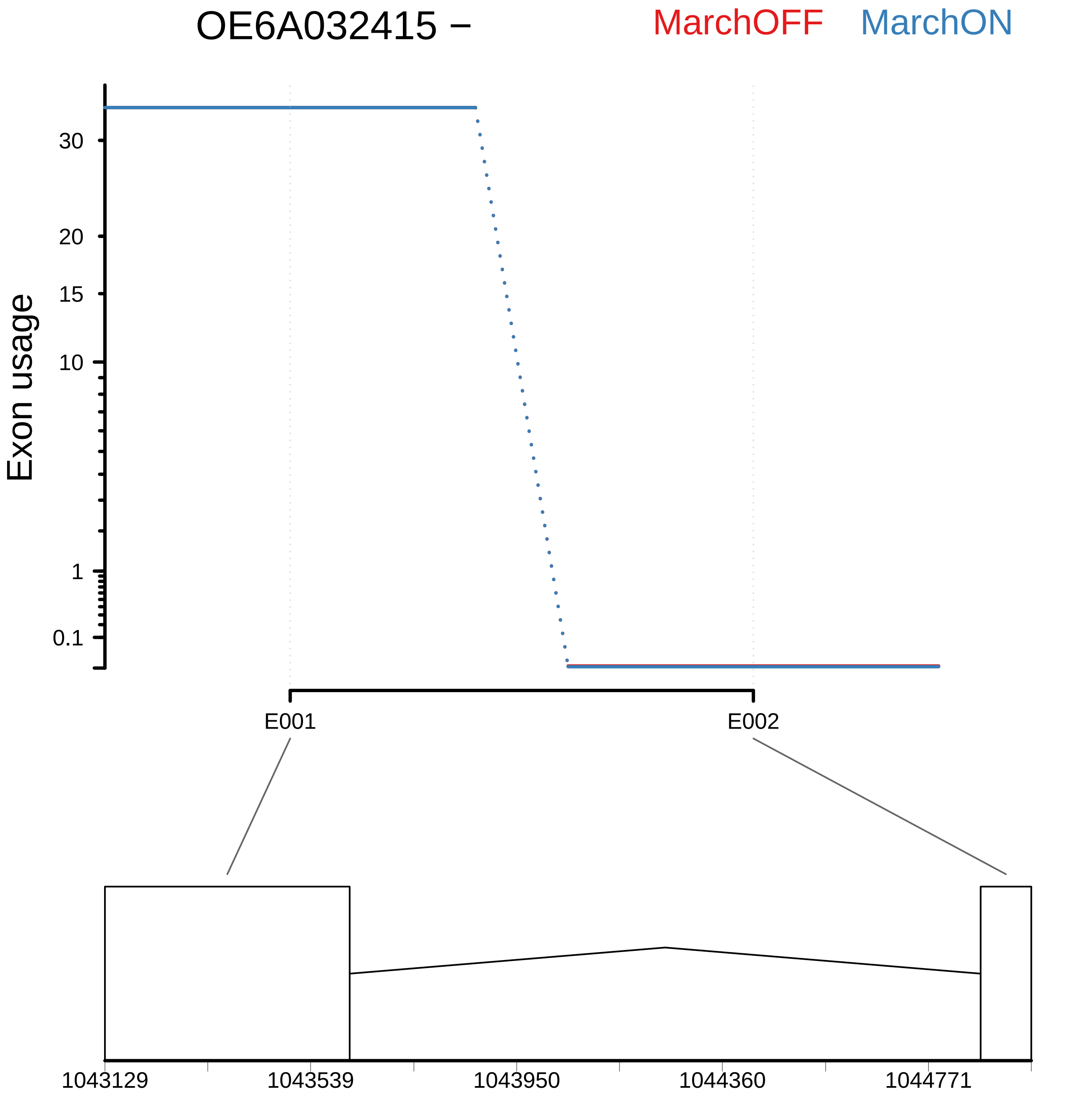
OE6A032415 −
MarchOFF
MarchON
Exon usage
30
20
15
10
1
0.1
E001
E002
1043129
1043539
1043950
1044360
1044771

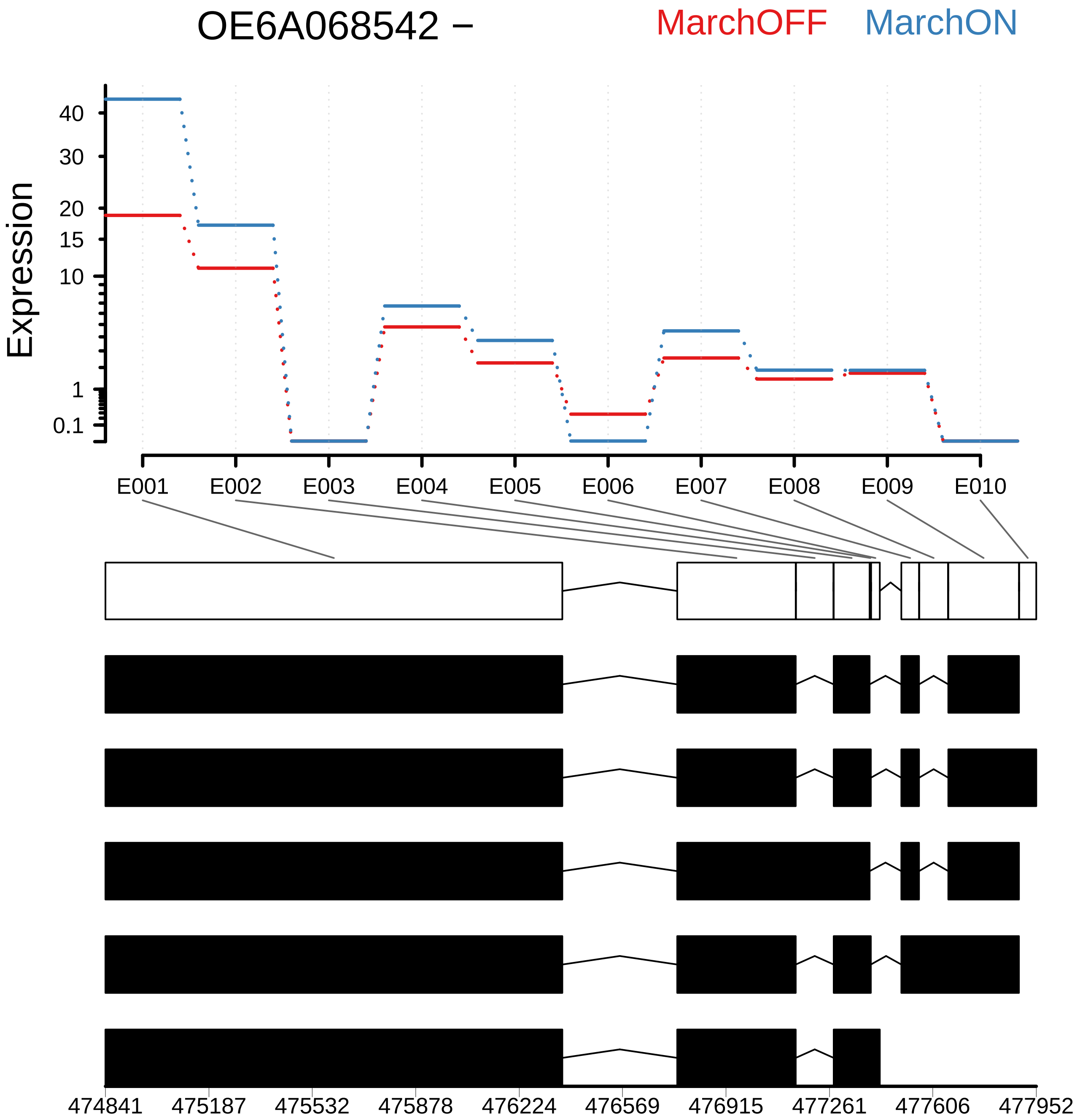

OE6A068542 −
MarchOFF
MarchON
Expression
40
30
20
15
10
1
0.1
E001
E002
E003
E004
E005
E006
E007
E008
E009
E010
474841
475187
475532
475878
476224
476569
476915
477261
477606
477952

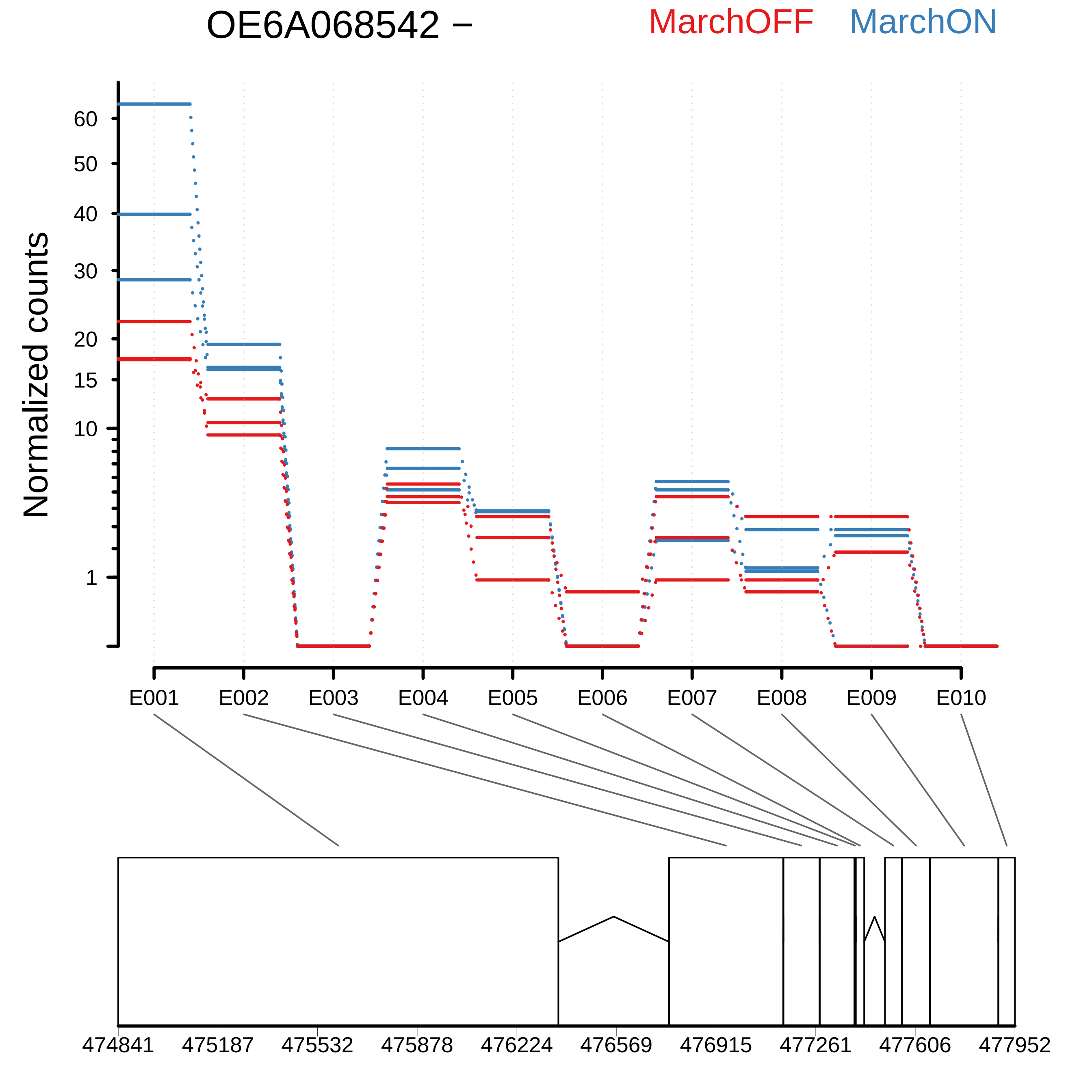

OE6A068542 −
MarchOFF
MarchON
Normalized counts
60
50
40
30
20
15
10
1
E001
E002
E003
E004
E005
E006
E007
E008
E009
E010
474841
475187
475532
475878
476224
476569
476915
477261
477606
477952

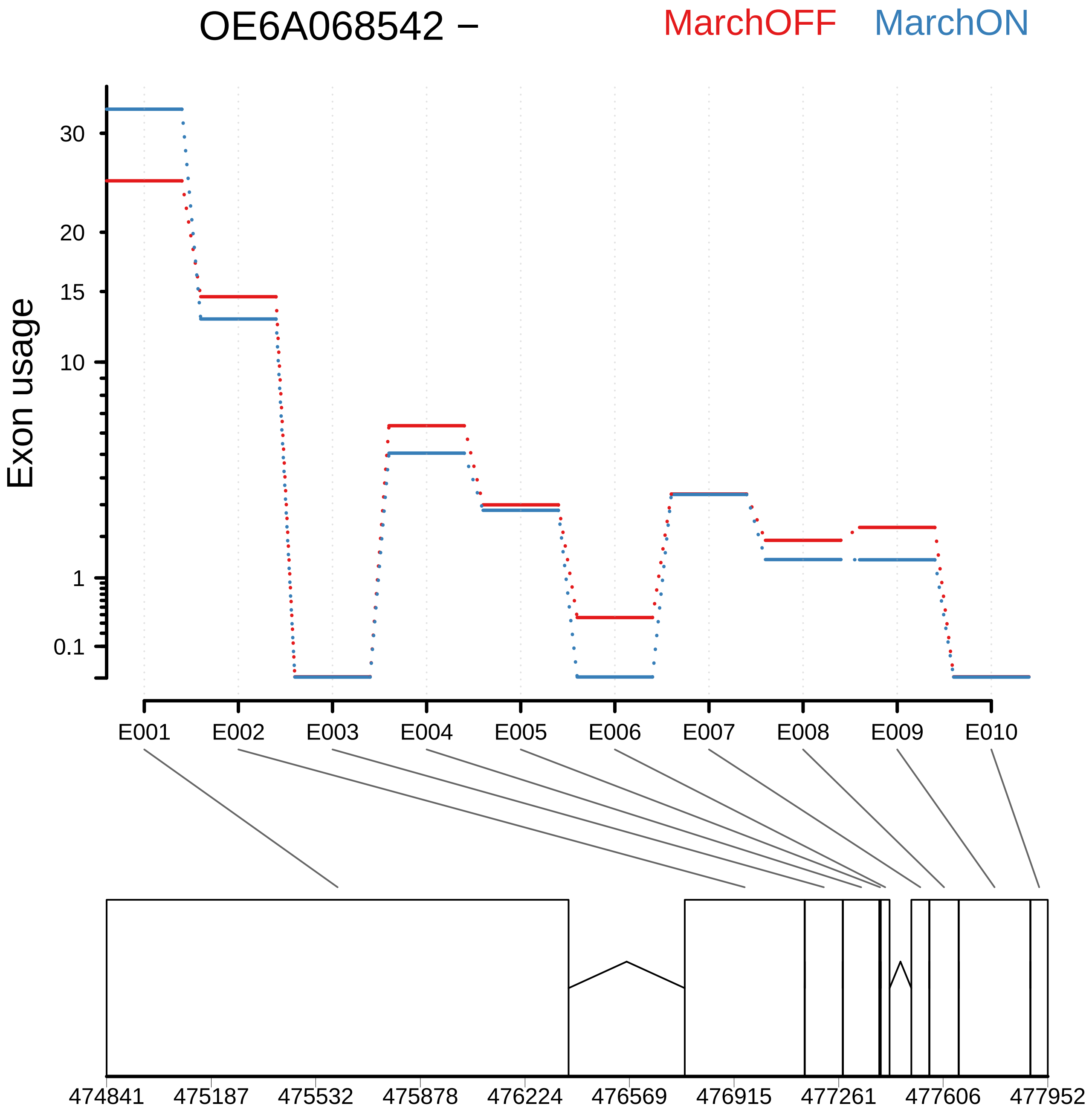

OE6A068542 −
MarchOFF
MarchON
Exon usage
30
20
15
10
1
0.1
E001
E002
E003
E004
E005
E006
E007
E008
E009
E010
474841
475187
475532
475878
476224
476569
476915
477261
477606
477952

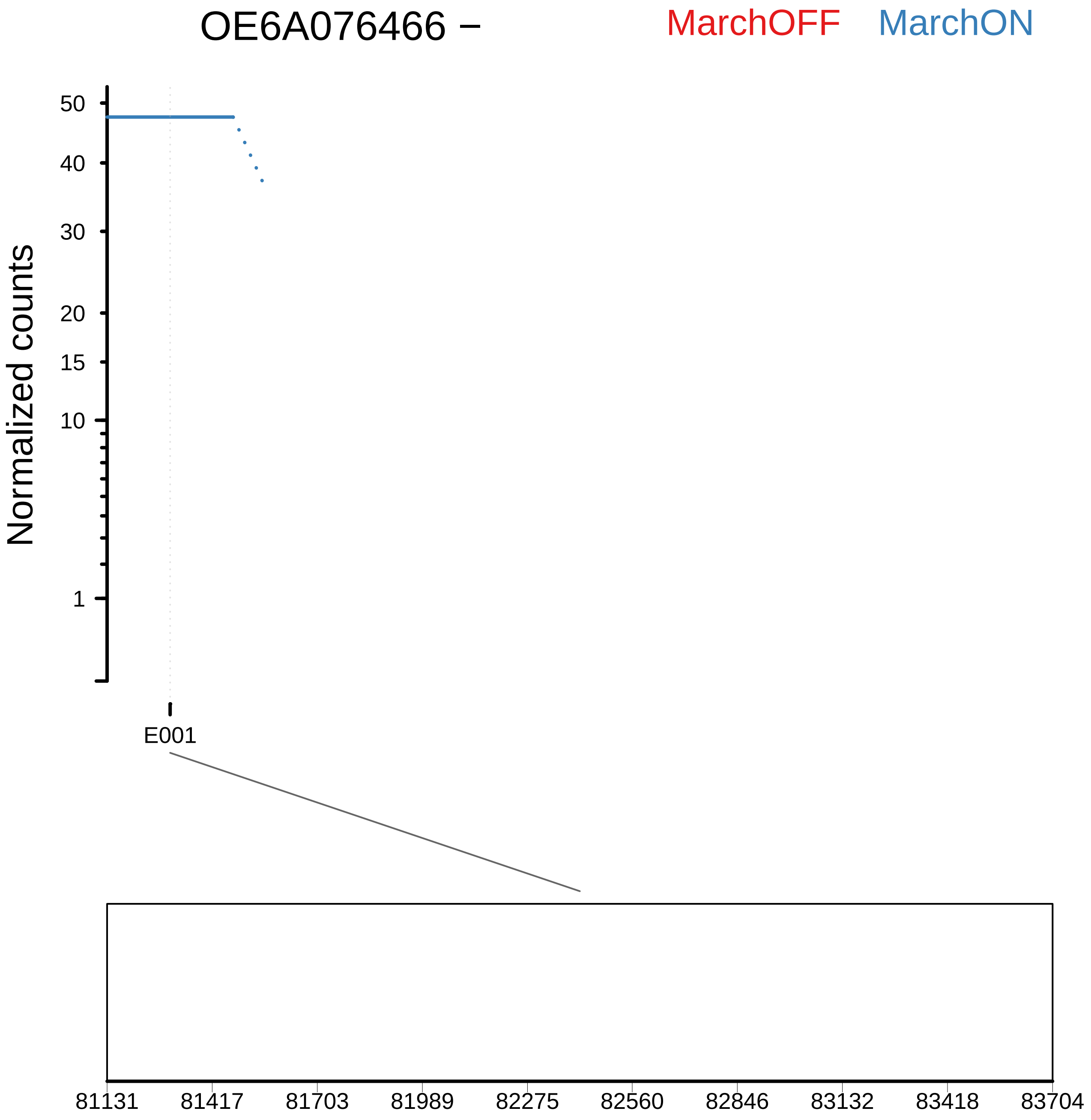

OE6A076466 −
MarchOFF
MarchON
Normalized counts
50
40
30
20
15
10
1
E001
81131
81417
81703
81989
82275
82560
82846
83132
83418
83704

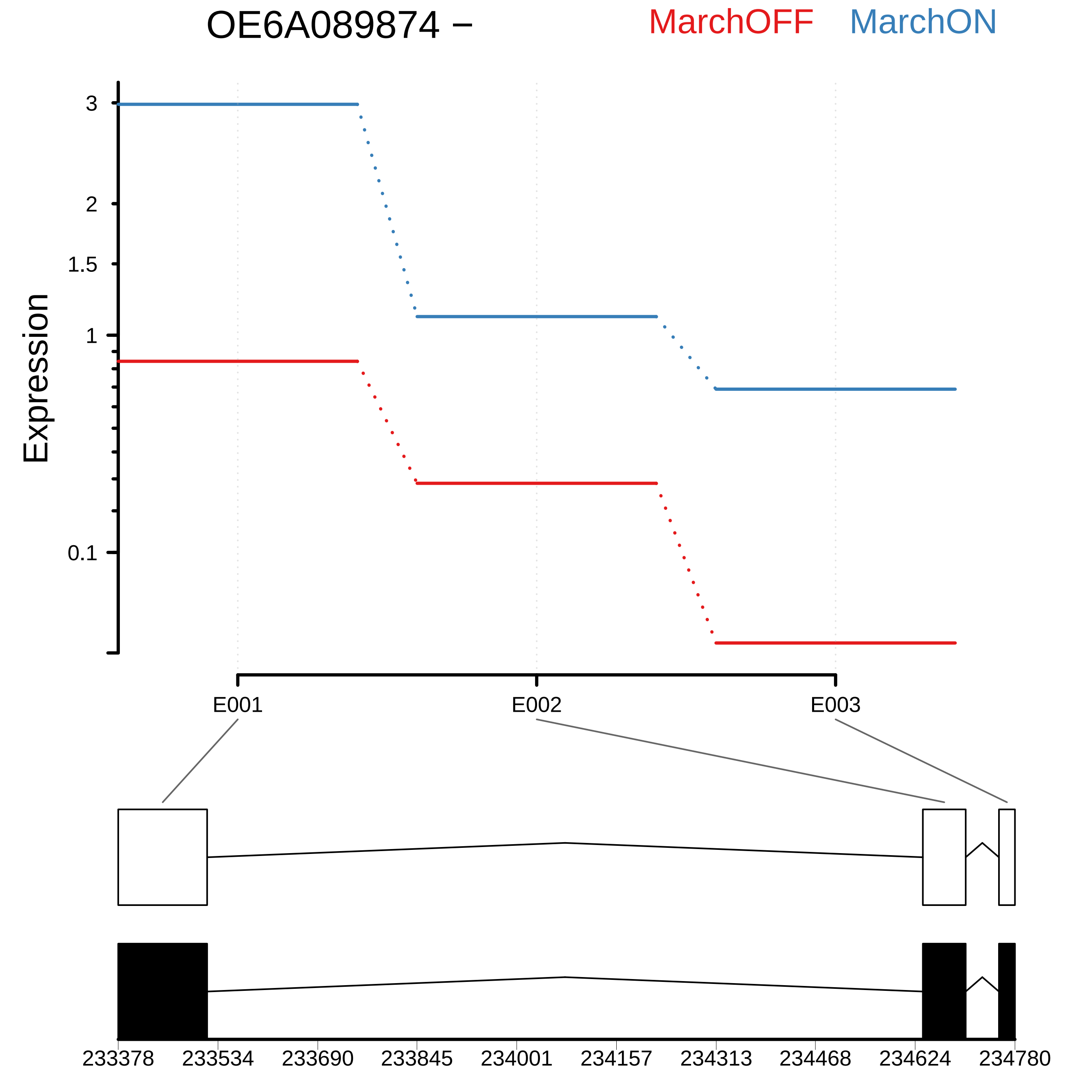

OE6A089874 −
MarchOFF
MarchON
Expression
3
2
1.5
1
0.1
E001
E002
E003
233378
233534
233690
233845
234001
234157
234313
234468
234624
234780

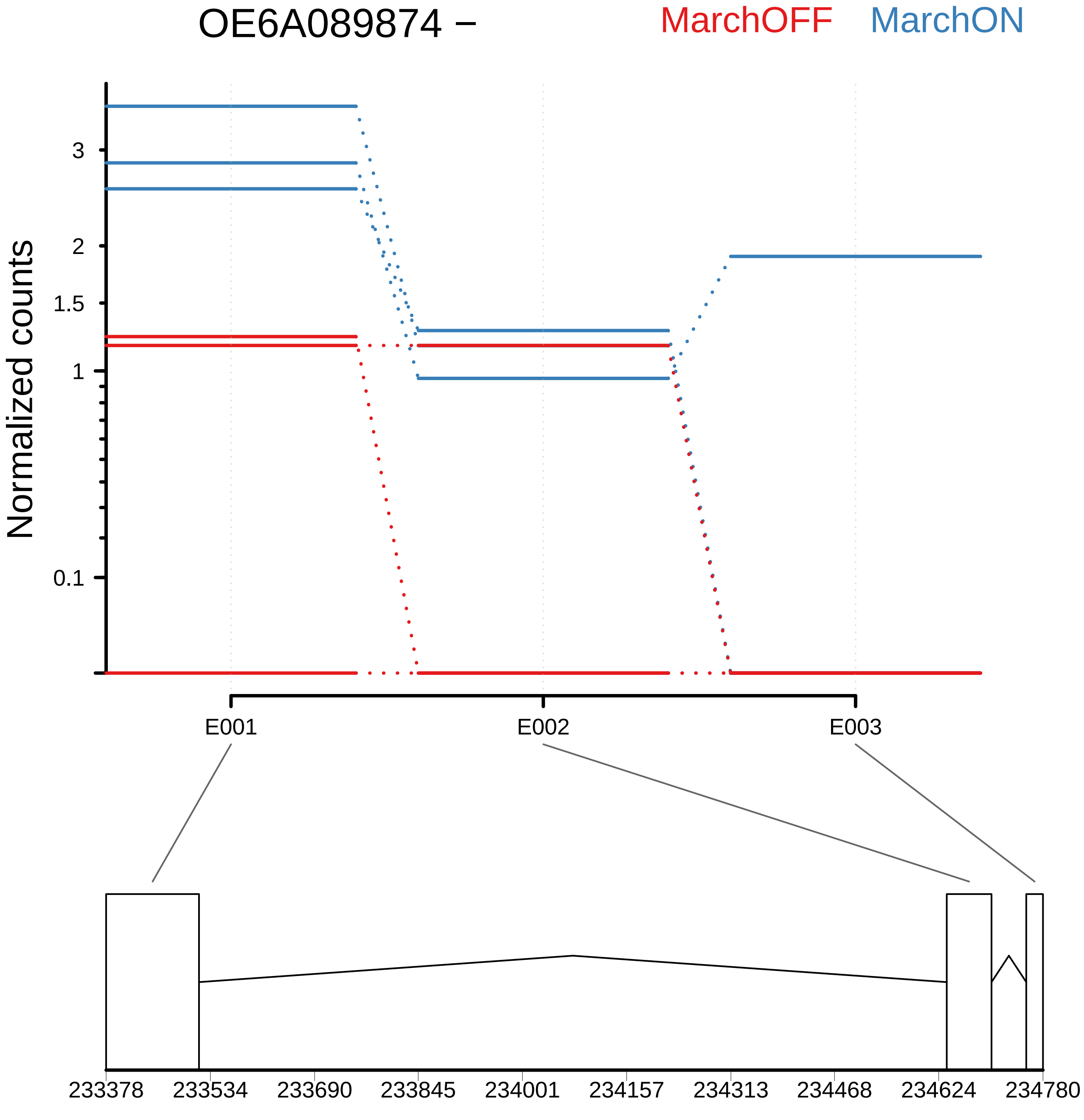
OE6A089874 −
MarchOFF
MarchON
Normalized counts
3
2
1.5
1
0.1
E001
E002
E003
233378
233534
233690
233845
234001
234157
234313
234468
234624
234780

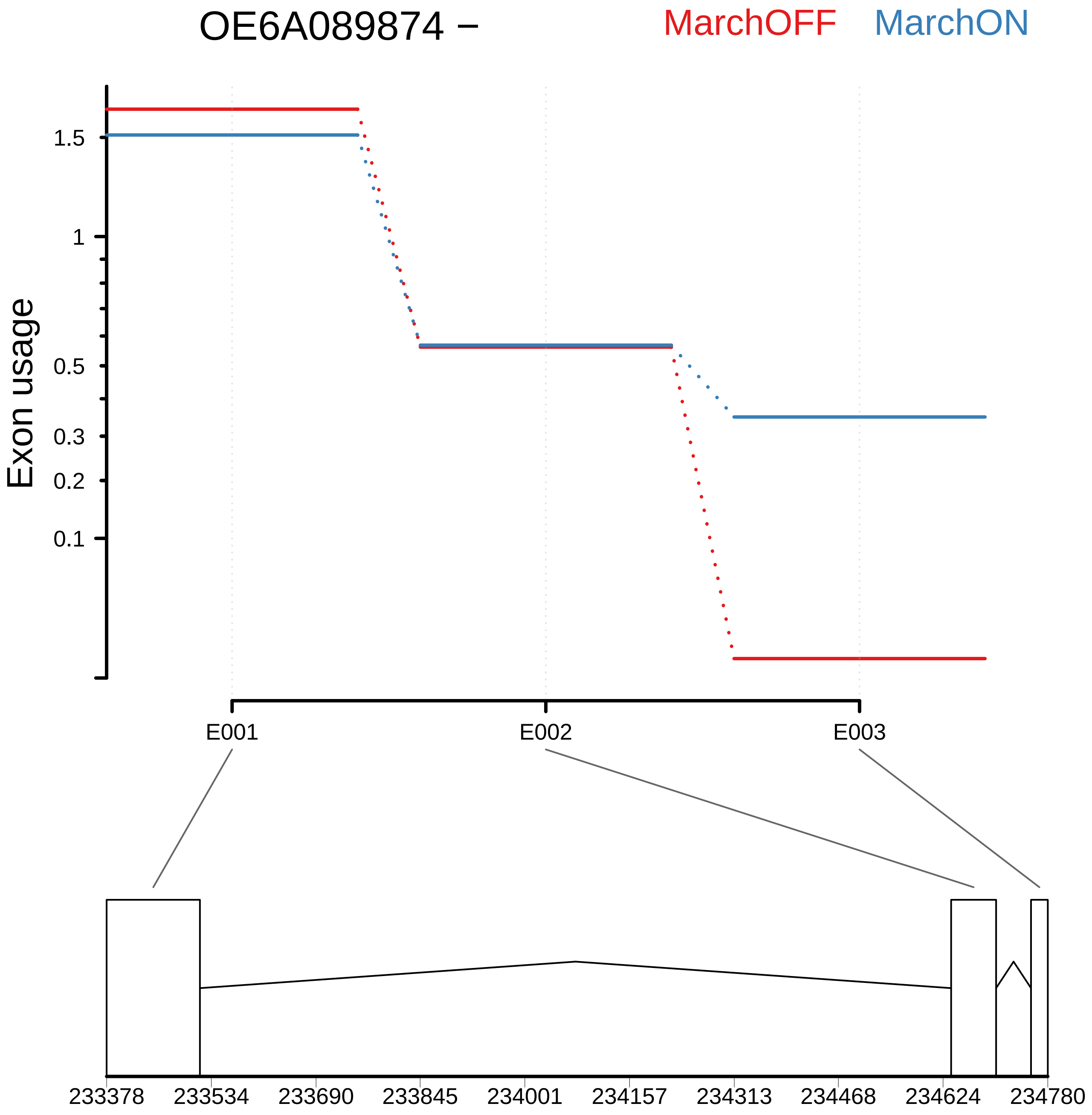

OE6A089874 −
MarchOFF
MarchON
Exon usage
1.5
1
0.5
0.3
0.2
0.1
E001
E002
E003
233378
233534
233690
233845
234001
234157
234313
234468
234624
234780

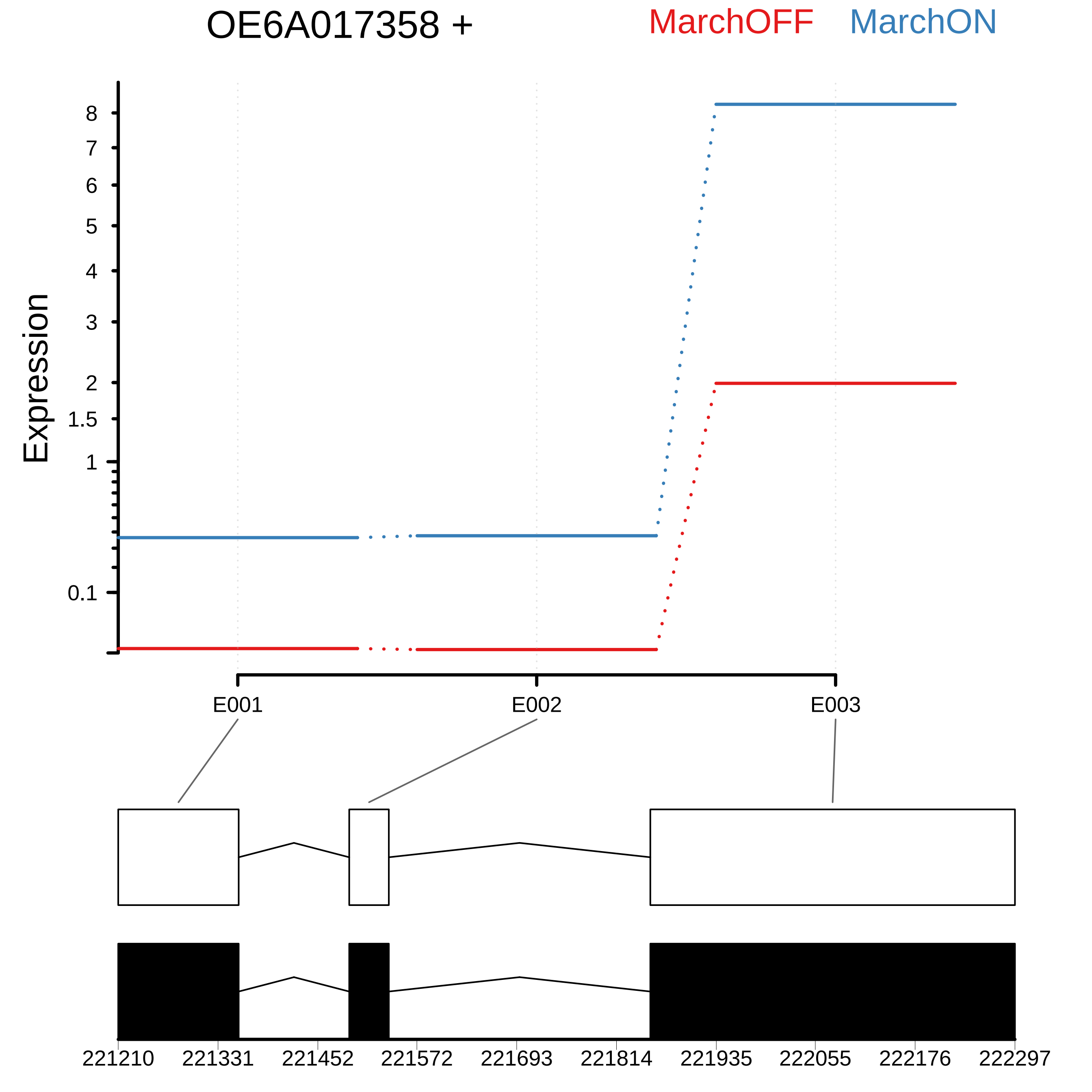

OE6A017358 +
MarchOFF
MarchON
Expression
8
7
6
5
4
3
2
1.5
1
0.1
E001
E002
E003
221210
221331
221452
221572
221693
221814
221935
222055
222176
222297

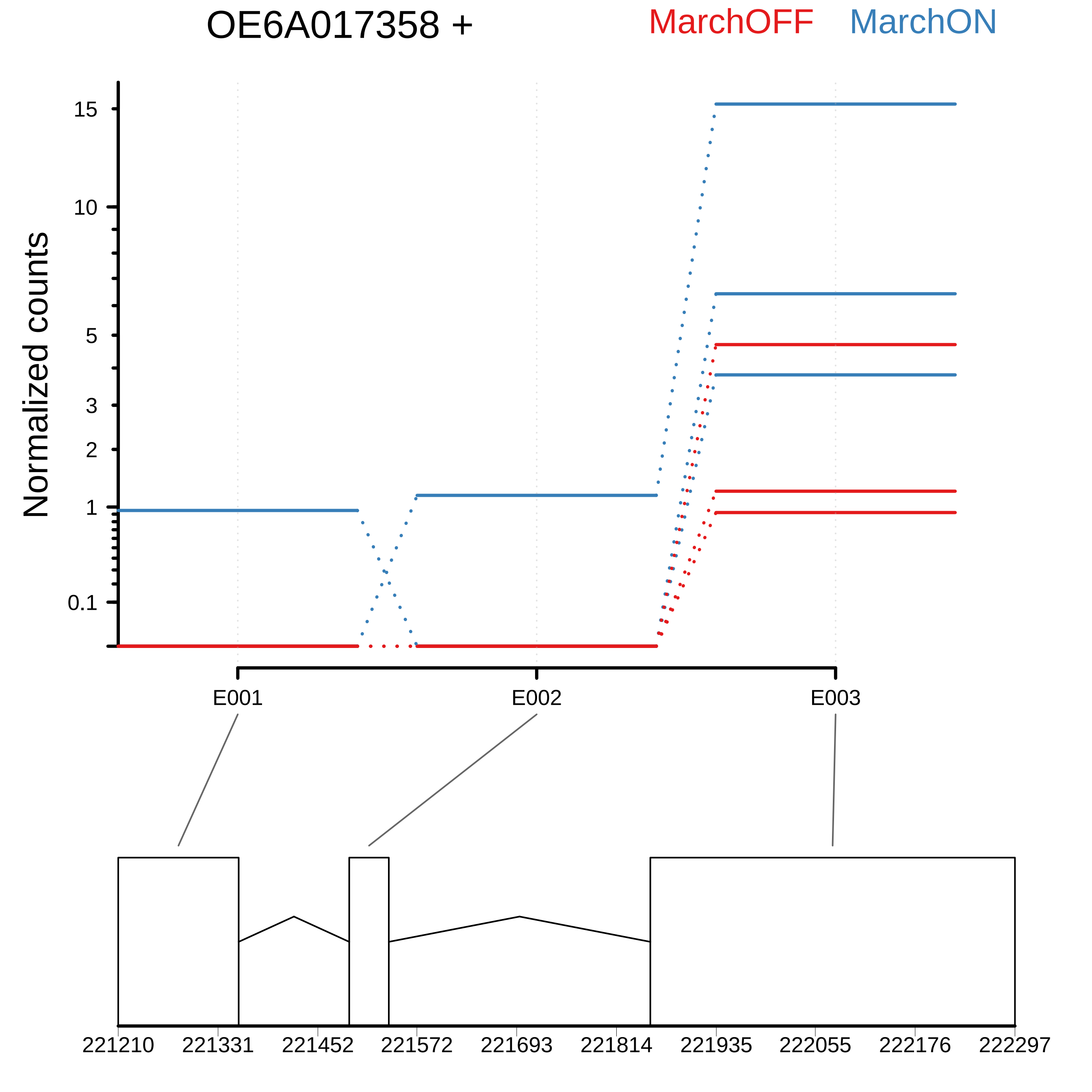

OE6A017358 +
MarchOFF
MarchON
Normalized counts
15
10
5
3
2
1
0.1
E001
E002
E003
221210
221331
221452
221572
221693
221814
221935
222055
222176
222297

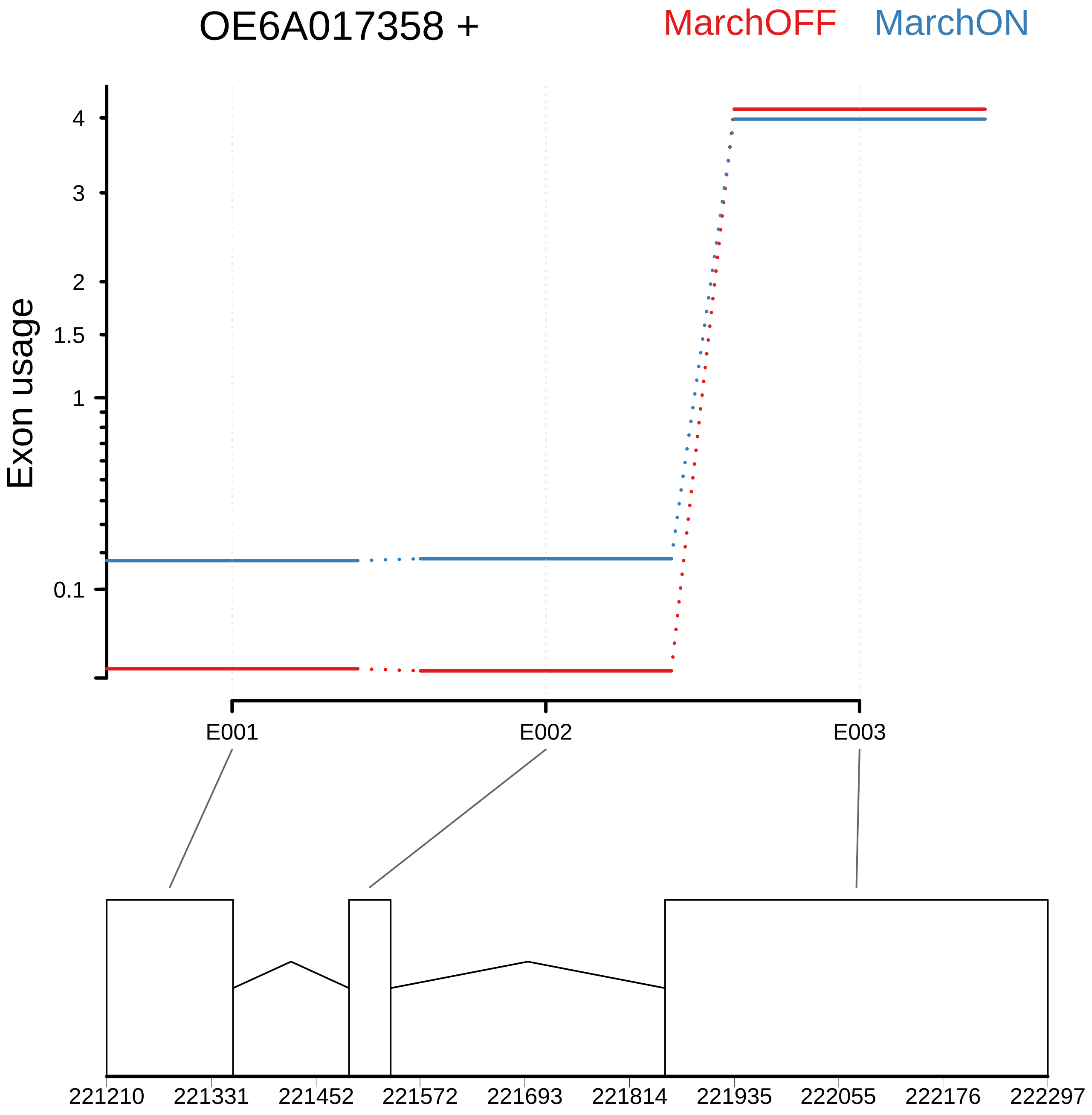
OE6A017358 +
MarchOFF
MarchON
Exon usage
4
3
2
1.5
1
0.1
E001
E002
E003
221210
221331
221452
221572
221693
221814
221935
222055
222176
222297

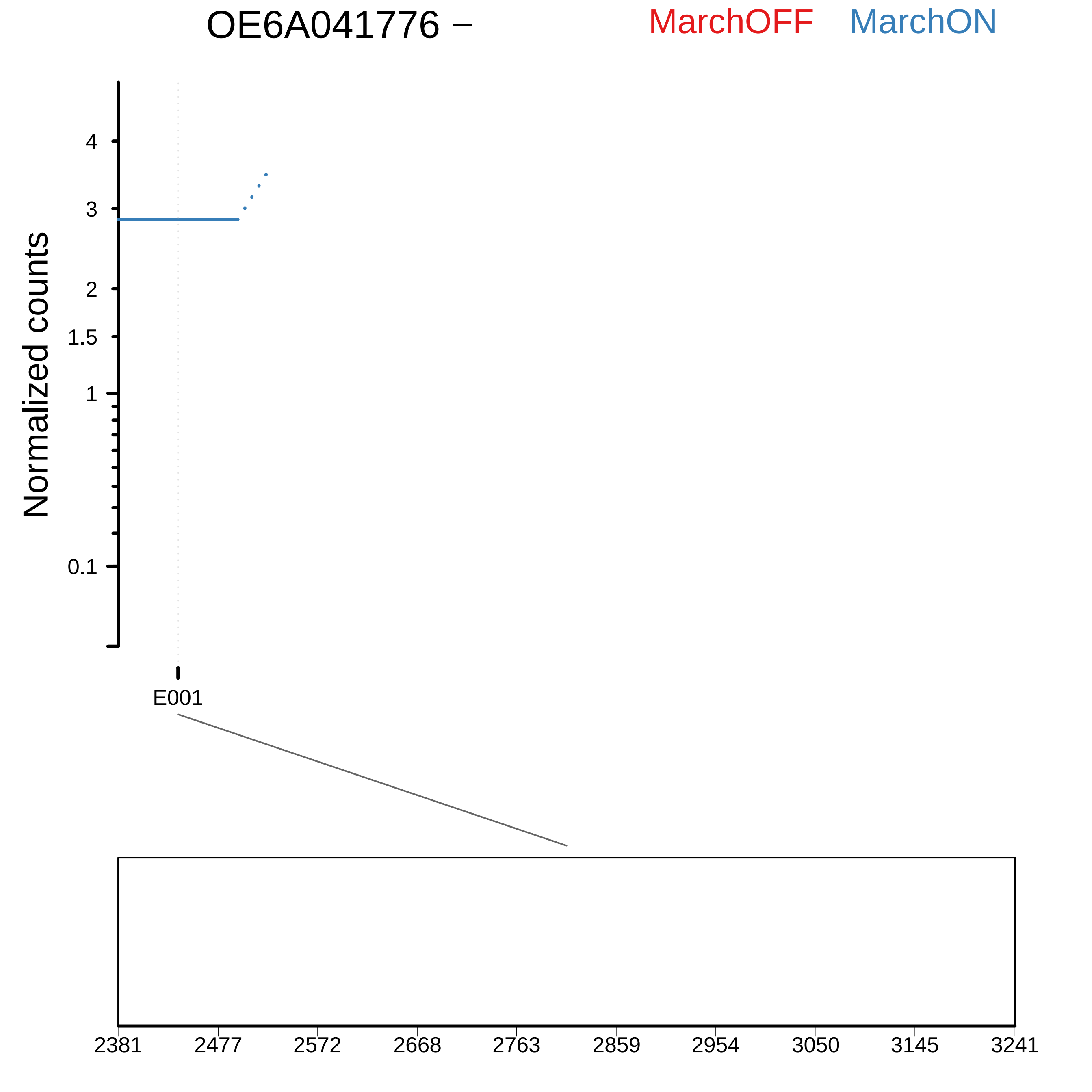

OE6A041776 −
MarchOFF
MarchON
Normalized counts
4
3
2
1.5
1
0.1
E001
2381
2477
2572
2668
2763
2859
2954
3050
3145
3241

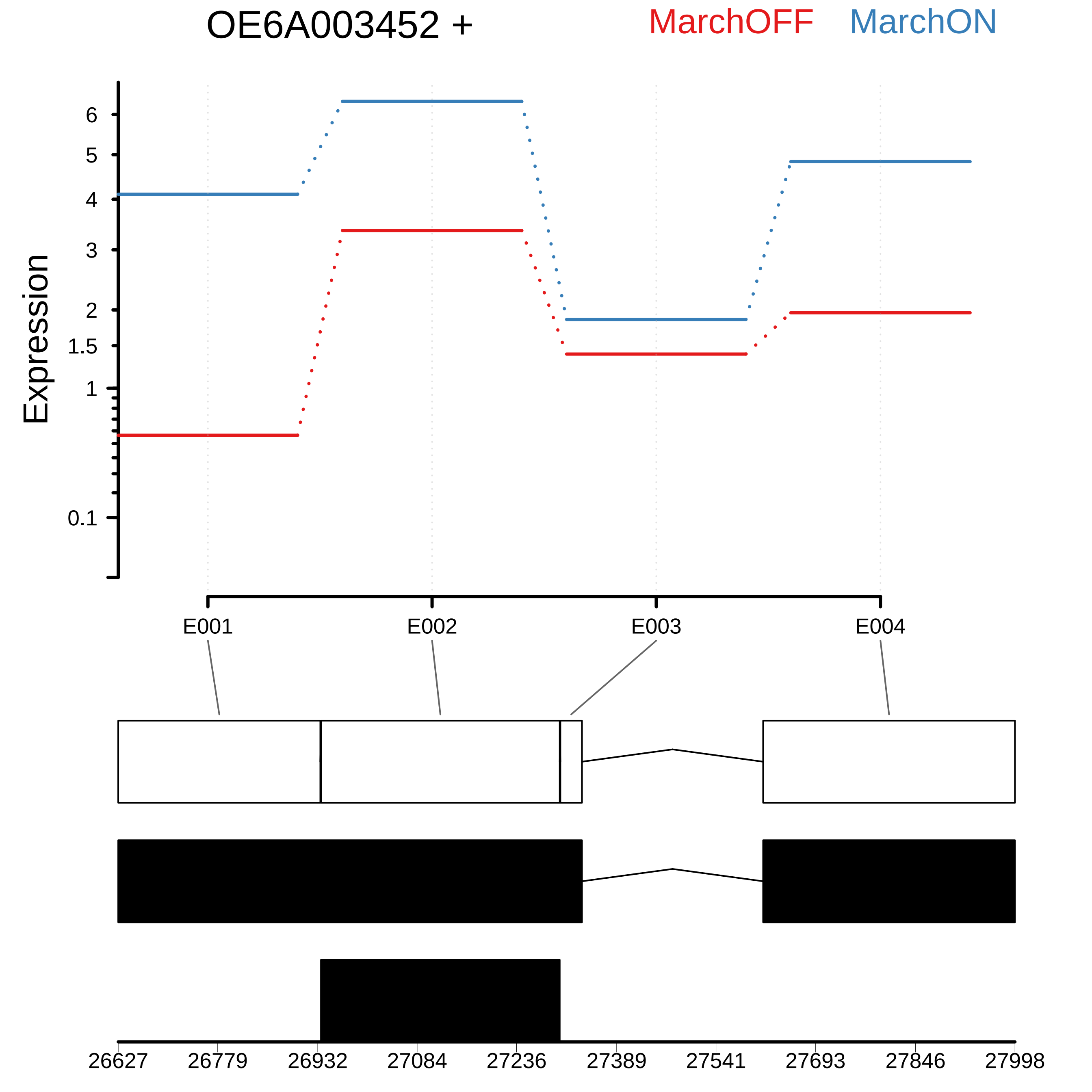

OE6A003452 +
MarchOFF
MarchON
Expression
6
5
4
3
2
1.5
1
0.1
E001
E002
E003
E004
26627
26779
26932
27084
27236
27389
27541
27693
27846
27998

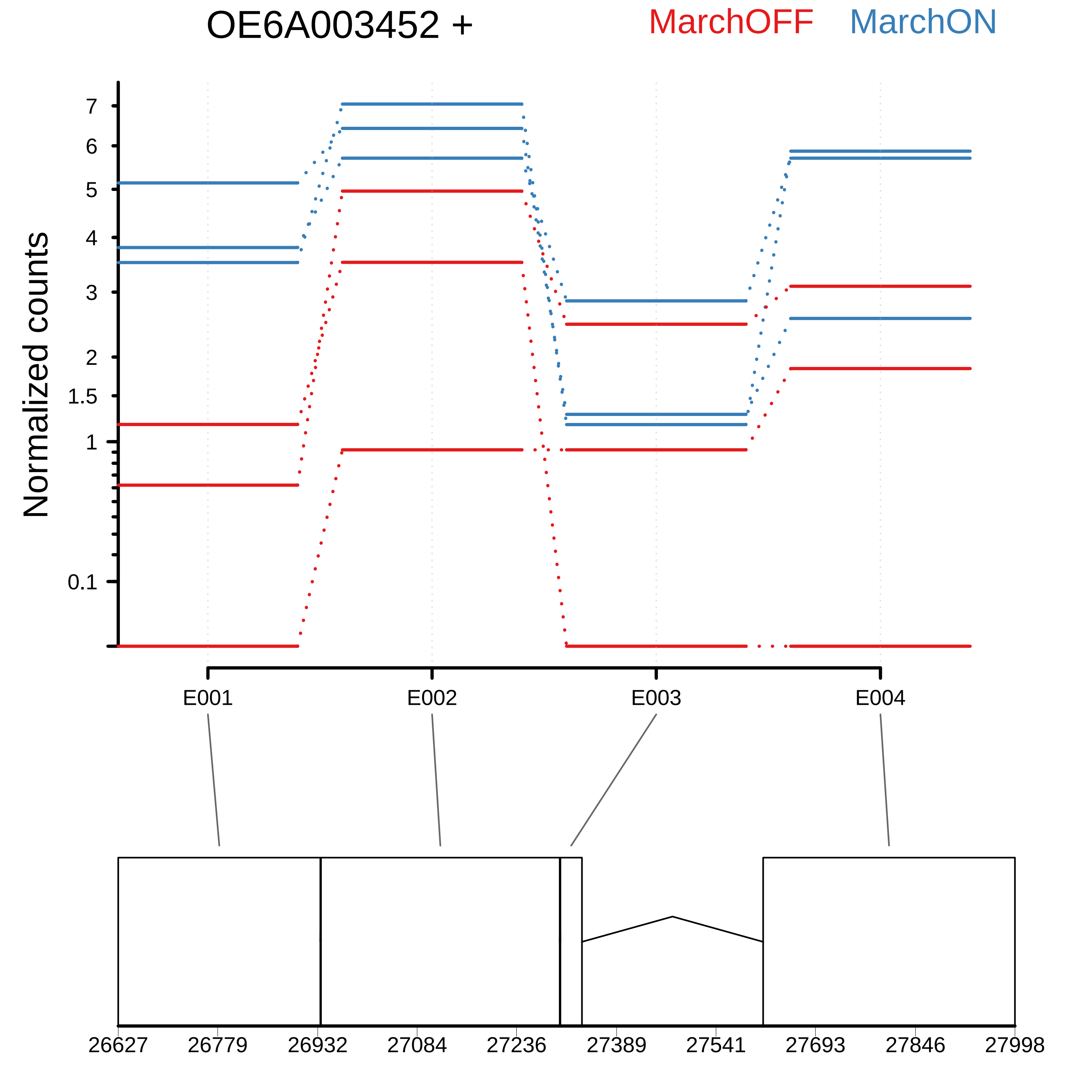

OE6A003452 +
MarchOFF
MarchON
Normalized counts
7
6
5
4
3
2
1.5
1
0.1
E001
E002
E003
E004
26627
26779
26932
27084
27236
27389
27541
27693
27846
27998

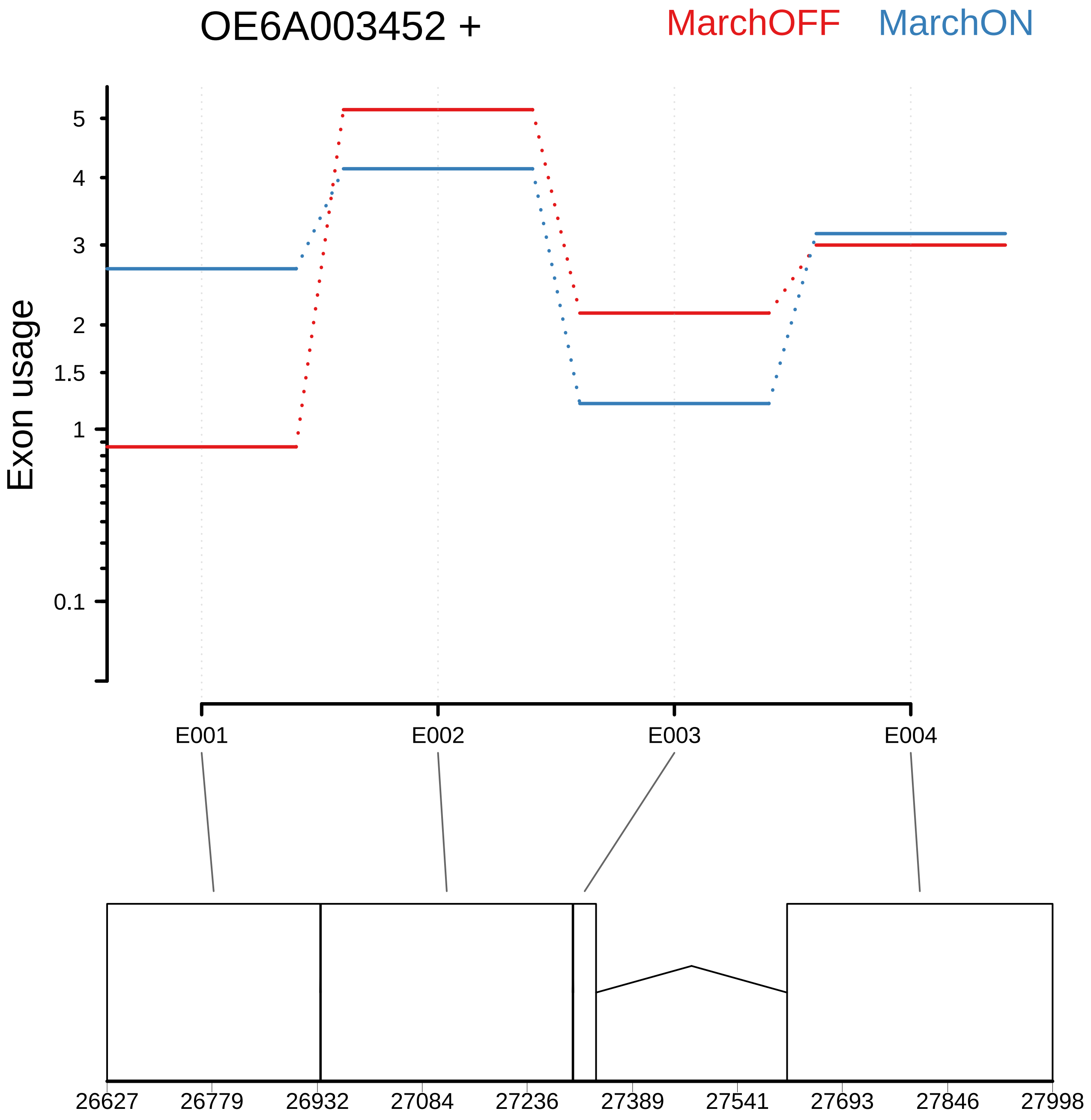

OE6A003452 +
MarchOFF
MarchON
Exon usage
5
4
3
2
1.5
1
0.1
E001
E002
E003
E004
26627
26779
26932
27084
27236
27389
27541
27693
27846
27998

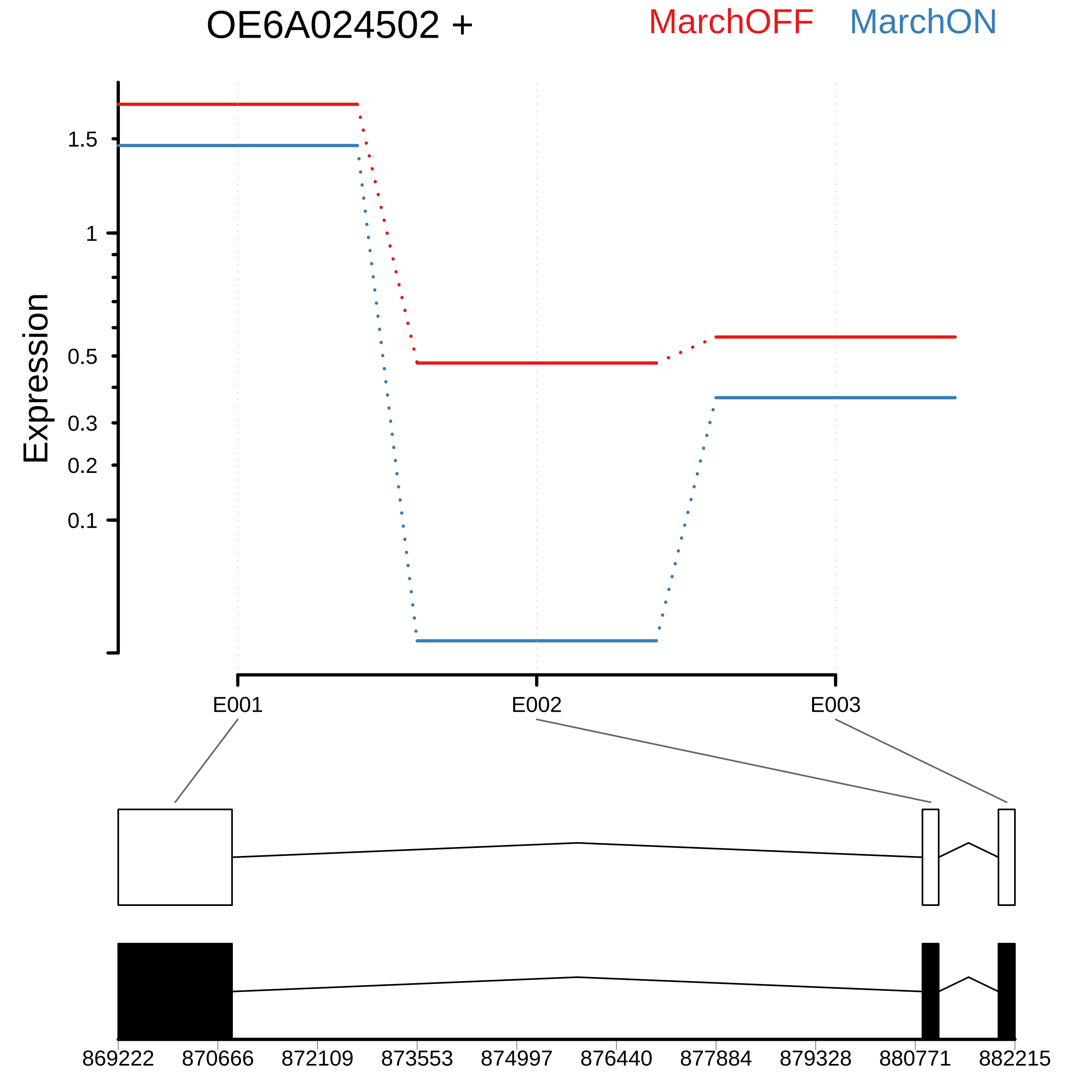

OE6A024502 +
MarchOFF
MarchON
Expression
1.5
1
0.5
0.3
0.2
0.1
E001
E002
E003
869222
870666
872109
873553
874997
876440
877884
879328
880771
882215

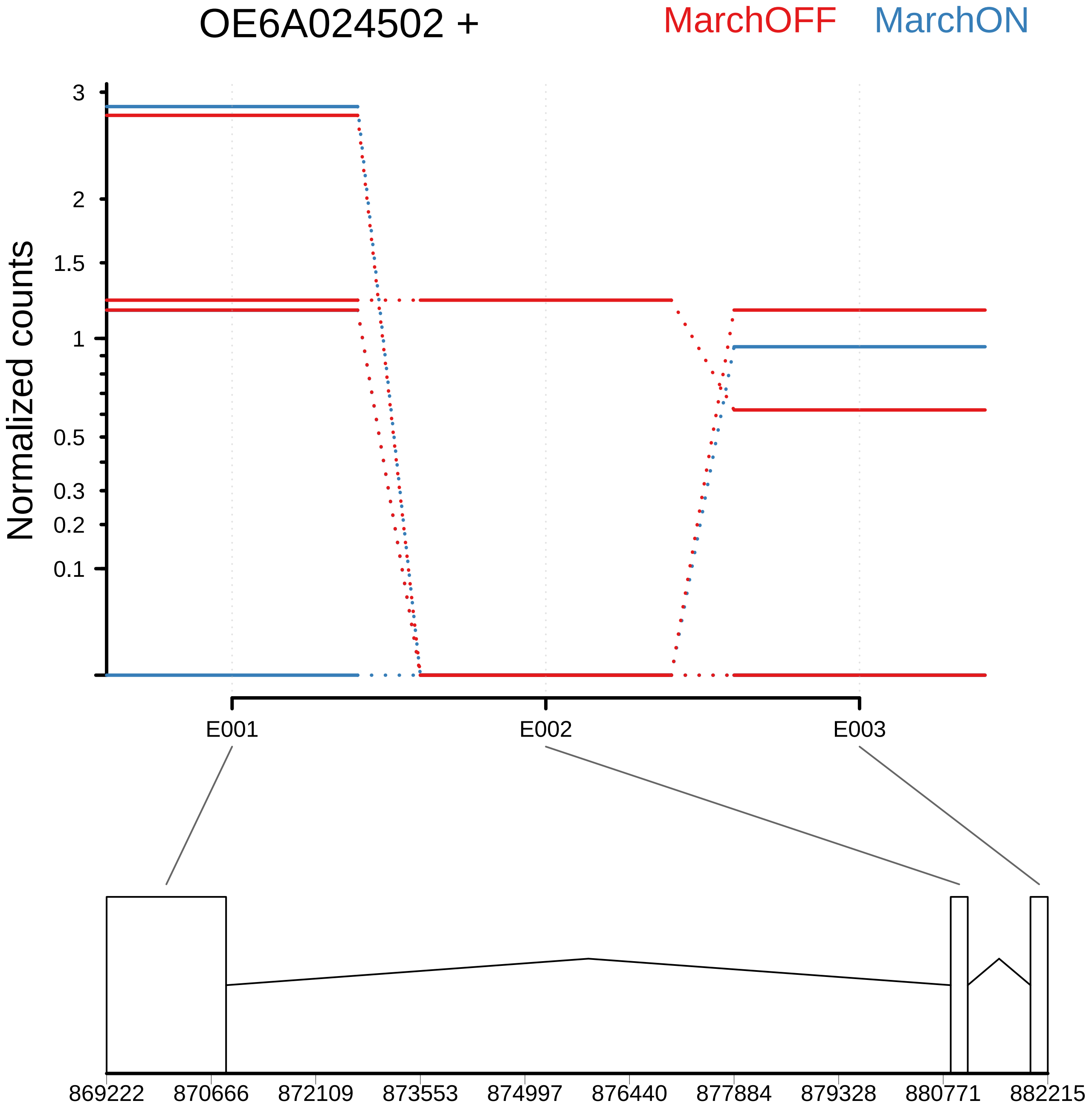

OE6A024502 +
MarchOFF
MarchON
Normalized counts
3
2
1.5
1
0.5
0.3
0.2
0.1
E001
E002
E003
869222
870666
872109
873553
874997
876440
877884
879328
880771
882215

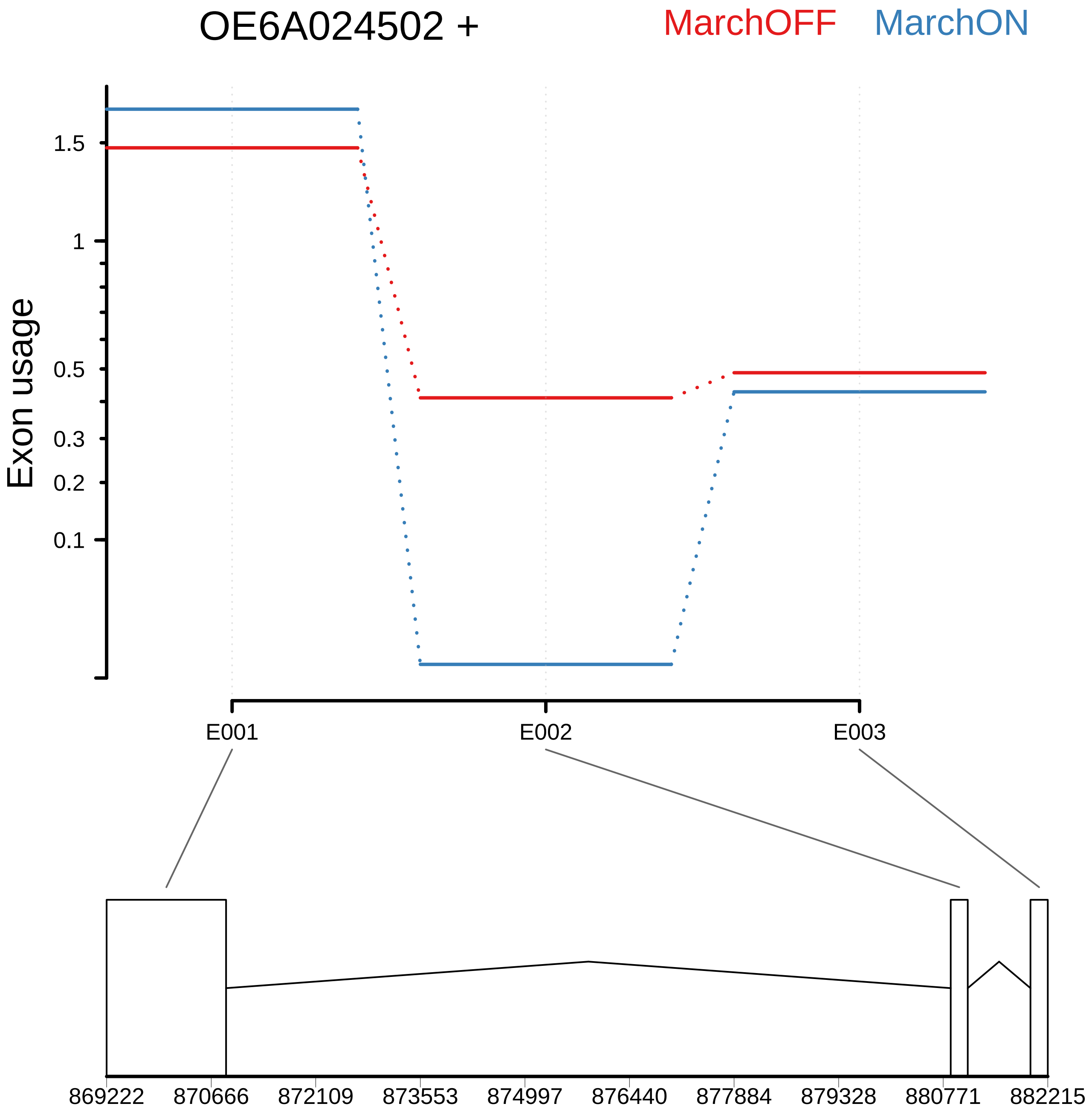

OE6A024502 +
MarchOFF
MarchON
Exon usage
1.5
1
0.5
0.3
0.2
0.1
E001
E002
E003
869222
870666
872109
873553
874997
876440
877884
879328
880771
882215

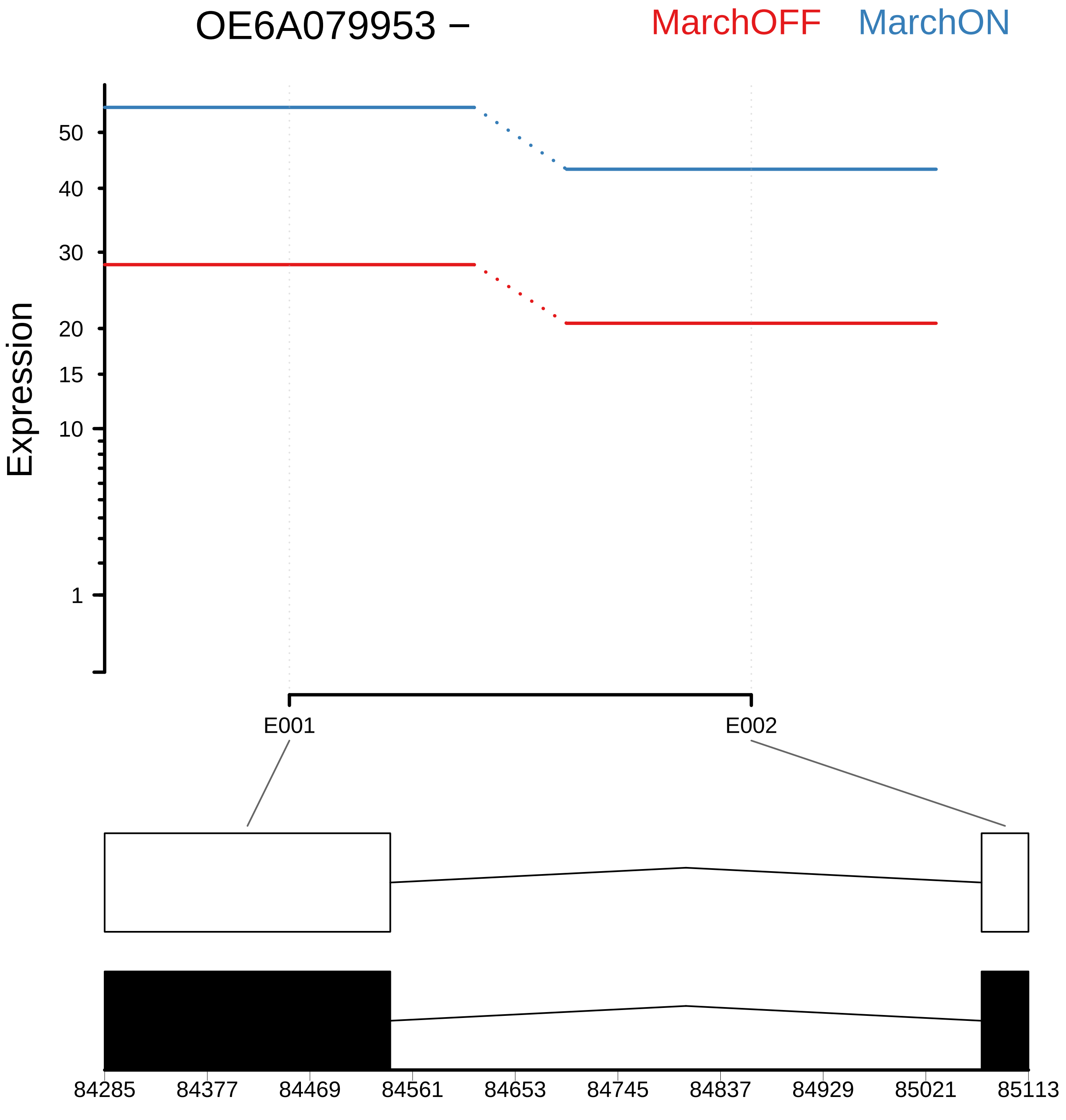

OE6A079953 −
MarchOFF
MarchON
Expression
50
40
30
20
15
10
1
E001
E002
84285
84377
84469
84561
84653
84745
84837
84929
85021
85113

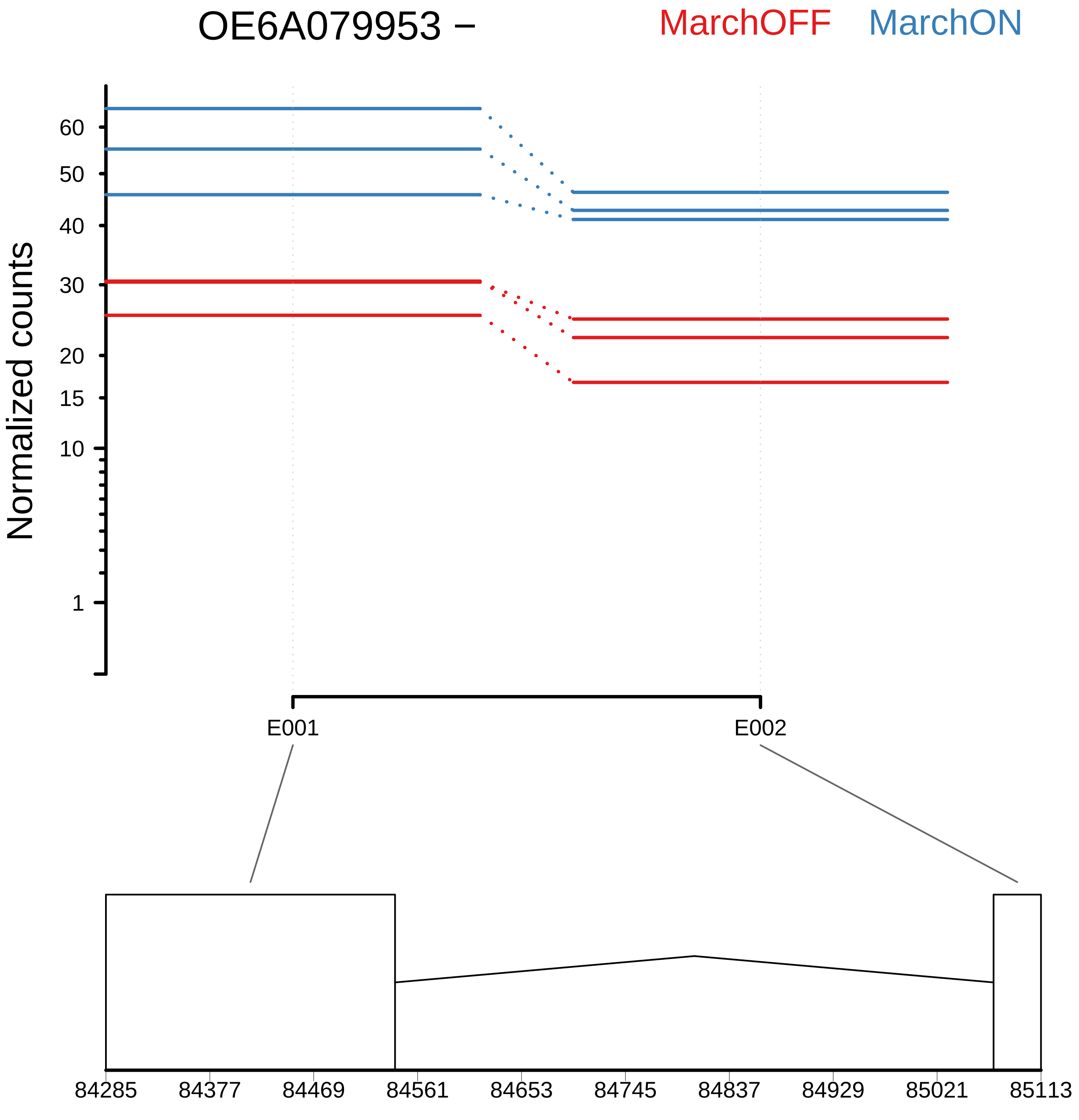
OE6A079953 −
MarchOFF
MarchON
Normalized counts
60
50
40
30
20
15
10
1
E001
E002
84285
84377
84469
84561
84653
84745
84837
84929
85021
85113

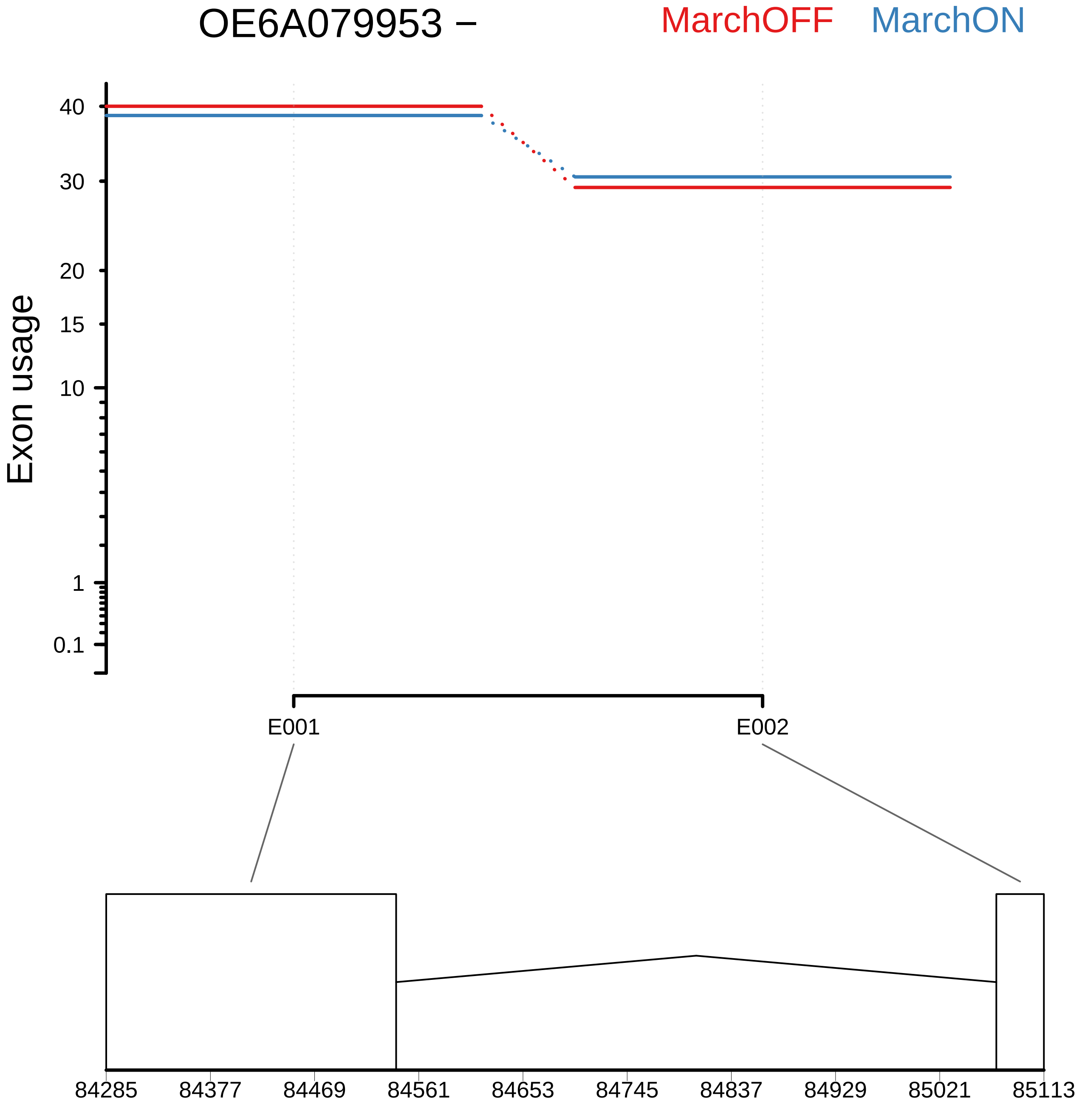

OE6A079953 −
MarchOFF
MarchON
Exon usage
40
30
20
15
10
1
0.1
E001
E002
84285
84377
84469
84561
84653
84745
84837
84929
85021
85113

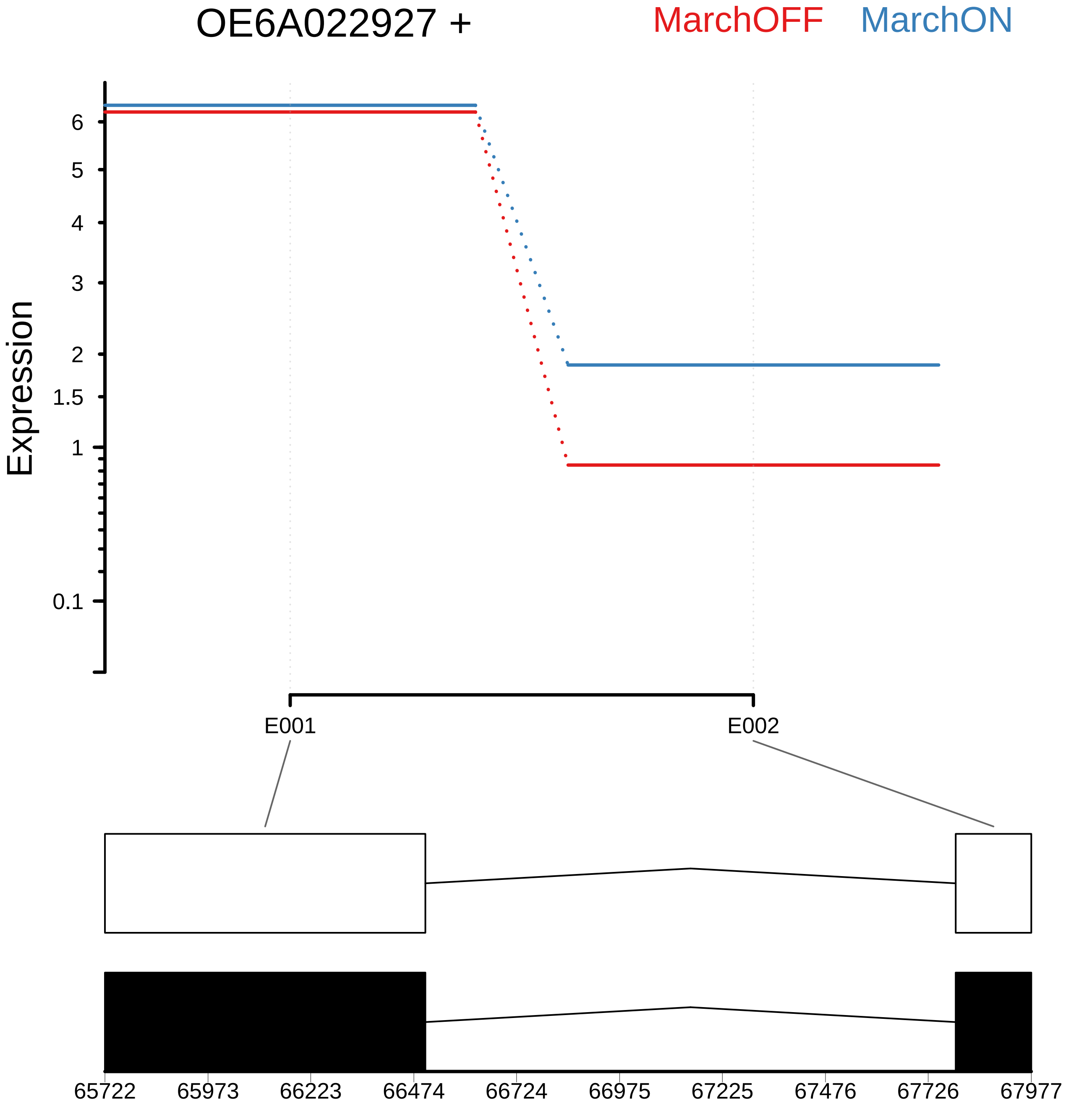

OE6A022927 +
MarchOFF
MarchON
Expression
6
5
4
3
2
1.5
1
0.1
E001
E002
65722
65973
66223
66474
66724
66975
67225
67476
67726
67977

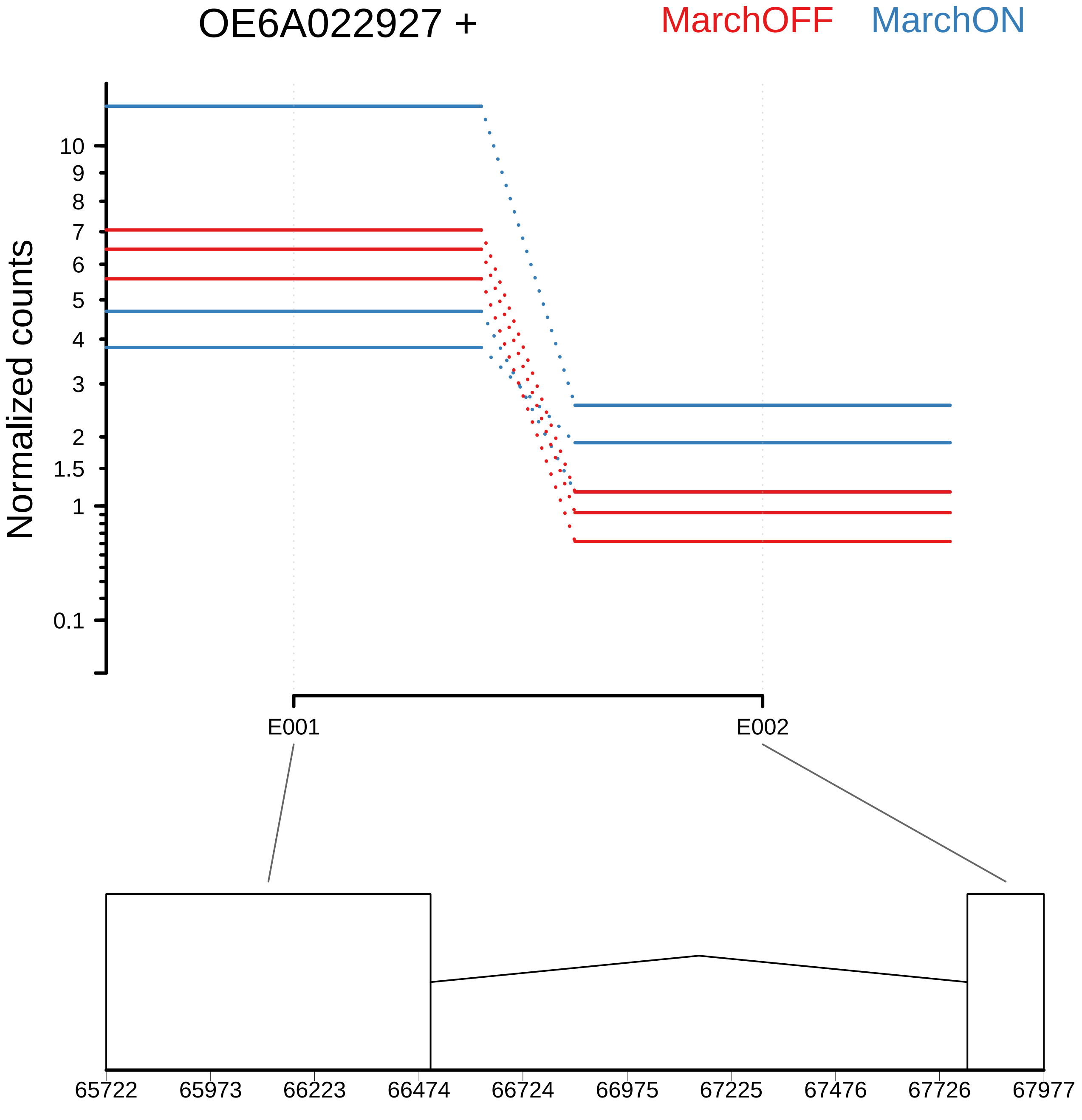

OE6A022927 +
MarchOFF
MarchON
Normalized counts
10
9
8
7
6
5
4
3
2
1.5
1
0.1
E001
E002
65722
65973
66223
66474
66724
66975
67225
67476
67726
67977

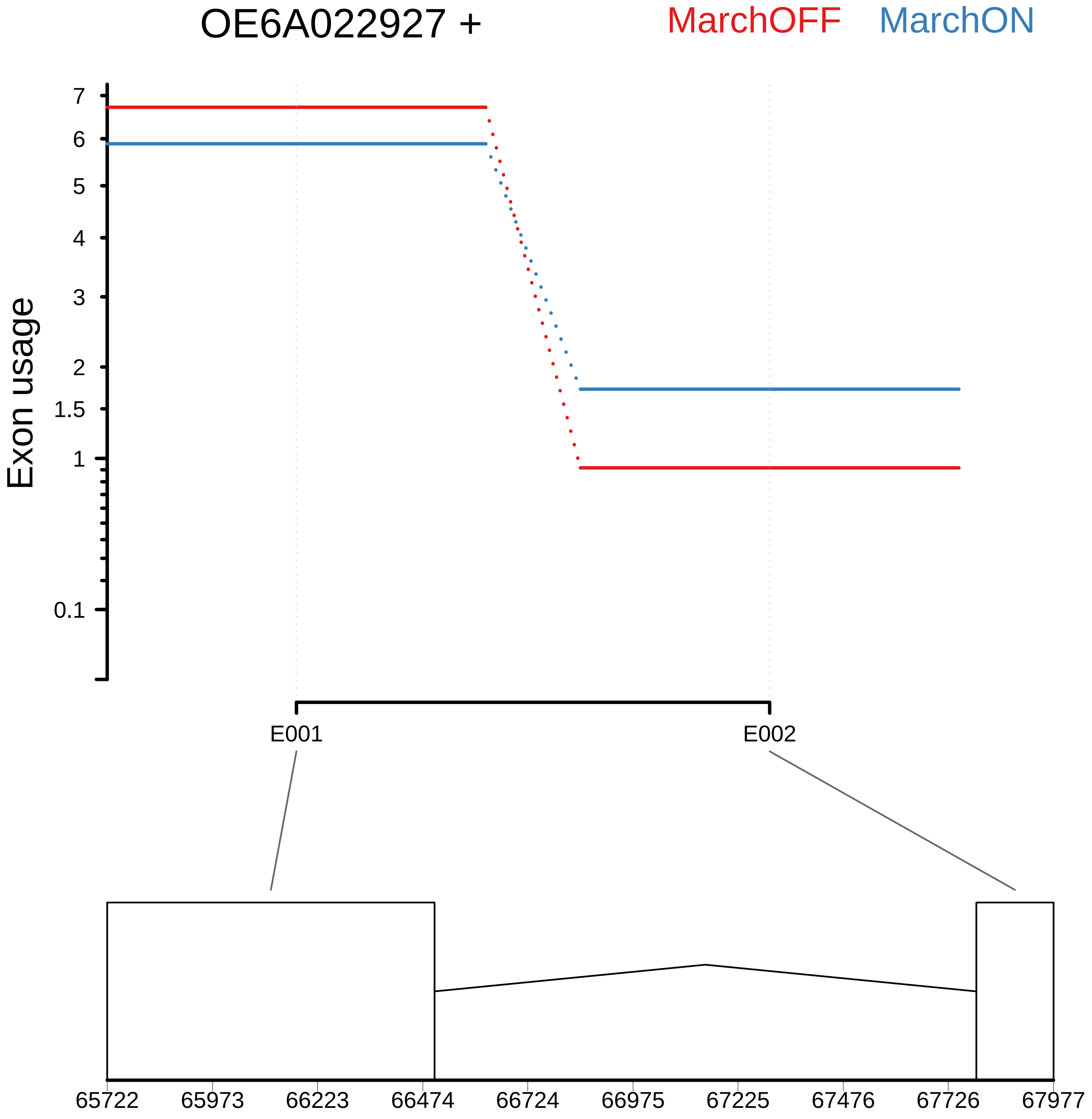

OE6A022927 +
MarchOFF
MarchON
Exon usage
7
6
5
4
3
2
1.5
1
0.1
E001
E002
65722
65973
66223
66474
66724
66975
67225
67476
67726
67977

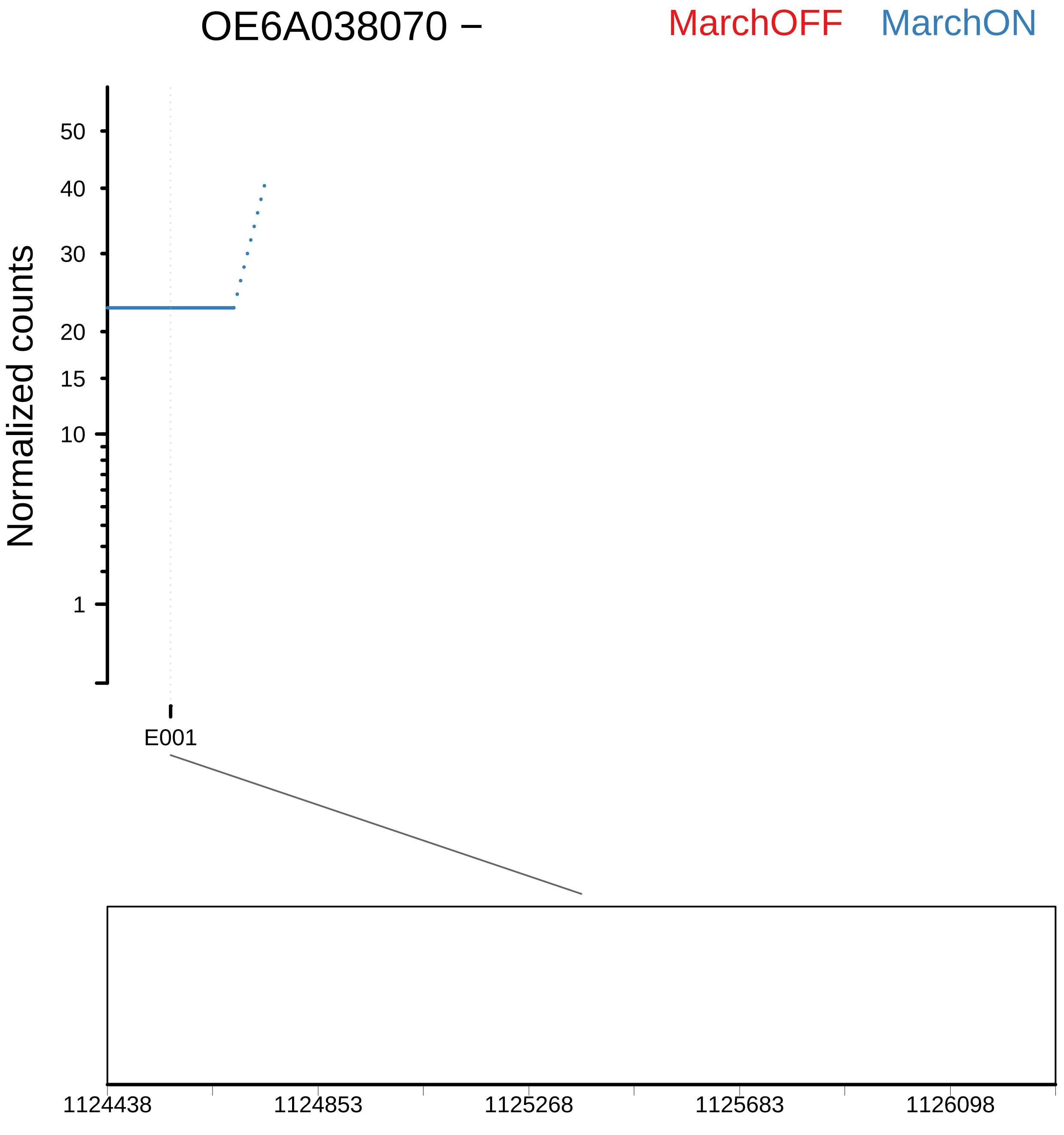

OE6A038070 −
MarchOFF
MarchON
Normalized counts
50
40
30
20
15
10
1
E001
1124438
1124853
1125268
1125683
1126098

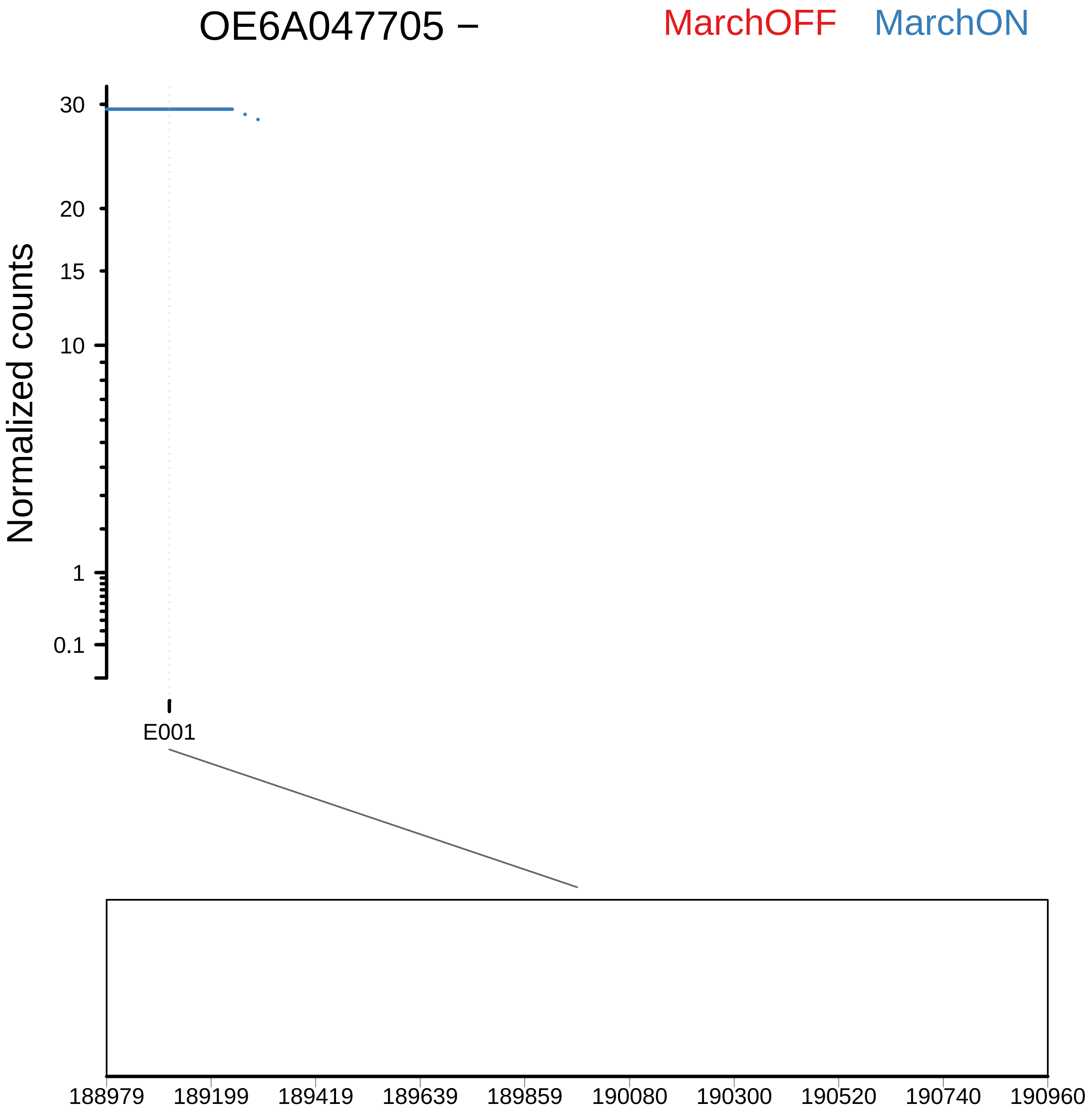

OE6A047705 −
MarchOFF
MarchON
Normalized counts
30
20
15
10
1
0.1
E001
188979
189199
189419
189639
189859
190080
190300
190520
190740
190960

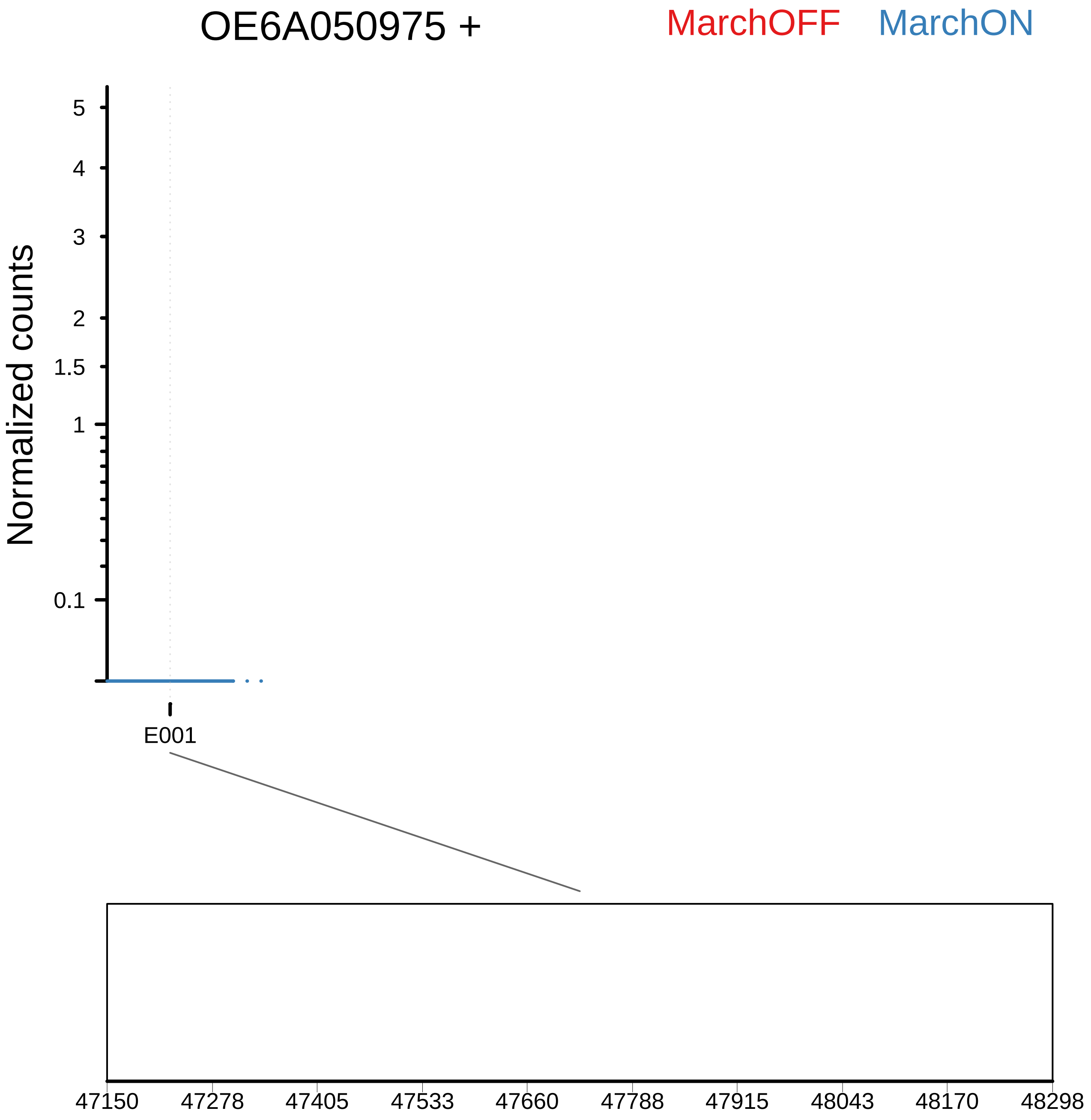

OE6A050975 +
MarchOFF
MarchON
Normalized counts
5
4
3
2
1.5
1
0.1
E001
47150
47278
47405
47533
47660
47788
47915
48043
48170
48298

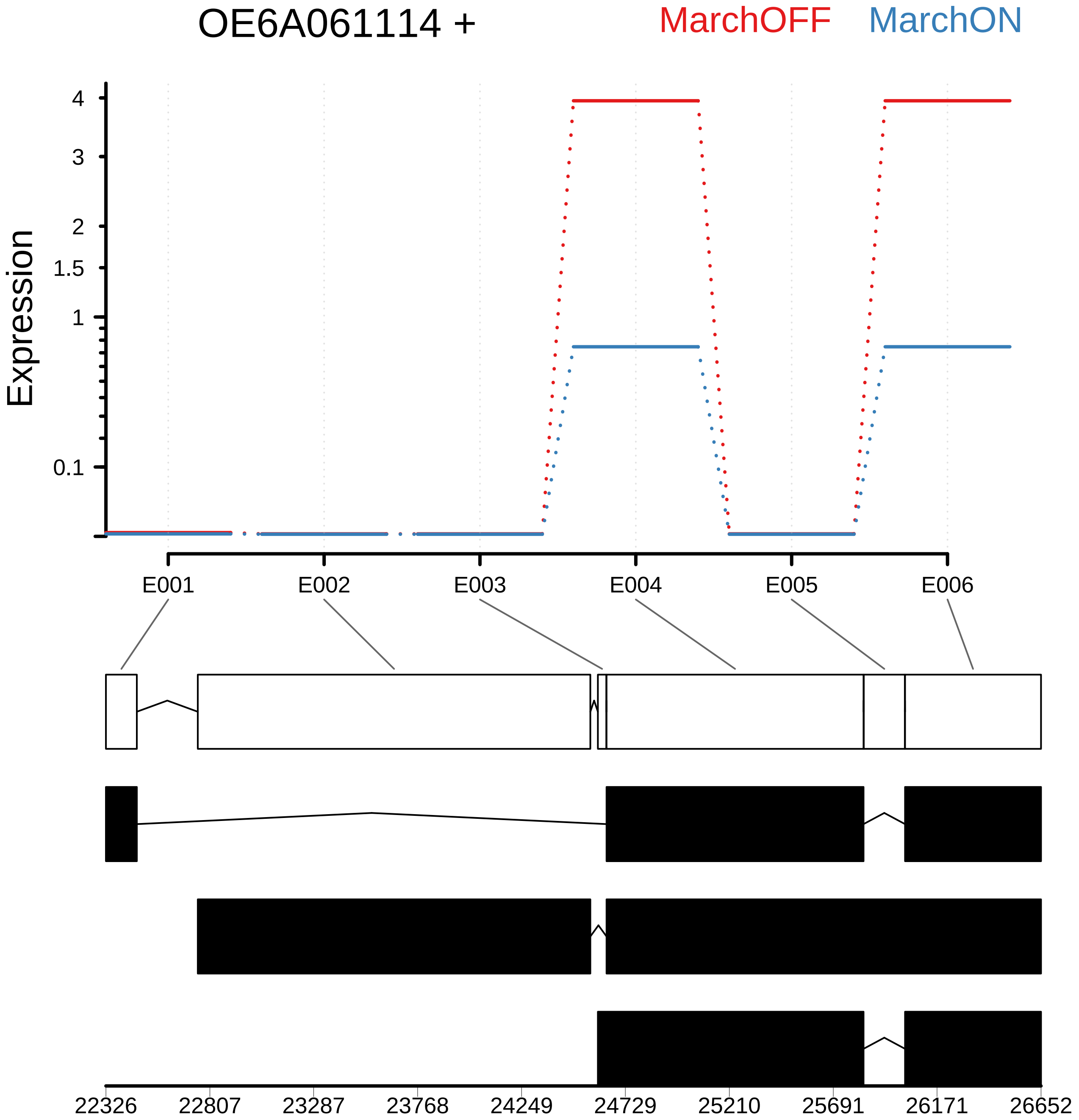

OE6A061114 +
MarchOFF
MarchON
Expression
4
3
2
1.5
1
0.1
E001
E002
E003
E004
E005
E006
22326
22807
23287
23768
24249
24729
25210
25691
26171
26652

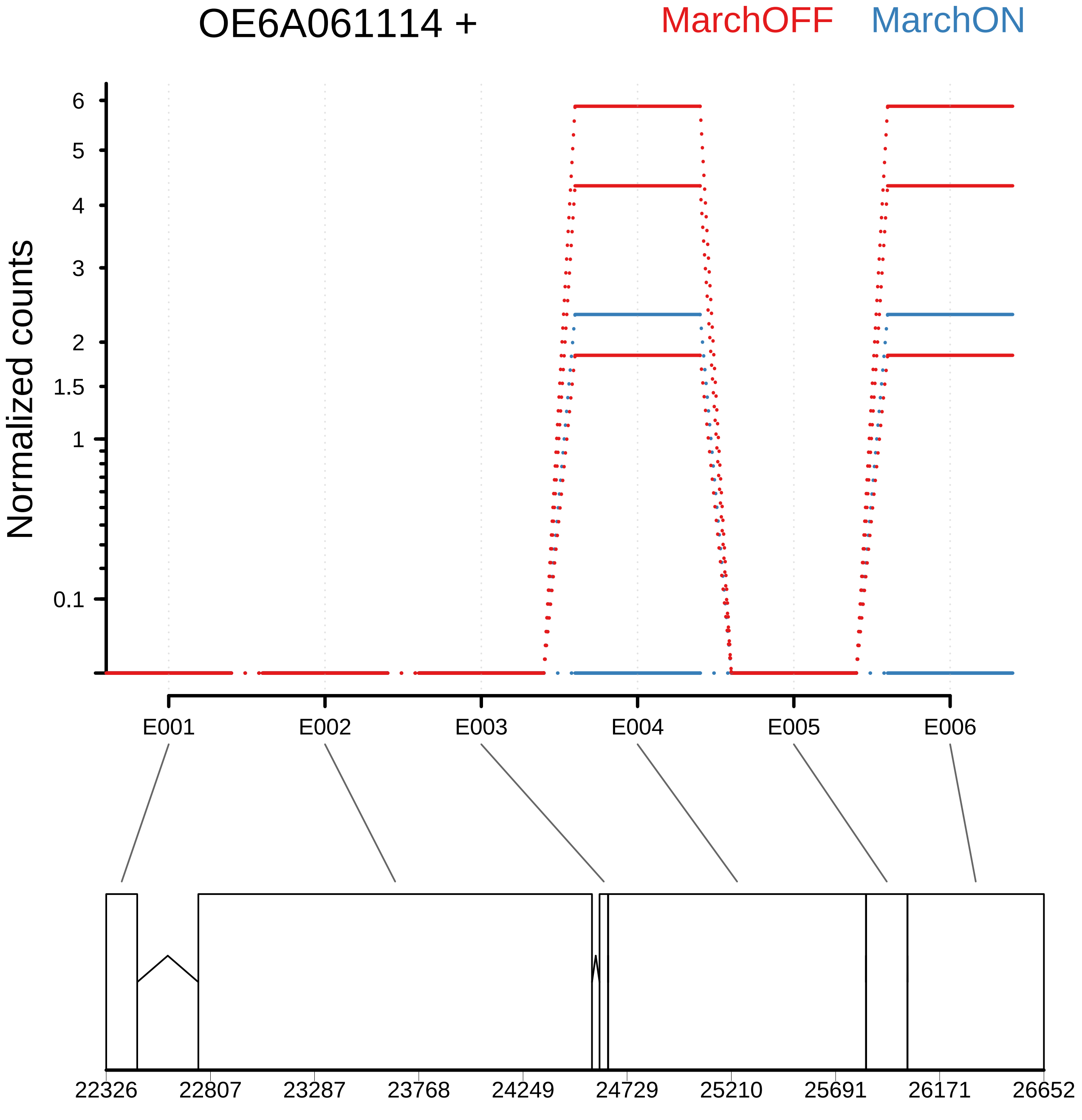
OE6A061114 +
MarchOFF
MarchON
Normalized counts
6
5
4
3
2
1.5
1
0.1
E001
E002
E003
E004
E005
E006
22326
22807
23287
23768
24249
24729
25210
25691
26171
26652

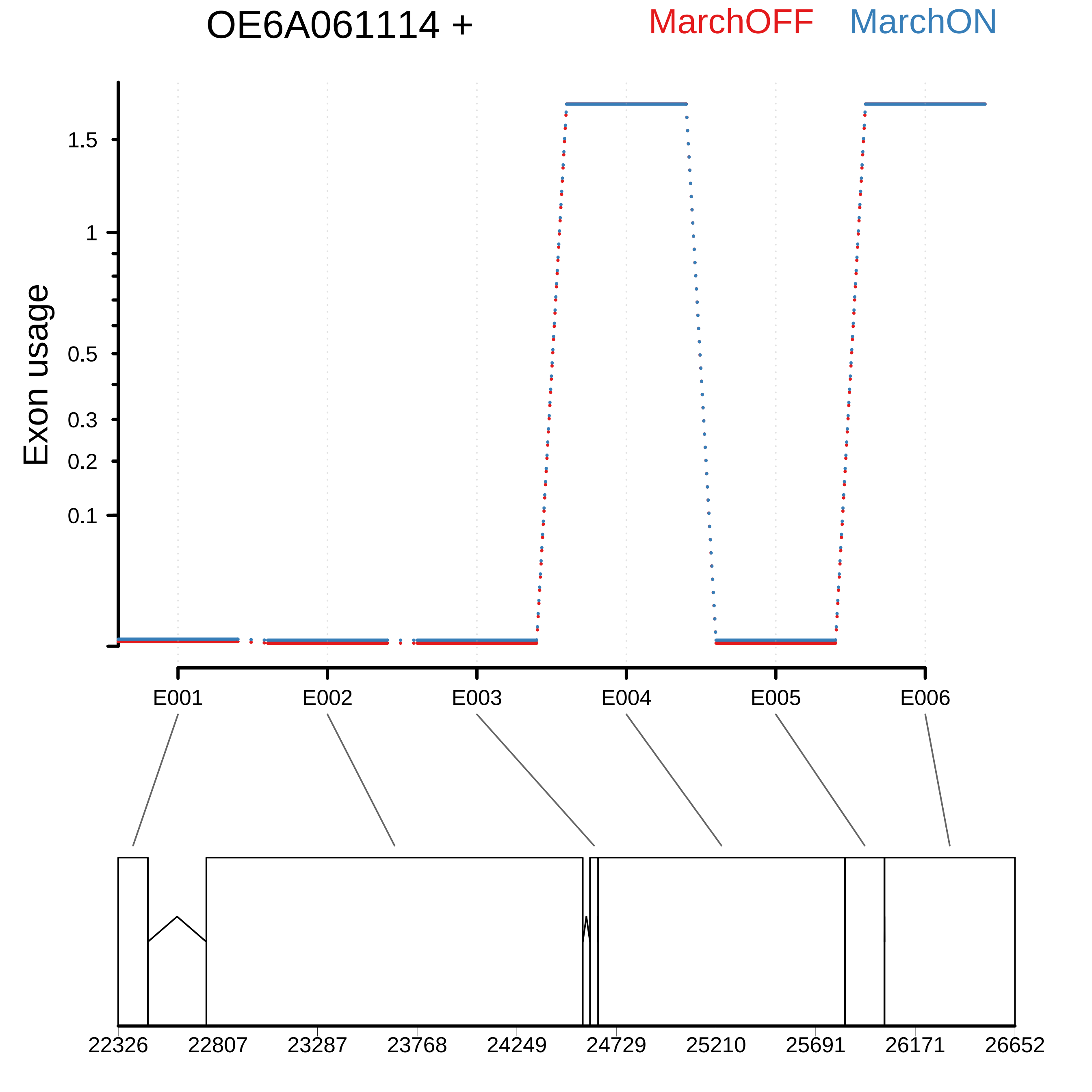
OE6A061114 +
MarchOFF
MarchON
Exon usage
1.5
1
0.5
0.3
0.2
0.1
E001
E002
E003
E004
E005
E006
22326
22807
23287
23768
24249
24729
25210
25691
26171
26652

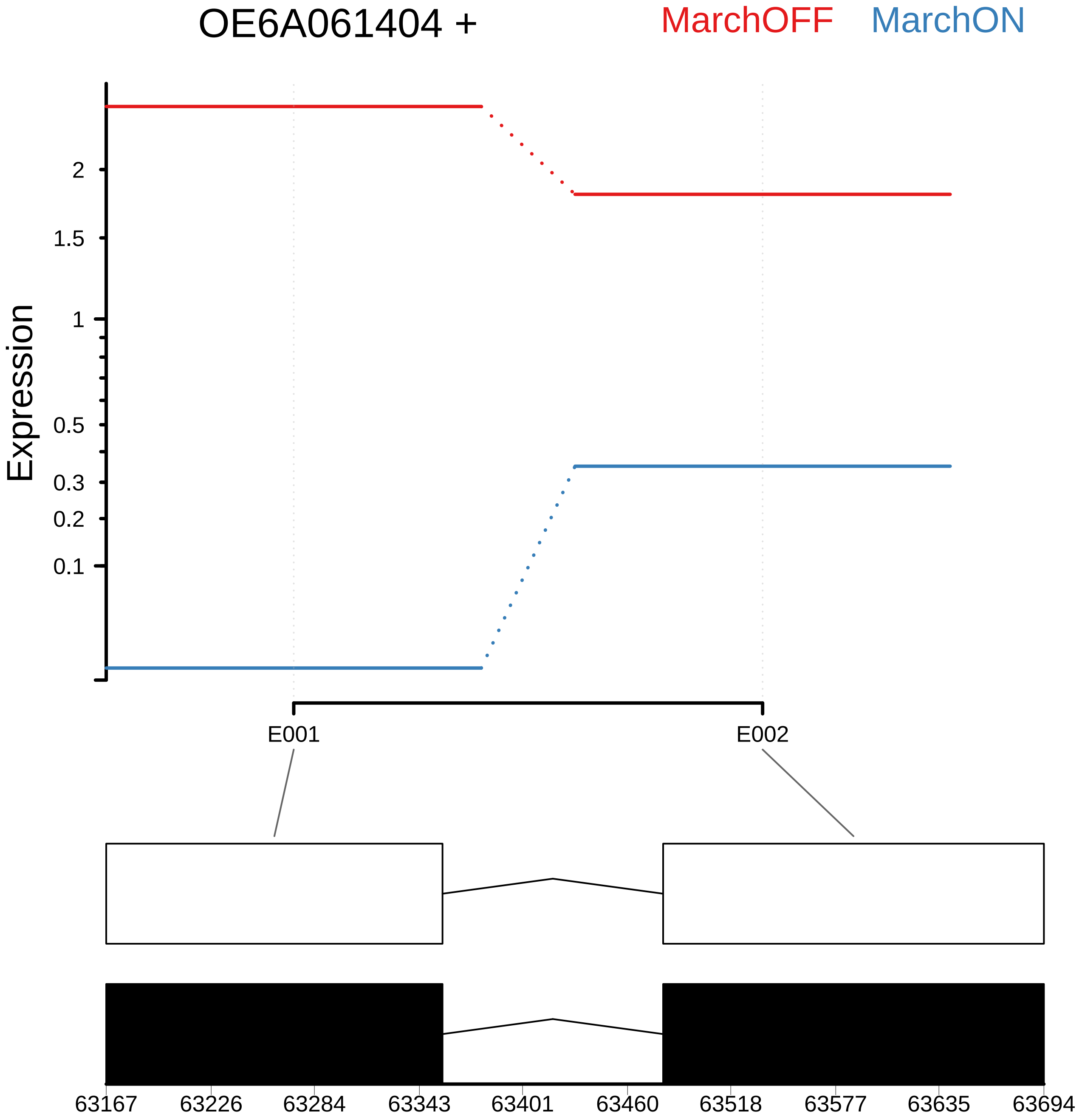
OE6A061404 +
MarchOFF
MarchON
Expression
2
1.5
1
0.5
0.3
0.2
0.1
E001
E002
63167
63226
63284
63343
63401
63460
63518
63577
63635
63694

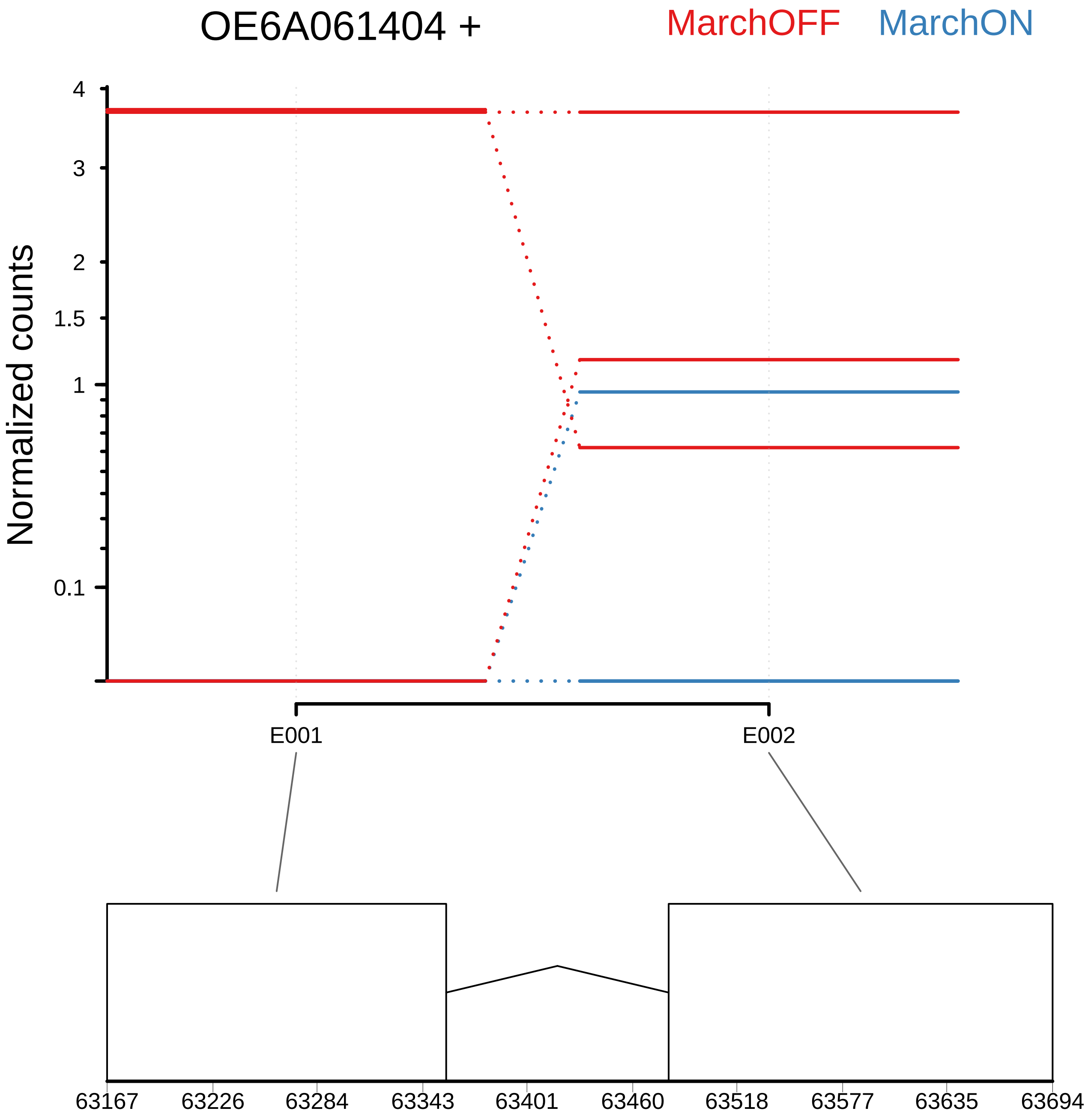
OE6A061404 +
MarchOFF
MarchON
Normalized counts
4
3
2
1.5
1
0.1
E001
E002
63167
63226
63284
63343
63401
63460
63518
63577
63635
63694

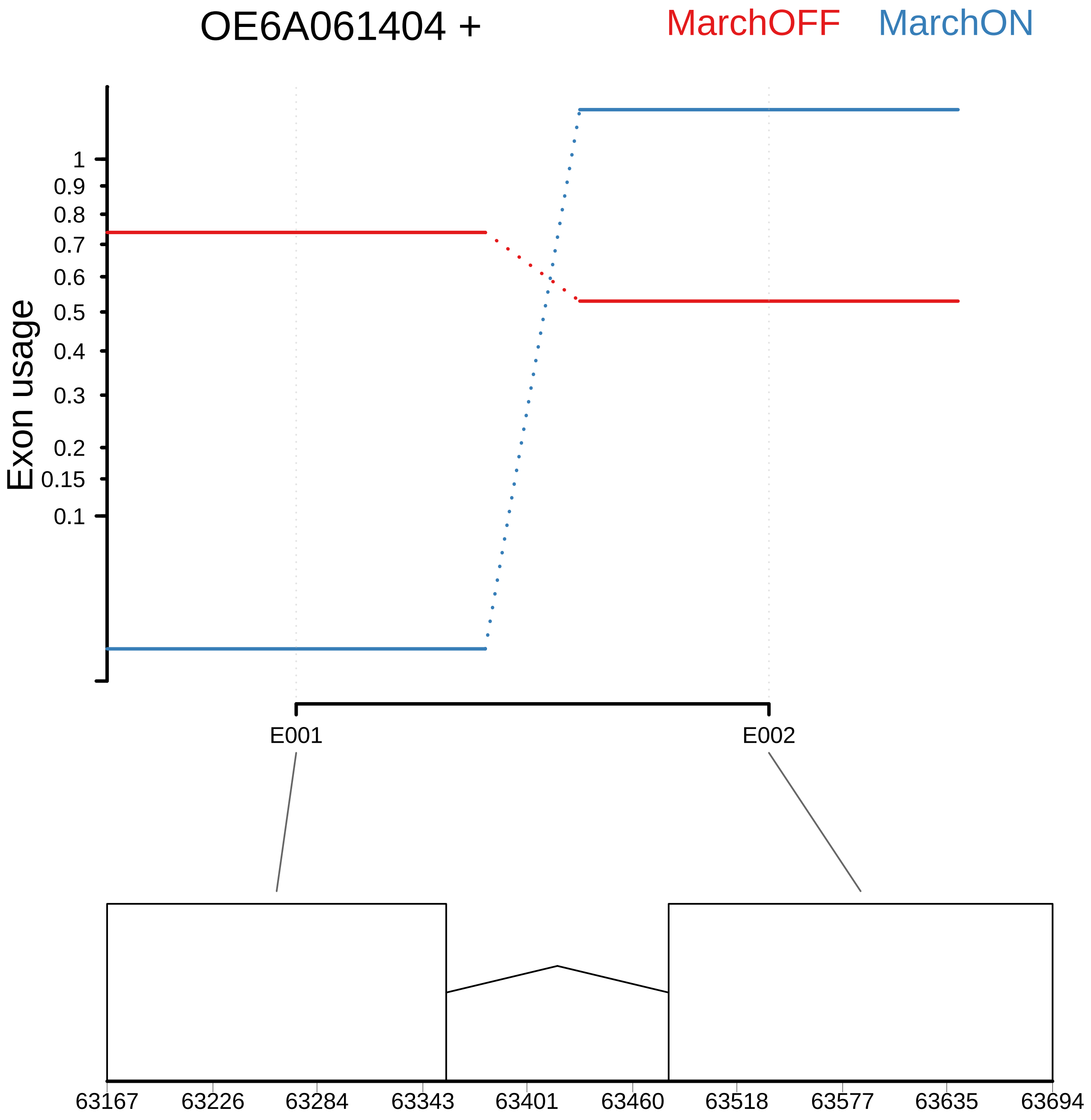

OE6A061404 +
MarchOFF
MarchON
Exon usage
1
0.9
0.8
0.7
0.6
0.5
0.4
0.3
0.2
0.15
0.1
E001
E002
63167
63226
63284
63343
63401
63460
63518
63577
63635
63694

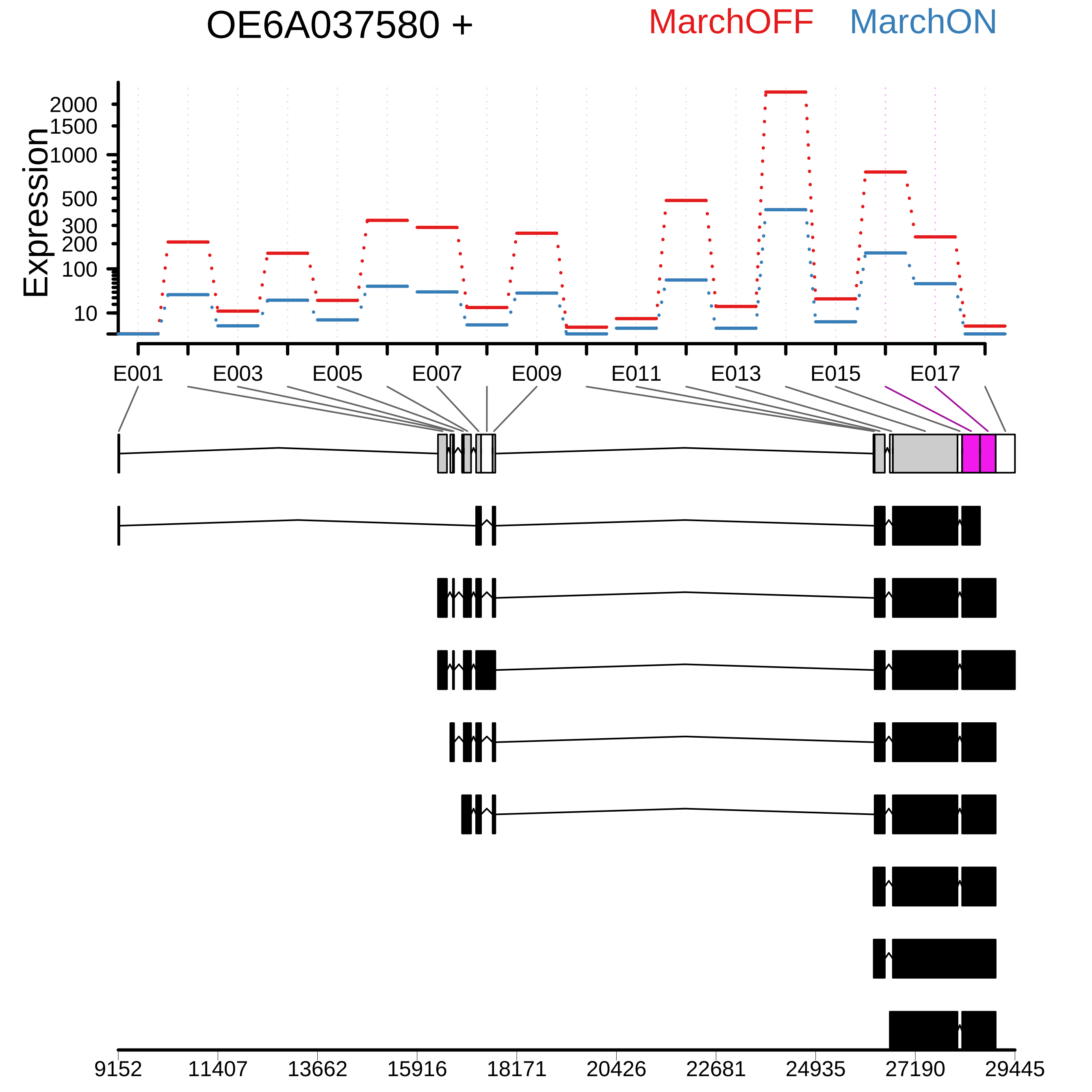
OE6A037580 +
MarchOFF
MarchON
Expression
2000
1500
1000
500
300
200
100
10
E001
E003
E005
E007
E009
E011
E013
E015
E017
9152
11407
13662
15916
18171
20426
22681
24935
27190
29445

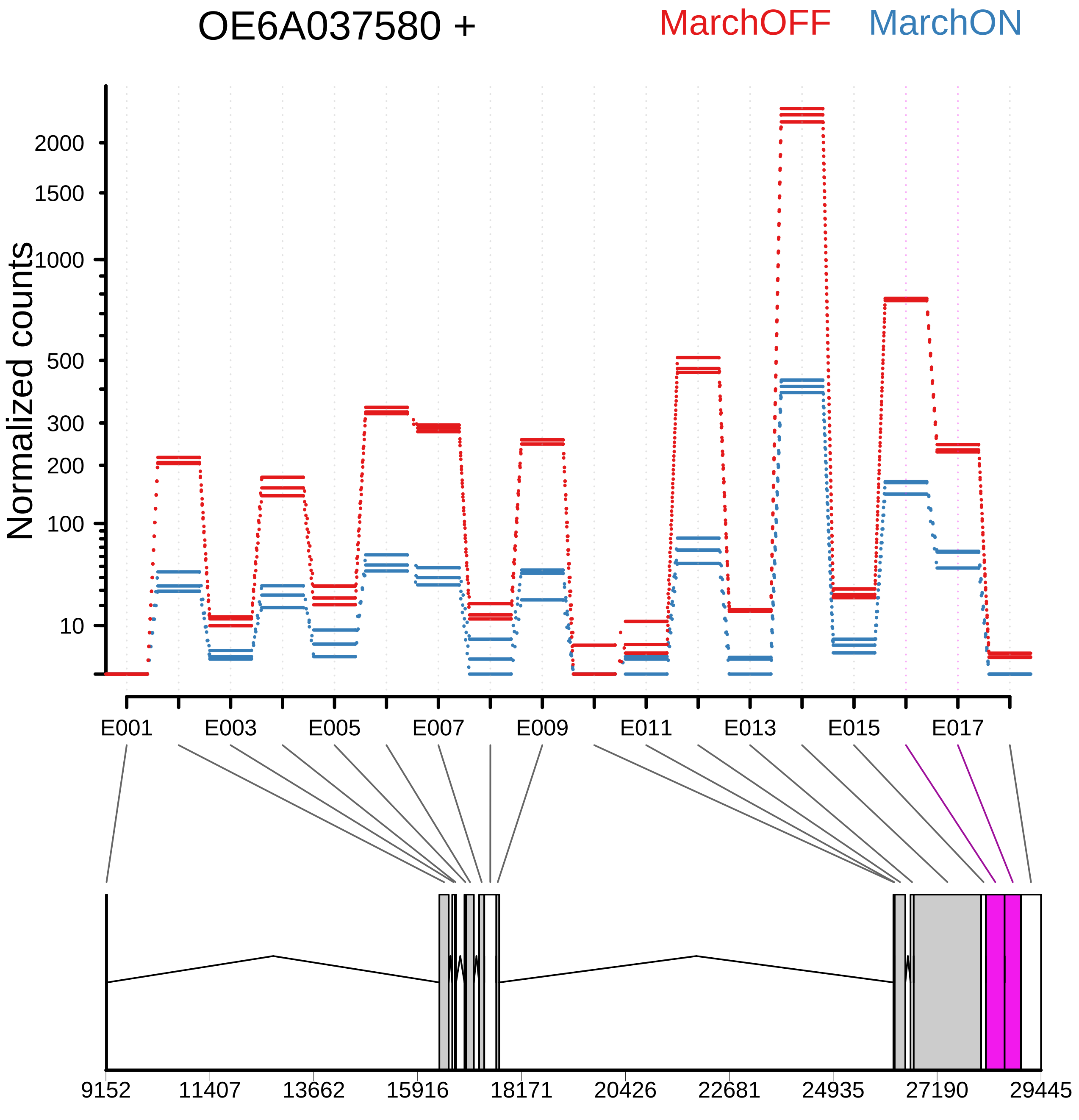

OE6A037580 +
MarchOFF
MarchON
Normalized counts
2000
1500
1000
500
300
200
100
10
E001
E003
E005
E007
E009
E011
E013
E015
E017
9152
11407
13662
15916
18171
20426
22681
24935
27190
29445

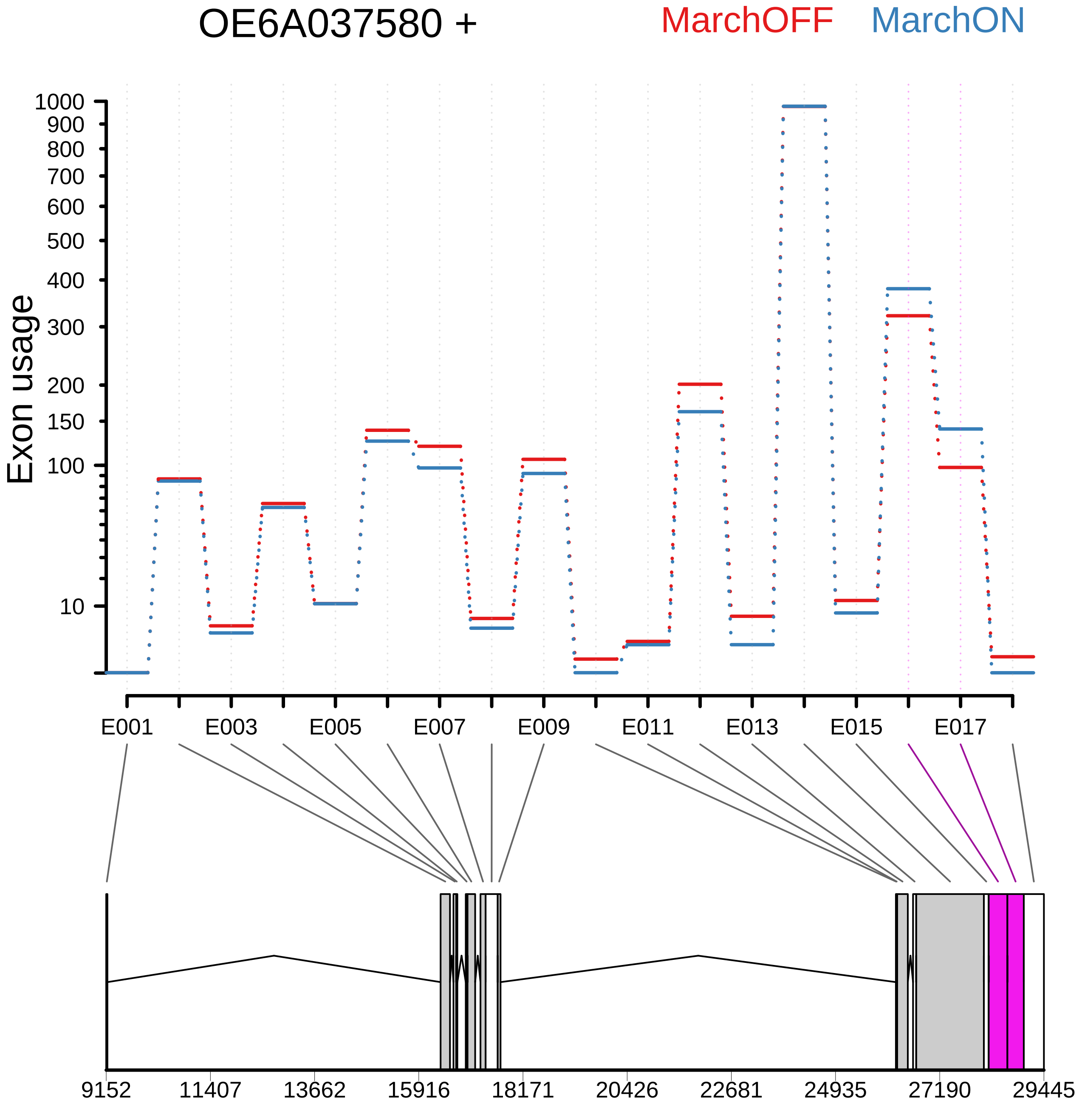

OE6A037580 +
MarchOFF
MarchON
Exon usage
1000
900
800
700
600
500
400
300
200
150
100
10
E001
E003
E005
E007
E009
E011
E013
E015
E017
9152
11407
13662
15916
18171
20426
22681
24935
27190
29445

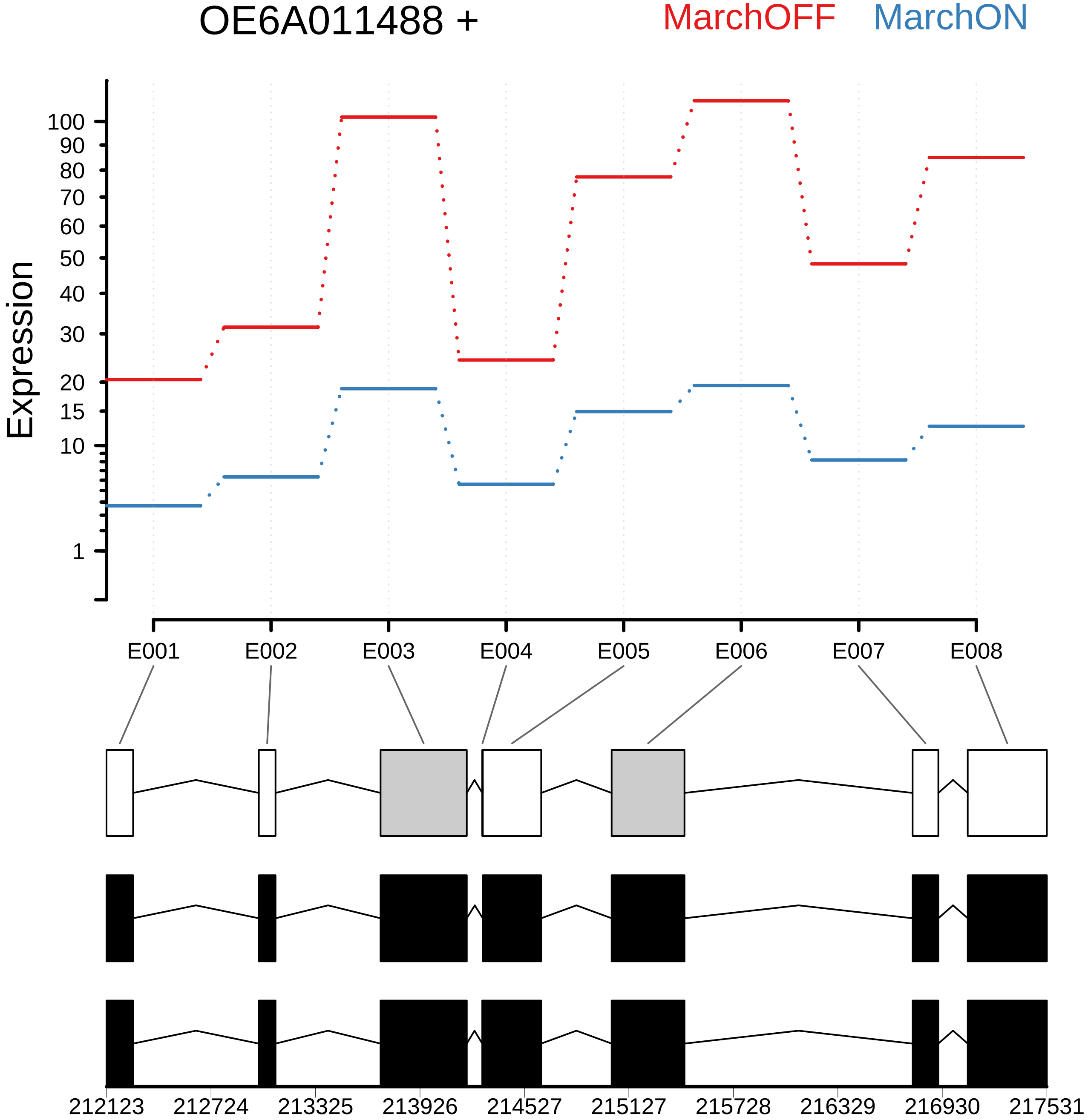

OE6A011488 +
MarchOFF
MarchON
Expression
100
90
80
70
60
50
40
30
20
15
10
1
E001
E002
E003
E004
E005
E006
E007
E008
212123
212724
213325
213926
214527
215127
215728
216329
216930
217531

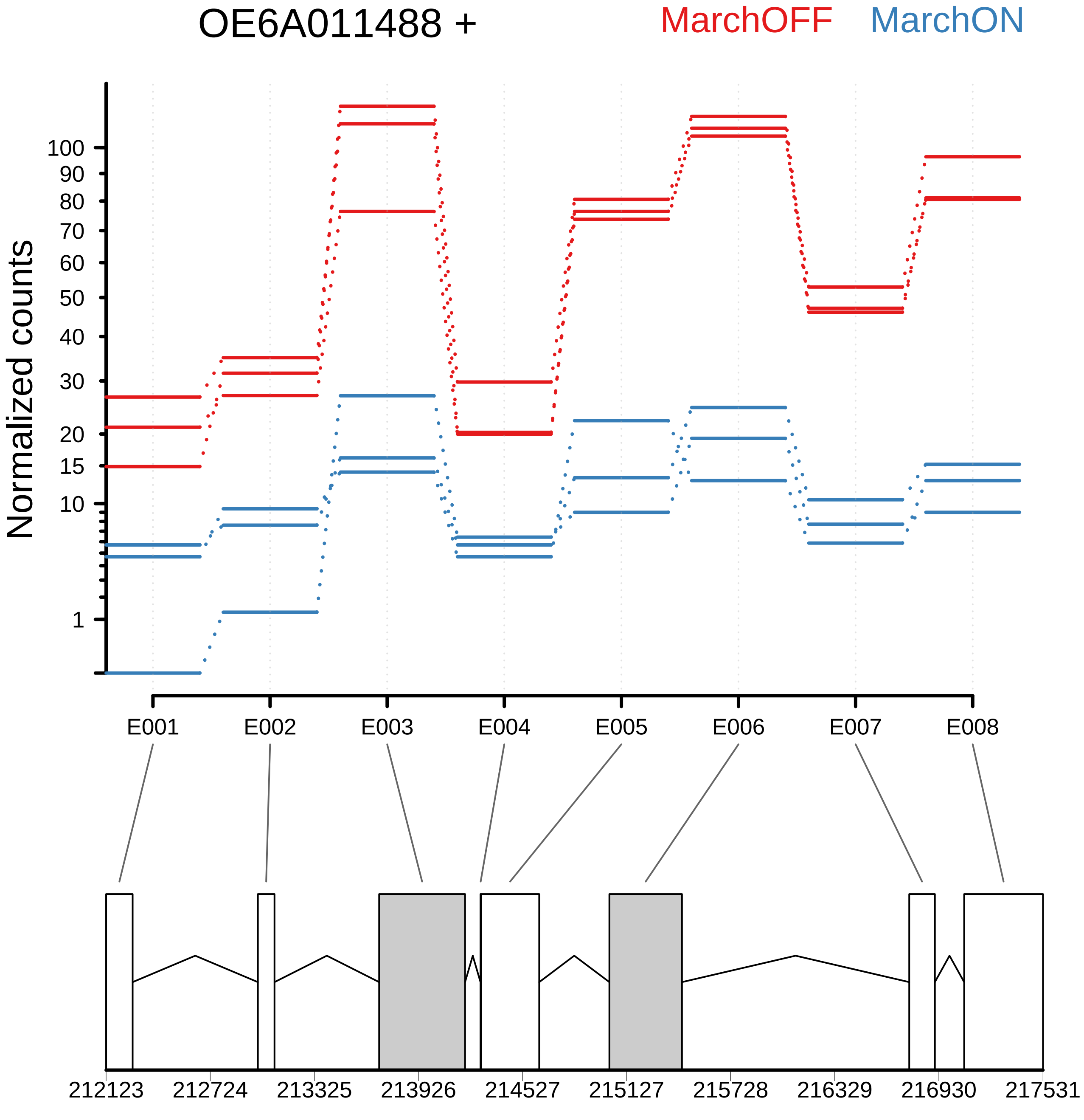

OE6A011488 +
MarchOFF
MarchON
Normalized counts
100
90
80
70
60
50
40
30
20
15
10
1
E001
E002
E003
E004
E005
E006
E007
E008
212123
212724
213325
213926
214527
215127
215728
216329
216930
217531

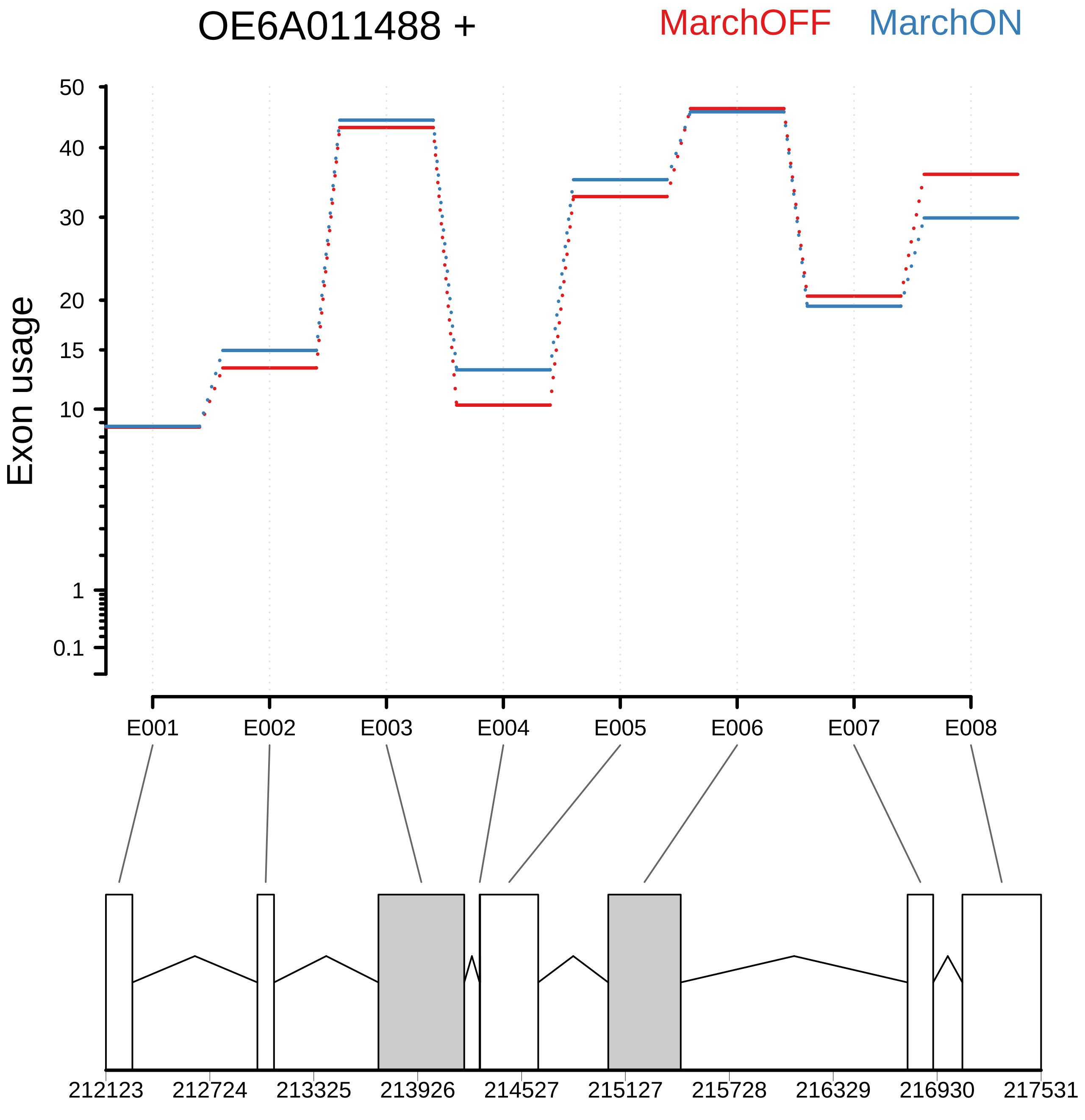

OE6A011488 +
MarchOFF
MarchON
Exon usage
50
40
30
20
15
10
1
0.1
E001
E002
E003
E004
E005
E006
E007
E008
212123
212724
213325
213926
214527
215127
215728
216329
216930
217531

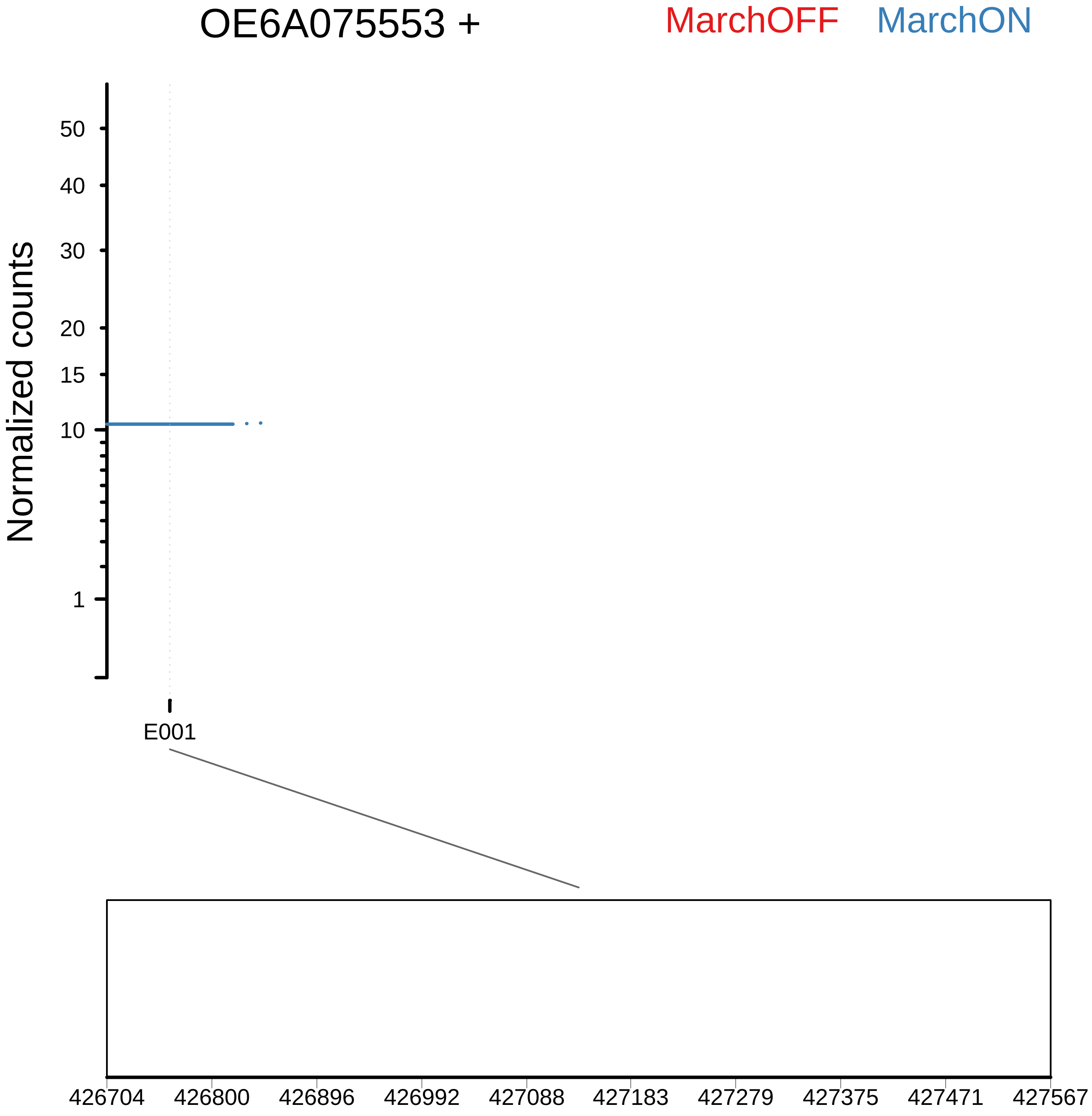
OE6A075553 +
MarchOFF
MarchON
Normalized counts
50
40
30
20
15
10
1
E001
426704
426800
426896
426992
427088
427183
427279
427375
427471
427567

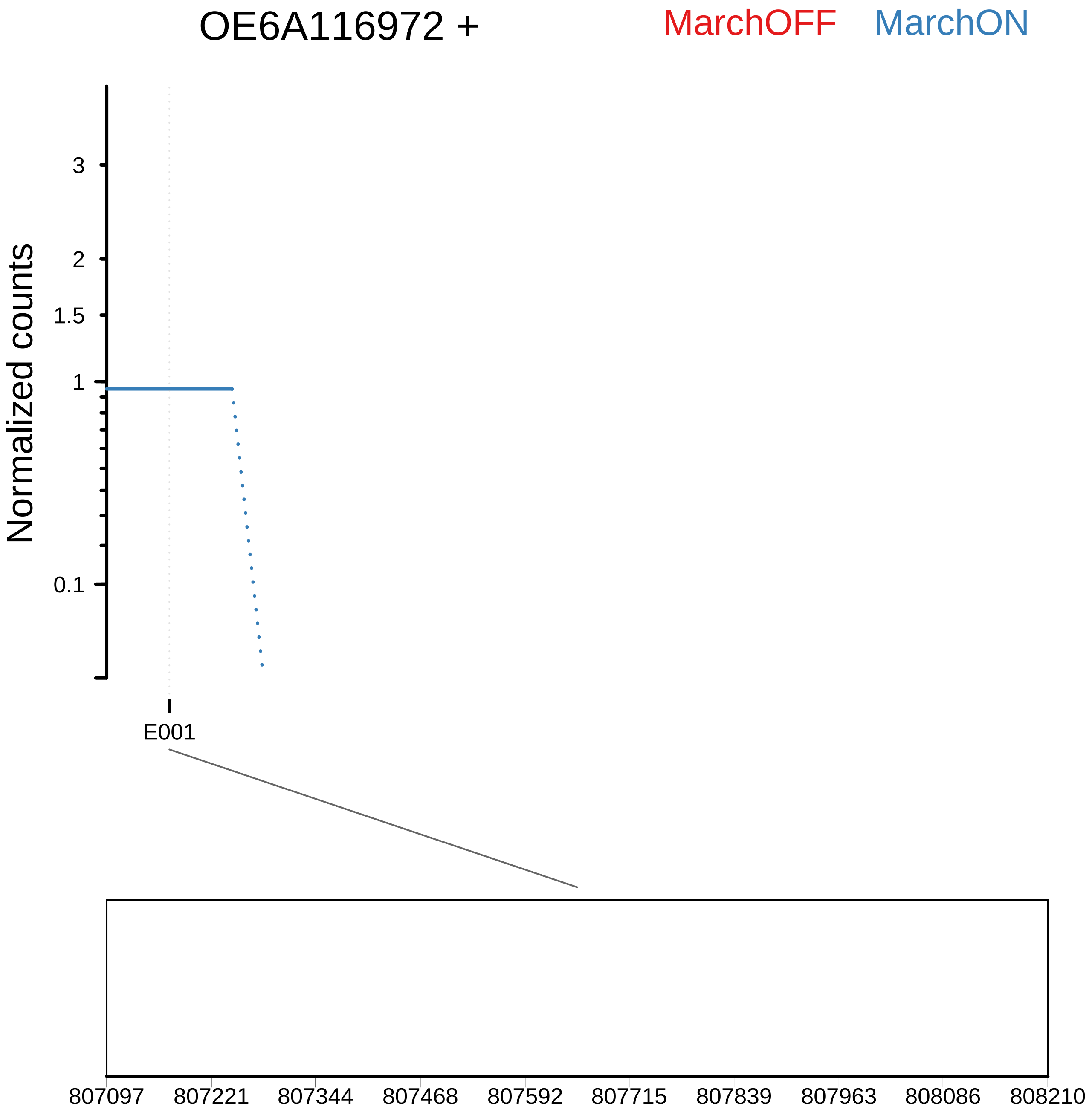

OE6A116972 +
MarchOFF
MarchON
Normalized counts
3
2
1.5
1
0.1
E001
807097
807221
807344
807468
807592
807715
807839
807963
808086
808210

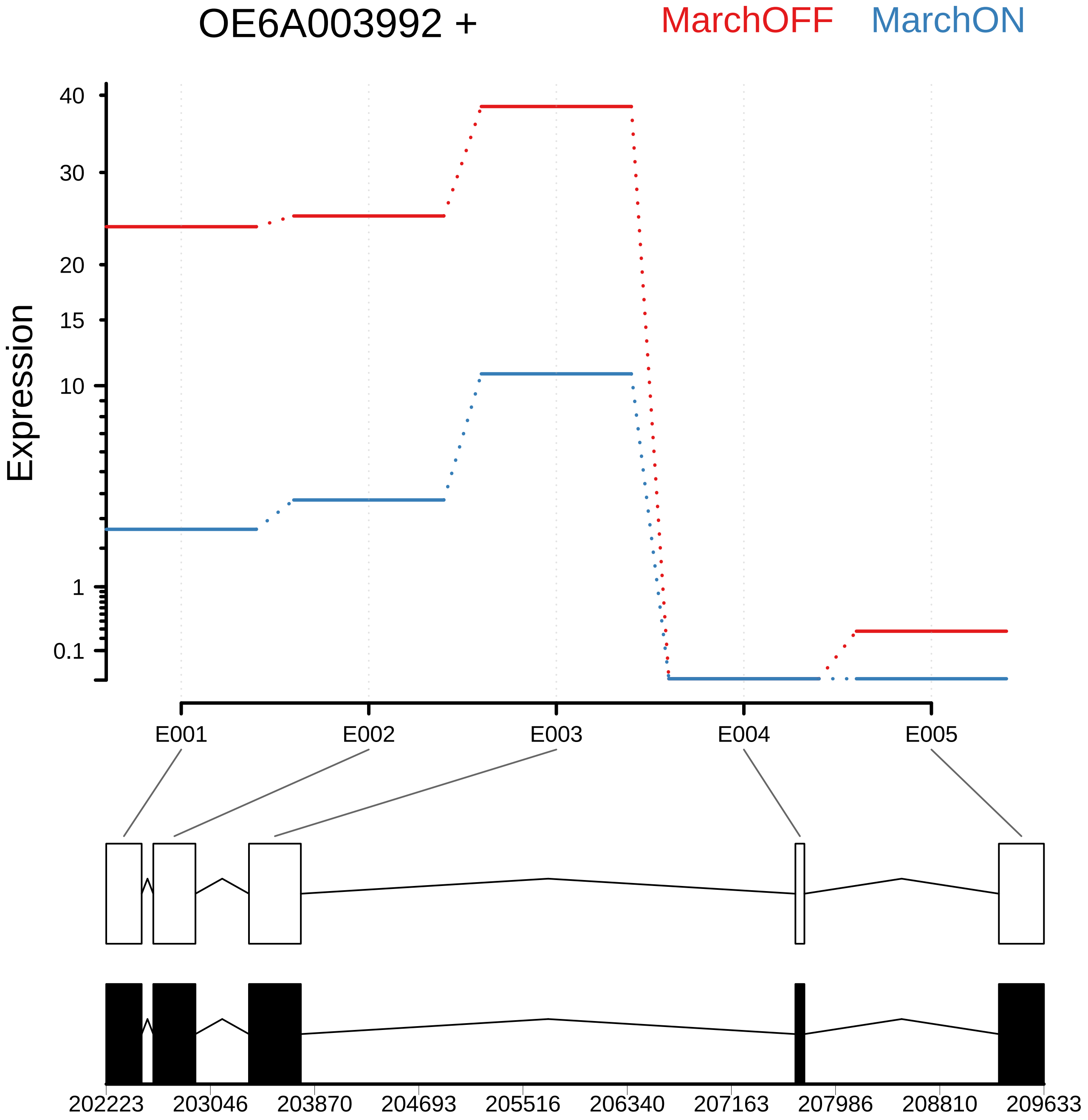

OE6A003992 +
MarchOFF
MarchON
Expression
40
30
20
15
10
1
0.1
E001
E002
E003
E004
E005
202223
203046
203870
204693
205516
206340
207163
207986
208810
209633

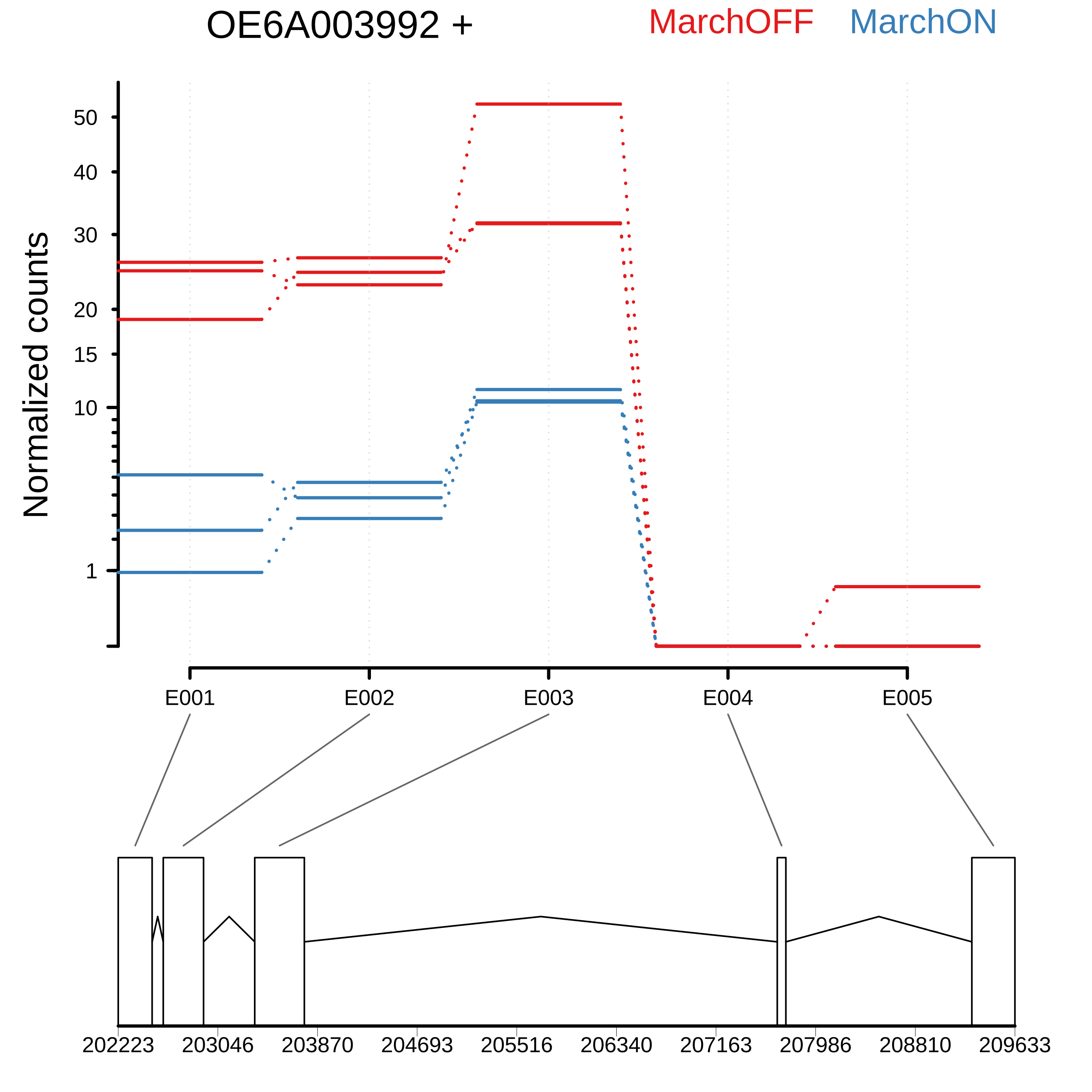

OE6A003992 +
MarchOFF
MarchON
Normalized counts
50
40
30
20
15
10
1
E001
E002
E003
E004
E005
202223
203046
203870
204693
205516
206340
207163
207986
208810
209633

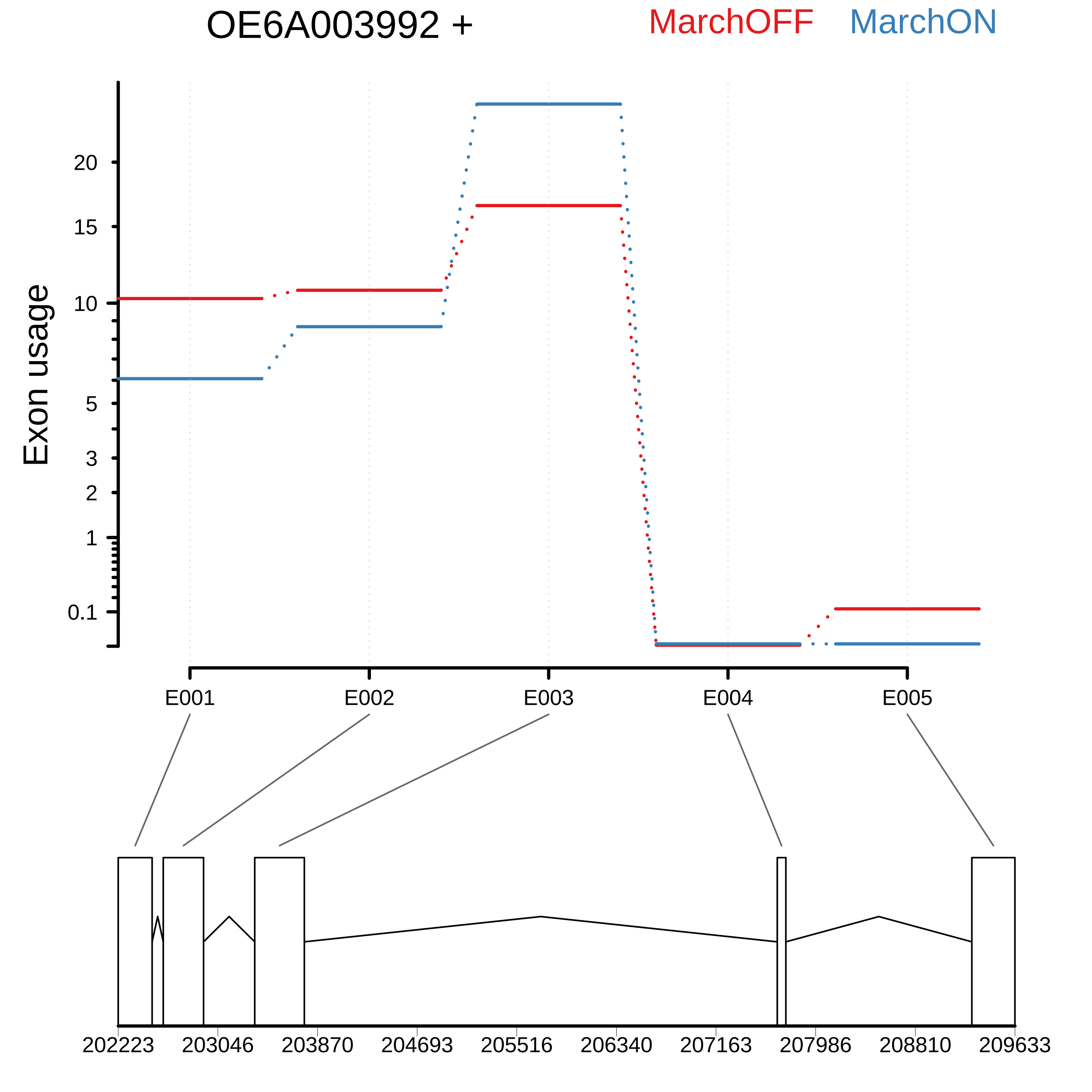

OE6A003992 +
MarchOFF
MarchON
Exon usage
20
15
10
5
3
2
1
0.1
E001
E002
E003
E004
E005
202223
203046
203870
204693
205516
206340
207163
207986
208810
209633

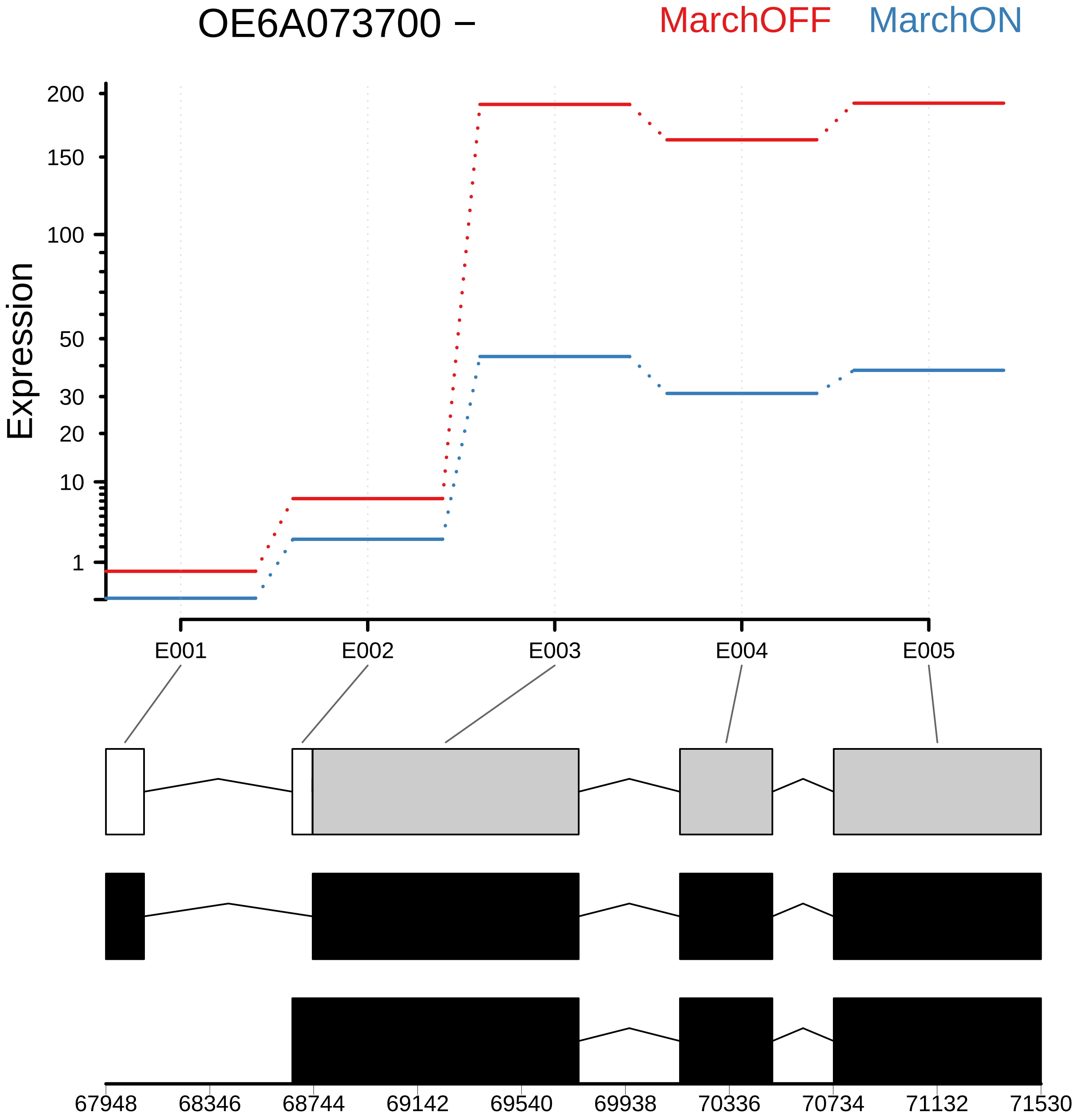

OE6A073700 –
MarchOFF
MarchON
Expression
200
150
100
50
30
20
10
1
E001
E002
E003
E004
E005
67948
68346
68744
69142
69540
69938
70336
70734
71132
71530

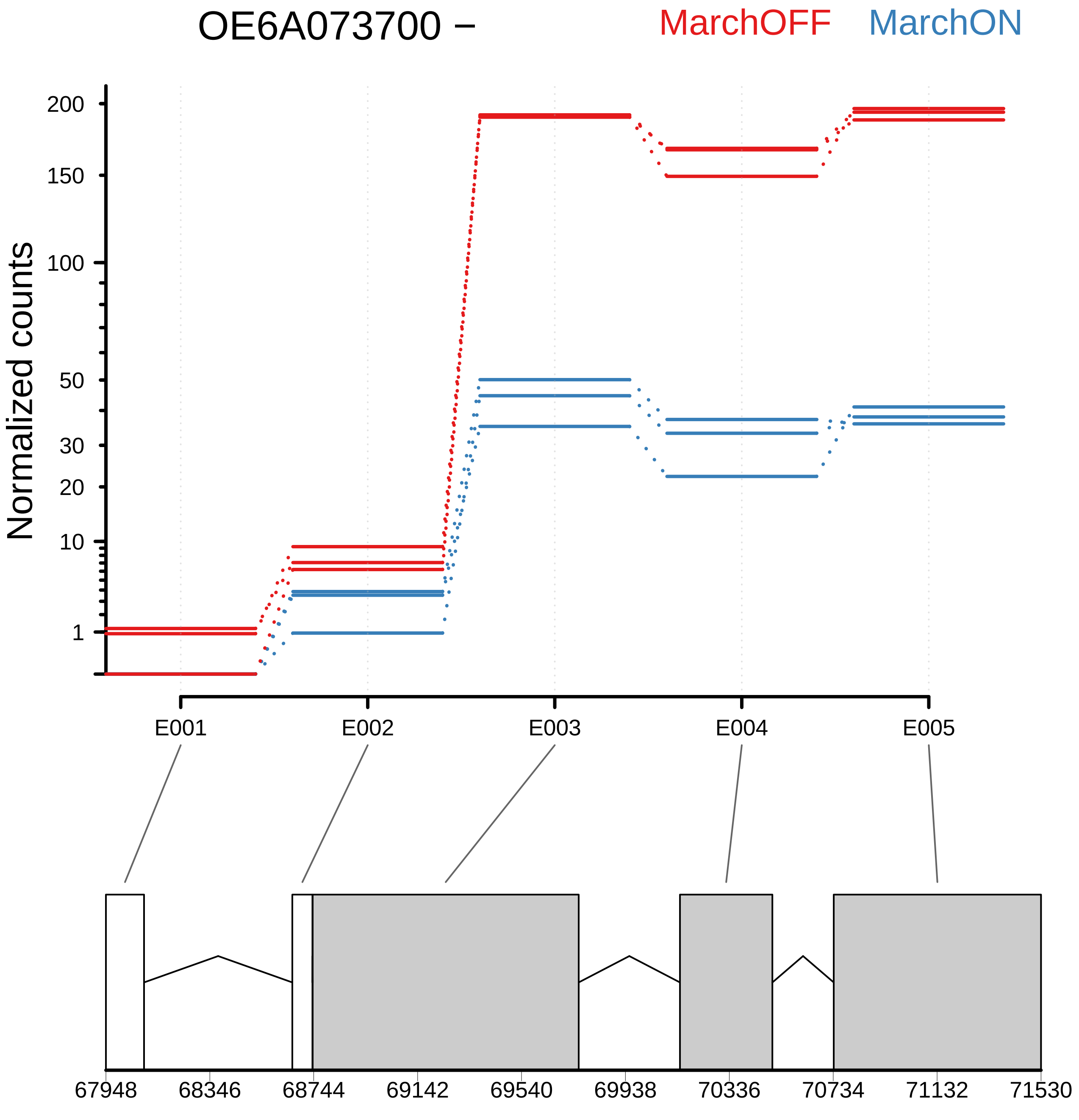

OE6A073700 −
MarchOFF
MarchON
Normalized counts
200
150
100
50
30
20
10
1
E001
E002
E003
E004
E005
67948
68346
68744
69142
69540
69938
70336
70734
71132
71530

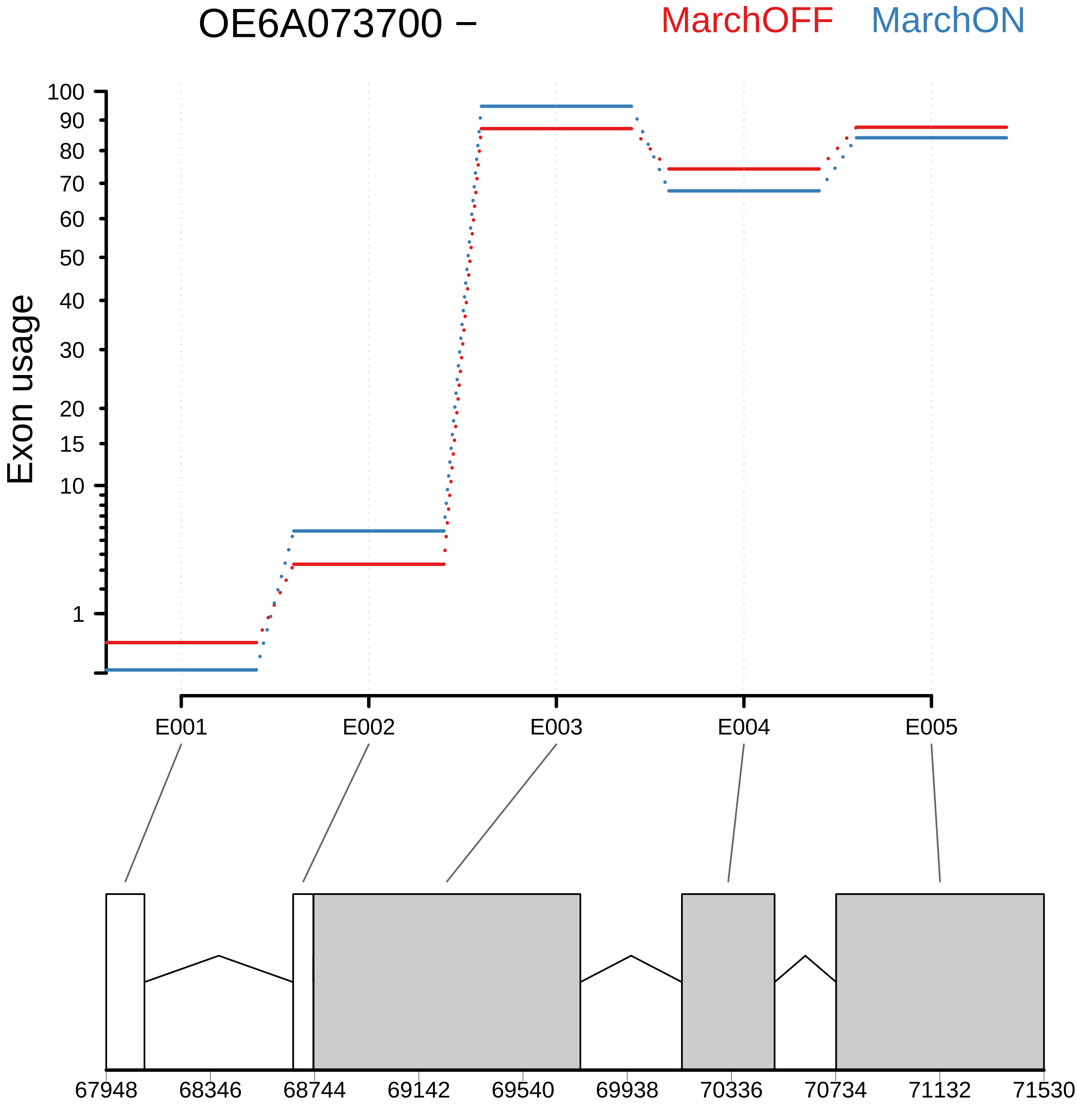

OE6A073700 −
MarchOFF
MarchON
Exon usage
100
90
80
70
60
50
40
30
20
15
10
1
E001
E002
E003
E004
E005
67948
68346
68744
69142
69540
69938
70336
70734
71132
71530

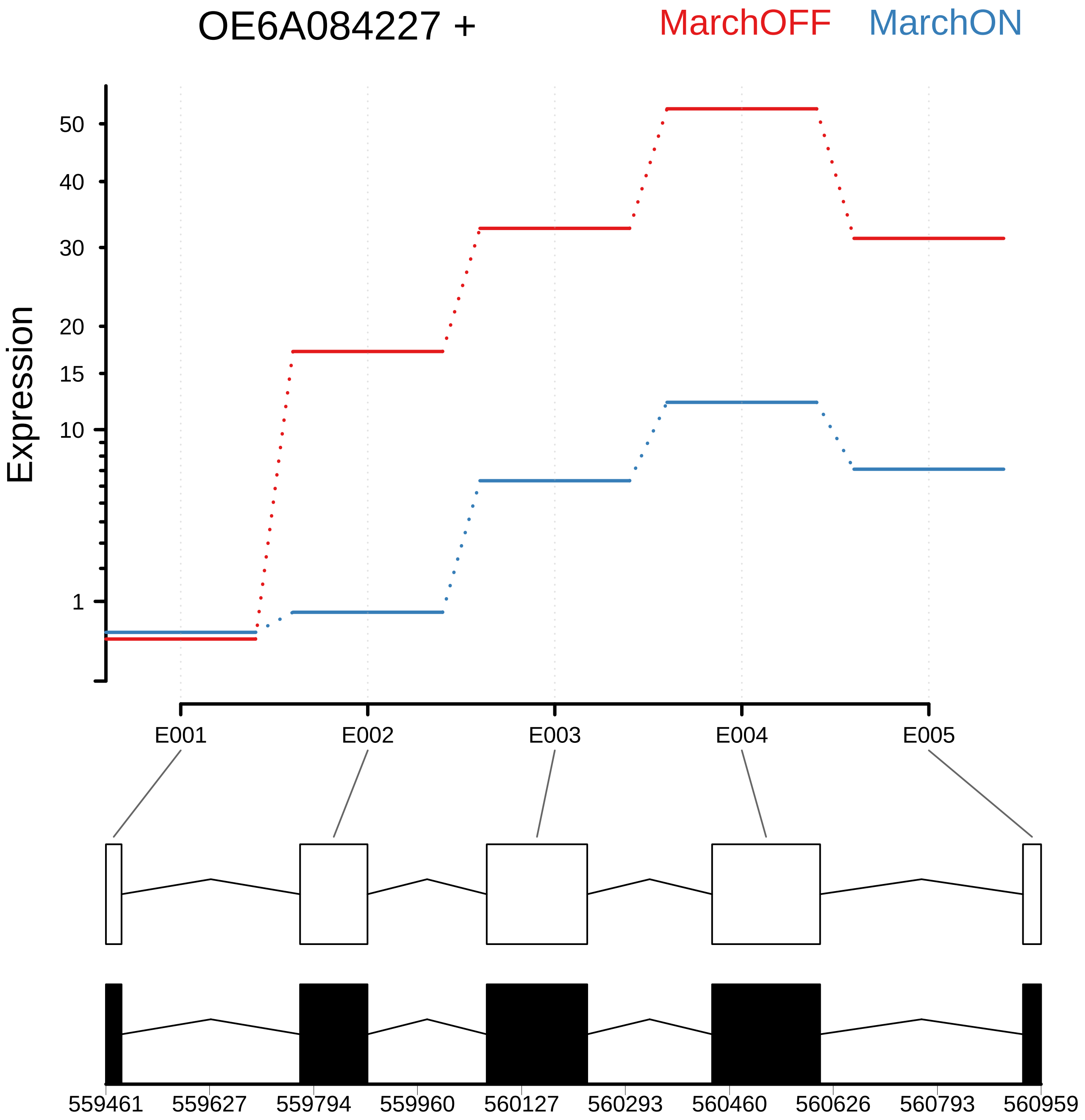
OE6A084227 +
MarchOFF
MarchON
Expression
50
40
30
20
15
10
1
E001
E002
E003
E004
E005
559461
559627
559794
559960
560127
560293
560460
560626
560793
560959

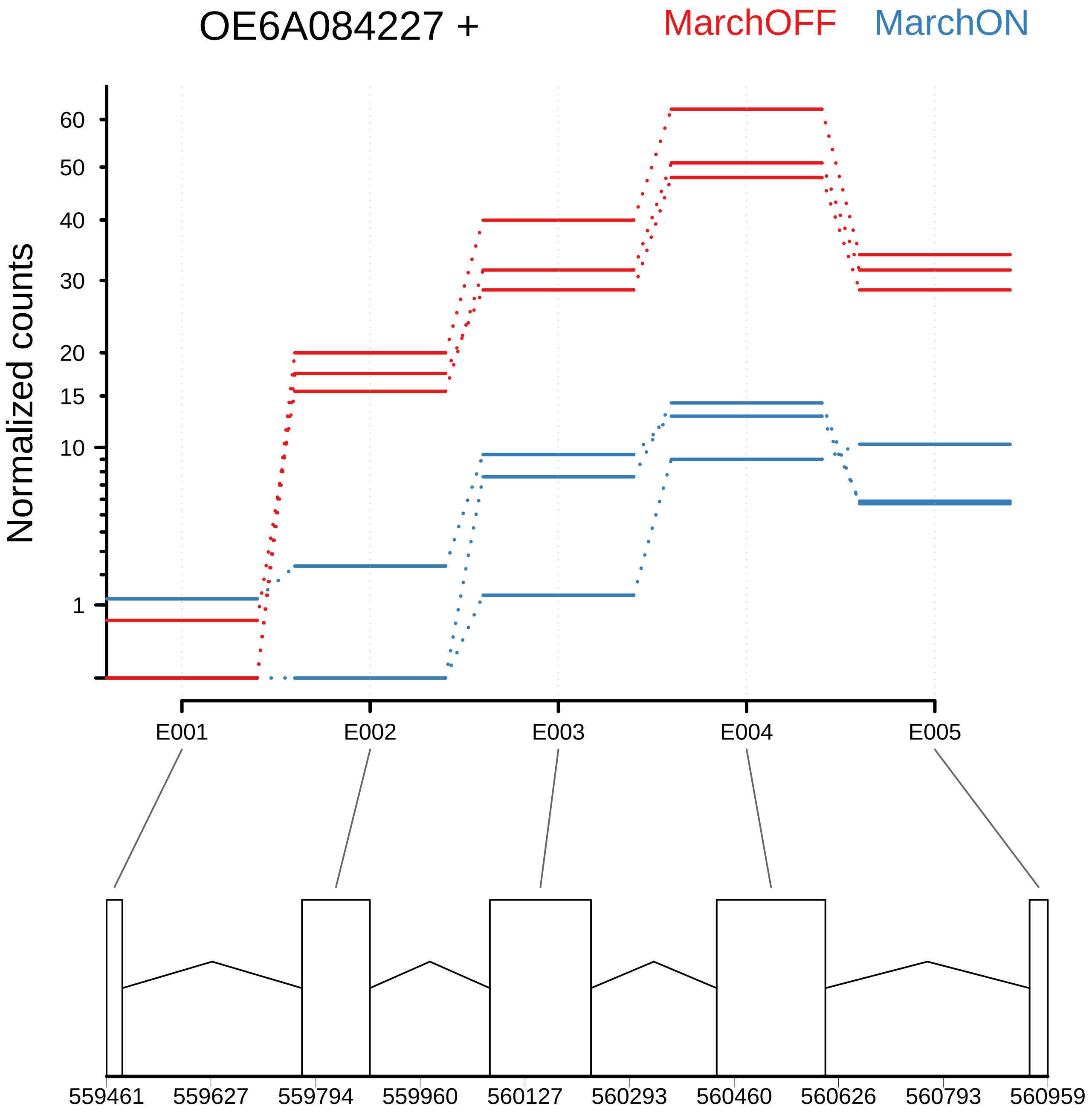
OE6A084227 +
MarchOFF
MarchON
Normalized counts
60
50
40
30
20
15
10
1
E001
E002
E003
E004
E005
559461
559627
559794
559960
560127
560293
560460
560626
560793
560959

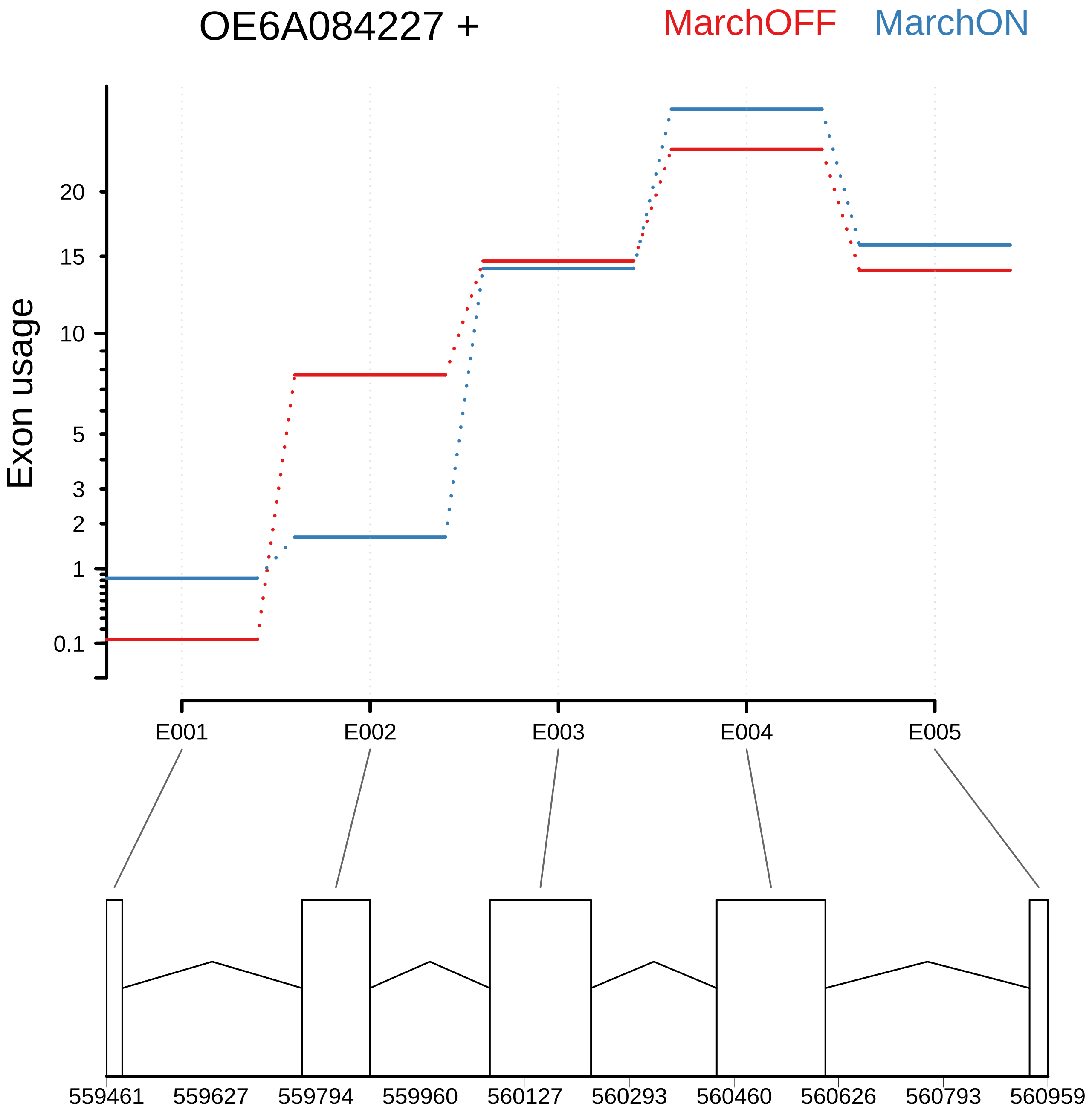
OE6A084227 +
MarchOFF
MarchON
Exon usage
20
15
10
5
3
2
1
0.1
E001
E002
E003
E004
E005
559461
559627
559794
559960
560127
560293
560460
560626
560793
560959

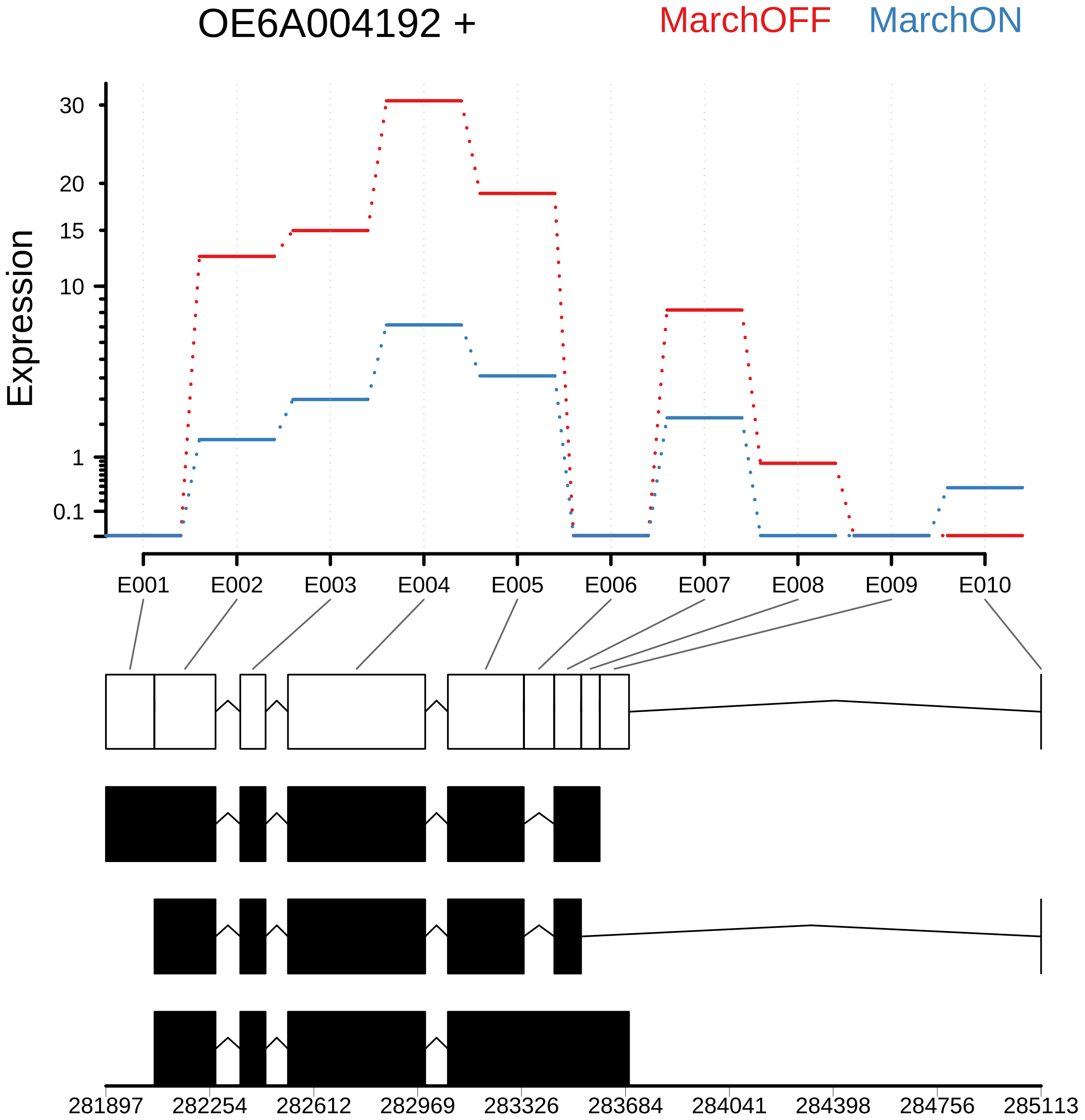

OE6A004192 +
MarchOFF
MarchON
Expression
30
20
15
10
1
0.1
E001
E002
E003
E004
E005
E006
E007
E008
E009
E010
281897
282254
282612
282969
283326
283684
284041
284398
284756
285113

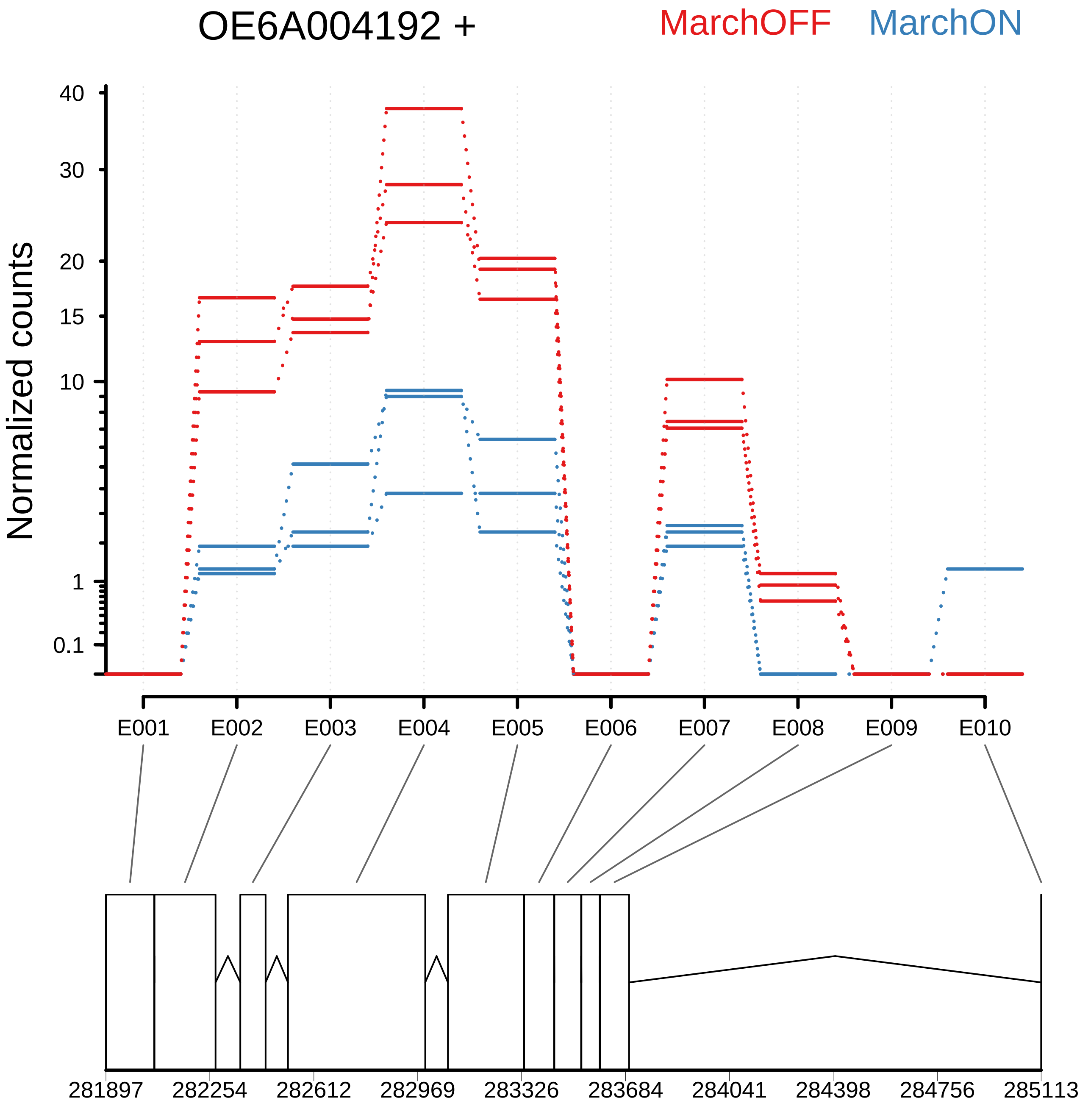
OE6A004192 +
MarchOFF
MarchON
Normalized counts
40
30
20
15
10
1
0.1
E001
E002
E003
E004
E005
E006
E007
E008
E009
E010
281897
282254
282612
282969
283326
283684
284041
284398
284756
285113

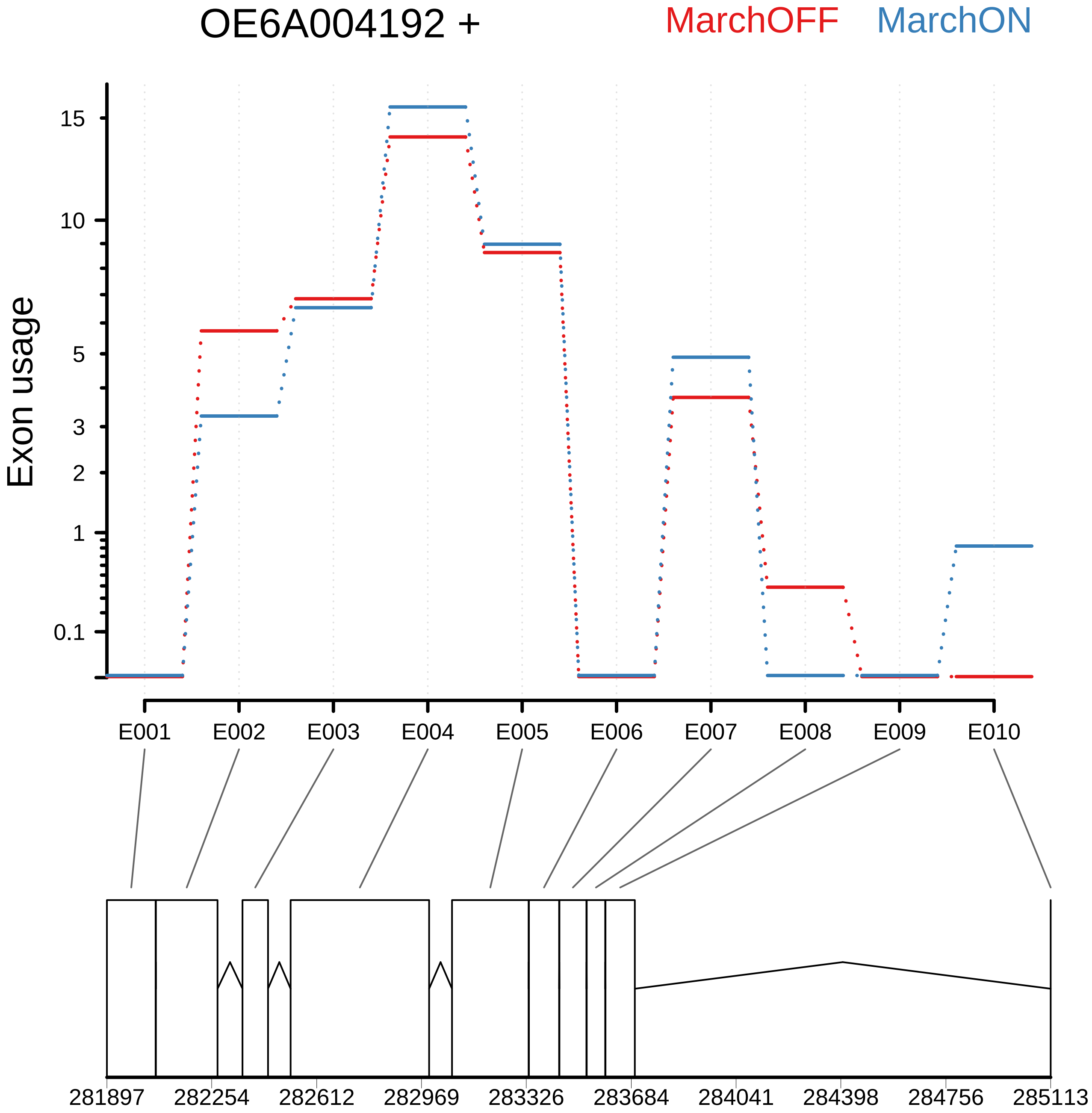

OE6A004192 +
MarchOFF
MarchON
Exon usage
15
10
5
3
2
1
0.1
E001
E002
E003
E004
E005
E006
E007
E008
E009
E010
281897
282254
282612
282969
283326
283684
284041
284398
284756
285113

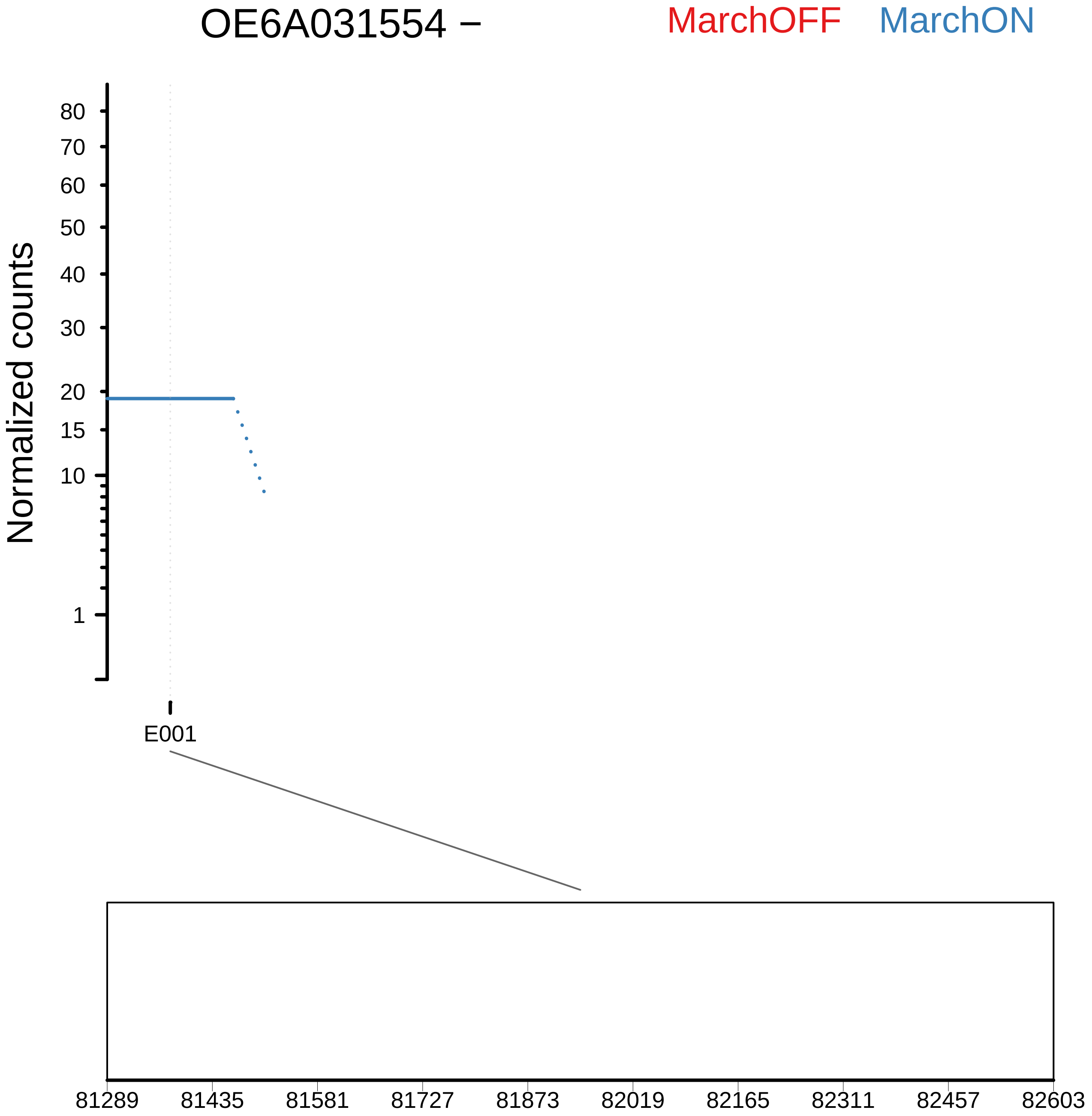

OE6A031554 −
MarchOFF
MarchON
Normalized counts
80
70
60
50
40
30
20
15
10
1
E001
81289
81435
81581
81727
81873
82019
82165
82311
82457
82603

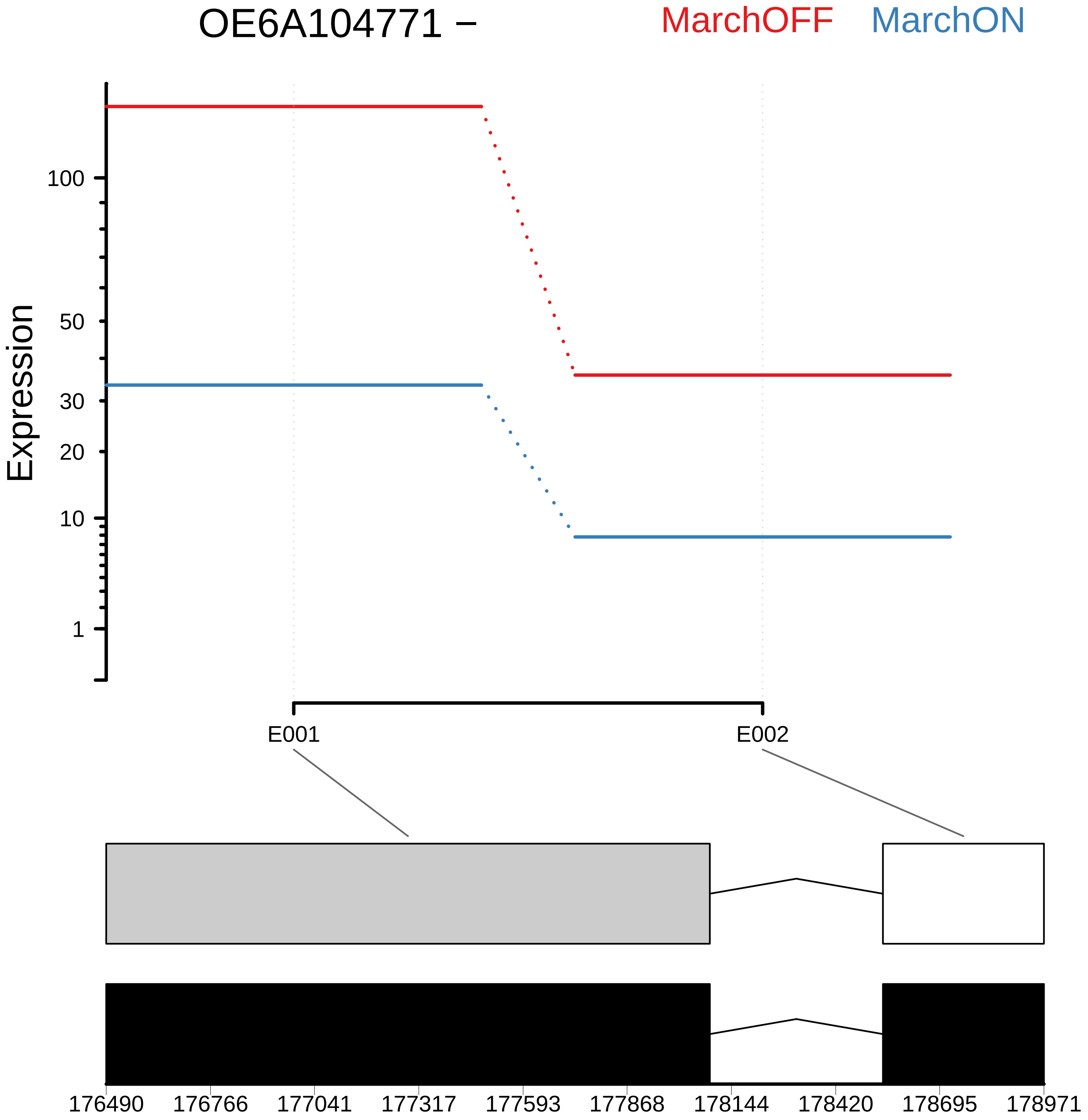
OE6A104771 −
MarchOFF
MarchON
Expression
100
50
30
20
10
1
E001
E002
176490
176766
177041
177317
177593
177868
178144
178420
178695
178971

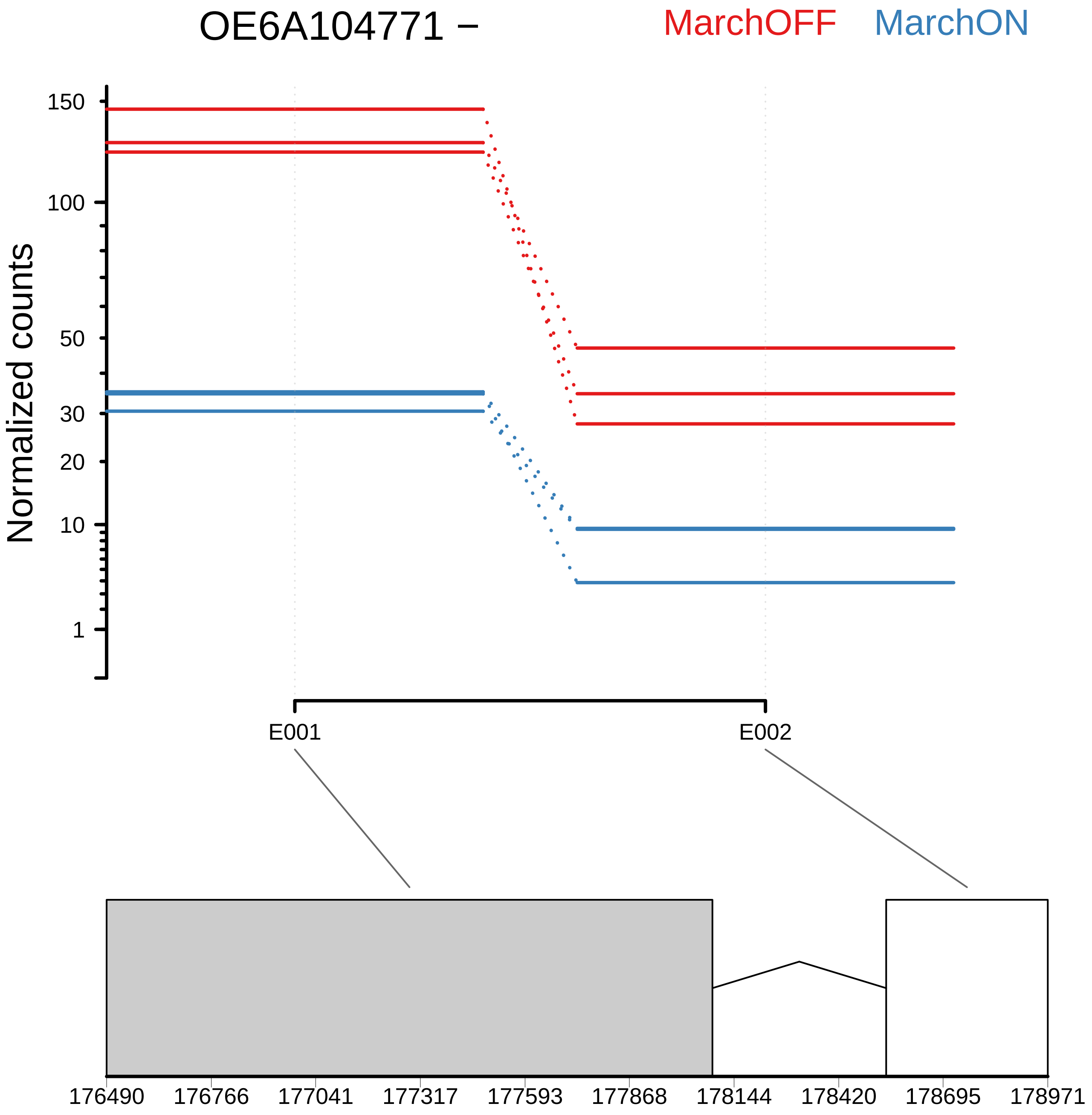

OE6A104771 −
MarchOFF
MarchON
Normalized counts
150
100
50
30
20
10
1
E001
E002
176490
176766
177041
177317
177593
177868
178144
178420
178695
178971

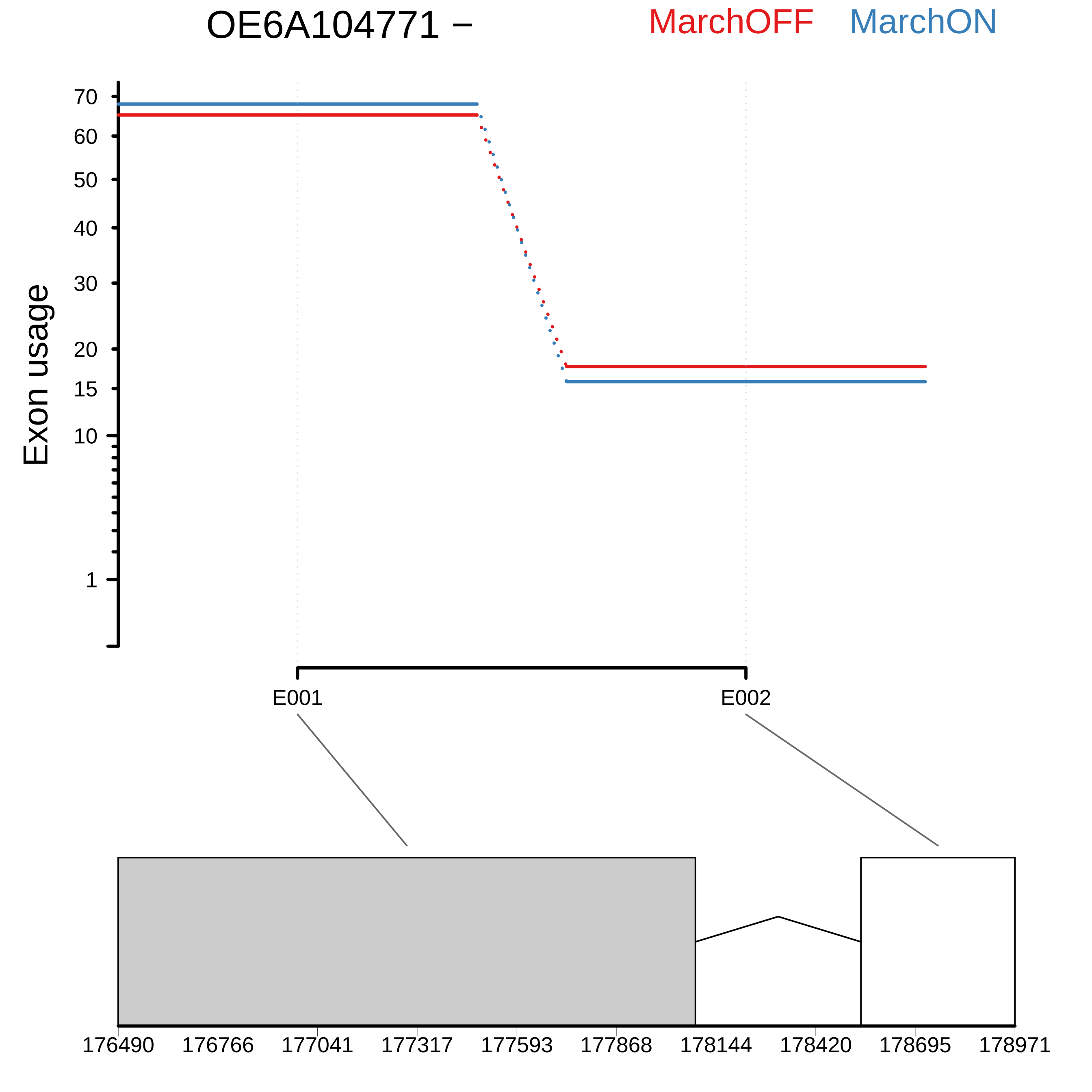

OE6A104771 −
MarchOFF
MarchON
Exon usage
70
60
50
40
30
20
15
10
1
E001
E002
176490
176766
177041
177317
177593
177868
178144
178420
178695
178971

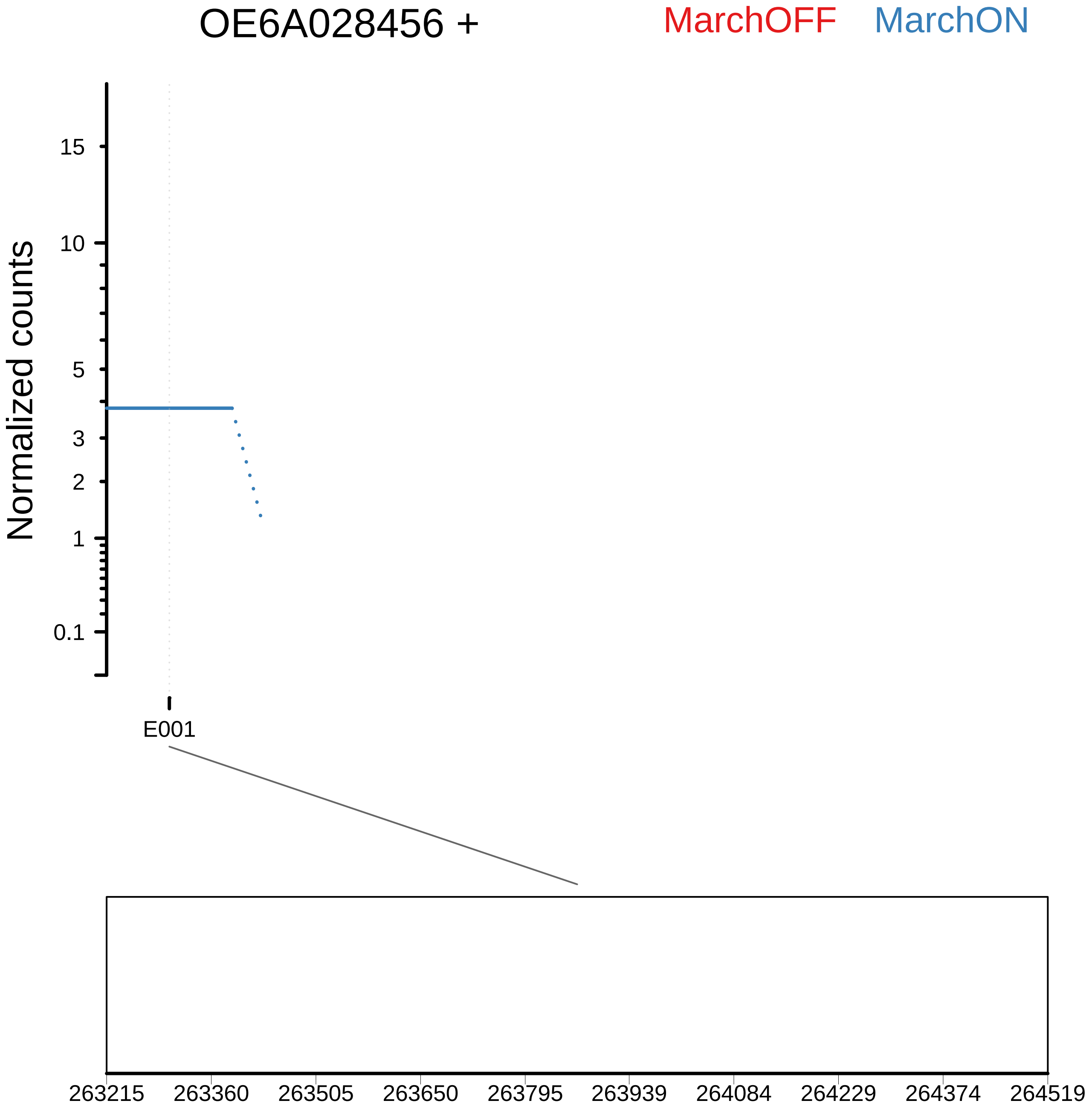
OE6A028456 +
MarchOFF
MarchON
Normalized counts
15
10
5
3
2
1
0.1
E001
263215
263360
263505
263650
263795
263939
264084
264229
264374
264519

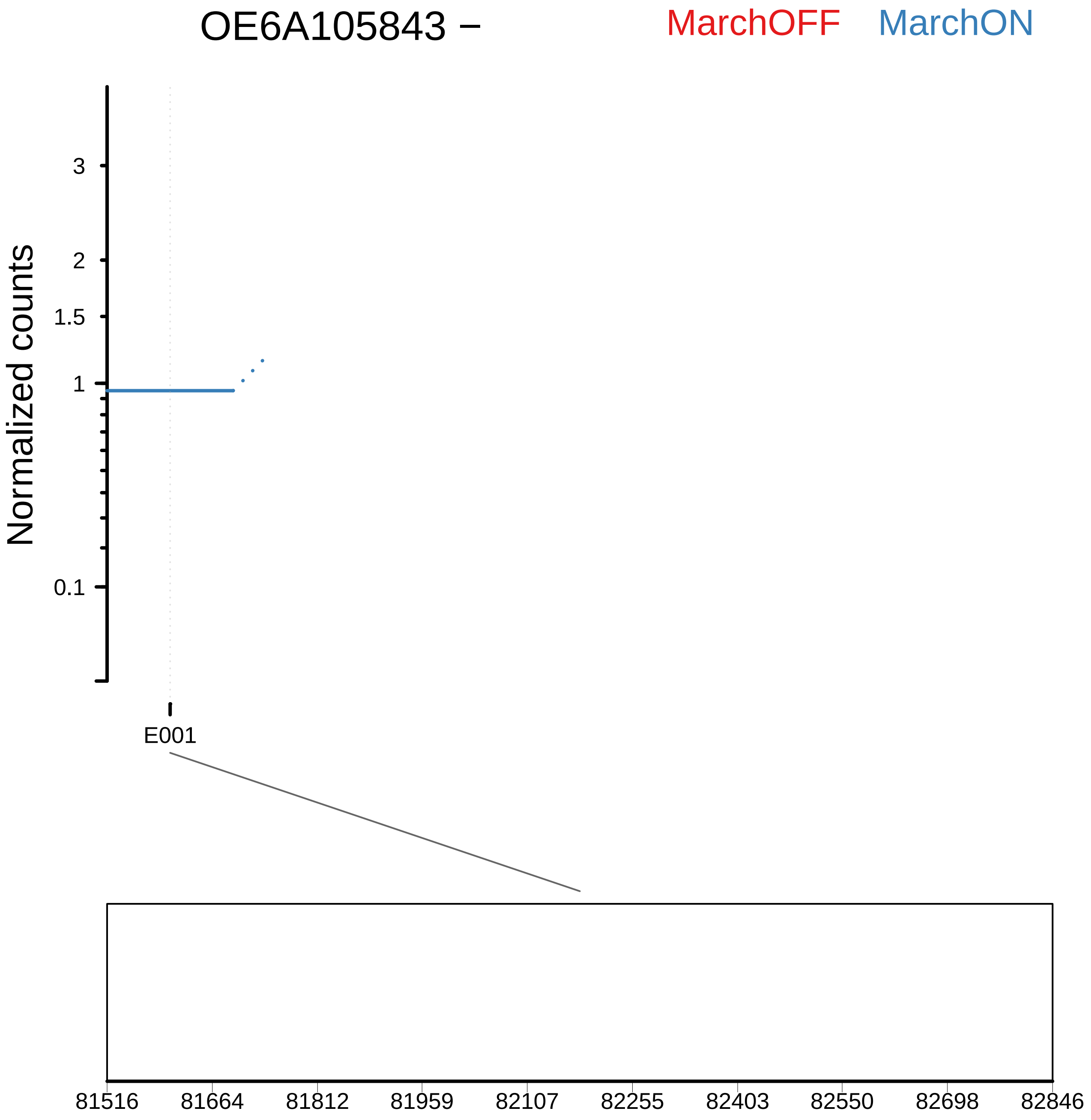

OE6A105843 −
MarchOFF
MarchON
Normalized counts
3
2
1.5
1
0.1
E001
81516
81664
81812
81959
82107
82255
82403
82550
82698
82846

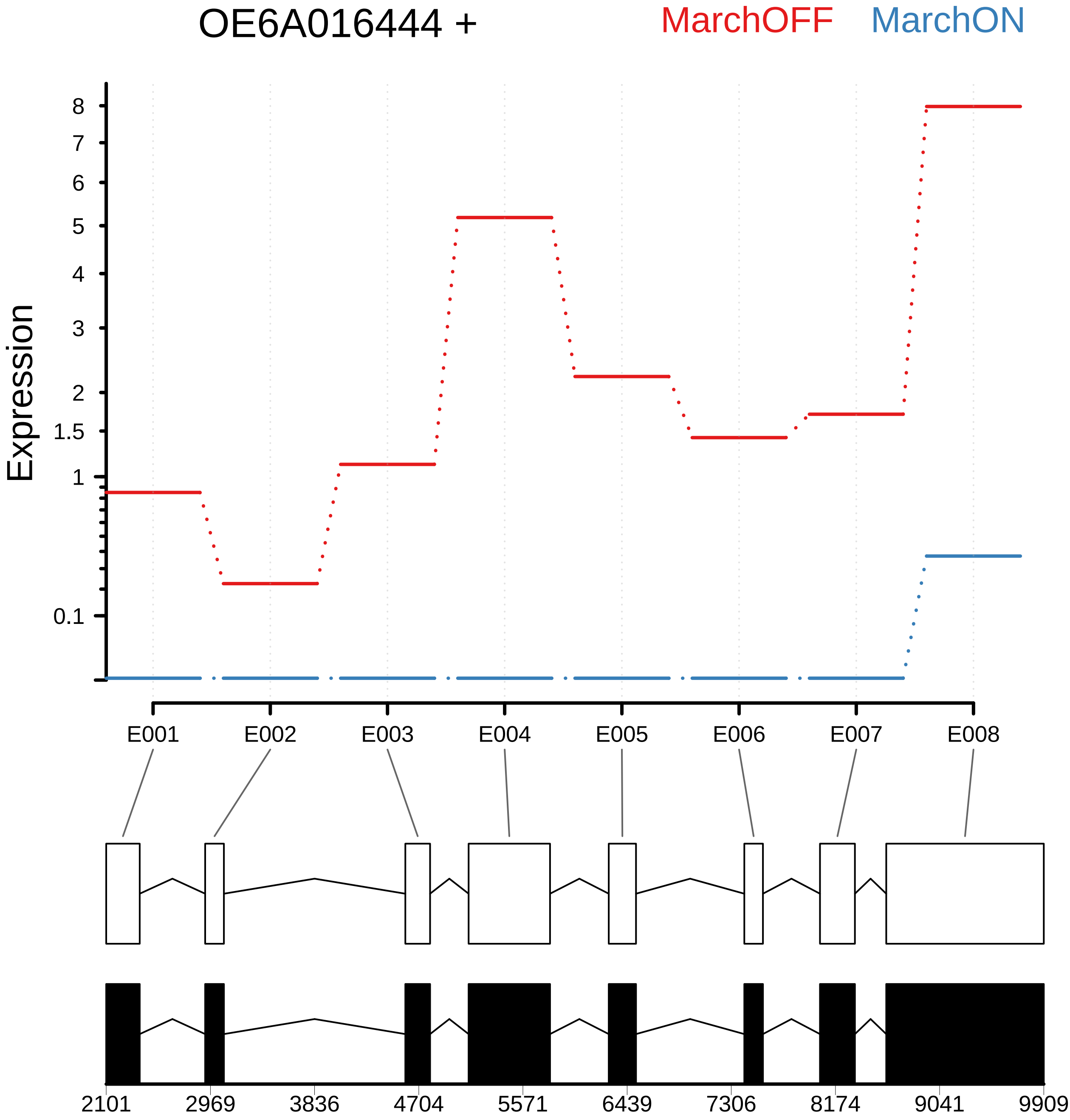
OE6A016444 +
MarchOFF
MarchON
Expression
8
7
6
5
4
3
2
1.5
1
0.1
E001
E002
E003
E004
E005
E006
E007
E008
2101
2969
3836
4704
5571
6439
7306
8174
9041
9909

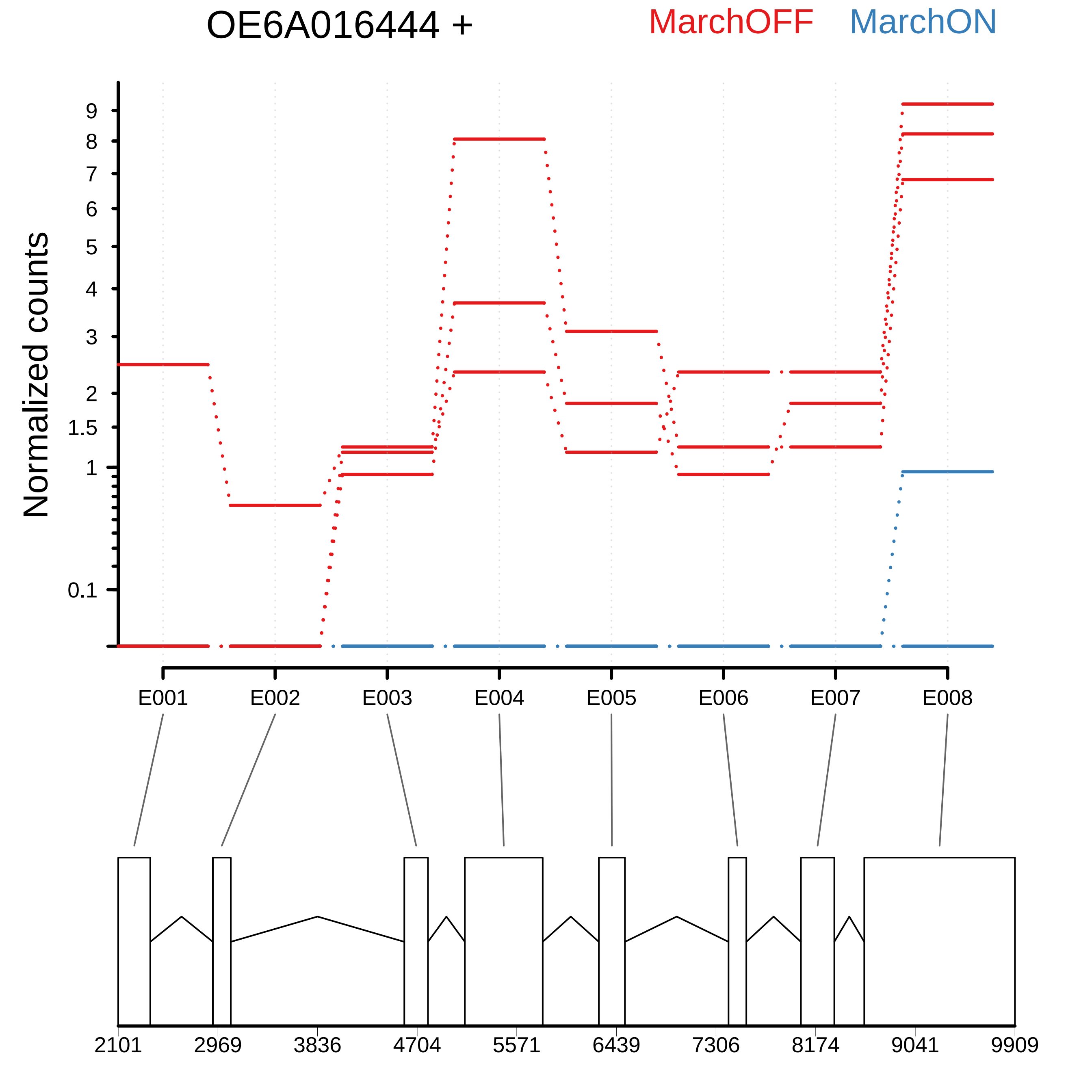

OE6A016444 +
MarchOFF
MarchON
Normalized counts
9
8
7
6
5
4
3
2
1.5
1
0.1
E001
E002
E003
E004
E005
E006
E007
E008
2101
2969
3836
4704
5571
6439
7306
8174
9041
9909

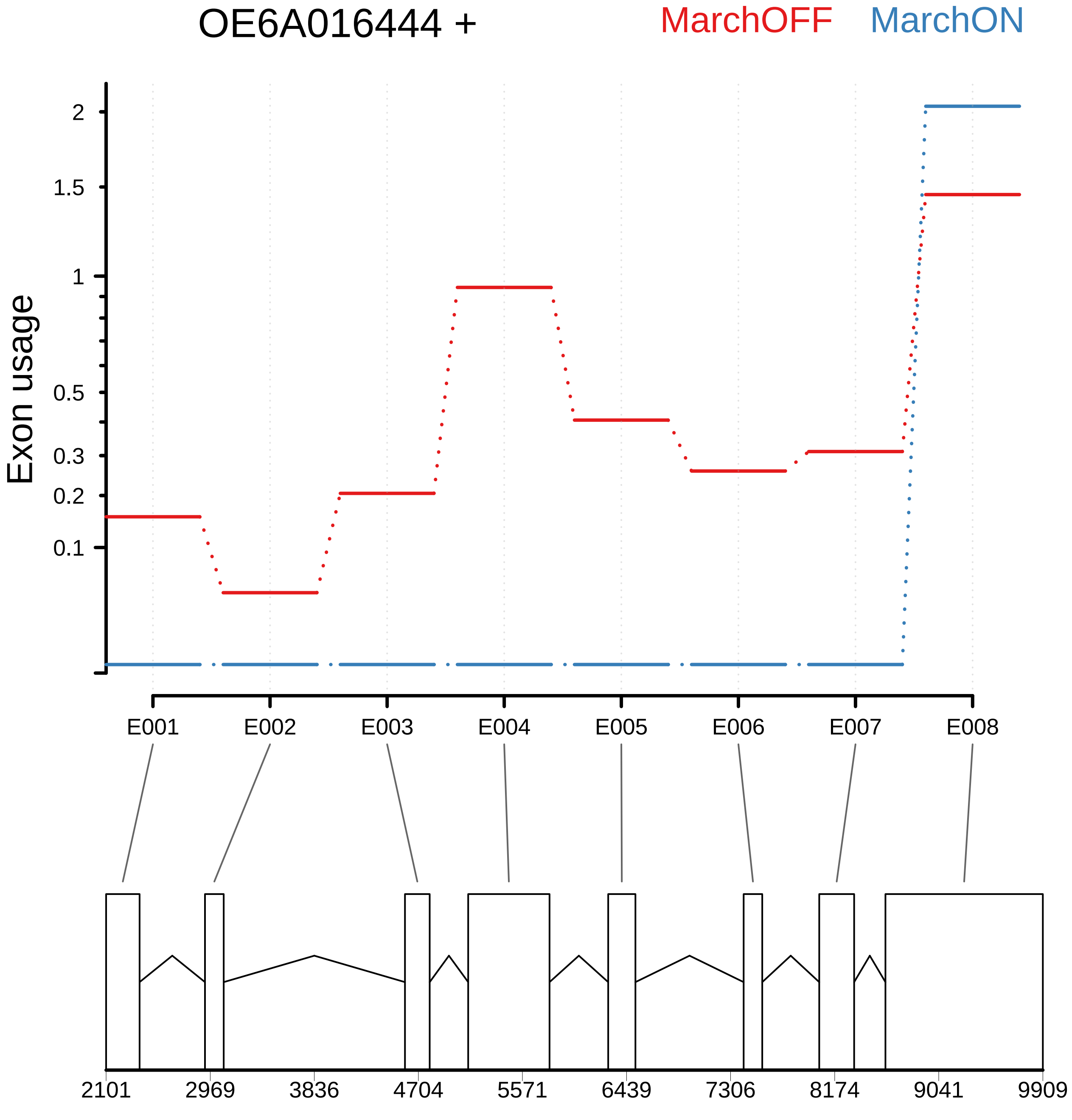
OE6A016444 +
MarchOFF
MarchON
Exon usage
2
1.5
1
0.5
0.3
0.2
0.1
E001
E002
E003
E004
E005
E006
E007
E008
2101
2969
3836
4704
5571
6439
7306
8174
9041
9909

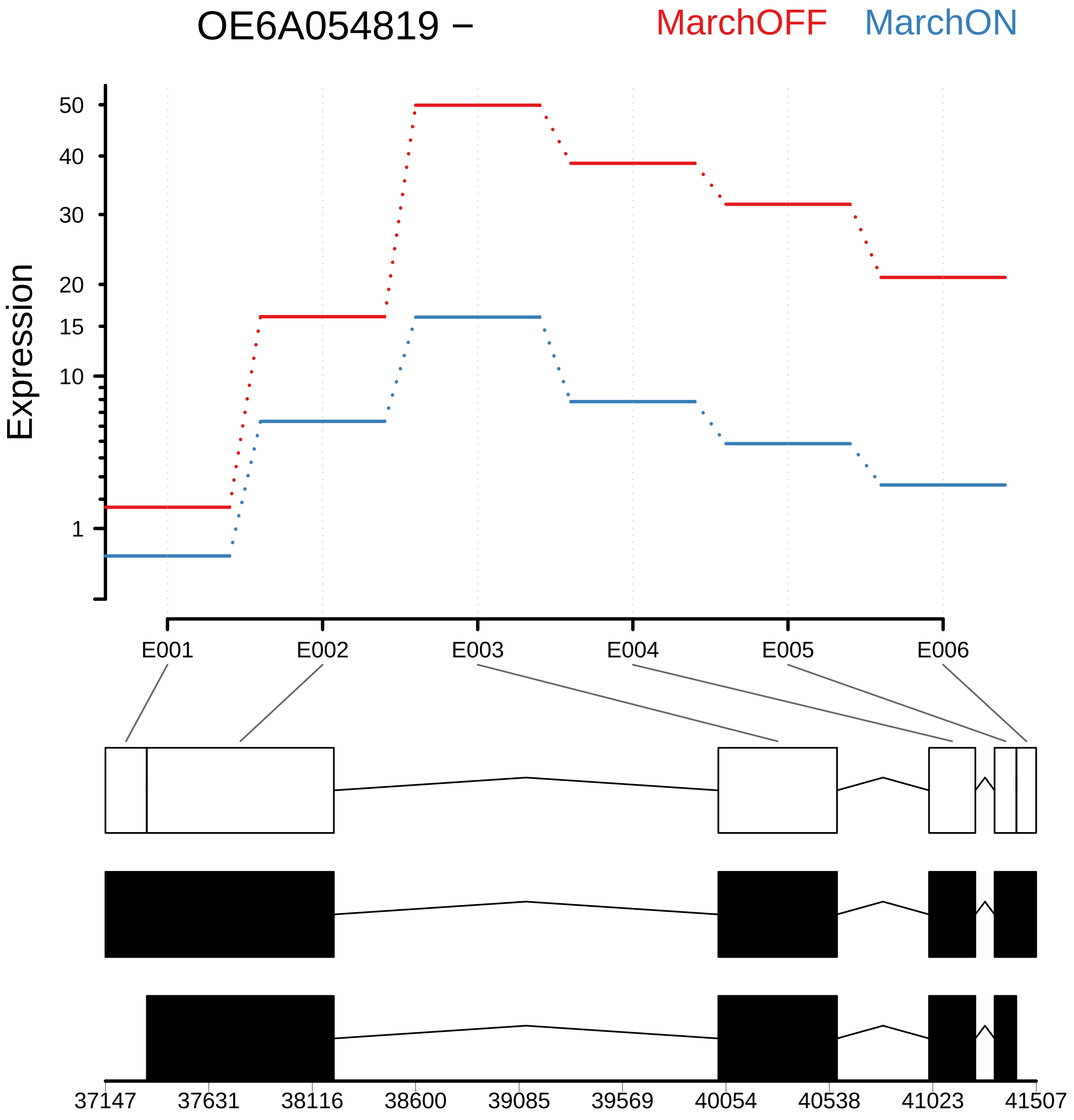

OE6A054819 –
MarchOFF
MarchON
Expression
50
40
30
20
15
10
1
E001
E002
E003
E004
E005
E006
37147
37631
38116
38600
39085
39569
40054
40538
41023
41507

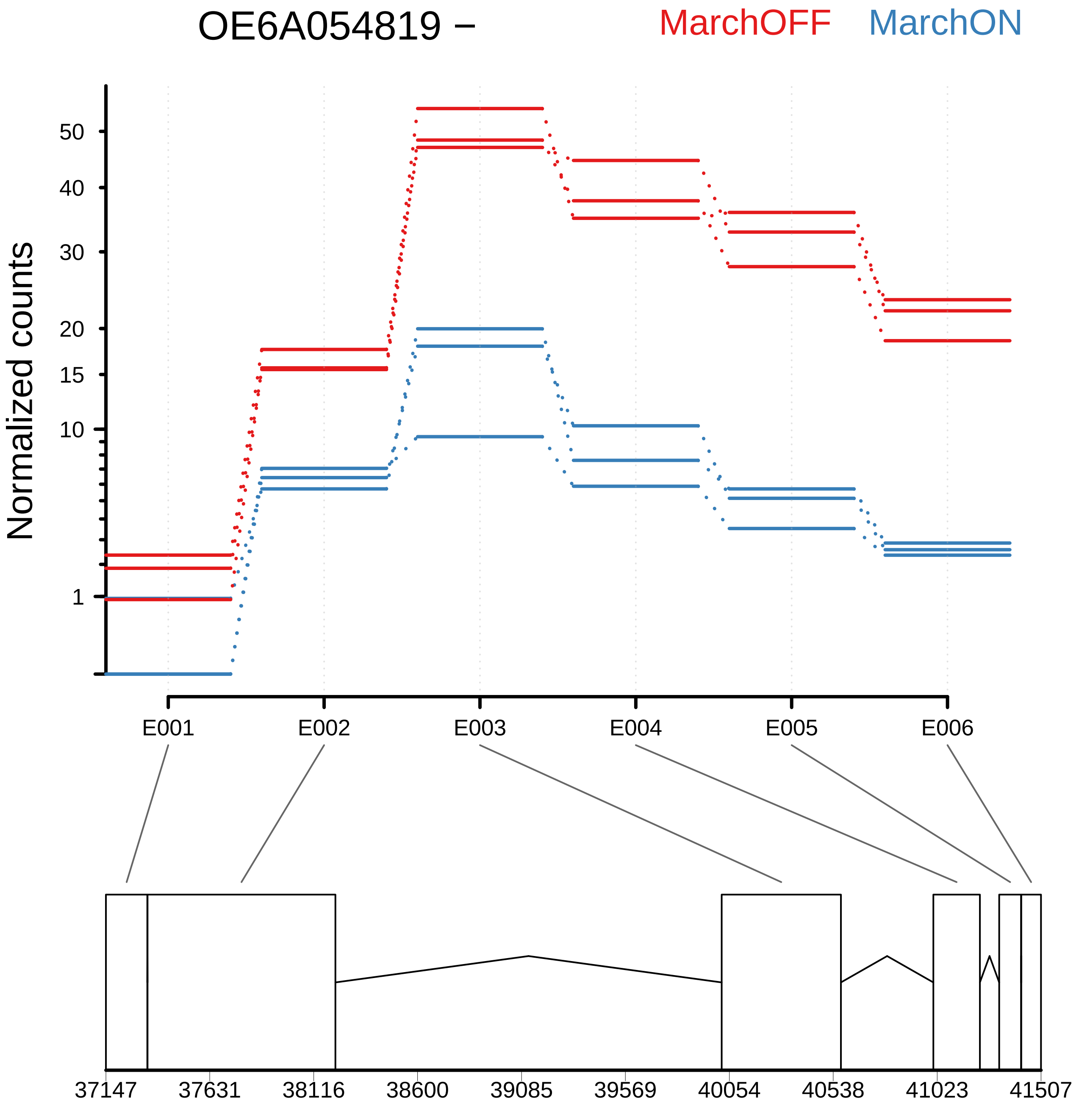

OE6A054819 –
MarchOFF
MarchON
Normalized counts
50
40
30
20
15
10
1
E001
E002
E003
E004
E005
E006
37147
37631
38116
38600
39085
39569
40054
40538
41023
41507

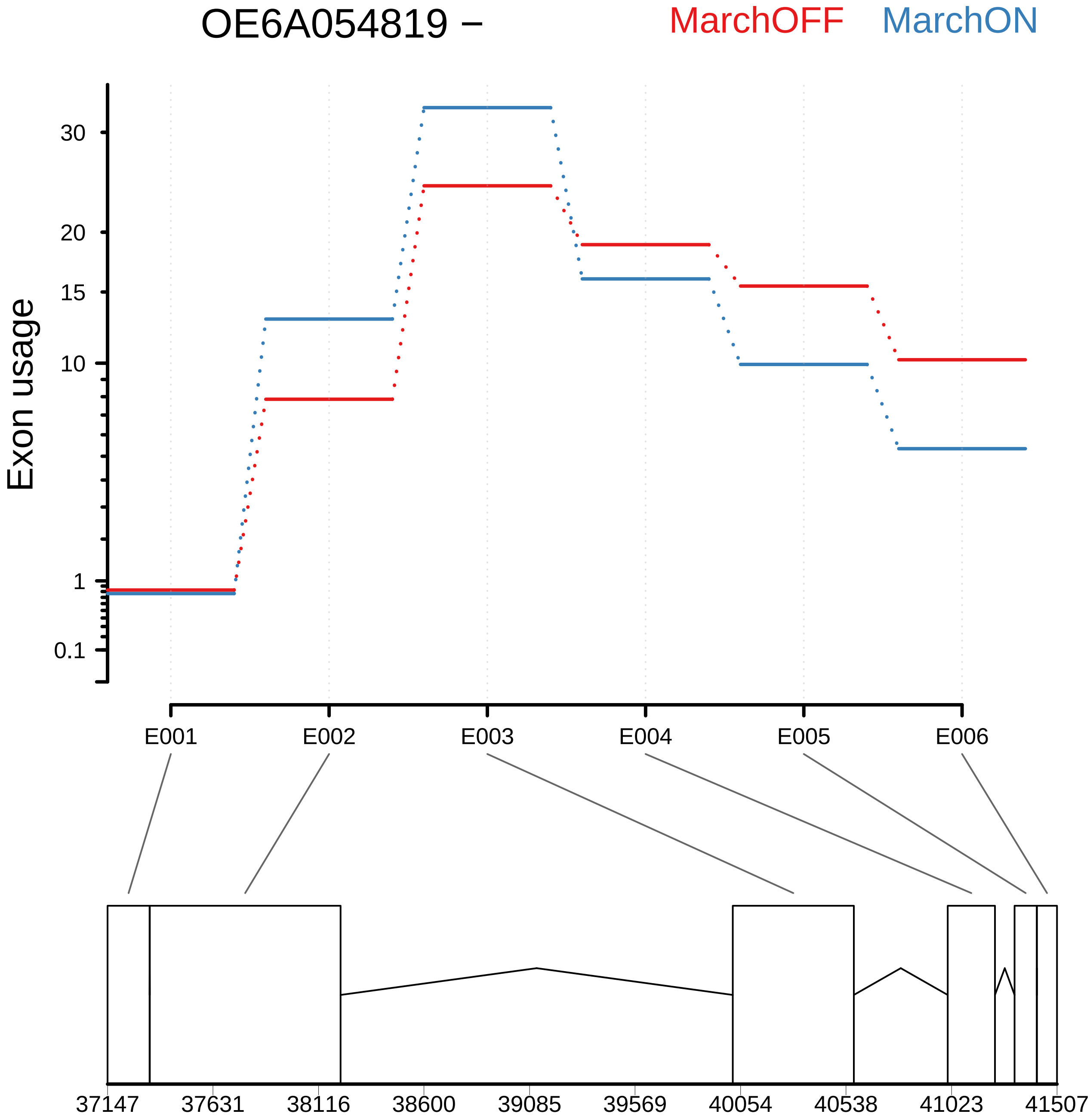

OE6A054819 −
MarchOFF
MarchON
Exon usage
30
20
15
10
1
0.1
E001
E002
E003
E004
E005
E006
37147
37631
38116
38600
39085
39569
40054
40538
41023
41507

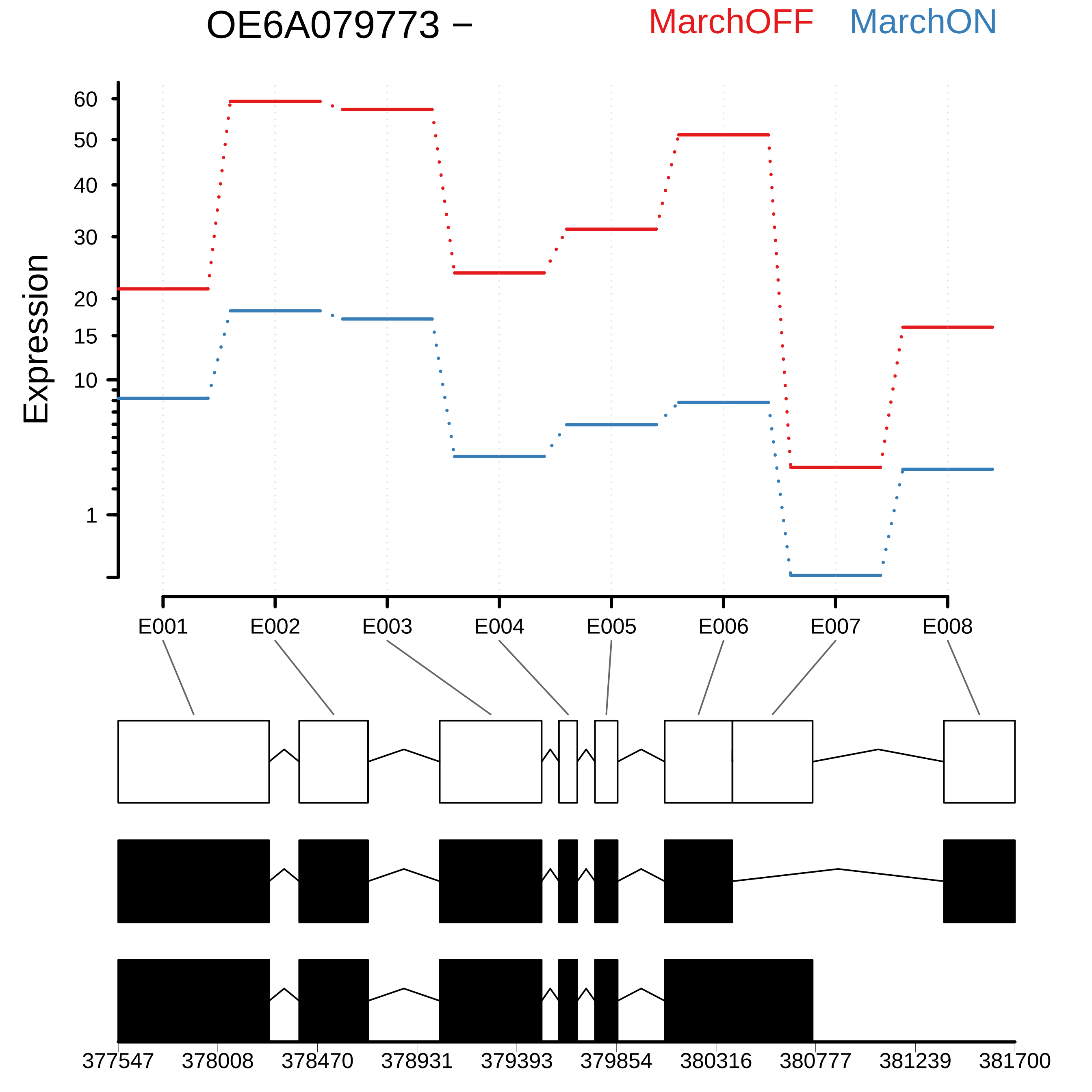
OE6A079773 −
MarchOFF
MarchON
Expression
60
50
40
30
20
15
10
1
E001
E002
E003
E004
E005
E006
E007
E008
377547
378008
378470
378931
379393
379854
380316
380777
381239
381700

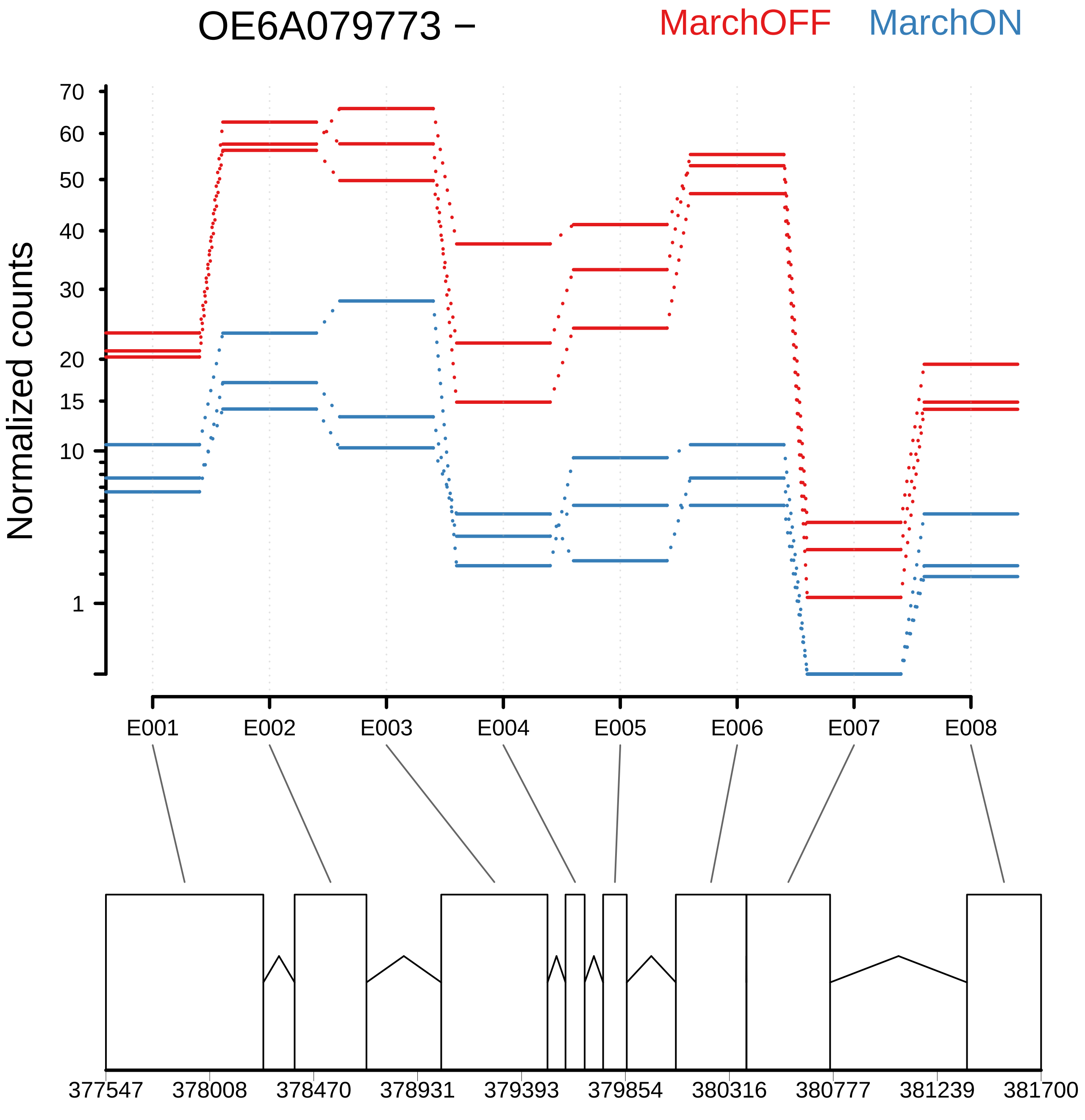

OE6A079773 −
MarchOFF
MarchON
Normalized counts
70
60
50
40
30
20
15
10
1
E001
E002
E003
E004
E005
E006
E007
E008
377547
378008
378470
378931
379393
379854
380316
380777
381239
381700

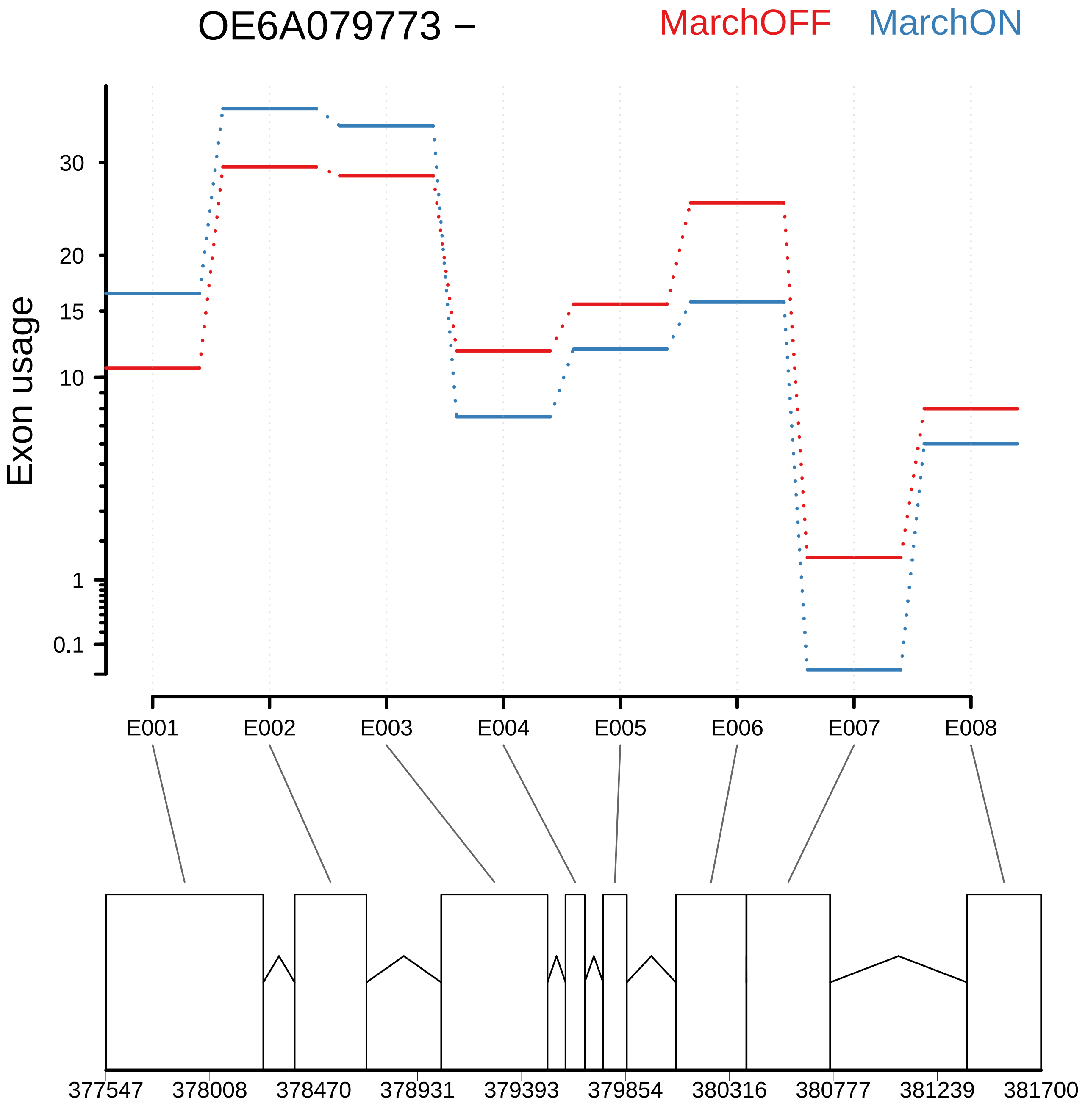

OE6A079773 −
MarchOFF
MarchON
Exon usage
30
20
15
10
1
0.1
E001
E002
E003
E004
E005
E006
E007
E008
377547
378008
378470
378931
379393
379854
380316
380777
381239
381700

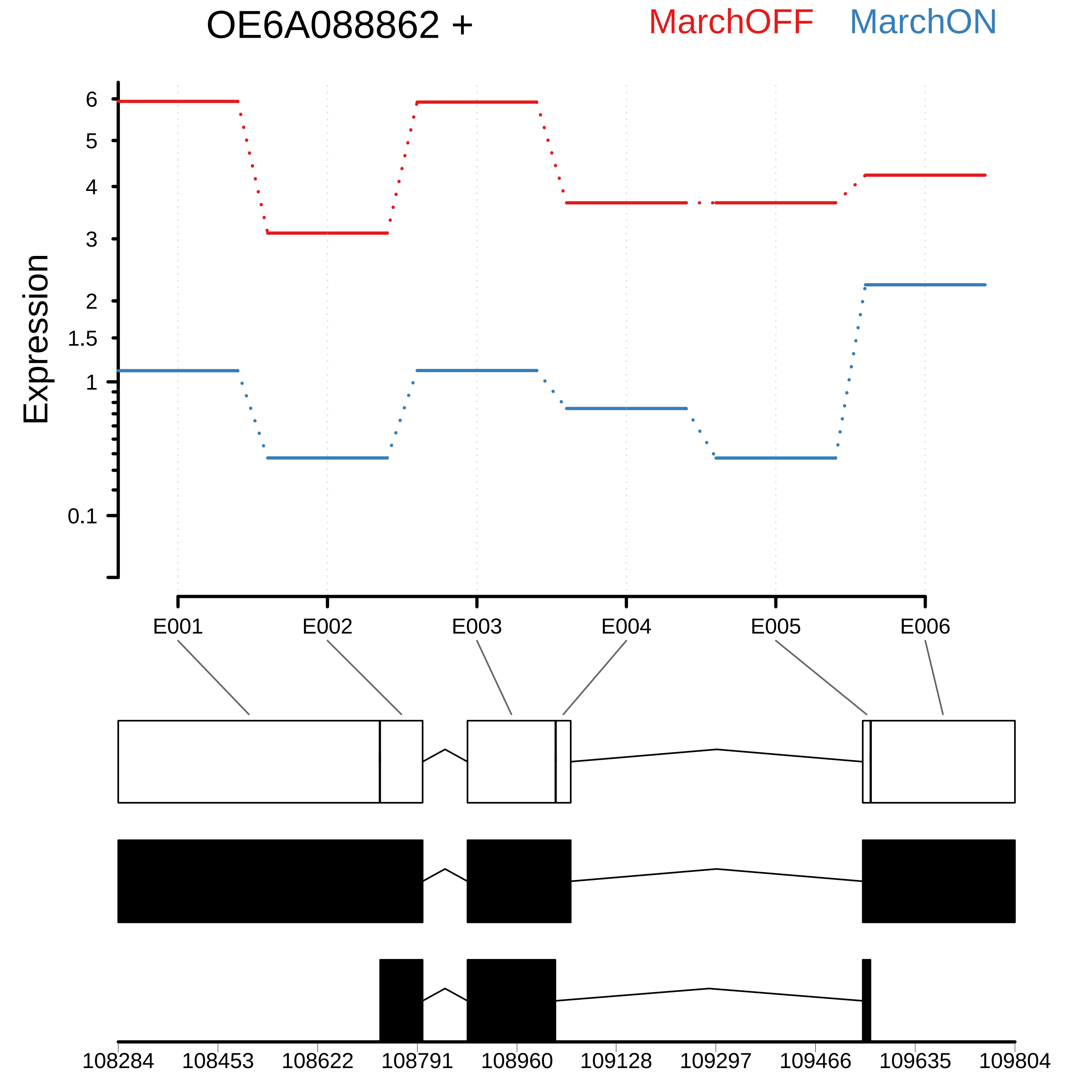

OE6A088862 +
MarchOFF
MarchON
Expression
6
5
4
3
2
1.5
1
0.1
E001
E002
E003
E004
E005
E006
108284
108453
108622
108791
108960
109128
109297
109466
109635
109804

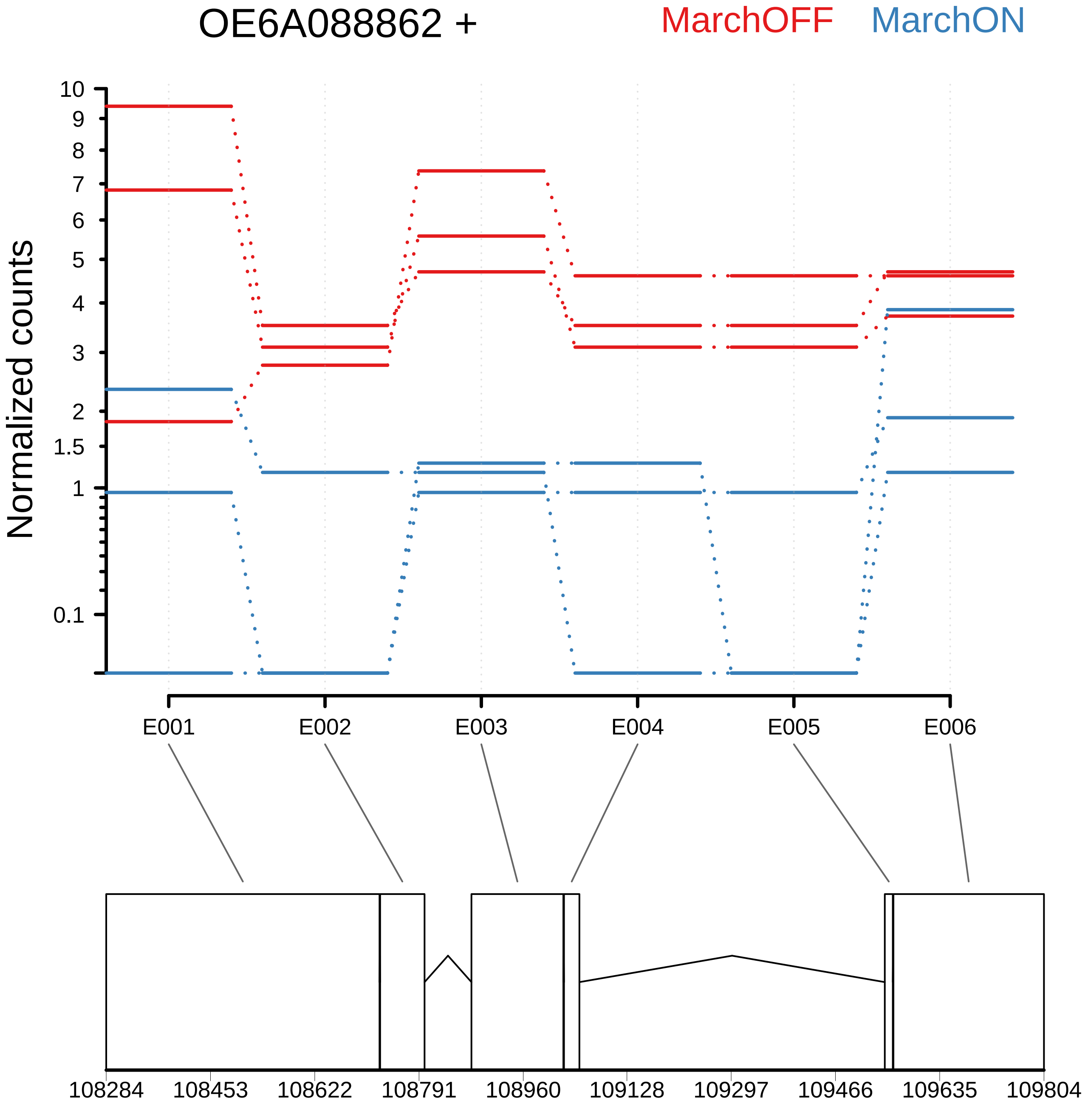
OE6A088862 +
MarchOFF
MarchON
Normalized counts
10
9
8
7
6
5
4
3
2
1.5
1
0.1
E001
E002
E003
E004
E005
E006
108284
108453
108622
108791
108960
109128
109297
109466
109635
109804

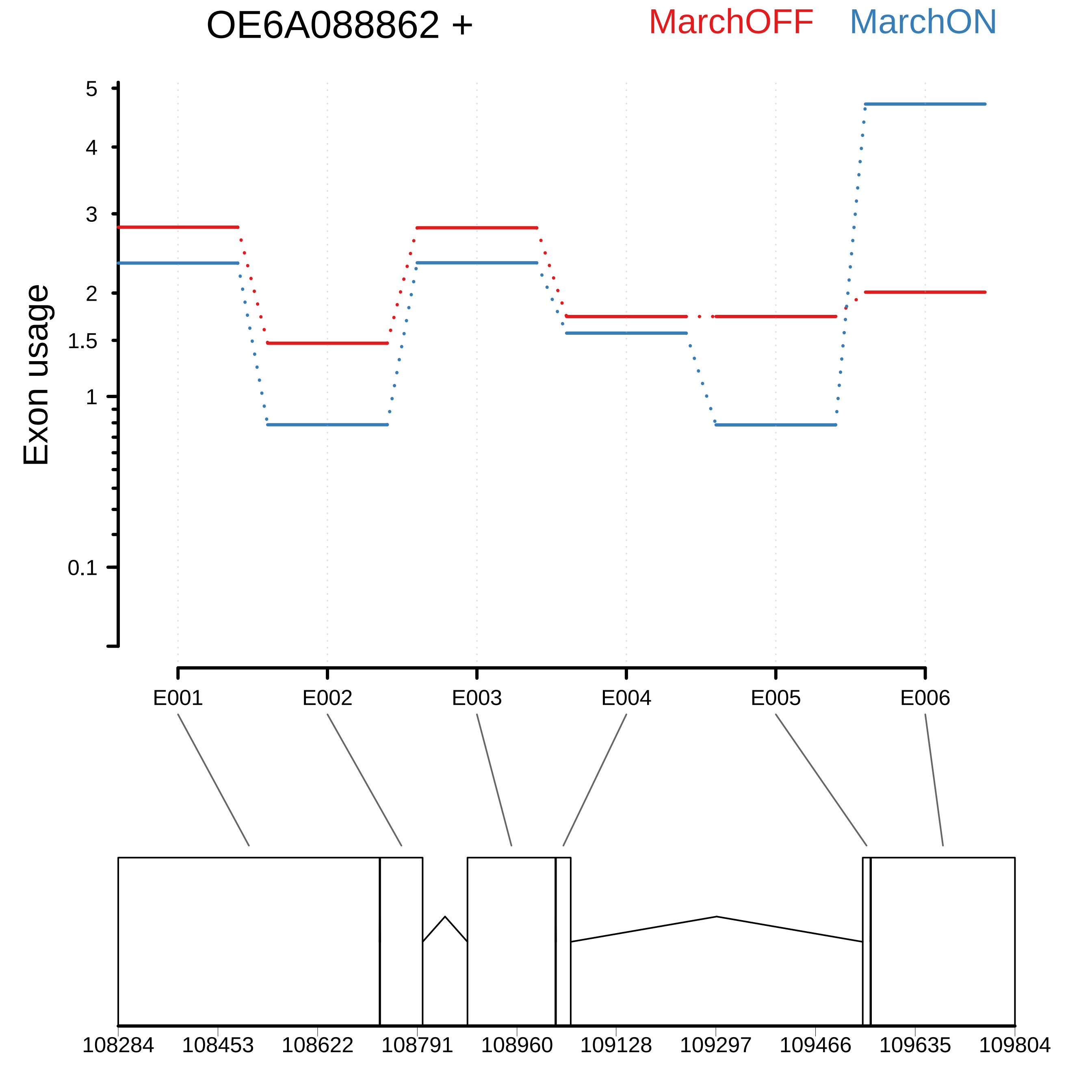

OE6A088862 +
MarchOFF
MarchON
Exon usage
5
4
3
2
1.5
1
0.1
E001
E002
E003
E004
E005
E006
108284
108453
108622
108791
108960
109128
109297
109466
109635
109804

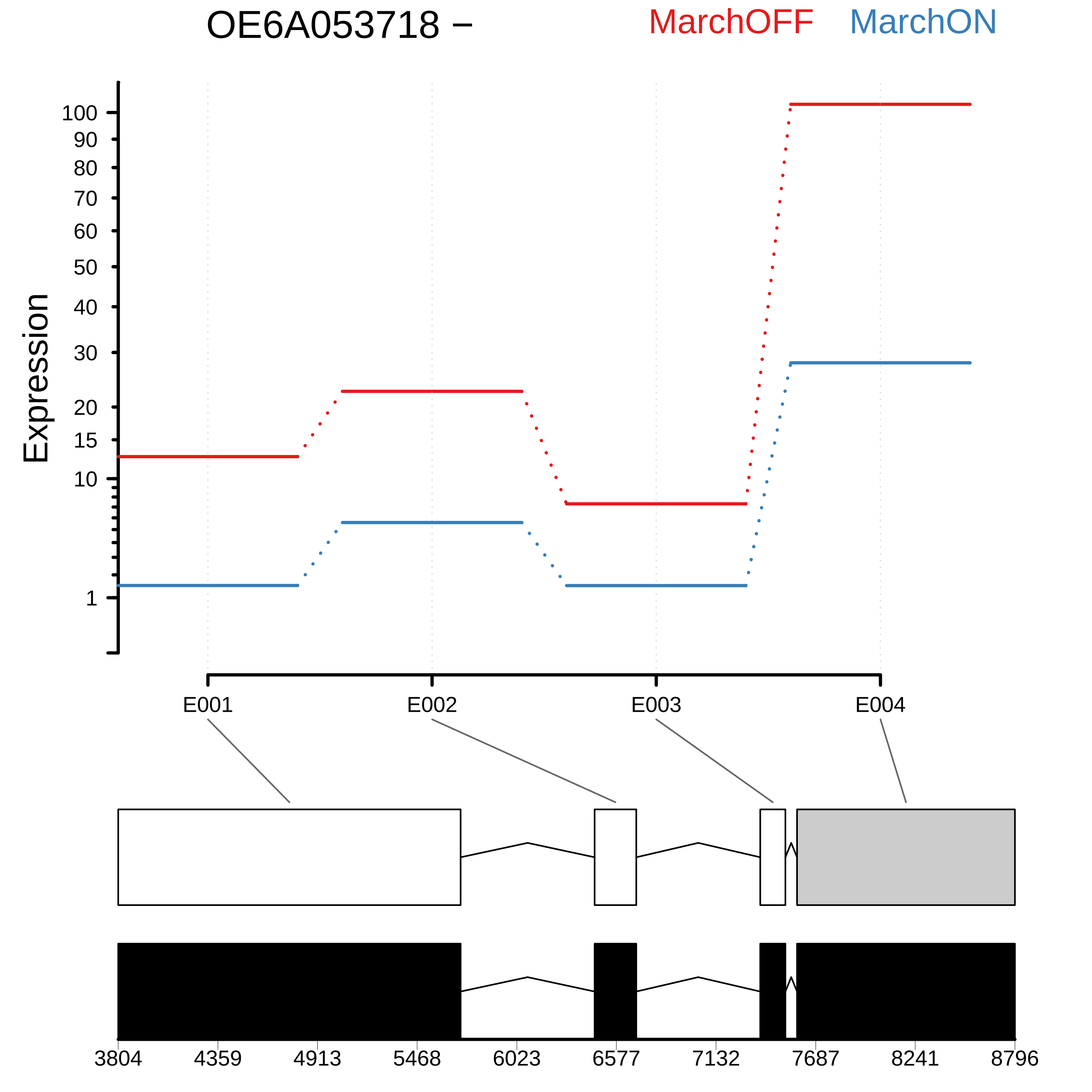

OE6A053718 −
MarchOFF
MarchON
Expression
100
90
80
70
60
50
40
30
20
15
10
1
E001
E002
E003
E004
3804
4359
4913
5468
6023
6577
7132
7687
8241
8796

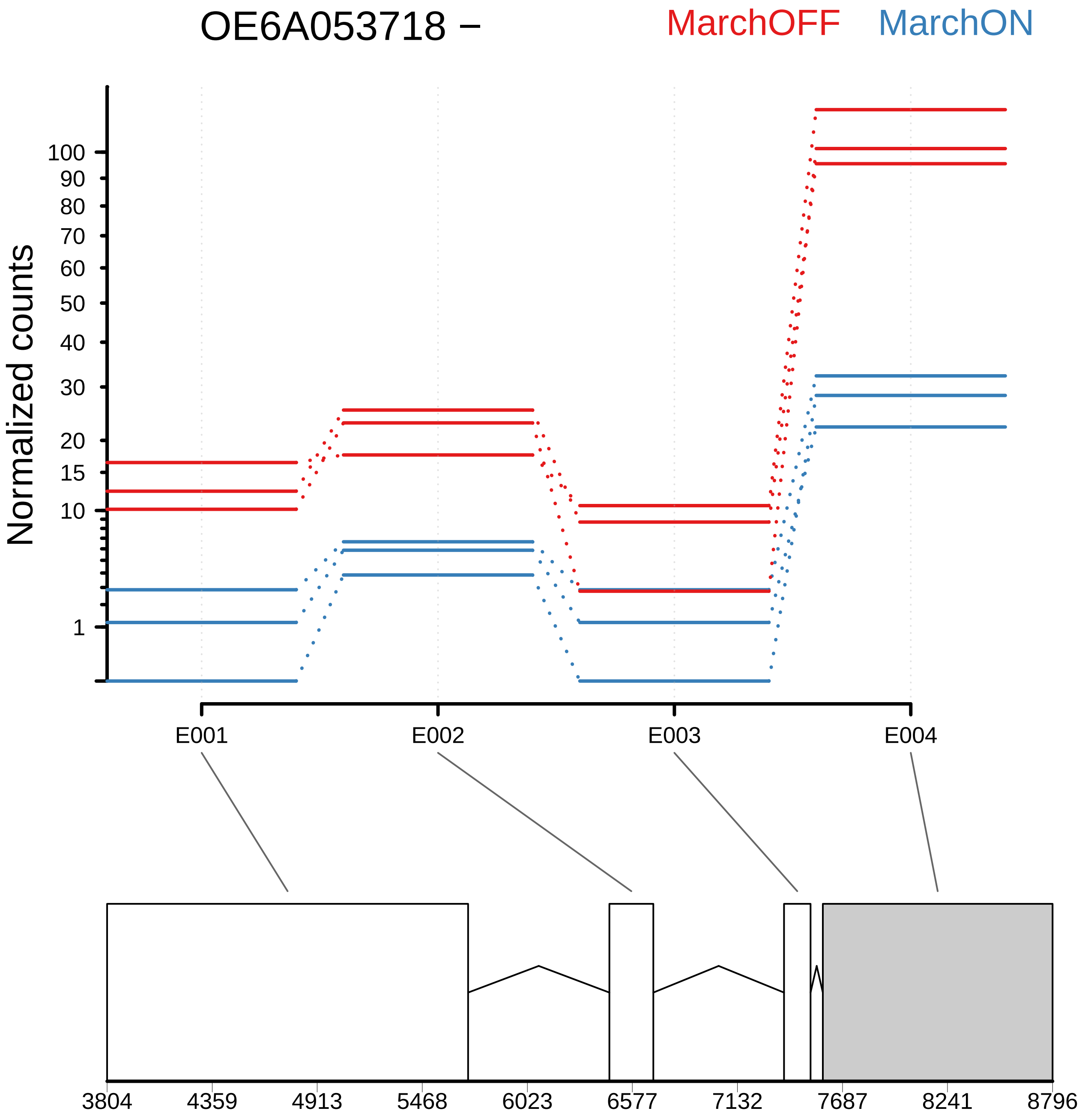

OE6A053718 −
MarchOFF
MarchON
Normalized counts
100
90
80
70
60
50
40
30
20
15
10
1
E001
E002
E003
E004
3804
4359
4913
5468
6023
6577
7132
7687
8241
8796

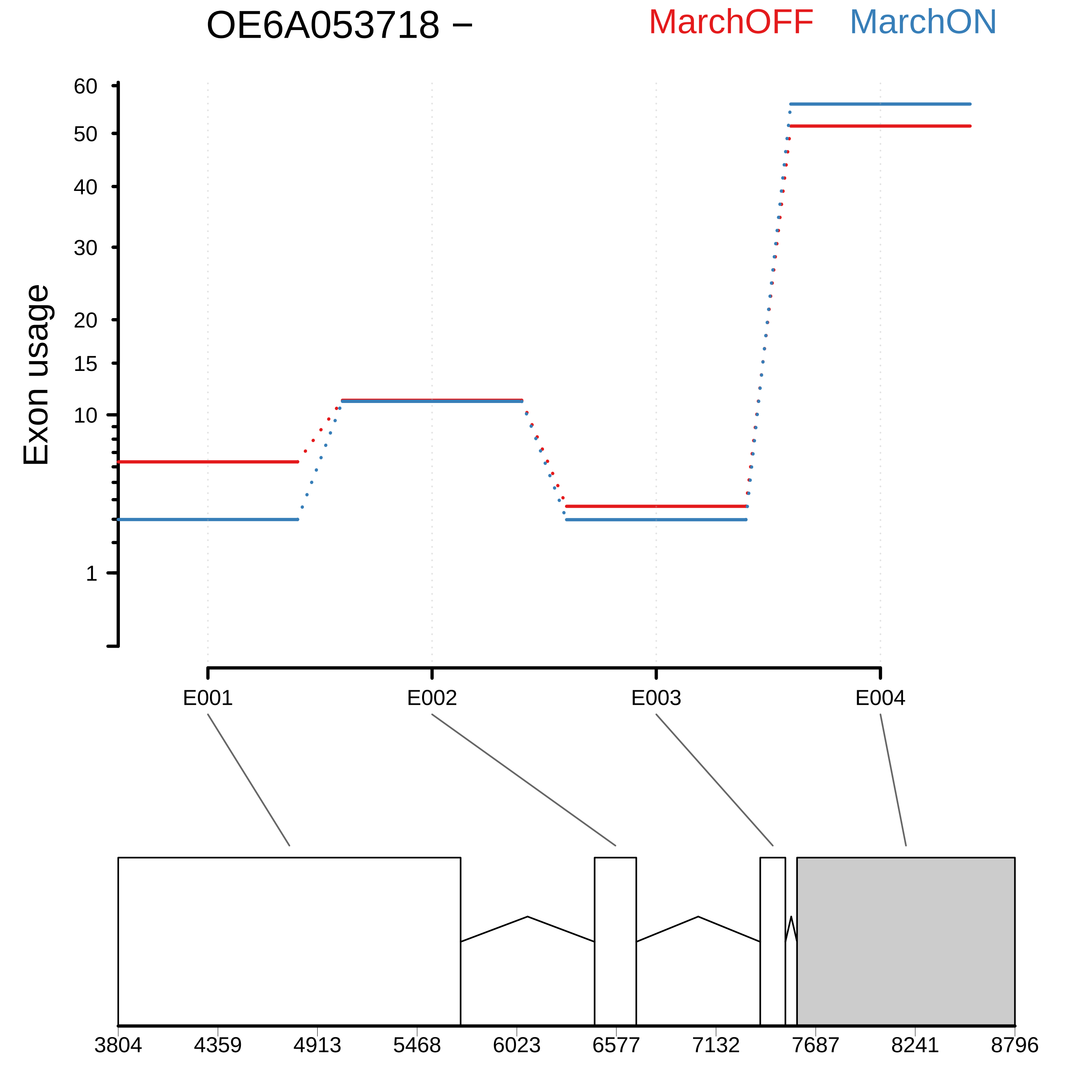

OE6A053718 −
MarchOFF
MarchON
Exon usage
60
50
40
30
20
15
10
1
E001
E002
E003
E004
3804
4359
4913
5468
6023
6577
7132
7687
8241
8796

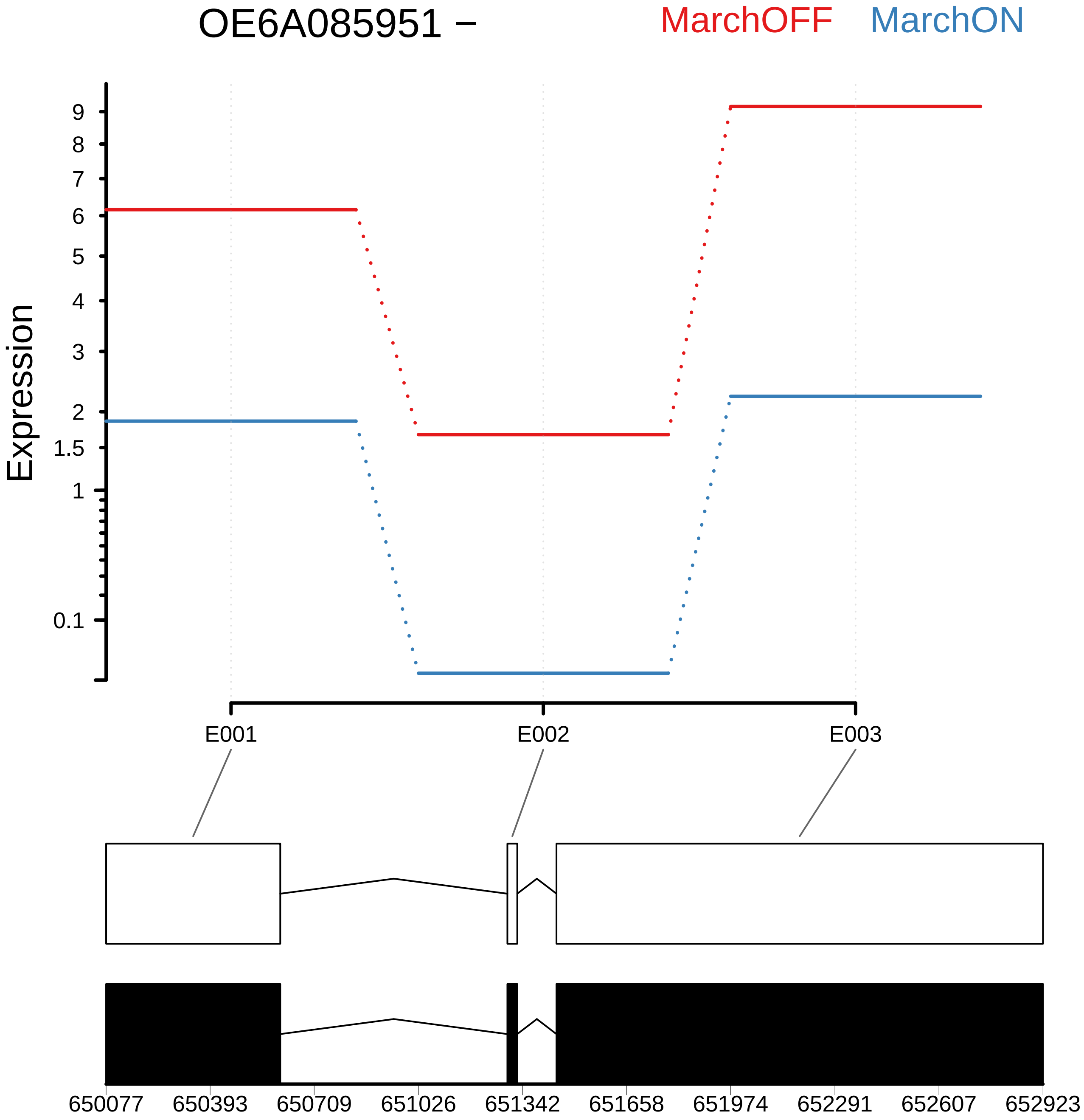
OE6A085951 −
MarchOFF
MarchON
Expression
9
8
7
6
5
4
3
2
1.5
1
0.1
E001
E002
E003
650077
650393
650709
651026
651342
651658
651974
652291
652607
652923

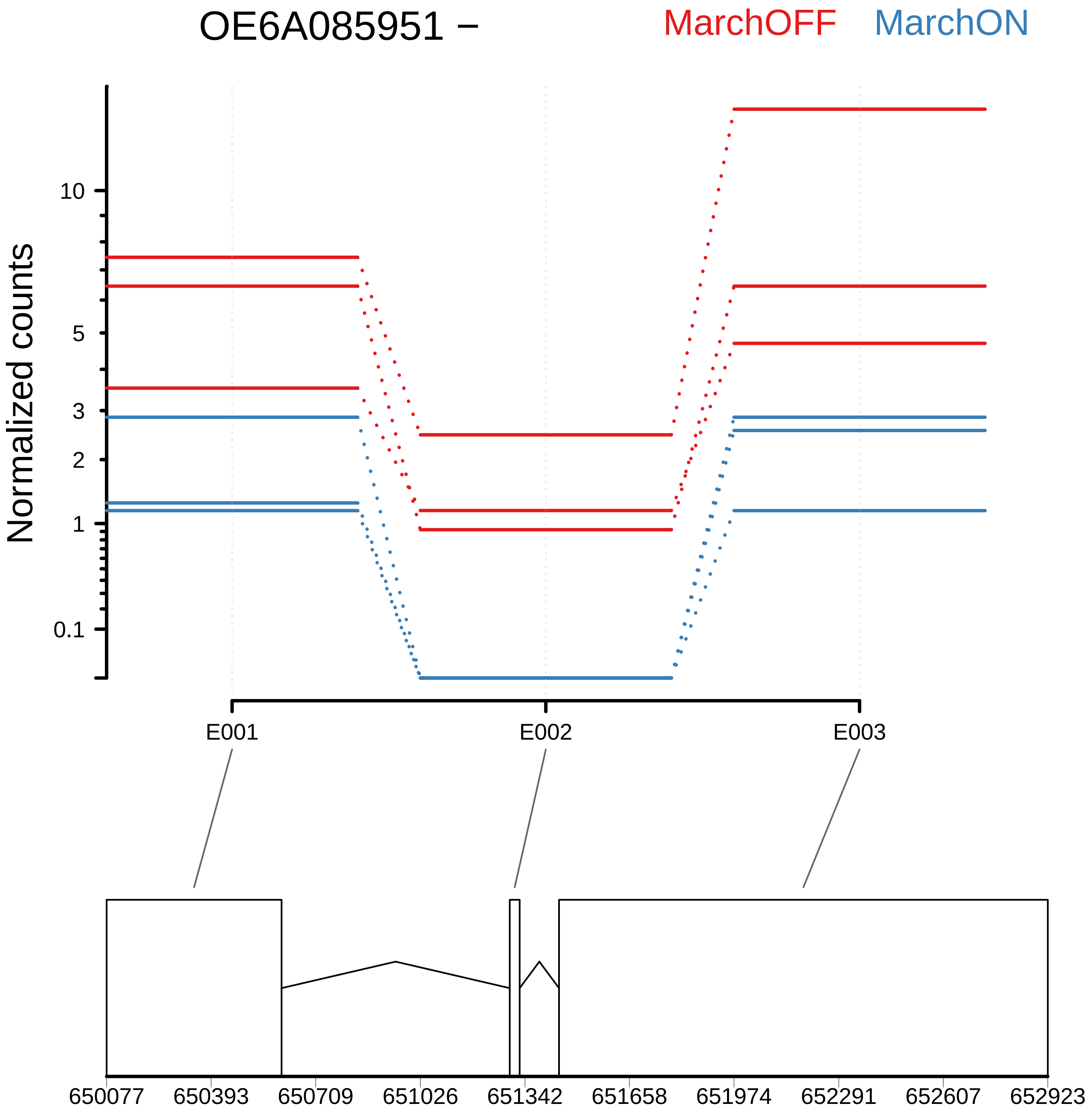

OE6A085951 −
MarchOFF
MarchON
Normalized counts
10
5
3
2
1
0.1
E001
E002
E003
650077
650393
650709
651026
651342
651658
651974
652291
652607
652923

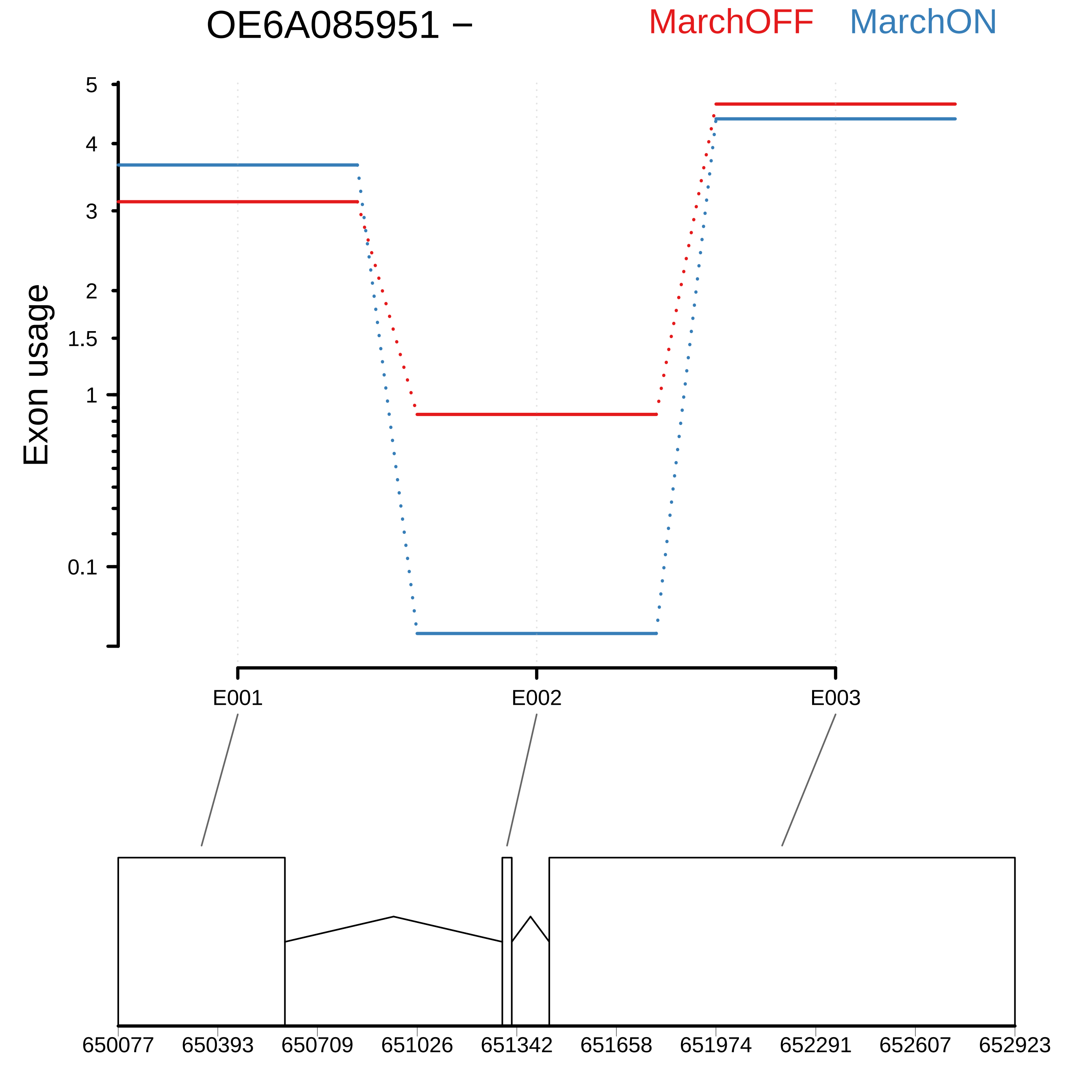

OE6A085951 −
MarchOFF
MarchON
Exon usage
5
4
3
2
1.5
1
0.1
E001
E002
E003
650077
650393
650709
651026
651342
651658
651974
652291
652607
652923

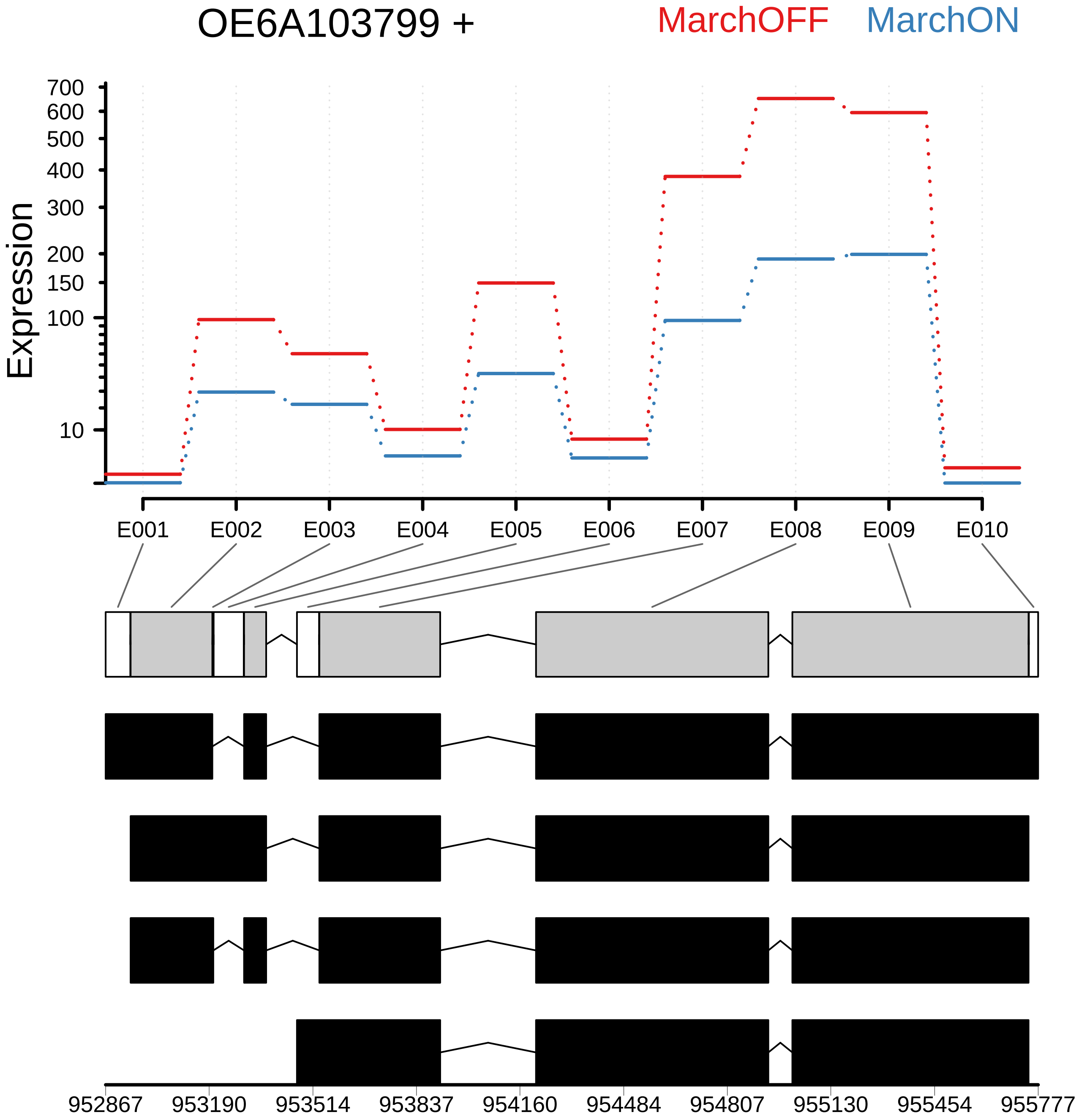
OE6A103799 +
MarchOFF
MarchON
Expression
700
600
500
400
300
200
150
100
10
E001
E002
E003
E004
E005
E006
E007
E008
E009
E010
952867
953190
953514
953837
954160
954484
954807
955130
955454
955777

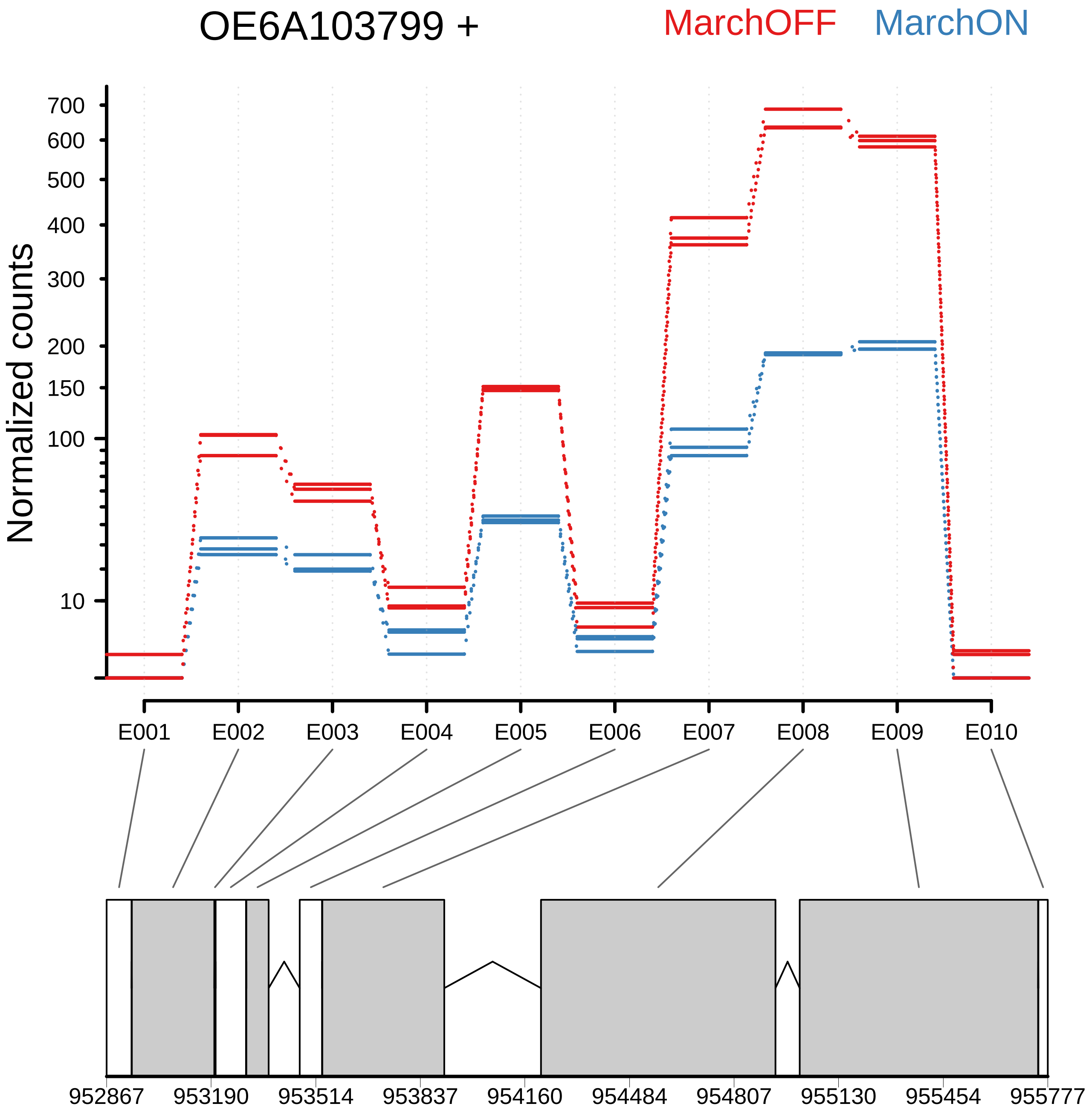

OE6A103799 +
MarchOFF
MarchON
Normalized counts
700
600
500
400
300
200
150
100
10
E001
E002
E003
E004
E005
E006
E007
E008
E009
E010
952867
953190
953514
953837
954160
954484
954807
955130
955454
955777

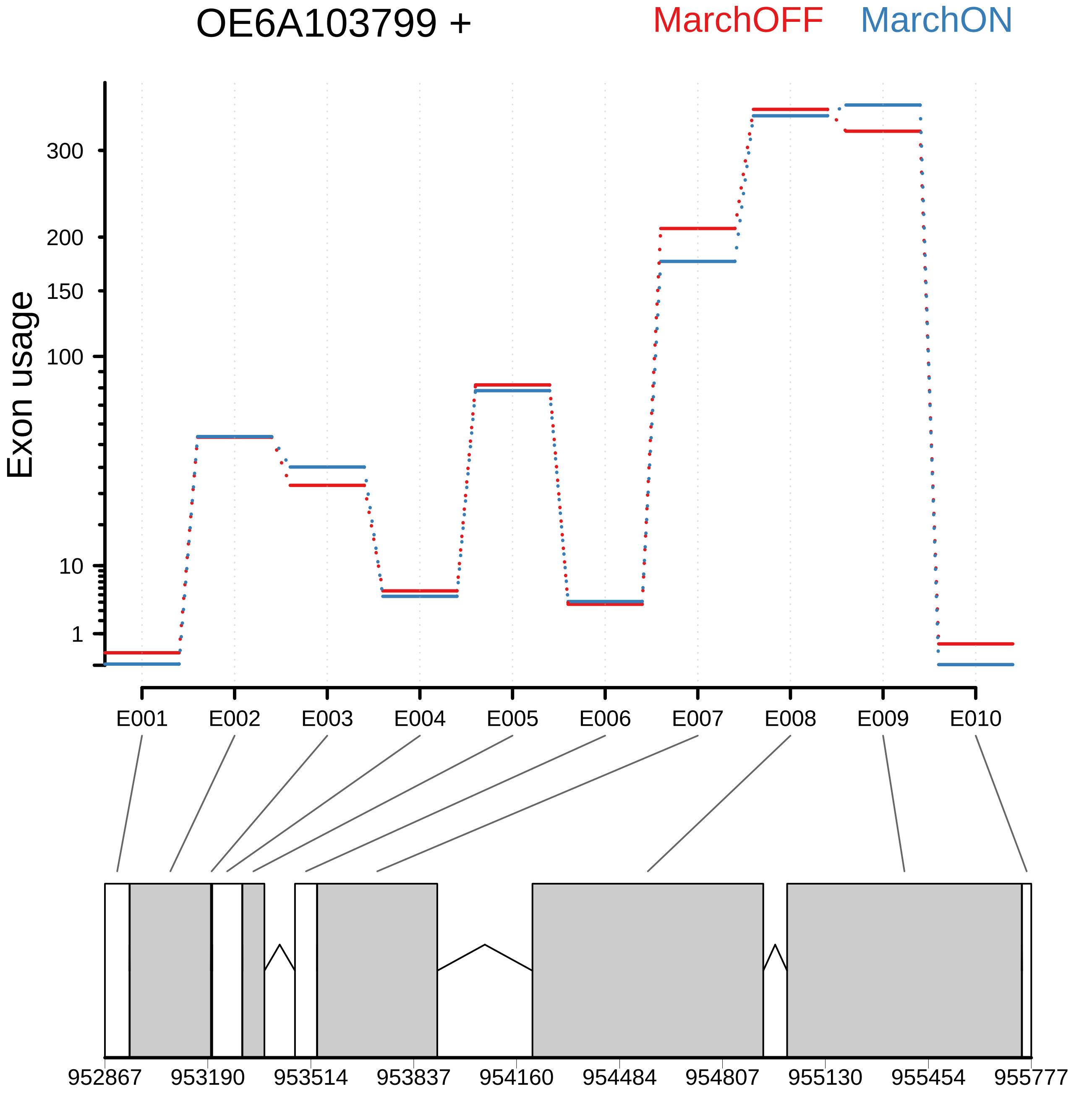
OE6A103799 +
MarchOFF
MarchON
Exon usage
300
200
150
100
10
1
E001
E002
E003
E004
E005
E006
E007
E008
E009
E010
952867
953190
953514
953837
954160
954484
954807
955130
955454
955777

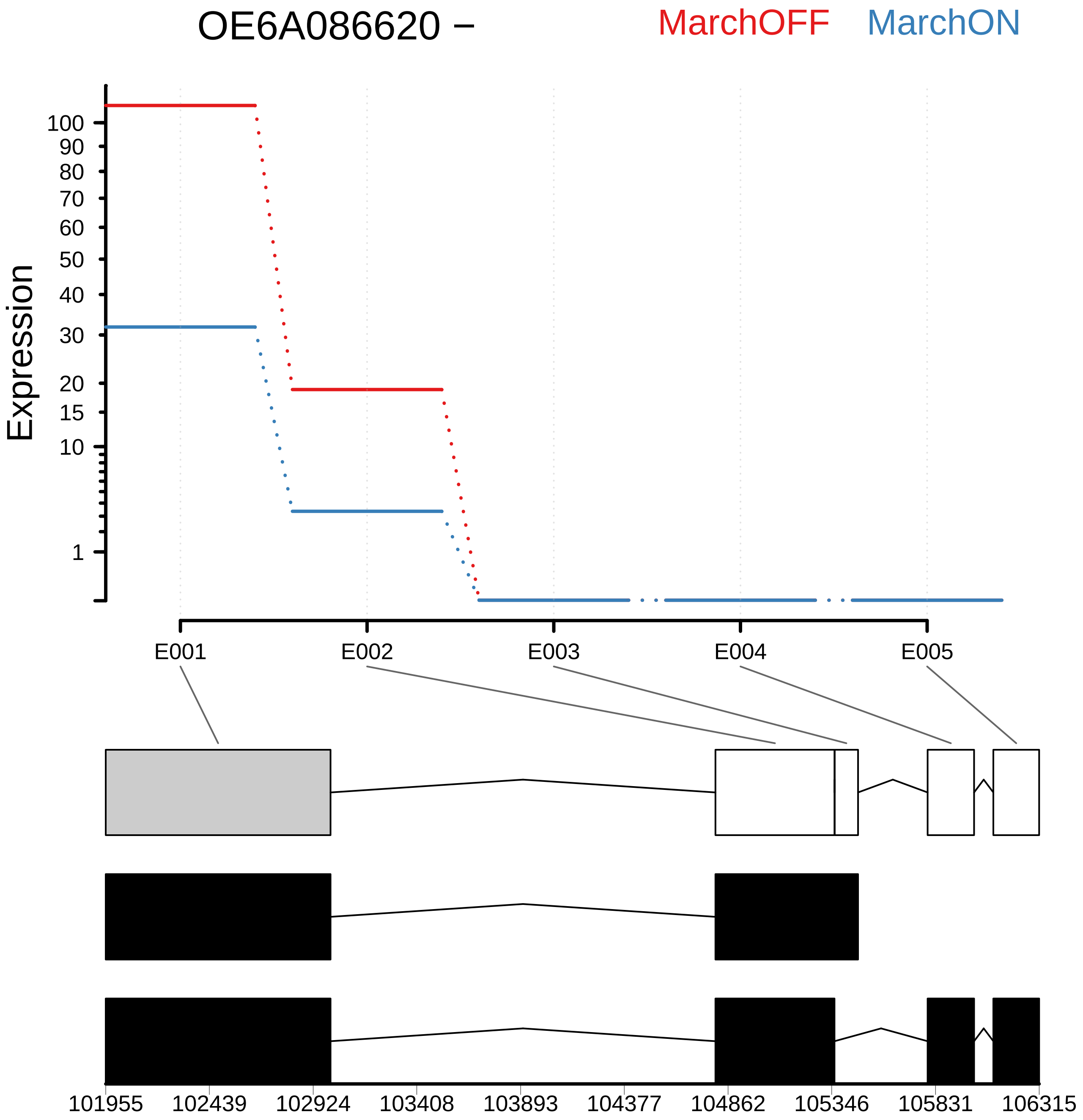

OE6A086620 –
MarchOFF
MarchON
Expression
100
90
80
70
60
50
40
30
20
15
10
1
E001
E002
E003
E004
E005
101955
102439
102924
103408
103893
104377
104862
105346
105831
106315

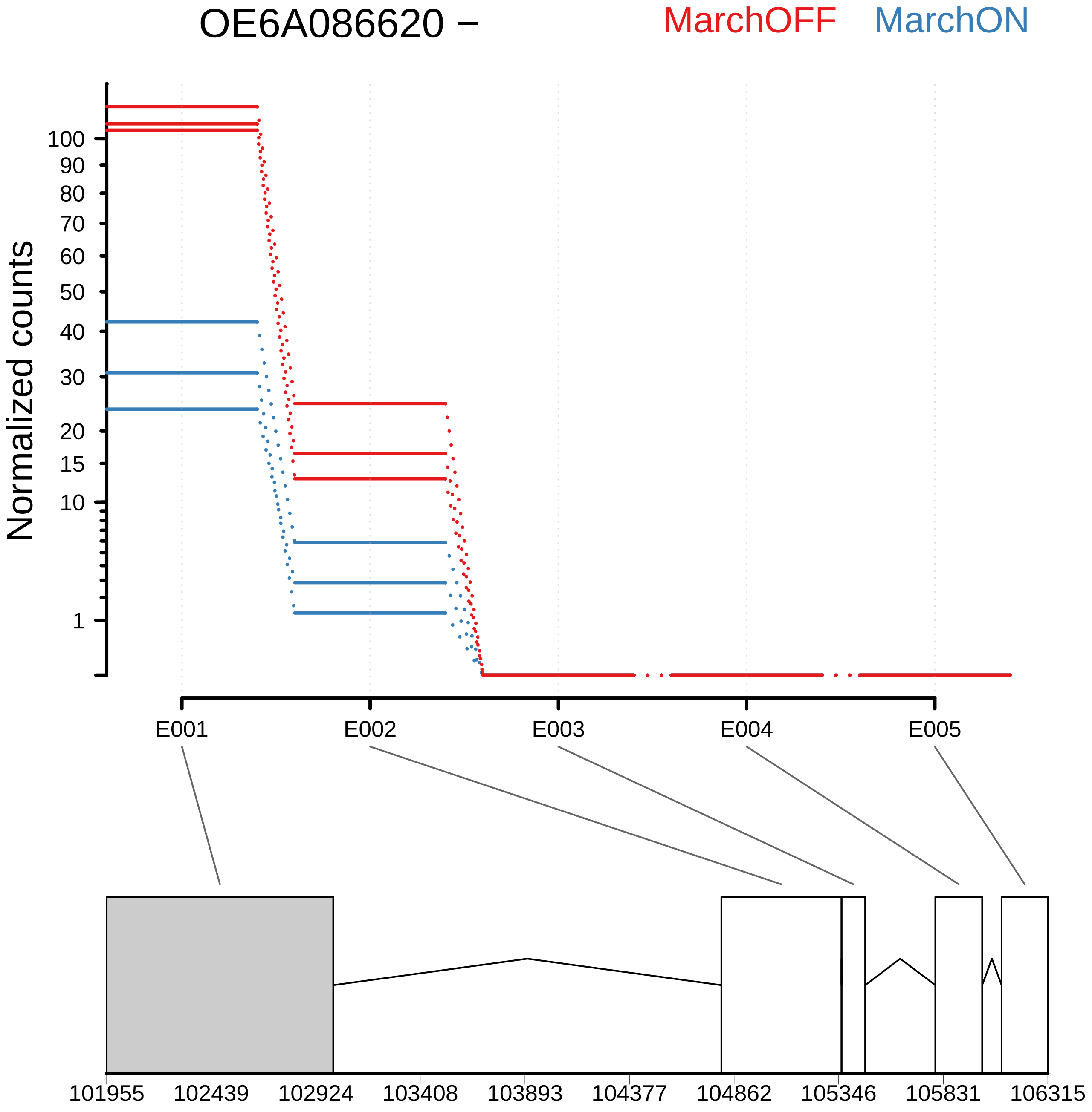

OE6A086620 −
MarchOFF
MarchON
Normalized counts
100
90
80
70
60
50
40
30
20
15
10
1
E001
E002
E003
E004
E005
101955
102439
102924
103408
103893
104377
104862
105346
105831
106315

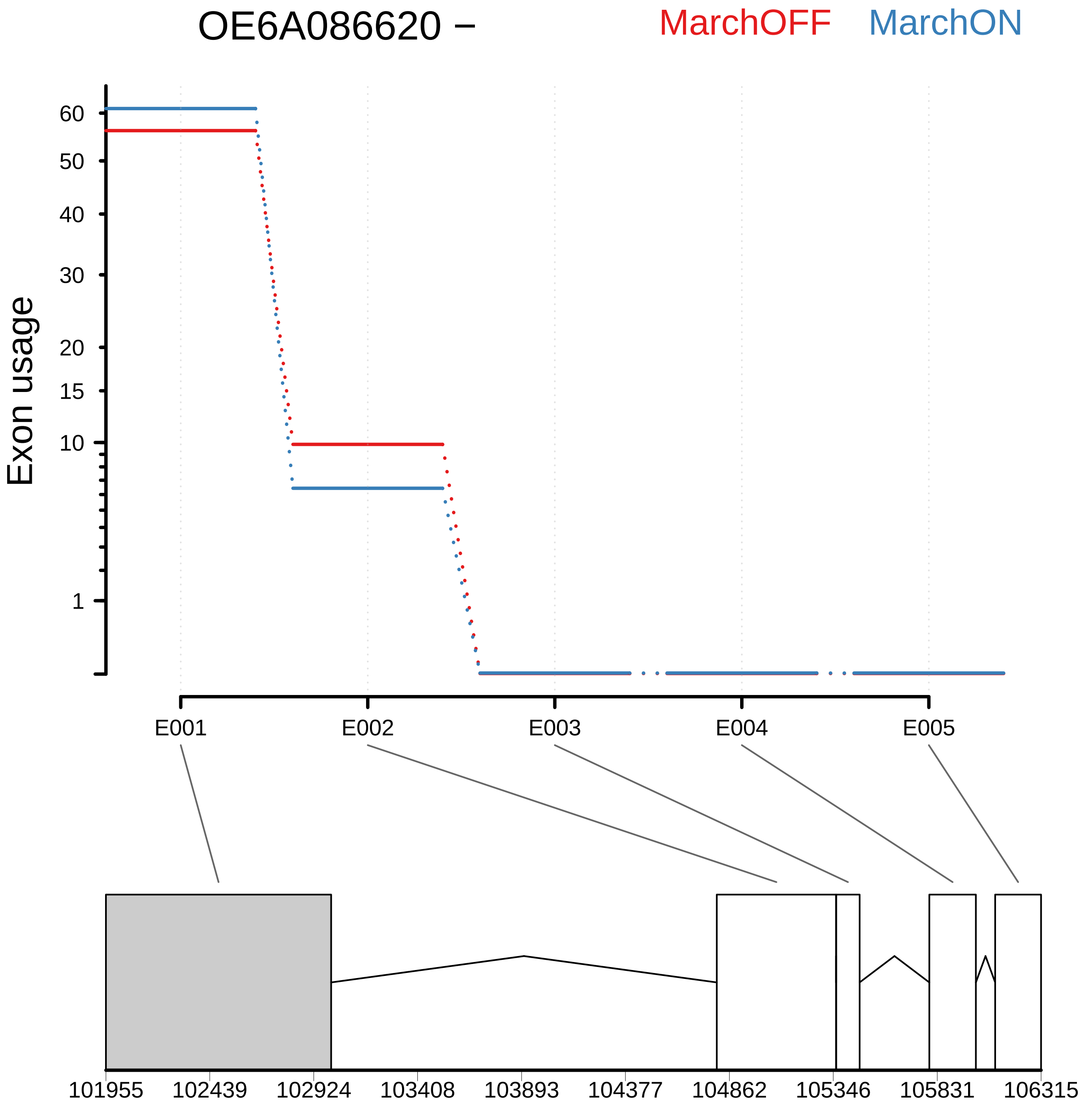

OE6A086620 −
MarchOFF
MarchON
Exon usage
60
50
40
30
20
15
10
1
E001
E002
E003
E004
E005
101955
102439
102924
103408
103893
104377
104862
105346
105831
106315

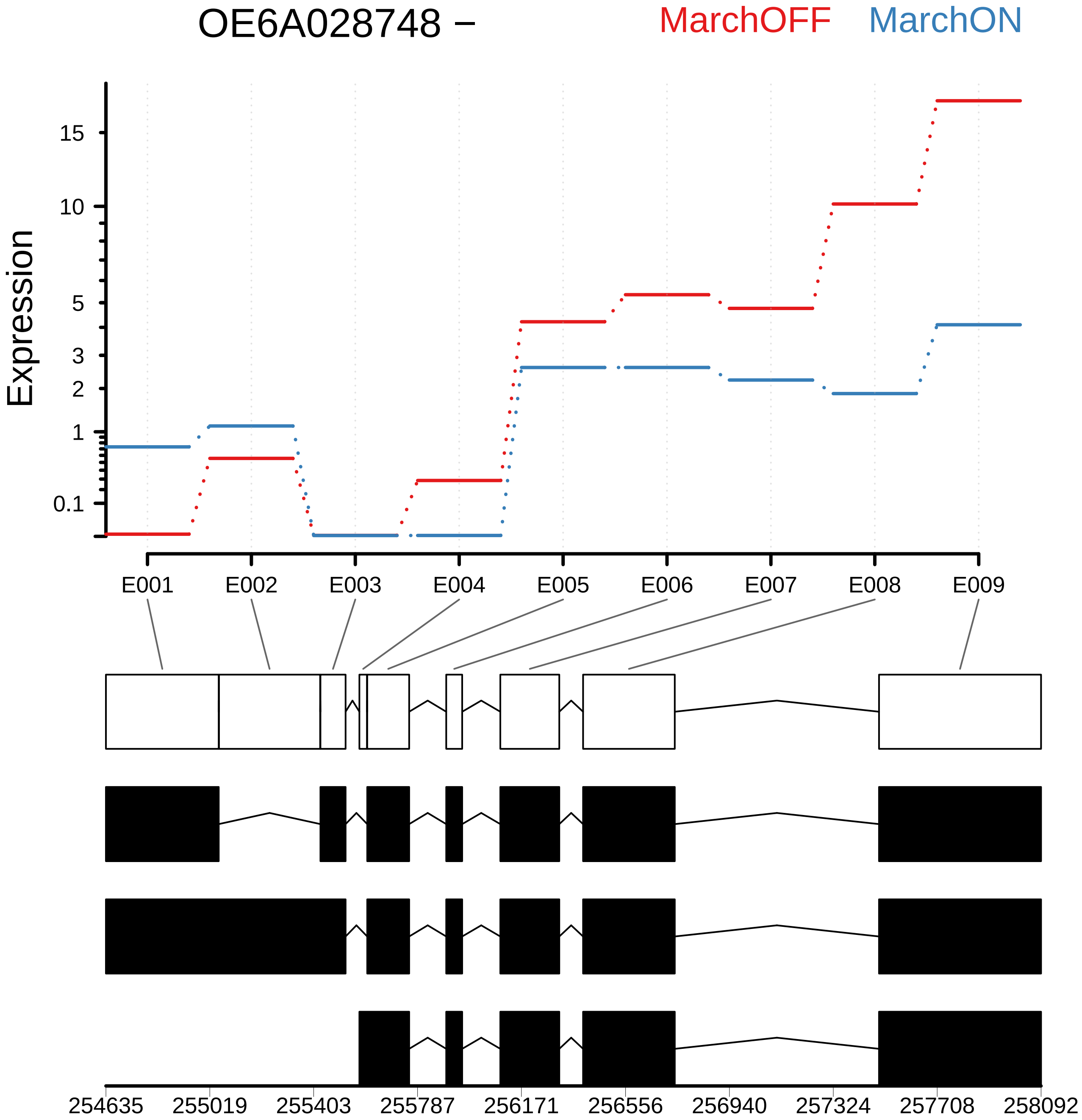

OE6A028748 –
MarchOFF
MarchON
Expression
15
10
5
3
2
1
0.1
E001
E002
E003
E004
E005
E006
E007
E008
E009
254635
255019
255403
255787
256171
256556
256940
257324
257708
258092

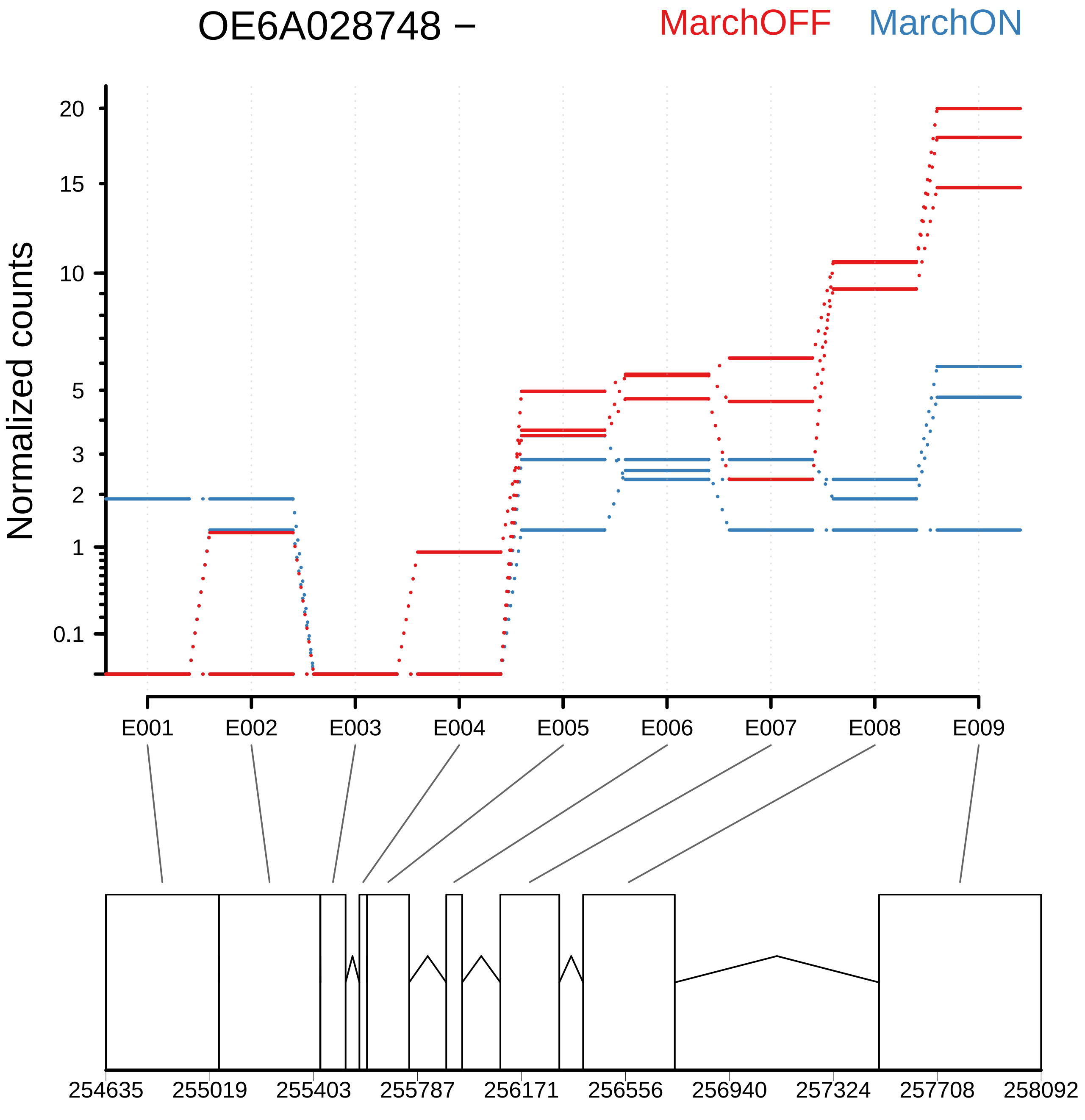

OE6A028748 −
MarchOFF
MarchON
Normalized counts
20
15
10
5
3
2
1
0.1
E001
E002
E003
E004
E005
E006
E007
E008
E009
254635
255019
255403
255787
256171
256556
256940
257324
257708
258092

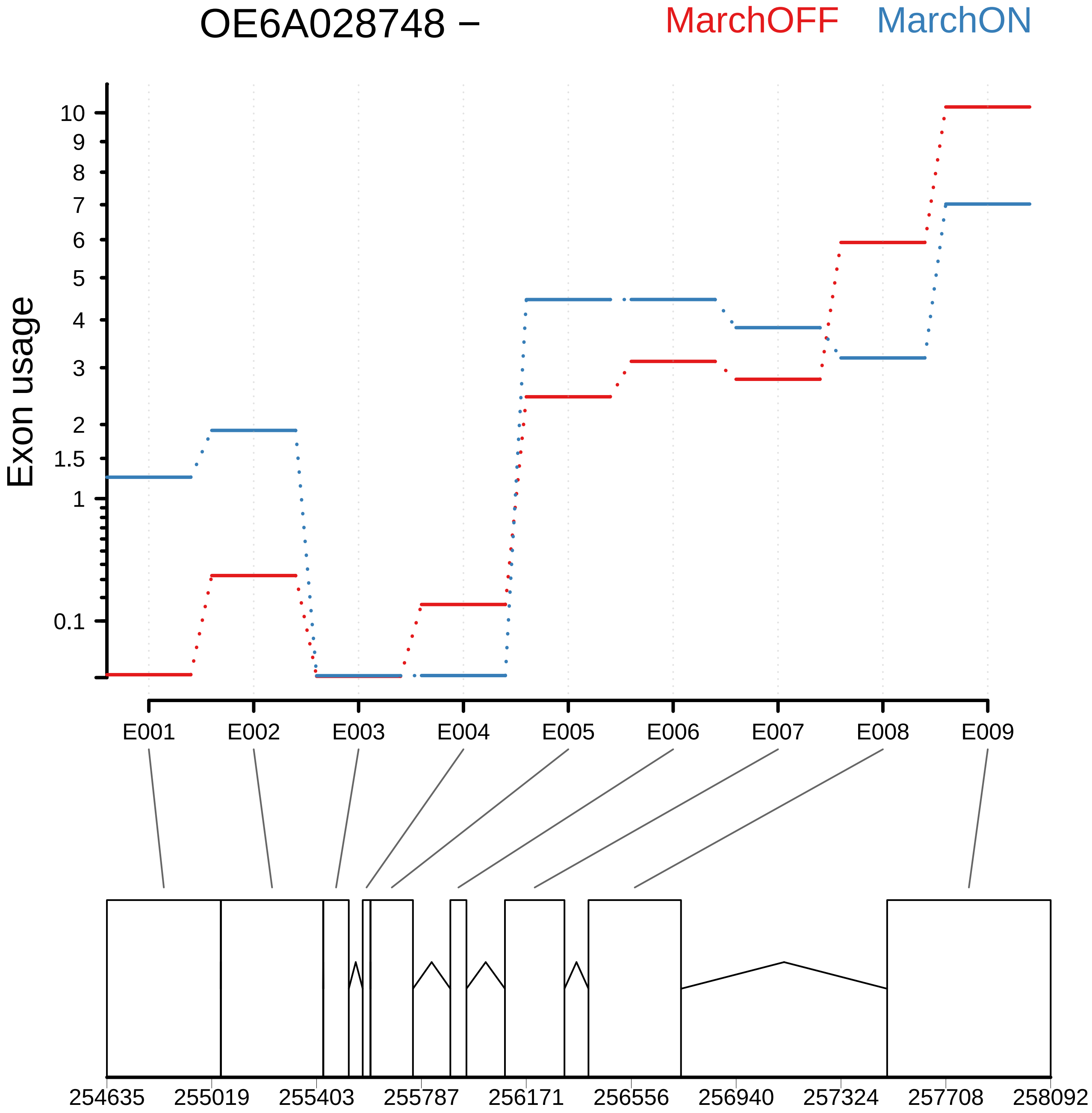

OE6A028748 −
MarchOFF
MarchON
Exon usage
10
9
8
7
6
5
4
3
2
1.5
1
0.1
E001
E002
E003
E004
E005
E006
E007
E008
E009
254635
255019
255403
255787
256171
256556
256940
257324
257708
258092

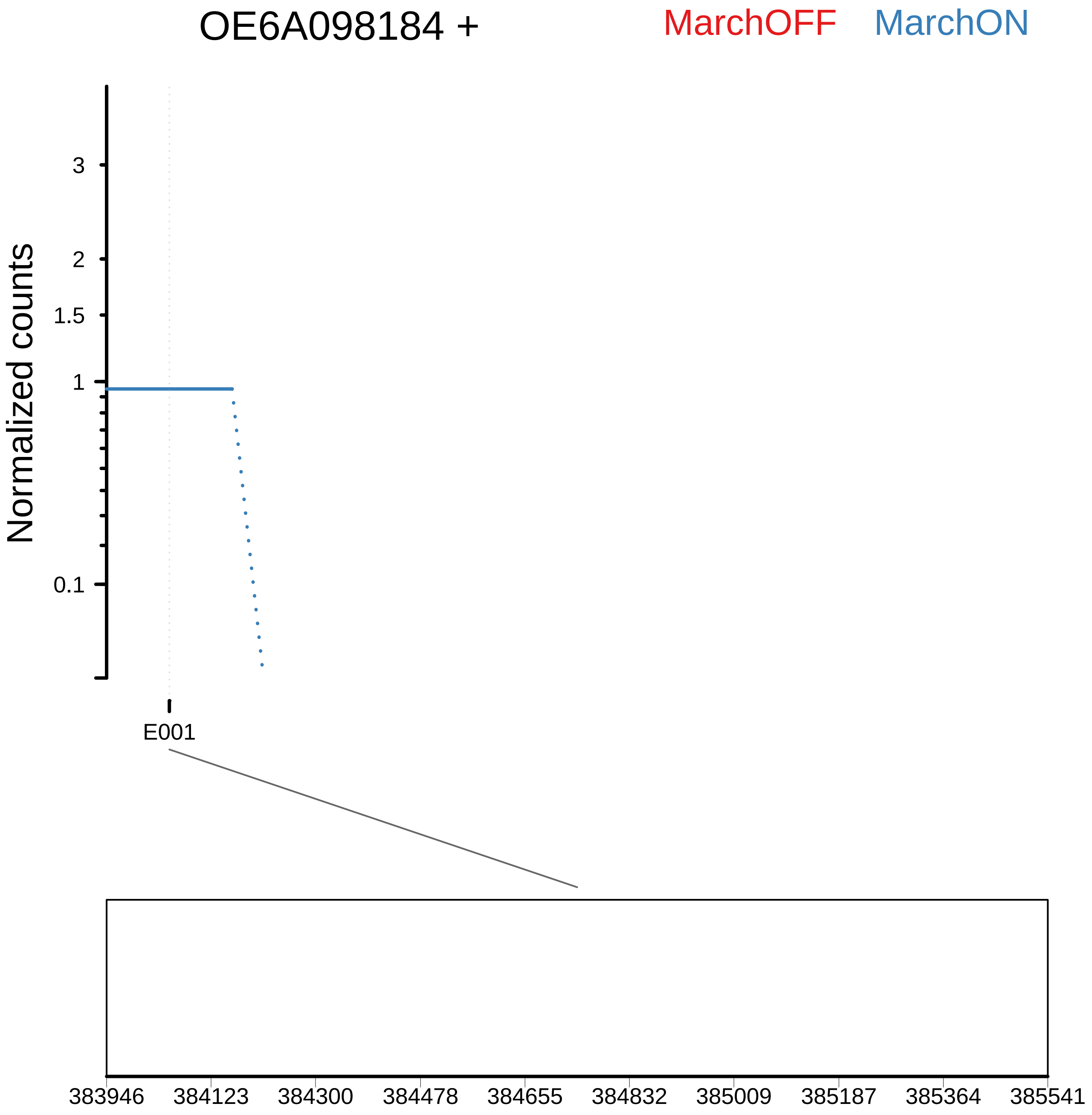
OE6A098184 +
MarchOFF
MarchON
Normalized counts
3
2
1.5
1
0.1
E001
383946
384123
384300
384478
384655
384832
385009
385187
385364
385541

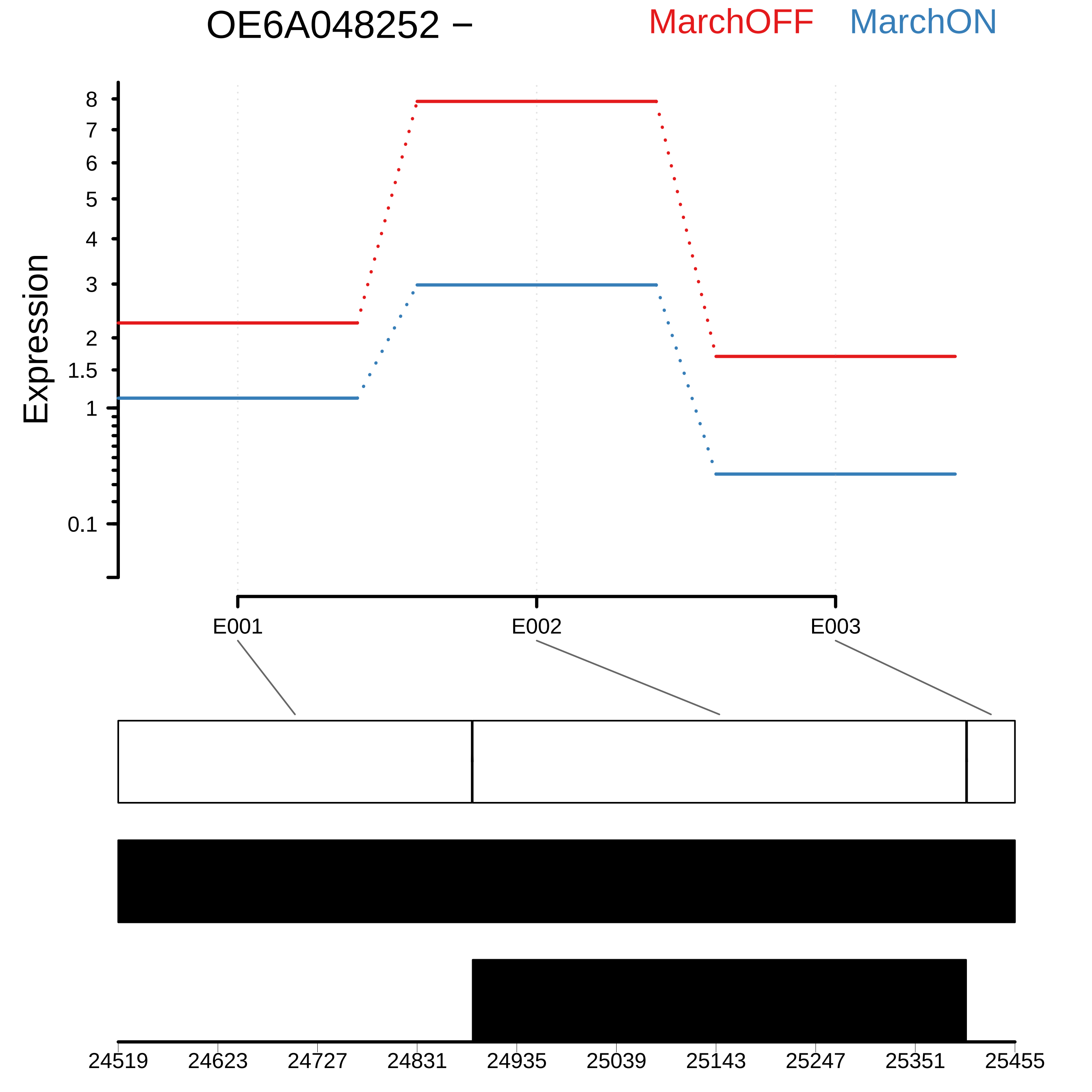
OE6A048252 −
MarchOFF
MarchON
Expression
8
7
6
5
4
3
2
1.5
1
0.1
E001
E002
E003
24519
24623
24727
24831
24935
25039
25143
25247
25351
25455

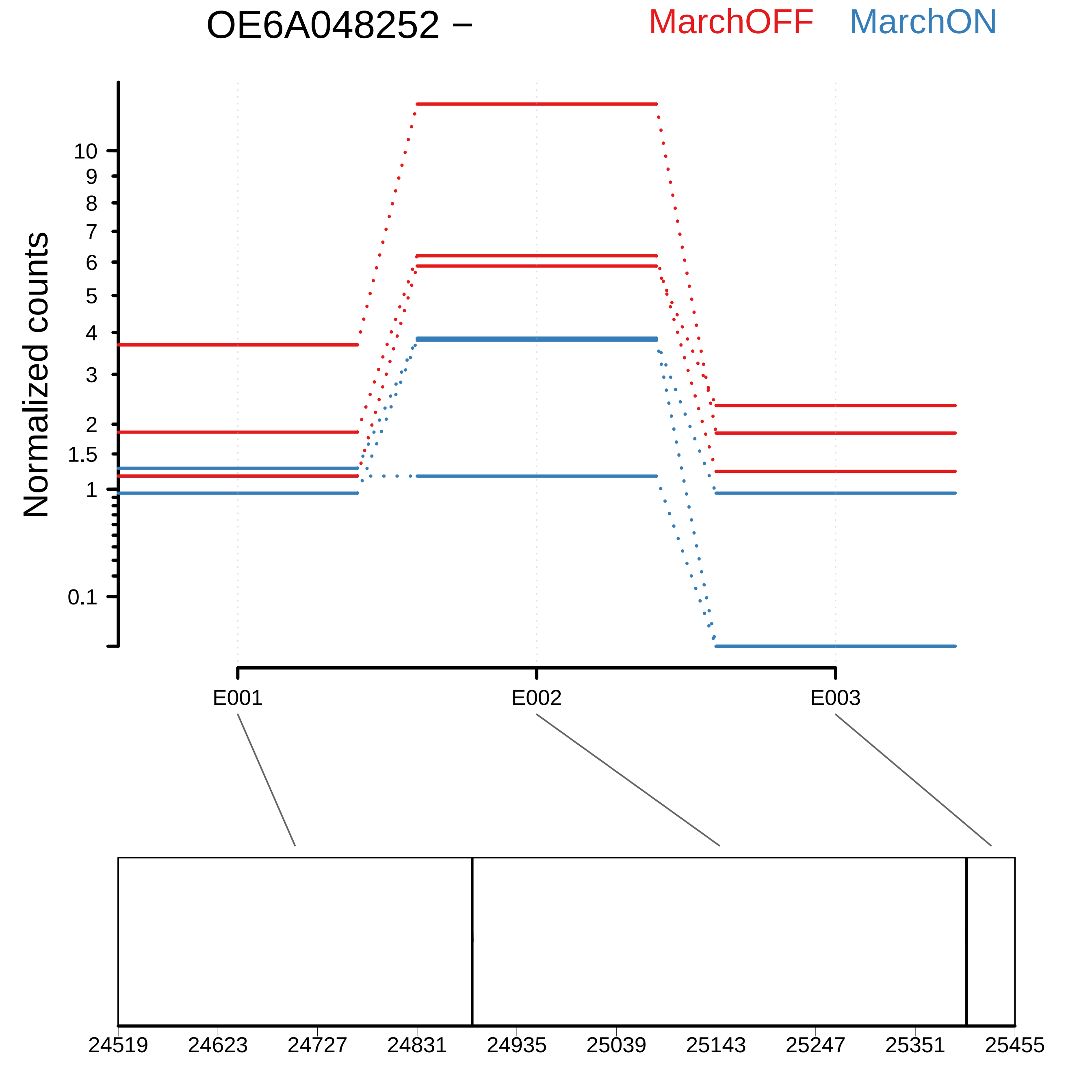

OE6A048252 −
MarchOFF
MarchON
Normalized counts
10
9
8
7
6
5
4
3
2
1.5
1
0.1
E001
E002
E003
24519
24623
24727
24831
24935
25039
25143
25247
25351
25455

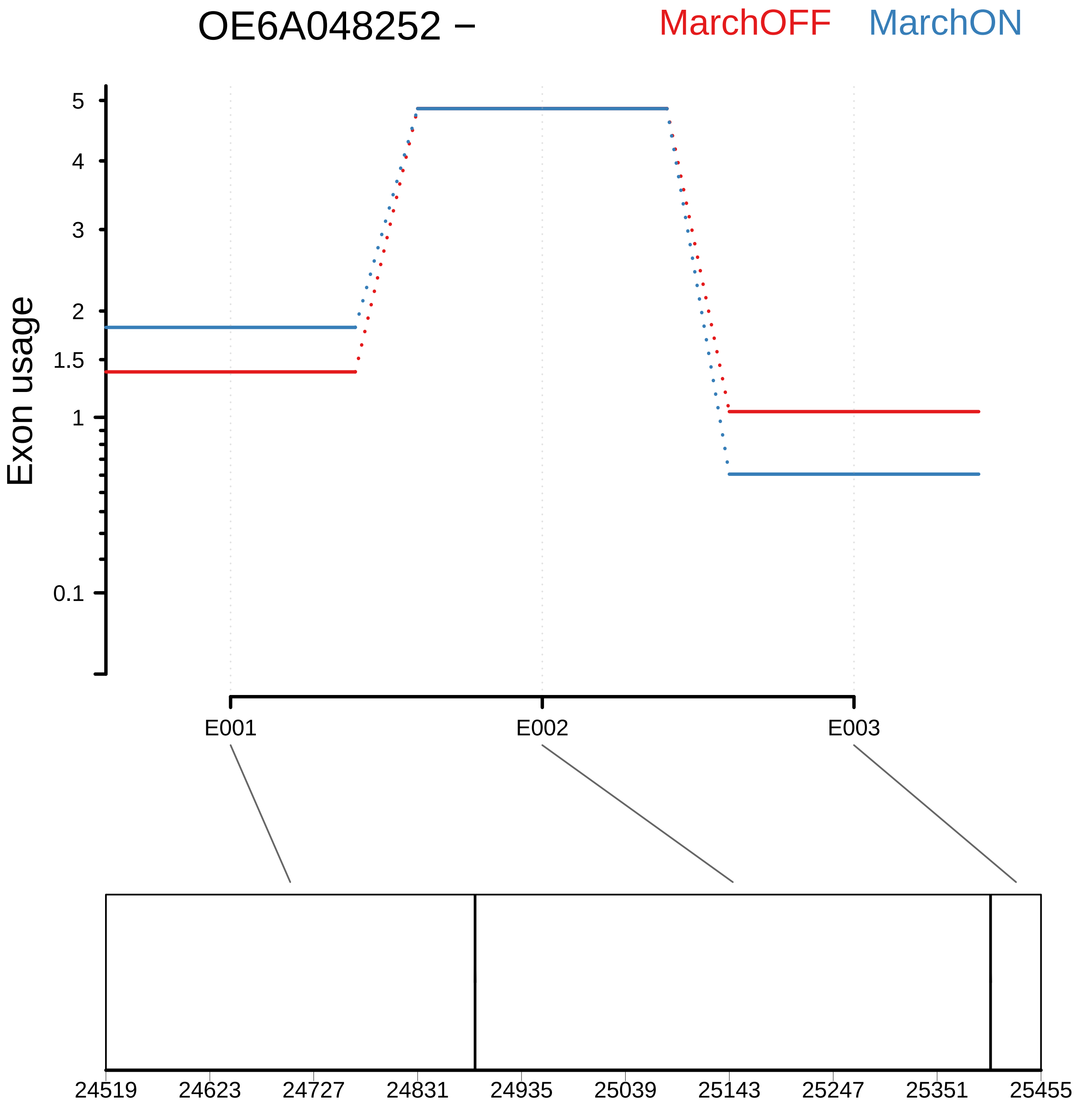

OE6A048252 −
MarchOFF
MarchON
Exon usage
5
4
3
2
1.5
1
0.1
E001
E002
E003
24519
24623
24727
24831
24935
25039
25143
25247
25351
25455

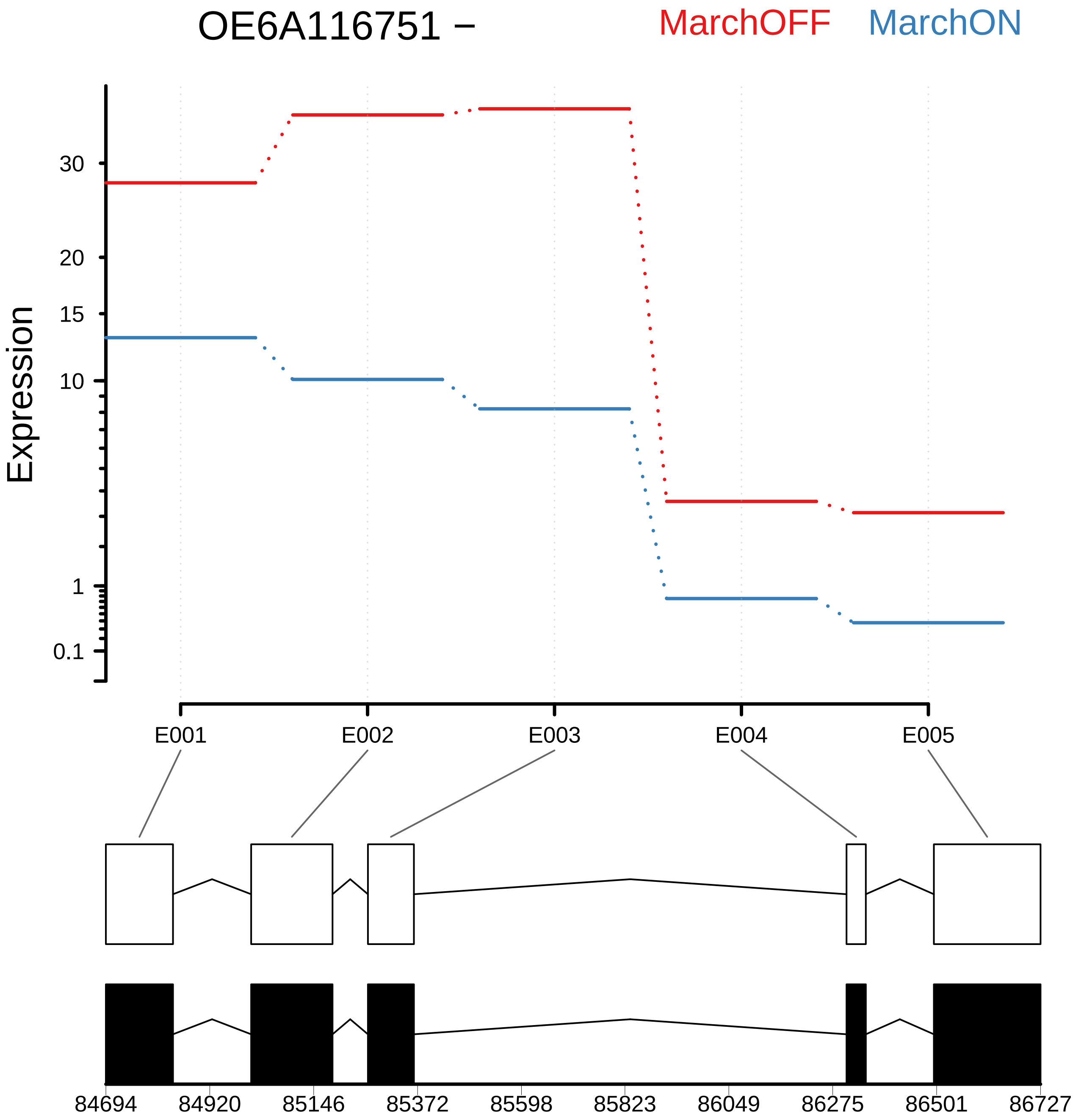
OE6A116751 −
MarchOFF
MarchON
Expression
30
20
15
10
1
0.1
E001
E002
E003
E004
E005
84694
84920
85146
85372
85598
85823
86049
86275
86501
86727

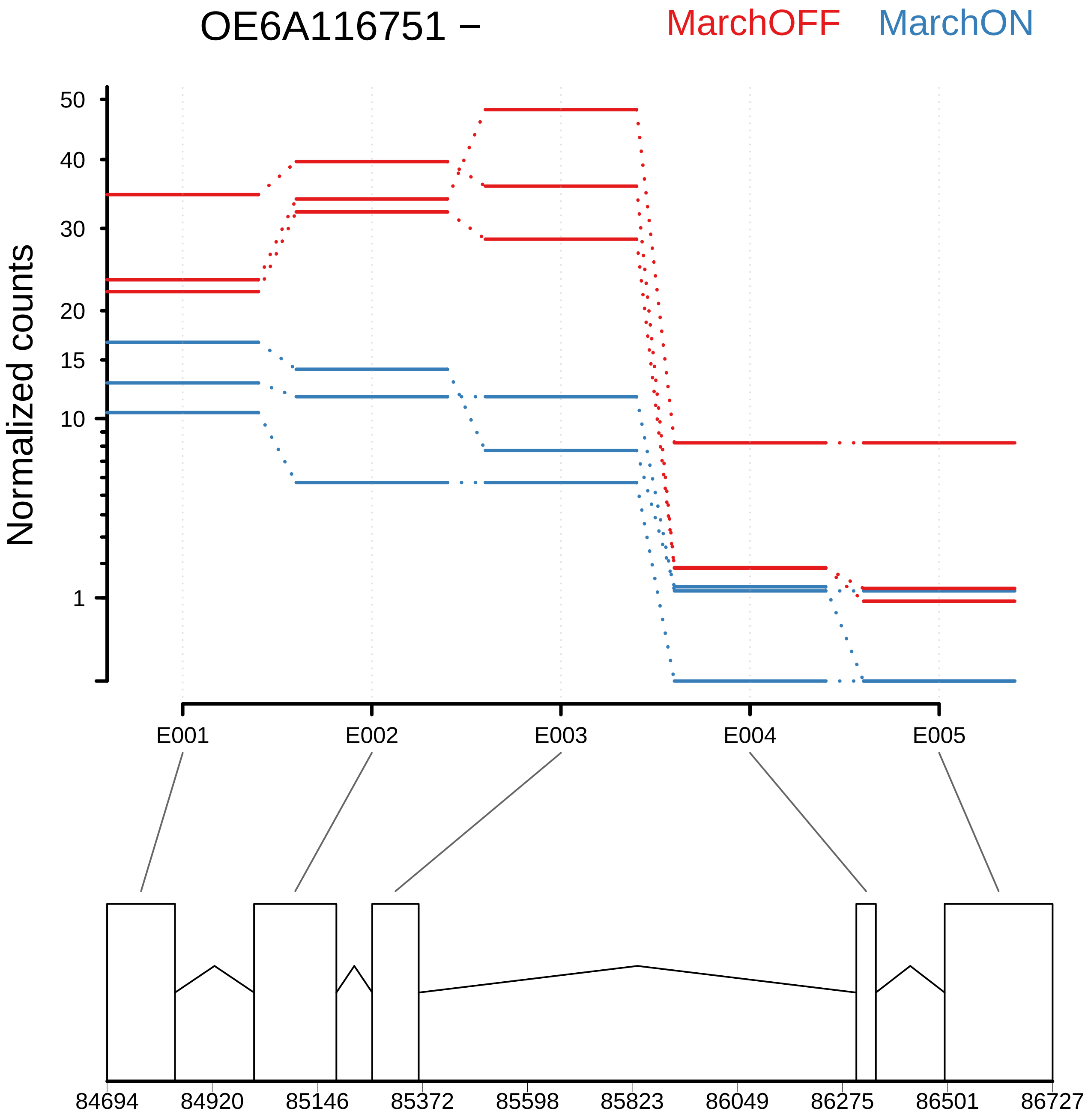

OE6A116751 −
MarchOFF
MarchON
Normalized counts
50
40
30
20
15
10
1
E001
E002
E003
E004
E005
84694
84920
85146
85372
85598
85823
86049
86275
86501
86727

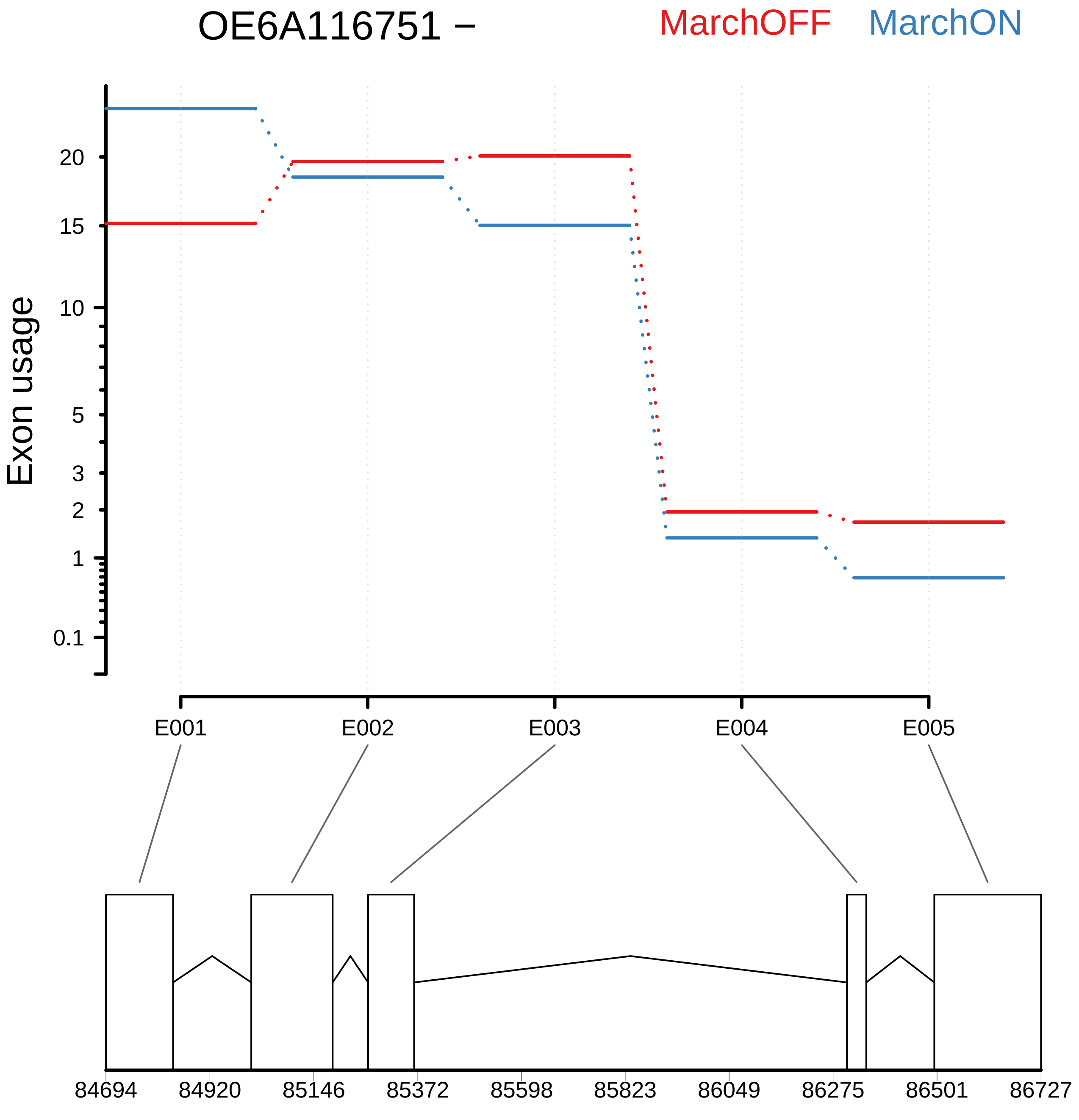

OE6A116751 −
MarchOFF
MarchON
Exon usage
20
15
10
5
3
2
1
0.1
E001
E002
E003
E004
E005
84694
84920
85146
85372
85598
85823
86049
86275
86501
86727

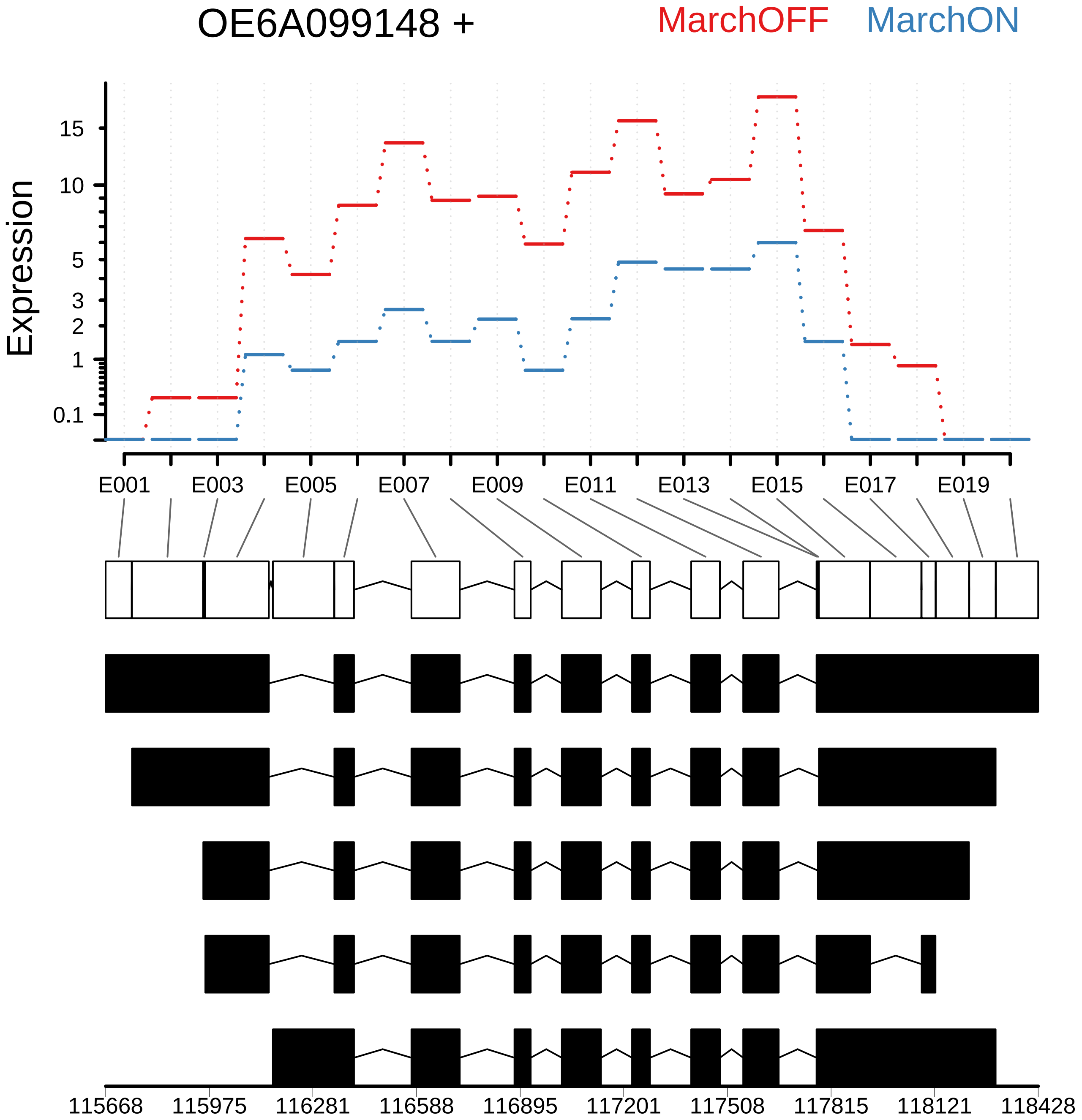

OE6A099148 +
MarchOFF
MarchON
Expression
15
10
5
3
2
1
0.1
E001
E003
E005
E007
E009
E011
E013
E015
E017
E019
115668
115975
116281
116588
116895
117201
117508
117815
118121
118428

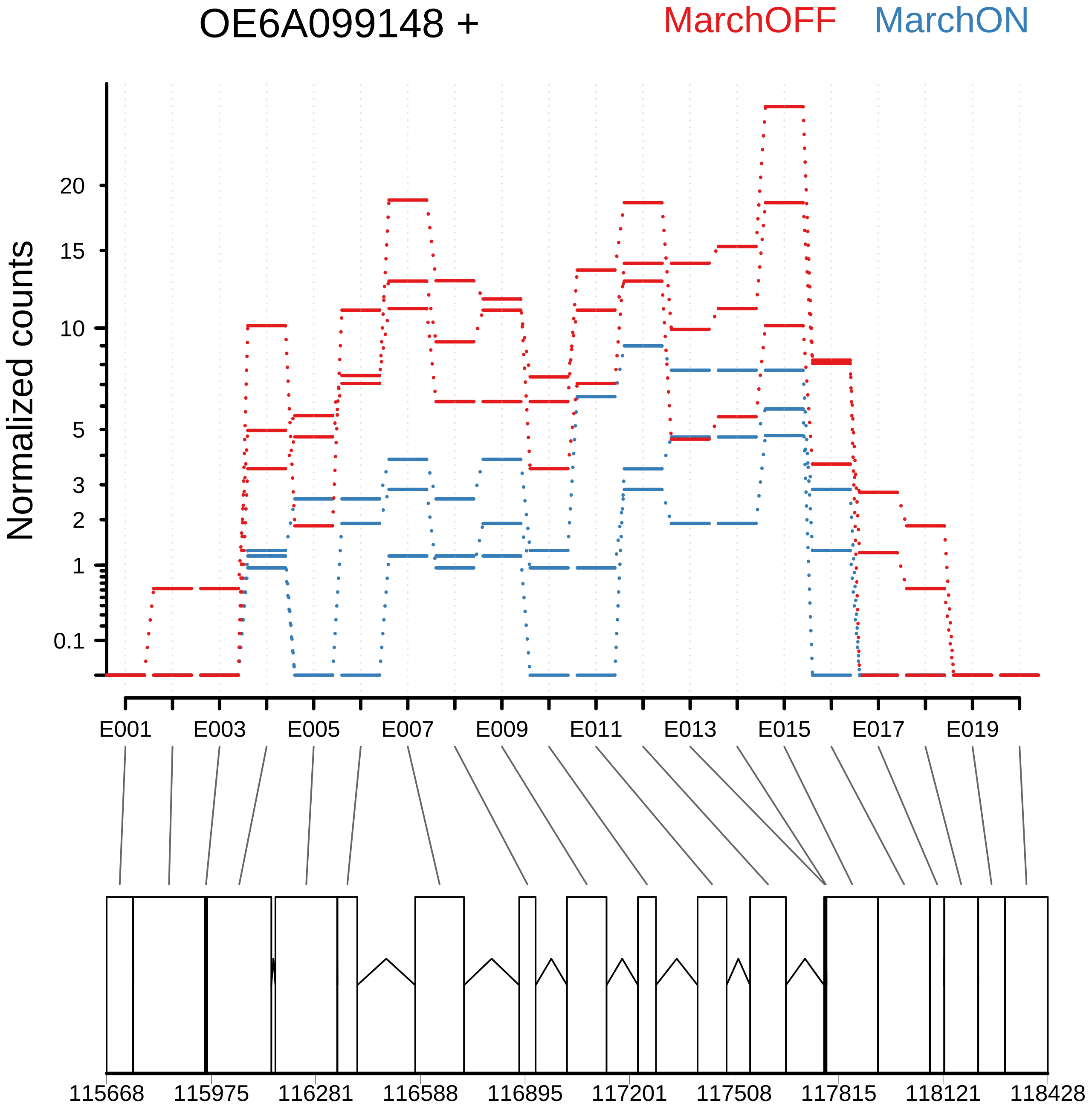

OE6A099148 +
MarchOFF
MarchON
Normalized counts
20
15
10
5
3
2
1
0.1
E001
E003
E005
E007
E009
E011
E013
E015
E017
E019
115668
115975
116281
116588
116895
117201
117508
117815
118121
118428

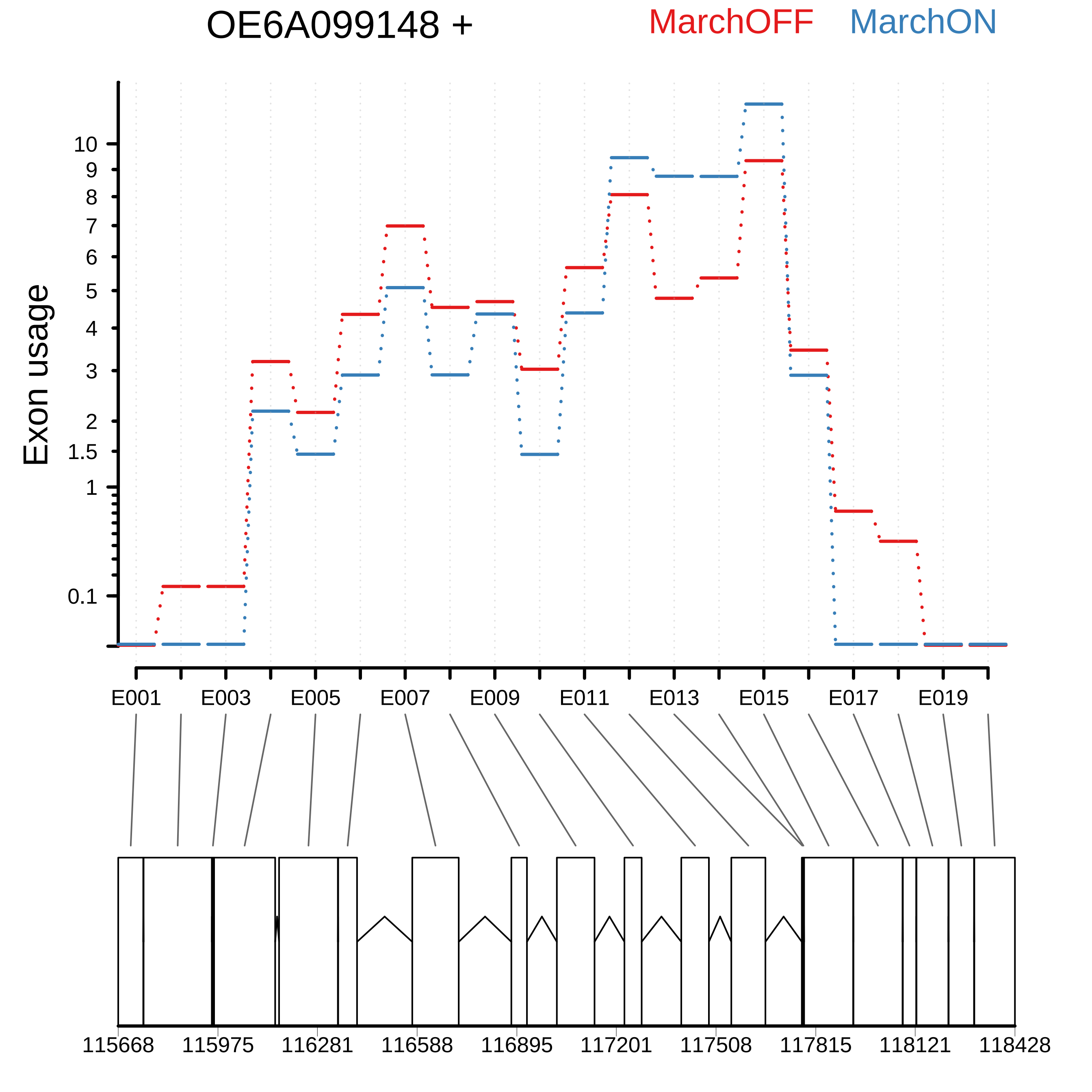

OE6A099148 +
MarchOFF
MarchON
Exon usage
10
9
8
7
6
5
4
3
2
1.5
1
0.1
E001
E003
E005
E007
E009
E011
E013
E015
E017
E019
115668
115975
116281
116588
116895
117201
117508
117815
118121
118428

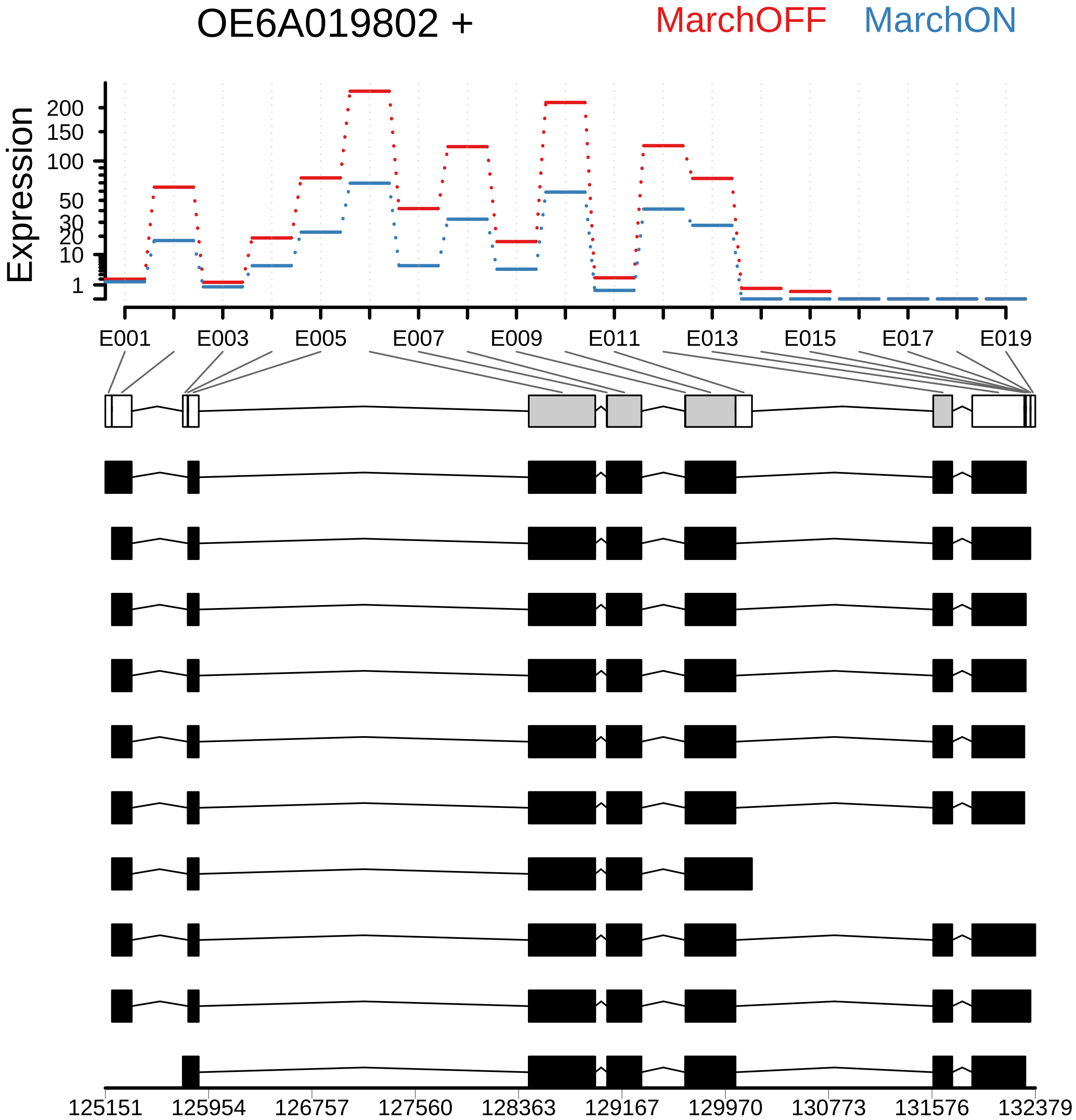

OE6A019802 +
MarchOFF
MarchON
Expression
200
150
100
50
30
20
10
1
E001
E003
E005
E007
E009
E011
E013
E015
E017
E019
125151
125954
126757
127560
128363
129167
129970
130773
131576
132379

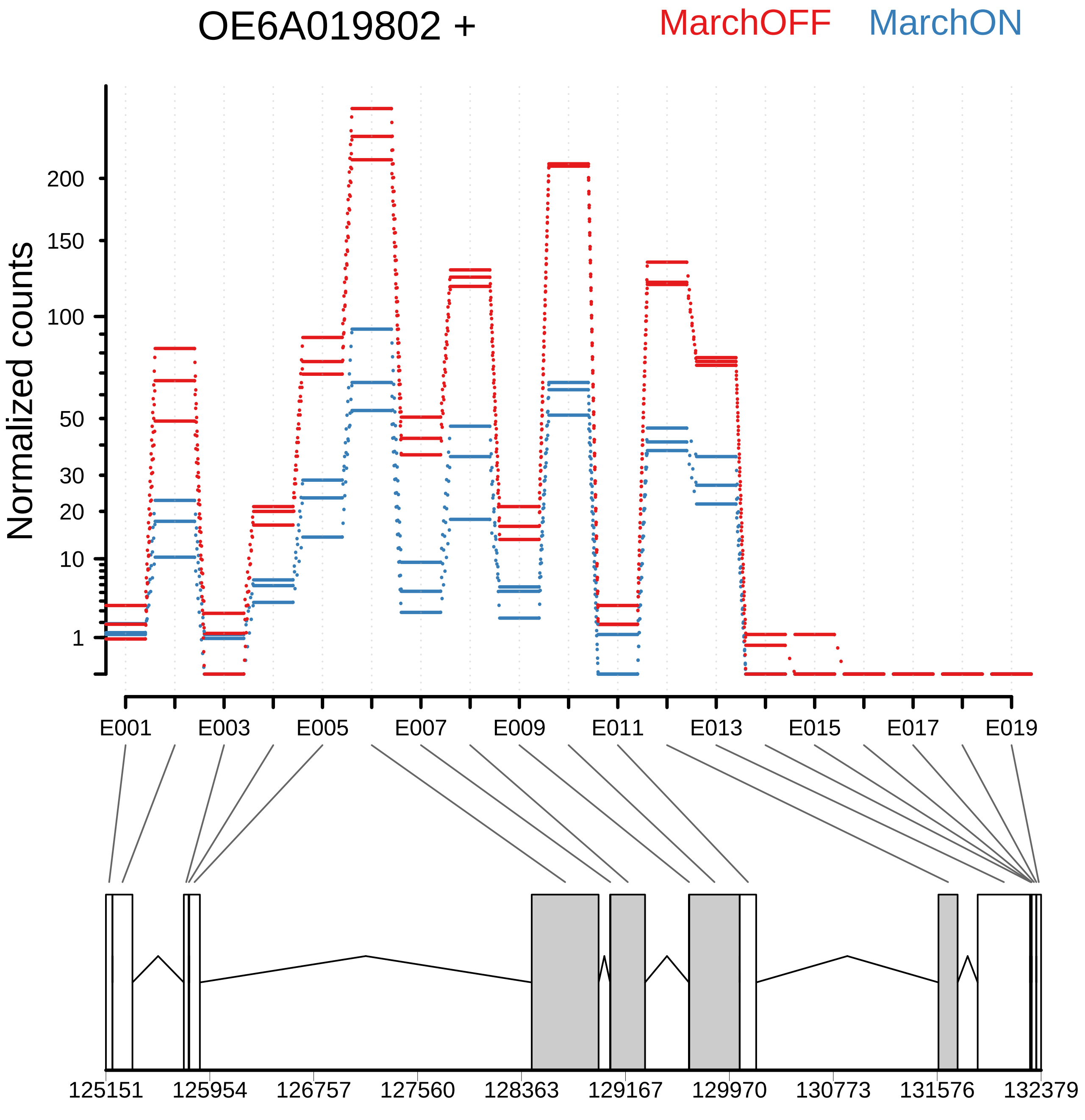
OE6A019802 +
MarchOFF
MarchON
Normalized counts
200
150
100
50
30
20
10
1
E001
E003
E005
E007
E009
E011
E013
E015
E017
E019
125151
125954
126757
127560
128363
129167
129970
130773
131576
132379

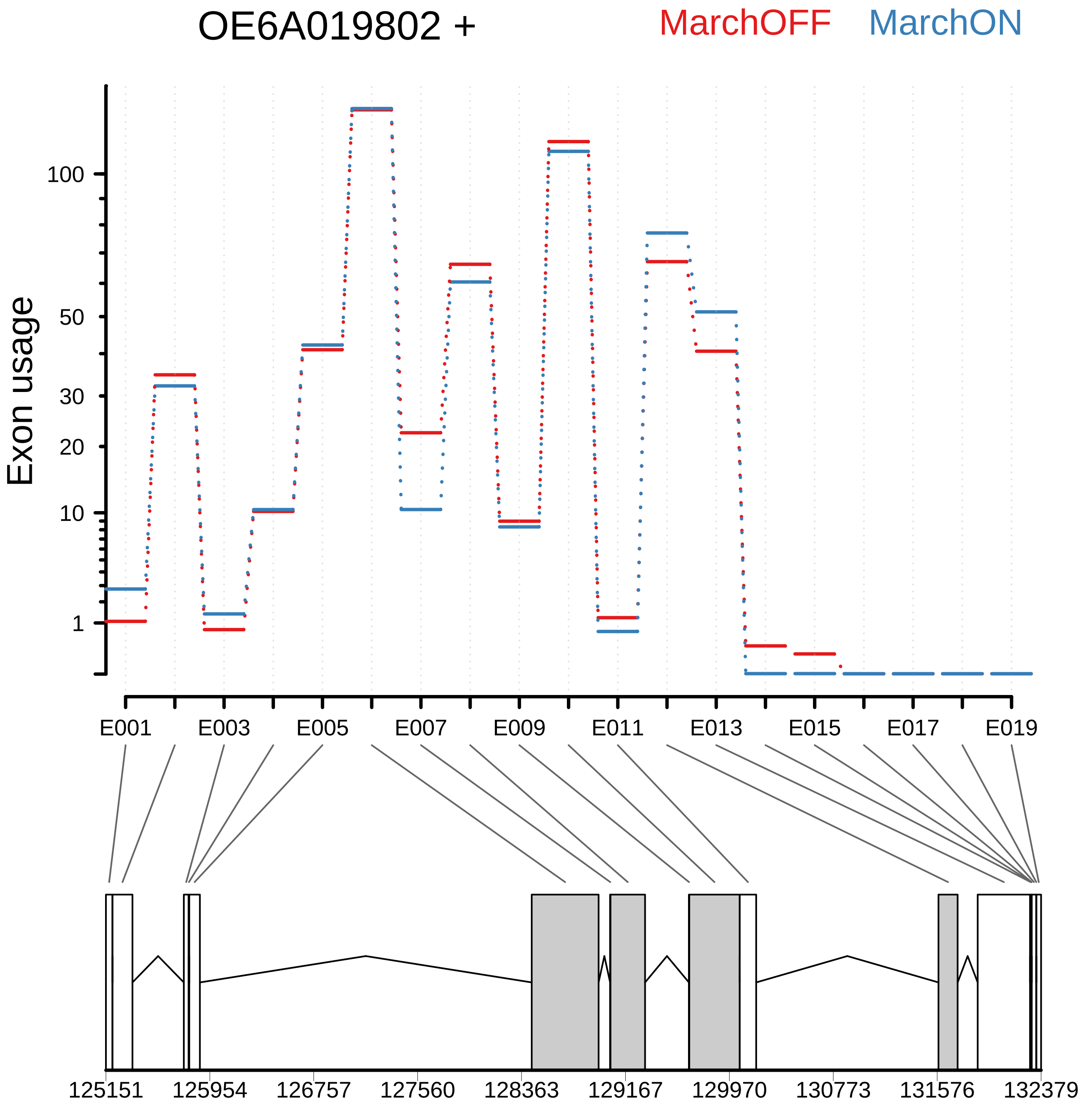

OE6A019802 +
MarchOFF
MarchON
Exon usage
100
50
30
20
10
1
E001
E003
E005
E007
E009
E011
E013
E015
E017
E019
125151
125954
126757
127560
128363
129167
129970
130773
131576
132379

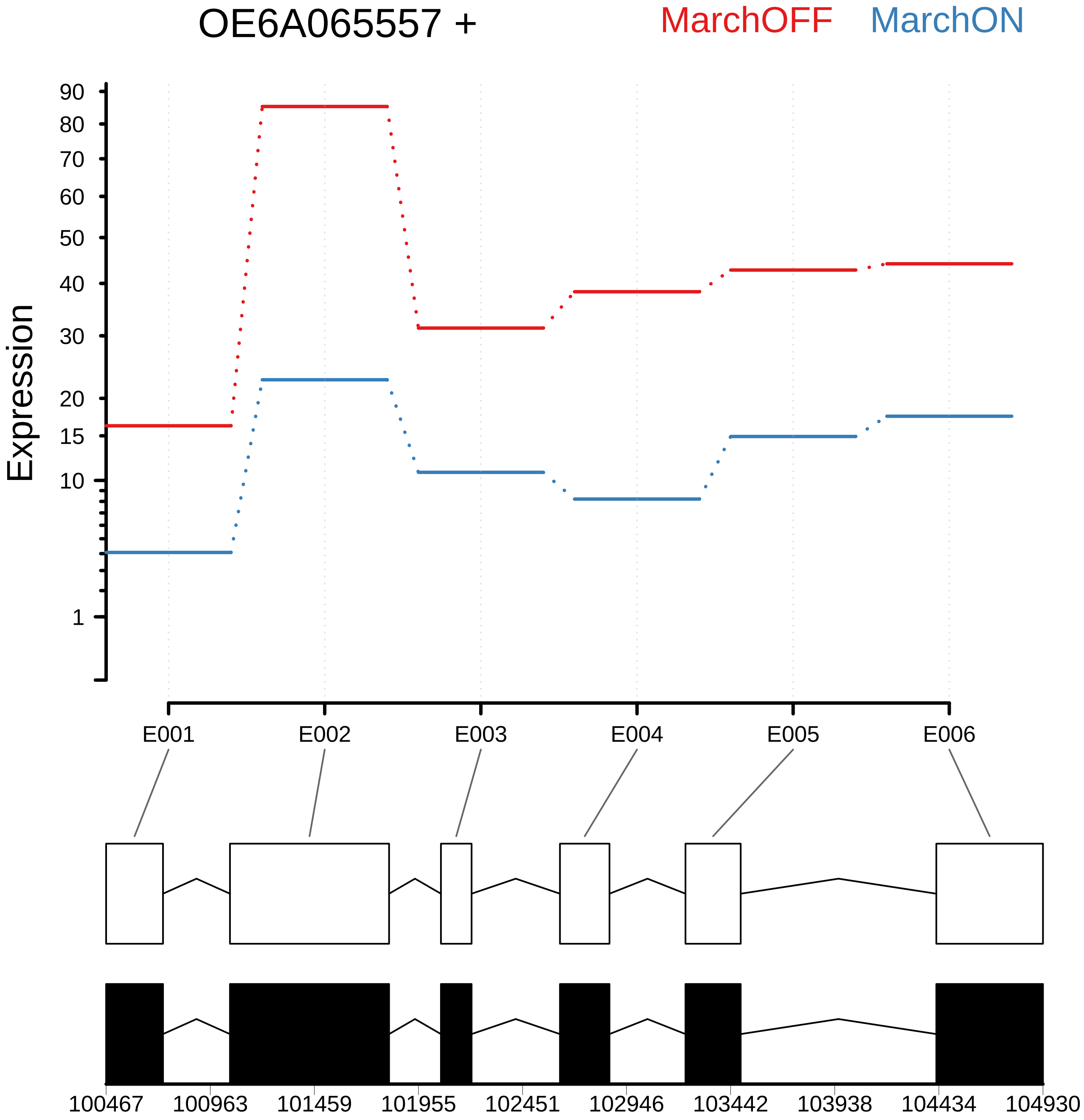

OE6A065557 +
MarchOFF
MarchON
Expression
90
80
70
60
50
40
30
20
15
10
1
E001
E002
E003
E004
E005
E006
100467
100963
101459
101955
102451
102946
103442
103938
104434
104930

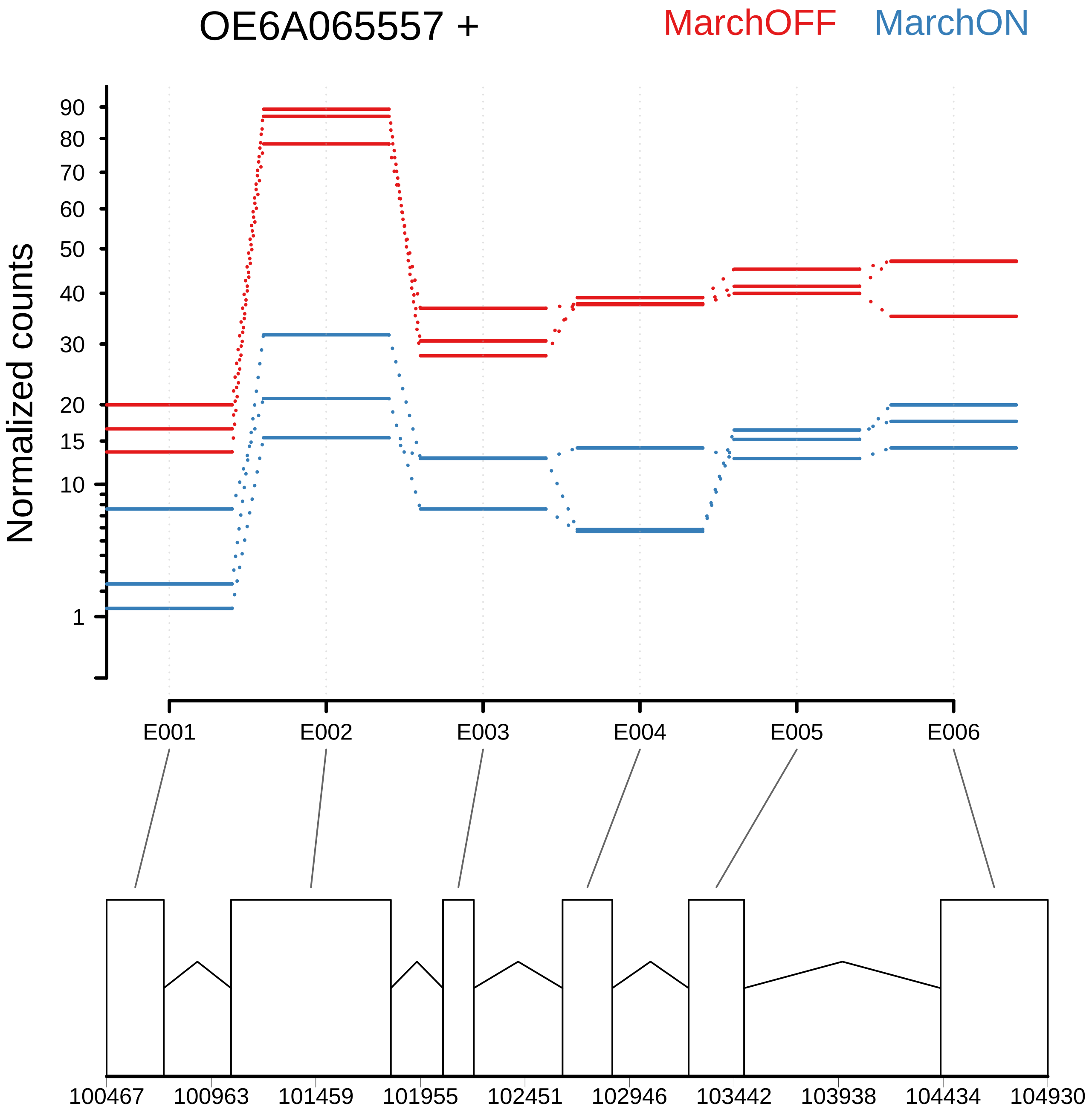

OE6A065557 +
MarchOFF
MarchON
Normalized counts
90
80
70
60
50
40
30
20
15
10
1
E001
E002
E003
E004
E005
E006
100467
100963
101459
101955
102451
102946
103442
103938
104434
104930

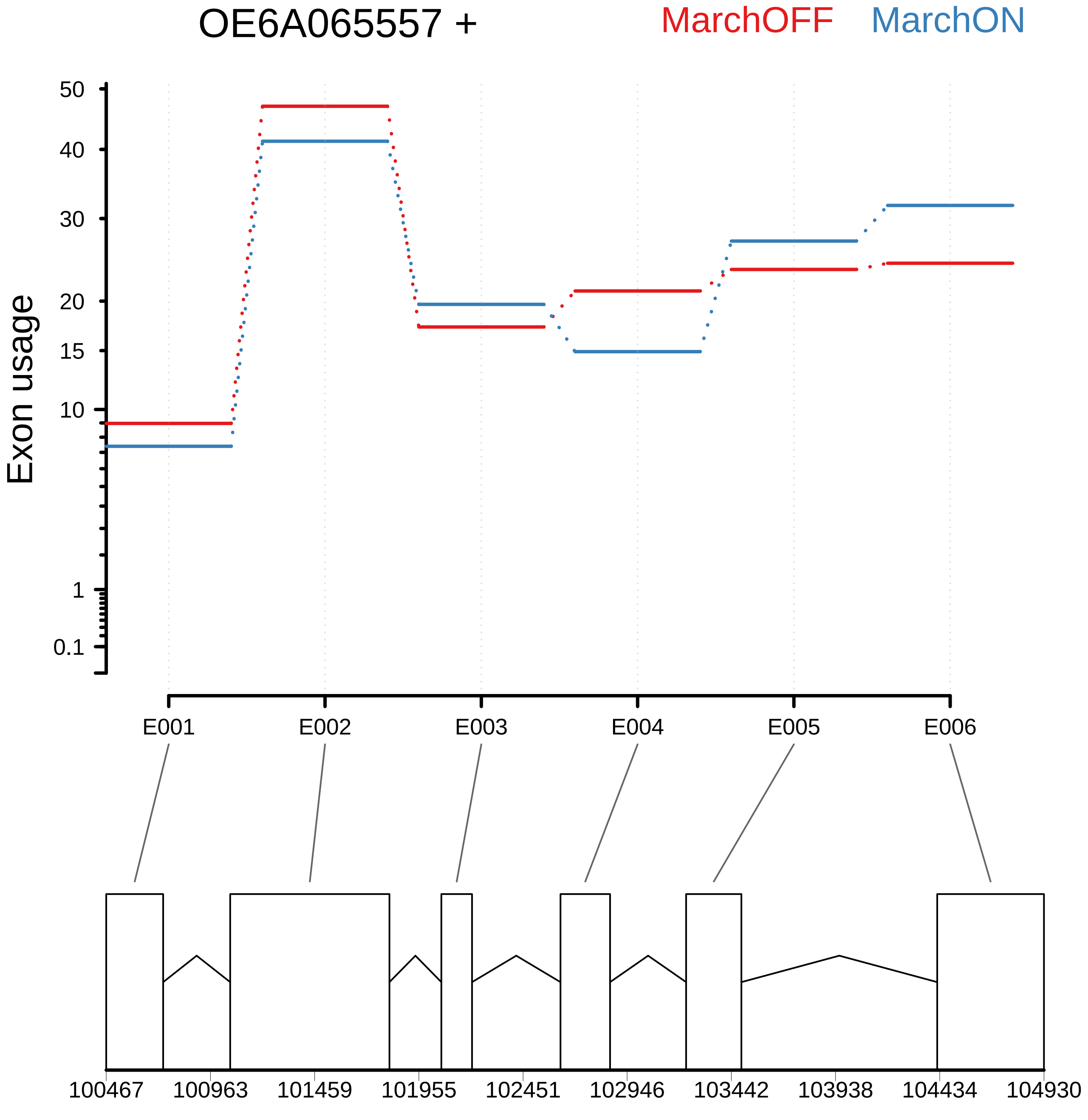

OE6A065557 +
MarchOFF
MarchON
Exon usage
50
40
30
20
15
10
1
0.1
E001
E002
E003
E004
E005
E006
100467
100963
101459
101955
102451
102946
103442
103938
104434
104930

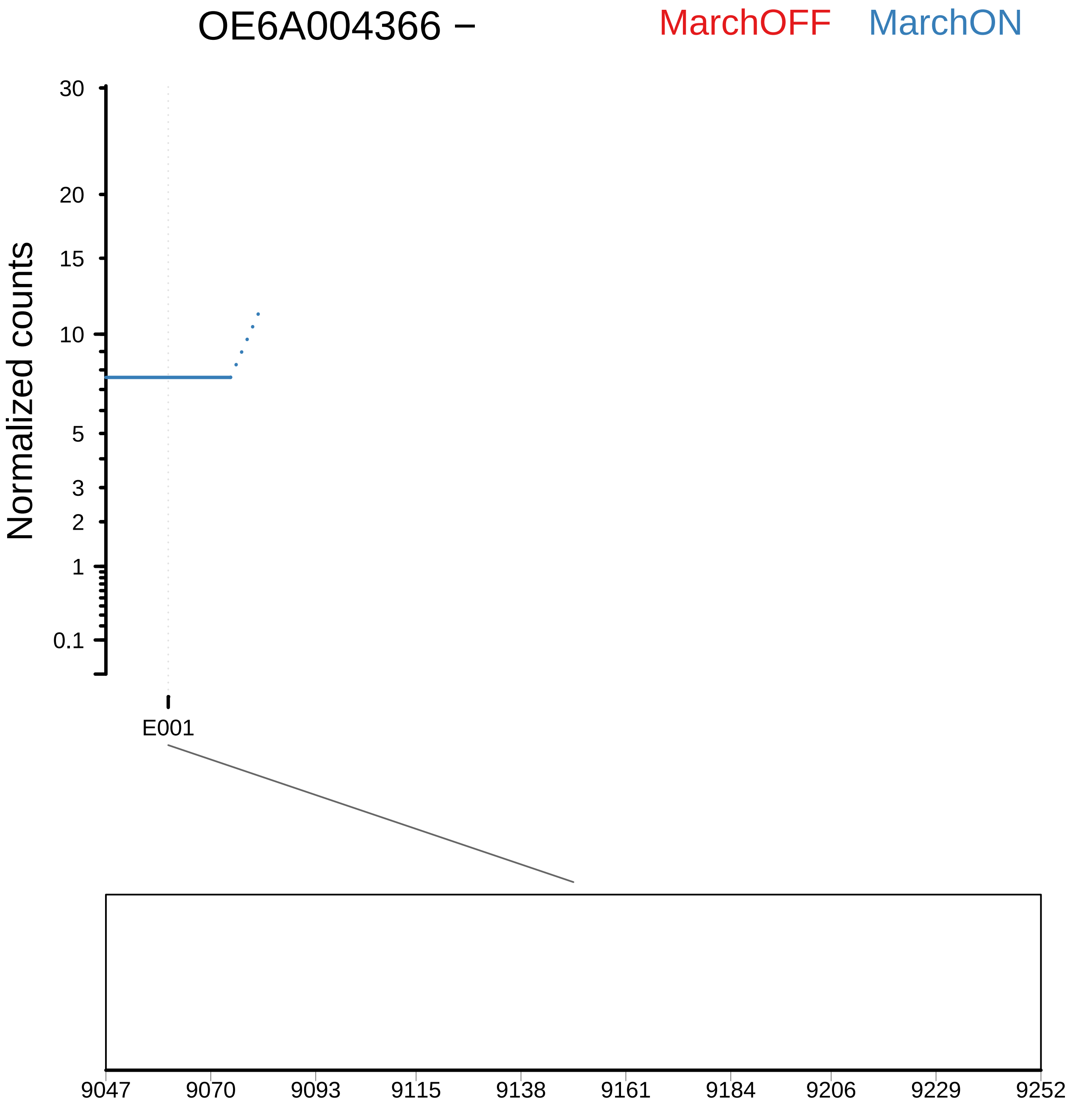

OE6A004366 −
MarchOFF
MarchON
Normalized counts
30
20
15
10
5
3
2
1
0.1
E001
9047
9070
9093
9115
9138
9161
9184
9206
9229
9252

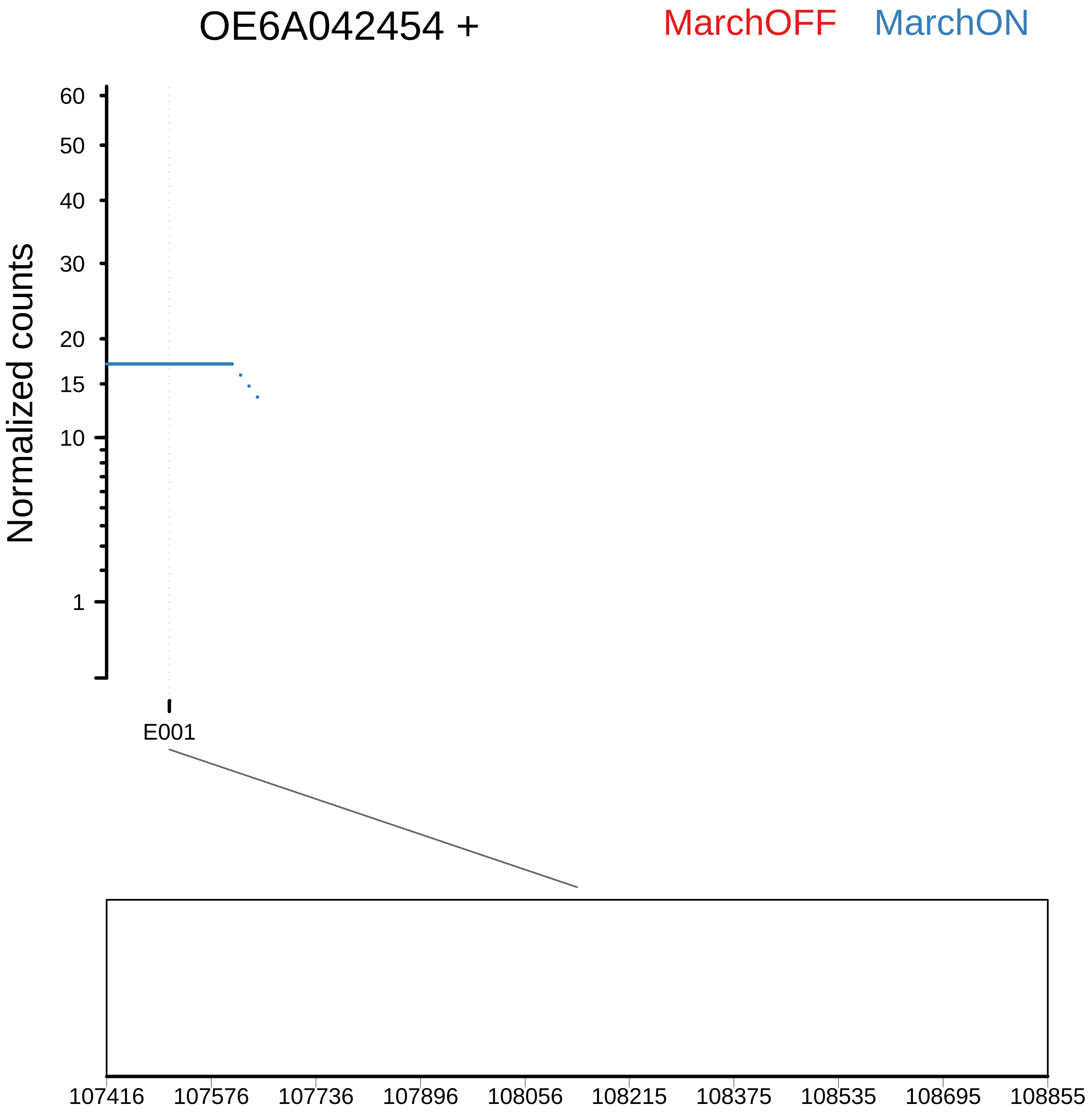
OE6A042454 +
MarchOFF
MarchON
Normalized counts
60
50
40
30
20
15
10
1
E001
107416
107576
107736
107896
108056
108215
108375
108535
108695
108855

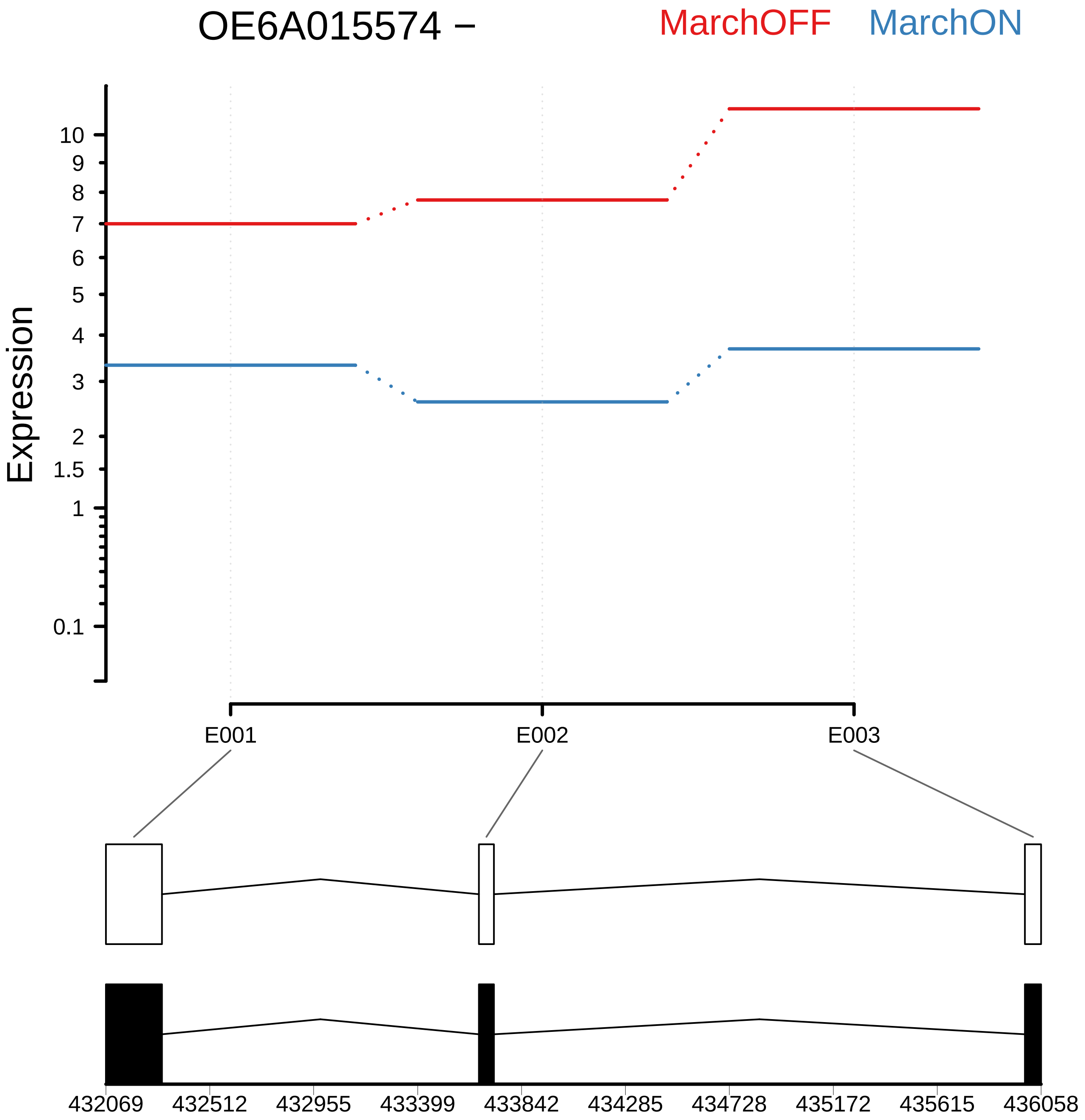
OE6A015574 −
MarchOFF
MarchON
Expression
10
9
8
7
6
5
4
3
2
1.5
1
0.1
E001
E002
E003
432069
432512
432955
433399
433842
434285
434728
435172
435615
436058

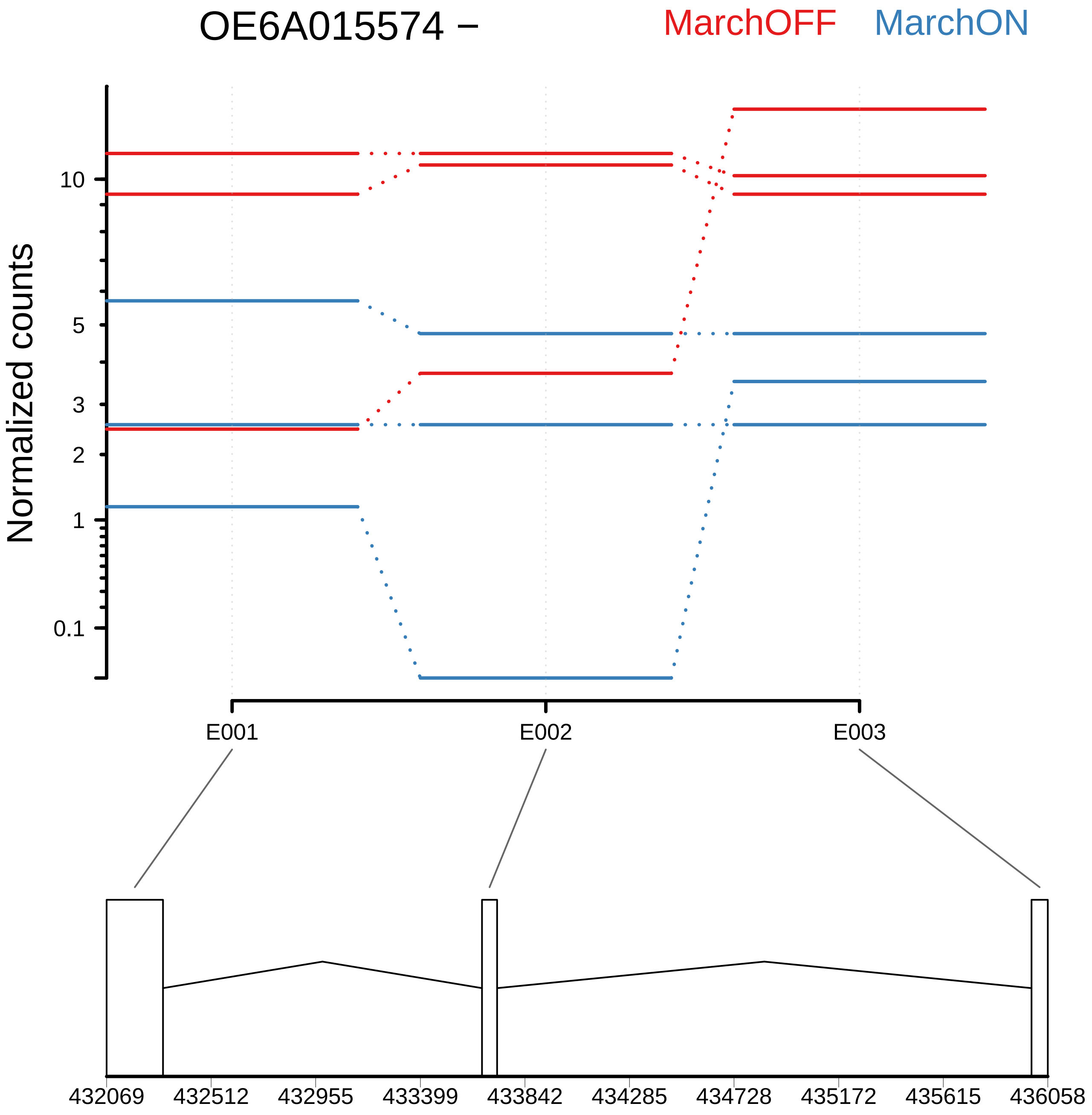

OE6A015574 −
MarchOFF
MarchON
Normalized counts
10
5
3
2
1
0.1
E001
E002
E003
432069
432512
432955
433399
433842
434285
434728
435172
435615
436058

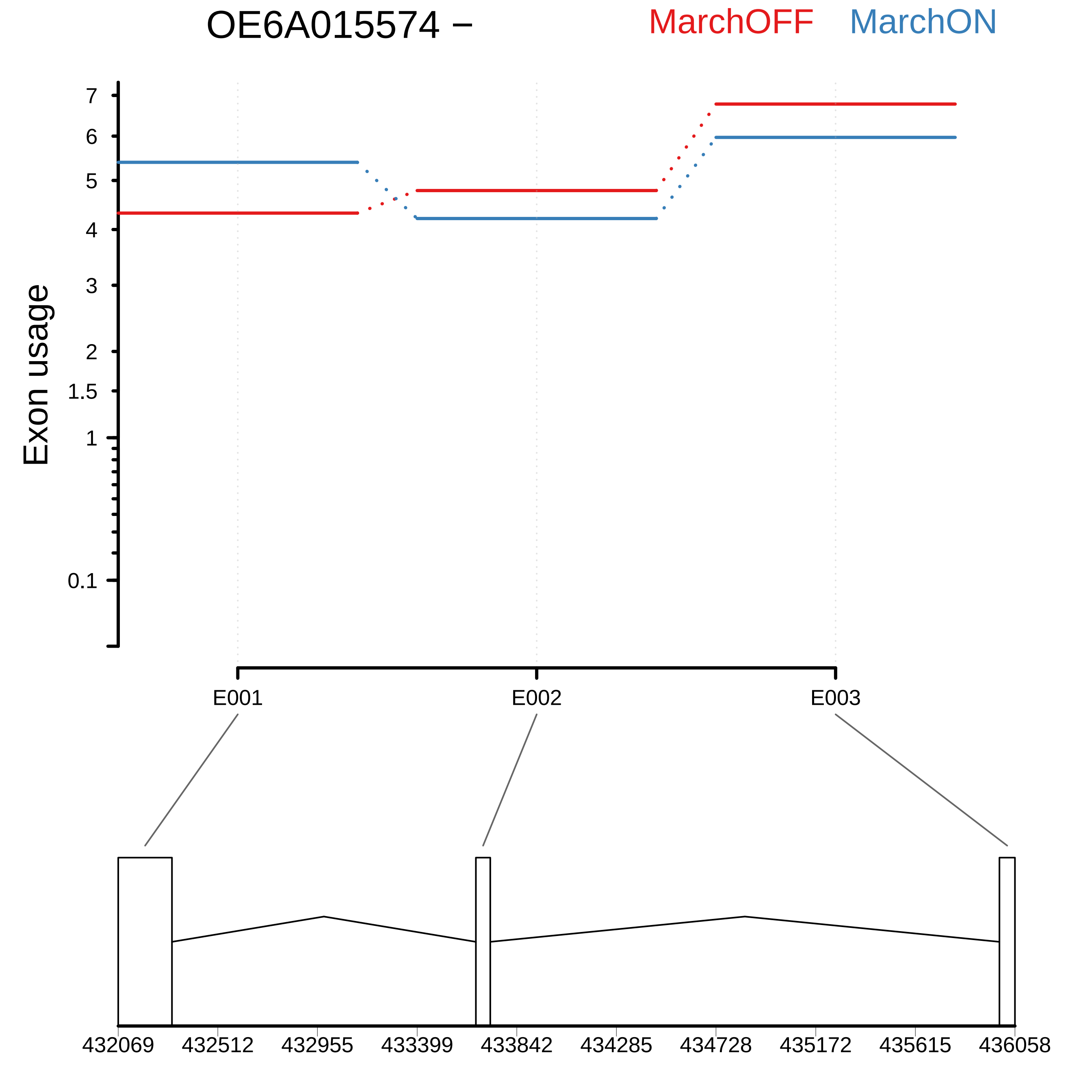

OE6A015574 −
MarchOFF
MarchON
Exon usage
7
6
5
4
3
2
1.5
1
0.1
E001
E002
E003
432069
432512
432955
433399
433842
434285
434728
435172
435615
436058

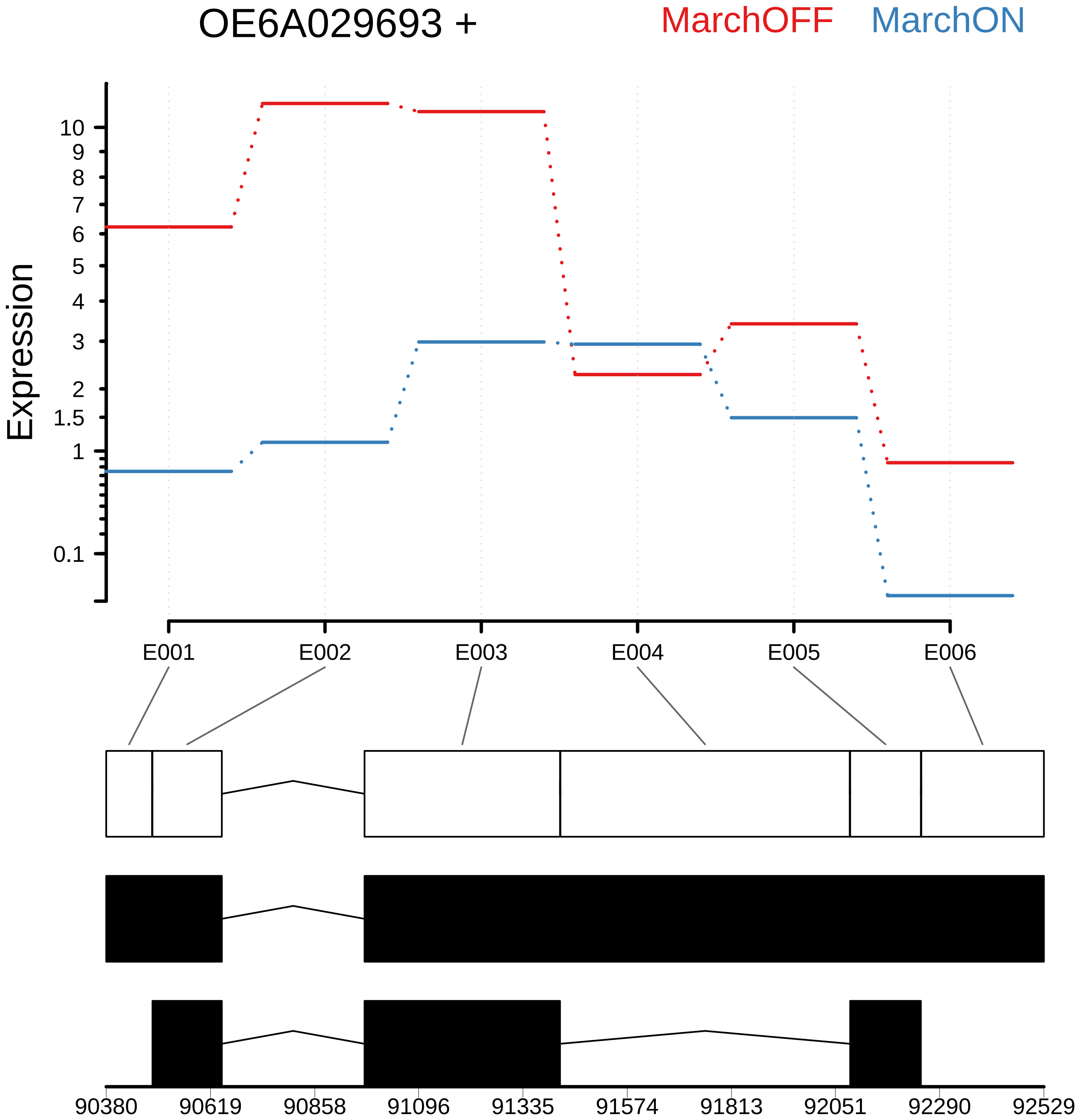

OE6A029693 +
MarchOFF
MarchON
Expression
10
9
8
7
6
5
4
3
2
1.5
1
0.1
E001
E002
E003
E004
E005
E006
90380
90619
90858
91096
91335
91574
91813
92051
92290
92529

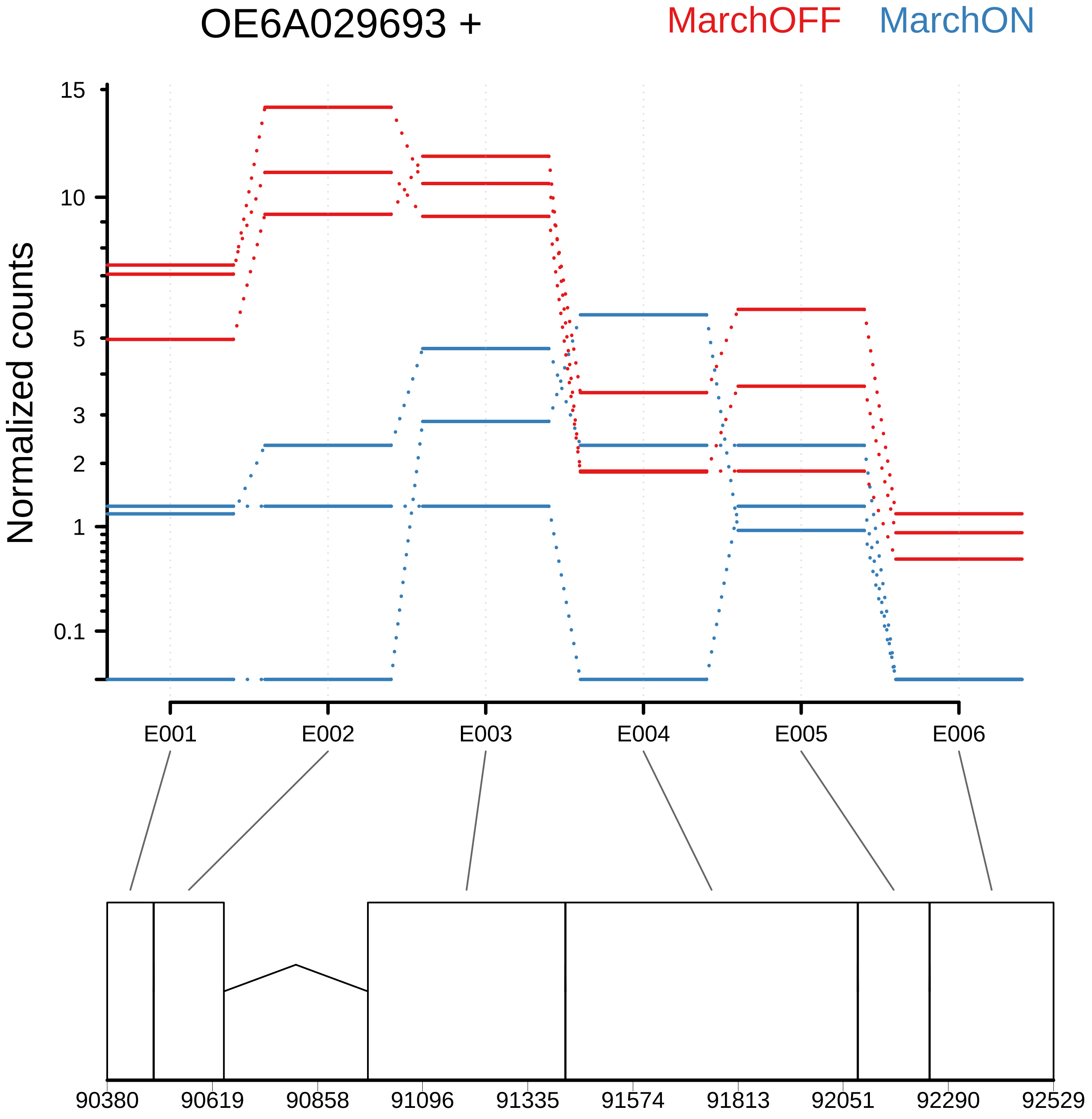
OE6A029693 +
MarchOFF
MarchON
Normalized counts
15
10
5
3
2
1
0.1
E001
E002
E003
E004
E005
E006
90380
90619
90858
91096
91335
91574
91813
92051
92290
92529

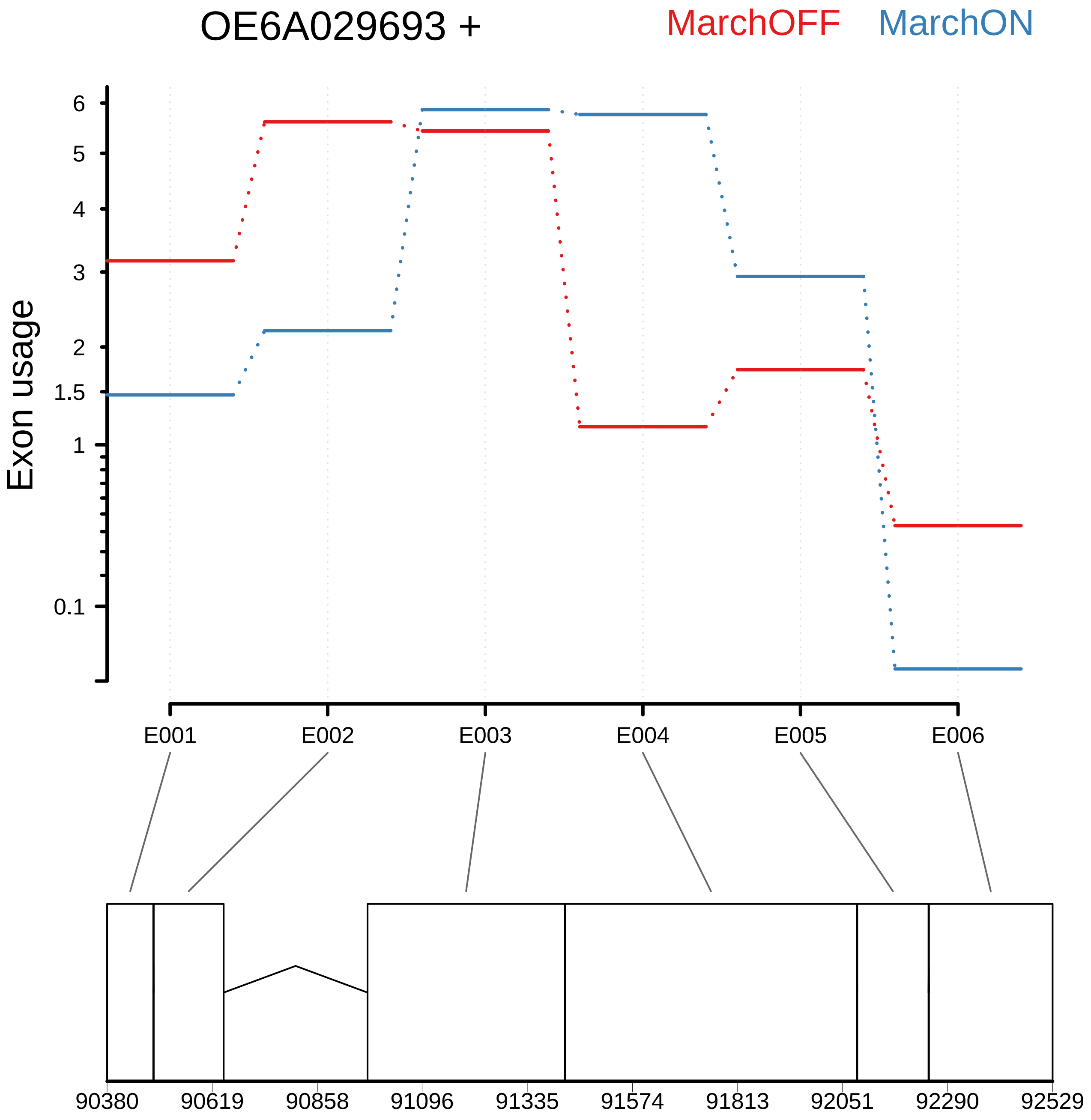
OE6A029693 +
MarchOFF
MarchON
Exon usage
6
5
4
3
2
1.5
1
0.1
E001
E002
E003
E004
E005
E006
90380
90619
90858
91096
91335
91574
91813
92051
92290
92529

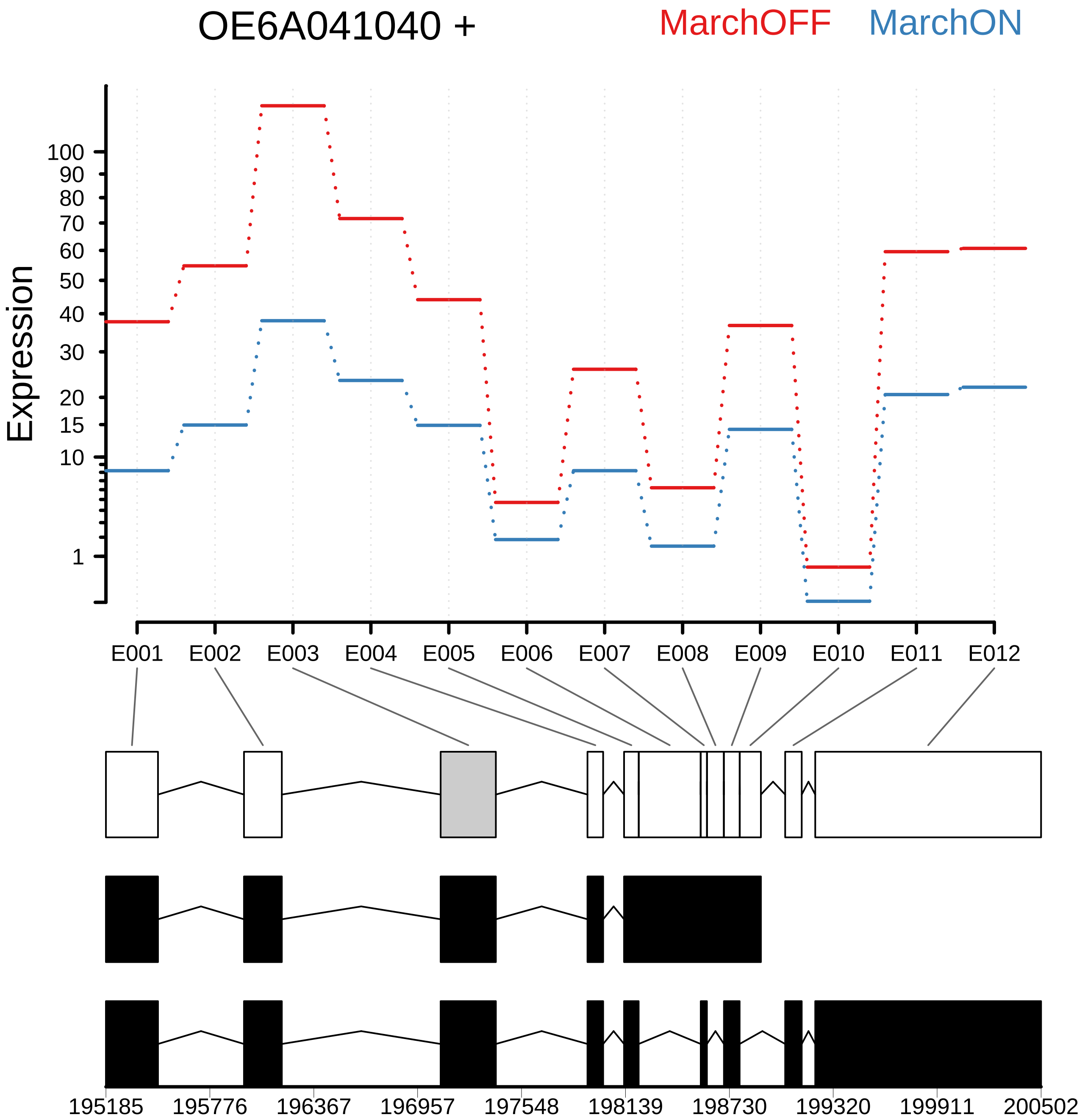

OE6A041040 +
MarchOFF
MarchON
Expression
100
90
80
70
60
50
40
30
20
15
10
1
E001
E002
E003
E004
E005
E006
E007
E008
E009
E010
E011
E012
195185
195776
196367
196957
197548
198139
198730
199320
199911
200502

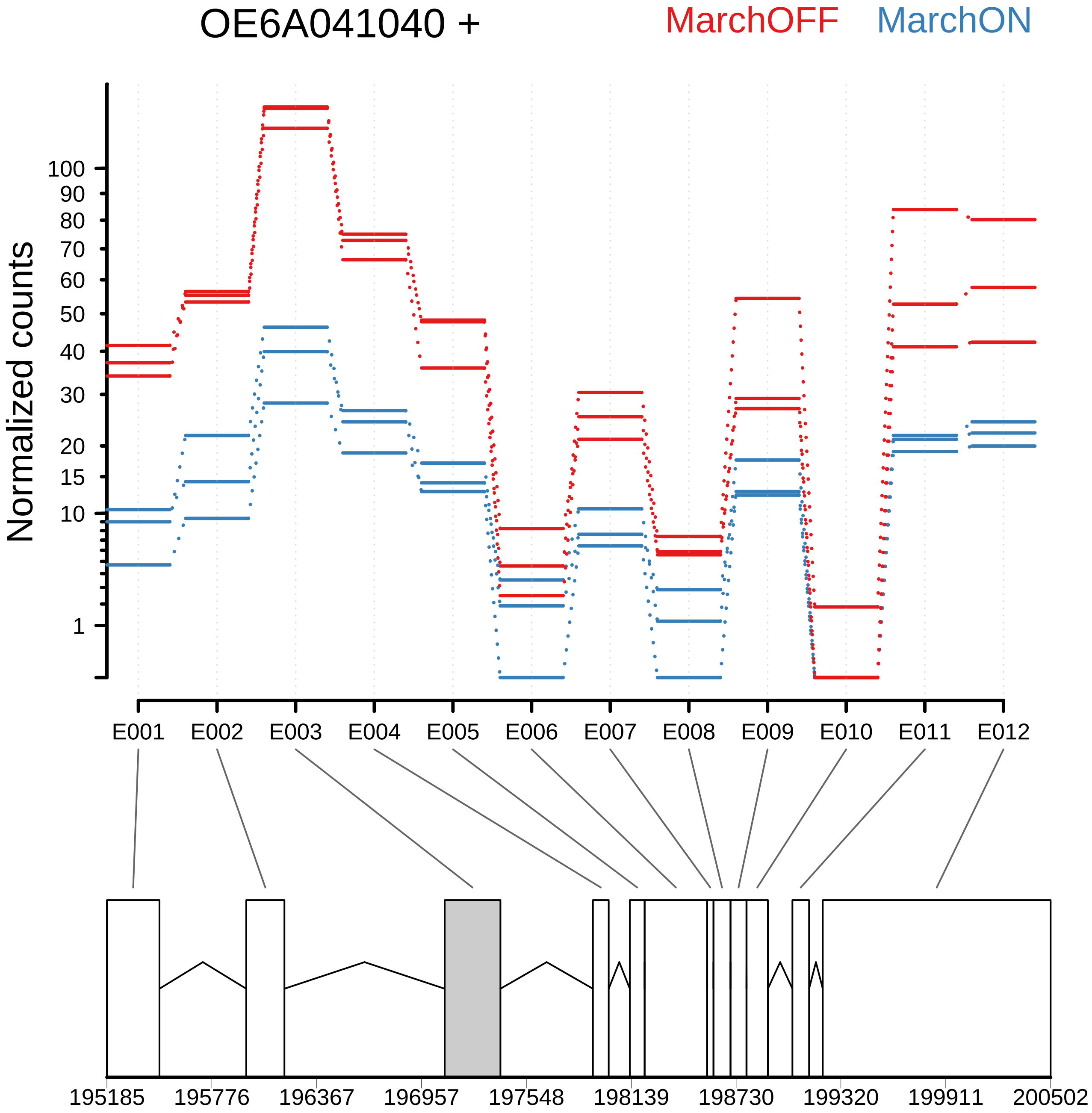

OE6A041040 +
MarchOFF
MarchON
Normalized counts
100
90
80
70
60
50
40
30
20
15
10
1
E001
E002
E003
E004
E005
E006
E007
E008
E009
E010
E011
E012
195185
195776
196367
196957
197548
198139
198730
199320
199911
200502

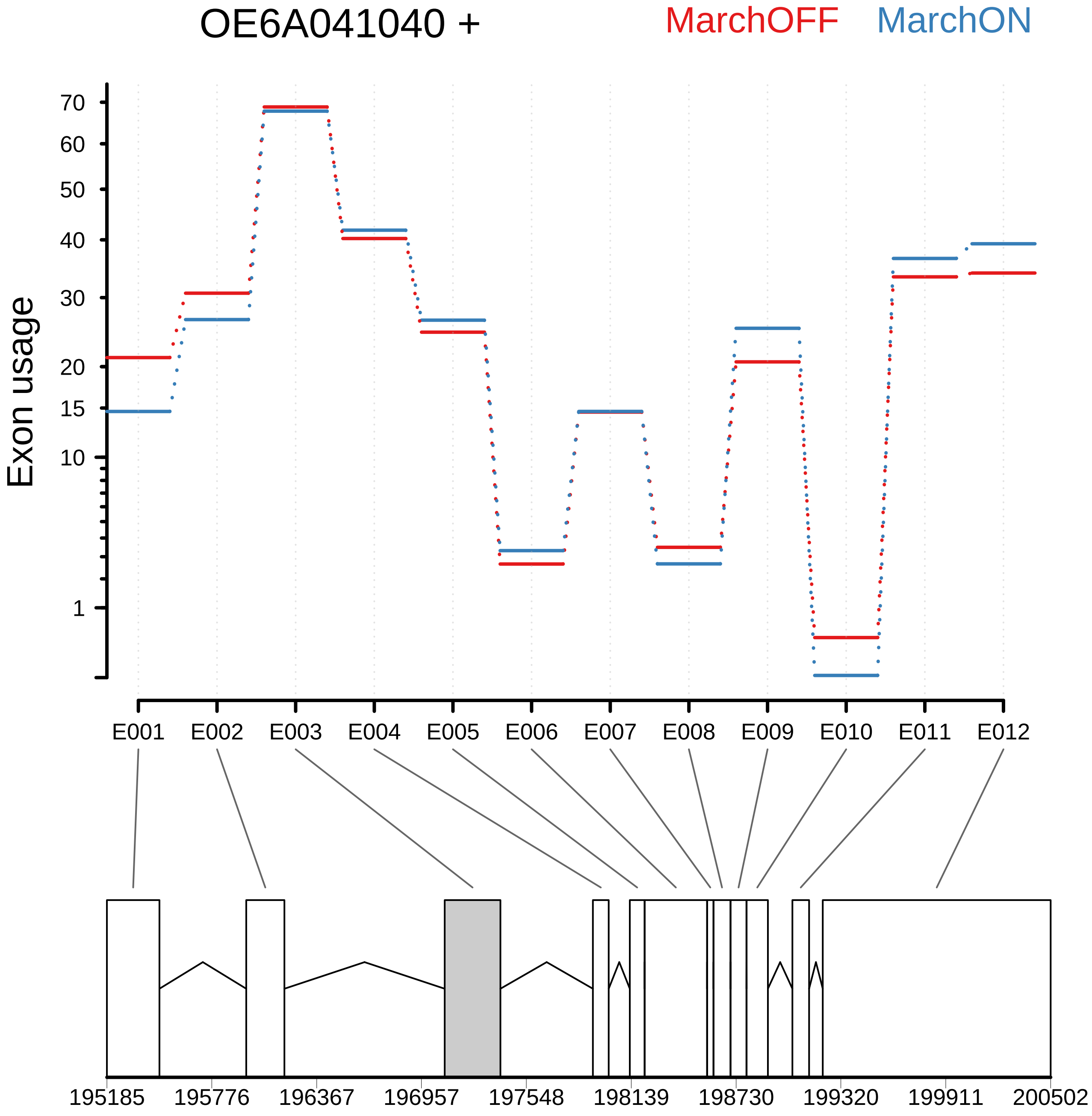

OE6A041040 +
MarchOFF
MarchON
Exon usage
70
60
50
40
30
20
15
10
1
E001
E002
E003
E004
E005
E006
E007
E008
E009
E010
E011
E012
195185
195776
196367
196957
197548
198139
198730
199320
199911
200502

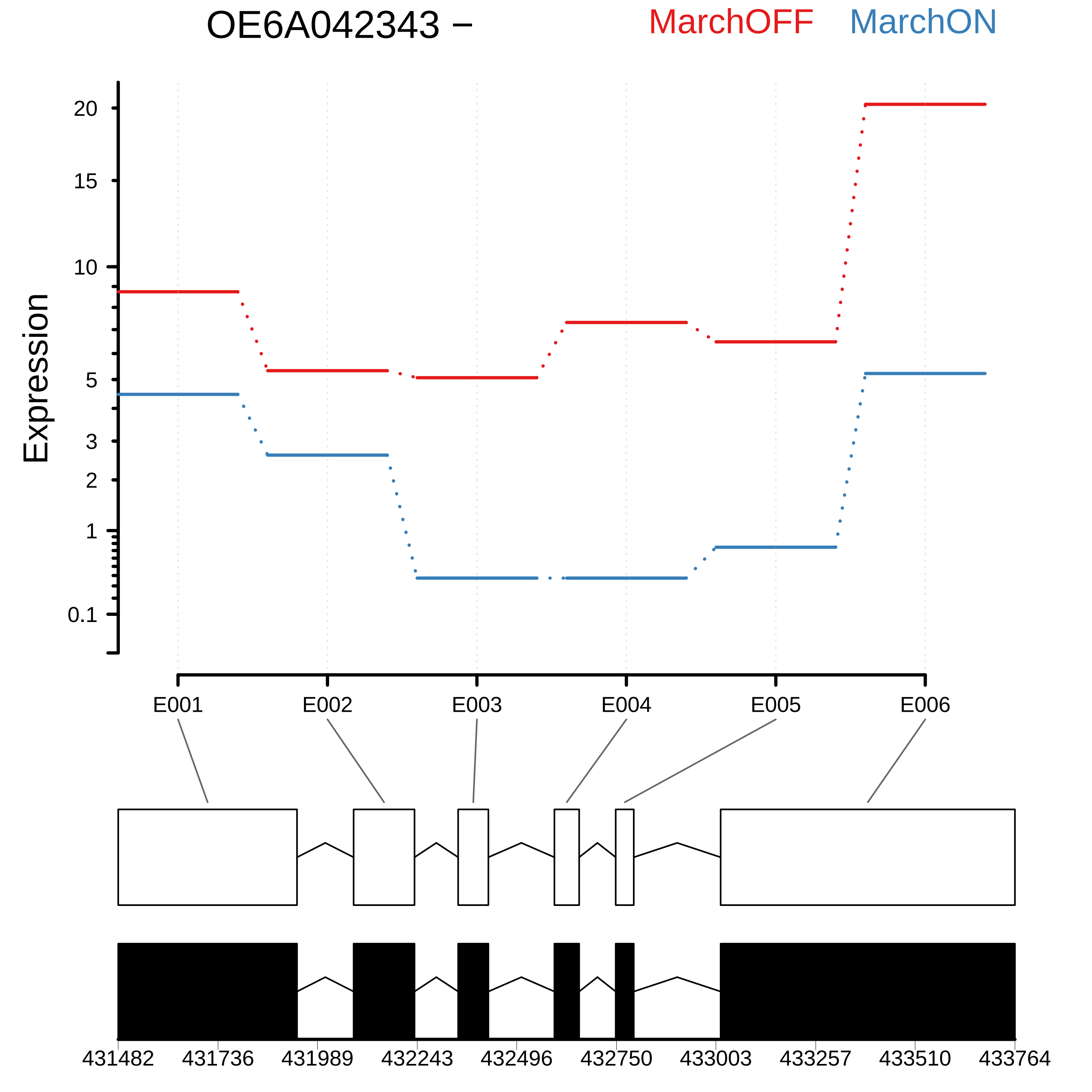

OE6A042343 −
MarchOFF
MarchON
Expression
20
15
10
5
3
2
1
0.1
E001
E002
E003
E004
E005
E006
431482
431736
431989
432243
432496
432750
433003
433257
433510
433764

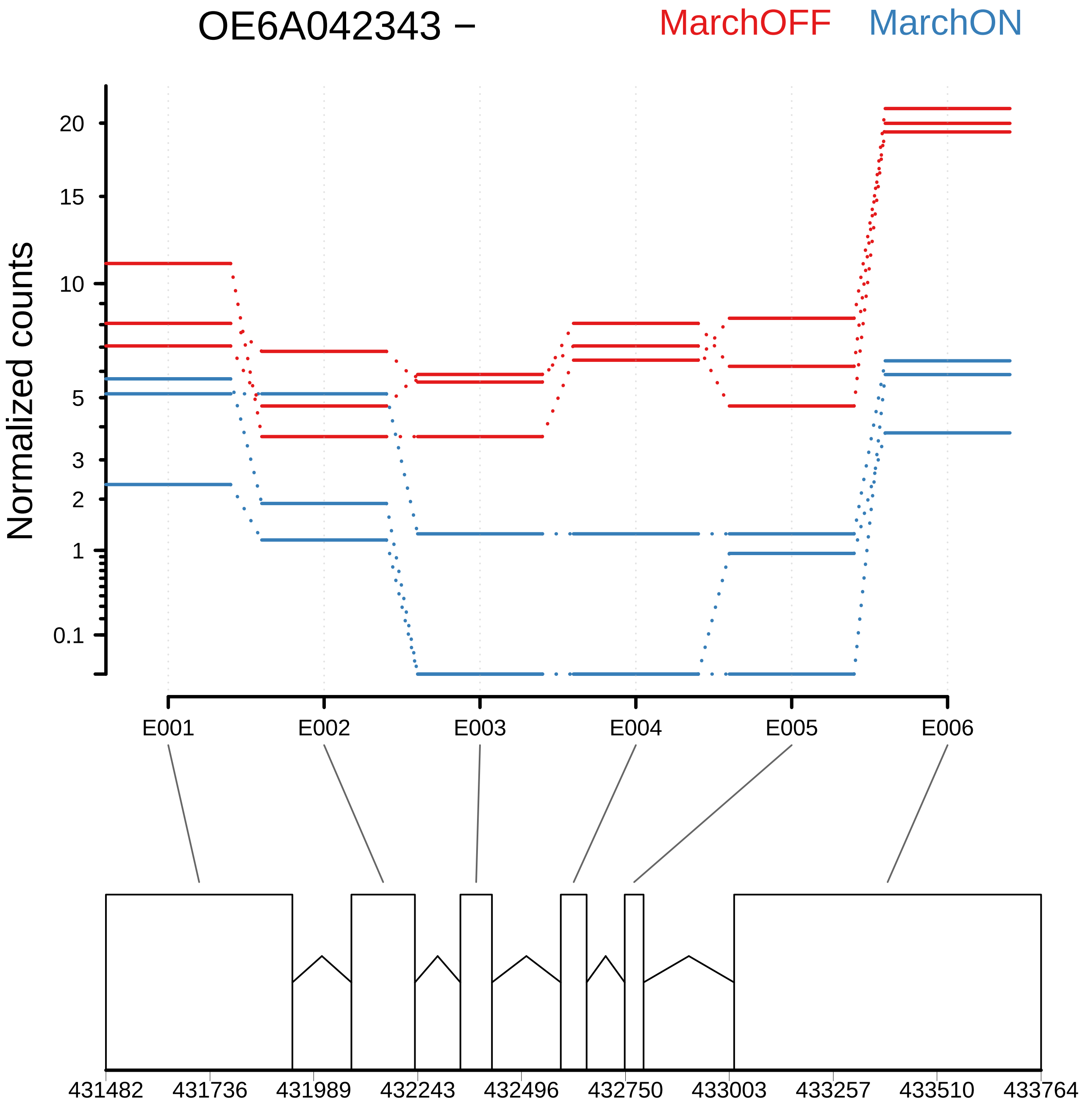
OE6A042343 −
MarchOFF
MarchON
Normalized counts
20
15
10
5
3
2
1
0.1
E001
E002
E003
E004
E005
E006
431482
431736
431989
432243
432496
432750
433003
433257
433510
433764

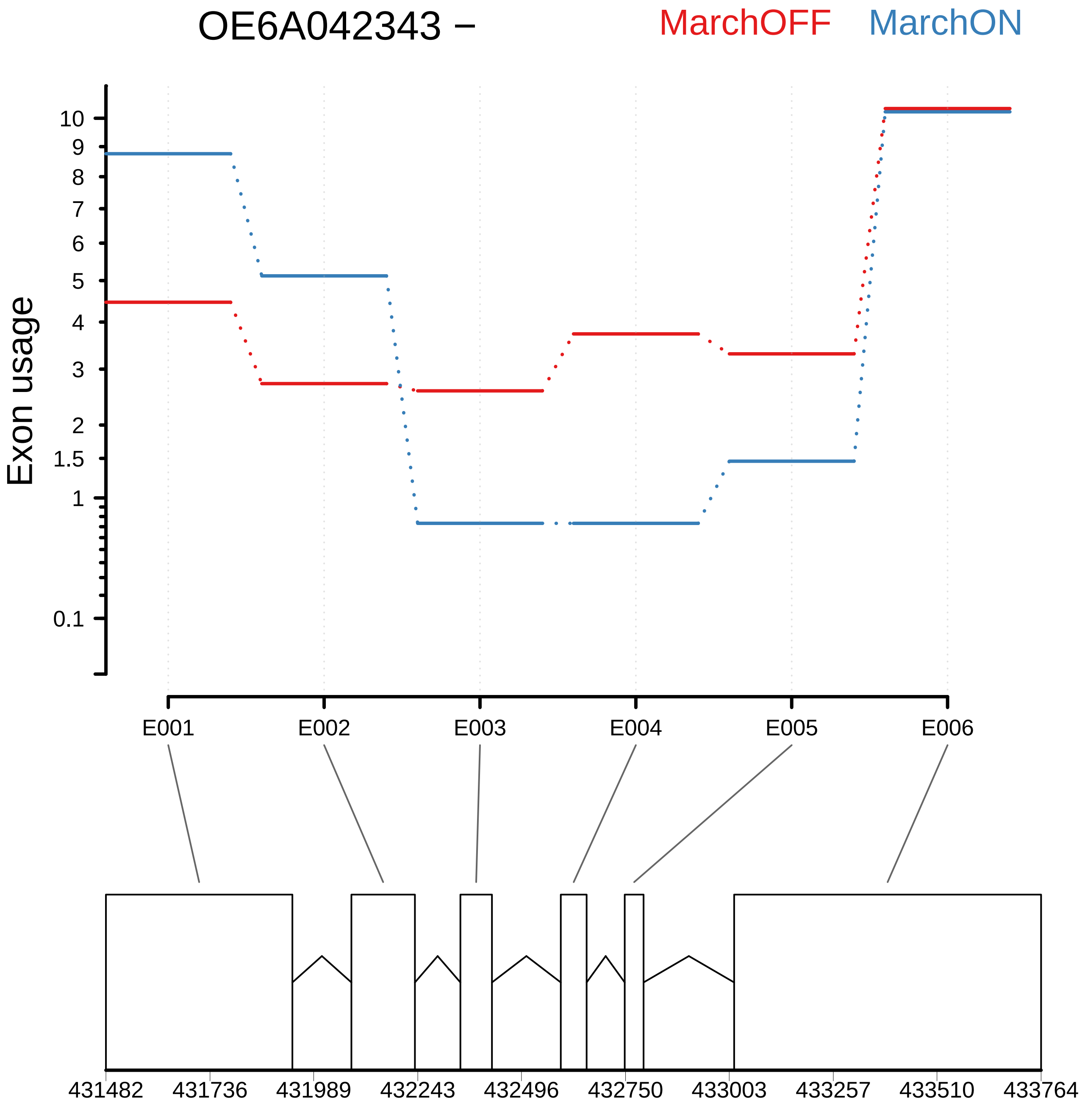

OE6A042343 −
MarchOFF
MarchON
Exon usage
10
9
8
7
6
5
4
3
2
1.5
1
0.1
E001
E002
E003
E004
E005
E006
431482
431736
431989
432243
432496
432750
433003
433257
433510
433764

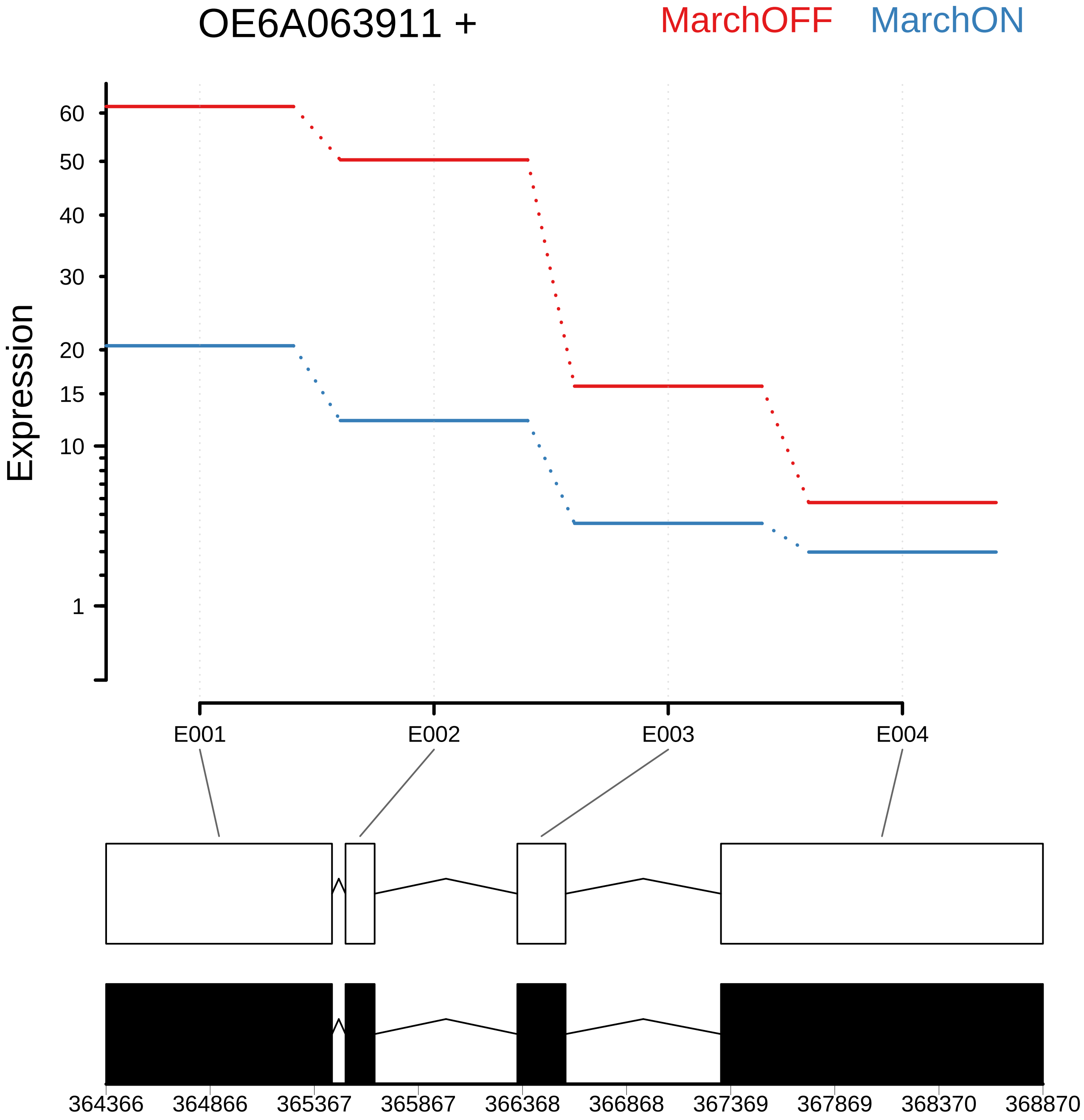

OE6A063911 +
MarchOFF
MarchON
Expression
60
50
40
30
20
15
10
1
E001
E002
E003
E004
364366
364866
365367
365867
366368
366868
367369
367869
368370
368870

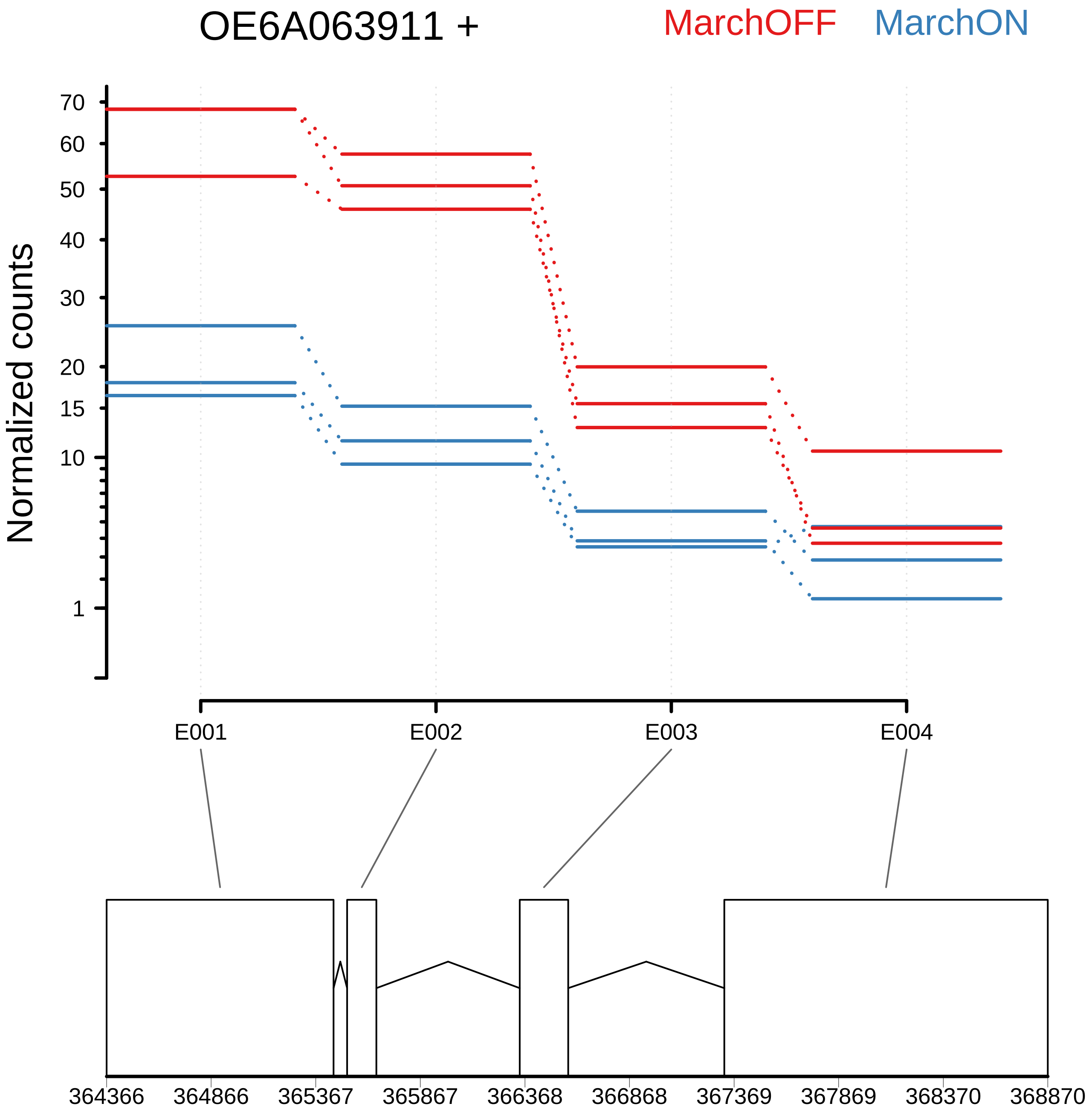
OE6A063911 +
MarchOFF
MarchON
Normalized counts
70
60
50
40
30
20
15
10
1
E001
E002
E003
E004
364366
364866
365367
365867
366368
366868
367369
367869
368370
368870

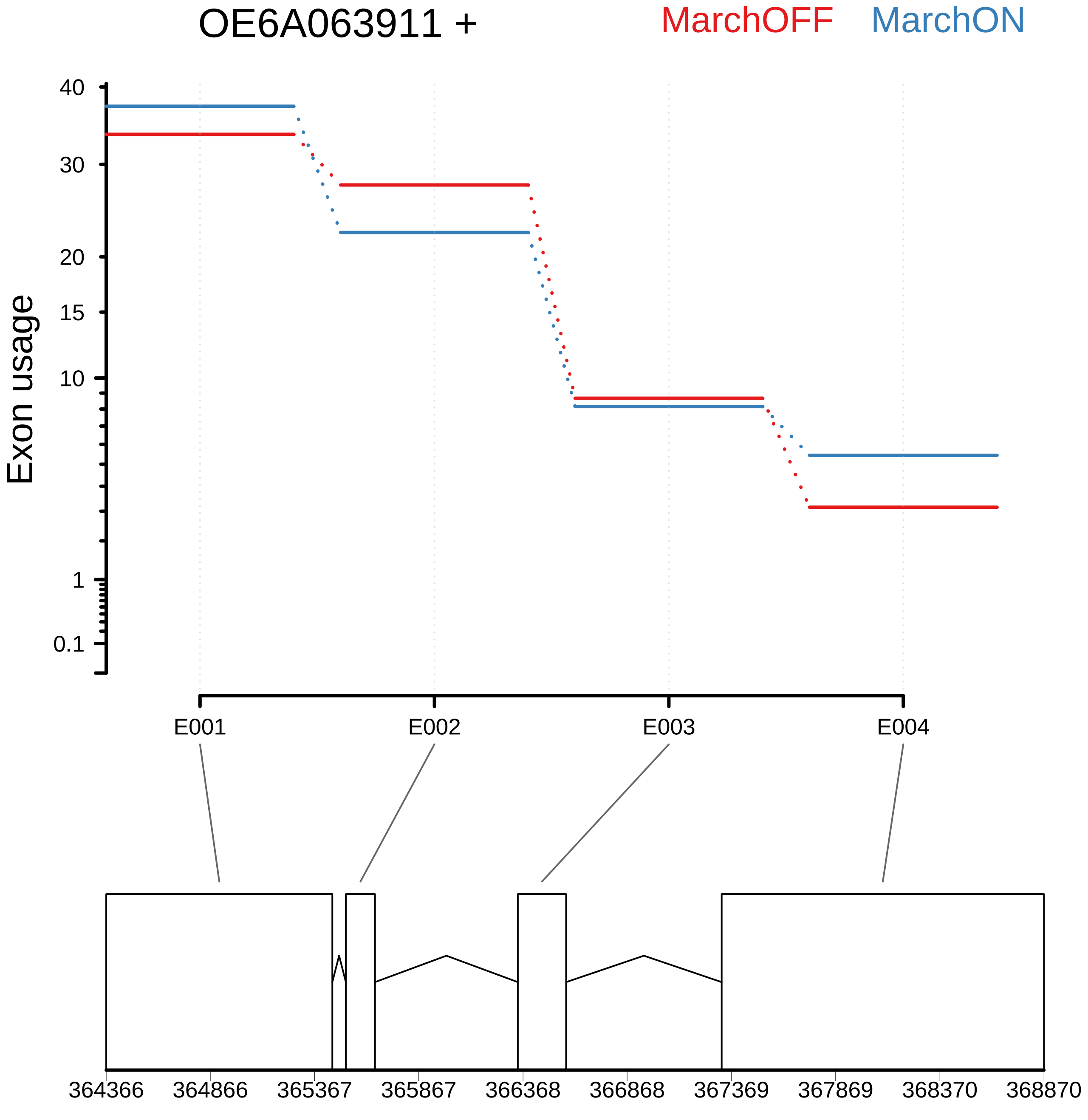

OE6A063911 +
MarchOFF
MarchON
Exon usage
40
30
20
15
10
1
0.1
E001
E002
E003
E004
364366
364866
365367
365867
366368
366868
367369
367869
368370
368870

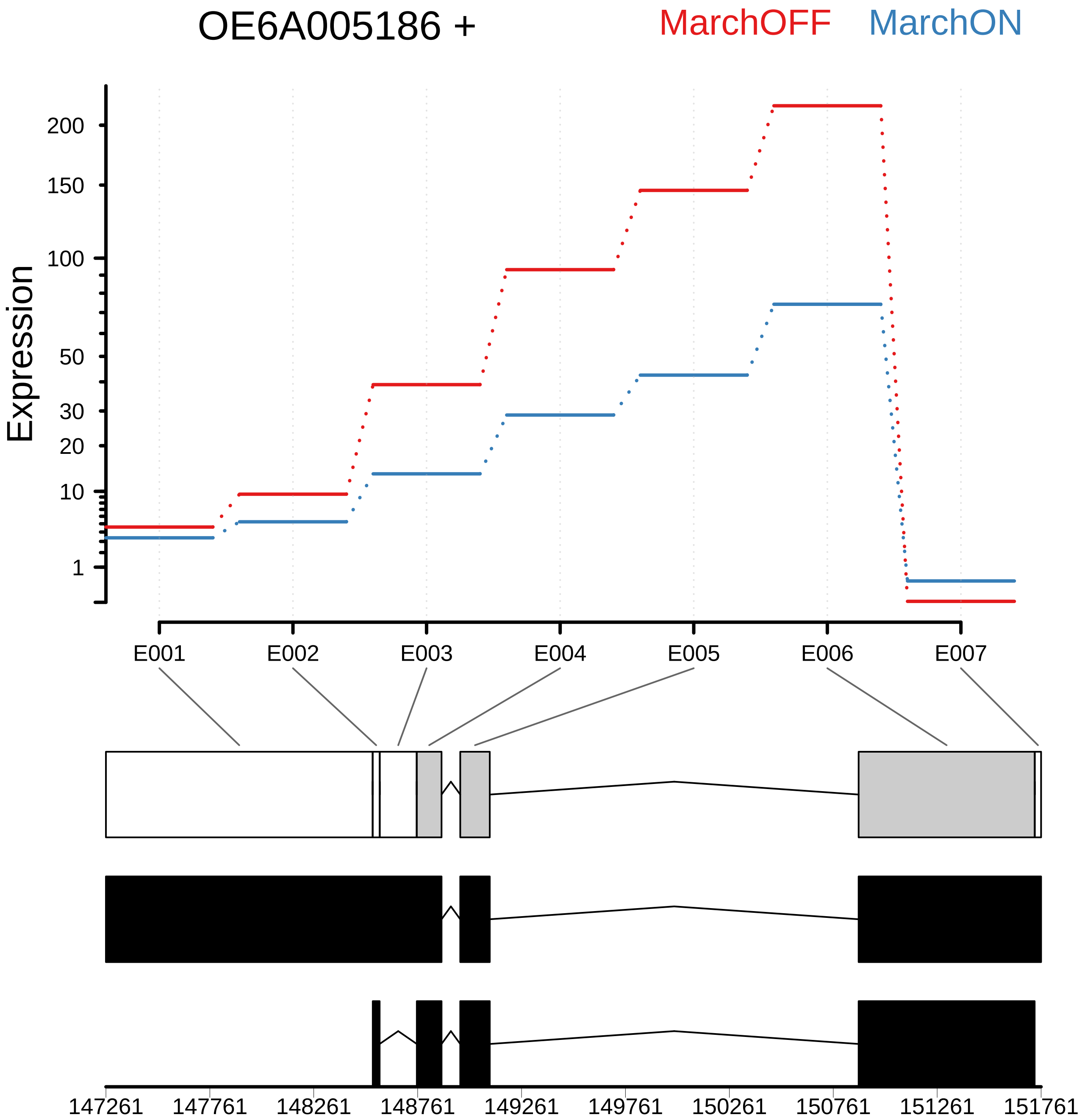

OE6A005186 +
MarchOFF
MarchON
Expression
200
150
100
50
30
20
10
1
E001
E002
E003
E004
E005
E006
E007
147261
147761
148261
148761
149261
149761
150261
150761
151261
151761

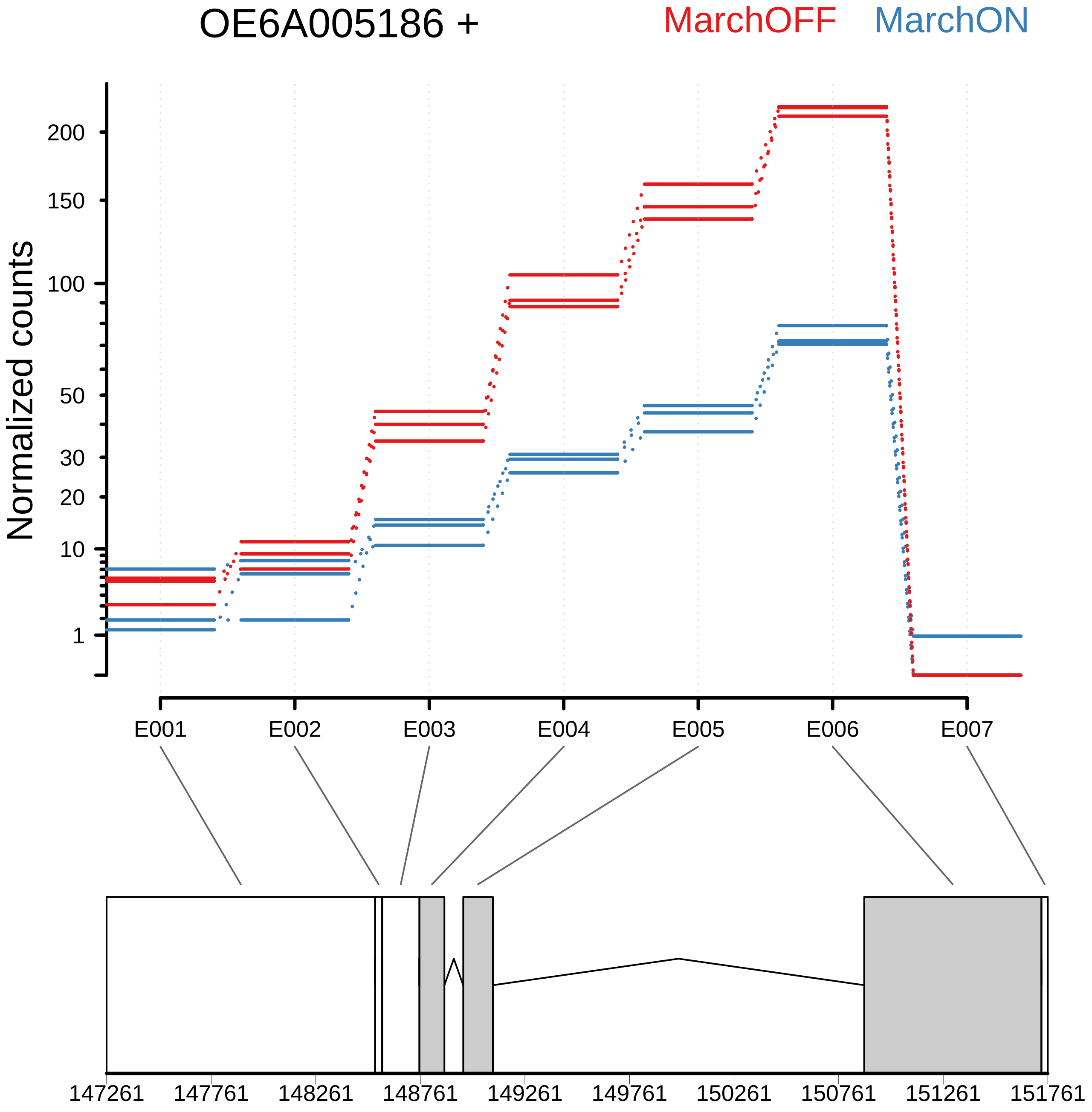
OE6A005186 +
MarchOFF
MarchON
Normalized counts
200
150
100
50
30
20
10
1
E001
E002
E003
E004
E005
E006
E007
147261
147761
148261
148761
149261
149761
150261
150761
151261
151761

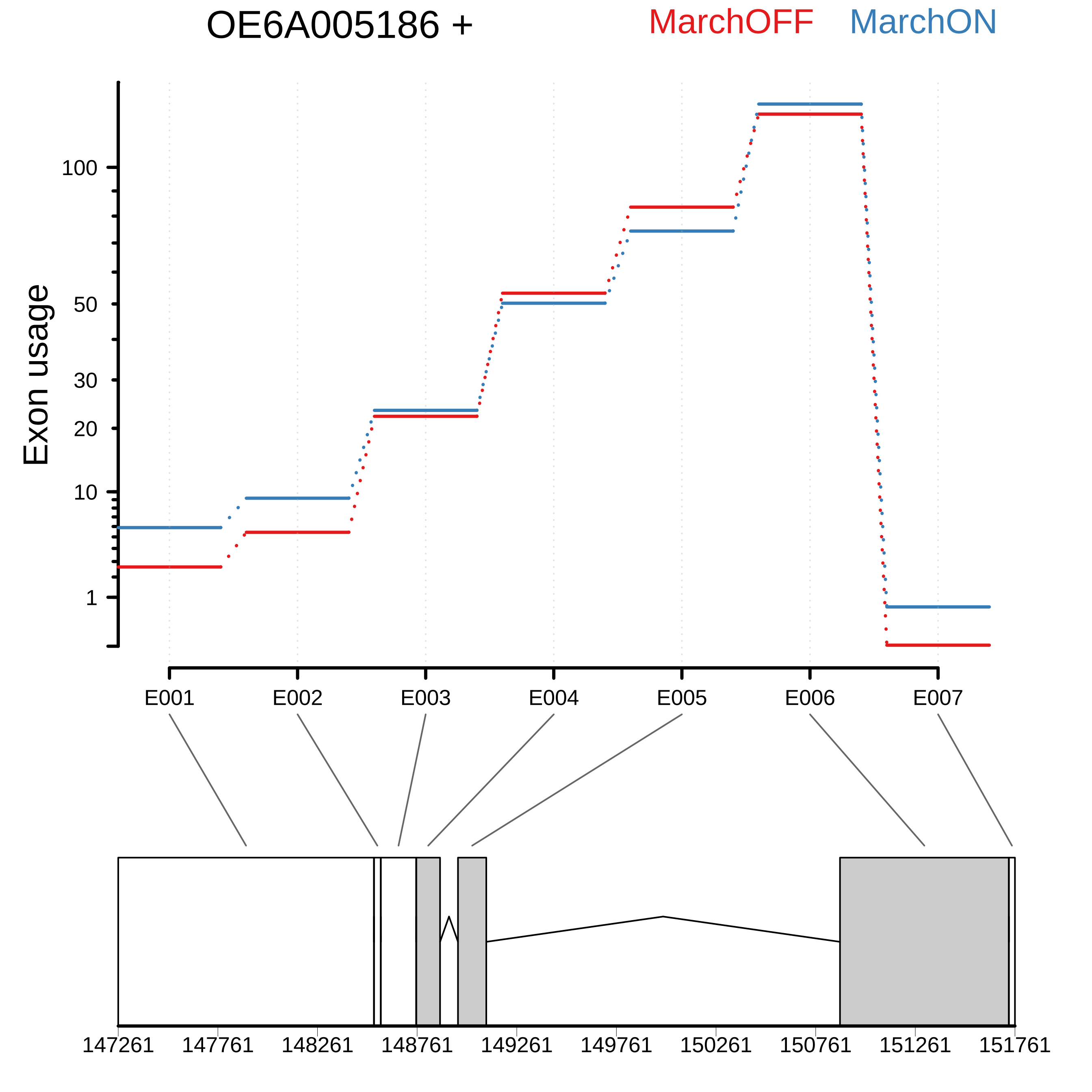

OE6A005186 +
MarchOFF
MarchON
Exon usage
100
50
30
20
10
1
E001
E002
E003
E004
E005
E006
E007
147261
147761
148261
148761
149261
149761
150261
150761
151261
151761

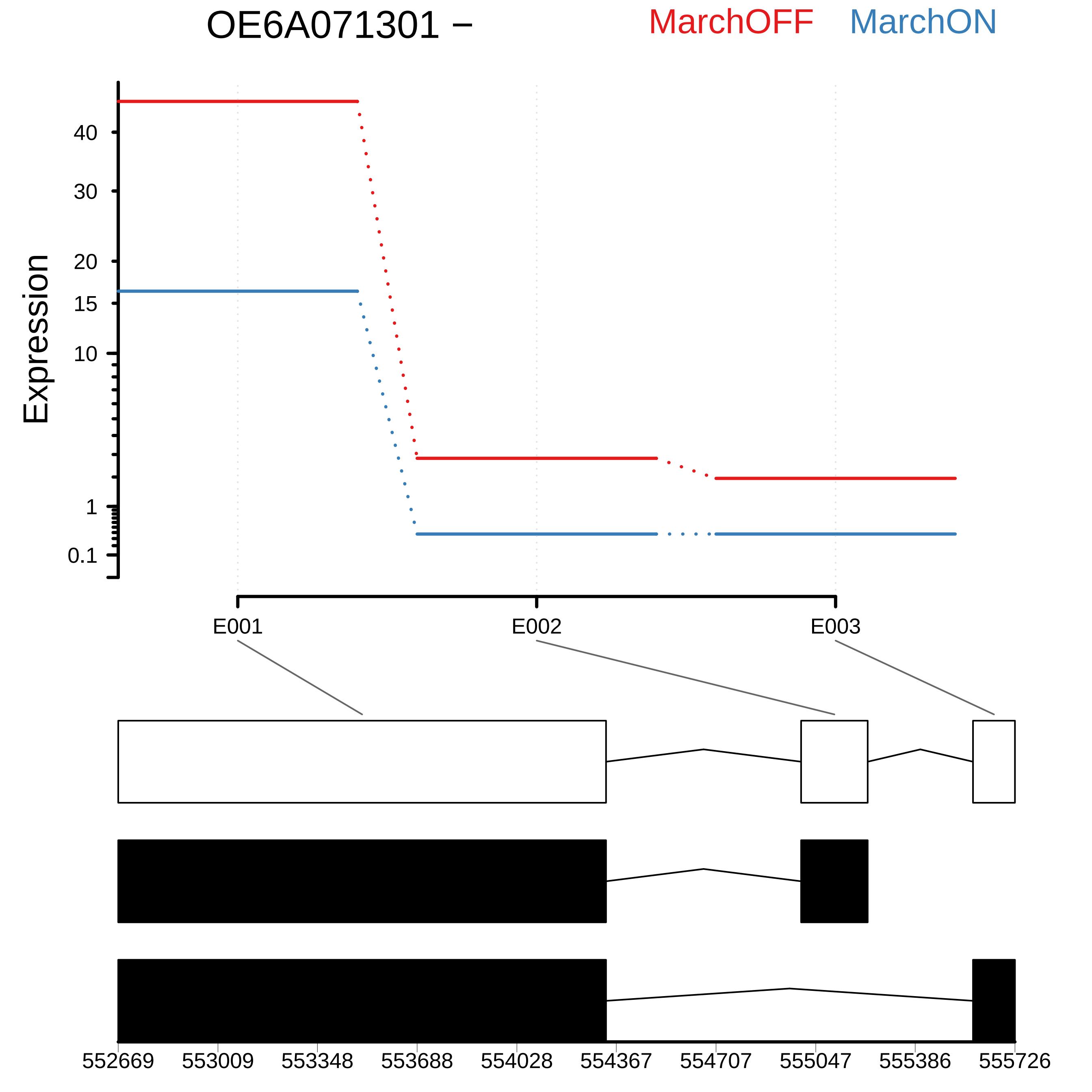

OE6A071301 −
MarchOFF
MarchON
Expression
40
30
20
15
10
1
0.1
E001
E002
E003
552669
553009
553348
553688
554028
554367
554707
555047
555386
555726

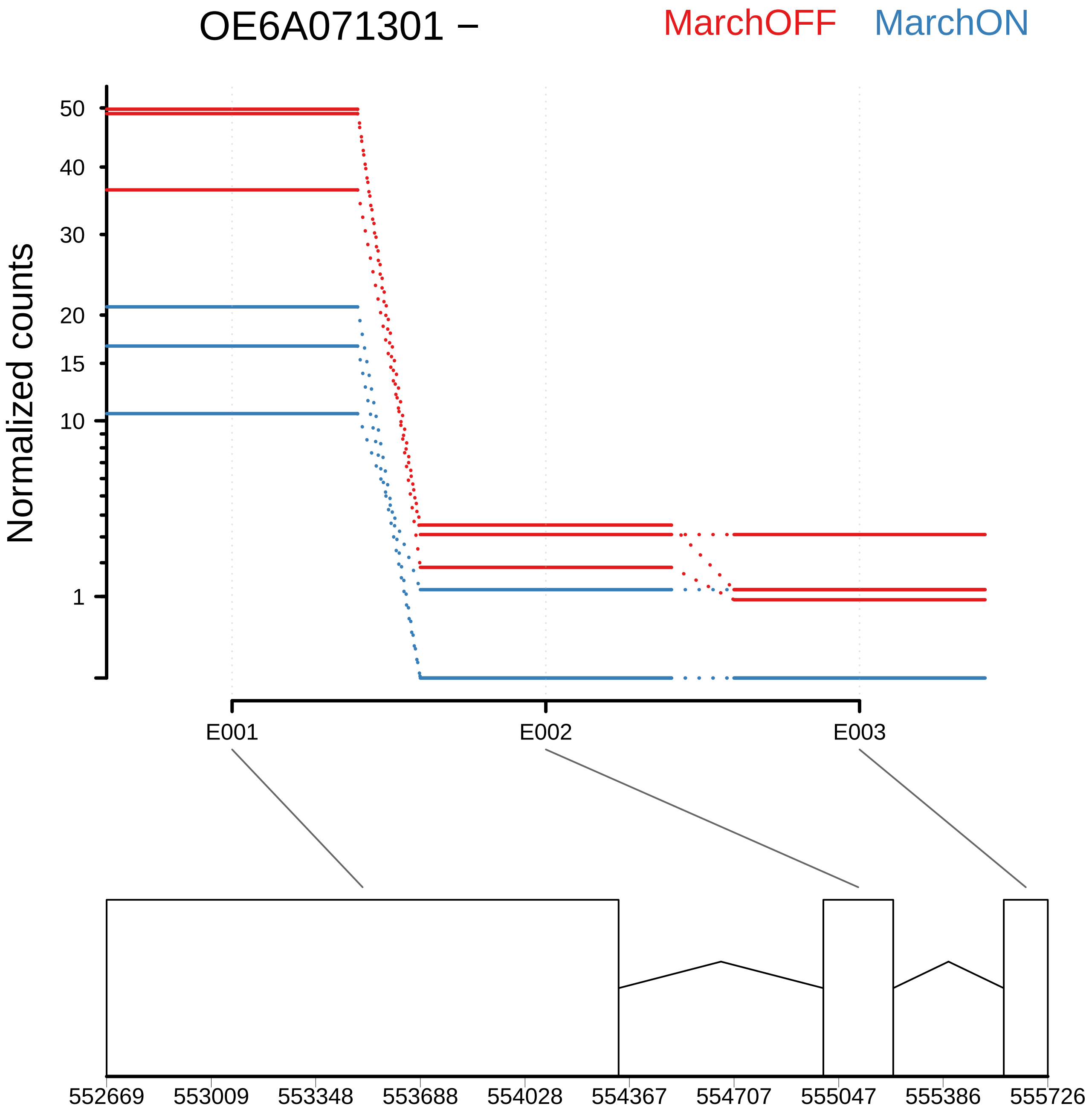
OE6A071301 −
MarchOFF
MarchON
Normalized counts
50
40
30
20
15
10
1
E001
E002
E003
552669
553009
553348
553688
554028
554367
554707
555047
555386
555726

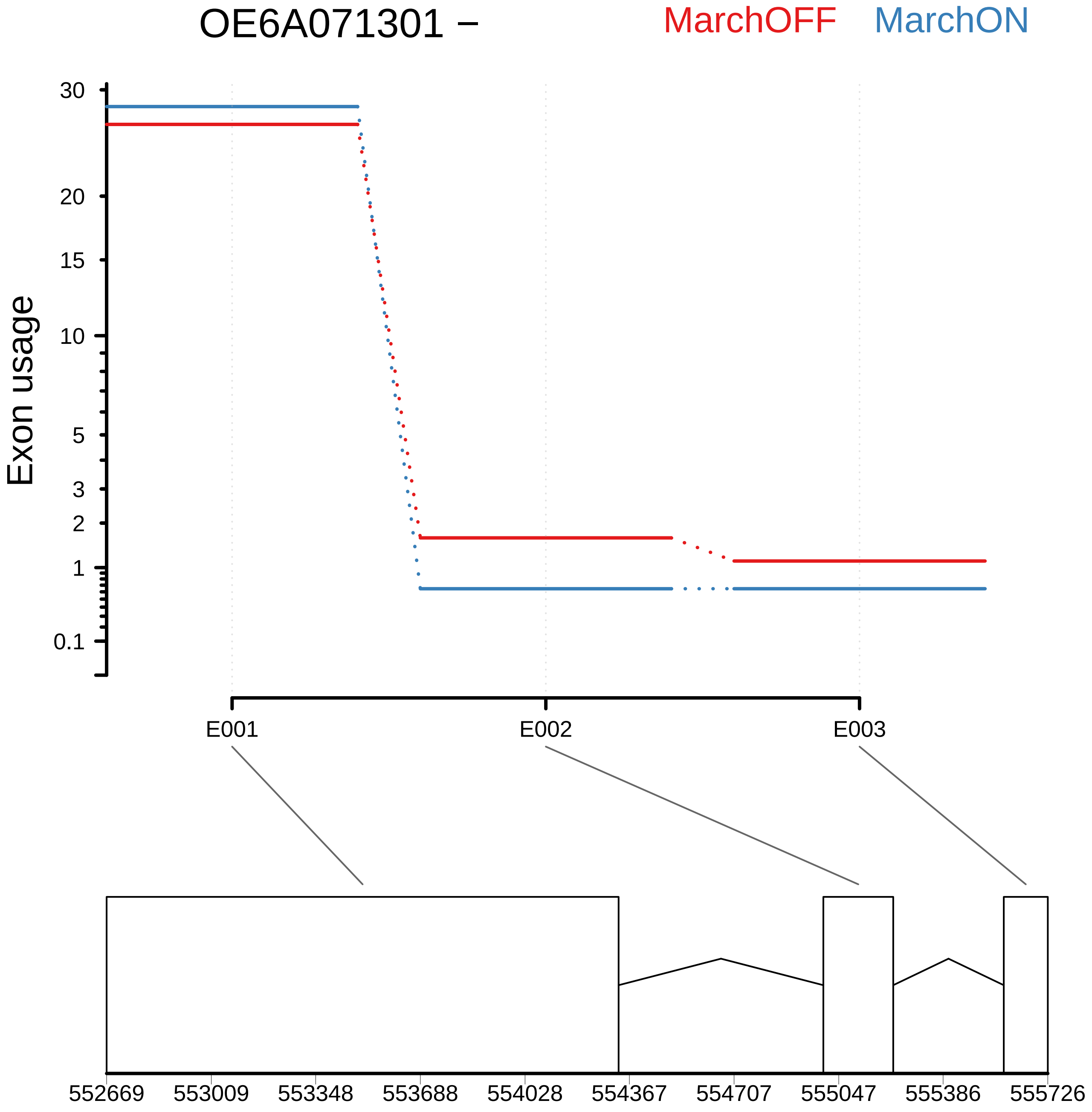

OE6A071301 −
MarchOFF
MarchON
Exon usage
30
20
15
10
5
3
2
1
0.1
E001
E002
E003
552669
553009
553348
553688
554028
554367
554707
555047
555386
555726

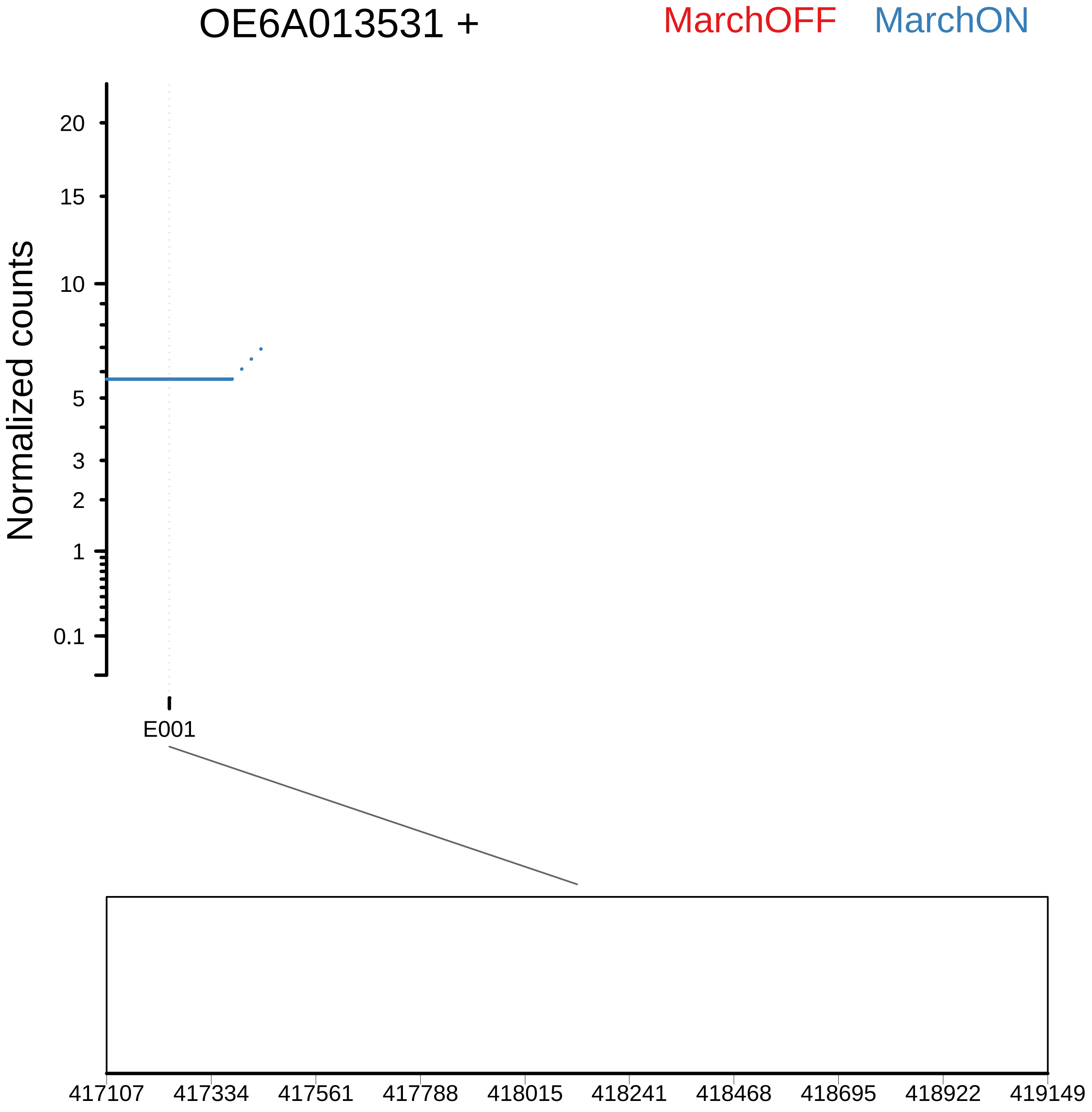
OE6A013531 +
MarchOFF
MarchON
Normalized counts
20
15
10
5
3
2
1
0.1
E001
417107
417334
417561
417788
418015
418241
418468
418695
418922
419149

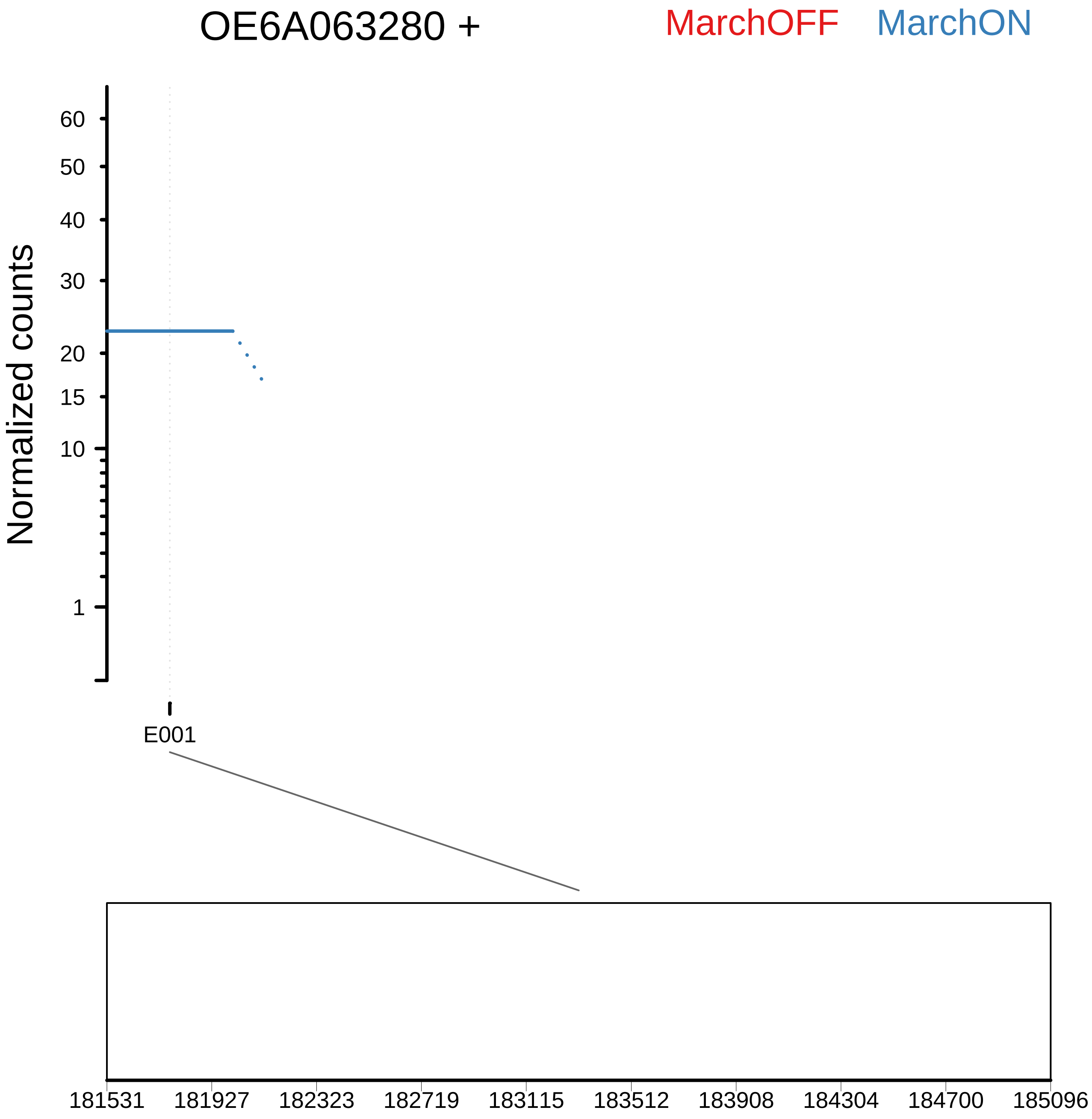
OE6A063280 +
MarchOFF
MarchON
Normalized counts
60
50
40
30
20
15
10
1
E001
181531
181927
182323
182719
183115
183512
183908
184304
184700
185096

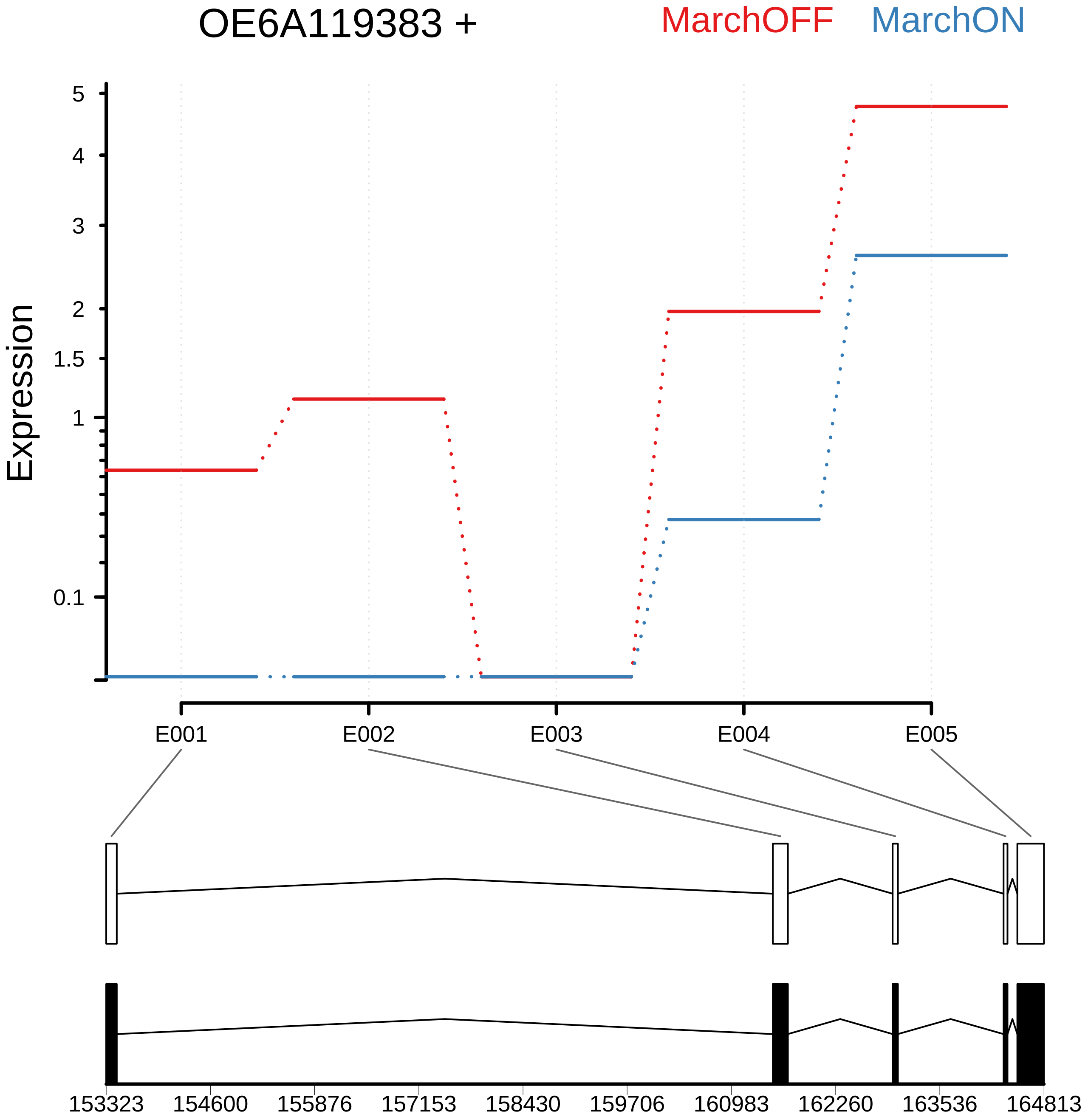

OE6A119383 +
MarchOFF
MarchON
Expression
5
4
3
2
1.5
1
0.1
E001
E002
E003
E004
E005
153323
154600
155876
157153
158430
159706
160983
162260
163536
164813

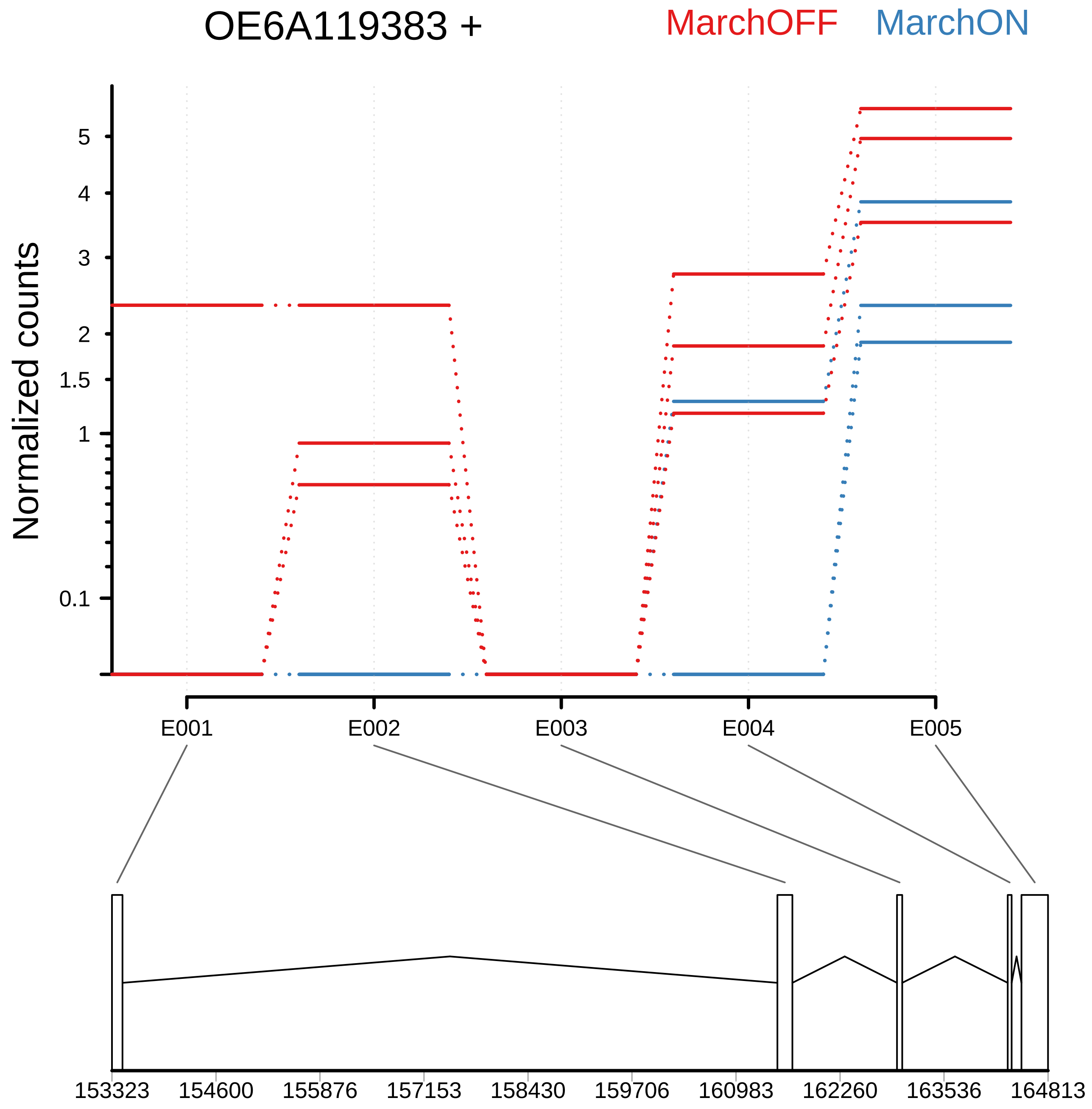

OE6A119383 +
MarchOFF
MarchON
Normalized counts
5
4
3
2
1.5
1
0.1
E001
E002
E003
E004
E005
153323
154600
155876
157153
158430
159706
160983
162260
163536
164813

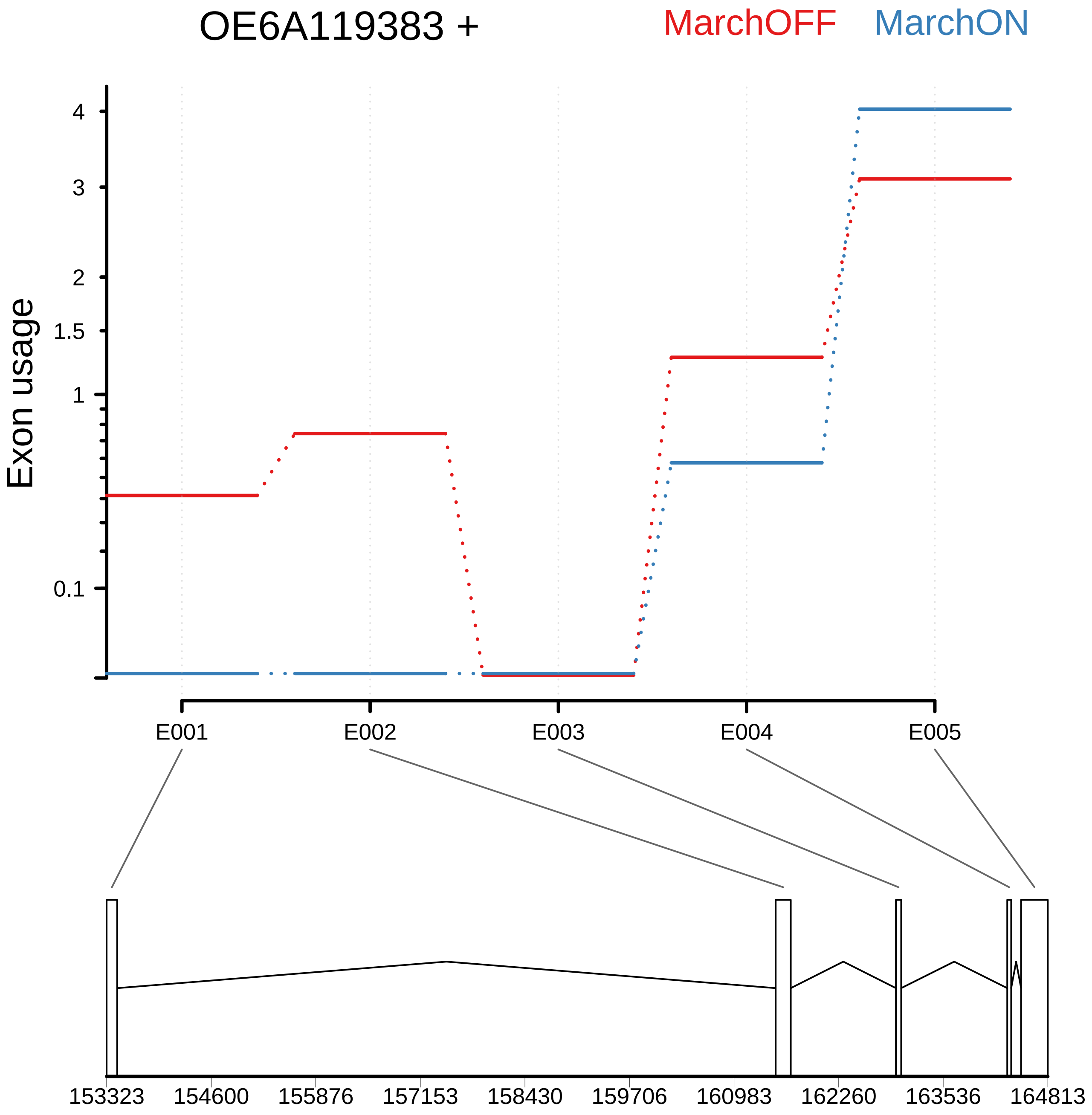
OE6A119383 +
MarchOFF
MarchON
Exon usage
4
3
2
1.5
1
0.1
E001
E002
E003
E004
E005
153323
154600
155876
157153
158430
159706
160983
162260
163536
164813

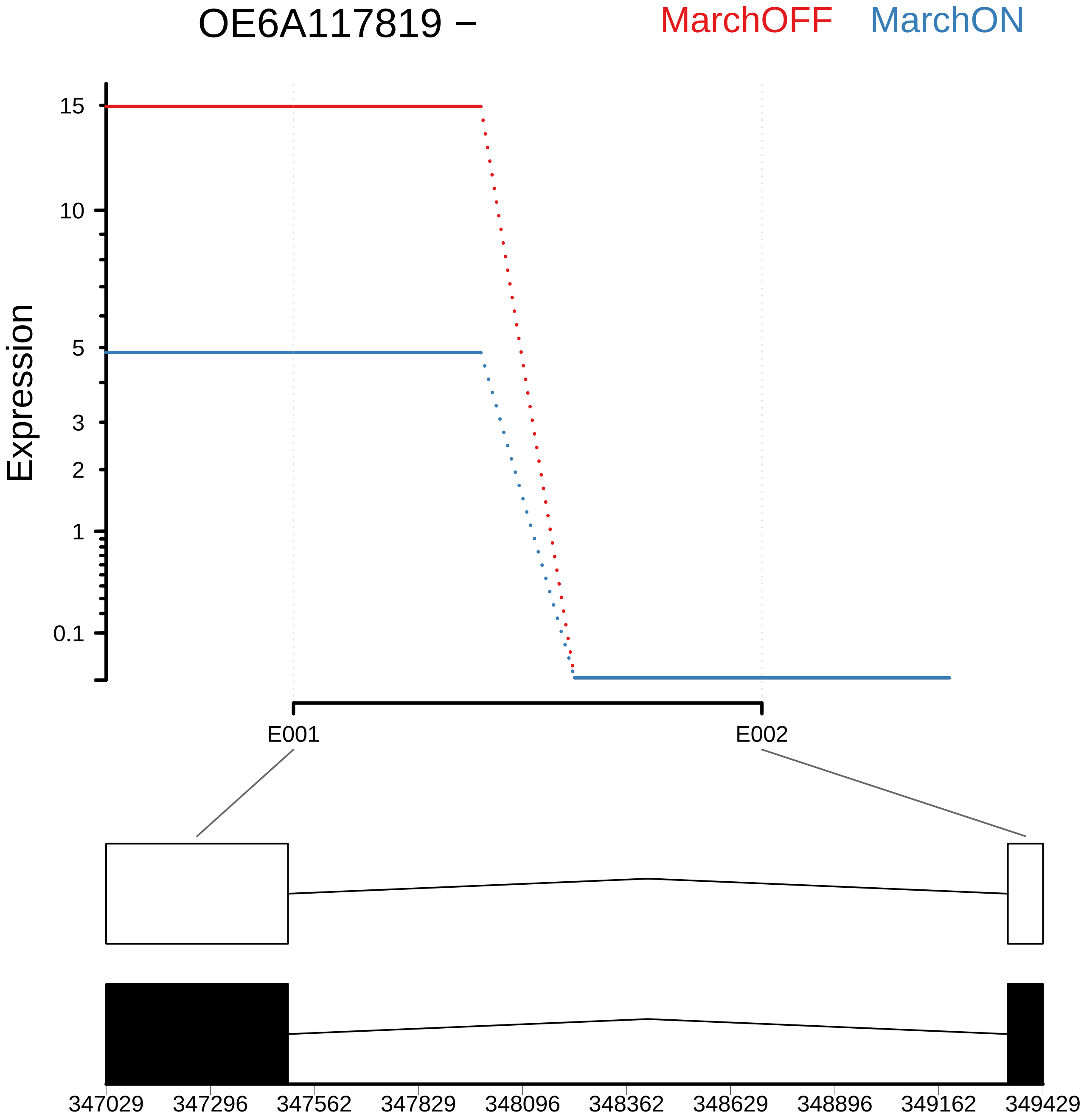
OE6A117819 −
MarchOFF
MarchON
Expression
15
10
5
3
2
1
0.1
E001
E002
347029
347296
347562
347829
348096
348362
348629
348896
349162
349429

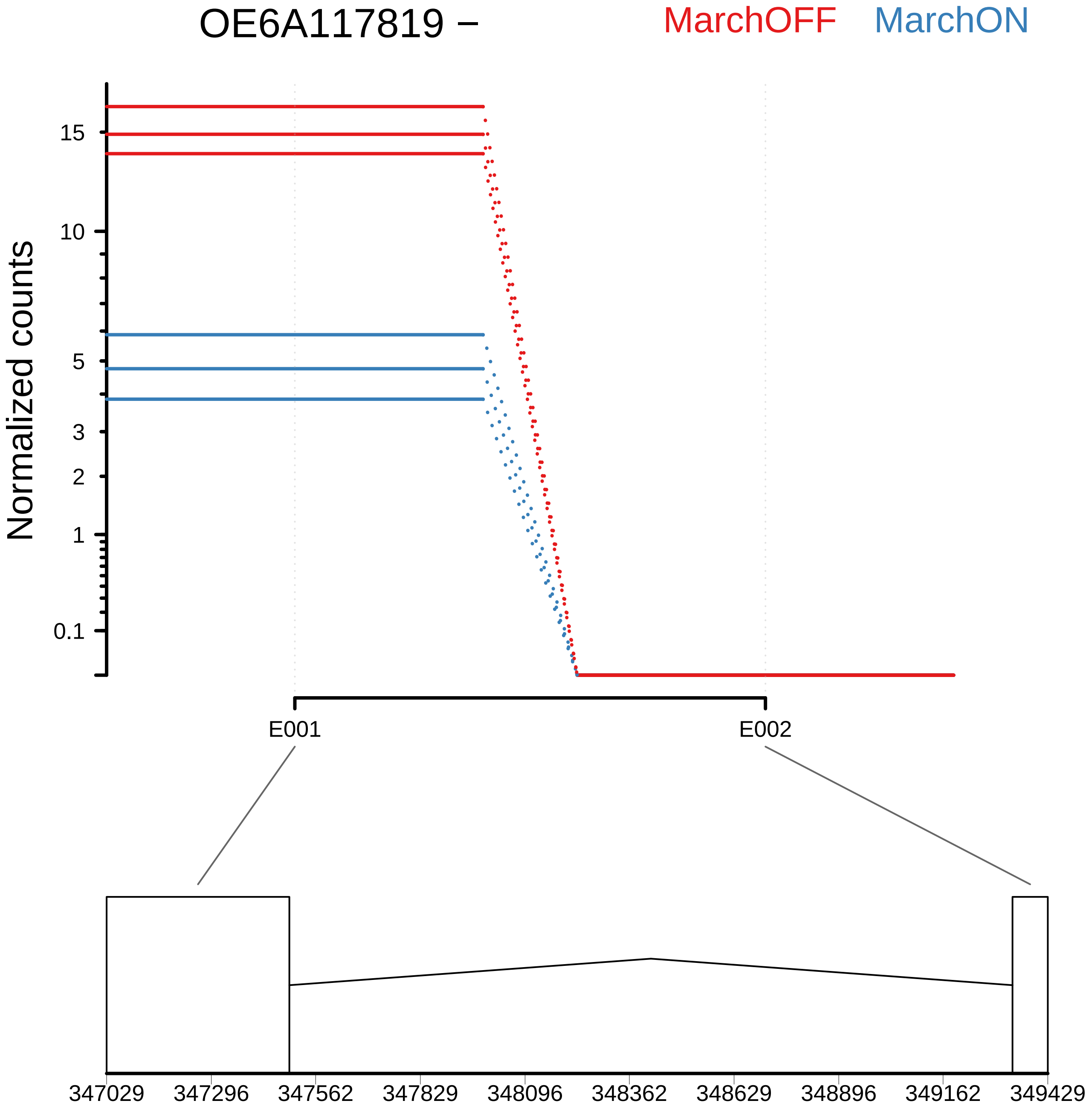

OE6A117819 −
MarchOFF
MarchON
Normalized counts
15
10
5
3
2
1
0.1
E001
E002
347029
347296
347562
347829
348096
348362
348629
348896
349162
349429

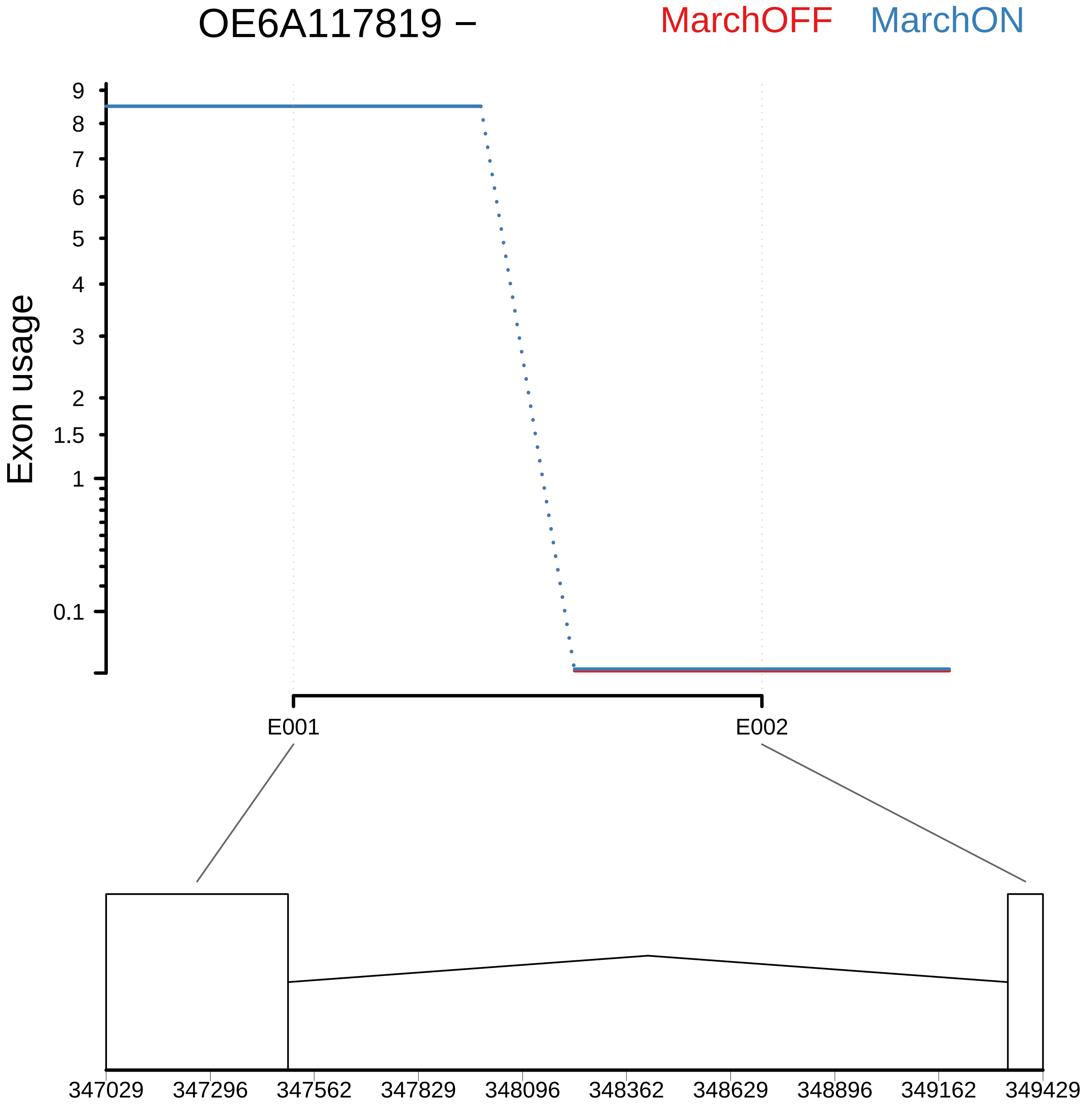
OE6A117819 −
MarchOFF
MarchON
Exon usage
9
8
7
6
5
4
3
2
1.5
1
0.1
E001
E002
347029
347296
347562
347829
348096
348362
348629
348896
349162
349429

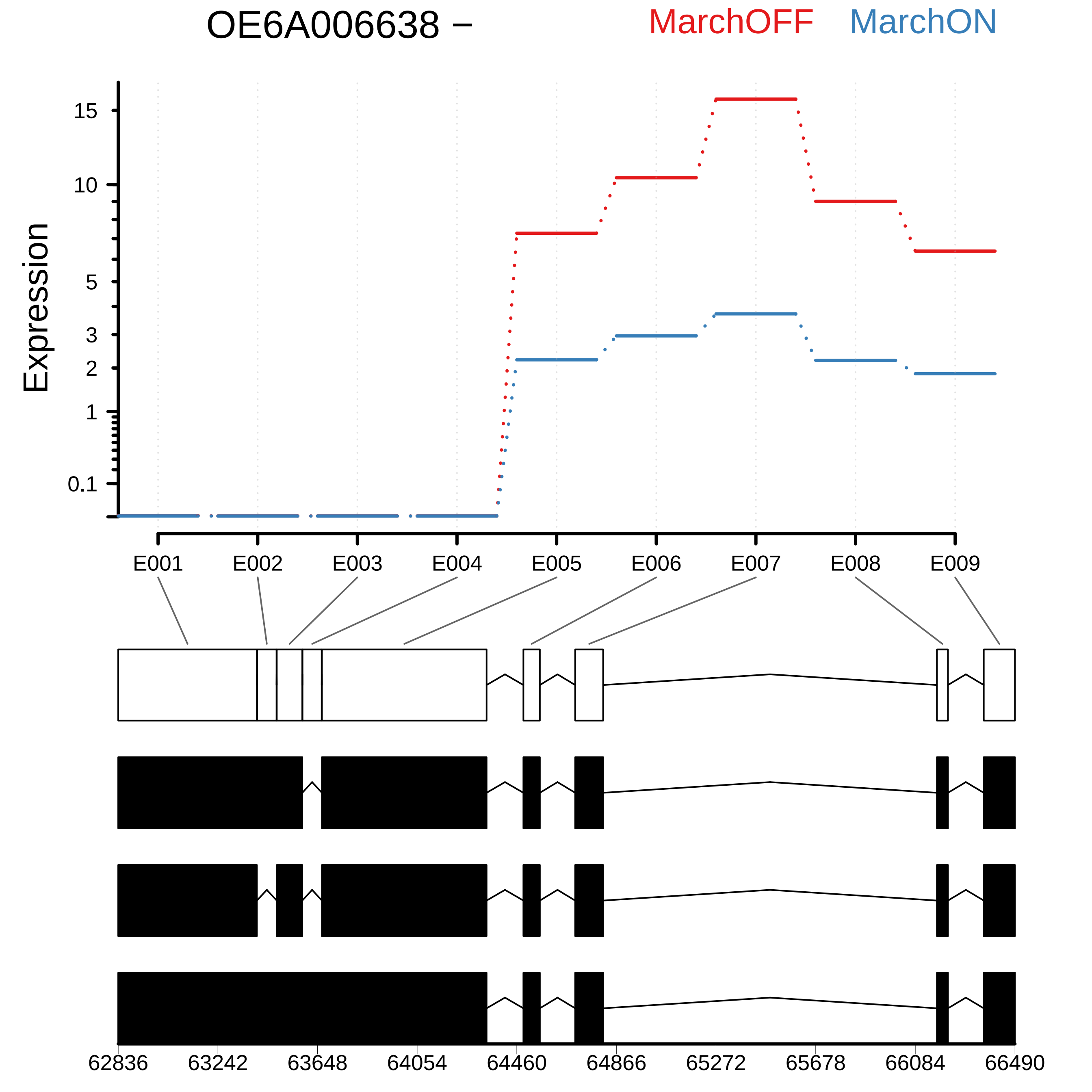

OE6A006638 –
MarchOFF
MarchON
Expression
15
10
5
3
2
1
0.1
E001
E002
E003
E004
E005
E006
E007
E008
E009
62836
63242
63648
64054
64460
64866
65272
65678
66084
66490

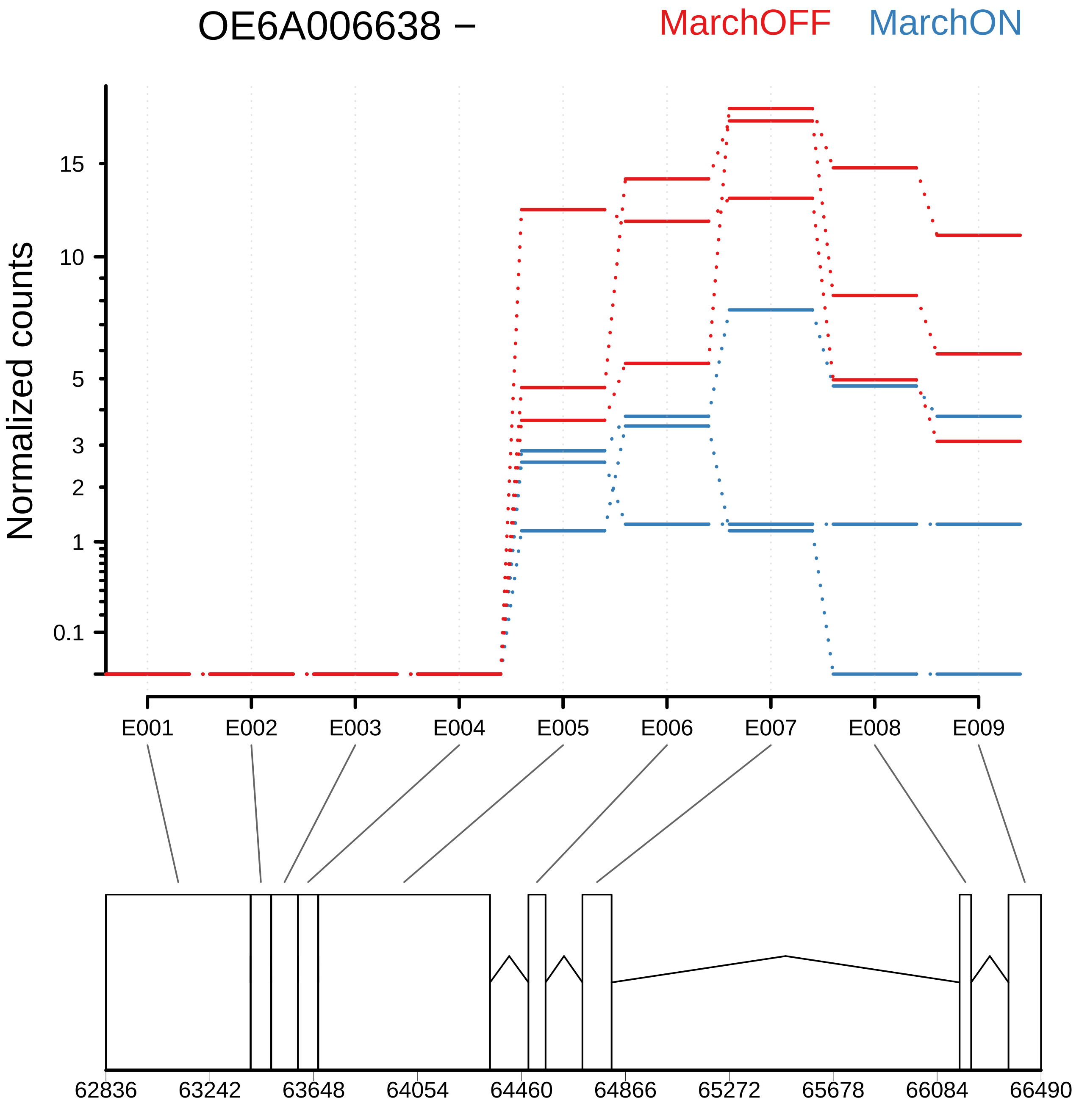

OE6A006638 −
MarchOFF
MarchON
Normalized counts
15
10
5
3
2
1
0.1
E001
E002
E003
E004
E005
E006
E007
E008
E009
62836
63242
63648
64054
64460
64866
65272
65678
66084
66490

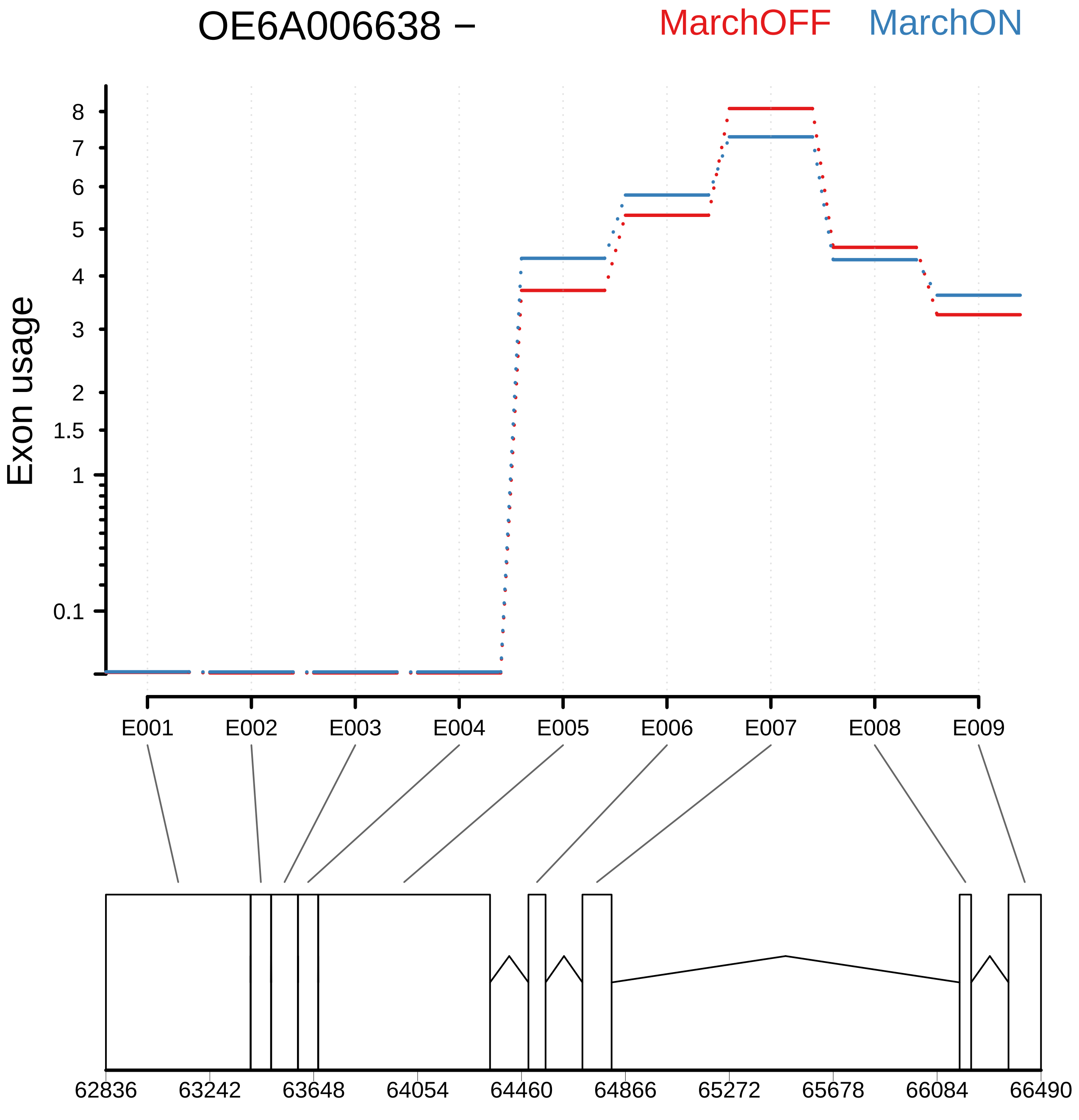

OE6A006638 −
MarchOFF
MarchON
Exon usage
8
7
6
5
4
3
2
1.5
1
0.1
E001
E002
E003
E004
E005
E006
E007
E008
E009
62836
63242
63648
64054
64460
64866
65272
65678
66084
66490

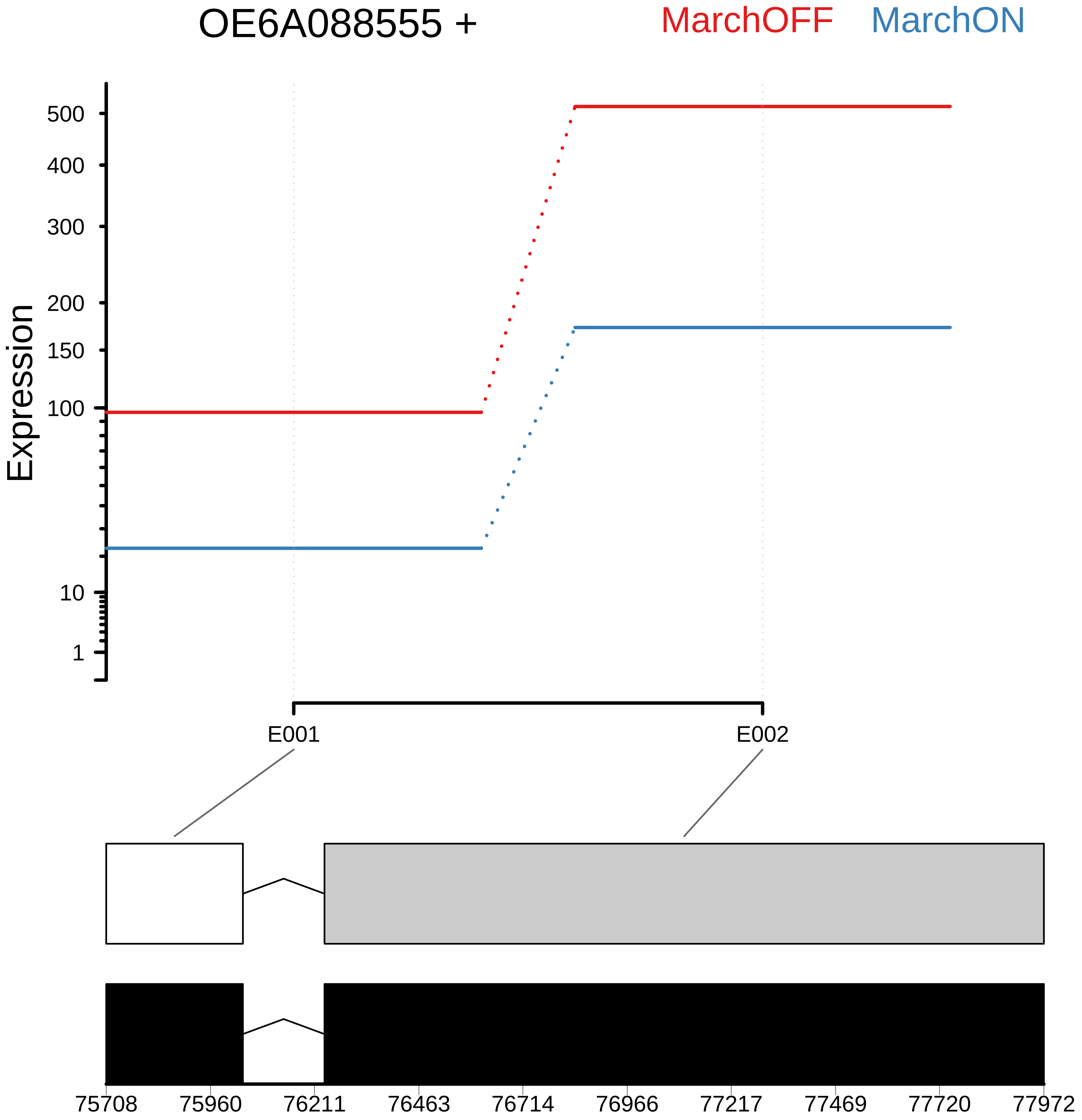

OE6A088555 +
MarchOFF
MarchON
Expression
500
400
300
200
150
100
10
1
E001
E002
75708
75960
76211
76463
76714
76966
77217
77469
77720
77972

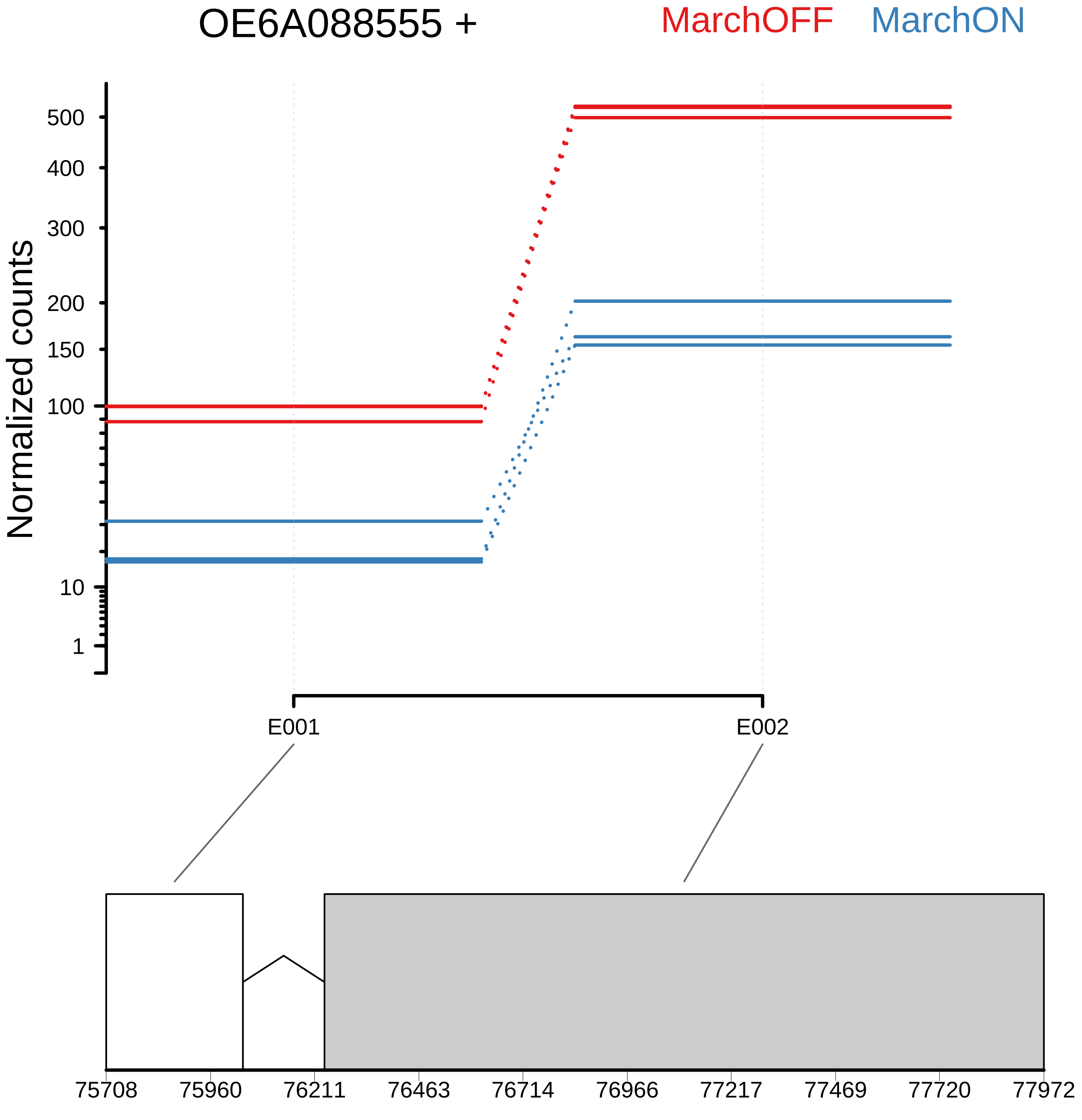
OE6A088555 +
MarchOFF
MarchON
Normalized counts
500
400
300
200
150
100
10
1
E001
E002
75708
75960
76211
76463
76714
76966
77217
77469
77720
77972

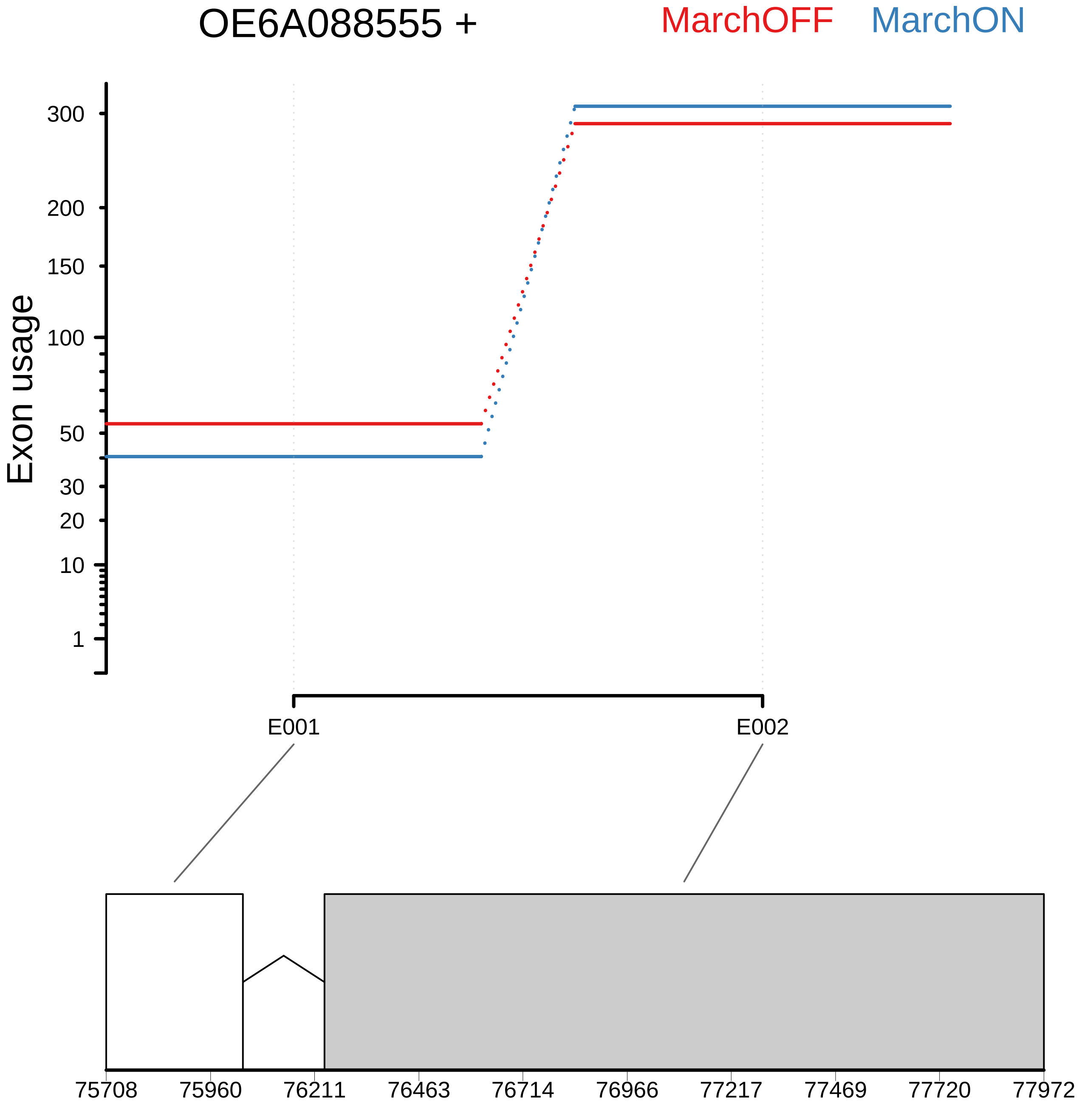

OE6A088555 +
MarchOFF
MarchON
Exon usage
300
200
150
100
50
30
20
10
1
E001
E002
75708
75960
76211
76463
76714
76966
77217
77469
77720
77972

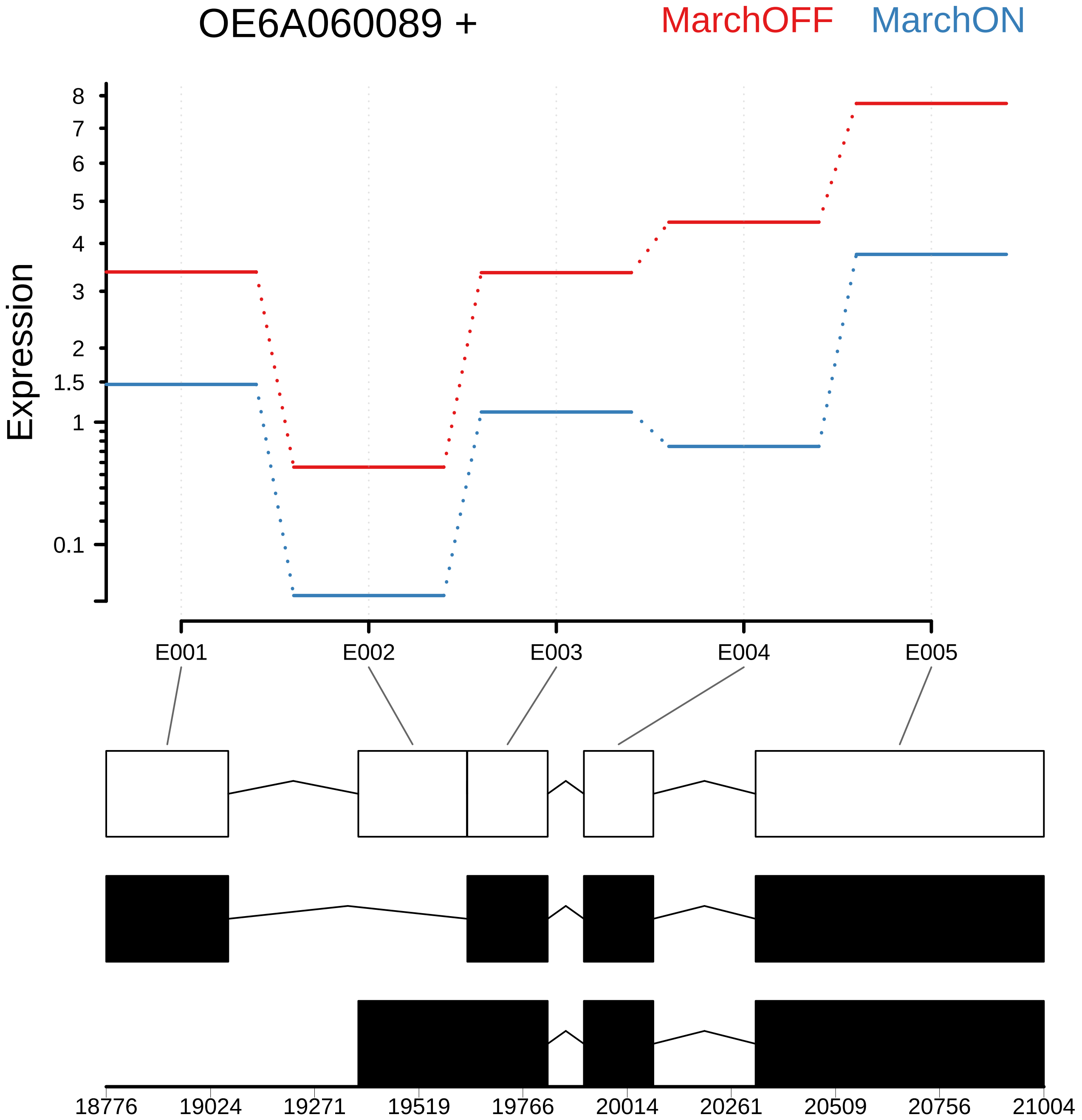

OE6A060089 +
MarchOFF
MarchON
Expression
8
7
6
5
4
3
2
1.5
1
0.1
E001
E002
E003
E004
E005
18776
19024
19271
19519
19766
20014
20261
20509
20756
21004

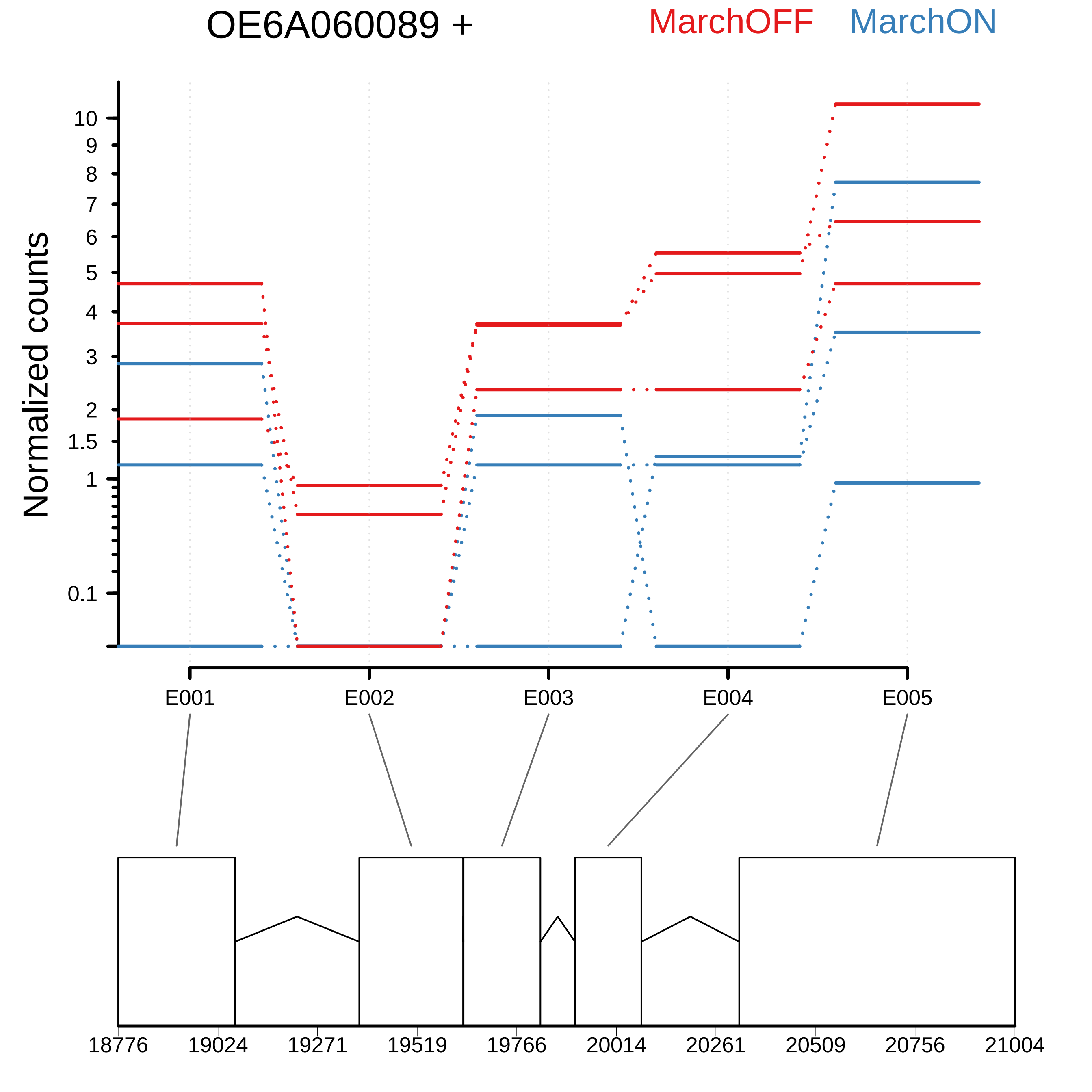
OE6A060089 +
MarchOFF
MarchON
Normalized counts
10
9
8
7
6
5
4
3
2
1.5
1
0.1
E001
E002
E003
E004
E005
18776
19024
19271
19519
19766
20014
20261
20509
20756
21004

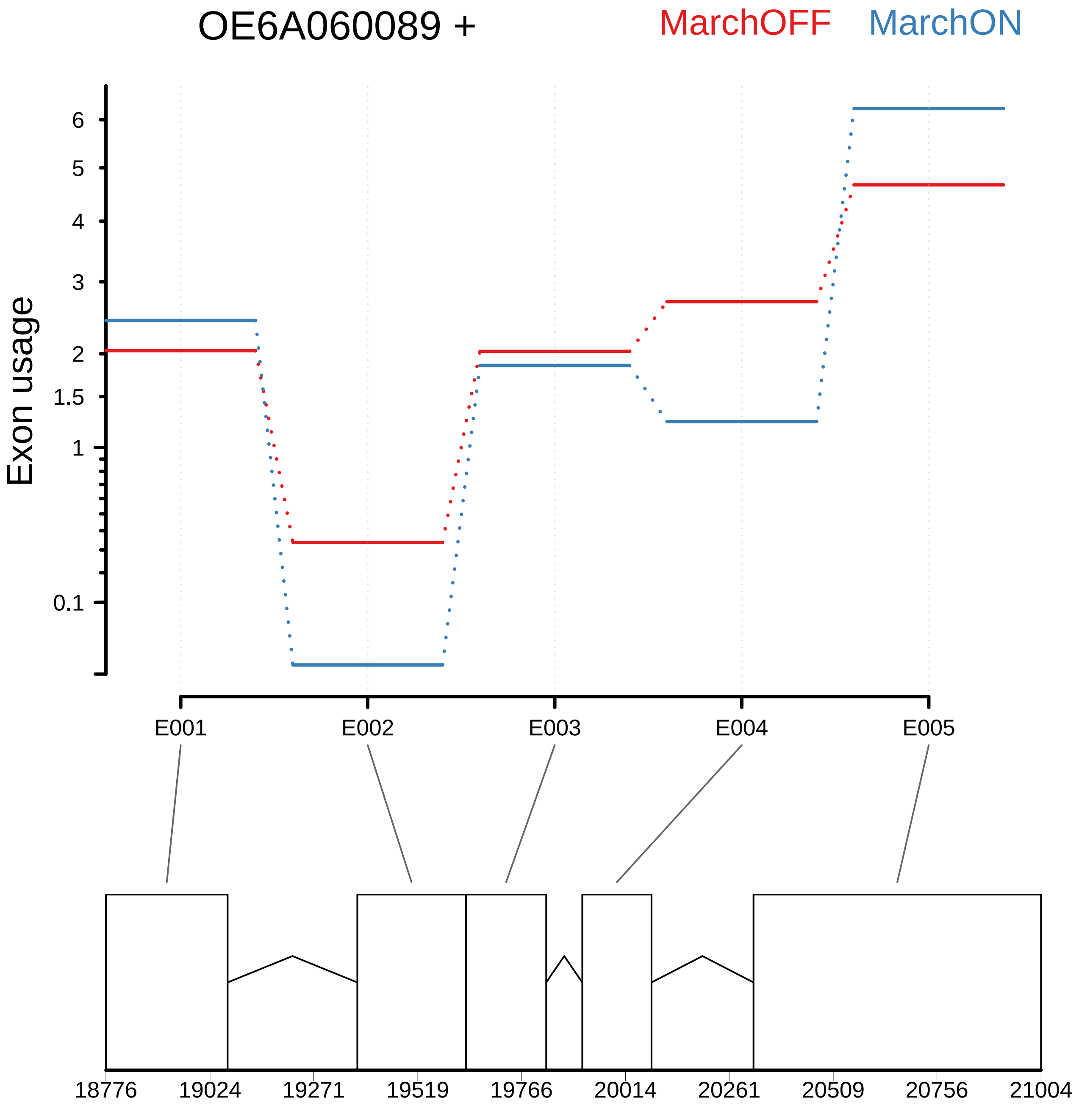

OE6A060089 +
MarchOFF
MarchON
Exon usage
6
5
4
3
2
1.5
1
0.1
E001
E002
E003
E004
E005
18776
19024
19271
19519
19766
20014
20261
20509
20756
21004

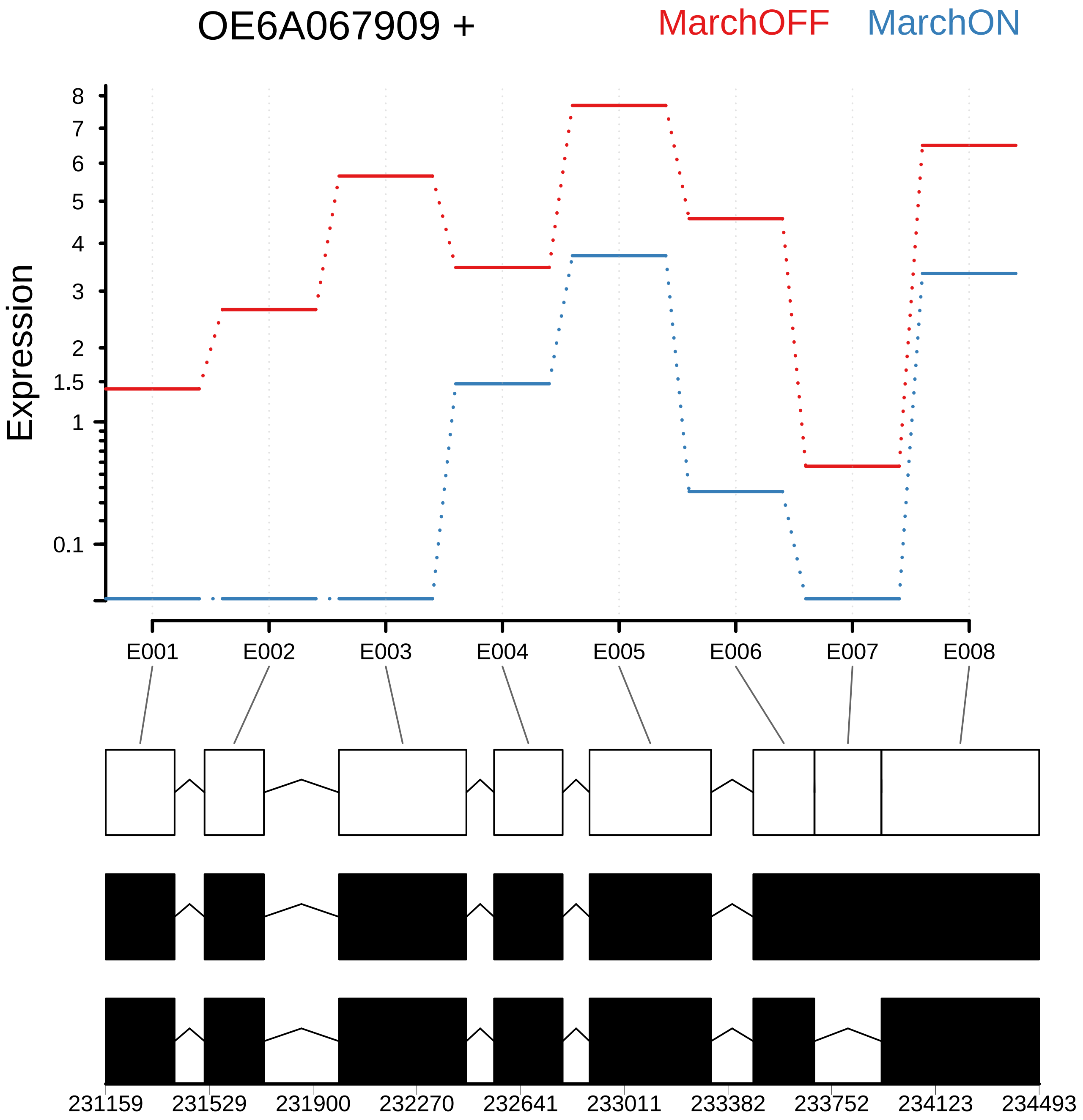

OE6A067909 +
MarchOFF
MarchON
Expression
8
7
6
5
4
3
2
1.5
1
0.1
E001
E002
E003
E004
E005
E006
E007
E008
231159
231529
231900
232270
232641
233011
233382
233752
234123
234493

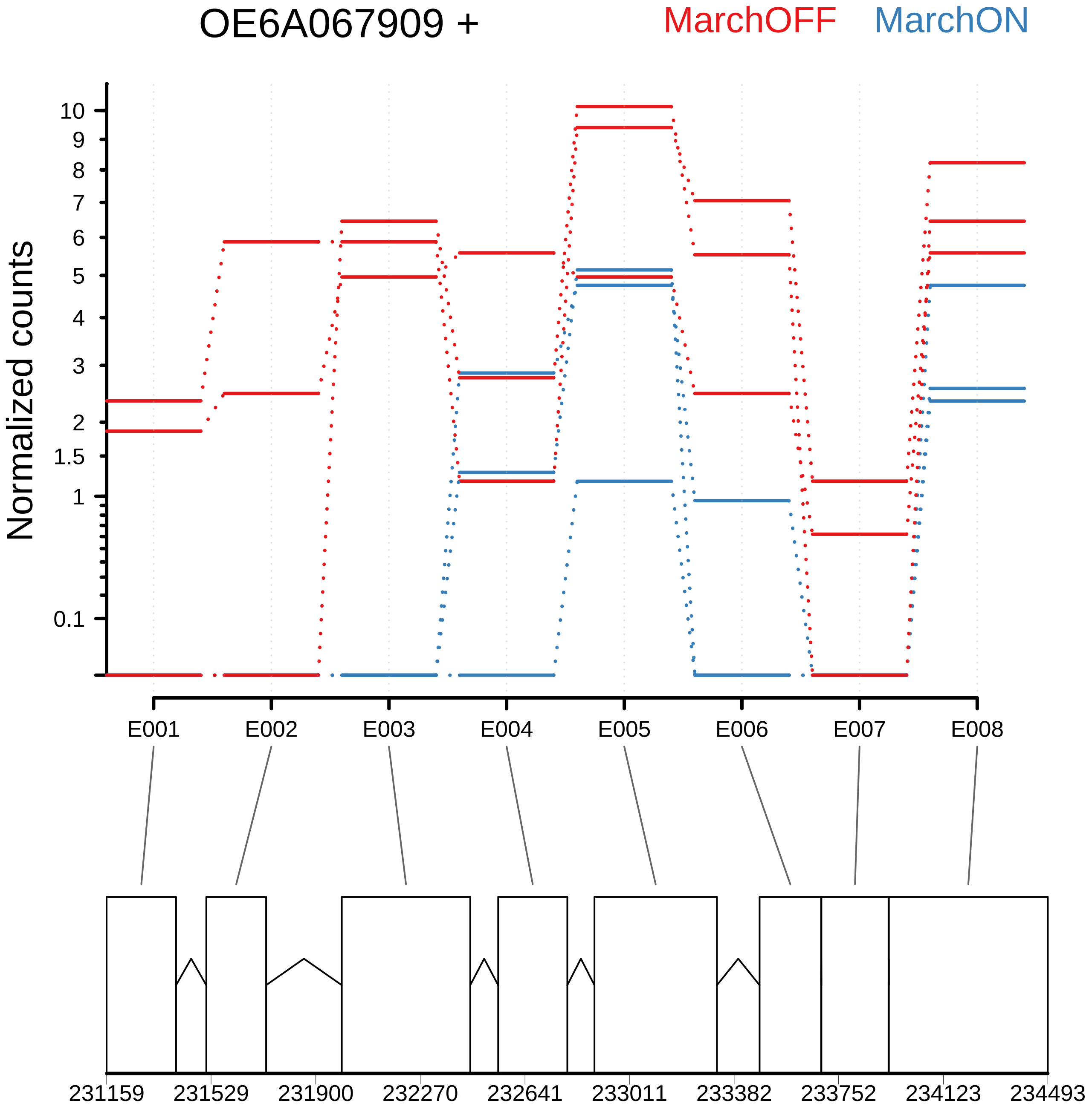

OE6A067909 +
MarchOFF
MarchON
Normalized counts
10
9
8
7
6
5
4
3
2
1.5
1
0.1
E001
E002
E003
E004
E005
E006
E007
E008
231159
231529
231900
232270
232641
233011
233382
233752
234123
234493

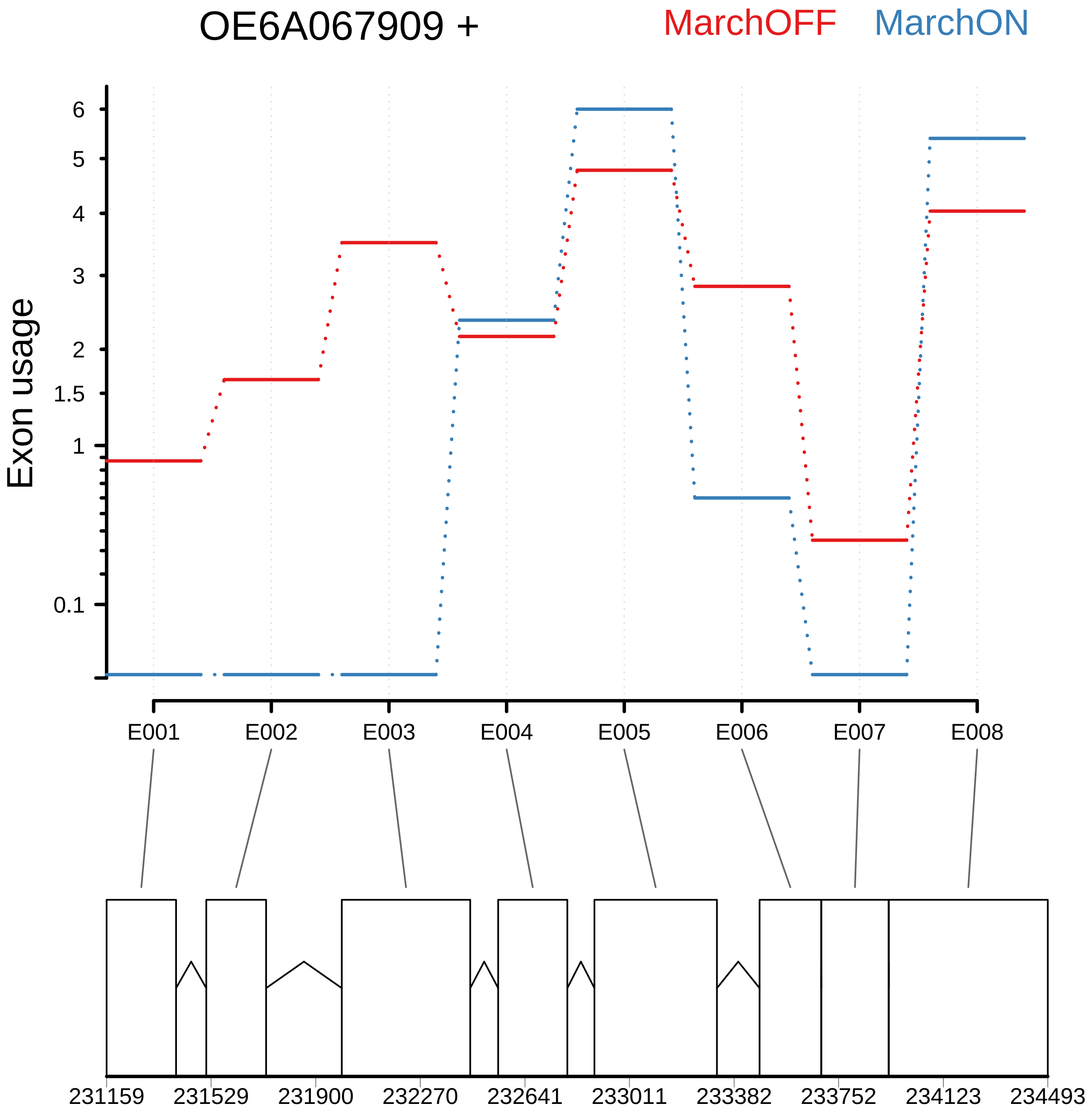

OE6A067909 +
MarchOFF
MarchON
Exon usage
6
5
4
3
2
1.5
1
0.1
E001
E002
E003
E004
E005
E006
E007
E008
231159
231529
231900
232270
232641
233011
233382
233752
234123
234493

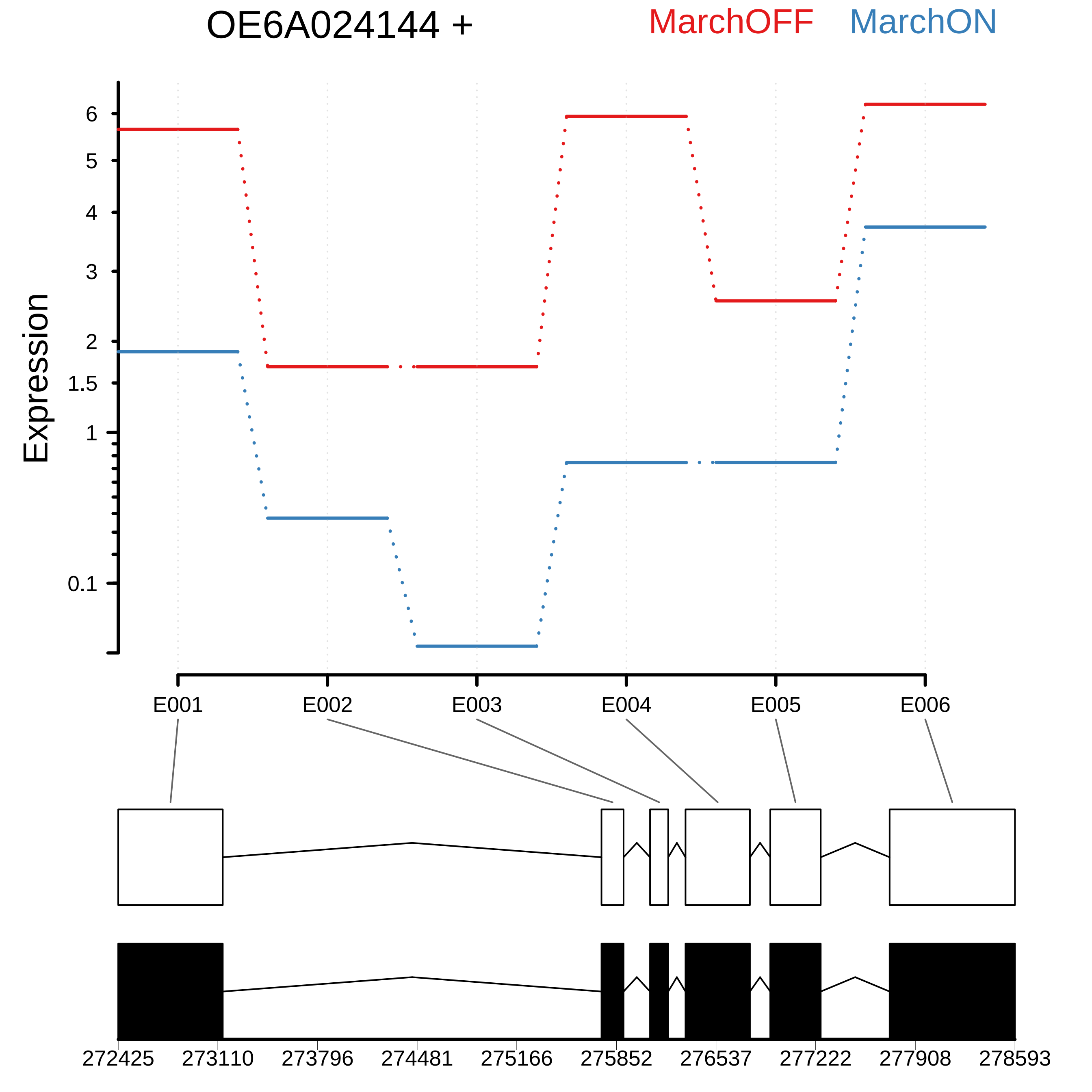

OE6A024144 +
MarchOFF
MarchON
Expression
6
5
4
3
2
1.5
1
0.1
E001
E002
E003
E004
E005
E006
272425
273110
273796
274481
275166
275852
276537
277222
277908
278593

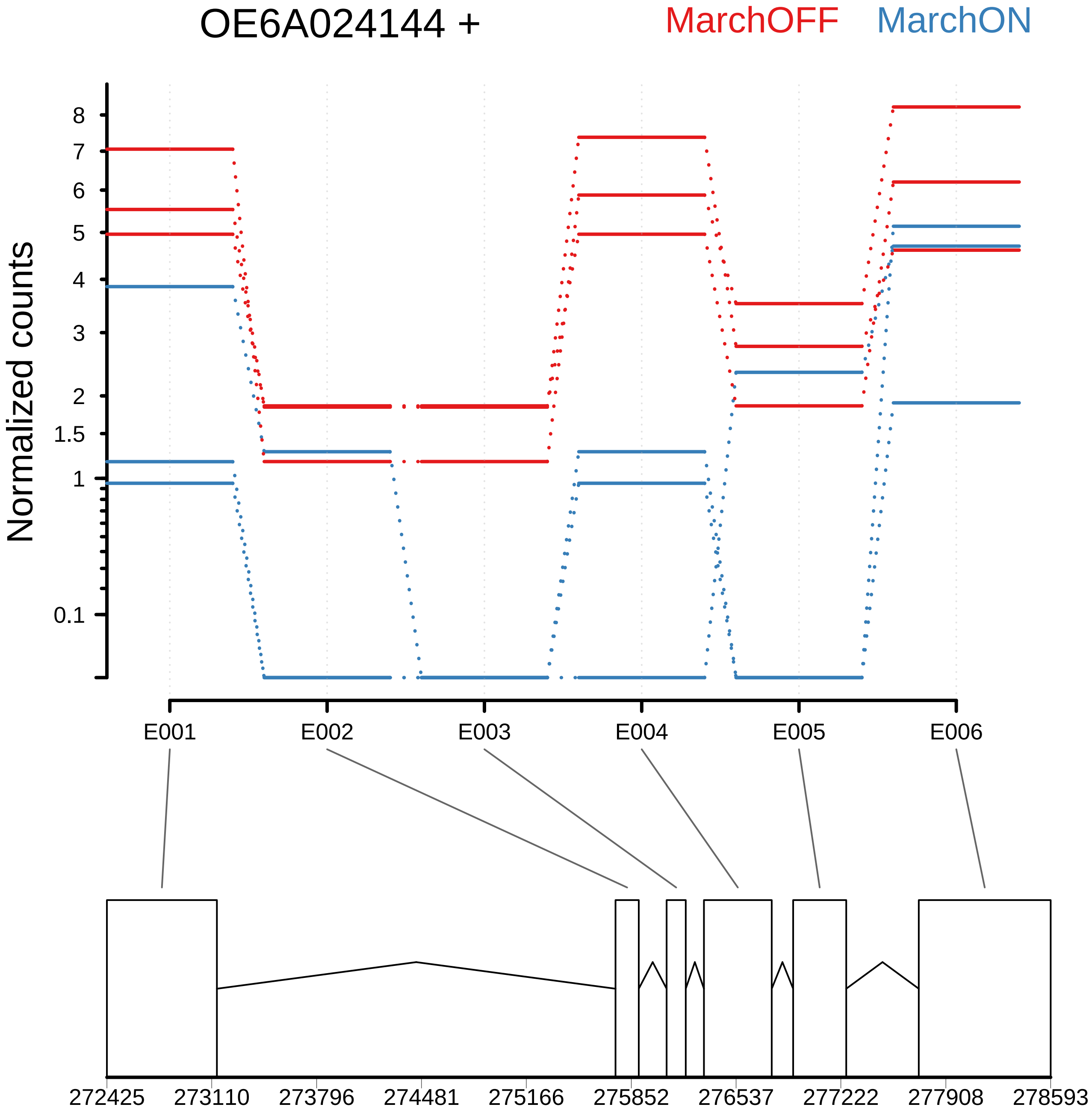

OE6A024144 +
MarchOFF
MarchON
Normalized counts
8
7
6
5
4
3
2
1.5
1
0.1
E001
E002
E003
E004
E005
E006
272425
273110
273796
274481
275166
275852
276537
277222
277908
278593

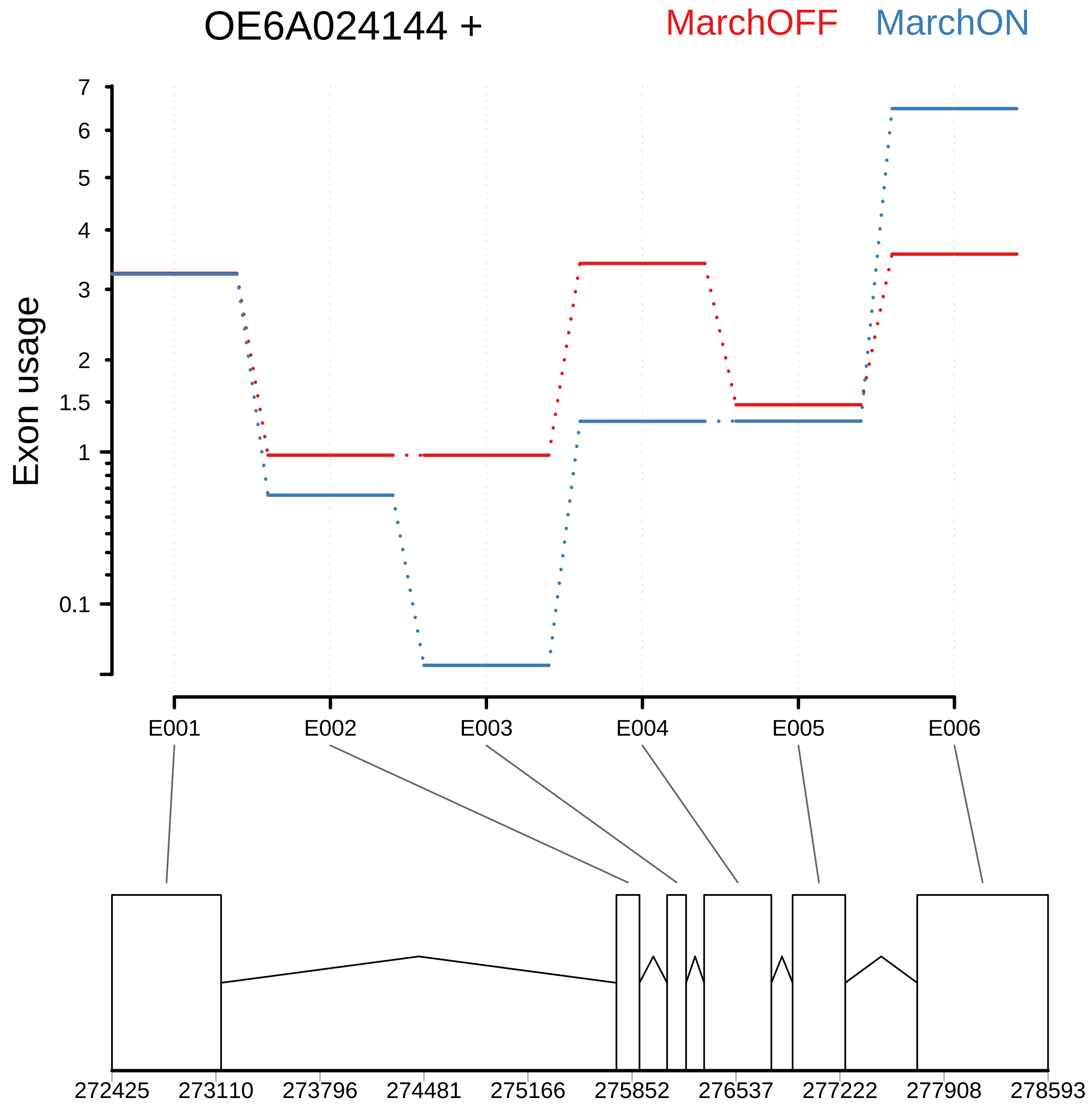

OE6A024144 +
MarchOFF
MarchON
Exon usage
7
6
5
4
3
2
1.5
1
0.1
E001
E002
E003
E004
E005
E006
272425
273110
273796
274481
275166
275852
276537
277222
277908
278593

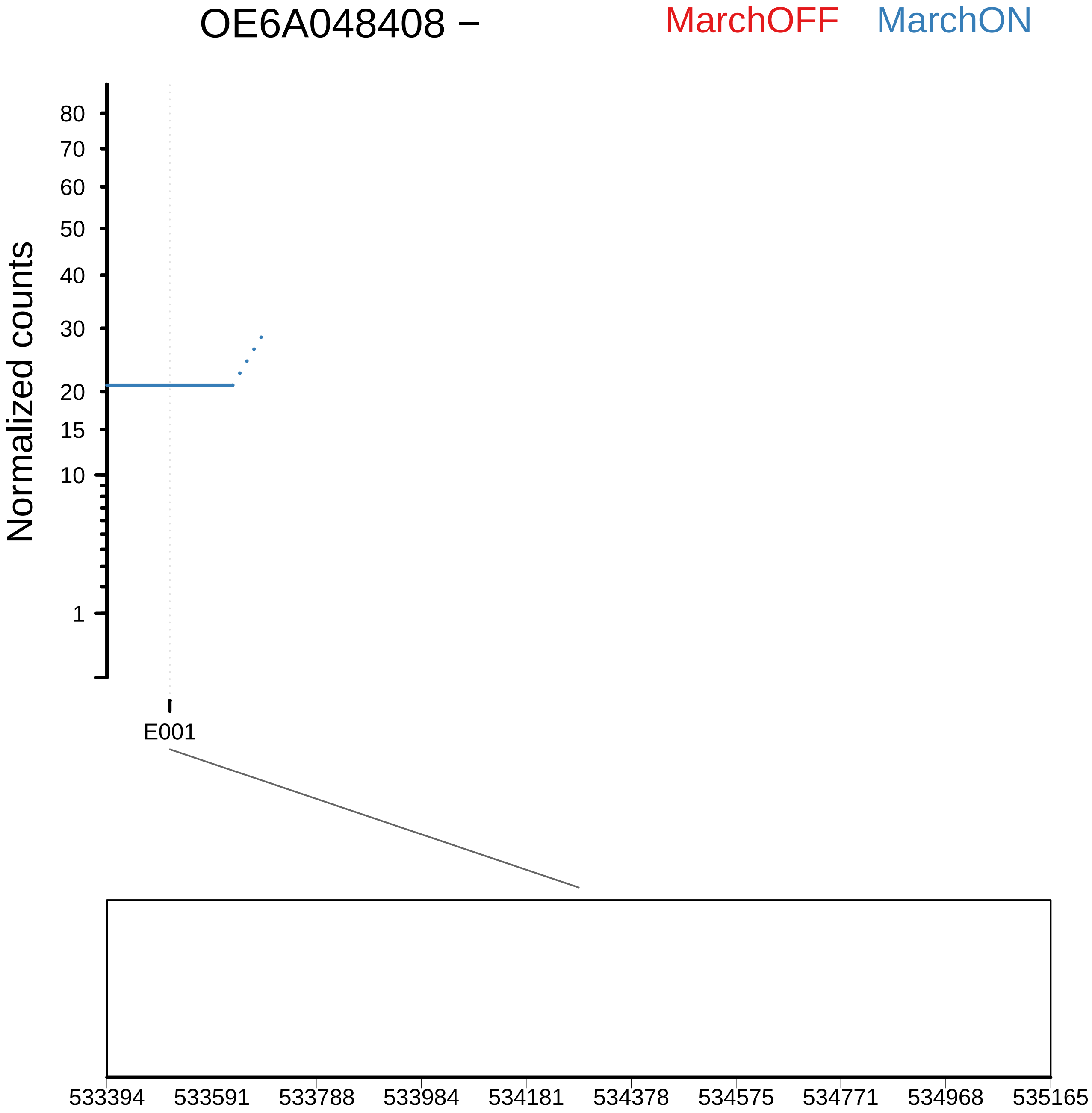
OE6A048408 −
MarchOFF
MarchON
Normalized counts
80
70
60
50
40
30
20
15
10
1
E001
533394
533591
533788
533984
534181
534378
534575
534771
534968
535165

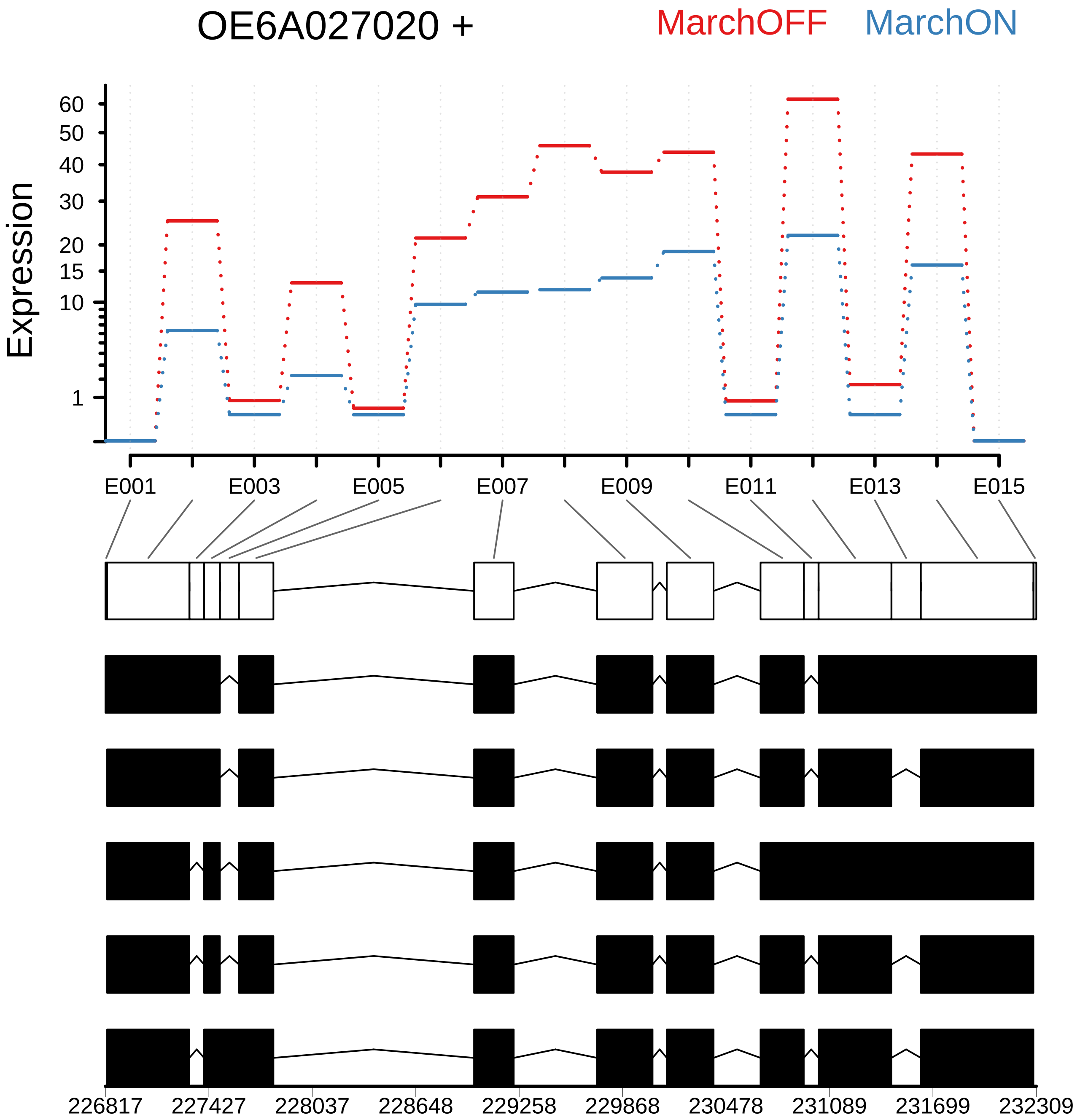

OE6A027020 +
MarchOFF
MarchON
Expression
60
50
40
30
20
15
10
1
E001
E003
E005
E007
E009
E011
E013
E015
226817
227427
228037
228648
229258
229868
230478
231089
231699
232309

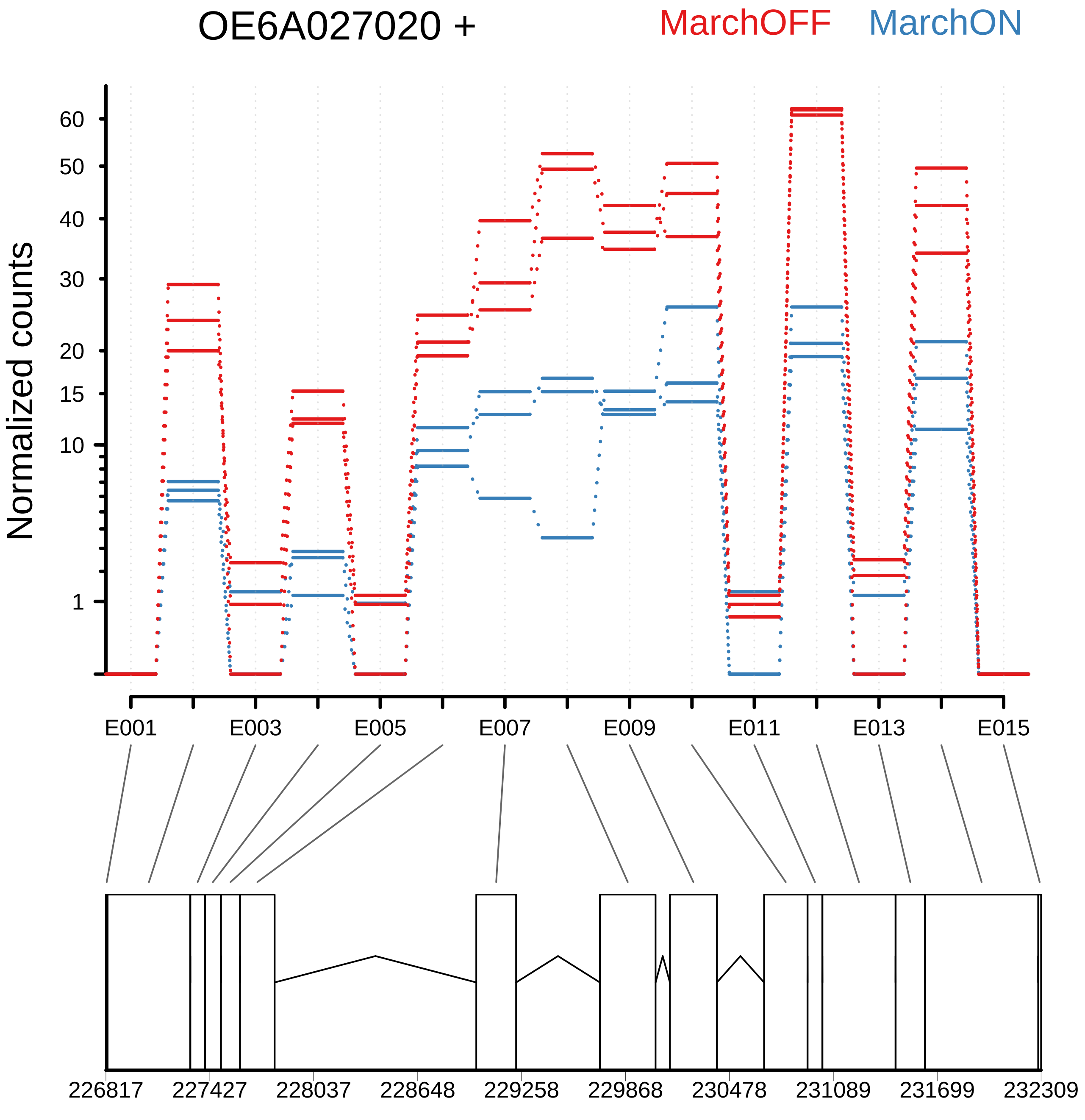

OE6A027020 +
MarchOFF
MarchON
Normalized counts
60
50
40
30
20
15
10
1
E001
E003
E005
E007
E009
E011
E013
E015
226817
227427
228037
228648
229258
229868
230478
231089
231699
232309

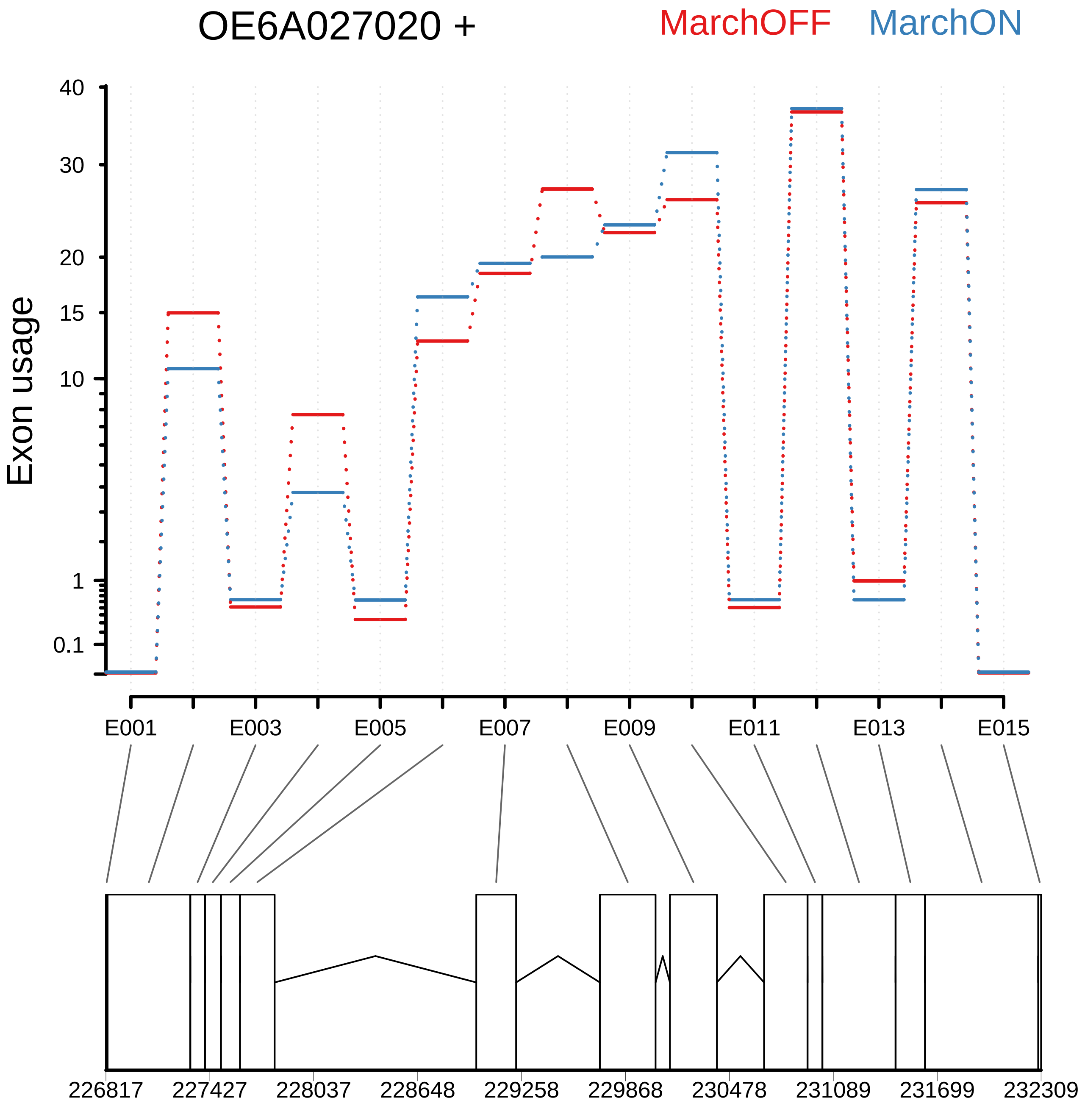

OE6A027020 +
MarchOFF
MarchON
Exon usage
40
30
20
15
10
1
0.1
E001
E003
E005
E007
E009
E011
E013
E015
226817
227427
228037
228648
229258
229868
230478
231089
231699
232309

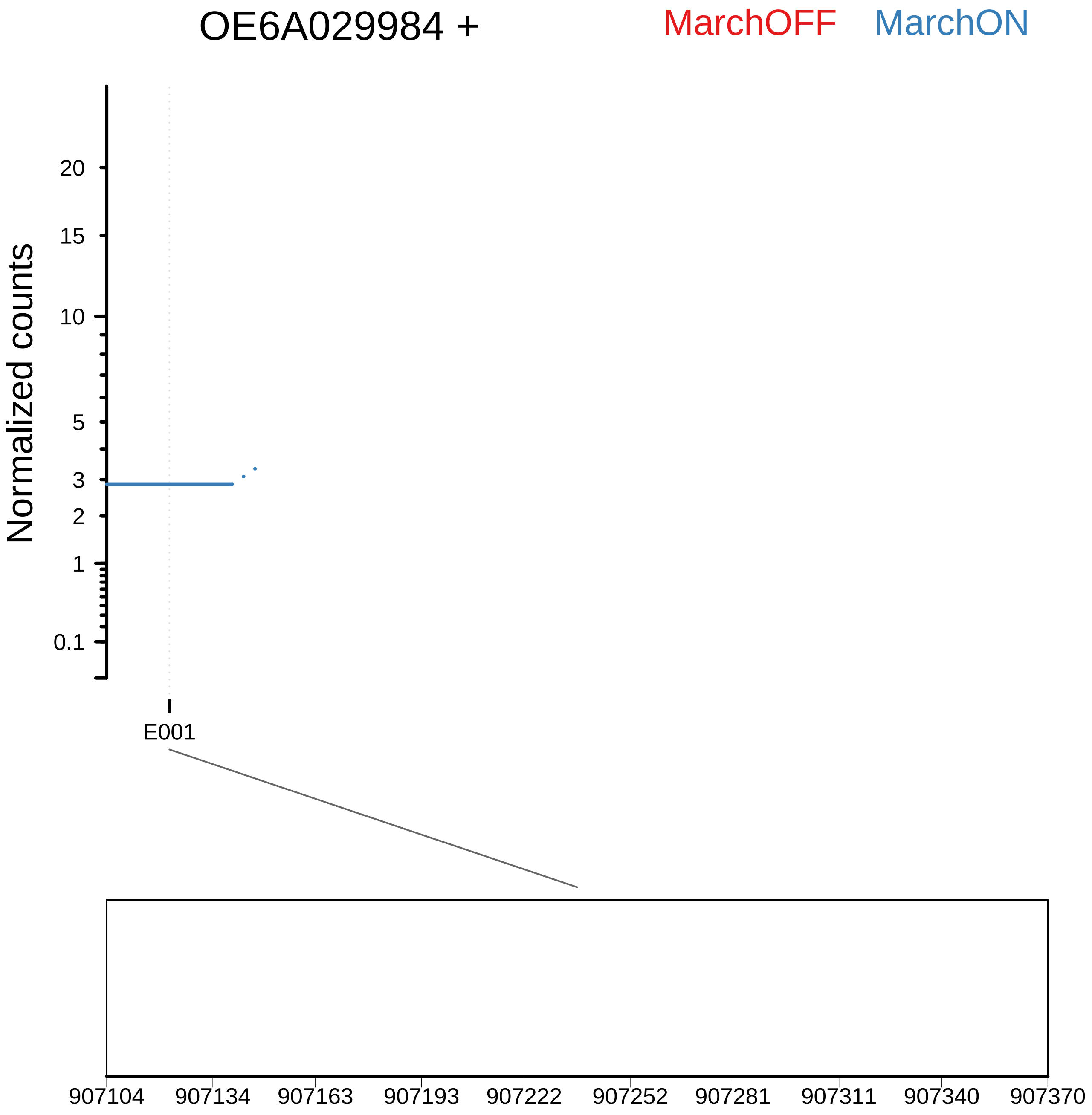

OE6A029984 +
MarchOFF
MarchON
Normalized counts
20
15
10
5
3
2
1
0.1
E001
907104
907134
907163
907193
907222
907252
907281
907311
907340
907370

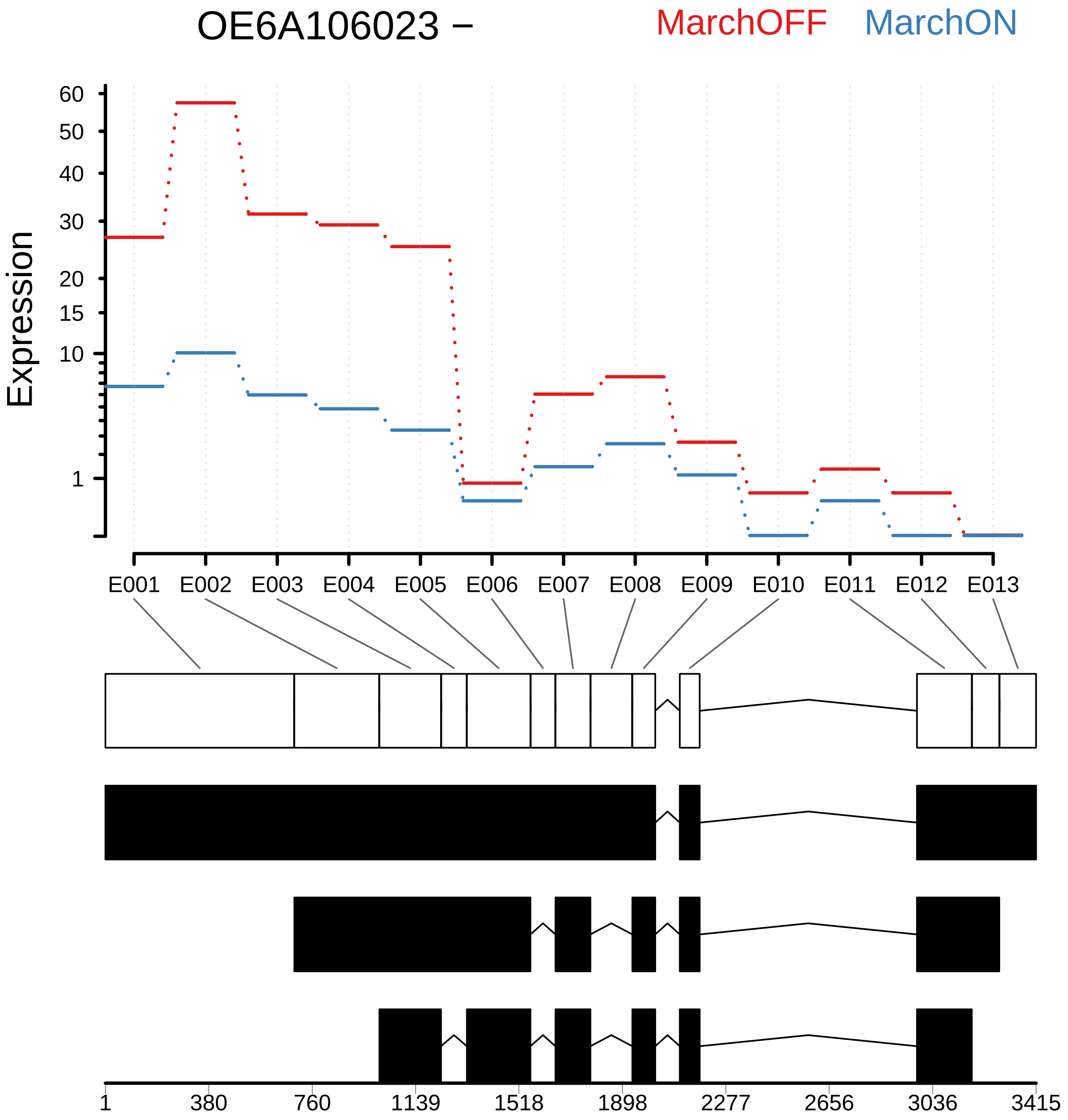

OE6A106023 −
MarchOFF
MarchON
Expression
60
50
40
30
20
15
10
1
E001
E002
E003
E004
E005
E006
E007
E008
E009
E010
E011
E012
E013
1
380
760
1139
1518
1898
2277
2656
3036
3415

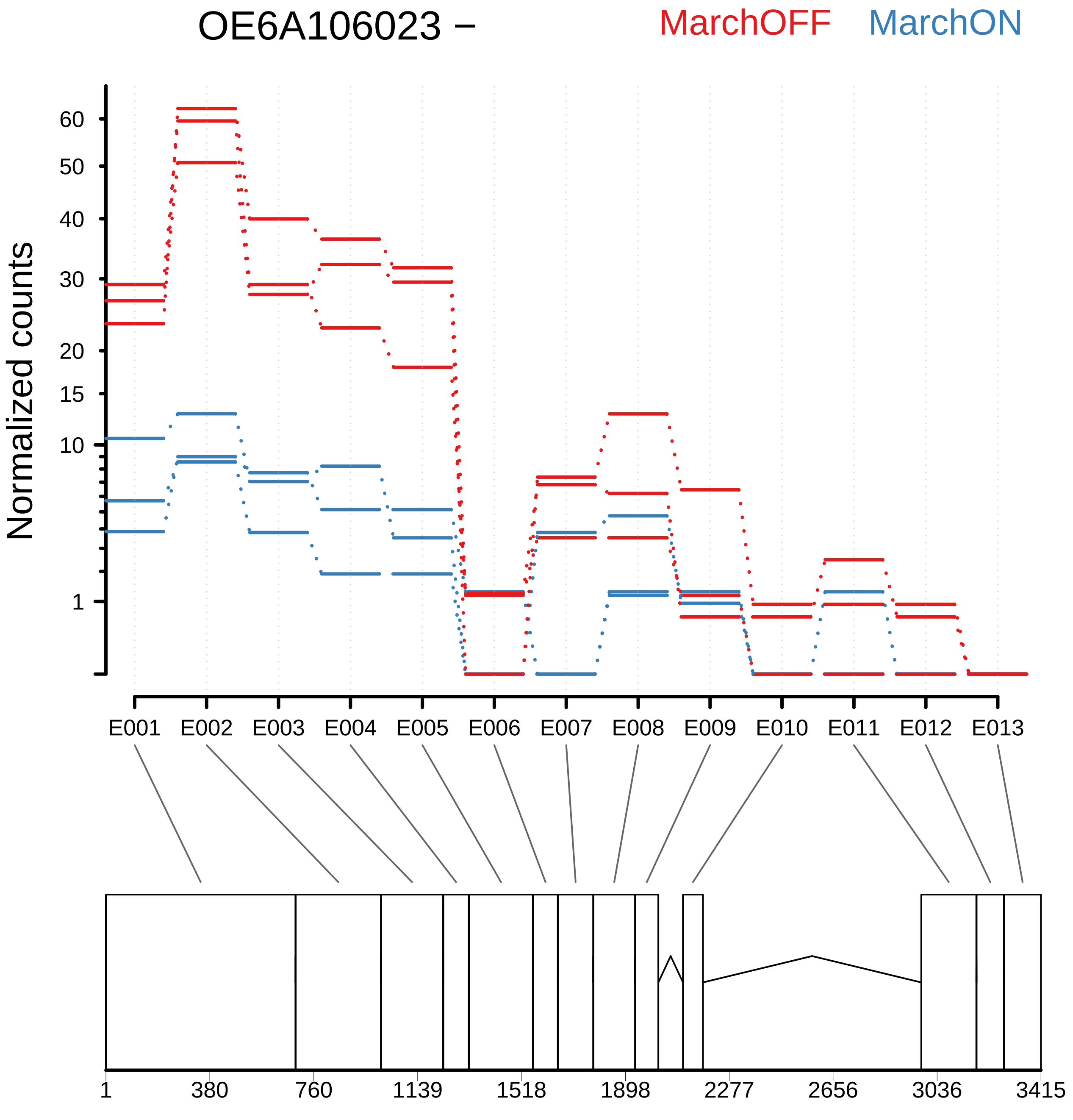

OE6A106023 −
MarchOFF
MarchON
Normalized counts
60
50
40
30
20
15
10
1
E001
E002
E003
E004
E005
E006
E007
E008
E009
E010
E011
E012
E013
1
380
760
1139
1518
1898
2277
2656
3036
3415

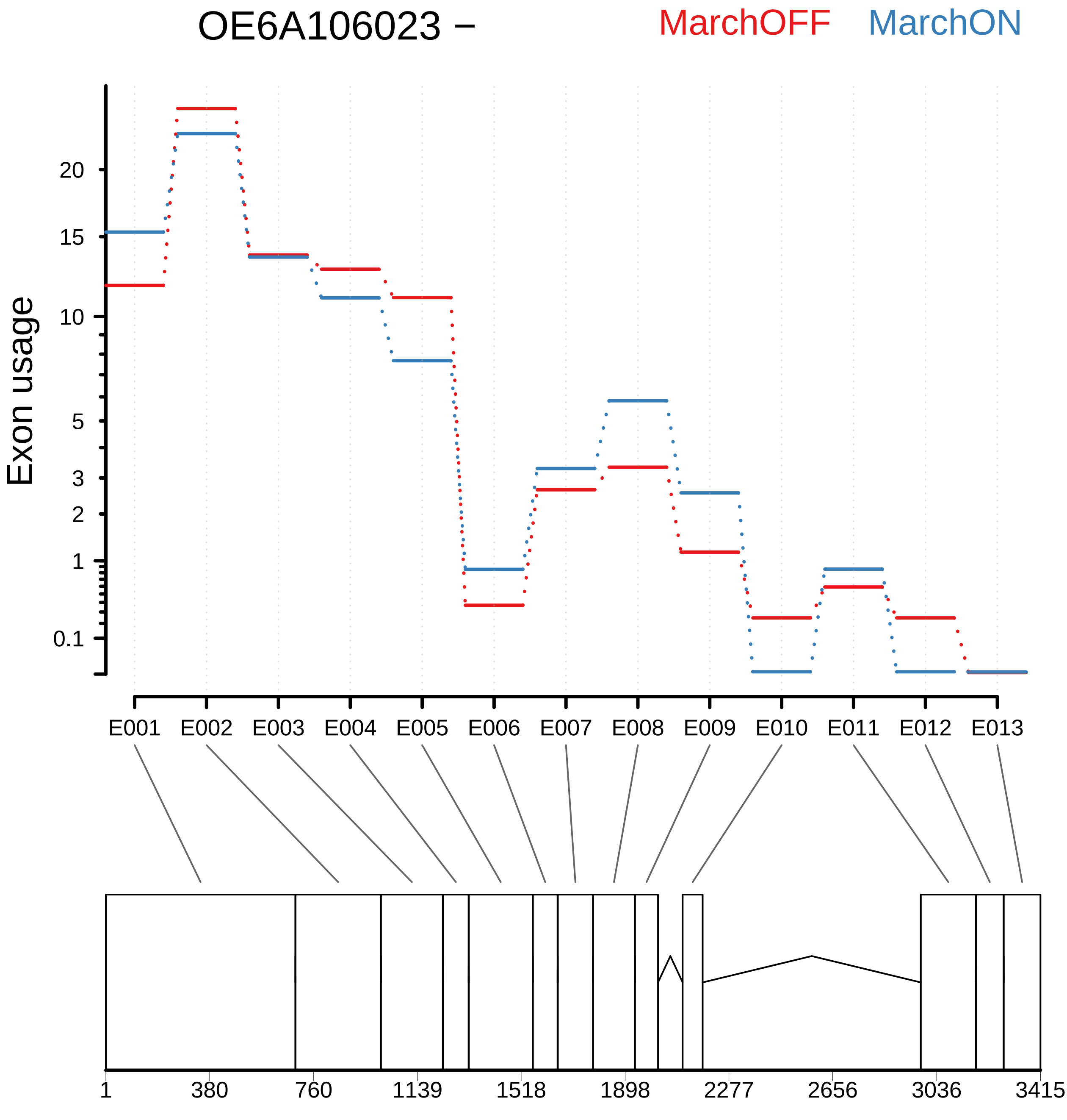

OE6A106023 −
MarchOFF
MarchON
Exon usage
20
15
10
5
3
2
1
0.1
E001
E002
E003
E004
E005
E006
E007
E008
E009
E010
E011
E012
E013
1
380
760
1139
1518
1898
2277
2656
3036
3415

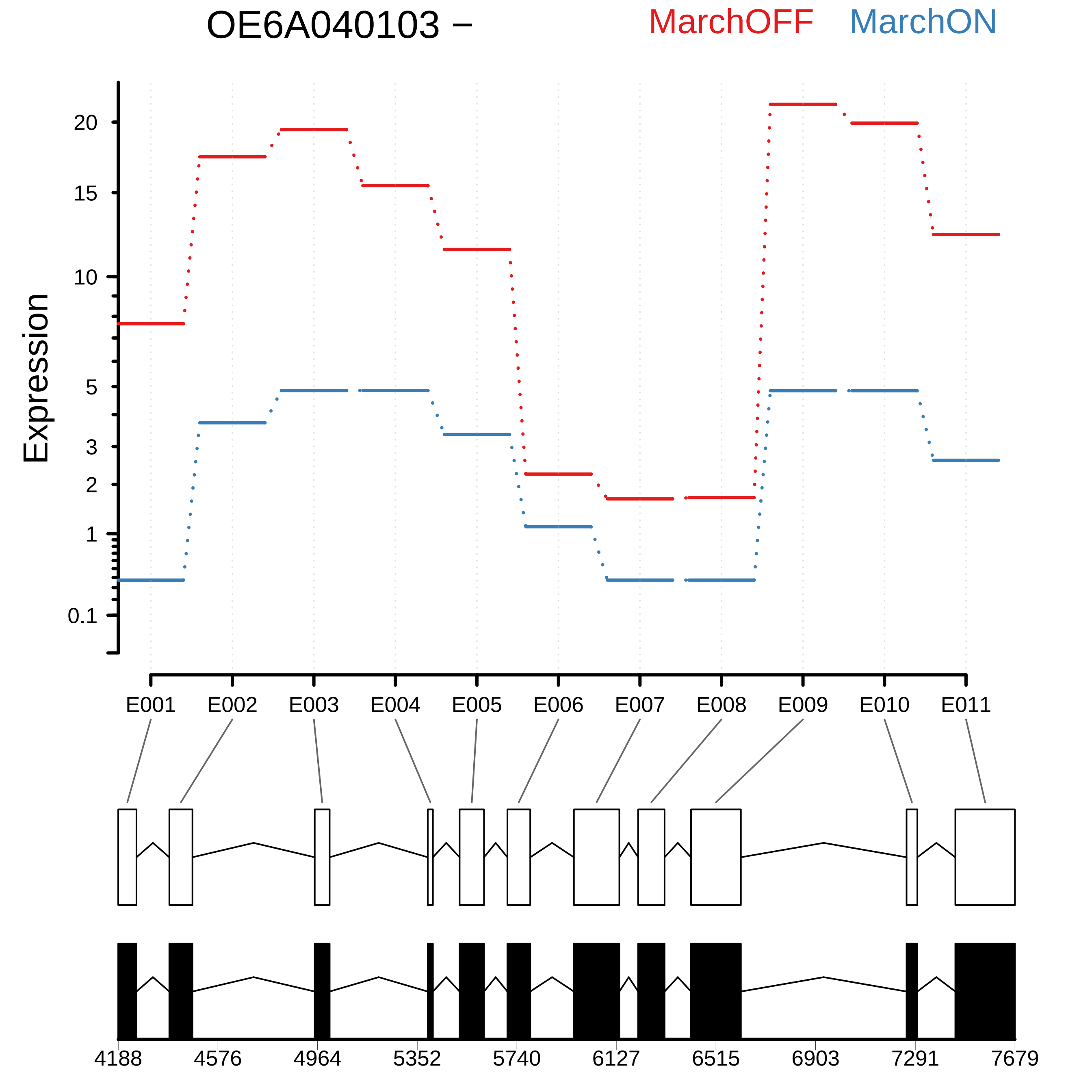

OE6A040103 −
MarchOFF
MarchON
Expression
20
15
10
5
3
2
1
0.1
E001
E002
E003
E004
E005
E006
E007
E008
E009
E010
E011
4188
4576
4964
5352
5740
6127
6515
6903
7291
7679

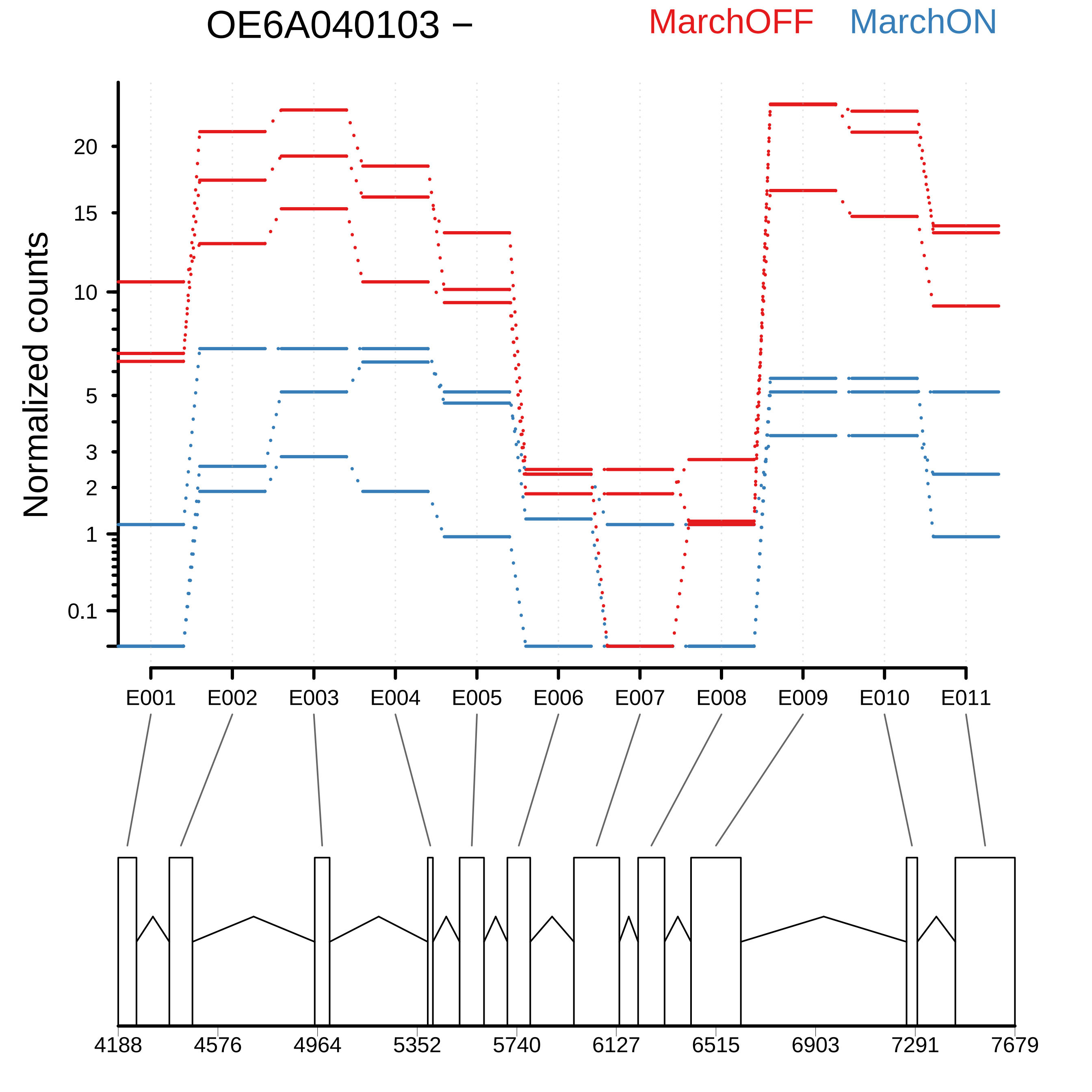

OE6A040103 −
MarchOFF
MarchON
Normalized counts
20
15
10
5
3
2
1
0.1
E001
E002
E003
E004
E005
E006
E007
E008
E009
E010
E011
4188
4576
4964
5352
5740
6127
6515
6903
7291
7679

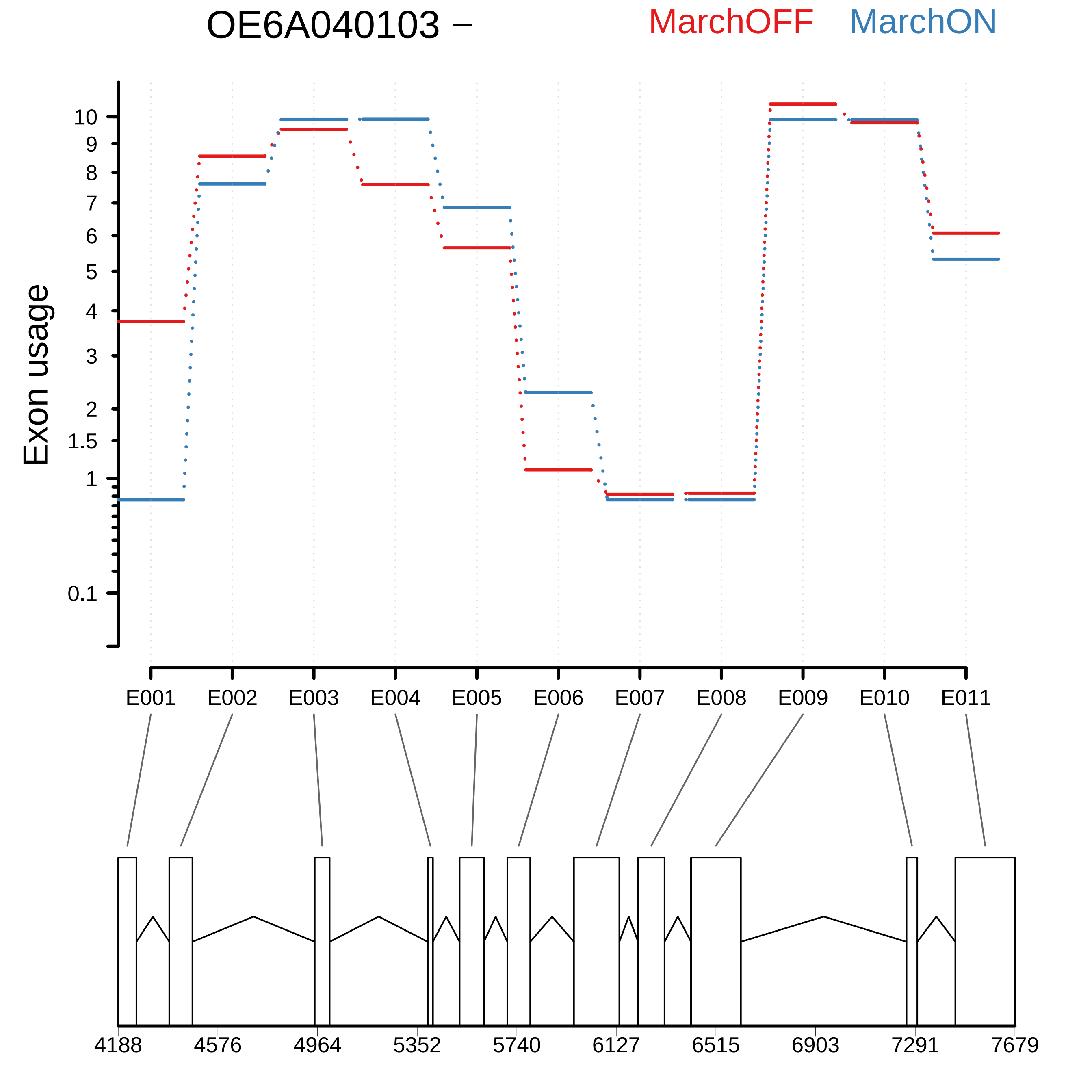
OE6A040103 −
MarchOFF
MarchON
Exon usage
10
9
8
7
6
5
4
3
2
1.5
1
0.1
E001
E002
E003
E004
E005
E006
E007
E008
E009
E010
E011
4188
4576
4964
5352
5740
6127
6515
6903
7291
7679

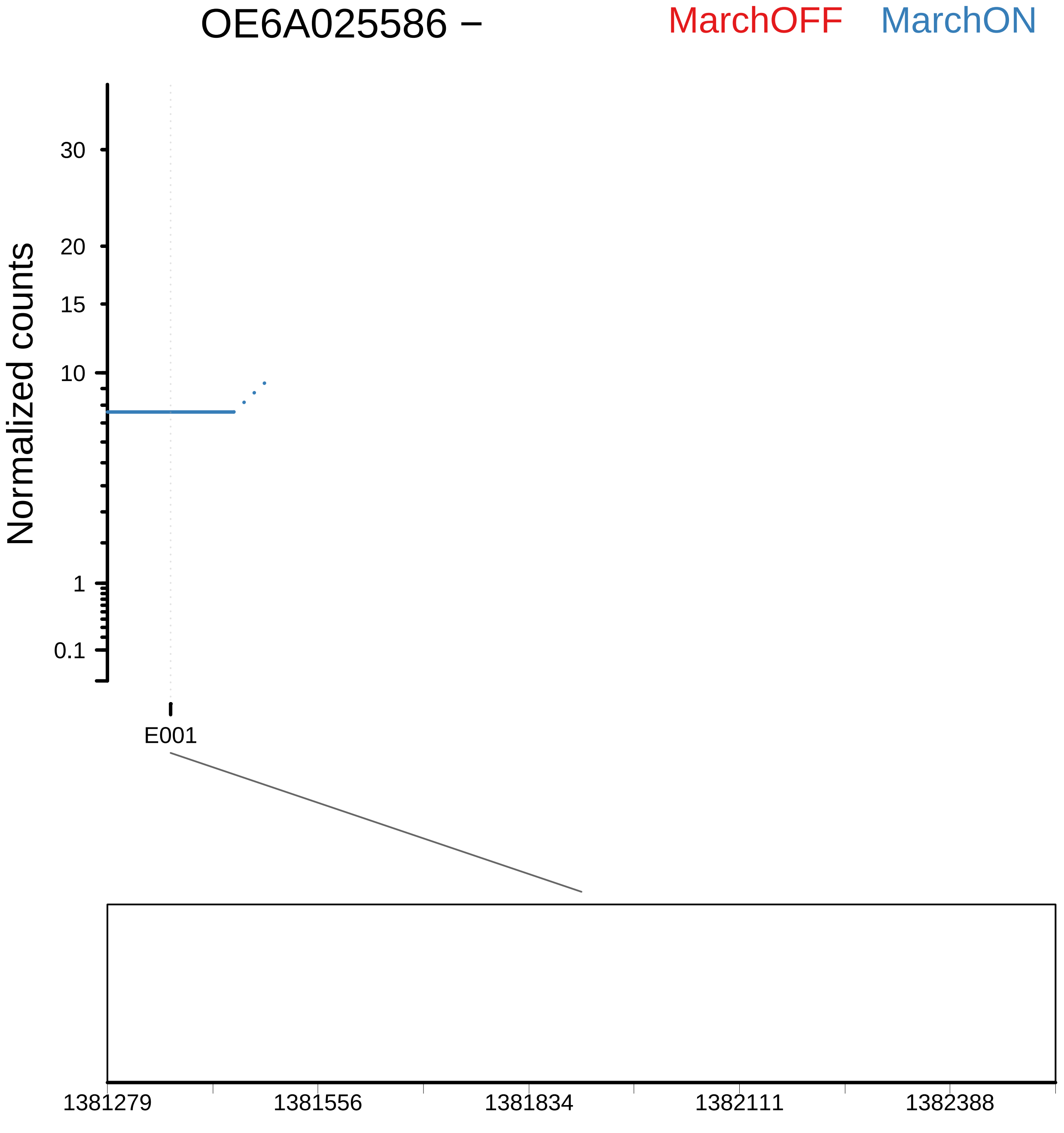

OE6A025586 −
MarchOFF
MarchON
Normalized counts
30
20
15
10
1
0.1
E001
1381279
1381556
1381834
1382111
1382388

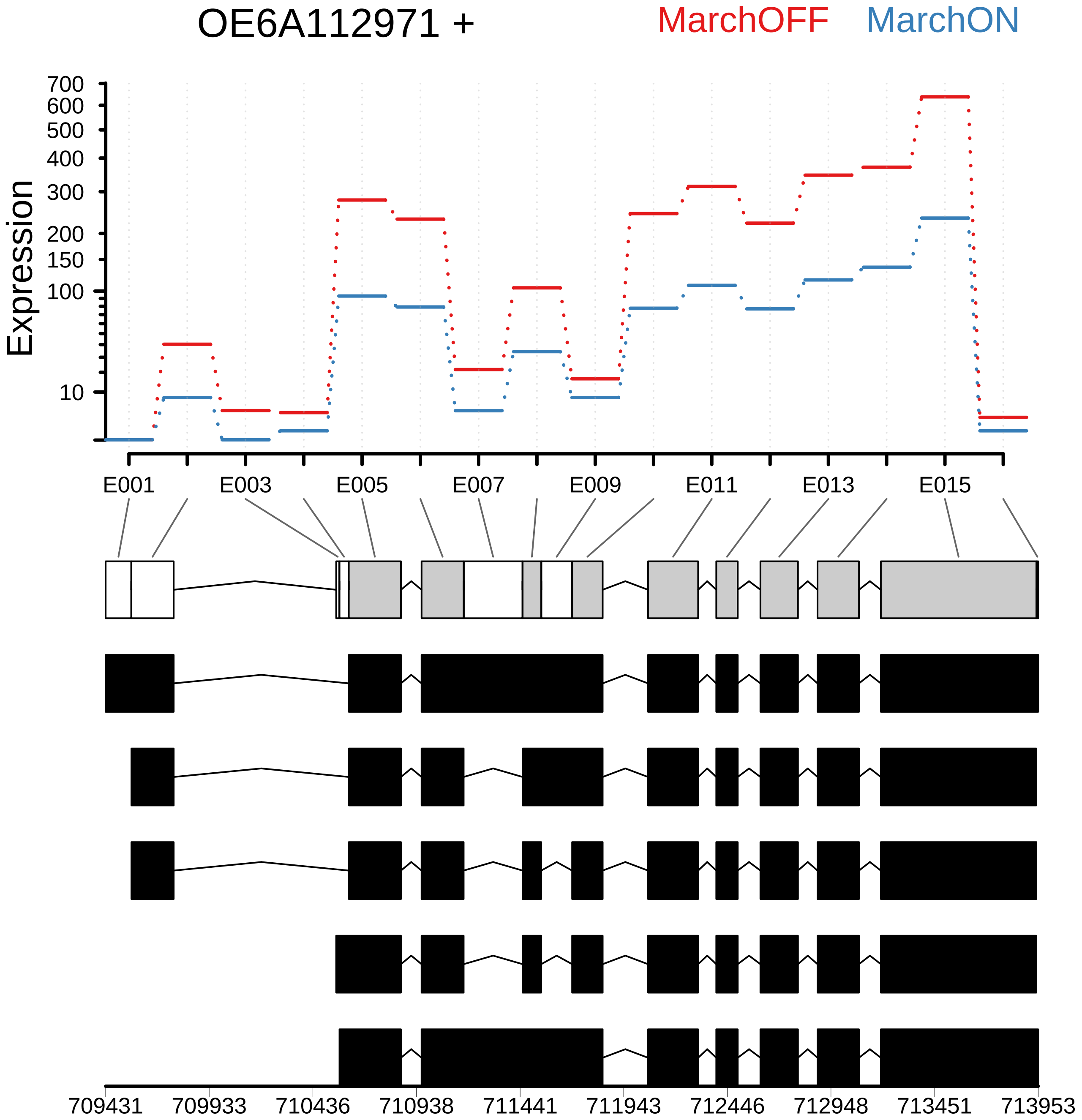

OE6A112971 +
MarchOFF
MarchON
Expression
700
600
500
400
300
200
150
100
10
E001
E003
E005
E007
E009
E011
E013
E015
709431
709933
710436
710938
711441
711943
712446
712948
713451
713953

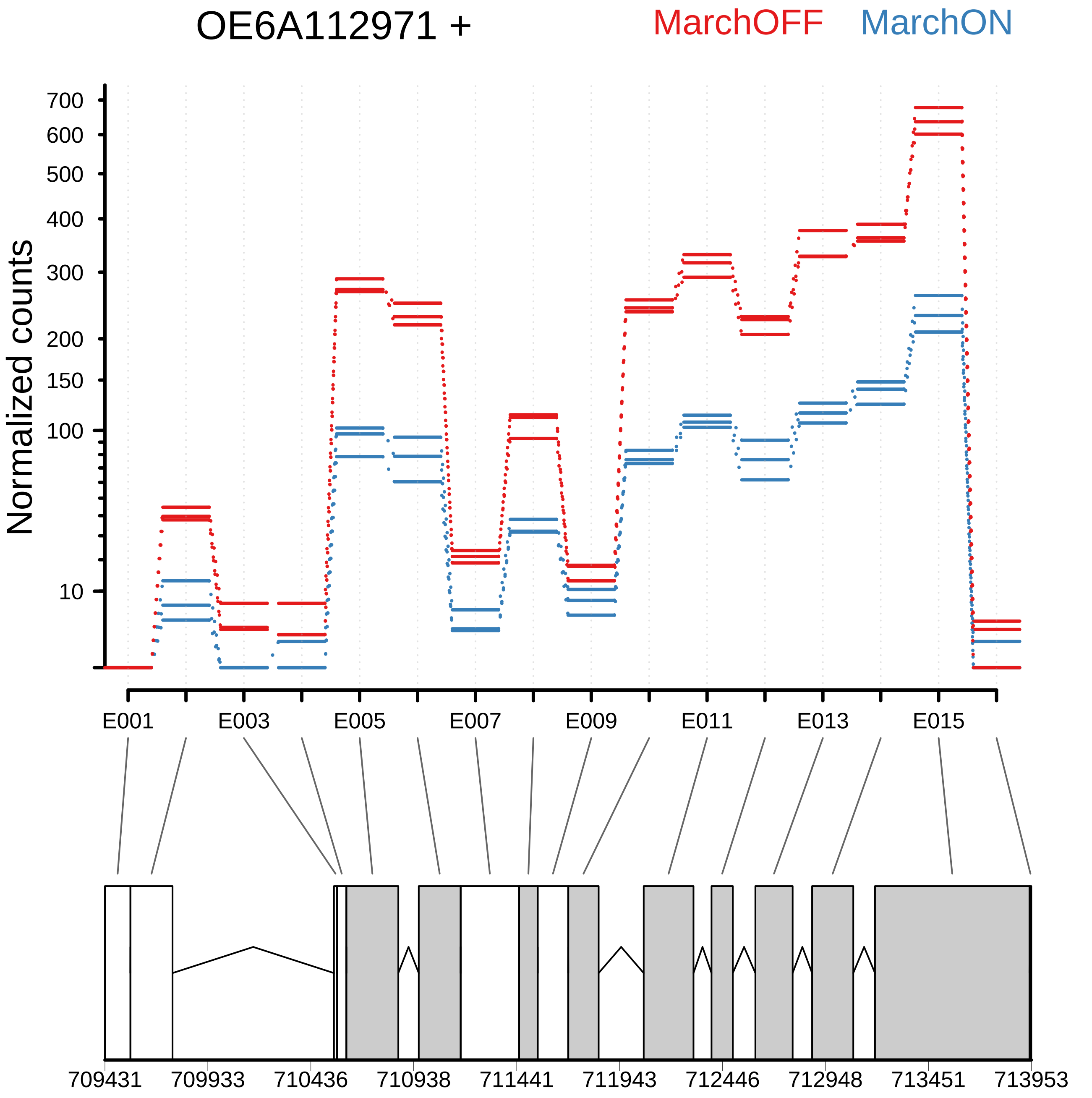

OE6A112971 +
MarchOFF
MarchON
Normalized counts
700
600
500
400
300
200
150
100
10
E001
E003
E005
E007
E009
E011
E013
E015
709431
709933
710436
710938
711441
711943
712446
712948
713451
713953

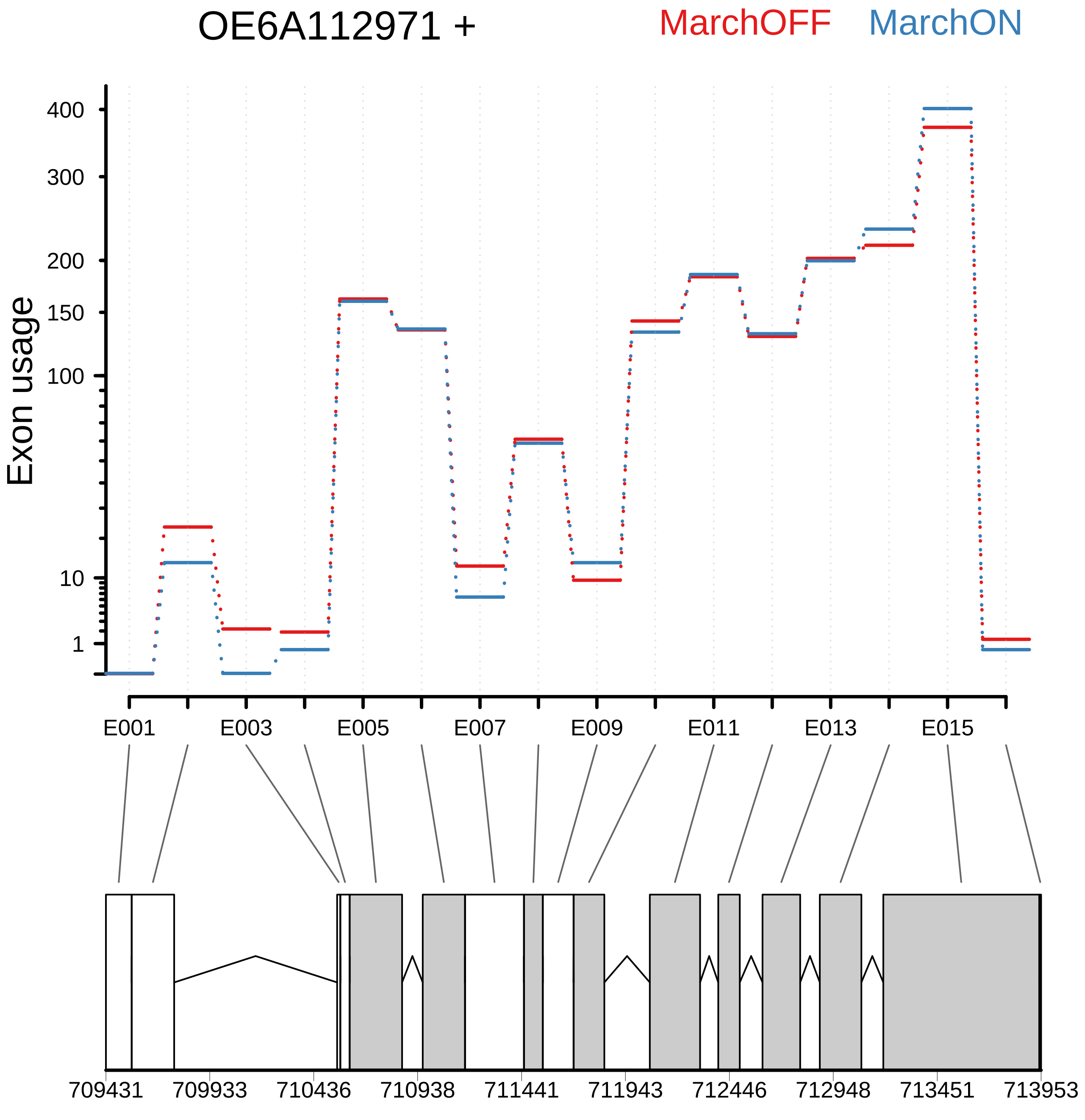
OE6A112971 +
MarchOFF
MarchON
Exon usage
400
300
200
150
100
10
1
E001
E003
E005
E007
E009
E011
E013
E015
709431
709933
710436
710938
711441
711943
712446
712948
713451
713953

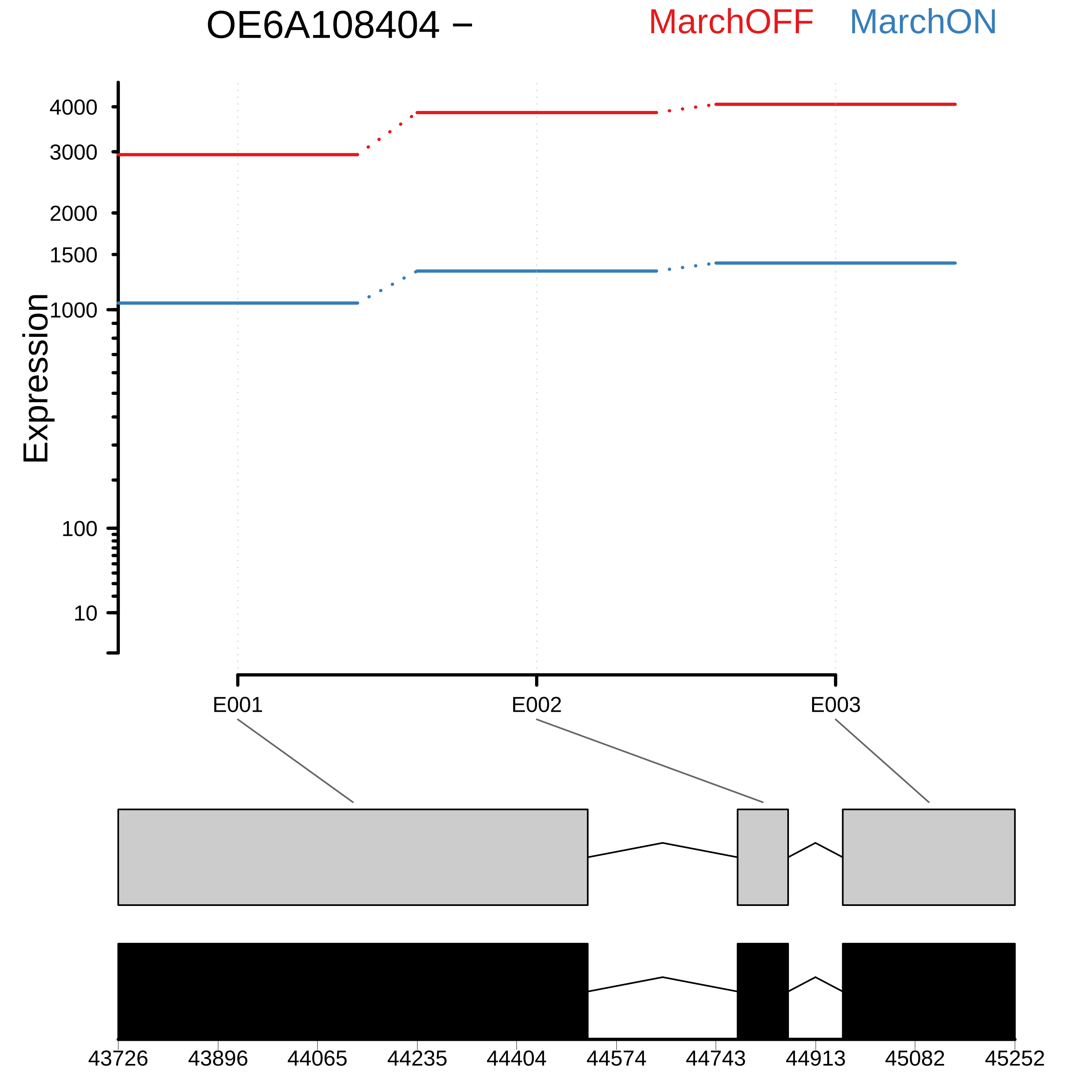

OE6A108404 −
MarchOFF
MarchON
Expression
4000
3000
2000
1500
1000
100
10
E001
E002
E003
43726
43896
44065
44235
44404
44574
44743
44913
45082
45252

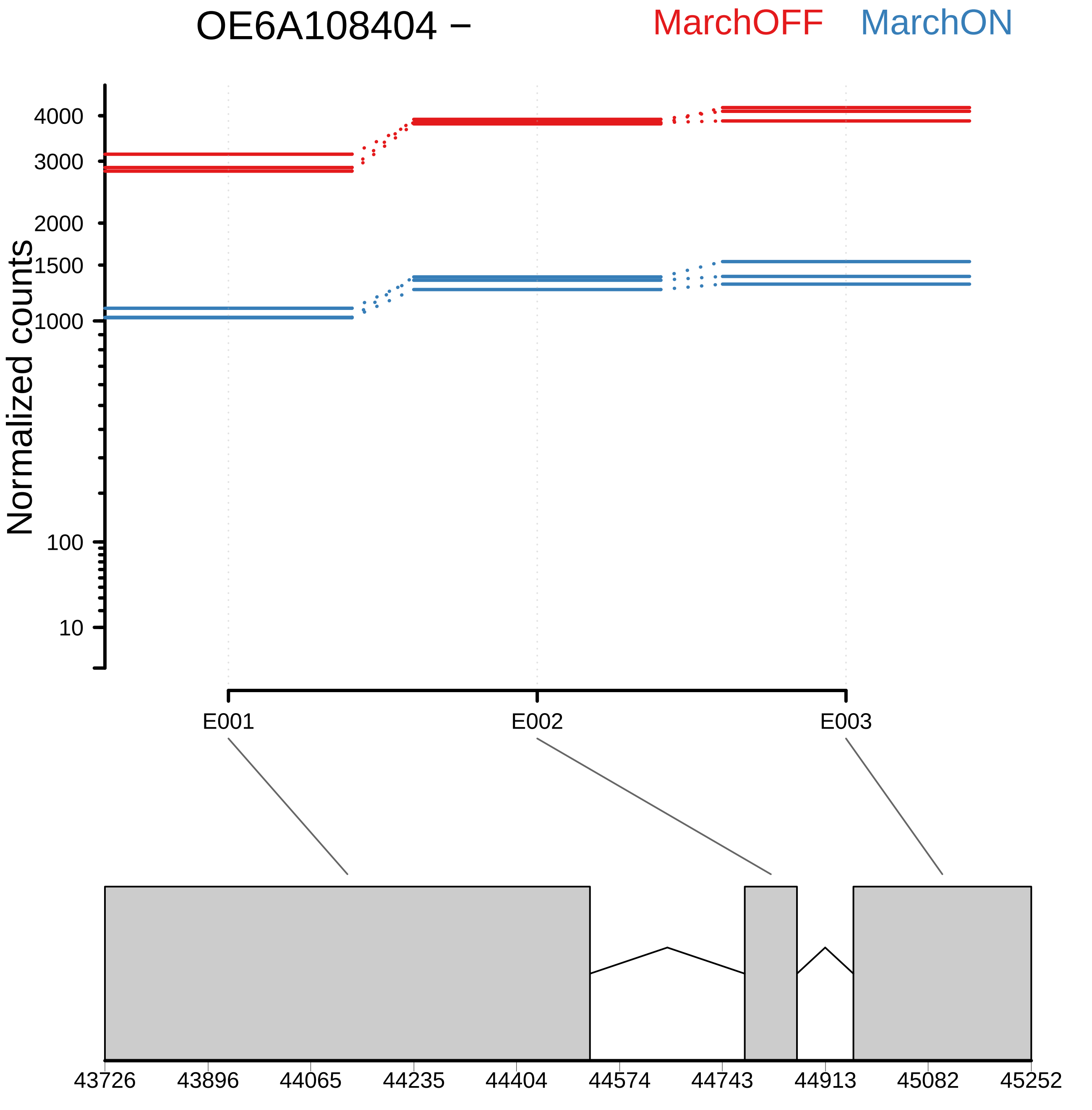

OE6A108404 −
MarchOFF
MarchON
Normalized counts
4000
3000
2000
1500
1000
100
10
E001
E002
E003
43726
43896
44065
44235
44404
44574
44743
44913
45082
45252

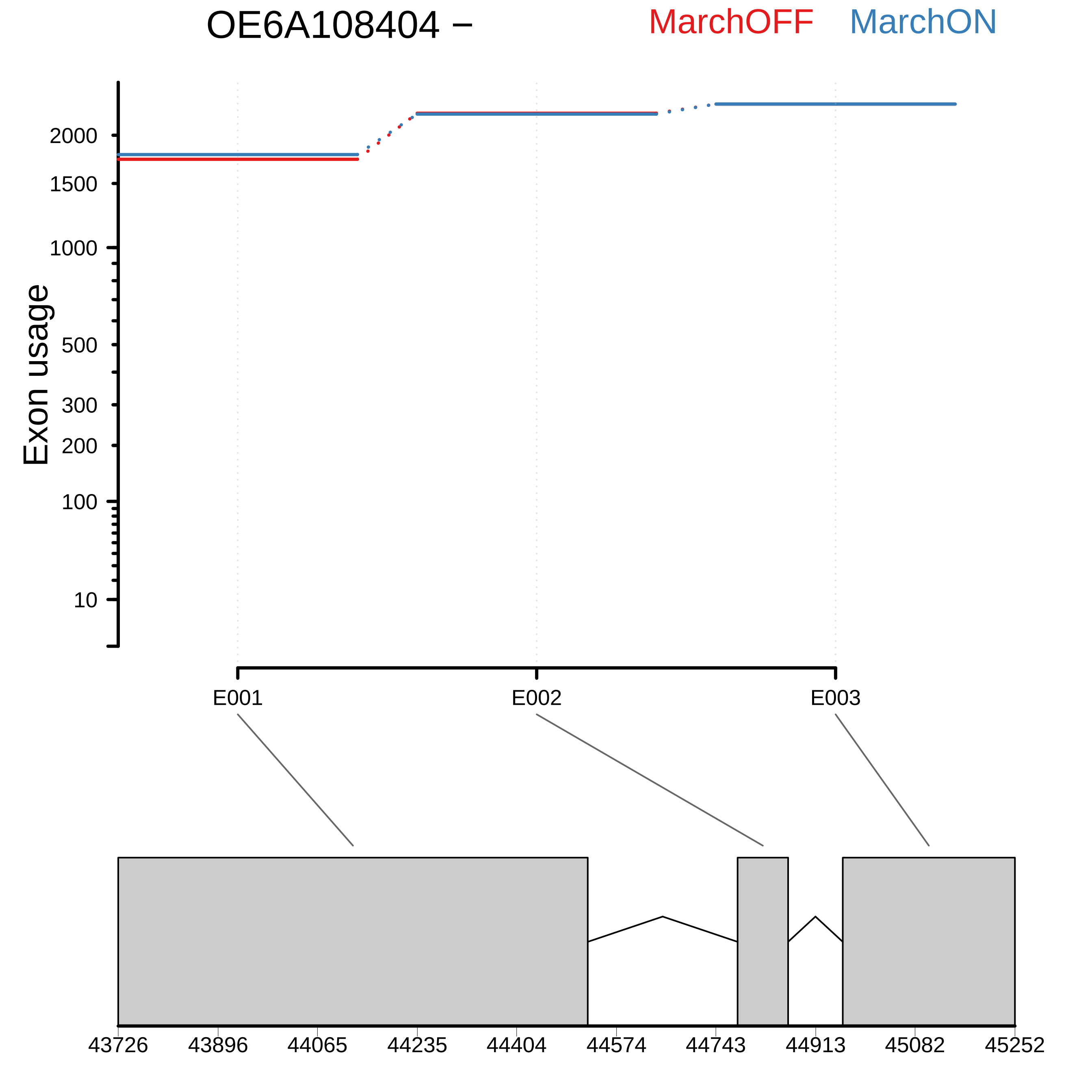

OE6A108404 −
MarchOFF
MarchON
Exon usage
2000
1500
1000
500
300
200
100
10
E001
E002
E003
43726
43896
44065
44235
44404
44574
44743
44913
45082
45252

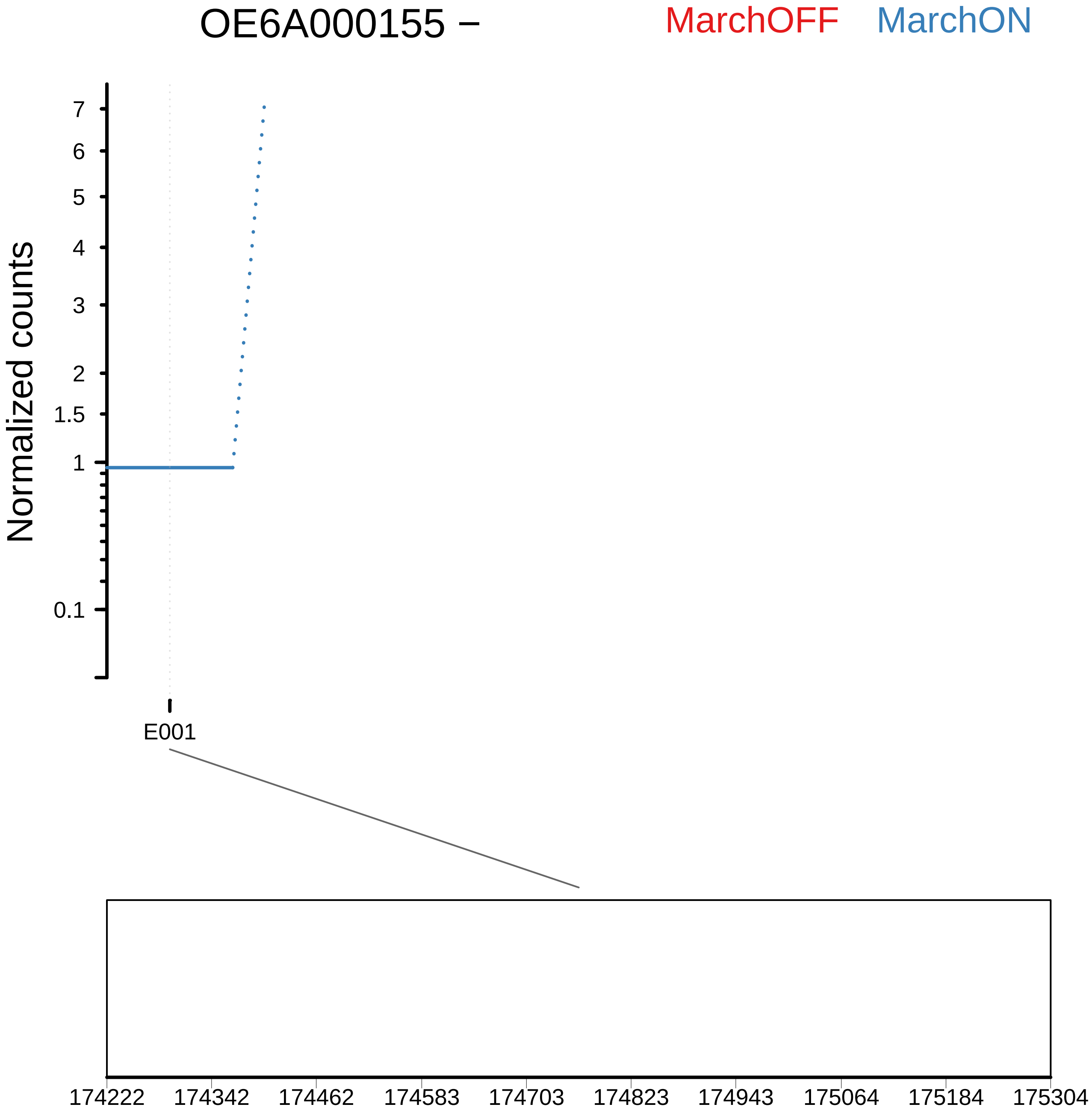
OE6A000155 −
MarchOFF
MarchON
Normalized counts
7
6
5
4
3
2
1.5
1
0.1
E001
174222
174342
174462
174583
174703
174823
174943
175064
175184
175304

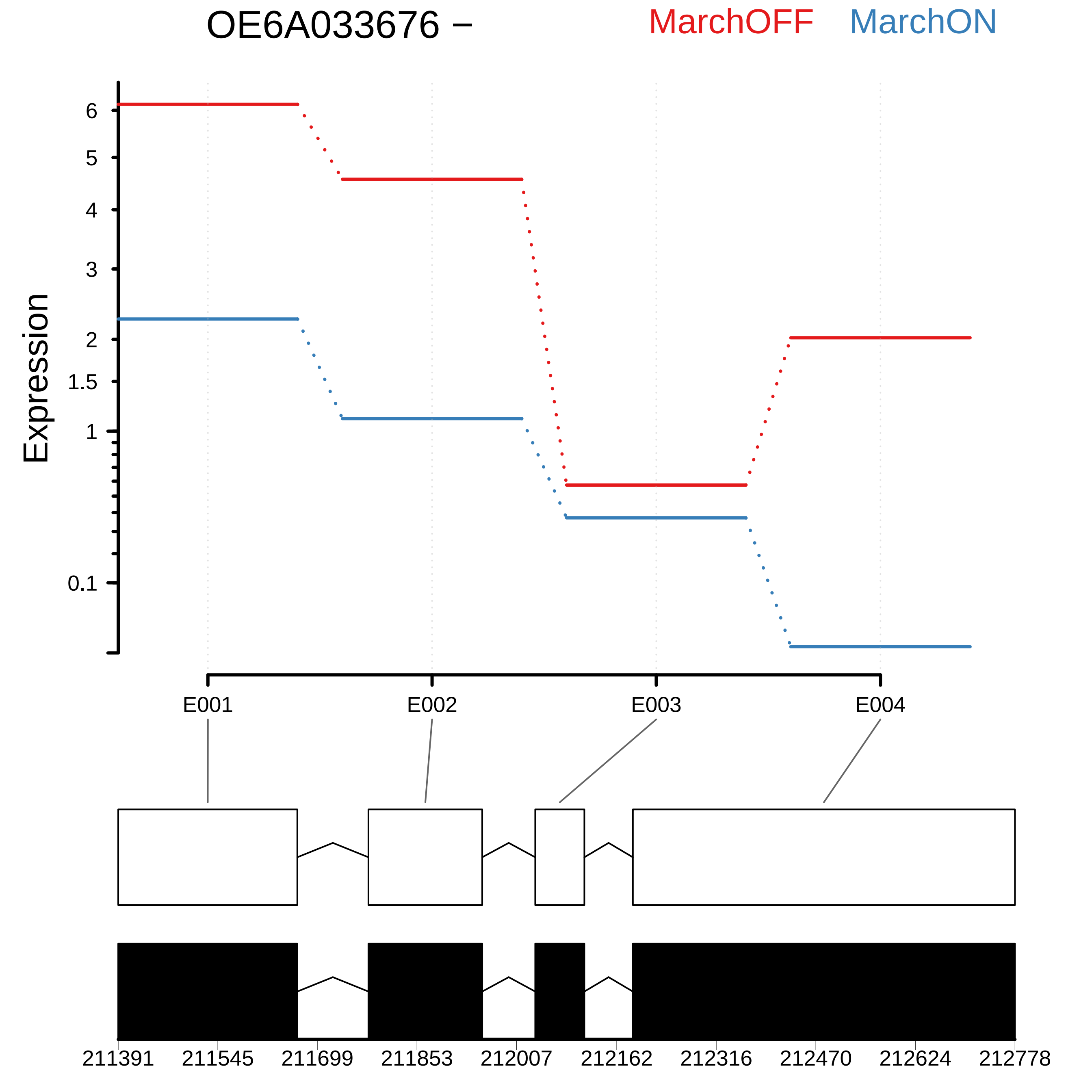

OE6A033676 −
MarchOFF
MarchON
Expression
6
5
4
3
2
1.5
1
0.1
E001
E002
E003
E004
211391
211545
211699
211853
212007
212162
212316
212470
212624
212778

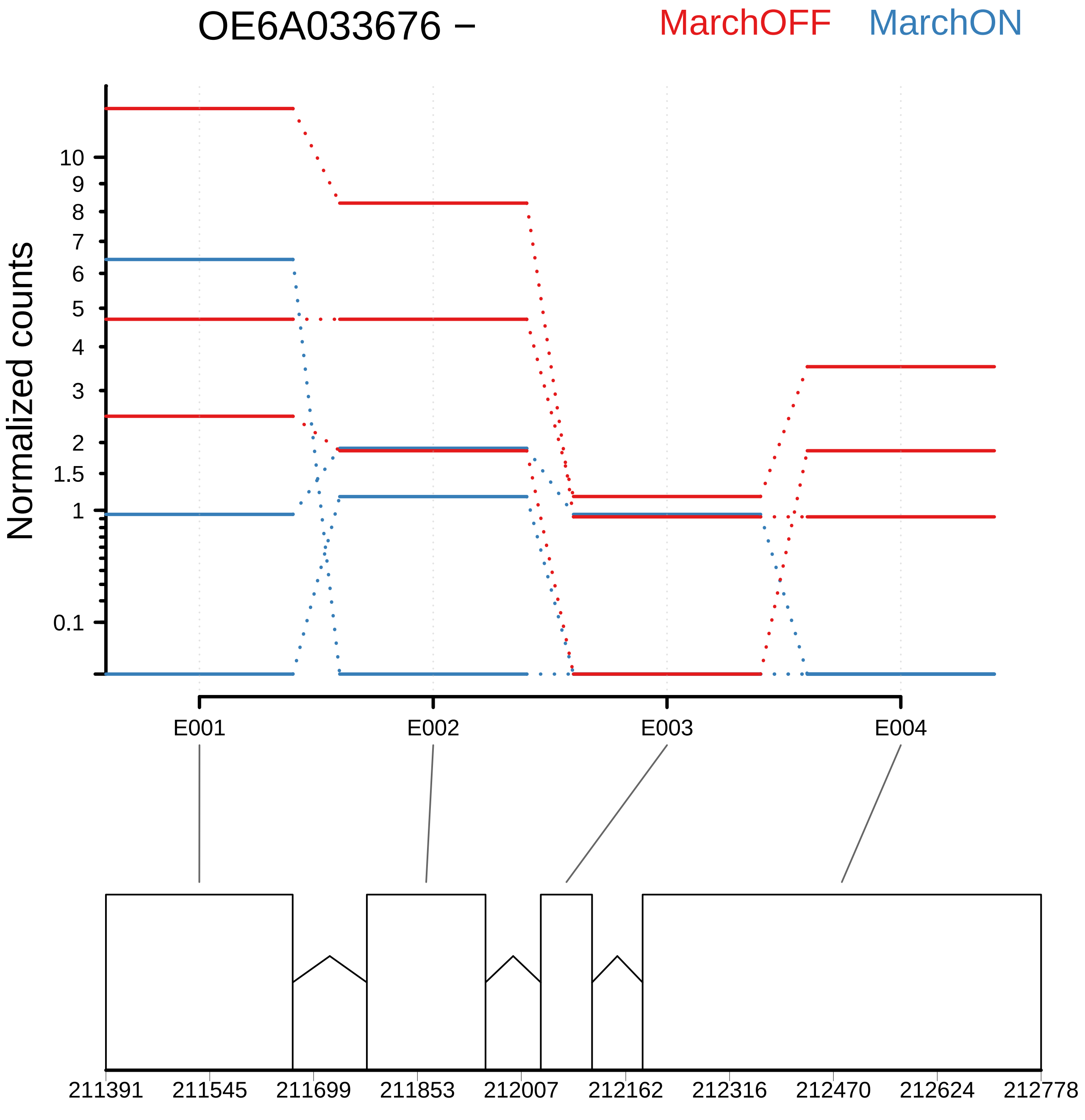

OE6A033676 −
MarchOFF
MarchON
Normalized counts
10
9
8
7
6
5
4
3
2
1.5
1
0.1
E001
E002
E003
E004
211391
211545
211699
211853
212007
212162
212316
212470
212624
212778

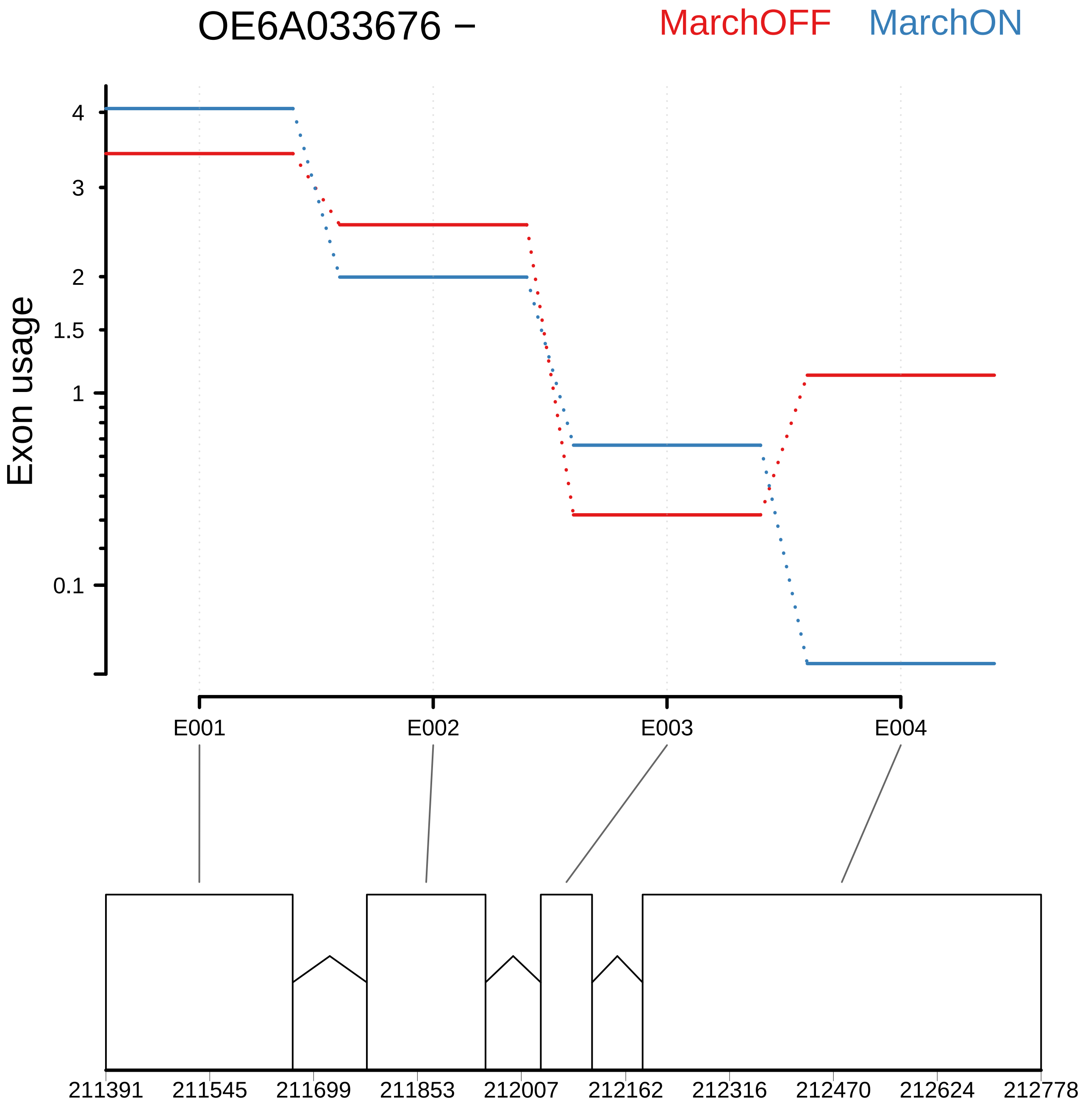

OE6A033676 −
MarchOFF
MarchON
Exon usage
4
3
2
1.5
1
0.1
E001
E002
E003
E004
211391
211545
211699
211853
212007
212162
212316
212470
212624
212778

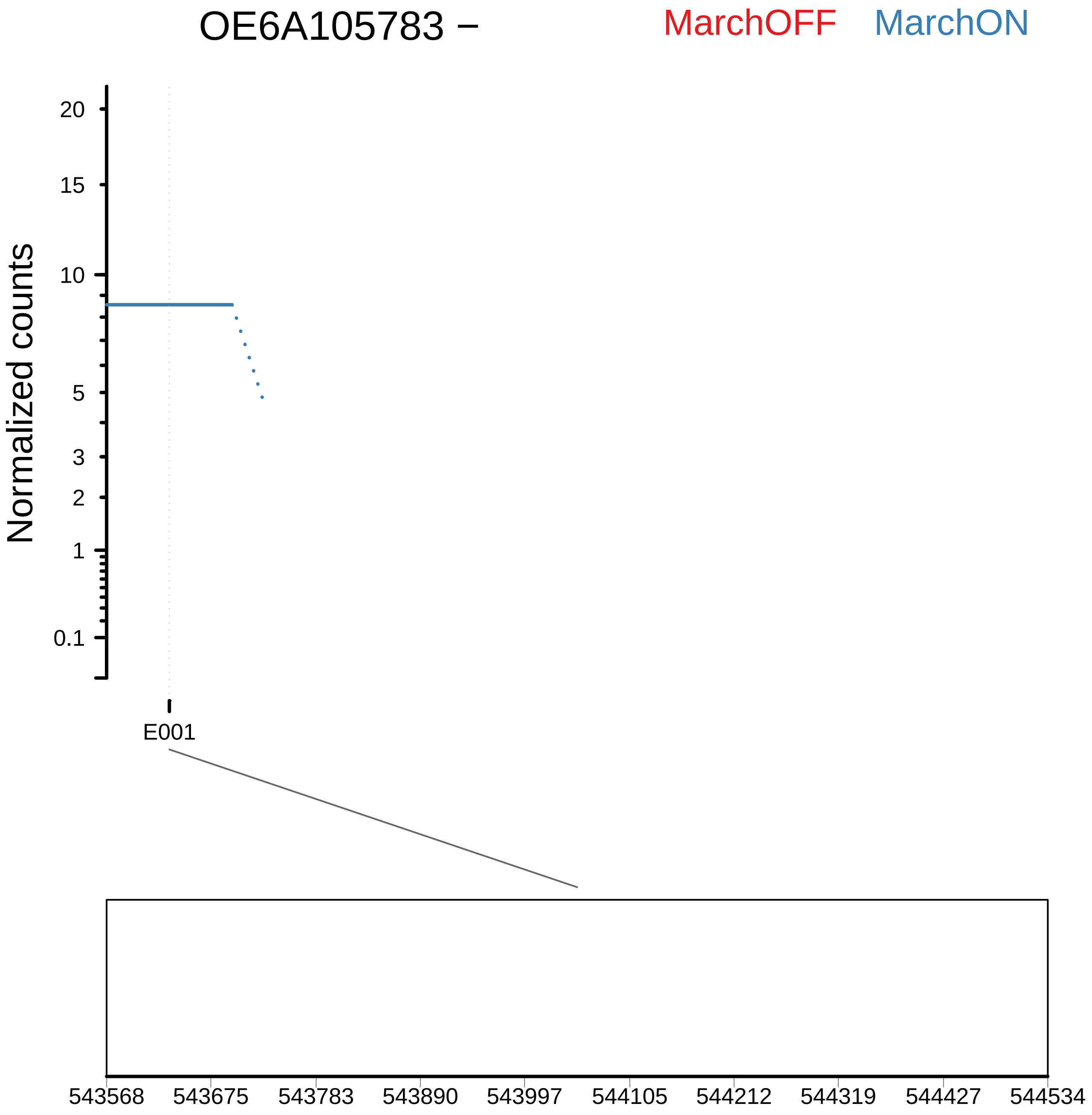

OE6A105783 −
MarchOFF
MarchON
Normalized counts
20
15
10
5
3
2
1
0.1
E001
543568
543675
543783
543890
543997
544105
544212
544319
544427
544534

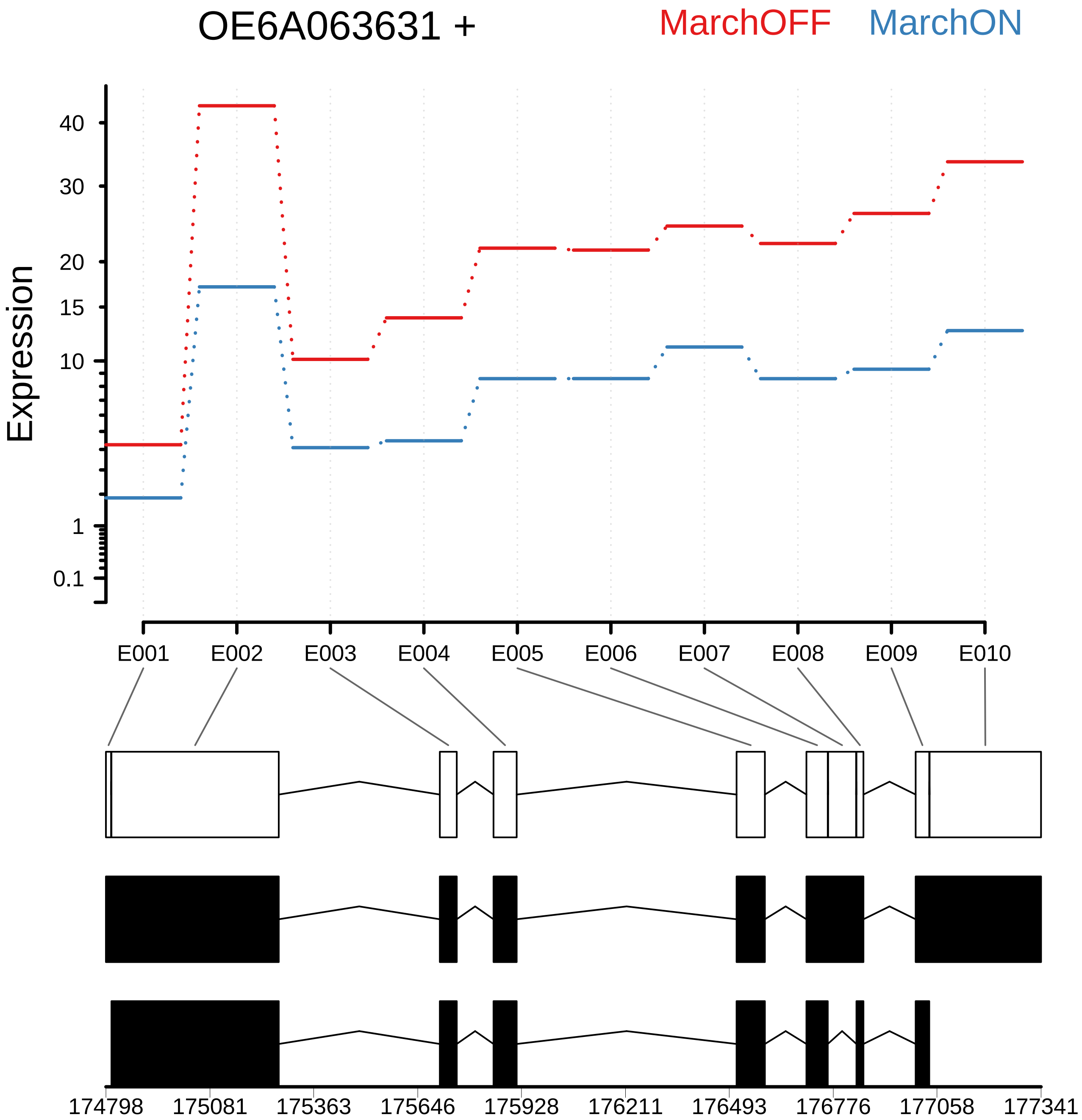

OE6A063631 +
MarchOFF
MarchON
Expression
40
30
20
15
10
1
0.1
E001
E002
E003
E004
E005
E006
E007
E008
E009
E010
174798
175081
175363
175646
175928
176211
176493
176776
177058
177341

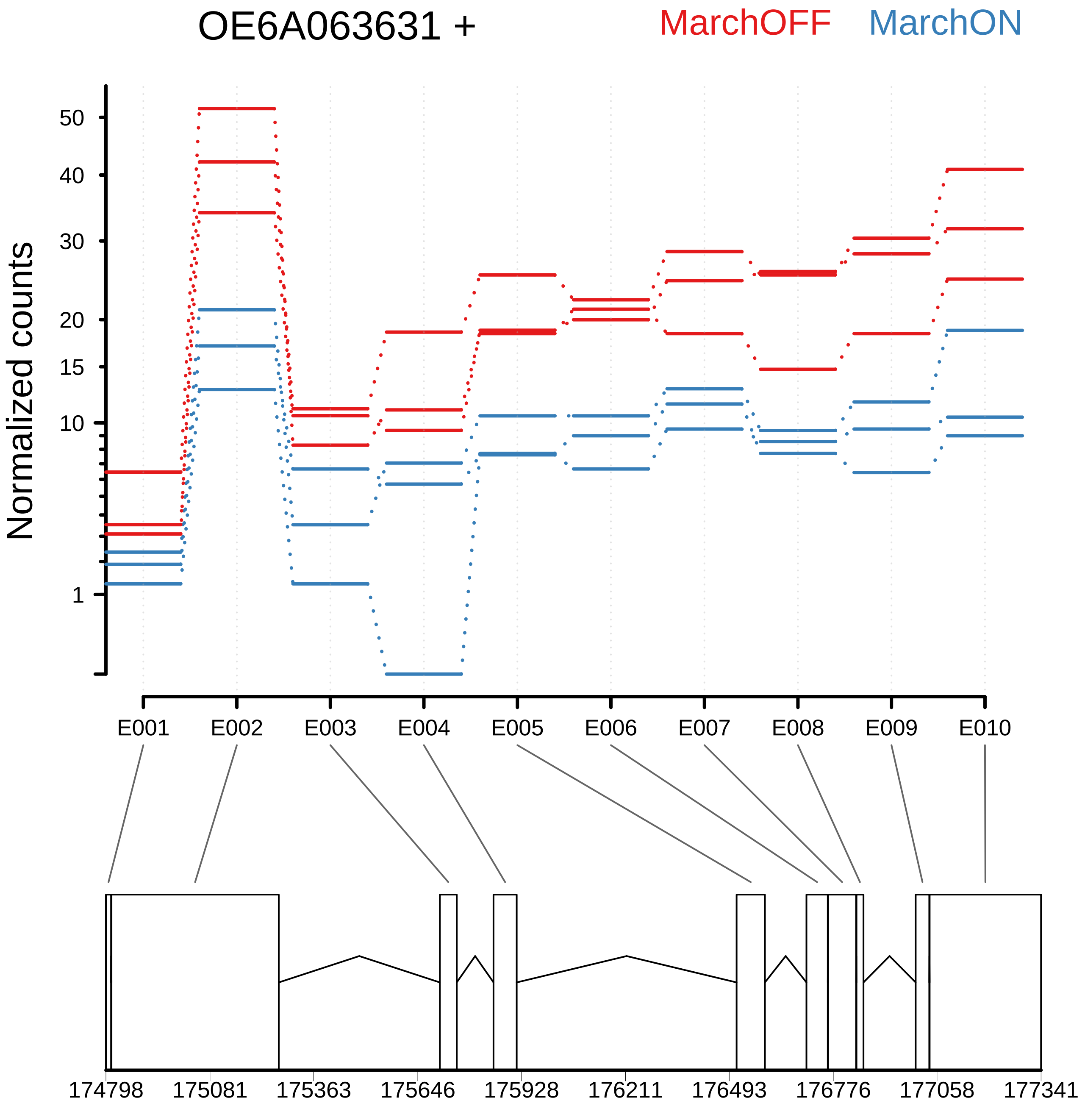
OE6A063631 +
MarchOFF
MarchON
Normalized counts
50
40
30
20
15
10
1
E001
E002
E003
E004
E005
E006
E007
E008
E009
E010
174798
175081
175363
175646
175928
176211
176493
176776
177058
177341

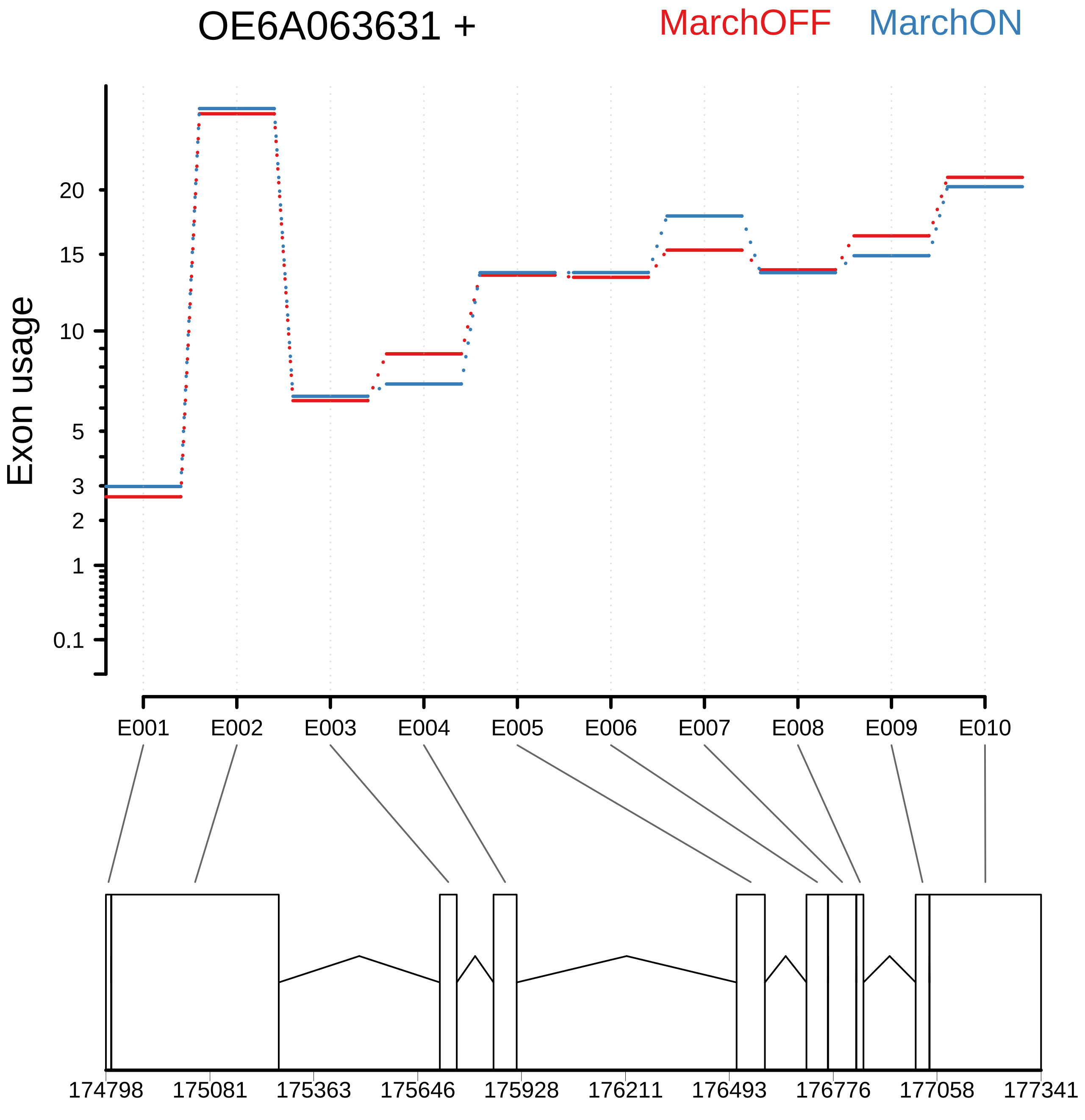
OE6A063631 +
MarchOFF
MarchON
Exon usage
20
15
10
5
3
2
1
0.1
E001
E002
E003
E004
E005
E006
E007
E008
E009
E010
174798
175081
175363
175646
175928
176211
176493
176776
177058
177341

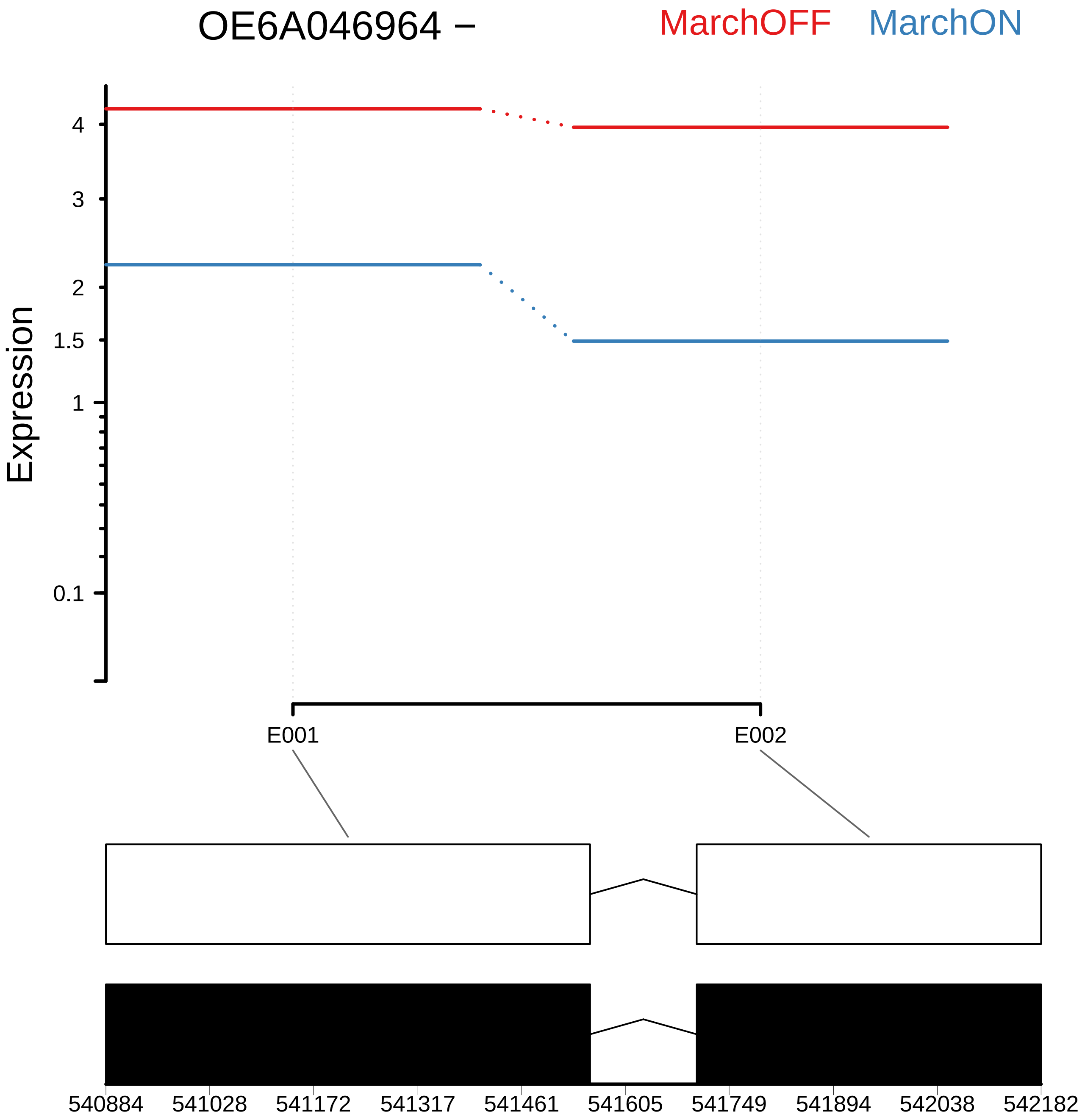
OE6A046964 −
MarchOFF
MarchON
Expression
4
3
2
1.5
1
0.1
E001
E002
540884
541028
541172
541317
541461
541605
541749
541894
542038
542182

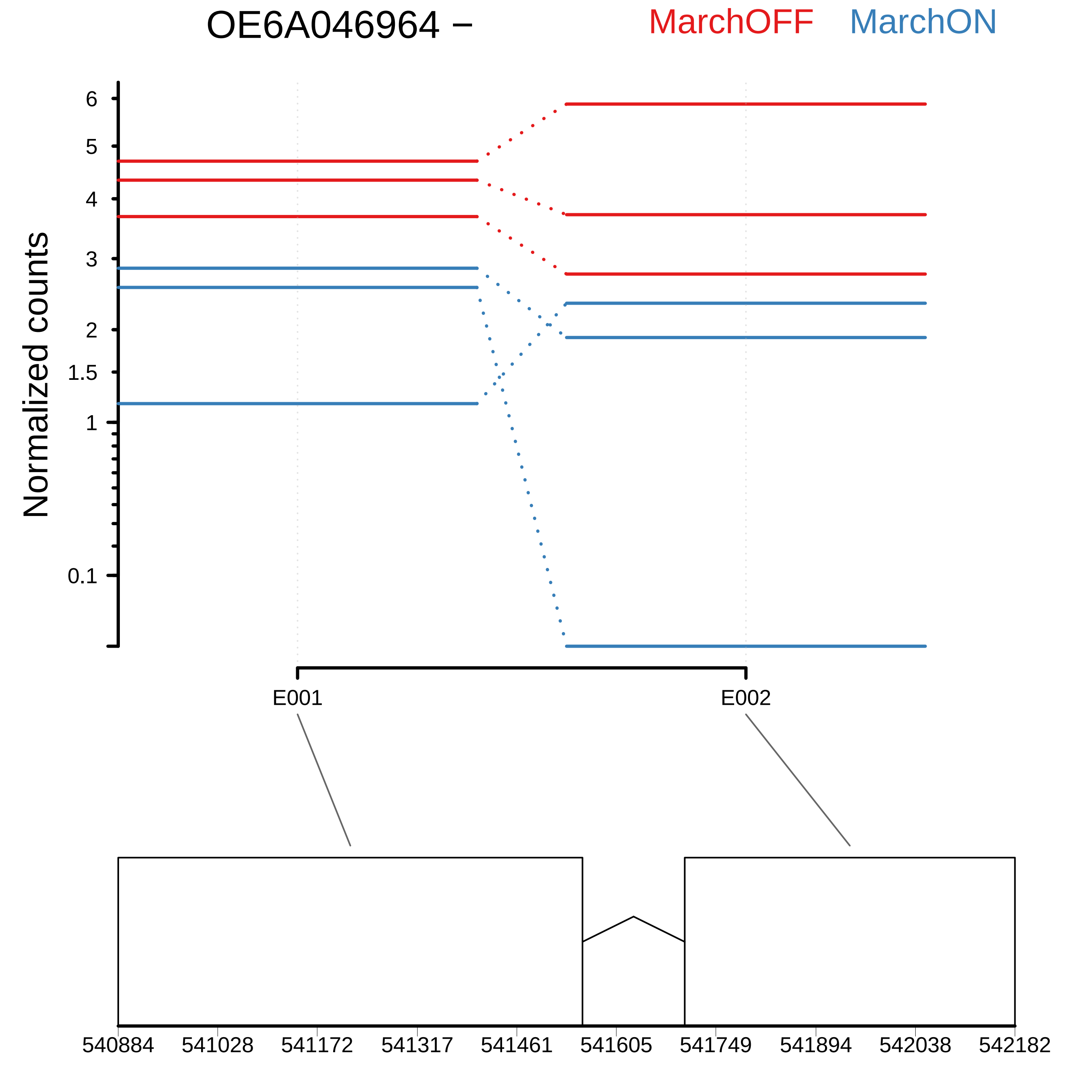
OE6A046964 −
MarchOFF
MarchON
Normalized counts
6
5
4
3
2
1.5
1
0.1
E001
E002
540884
541028
541172
541317
541461
541605
541749
541894
542038
542182

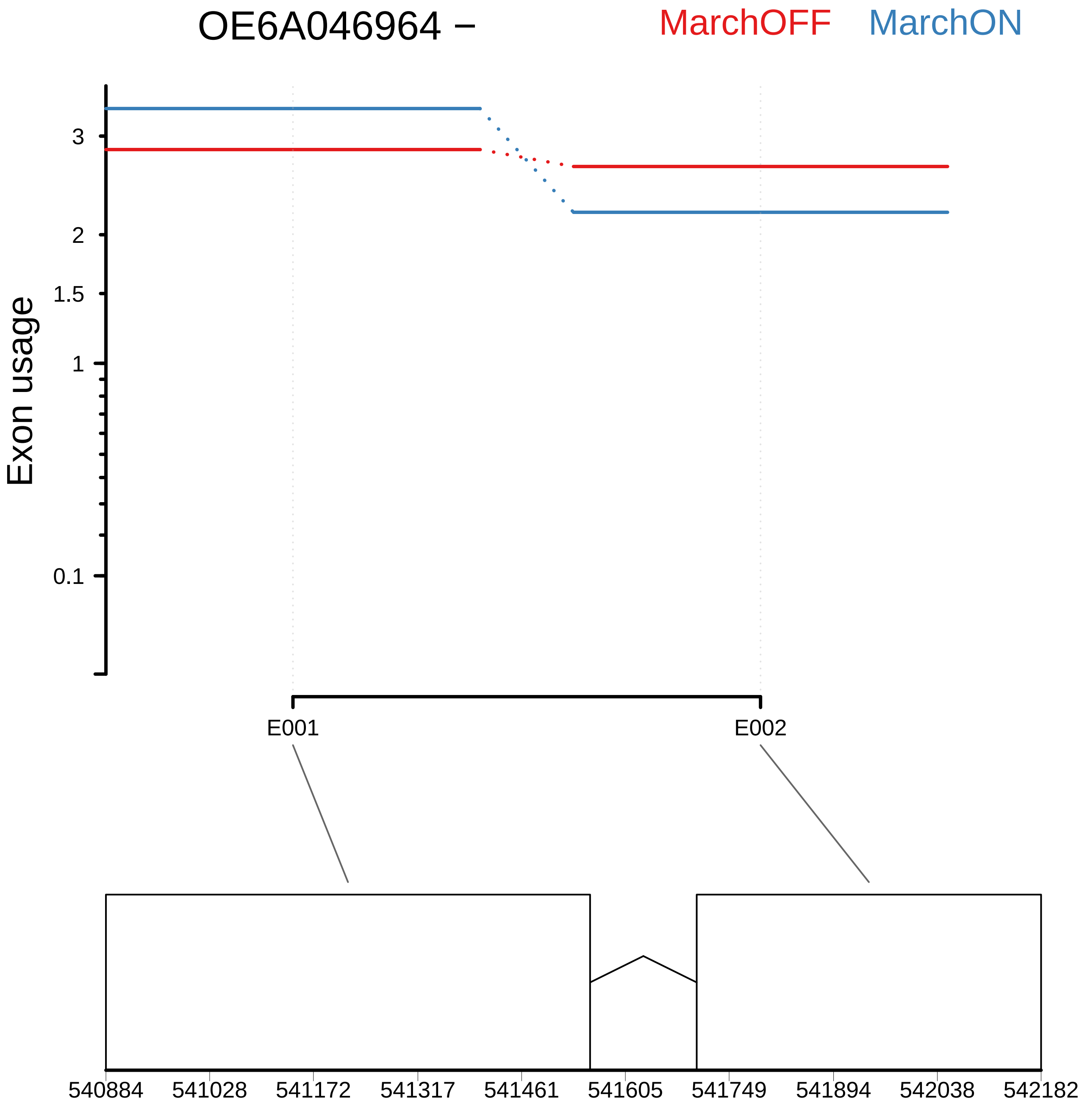

OE6A046964 −
MarchOFF
MarchON
Exon usage
3
2
1.5
1
0.1
E001
E002
540884
541028
541172
541317
541461
541605
541749
541894
542038
542182

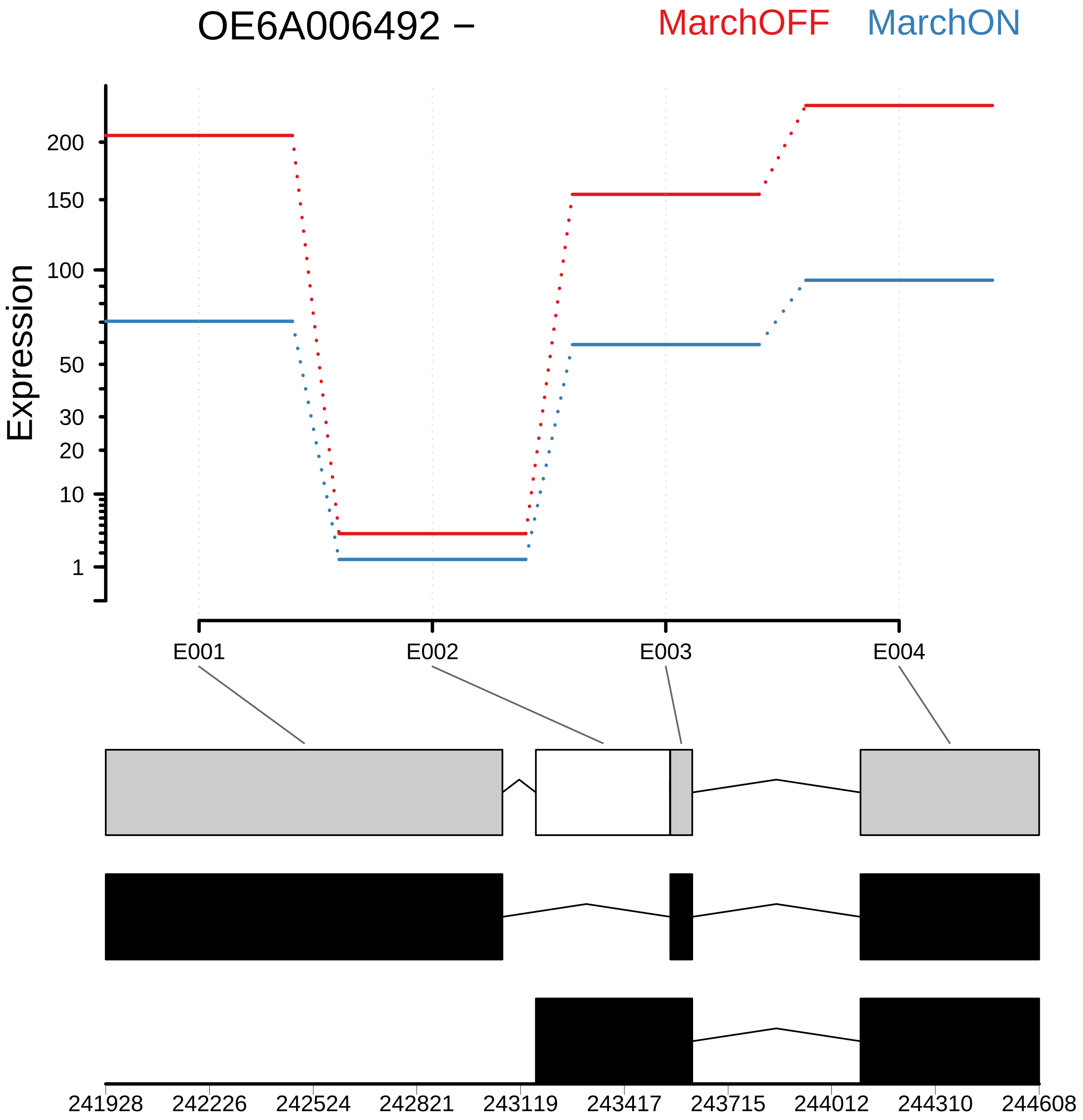
OE6A006492 −
MarchOFF
MarchON
Expression
200
150
100
50
30
20
10
1
E001
E002
E003
E004
241928
242226
242524
242821
243119
243417
243715
244012
244310
244608

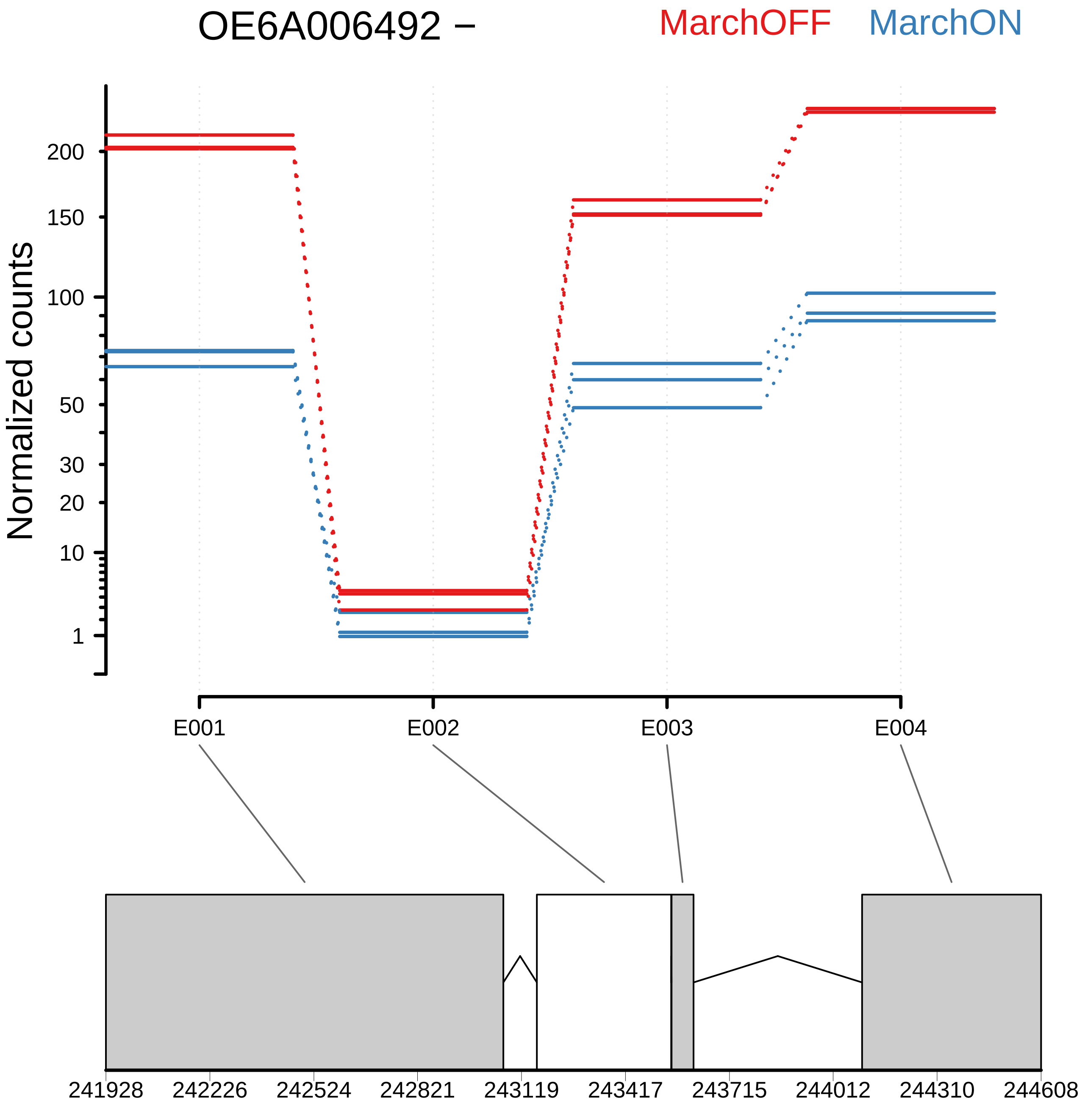

OE6A006492 −
MarchOFF
MarchON
Normalized counts
200
150
100
50
30
20
10
1
E001
E002
E003
E004
241928
242226
242524
242821
243119
243417
243715
244012
244310
244608

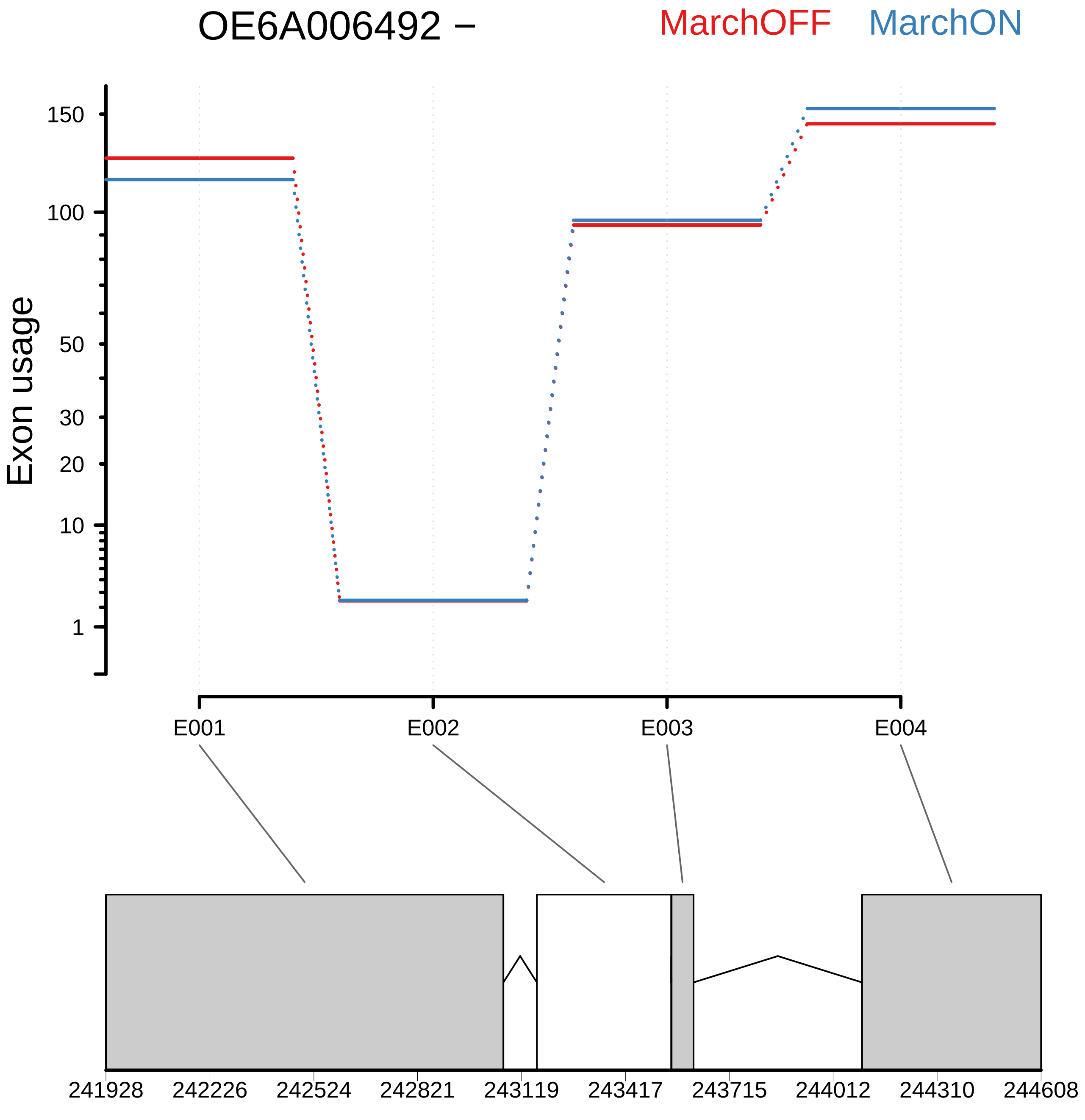

OE6A006492 −
MarchOFF
MarchON
Exon usage
150
100
50
30
20
10
1
E001
E002
E003
E004
241928
242226
242524
242821
243119
243417
243715
244012
244310
244608

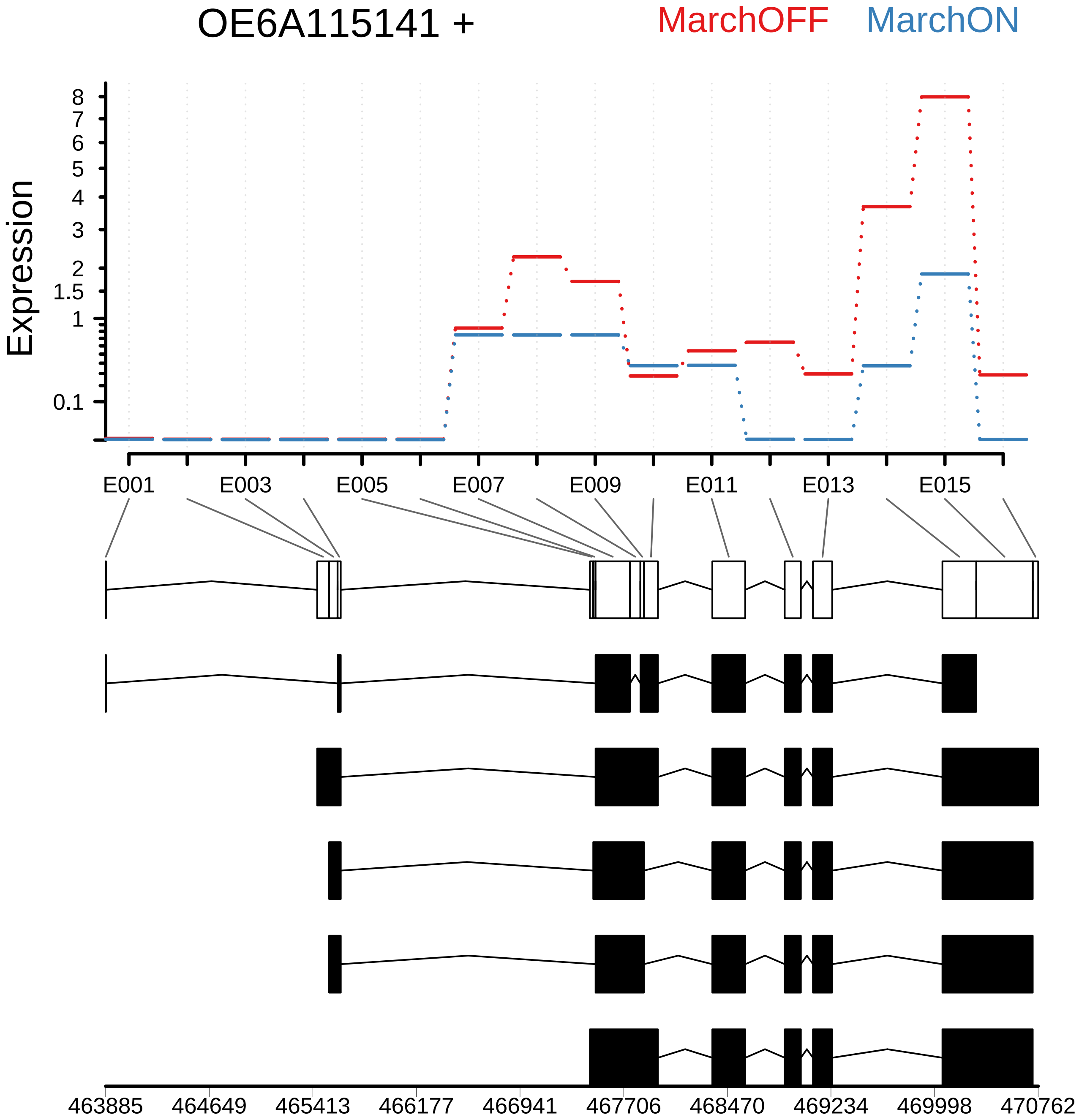
OE6A115141 +
MarchOFF
MarchON
Expression
8
7
6
5
4
3
2
1.5
1
0.1
E001
E003
E005
E007
E009
E011
E013
E015
463885
464649
465413
466177
466941
467706
468470
469234
469998
470762

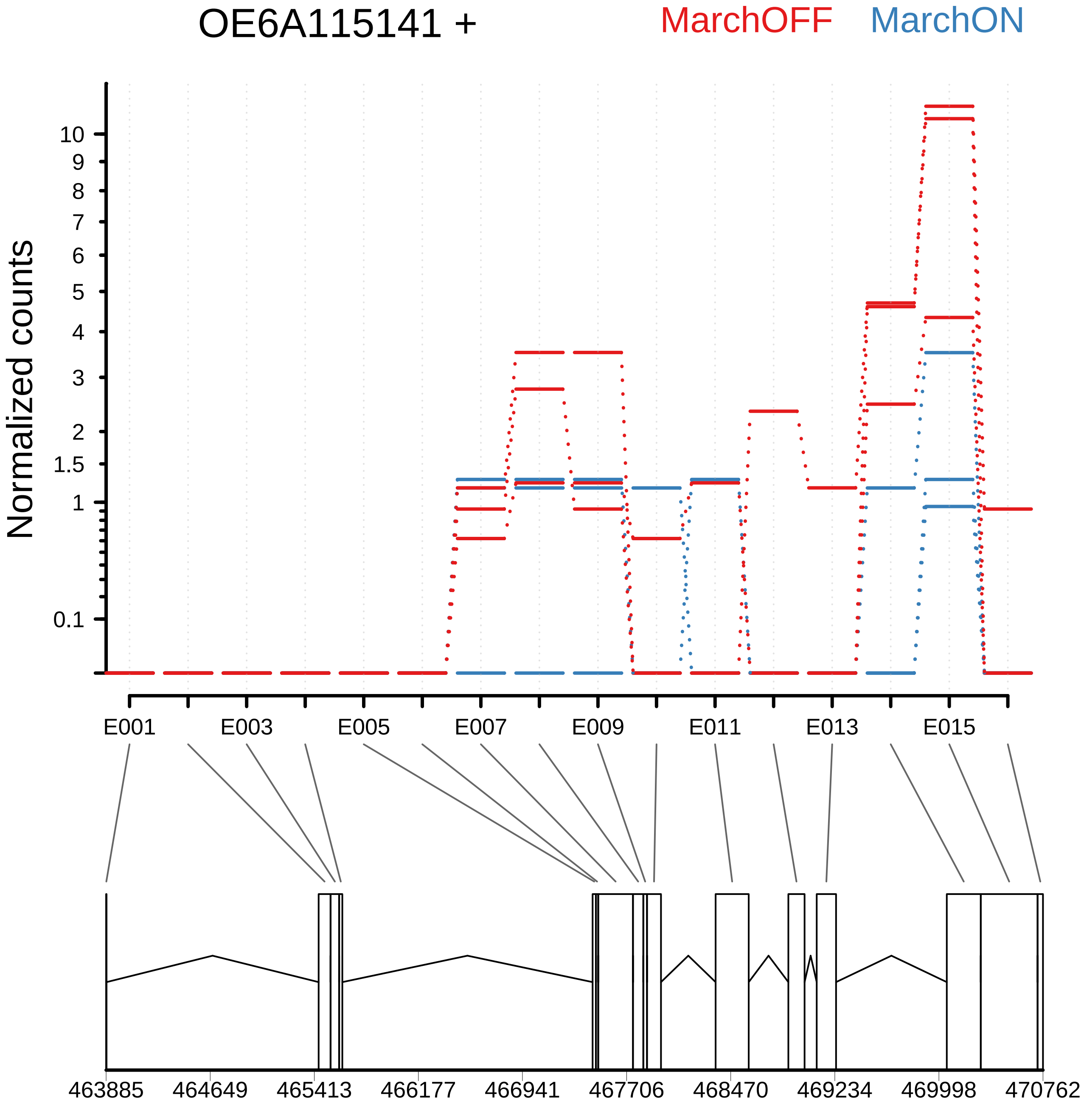

OE6A115141 +
MarchOFF
MarchON
Normalized counts
10
9
8
7
6
5
4
3
2
1.5
1
0.1
E001
E003
E005
E007
E009
E011
E013
E015
463885
464649
465413
466177
466941
467706
468470
469234
469998
470762

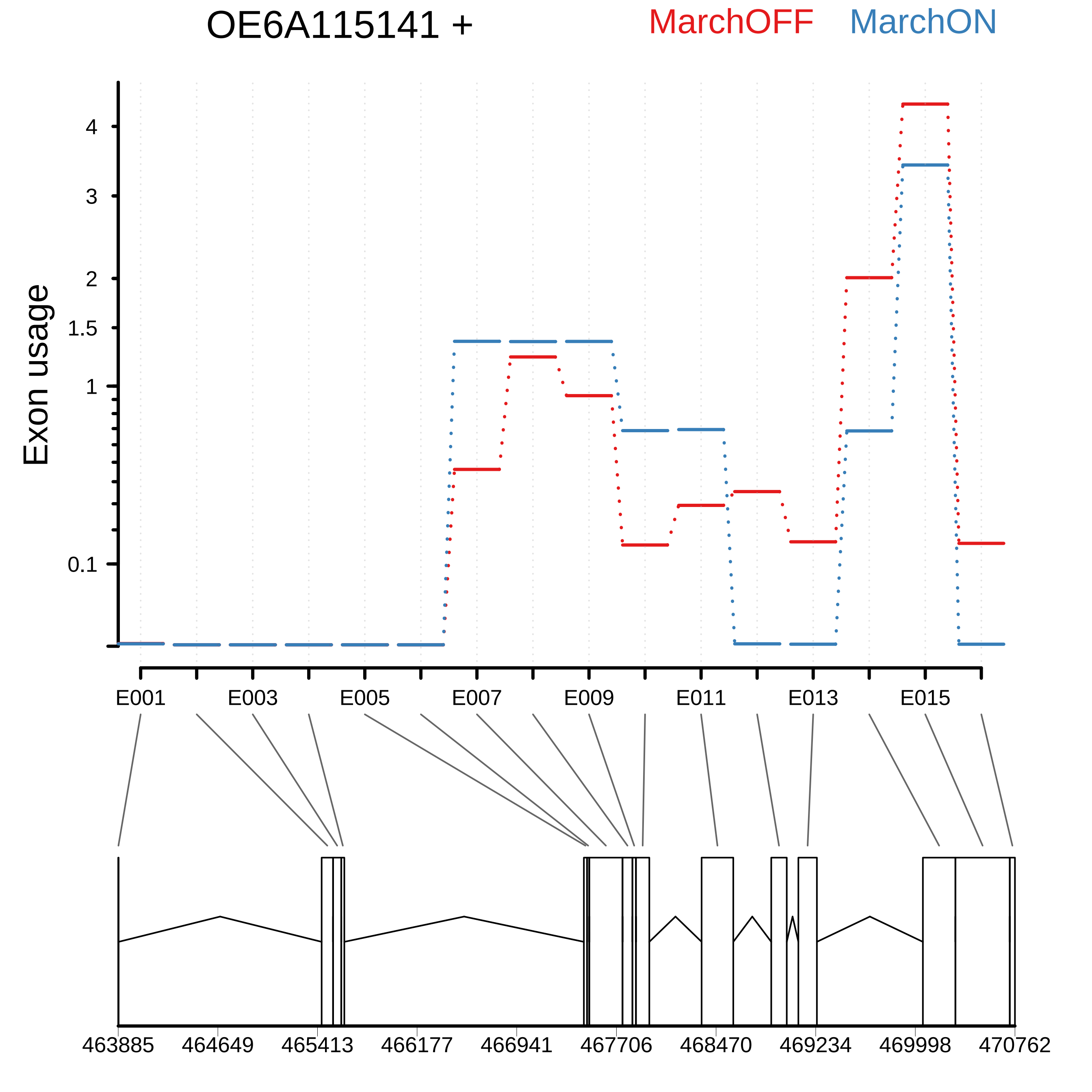

OE6A115141 +
MarchOFF
MarchON
Exon usage
4
3
2
1.5
1
0.1
E001
E003
E005
E007
E009
E011
E013
E015
463885
464649
465413
466177
466941
467706
468470
469234
469998
470762

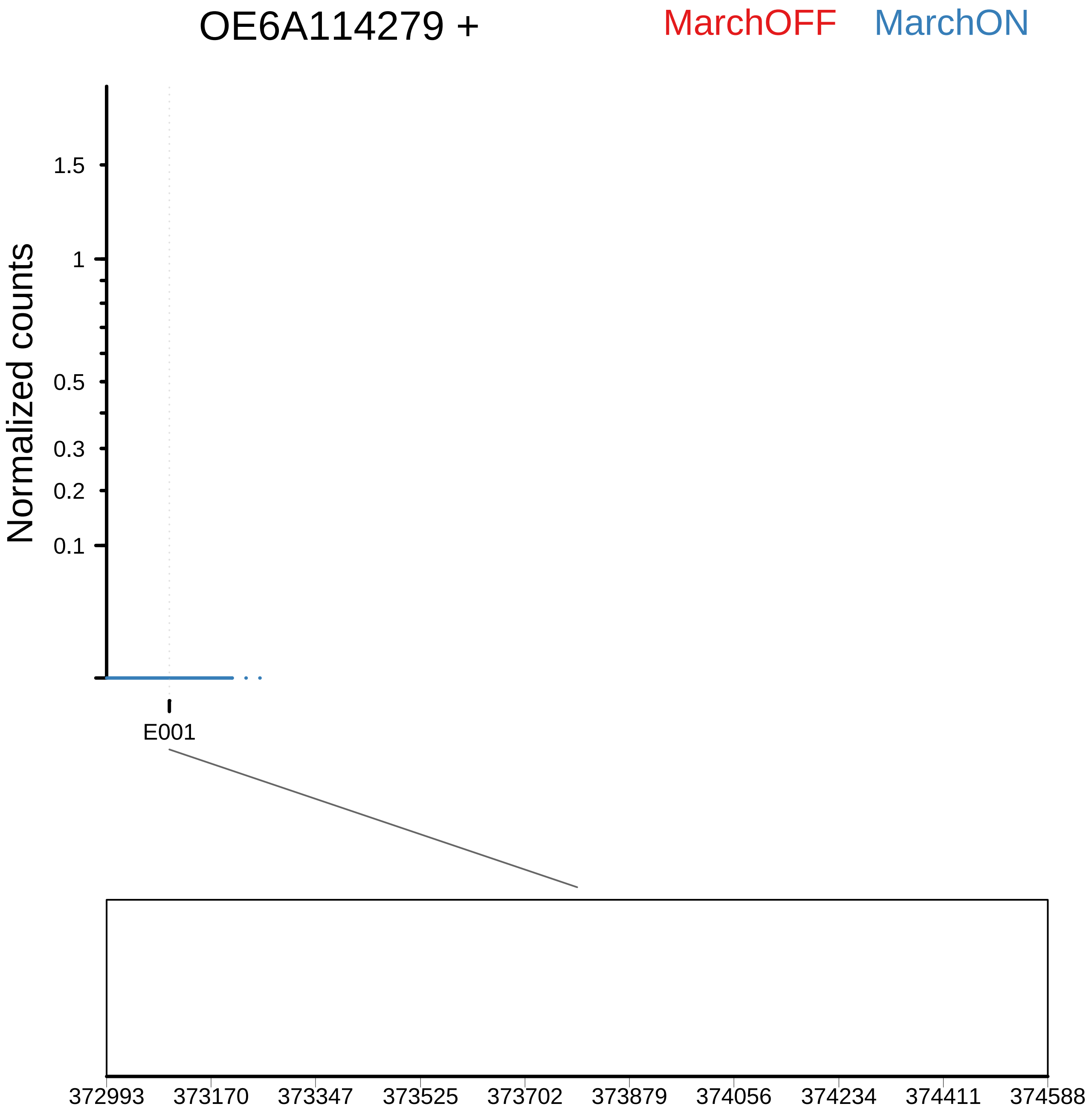
OE6A114279 +
MarchOFF
MarchON
Normalized counts
1.5
1
0.5
0.3
0.2
0.1
E001
372993
373170
373347
373525
373702
373879
374056
374234
374411
374588

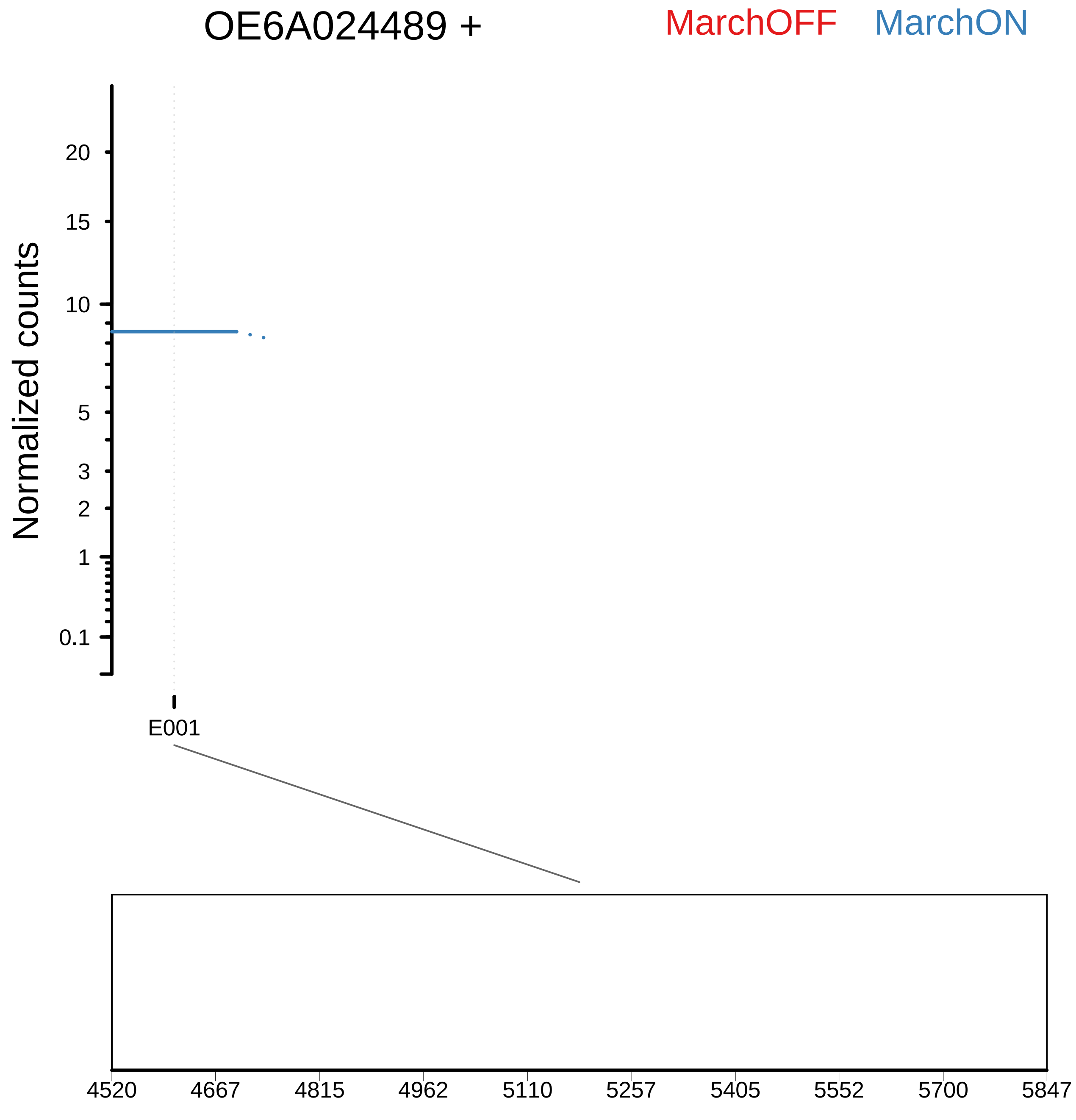

OE6A024489 +
MarchOFF
MarchON
Normalized counts
20
15
10
5
3
2
1
0.1
E001
4520
4667
4815
4962
5110
5257
5405
5552
5700
5847

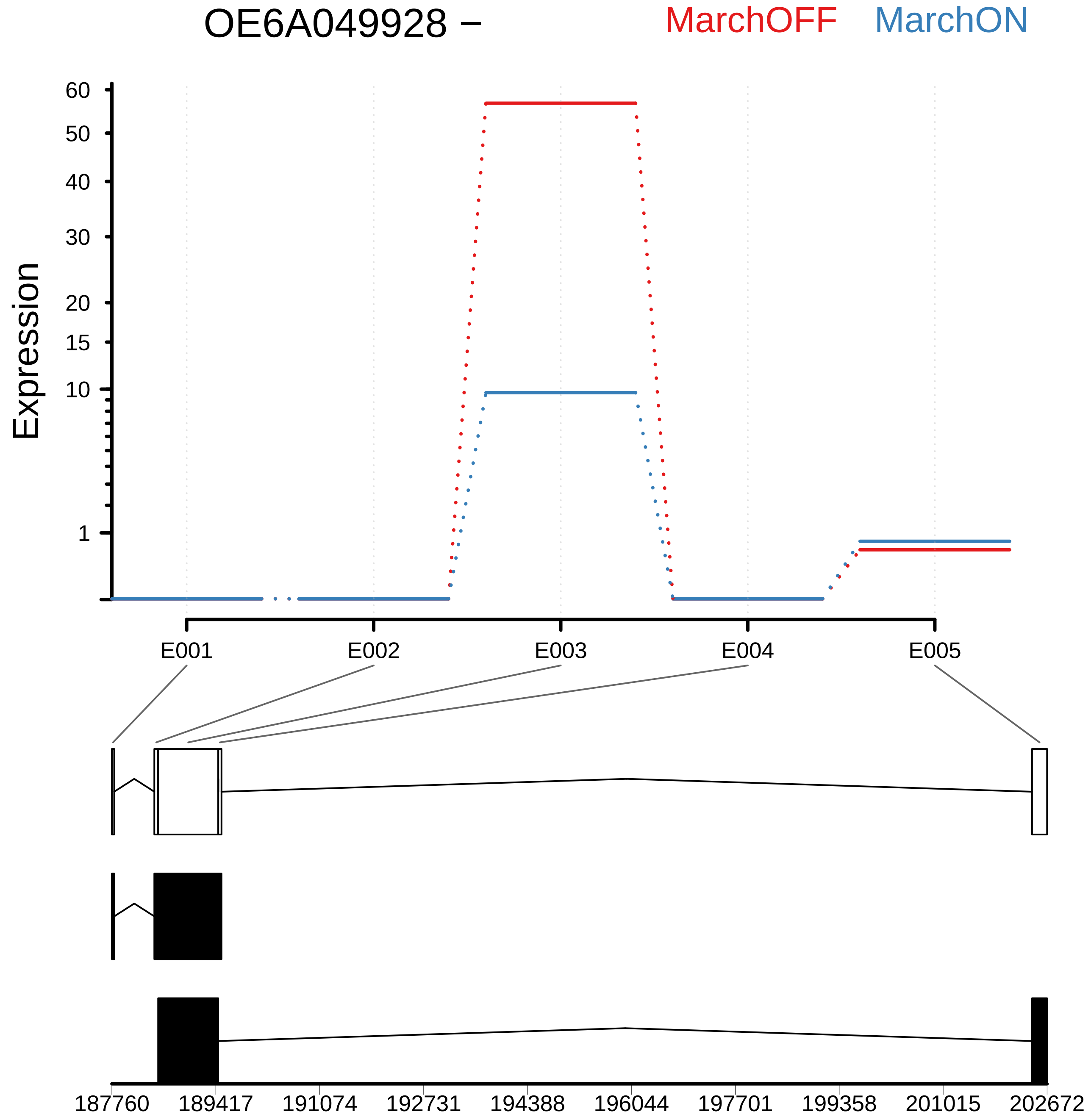

OE6A049928 −
MarchOFF
MarchON
Expression
60
50
40
30
20
15
10
1
E001
E002
E003
E004
E005
187760
189417
191074
192731
194388
196044
197701
199358
201015
202672

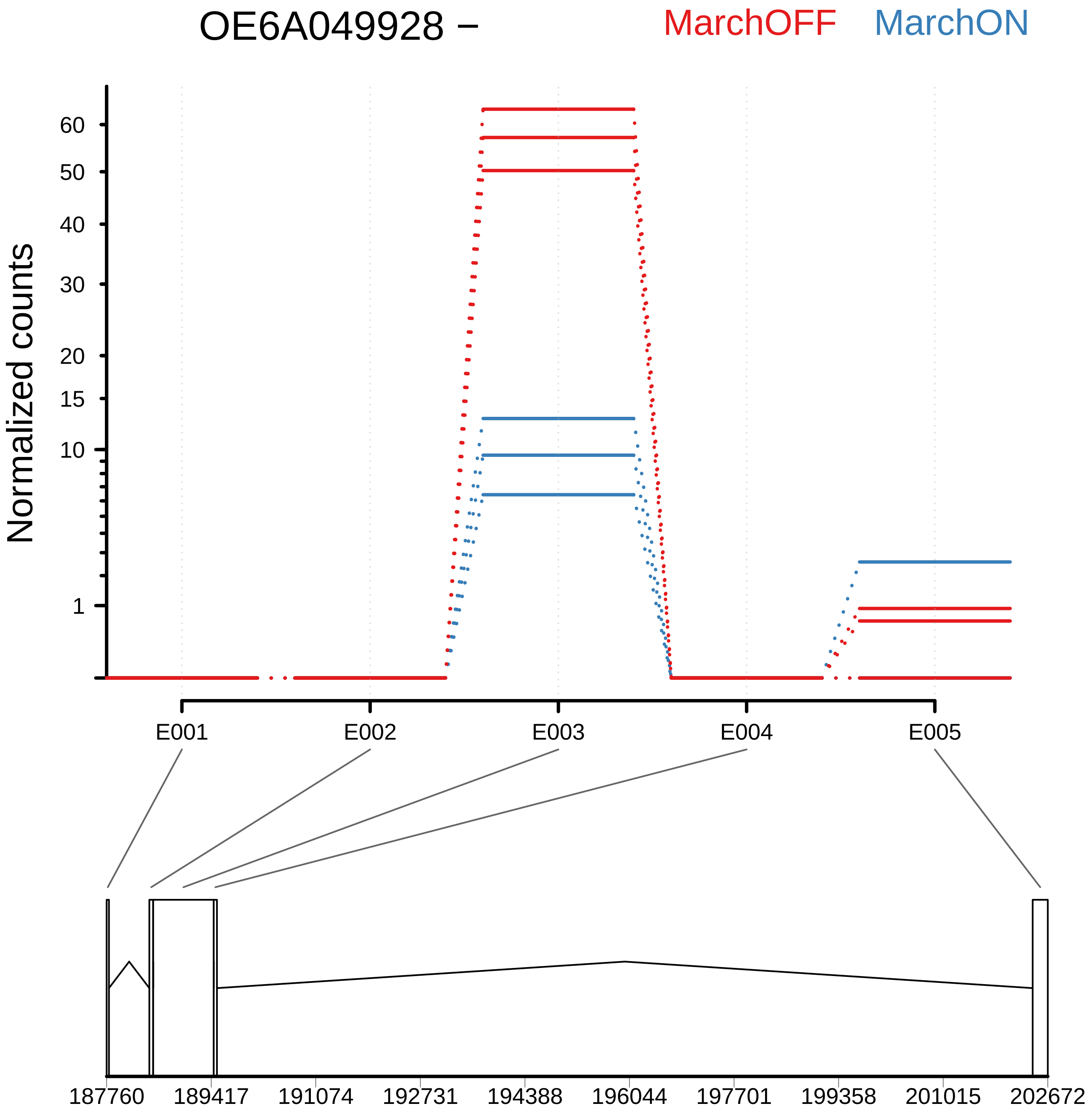
OE6A049928 −
MarchOFF
MarchON
Normalized counts
60
50
40
30
20
15
10
1
E001
E002
E003
E004
E005
187760
189417
191074
192731
194388
196044
197701
199358
201015
202672

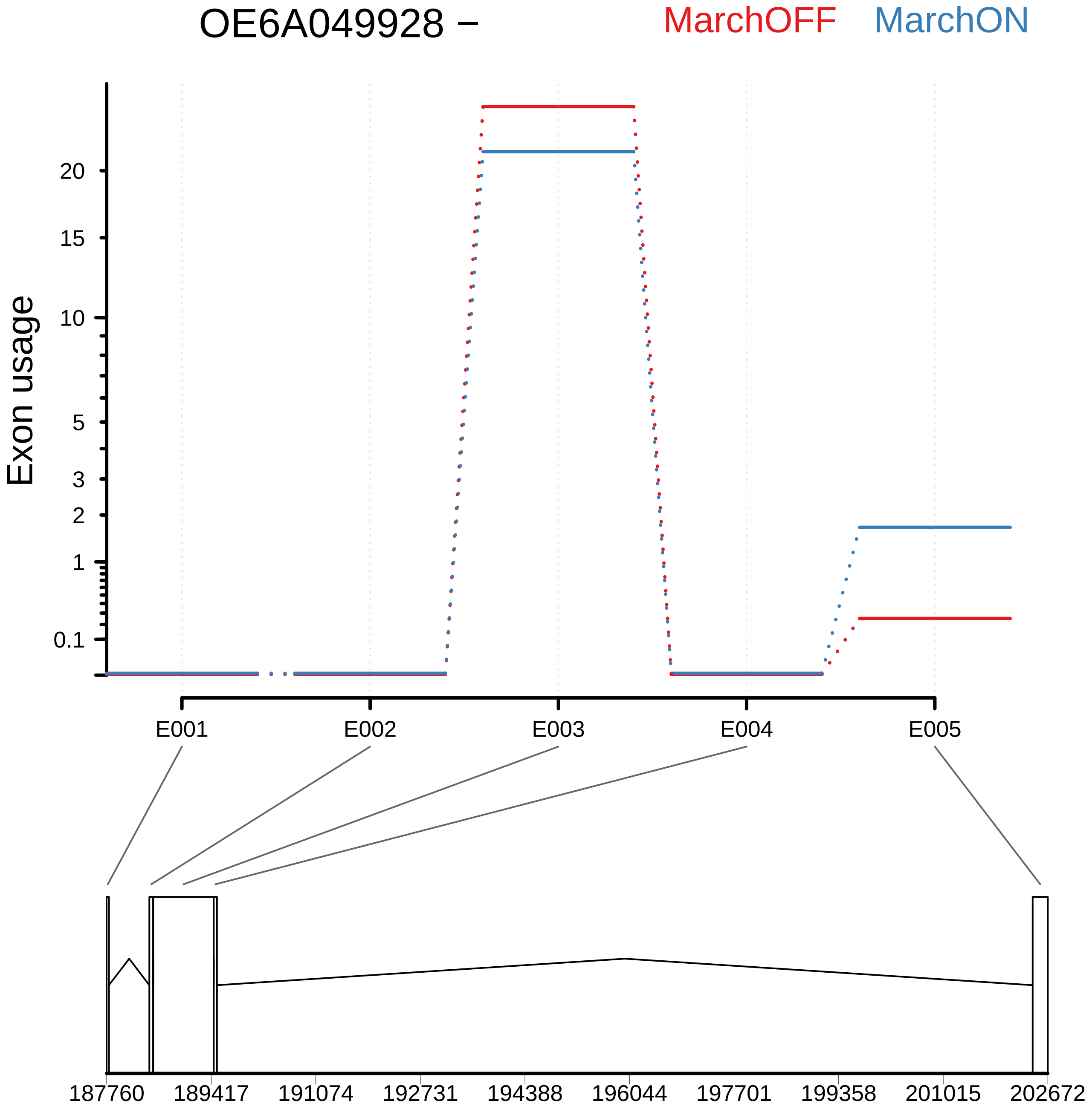

OE6A049928 −
MarchOFF
MarchON
Exon usage
20
15
10
5
3
2
1
0.1
E001
E002
E003
E004
E005
187760
189417
191074
192731
194388
196044
197701
199358
201015
202672

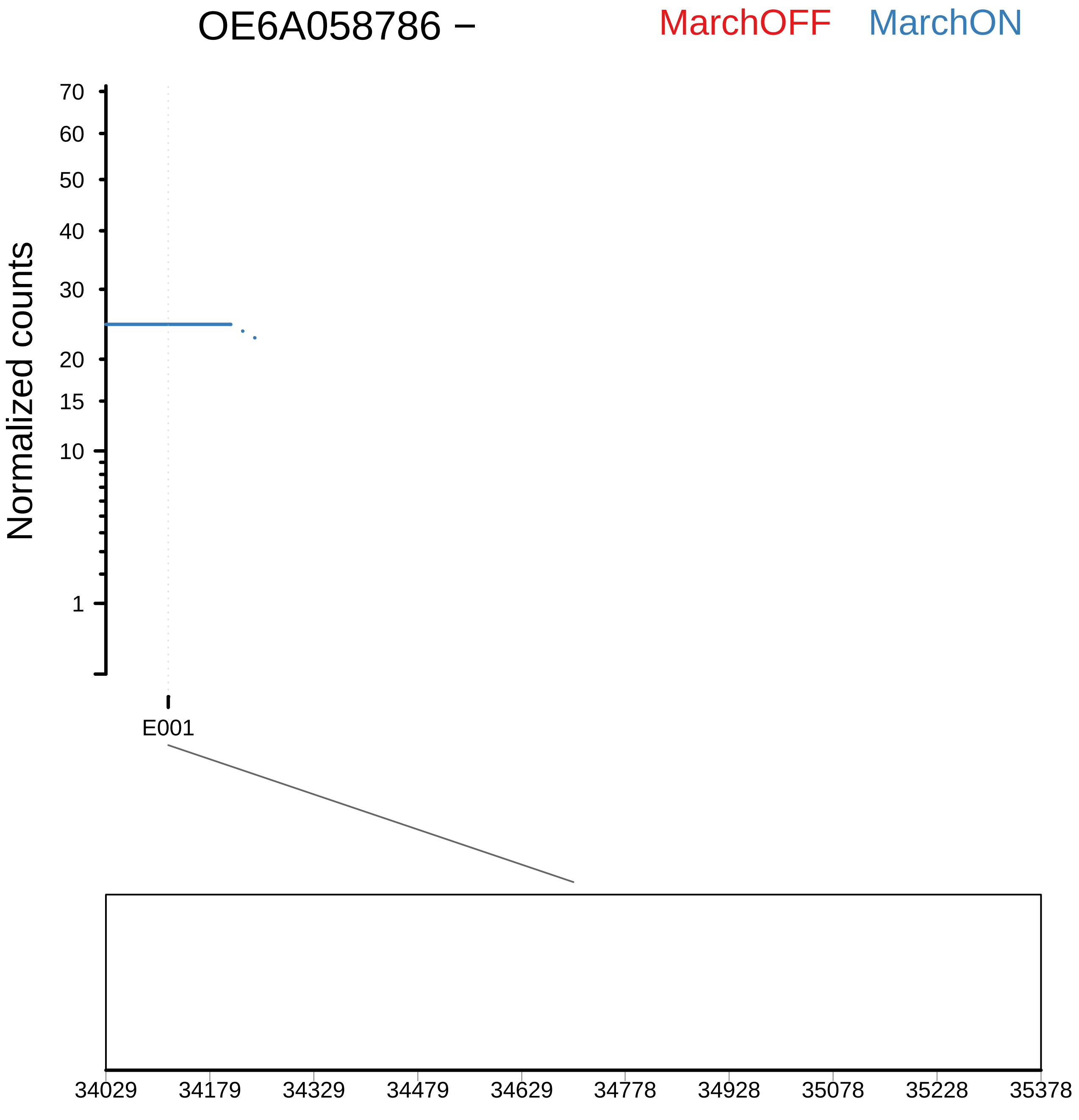

OE6A058786 −
MarchOFF
MarchON
Normalized counts
70
60
50
40
30
20
15
10
1
E001
34029
34179
34329
34479
34629
34778
34928
35078
35228
35378

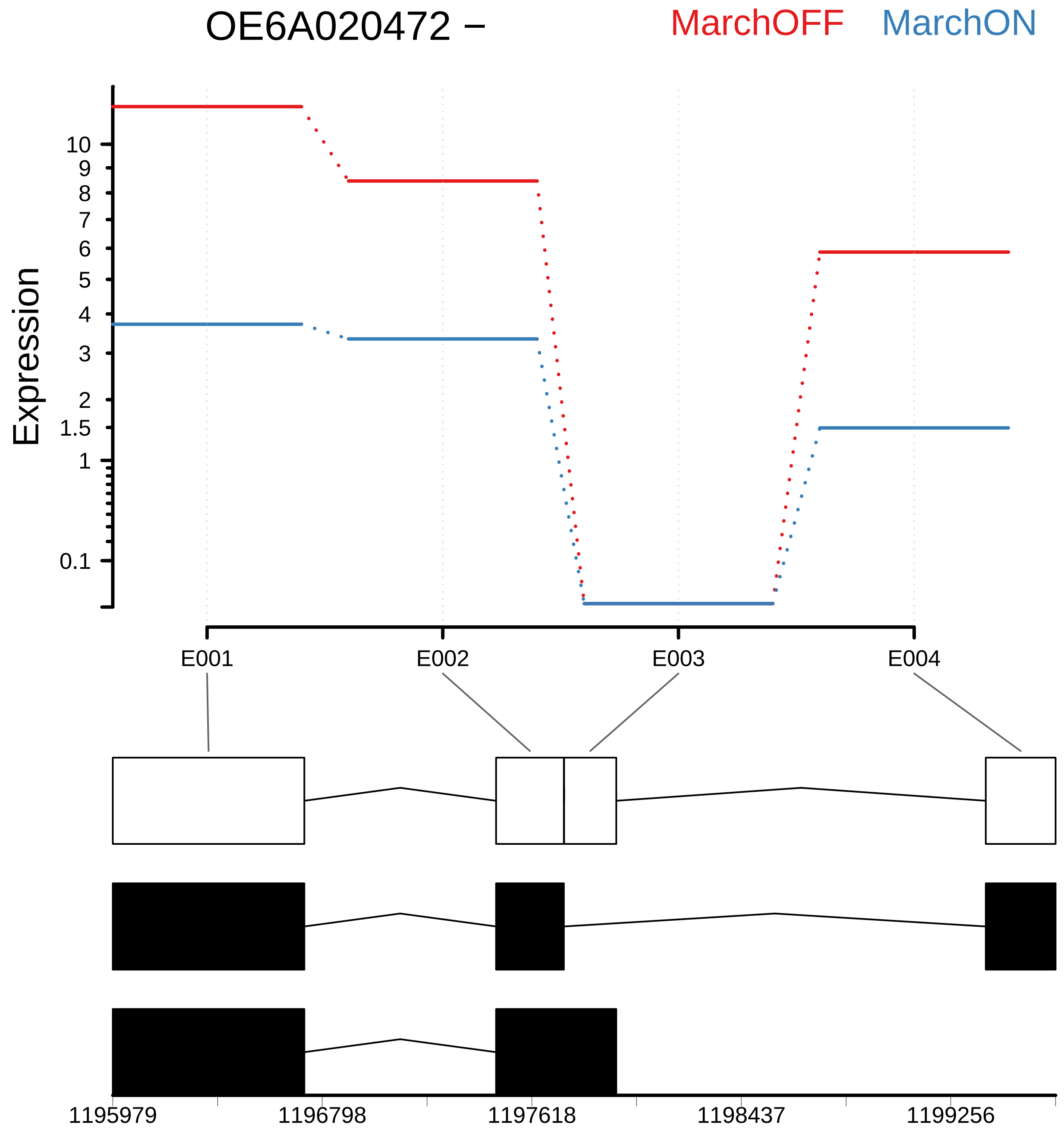
OE6A020472 −
MarchOFF
MarchON
Expression
10
9
8
7
6
5
4
3
2
1.5
1
0.1
E001
E002
E003
E004
1195979
1196798
1197618
1198437
1199256

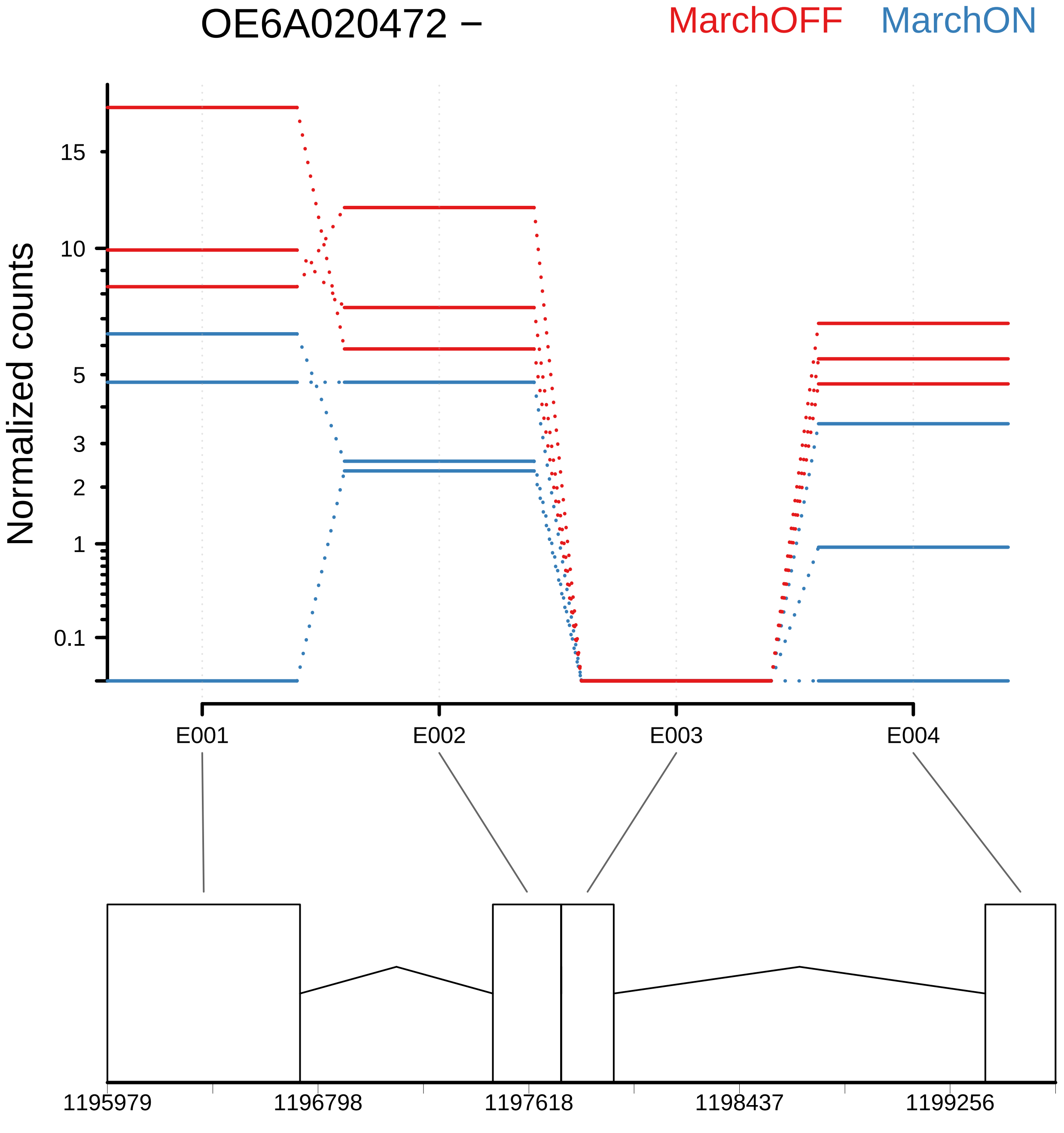

OE6A020472 −
MarchOFF
MarchON
Normalized counts
15
10
5
3
2
1
0.1
E001
E002
E003
E004
1195979
1196798
1197618
1198437
1199256

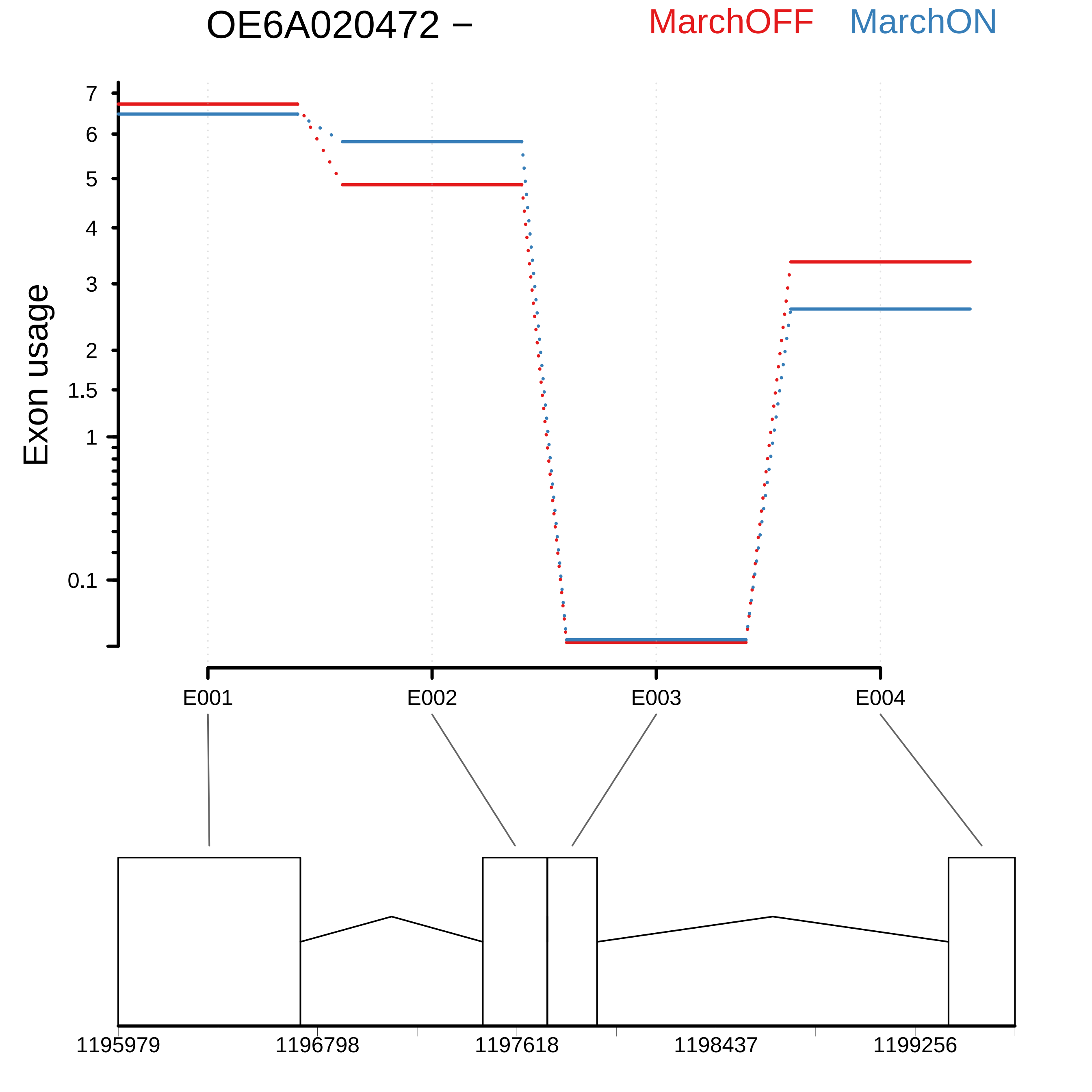

OE6A020472 −
MarchOFF
MarchON
Exon usage
7
6
5
4
3
2
1.5
1
0.1
E001
E002
E003
E004
1195979
1196798
1197618
1198437
1199256

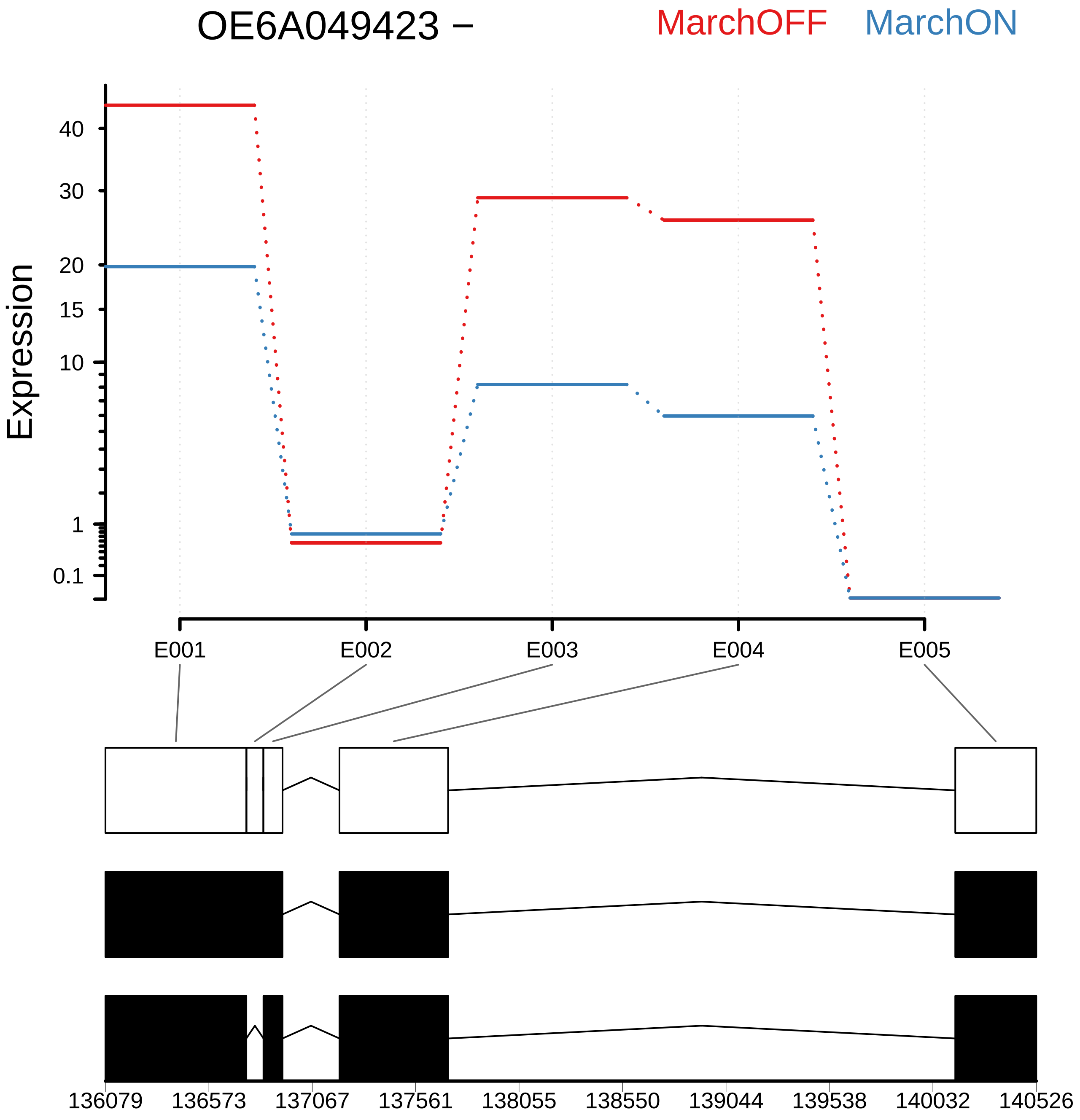
OE6A049423 −
MarchOFF
MarchON
Expression
40
30
20
15
10
1
0.1
E001
E002
E003
E004
E005
136079
136573
137067
137561
138055
138550
139044
139538
140032
140526

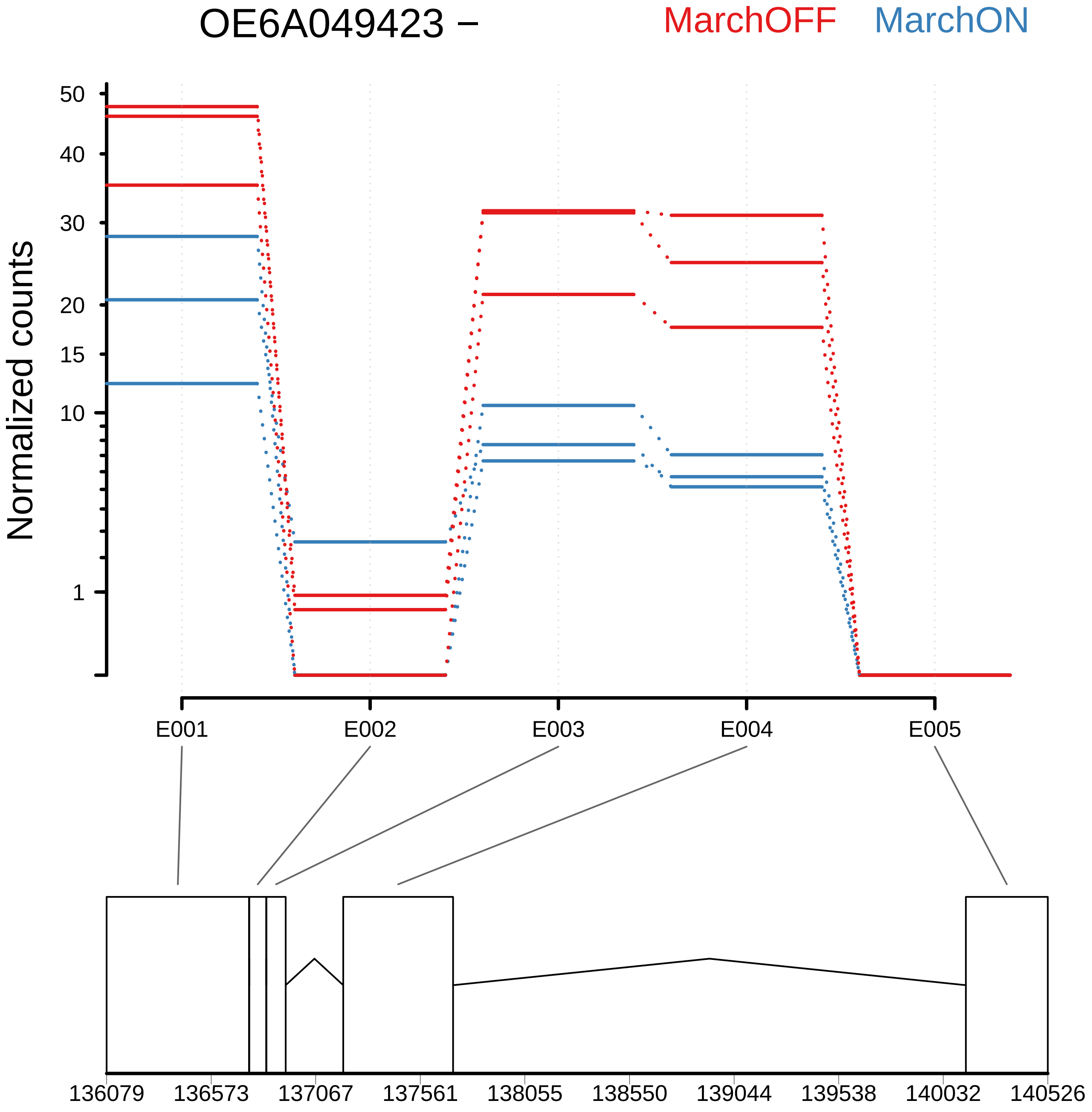

OE6A049423 −
MarchOFF
MarchON
Normalized counts
50
40
30
20
15
10
1
E001
E002
E003
E004
E005
136079
136573
137067
137561
138055
138550
139044
139538
140032
140526

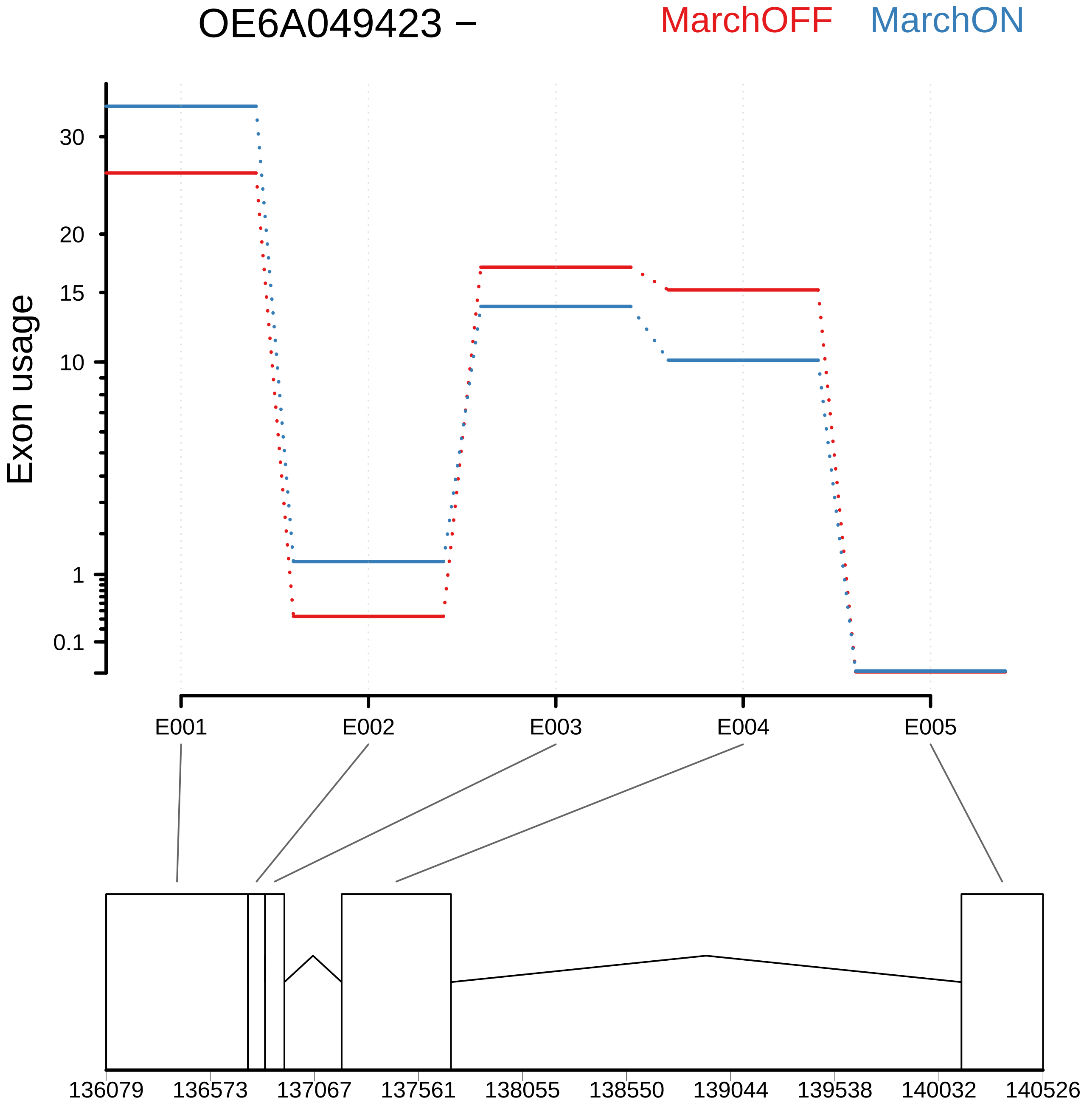
OE6A049423 –
MarchOFF
MarchON
Exon usage
30
20
15
10
1
0.1
E001
E002
E003
E004
E005
136079
136573
137067
137561
138055
138550
139044
139538
140032
140526

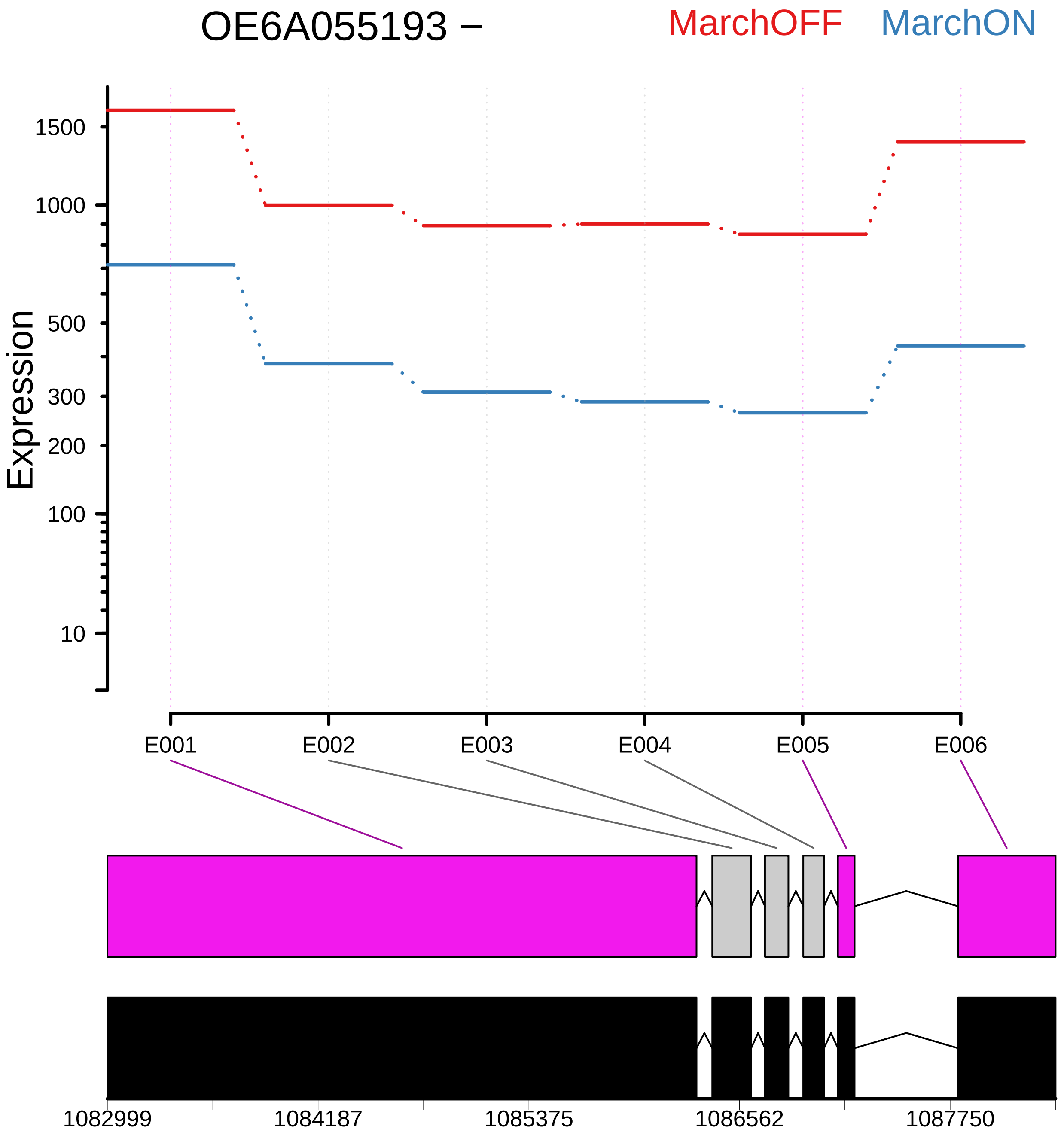
OE6A055193 −
MarchOFF
MarchON
Expression
1500
1000
500
300
200
100
10
E001
E002
E003
E004
E005
E006
1082999
1084187
1085375
1086562
1087750

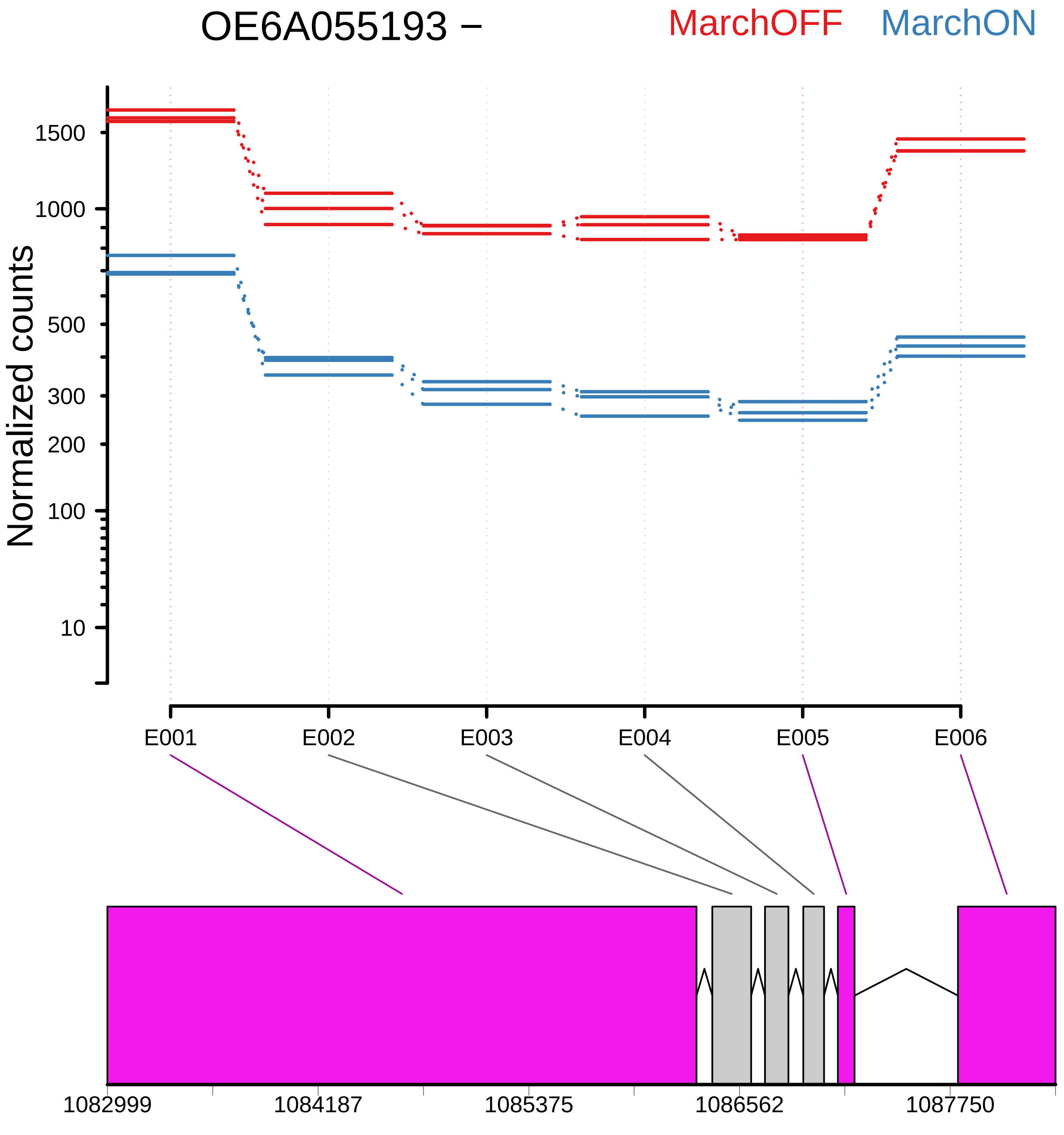

OE6A055193 −
MarchOFF
MarchON
Normalized counts
1500
1000
500
300
200
100
10
E001
E002
E003
E004
E005
E006
1082999
1084187
1085375
1086562
1087750

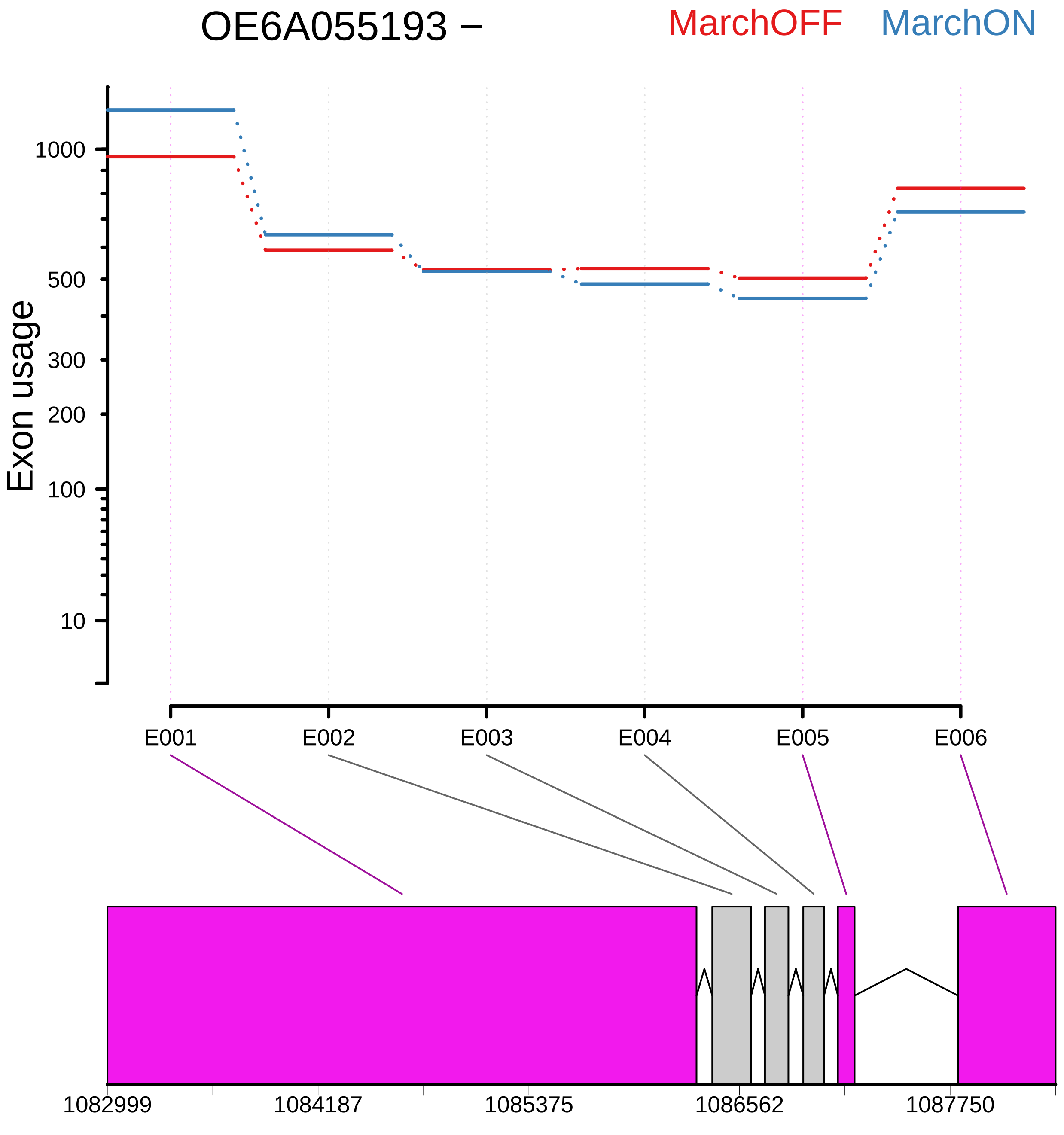
OE6A055193 −
MarchOFF
MarchON
Exon usage
1000
500
300
200
100
10
E001
E002
E003
E004
E005
E006
1082999
1084187
1085375
1086562
1087750

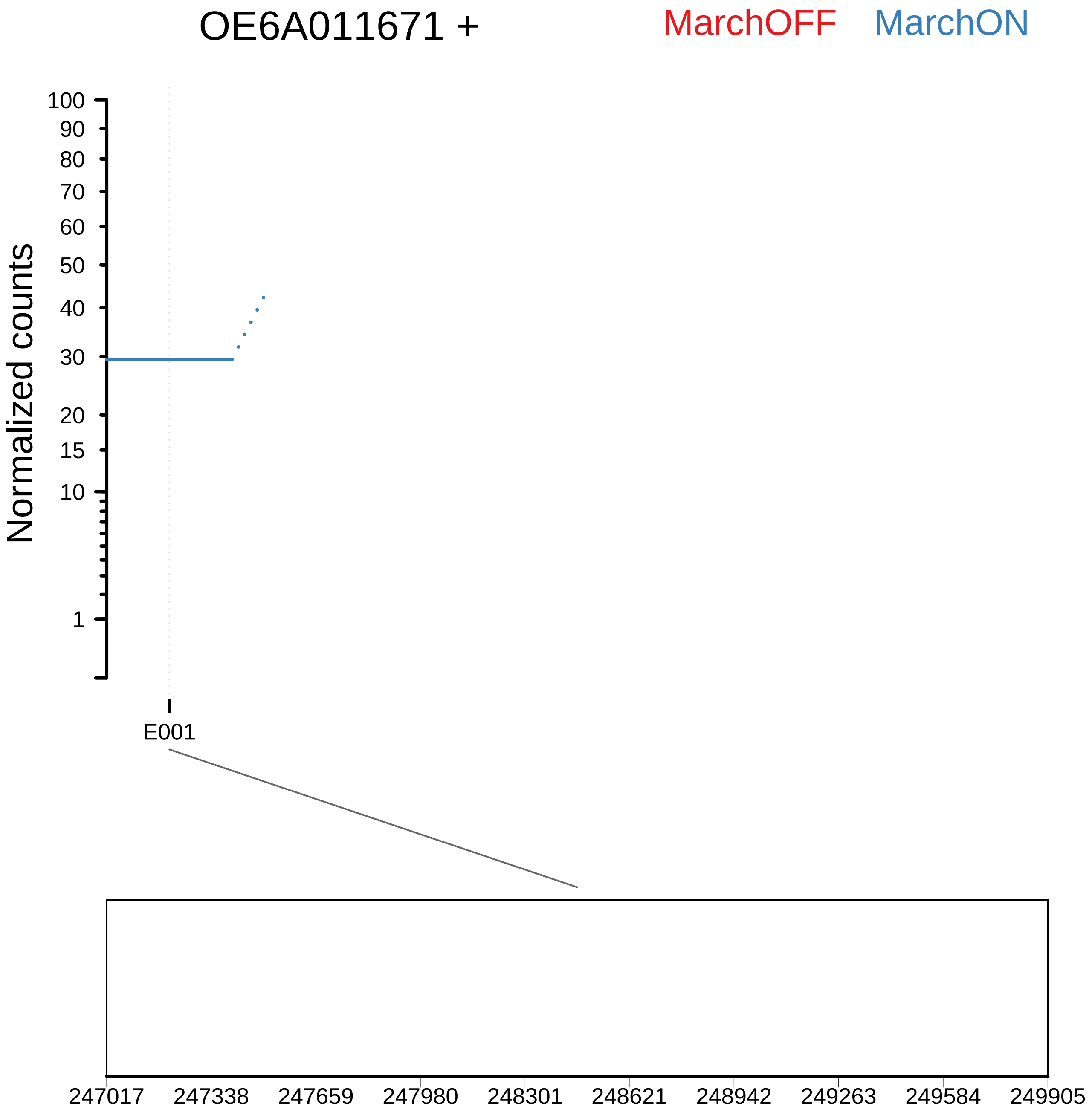

OE6A011671 +
MarchOFF
MarchON
Normalized counts
100
90
80
70
60
50
40
30
20
15
10
1
E001
247017
247338
247659
247980
248301
248621
248942
249263
249584
249905

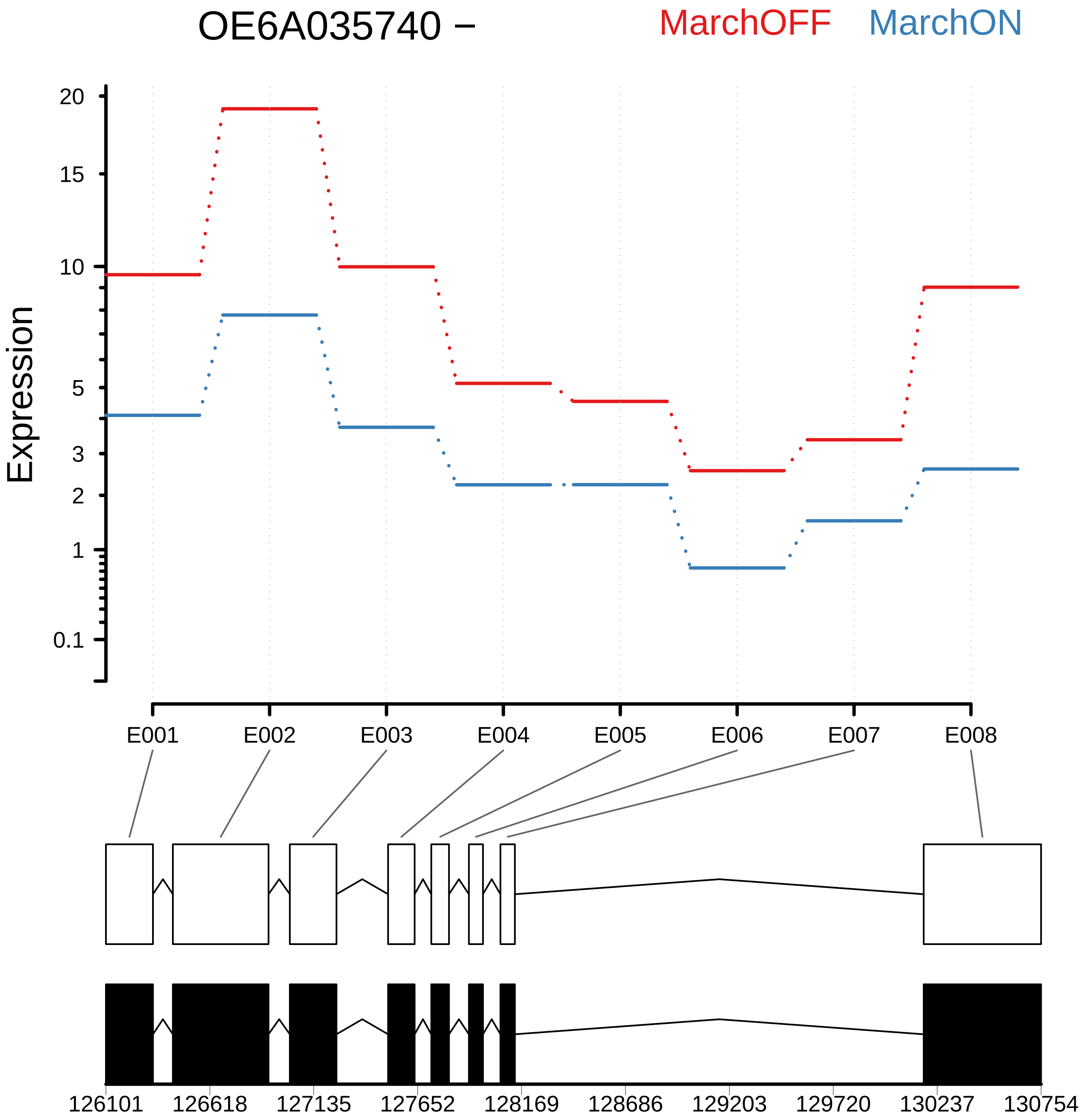

OE6A035740 −
MarchOFF
MarchON
Expression
20
15
10
5
3
2
1
0.1
E001
E002
E003
E004
E005
E006
E007
E008
126101
126618
127135
127652
128169
128686
129203
129720
130237
130754

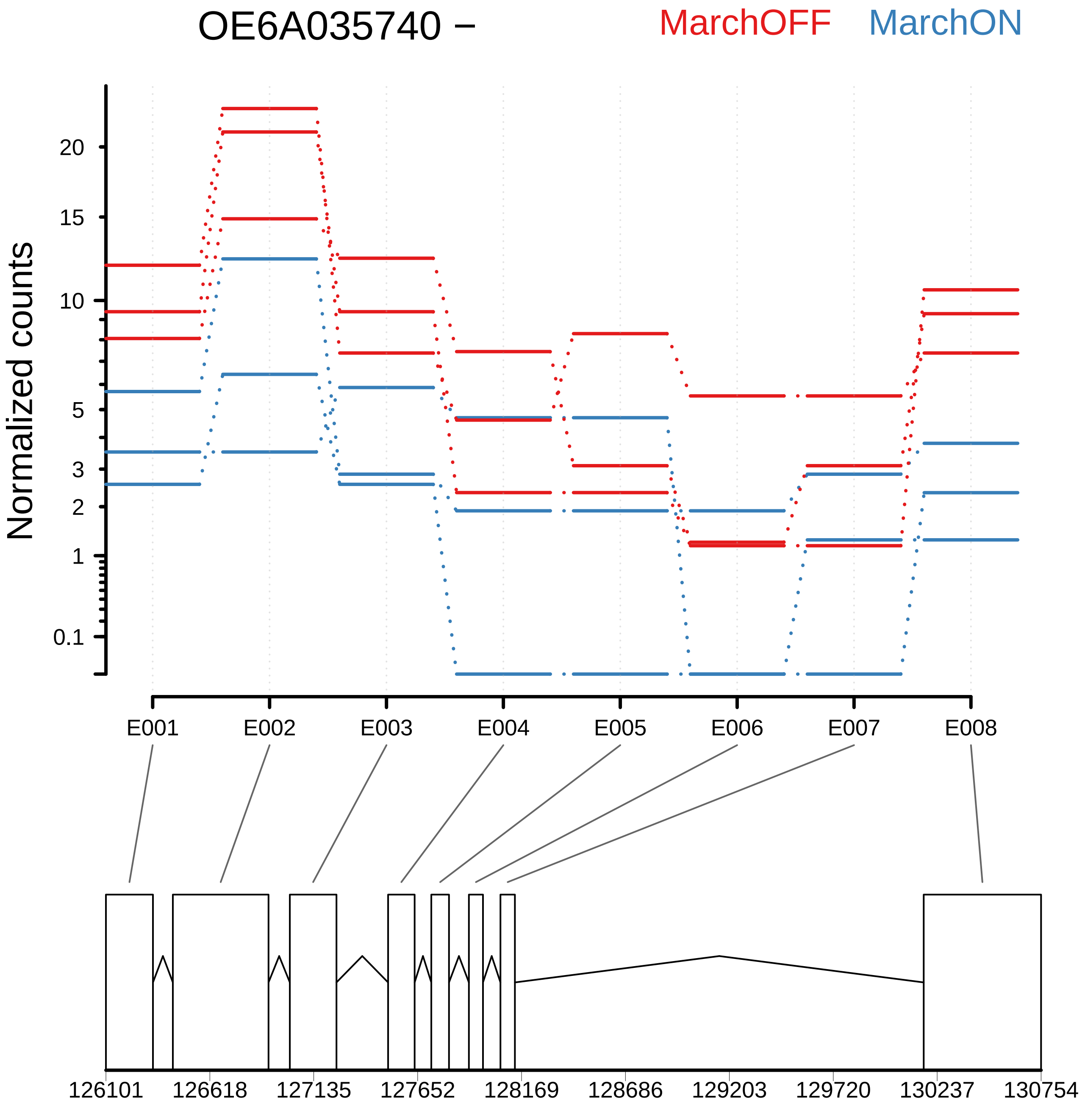
OE6A035740 −
MarchOFF
MarchON
Normalized counts
20
15
10
5
3
2
1
0.1
E001
E002
E003
E004
E005
E006
E007
E008
126101
126618
127135
127652
128169
128686
129203
129720
130237
130754

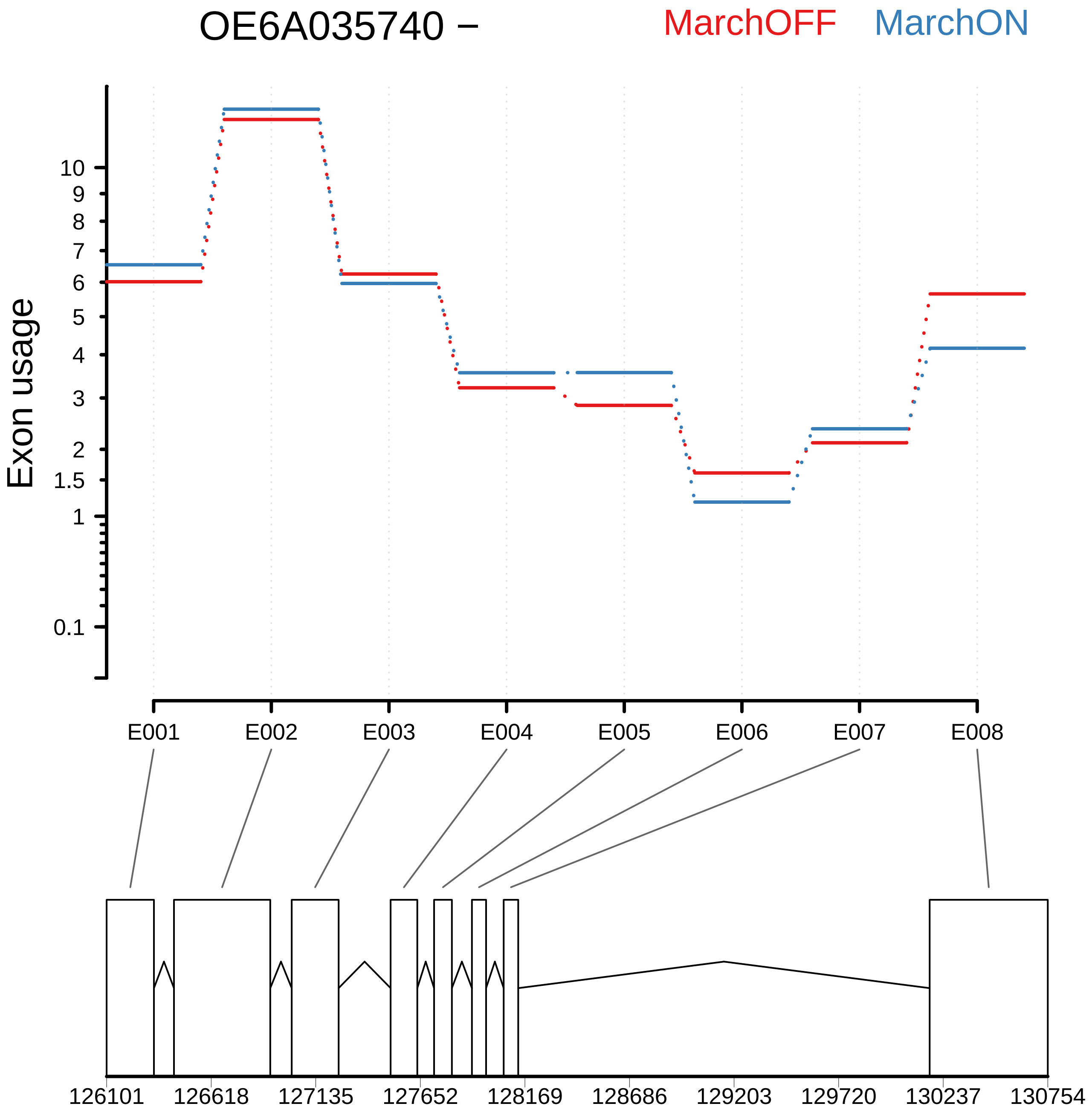
OE6A035740 −
MarchOFF
MarchON
Exon usage
10
9
8
7
6
5
4
3
2
1.5
1
0.1
E001
E002
E003
E004
E005
E006
E007
E008
126101
126618
127135
127652
128169
128686
129203
129720
130237
130754

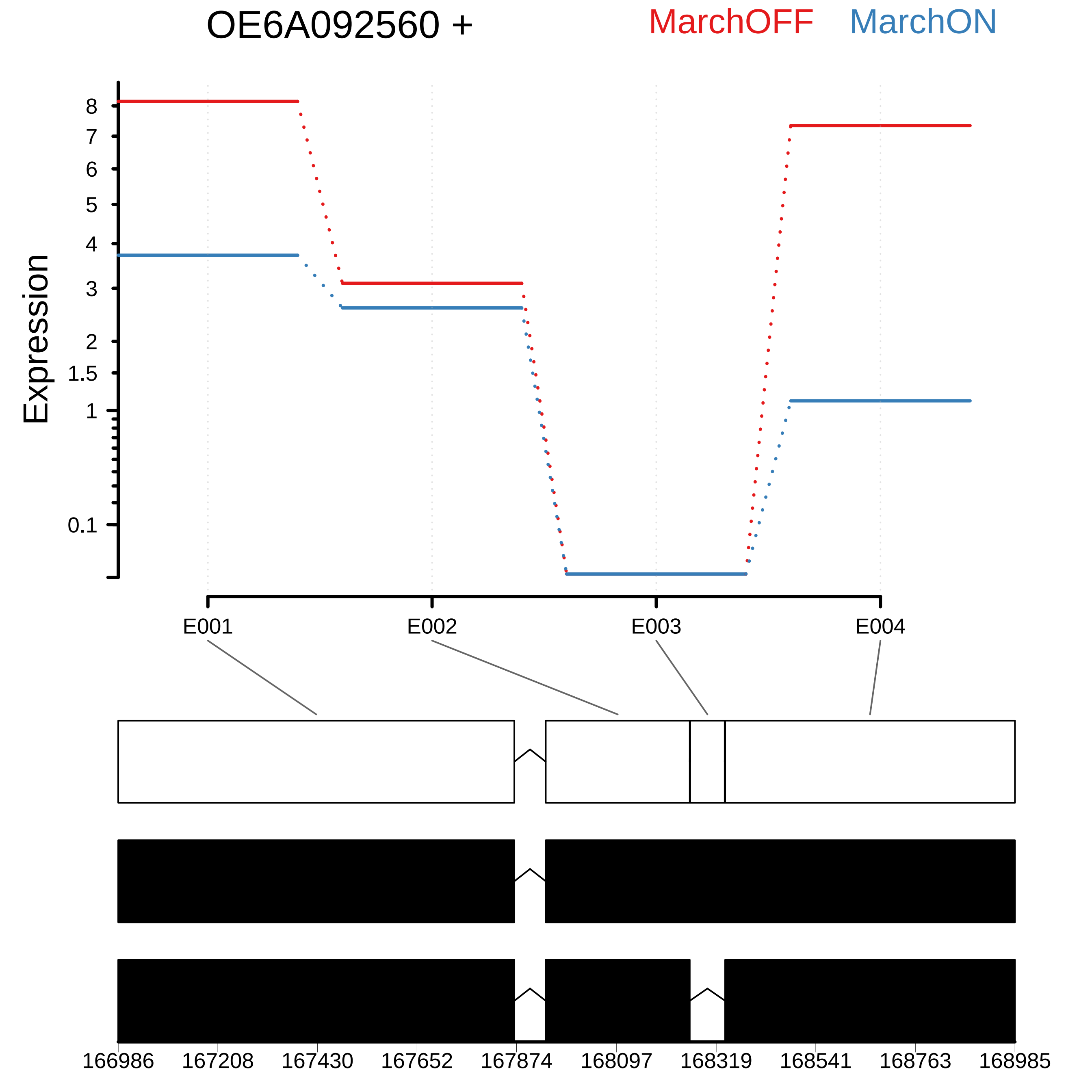

OE6A092560 +
MarchOFF
MarchON
Expression
8
7
6
5
4
3
2
1.5
1
0.1
E001
E002
E003
E004
166986
167208
167430
167652
167874
168097
168319
168541
168763
168985

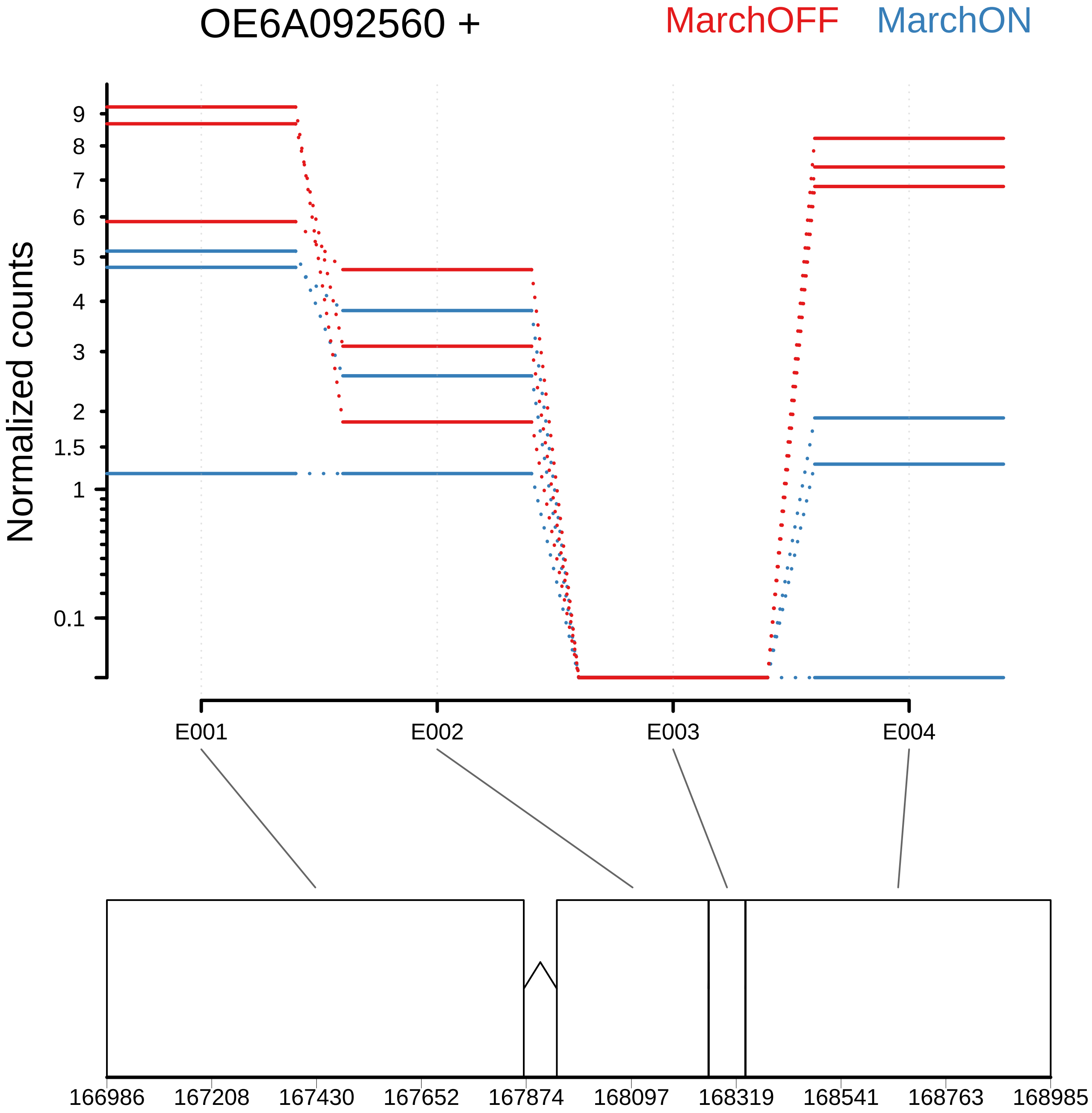

OE6A092560 +
MarchOFF
MarchON
Normalized counts
9
8
7
6
5
4
3
2
1.5
1
0.1
E001
E002
E003
E004
166986
167208
167430
167652
167874
168097
168319
168541
168763
168985

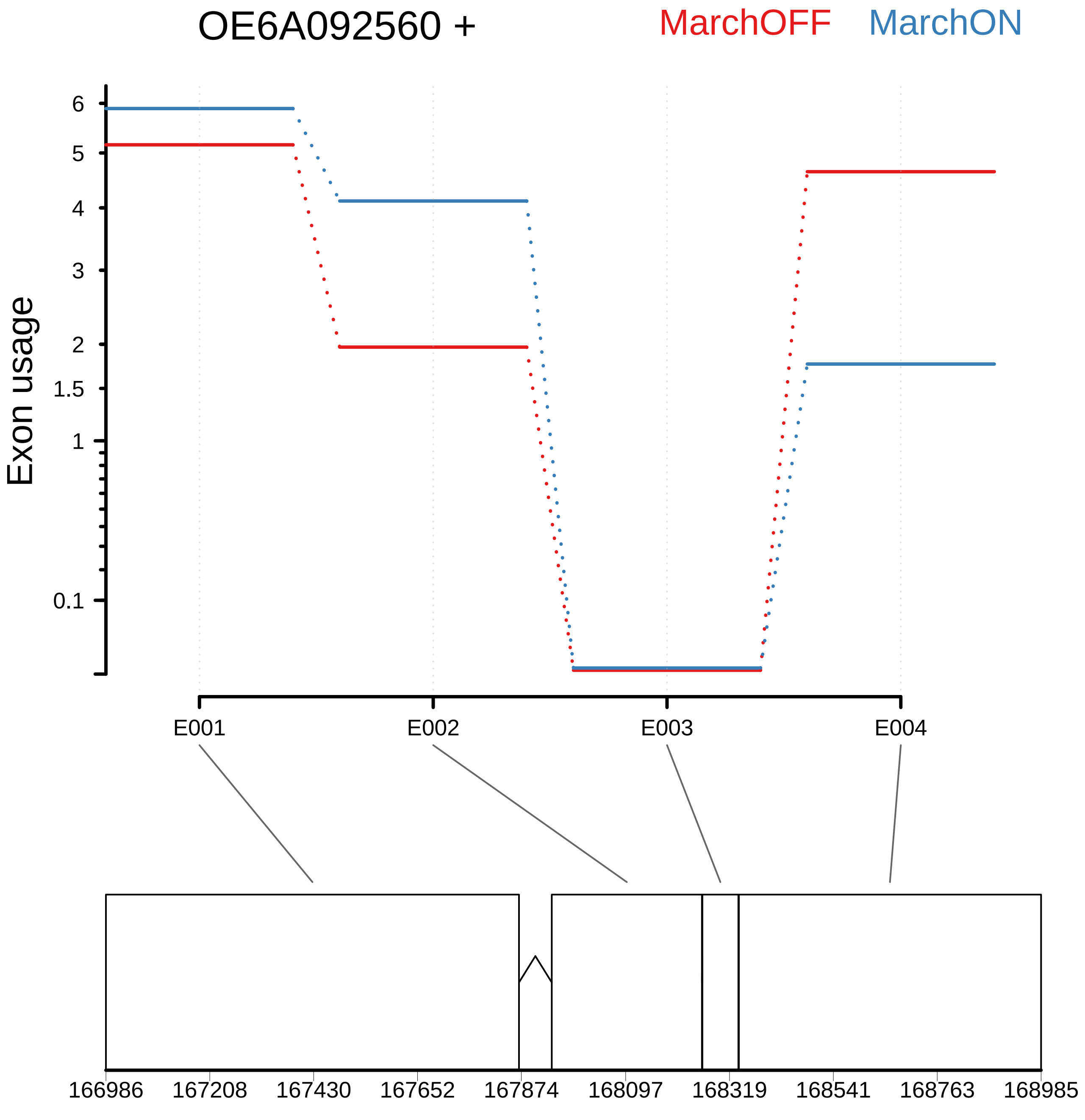

OE6A092560 +
MarchOFF
MarchON
Exon usage
6
5
4
3
2
1.5
1
0.1
E001
E002
E003
E004
166986
167208
167430
167652
167874
168097
168319
168541
168763
168985

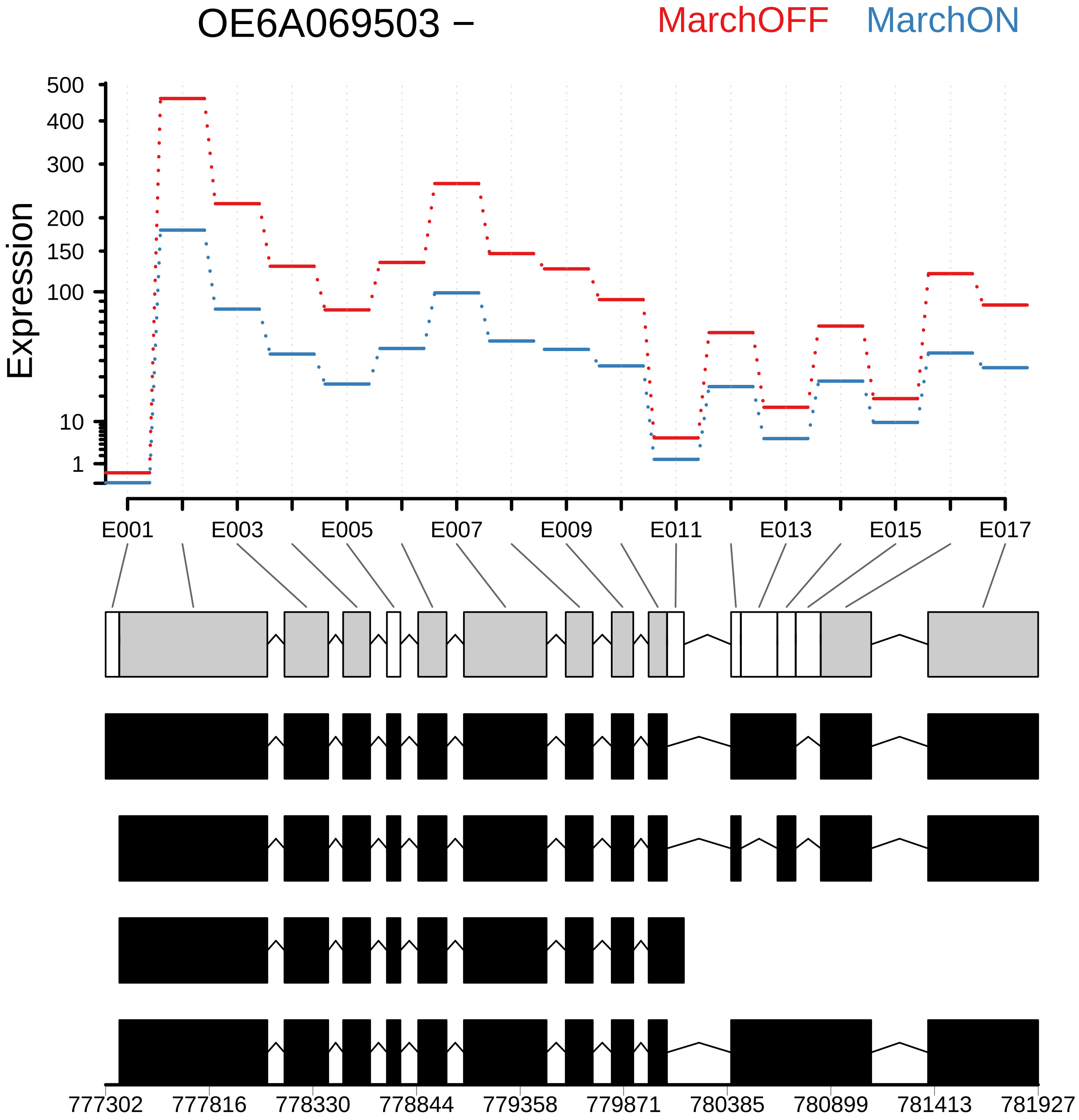

OE6A069503 –
MarchOFF
MarchON
Expression
500
400
300
200
150
100
10
1
E001
E003
E005
E007
E009
E011
E013
E015
E017
777302
777816
778330
778844
779358
779871
780385
780899
781413
781927

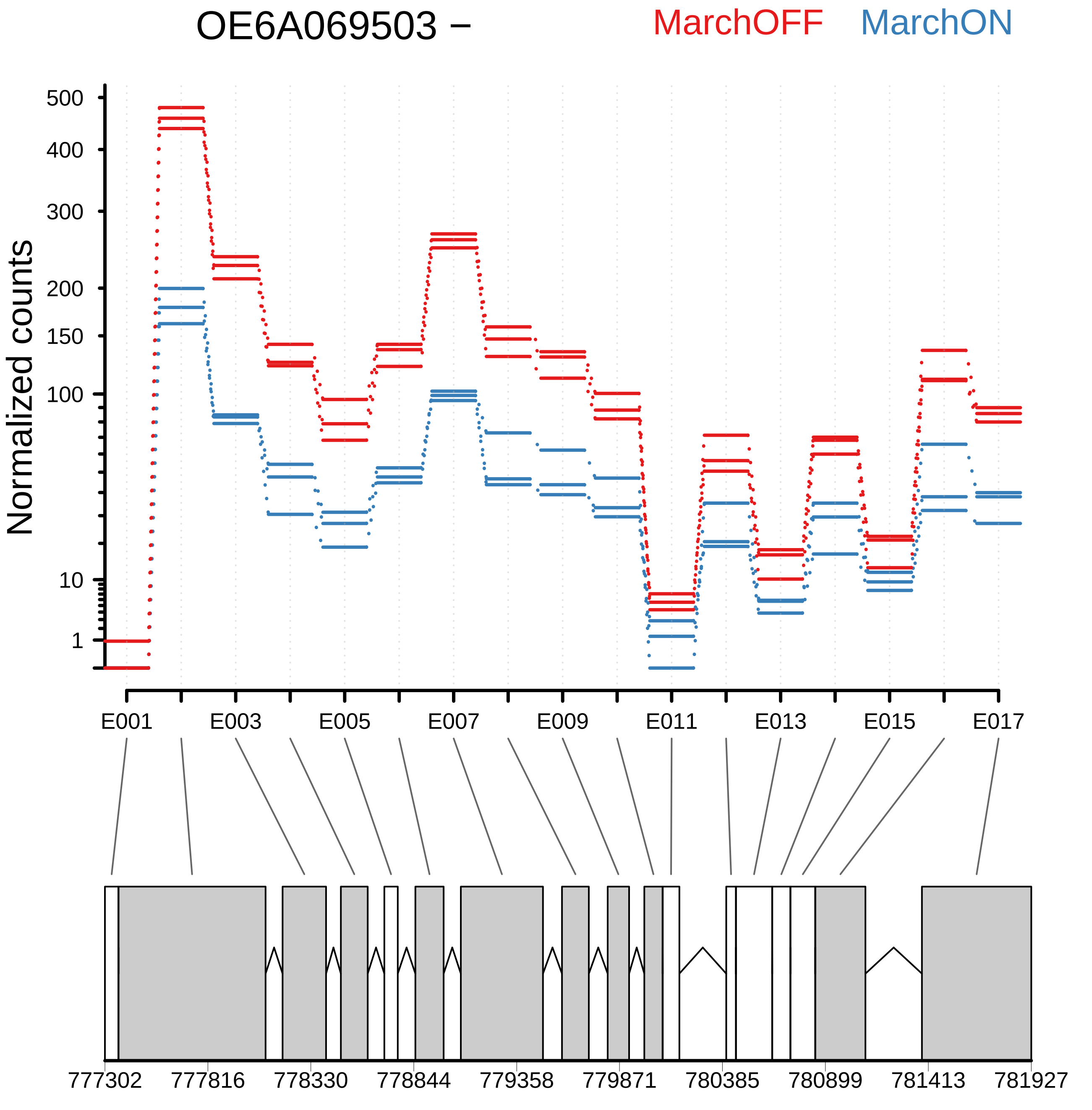
OE6A069503 −
MarchOFF
MarchON
Normalized counts
500
400
300
200
150
100
10
1
E001
E003
E005
E007
E009
E011
E013
E015
E017
777302
777816
778330
778844
779358
779871
780385
780899
781413
781927

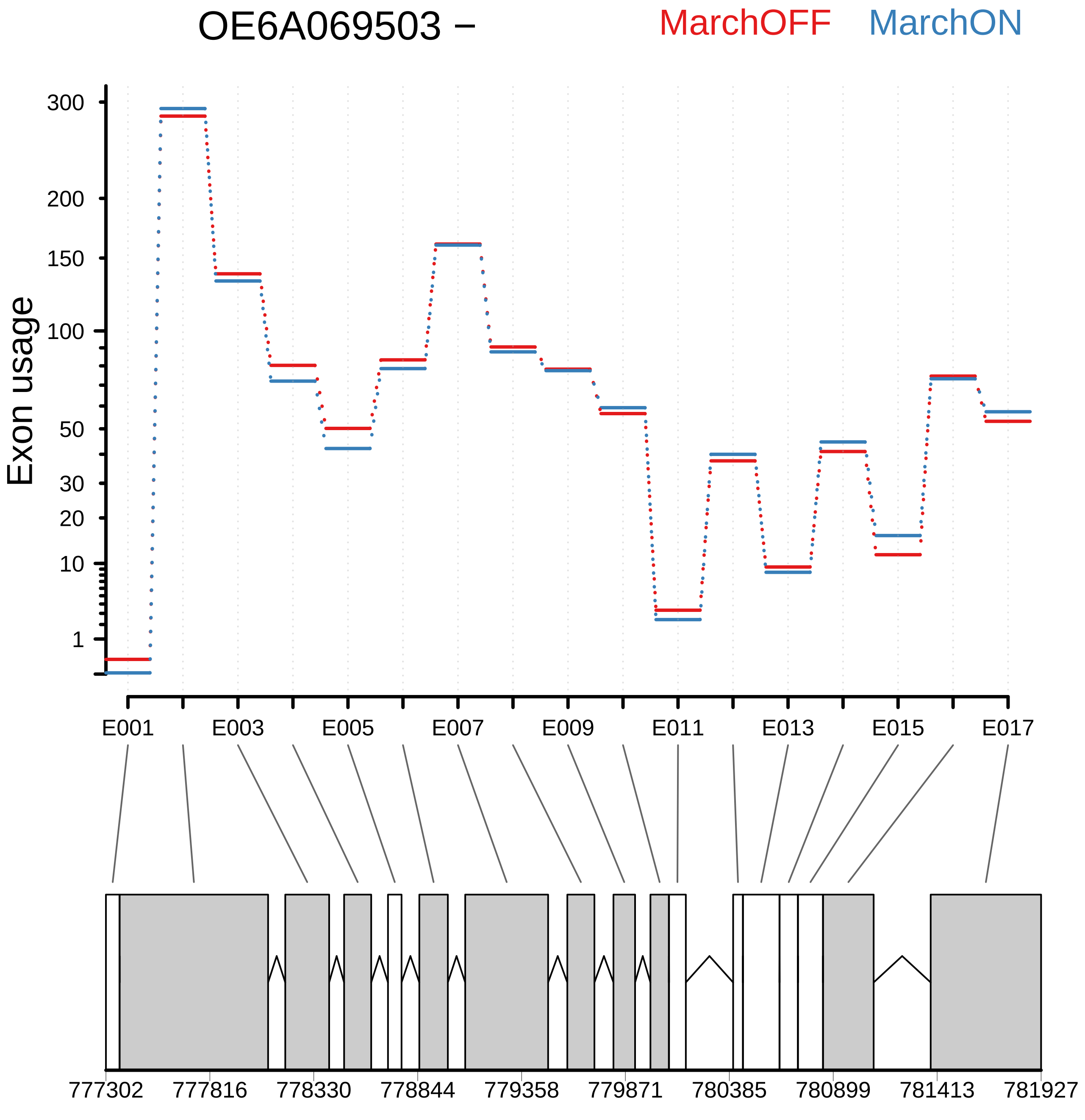

OE6A069503 −
MarchOFF
MarchON
Exon usage
300
200
150
100
50
30
20
10
1
E001
E003
E005
E007
E009
E011
E013
E015
E017
777302
777816
778330
778844
779358
779871
780385
780899
781413
781927

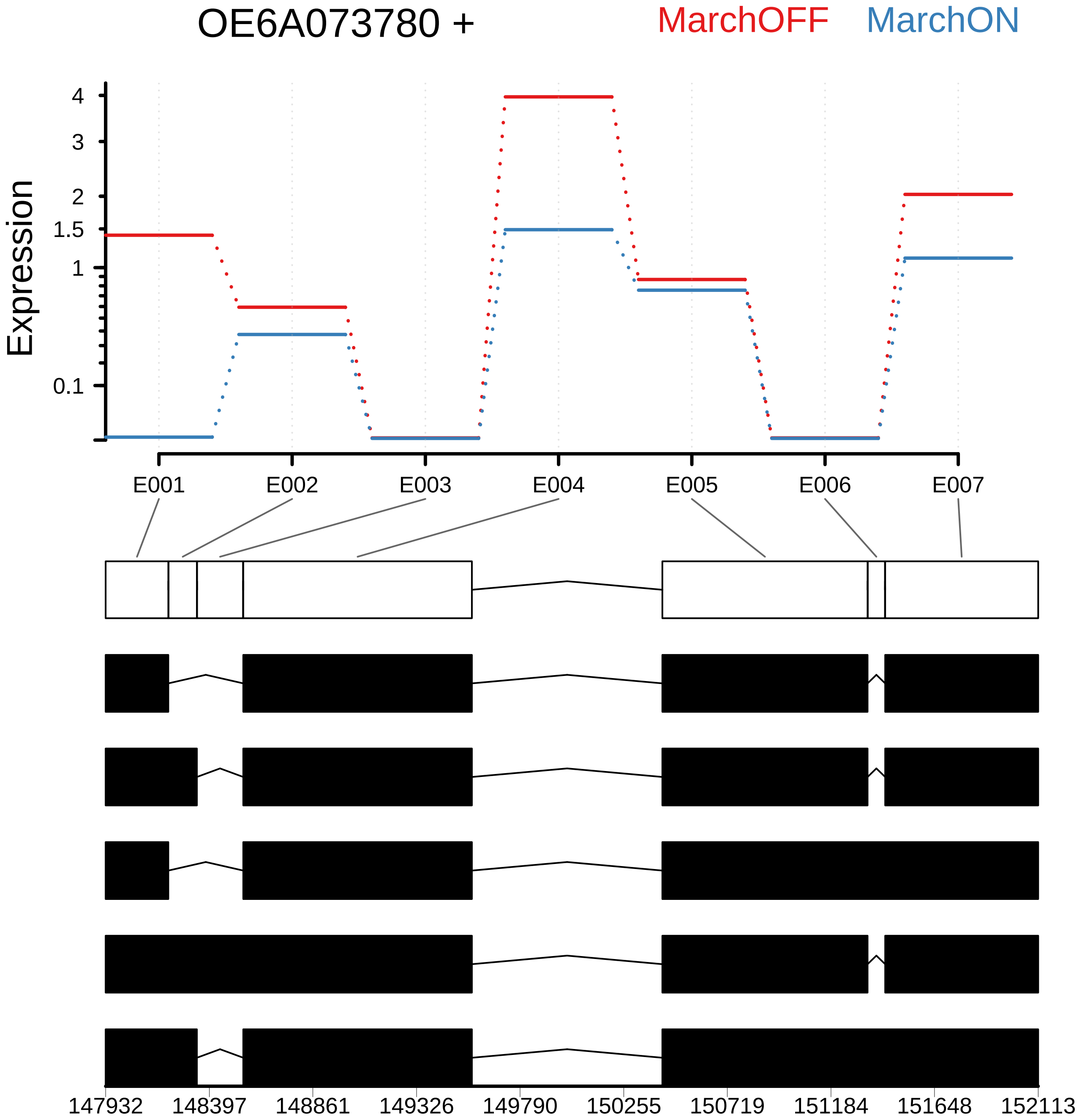

OE6A073780 +
MarchOFF
MarchON
Expression
4
3
2
1.5
1
0.1
E001
E002
E003
E004
E005
E006
E007
147932
148397
148861
149326
149790
150255
150719
151184
151648
152113

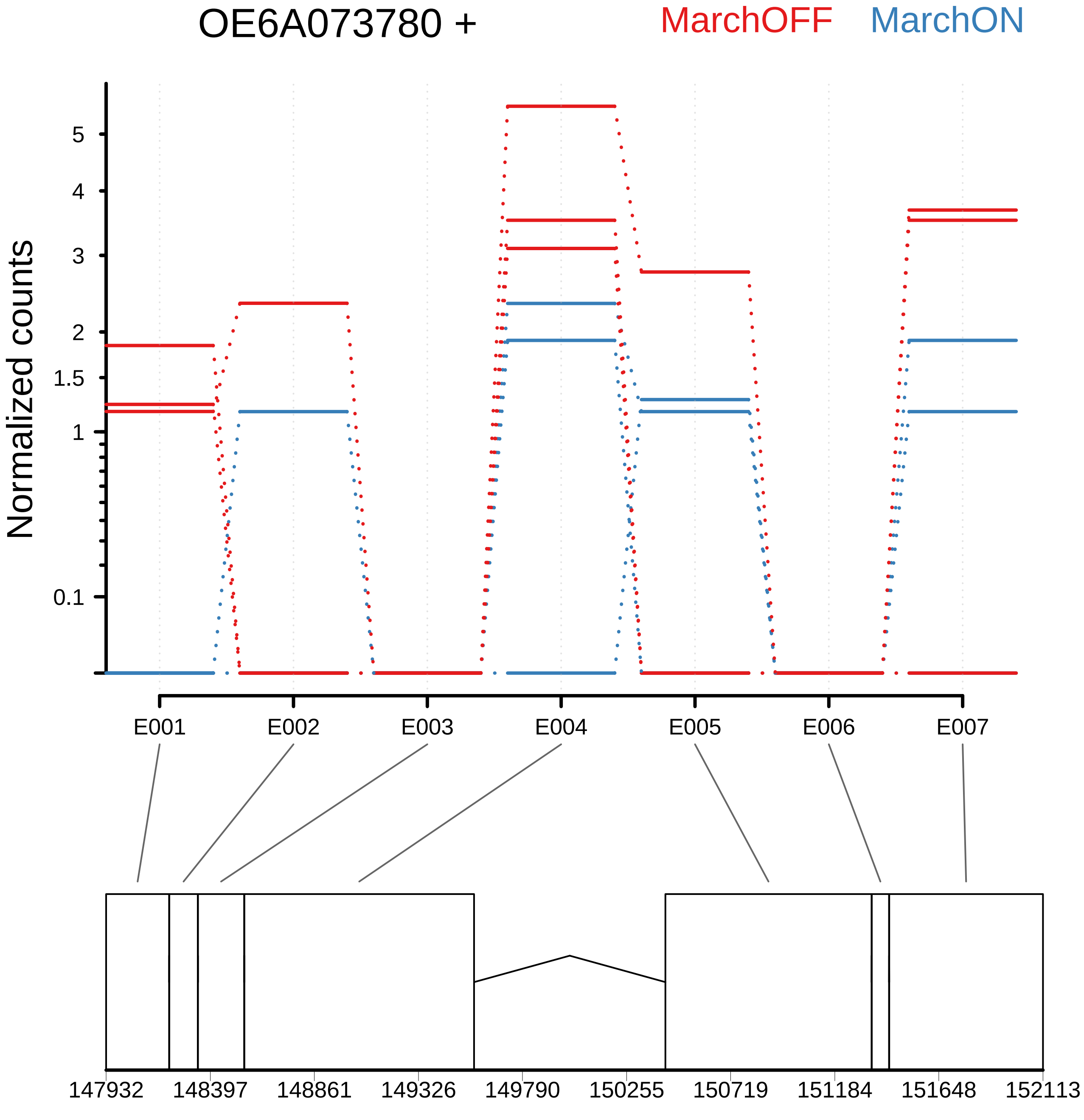
OE6A073780 +
MarchOFF
MarchON
Normalized counts
5
4
3
2
1.5
1
0.1
E001
E002
E003
E004
E005
E006
E007
147932
148397
148861
149326
149790
150255
150719
151184
151648
152113

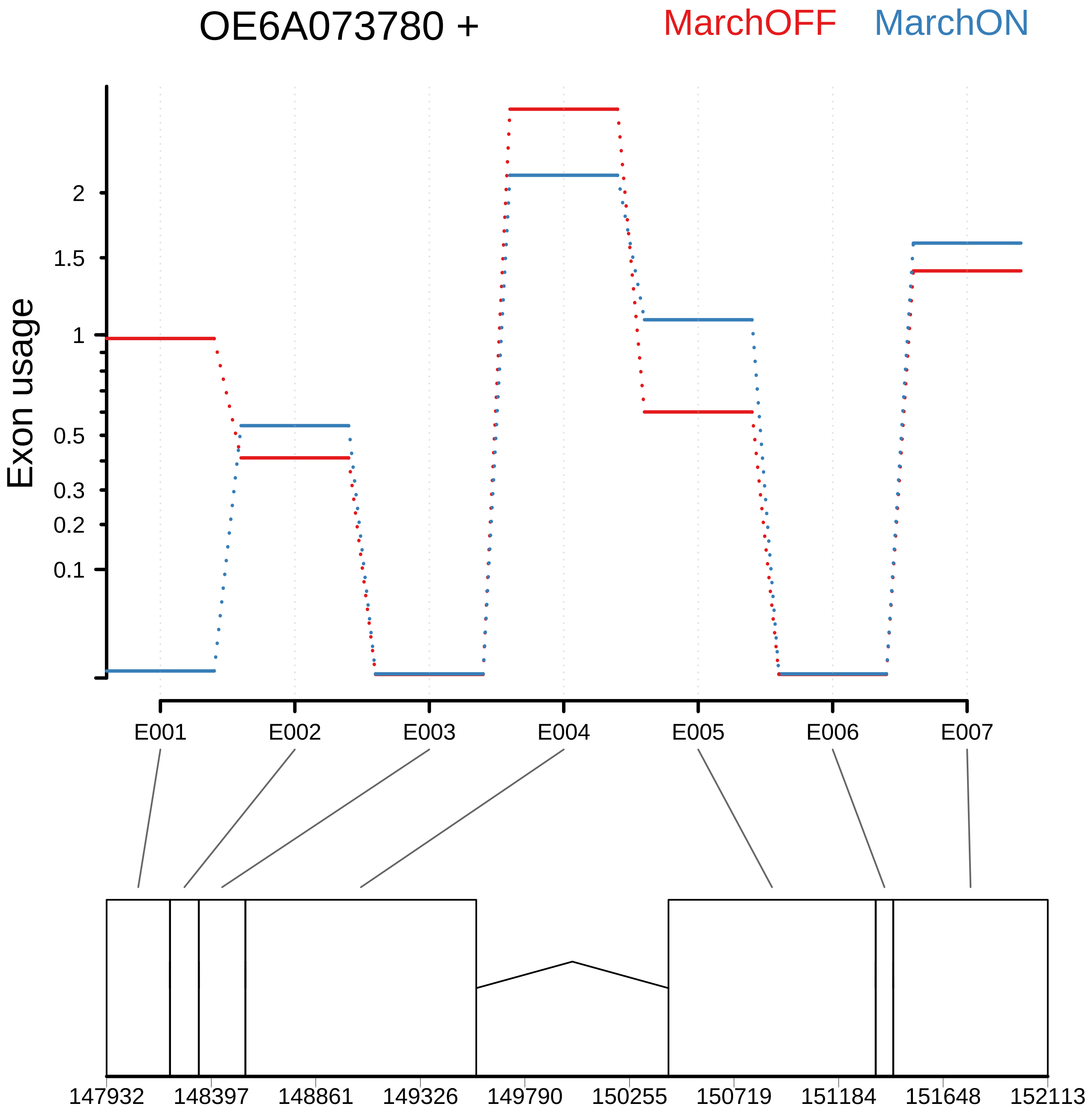
OE6A073780 +
MarchOFF
MarchON
Exon usage
2
1.5
1
0.5
0.3
0.2
0.1
E001
E002
E003
E004
E005
E006
E007
147932
148397
148861
149326
149790
150255
150719
151184
151648
152113

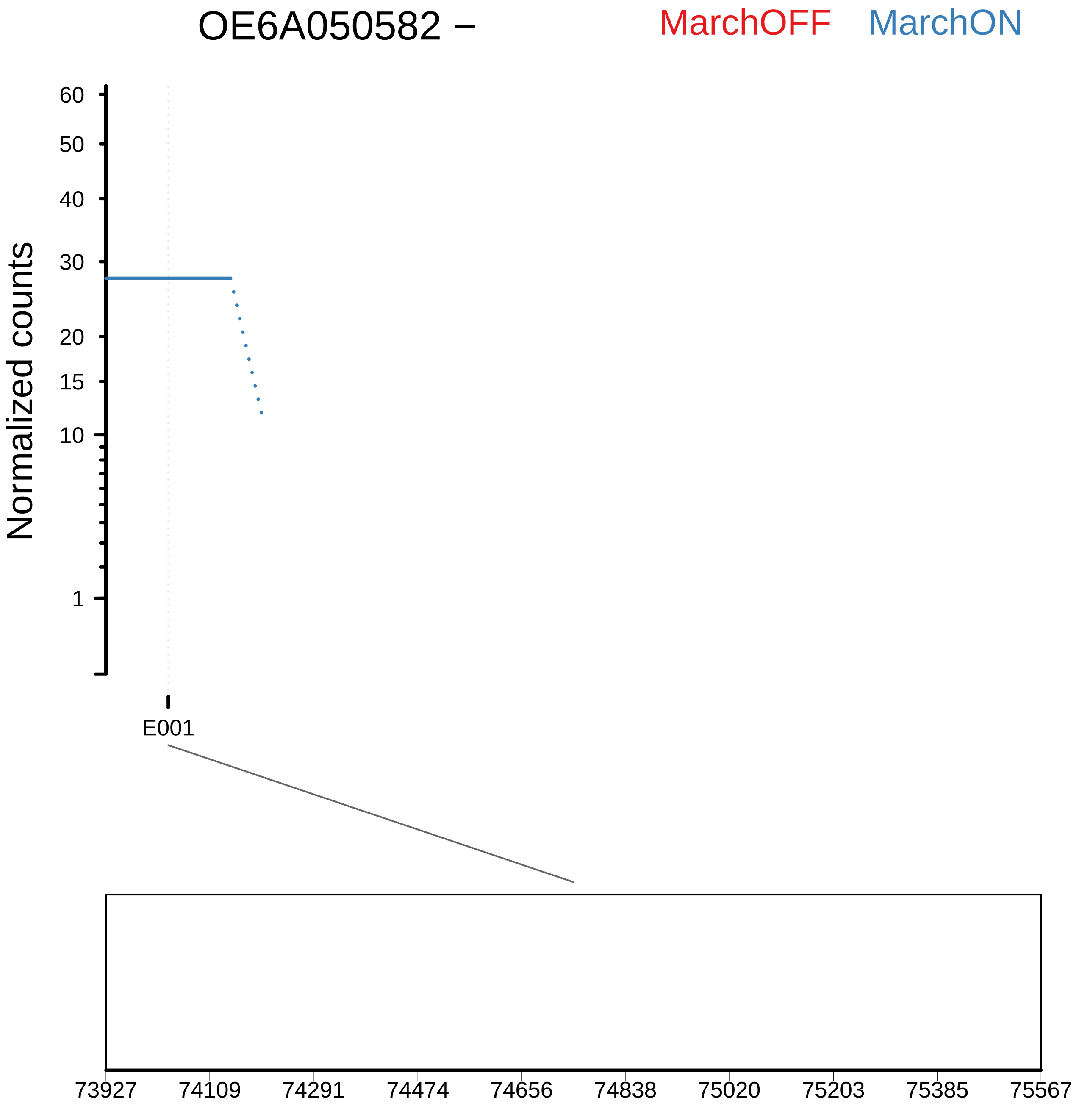
OE6A050582 −
MarchOFF
MarchON
Normalized counts
60
50
40
30
20
15
10
1
E001
73927
74109
74291
74474
74656
74838
75020
75203
75385
75567

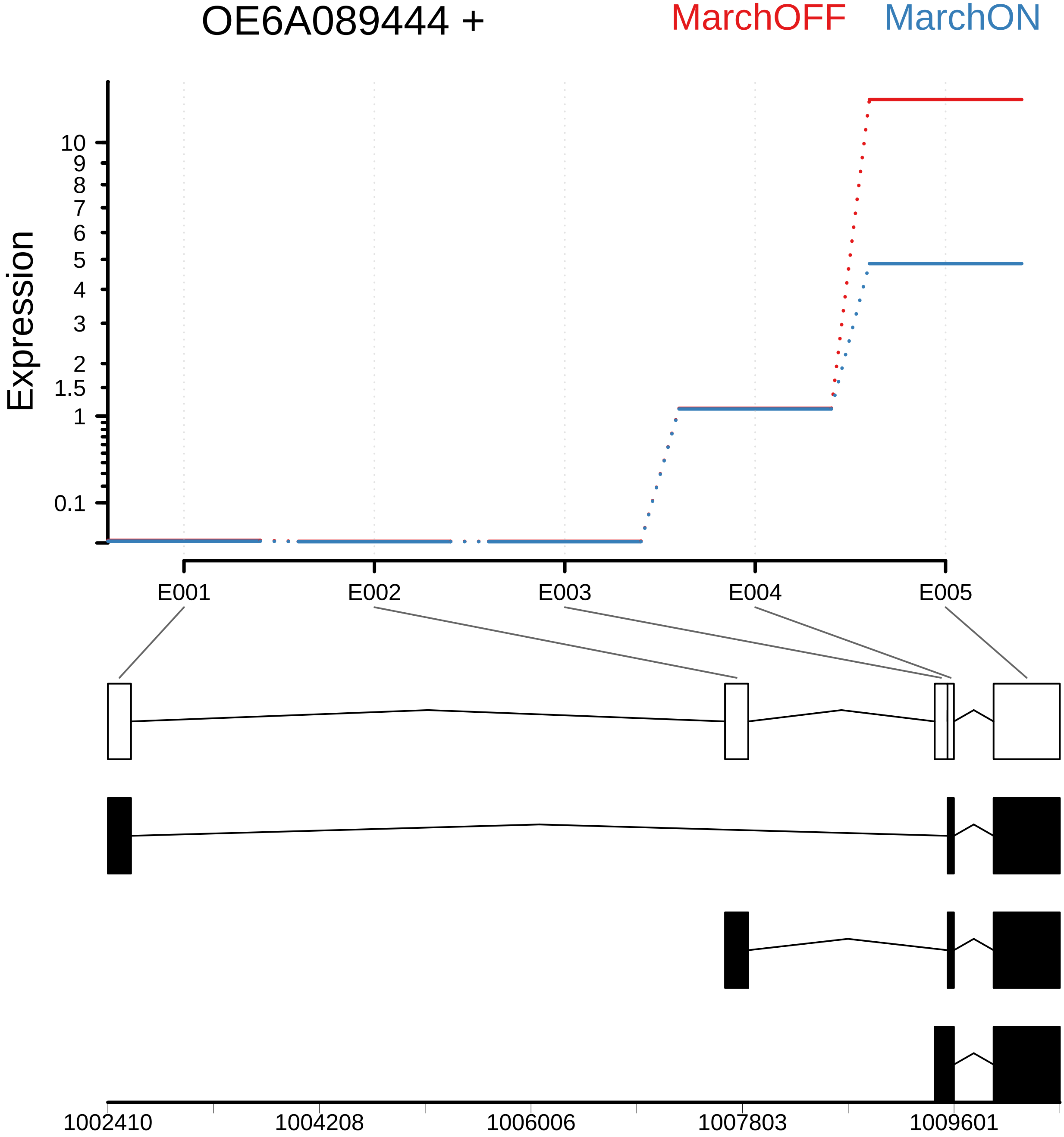

OE6A089444 +
MarchOFF
MarchON
Expression
10
9
8
7
6
5
4
3
2
1.5
1
0.1
E001
E002
E003
E004
E005
1002410
1004208
1006006
1007803
1009601

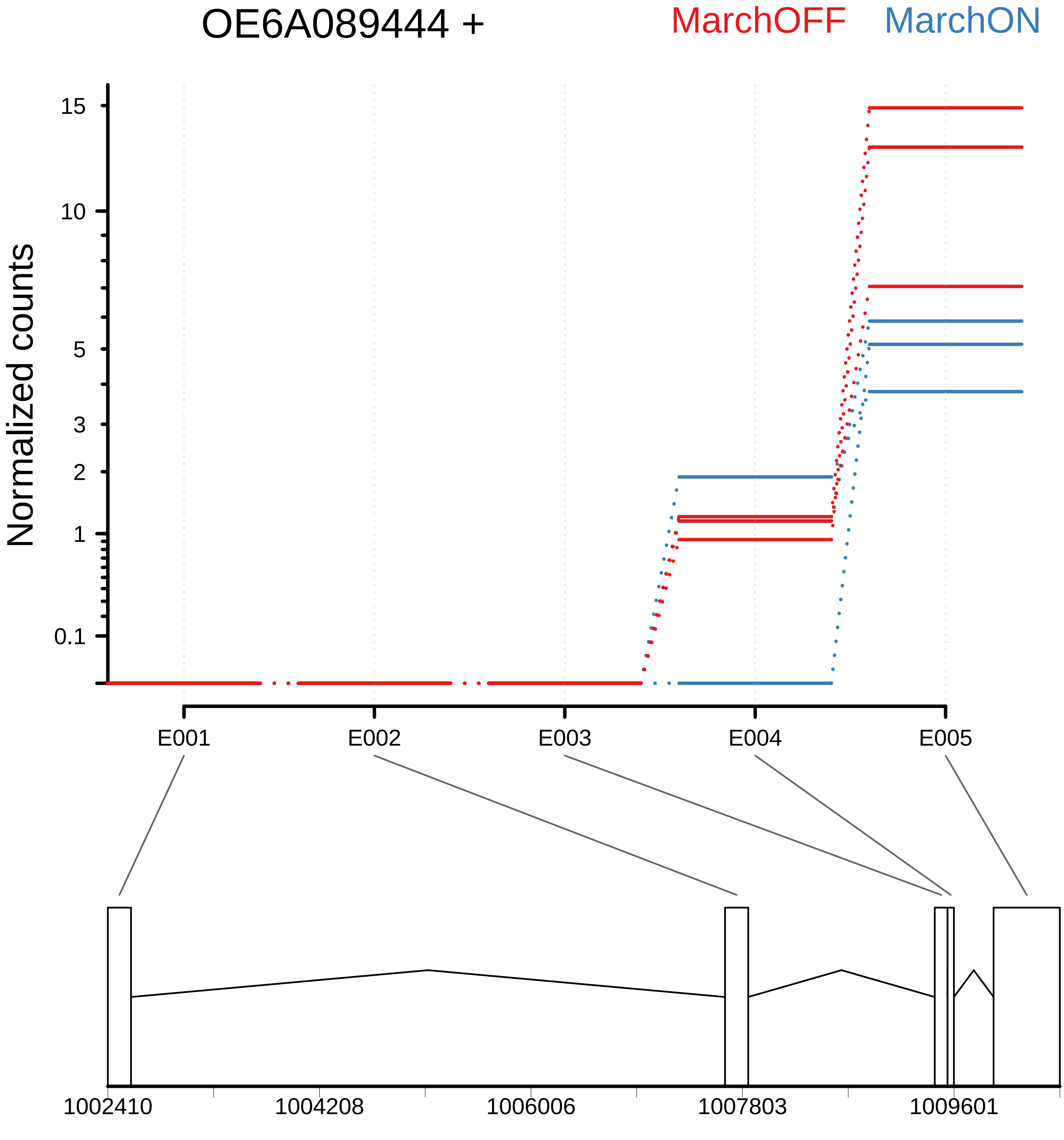

OE6A089444 +
MarchOFF
MarchON
Normalized counts
15
10
5
3
2
1
0.1
E001
E002
E003
E004
E005
1002410
1004208
1006006
1007803
1009601

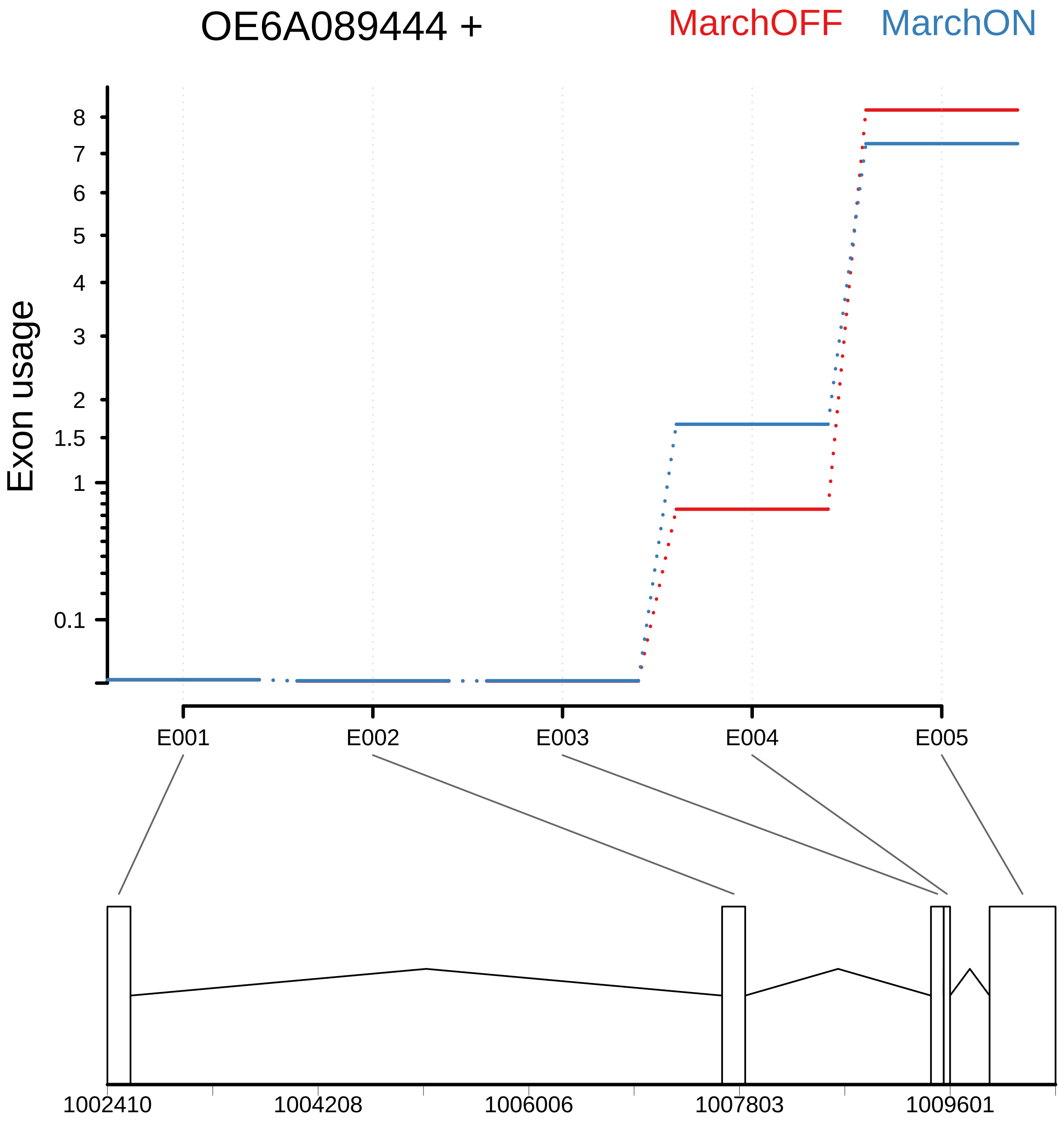

OE6A089444 +
MarchOFF
MarchON
Exon usage
8
7
6
5
4
3
2
1.5
1
0.1
E001
E002
E003
E004
E005
1002410
1004208
1006006
1007803
1009601

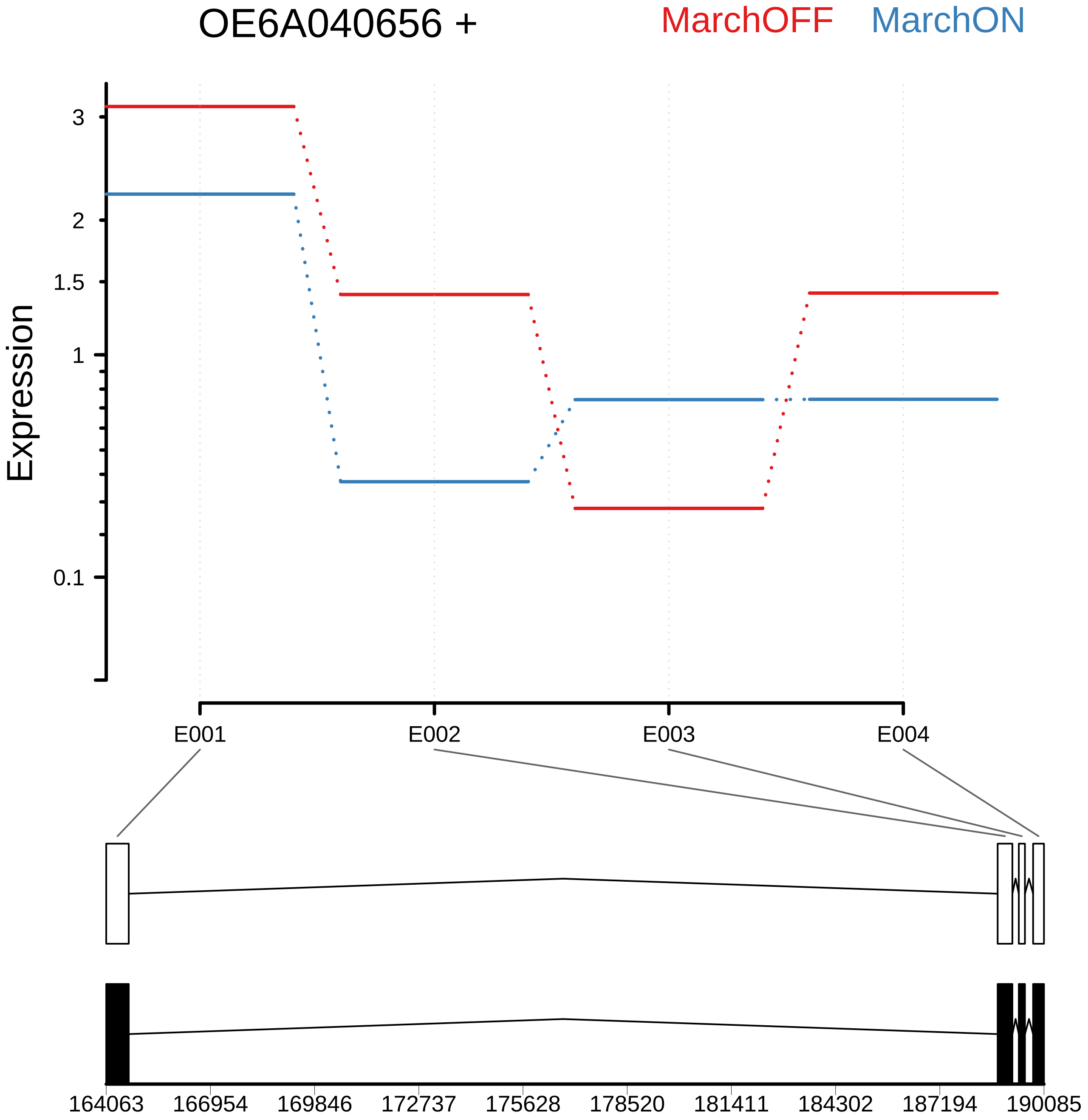
OE6A040656 +
MarchOFF
MarchON
Expression
3
2
1.5
1
0.1
E001
E002
E003
E004
164063
166954
169846
172737
175628
178520
181411
184302
187194
190085

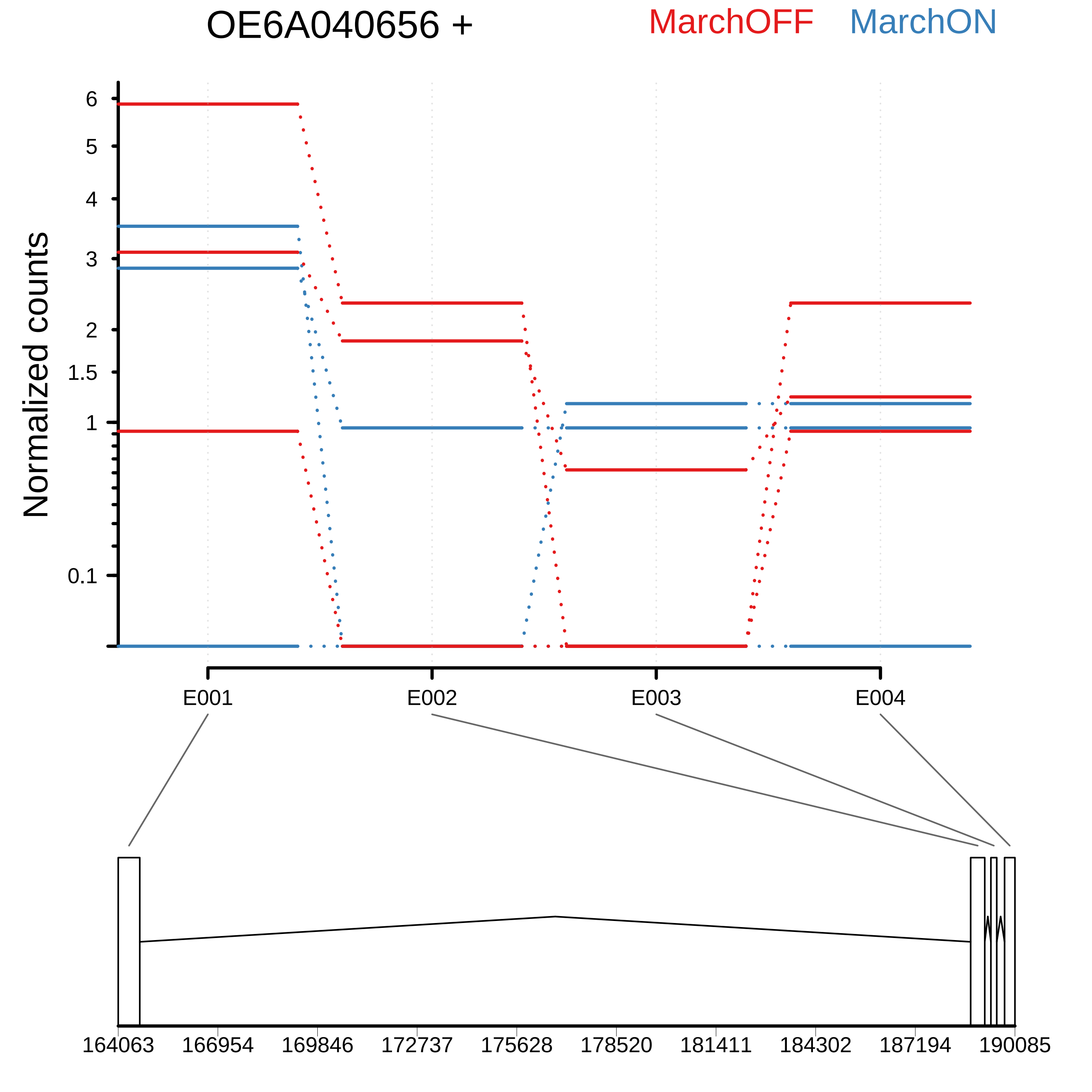
OE6A040656 +
MarchOFF
MarchON
Normalized counts
6
5
4
3
2
1.5
1
0.1
E001
E002
E003
E004
164063
166954
169846
172737
175628
178520
181411
184302
187194
190085

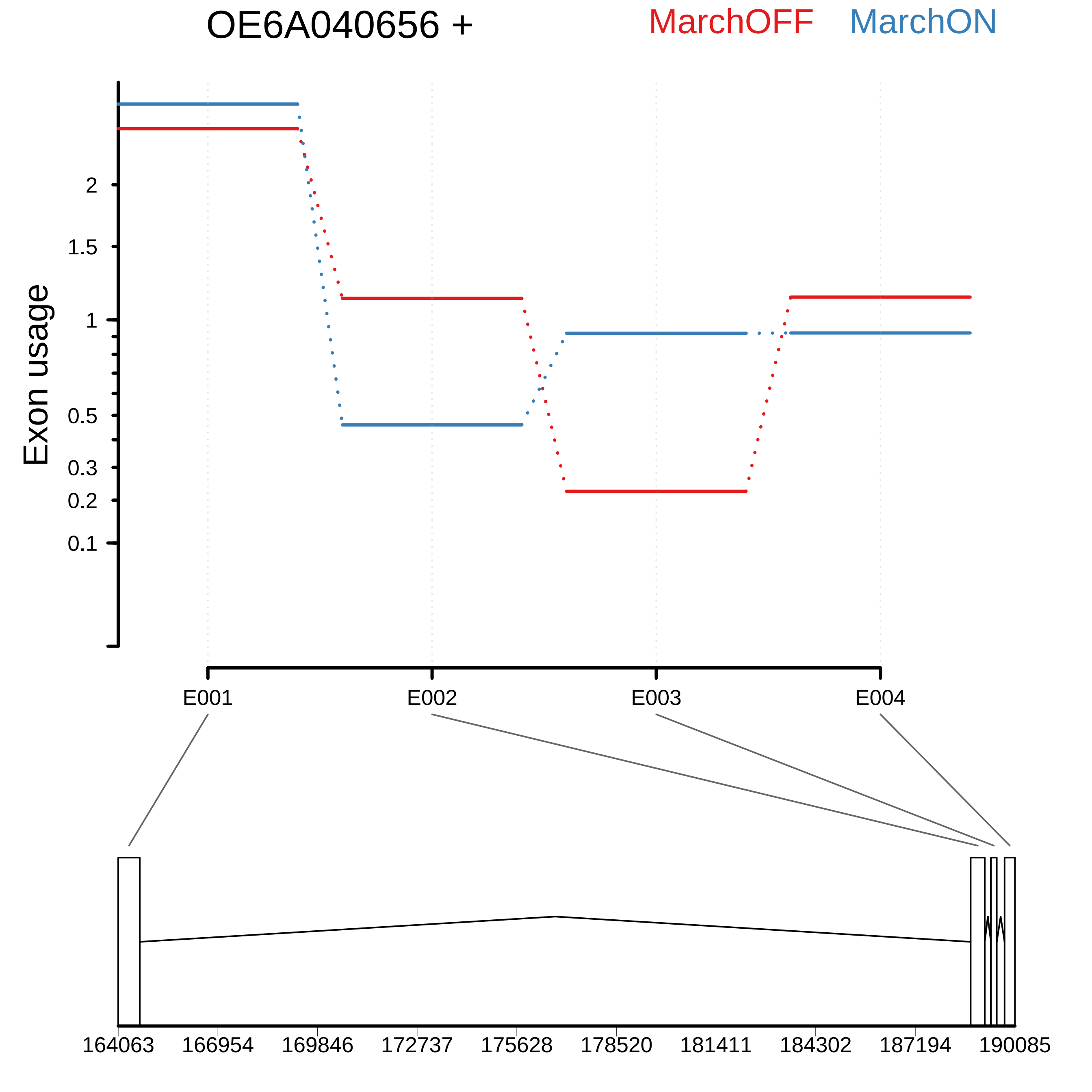

OE6A040656 +
MarchOFF
MarchON
Exon usage
2
1.5
1
0.5
0.3
0.2
0.1
E001
E002
E003
E004
164063
166954
169846
172737
175628
178520
181411
184302
187194
190085

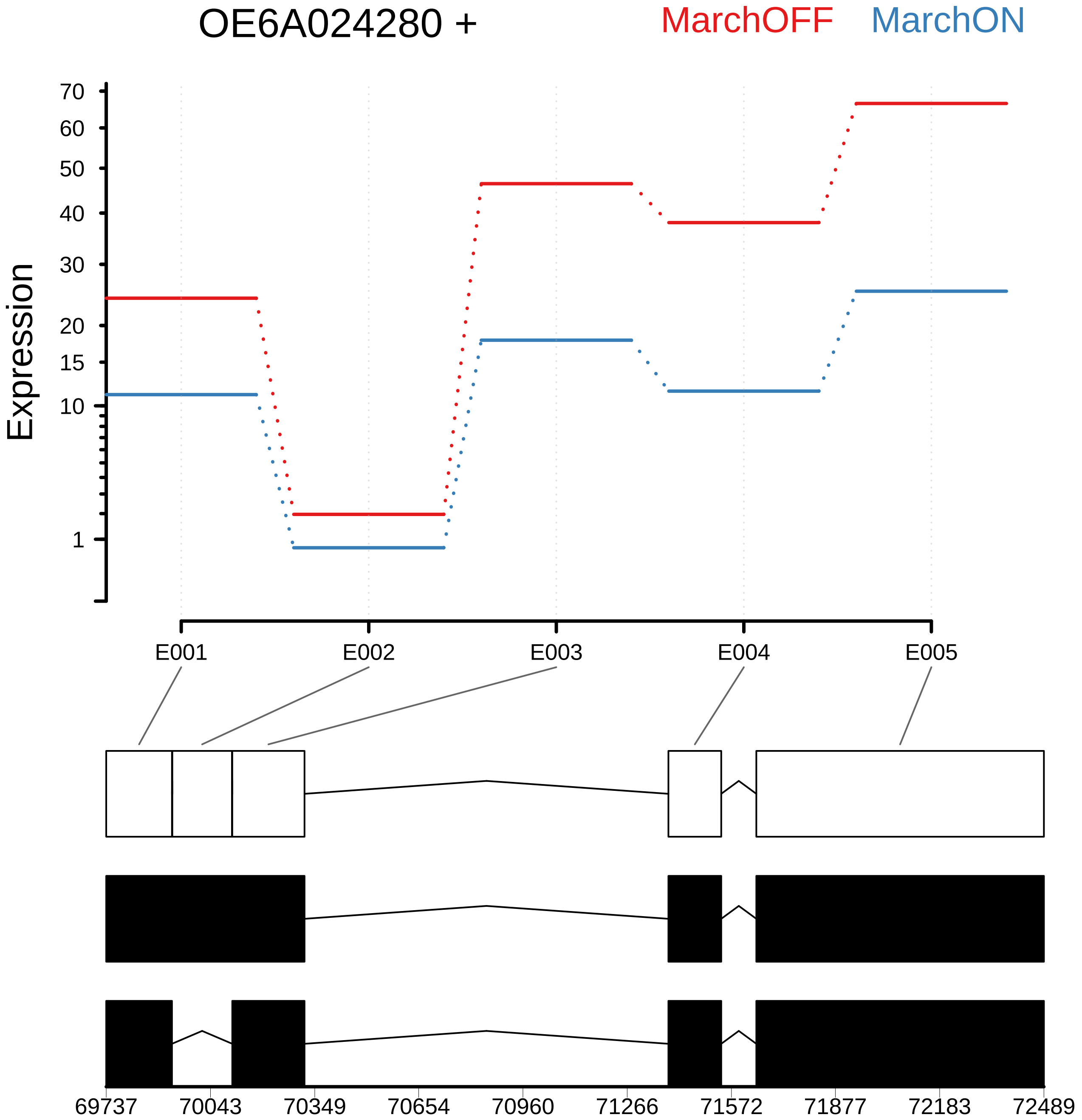

OE6A024280 +
MarchOFF
MarchON
Expression
70
60
50
40
30
20
15
10
1
E001
E002
E003
E004
E005
69737
70043
70349
70654
70960
71266
71572
71877
72183
72489

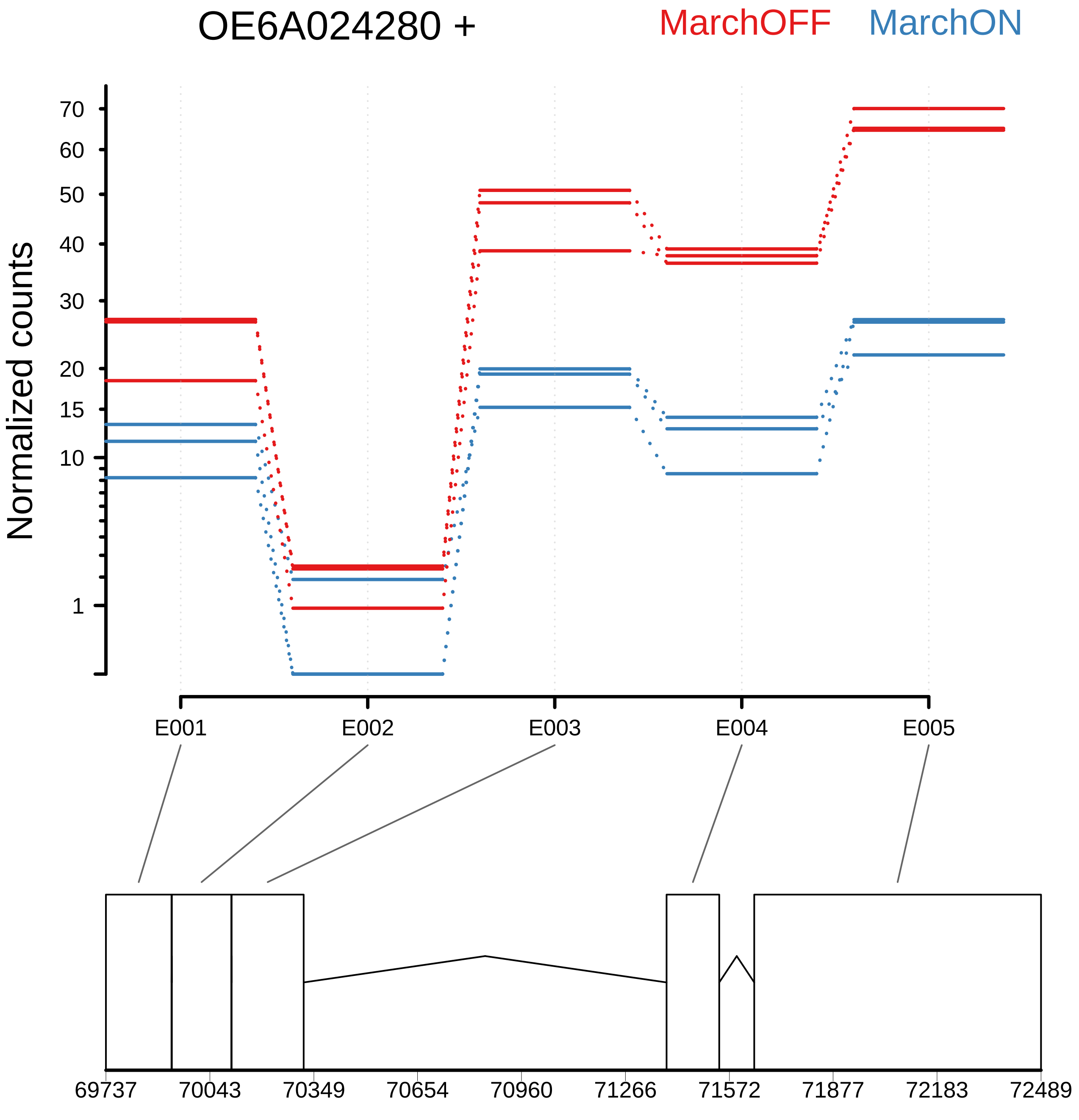

OE6A024280 +
MarchOFF
MarchON
Normalized counts
70
60
50
40
30
20
15
10
1
E001
E002
E003
E004
E005
69737
70043
70349
70654
70960
71266
71572
71877
72183
72489

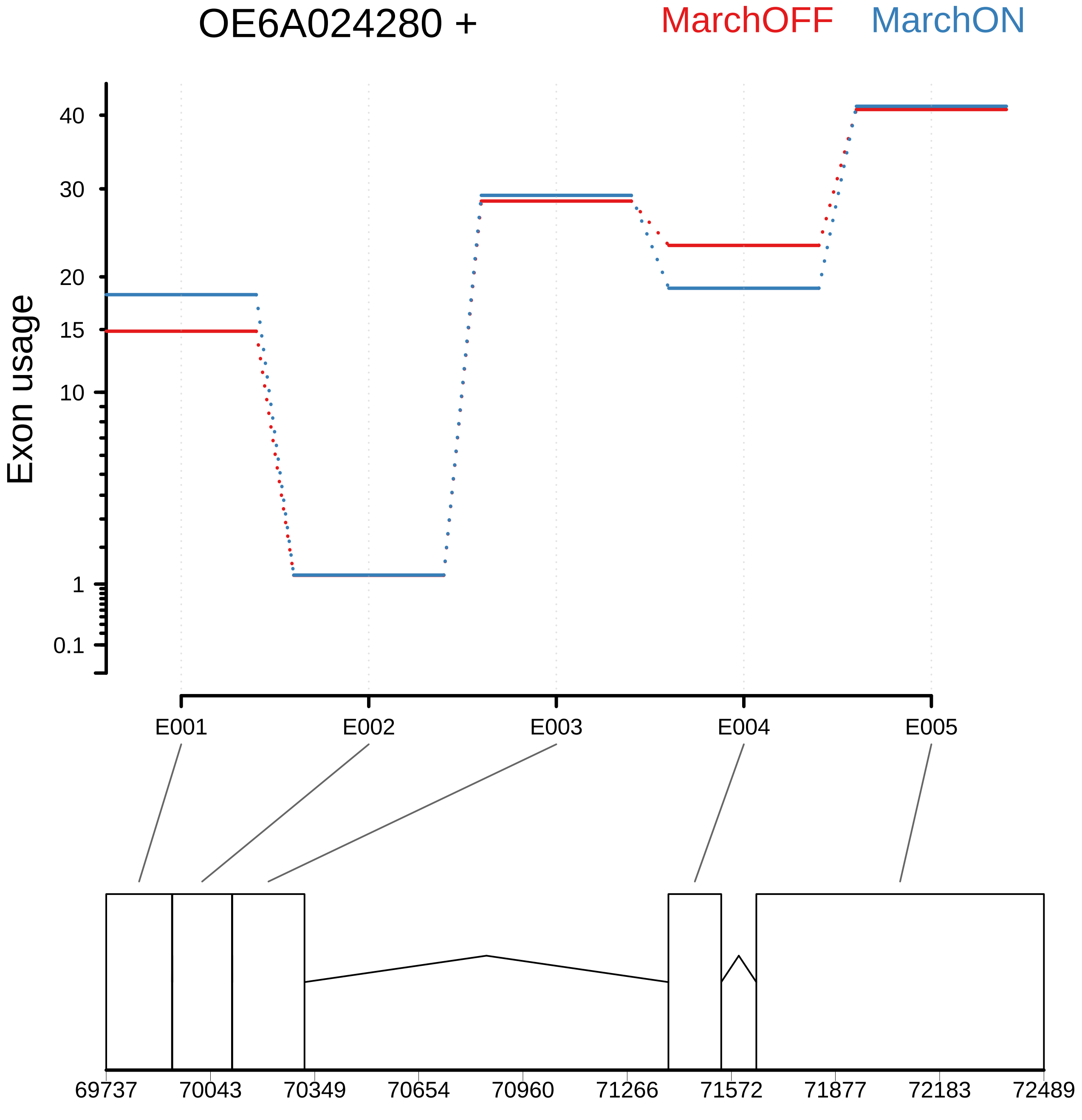

OE6A024280 +
MarchOFF
MarchON
Exon usage
40
30
20
15
10
1
0.1
E001
E002
E003
E004
E005
69737
70043
70349
70654
70960
71266
71572
71877
72183
72489

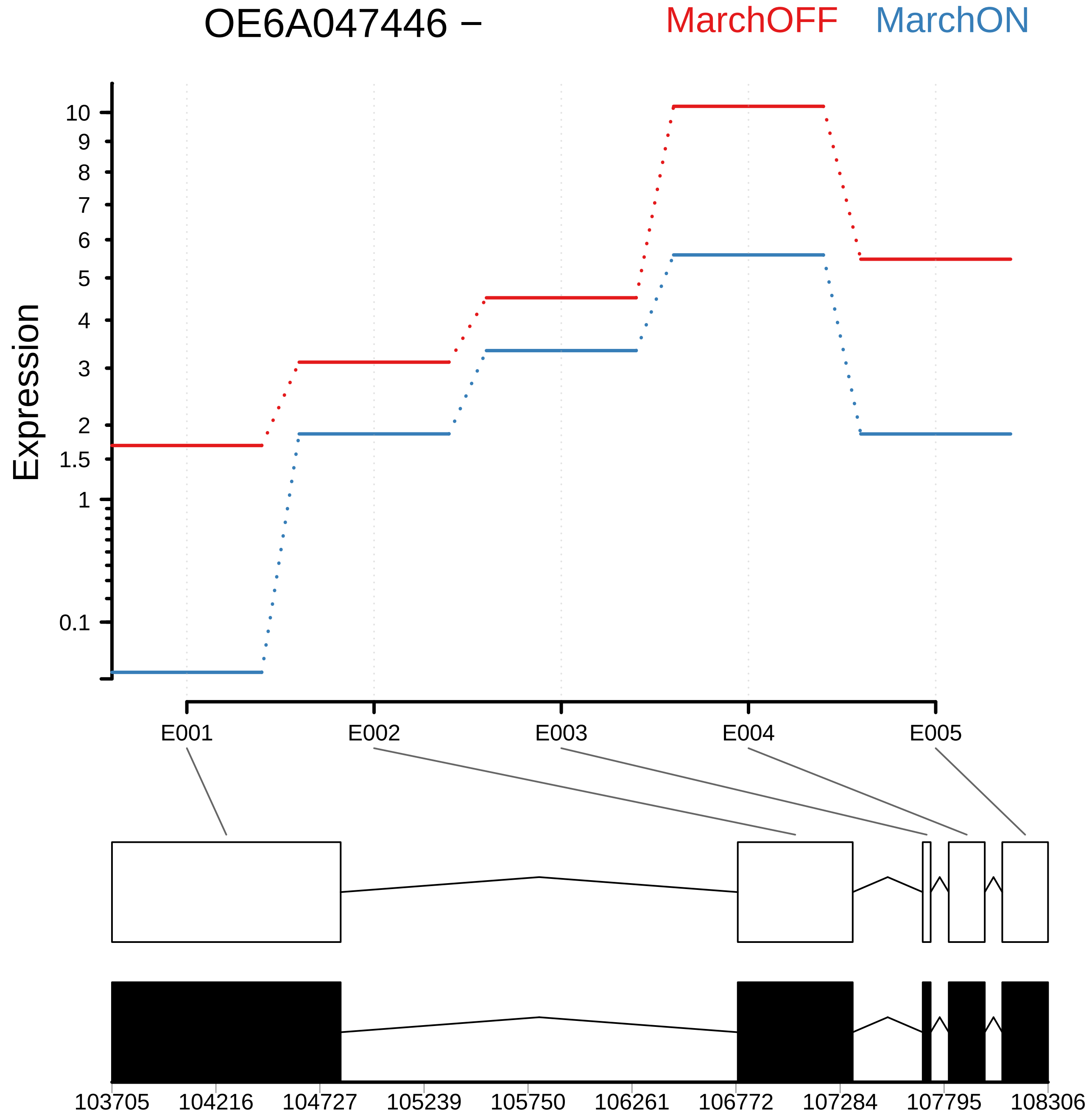
OE6A047446 −
MarchOFF
MarchON
Expression
10
9
8
7
6
5
4
3
2
1.5
1
0.1
E001
E002
E003
E004
E005
103705
104216
104727
105239
105750
106261
106772
107284
107795
108306

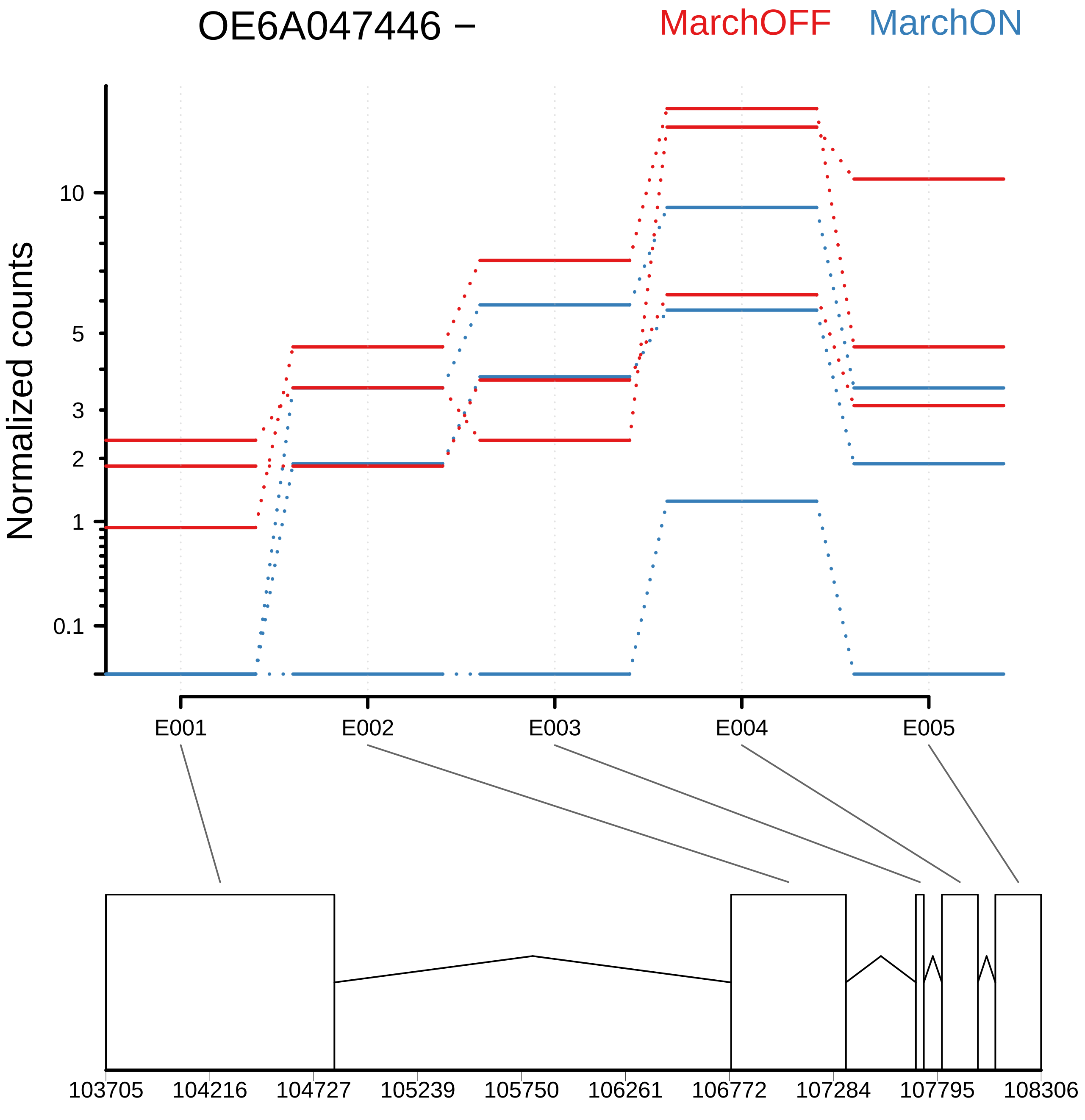

OE6A047446 −
MarchOFF
MarchON
Normalized counts
10
5
3
2
1
0.1
E001
E002
E003
E004
E005
103705
104216
104727
105239
105750
106261
106772
107284
107795
108306

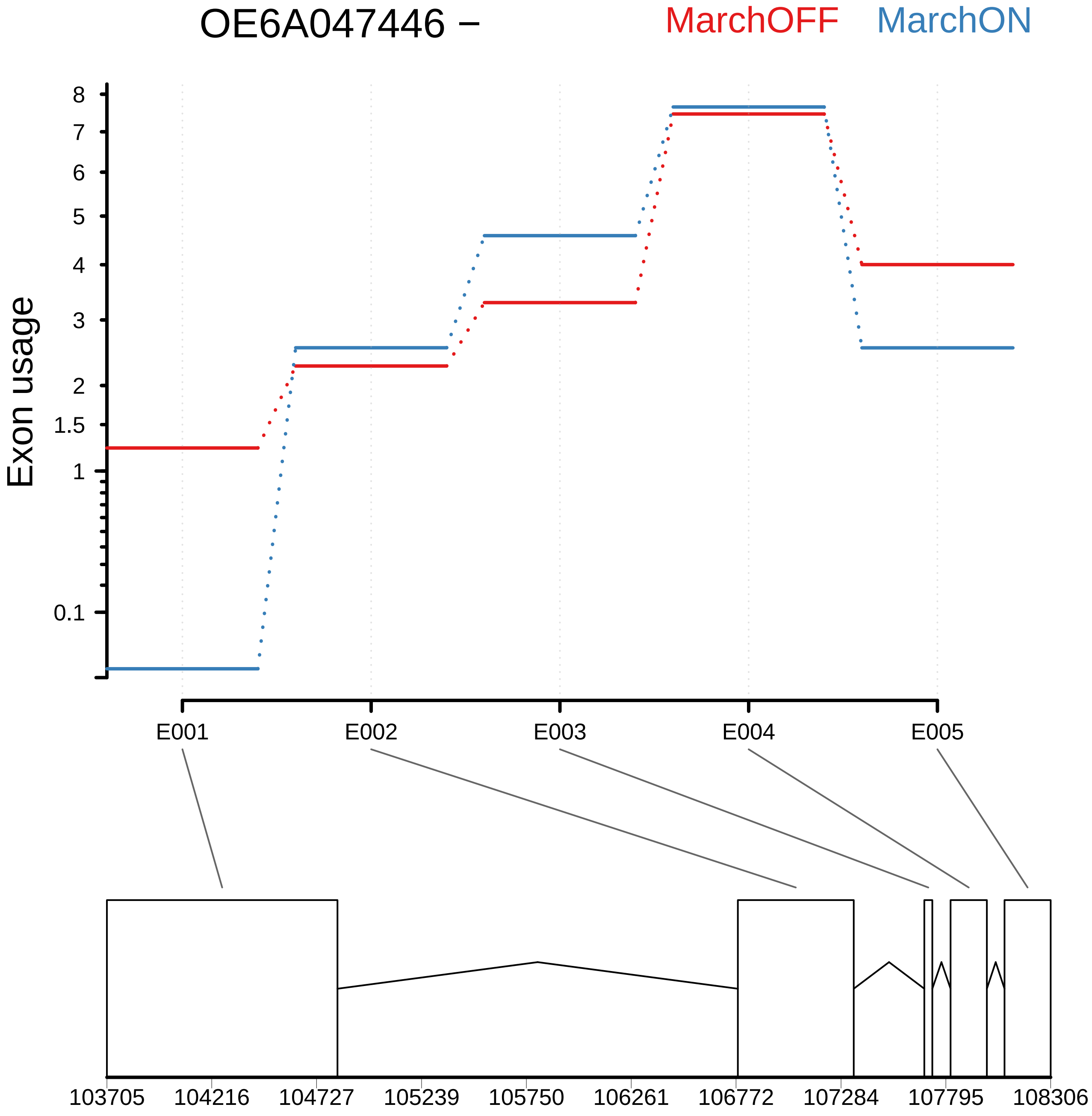

OE6A047446 −
MarchOFF
MarchON
Exon usage
8
7
6
5
4
3
2
1.5
1
0.1
E001
E002
E003
E004
E005
103705
104216
104727
105239
105750
106261
106772
107284
107795
108306

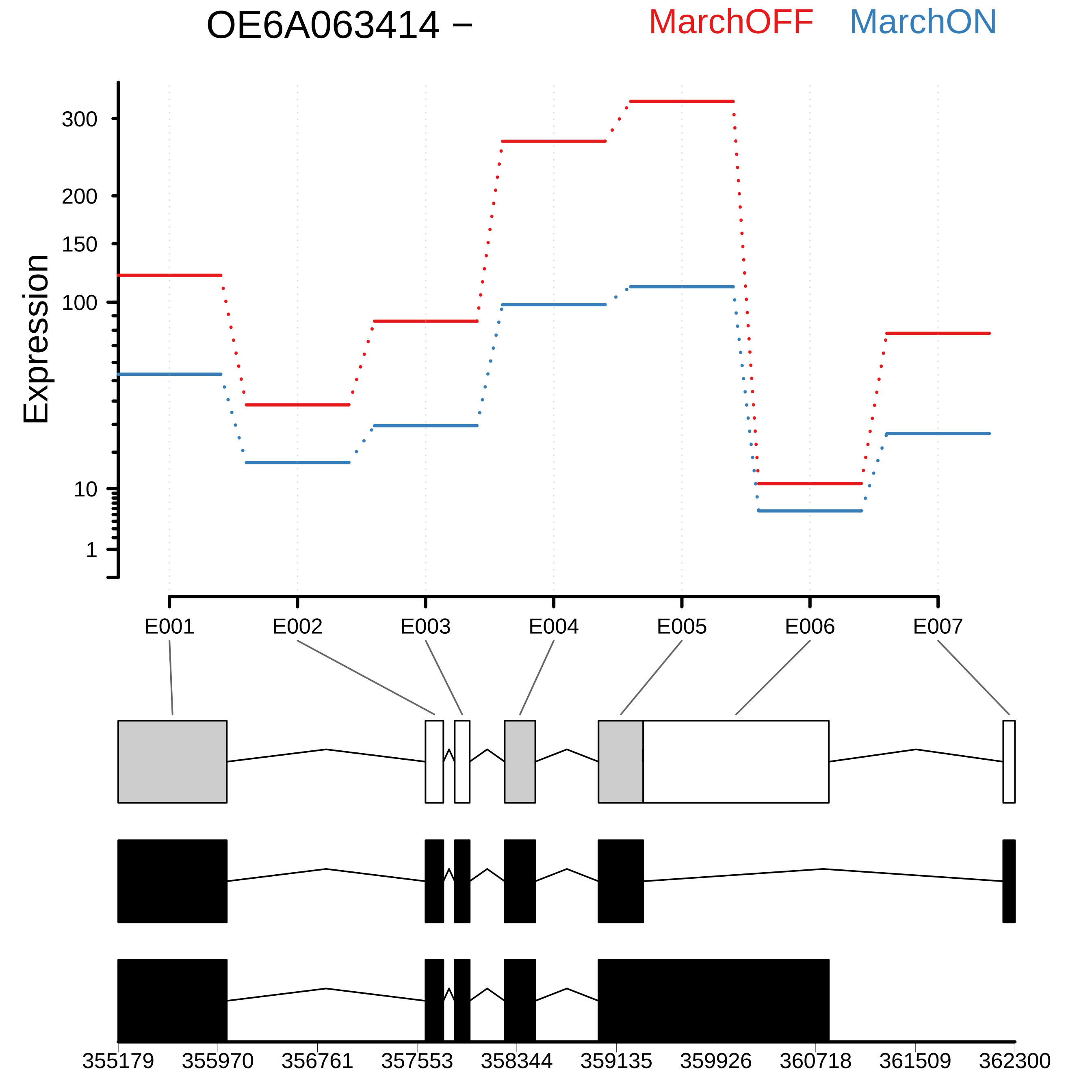

OE6A063414 –
MarchOFF
MarchON
Expression
300
200
150
100
10
1
E001
E002
E003
E004
E005
E006
E007
355179
355970
356761
357553
358344
359135
359926
360718
361509
362300

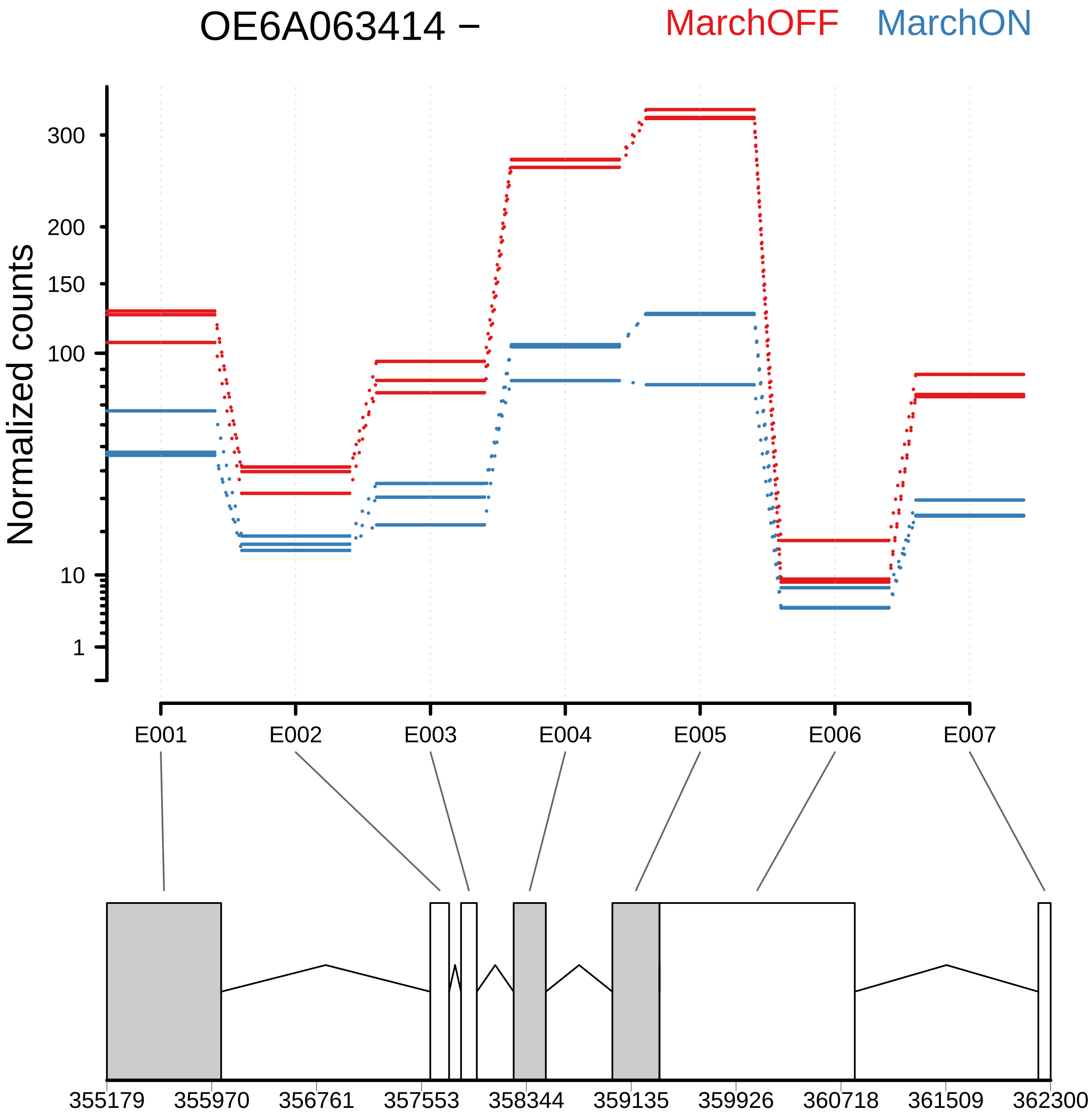

OE6A063414 −
MarchOFF
MarchON
Normalized counts
300
200
150
100
10
1
E001
E002
E003
E004
E005
E006
E007
355179
355970
356761
357553
358344
359135
359926
360718
361509
362300

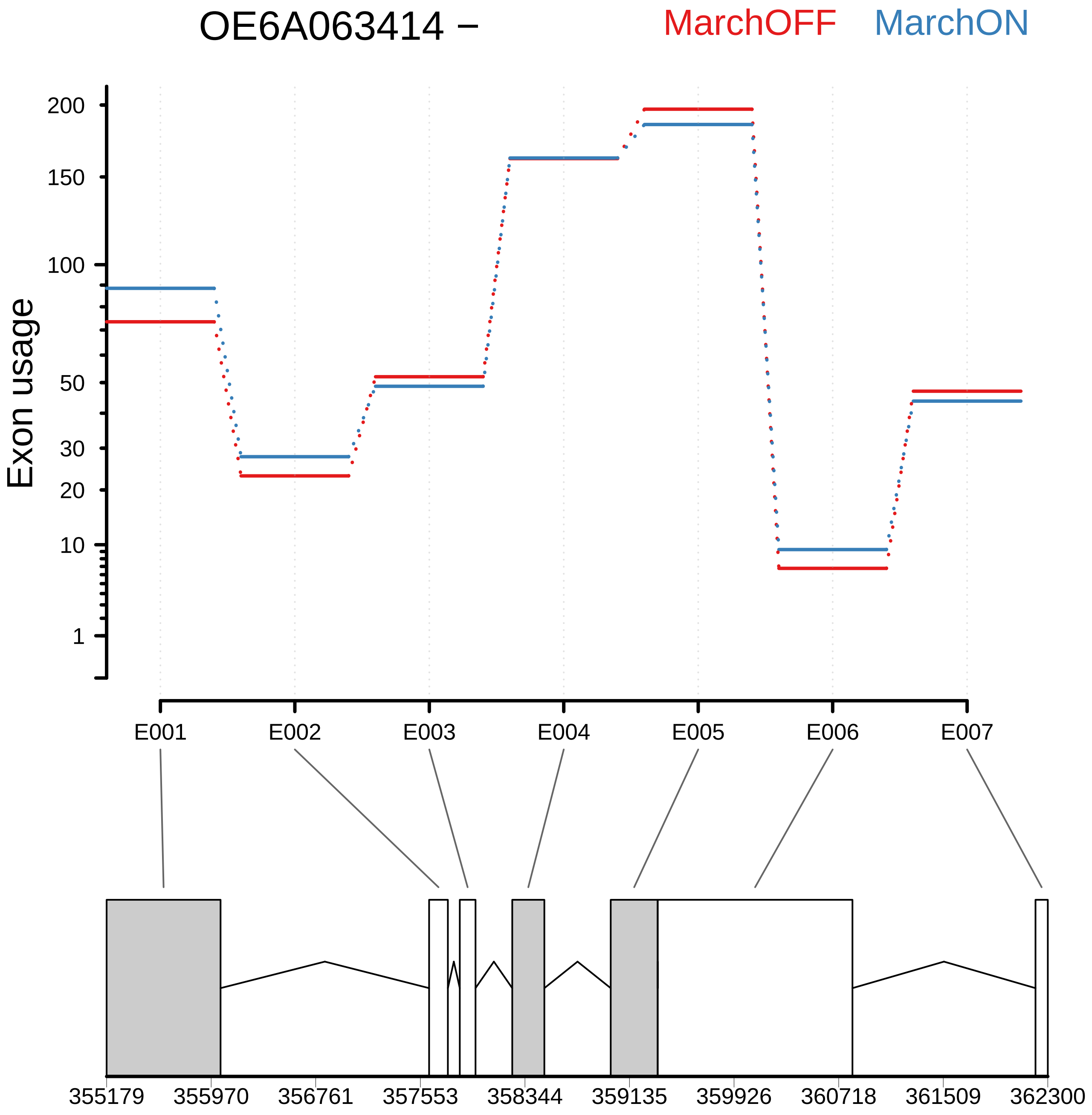

OE6A063414 −
MarchOFF
MarchON
Exon usage
200
150
100
50
30
20
10
1
E001
E002
E003
E004
E005
E006
E007
355179
355970
356761
357553
358344
359135
359926
360718
361509
362300

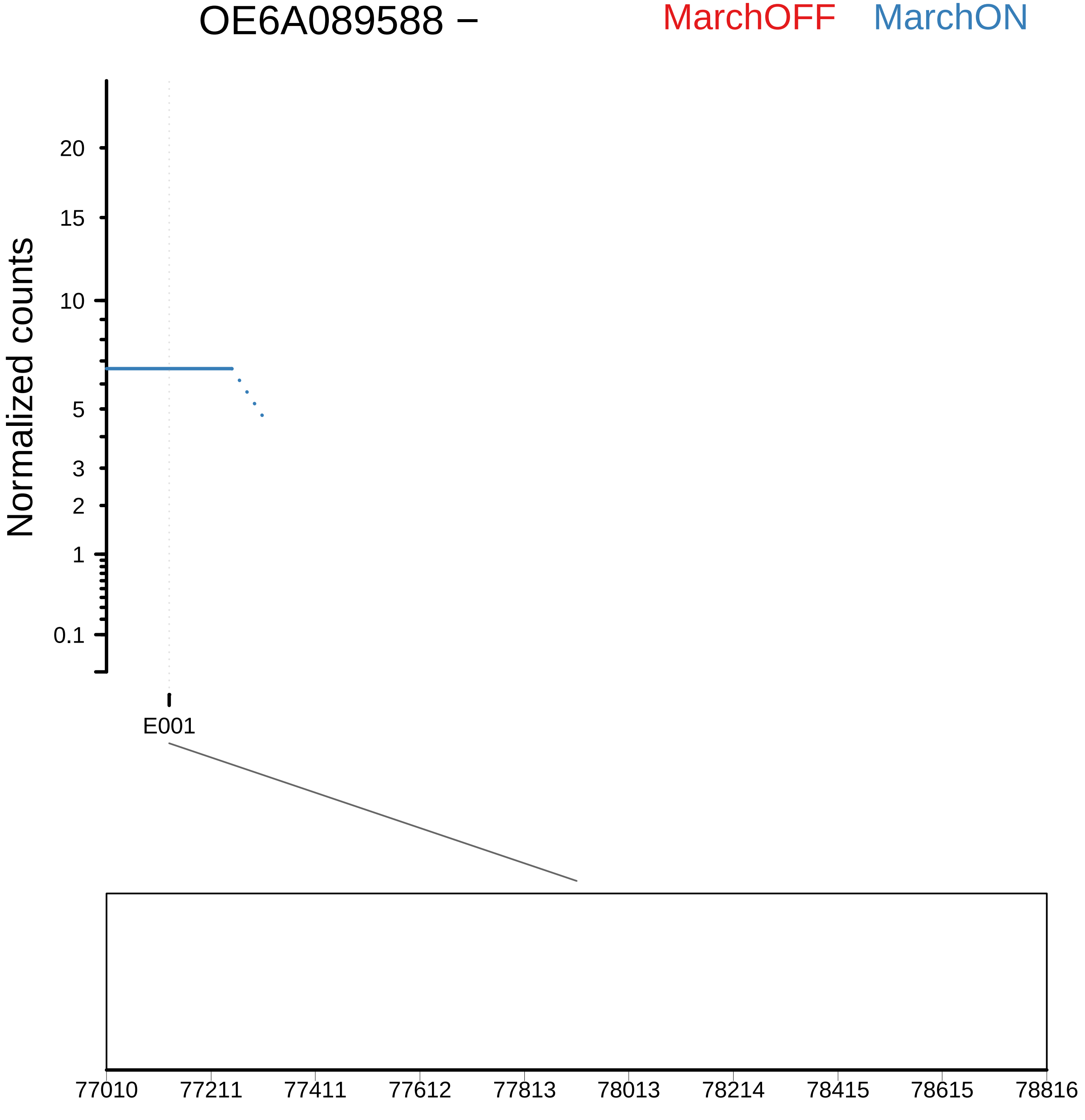
OE6A089588 −
MarchOFF
MarchON
Normalized counts
20
15
10
5
3
2
1
0.1
E001
77010
77211
77411
77612
77813
78013
78214
78415
78615
78816

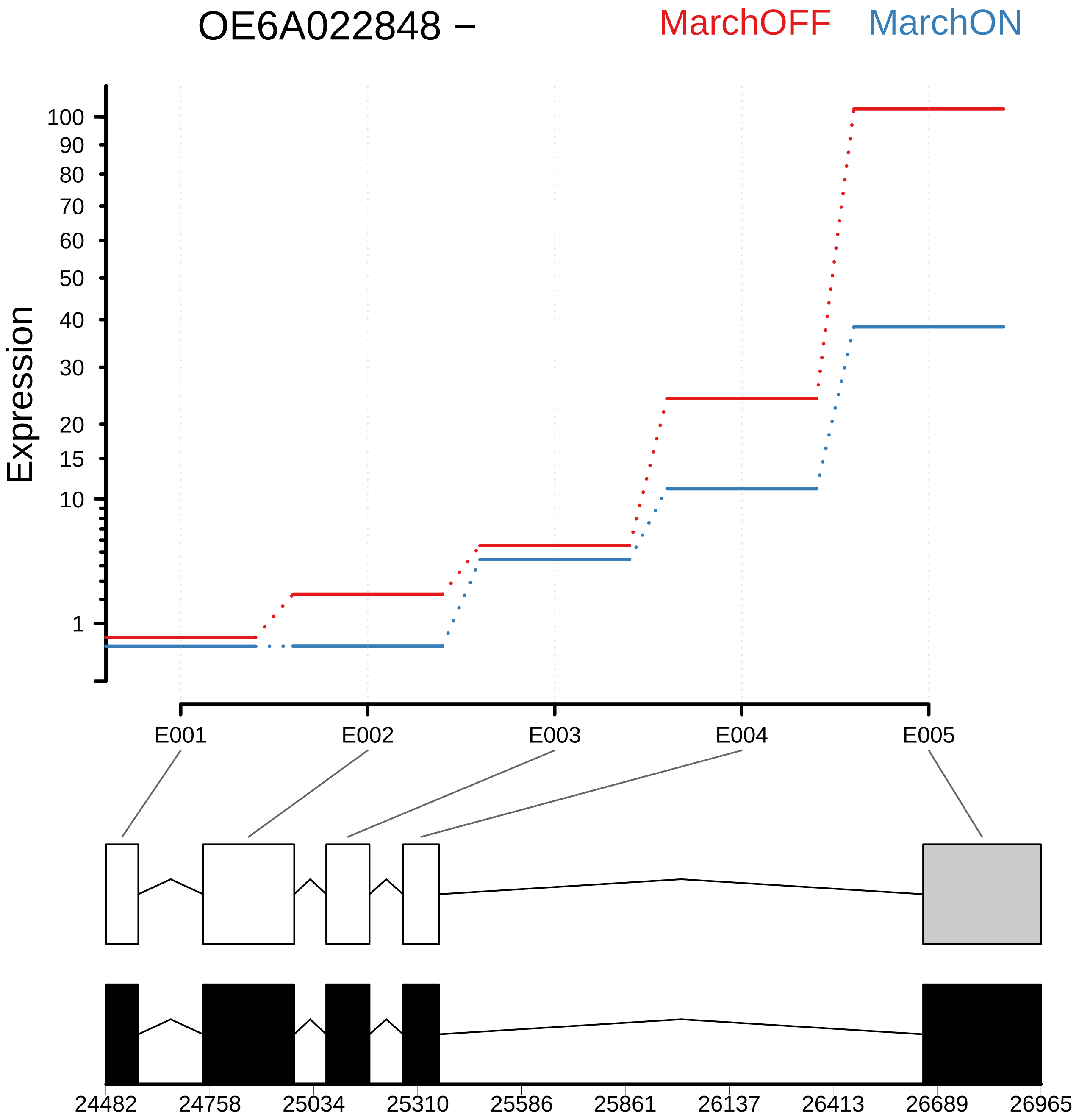

OE6A022848 −
MarchOFF
MarchON
Expression
100
90
80
70
60
50
40
30
20
15
10
1
E001
E002
E003
E004
E005
24482
24758
25034
25310
25586
25861
26137
26413
26689
26965

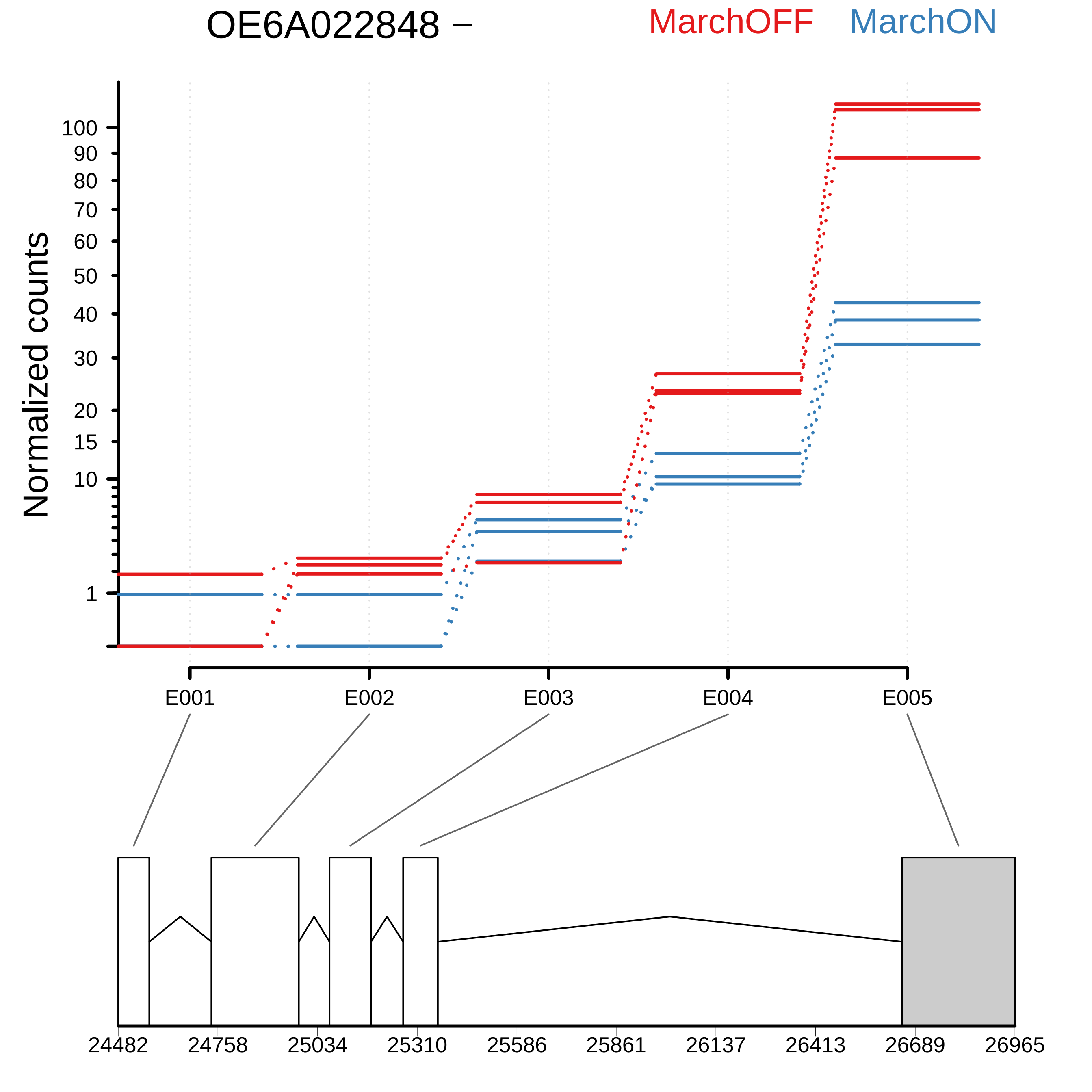
OE6A022848 −
MarchOFF
MarchON
Normalized counts
100
90
80
70
60
50
40
30
20
15
10
1
E001
E002
E003
E004
E005
24482
24758
25034
25310
25586
25861
26137
26413
26689
26965

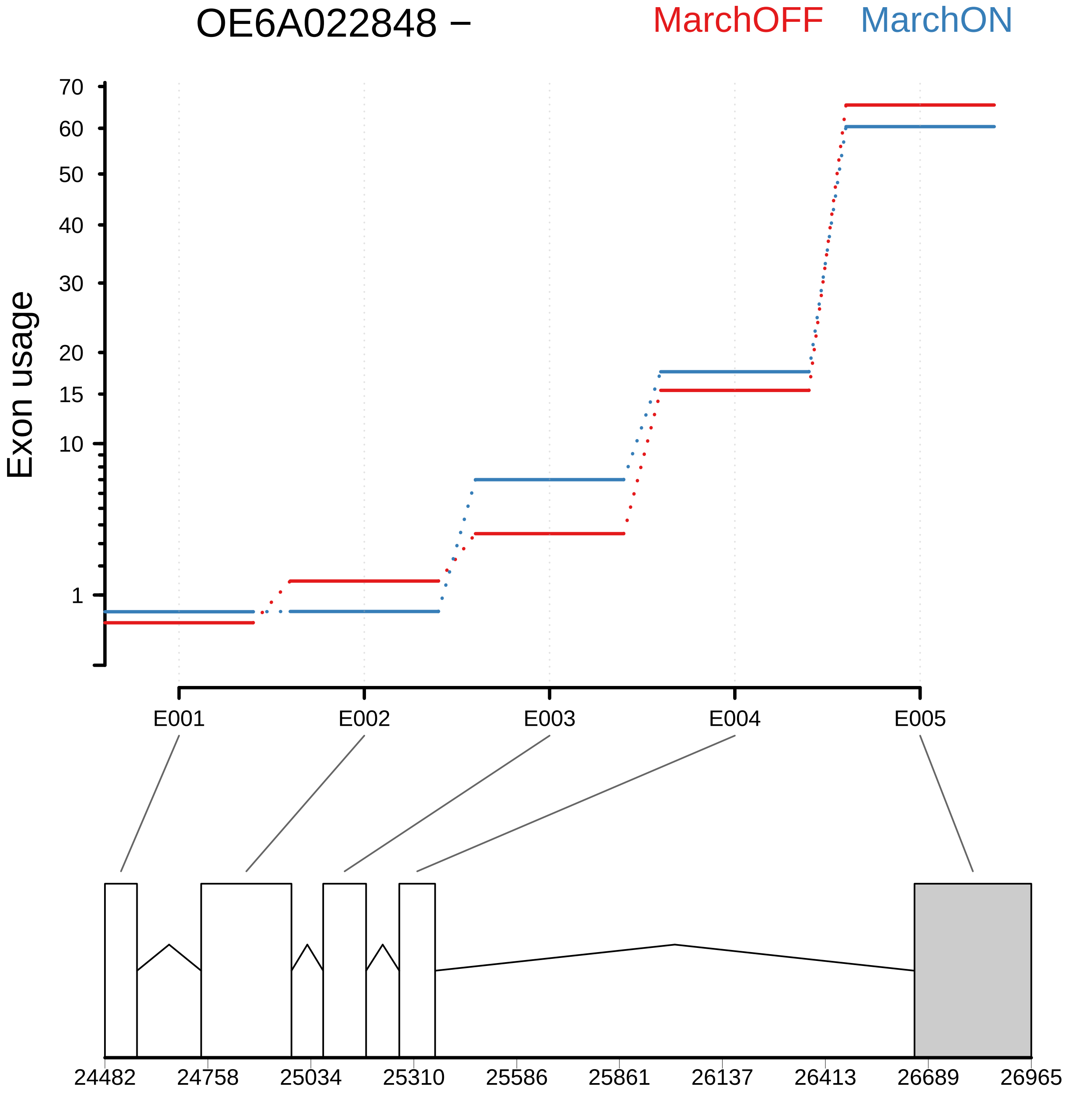

OE6A022848 −
MarchOFF
MarchON
Exon usage
70
60
50
40
30
20
15
10
1
E001
E002
E003
E004
E005
24482
24758
25034
25310
25586
25861
26137
26413
26689
26965

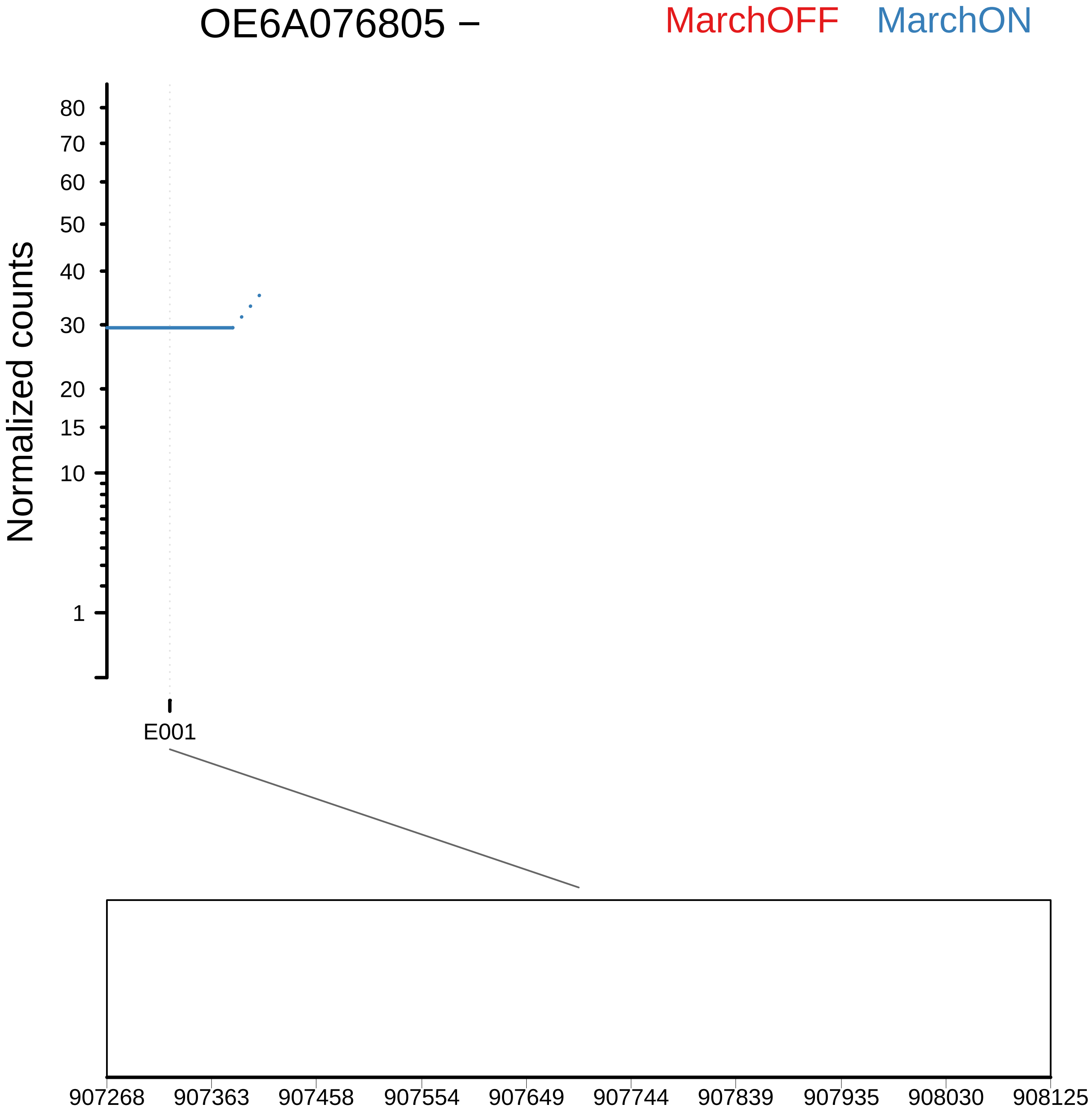
OE6A076805 −
MarchOFF
MarchON
Normalized counts
80
70
60
50
40
30
20
15
10
1
E001
907268
907363
907458
907554
907649
907744
907839
907935
908030
908125

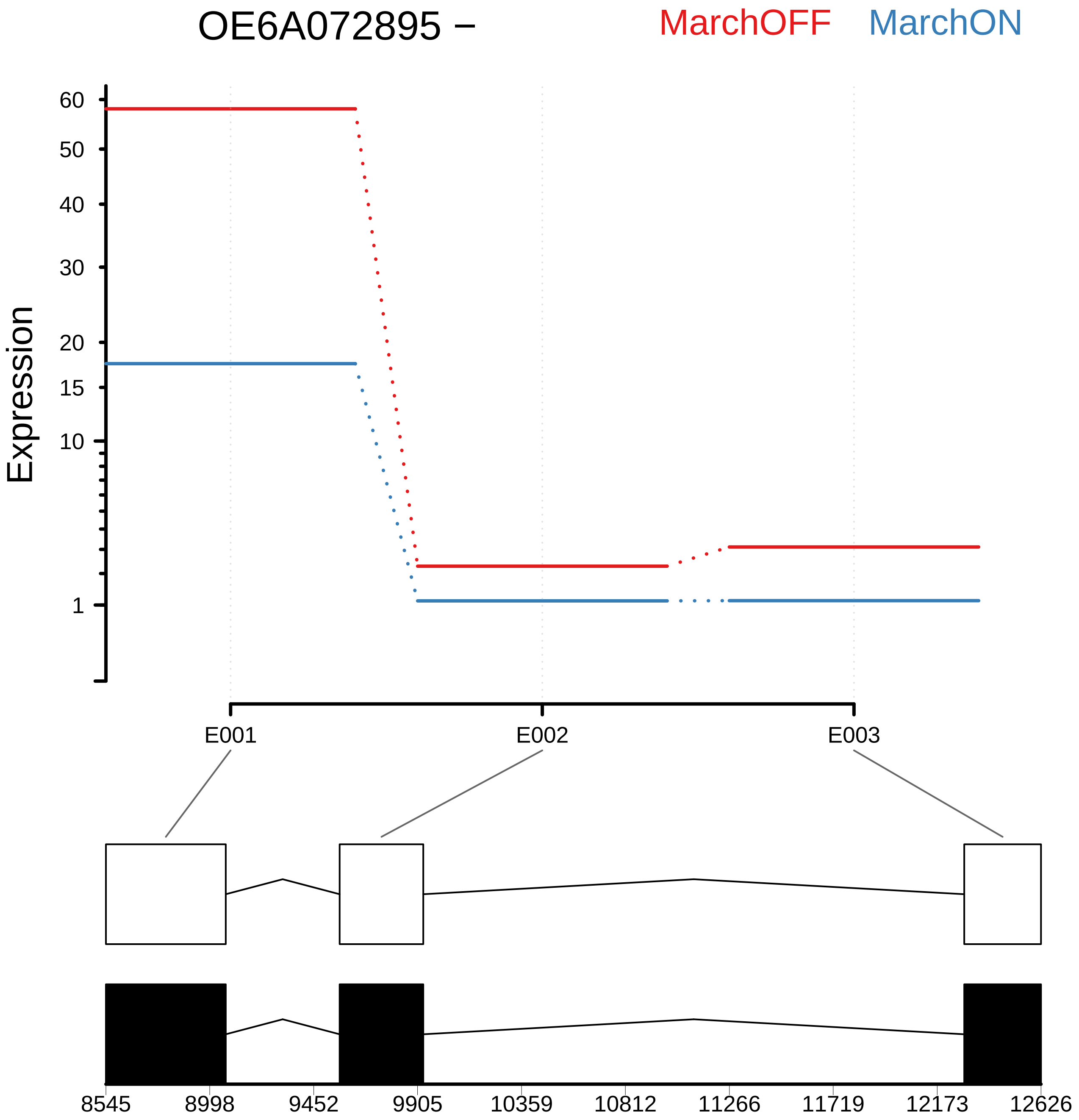

OE6A072895 −
MarchOFF
MarchON
Expression
60
50
40
30
20
15
10
1
E001
E002
E003
8545
8998
9452
9905
10359
10812
11266
11719
12173
12626

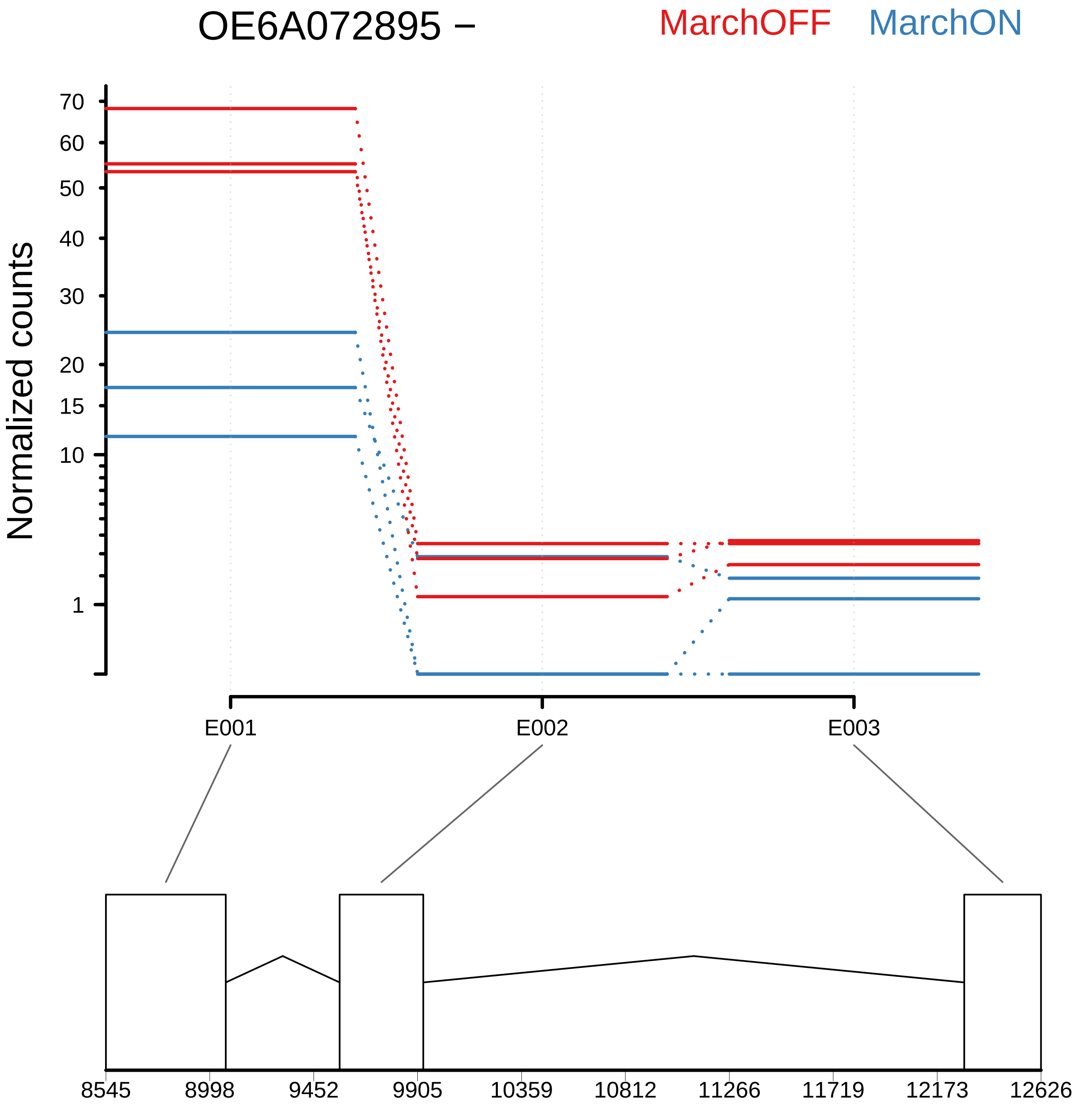

OE6A072895 −
MarchOFF
MarchON
Normalized counts
70
60
50
40
30
20
15
10
1
E001
E002
E003
8545
8998
9452
9905
10359
10812
11266
11719
12173
12626

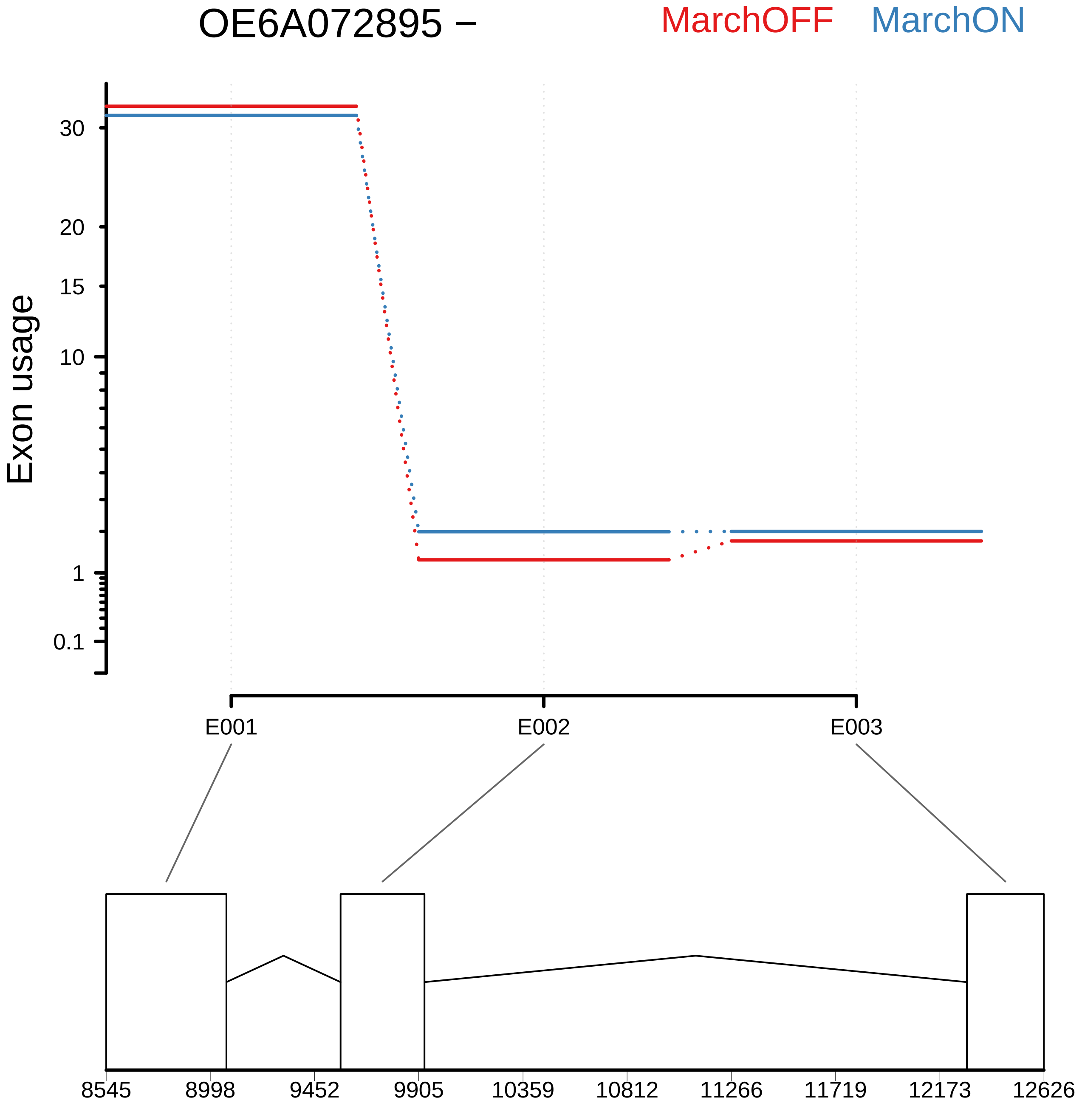
OE6A072895 −
MarchOFF
MarchON
Exon usage
30
20
15
10
1
0.1
E001
E002
E003
8545
8998
9452
9905
10359
10812
11266
11719
12173
12626

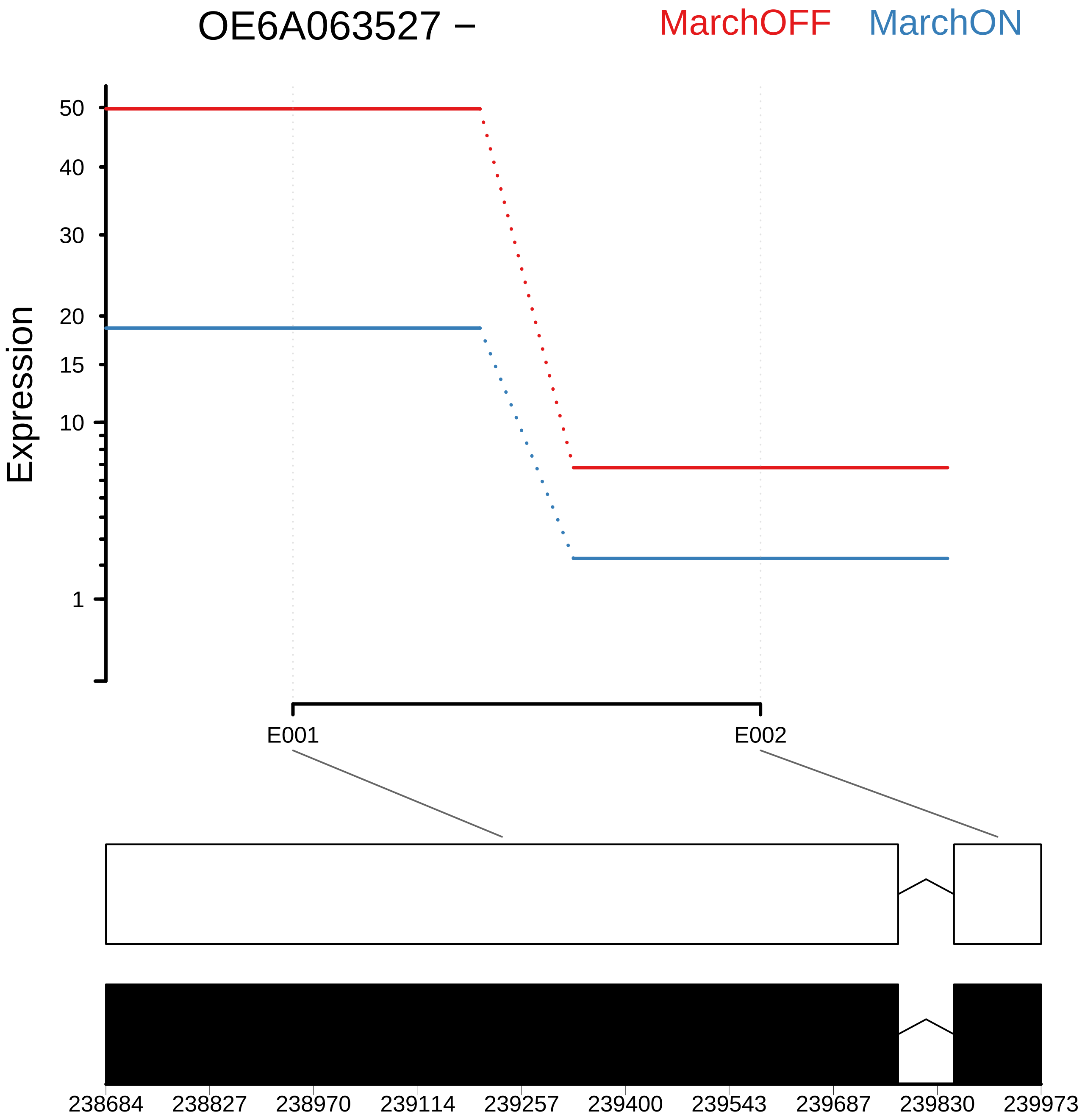

OE6A063527 −
MarchOFF
MarchON
Expression
50
40
30
20
15
10
1
E001
E002
238684
238827
238970
239114
239257
239400
239543
239687
239830
239973

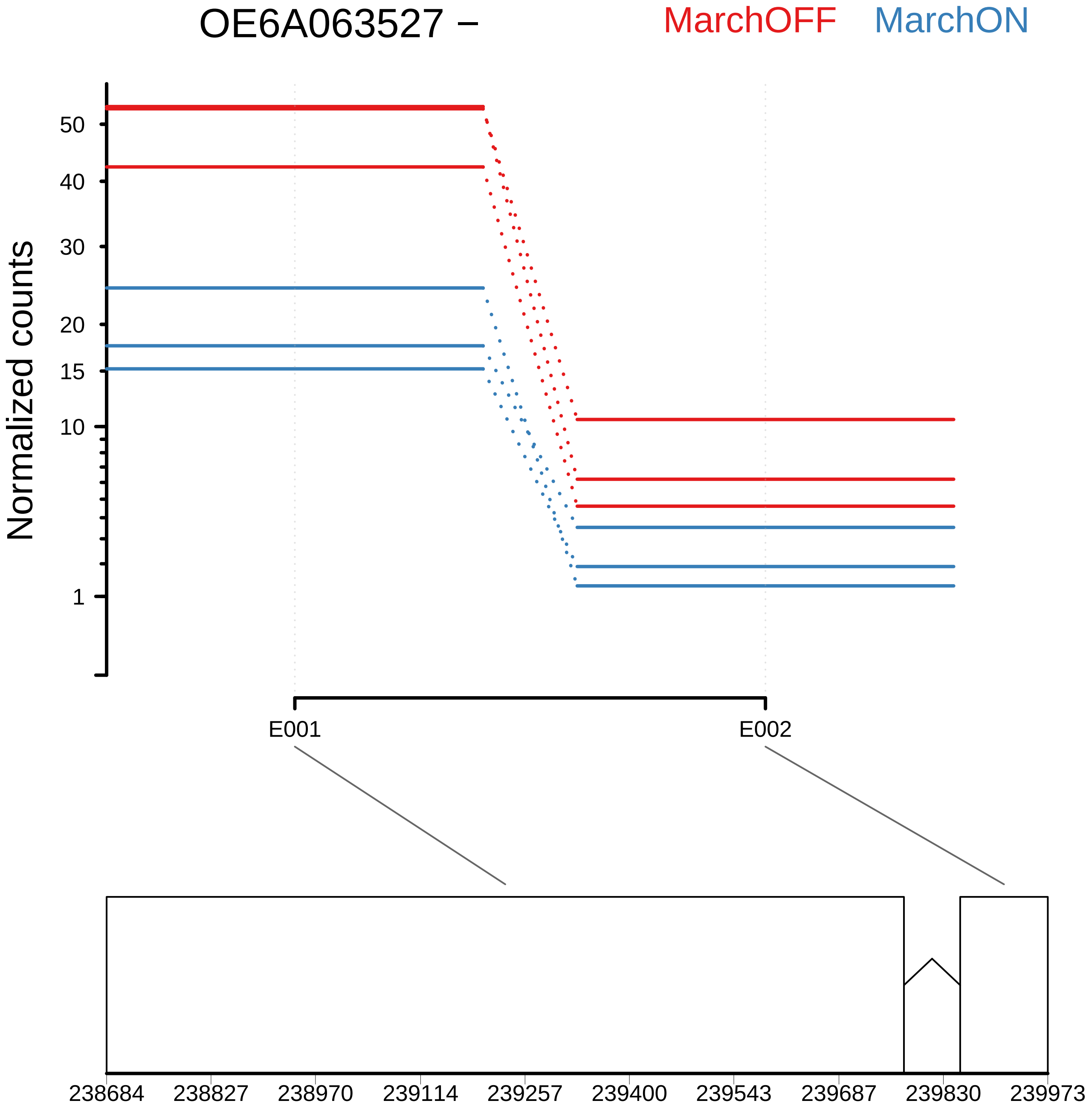
OE6A063527 −
MarchOFF
MarchON
Normalized counts
50
40
30
20
15
10
1
E001
E002
238684
238827
238970
239114
239257
239400
239543
239687
239830
239973

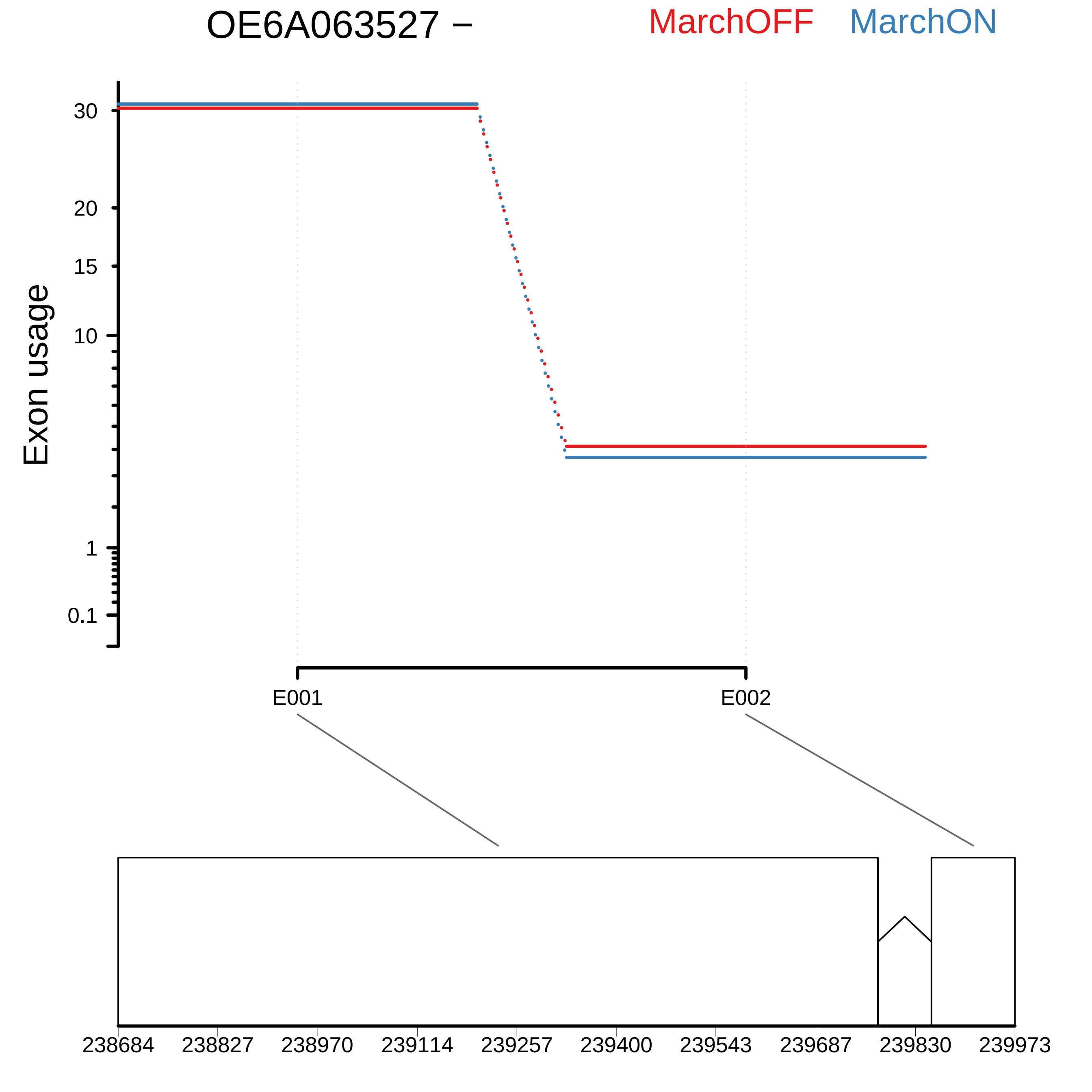

OE6A063527 −
MarchOFF
MarchON
Exon usage
30
20
15
10
1
0.1
E001
E002
238684
238827
238970
239114
239257
239400
239543
239687
239830
239973

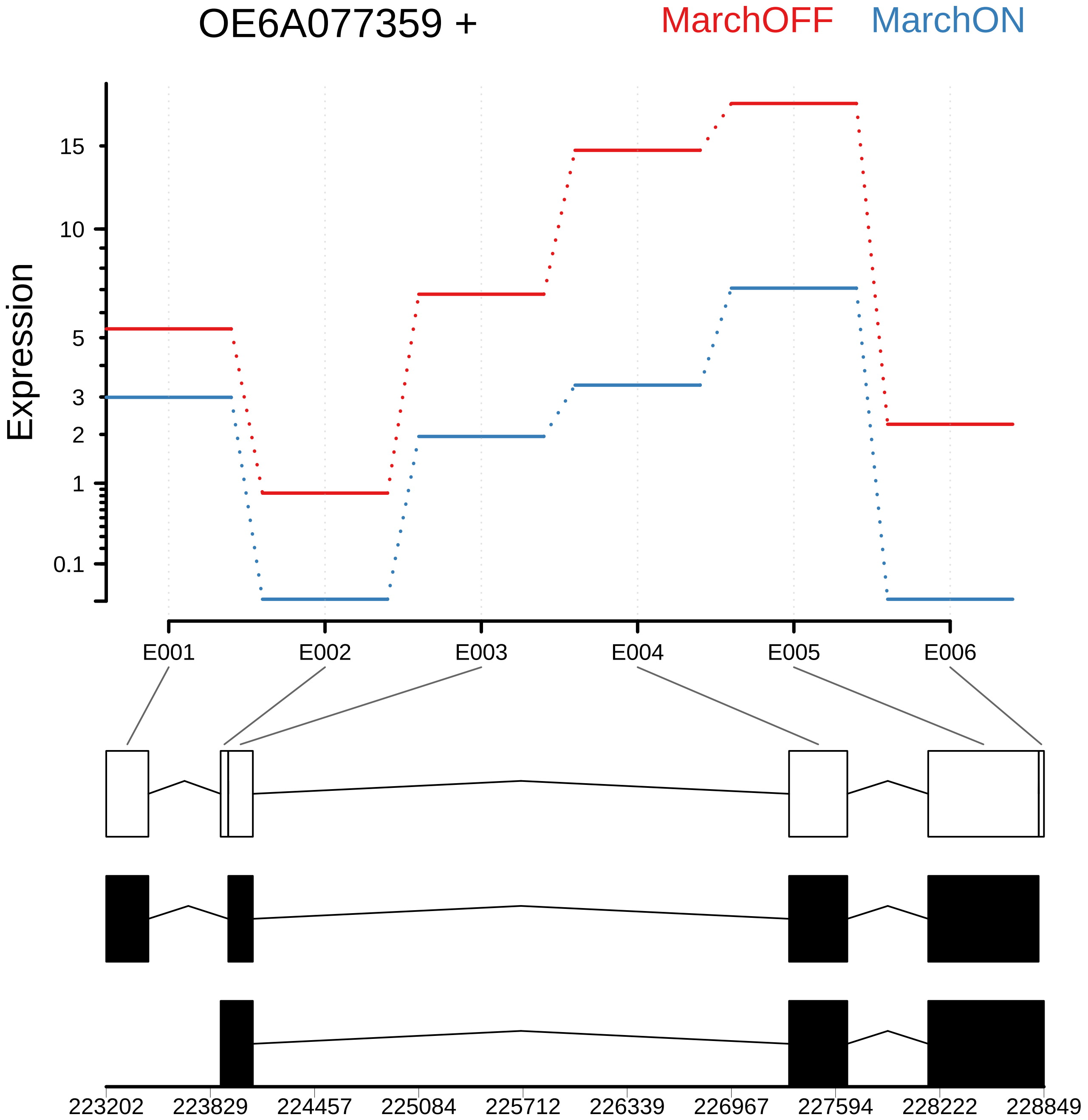

OE6A077359 +
MarchOFF
MarchON
Expression
15
10
5
3
2
1
0.1
E001
E002
E003
E004
E005
E006
223202
223829
224457
225084
225712
226339
226967
227594
228222
228849

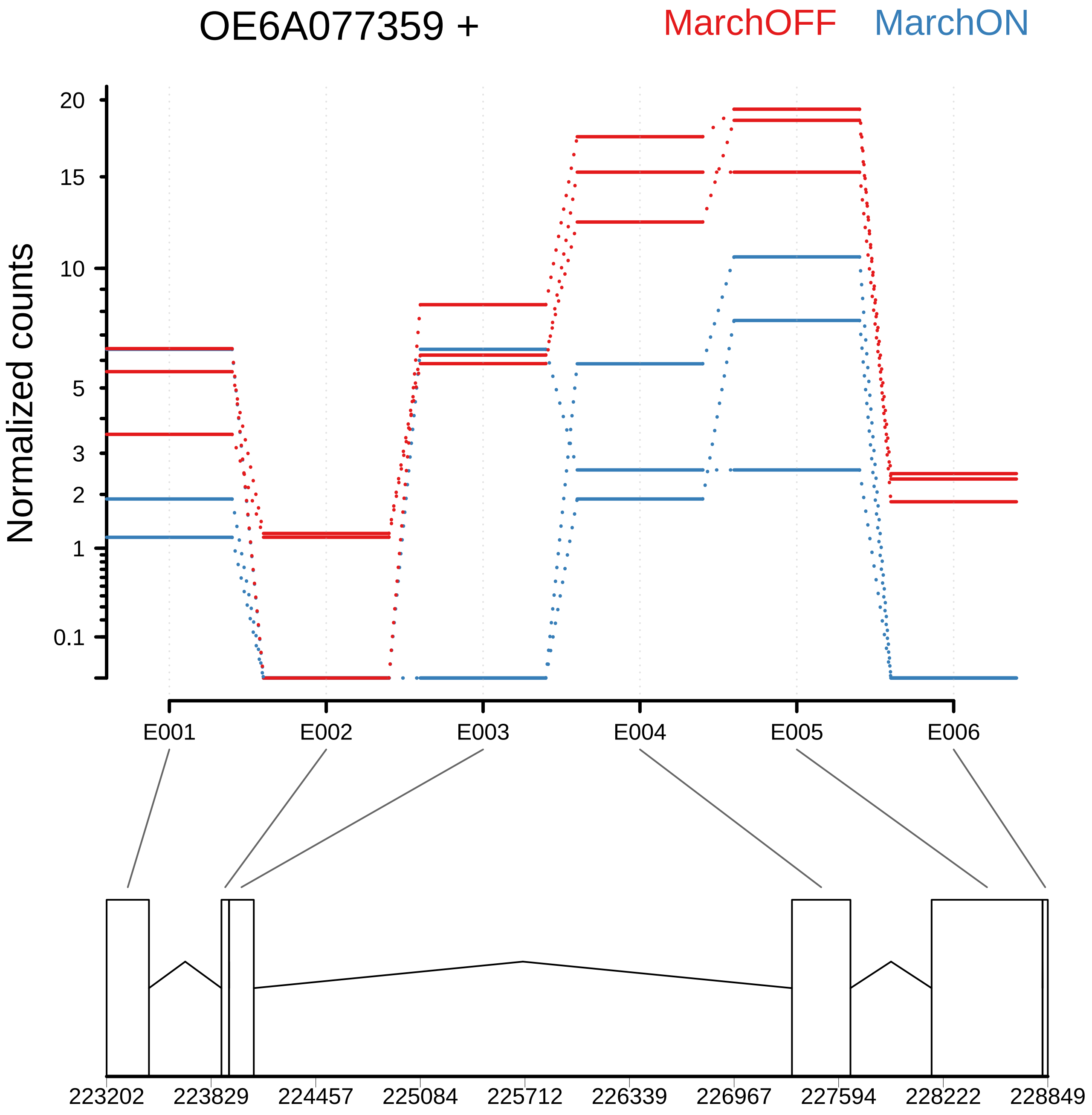
OE6A077359 +
MarchOFF
MarchON
Normalized counts
20
15
10
5
3
2
1
0.1
E001
E002
E003
E004
E005
E006
223202
223829
224457
225084
225712
226339
226967
227594
228222
228849

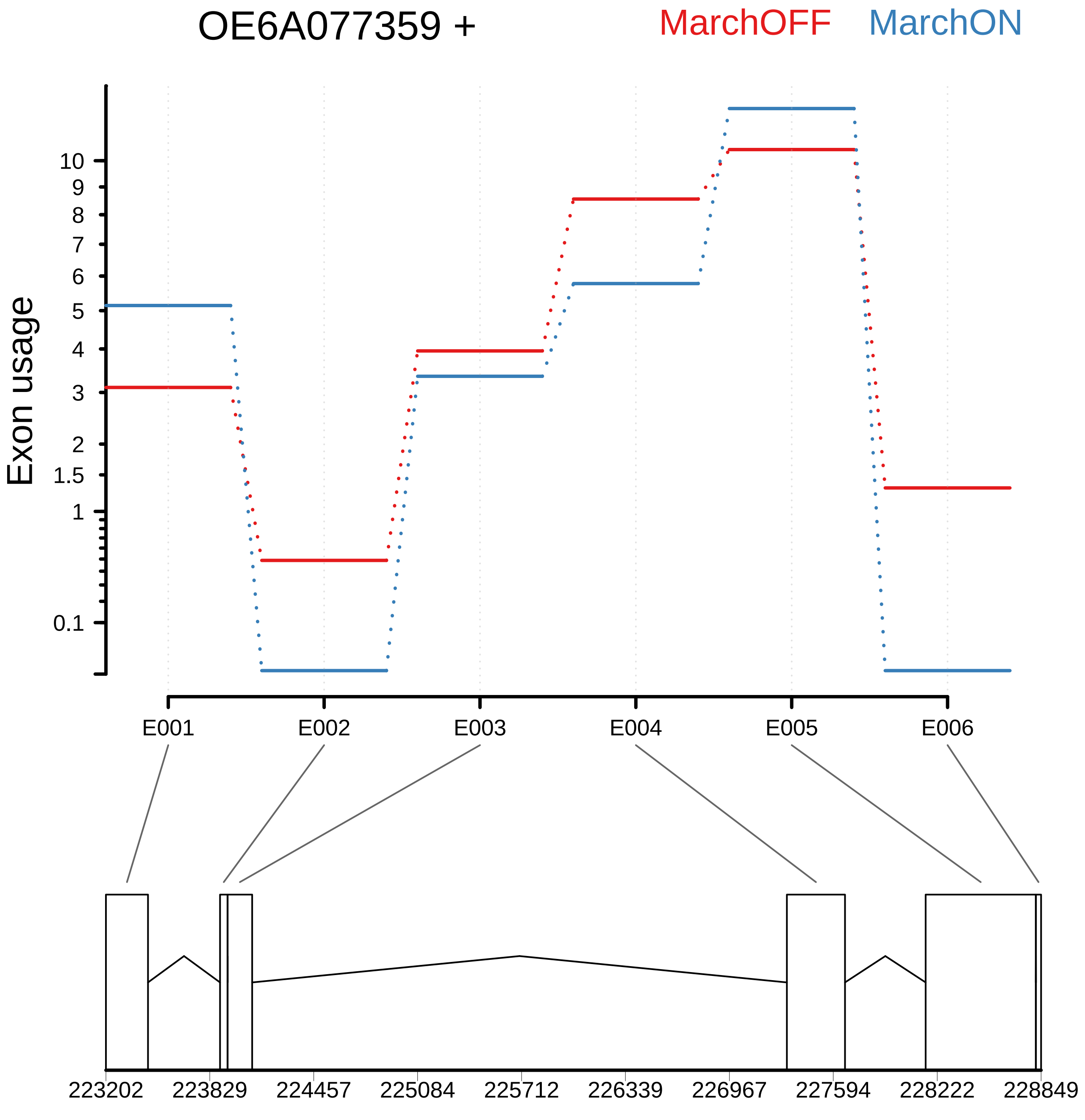

OE6A077359 +
MarchOFF
MarchON
Exon usage
10
9
8
7
6
5
4
3
2
1.5
1
0.1
E001
E002
E003
E004
E005
E006
223202
223829
224457
225084
225712
226339
226967
227594
228222
228849

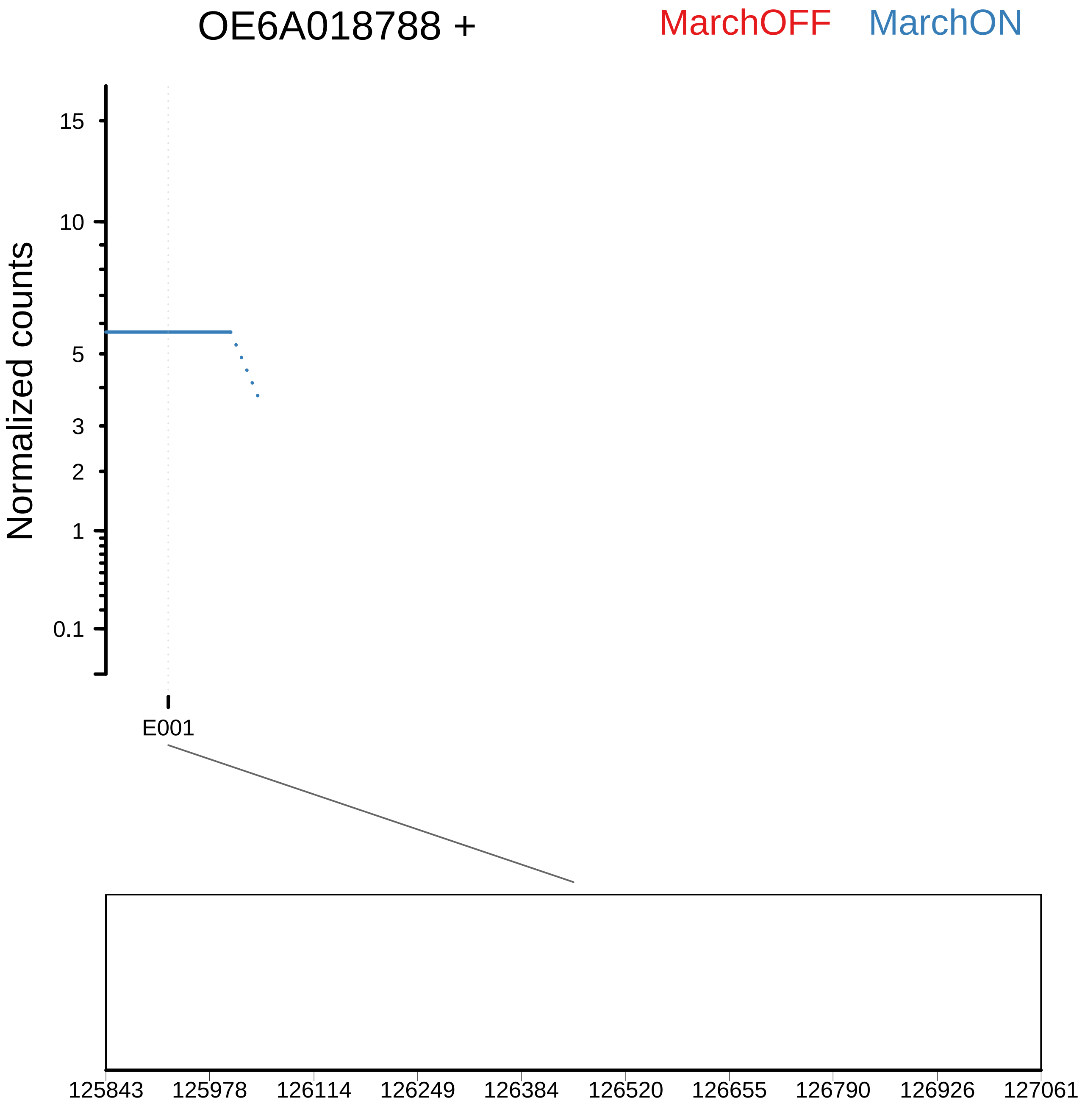

OE6A018788 +
MarchOFF
MarchON
Normalized counts
15
10
5
3
2
1
0.1
E001
125843
125978
126114
126249
126384
126520
126655
126790
126926
127061

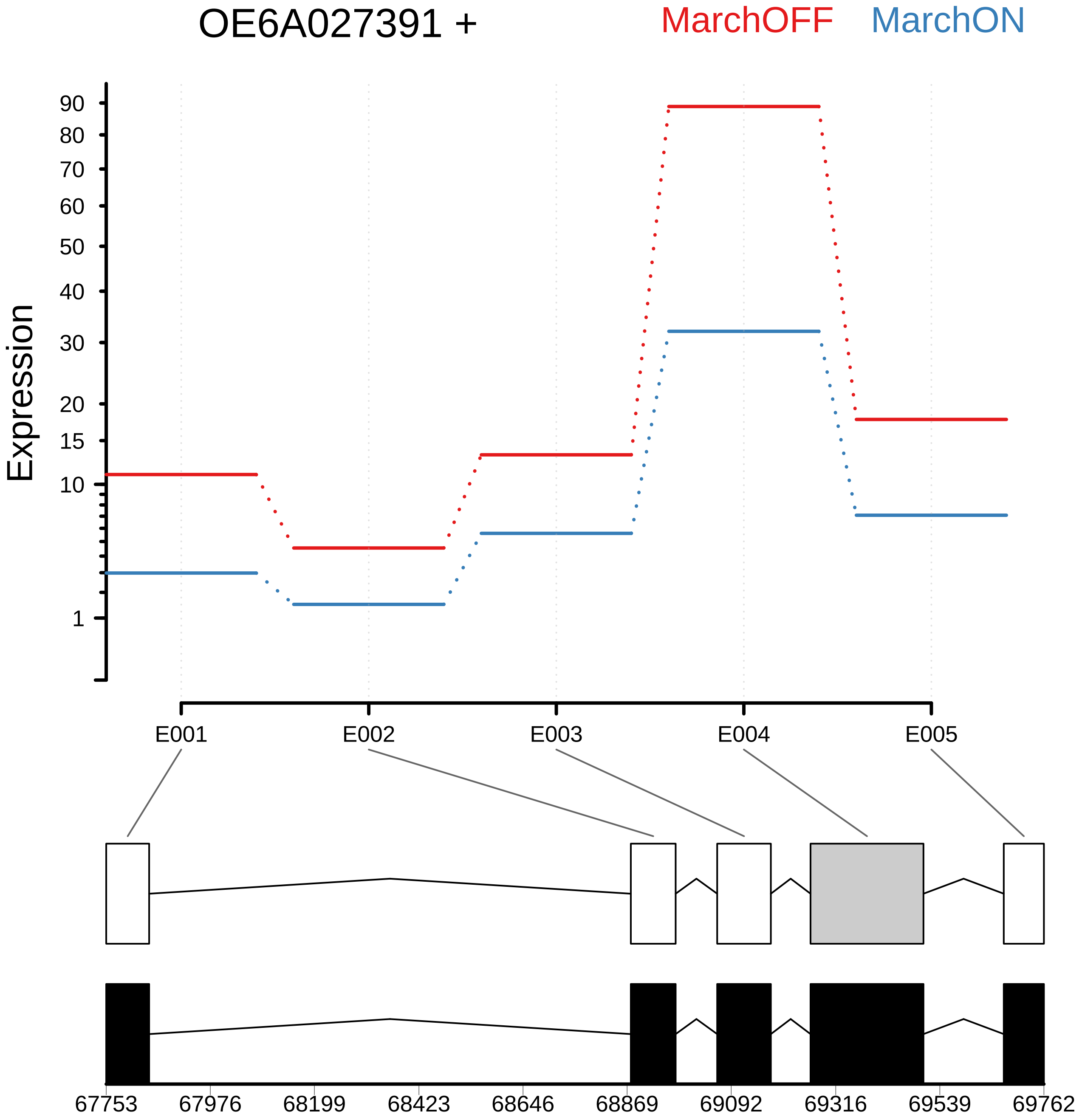
OE6A027391 +
MarchOFF
MarchON
Expression
90
80
70
60
50
40
30
20
15
10
1
E001
E002
E003
E004
E005
67753
67976
68199
68423
68646
68869
69092
69316
69539
69762

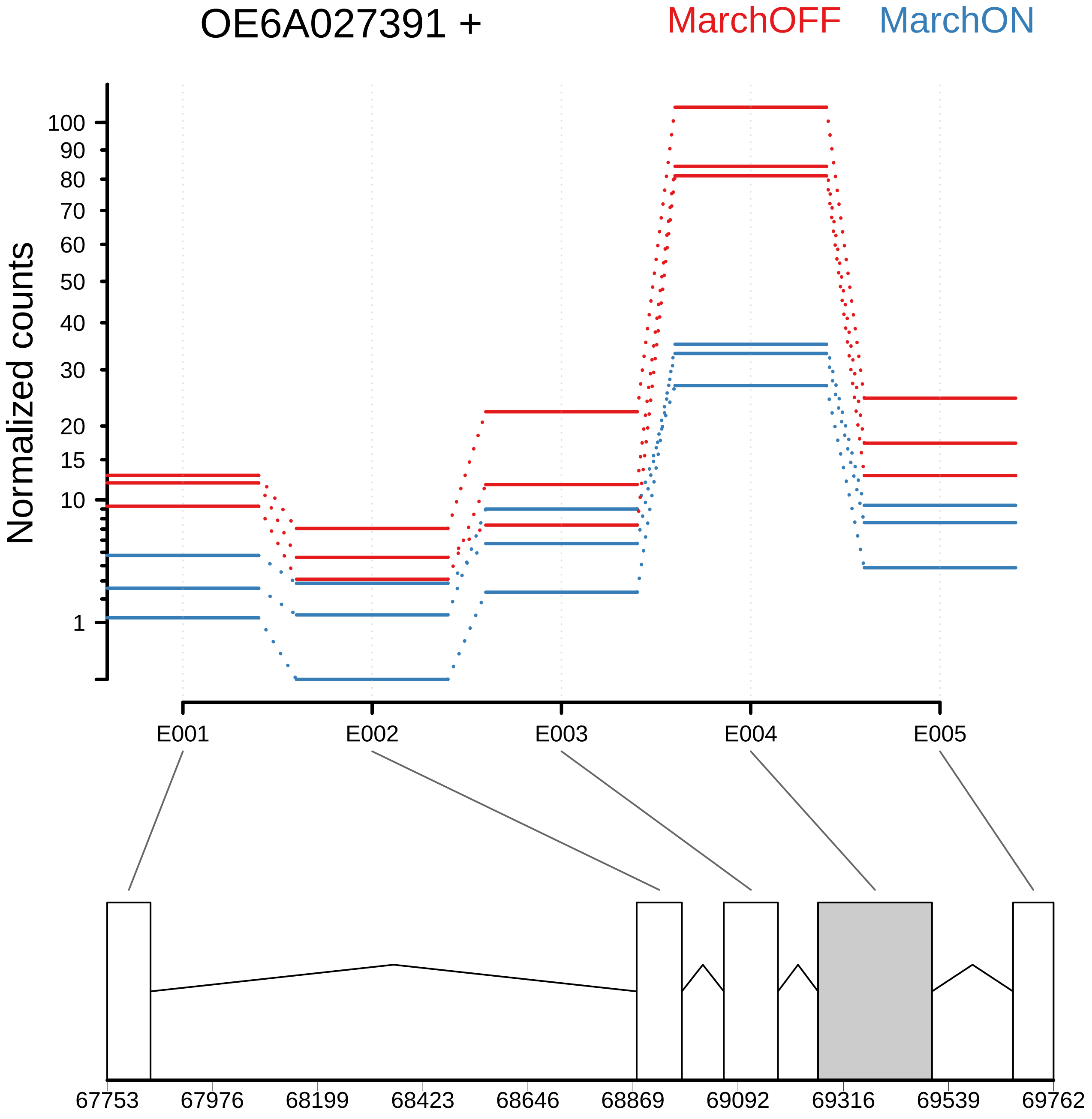
OE6A027391 +
MarchOFF
MarchON
Normalized counts
100
90
80
70
60
50
40
30
20
15
10
1
E001
E002
E003
E004
E005
67753
67976
68199
68423
68646
68869
69092
69316
69539
69762

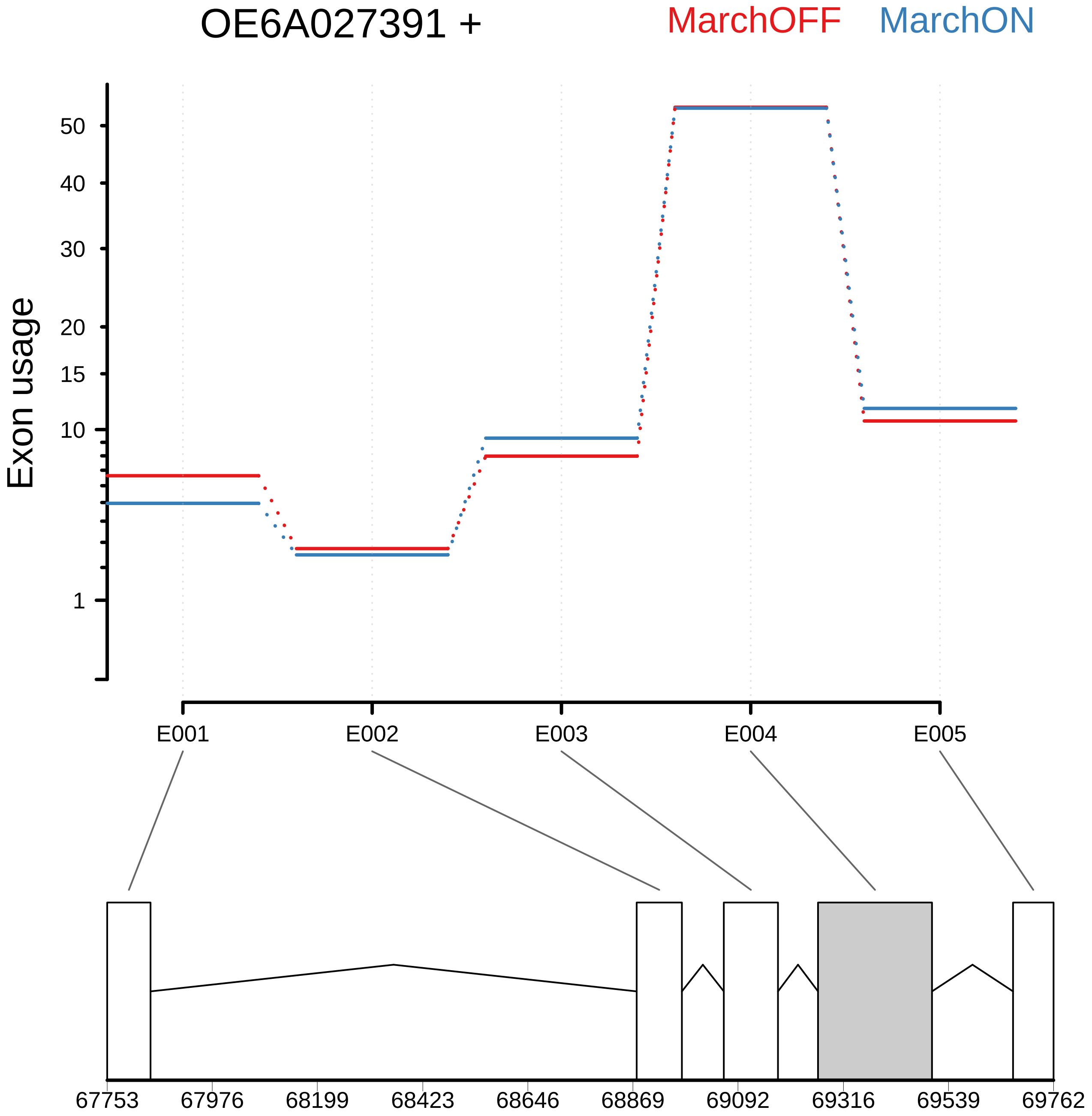

OE6A027391 +
MarchOFF
MarchON
Exon usage
50
40
30
20
15
10
1
E001
E002
E003
E004
E005
67753
67976
68199
68423
68646
68869
69092
69316
69539
69762

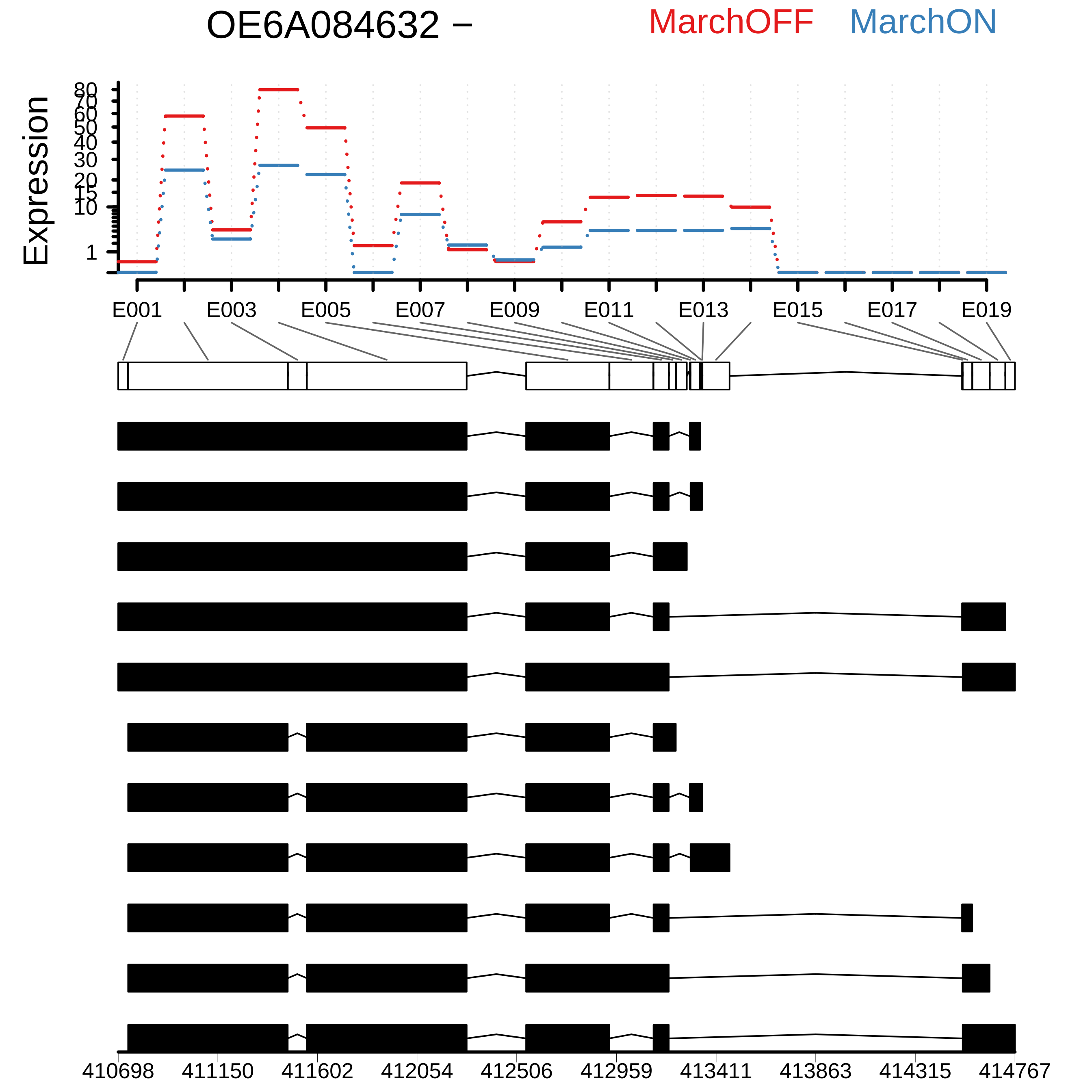

OE6A084632 −
MarchOFF
MarchON
Expression
80
70
60
50
40
30
20
15
10
1
E001
E003
E005
E007
E009
E011
E013
E015
E017
E019
410698
411150
411602
412054
412506
412959
413411
413863
414315
414767

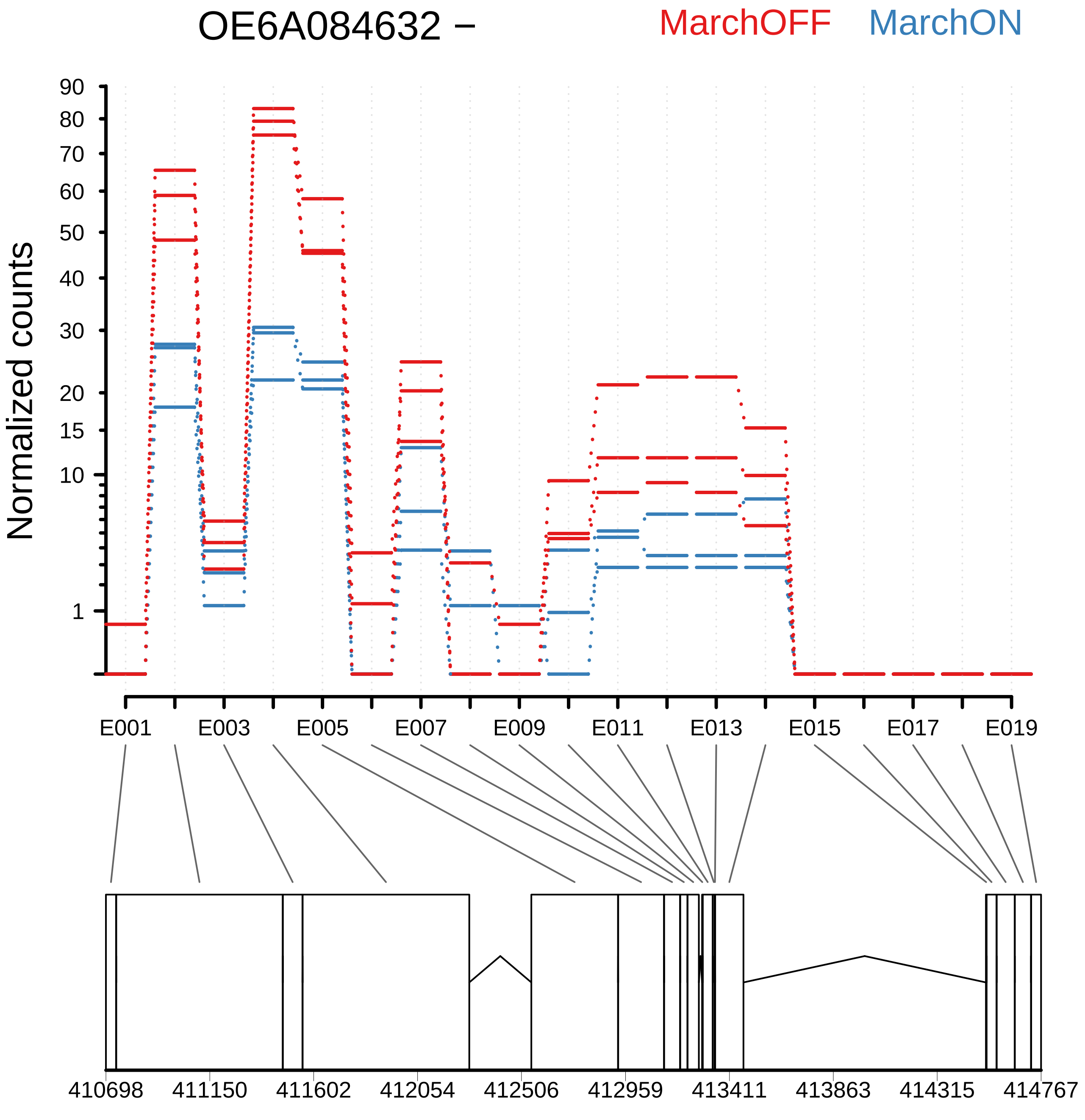
OE6A084632 −
MarchOFF
MarchON
Normalized counts
90
80
70
60
50
40
30
20
15
10
1
E001
E003
E005
E007
E009
E011
E013
E015
E017
E019
410698
411150
411602
412054
412506
412959
413411
413863
414315
414767

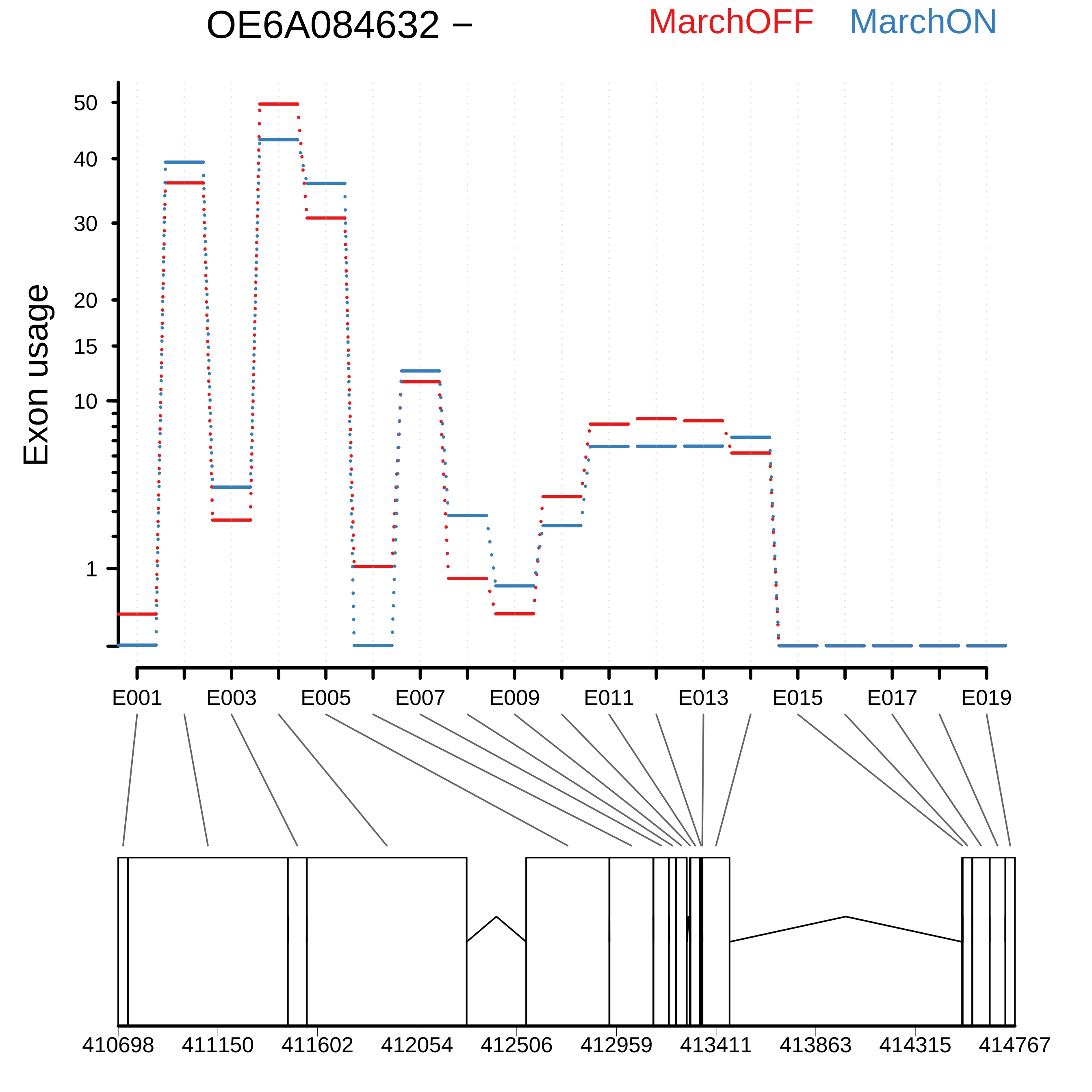

OE6A084632 −
MarchOFF
MarchON
Exon usage
50
40
30
20
15
10
1
E001
E003
E005
E007
E009
E011
E013
E015
E017
E019
410698
411150
411602
412054
412506
412959
413411
413863
414315
414767

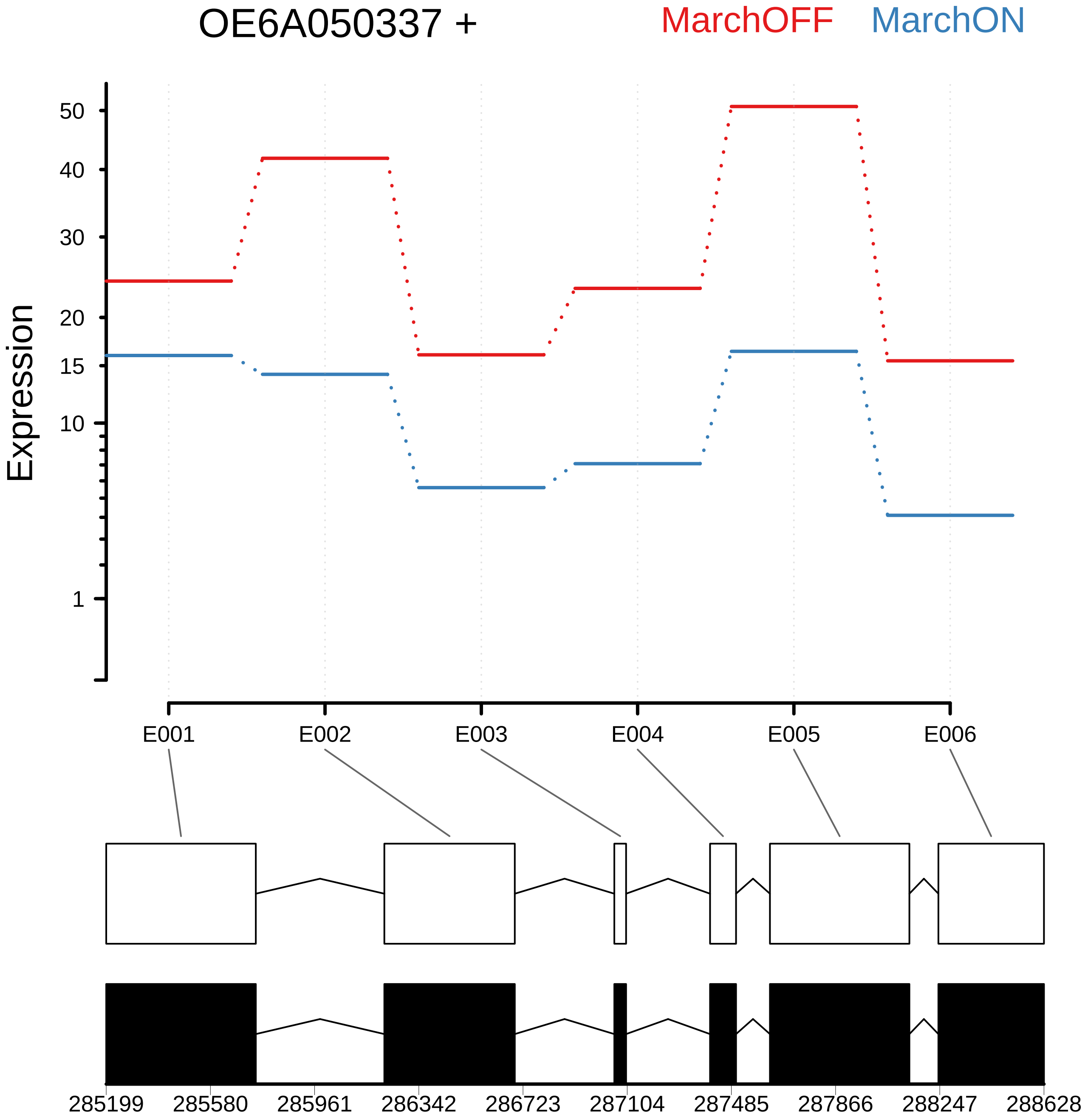

OE6A050337 +
MarchOFF
MarchON
Expression
50
40
30
20
15
10
1
E001
E002
E003
E004
E005
E006
285199
285580
285961
286342
286723
287104
287485
287866
288247
288628

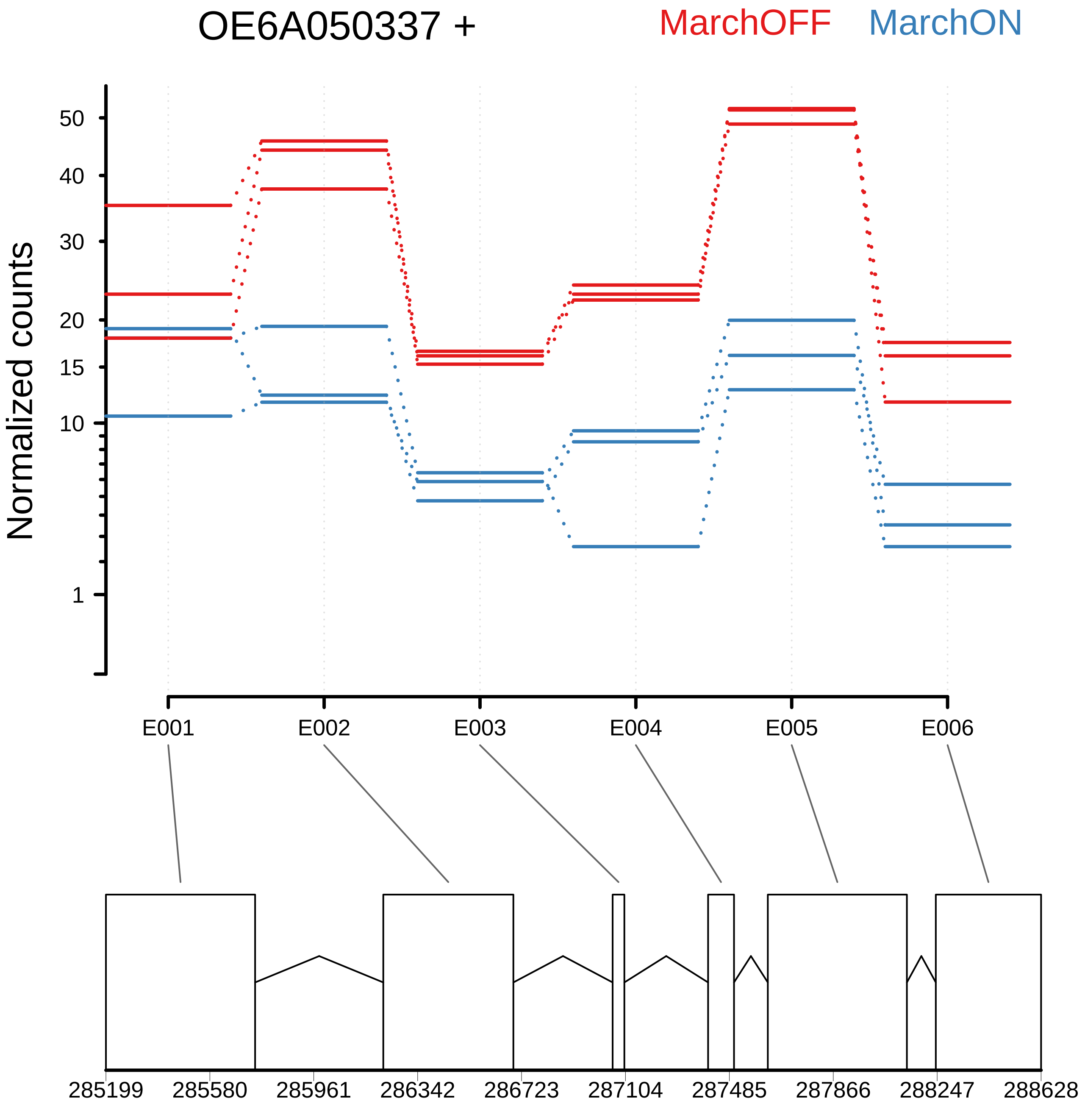

OE6A050337 +
MarchOFF
MarchON
Normalized counts
50
40
30
20
15
10
1
E001
E002
E003
E004
E005
E006
285199
285580
285961
286342
286723
287104
287485
287866
288247
288628

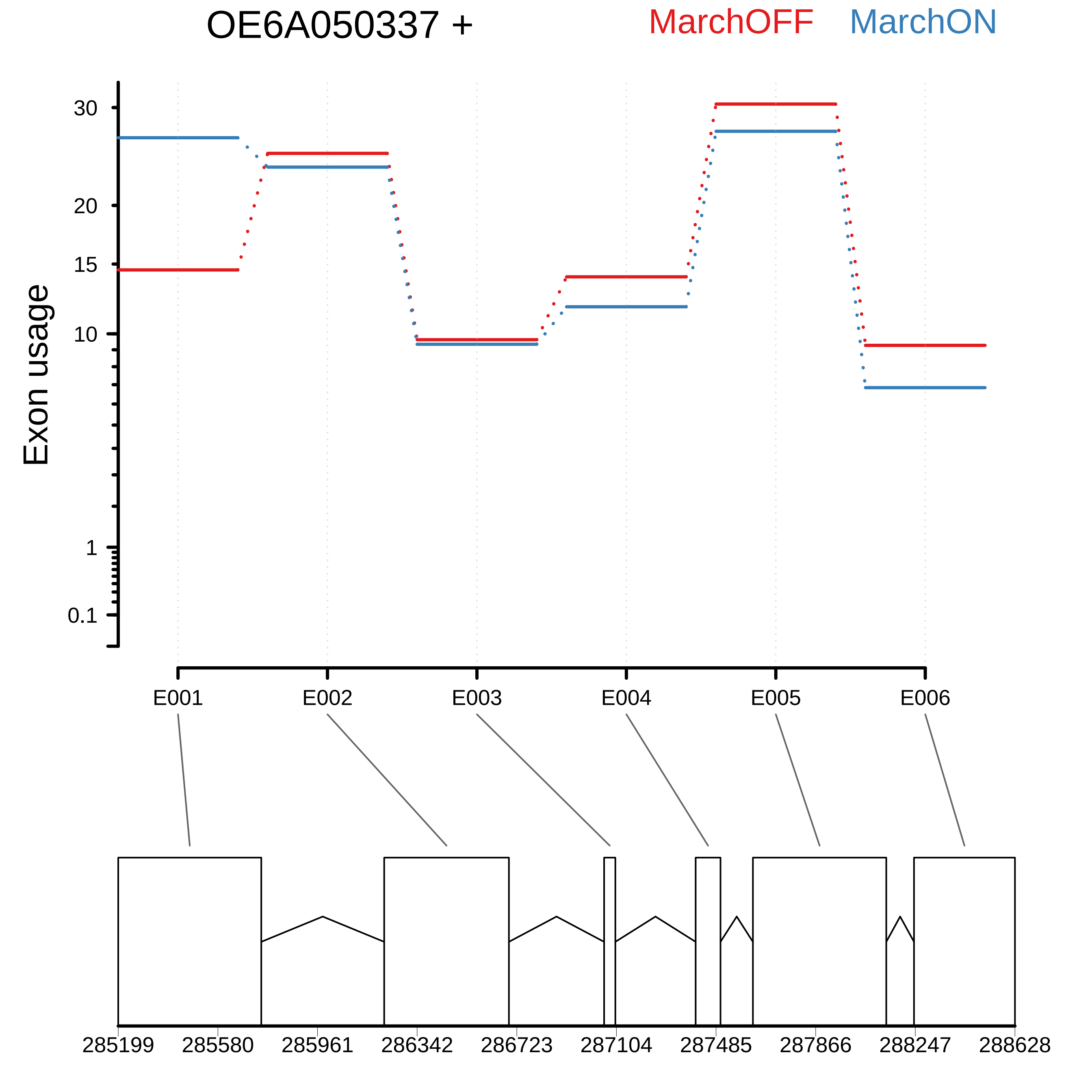

OE6A050337 +
MarchOFF
MarchON
Exon usage
30
20
15
10
1
0.1
E001
E002
E003
E004
E005
E006
285199
285580
285961
286342
286723
287104
287485
287866
288247
288628

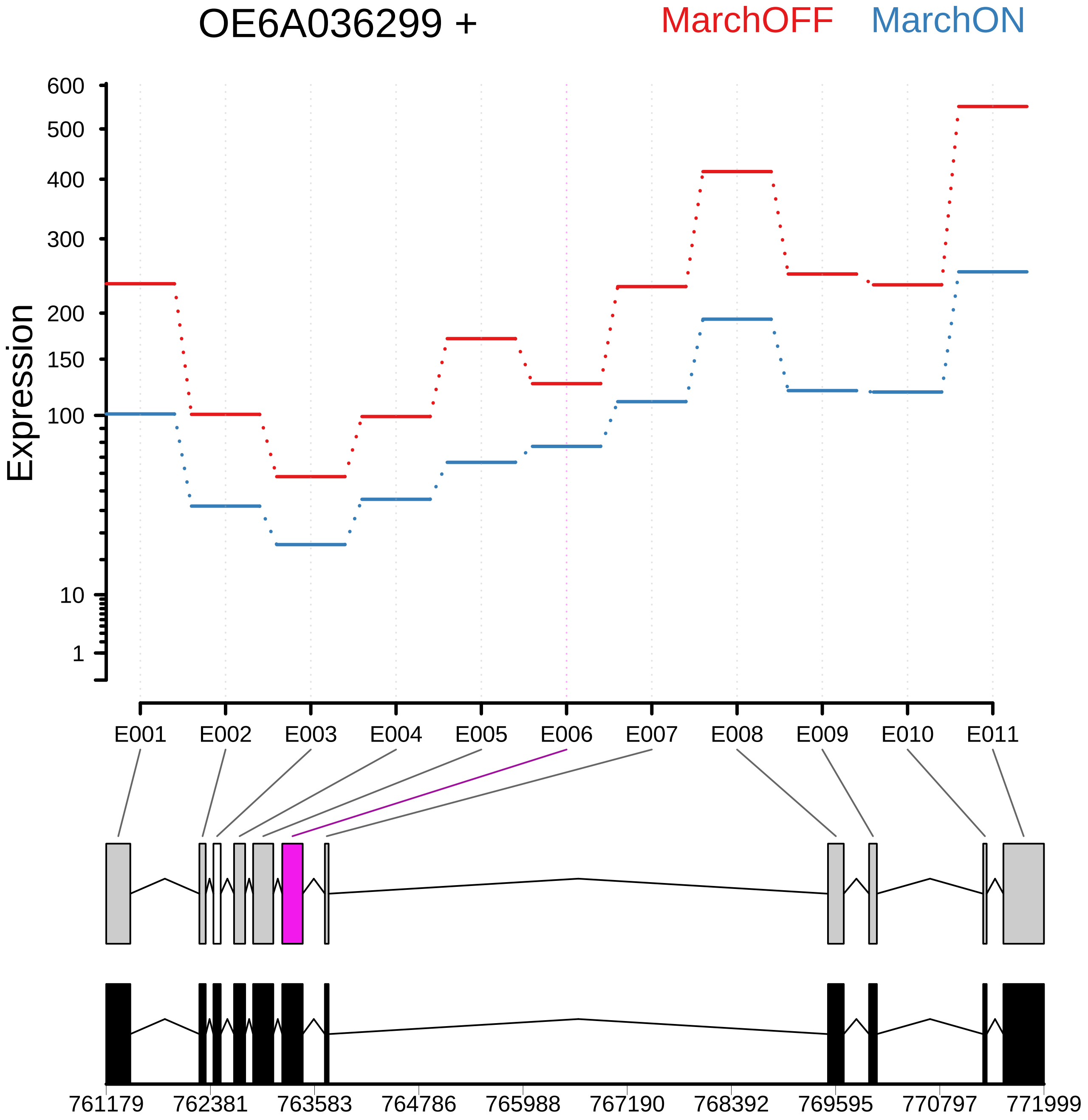

OE6A036299 +
MarchOFF
MarchON
Expression
600
500
400
300
200
150
100
10
1
E001
E002
E003
E004
E005
E006
E007
E008
E009
E010
E011
761179
762381
763583
764786
765988
767190
768392
769595
770797
771999

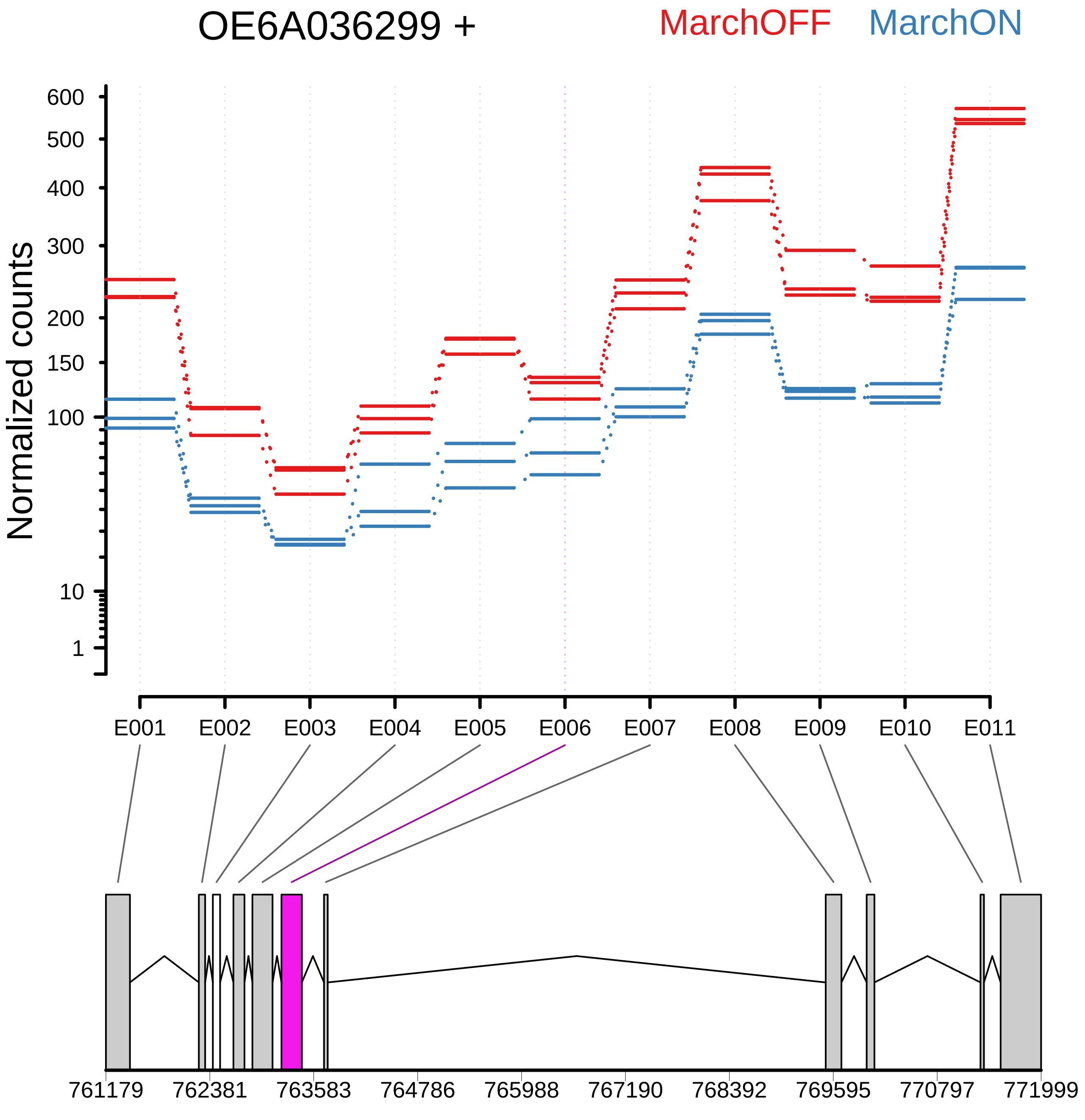

OE6A036299 +
MarchOFF
MarchON
Normalized counts
600
500
400
300
200
150
100
10
1
E001
E002
E003
E004
E005
E006
E007
E008
E009
E010
E011
761179
762381
763583
764786
765988
767190
768392
769595
770797
771999

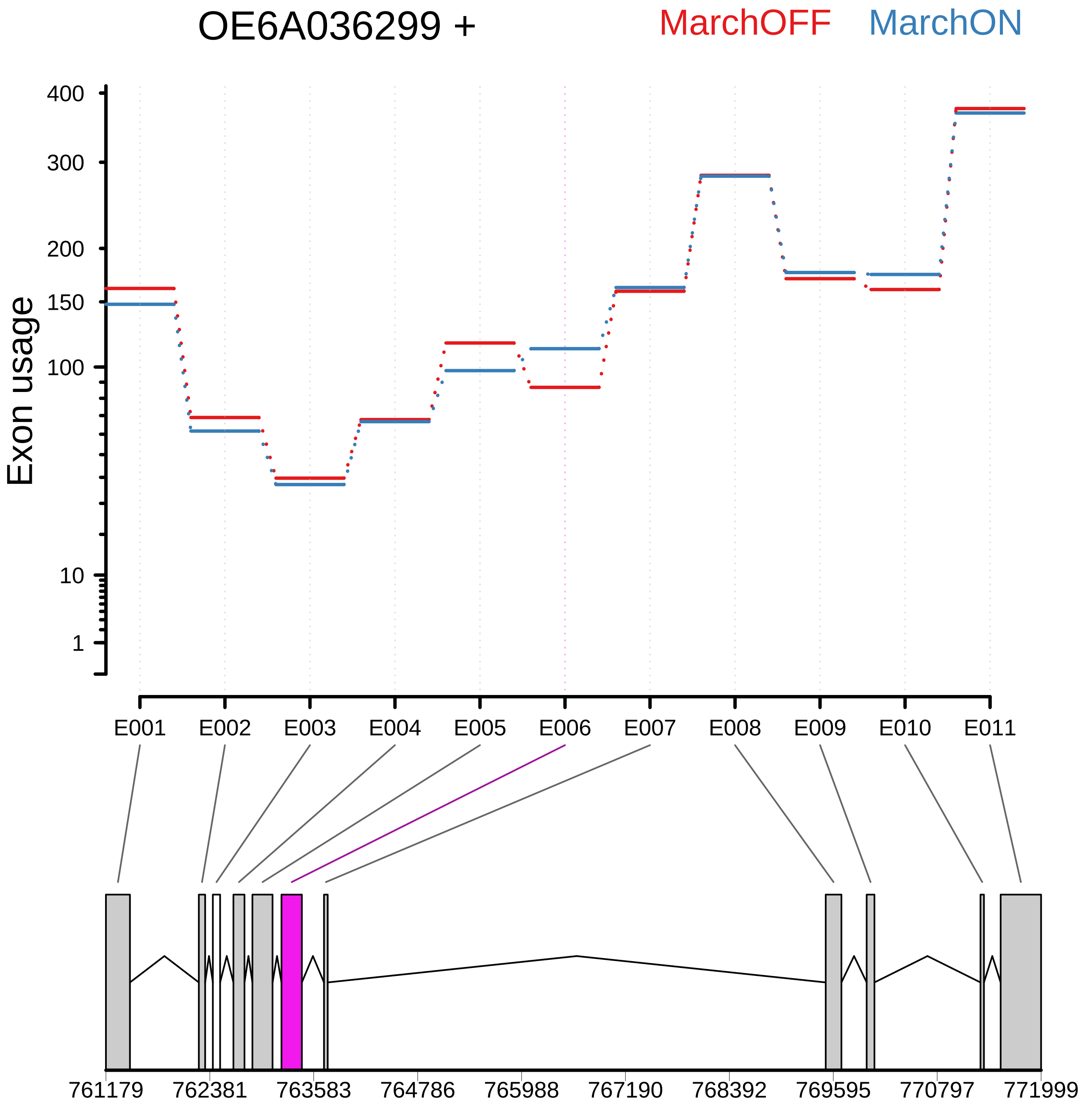
OE6A036299 +
MarchOFF
MarchON
Exon usage
400
300
200
150
100
10
1
E001
E002
E003
E004
E005
E006
E007
E008
E009
E010
E011
761179
762381
763583
764786
765988
767190
768392
769595
770797
771999

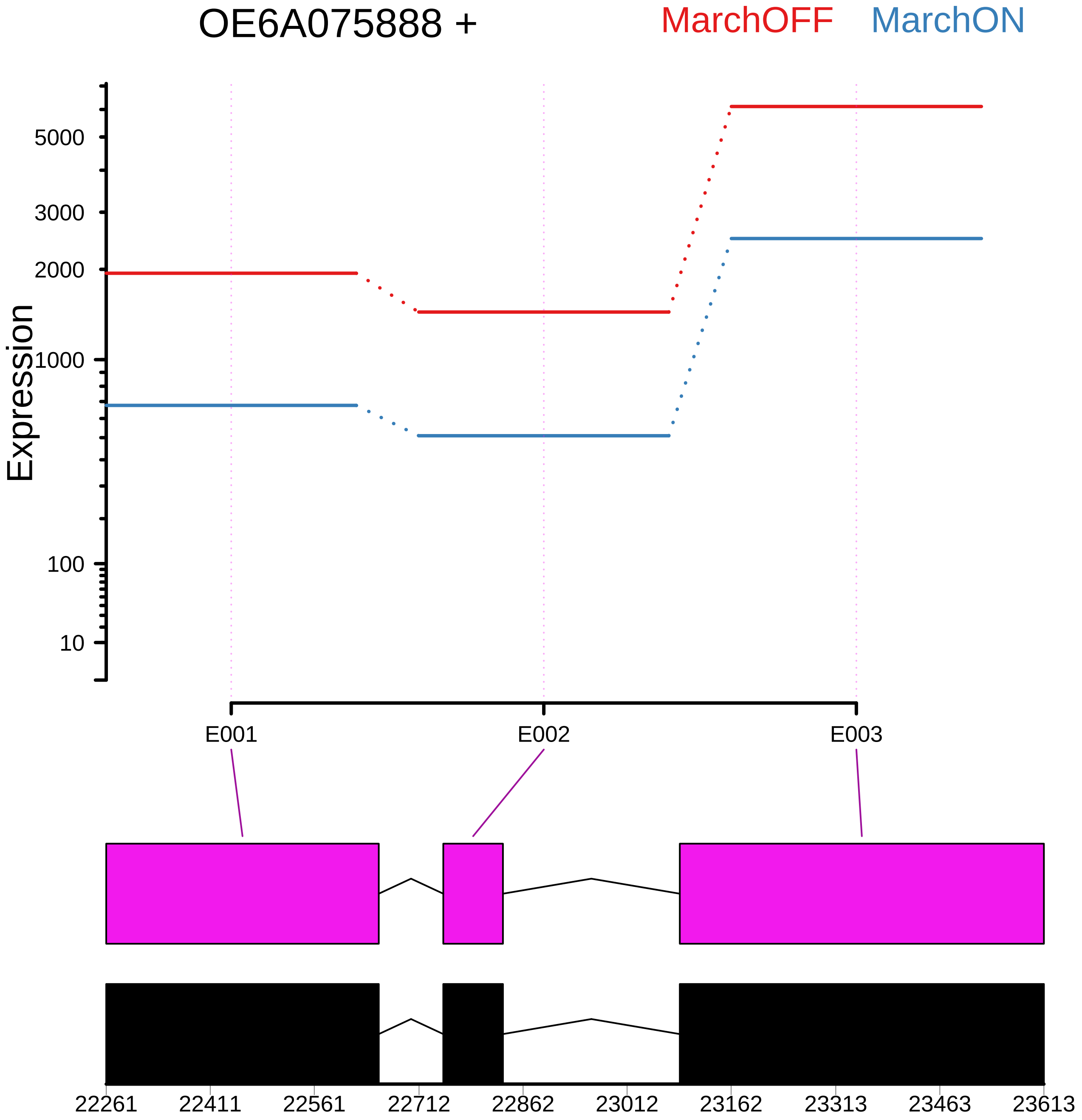
OE6A075888 +
MarchOFF
MarchON
Expression
5000
3000
2000
1000
100
10
E001
E002
E003
22261
22411
22561
22712
22862
23012
23162
23313
23463
23613

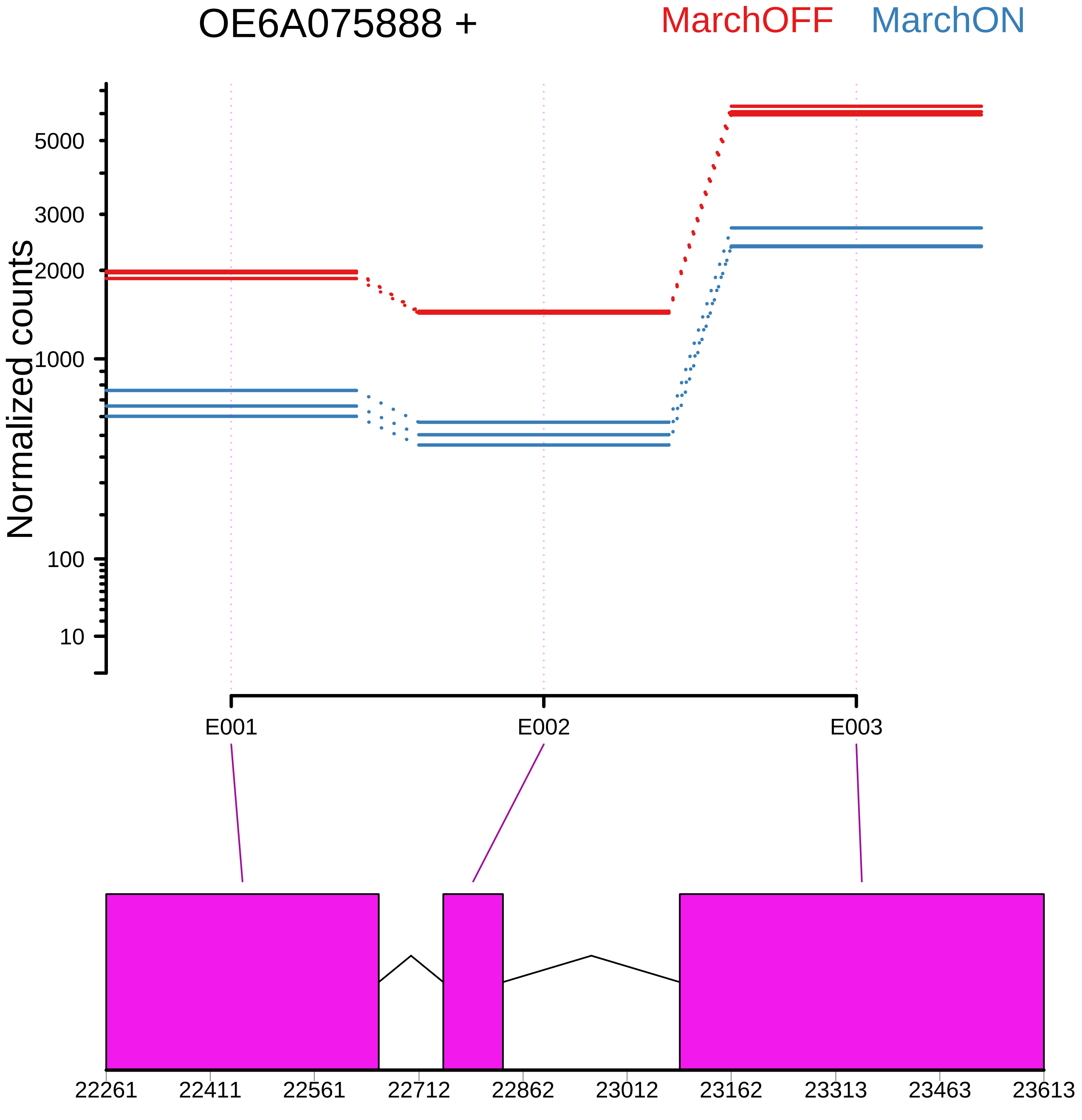
OE6A075888 +
MarchOFF
MarchON
Normalized counts
5000
3000
2000
1000
100
10
E001
E002
E003
22261
22411
22561
22712
22862
23012
23162
23313
23463
23613

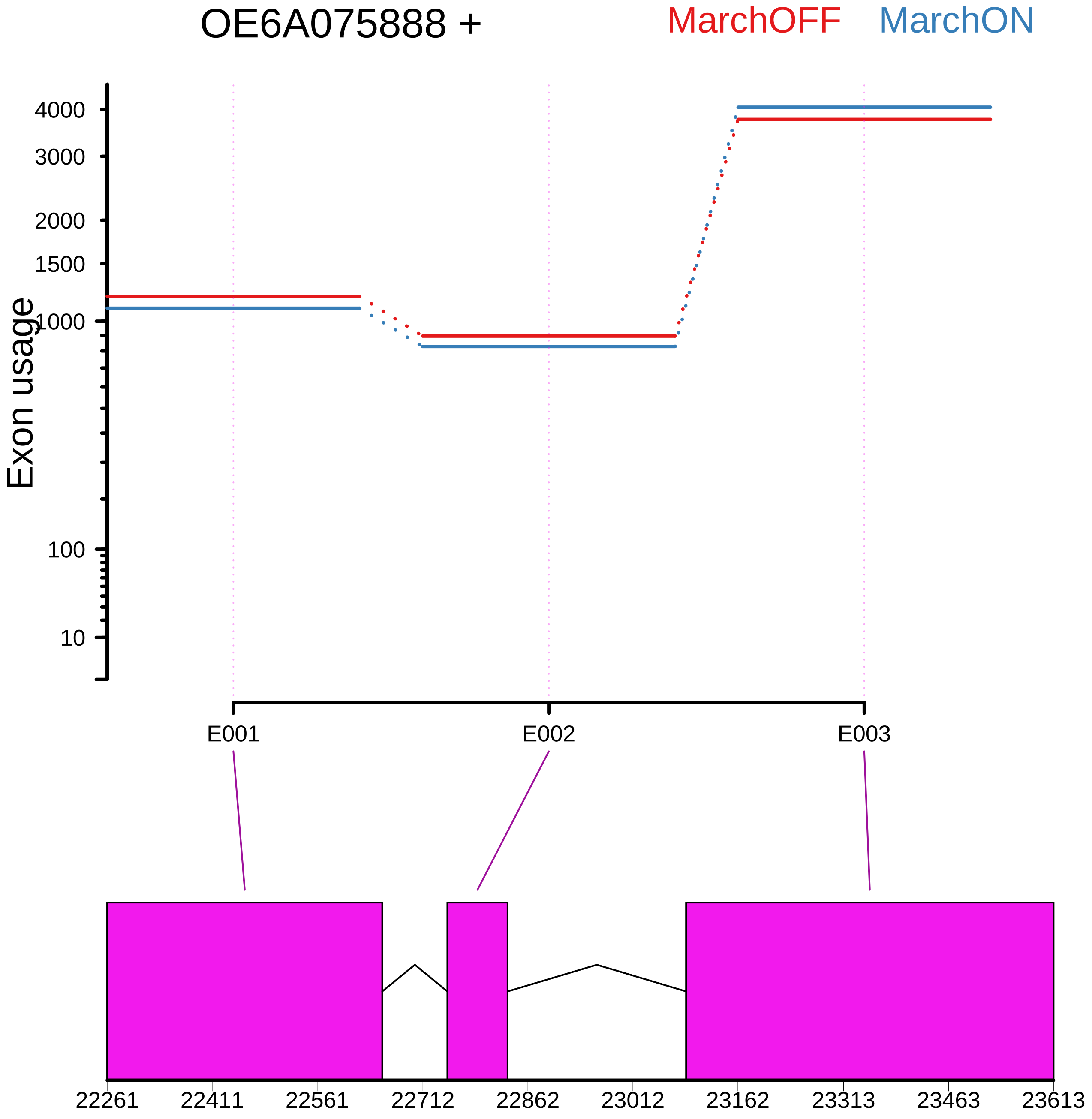

OE6A075888 +
MarchOFF
MarchON
Exon usage
4000
3000
2000
1500
1000
100
10
E001
E002
E003
22261
22411
22561
22712
22862
23012
23162
23313
23463
23613

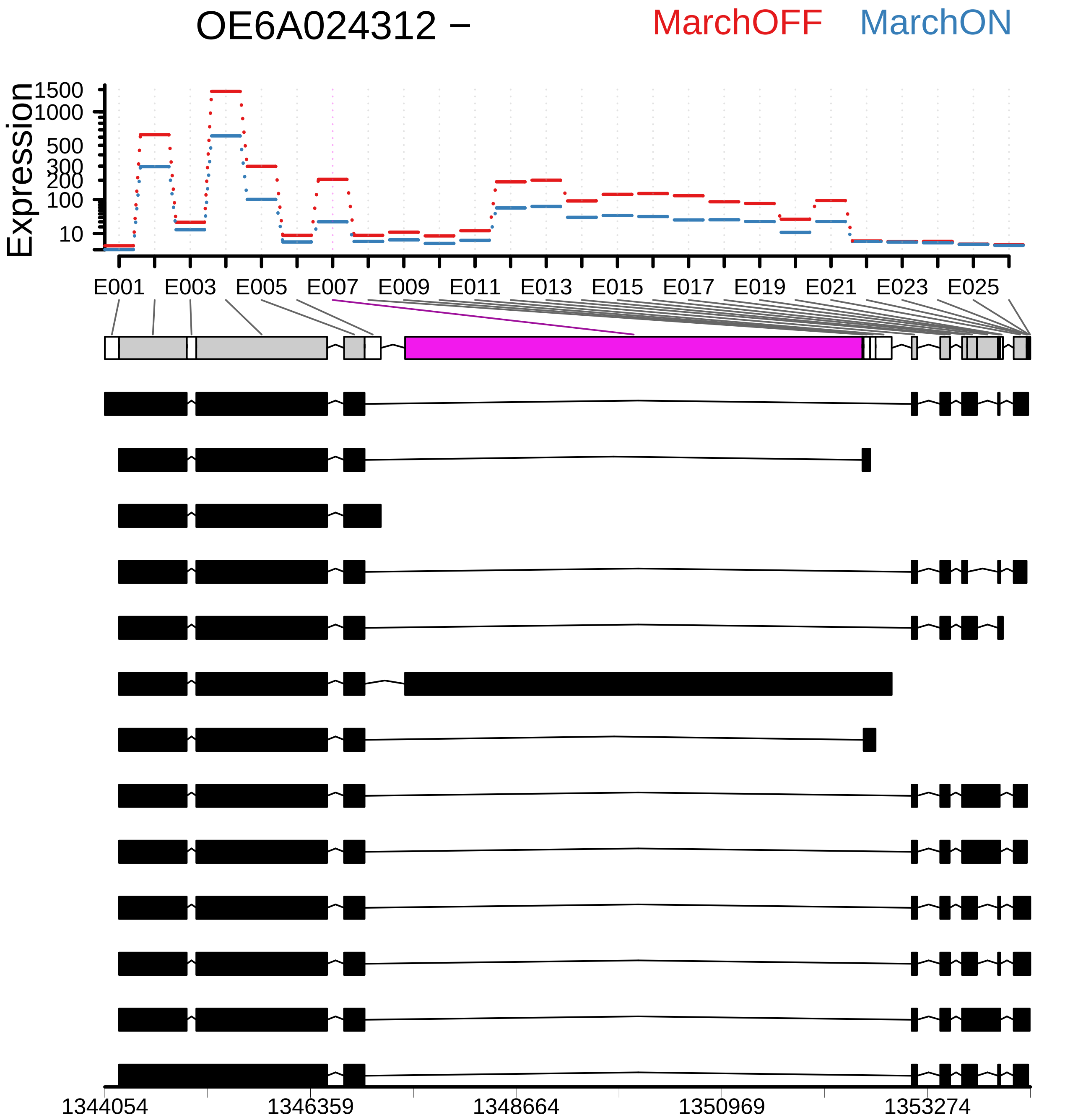

OE6A024312 −
MarchOFF
MarchON
Expression
1500
1000
500
300
200
100
10
E001
E003
E005
E007
E009
E011
E013
E015
E017
E019
E021
E023
E025
1344054
1346359
1348664
1350969
1353274

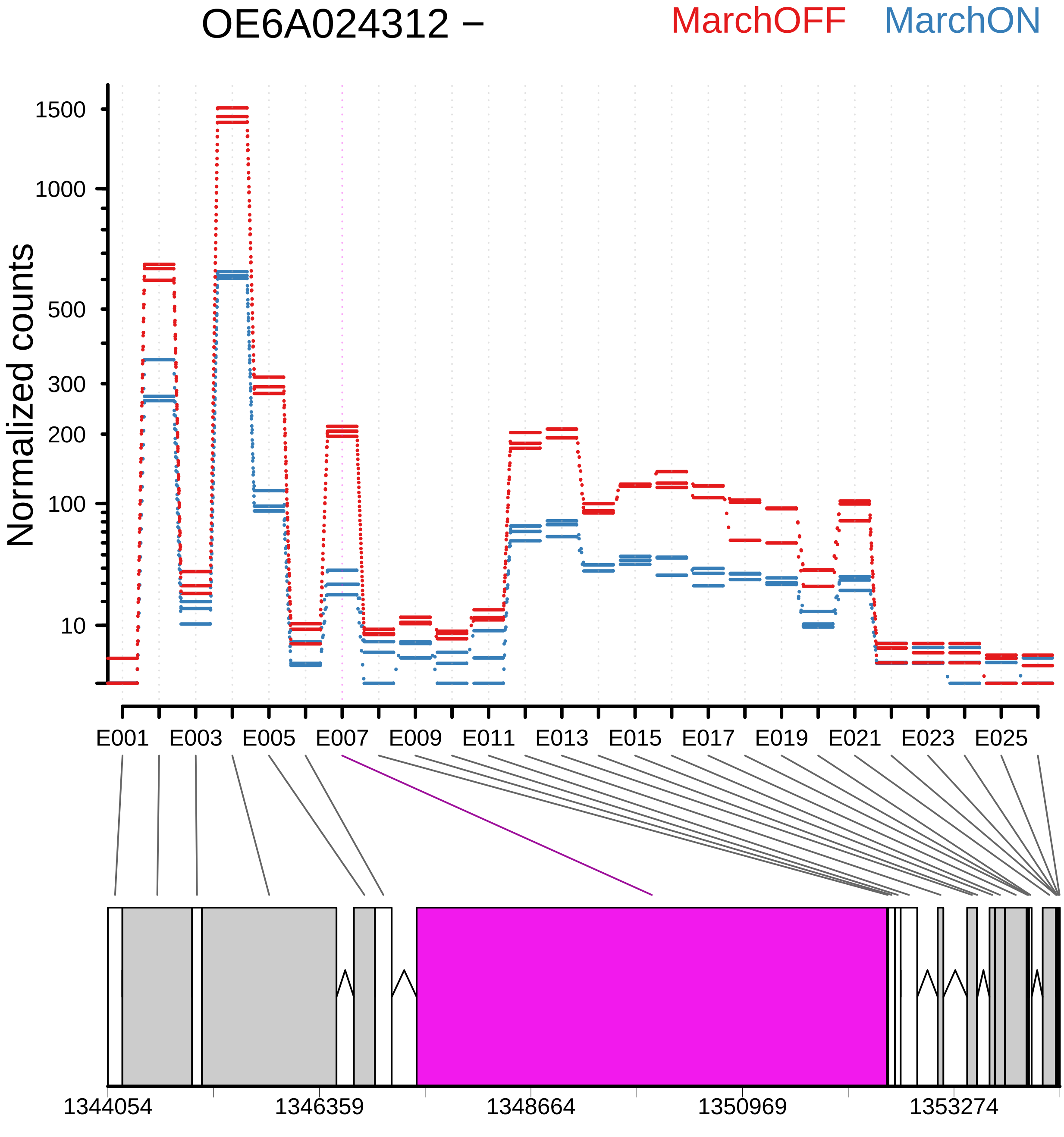
OE6A024312 −
MarchOFF
MarchON
Normalized counts
1500
1000
500
300
200
100
10
E001
E003
E005
E007
E009
E011
E013
E015
E017
E019
E021
E023
E025
1344054
1346359
1348664
1350969
1353274

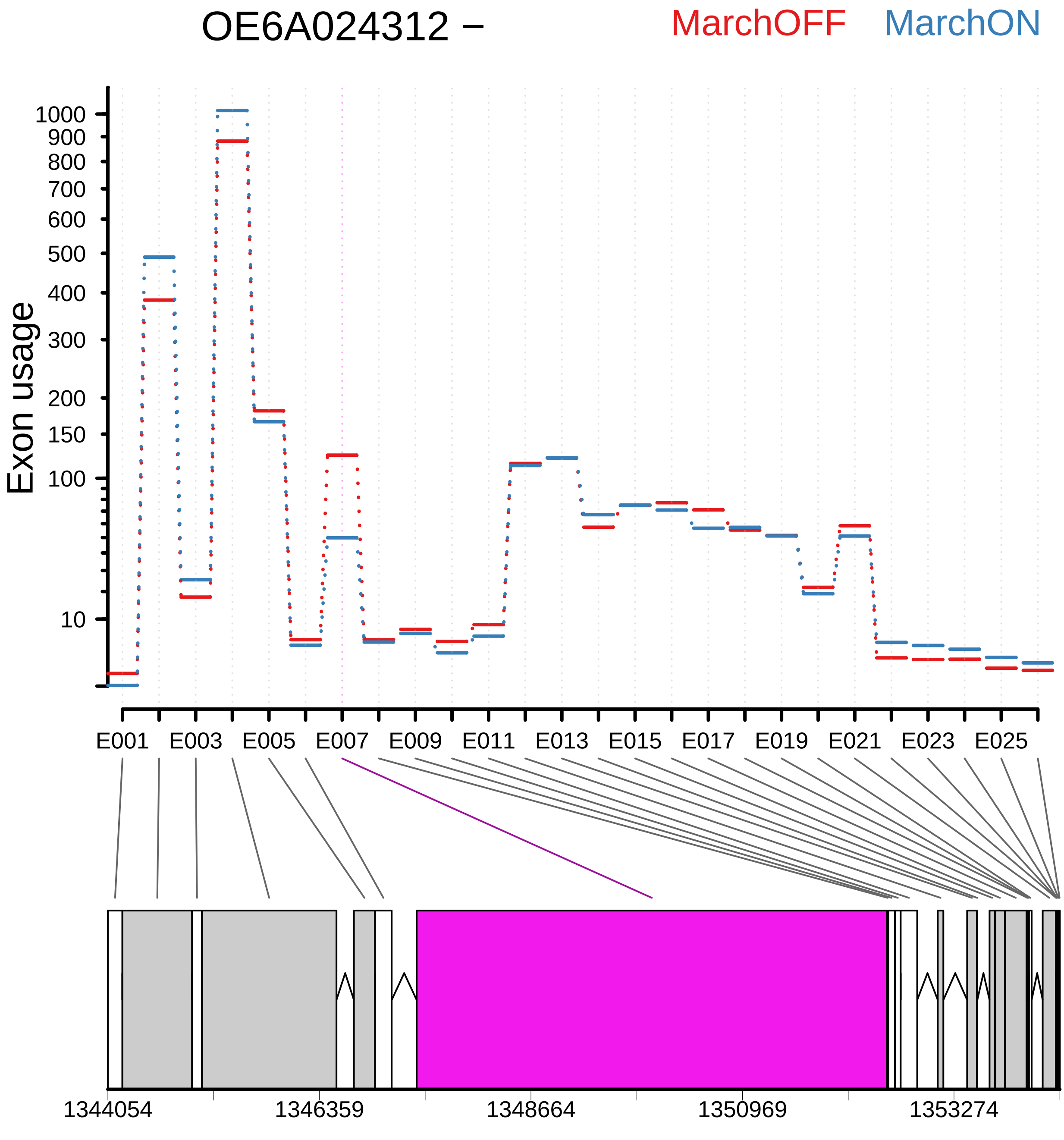

OE6A024312 −
MarchOFF
MarchON
Exon usage
1000
900
800
700
600
500
400
300
200
150
100
10
E001
E003
E005
E007
E009
E011
E013
E015
E017
E019
E021
E023
E025
1344054
1346359
1348664
1350969
1353274

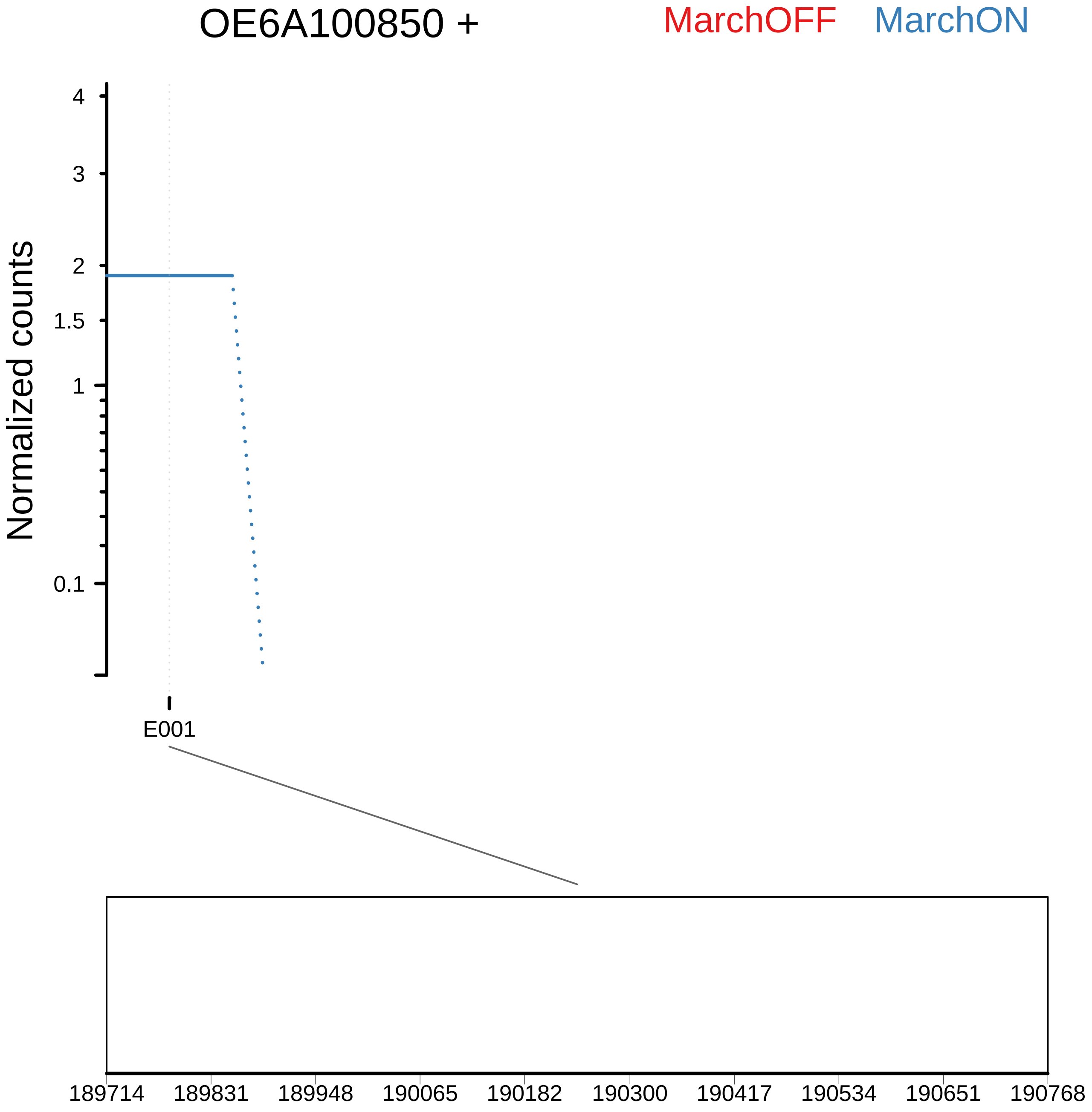

OE6A100850 +
MarchOFF
MarchON
Normalized counts
4
3
2
1.5
1
0.1
E001
189714
189831
189948
190065
190182
190300
190417
190534
190651
190768

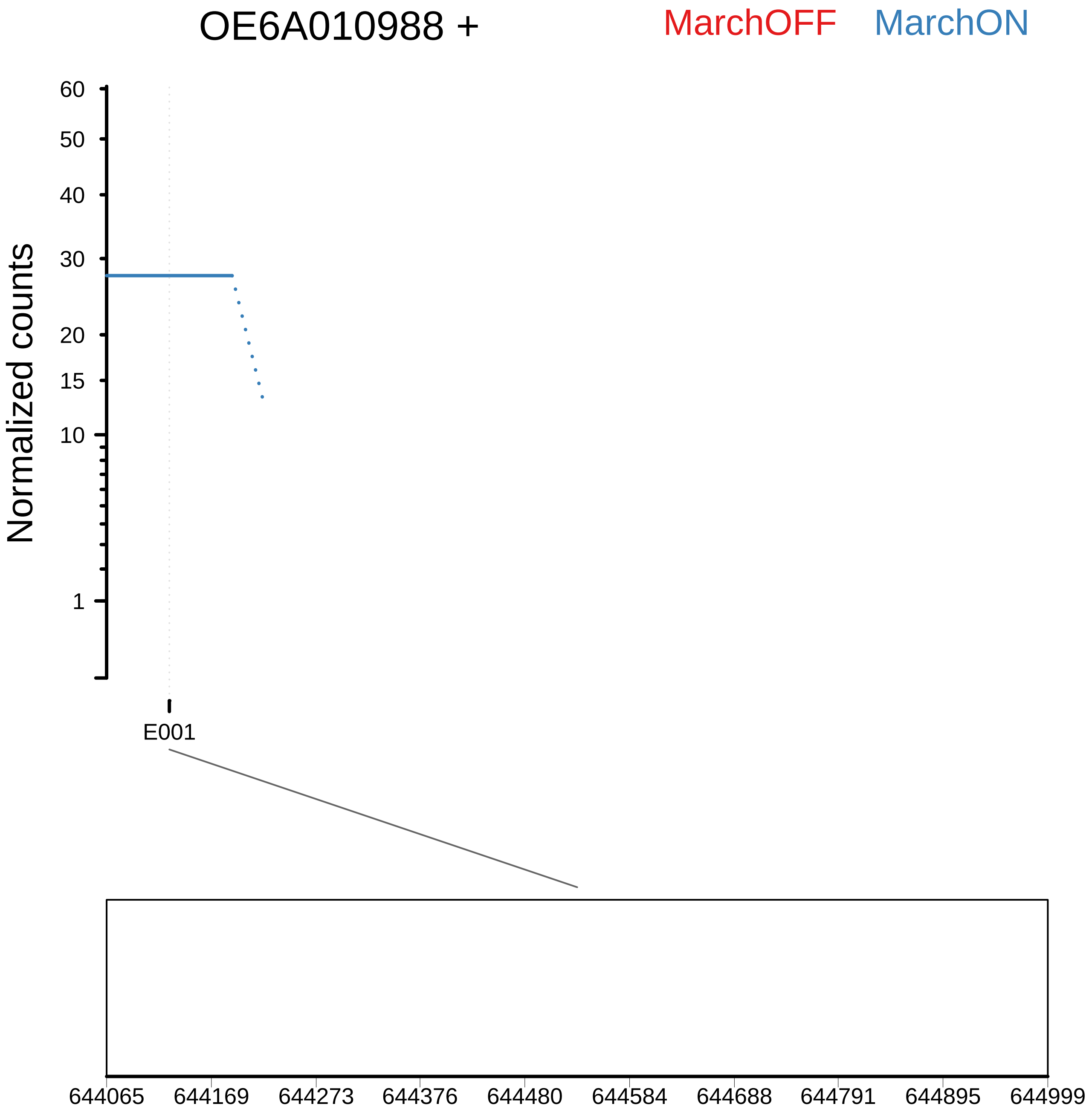
OE6A010988 +
MarchOFF
MarchON
Normalized counts
60
50
40
30
20
15
10
1
E001
644065
644169
644273
644376
644480
644584
644688
644791
644895
644999

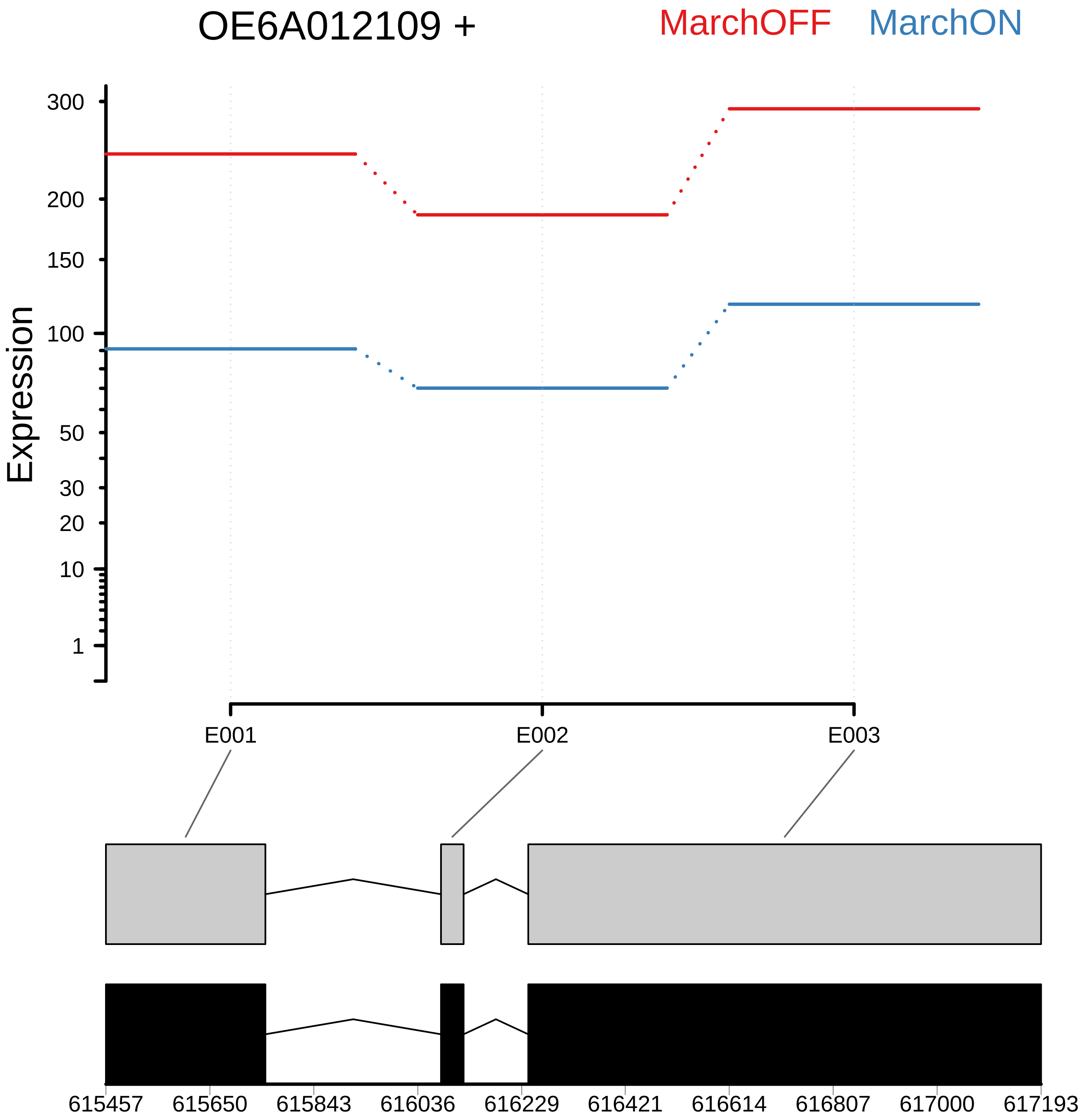

OE6A012109 +
MarchOFF
MarchON
Expression
300
200
150
100
50
30
20
10
1
E001
E002
E003
615457
615650
615843
616036
616229
616421
616614
616807
617000
617193

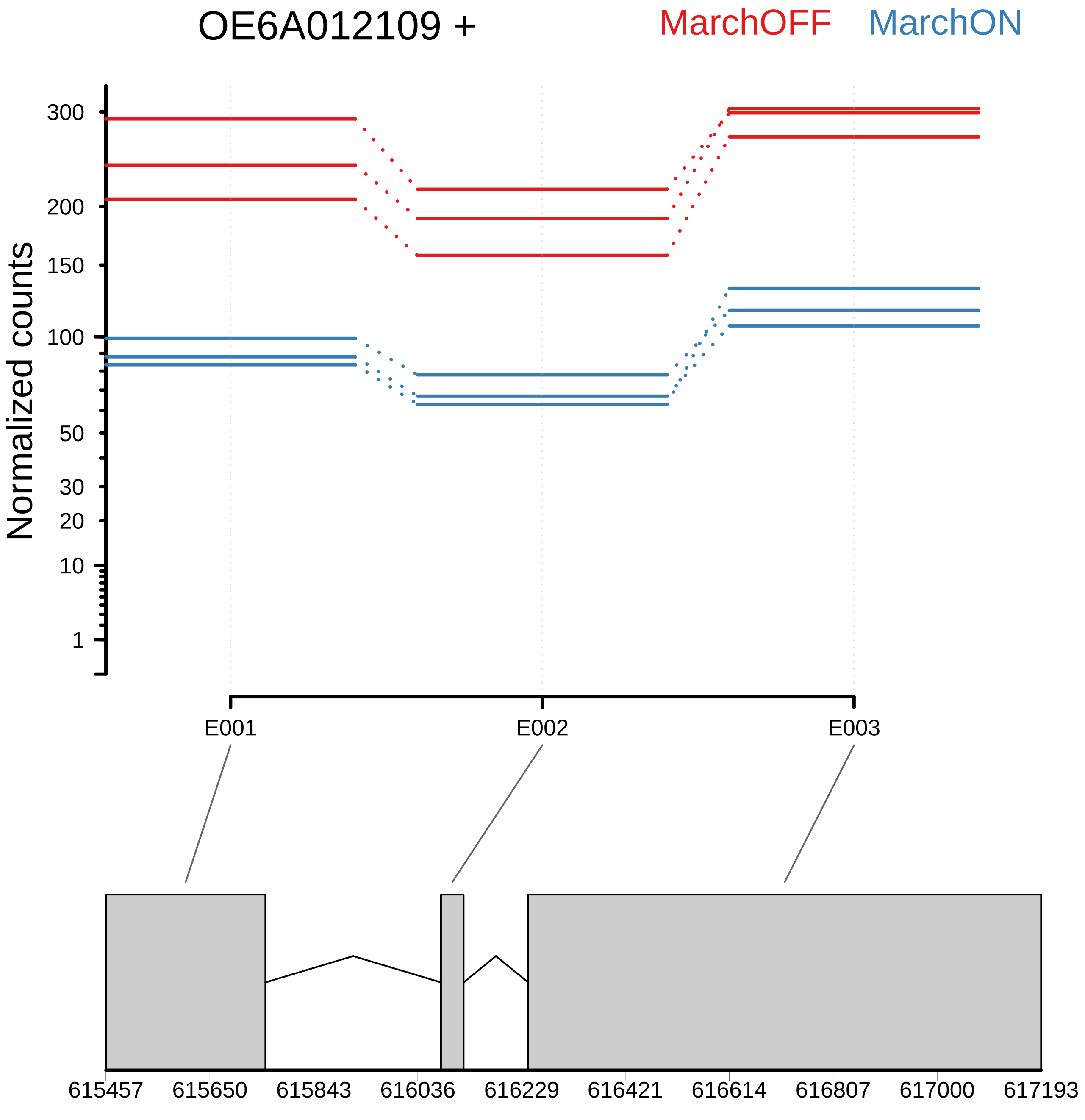

OE6A012109 +
MarchOFF
MarchON
Normalized counts
300
200
150
100
50
30
20
10
1
E001
E002
E003
615457
615650
615843
616036
616229
616421
616614
616807
617000
617193

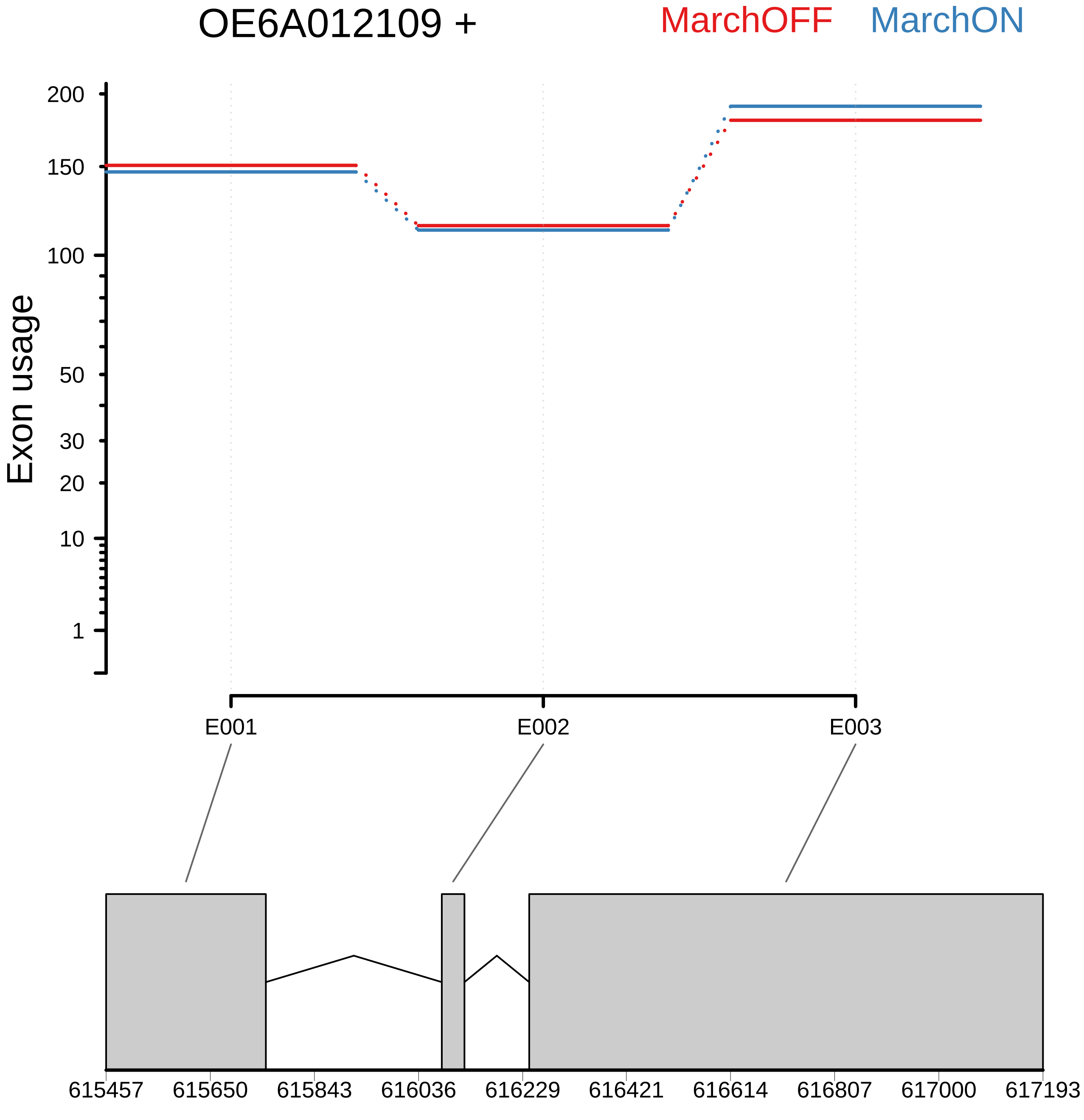

OE6A012109 +
MarchOFF
MarchON
Exon usage
200
150
100
50
30
20
10
1
E001
E002
E003
615457
615650
615843
616036
616229
616421
616614
616807
617000
617193

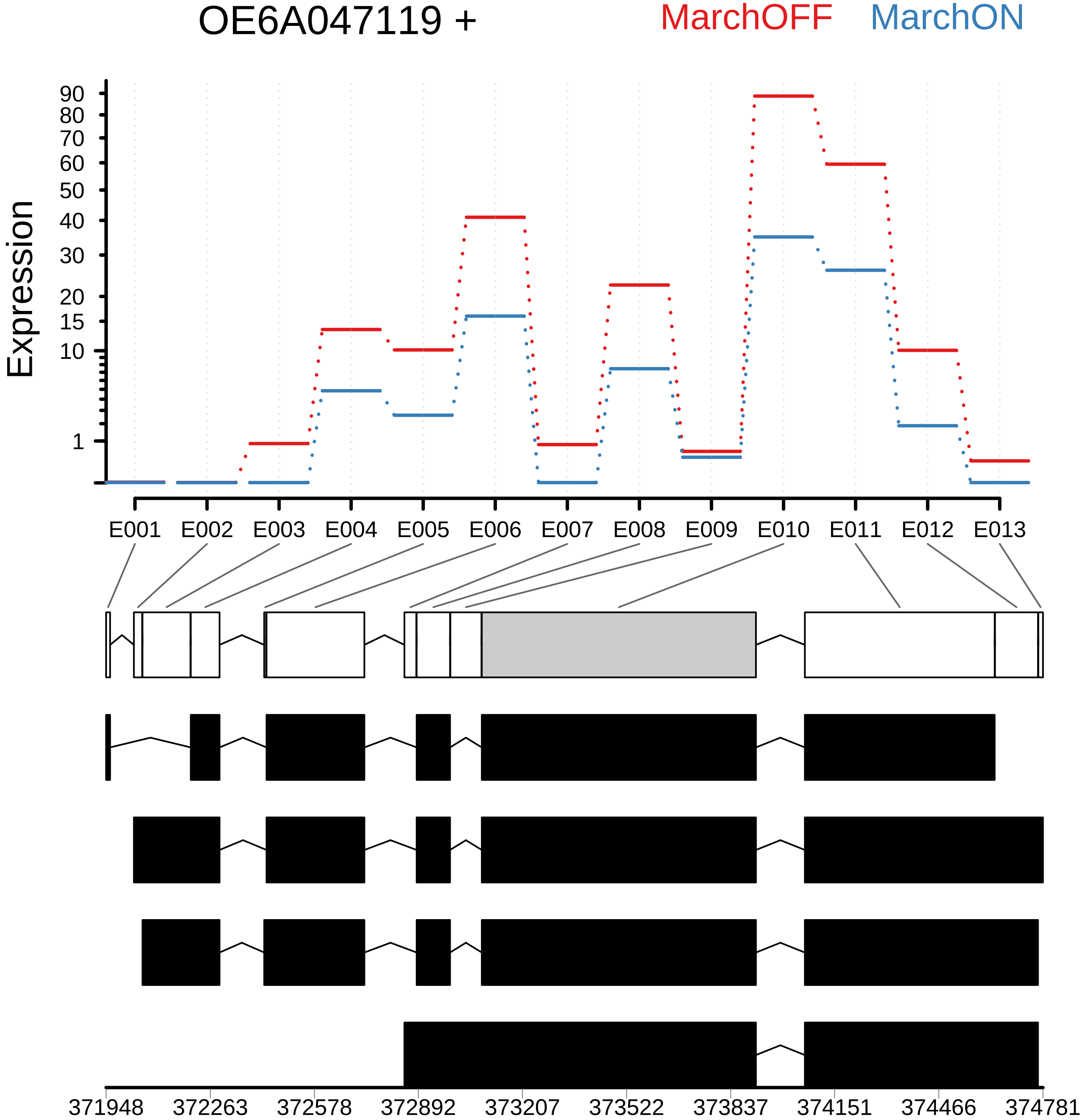
OE6A047119 +
MarchOFF
MarchON
Expression
90
80
70
60
50
40
30
20
15
10
1
E001
E002
E003
E004
E005
E006
E007
E008
E009
E010
E011
E012
E013
371948
372263
372578
372892
373207
373522
373837
374151
374466
374781

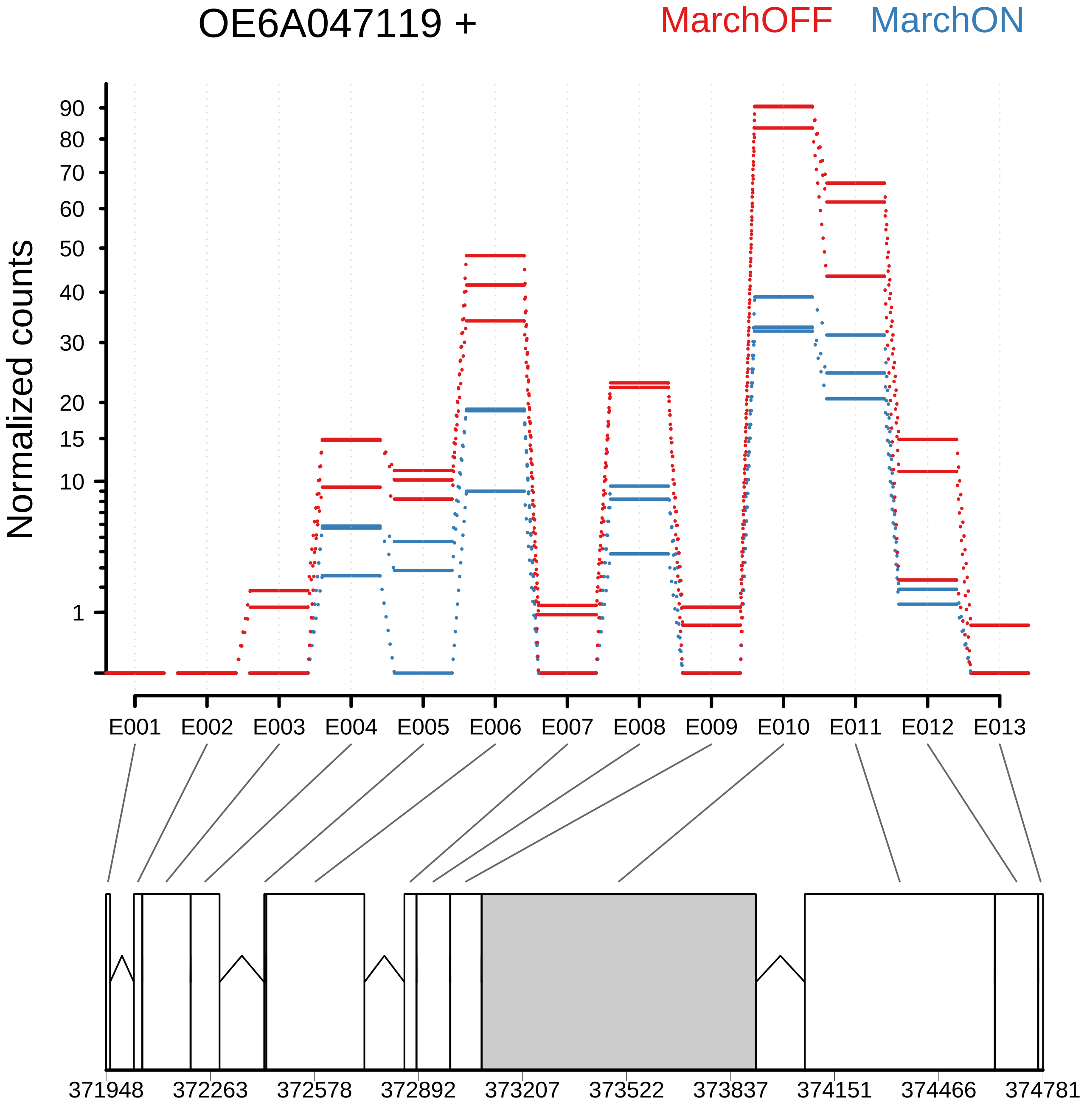

OE6A047119 +
MarchOFF
MarchON
Normalized counts
90
80
70
60
50
40
30
20
15
10
1
E001
E002
E003
E004
E005
E006
E007
E008
E009
E010
E011
E012
E013
371948
372263
372578
372892
373207
373522
373837
374151
374466
374781

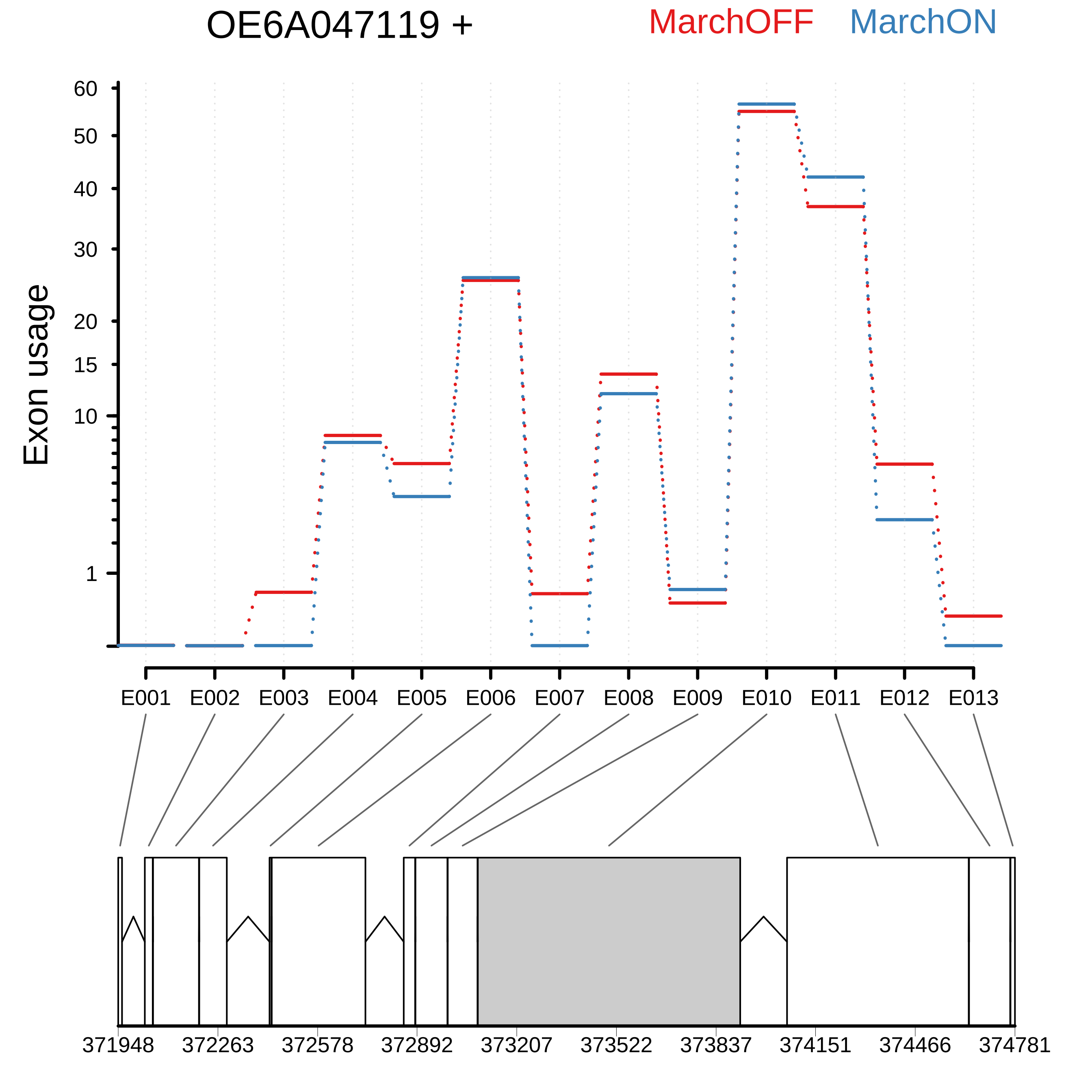
OE6A047119 +
MarchOFF
MarchON
Exon usage
60
50
40
30
20
15
10
1
E001
E002
E003
E004
E005
E006
E007
E008
E009
E010
E011
E012
E013
371948
372263
372578
372892
373207
373522
373837
374151
374466
374781

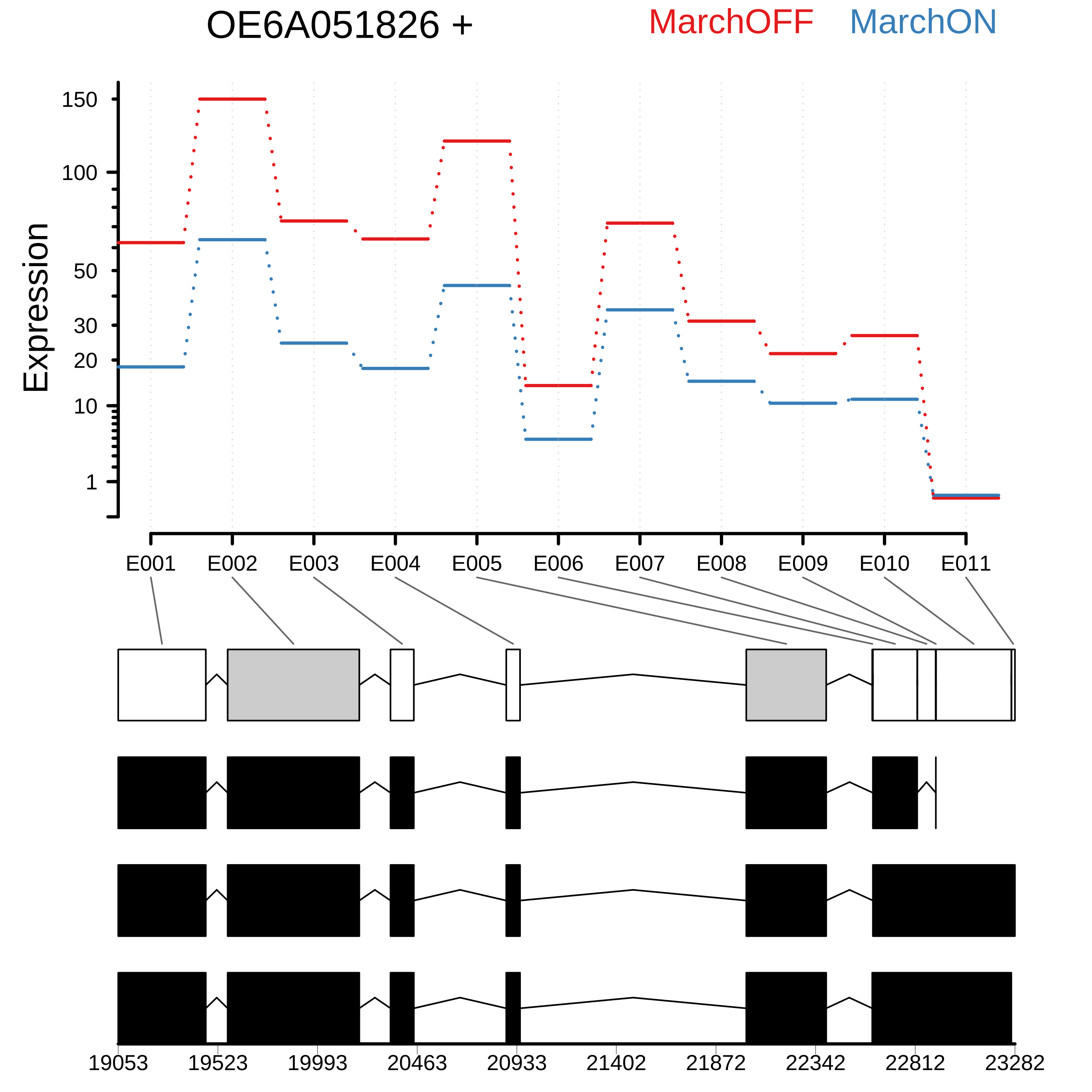
OE6A051826 +
MarchOFF
MarchON
Expression
150
100
50
30
20
10
1
E001
E002
E003
E004
E005
E006
E007
E008
E009
E010
E011
19053
19523
19993
20463
20933
21402
21872
22342
22812
23282

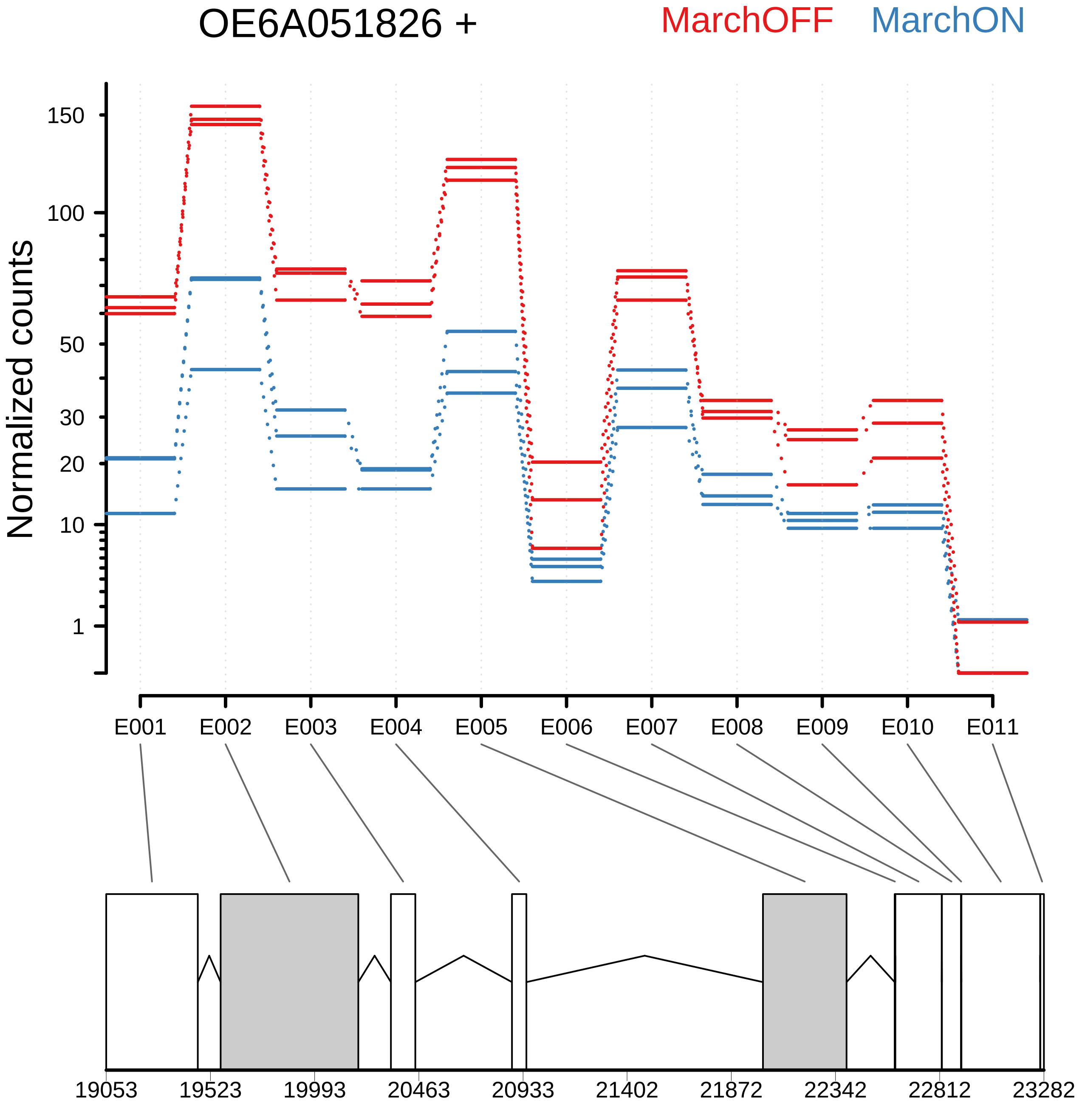

OE6A051826 +
MarchOFF
MarchON
Normalized counts
150
100
50
30
20
10
1
E001
E002
E003
E004
E005
E006
E007
E008
E009
E010
E011
19053
19523
19993
20463
20933
21402
21872
22342
22812
23282

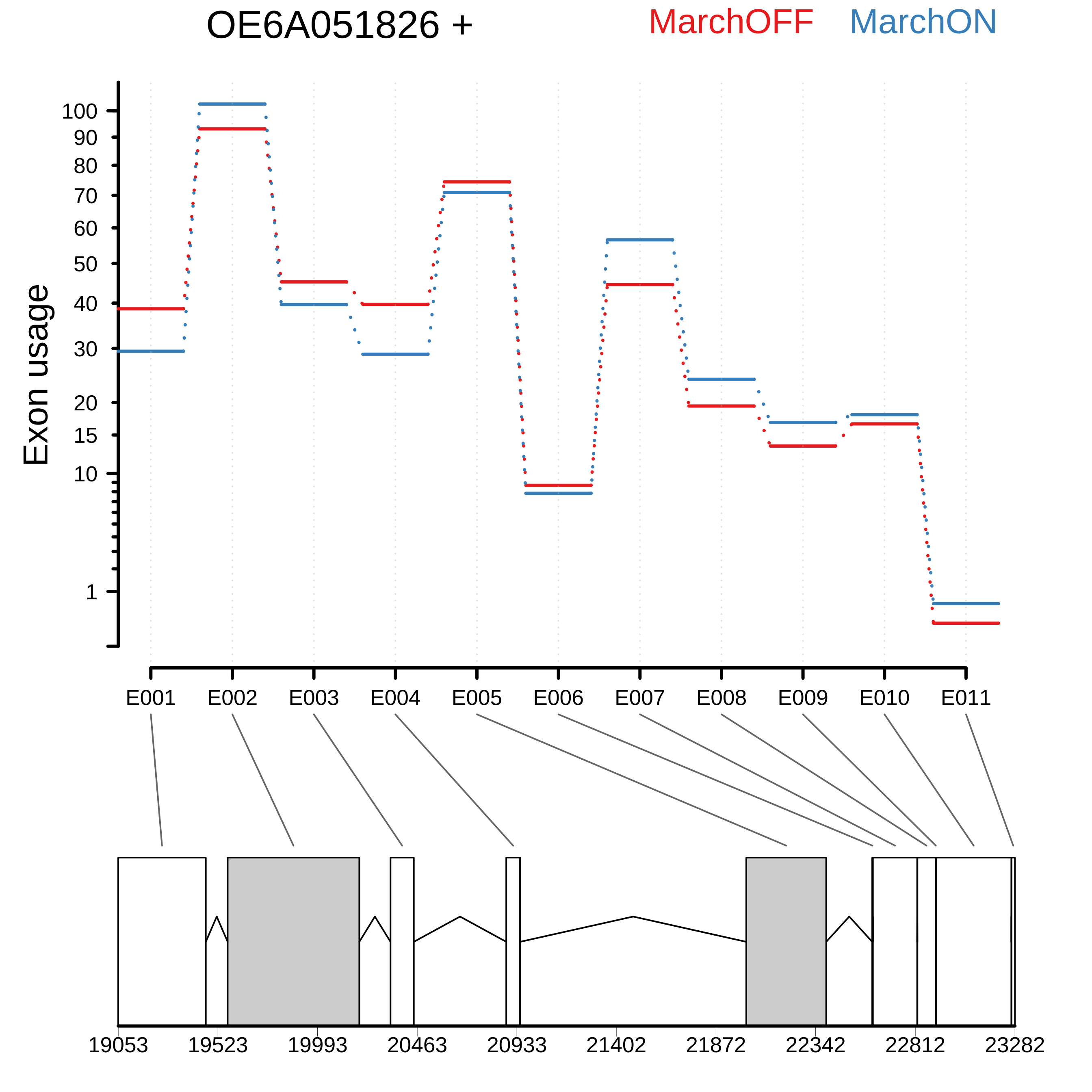
OE6A051826 +
MarchOFF
MarchON
Exon usage
100
90
80
70
60
50
40
30
20
15
10
1
E001
E002
E003
E004
E005
E006
E007
E008
E009
E010
E011
19053
19523
19993
20463
20933
21402
21872
22342
22812
23282

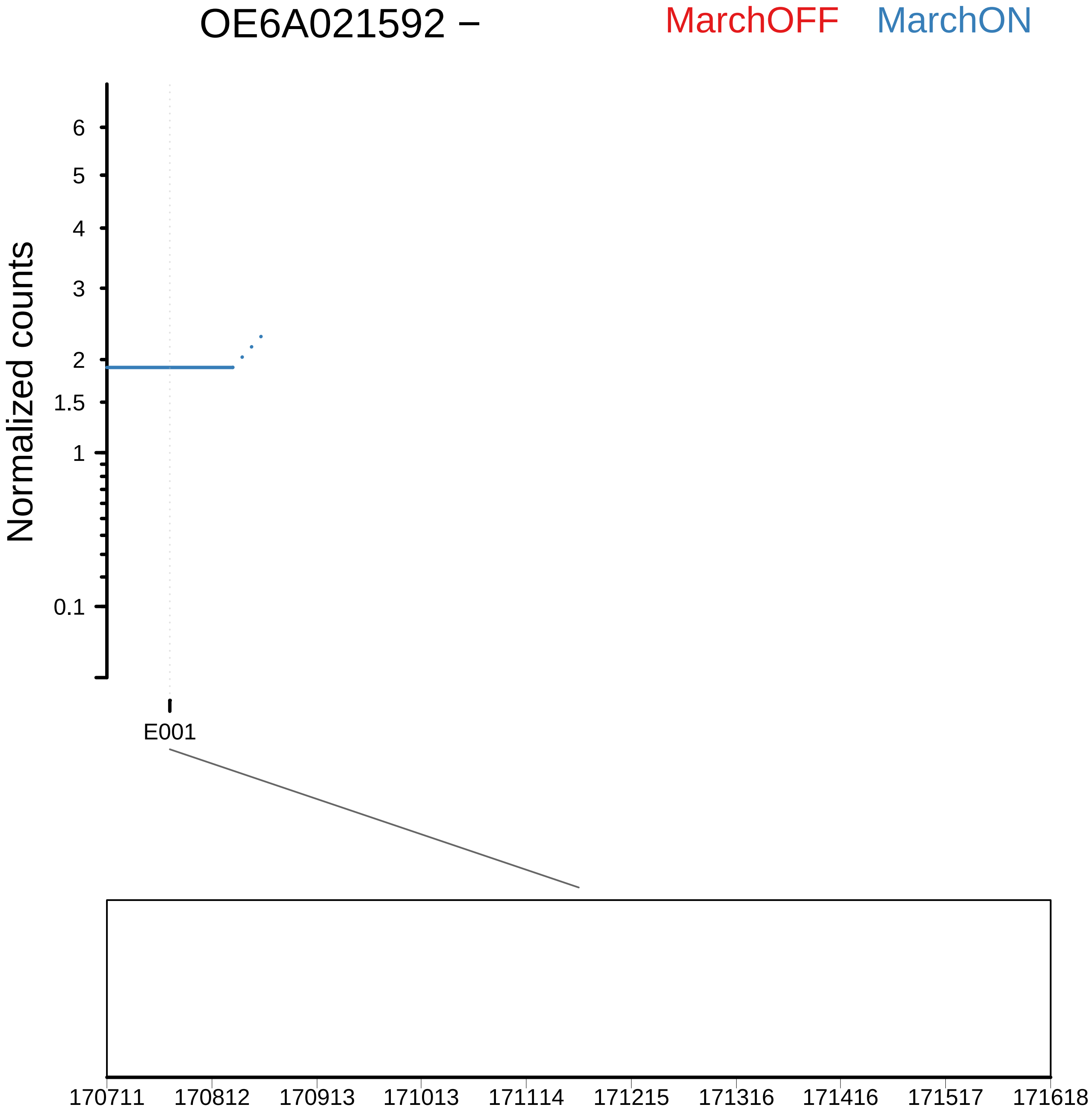

OE6A021592 −
MarchOFF
MarchON
Normalized counts
6
5
4
3
2
1.5
1
0.1
E001
170711
170812
170913
171013
171114
171215
171316
171416
171517
171618

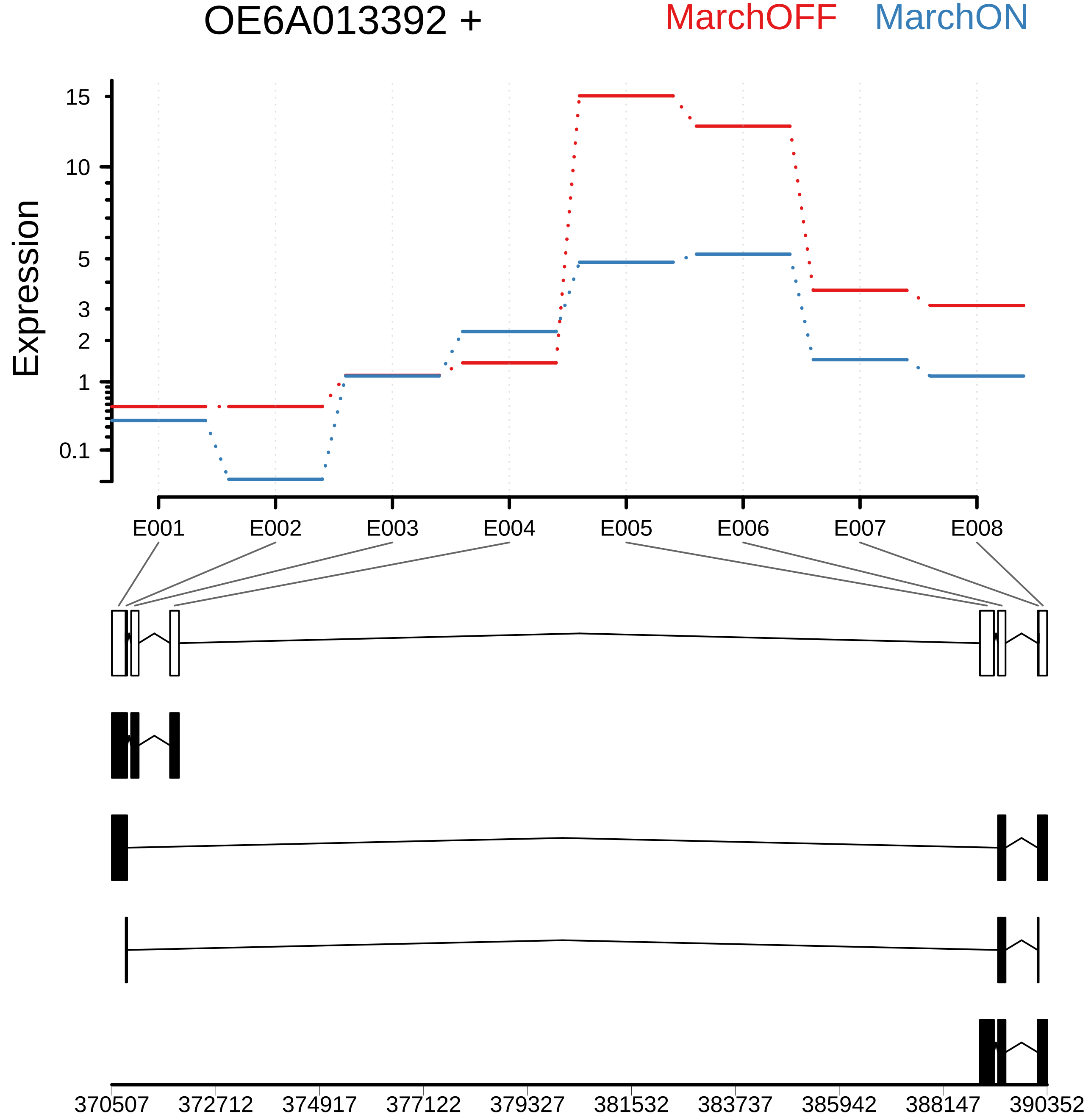
OE6A013392 +
MarchOFF
MarchON
Expression
15
10
5
3
2
1
0.1
E001
E002
E003
E004
E005
E006
E007
E008
370507
372712
374917
377122
379327
381532
383737
385942
388147
390352

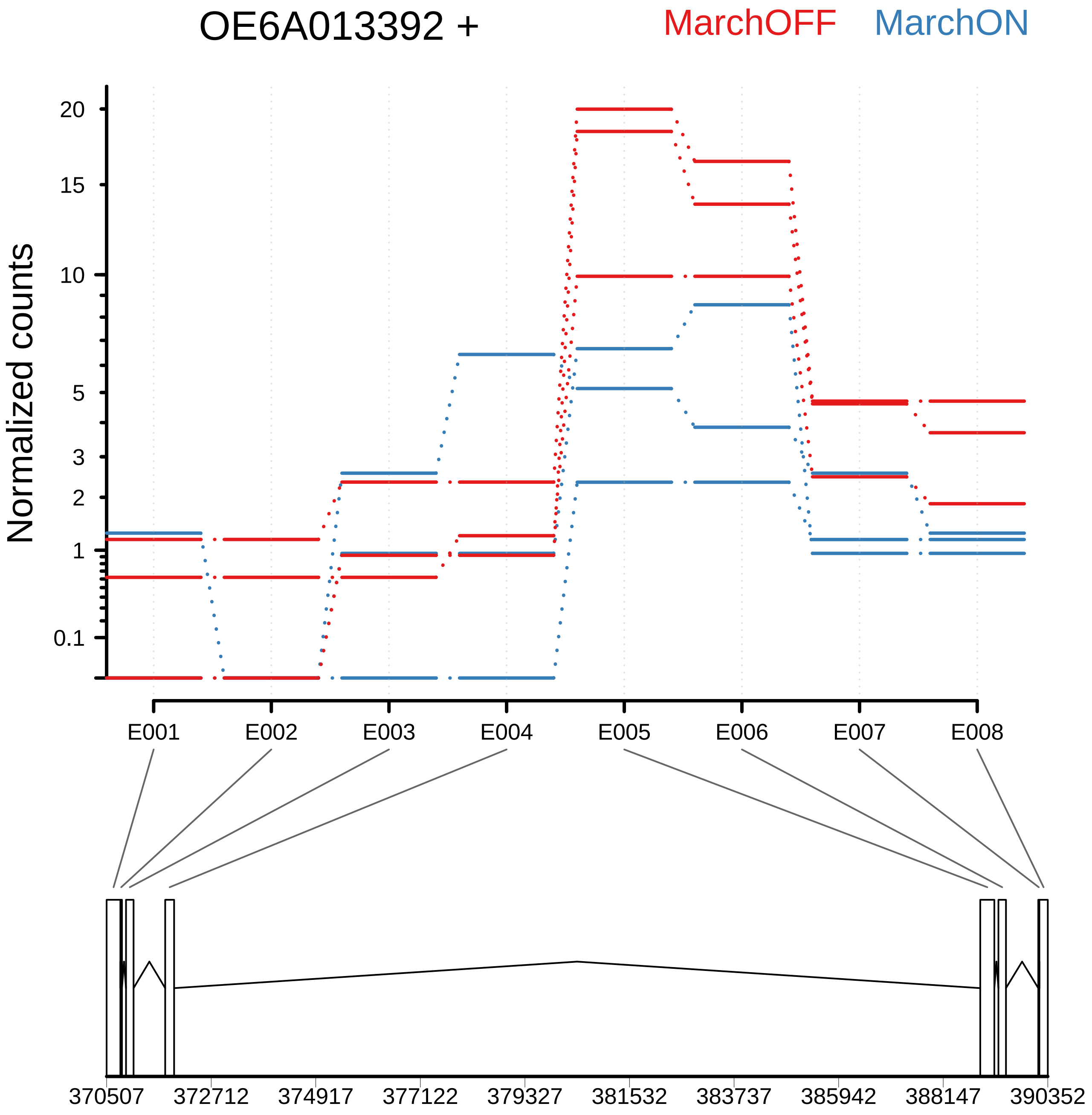

OE6A013392 +
MarchOFF
MarchON
Normalized counts
20
15
10
5
3
2
1
0.1
E001
E002
E003
E004
E005
E006
E007
E008
370507
372712
374917
377122
379327
381532
383737
385942
388147
390352

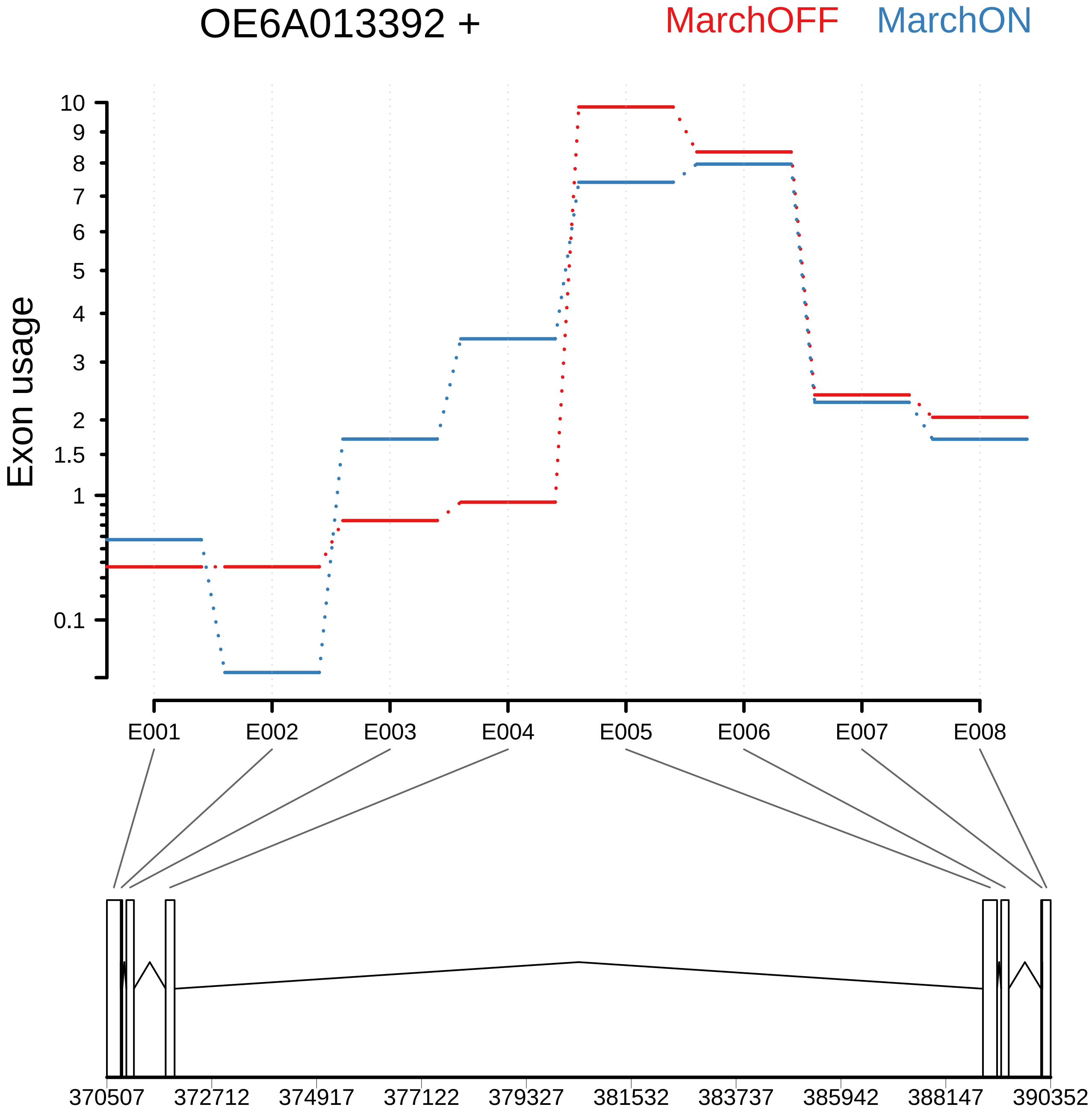

OE6A013392 +
MarchOFF
MarchON
Exon usage
10
9
8
7
6
5
4
3
2
1.5
1
0.1
E001
E002
E003
E004
E005
E006
E007
E008
370507
372712
374917
377122
379327
381532
383737
385942
388147
390352

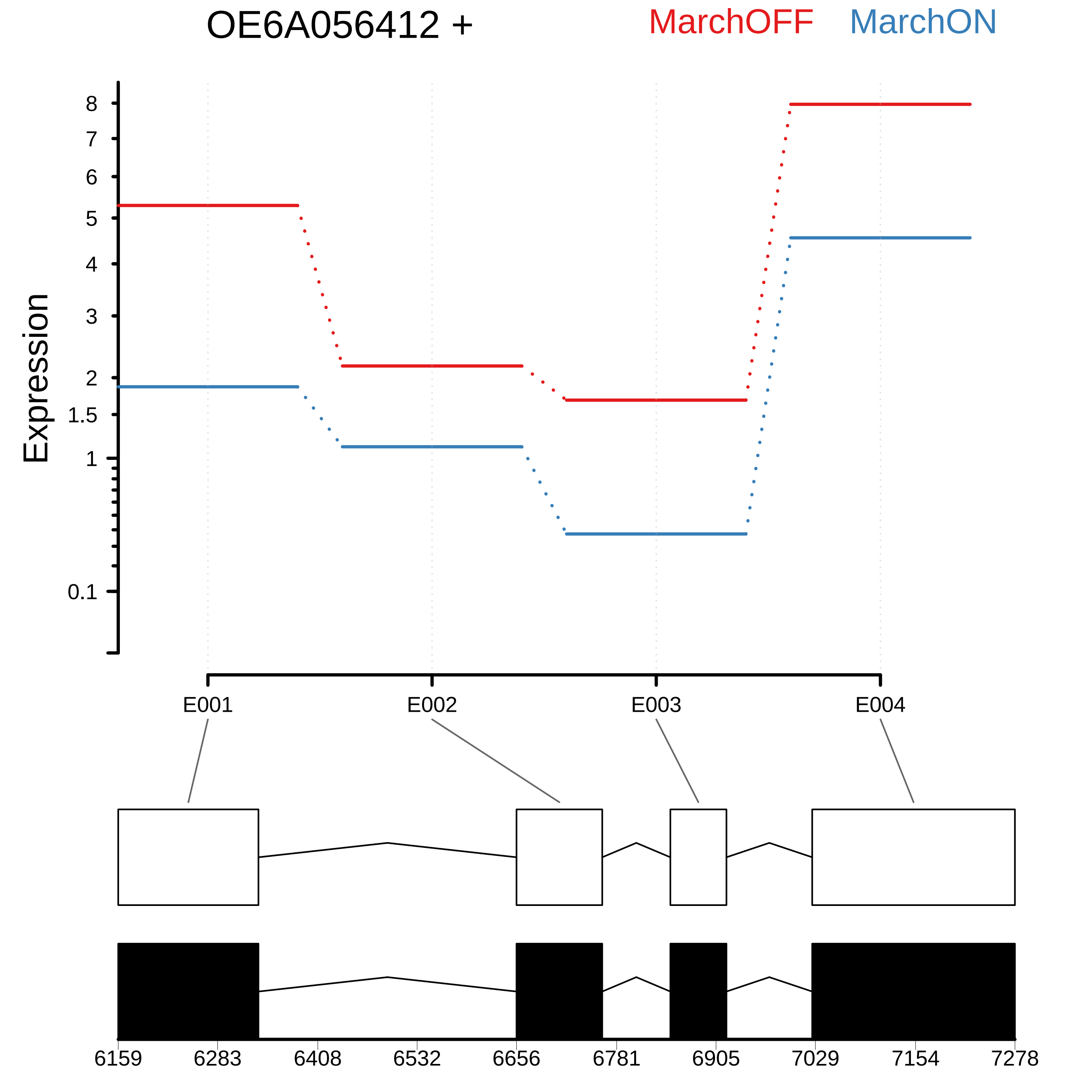
OE6A056412 +
MarchOFF
MarchON
Expression
8
7
6
5
4
3
2
1.5
1
0.1
E001
E002
E003
E004
6159
6283
6408
6532
6656
6781
6905
7029
7154
7278

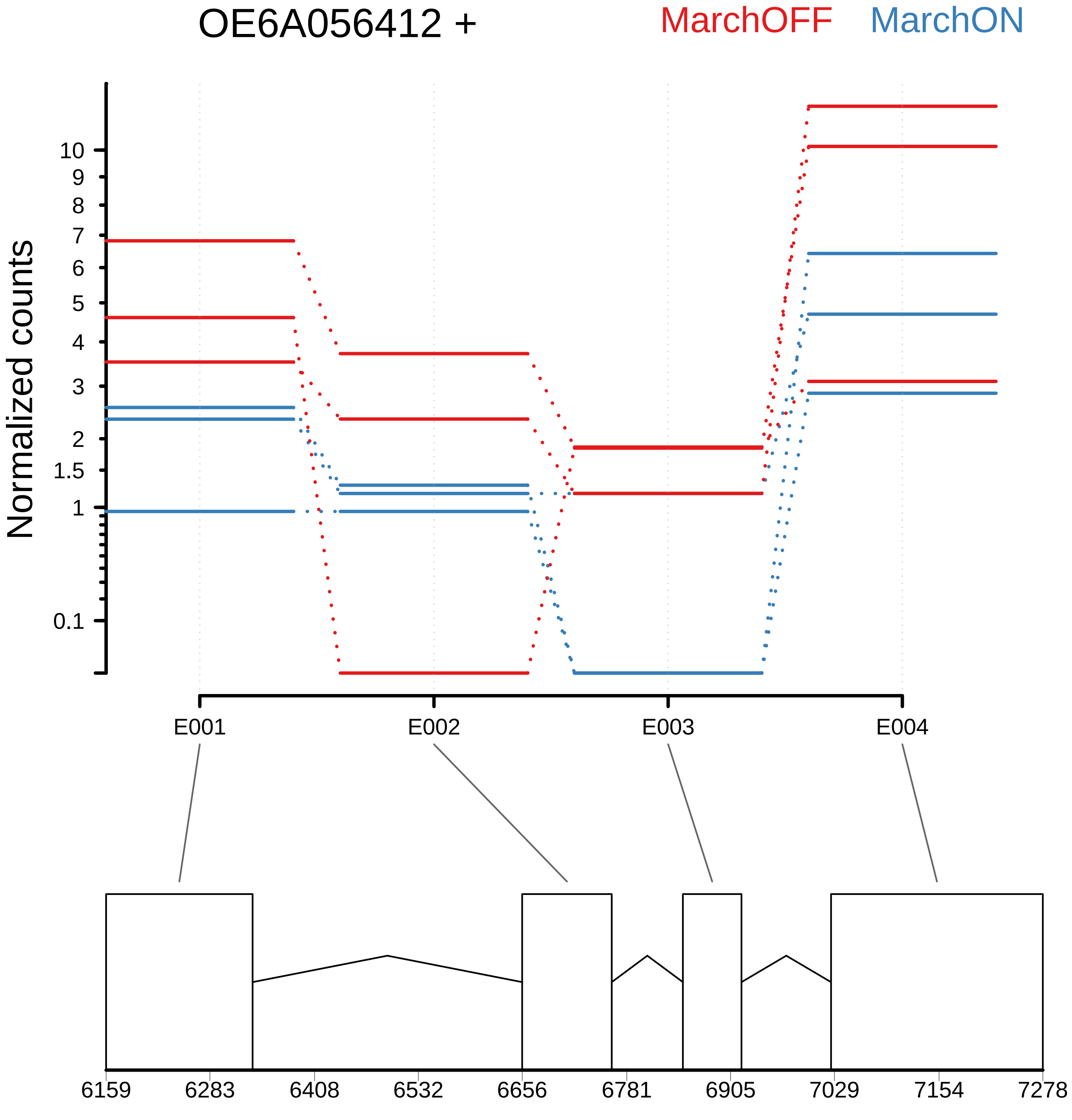
OE6A056412 +
MarchOFF
MarchON
Normalized counts
10
9
8
7
6
5
4
3
2
1.5
1
0.1
E001
E002
E003
E004
6159
6283
6408
6532
6656
6781
6905
7029
7154
7278

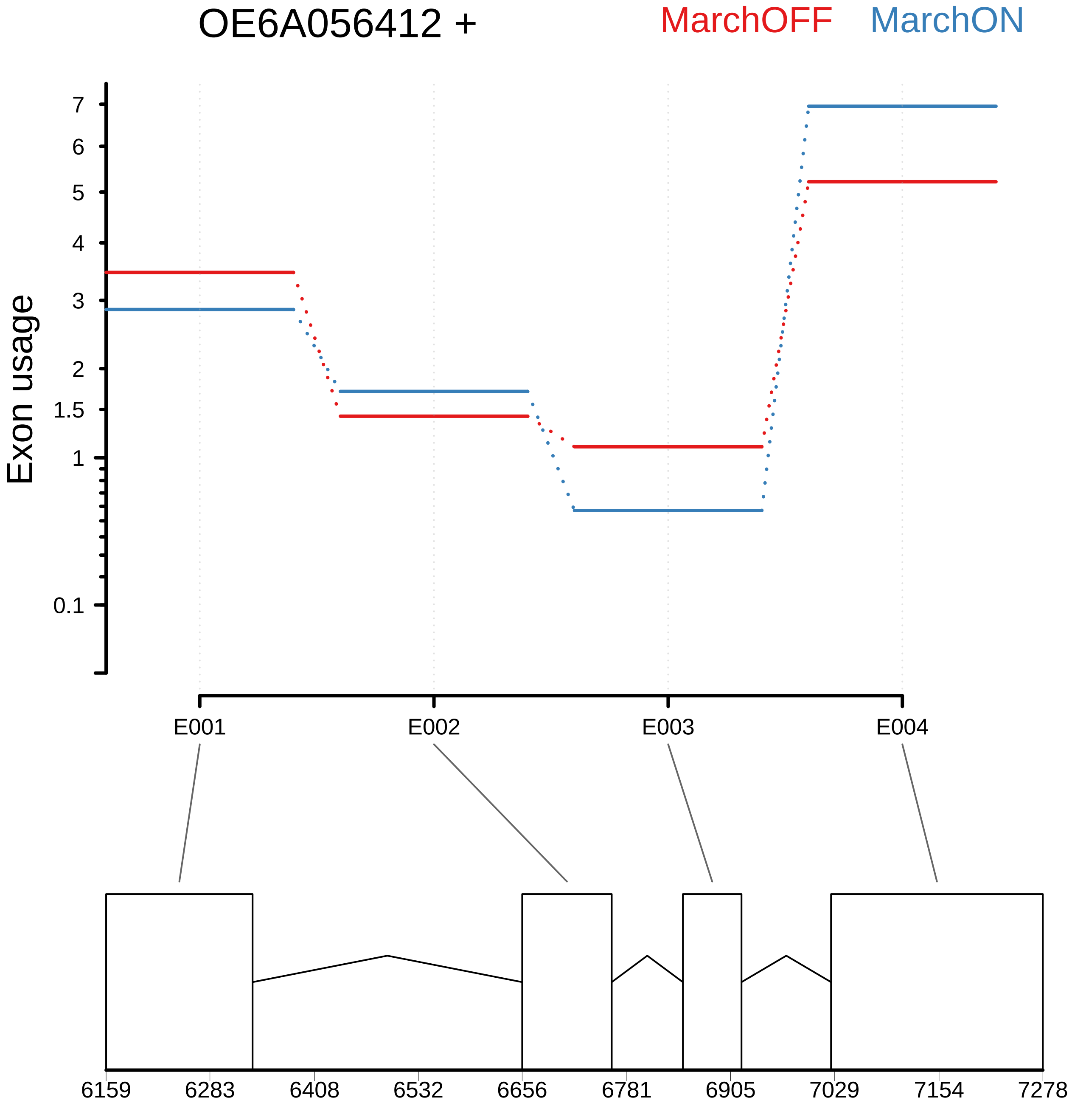

OE6A056412 +
MarchOFF
MarchON
Exon usage
7
6
5
4
3
2
1.5
1
0.1
E001
E002
E003
E004
6159
6283
6408
6532
6656
6781
6905
7029
7154
7278

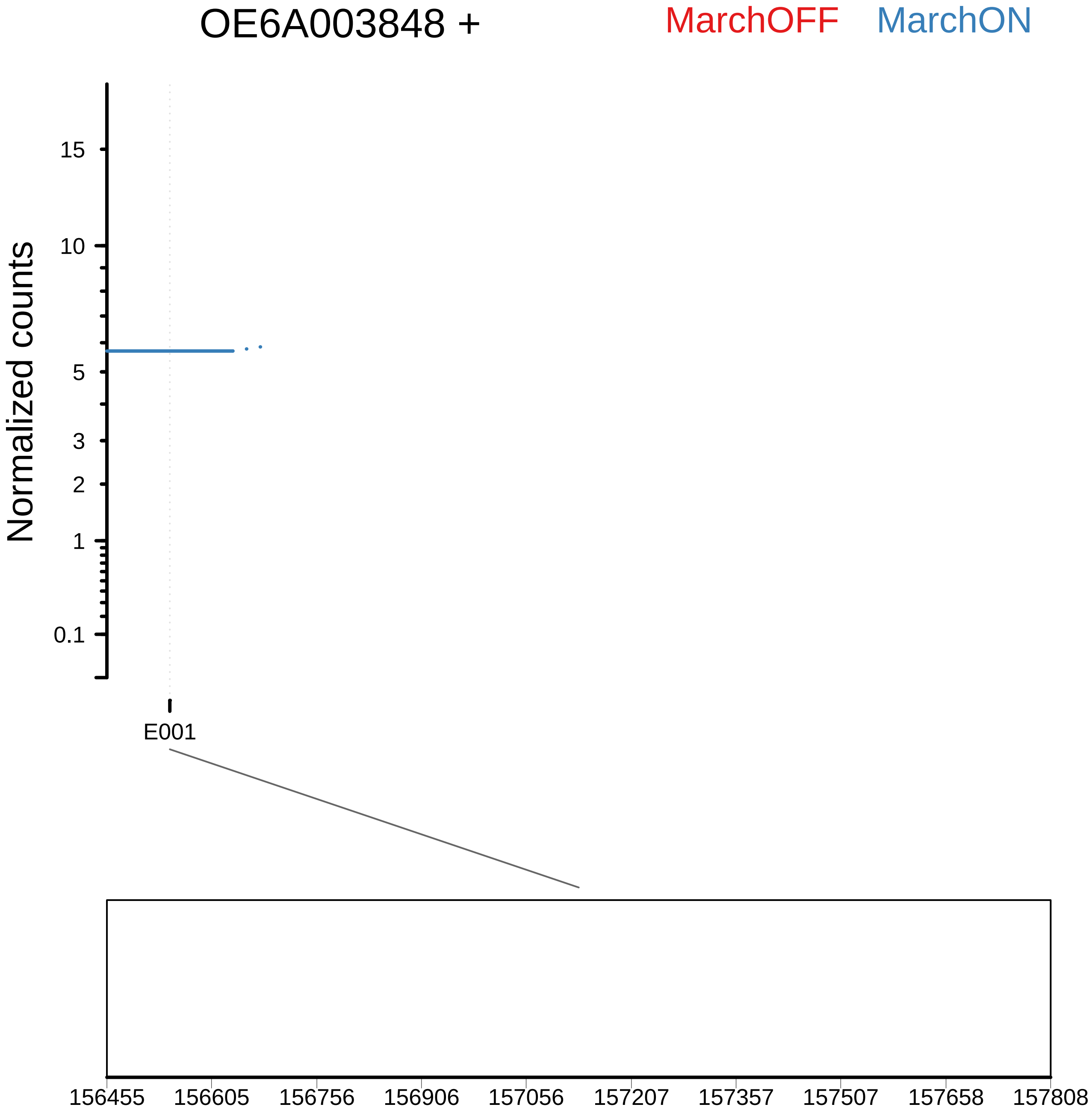

OE6A003848 +
MarchOFF
MarchON
Normalized counts
15
10
5
3
2
1
0.1
E001
156455
156605
156756
156906
157056
157207
157357
157507
157658
157808

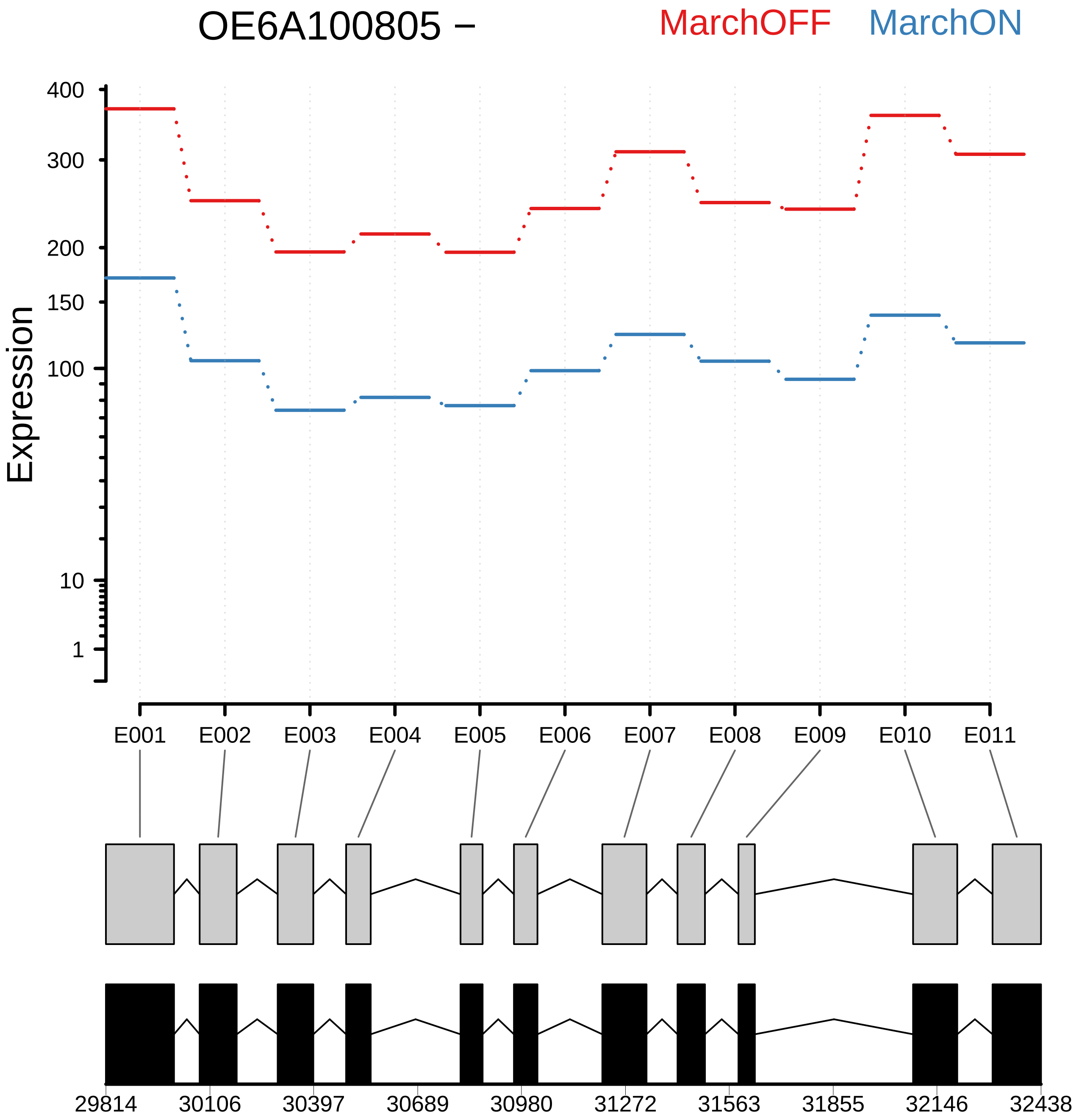
OE6A100805 –
MarchOFF
MarchON
Expression
400
300
200
150
100
10
1
E001
E002
E003
E004
E005
E006
E007
E008
E009
E010
E011
29814
30106
30397
30689
30980
31272
31563
31855
32146
32438

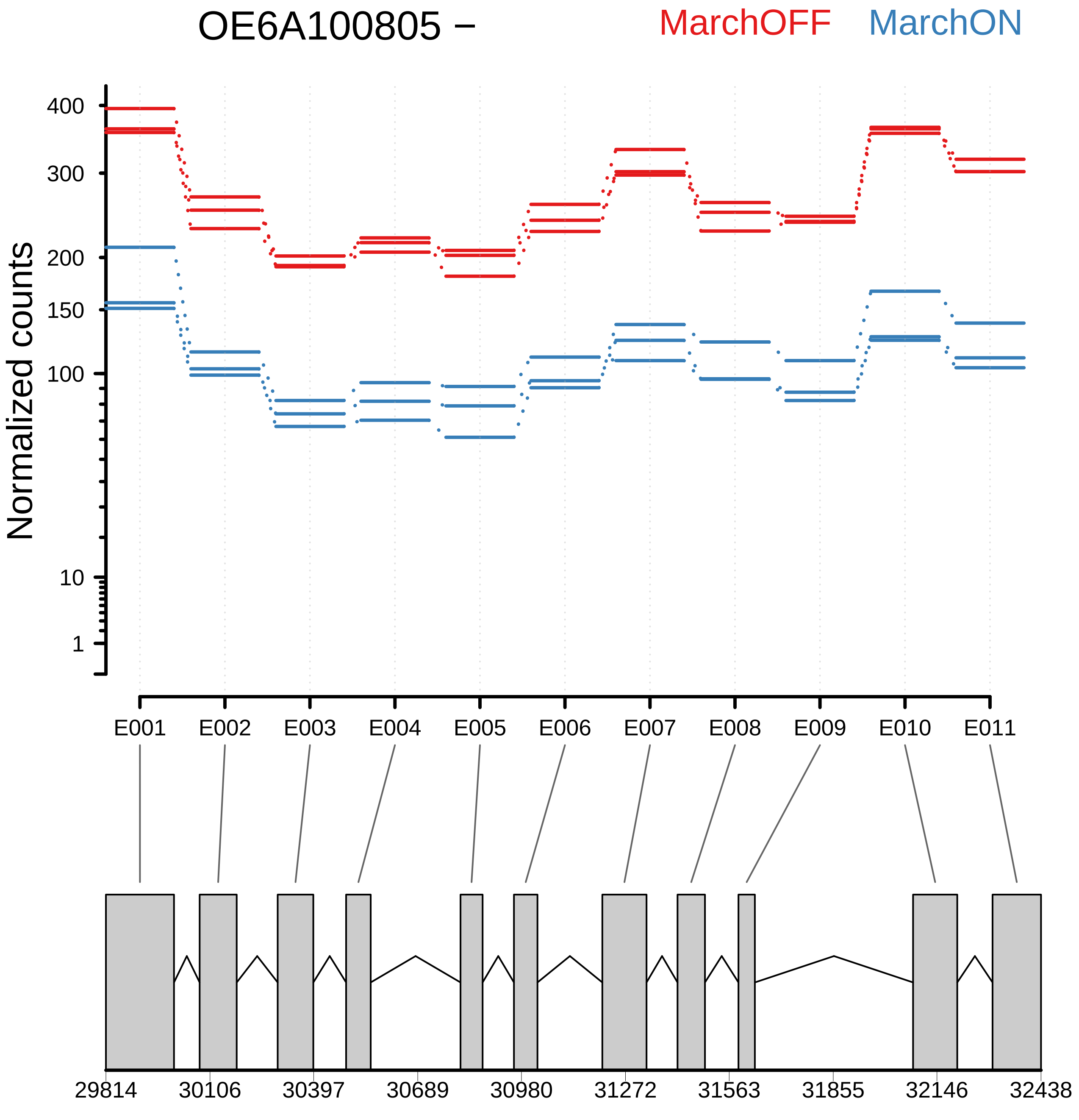
OE6A100805 −
MarchOFF
MarchON
Normalized counts
400
300
200
150
100
10
1
E001
E002
E003
E004
E005
E006
E007
E008
E009
E010
E011
29814
30106
30397
30689
30980
31272
31563
31855
32146
32438

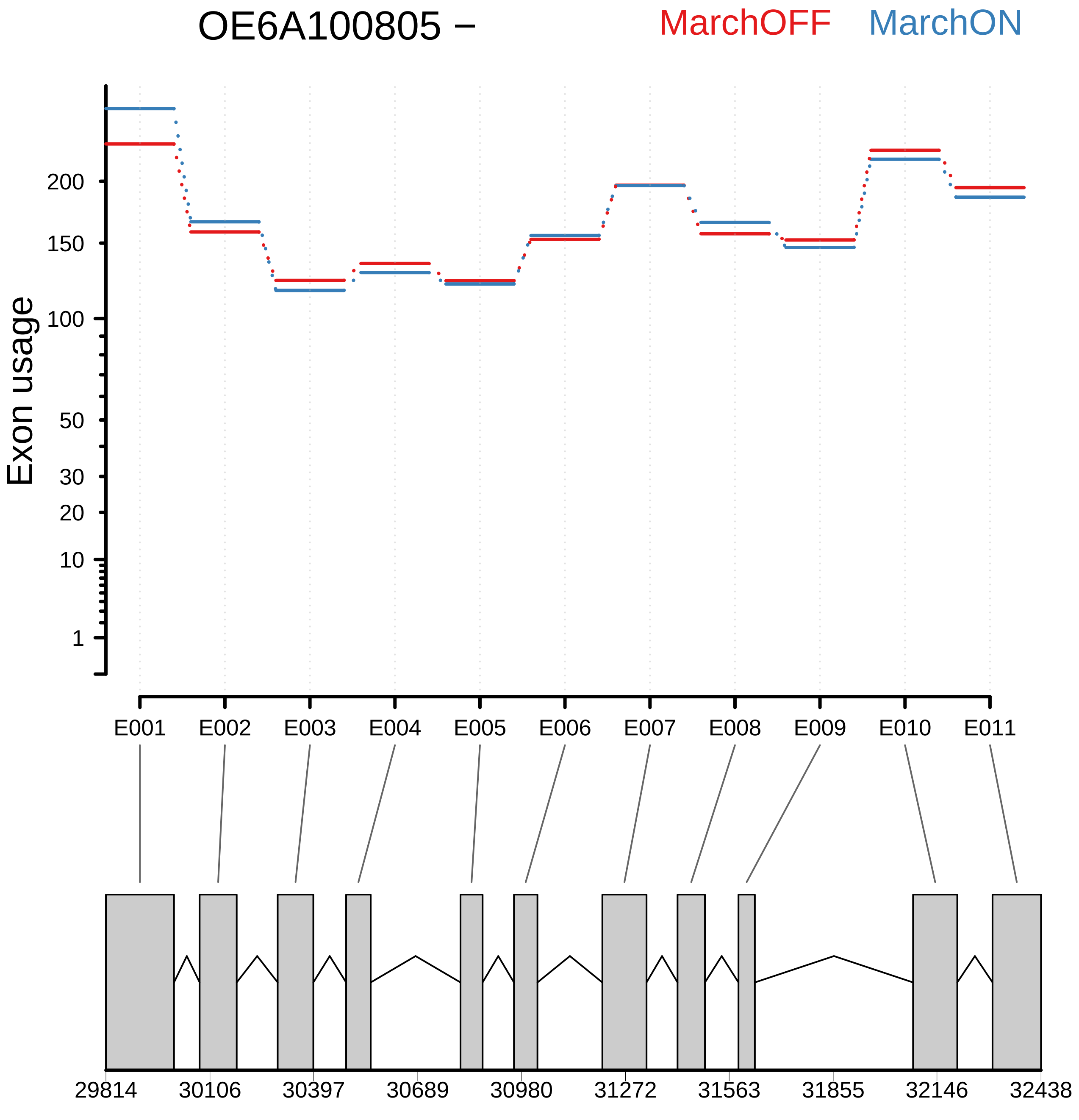
OE6A100805 −
MarchOFF
MarchON
Exon usage
200
150
100
50
30
20
10
1
E001
E002
E003
E004
E005
E006
E007
E008
E009
E010
E011
29814
30106
30397
30689
30980
31272
31563
31855
32146
32438

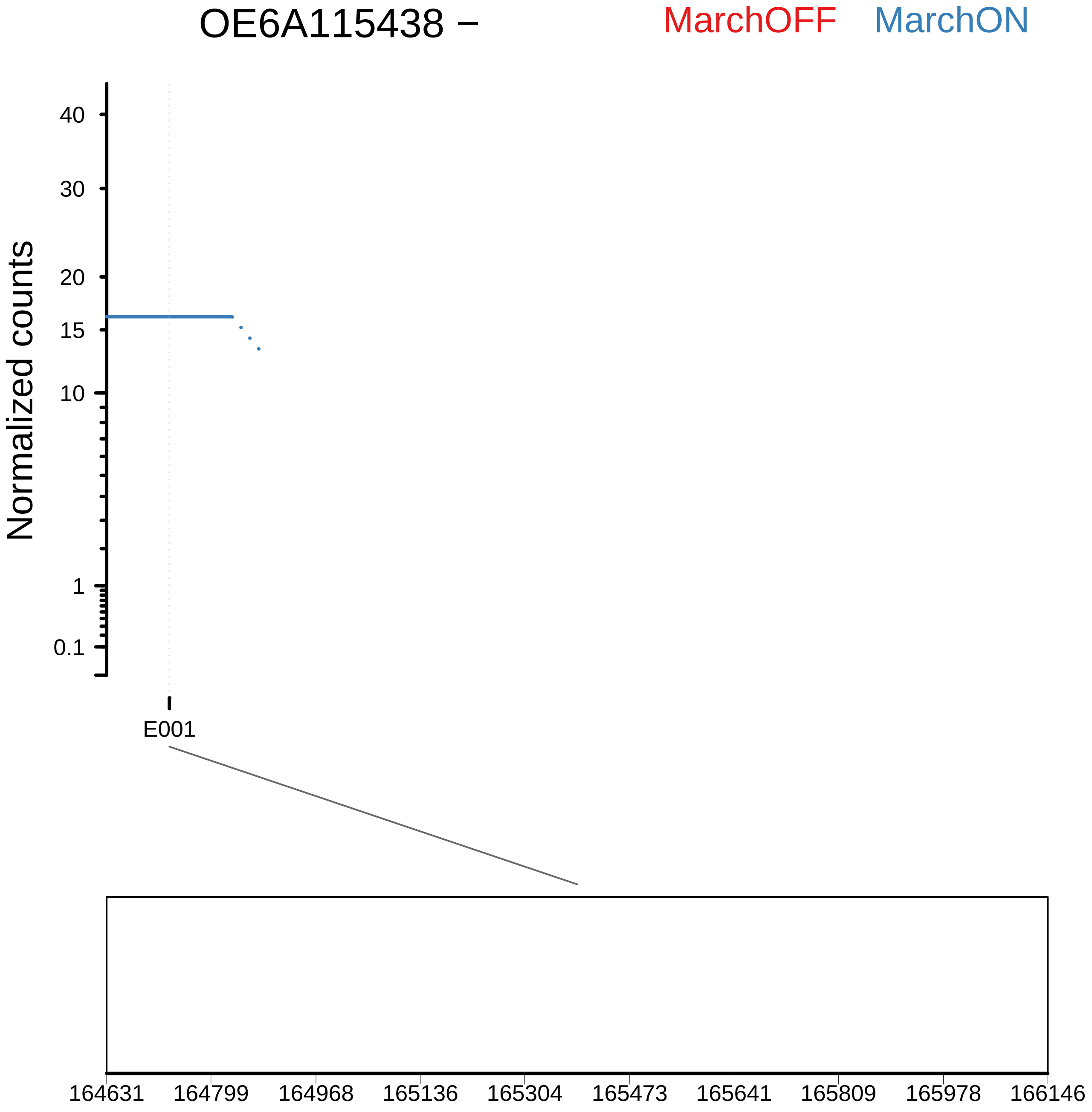

OE6A115438 −
MarchOFF
MarchON
Normalized counts
40
30
20
15
10
1
0.1
E001
164631
164799
164968
165136
165304
165473
165641
165809
165978
166146

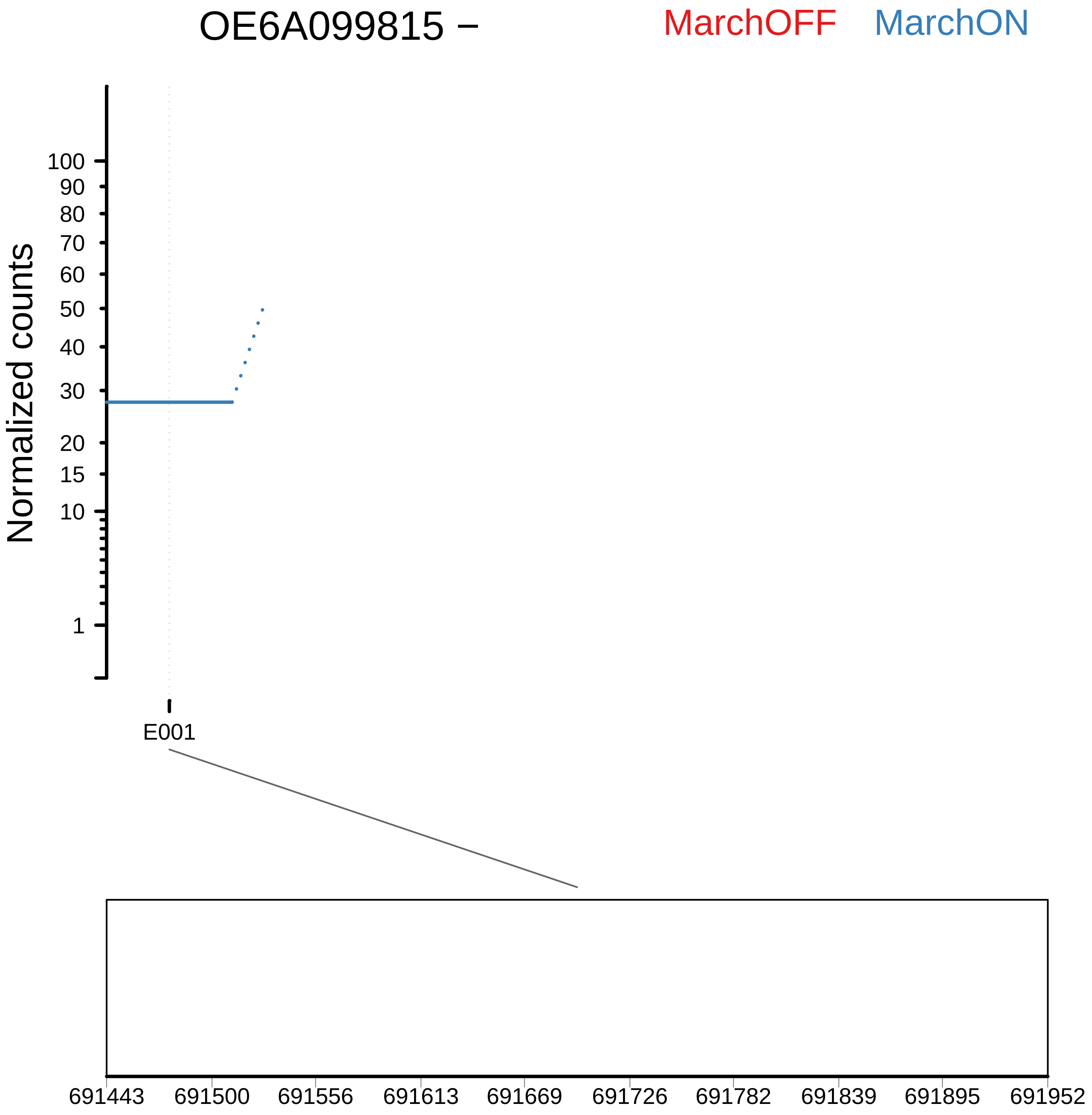
OE6A099815 −
MarchOFF
MarchON
Normalized counts
100
90
80
70
60
50
40
30
20
15
10
1
E001
691443
691500
691556
691613
691669
691726
691782
691839
691895
691952

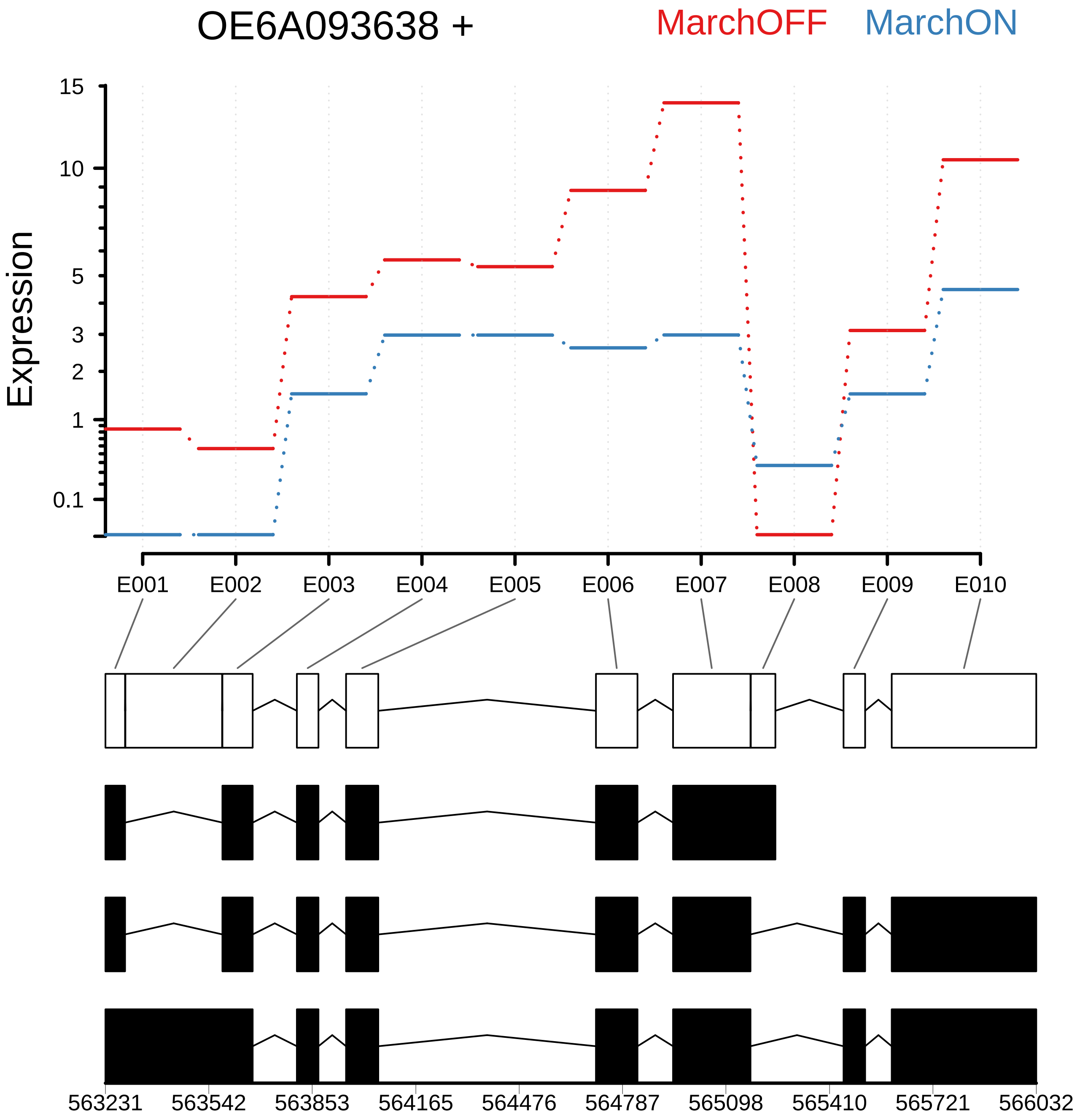
OE6A093638 +
MarchOFF
MarchON
Expression
15
10
5
3
2
1
0.1
E001
E002
E003
E004
E005
E006
E007
E008
E009
E010
563231
563542
563853
564165
564476
564787
565098
565410
565721
566032

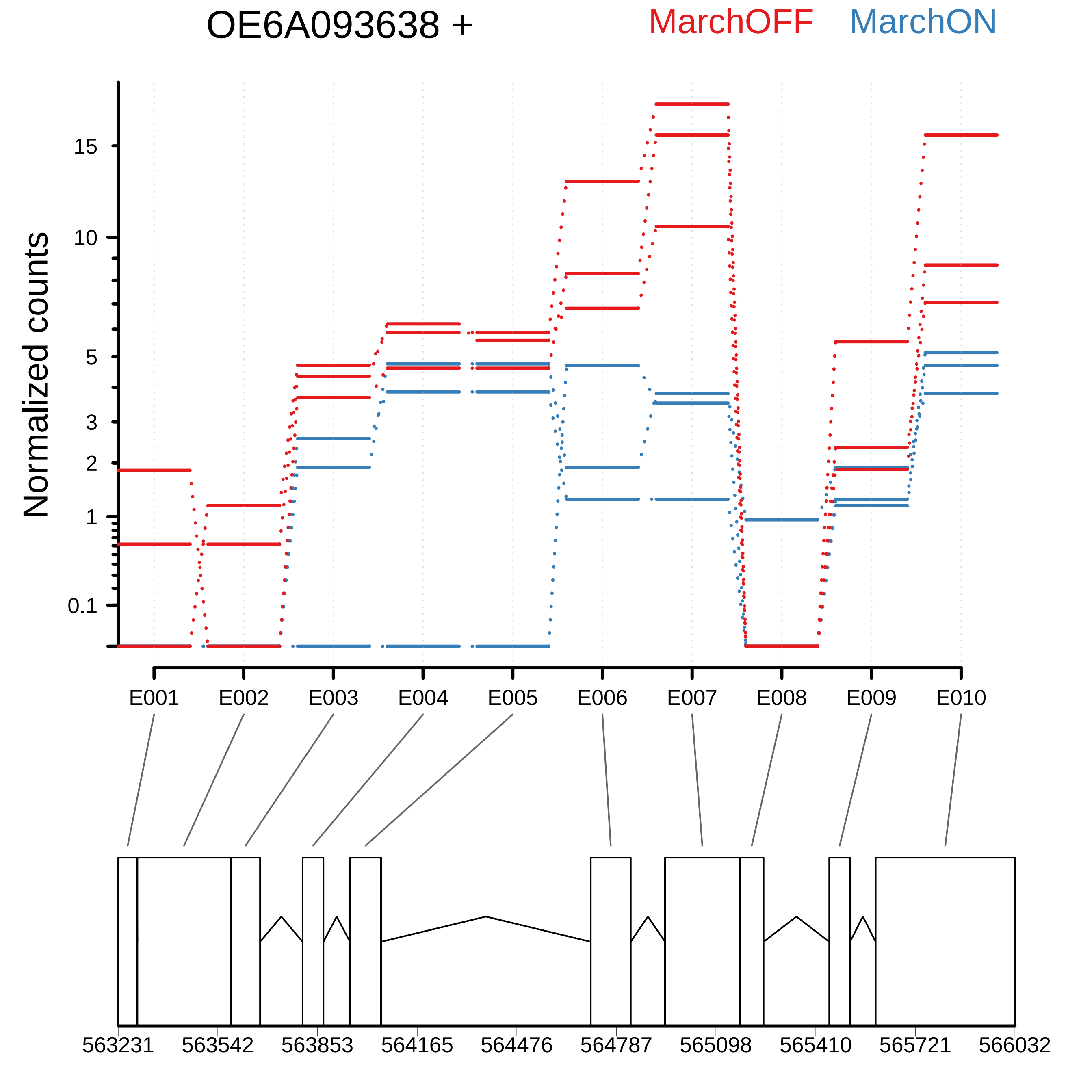
OE6A093638 +
MarchOFF
MarchON
Normalized counts
15
10
5
3
2
1
0.1
E001
E002
E003
E004
E005
E006
E007
E008
E009
E010
563231
563542
563853
564165
564476
564787
565098
565410
565721
566032

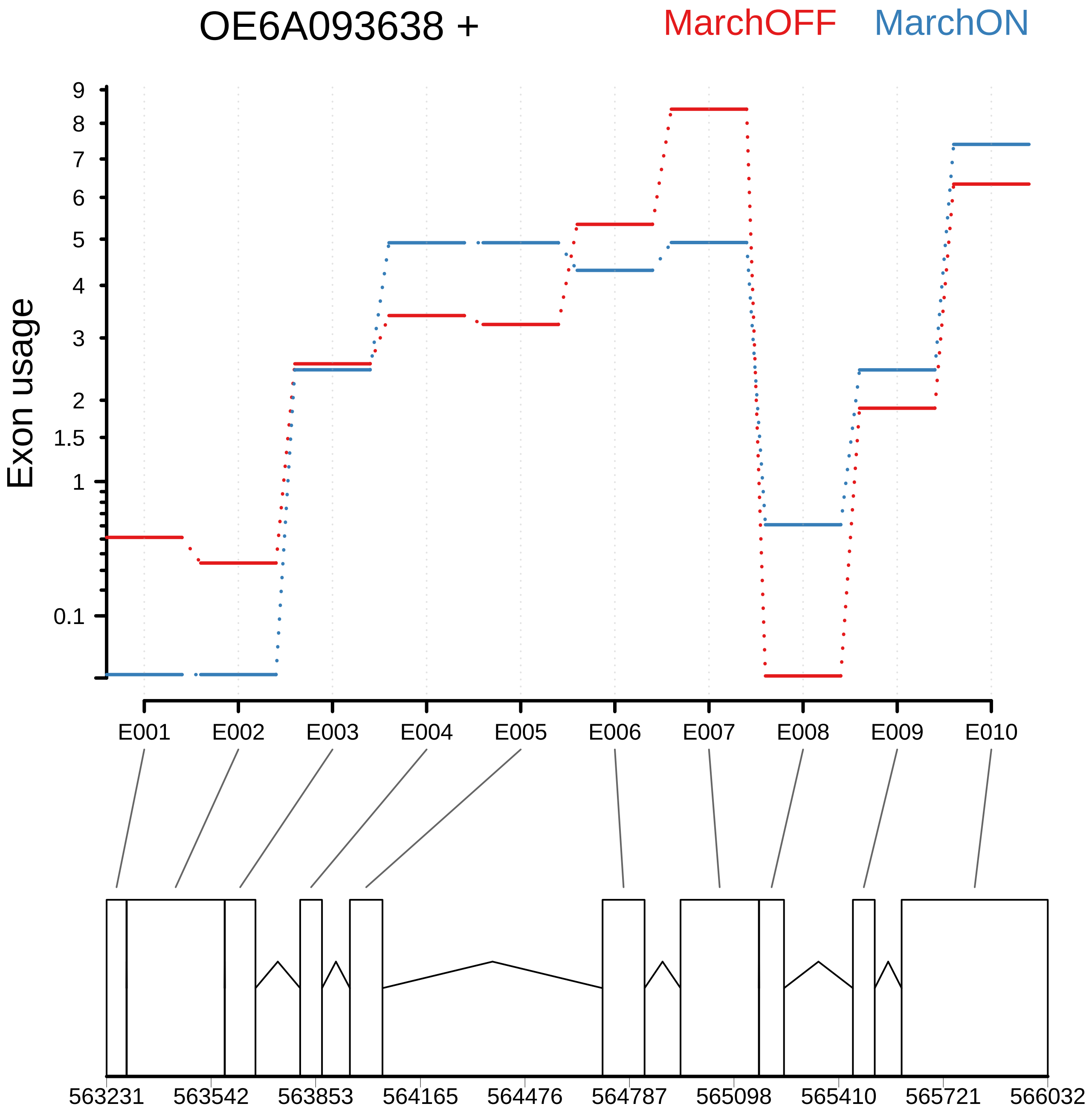

OE6A093638 +
MarchOFF
MarchON
Exon usage
9
8
7
6
5
4
3
2
1.5
1
0.1
E001
E002
E003
E004
E005
E006
E007
E008
E009
E010
563231
563542
563853
564165
564476
564787
565098
565410
565721
566032

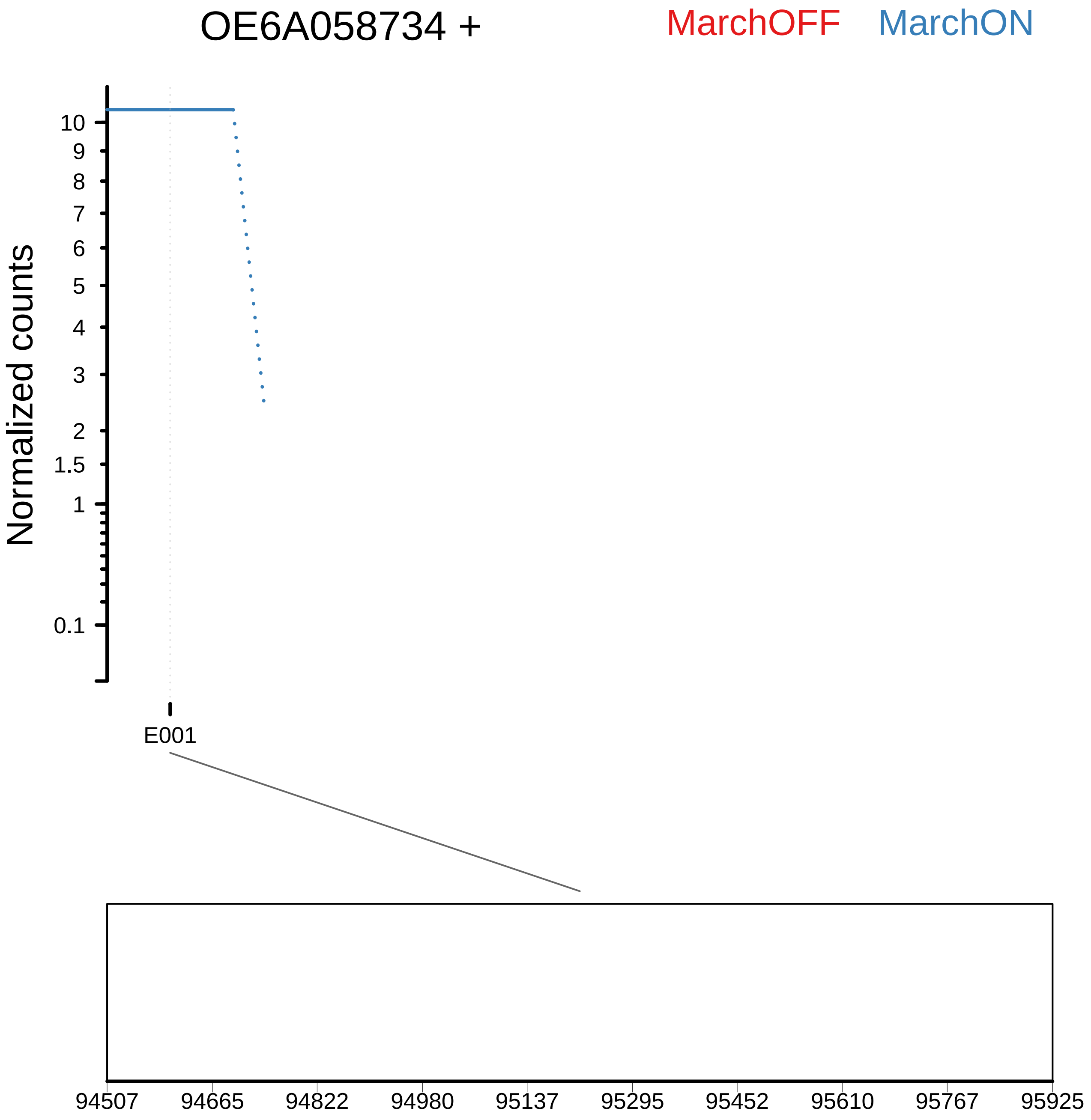
OE6A058734 +
MarchOFF
MarchON
Normalized counts
10
9
8
7
6
5
4
3
2
1.5
1
0.1
E001
94507
94665
94822
94980
95137
95295
95452
95610
95767
95925

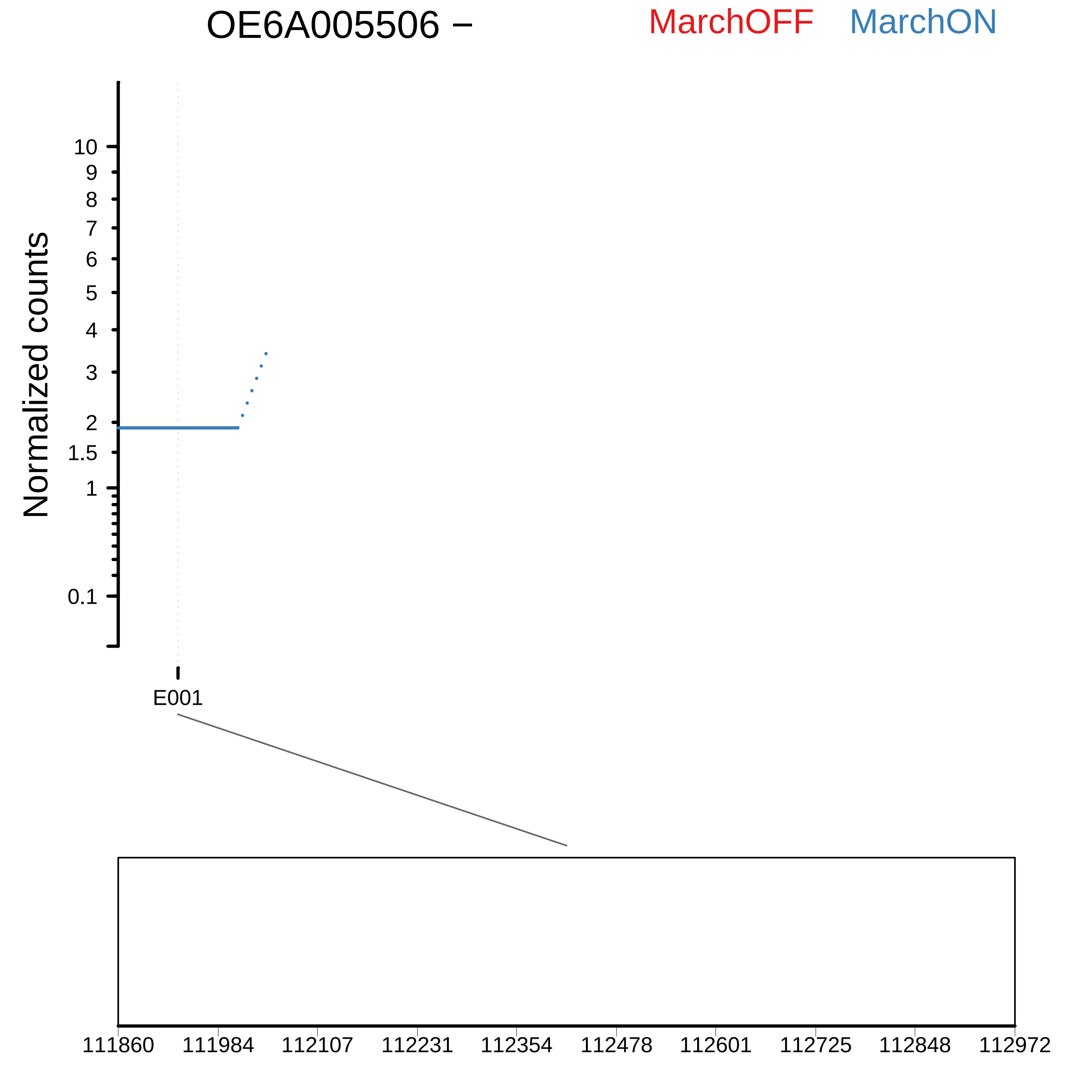
OE6A005506 −
MarchOFF
MarchON
Normalized counts
10
9
8
7
6
5
4
3
2
1.5
1
0.1
E001
111860
111984
112107
112231
112354
112478
112601
112725
112848
112972

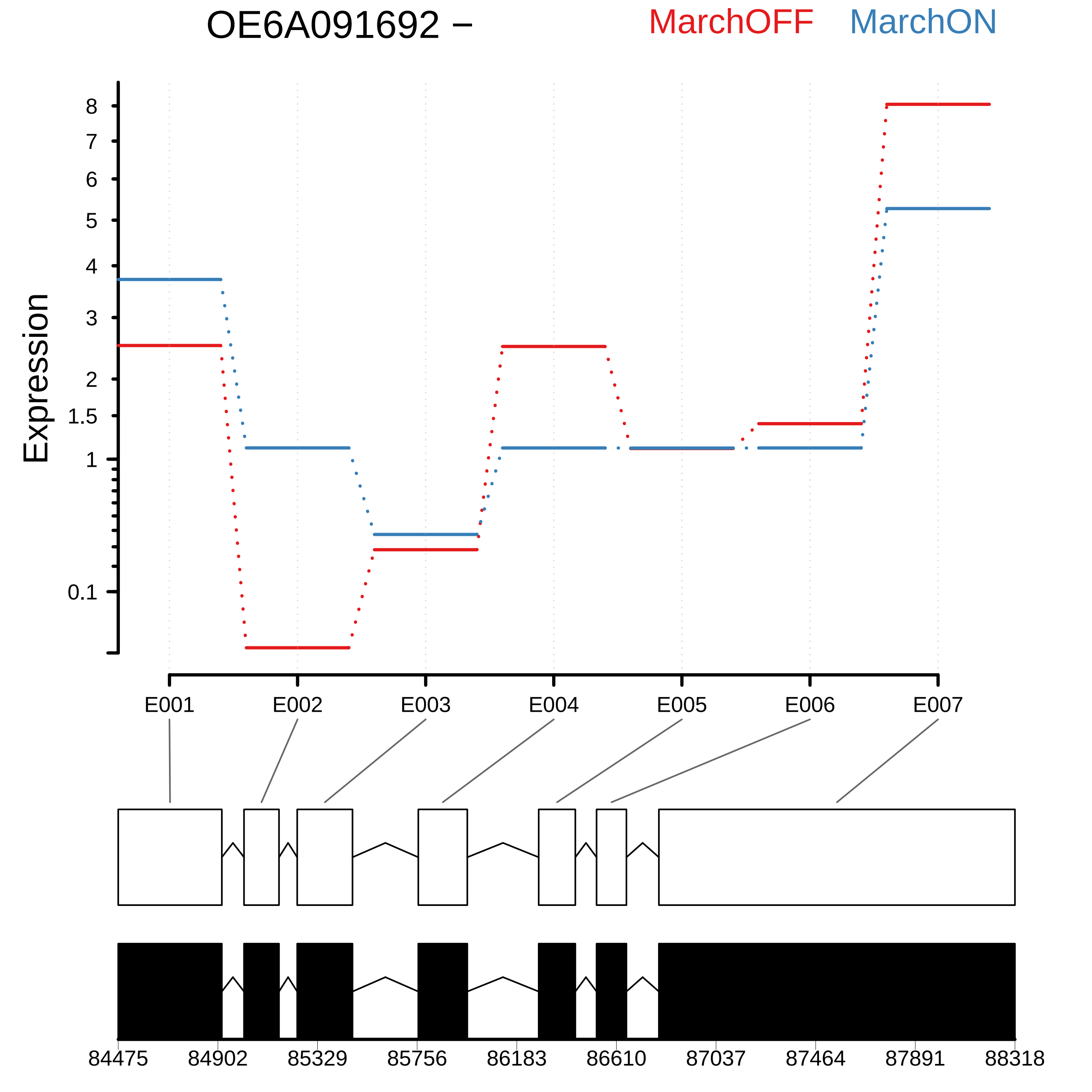

OE6A091692 −
MarchOFF
MarchON
Expression
8
7
6
5
4
3
2
1.5
1
0.1
E001
E002
E003
E004
E005
E006
E007
84475
84902
85329
85756
86183
86610
87037
87464
87891
88318

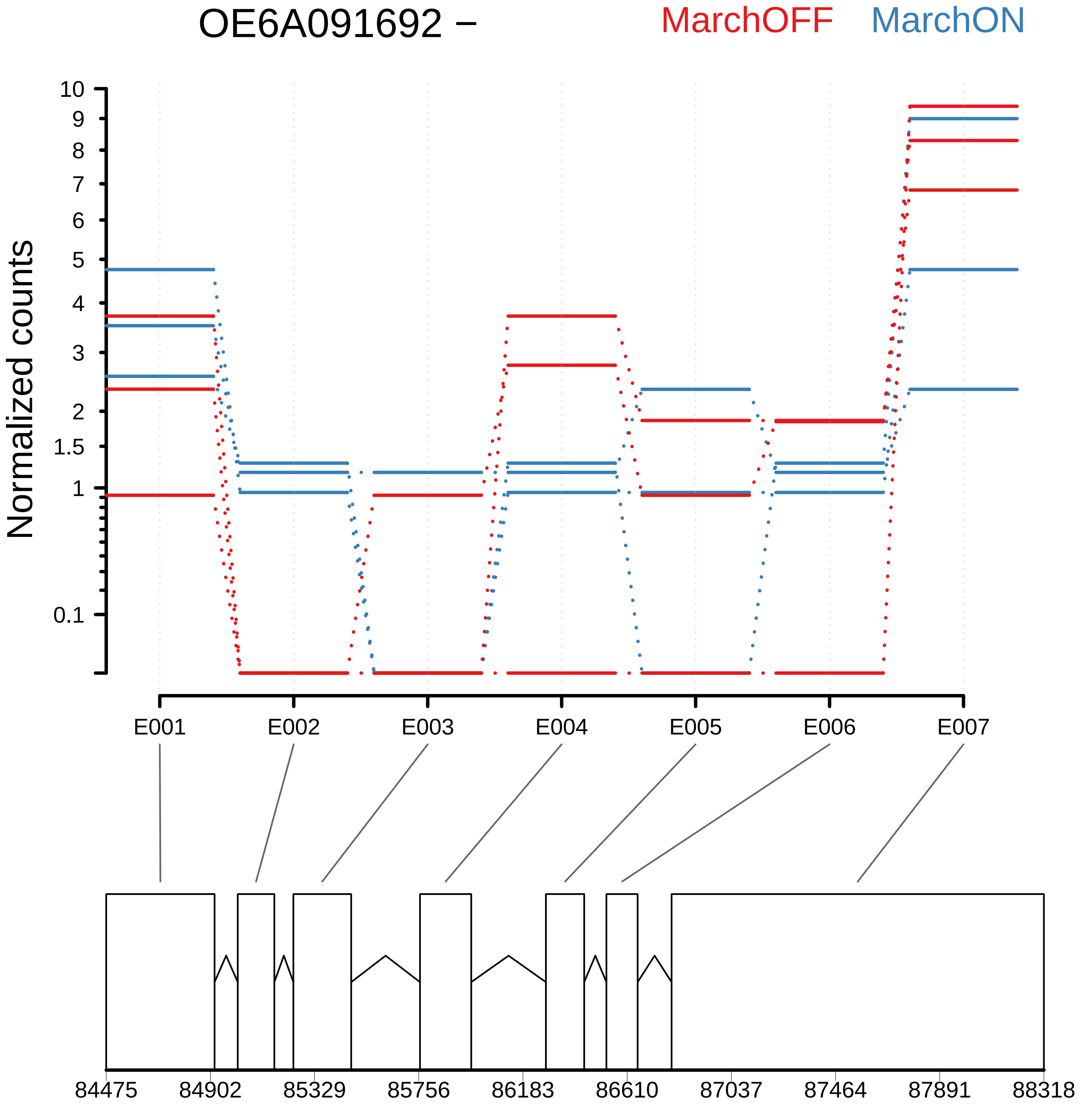

OE6A091692 −
MarchOFF
MarchON
Normalized counts
10
9
8
7
6
5
4
3
2
1.5
1
0.1
E001
E002
E003
E004
E005
E006
E007
84475
84902
85329
85756
86183
86610
87037
87464
87891
88318

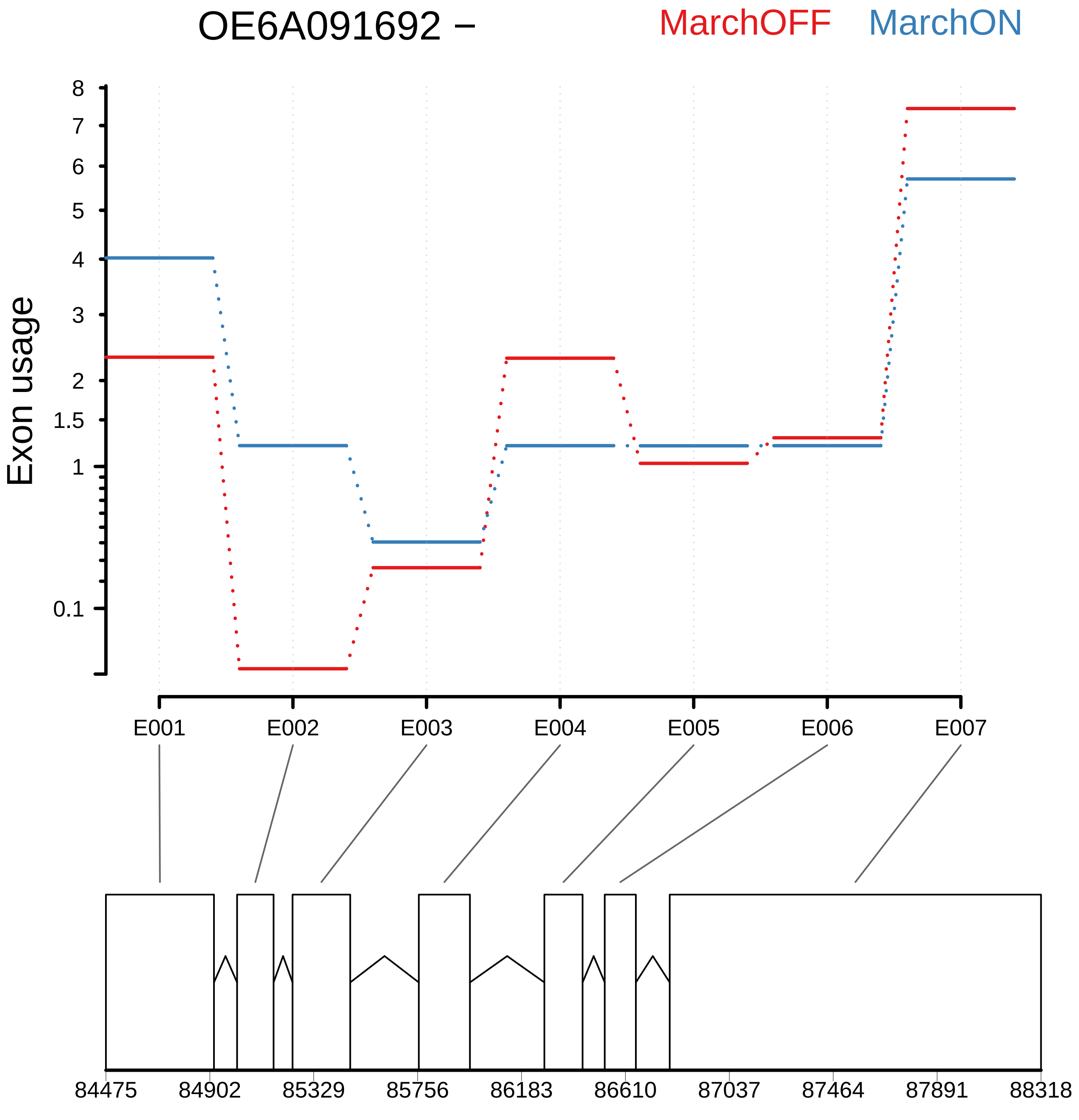
OE6A091692 −
MarchOFF
MarchON
Exon usage
8
7
6
5
4
3
2
1.5
1
0.1
E001
E002
E003
E004
E005
E006
E007
84475
84902
85329
85756
86183
86610
87037
87464
87891
88318

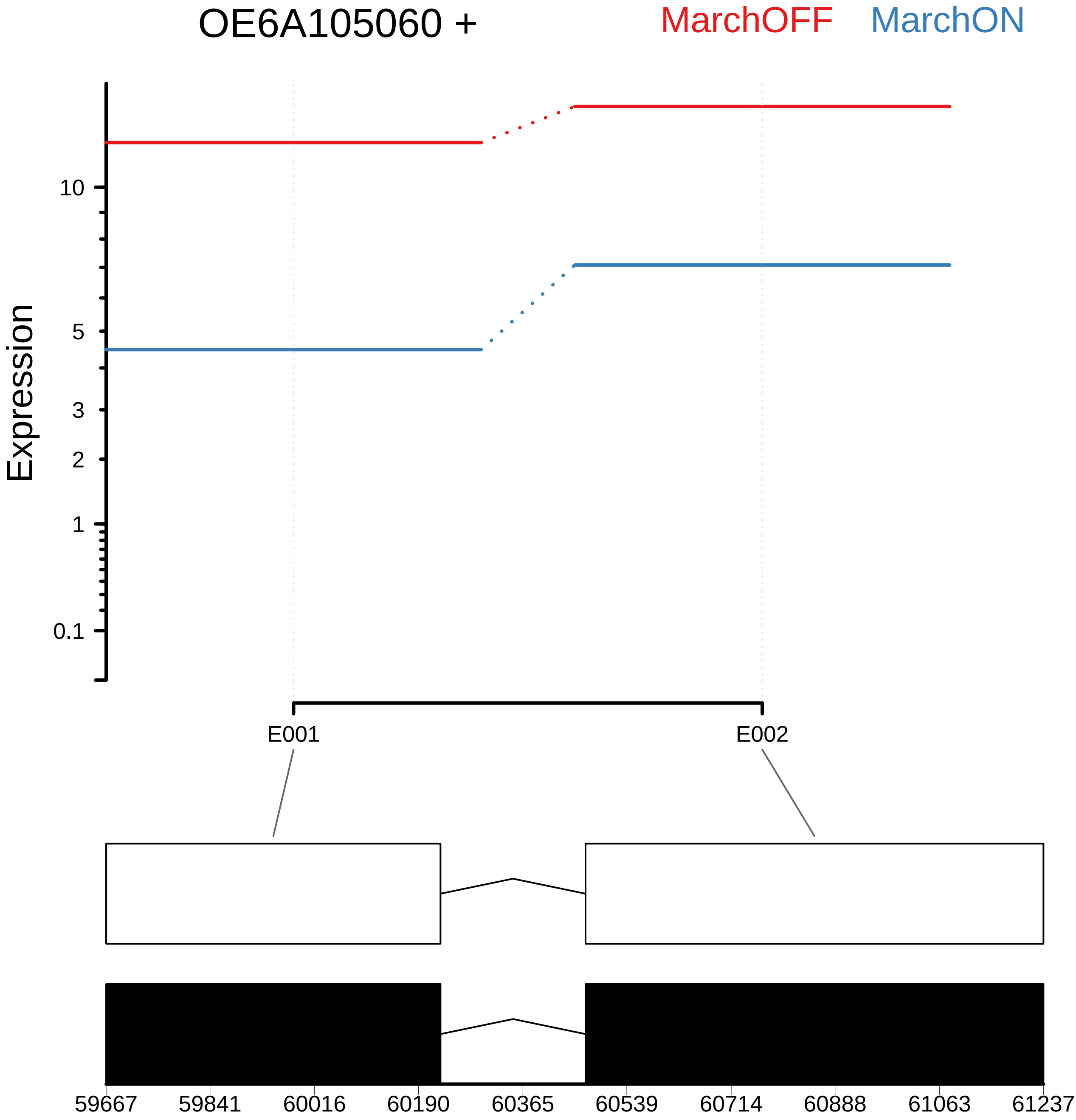

OE6A105060 +
MarchOFF
MarchON
Expression
10
5
3
2
1
0.1
E001
E002
59667
59841
60016
60190
60365
60539
60714
60888
61063
61237

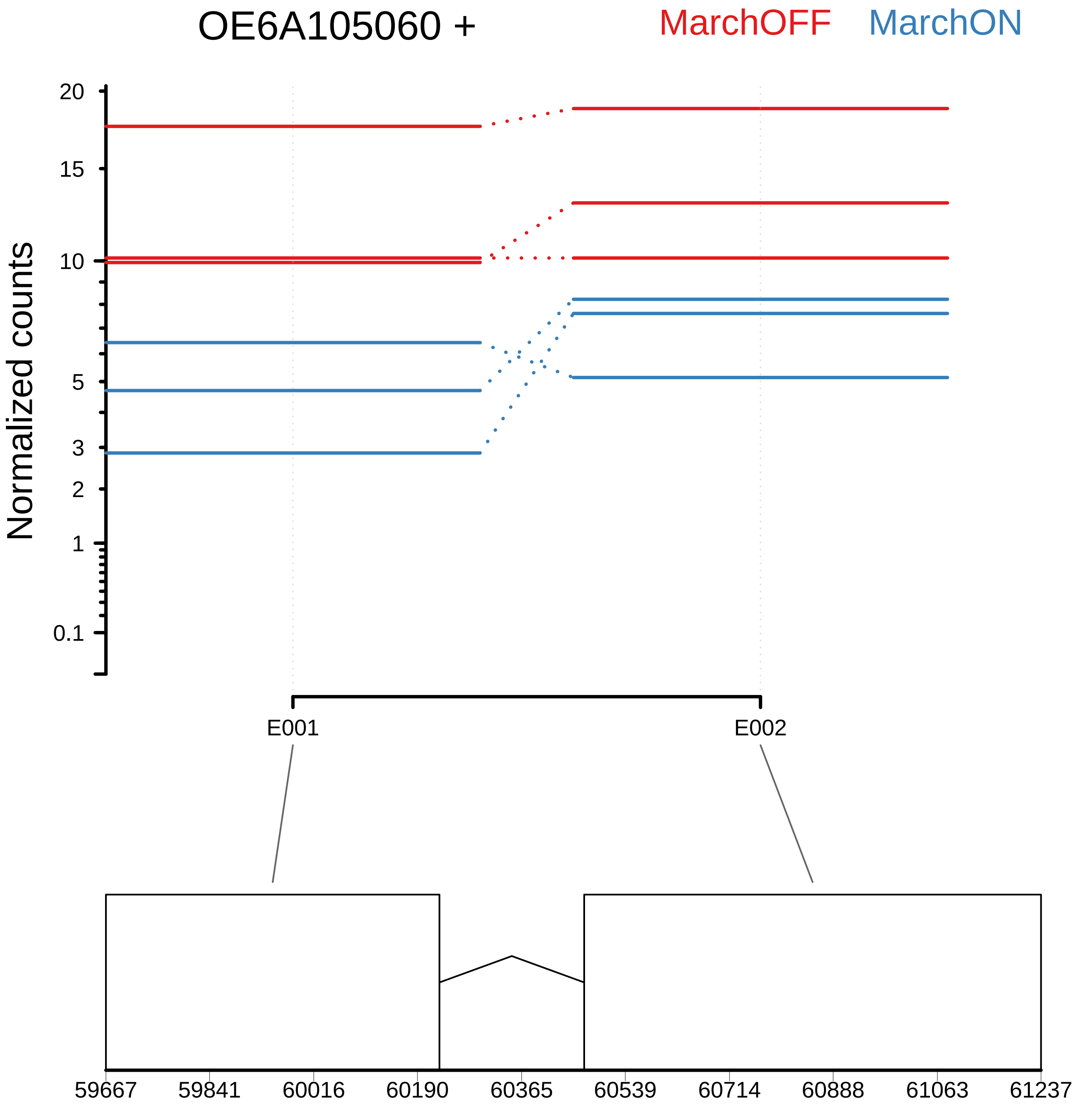

OE6A105060 +
MarchOFF
MarchON
Normalized counts
20
15
10
5
3
2
1
0.1
E001
E002
59667
59841
60016
60190
60365
60539
60714
60888
61063
61237

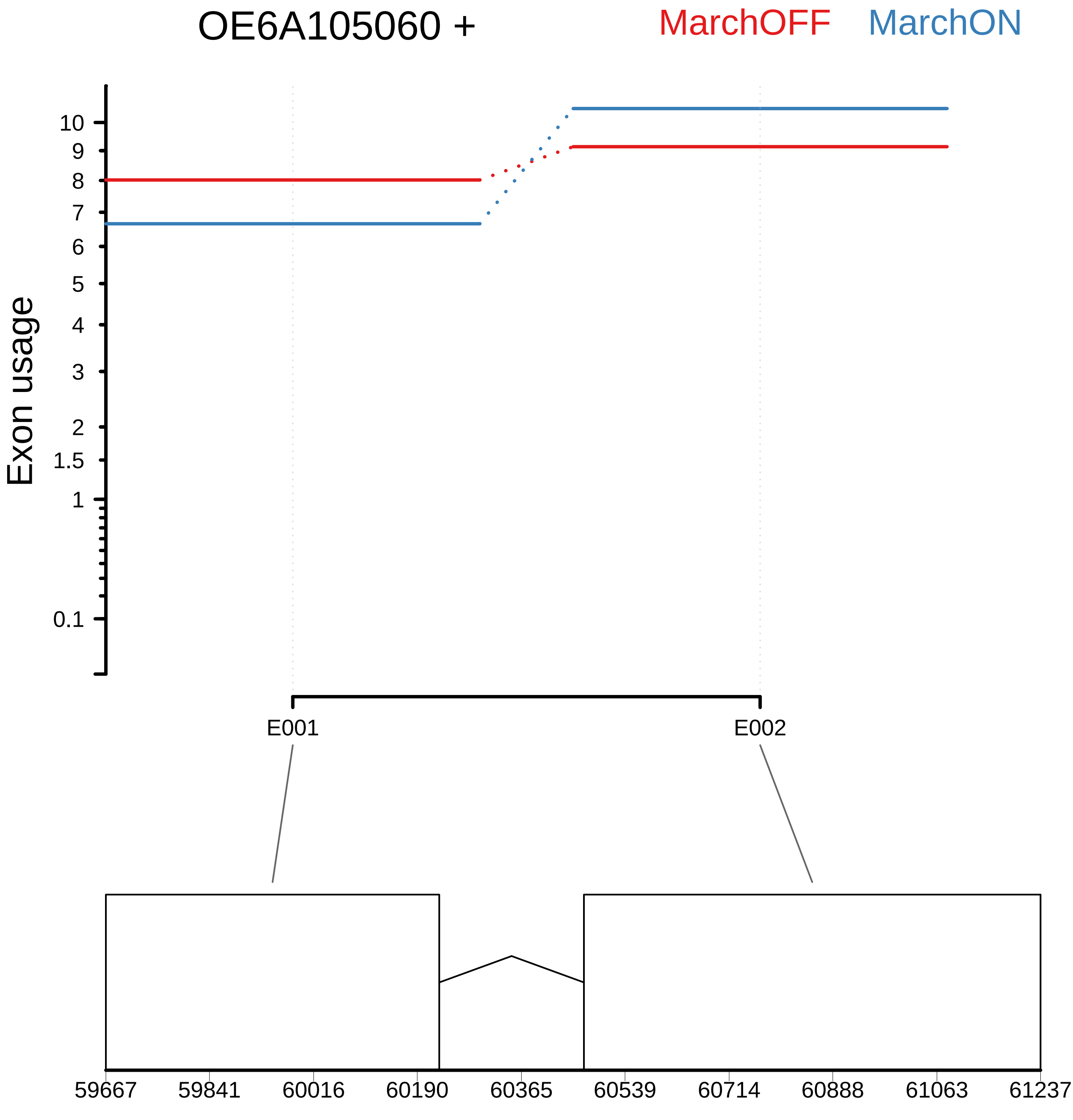

OE6A105060 +
MarchOFF
MarchON
Exon usage
10
9
8
7
6
5
4
3
2
1.5
1
0.1
E001
E002
59667
59841
60016
60190
60365
60539
60714
60888
61063
61237

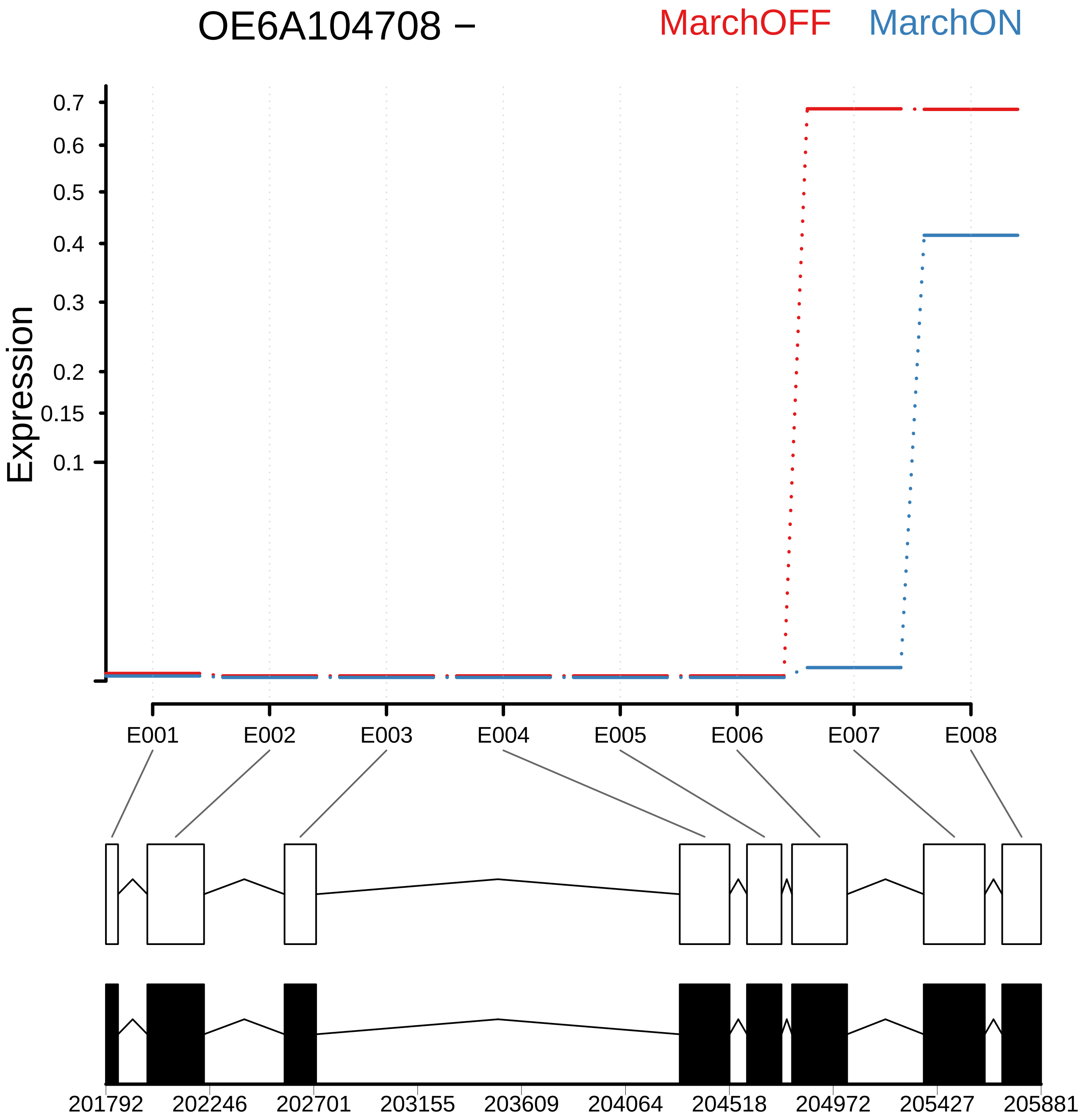

OE6A104708 −
MarchOFF
MarchON
Expression
0.7
0.6
0.5
0.4
0.3
0.2
0.15
0.1
E001
E002
E003
E004
E005
E006
E007
E008
201792
202246
202701
203155
203609
204064
204518
204972
205427
205881

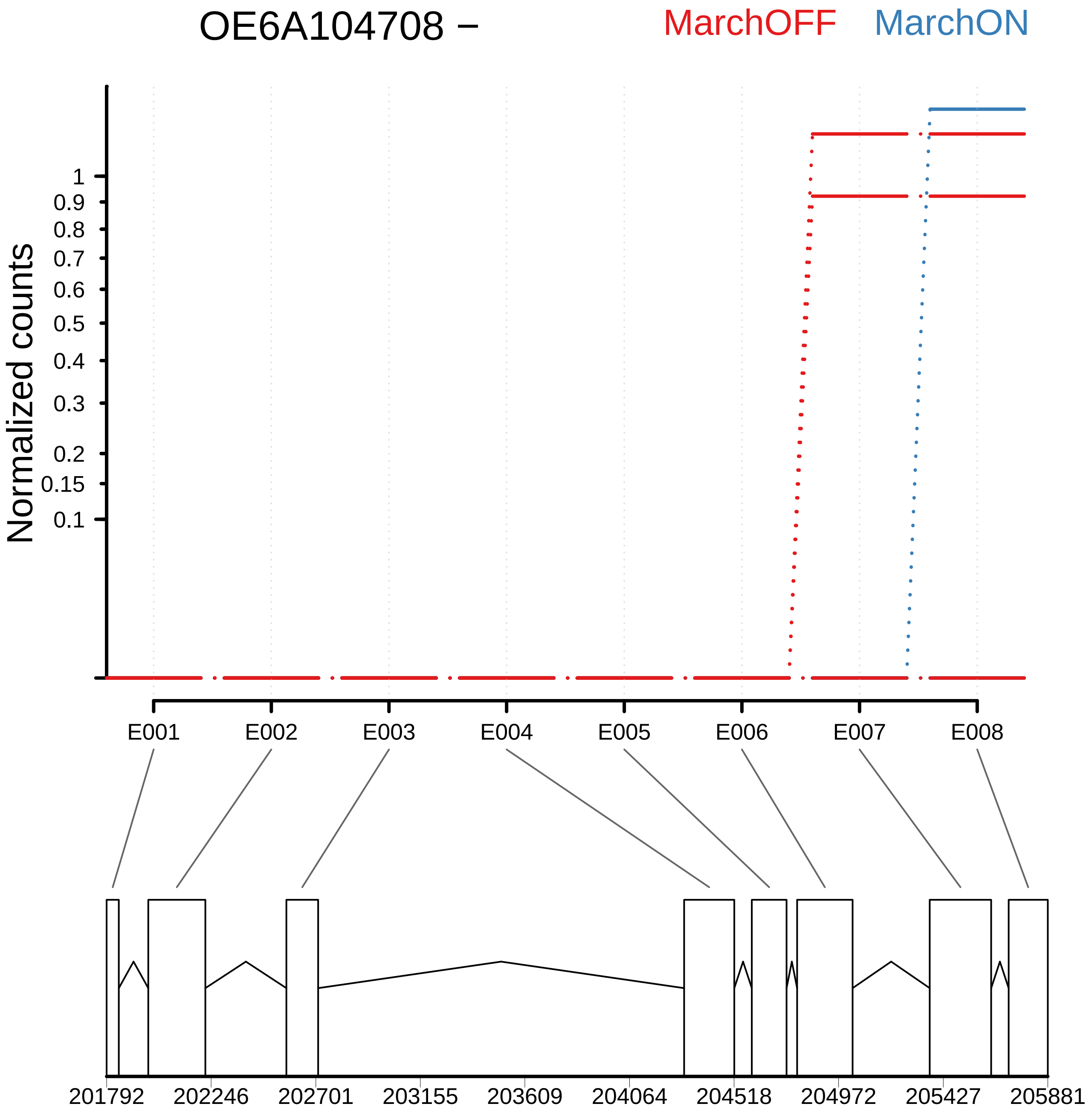
OE6A104708 −
MarchOFF
MarchON
Normalized counts
1
0.9
0.8
0.7
0.6
0.5
0.4
0.3
0.2
0.15
0.1
E001
E002
E003
E004
E005
E006
E007
E008
201792
202246
202701
203155
203609
204064
204518
204972
205427
205881

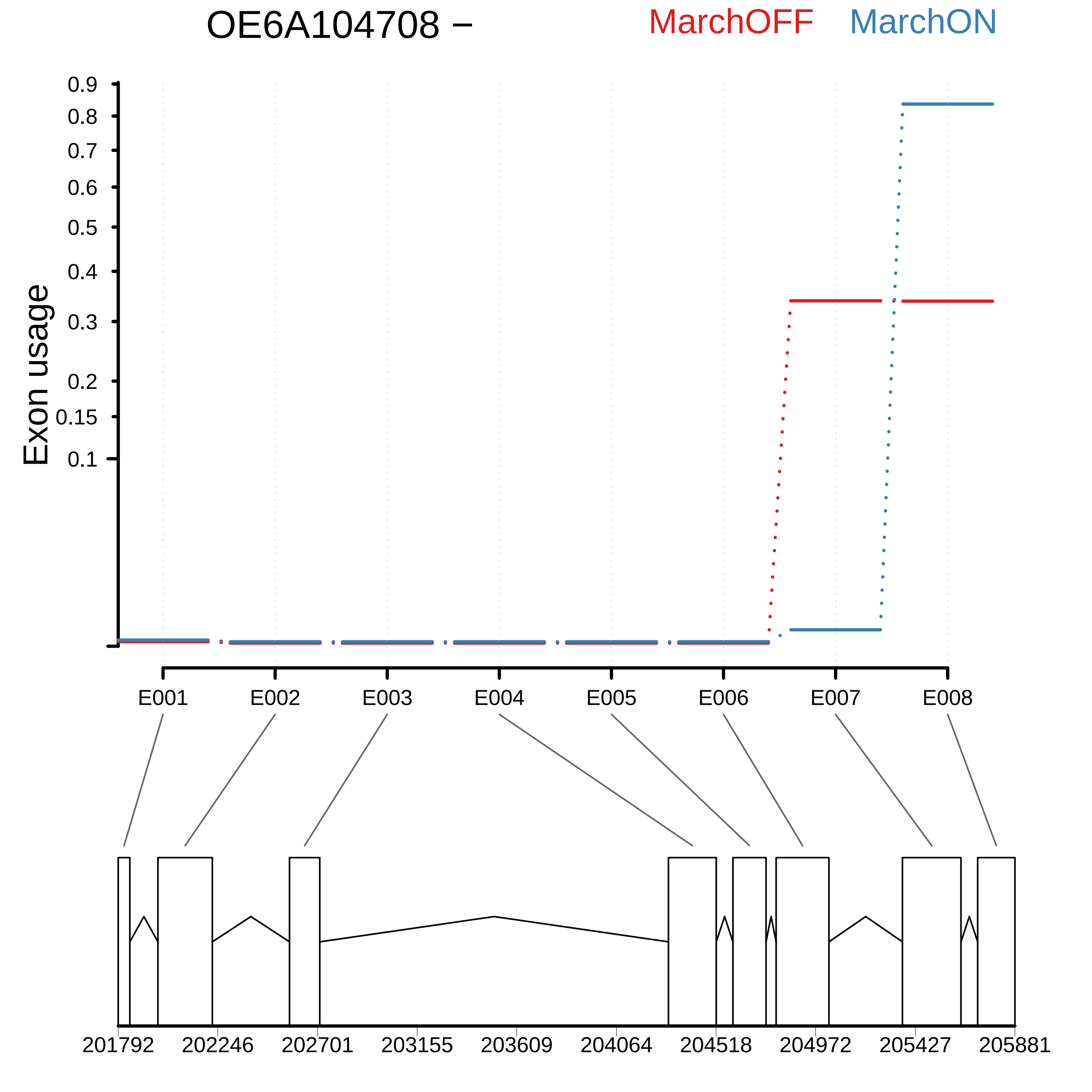
OE6A104708 −
MarchOFF
MarchON
Exon usage
0.9
0.8
0.7
0.6
0.5
0.4
0.3
0.2
0.15
0.1
E001
E002
E003
E004
E005
E006
E007
E008
201792
202246
202701
203155
203609
204064
204518
204972
205427
205881

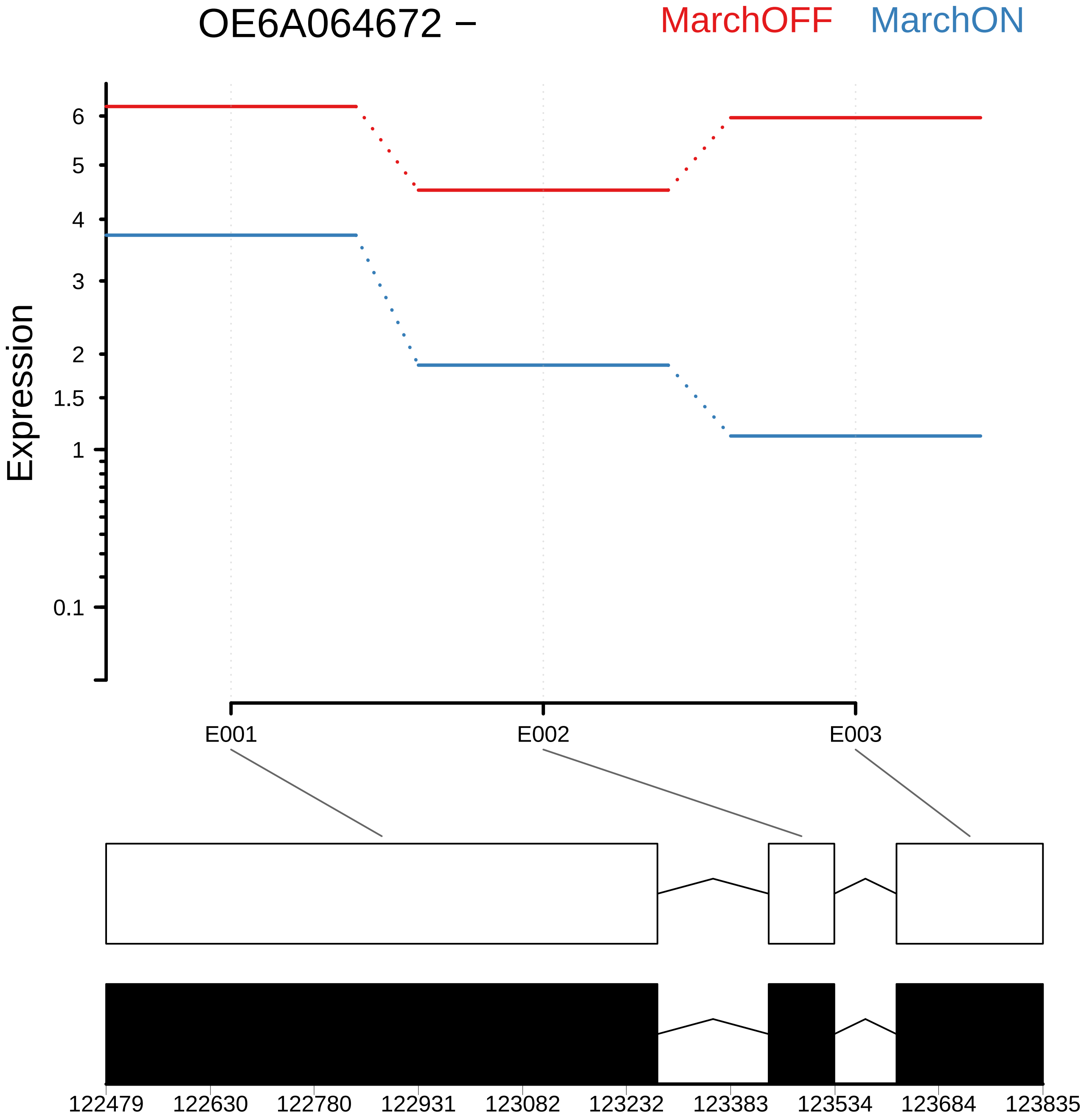

OE6A064672 −
MarchOFF
MarchON
Expression
6
5
4
3
2
1.5
1
0.1
E001
E002
E003
122479
122630
122780
122931
123082
123232
123383
123534
123684
123835

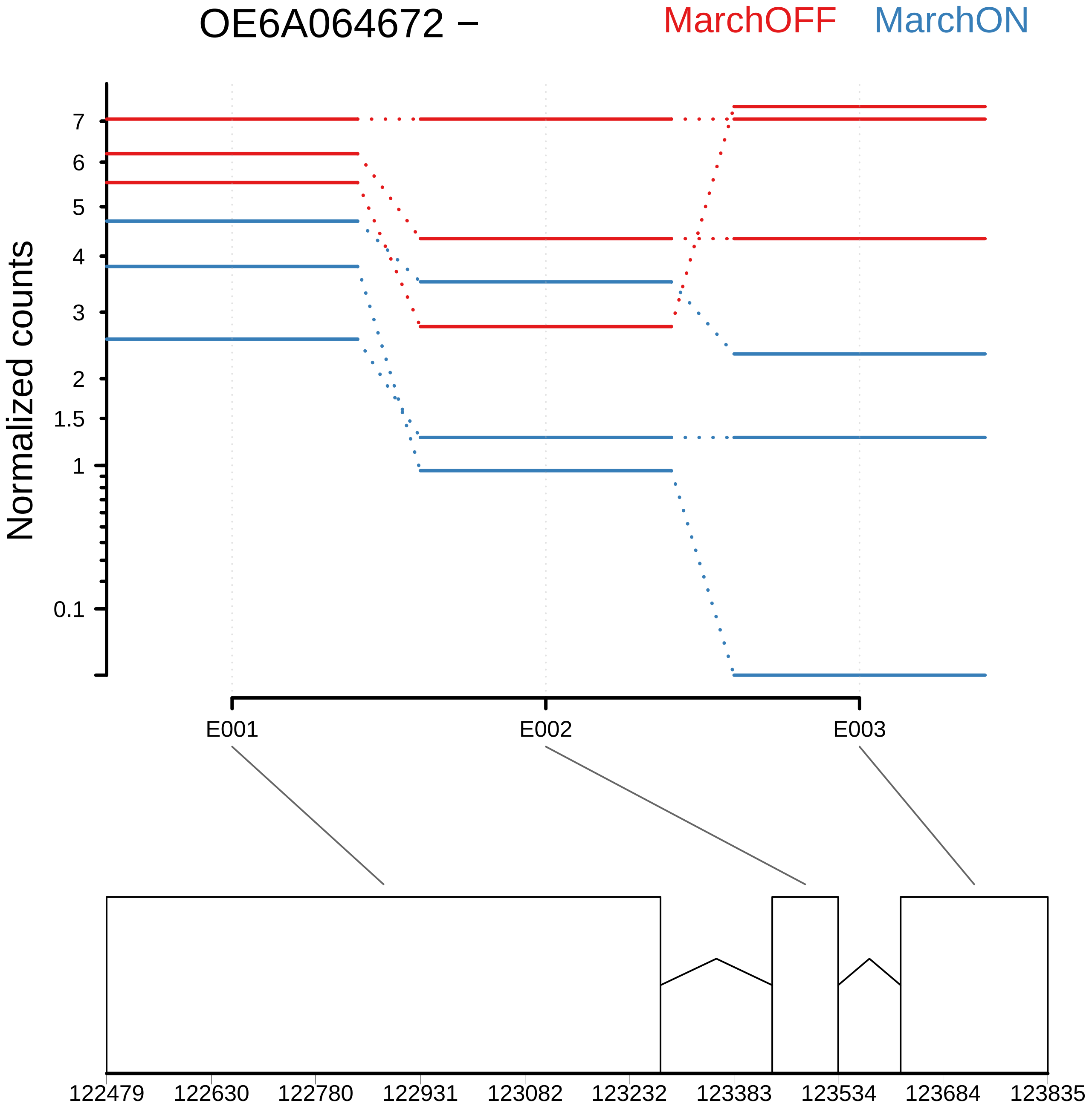
OE6A064672 −
MarchOFF
MarchON
Normalized counts
7
6
5
4
3
2
1.5
1
0.1
E001
E002
E003
122479
122630
122780
122931
123082
123232
123383
123534
123684
123835

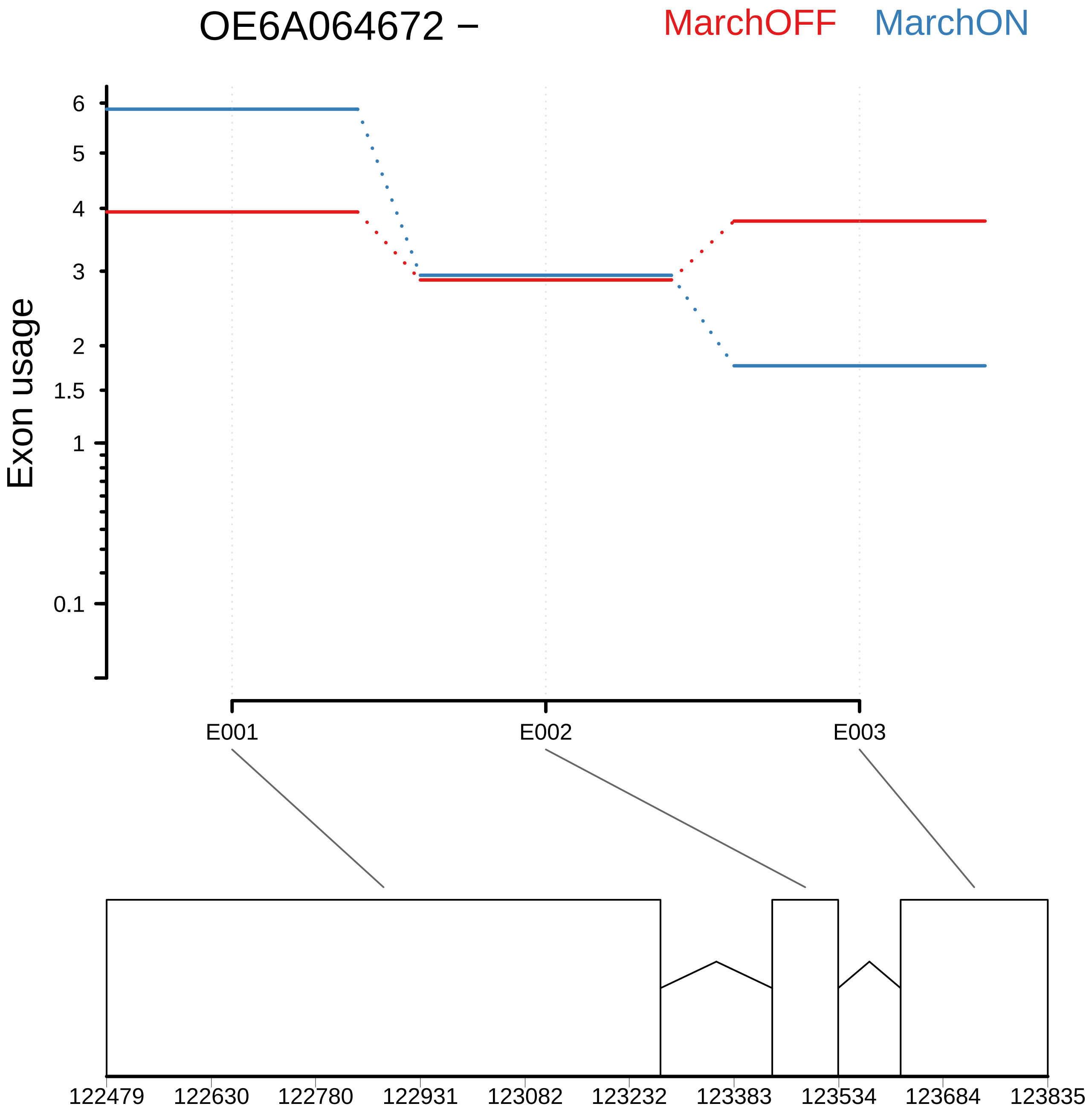

OE6A064672 −
MarchOFF
MarchON
Exon usage
6
5
4
3
2
1.5
1
0.1
E001
E002
E003
122479
122630
122780
122931
123082
123232
123383
123534
123684
123835

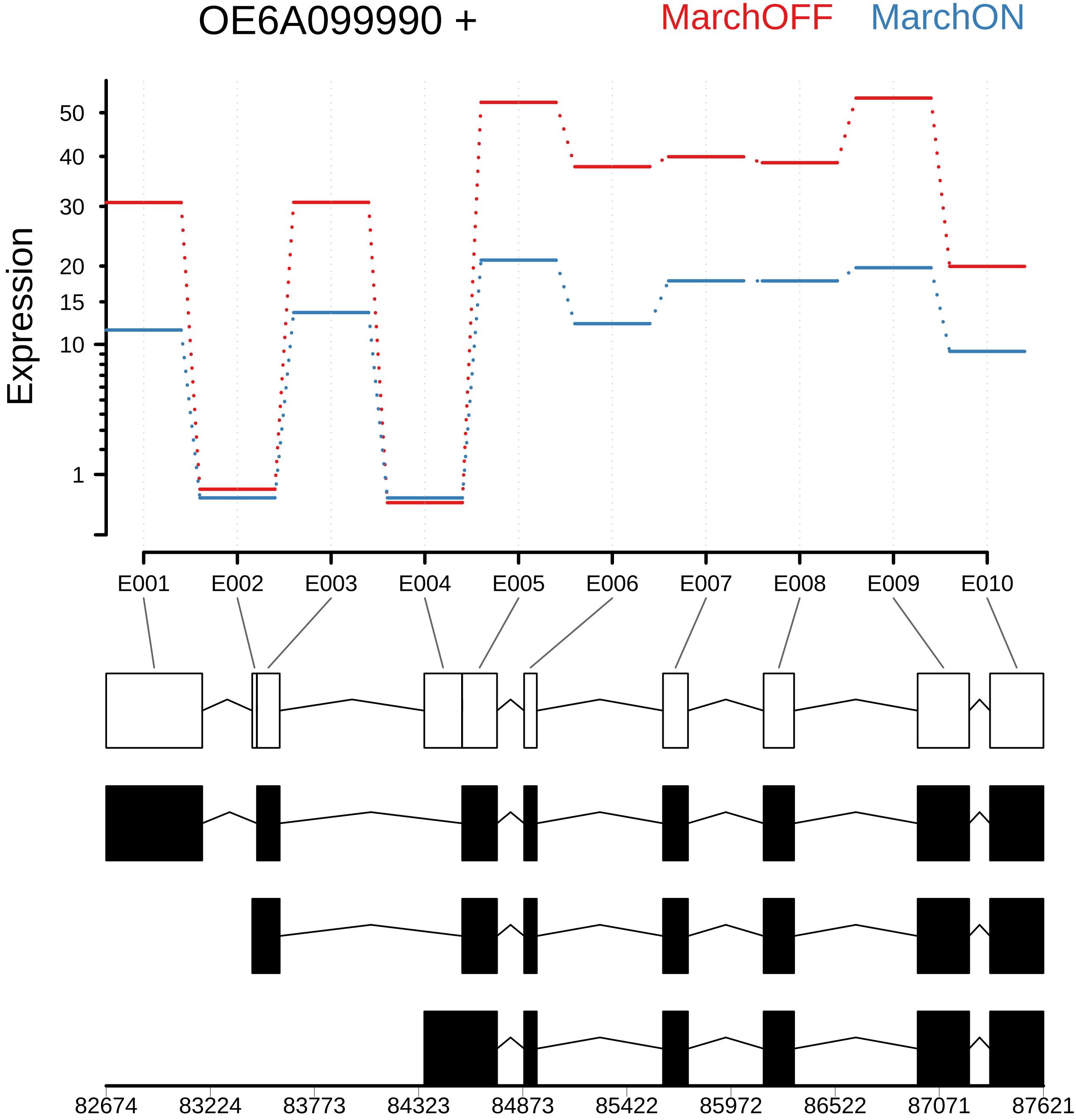
OE6A099990 +
MarchOFF
MarchON
Expression
50
40
30
20
15
10
1
E001
E002
E003
E004
E005
E006
E007
E008
E009
E010
82674
83224
83773
84323
84873
85422
85972
86522
87071
87621

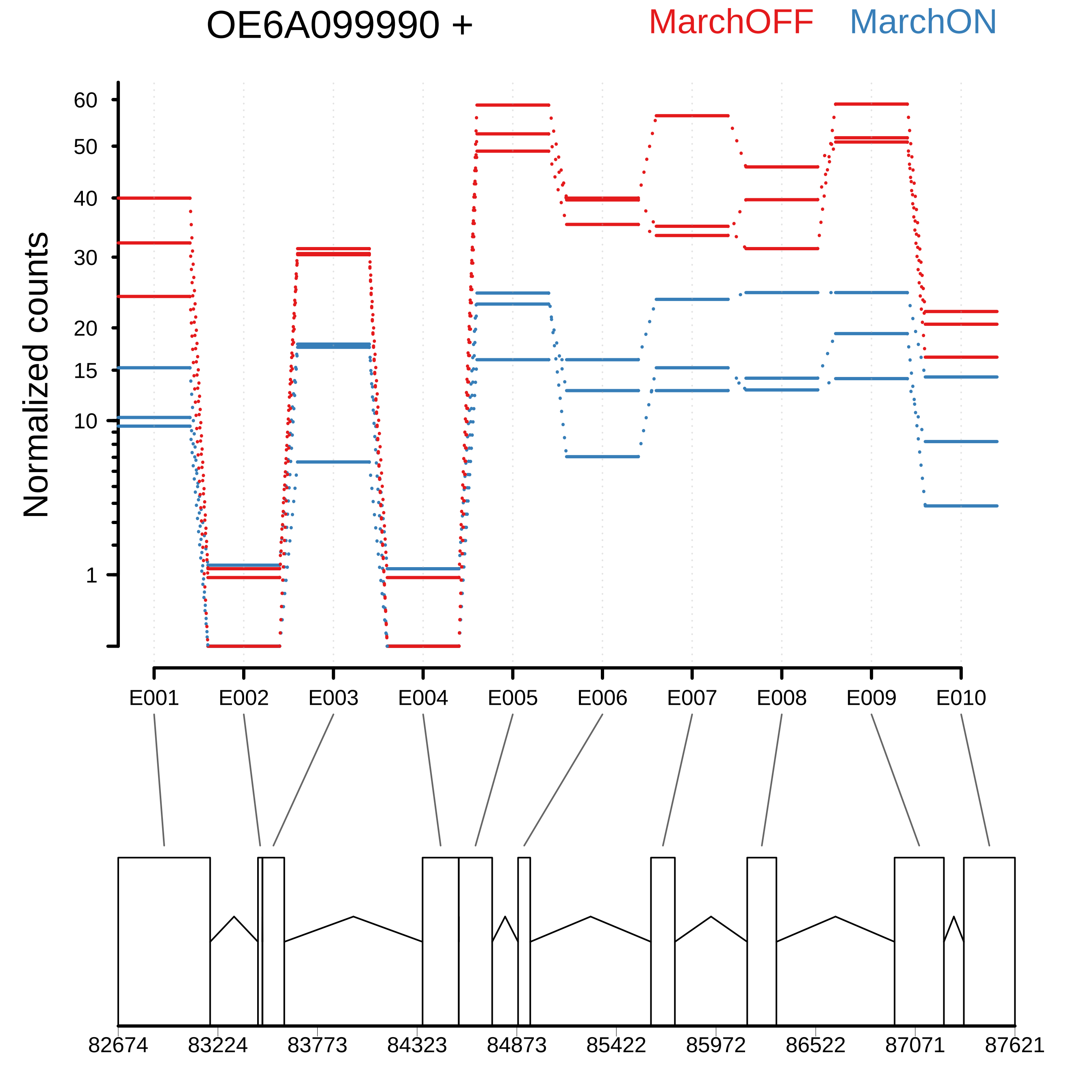

OE6A099990 +
MarchOFF
MarchON
Normalized counts
60
50
40
30
20
15
10
1
E001
E002
E003
E004
E005
E006
E007
E008
E009
E010
82674
83224
83773
84323
84873
85422
85972
86522
87071
87621

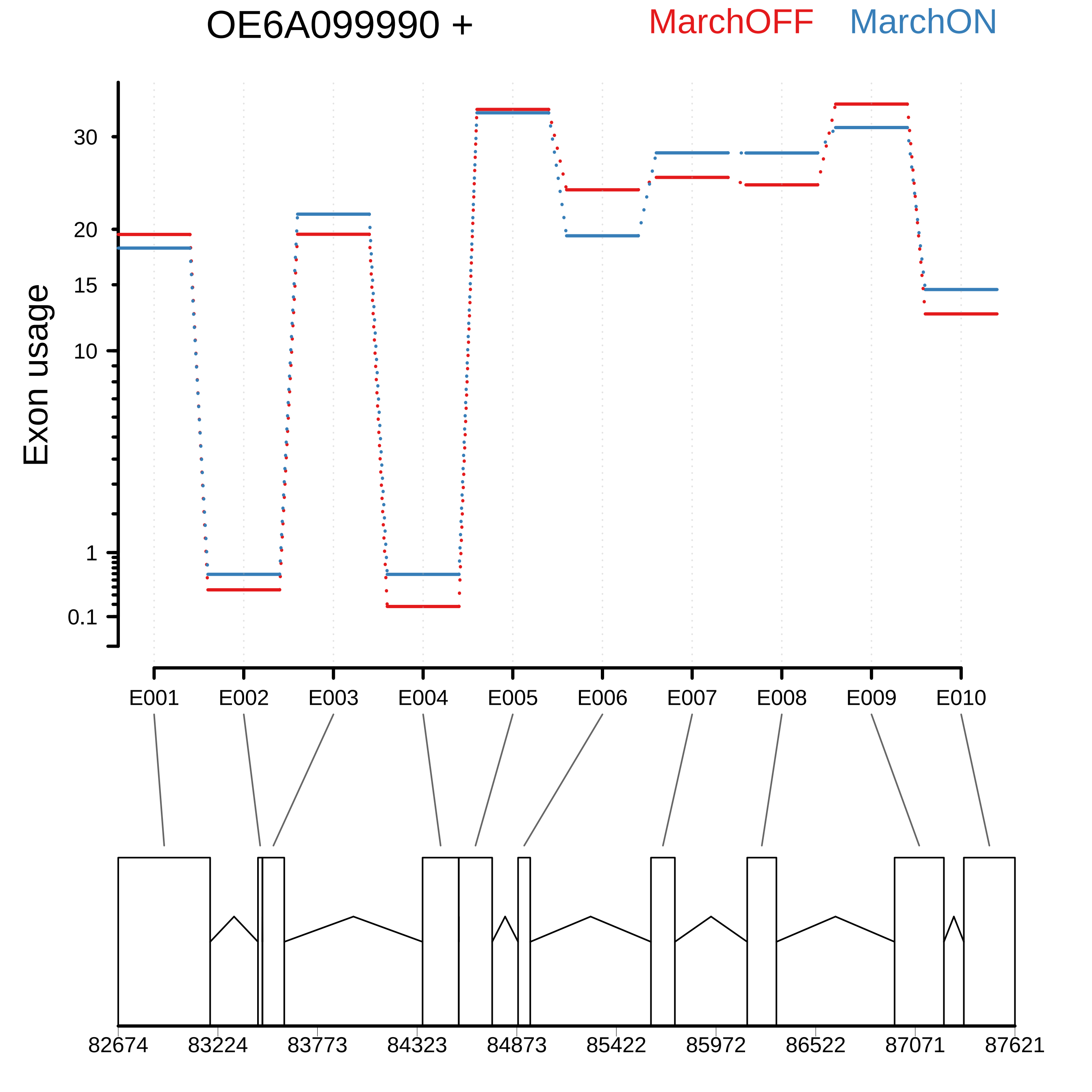

OE6A099990 +
MarchOFF
MarchON
Exon usage
30
20
15
10
1
0.1
E001
E002
E003
E004
E005
E006
E007
E008
E009
E010
82674
83224
83773
84323
84873
85422
85972
86522
87071
87621

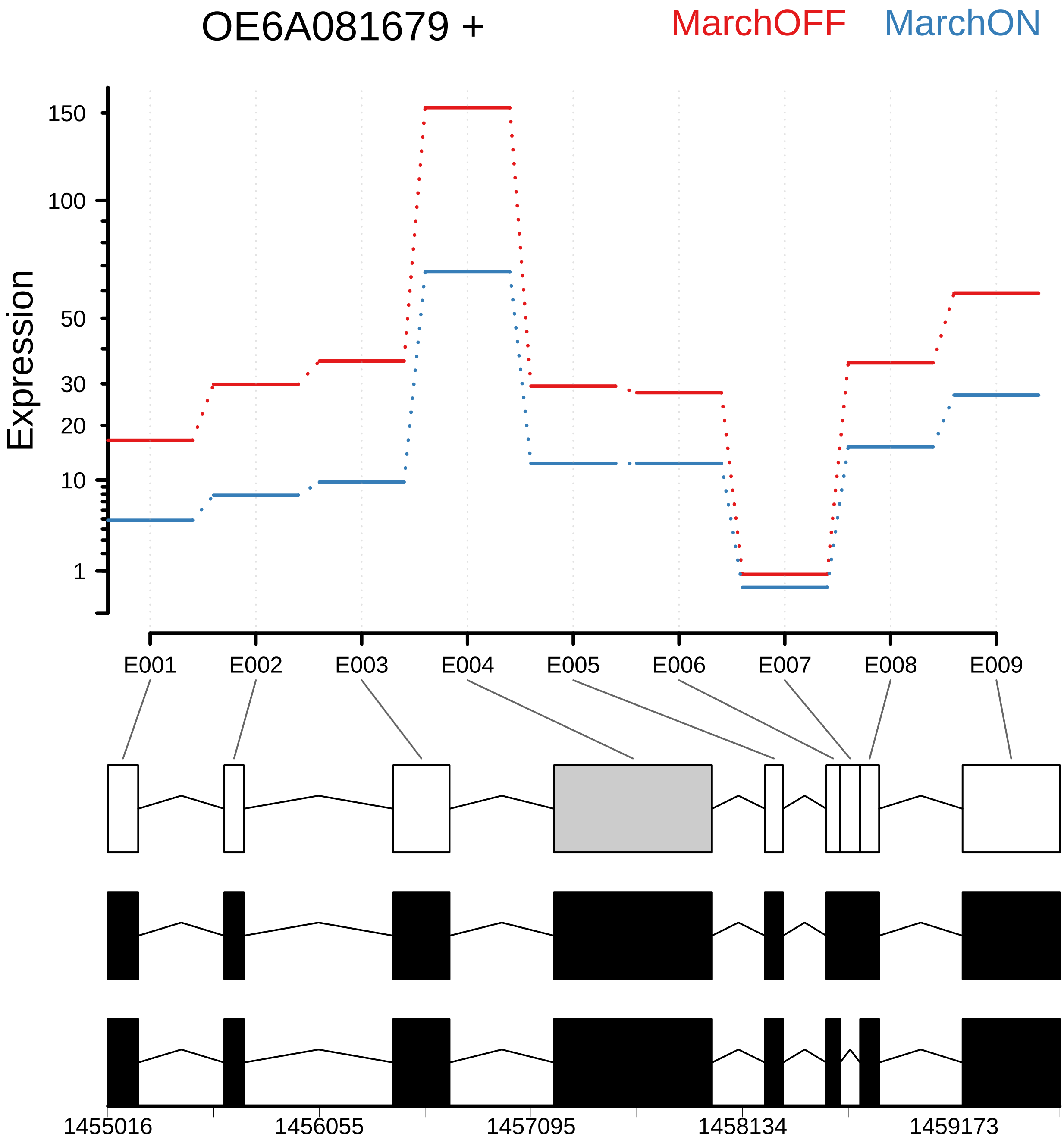
OE6A081679 +
MarchOFF
MarchON
Expression
150
100
50
30
20
10
1
E001
E002
E003
E004
E005
E006
E007
E008
E009
1455016
1456055
1457095
1458134
1459173

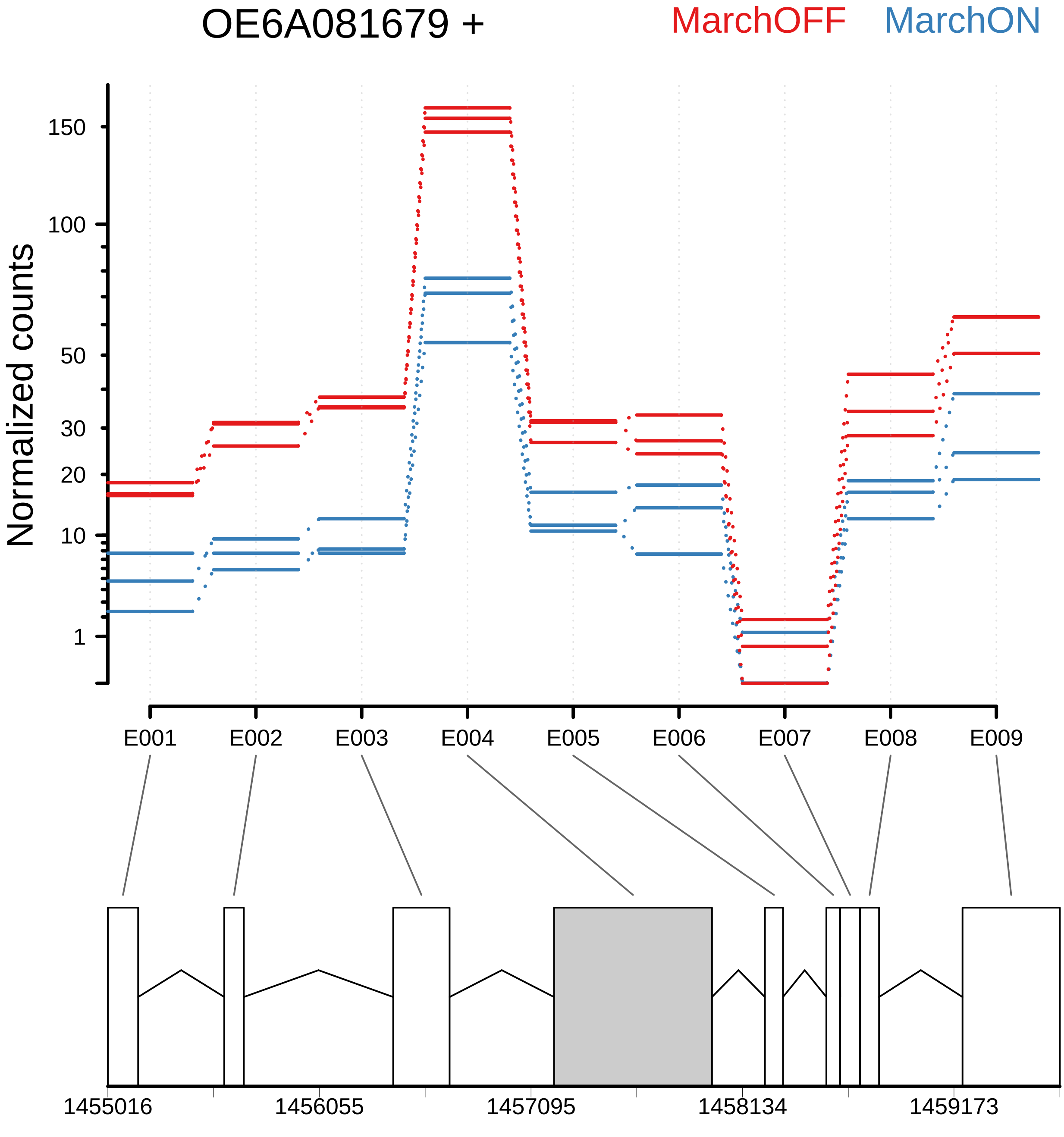
OE6A081679 +
MarchOFF
MarchON
Normalized counts
150
100
50
30
20
10
1
E001
E002
E003
E004
E005
E006
E007
E008
E009
1455016
1456055
1457095
1458134
1459173

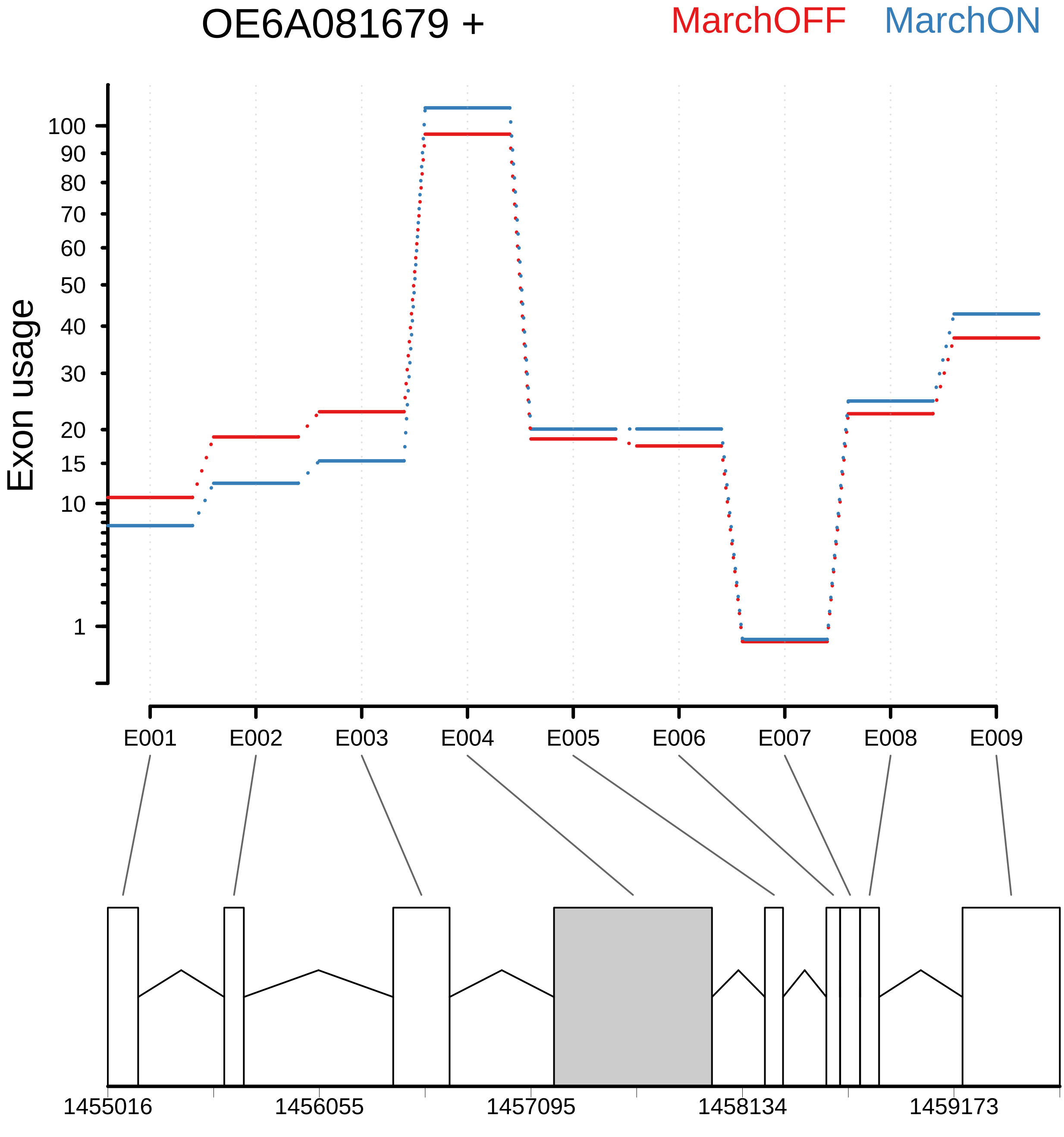
OE6A081679 +
MarchOFF
MarchON
Exon usage
100
90
80
70
60
50
40
30
20
15
10
1
E001
E002
E003
E004
E005
E006
E007
E008
E009
1455016
1456055
1457095
1458134
1459173

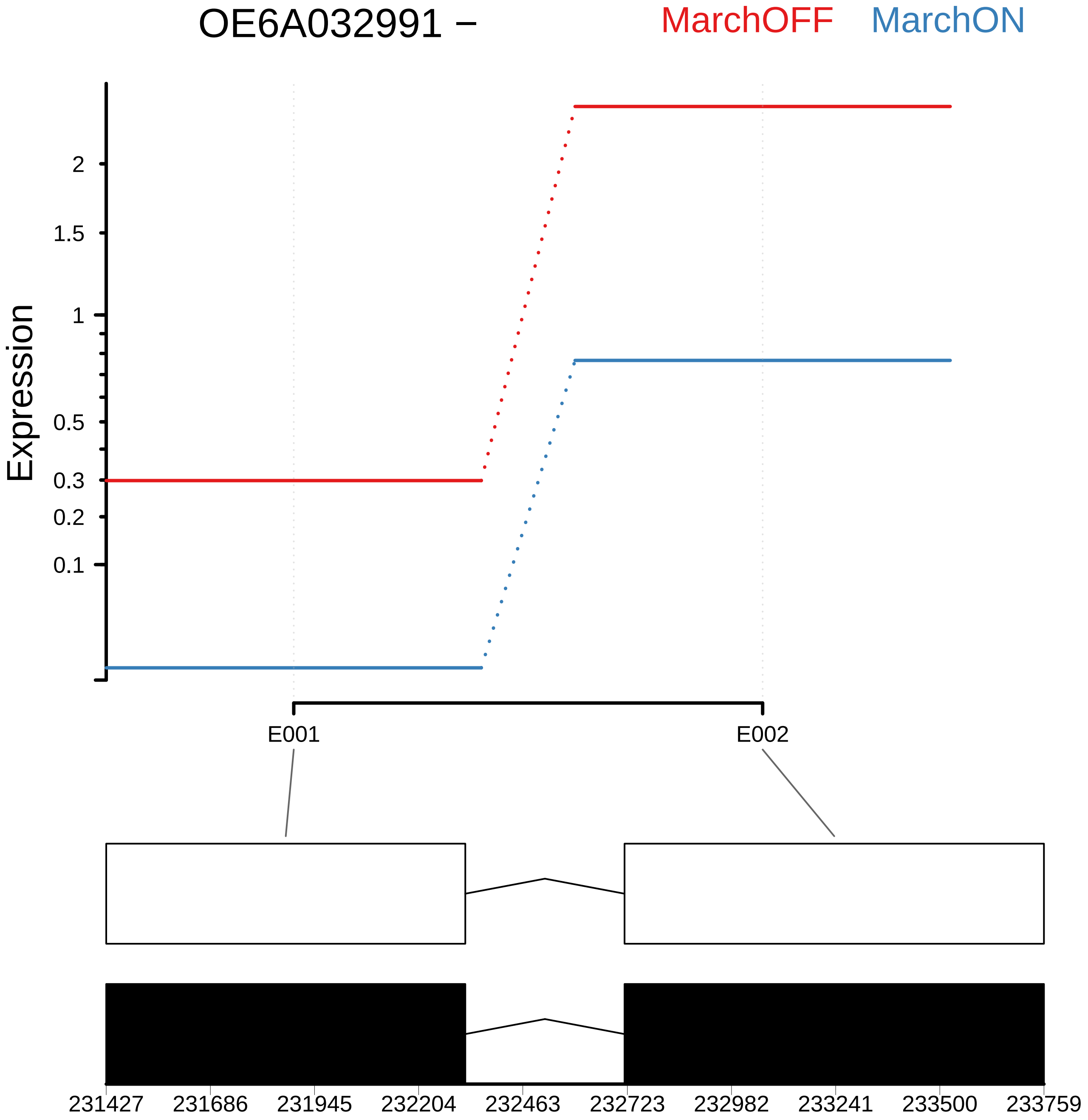

OE6A032991 −
MarchOFF
MarchON
Expression
2
1.5
1
0.5
0.3
0.2
0.1
E001
E002
231427
231686
231945
232204
232463
232723
232982
233241
233500
233759

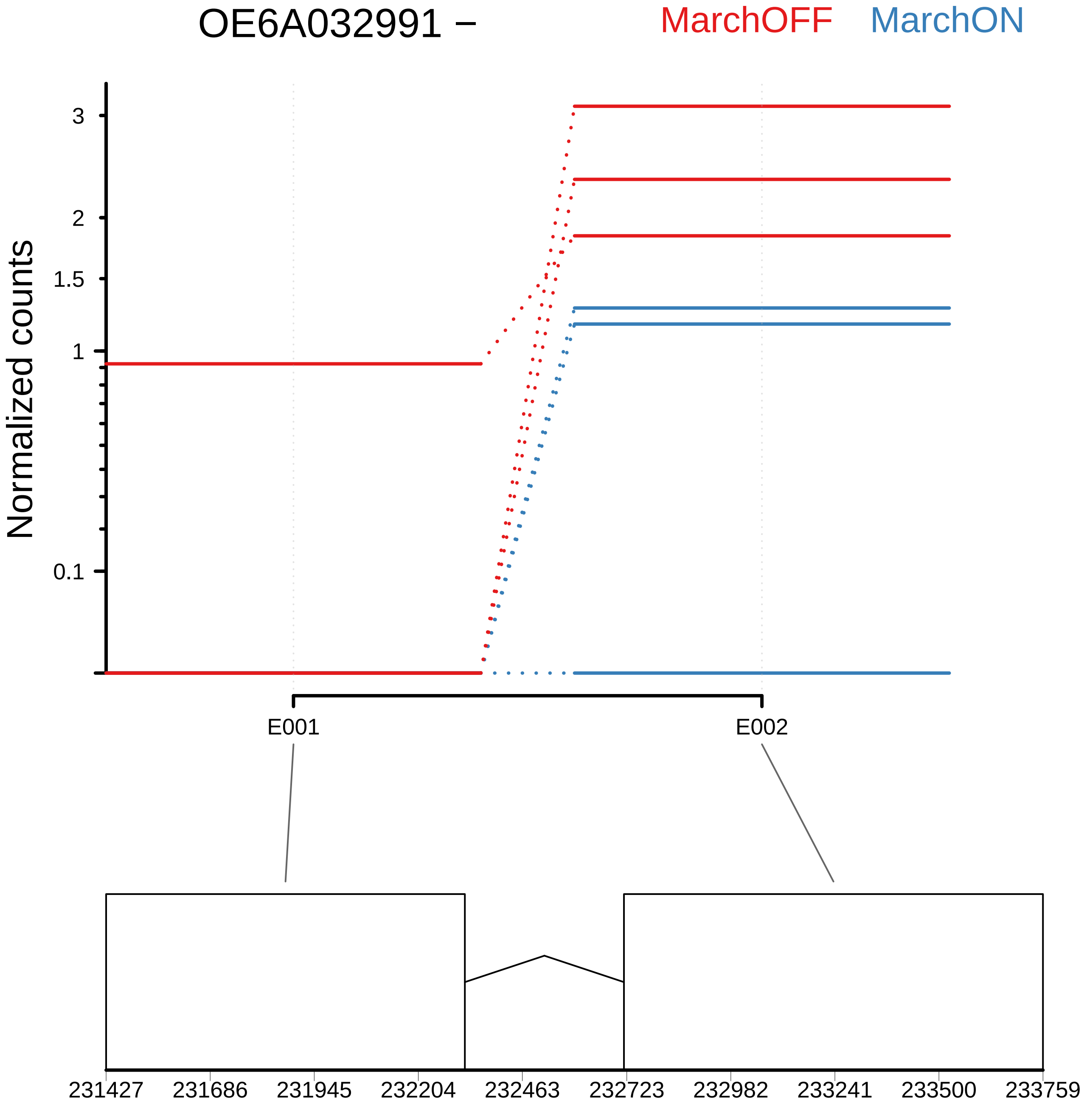

OE6A032991 −
MarchOFF
MarchON
Normalized counts
3
2
1.5
1
0.1
E001
E002
231427
231686
231945
232204
232463
232723
232982
233241
233500
233759

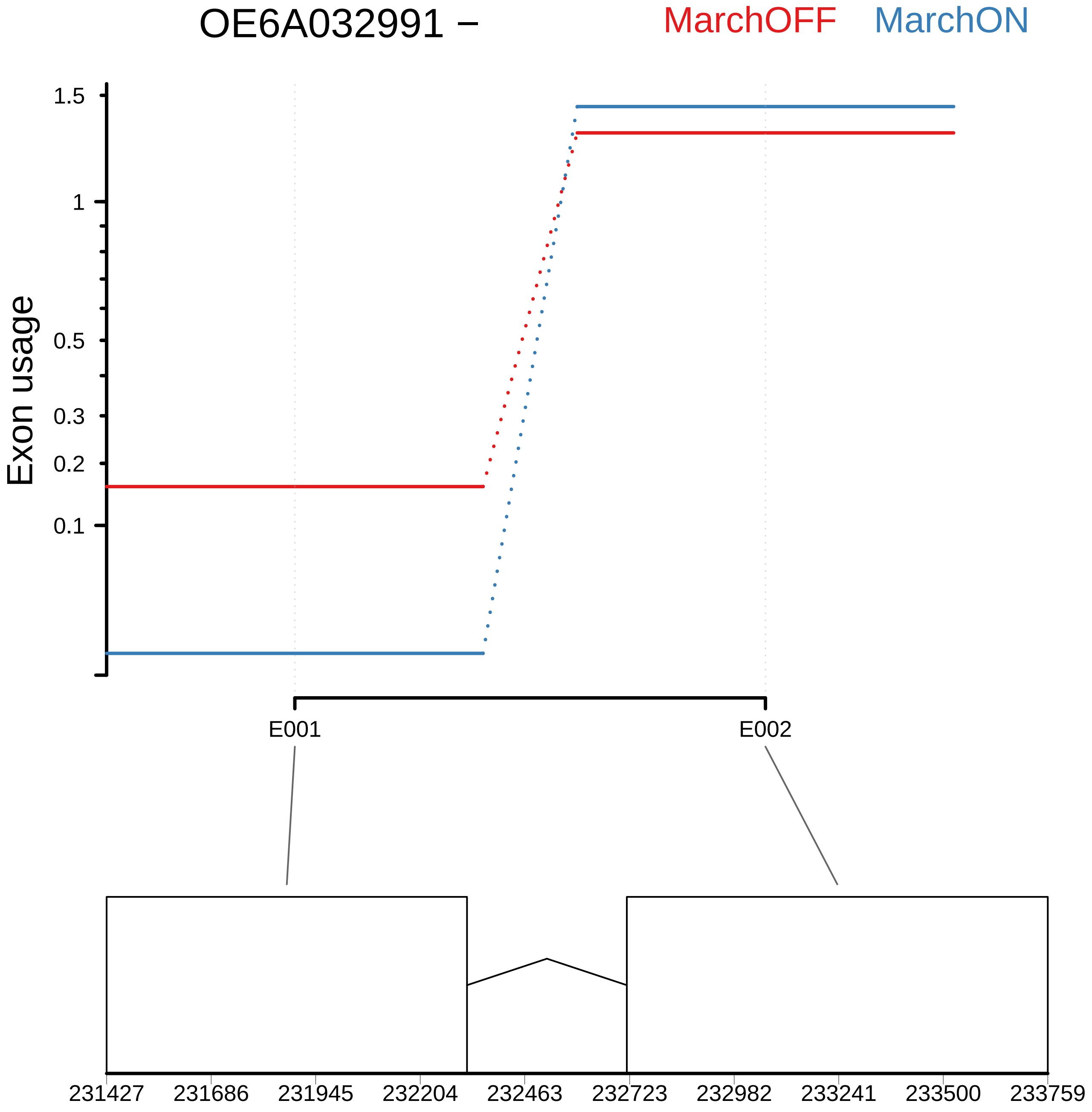

OE6A032991 −
MarchOFF
MarchON
Exon usage
1.5
1
0.5
0.3
0.2
0.1
E001
E002
231427
231686
231945
232204
232463
232723
232982
233241
233500
233759

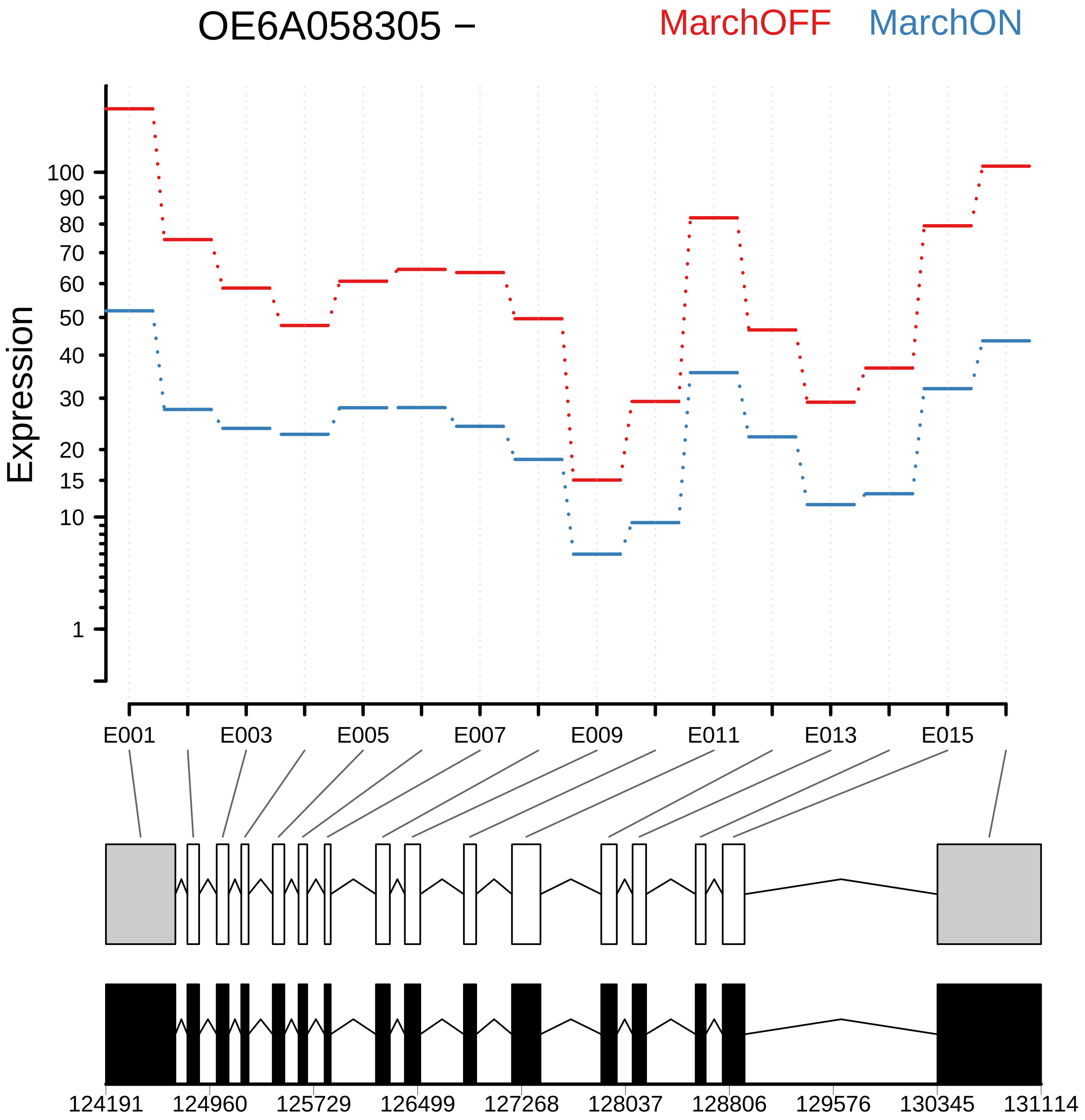

OE6A058305 −
MarchOFF
MarchON
Expression
100
90
80
70
60
50
40
30
20
15
10
1
E001
E003
E005
E007
E009
E011
E013
E015
124191
124960
125729
126499
127268
128037
128806
129576
130345
131114

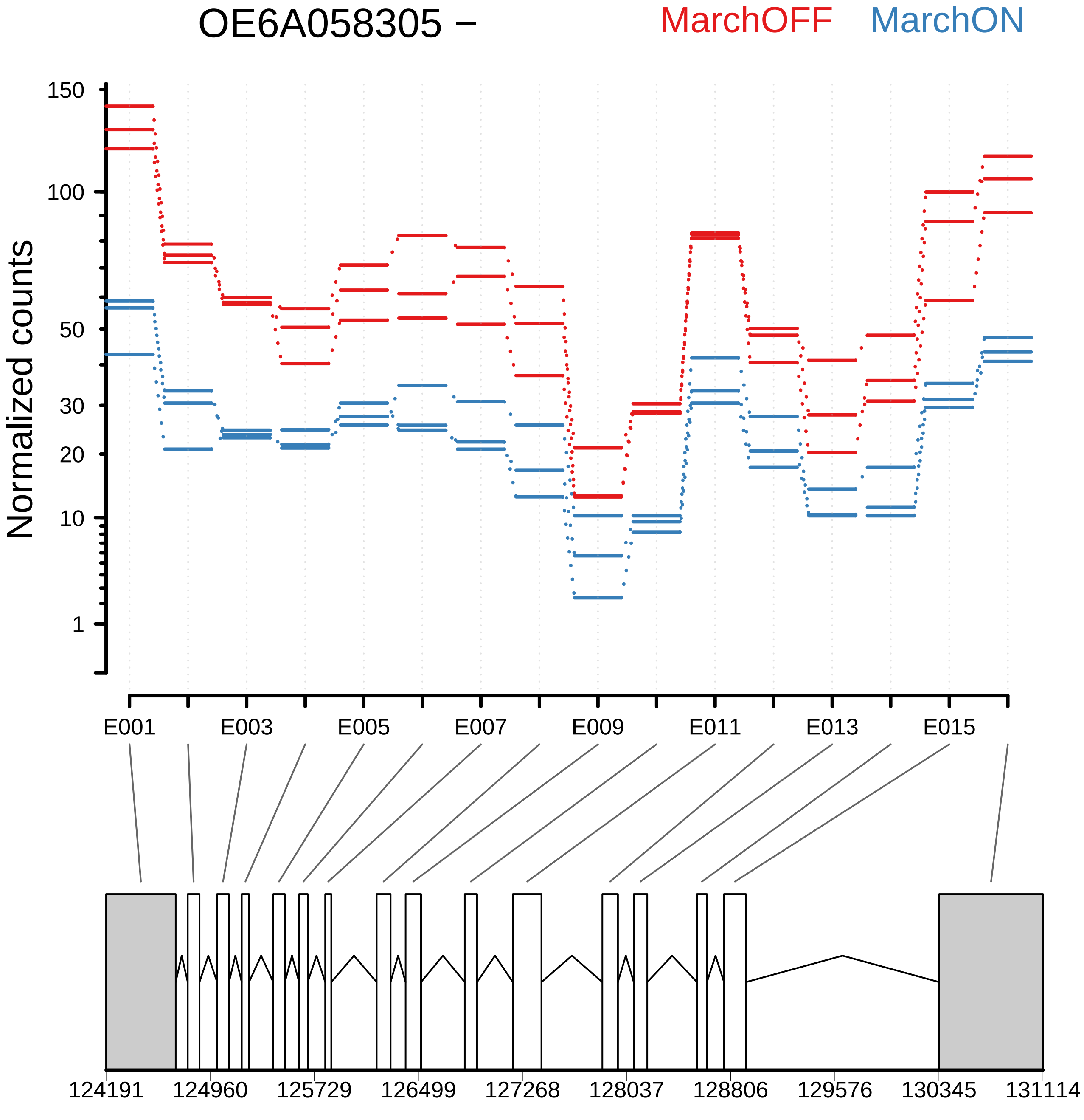

OE6A058305 −
MarchOFF
MarchON
Normalized counts
150
100
50
30
20
10
1
E001
E003
E005
E007
E009
E011
E013
E015
124191
124960
125729
126499
127268
128037
128806
129576
130345
131114

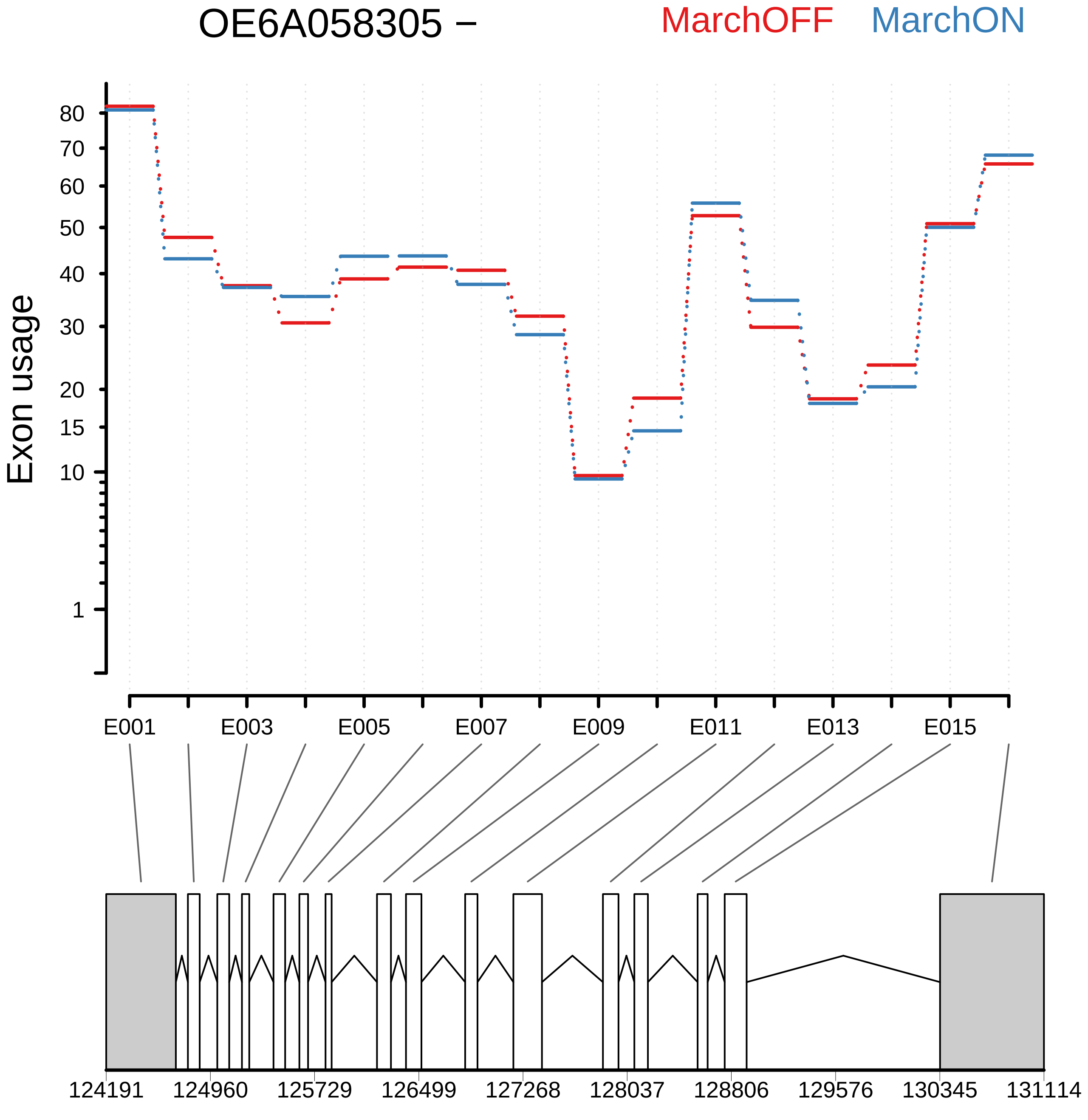

OE6A058305 −
MarchOFF
MarchON
Exon usage
80
70
60
50
40
30
20
15
10
1
E001
E003
E005
E007
E009
E011
E013
E015
124191
124960
125729
126499
127268
128037
128806
129576
130345
131114

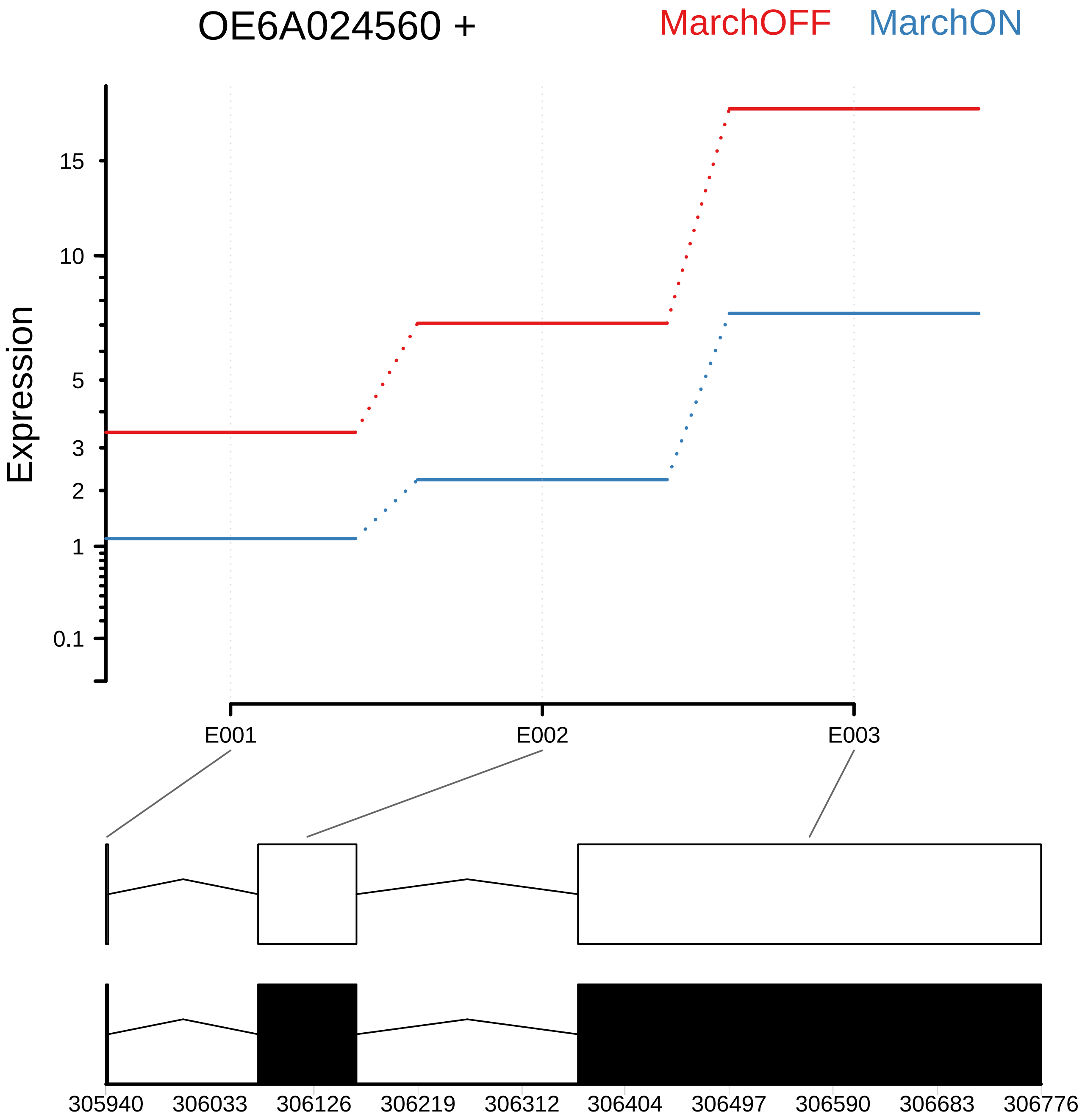
OE6A024560 +
MarchOFF
MarchON
Expression
15
10
5
3
2
1
0.1
E001
E002
E003
305940
306033
306126
306219
306312
306404
306497
306590
306683
306776

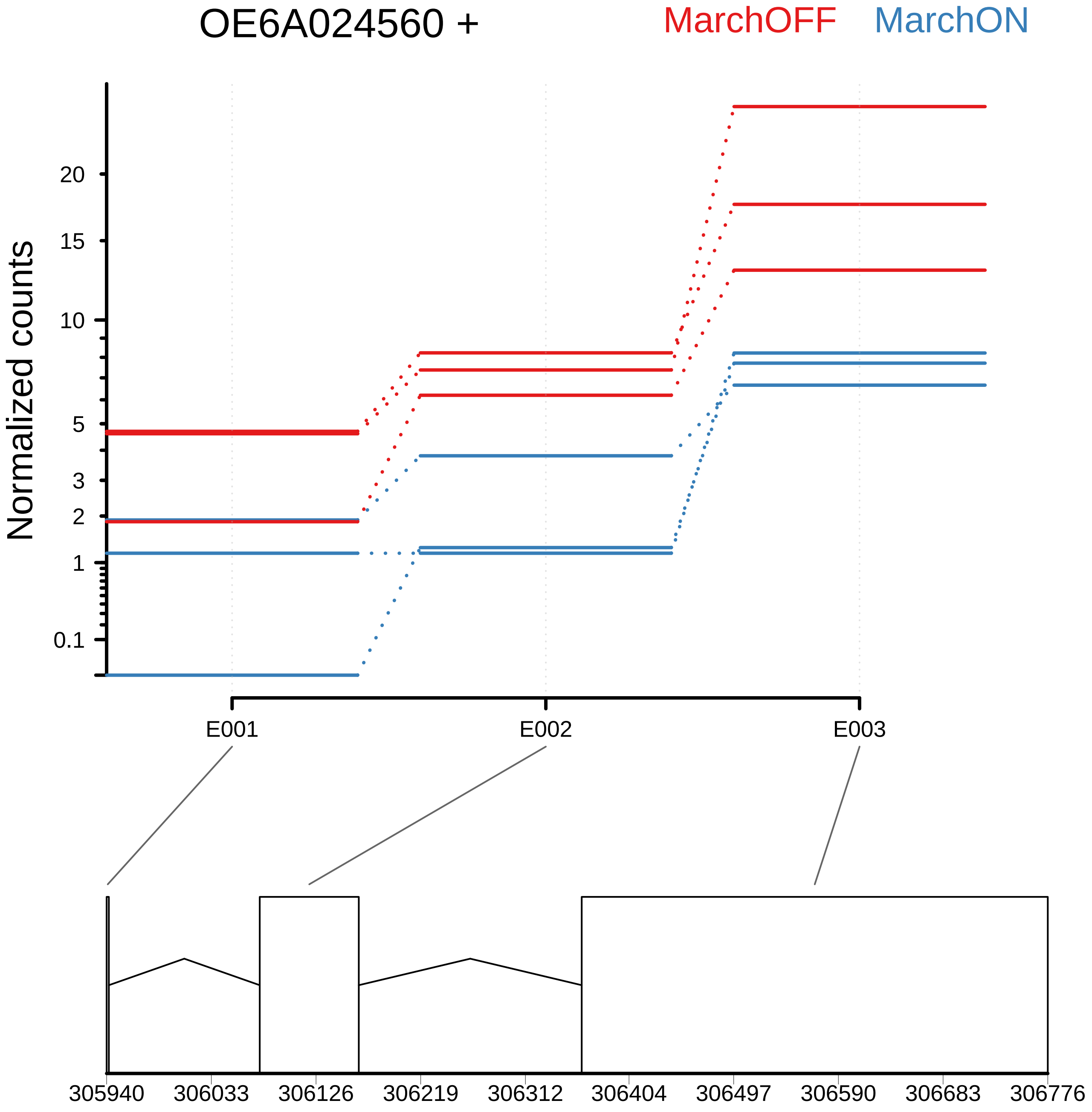
OE6A024560 +
MarchOFF
MarchON
Normalized counts
20
15
10
5
3
2
1
0.1
E001
E002
E003
305940
306033
306126
306219
306312
306404
306497
306590
306683
306776

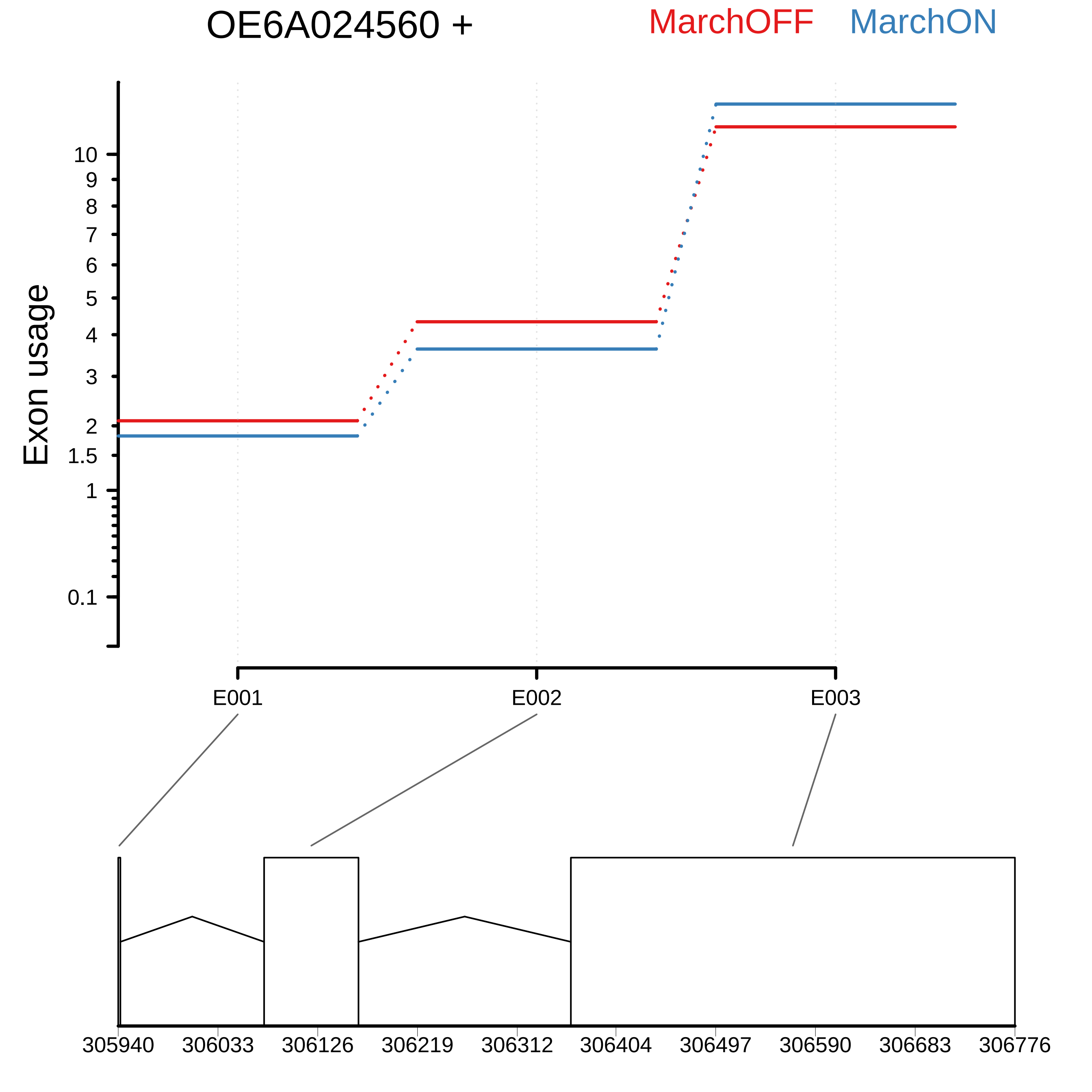

OE6A024560 +
MarchOFF
MarchON
Exon usage
10
9
8
7
6
5
4
3
2
1.5
1
0.1
E001
E002
E003
305940
306033
306126
306219
306312
306404
306497
306590
306683
306776

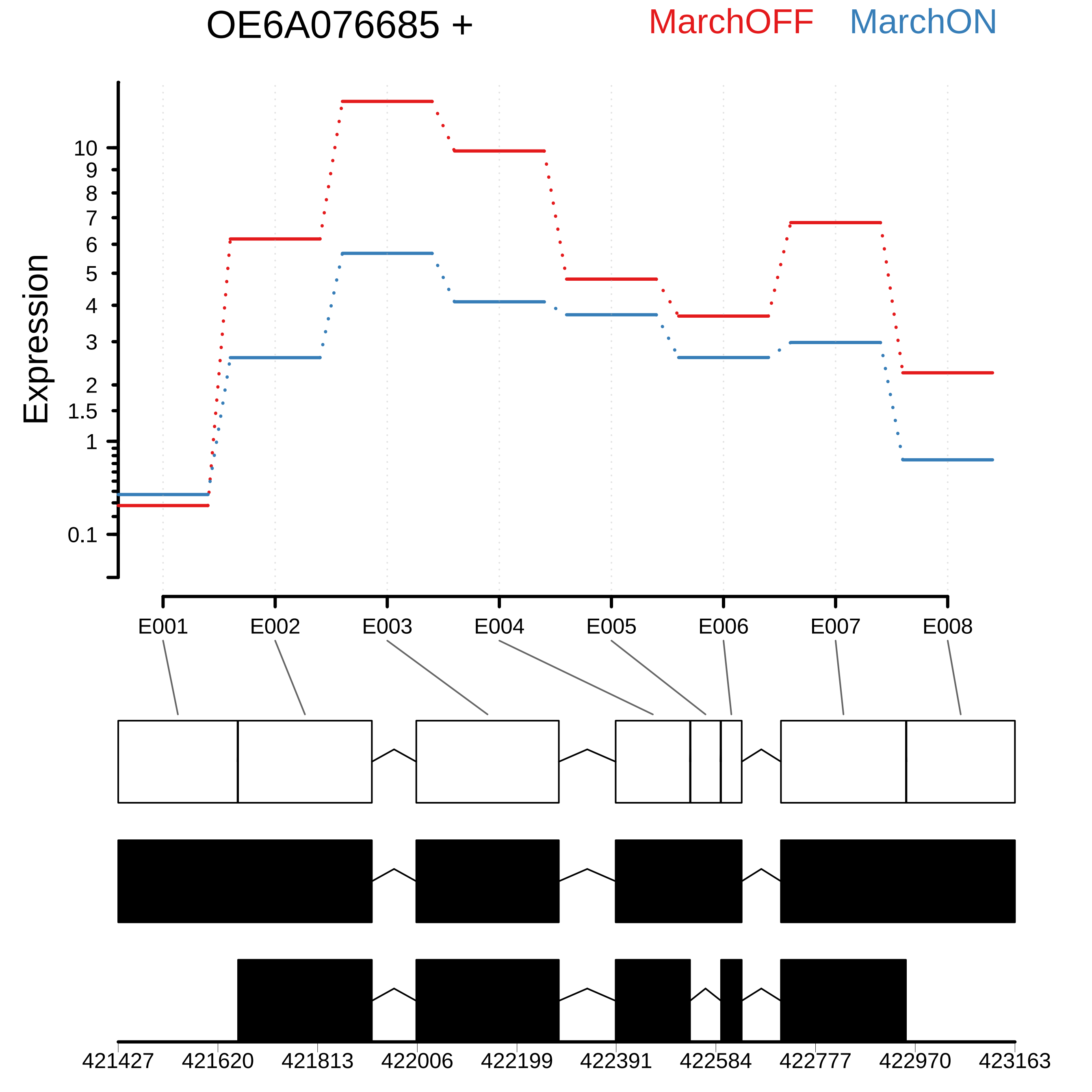

OE6A076685 +
MarchOFF
MarchON
Expression
10
9
8
7
6
5
4
3
2
1.5
1
0.1
E001
E002
E003
E004
E005
E006
E007
E008
421427
421620
421813
422006
422199
422391
422584
422777
422970
423163

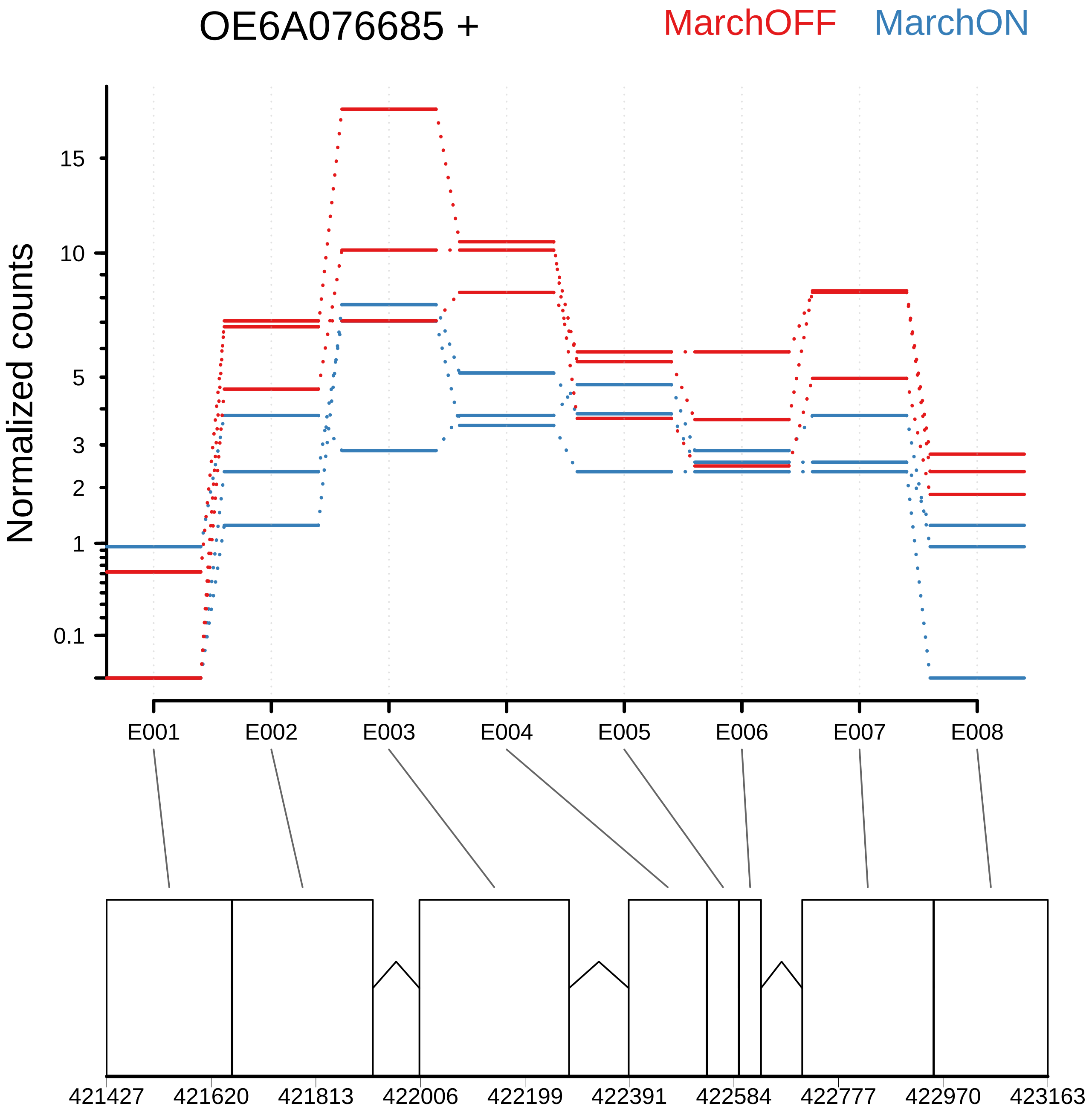

OE6A076685 +
MarchOFF
MarchON
Normalized counts
15
10
5
3
2
1
0.1
E001
E002
E003
E004
E005
E006
E007
E008
421427
421620
421813
422006
422199
422391
422584
422777
422970
423163

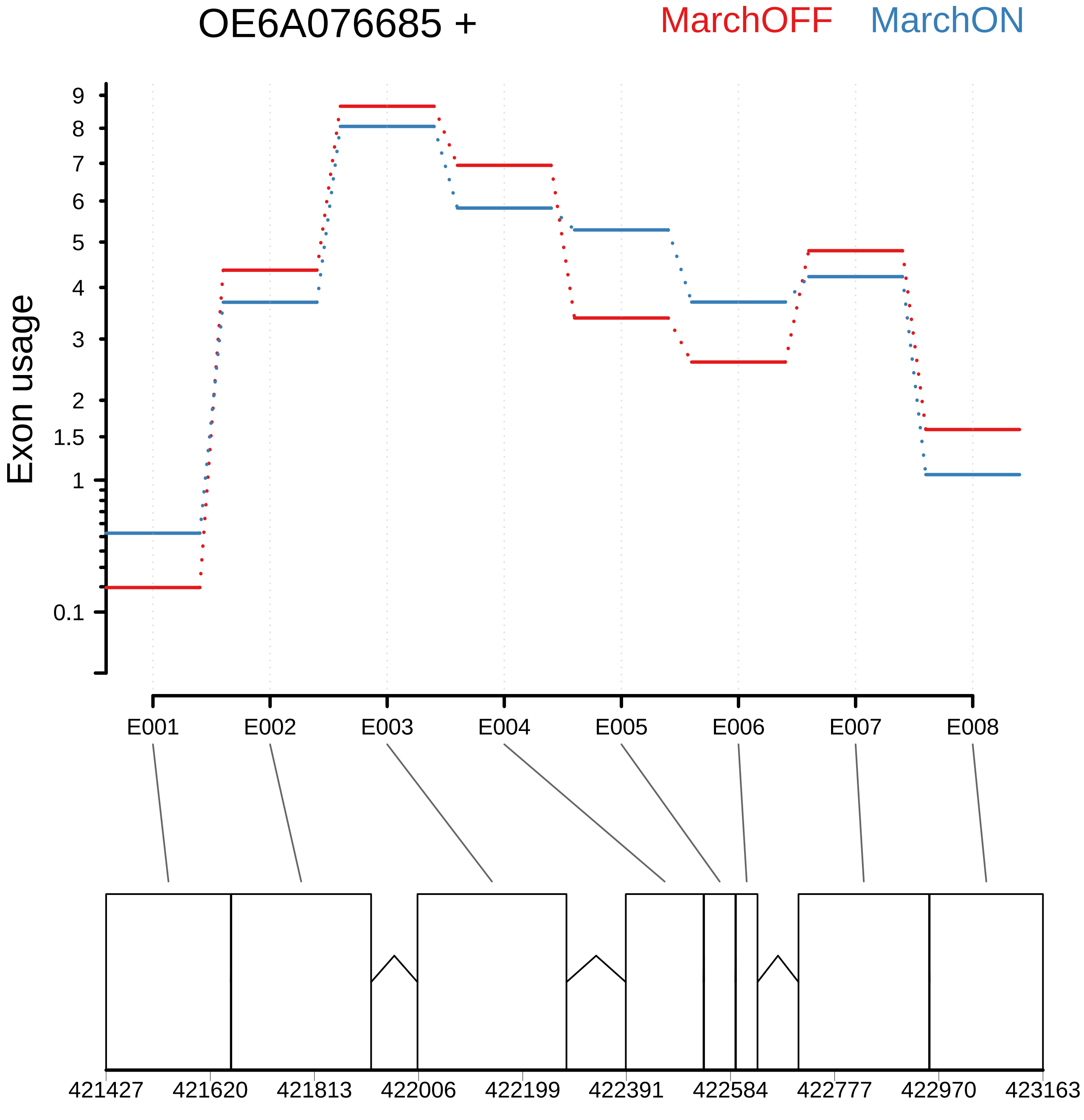
OE6A076685 +
MarchOFF
MarchON
Exon usage
9
8
7
6
5
4
3
2
1.5
1
0.1
E001
E002
E003
E004
E005
E006
E007
E008
421427
421620
421813
422006
422199
422391
422584
422777
422970
423163

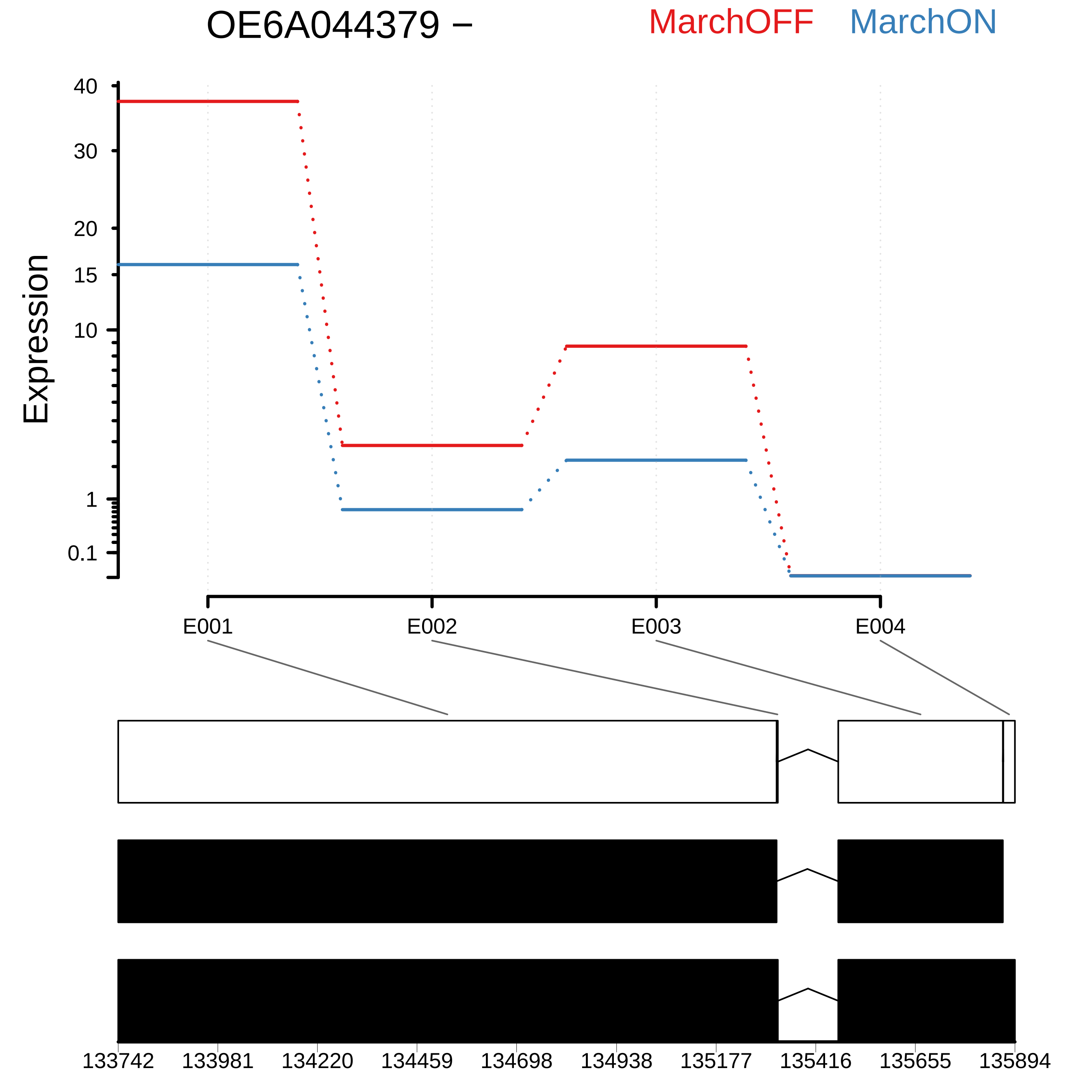
OE6A044379 –
MarchOFF
MarchON
Expression
40
30
20
15
10
1
0.1
E001
E002
E003
E004
133742
133981
134220
134459
134698
134938
135177
135416
135655
135894

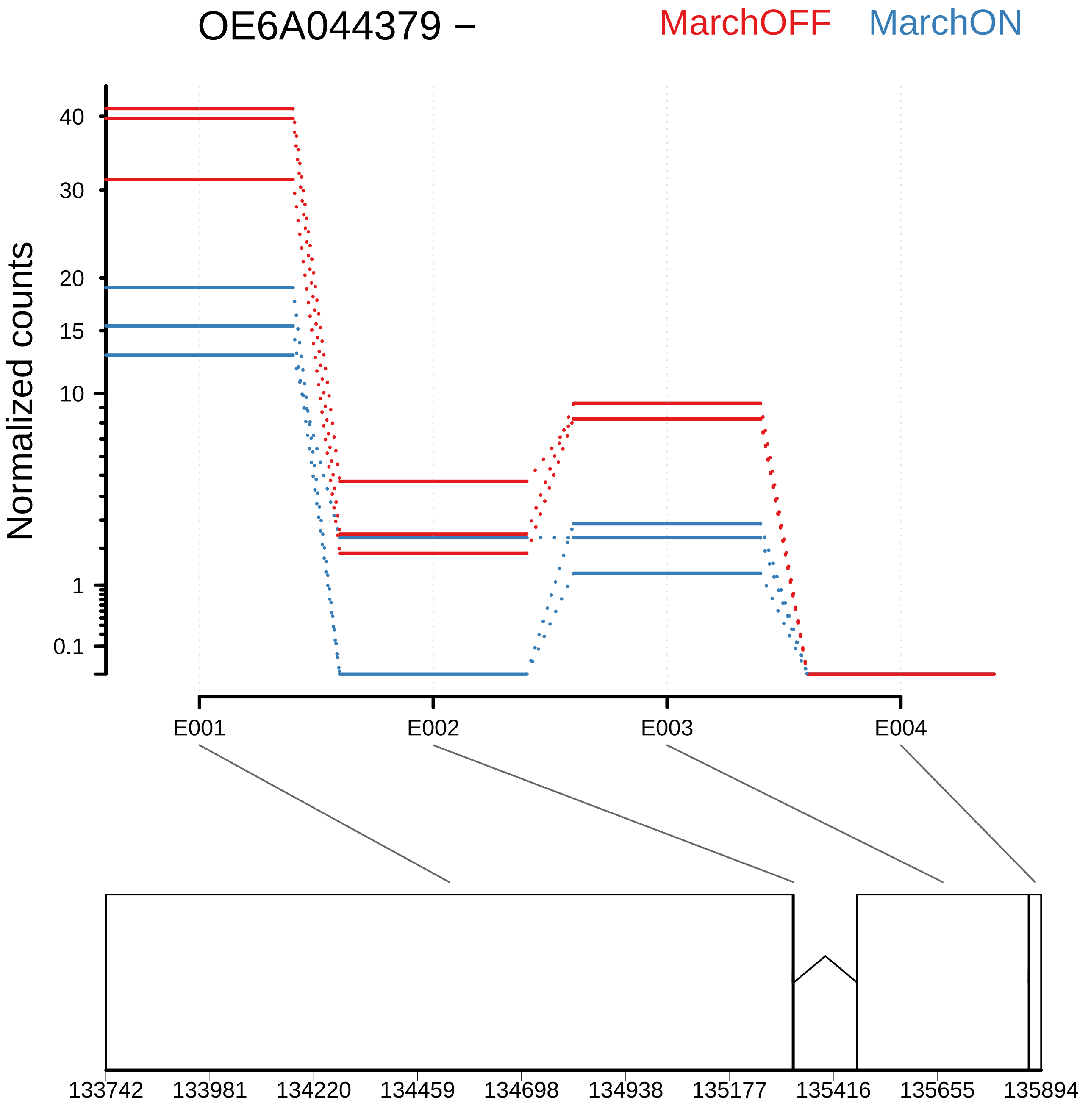
OE6A044379 −
MarchOFF
MarchON
Normalized counts
40
30
20
15
10
1
0.1
E001
E002
E003
E004
133742
133981
134220
134459
134698
134938
135177
135416
135655
135894

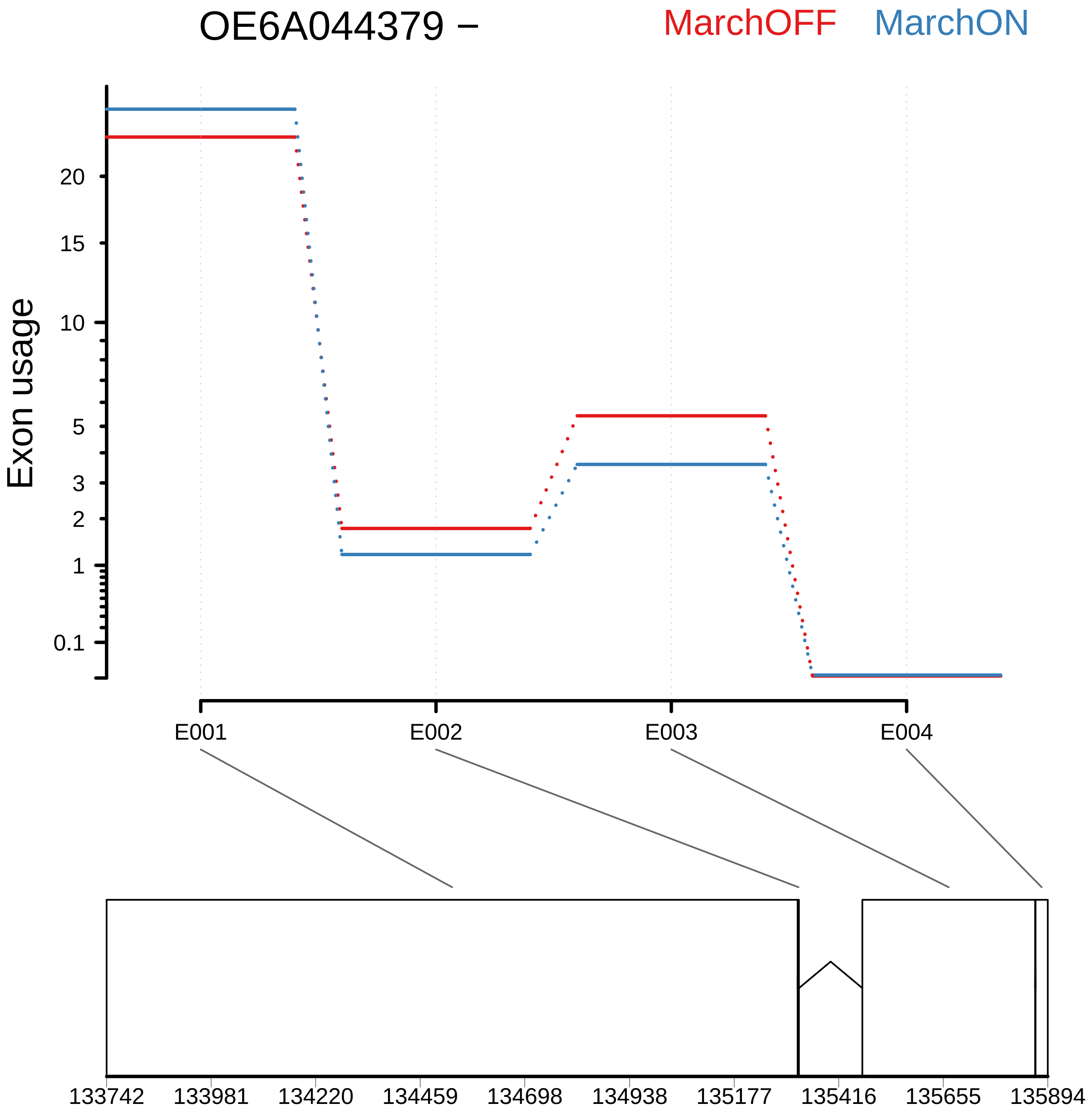

OE6A044379 −
MarchOFF
MarchON
Exon usage
20
15
10
5
3
2
1
0.1
E001
E002
E003
E004
133742
133981
134220
134459
134698
134938
135177
135416
135655
135894

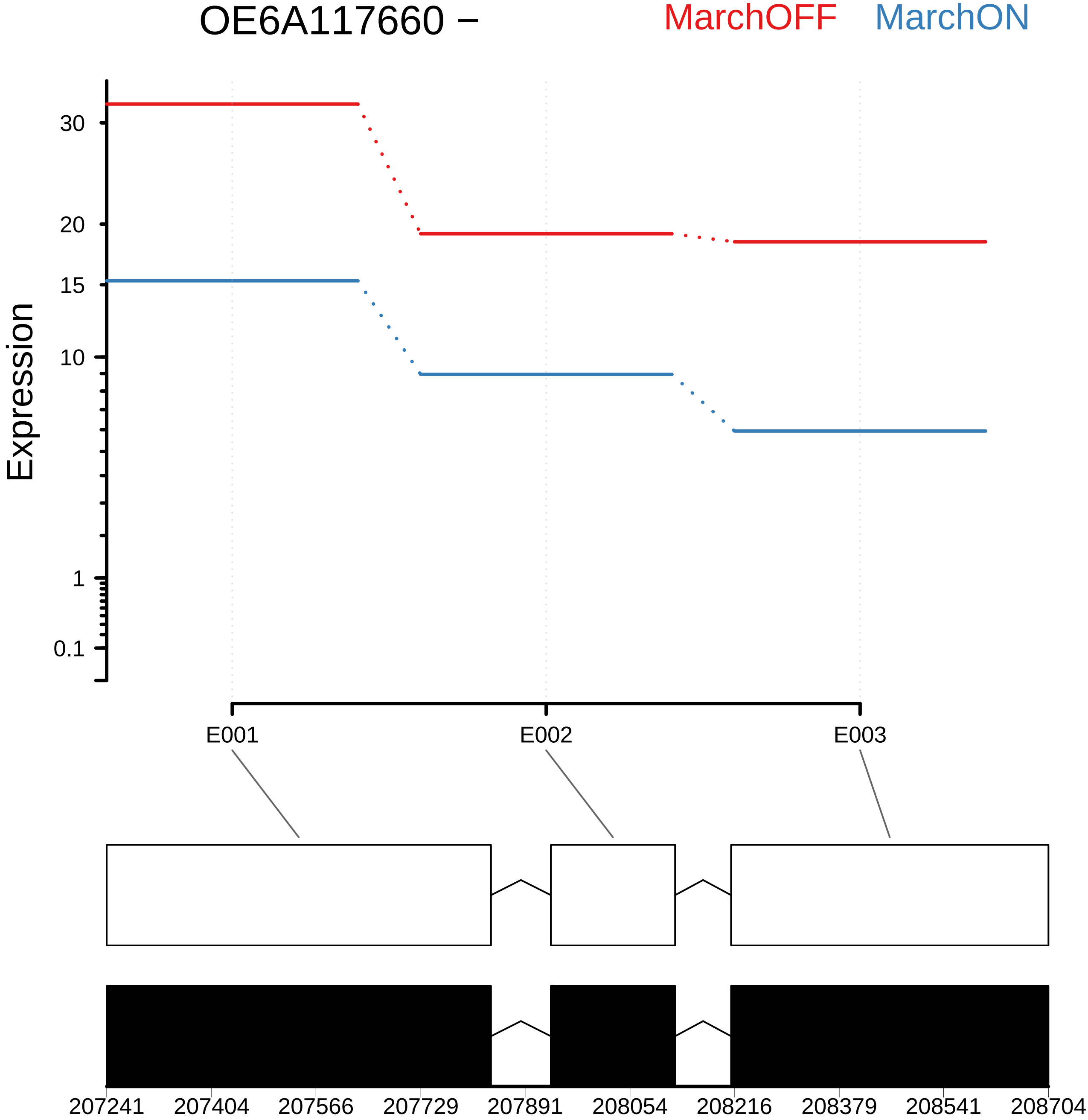

OE6A117660 −
MarchOFF
MarchON
Expression
30
20
15
10
1
0.1
E001
E002
E003
207241
207404
207566
207729
207891
208054
208216
208379
208541
208704

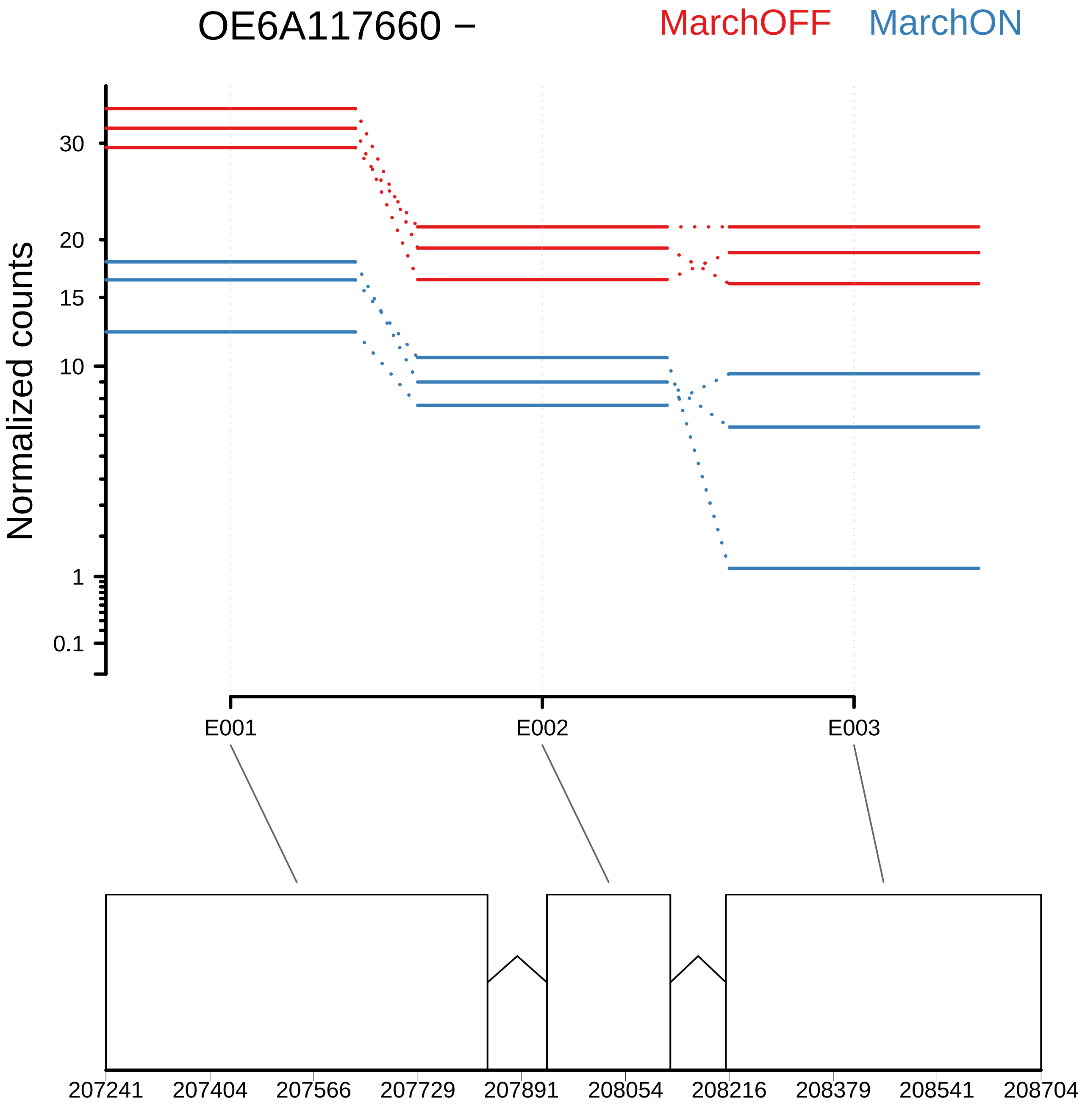

OE6A117660 −
MarchOFF
MarchON
Normalized counts
30
20
15
10
1
0.1
E001
E002
E003
207241
207404
207566
207729
207891
208054
208216
208379
208541
208704

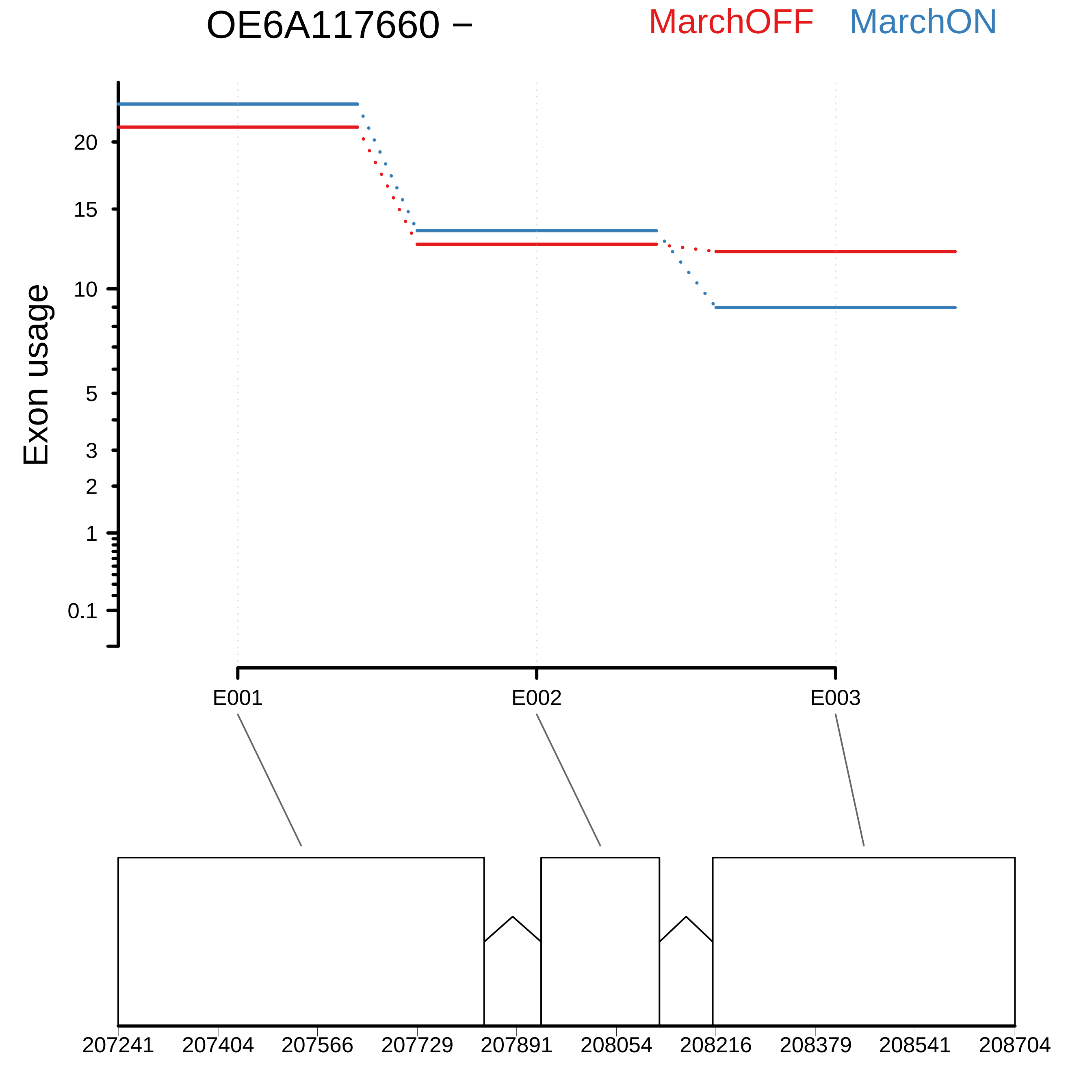

OE6A117660 −
MarchOFF
MarchON
Exon usage
20
15
10
5
3
2
1
0.1
E001
E002
E003
207241
207404
207566
207729
207891
208054
208216
208379
208541
208704

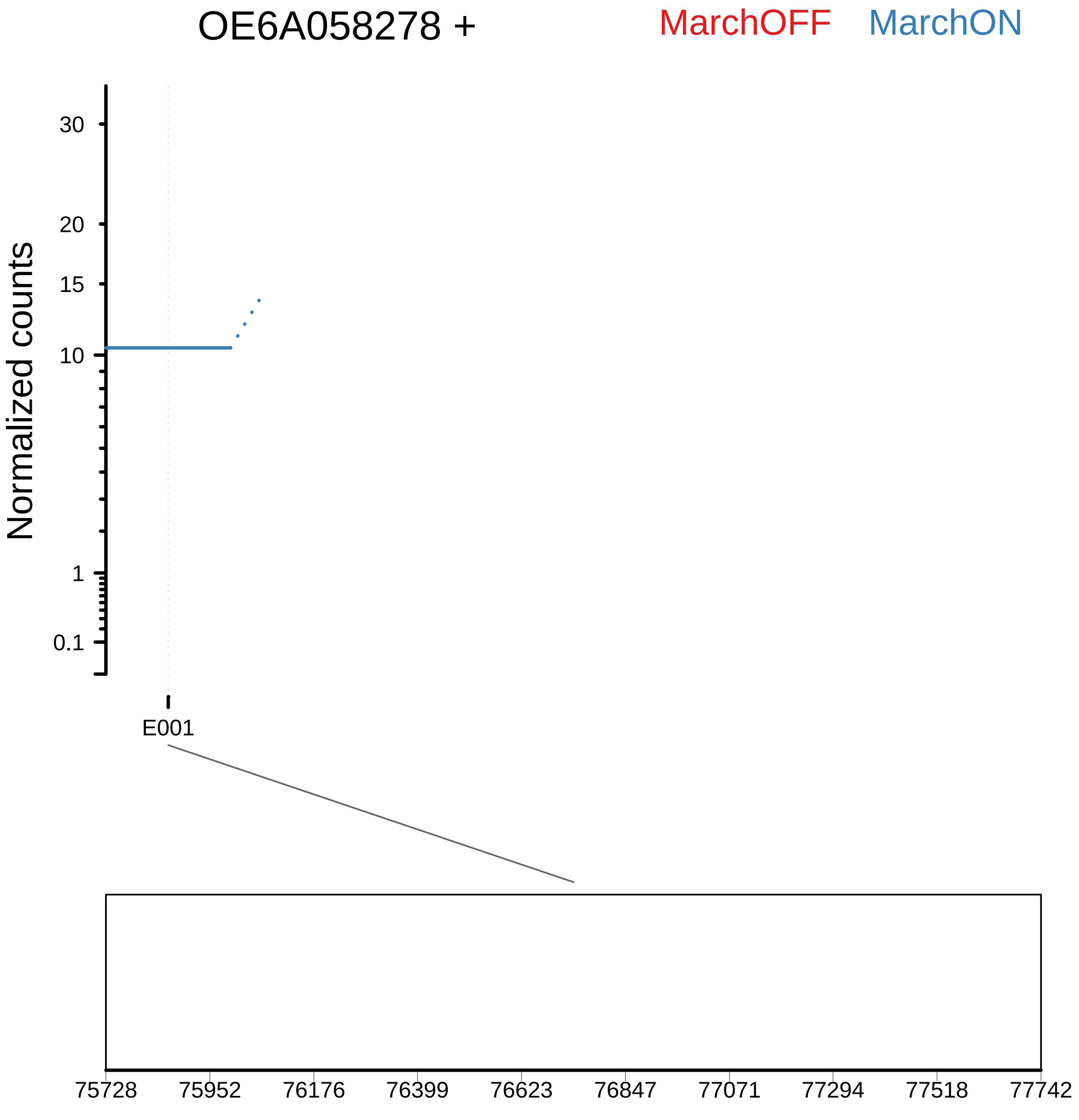
OE6A058278 +
MarchOFF
MarchON
Normalized counts
30
20
15
10
1
0.1
E001
75728
75952
76176
76399
76623
76847
77071
77294
77518
77742

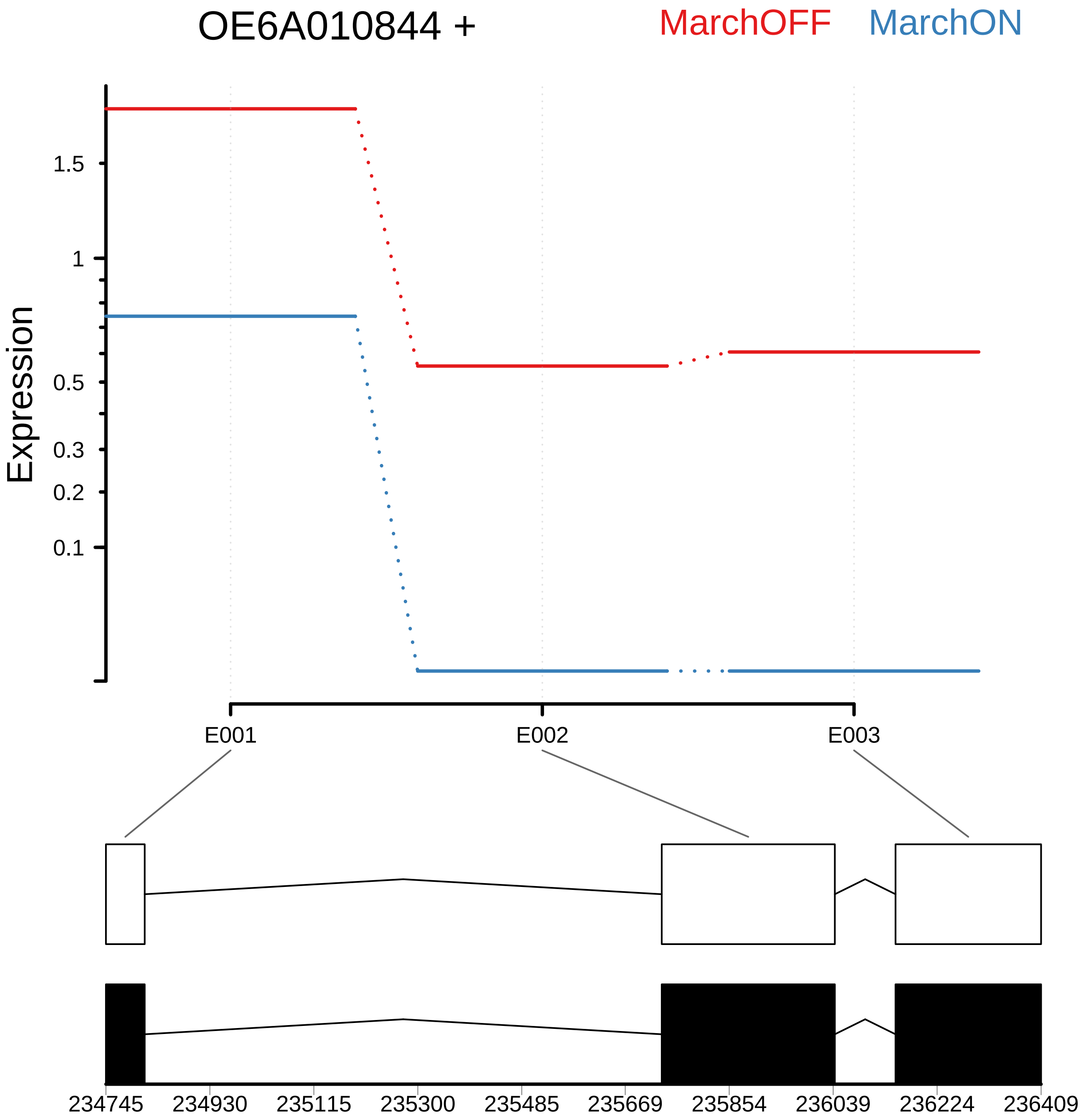

OE6A010844 +
MarchOFF
MarchON
Expression
1.5
1
0.5
0.3
0.2
0.1
E001
E002
E003
234745
234930
235115
235300
235485
235669
235854
236039
236224
236409

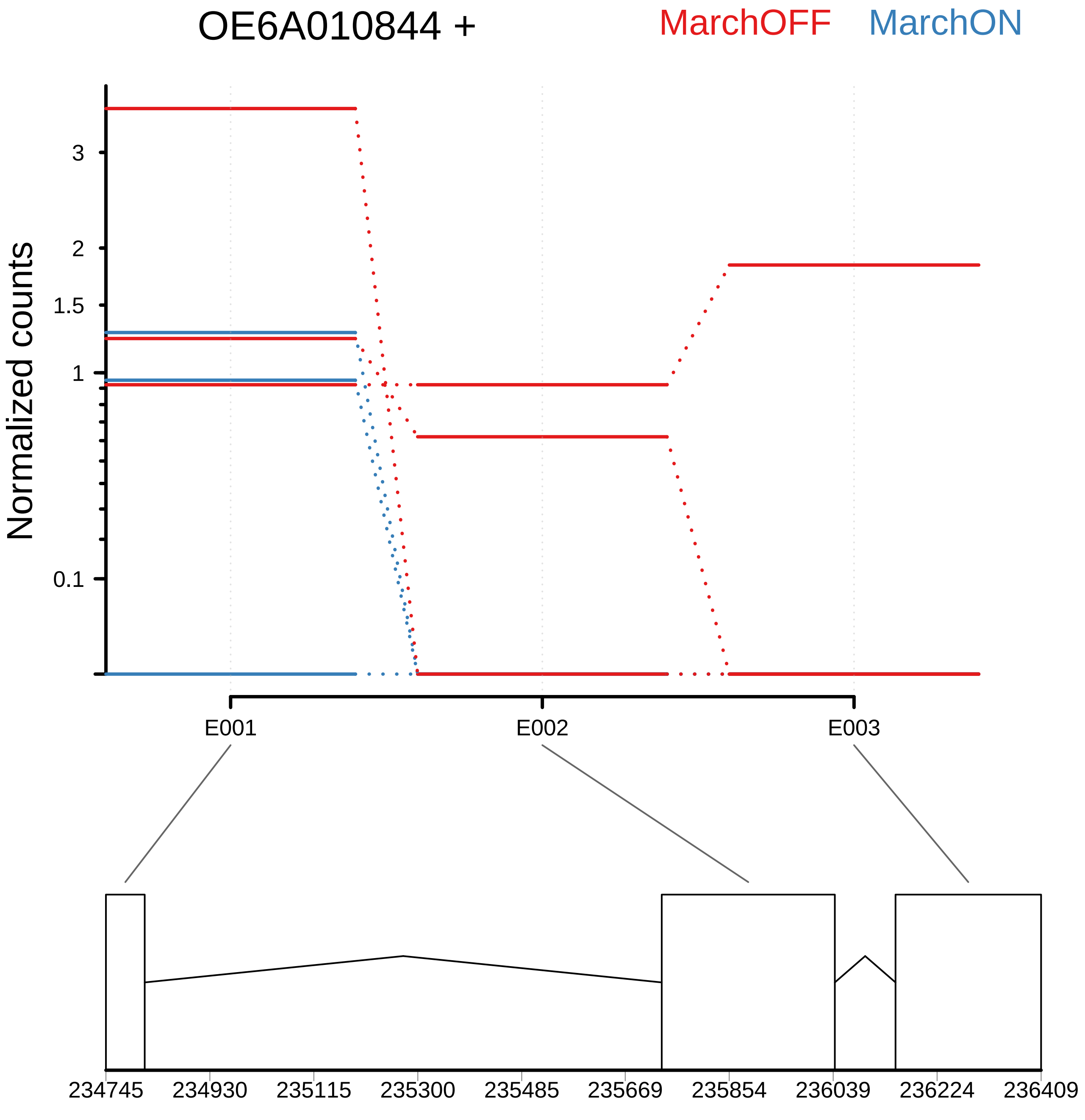
OE6A010844 +
MarchOFF
MarchON
Normalized counts
3
2
1.5
1
0.1
E001
E002
E003
234745
234930
235115
235300
235485
235669
235854
236039
236224
236409

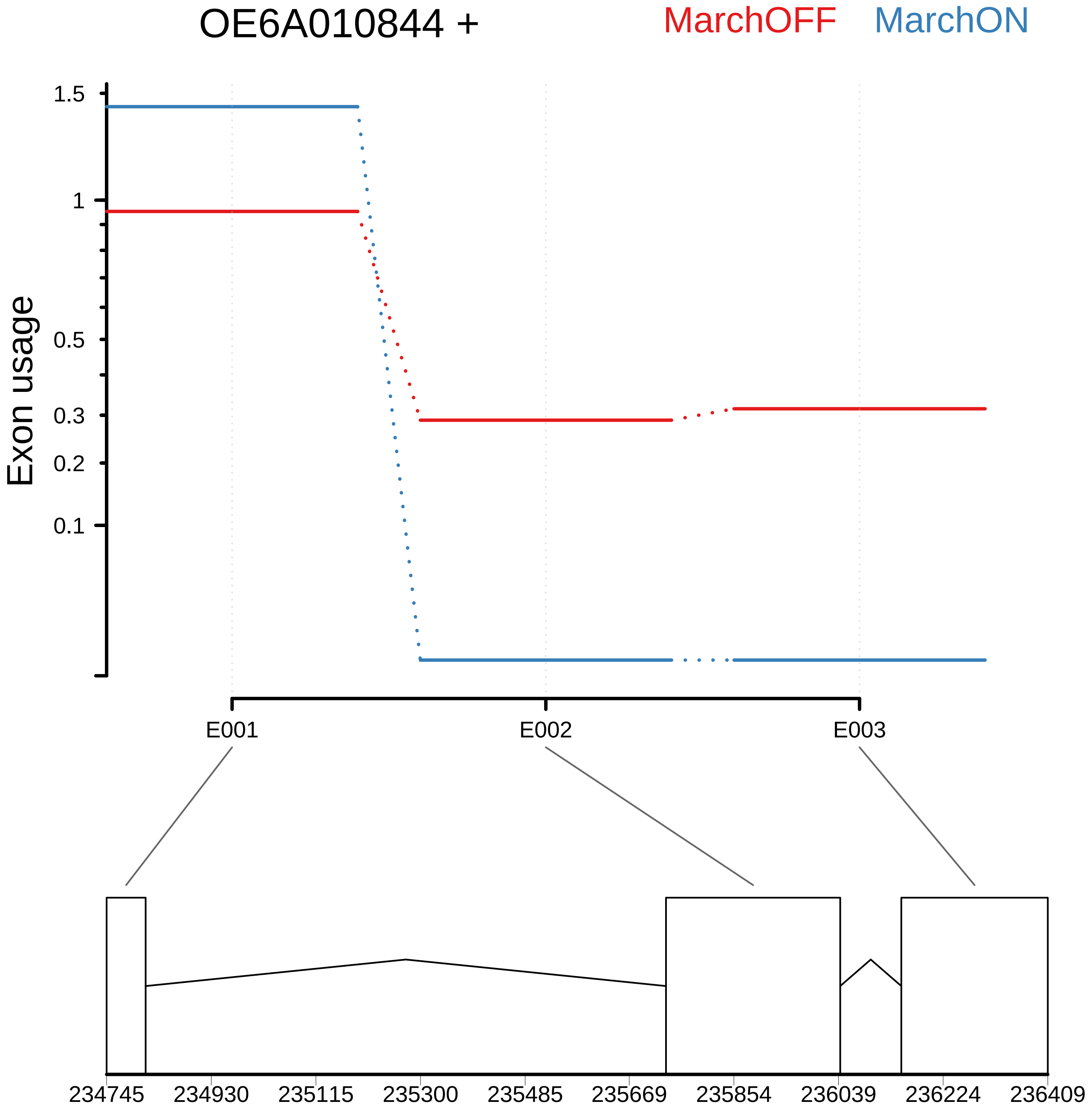

OE6A010844 +
MarchOFF
MarchON
Exon usage
1.5
1
0.5
0.3
0.2
0.1
E001
E002
E003
234745
234930
235115
235300
235485
235669
235854
236039
236224
236409

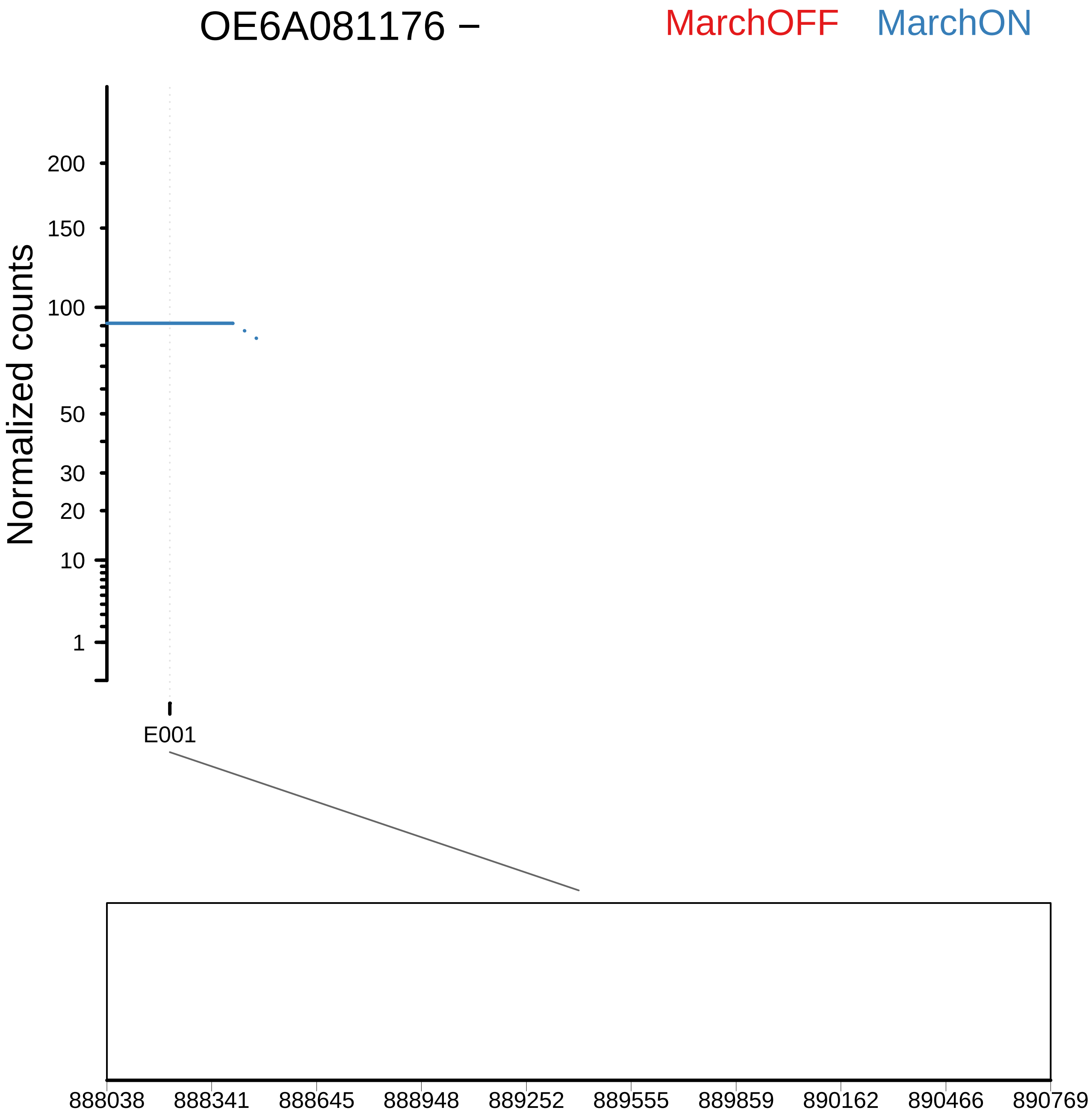
OE6A081176 −
MarchOFF
MarchON
Normalized counts
200
150
100
50
30
20
10
1
E001
888038
888341
888645
888948
889252
889555
889859
890162
890466
890769

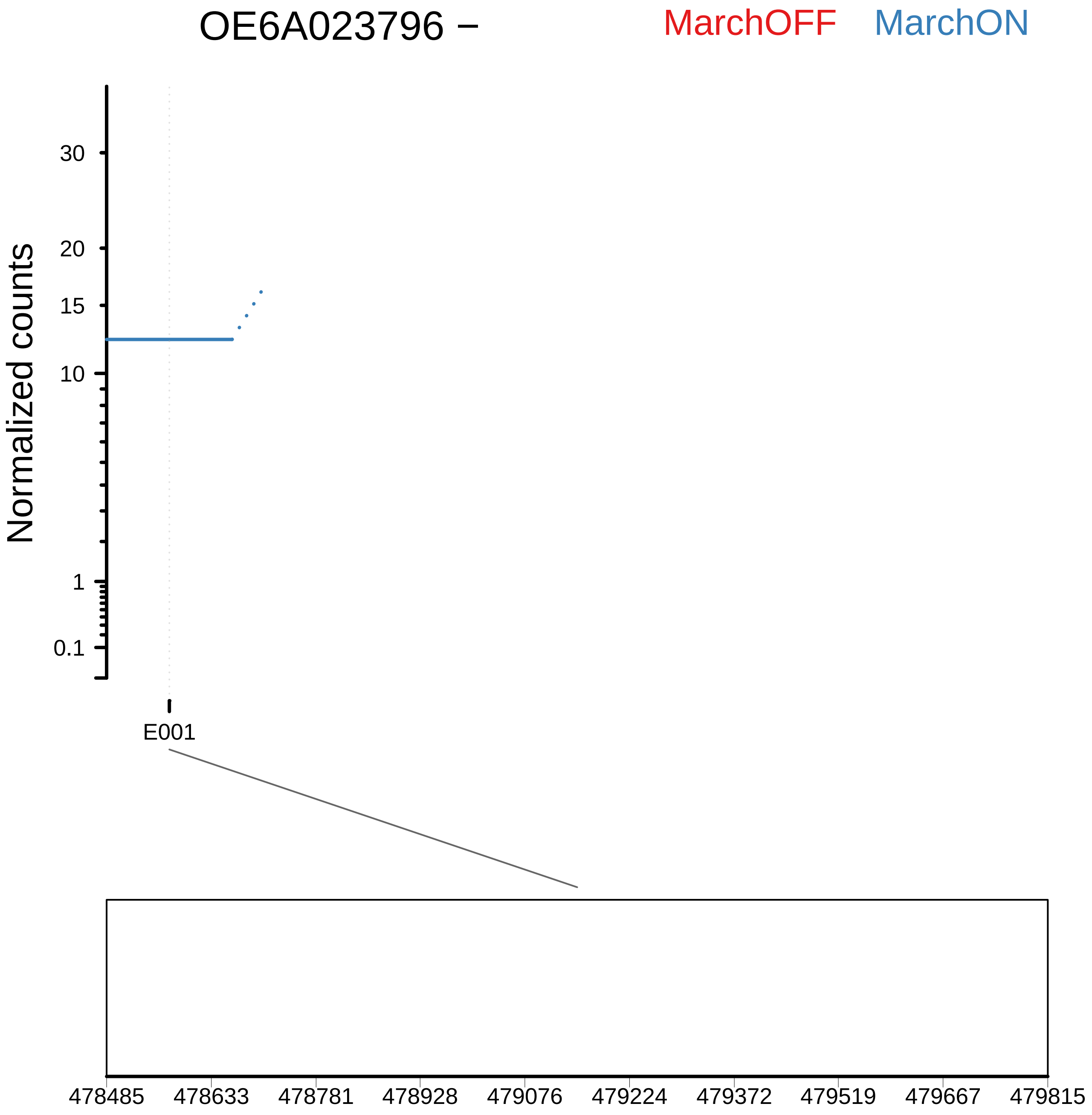

OE6A023796 −
MarchOFF
MarchON
Normalized counts
30
20
15
10
1
0.1
E001
478485
478633
478781
478928
479076
479224
479372
479519
479667
479815

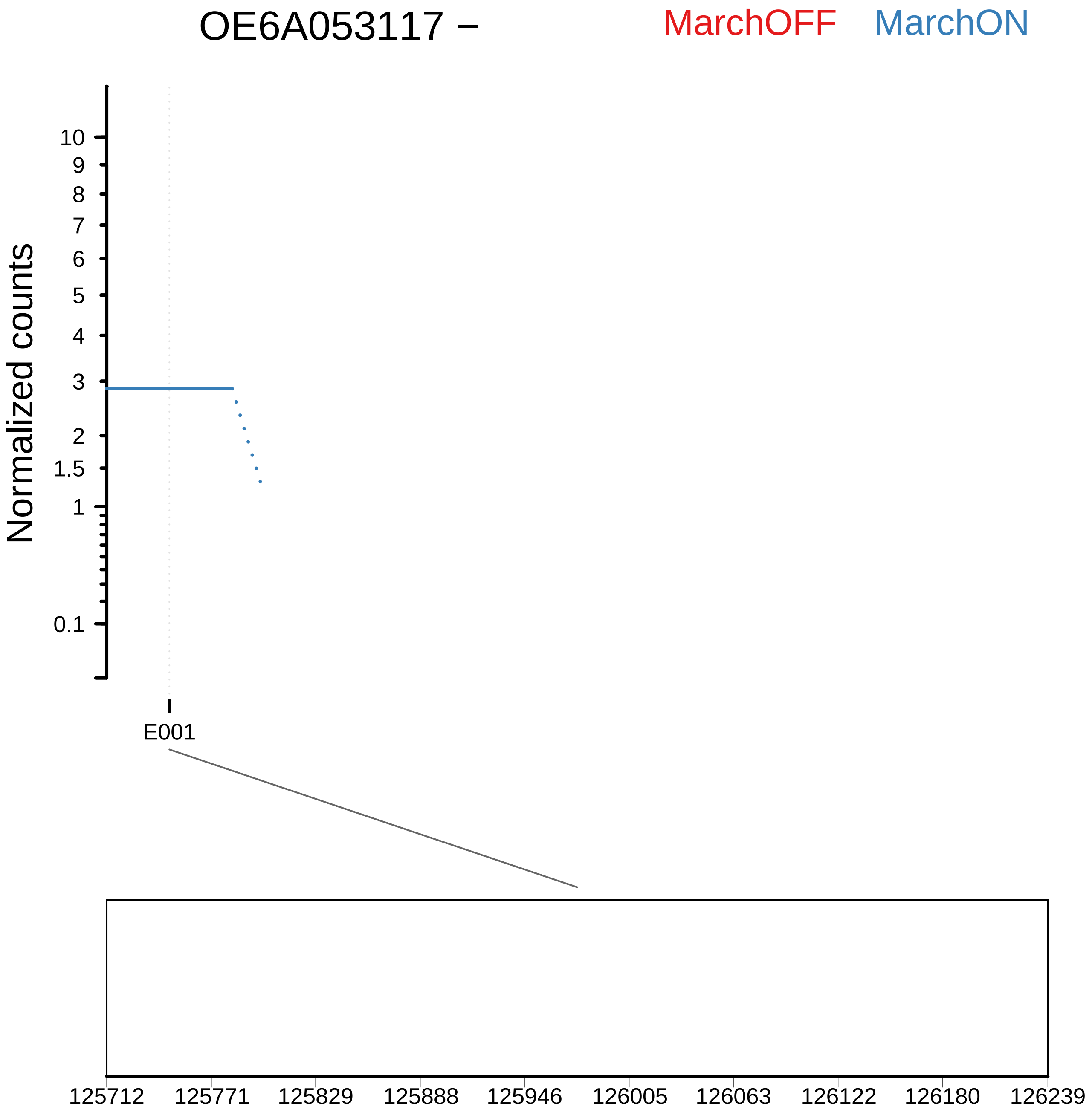

OE6A053117 −
MarchOFF
MarchON
Normalized counts
10
9
8
7
6
5
4
3
2
1.5
1
0.1
E001
125712
125771
125829
125888
125946
126005
126063
126122
126180
126239

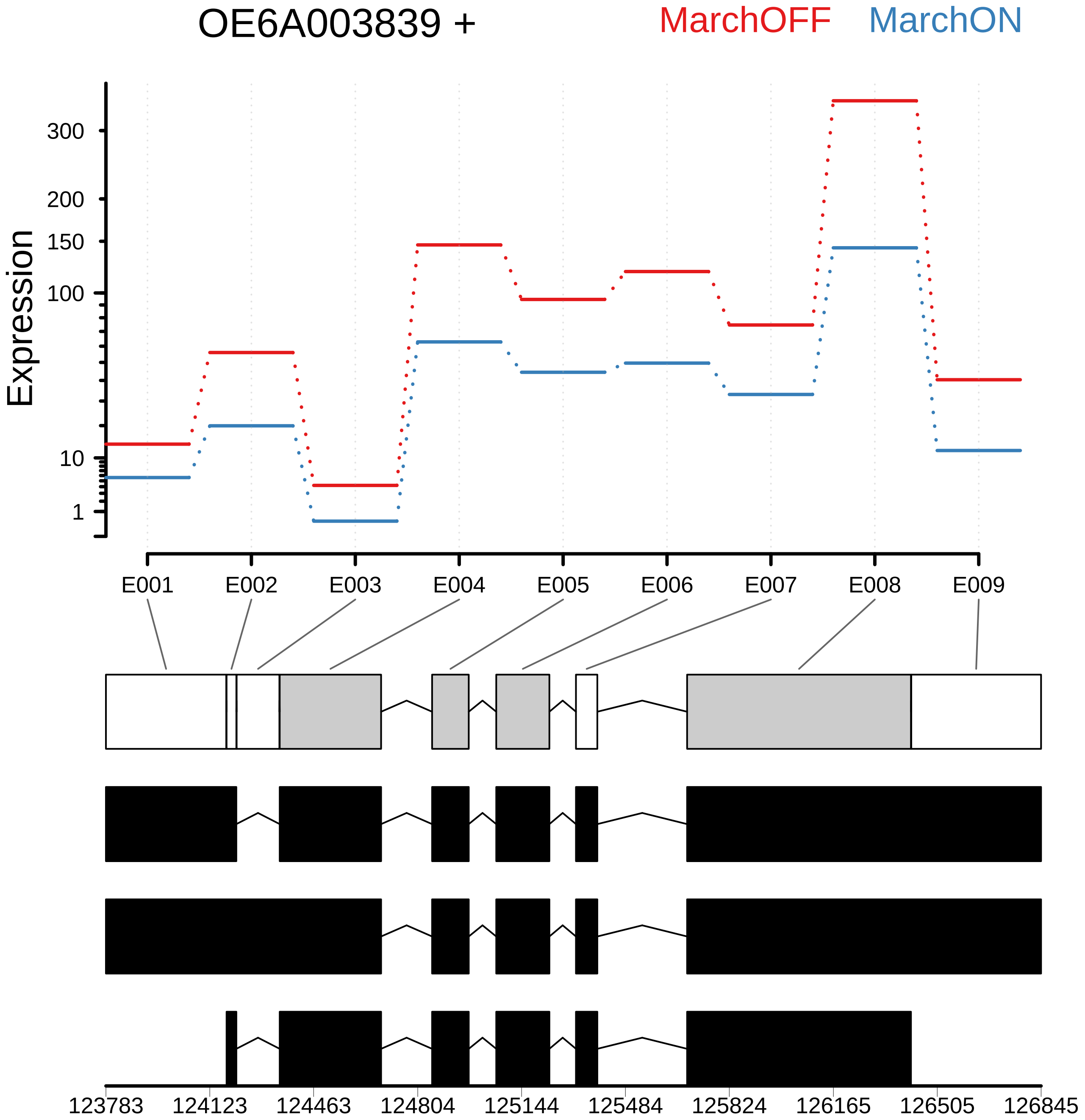

OE6A003839 +
MarchOFF
MarchON
Expression
300
200
150
100
10
1
E001
E002
E003
E004
E005
E006
E007
E008
E009
123783
124123
124463
124804
125144
125484
125824
126165
126505
126845

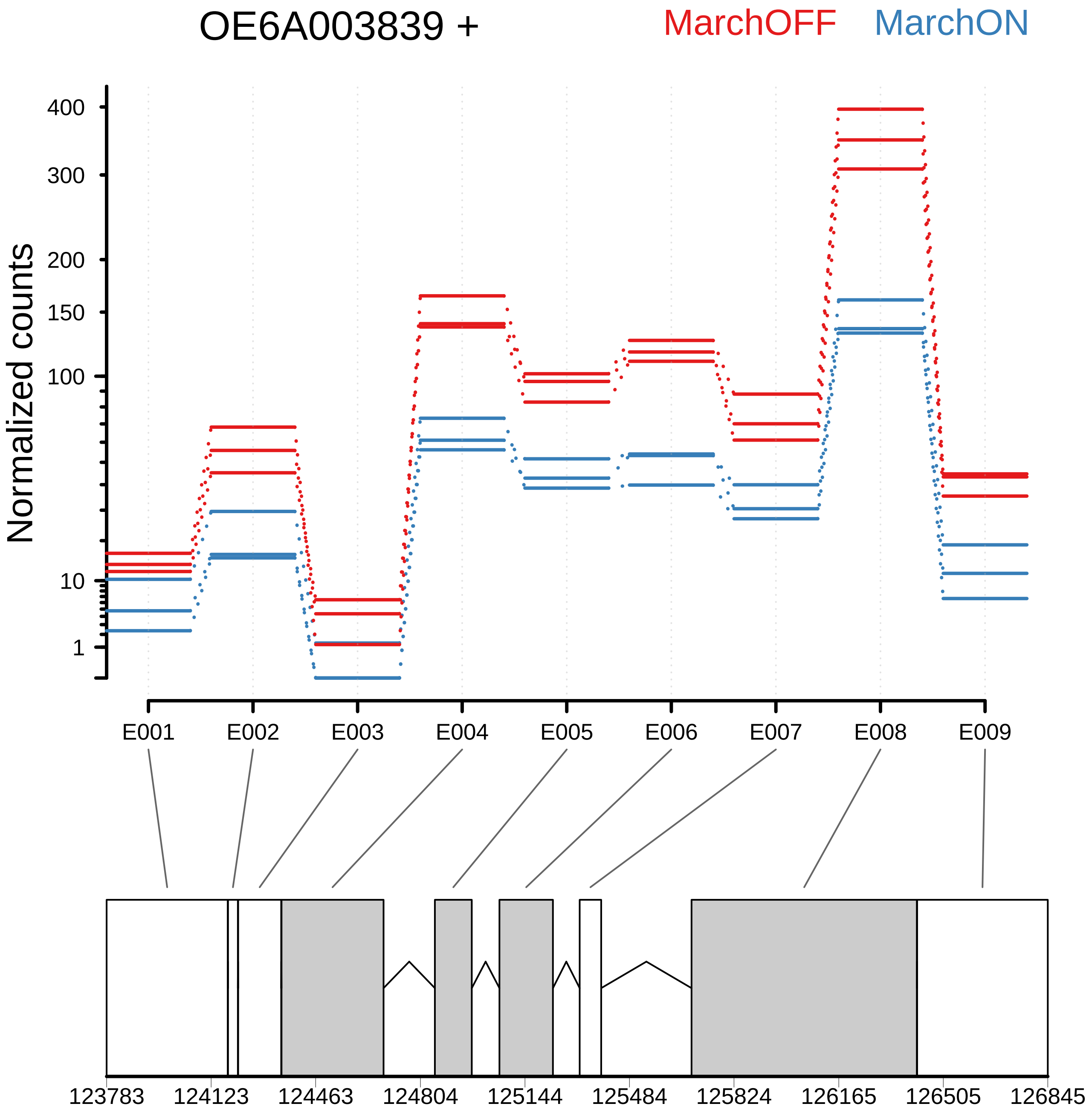

OE6A003839 +
MarchOFF
MarchON
Normalized counts
400
300
200
150
100
10
1
E001
E002
E003
E004
E005
E006
E007
E008
E009
123783
124123
124463
124804
125144
125484
125824
126165
126505
126845

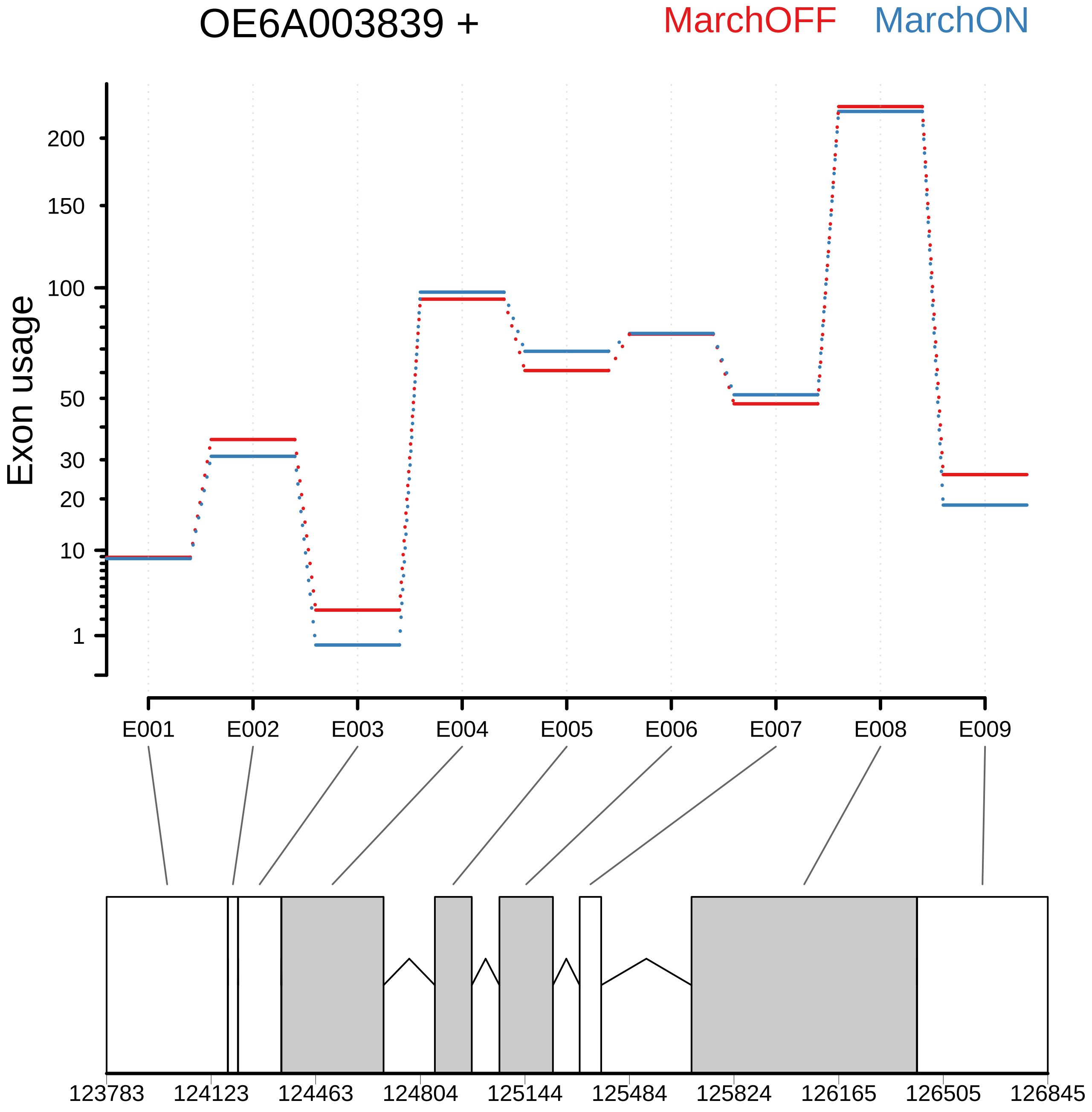

OE6A003839 +
MarchOFF
MarchON
Exon usage
1
10
20
30
50
100
150
200
E001
E002
E003
E004
E005
E006
E007
E008
E009
123783
124123
124463
124804
125144
125484
125824
126165
126505
126845

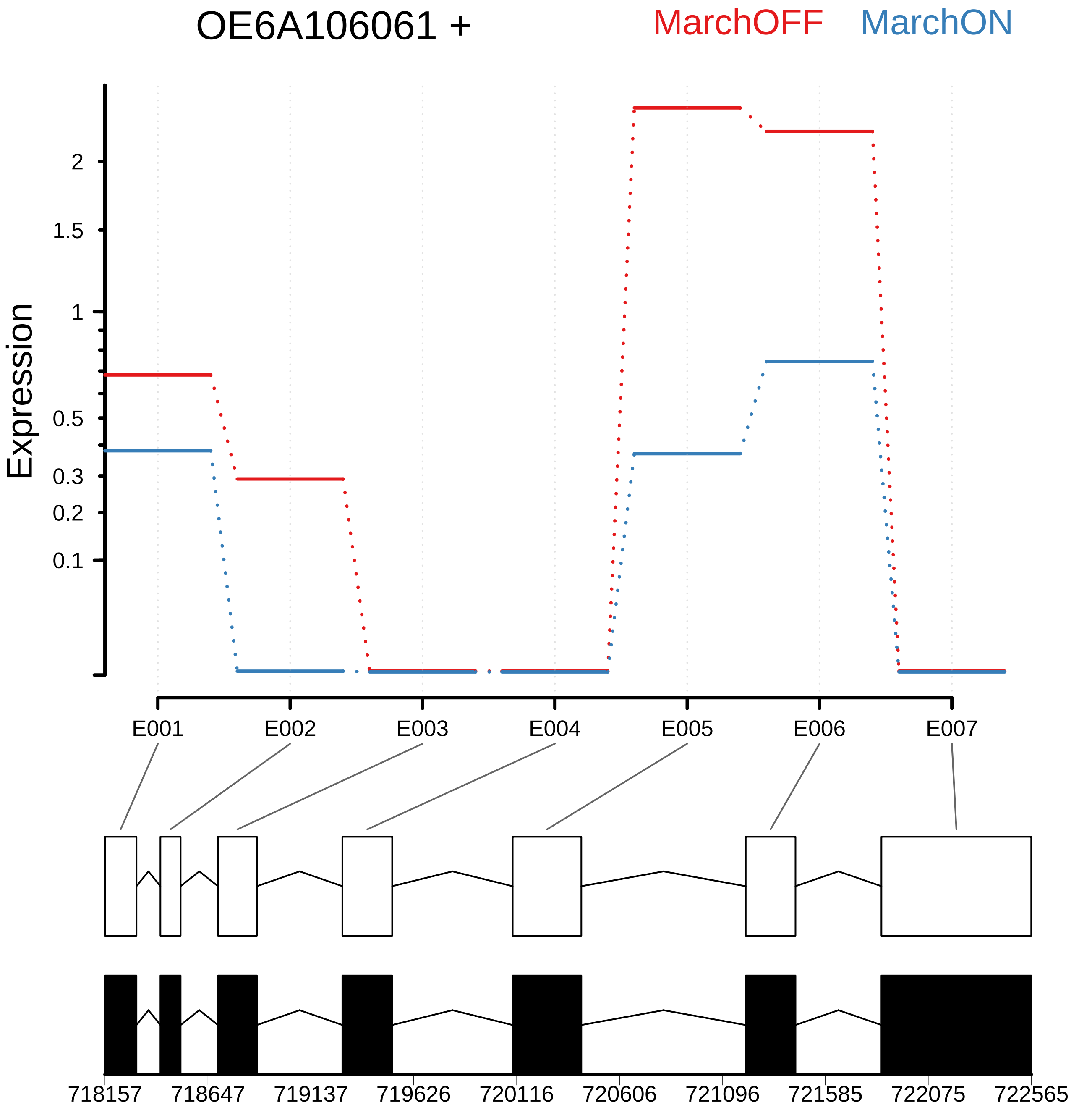

OE6A106061 +
MarchOFF
MarchON
Expression
2
1.5
1
0.5
0.3
0.2
0.1
E001
E002
E003
E004
E005
E006
E007
718157
718647
719137
719626
720116
720606
721096
721585
722075
722565

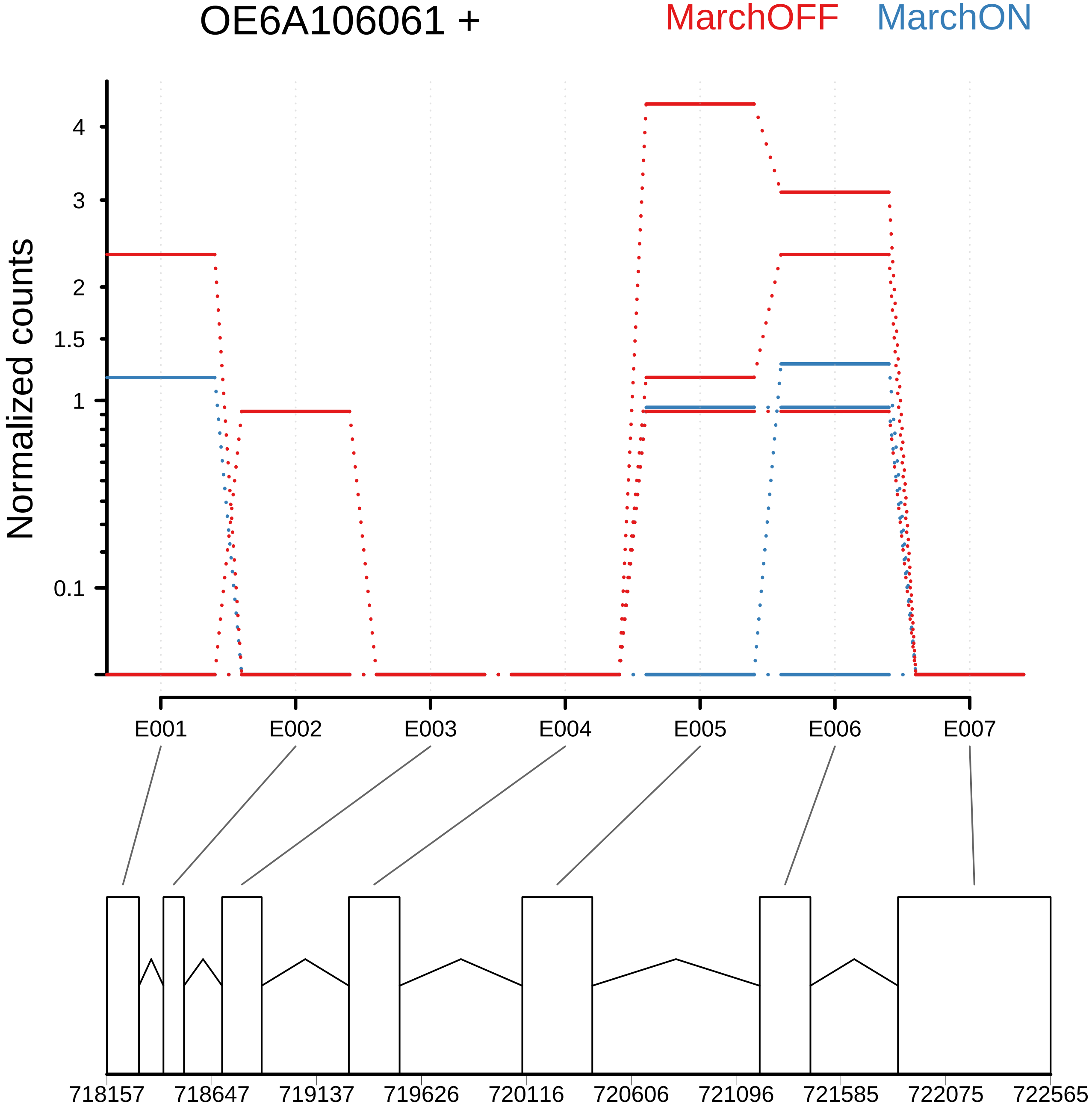

OE6A106061 +
MarchOFF
MarchON
Normalized counts
4
3
2
1.5
1
0.1
E001
E002
E003
E004
E005
E006
E007
718157
718647
719137
719626
720116
720606
721096
721585
722075
722565

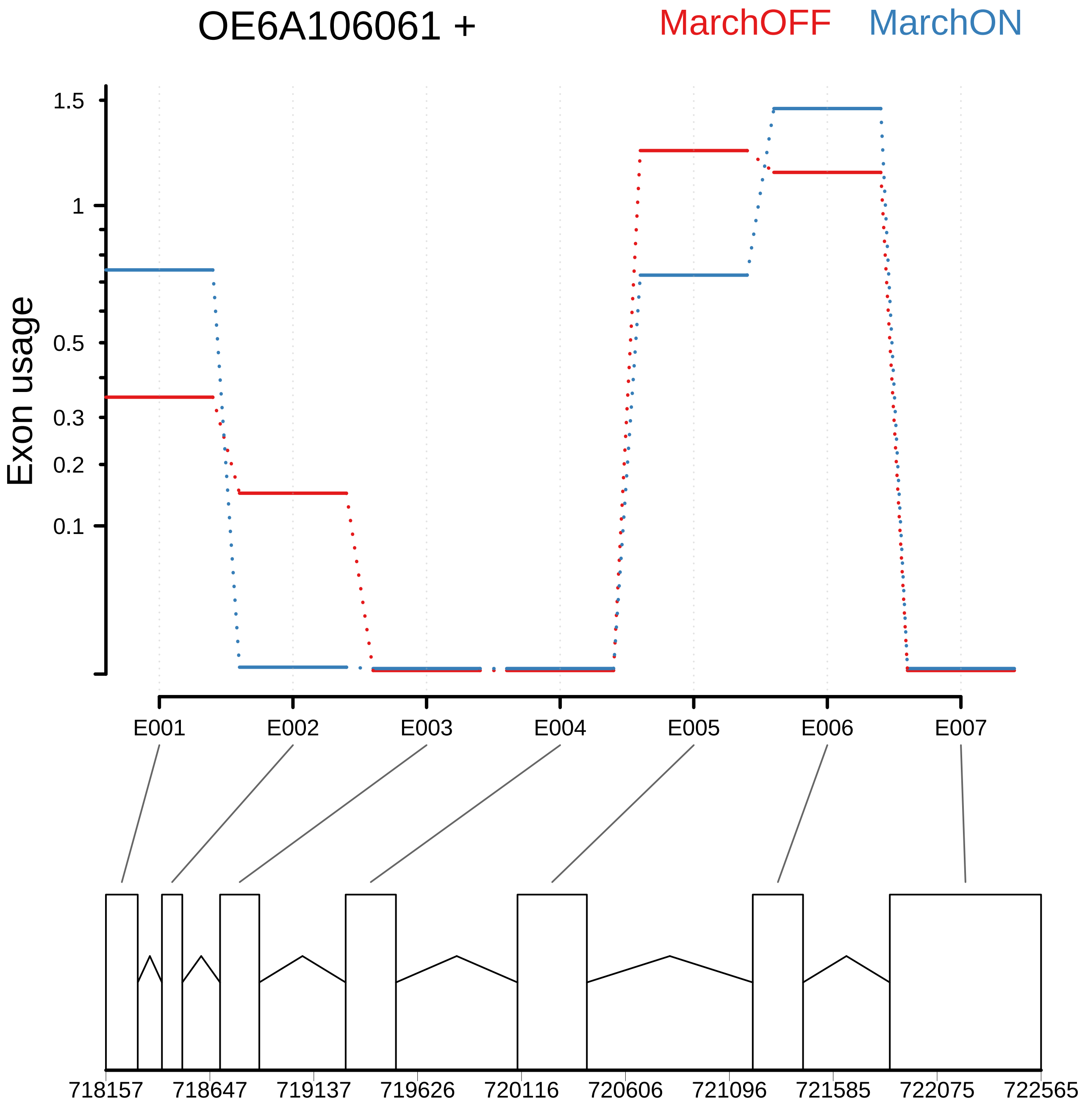

OE6A106061 +
MarchOFF
MarchON
Exon usage
1.5
1
0.5
0.3
0.2
0.1
E001
E002
E003
E004
E005
E006
E007
718157
718647
719137
719626
720116
720606
721096
721585
722075
722565

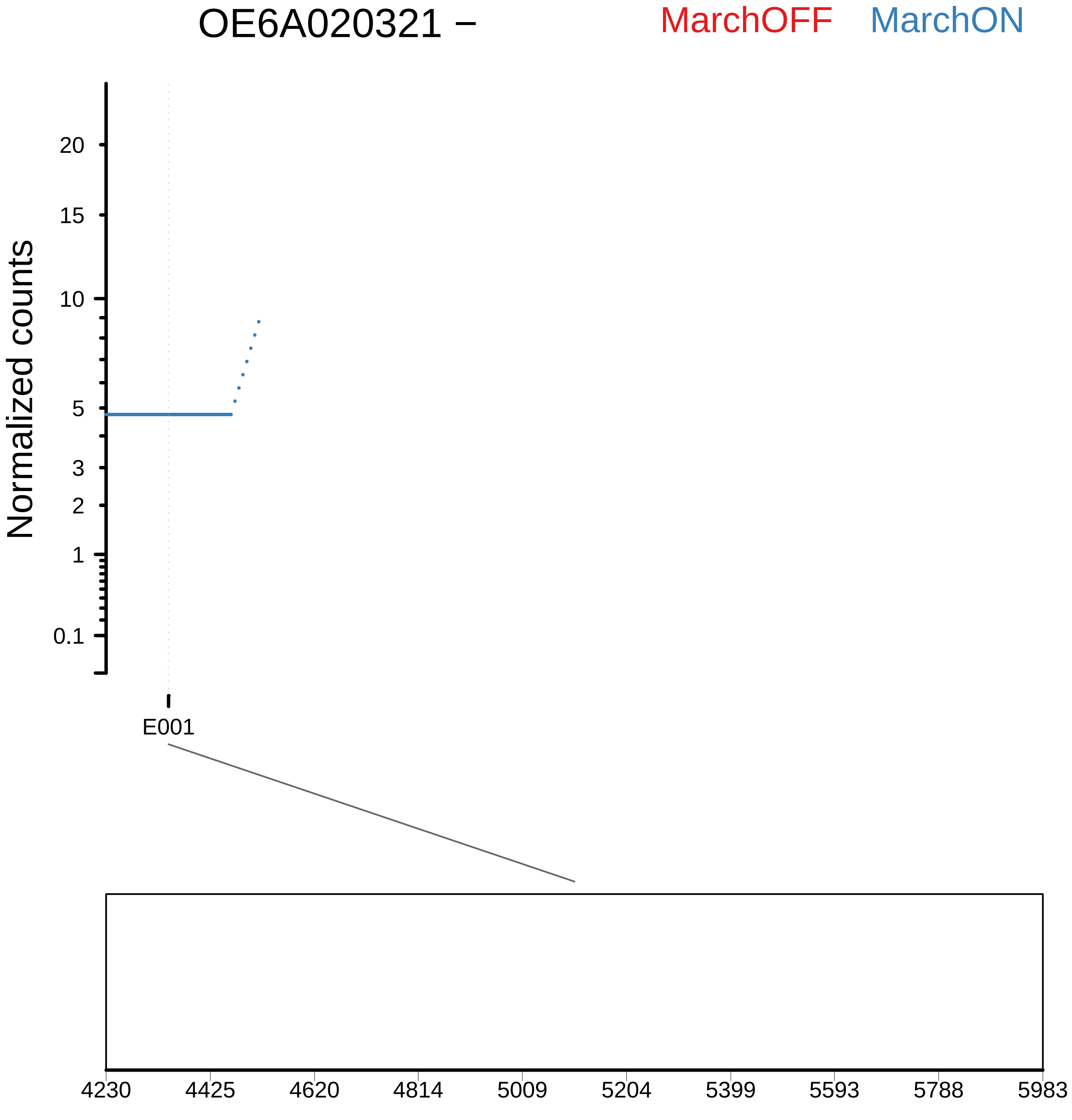
OE6A020321 −
MarchOFF
MarchON
Normalized counts
20
15
10
5
3
2
1
0.1
E001
4230
4425
4620
4814
5009
5204
5399
5593
5788
5983

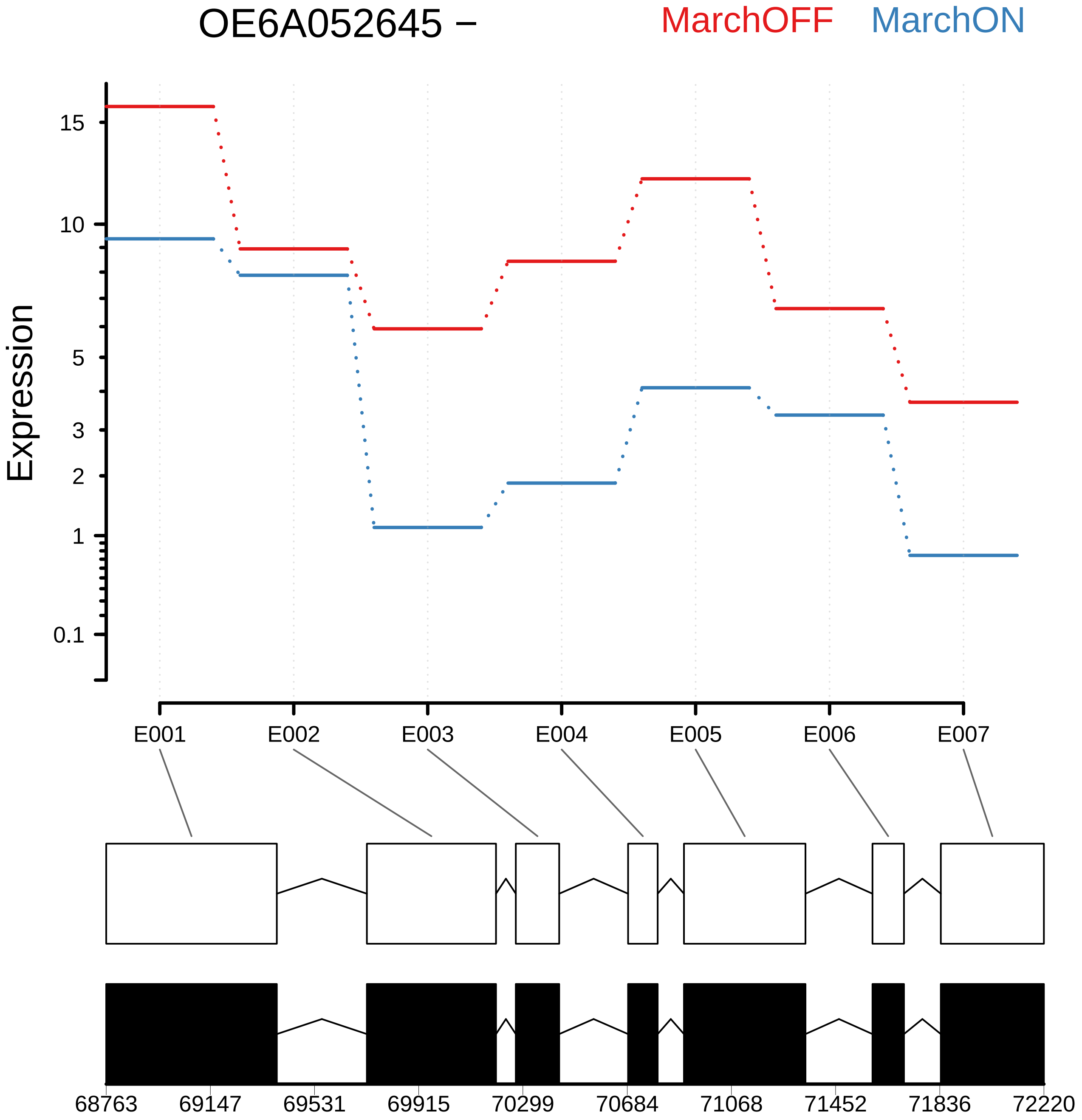
OE6A052645 −
MarchOFF
MarchON
Expression
15
10
5
3
2
1
0.1
E001
E002
E003
E004
E005
E006
E007
68763
69147
69531
69915
70299
70684
71068
71452
71836
72220

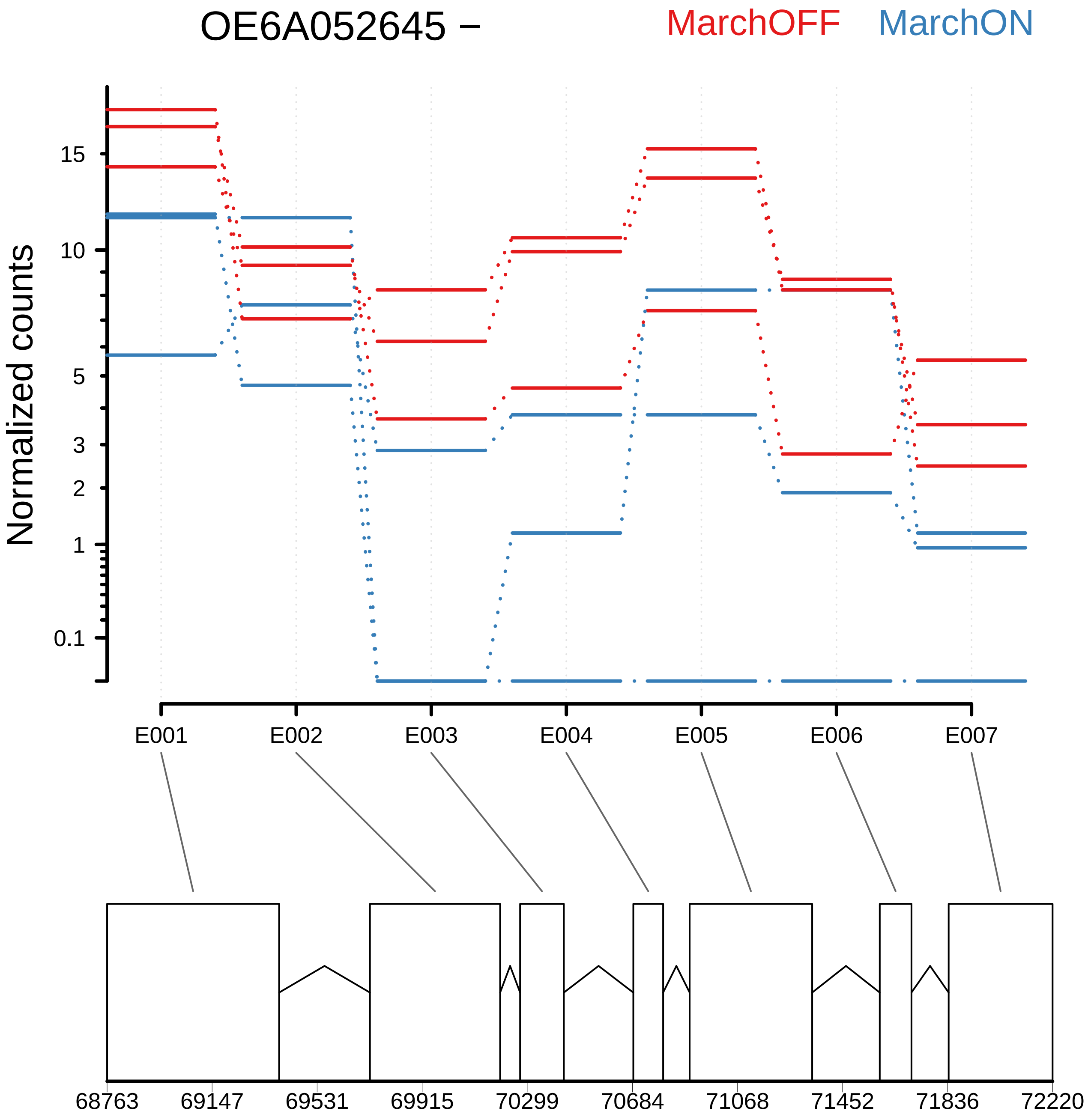
OE6A052645 −
MarchOFF
MarchON
Normalized counts
15
10
5
3
2
1
0.1
E001
E002
E003
E004
E005
E006
E007
68763
69147
69531
69915
70299
70684
71068
71452
71836
72220

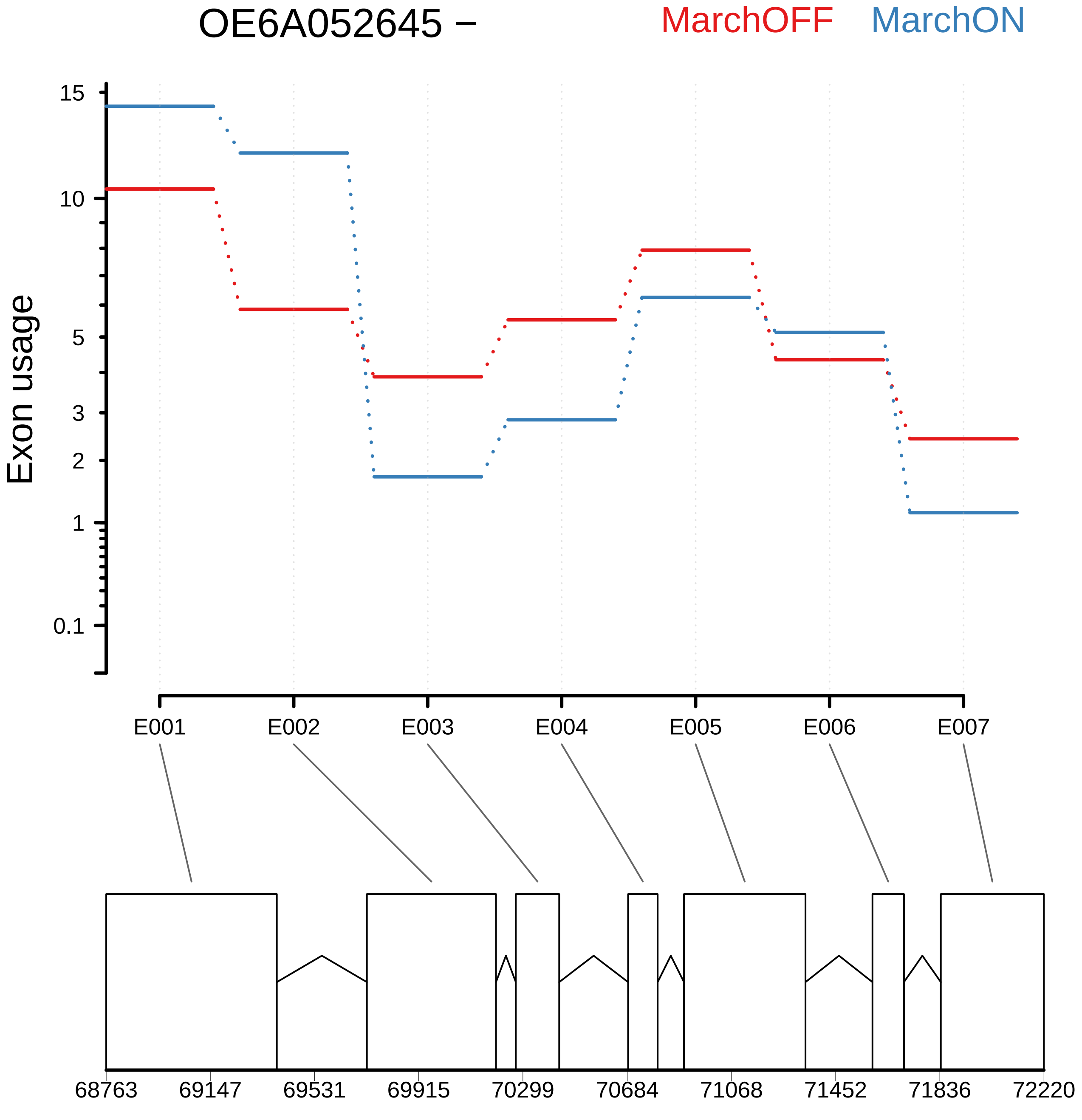

OE6A052645 −
MarchOFF
MarchON
Exon usage
15
10
5
3
2
1
0.1
E001
E002
E003
E004
E005
E006
E007
68763
69147
69531
69915
70299
70684
71068
71452
71836
72220

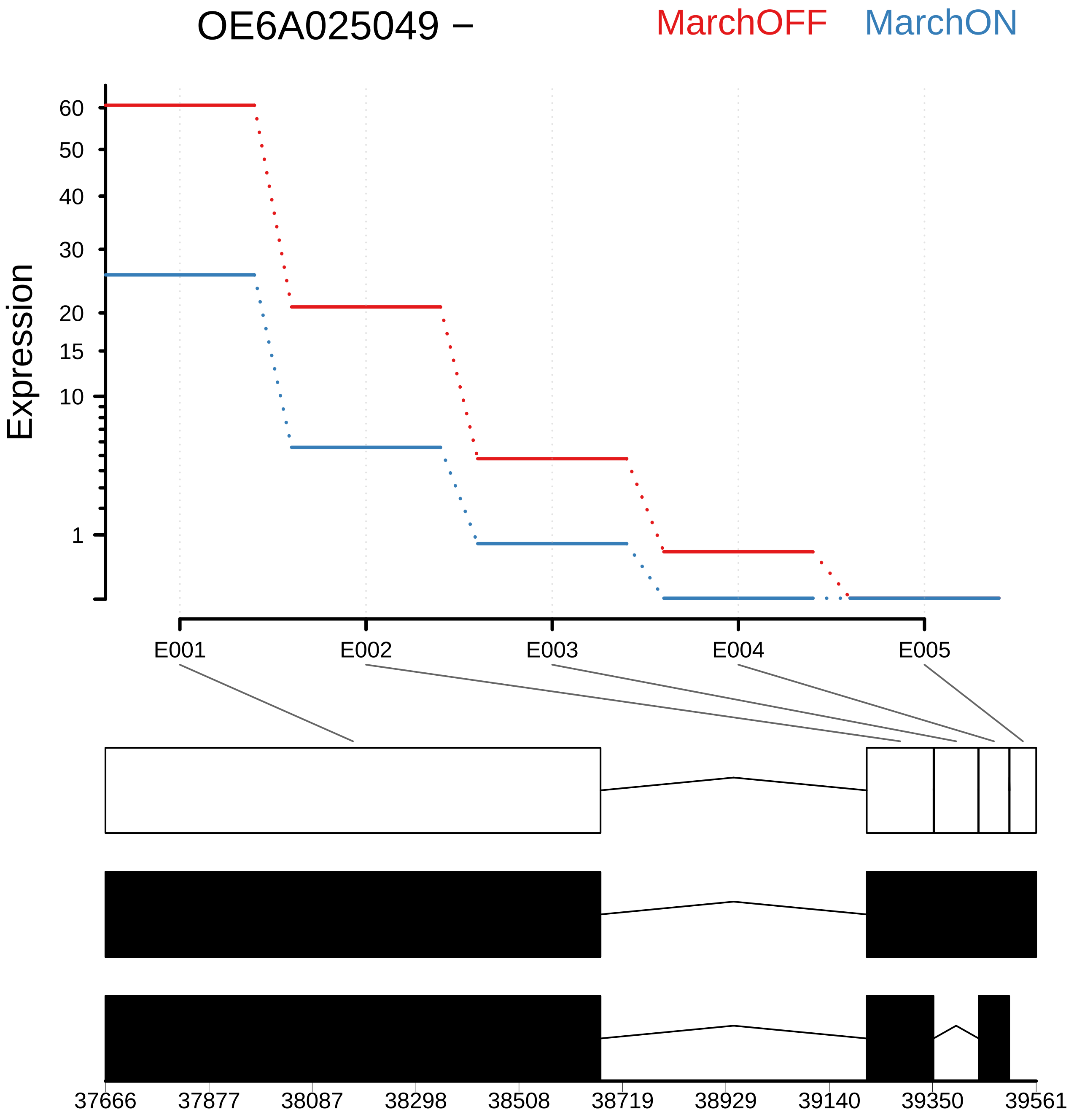

OE6A025049 −
MarchOFF
MarchON
Expression
60
50
40
30
20
15
10
1
E001
E002
E003
E004
E005
37666
37877
38087
38298
38508
38719
38929
39140
39350
39561

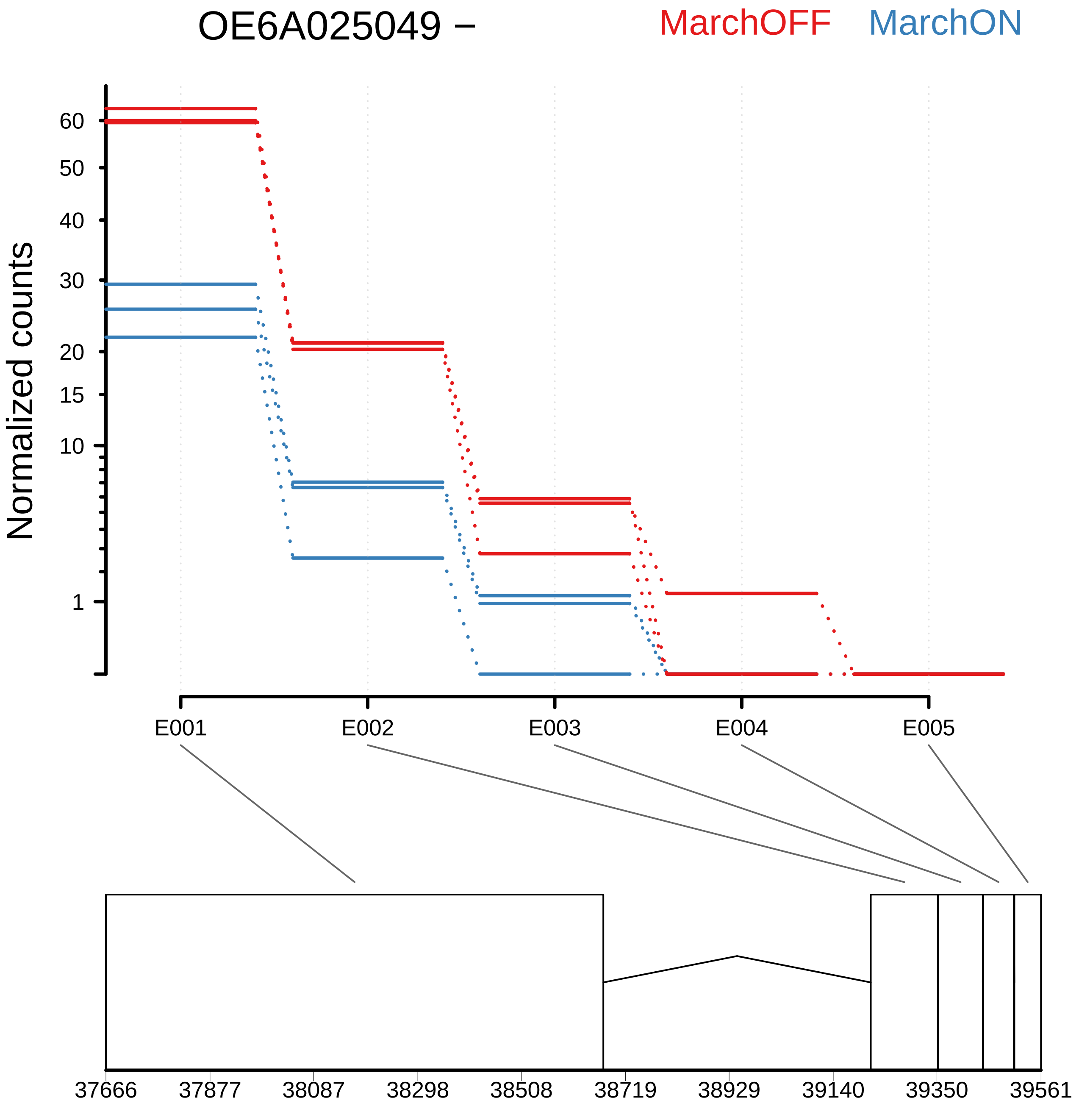

OE6A025049 −
MarchOFF
MarchON
Normalized counts
60
50
40
30
20
15
10
1
E001
E002
E003
E004
E005
37666
37877
38087
38298
38508
38719
38929
39140
39350
39561

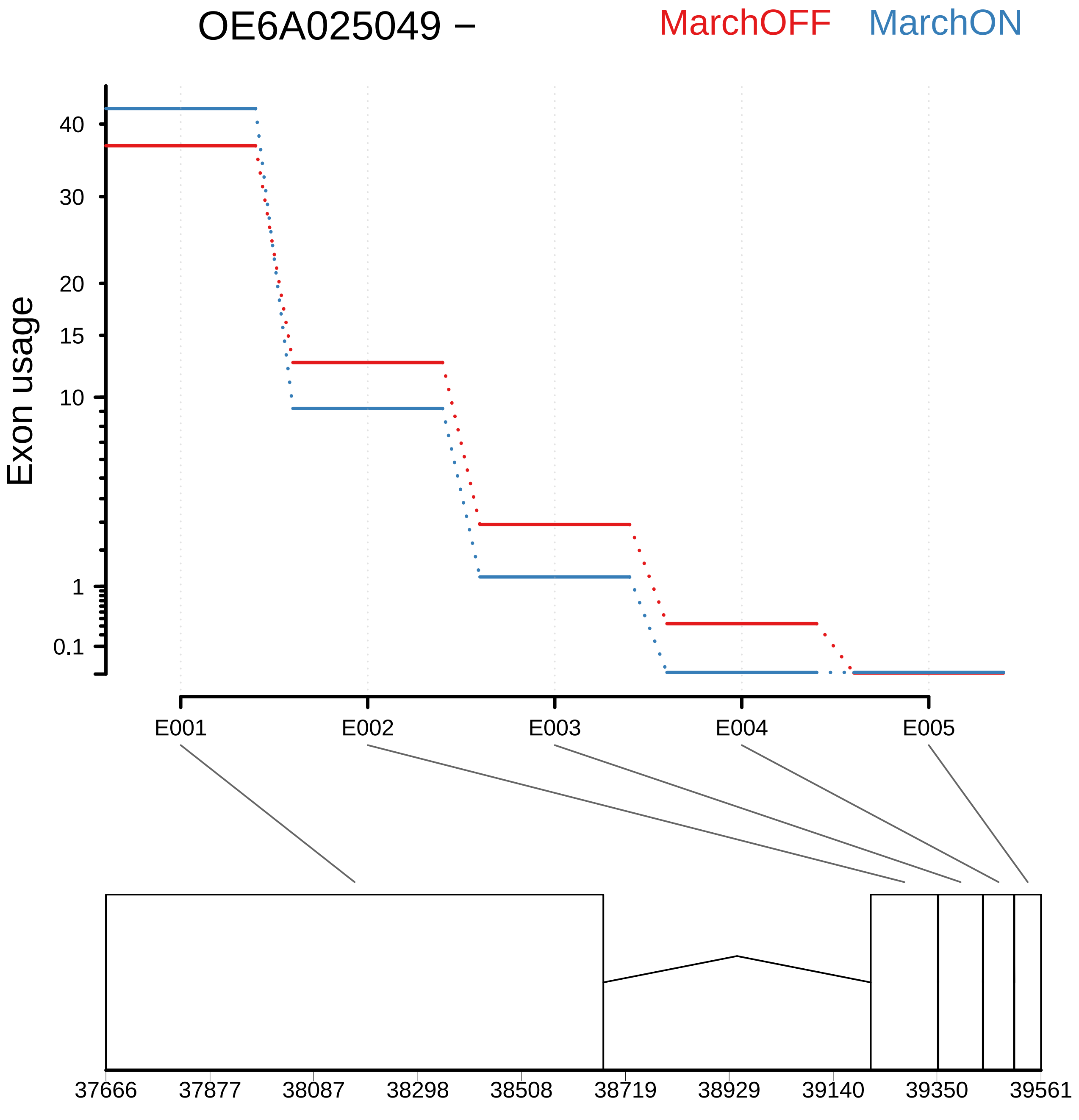

OE6A025049 −
MarchOFF
MarchON
Exon usage
40
30
20
15
10
1
0.1
E001
E002
E003
E004
E005
37666
37877
38087
38298
38508
38719
38929
39140
39350
39561

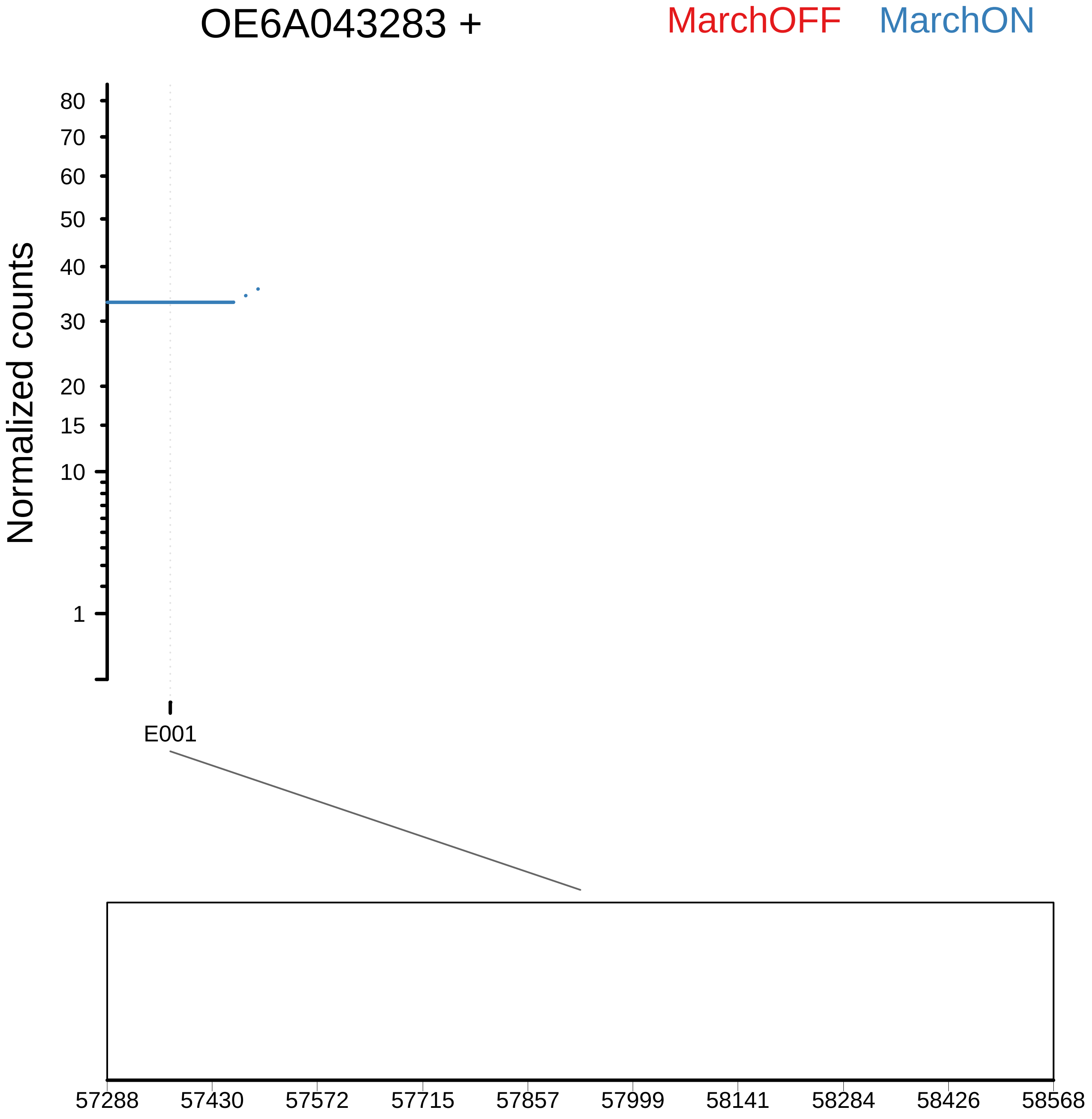

OE6A043283 +
MarchOFF
MarchON
Normalized counts
80
70
60
50
40
30
20
15
10
1
E001
57288
57430
57572
57715
57857
57999
58141
58284
58426
58568

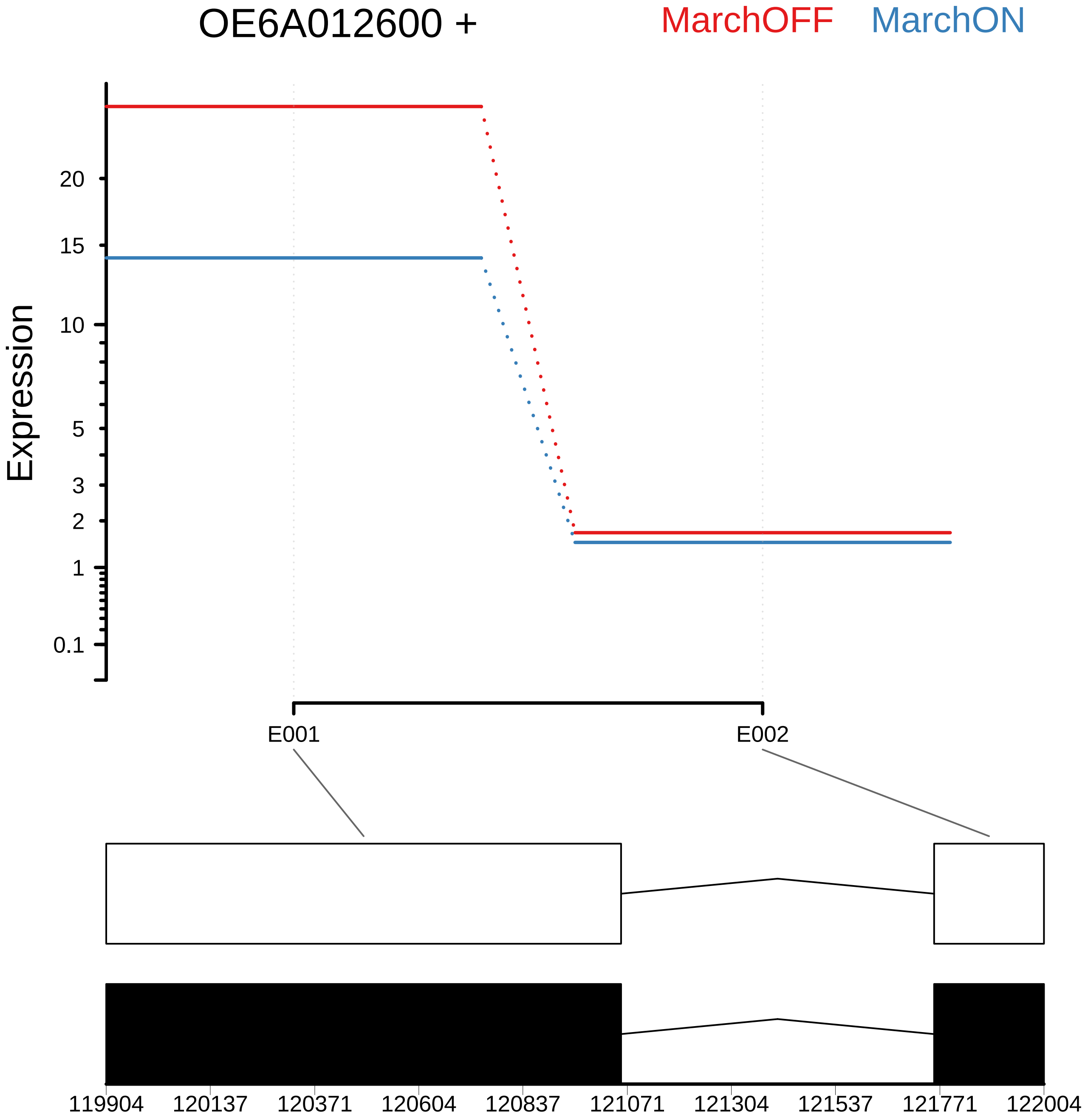
OE6A012600 +
MarchOFF
MarchON
Expression
20
15
10
5
3
2
1
0.1
E001
E002
119904
120137
120371
120604
120837
121071
121304
121537
121771
122004

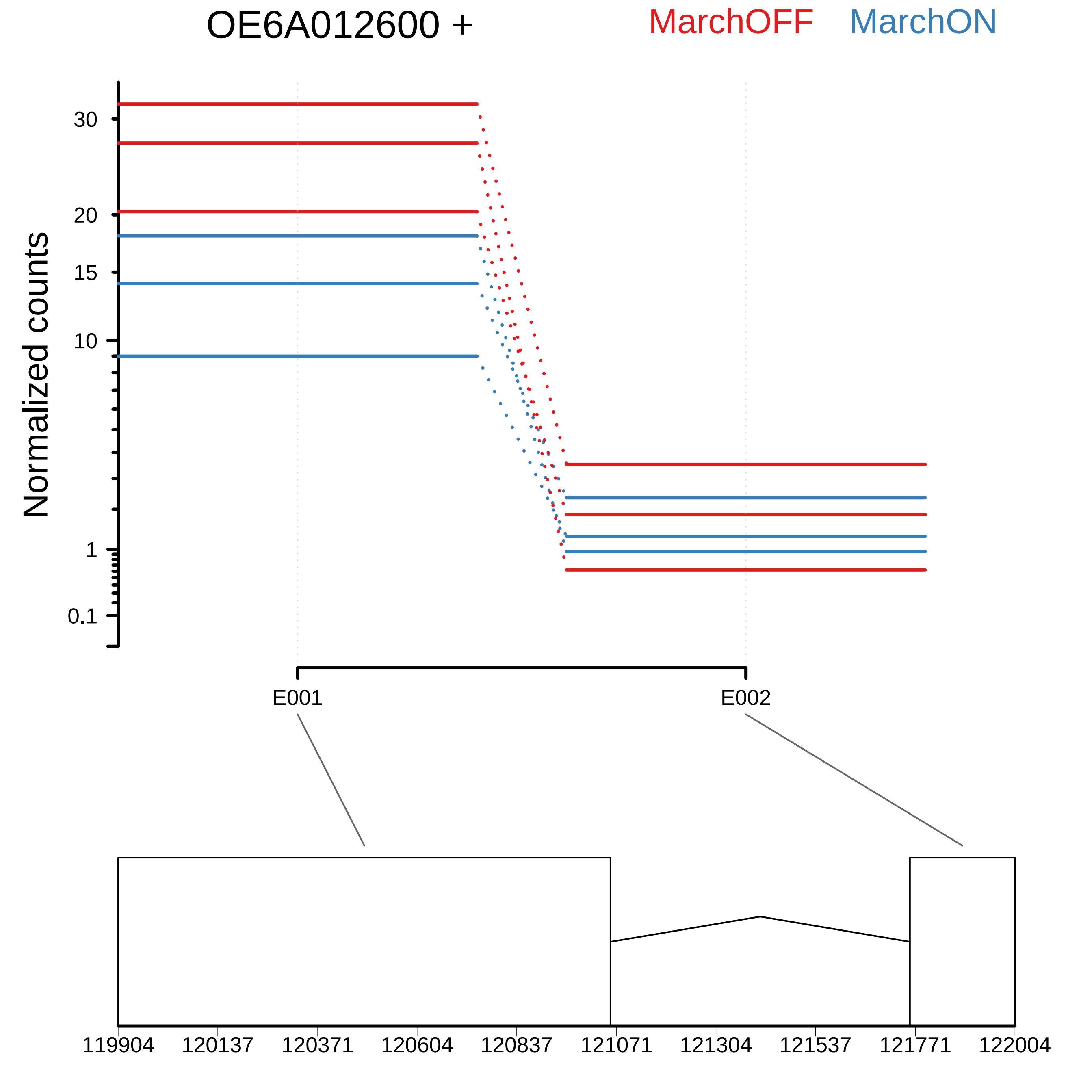
OE6A012600 +
MarchOFF
MarchON
Normalized counts
30
20
15
10
1
0.1
E001
E002
119904
120137
120371
120604
120837
121071
121304
121537
121771
122004

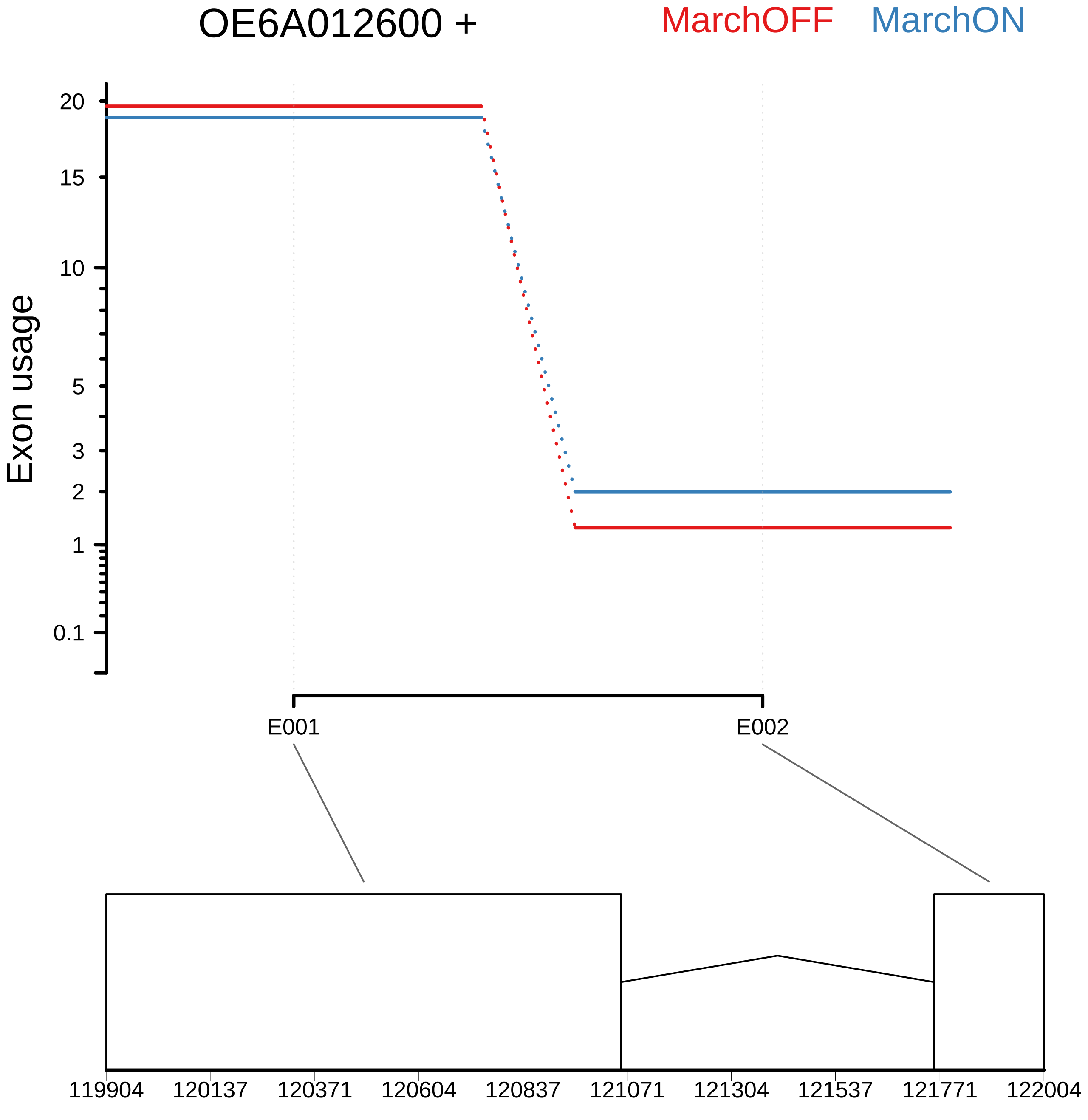
OE6A012600 +
MarchOFF
MarchON
Exon usage
20
15
10
5
3
2
1
0.1
E001
E002
119904
120137
120371
120604
120837
121071
121304
121537
121771
122004

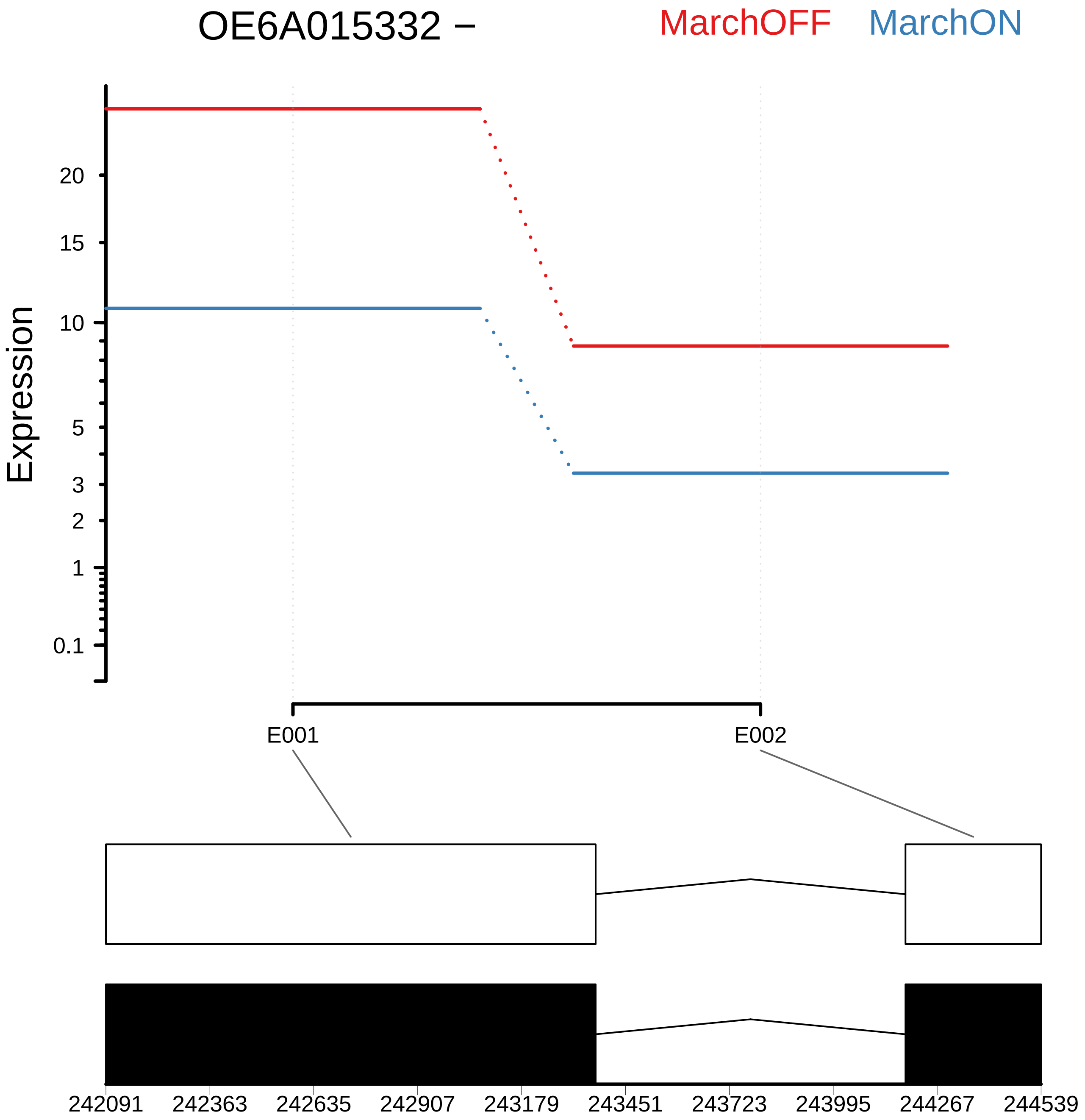

OE6A015332 −
MarchOFF
MarchON
Expression
20
15
10
5
3
2
1
0.1
E001
E002
242091
242363
242635
242907
243179
243451
243723
243995
244267
244539

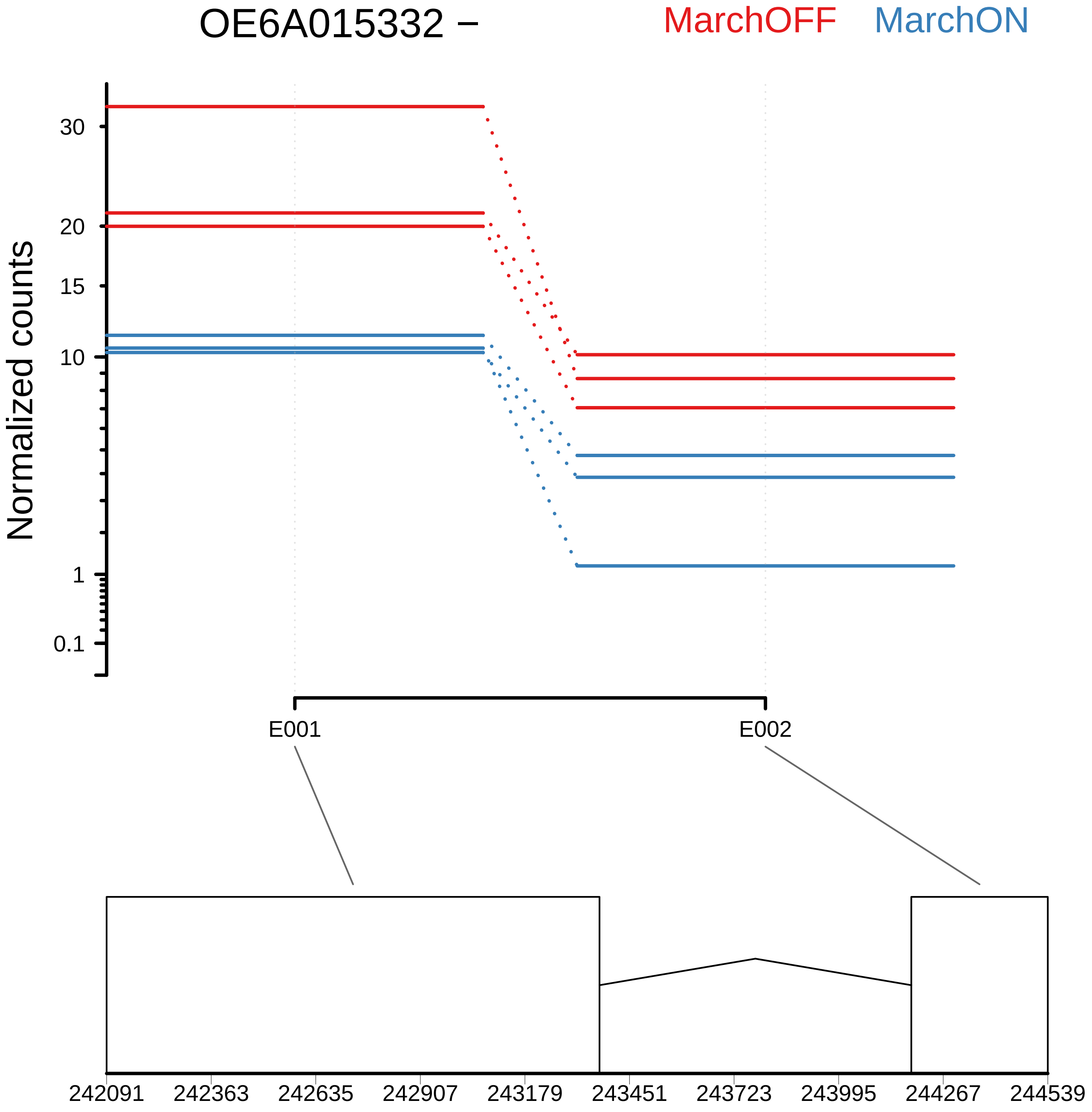
OE6A015332 −
MarchOFF
MarchON
Normalized counts
30
20
15
10
1
0.1
E001
E002
242091
242363
242635
242907
243179
243451
243723
243995
244267
244539

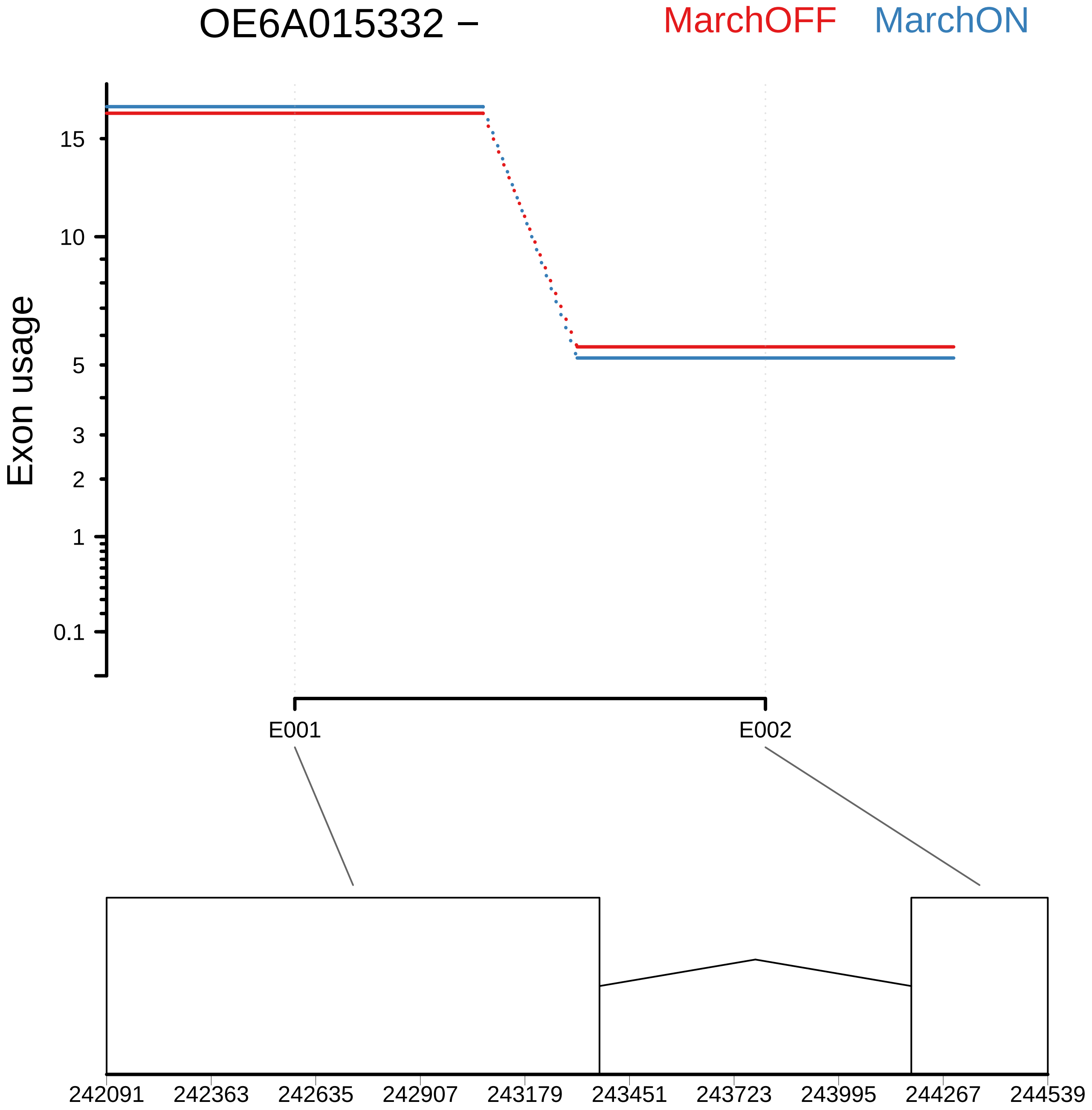

OE6A015332 −
MarchOFF
MarchON
Exon usage
15
10
5
3
2
1
0.1
E001
E002
242091
242363
242635
242907
243179
243451
243723
243995
244267
244539

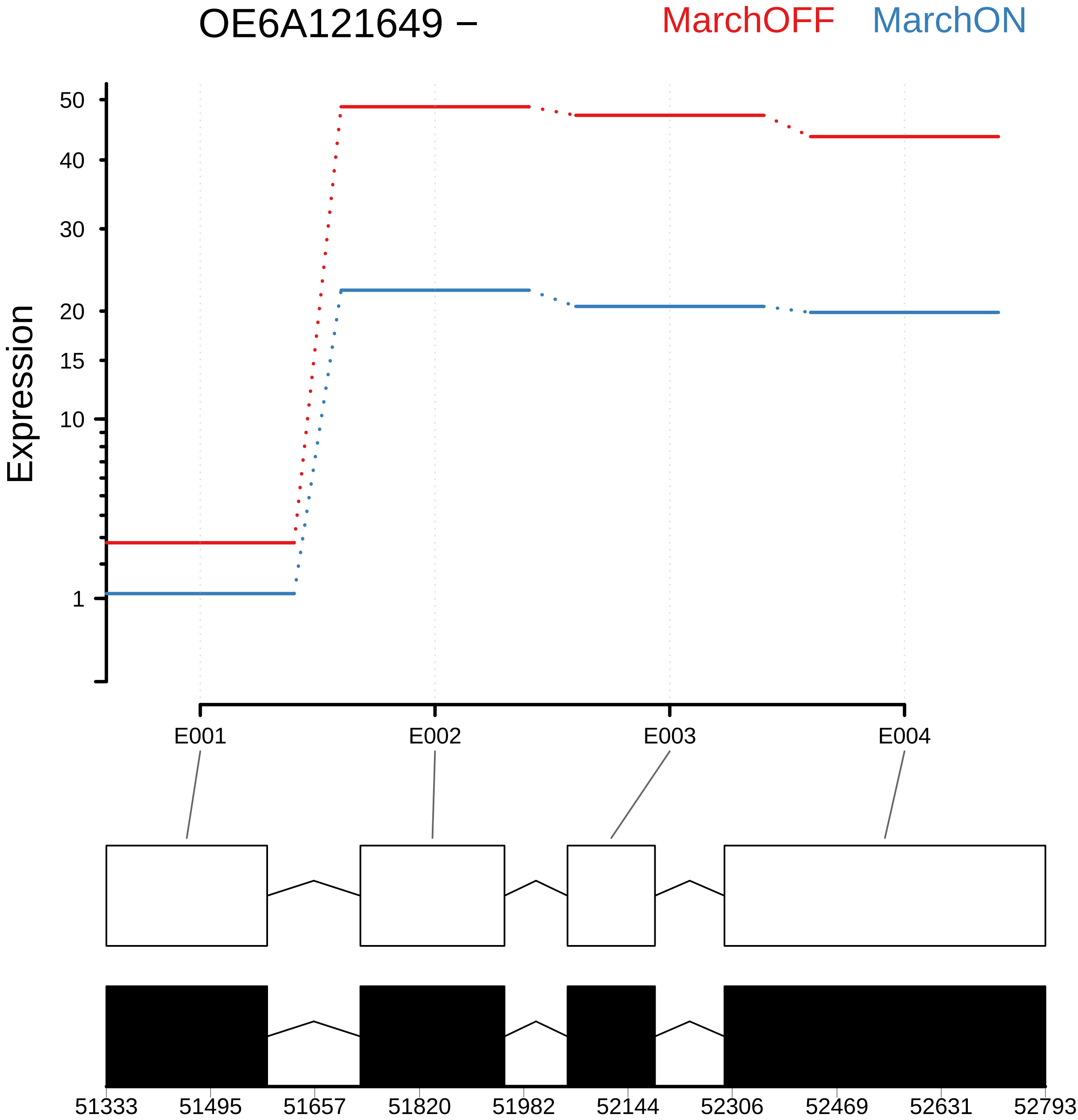

OE6A121649 −
MarchOFF
MarchON
Expression
50
40
30
20
15
10
1
E001
E002
E003
E004
51333
51495
51657
51820
51982
52144
52306
52469
52631
52793

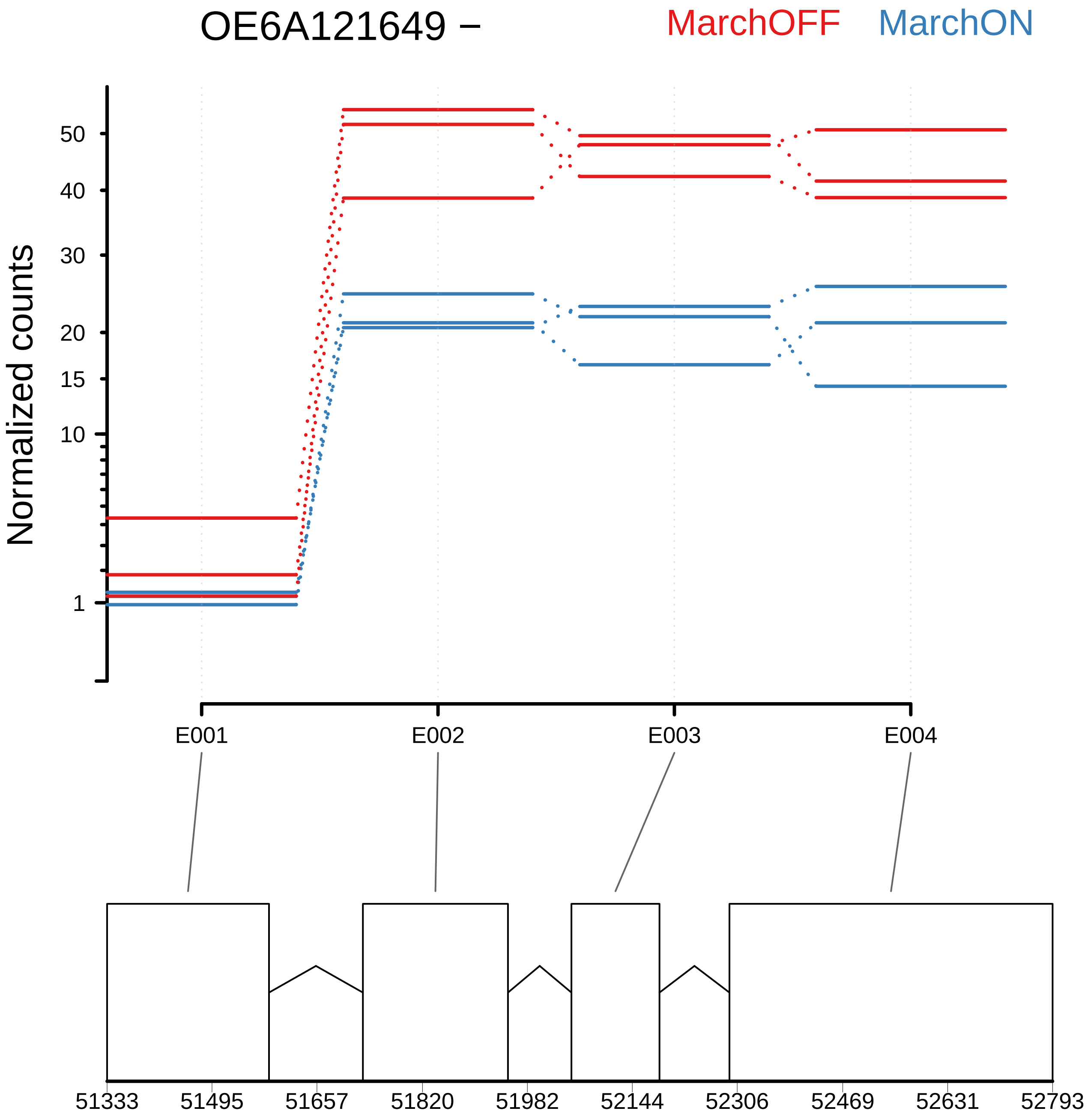

OE6A121649 −
MarchOFF
MarchON
Normalized counts
50
40
30
20
15
10
1
E001
E002
E003
E004
51333
51495
51657
51820
51982
52144
52306
52469
52631
52793

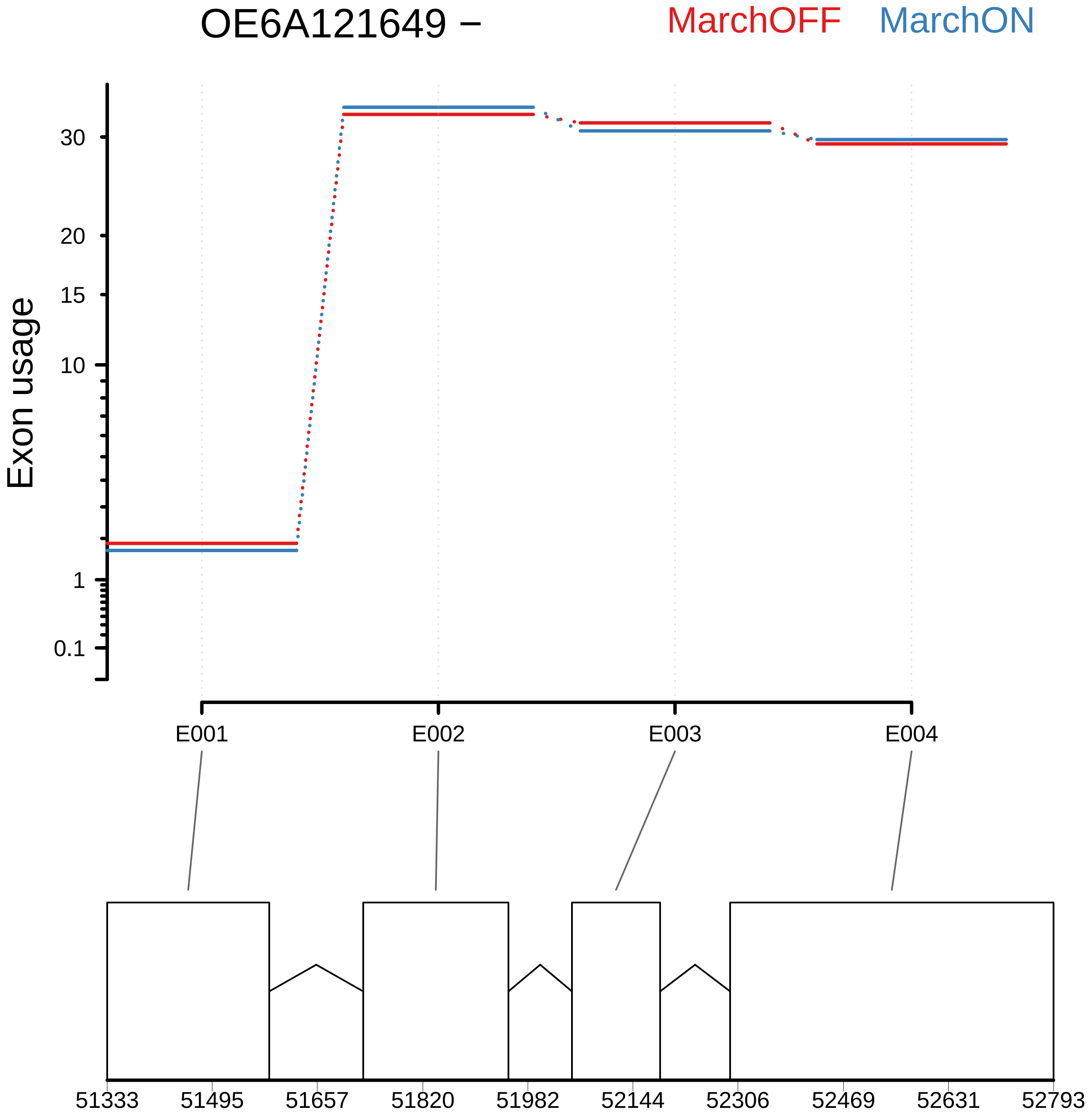

OE6A121649 −
MarchOFF
MarchON
Exon usage
30
20
15
10
1
0.1
E001
E002
E003
E004
51333
51495
51657
51820
51982
52144
52306
52469
52631
52793

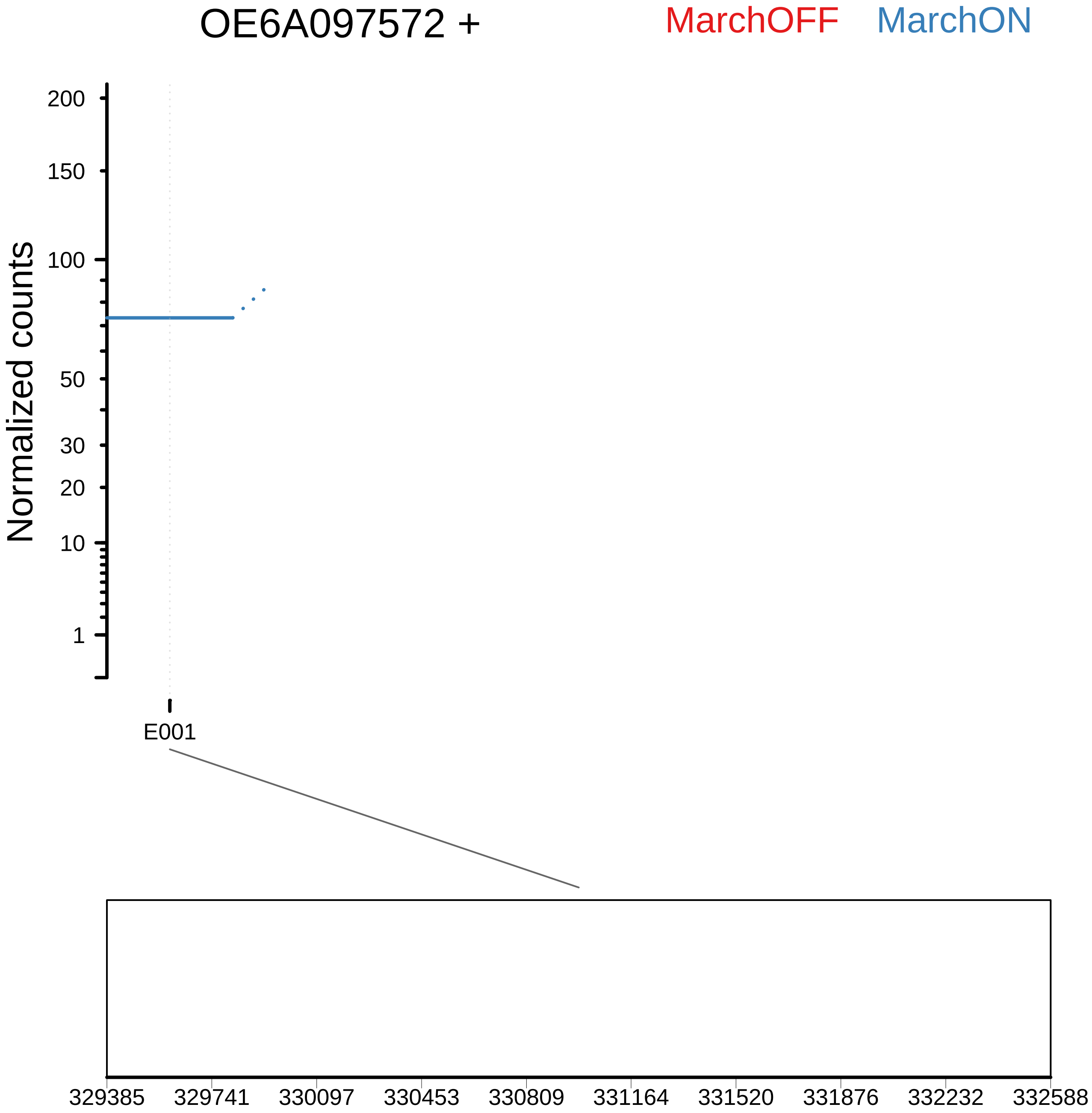

OE6A097572 +
MarchOFF
MarchON
Normalized counts
200
150
100
50
30
20
10
1
E001
329385
329741
330097
330453
330809
331164
331520
331876
332232
332588

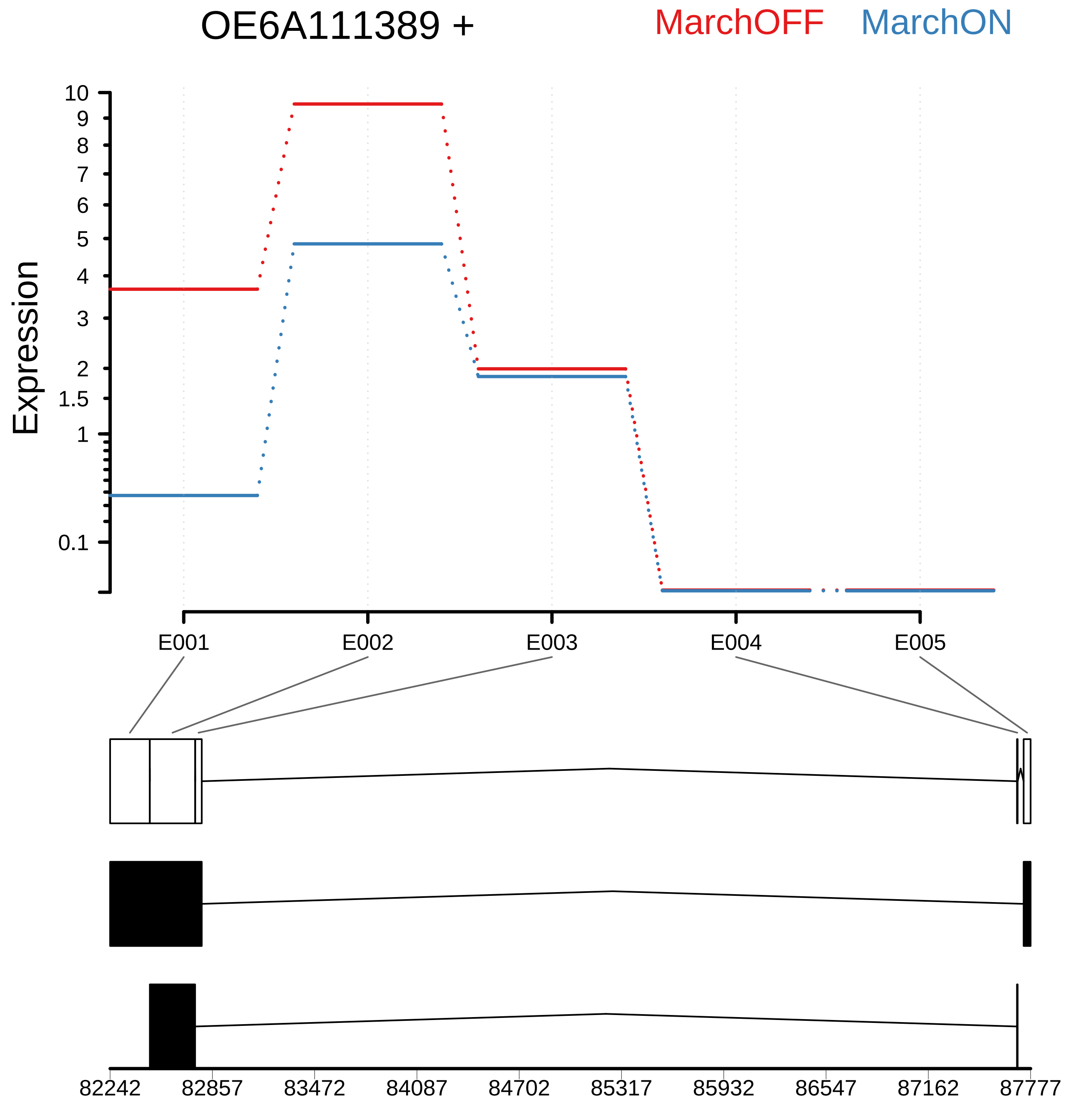

OE6A111389 +
MarchOFF
MarchON
Expression
10
9
8
7
6
5
4
3
2
1.5
1
0.1
E001
E002
E003
E004
E005
82242
82857
83472
84087
84702
85317
85932
86547
87162
87777

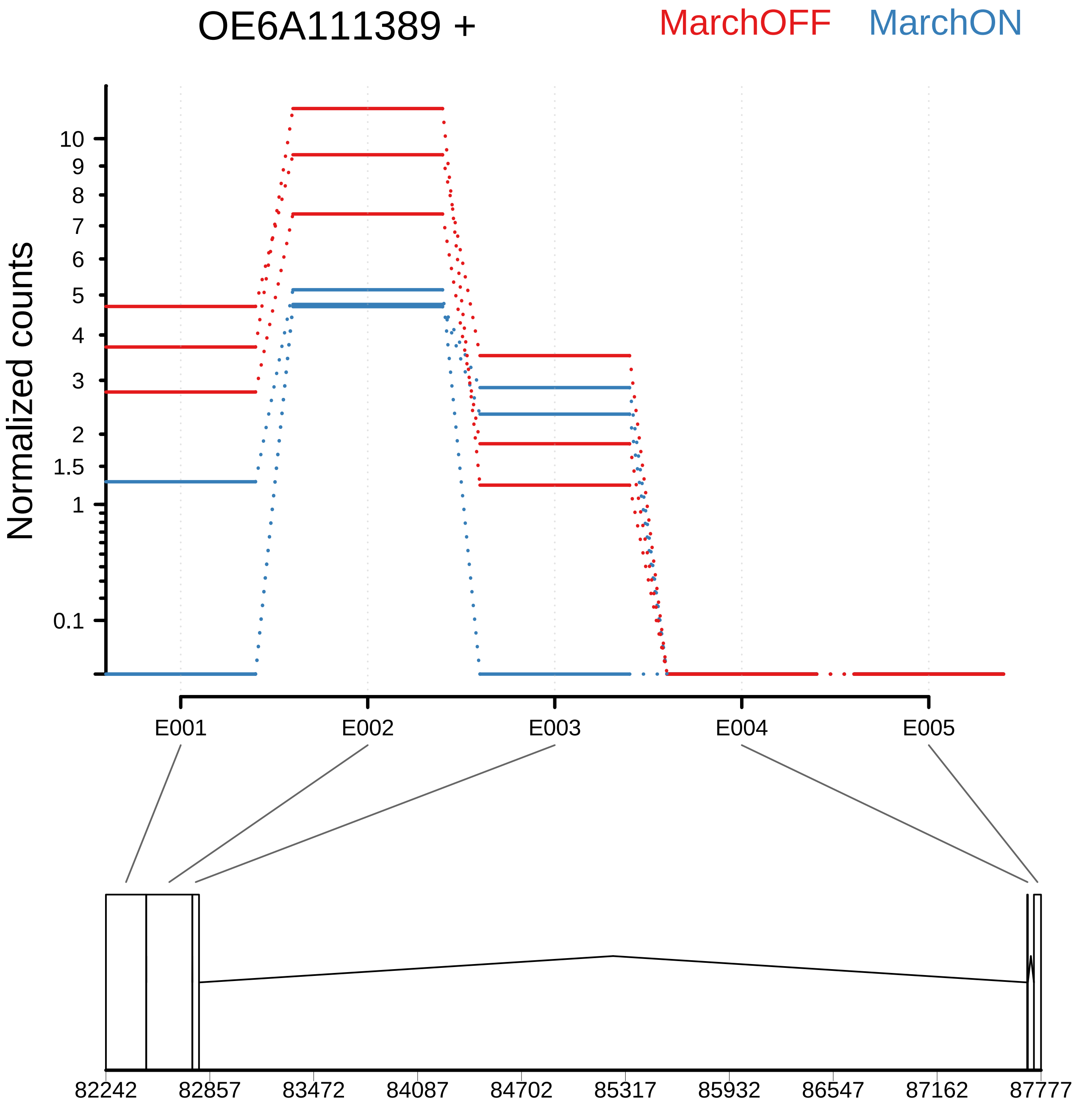
OE6A111389 +
MarchOFF
MarchON
Normalized counts
10
9
8
7
6
5
4
3
2
1.5
1
0.1
E001
E002
E003
E004
E005
82242
82857
83472
84087
84702
85317
85932
86547
87162
87777

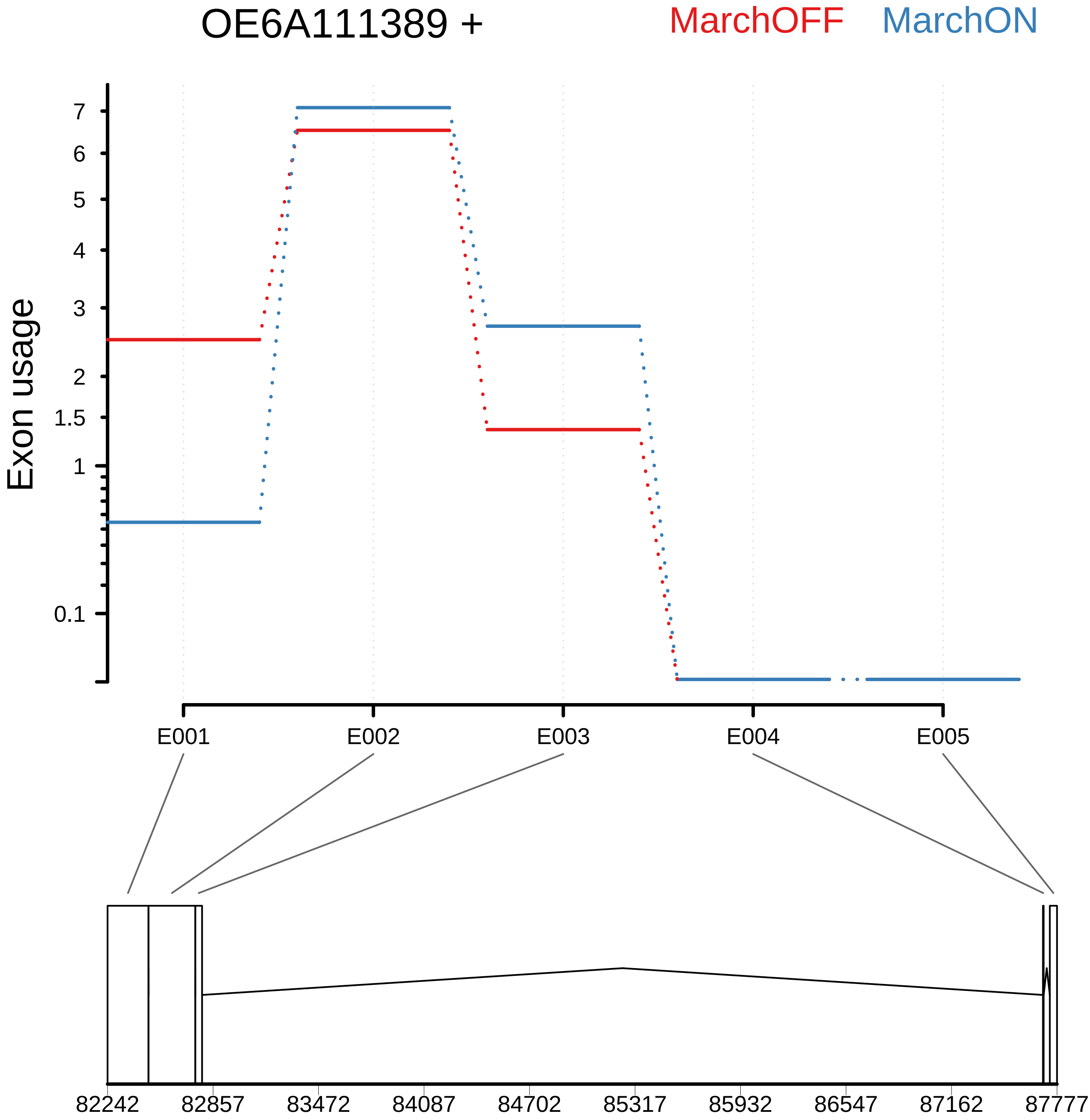
OE6A111389 +
MarchOFF
MarchON
Exon usage
7
6
5
4
3
2
1.5
1
0.1
E001
E002
E003
E004
E005
82242
82857
83472
84087
84702
85317
85932
86547
87162
87777

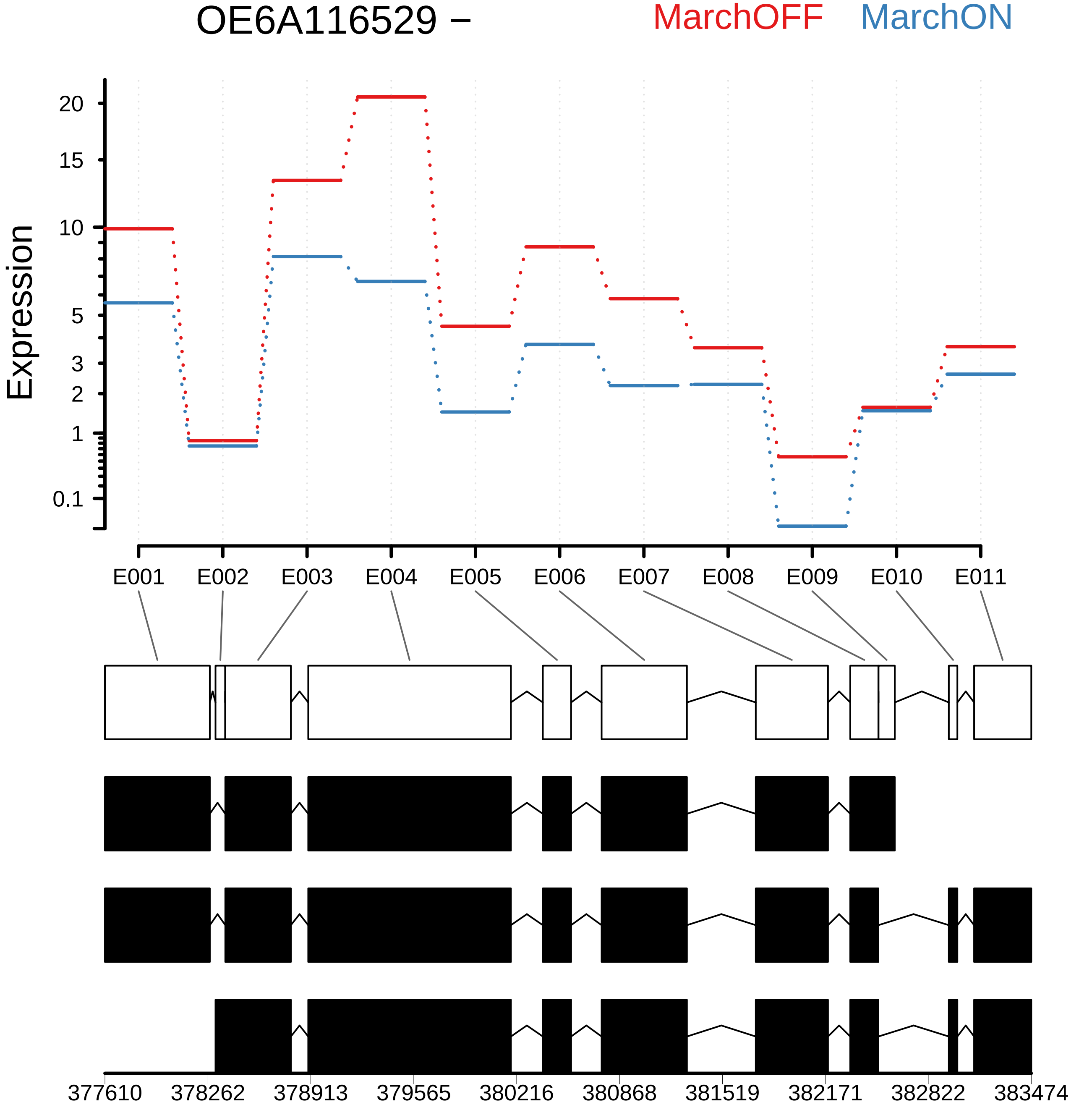
OE6A116529 –
MarchOFF
MarchON
Expression
20
15
10
5
3
2
1
0.1
E001
E002
E003
E004
E005
E006
E007
E008
E009
E010
E011
377610
378262
378913
379565
380216
380868
381519
382171
382822
383474

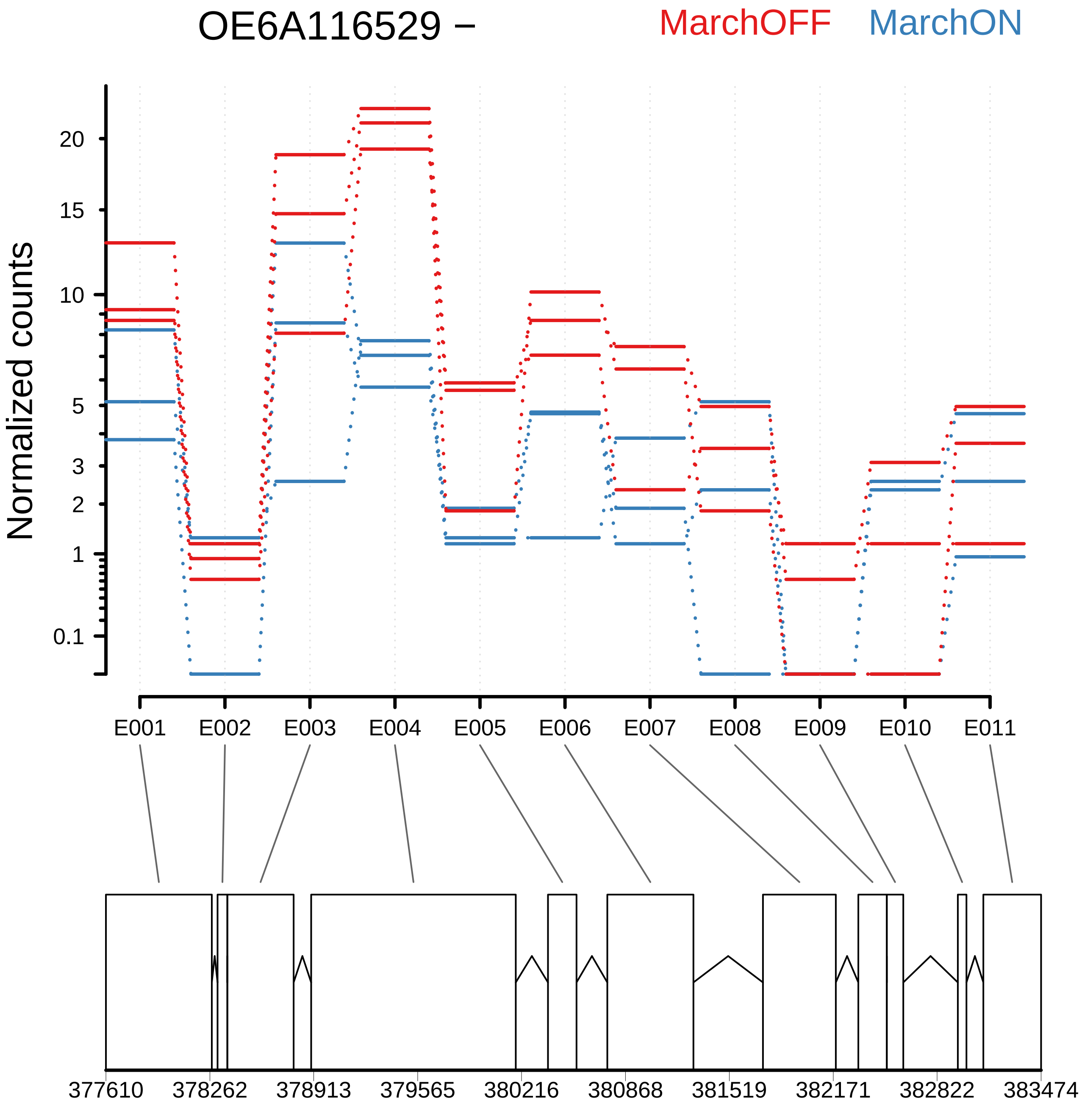
OE6A116529 −
MarchOFF
MarchON
Normalized counts
20
15
10
5
3
2
1
0.1
E001
E002
E003
E004
E005
E006
E007
E008
E009
E010
E011
377610
378262
378913
379565
380216
380868
381519
382171
382822
383474

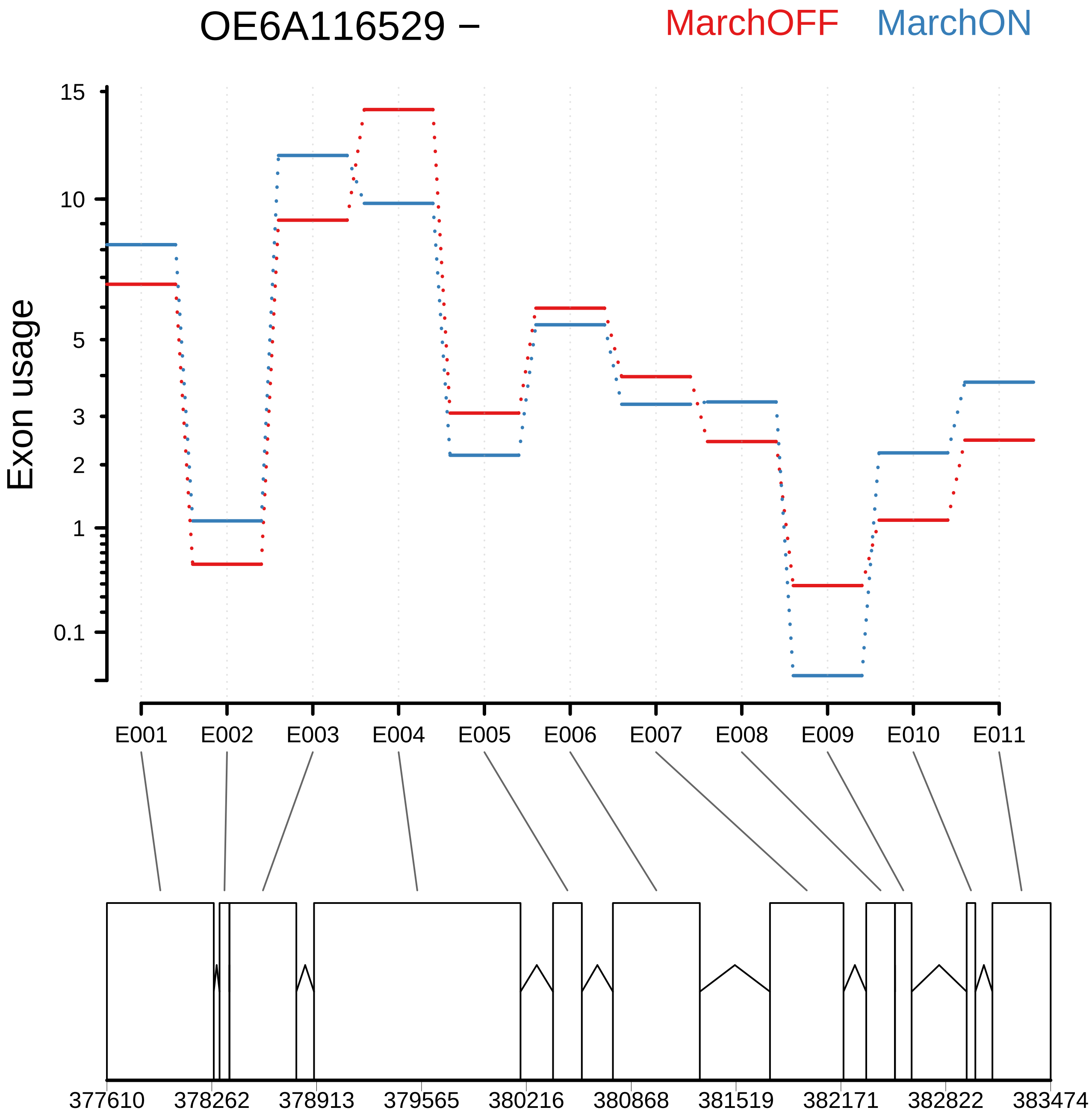

OE6A116529 −
MarchOFF
MarchON
Exon usage
15
10
5
3
2
1
0.1
E001
E002
E003
E004
E005
E006
E007
E008
E009
E010
E011
377610
378262
378913
379565
380216
380868
381519
382171
382822
383474

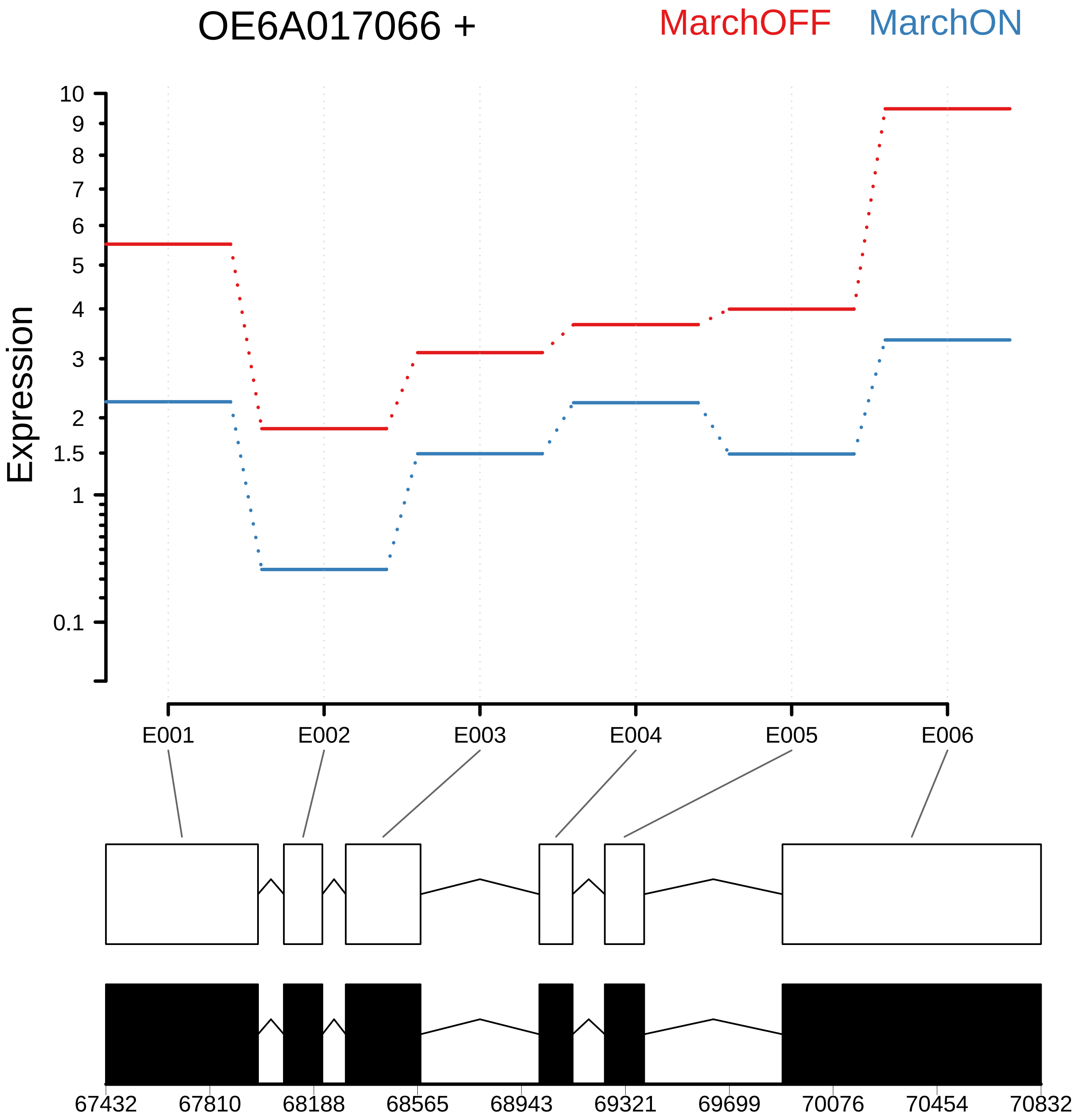
OE6A017066 +
MarchOFF
MarchON
Expression
10
9
8
7
6
5
4
3
2
1.5
1
0.1
E001
E002
E003
E004
E005
E006
67432
67810
68188
68565
68943
69321
69699
70076
70454
70832

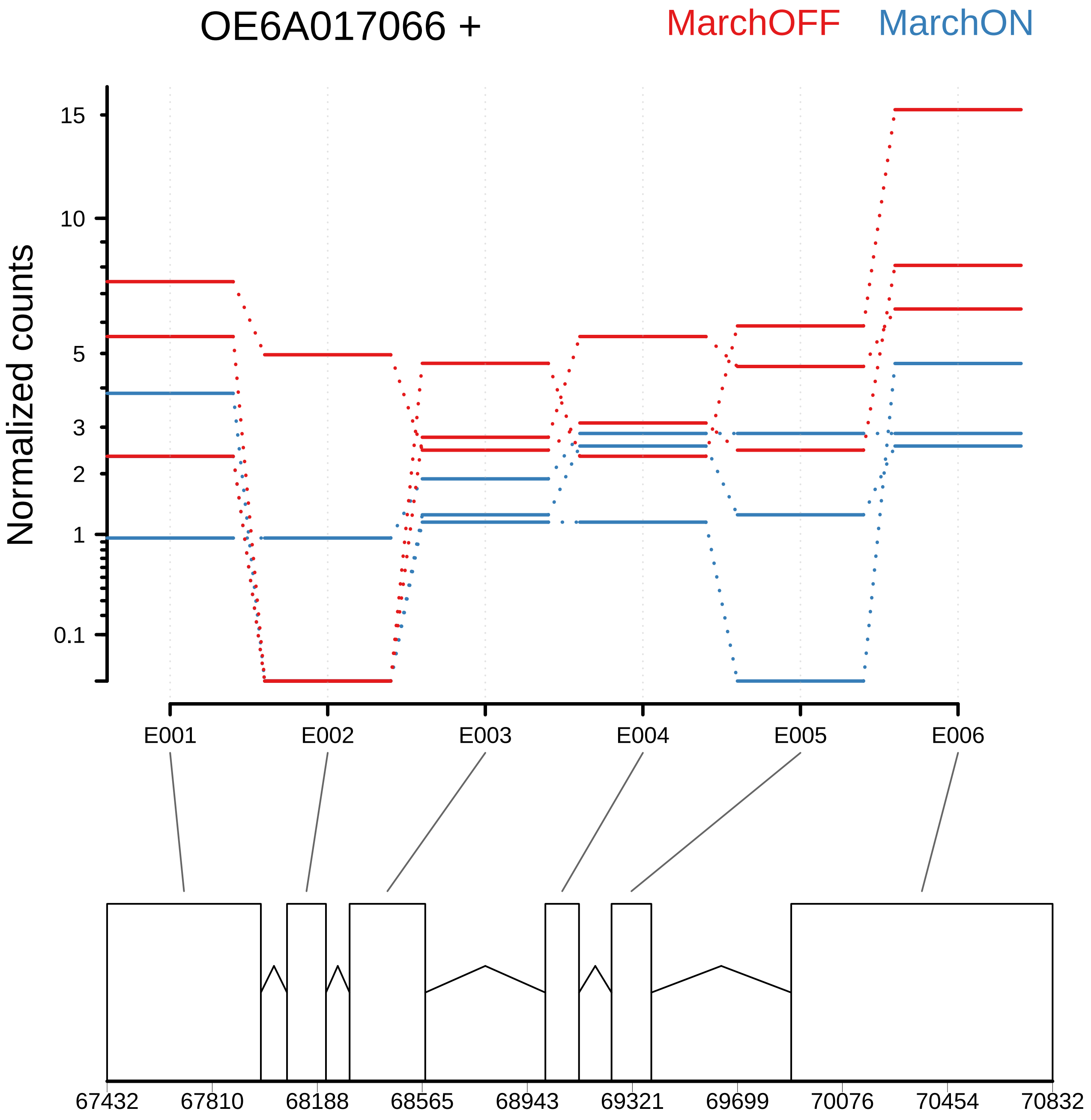

OE6A017066 +
MarchOFF
MarchON
Normalized counts
15
10
5
3
2
1
0.1
E001
E002
E003
E004
E005
E006
67432
67810
68188
68565
68943
69321
69699
70076
70454
70832

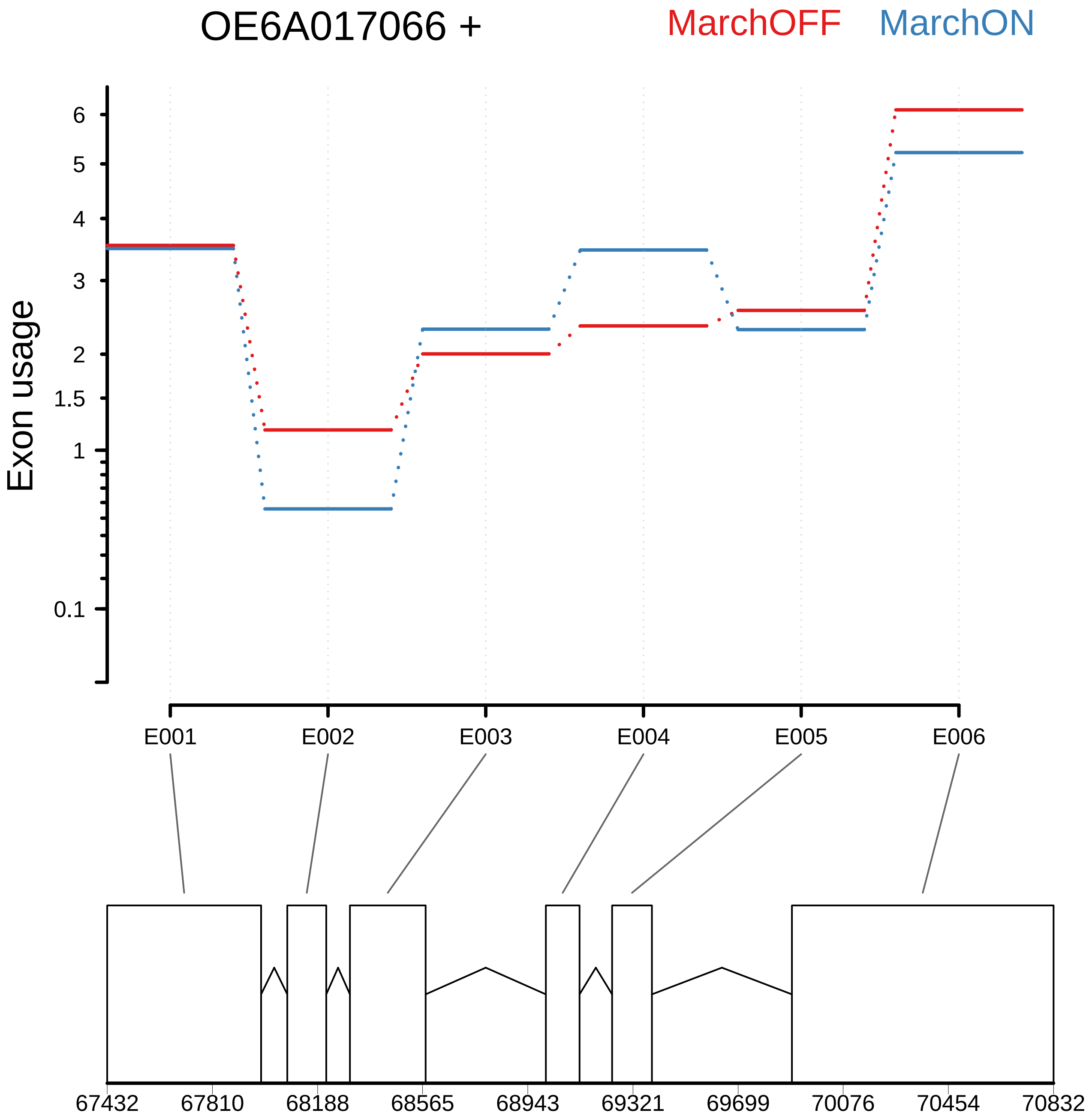

OE6A017066 +
MarchOFF
MarchON
Exon usage
6
5
4
3
2
1.5
1
0.1
E001
E002
E003
E004
E005
E006
67432
67810
68188
68565
68943
69321
69699
70076
70454
70832

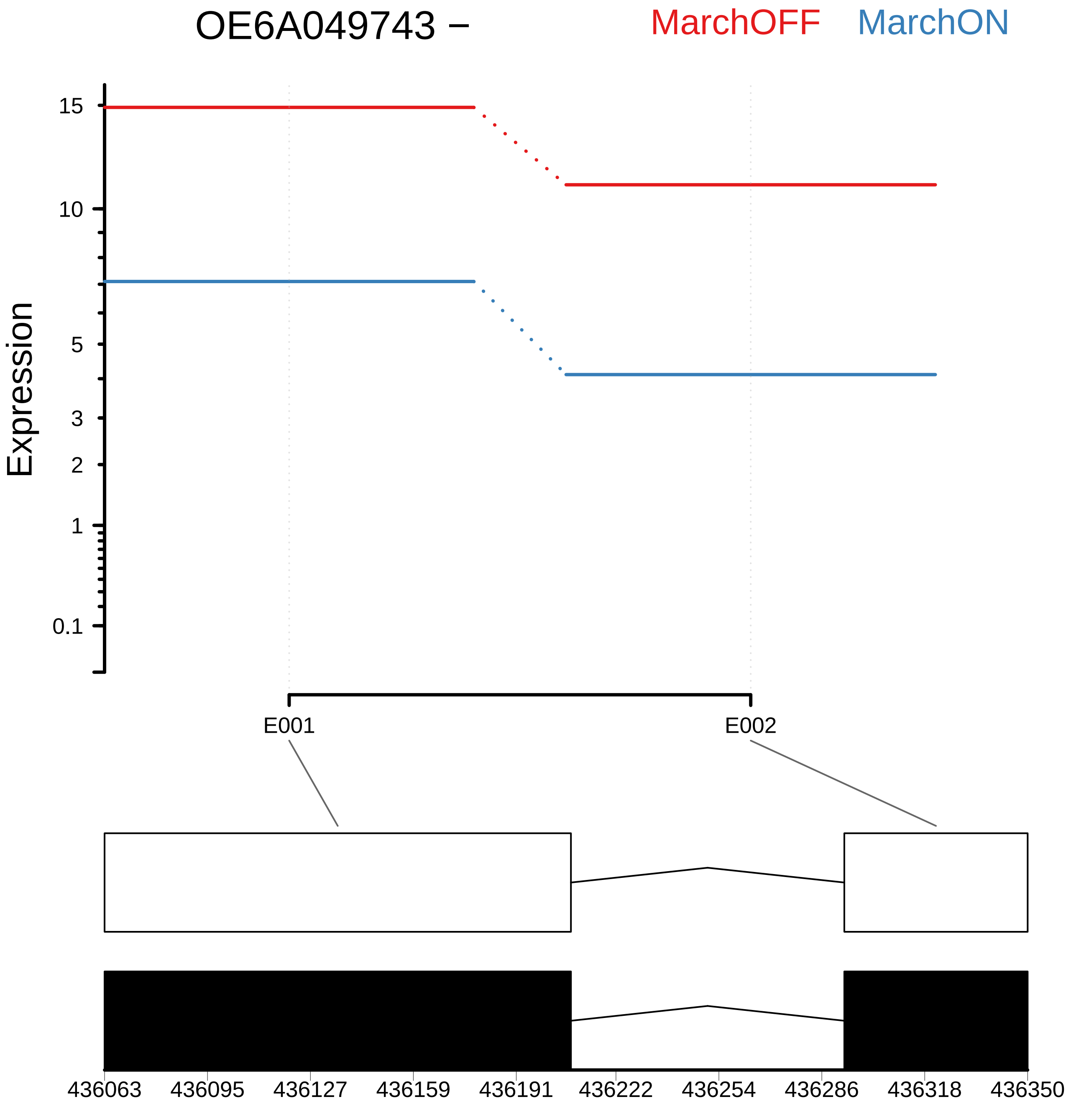
OE6A049743 −
MarchOFF
MarchON
Expression
15
10
5
3
2
1
0.1
E001
E002
436063
436095
436127
436159
436191
436222
436254
436286
436318
436350

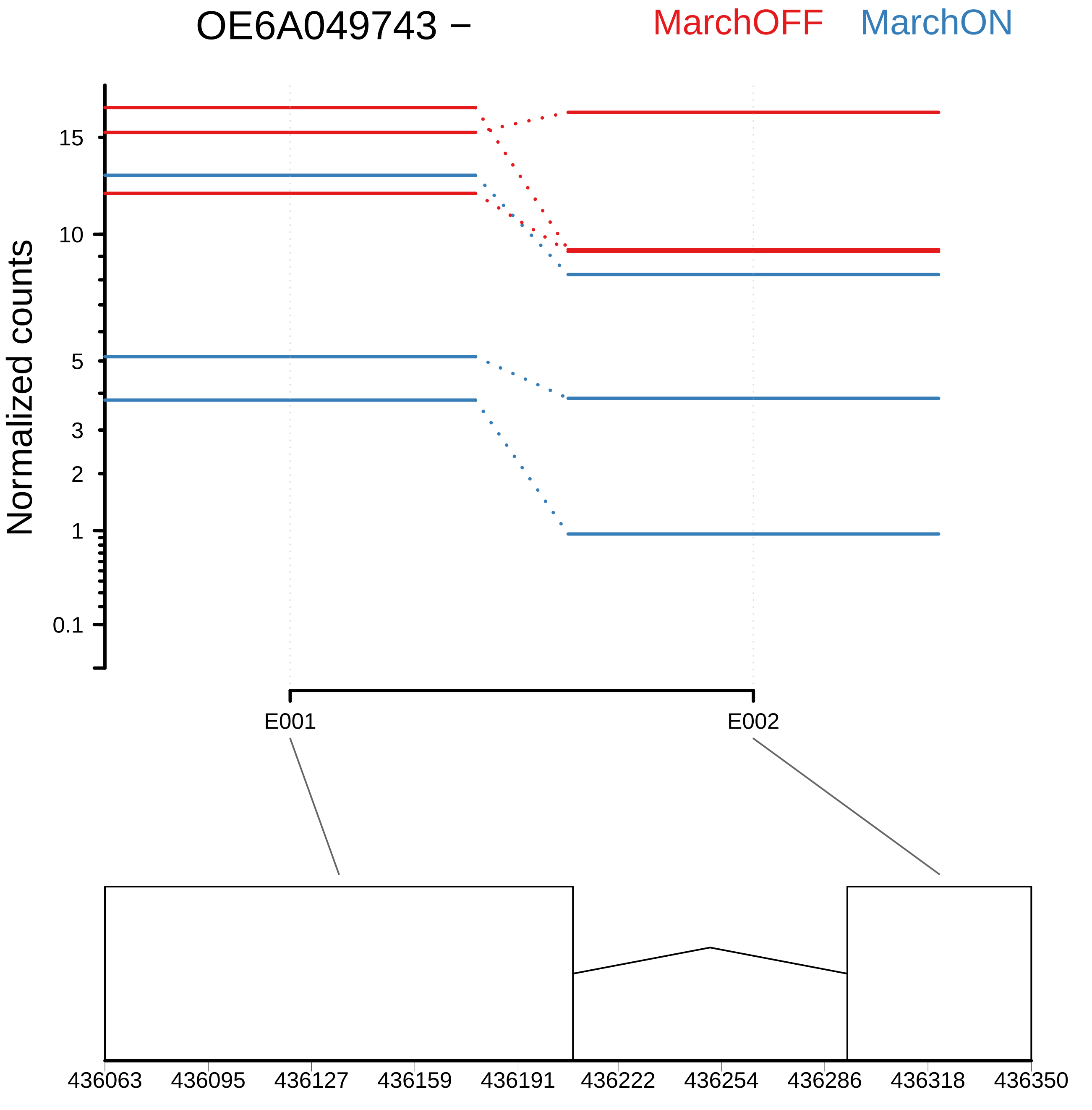
OE6A049743 −
MarchOFF
MarchON
Normalized counts
15
10
5
3
2
1
0.1
E001
E002
436063
436095
436127
436159
436191
436222
436254
436286
436318
436350

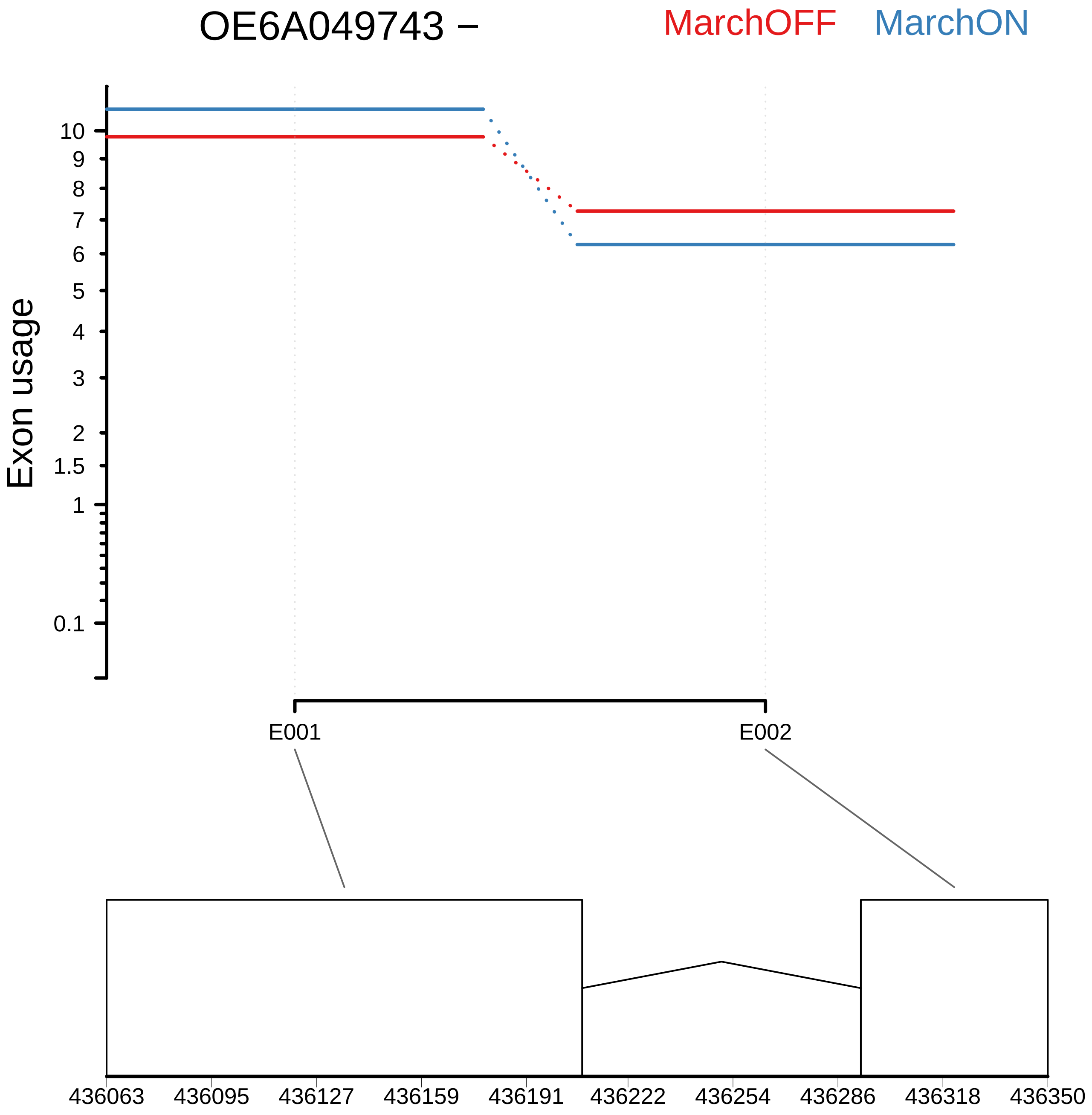

OE6A049743 −
MarchOFF
MarchON
Exon usage
10
9
8
7
6
5
4
3
2
1.5
1
0.1
E001
E002
436063
436095
436127
436159
436191
436222
436254
436286
436318
436350

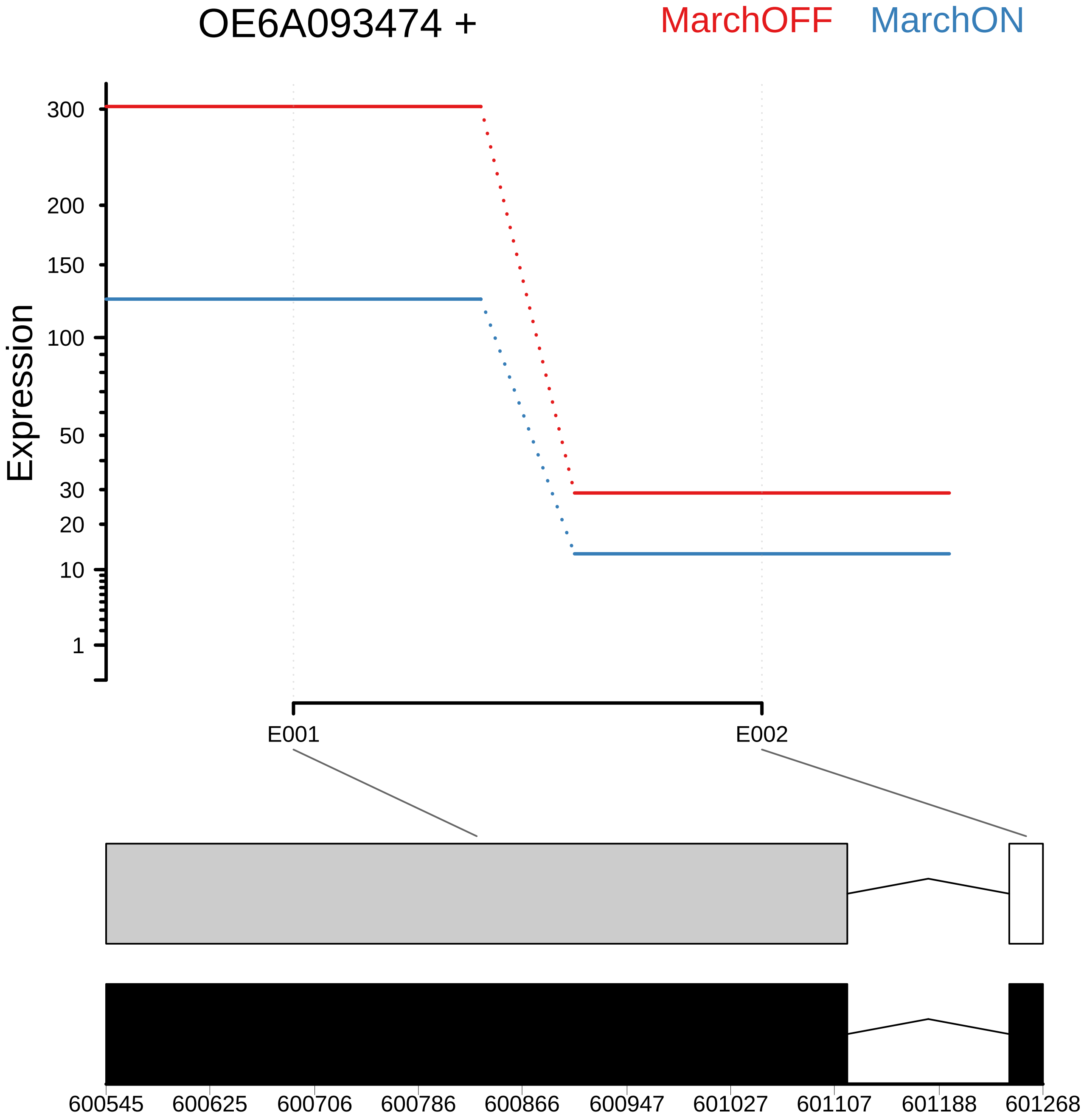

OE6A093474 +
MarchOFF
MarchON
Expression
300
200
150
100
50
30
20
10
1
E001
E002
600545
600625
600706
600786
600866
600947
601027
601107
601188
601268

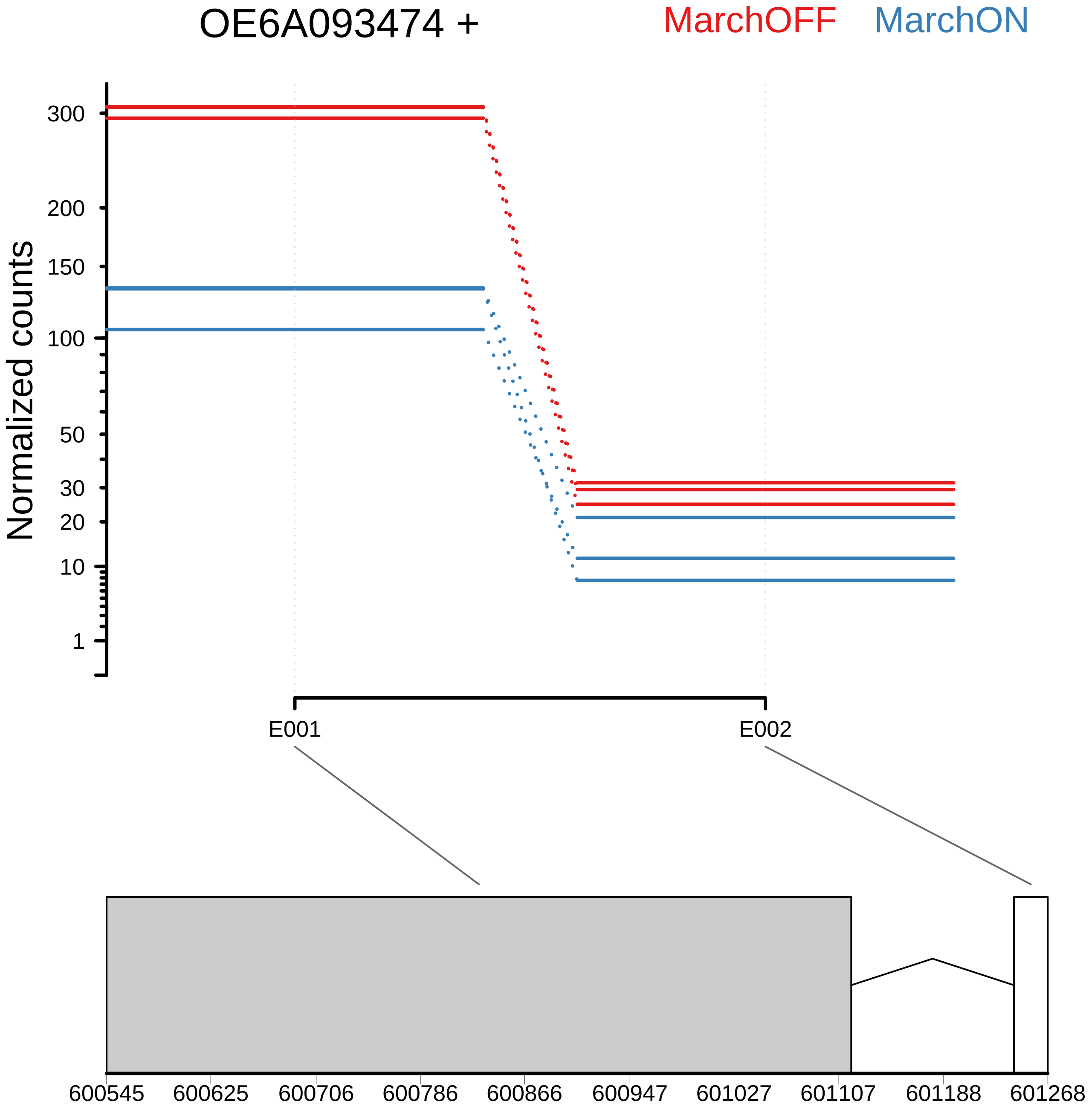

OE6A093474 +
MarchOFF
MarchON
Normalized counts
300
200
150
100
50
30
20
10
1
E001
E002
600545
600625
600706
600786
600866
600947
601027
601107
601188
601268

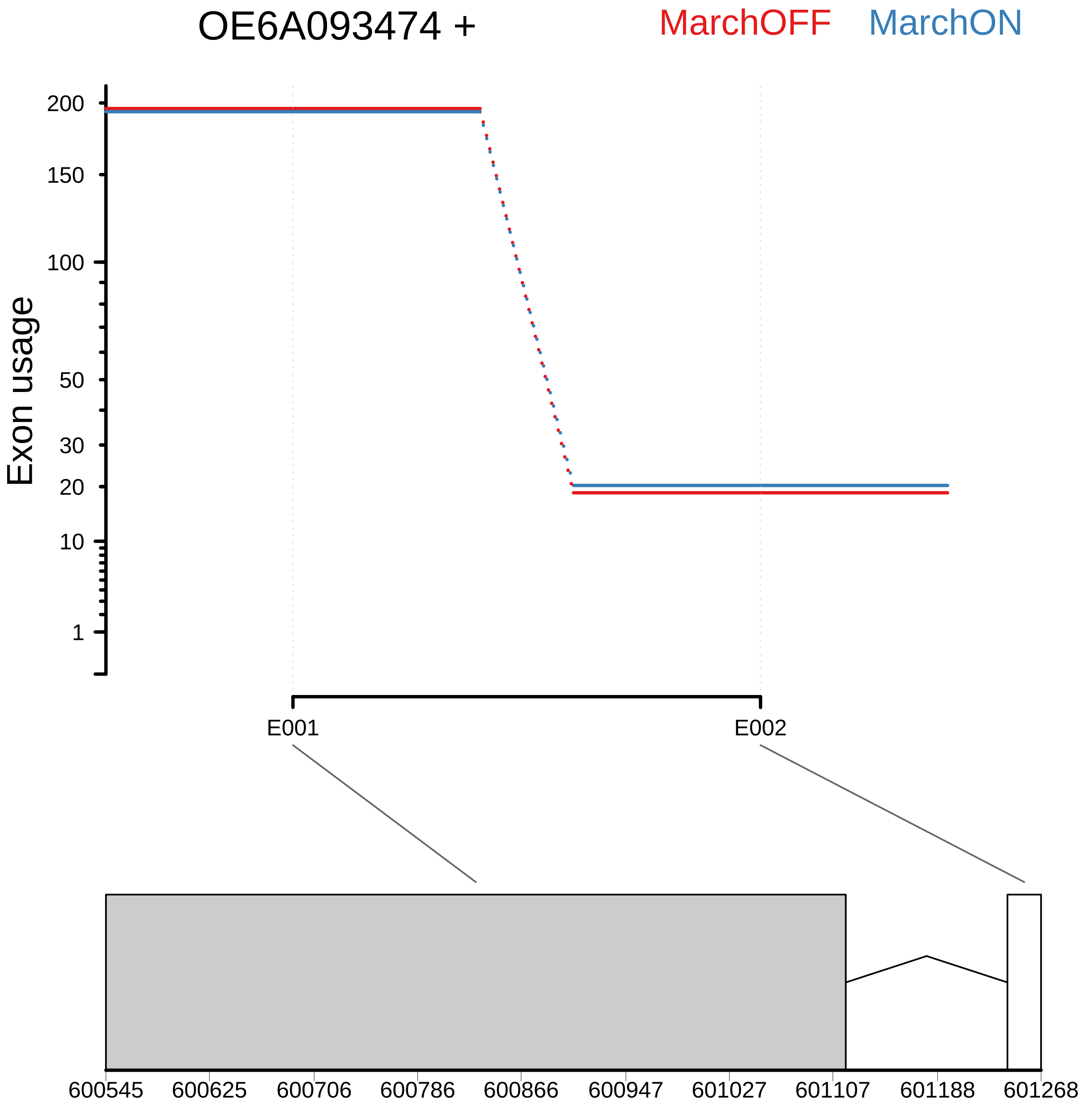

OE6A093474 +
MarchOFF
MarchON
Exon usage
200
150
100
50
30
20
10
1
E001
E002
600545
600625
600706
600786
600866
600947
601027
601107
601188
601268

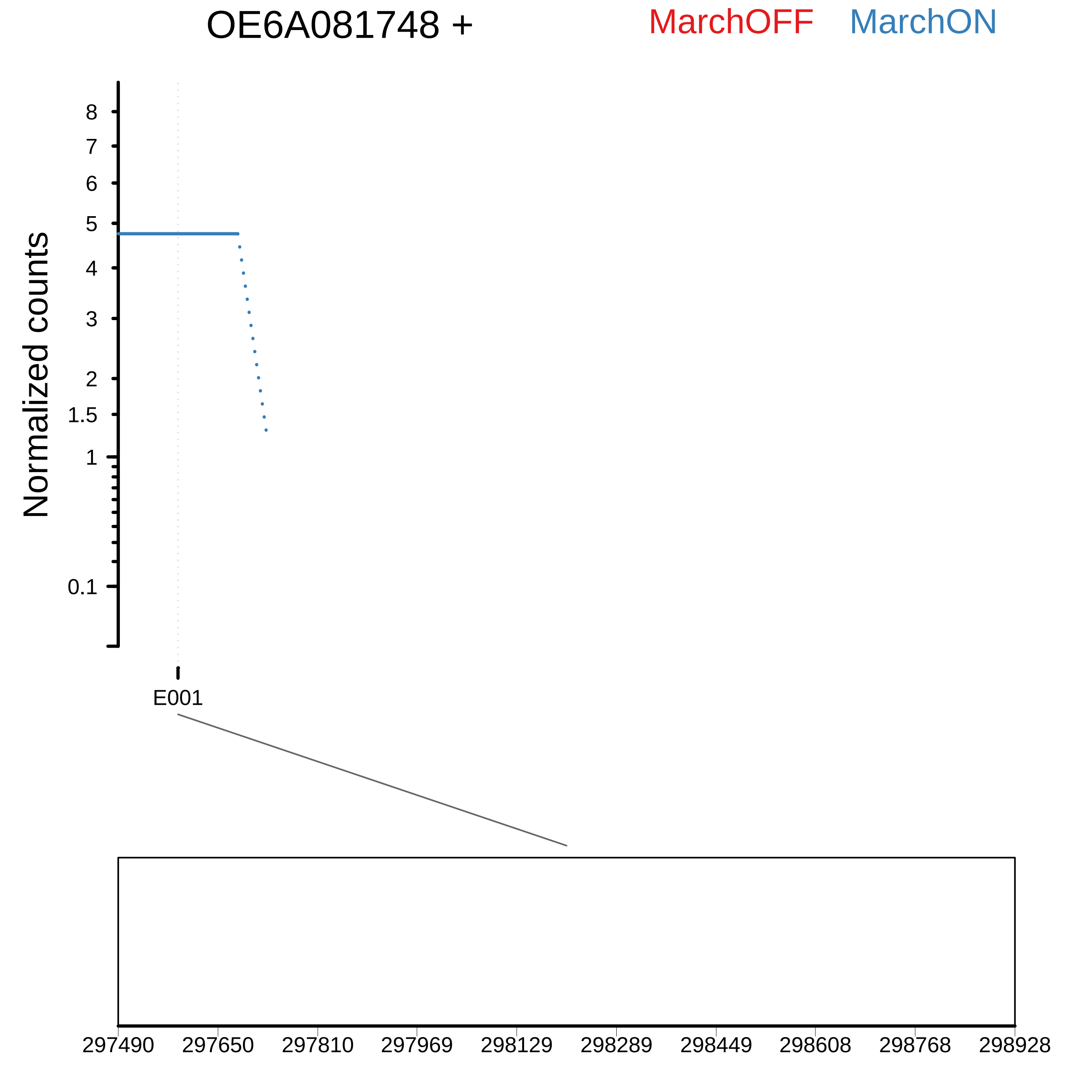
OE6A081748 +
MarchOFF
MarchON
Normalized counts
8
7
6
5
4
3
2
1.5
1
0.1
E001
297490
297650
297810
297969
298129
298289
298449
298608
298768
298928

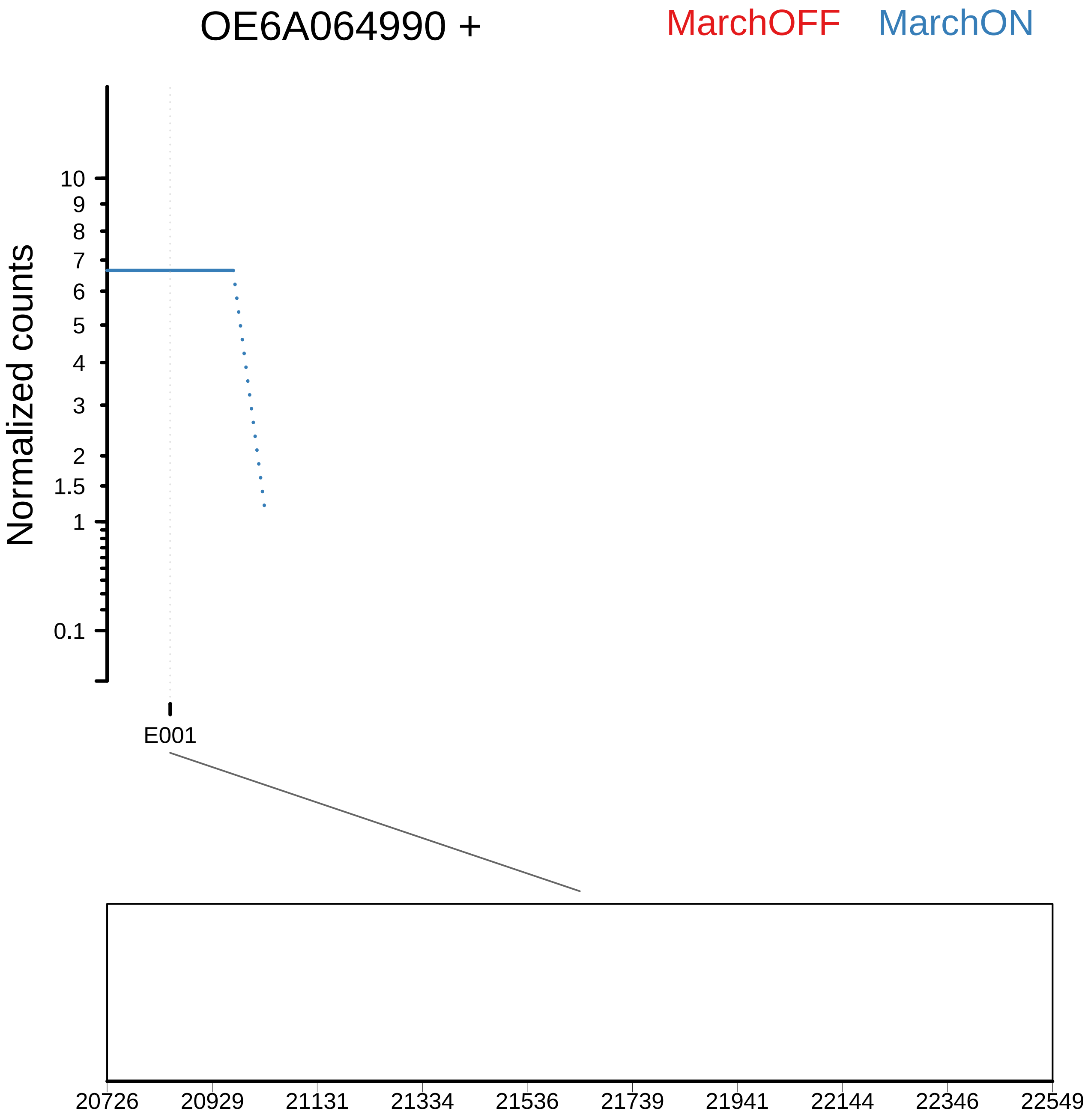

OE6A064990 +
MarchOFF
MarchON
Normalized counts
10
9
8
7
6
5
4
3
2
1.5
1
0.1
E001
20726
20929
21131
21334
21536
21739
21941
22144
22346
22549

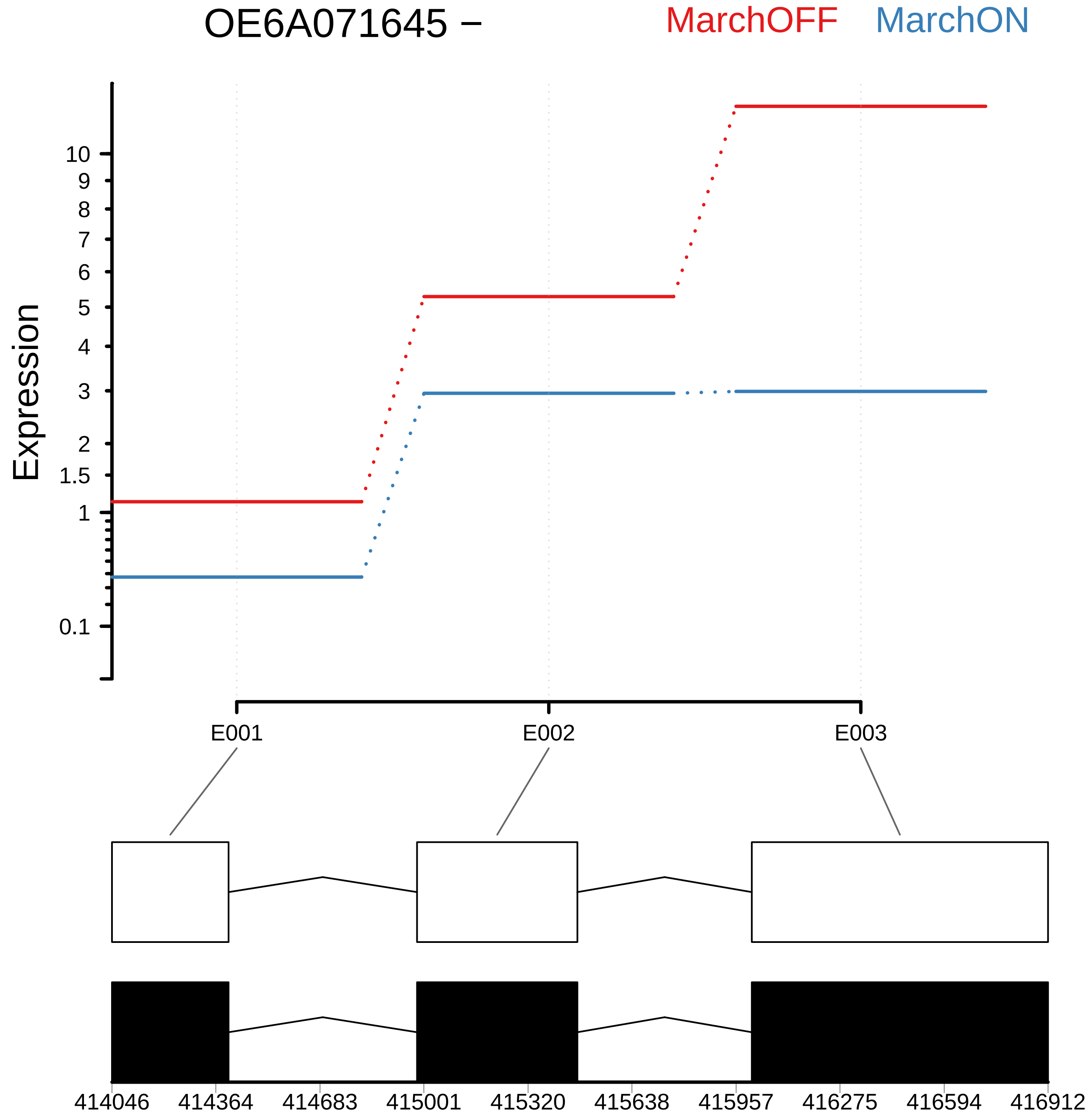

OE6A071645 −
MarchOFF
MarchON
Expression
10
9
8
7
6
5
4
3
2
1.5
1
0.1
E001
E002
E003
414046
414364
414683
415001
415320
415638
415957
416275
416594
416912

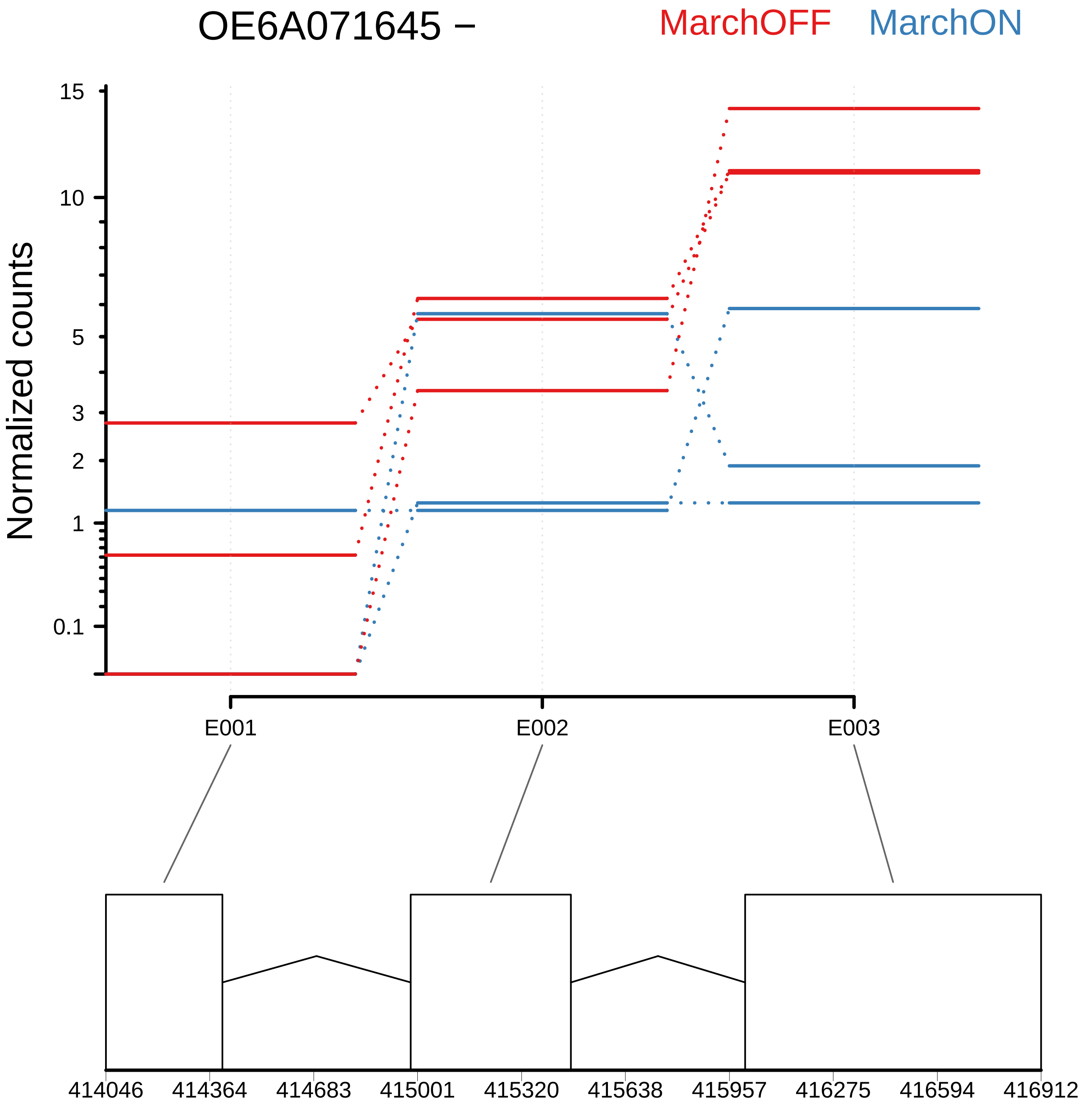

OE6A071645 −
MarchOFF
MarchON
Normalized counts
15
10
5
3
2
1
0.1
E001
E002
E003
414046
414364
414683
415001
415320
415638
415957
416275
416594
416912

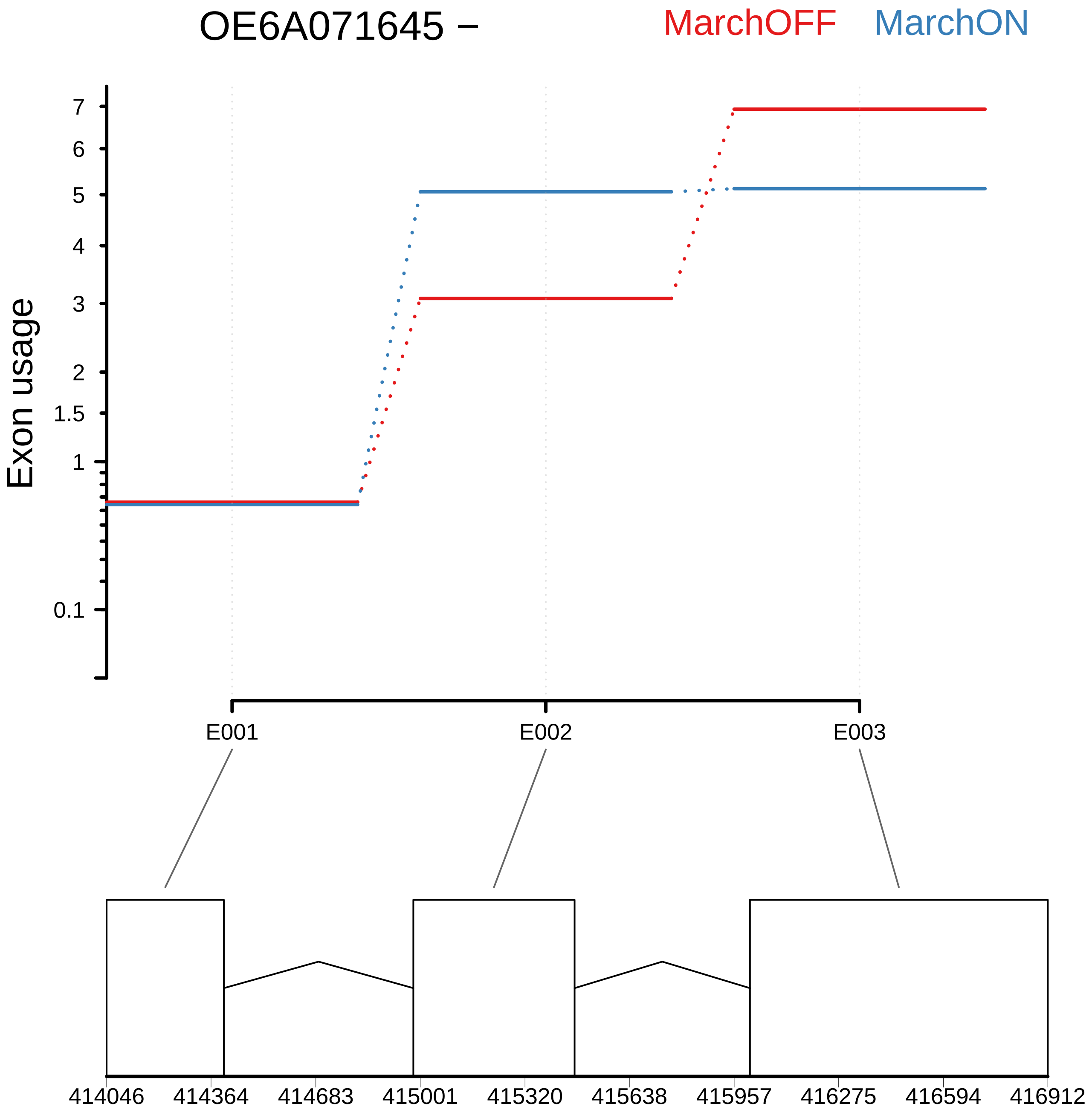
OE6A071645 −
MarchOFF
MarchON
Exon usage
7
6
5
4
3
2
1.5
1
0.1
E001
E002
E003
414046
414364
414683
415001
415320
415638
415957
416275
416594
416912

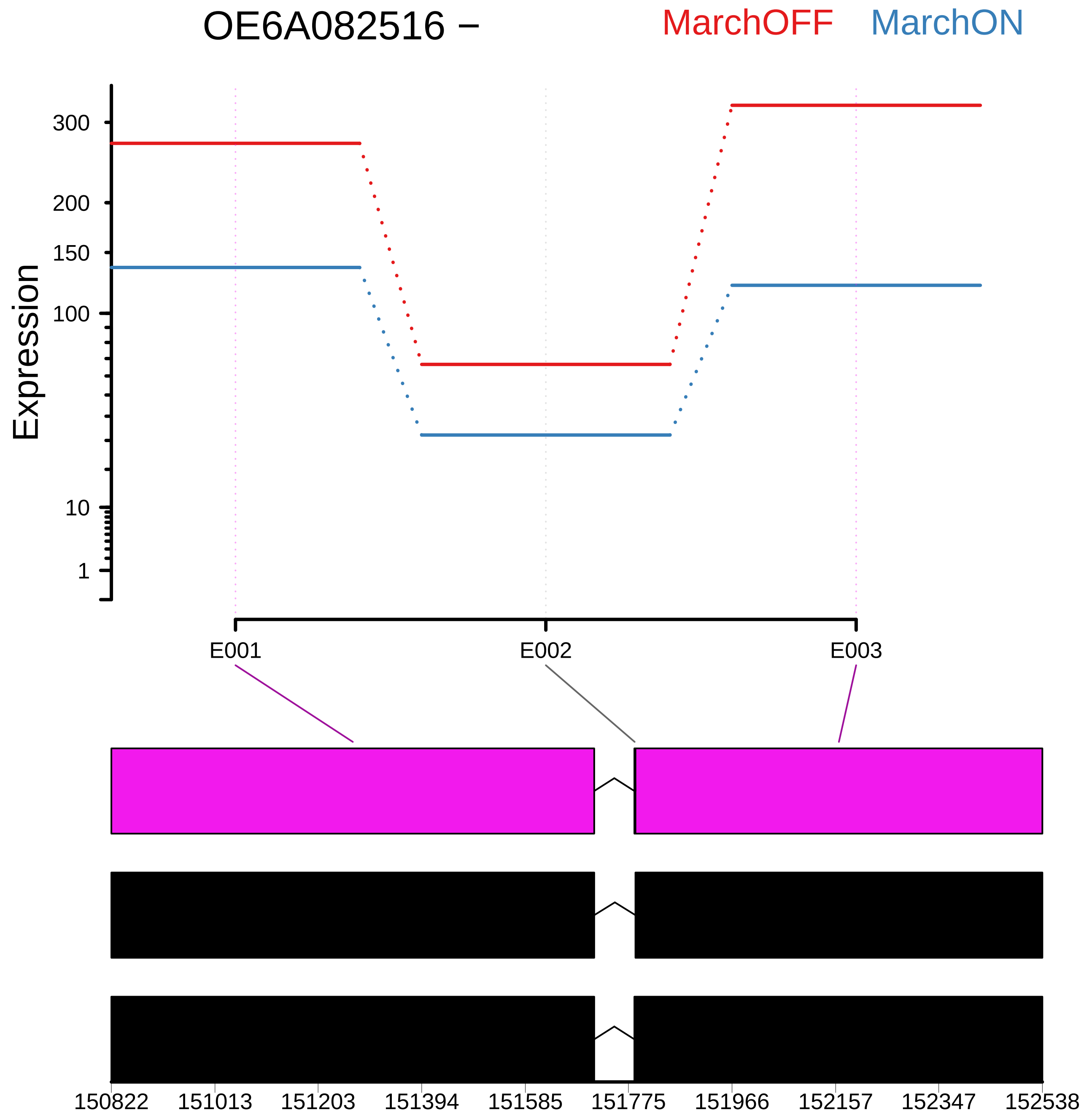
OE6A082516 −
MarchOFF
MarchON
Expression
300
200
150
100
10
1
E001
E002
E003
150822
151013
151203
151394
151585
151775
151966
152157
152347
152538

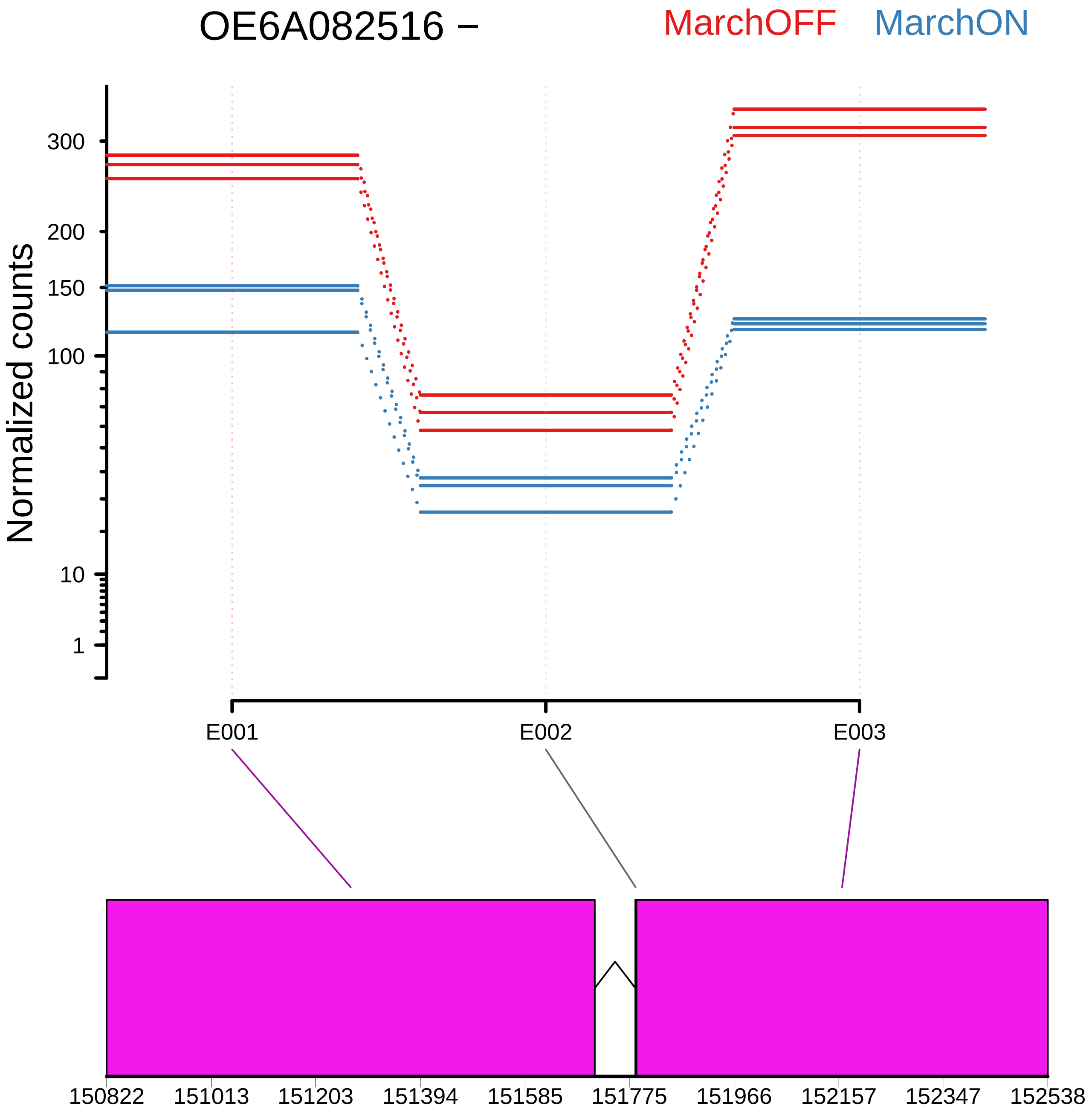

OE6A082516 −
MarchOFF
MarchON
Normalized counts
300
200
150
100
10
1
E001
E002
E003
150822
151013
151203
151394
151585
151775
151966
152157
152347
152538

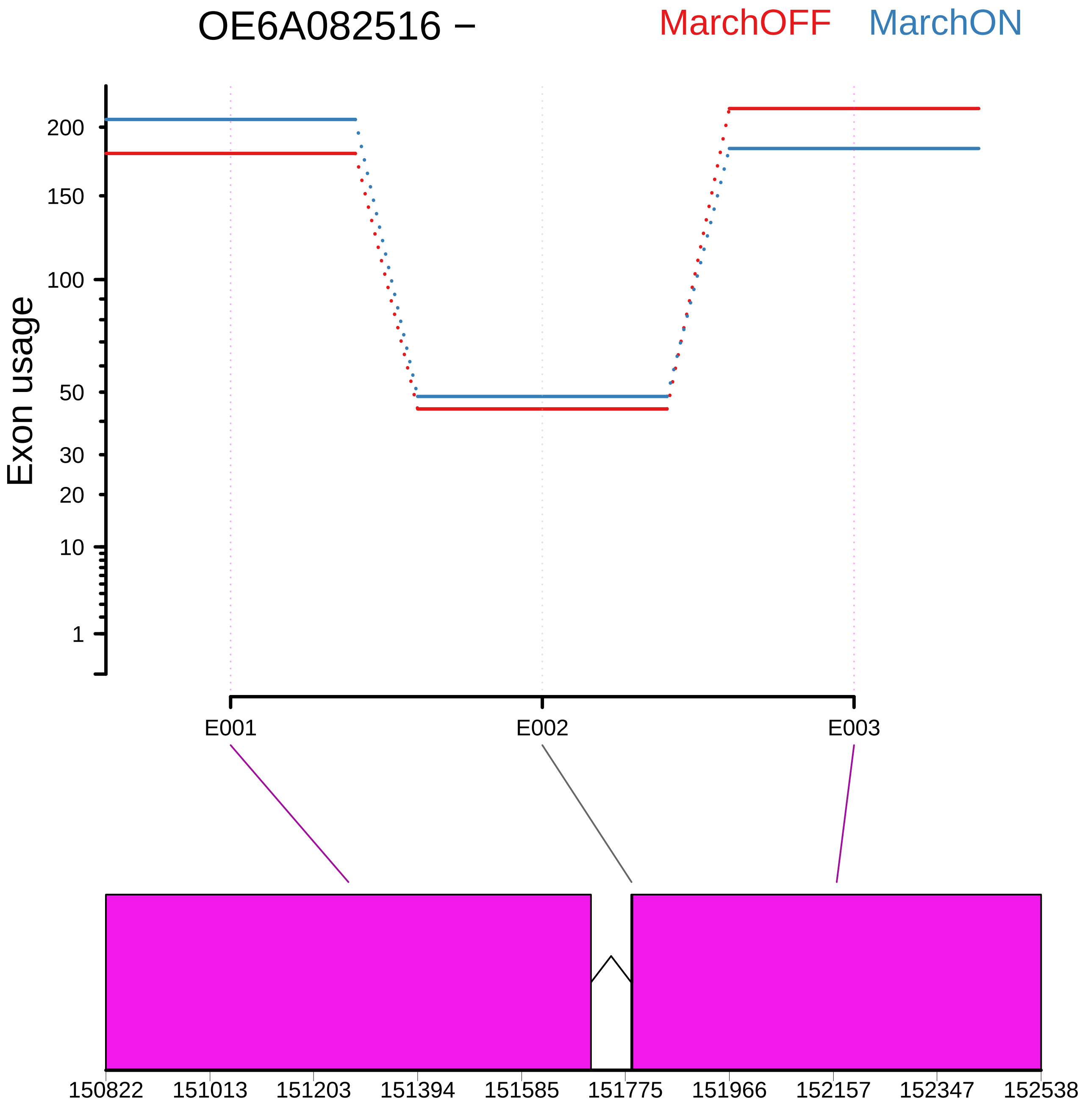
OE6A082516 −
MarchOFF
MarchON
Exon usage
200
150
100
50
30
20
10
1
E001
E002
E003
150822
151013
151203
151394
151585
151775
151966
152157
152347
152538

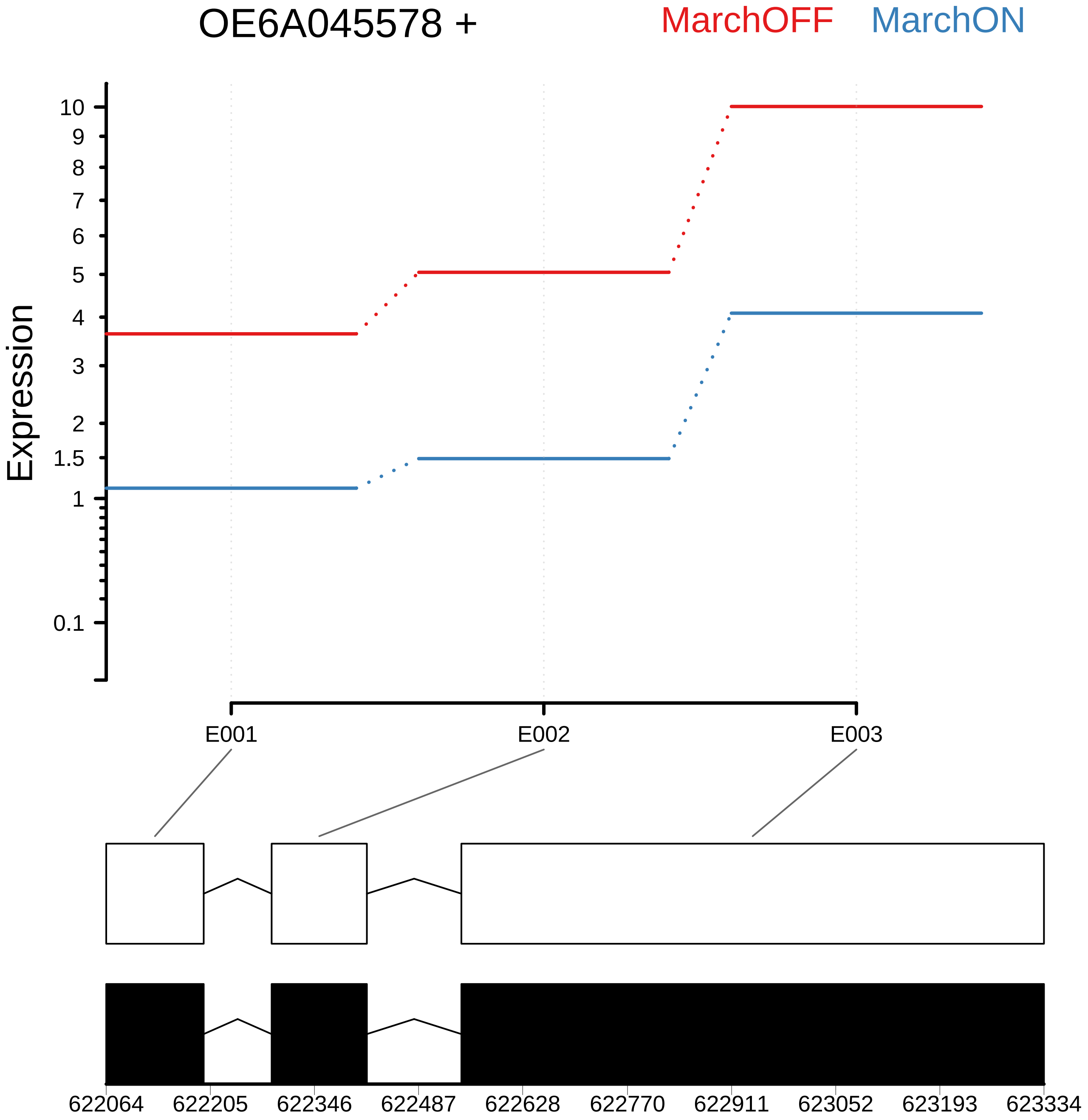
OE6A045578 +
MarchOFF
MarchON
Expression
10
9
8
7
6
5
4
3
2
1.5
1
0.1
E001
E002
E003
622064
622205
622346
622487
622628
622770
622911
623052
623193
623334

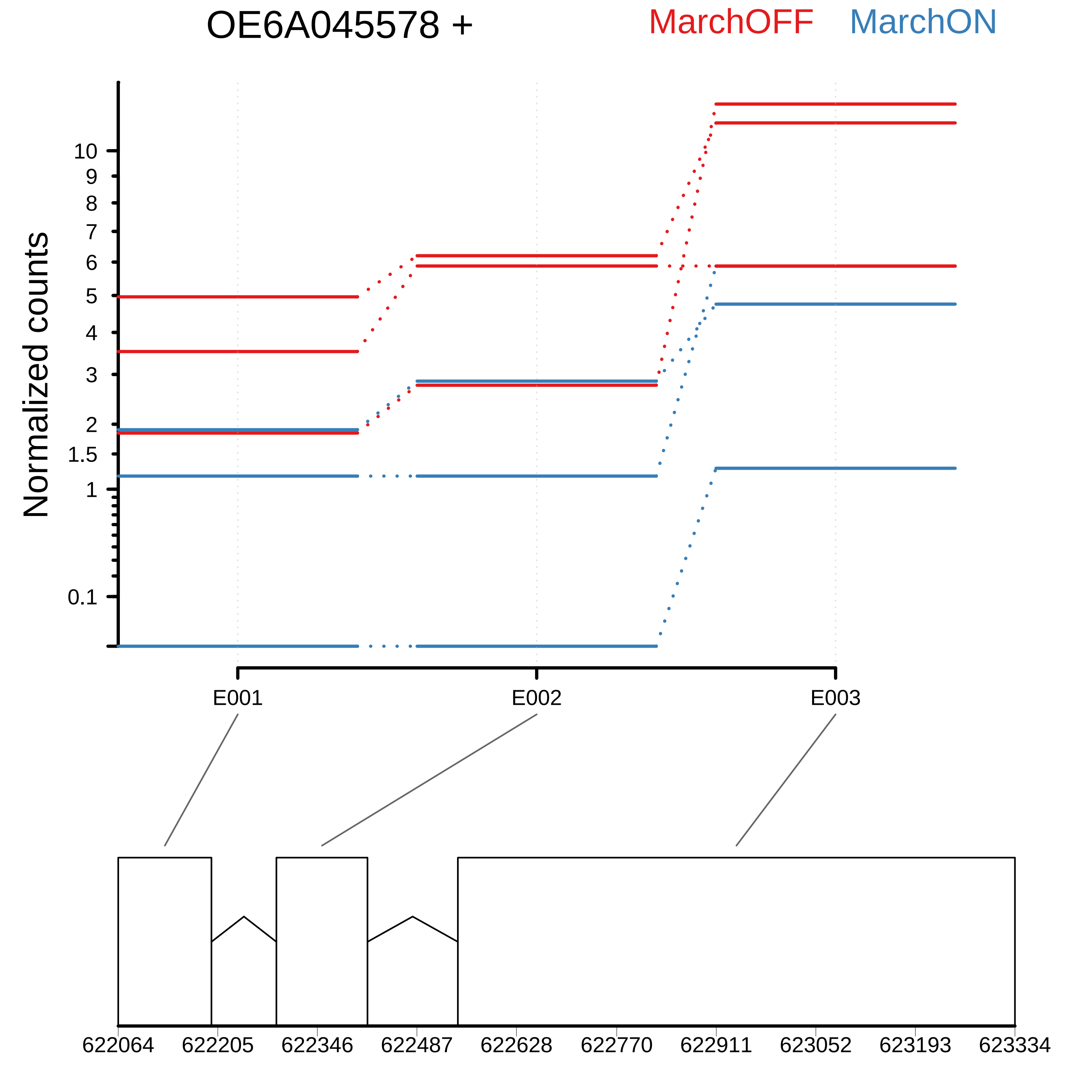
OE6A045578 +
MarchOFF
MarchON
Normalized counts
10
9
8
7
6
5
4
3
2
1.5
1
0.1
E001
E002
E003
622064
622205
622346
622487
622628
622770
622911
623052
623193
623334

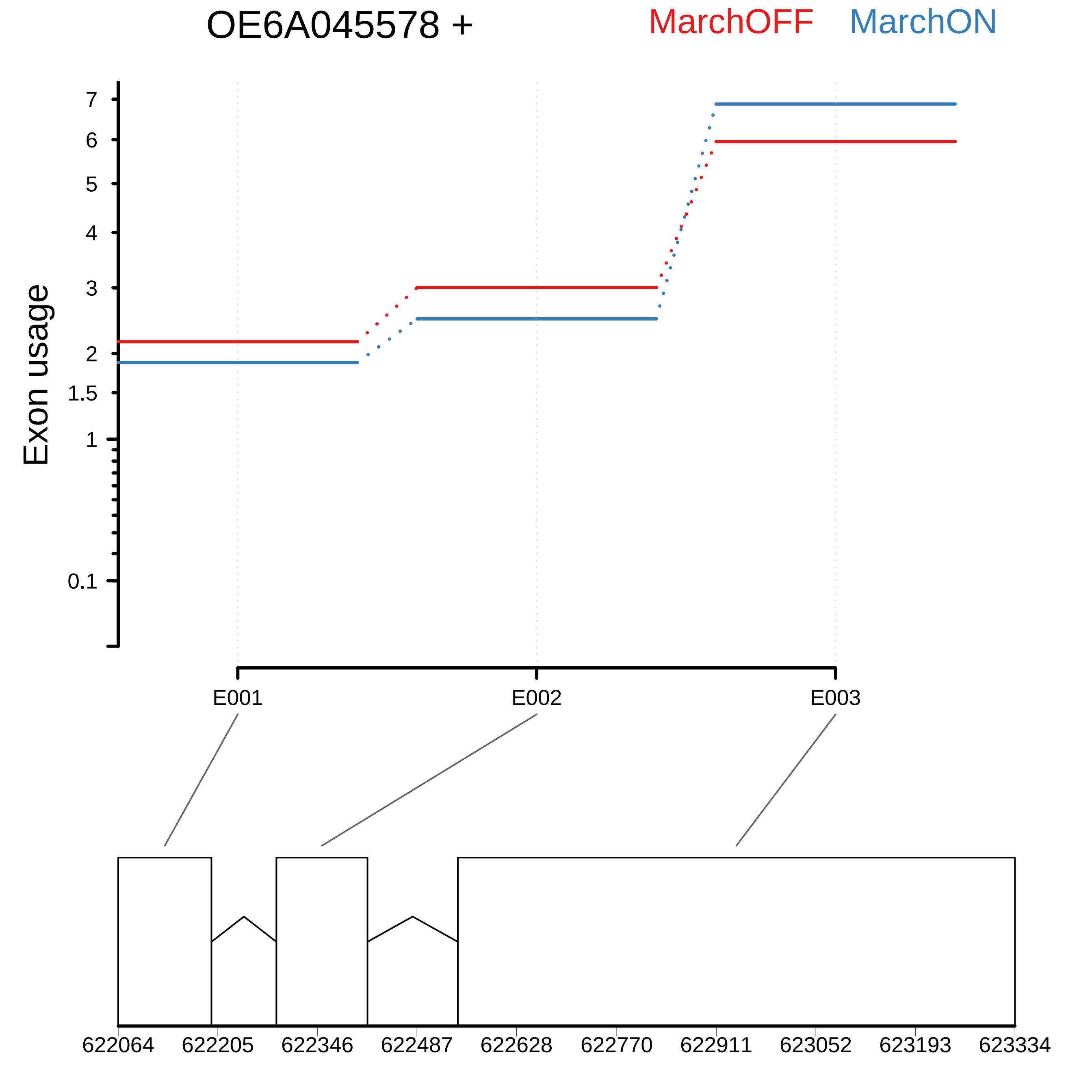

OE6A045578 +
MarchOFF
MarchON
Exon usage
7
6
5
4
3
2
1.5
1
0.1
E001
E002
E003
622064
622205
622346
622487
622628
622770
622911
623052
623193
623334

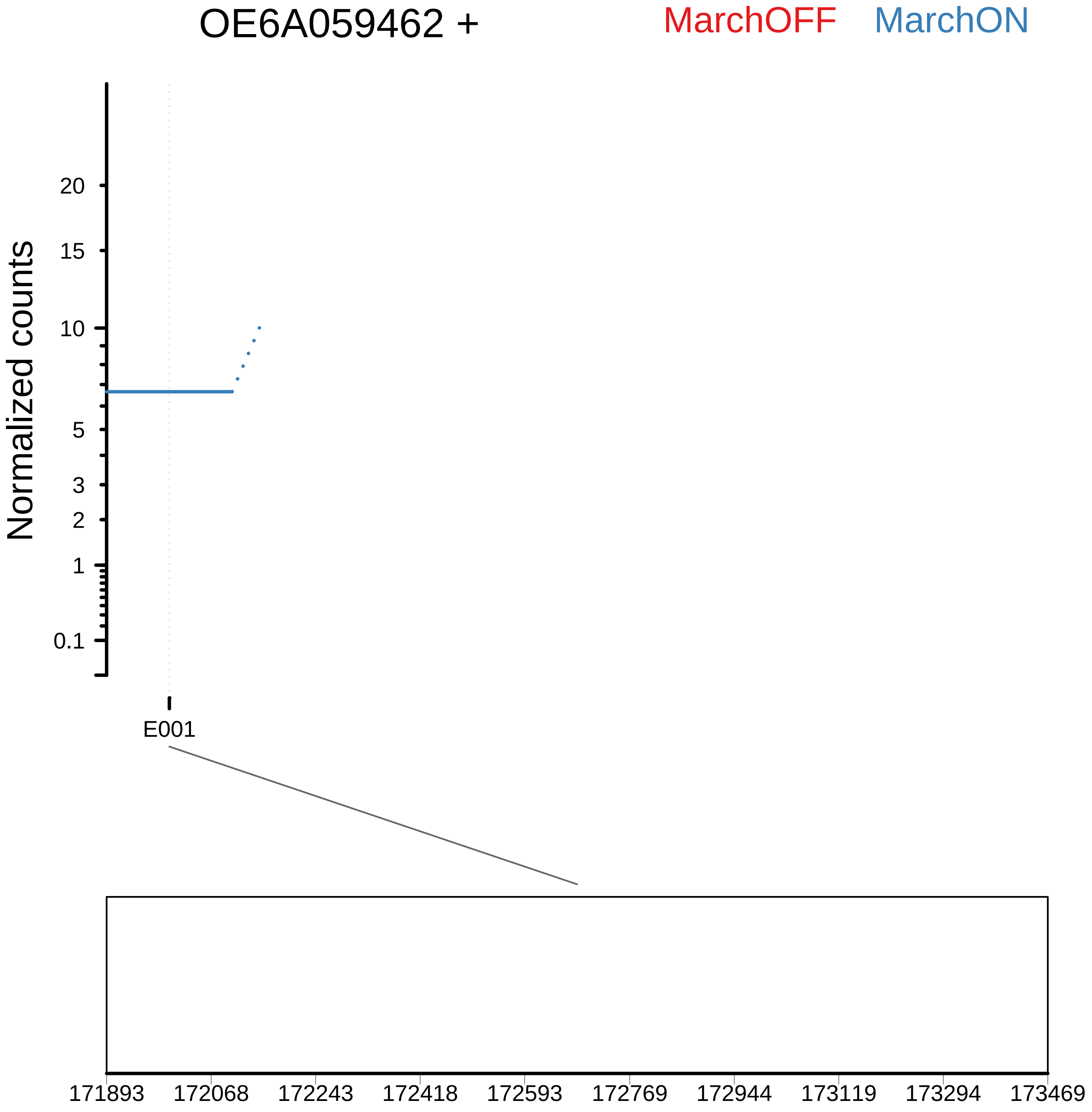
OE6A059462 +
MarchOFF
MarchON
Normalized counts
20
15
10
5
3
2
1
0.1
E001
171893
172068
172243
172418
172593
172769
172944
173119
173294
173469

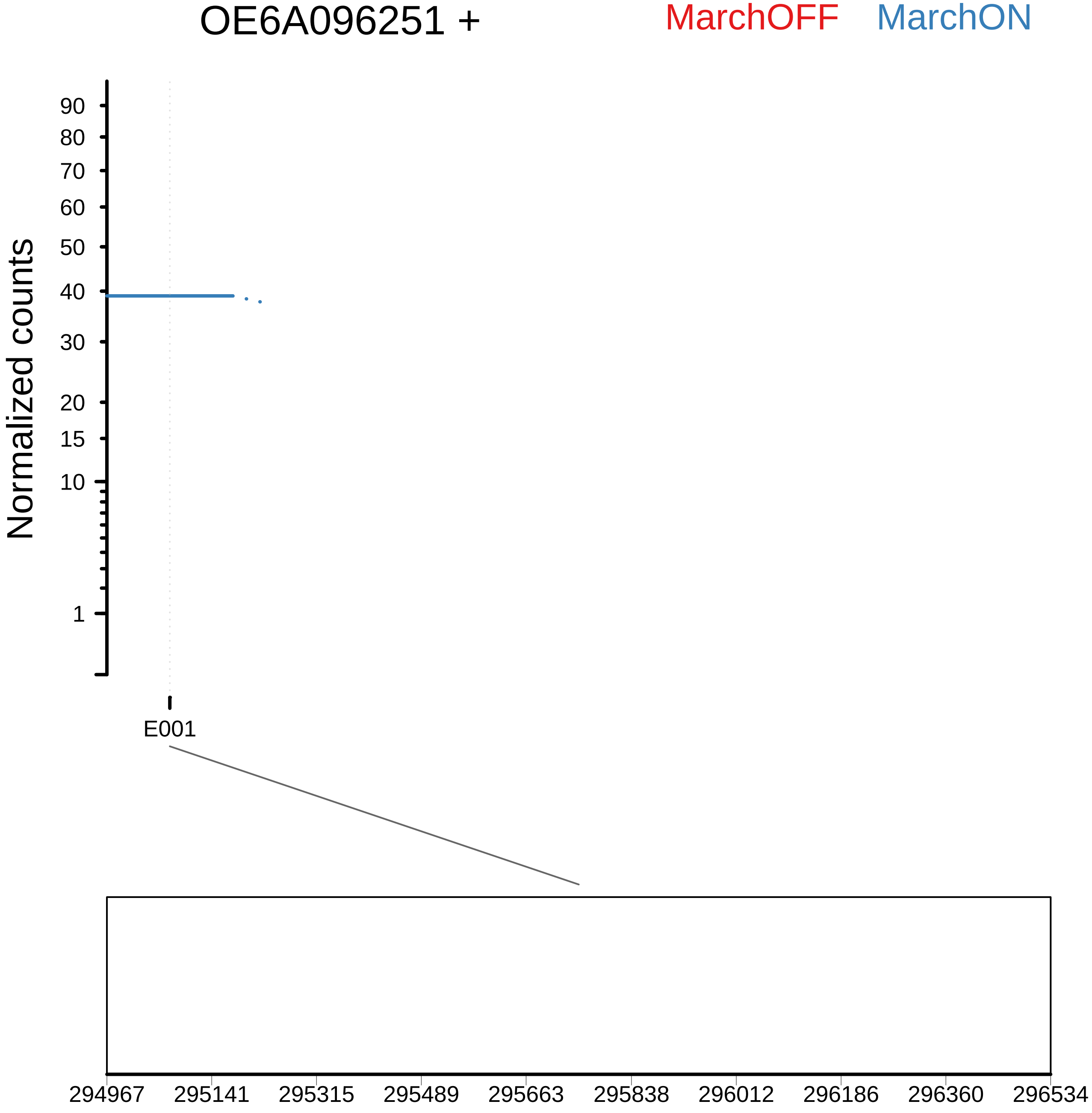

OE6A096251 +
MarchOFF
MarchON
Normalized counts
90
80
70
60
50
40
30
20
15
10
1
E001
294967
295141
295315
295489
295663
295838
296012
296186
296360
296534

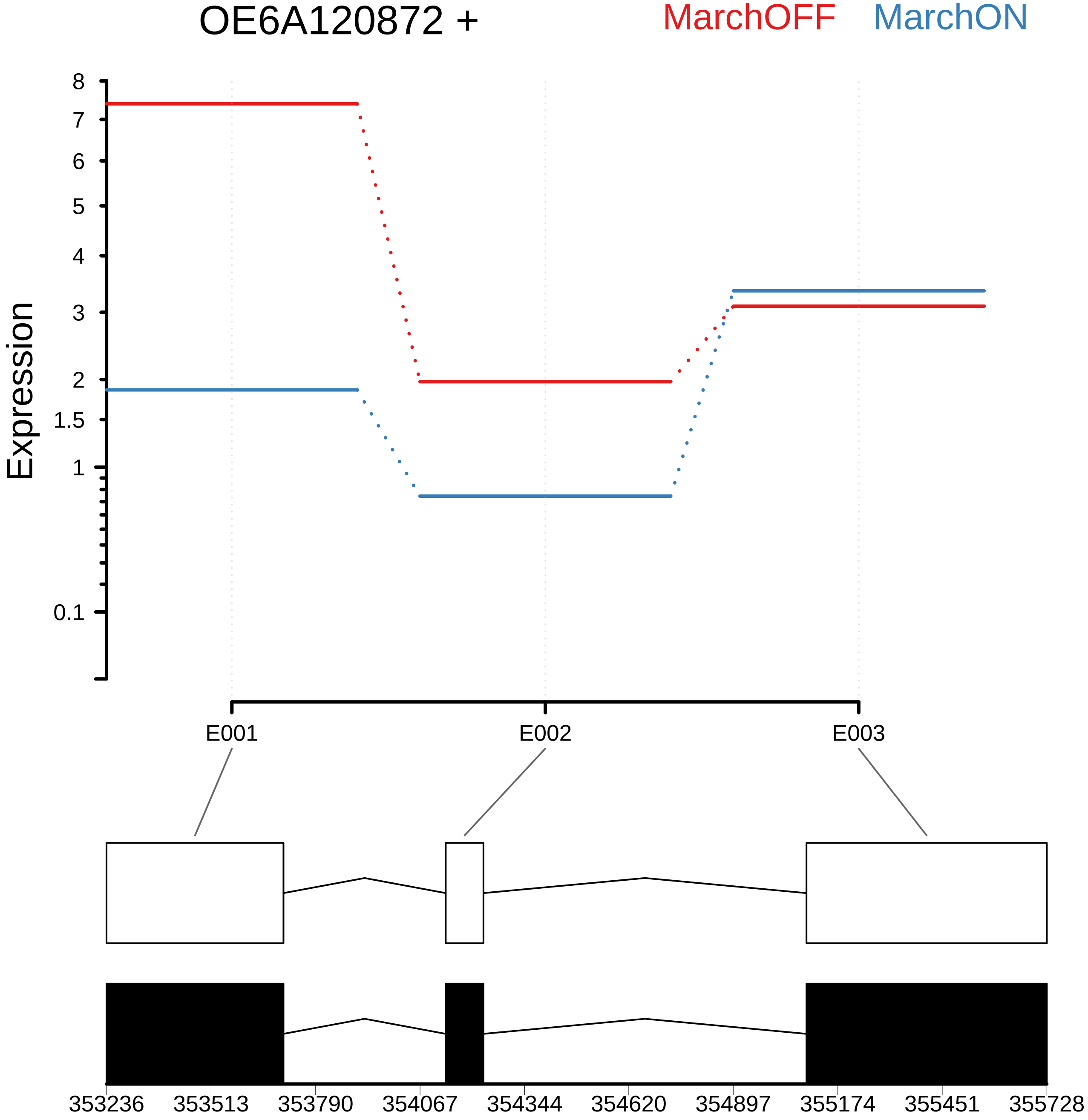
OE6A120872 +
MarchOFF
MarchON
Expression
8
7
6
5
4
3
2
1.5
1
0.1
E001
E002
E003
353236
353513
353790
354067
354344
354620
354897
355174
355451
355728

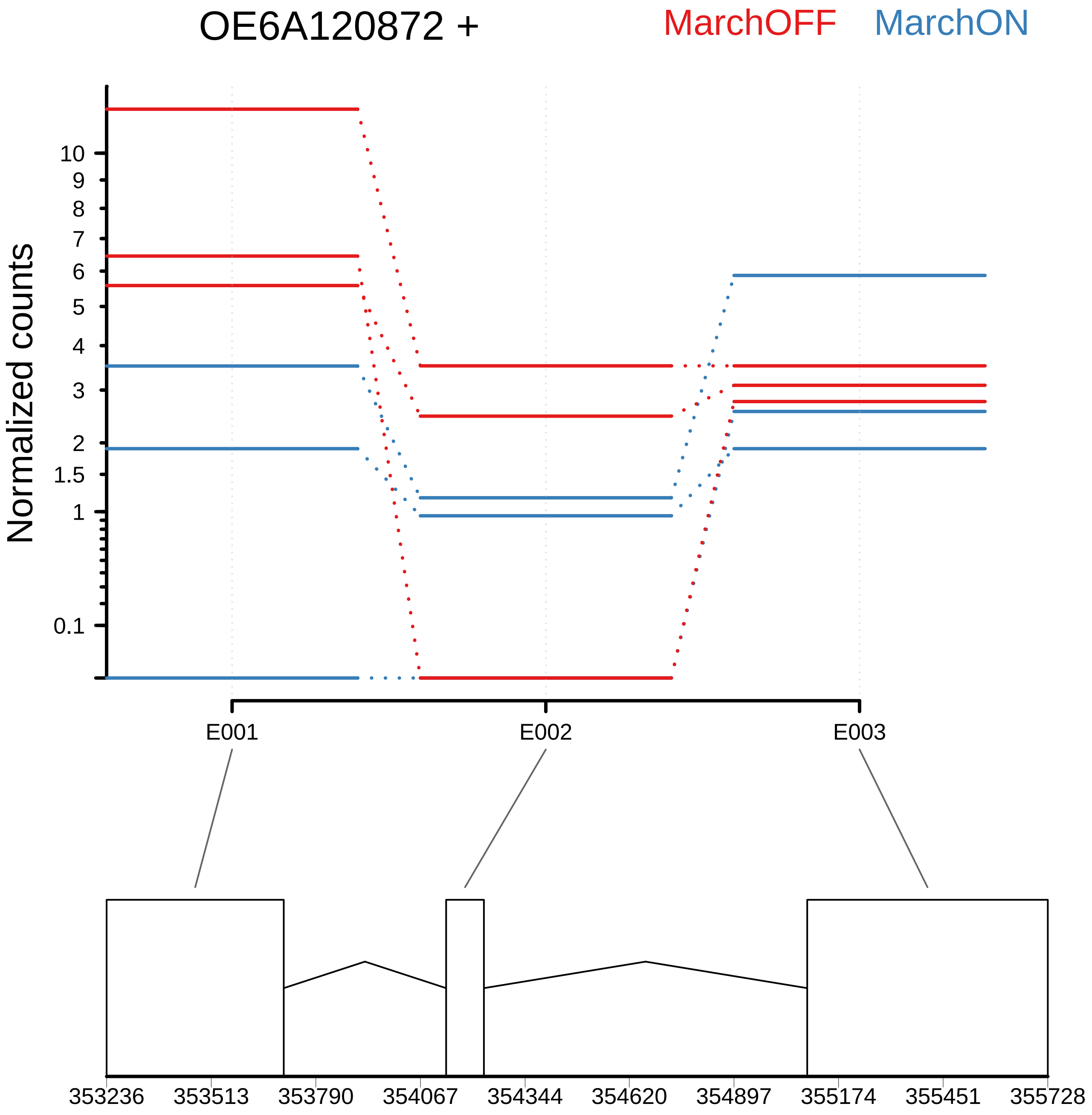

OE6A120872 +
MarchOFF
MarchON
Normalized counts
10
9
8
7
6
5
4
3
2
1.5
1
0.1
E001
E002
E003
353236
353513
353790
354067
354344
354620
354897
355174
355451
355728

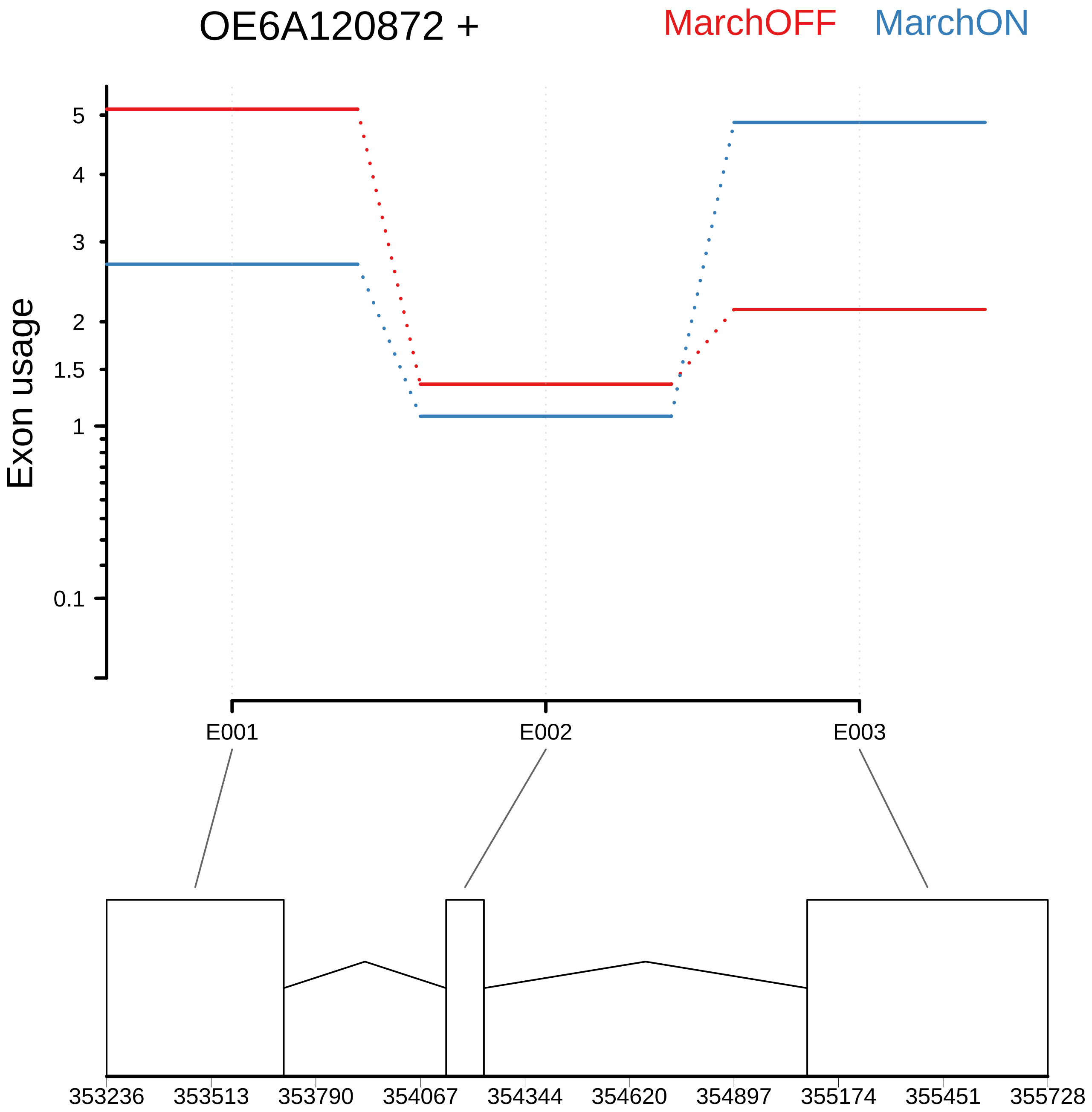
OE6A120872 +
MarchOFF
MarchON
Exon usage
5
4
3
2
1.5
1
0.1
E001
E002
E003
353236
353513
353790
354067
354344
354620
354897
355174
355451
355728

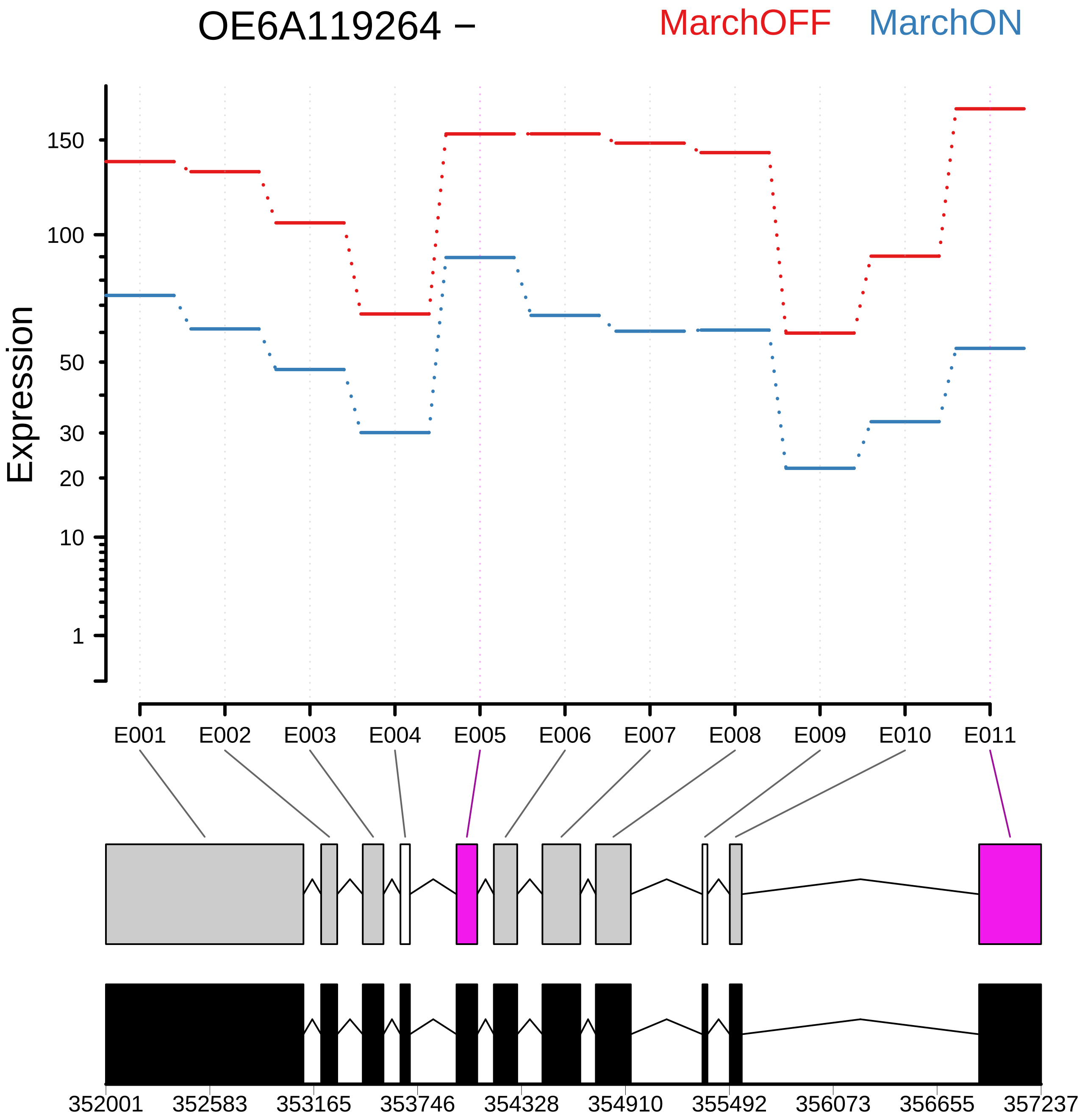
OE6A119264 −
MarchOFF
MarchON
Expression
150
100
50
30
20
10
1
E001
E002
E003
E004
E005
E006
E007
E008
E009
E010
E011
352001
352583
353165
353746
354328
354910
355492
356073
356655
357237

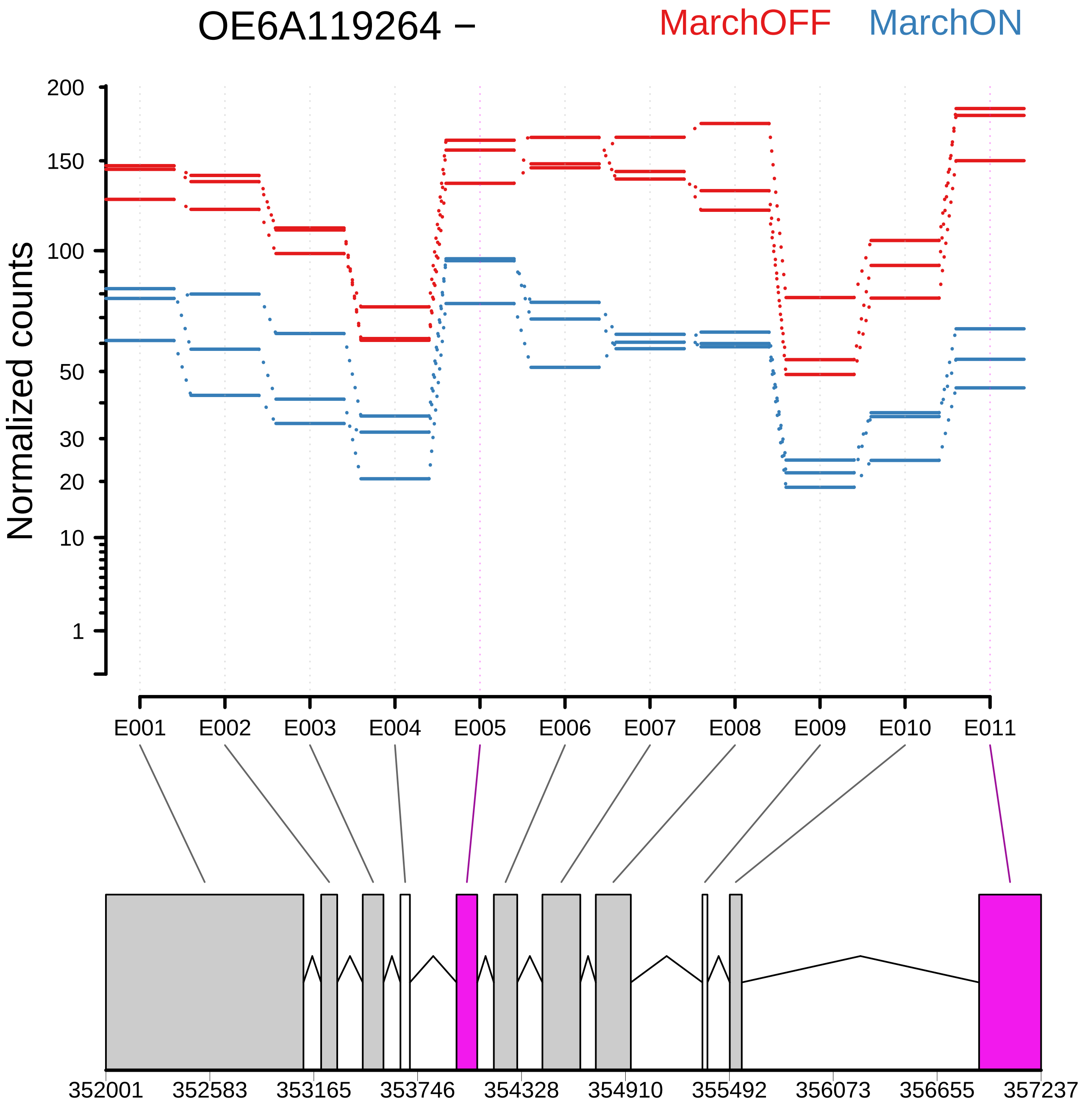

OE6A119264 −
MarchOFF
MarchON
Normalized counts
200
150
100
50
30
20
10
1
E001
E002
E003
E004
E005
E006
E007
E008
E009
E010
E011
352001
352583
353165
353746
354328
354910
355492
356073
356655
357237

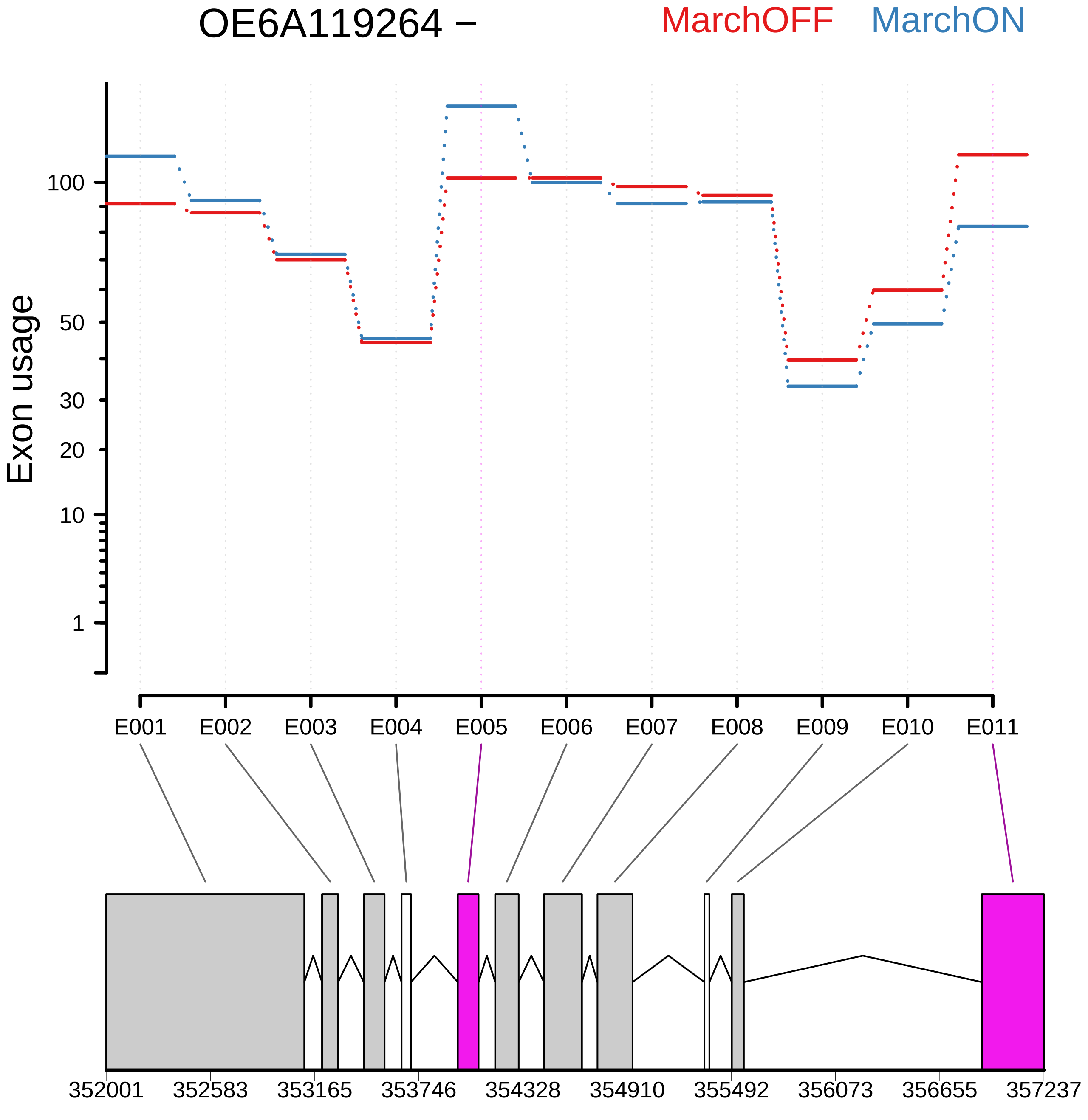
OE6A119264 −
MarchOFF
MarchON
Exon usage
100
50
30
20
10
1
E001
E002
E003
E004
E005
E006
E007
E008
E009
E010
E011
352001
352583
353165
353746
354328
354910
355492
356073
356655
357237

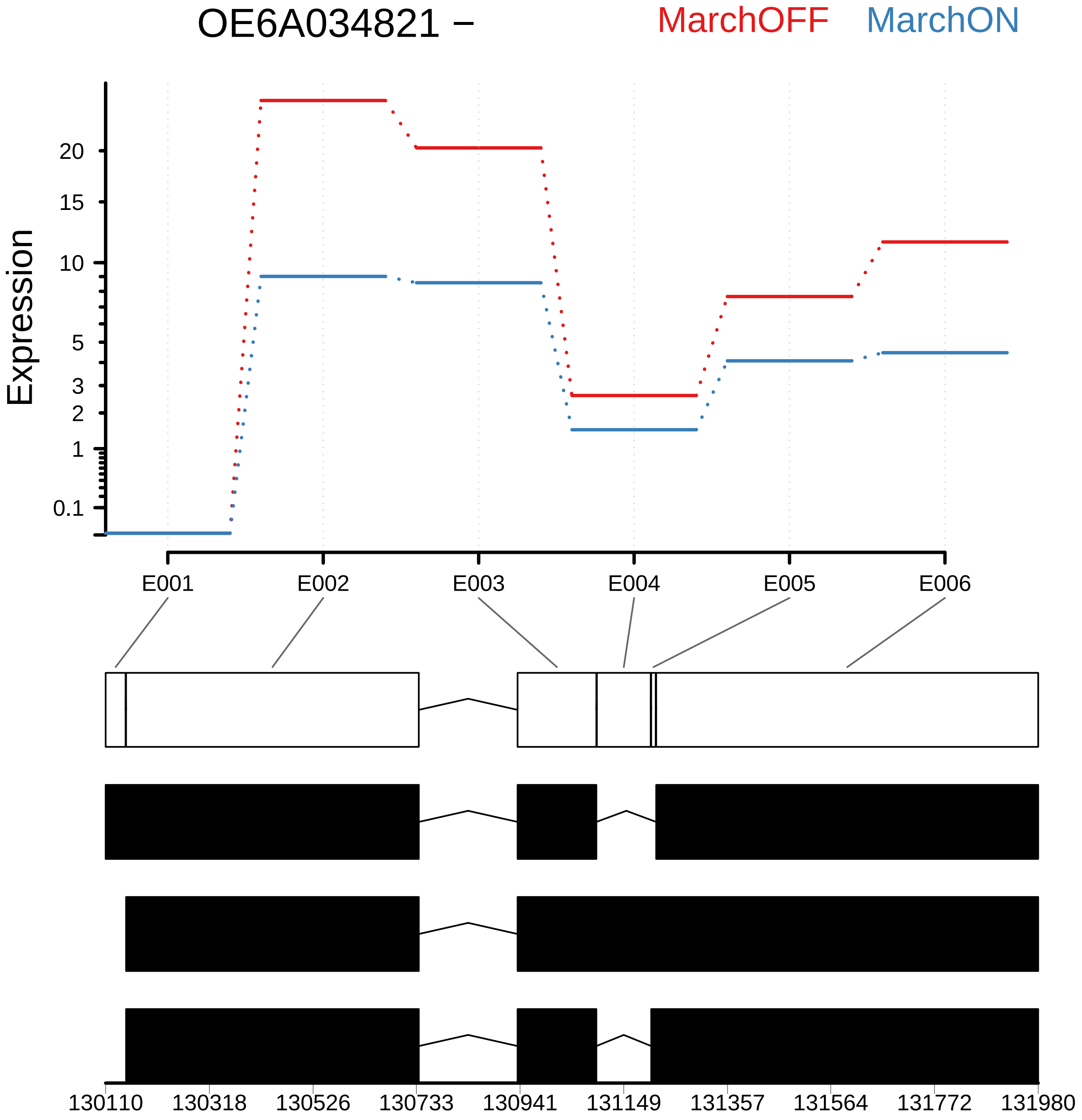

OE6A034821 −
MarchOFF
MarchON
Expression
20
15
10
5
3
2
1
0.1
E001
E002
E003
E004
E005
E006
130110
130318
130526
130733
130941
131149
131357
131564
131772
131980

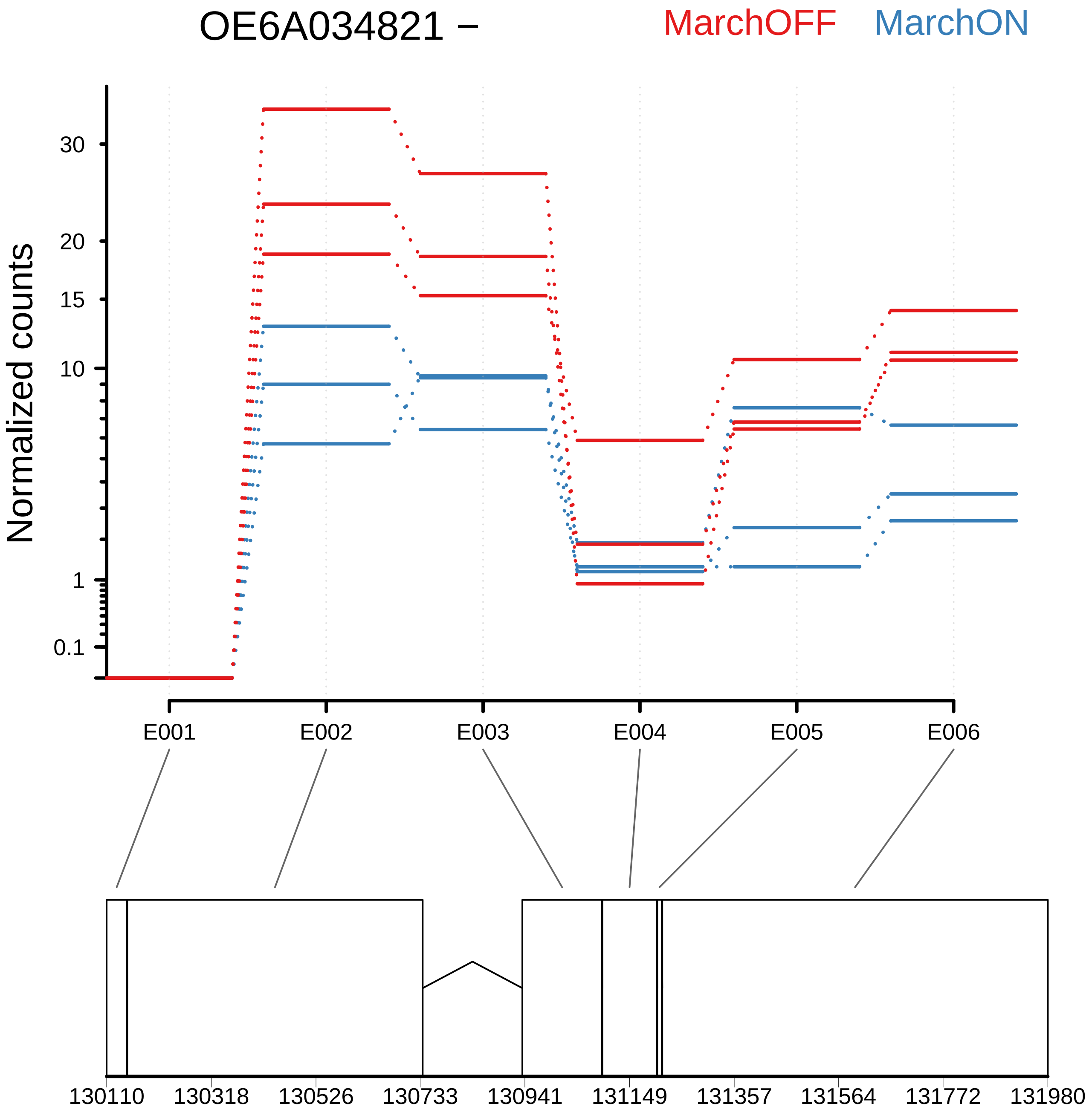

OE6A034821 −
MarchOFF
MarchON
Normalized counts
30
20
15
10
1
0.1
E001
E002
E003
E004
E005
E006
130110
130318
130526
130733
130941
131149
131357
131564
131772
131980

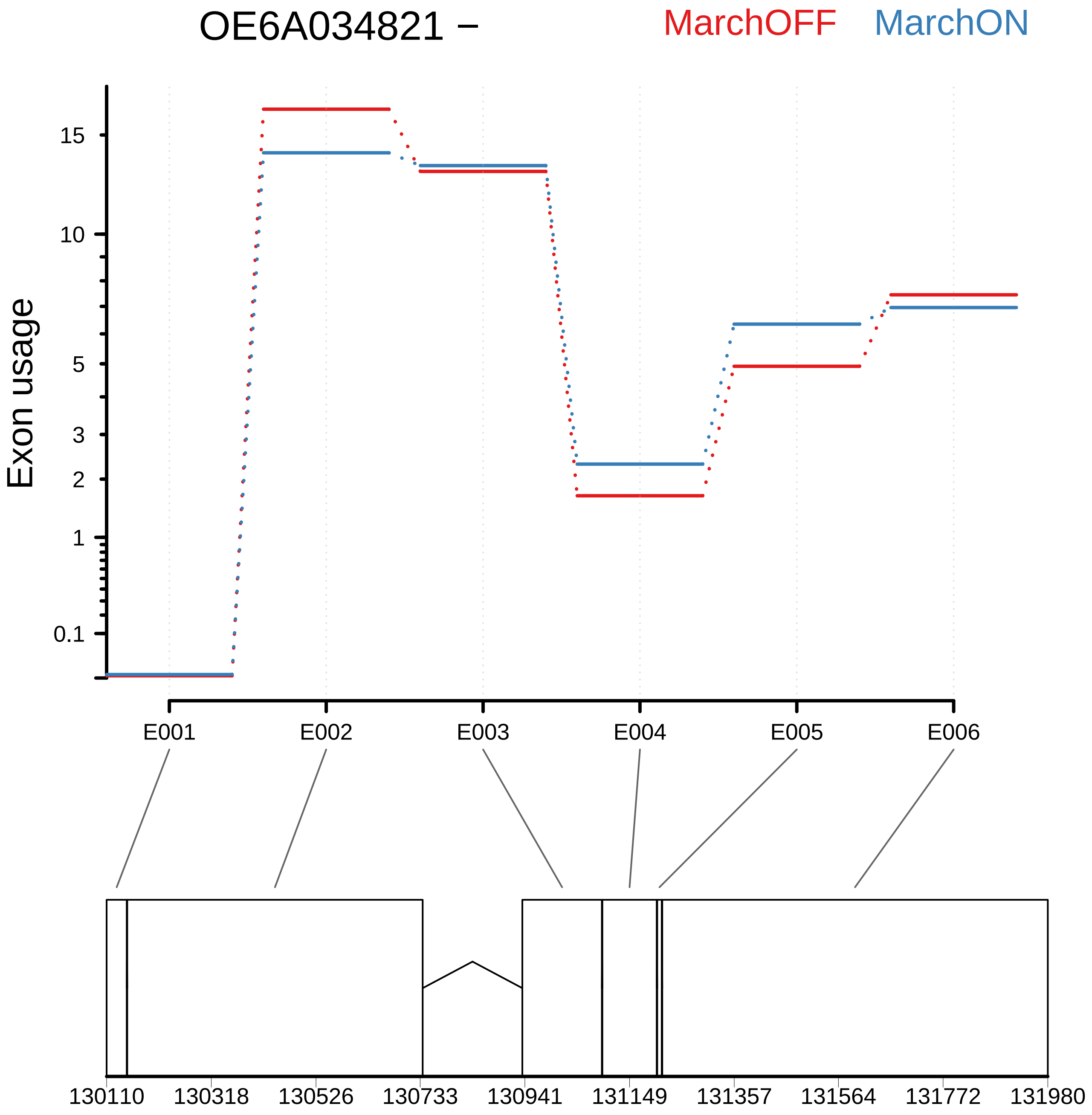
OE6A034821 −
MarchOFF
MarchON
Exon usage
15
10
5
3
2
1
0.1
E001
E002
E003
E004
E005
E006
130110
130318
130526
130733
130941
131149
131357
131564
131772
131980

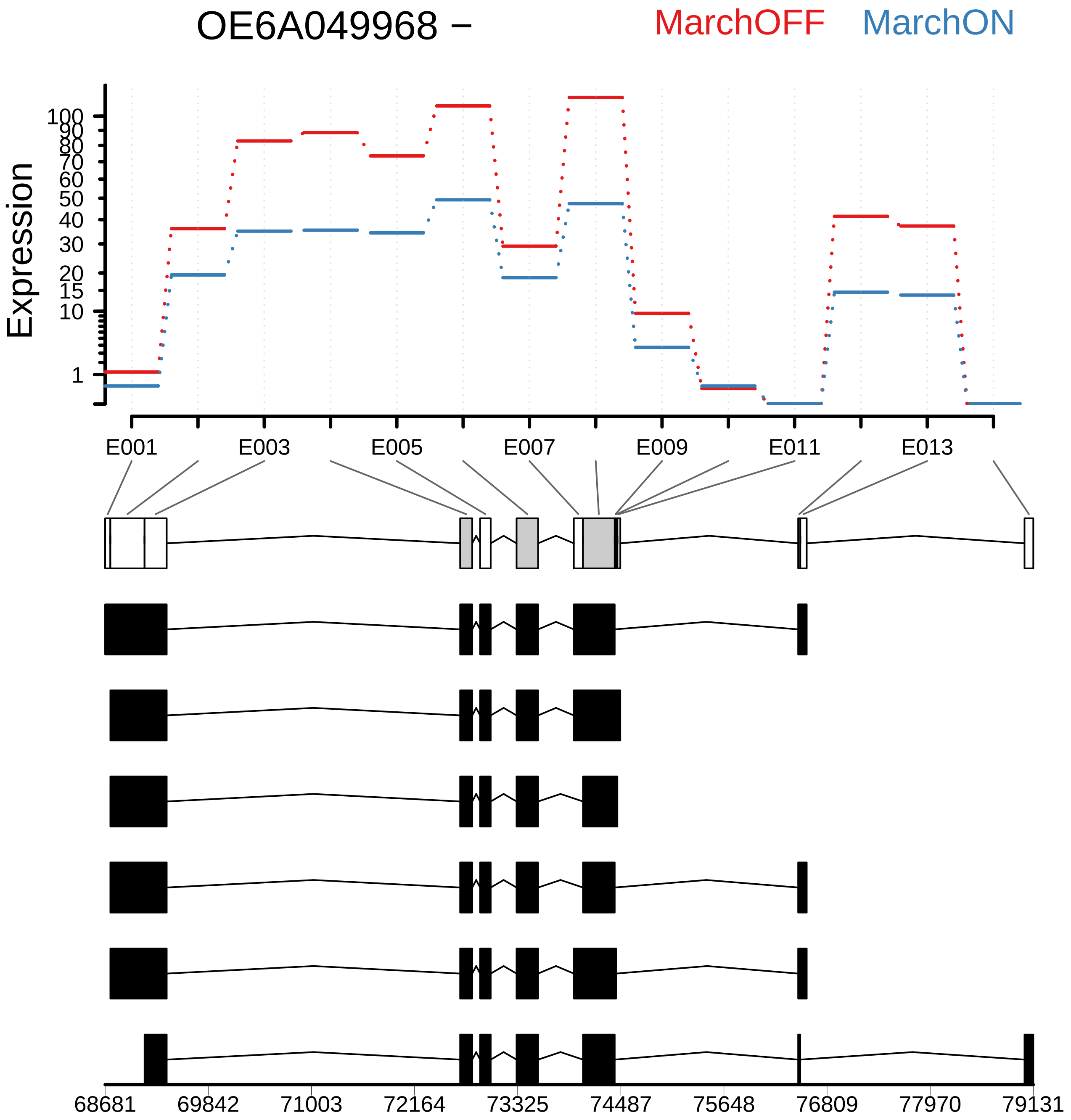
OE6A049968 –
MarchOFF
MarchON
Expression
100
90
80
70
60
50
40
30
20
15
10
1
E001
E003
E005
E007
E009
E011
E013
68681
69842
71003
72164
73325
74487
75648
76809
77970
79131

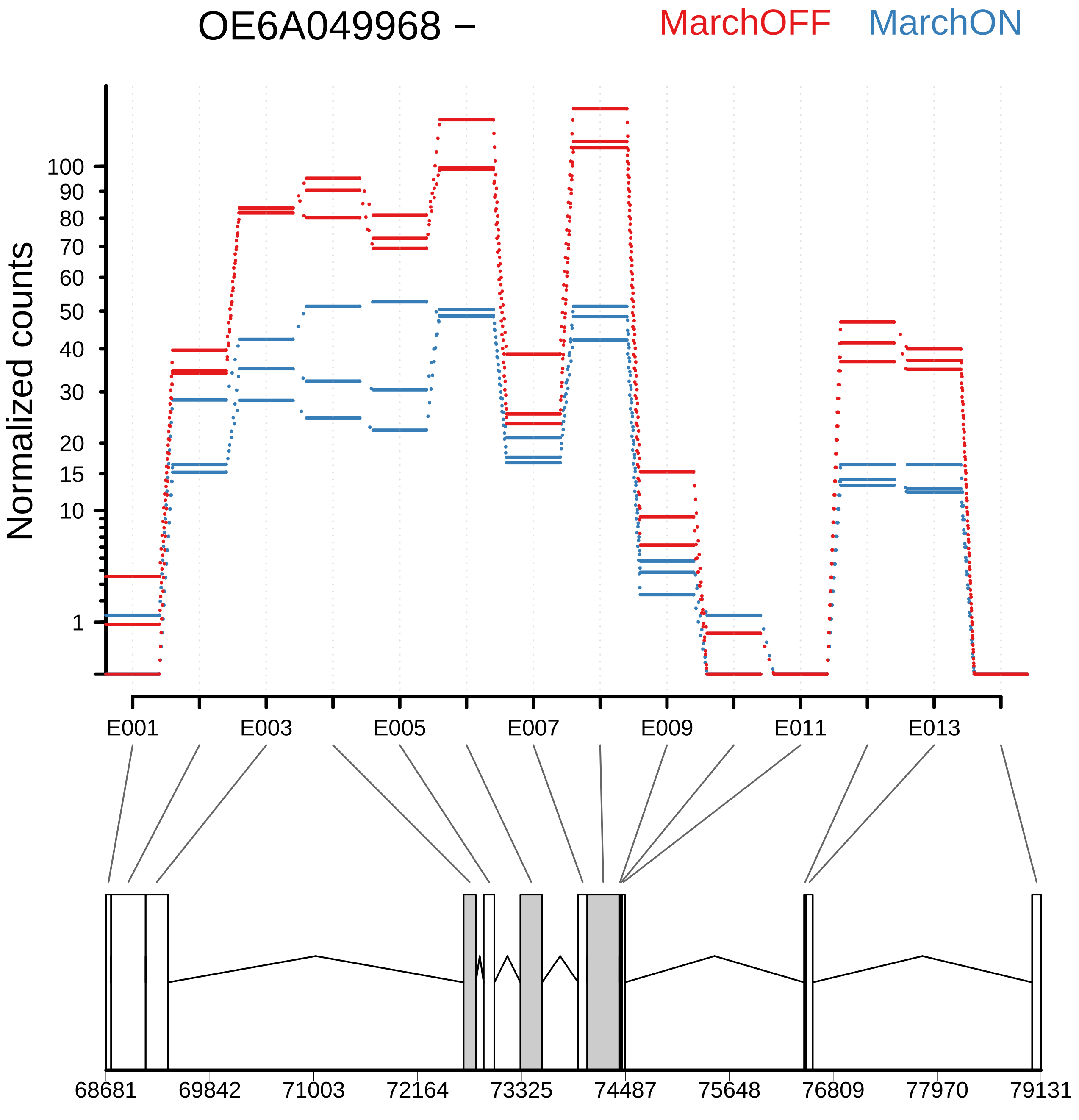

OE6A049968 –
MarchOFF
MarchON
Normalized counts
100
90
80
70
60
50
40
30
20
15
10
1
E001
E003
E005
E007
E009
E011
E013
68681
69842
71003
72164
73325
74487
75648
76809
77970
79131

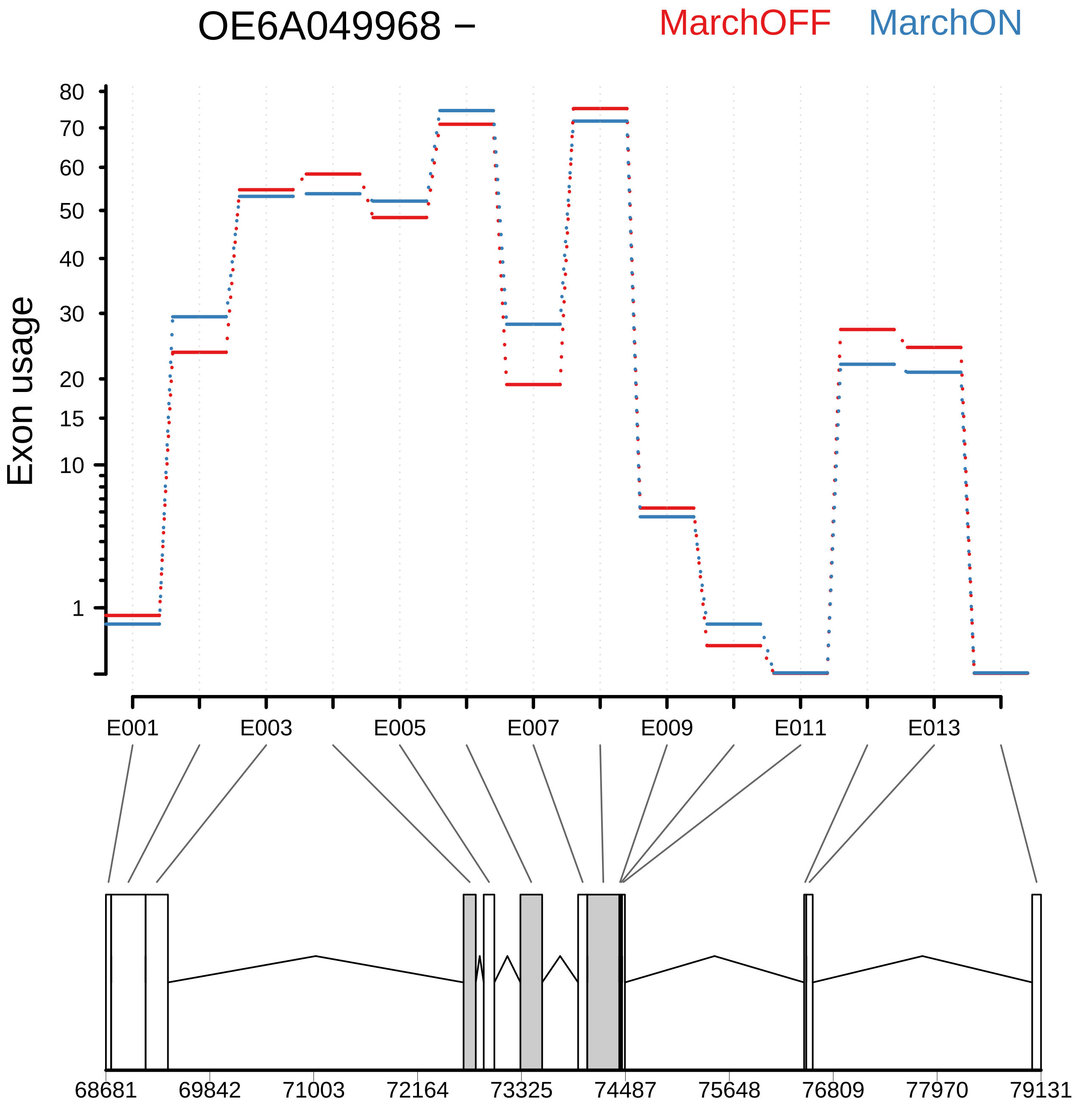
OE6A049968 –
MarchOFF
MarchON
Exon usage
80
70
60
50
40
30
20
15
10
1
E001
E003
E005
E007
E009
E011
E013
68681
69842
71003
72164
73325
74487
75648
76809
77970
79131

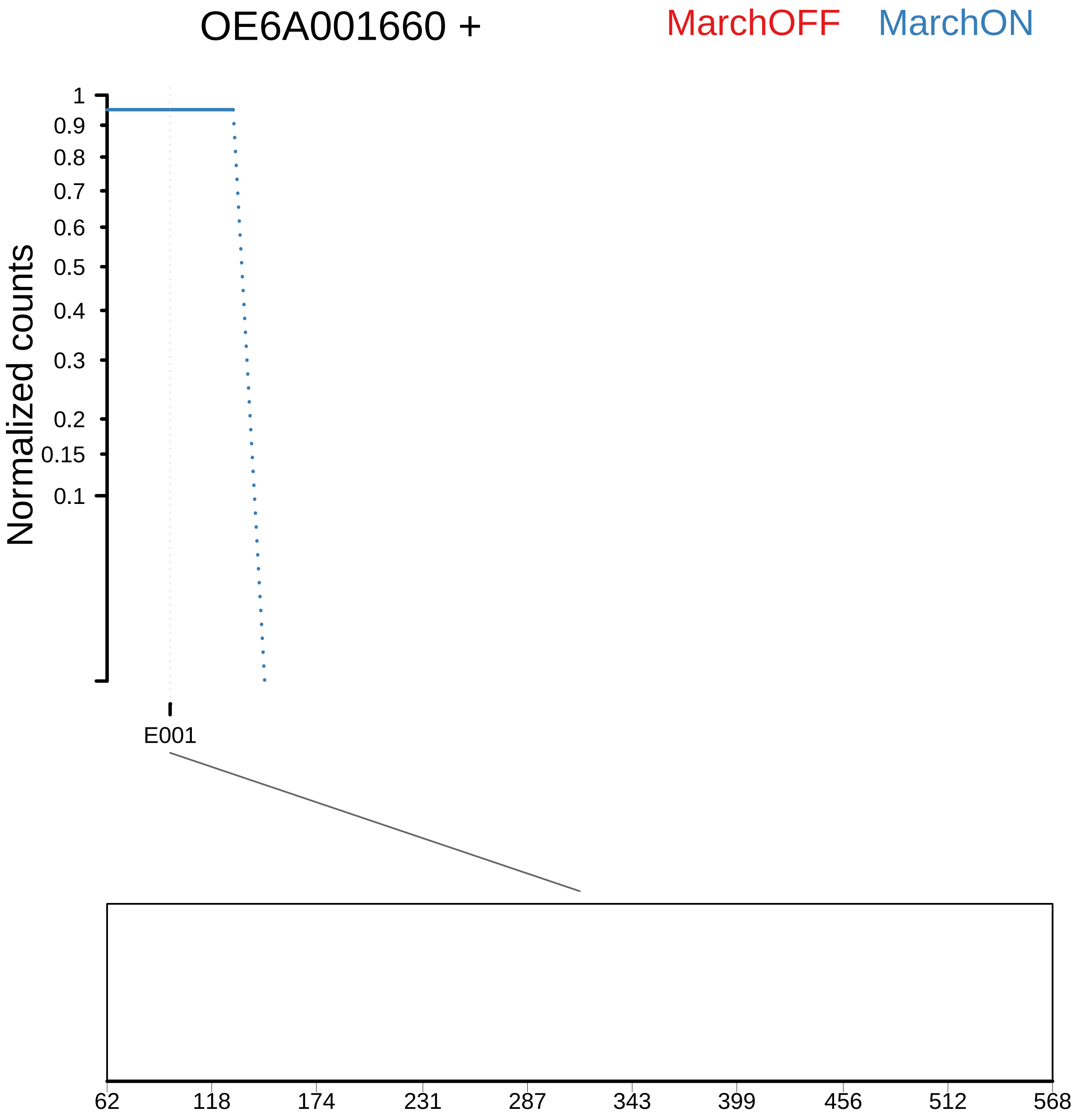
OE6A001660 +
MarchOFF
MarchON
Normalized counts
1
0.9
0.8
0.7
0.6
0.5
0.4
0.3
0.2
0.15
0.1
E001
62
118
174
231
287
343
399
456
512
568

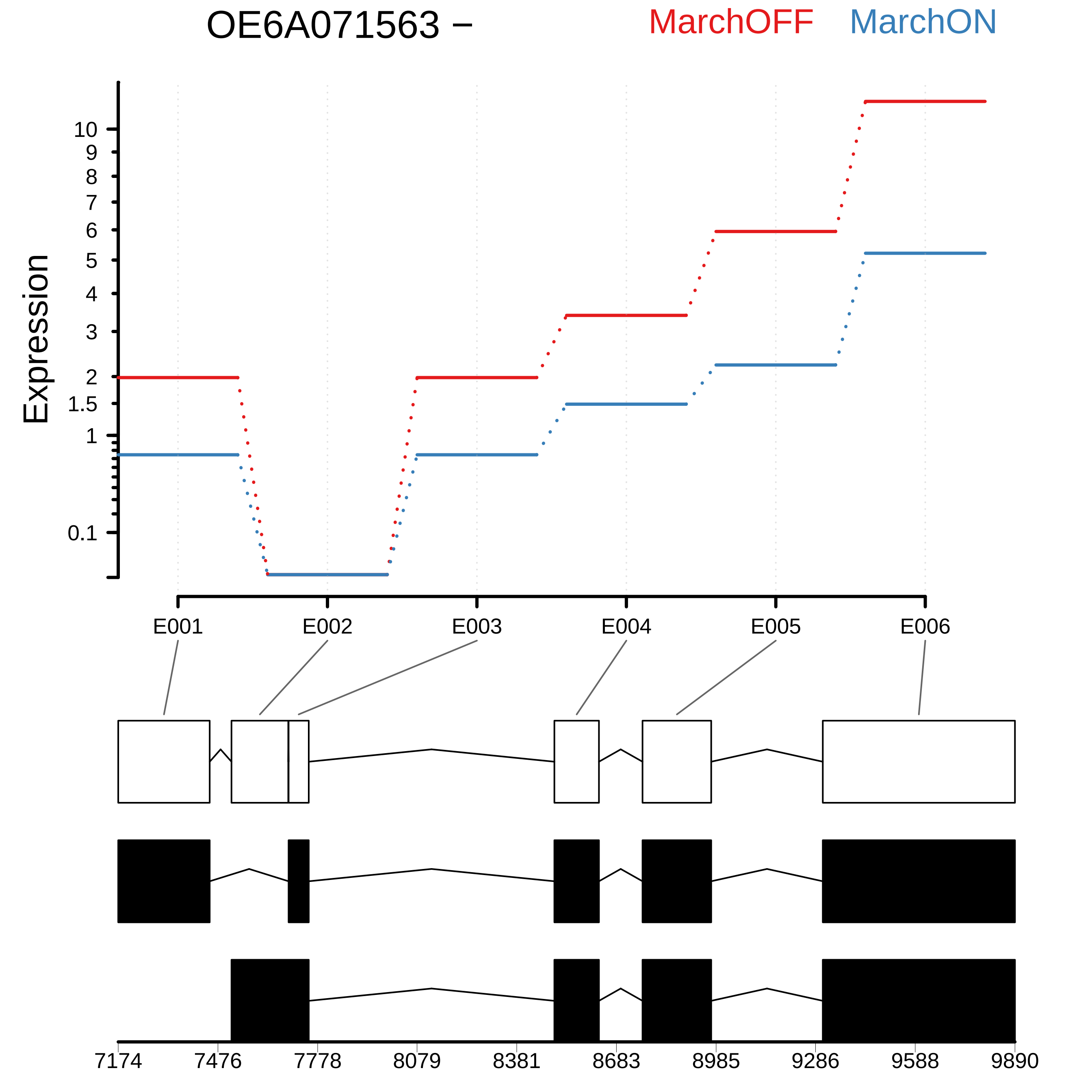

OE6A071563 −
MarchOFF
MarchON
Expression
10
9
8
7
6
5
4
3
2
1.5
1
0.1
E001
E002
E003
E004
E005
E006
7174
7476
7778
8079
8381
8683
8985
9286
9588
9890

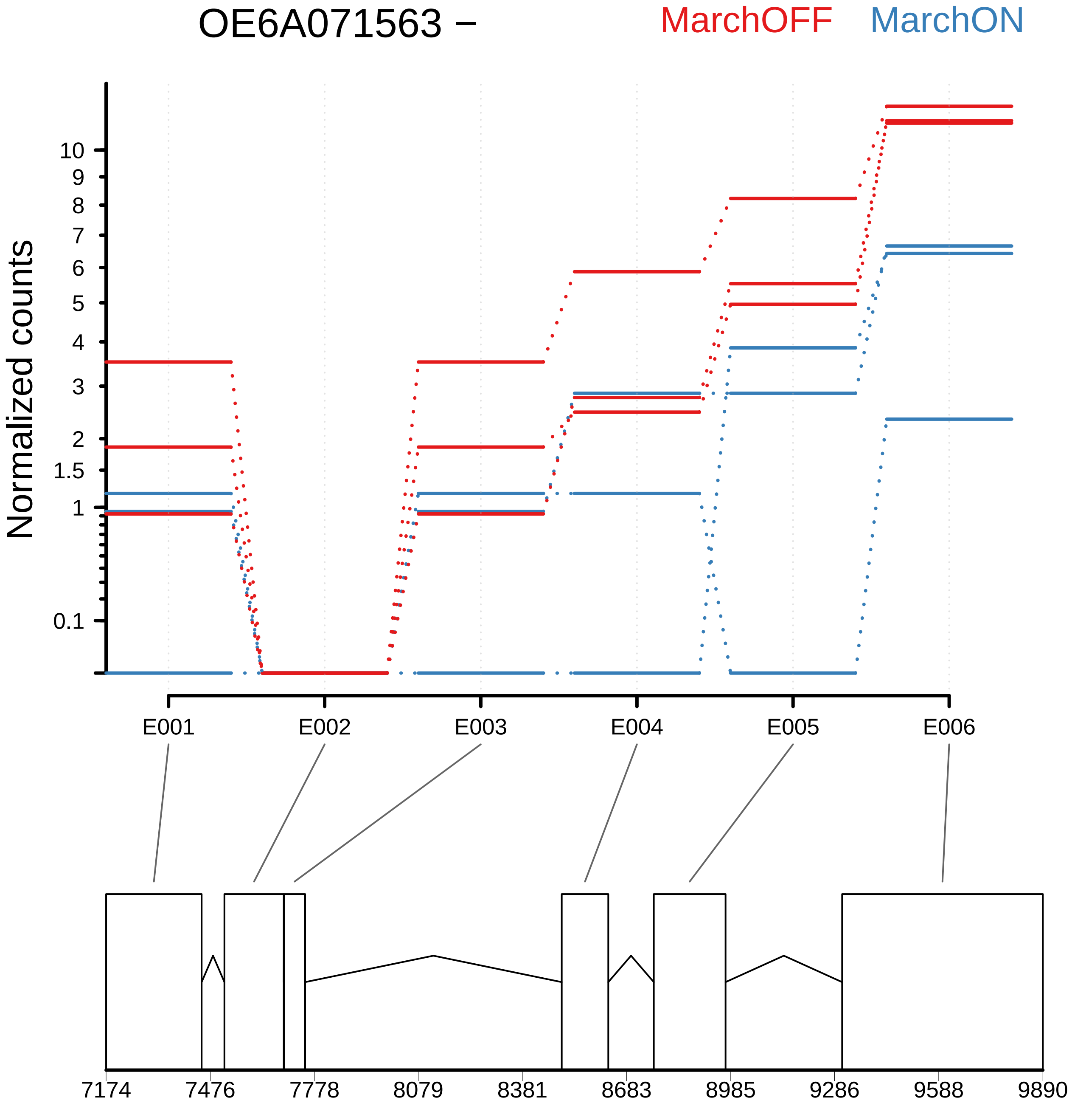
OE6A071563 −
MarchOFF
MarchON
Normalized counts
10
9
8
7
6
5
4
3
2
1.5
1
0.1
E001
E002
E003
E004
E005
E006
7174
7476
7778
8079
8381
8683
8985
9286
9588
9890

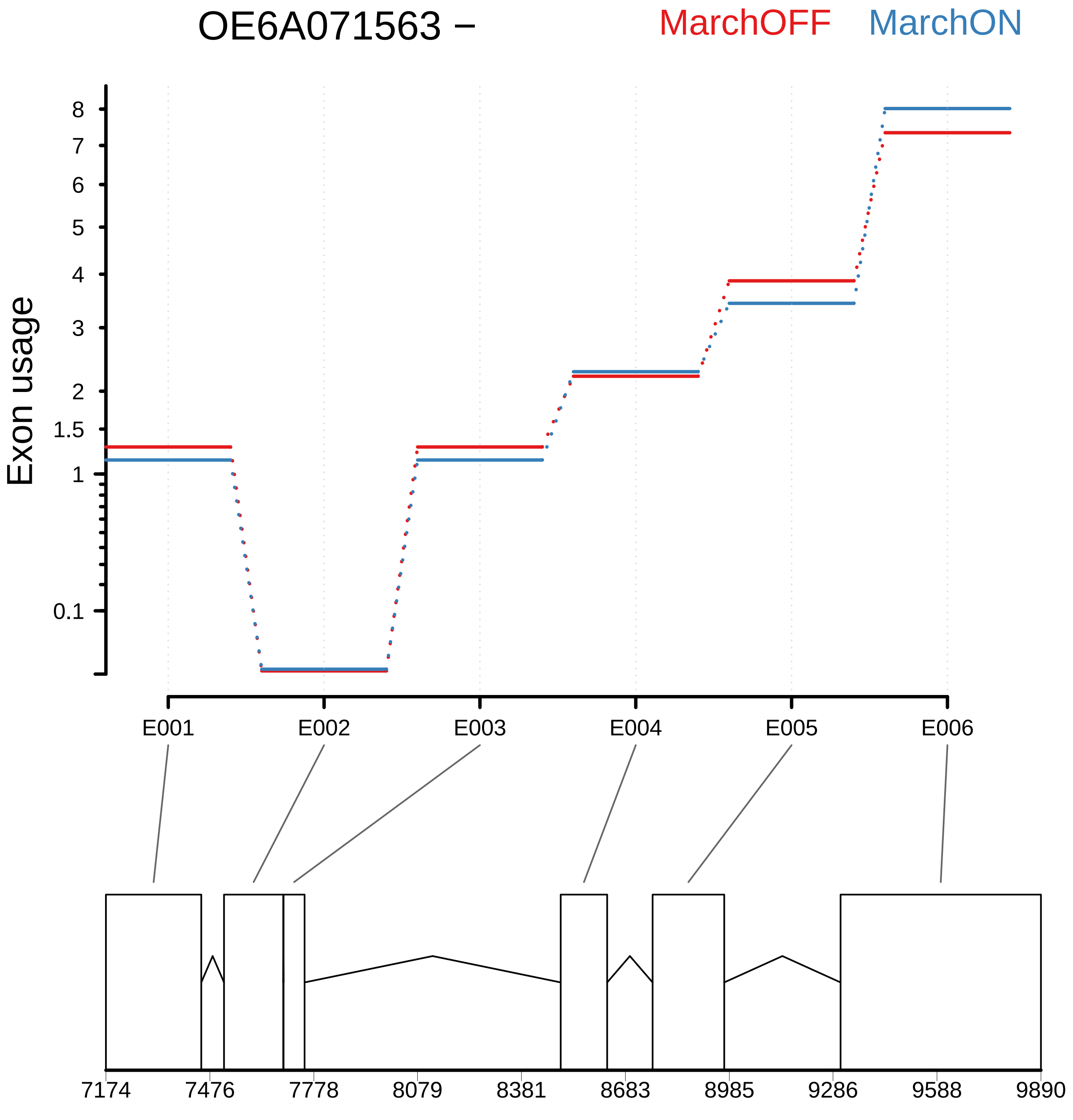
OE6A071563 −
MarchOFF
MarchON
Exon usage
8
7
6
5
4
3
2
1.5
1
0.1
E001
E002
E003
E004
E005
E006
7174
7476
7778
8079
8381
8683
8985
9286
9588
9890

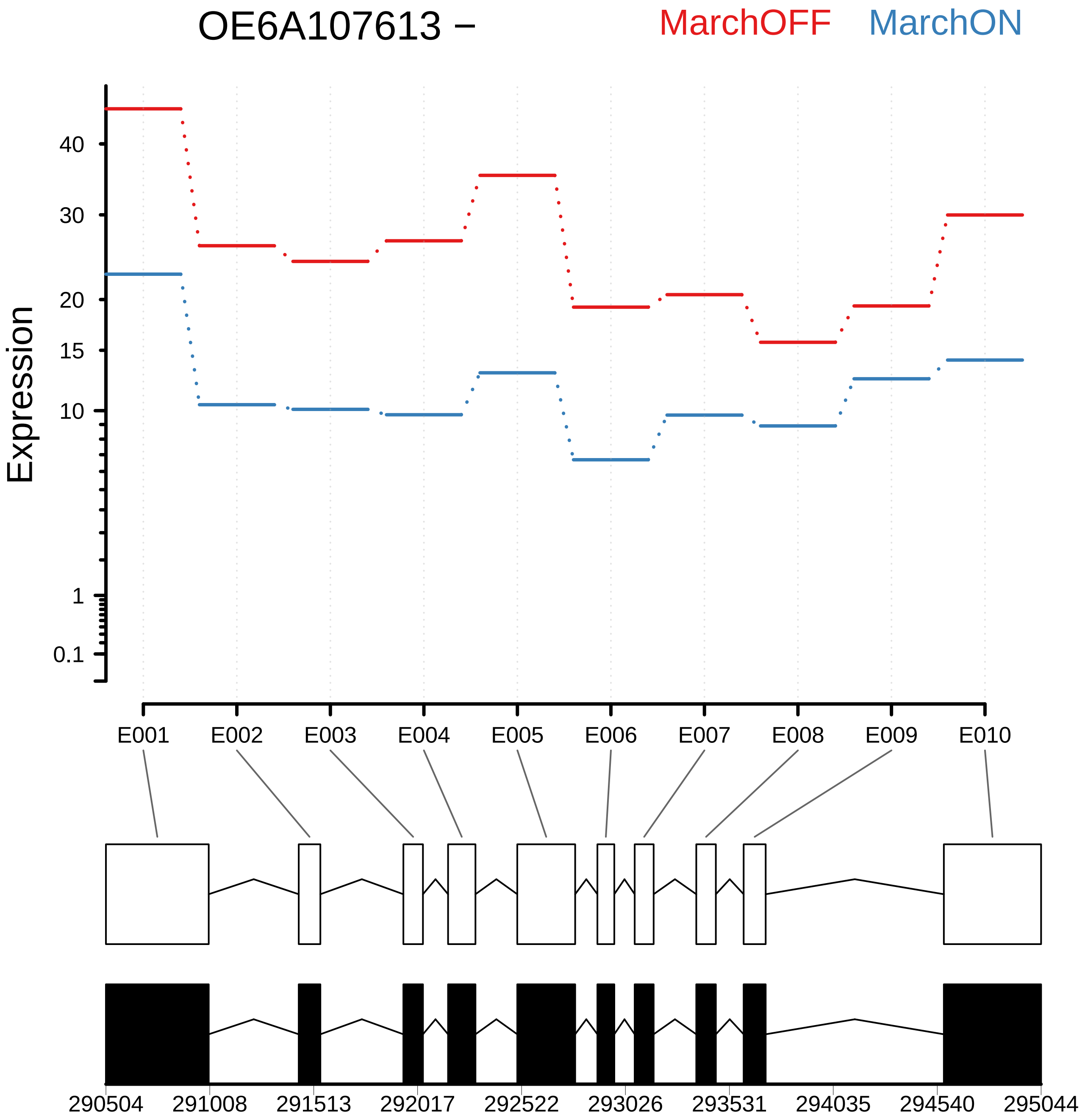
OE6A107613 −
MarchOFF
MarchON
Expression
40
30
20
15
10
1
0.1
E001
E002
E003
E004
E005
E006
E007
E008
E009
E010
290504
291008
291513
292017
292522
293026
293531
294035
294540
295044

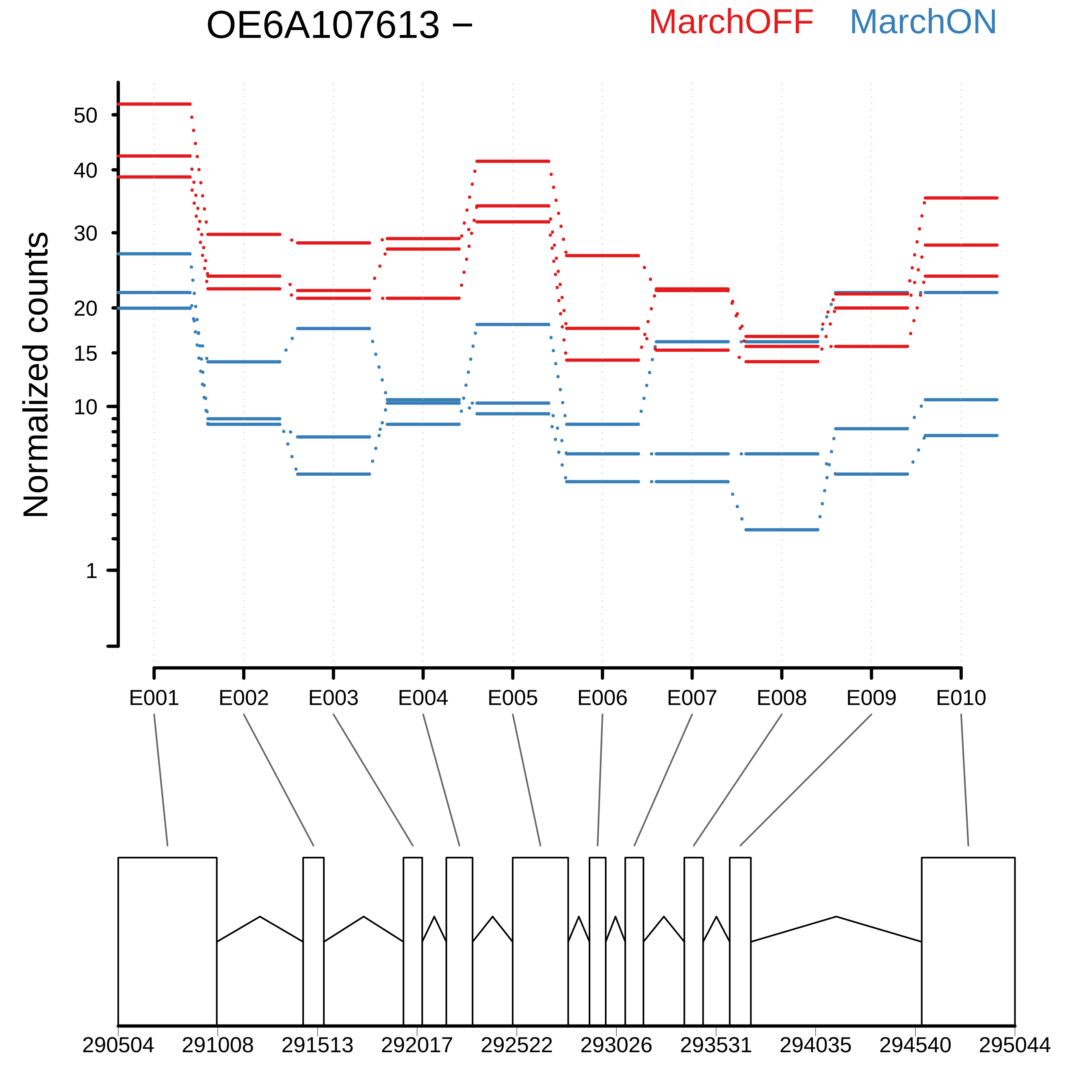

OE6A107613 −
MarchOFF
MarchON
Normalized counts
50
40
30
20
15
10
1
E001
E002
E003
E004
E005
E006
E007
E008
E009
E010
290504
291008
291513
292017
292522
293026
293531
294035
294540
295044

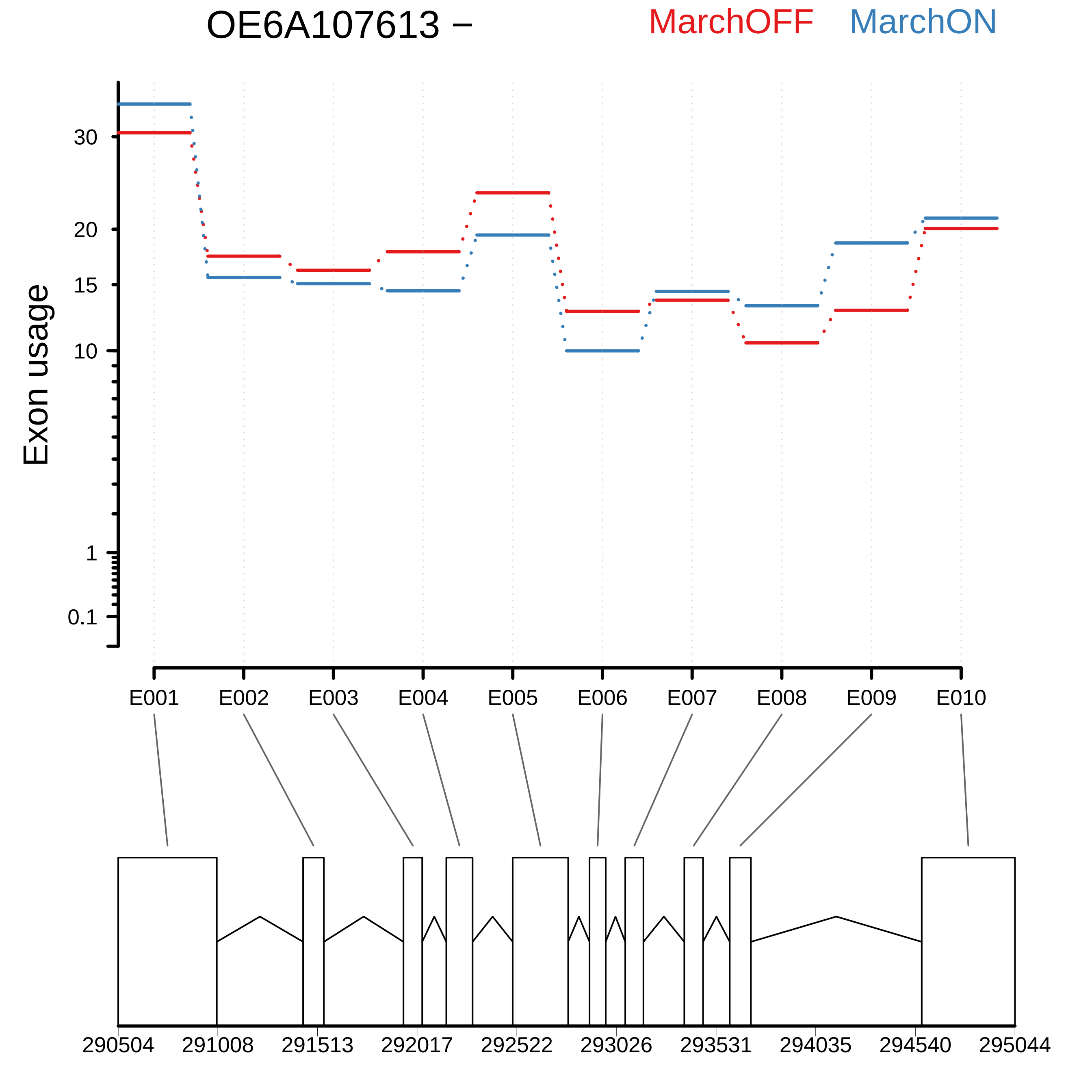
OE6A107613 −
MarchOFF
MarchON
Exon usage
30
20
15
10
1
0.1
E001
E002
E003
E004
E005
E006
E007
E008
E009
E010
290504
291008
291513
292017
292522
293026
293531
294035
294540
295044

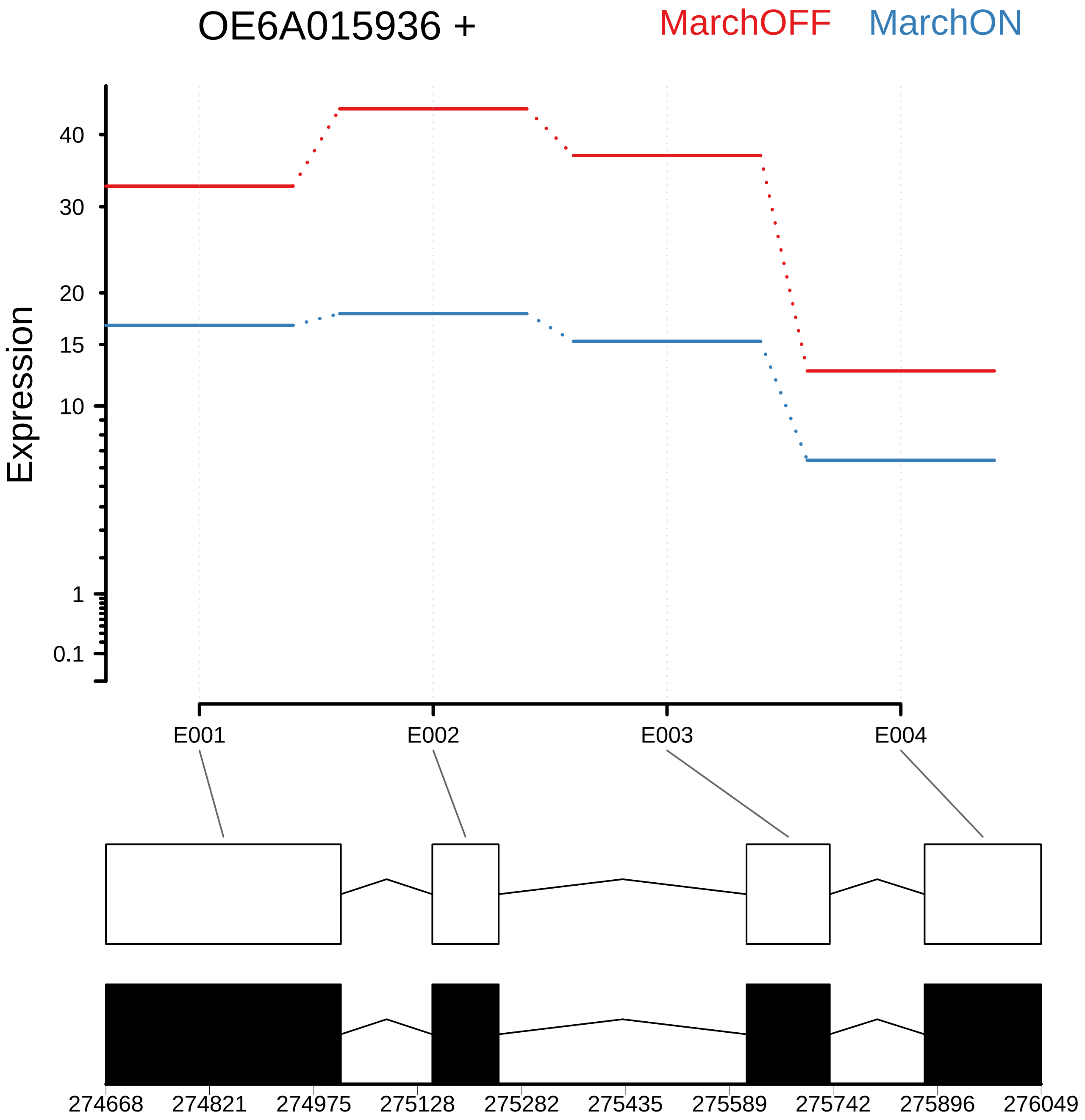

OE6A015936 +
MarchOFF
MarchON
Expression
40
30
20
15
10
1
0.1
E001
E002
E003
E004
274668
274821
274975
275128
275282
275435
275589
275742
275896
276049

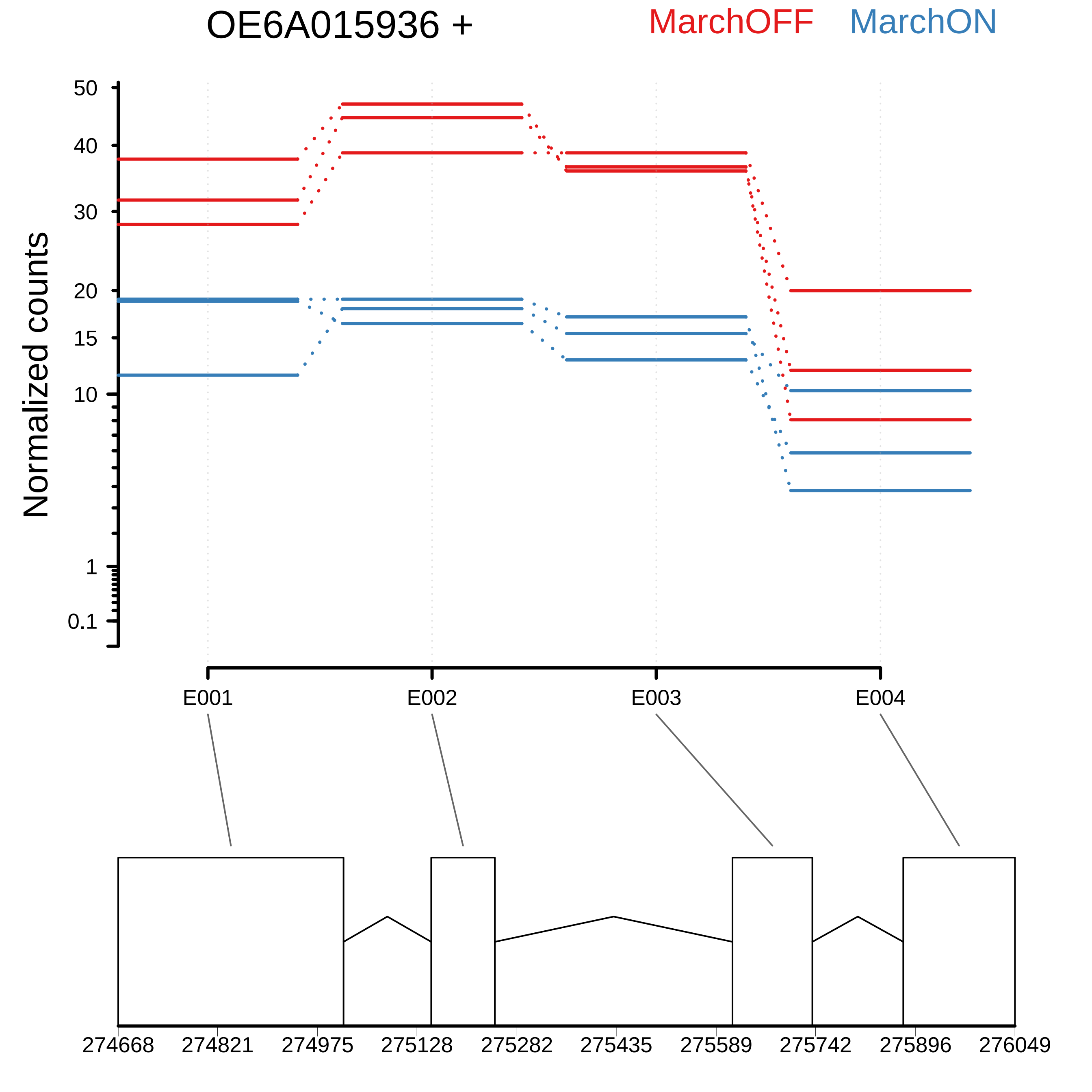

OE6A015936 +
MarchOFF
MarchON
Normalized counts
50
40
30
20
15
10
1
0.1
E001
E002
E003
E004
274668
274821
274975
275128
275282
275435
275589
275742
275896
276049

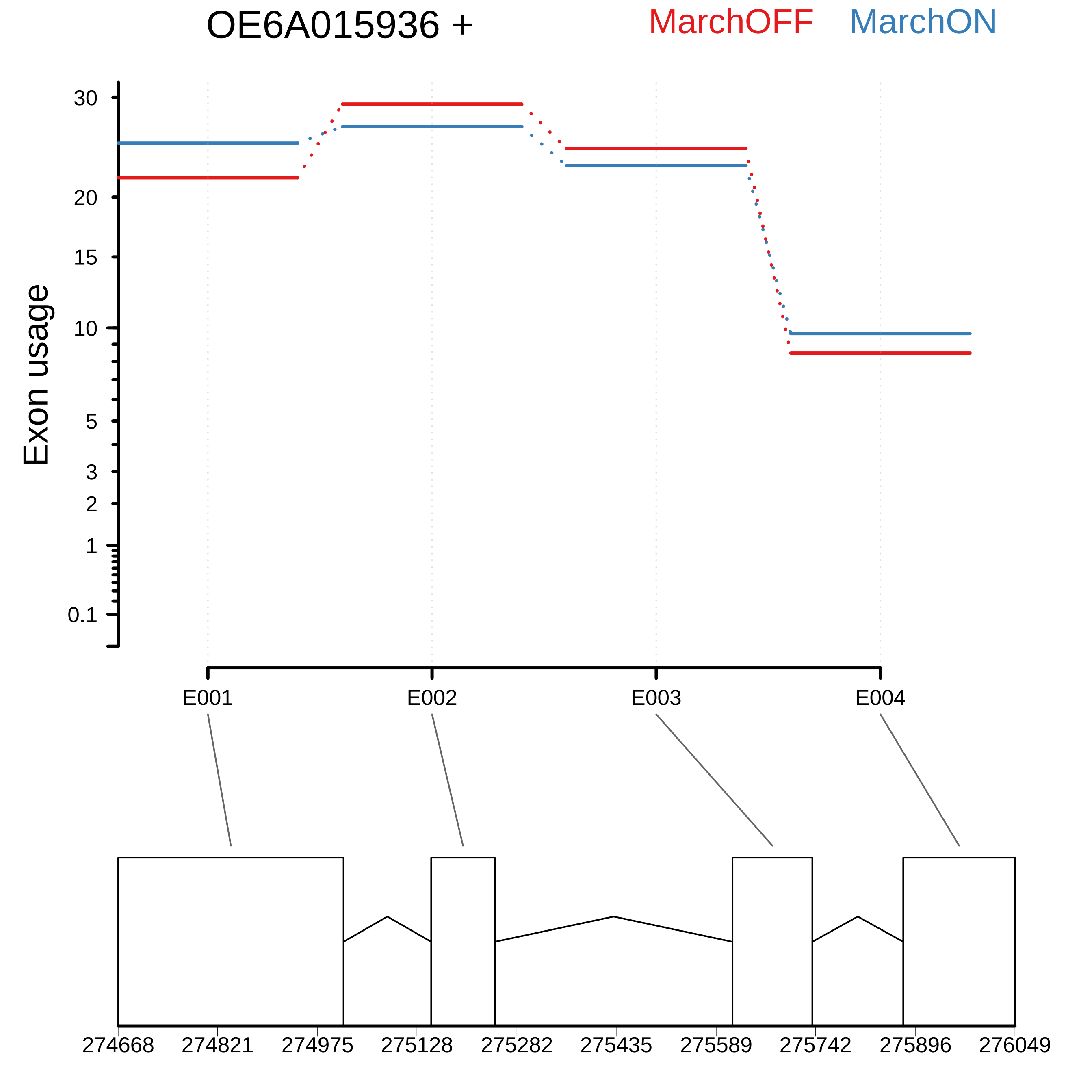

OE6A015936 +
MarchOFF
MarchON
Exon usage
30
20
15
10
5
3
2
1
0.1
E001
E002
E003
E004
274668
274821
274975
275128
275282
275435
275589
275742
275896
276049

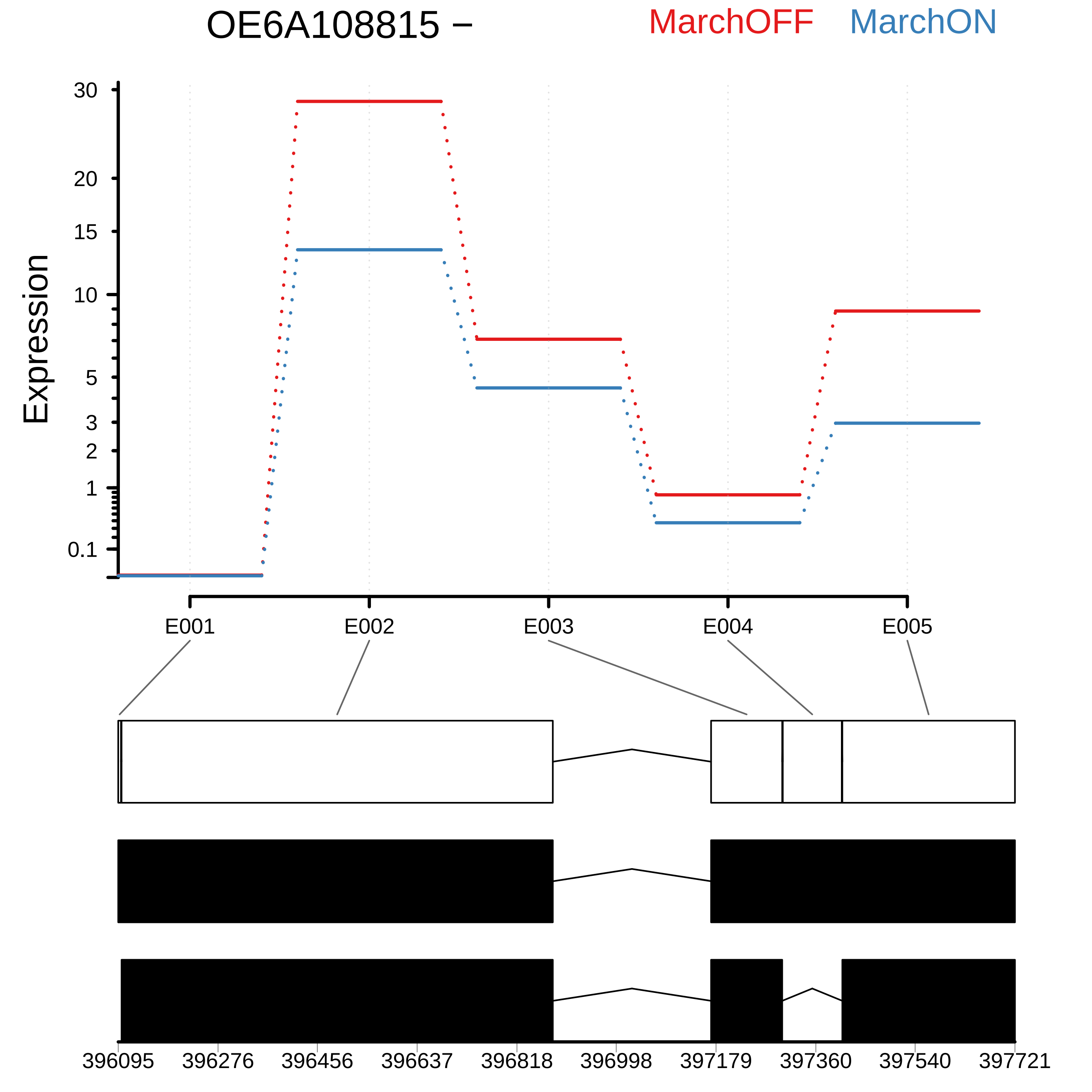

OE6A108815 –
MarchOFF
MarchON
Expression
30
20
15
10
5
3
2
1
0.1
E001
E002
E003
E004
E005
396095
396276
396456
396637
396818
396998
397179
397360
397540
397721

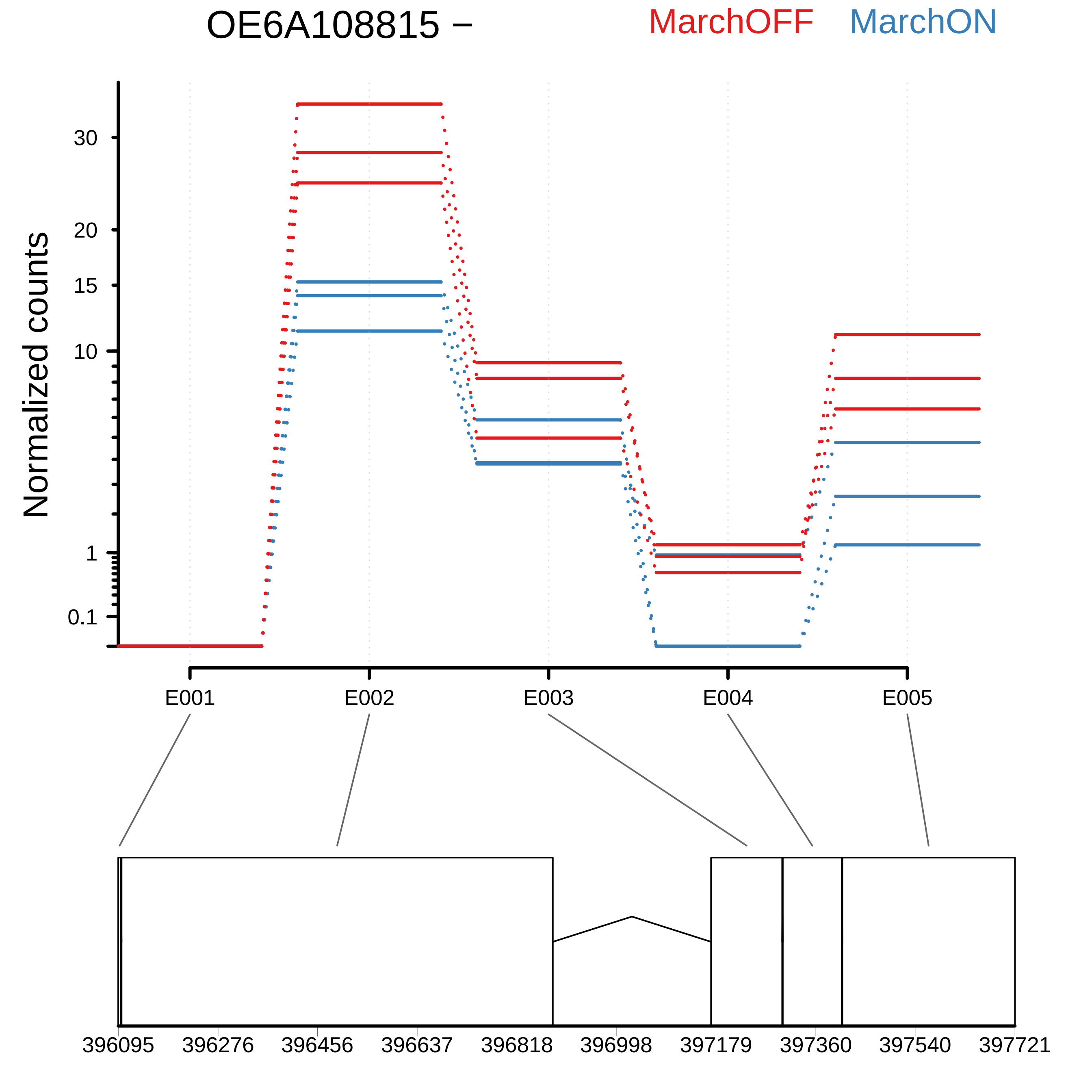

OE6A108815 −
MarchOFF
MarchON
Normalized counts
30
20
15
10
1
0.1
E001
E002
E003
E004
E005
396095
396276
396456
396637
396818
396998
397179
397360
397540
397721

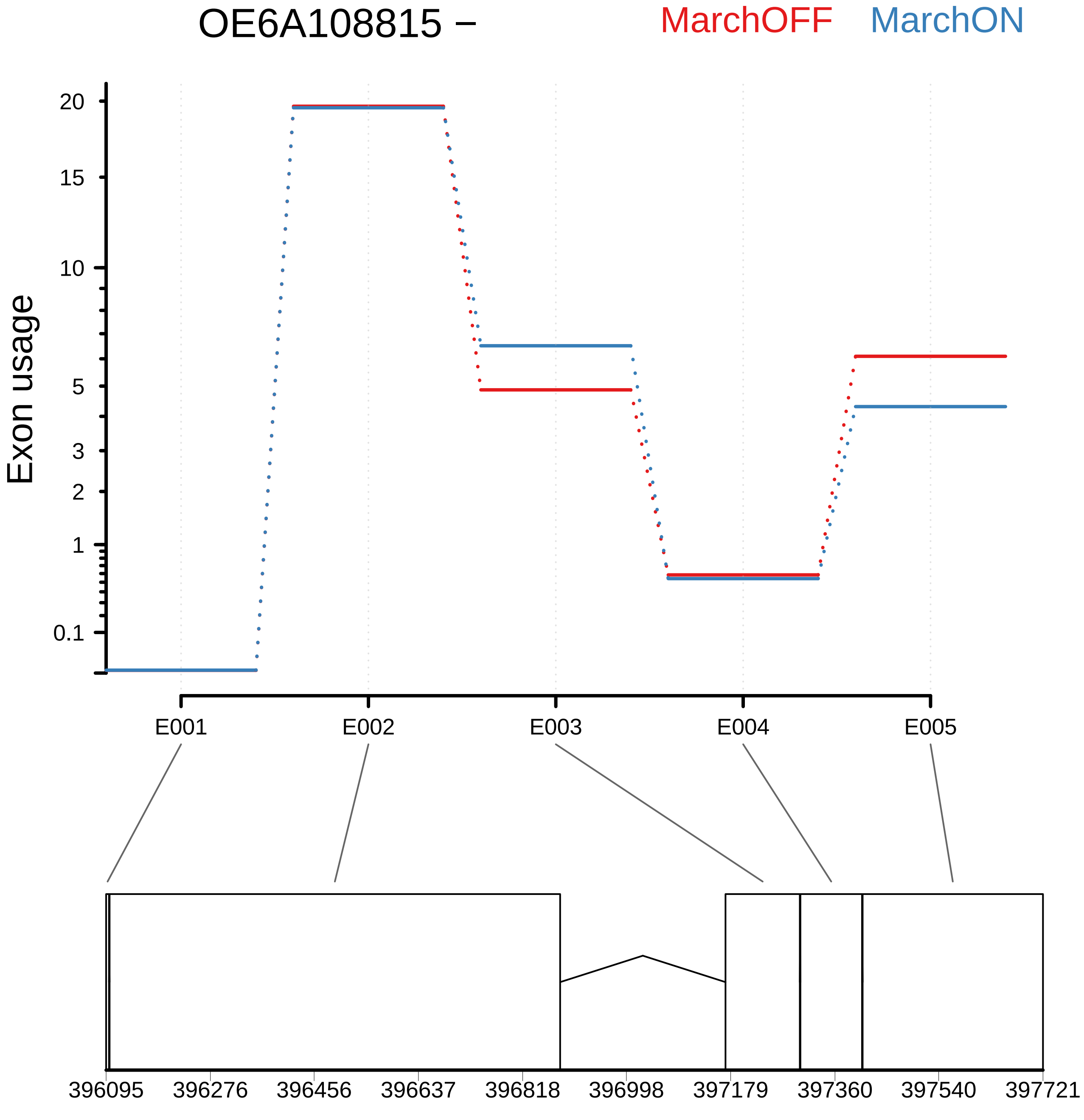

OE6A108815 –
MarchOFF
MarchON
Exon usage
20
15
10
5
3
2
1
0.1
E001
E002
E003
E004
E005
396095
396276
396456
396637
396818
396998
397179
397360
397540
397721

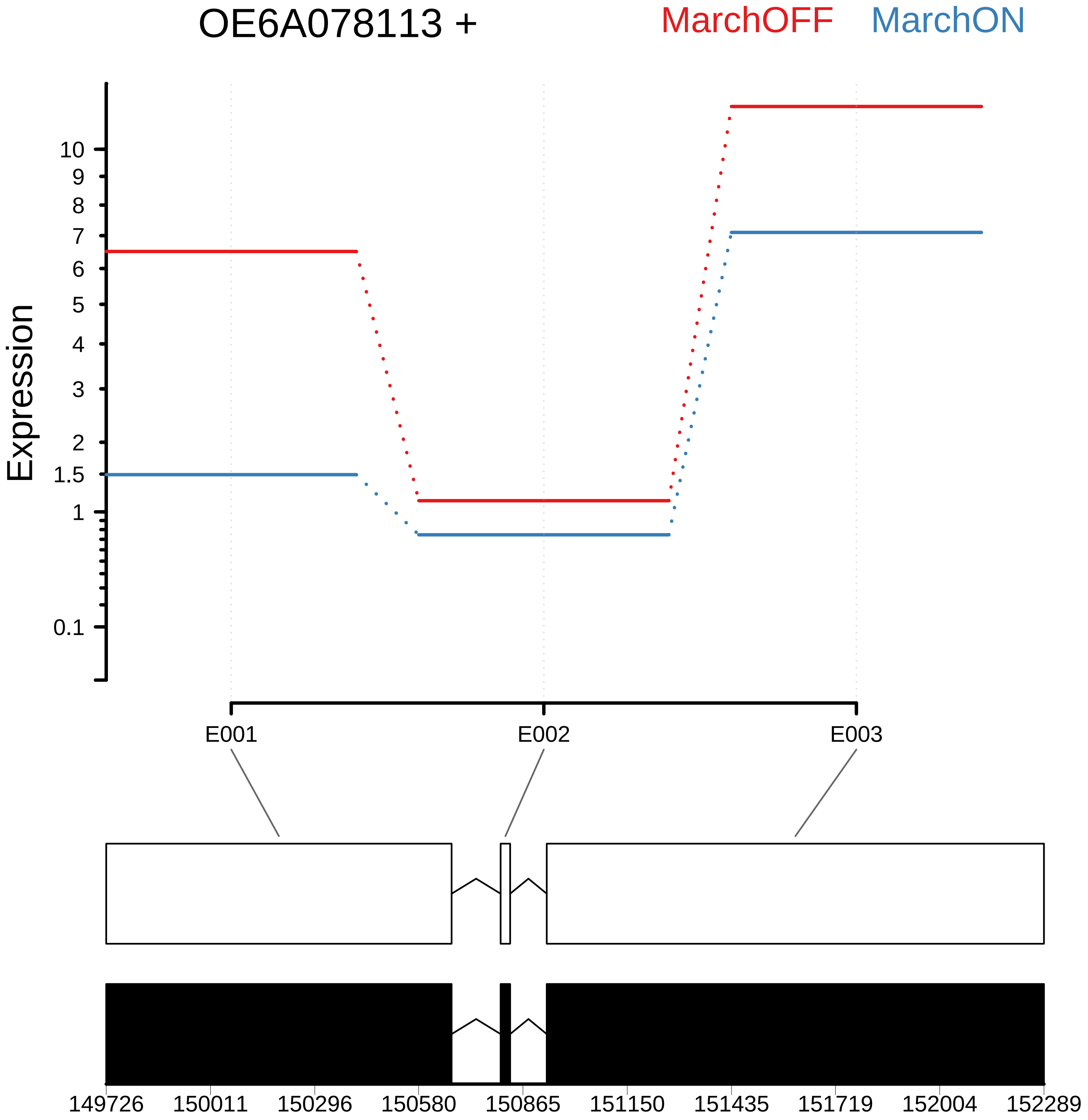
OE6A078113 +
MarchOFF
MarchON
Expression
10
9
8
7
6
5
4
3
2
1.5
1
0.1
E001
E002
E003
149726
150011
150296
150580
150865
151150
151435
151719
152004
152289

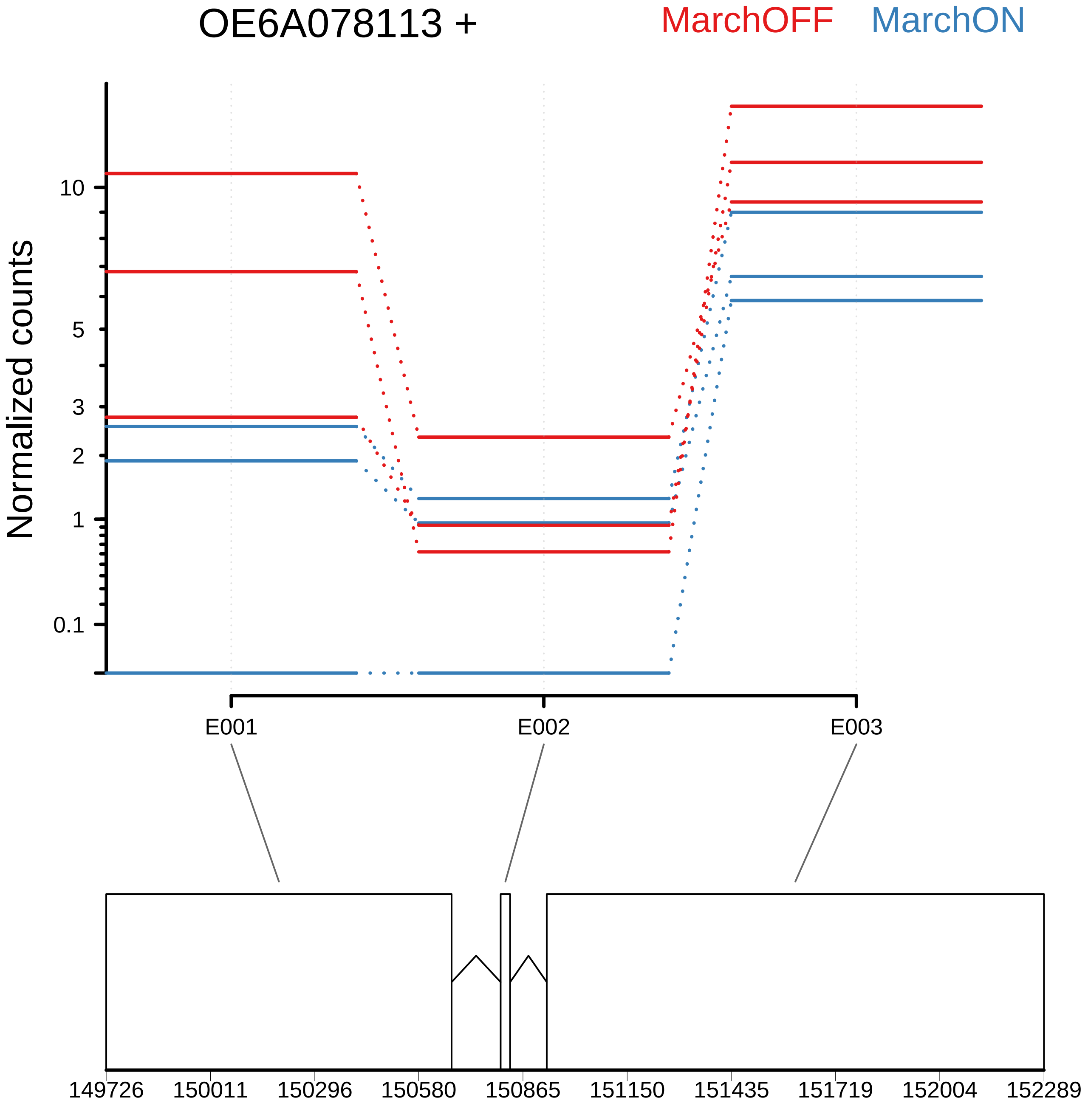

OE6A078113 +
MarchOFF
MarchON
Normalized counts
10
5
3
2
1
0.1
E001
E002
E003
149726
150011
150296
150580
150865
151150
151435
151719
152004
152289

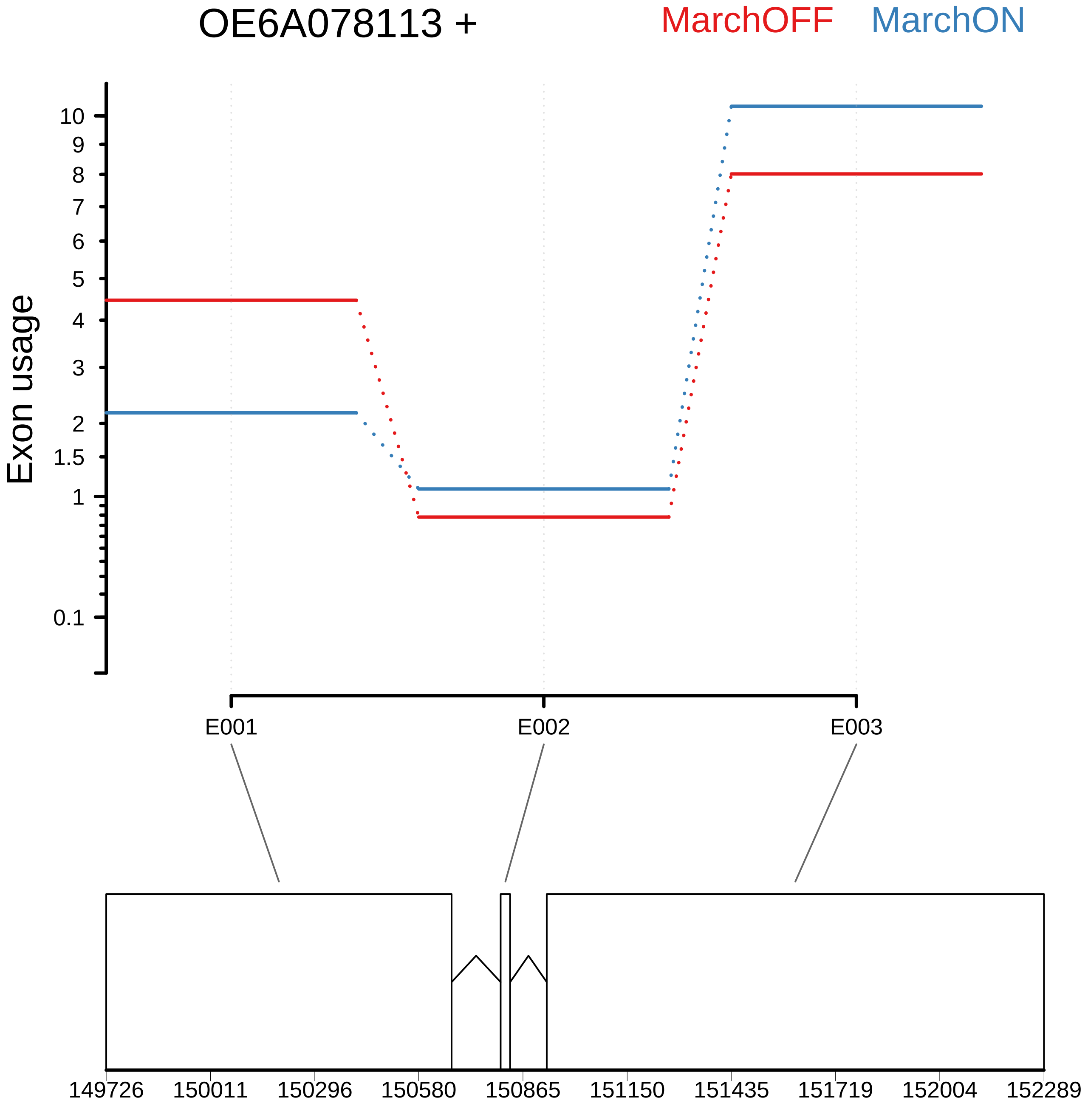
OE6A078113 +
MarchOFF
MarchON
Exon usage
10
9
8
7
6
5
4
3
2
1.5
1
0.1
E001
E002
E003
149726
150011
150296
150580
150865
151150
151435
151719
152004
152289

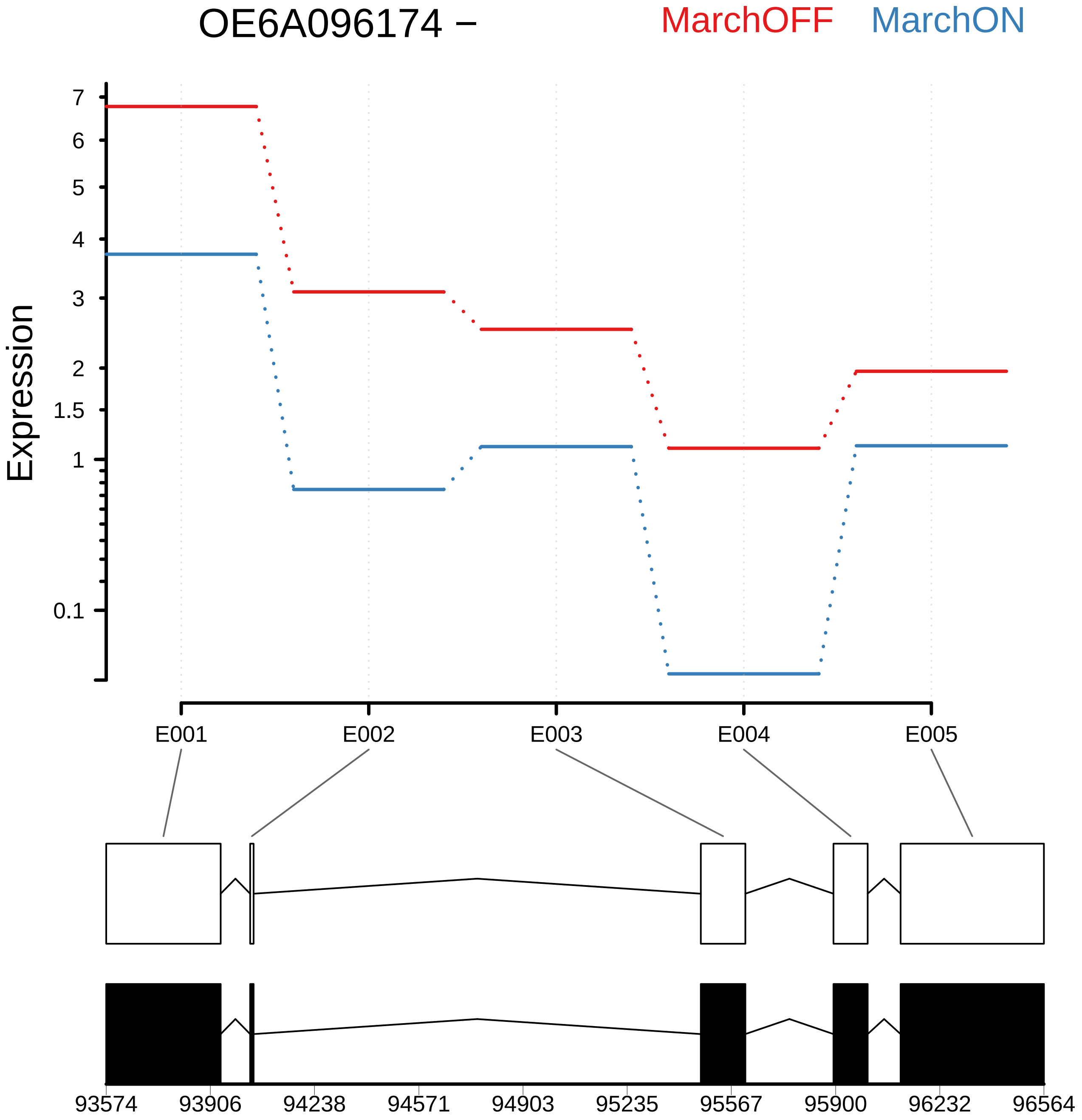

OE6A096174 −
MarchOFF
MarchON
Expression
7
6
5
4
3
2
1.5
1
0.1
E001
E002
E003
E004
E005
93574
93906
94238
94571
94903
95235
95567
95900
96232
96564

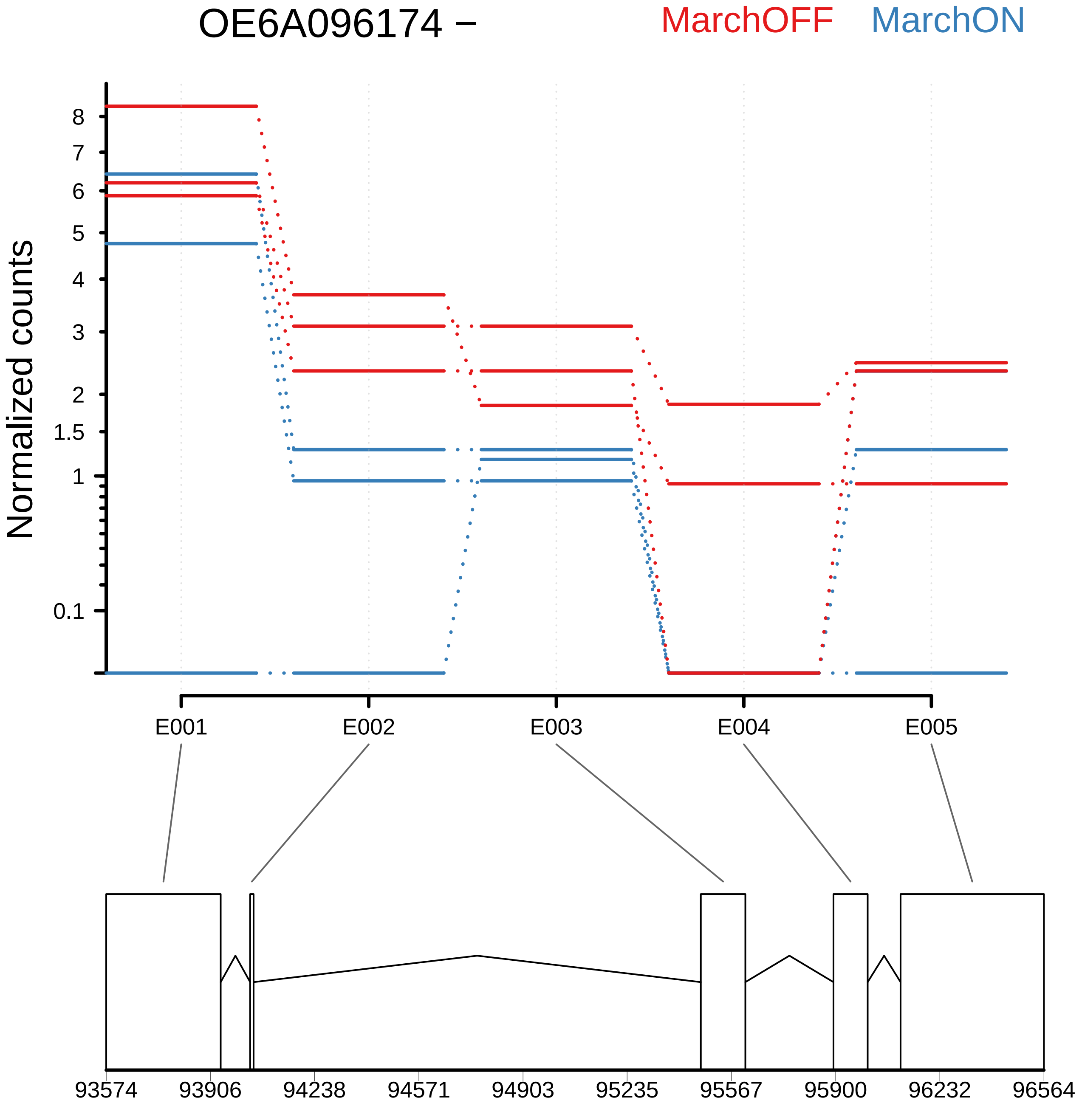

OE6A096174 −
MarchOFF
MarchON
Normalized counts
8
7
6
5
4
3
2
1.5
1
0.1
E001
E002
E003
E004
E005
93574
93906
94238
94571
94903
95235
95567
95900
96232
96564

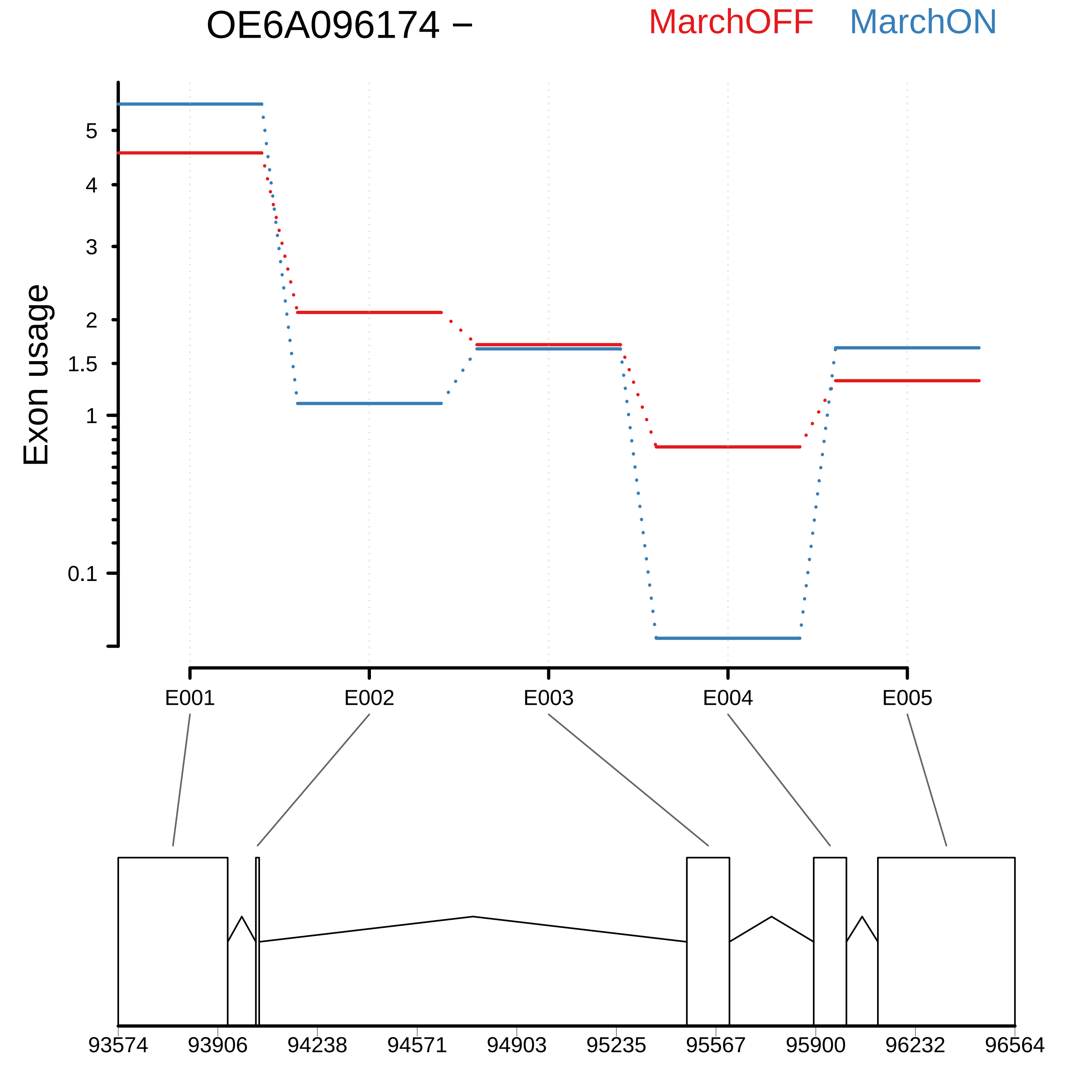

OE6A096174 −
MarchOFF
MarchON
Exon usage
5
4
3
2
1.5
1
0.1
E001
E002
E003
E004
E005
93574
93906
94238
94571
94903
95235
95567
95900
96232
96564

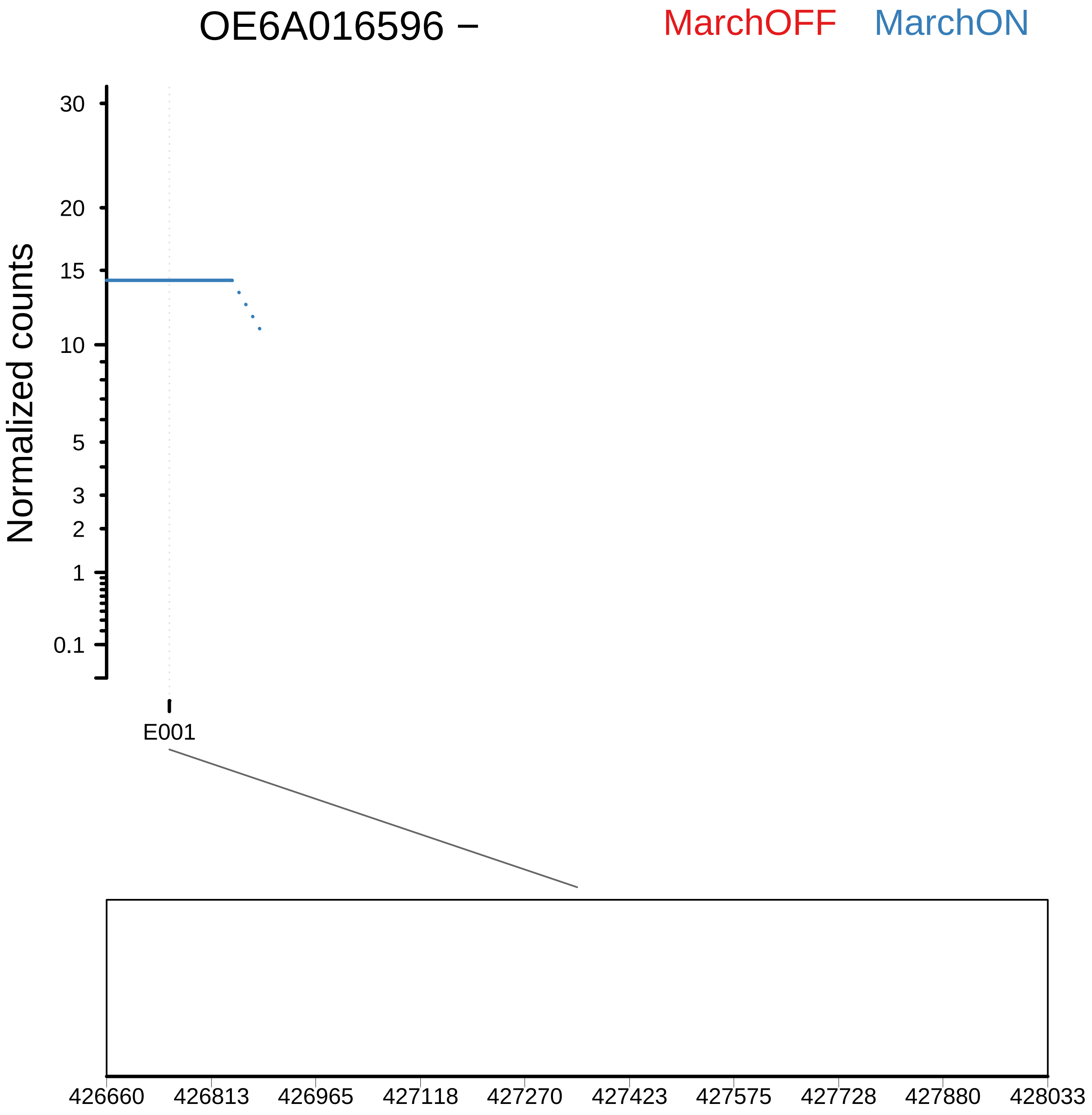

OE6A016596 −
MarchOFF
MarchON
Normalized counts
30
20
15
10
5
3
2
1
0.1
E001
426660
426813
426965
427118
427270
427423
427575
427728
427880
428033

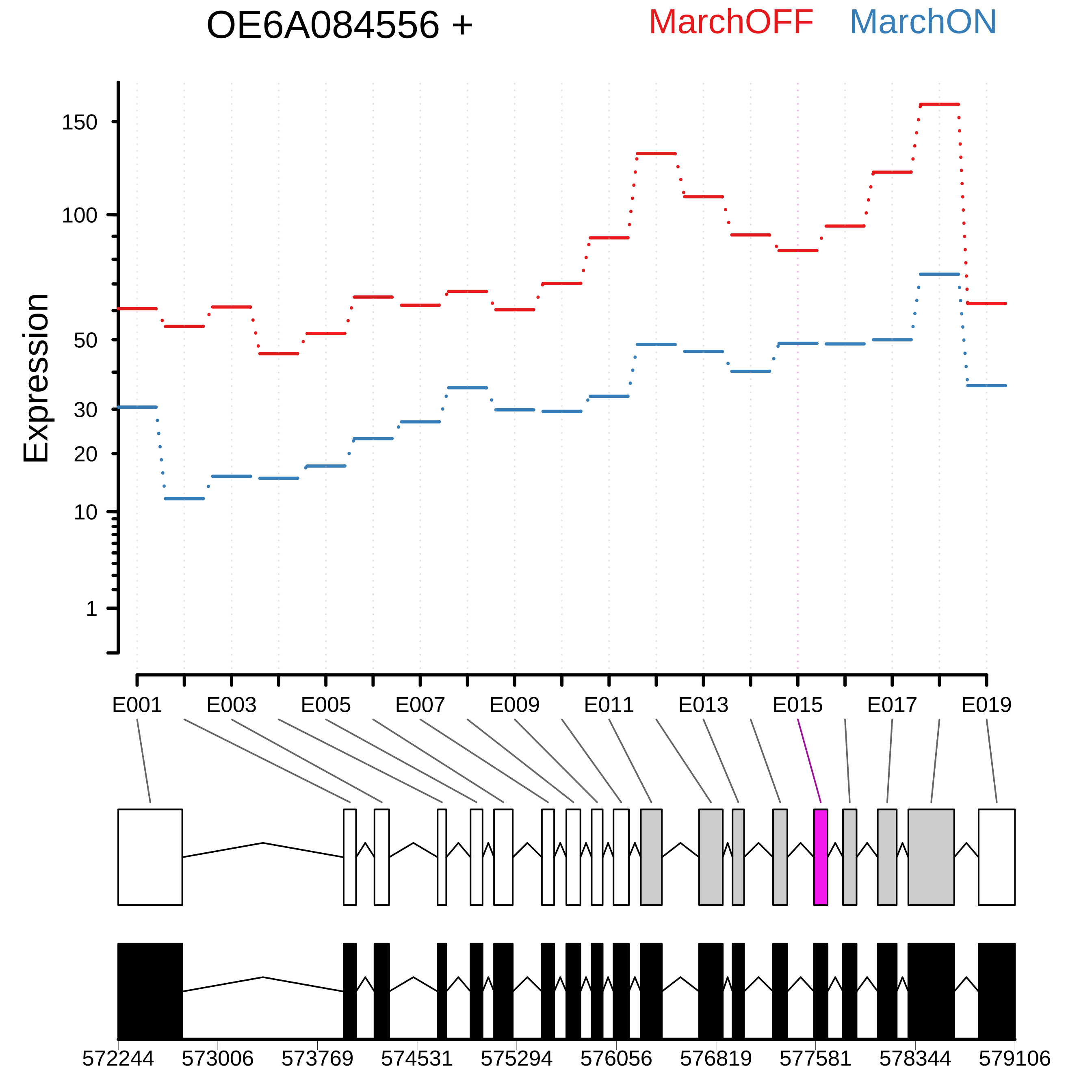

OE6A084556 +
MarchOFF
MarchON
Expression
150
100
50
30
20
10
1
E001
E003
E005
E007
E009
E011
E013
E015
E017
E019
572244
573006
573769
574531
575294
576056
576819
577581
578344
579106

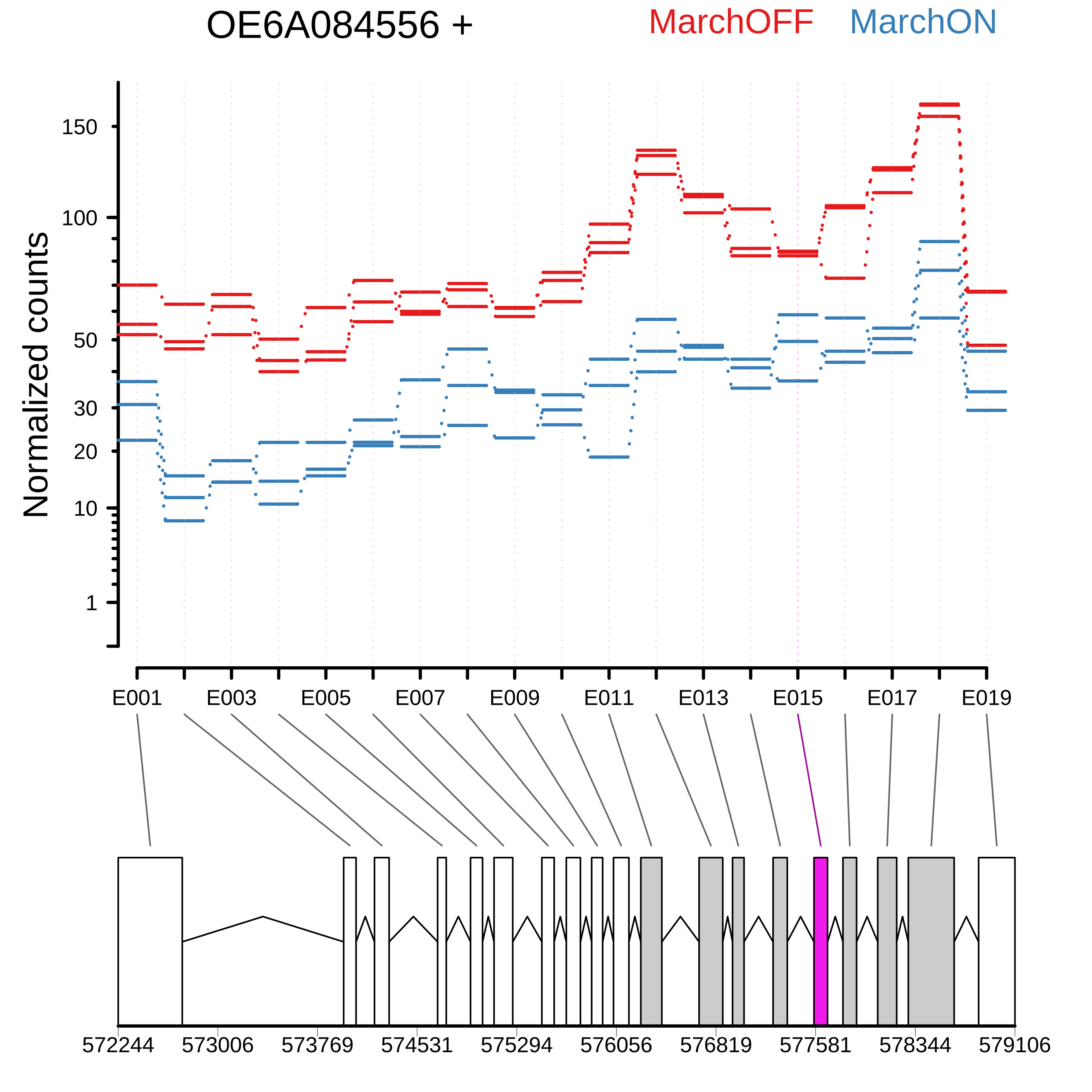

OE6A084556 +
MarchOFF
MarchON
Normalized counts
150
100
50
30
20
10
1
E001
E003
E005
E007
E009
E011
E013
E015
E017
E019
572244
573006
573769
574531
575294
576056
576819
577581
578344
579106

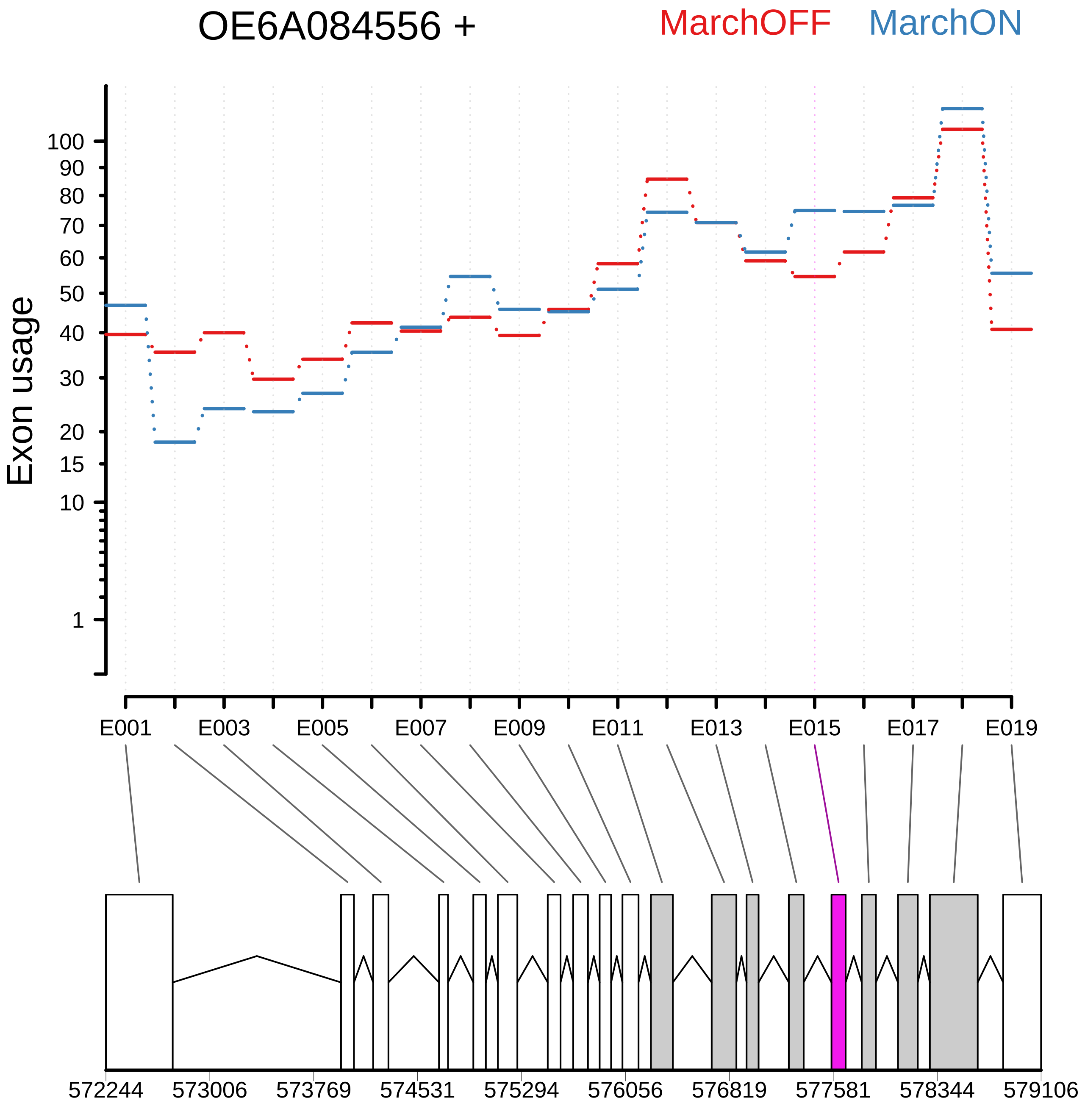

OE6A084556 +
MarchOFF
MarchON
Exon usage
100
90
80
70
60
50
40
30
20
15
10
1
E001
E003
E005
E007
E009
E011
E013
E015
E017
E019
572244
573006
573769
574531
575294
576056
576819
577581
578344
579106

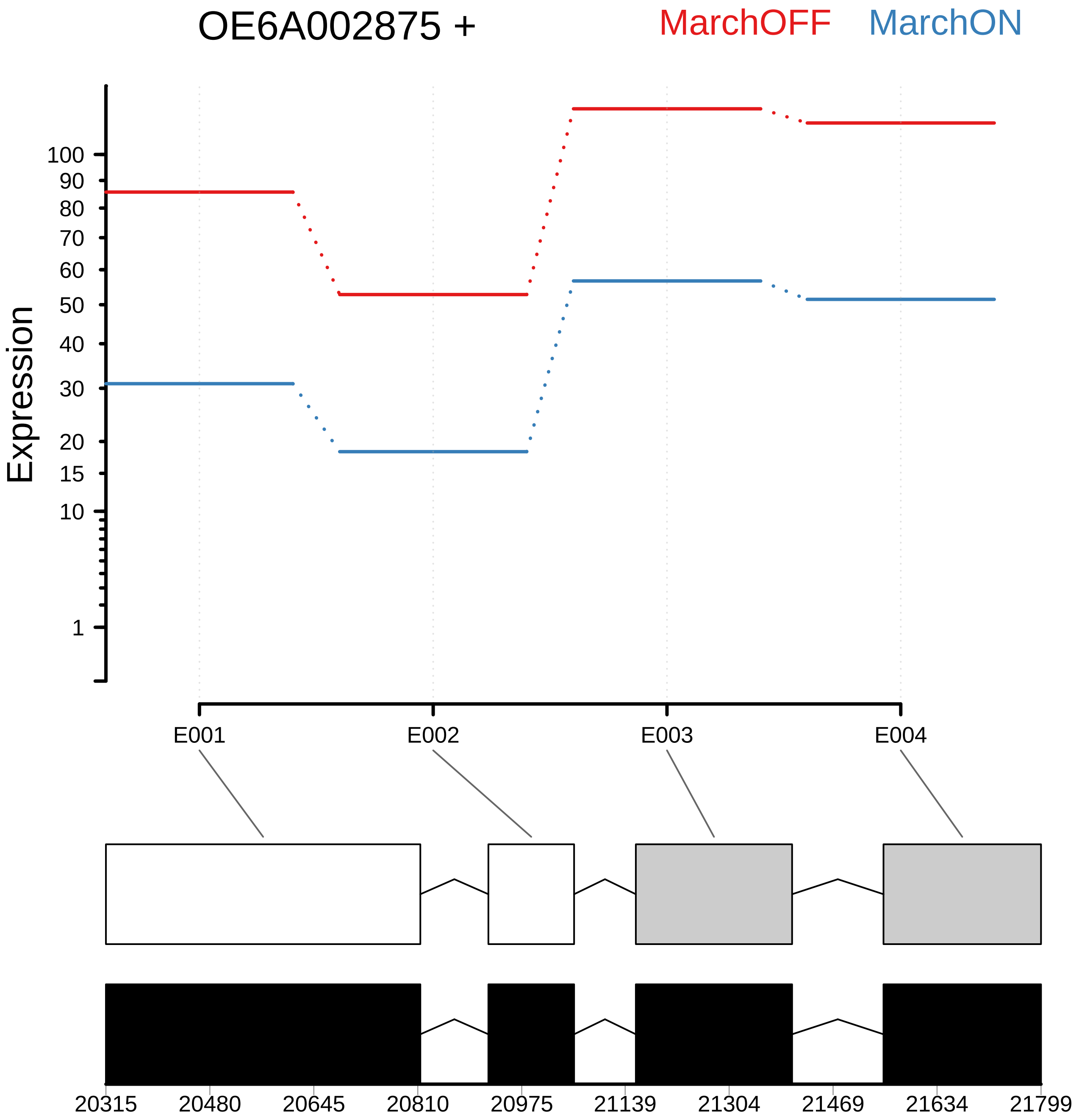

OE6A002875 +
MarchOFF
MarchON
Expression
100
90
80
70
60
50
40
30
20
15
10
1
E001
E002
E003
E004
20315
20480
20645
20810
20975
21139
21304
21469
21634
21799

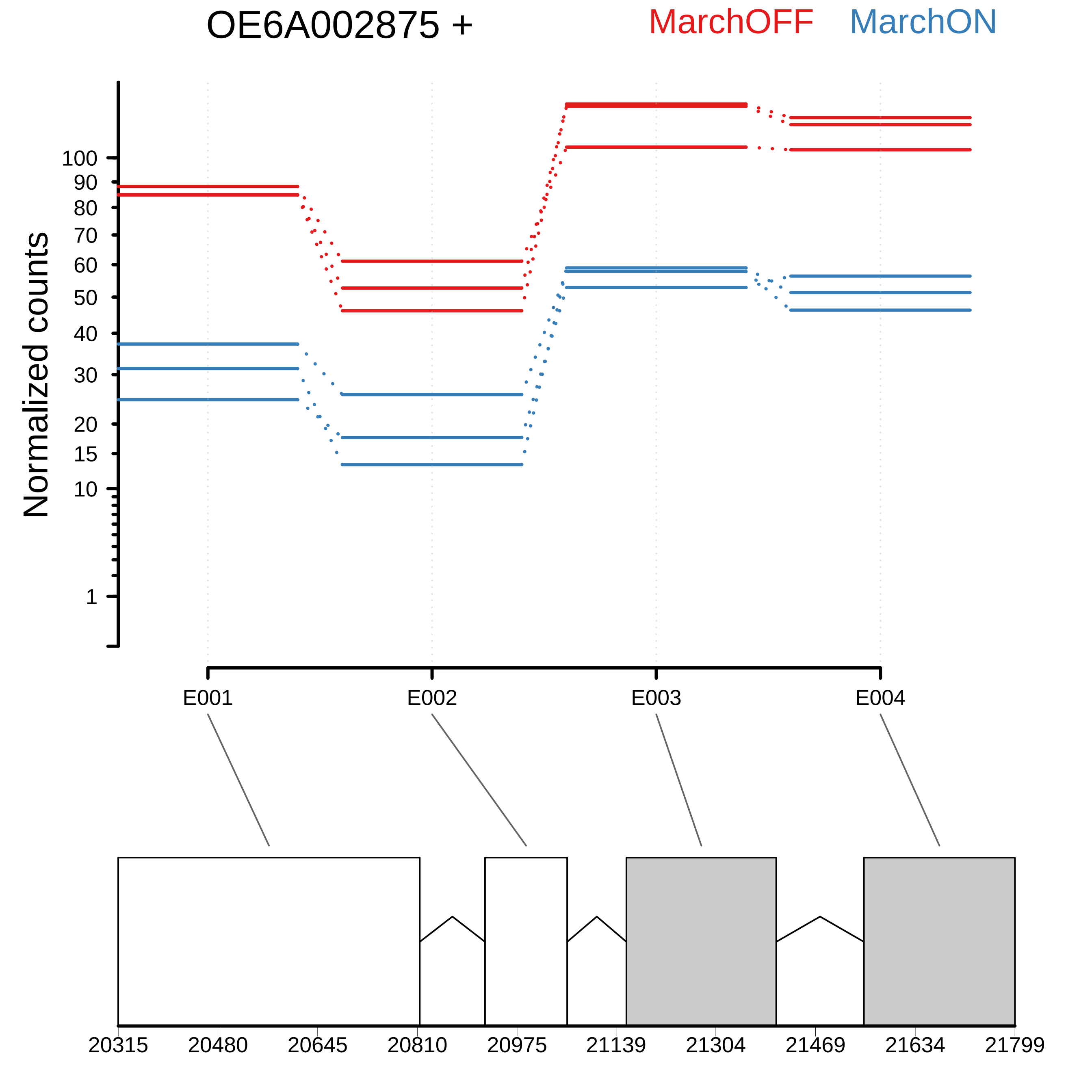

OE6A002875 +
MarchOFF
MarchON
Normalized counts
100
90
80
70
60
50
40
30
20
15
10
1
E001
E002
E003
E004
20315
20480
20645
20810
20975
21139
21304
21469
21634
21799

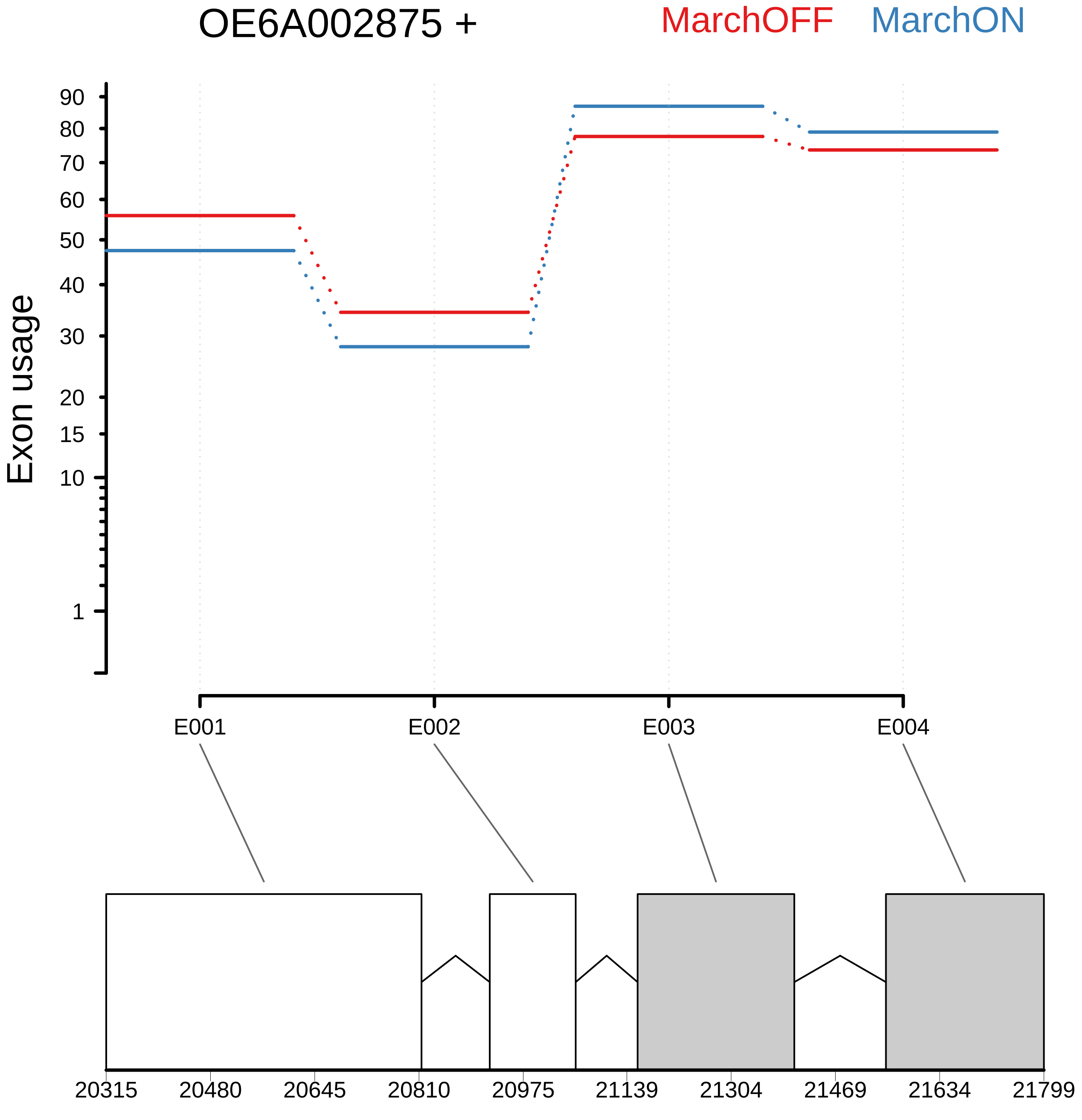

OE6A002875 +
MarchOFF
MarchON
Exon usage
90
80
70
60
50
40
30
20
15
10
1
E001
E002
E003
E004
20315
20480
20645
20810
20975
21139
21304
21469
21634
21799

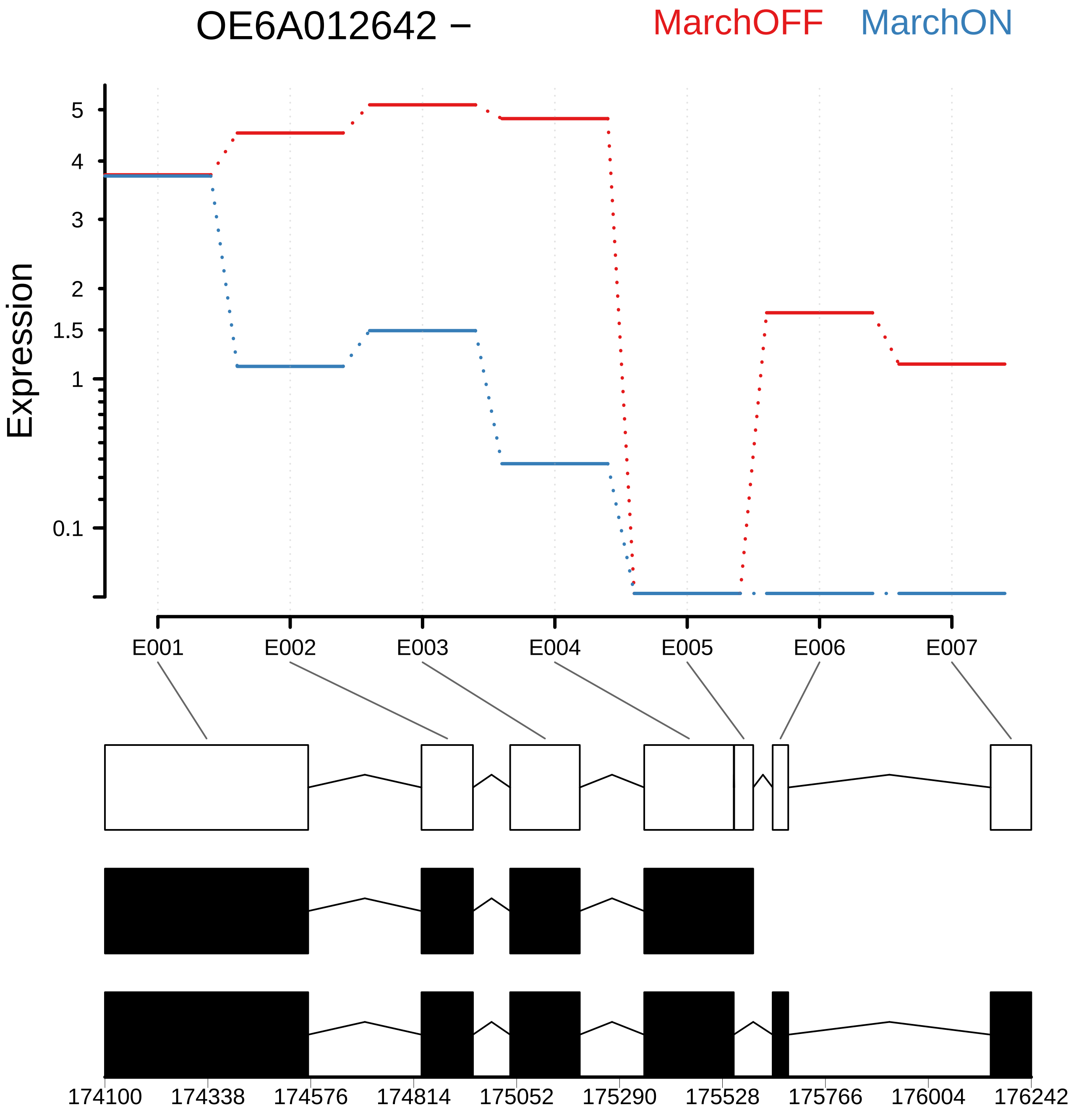

OE6A012642 –
MarchOFF
MarchON
Expression
5
4
3
2
1.5
1
0.1
E001
E002
E003
E004
E005
E006
E007
174100
174338
174576
174814
175052
175290
175528
175766
176004
176242

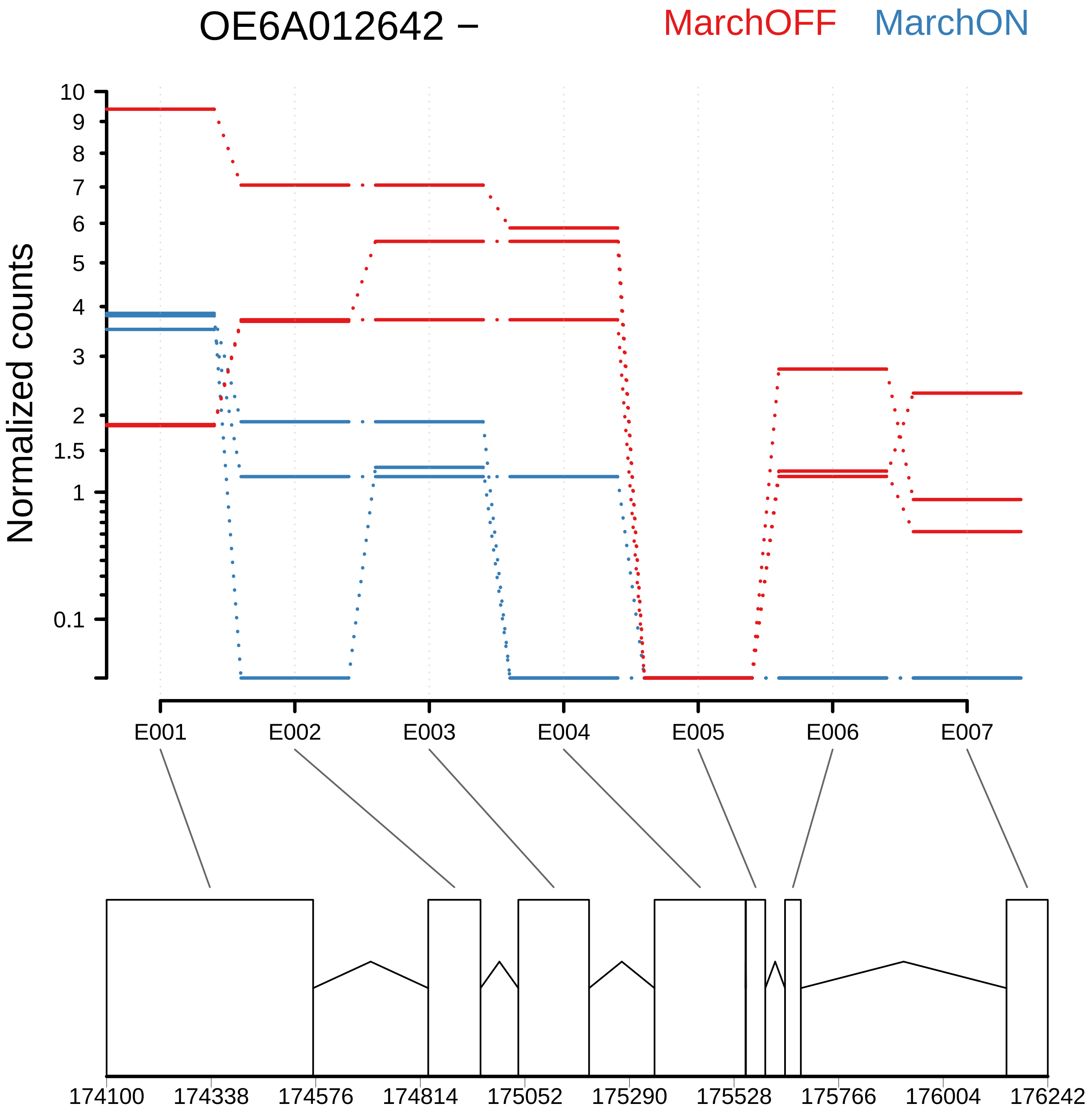

OE6A012642 −
MarchOFF
MarchON
Normalized counts
10
9
8
7
6
5
4
3
2
1.5
1
0.1
E001
E002
E003
E004
E005
E006
E007
174100
174338
174576
174814
175052
175290
175528
175766
176004
176242

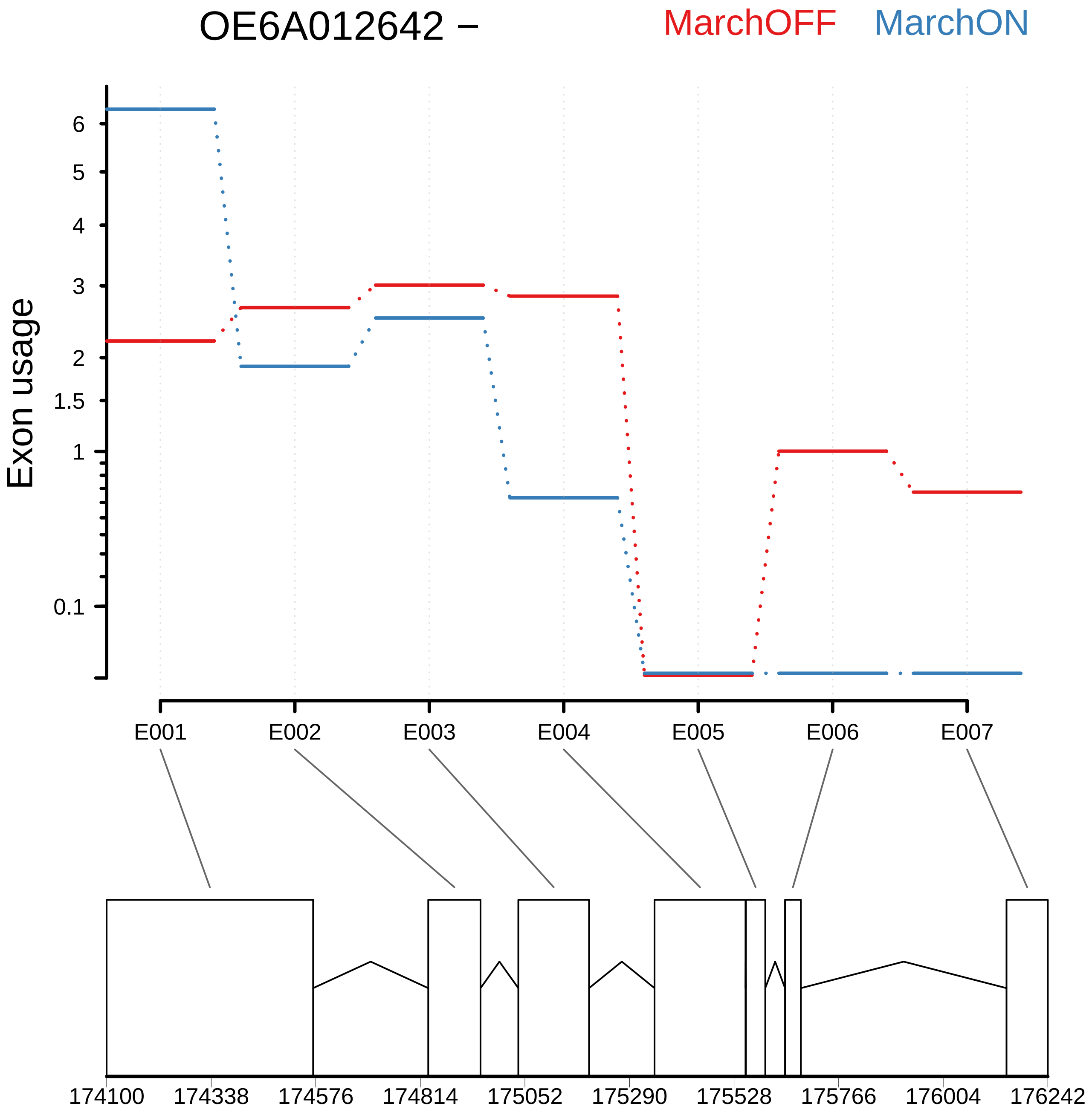
OE6A012642 −
MarchOFF
MarchON
Exon usage
6
5
4
3
2
1.5
1
0.1
E001
E002
E003
E004
E005
E006
E007
174100
174338
174576
174814
175052
175290
175528
175766
176004
176242

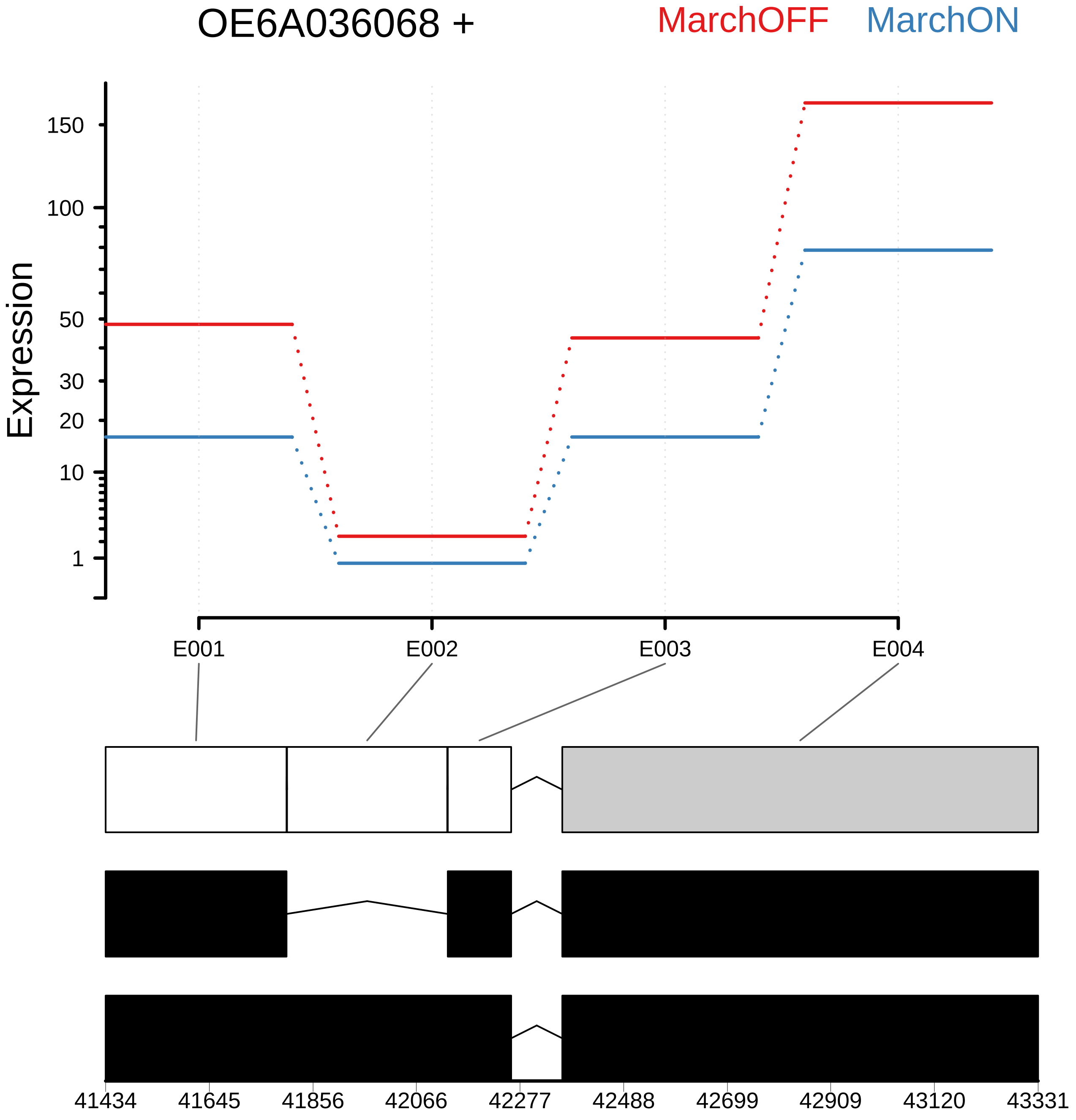

OE6A036068 +
MarchOFF
MarchON
Expression
150
100
50
30
20
10
1
E001
E002
E003
E004
41434
41645
41856
42066
42277
42488
42699
42909
43120
43331

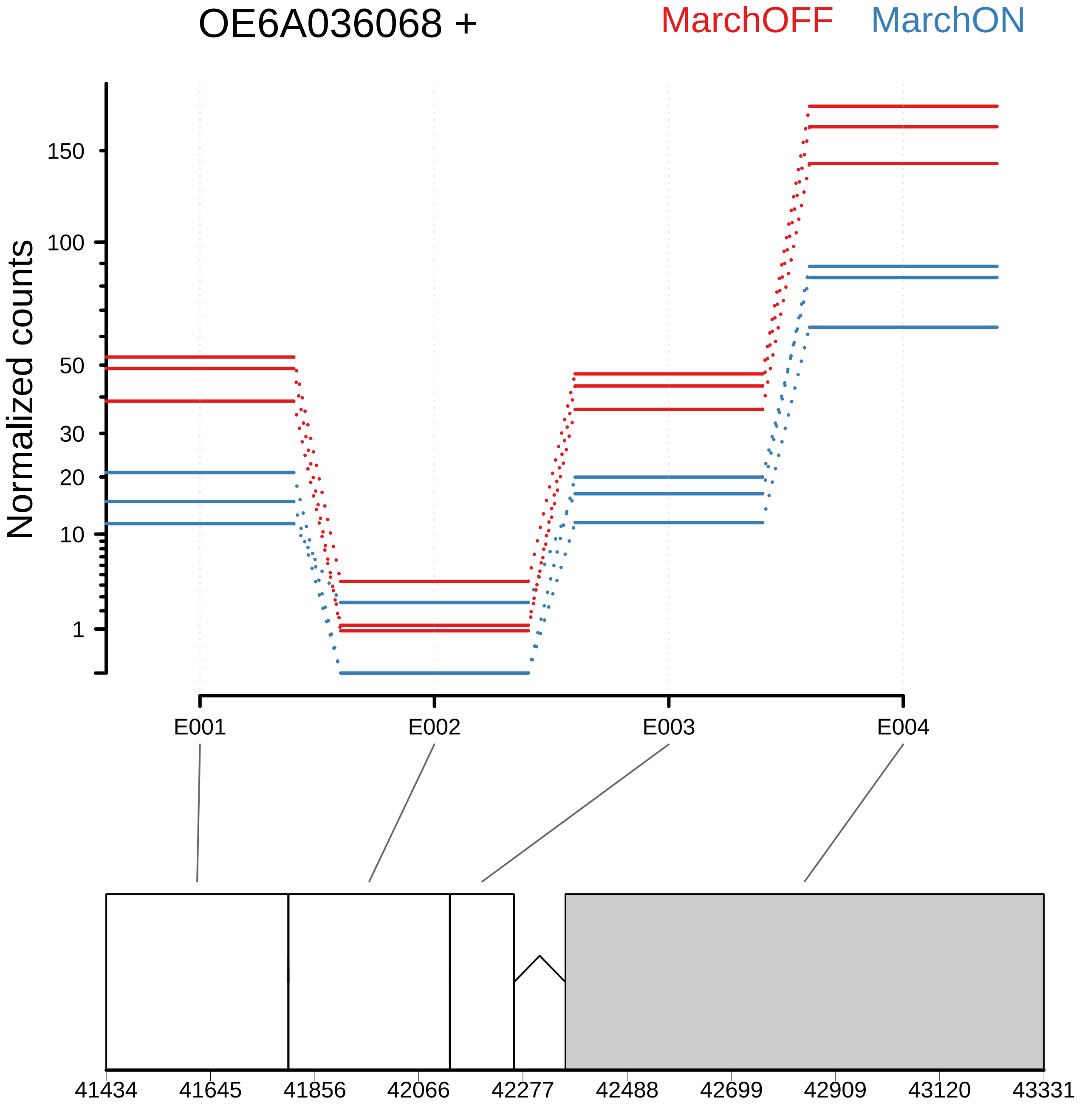

OE6A036068 +
MarchOFF
MarchON
Normalized counts
150
100
50
30
20
10
1
E001
E002
E003
E004
41434
41645
41856
42066
42277
42488
42699
42909
43120
43331

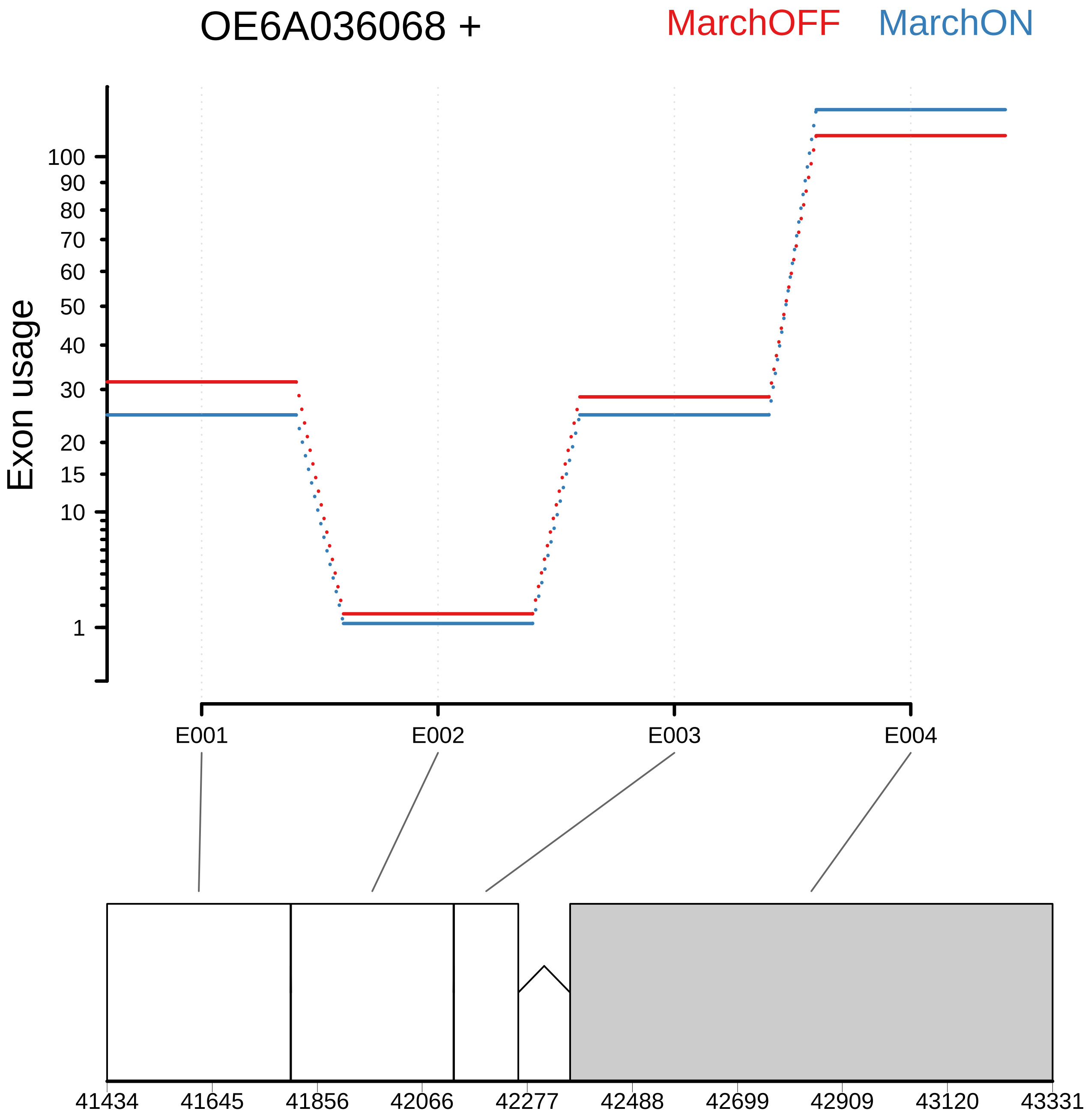

OE6A036068 +
MarchOFF
MarchON
Exon usage
100
90
80
70
60
50
40
30
20
15
10
1
E001
E002
E003
E004
41434
41645
41856
42066
42277
42488
42699
42909
43120
43331

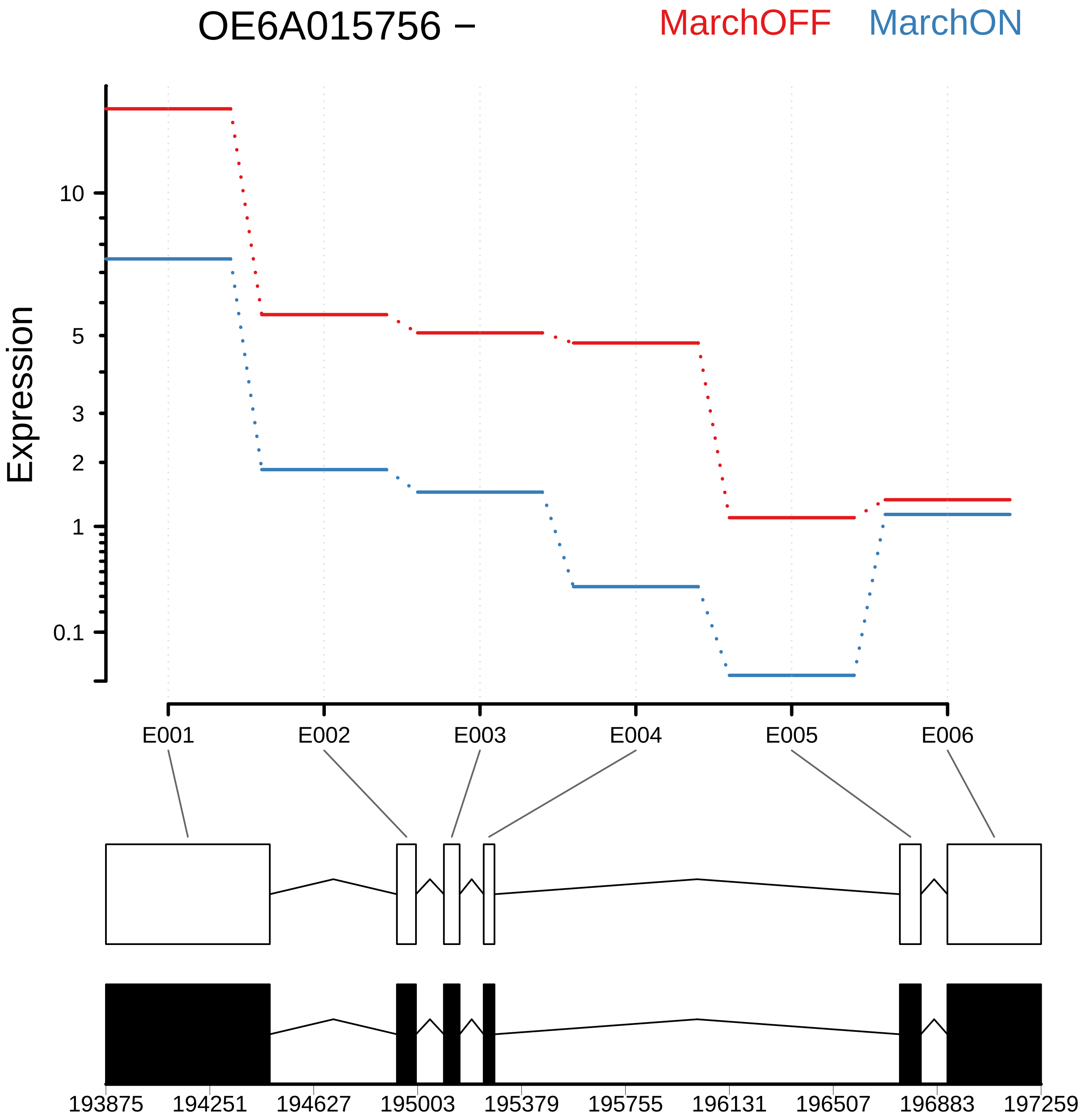

OE6A015756 −
MarchOFF
MarchON
Expression
10
5
3
2
1
0.1
E001
E002
E003
E004
E005
E006
193875
194251
194627
195003
195379
195755
196131
196507
196883
197259

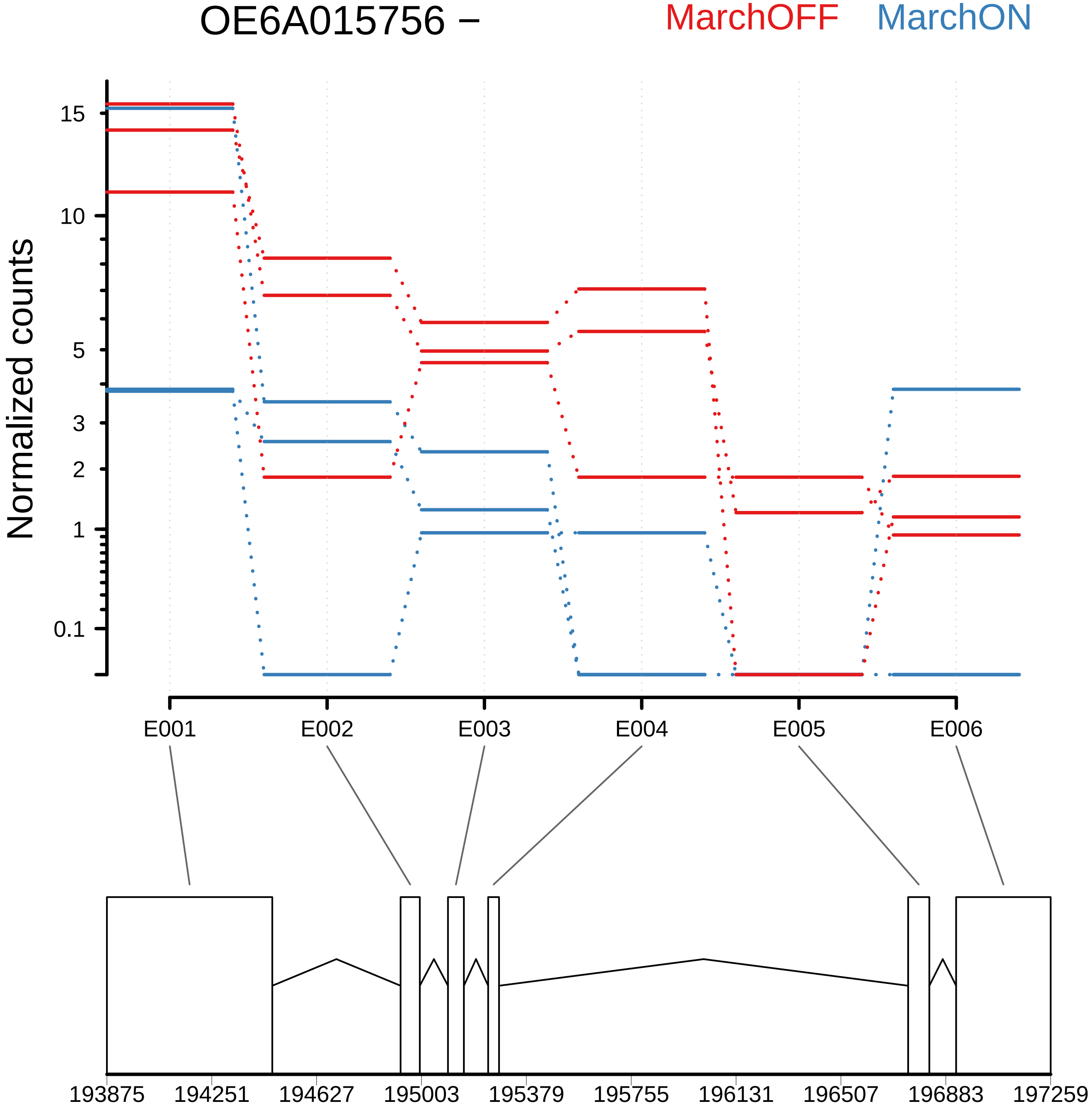
OE6A015756 −
MarchOFF
MarchON
Normalized counts
15
10
5
3
2
1
0.1
E001
E002
E003
E004
E005
E006
193875
194251
194627
195003
195379
195755
196131
196507
196883
197259

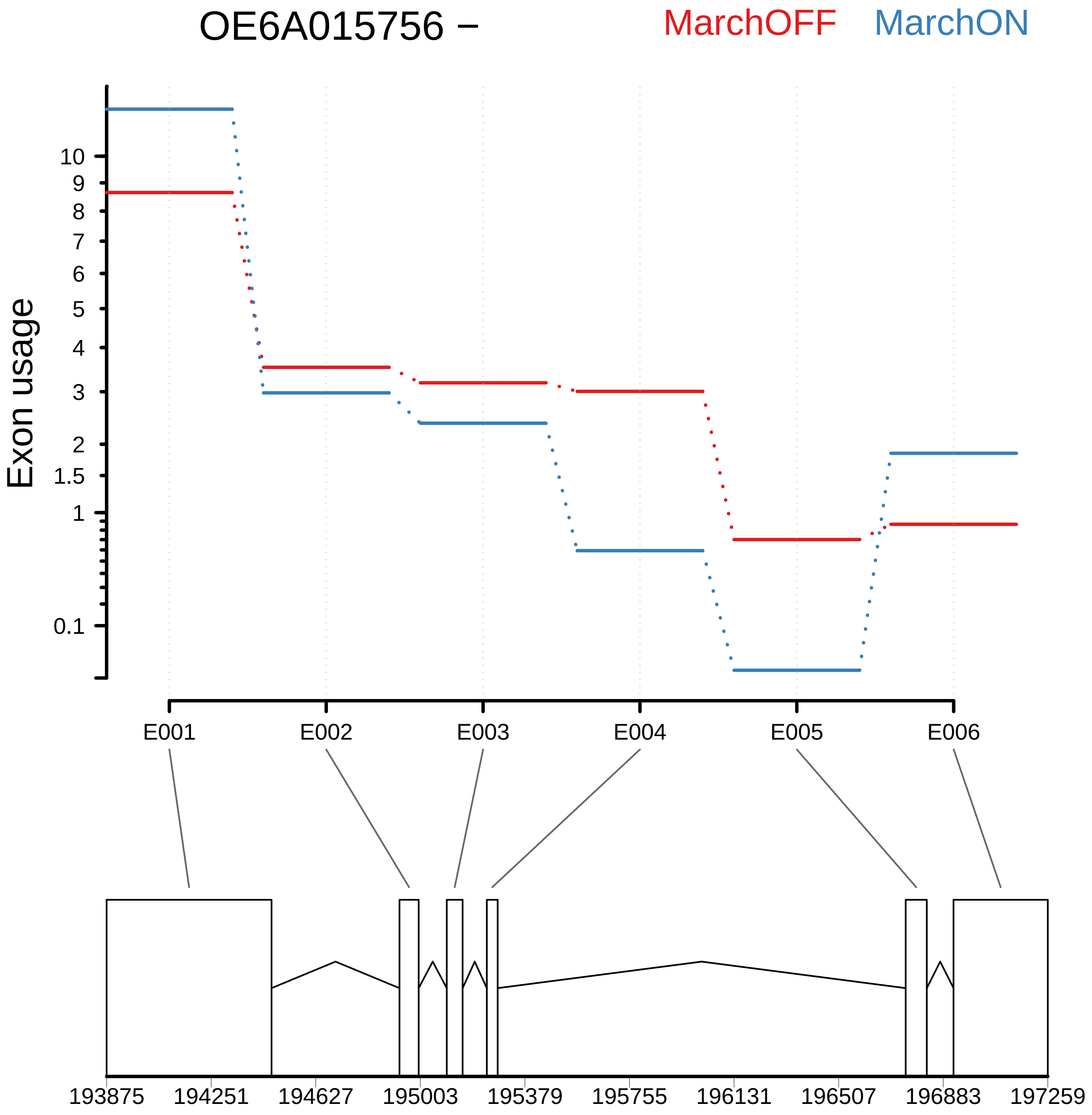

OE6A015756 −
MarchOFF
MarchON
Exon usage
10
9
8
7
6
5
4
3
2
1.5
1
0.1
E001
E002
E003
E004
E005
E006
193875
194251
194627
195003
195379
195755
196131
196507
196883
197259

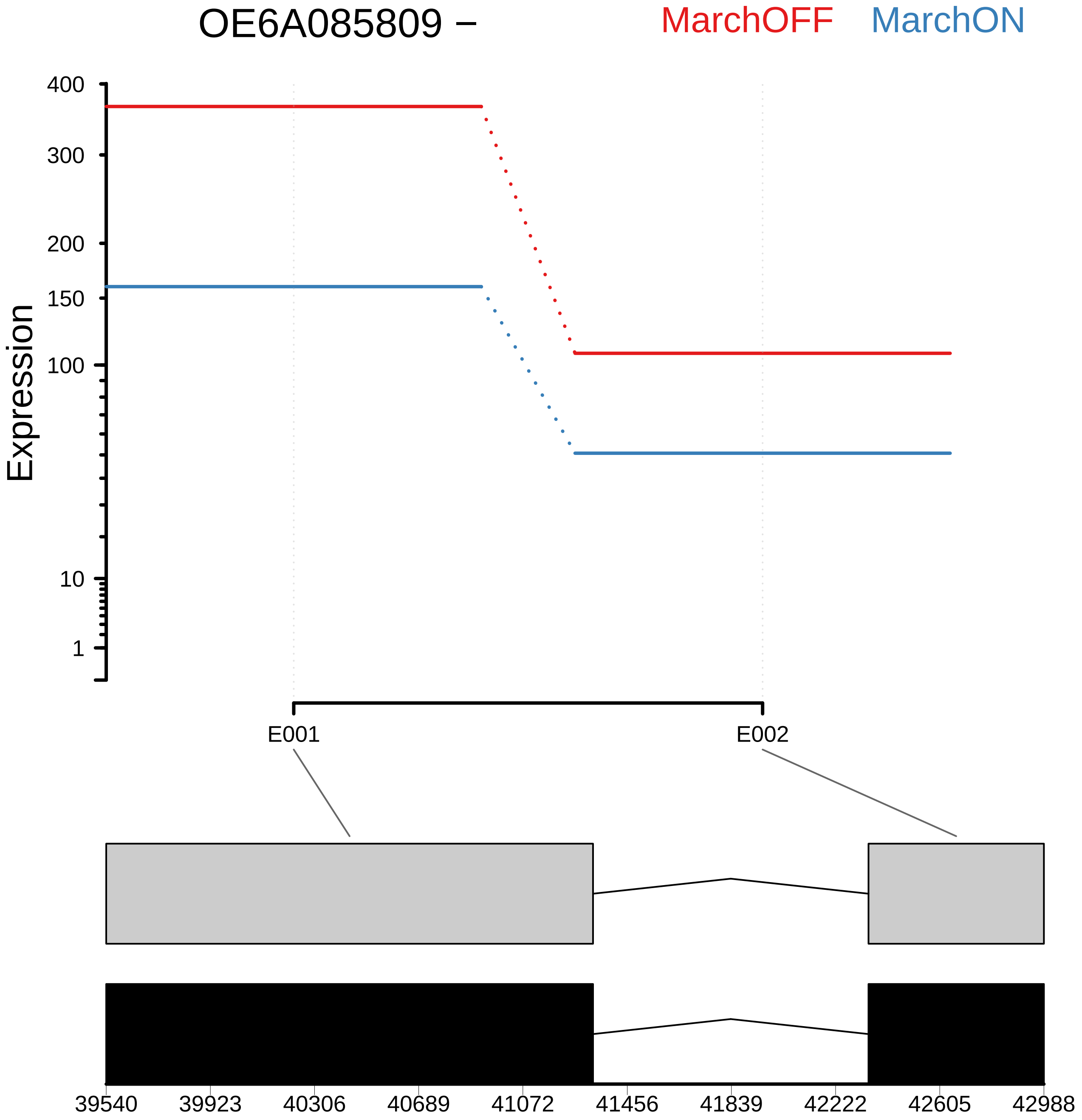

OE6A085809 −
MarchOFF
MarchON
Expression
400
300
200
150
100
10
1
E001
E002
39540
39923
40306
40689
41072
41456
41839
42222
42605
42988

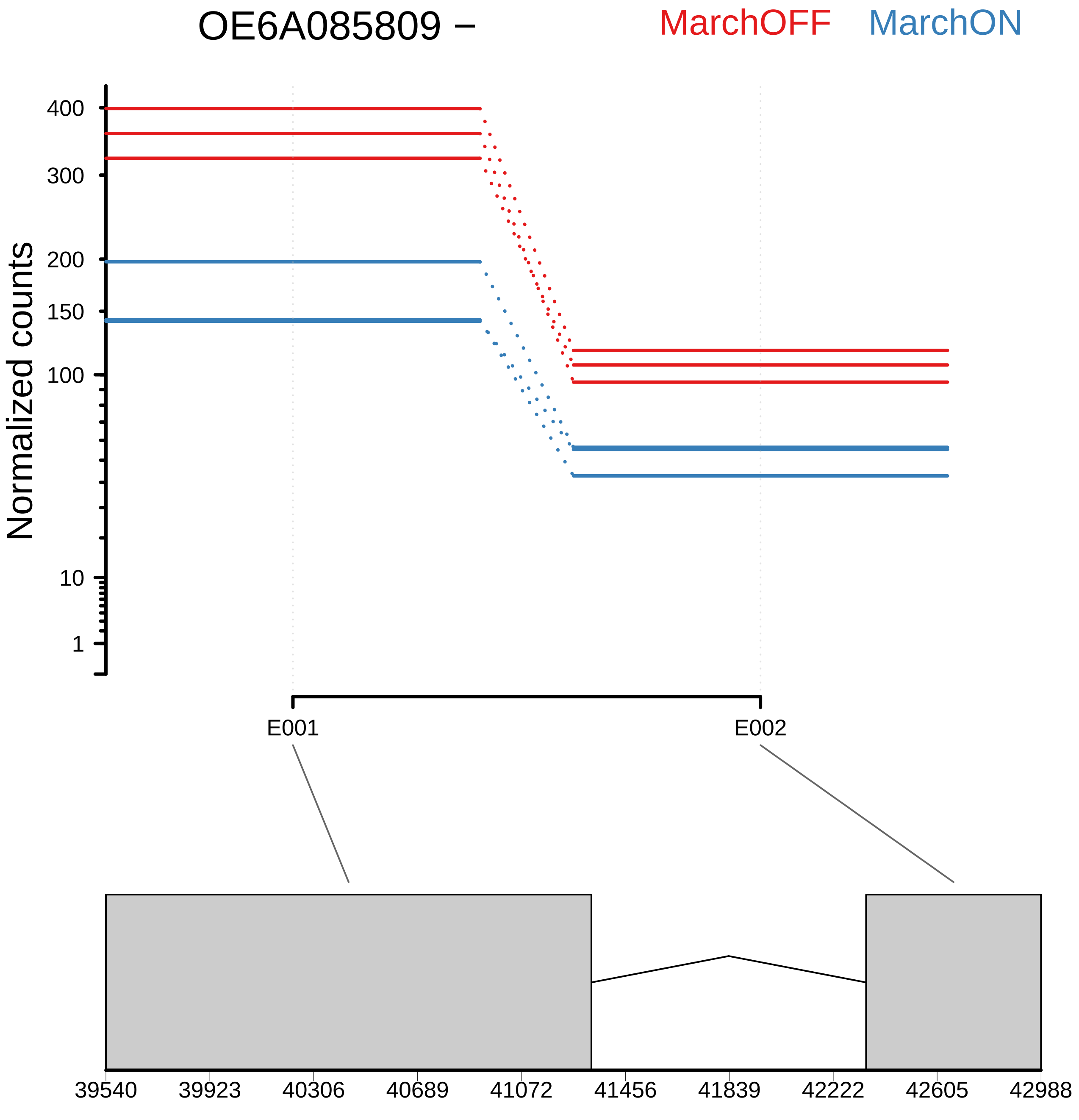

OE6A085809 −
MarchOFF
MarchON
Normalized counts
400
300
200
150
100
10
1
E001
E002
39540
39923
40306
40689
41072
41456
41839
42222
42605
42988

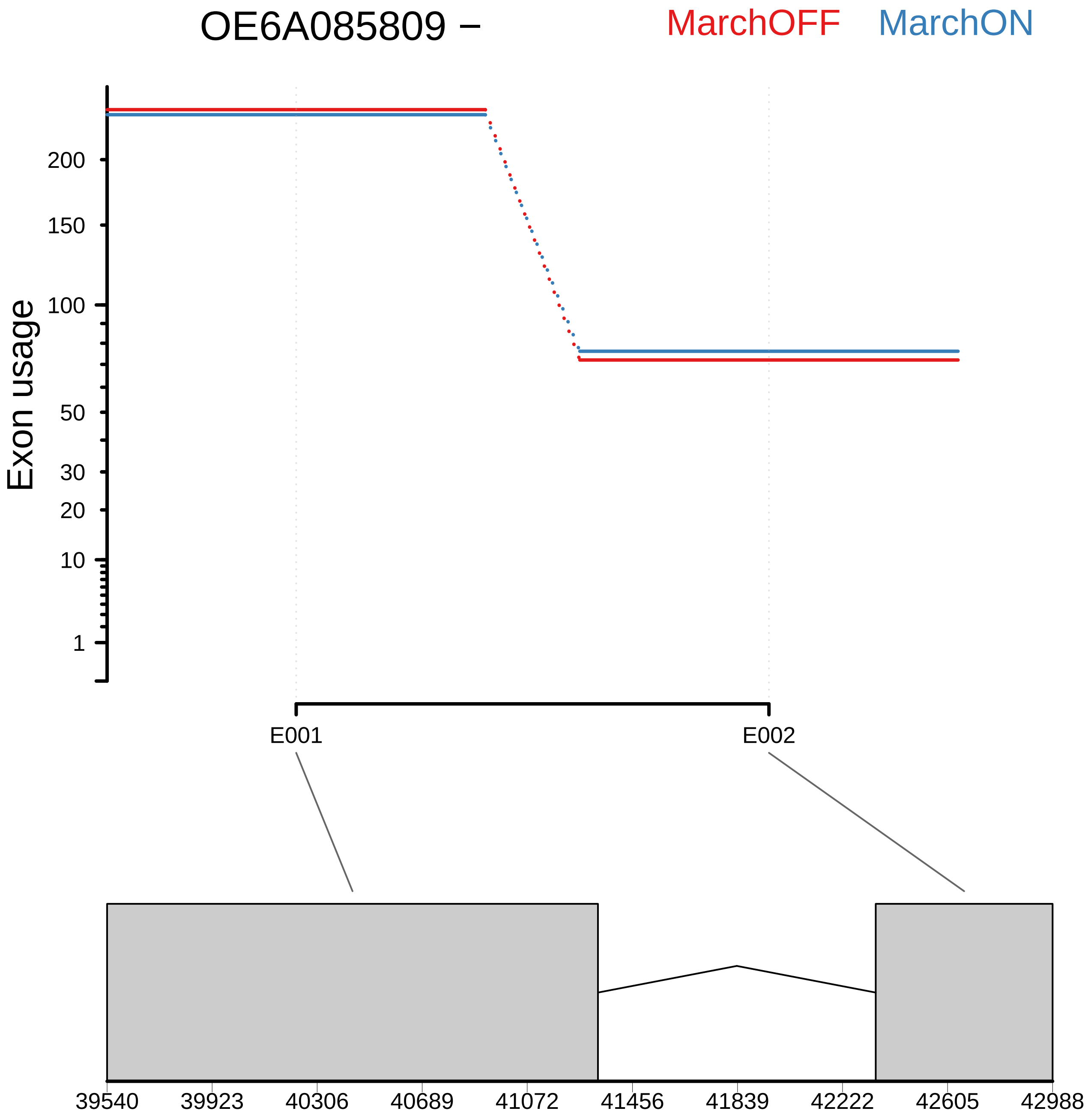

OE6A085809 −
MarchOFF
MarchON
Exon usage
200
150
100
50
30
20
10
1
E001
E002
39540
39923
40306
40689
41072
41456
41839
42222
42605
42988

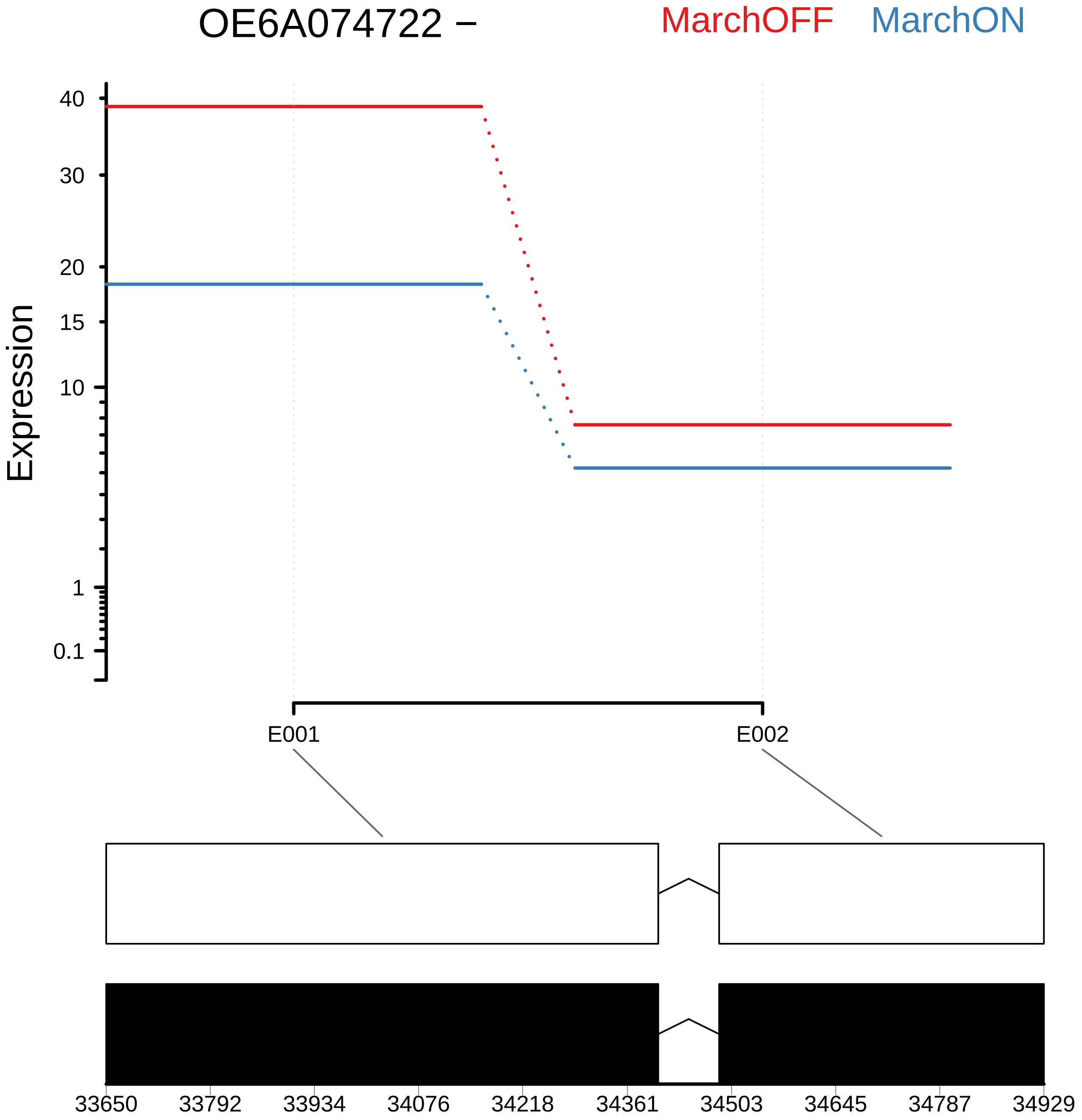
OE6A074722 −
MarchOFF
MarchON
Expression
40
30
20
15
10
1
0.1
E001
E002
33650
33792
33934
34076
34218
34361
34503
34645
34787
34929

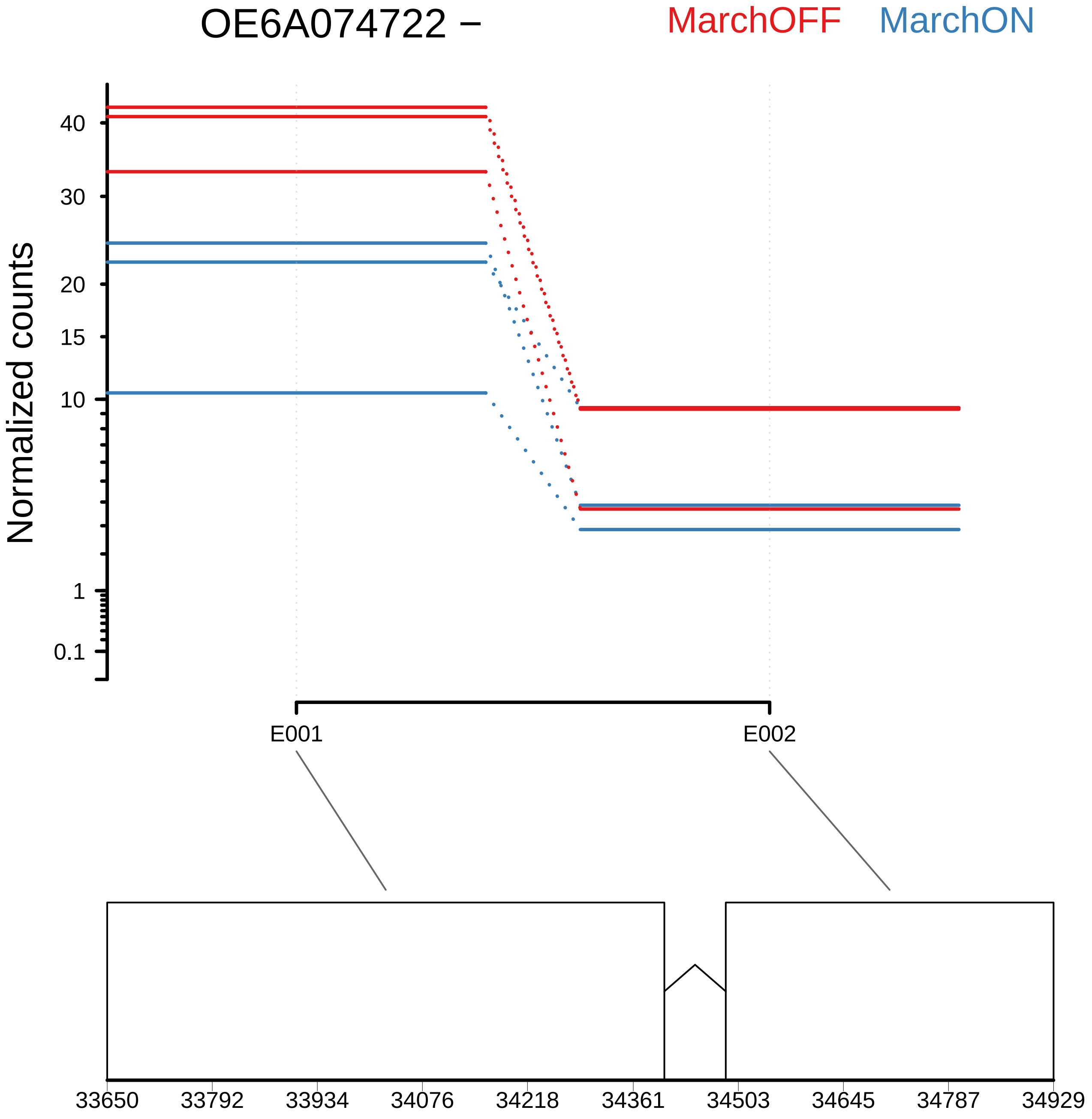

OE6A074722 −
MarchOFF
MarchON
Normalized counts
40
30
20
15
10
1
0.1
E001
E002
33650
33792
33934
34076
34218
34361
34503
34645
34787
34929

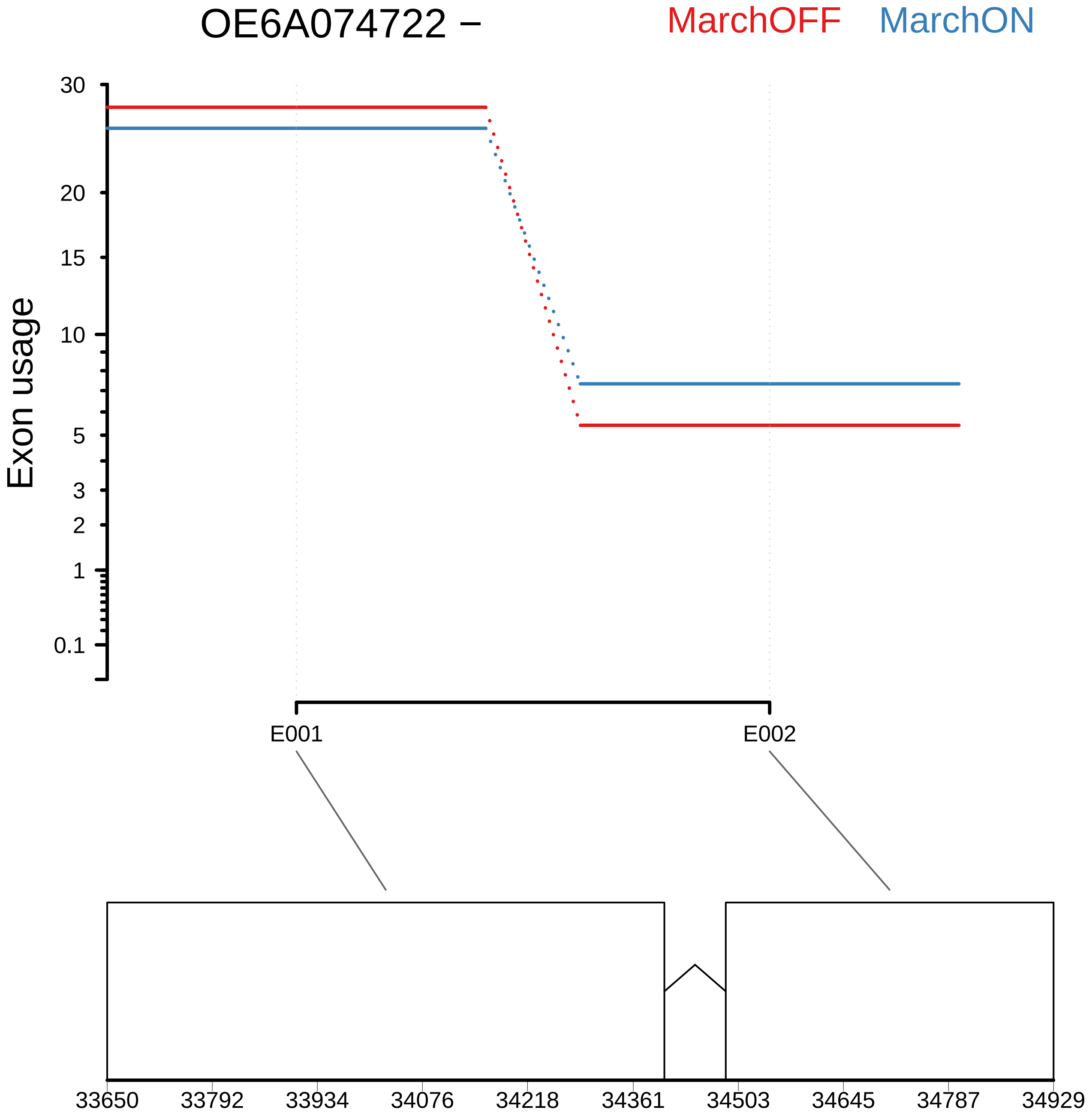

OE6A074722 −
MarchOFF
MarchON
Exon usage
30
20
15
10
5
3
2
1
0.1
E001
E002
33650
33792
33934
34076
34218
34361
34503
34645
34787
34929

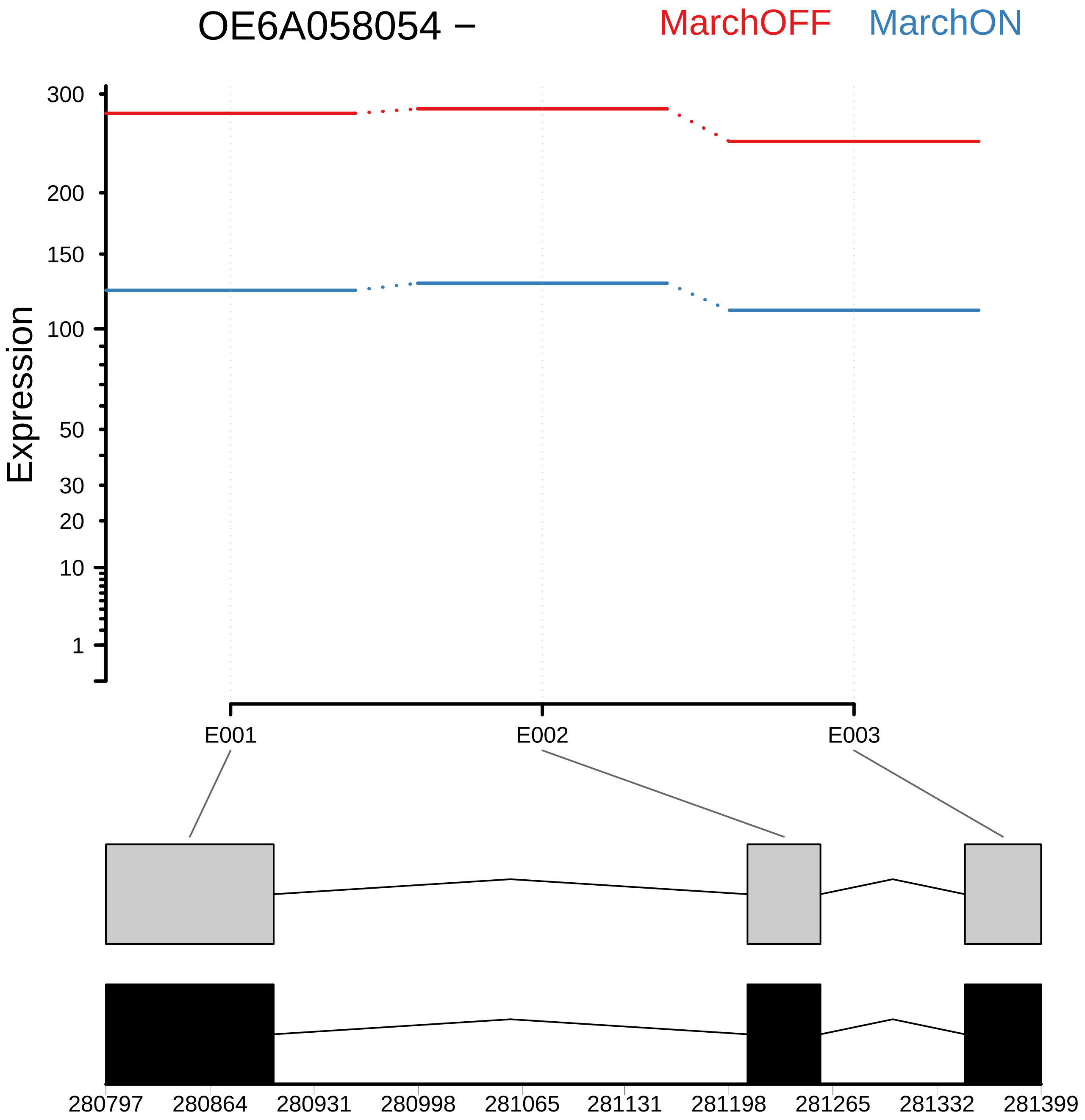

OE6A058054 −
MarchOFF
MarchON
Expression
300
200
150
100
50
30
20
10
1
E001
E002
E003
280797
280864
280931
280998
281065
281131
281198
281265
281332
281399

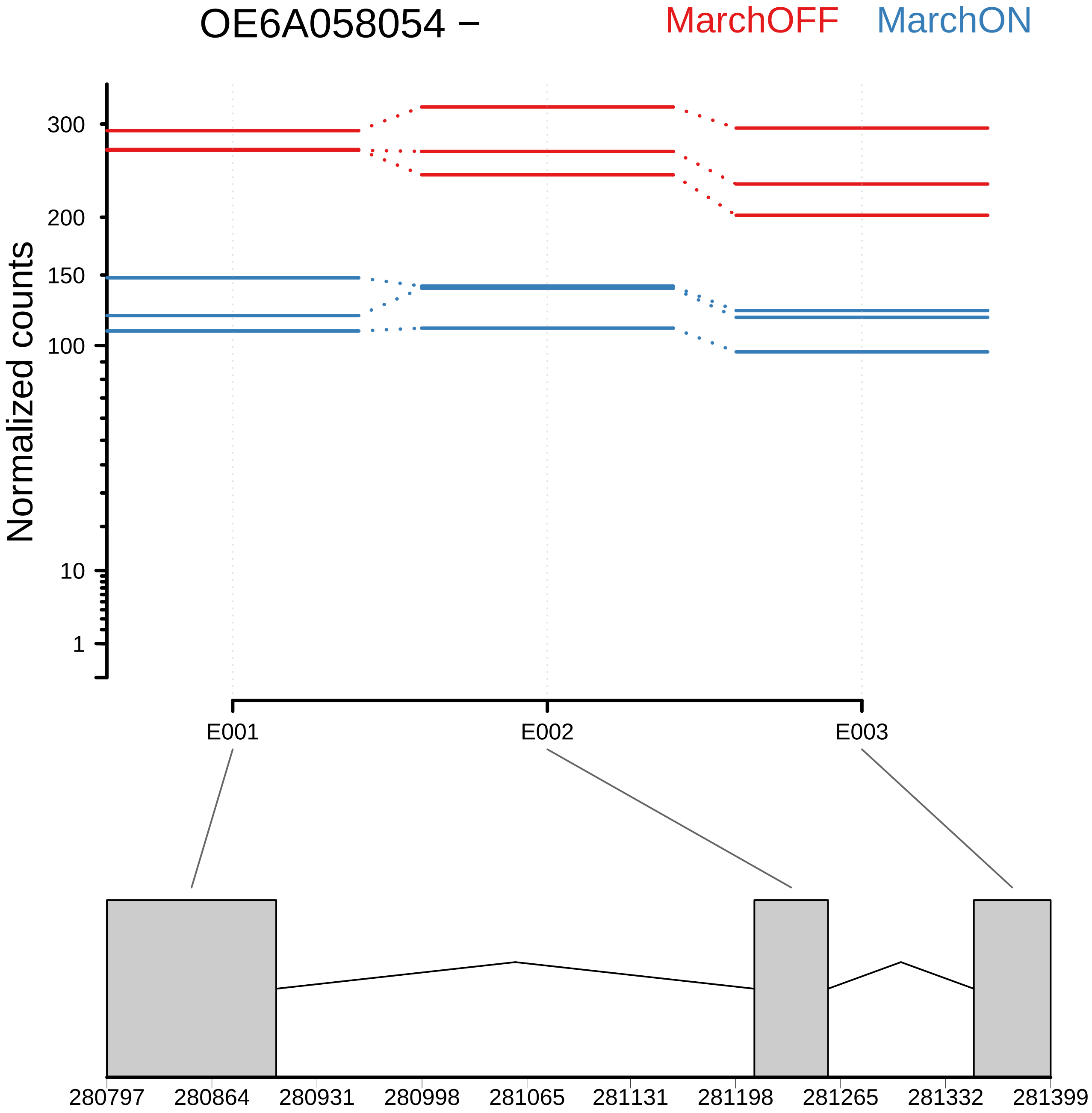

OE6A058054 −
MarchOFF
MarchON
Normalized counts
300
200
150
100
10
1
E001
E002
E003
280797
280864
280931
280998
281065
281131
281198
281265
281332
281399

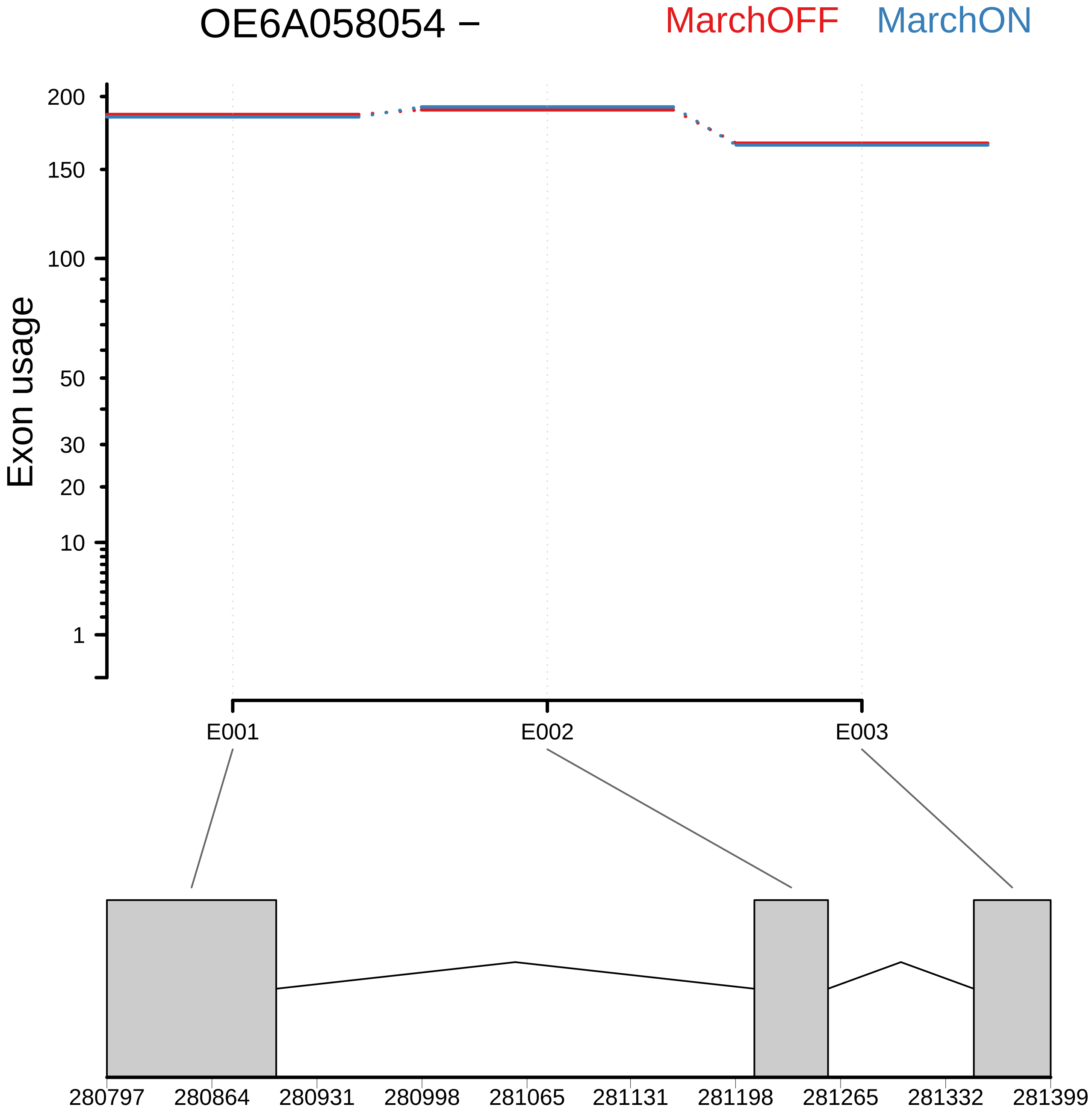
OE6A058054 −
MarchOFF
MarchON
Exon usage
200
150
100
50
30
20
10
1
E001
E002
E003
280797
280864
280931
280998
281065
281131
281198
281265
281332
281399

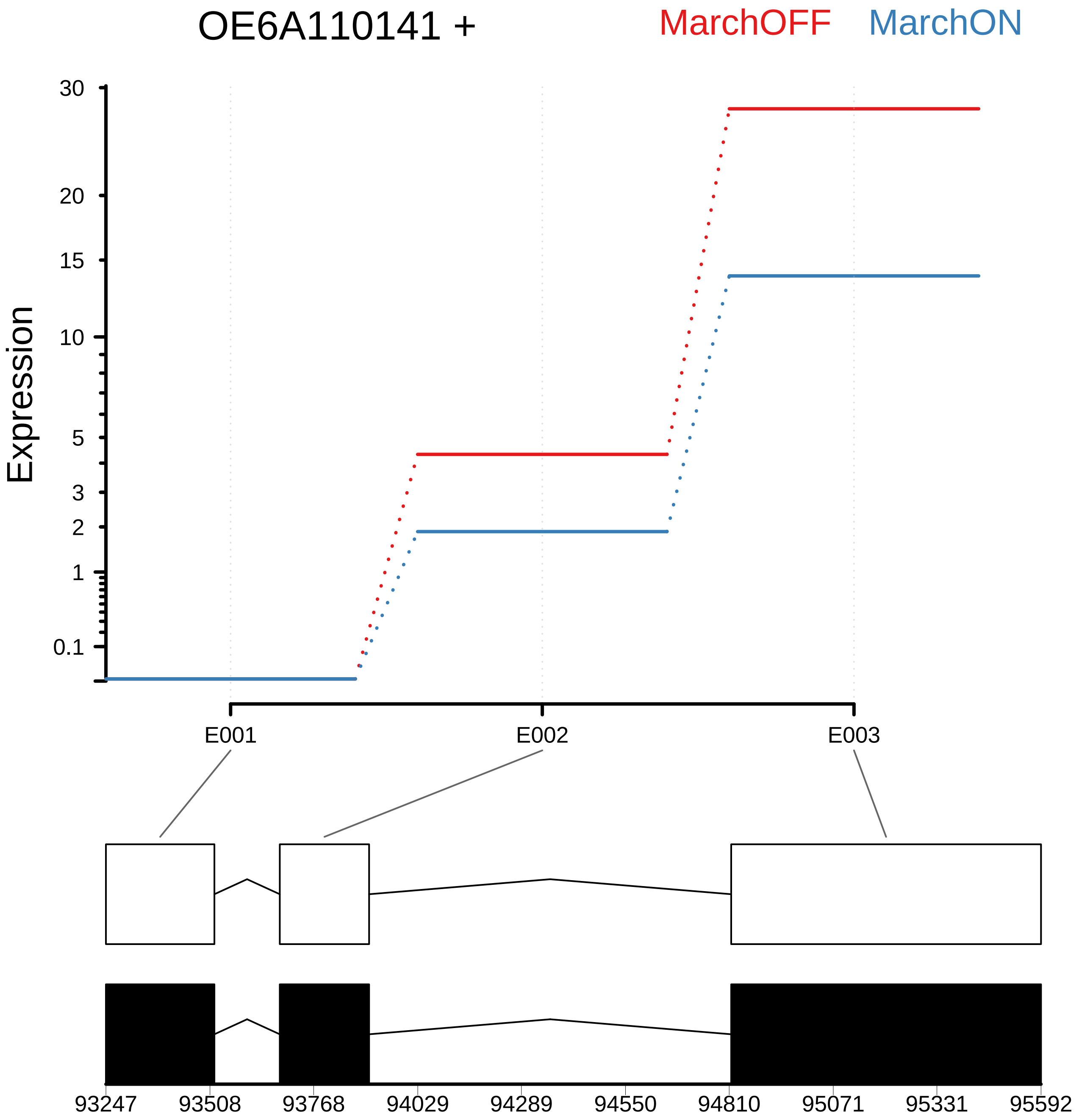
OE6A110141 +
MarchOFF
MarchON
Expression
30
20
15
10
5
3
2
1
0.1
E001
E002
E003
93247
93508
93768
94029
94289
94550
94810
95071
95331
95592

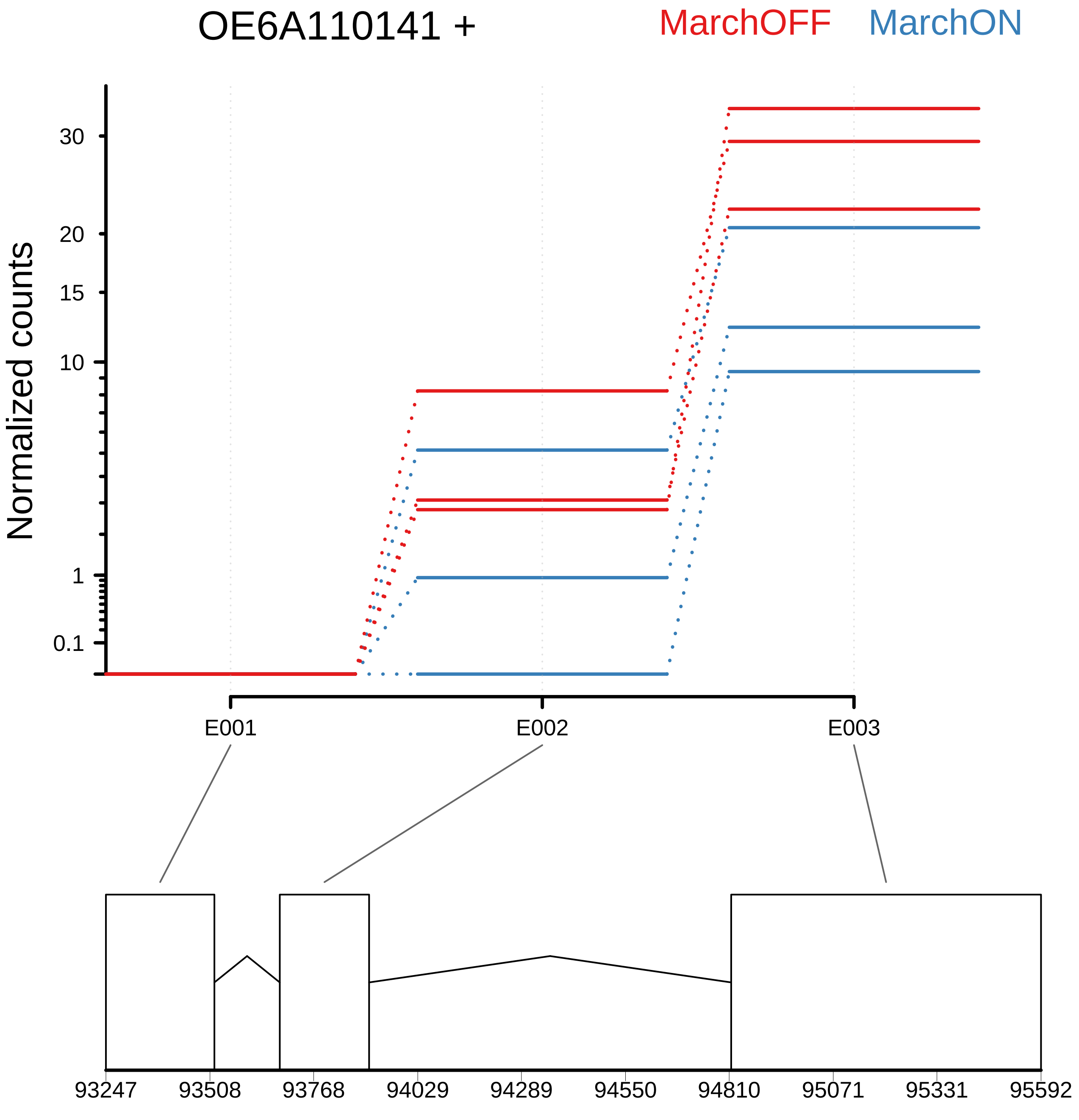

OE6A110141 +
MarchOFF
MarchON
Normalized counts
30
20
15
10
1
0.1
E001
E002
E003
93247
93508
93768
94029
94289
94550
94810
95071
95331
95592

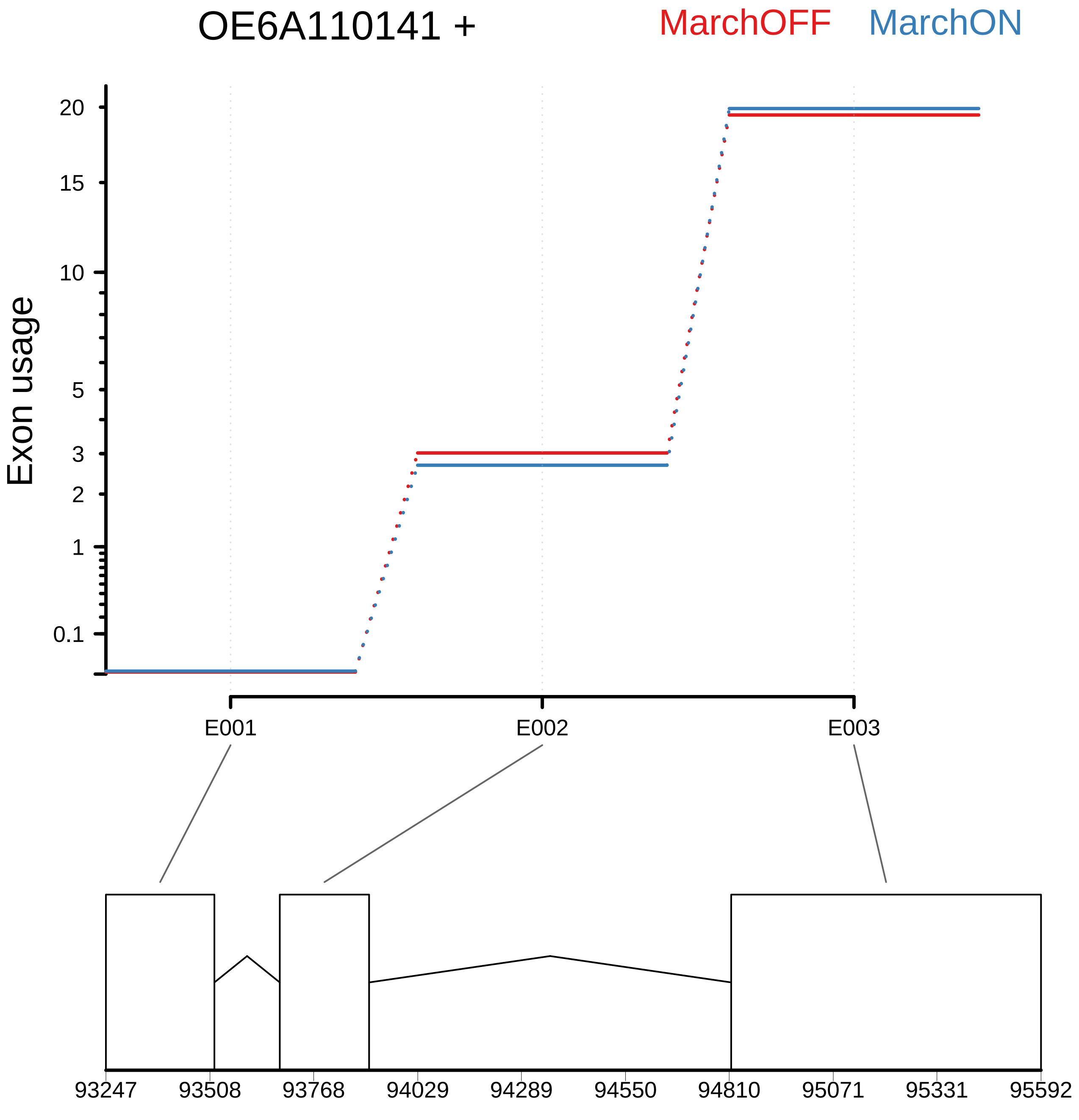
OE6A110141 +
MarchOFF
MarchON
Exon usage
20
15
10
5
3
2
1
0.1
E001
E002
E003
93247
93508
93768
94029
94289
94550
94810
95071
95331
95592

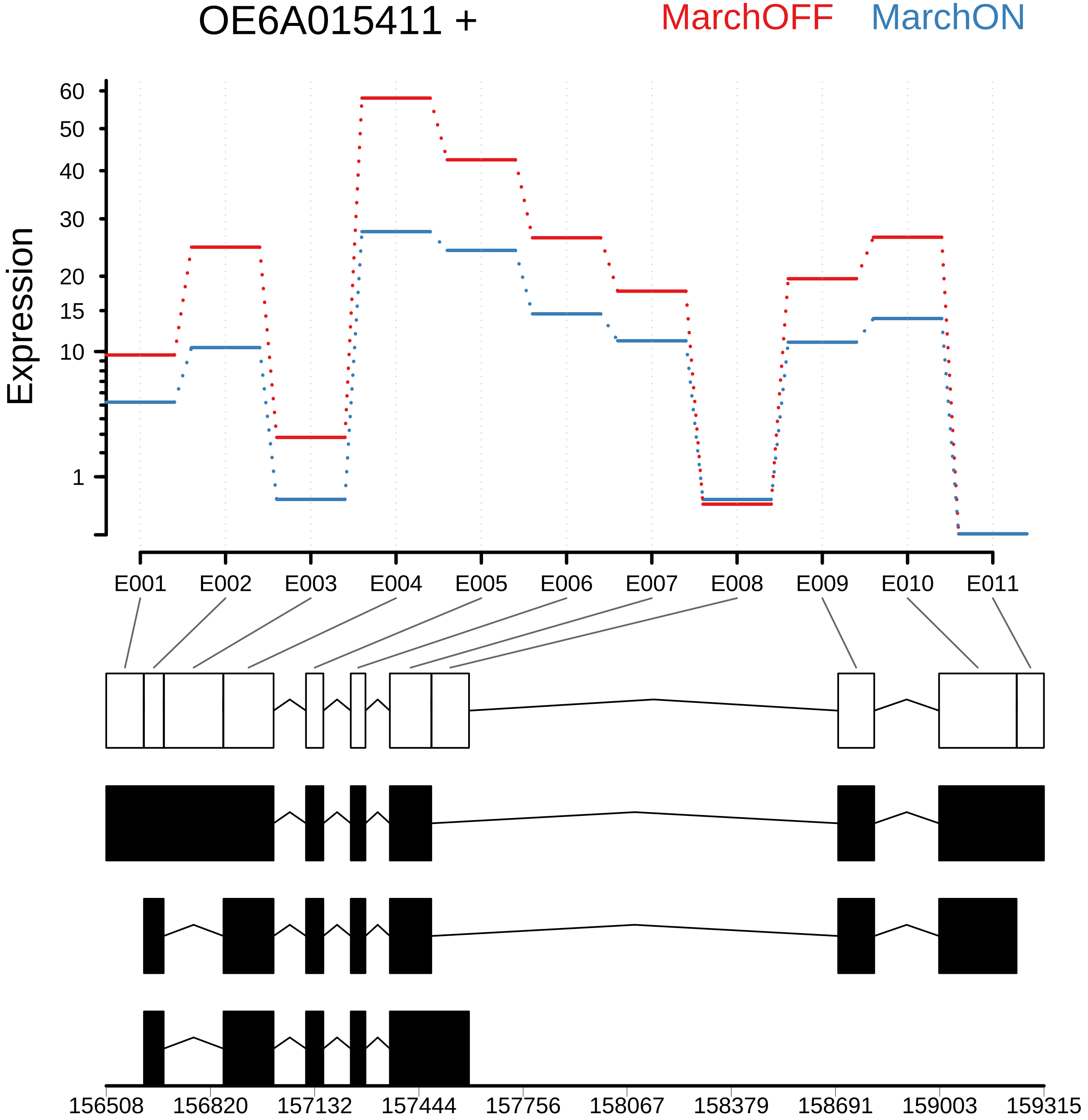
OE6A015411 +
MarchOFF
MarchON
Expression
60
50
40
30
20
15
10
1
E001
E002
E003
E004
E005
E006
E007
E008
E009
E010
E011
156508
156820
157132
157444
157756
158067
158379
158691
159003
159315

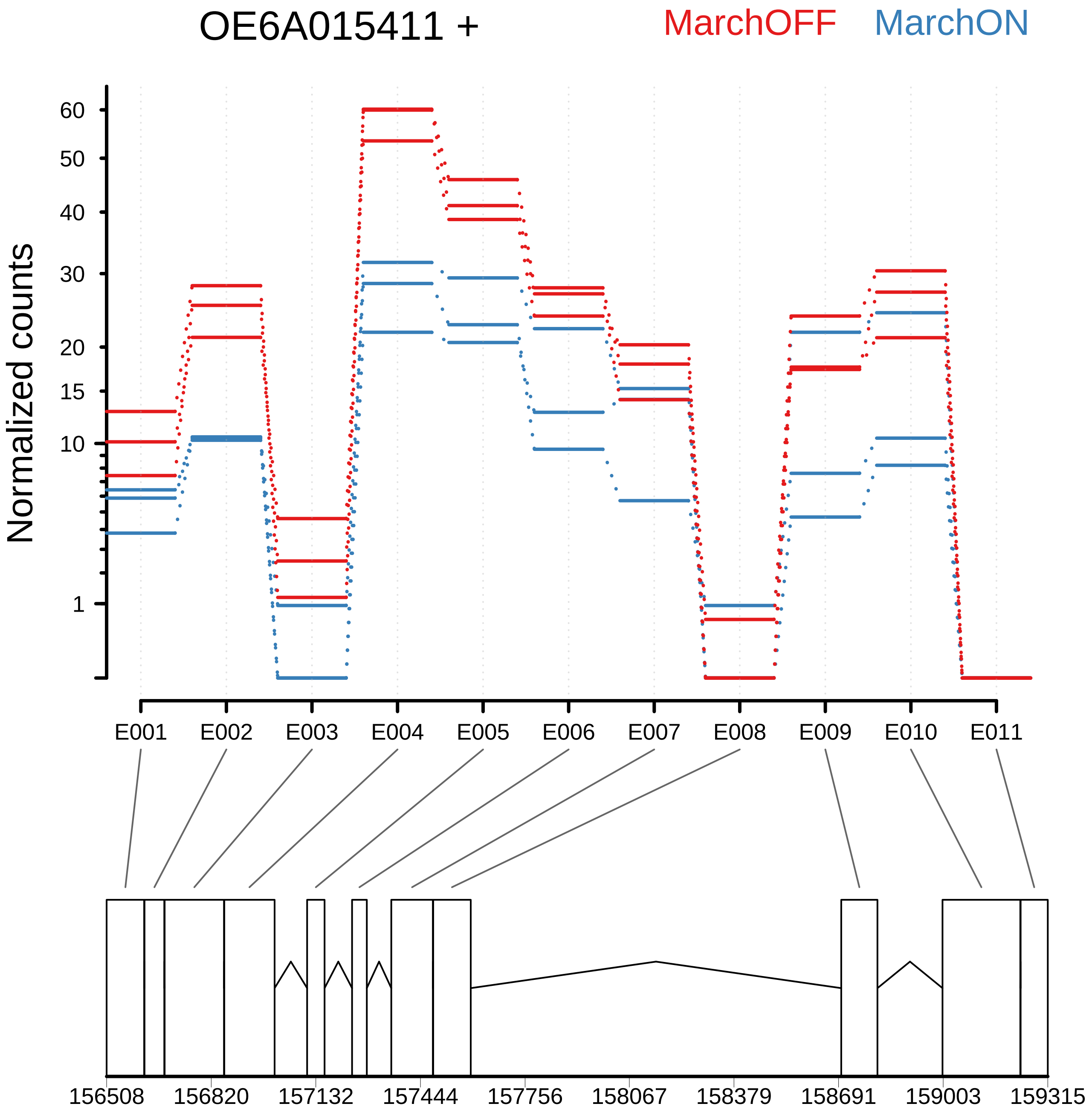

OE6A015411 +
MarchOFF
MarchON
Normalized counts
60
50
40
30
20
15
10
1
E001
E002
E003
E004
E005
E006
E007
E008
E009
E010
E011
156508
156820
157132
157444
157756
158067
158379
158691
159003
159315

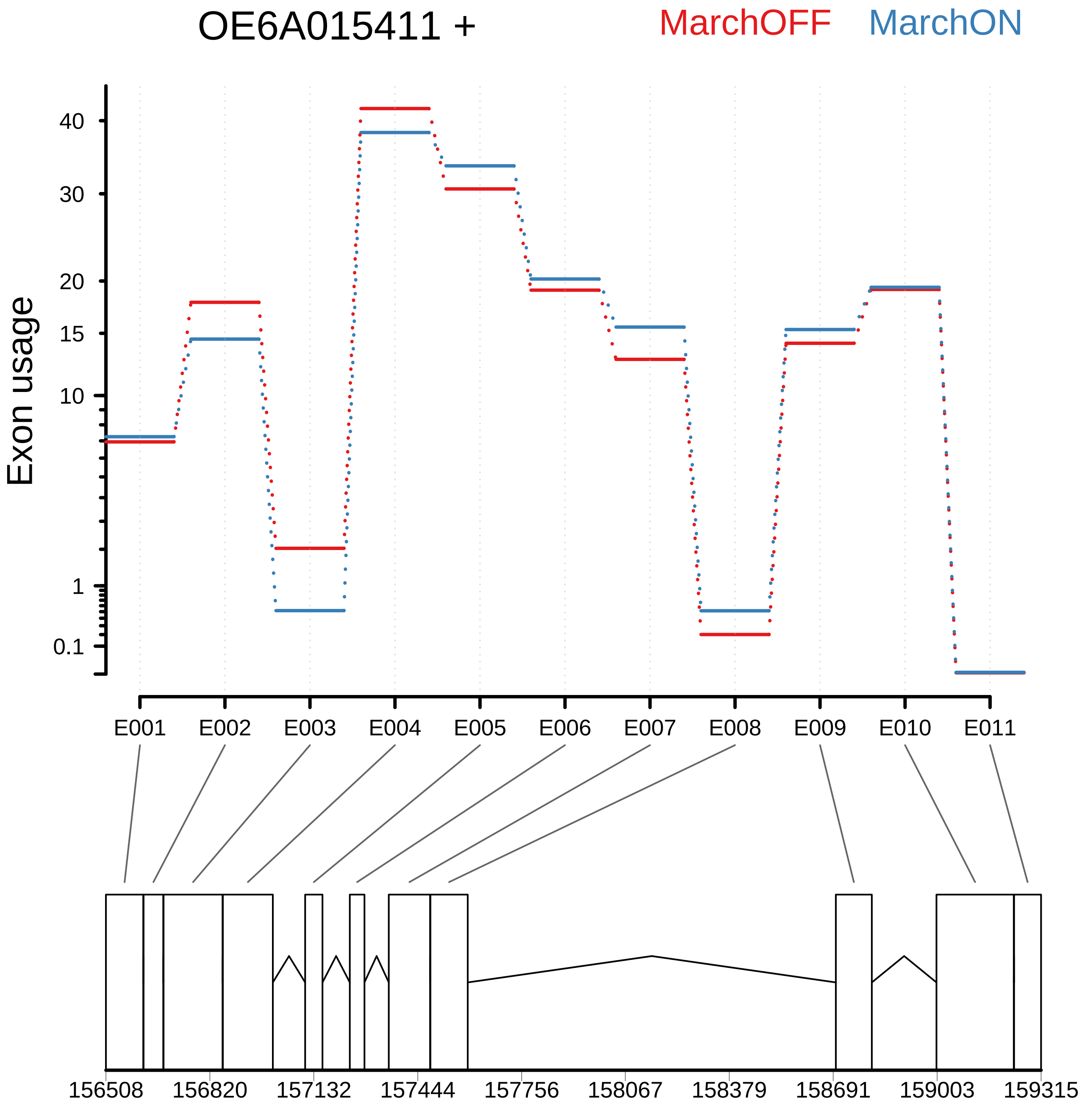
OE6A015411 +
MarchOFF
MarchON
Exon usage
40
30
20
15
10
1
0.1
E001
E002
E003
E004
E005
E006
E007
E008
E009
E010
E011
156508
156820
157132
157444
157756
158067
158379
158691
159003
159315

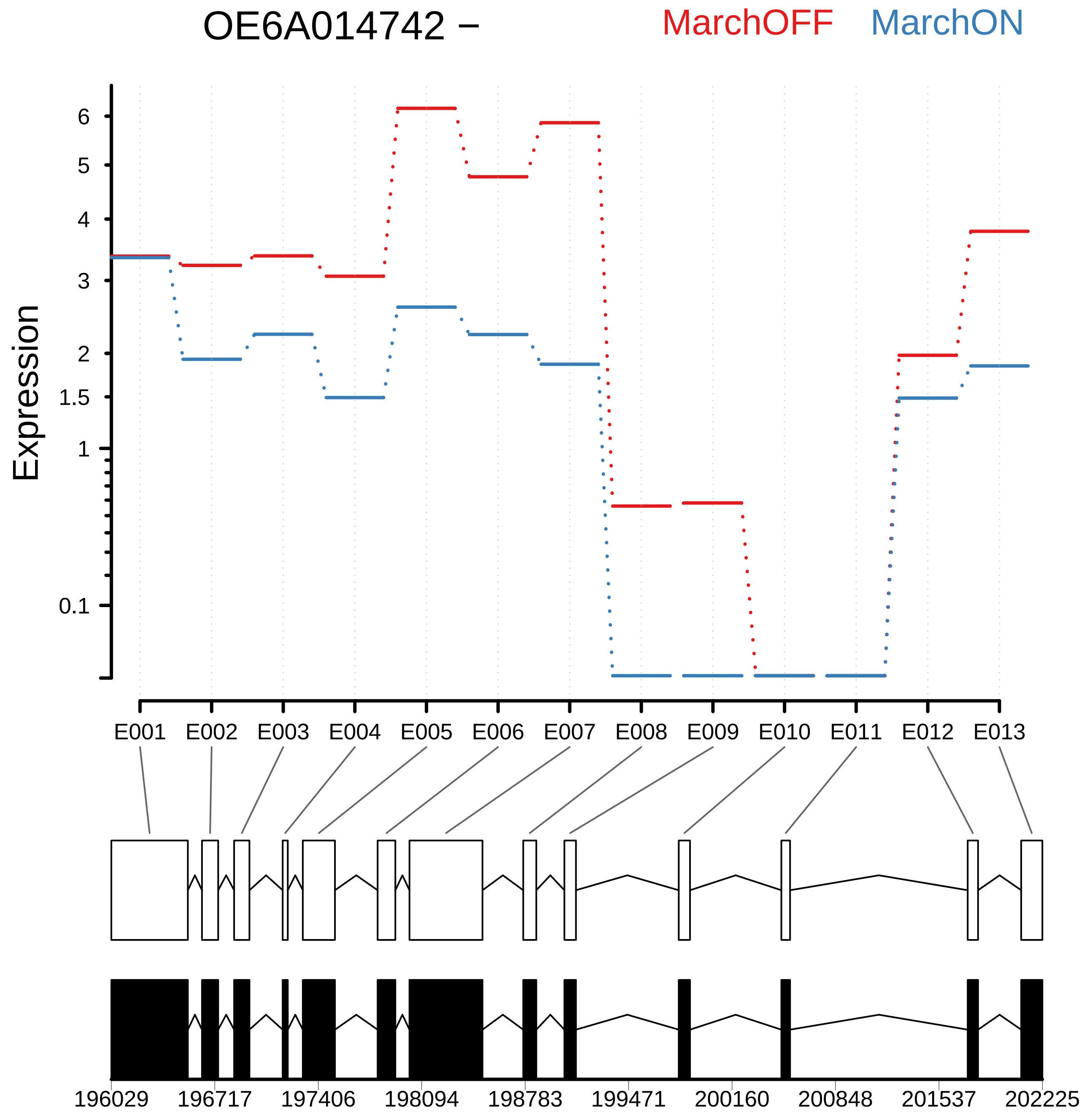

OE6A014742 −
MarchOFF
MarchON
Expression
6
5
4
3
2
1.5
1
0.1
E001
E002
E003
E004
E005
E006
E007
E008
E009
E010
E011
E012
E013
196029
196717
197406
198094
198783
199471
200160
200848
201537
202225

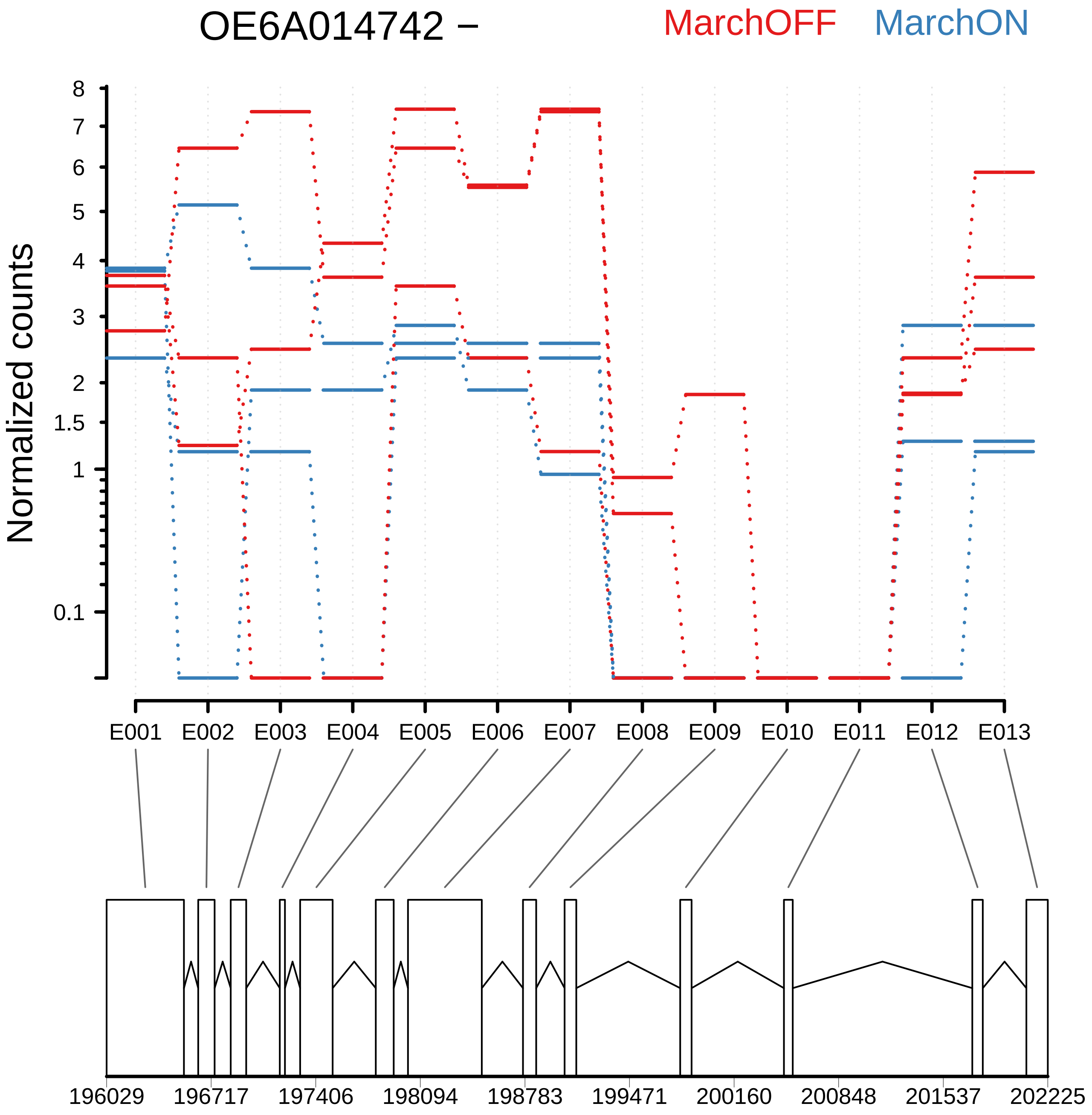

OE6A014742 −
MarchOFF
MarchON
Normalized counts
8
7
6
5
4
3
2
1.5
1
0.1
E001
E002
E003
E004
E005
E006
E007
E008
E009
E010
E011
E012
E013
196029
196717
197406
198094
198783
199471
200160
200848
201537
202225

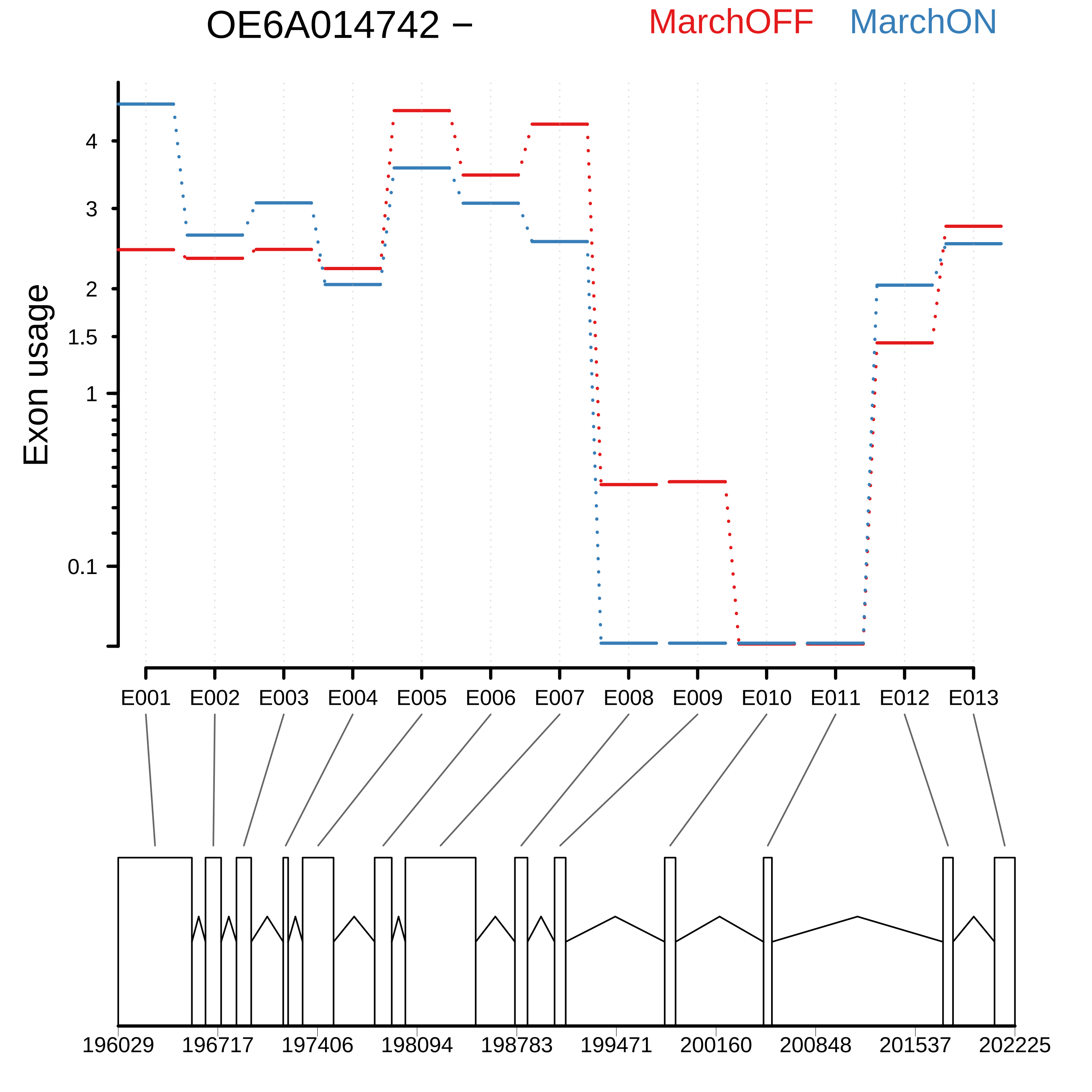

OE6A014742 −
MarchOFF
MarchON
Exon usage
4
3
2
1.5
1
0.1
E001
E002
E003
E004
E005
E006
E007
E008
E009
E010
E011
E012
E013
196029
196717
197406
198094
198783
199471
200160
200848
201537
202225

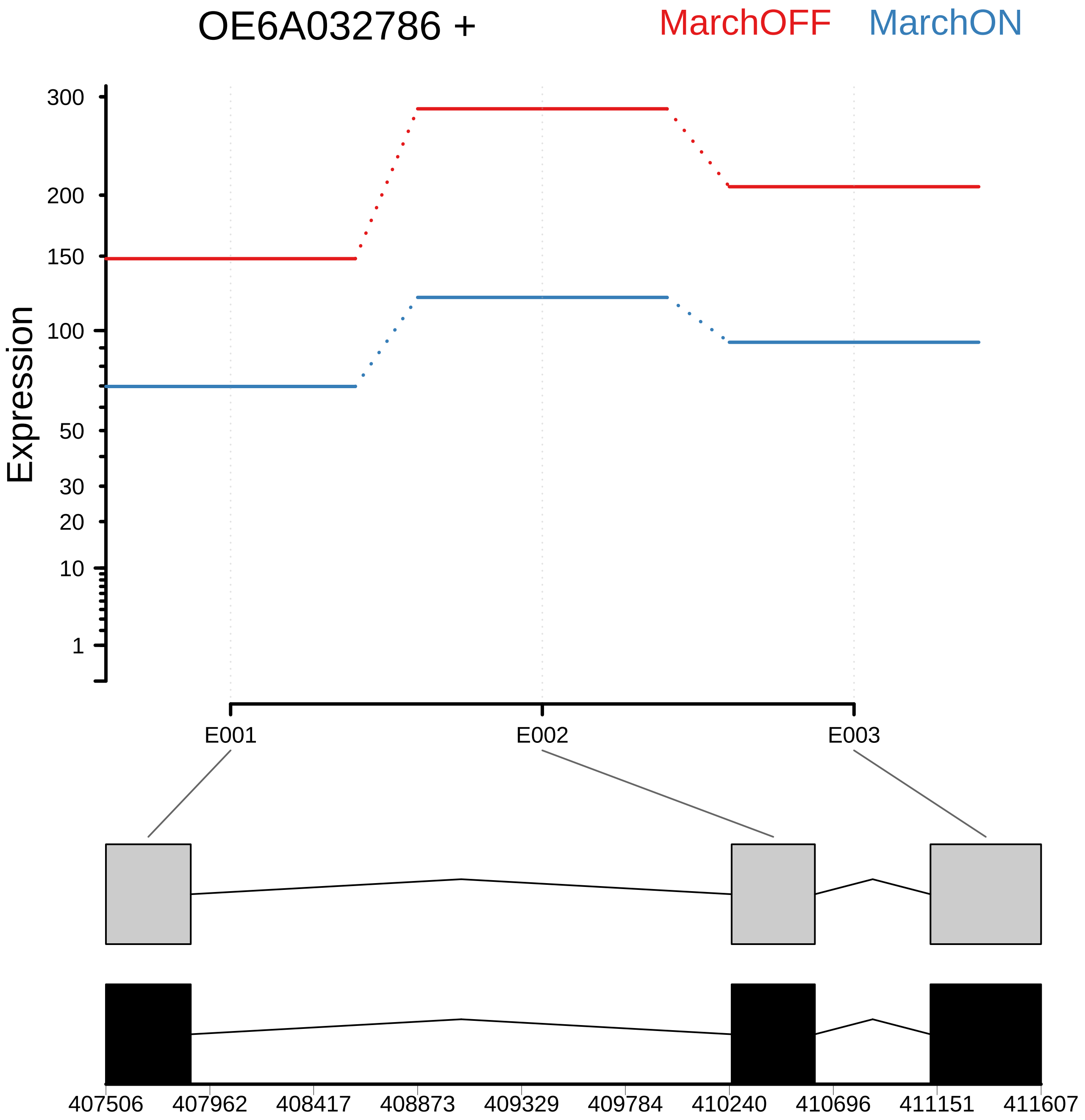

OE6A032786 +
MarchOFF
MarchON
Expression
300
200
150
100
50
30
20
10
1
E001
E002
E003
407506
407962
408417
408873
409329
409784
410240
410696
411151
411607

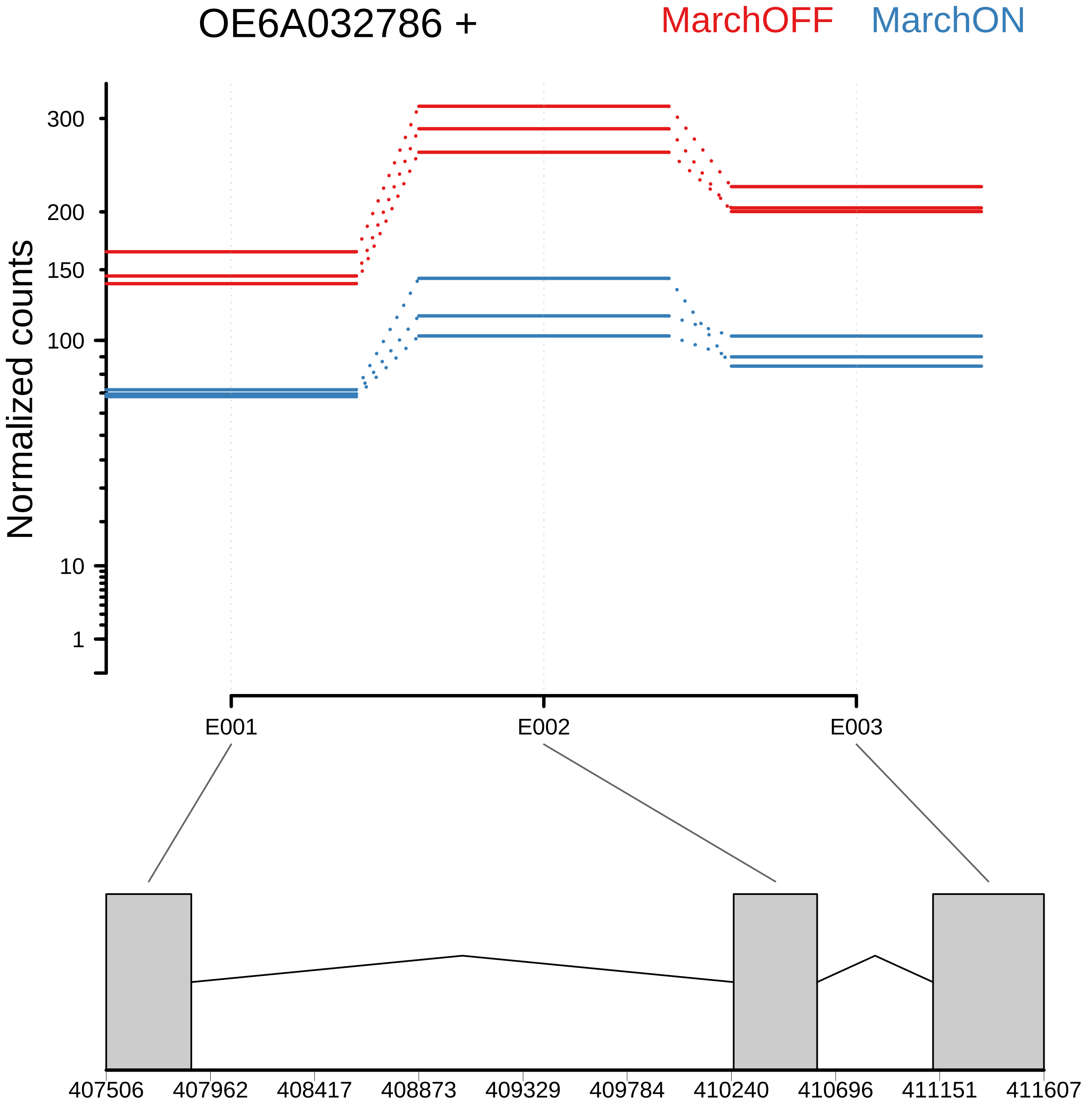

OE6A032786 +
MarchOFF
MarchON
Normalized counts
300
200
150
100
10
1
E001
E002
E003
407506
407962
408417
408873
409329
409784
410240
410696
411151
411607

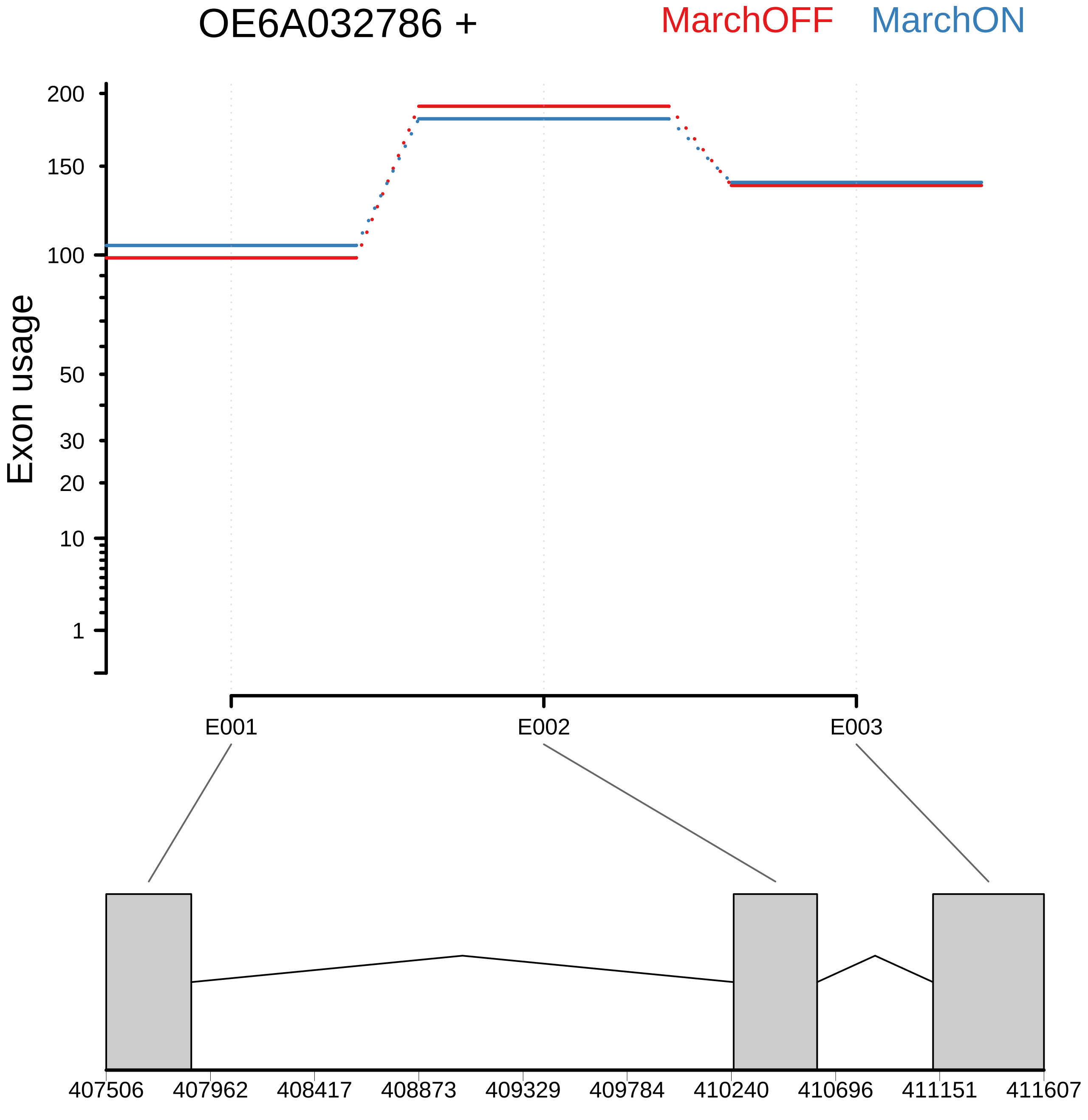
OE6A032786 +
MarchOFF
MarchON
Exon usage
200
150
100
50
30
20
10
1
E001
E002
E003
407506
407962
408417
408873
409329
409784
410240
410696
411151
411607

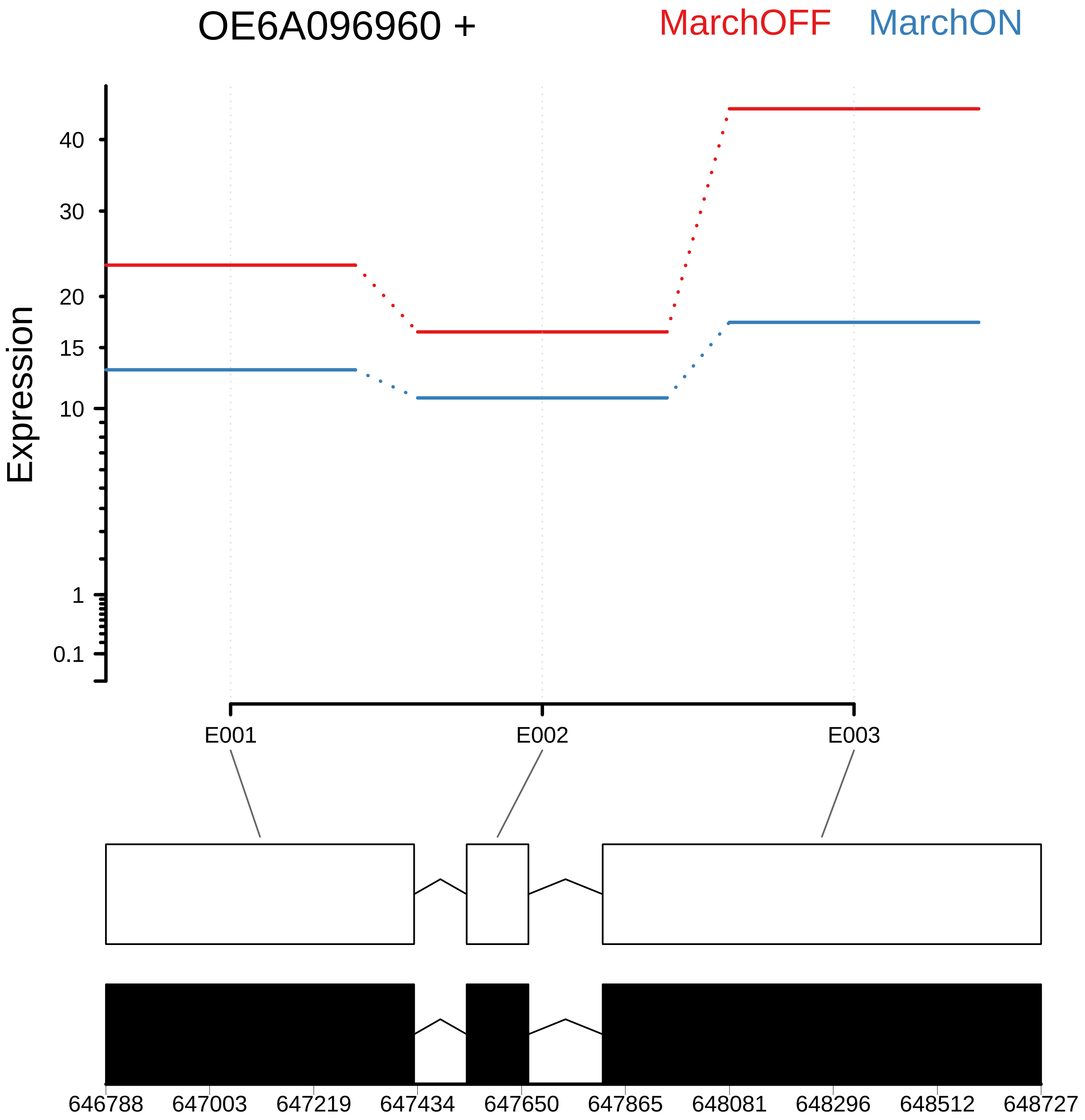

OE6A096960 +
MarchOFF
MarchON
Expression
40
30
20
15
10
1
0.1
E001
E002
E003
646788
647003
647219
647434
647650
647865
648081
648296
648512
648727

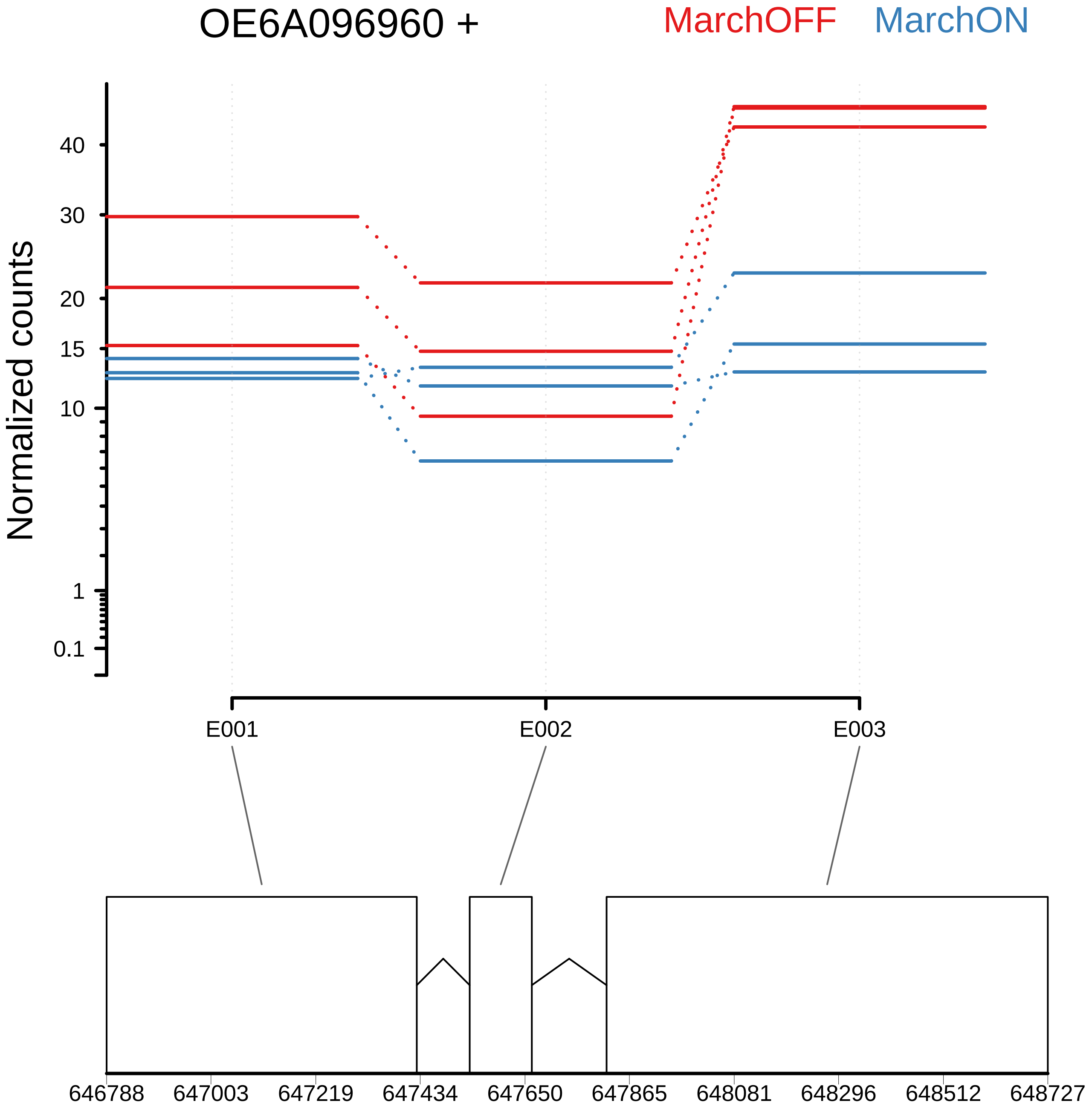

OE6A096960 +
MarchOFF
MarchON
Normalized counts
40
30
20
15
10
1
0.1
E001
E002
E003
646788
647003
647219
647434
647650
647865
648081
648296
648512
648727

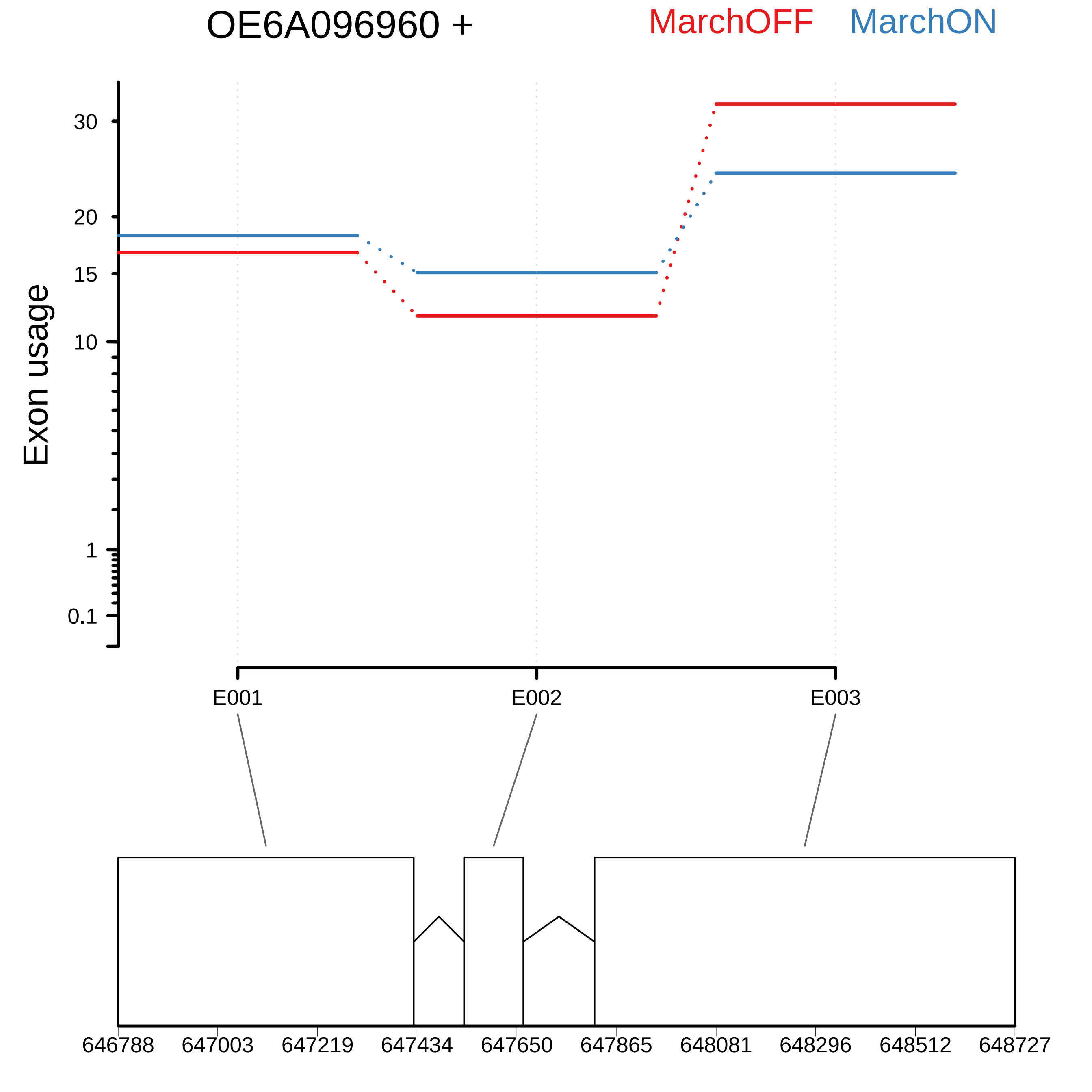
OE6A096960 +
MarchOFF
MarchON
Exon usage
30
20
15
10
1
0.1
E001
E002
E003
646788
647003
647219
647434
647650
647865
648081
648296
648512
648727

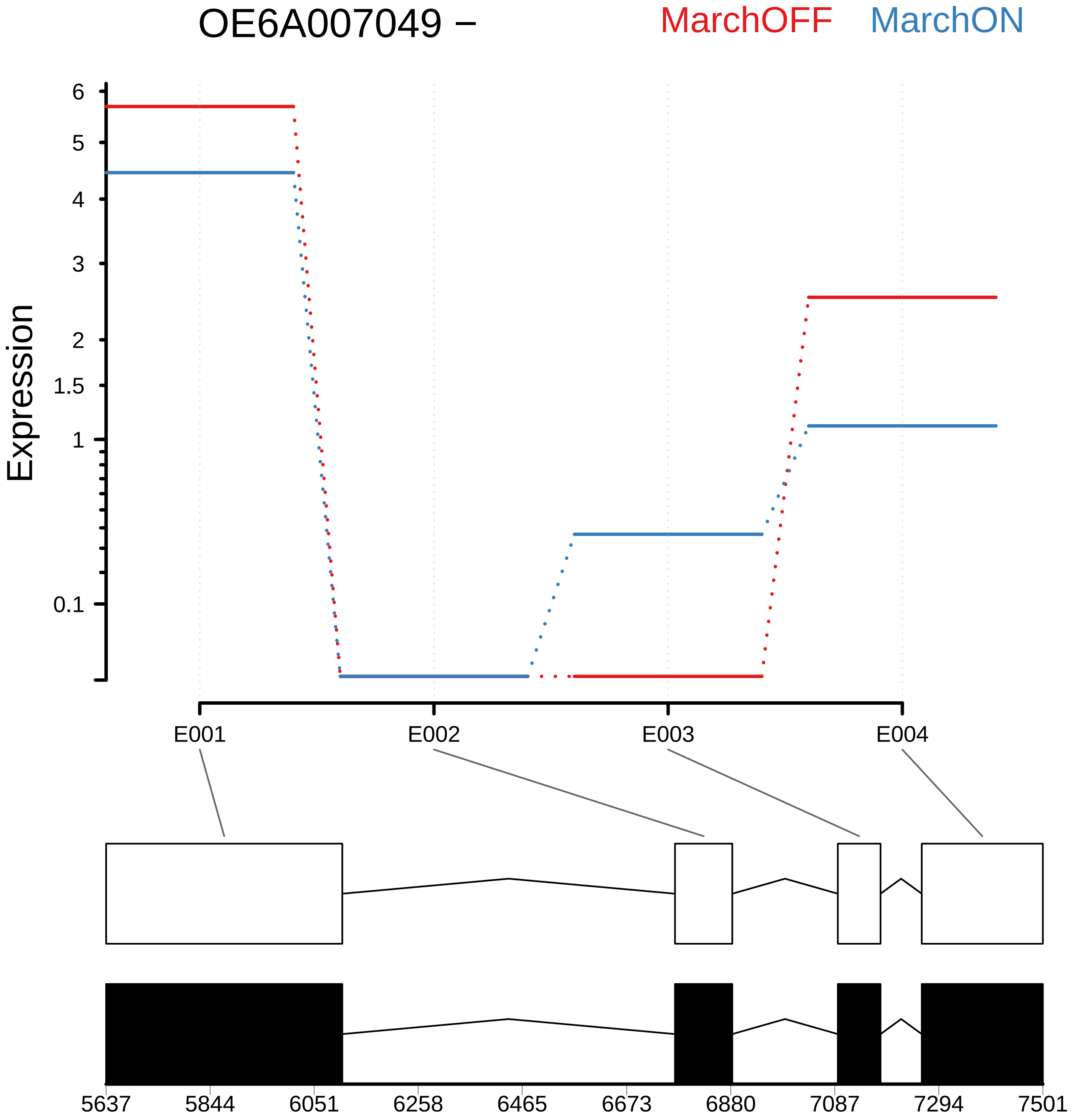

OE6A007049 −
MarchOFF
MarchON
Expression
6
5
4
3
2
1.5
1
0.1
E001
E002
E003
E004
5637
5844
6051
6258
6465
6673
6880
7087
7294
7501

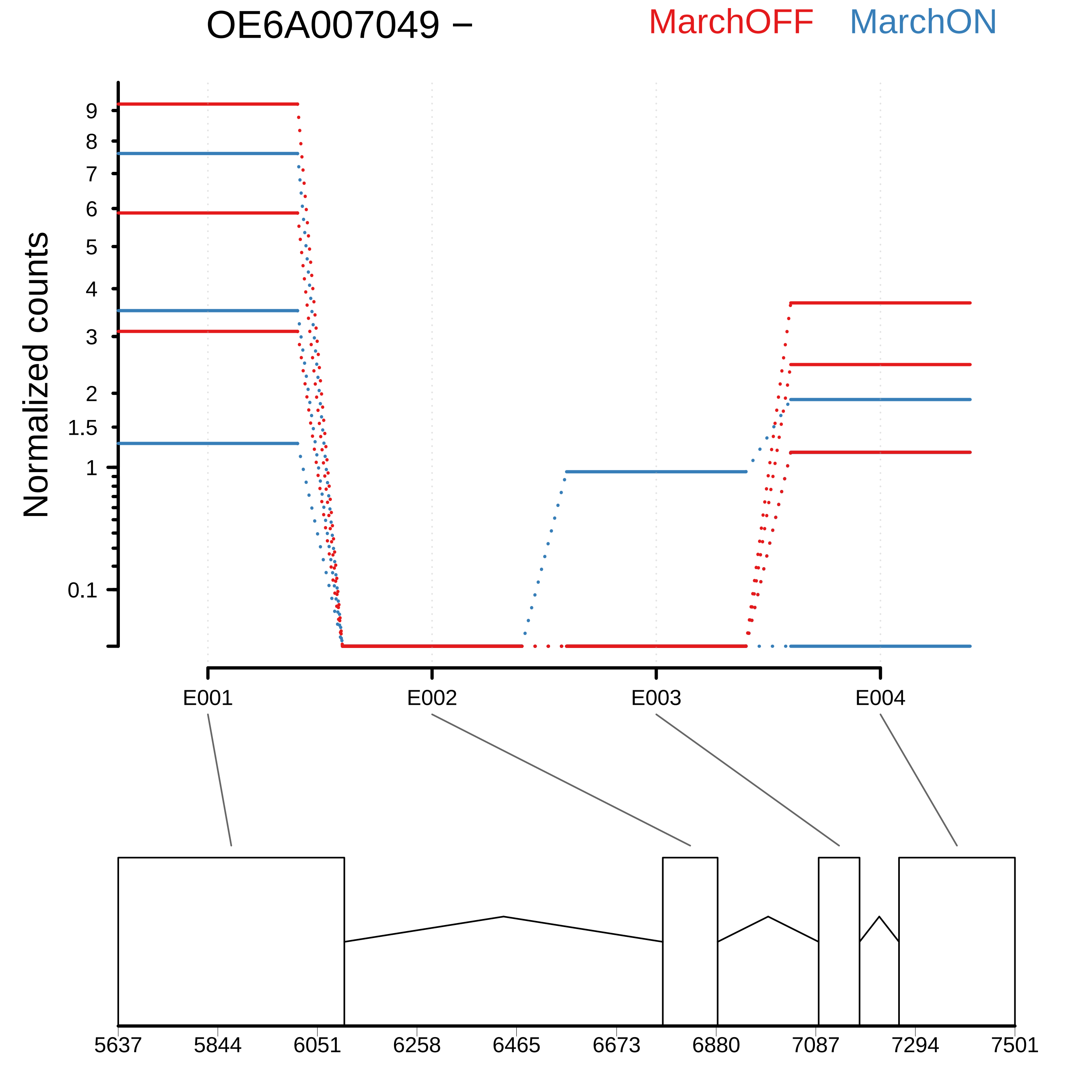

OE6A007049 −
MarchOFF
MarchON
Normalized counts
9
8
7
6
5
4
3
2
1.5
1
0.1
E001
E002
E003
E004
5637
5844
6051
6258
6465
6673
6880
7087
7294
7501

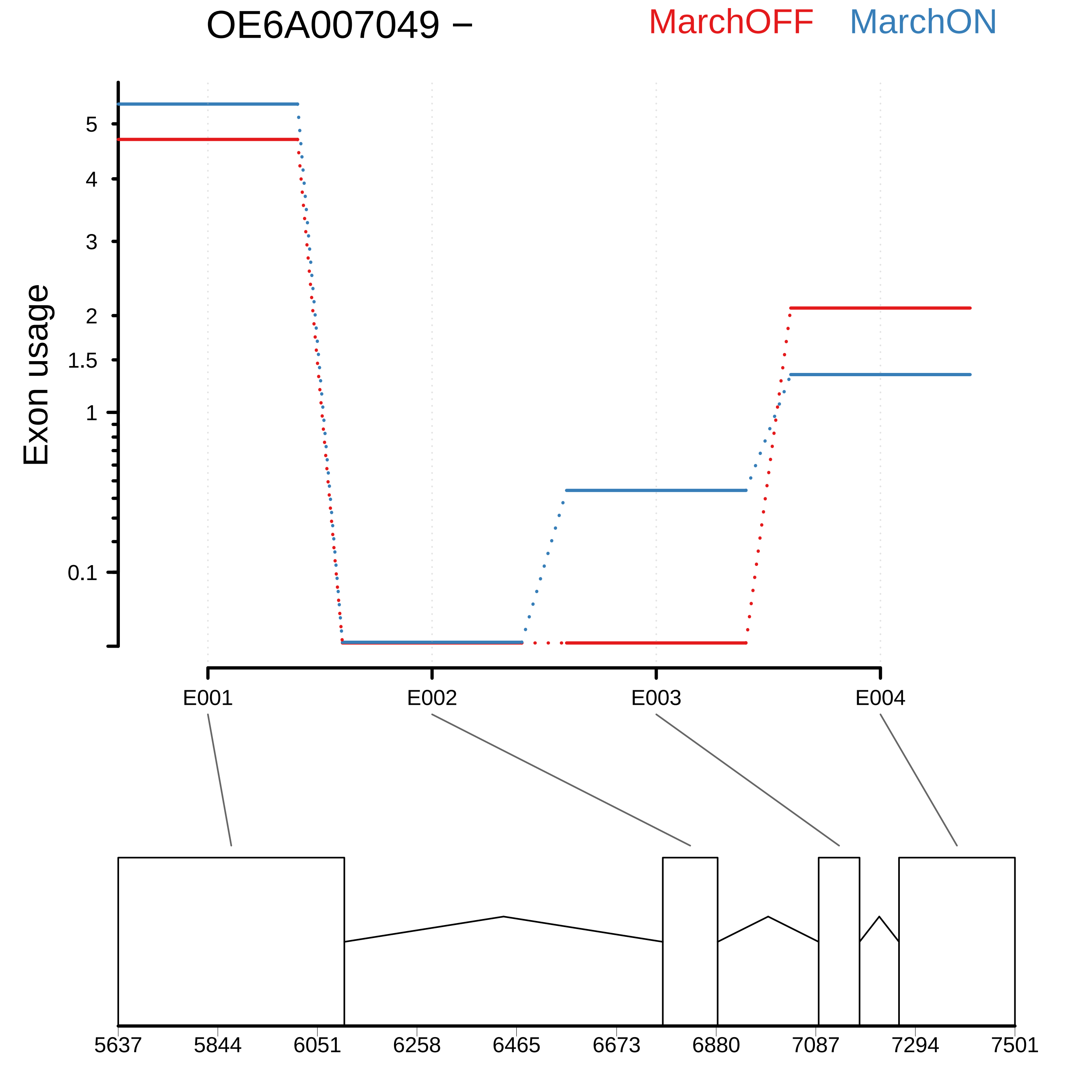

OE6A007049 −
MarchOFF
MarchON
Exon usage
5
4
3
2
1.5
1
0.1
E001
E002
E003
E004
5637
5844
6051
6258
6465
6673
6880
7087
7294
7501

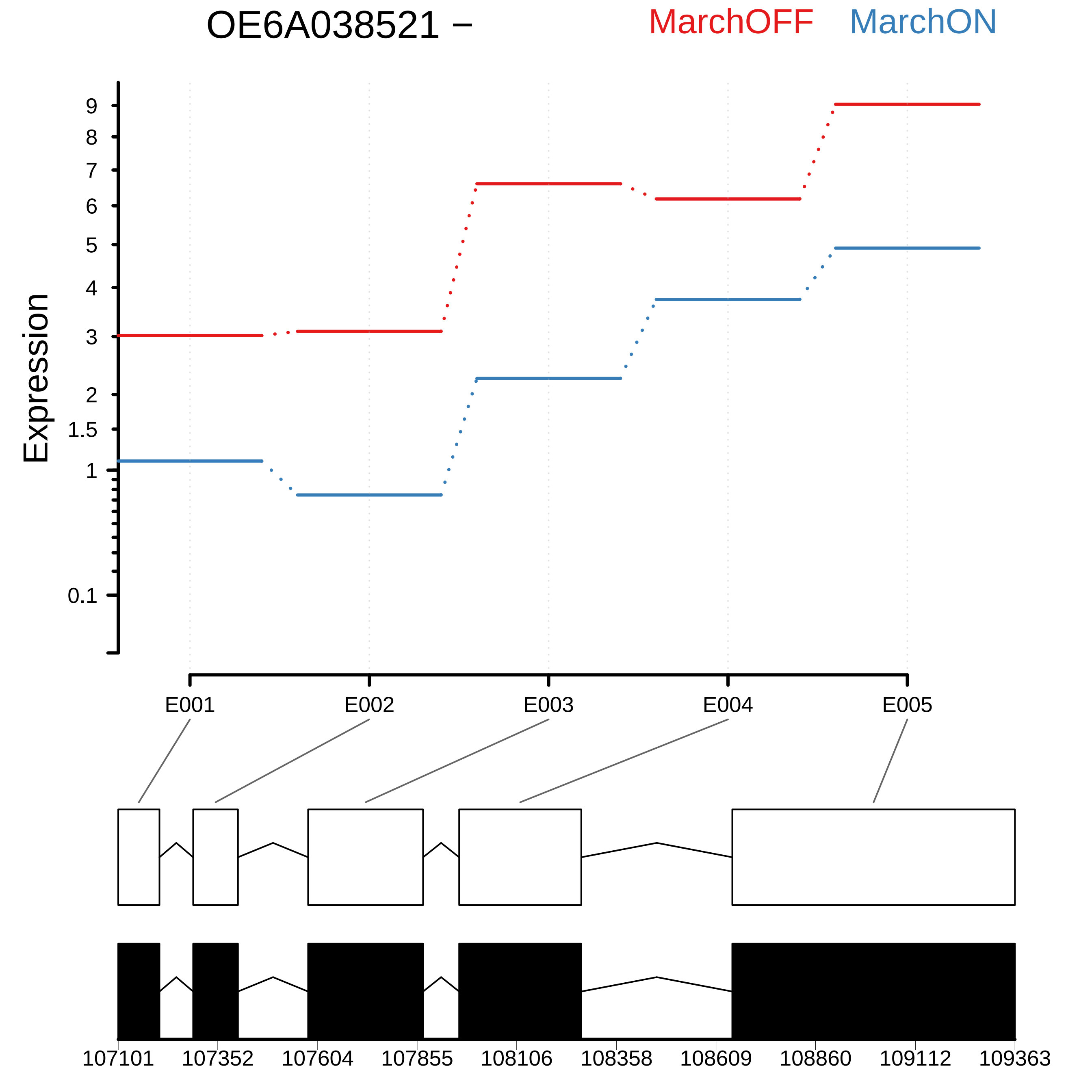

OE6A038521 −
MarchOFF
MarchON
Expression
9
8
7
6
5
4
3
2
1.5
1
0.1
E001
E002
E003
E004
E005
107101
107352
107604
107855
108106
108358
108609
108860
109112
109363

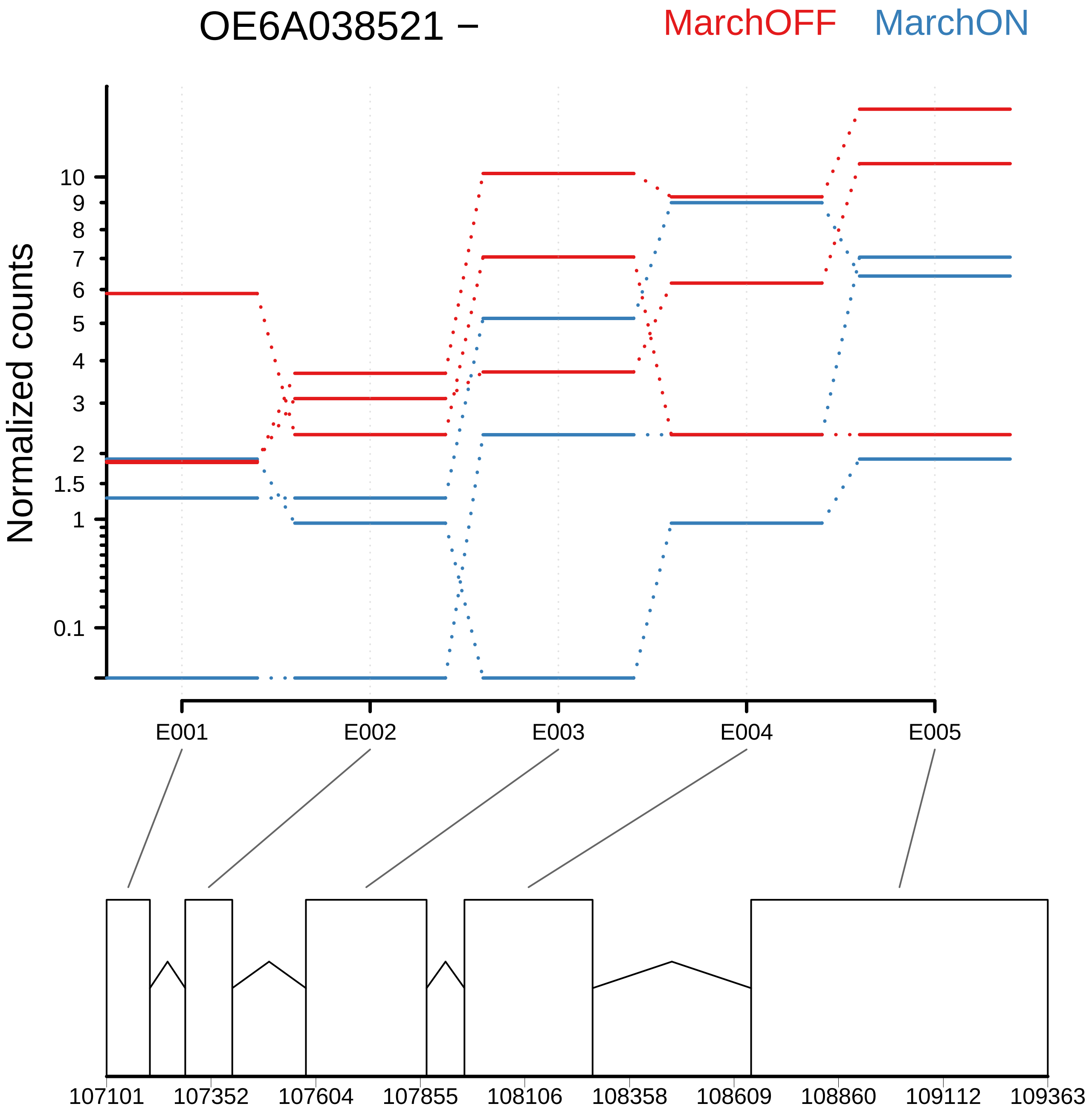

OE6A038521 −
MarchOFF
MarchON
Normalized counts
10
9
8
7
6
5
4
3
2
1.5
1
0.1
E001
E002
E003
E004
E005
107101
107352
107604
107855
108106
108358
108609
108860
109112
109363

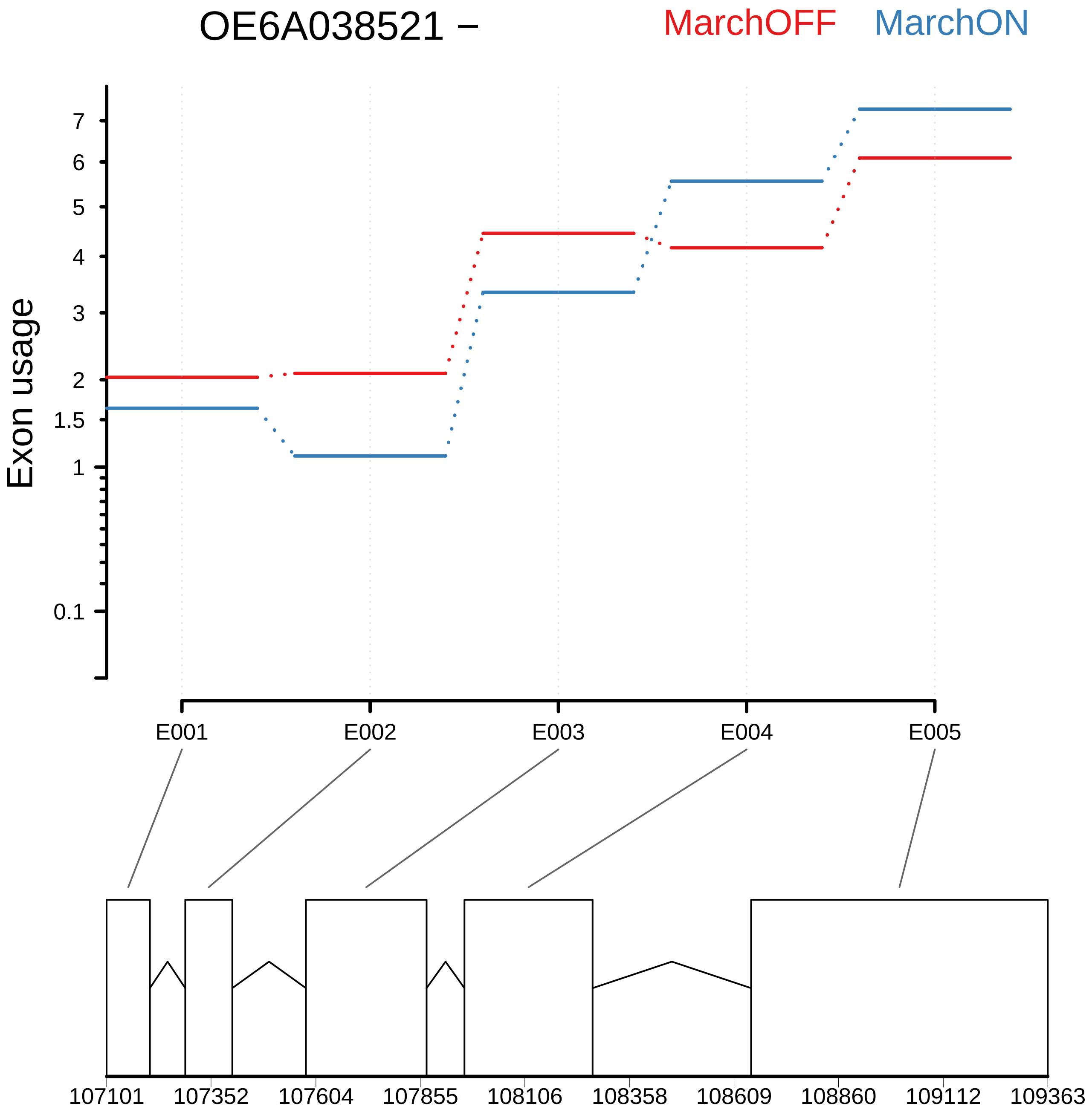

OE6A038521 −
MarchOFF
MarchON
Exon usage
7
6
5
4
3
2
1.5
1
0.1
E001
E002
E003
E004
E005
107101
107352
107604
107855
108106
108358
108609
108860
109112
109363

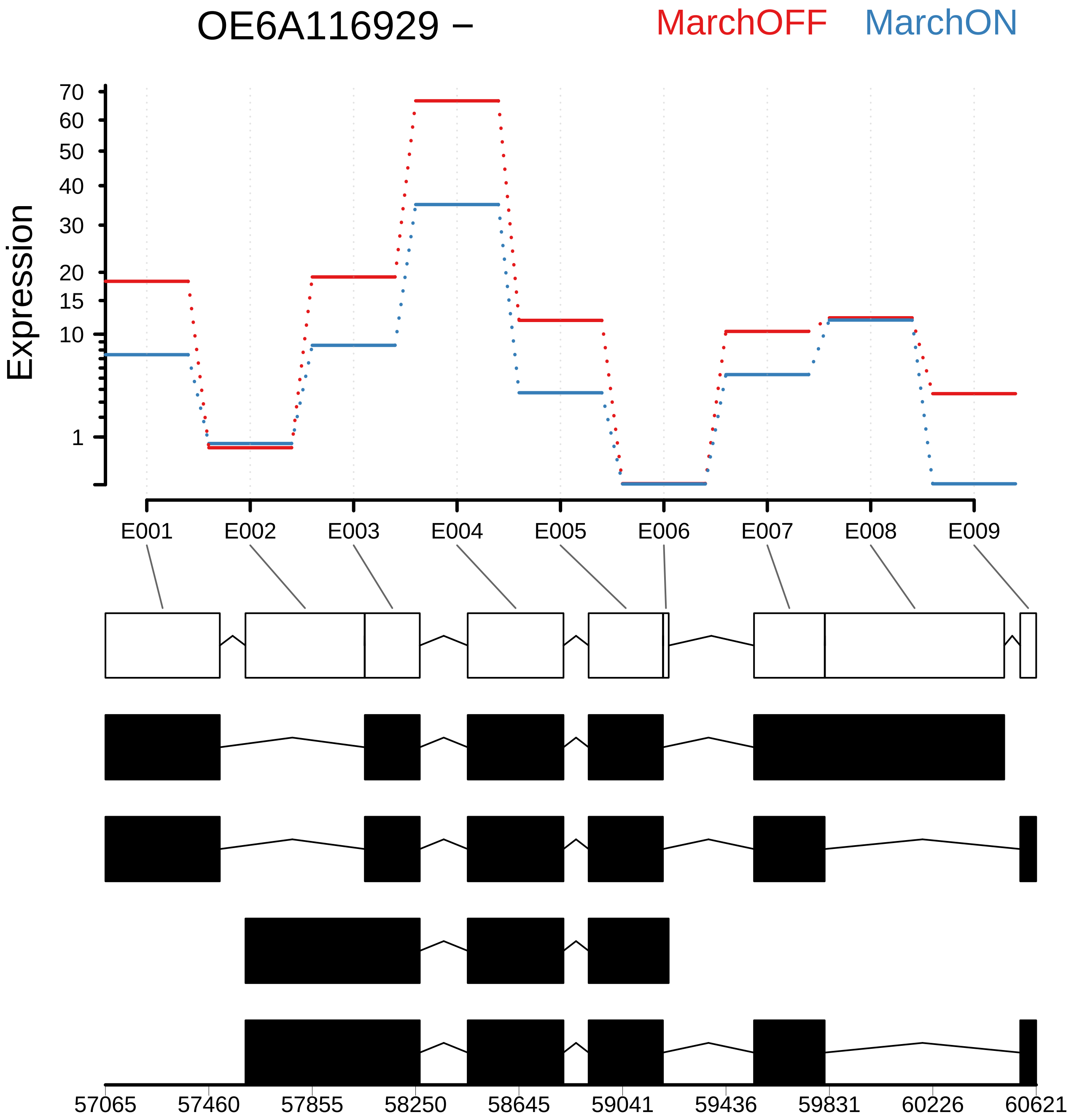
OE6A116929 –
MarchOFF
MarchON
Expression
70
60
50
40
30
20
15
10
1
E001
E002
E003
E004
E005
E006
E007
E008
E009
57065
57460
57855
58250
58645
59041
59436
59831
60226
60621

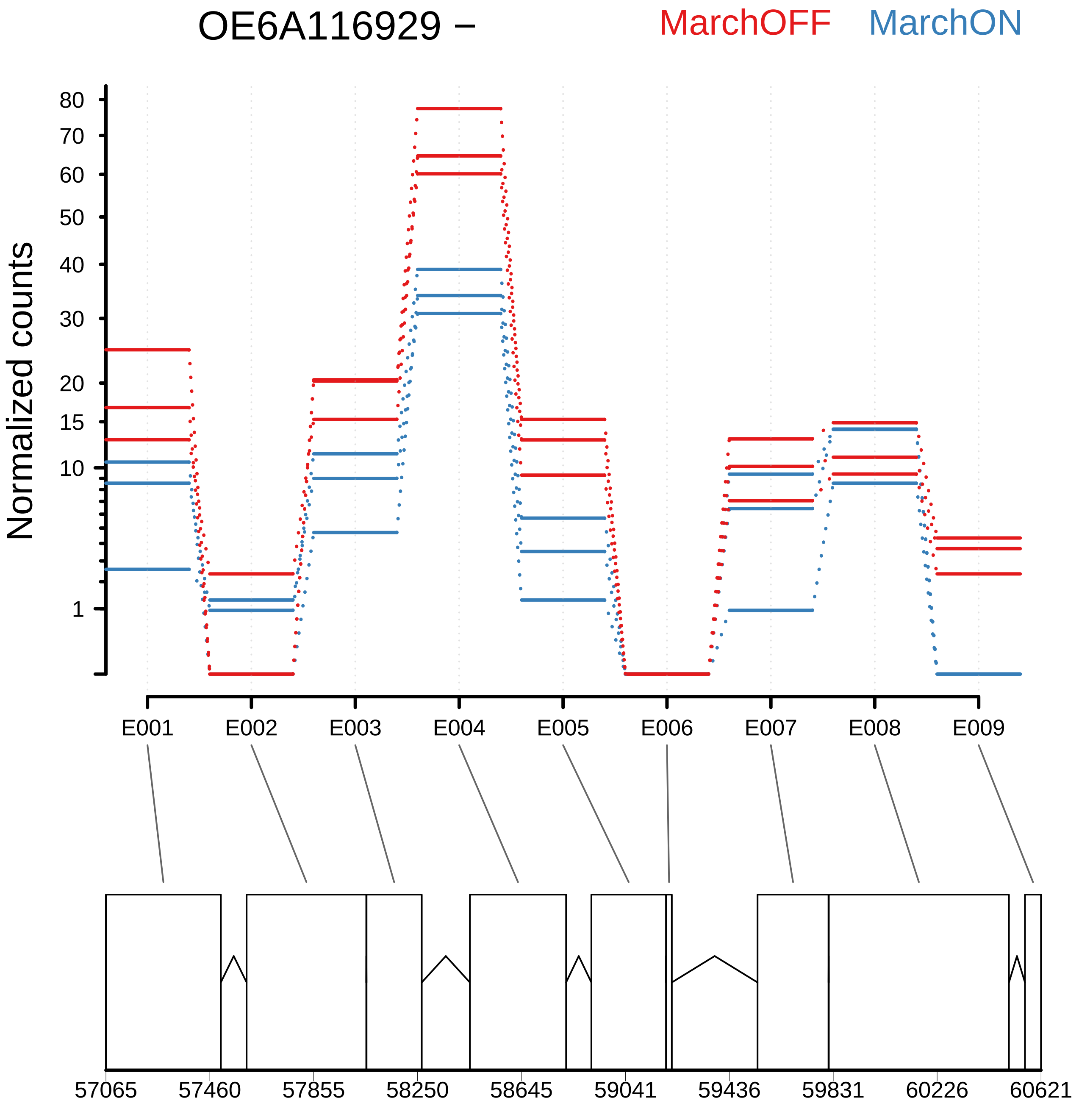

OE6A116929 −
MarchOFF
MarchON
Normalized counts
80
70
60
50
40
30
20
15
10
1
E001
E002
E003
E004
E005
E006
E007
E008
E009
57065
57460
57855
58250
58645
59041
59436
59831
60226
60621

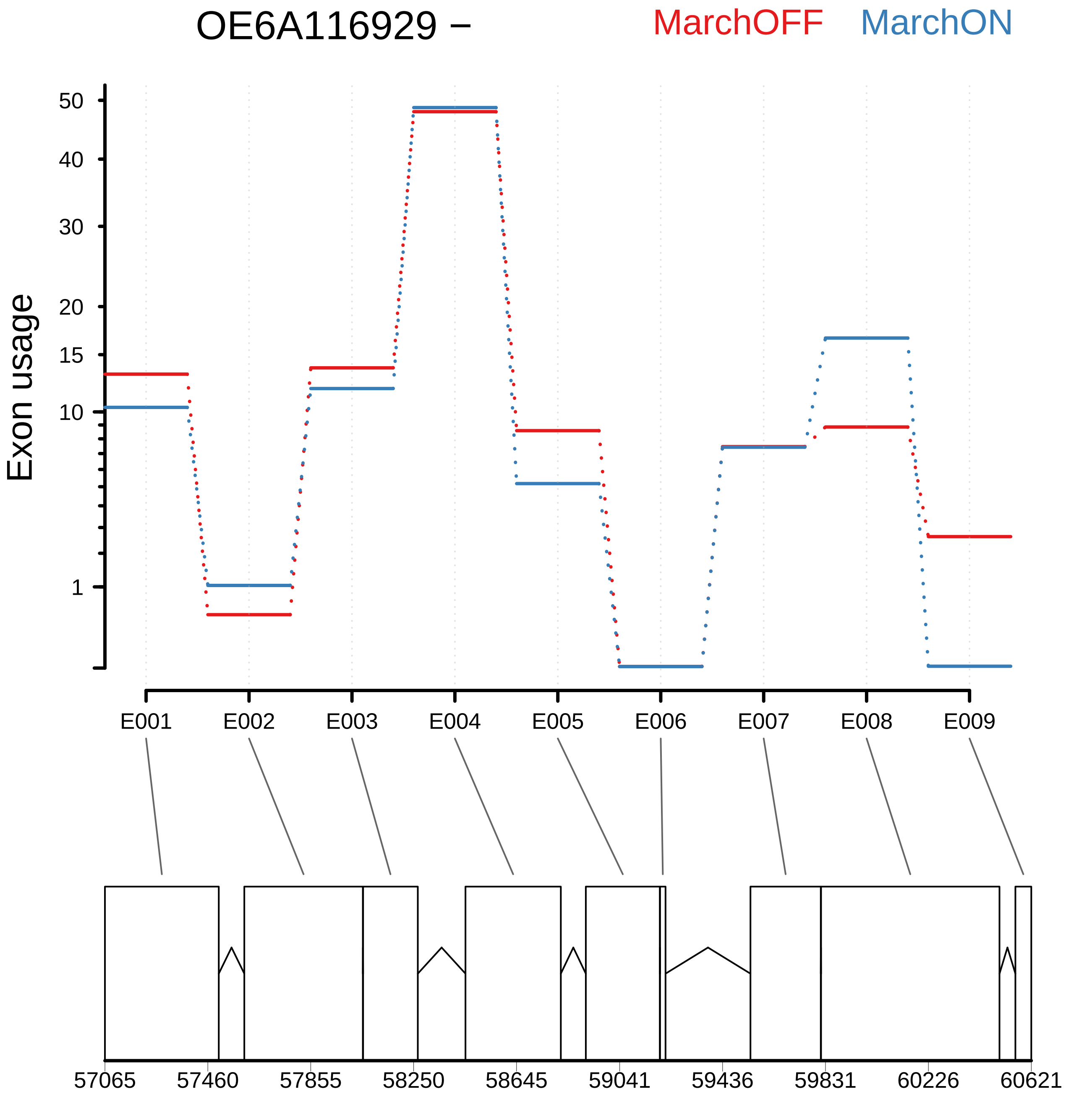
OE6A116929 −
MarchOFF
MarchON
Exon usage
50
40
30
20
15
10
1
E001
E002
E003
E004
E005
E006
E007
E008
E009
57065
57460
57855
58250
58645
59041
59436
59831
60226
60621

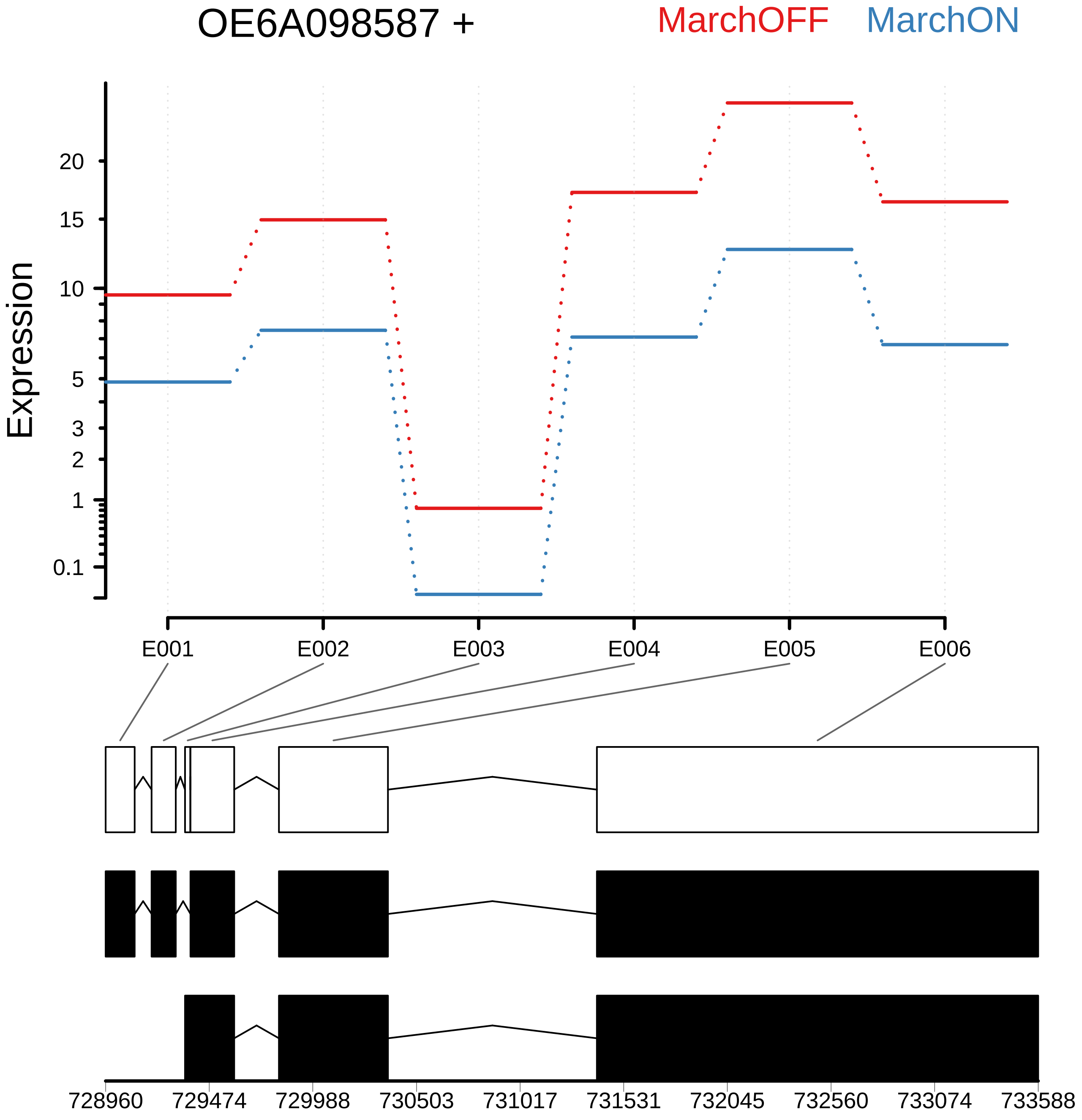
OE6A098587 +
MarchOFF
MarchON
Expression
20
15
10
5
3
2
1
0.1
E001
E002
E003
E004
E005
E006
728960
729474
729988
730503
731017
731531
732045
732560
733074
733588

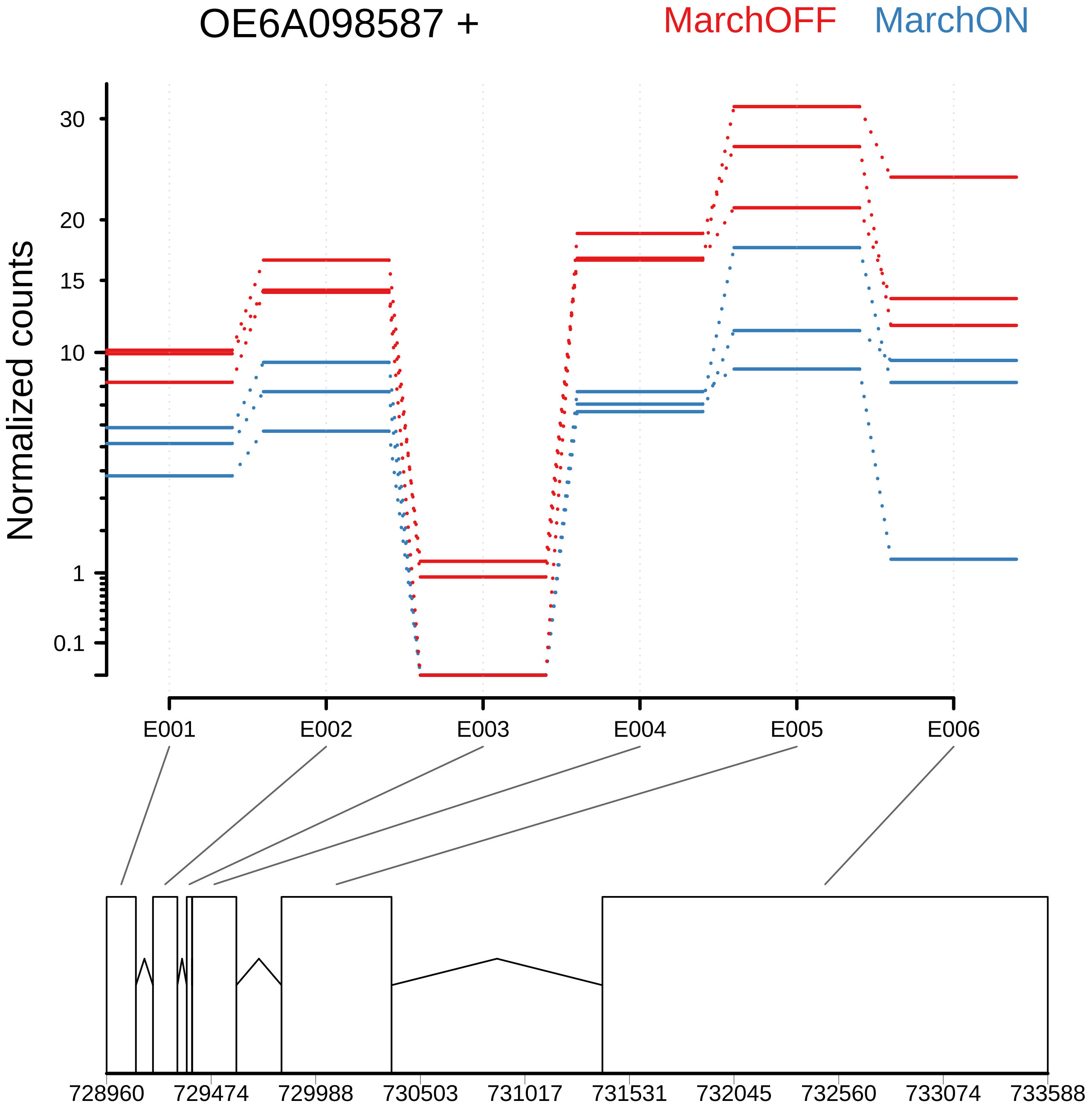

OE6A098587 +
MarchOFF
MarchON
Normalized counts
30
20
15
10
1
0.1
E001
E002
E003
E004
E005
E006
728960
729474
729988
730503
731017
731531
732045
732560
733074
733588

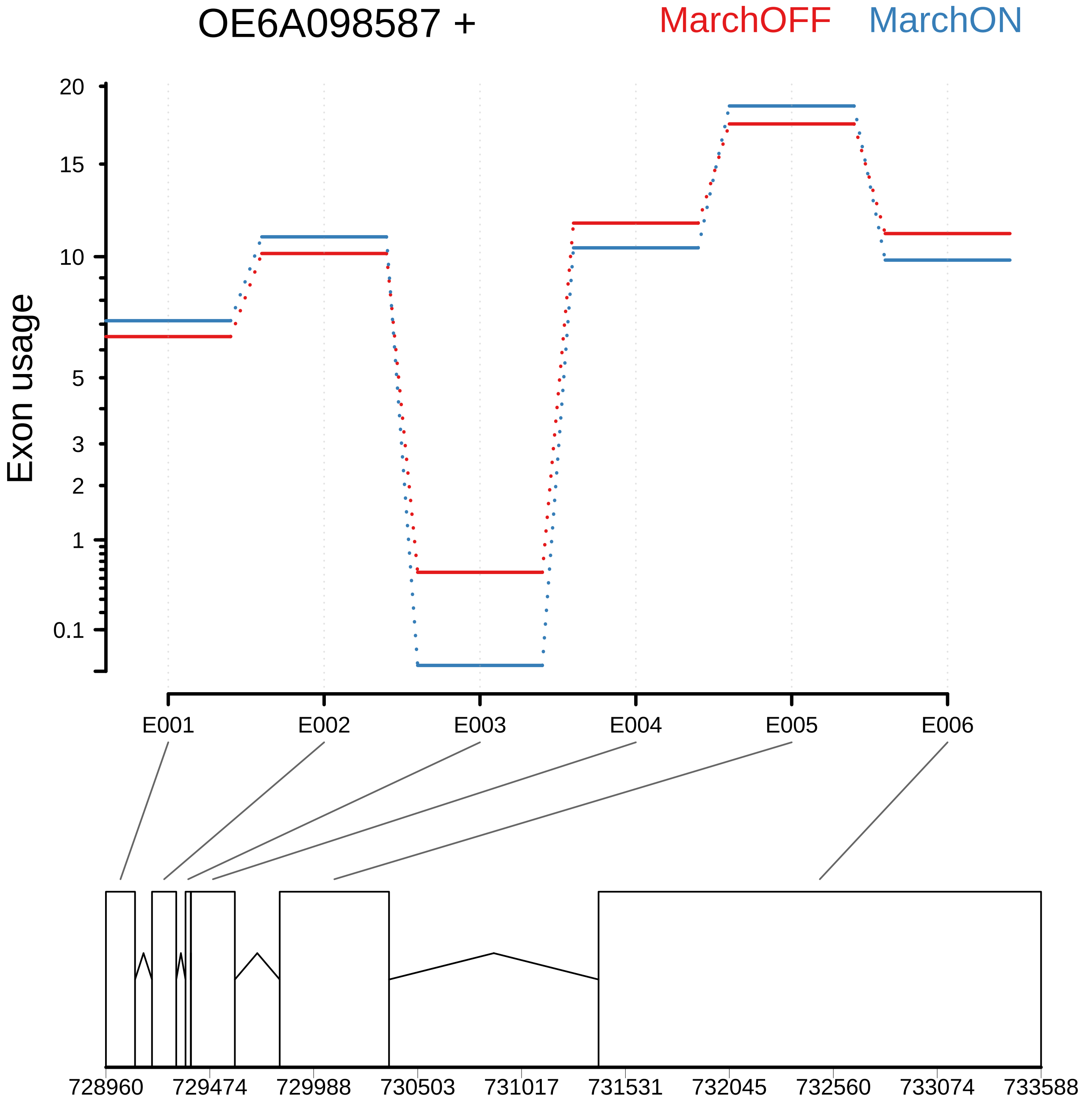

OE6A098587 +
MarchOFF
MarchON
Exon usage
20
15
10
5
3
2
1
0.1
E001
E002
E003
E004
E005
E006
728960
729474
729988
730503
731017
731531
732045
732560
733074
733588

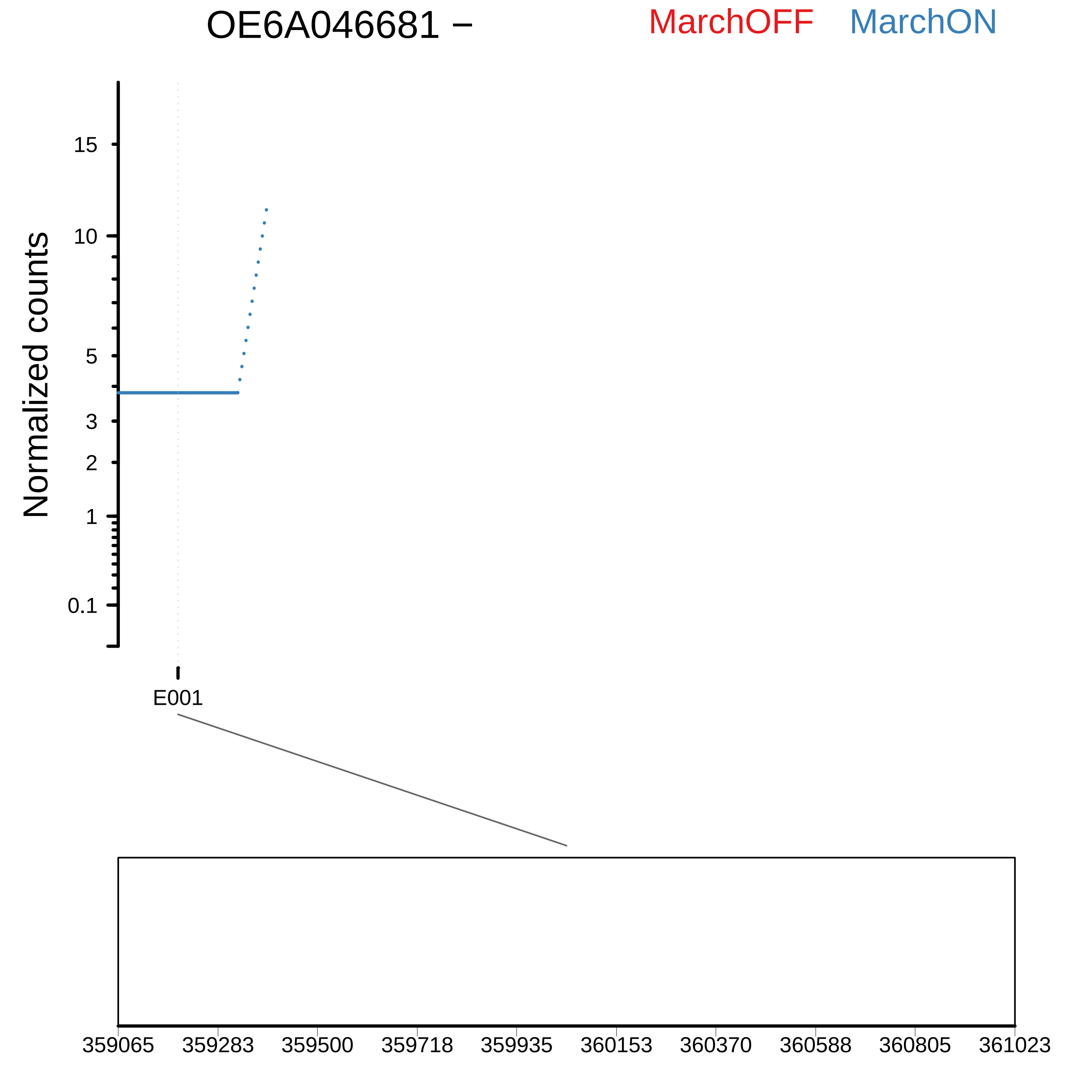
OE6A046681 −
MarchOFF
MarchON
Normalized counts
15
10
5
3
2
1
0.1
E001
359065
359283
359500
359718
359935
360153
360370
360588
360805
361023

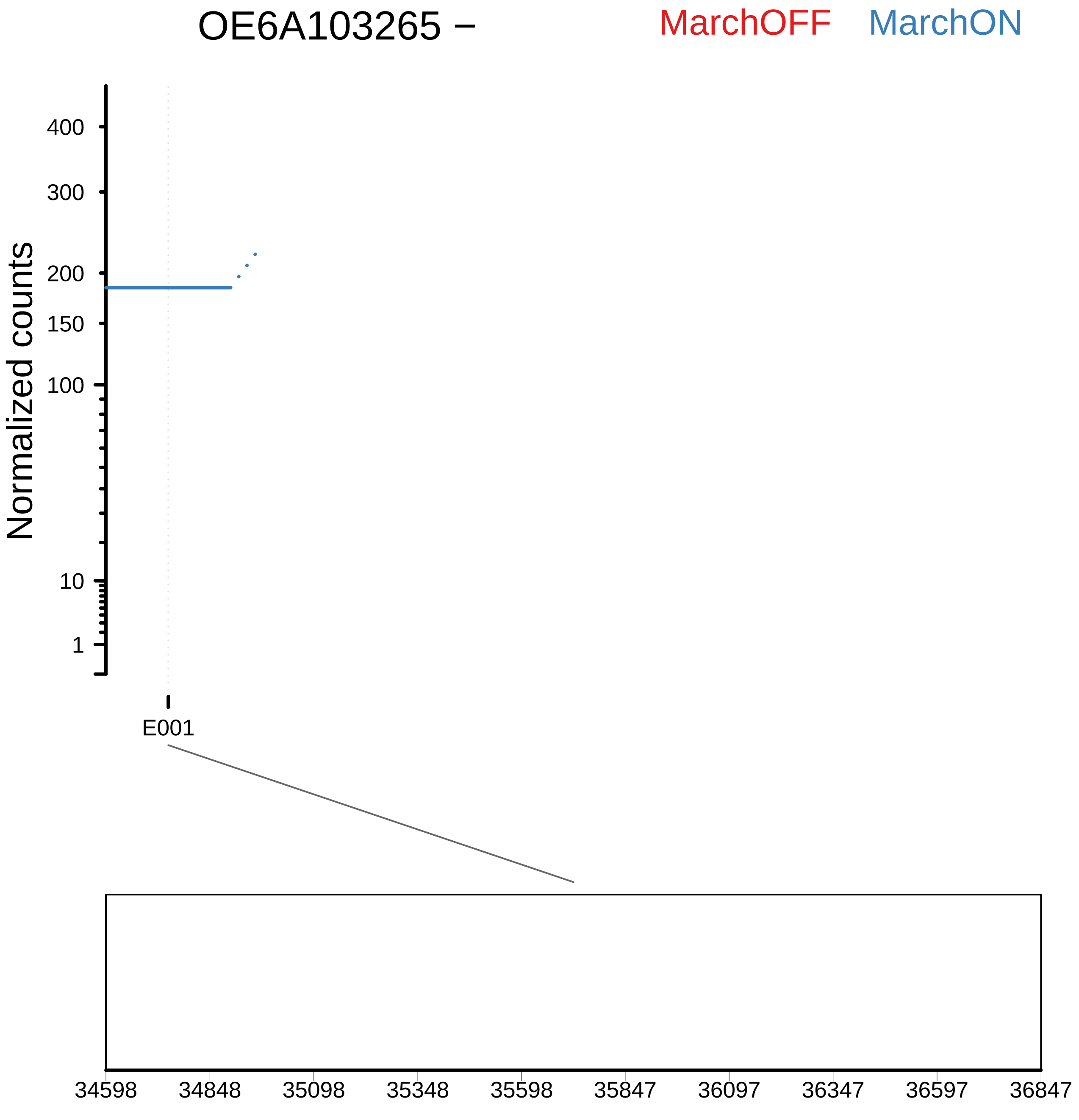
OE6A103265 −
MarchOFF
MarchON
Normalized counts
400
300
200
150
100
10
1
E001
34598
34848
35098
35348
35598
35847
36097
36347
36597
36847

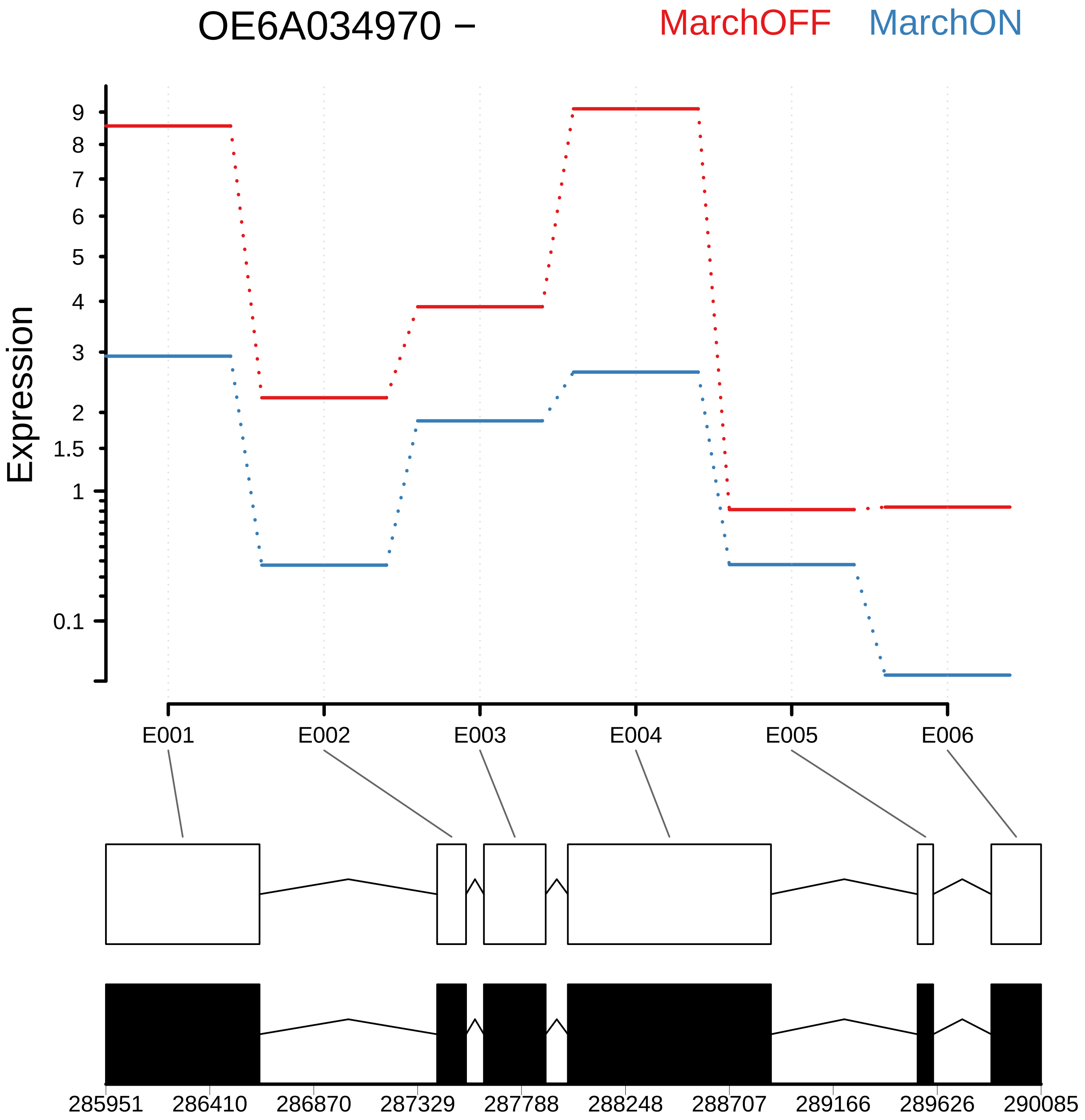
OE6A034970 −
MarchOFF
MarchON
Expression
9
8
7
6
5
4
3
2
1.5
1
0.1
E001
E002
E003
E004
E005
E006
285951
286410
286870
287329
287788
288248
288707
289166
289626
290085

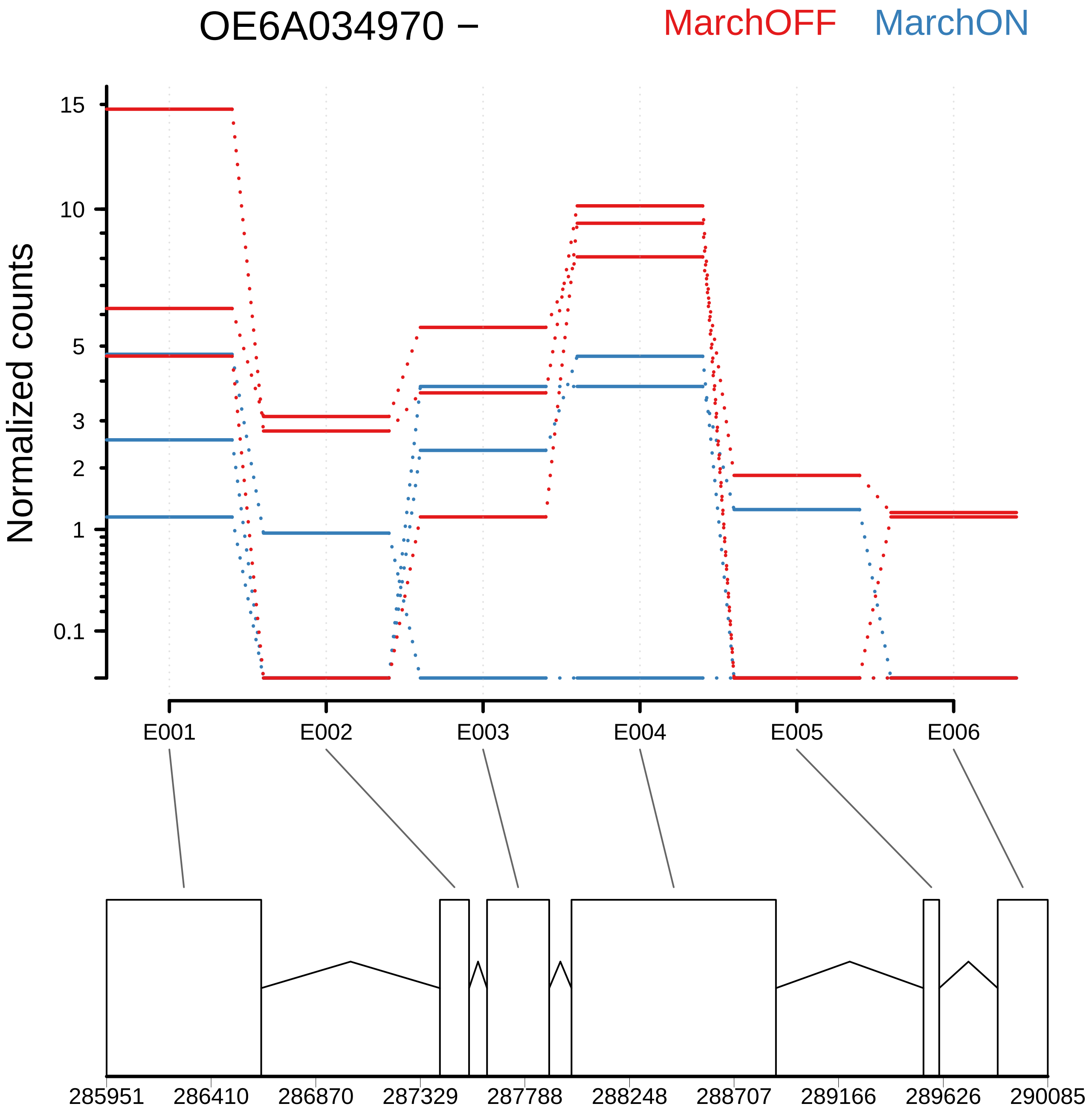
OE6A034970 −
MarchOFF
MarchON
Normalized counts
15
10
5
3
2
1
0.1
E001
E002
E003
E004
E005
E006
285951
286410
286870
287329
287788
288248
288707
289166
289626
290085

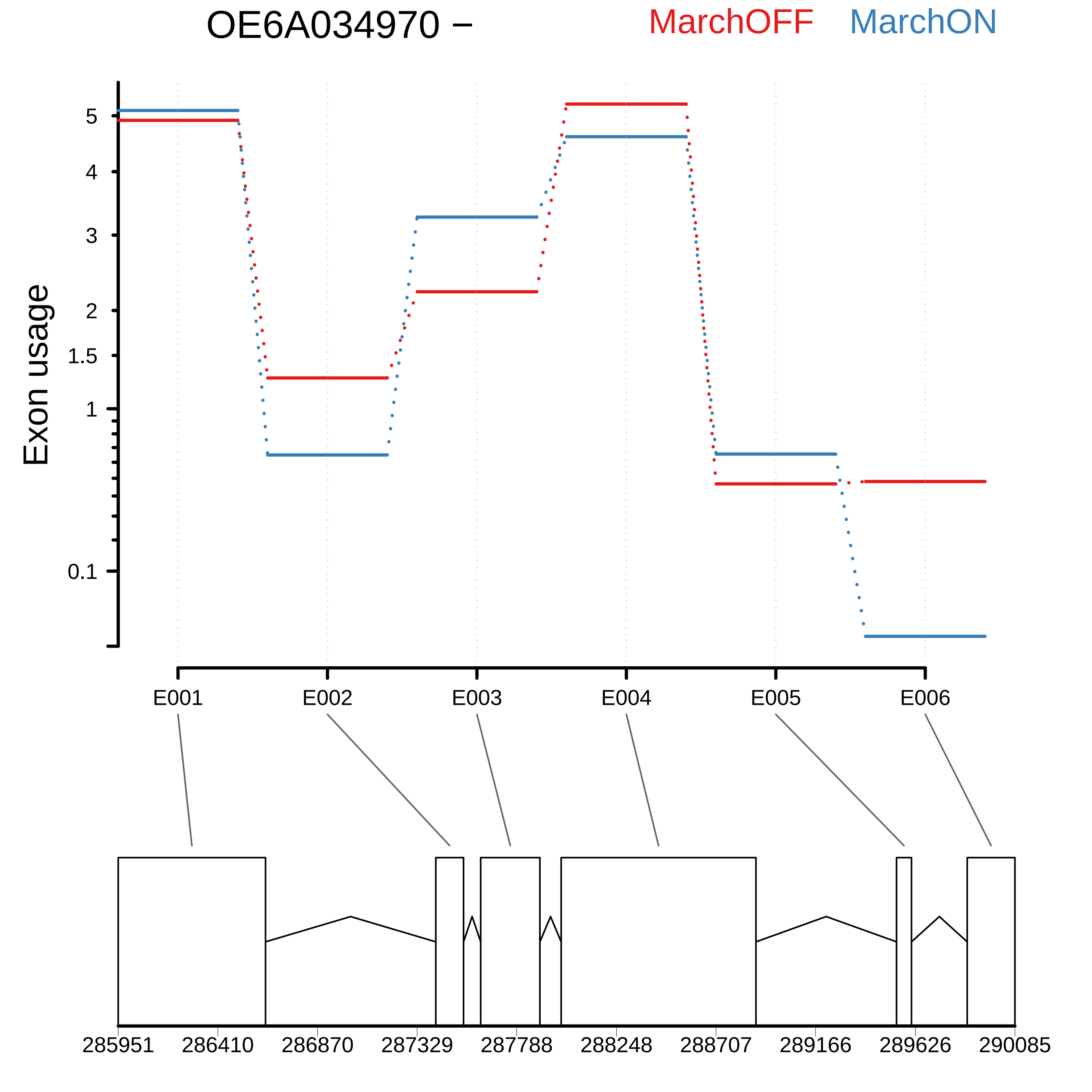
OE6A034970 −
MarchOFF
MarchON
Exon usage
5
4
3
2
1.5
1
0.1
E001
E002
E003
E004
E005
E006
285951
286410
286870
287329
287788
288248
288707
289166
289626
290085

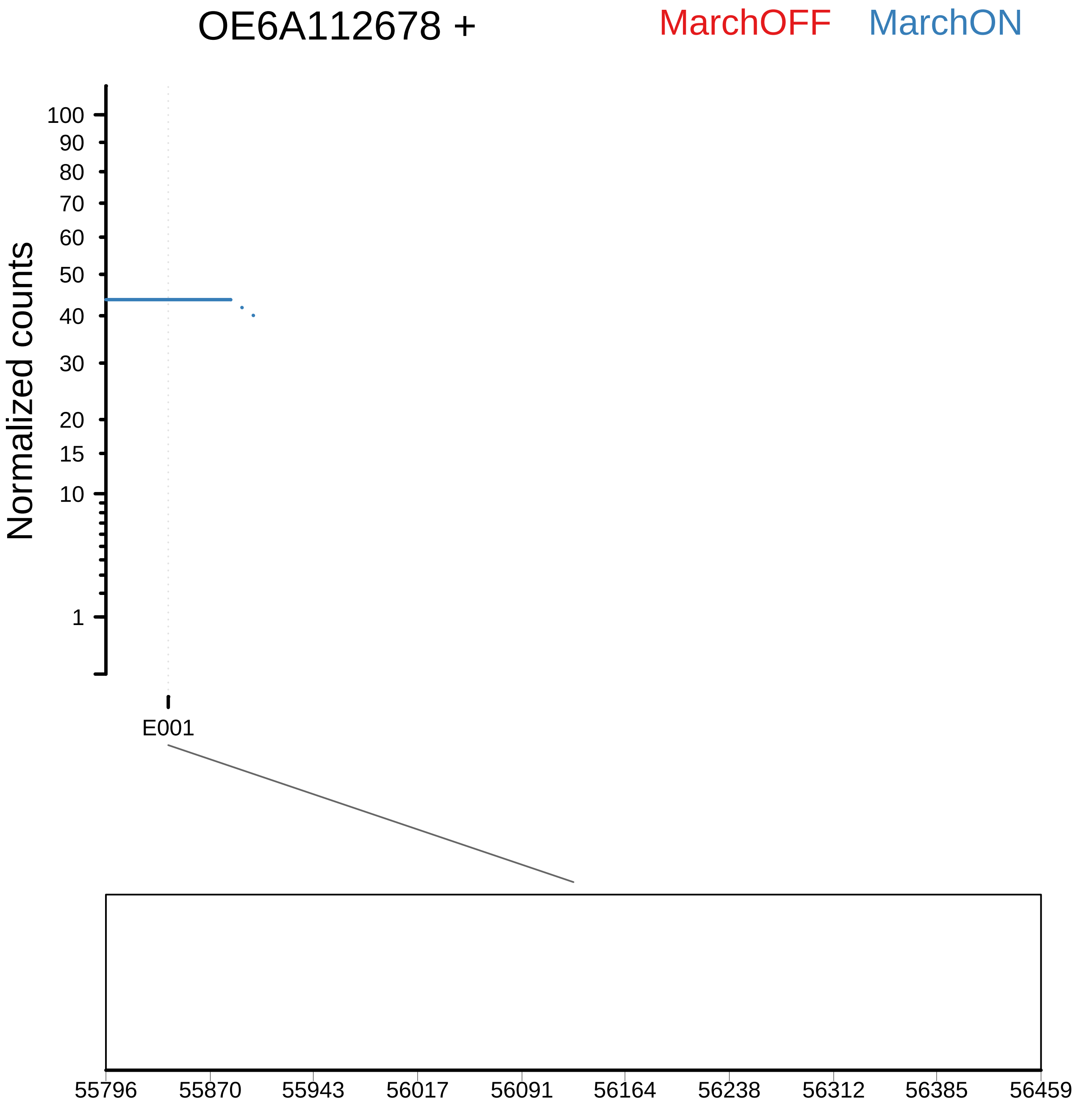
OE6A112678 +
MarchOFF
MarchON
Normalized counts
100
90
80
70
60
50
40
30
20
15
10
1
E001
55796
55870
55943
56017
56091
56164
56238
56312
56385
56459

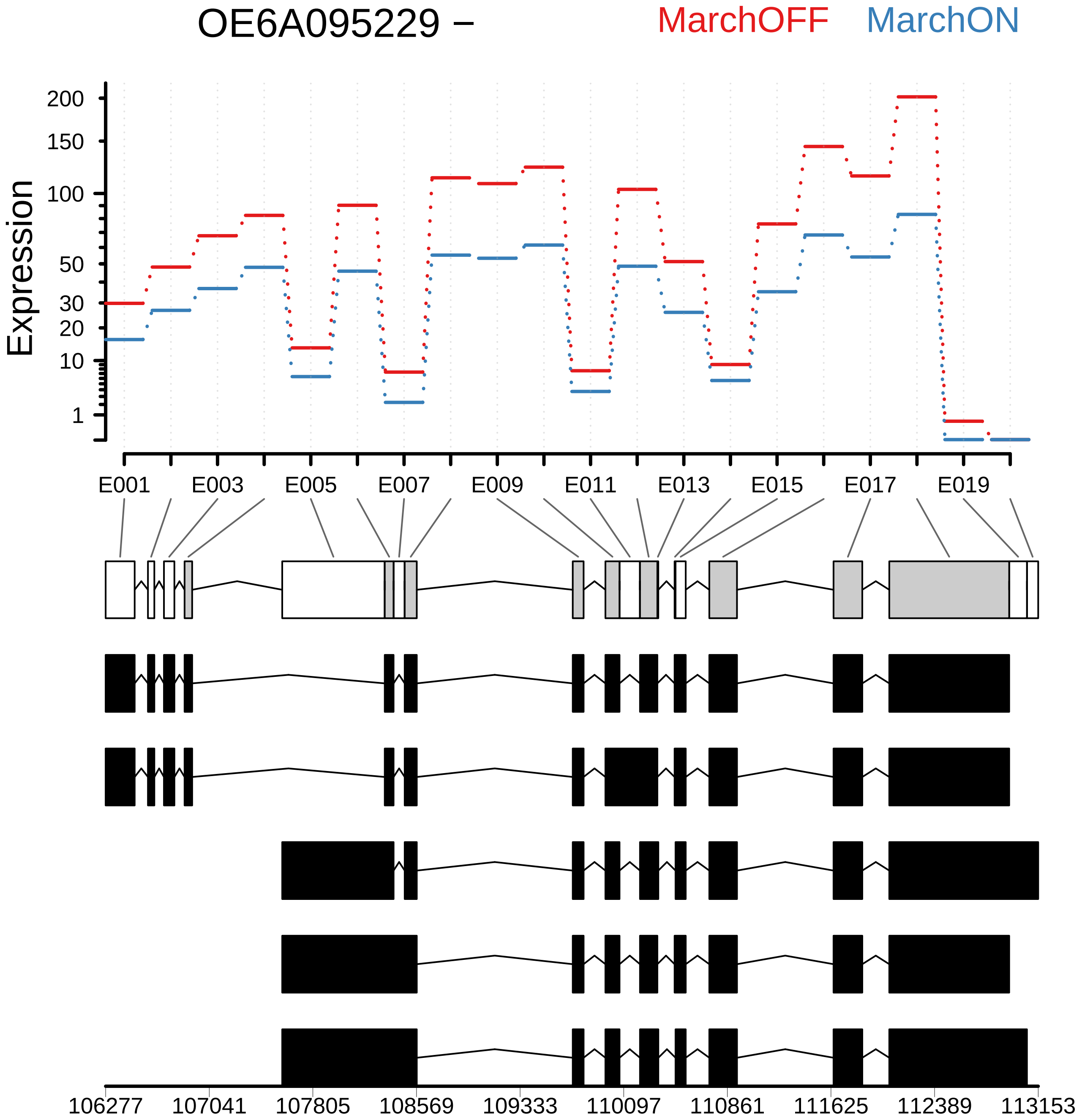

OE6A095229 –
MarchOFF
MarchON
Expression
200
150
100
50
30
20
10
1
E001
E003
E005
E007
E009
E011
E013
E015
E017
E019
106277
107041
107805
108569
109333
110097
110861
111625
112389
113153

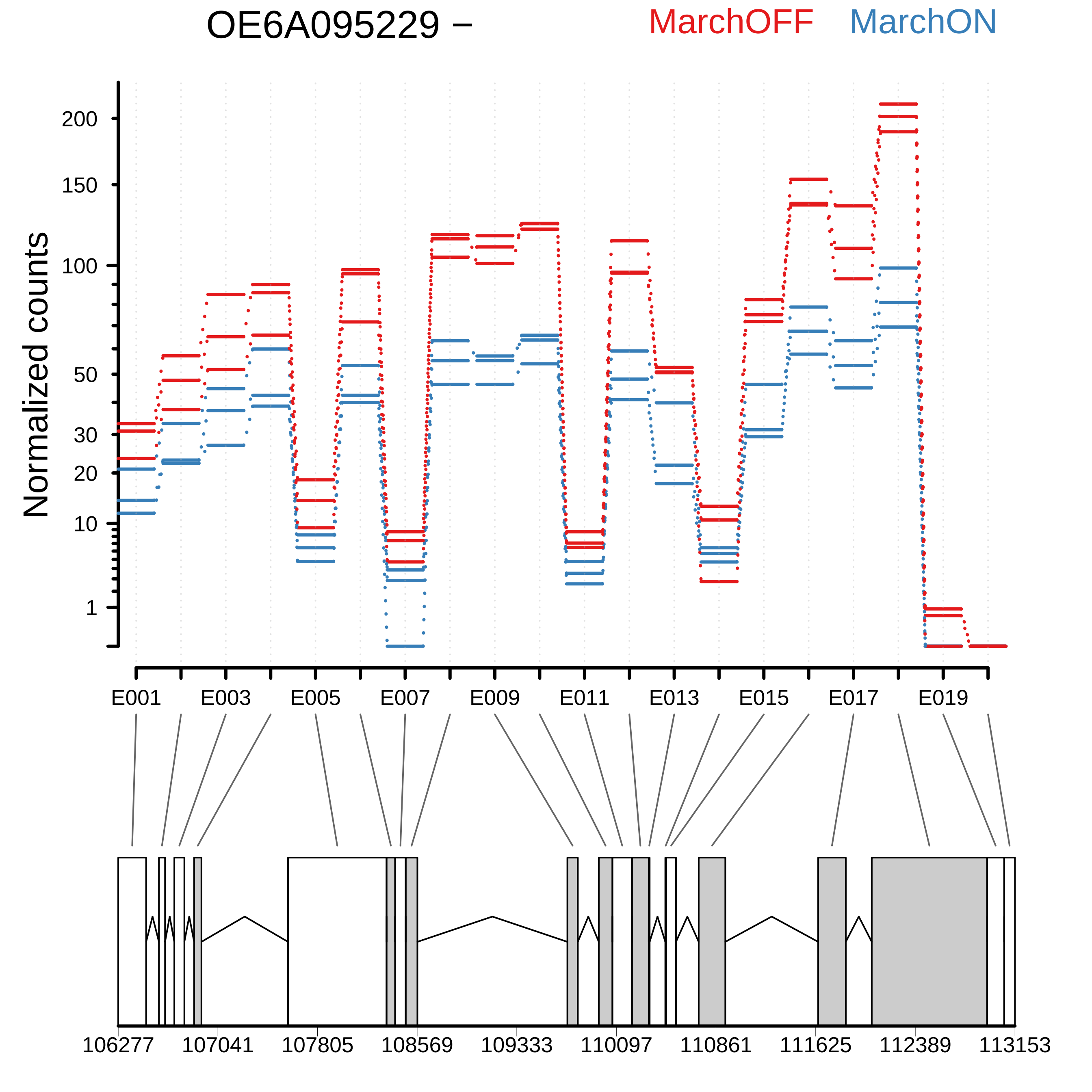

OE6A095229 −
MarchOFF
MarchON
Normalized counts
200
150
100
50
30
20
10
1
E001
E003
E005
E007
E009
E011
E013
E015
E017
E019
106277
107041
107805
108569
109333
110097
110861
111625
112389
113153

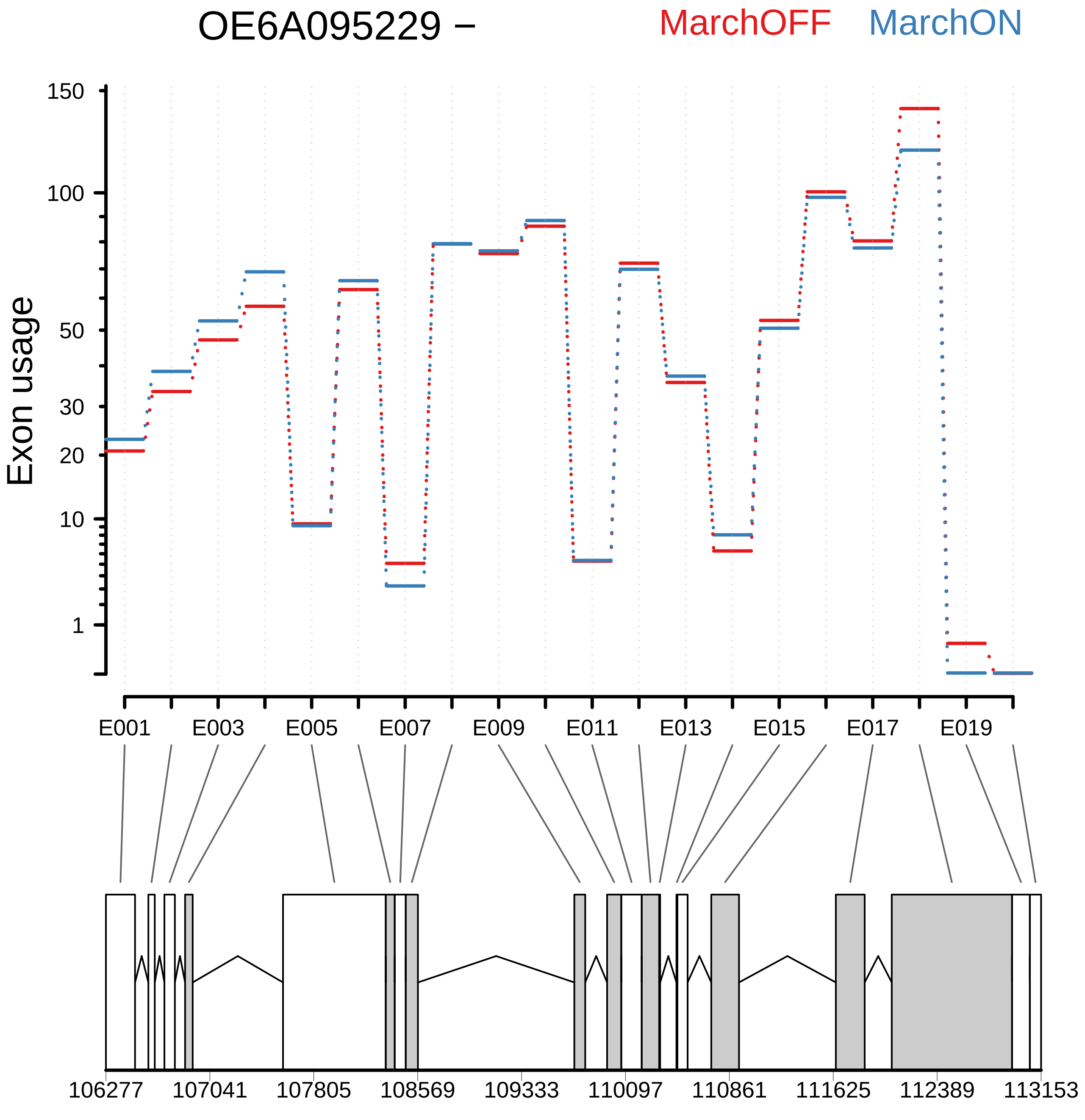
OE6A095229 −
MarchOFF
MarchON
Exon usage
150
100
50
30
20
10
1
E001
E003
E005
E007
E009
E011
E013
E015
E017
E019
106277
107041
107805
108569
109333
110097
110861
111625
112389
113153

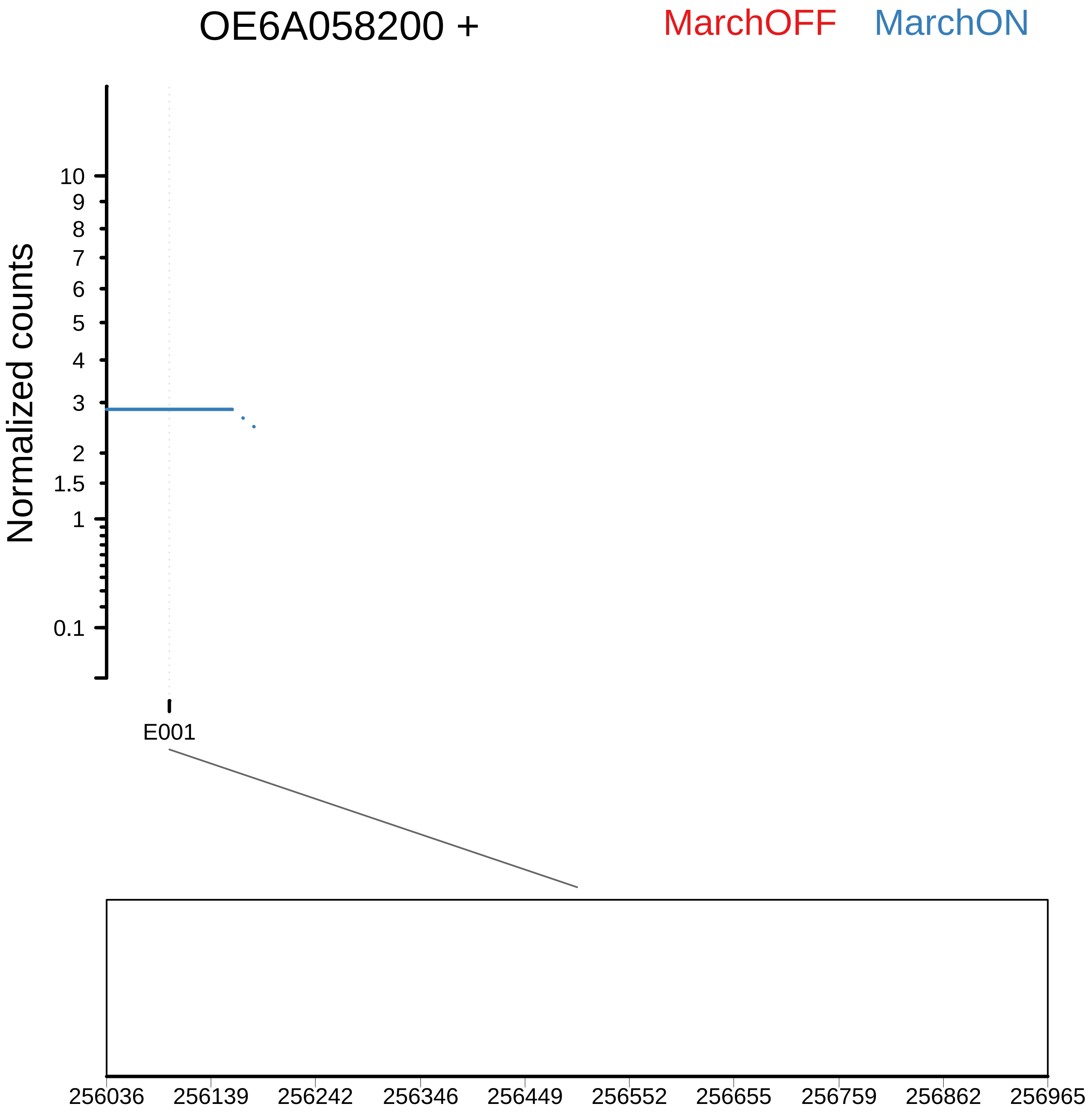

OE6A058200 +
MarchOFF
MarchON
Normalized counts
10
9
8
7
6
5
4
3
2
1.5
1
0.1
E001
256036
256139
256242
256346
256449
256552
256655
256759
256862
256965

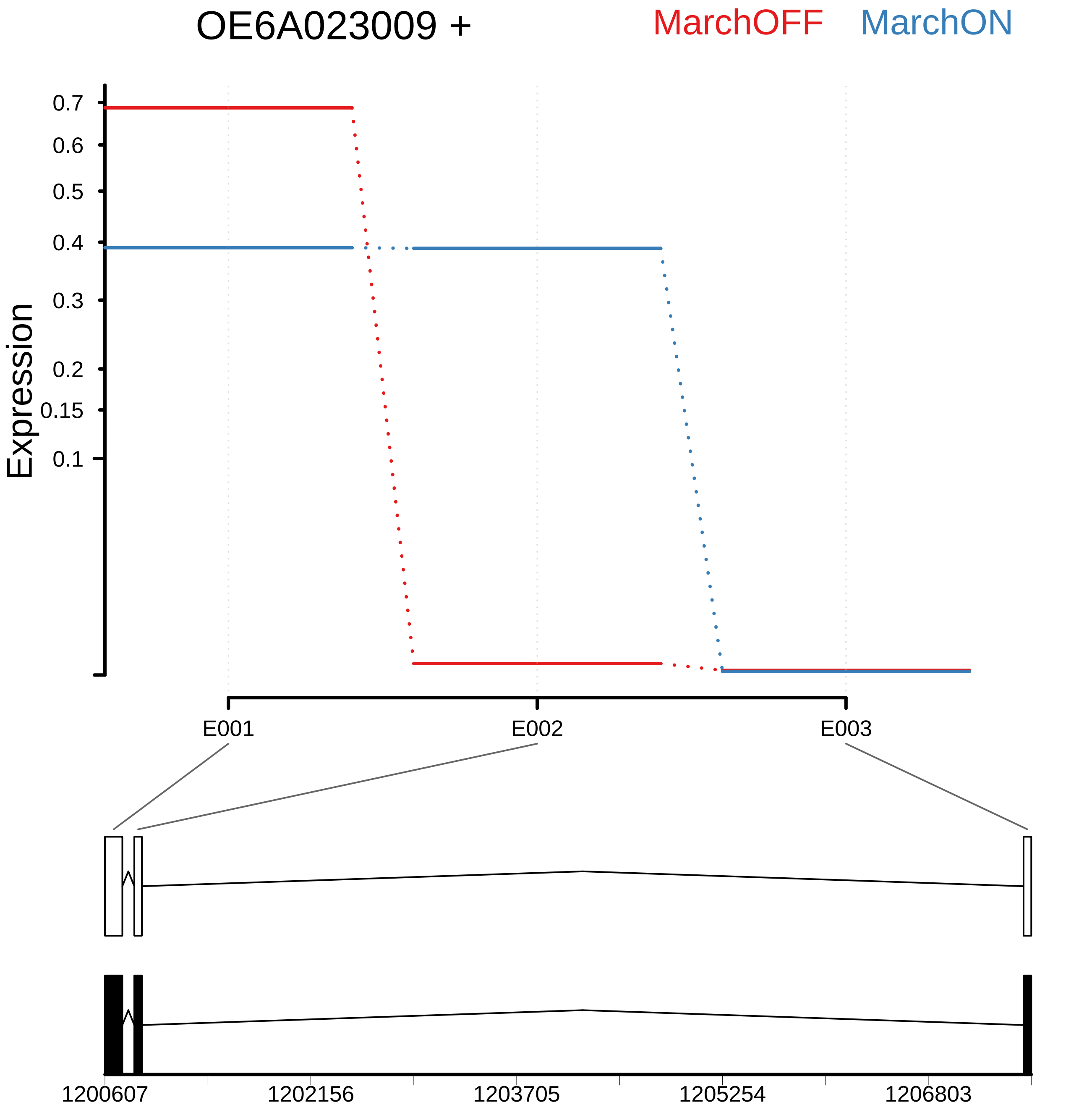

OE6A023009 +
MarchOFF
MarchON
Expression
0.7
0.6
0.5
0.4
0.3
0.2
0.15
0.1
E001
E002
E003
1200607
1202156
1203705
1205254
1206803

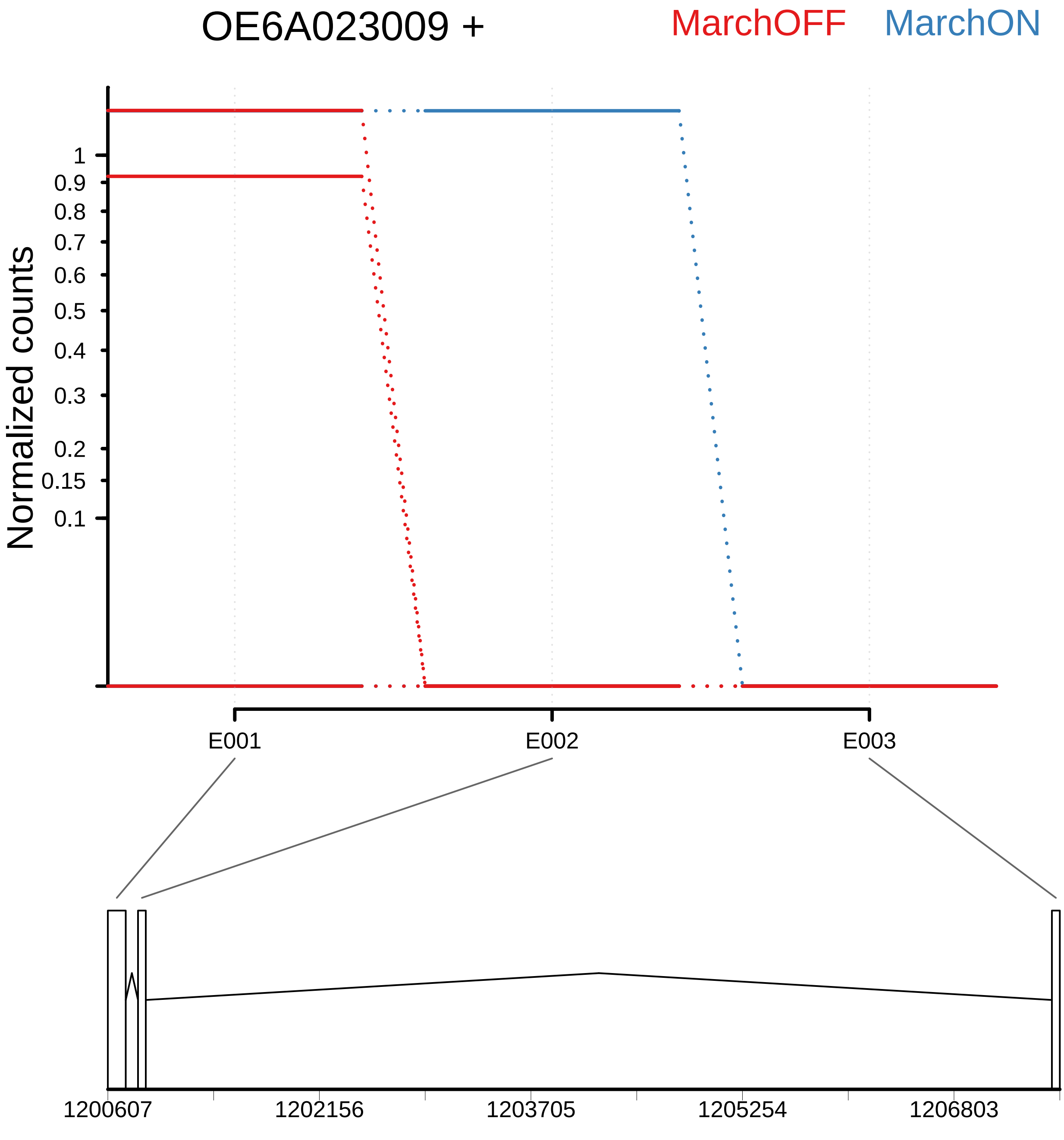
OE6A023009 +
MarchOFF
MarchON
Normalized counts
1
0.9
0.8
0.7
0.6
0.5
0.4
0.3
0.2
0.15
0.1
E001
E002
E003
1200607
1202156
1203705
1205254
1206803

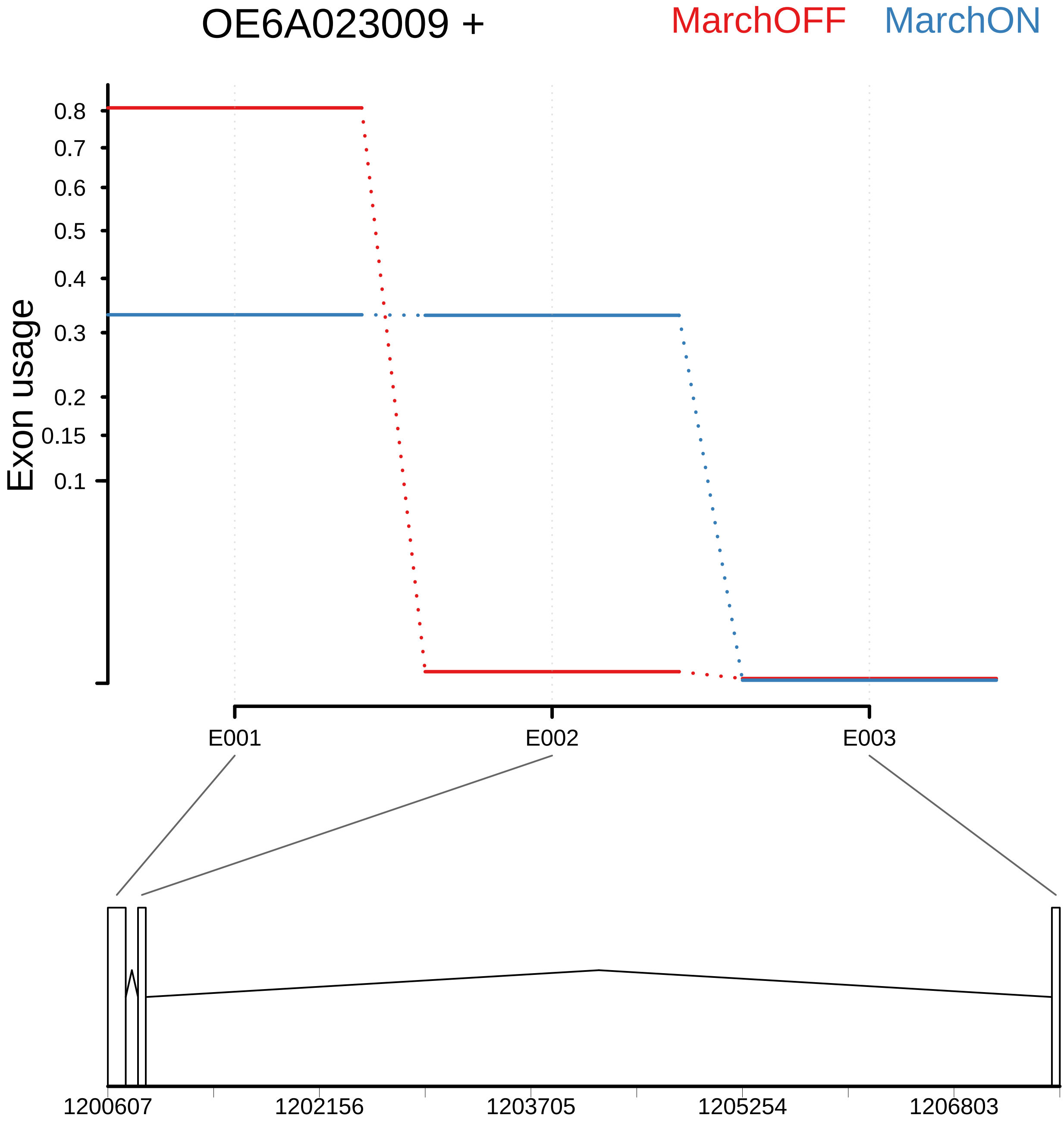

OE6A023009 +
MarchOFF
MarchON
Exon usage
0.8
0.7
0.6
0.5
0.4
0.3
0.2
0.15
0.1
E001
E002
E003
1200607
1202156
1203705
1205254
1206803

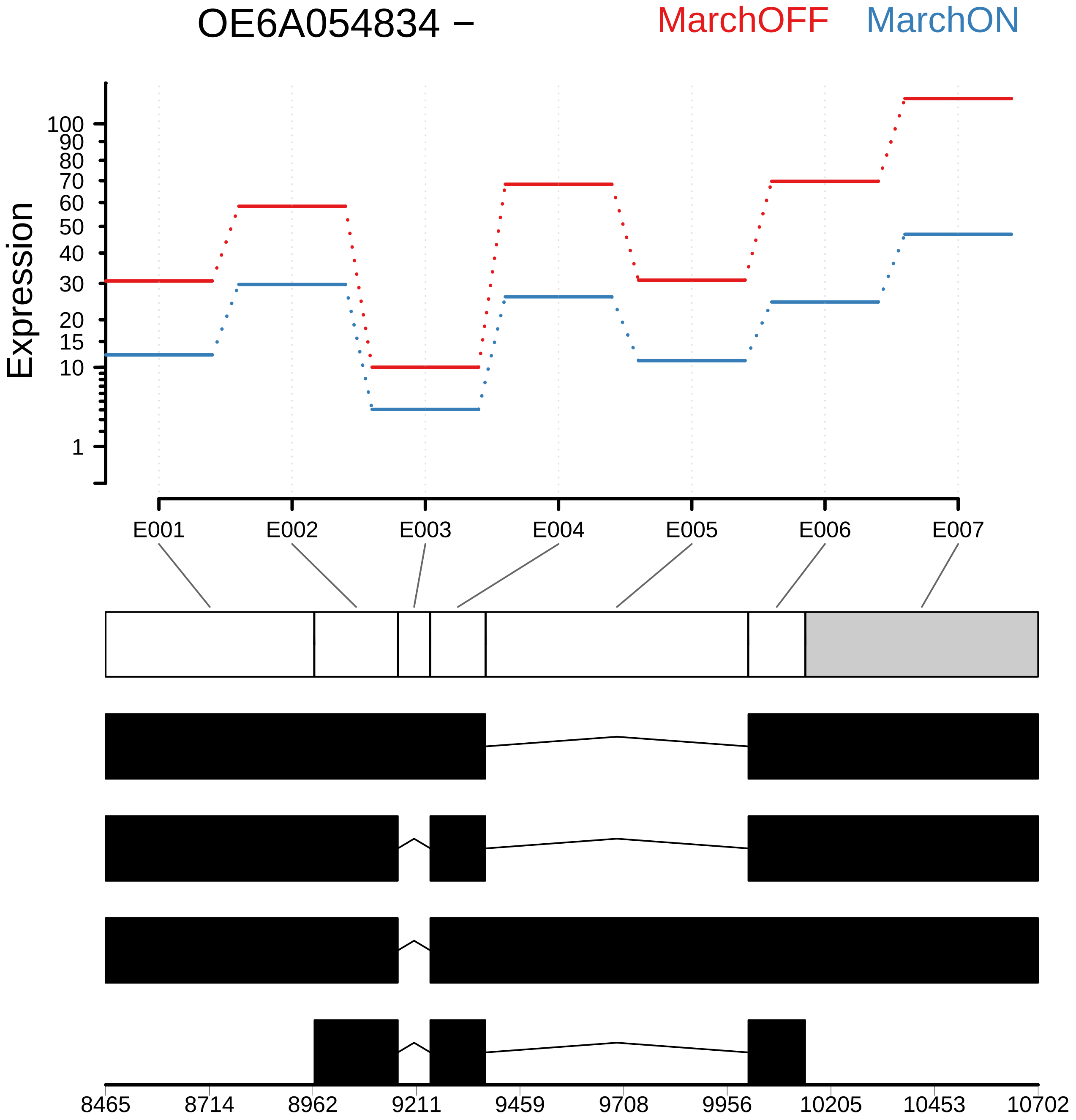
OE6A054834 −
MarchOFF
MarchON
Expression
100
90
80
70
60
50
40
30
20
15
10
1
E001
E002
E003
E004
E005
E006
E007
8465
8714
8962
9211
9459
9708
9956
10205
10453
10702

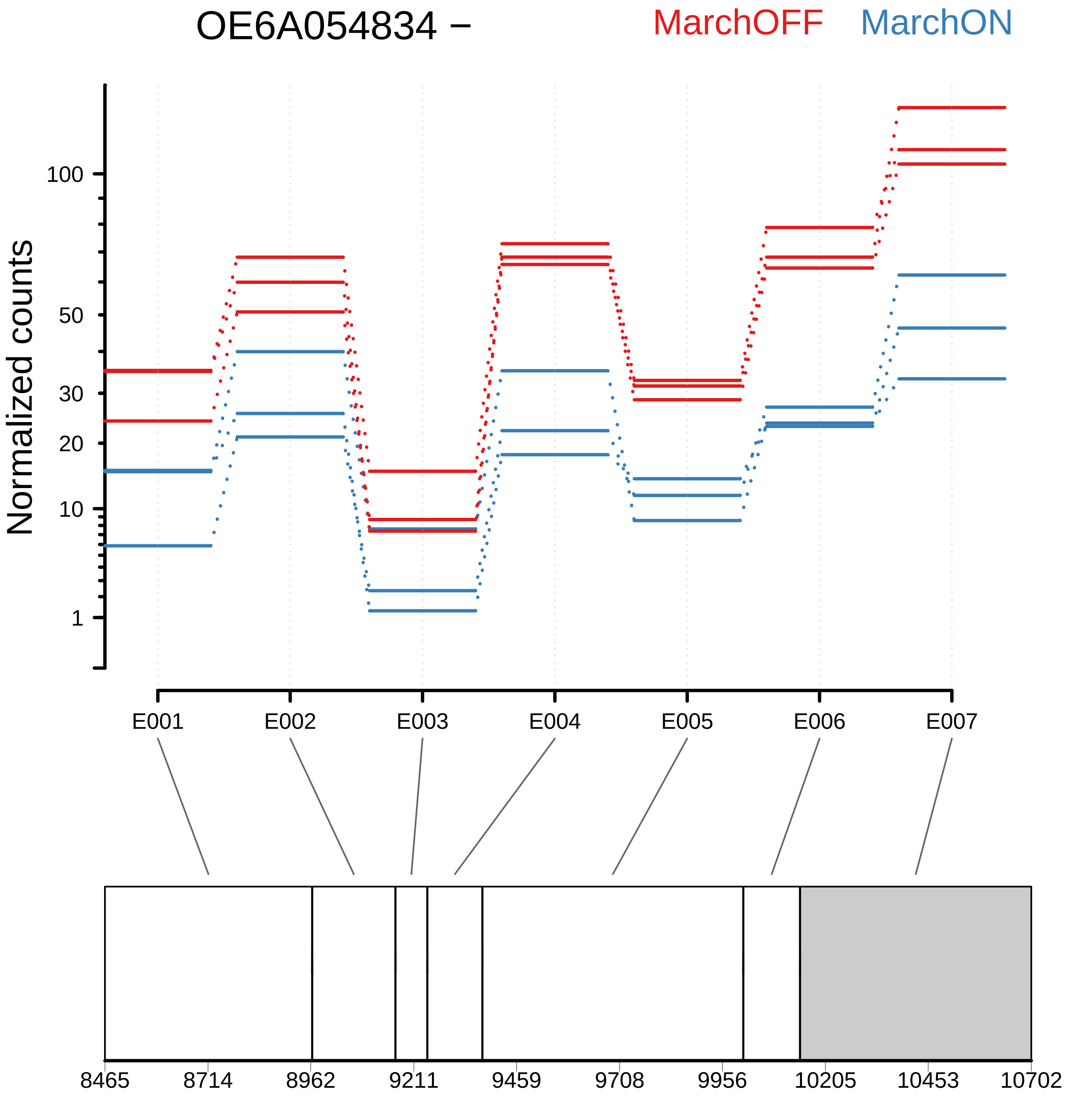

OE6A054834 −
MarchOFF
MarchON
Normalized counts
100
50
30
20
10
1
E001
E002
E003
E004
E005
E006
E007
8465
8714
8962
9211
9459
9708
9956
10205
10453
10702

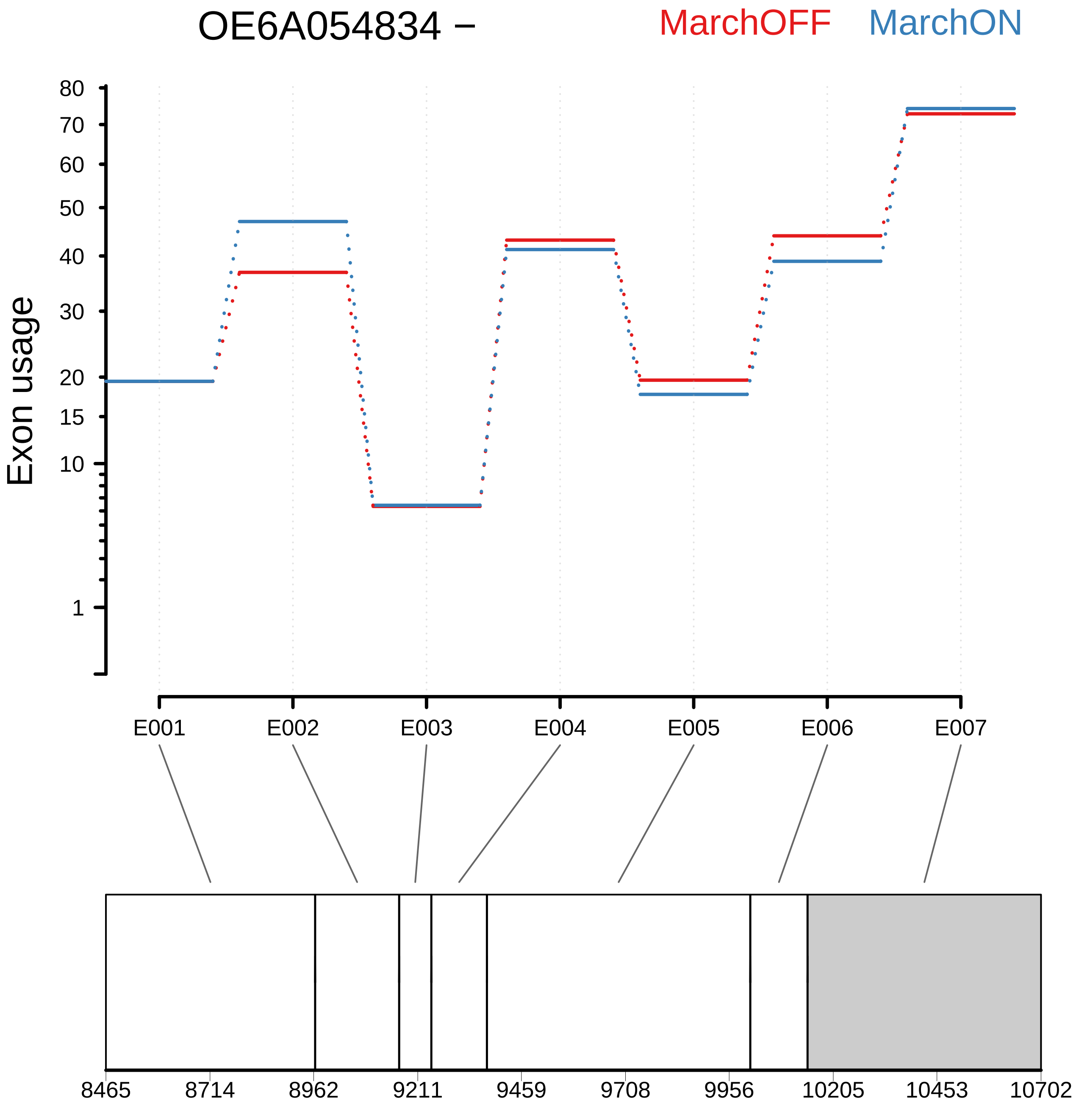

OE6A054834 −
MarchOFF
MarchON
Exon usage
80
70
60
50
40
30
20
15
10
1
E001
E002
E003
E004
E005
E006
E007
8465
8714
8962
9211
9459
9708
9956
10205
10453
10702

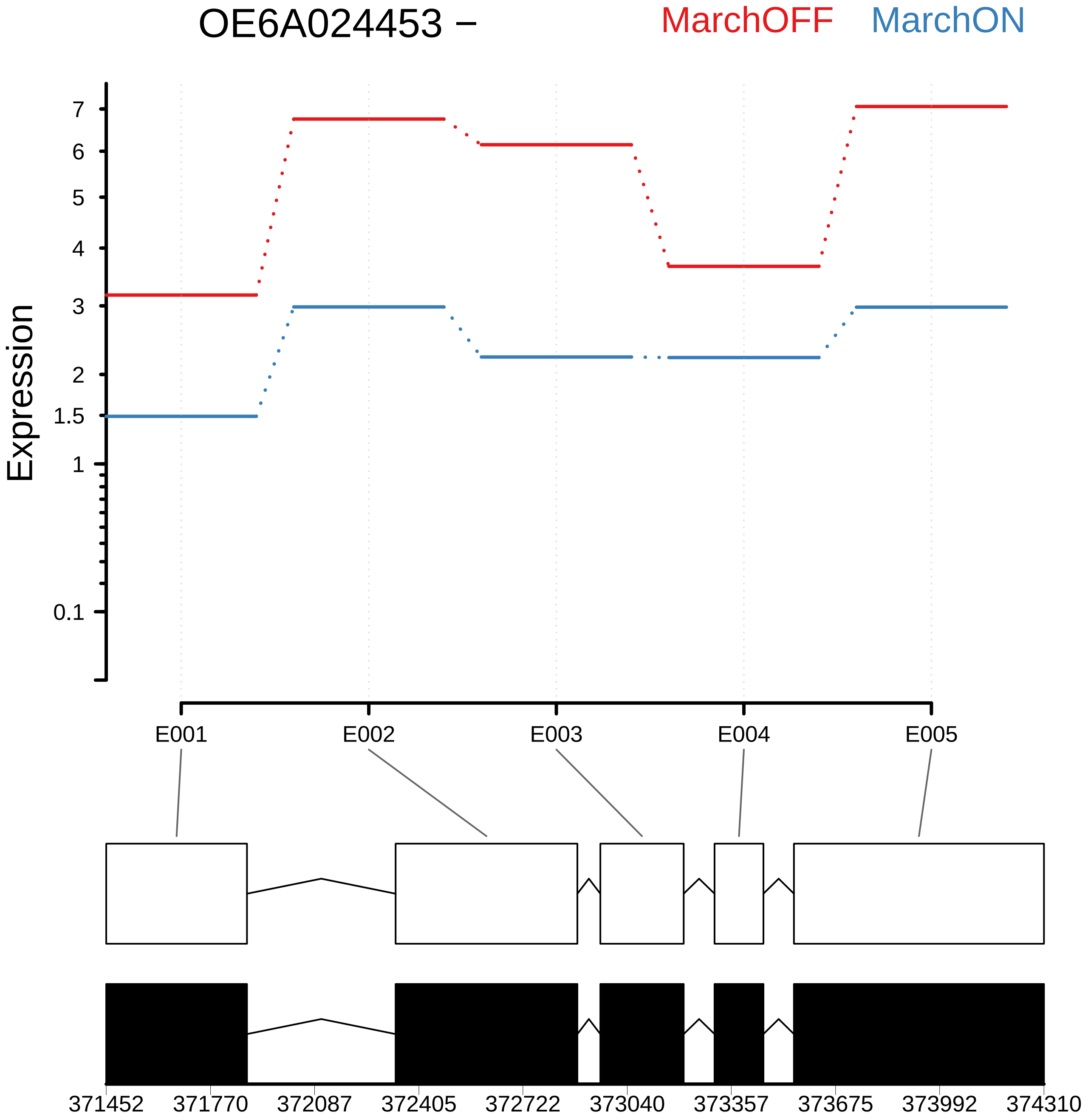

OE6A024453 −
MarchOFF
MarchON
Expression
7
6
5
4
3
2
1.5
1
0.1
E001
E002
E003
E004
E005
371452
371770
372087
372405
372722
373040
373357
373675
373992
374310

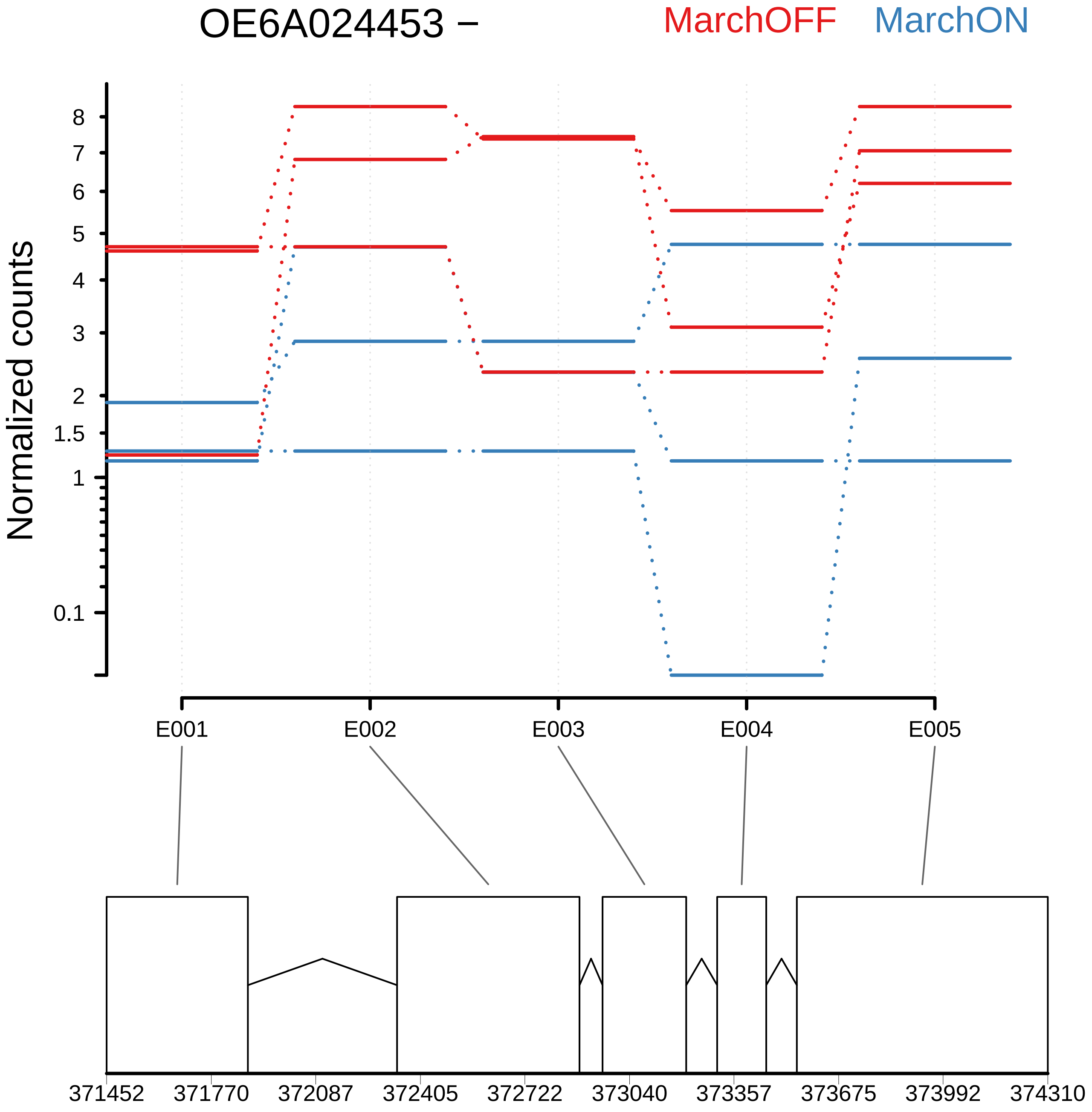

OE6A024453 −
MarchOFF
MarchON
Normalized counts
8
7
6
5
4
3
2
1.5
1
0.1
E001
E002
E003
E004
E005
371452
371770
372087
372405
372722
373040
373357
373675
373992
374310

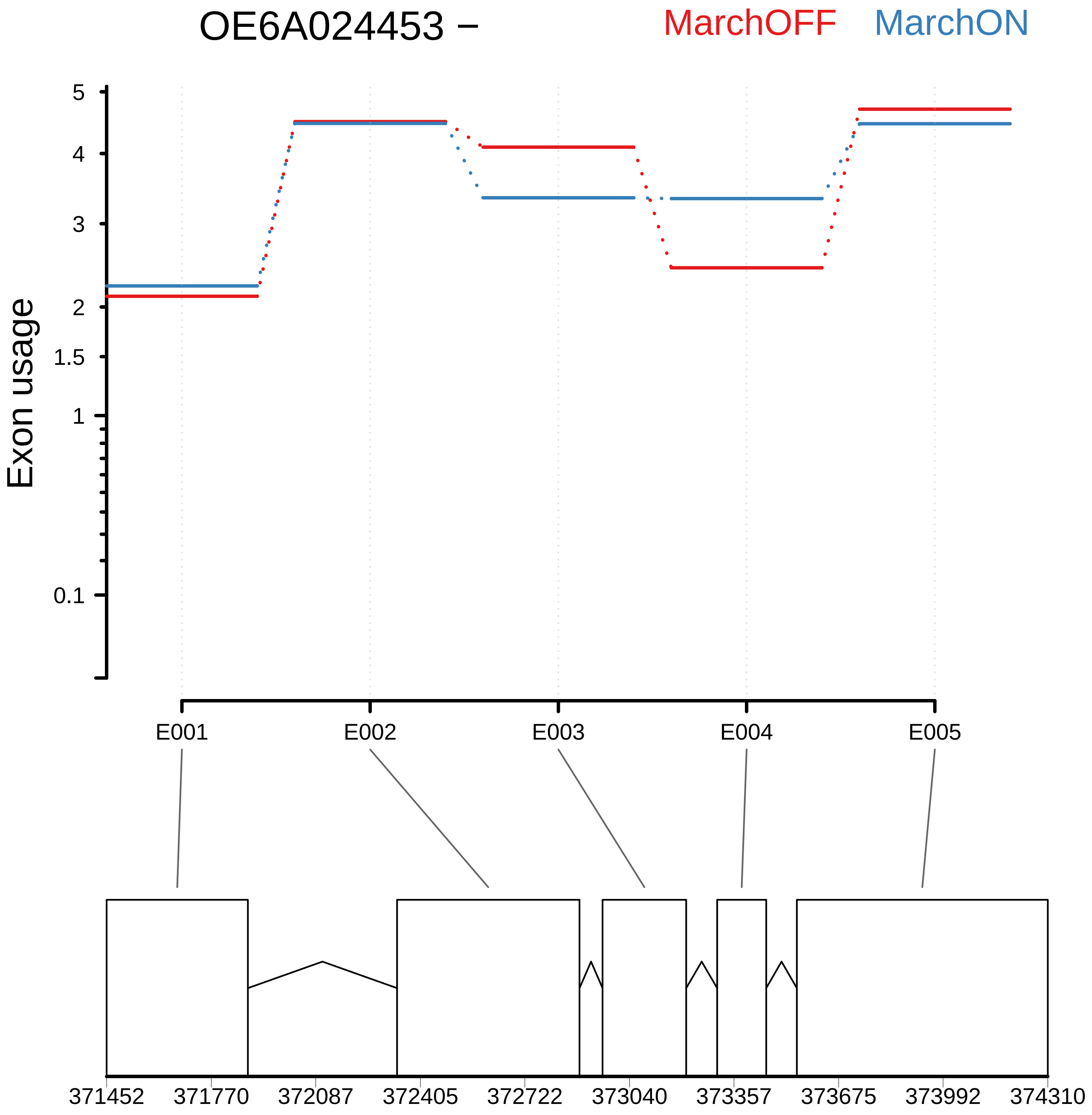
OE6A024453 −
MarchOFF
MarchON
Exon usage
5
4
3
2
1.5
1
0.1
E001
E002
E003
E004
E005
371452
371770
372087
372405
372722
373040
373357
373675
373992
374310

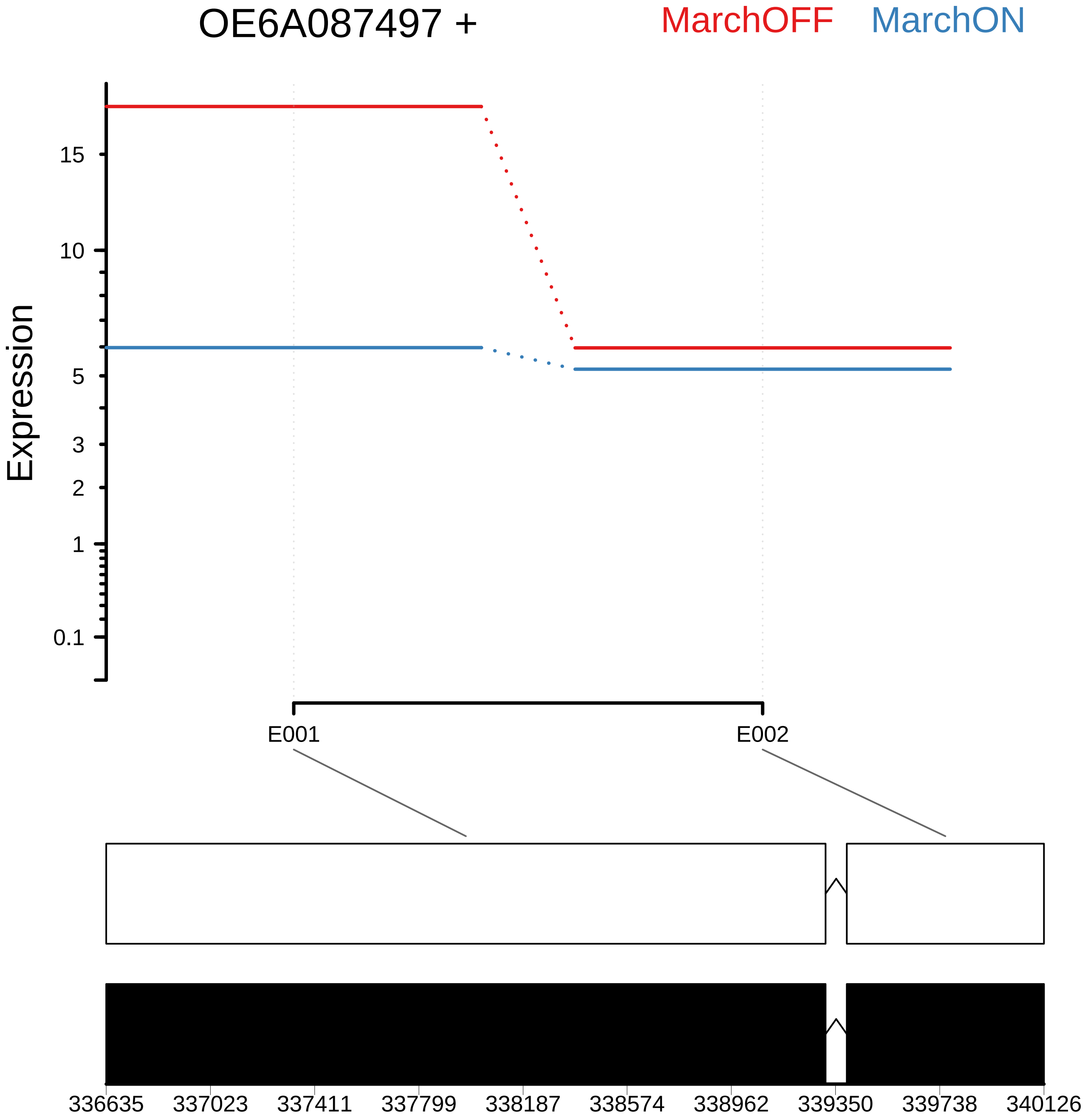

OE6A087497 +
MarchOFF
MarchON
Expression
15
10
5
3
2
1
0.1
E001
E002
336635
337023
337411
337799
338187
338574
338962
339350
339738
340126

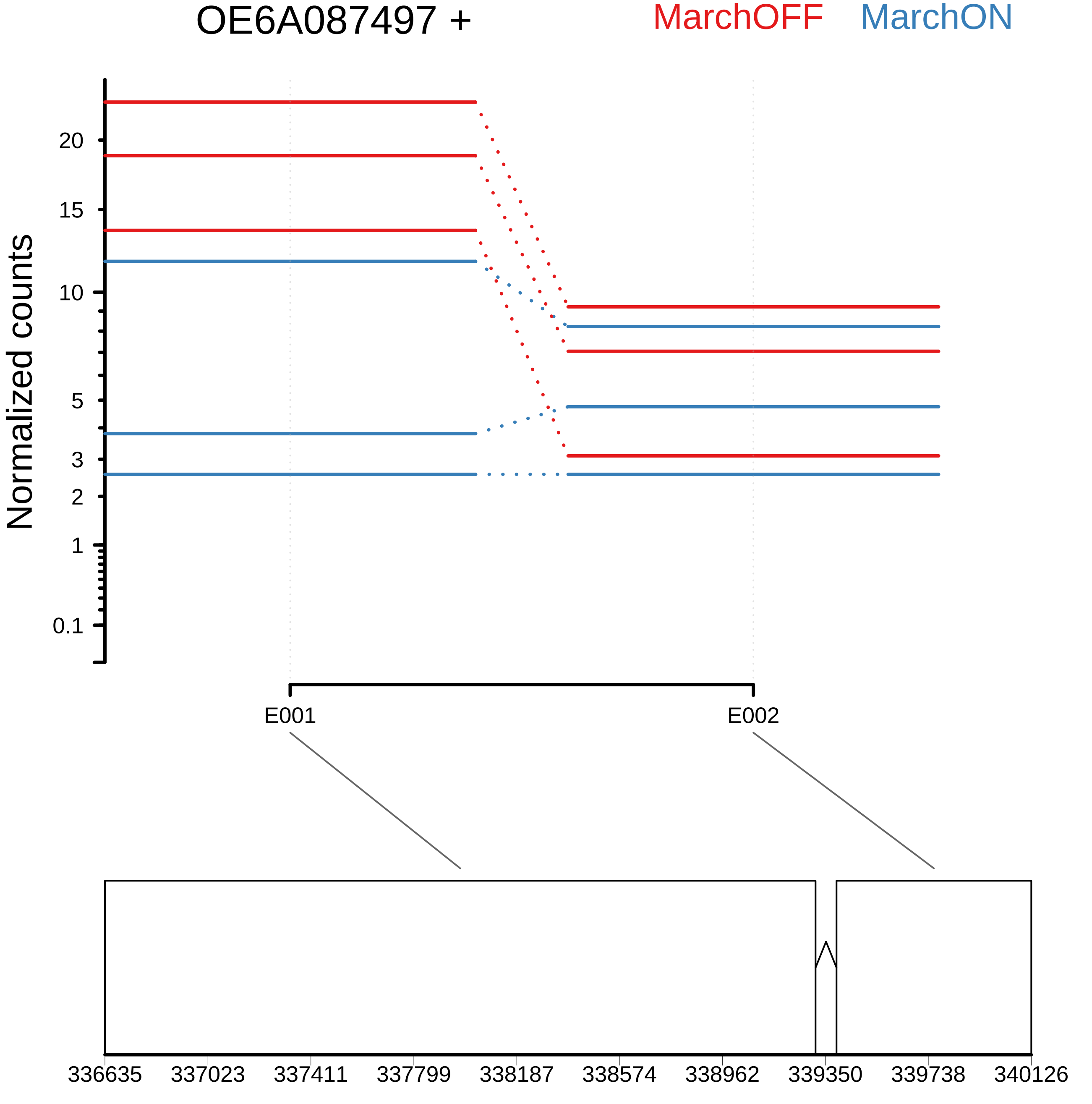

OE6A087497 +
MarchOFF
MarchON
Normalized counts
20
15
10
5
3
2
1
0.1
E001
E002
336635
337023
337411
337799
338187
338574
338962
339350
339738
340126

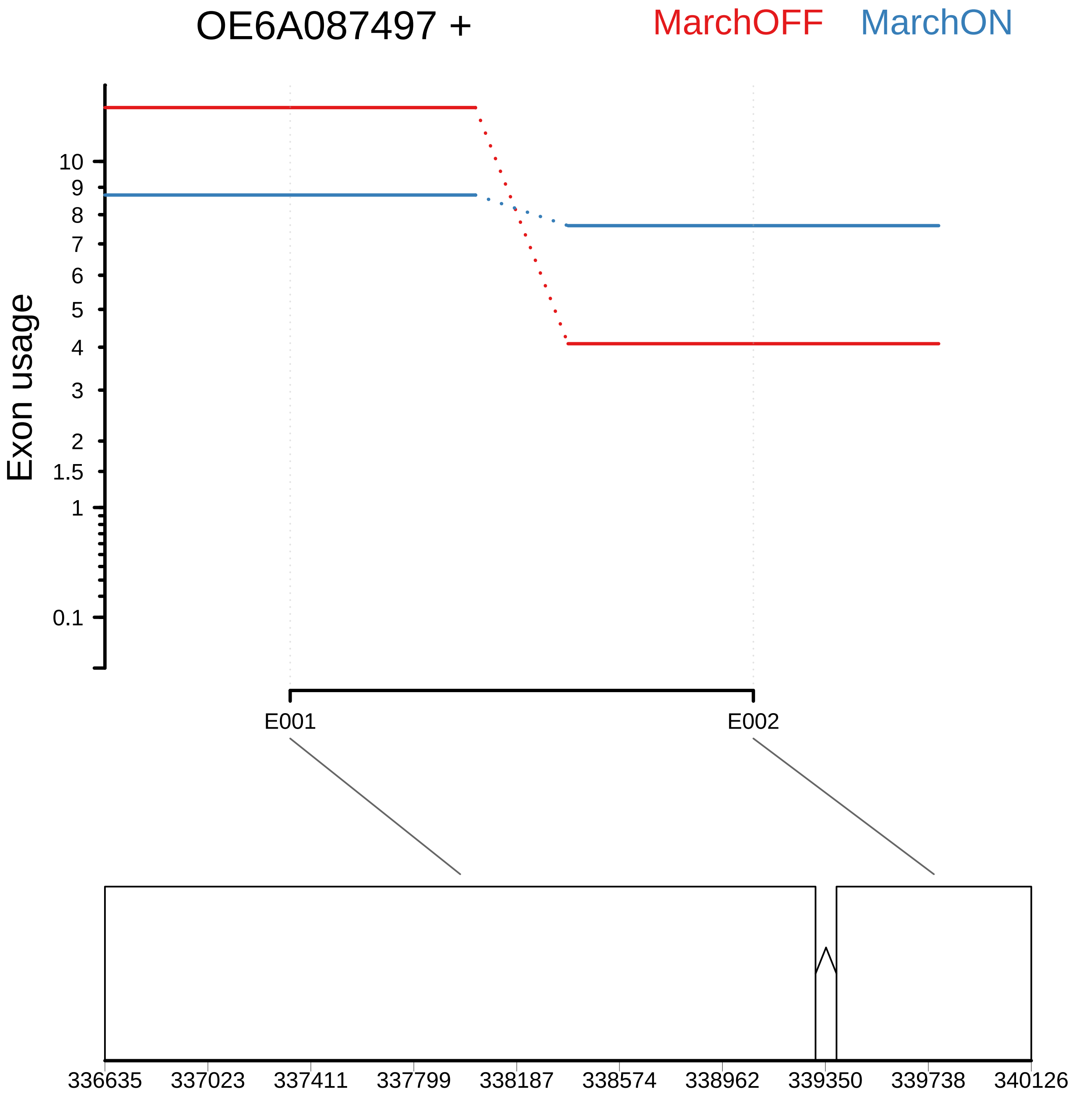

OE6A087497 +
MarchOFF
MarchON
Exon usage
10
9
8
7
6
5
4
3
2
1.5
1
0.1
E001
E002
336635
337023
337411
337799
338187
338574
338962
339350
339738
340126

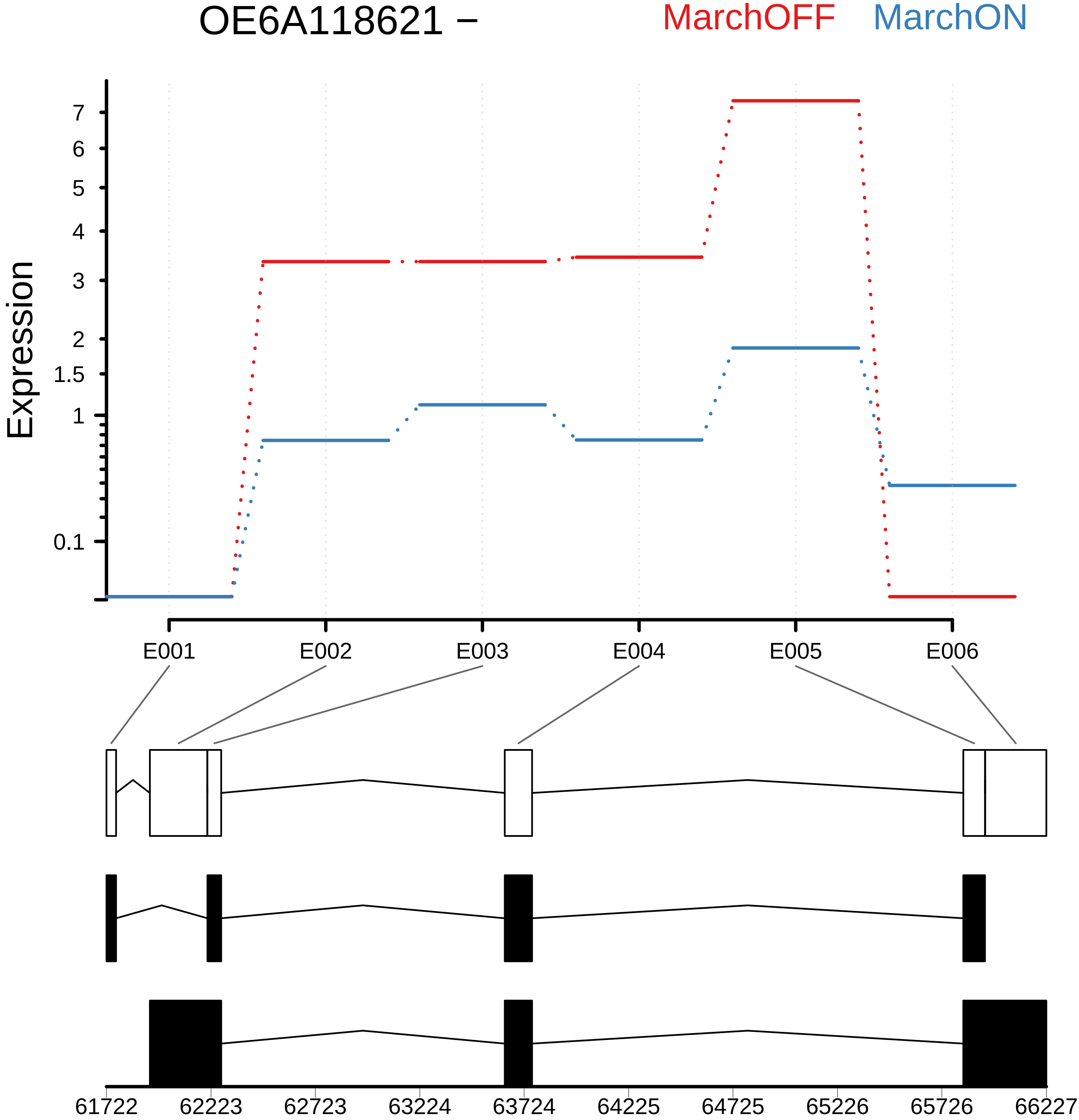
OE6A118621 −
MarchOFF
MarchON
Expression
7
6
5
4
3
2
1.5
1
0.1
E001
E002
E003
E004
E005
E006
61722
62223
62723
63224
63724
64225
64725
65226
65726
66227

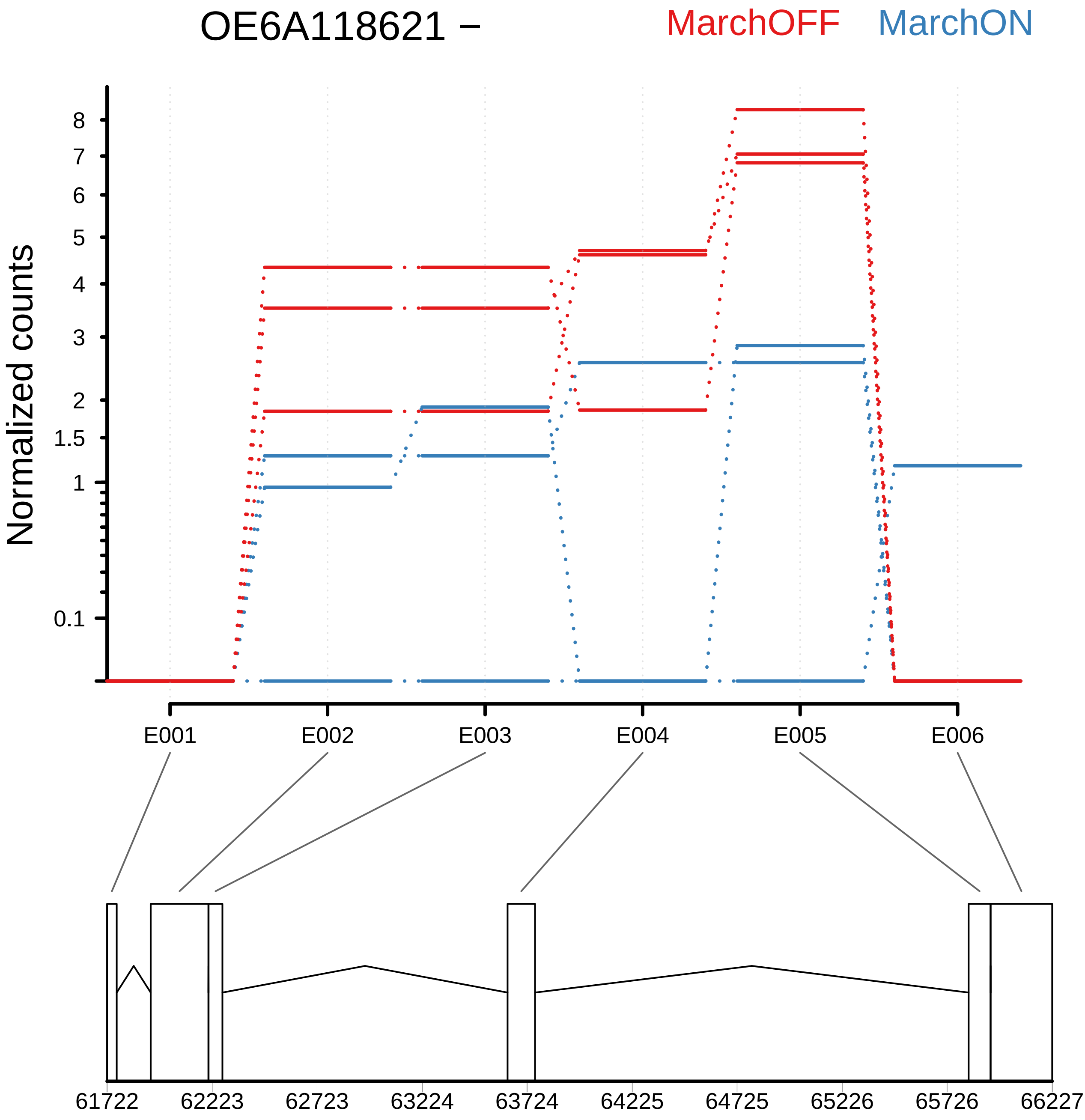

OE6A118621 −
MarchOFF
MarchON
Normalized counts
8
7
6
5
4
3
2
1.5
1
0.1
E001
E002
E003
E004
E005
E006
61722
62223
62723
63224
63724
64225
64725
65226
65726
66227

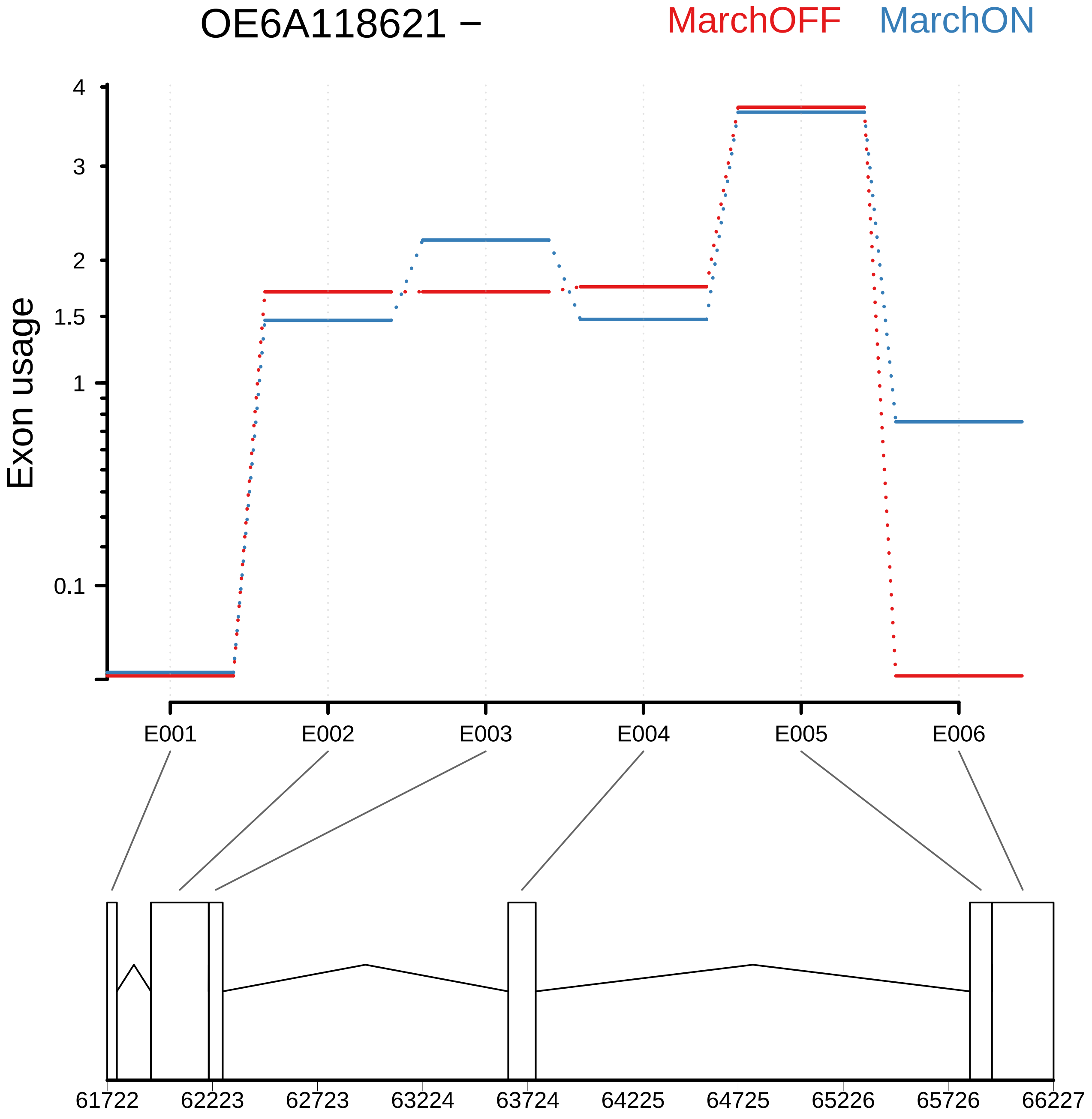
OE6A118621 −
MarchOFF
MarchON
Exon usage
4
3
2
1.5
1
0.1
E001
E002
E003
E004
E005
E006
61722
62223
62723
63224
63724
64225
64725
65226
65726
66227

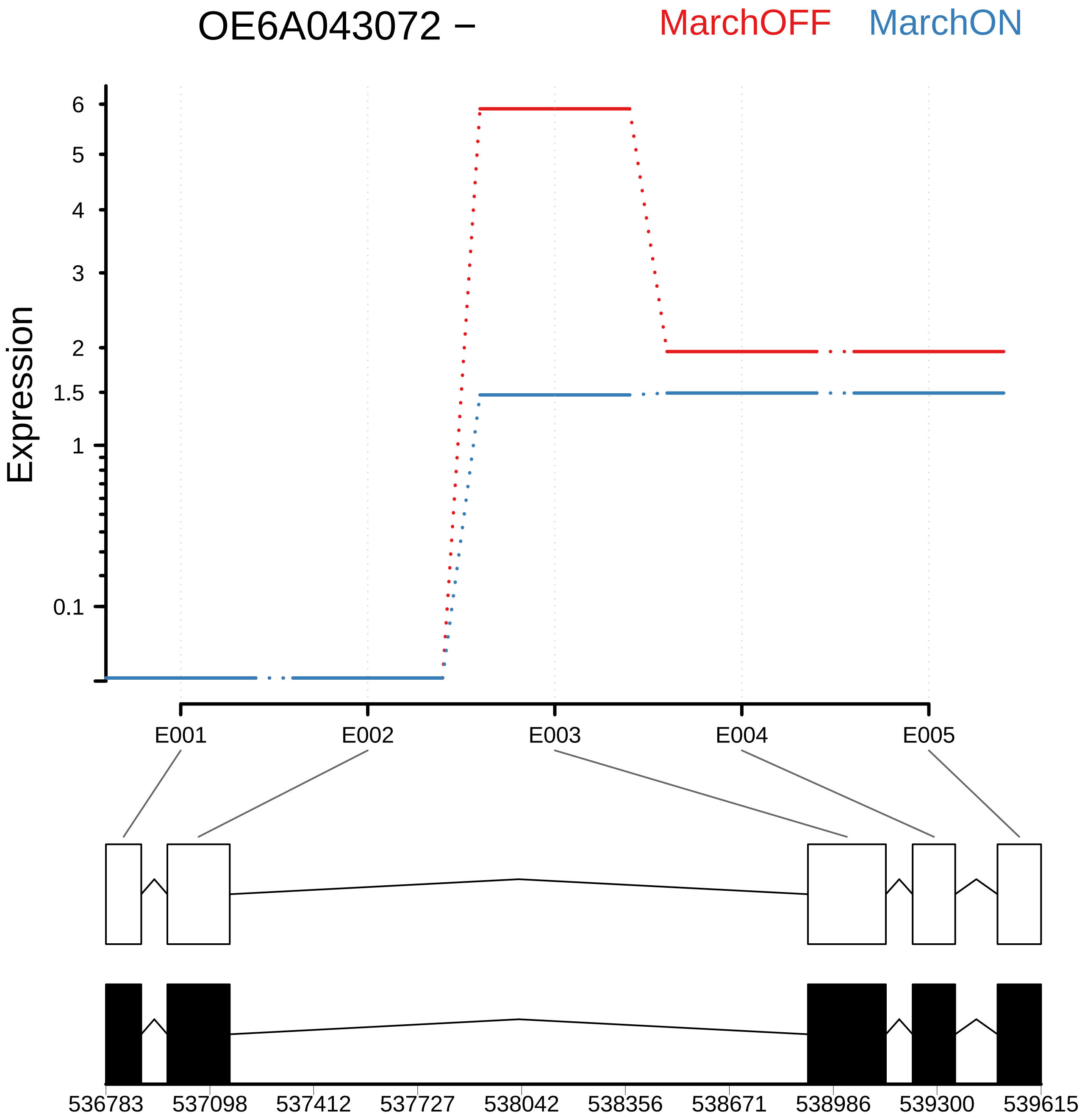

OE6A043072 −
MarchOFF
MarchON
Expression
6
5
4
3
2
1.5
1
0.1
E001
E002
E003
E004
E005
536783
537098
537412
537727
538042
538356
538671
538986
539300
539615

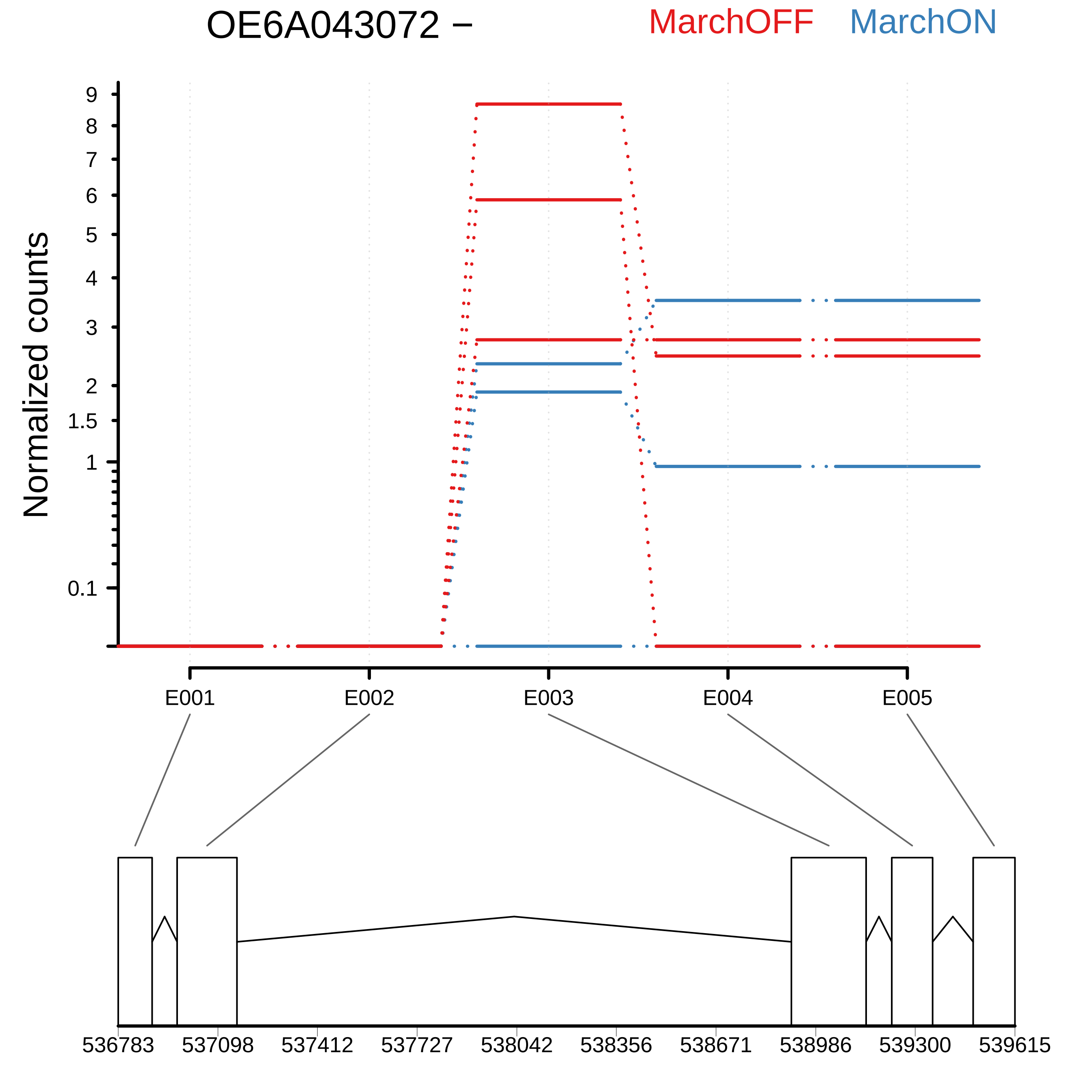
OE6A043072 −
MarchOFF
MarchON
Normalized counts
9
8
7
6
5
4
3
2
1.5
1
0.1
E001
E002
E003
E004
E005
536783
537098
537412
537727
538042
538356
538671
538986
539300
539615

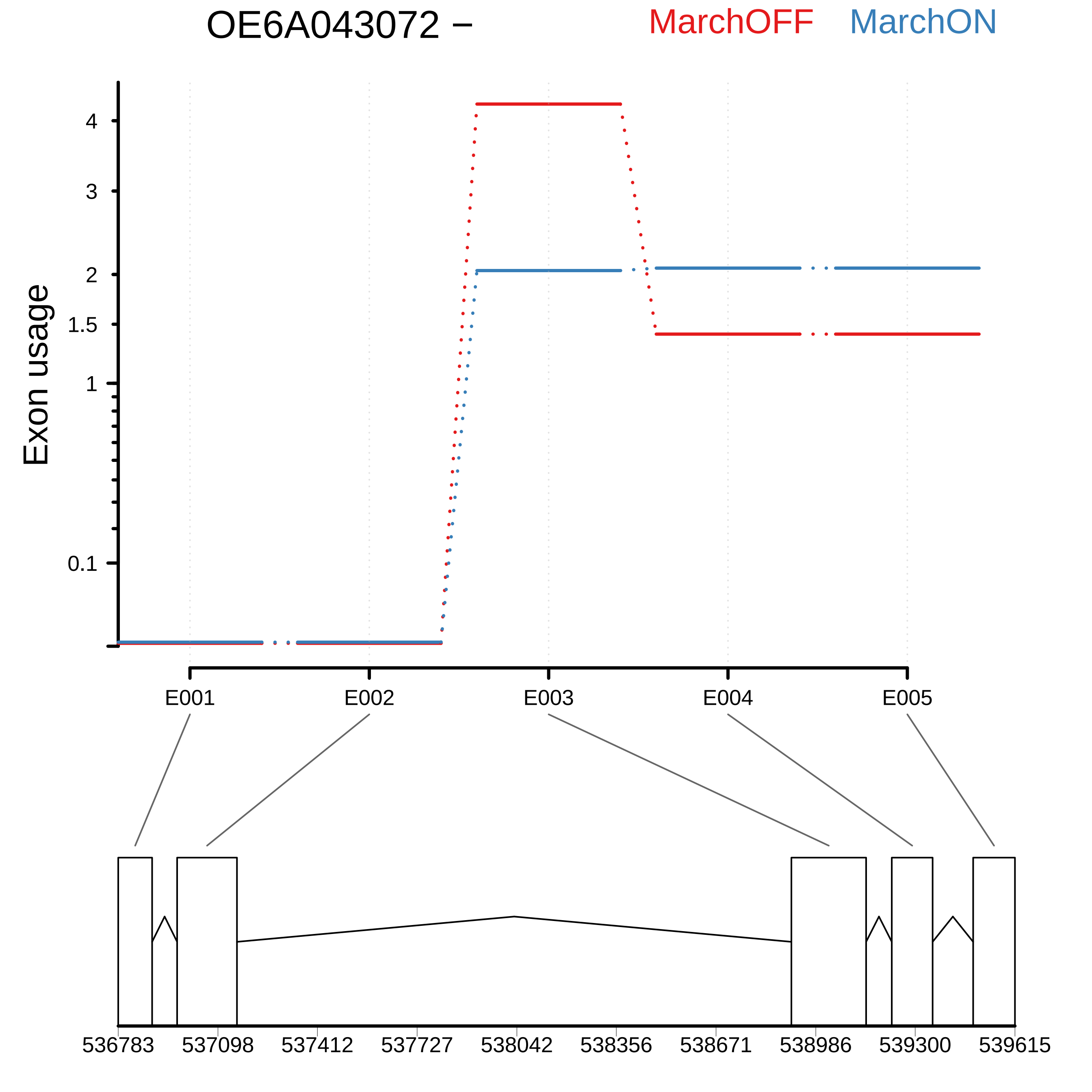

OE6A043072 −
MarchOFF
MarchON
Exon usage
4
3
2
1.5
1
0.1
E001
E002
E003
E004
E005
536783
537098
537412
537727
538042
538356
538671
538986
539300
539615

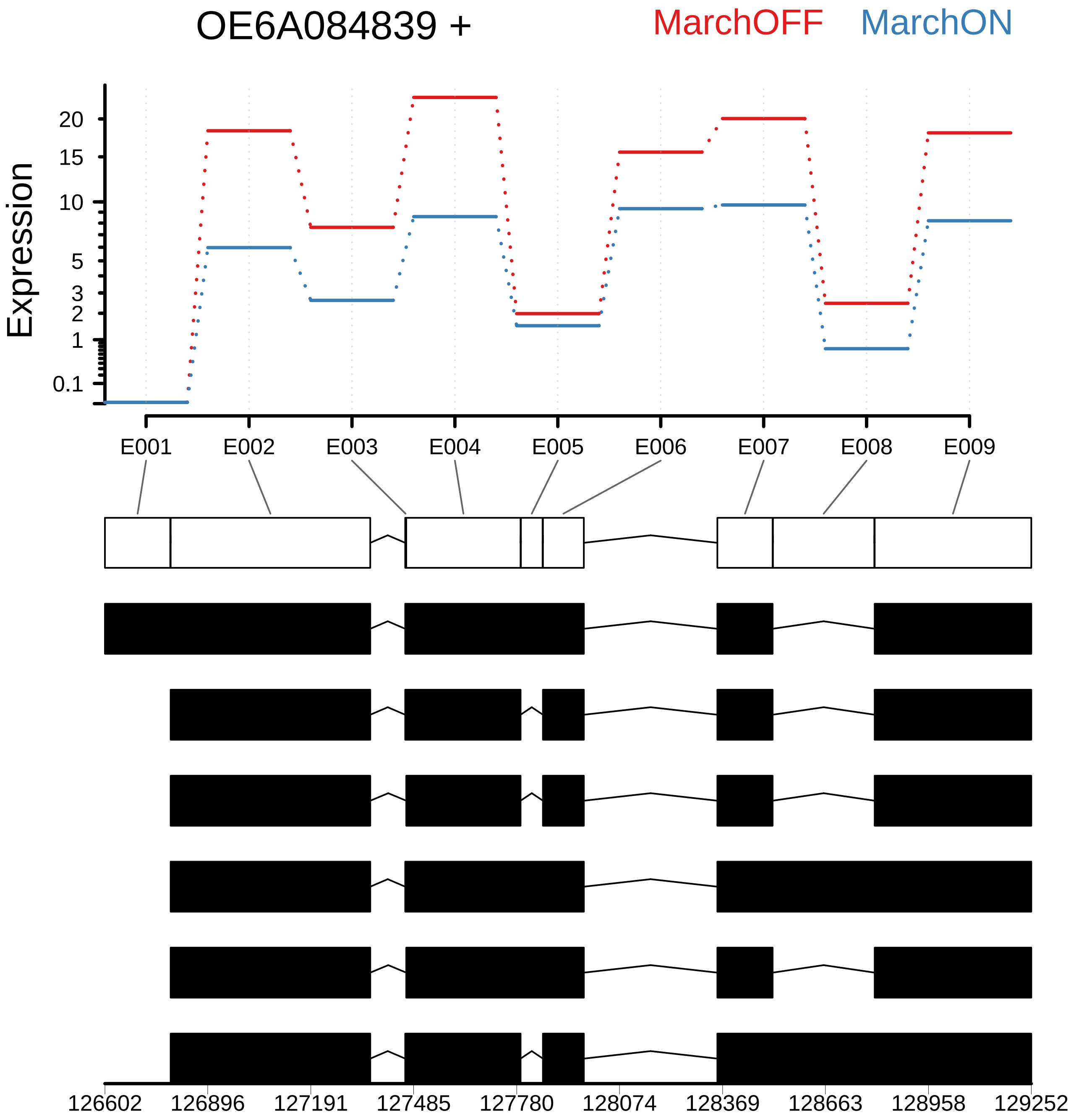

OE6A084839 +
MarchOFF
MarchON
Expression
20
15
10
5
3
2
1
0.1
E001
E002
E003
E004
E005
E006
E007
E008
E009
126602
126896
127191
127485
127780
128074
128369
128663
128958
129252

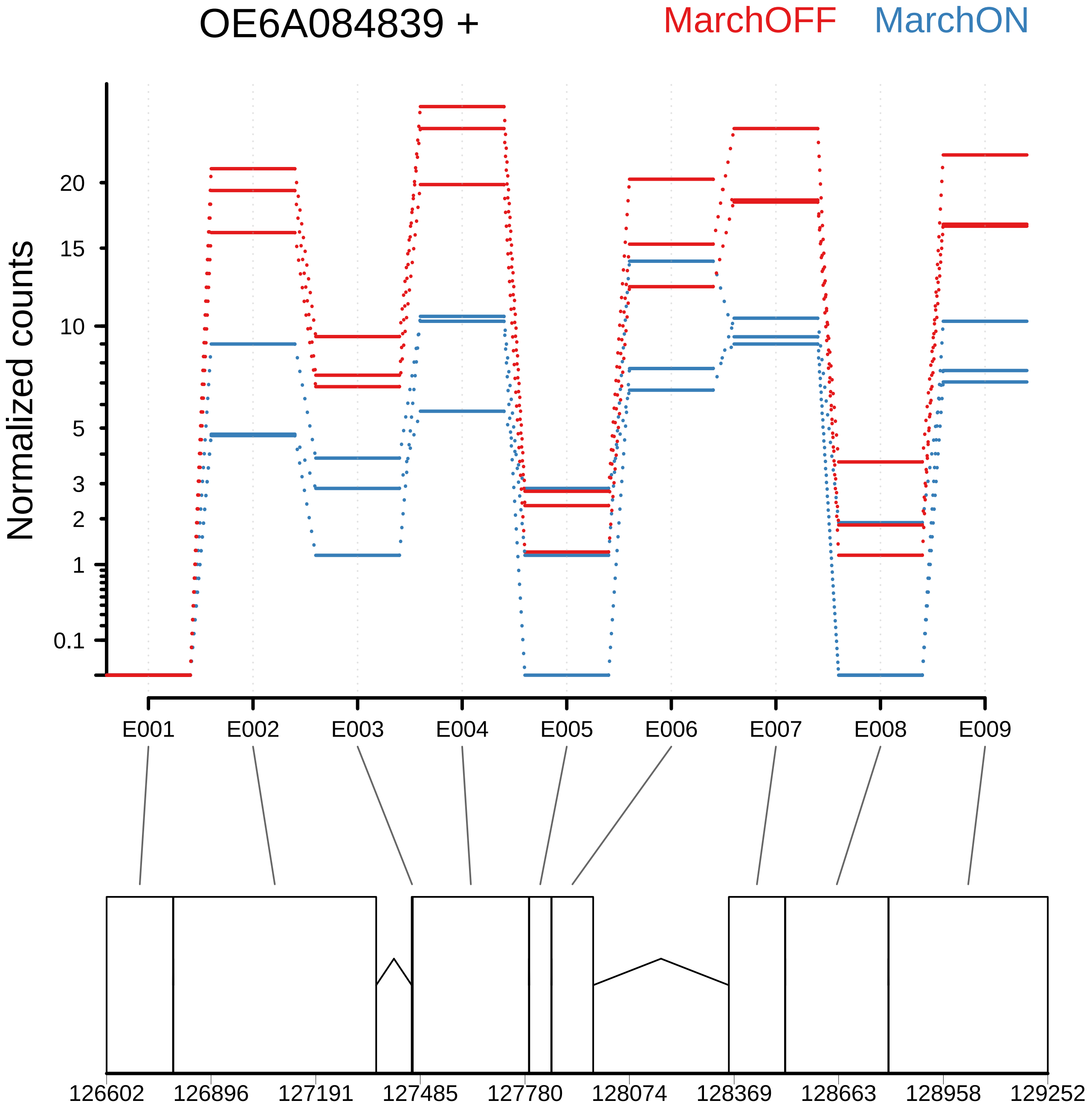

OE6A084839 +
MarchOFF
MarchON
Normalized counts
20
15
10
5
3
2
1
0.1
E001
E002
E003
E004
E005
E006
E007
E008
E009
126602
126896
127191
127485
127780
128074
128369
128663
128958
129252

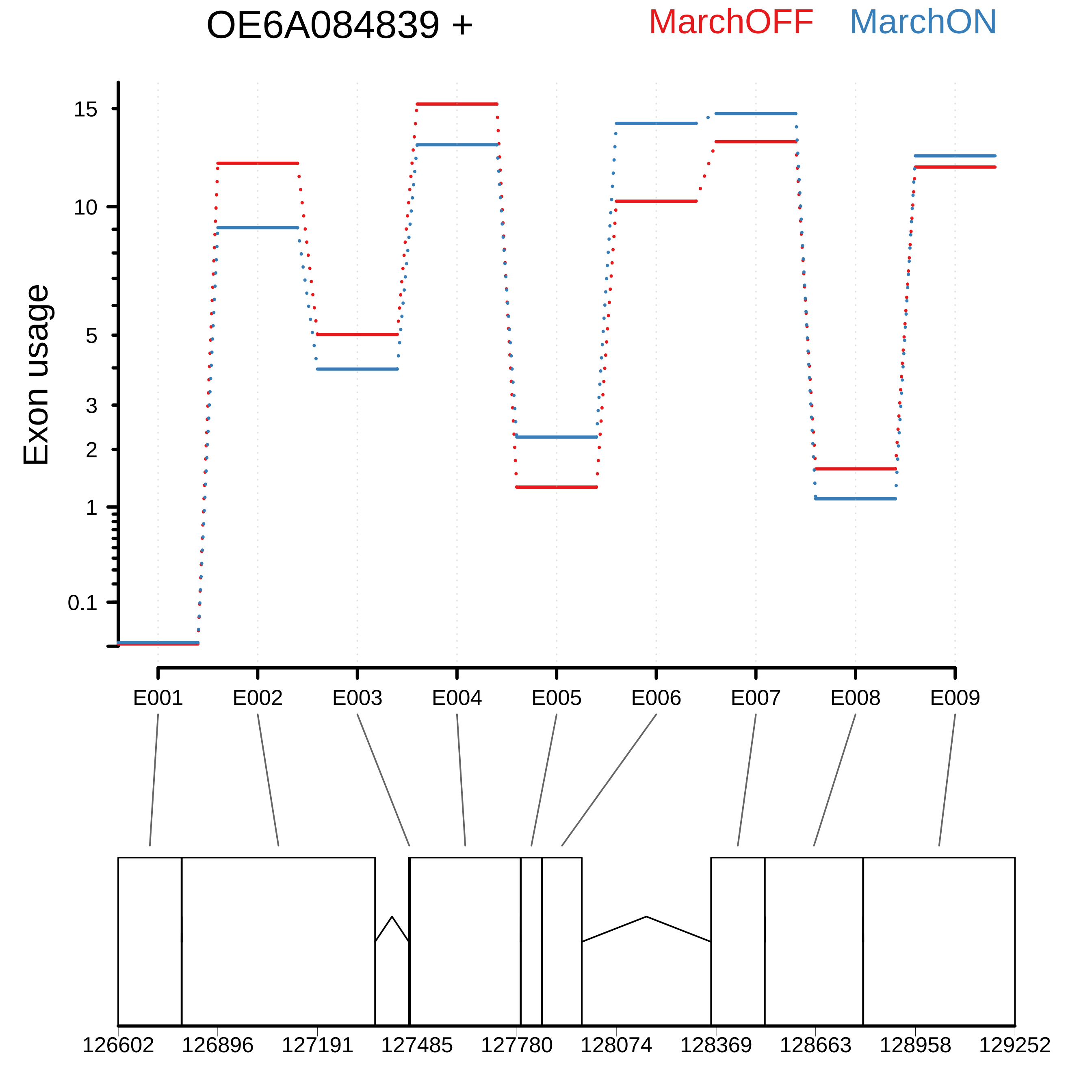

OE6A084839 +
MarchOFF
MarchON
Exon usage
15
10
5
3
2
1
0.1
E001
E002
E003
E004
E005
E006
E007
E008
E009
126602
126896
127191
127485
127780
128074
128369
128663
128958
129252

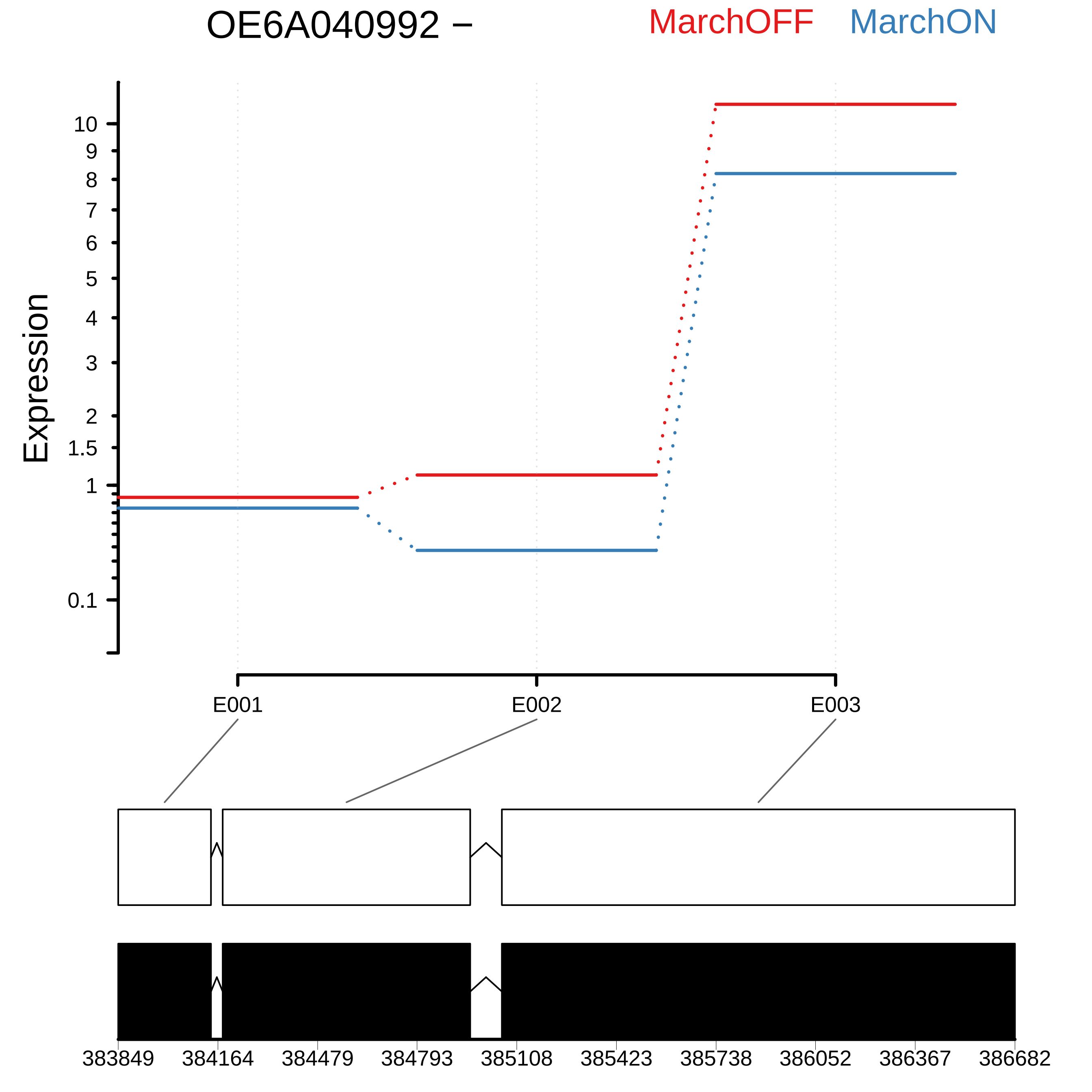
OE6A040992 −
MarchOFF
MarchON
Expression
10
9
8
7
6
5
4
3
2
1.5
1
0.1
E001
E002
E003
383849
384164
384479
384793
385108
385423
385738
386052
386367
386682

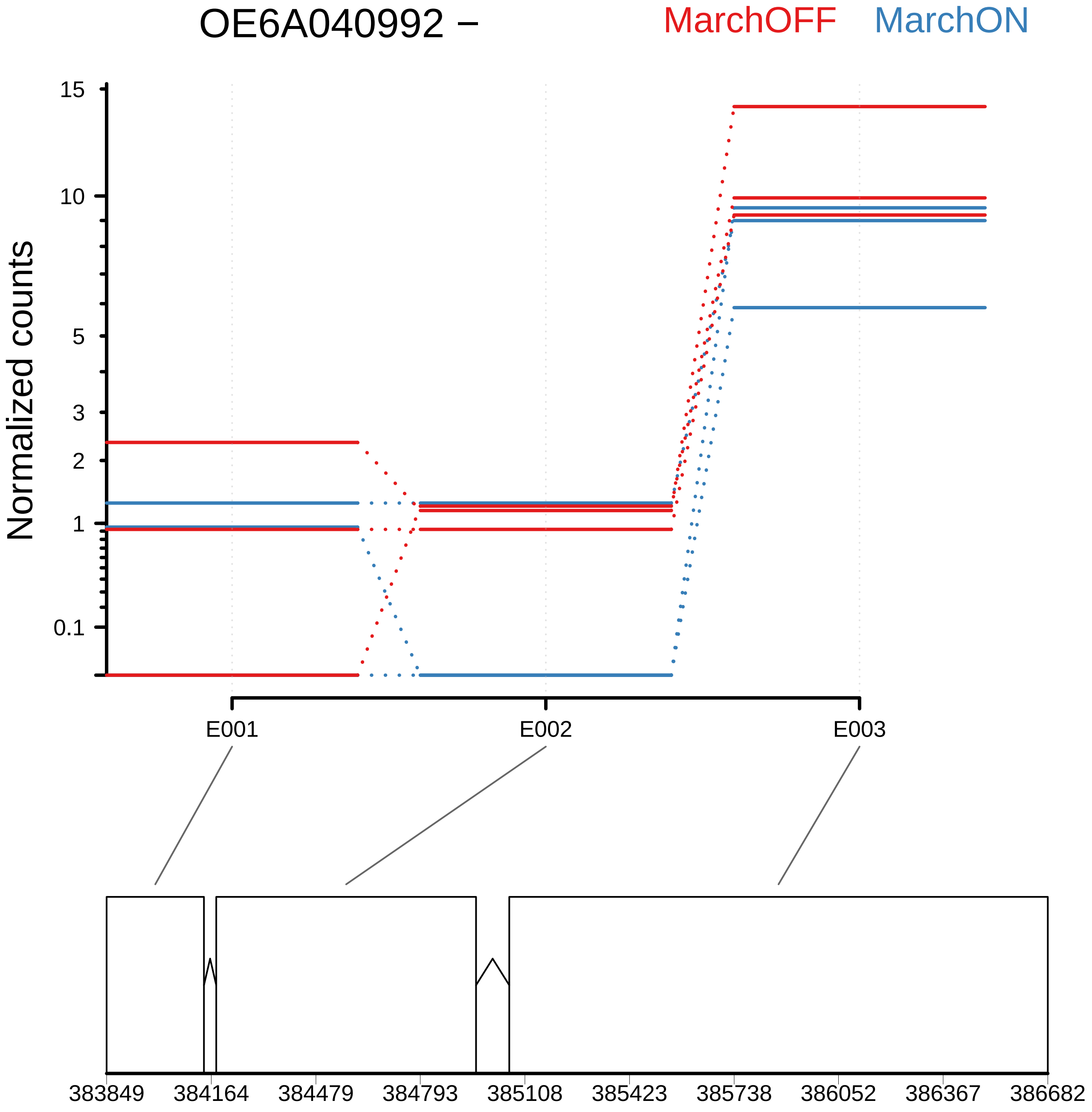
OE6A040992 −
MarchOFF
MarchON
Normalized counts
15
10
5
3
2
1
0.1
E001
E002
E003
383849
384164
384479
384793
385108
385423
385738
386052
386367
386682

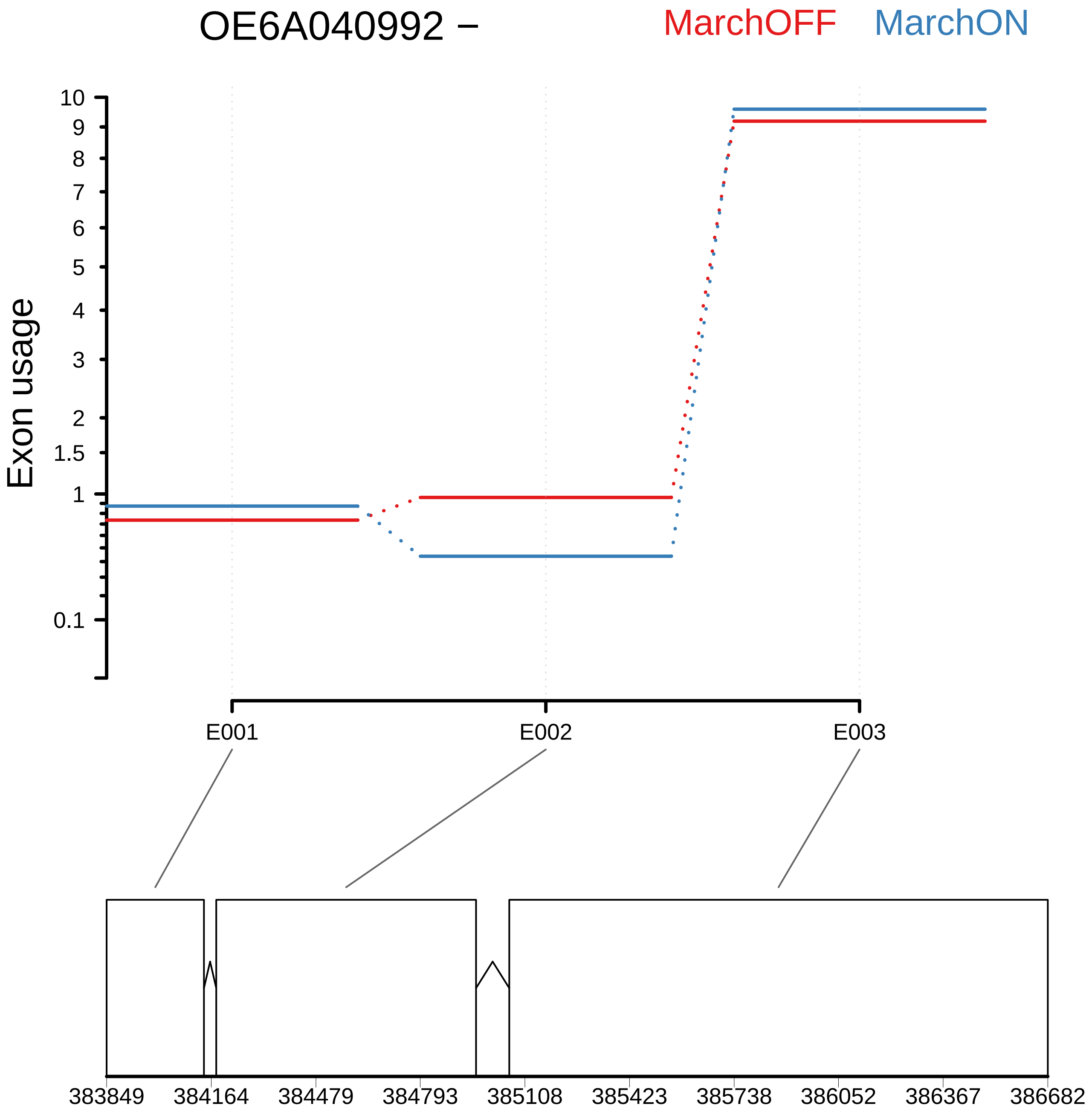

OE6A040992 −
MarchOFF
MarchON
Exon usage
10
9
8
7
6
5
4
3
2
1.5
1
0.1
E001
E002
E003
383849
384164
384479
384793
385108
385423
385738
386052
386367
386682

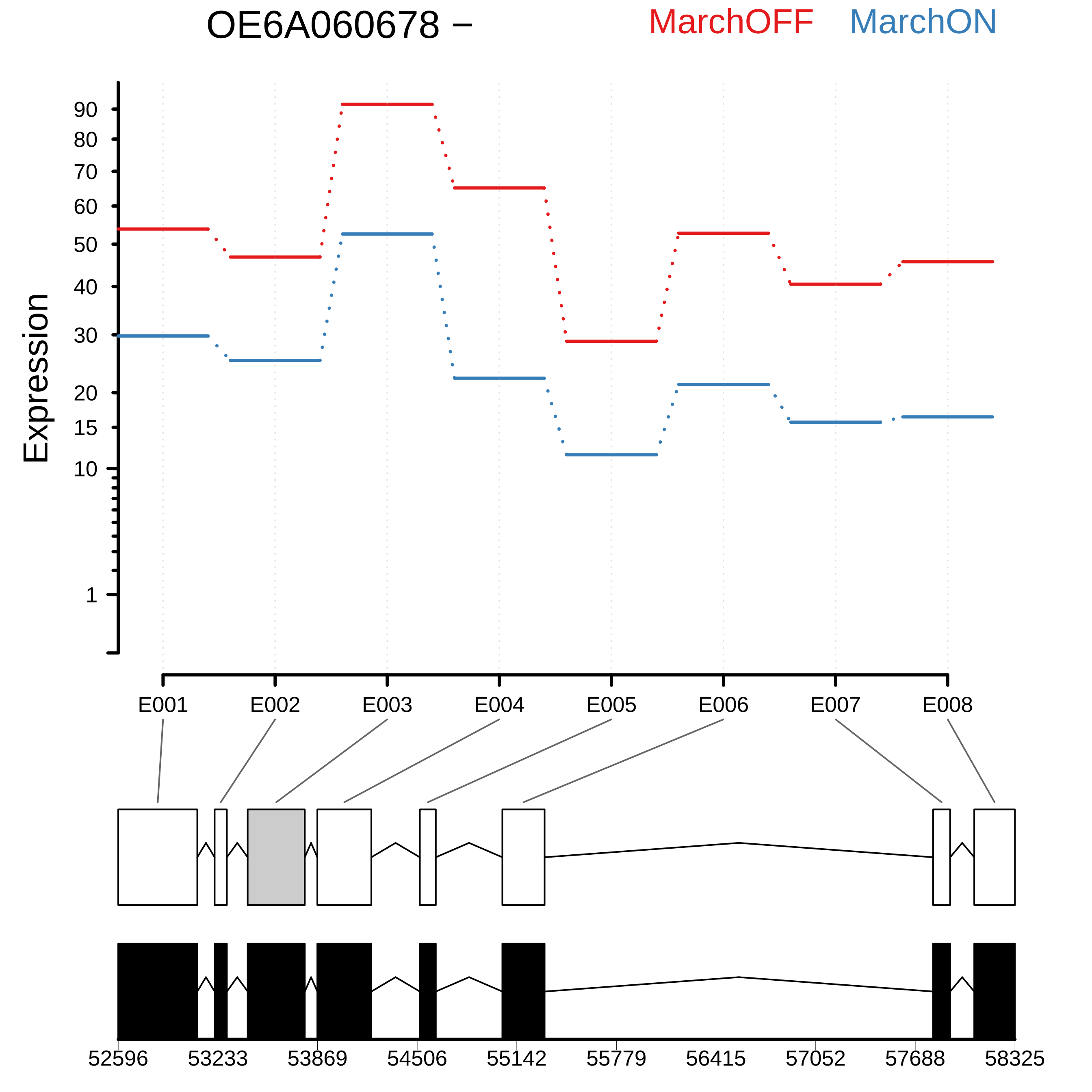

OE6A060678 −
MarchOFF
MarchON
Expression
90
80
70
60
50
40
30
20
15
10
1
E001
E002
E003
E004
E005
E006
E007
E008
52596
53233
53869
54506
55142
55779
56415
57052
57688
58325

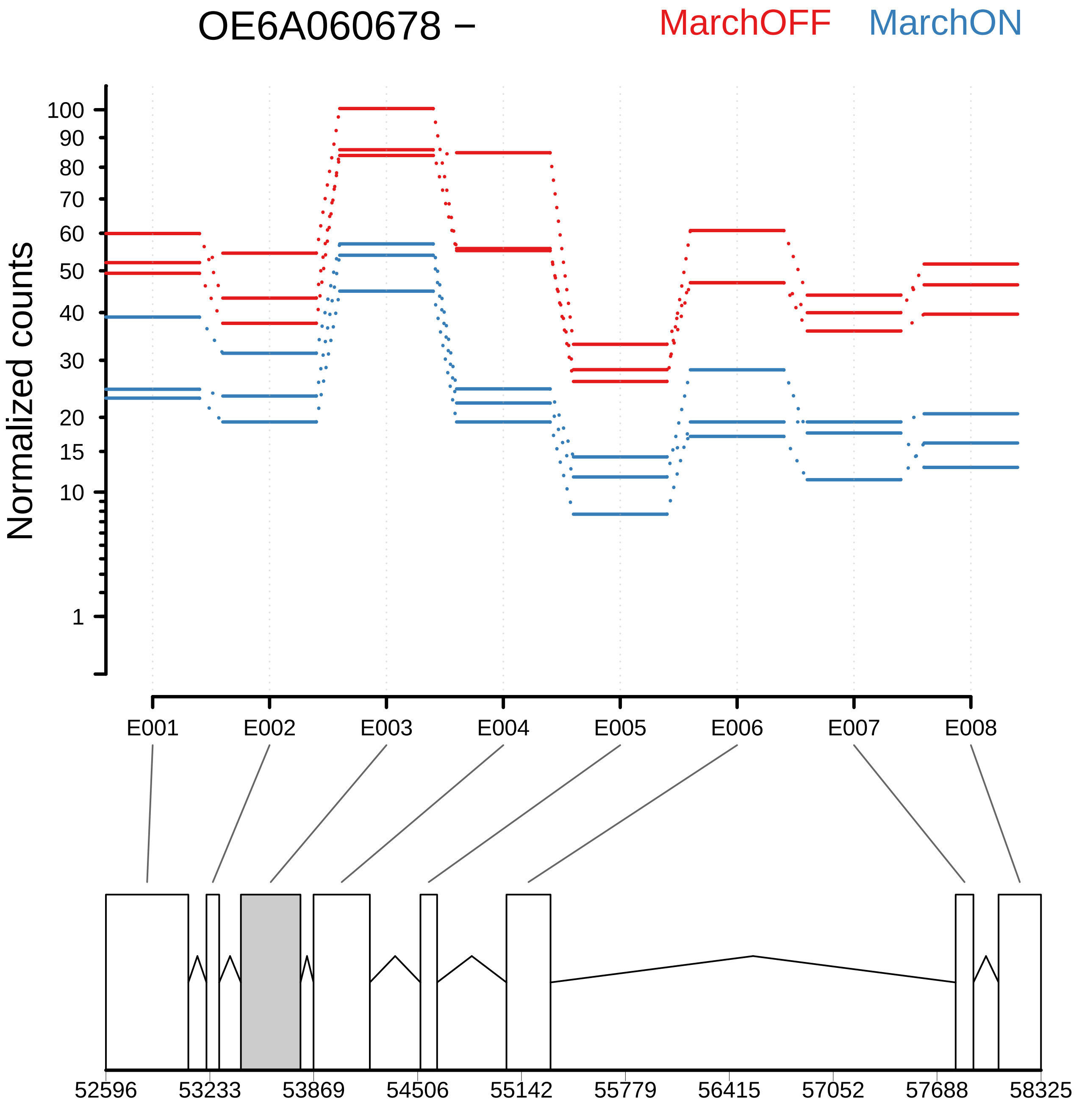

OE6A060678 −
MarchOFF
MarchON
Normalized counts
100
90
80
70
60
50
40
30
20
15
10
1
E001
E002
E003
E004
E005
E006
E007
E008
52596
53233
53869
54506
55142
55779
56415
57052
57688
58325

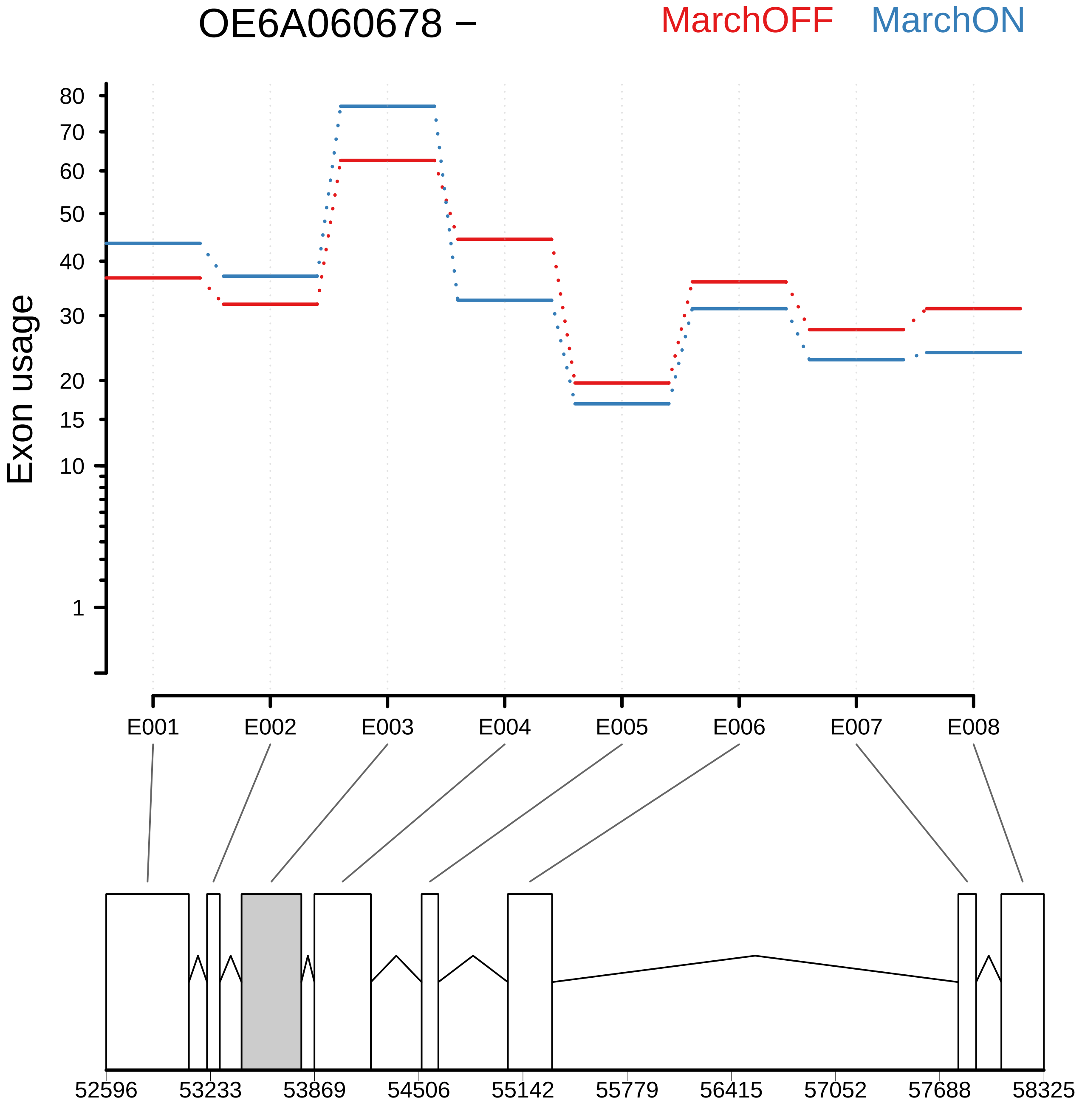

OE6A060678 −
MarchOFF
MarchON
Exon usage
80
70
60
50
40
30
20
15
10
1
E001
E002
E003
E004
E005
E006
E007
E008
52596
53233
53869
54506
55142
55779
56415
57052
57688
58325

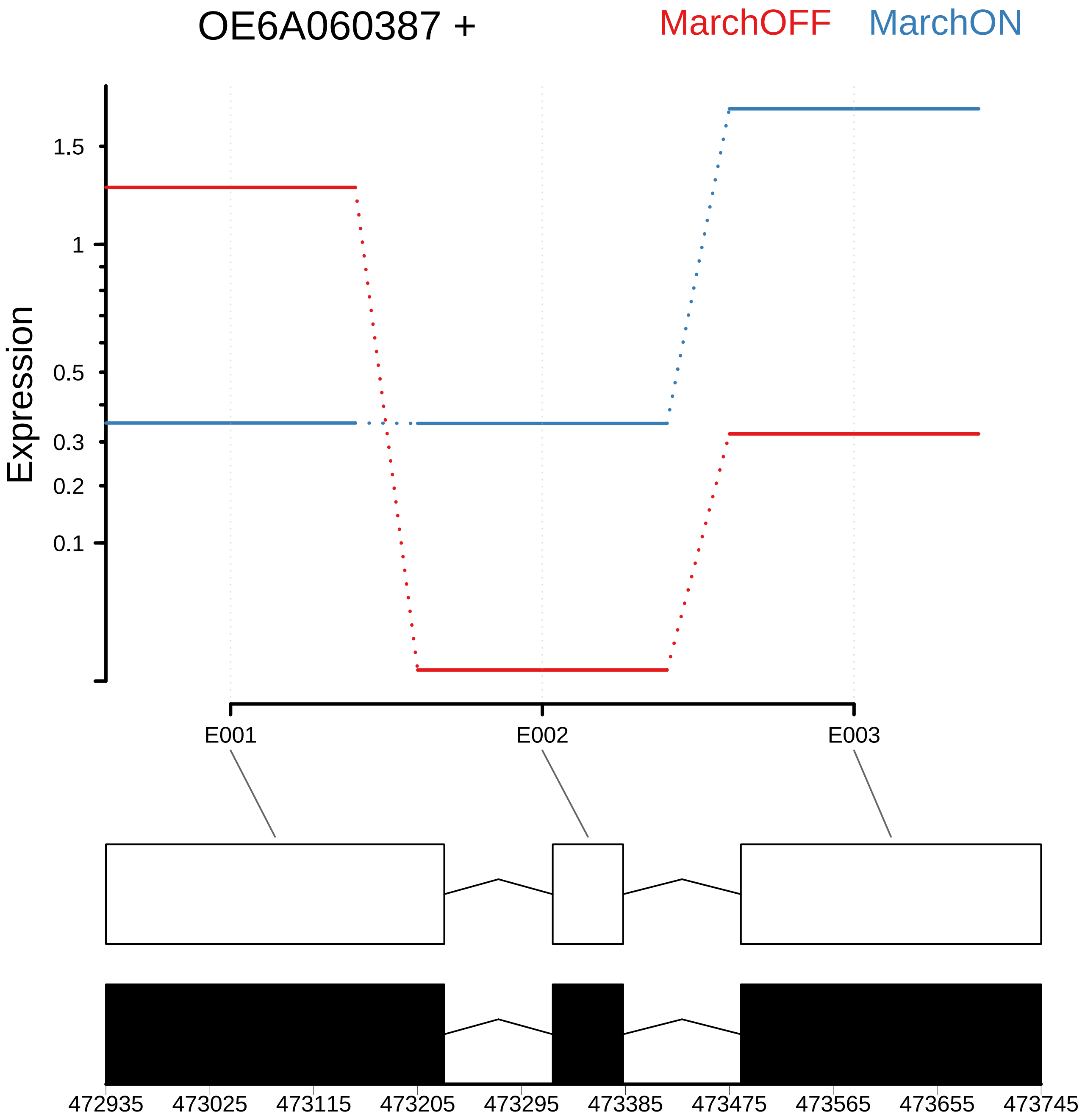

OE6A060387 +
MarchOFF
MarchON
Expression
1.5
1
0.5
0.3
0.2
0.1
E001
E002
E003
472935
473025
473115
473205
473295
473385
473475
473565
473655
473745

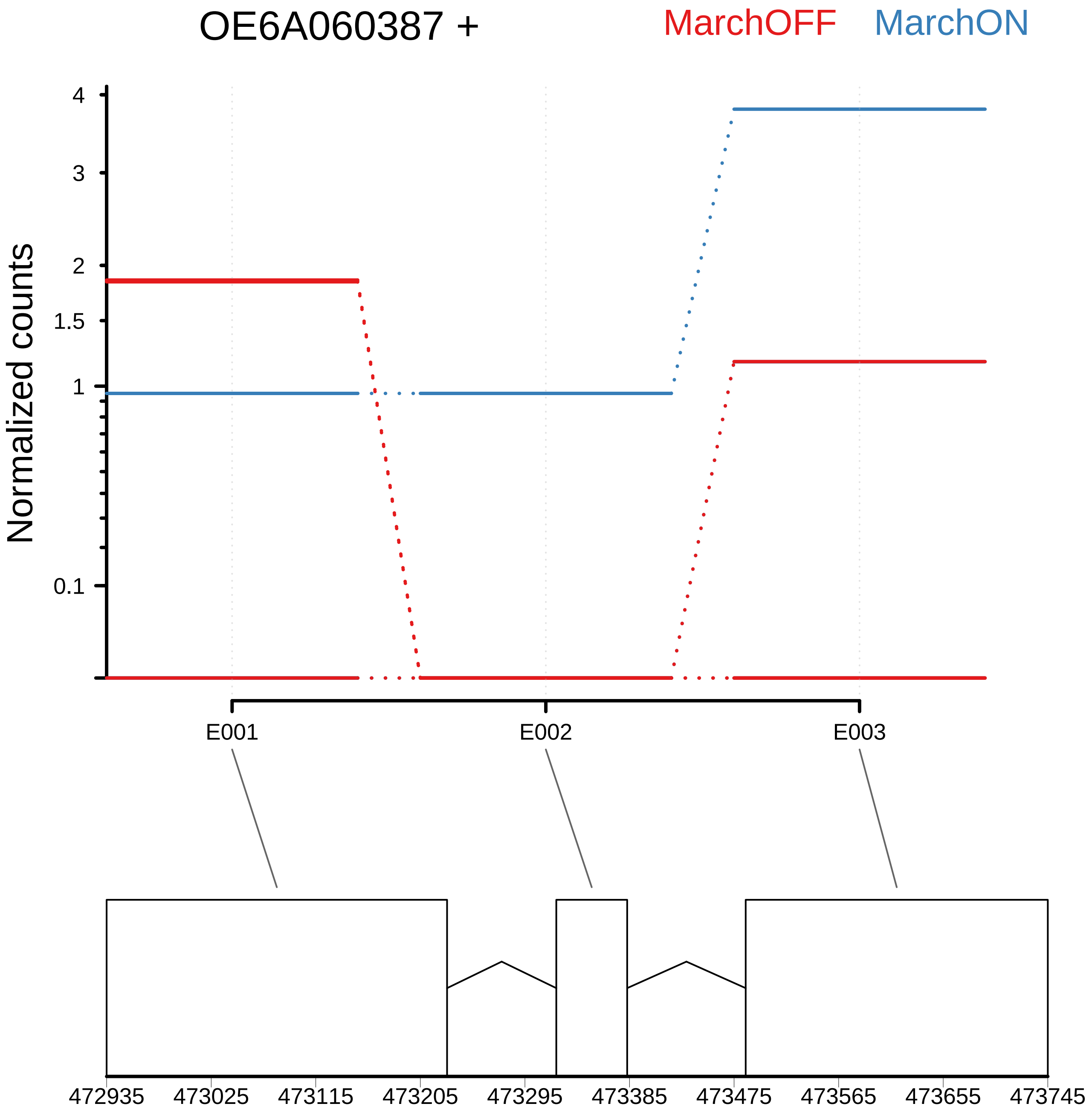

OE6A060387 +
MarchOFF
MarchON
Normalized counts
4
3
2
1.5
1
0.1
E001
E002
E003
472935
473025
473115
473205
473295
473385
473475
473565
473655
473745

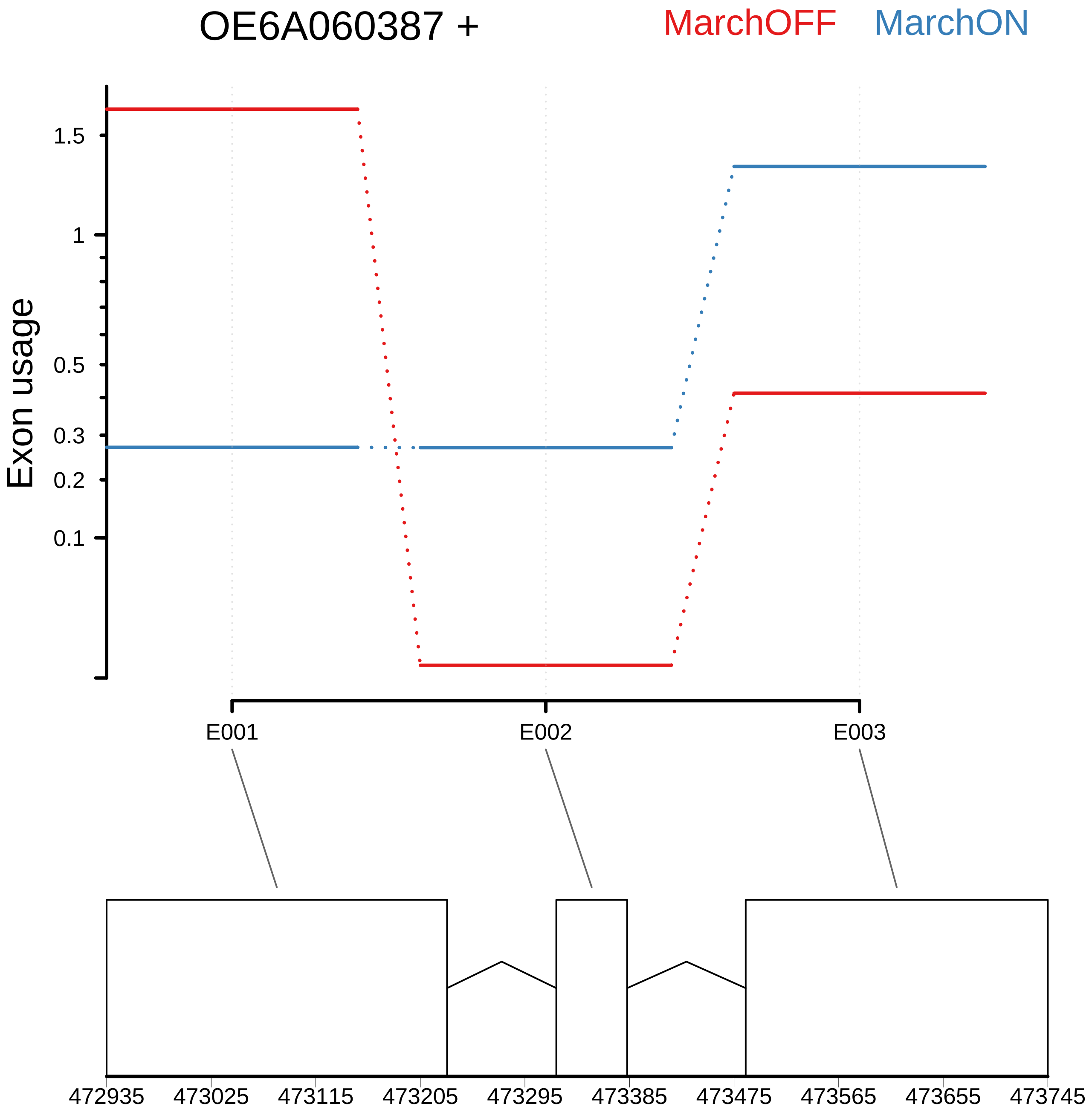
OE6A060387 +
MarchOFF
MarchON
Exon usage
1.5
1
0.5
0.3
0.2
0.1
E001
E002
E003
472935
473025
473115
473205
473295
473385
473475
473565
473655
473745

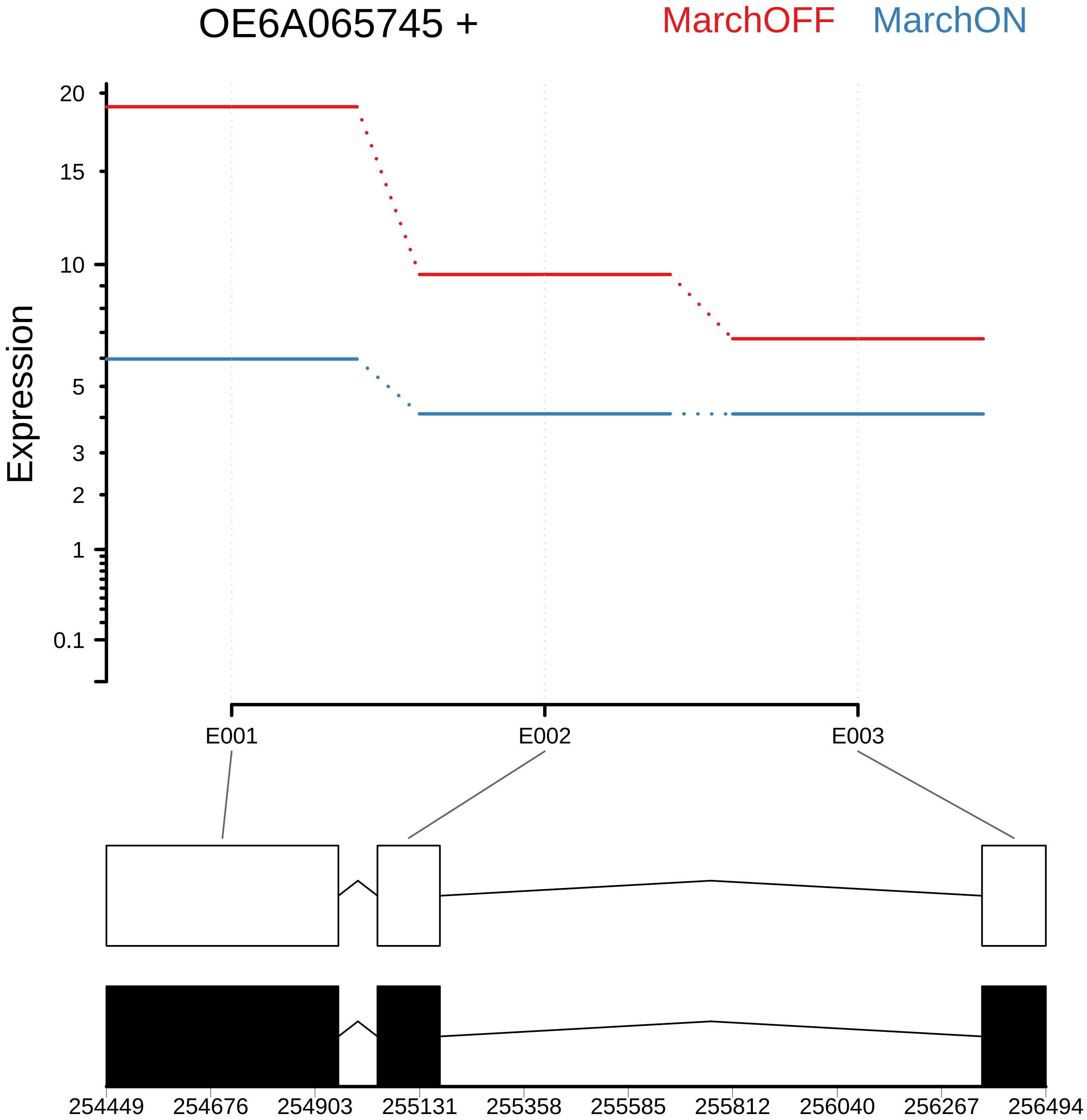

OE6A065745 +
MarchOFF
MarchON
Expression
20
15
10
5
3
2
1
0.1
E001
E002
E003
254449
254676
254903
255131
255358
255585
255812
256040
256267
256494

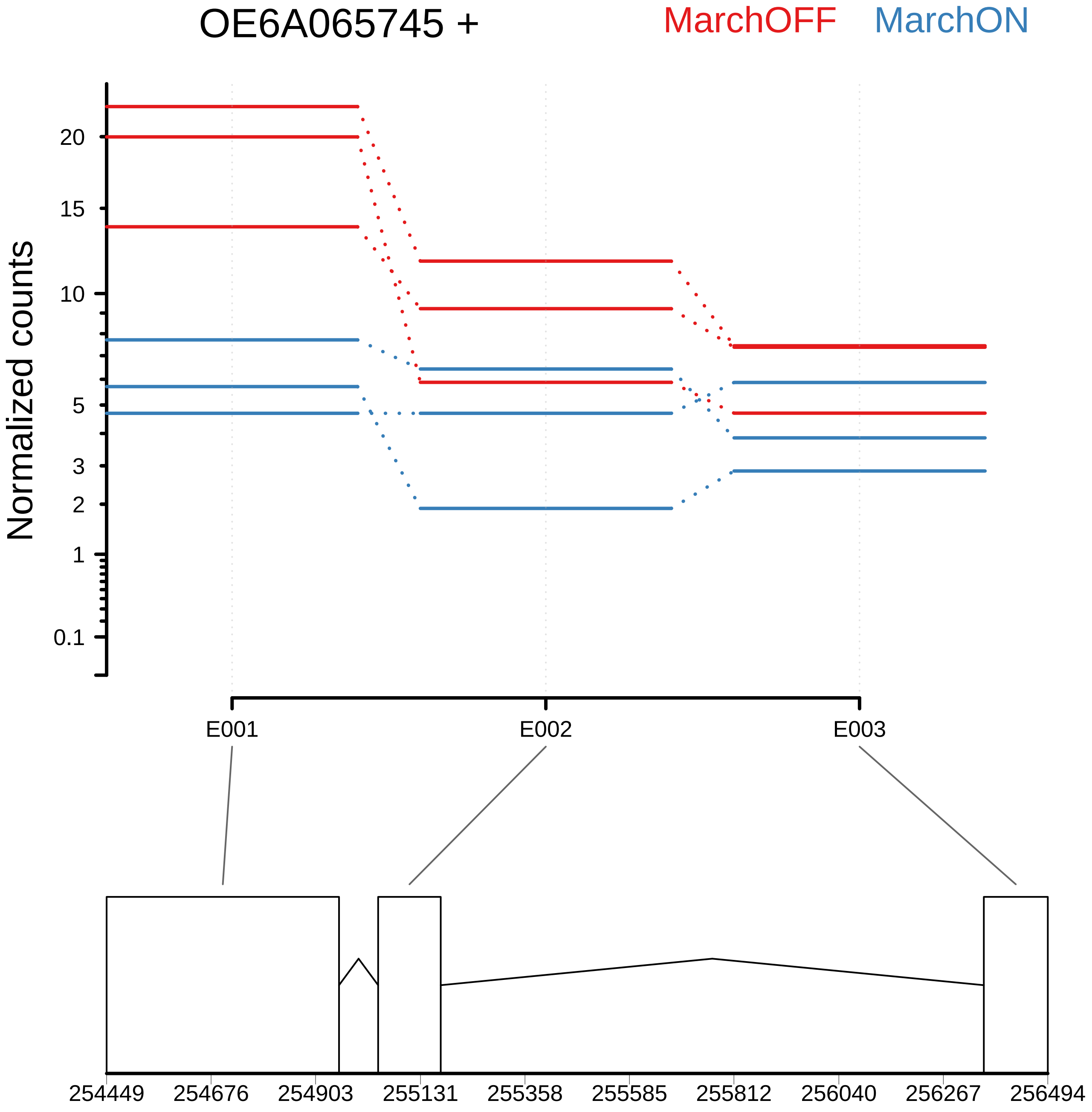
OE6A065745 +
MarchOFF
MarchON
Normalized counts
20
15
10
5
3
2
1
0.1
E001
E002
E003
254449
254676
254903
255131
255358
255585
255812
256040
256267
256494

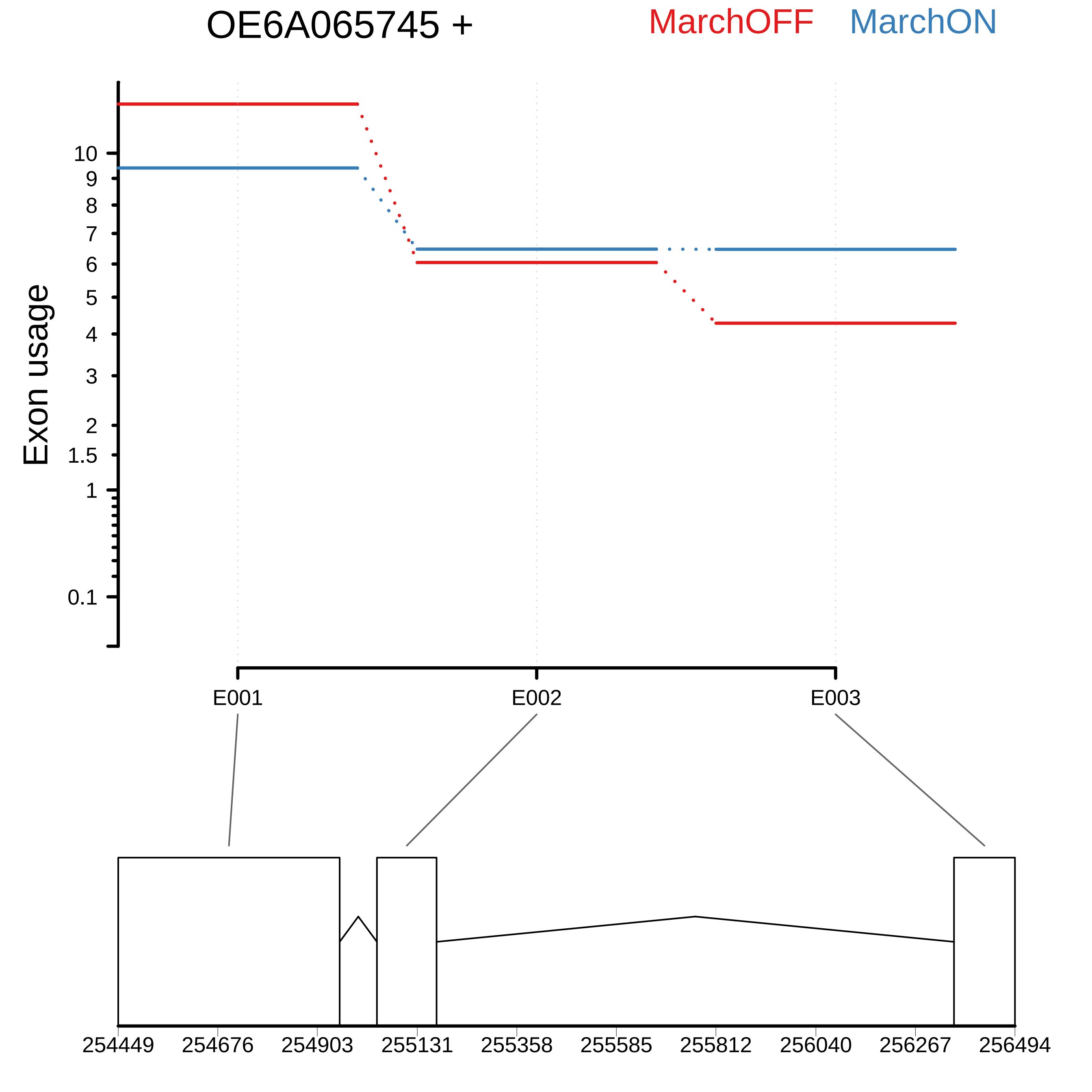
OE6A065745 +
MarchOFF
MarchON
Exon usage
10
9
8
7
6
5
4
3
2
1.5
1
0.1
E001
E002
E003
254449
254676
254903
255131
255358
255585
255812
256040
256267
256494

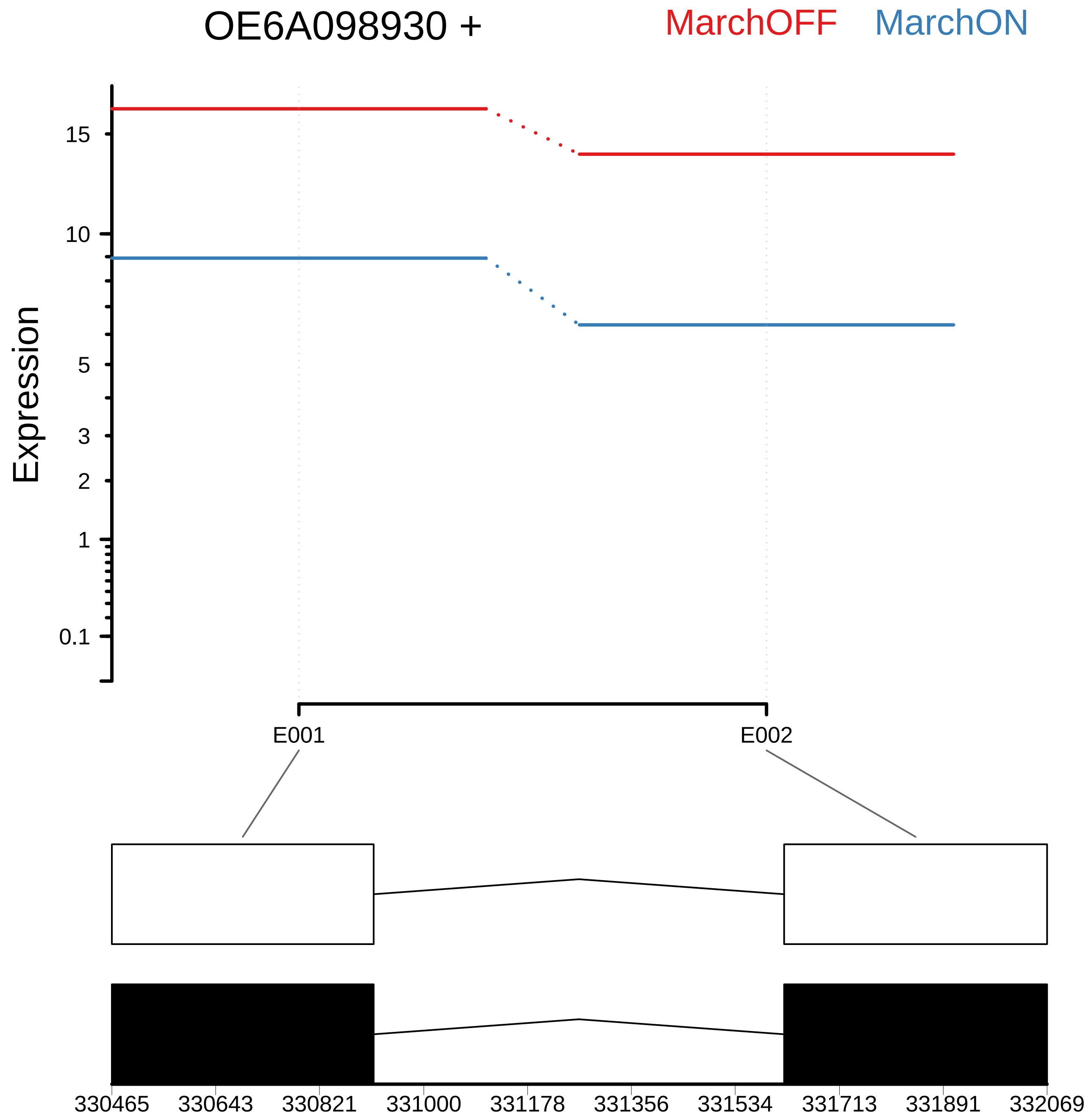

OE6A098930 +
MarchOFF
MarchON
Expression
15
10
5
3
2
1
0.1
E001
E002
330465
330643
330821
331000
331178
331356
331534
331713
331891
332069

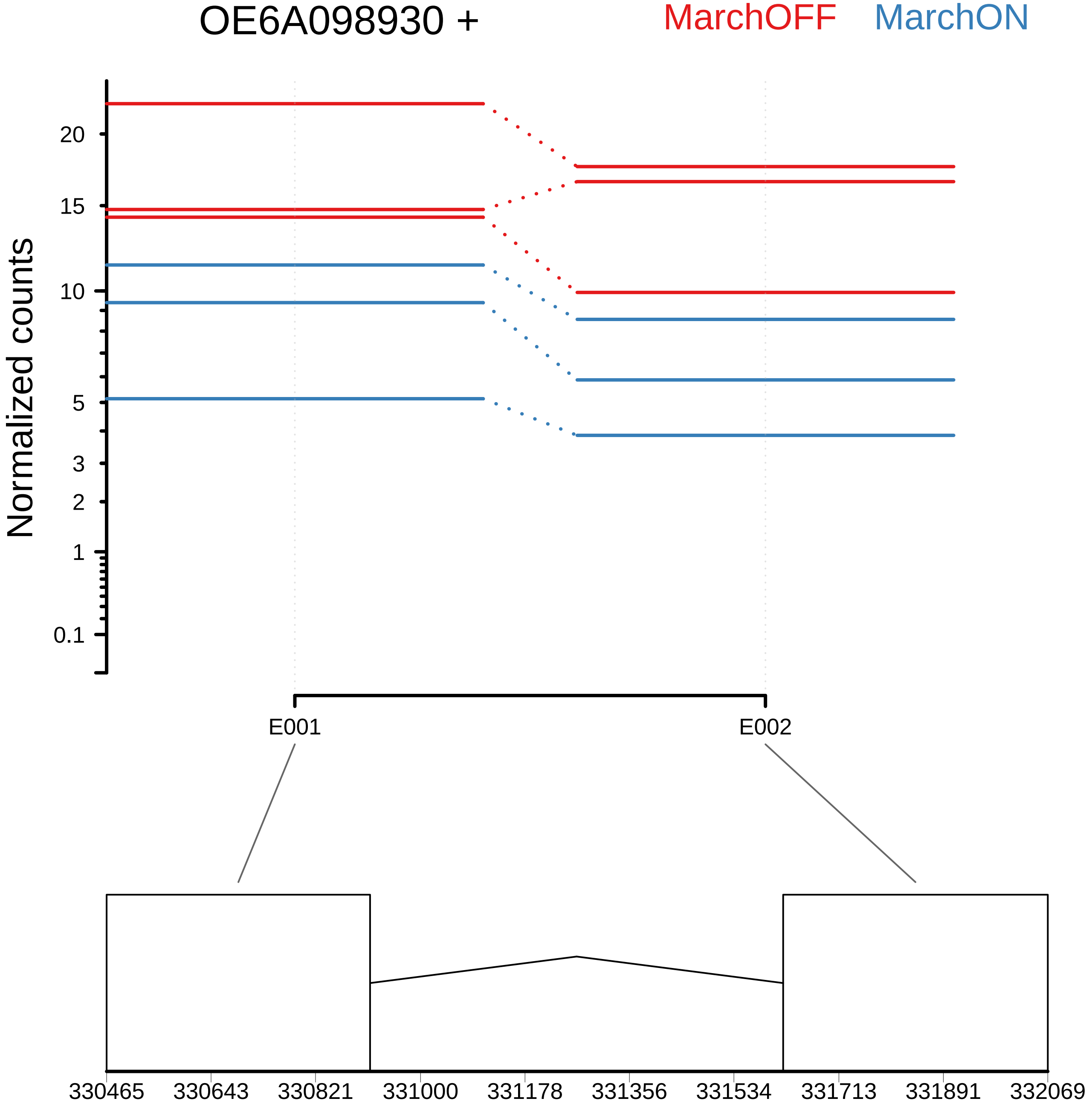

OE6A098930 +
MarchOFF
MarchON
Normalized counts
20
15
10
5
3
2
1
0.1
E001
E002
330465
330643
330821
331000
331178
331356
331534
331713
331891
332069

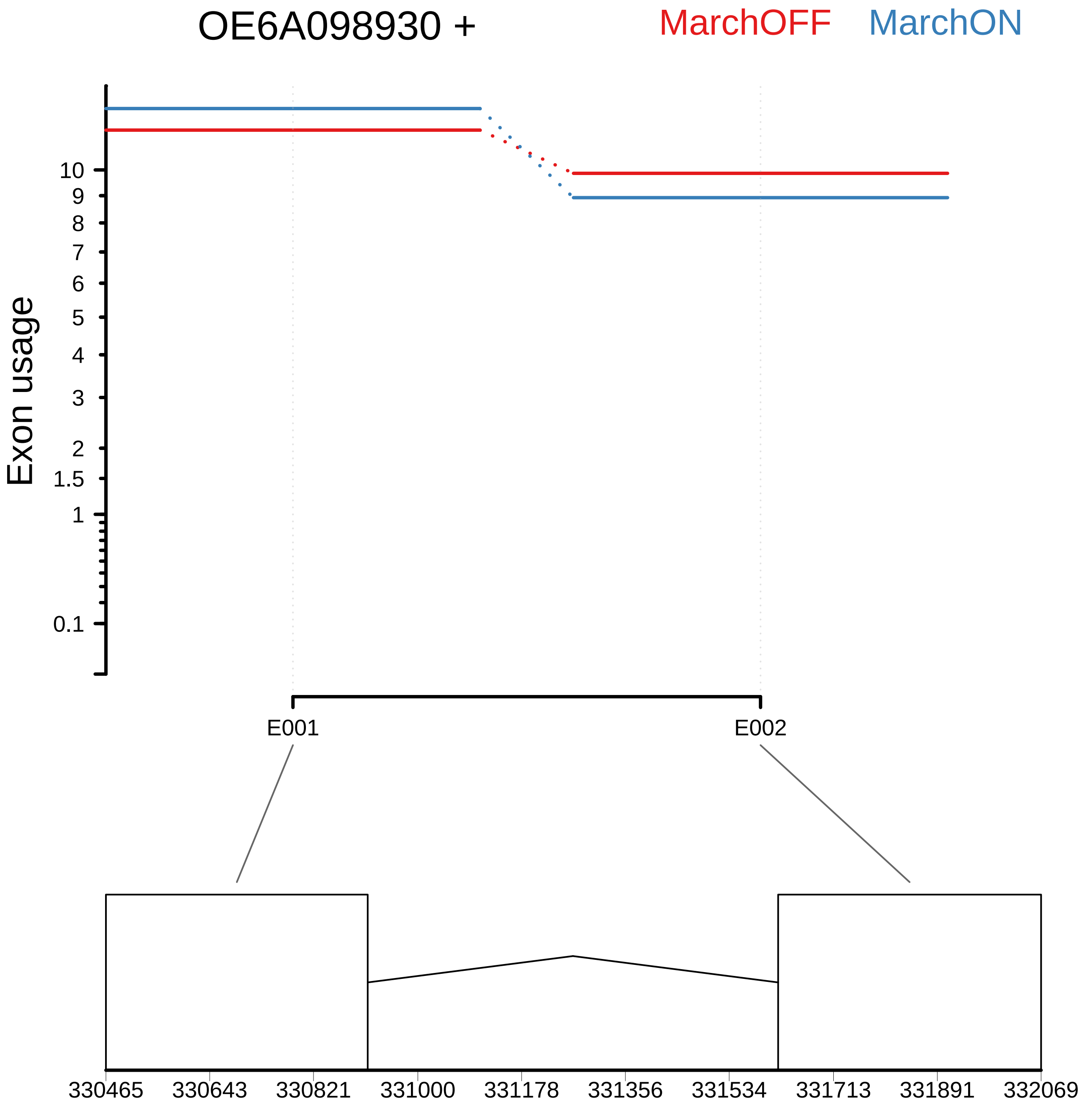
OE6A098930 +
MarchOFF
MarchON
Exon usage
10
9
8
7
6
5
4
3
2
1.5
1
0.1
E001
E002
330465
330643
330821
331000
331178
331356
331534
331713
331891
332069

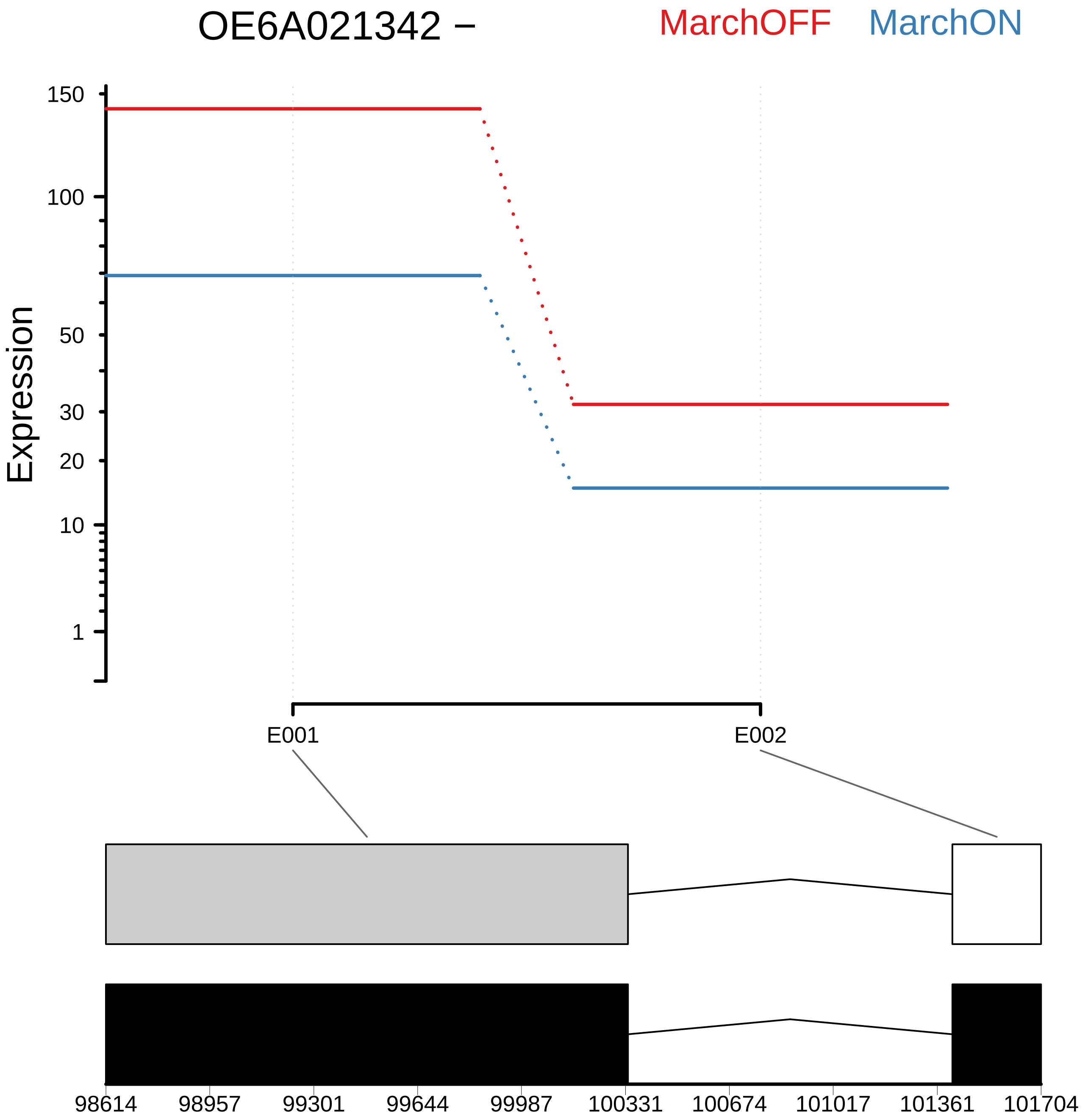
OE6A021342 −
MarchOFF
MarchON
Expression
150
100
50
30
20
10
1
E001
E002
98614
98957
99301
99644
99987
100331
100674
101017
101361
101704

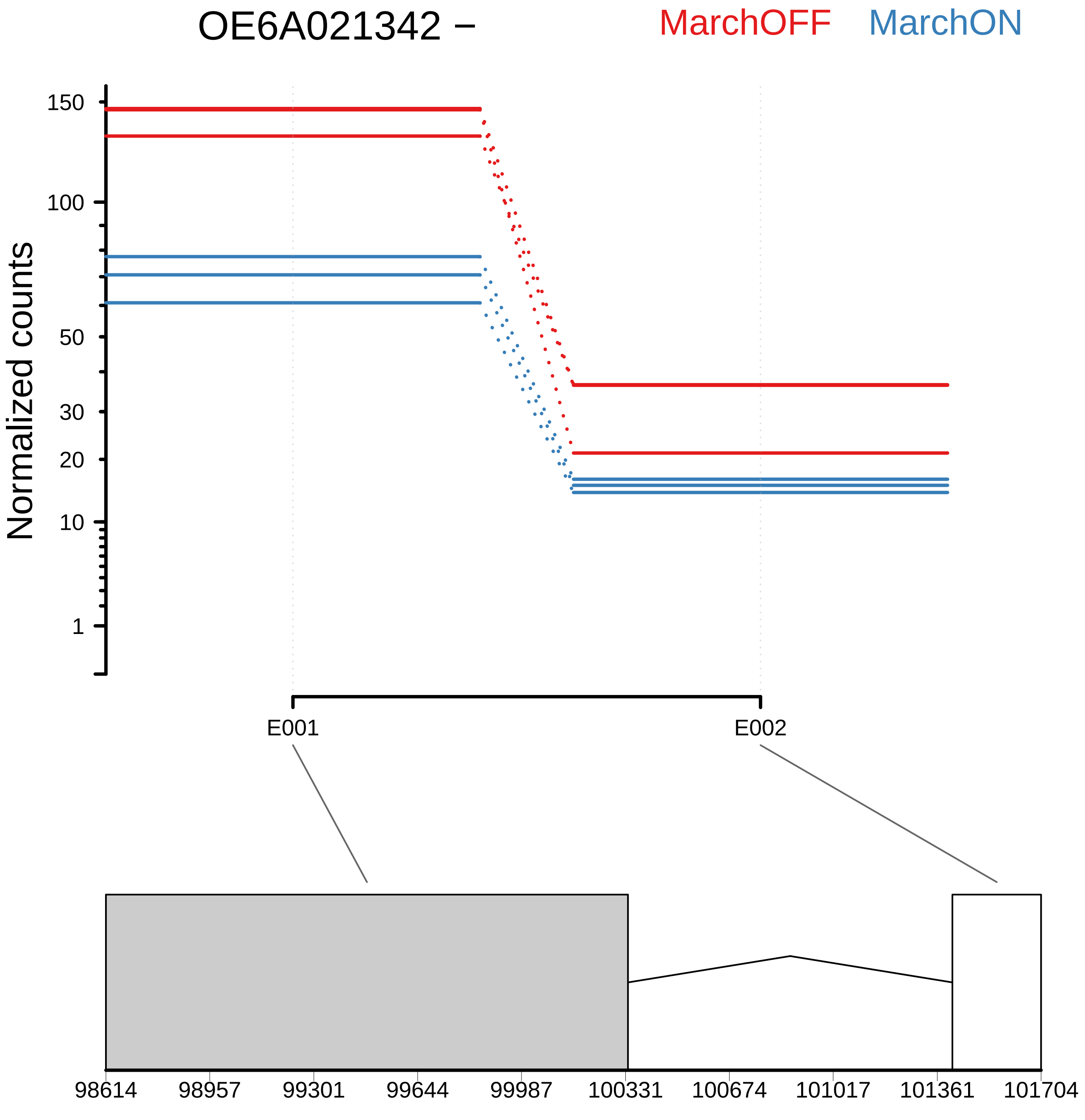
OE6A021342 −
MarchOFF
MarchON
Normalized counts
150
100
50
30
20
10
1
E001
E002
98614
98957
99301
99644
99987
100331
100674
101017
101361
101704

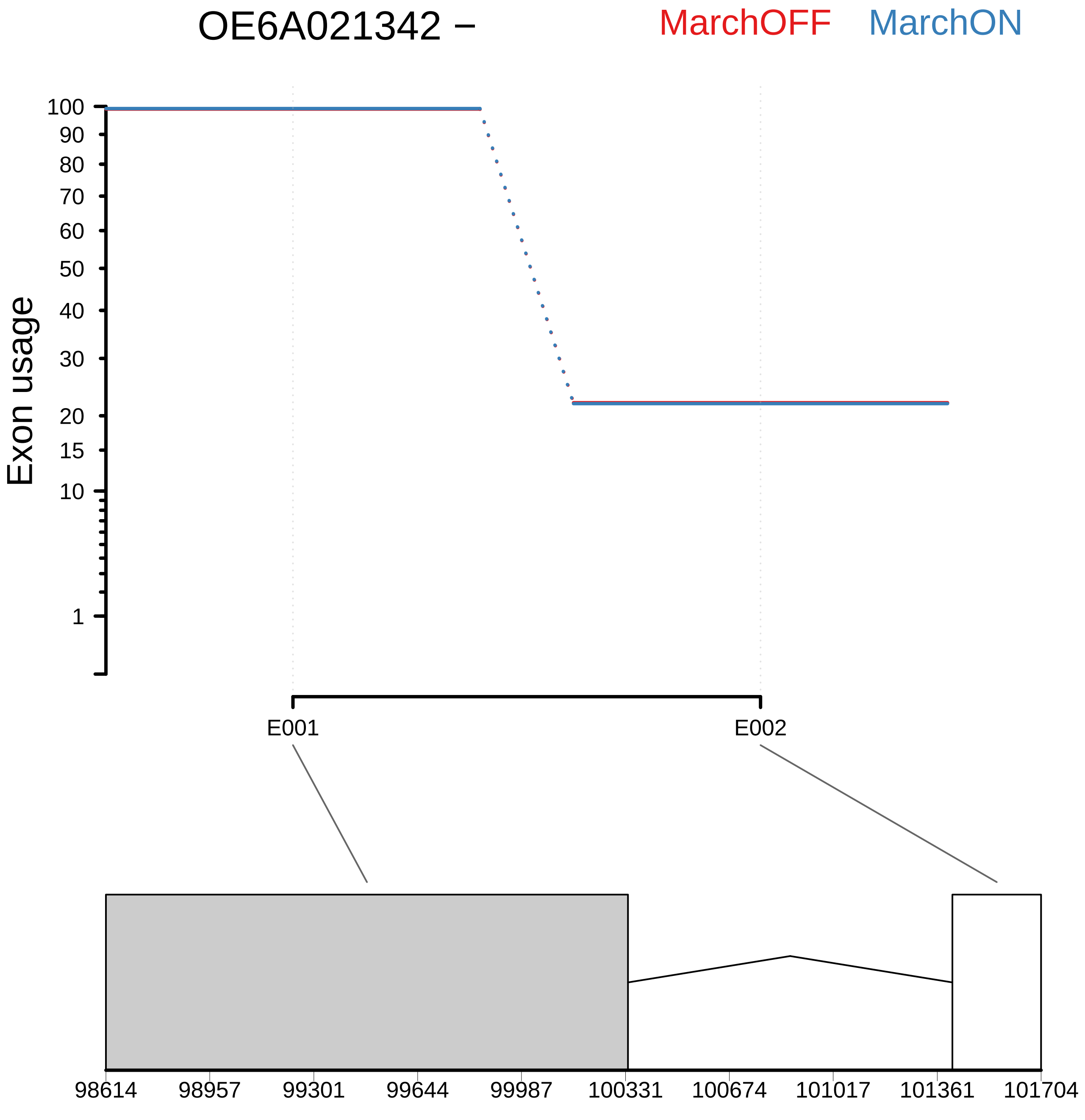

OE6A021342 −
MarchOFF
MarchON
Exon usage
100
90
80
70
60
50
40
30
20
15
10
1
E001
E002
98614
98957
99301
99644
99987
100331
100674
101017
101361
101704

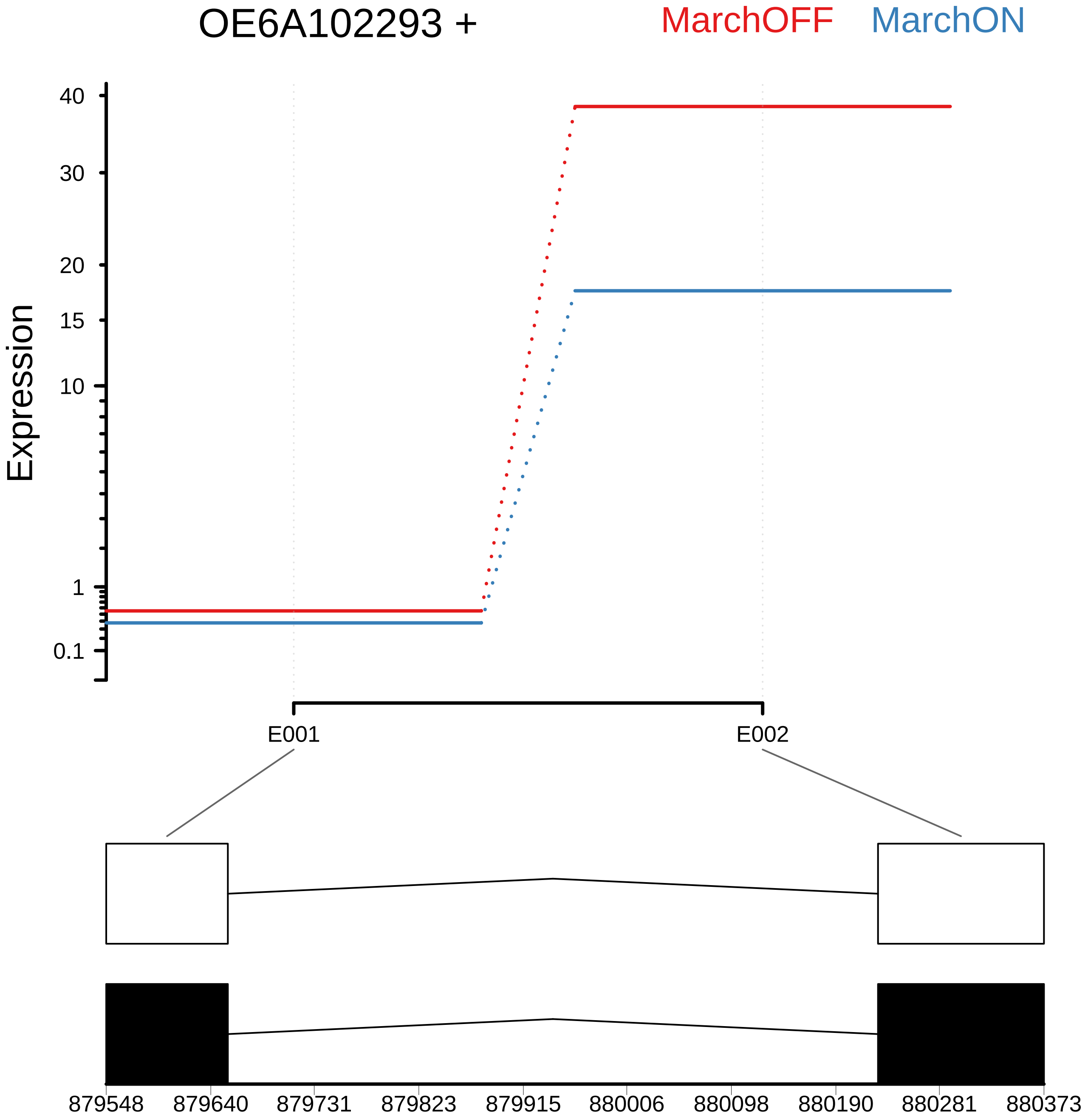

OE6A102293 +
MarchOFF
MarchON
Expression
40
30
20
15
10
1
0.1
E001
E002
879548
879640
879731
879823
879915
880006
880098
880190
880281
880373

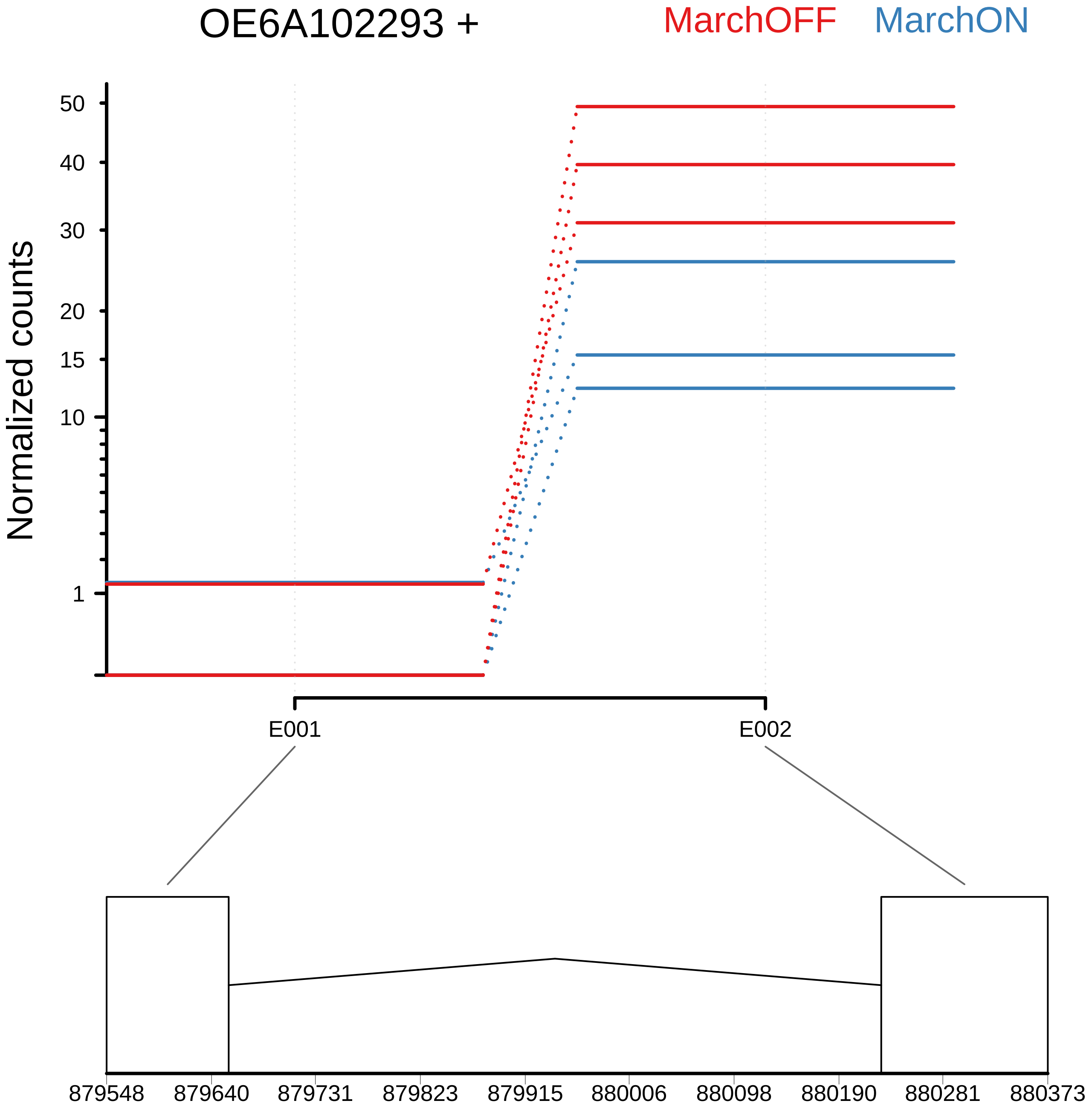

OE6A102293 +
MarchOFF
MarchON
Normalized counts
50
40
30
20
15
10
1
E001
E002
879548
879640
879731
879823
879915
880006
880098
880190
880281
880373

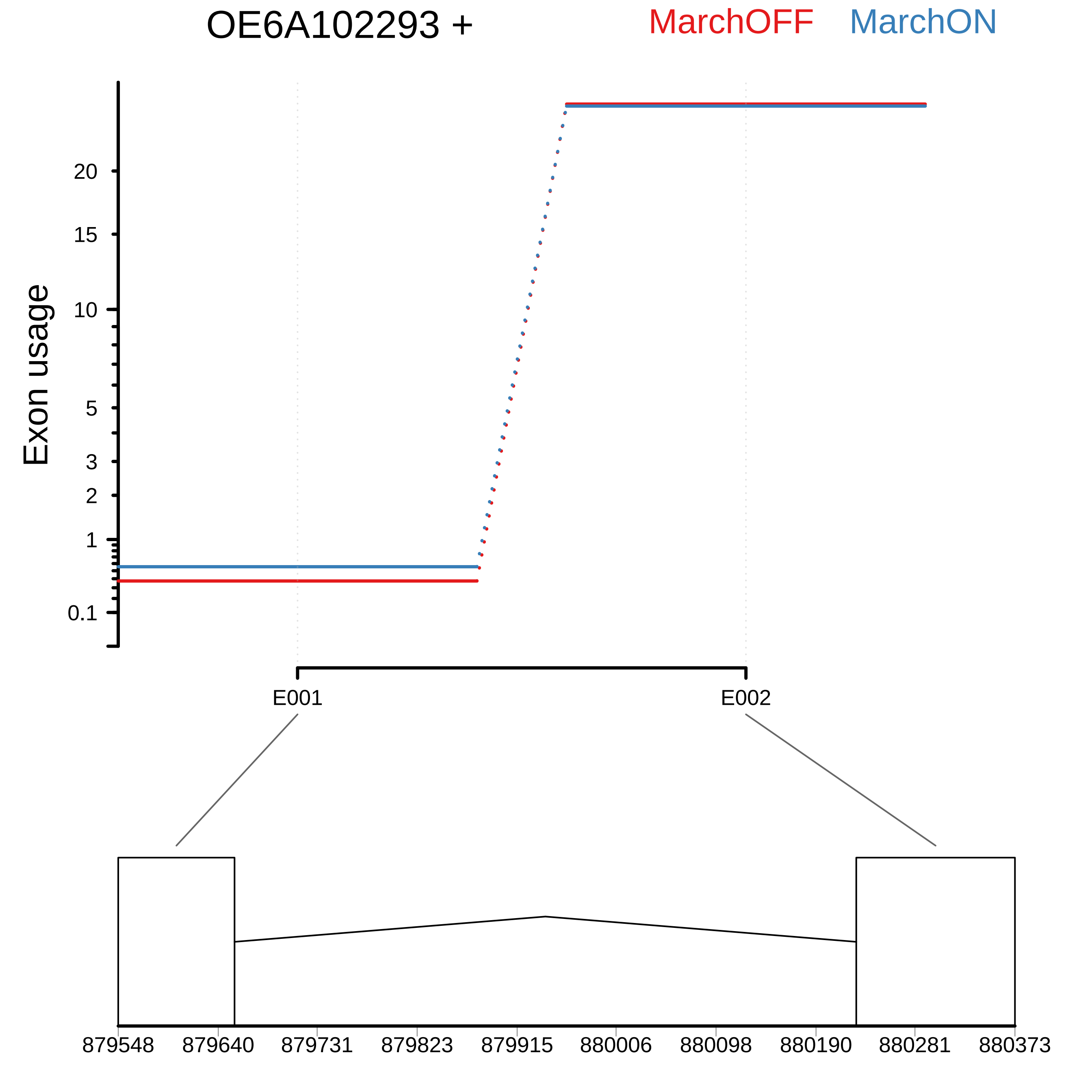
OE6A102293 +
MarchOFF
MarchON
Exon usage
20
15
10
5
3
2
1
0.1
E001
E002
879548
879640
879731
879823
879915
880006
880098
880190
880281
880373

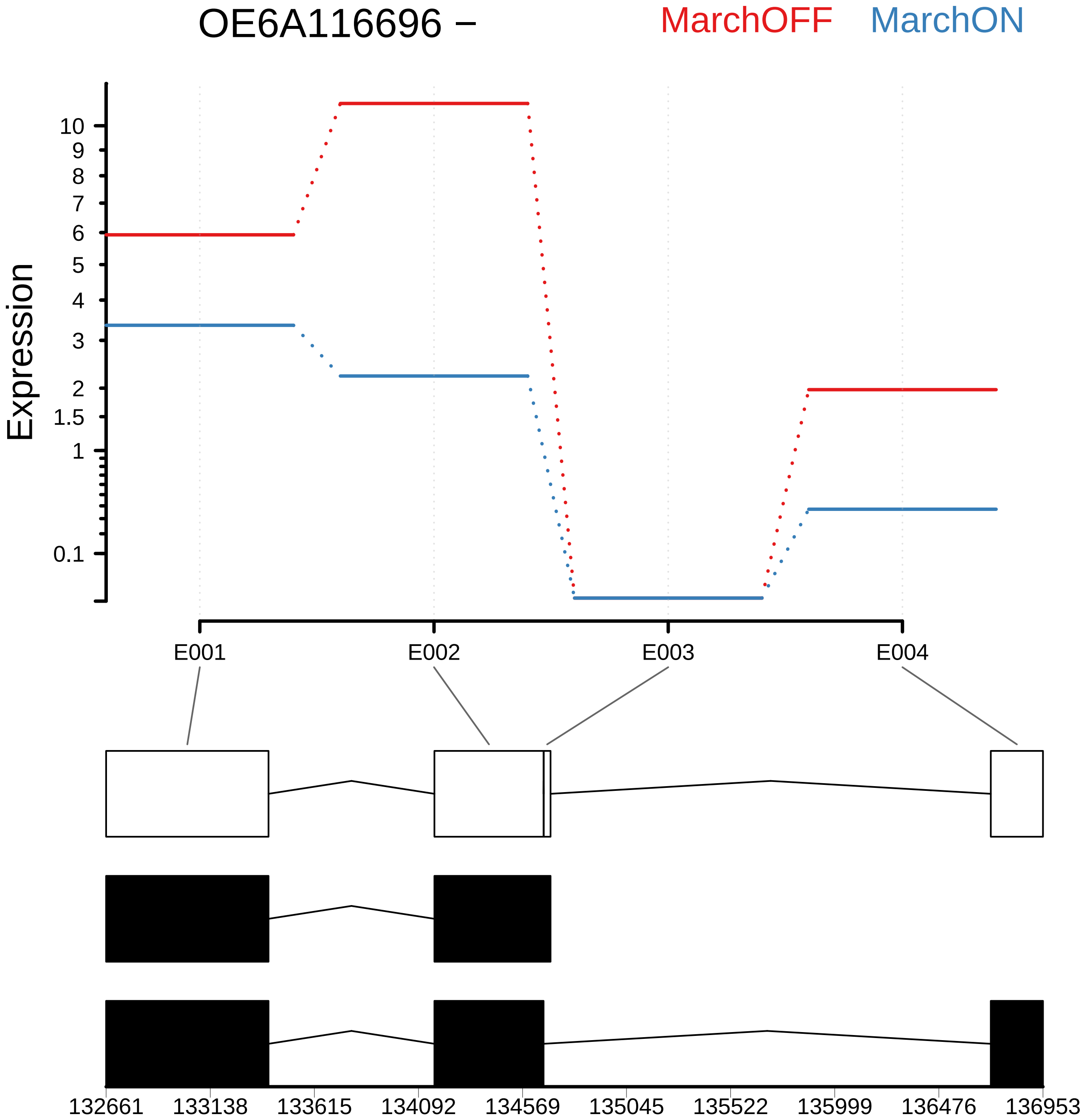

OE6A116696 −
MarchOFF
MarchON
Expression
10
9
8
7
6
5
4
3
2
1.5
1
0.1
E001
E002
E003
E004
132661
133138
133615
134092
134569
135045
135522
135999
136476
136953

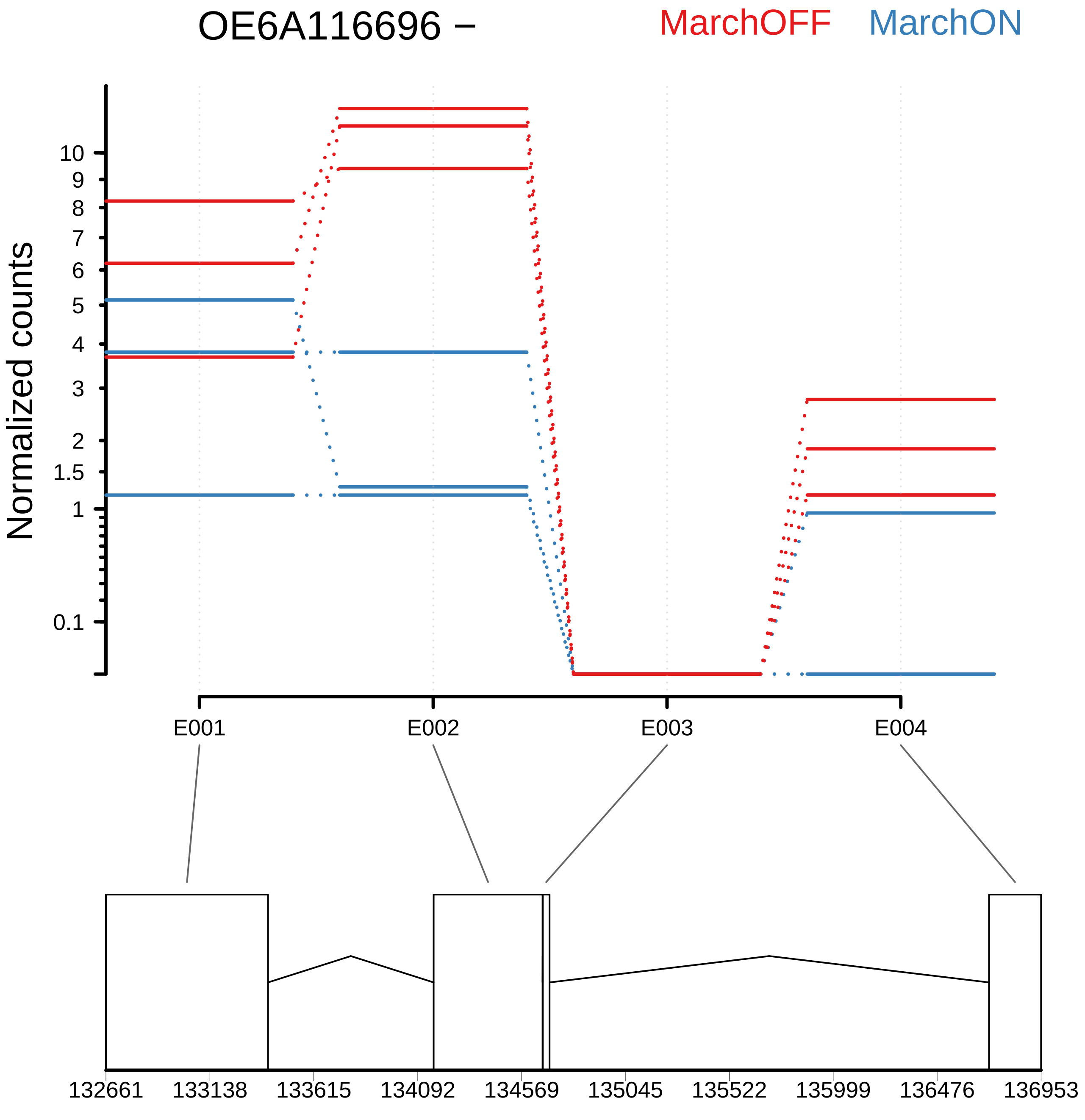

OE6A116696 −
MarchOFF
MarchON
Normalized counts
10
9
8
7
6
5
4
3
2
1.5
1
0.1
E001
E002
E003
E004
132661
133138
133615
134092
134569
135045
135522
135999
136476
136953

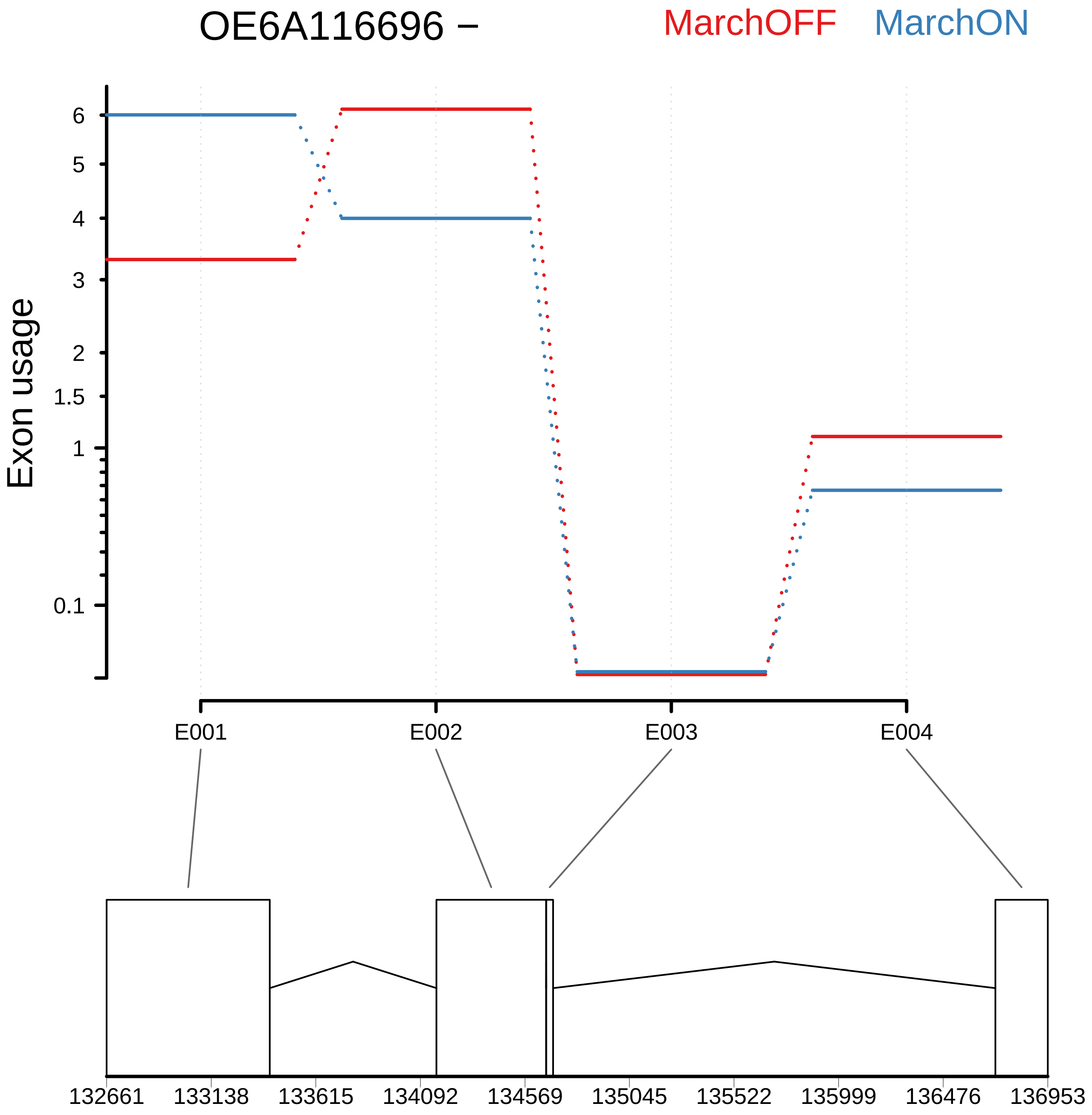

OE6A116696 −
MarchOFF
MarchON
Exon usage
6
5
4
3
2
1.5
1
0.1
E001
E002
E003
E004
132661
133138
133615
134092
134569
135045
135522
135999
136476
136953

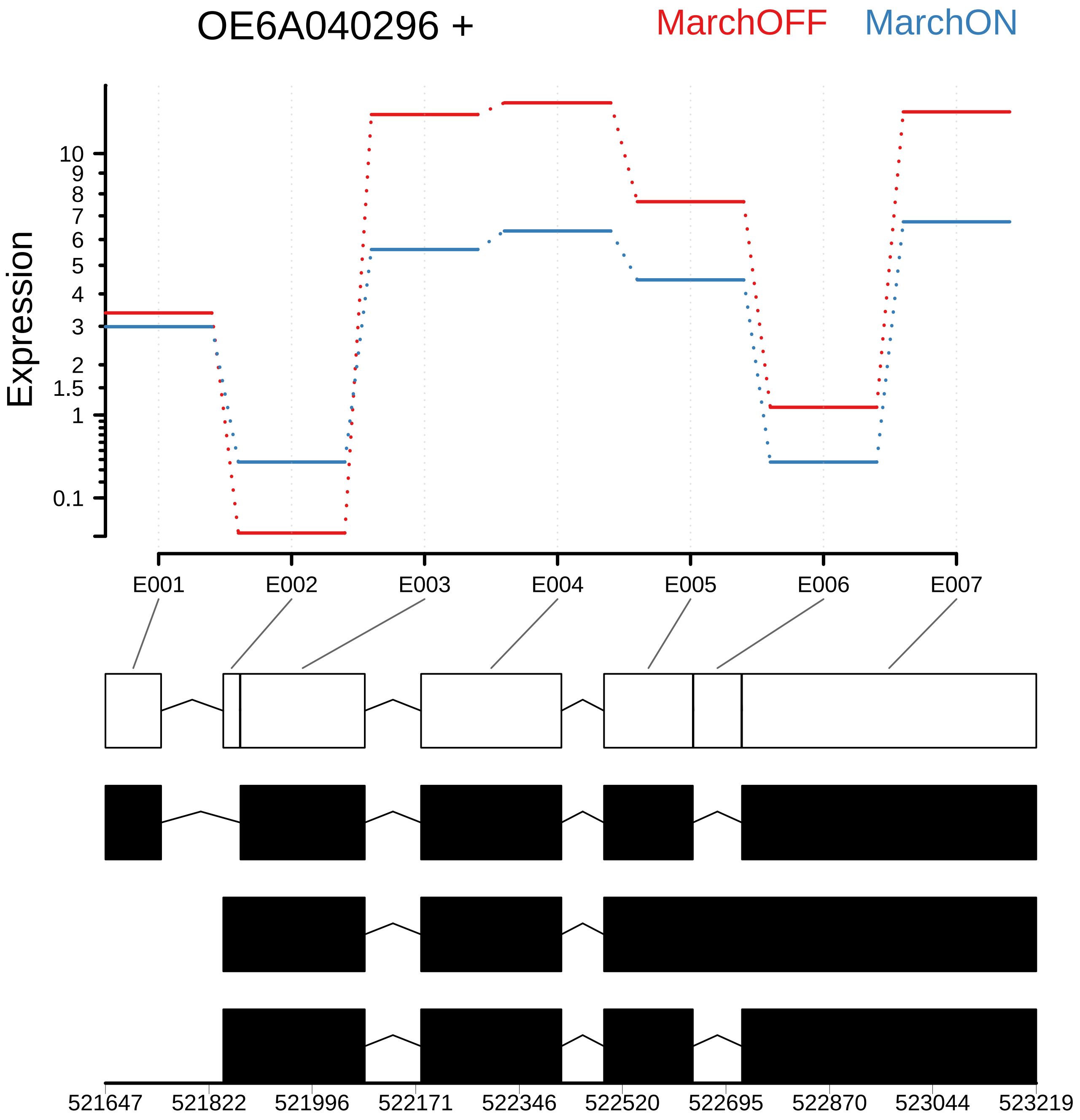

OE6A040296 +
MarchOFF
MarchON
Expression
10
9
8
7
6
5
4
3
2
1.5
1
0.1
E001
E002
E003
E004
E005
E006
E007
521647
521822
521996
522171
522346
522520
522695
522870
523044
523219

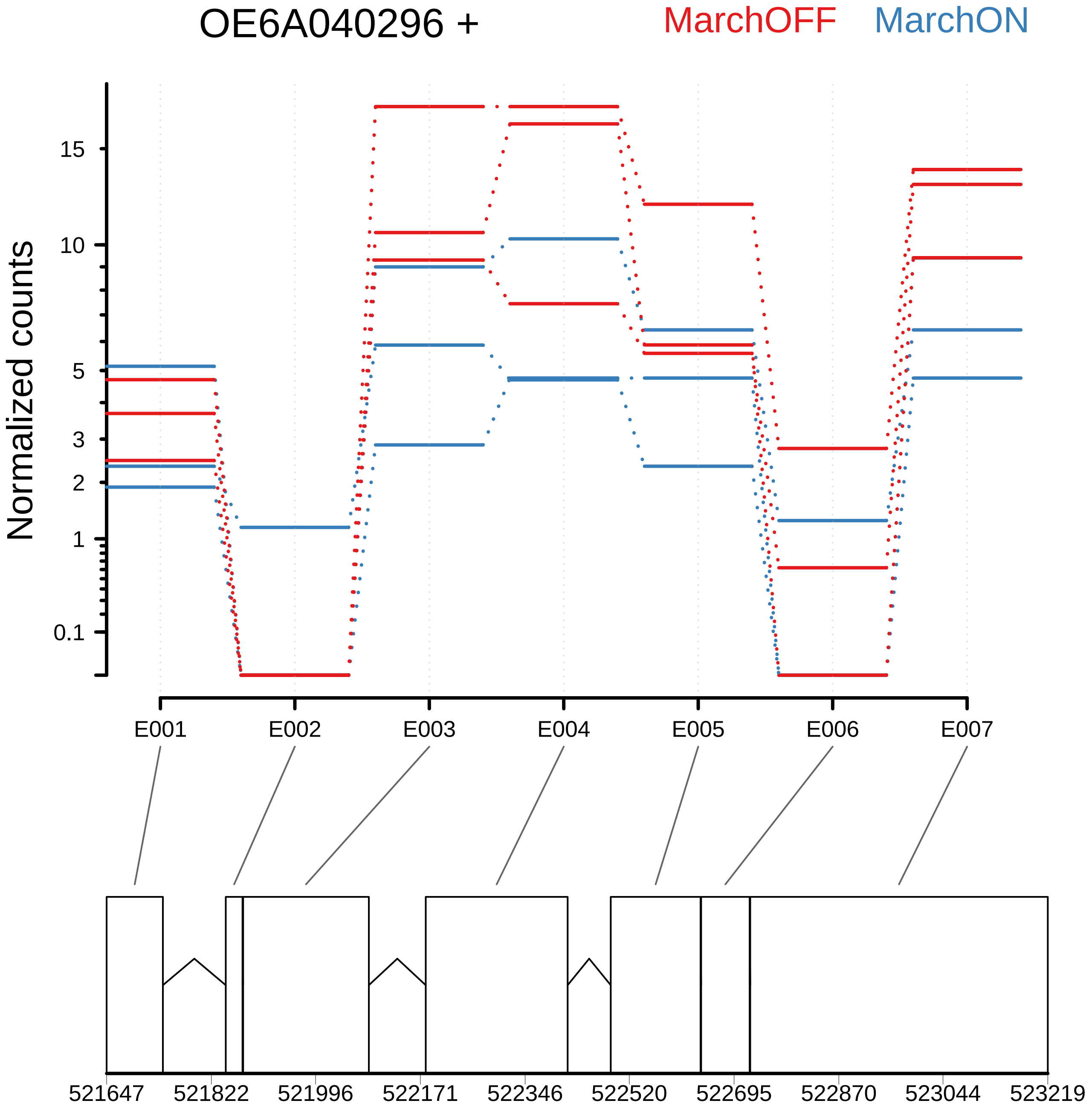

OE6A040296 +
MarchOFF
MarchON
Normalized counts
15
10
5
3
2
1
0.1
E001
E002
E003
E004
E005
E006
E007
521647
521822
521996
522171
522346
522520
522695
522870
523044
523219

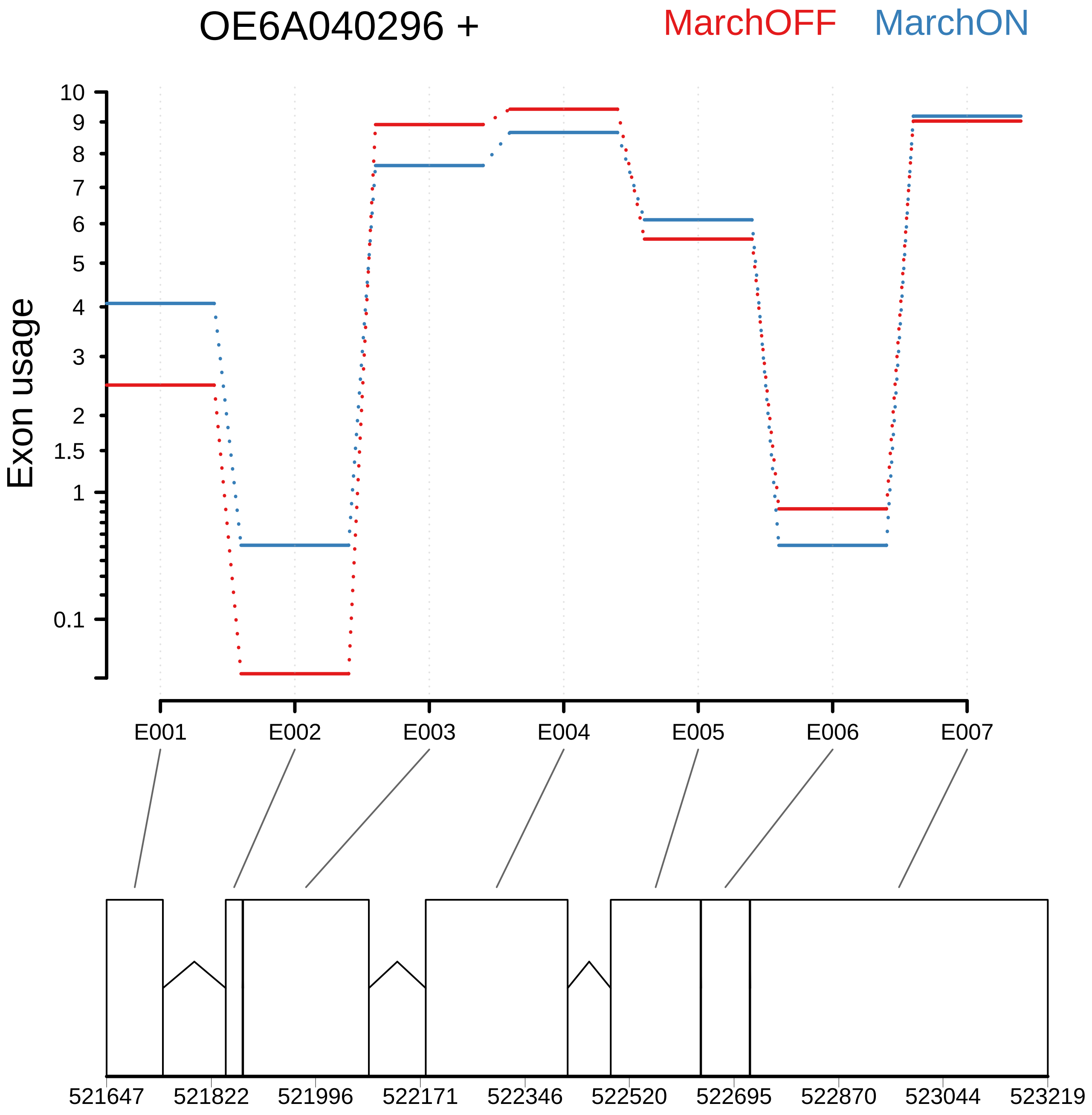

OE6A040296 +
MarchOFF
MarchON
Exon usage
10
9
8
7
6
5
4
3
2
1.5
1
0.1
E001
E002
E003
E004
E005
E006
E007
521647
521822
521996
522171
522346
522520
522695
522870
523044
523219

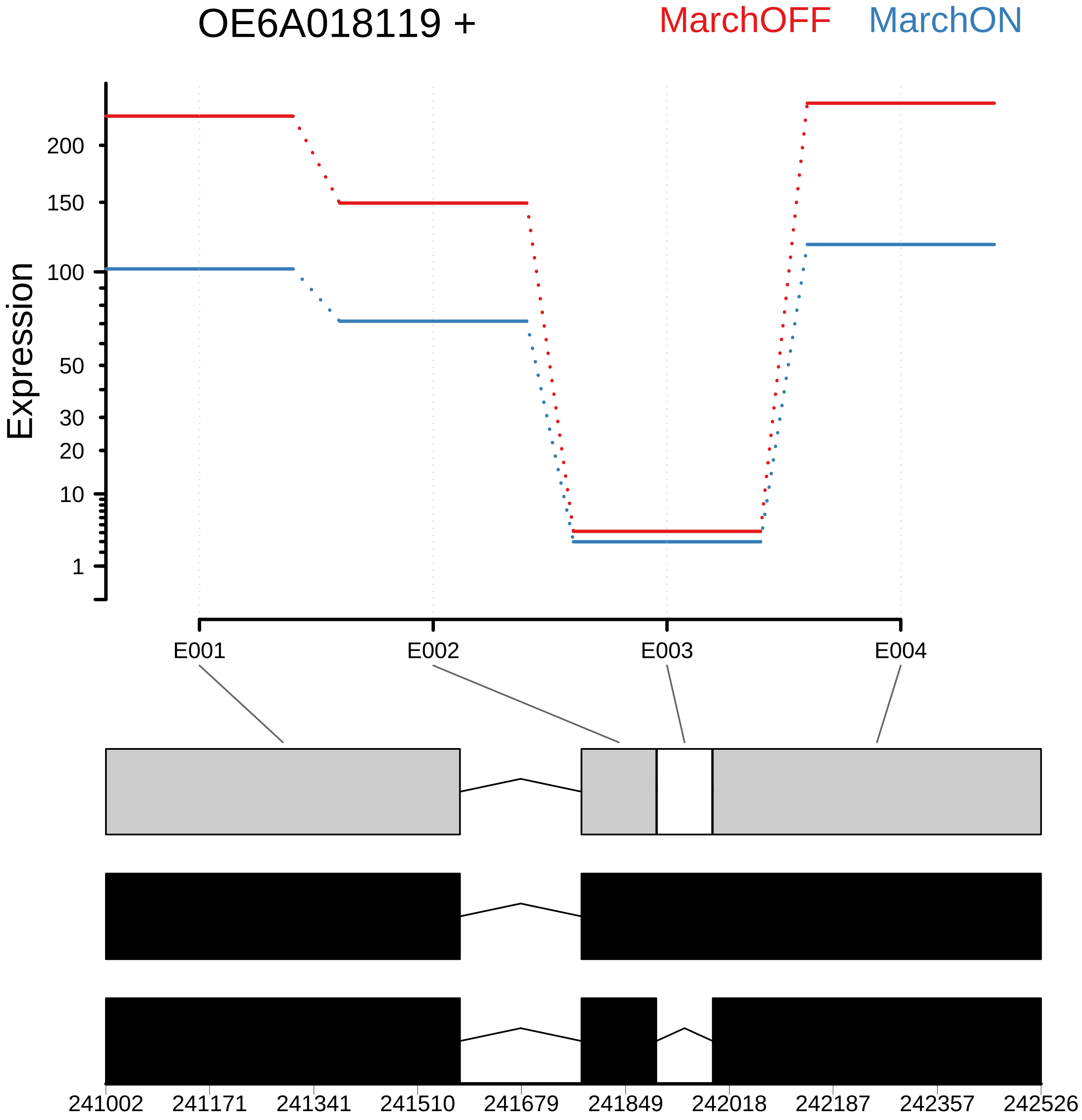

OE6A018119 +
MarchOFF
MarchON
Expression
200
150
100
50
30
20
10
1
E001
E002
E003
E004
241002
241171
241341
241510
241679
241849
242018
242187
242357
242526

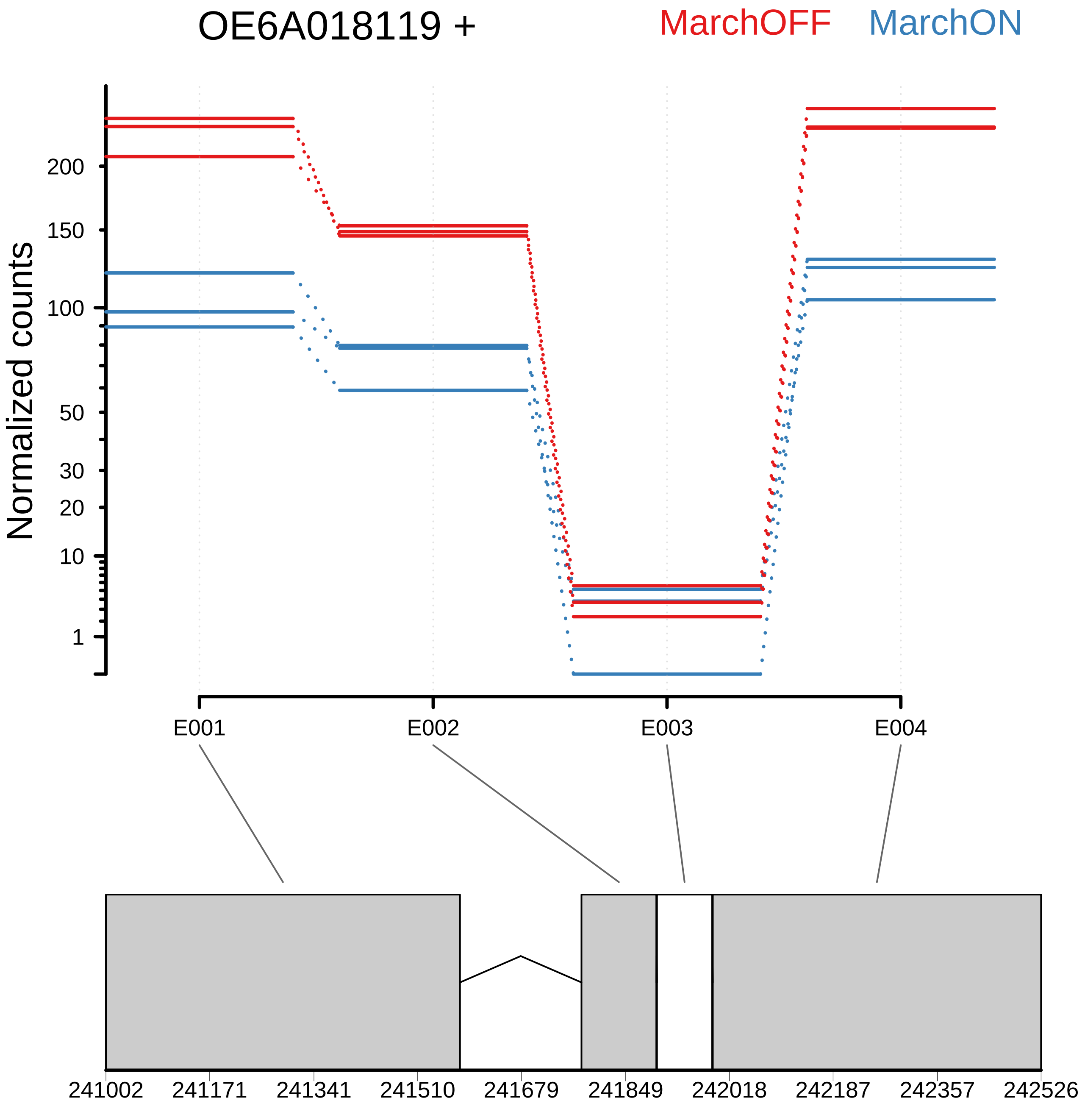

OE6A018119 +
MarchOFF
MarchON
Normalized counts
200
150
100
50
30
20
10
1
E001
E002
E003
E004
241002
241171
241341
241510
241679
241849
242018
242187
242357
242526

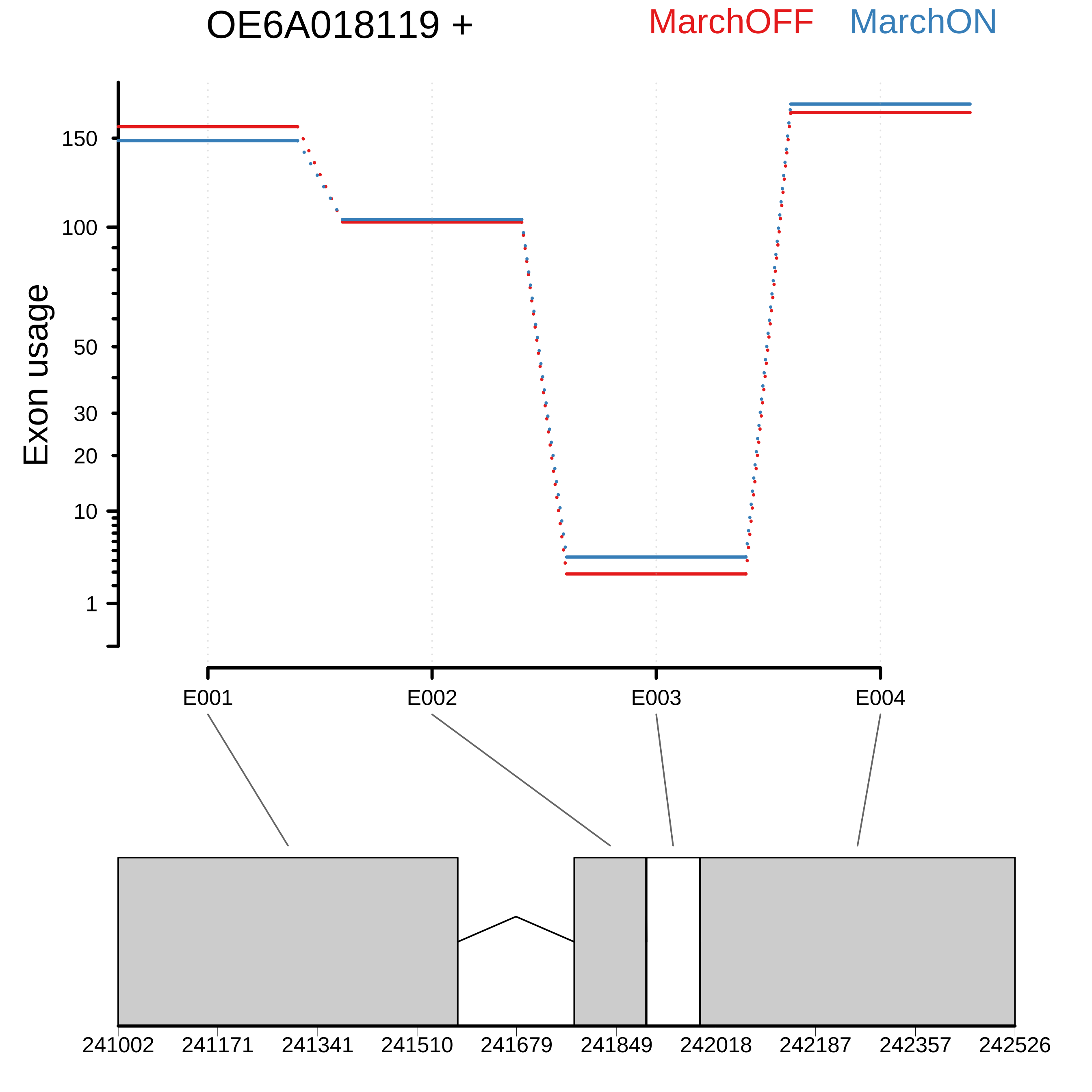

OE6A018119 +
MarchOFF
MarchON
Exon usage
150
100
50
30
20
10
1
E001
E002
E003
E004
241002
241171
241341
241510
241679
241849
242018
242187
242357
242526

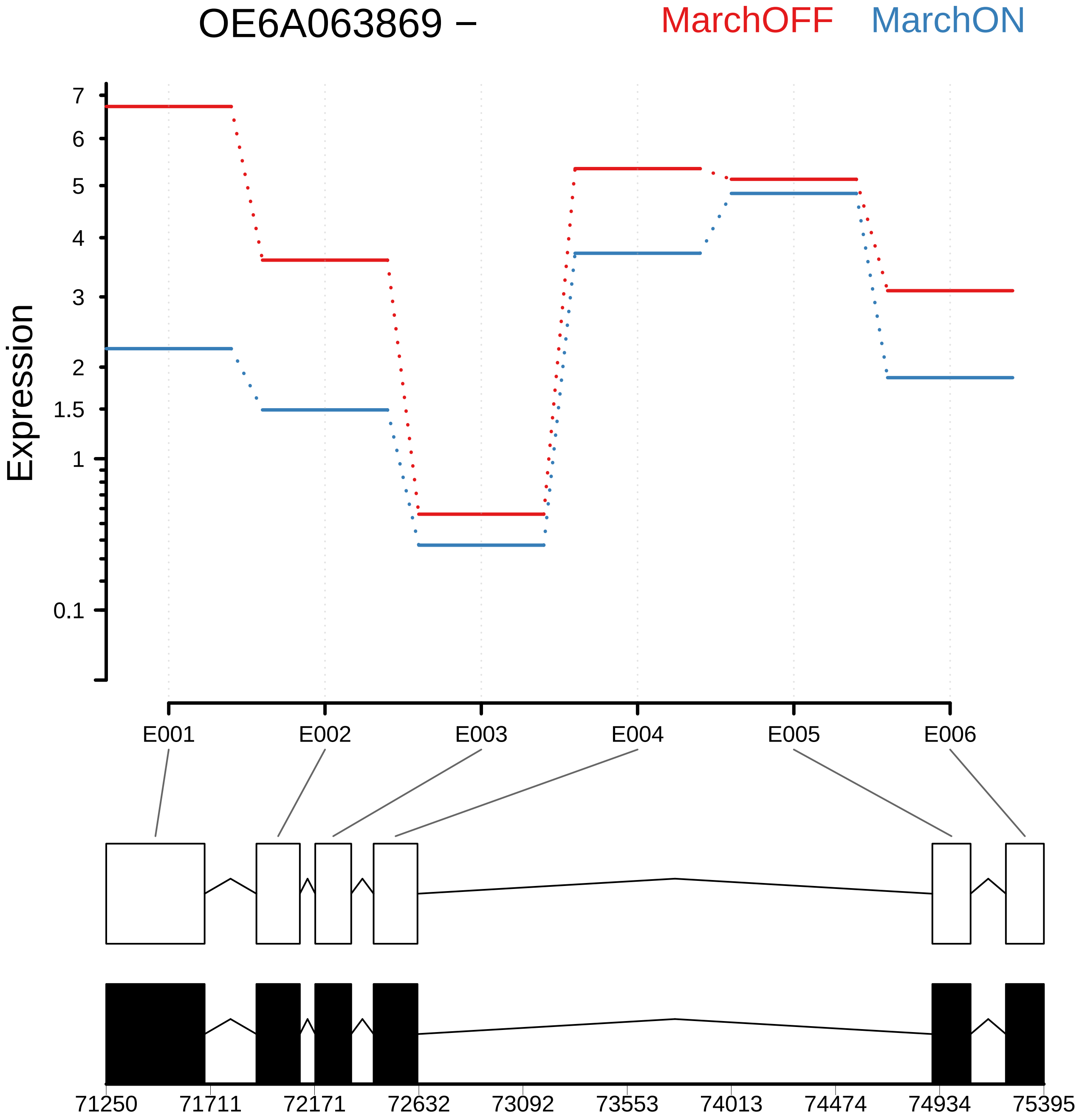
OE6A063869 –
MarchOFF
MarchON
Expression
7
6
5
4
3
2
1.5
1
0.1
E001
E002
E003
E004
E005
E006
71250
71711
72171
72632
73092
73553
74013
74474
74934
75395

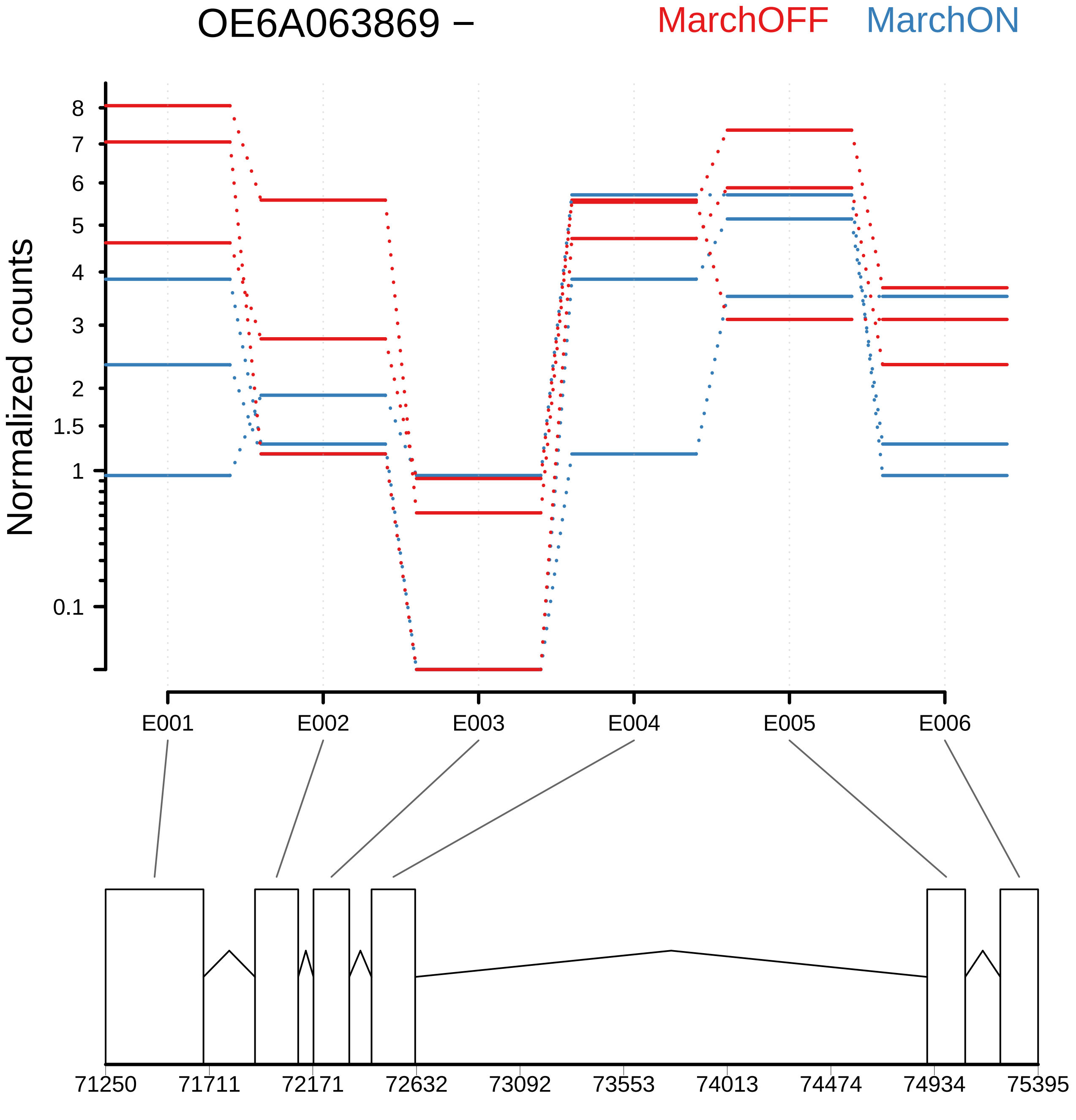

OE6A063869 −
MarchOFF
MarchON
Normalized counts
8
7
6
5
4
3
2
1.5
1
0.1
E001
E002
E003
E004
E005
E006
71250
71711
72171
72632
73092
73553
74013
74474
74934
75395

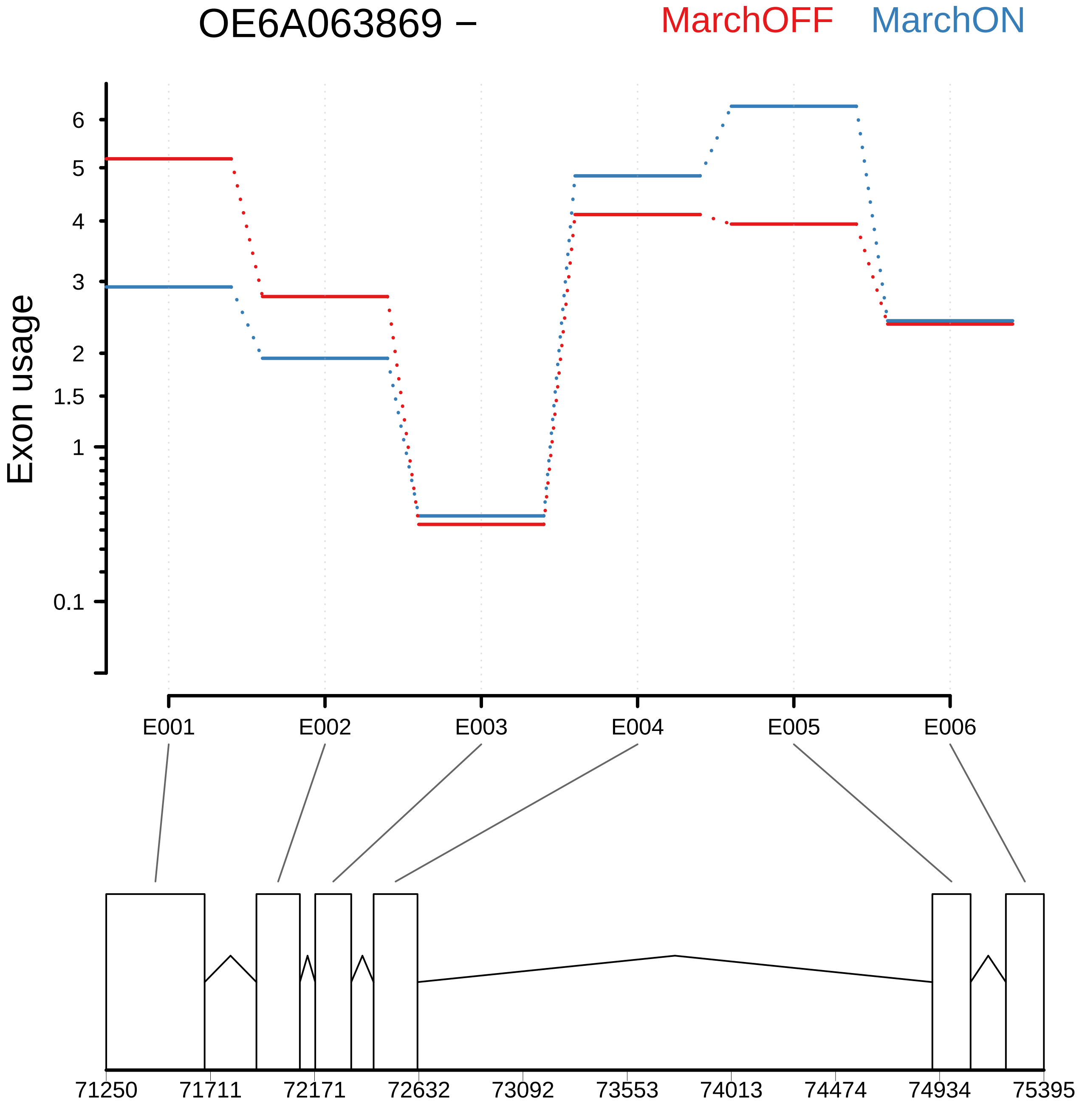
OE6A063869 −
MarchOFF
MarchON
Exon usage
6
5
4
3
2
1.5
1
0.1
E001
E002
E003
E004
E005
E006
71250
71711
72171
72632
73092
73553
74013
74474
74934
75395

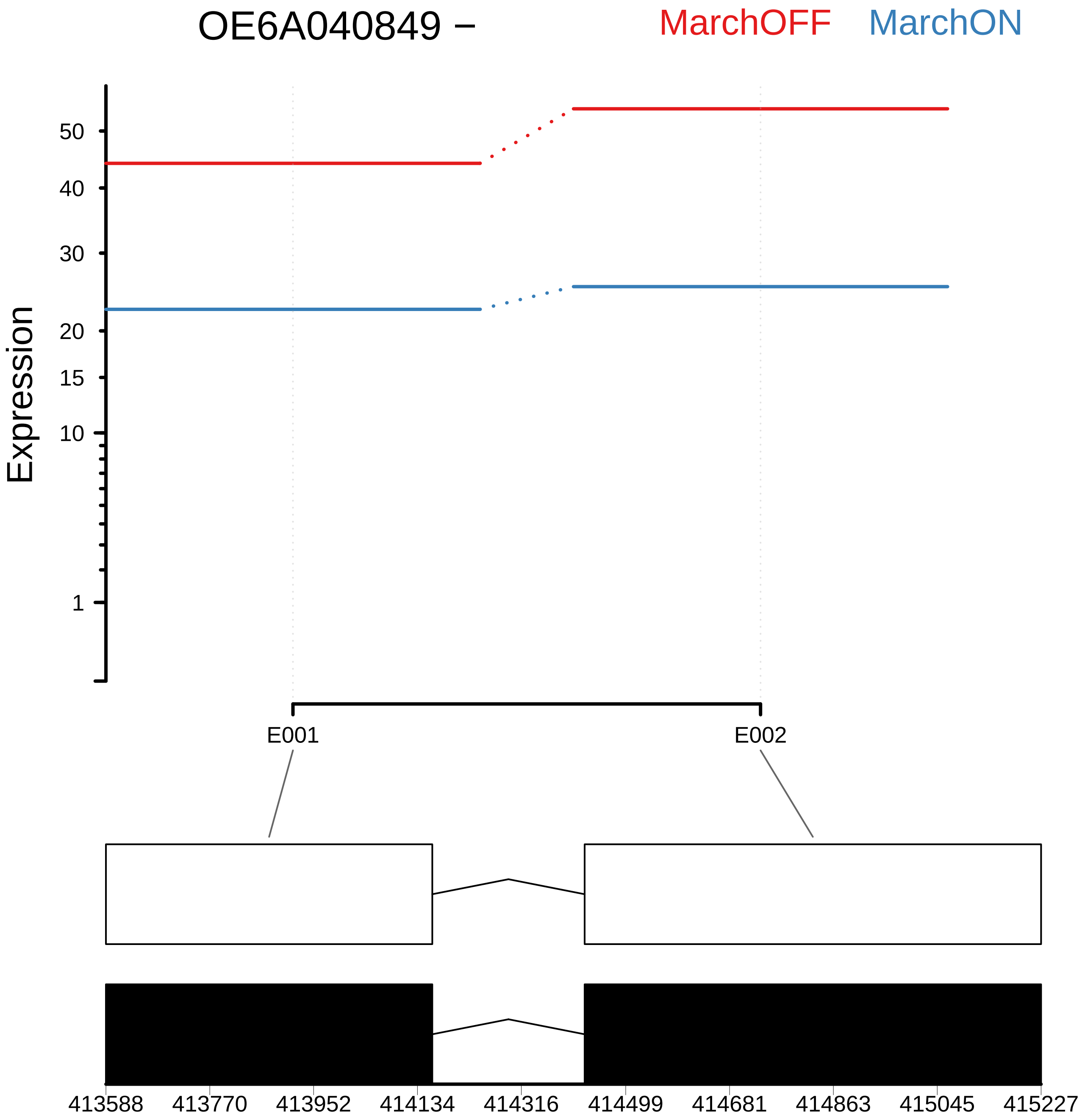
OE6A040849 −
MarchOFF
MarchON
Expression
50
40
30
20
15
10
1
E001
E002
413588
413770
413952
414134
414316
414499
414681
414863
415045
415227

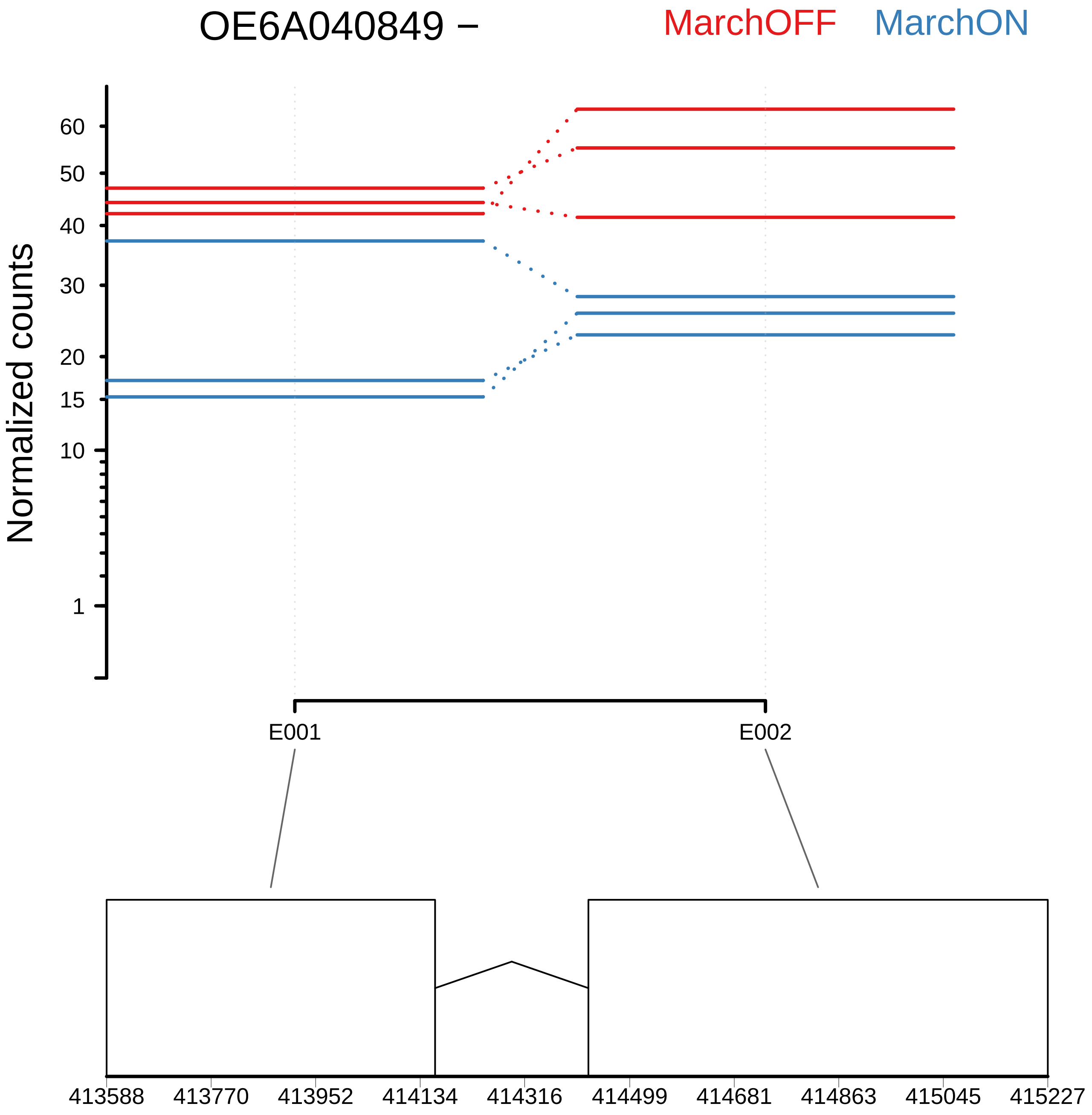

OE6A040849 −
MarchOFF
MarchON
Normalized counts
60
50
40
30
20
15
10
1
E001
E002
413588
413770
413952
414134
414316
414499
414681
414863
415045
415227

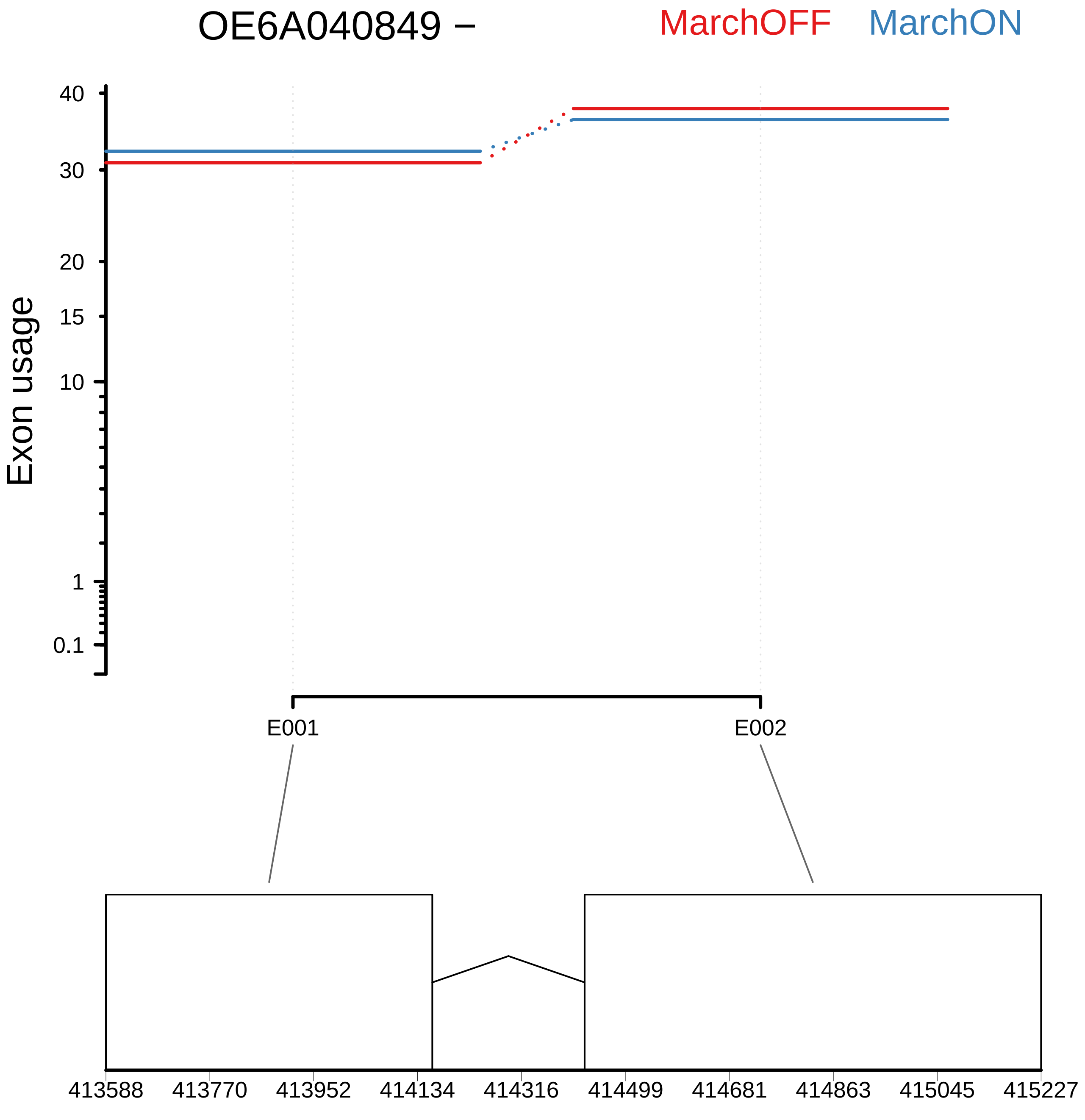

OE6A040849 −
MarchOFF
MarchON
Exon usage
40
30
20
15
10
1
0.1
E001
E002
413588
413770
413952
414134
414316
414499
414681
414863
415045
415227

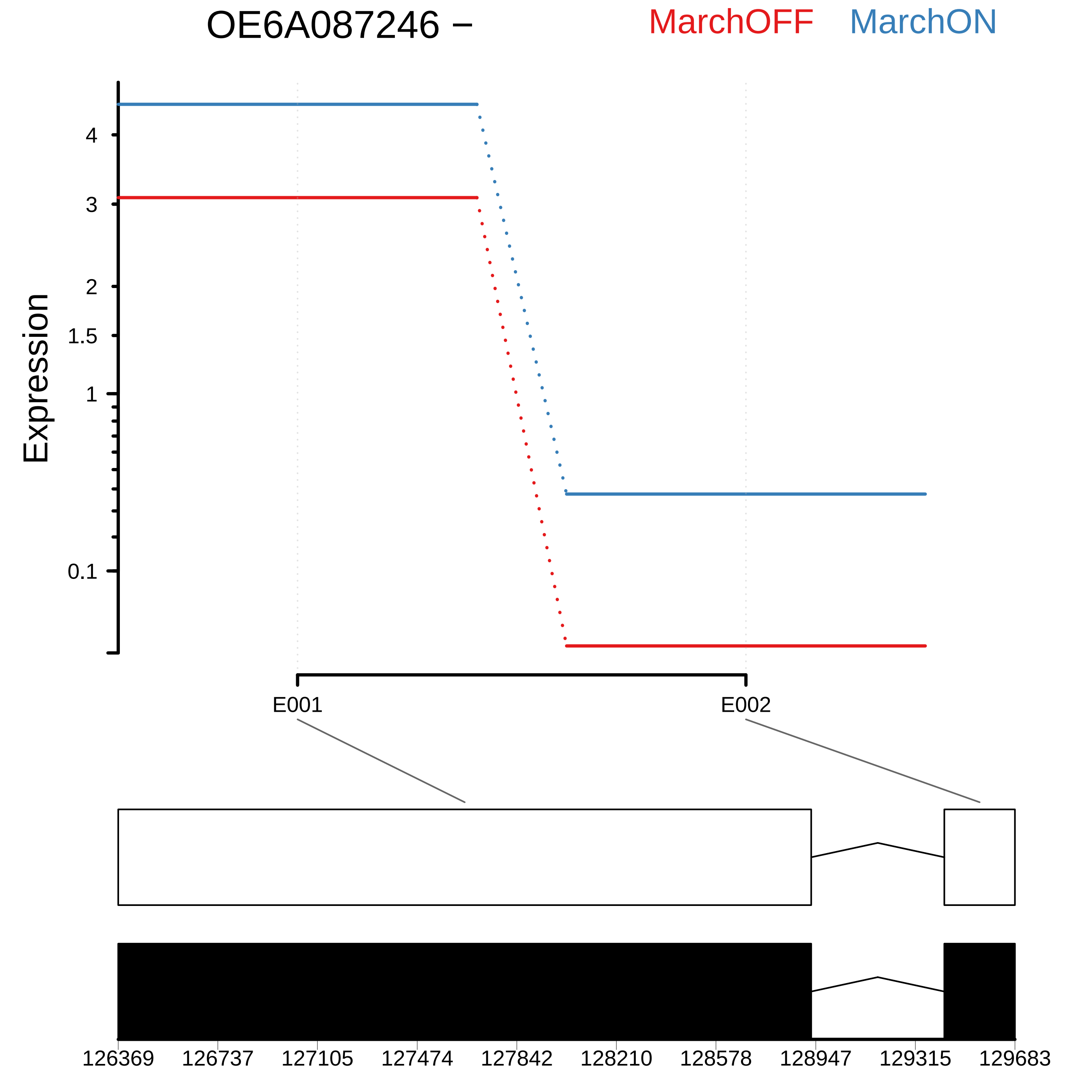
OE6A087246 −
MarchOFF
MarchON
Expression
4
3
2
1.5
1
0.1
E001
E002
126369
126737
127105
127474
127842
128210
128578
128947
129315
129683

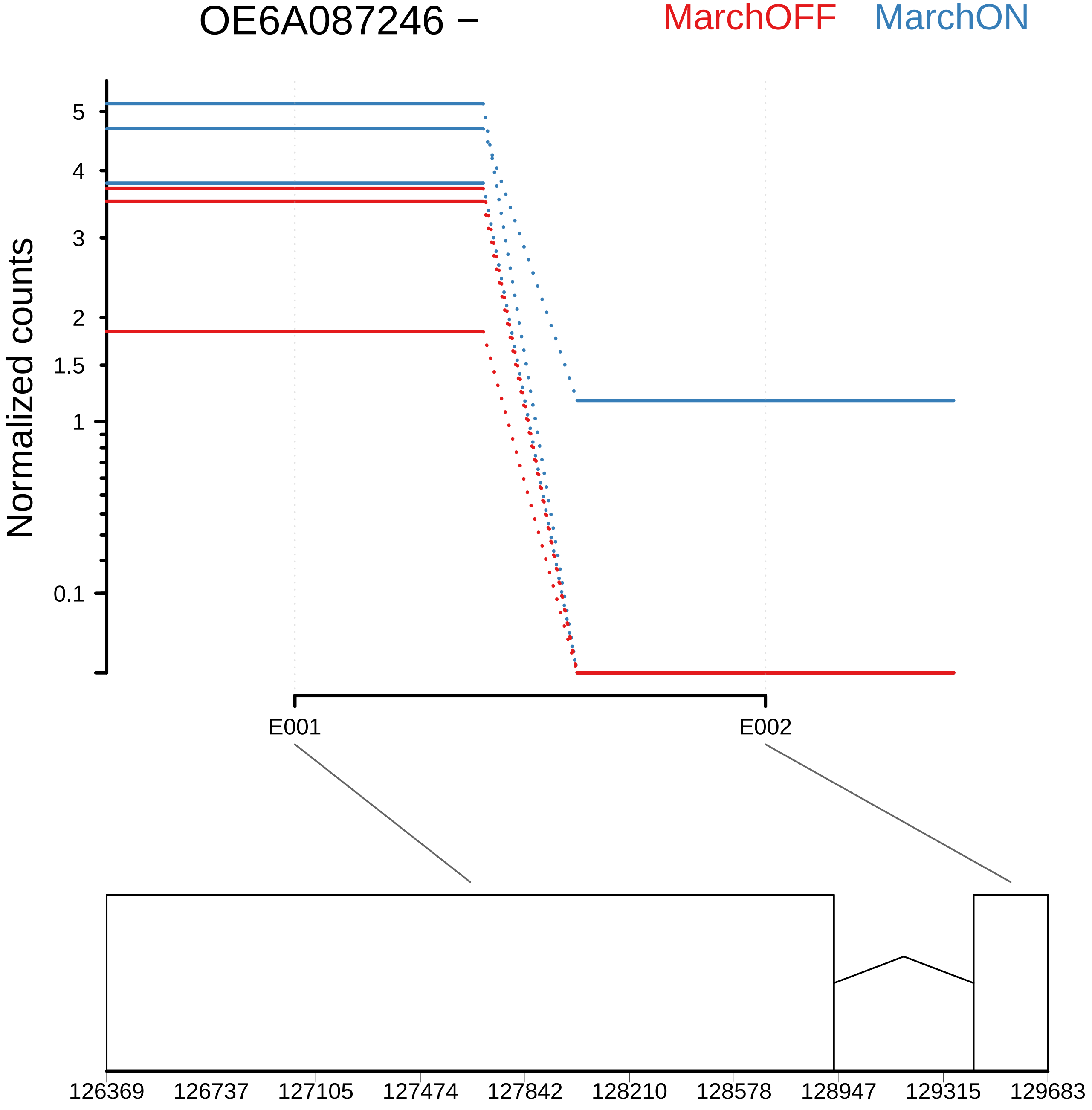

OE6A087246 −
MarchOFF
MarchON
Normalized counts
5
4
3
2
1.5
1
0.1
E001
E002
126369
126737
127105
127474
127842
128210
128578
128947
129315
129683

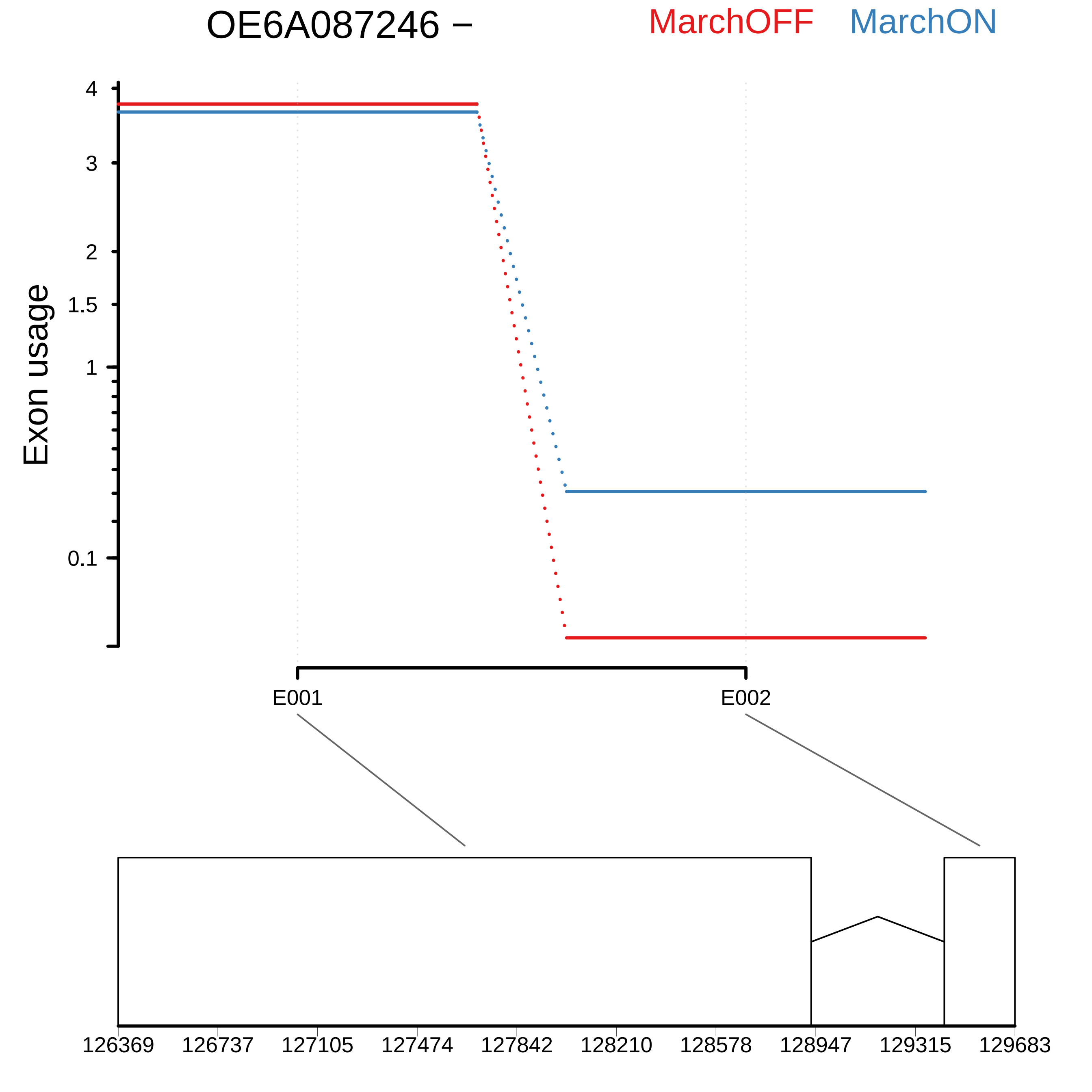
OE6A087246 −
MarchOFF
MarchON
Exon usage
4
3
2
1.5
1
0.1
E001
E002
126369
126737
127105
127474
127842
128210
128578
128947
129315
129683

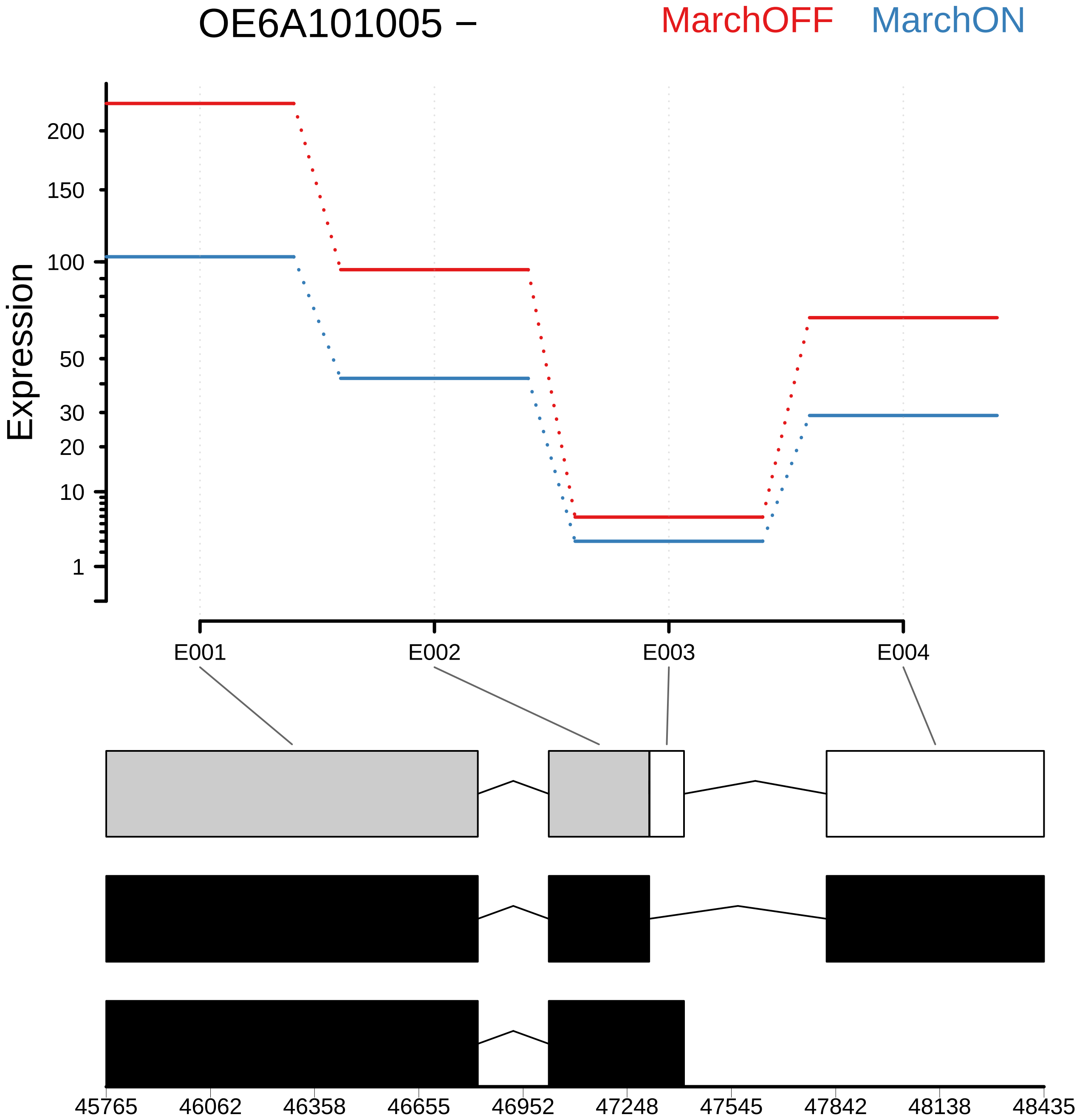

OE6A101005 −
MarchOFF
MarchON
Expression
200
150
100
50
30
20
10
1
E001
E002
E003
E004
45765
46062
46358
46655
46952
47248
47545
47842
48138
48435

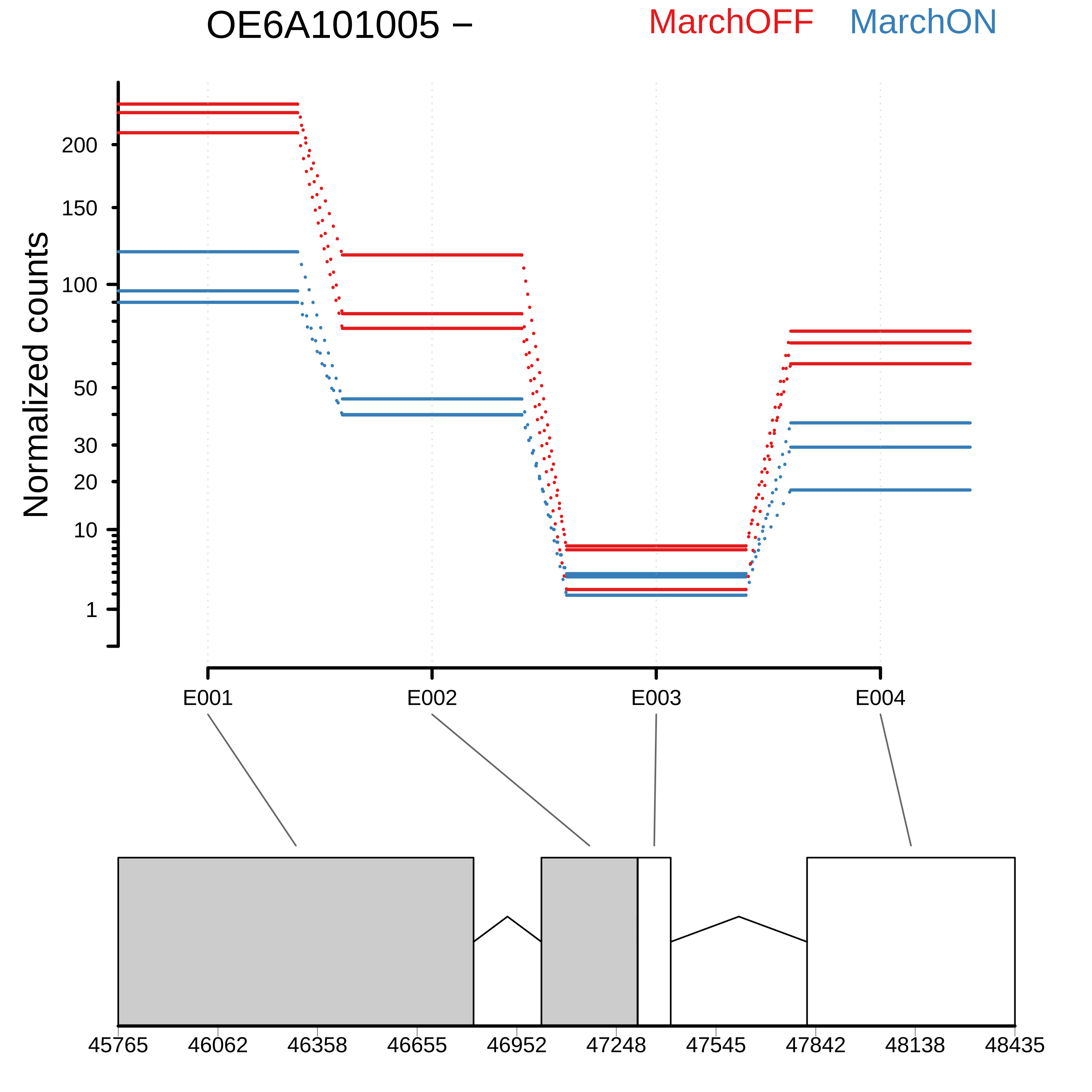

OE6A101005 −
MarchOFF
MarchON
Normalized counts
200
150
100
50
30
20
10
1
E001
E002
E003
E004
45765
46062
46358
46655
46952
47248
47545
47842
48138
48435

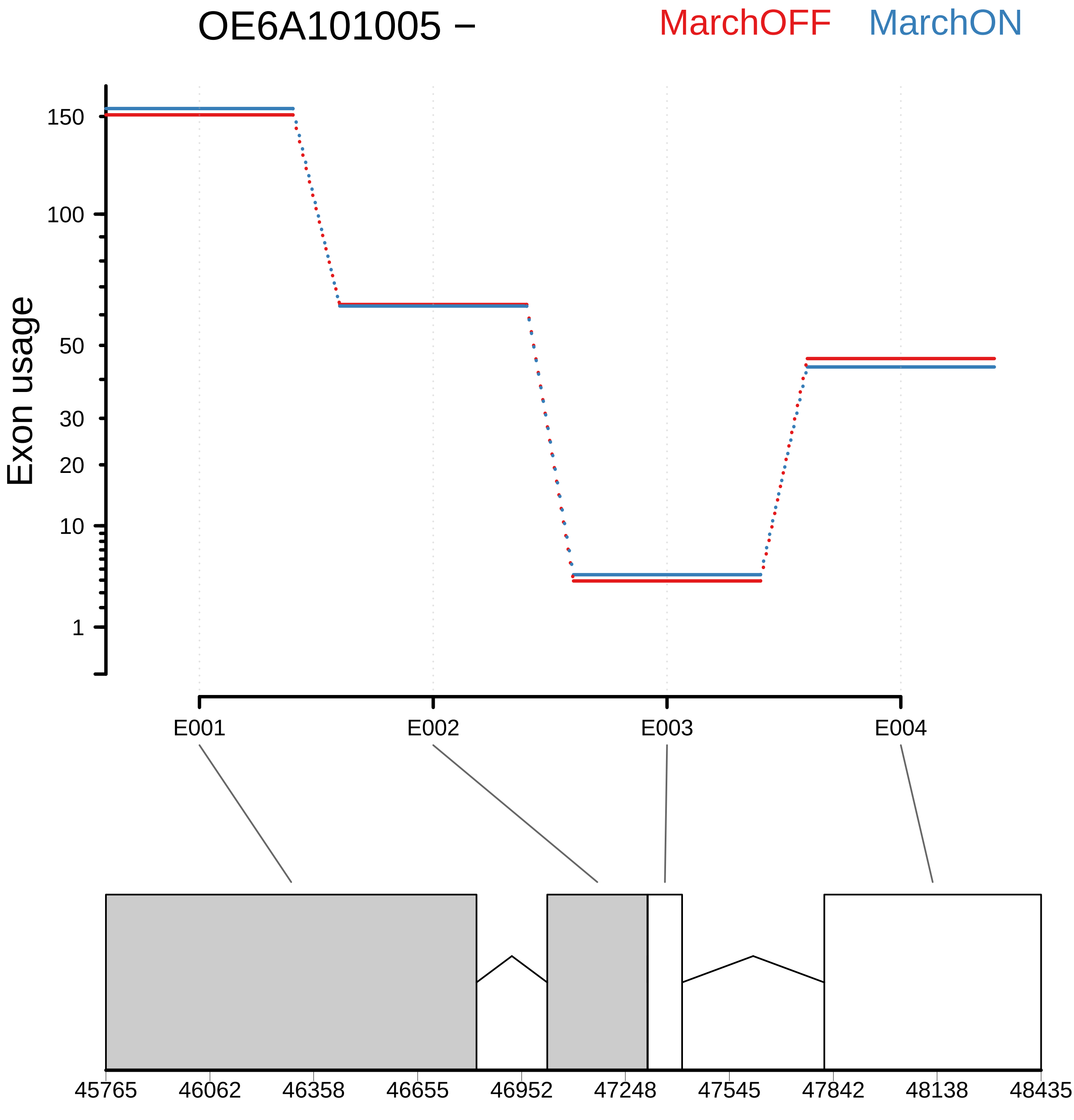

OE6A101005 −
MarchOFF
MarchON
Exon usage
150
100
50
30
20
10
1
E001
E002
E003
E004
45765
46062
46358
46655
46952
47248
47545
47842
48138
48435

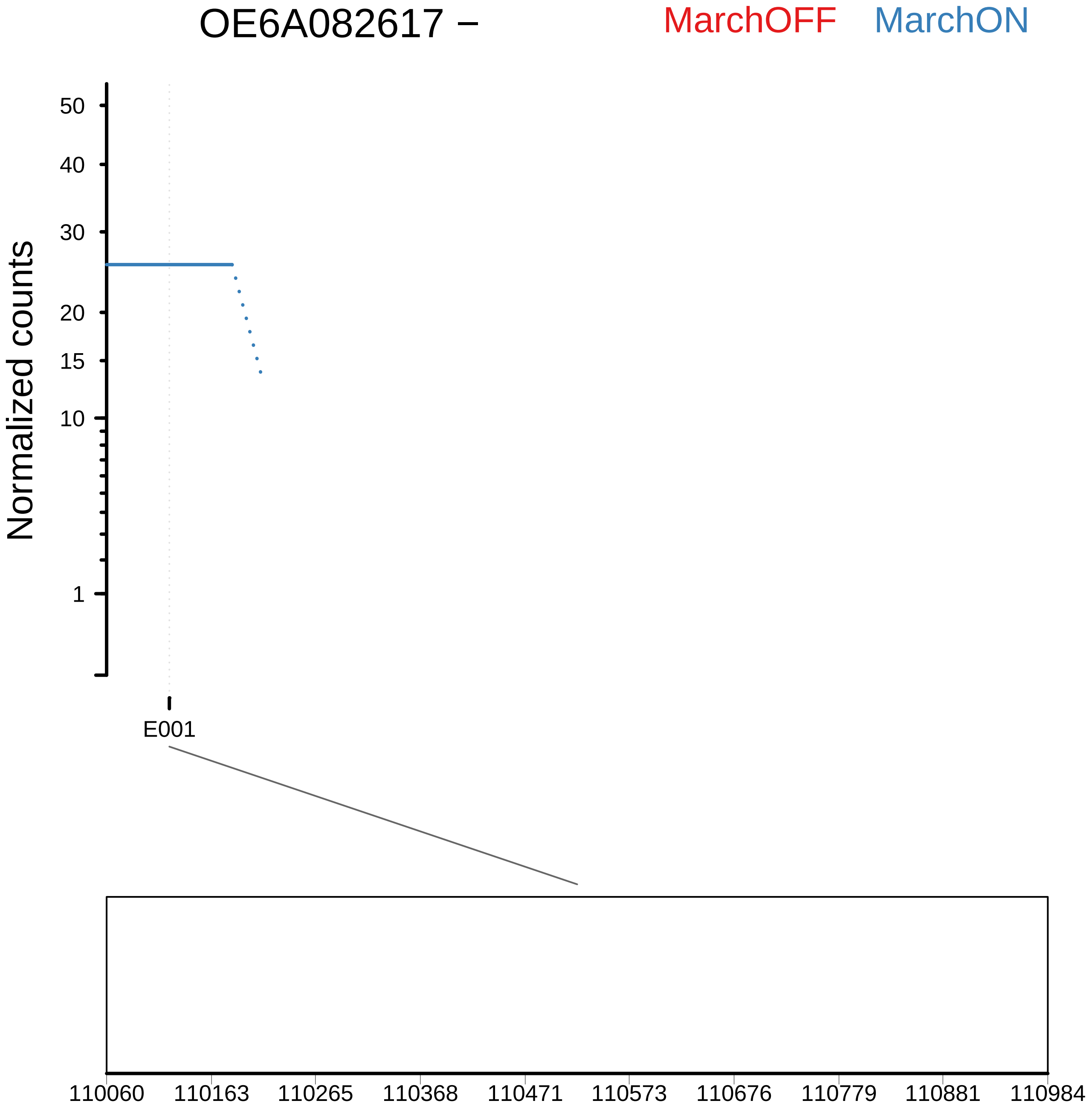
OE6A082617 −
MarchOFF
MarchON
Normalized counts
50
40
30
20
15
10
1
E001
110060
110163
110265
110368
110471
110573
110676
110779
110881
110984

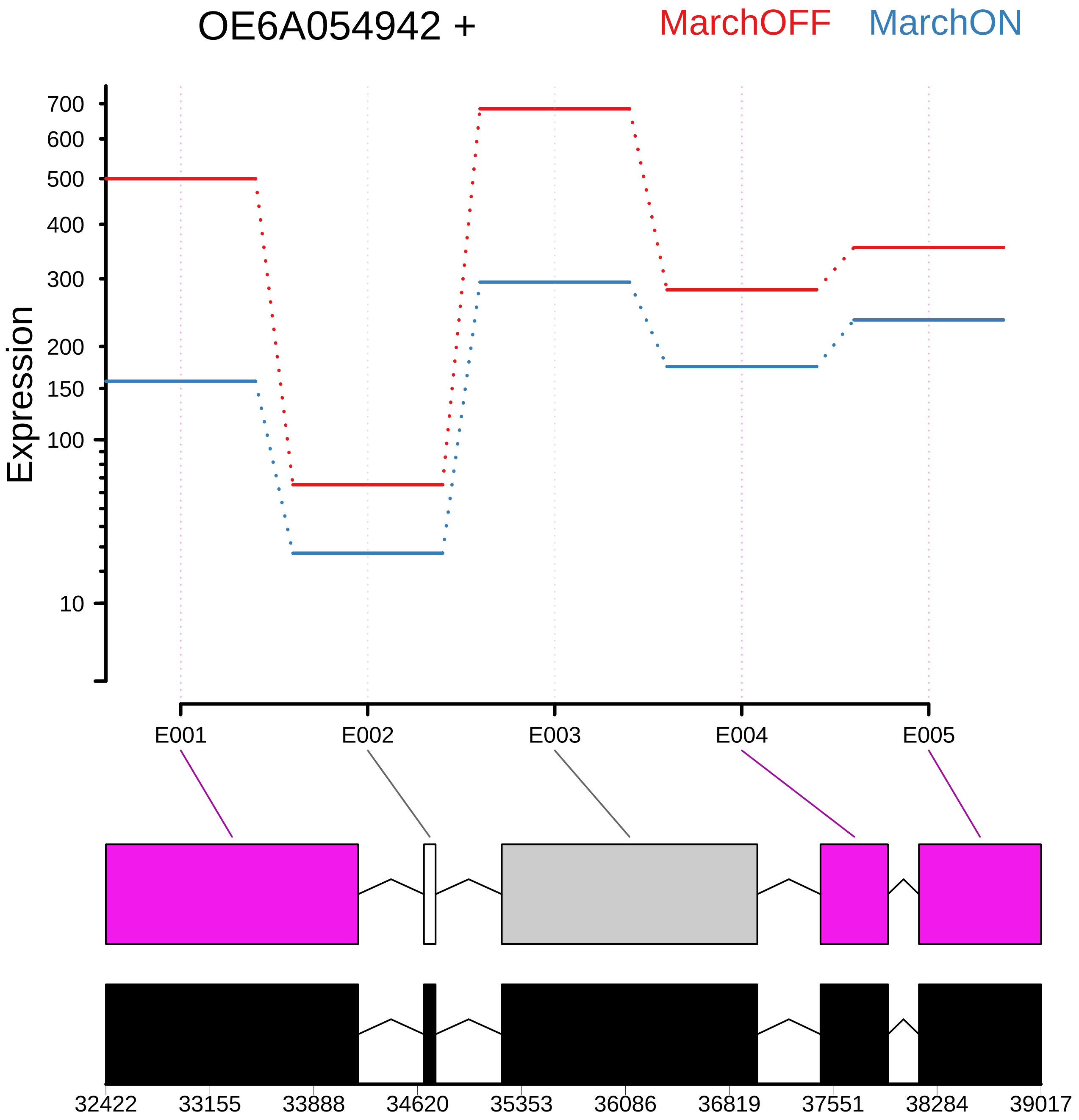
OE6A054942 +
MarchOFF
MarchON
Expression
700
600
500
400
300
200
150
100
10
E001
E002
E003
E004
E005
32422
33155
33888
34620
35353
36086
36819
37551
38284
39017

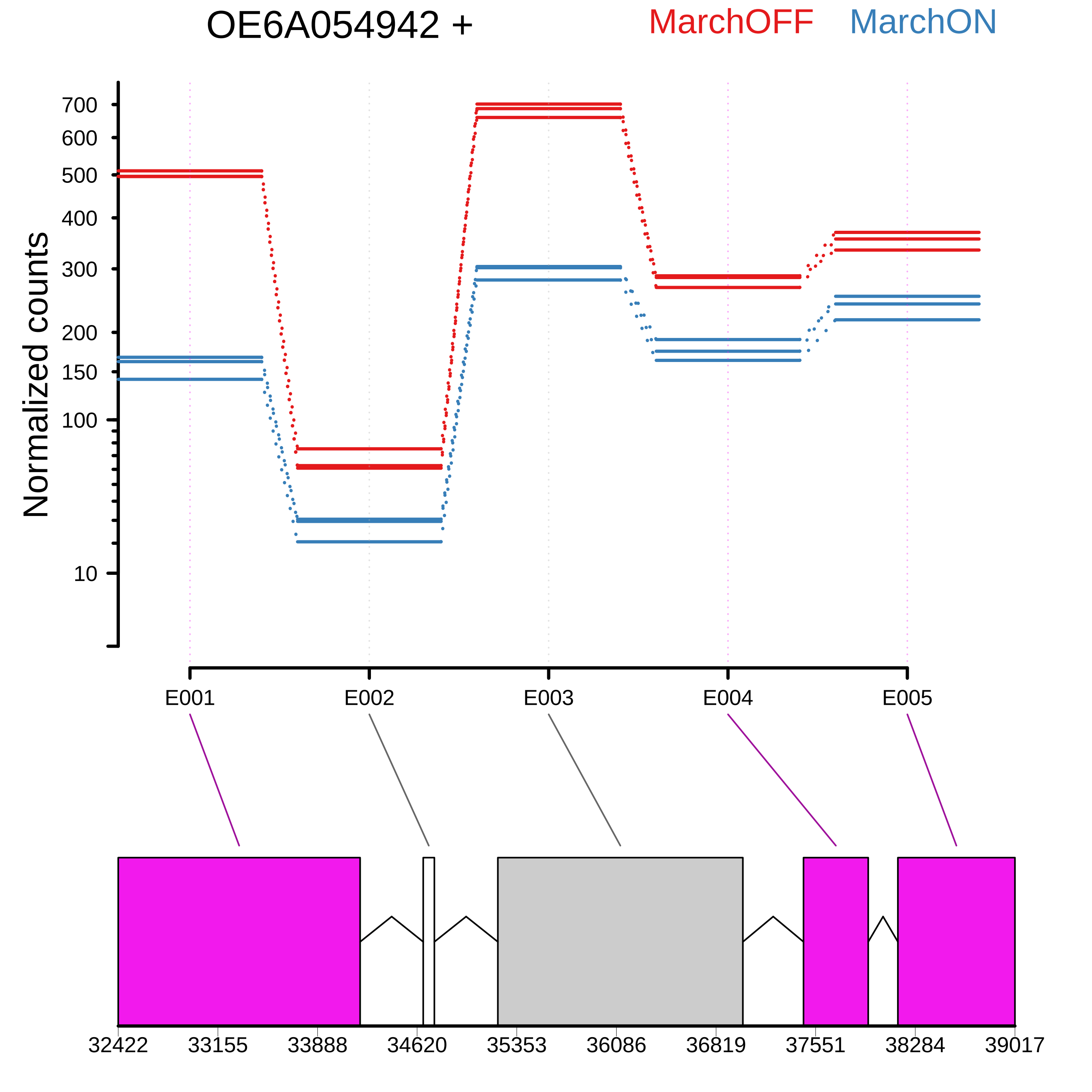
OE6A054942 +
MarchOFF
MarchON
Normalized counts
700
600
500
400
300
200
150
100
10
E001
E002
E003
E004
E005
32422
33155
33888
34620
35353
36086
36819
37551
38284
39017

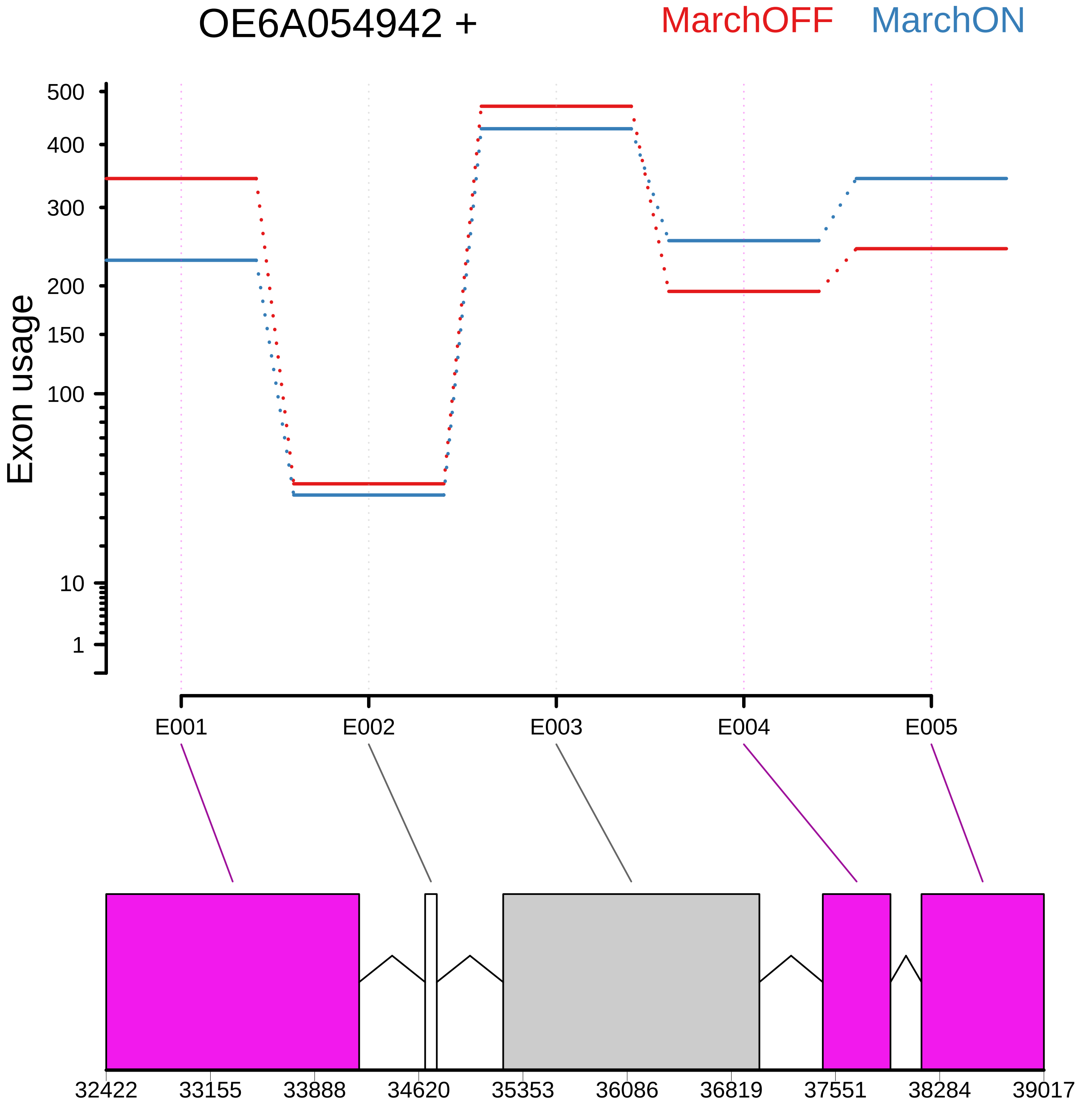
OE6A054942 +
MarchOFF
MarchON
Exon usage
500
400
300
200
150
100
10
1
E001
E002
E003
E004
E005
32422
33155
33888
34620
35353
36086
36819
37551
38284
39017

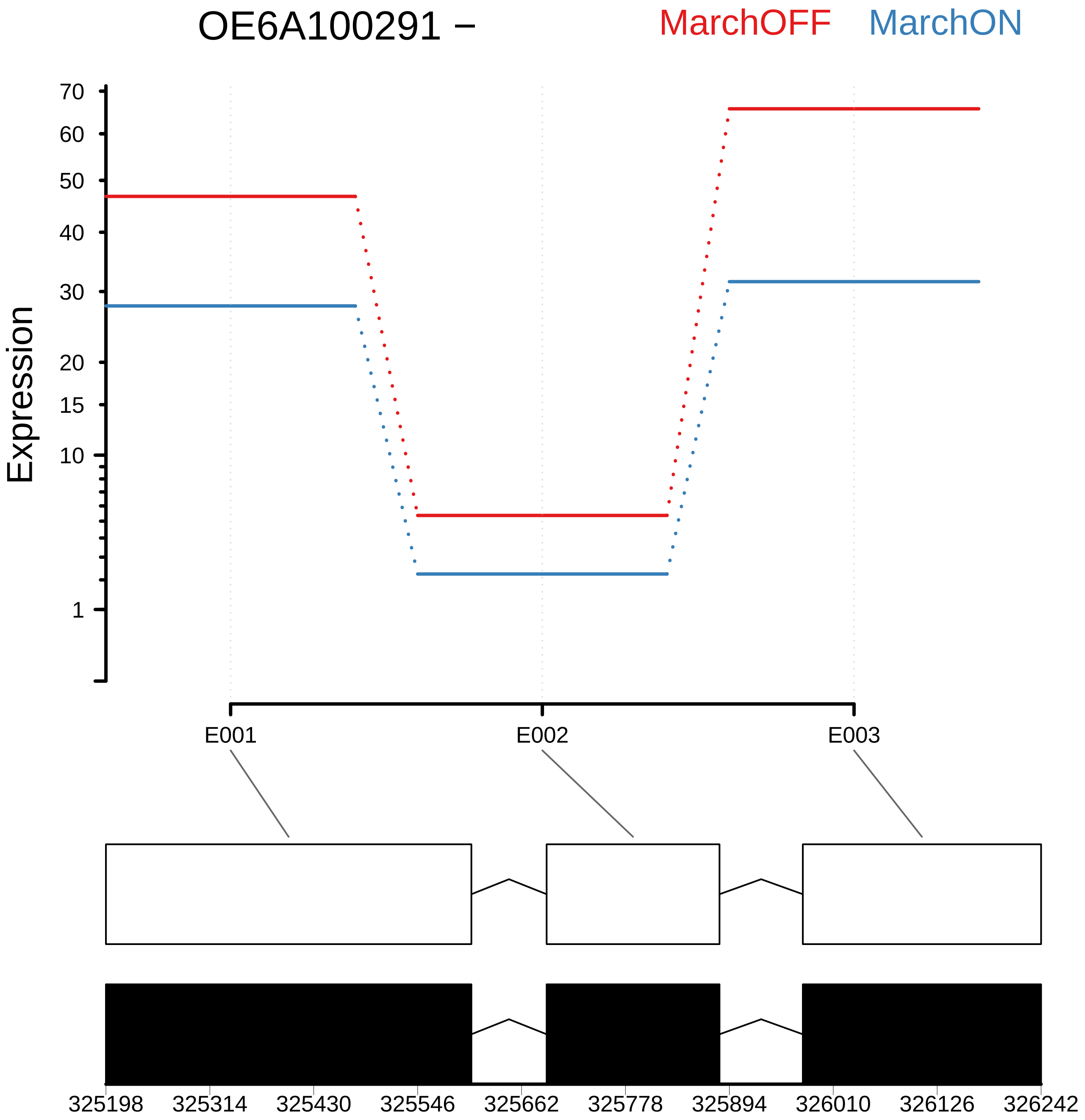

OE6A100291 −
MarchOFF
MarchON
Expression
70
60
50
40
30
20
15
10
1
E001
E002
E003
325198
325314
325430
325546
325662
325778
325894
326010
326126
326242

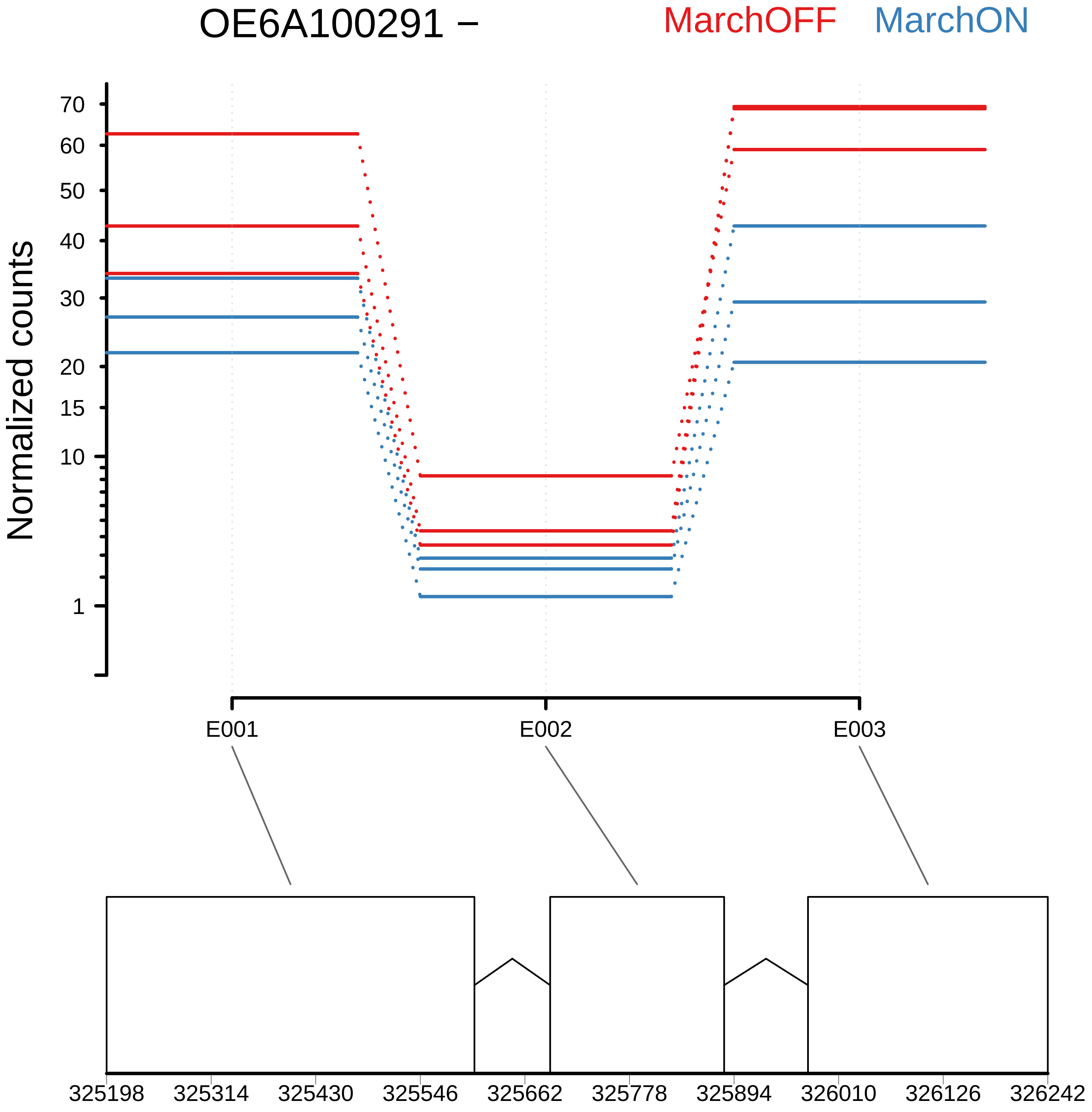

OE6A100291 −
MarchOFF
MarchON
Normalized counts
70
60
50
40
30
20
15
10
1
E001
E002
E003
325198
325314
325430
325546
325662
325778
325894
326010
326126
326242

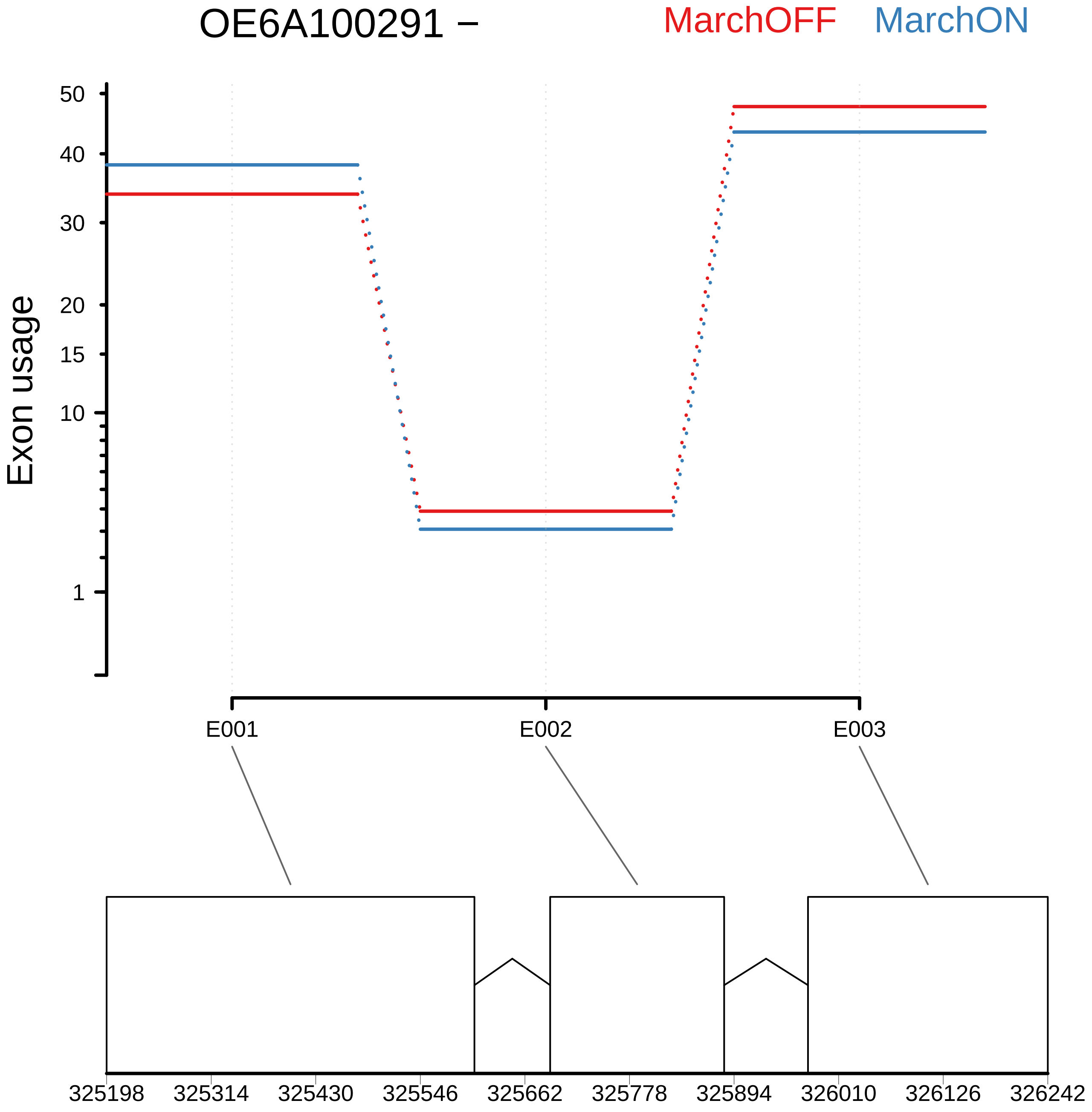

OE6A100291 −
MarchOFF
MarchON
Exon usage
50
40
30
20
15
10
1
E001
E002
E003
325198
325314
325430
325546
325662
325778
325894
326010
326126
326242

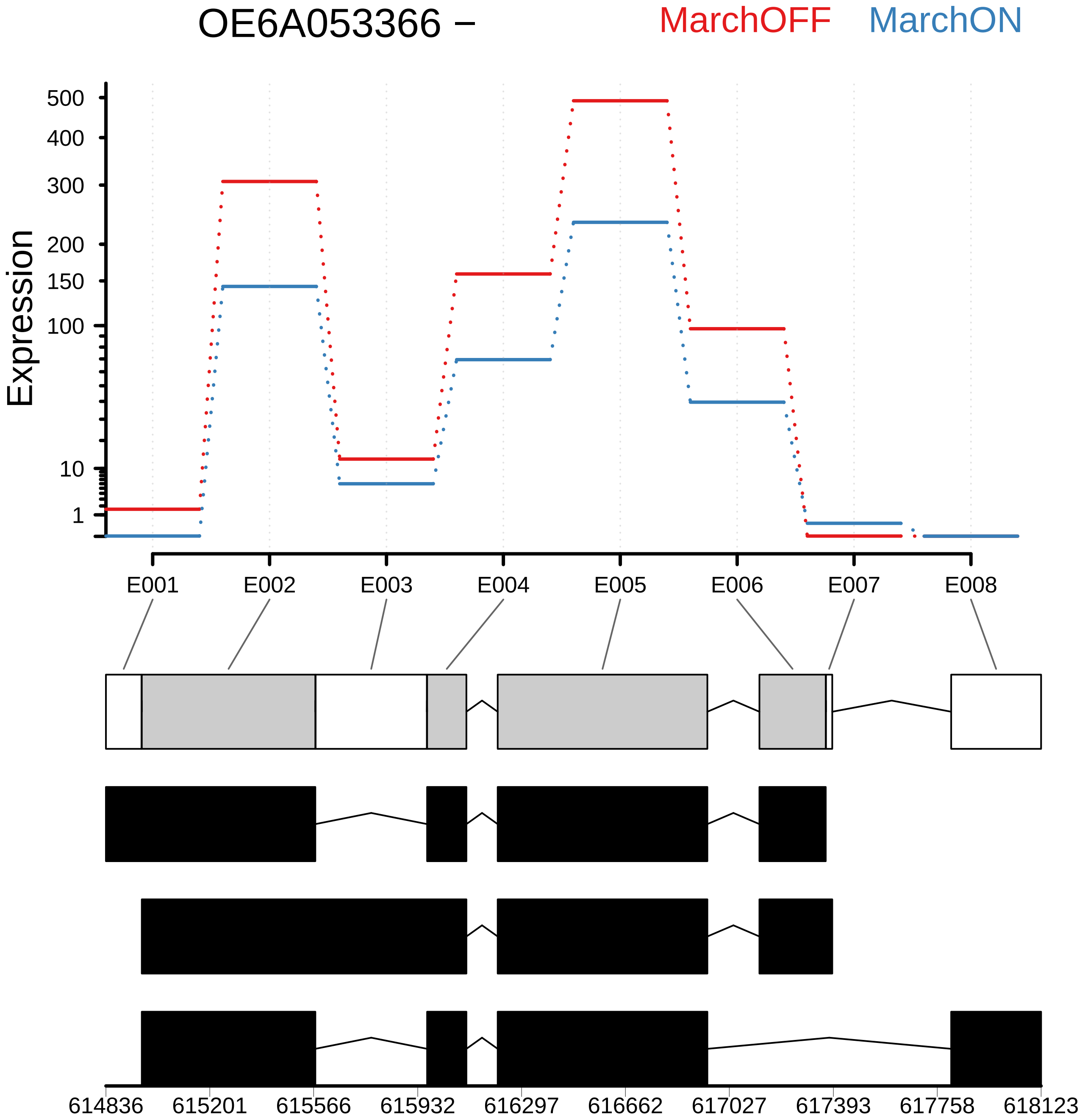
OE6A053366 −
MarchOFF
MarchON
Expression
500
400
300
200
150
100
10
1
E001
E002
E003
E004
E005
E006
E007
E008
614836
615201
615566
615932
616297
616662
617027
617393
617758
618123

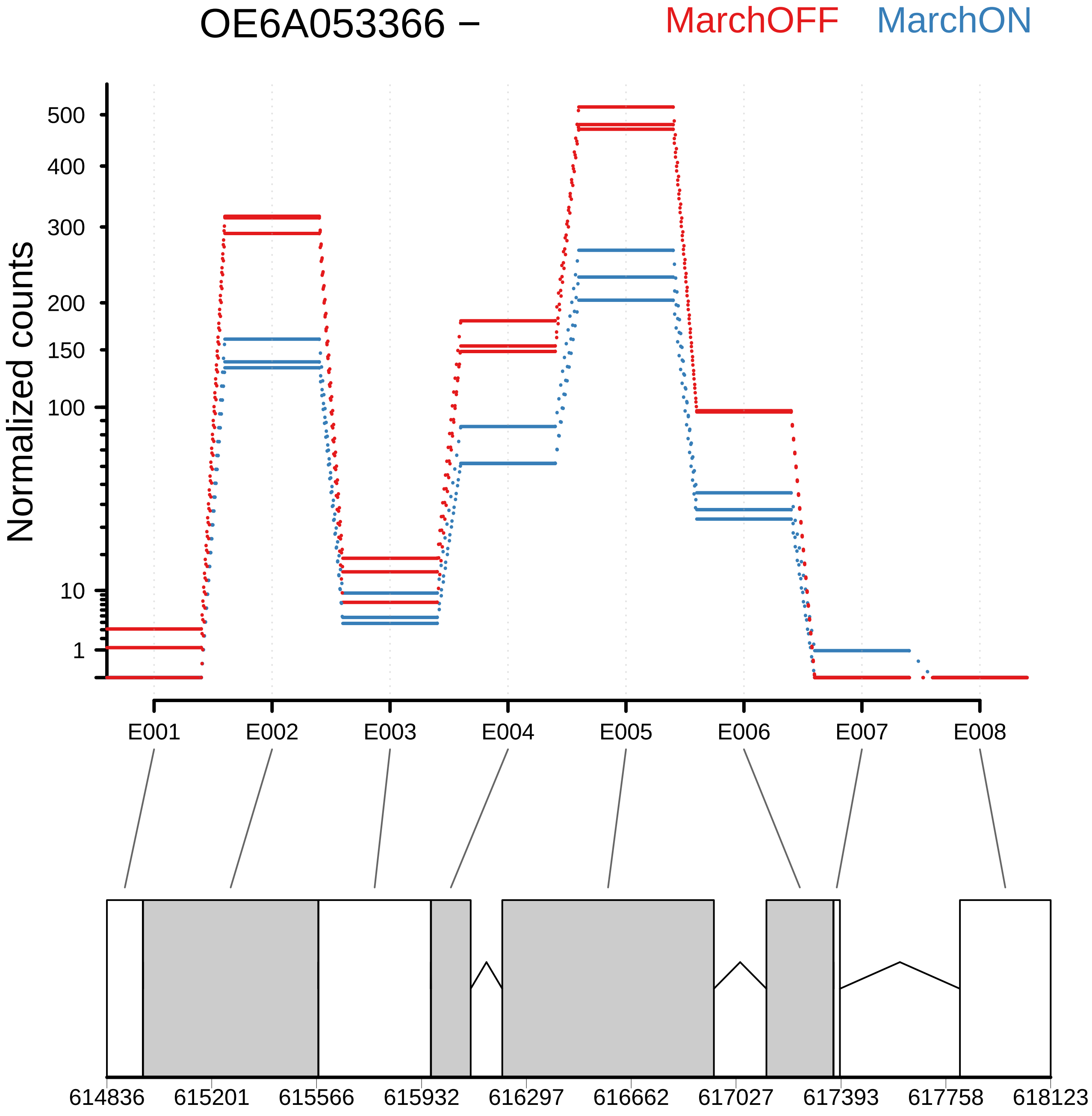

OE6A053366 −
MarchOFF
MarchON
Normalized counts
500
400
300
200
150
100
10
1
E001
E002
E003
E004
E005
E006
E007
E008
614836
615201
615566
615932
616297
616662
617027
617393
617758
618123

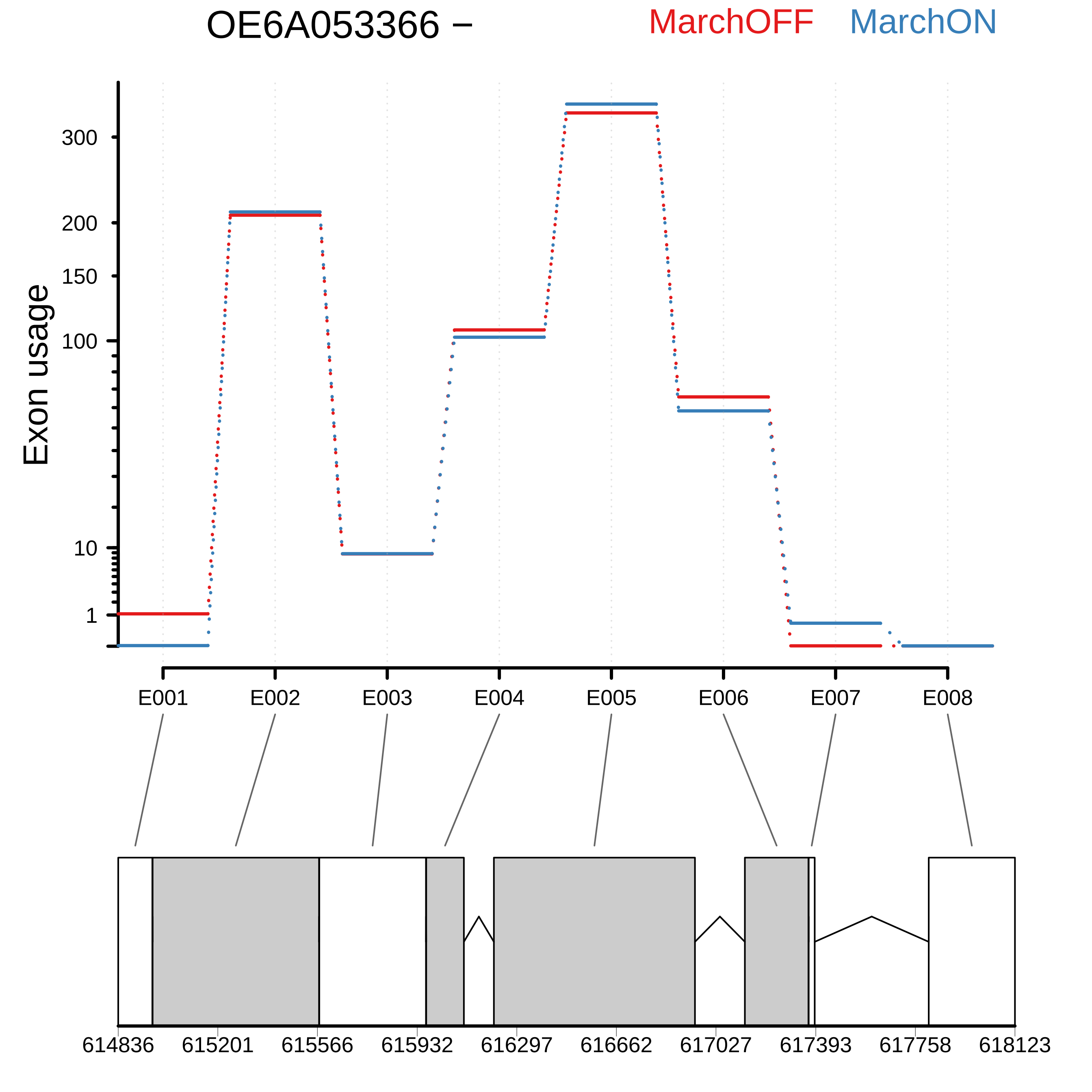

OE6A053366 −
MarchOFF
MarchON
Exon usage
300
200
150
100
10
1
E001
E002
E003
E004
E005
E006
E007
E008
614836
615201
615566
615932
616297
616662
617027
617393
617758
618123

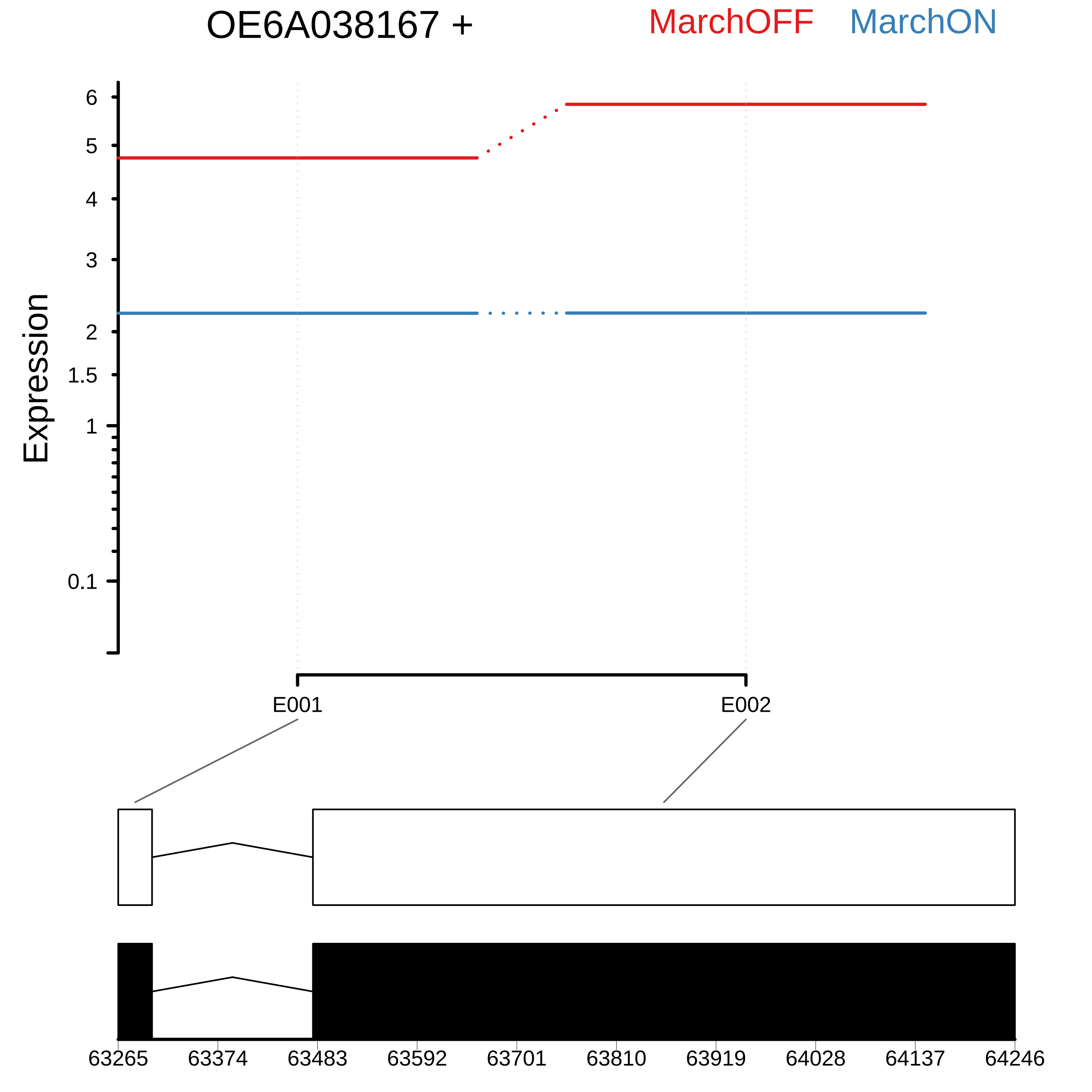
OE6A038167 +
MarchOFF
MarchON
Expression
6
5
4
3
2
1.5
1
0.1
E001
E002
63265
63374
63483
63592
63701
63810
63919
64028
64137
64246

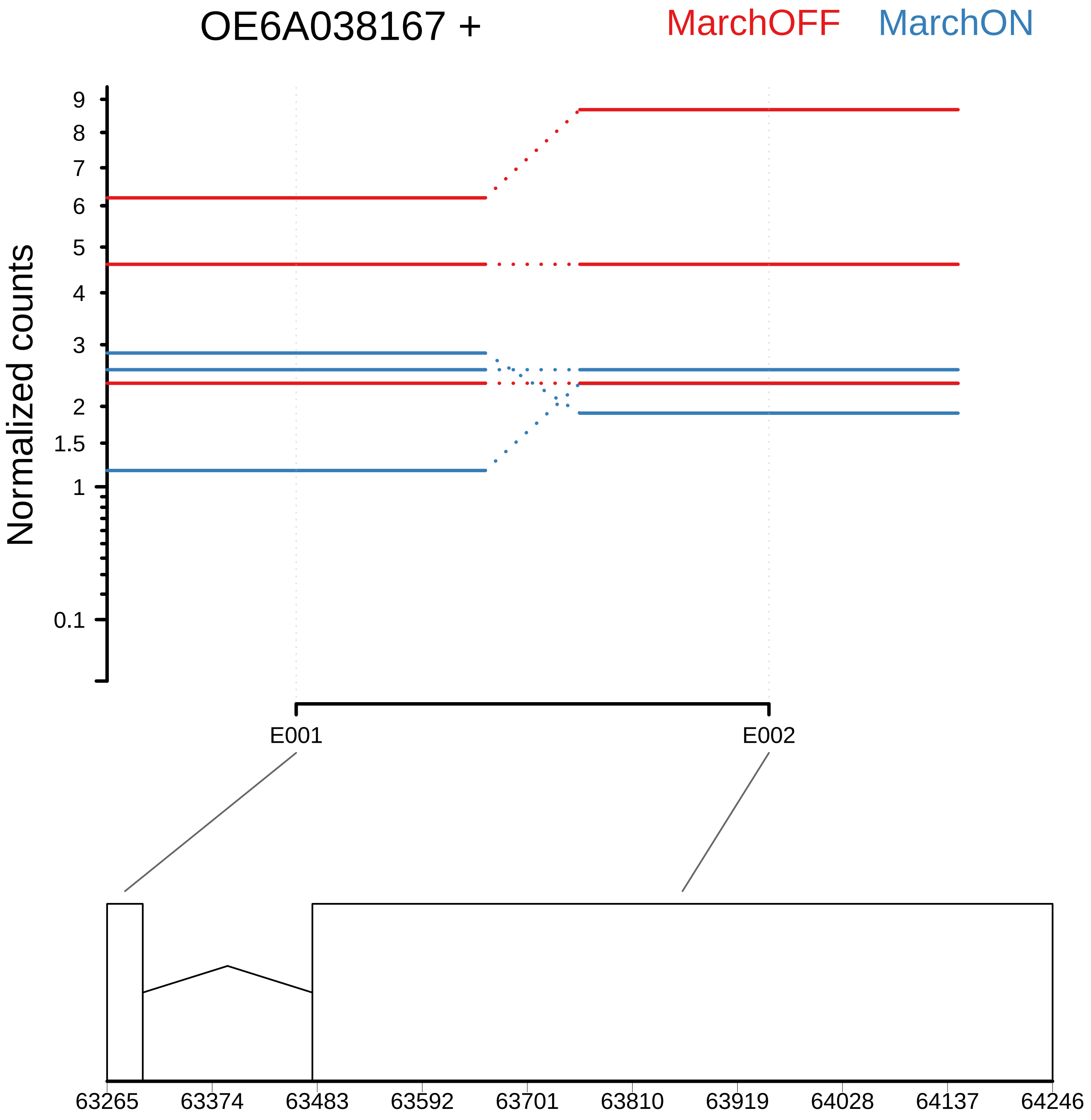

OE6A038167 +
MarchOFF
MarchON
Normalized counts
9
8
7
6
5
4
3
2
1.5
1
0.1
E001
E002
63265
63374
63483
63592
63701
63810
63919
64028
64137
64246

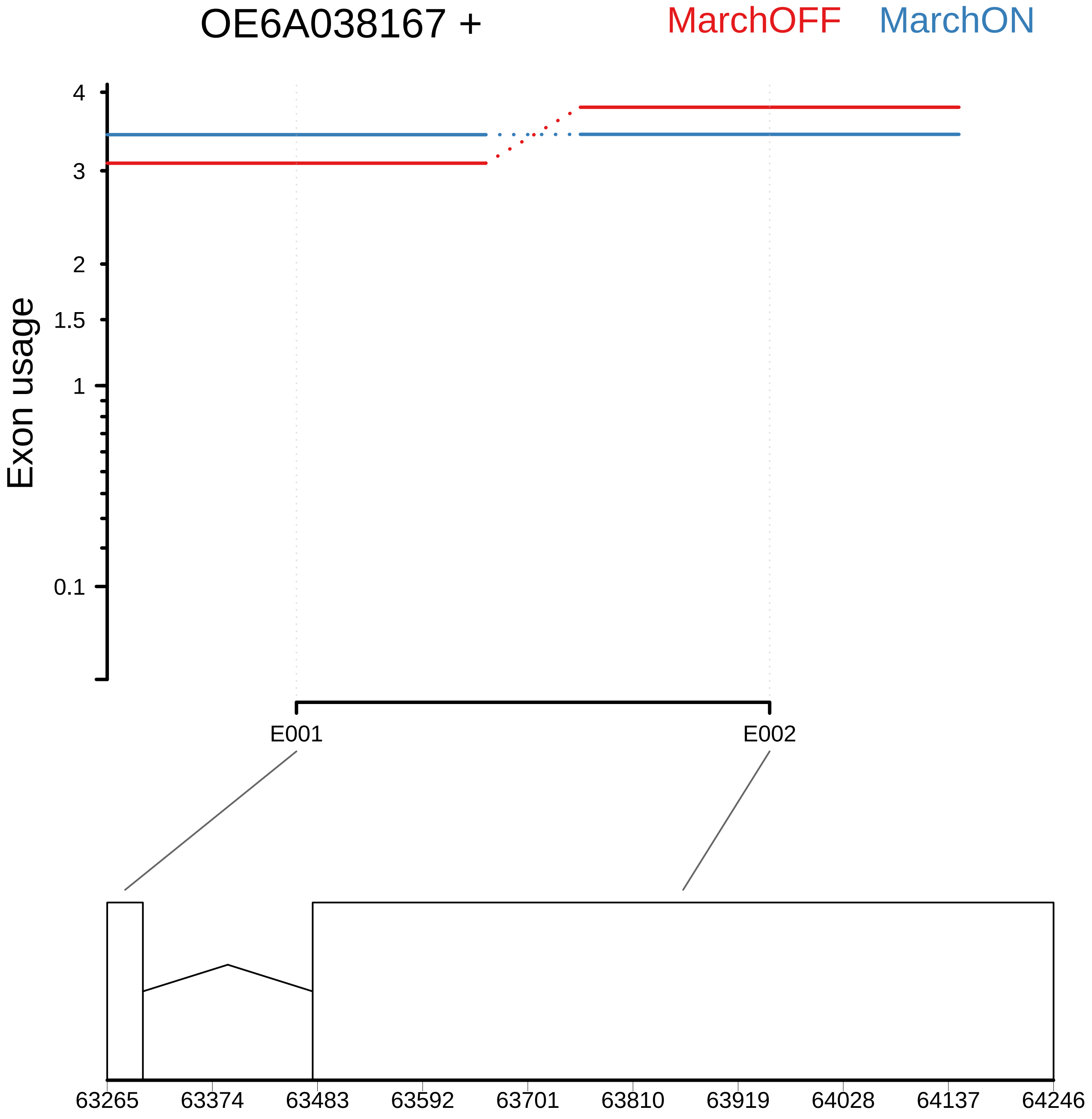
OE6A038167 +
MarchOFF
MarchON
Exon usage
4
3
2
1.5
1
0.1
E001
E002
63265
63374
63483
63592
63701
63810
63919
64028
64137
64246

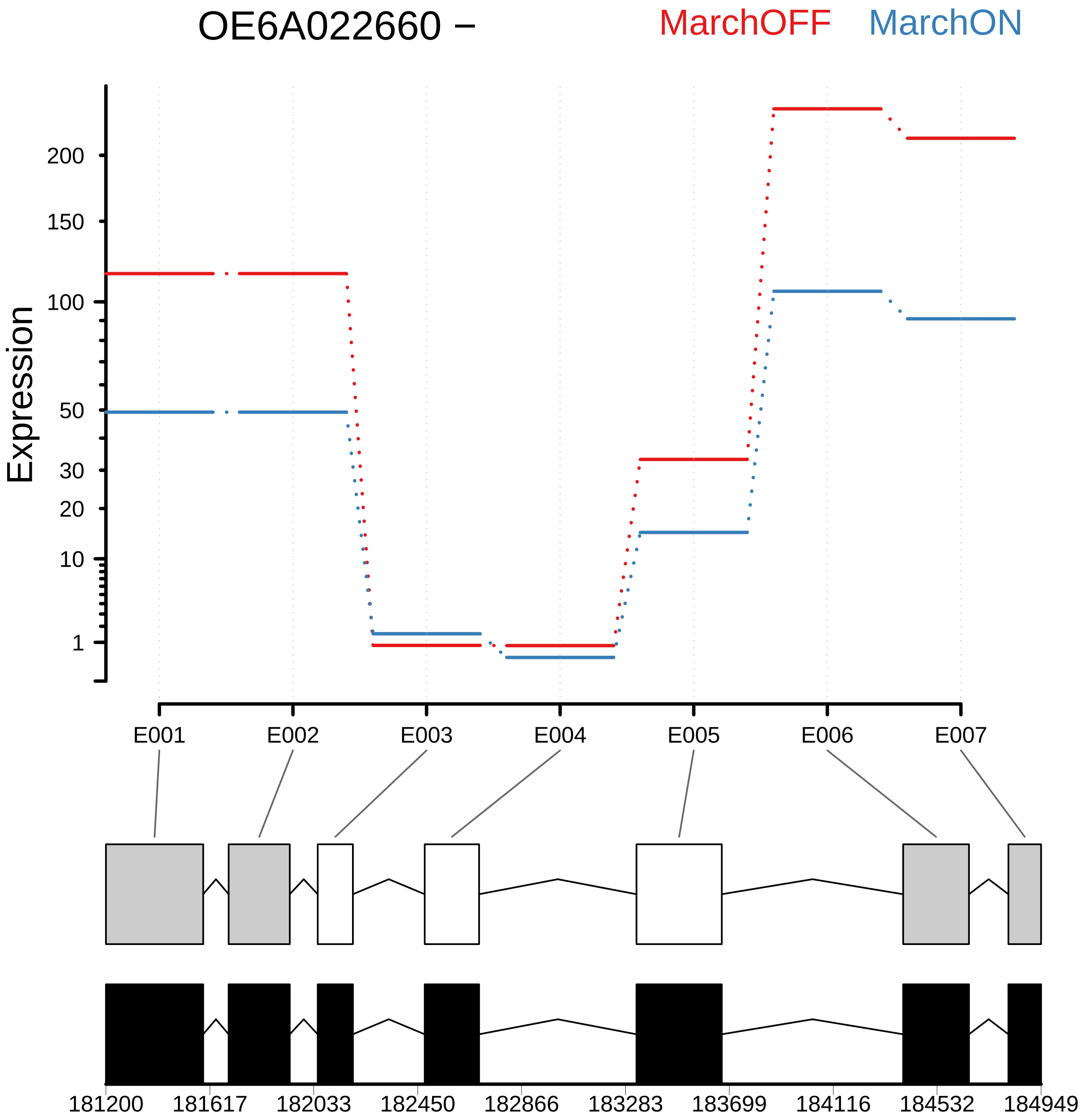
OE6A022660 −
MarchOFF
MarchON
Expression
200
150
100
50
30
20
10
1
E001
E002
E003
E004
E005
E006
E007
181200
181617
182033
182450
182866
183283
183699
184116
184532
184949

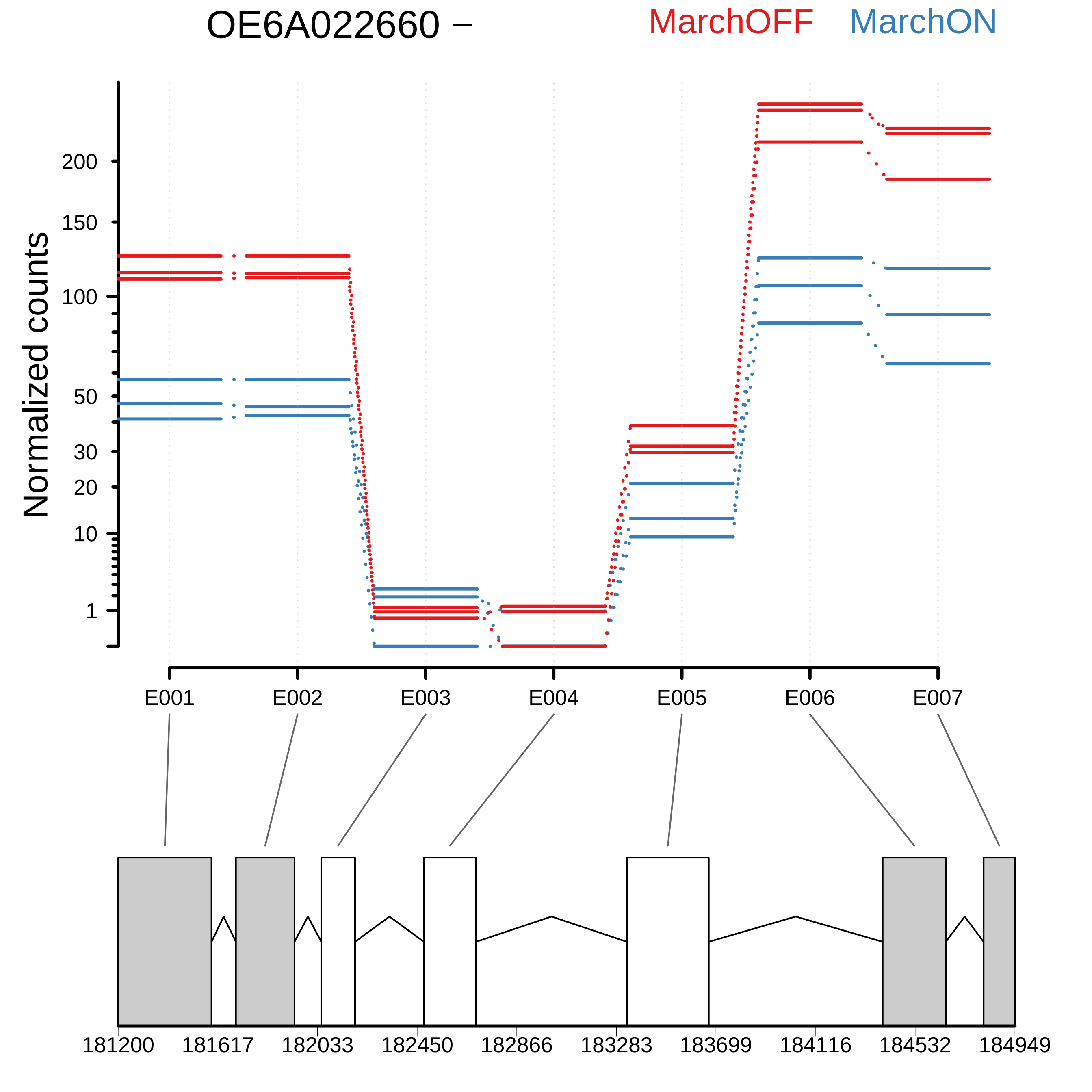
OE6A022660 −
MarchOFF
MarchON
Normalized counts
200
150
100
50
30
20
10
1
E001
E002
E003
E004
E005
E006
E007
181200
181617
182033
182450
182866
183283
183699
184116
184532
184949

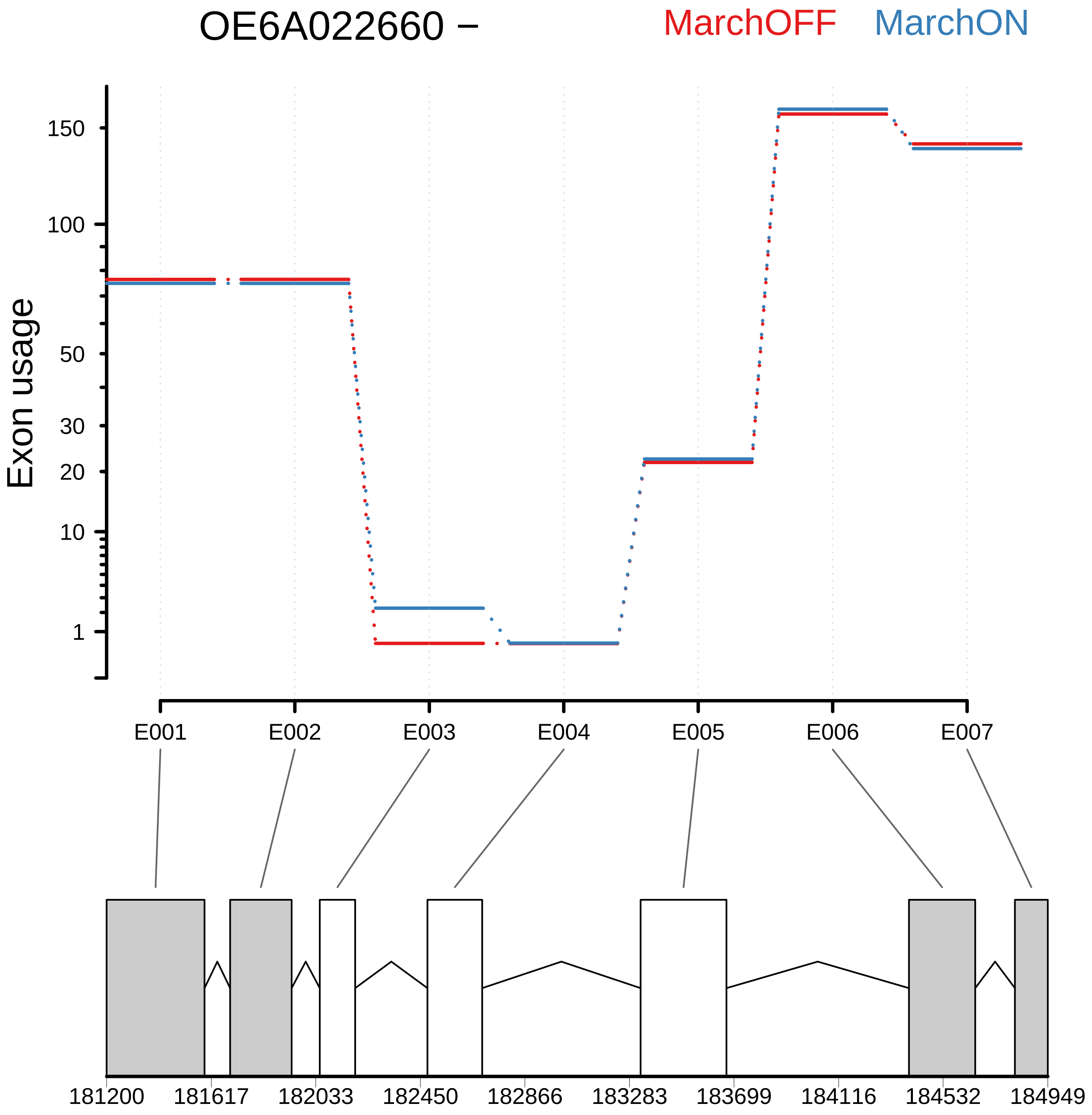

OE6A022660 −
MarchOFF
MarchON
Exon usage
150
100
50
30
20
10
1
E001
E002
E003
E004
E005
E006
E007
181200
181617
182033
182450
182866
183283
183699
184116
184532
184949

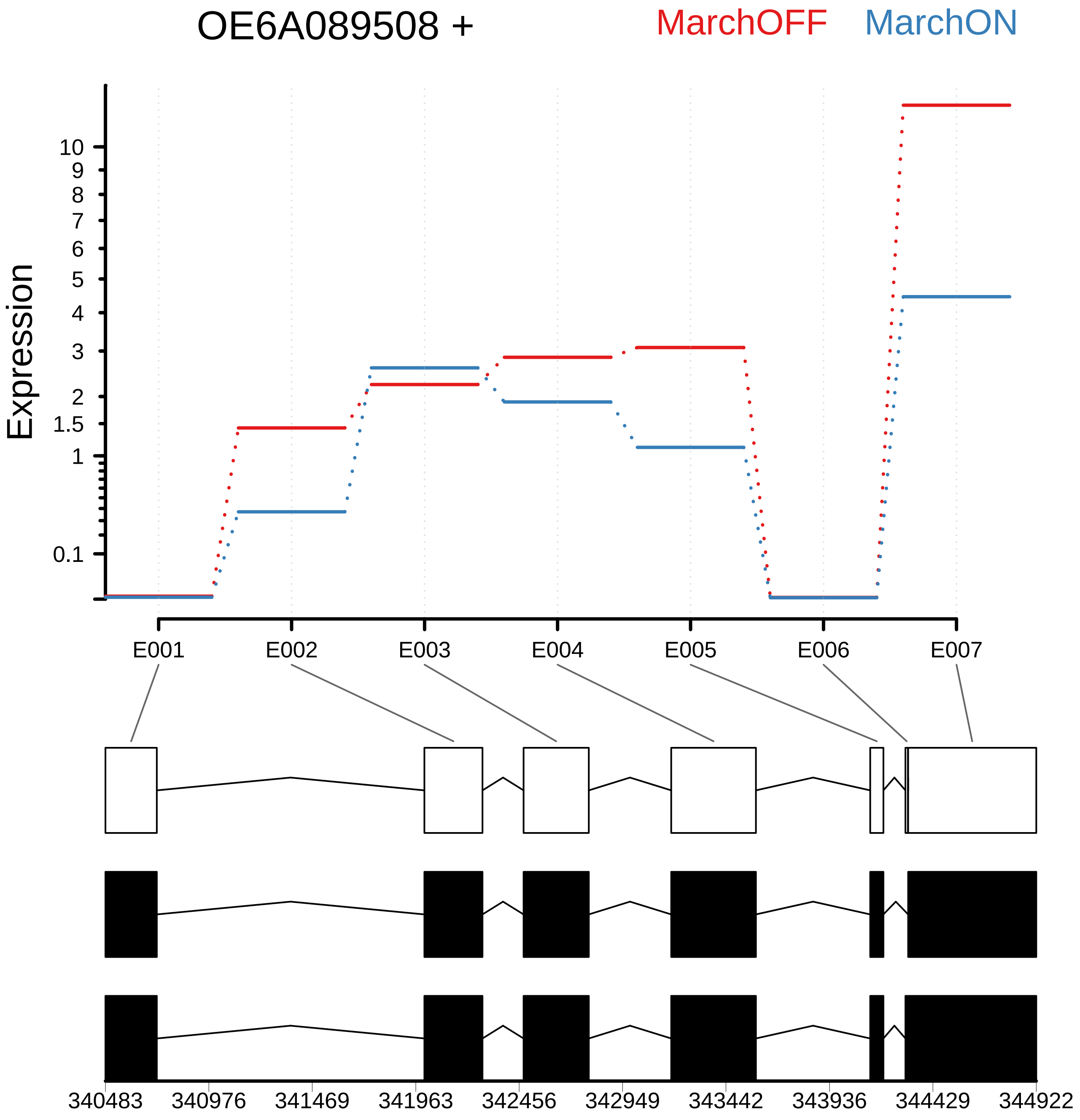

OE6A089508 +
MarchOFF
MarchON
Expression
10
9
8
7
6
5
4
3
2
1.5
1
0.1
E001
E002
E003
E004
E005
E006
E007
340483
340976
341469
341963
342456
342949
343442
343936
344429
344922

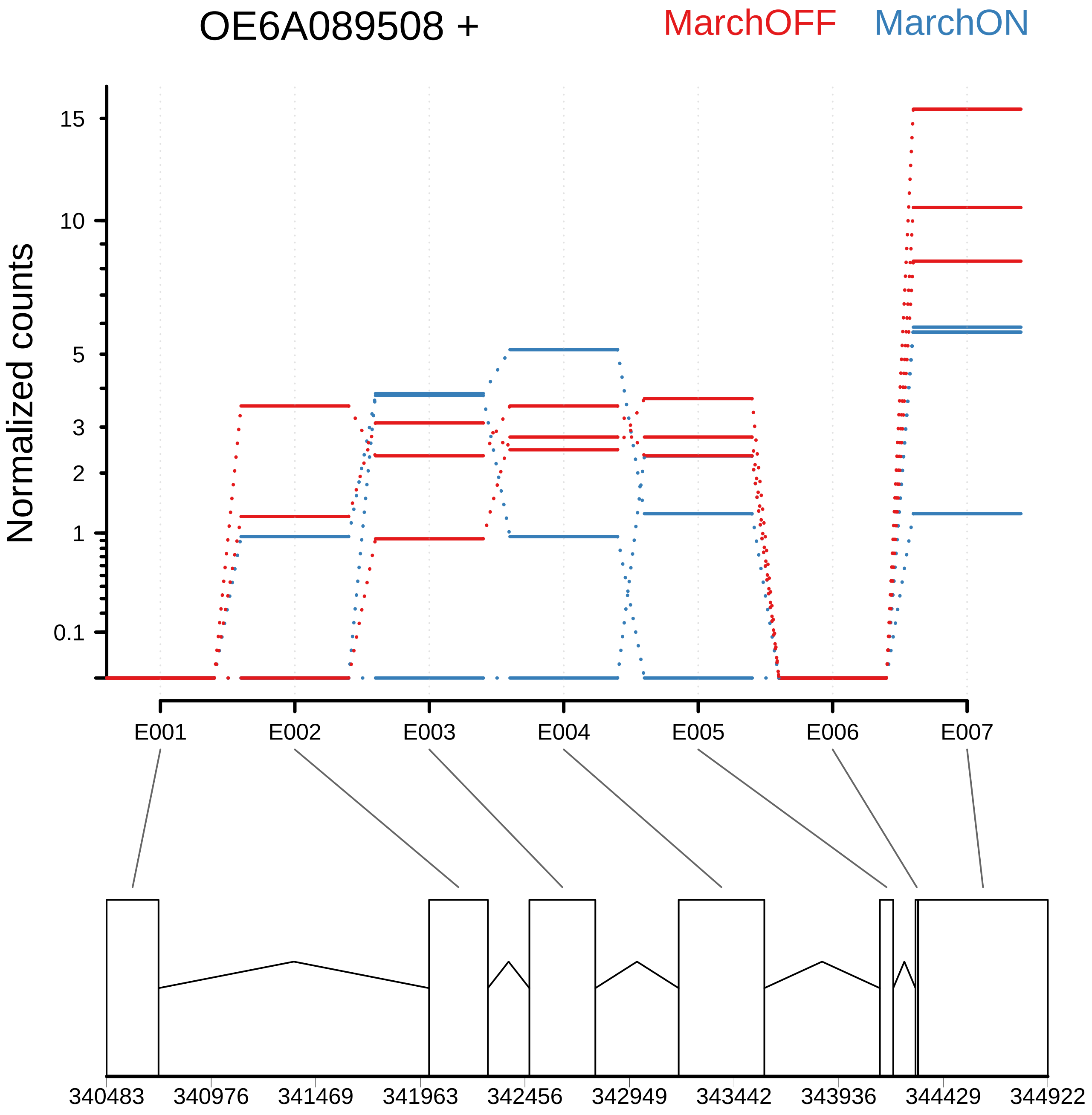
OE6A089508 +
MarchOFF
MarchON
Normalized counts
15
10
5
3
2
1
0.1
E001
E002
E003
E004
E005
E006
E007
340483
340976
341469
341963
342456
342949
343442
343936
344429
344922

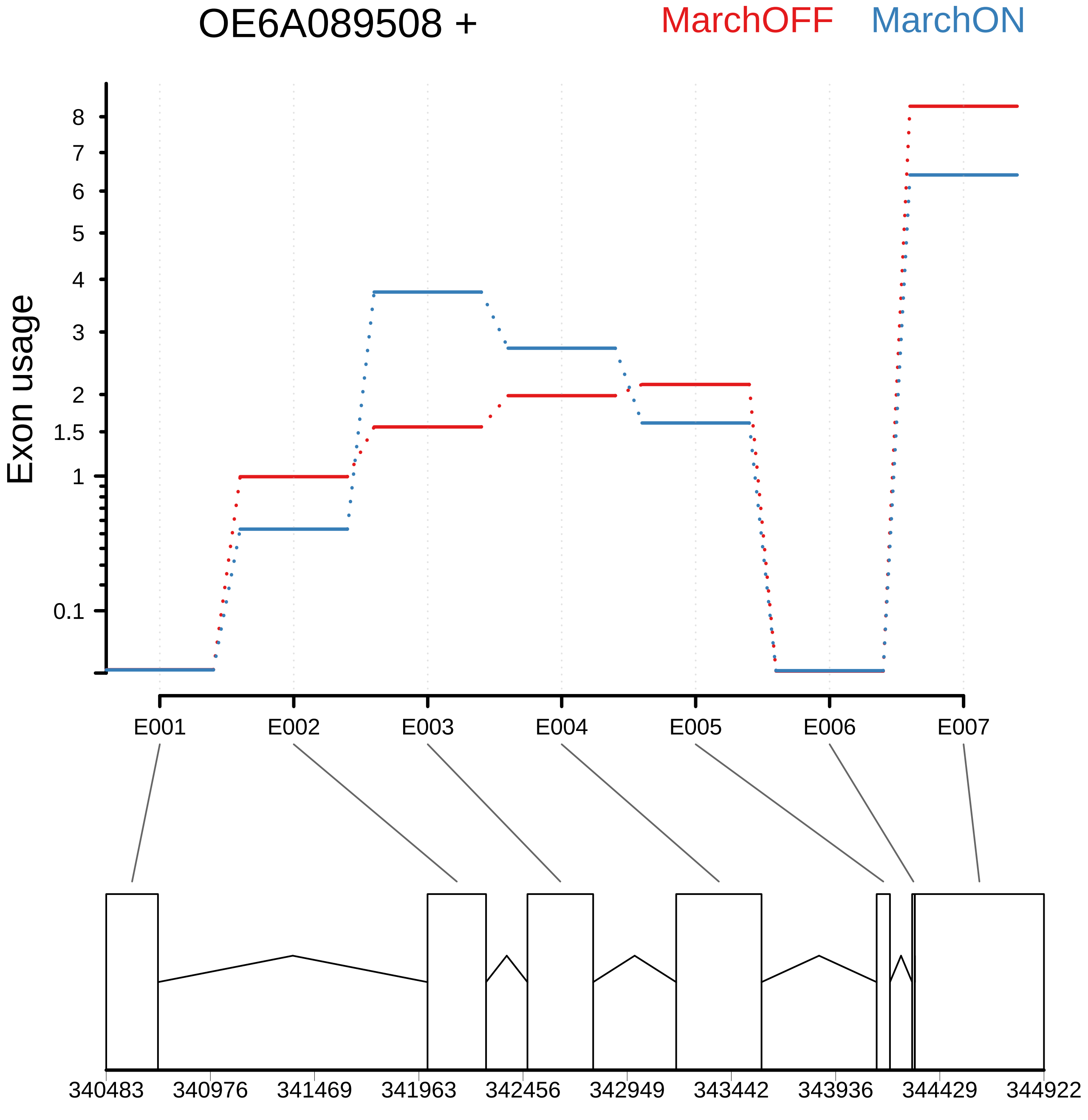

OE6A089508 +
MarchOFF
MarchON
Exon usage
8
7
6
5
4
3
2
1.5
1
0.1
E001
E002
E003
E004
E005
E006
E007
340483
340976
341469
341963
342456
342949
343442
343936
344429
344922

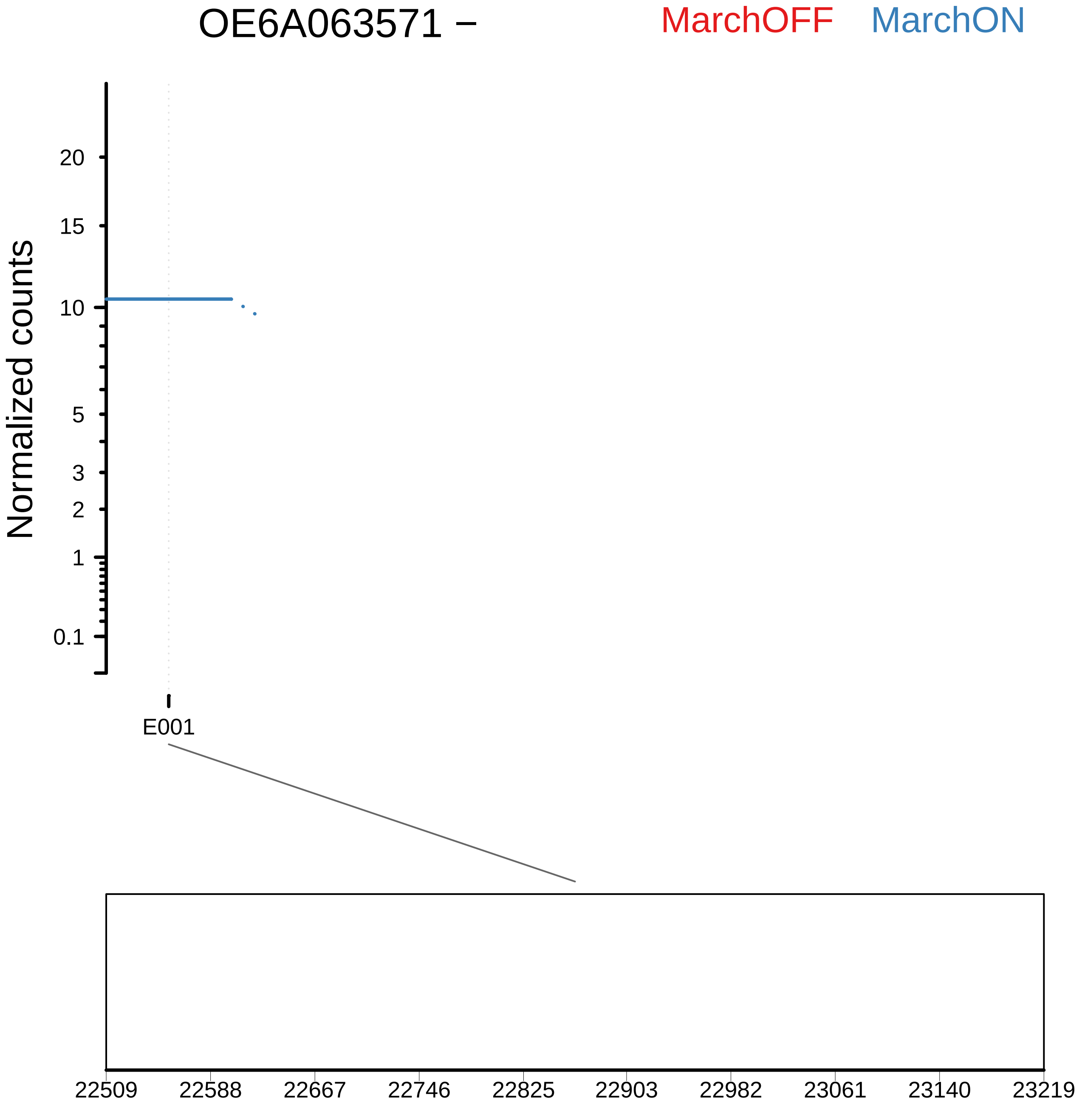

OE6A063571 −
MarchOFF
MarchON
Normalized counts
20
15
10
5
3
2
1
0.1
E001
22509
22588
22667
22746
22825
22903
22982
23061
23140
23219

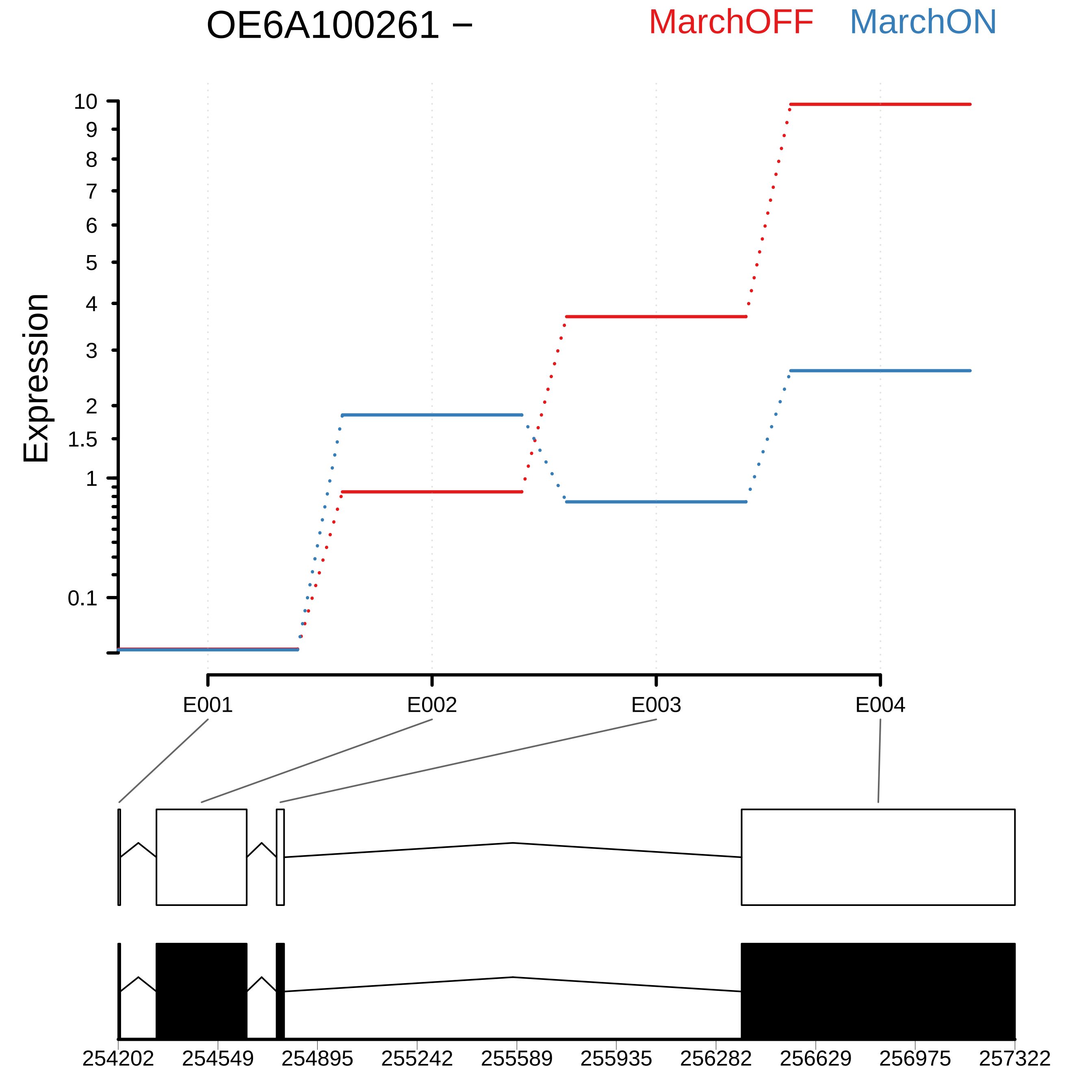

OE6A100261 −
MarchOFF
MarchON
Expression
10
9
8
7
6
5
4
3
2
1.5
1
0.1
E001
E002
E003
E004
254202
254549
254895
255242
255589
255935
256282
256629
256975
257322

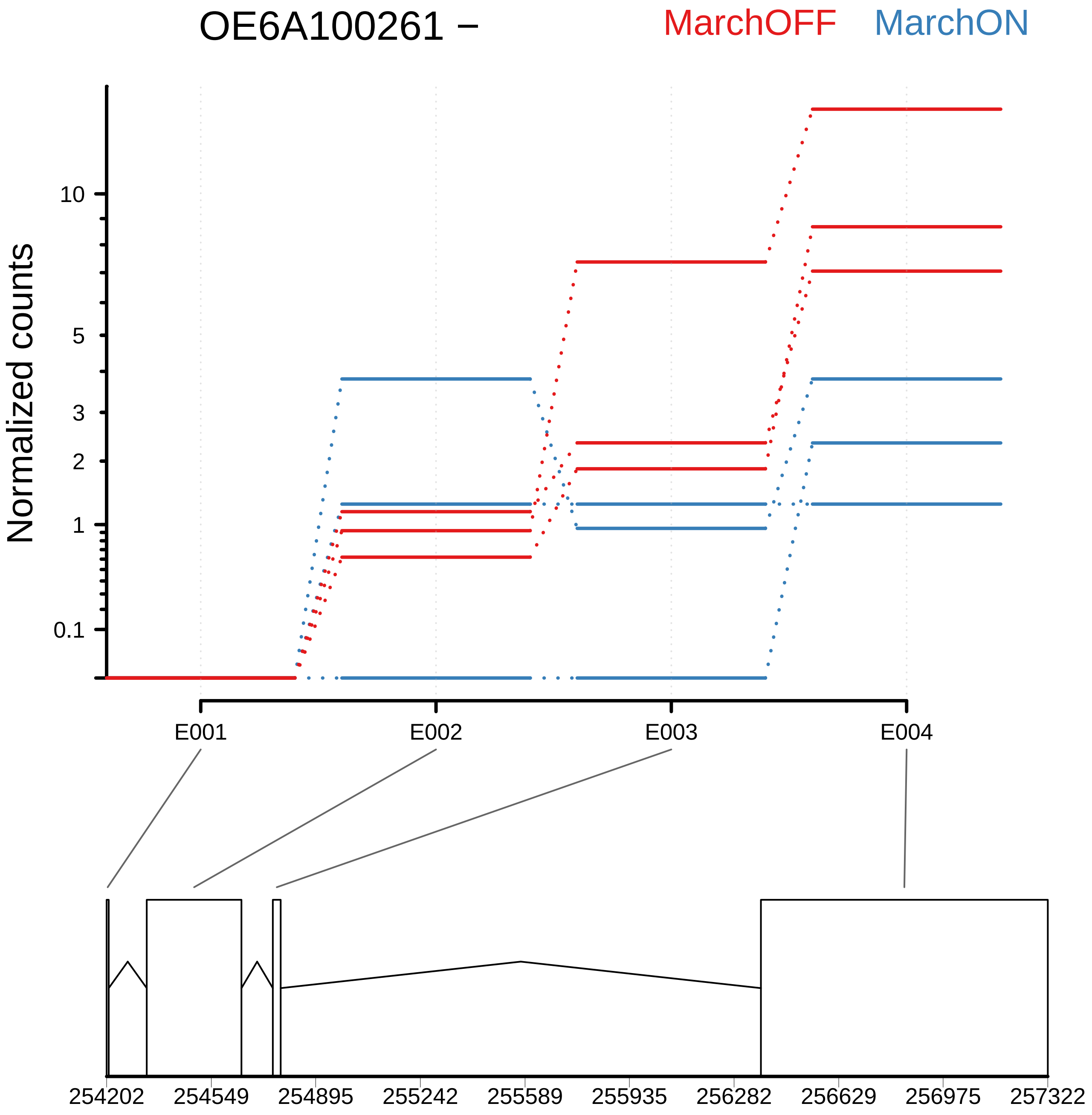
OE6A100261 −
MarchOFF
MarchON
Normalized counts
10
5
3
2
1
0.1
E001
E002
E003
E004
254202
254549
254895
255242
255589
255935
256282
256629
256975
257322

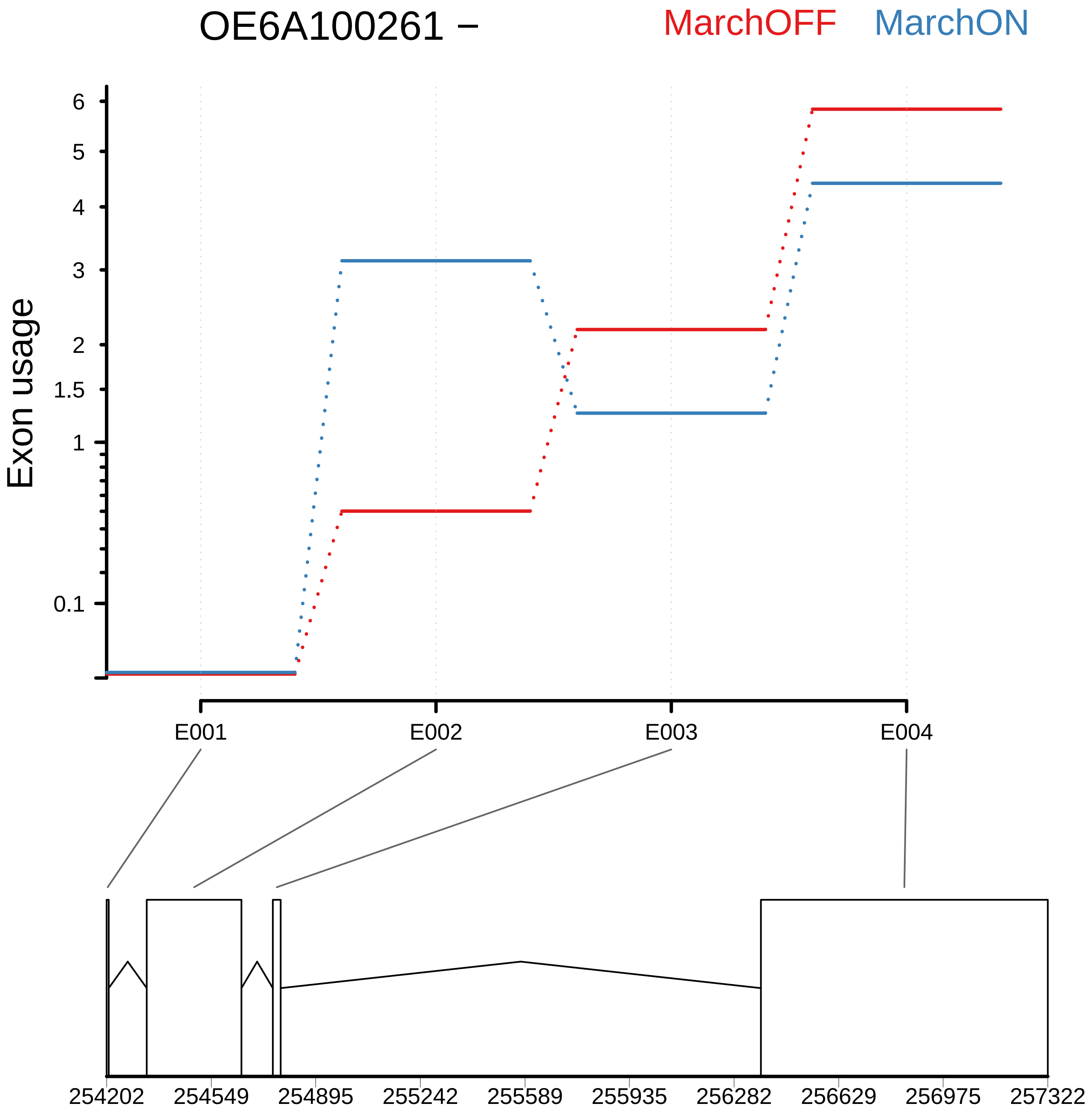

OE6A100261 −
MarchOFF
MarchON
Exon usage
6
5
4
3
2
1.5
1
0.1
E001
E002
E003
E004
254202
254549
254895
255242
255589
255935
256282
256629
256975
257322

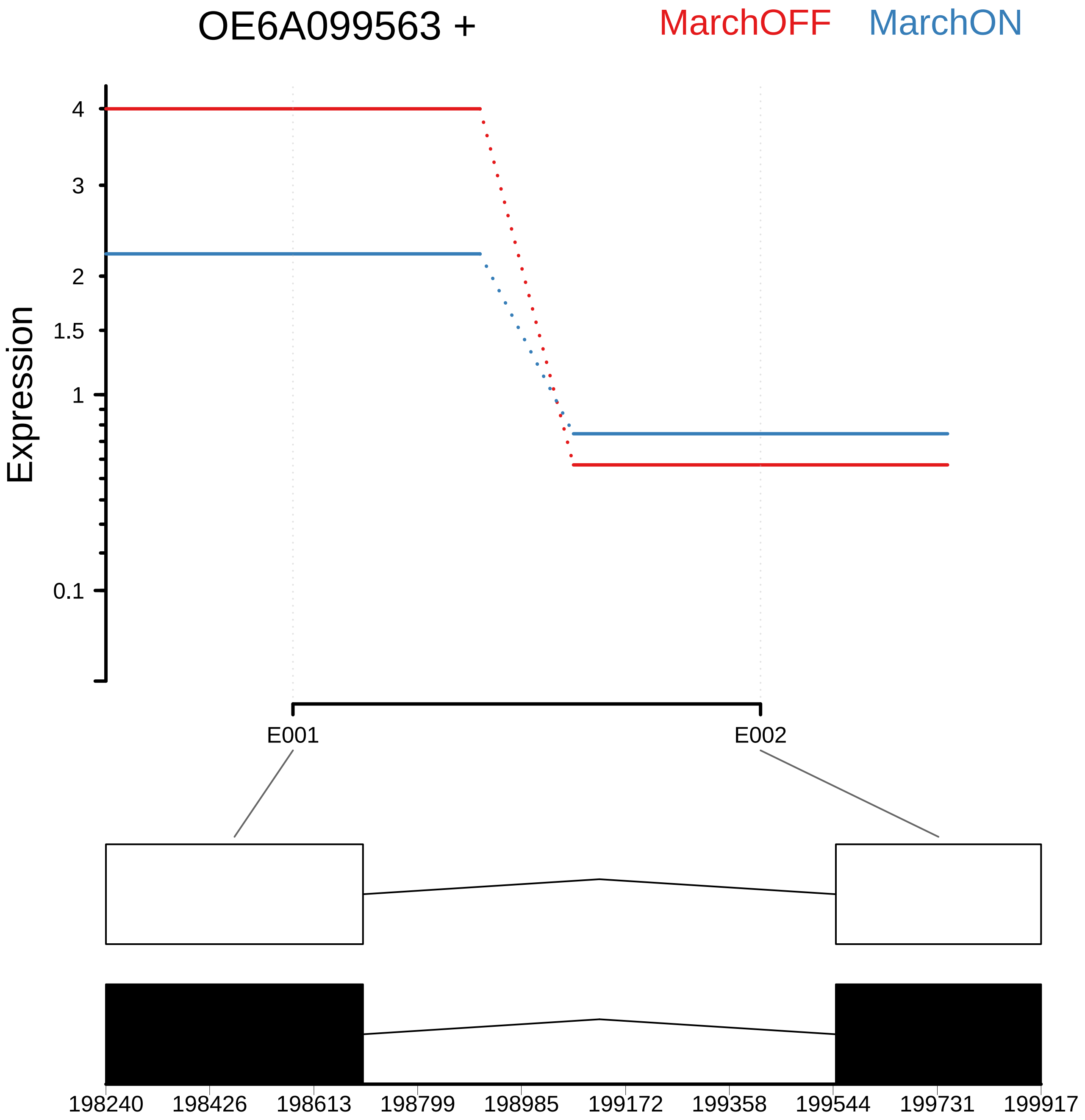
OE6A099563 +
MarchOFF
MarchON
Expression
4
3
2
1.5
1
0.1
E001
E002
198240
198426
198613
198799
198985
199172
199358
199544
199731
199917

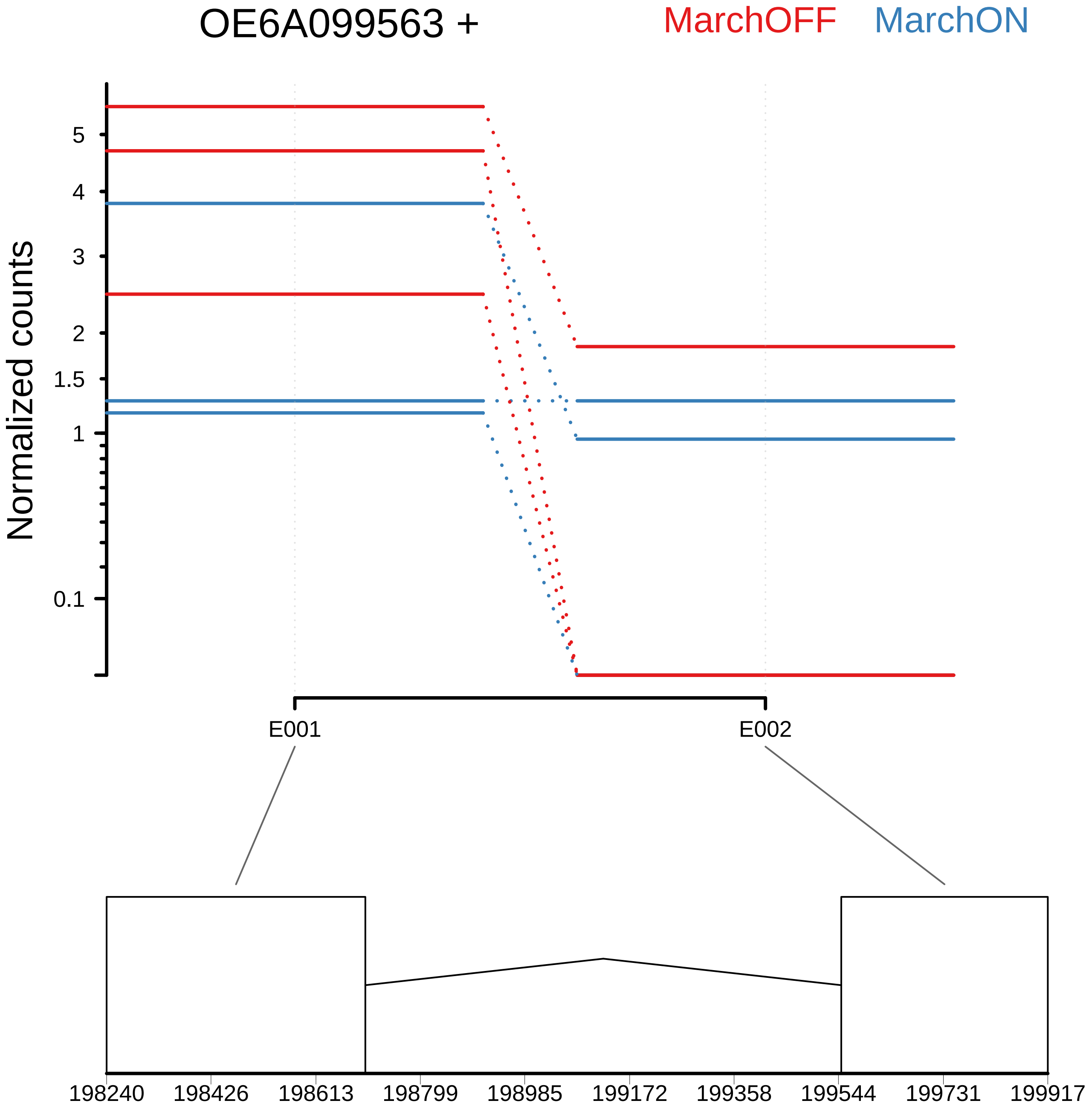

OE6A099563 +
MarchOFF
MarchON
Normalized counts
5
4
3
2
1.5
1
0.1
E001
E002
198240
198426
198613
198799
198985
199172
199358
199544
199731
199917

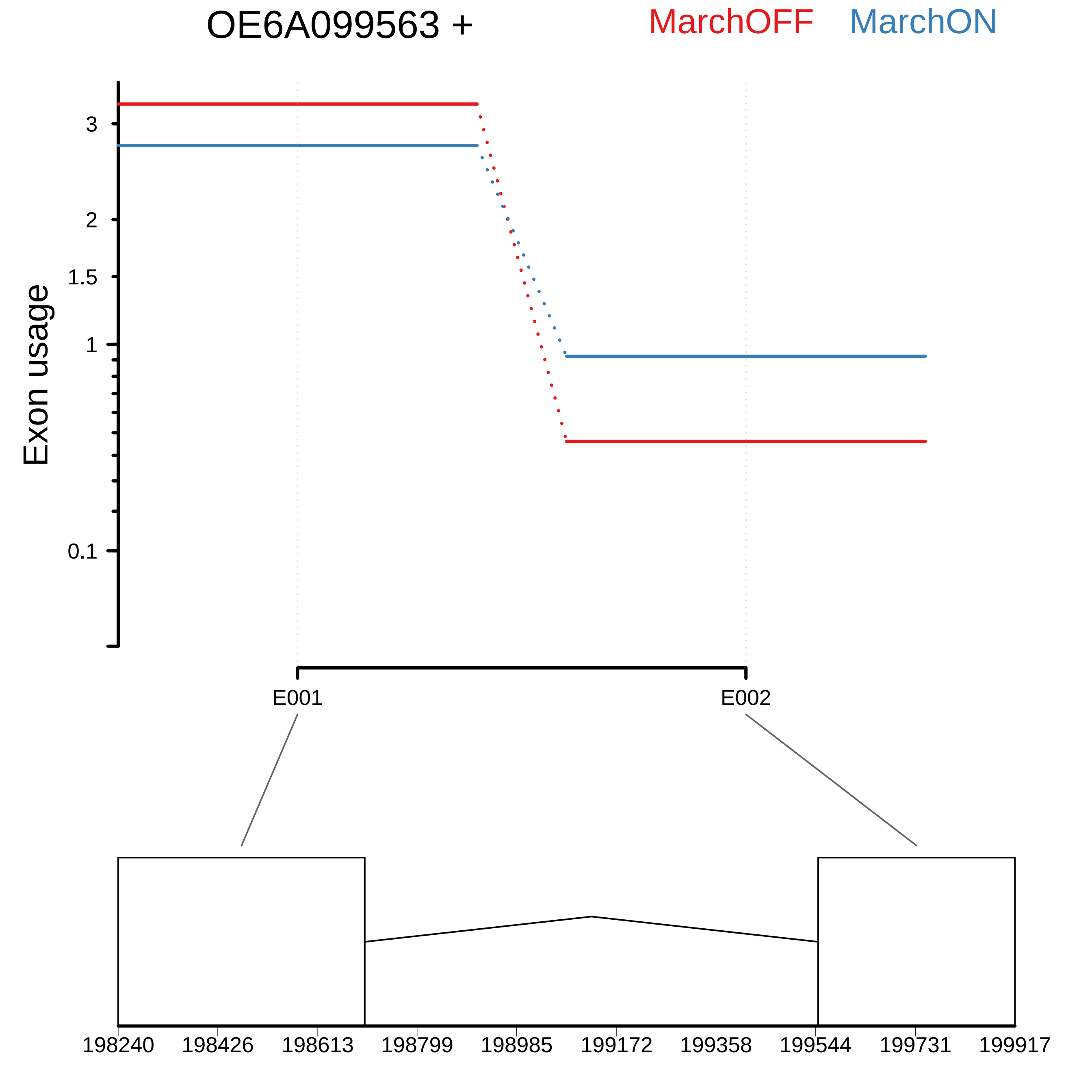

OE6A099563 +
MarchOFF
MarchON
Exon usage
3
2
1.5
1
0.1
E001
E002
198240
198426
198613
198799
198985
199172
199358
199544
199731
199917

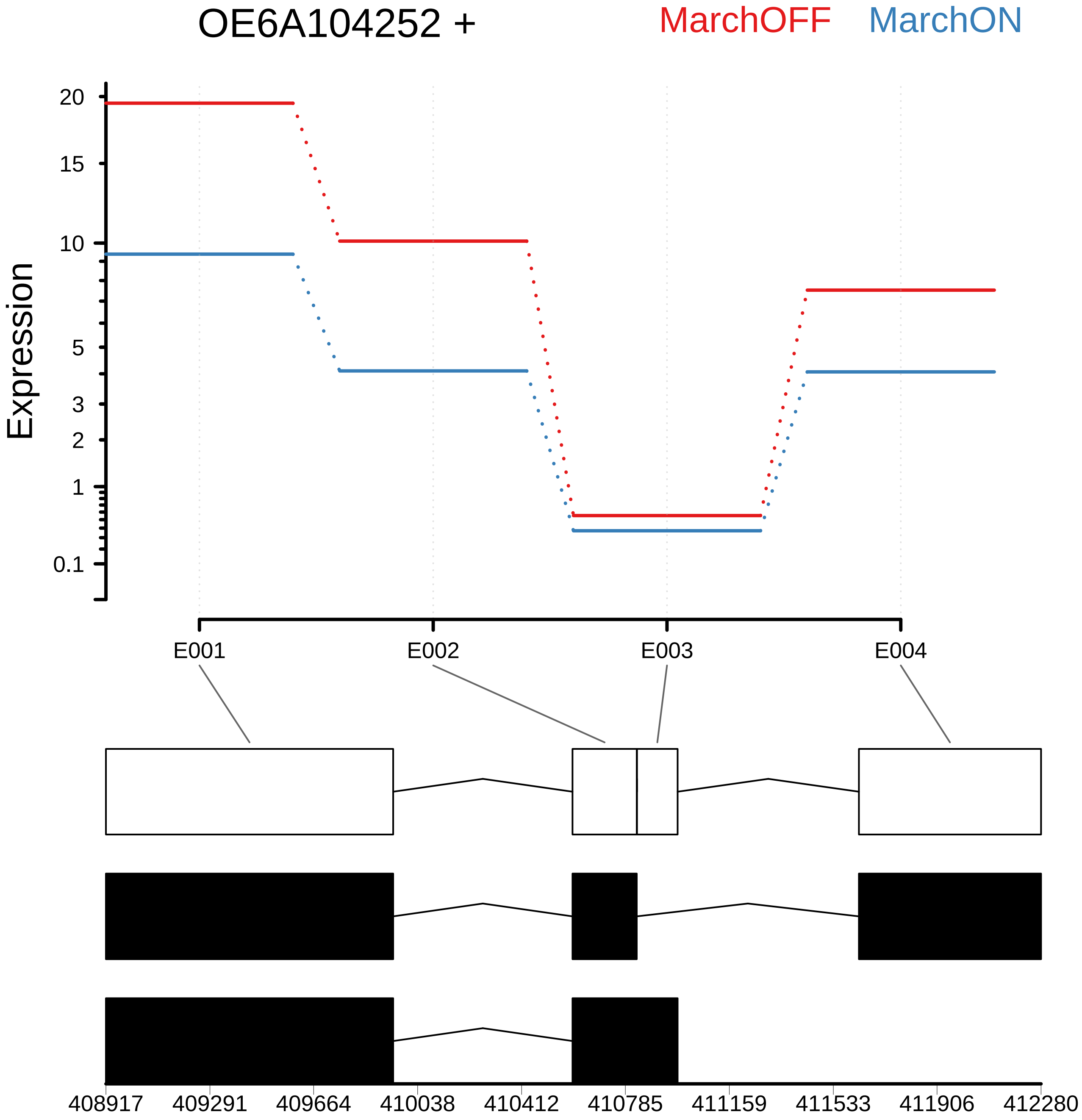

OE6A104252 +
MarchOFF
MarchON
Expression
20
15
10
5
3
2
1
0.1
E001
E002
E003
E004
408917
409291
409664
410038
410412
410785
411159
411533
411906
412280

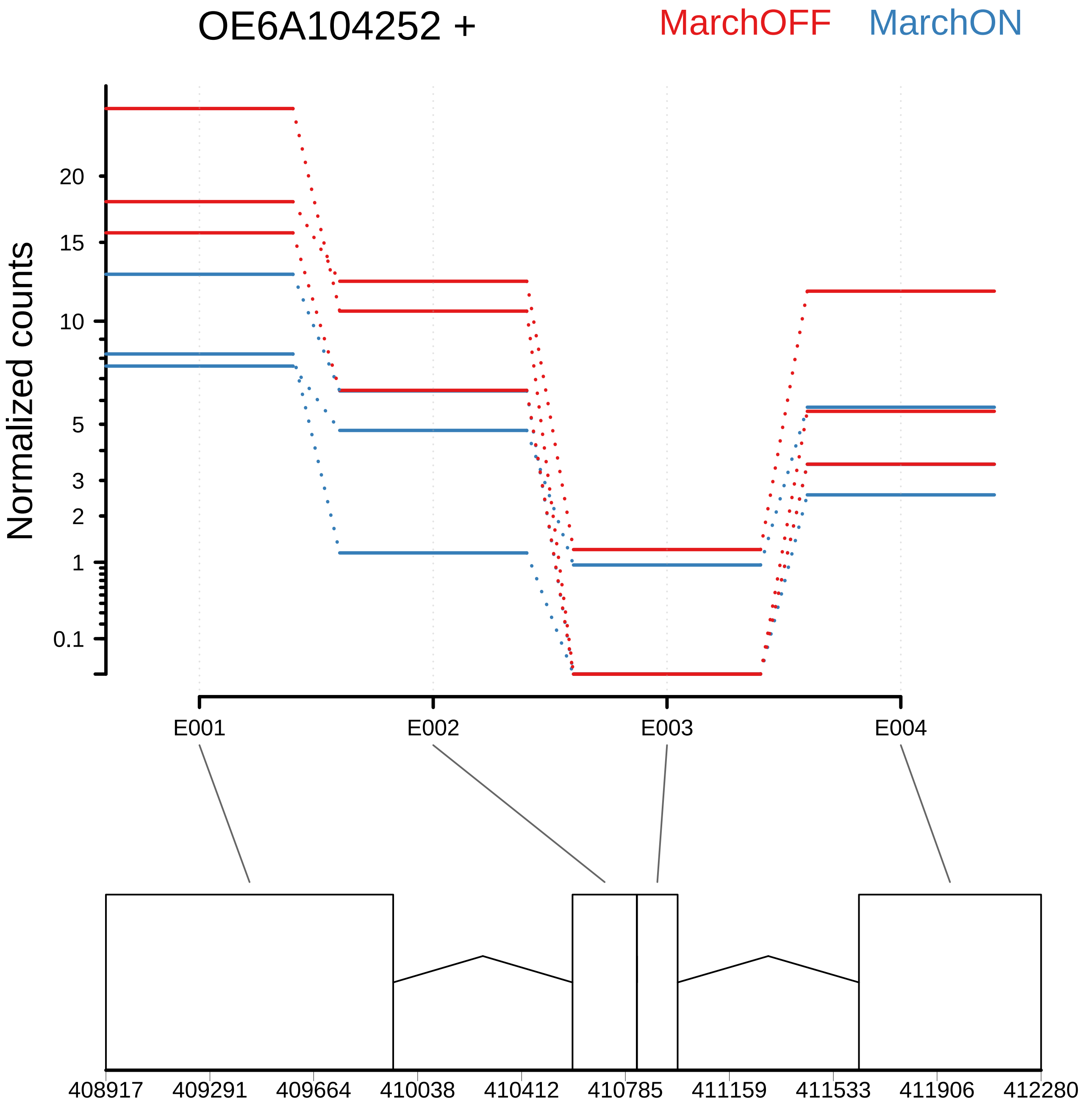
OE6A104252 +
MarchOFF
MarchON
Normalized counts
20
15
10
5
3
2
1
0.1
E001
E002
E003
E004
408917
409291
409664
410038
410412
410785
411159
411533
411906
412280

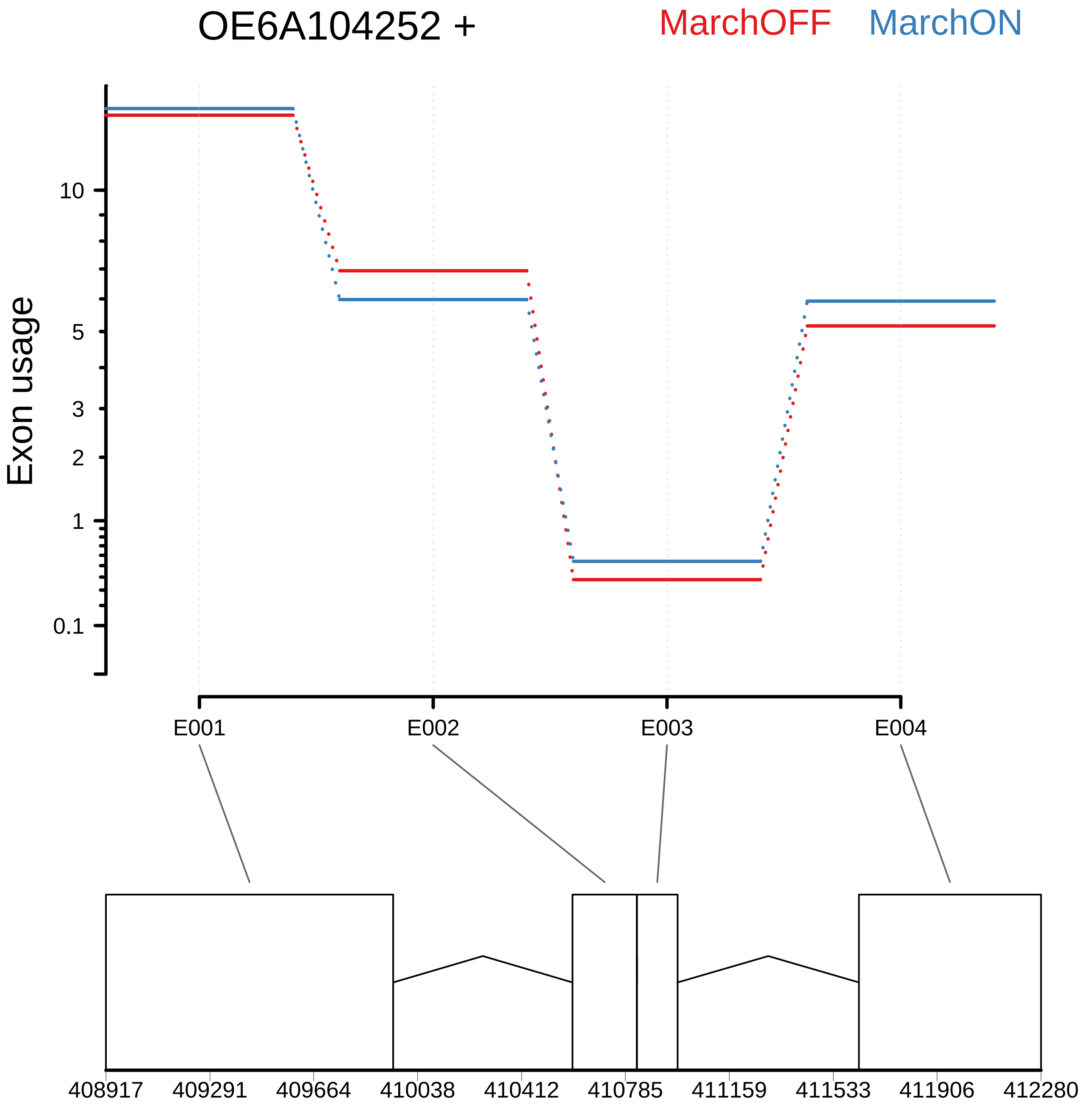

OE6A104252 +
MarchOFF
MarchON
Exon usage
10
5
3
2
1
0.1
E001
E002
E003
E004
408917
409291
409664
410038
410412
410785
411159
411533
411906
412280

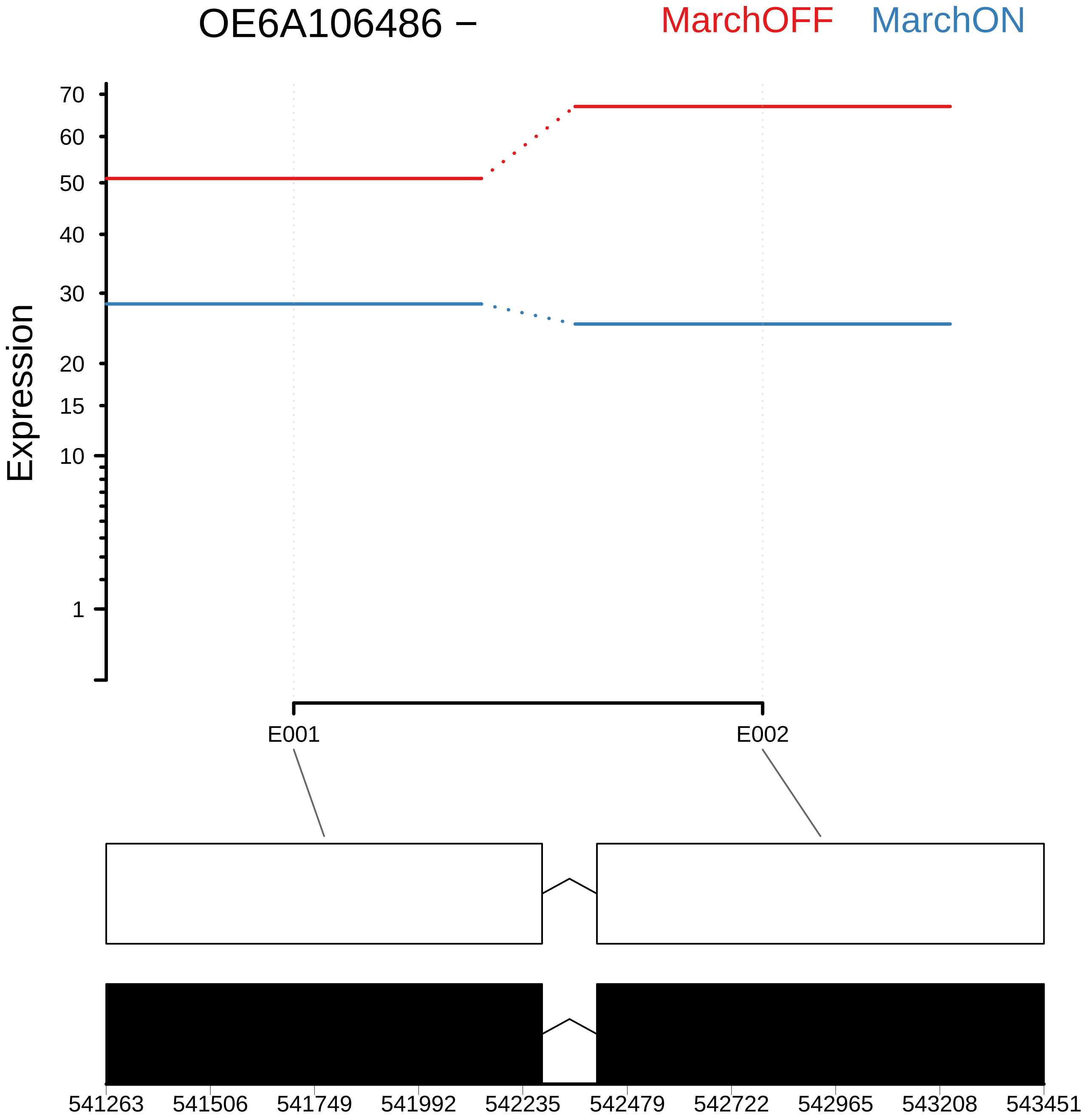

OE6A106486 −
MarchOFF
MarchON
Expression
70
60
50
40
30
20
15
10
1
E001
E002
541263
541506
541749
541992
542235
542479
542722
542965
543208
543451

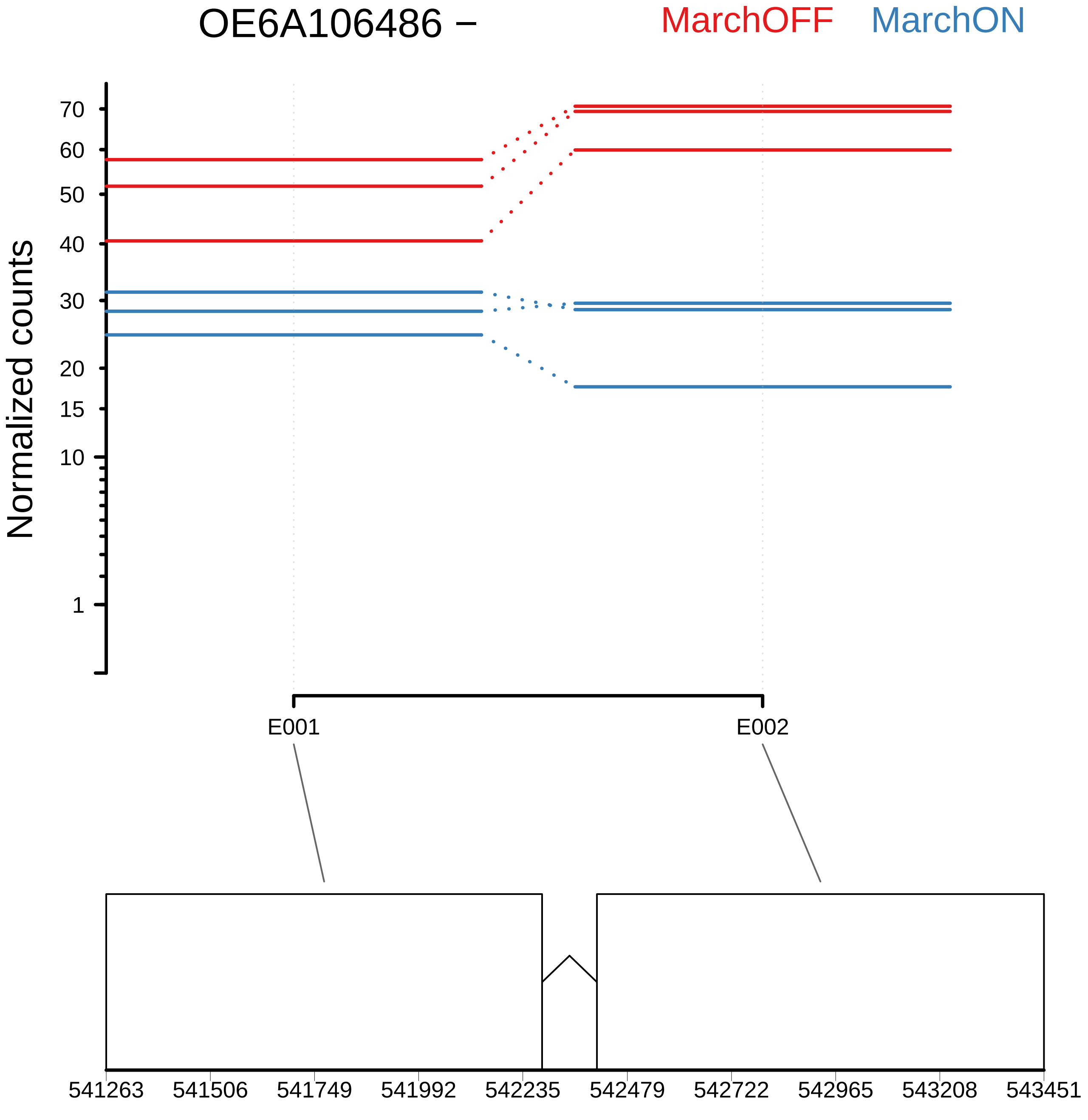
OE6A106486 −
MarchOFF
MarchON
Normalized counts
70
60
50
40
30
20
15
10
1
E001
E002
541263
541506
541749
541992
542235
542479
542722
542965
543208
543451

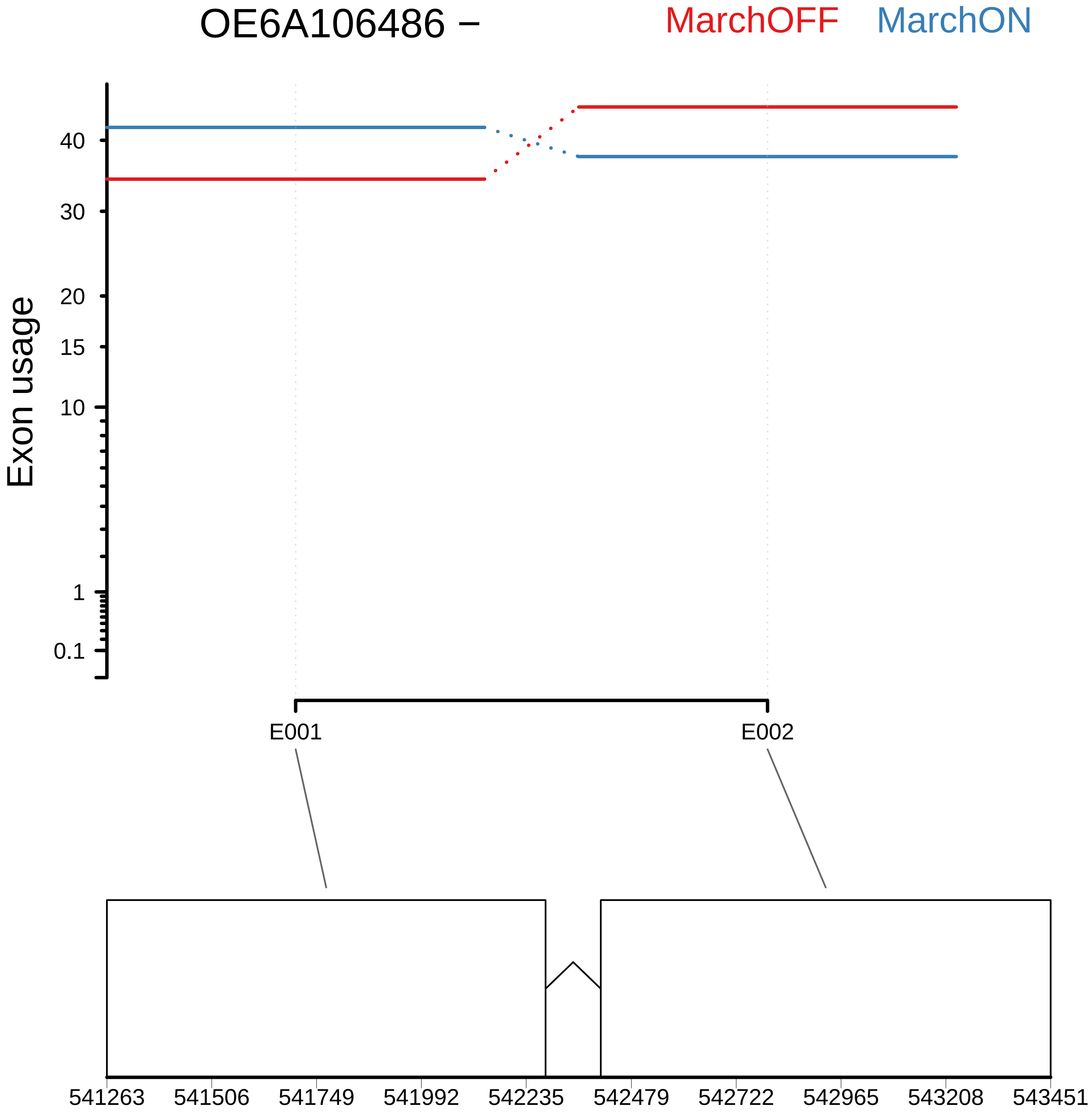

OE6A106486 −
MarchOFF
MarchON
Exon usage
40
30
20
15
10
1
0.1
E001
E002
541263
541506
541749
541992
542235
542479
542722
542965
543208
543451

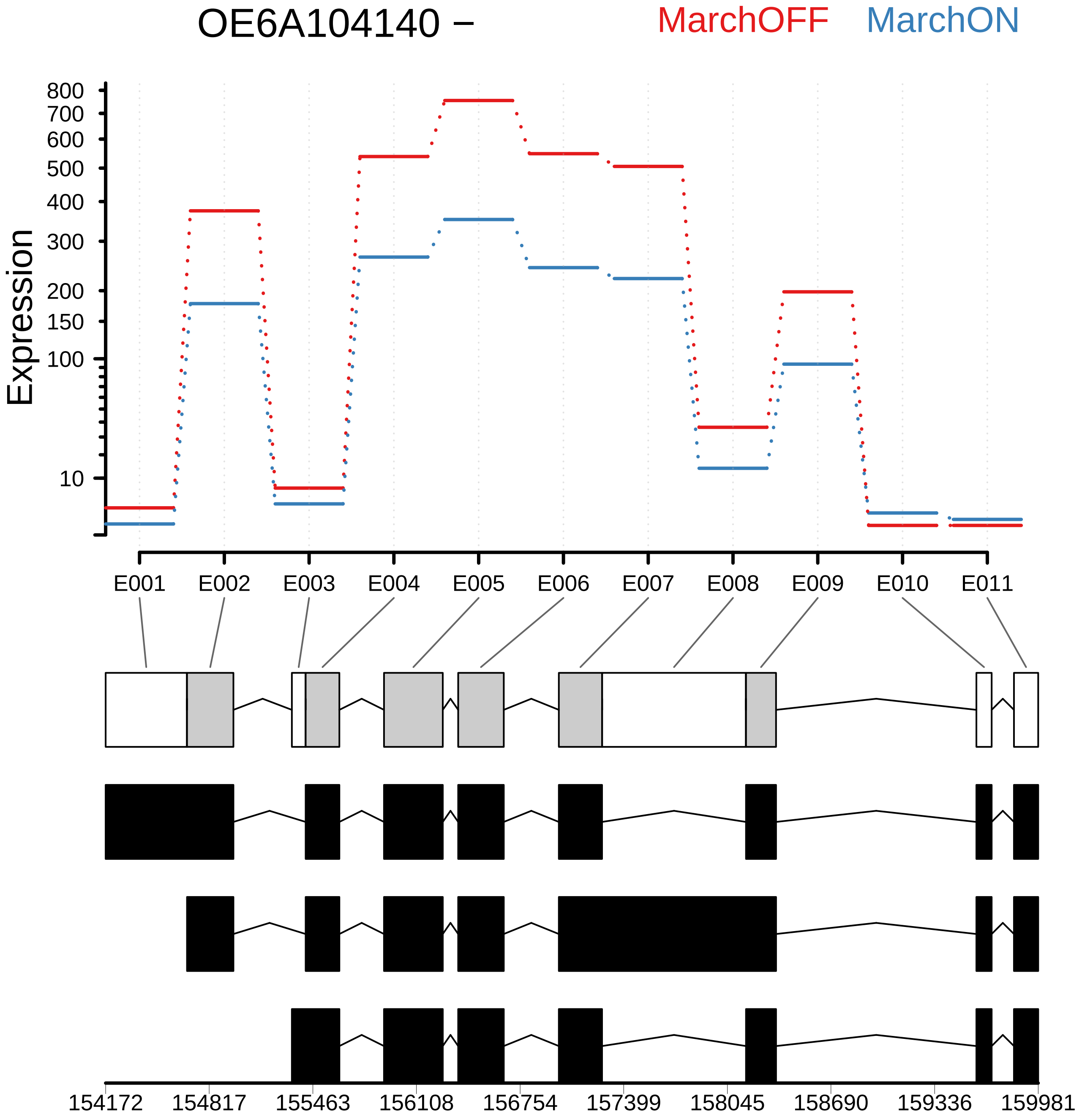

OE6A104140 −
MarchOFF
MarchON
Expression
800
700
600
500
400
300
200
150
100
10
E001
E002
E003
E004
E005
E006
E007
E008
E009
E010
E011
154172
154817
155463
156108
156754
157399
158045
158690
159336
159981

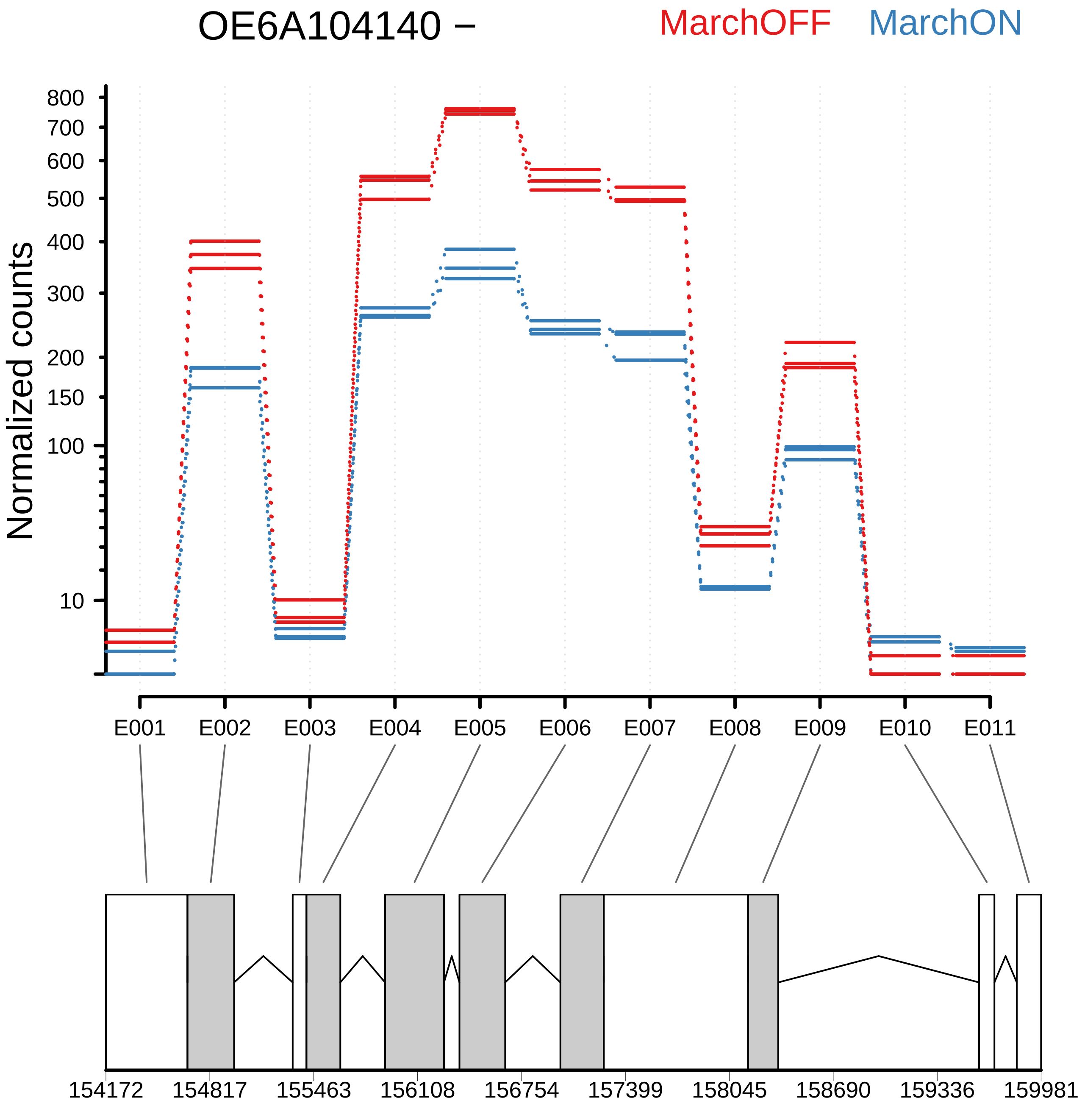

OE6A104140 −
MarchOFF
MarchON
Normalized counts
800
700
600
500
400
300
200
150
100
10
E001
E002
E003
E004
E005
E006
E007
E008
E009
E010
E011
154172
154817
155463
156108
156754
157399
158045
158690
159336
159981

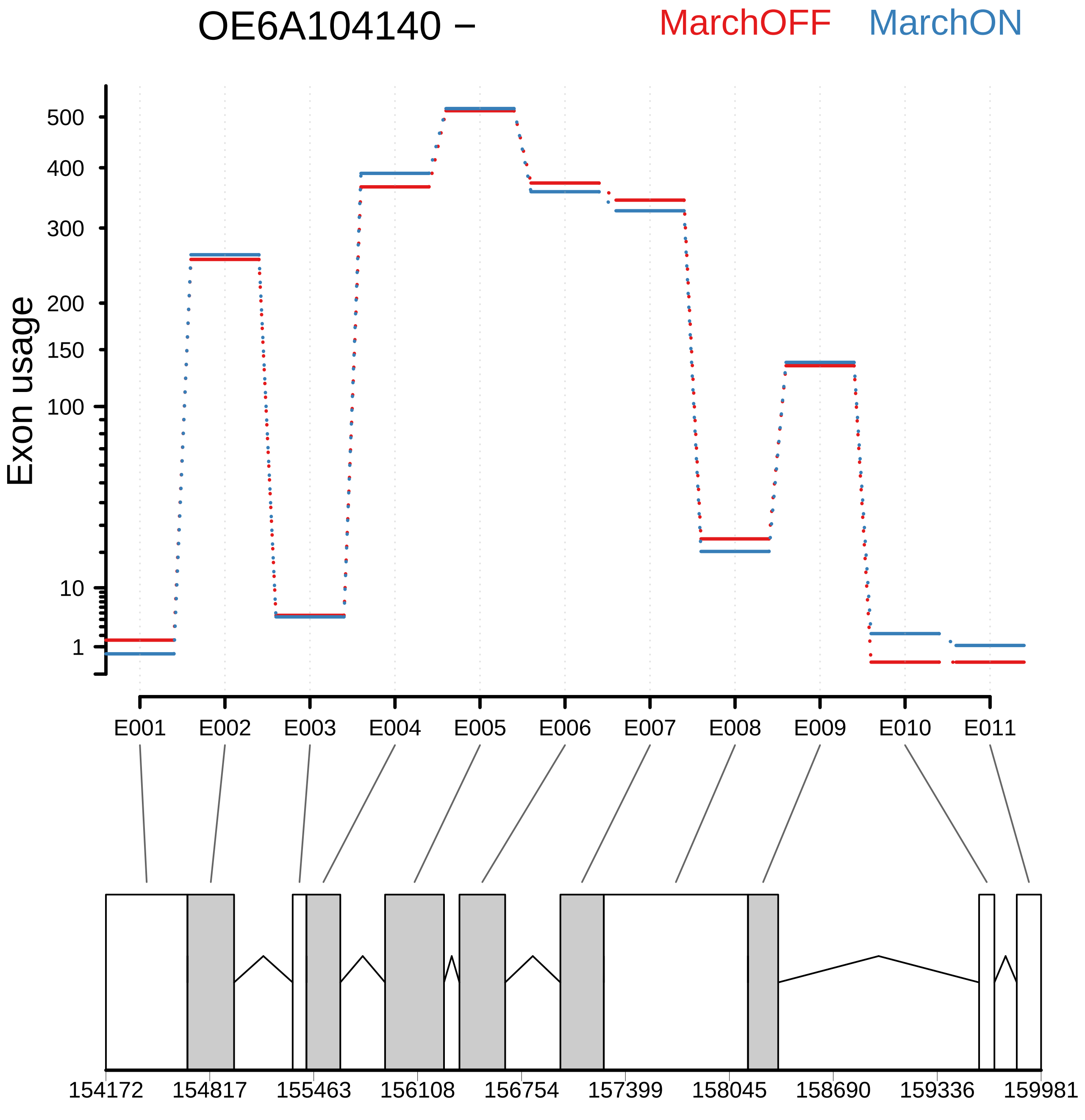

OE6A104140 −
MarchOFF
MarchON
Exon usage
500
400
300
200
150
100
10
1
E001
E002
E003
E004
E005
E006
E007
E008
E009
E010
E011
154172
154817
155463
156108
156754
157399
158045
158690
159336
159981

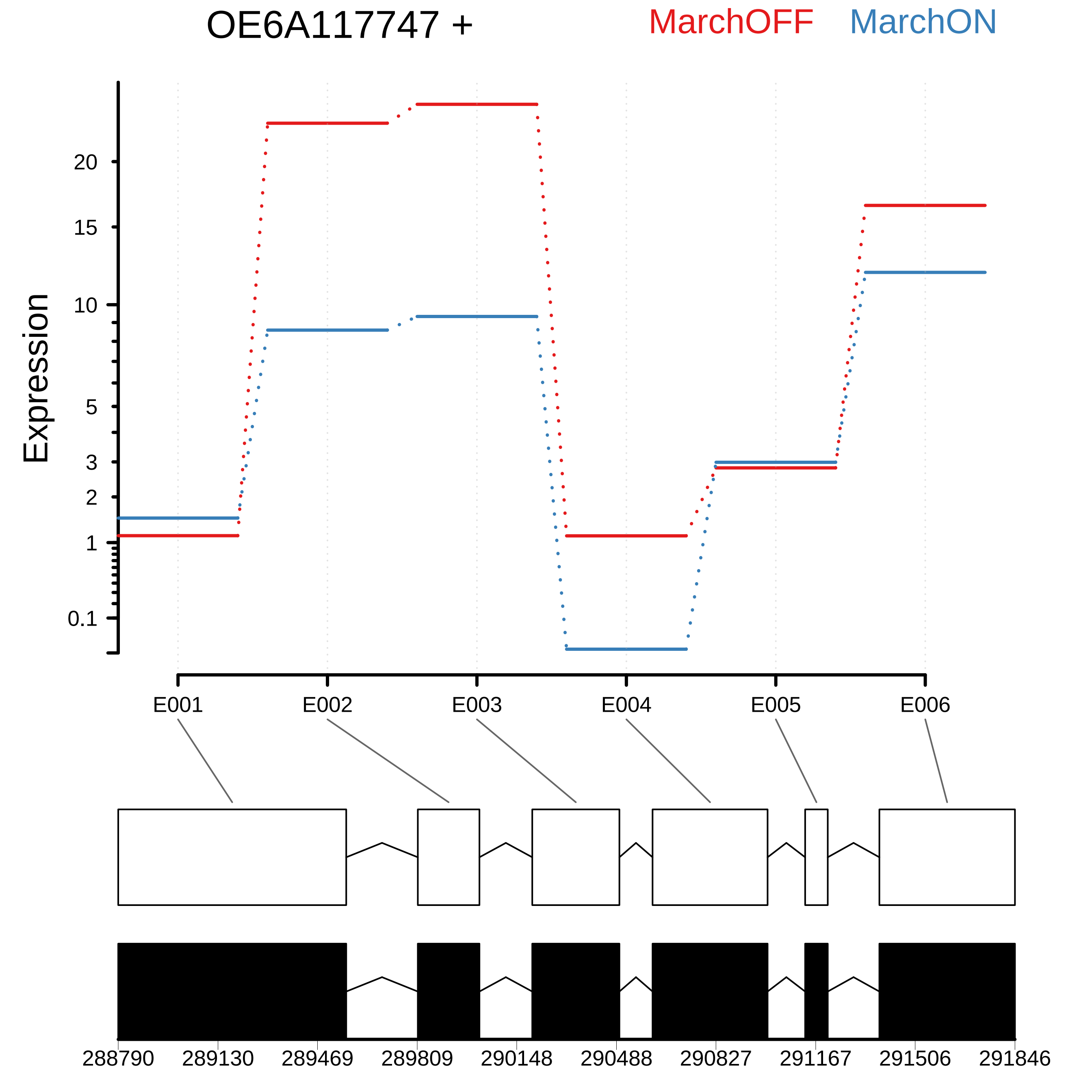
OE6A117747 +
MarchOFF
MarchON
Expression
20
15
10
5
3
2
1
0.1
E001
E002
E003
E004
E005
E006
288790
289130
289469
289809
290148
290488
290827
291167
291506
291846

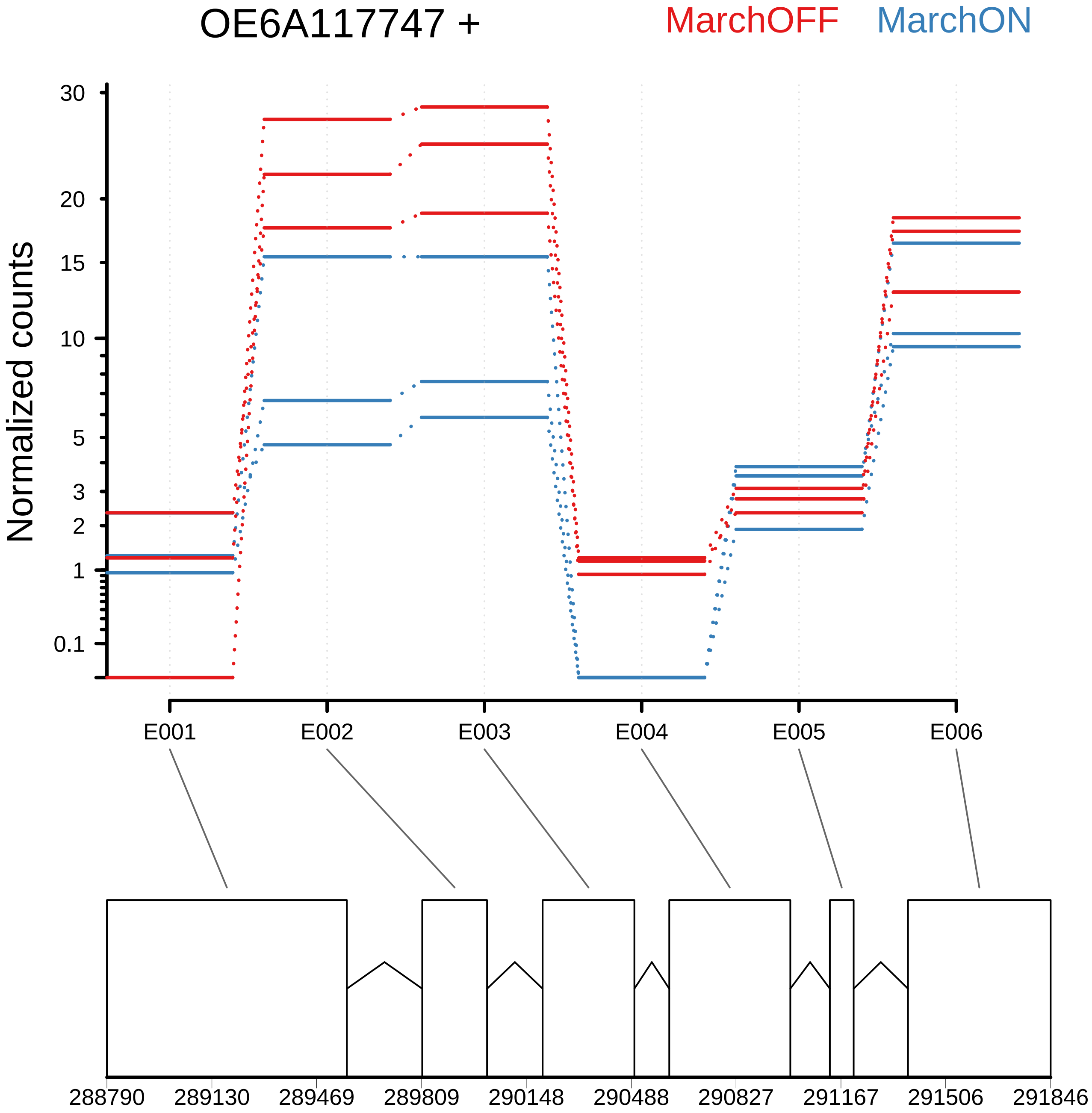

OE6A117747 +
MarchOFF
MarchON
Normalized counts
30
20
15
10
5
3
2
1
0.1
E001
E002
E003
E004
E005
E006
288790
289130
289469
289809
290148
290488
290827
291167
291506
291846

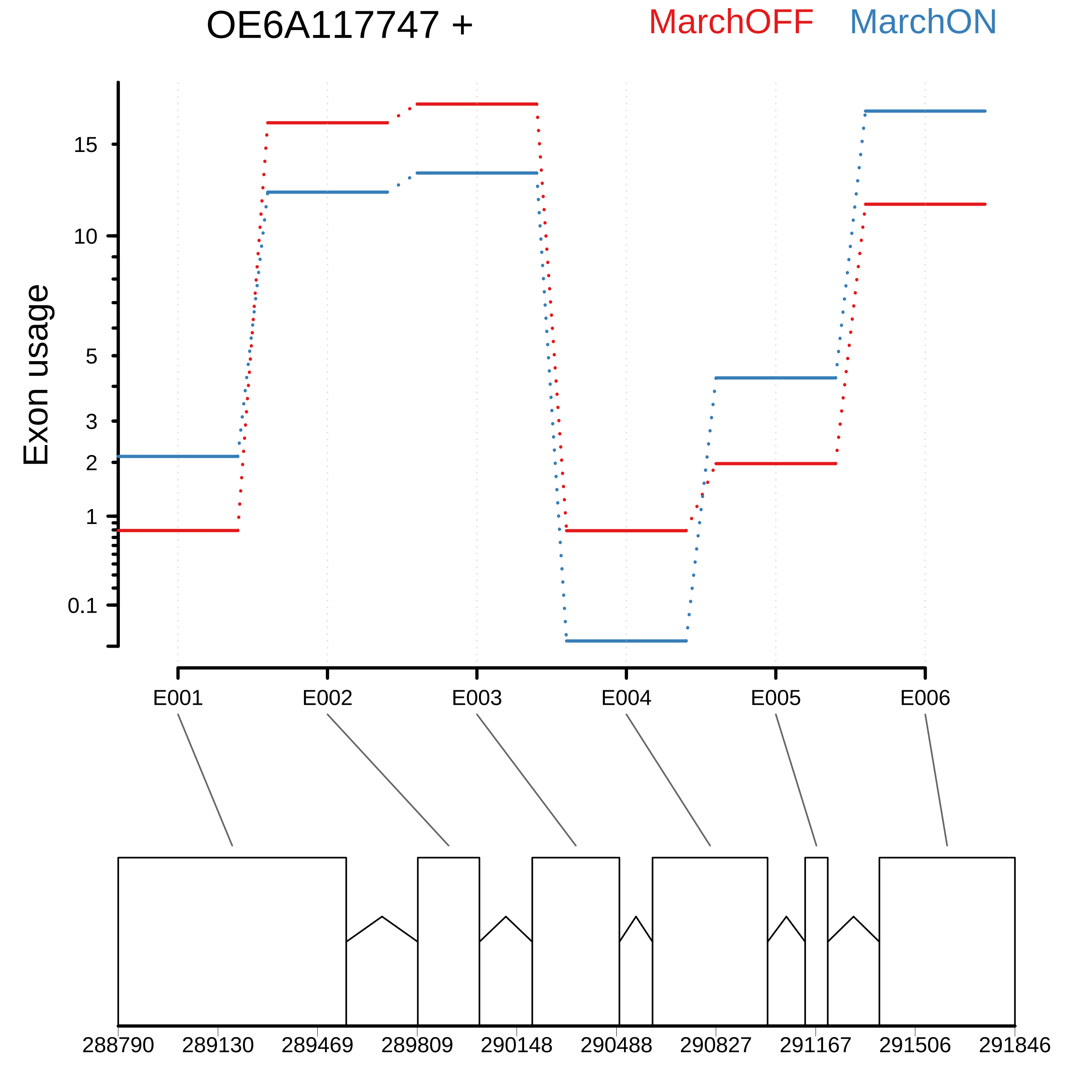

OE6A117747 +
MarchOFF
MarchON
Exon usage
15
10
5
3
2
1
0.1
E001
E002
E003
E004
E005
E006
288790
289130
289469
289809
290148
290488
290827
291167
291506
291846

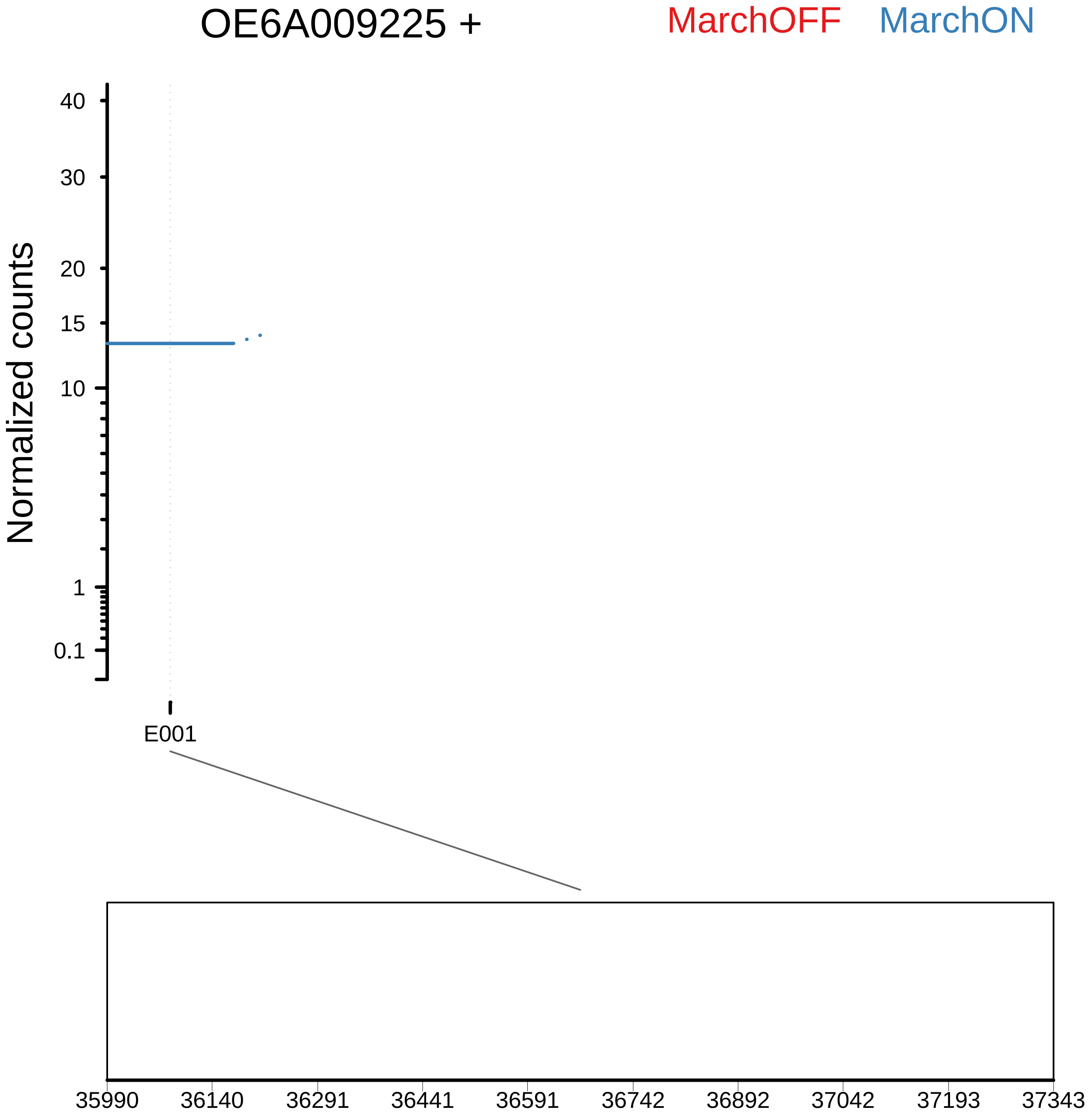
OE6A009225 +
MarchOFF
MarchON
Normalized counts
40
30
20
15
10
1
0.1
E001
35990
36140
36291
36441
36591
36742
36892
37042
37193
37343

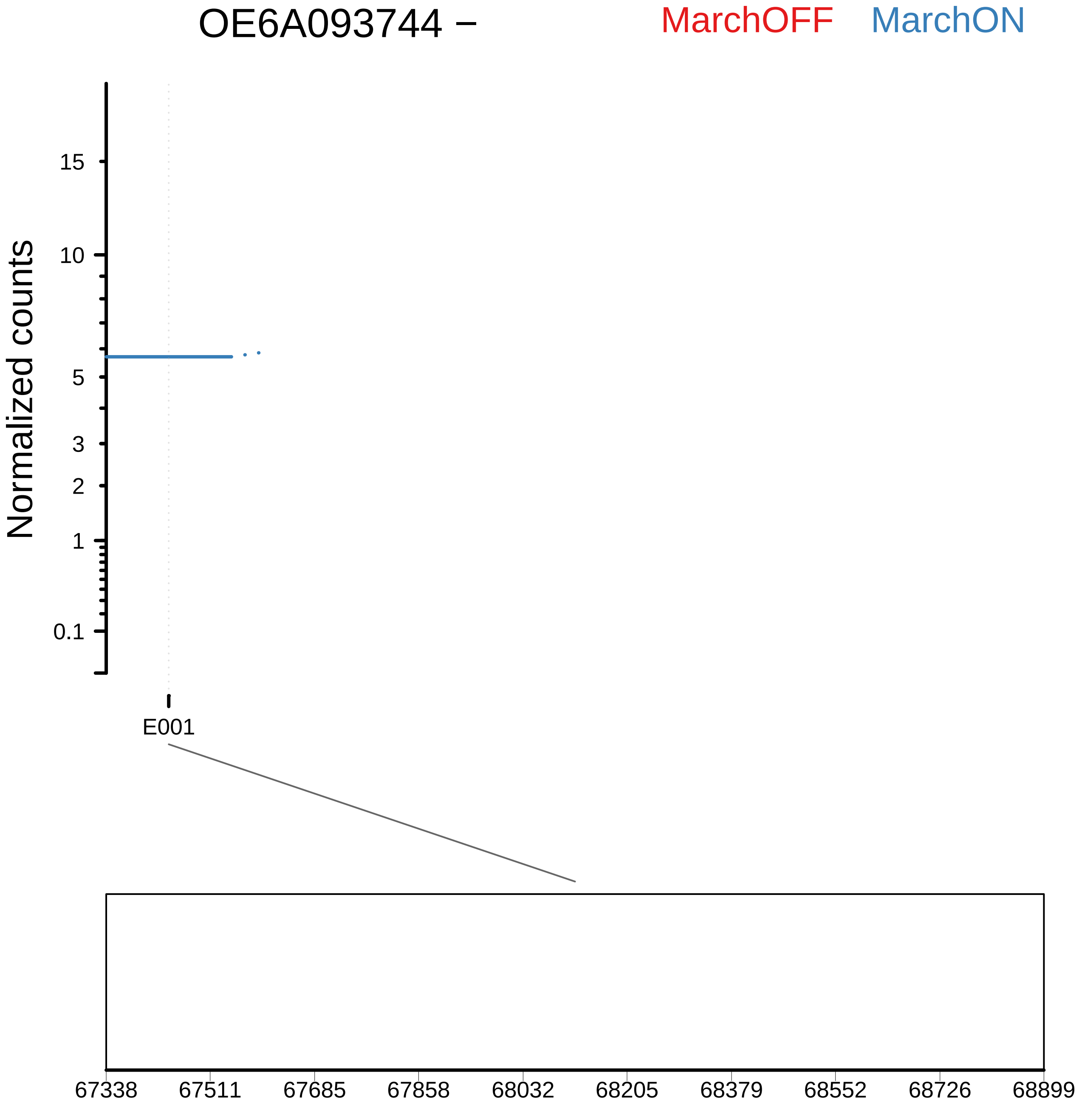
OE6A093744 −
MarchOFF
MarchON
Normalized counts
15
10
5
3
2
1
0.1
E001
67338
67511
67685
67858
68032
68205
68379
68552
68726
68899

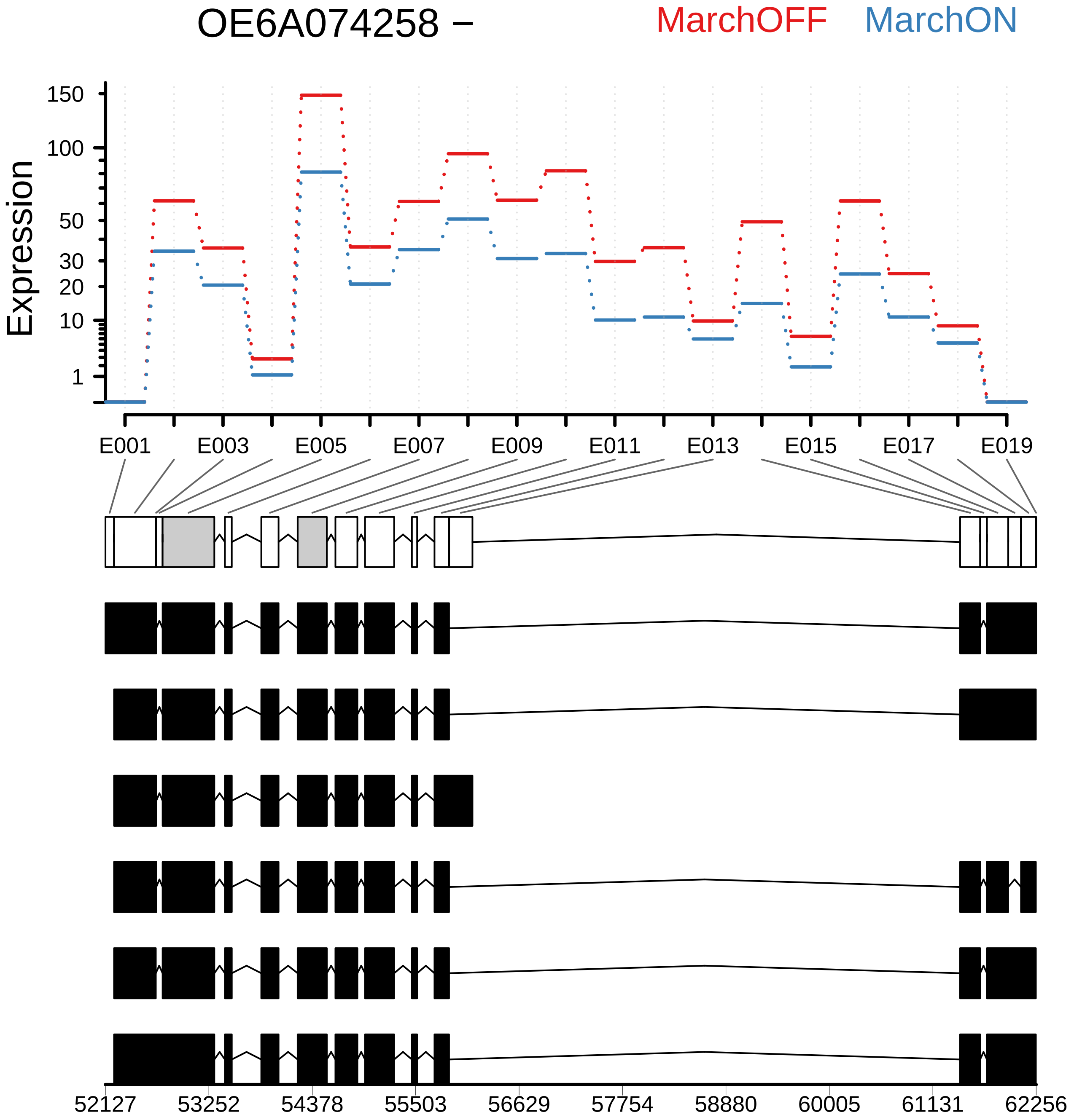

OE6A074258 –
MarchOFF
MarchON
Expression
150
100
50
30
20
10
1
E001
E003
E005
E007
E009
E011
E013
E015
E017
E019
52127
53252
54378
55503
56629
57754
58880
60005
61131
62256

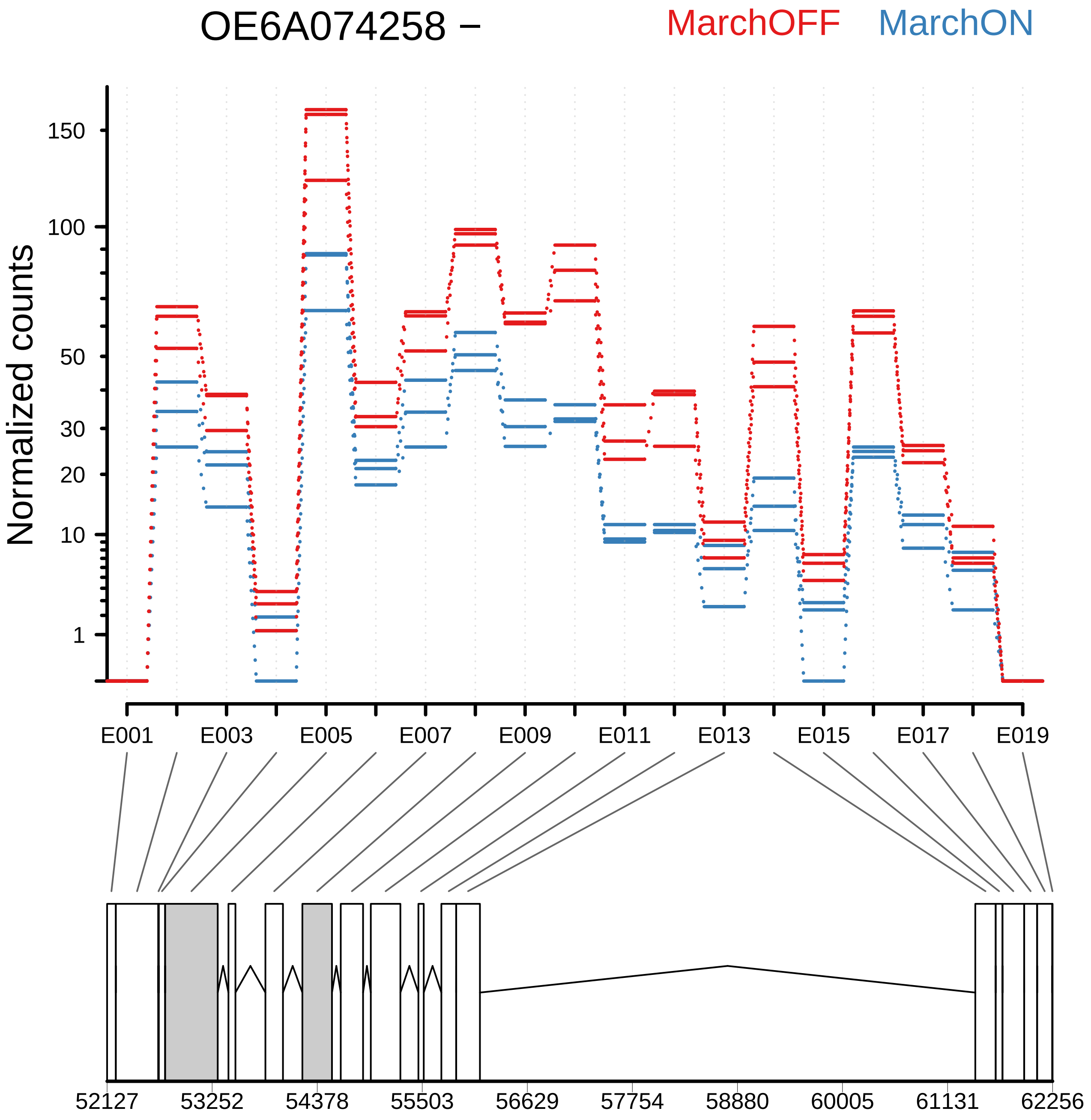
OE6A074258 −
MarchOFF
MarchON
Normalized counts
150
100
50
30
20
10
1
E001
E003
E005
E007
E009
E011
E013
E015
E017
E019
52127
53252
54378
55503
56629
57754
58880
60005
61131
62256

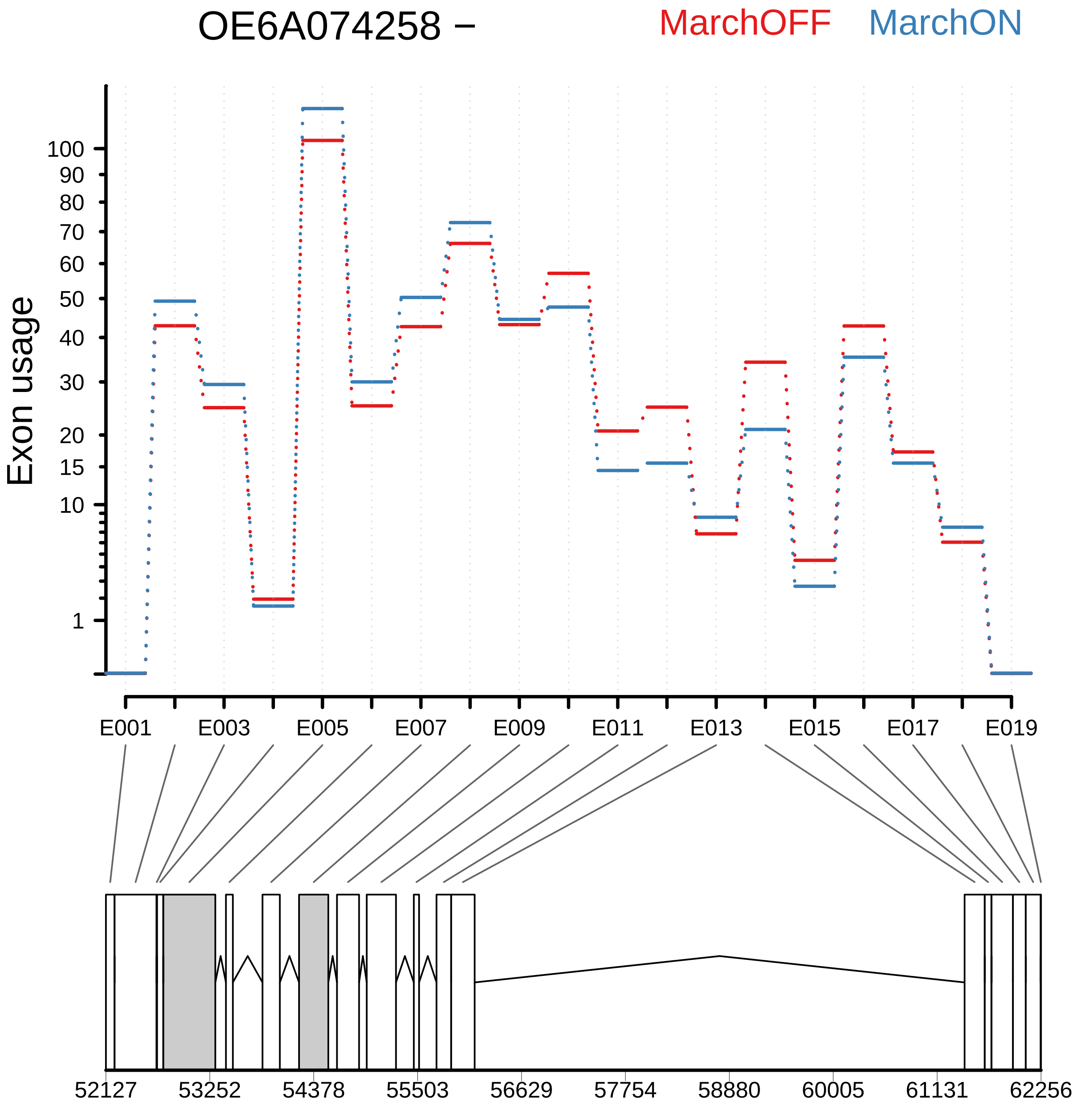
OE6A074258 −
MarchOFF
MarchON
Exon usage
100
90
80
70
60
50
40
30
20
15
10
1
E001
E003
E005
E007
E009
E011
E013
E015
E017
E019
52127
53252
54378
55503
56629
57754
58880
60005
61131
62256

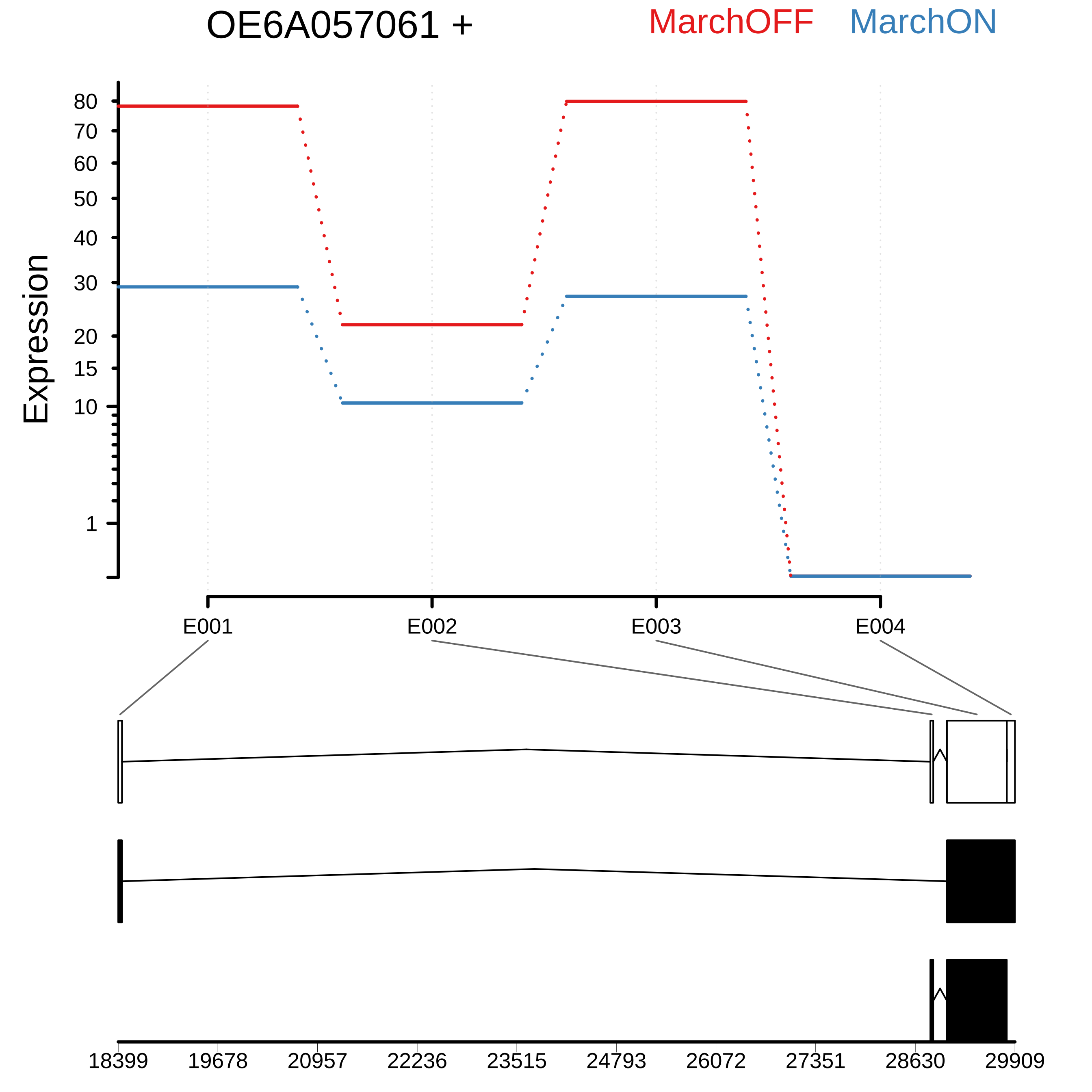
OE6A057061 +
MarchOFF
MarchON
Expression
80
70
60
50
40
30
20
15
10
1
E001
E002
E003
E004
18399
19678
20957
22236
23515
24793
26072
27351
28630
29909

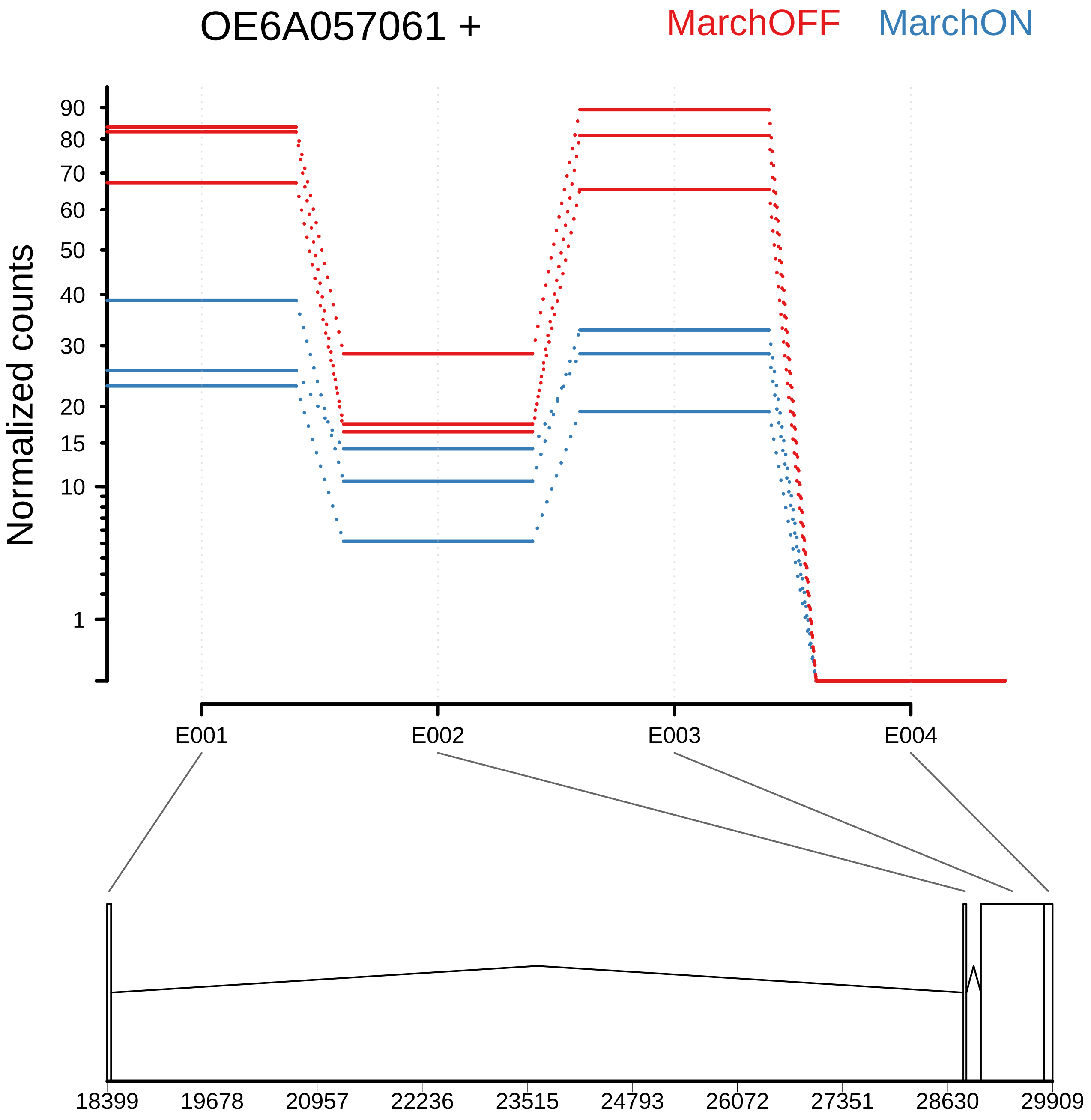

OE6A057061 +
MarchOFF
MarchON
Normalized counts
90
80
70
60
50
40
30
20
15
10
1
E001
E002
E003
E004
18399
19678
20957
22236
23515
24793
26072
27351
28630
29909

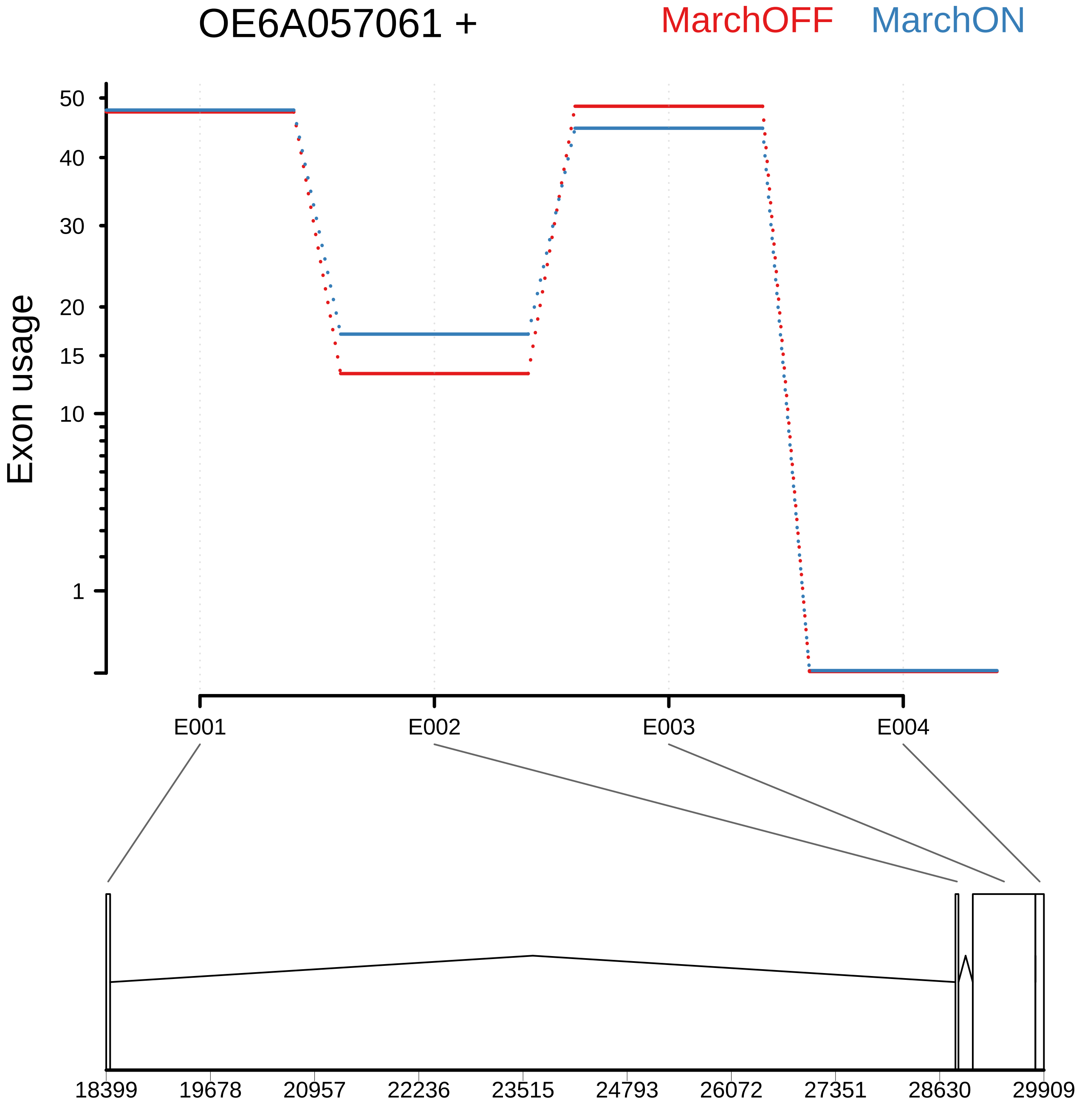

OE6A057061 +
MarchOFF
MarchON
Exon usage
50
40
30
20
15
10
1
E001
E002
E003
E004
18399
19678
20957
22236
23515
24793
26072
27351
28630
29909

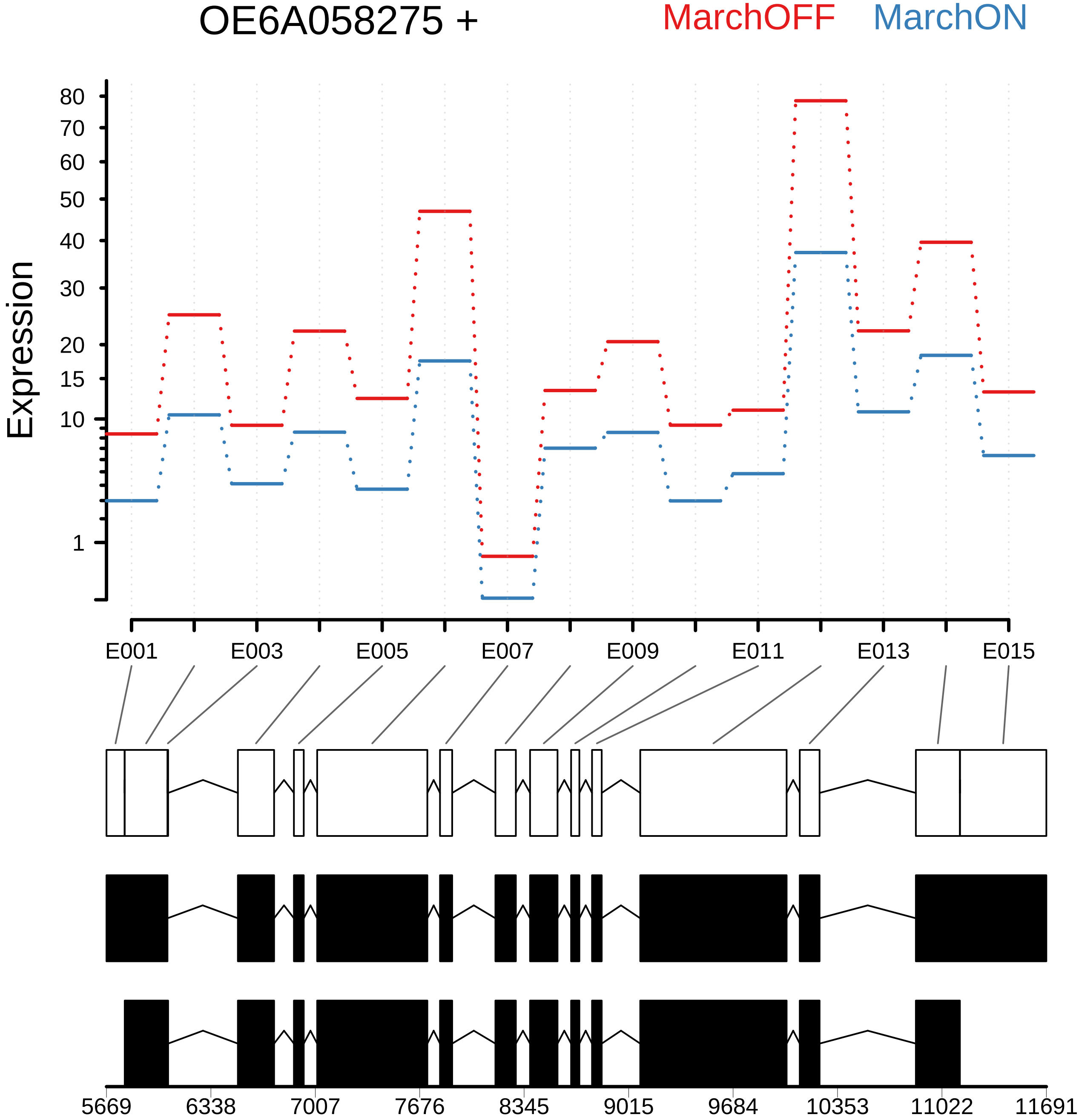
OE6A058275 +
MarchOFF
MarchON
Expression
80
70
60
50
40
30
20
15
10
1
E001
E003
E005
E007
E009
E011
E013
E015
5669
6338
7007
7676
8345
9015
9684
10353
11022
11691

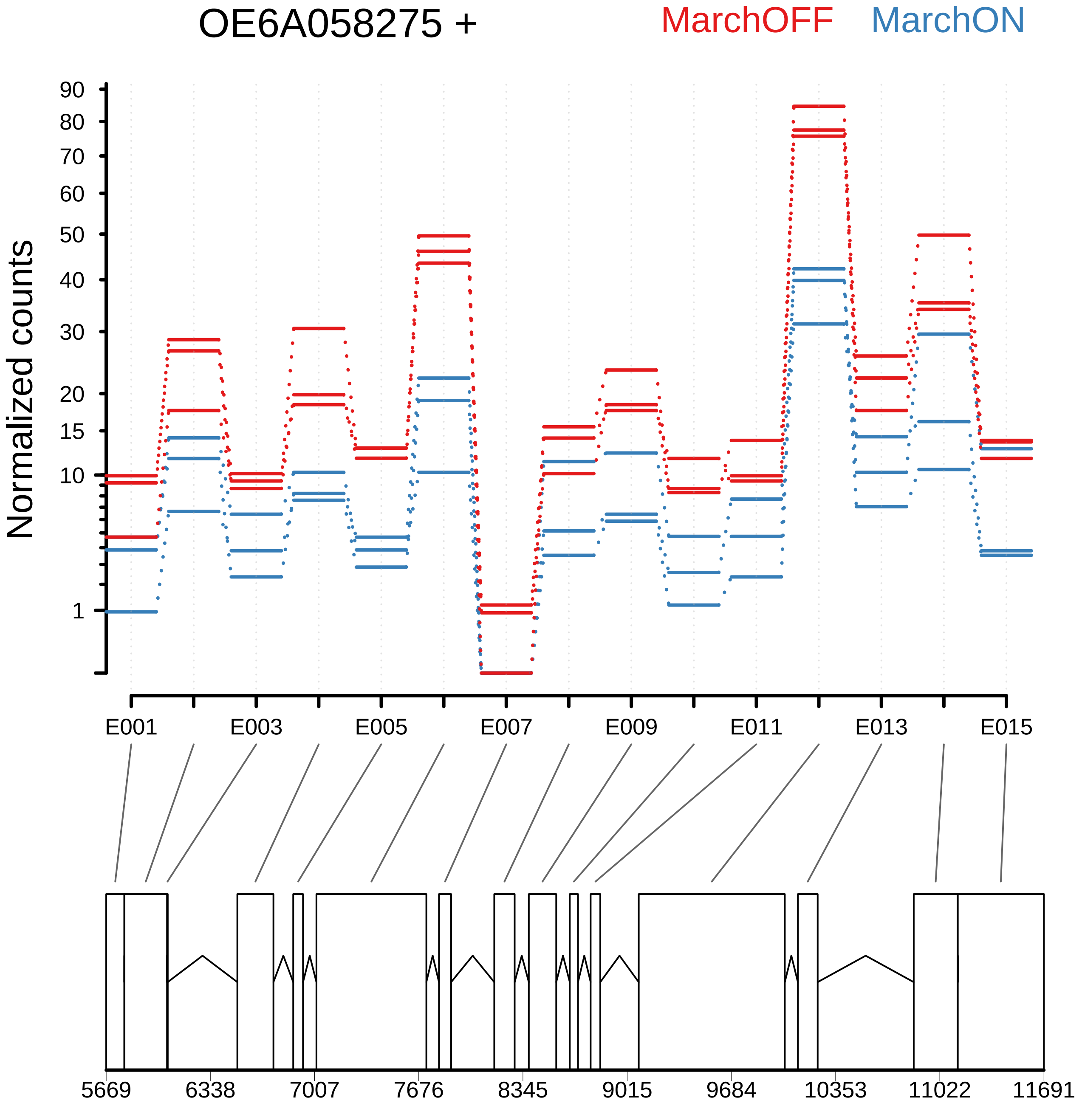

OE6A058275 +
MarchOFF
MarchON
Normalized counts
90
80
70
60
50
40
30
20
15
10
1
E001
E003
E005
E007
E009
E011
E013
E015
5669
6338
7007
7676
8345
9015
9684
10353
11022
11691

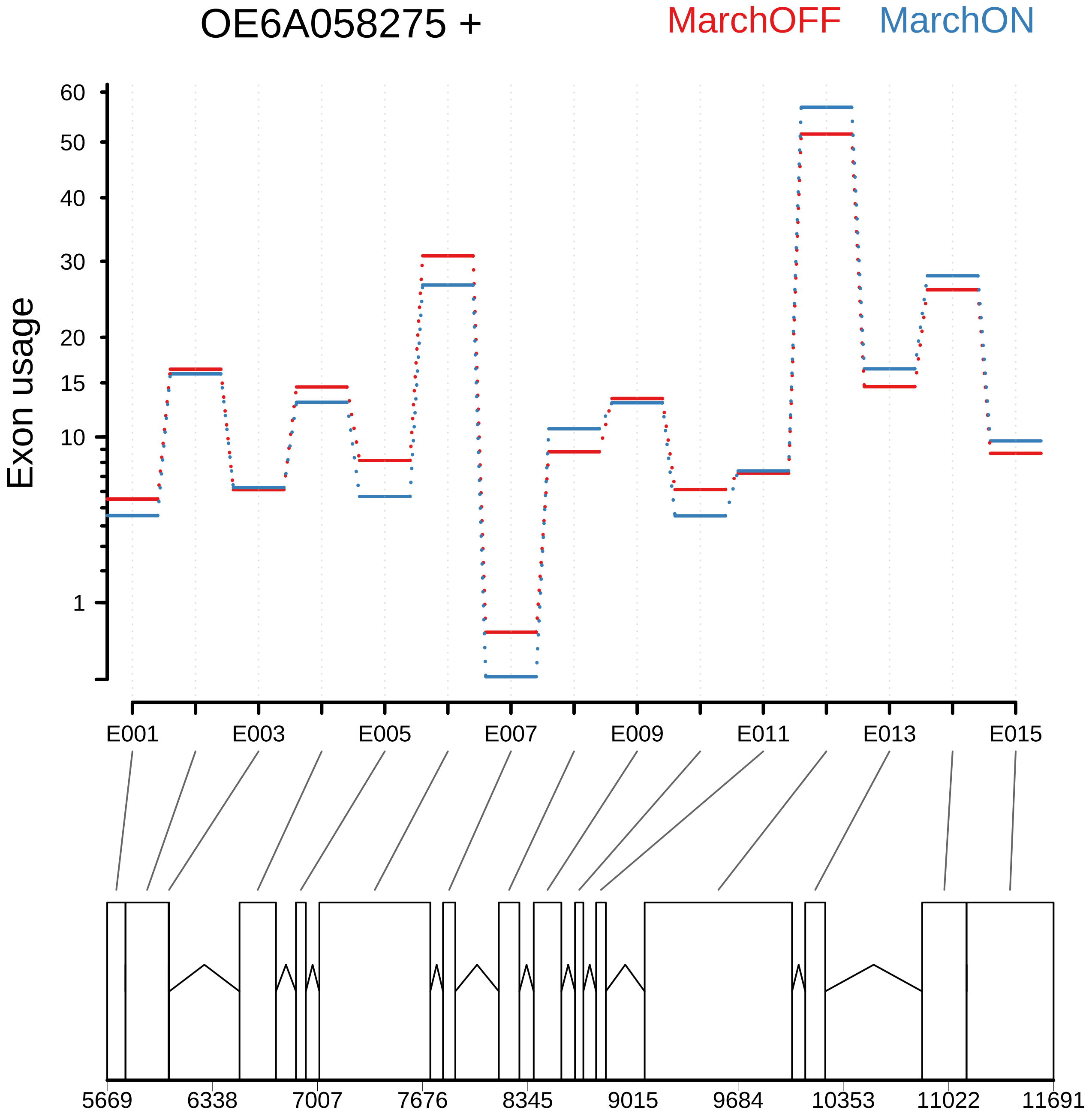
OE6A058275 +
MarchOFF
MarchON
Exon usage
60
50
40
30
20
15
10
1
E001
E003
E005
E007
E009
E011
E013
E015
5669
6338
7007
7676
8345
9015
9684
10353
11022
11691

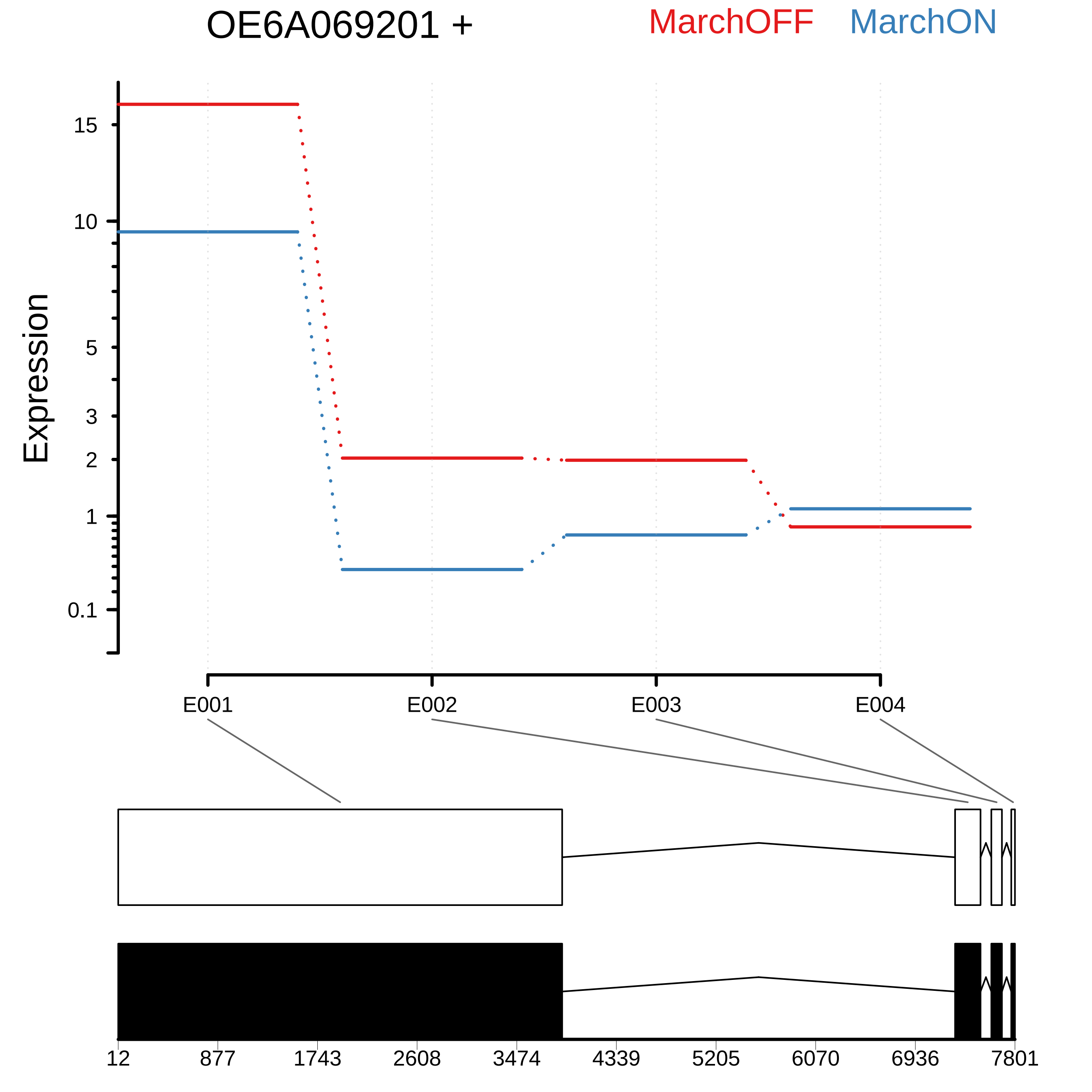

OE6A069201 +
MarchOFF
MarchON
Expression
15
10
5
3
2
1
0.1
E001
E002
E003
E004
12
877
1743
2608
3474
4339
5205
6070
6936
7801

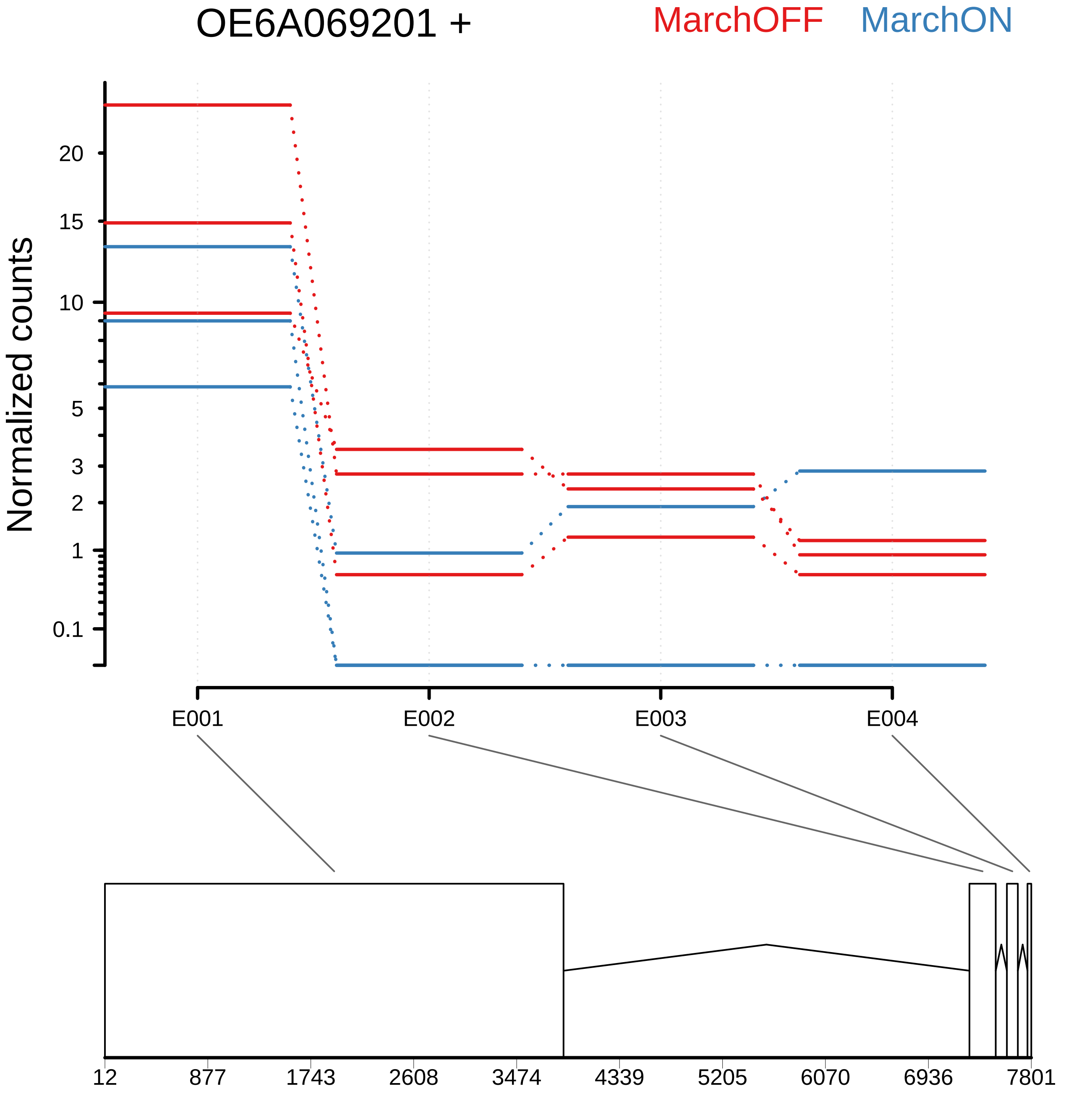

OE6A069201 +
MarchOFF
MarchON
Normalized counts
20
15
10
5
3
2
1
0.1
E001
E002
E003
E004
12
877
1743
2608
3474
4339
5205
6070
6936
7801

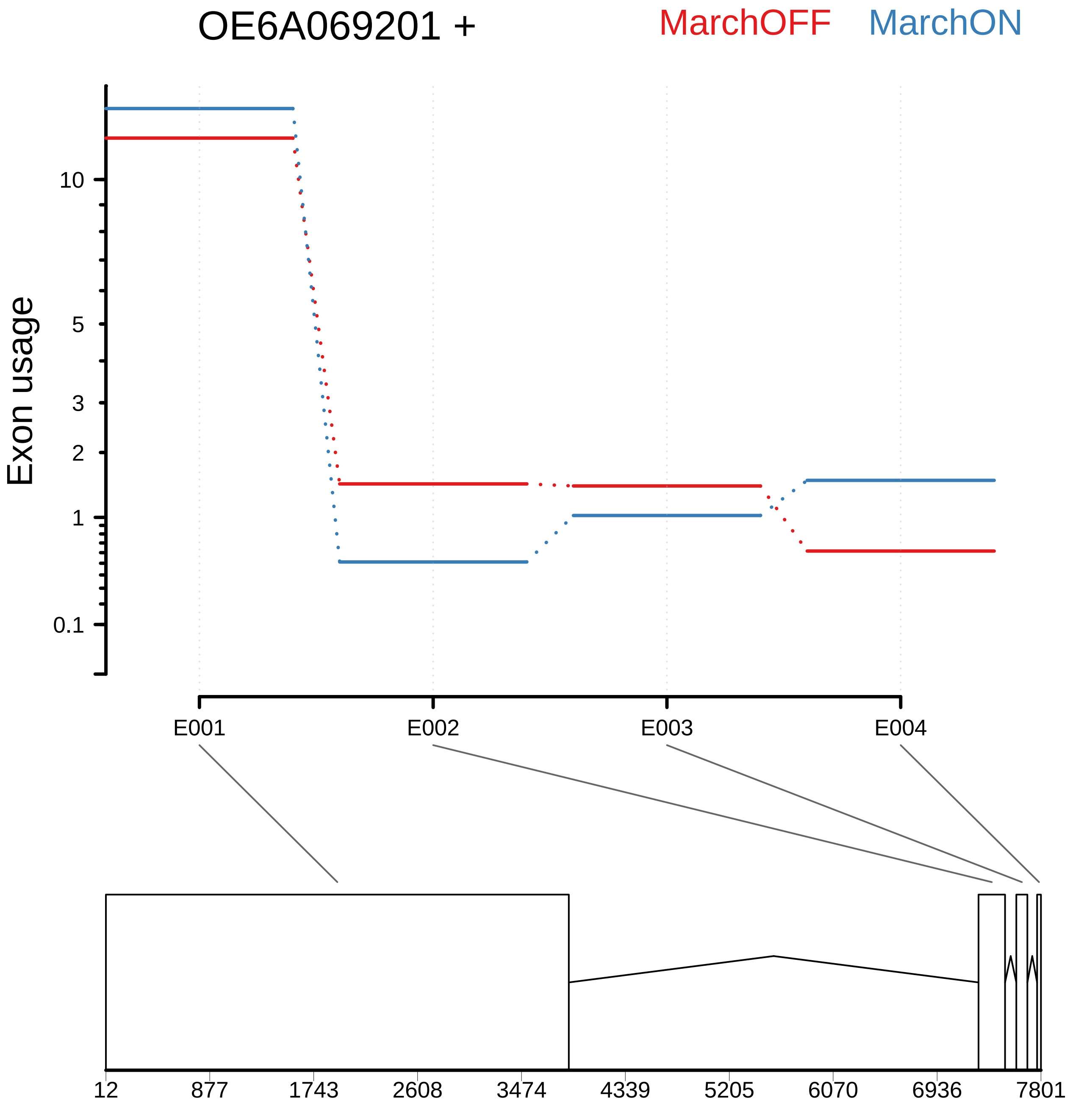

OE6A069201 +
MarchOFF
MarchON
Exon usage
10
5
3
2
1
0.1
E001
E002
E003
E004
12
877
1743
2608
3474
4339
5205
6070
6936
7801

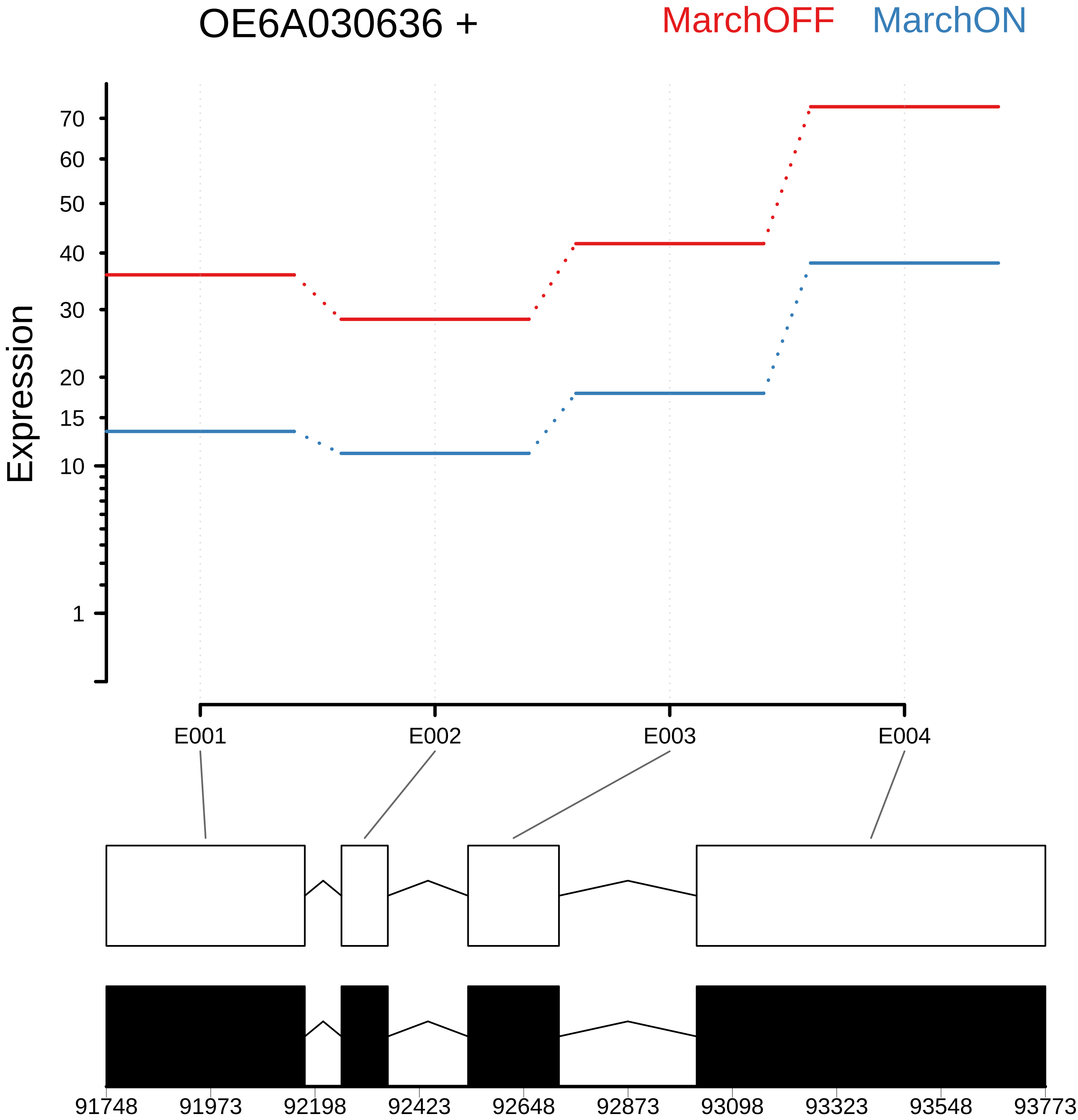

OE6A030636 +
MarchOFF
MarchON
Expression
70
60
50
40
30
20
15
10
1
E001
E002
E003
E004
91748
91973
92198
92423
92648
92873
93098
93323
93548
93773

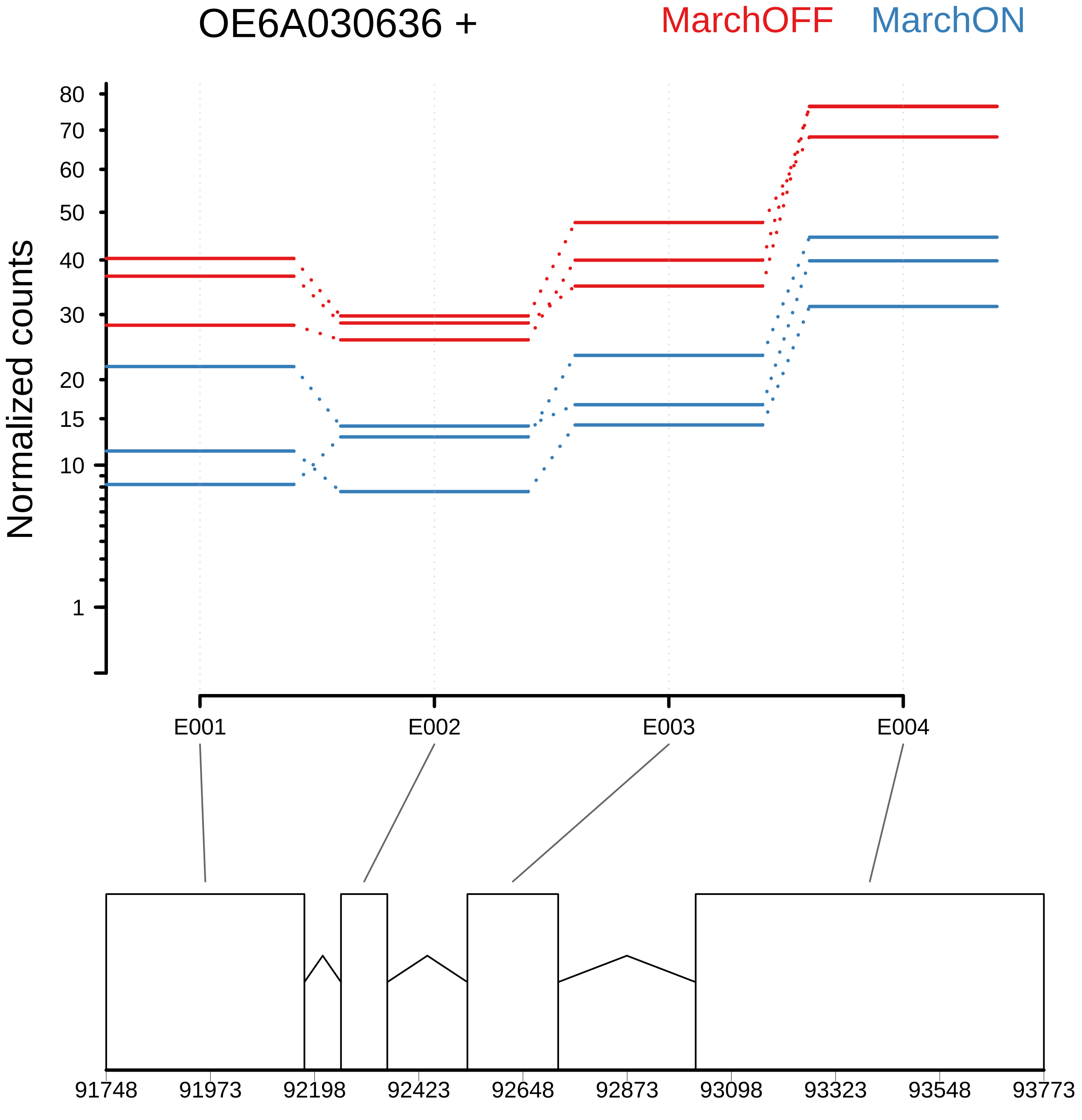

OE6A030636 +
MarchOFF
MarchON
Normalized counts
80
70
60
50
40
30
20
15
10
1
E001
E002
E003
E004
91748
91973
92198
92423
92648
92873
93098
93323
93548
93773

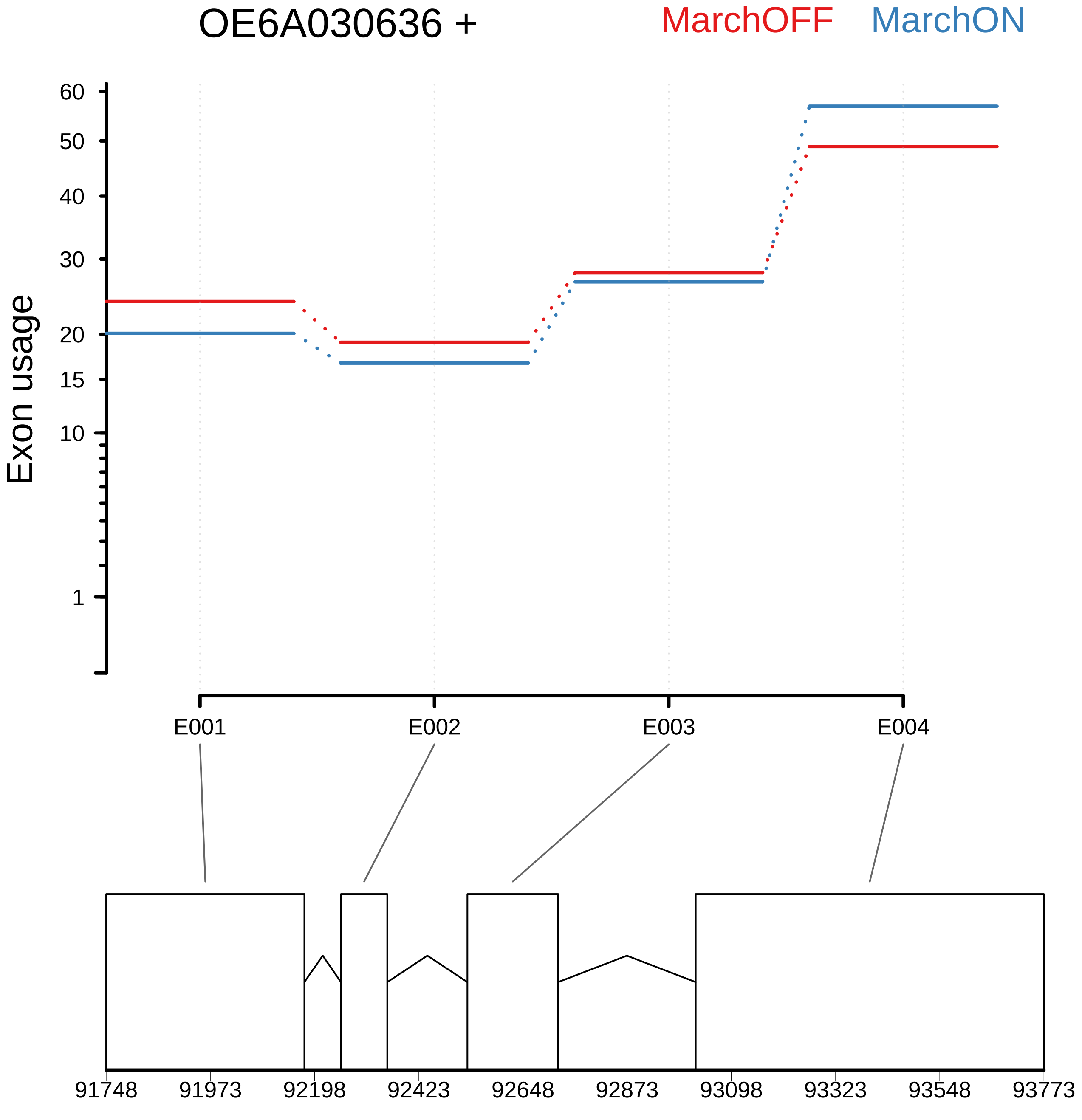

OE6A030636 +
MarchOFF
MarchON
Exon usage
60
50
40
30
20
15
10
1
E001
E002
E003
E004
91748
91973
92198
92423
92648
92873
93098
93323
93548
93773

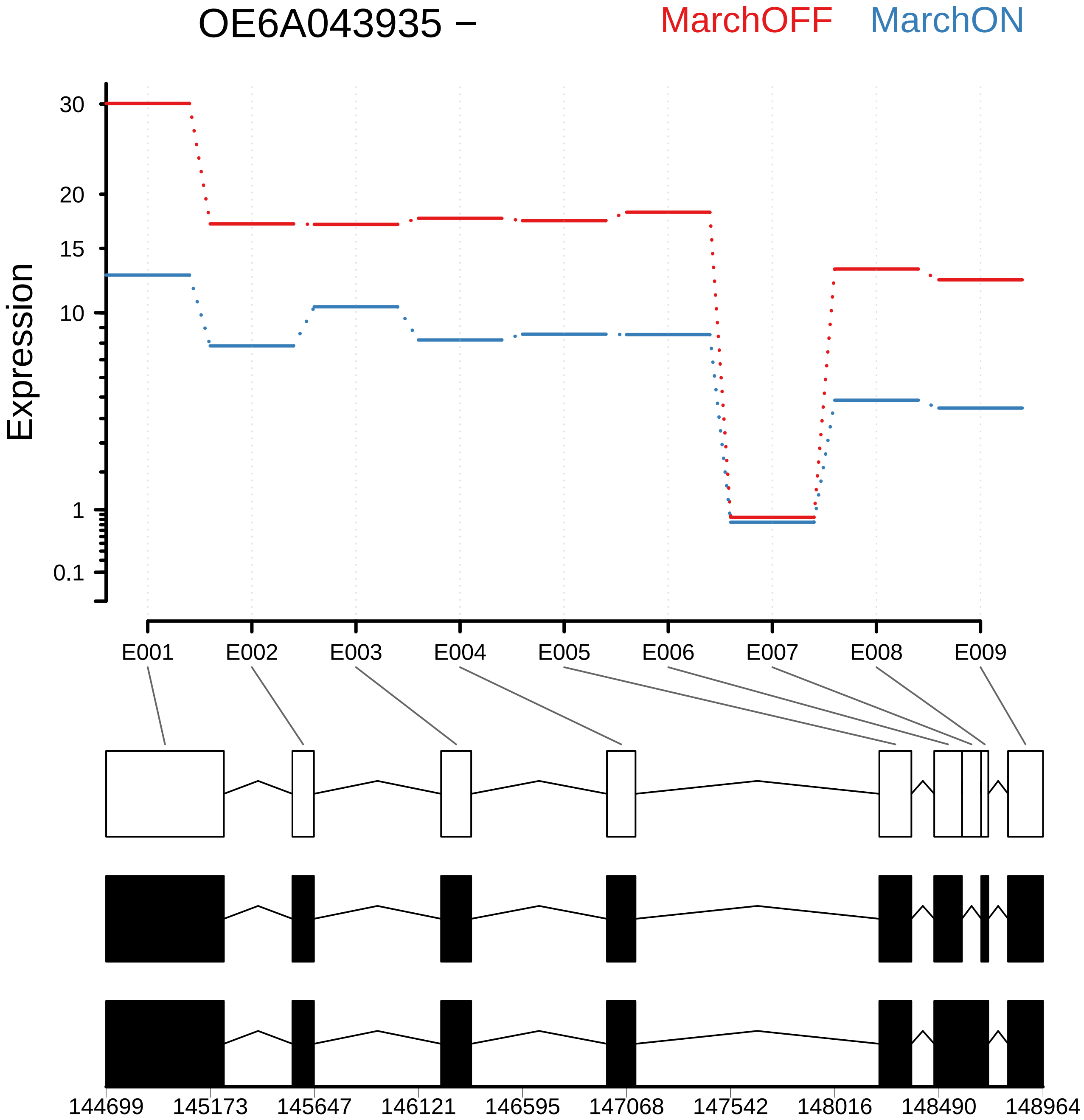

OE6A043935 −
MarchOFF
MarchON
Expression
30
20
15
10
1
0.1
E001
E002
E003
E004
E005
E006
E007
E008
E009
144699
145173
145647
146121
146595
147068
147542
148016
148490
148964

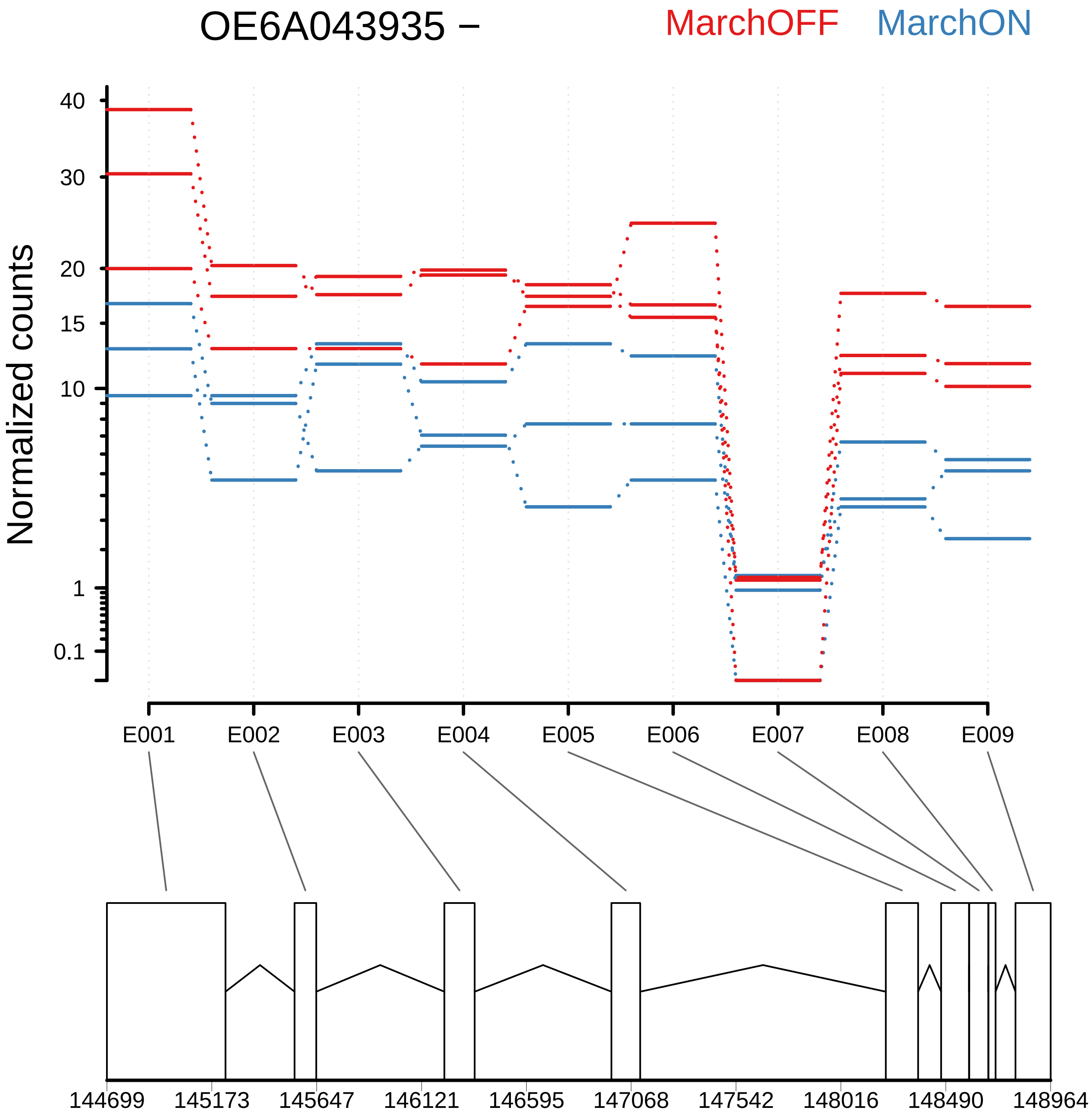

OE6A043935 −
MarchOFF
MarchON
Normalized counts
40
30
20
15
10
1
0.1
E001
E002
E003
E004
E005
E006
E007
E008
E009
144699
145173
145647
146121
146595
147068
147542
148016
148490
148964

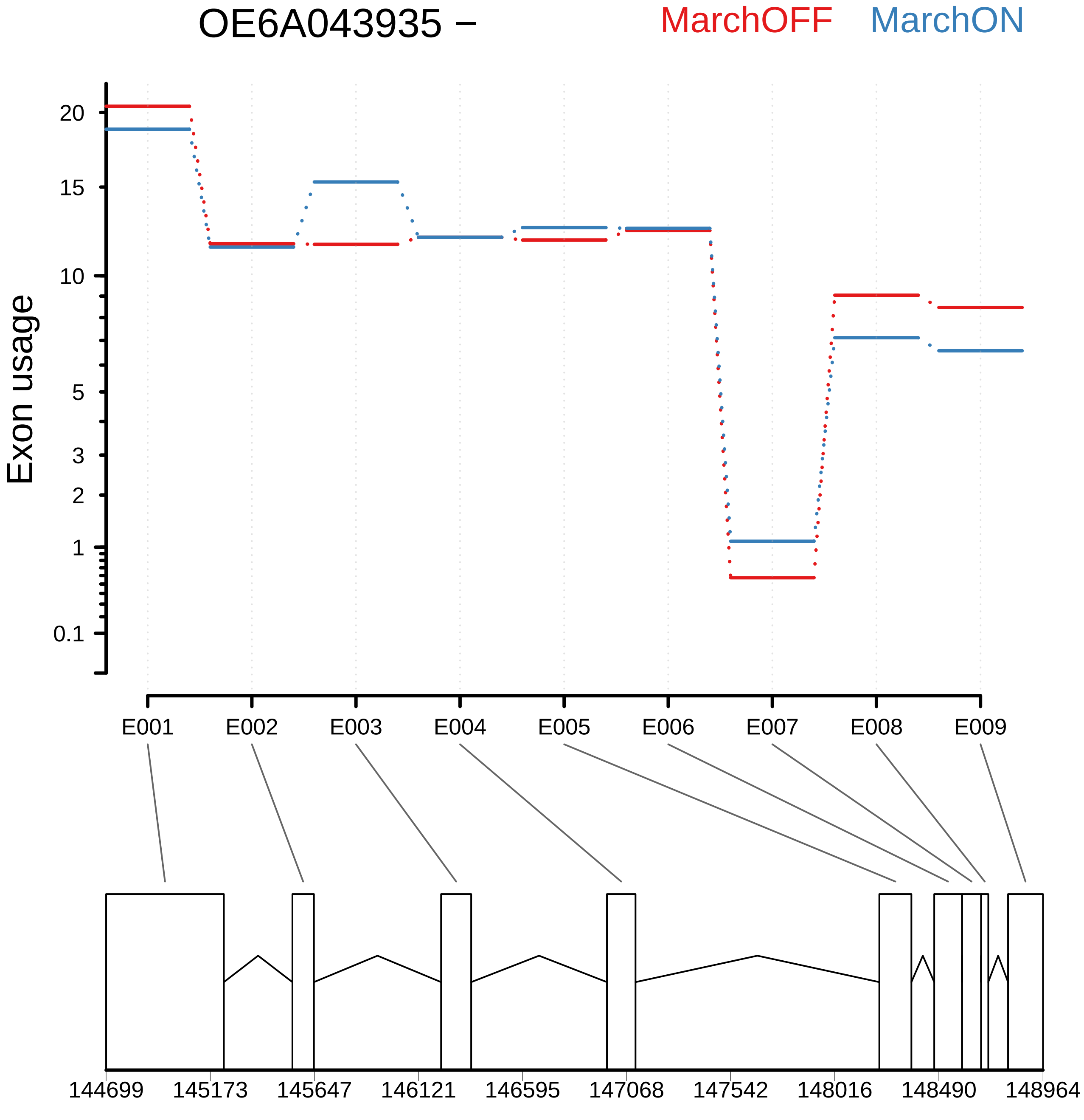

OE6A043935 −
MarchOFF
MarchON
Exon usage
20
15
10
5
3
2
1
0.1
E001
E002
E003
E004
E005
E006
E007
E008
E009
144699
145173
145647
146121
146595
147068
147542
148016
148490
148964

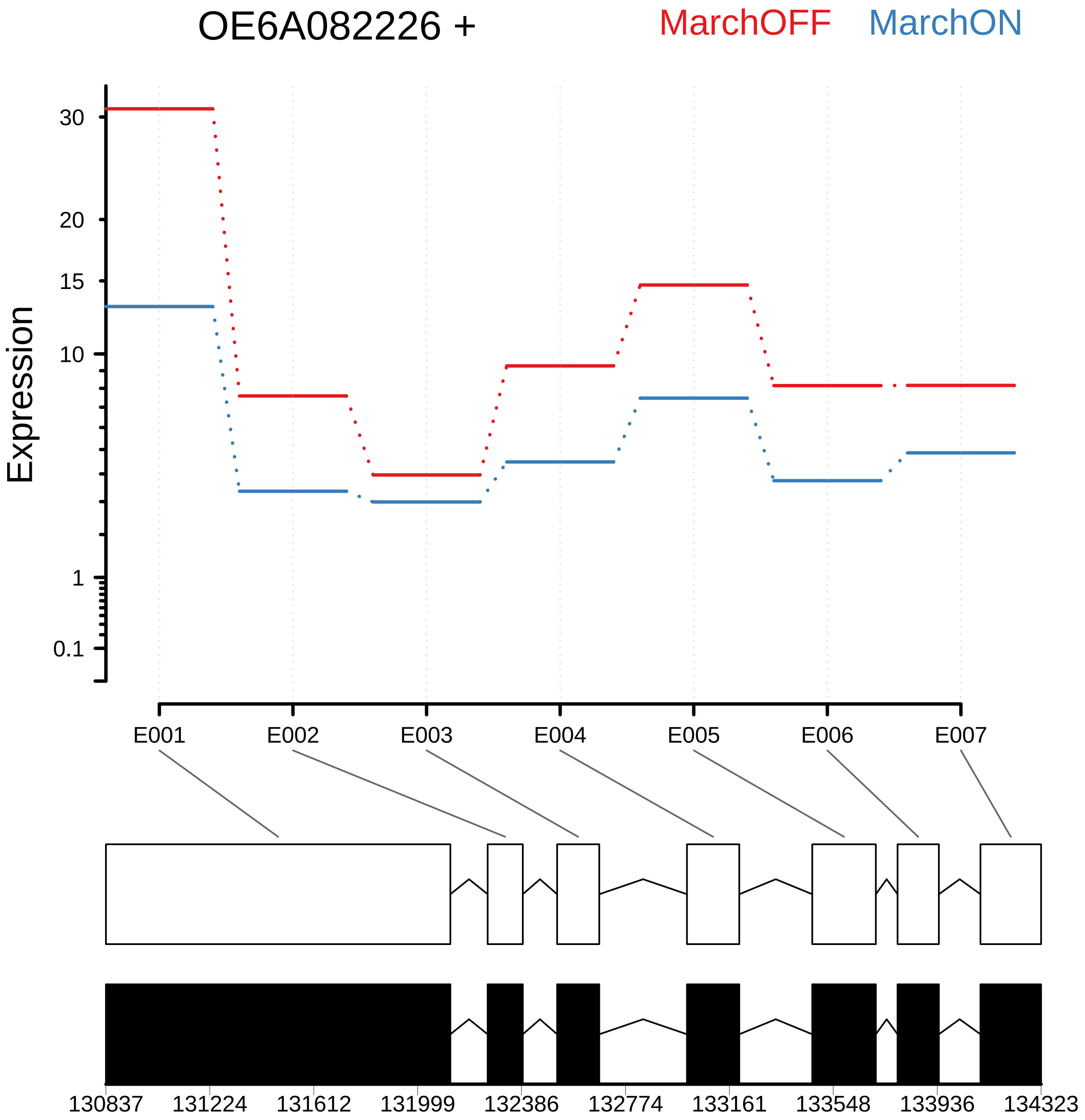

OE6A082226 +
MarchOFF
MarchON
Expression
30
20
15
10
1
0.1
E001
E002
E003
E004
E005
E006
E007
130837
131224
131612
131999
132386
132774
133161
133548
133936
134323

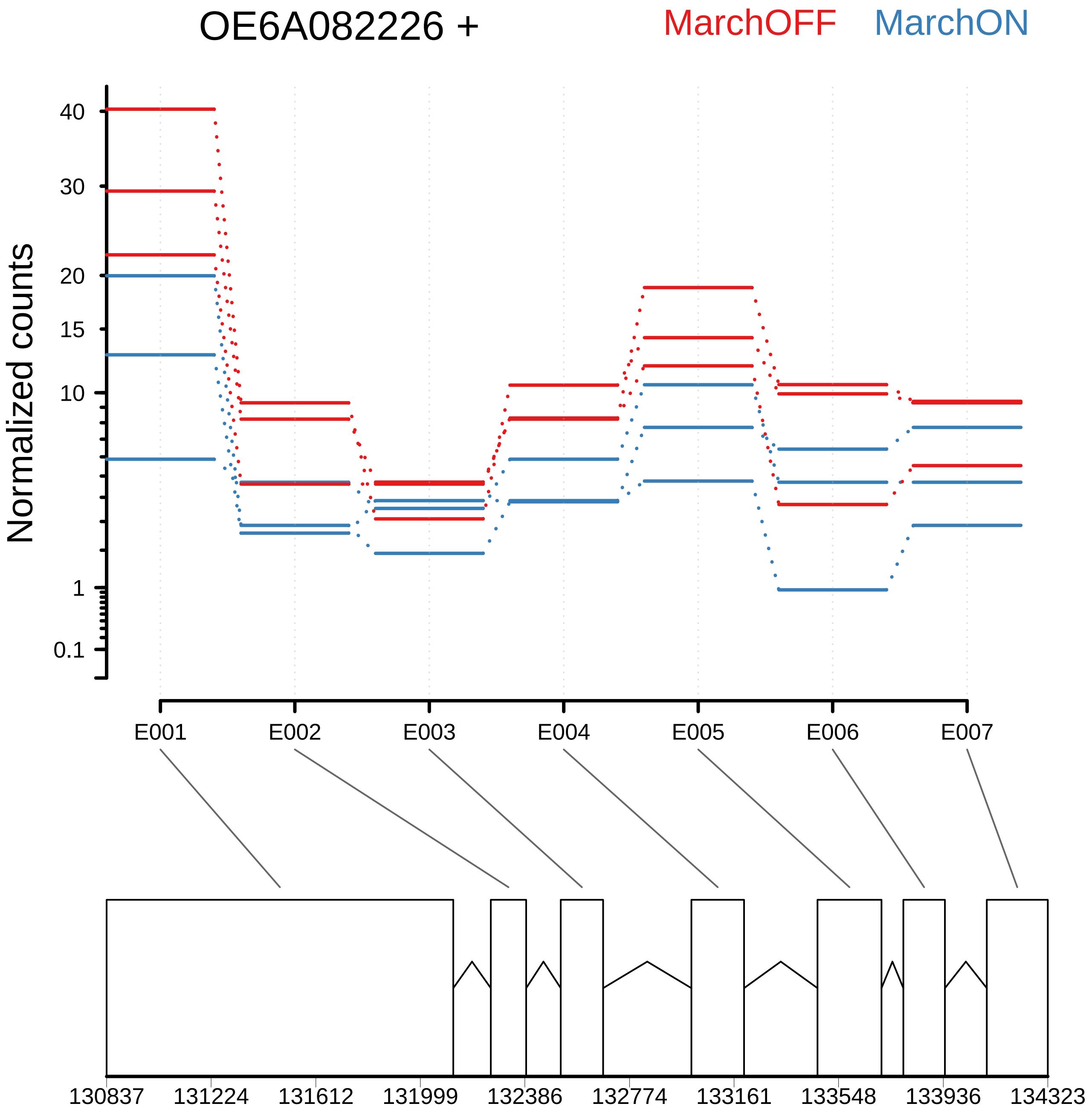

OE6A082226 +
MarchOFF
MarchON
Normalized counts
40
30
20
15
10
1
0.1
E001
E002
E003
E004
E005
E006
E007
130837
131224
131612
131999
132386
132774
133161
133548
133936
134323

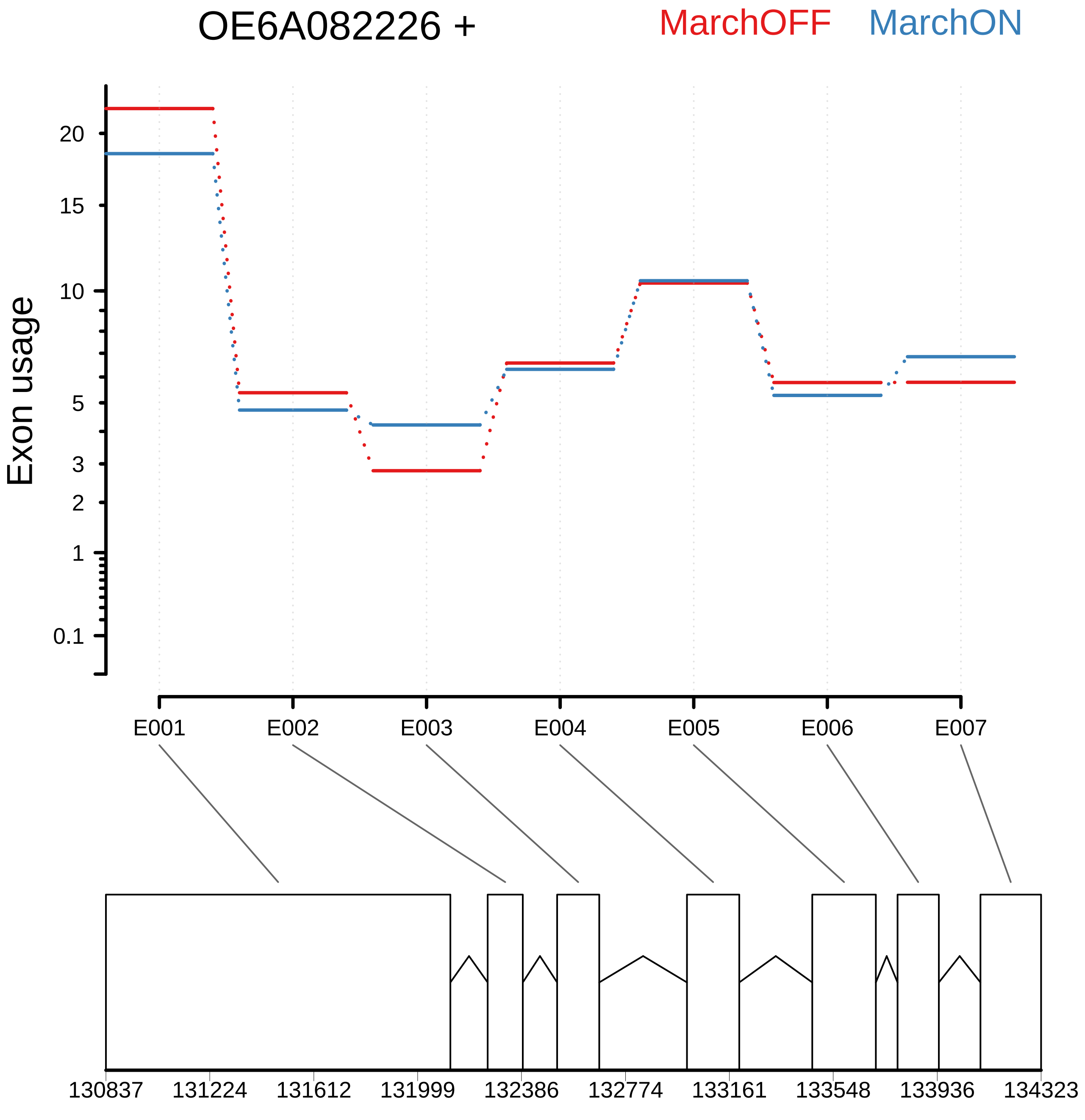

OE6A082226 +
MarchOFF
MarchON
Exon usage
20
15
10
5
3
2
1
0.1
E001
E002
E003
E004
E005
E006
E007
130837
131224
131612
131999
132386
132774
133161
133548
133936
134323

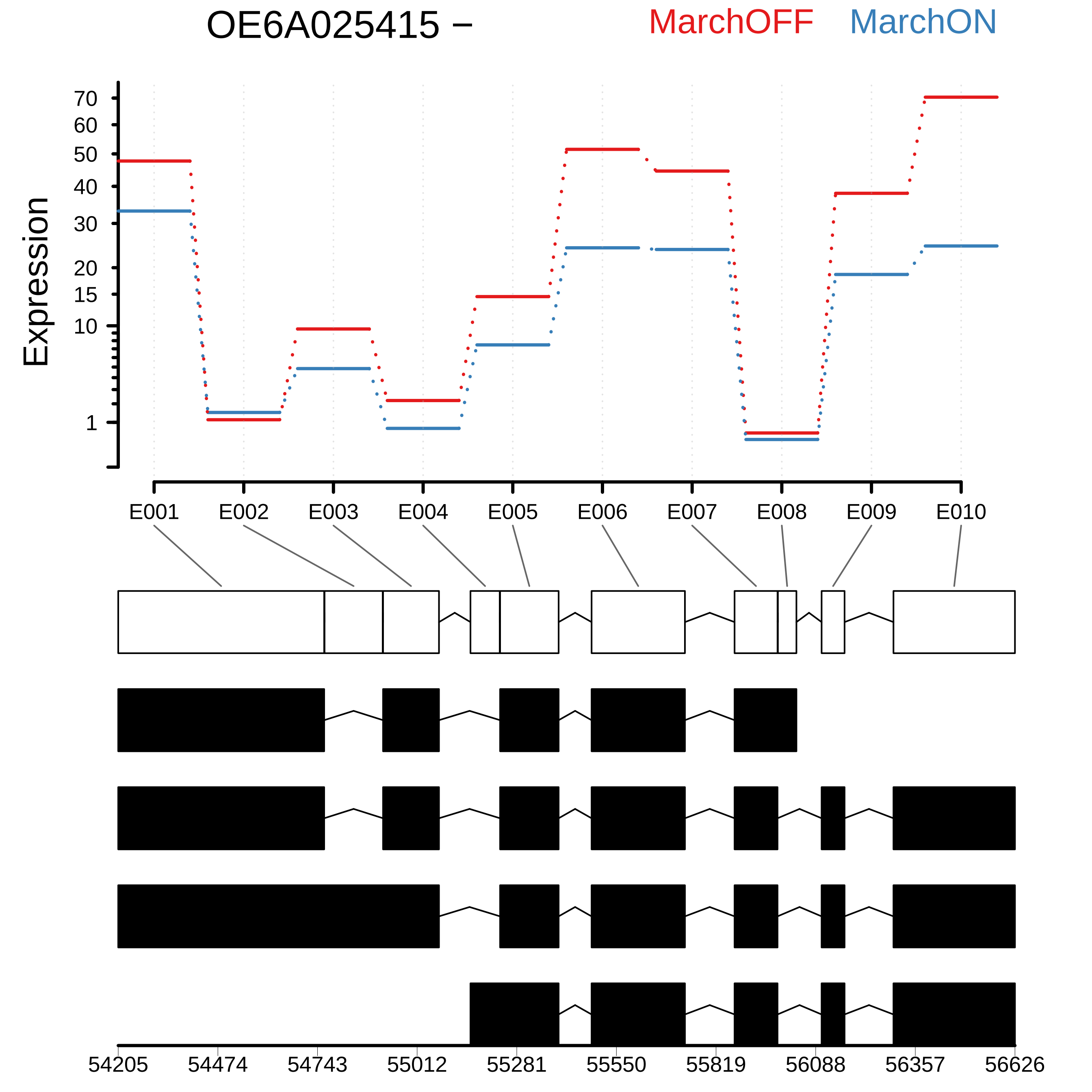

OE6A025415 −
MarchOFF
MarchON
Expression
70
60
50
40
30
20
15
10
1
E001
E002
E003
E004
E005
E006
E007
E008
E009
E010
54205
54474
54743
55012
55281
55550
55819
56088
56357
56626

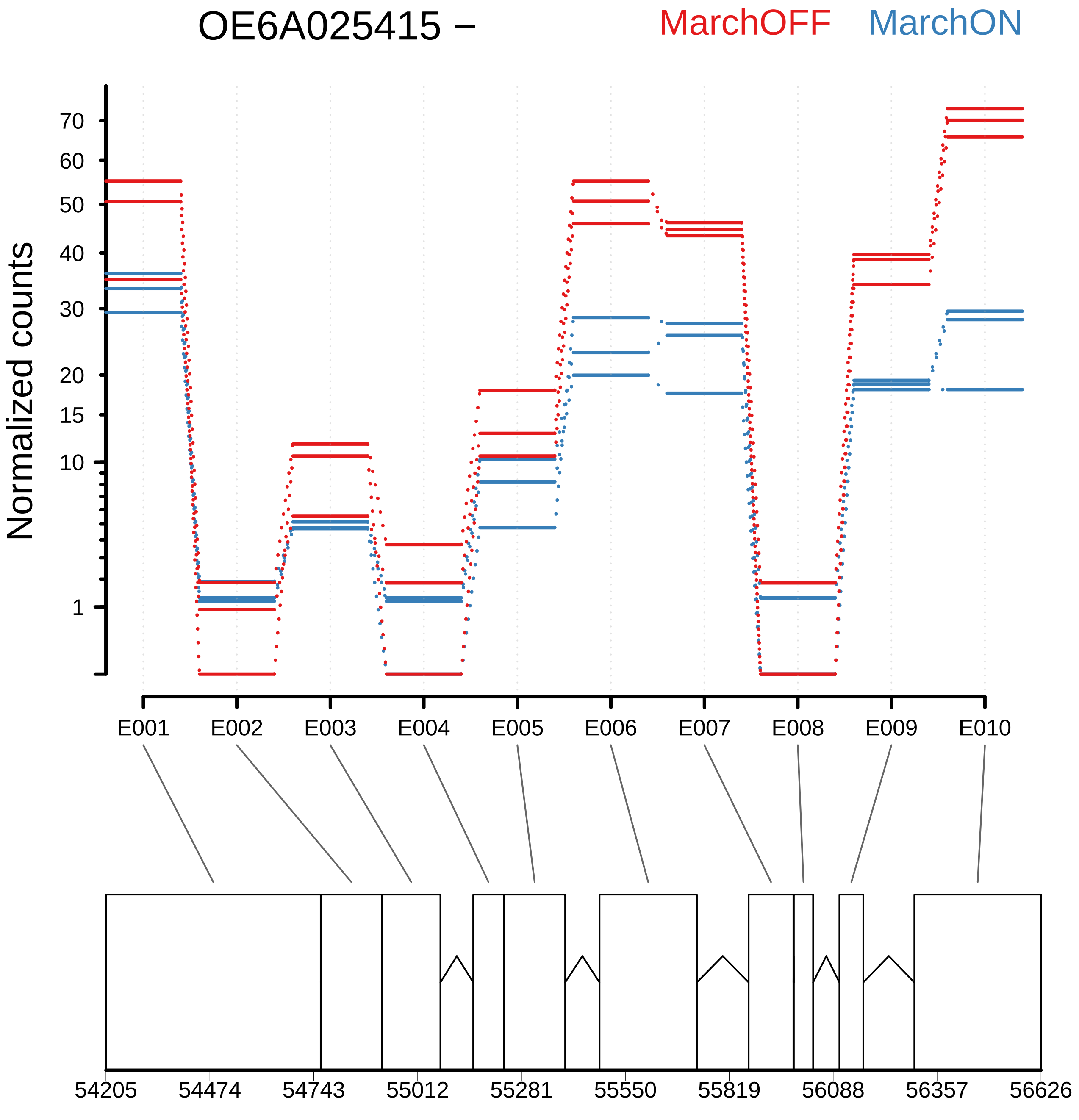
OE6A025415 −
MarchOFF
MarchON
Normalized counts
70
60
50
40
30
20
15
10
1
E001
E002
E003
E004
E005
E006
E007
E008
E009
E010
54205
54474
54743
55012
55281
55550
55819
56088
56357
56626

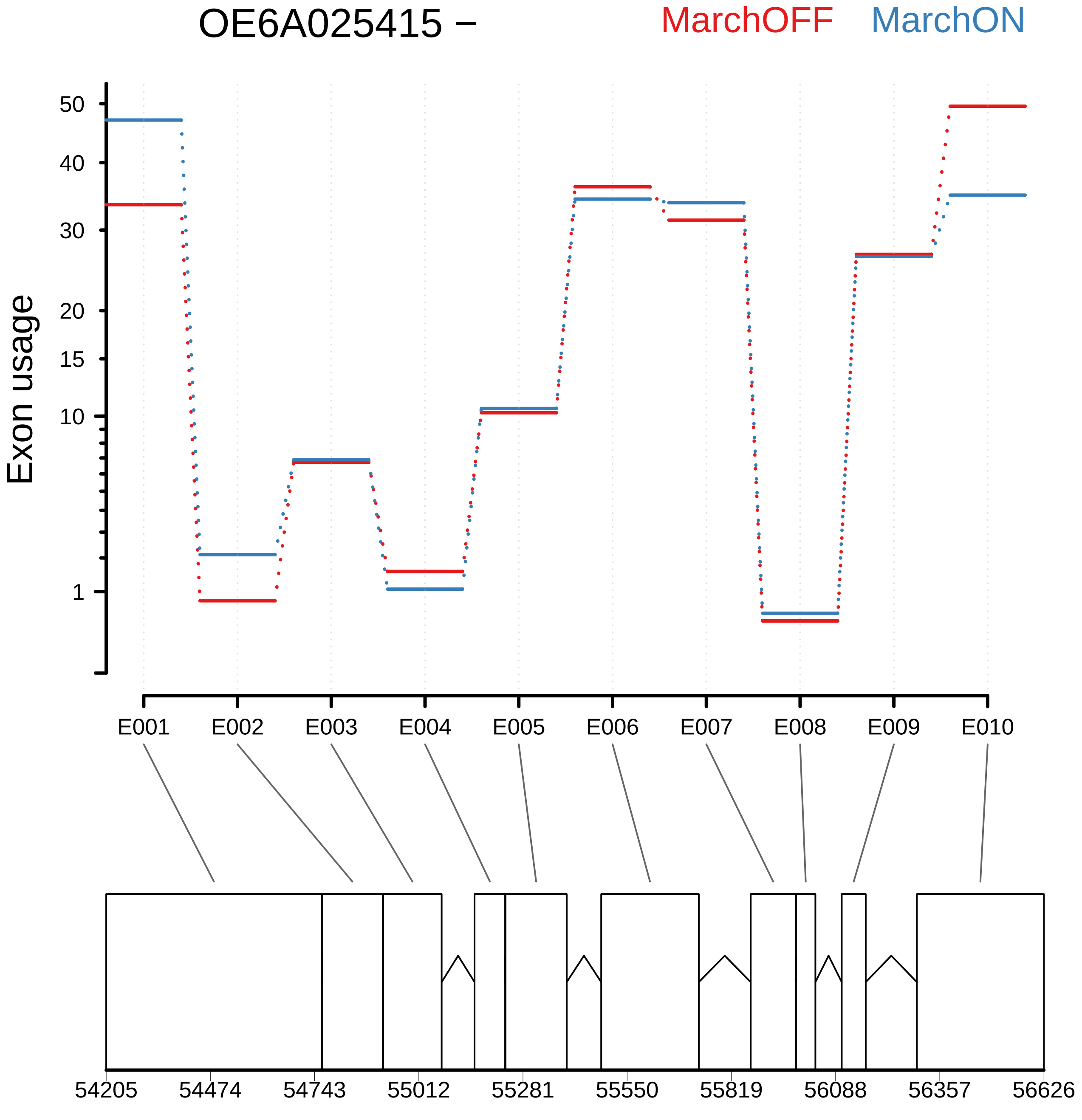

OE6A025415 −
MarchOFF
MarchON
Exon usage
50
40
30
20
15
10
1
E001
E002
E003
E004
E005
E006
E007
E008
E009
E010
54205
54474
54743
55012
55281
55550
55819
56088
56357
56626

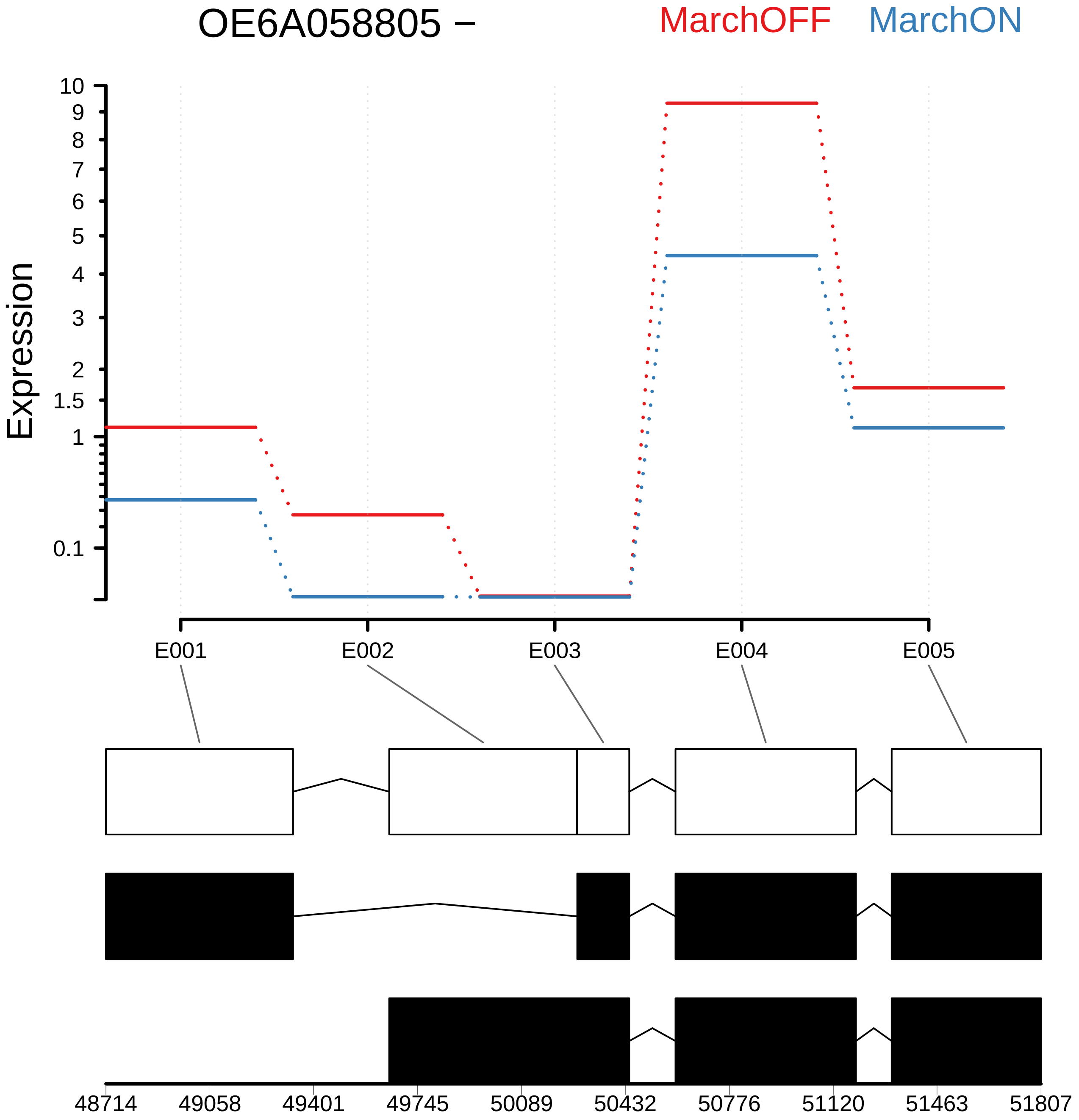

OE6A058805 –
MarchOFF
MarchON
Expression
10
9
8
7
6
5
4
3
2
1.5
1
0.1
E001
E002
E003
E004
E005
48714
49058
49401
49745
50089
50432
50776
51120
51463
51807

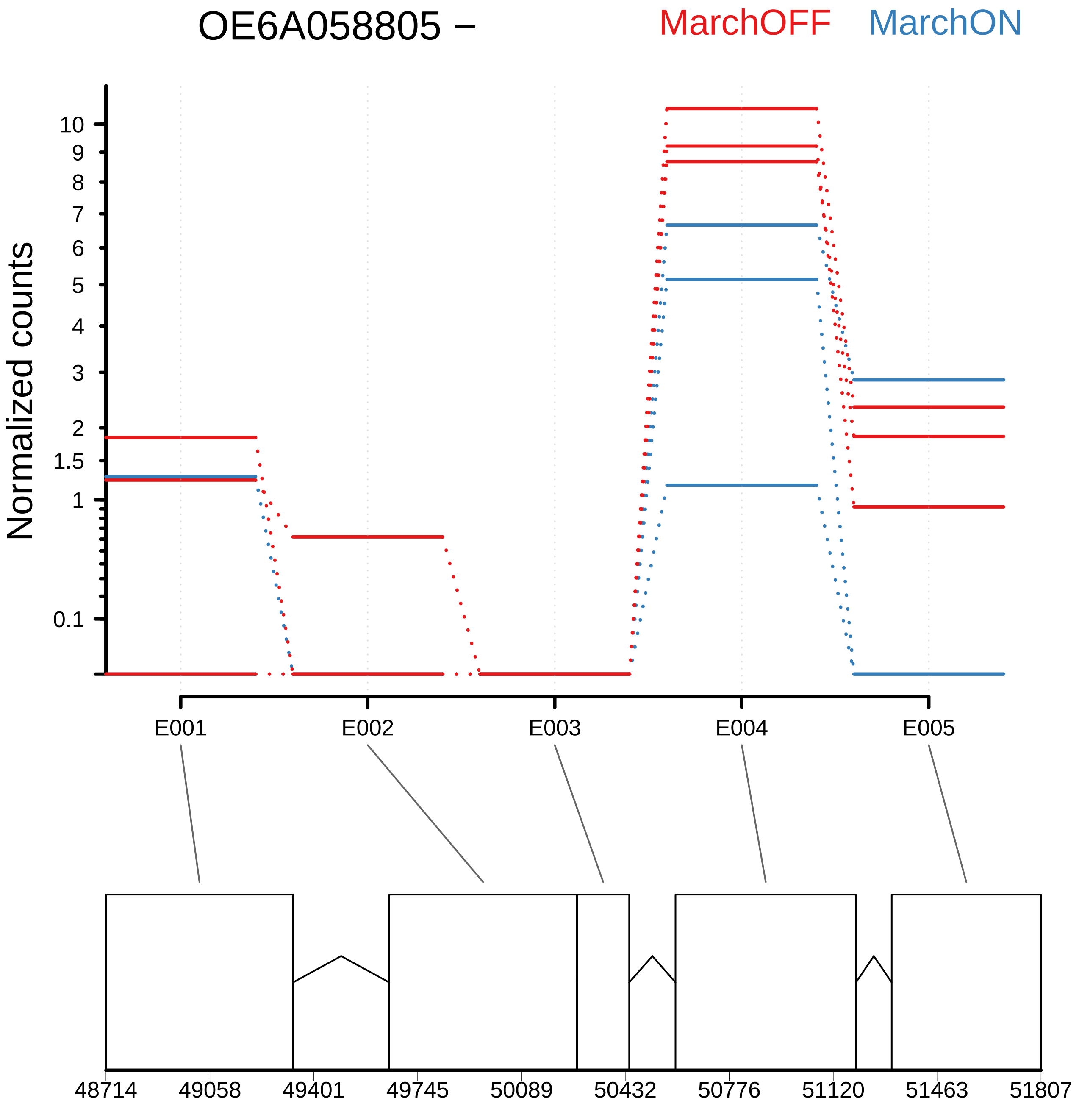
OE6A058805 –
MarchOFF
MarchON
Normalized counts
10
9
8
7
6
5
4
3
2
1.5
1
0.1
E001
E002
E003
E004
E005
48714
49058
49401
49745
50089
50432
50776
51120
51463
51807

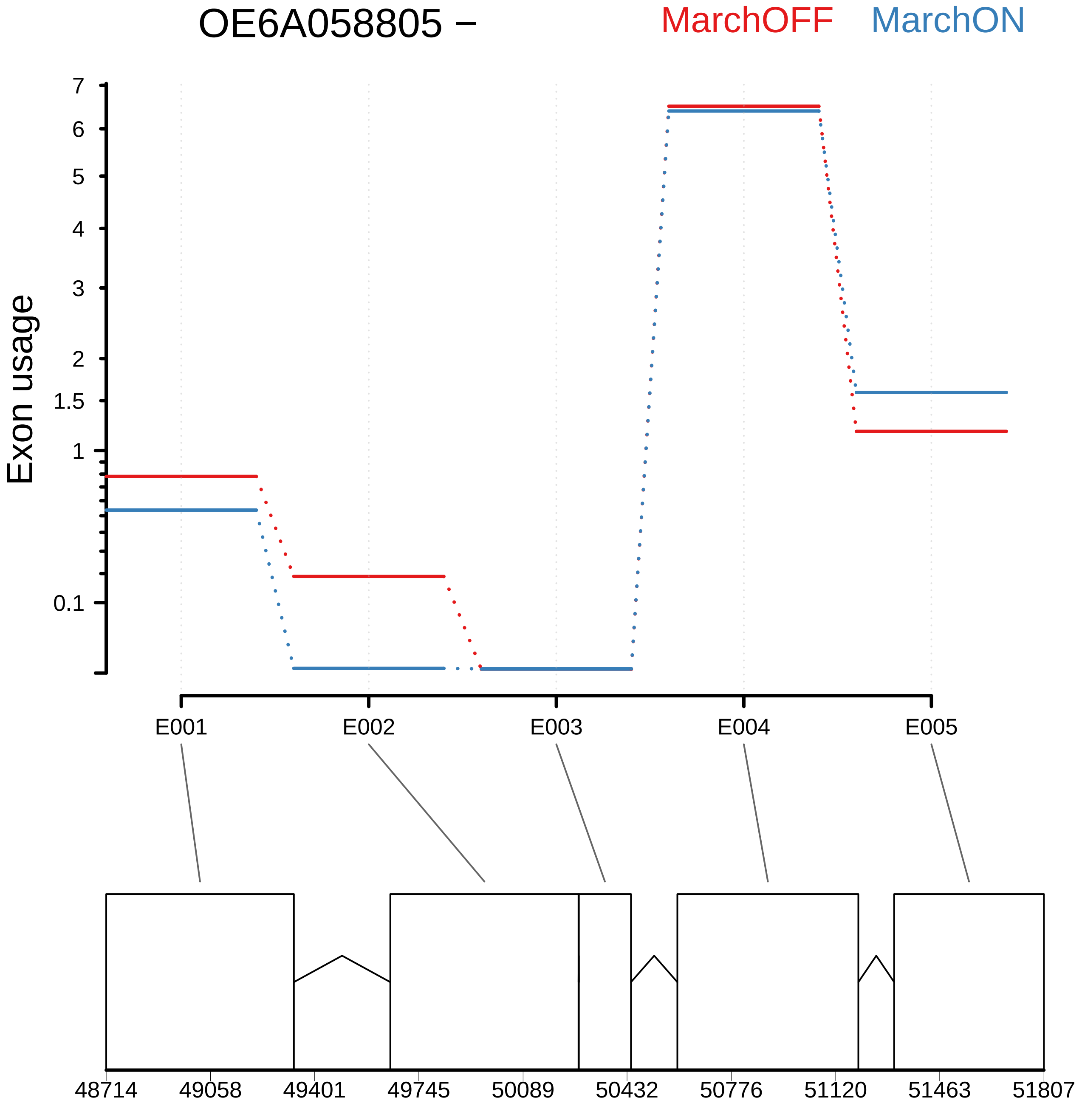

OE6A058805 −
MarchOFF
MarchON
Exon usage
7
6
5
4
3
2
1.5
1
0.1
E001
E002
E003
E004
E005
48714
49058
49401
49745
50089
50432
50776
51120
51463
51807

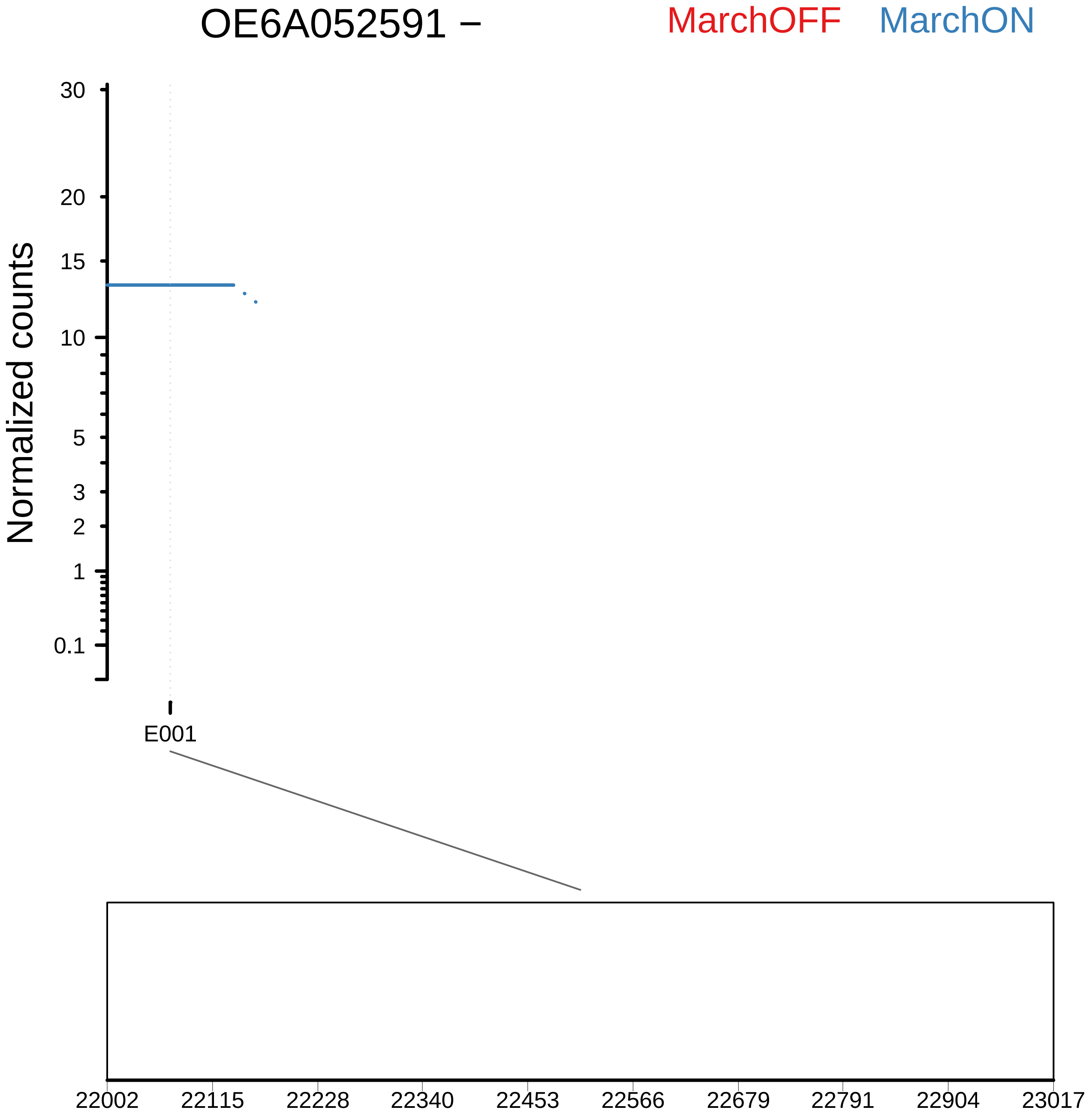

OE6A052591 −
MarchOFF
MarchON
Normalized counts
30
20
15
10
5
3
2
1
0.1
E001
22002
22115
22228
22340
22453
22566
22679
22791
22904
23017

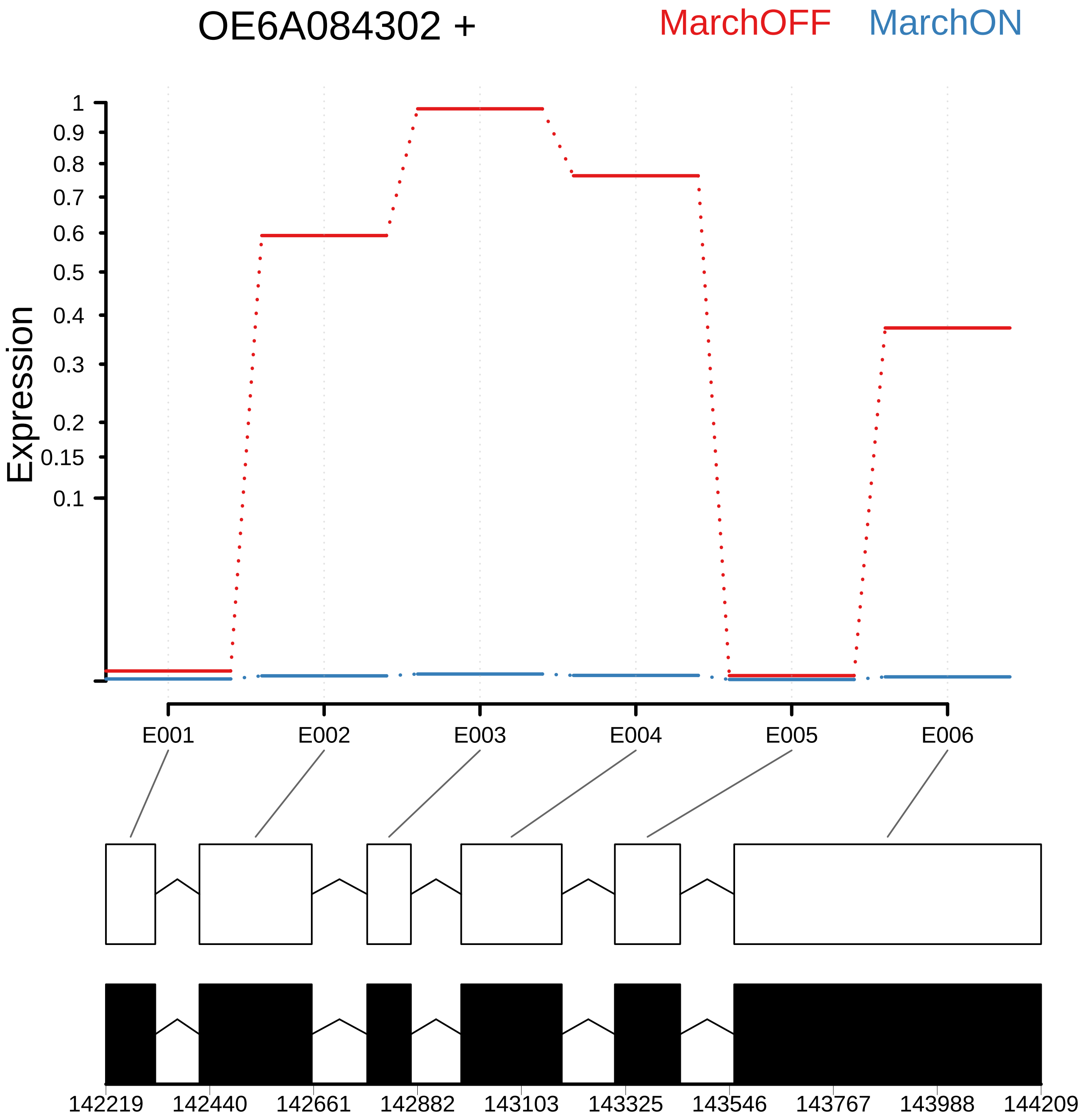

OE6A084302 +
MarchOFF
MarchON
Expression
1
0.9
0.8
0.7
0.6
0.5
0.4
0.3
0.2
0.15
0.1
E001
E002
E003
E004
E005
E006
142219
142440
142661
142882
143103
143325
143546
143767
143988
144209

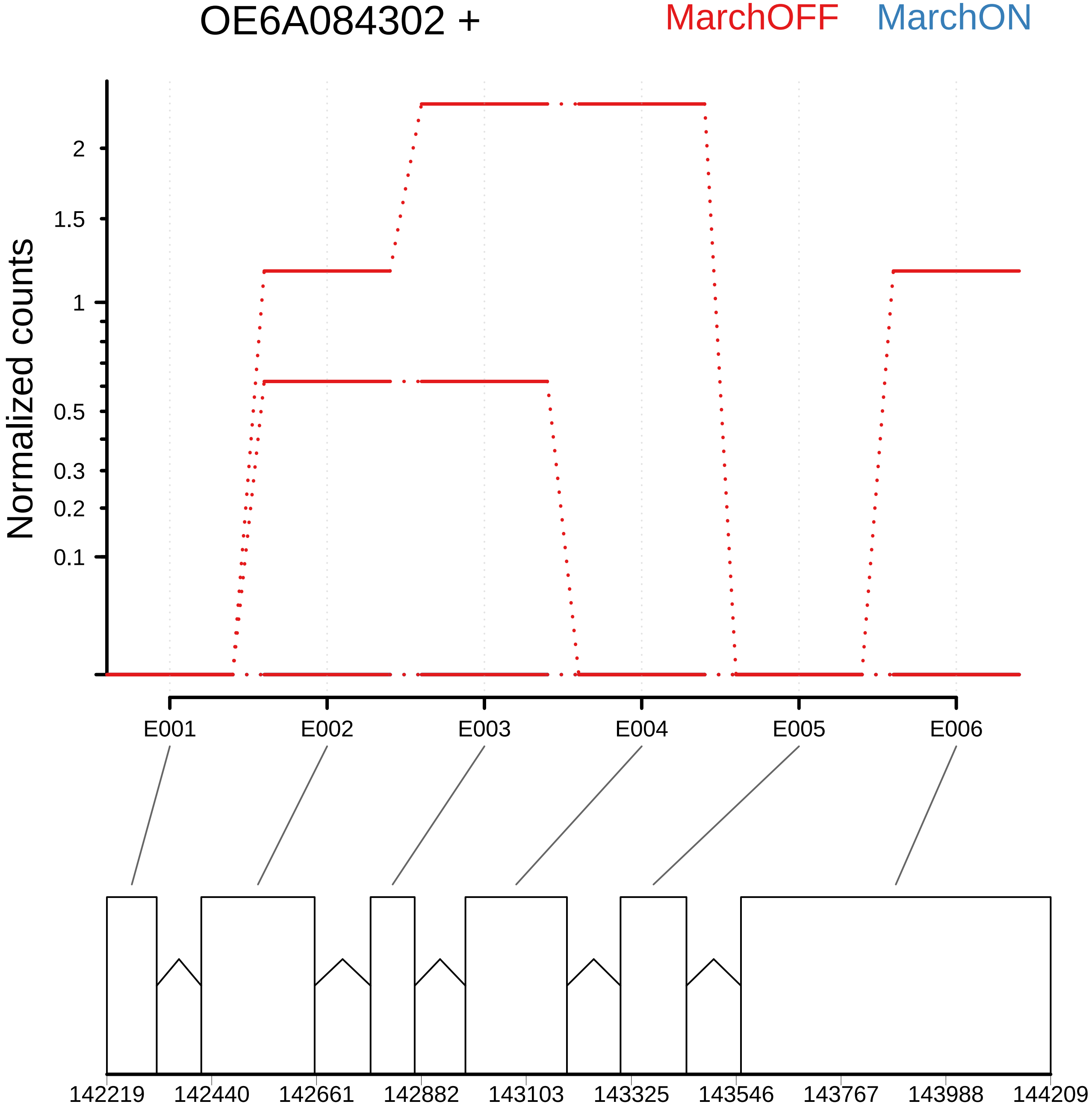
OE6A084302 +
MarchOFF
MarchON
Normalized counts
2
1.5
1
0.5
0.3
0.2
0.1
E001
E002
E003
E004
E005
E006
142219
142440
142661
142882
143103
143325
143546
143767
143988
144209

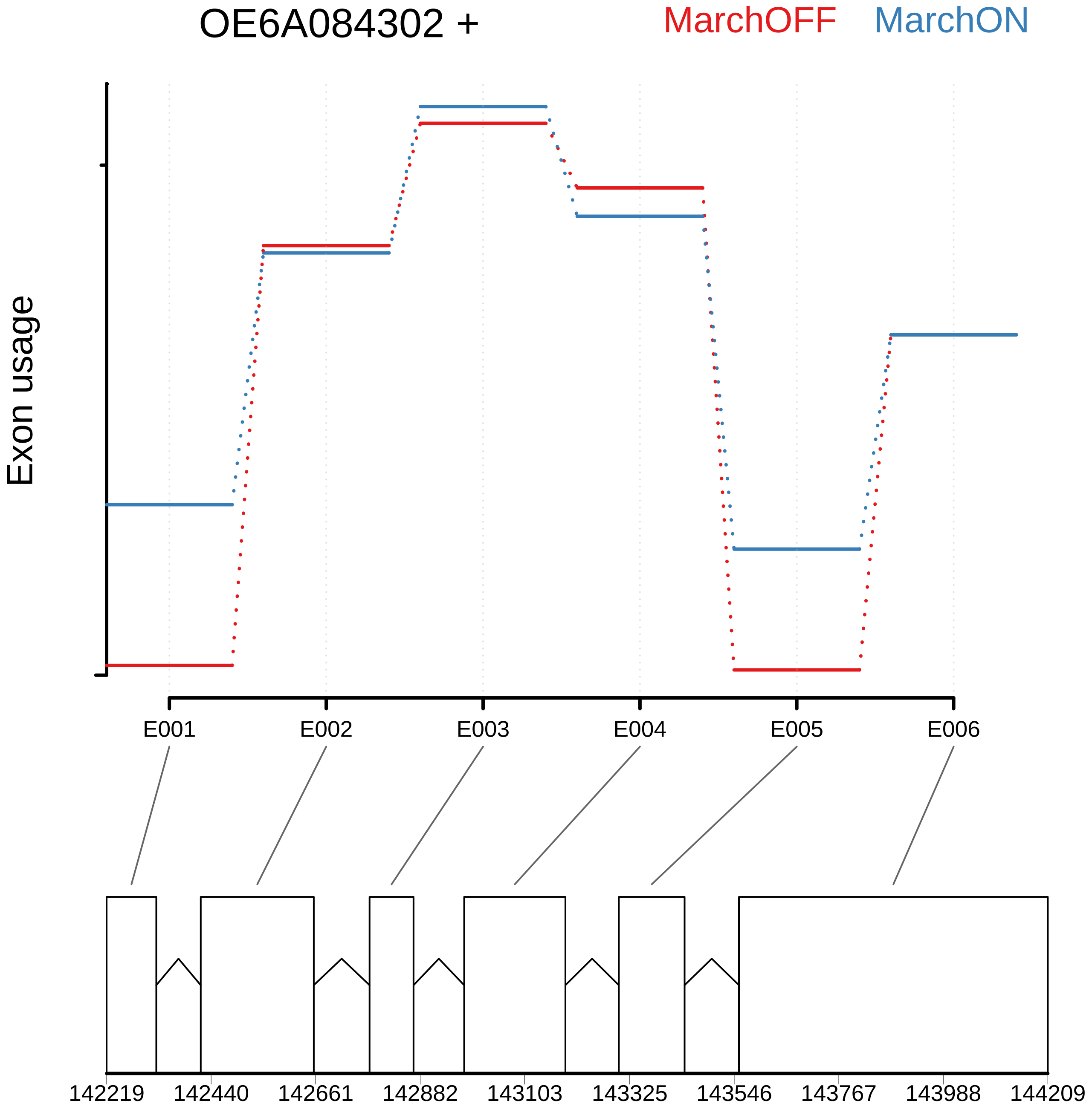

OE6A084302 +
MarchOFF
MarchON
Exon usage
E001
E002
E003
E004
E005
E006
142219
142440
142661
142882
143103
143325
143546
143767
143988
144209

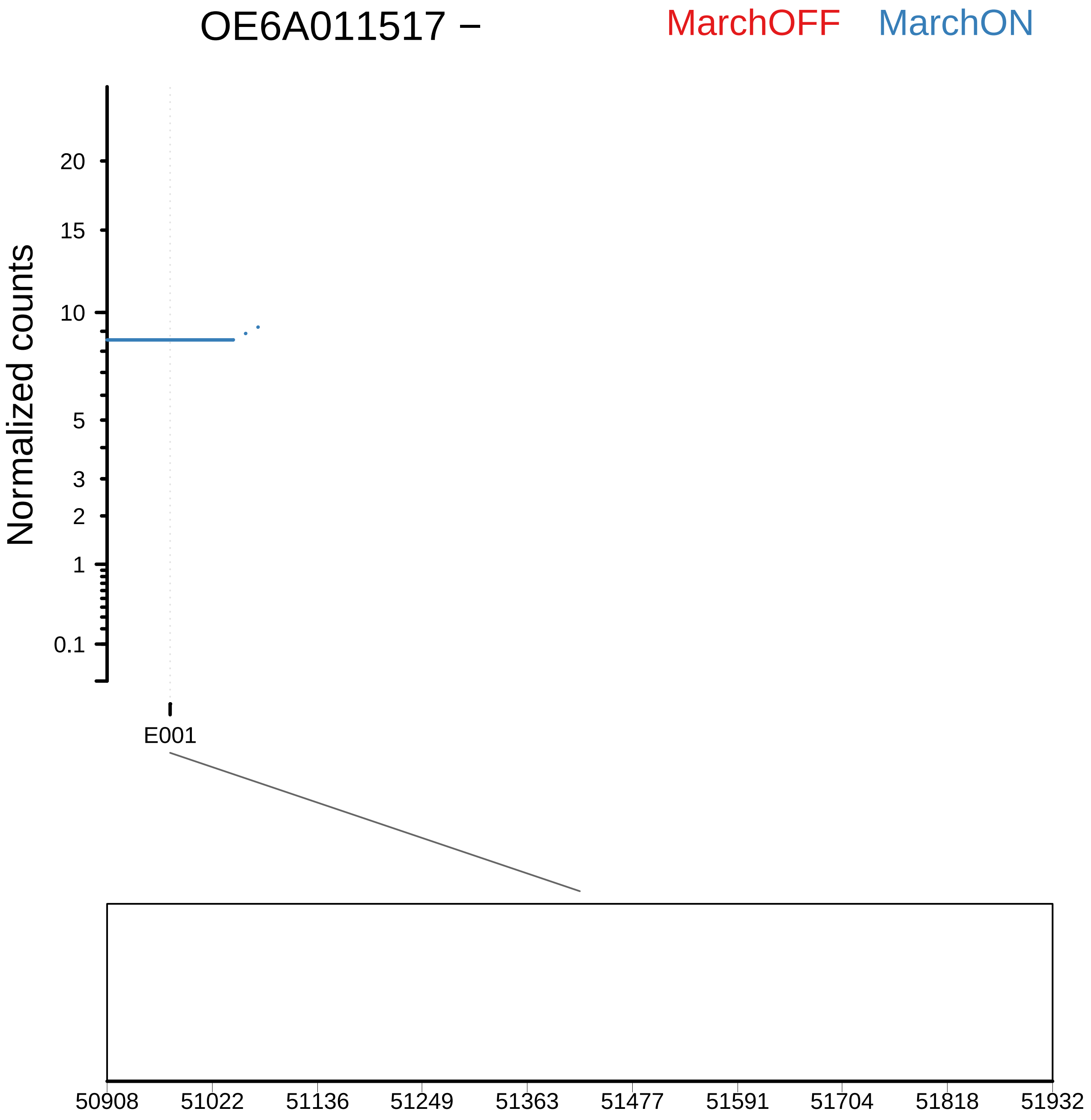
OE6A011517 −
MarchOFF
MarchON
Normalized counts
20
15
10
5
3
2
1
0.1
E001
50908
51022
51136
51249
51363
51477
51591
51704
51818
51932

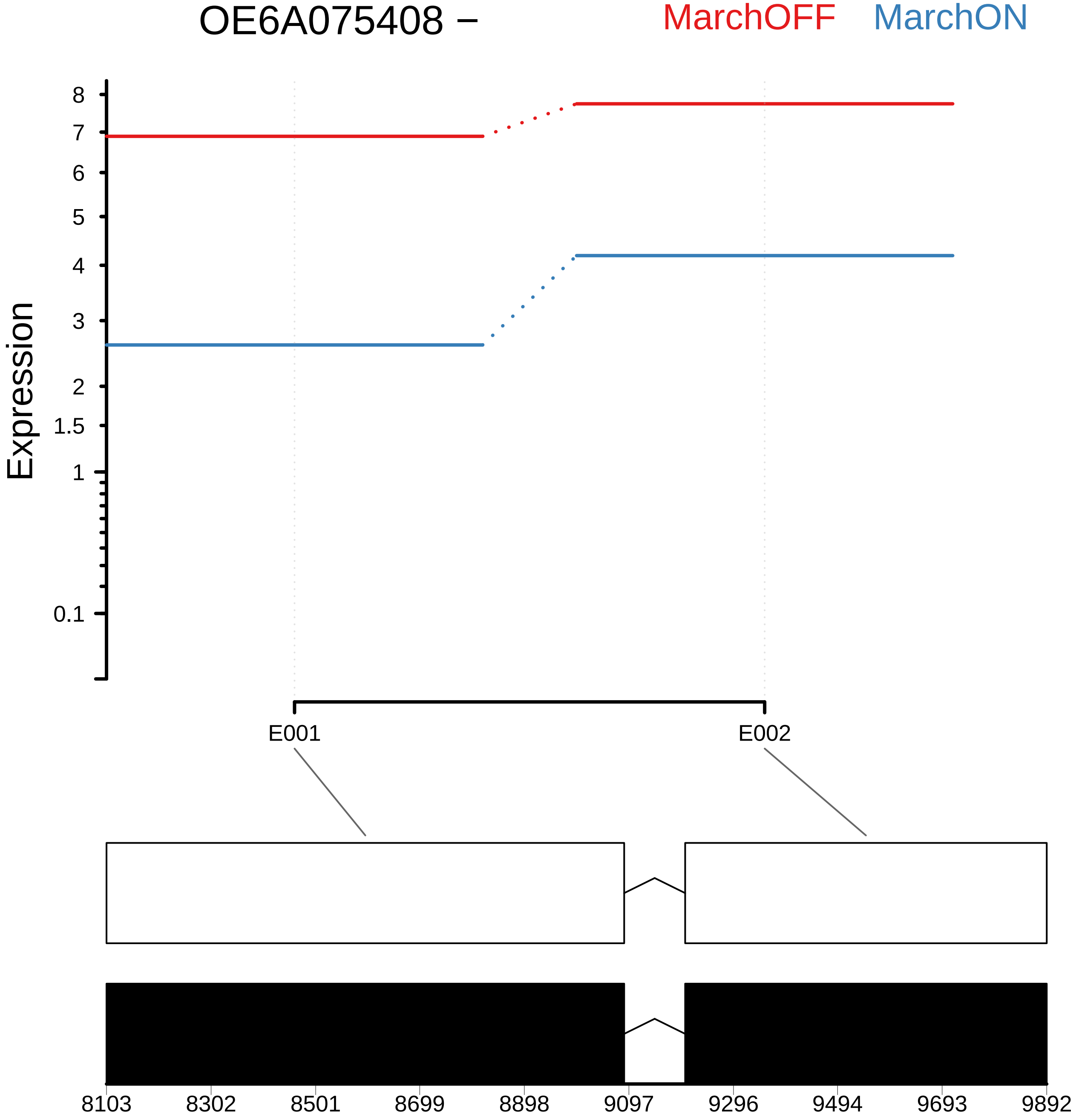

OE6A075408 −
MarchOFF
MarchON
Expression
8
7
6
5
4
3
2
1.5
1
0.1
E001
E002
8103
8302
8501
8699
8898
9097
9296
9494
9693
9892

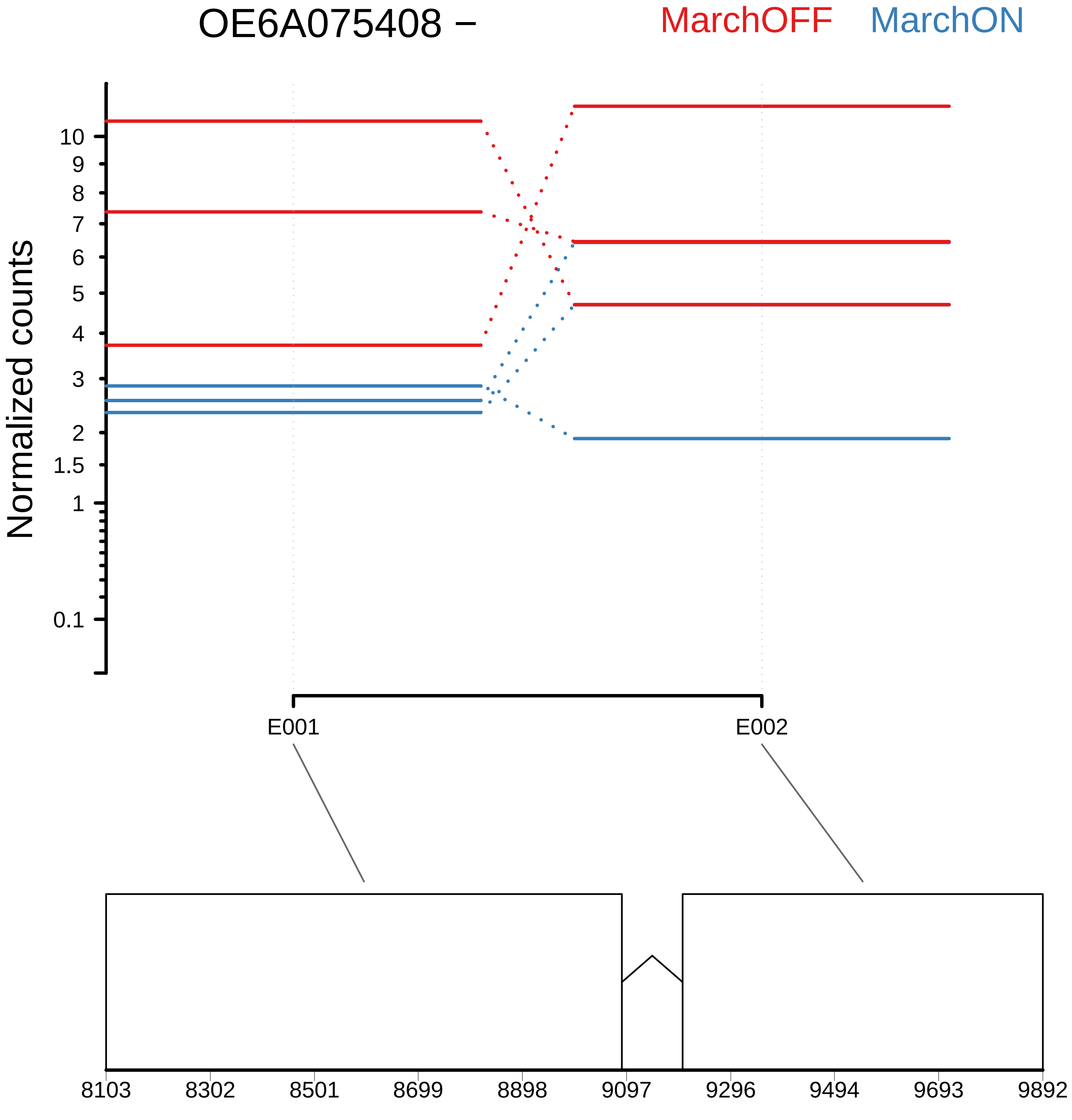
OE6A075408 −
MarchOFF
MarchON
Normalized counts
10
9
8
7
6
5
4
3
2
1.5
1
0.1
E001
E002
8103
8302
8501
8699
8898
9097
9296
9494
9693
9892

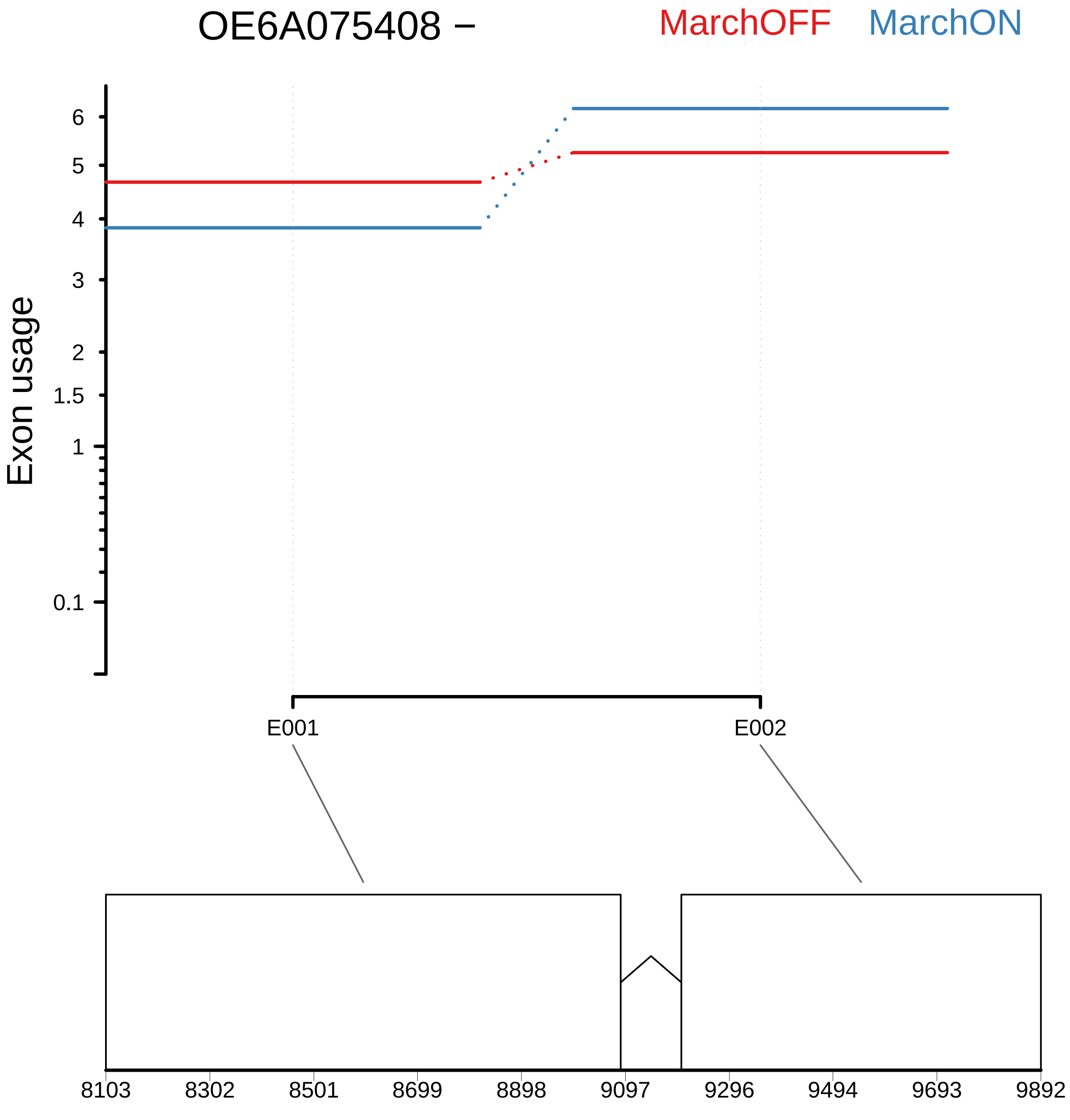

OE6A075408 −
MarchOFF
MarchON
Exon usage
6
5
4
3
2
1.5
1
0.1
E001
E002
8103
8302
8501
8699
8898
9097
9296
9494
9693
9892

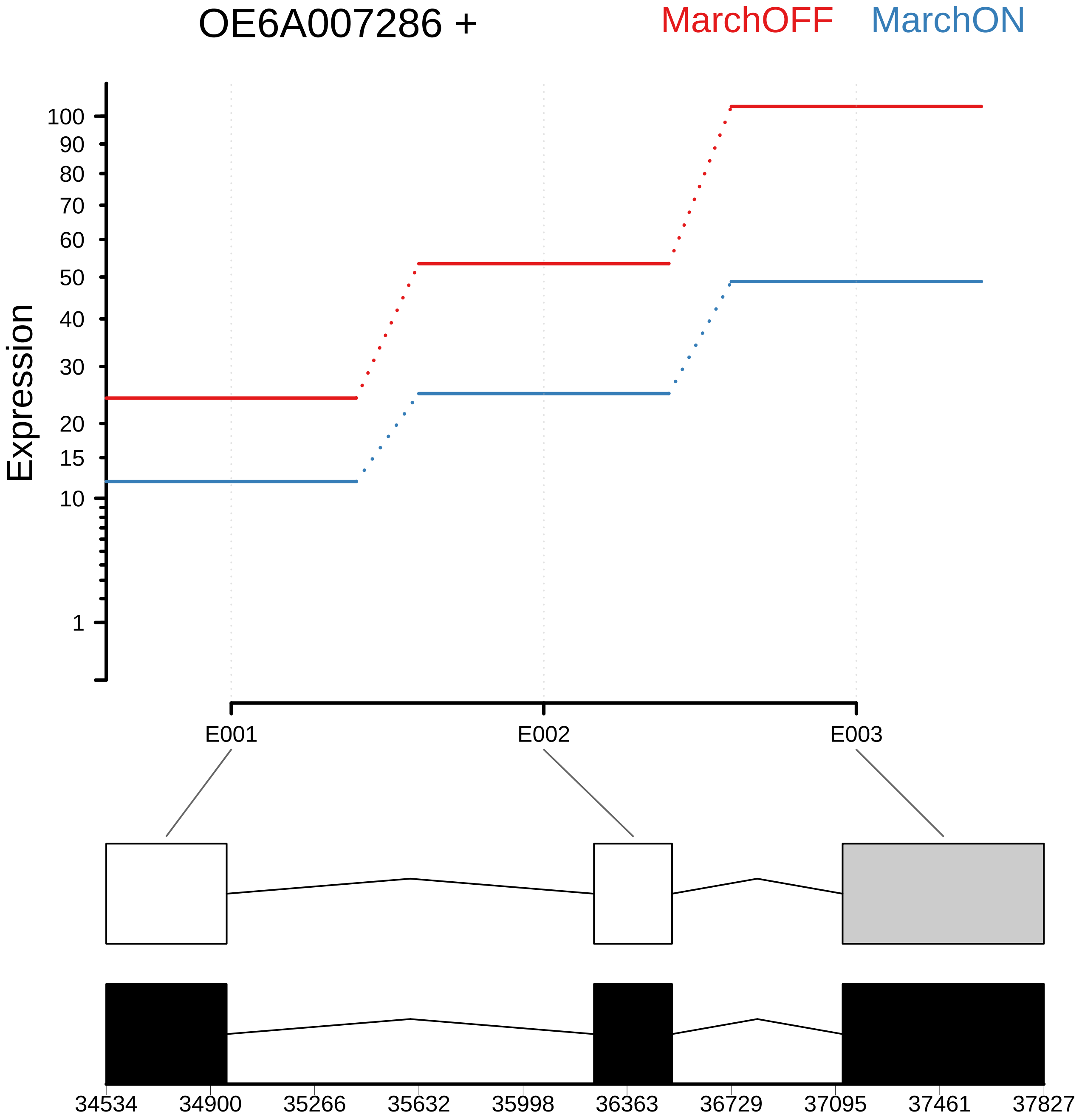
OE6A007286 +
MarchOFF
MarchON
Expression
100
90
80
70
60
50
40
30
20
15
10
1
E001
E002
E003
34534
34900
35266
35632
35998
36363
36729
37095
37461
37827

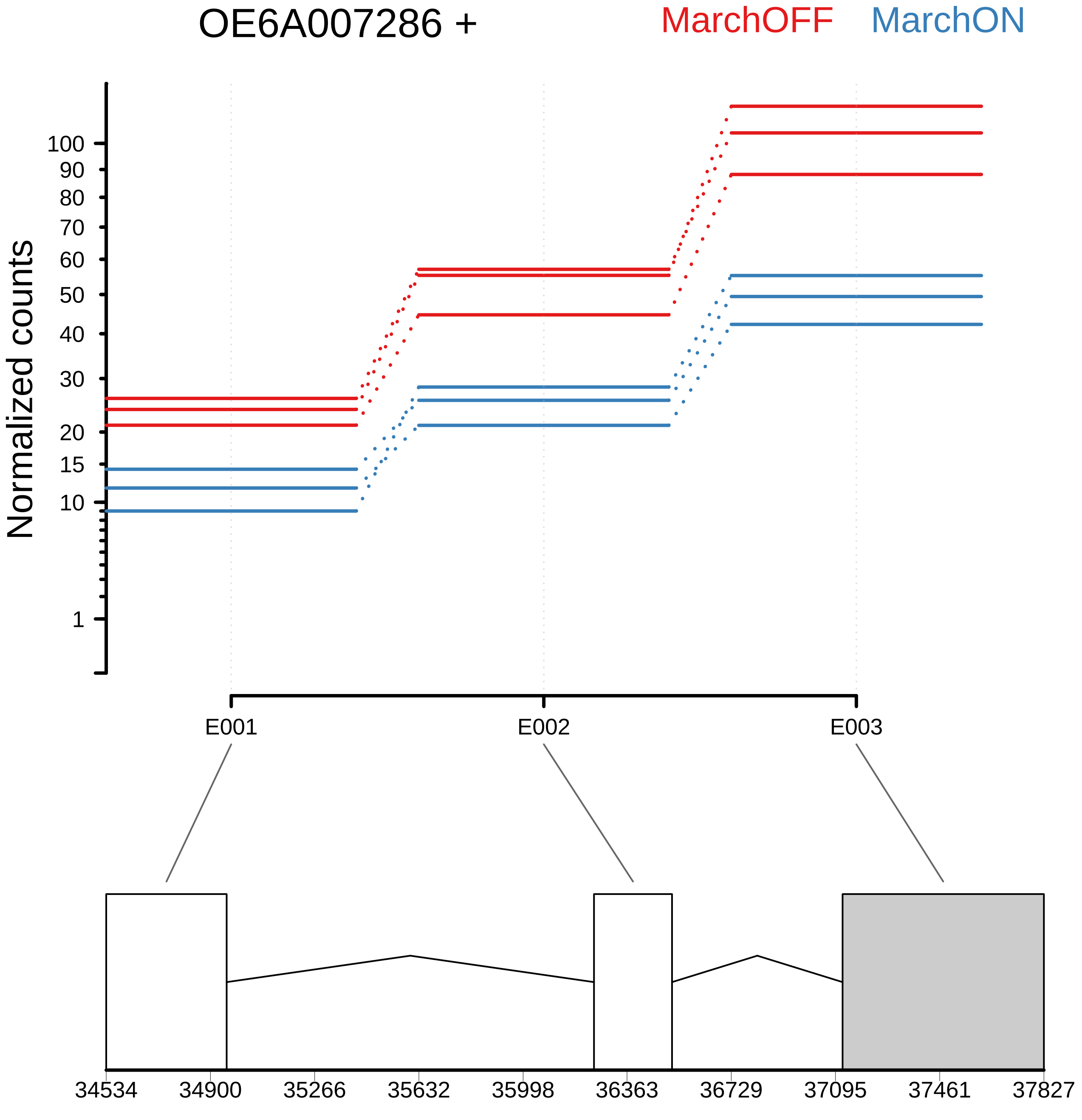
OE6A007286 +
MarchOFF
MarchON
Normalized counts
100
90
80
70
60
50
40
30
20
15
10
1
E001
E002
E003
34534
34900
35266
35632
35998
36363
36729
37095
37461
37827

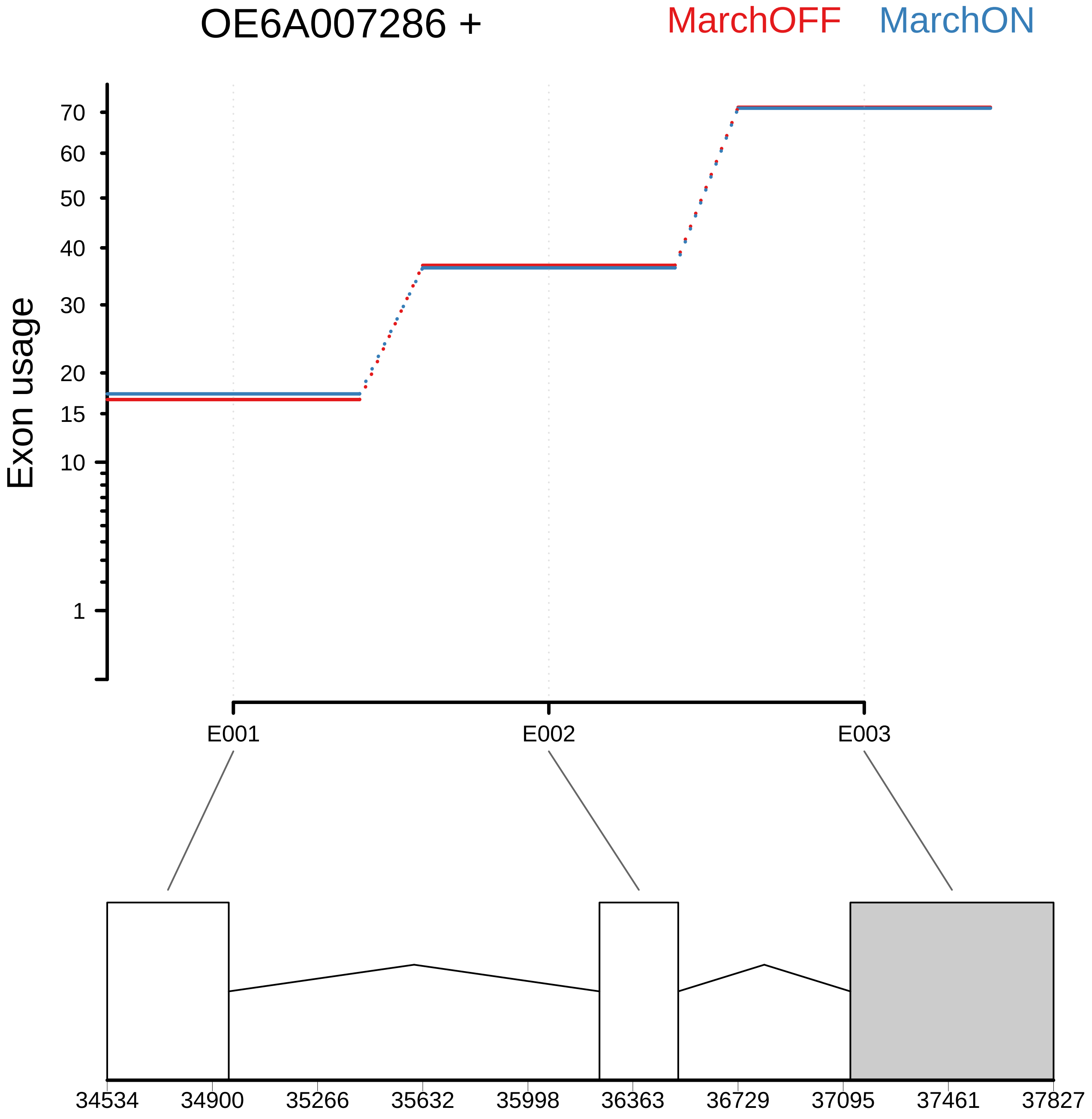

OE6A007286 +
MarchOFF
MarchON
Exon usage
70
60
50
40
30
20
15
10
1
E001
E002
E003
34534
34900
35266
35632
35998
36363
36729
37095
37461
37827

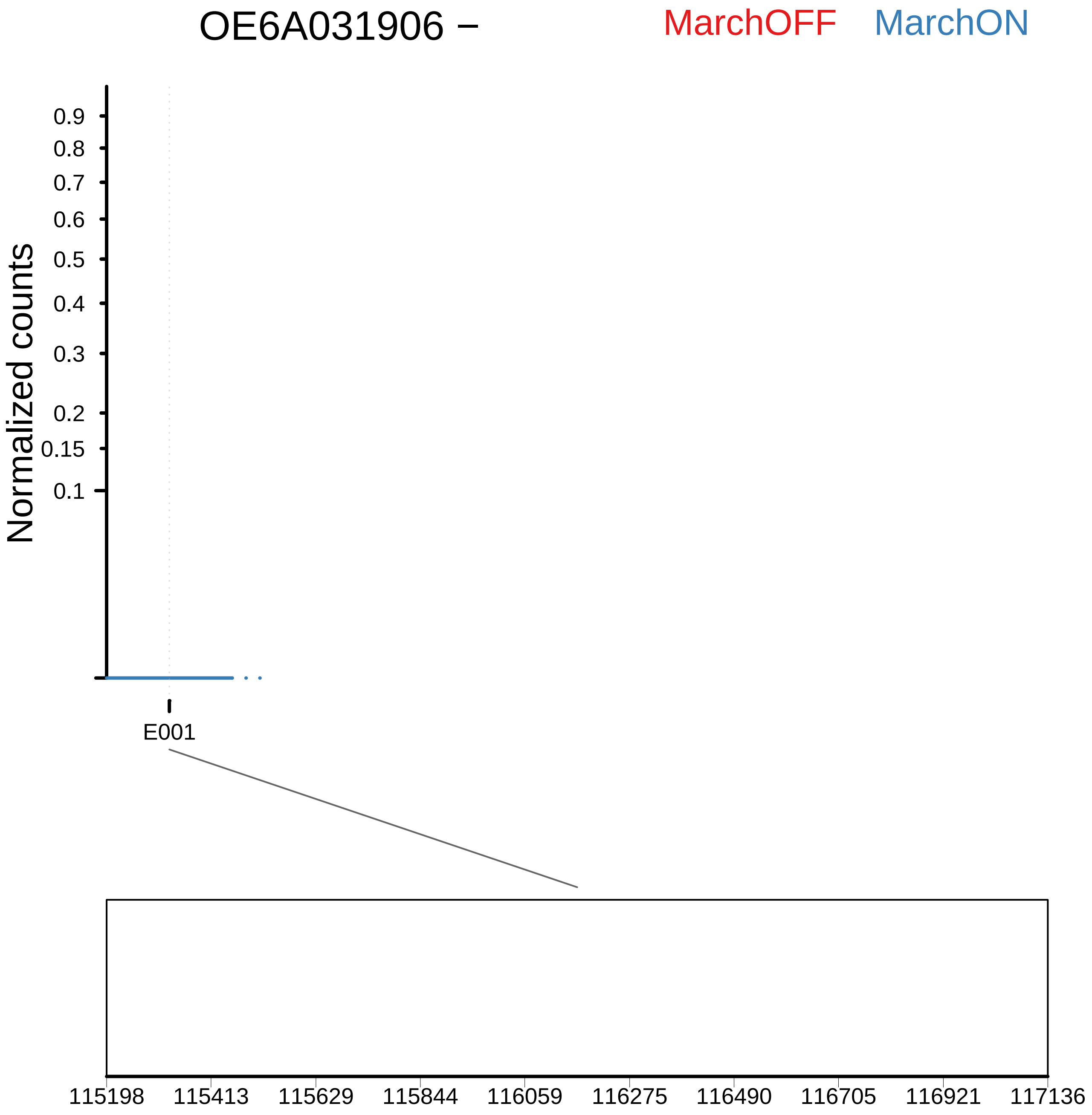

OE6A031906 −
MarchOFF
MarchON
Normalized counts
0.9
0.8
0.7
0.6
0.5
0.4
0.3
0.2
0.15
0.1
E001
115198
115413
115629
115844
116059
116275
116490
116705
116921
117136

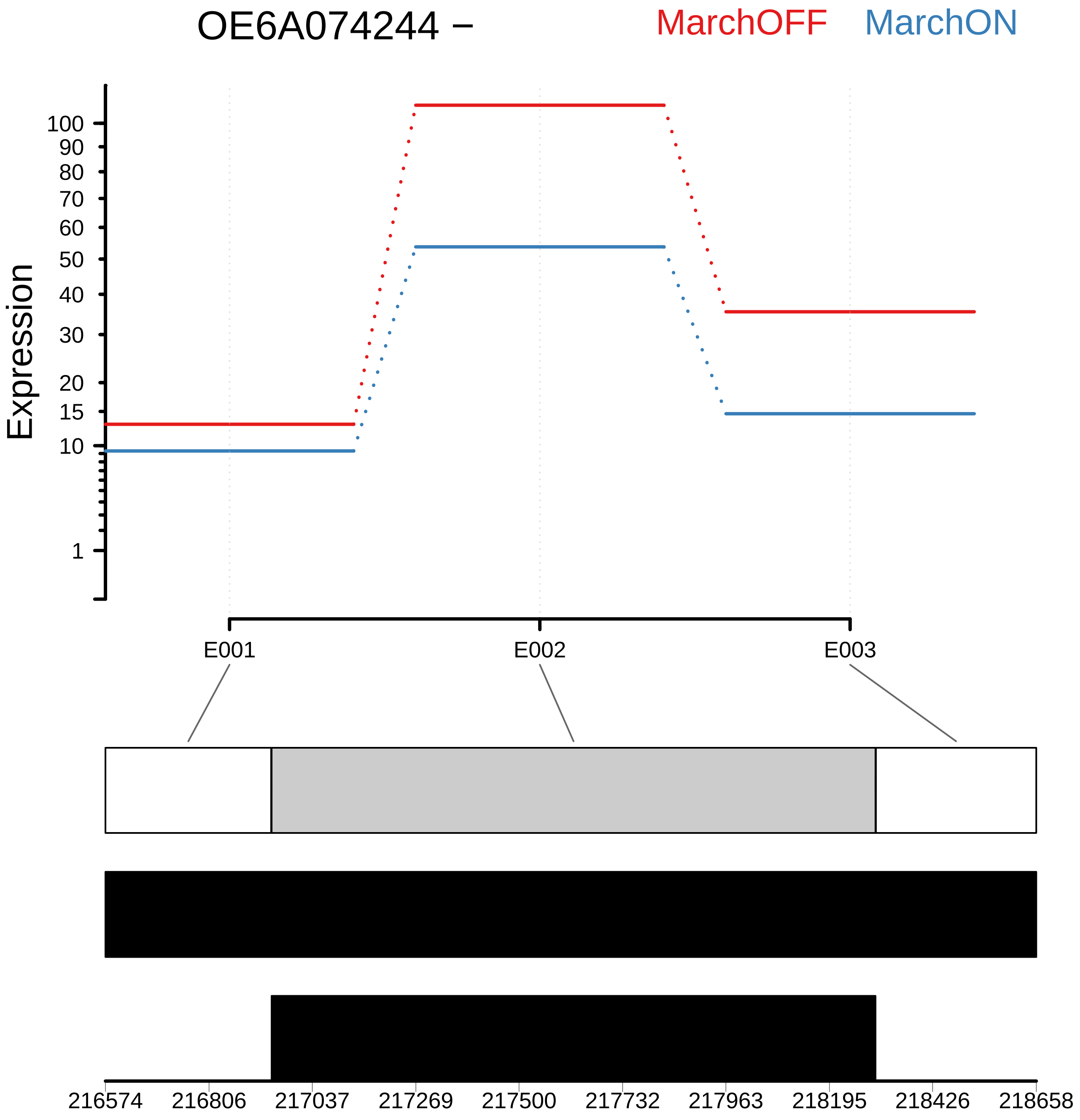

OE6A074244 −
MarchOFF
MarchON
Expression
100
90
80
70
60
50
40
30
20
15
10
1
E001
E002
E003
216574
216806
217037
217269
217500
217732
217963
218195
218426
218658

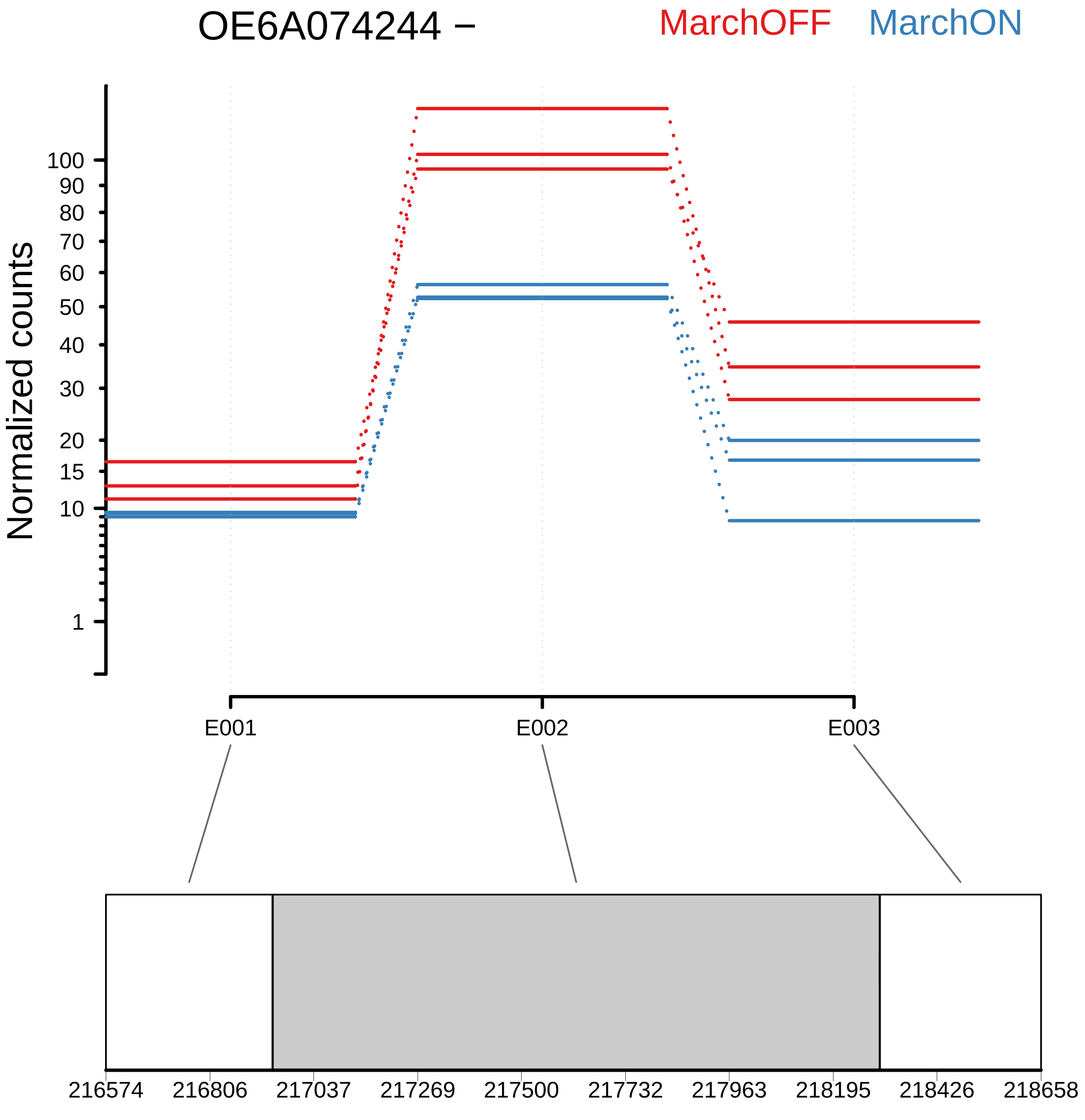

OE6A074244 −
MarchOFF
MarchON
Normalized counts
100
90
80
70
60
50
40
30
20
15
10
1
E001
E002
E003
216574
216806
217037
217269
217500
217732
217963
218195
218426
218658

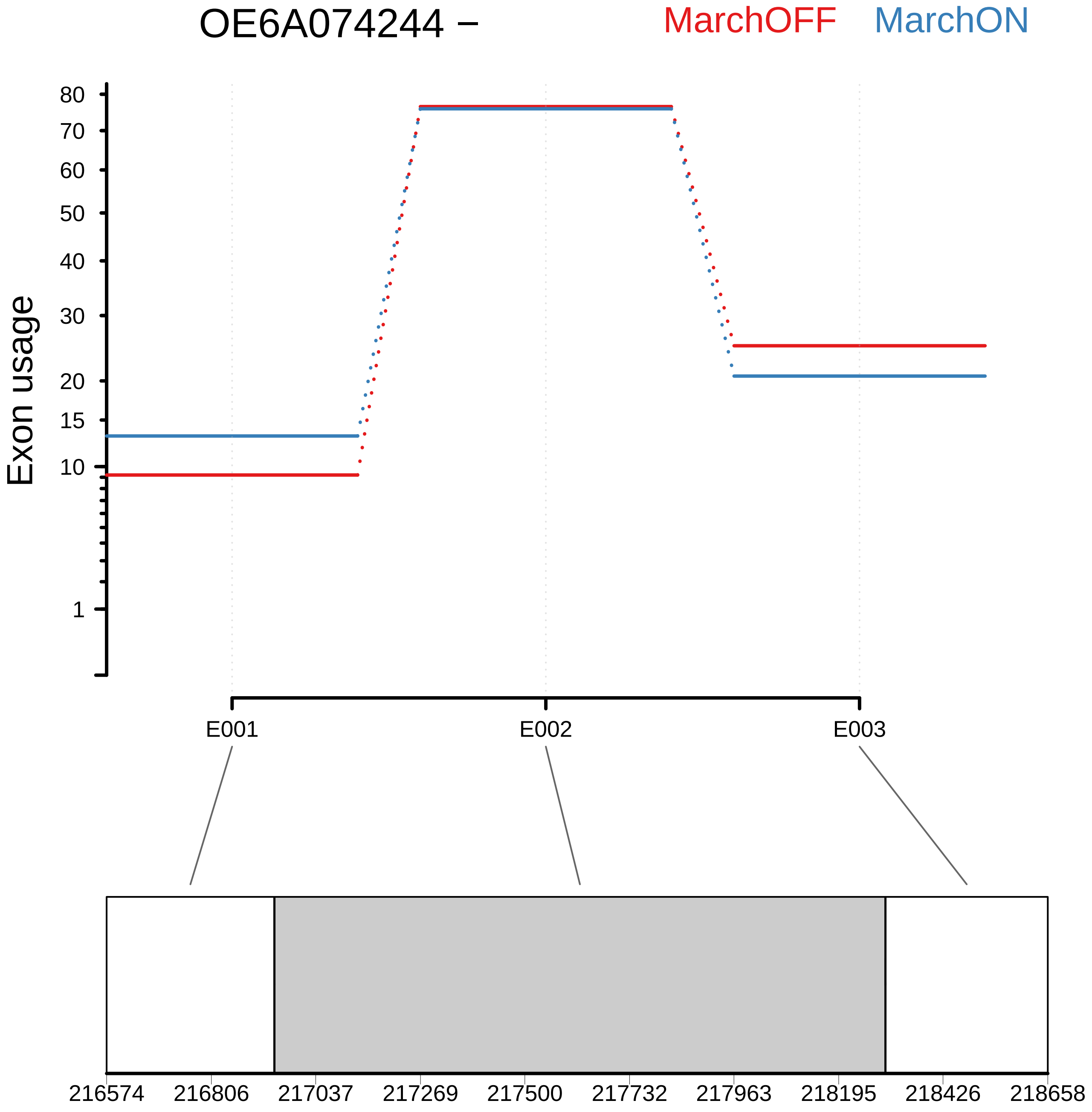
OE6A074244 −
MarchOFF
MarchON
Exon usage
80
70
60
50
40
30
20
15
10
1
E001
E002
E003
216574
216806
217037
217269
217500
217732
217963
218195
218426
218658

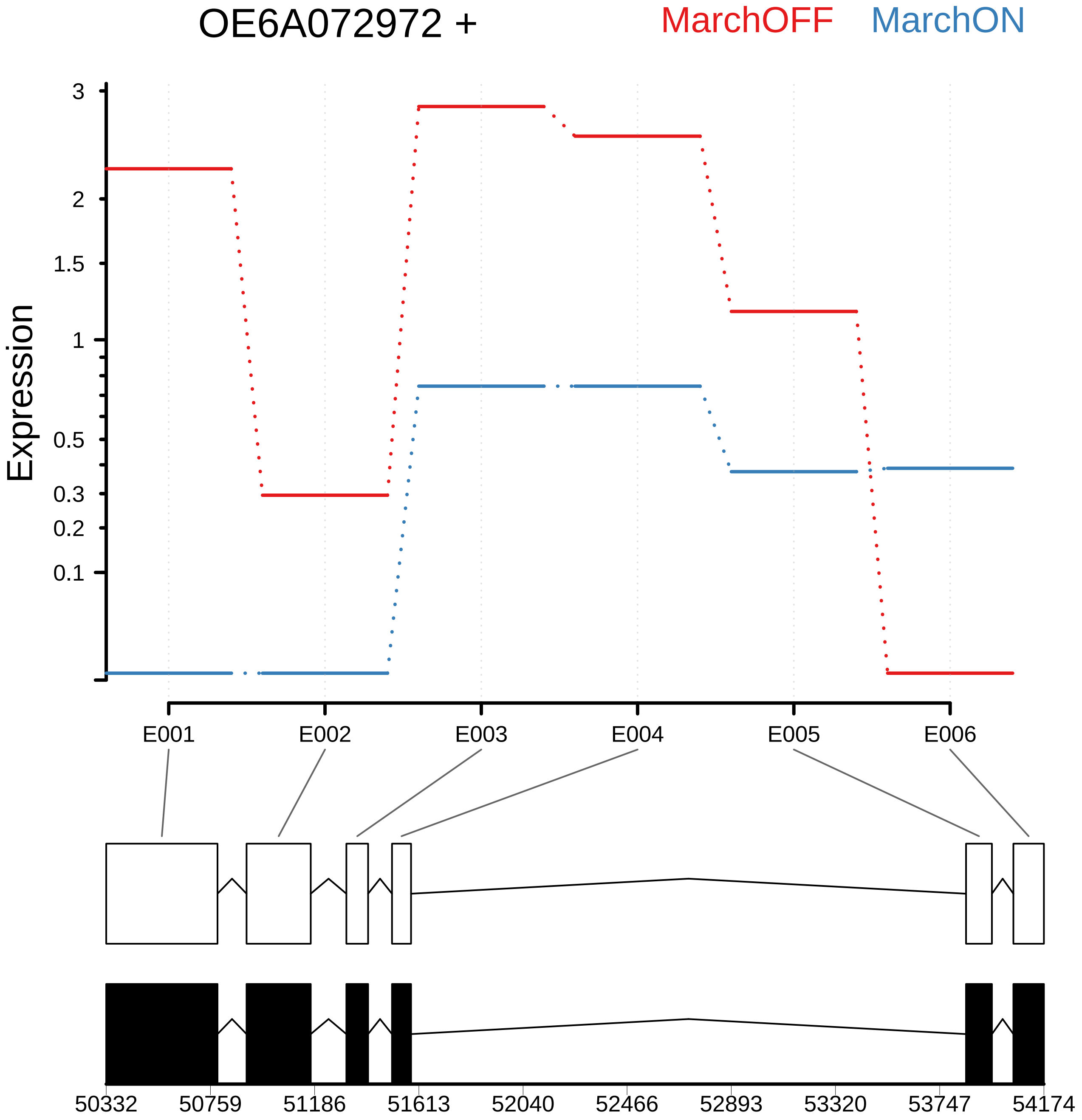
OE6A072972 +
MarchOFF
MarchON
Expression
3
2
1.5
1
0.5
0.3
0.2
0.1
E001
E002
E003
E004
E005
E006
50332
50759
51186
51613
52040
52466
52893
53320
53747
54174

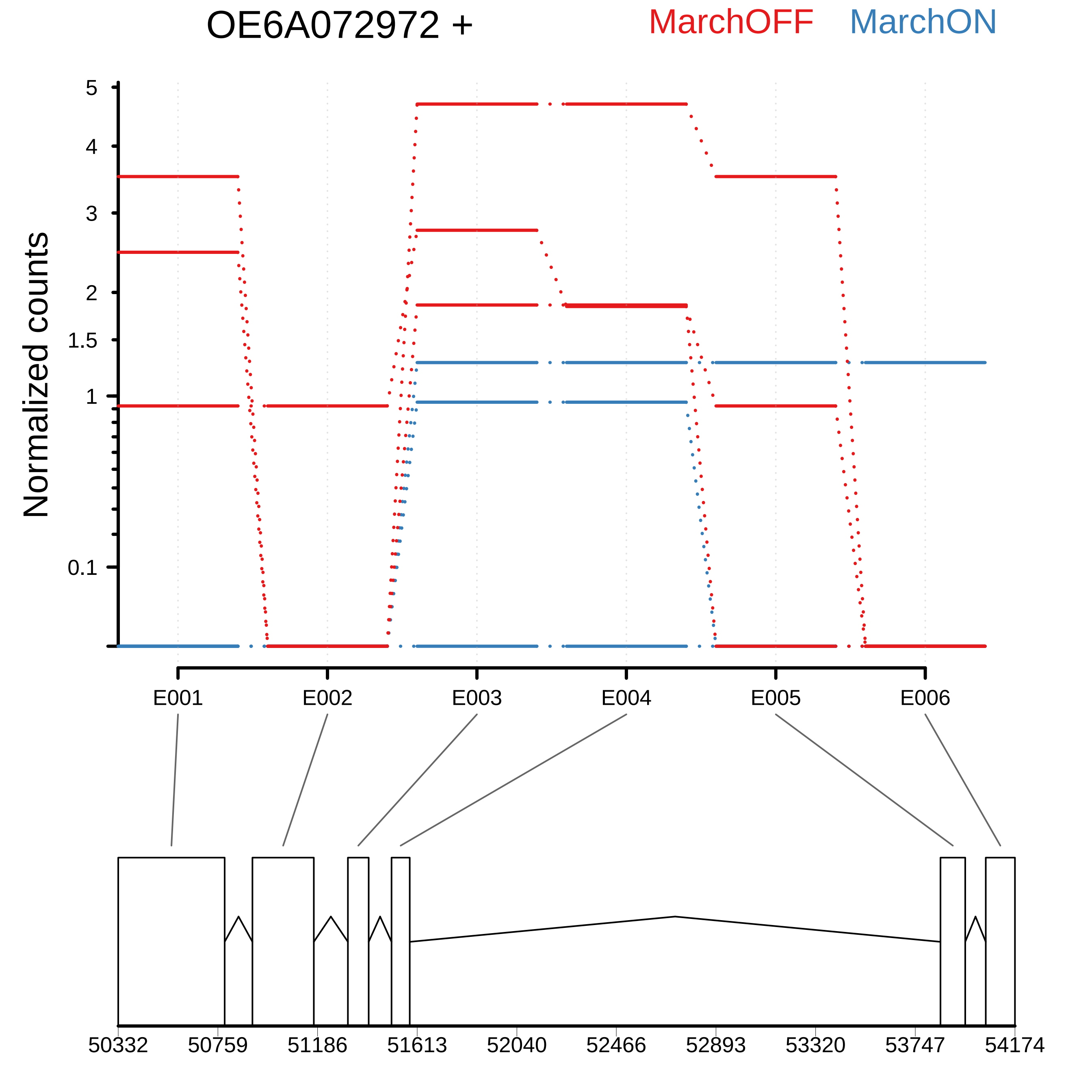
OE6A072972 +
MarchOFF
MarchON
Normalized counts
5
4
3
2
1.5
1
0.1
E001
E002
E003
E004
E005
E006
50332
50759
51186
51613
52040
52466
52893
53320
53747
54174

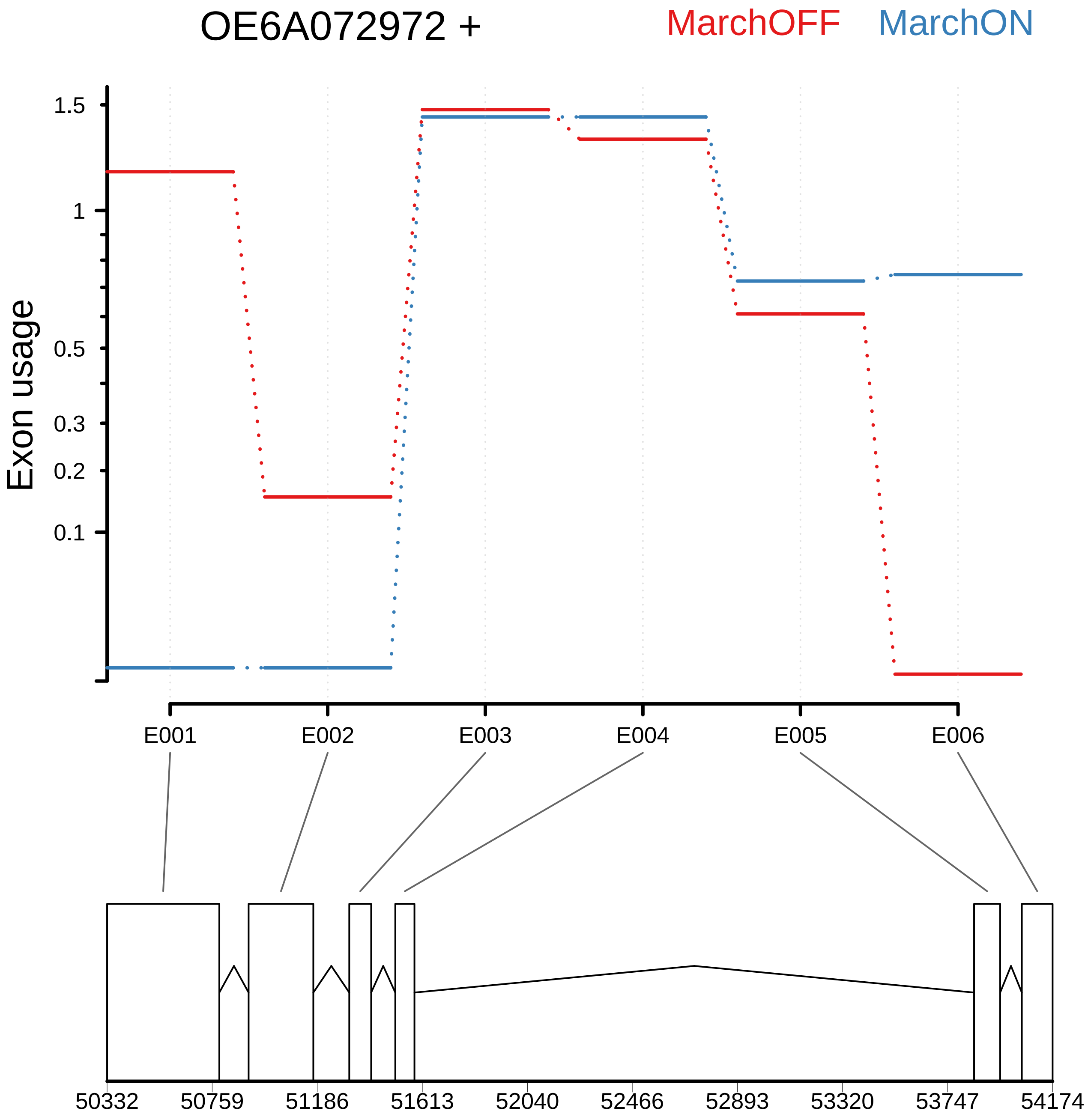
OE6A072972 +
MarchOFF
MarchON
Exon usage
1.5
1
0.5
0.3
0.2
0.1
E001
E002
E003
E004
E005
E006
50332
50759
51186
51613
52040
52466
52893
53320
53747
54174

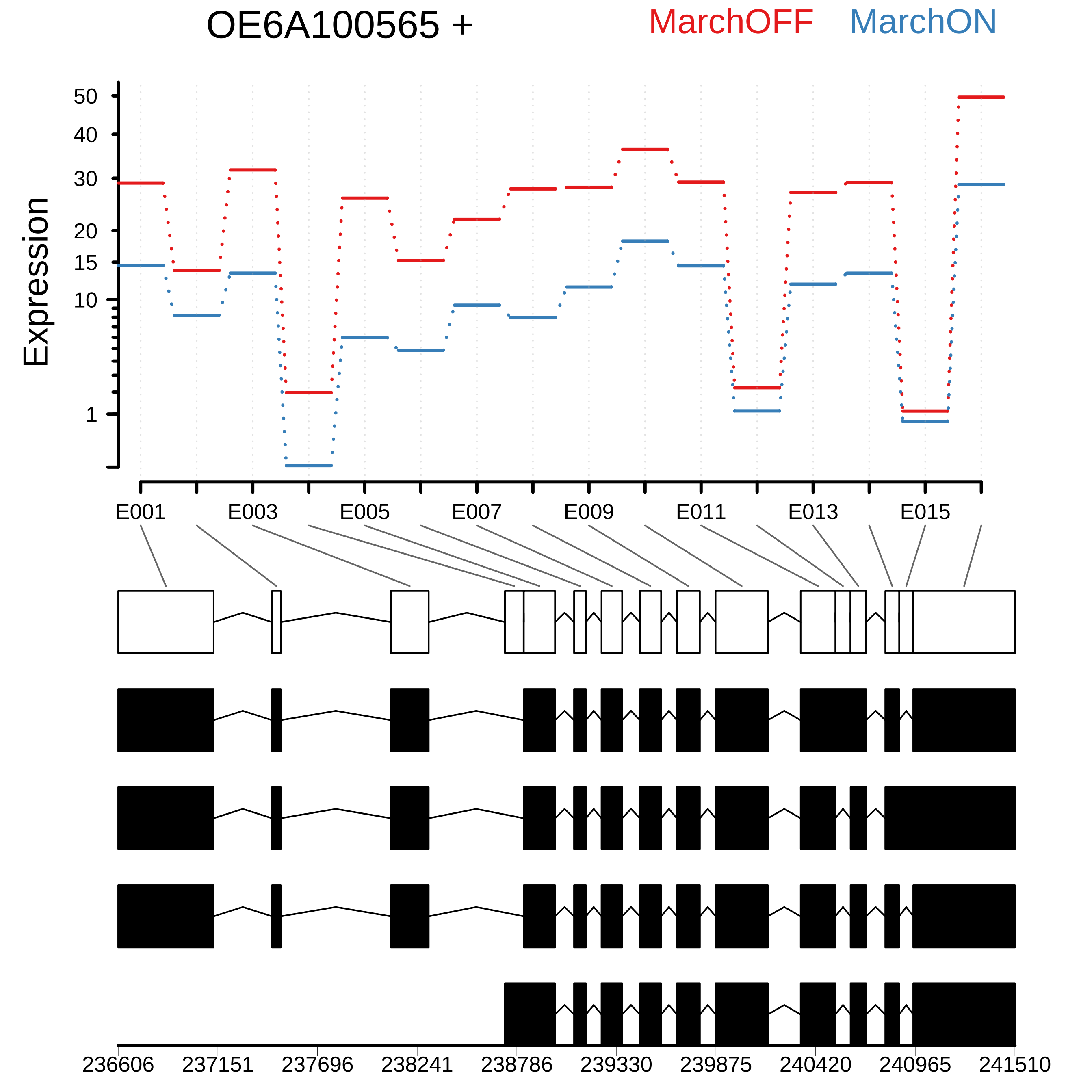

OE6A100565 +
MarchOFF
MarchON
Expression
50
40
30
20
15
10
1
E001
E003
E005
E007
E009
E011
E013
E015
236606
237151
237696
238241
238786
239330
239875
240420
240965
241510

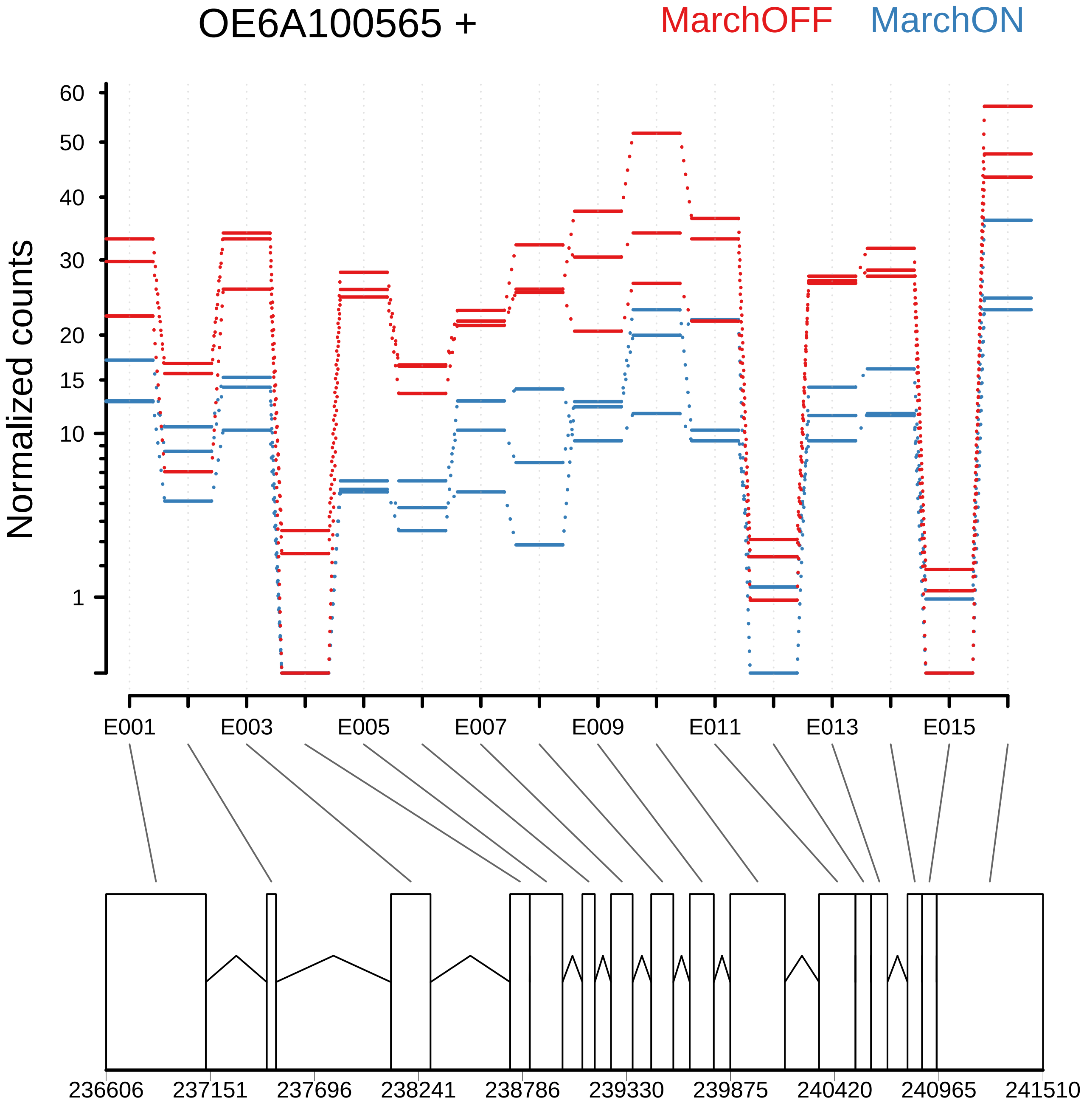

OE6A100565 +
MarchOFF
MarchON
Normalized counts
60
50
40
30
20
15
10
1
E001
E003
E005
E007
E009
E011
E013
E015
236606
237151
237696
238241
238786
239330
239875
240420
240965
241510

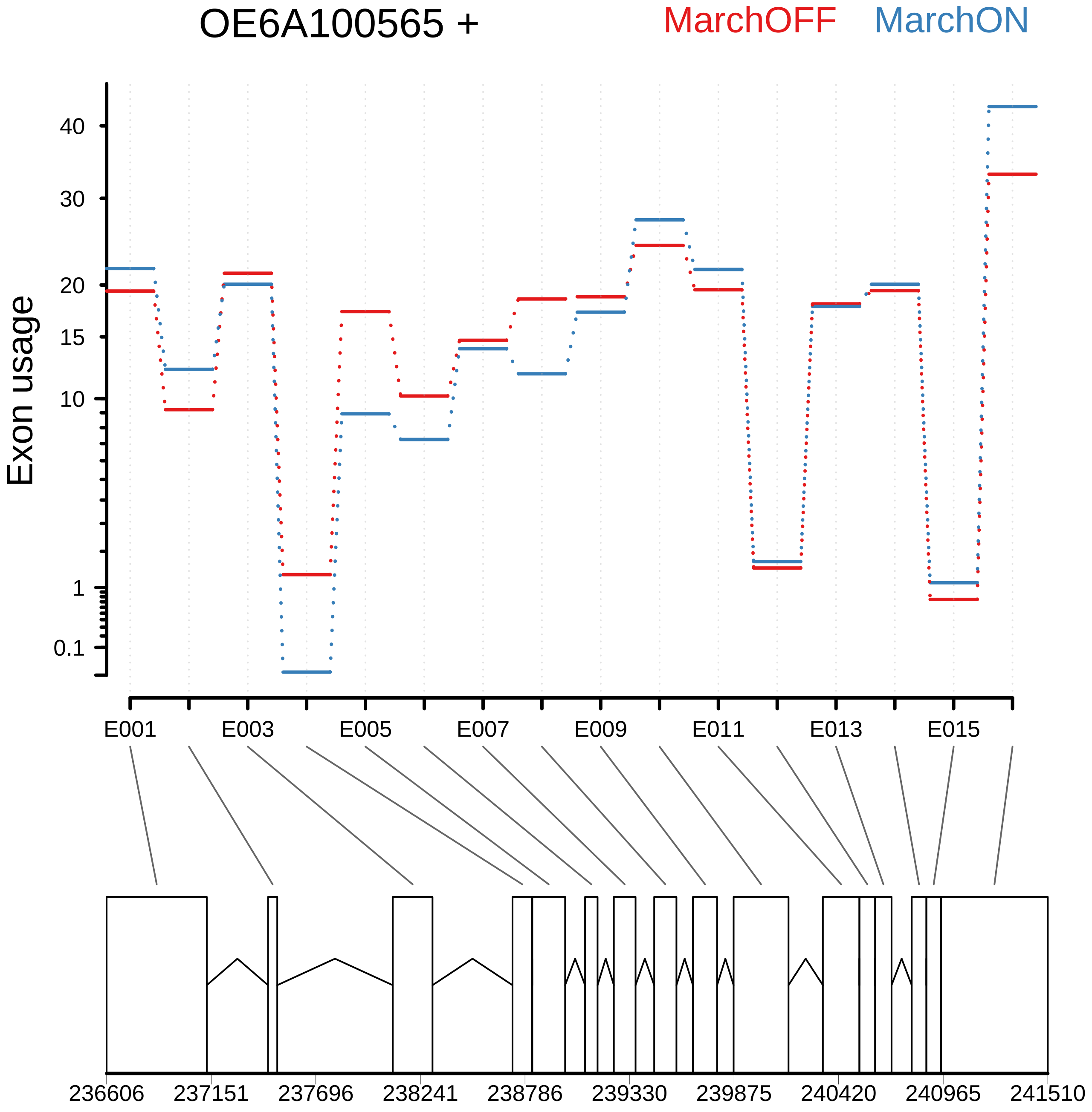
OE6A100565 +
MarchOFF
MarchON
Exon usage
40
30
20
15
10
1
0.1
E001
E003
E005
E007
E009
E011
E013
E015
236606
237151
237696
238241
238786
239330
239875
240420
240965
241510

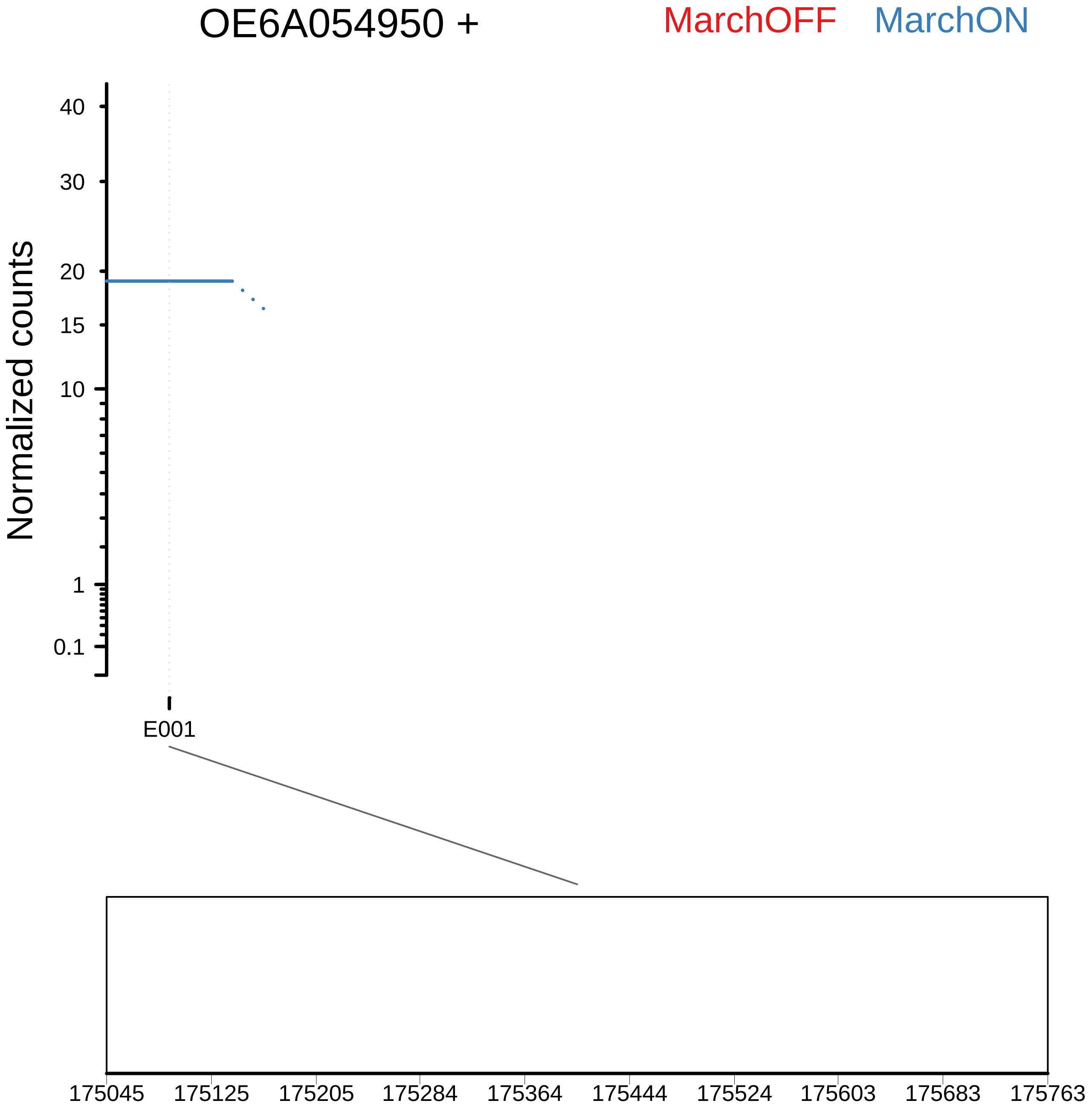

OE6A054950 +
MarchOFF
MarchON
Normalized counts
40
30
20
15
10
1
0.1
E001
175045
175125
175205
175284
175364
175444
175524
175603
175683
175763

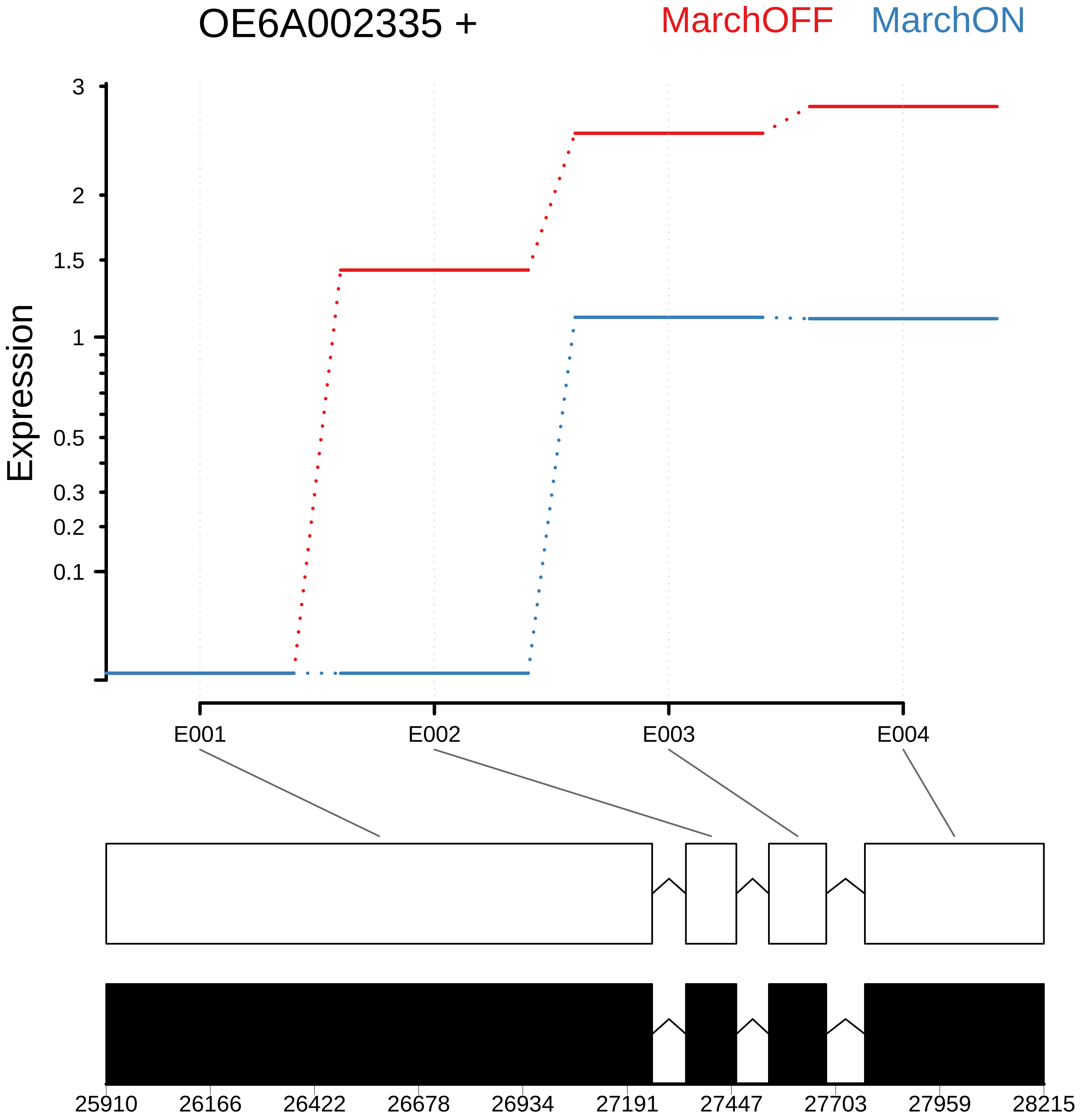

OE6A002335 +
MarchOFF
MarchON
Expression
3
2
1.5
1
0.5
0.3
0.2
0.1
E001
E002
E003
E004
25910
26166
26422
26678
26934
27191
27447
27703
27959
28215

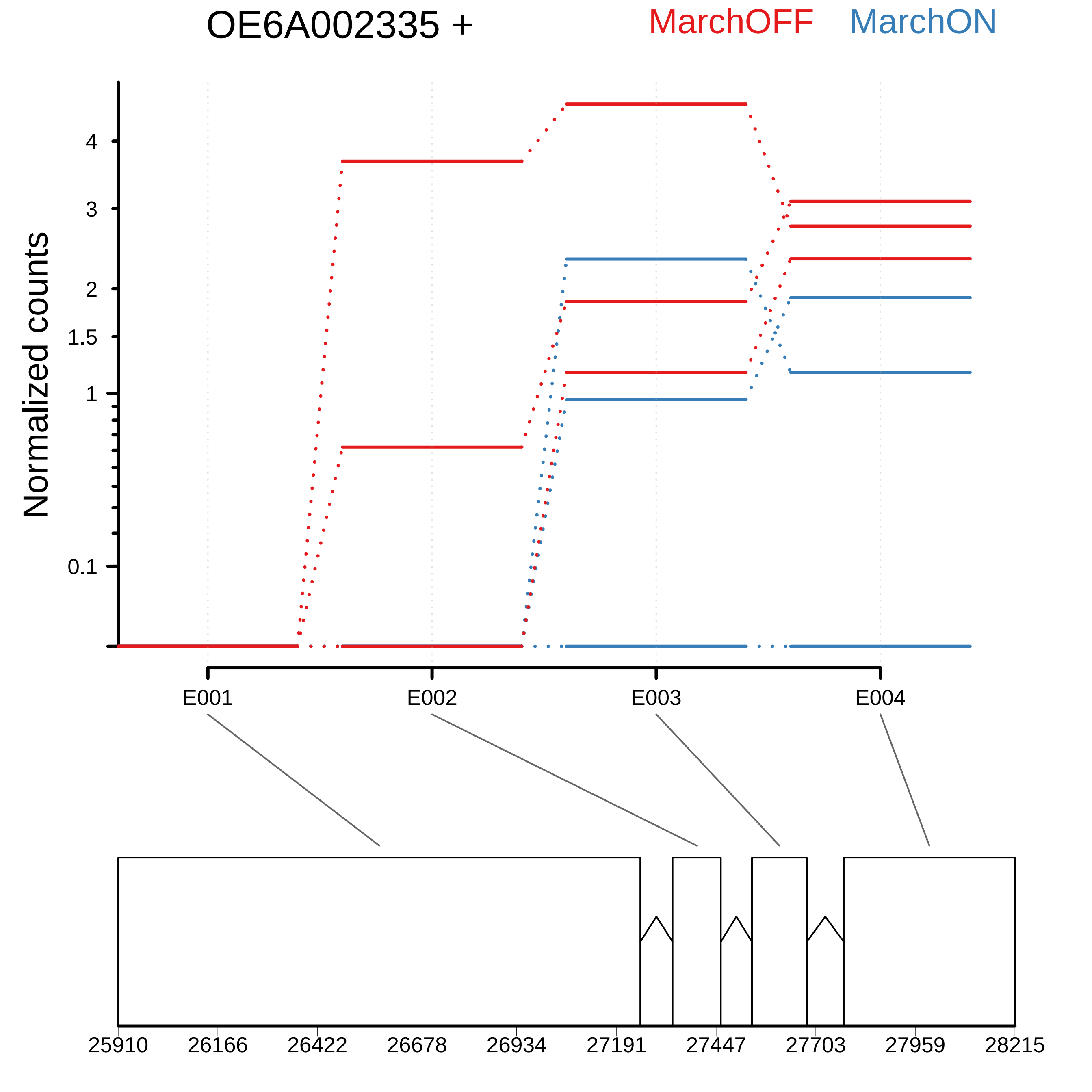

OE6A002335 +
MarchOFF
MarchON
Normalized counts
4
3
2
1.5
1
0.1
E001
E002
E003
E004
25910
26166
26422
26678
26934
27191
27447
27703
27959
28215

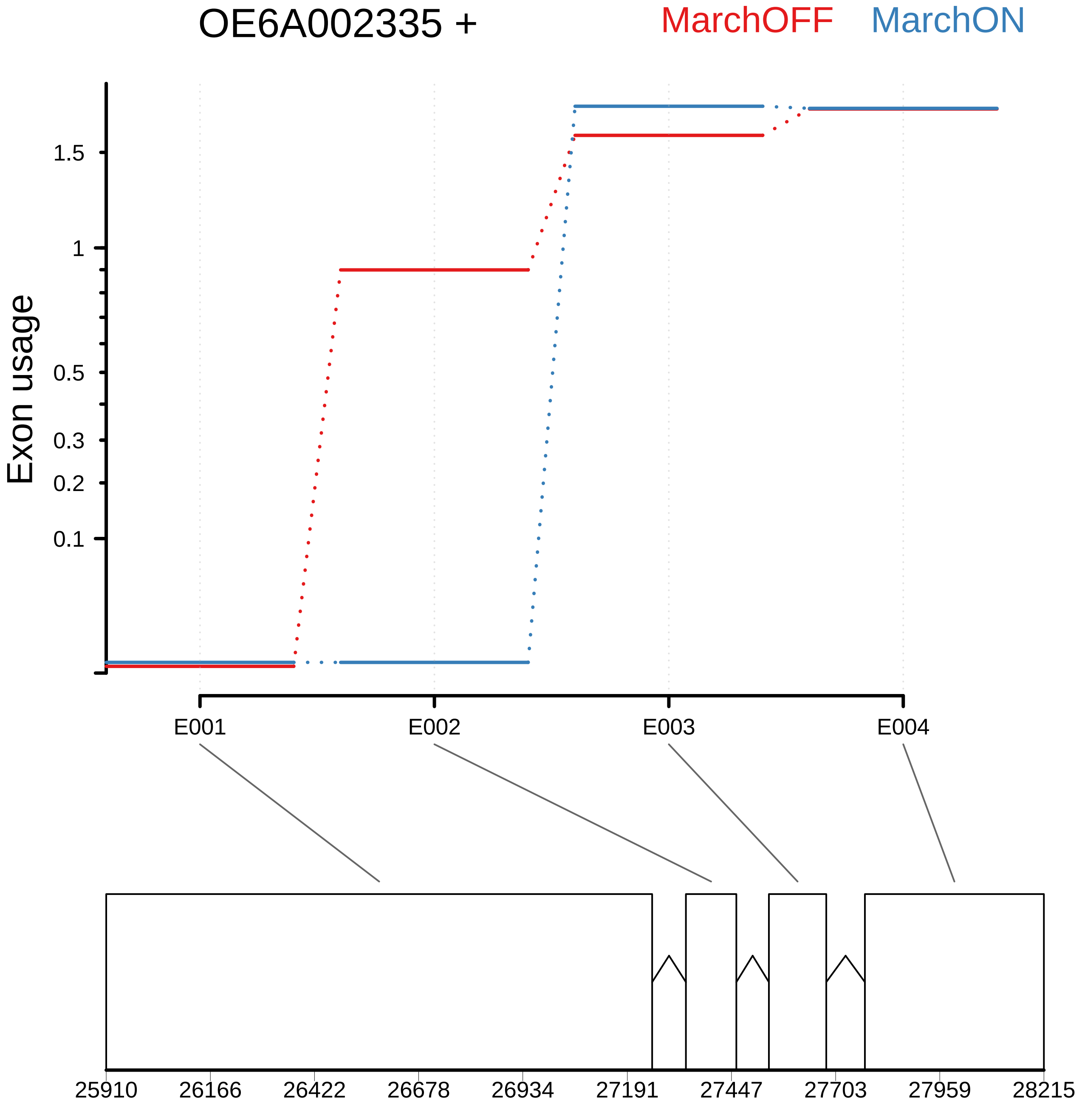
OE6A002335 +
MarchOFF
MarchON
Exon usage
1.5
1
0.5
0.3
0.2
0.1
E001
E002
E003
E004
25910
26166
26422
26678
26934
27191
27447
27703
27959
28215

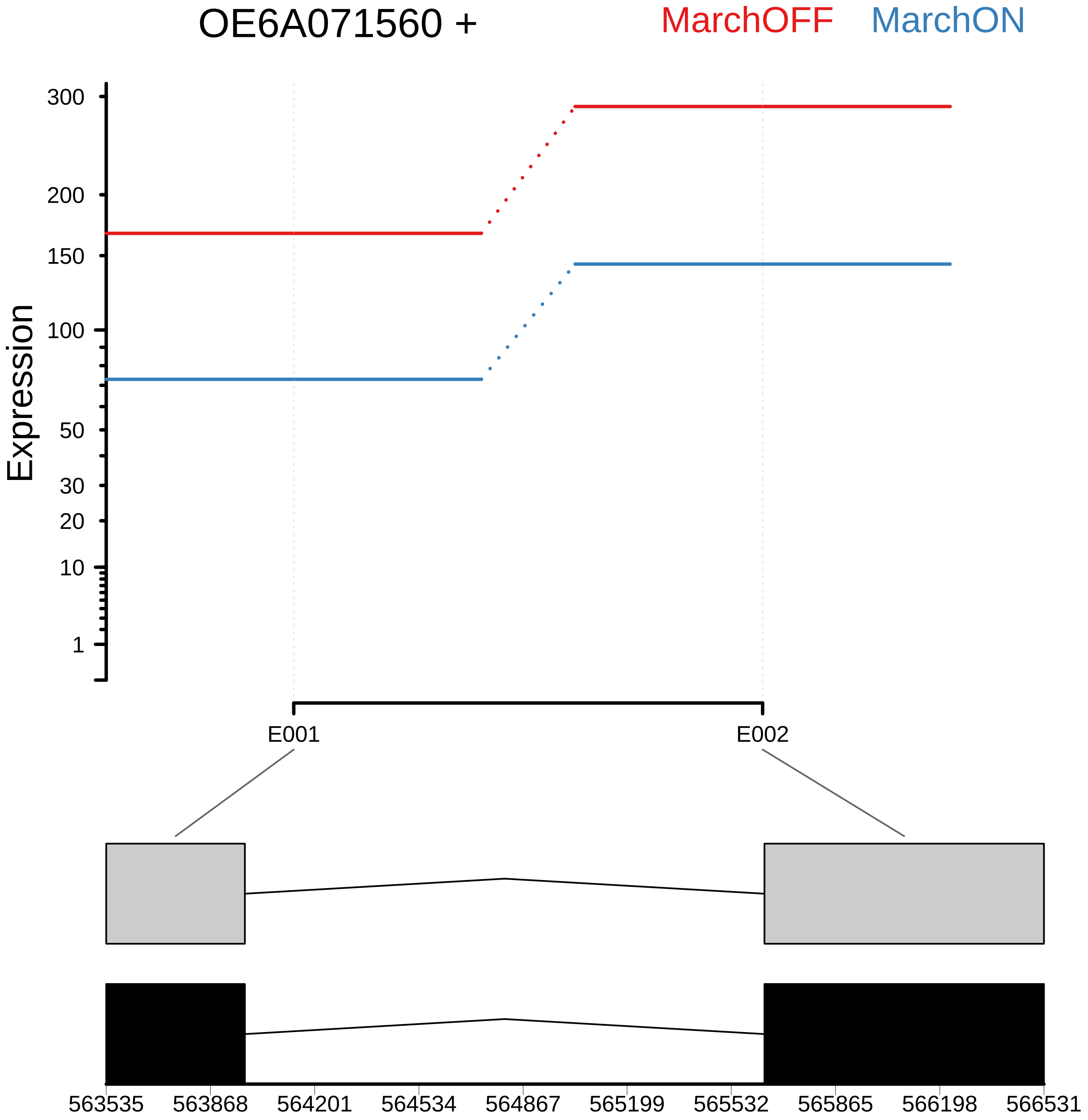

OE6A071560 +
MarchOFF
MarchON
Expression
300
200
150
100
50
30
20
10
1
E001
E002
563535
563868
564201
564534
564867
565199
565532
565865
566198
566531

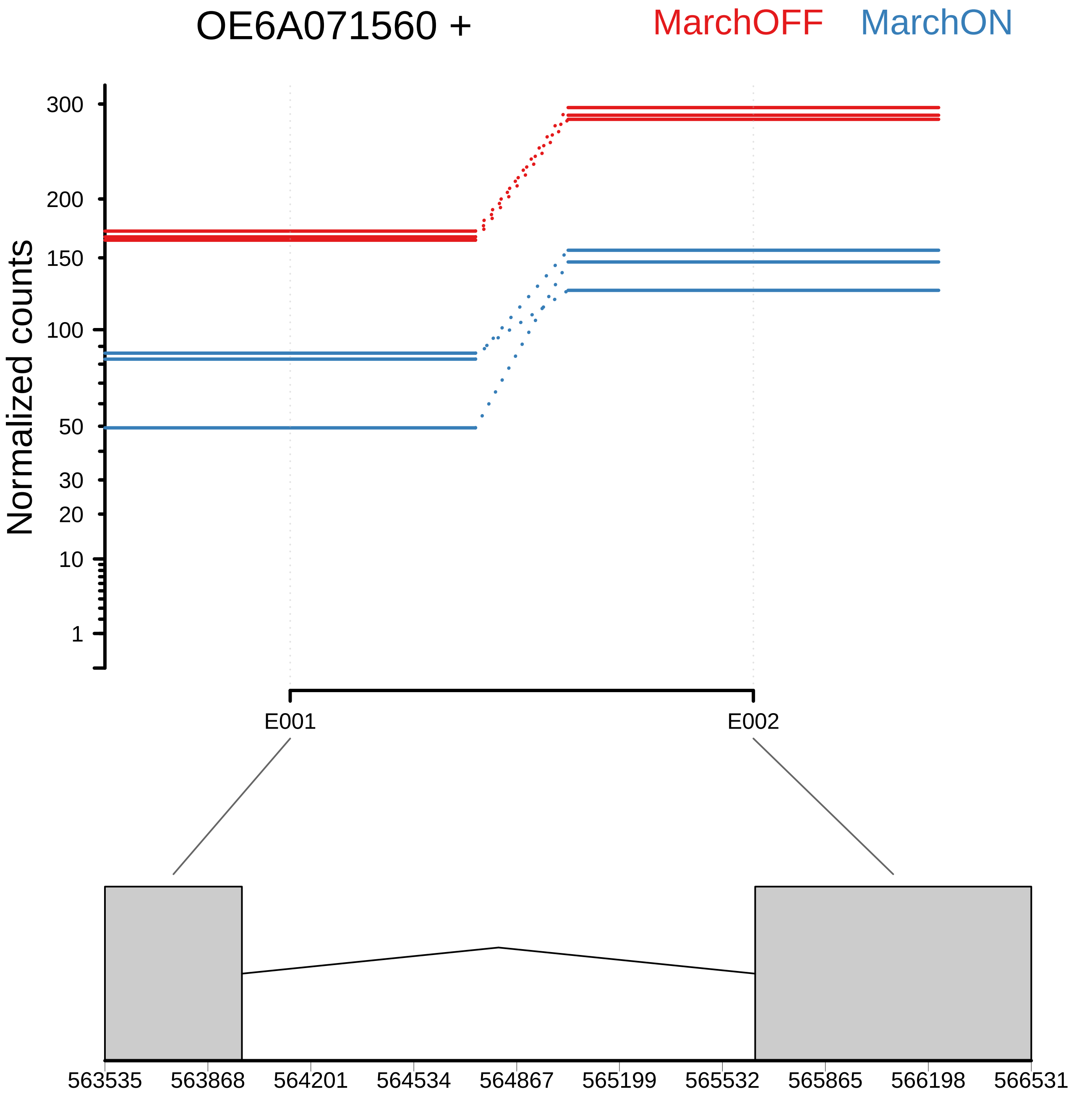
OE6A071560 +
MarchOFF
MarchON
Normalized counts
300
200
150
100
50
30
20
10
1
E001
E002
563535
563868
564201
564534
564867
565199
565532
565865
566198
566531

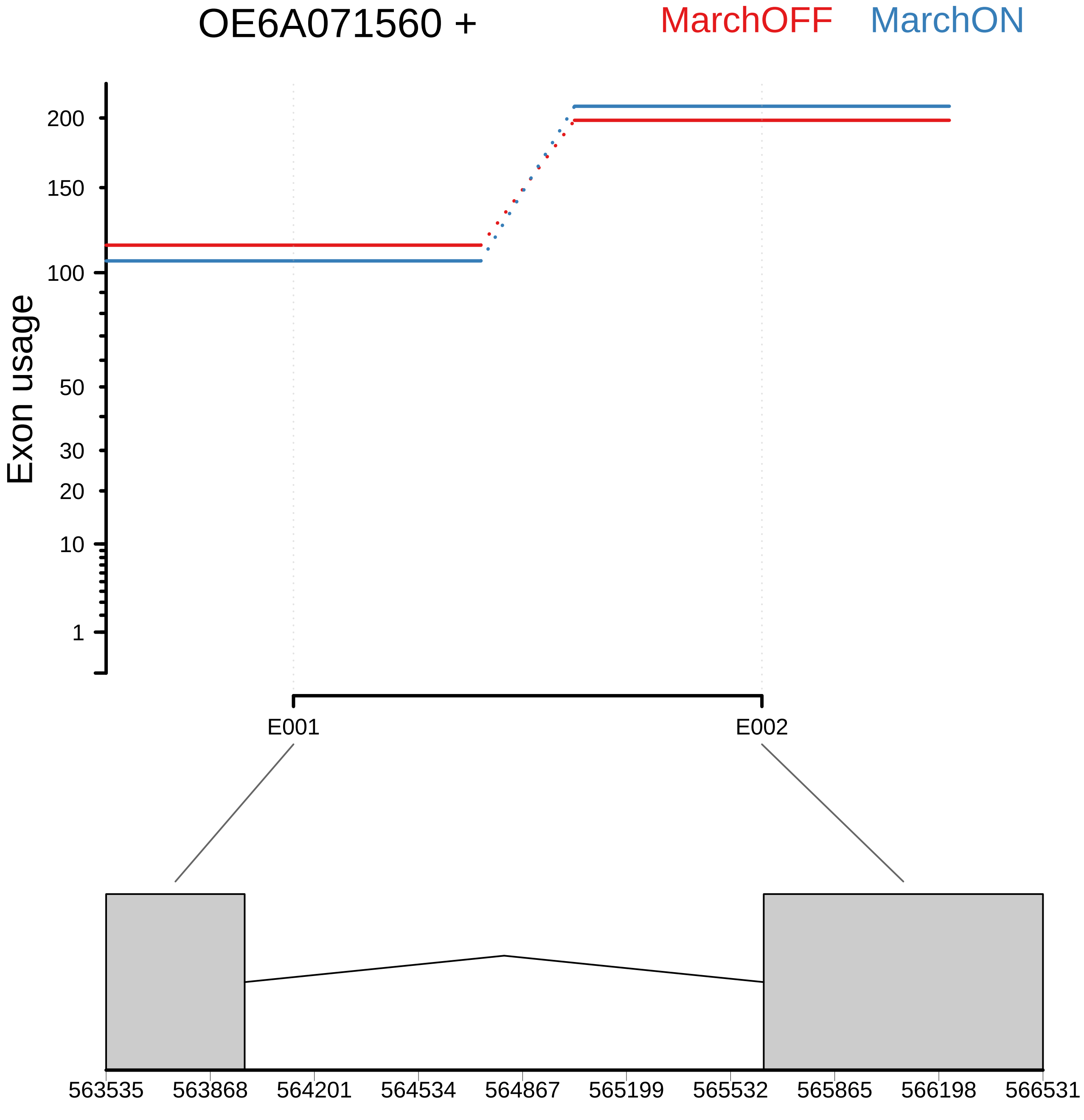

OE6A071560 +
MarchOFF
MarchON
Exon usage
200
150
100
50
30
20
10
1
E001
E002
563535
563868
564201
564534
564867
565199
565532
565865
566198
566531

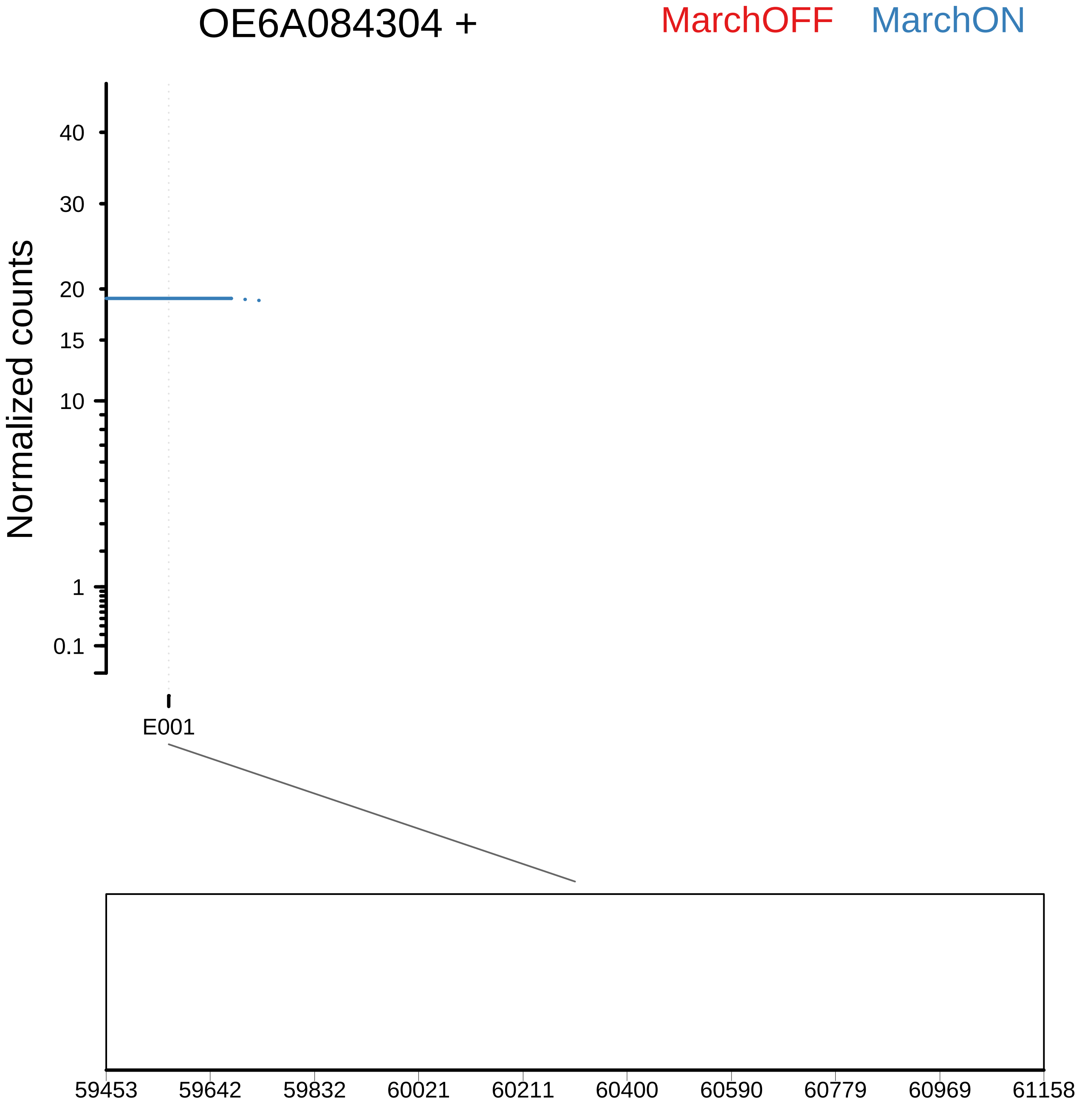
OE6A084304 +
MarchOFF
MarchON
Normalized counts
40
30
20
15
10
1
0.1
E001
59453
59642
59832
60021
60211
60400
60590
60779
60969
61158

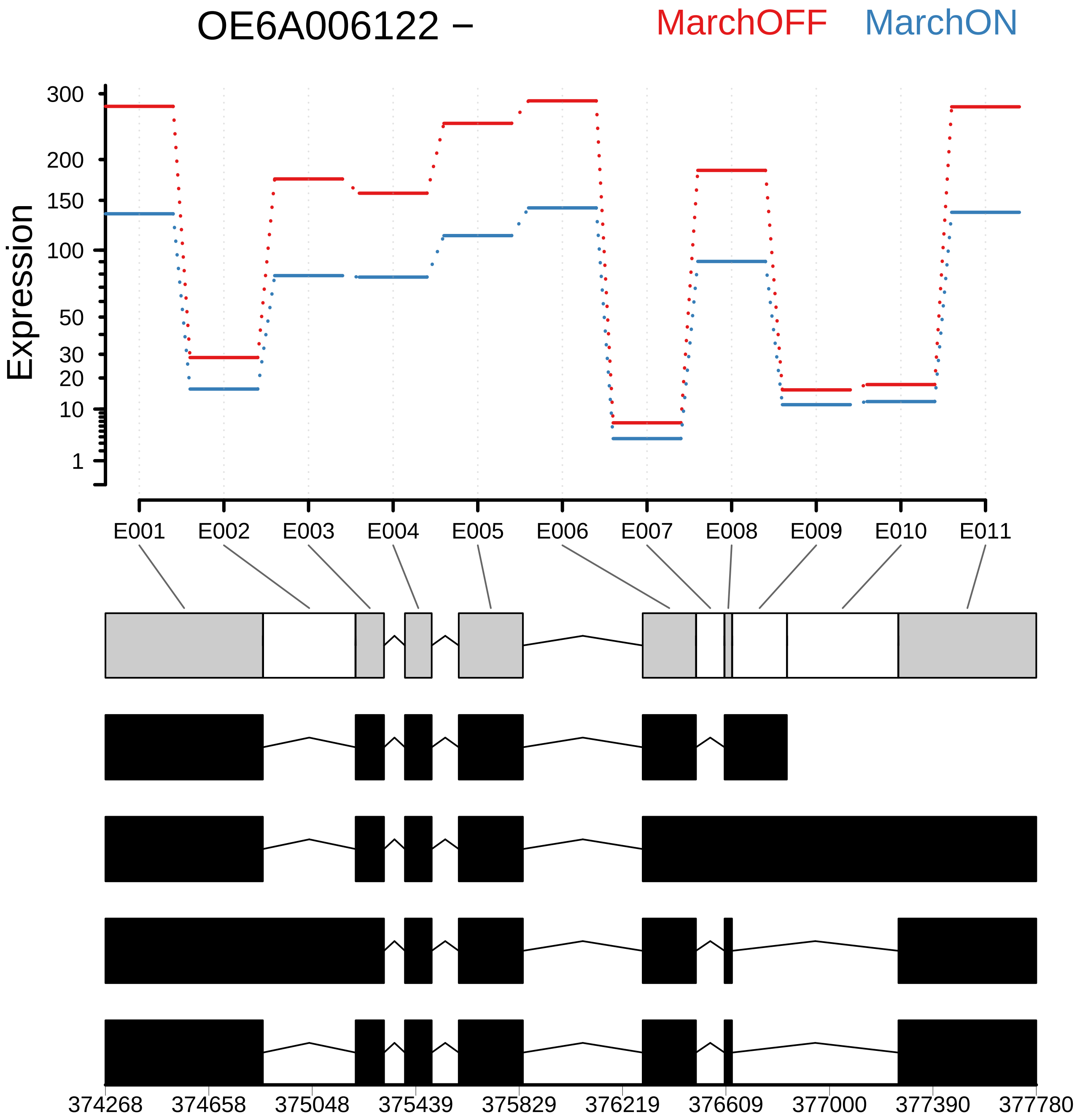

OE6A006122 −
MarchOFF
MarchON
Expression
300
200
150
100
50
30
20
10
1
E001
E002
E003
E004
E005
E006
E007
E008
E009
E010
E011
374268
374658
375048
375439
375829
376219
376609
377000
377390
377780

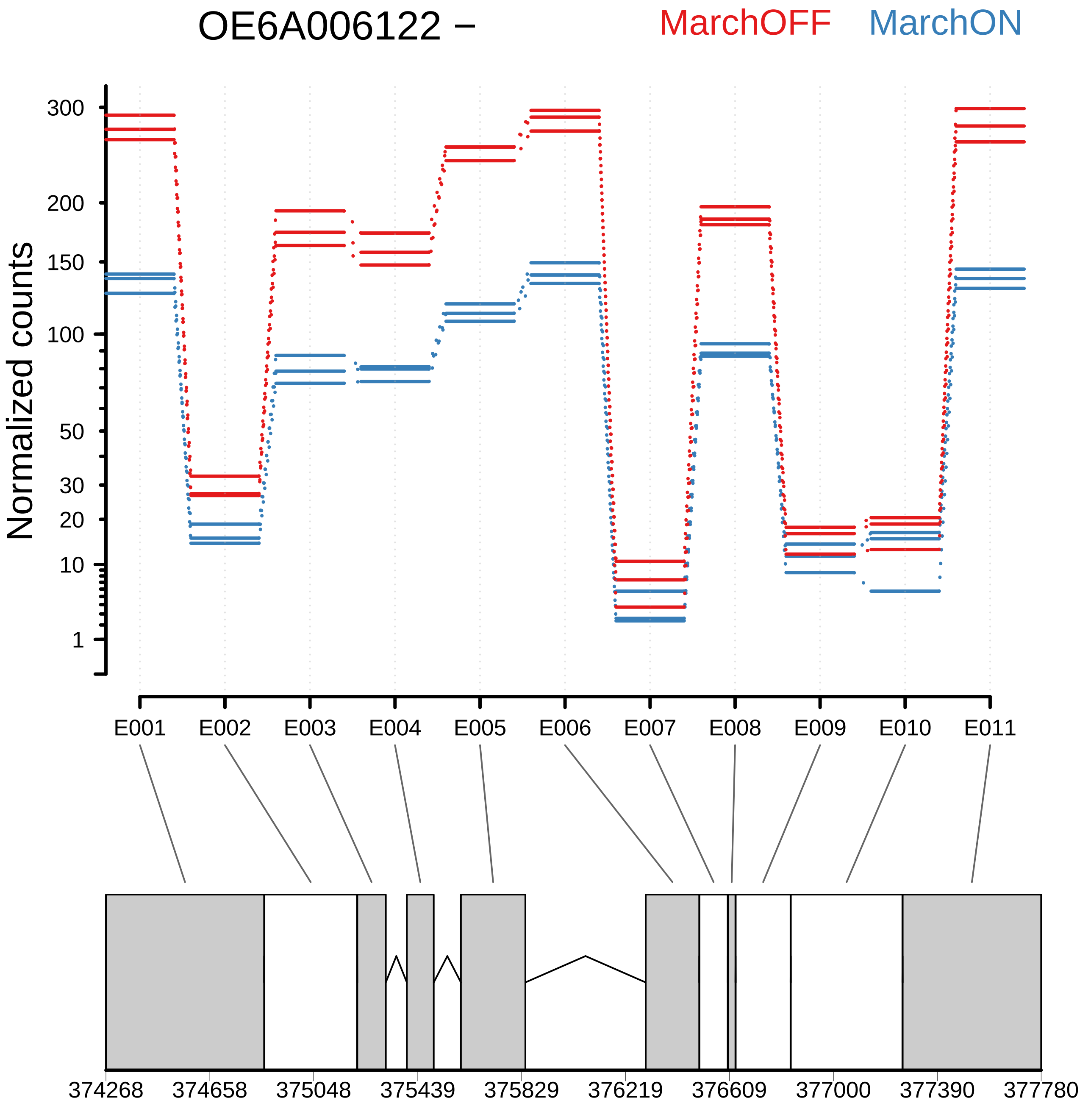

OE6A006122 −
MarchOFF
MarchON
Normalized counts
300
200
150
100
50
30
20
10
1
E001
E002
E003
E004
E005
E006
E007
E008
E009
E010
E011
374268
374658
375048
375439
375829
376219
376609
377000
377390
377780

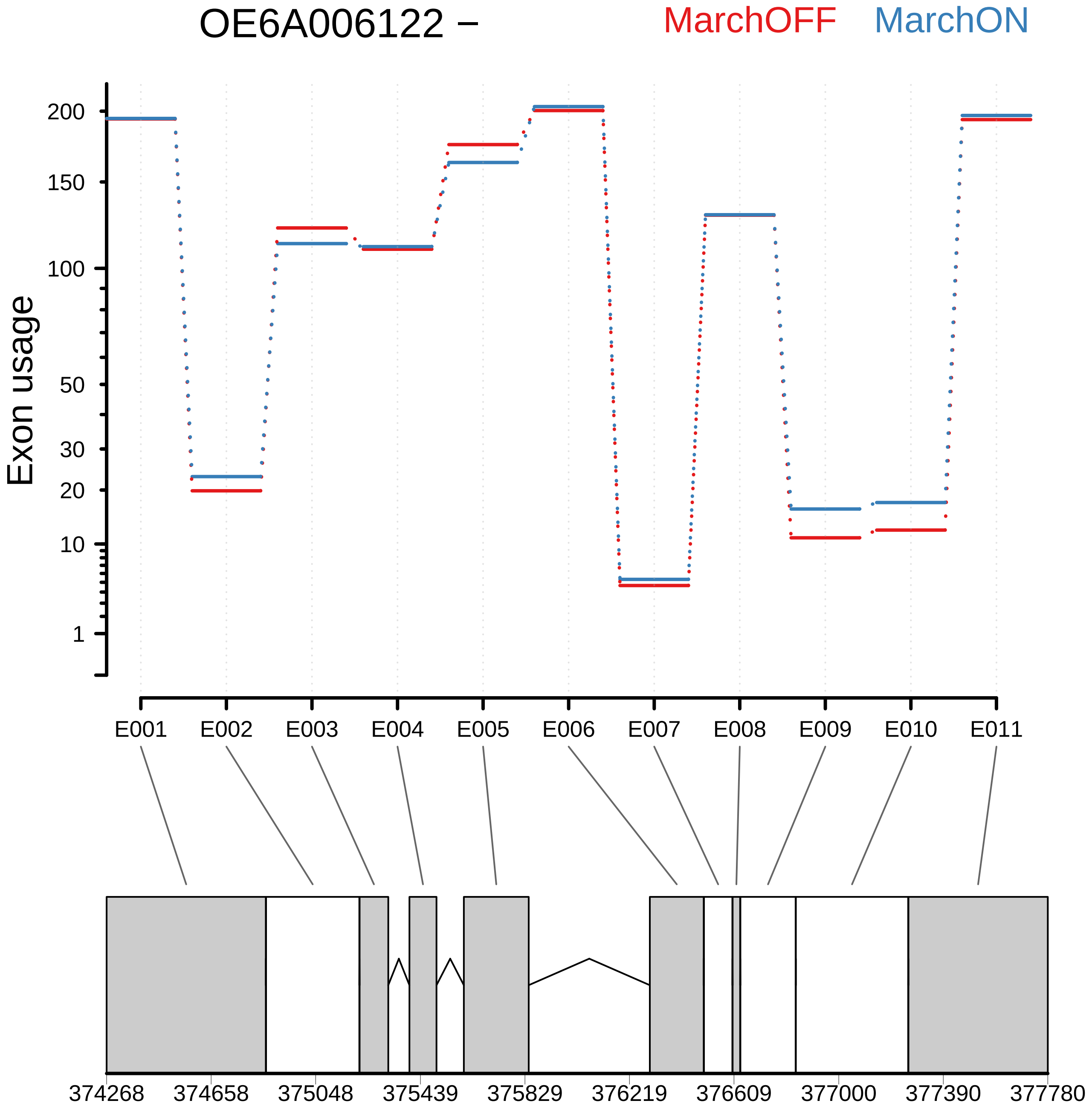

OE6A006122 −
MarchOFF
MarchON
Exon usage
200
150
100
50
30
20
10
1
E001
E002
E003
E004
E005
E006
E007
E008
E009
E010
E011
374268
374658
375048
375439
375829
376219
376609
377000
377390
377780

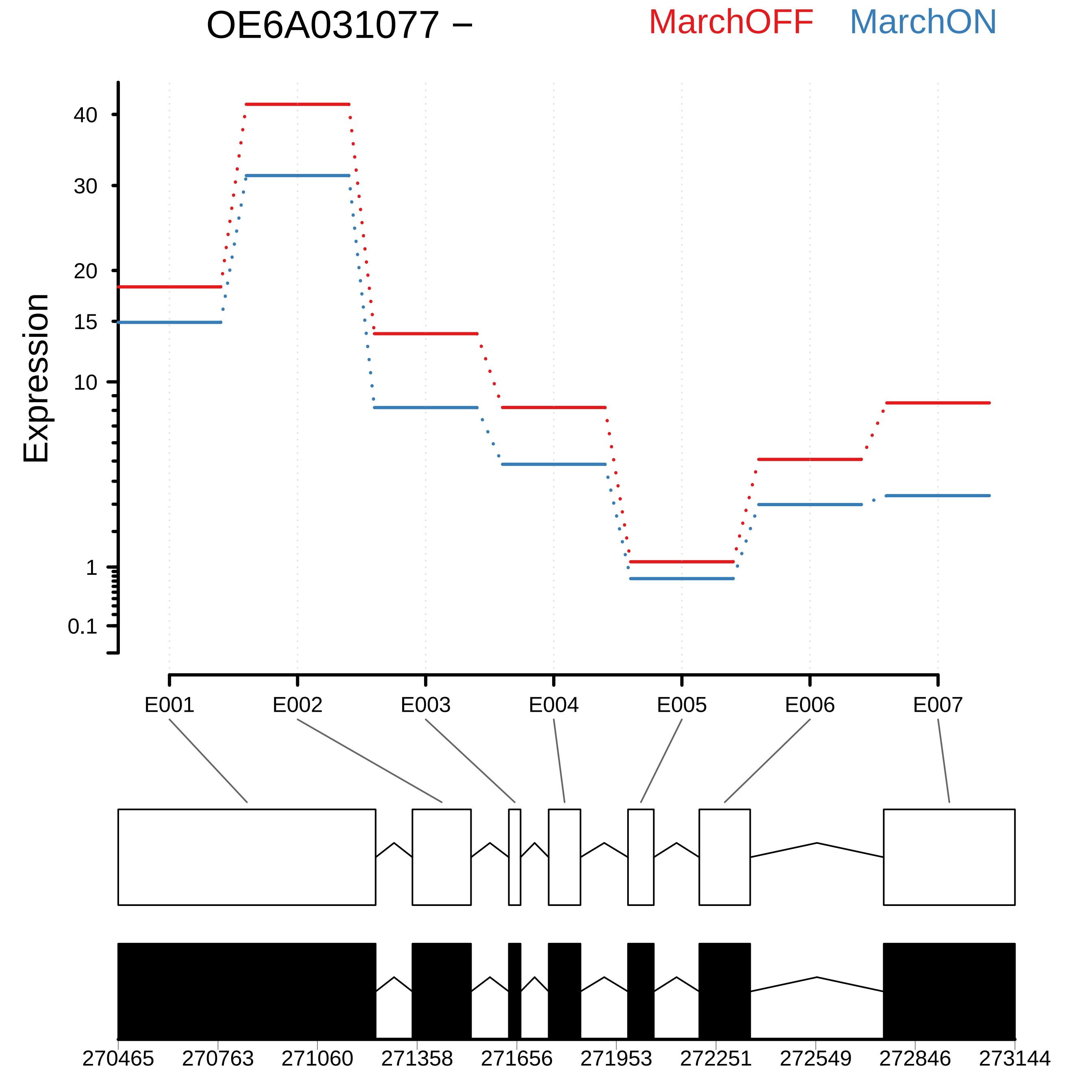
OE6A031077 −
MarchOFF
MarchON
Expression
40
30
20
15
10
1
0.1
E001
E002
E003
E004
E005
E006
E007
270465
270763
271060
271358
271656
271953
272251
272549
272846
273144

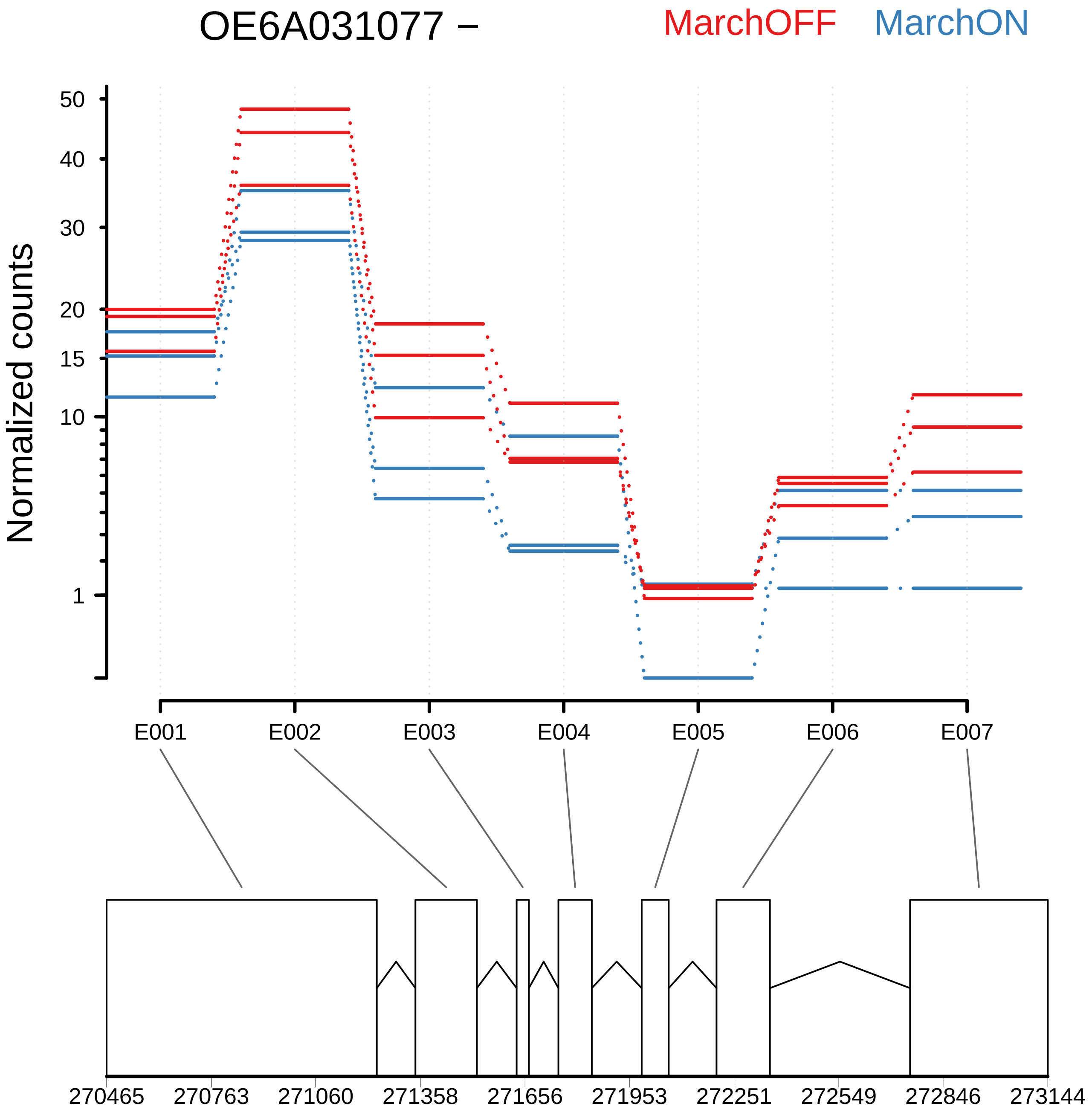
OE6A031077 −
MarchOFF
MarchON
Normalized counts
50
40
30
20
15
10
1
E001
E002
E003
E004
E005
E006
E007
270465
270763
271060
271358
271656
271953
272251
272549
272846
273144

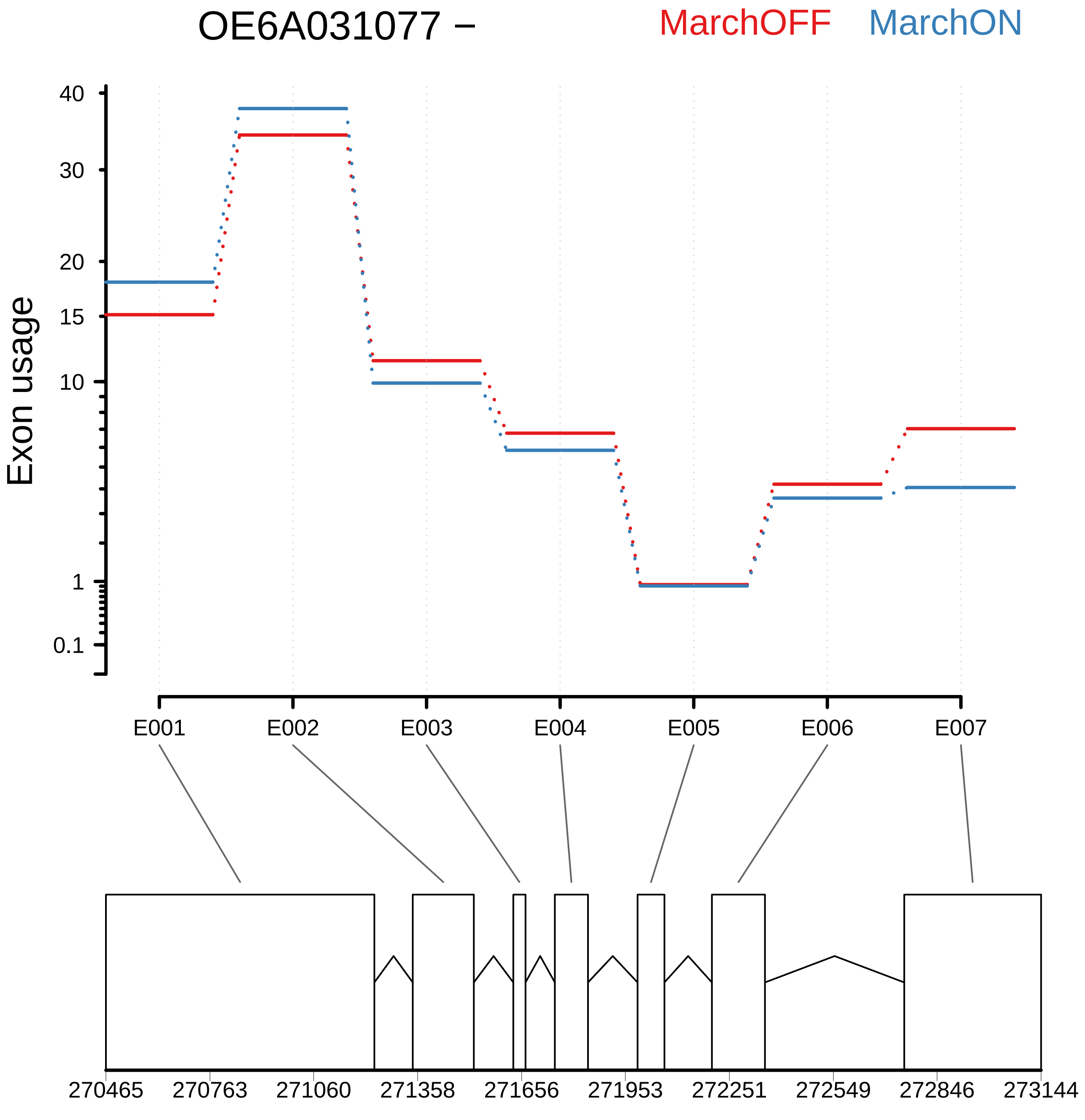
OE6A031077 −
MarchOFF
MarchON
Exon usage
40
30
20
15
10
1
0.1
E001
E002
E003
E004
E005
E006
E007
270465
270763
271060
271358
271656
271953
272251
272549
272846
273144

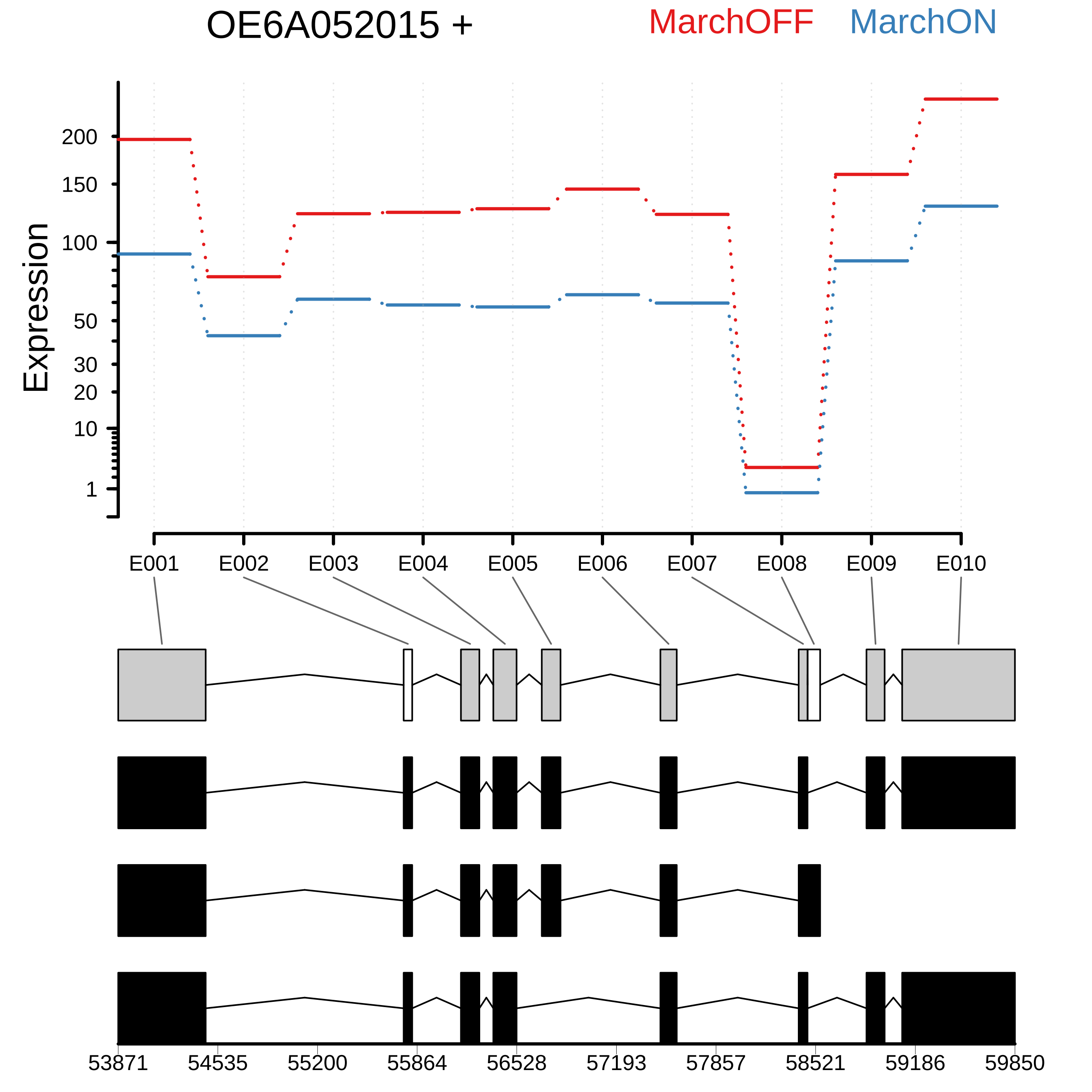

OE6A052015 +
MarchOFF
MarchON
Expression
200
150
100
50
30
20
10
1
E001
E002
E003
E004
E005
E006
E007
E008
E009
E010
53871
54535
55200
55864
56528
57193
57857
58521
59186
59850

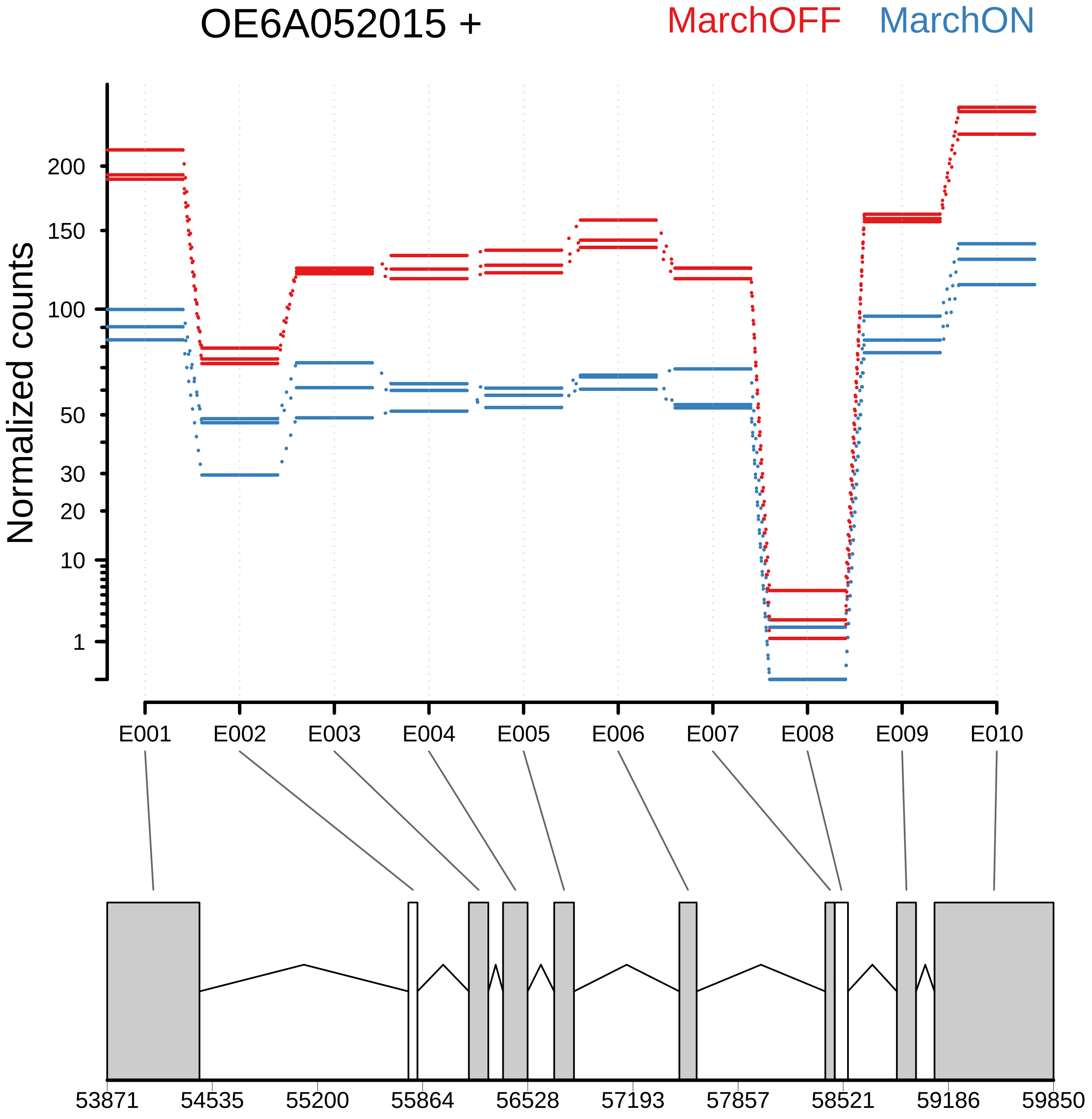

OE6A052015 +
MarchOFF
MarchON
Normalized counts
200
150
100
50
30
20
10
1
E001
E002
E003
E004
E005
E006
E007
E008
E009
E010
53871
54535
55200
55864
56528
57193
57857
58521
59186
59850

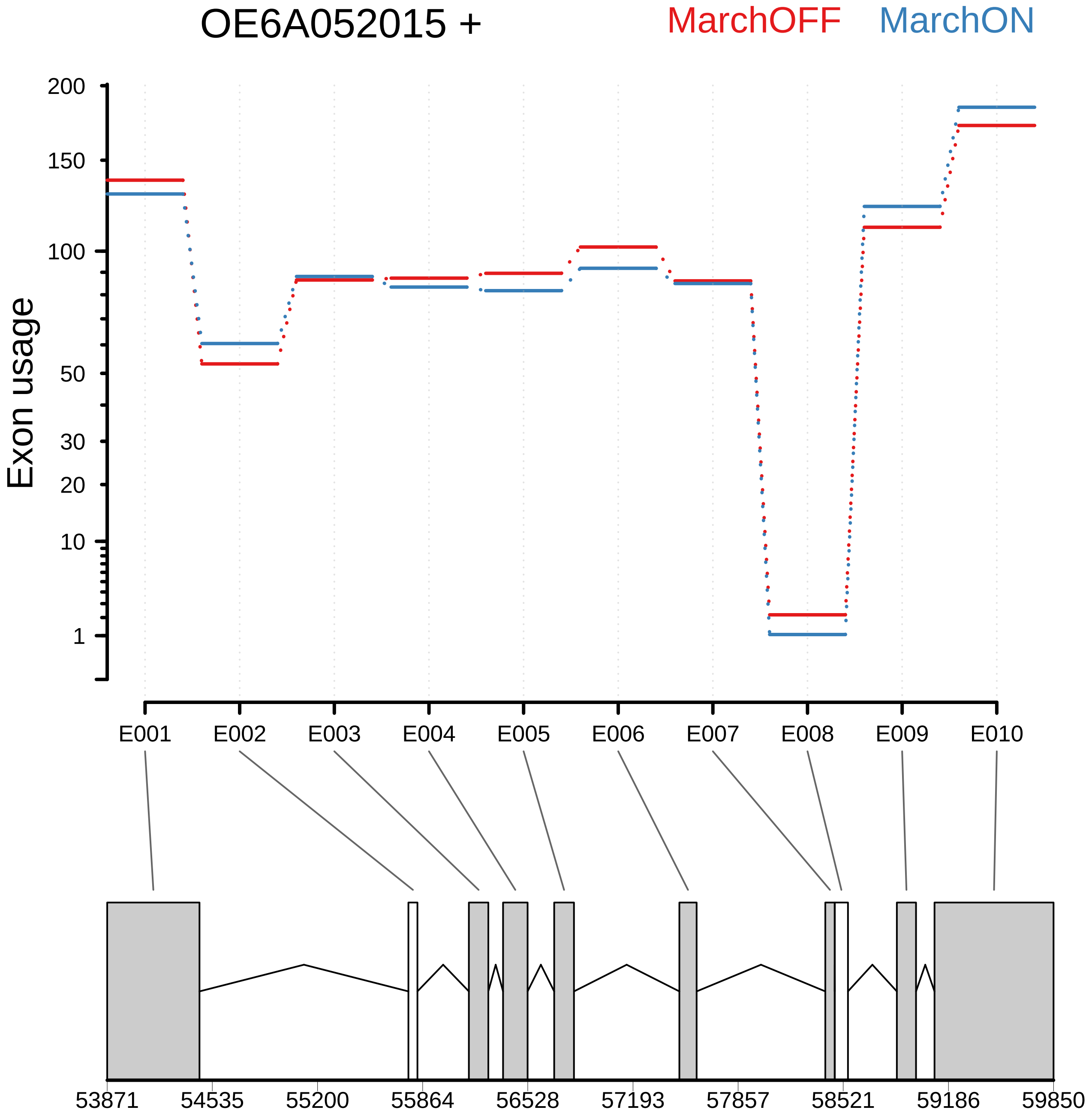

OE6A052015 +
MarchOFF
MarchON
Exon usage
200
150
100
50
30
20
10
1
E001
E002
E003
E004
E005
E006
E007
E008
E009
E010
53871
54535
55200
55864
56528
57193
57857
58521
59186
59850

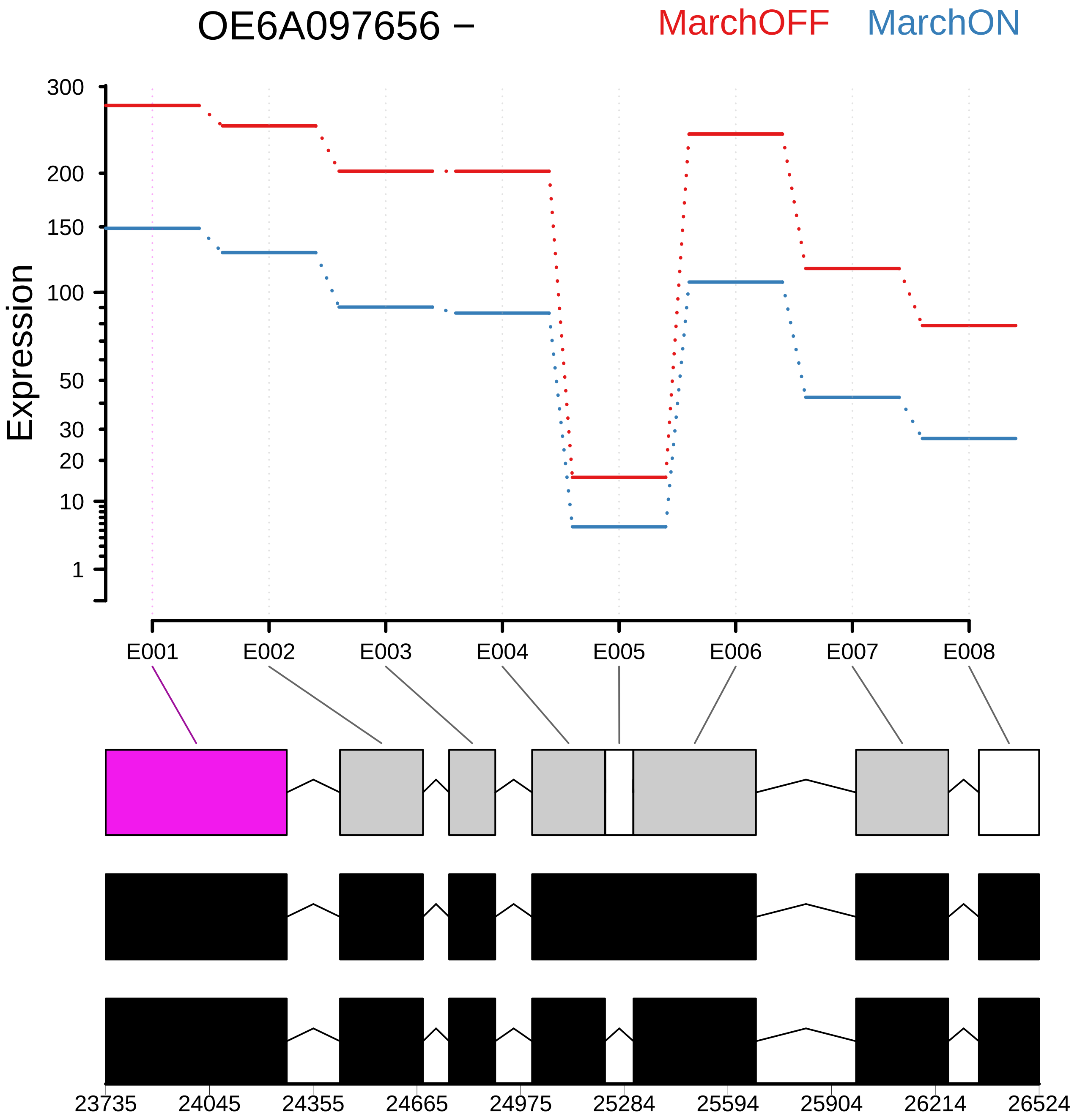

OE6A097656 −
MarchOFF
MarchON
Expression
300
200
150
100
50
30
20
10
1
E001
E002
E003
E004
E005
E006
E007
E008
23735
24045
24355
24665
24975
25284
25594
25904
26214
26524

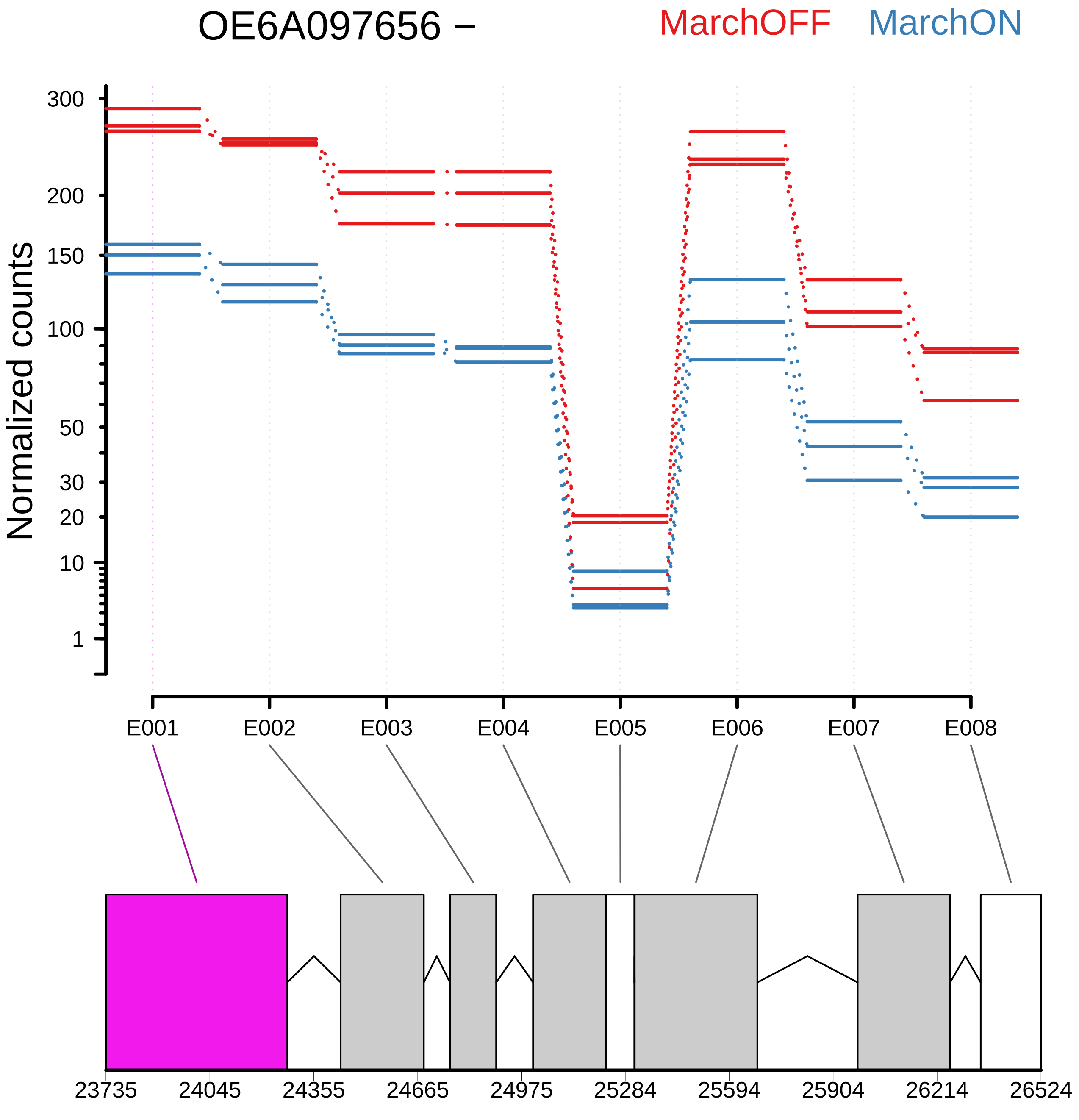

OE6A097656 −
MarchOFF
MarchON
Normalized counts
300
200
150
100
50
30
20
10
1
E001
E002
E003
E004
E005
E006
E007
E008
23735
24045
24355
24665
24975
25284
25594
25904
26214
26524

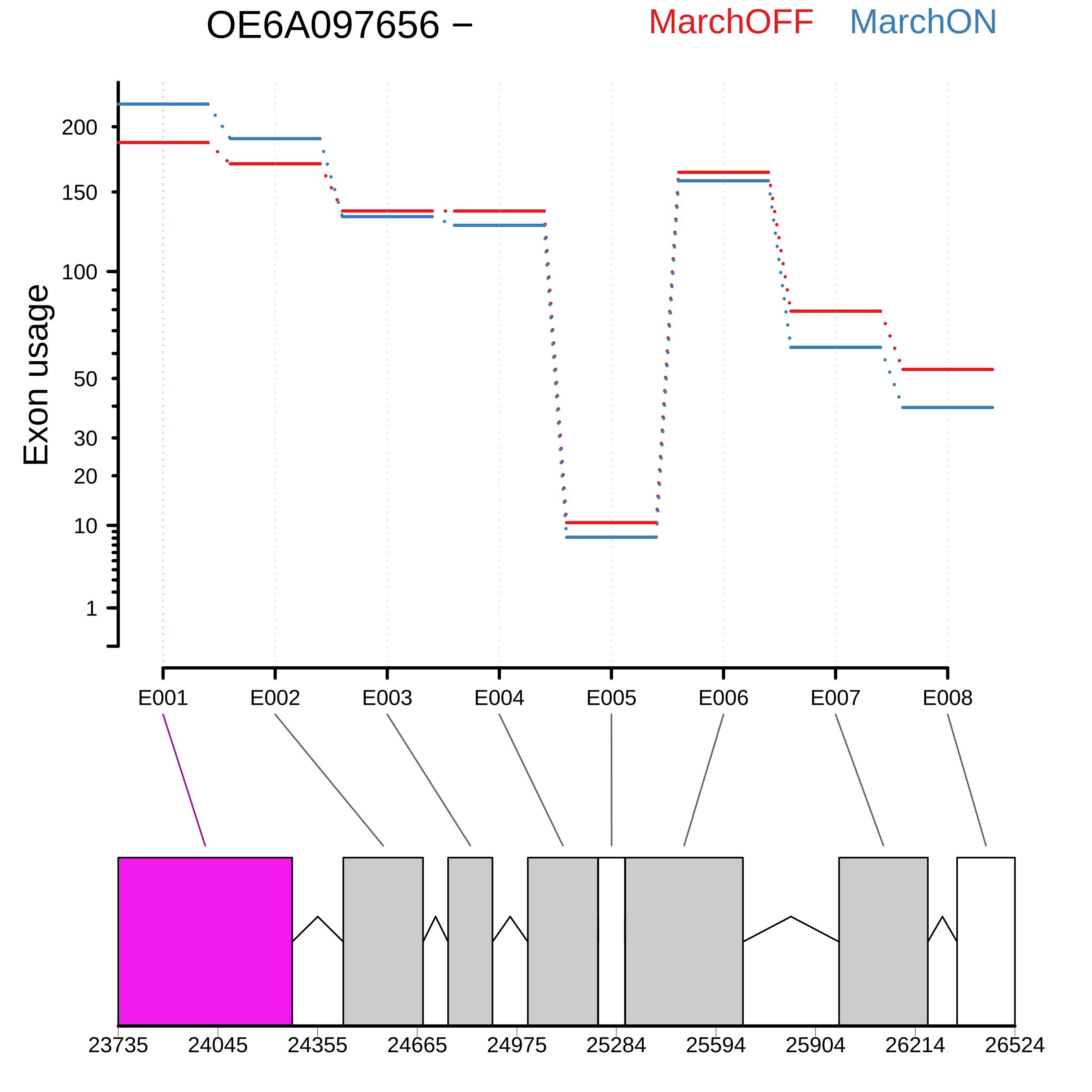
OE6A097656 −
MarchOFF
MarchON
Exon usage
200
150
100
50
30
20
10
1
E001
E002
E003
E004
E005
E006
E007
E008
23735
24045
24355
24665
24975
25284
25594
25904
26214
26524

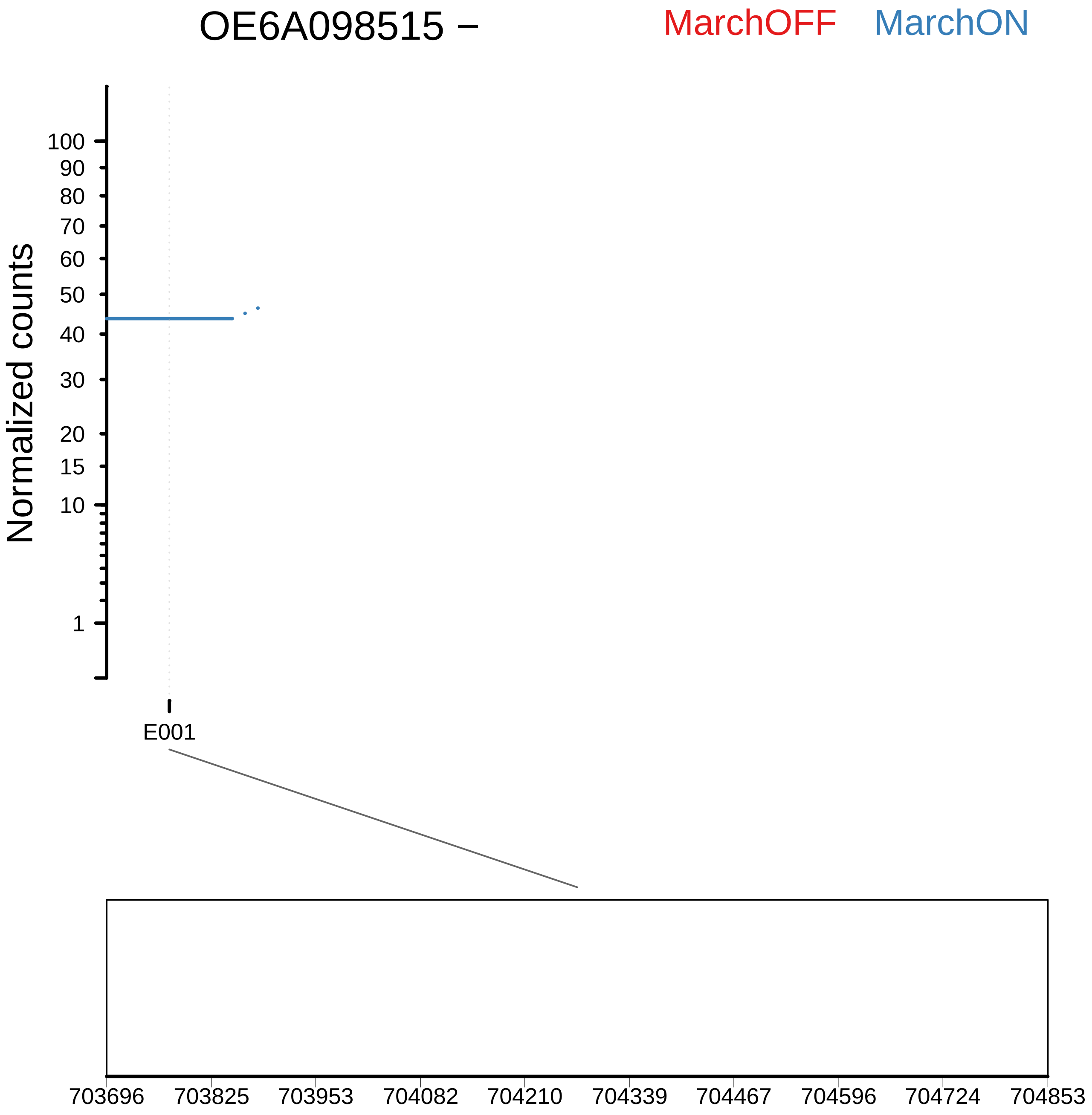
OE6A098515 −
MarchOFF
MarchON
Normalized counts
100
90
80
70
60
50
40
30
20
15
10
1
E001
703696
703825
703953
704082
704210
704339
704467
704596
704724
704853

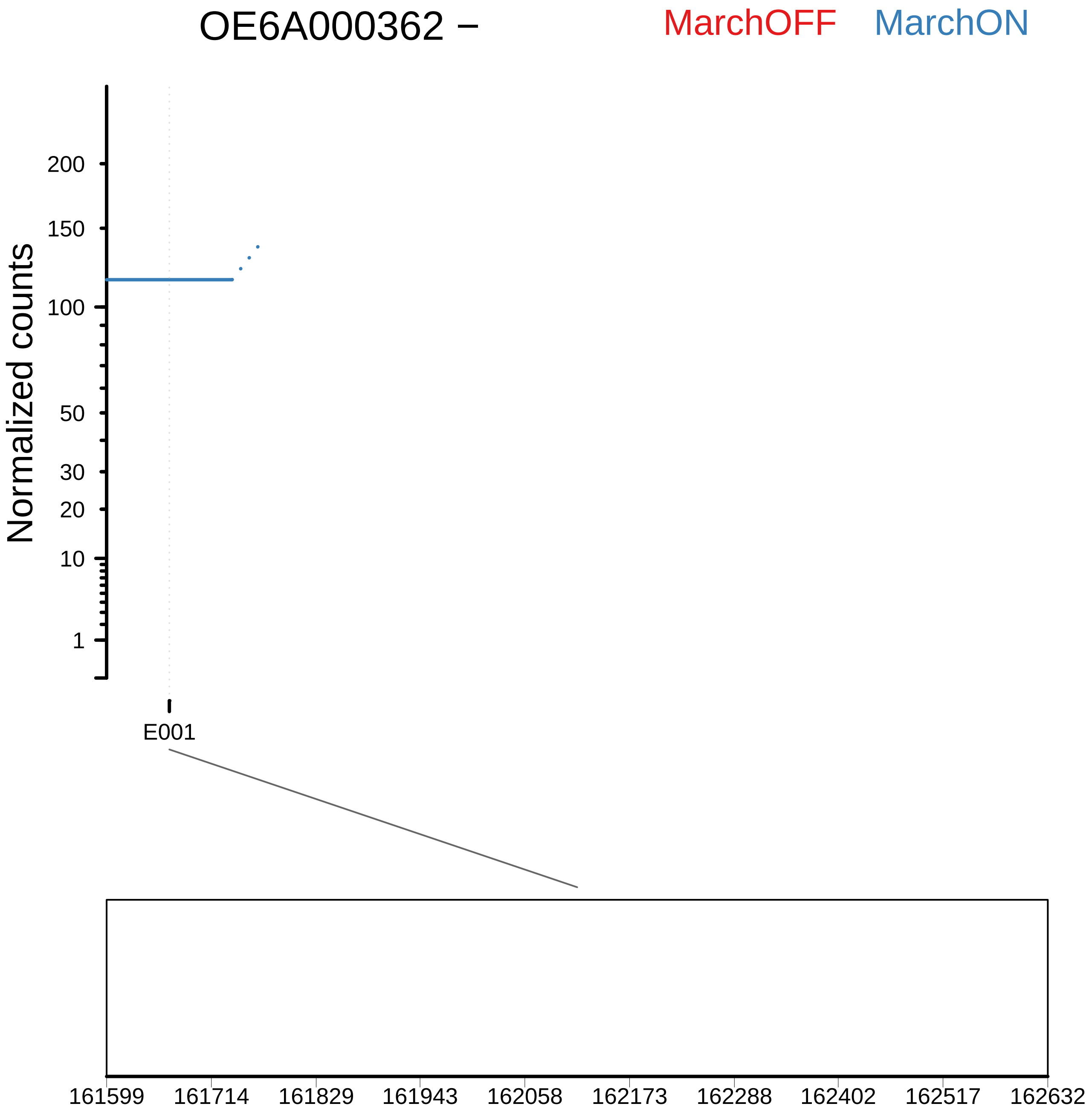

OE6A000362 −
MarchOFF
MarchON
Normalized counts
200
150
100
50
30
20
10
1
E001
161599
161714
161829
161943
162058
162173
162288
162402
162517
162632

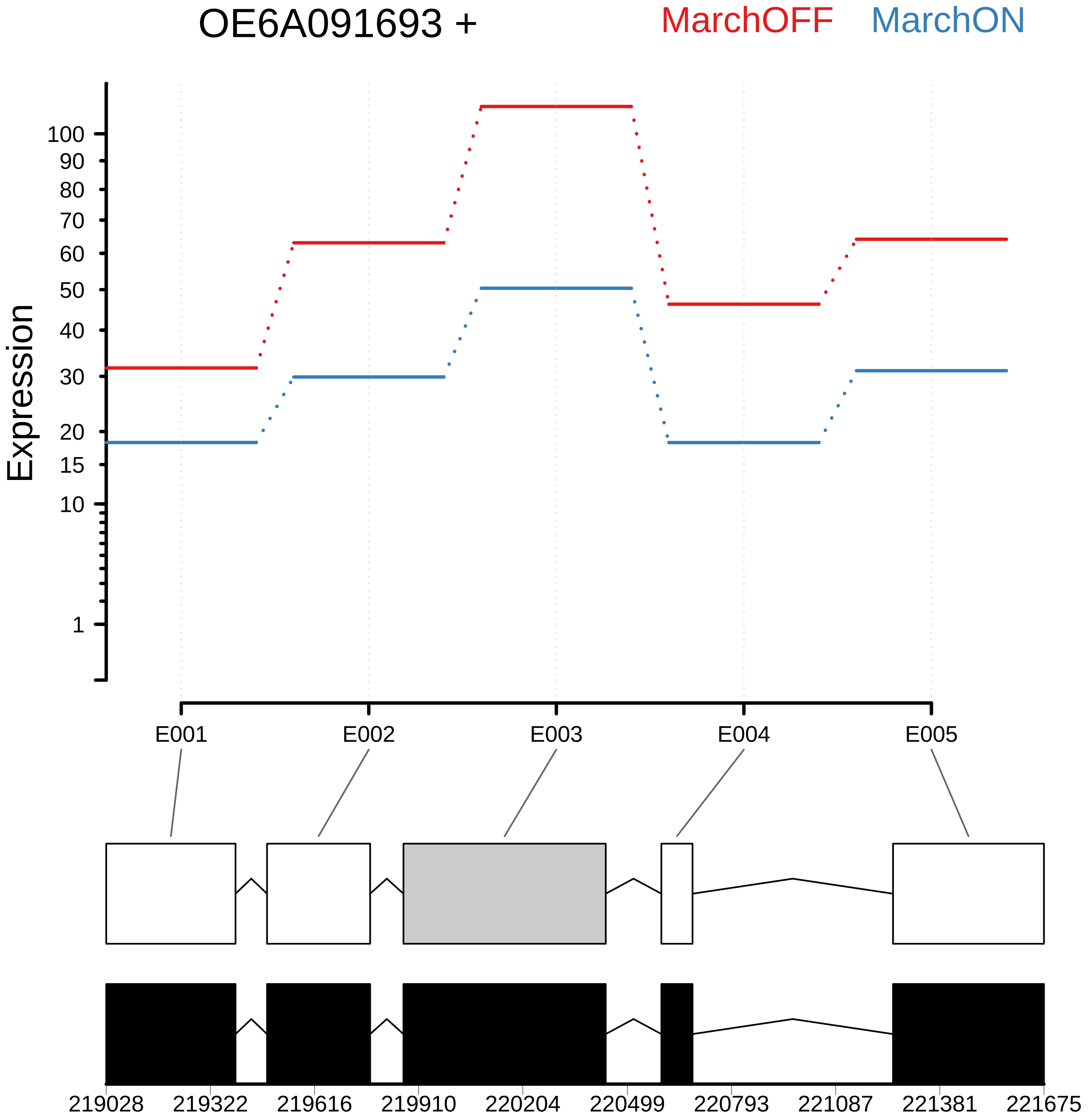

OE6A091693 +
MarchOFF
MarchON
Expression
100
90
80
70
60
50
40
30
20
15
10
1
E001
E002
E003
E004
E005
219028
219322
219616
219910
220204
220499
220793
221087
221381
221675

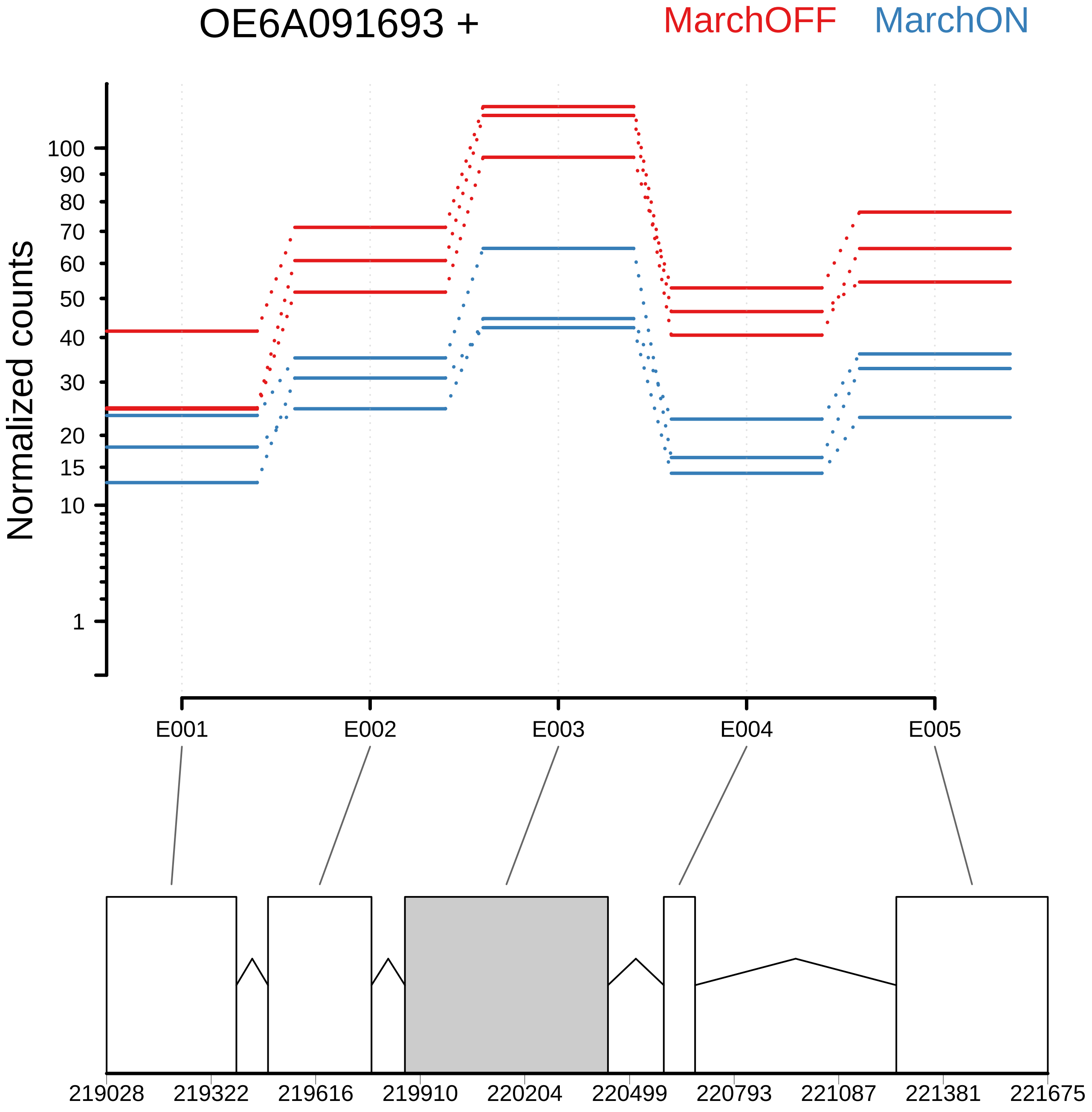
OE6A091693 +
MarchOFF
MarchON
Normalized counts
100
90
80
70
60
50
40
30
20
15
10
1
E001
E002
E003
E004
E005
219028
219322
219616
219910
220204
220499
220793
221087
221381
221675

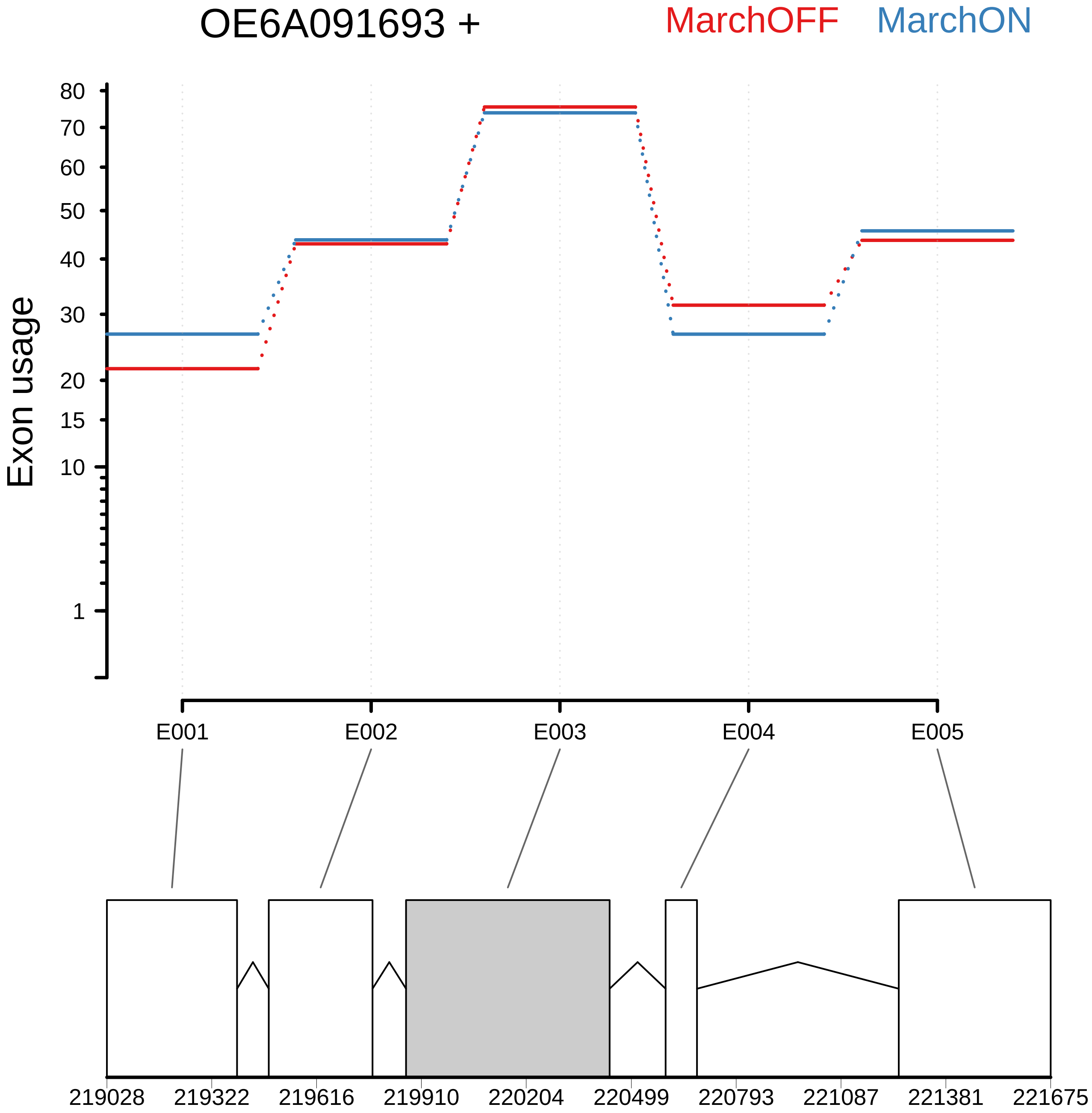

OE6A091693 +
MarchOFF
MarchON
Exon usage
80
70
60
50
40
30
20
15
10
1
E001
E002
E003
E004
E005
219028
219322
219616
219910
220204
220499
220793
221087
221381
221675

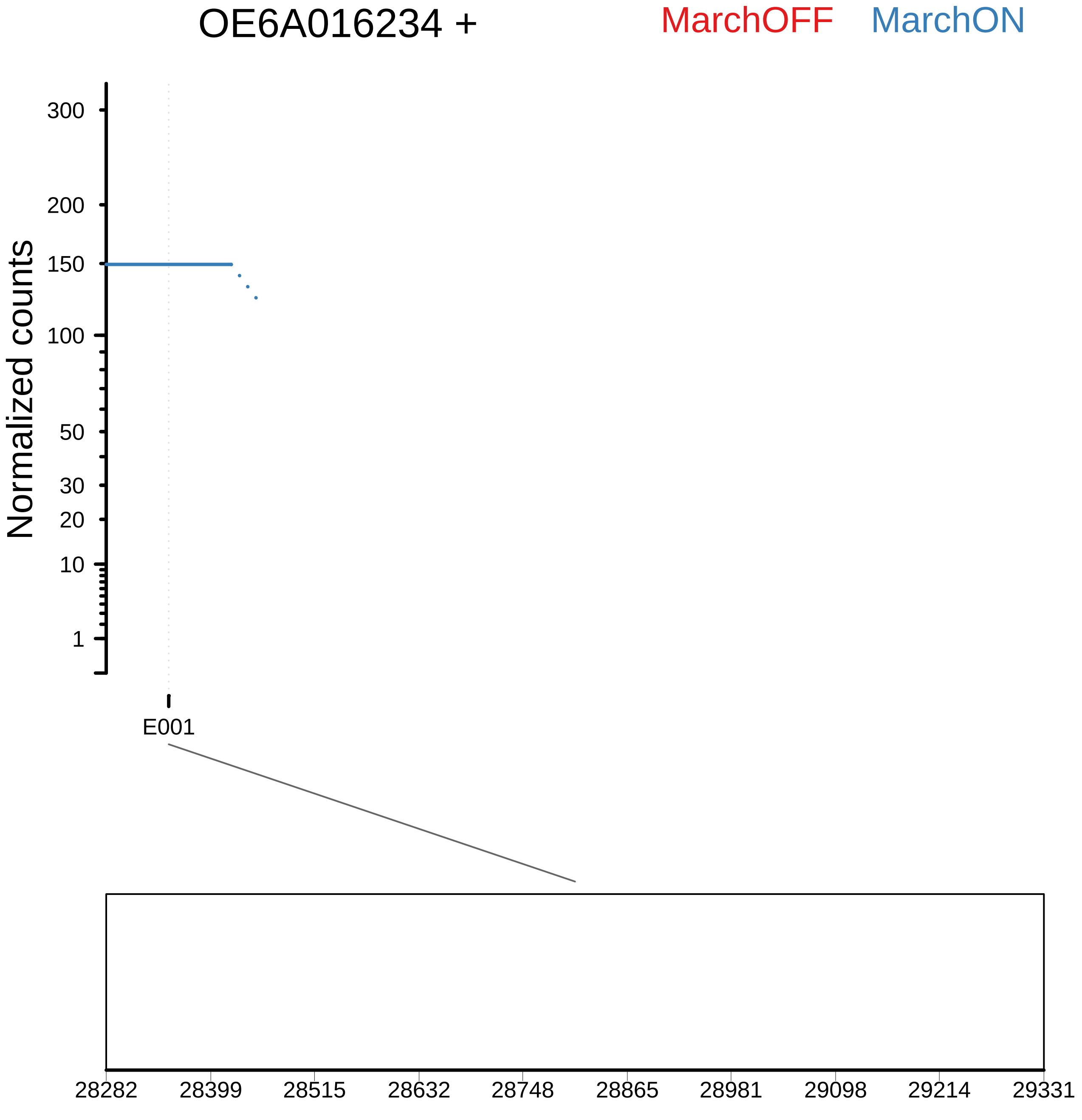

OE6A016234 +
MarchOFF
MarchON
Normalized counts
300
200
150
100
50
30
20
10
1
E001
28282
28399
28515
28632
28748
28865
28981
29098
29214
29331

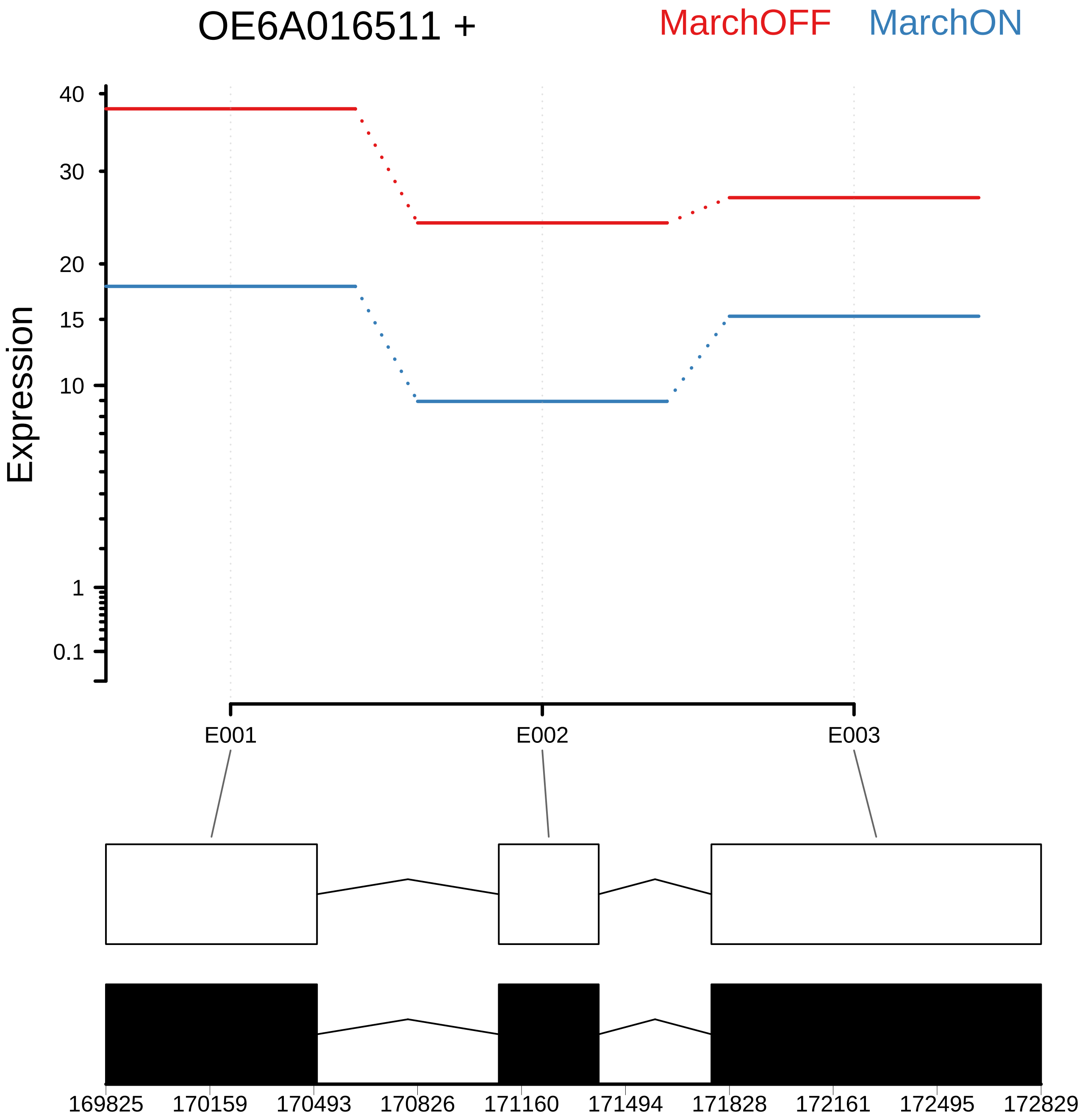

OE6A016511 +
MarchOFF
MarchON
Expression
40
30
20
15
10
1
0.1
E001
E002
E003
169825
170159
170493
170826
171160
171494
171828
172161
172495
172829

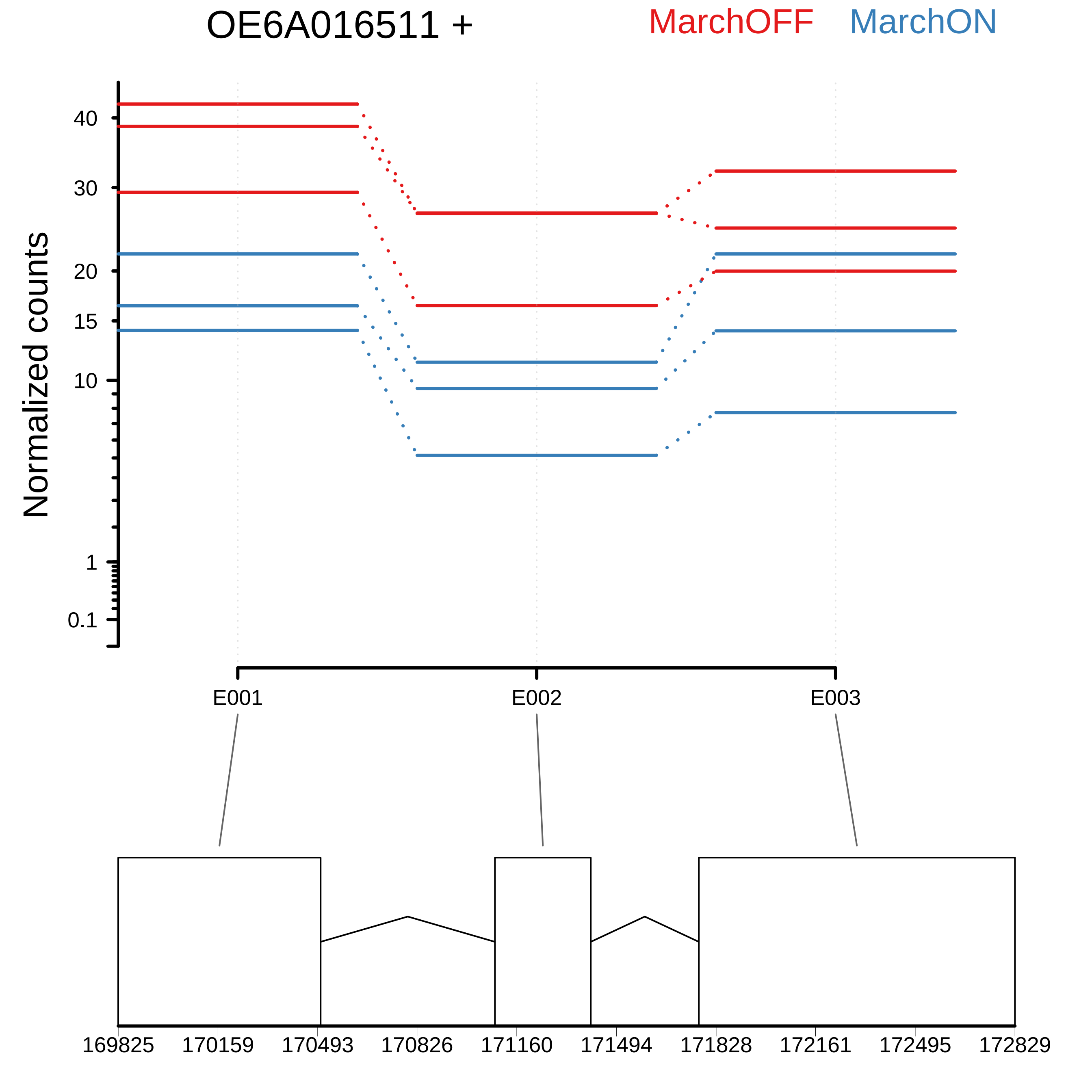

OE6A016511 +
MarchOFF
MarchON
Normalized counts
40
30
20
15
10
1
0.1
E001
E002
E003
169825
170159
170493
170826
171160
171494
171828
172161
172495
172829

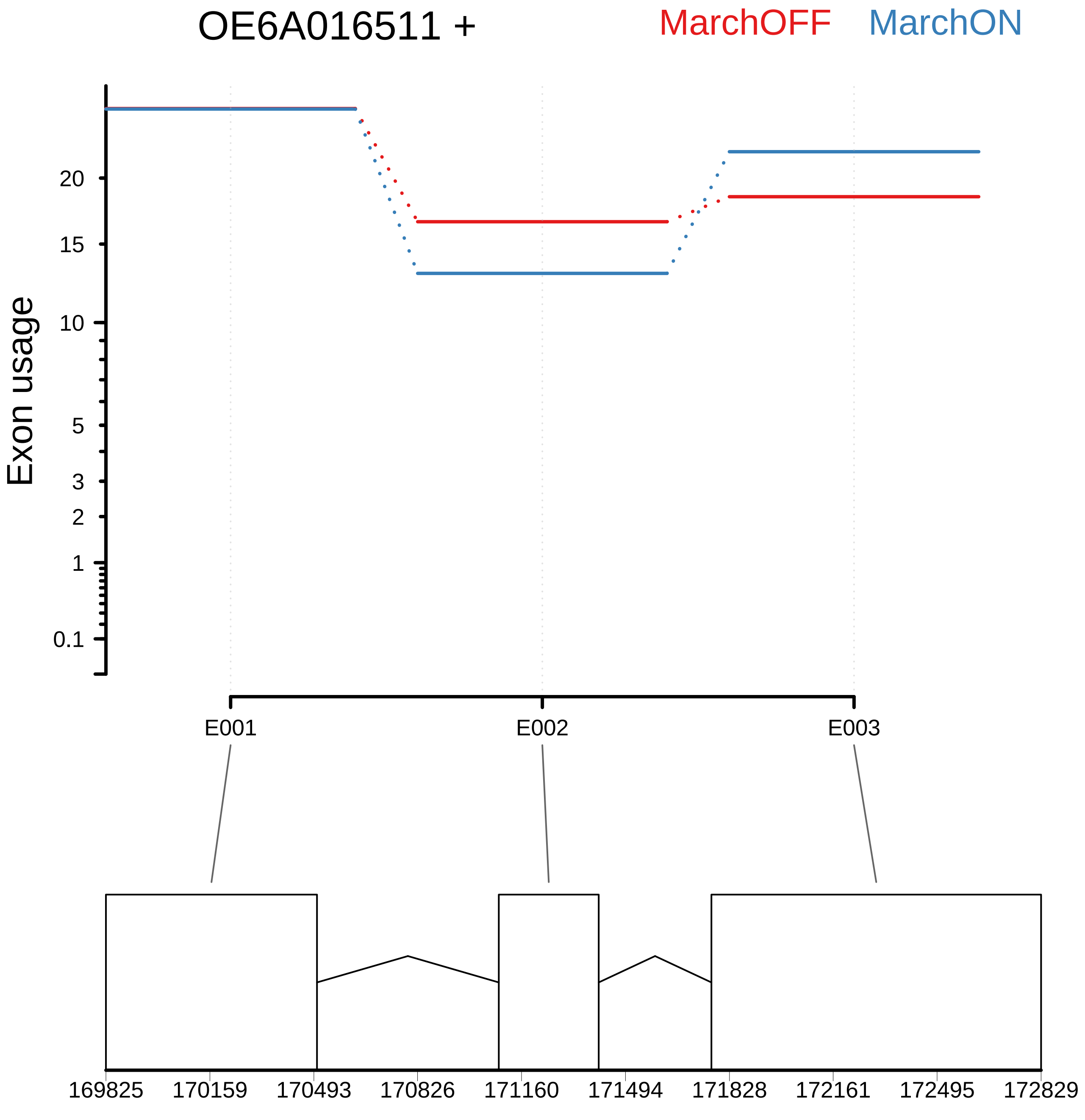
OE6A016511 +
MarchOFF
MarchON
Exon usage
20
15
10
5
3
2
1
0.1
E001
E002
E003
169825
170159
170493
170826
171160
171494
171828
172161
172495
172829

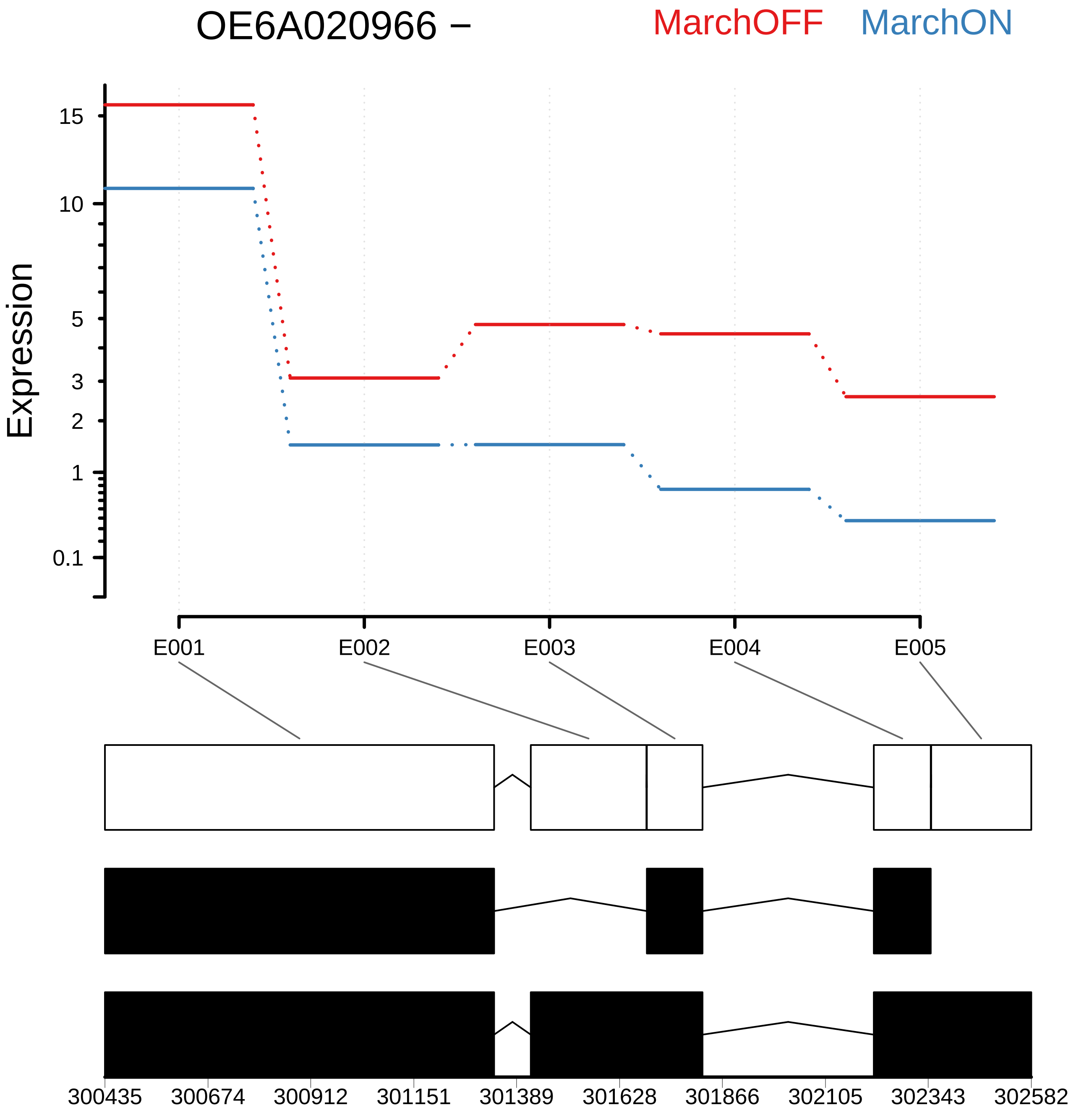
OE6A020966 –
MarchOFF
MarchON
Expression
15
10
5
3
2
1
0.1
E001
E002
E003
E004
E005
300435
300674
300912
301151
301389
301628
301866
302105
302343
302582

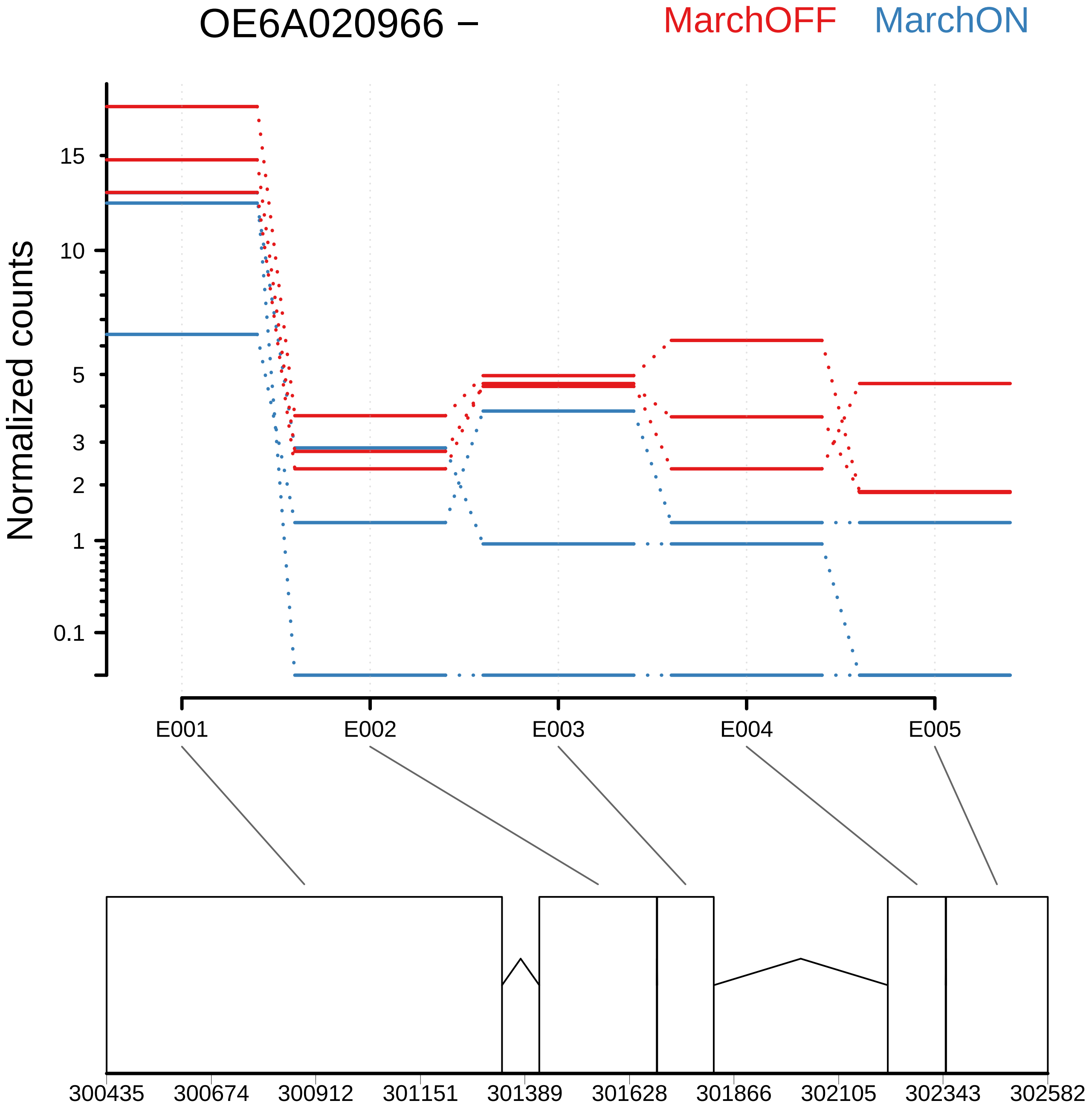
OE6A020966 −
MarchOFF
MarchON
Normalized counts
15
10
5
3
2
1
0.1
E001
E002
E003
E004
E005
300435
300674
300912
301151
301389
301628
301866
302105
302343
302582

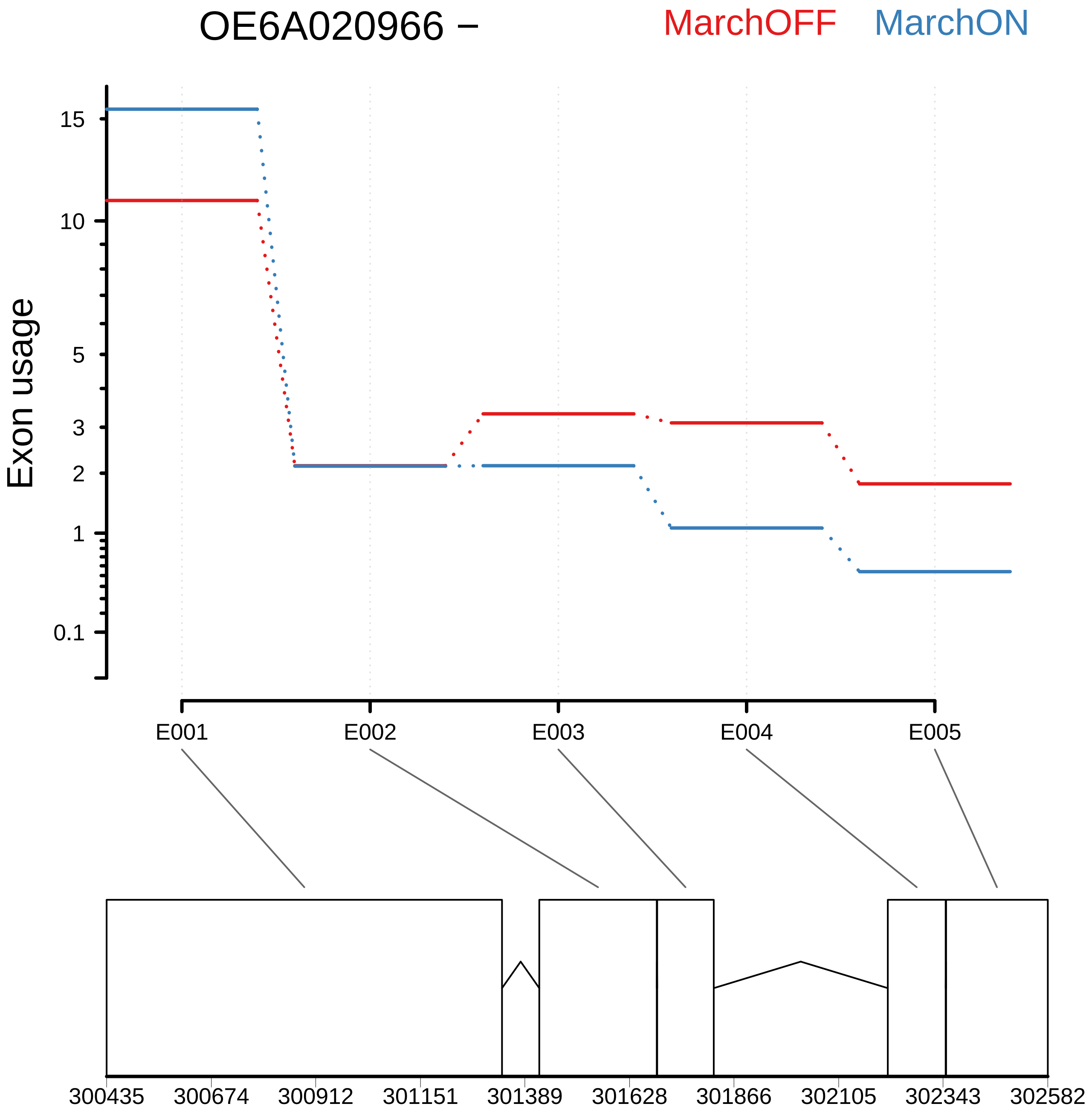

OE6A020966 −
MarchOFF
MarchON
Exon usage
15
10
5
3
2
1
0.1
E001
E002
E003
E004
E005
300435
300674
300912
301151
301389
301628
301866
302105
302343
302582

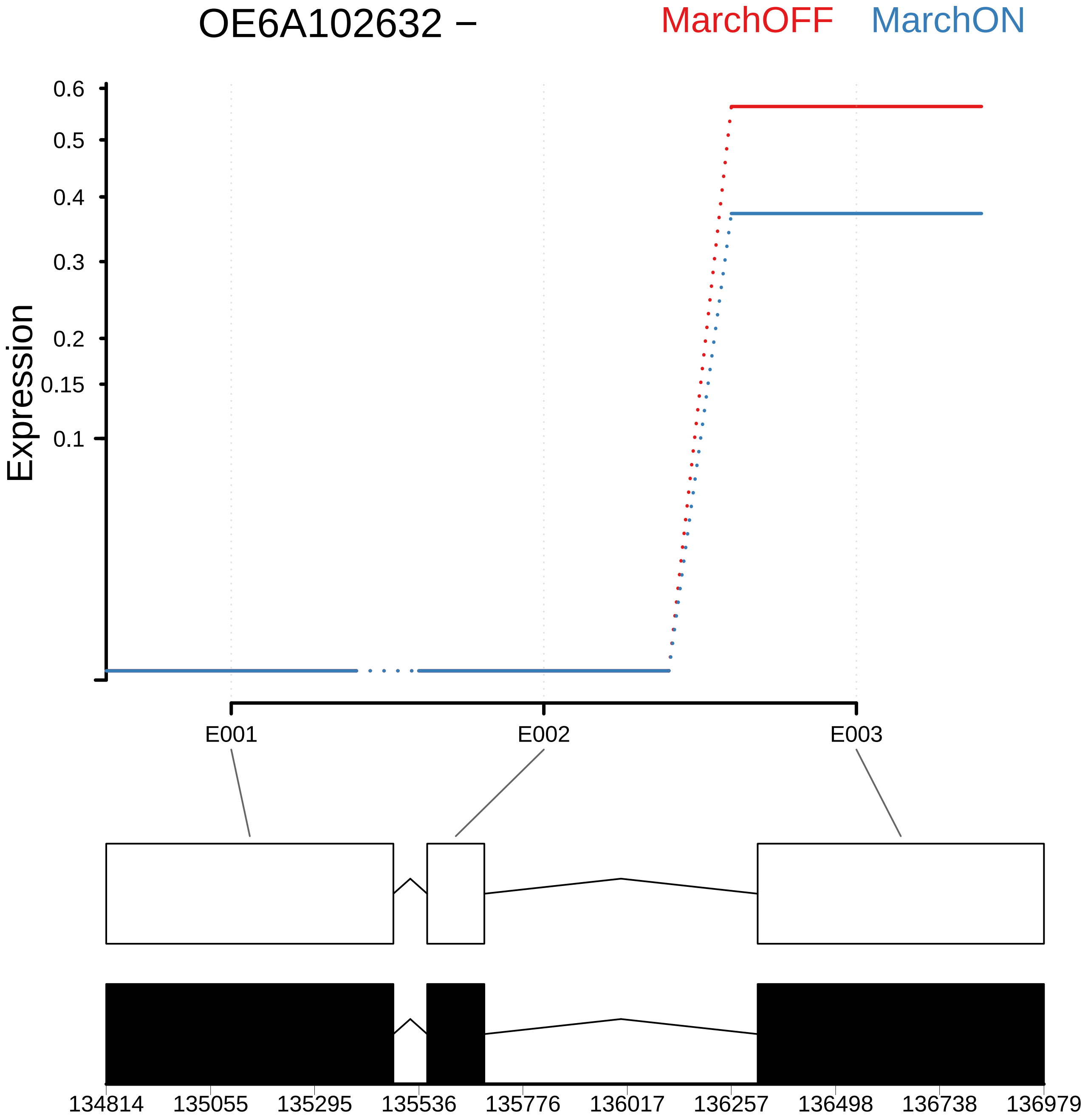
OE6A102632 −
MarchOFF
MarchON
Expression
0.6
0.5
0.4
0.3
0.2
0.15
0.1
E001
E002
E003
134814
135055
135295
135536
135776
136017
136257
136498
136738
136979

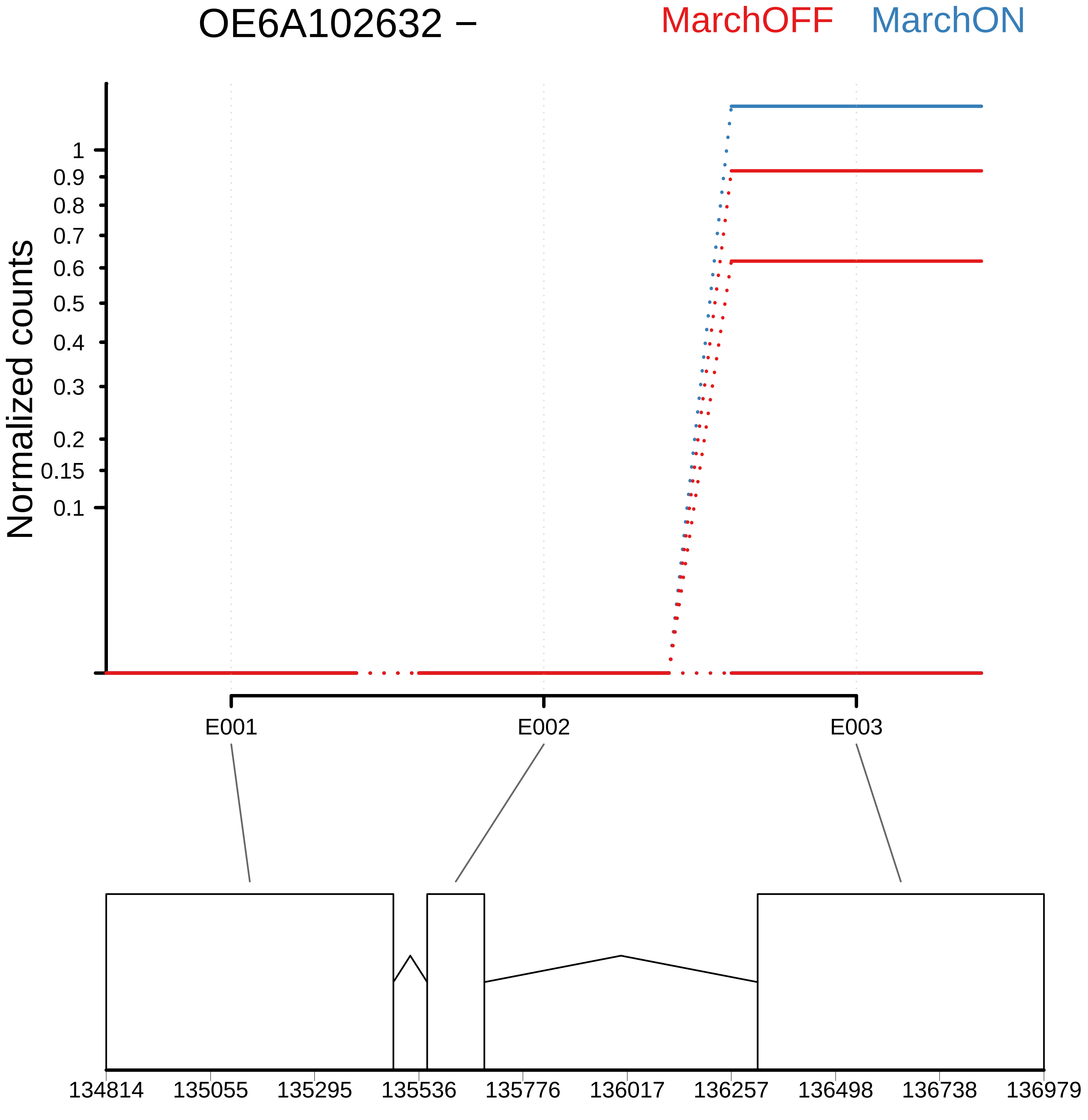

OE6A102632 −
MarchOFF
MarchON
Normalized counts
1
0.9
0.8
0.7
0.6
0.5
0.4
0.3
0.2
0.15
0.1
E001
E002
E003
134814
135055
135295
135536
135776
136017
136257
136498
136738
136979

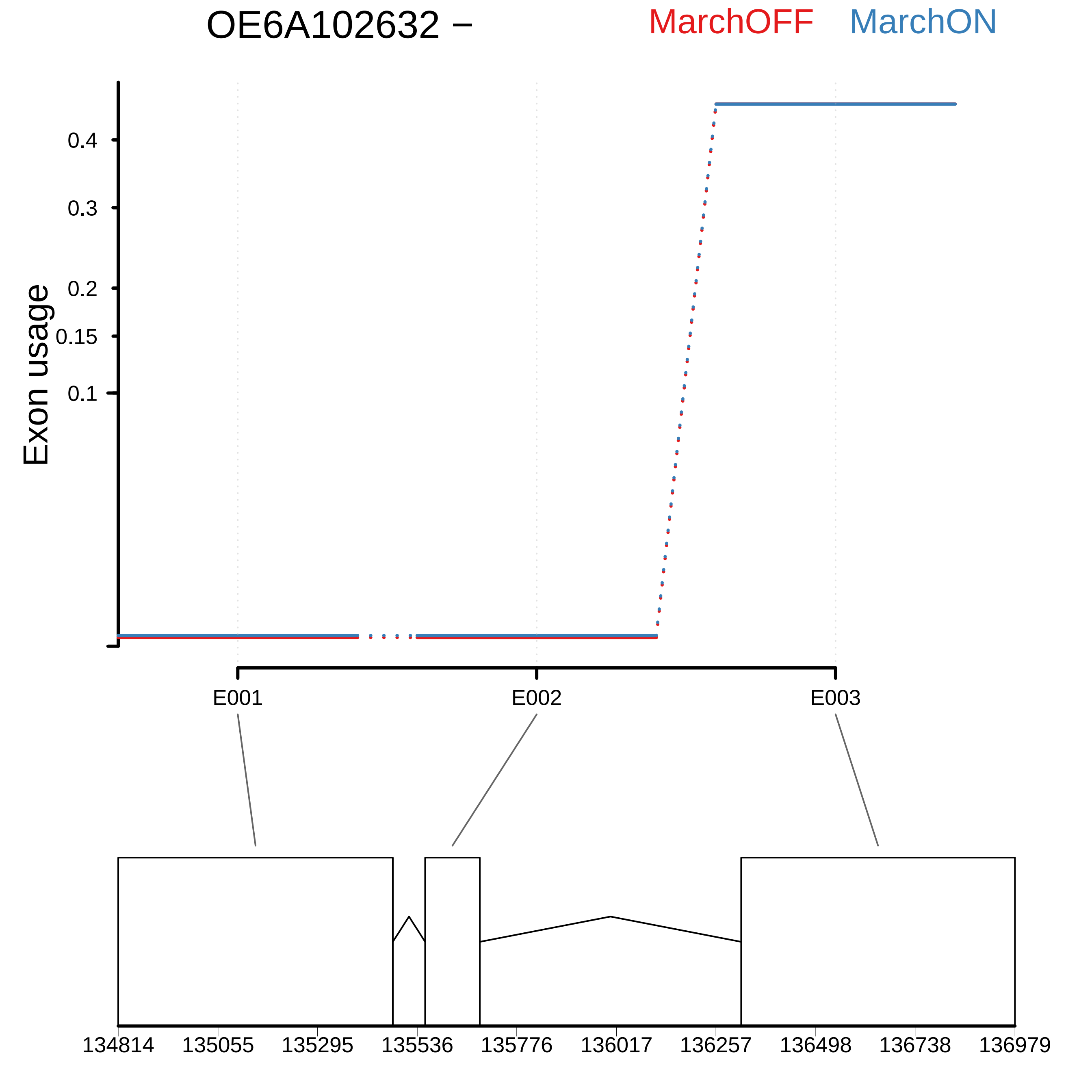

OE6A102632 −
MarchOFF
MarchON
Exon usage
0.4
0.3
0.2
0.15
0.1
E001
E002
E003
134814
135055
135295
135536
135776
136017
136257
136498
136738
136979

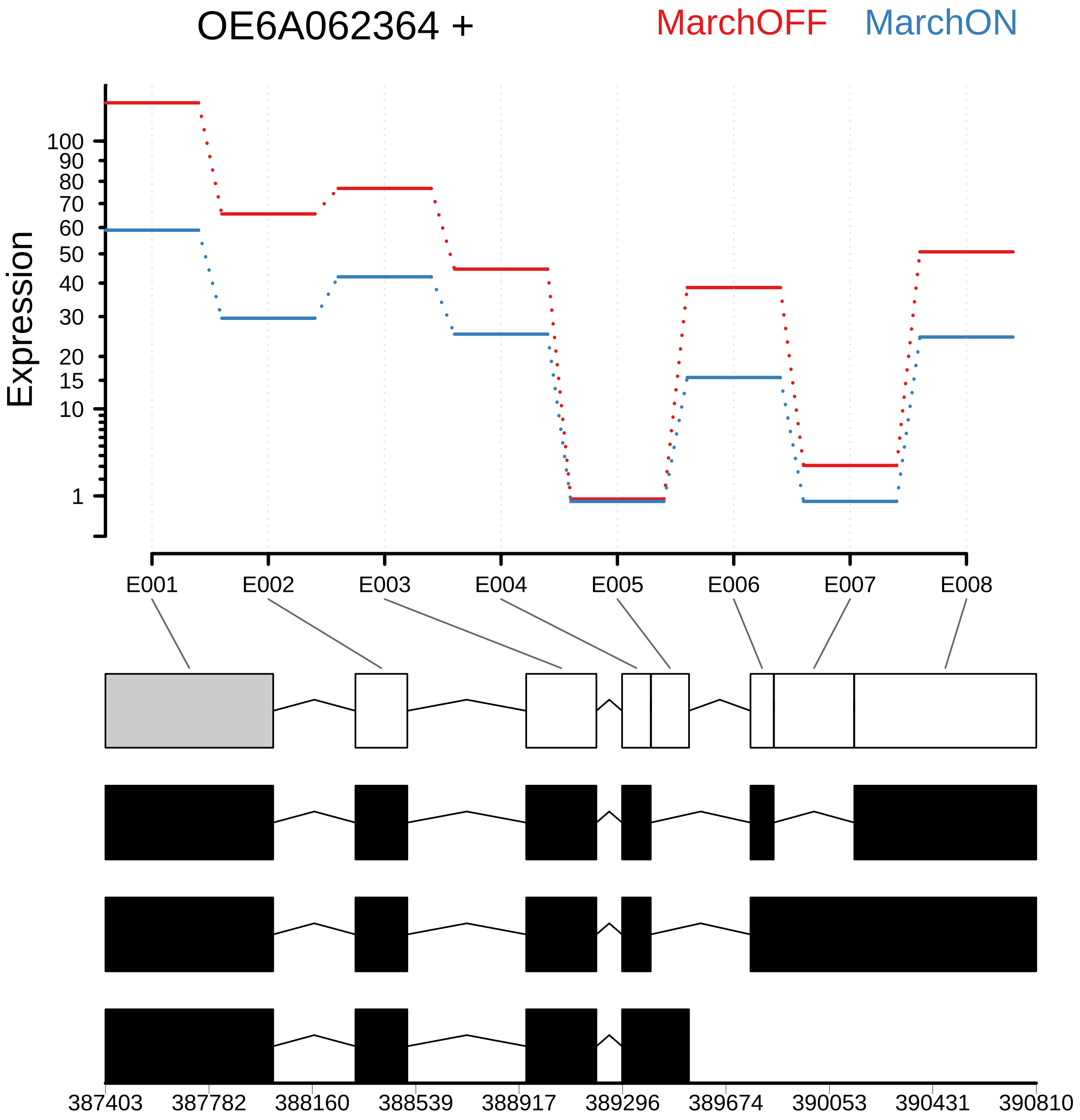

OE6A062364 +
MarchOFF
MarchON
Expression
100
90
80
70
60
50
40
30
20
15
10
1
E001
E002
E003
E004
E005
E006
E007
E008
387403
387782
388160
388539
388917
389296
389674
390053
390431
390810

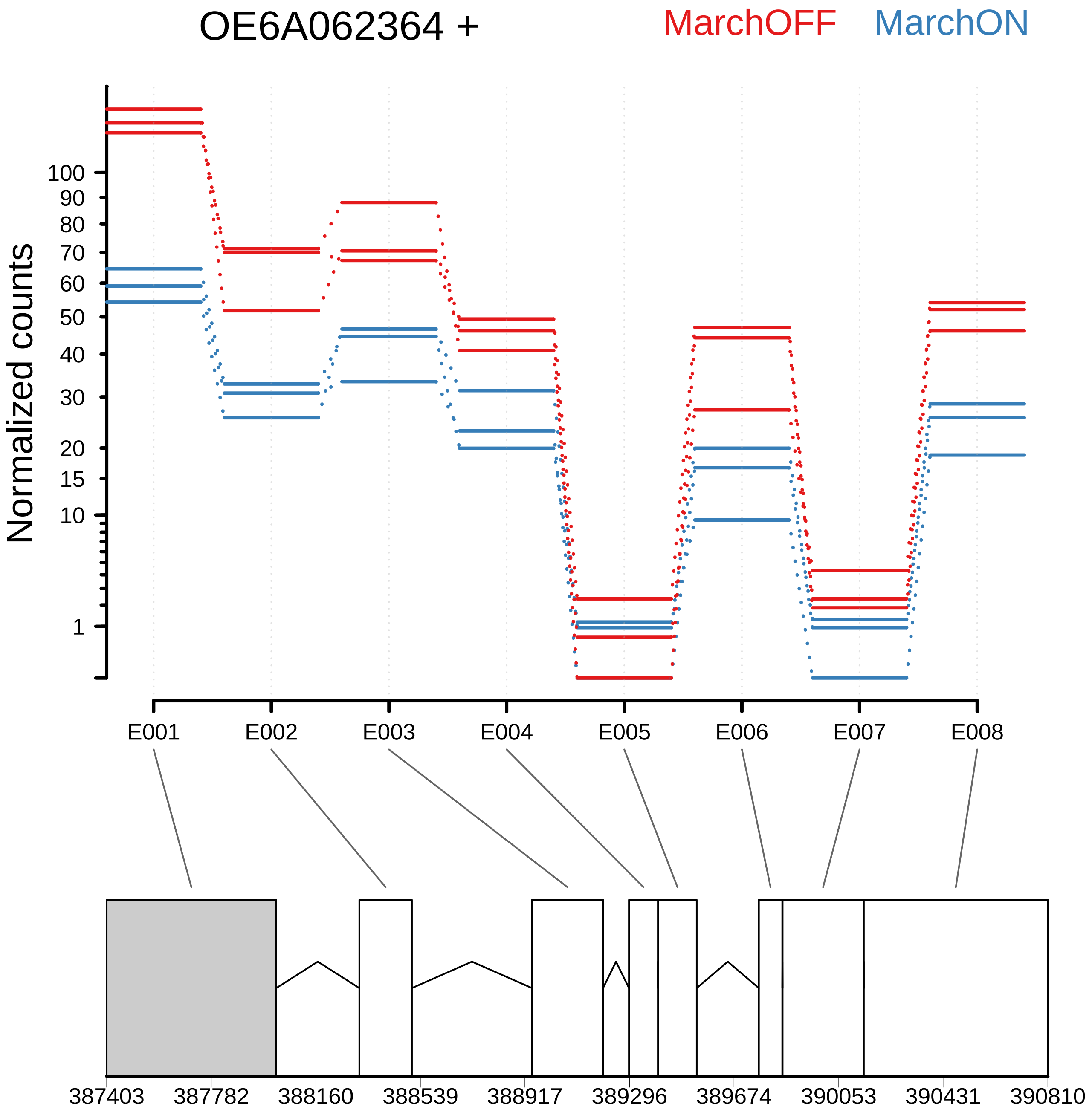

OE6A062364 +
MarchOFF
MarchON
Normalized counts
100
90
80
70
60
50
40
30
20
15
10
1
E001
E002
E003
E004
E005
E006
E007
E008
387403
387782
388160
388539
388917
389296
389674
390053
390431
390810

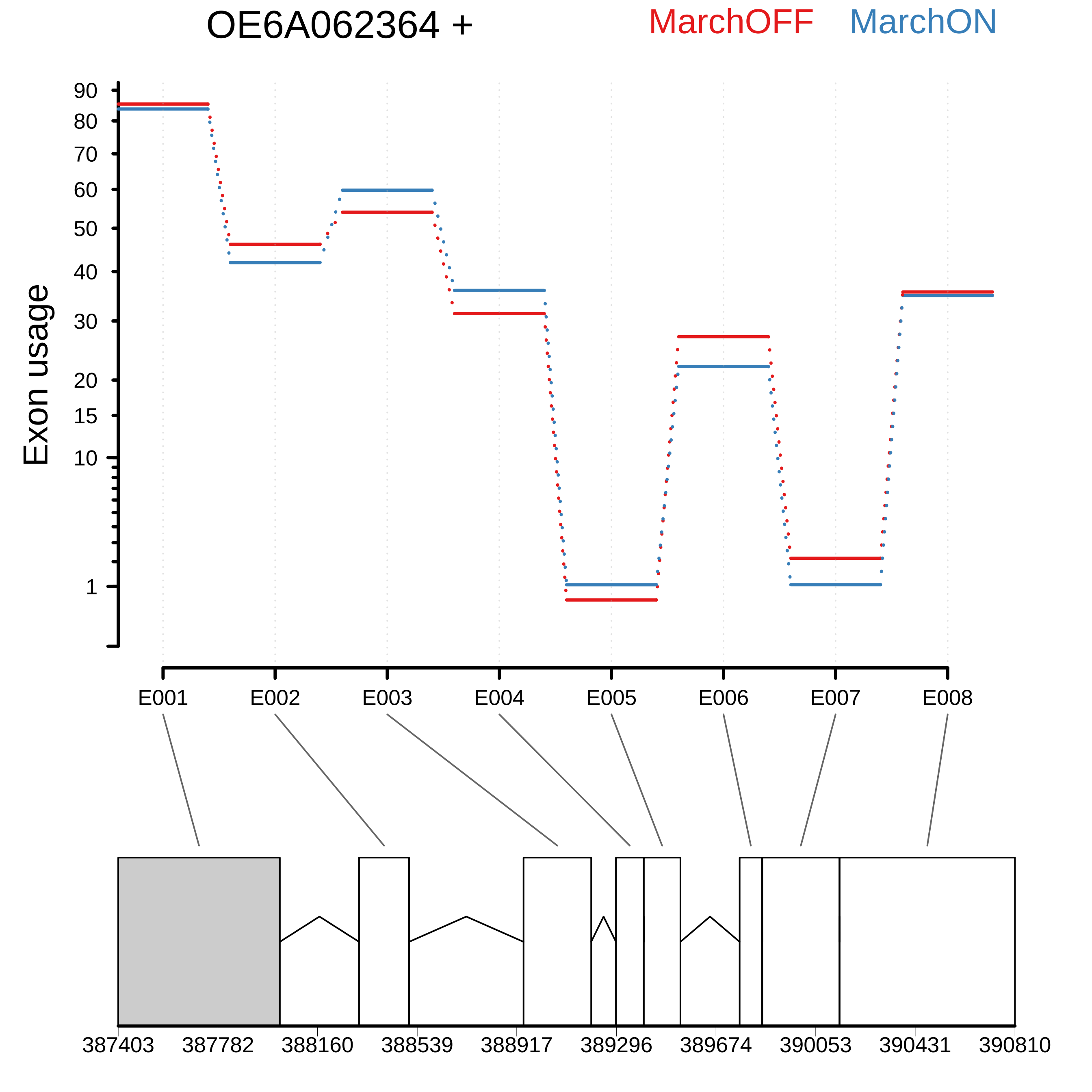

OE6A062364 +
MarchOFF
MarchON
Exon usage
90
80
70
60
50
40
30
20
15
10
1
E001
E002
E003
E004
E005
E006
E007
E008
387403
387782
388160
388539
388917
389296
389674
390053
390431
390810

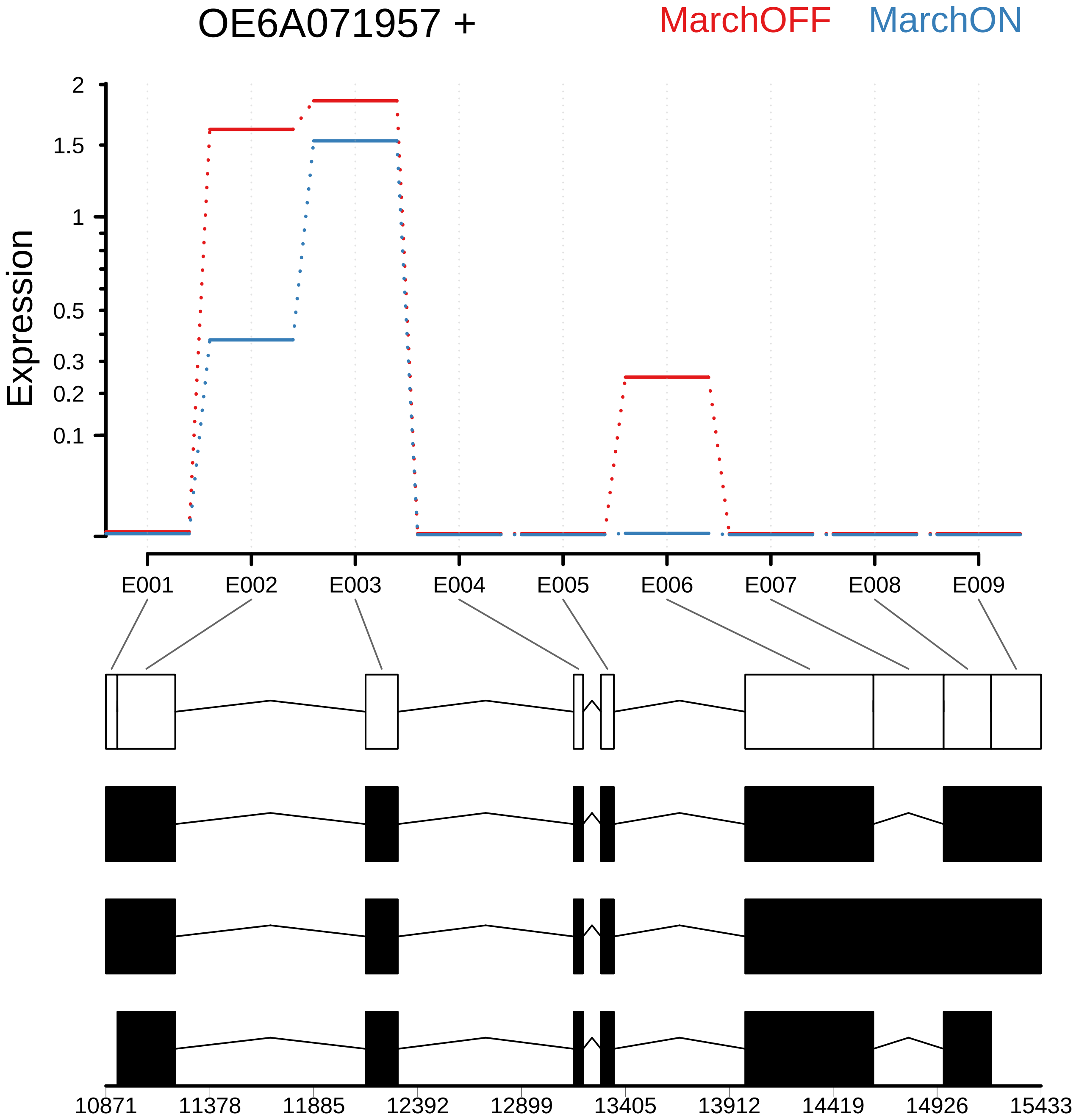

OE6A071957 +
MarchOFF
MarchON
Expression
2
1.5
1
0.5
0.3
0.2
0.1
E001
E002
E003
E004
E005
E006
E007
E008
E009
10871
11378
11885
12392
12899
13405
13912
14419
14926
15433

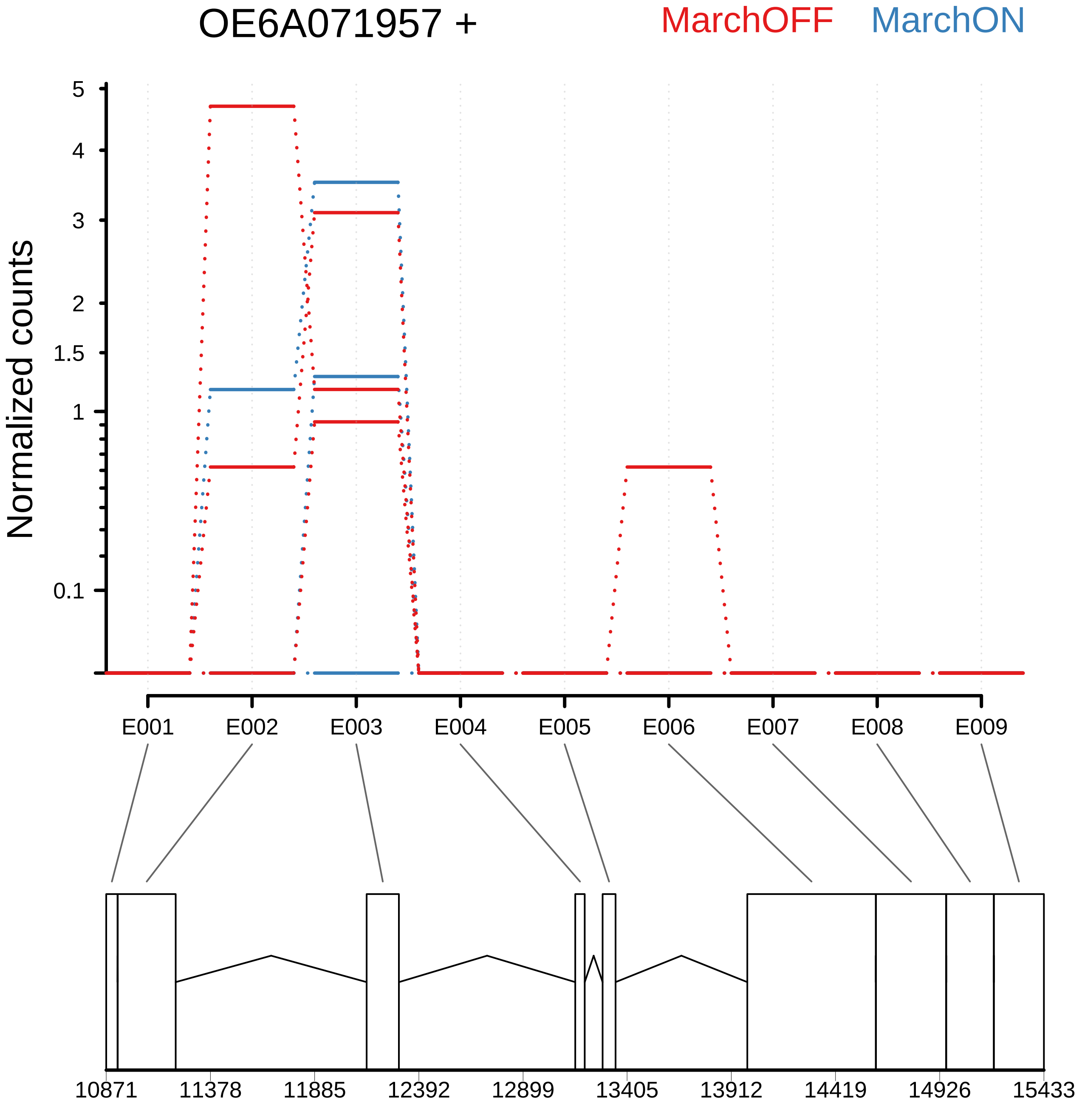
OE6A071957 +
MarchOFF
MarchON
Normalized counts
5
4
3
2
1.5
1
0.1
E001
E002
E003
E004
E005
E006
E007
E008
E009
10871
11378
11885
12392
12899
13405
13912
14419
14926
15433

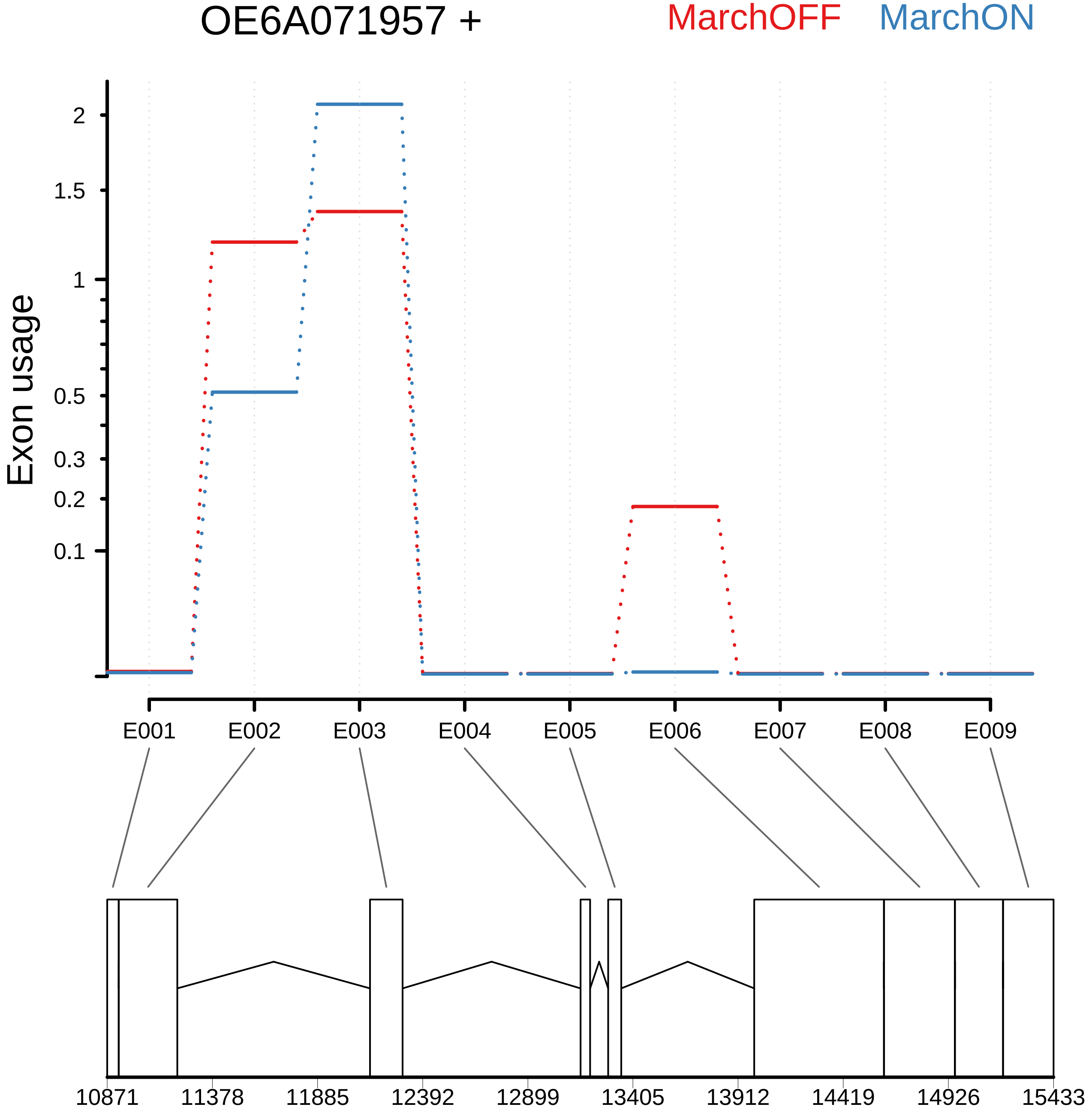

OE6A071957 +
MarchOFF
MarchON
Exon usage
2
1.5
1
0.5
0.3
0.2
0.1
E001
E002
E003
E004
E005
E006
E007
E008
E009
10871
11378
11885
12392
12899
13405
13912
14419
14926
15433

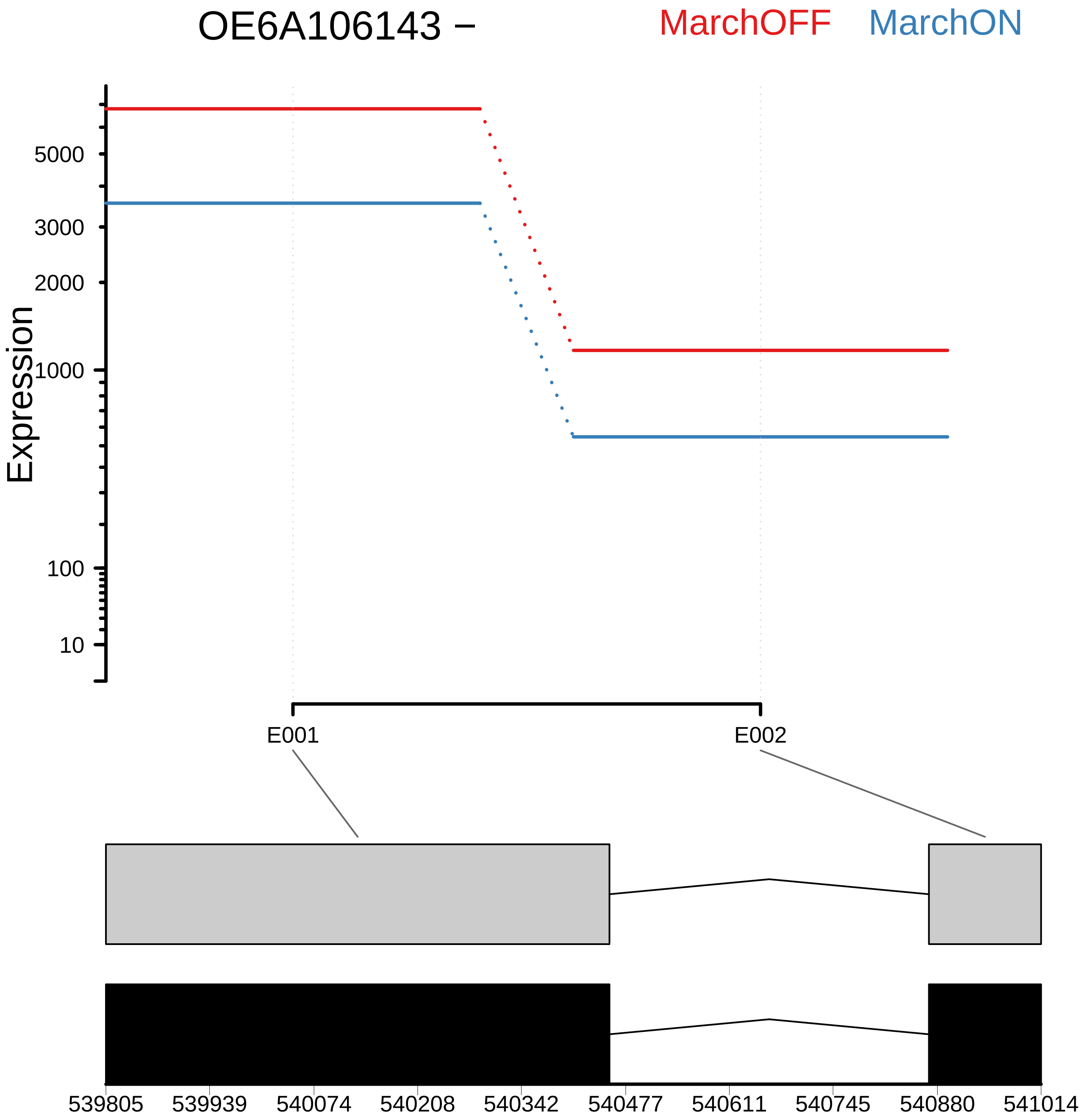

OE6A106143 −
MarchOFF
MarchON
Expression
5000
3000
2000
1000
100
10
E001
E002
539805
539939
540074
540208
540342
540477
540611
540745
540880
541014

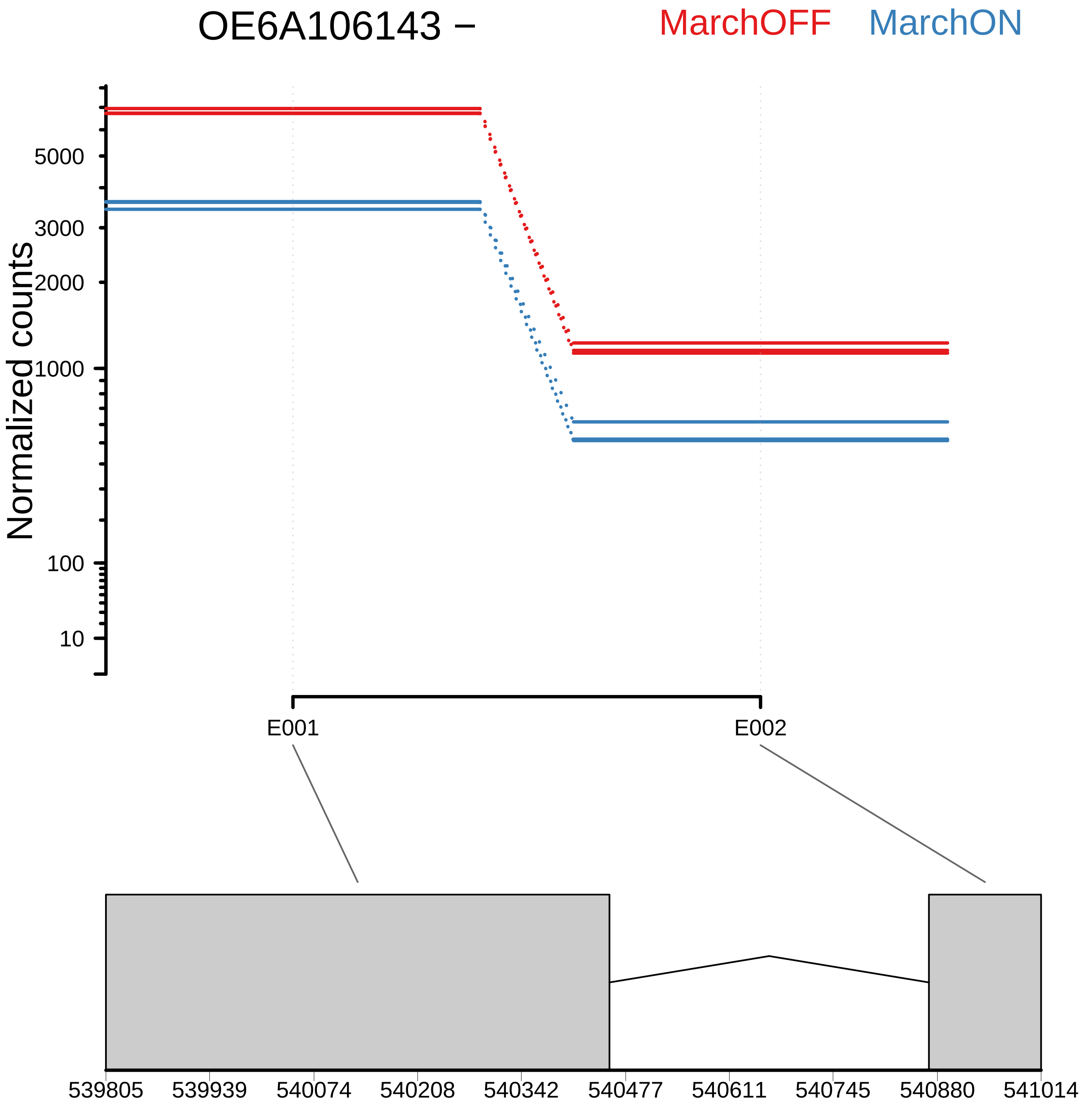

OE6A106143 −
MarchOFF
MarchON
Normalized counts
5000
3000
2000
1000
100
10
E001
E002
539805
539939
540074
540208
540342
540477
540611
540745
540880
541014

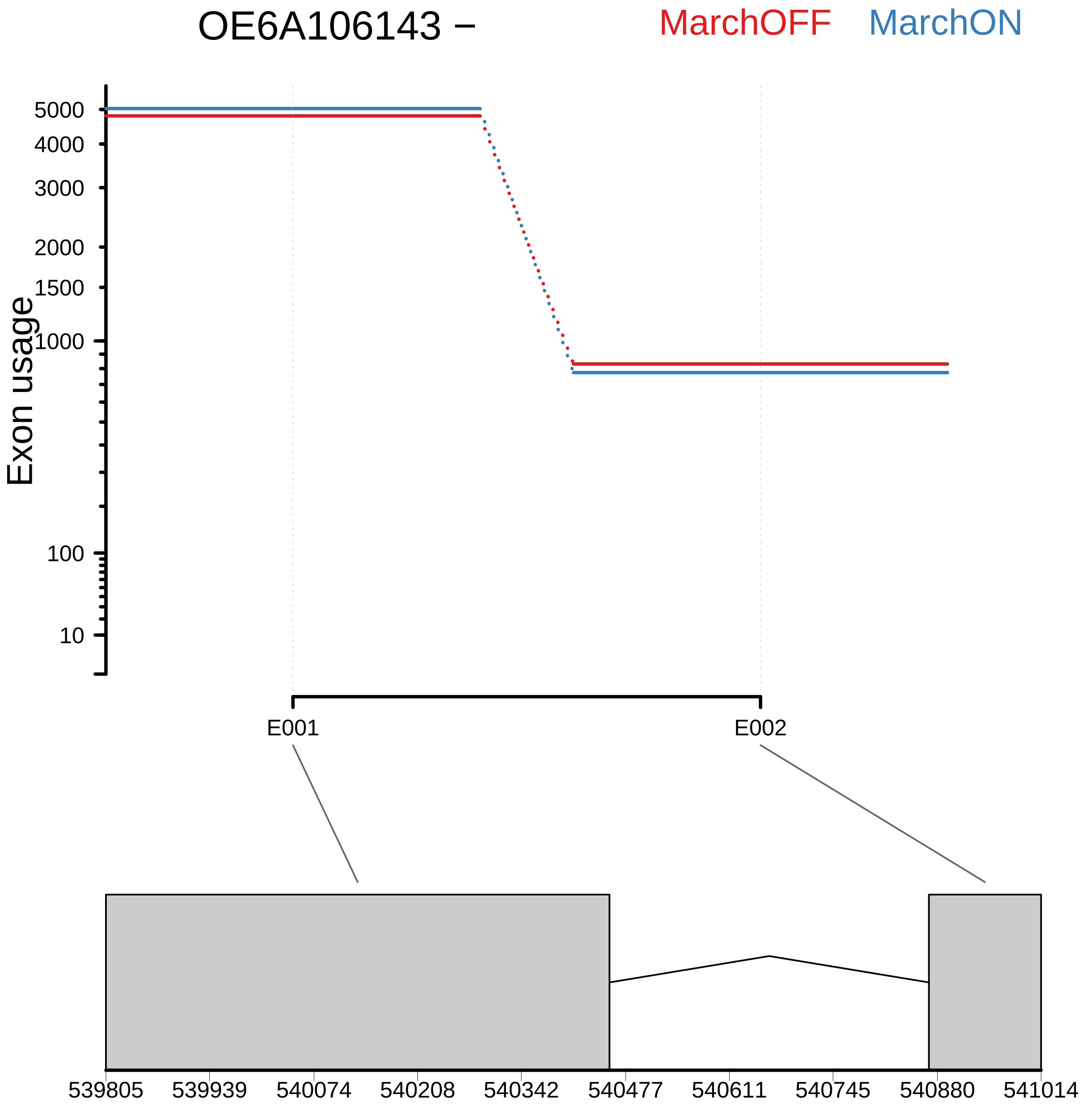
OE6A106143 −
MarchOFF
MarchON
Exon usage
5000
4000
3000
2000
1500
1000
100
10
E001
E002
539805
539939
540074
540208
540342
540477
540611
540745
540880
541014

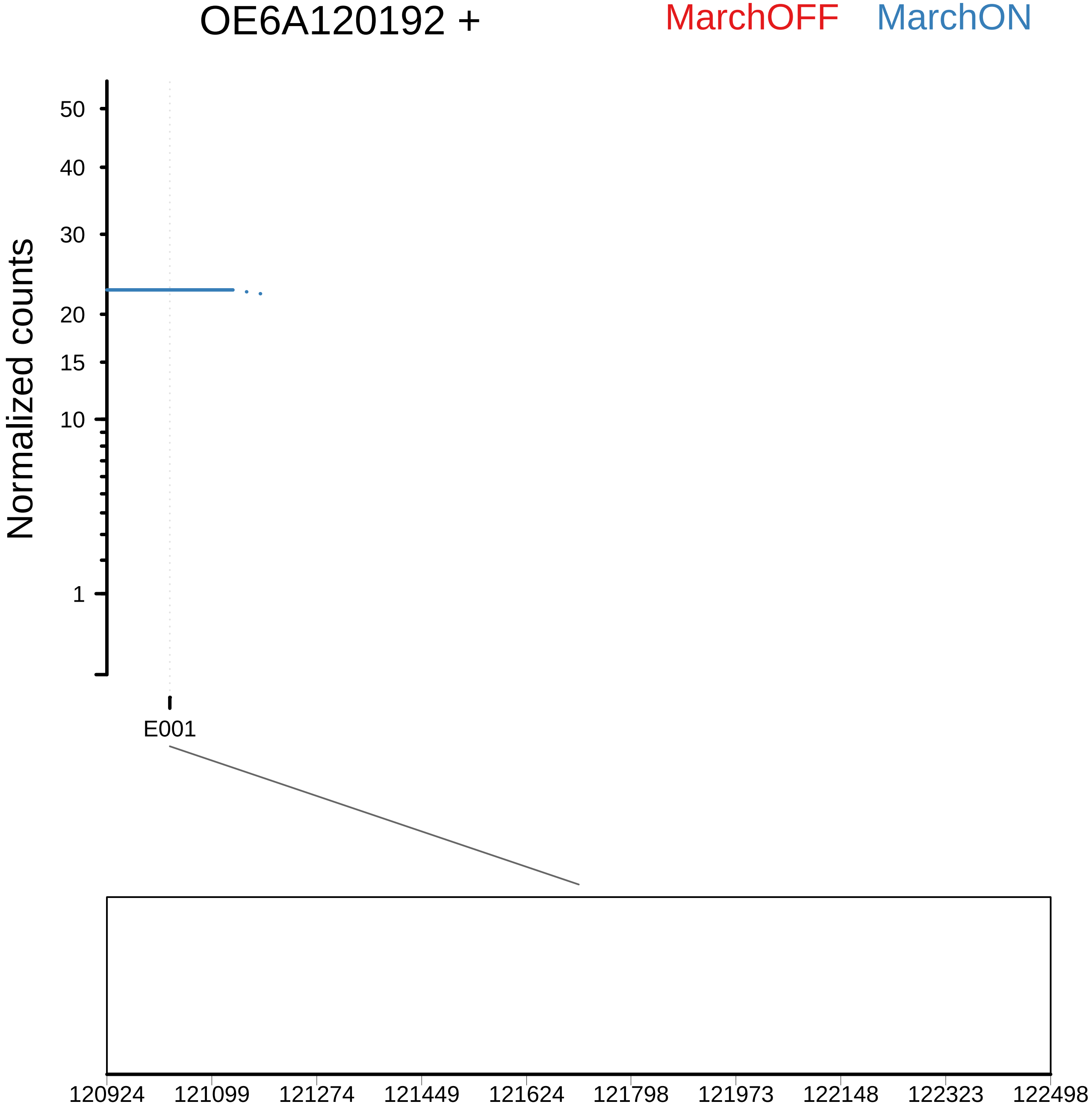

OE6A120192 +
MarchOFF
MarchON
Normalized counts
50
40
30
20
15
10
1
E001
120924
121099
121274
121449
121624
121798
121973
122148
122323
122498

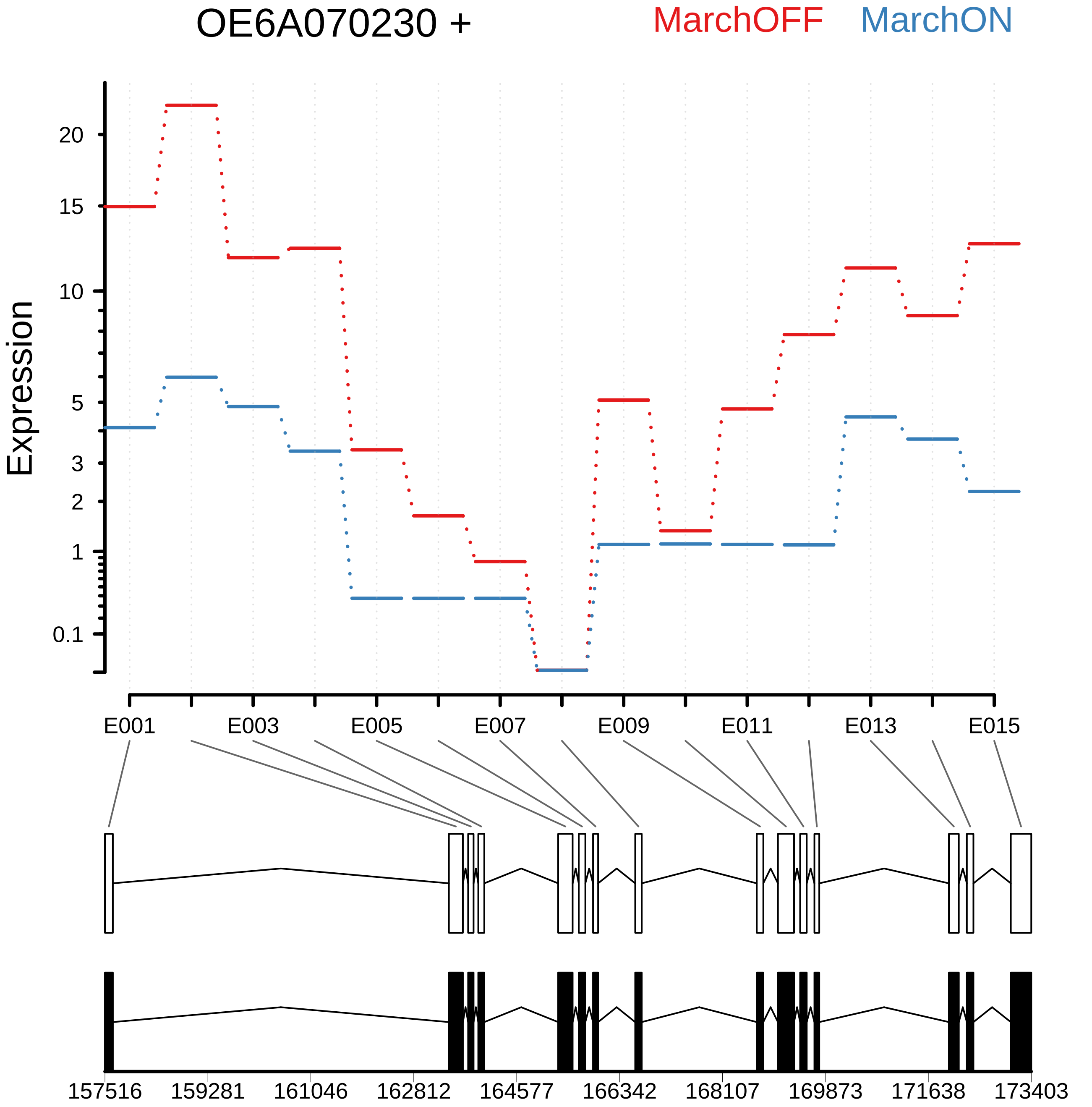

OE6A070230 +
MarchOFF
MarchON
Expression
20
15
10
5
3
2
1
0.1
E001
E003
E005
E007
E009
E011
E013
E015
157516
159281
161046
162812
164577
166342
168107
169873
171638
173403

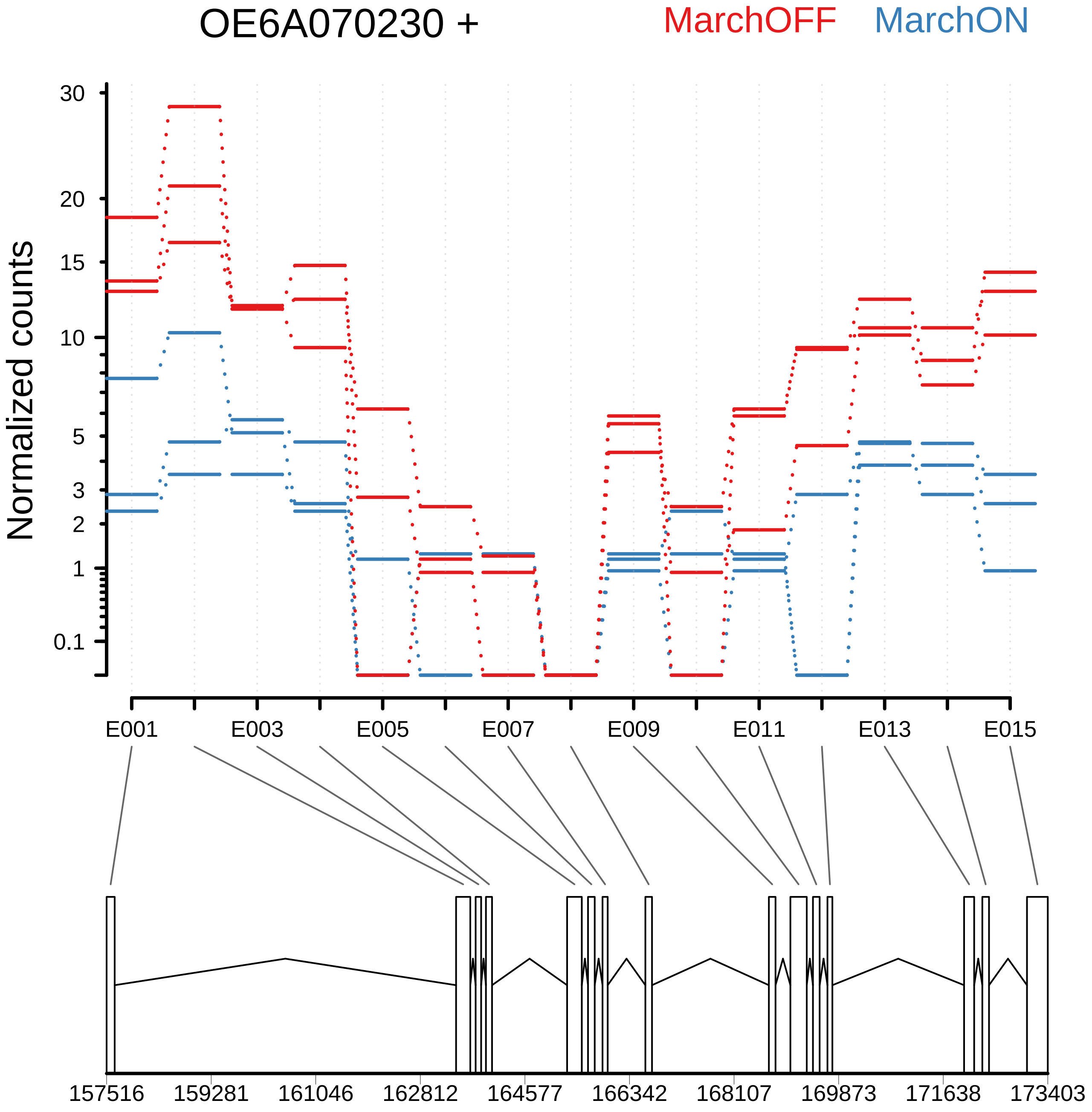

OE6A070230 +
MarchOFF
MarchON
Normalized counts
30
20
15
10
5
3
2
1
0.1
E001
E003
E005
E007
E009
E011
E013
E015
157516
159281
161046
162812
164577
166342
168107
169873
171638
173403

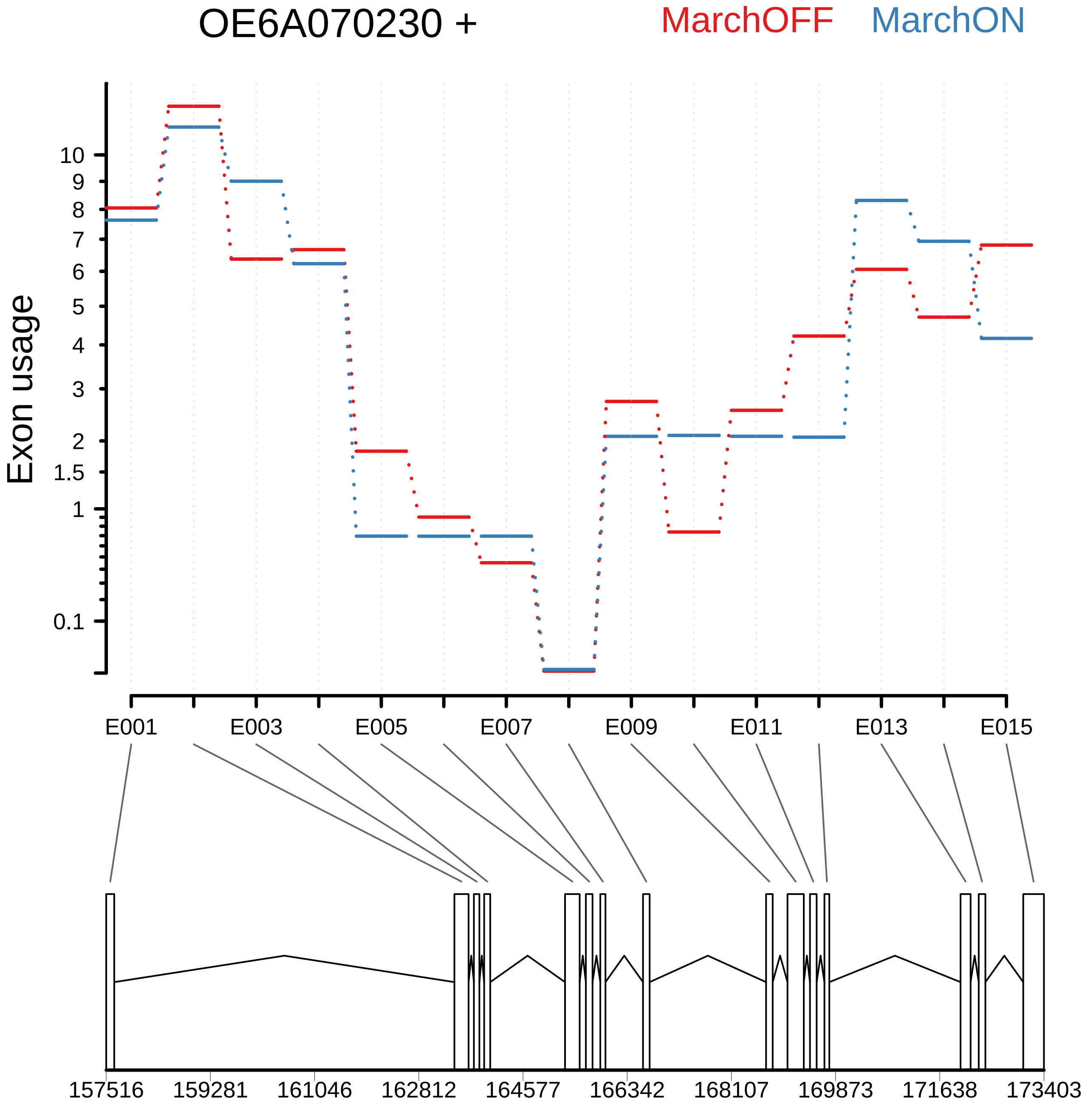

OE6A070230 +
MarchOFF
MarchON
Exon usage
10
9
8
7
6
5
4
3
2
1.5
1
0.1
E001
E003
E005
E007
E009
E011
E013
E015
157516
159281
161046
162812
164577
166342
168107
169873
171638
173403

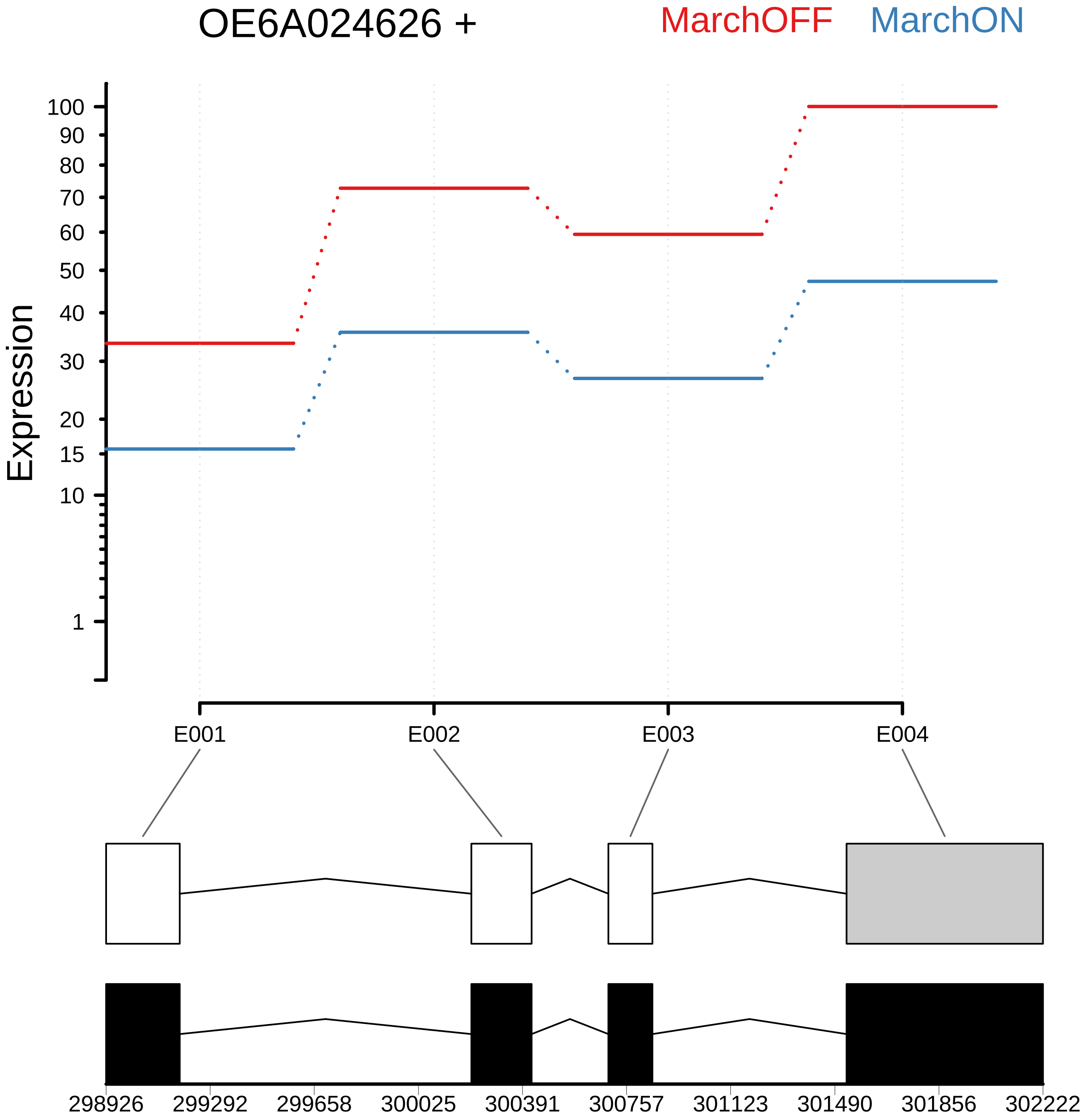

OE6A024626 +
MarchOFF
MarchON
Expression
100
90
80
70
60
50
40
30
20
15
10
1
E001
E002
E003
E004
298926
299292
299658
300025
300391
300757
301123
301490
301856
302222

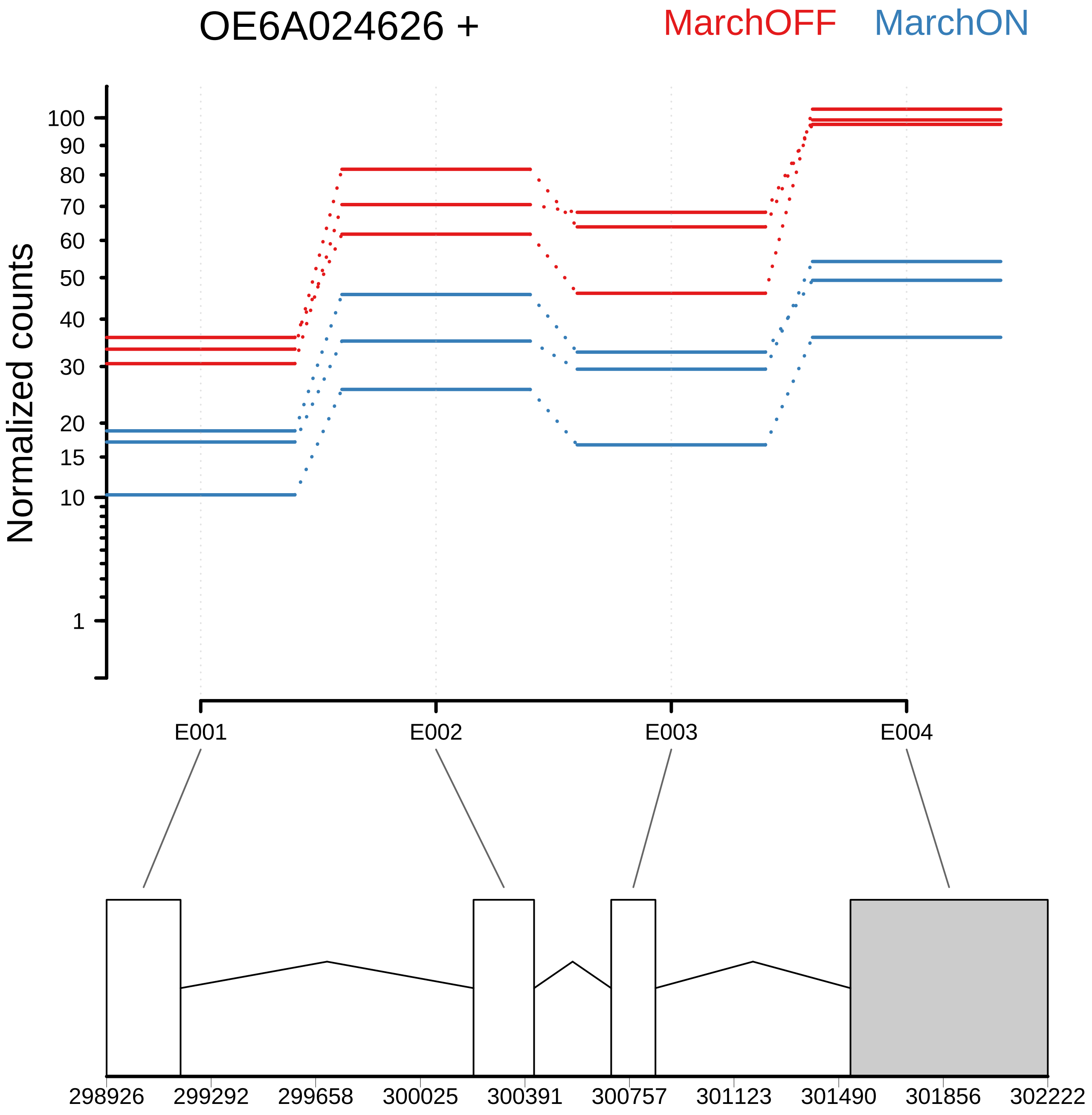

OE6A024626 +
MarchOFF
MarchON
Normalized counts
100
90
80
70
60
50
40
30
20
15
10
1
E001
E002
E003
E004
298926
299292
299658
300025
300391
300757
301123
301490
301856
302222

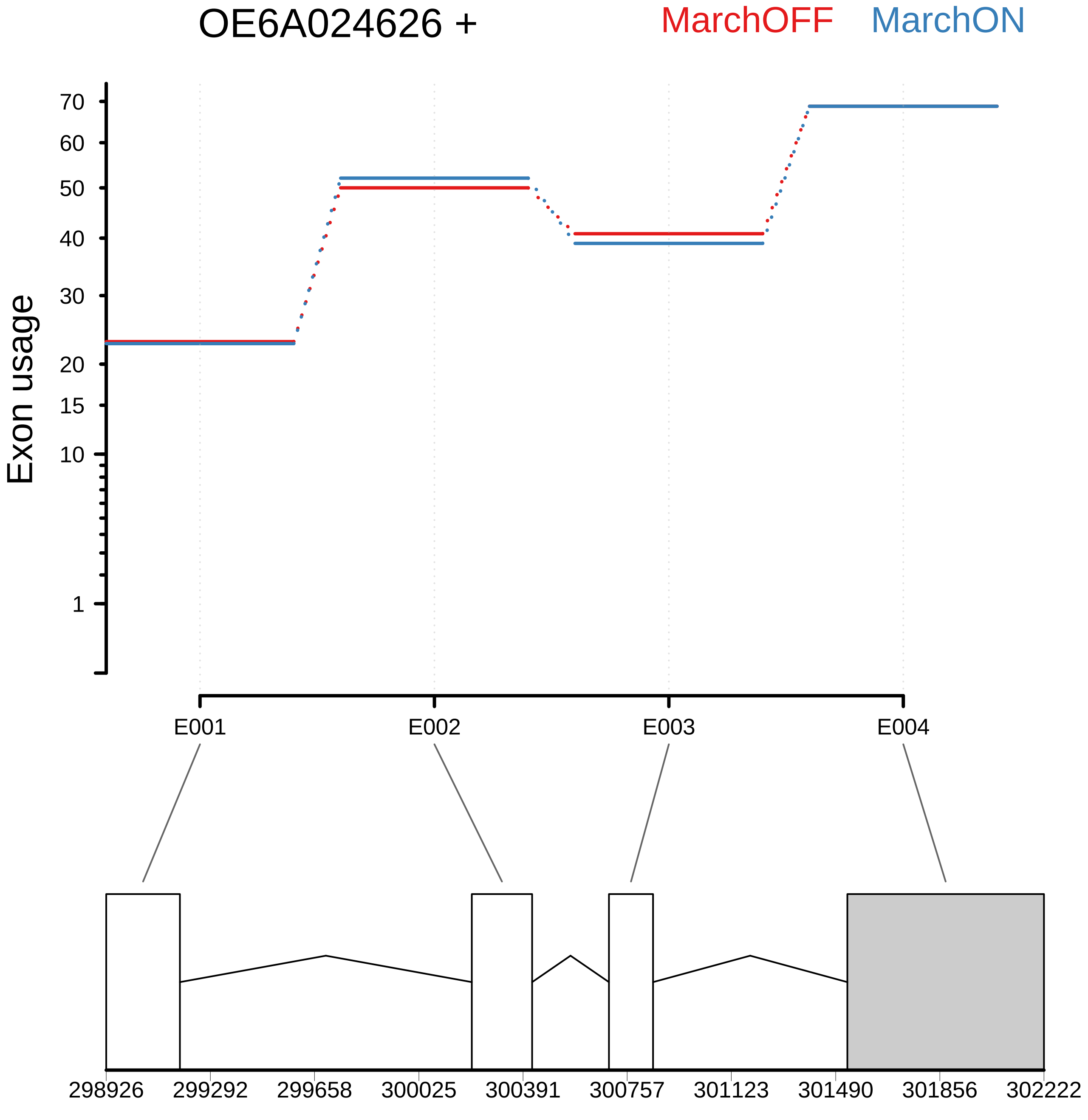

OE6A024626 +
MarchOFF
MarchON
Exon usage
70
60
50
40
30
20
15
10
1
E001
E002
E003
E004
298926
299292
299658
300025
300391
300757
301123
301490
301856
302222

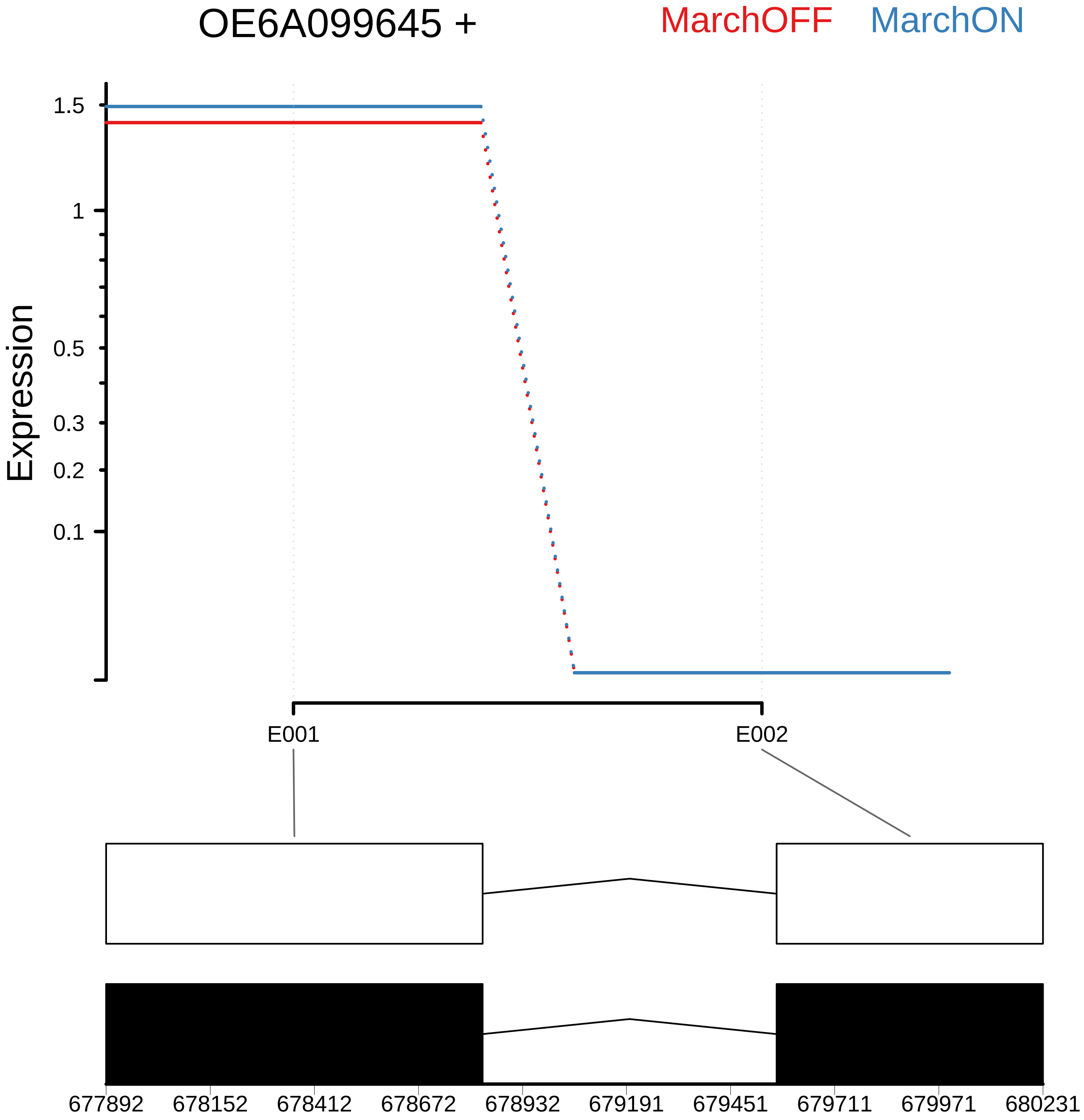

OE6A099645 +
MarchOFF
MarchON
Expression
1.5
1
0.5
0.3
0.2
0.1
E001
E002
677892
678152
678412
678672
678932
679191
679451
679711
679971
680231

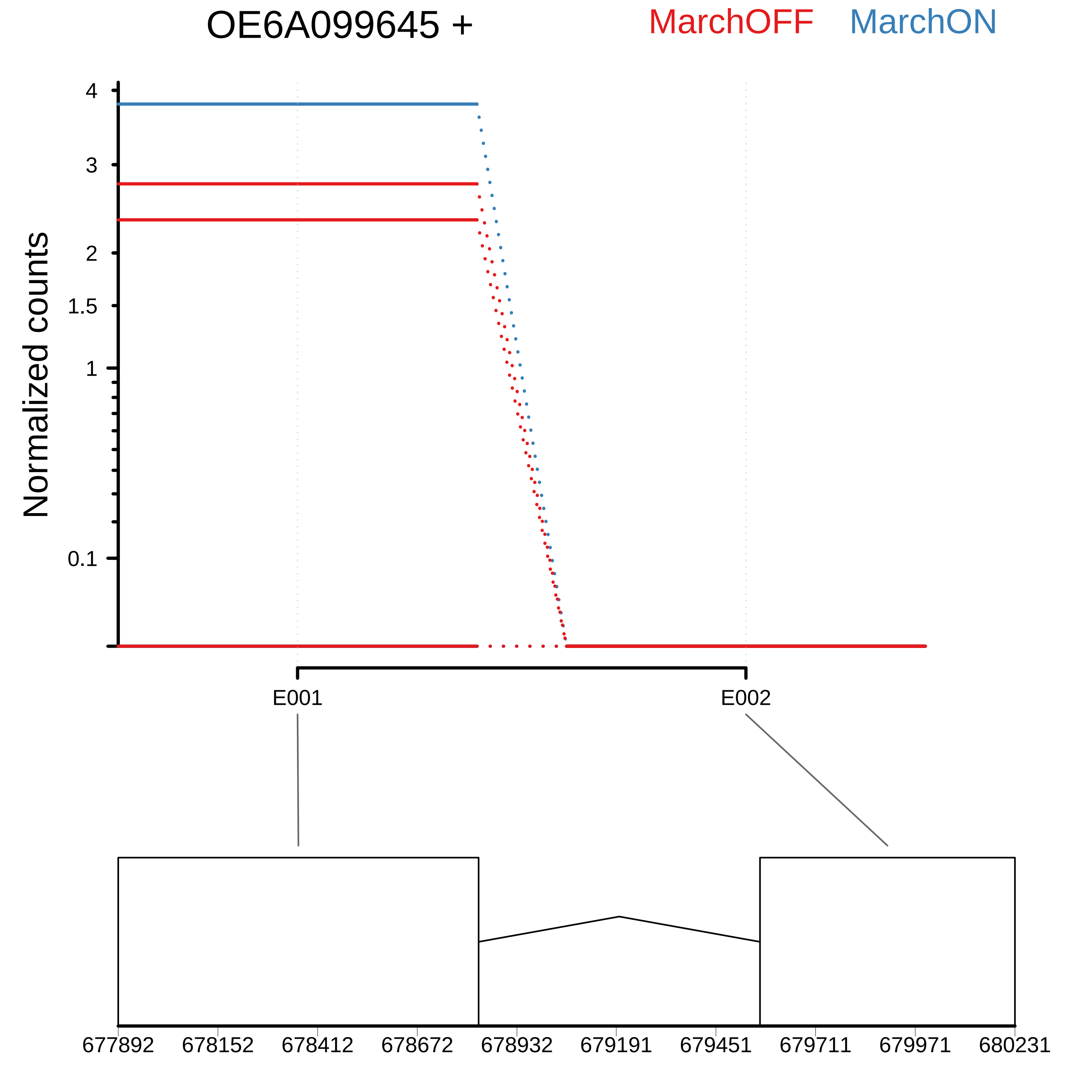

OE6A099645 +
MarchOFF
MarchON
Normalized counts
4
3
2
1.5
1
0.1
E001
E002
677892
678152
678412
678672
678932
679191
679451
679711
679971
680231

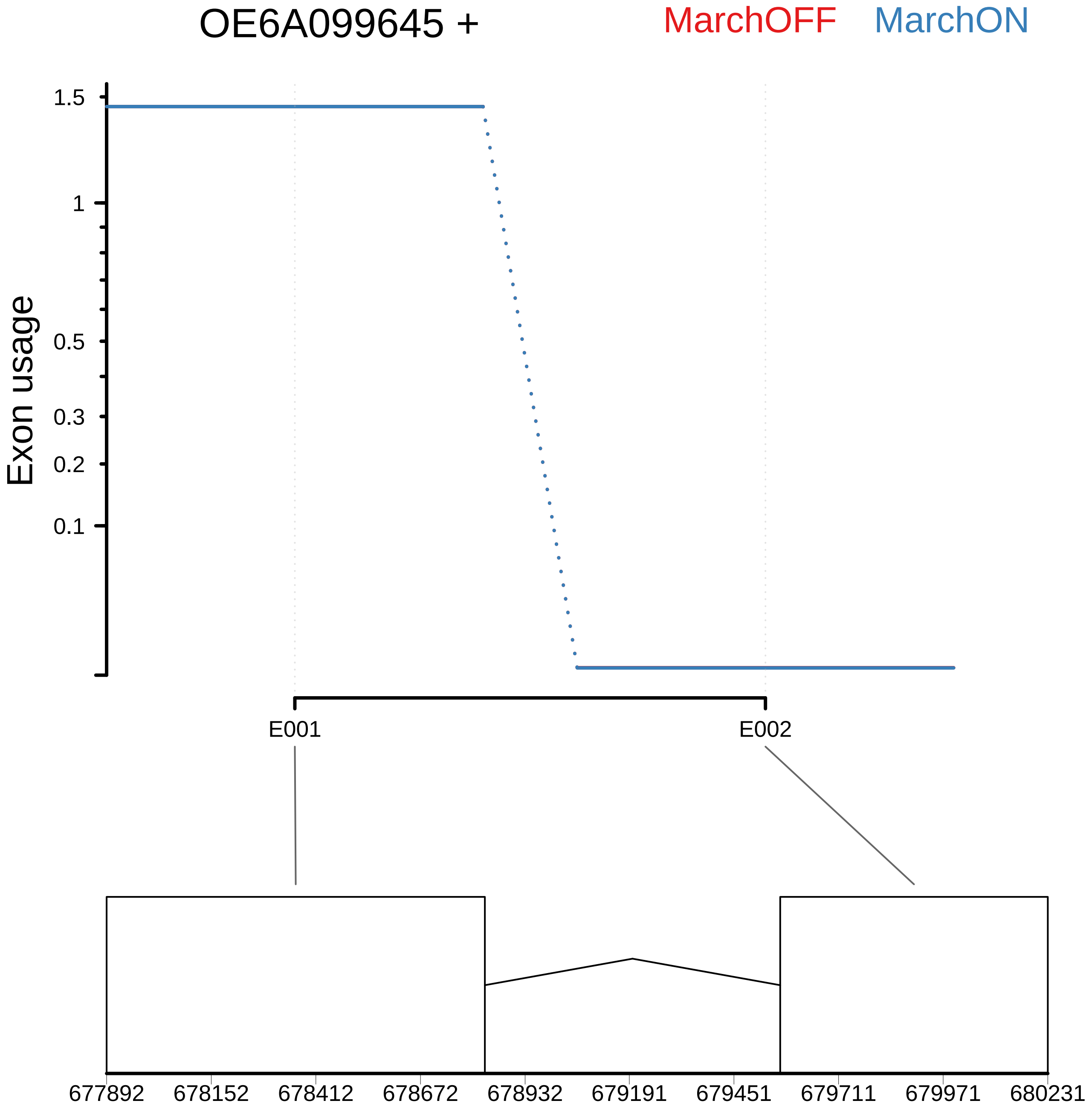

OE6A099645 +
MarchOFF
MarchON
Exon usage
1.5
1
0.5
0.3
0.2
0.1
E001
E002
677892
678152
678412
678672
678932
679191
679451
679711
679971
680231

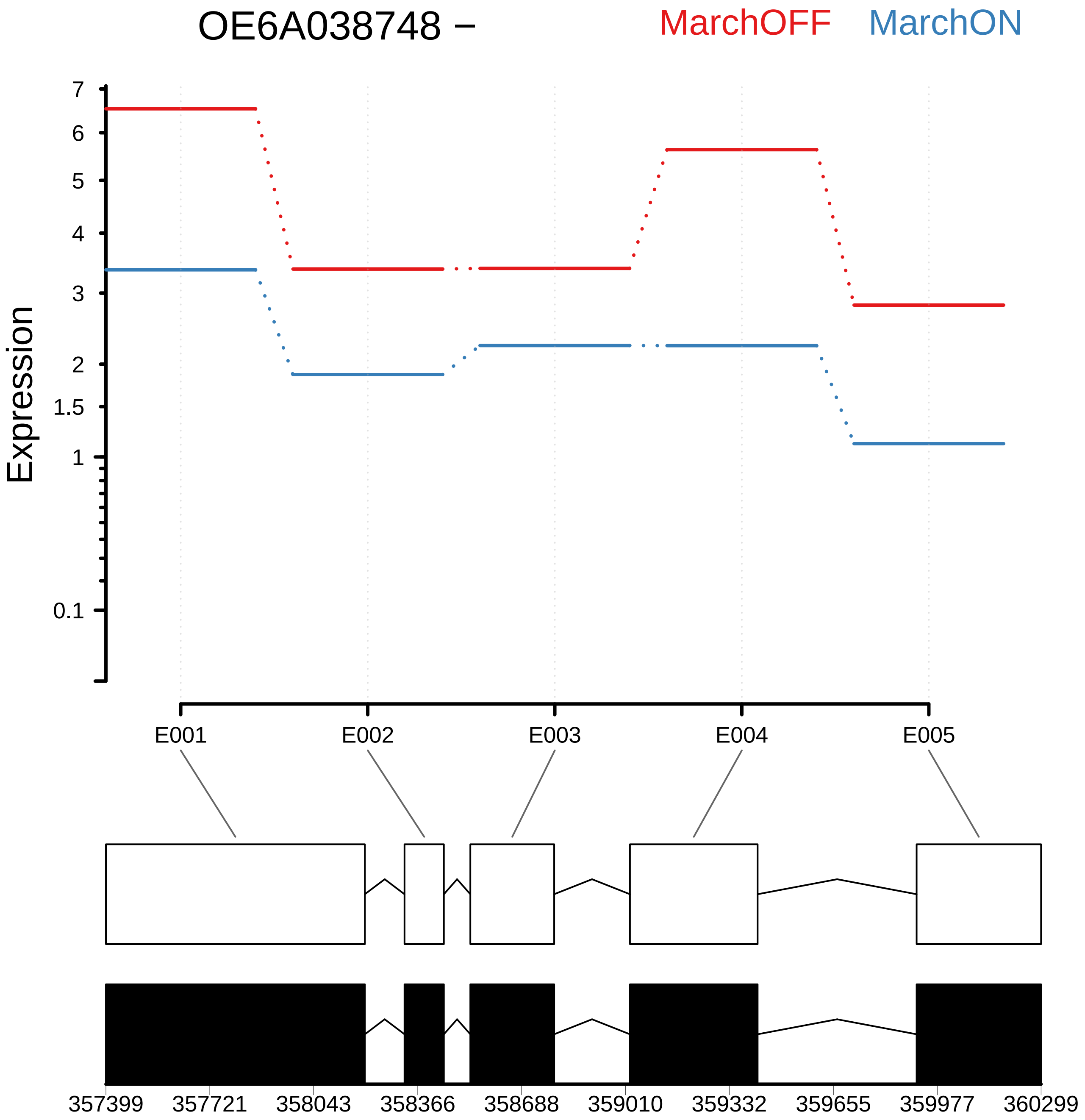

OE6A038748 –
MarchOFF
MarchON
Expression
7
6
5
4
3
2
1.5
1
0.1
E001
E002
E003
E004
E005
357399
357721
358043
358366
358688
359010
359332
359655
359977
360299

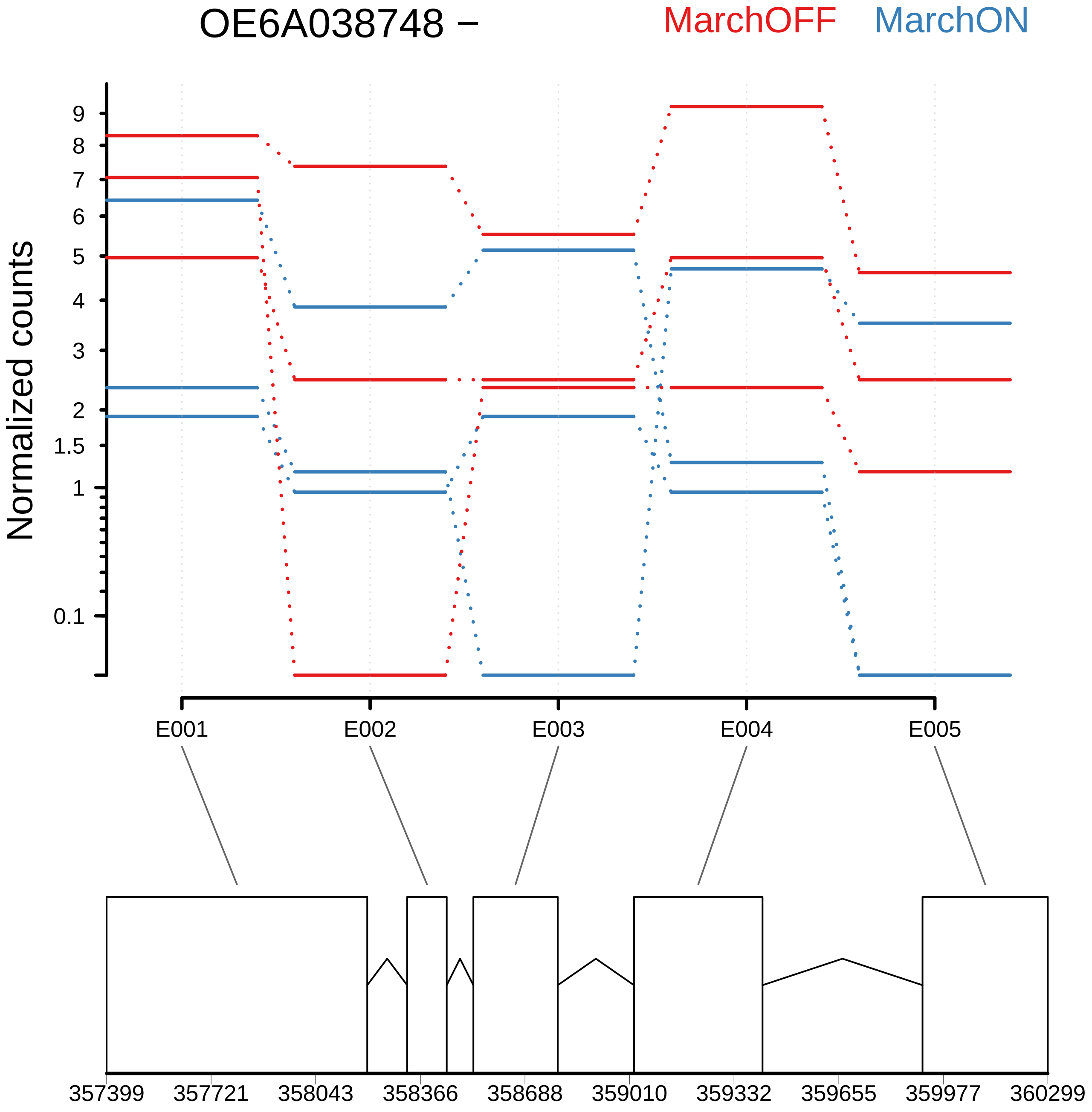
OE6A038748 −
MarchOFF
MarchON
Normalized counts
9
8
7
6
5
4
3
2
1.5
1
0.1
E001
E002
E003
E004
E005
357399
357721
358043
358366
358688
359010
359332
359655
359977
360299

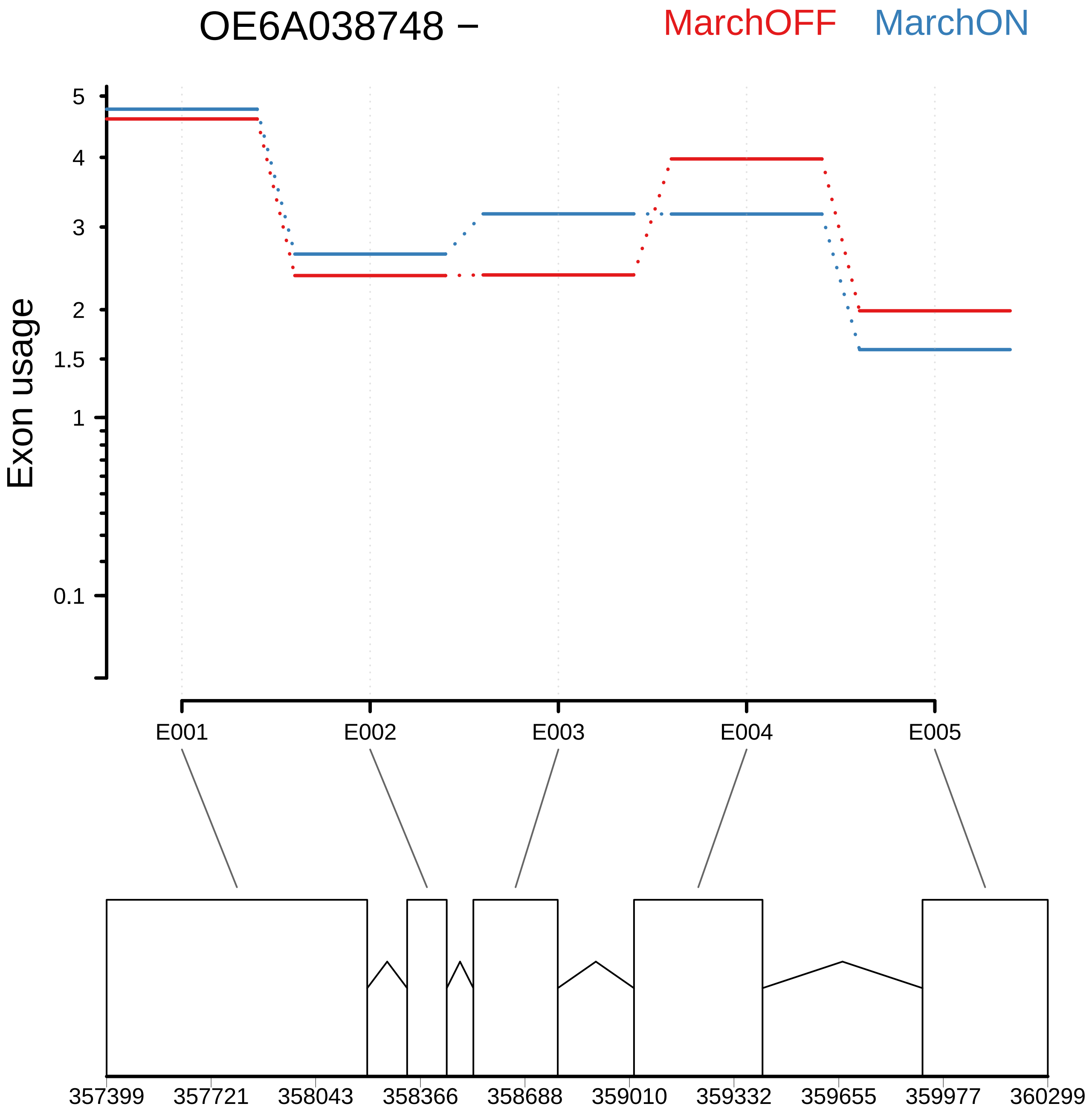

OE6A038748 −
MarchOFF
MarchON
Exon usage
5
4
3
2
1.5
1
0.1
E001
E002
E003
E004
E005
357399
357721
358043
358366
358688
359010
359332
359655
359977
360299

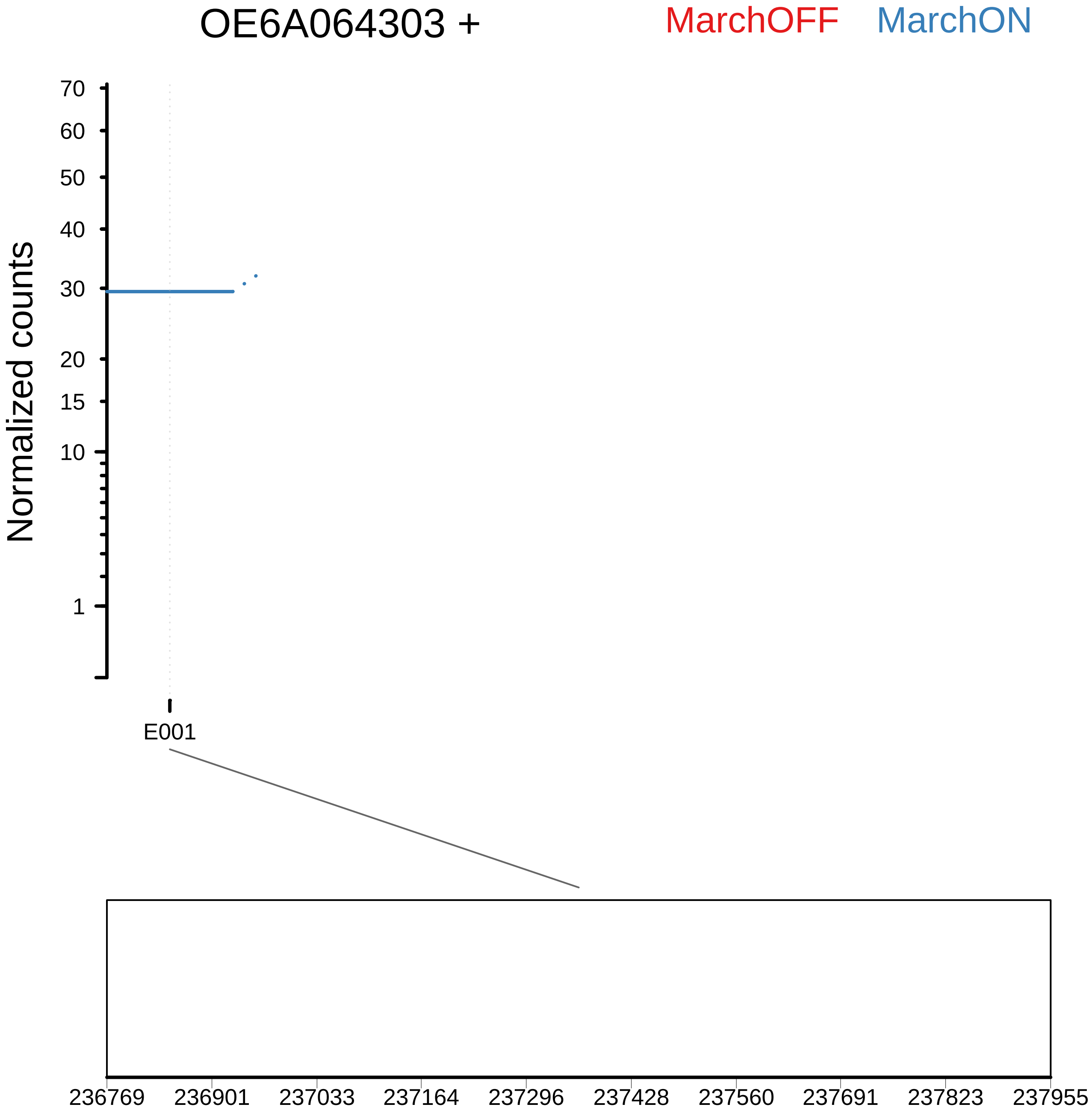
OE6A064303 +
MarchOFF
MarchON
Normalized counts
70
60
50
40
30
20
15
10
1
E001
236769
236901
237033
237164
237296
237428
237560
237691
237823
237955

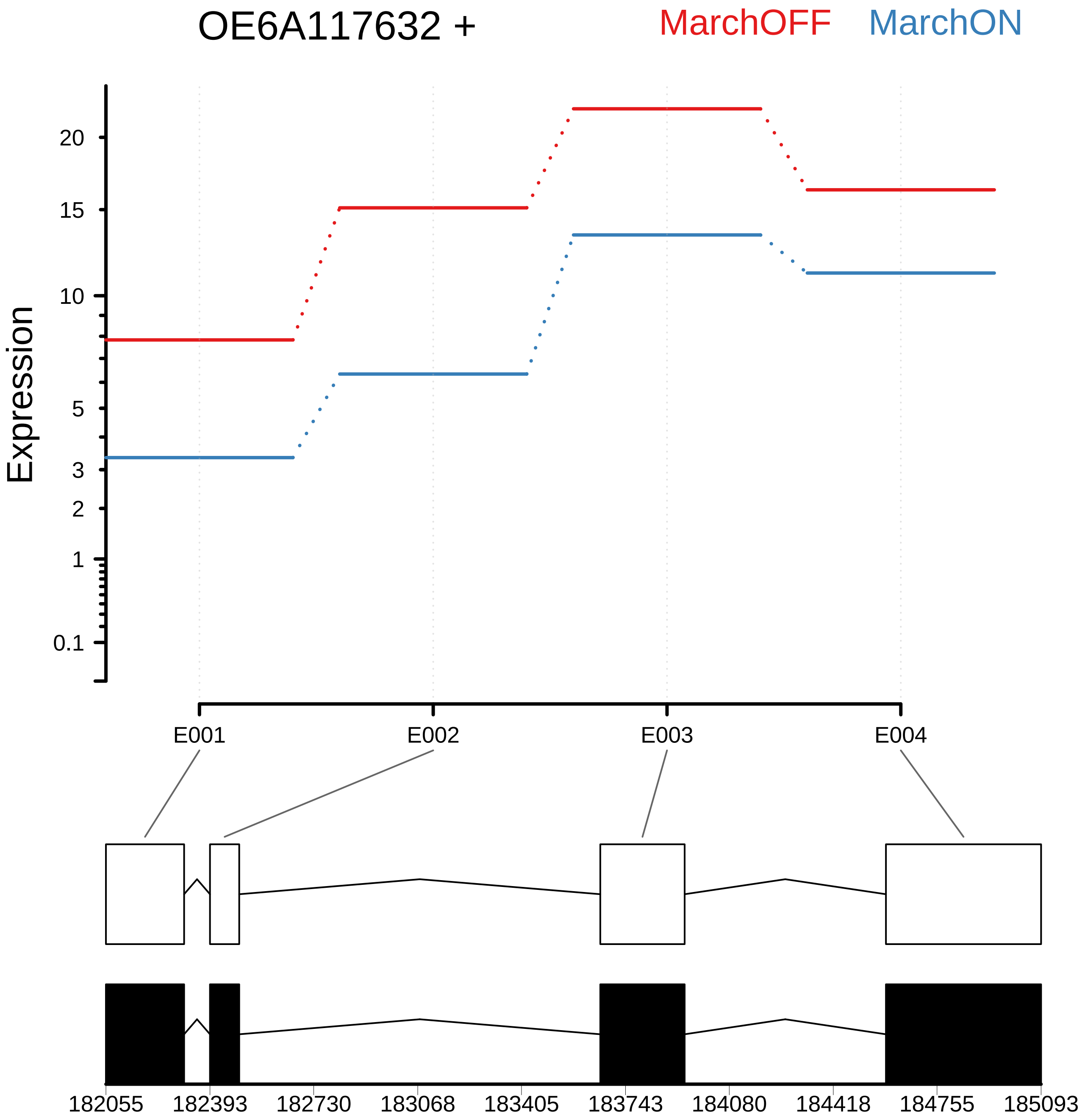
OE6A117632 +
MarchOFF
MarchON
Expression
20
15
10
5
3
2
1
0.1
E001
E002
E003
E004
182055
182393
182730
183068
183405
183743
184080
184418
184755
185093

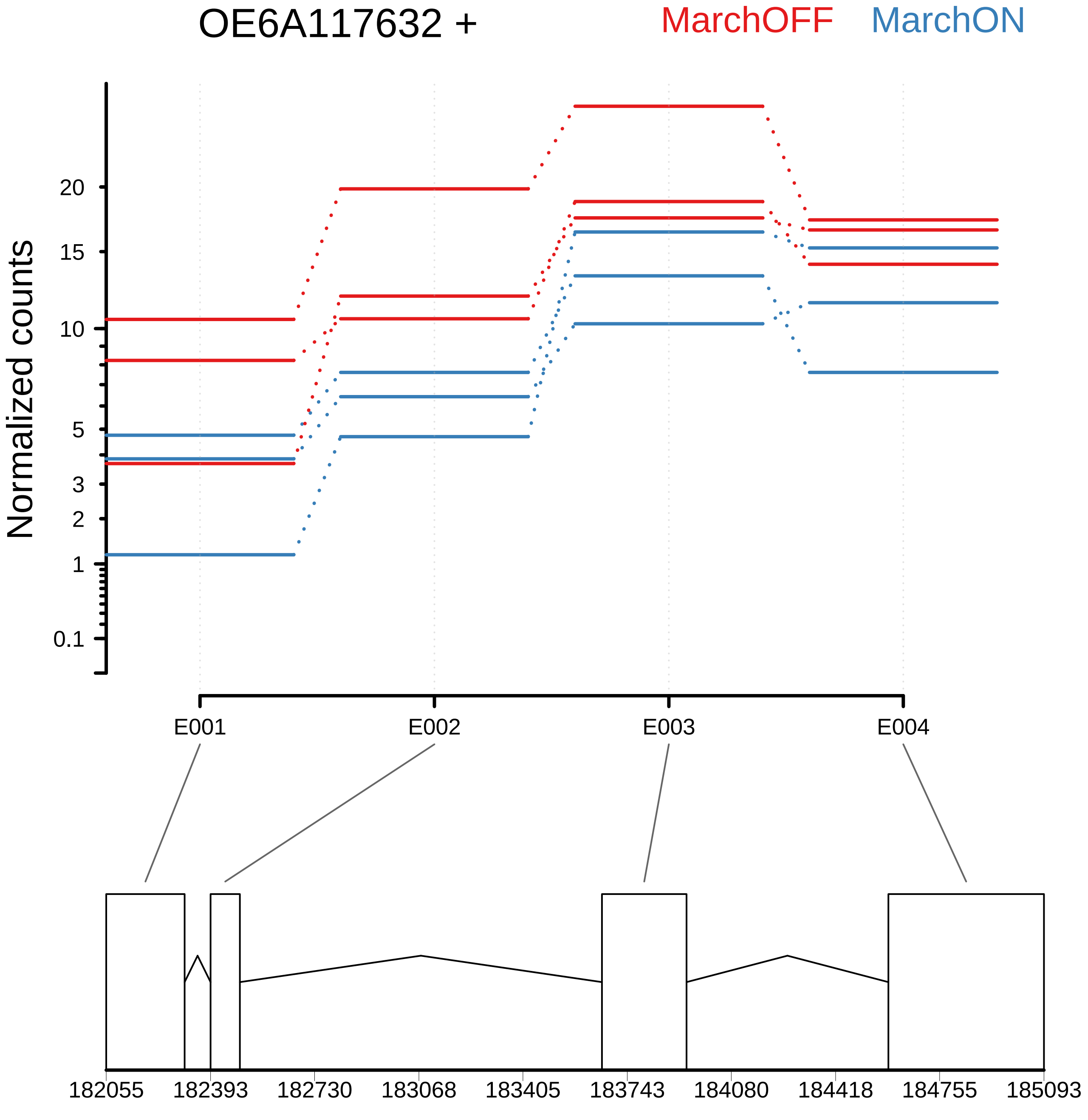
OE6A117632 +
MarchOFF
MarchON
Normalized counts
20
15
10
5
3
2
1
0.1
E001
E002
E003
E004
182055
182393
182730
183068
183405
183743
184080
184418
184755
185093

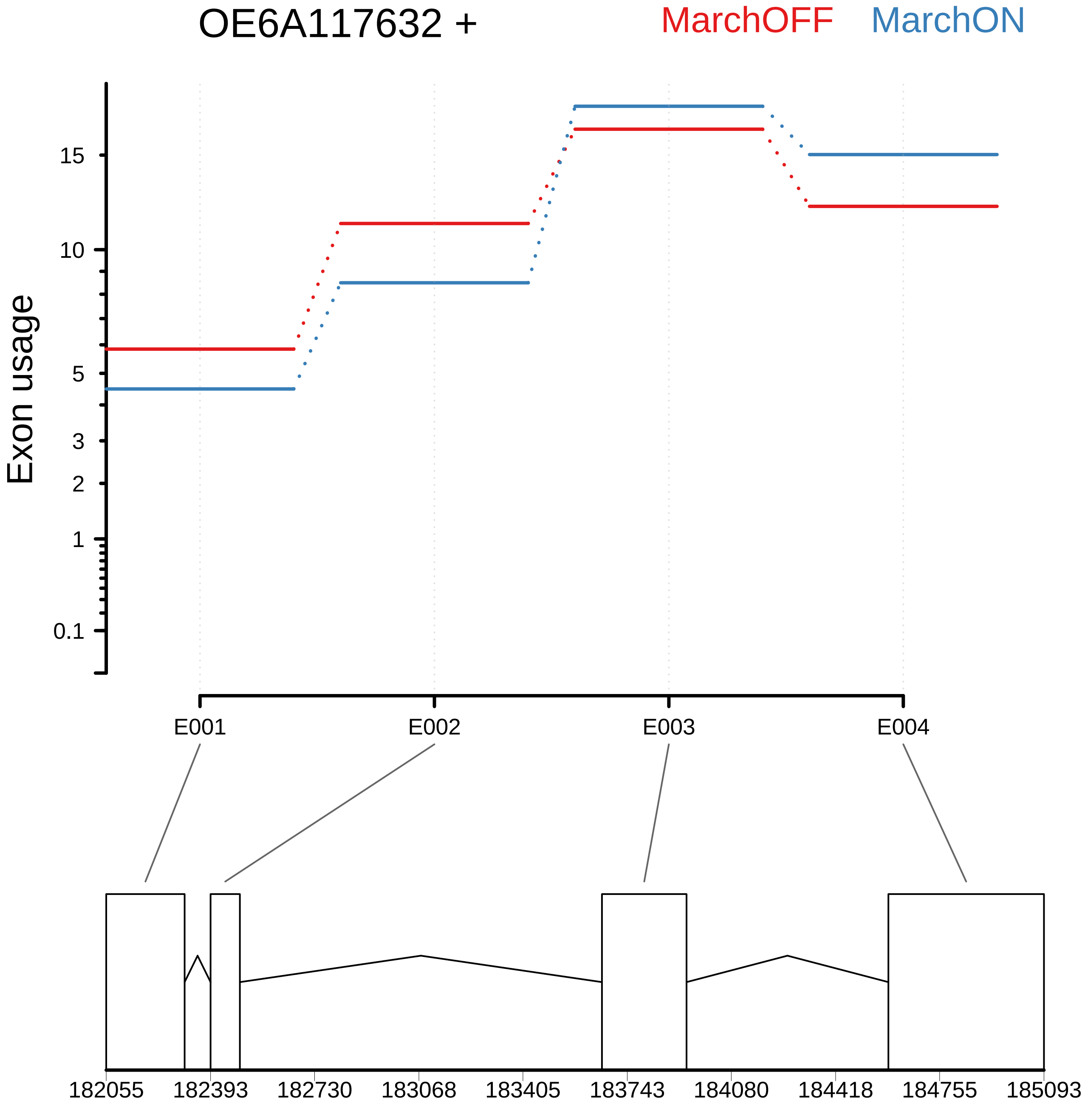
OE6A117632 +
MarchOFF
MarchON
Exon usage
15
10
5
3
2
1
0.1
E001
E002
E003
E004
182055
182393
182730
183068
183405
183743
184080
184418
184755
185093

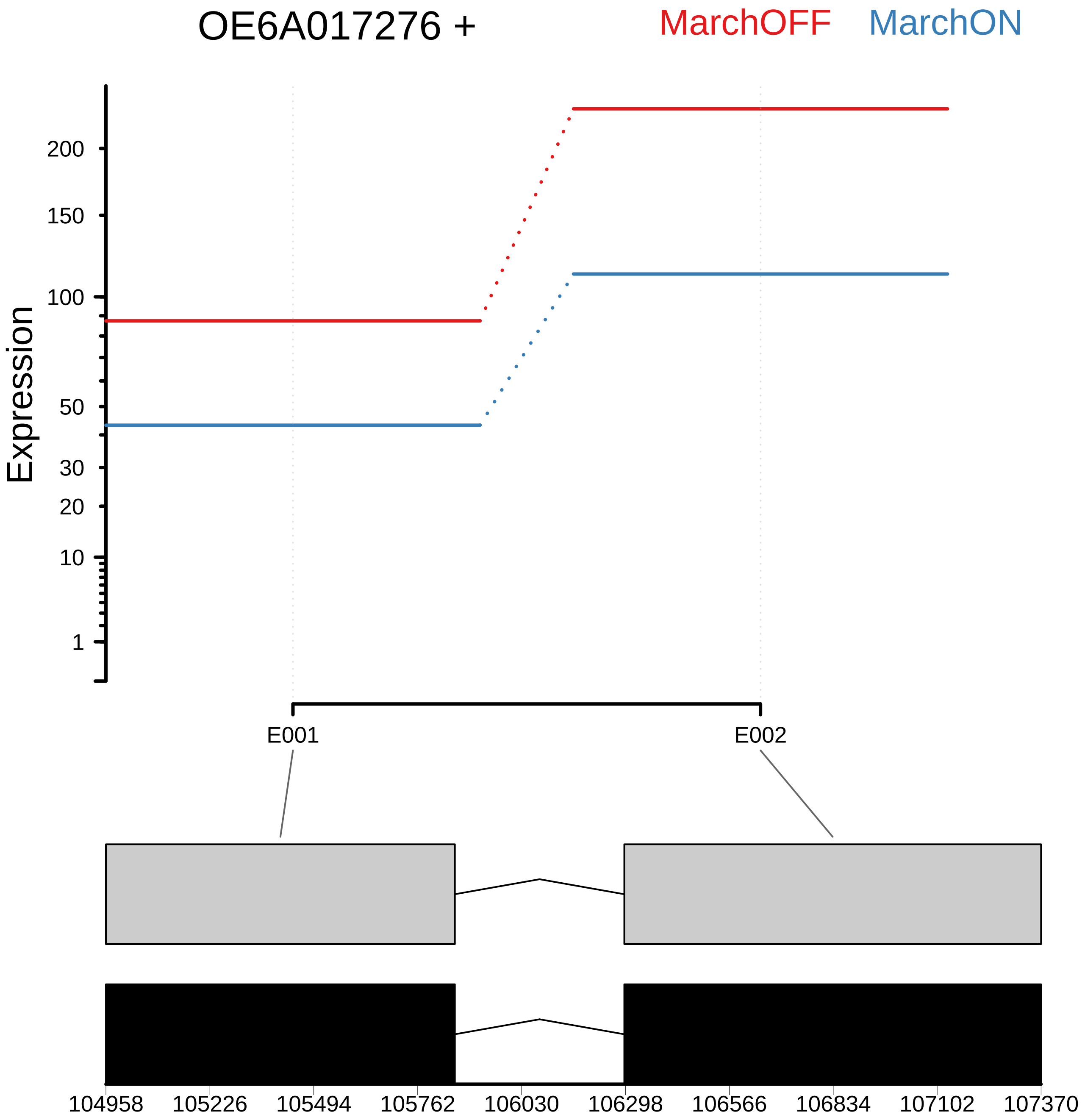
OE6A017276 +
MarchOFF
MarchON
Expression
200
150
100
50
30
20
10
1
E001
E002
104958
105226
105494
105762
106030
106298
106566
106834
107102
107370

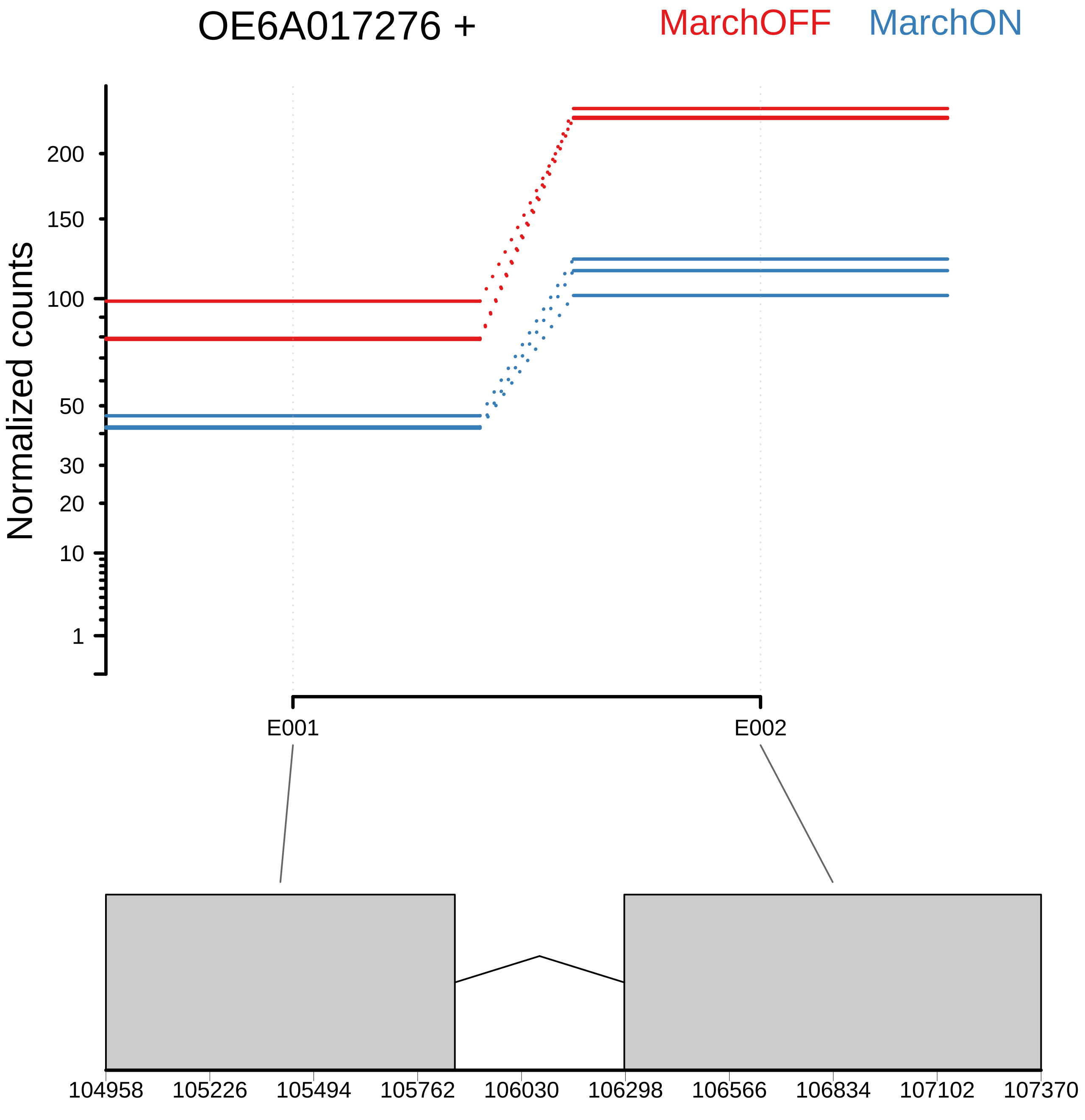

OE6A017276 +
MarchOFF
MarchON
Normalized counts
200
150
100
50
30
20
10
1
E001
E002
104958
105226
105494
105762
106030
106298
106566
106834
107102
107370

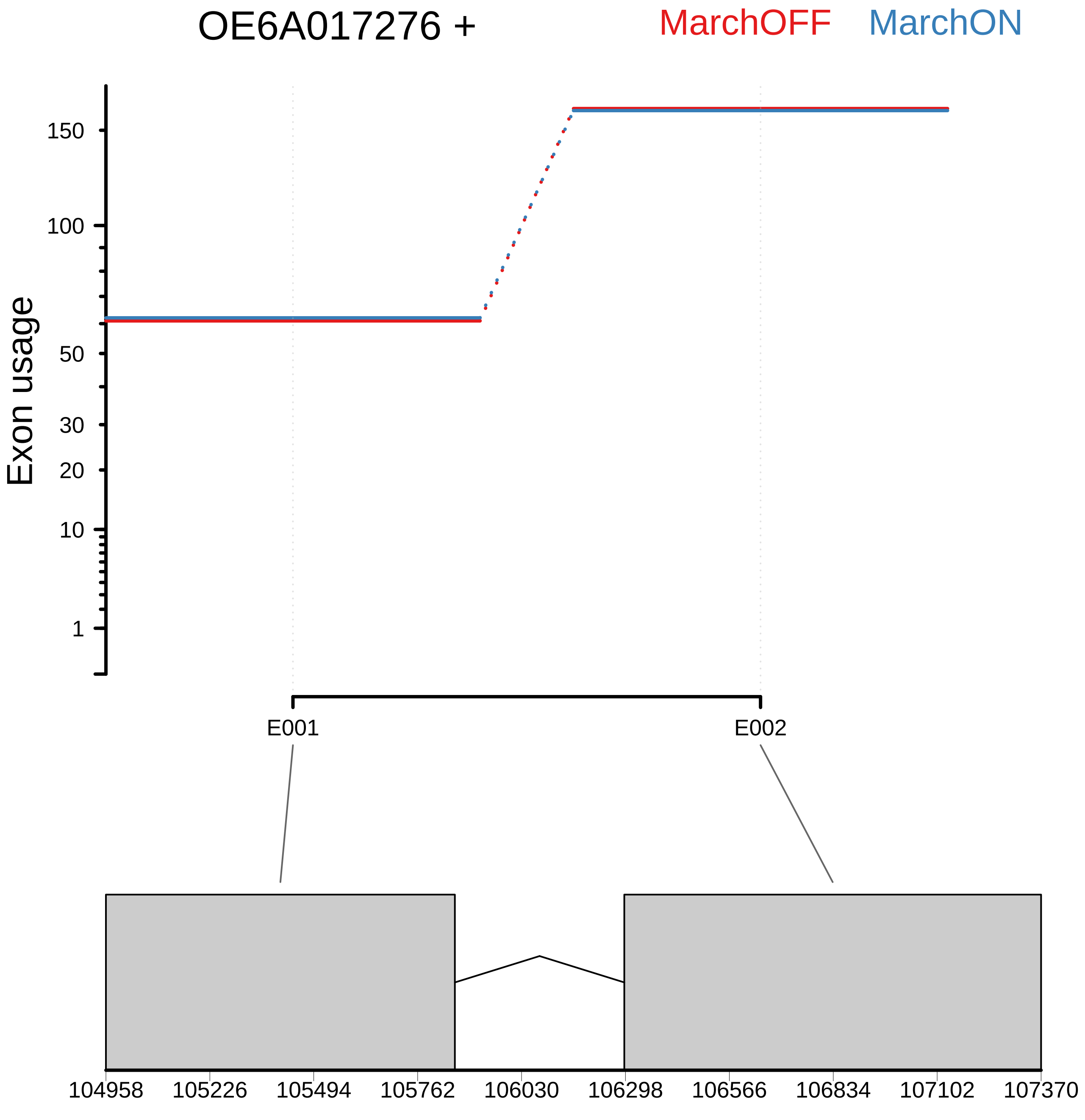
OE6A017276 +
MarchOFF
MarchON
Exon usage
150
100
50
30
20
10
1
E001
E002
104958
105226
105494
105762
106030
106298
106566
106834
107102
107370

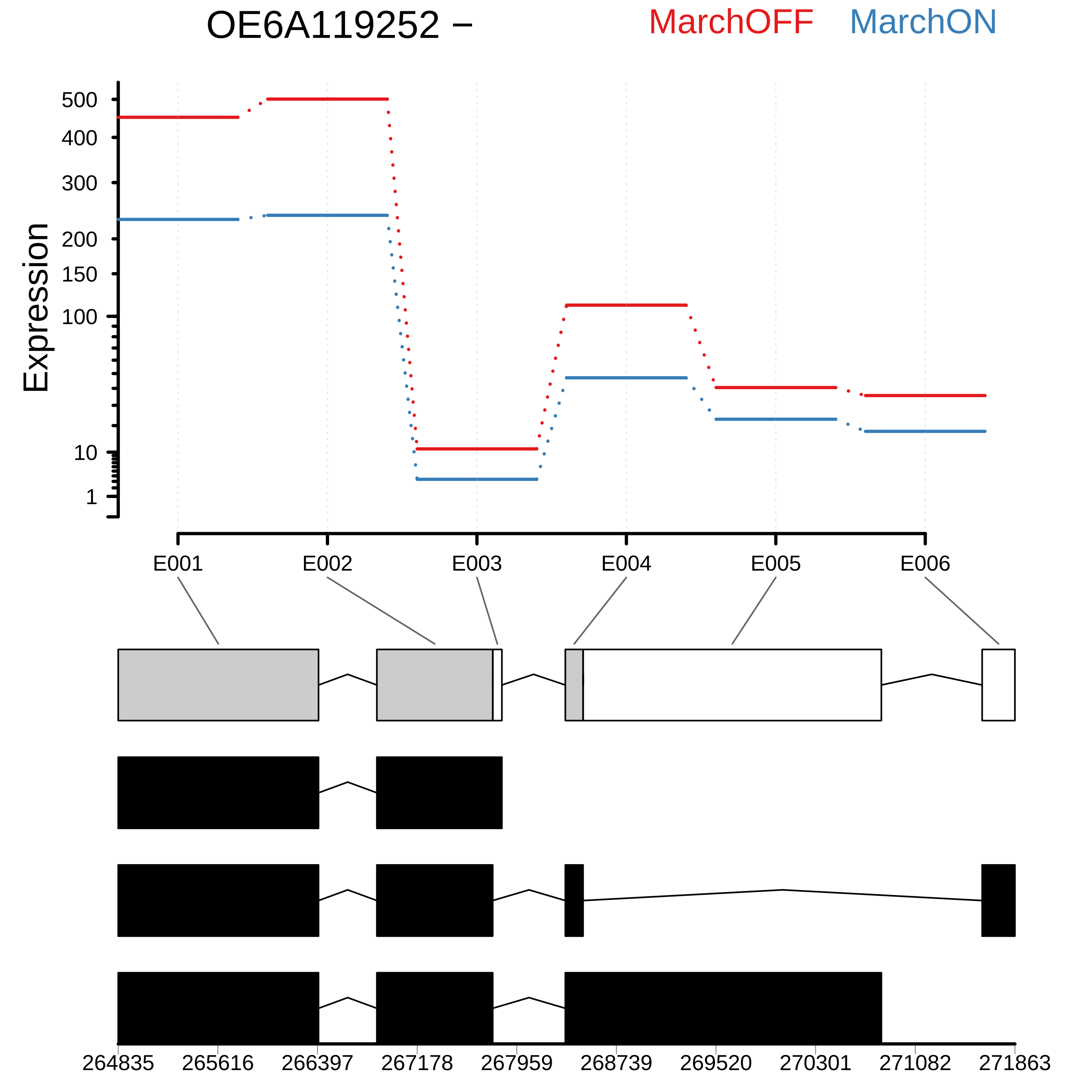

OE6A119252 –
MarchOFF
MarchON
Expression
500
400
300
200
150
100
10
1
E001
E002
E003
E004
E005
E006
264835
265616
266397
267178
267959
268739
269520
270301
271082
271863

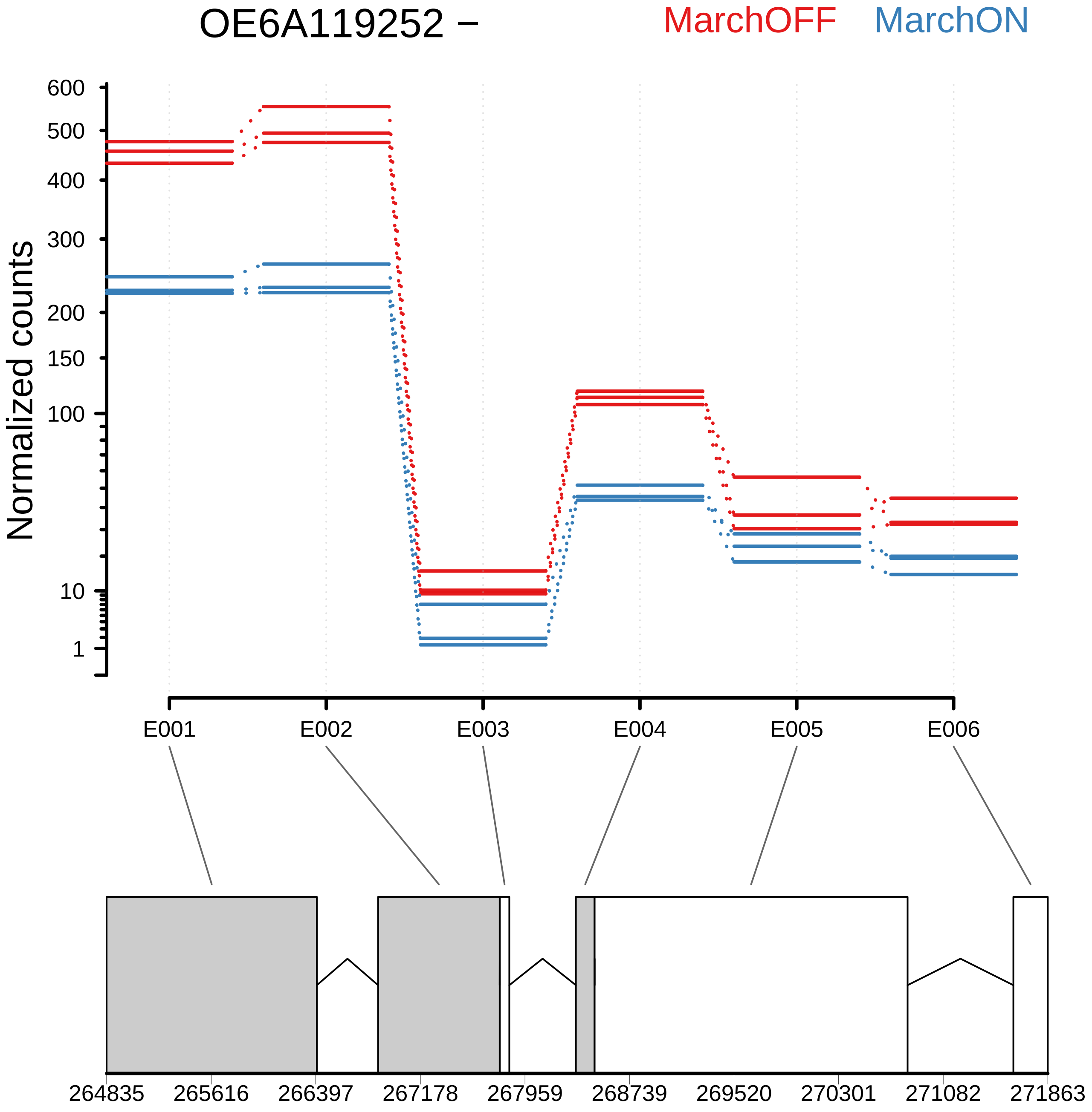

OE6A119252 −
MarchOFF
MarchON
Normalized counts
600
500
400
300
200
150
100
10
1
E001
E002
E003
E004
E005
E006
264835
265616
266397
267178
267959
268739
269520
270301
271082
271863

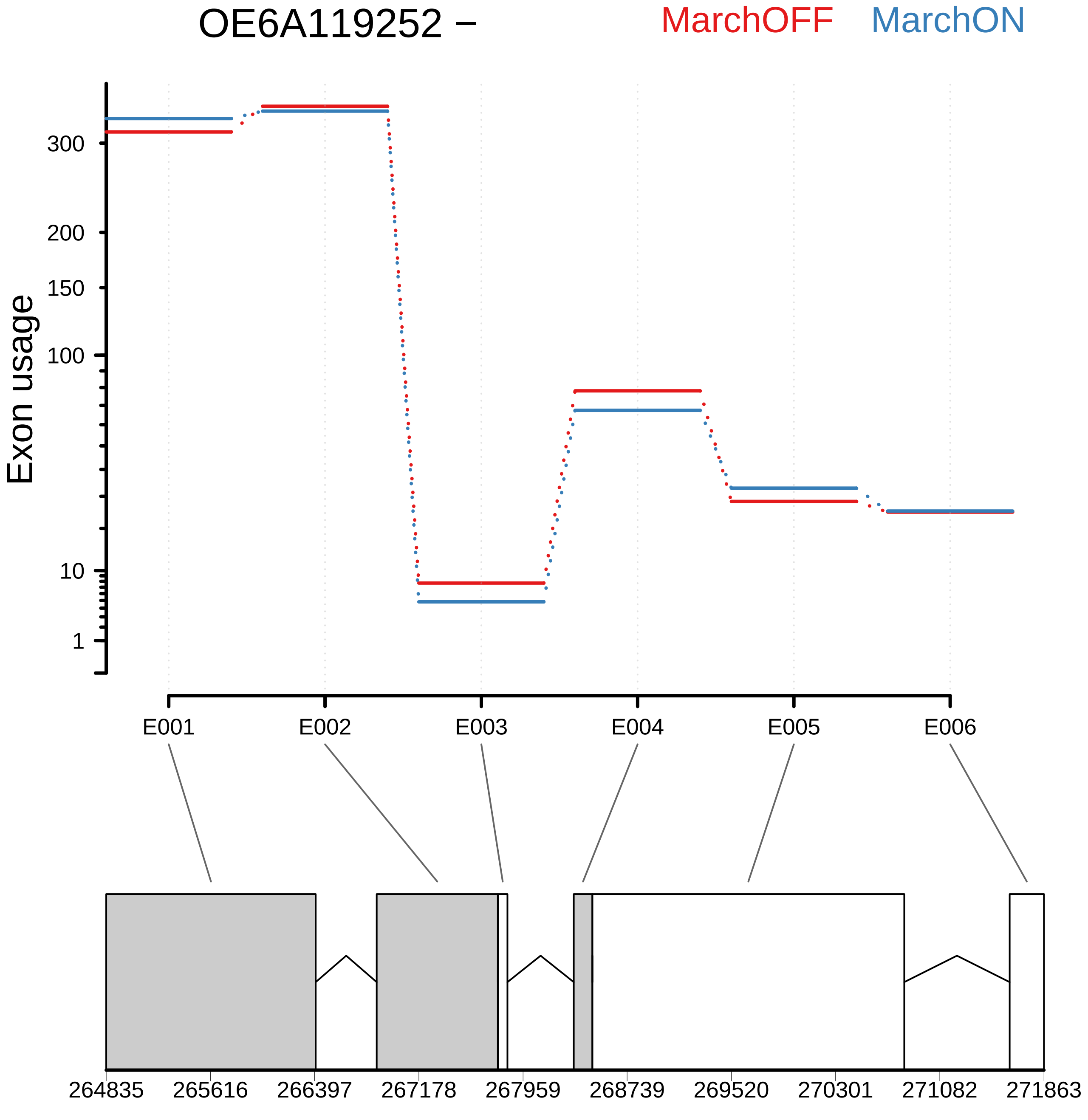

OE6A119252 −
MarchOFF
MarchON
Exon usage
300
200
150
100
10
1
E001
E002
E003
E004
E005
E006
264835
265616
266397
267178
267959
268739
269520
270301
271082
271863

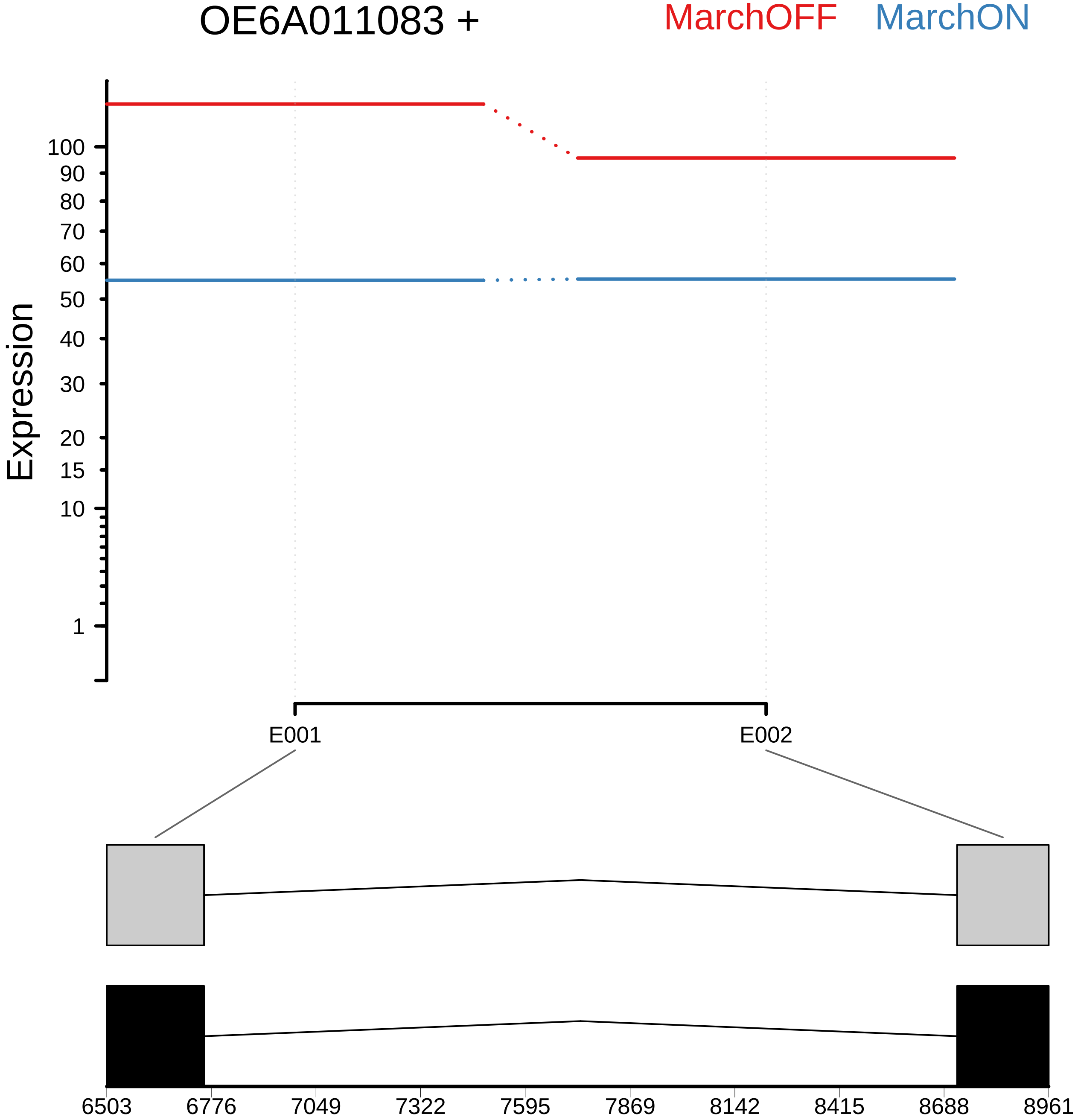

OE6A011083 +
MarchOFF
MarchON
Expression
100
90
80
70
60
50
40
30
20
15
10
1
E001
E002
6503
6776
7049
7322
7595
7869
8142
8415
8688
8961

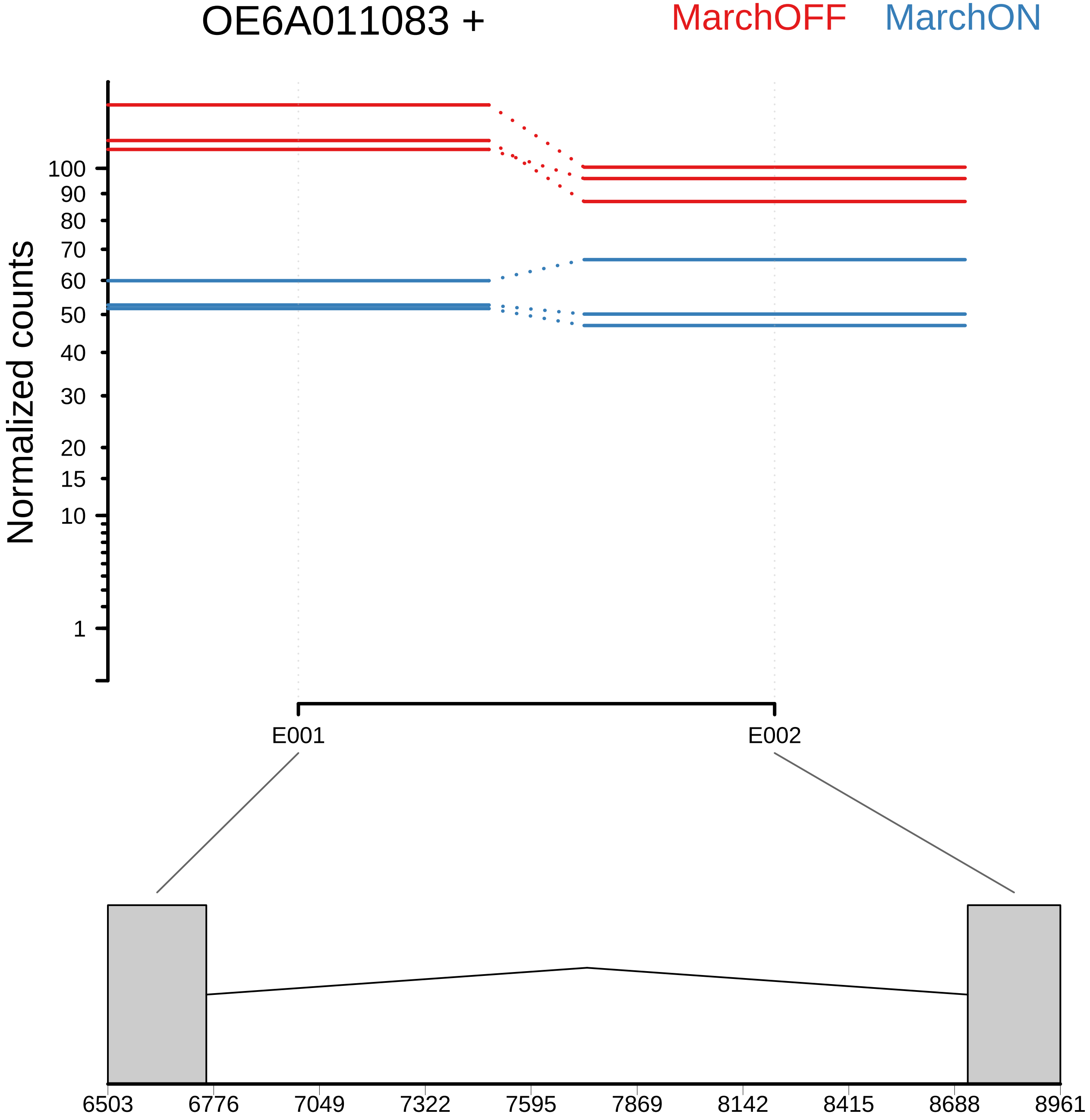
OE6A011083 +
MarchOFF
MarchON
Normalized counts
100
90
80
70
60
50
40
30
20
15
10
1
E001
E002
6503
6776
7049
7322
7595
7869
8142
8415
8688
8961

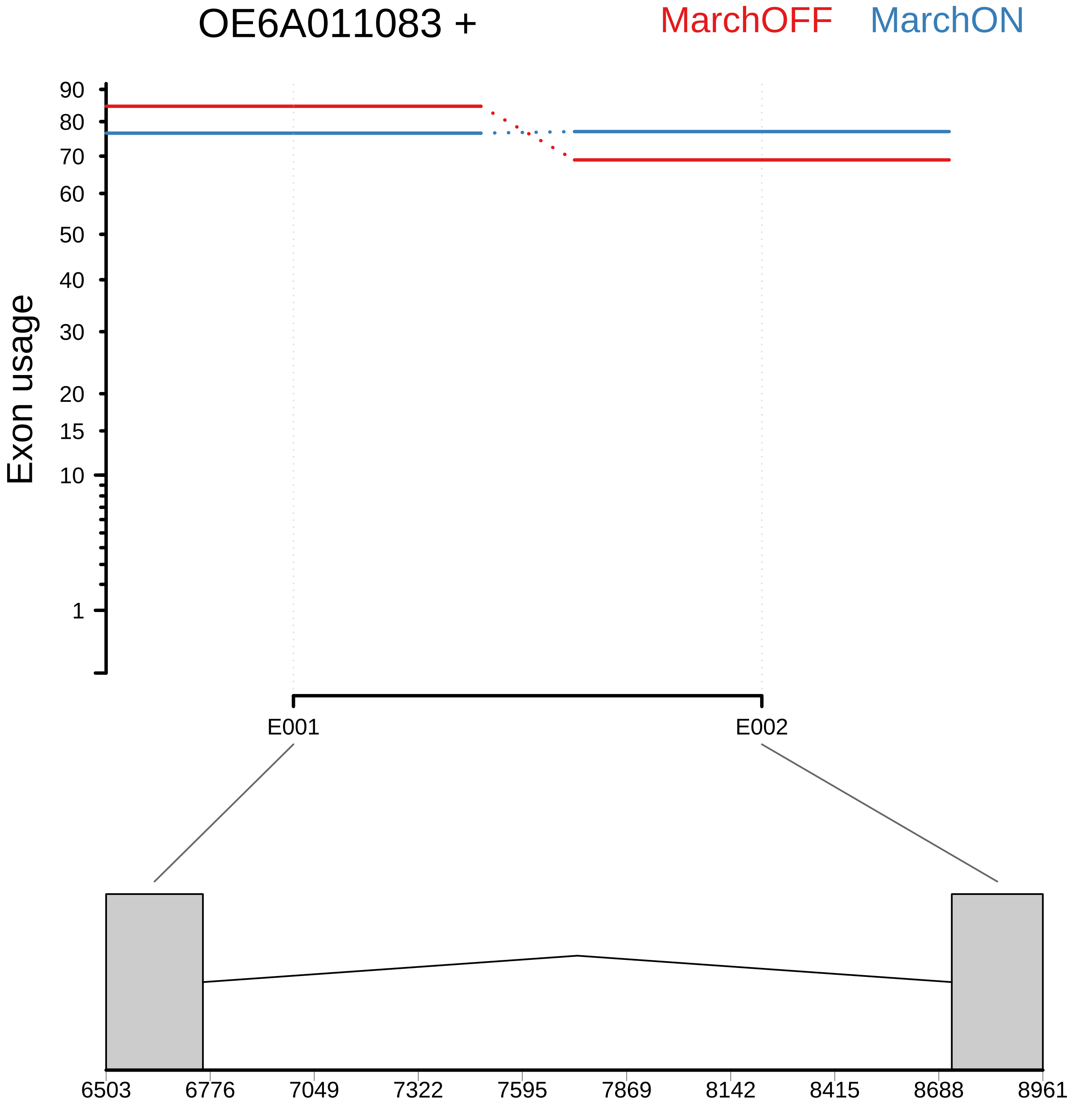
OE6A011083 +
MarchOFF
MarchON
Exon usage
90
80
70
60
50
40
30
20
15
10
1
E001
E002
6503
6776
7049
7322
7595
7869
8142
8415
8688
8961

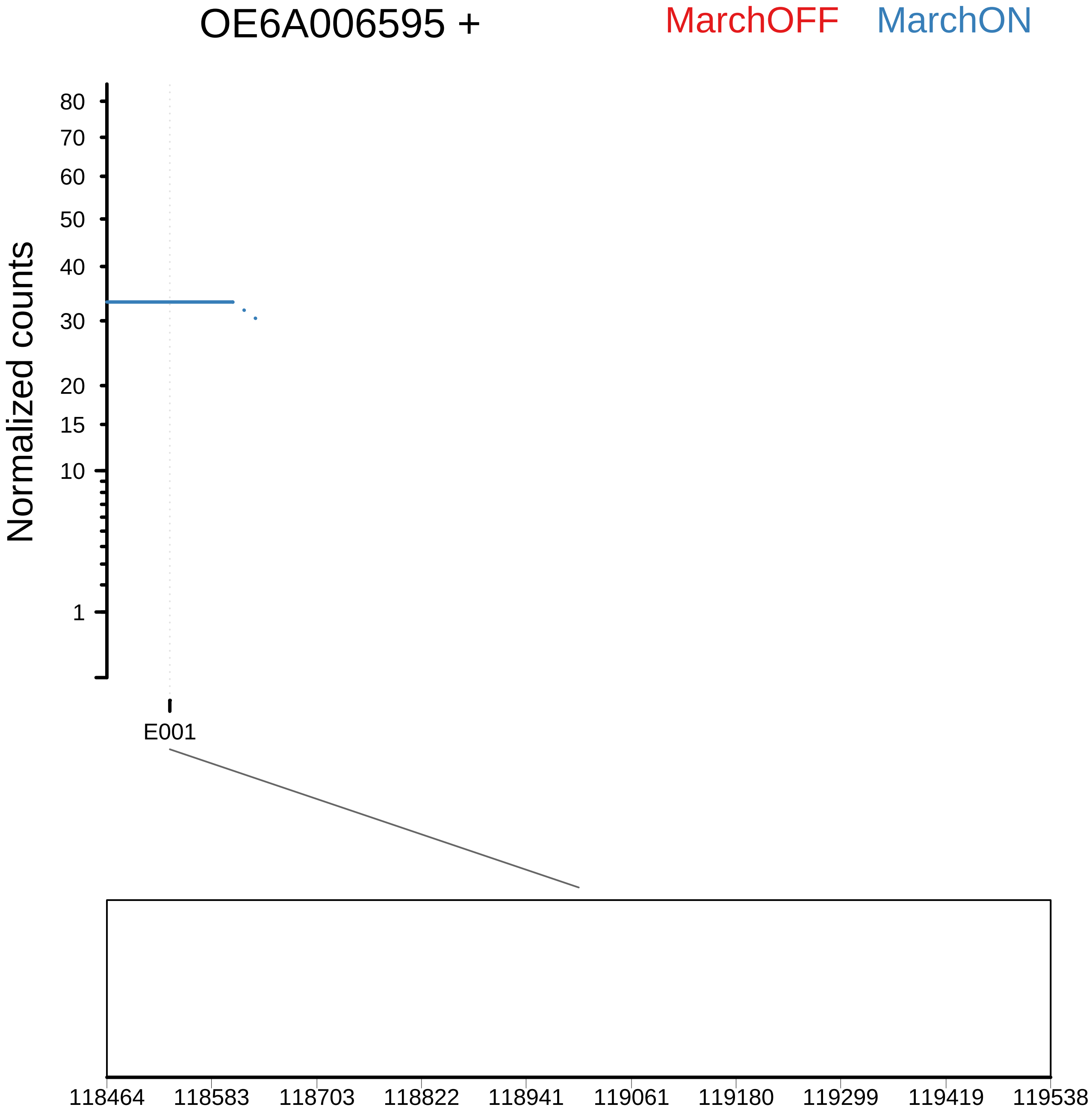

OE6A006595 +
MarchOFF
MarchON
Normalized counts
80
70
60
50
40
30
20
15
10
1
E001
118464
118583
118703
118822
118941
119061
119180
119299
119419
119538

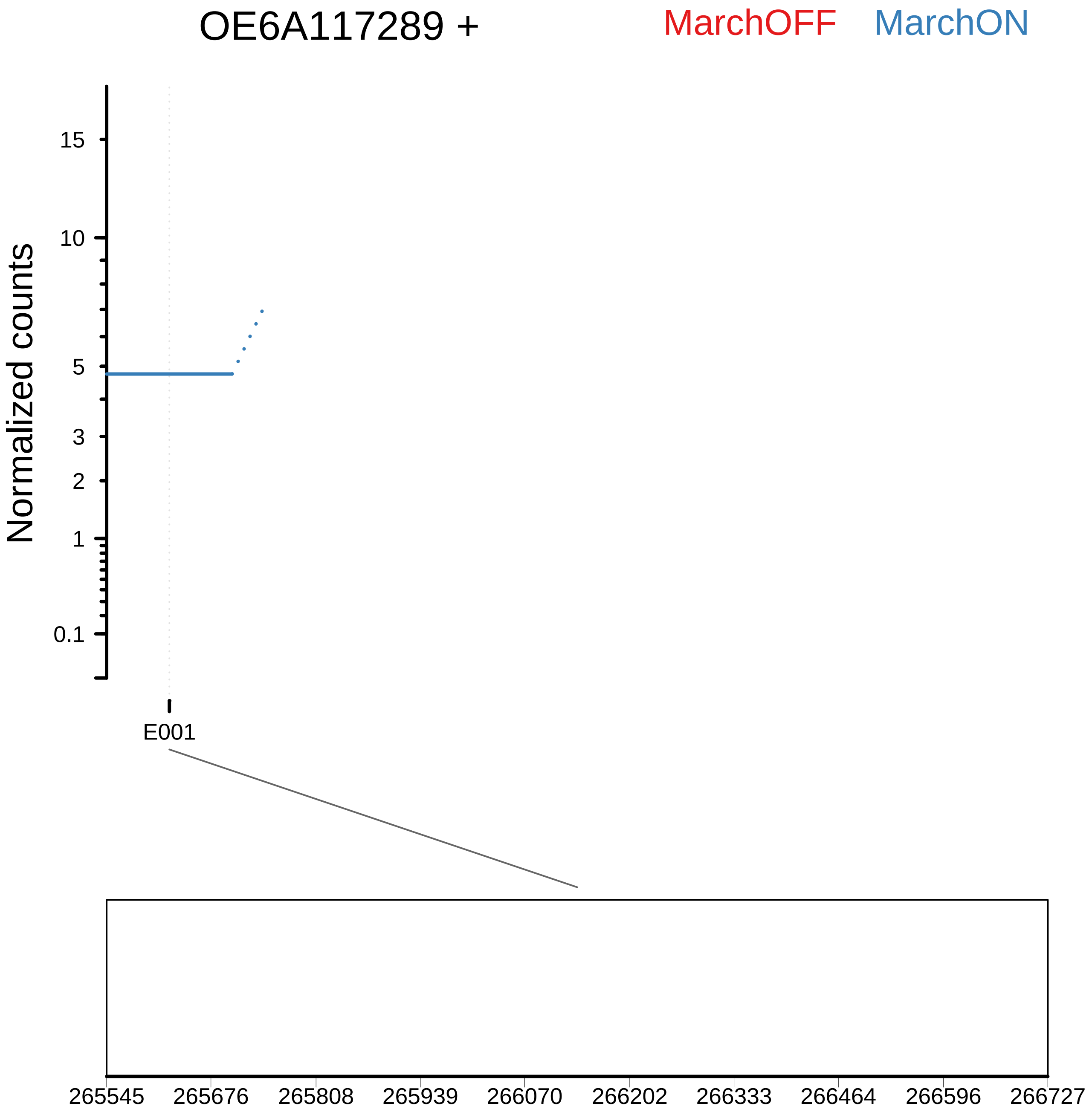
OE6A117289 +
MarchOFF
MarchON
Normalized counts
15
10
5
3
2
1
0.1
E001
265545
265676
265808
265939
266070
266202
266333
266464
266596
266727

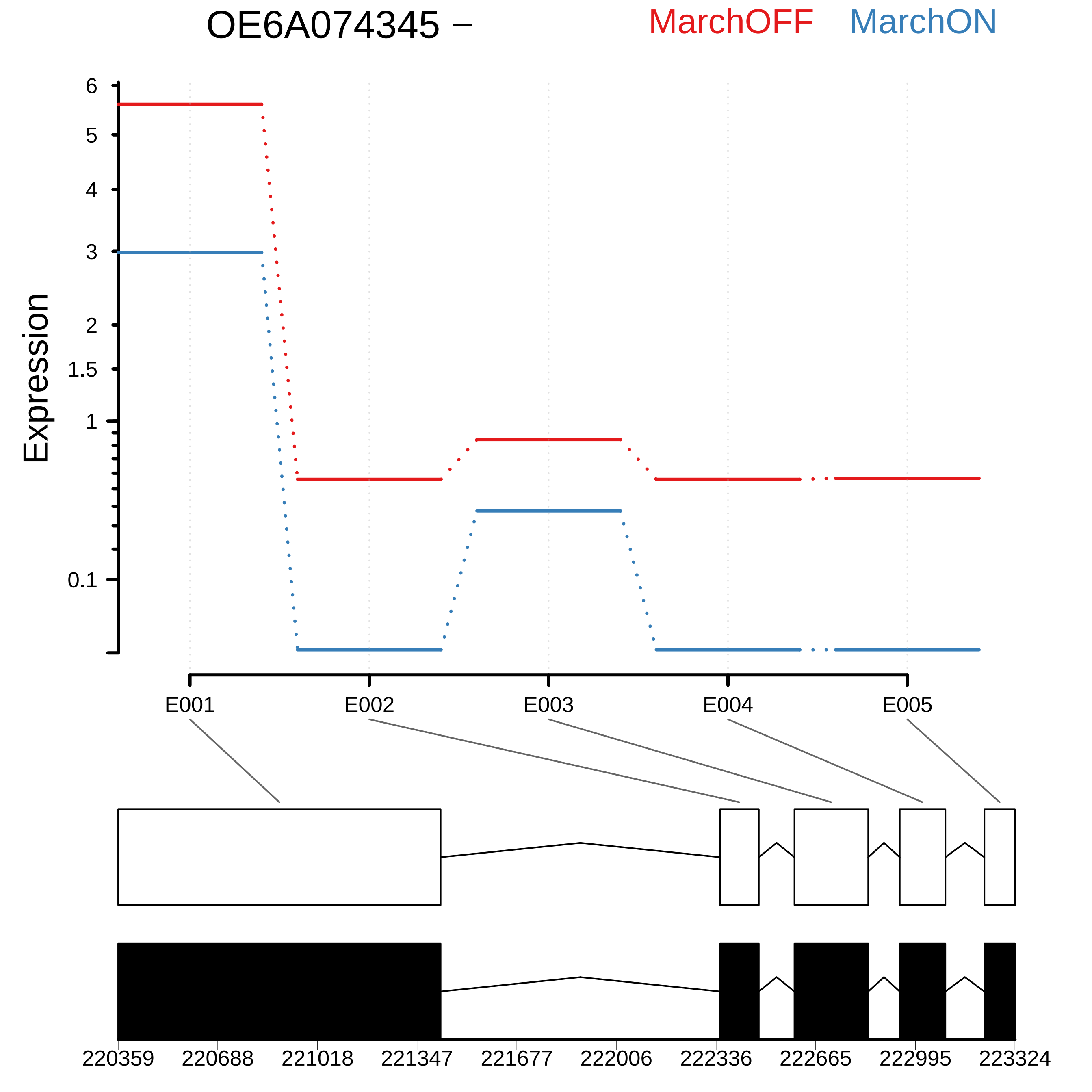

OE6A074345 −
MarchOFF
MarchON
Expression
6
5
4
3
2
1.5
1
0.1
E001
E002
E003
E004
E005
220359
220688
221018
221347
221677
222006
222336
222665
222995
223324

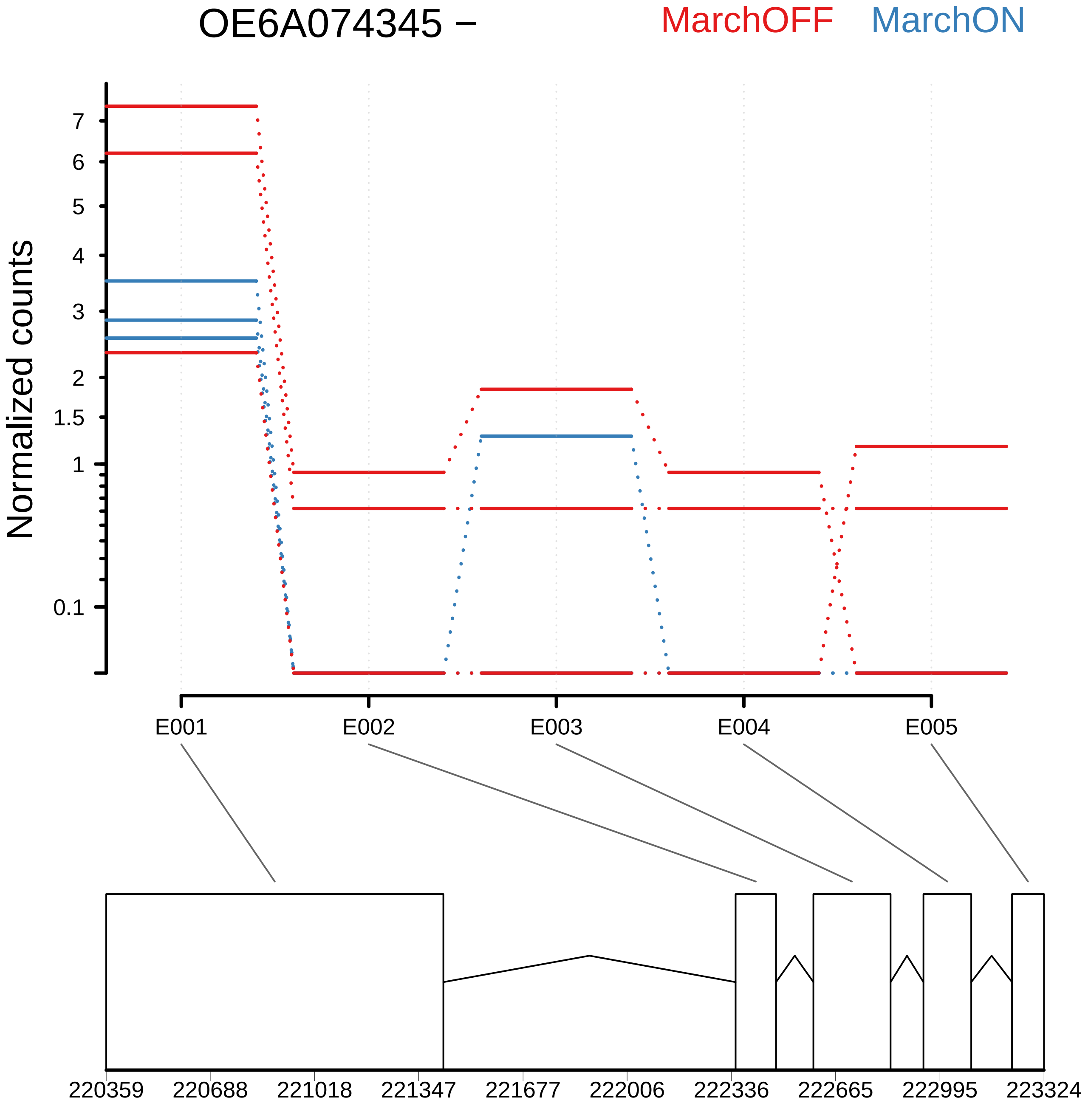

OE6A074345 −
MarchOFF
MarchON
Normalized counts
7
6
5
4
3
2
1.5
1
0.1
E001
E002
E003
E004
E005
220359
220688
221018
221347
221677
222006
222336
222665
222995
223324

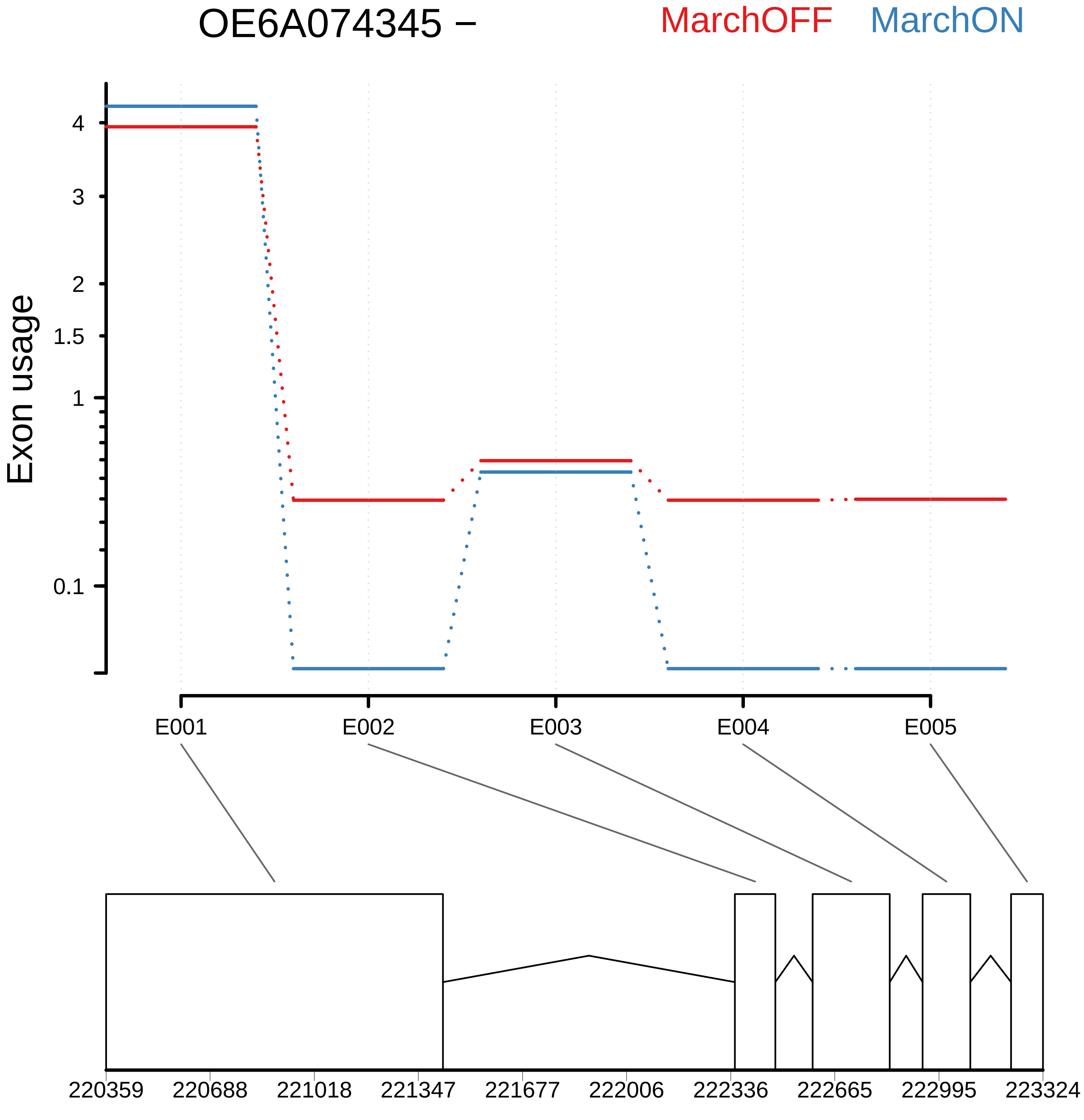

OE6A074345 −
MarchOFF
MarchON
Exon usage
4
3
2
1.5
1
0.1
E001
E002
E003
E004
E005
220359
220688
221018
221347
221677
222006
222336
222665
222995
223324

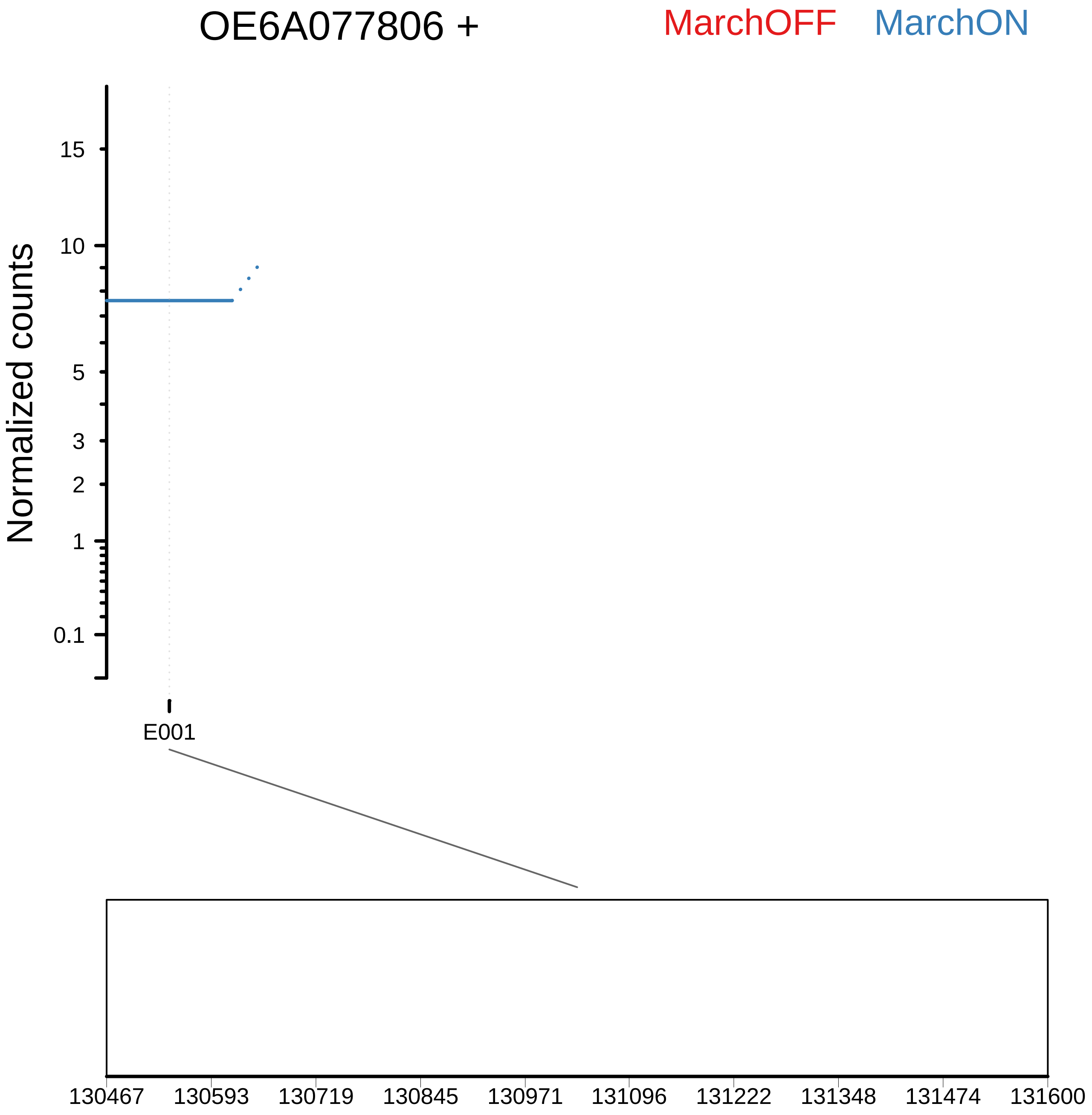
OE6A077806 +
MarchOFF
MarchON
Normalized counts
15
10
5
3
2
1
0.1
E001
130467
130593
130719
130845
130971
131096
131222
131348
131474
131600

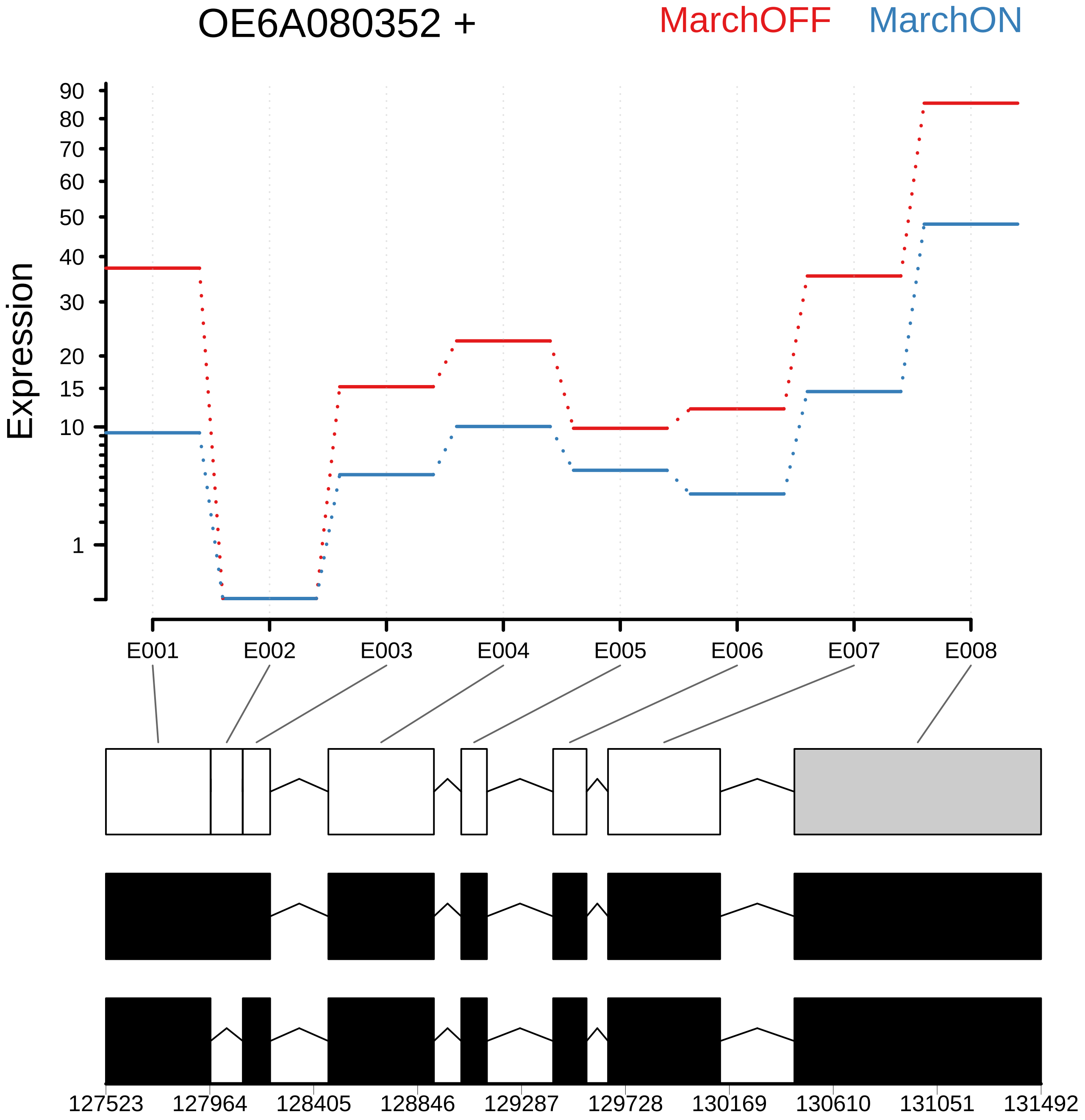

OE6A080352 +
MarchOFF
MarchON
Expression
90
80
70
60
50
40
30
20
15
10
1
E001
E002
E003
E004
E005
E006
E007
E008
127523
127964
128405
128846
129287
129728
130169
130610
131051
131492

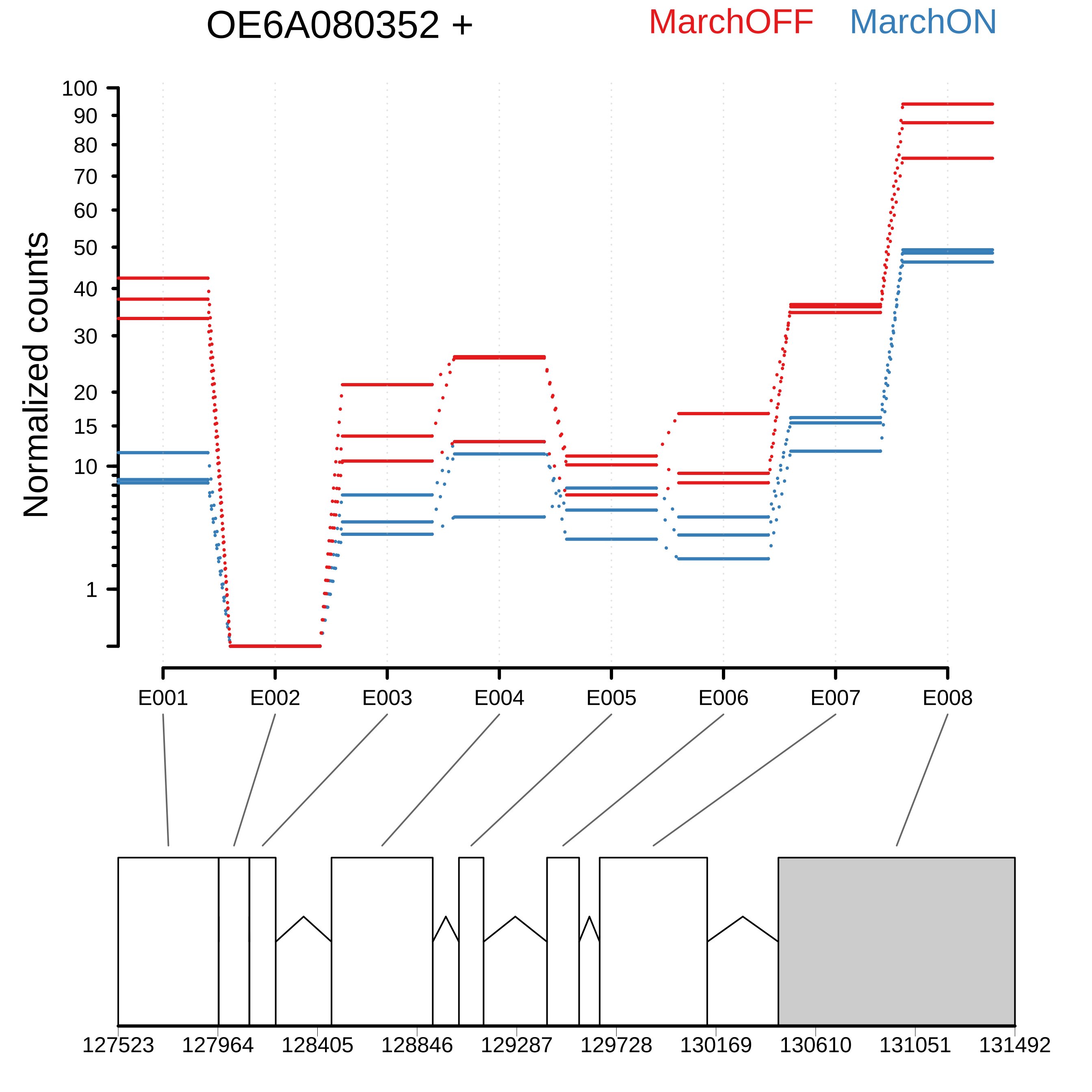

OE6A080352 +
MarchOFF
MarchON
Normalized counts
100
90
80
70
60
50
40
30
20
15
10
1
E001
E002
E003
E004
E005
E006
E007
E008
127523
127964
128405
128846
129287
129728
130169
130610
131051
131492

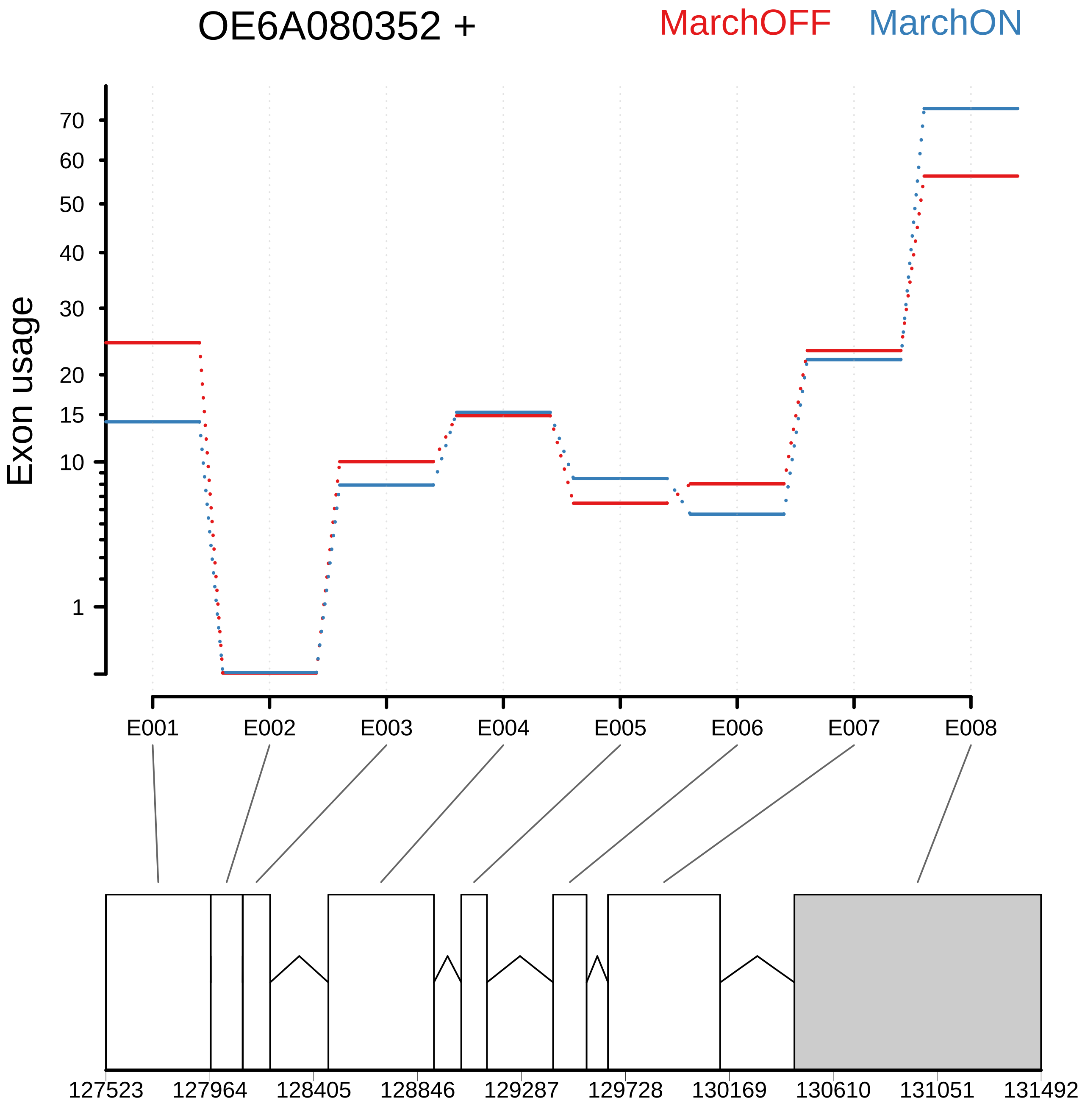

OE6A080352 +
MarchOFF
MarchON
Exon usage
70
60
50
40
30
20
15
10
1
E001
E002
E003
E004
E005
E006
E007
E008
127523
127964
128405
128846
129287
129728
130169
130610
131051
131492

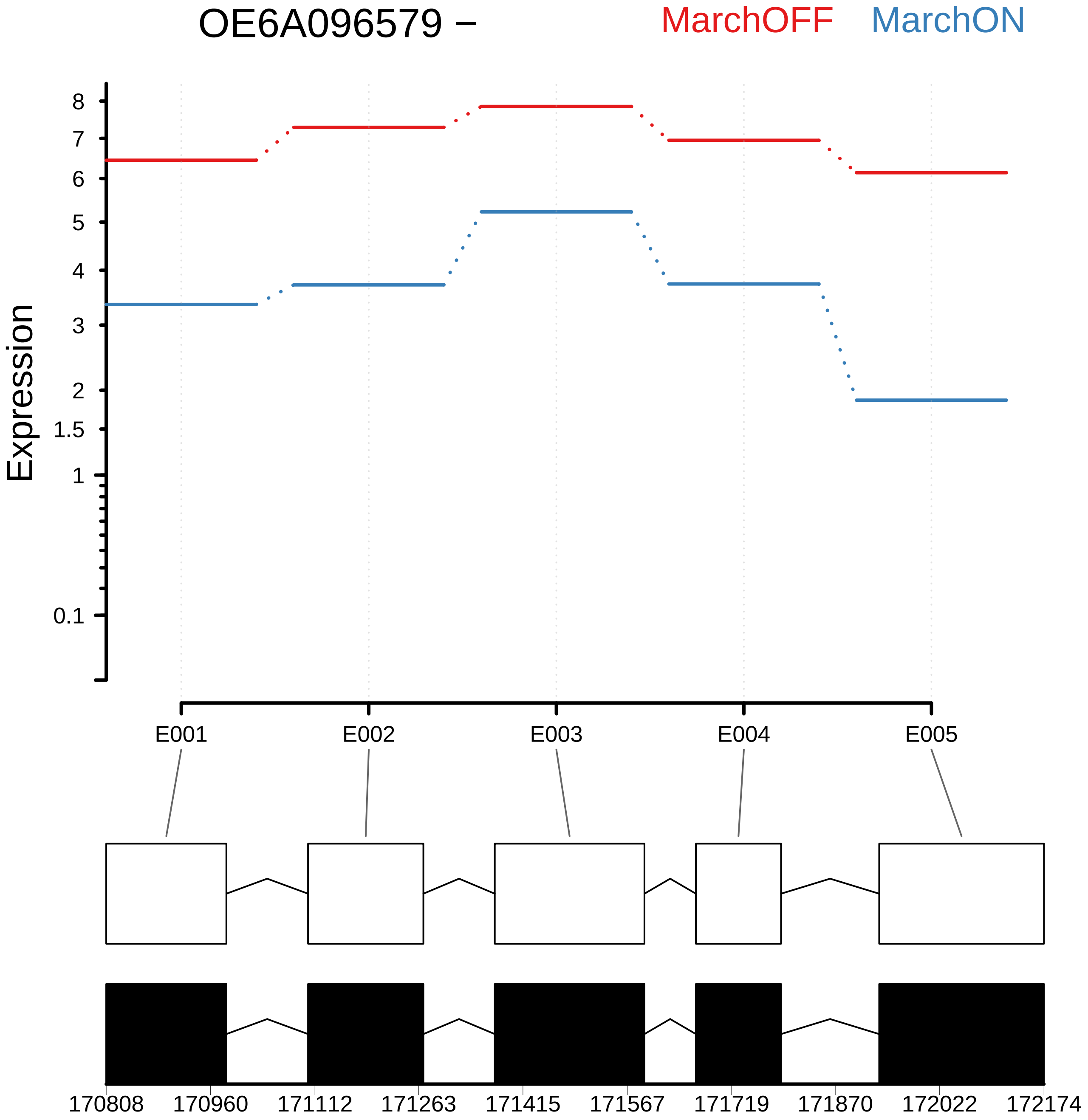

OE6A096579 –
MarchOFF
MarchON
Expression
8
7
6
5
4
3
2
1.5
1
0.1
E001
E002
E003
E004
E005
170808
170960
171112
171263
171415
171567
171719
171870
172022
172174

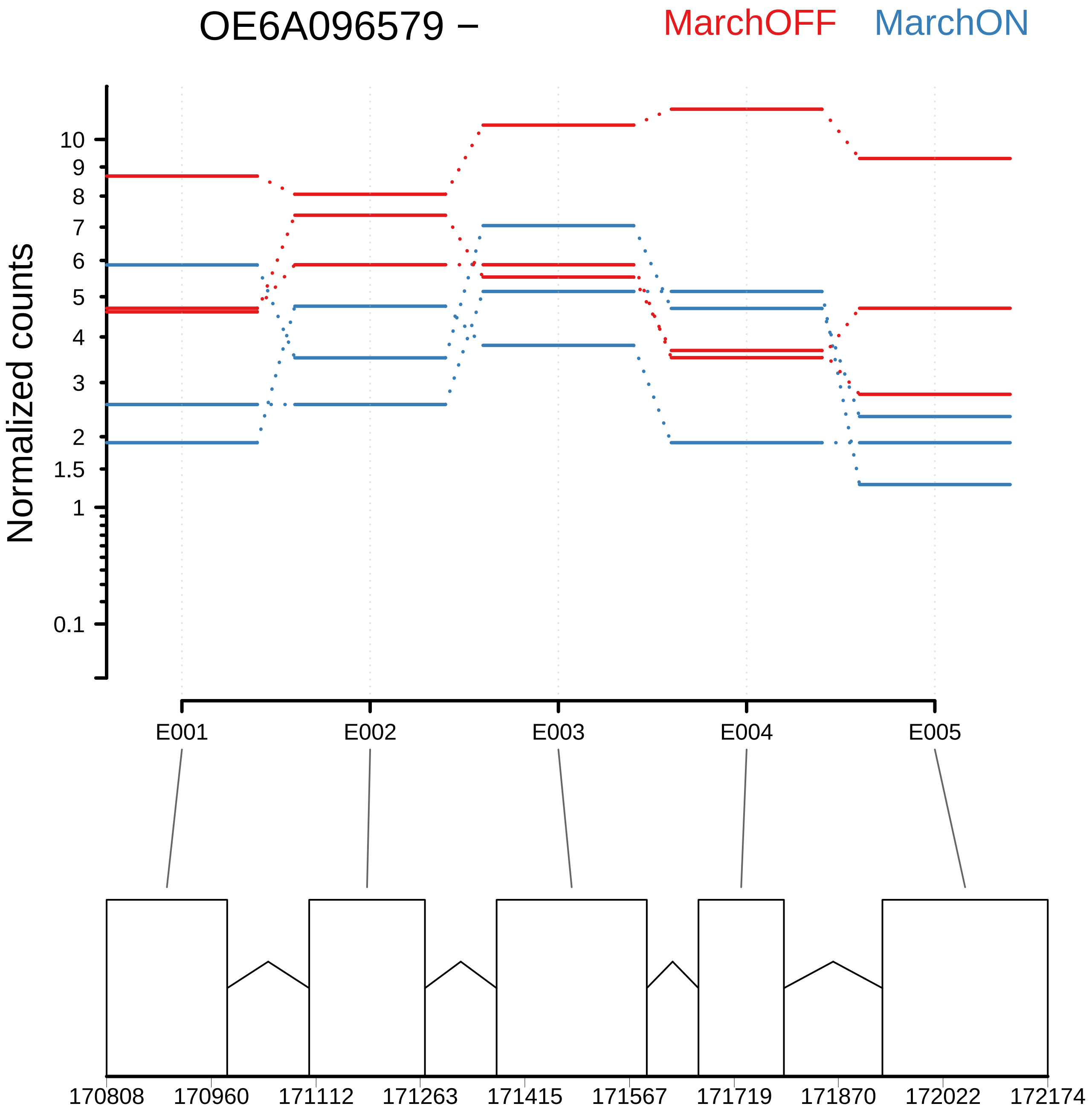

OE6A096579 −
MarchOFF
MarchON
Normalized counts
10
9
8
7
6
5
4
3
2
1.5
1
0.1
E001
E002
E003
E004
E005
170808
170960
171112
171263
171415
171567
171719
171870
172022
172174

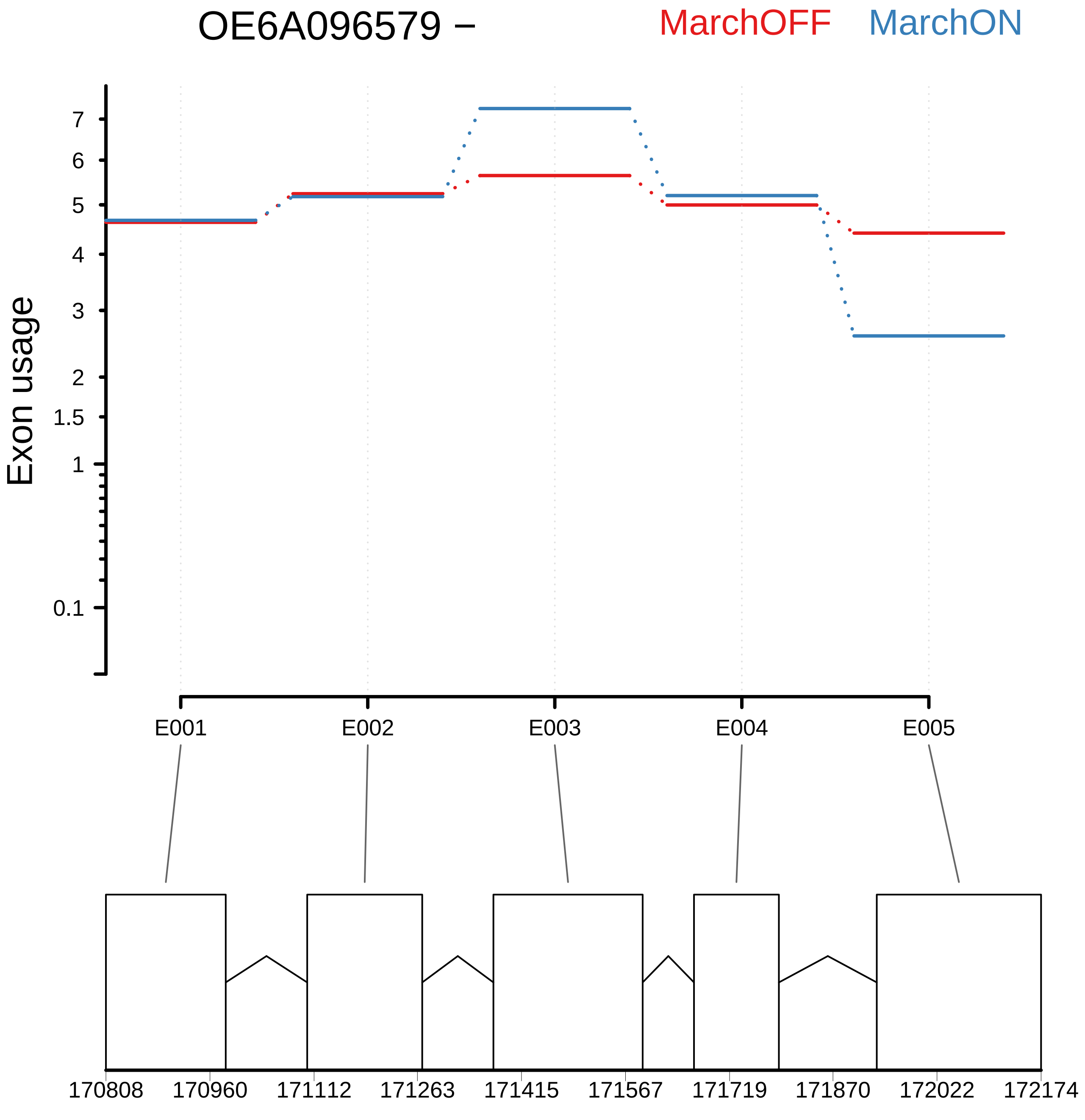
OE6A096579 −
MarchOFF
MarchON
Exon usage
7
6
5
4
3
2
1.5
1
0.1
E001
E002
E003
E004
E005
170808
170960
171112
171263
171415
171567
171719
171870
172022
172174

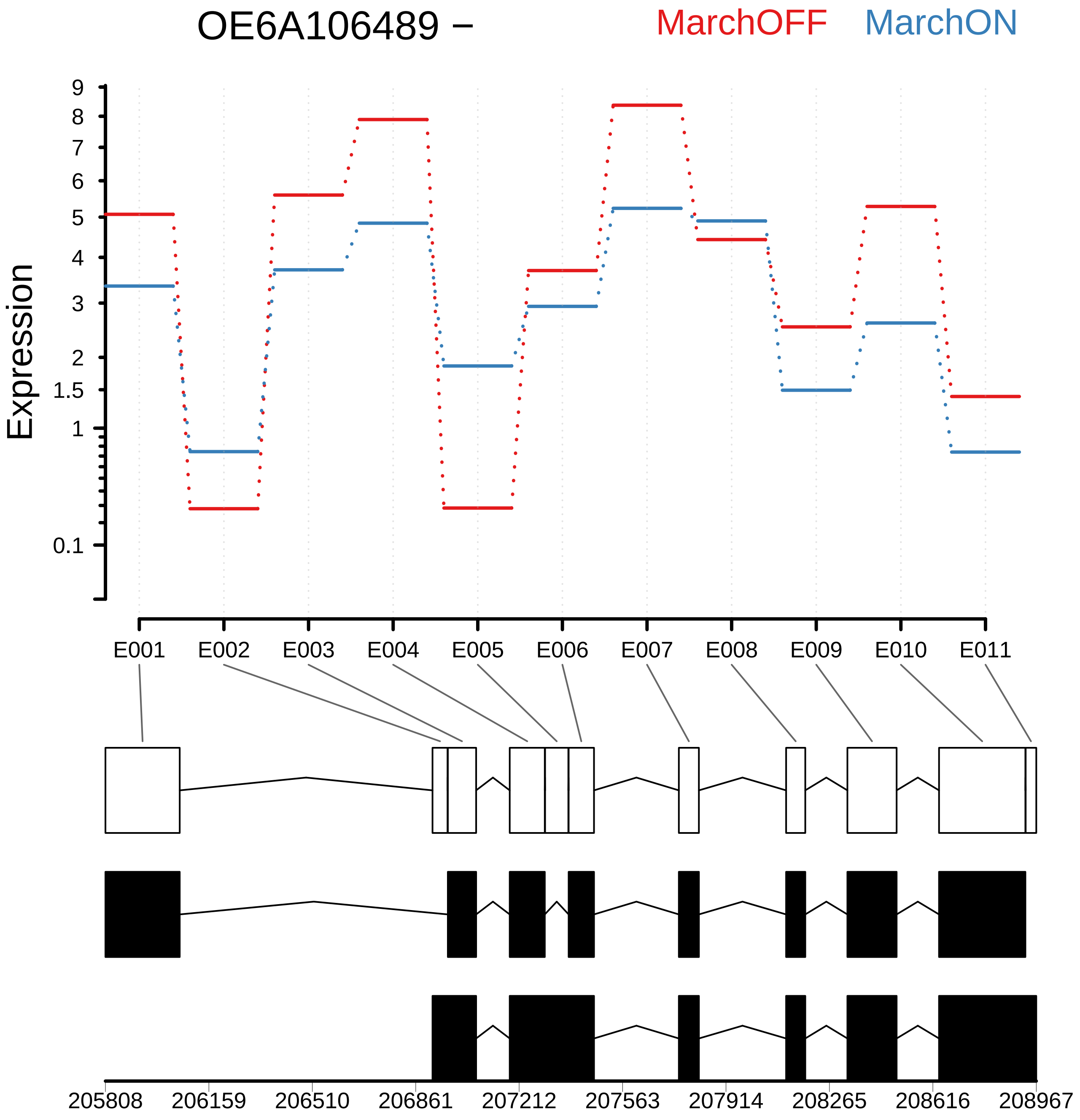

OE6A106489 –
MarchOFF
MarchON
Expression
9
8
7
6
5
4
3
2
1.5
1
0.1
E001
E002
E003
E004
E005
E006
E007
E008
E009
E010
E011
205808
206159
206510
206861
207212
207563
207914
208265
208616
208967

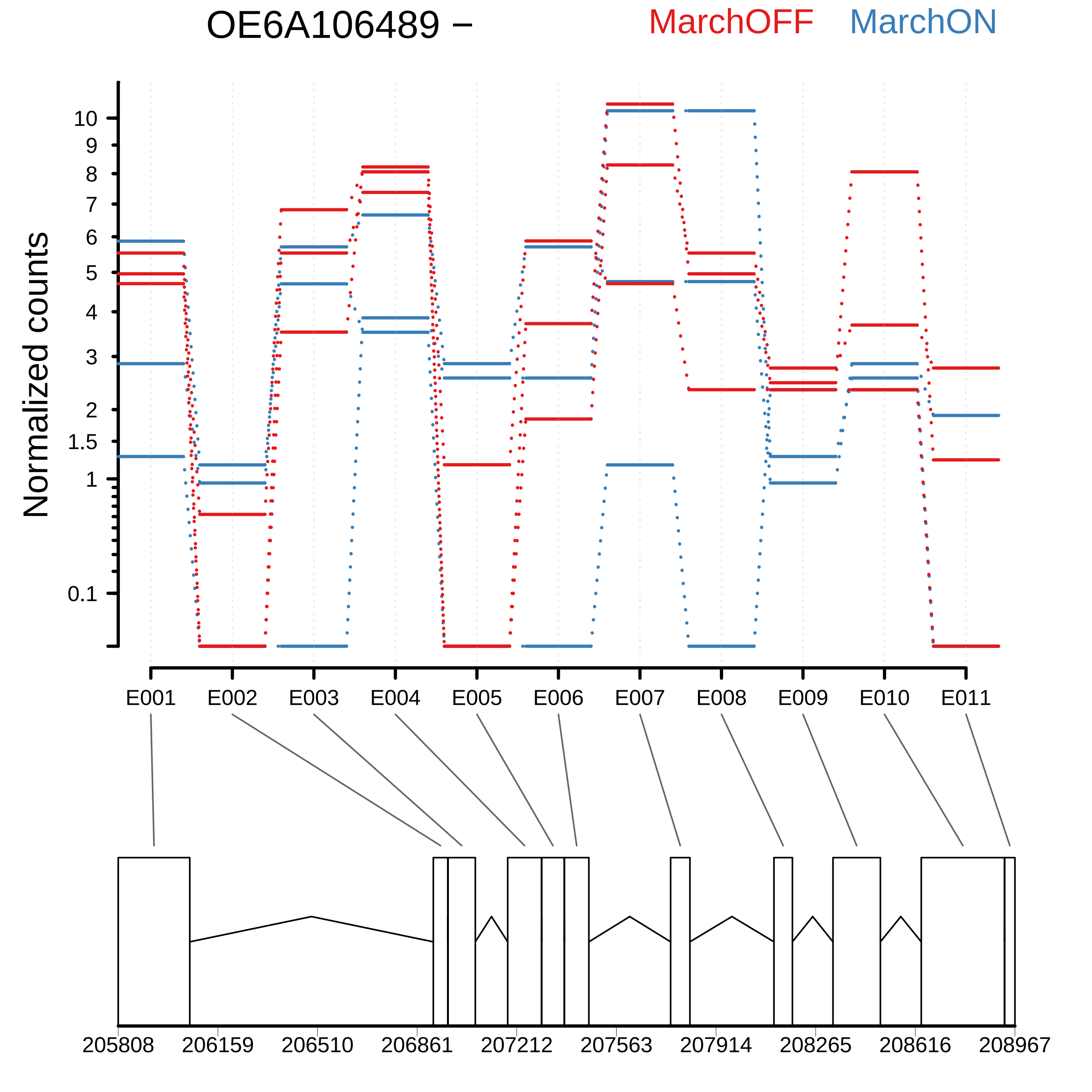
OE6A106489 −
MarchOFF
MarchON
Normalized counts
10
9
8
7
6
5
4
3
2
1.5
1
0.1
E001
E002
E003
E004
E005
E006
E007
E008
E009
E010
E011
205808
206159
206510
206861
207212
207563
207914
208265
208616
208967

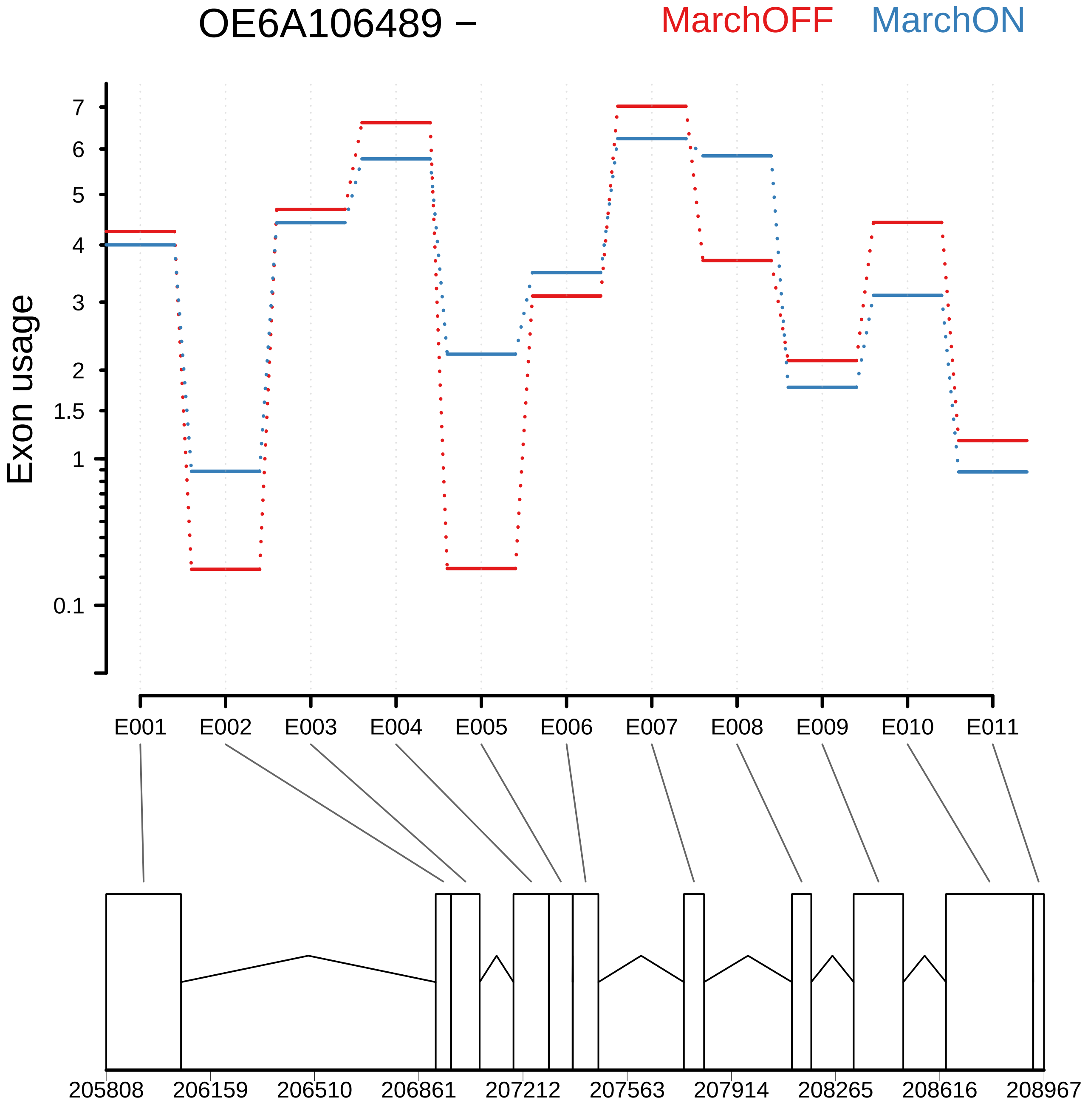

OE6A106489 −
MarchOFF
MarchON
Exon usage
7
6
5
4
3
2
1.5
1
0.1
E001
E002
E003
E004
E005
E006
E007
E008
E009
E010
E011
205808
206159
206510
206861
207212
207563
207914
208265
208616
208967

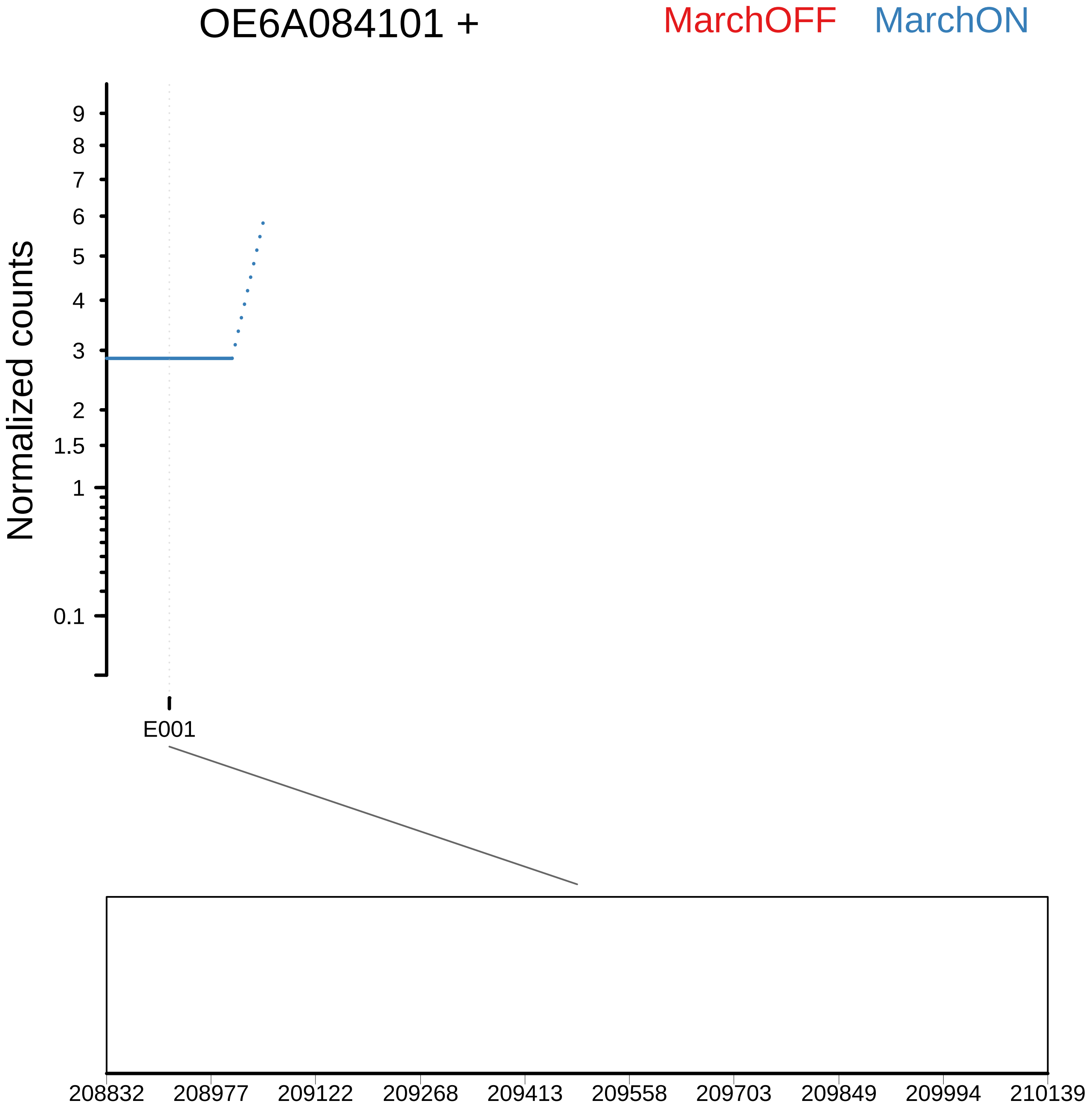

OE6A084101 +
MarchOFF
MarchON
Normalized counts
9
8
7
6
5
4
3
2
1.5
1
0.1
E001
208832
208977
209122
209268
209413
209558
209703
209849
209994
210139

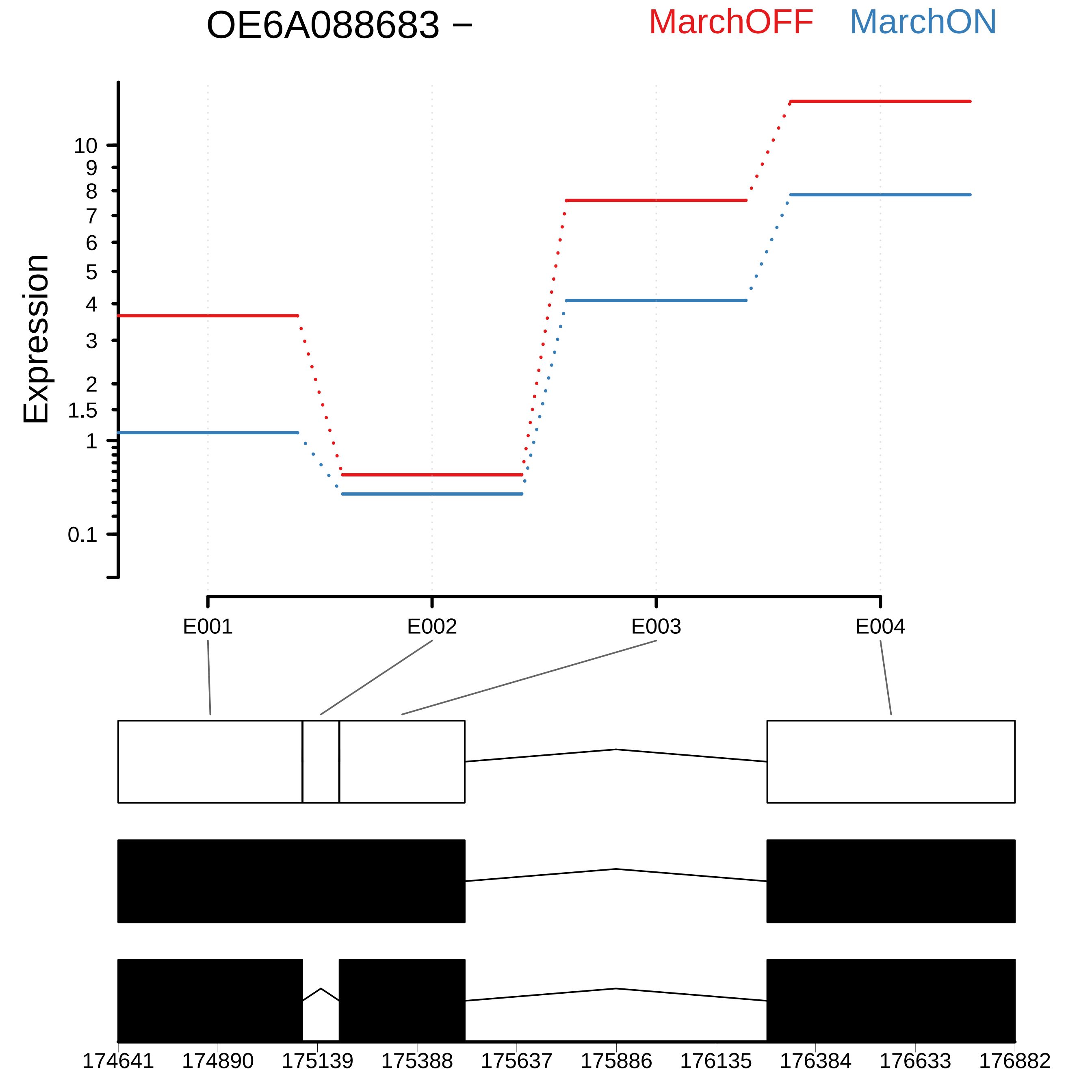

OE6A088683 −
MarchOFF
MarchON
Expression
10
9
8
7
6
5
4
3
2
1.5
1
0.1
E001
E002
E003
E004
174641
174890
175139
175388
175637
175886
176135
176384
176633
176882

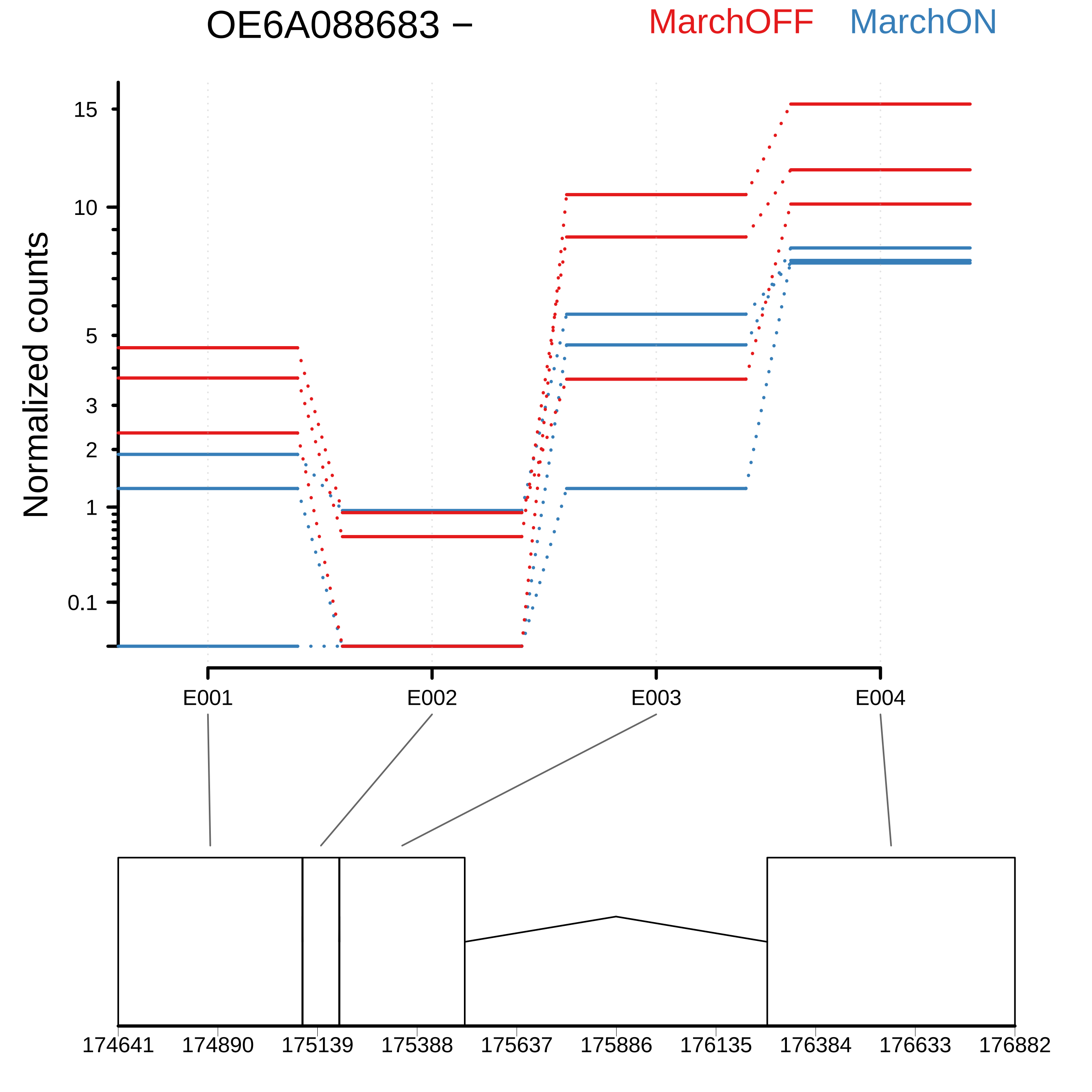
OE6A088683 −
MarchOFF
MarchON
Normalized counts
15
10
5
3
2
1
0.1
E001
E002
E003
E004
174641
174890
175139
175388
175637
175886
176135
176384
176633
176882

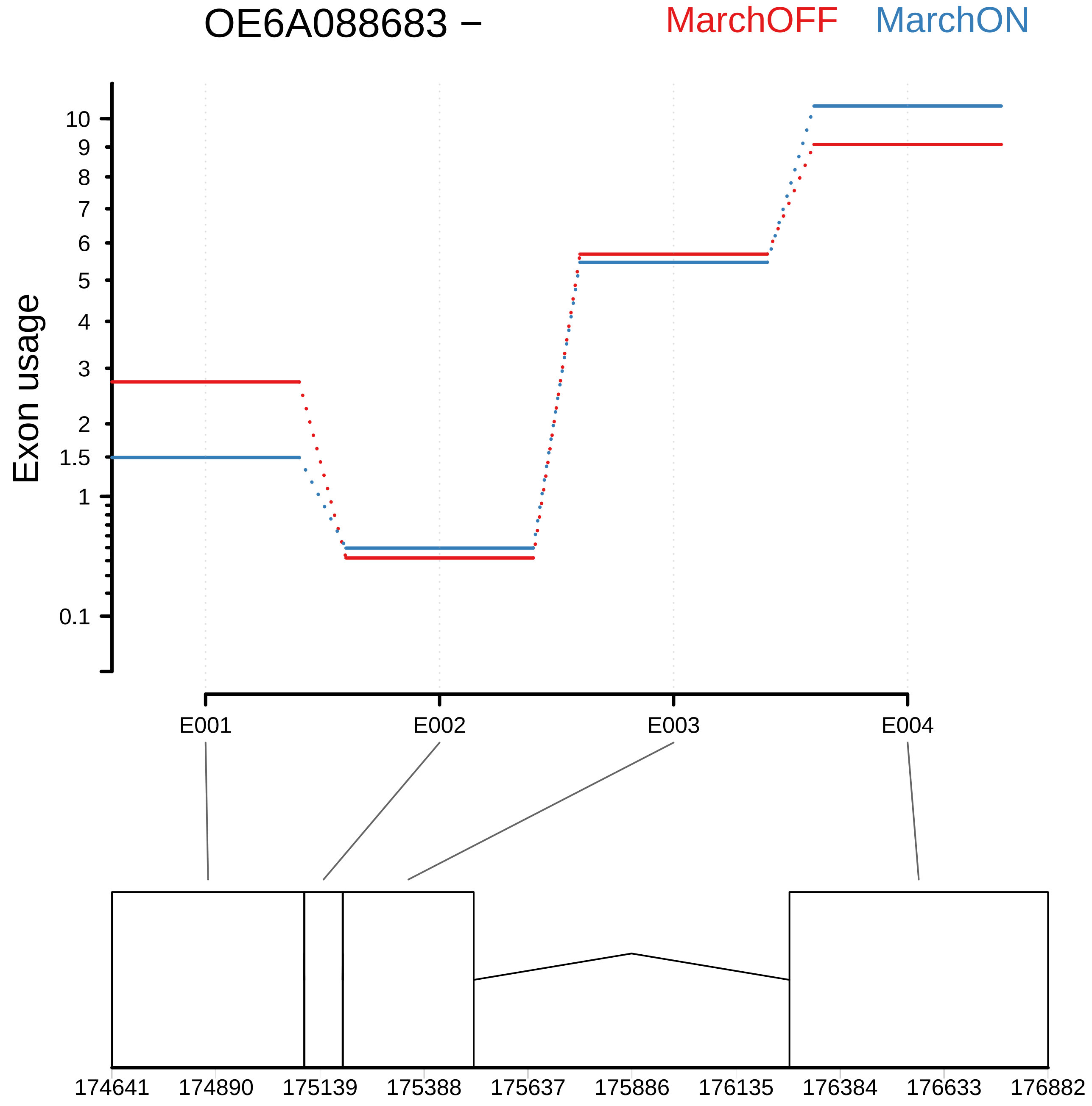

OE6A088683 −
MarchOFF
MarchON
Exon usage
10
9
8
7
6
5
4
3
2
1.5
1
0.1
E001
E002
E003
E004
174641
174890
175139
175388
175637
175886
176135
176384
176633
176882

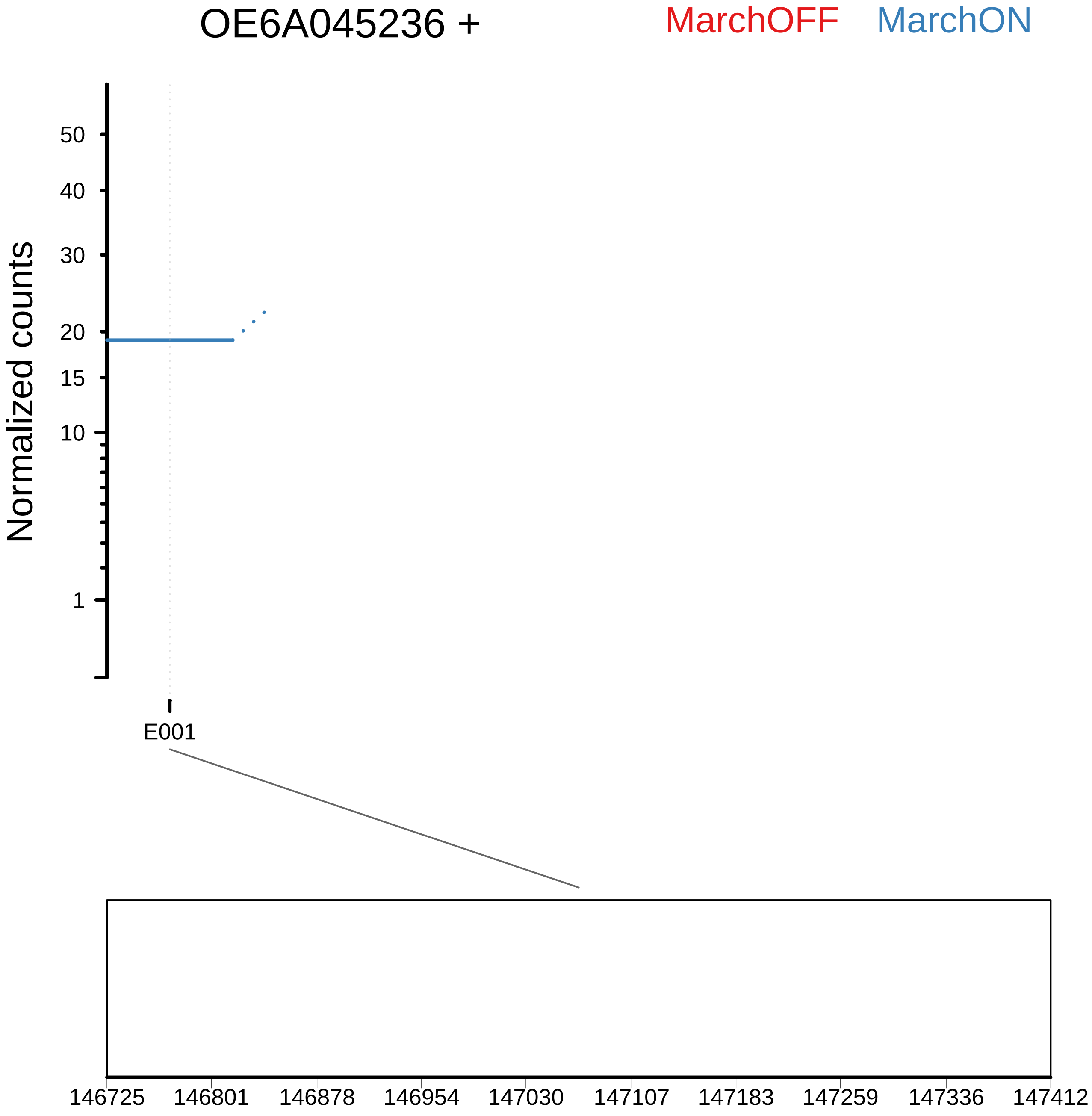
OE6A045236 +
MarchOFF
MarchON
Normalized counts
50
40
30
20
15
10
1
E001
146725
146801
146878
146954
147030
147107
147183
147259
147336
147412

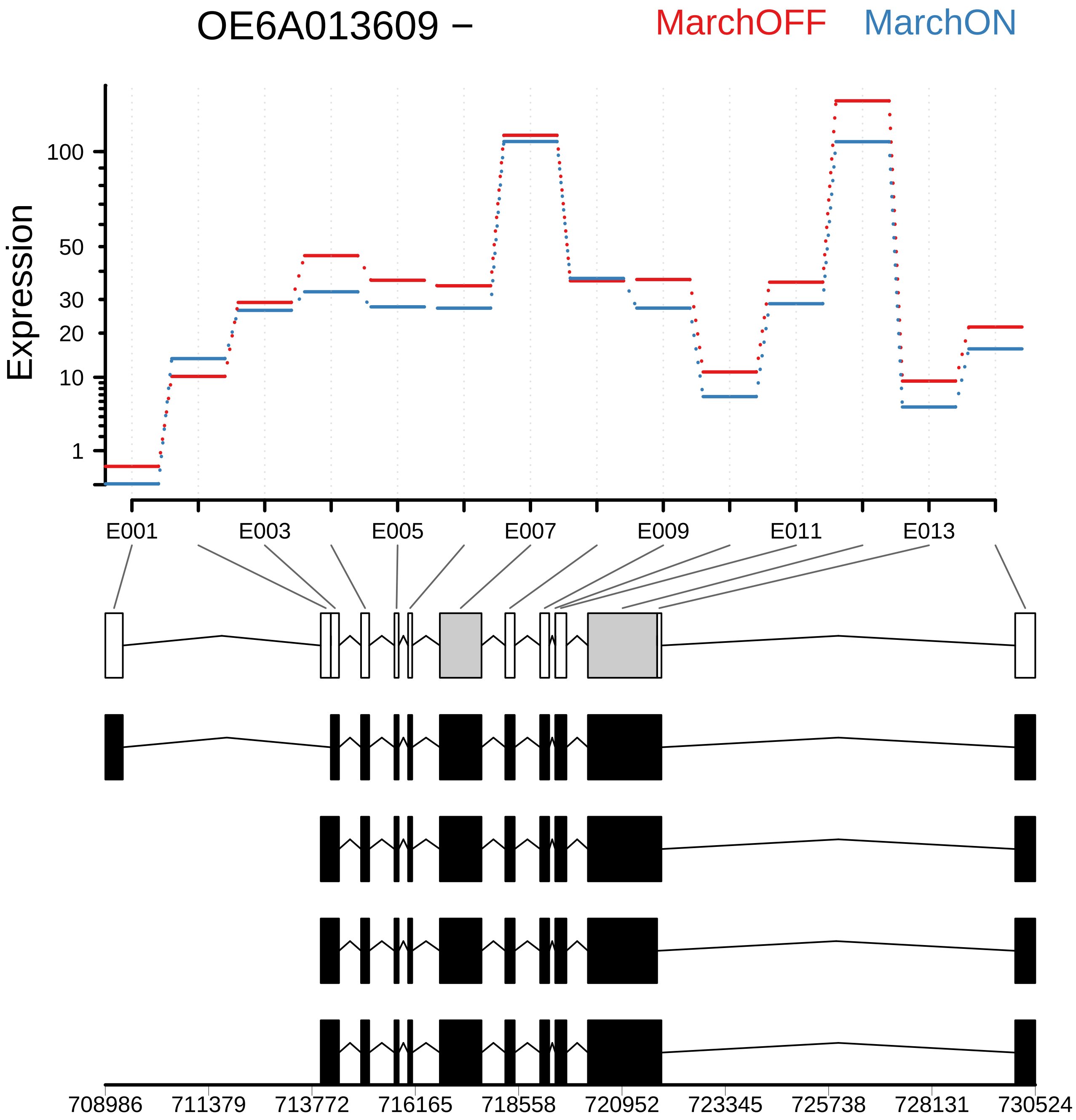

OE6A013609 –
MarchOFF
MarchON
Expression
100
50
30
20
10
1
E001
E003
E005
E007
E009
E011
E013
708986
711379
713772
716165
718558
720952
723345
725738
728131
730524

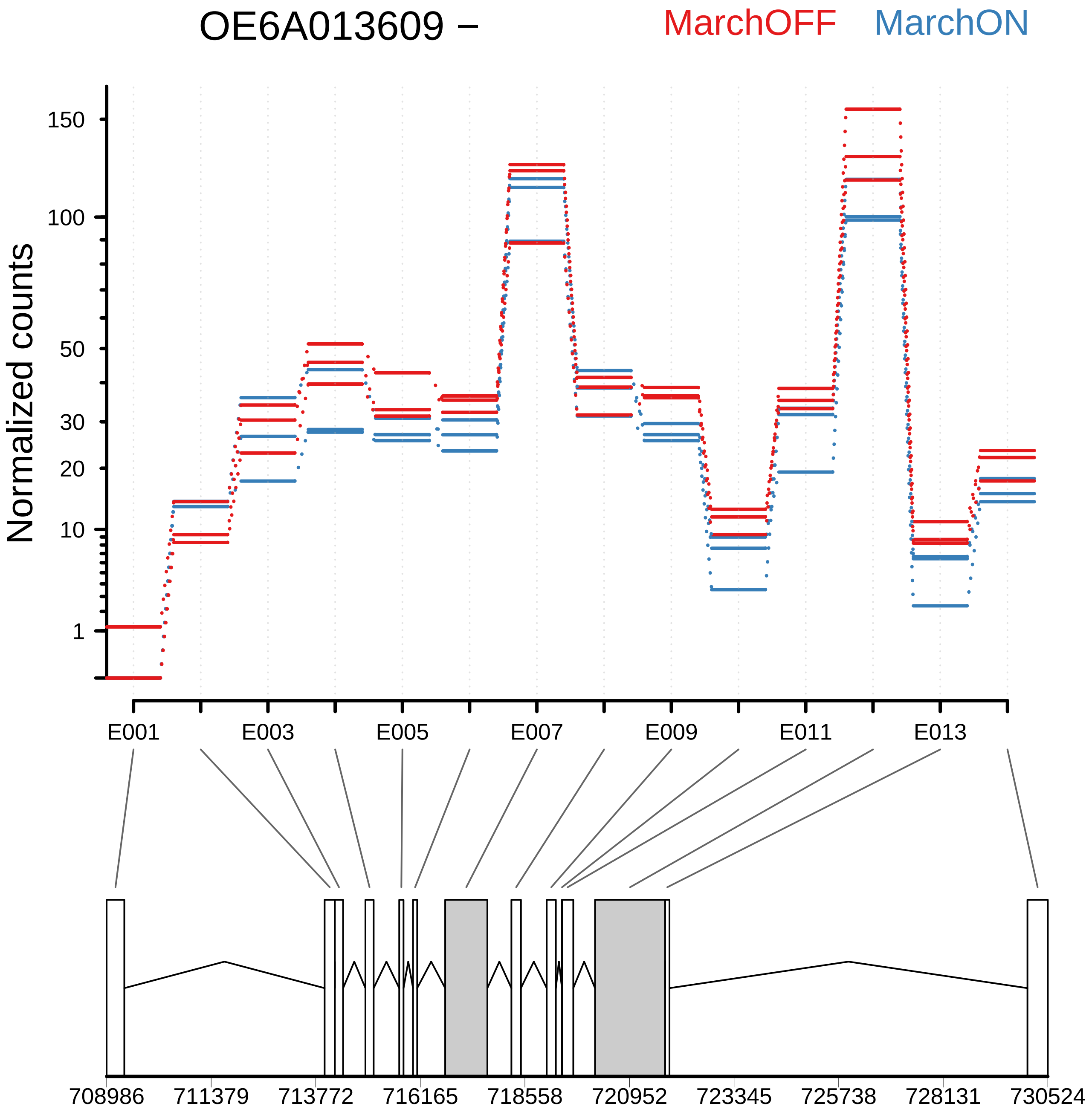

OE6A013609 −
MarchOFF
MarchON
Normalized counts
150
100
50
30
20
10
1
E001
E003
E005
E007
E009
E011
E013
708986
711379
713772
716165
718558
720952
723345
725738
728131
730524

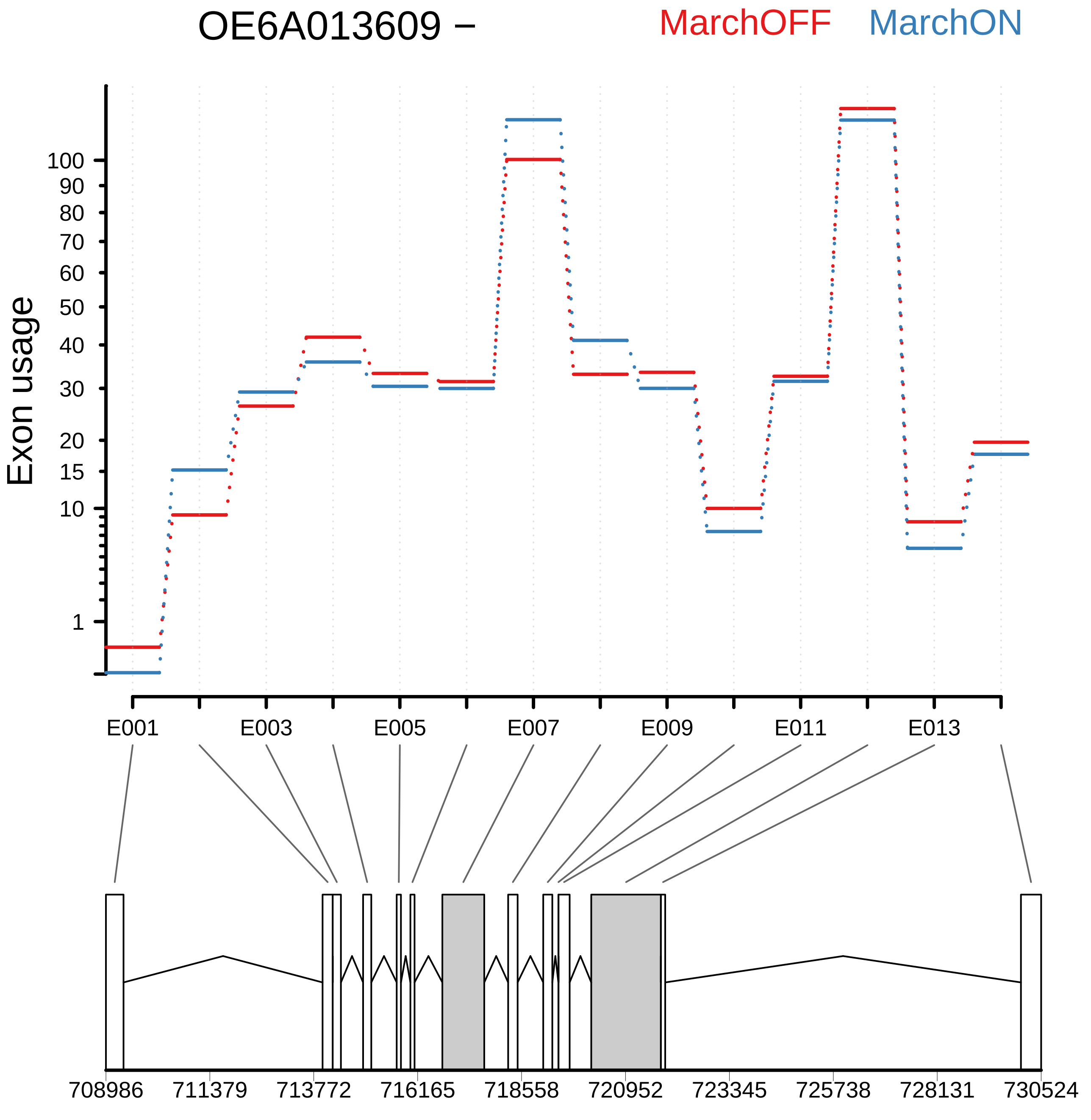

OE6A013609 −
MarchOFF
MarchON
Exon usage
100
90
80
70
60
50
40
30
20
15
10
1
E001
E003
E005
E007
E009
E011
E013
708986
711379
713772
716165
718558
720952
723345
725738
728131
730524

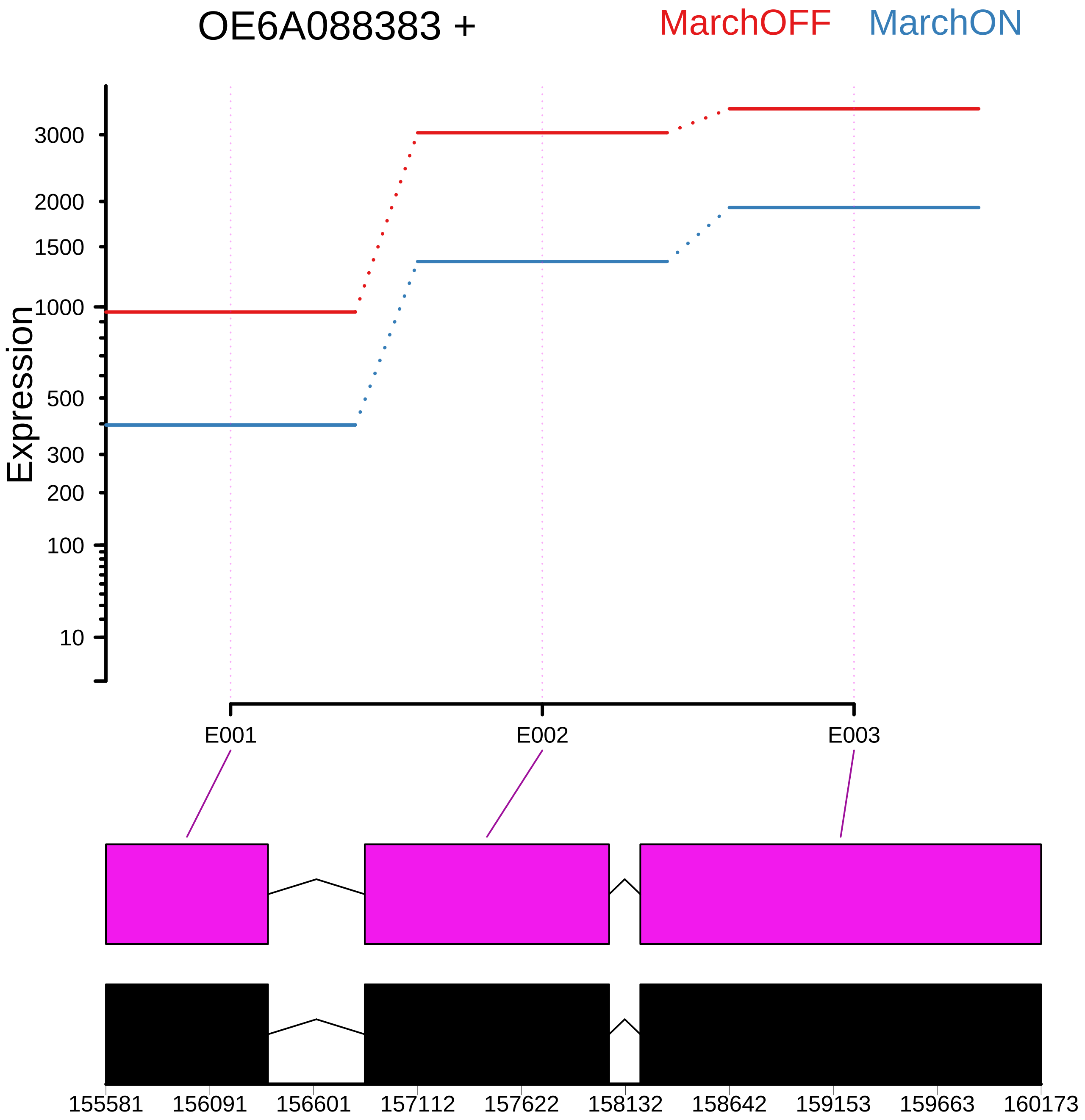

OE6A088383 +
MarchOFF
MarchON
Expression
3000
2000
1500
1000
500
300
200
100
10
E001
E002
E003
155581
156091
156601
157112
157622
158132
158642
159153
159663
160173

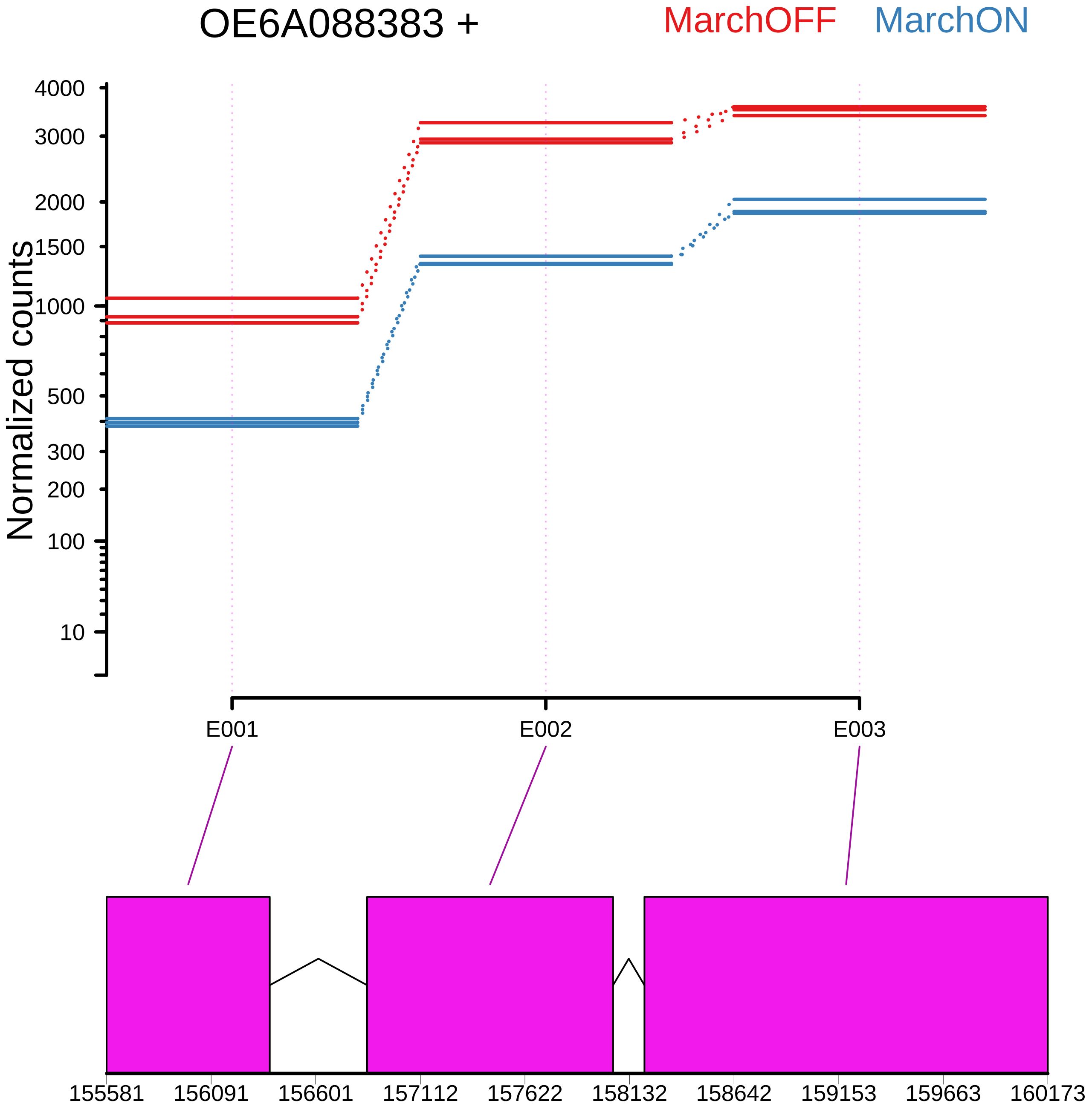

OE6A088383 +
MarchOFF
MarchON
Normalized counts
4000
3000
2000
1500
1000
500
300
200
100
10
E001
E002
E003
155581
156091
156601
157112
157622
158132
158642
159153
159663
160173

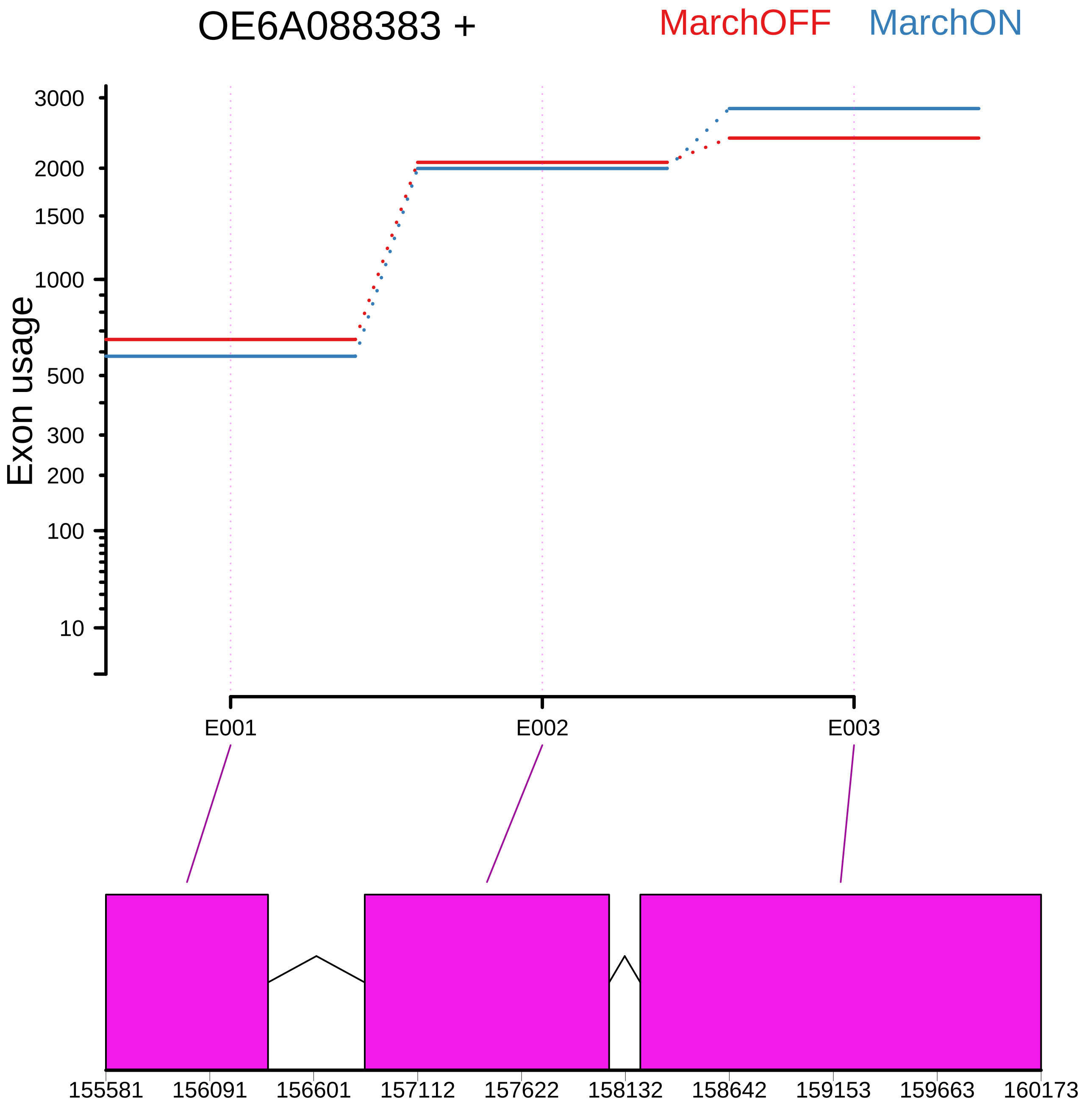

OE6A088383 +
MarchOFF
MarchON
Exon usage
3000
2000
1500
1000
500
300
200
100
10
E001
E002
E003
155581
156091
156601
157112
157622
158132
158642
159153
159663
160173

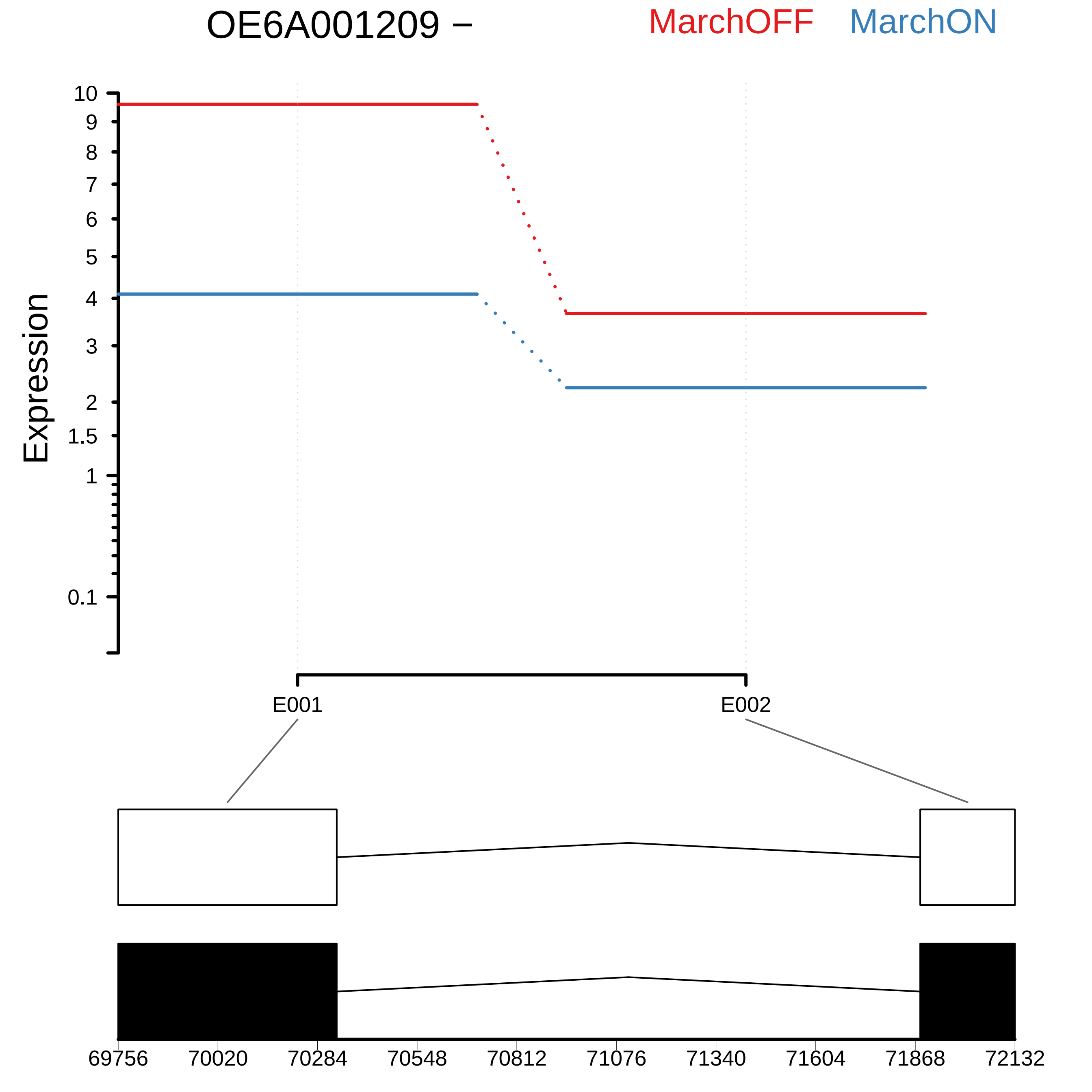

OE6A001209 −
MarchOFF
MarchON
Expression
10
9
8
7
6
5
4
3
2
1.5
1
0.1
E001
E002
69756
70020
70284
70548
70812
71076
71340
71604
71868
72132

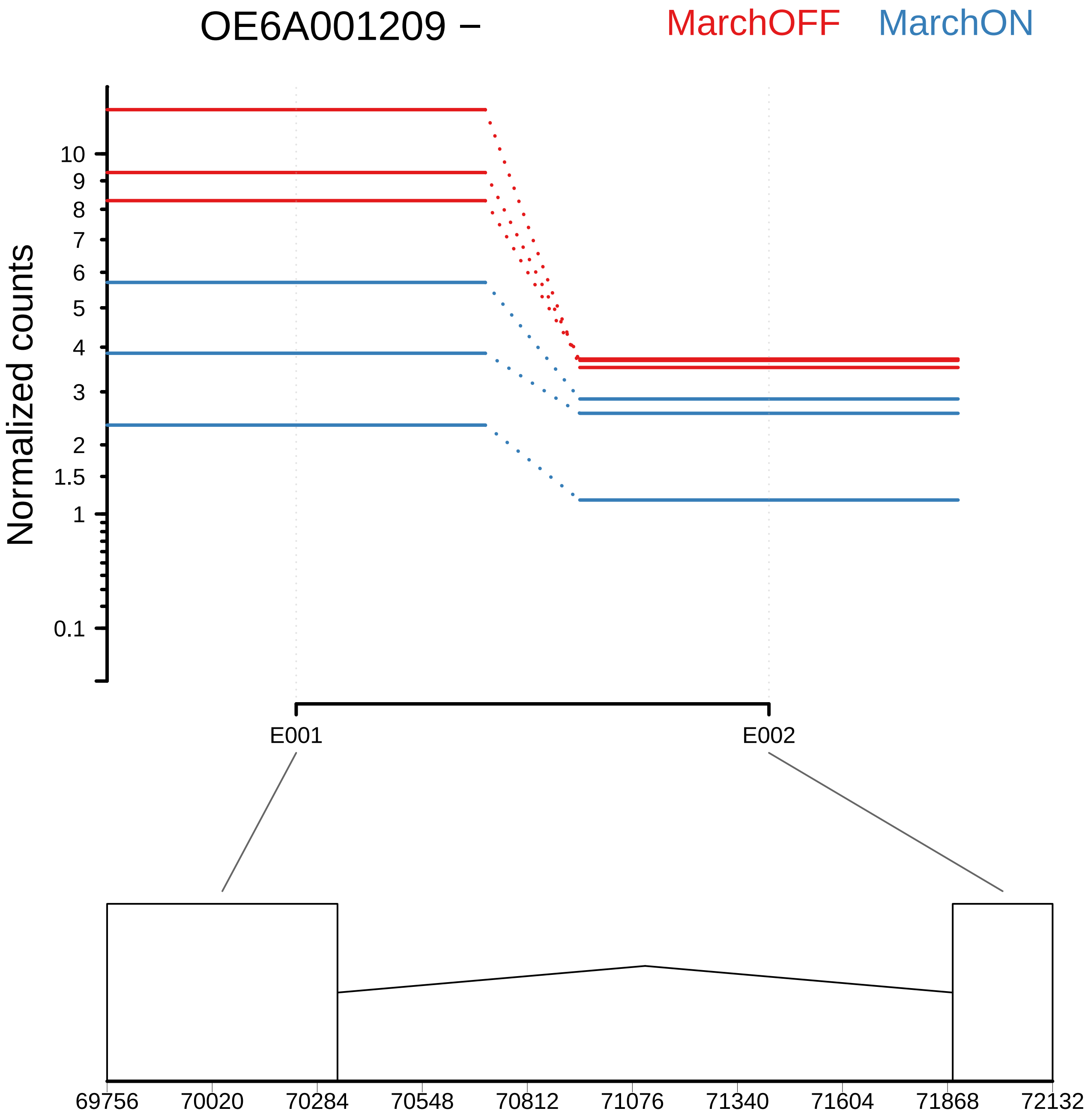
OE6A001209 −
MarchOFF
MarchON
Normalized counts
10
9
8
7
6
5
4
3
2
1.5
1
0.1
E001
E002
69756
70020
70284
70548
70812
71076
71340
71604
71868
72132

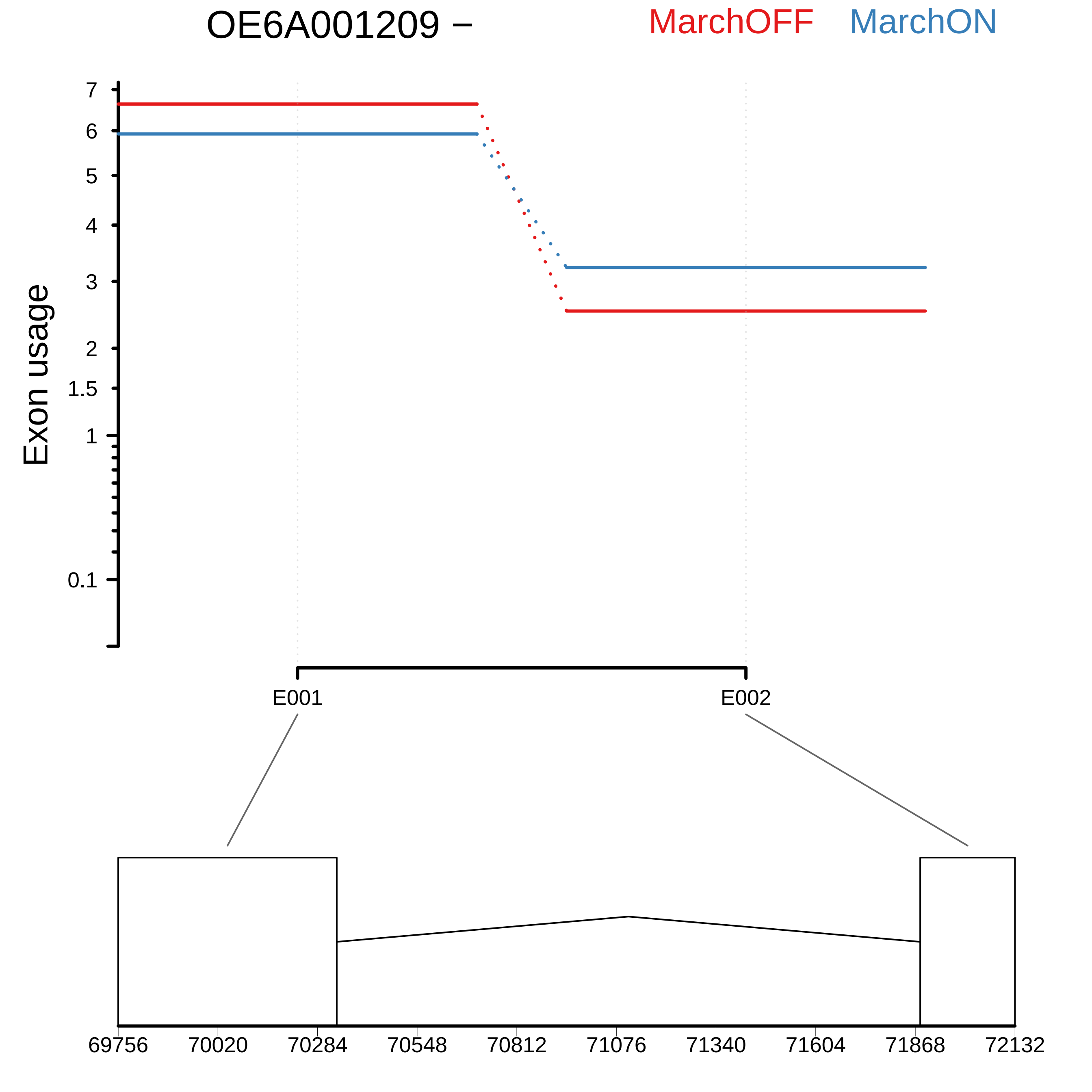
OE6A001209 −
MarchOFF
MarchON
Exon usage
7
6
5
4
3
2
1.5
1
0.1
E001
E002
69756
70020
70284
70548
70812
71076
71340
71604
71868
72132

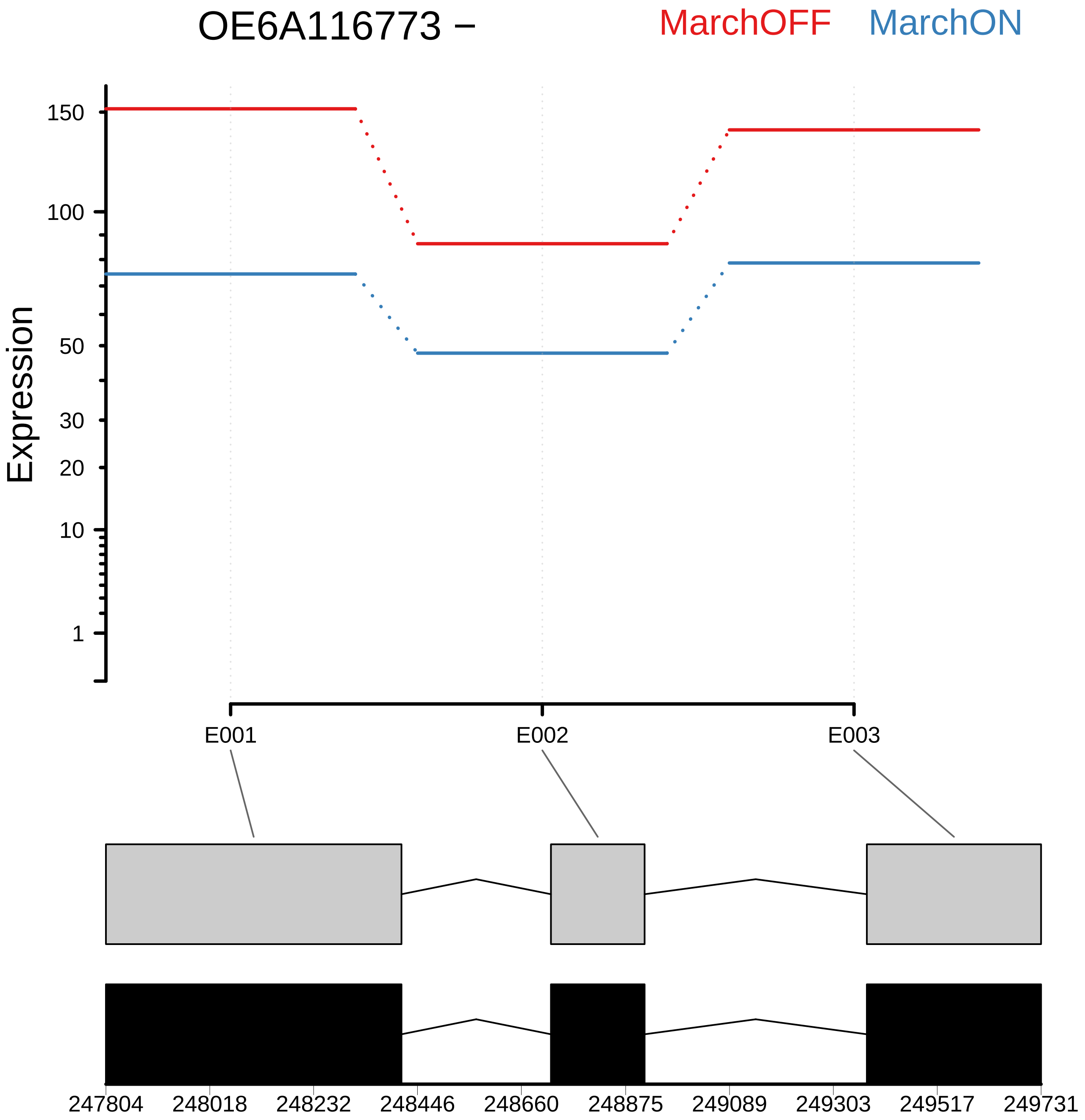

OE6A116773 −
MarchOFF
MarchON
Expression
150
100
50
30
20
10
1
E001
E002
E003
247804
248018
248232
248446
248660
248875
249089
249303
249517
249731

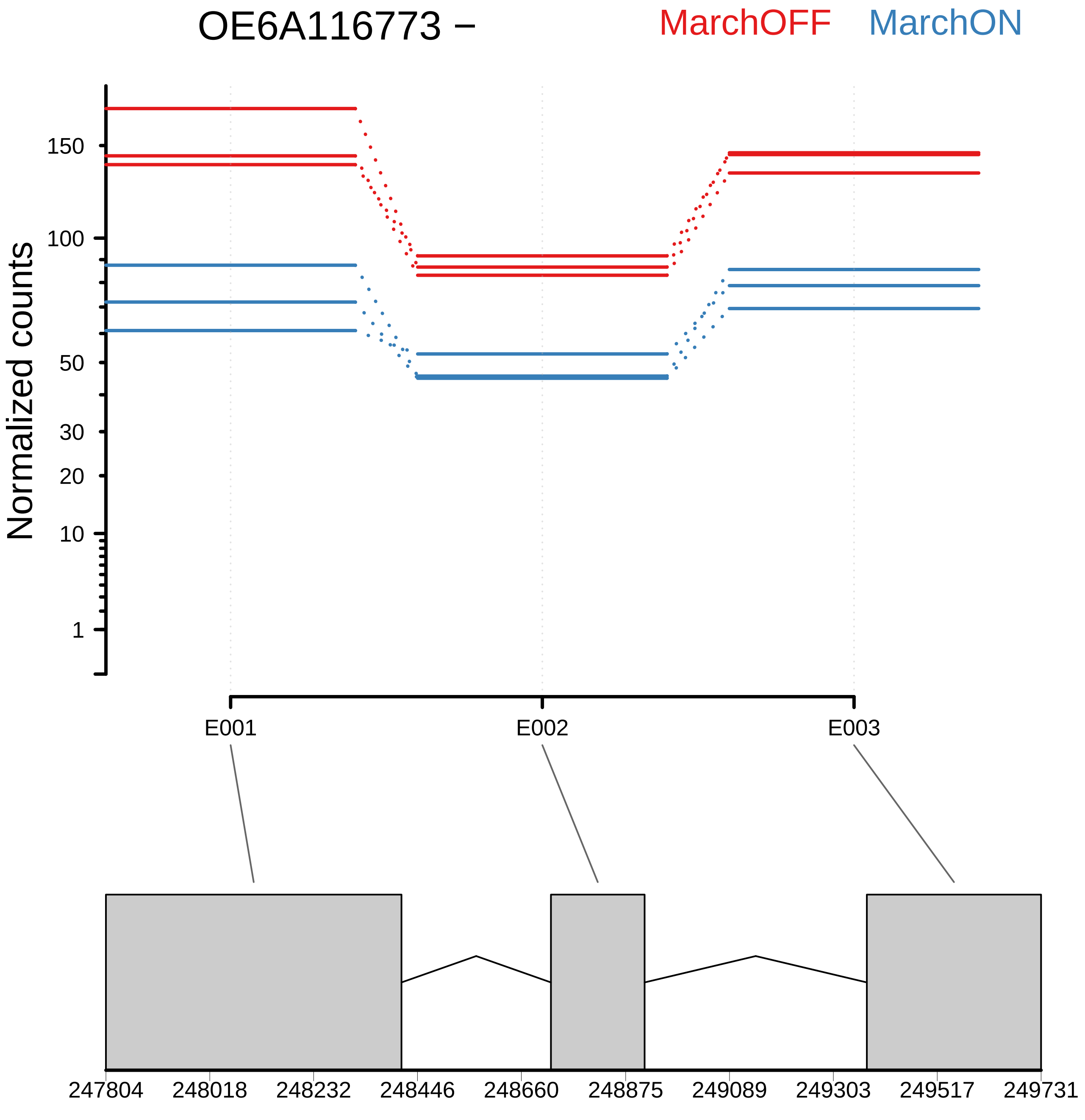

OE6A116773 −
MarchOFF
MarchON
Normalized counts
150
100
50
30
20
10
1
E001
E002
E003
247804
248018
248232
248446
248660
248875
249089
249303
249517
249731

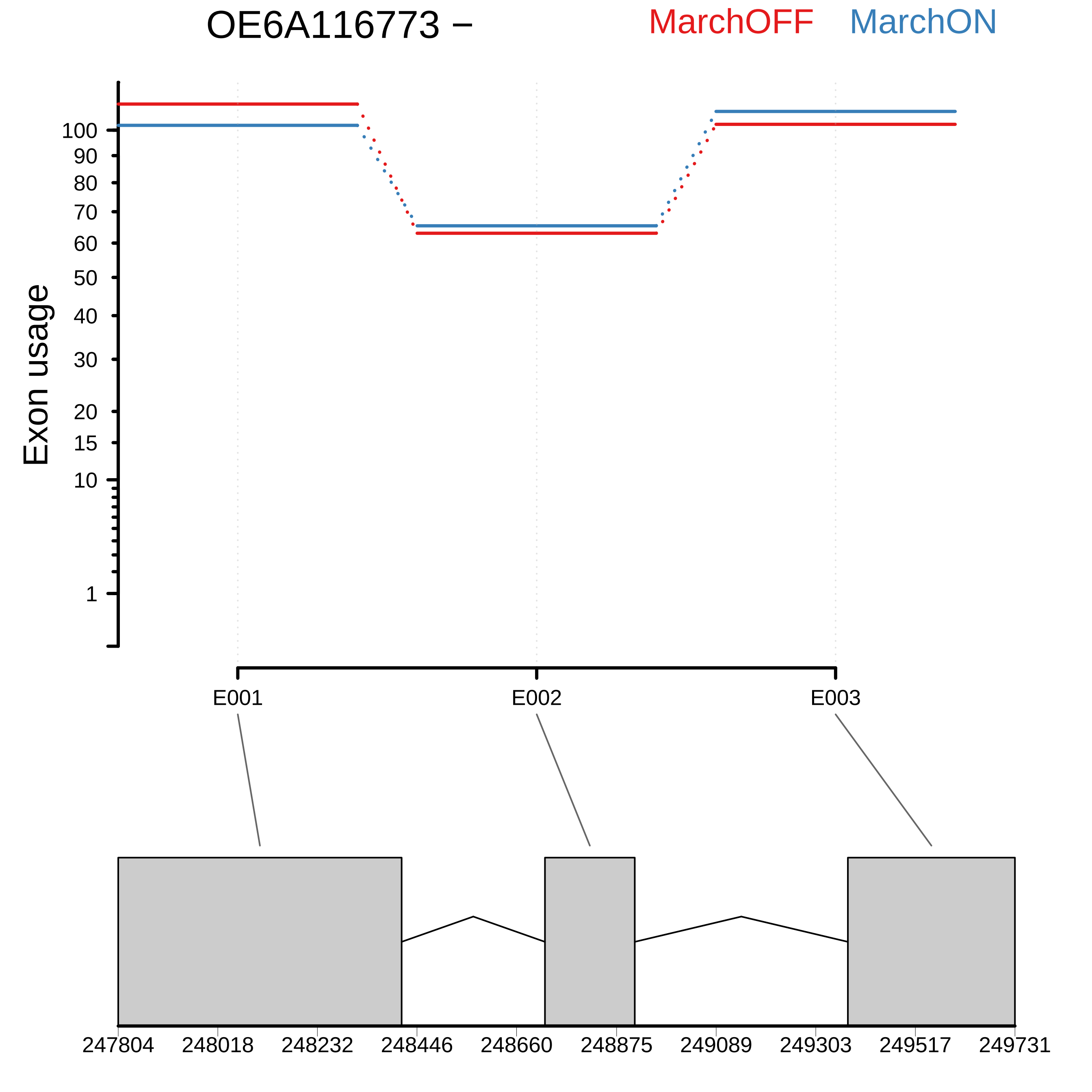

OE6A116773 −
MarchOFF
MarchON
Exon usage
100
90
80
70
60
50
40
30
20
15
10
1
E001
E002
E003
247804
248018
248232
248446
248660
248875
249089
249303
249517
249731

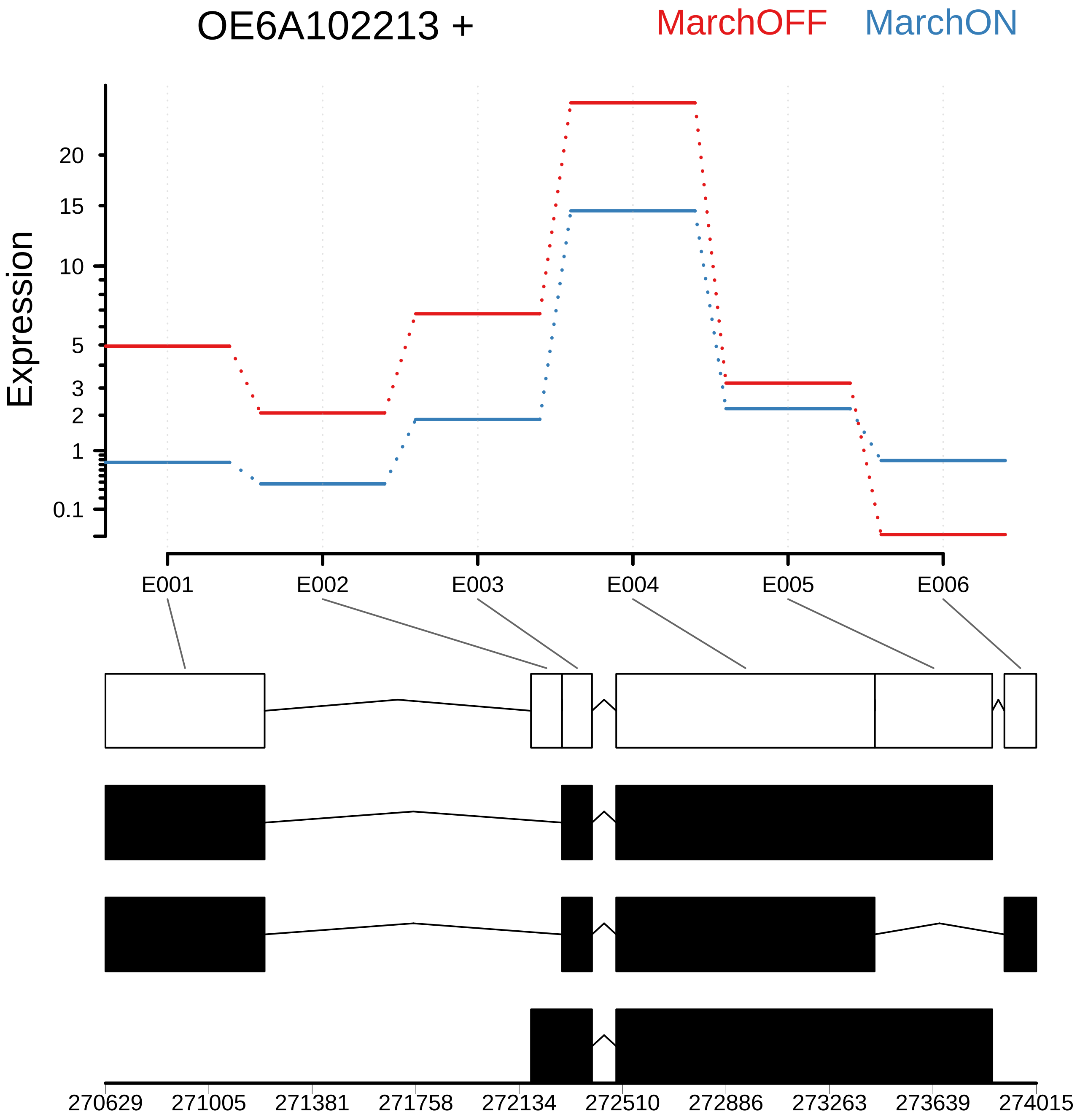
OE6A102213 +
MarchOFF
MarchON
Expression
20
15
10
5
3
2
1
0.1
E001
E002
E003
E004
E005
E006
270629
271005
271381
271758
272134
272510
272886
273263
273639
274015

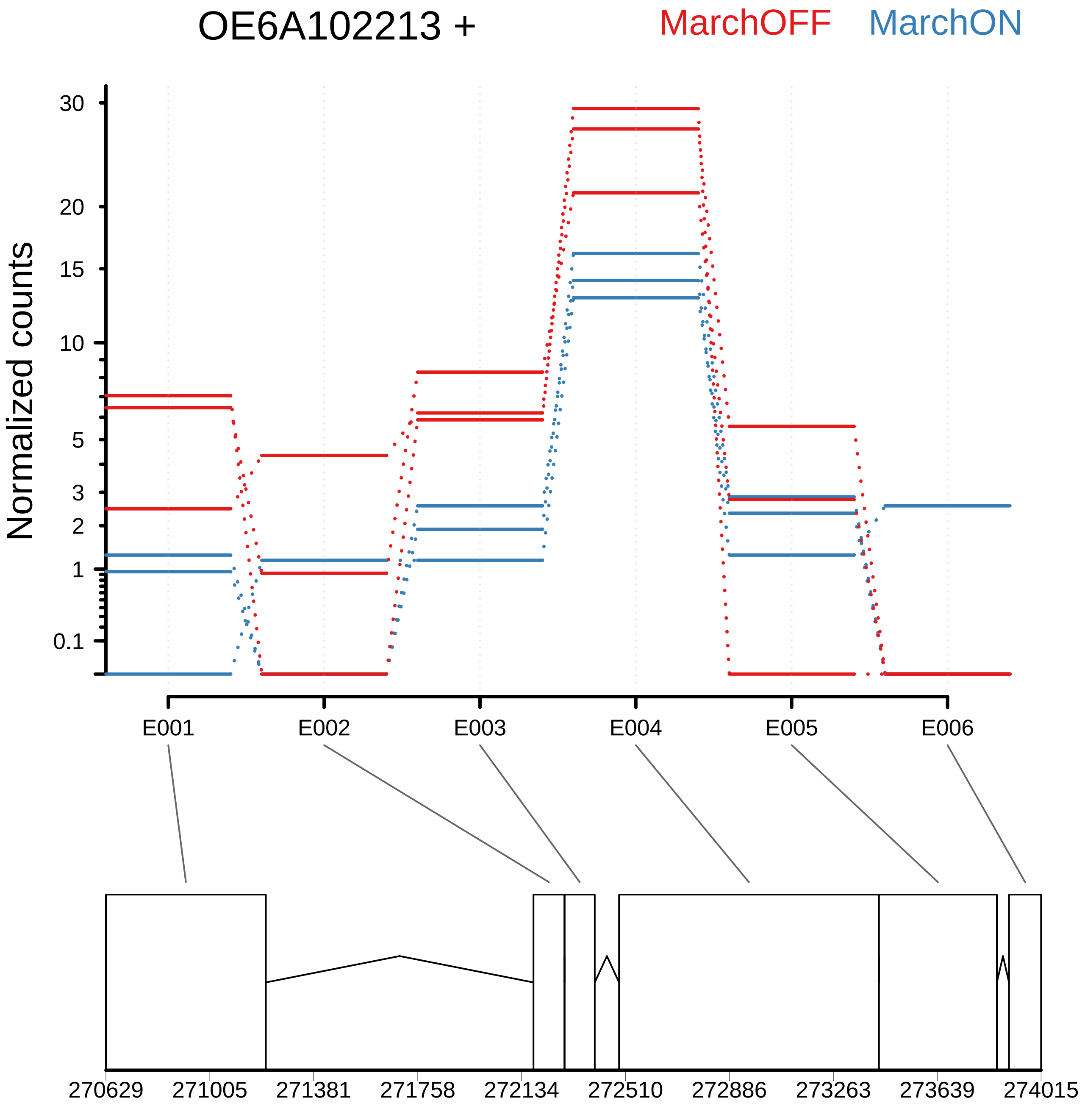
OE6A102213 +
MarchOFF
MarchON
Normalized counts
30
20
15
10
5
3
2
1
0.1
E001
E002
E003
E004
E005
E006
270629
271005
271381
271758
272134
272510
272886
273263
273639
274015

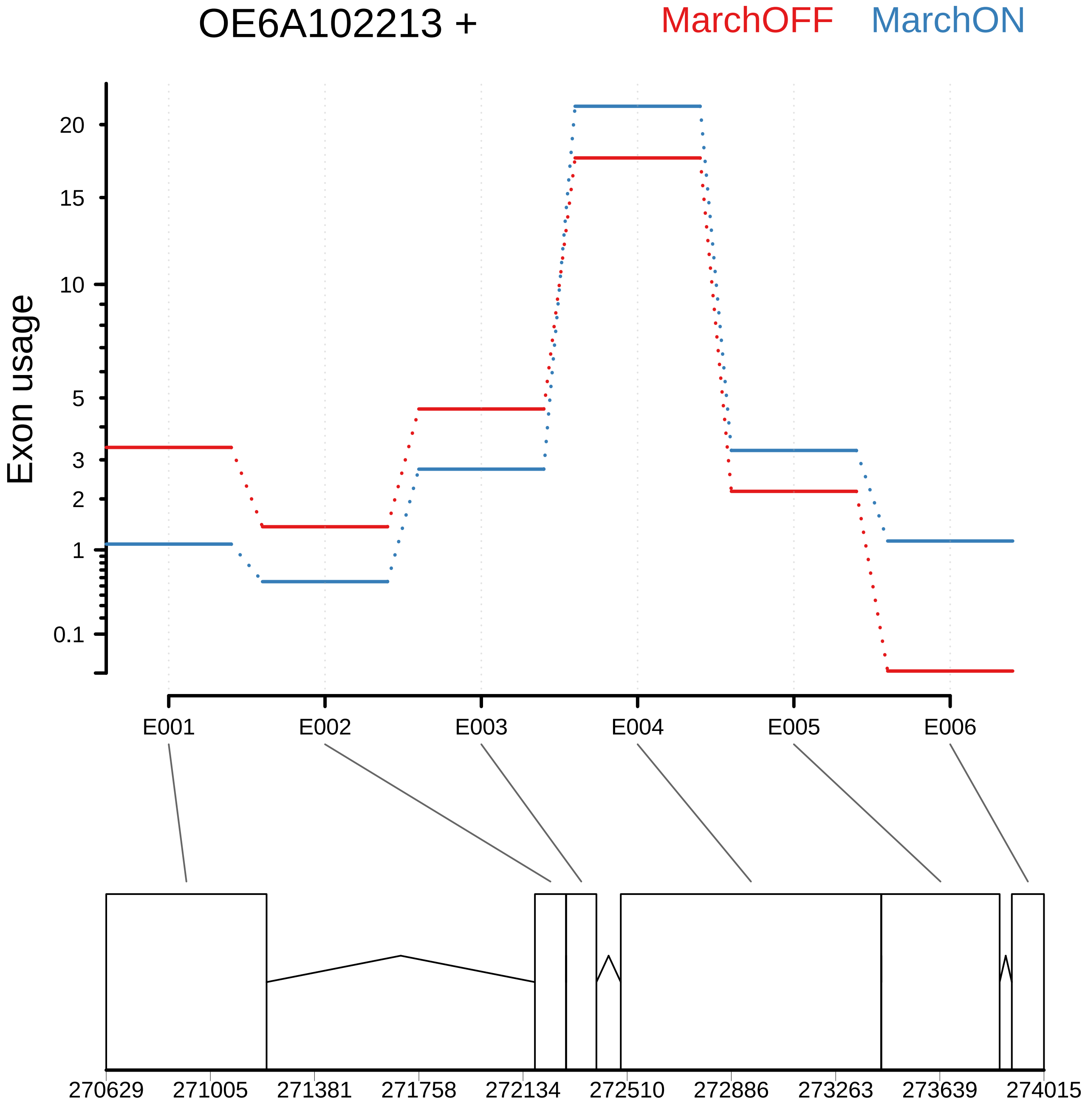

OE6A102213 +
MarchOFF
MarchON
Exon usage
20
15
10
5
3
2
1
0.1
E001
E002
E003
E004
E005
E006
270629
271005
271381
271758
272134
272510
272886
273263
273639
274015

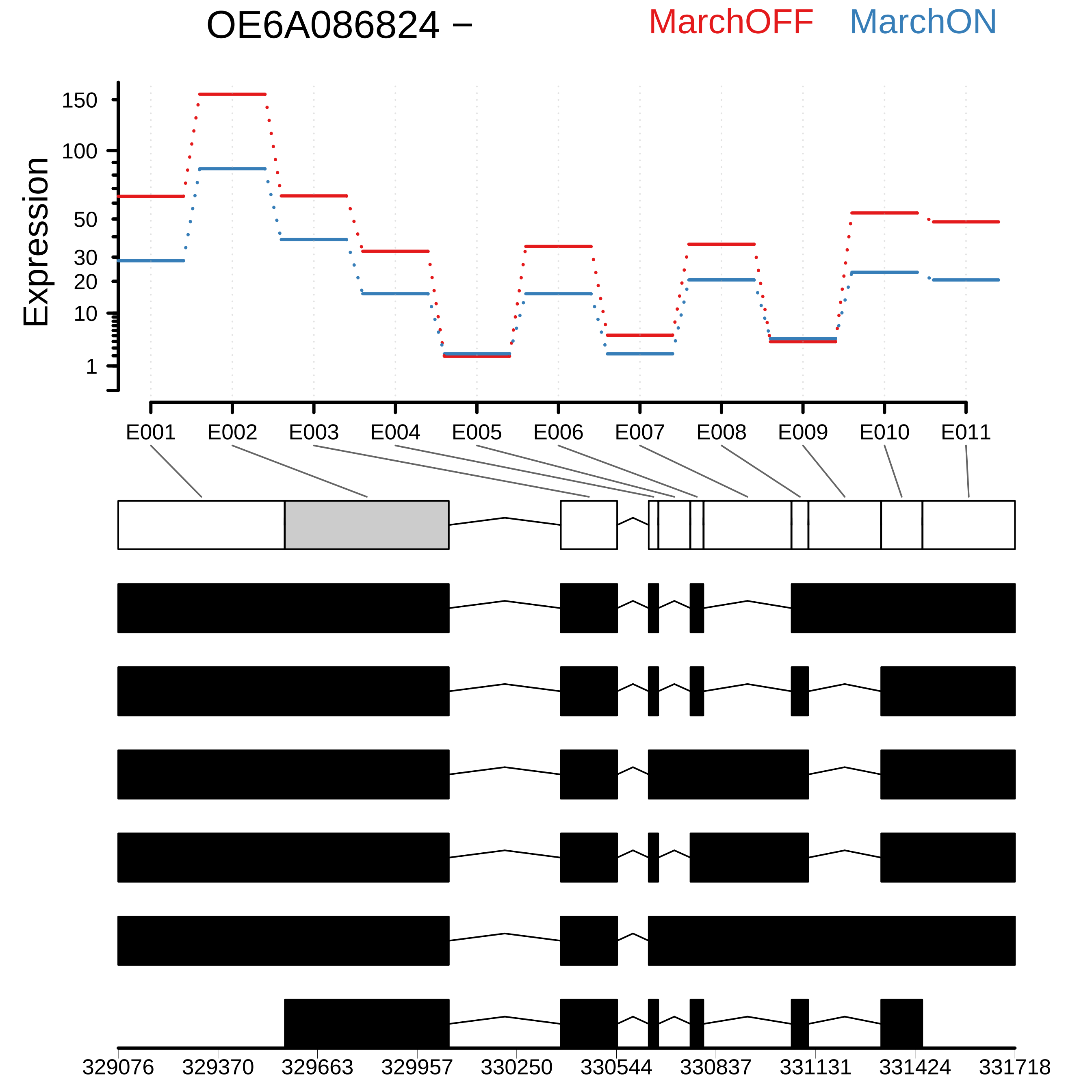
OE6A086824 –
MarchOFF
MarchON
Expression
150
100
50
30
20
10
1
E001
E002
E003
E004
E005
E006
E007
E008
E009
E010
E011
329076
329370
329663
329957
330250
330544
330837
331131
331424
331718

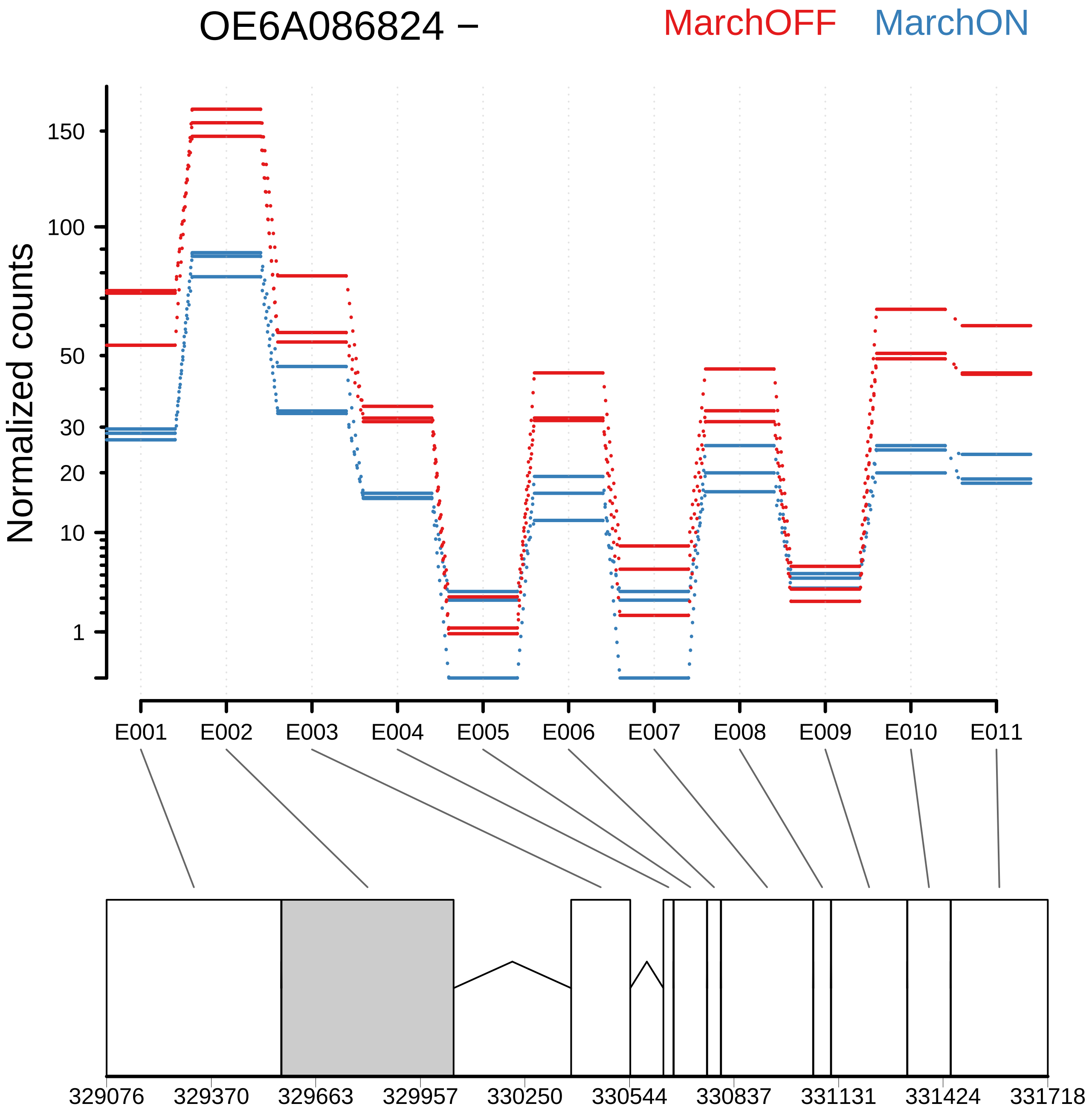

OE6A086824 −
MarchOFF
MarchON
Normalized counts
150
100
50
30
20
10
1
E001
E002
E003
E004
E005
E006
E007
E008
E009
E010
E011
329076
329370
329663
329957
330250
330544
330837
331131
331424
331718

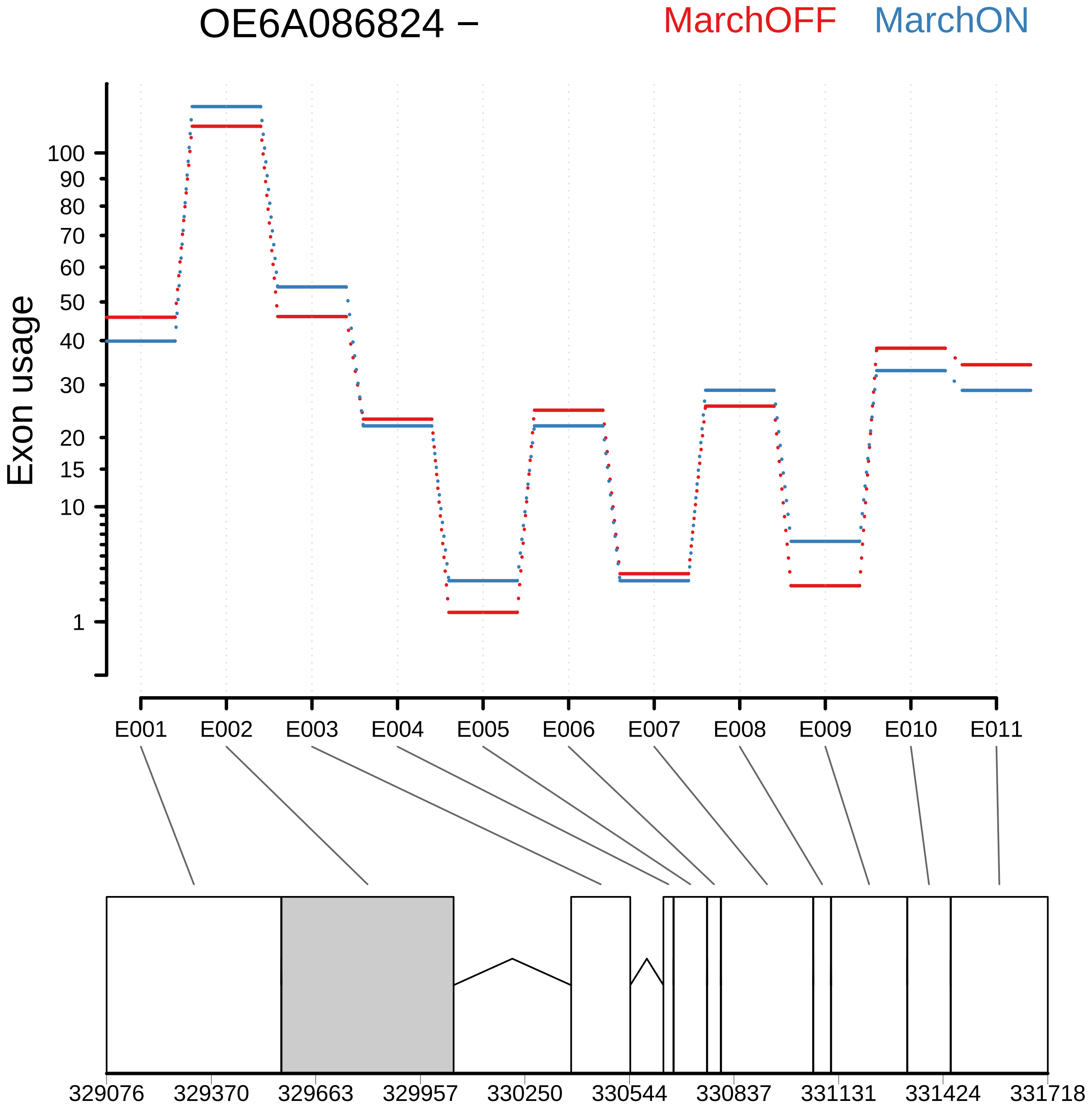

OE6A086824 −
MarchOFF
MarchON
Exon usage
100
90
80
70
60
50
40
30
20
15
10
1
E001
E002
E003
E004
E005
E006
E007
E008
E009
E010
E011
329076
329370
329663
329957
330250
330544
330837
331131
331424
331718

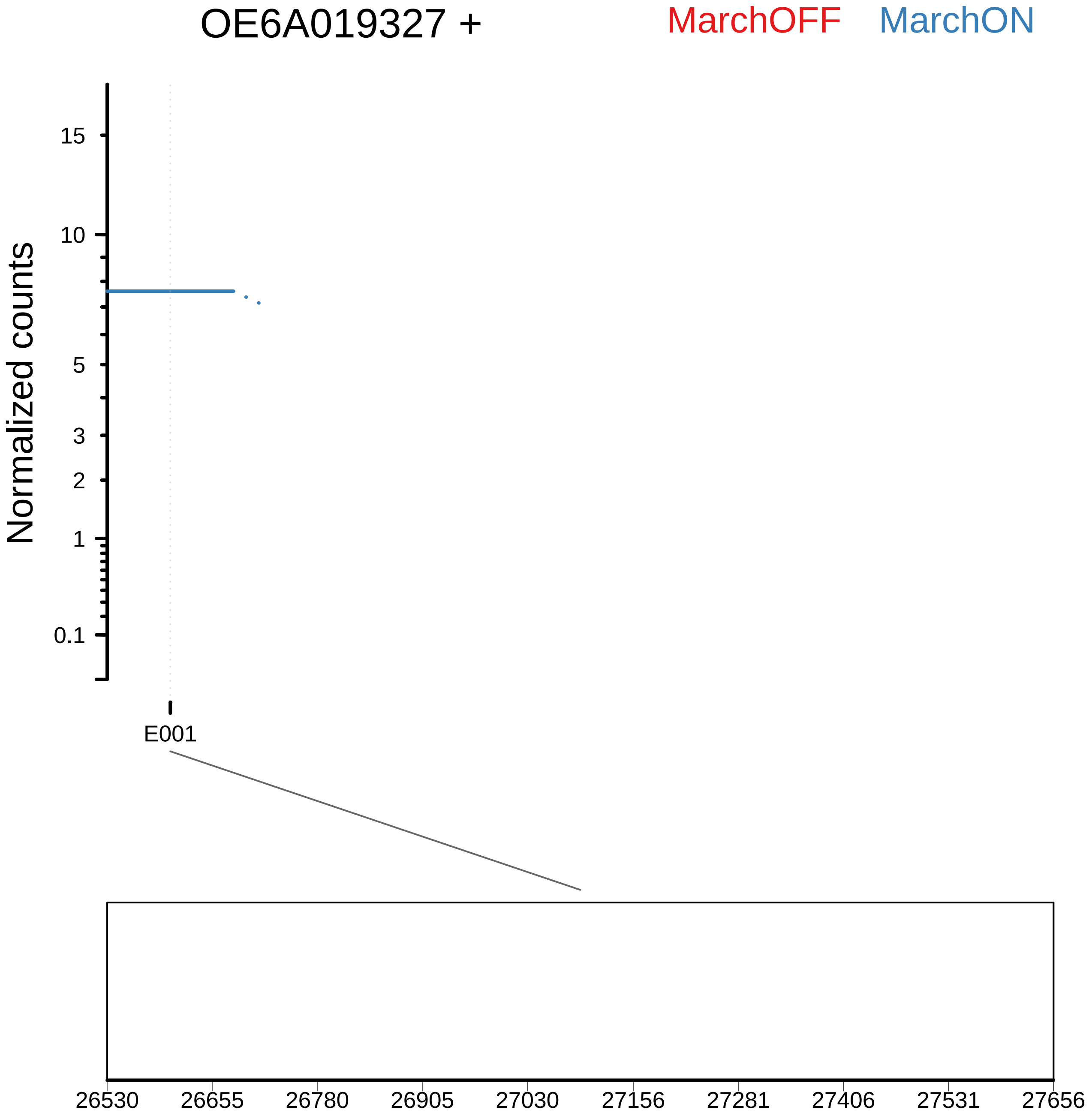
OE6A019327 +
MarchOFF
MarchON
Normalized counts
15
10
5
3
2
1
0.1
E001
26530
26655
26780
26905
27030
27156
27281
27406
27531
27656

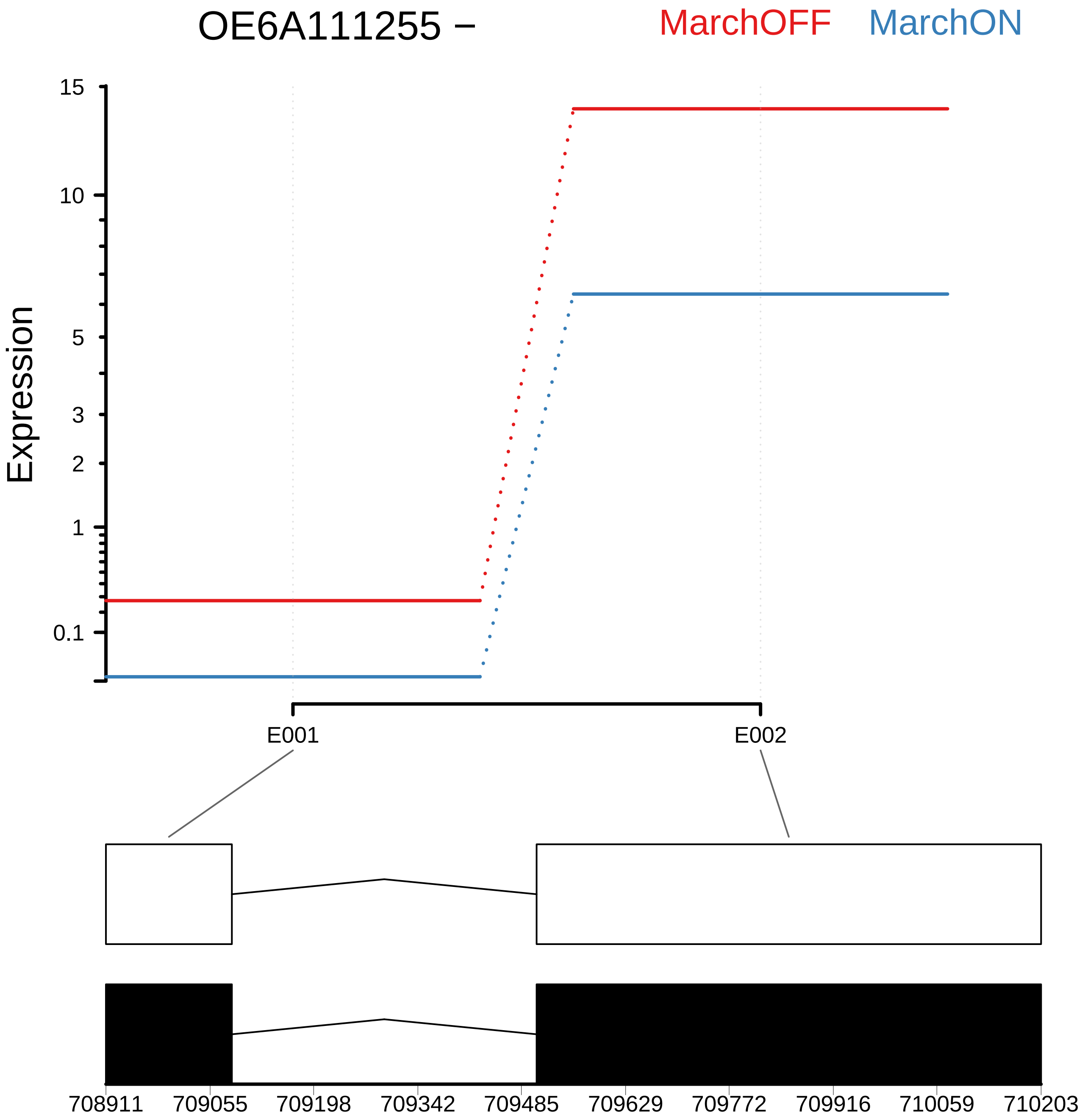

OE6A111255 −
MarchOFF
MarchON
Expression
15
10
5
3
2
1
0.1
E001
E002
708911
709055
709198
709342
709485
709629
709772
709916
710059
710203

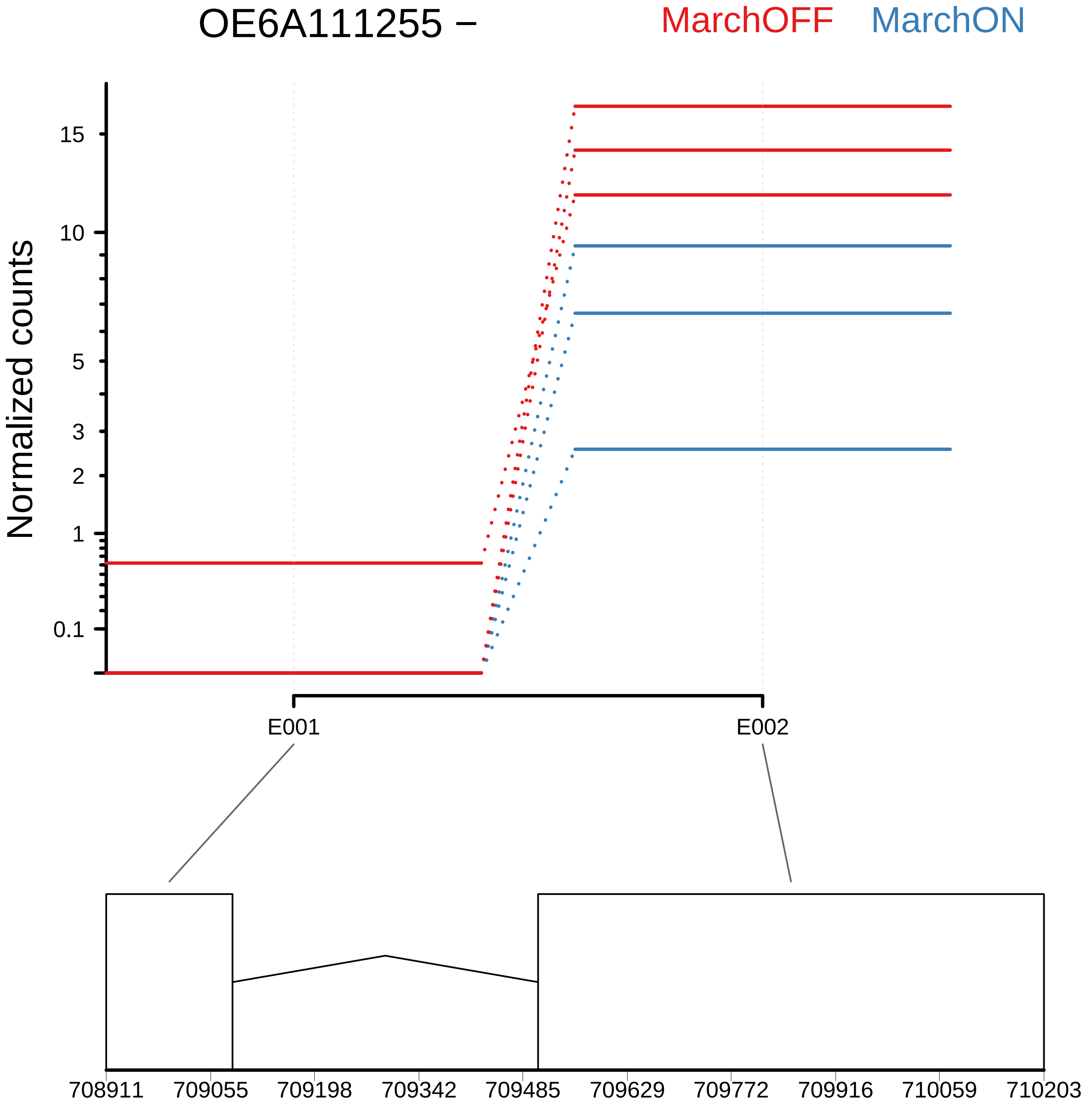

OE6A111255 −
MarchOFF
MarchON
Normalized counts
15
10
5
3
2
1
0.1
E001
E002
708911
709055
709198
709342
709485
709629
709772
709916
710059
710203

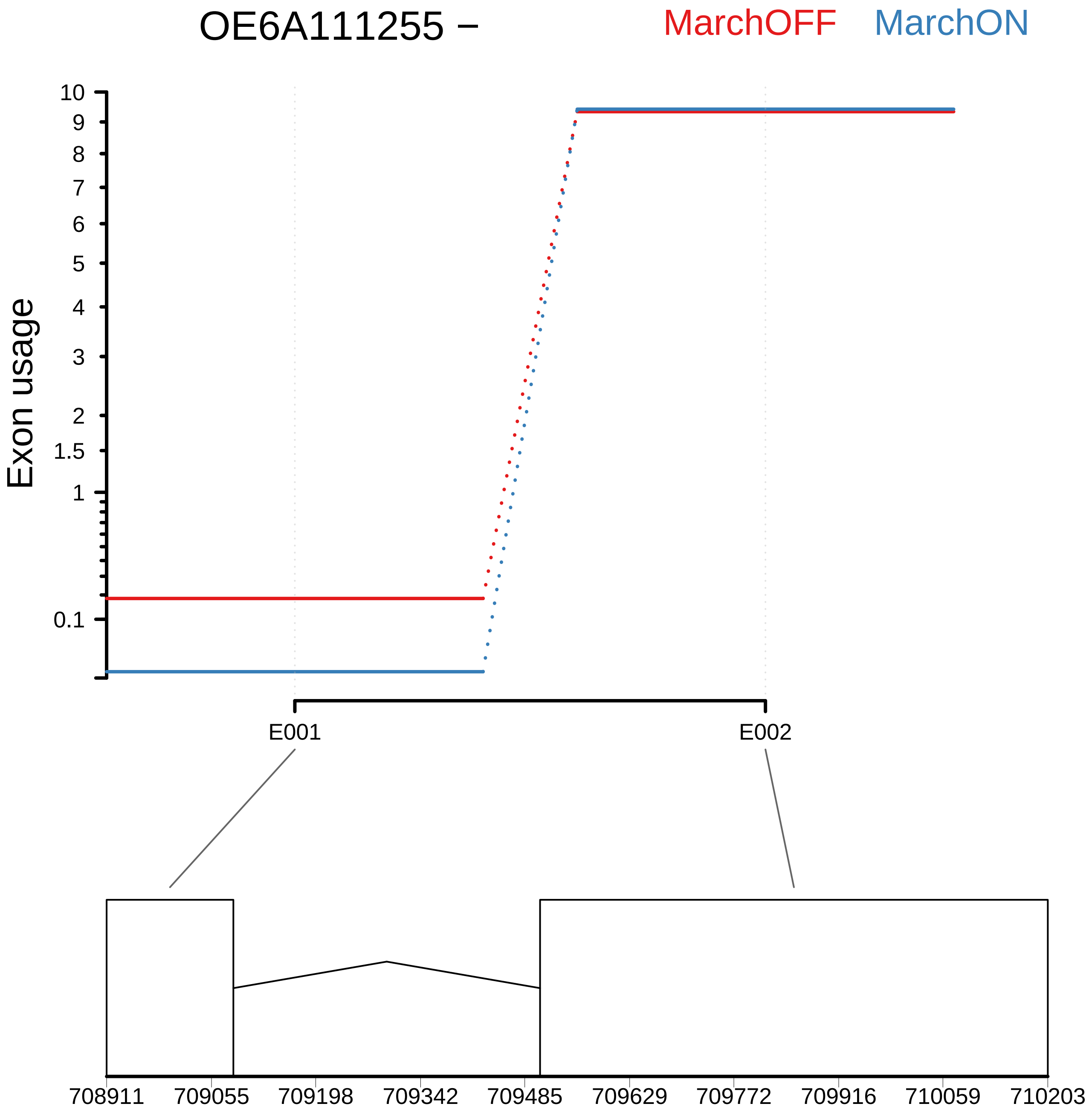
OE6A111255 −
MarchOFF
MarchON
Exon usage
10
9
8
7
6
5
4
3
2
1.5
1
0.1
E001
E002
708911
709055
709198
709342
709485
709629
709772
709916
710059
710203

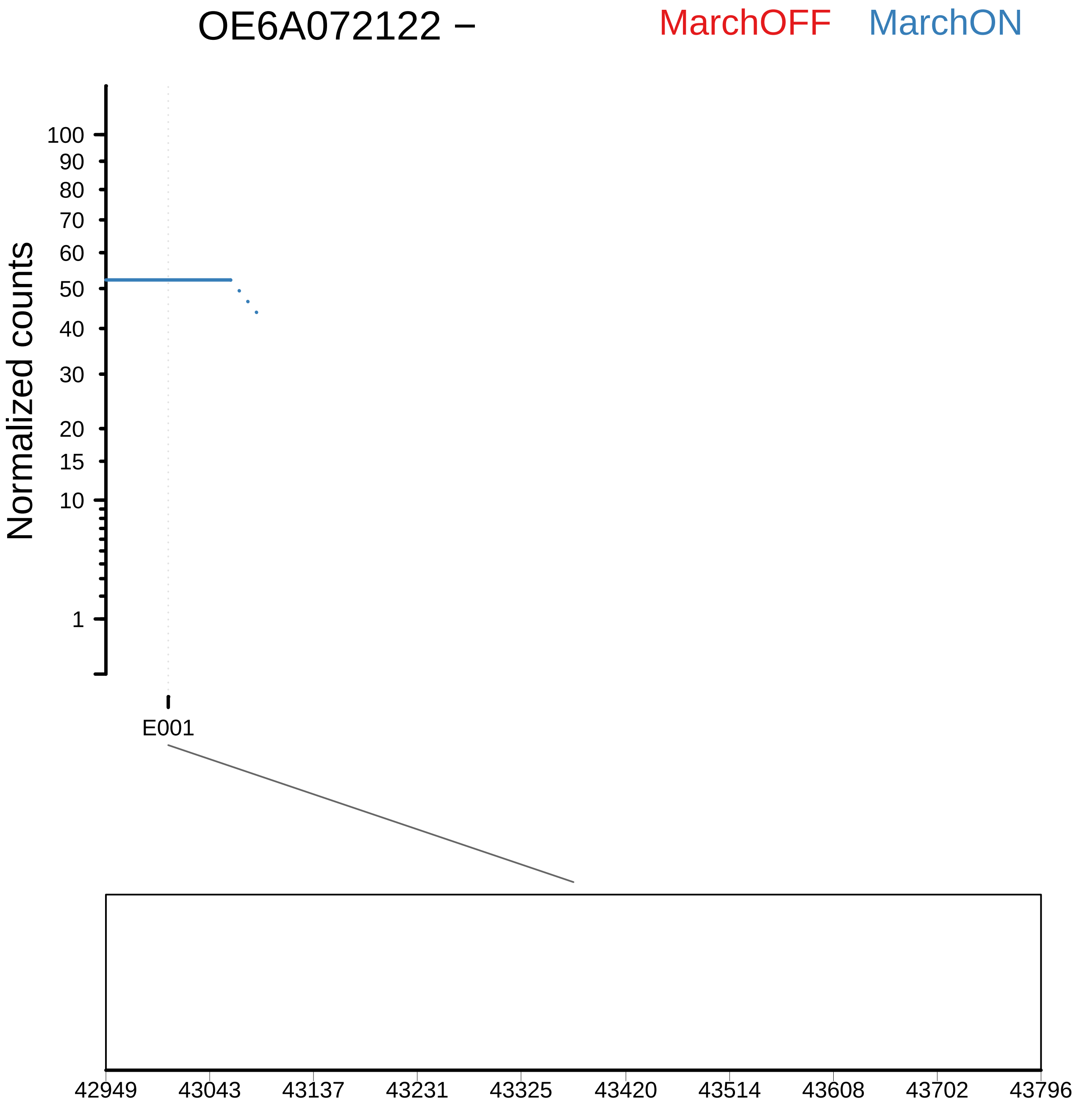

OE6A072122 −
MarchOFF
MarchON
Normalized counts
100
90
80
70
60
50
40
30
20
15
10
1
E001
42949
43043
43137
43231
43325
43420
43514
43608
43702
43796

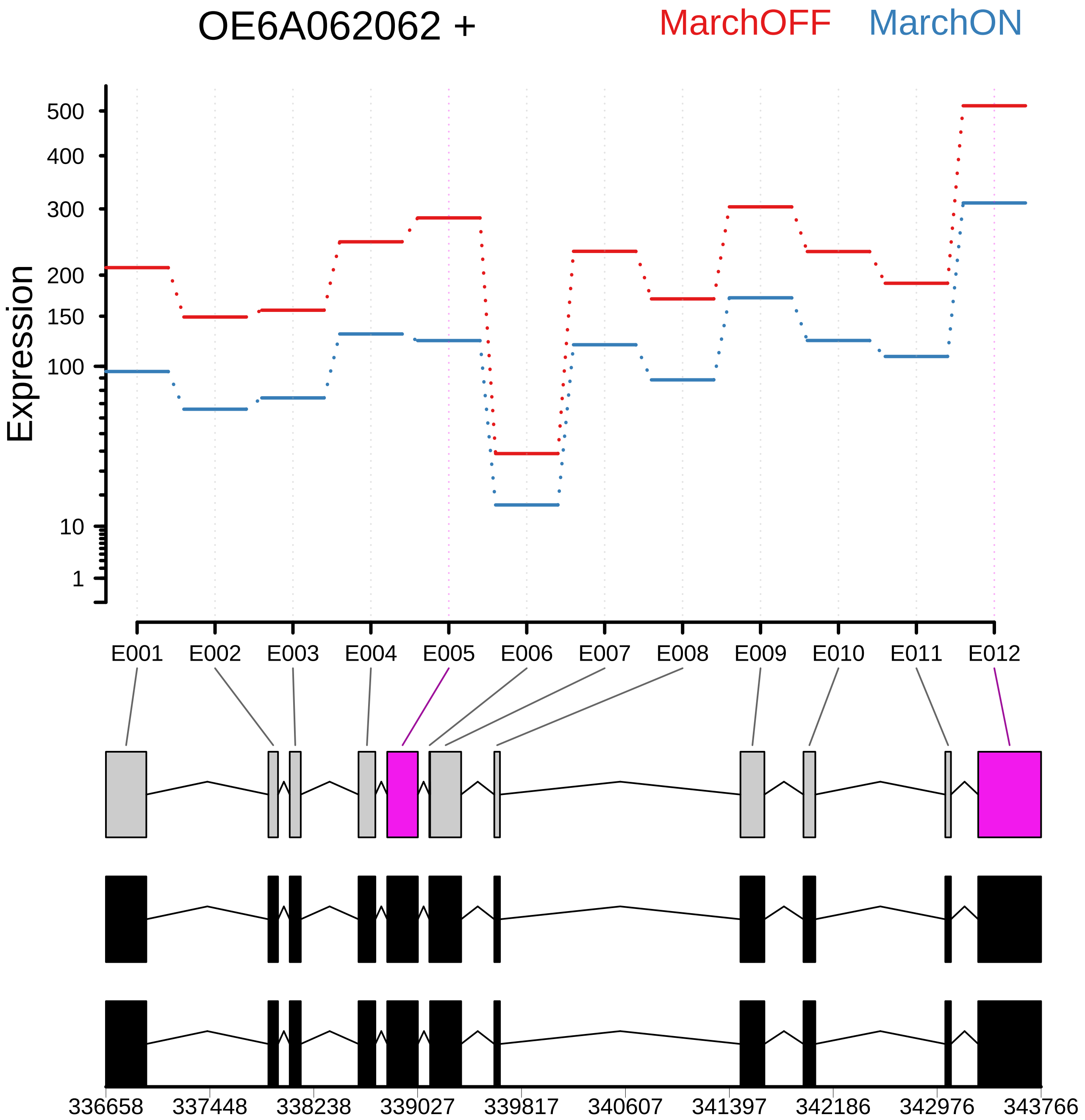

OE6A062062 +
MarchOFF
MarchON
Expression
500
400
300
200
150
100
10
1
E001
E002
E003
E004
E005
E006
E007
E008
E009
E010
E011
E012
336658
337448
338238
339027
339817
340607
341397
342186
342976
343766

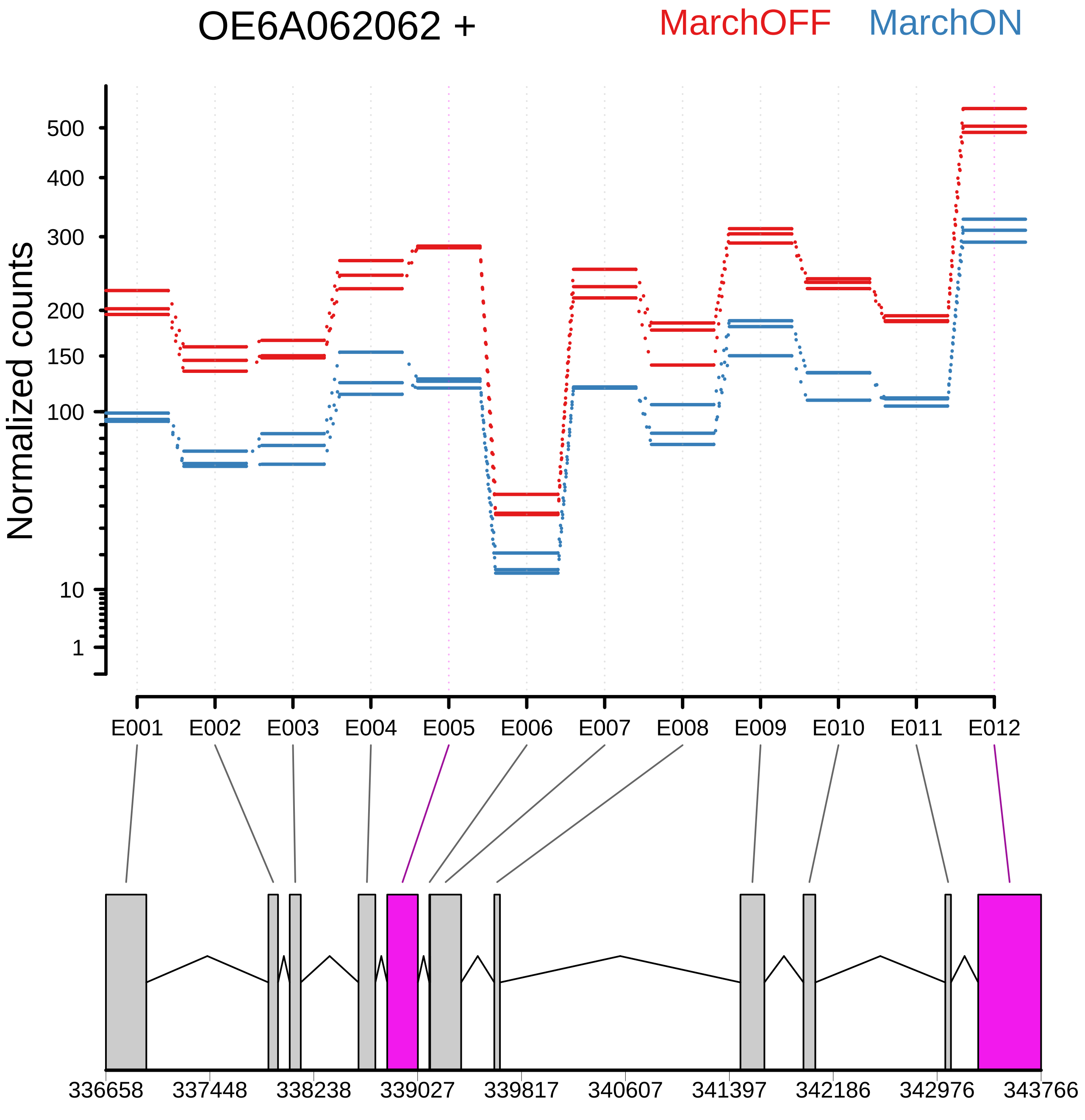
OE6A062062 +
MarchOFF
MarchON
Normalized counts
500
400
300
200
150
100
10
1
E001
E002
E003
E004
E005
E006
E007
E008
E009
E010
E011
E012
336658
337448
338238
339027
339817
340607
341397
342186
342976
343766

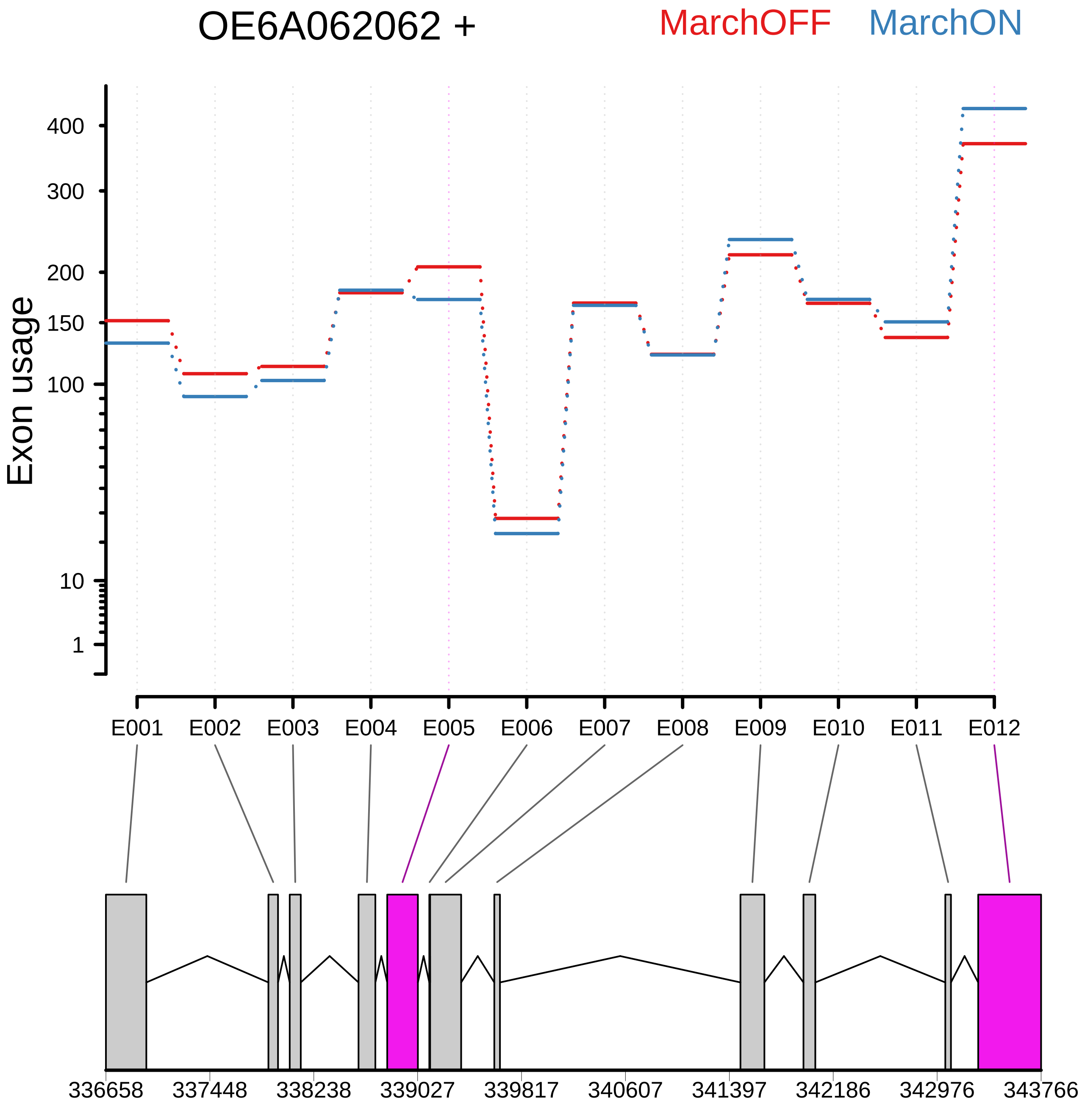
OE6A062062 +
MarchOFF
MarchON
Exon usage
400
300
200
150
100
10
1
E001
E002
E003
E004
E005
E006
E007
E008
E009
E010
E011
E012
336658
337448
338238
339027
339817
340607
341397
342186
342976
343766

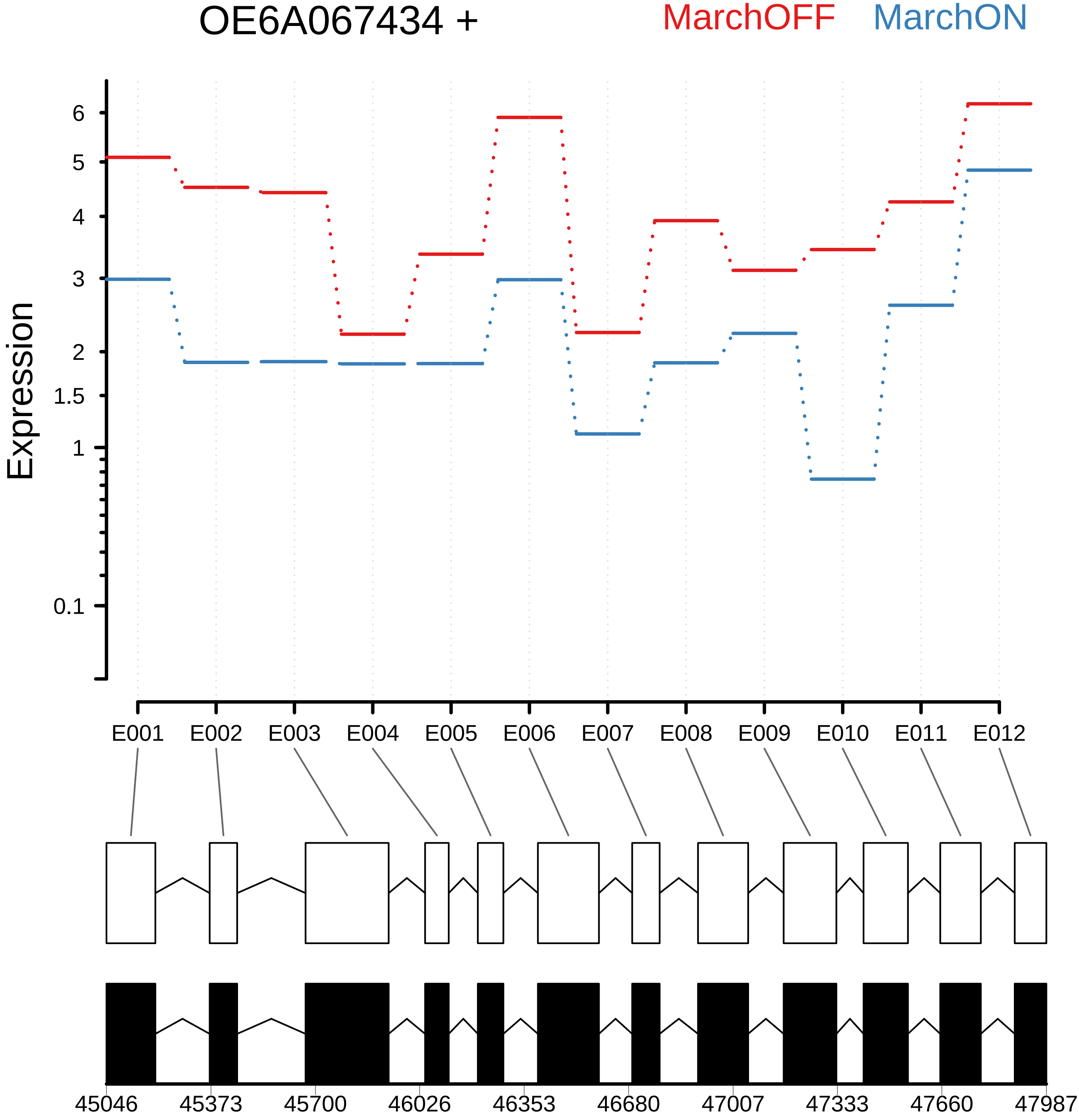

OE6A067434 +
MarchOFF
MarchON
Expression
6
5
4
3
2
1.5
1
0.1
E001
E002
E003
E004
E005
E006
E007
E008
E009
E010
E011
E012
45046
45373
45700
46026
46353
46680
47007
47333
47660
47987

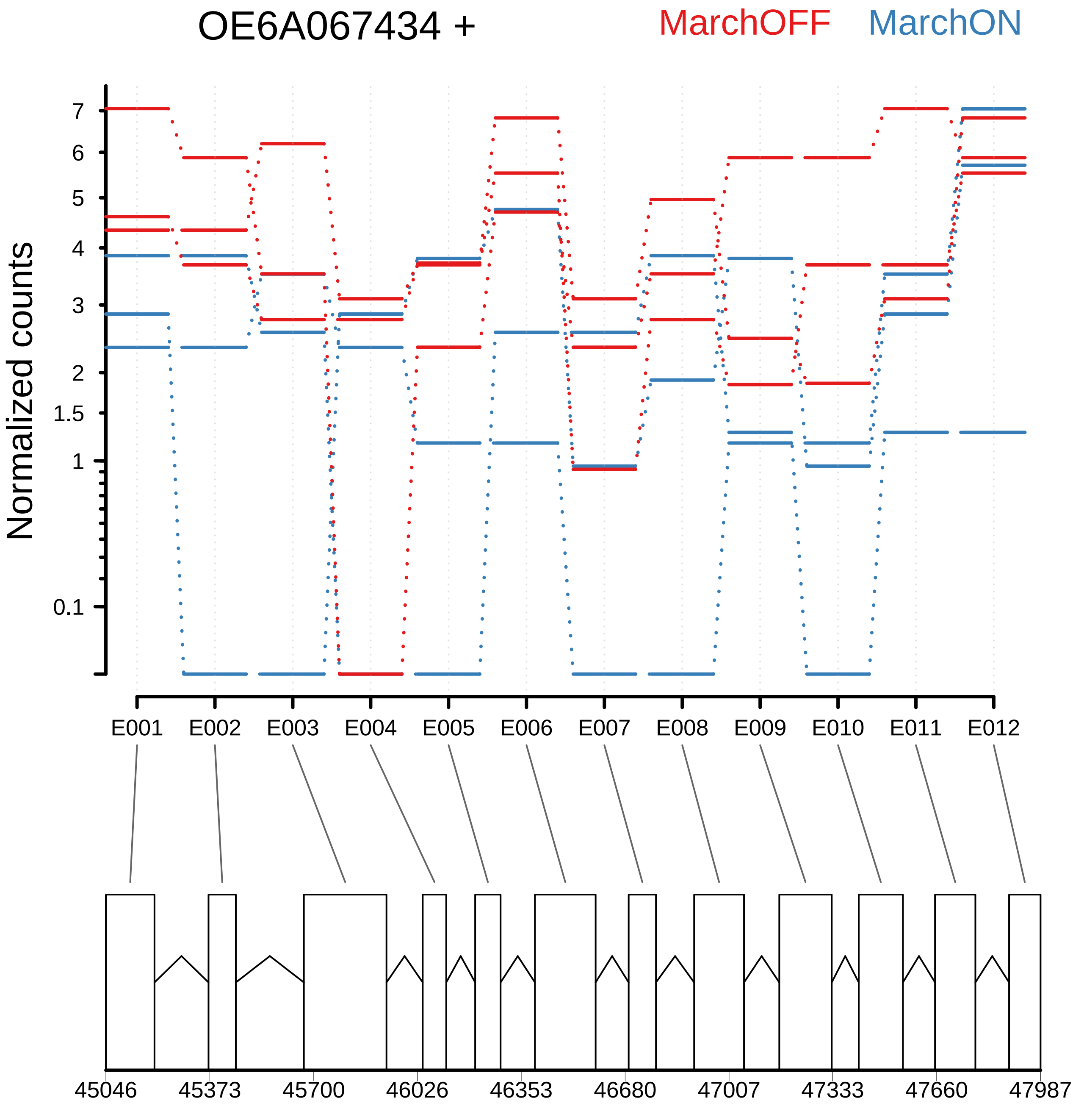
OE6A067434 +
MarchOFF
MarchON
Normalized counts
7
6
5
4
3
2
1.5
1
0.1
E001
E002
E003
E004
E005
E006
E007
E008
E009
E010
E011
E012
45046
45373
45700
46026
46353
46680
47007
47333
47660
47987

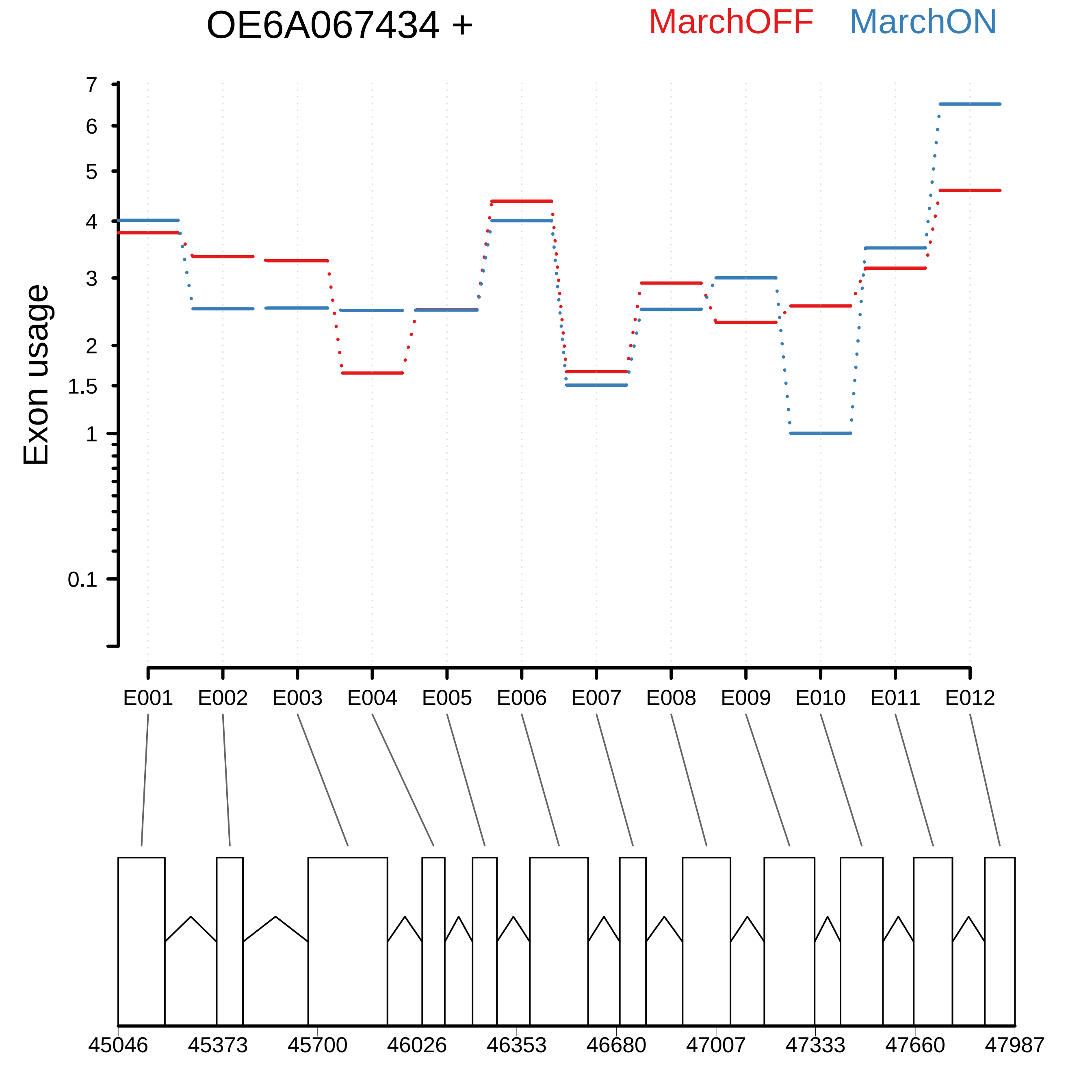

OE6A067434 +
MarchOFF
MarchON
Exon usage
7
6
5
4
3
2
1.5
1
0.1
E001
E002
E003
E004
E005
E006
E007
E008
E009
E010
E011
E012
45046
45373
45700
46026
46353
46680
47007
47333
47660
47987

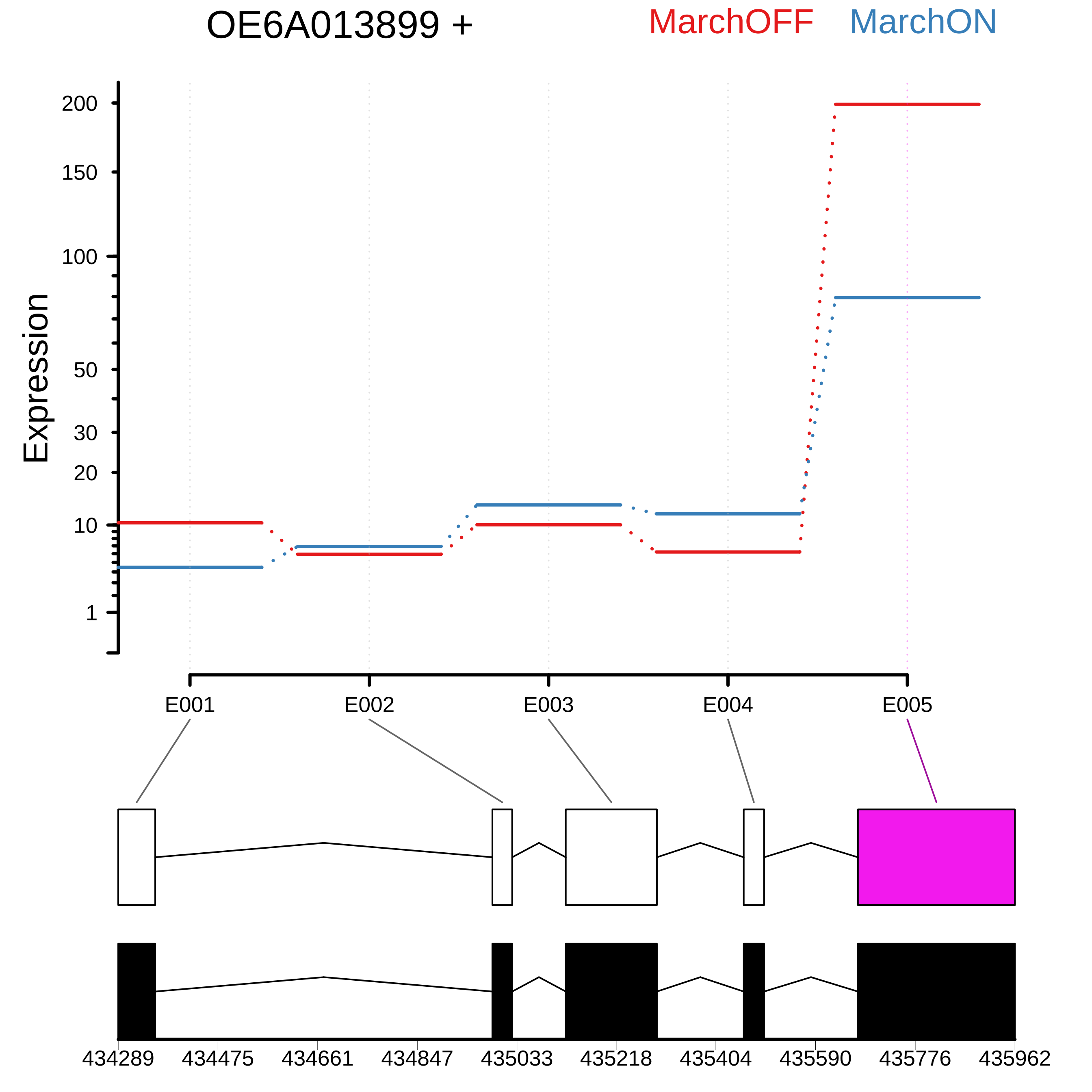

OE6A013899 +
MarchOFF
MarchON
Expression
200
150
100
50
30
20
10
1
E001
E002
E003
E004
E005
434289
434475
434661
434847
435033
435218
435404
435590
435776
435962

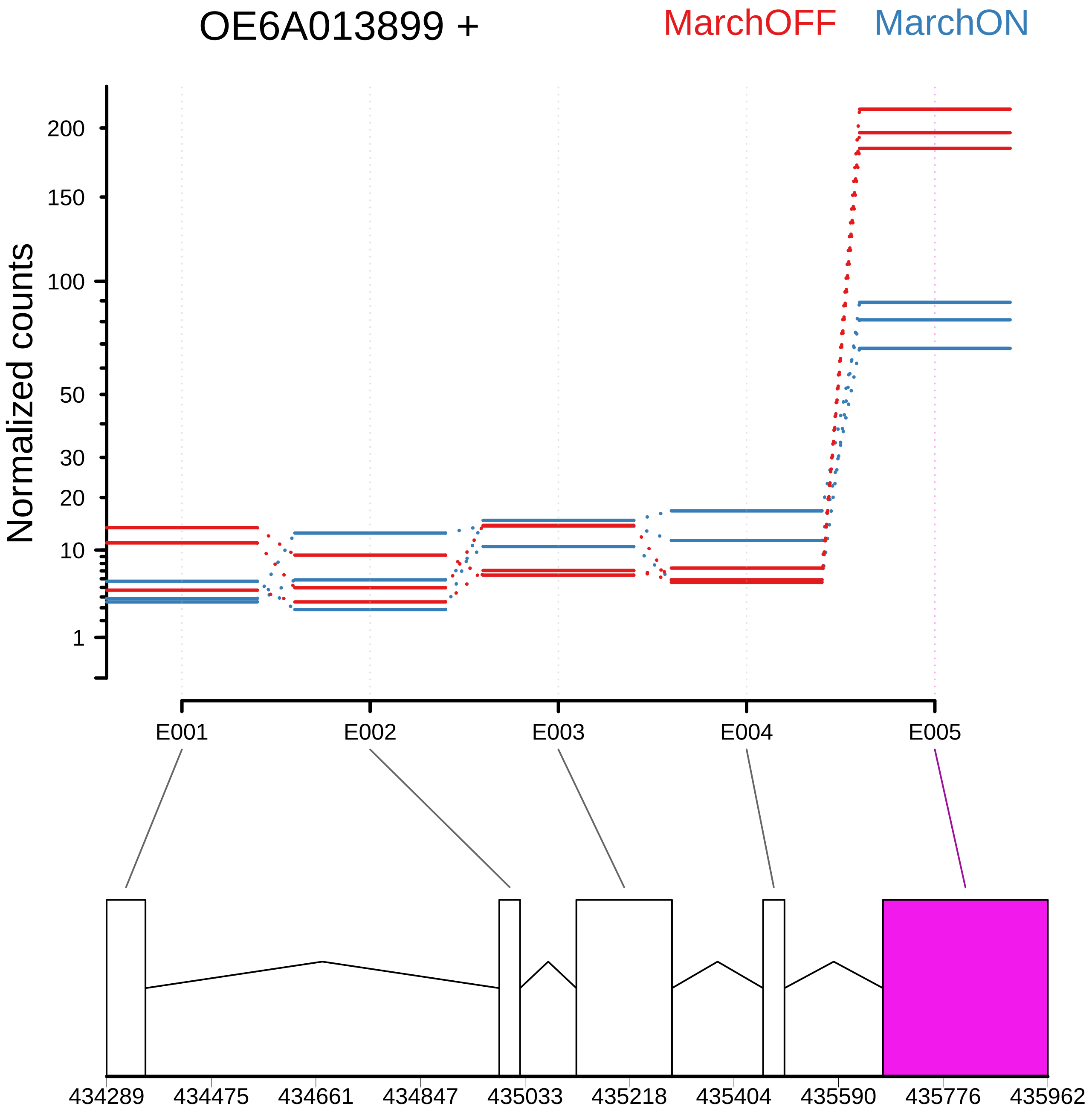

OE6A013899 +
MarchOFF
MarchON
Normalized counts
200
150
100
50
30
20
10
1
E001
E002
E003
E004
E005
434289
434475
434661
434847
435033
435218
435404
435590
435776
435962

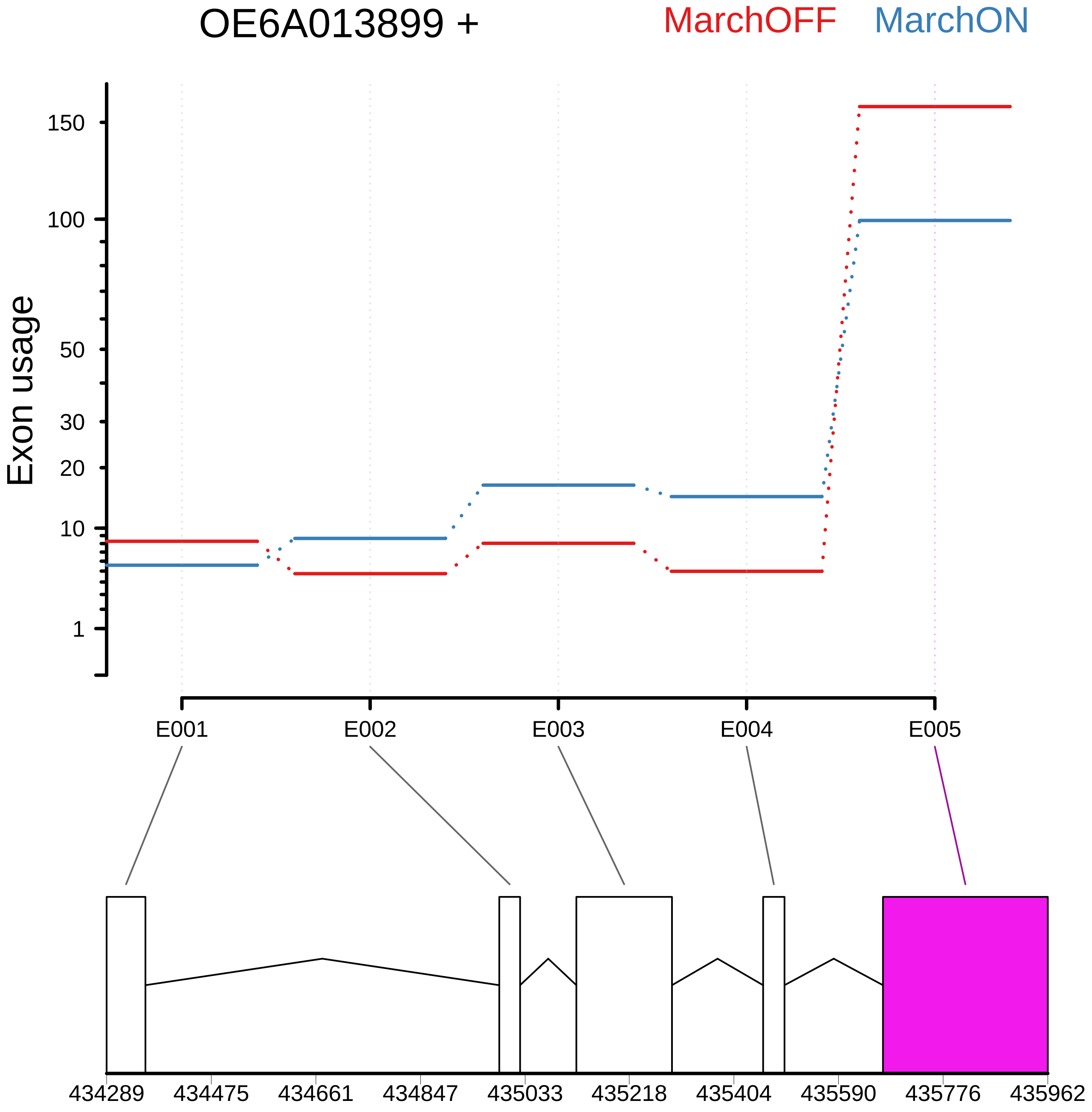
OE6A013899 +
MarchOFF
MarchON
Exon usage
150
100
50
30
20
10
1
E001
E002
E003
E004
E005
434289
434475
434661
434847
435033
435218
435404
435590
435776
435962

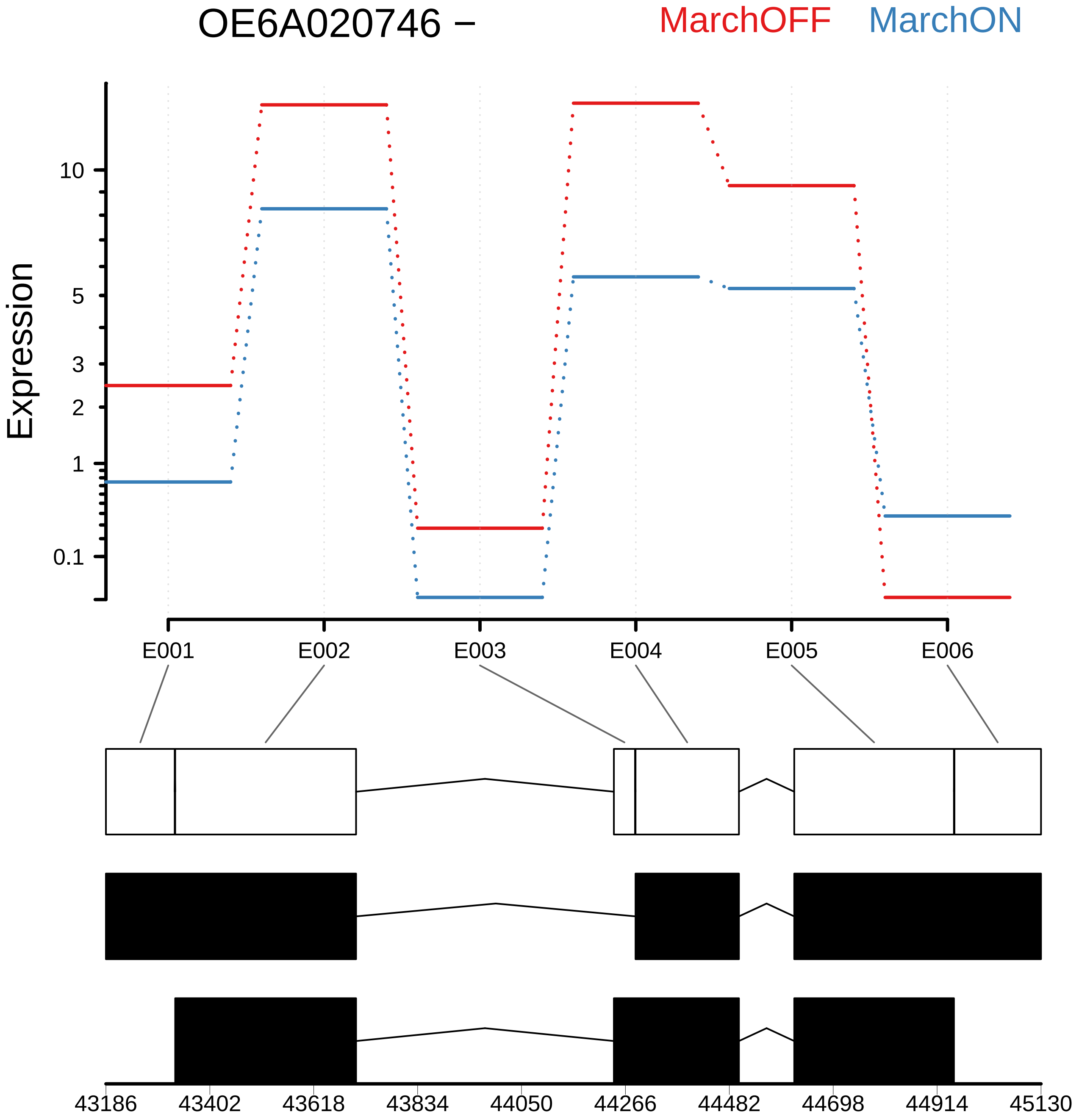

OE6A020746 −
MarchOFF
MarchON
Expression
10
5
3
2
1
0.1
E001
E002
E003
E004
E005
E006
43186
43402
43618
43834
44050
44266
44482
44698
44914
45130

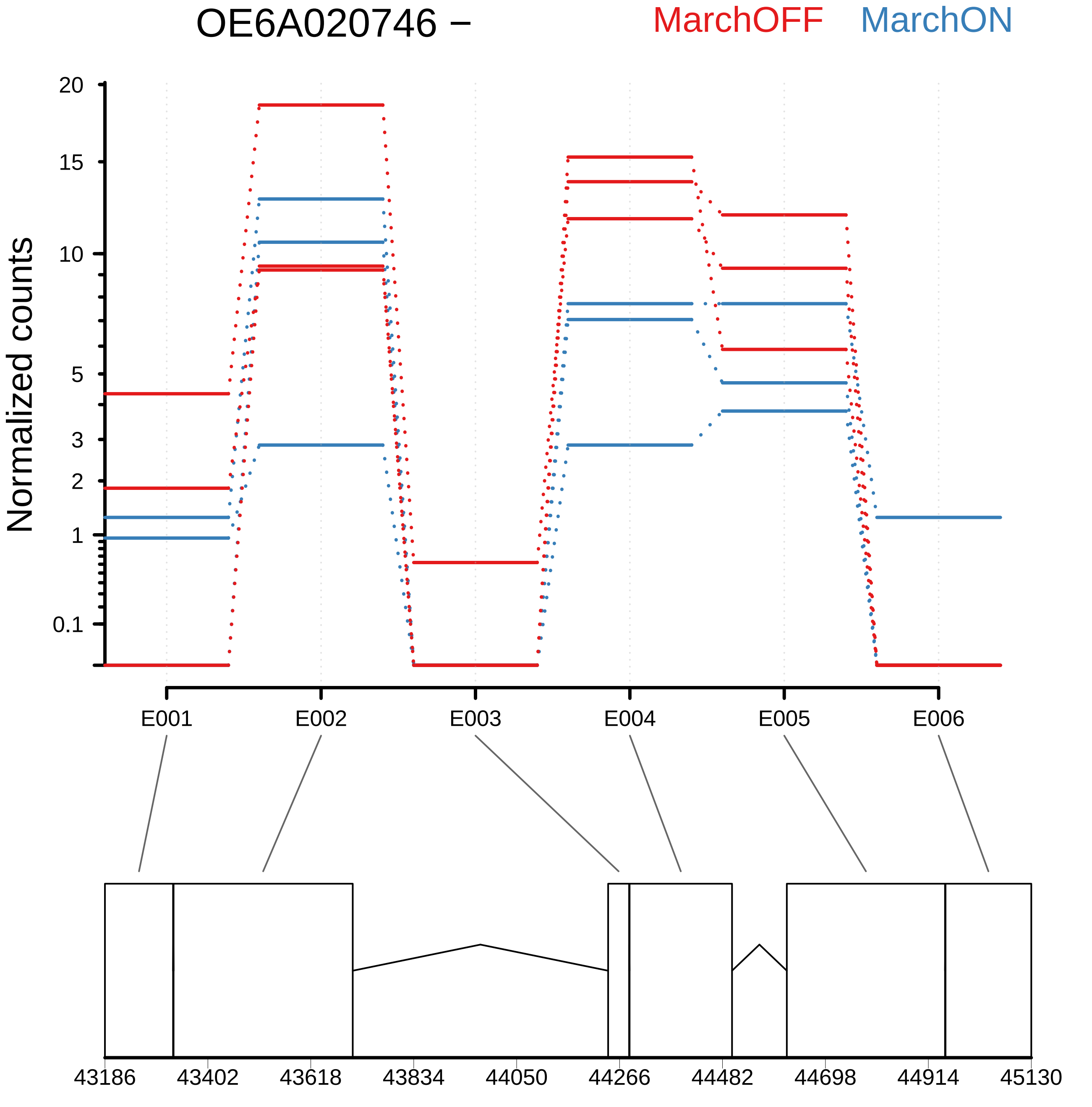

OE6A020746 −
MarchOFF
MarchON
Normalized counts
20
15
10
5
3
2
1
0.1
E001
E002
E003
E004
E005
E006
43186
43402
43618
43834
44050
44266
44482
44698
44914
45130

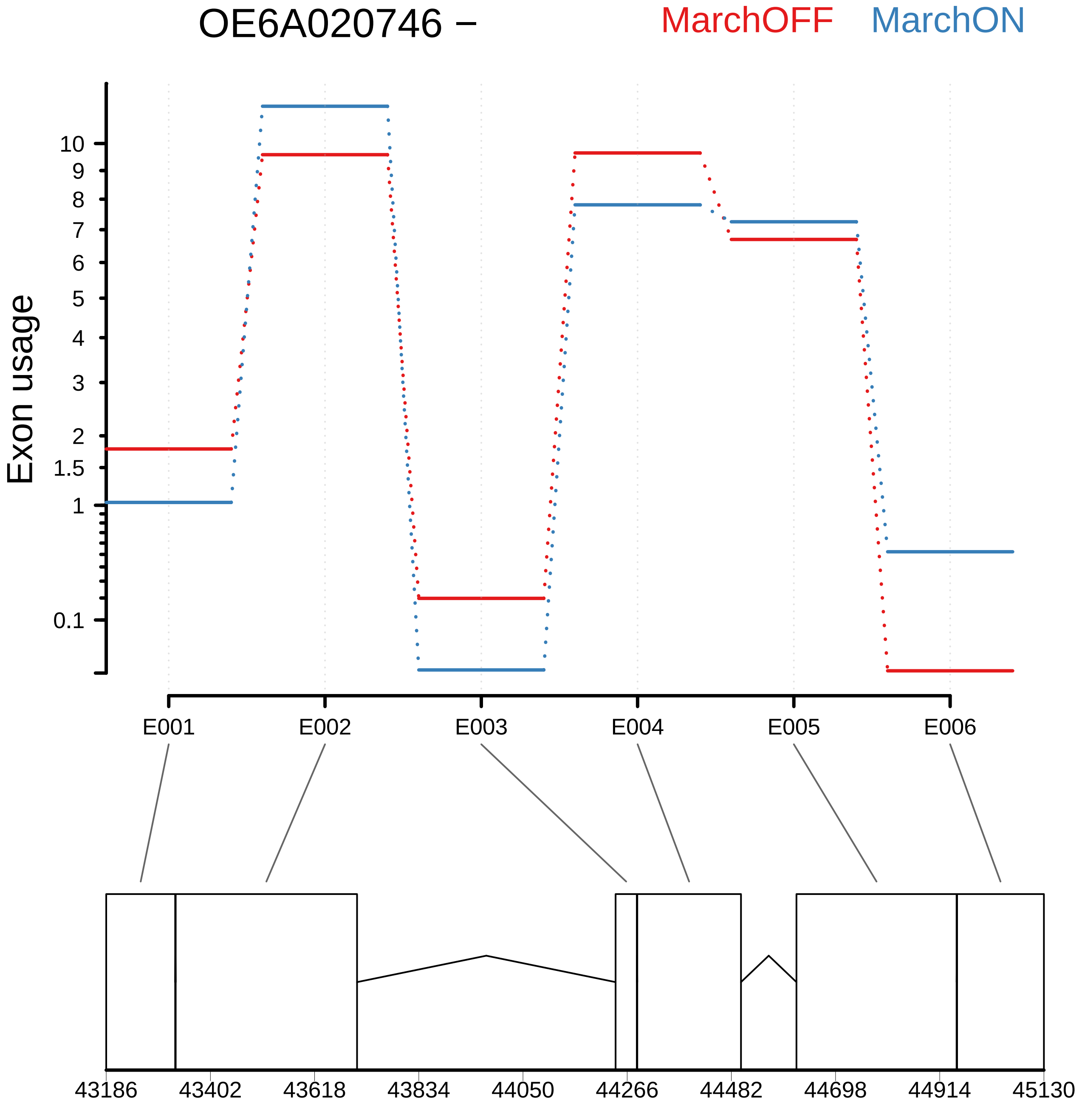

OE6A020746 −
MarchOFF
MarchON
Exon usage
10
9
8
7
6
5
4
3
2
1.5
1
0.1
E001
E002
E003
E004
E005
E006
43186
43402
43618
43834
44050
44266
44482
44698
44914
45130

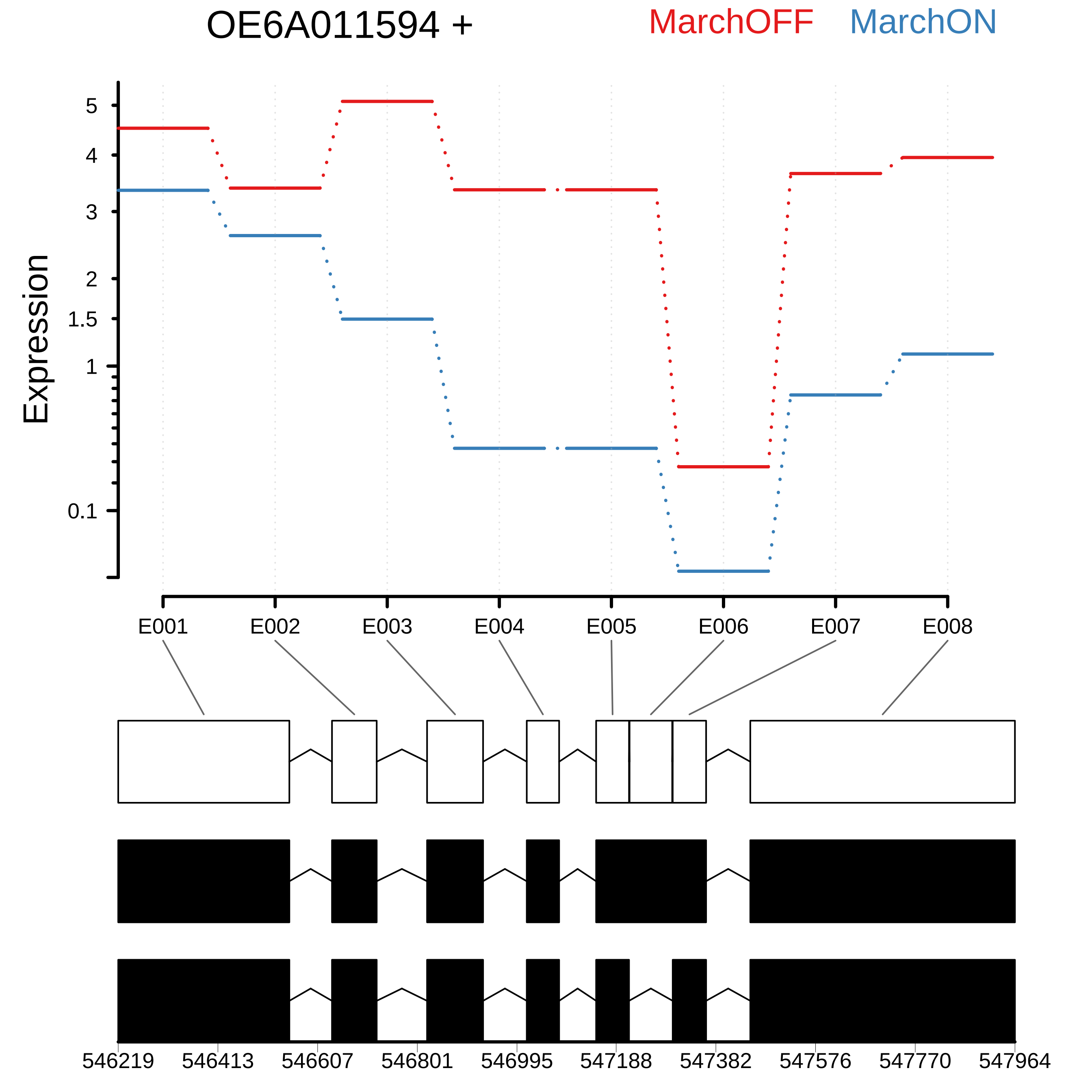

OE6A011594 +
MarchOFF
MarchON
Expression
5
4
3
2
1.5
1
0.1
E001
E002
E003
E004
E005
E006
E007
E008
546219
546413
546607
546801
546995
547188
547382
547576
547770
547964

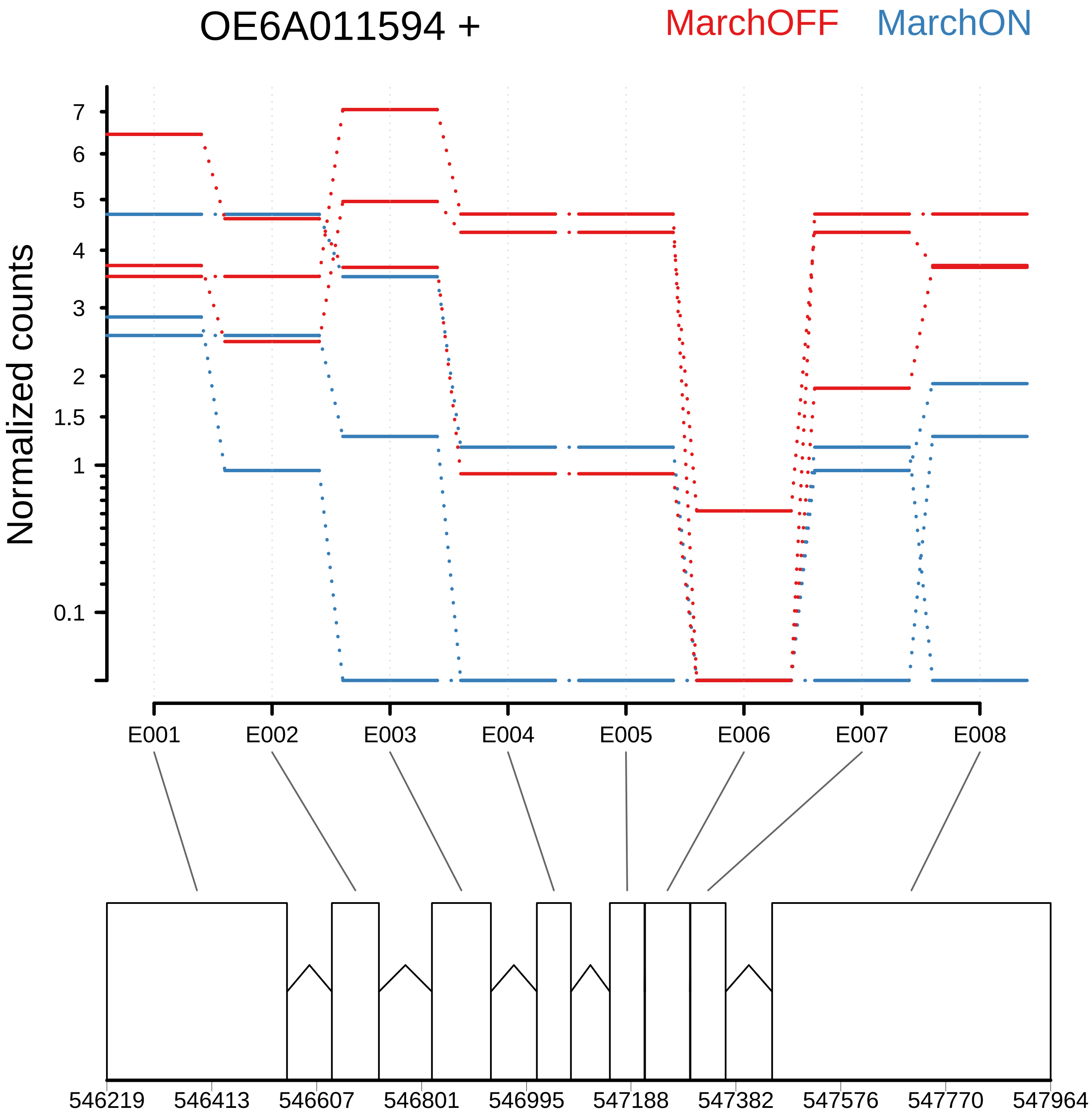

OE6A011594 +
MarchOFF
MarchON
Normalized counts
7
6
5
4
3
2
1.5
1
0.1
E001
E002
E003
E004
E005
E006
E007
E008
546219
546413
546607
546801
546995
547188
547382
547576
547770
547964

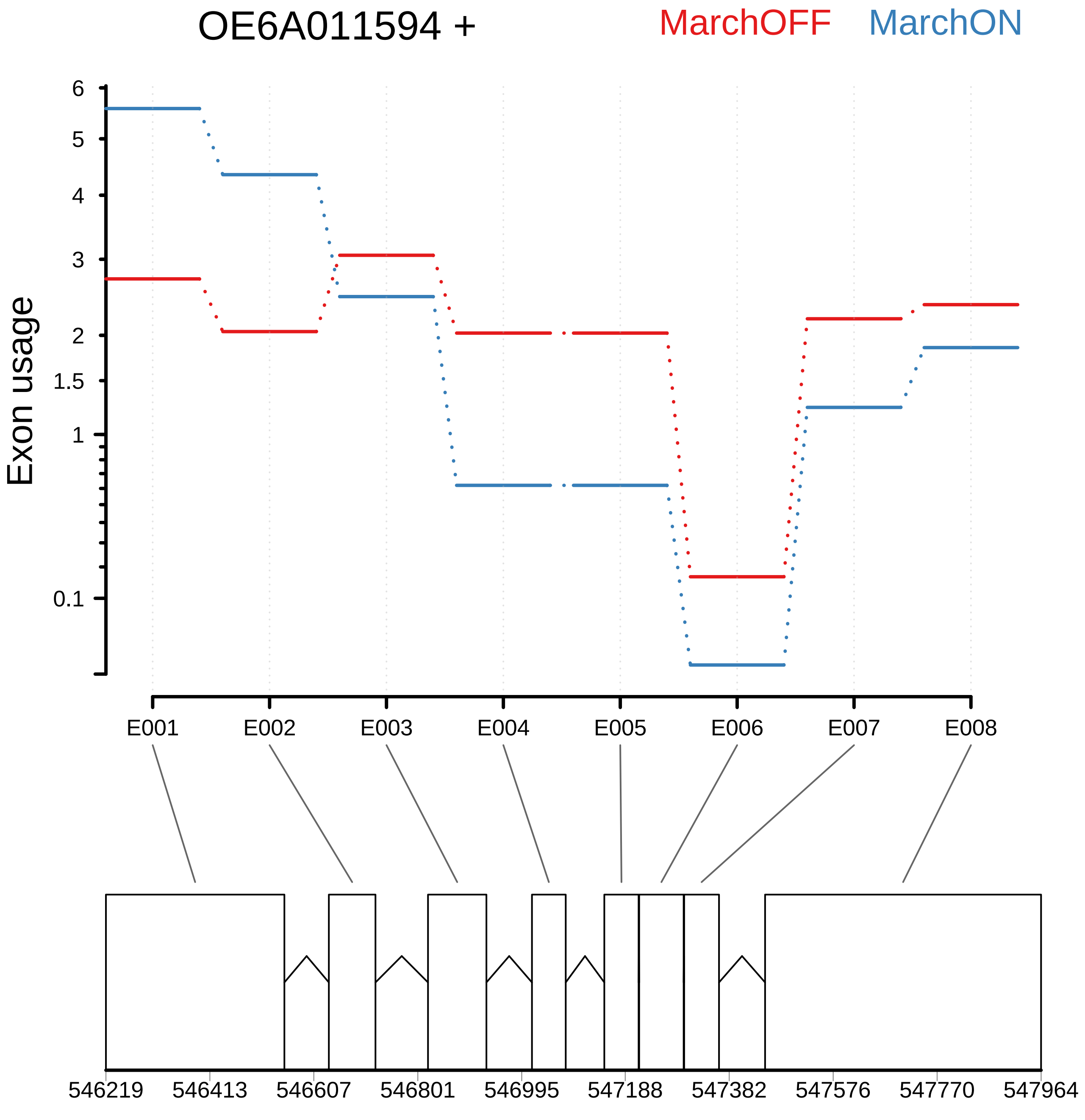

OE6A011594 +
MarchOFF
MarchON
Exon usage
6
5
4
3
2
1.5
1
0.1
E001
E002
E003
E004
E005
E006
E007
E008
546219
546413
546607
546801
546995
547188
547382
547576
547770
547964

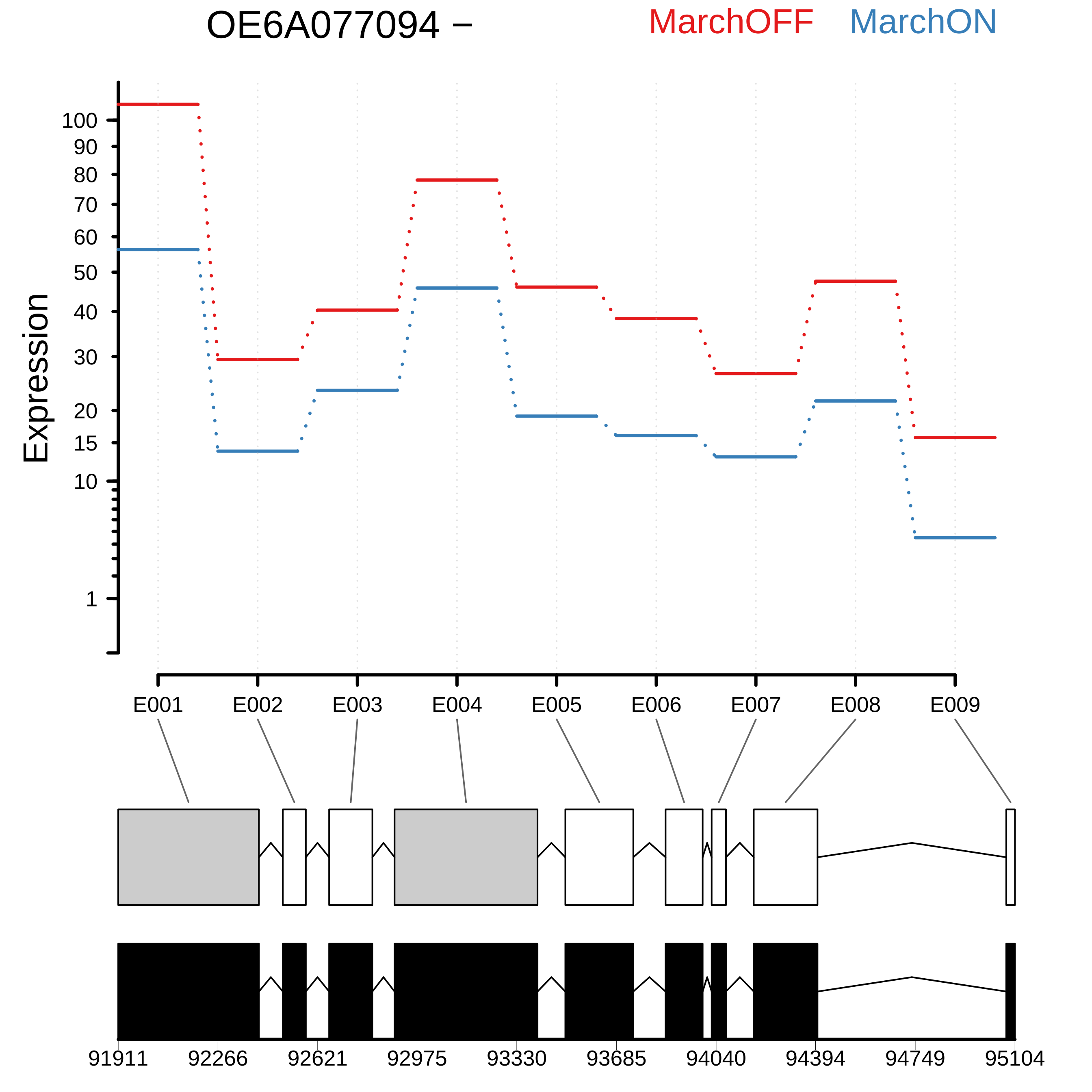
OE6A077094 −
MarchOFF
MarchON
Expression
100
90
80
70
60
50
40
30
20
15
10
1
E001
E002
E003
E004
E005
E006
E007
E008
E009
91911
92266
92621
92975
93330
93685
94040
94394
94749
95104

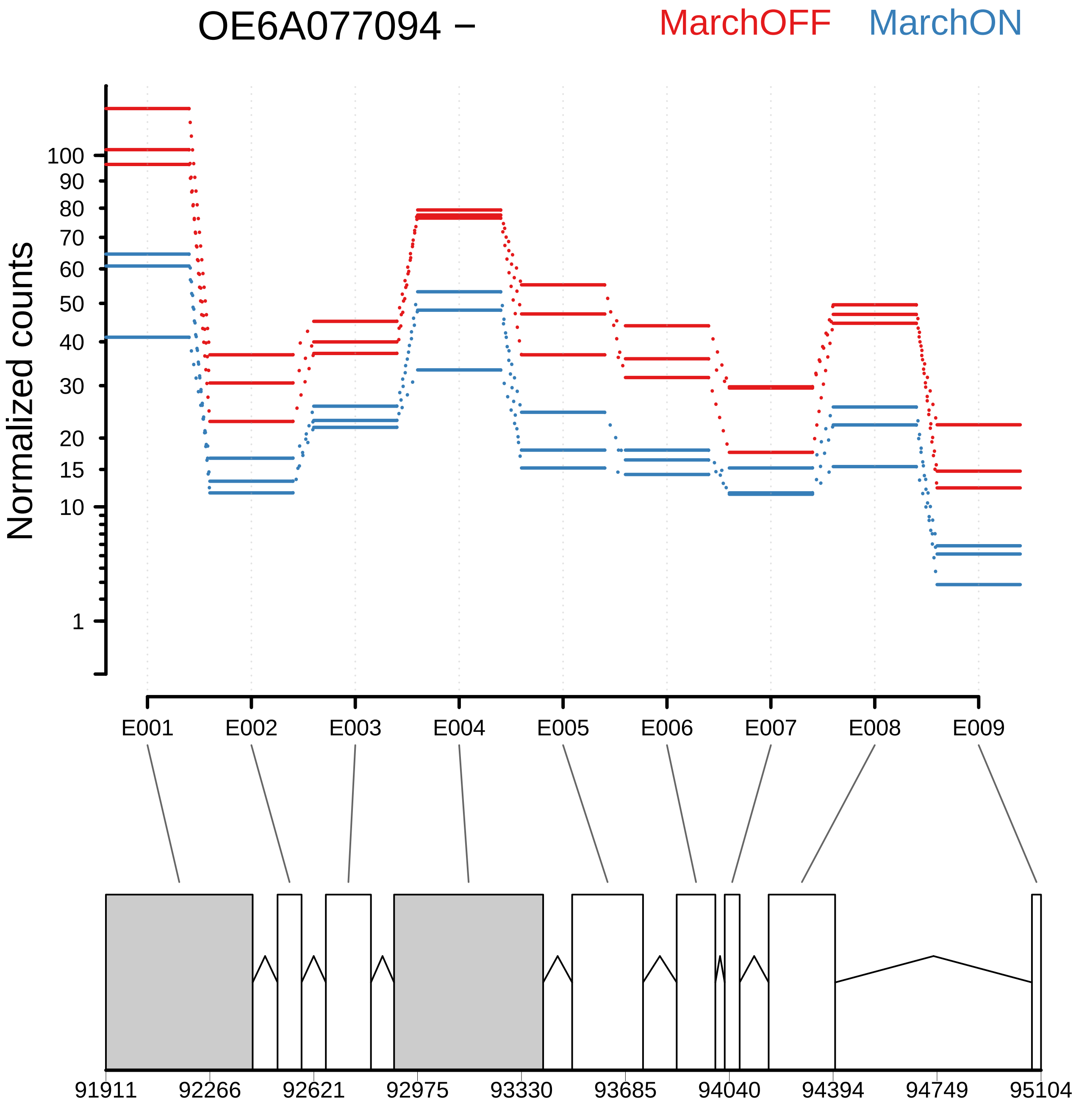

OE6A077094 −
MarchOFF
MarchON
Normalized counts
100
90
80
70
60
50
40
30
20
15
10
1
E001
E002
E003
E004
E005
E006
E007
E008
E009
91911
92266
92621
92975
93330
93685
94040
94394
94749
95104

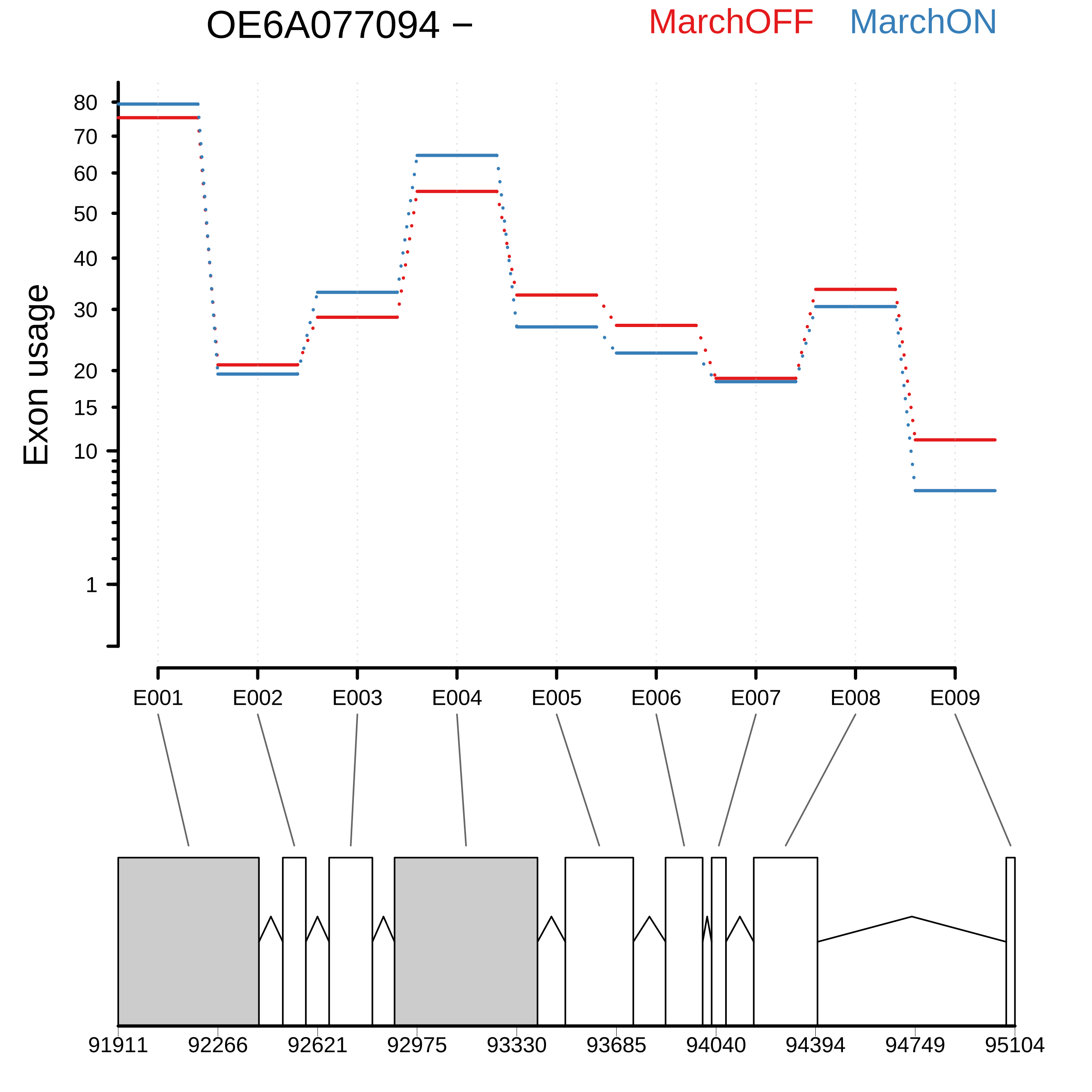
OE6A077094 −
MarchOFF
MarchON
Exon usage
80
70
60
50
40
30
20
15
10
1
E001
E002
E003
E004
E005
E006
E007
E008
E009
91911
92266
92621
92975
93330
93685
94040
94394
94749
95104

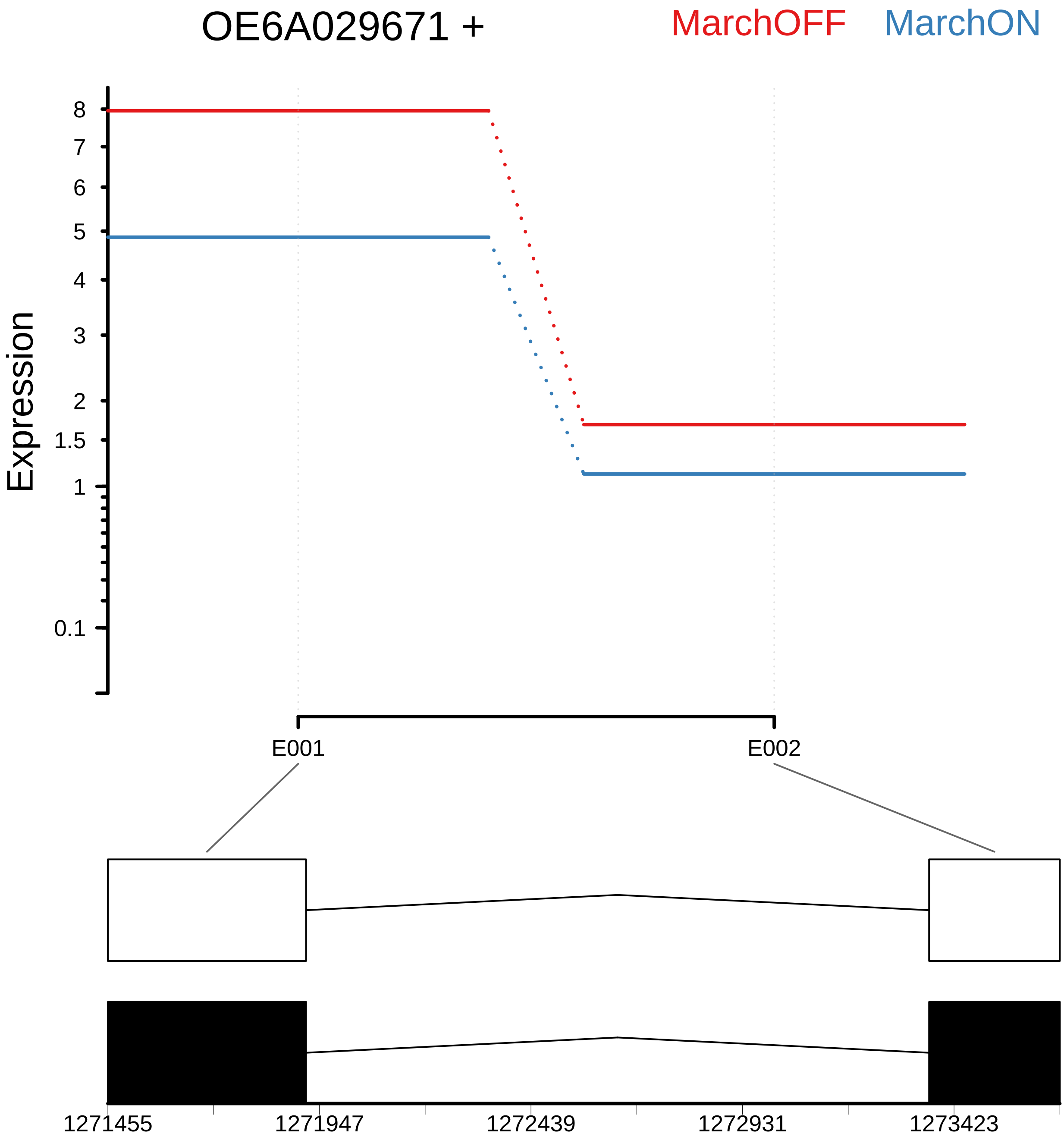

OE6A029671 +
MarchOFF
MarchON
Expression
8
7
6
5
4
3
2
1.5
1
0.1
E001
E002
1271455
1271947
1272439
1272931
1273423

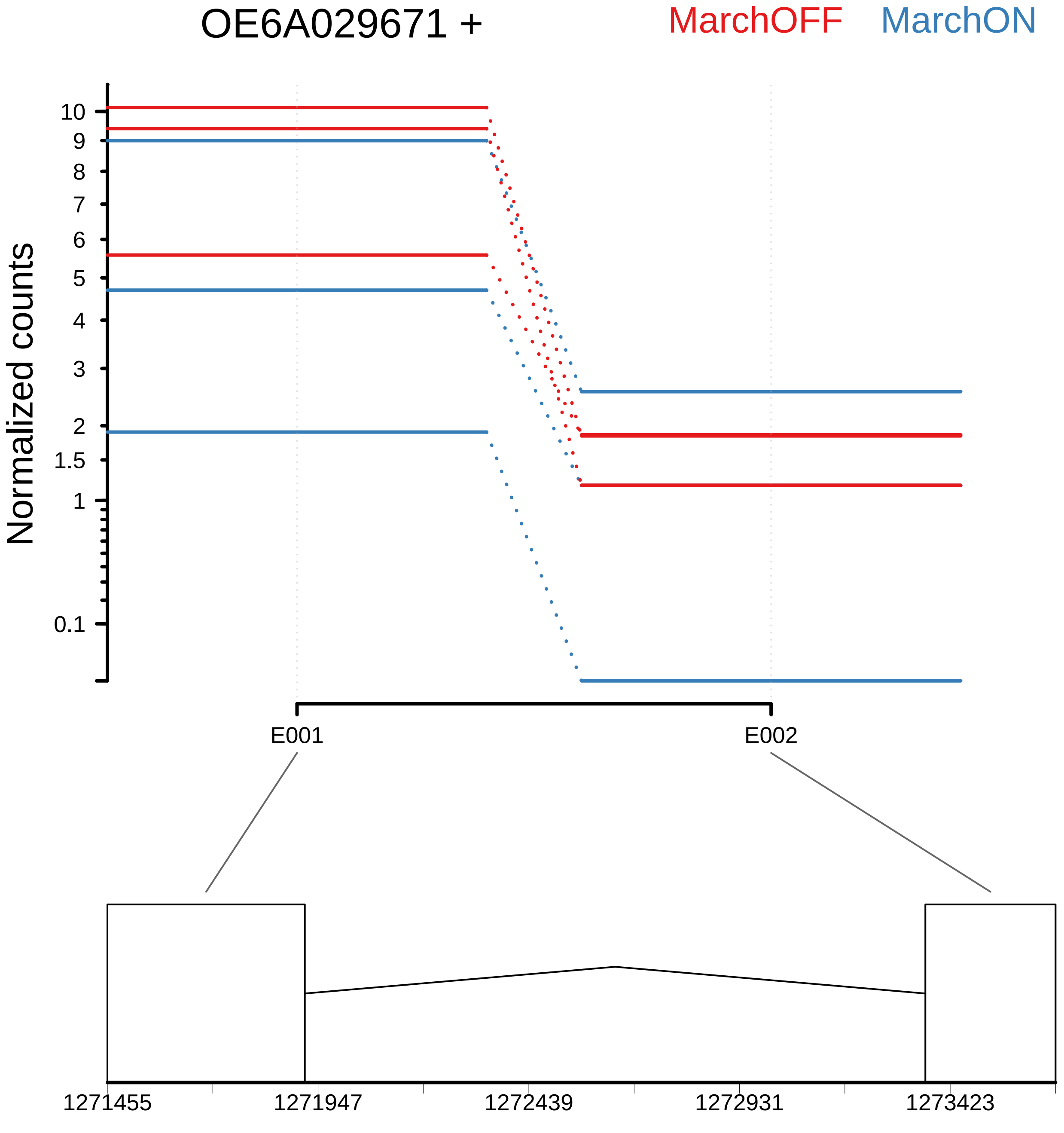

OE6A029671 +
MarchOFF
MarchON
Normalized counts
10
9
8
7
6
5
4
3
2
1.5
1
0.1
E001
E002
1271455
1271947
1272439
1272931
1273423

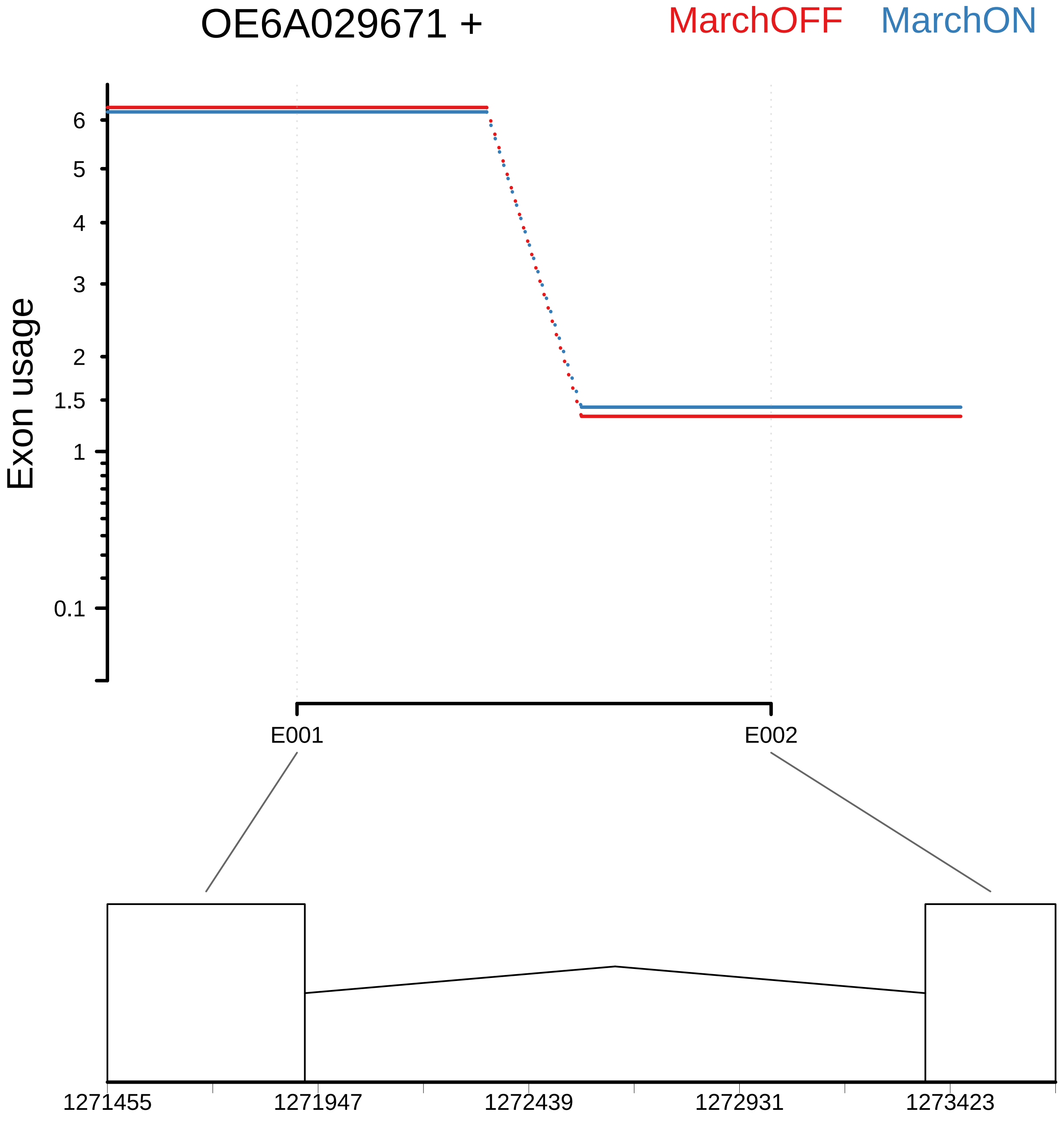
OE6A029671 +
MarchOFF
MarchON
Exon usage
6
5
4
3
2
1.5
1
0.1
E001
E002
1271455
1271947
1272439
1272931
1273423

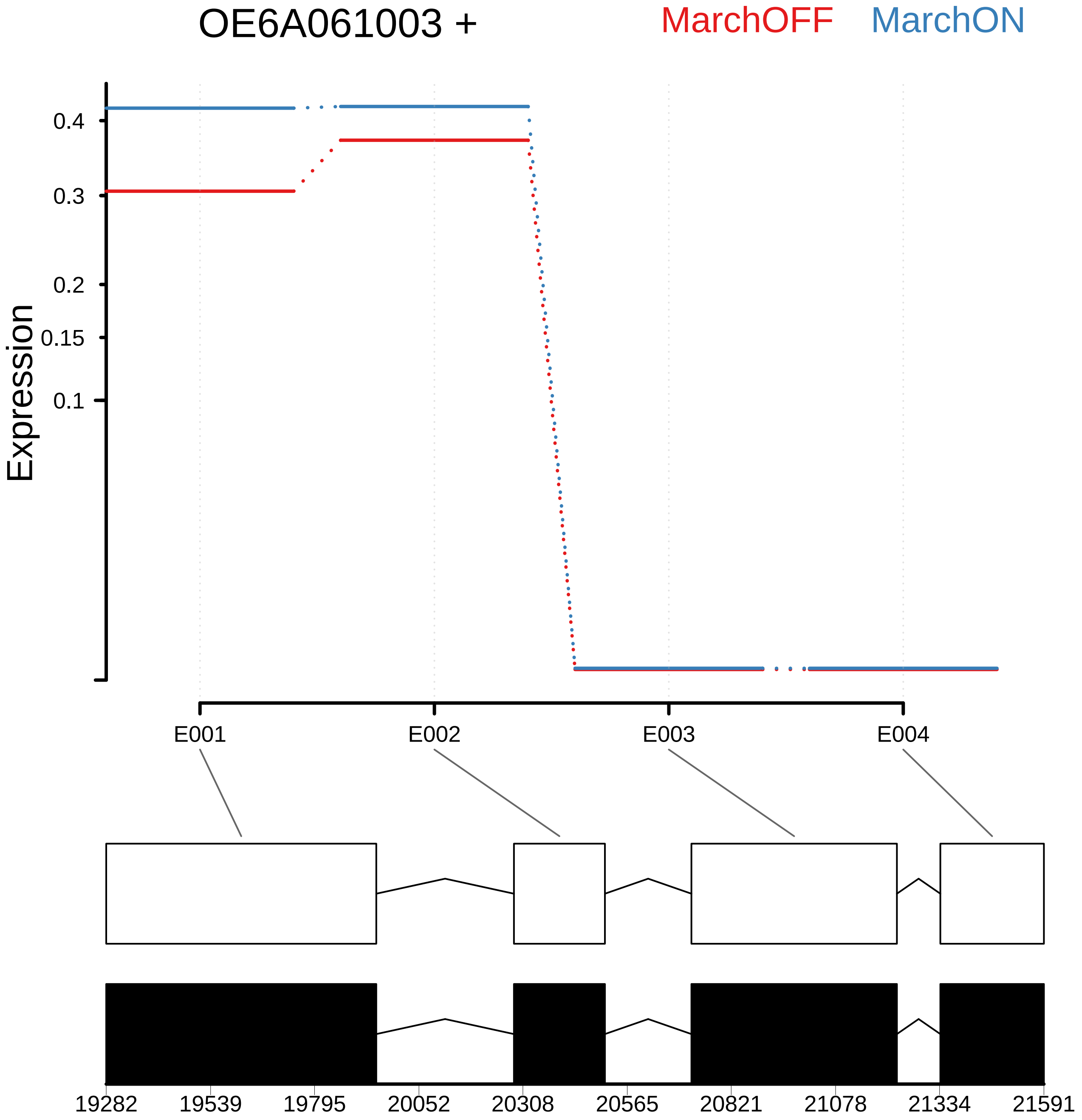
OE6A061003 +
MarchOFF
MarchON
Expression
0.4
0.3
0.2
0.15
0.1
E001
E002
E003
E004
19282
19539
19795
20052
20308
20565
20821
21078
21334
21591

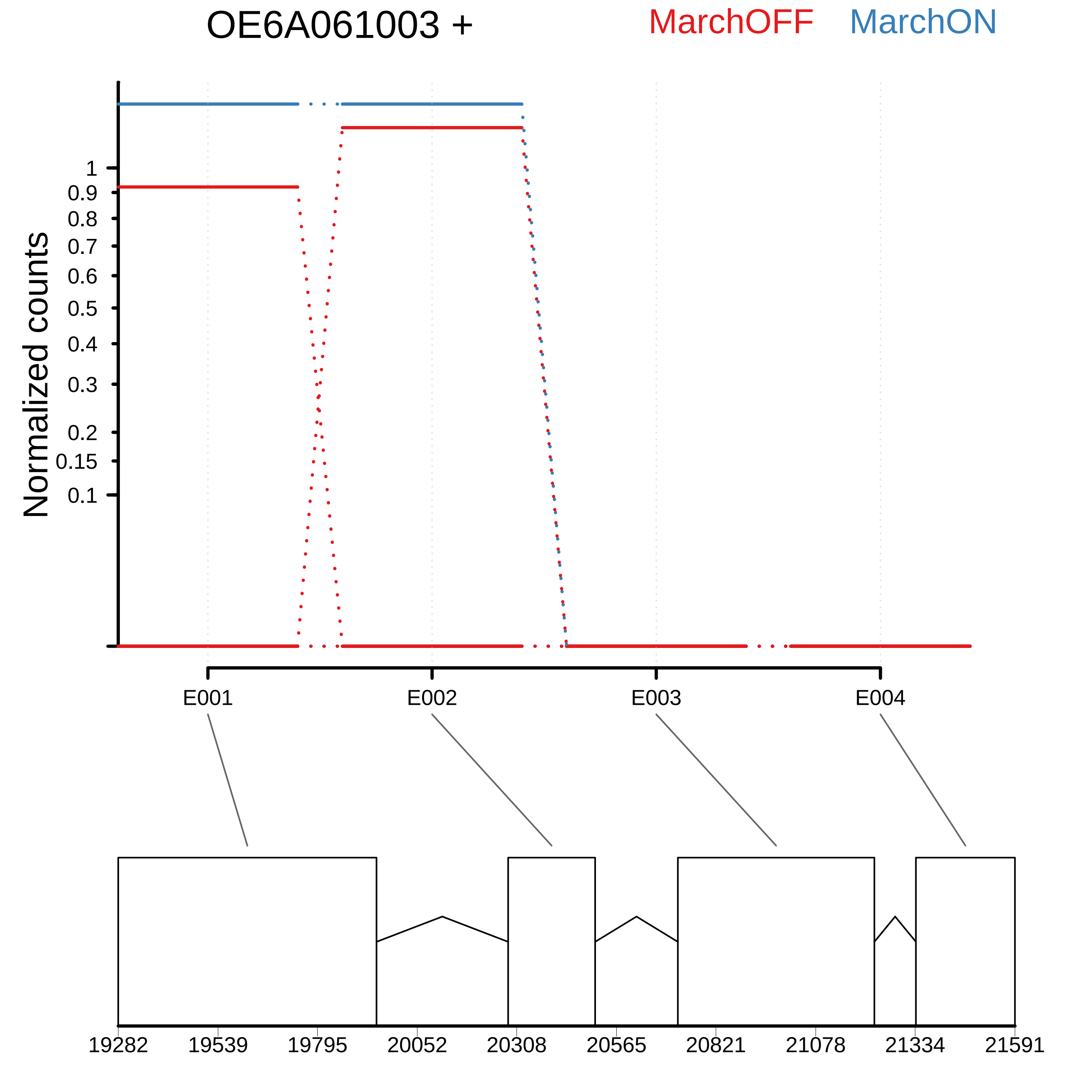

OE6A061003 +
MarchOFF
MarchON
Normalized counts
1
0.9
0.8
0.7
0.6
0.5
0.4
0.3
0.2
0.15
0.1
E001
E002
E003
E004
19282
19539
19795
20052
20308
20565
20821
21078
21334
21591

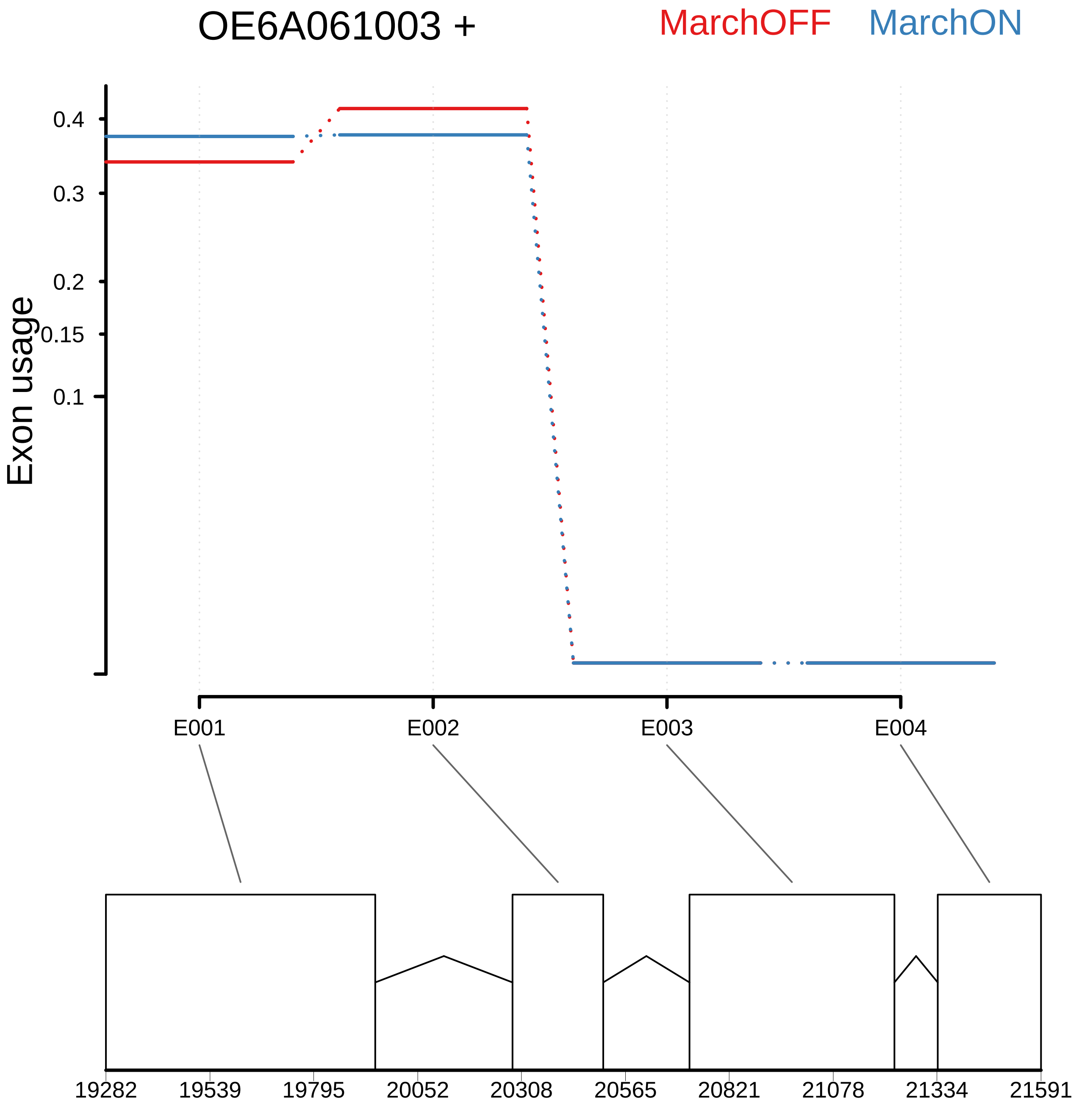

OE6A061003 +
MarchOFF
MarchON
Exon usage
0.4
0.3
0.2
0.15
0.1
E001
E002
E003
E004
19282
19539
19795
20052
20308
20565
20821
21078
21334
21591

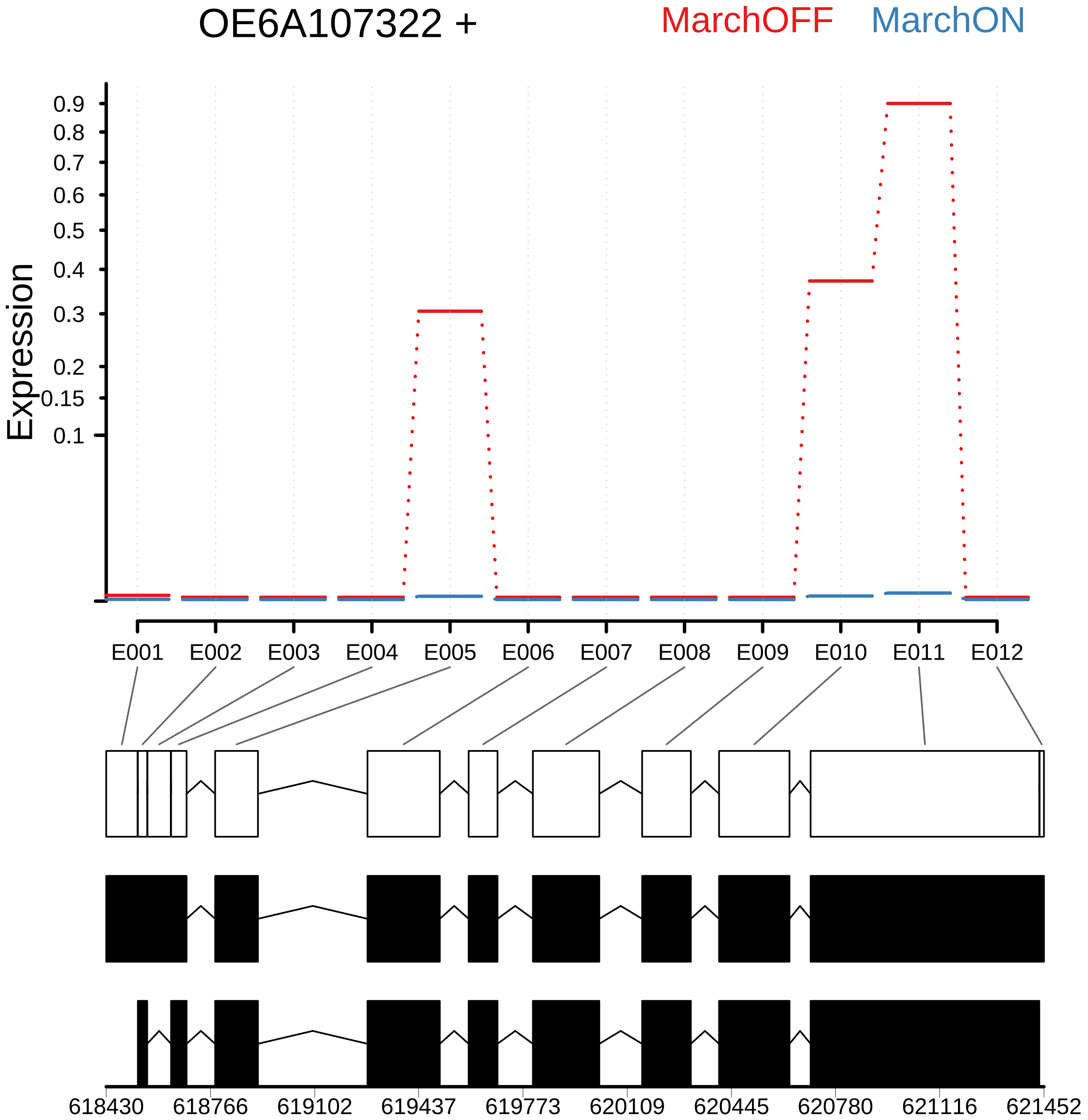

OE6A107322 +
MarchOFF
MarchON
Expression
0.9
0.8
0.7
0.6
0.5
0.4
0.3
0.2
0.15
0.1
E001
E002
E003
E004
E005
E006
E007
E008
E009
E010
E011
E012
618430
618766
619102
619437
619773
620109
620445
620780
621116
621452

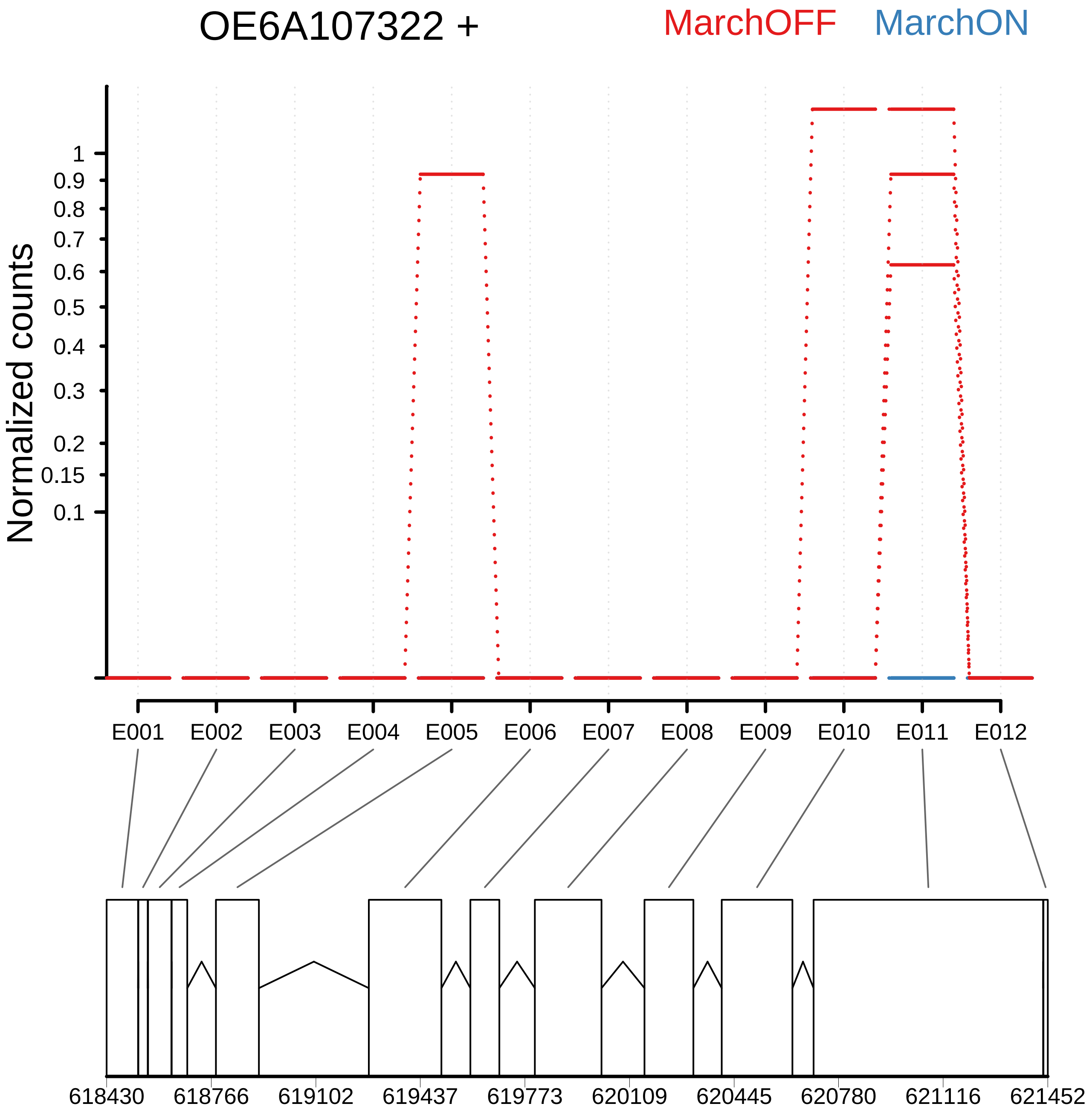
OE6A107322 +
MarchOFF
MarchON
Normalized counts
1
0.9
0.8
0.7
0.6
0.5
0.4
0.3
0.2
0.15
0.1
E001
E002
E003
E004
E005
E006
E007
E008
E009
E010
E011
E012
618430
618766
619102
619437
619773
620109
620445
620780
621116
621452

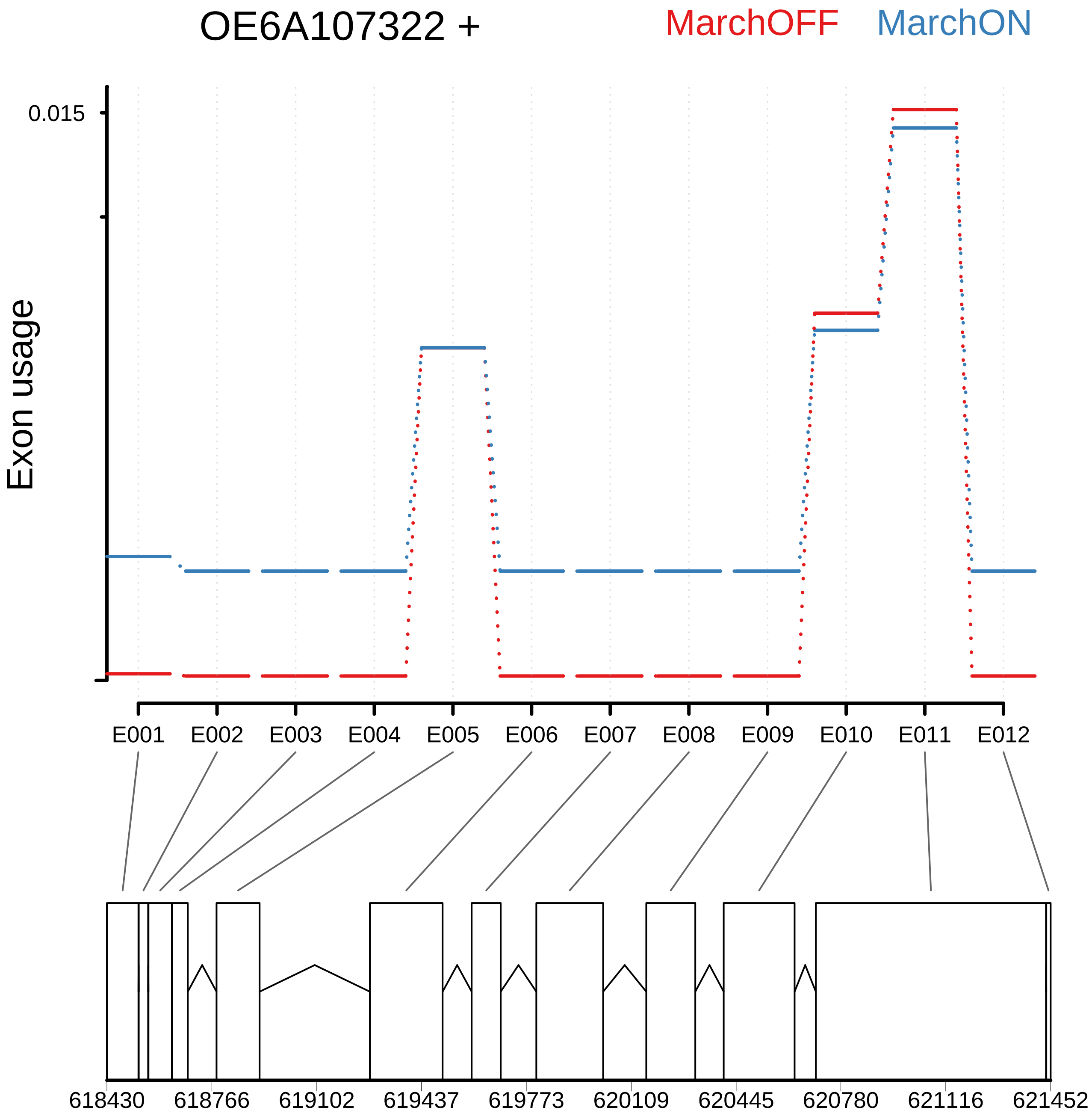
OE6A107322 +
MarchOFF
MarchON
Exon usage
0.015
E001
E002
E003
E004
E005
E006
E007
E008
E009
E010
E011
E012
618430
618766
619102
619437
619773
620109
620445
620780
621116
621452

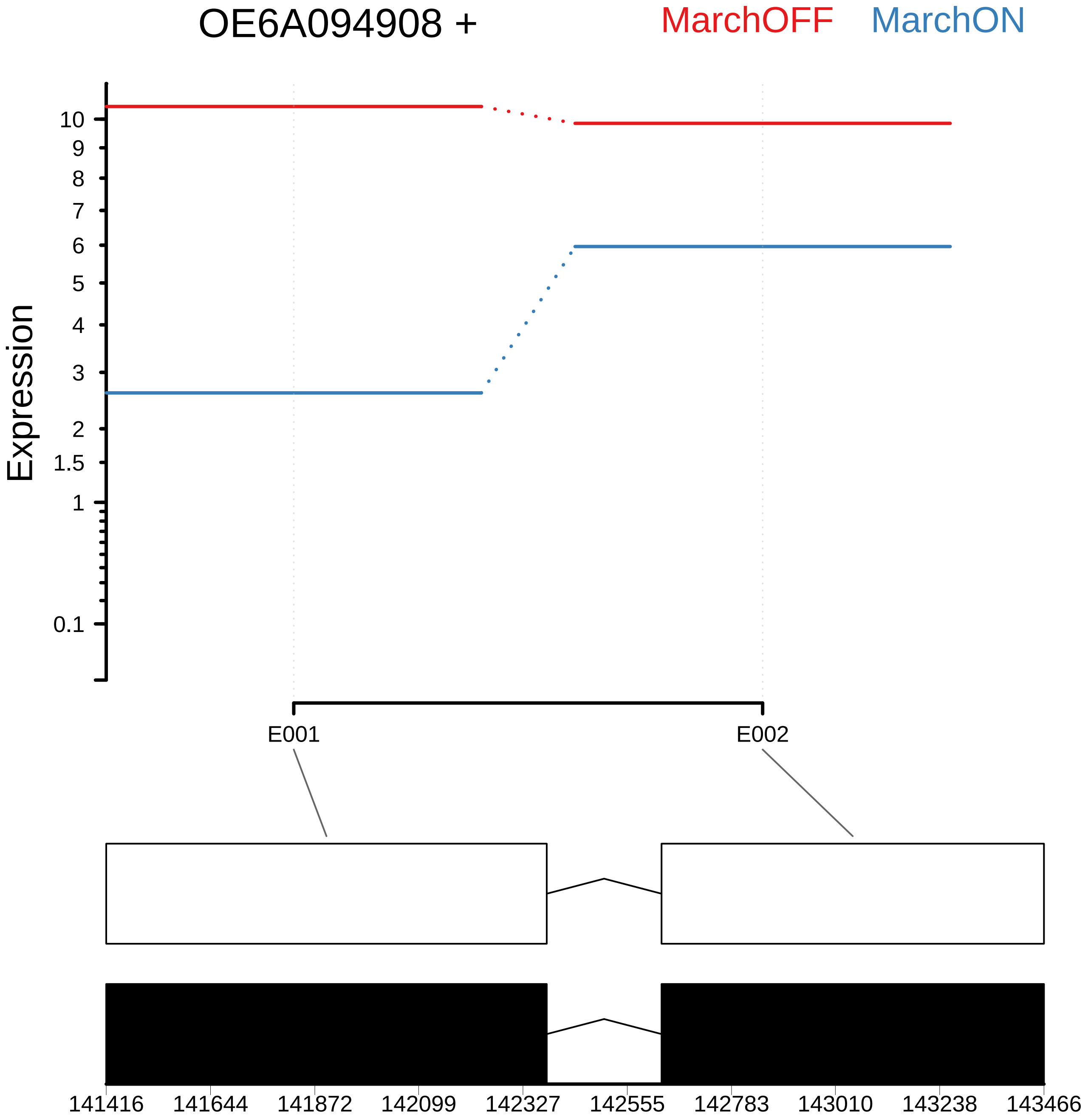

OE6A094908 +
MarchOFF
MarchON
Expression
10
9
8
7
6
5
4
3
2
1.5
1
0.1
E001
E002
141416
141644
141872
142099
142327
142555
142783
143010
143238
143466

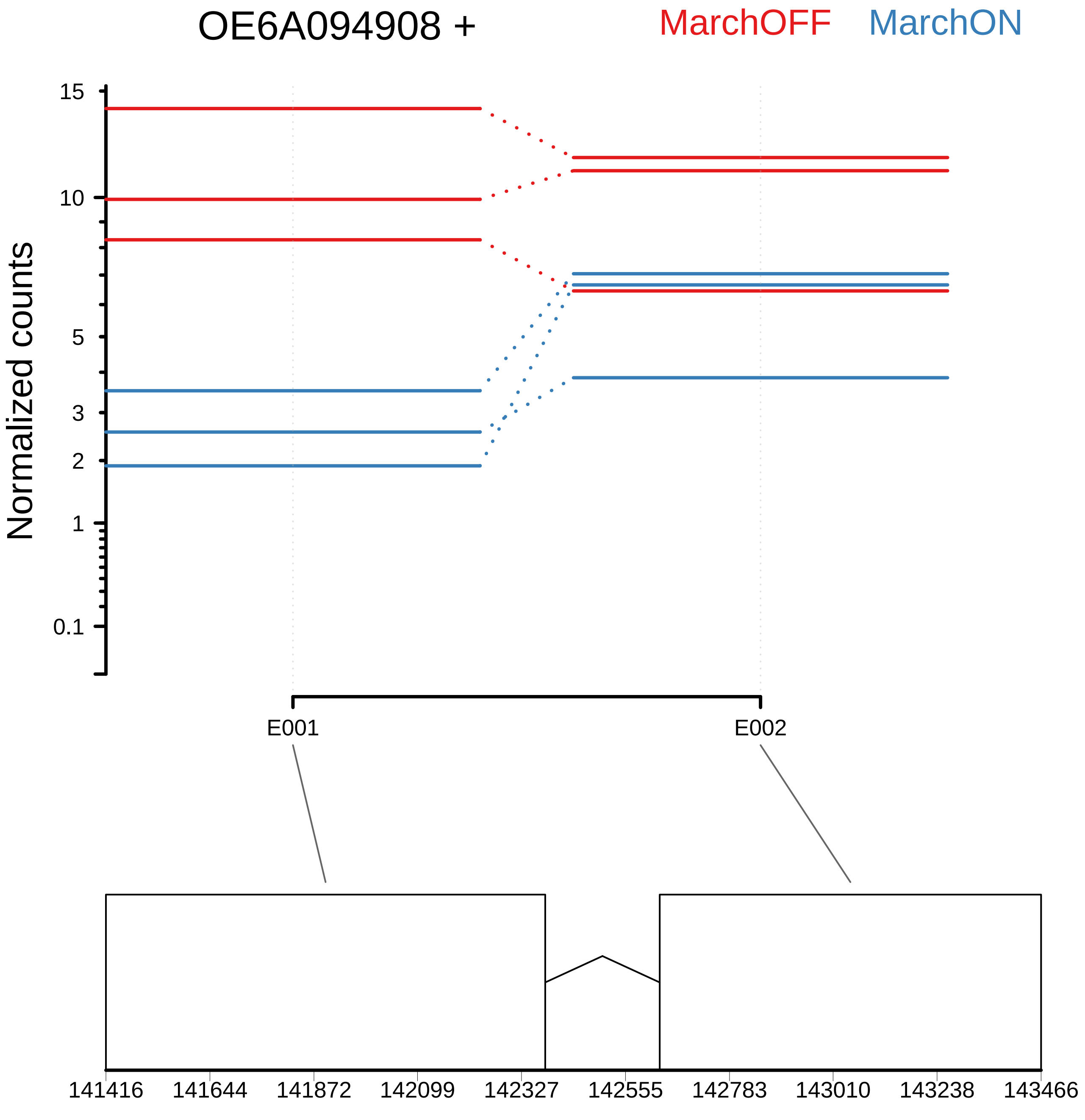
OE6A094908 +
MarchOFF
MarchON
Normalized counts
15
10
5
3
2
1
0.1
E001
E002
141416
141644
141872
142099
142327
142555
142783
143010
143238
143466

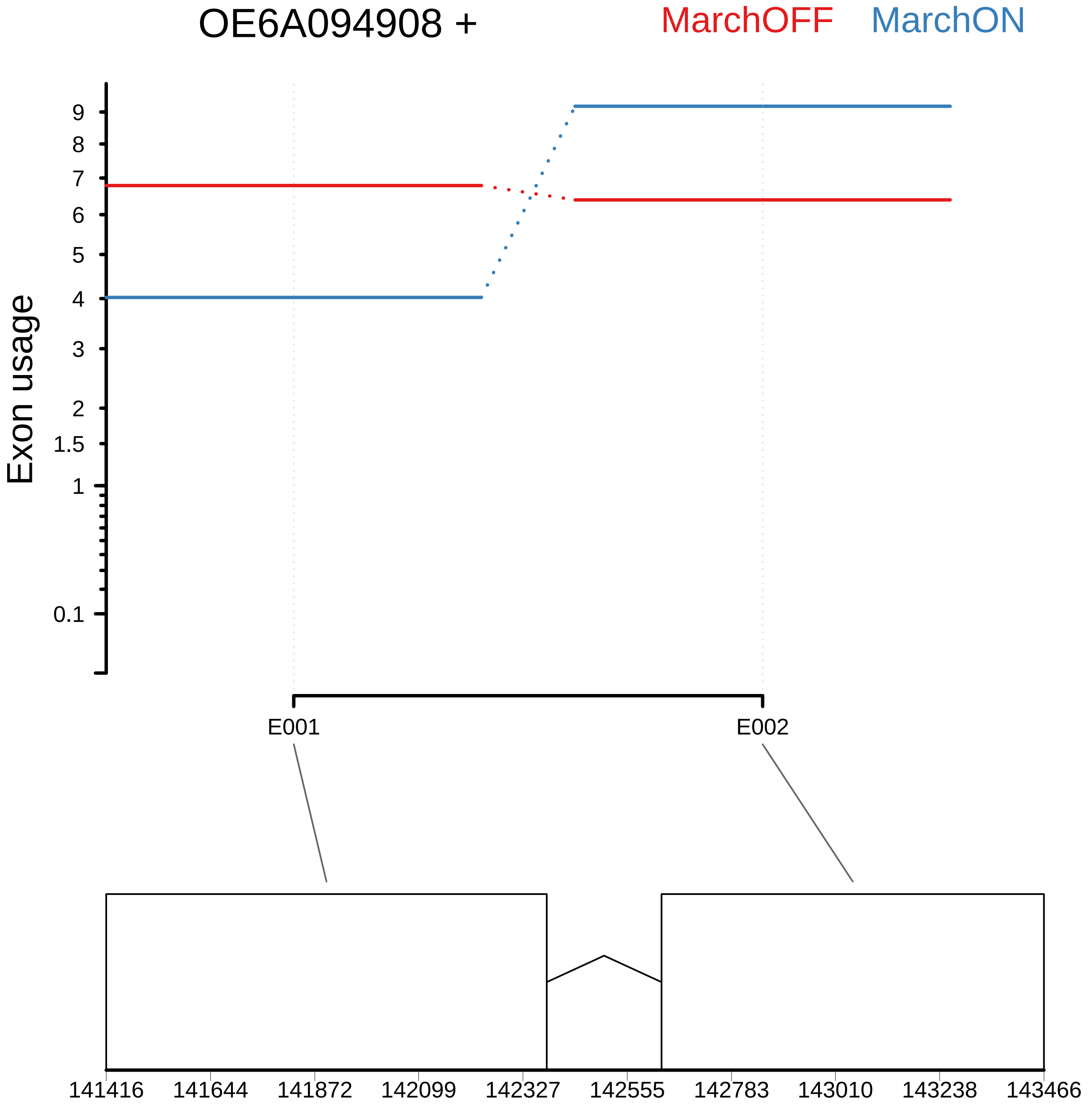
OE6A094908 +
MarchOFF
MarchON
Exon usage
9
8
7
6
5
4
3
2
1.5
1
0.1
E001
E002
141416
141644
141872
142099
142327
142555
142783
143010
143238
143466

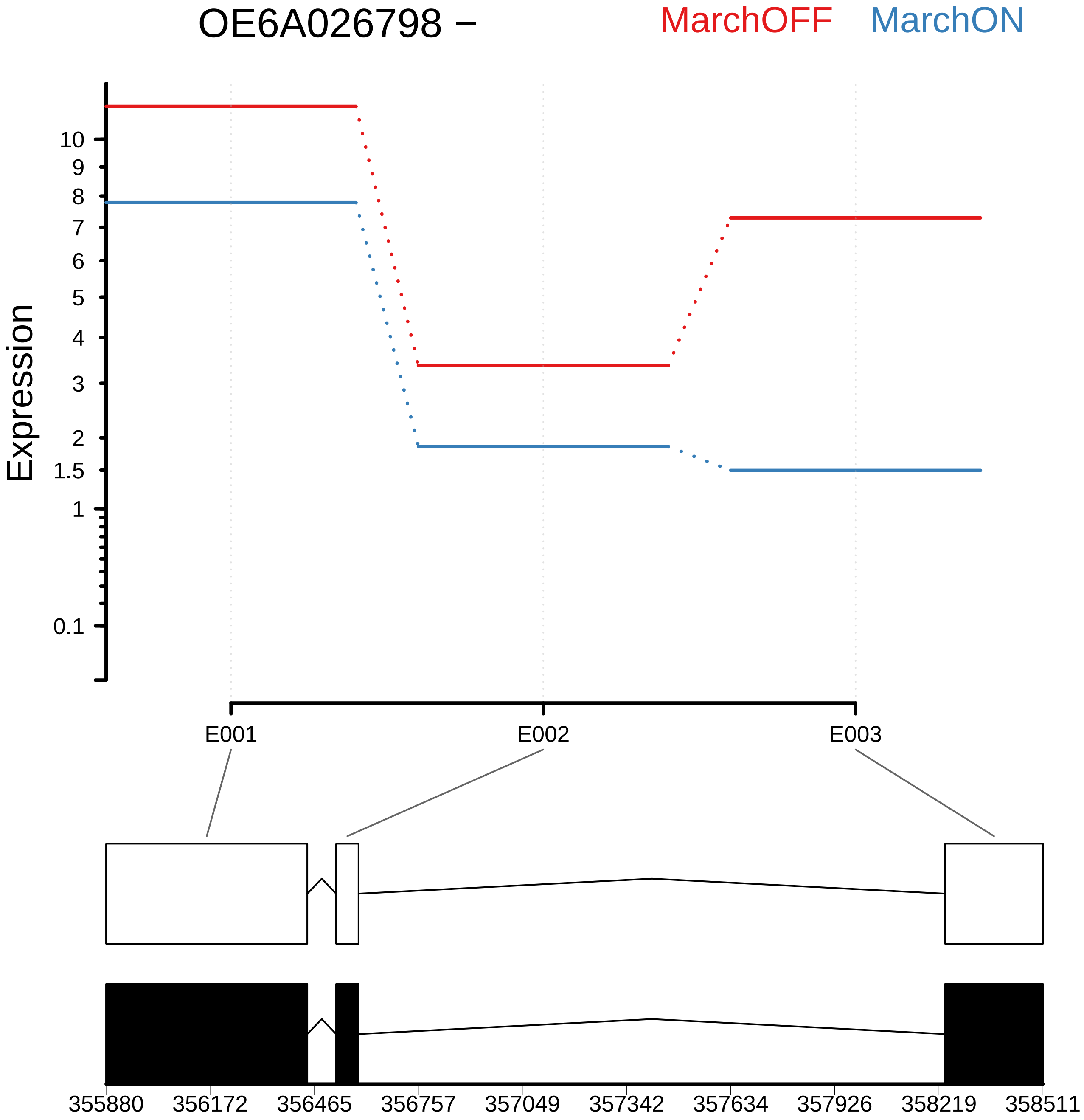
OE6A026798 −
MarchOFF
MarchON
Expression
10
9
8
7
6
5
4
3
2
1.5
1
0.1
E001
E002
E003
355880
356172
356465
356757
357049
357342
357634
357926
358219
358511

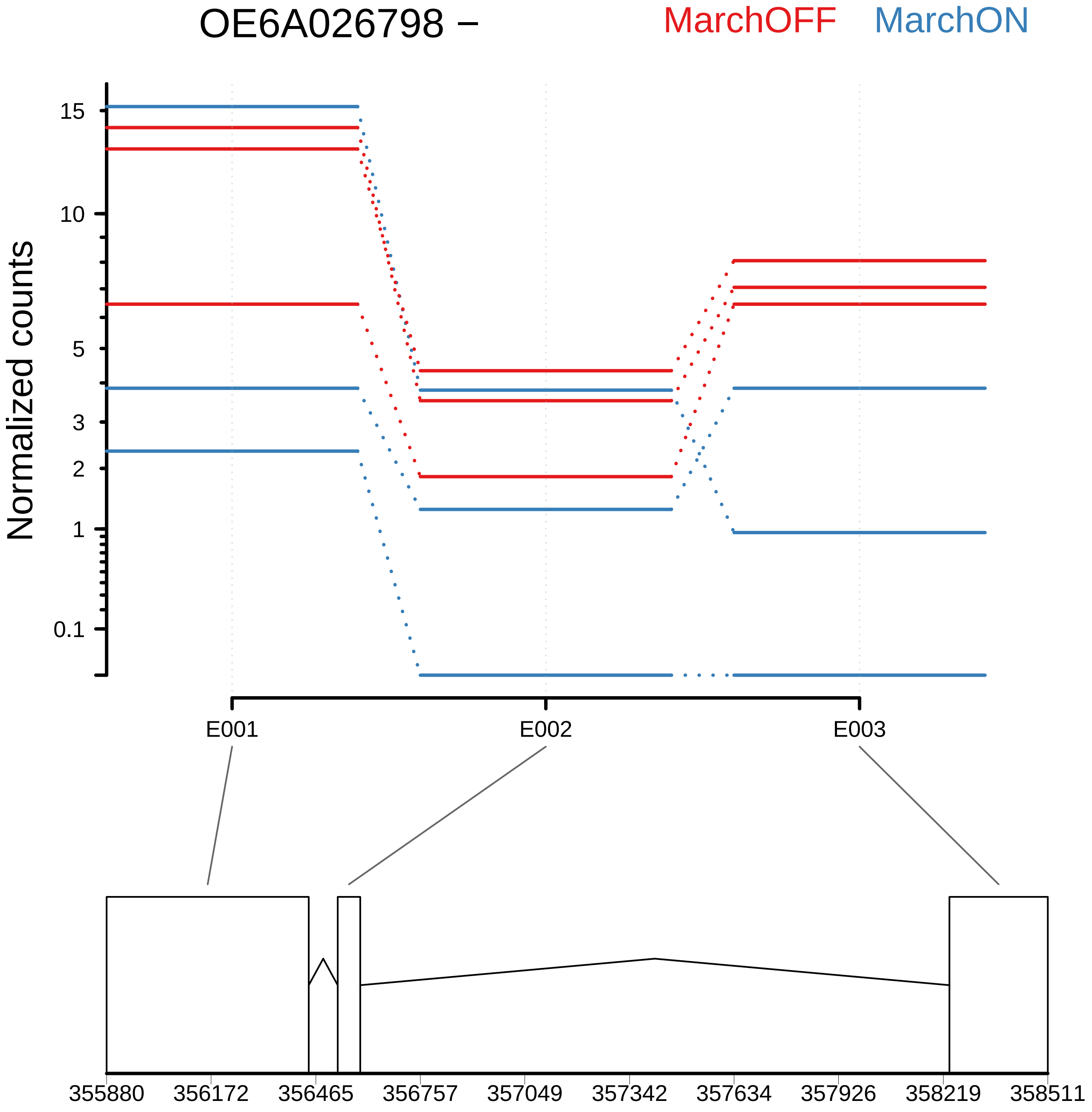

OE6A026798 −
MarchOFF
MarchON
Normalized counts
15
10
5
3
2
1
0.1
E001
E002
E003
355880
356172
356465
356757
357049
357342
357634
357926
358219
358511

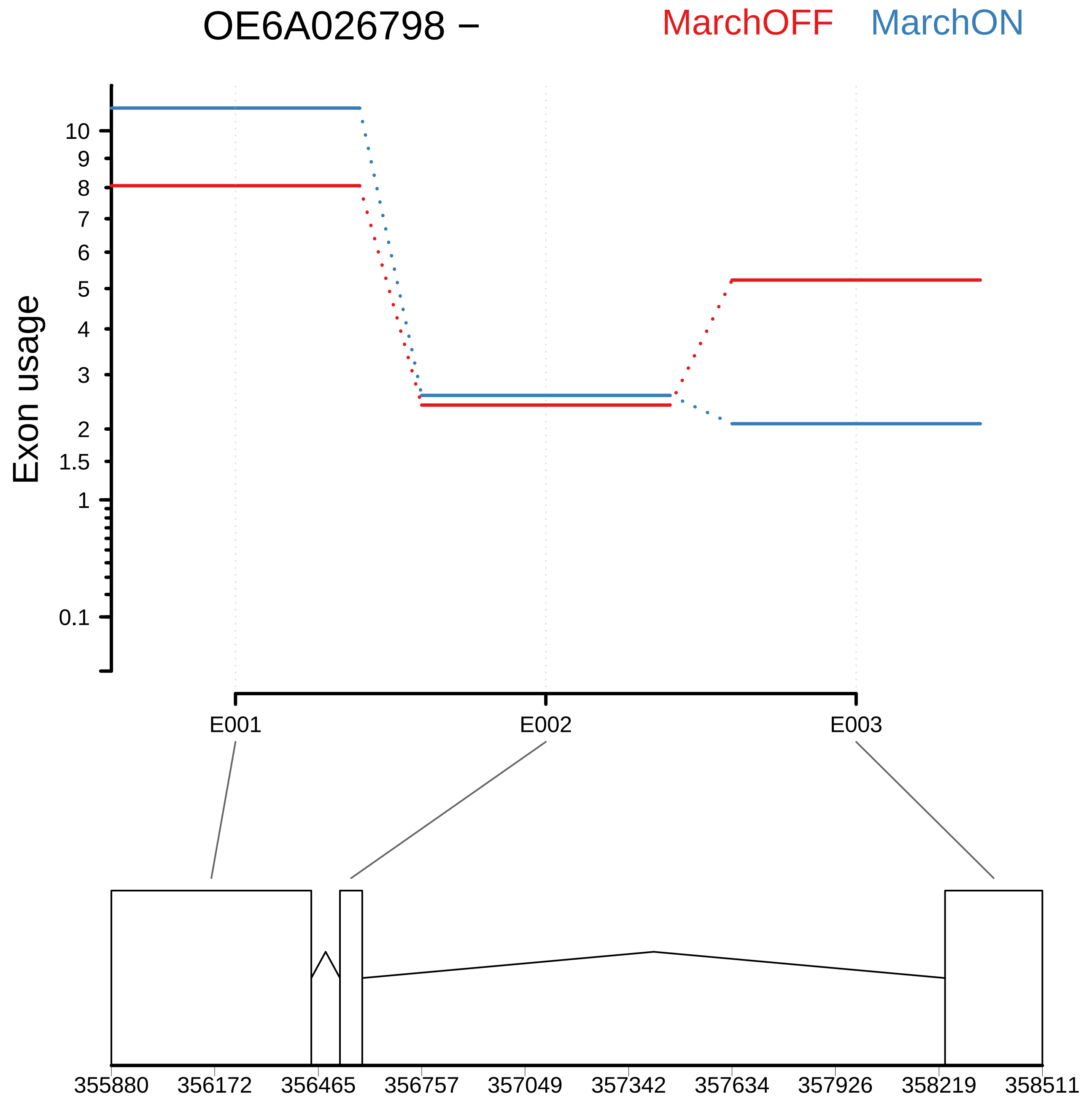

OE6A026798 −
MarchOFF
MarchON
Exon usage
10
9
8
7
6
5
4
3
2
1.5
1
0.1
E001
E002
E003
355880
356172
356465
356757
357049
357342
357634
357926
358219
358511

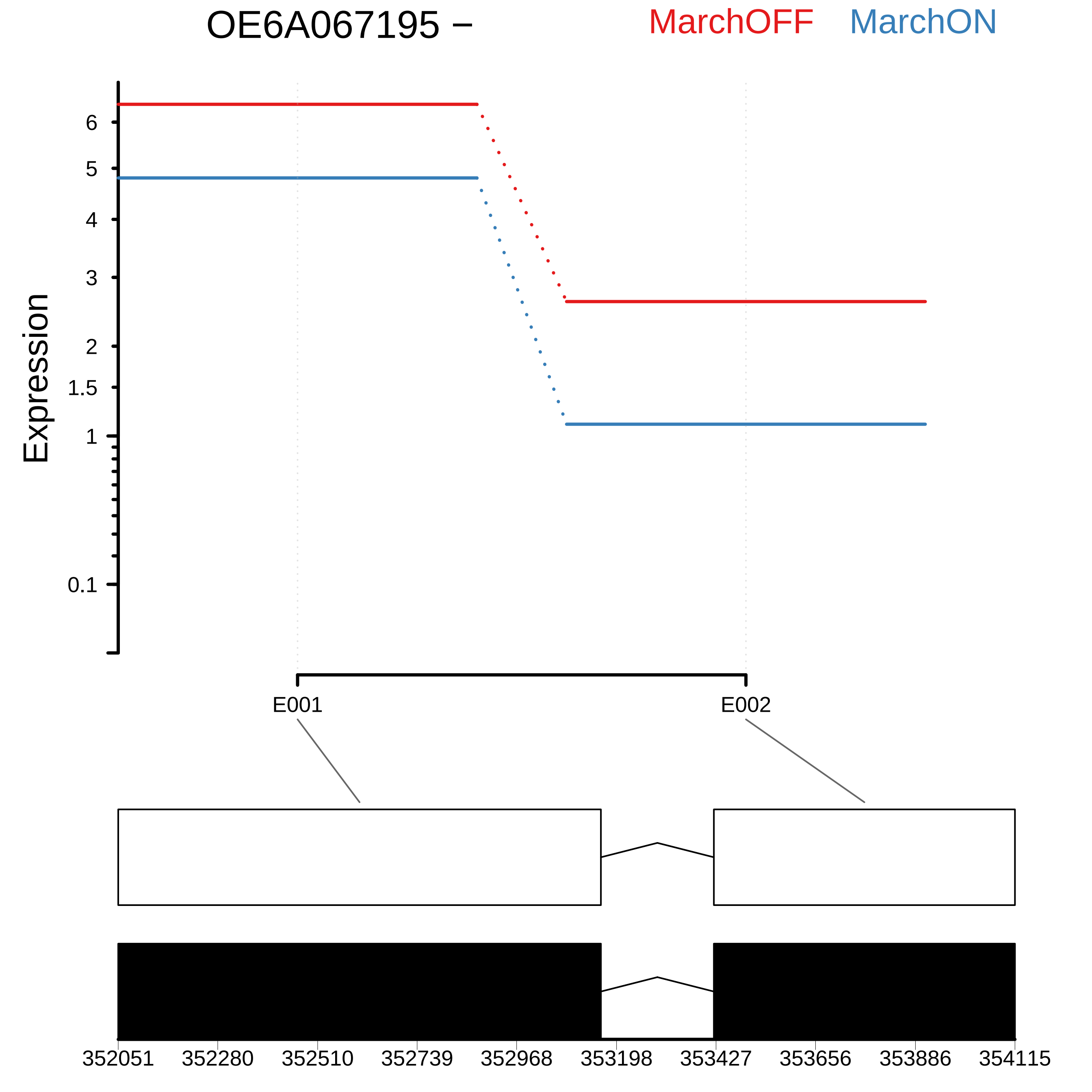
OE6A067195 −
MarchOFF
MarchON
Expression
6
5
4
3
2
1.5
1
0.1
E001
E002
352051
352280
352510
352739
352968
353198
353427
353656
353886
354115

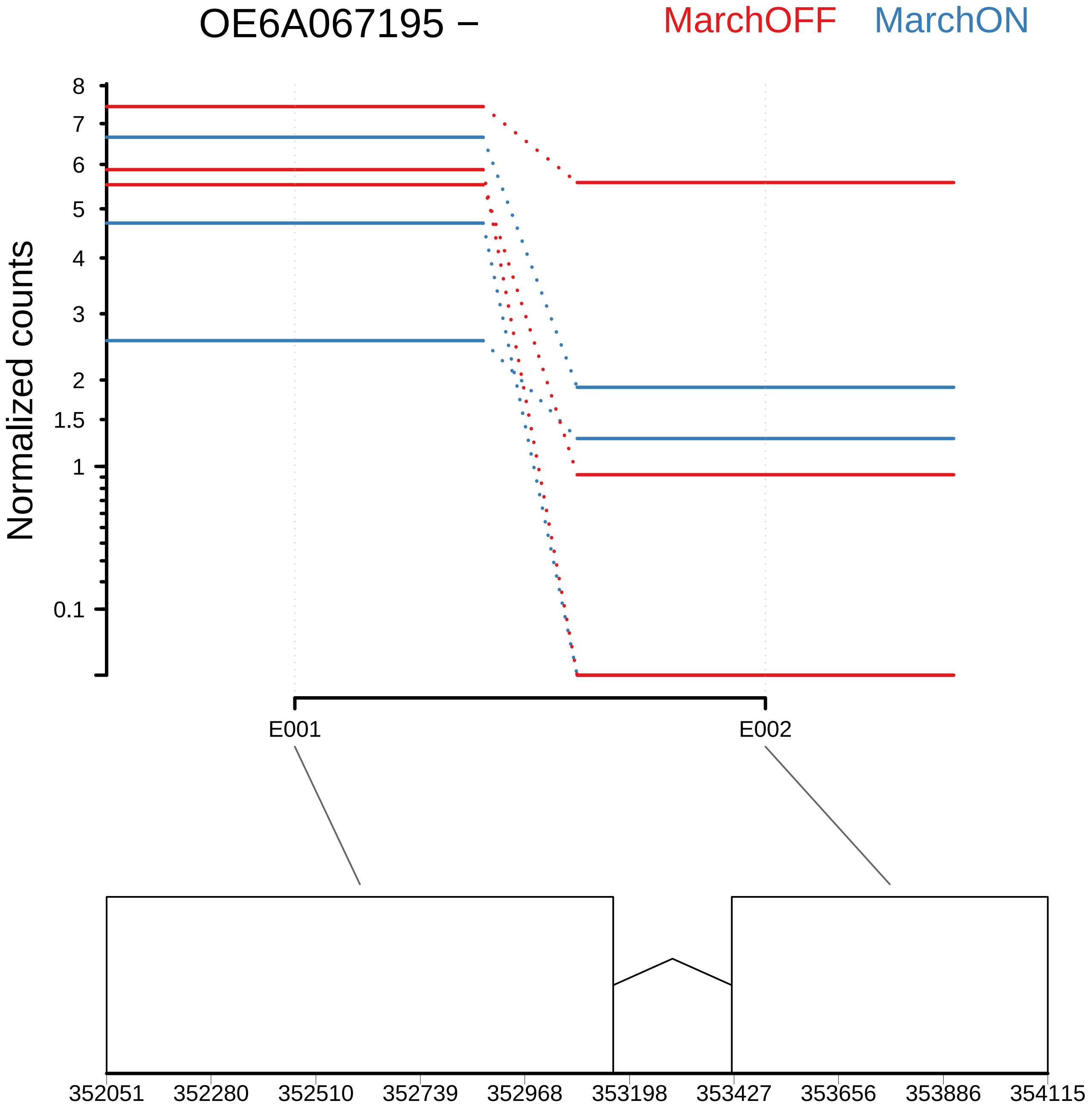

OE6A067195 −
MarchOFF
MarchON
Normalized counts
8
7
6
5
4
3
2
1.5
1
0.1
E001
E002
352051
352280
352510
352739
352968
353198
353427
353656
353886
354115

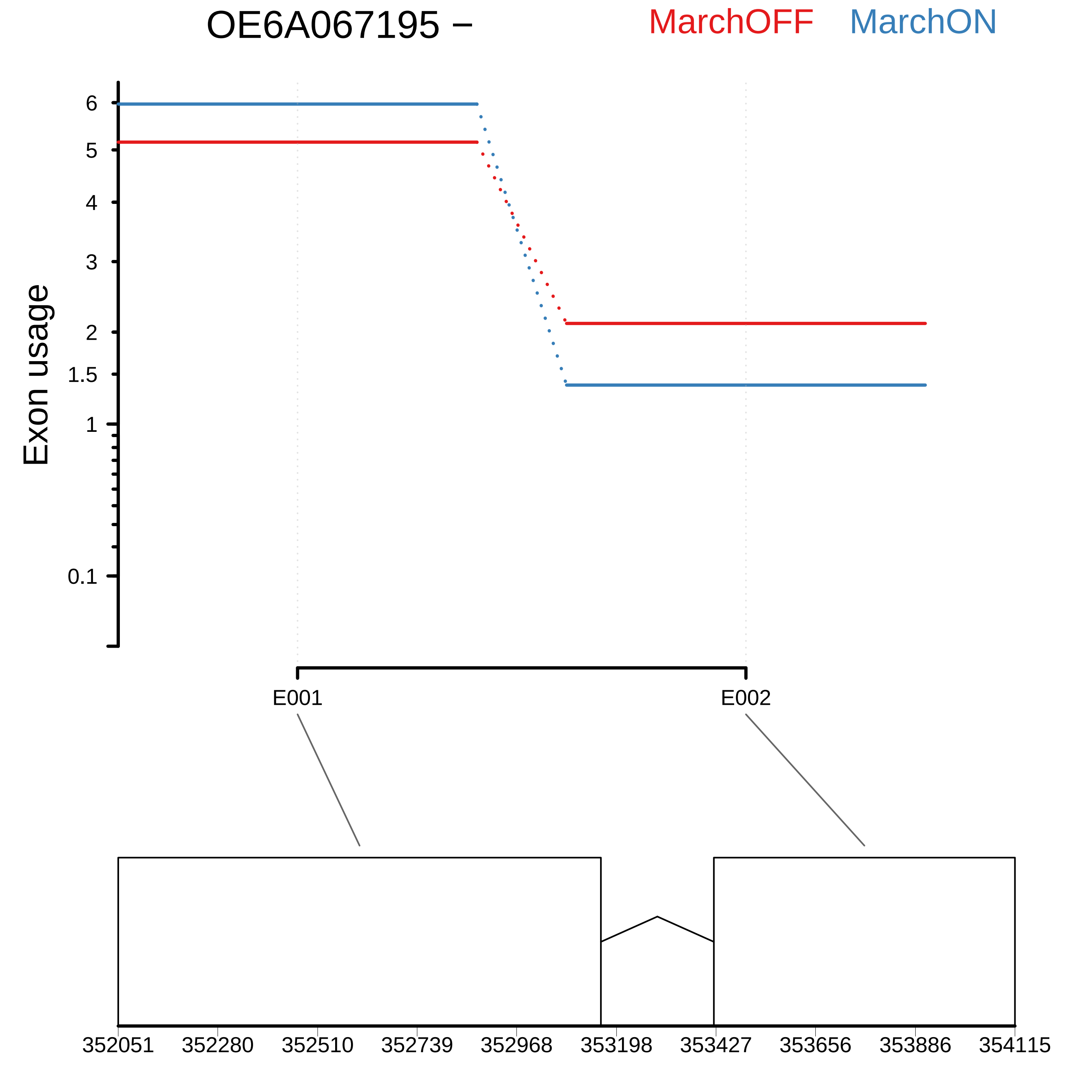
OE6A067195 −
MarchOFF
MarchON
Exon usage
6
5
4
3
2
1.5
1
0.1
E001
E002
352051
352280
352510
352739
352968
353198
353427
353656
353886
354115

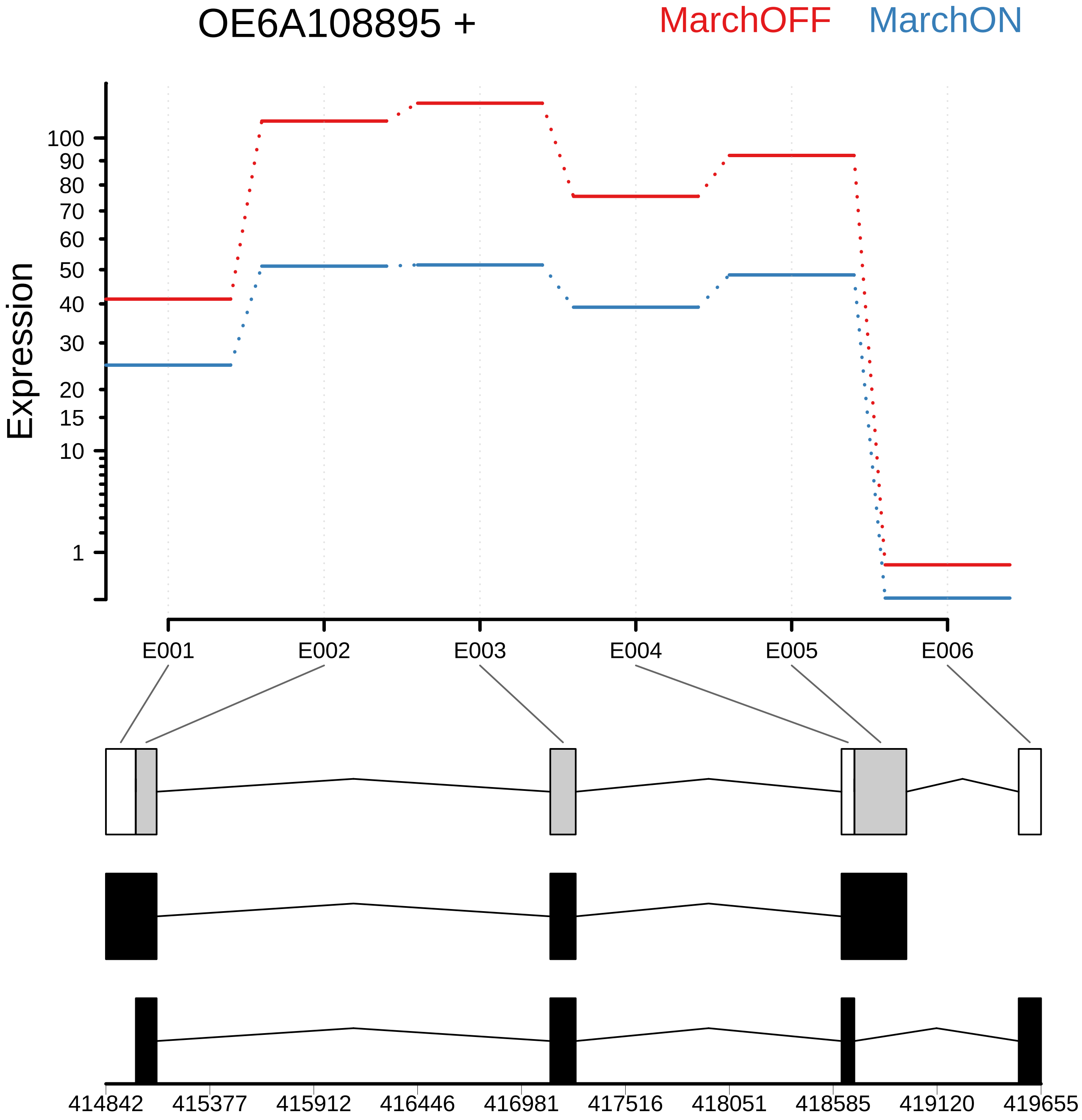
OE6A108895 +
MarchOFF
MarchON
Expression
100
90
80
70
60
50
40
30
20
15
10
1
E001
E002
E003
E004
E005
E006
414842
415377
415912
416446
416981
417516
418051
418585
419120
419655

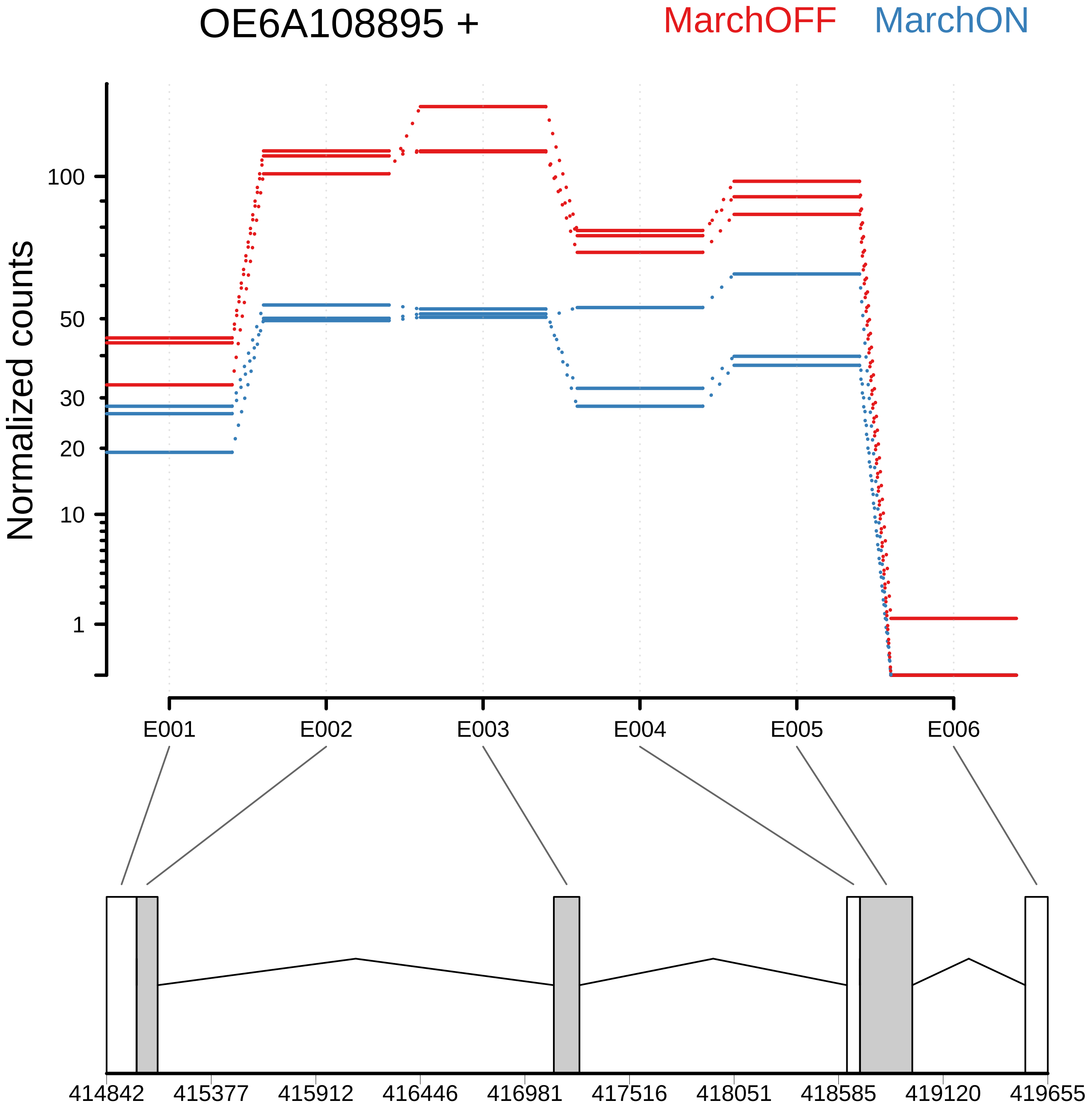

OE6A108895 +
MarchOFF
MarchON
Normalized counts
100
50
30
20
10
1
E001
E002
E003
E004
E005
E006
414842
415377
415912
416446
416981
417516
418051
418585
419120
419655

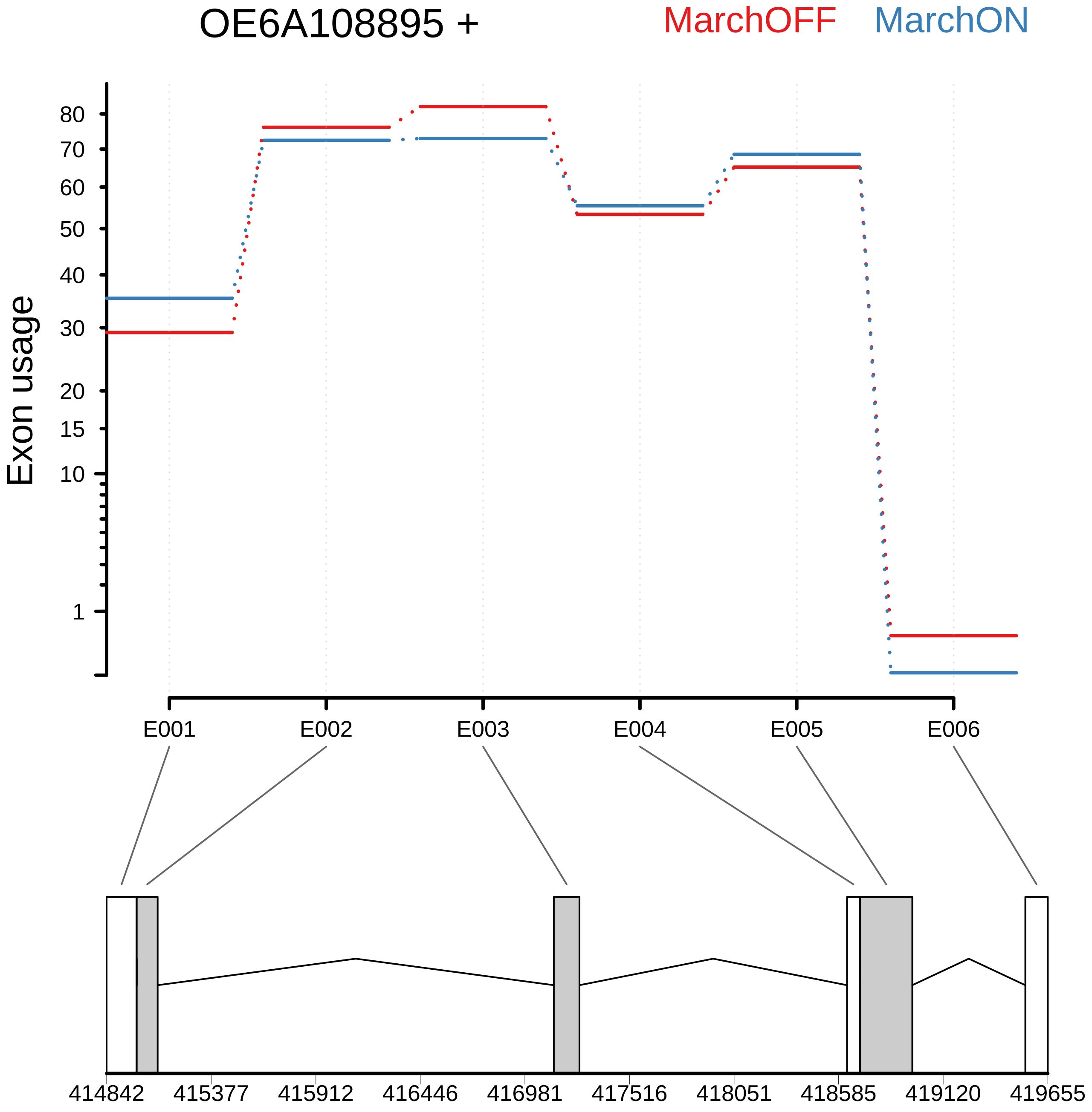

OE6A108895 +
MarchOFF
MarchON
Exon usage
80
70
60
50
40
30
20
15
10
1
E001
E002
E003
E004
E005
E006
414842
415377
415912
416446
416981
417516
418051
418585
419120
419655

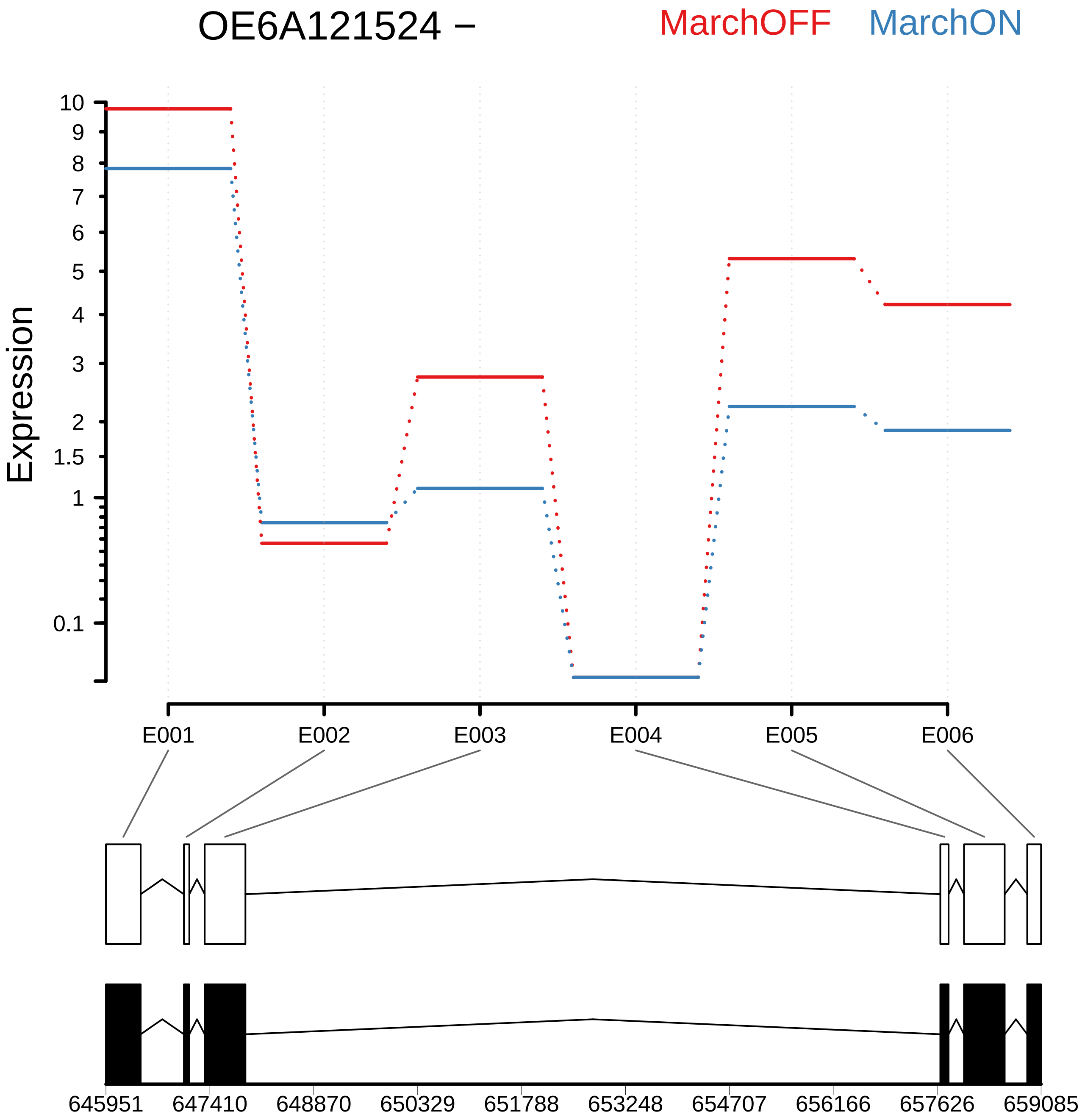

OE6A121524 −
MarchOFF
MarchON
Expression
10
9
8
7
6
5
4
3
2
1.5
1
0.1
E001
E002
E003
E004
E005
E006
645951
647410
648870
650329
651788
653248
654707
656166
657626
659085

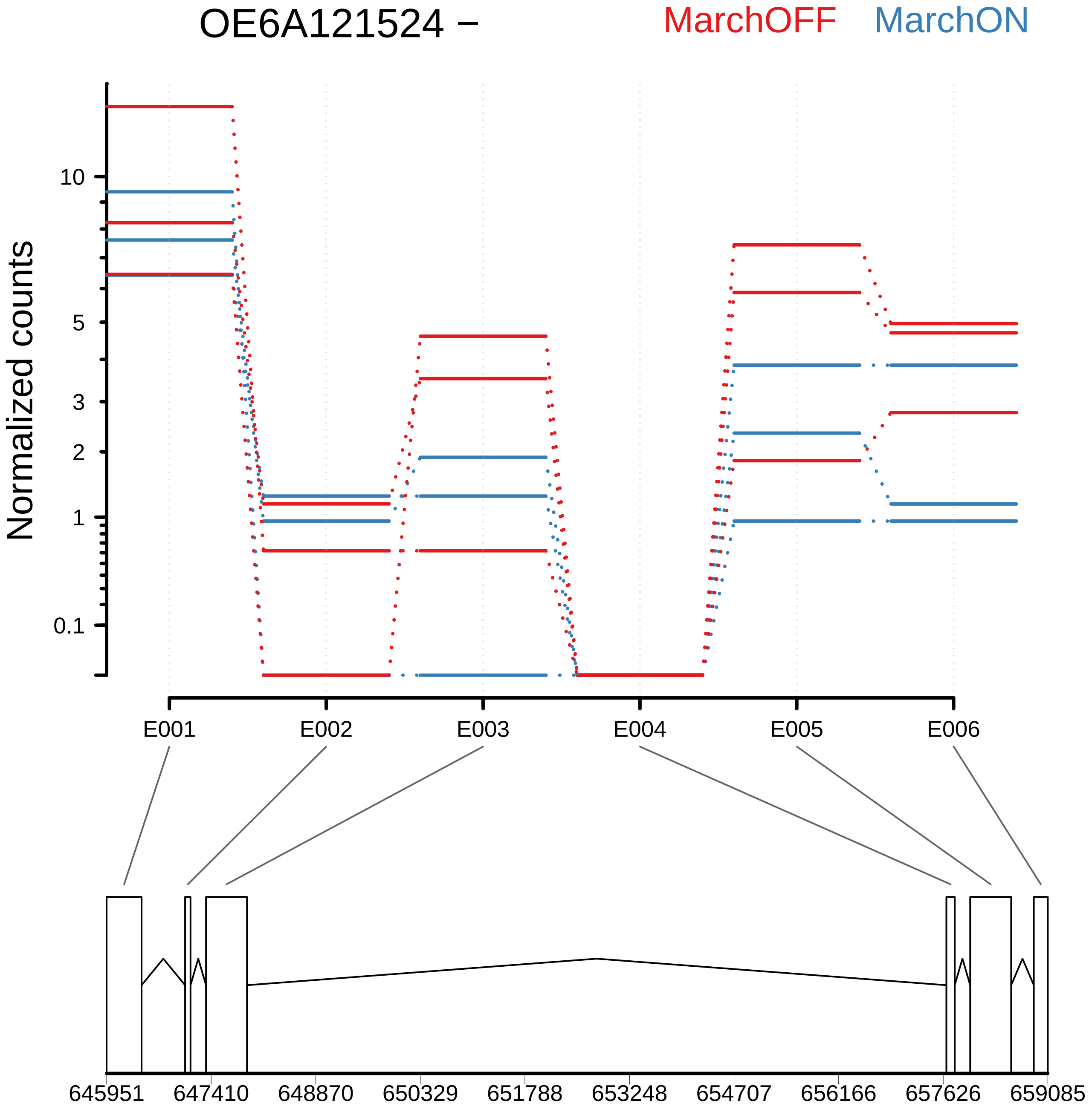
OE6A121524 −
MarchOFF
MarchON
Normalized counts
10
5
3
2
1
0.1
E001
E002
E003
E004
E005
E006
645951
647410
648870
650329
651788
653248
654707
656166
657626
659085

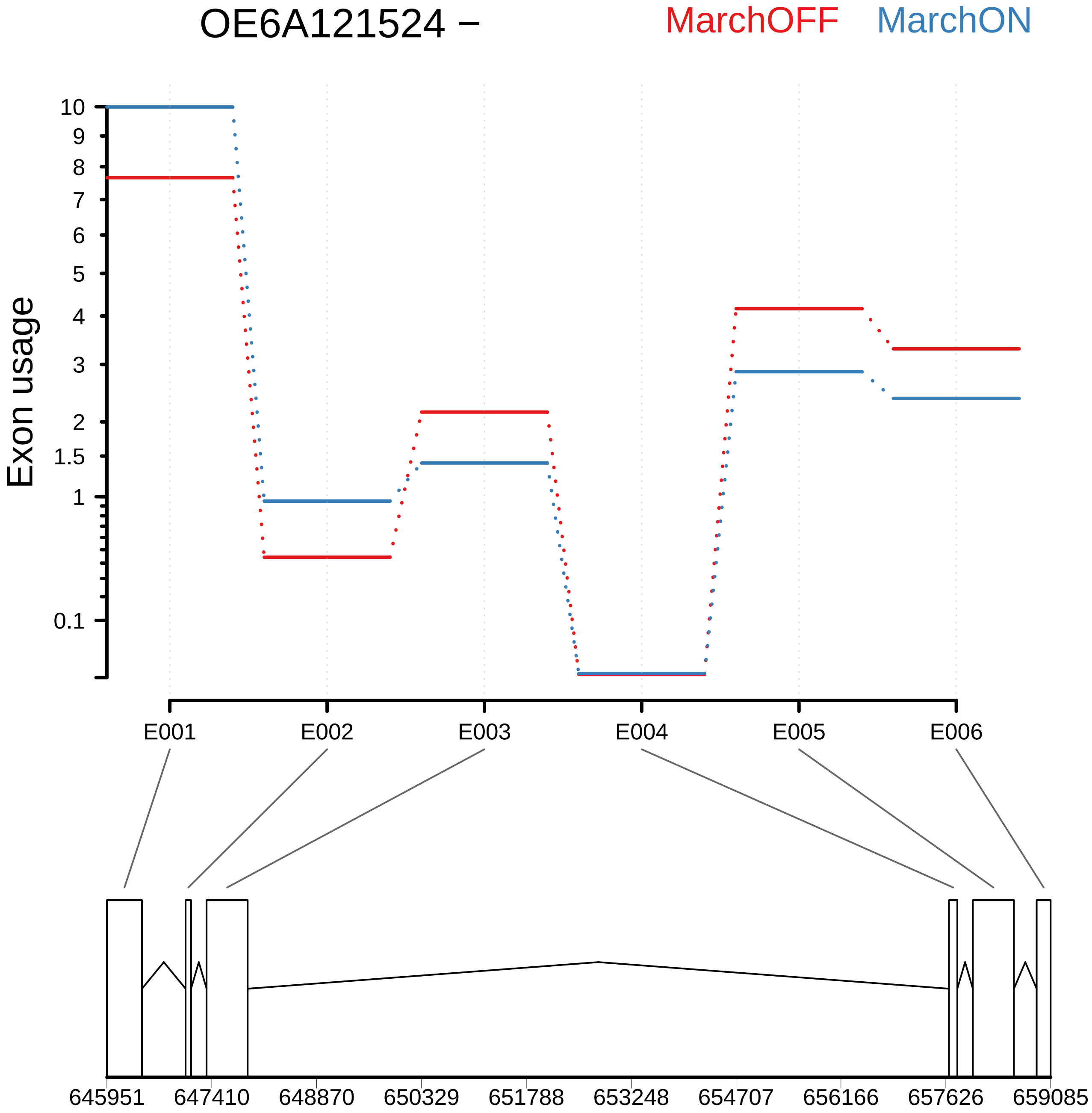

OE6A121524 −
MarchOFF
MarchON
Exon usage
10
9
8
7
6
5
4
3
2
1.5
1
0.1
E001
E002
E003
E004
E005
E006
645951
647410
648870
650329
651788
653248
654707
656166
657626
659085

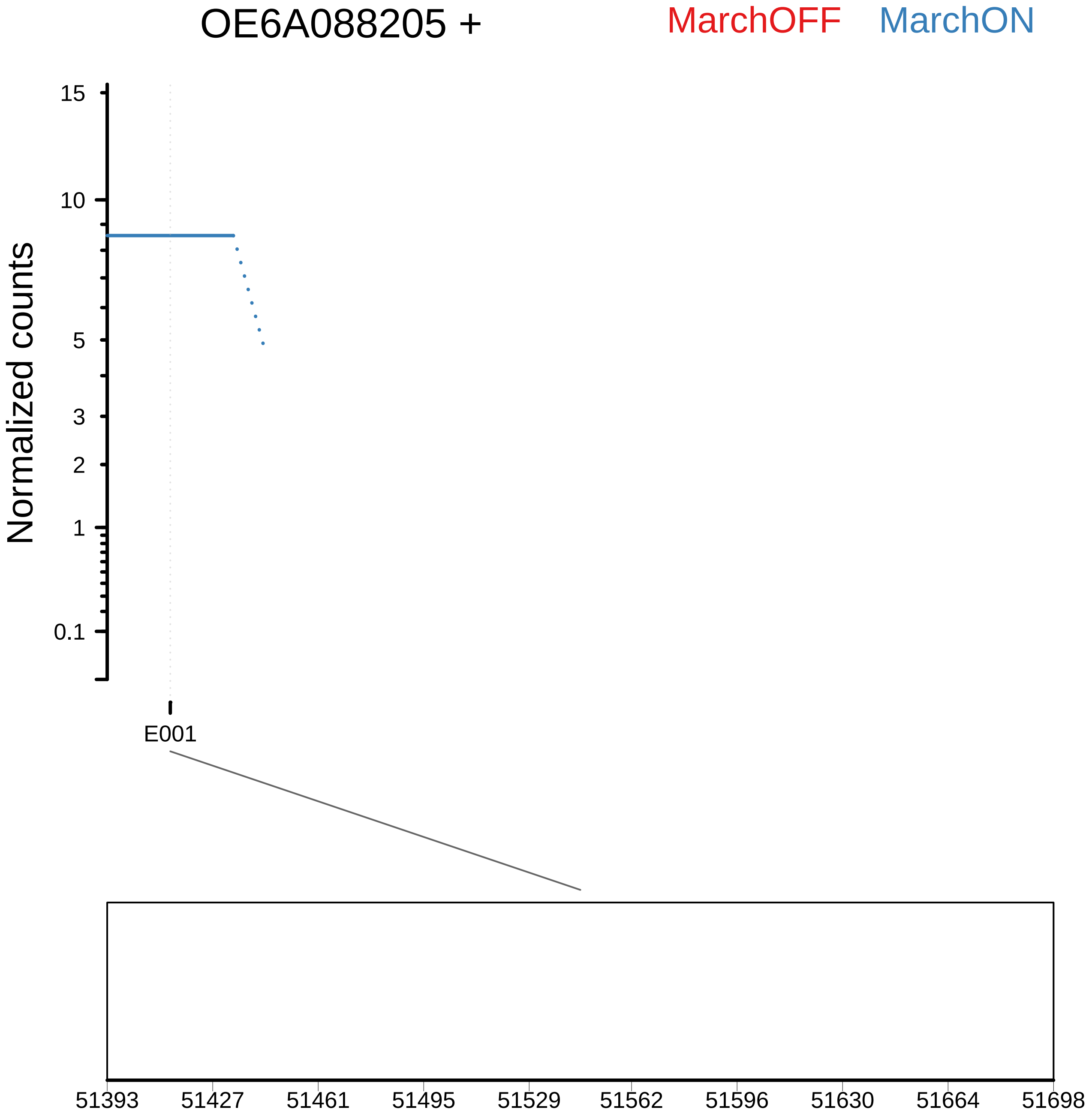

OE6A088205 +
MarchOFF
MarchON
Normalized counts
15
10
5
3
2
1
0.1
E001
51393
51427
51461
51495
51529
51562
51596
51630
51664
51698

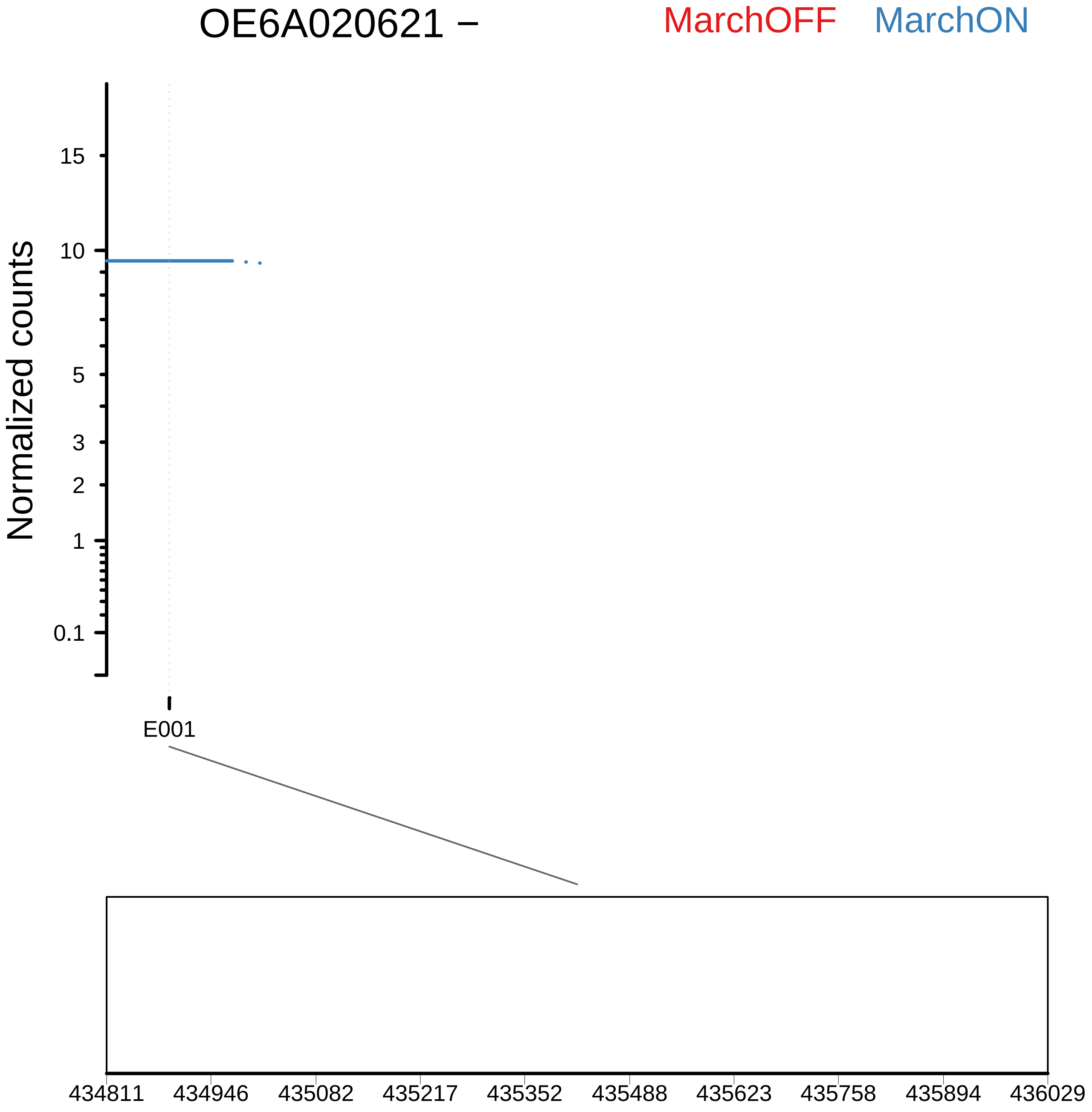
OE6A020621 −
MarchOFF
MarchON
Normalized counts
15
10
5
3
2
1
0.1
E001
434811
434946
435082
435217
435352
435488
435623
435758
435894
436029

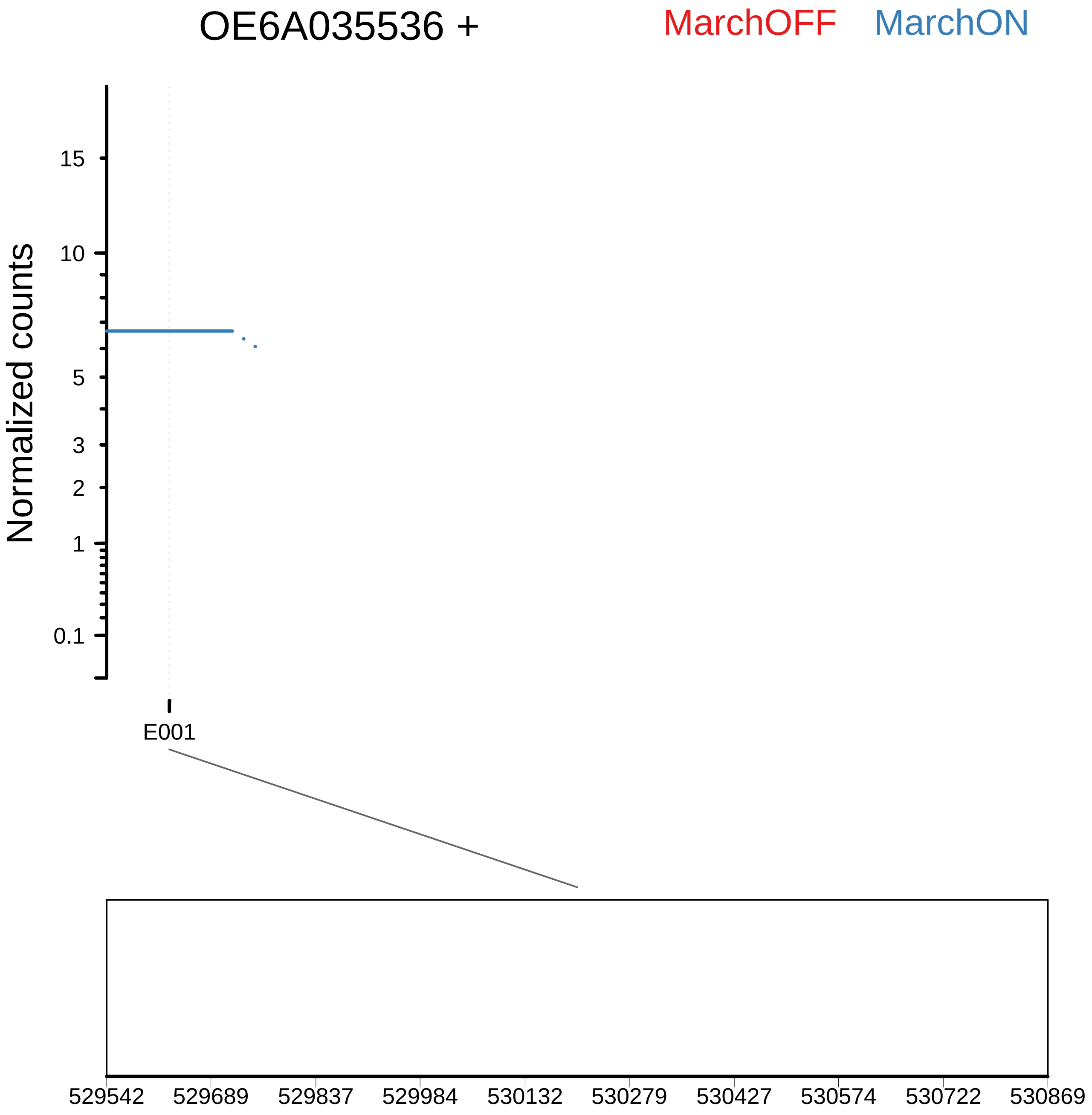
OE6A035536 +
MarchOFF
MarchON
Normalized counts
15
10
5
3
2
1
0.1
E001
529542
529689
529837
529984
530132
530279
530427
530574
530722
530869

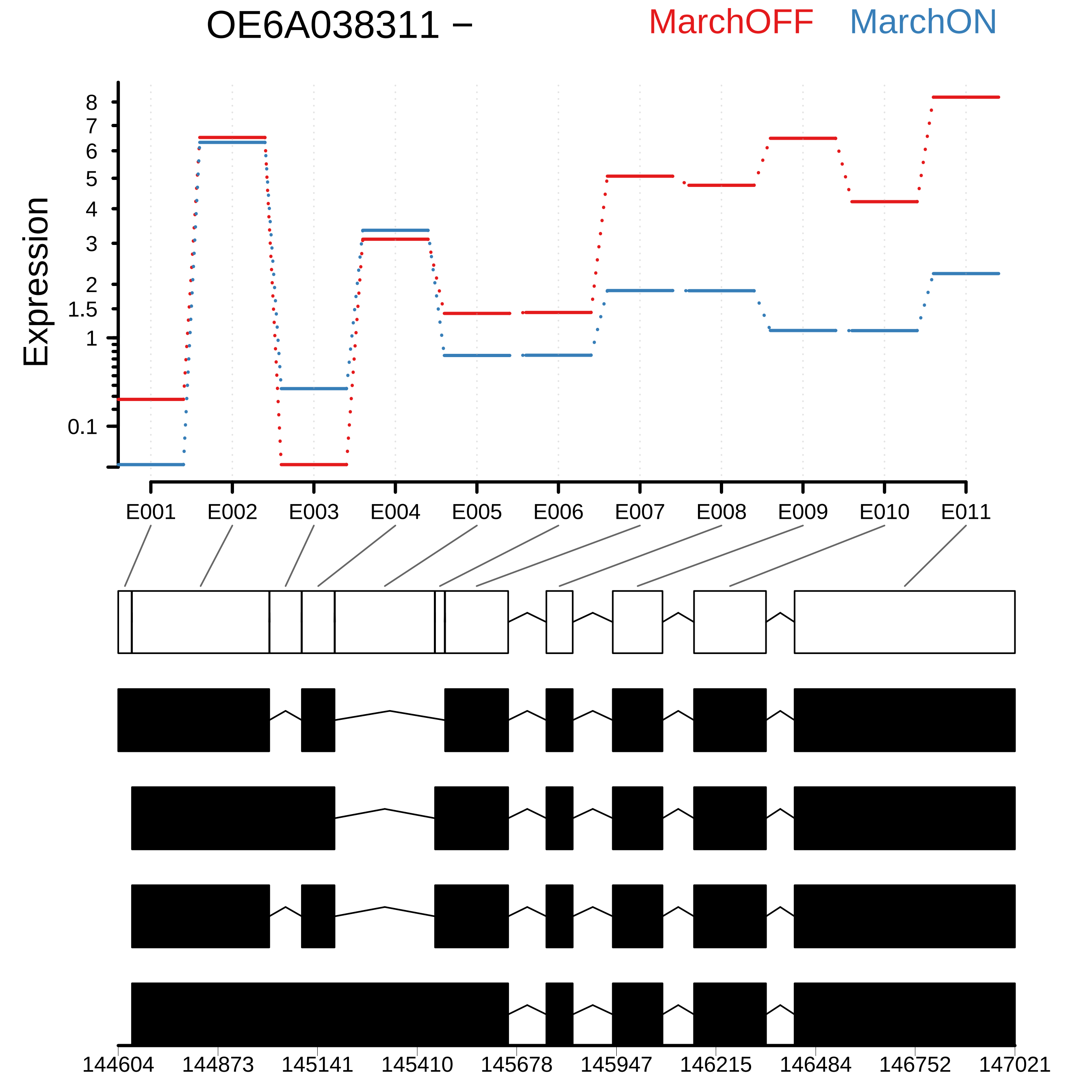

OE6A038311 −
MarchOFF
MarchON
Expression
8
7
6
5
4
3
2
1.5
1
0.1
E001
E002
E003
E004
E005
E006
E007
E008
E009
E010
E011
144604
144873
145141
145410
145678
145947
146215
146484
146752
147021

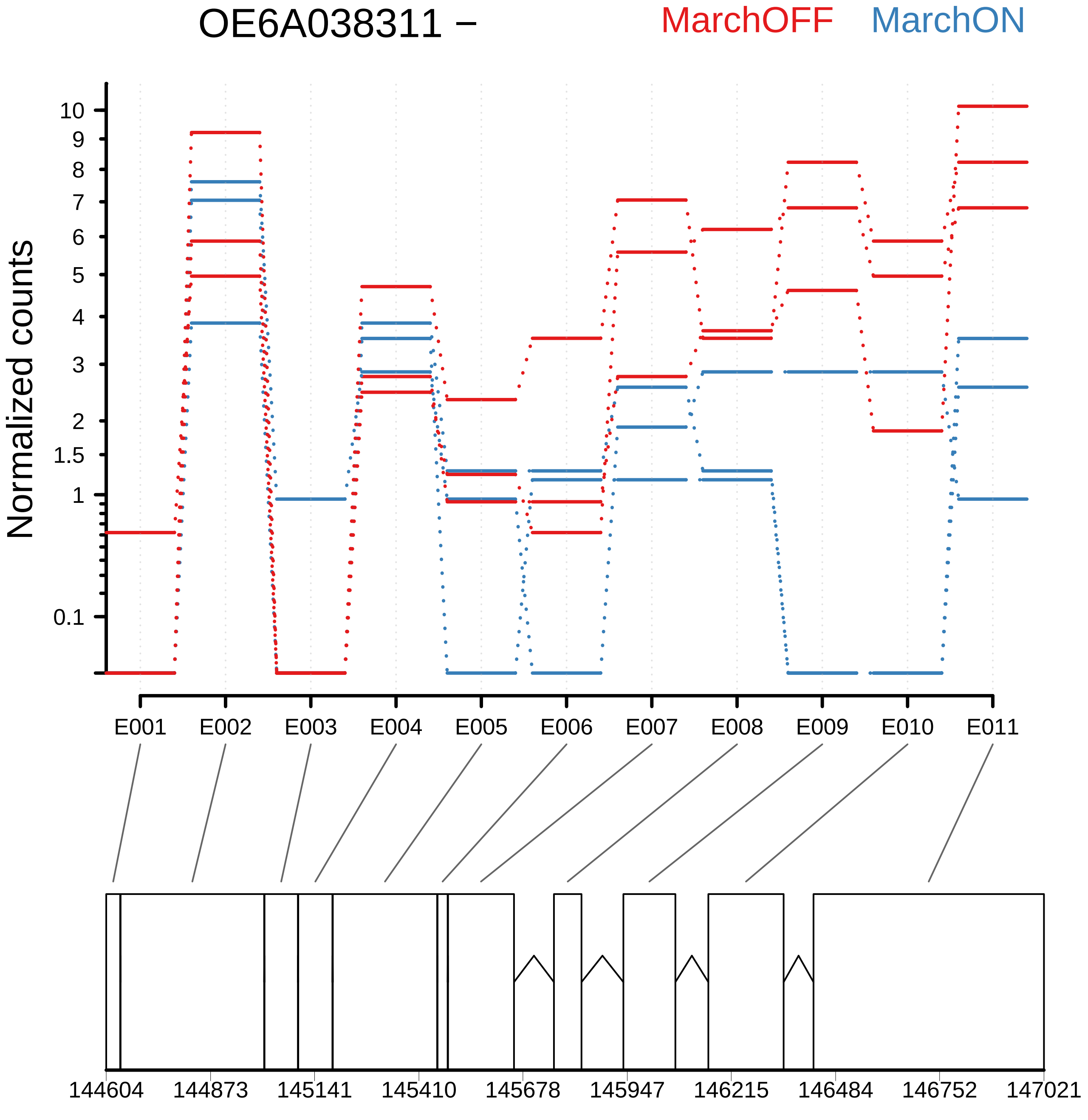

OE6A038311 −
MarchOFF
MarchON
Normalized counts
10
9
8
7
6
5
4
3
2
1.5
1
0.1
E001
E002
E003
E004
E005
E006
E007
E008
E009
E010
E011
144604
144873
145141
145410
145678
145947
146215
146484
146752
147021

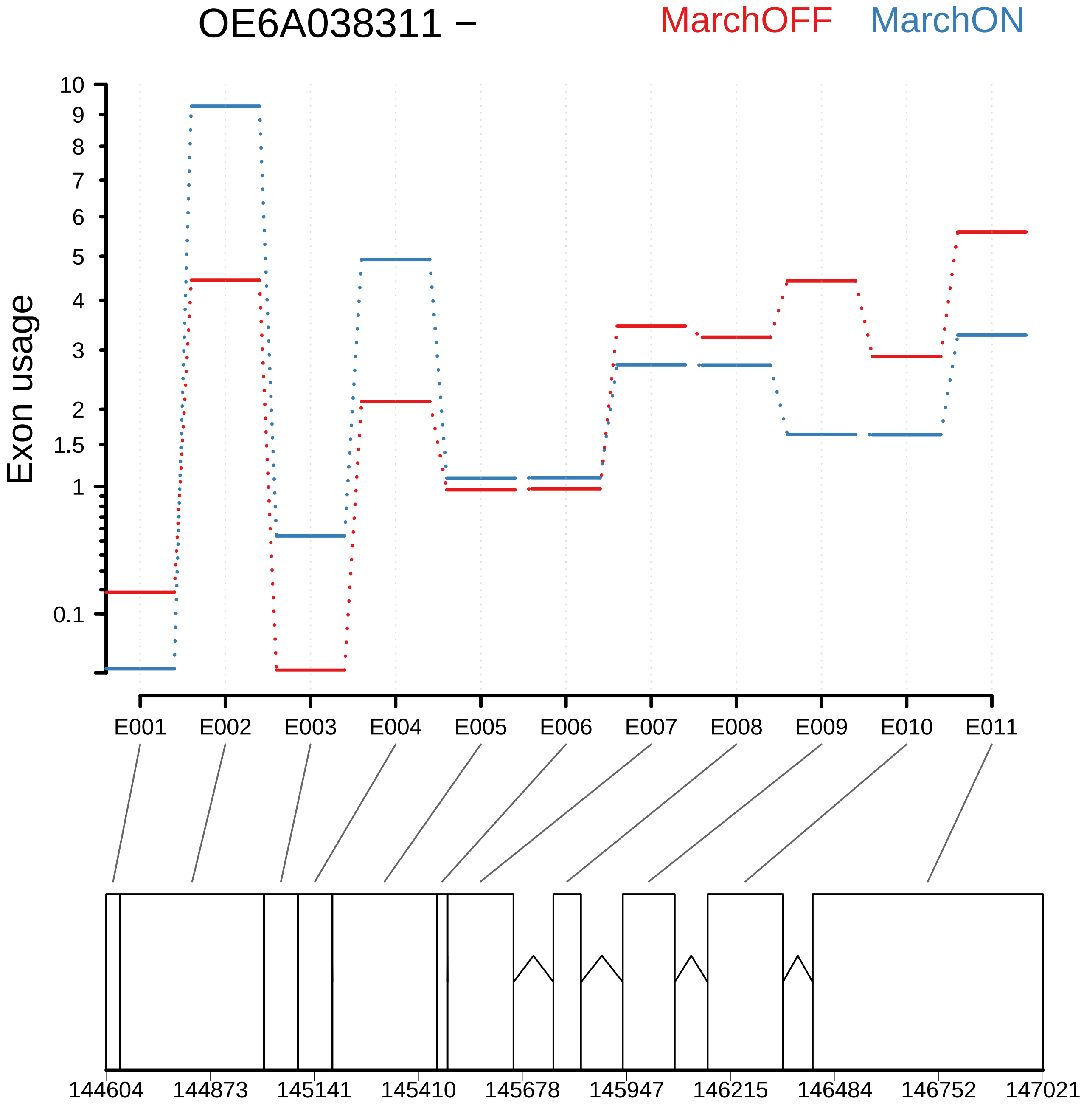
OE6A038311 −
MarchOFF
MarchON
Exon usage
10
9
8
7
6
5
4
3
2
1.5
1
0.1
E001
E002
E003
E004
E005
E006
E007
E008
E009
E010
E011
144604
144873
145141
145410
145678
145947
146215
146484
146752
147021

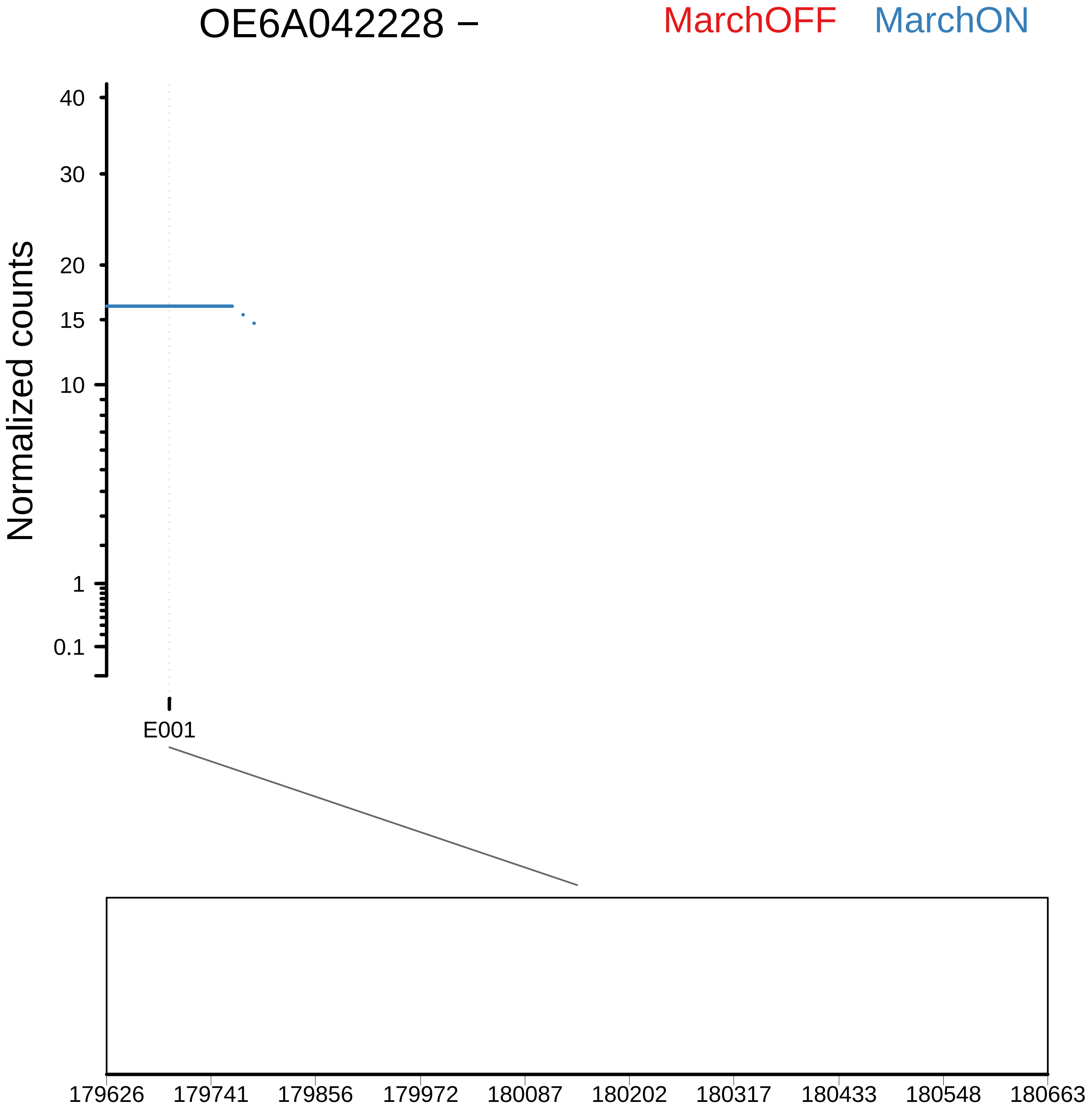

OE6A042228 −
MarchOFF
MarchON
Normalized counts
40
30
20
15
10
1
0.1
E001
179626
179741
179856
179972
180087
180202
180317
180433
180548
180663

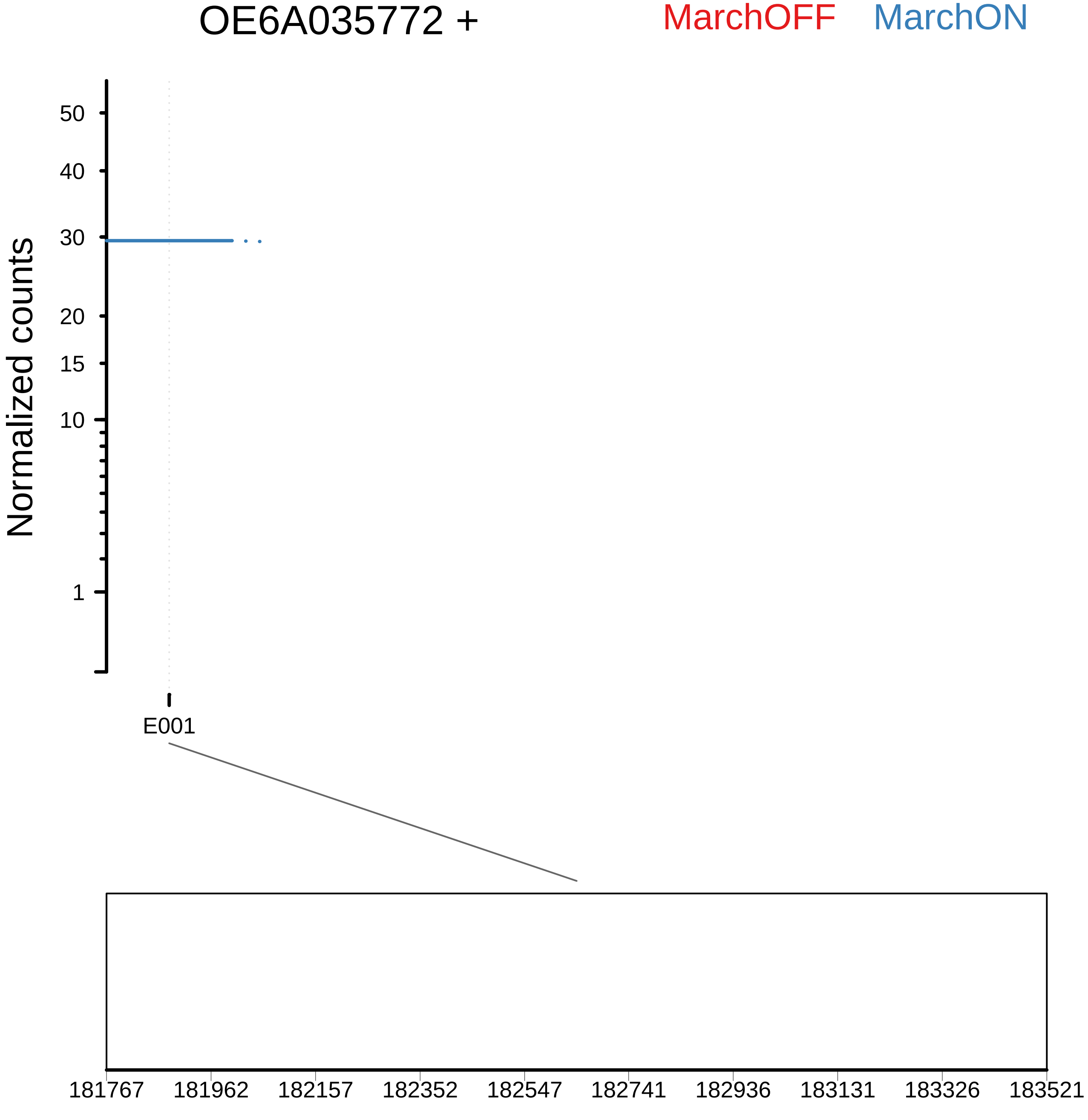

OE6A035772 +
MarchOFF
MarchON
Normalized counts
50
40
30
20
15
10
1
E001
181767
181962
182157
182352
182547
182741
182936
183131
183326
183521

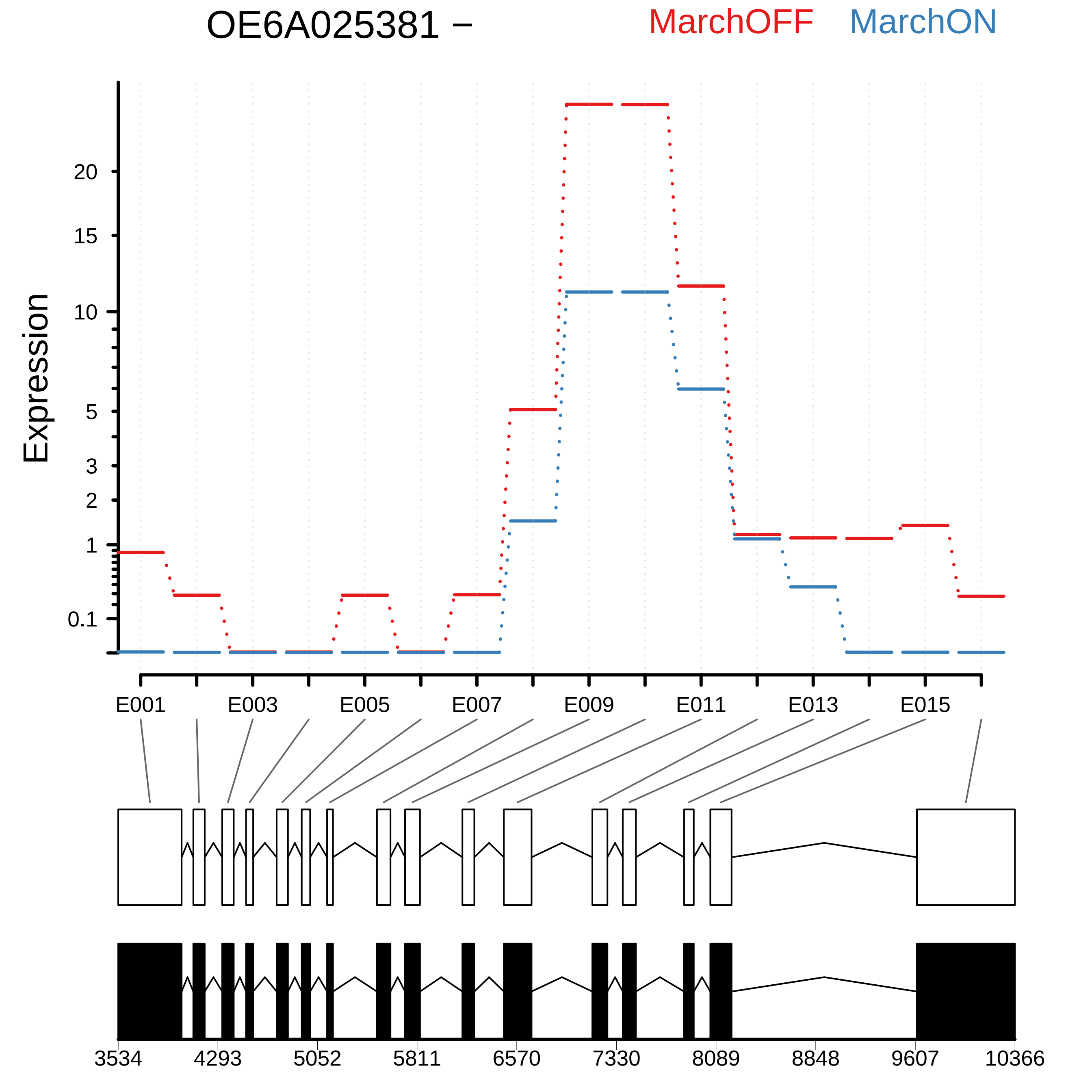

OE6A025381 −
MarchOFF
MarchON
Expression
20
15
10
5
3
2
1
0.1
E001
E003
E005
E007
E009
E011
E013
E015
3534
4293
5052
5811
6570
7330
8089
8848
9607
10366

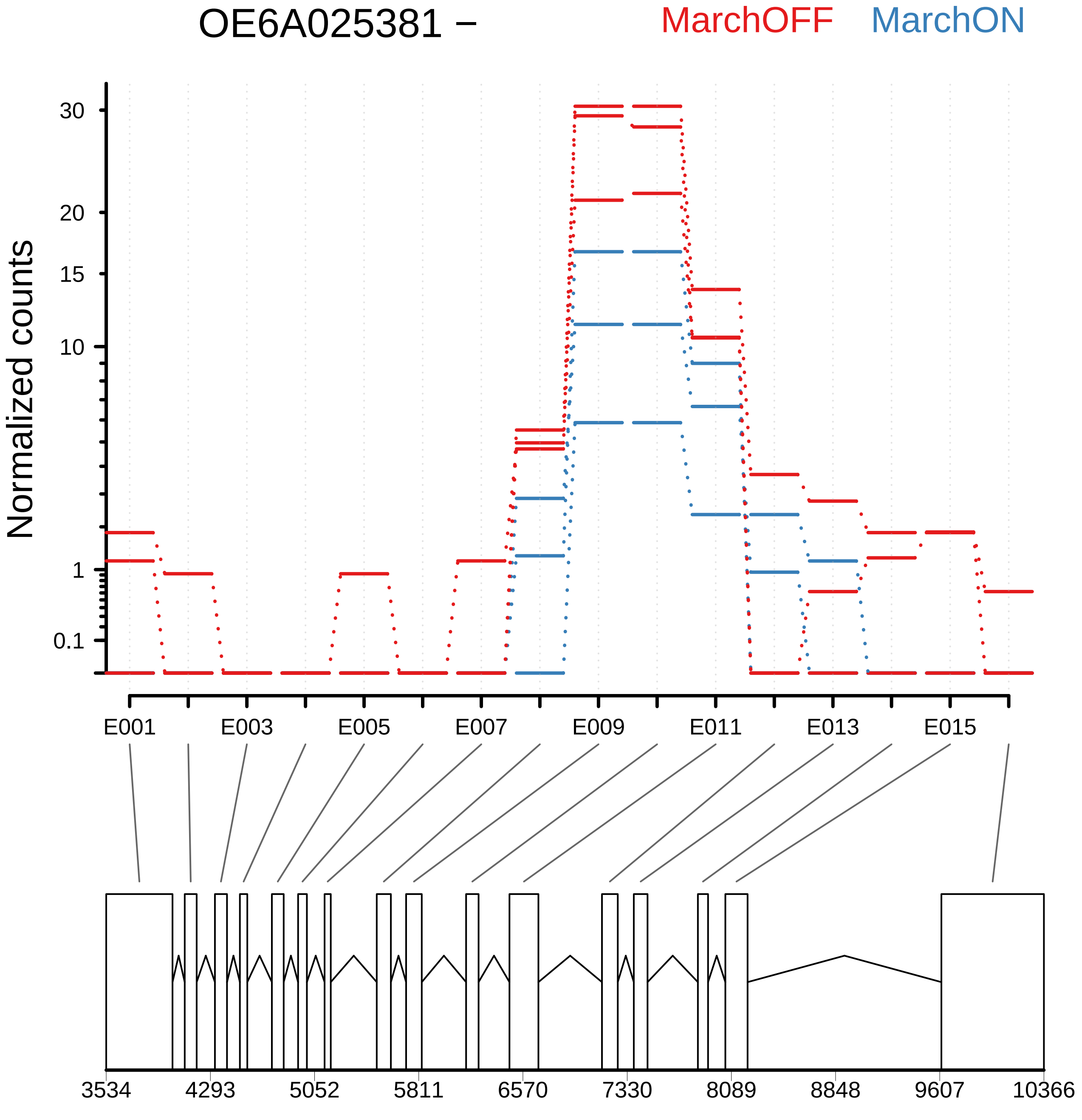
OE6A025381 −
MarchOFF
MarchON
Normalized counts
30
20
15
10
1
0.1
E001
E003
E005
E007
E009
E011
E013
E015
3534
4293
5052
5811
6570
7330
8089
8848
9607
10366

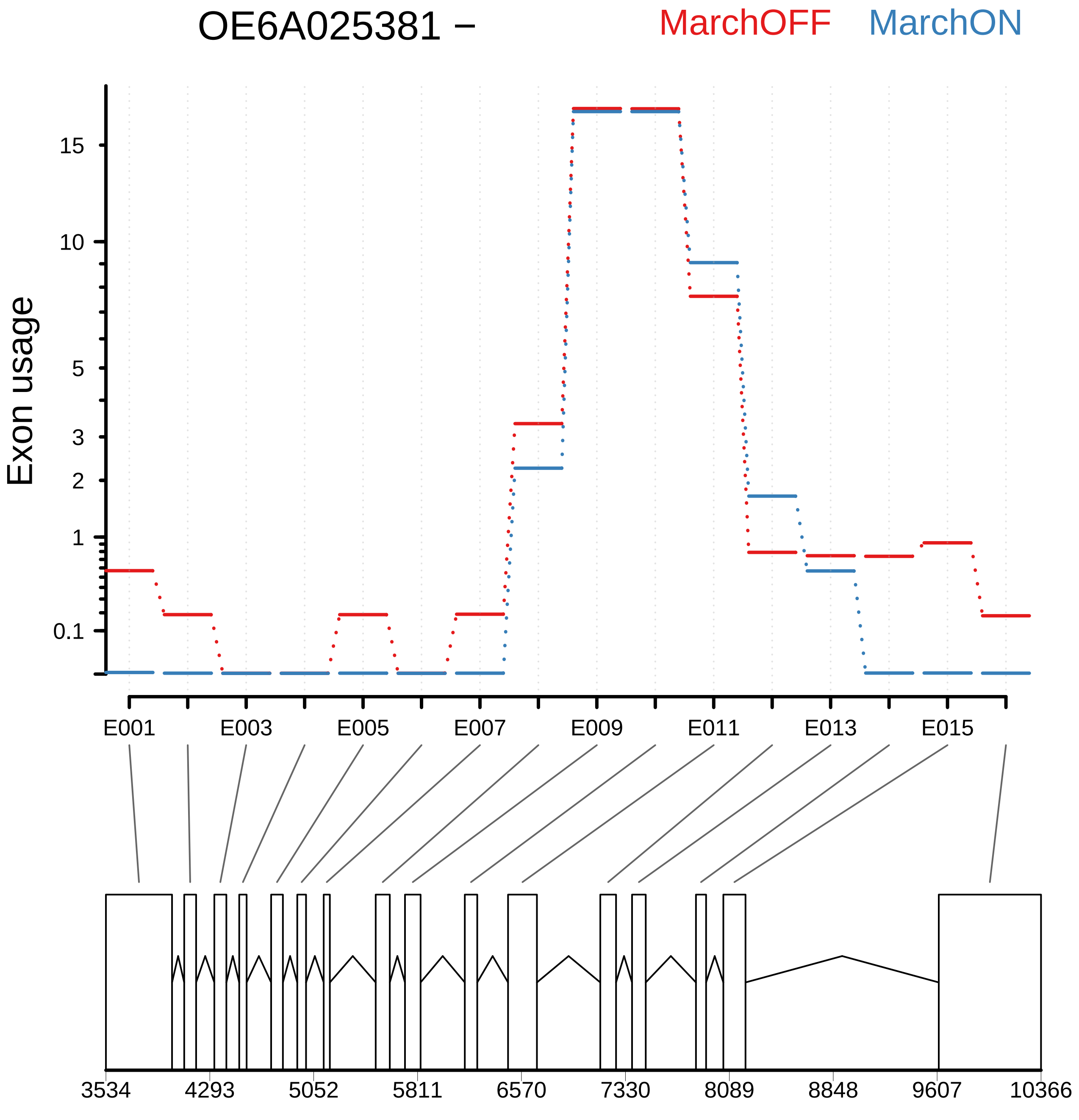

OE6A025381 −
MarchOFF
MarchON
Exon usage
15
10
5
3
2
1
0.1
E001
E003
E005
E007
E009
E011
E013
E015
3534
4293
5052
5811
6570
7330
8089
8848
9607
10366

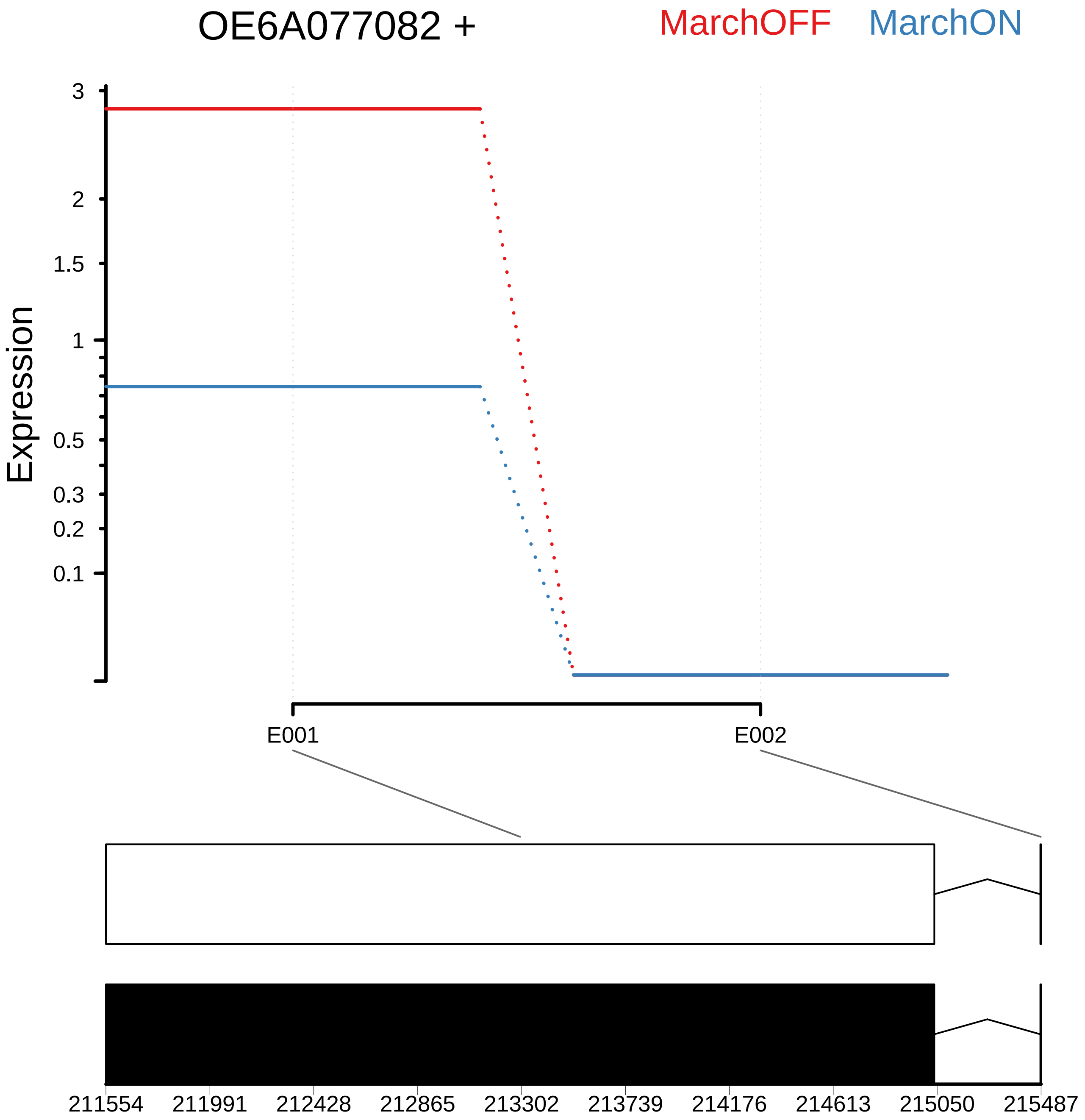
OE6A077082 +
MarchOFF
MarchON
Expression
3
2
1.5
1
0.5
0.3
0.2
0.1
E001
E002
211554
211991
212428
212865
213302
213739
214176
214613
215050
215487

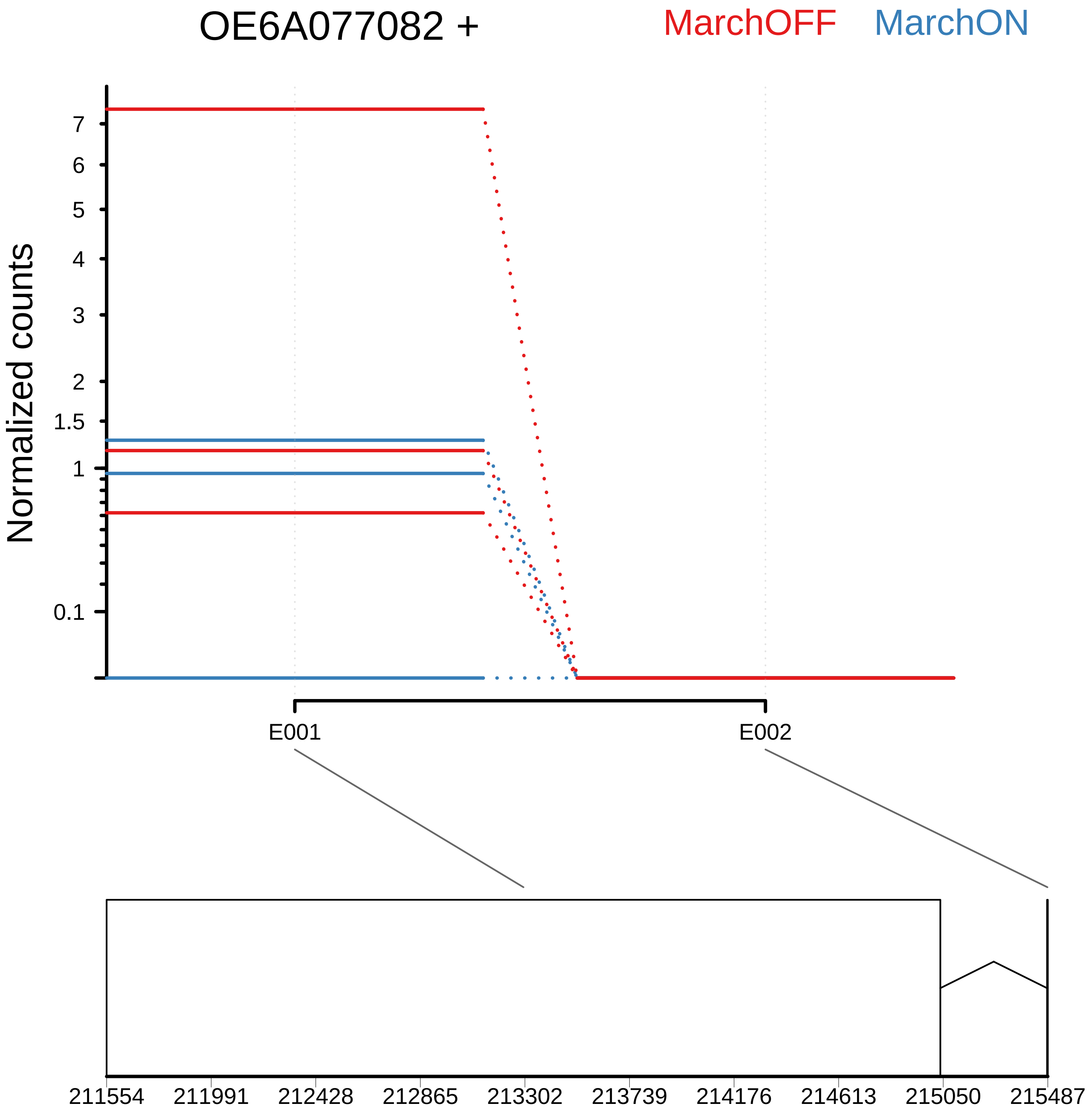

OE6A077082 +
MarchOFF
MarchON
Normalized counts
7
6
5
4
3
2
1.5
1
0.1
E001
E002
211554
211991
212428
212865
213302
213739
214176
214613
215050
215487

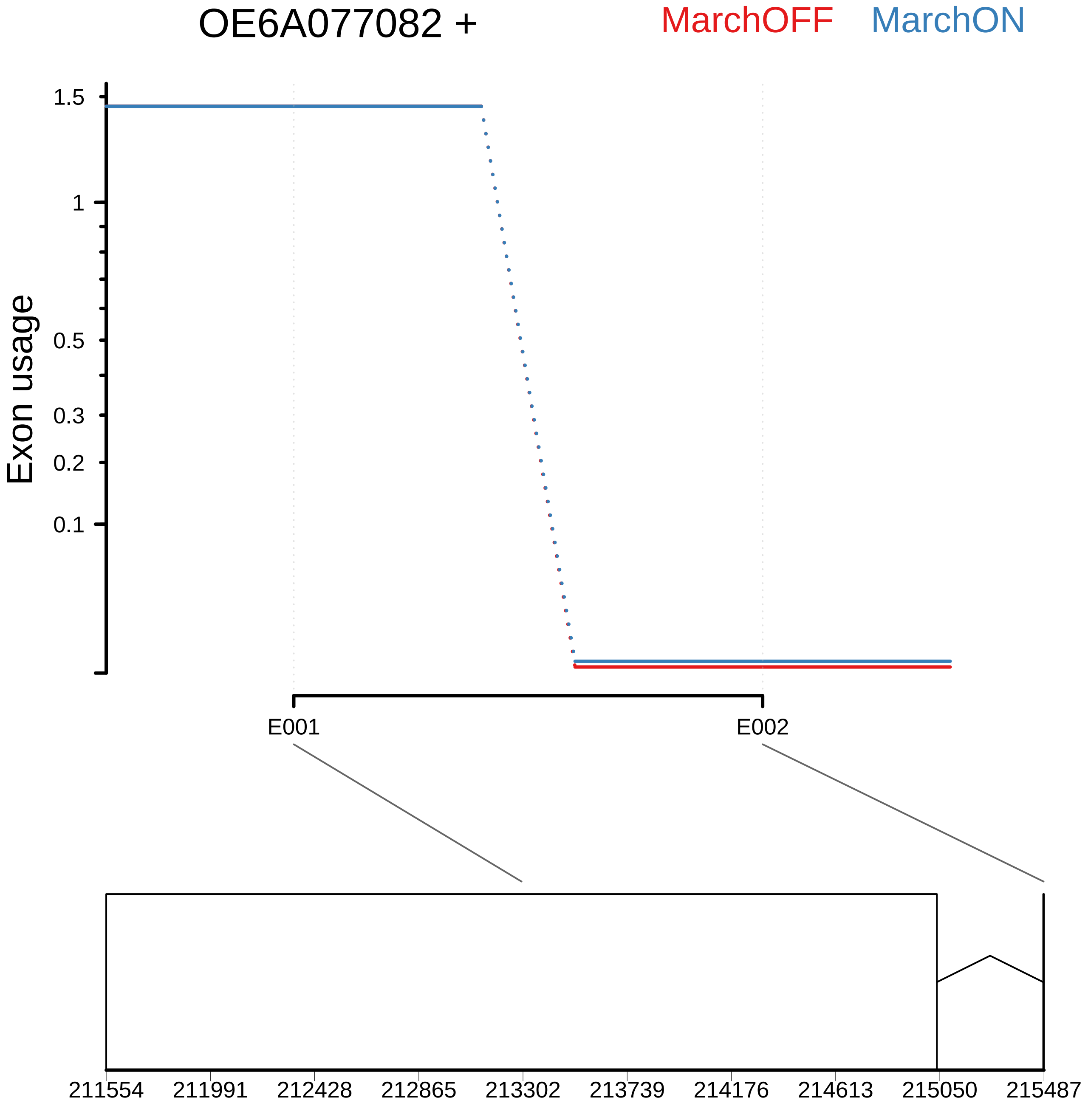
OE6A077082 +
MarchOFF
MarchON
Exon usage
1.5
1
0.5
0.3
0.2
0.1
E001
E002
211554
211991
212428
212865
213302
213739
214176
214613
215050
215487

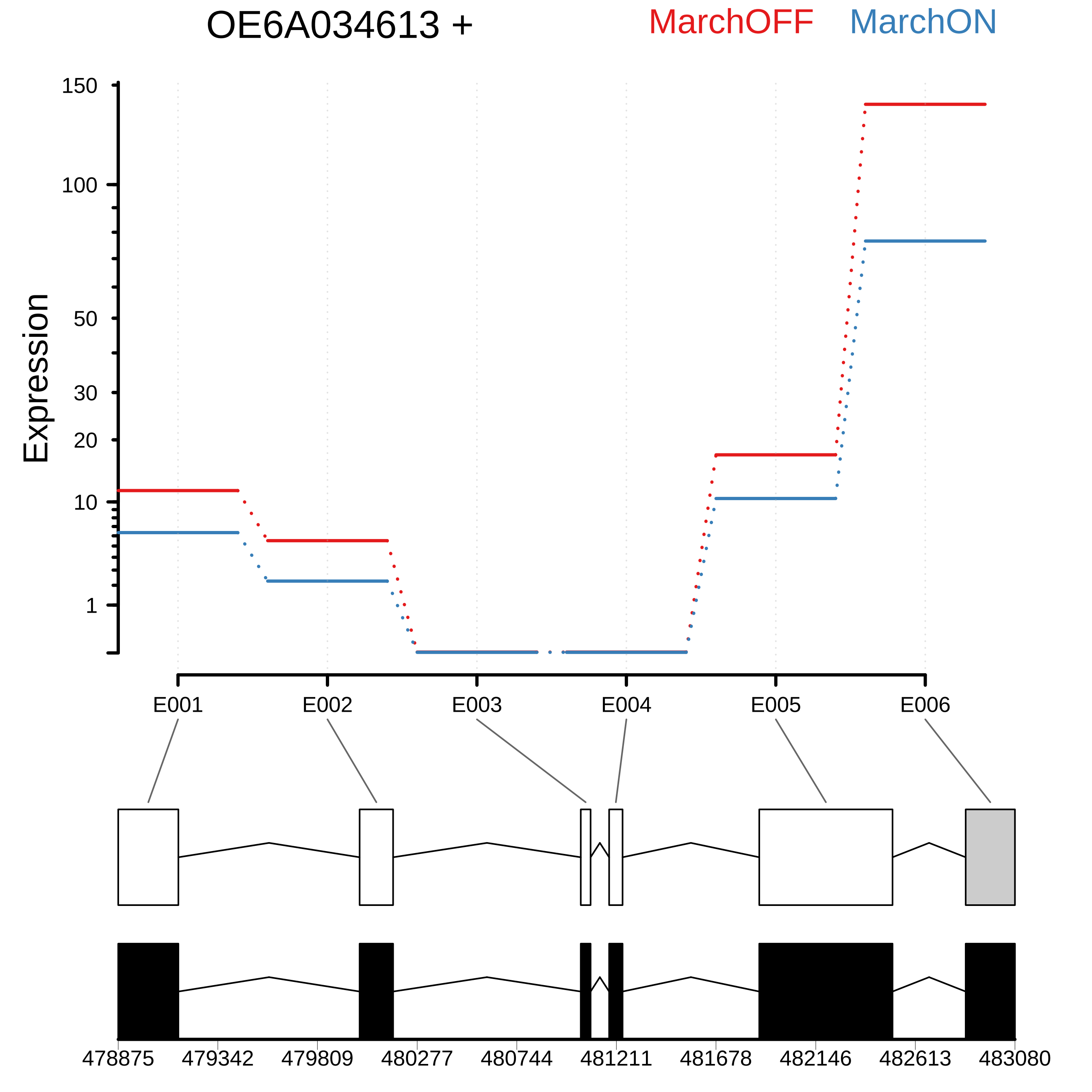

OE6A034613 +
MarchOFF
MarchON
Expression
150
100
50
30
20
10
1
E001
E002
E003
E004
E005
E006
478875
479342
479809
480277
480744
481211
481678
482146
482613
483080

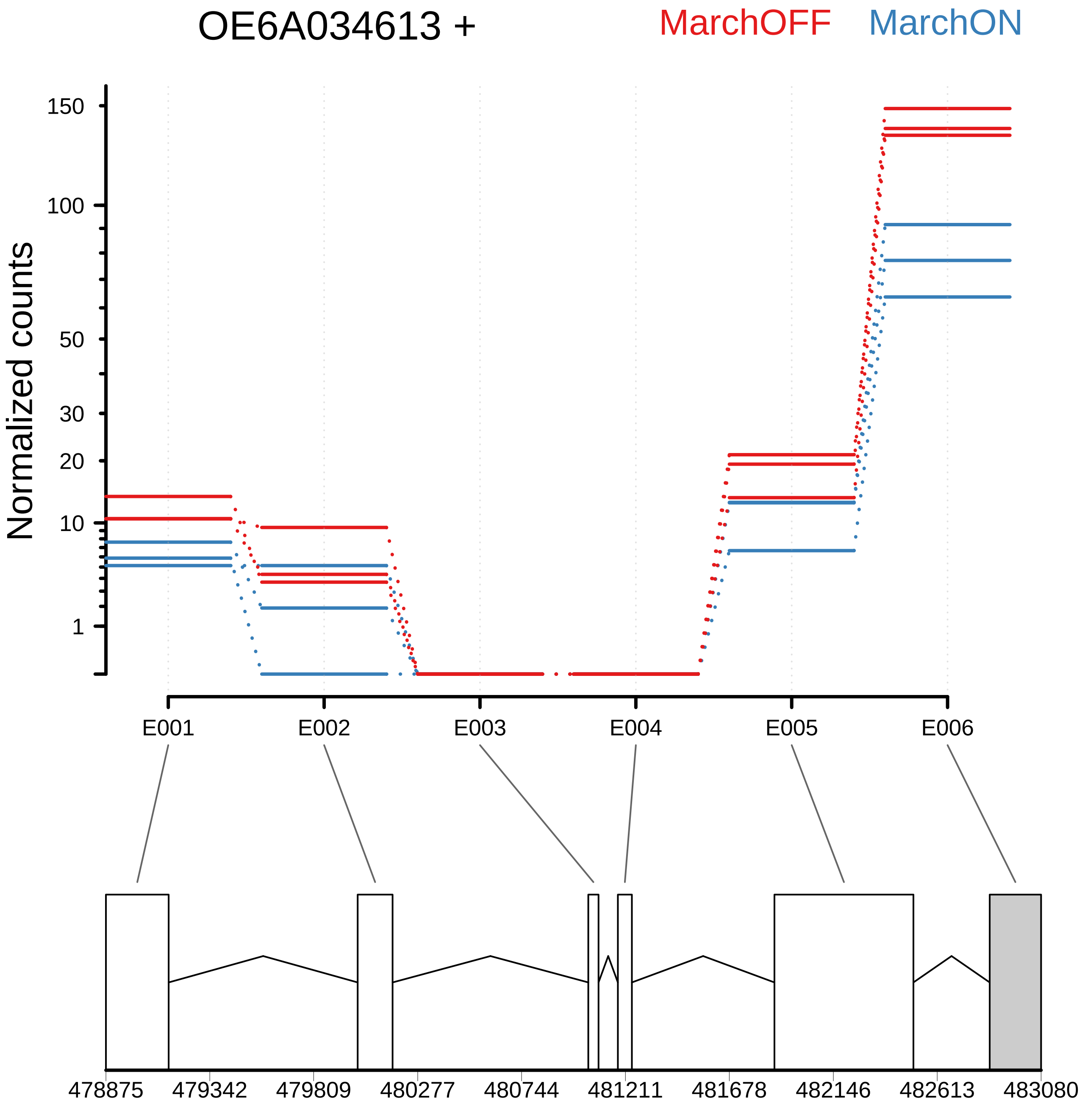
OE6A034613 +
MarchOFF
MarchON
Normalized counts
150
100
50
30
20
10
1
E001
E002
E003
E004
E005
E006
478875
479342
479809
480277
480744
481211
481678
482146
482613
483080

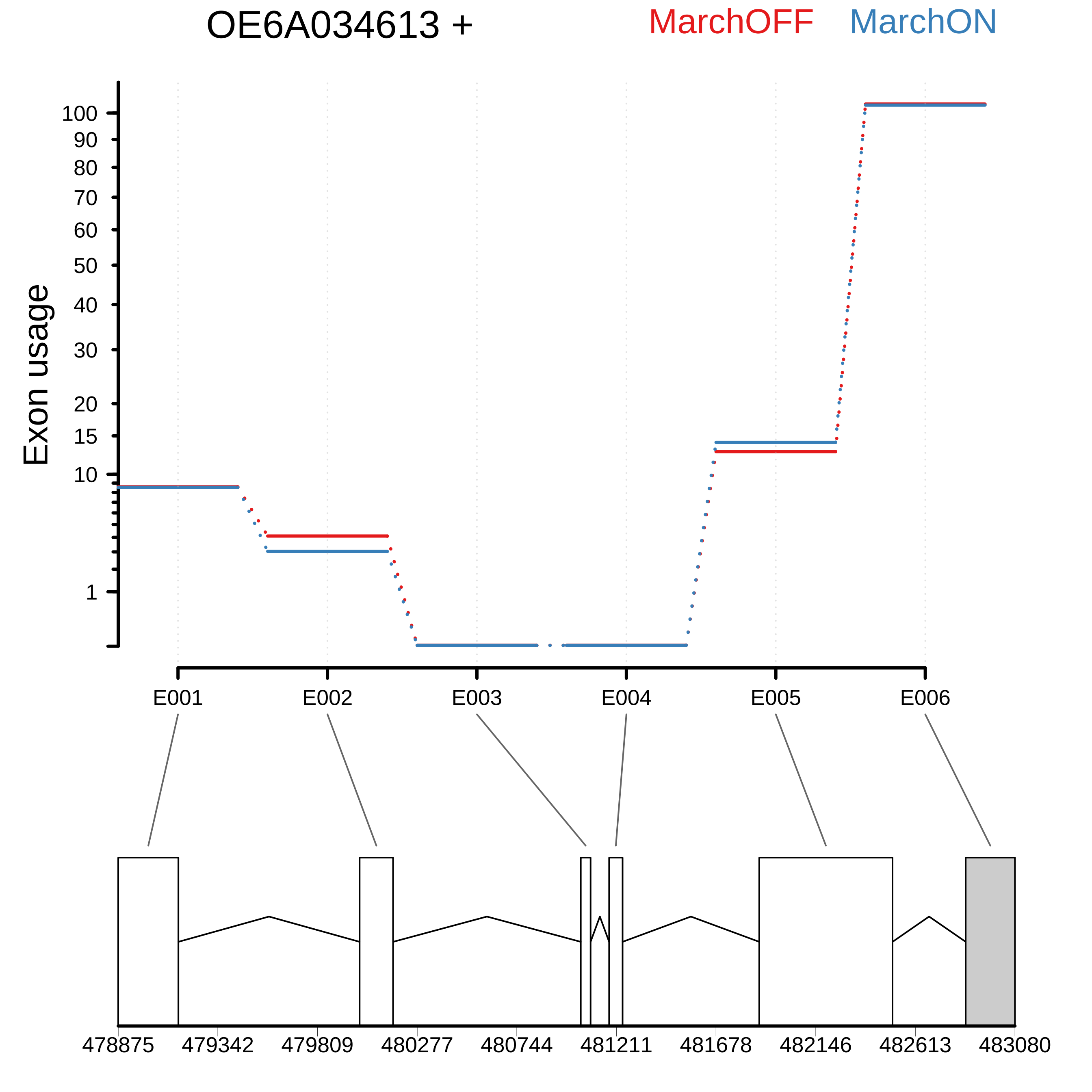

OE6A034613 +
MarchOFF
MarchON
Exon usage
100
90
80
70
60
50
40
30
20
15
10
1
E001
E002
E003
E004
E005
E006
478875
479342
479809
480277
480744
481211
481678
482146
482613
483080

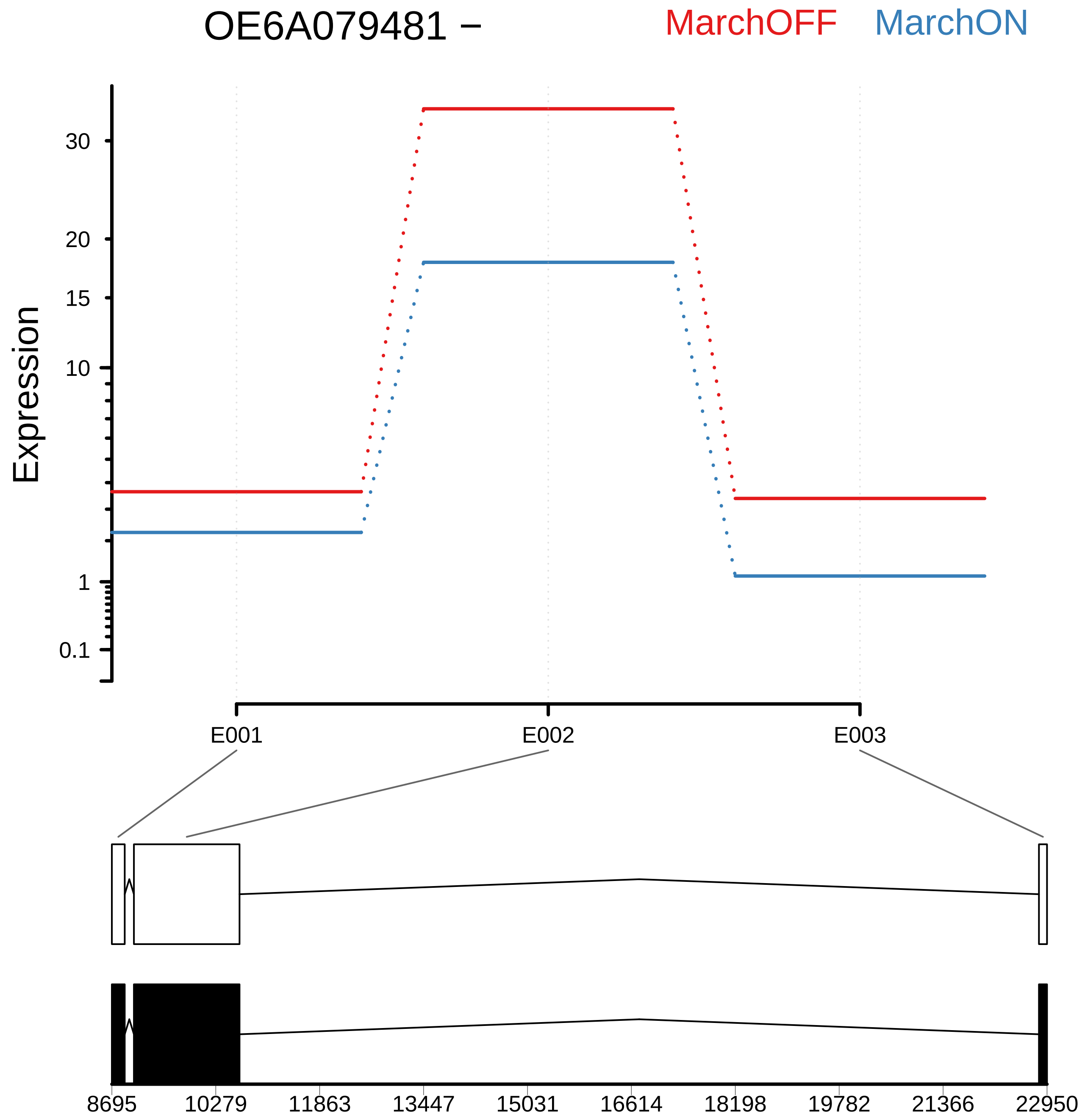

OE6A079481 −
MarchOFF
MarchON
Expression
30
20
15
10
1
0.1
E001
E002
E003
8695
10279
11863
13447
15031
16614
18198
19782
21366
22950

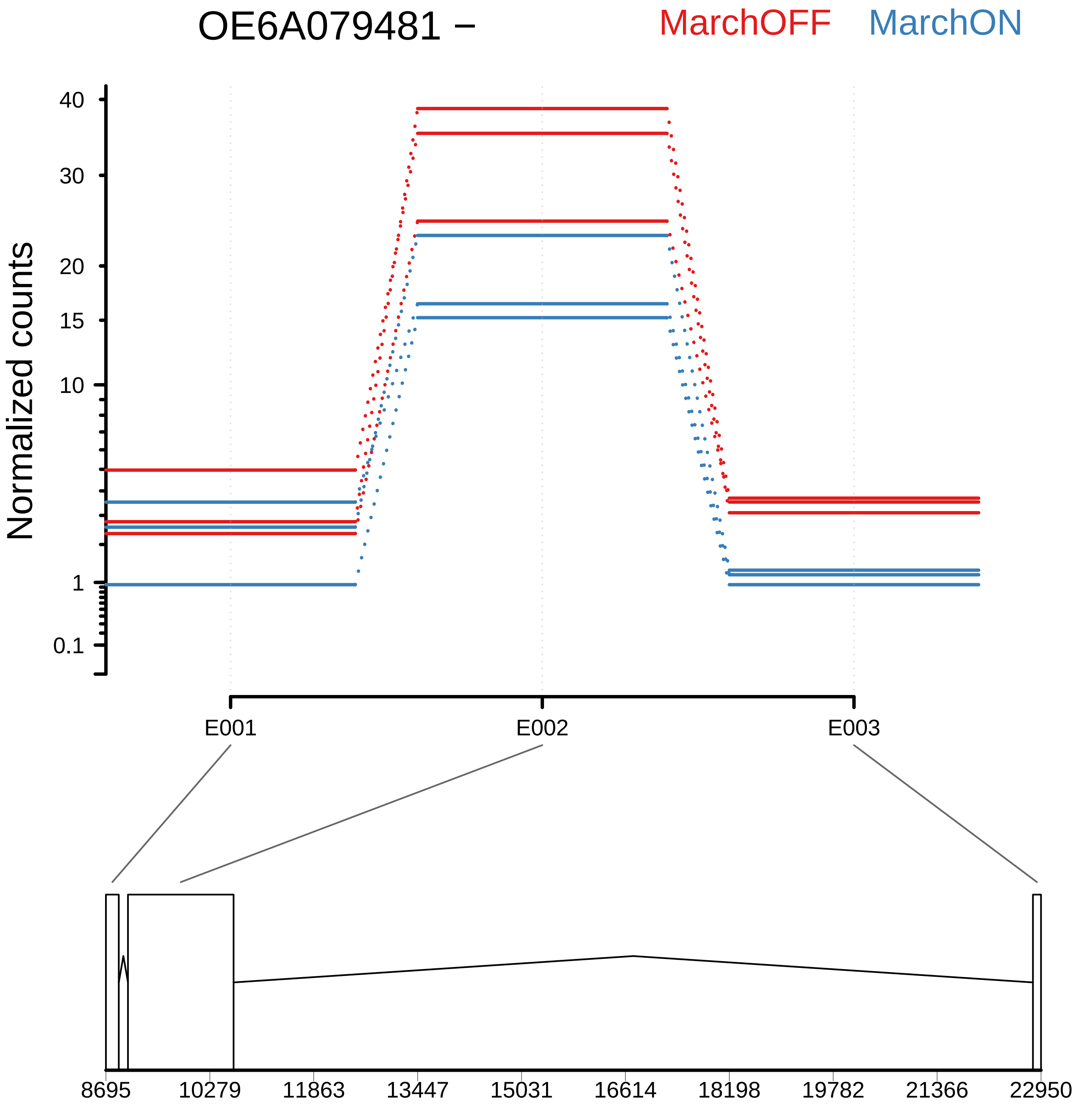
OE6A079481 −
MarchOFF
MarchON
Normalized counts
40
30
20
15
10
1
0.1
E001
E002
E003
8695
10279
11863
13447
15031
16614
18198
19782
21366
22950

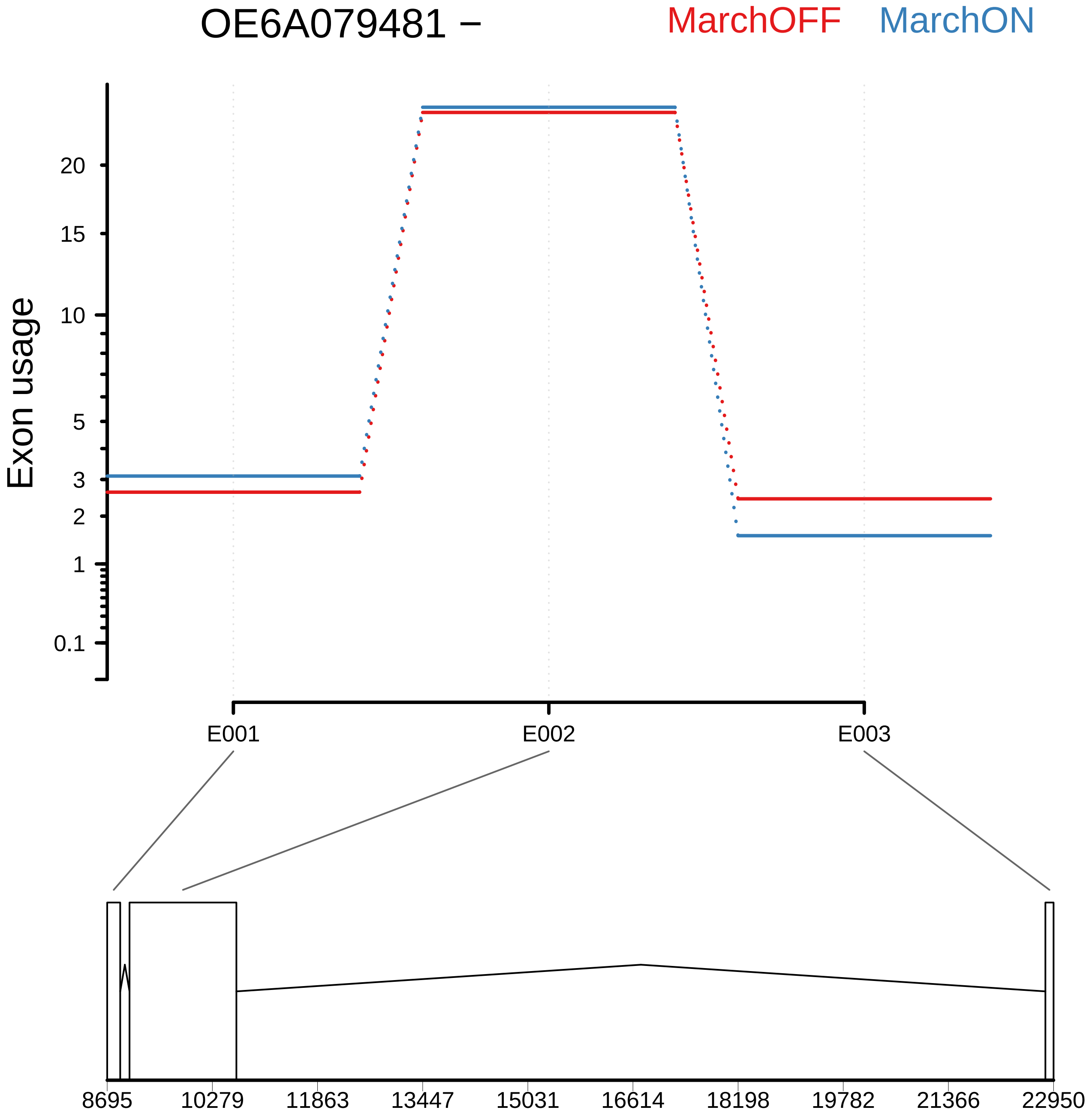
OE6A079481 −
MarchOFF
MarchON
Exon usage
20
15
10
5
3
2
1
0.1
E001
E002
E003
8695
10279
11863
13447
15031
16614
18198
19782
21366
22950

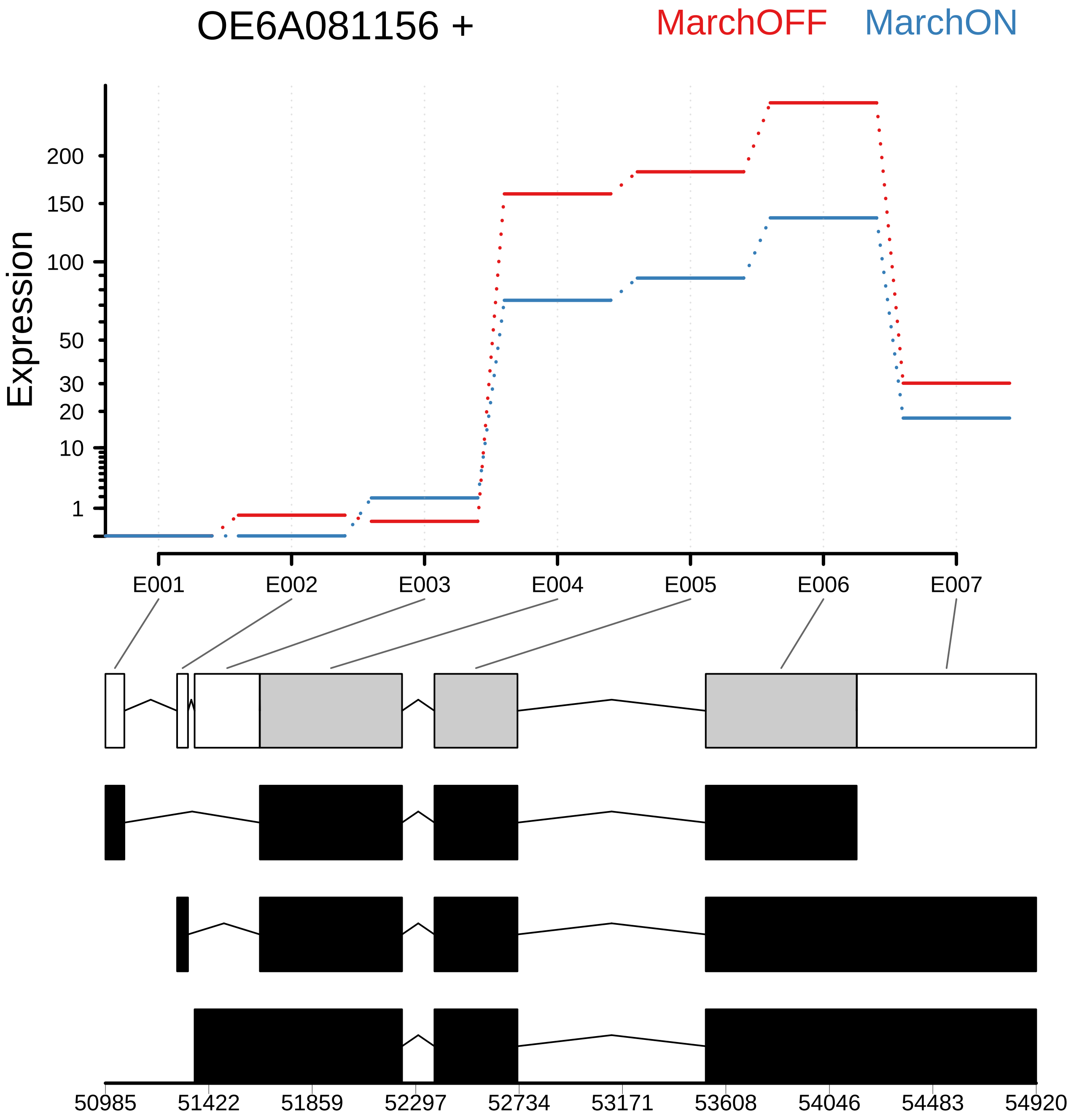

OE6A081156 +
MarchOFF
MarchON
Expression
200
150
100
50
30
20
10
1
E001
E002
E003
E004
E005
E006
E007
50985
51422
51859
52297
52734
53171
53608
54046
54483
54920

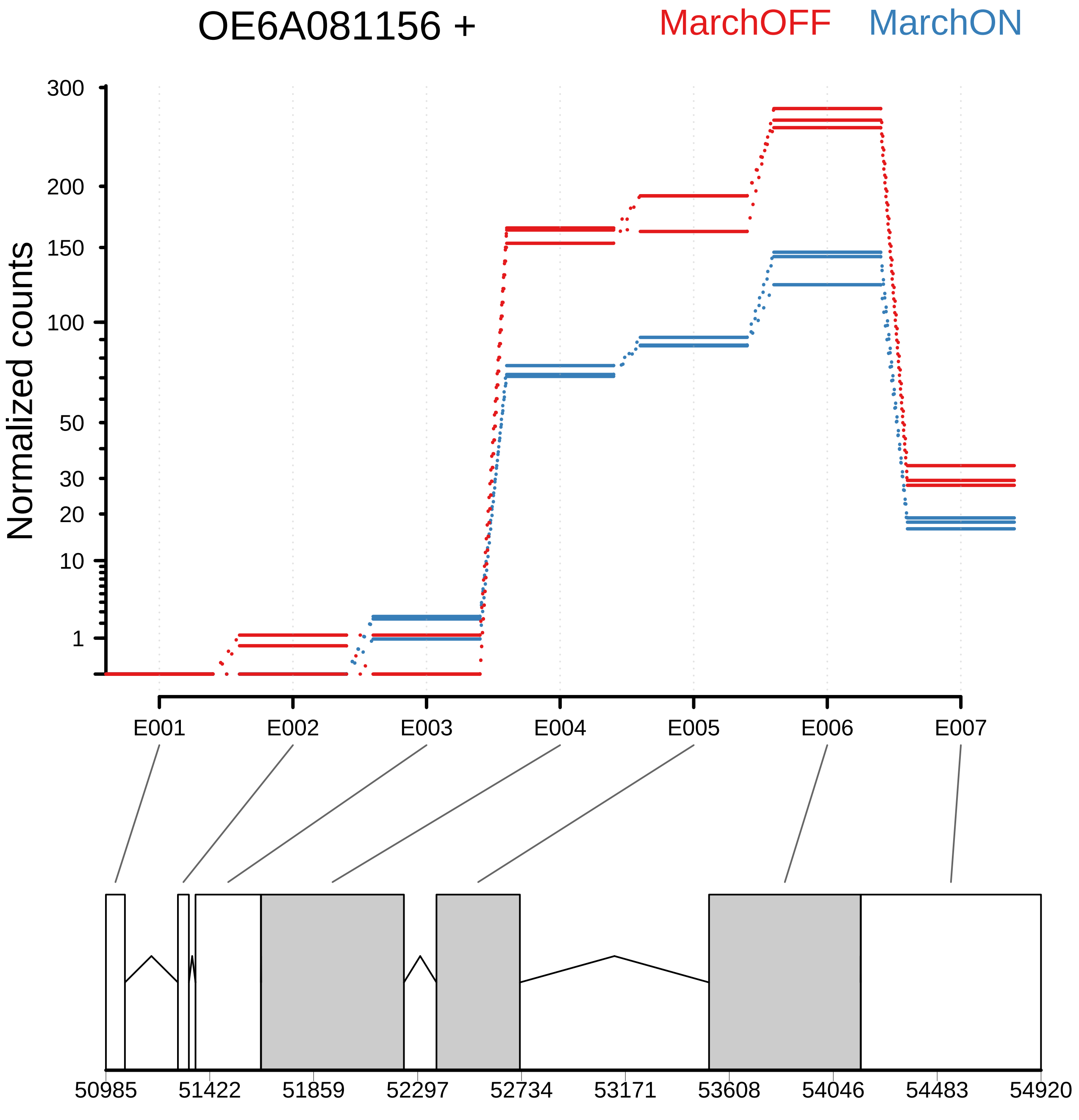
OE6A081156 +
MarchOFF
MarchON
Normalized counts
300
200
150
100
50
30
20
10
1
E001
E002
E003
E004
E005
E006
E007
50985
51422
51859
52297
52734
53171
53608
54046
54483
54920

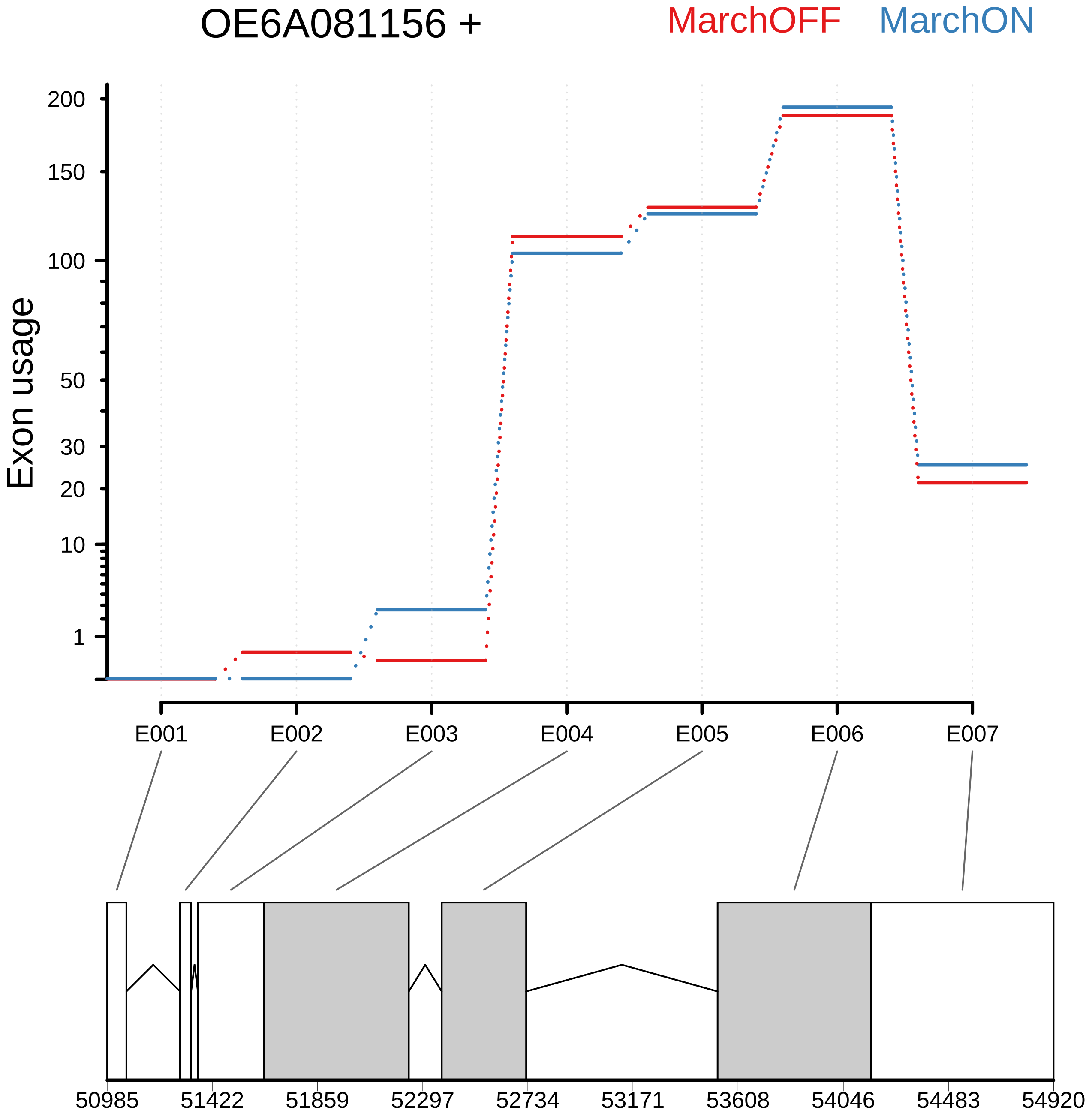

OE6A081156 +
MarchOFF
MarchON
Exon usage
200
150
100
50
30
20
10
1
E001
E002
E003
E004
E005
E006
E007
50985
51422
51859
52297
52734
53171
53608
54046
54483
54920

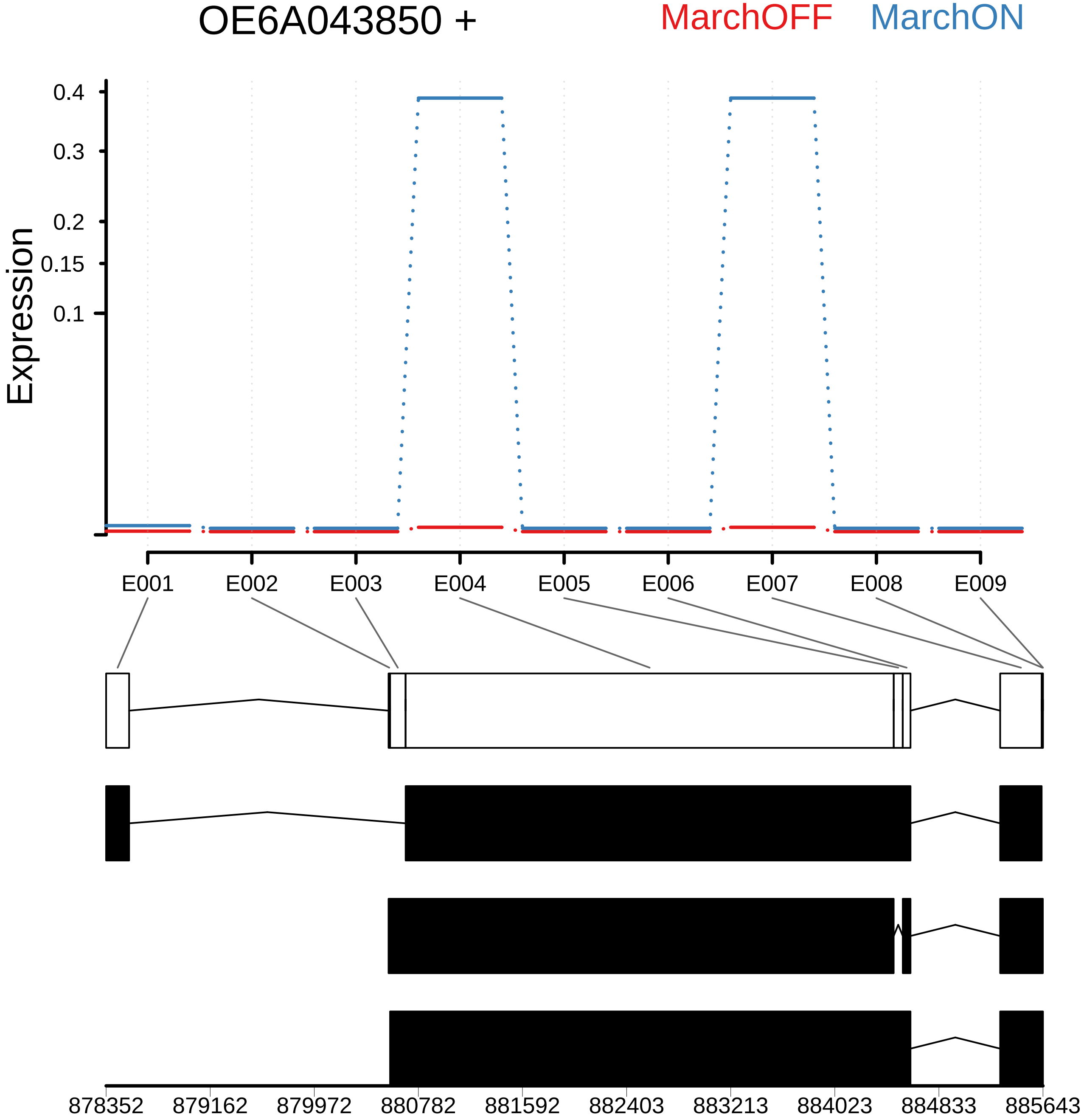

OE6A043850 +
MarchOFF
MarchON
Expression
0.4
0.3
0.2
0.15
0.1
E001
E002
E003
E004
E005
E006
E007
E008
E009
878352
879162
879972
880782
881592
882403
883213
884023
884833
885643

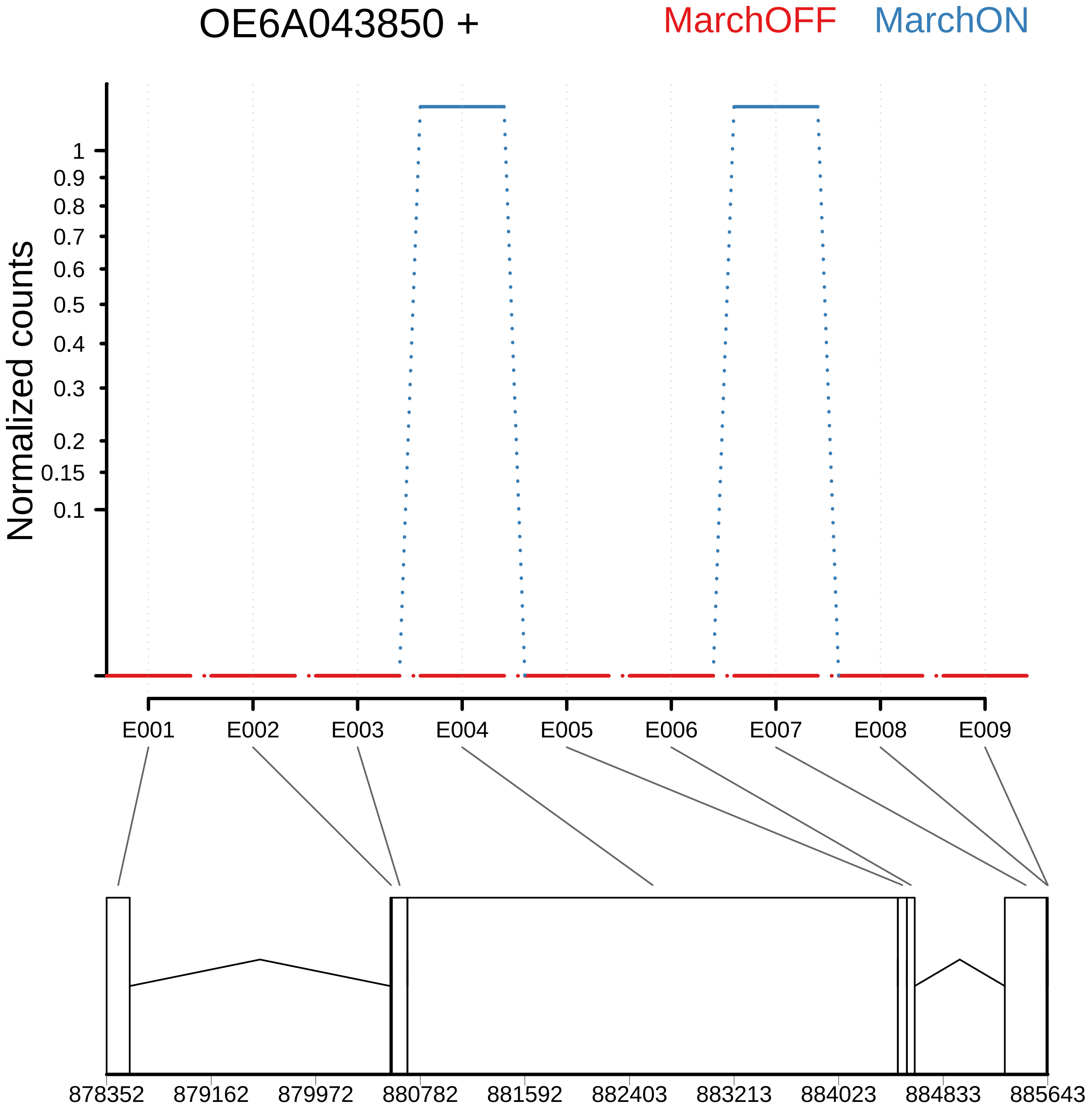

OE6A043850 +
MarchOFF
MarchON
Normalized counts
1
0.9
0.8
0.7
0.6
0.5
0.4
0.3
0.2
0.15
0.1
E001
E002
E003
E004
E005
E006
E007
E008
E009
878352
879162
879972
880782
881592
882403
883213
884023
884833
885643

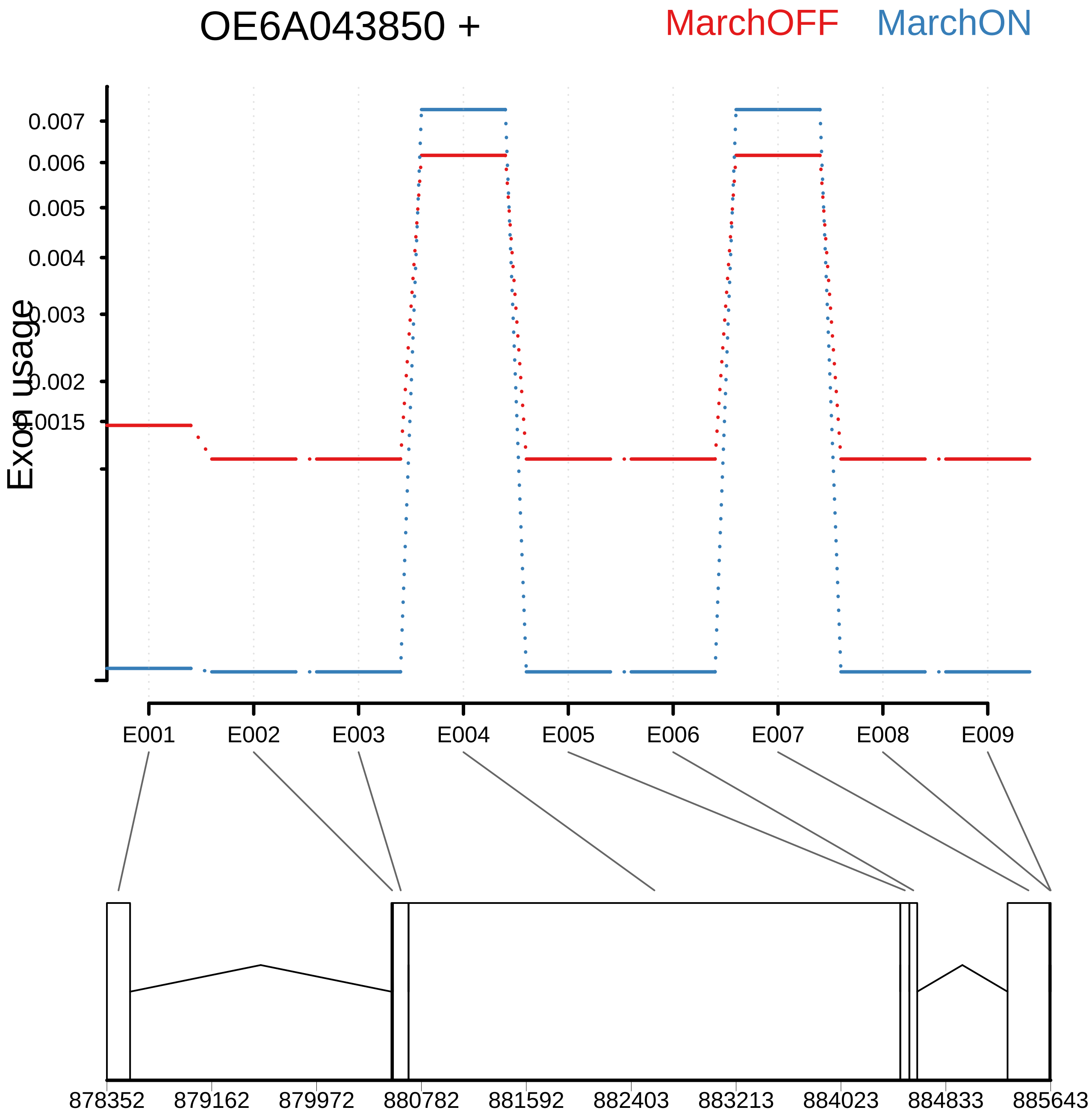
OE6A043850 +
MarchOFF
MarchON
Exon usage
0.007
0.006
0.005
0.004
0.003
0.002
0.0015
E001
E002
E003
E004
E005
E006
E007
E008
E009
878352
879162
879972
880782
881592
882403
883213
884023
884833
885643

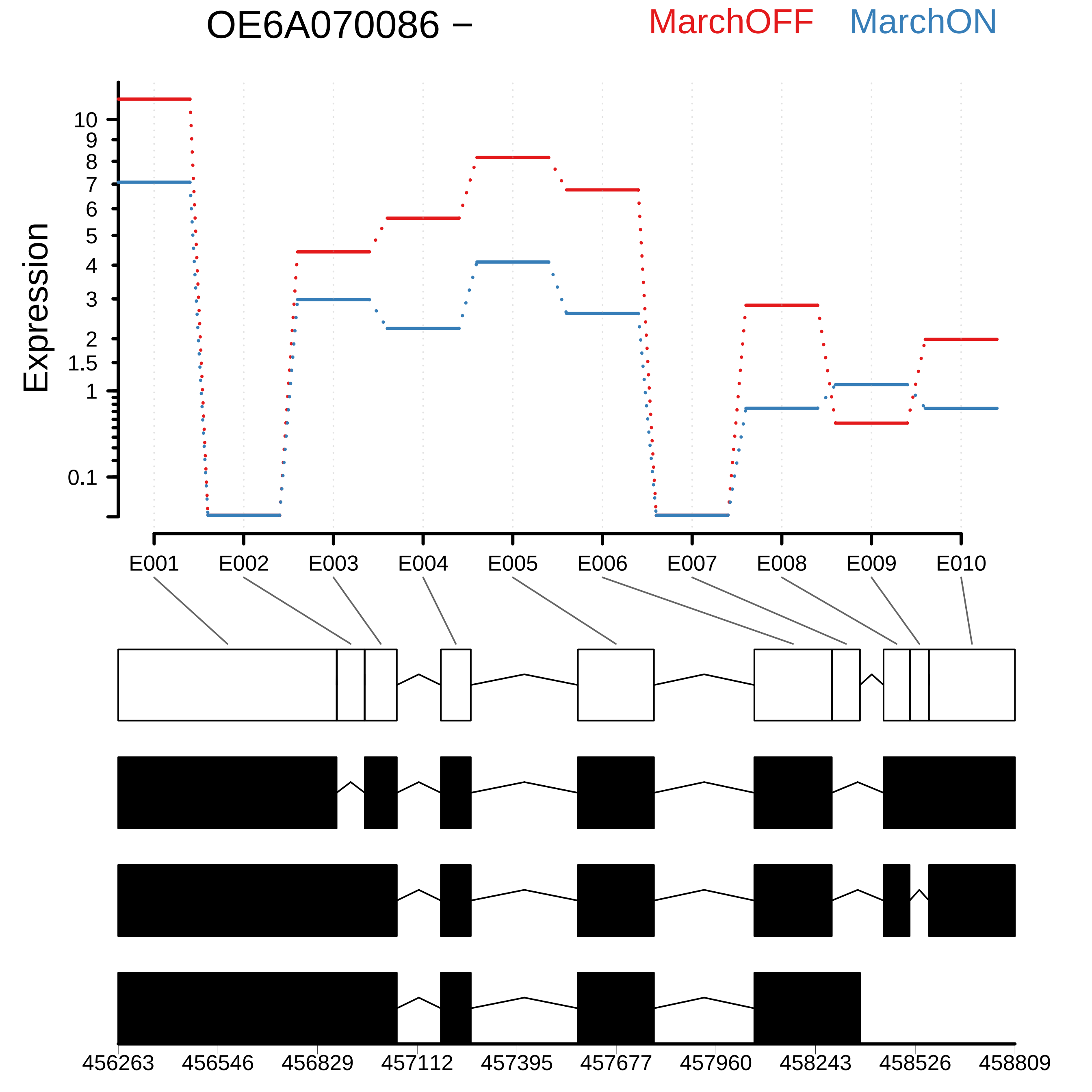

OE6A070086 –
MarchOFF
MarchON
Expression
10
9
8
7
6
5
4
3
2
1.5
1
0.1
E001
E002
E003
E004
E005
E006
E007
E008
E009
E010
456263
456546
456829
457112
457395
457677
457960
458243
458526
458809

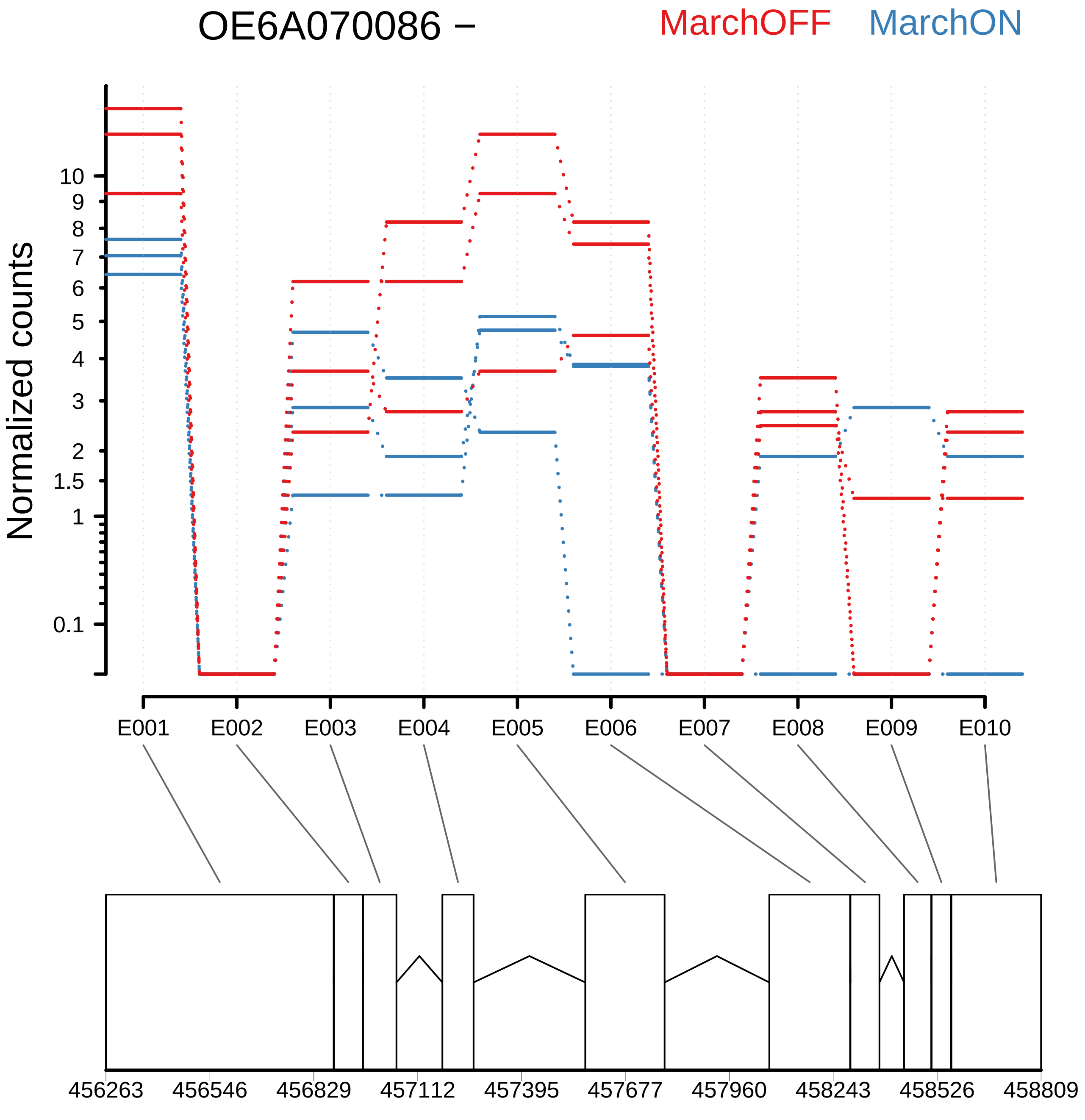

OE6A070086 −
MarchOFF
MarchON
Normalized counts
10
9
8
7
6
5
4
3
2
1.5
1
0.1
E001
E002
E003
E004
E005
E006
E007
E008
E009
E010
456263
456546
456829
457112
457395
457677
457960
458243
458526
458809

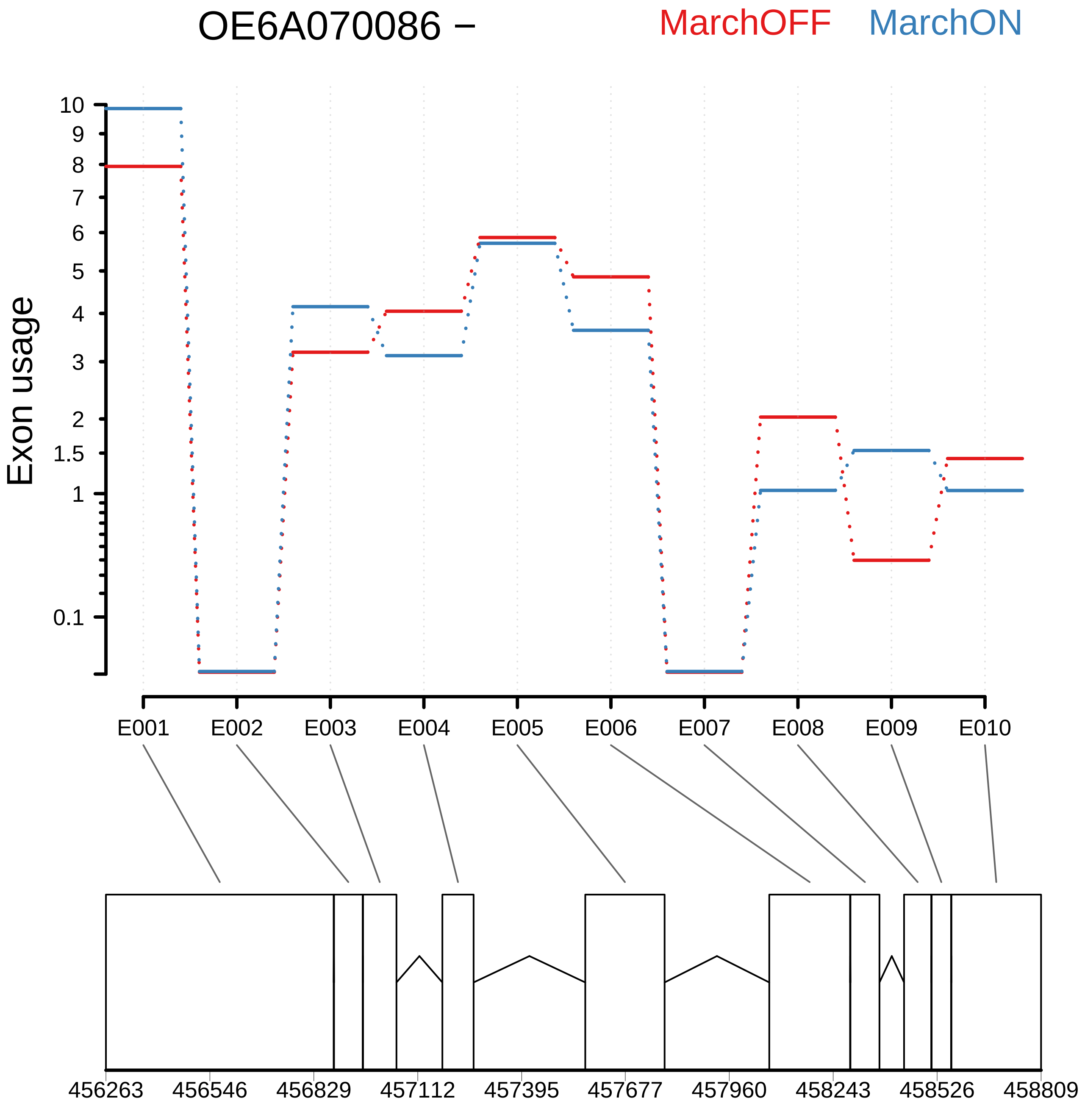
OE6A070086 −
MarchOFF
MarchON
Exon usage
10
9
8
7
6
5
4
3
2
1.5
1
0.1
E001
E002
E003
E004
E005
E006
E007
E008
E009
E010
456263
456546
456829
457112
457395
457677
457960
458243
458526
458809

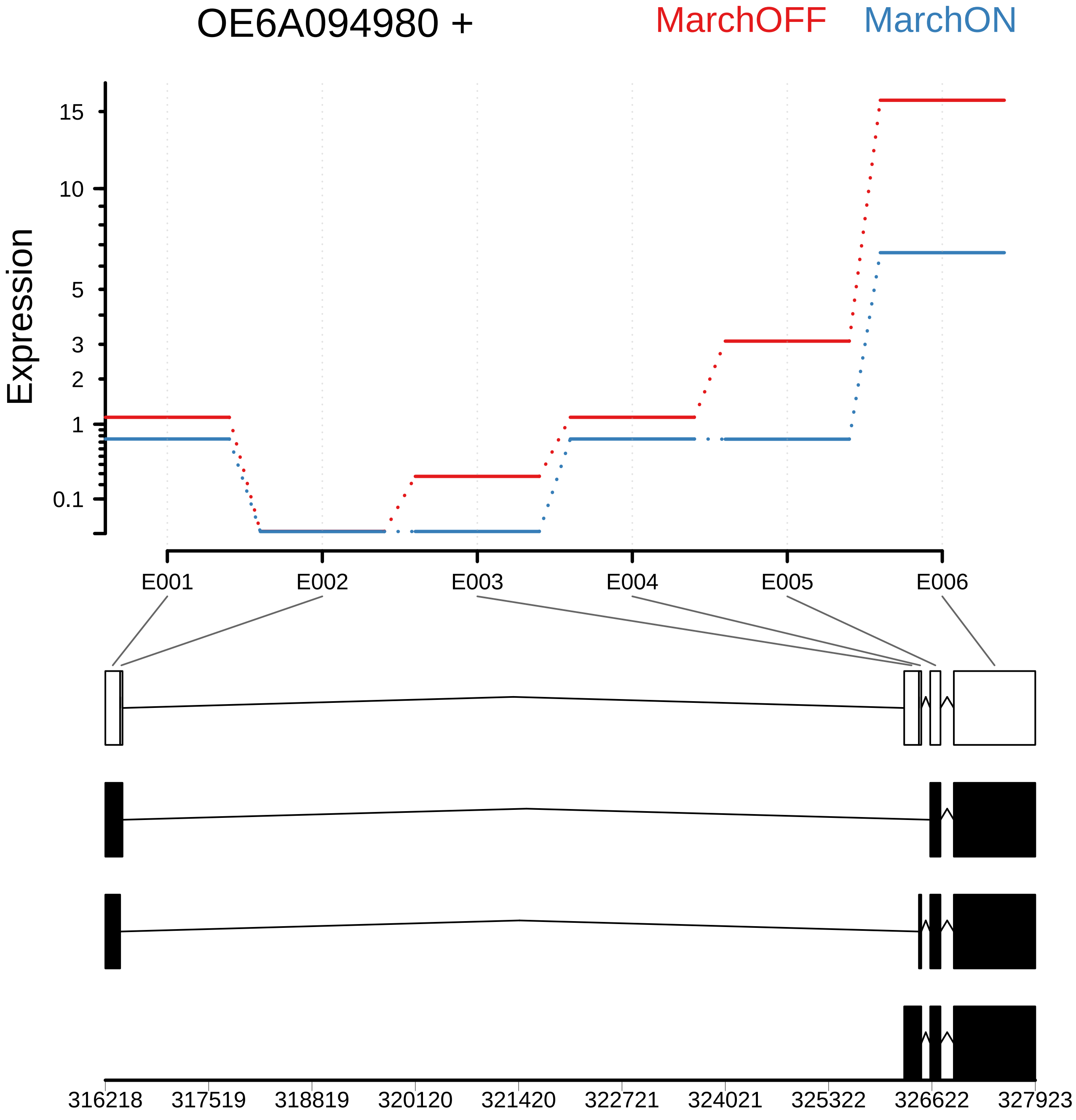

OE6A094980 +
MarchOFF
MarchON
Expression
15
10
5
3
2
1
0.1
E001
E002
E003
E004
E005
E006
316218
317519
318819
320120
321420
322721
324021
325322
326622
327923

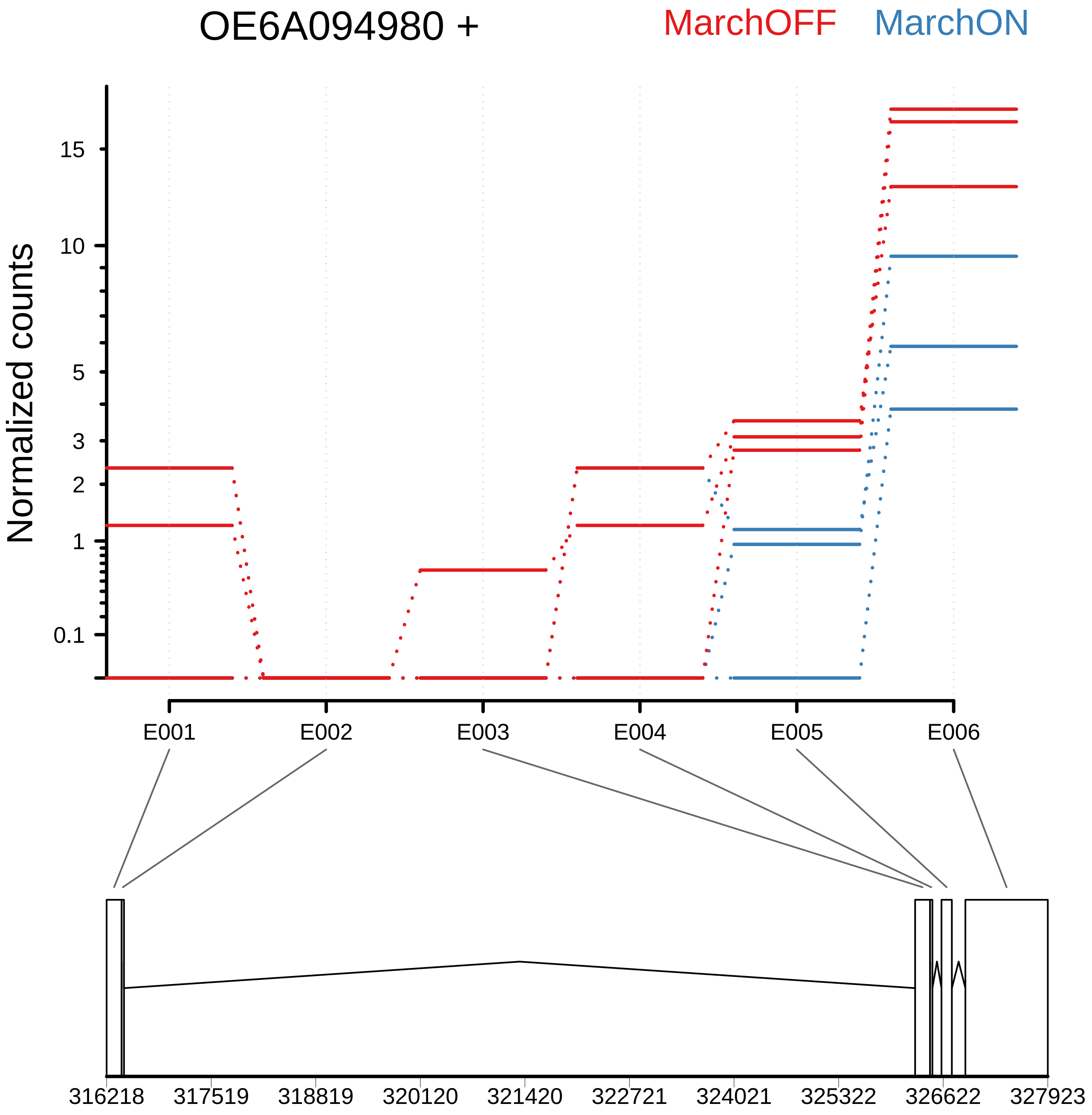

OE6A094980 +
MarchOFF
MarchON
Normalized counts
15
10
5
3
2
1
0.1
E001
E002
E003
E004
E005
E006
316218
317519
318819
320120
321420
322721
324021
325322
326622
327923

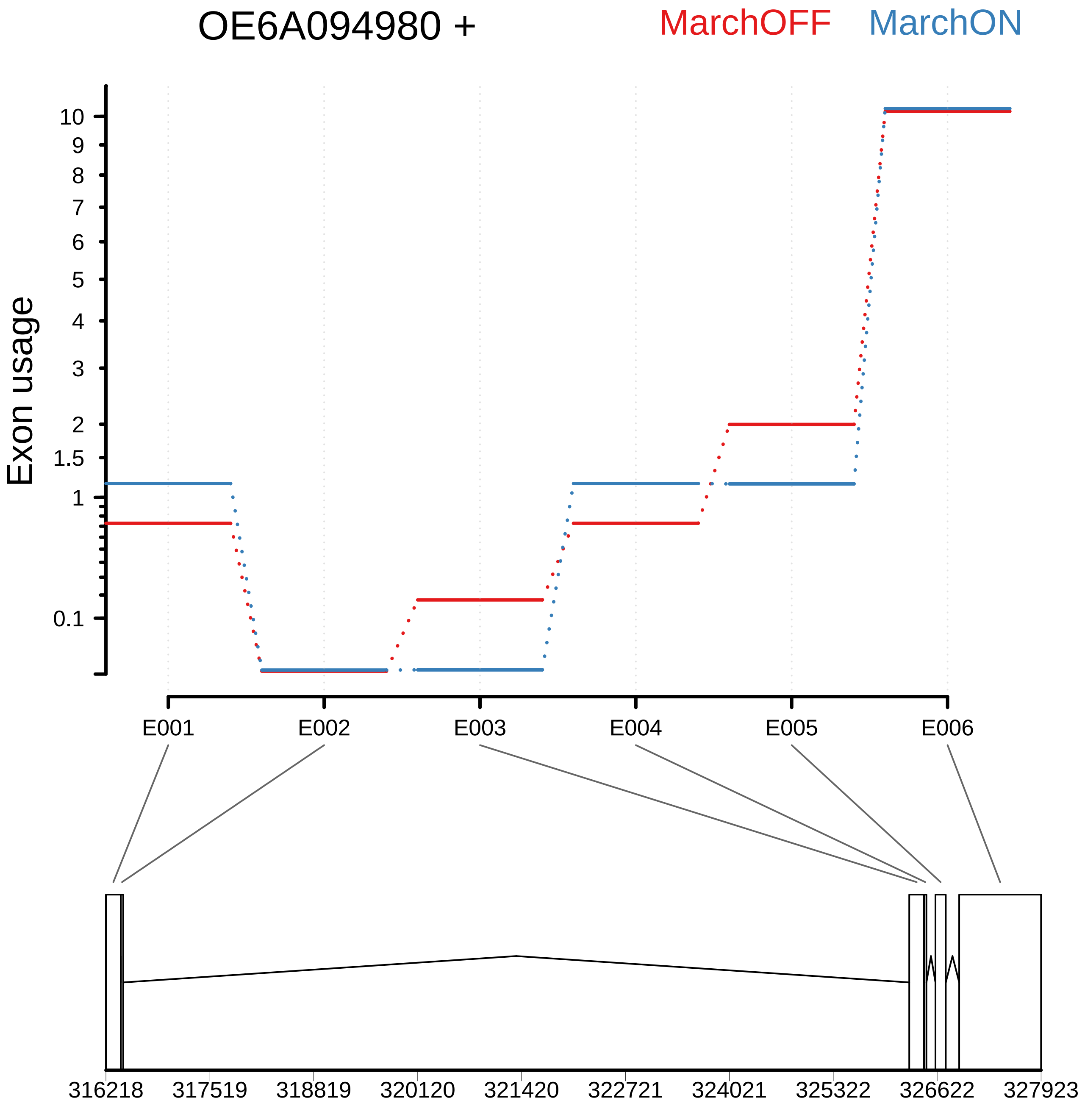
OE6A094980 +
MarchOFF
MarchON
Exon usage
10
9
8
7
6
5
4
3
2
1.5
1
0.1
E001
E002
E003
E004
E005
E006
316218
317519
318819
320120
321420
322721
324021
325322
326622
327923

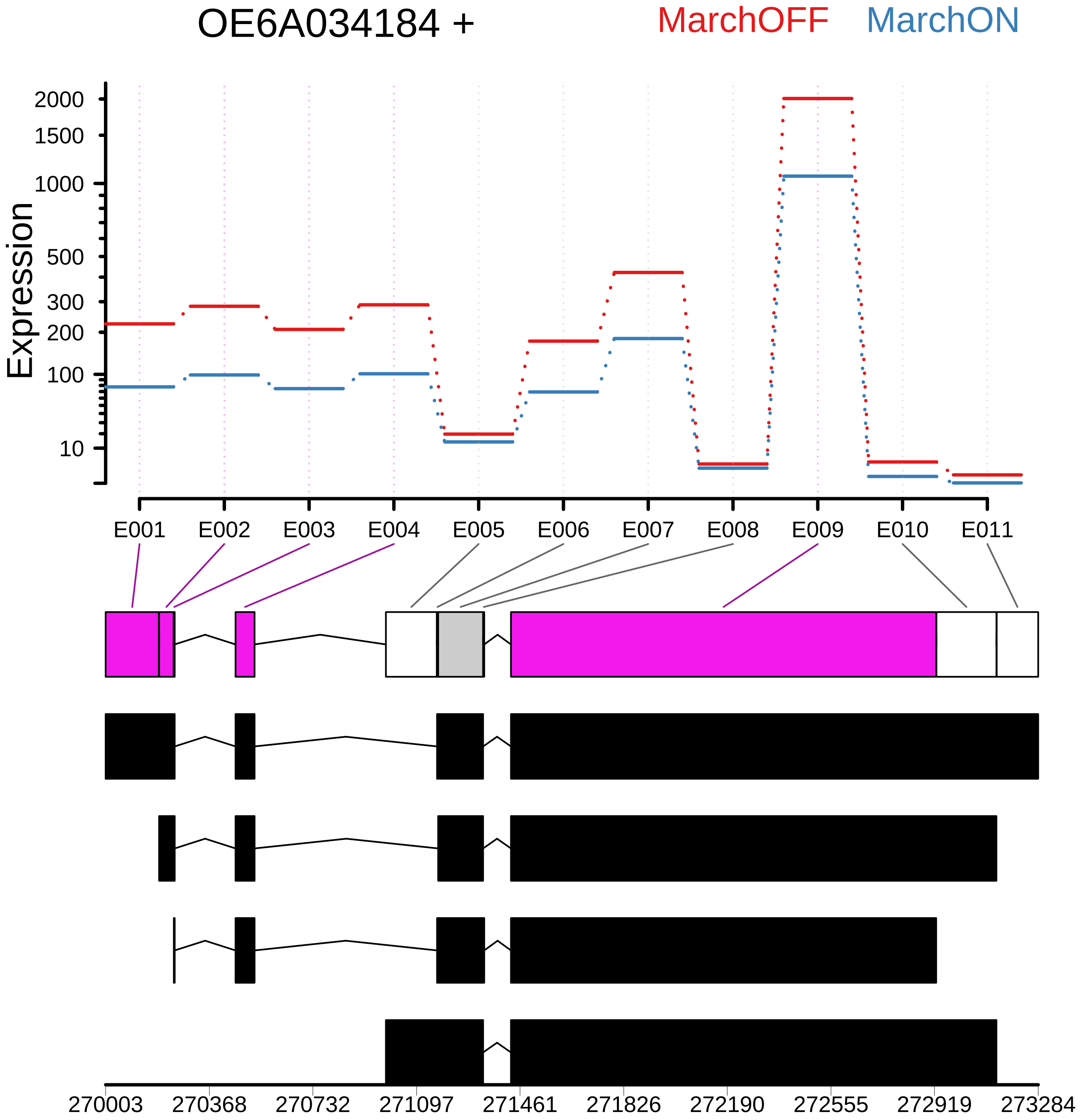

OE6A034184 +
MarchOFF
MarchON
Expression
2000
1500
1000
500
300
200
100
10
E001
E002
E003
E004
E005
E006
E007
E008
E009
E010
E011
270003
270368
270732
271097
271461
271826
272190
272555
272919
273284

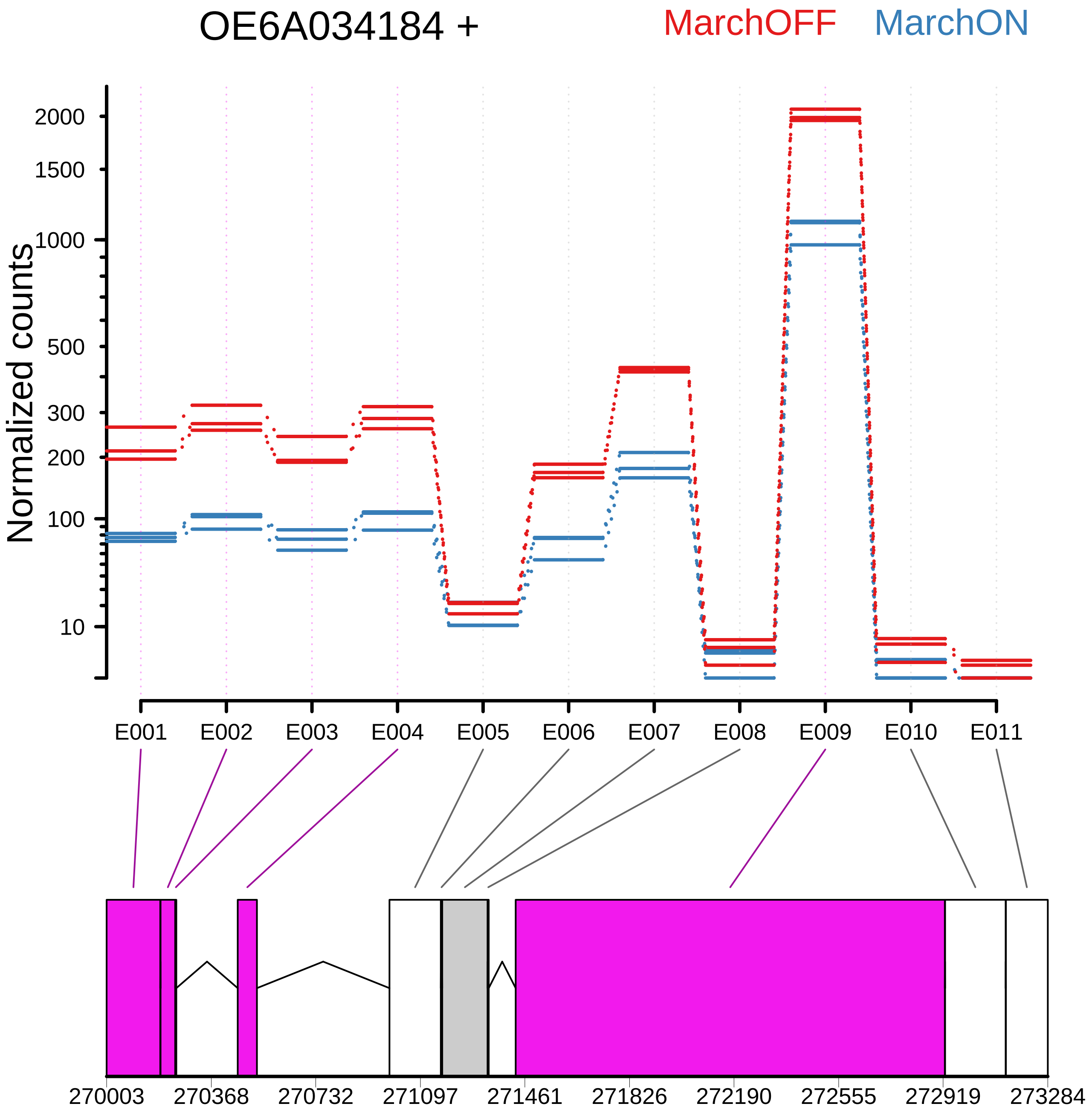

OE6A034184 +
MarchOFF
MarchON
Normalized counts
2000
1500
1000
500
300
200
100
10
E001
E002
E003
E004
E005
E006
E007
E008
E009
E010
E011
270003
270368
270732
271097
271461
271826
272190
272555
272919
273284

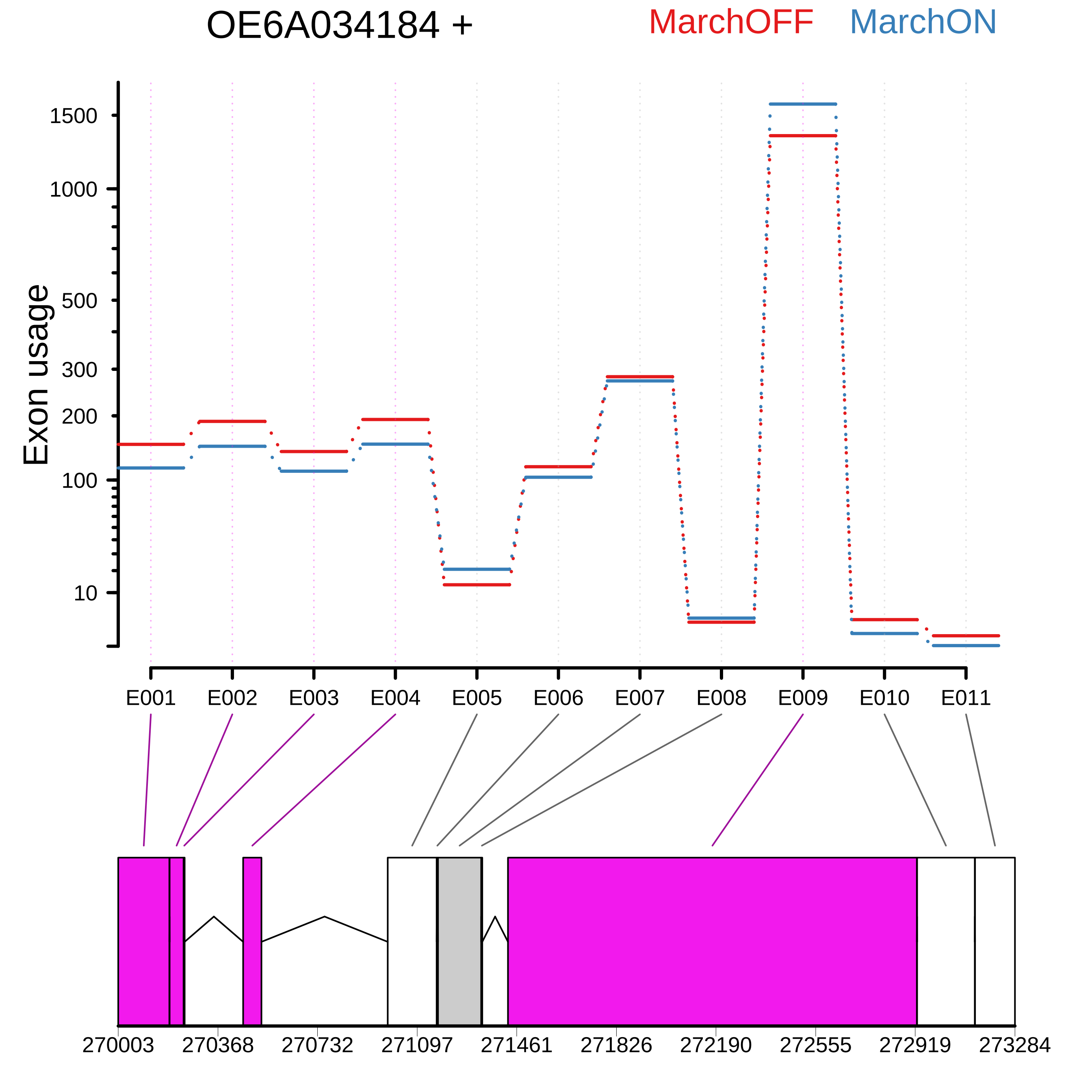

OE6A034184 +
MarchOFF
MarchON
Exon usage
1500
1000
500
300
200
100
10
E001
E002
E003
E004
E005
E006
E007
E008
E009
E010
E011
270003
270368
270732
271097
271461
271826
272190
272555
272919
273284

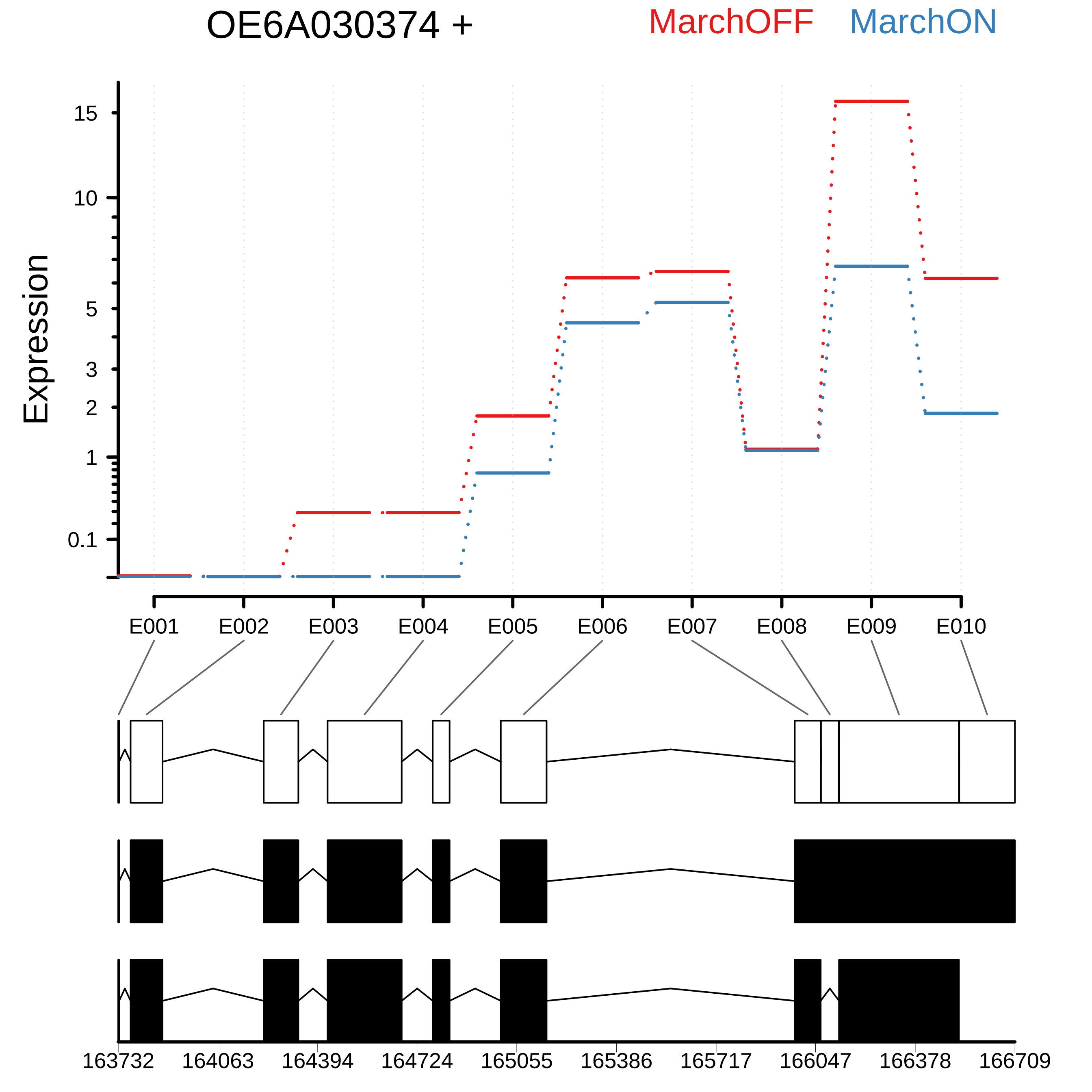

OE6A030374 +
MarchOFF
MarchON
Expression
15
10
5
3
2
1
0.1
E001
E002
E003
E004
E005
E006
E007
E008
E009
E010
163732
164063
164394
164724
165055
165386
165717
166047
166378
166709

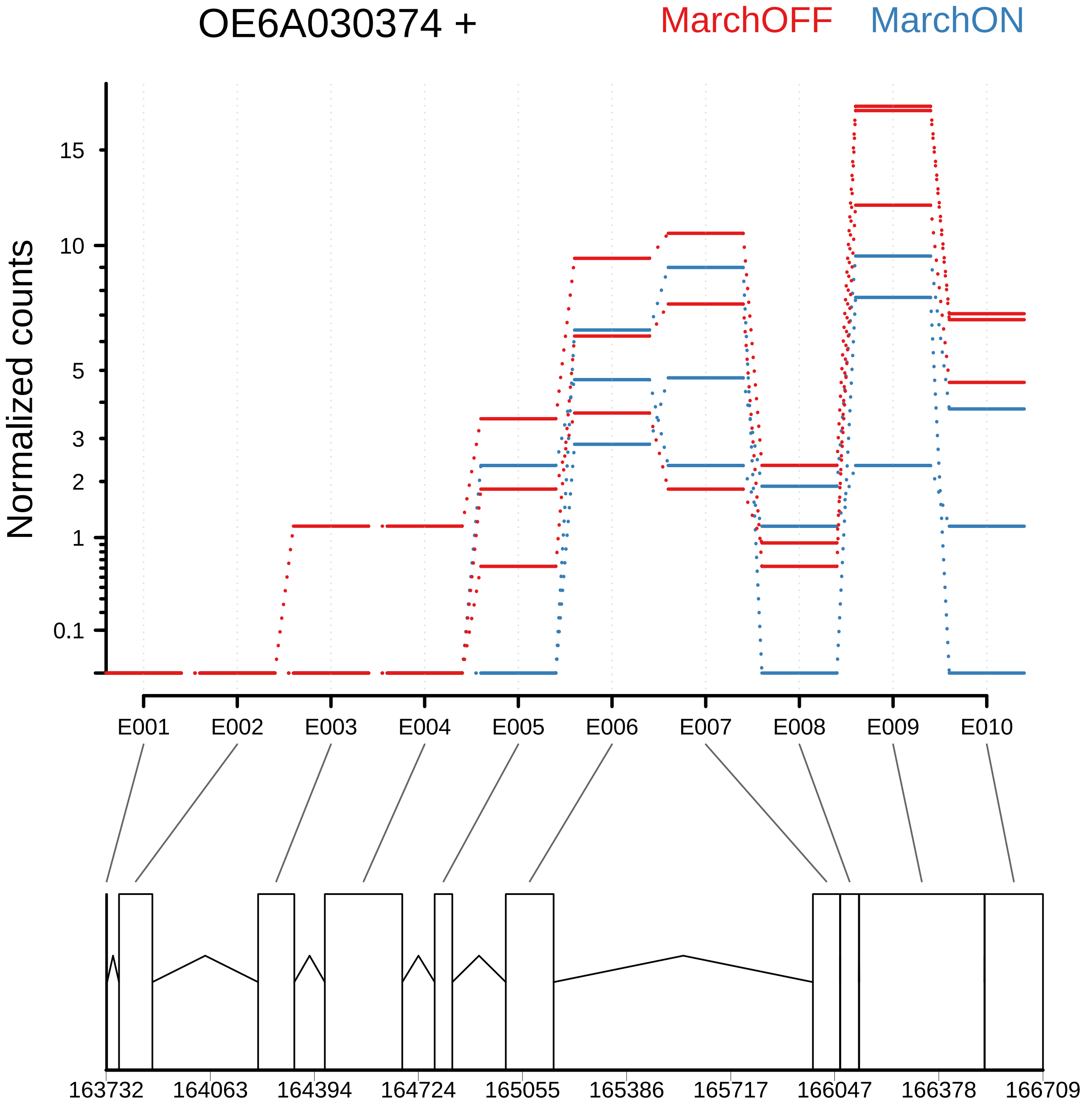
OE6A030374 +
MarchOFF
MarchON
Normalized counts
15
10
5
3
2
1
0.1
E001
E002
E003
E004
E005
E006
E007
E008
E009
E010
163732
164063
164394
164724
165055
165386
165717
166047
166378
166709

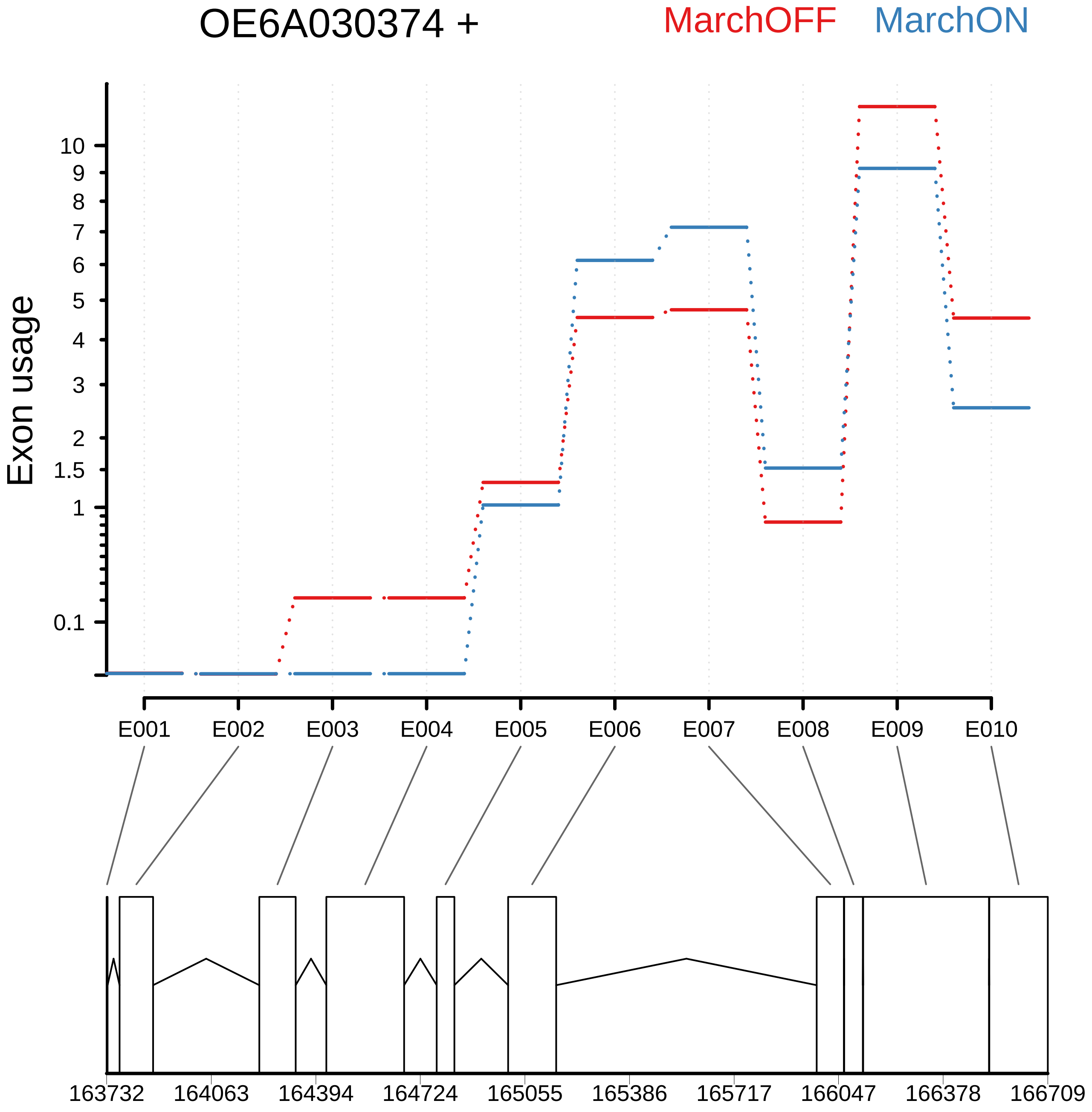
OE6A030374 +
MarchOFF
MarchON
Exon usage
10
9
8
7
6
5
4
3
2
1.5
1
0.1
E001
E002
E003
E004
E005
E006
E007
E008
E009
E010
163732
164063
164394
164724
165055
165386
165717
166047
166378
166709

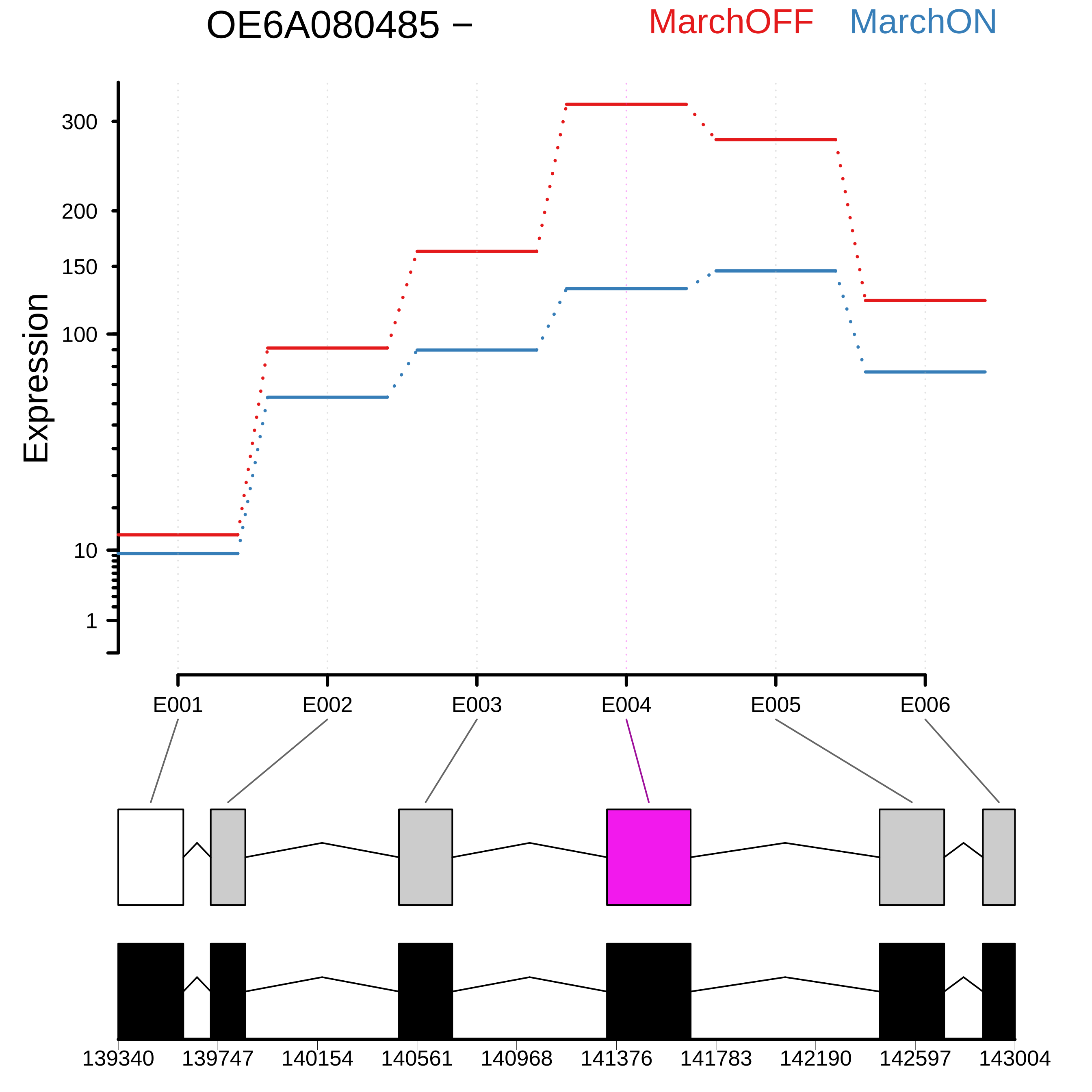

OE6A080485 –
MarchOFF
MarchON
Expression
300
200
150
100
10
1
E001
E002
E003
E004
E005
E006
139340
139747
140154
140561
140968
141376
141783
142190
142597
143004

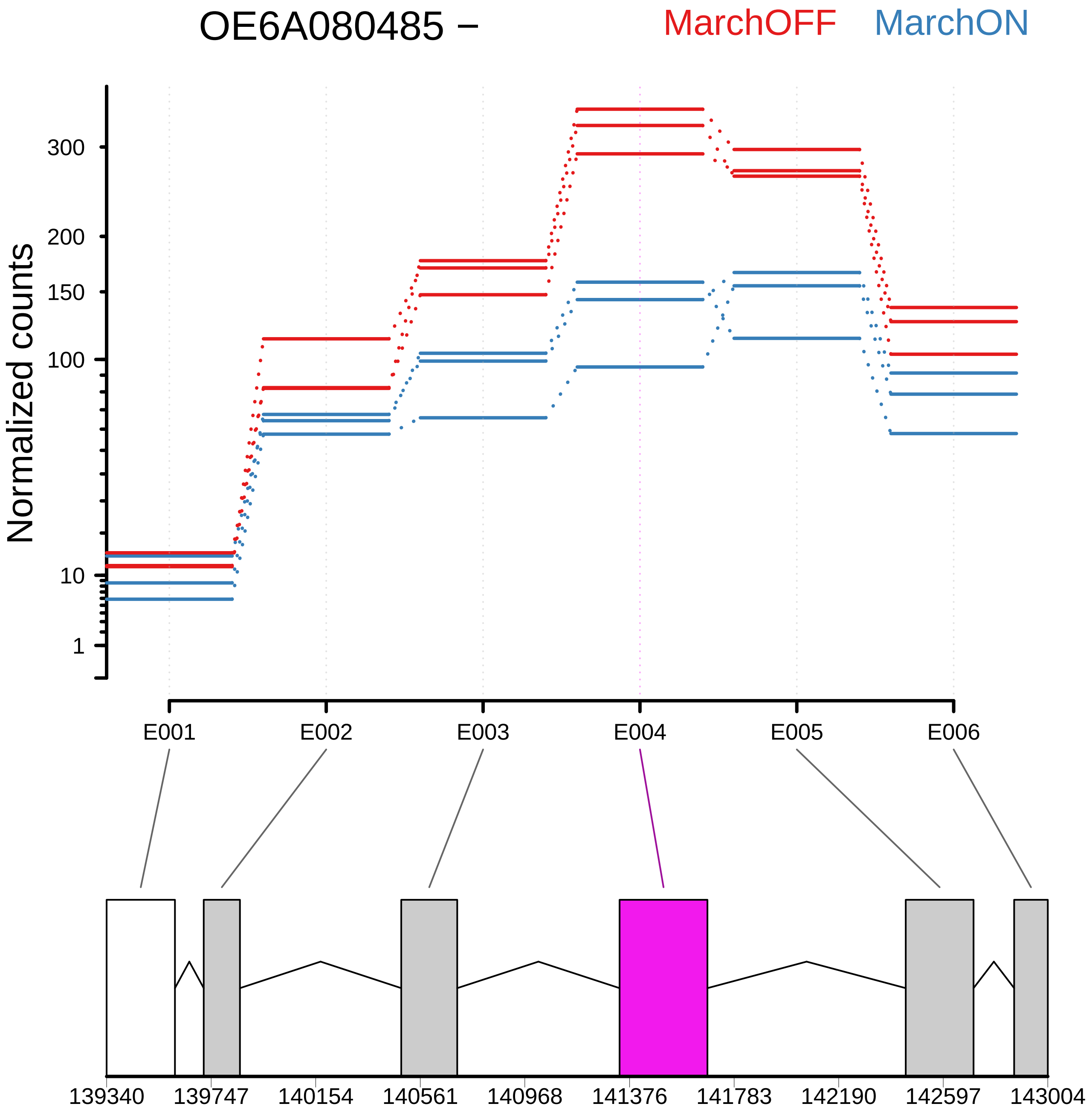
OE6A080485 −
MarchOFF
MarchON
Normalized counts
300
200
150
100
10
1
E001
E002
E003
E004
E005
E006
139340
139747
140154
140561
140968
141376
141783
142190
142597
143004

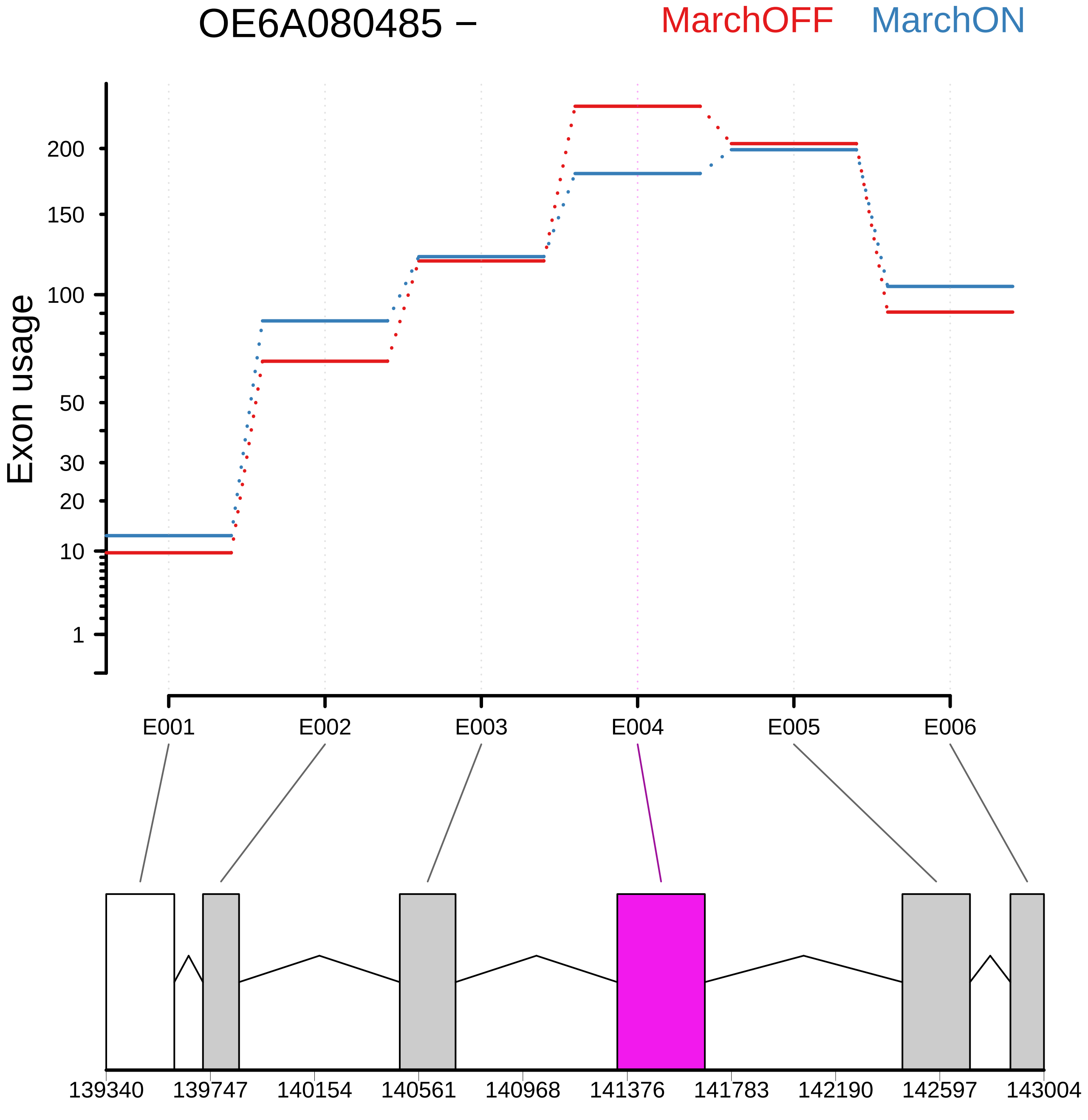

OE6A080485 −
MarchOFF
MarchON
Exon usage
200
150
100
50
30
20
10
1
E001
E002
E003
E004
E005
E006
139340
139747
140154
140561
140968
141376
141783
142190
142597
143004

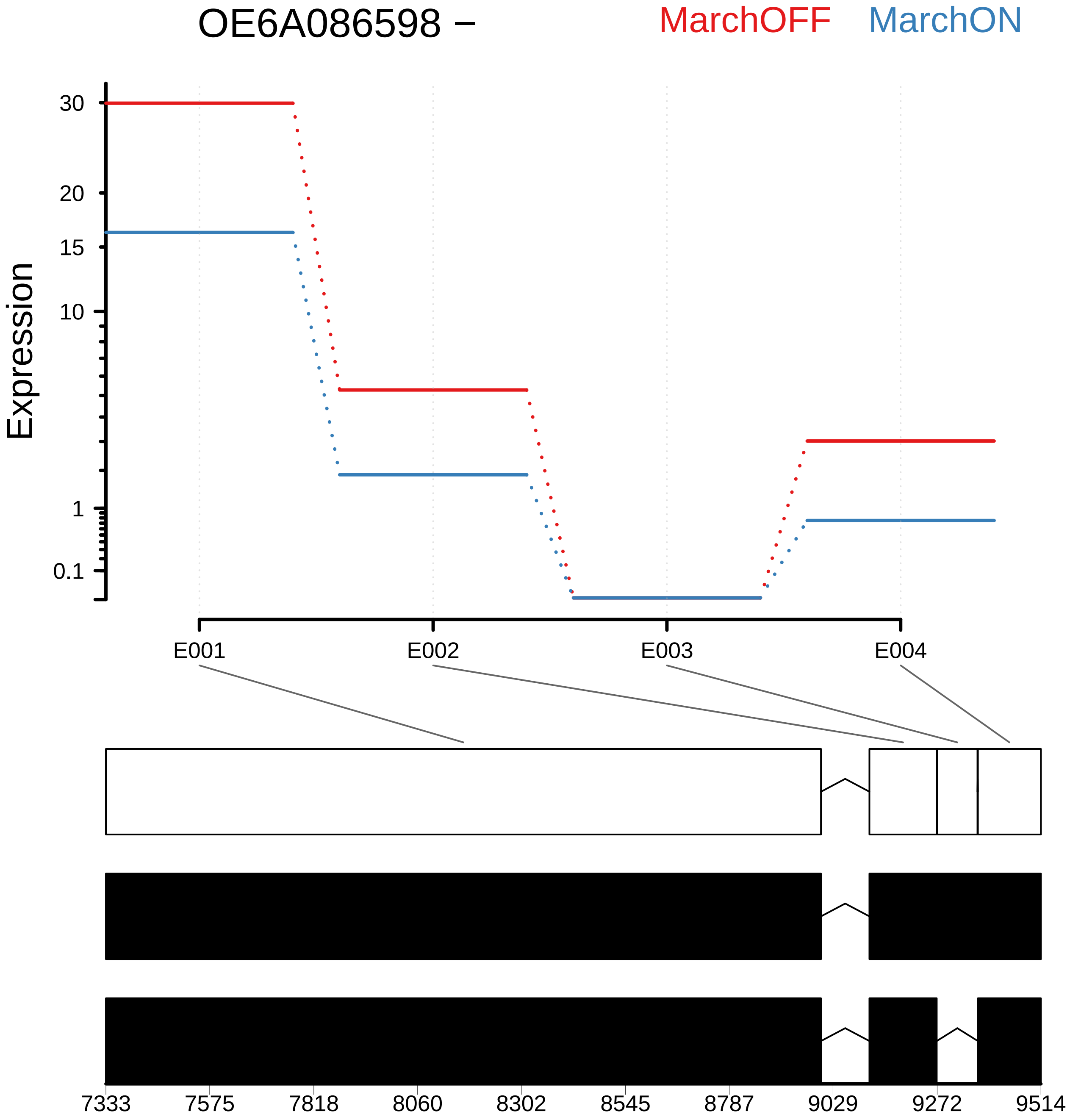
OE6A086598 −
MarchOFF
MarchON
Expression
30
20
15
10
1
0.1
E001
E002
E003
E004
7333
7575
7818
8060
8302
8545
8787
9029
9272
9514

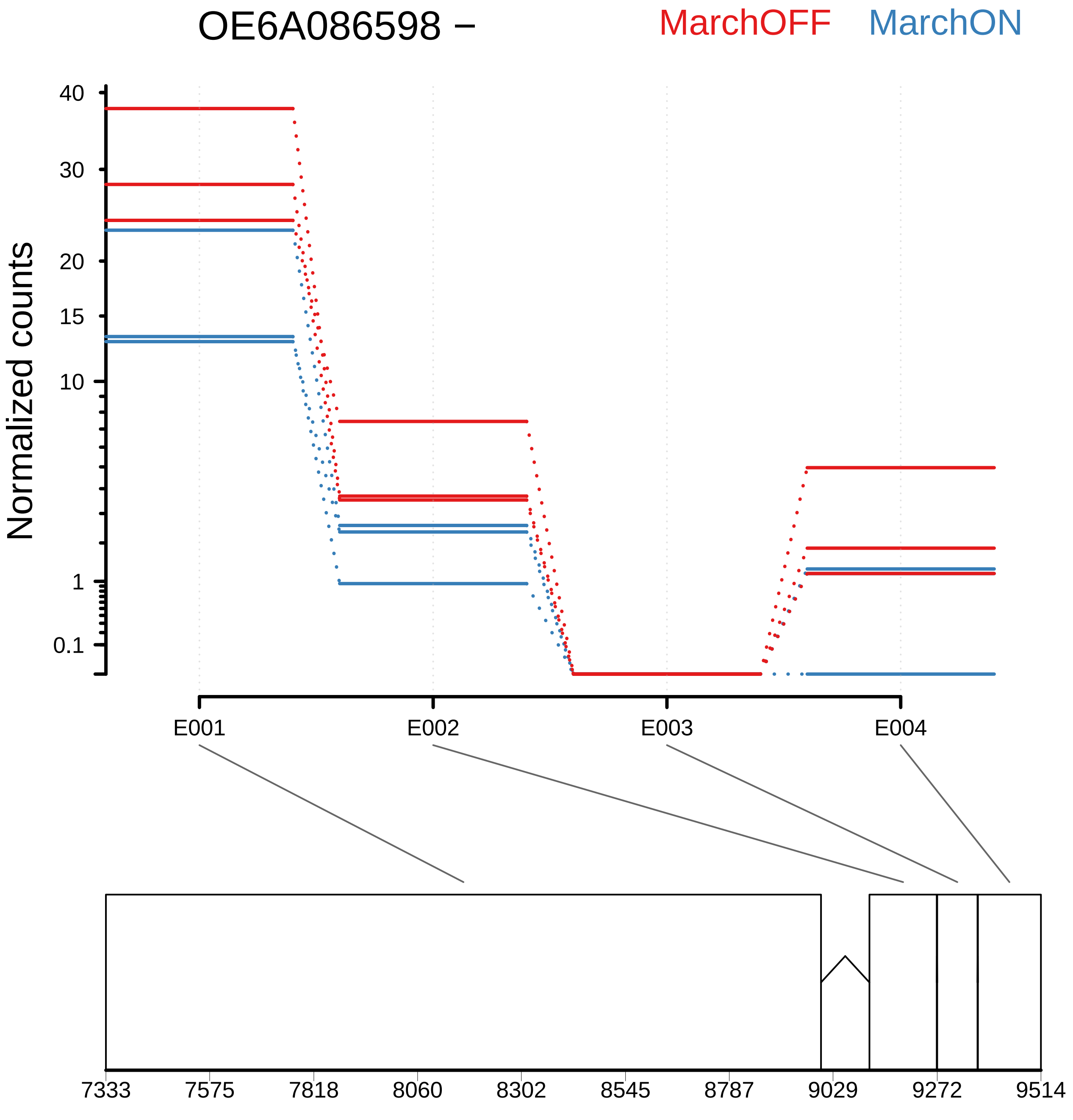

OE6A086598 −
MarchOFF
MarchON
Normalized counts
40
30
20
15
10
1
0.1
E001
E002
E003
E004
7333
7575
7818
8060
8302
8545
8787
9029
9272
9514

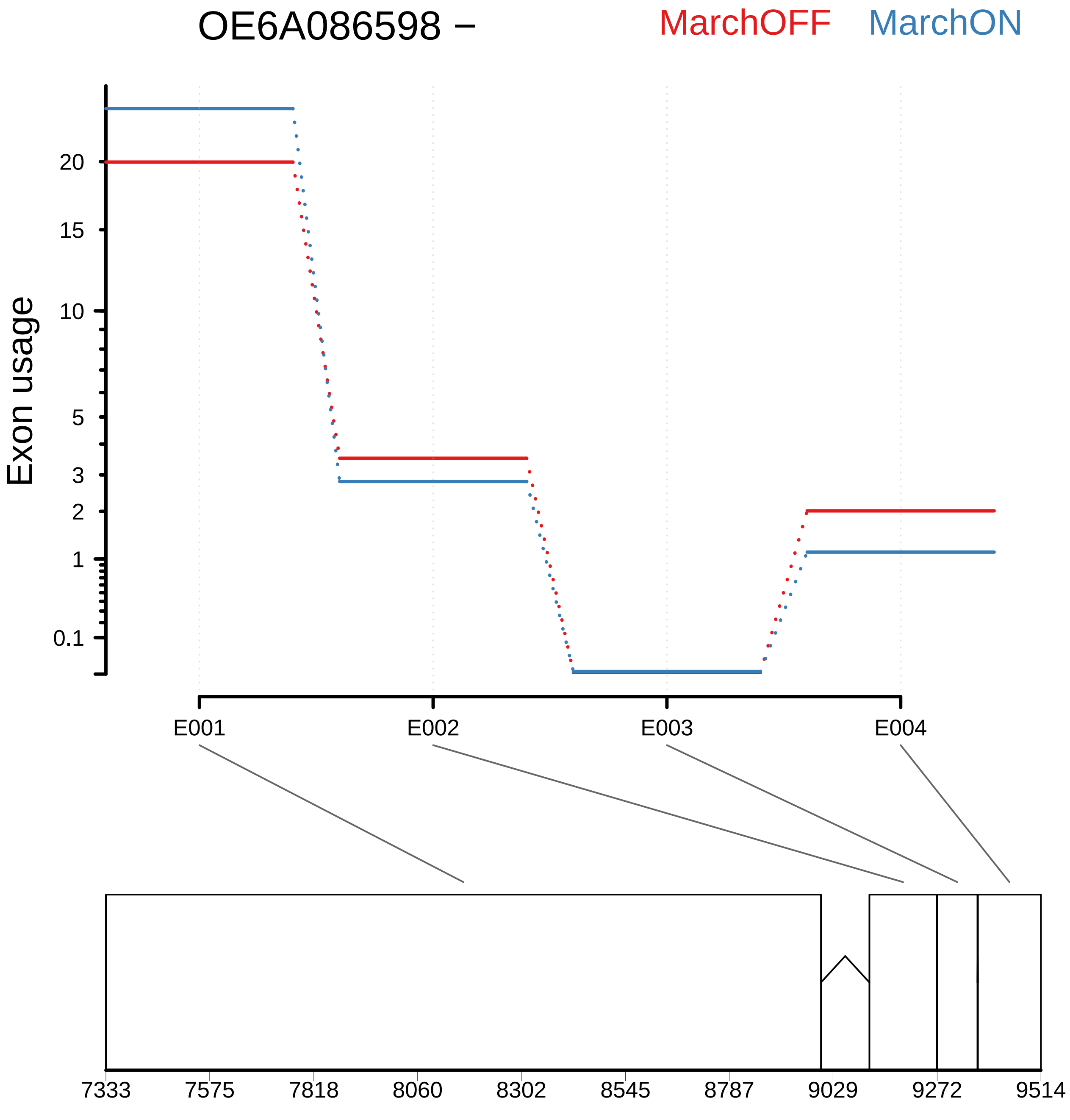

OE6A086598 −
MarchOFF
MarchON
Exon usage
20
15
10
5
3
2
1
0.1
E001
E002
E003
E004
7333
7575
7818
8060
8302
8545
8787
9029
9272
9514

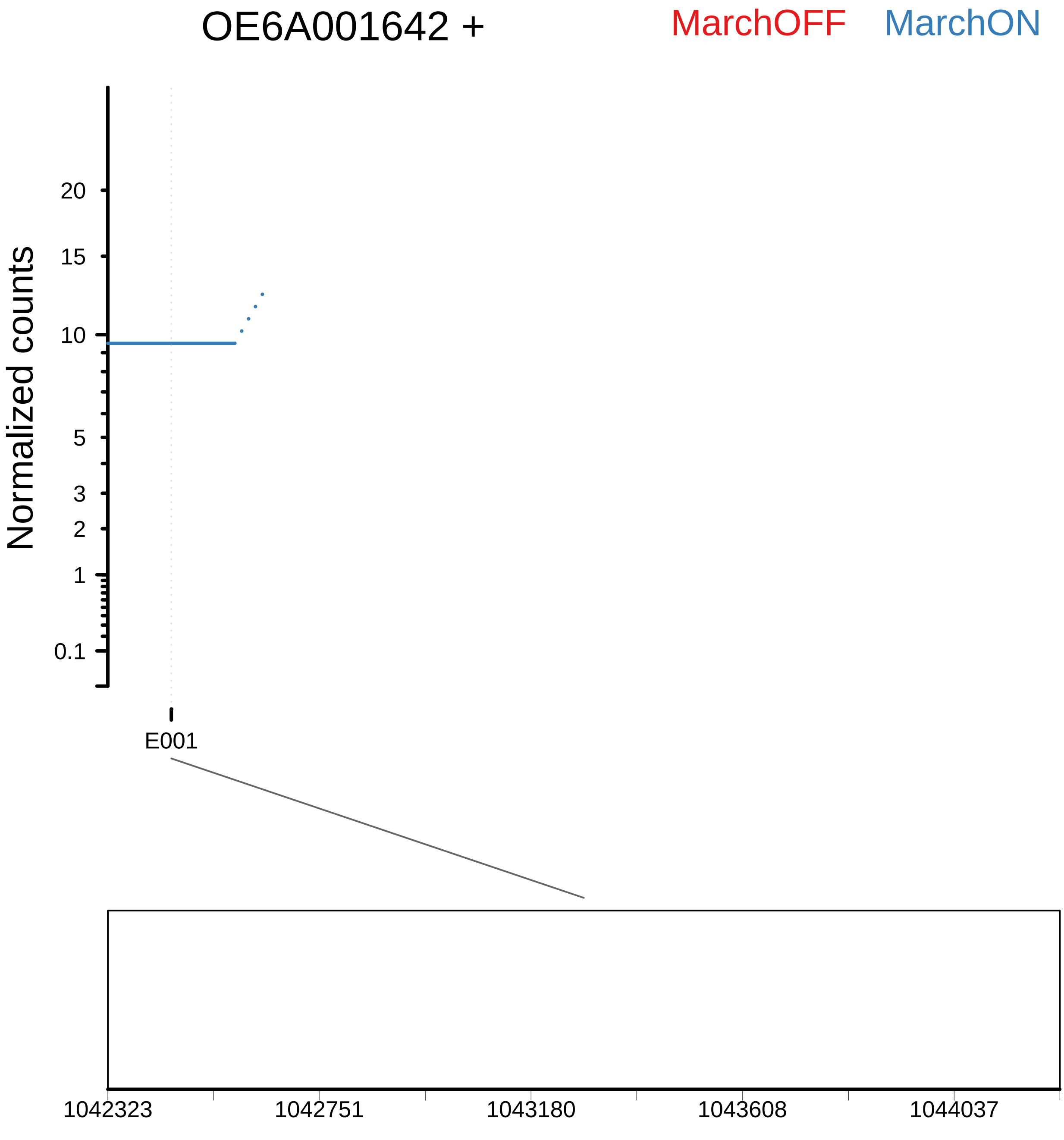
OE6A001642 +
MarchOFF
MarchON
Normalized counts
20
15
10
5
3
2
1
0.1
E001
1042323
1042751
1043180
1043608
1044037

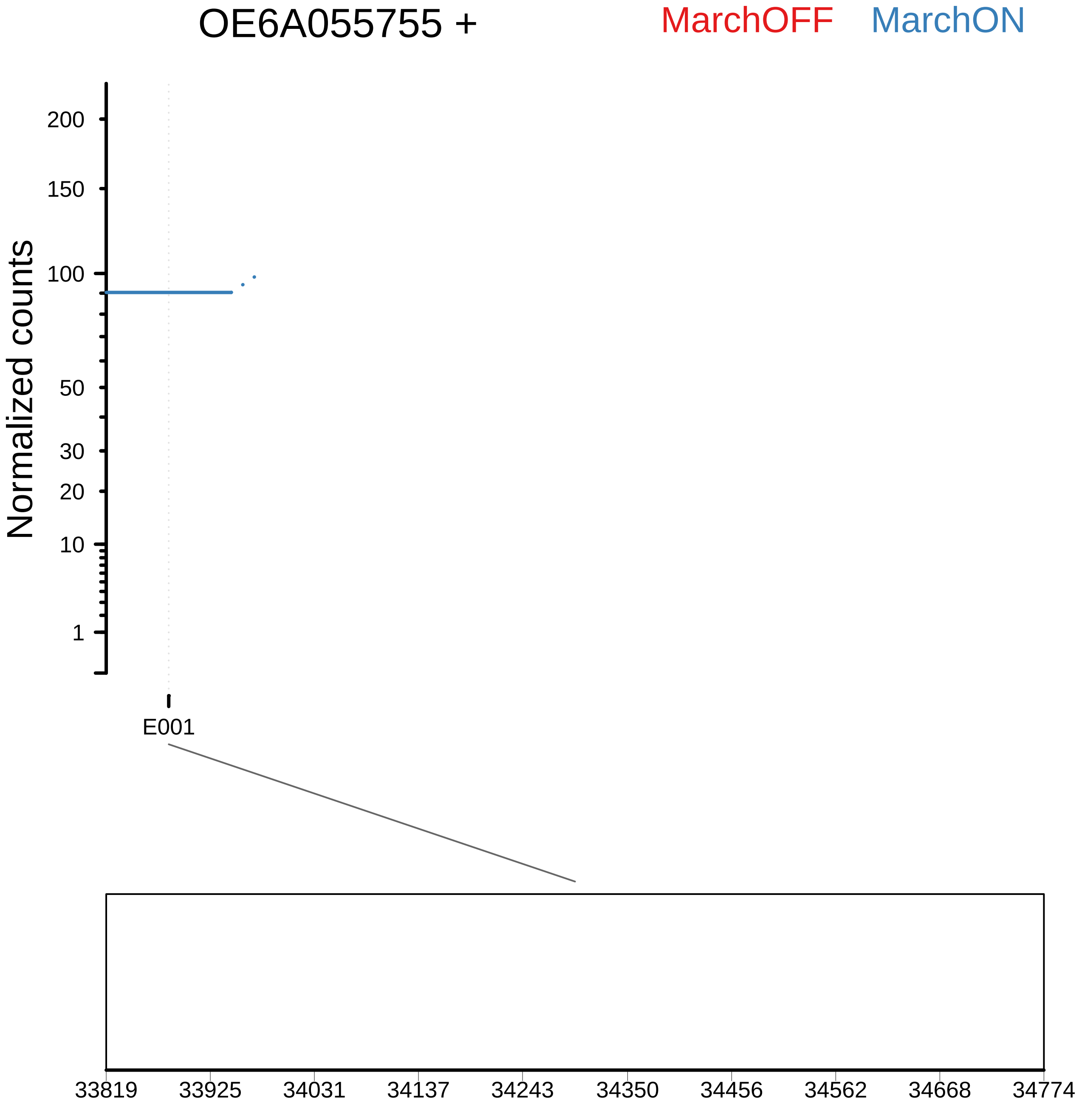

OE6A055755 +
MarchOFF
MarchON
Normalized counts
200
150
100
50
30
20
10
1
E001
33819
33925
34031
34137
34243
34350
34456
34562
34668
34774

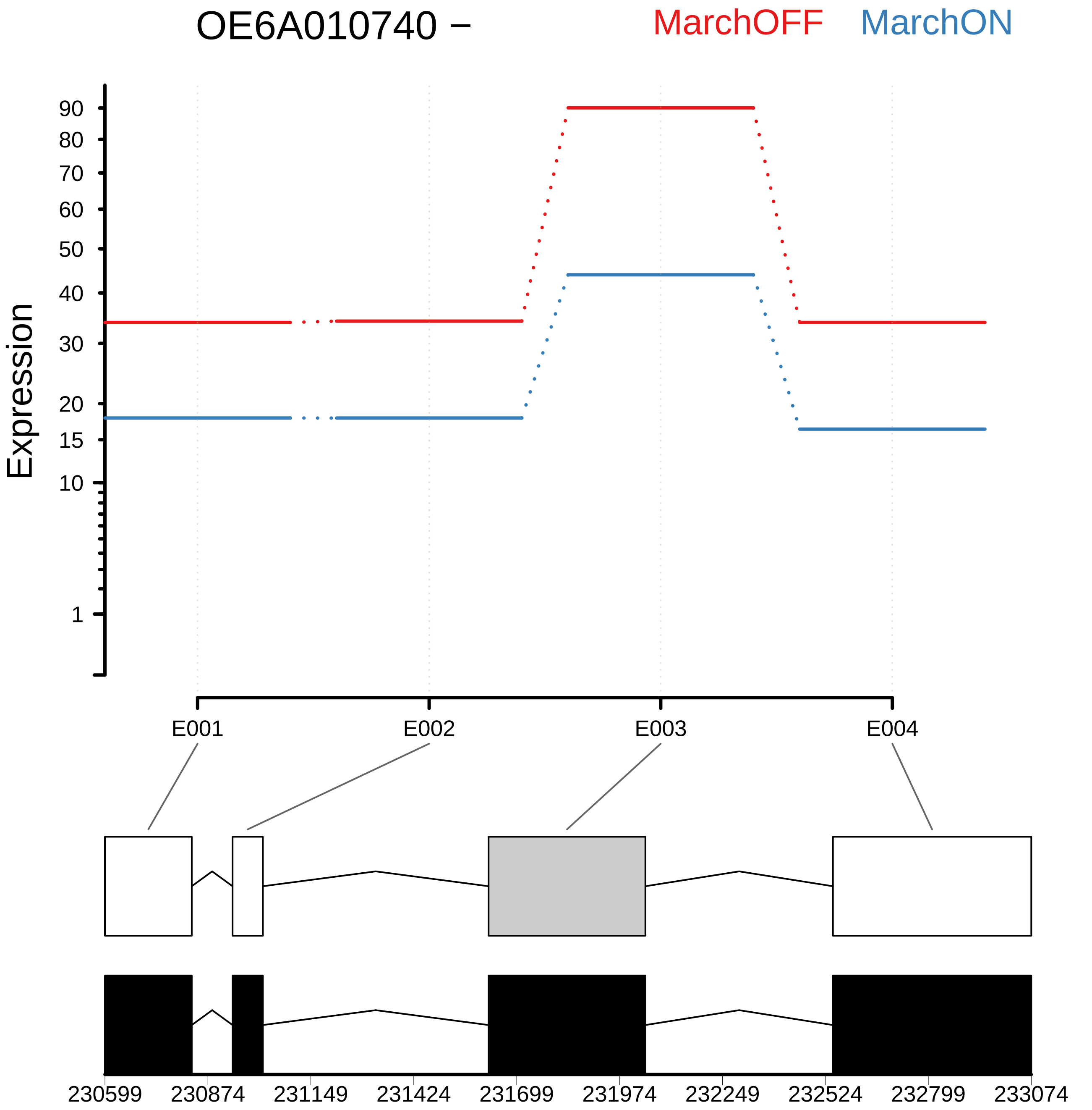

OE6A010740 −
MarchOFF
MarchON
Expression
90
80
70
60
50
40
30
20
15
10
1
E001
E002
E003
E004
230599
230874
231149
231424
231699
231974
232249
232524
232799
233074

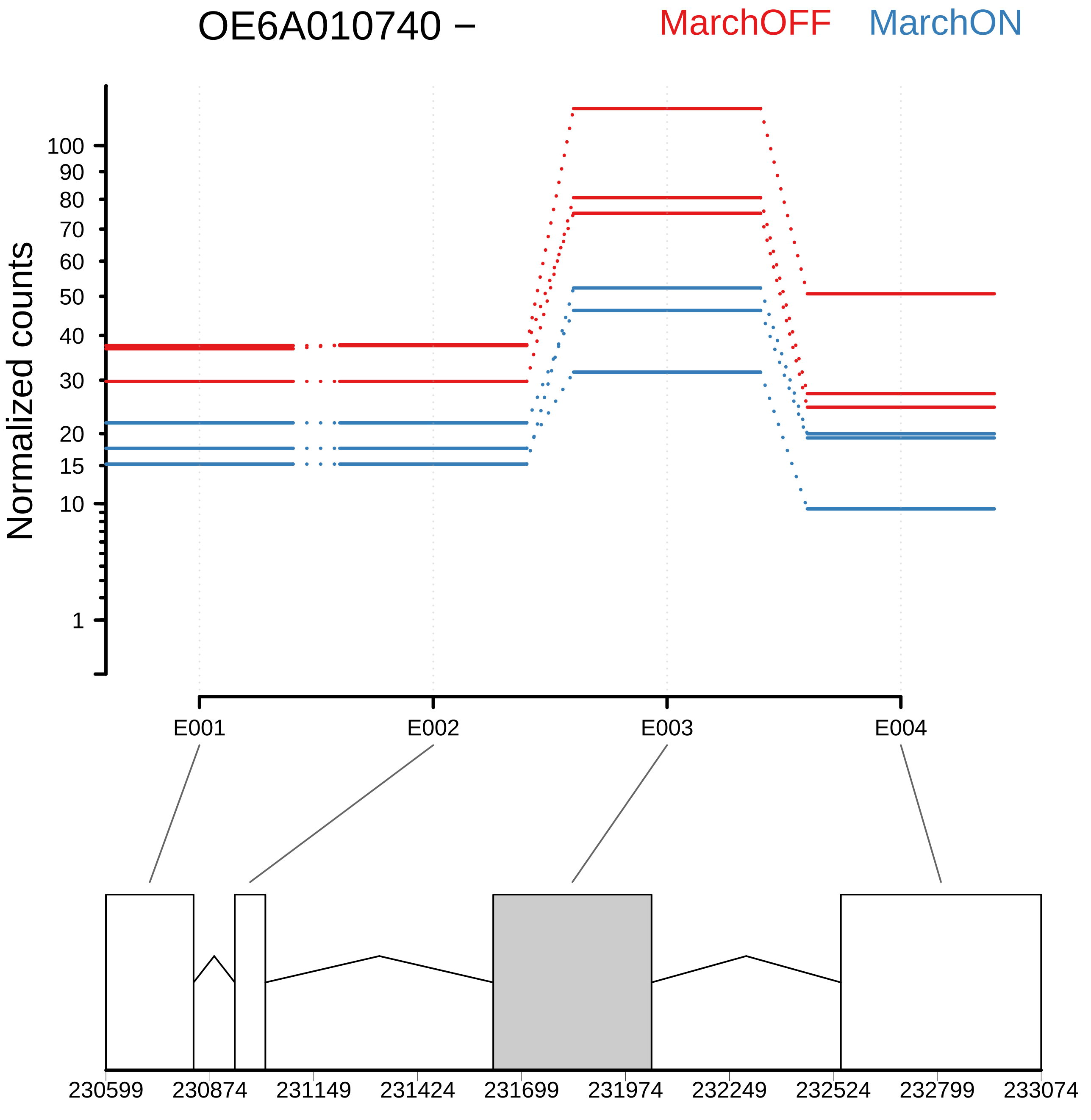

OE6A010740 −
MarchOFF
MarchON
Normalized counts
100
90
80
70
60
50
40
30
20
15
10
1
E001
E002
E003
E004
230599
230874
231149
231424
231699
231974
232249
232524
232799
233074

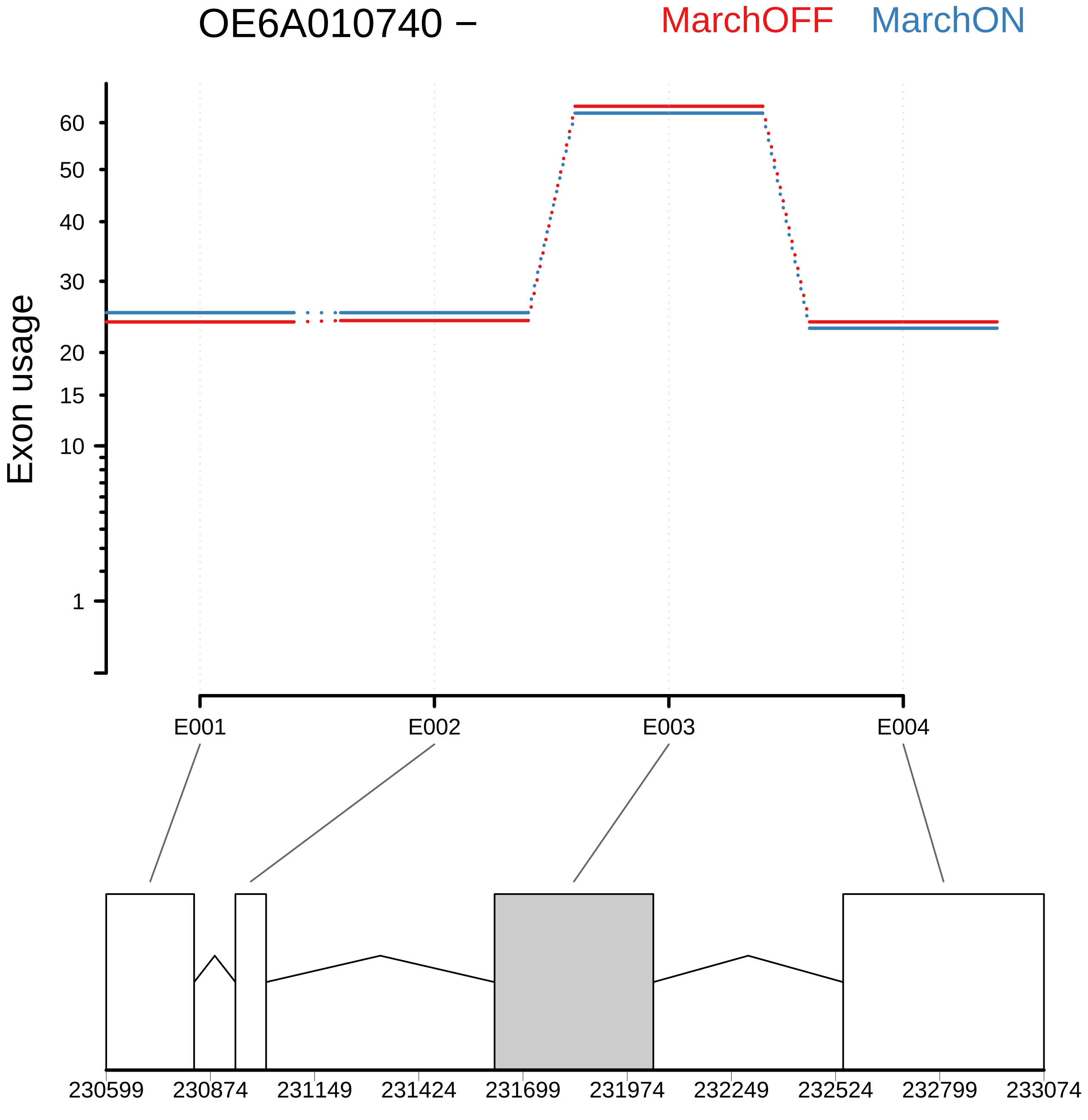
OE6A010740 −
MarchOFF
MarchON
Exon usage
60
50
40
30
20
15
10
1
E001
E002
E003
E004
230599
230874
231149
231424
231699
231974
232249
232524
232799
233074

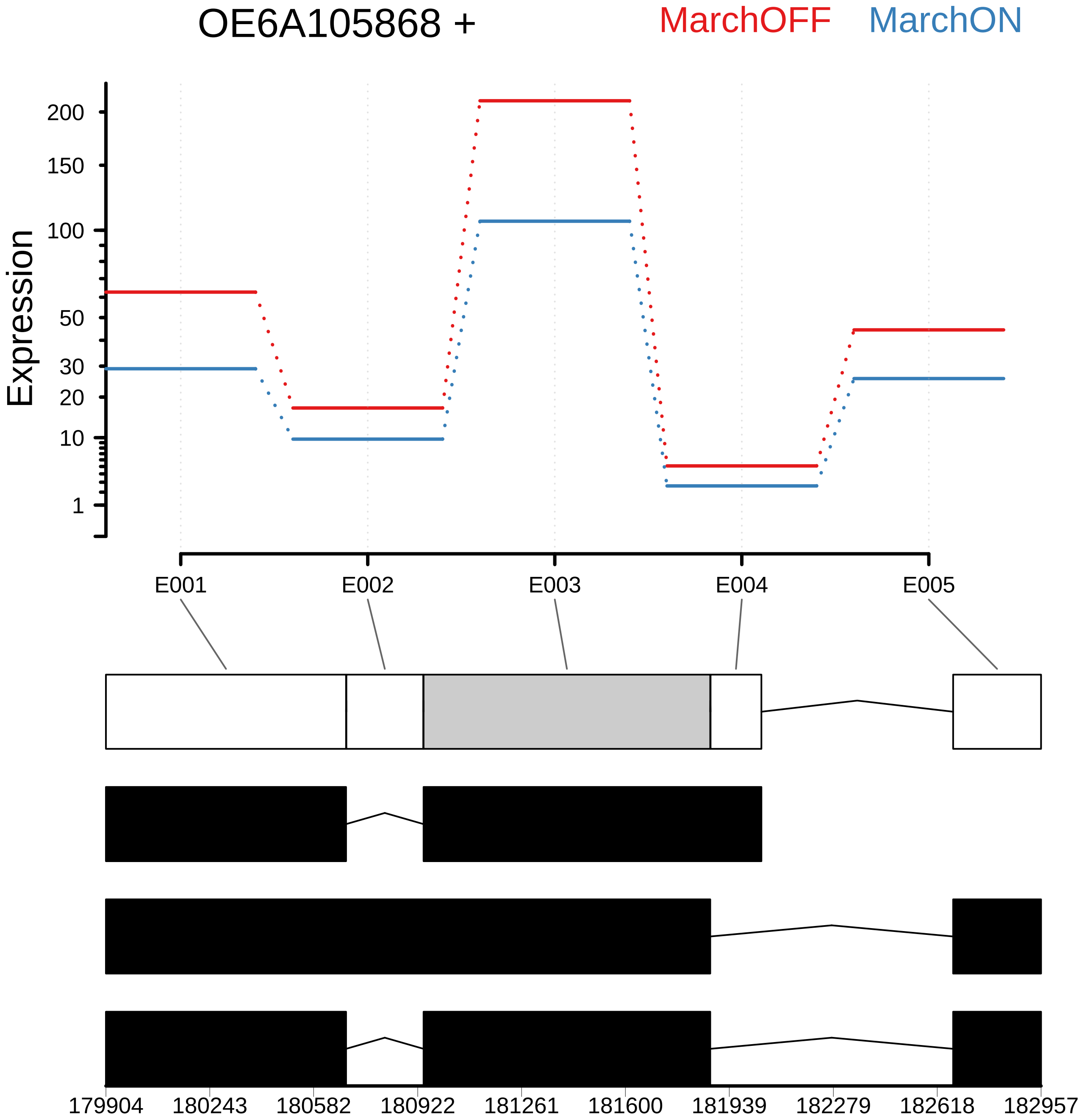

OE6A105868 +
MarchOFF
MarchON
Expression
200
150
100
50
30
20
10
1
E001
E002
E003
E004
E005
179904
180243
180582
180922
181261
181600
181939
182279
182618
182957

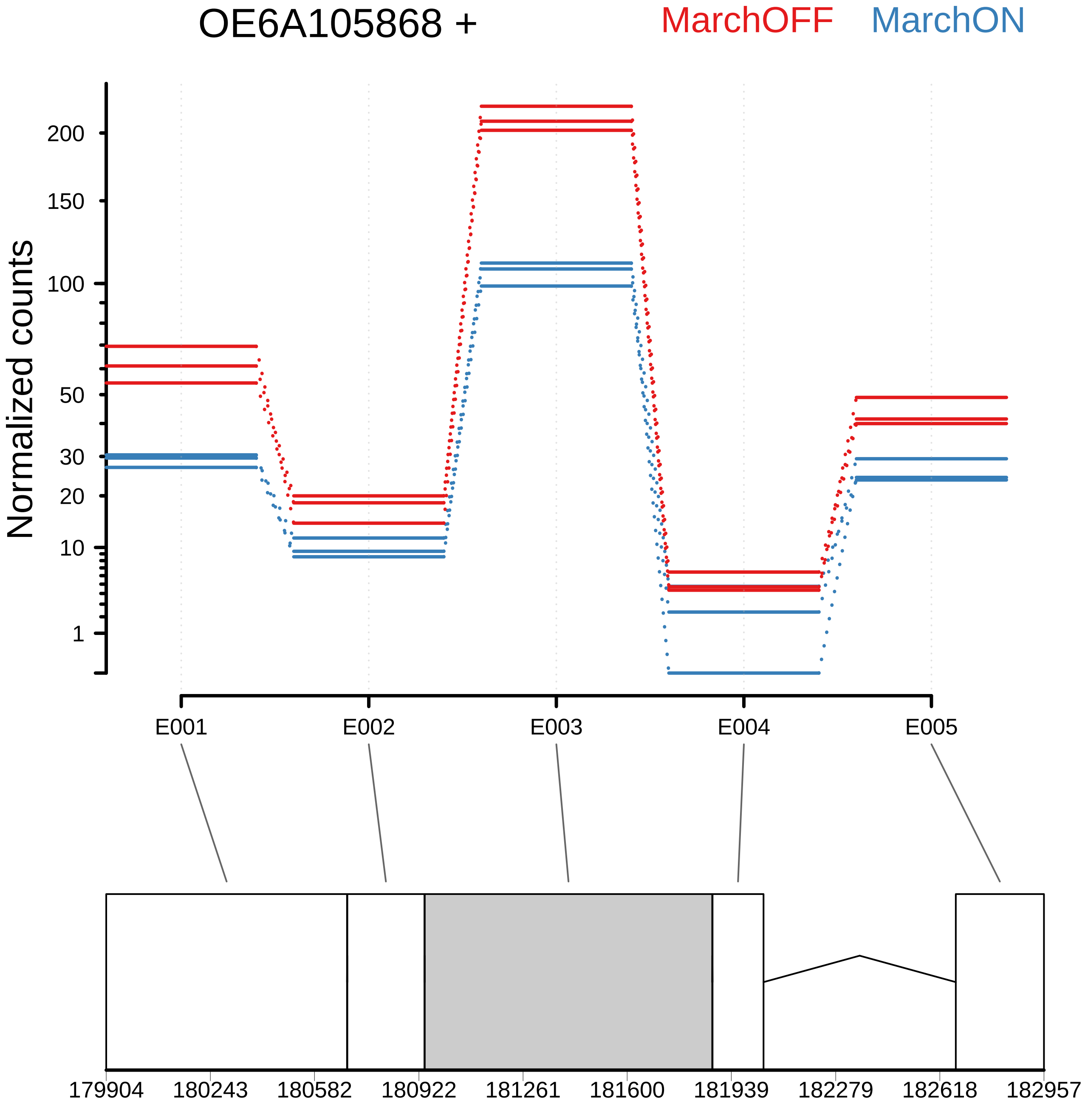

OE6A105868 +
MarchOFF
MarchON
Normalized counts
200
150
100
50
30
20
10
1
E001
E002
E003
E004
E005
179904
180243
180582
180922
181261
181600
181939
182279
182618
182957

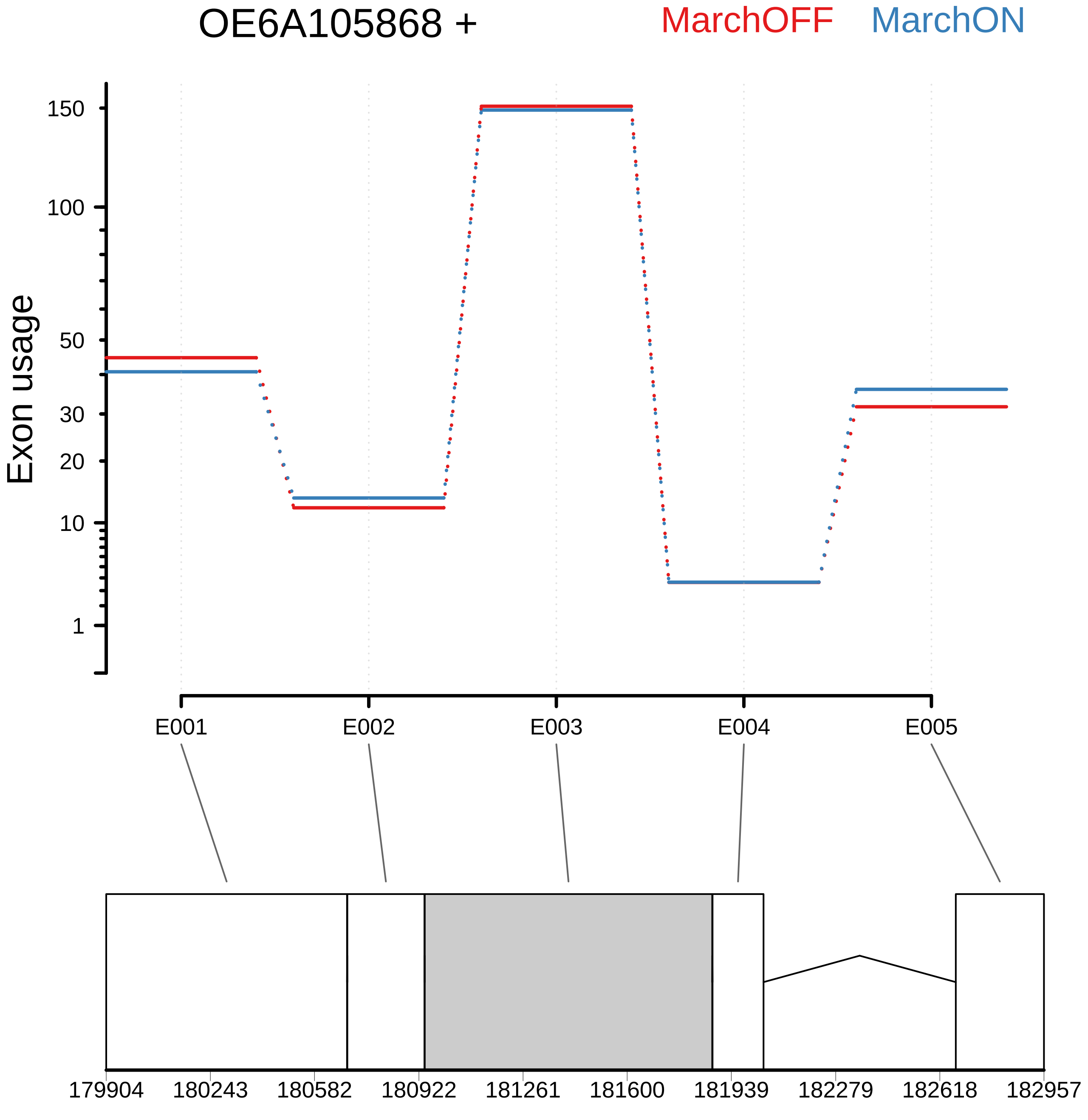
OE6A105868 +
MarchOFF
MarchON
Exon usage
150
100
50
30
20
10
1
E001
E002
E003
E004
E005
179904
180243
180582
180922
181261
181600
181939
182279
182618
182957

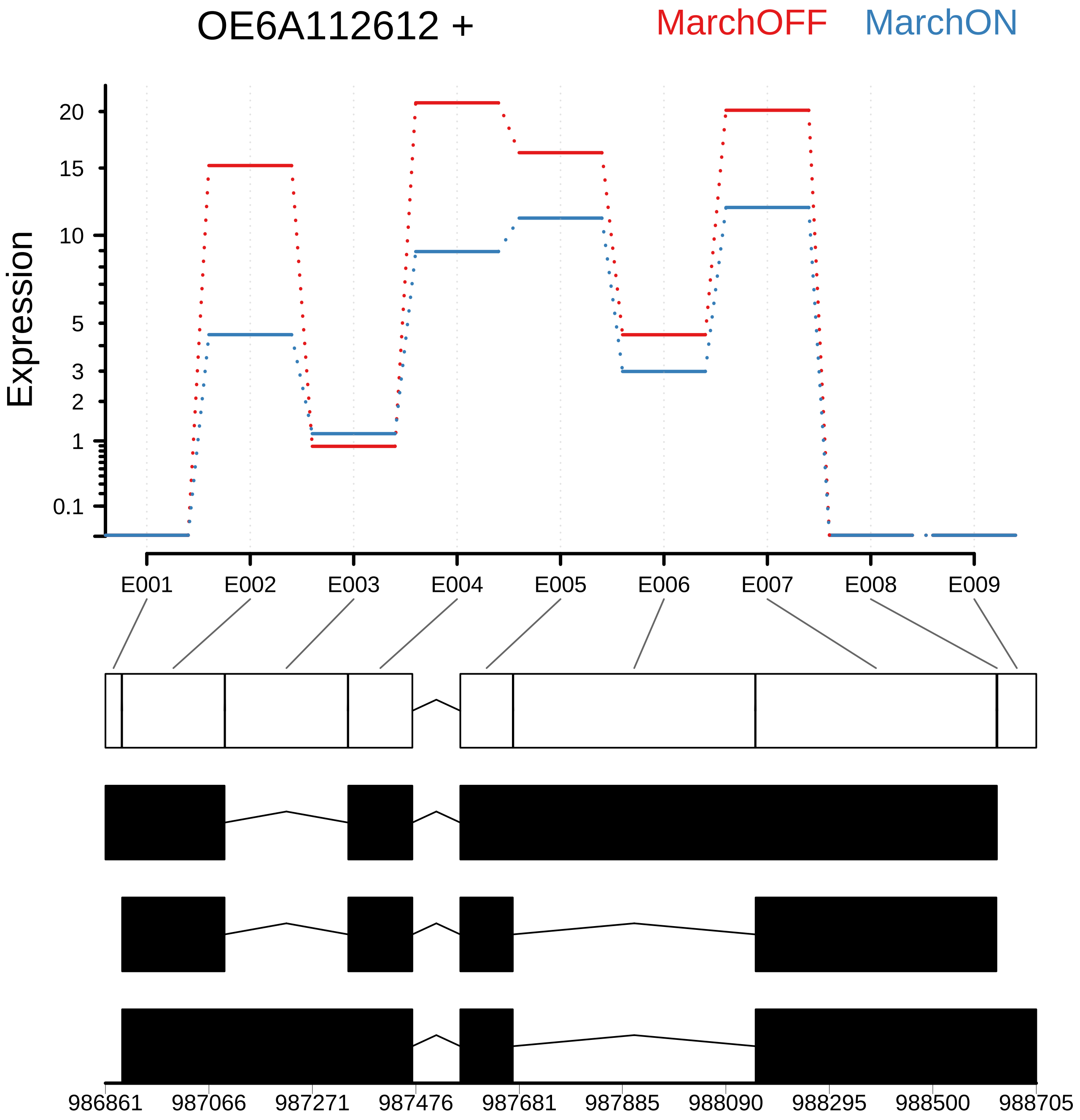

OE6A112612 +
MarchOFF
MarchON
Expression
20
15
10
5
3
2
1
0.1
E001
E002
E003
E004
E005
E006
E007
E008
E009
986861
987066
987271
987476
987681
987885
988090
988295
988500
988705

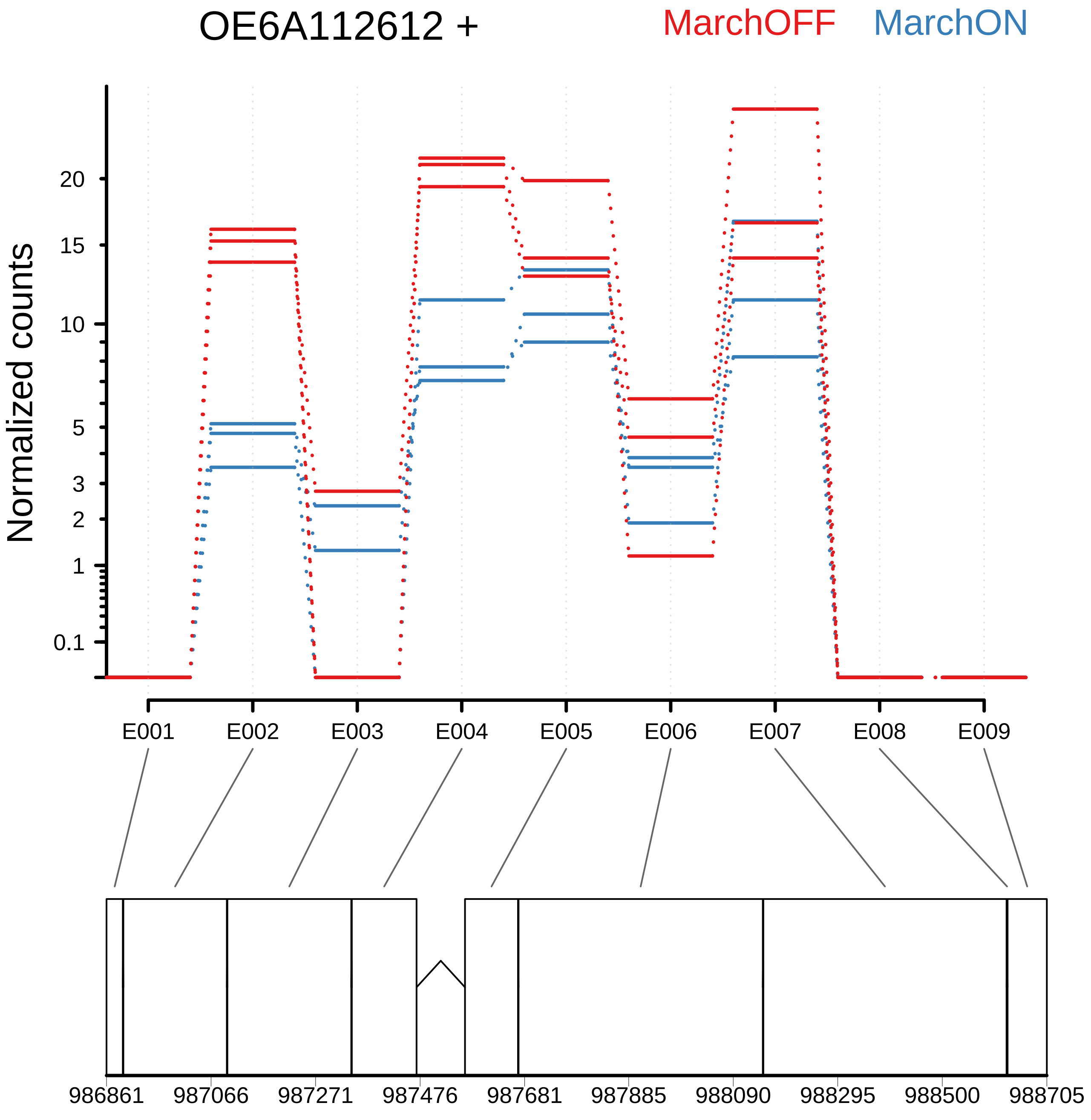
OE6A112612 +
MarchOFF
MarchON
Normalized counts
20
15
10
5
3
2
1
0.1
E001
E002
E003
E004
E005
E006
E007
E008
E009
986861
987066
987271
987476
987681
987885
988090
988295
988500
988705

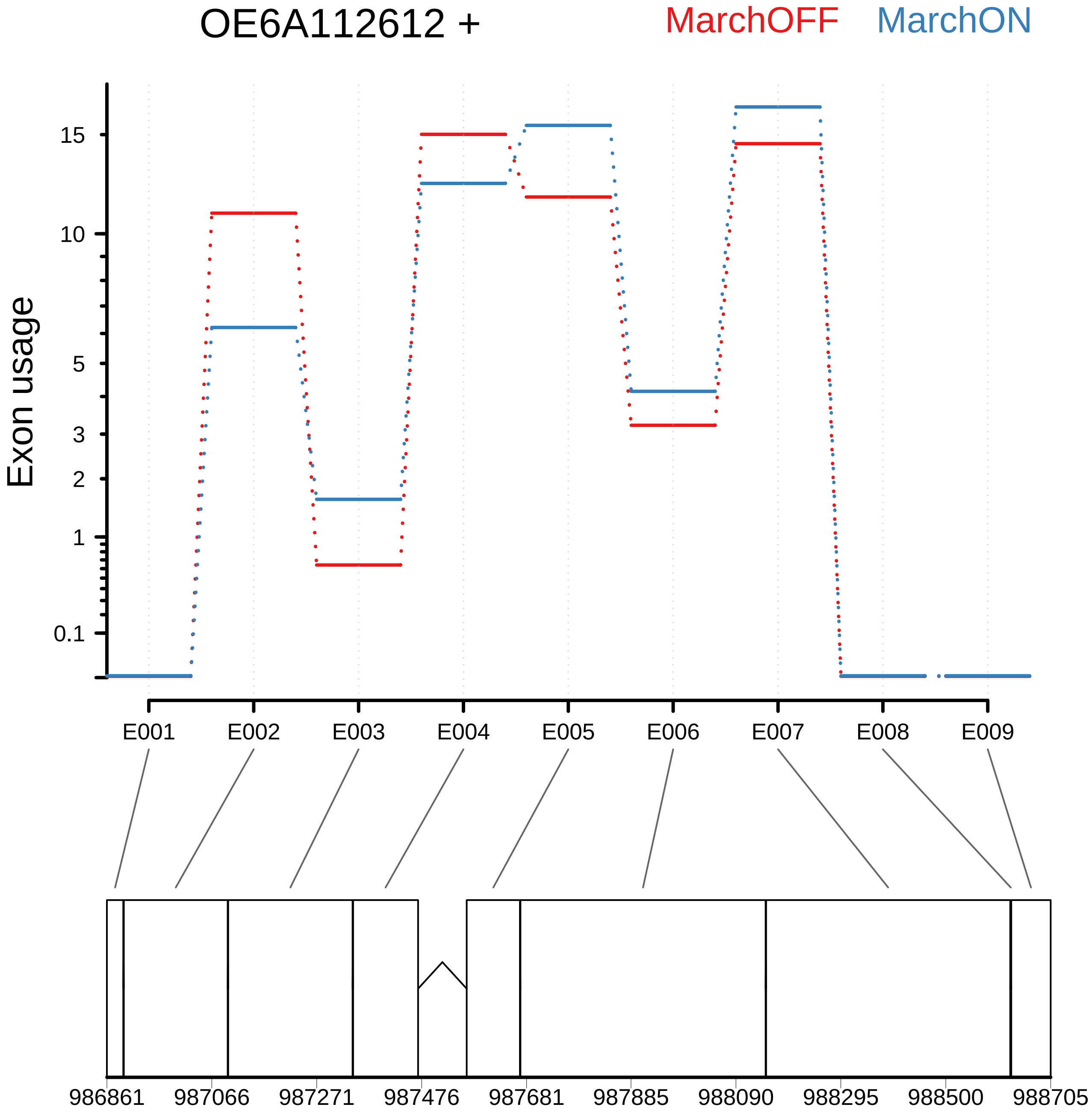
OE6A112612 +
MarchOFF
MarchON
Exon usage
15
10
5
3
2
1
0.1
E001
E002
E003
E004
E005
E006
E007
E008
E009
986861
987066
987271
987476
987681
987885
988090
988295
988500
988705

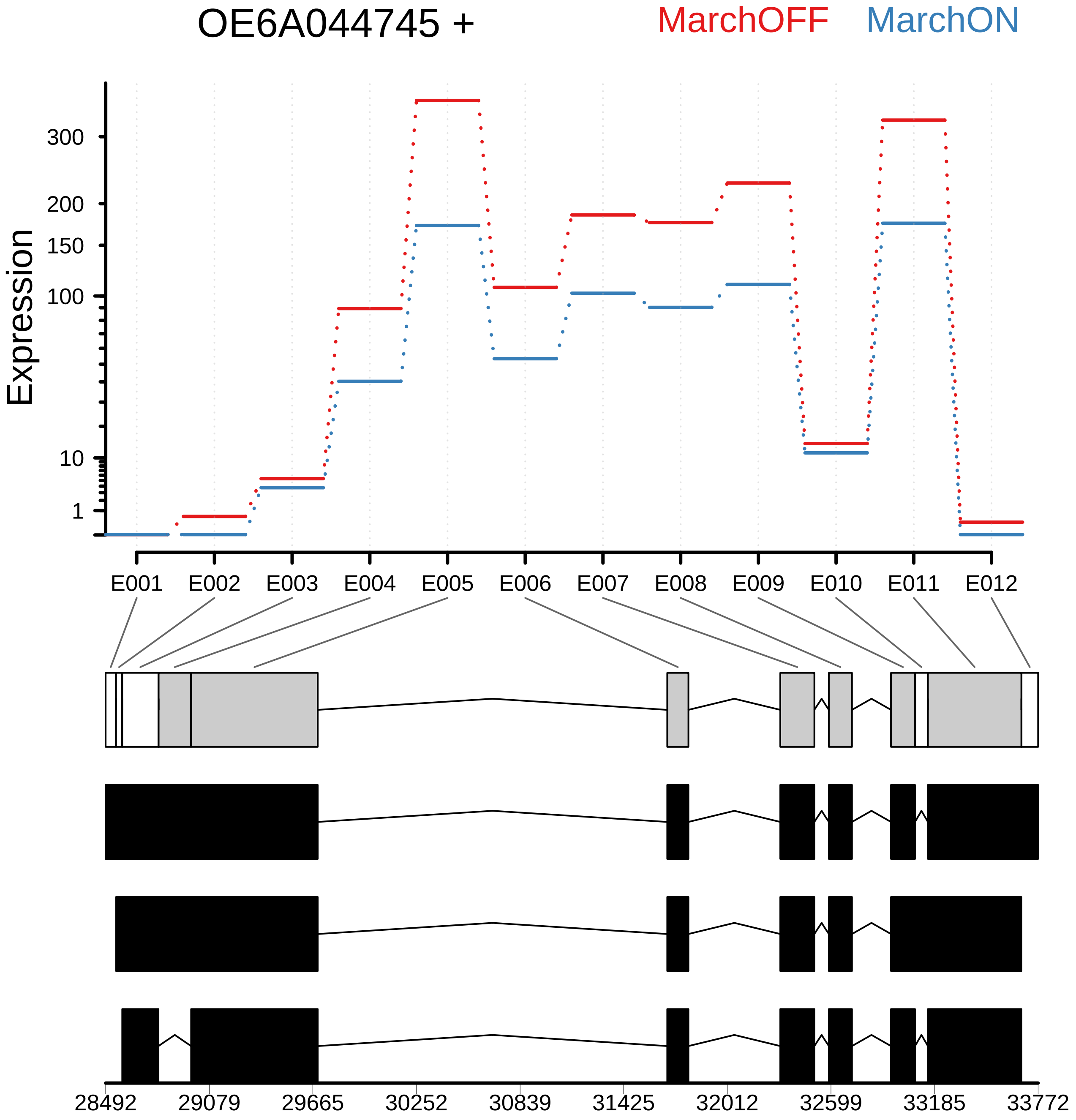

OE6A044745 +
MarchOFF
MarchON
Expression
300
200
150
100
10
1
E001
E002
E003
E004
E005
E006
E007
E008
E009
E010
E011
E012
28492
29079
29665
30252
30839
31425
32012
32599
33185
33772

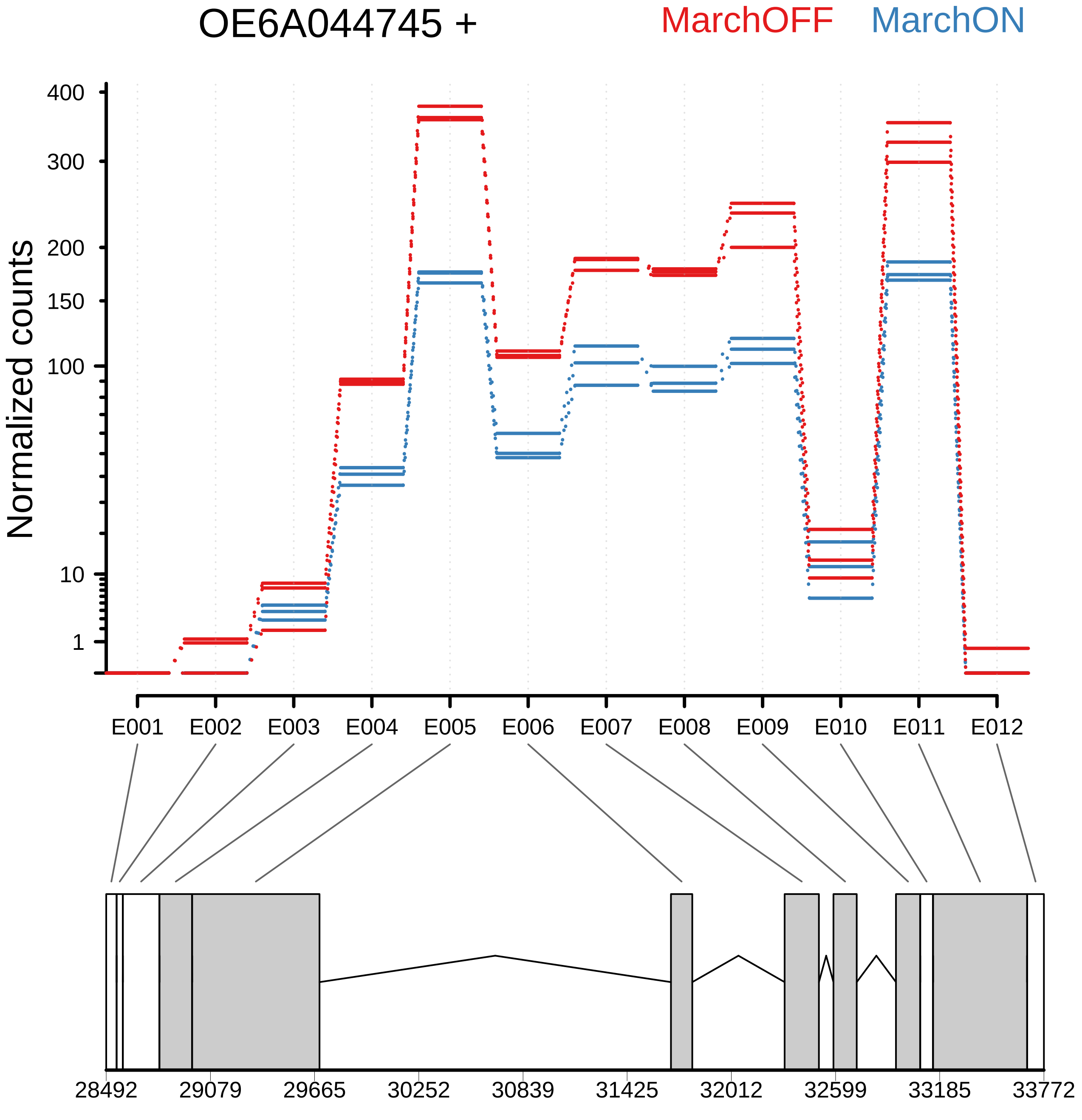

OE6A044745 +
MarchOFF
MarchON
Normalized counts
400
300
200
150
100
10
1
E001
E002
E003
E004
E005
E006
E007
E008
E009
E010
E011
E012
28492
29079
29665
30252
30839
31425
32012
32599
33185
33772

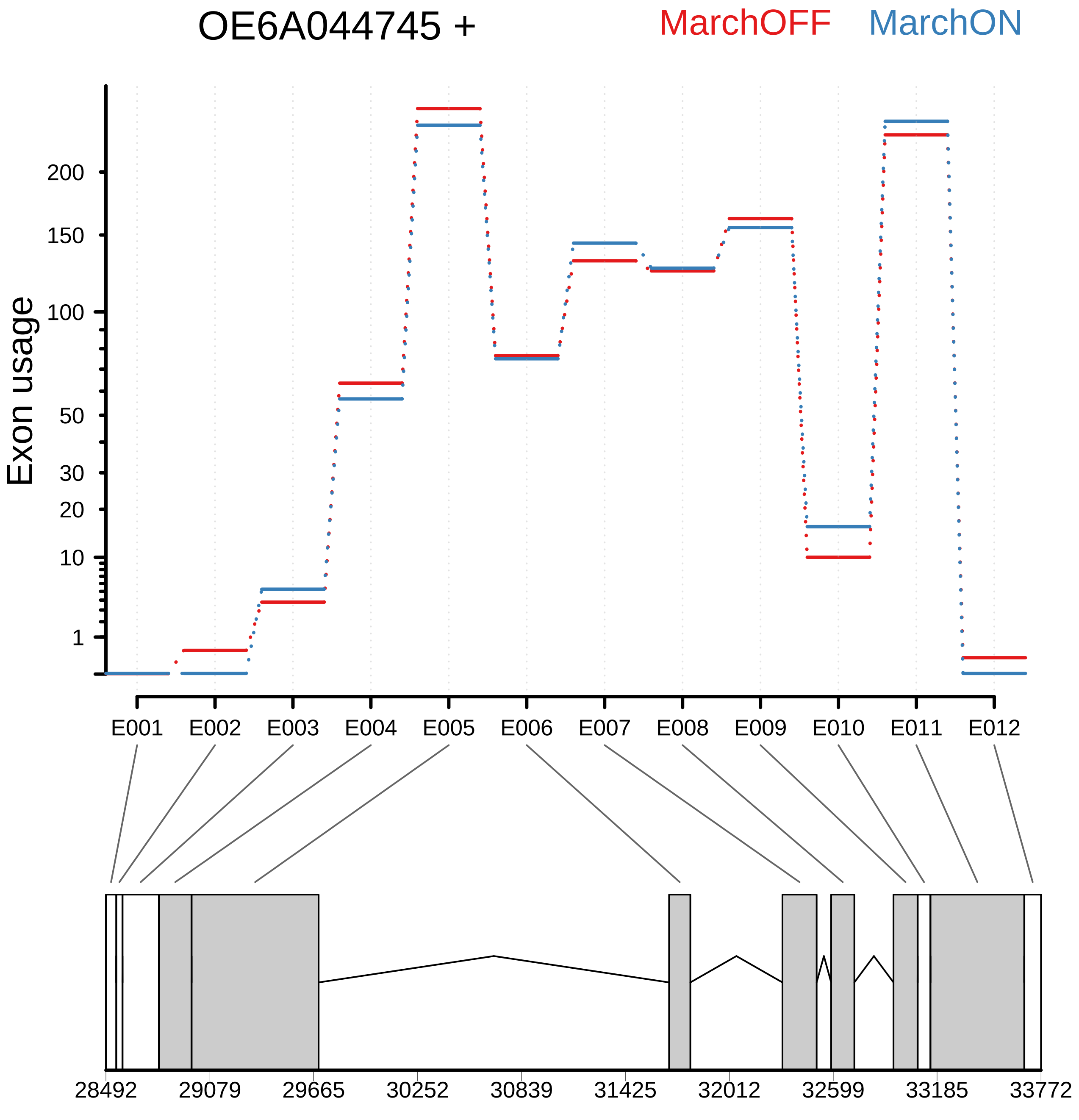

OE6A044745 +
MarchOFF
MarchON
Exon usage
200
150
100
50
30
20
10
1
E001
E002
E003
E004
E005
E006
E007
E008
E009
E010
E011
E012
28492
29079
29665
30252
30839
31425
32012
32599
33185
33772

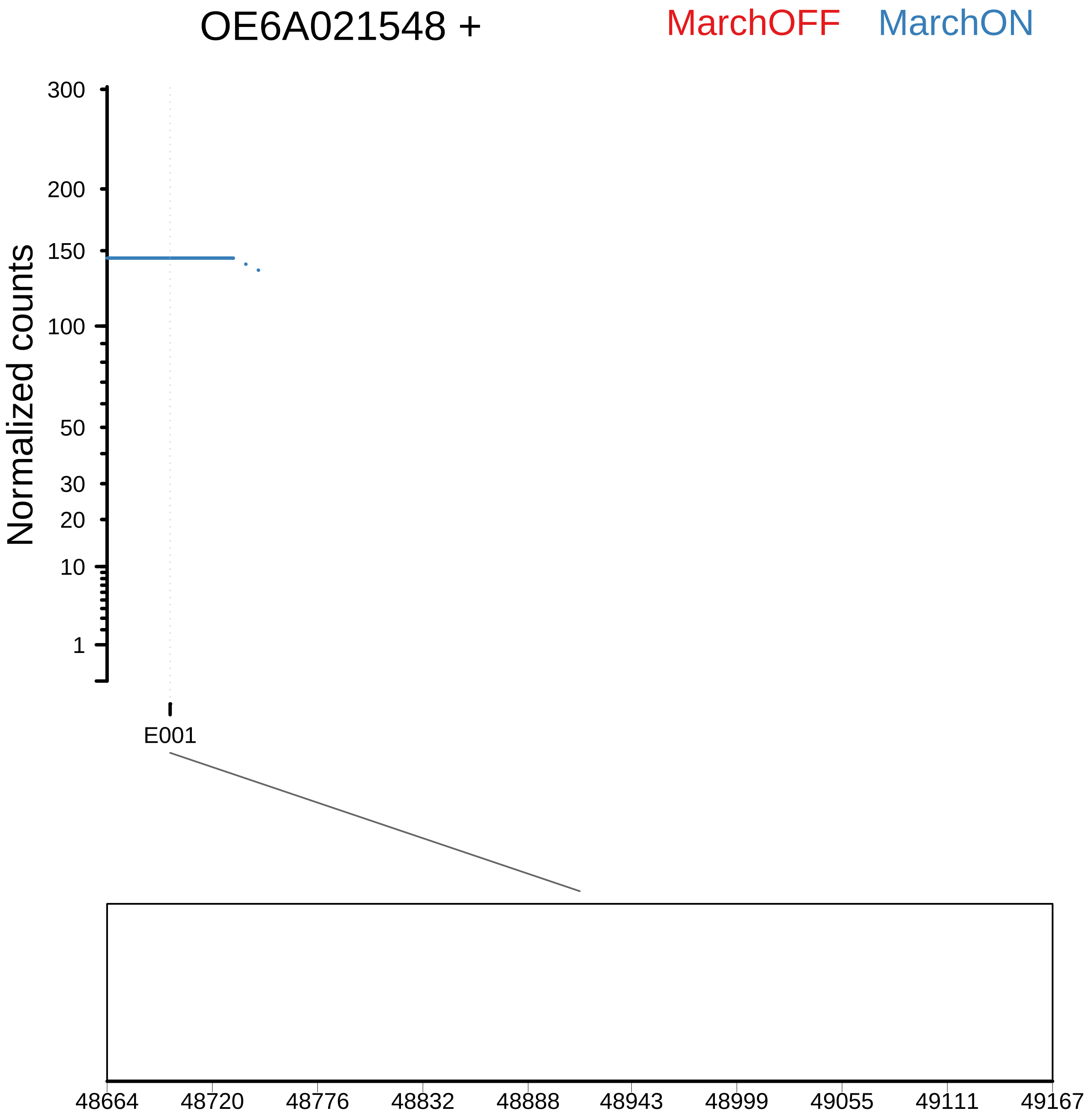

OE6A021548 +
MarchOFF
MarchON
Normalized counts
300
200
150
100
50
30
20
10
1
E001
48664
48720
48776
48832
48888
48943
48999
49055
49111
49167

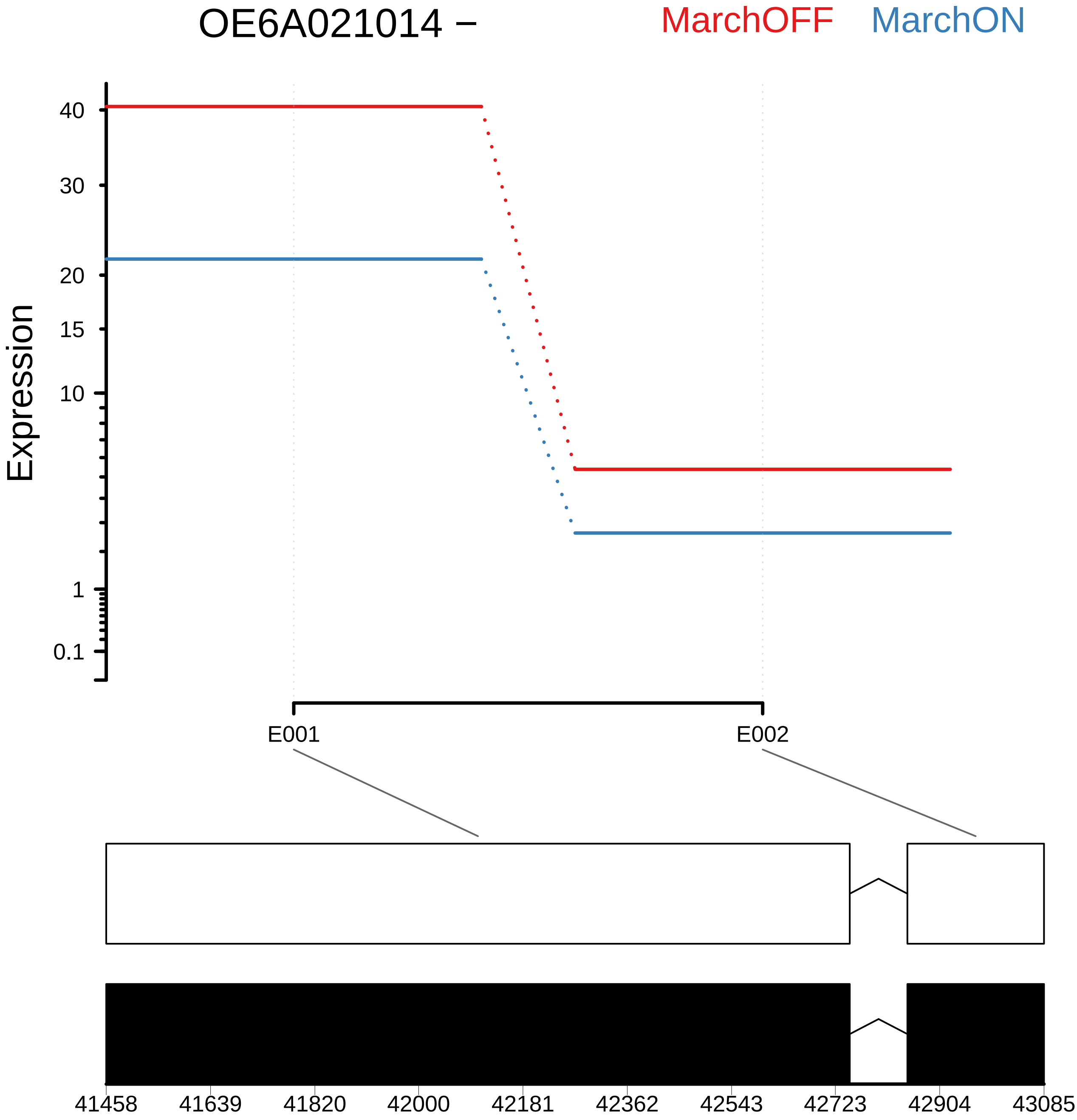

OE6A021014 −
MarchOFF
MarchON
Expression
40
30
20
15
10
1
0.1
E001
E002
41458
41639
41820
42000
42181
42362
42543
42723
42904
43085

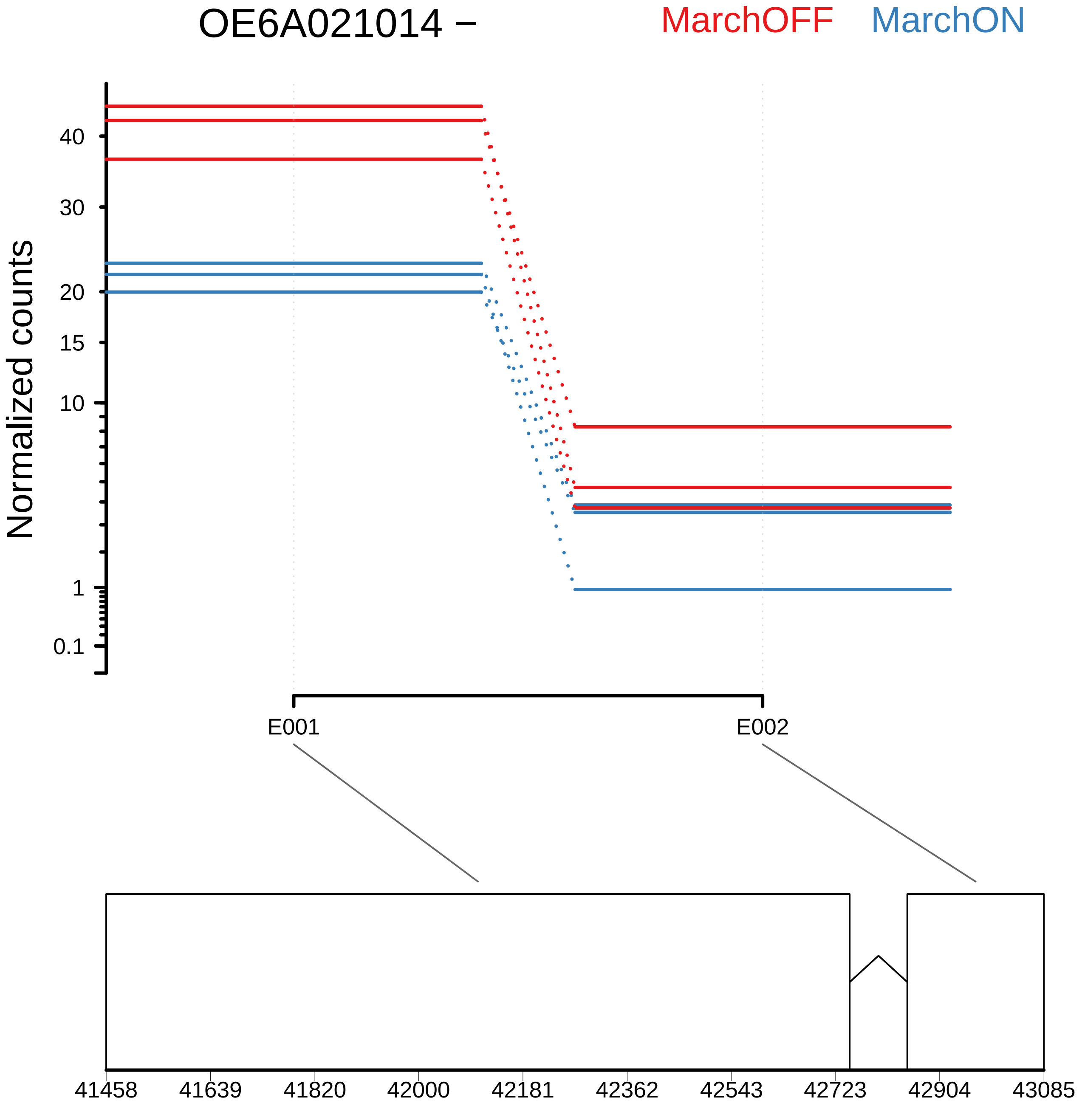
OE6A021014 −
MarchOFF
MarchON
Normalized counts
40
30
20
15
10
1
0.1
E001
E002
41458
41639
41820
42000
42181
42362
42543
42723
42904
43085

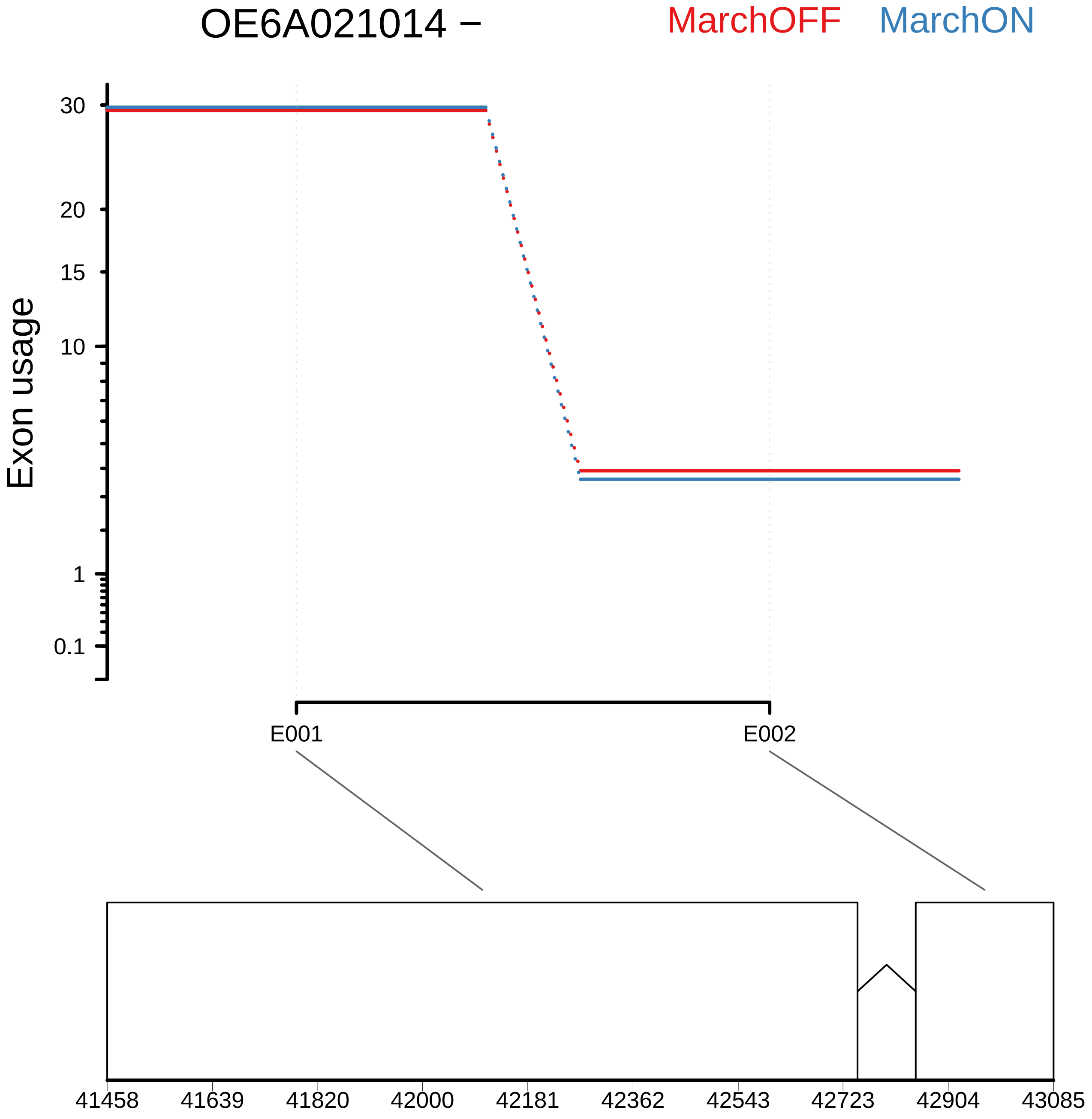

OE6A021014 −
MarchOFF
MarchON
Exon usage
30
20
15
10
1
0.1
E001
E002
41458
41639
41820
42000
42181
42362
42543
42723
42904
43085

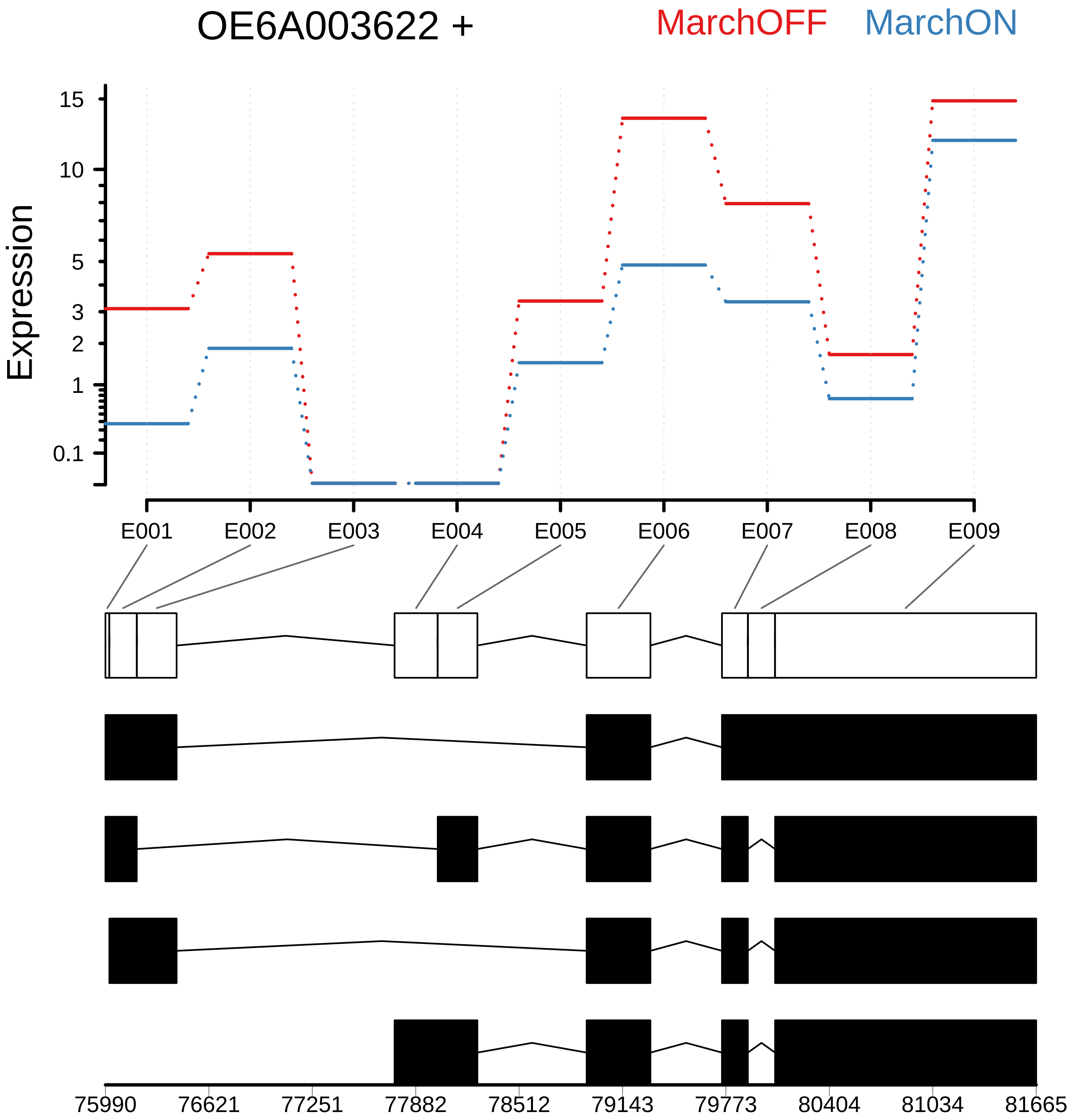

OE6A003622 +
MarchOFF
MarchON
Expression
15
10
5
3
2
1
0.1
E001
E002
E003
E004
E005
E006
E007
E008
E009
75990
76621
77251
77882
78512
79143
79773
80404
81034
81665

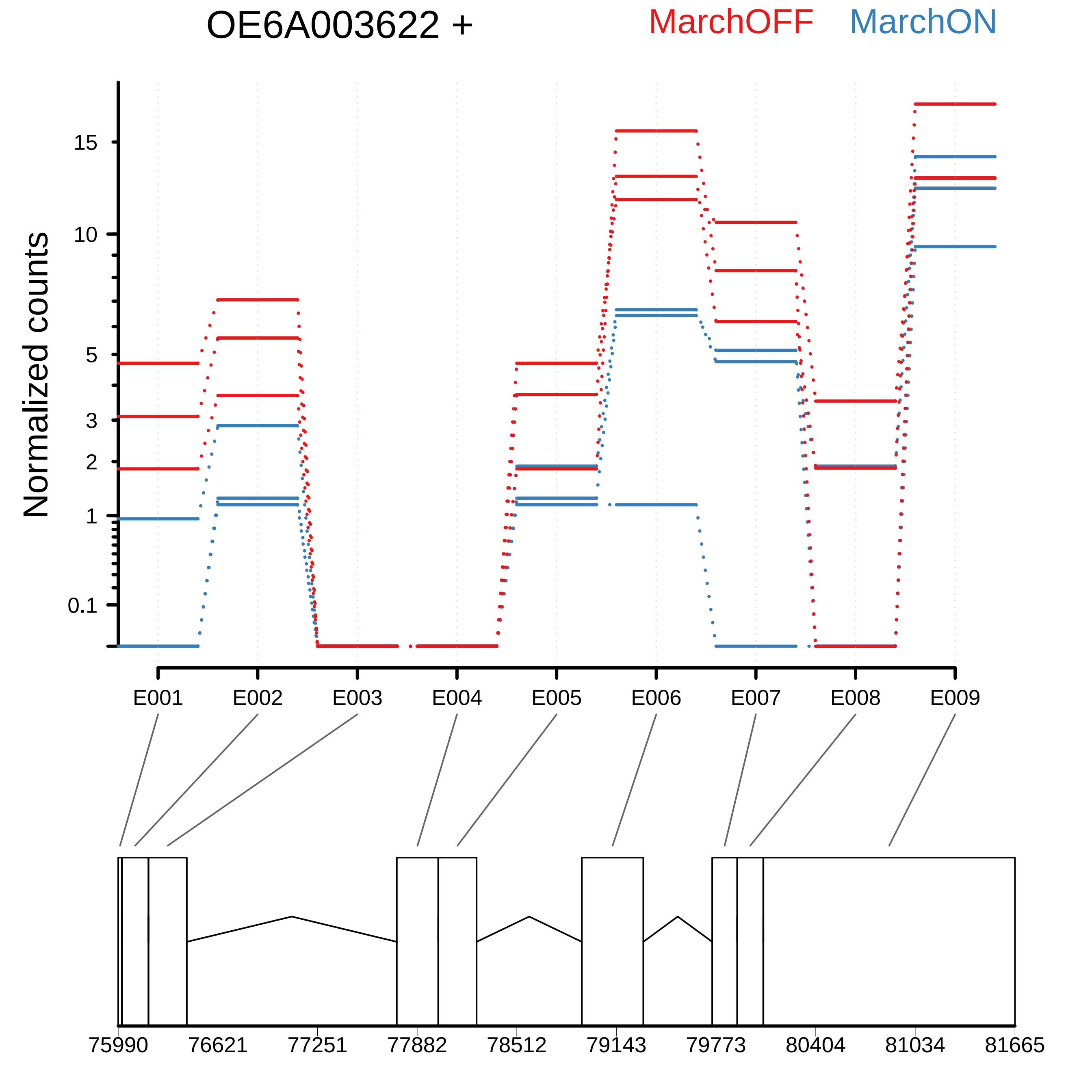

OE6A003622 +
MarchOFF
MarchON
Normalized counts
15
10
5
3
2
1
0.1
E001
E002
E003
E004
E005
E006
E007
E008
E009
75990
76621
77251
77882
78512
79143
79773
80404
81034
81665

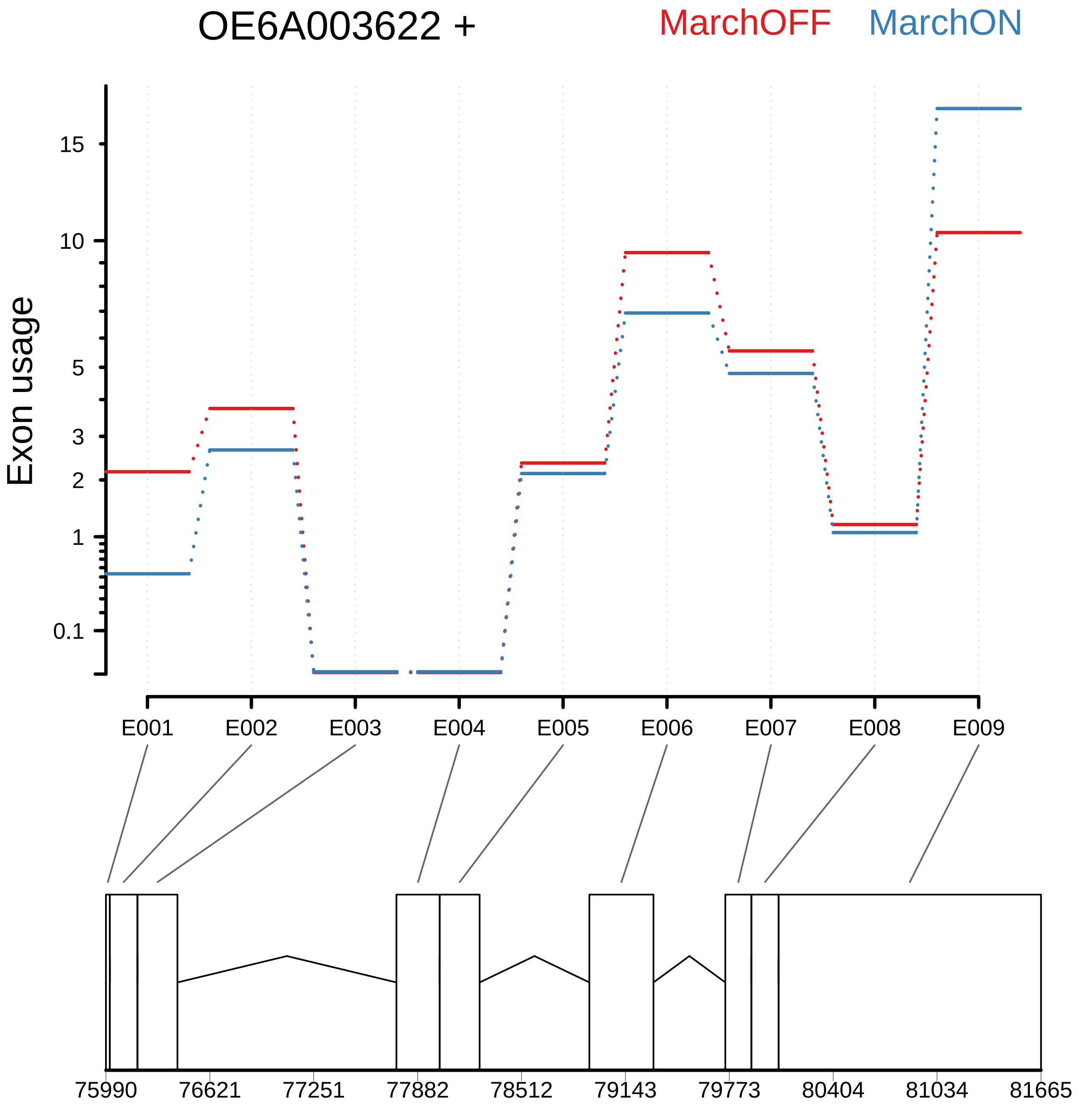

OE6A003622 +
MarchOFF
MarchON
Exon usage
15
10
5
3
2
1
0.1
E001
E002
E003
E004
E005
E006
E007
E008
E009
75990
76621
77251
77882
78512
79143
79773
80404
81034
81665

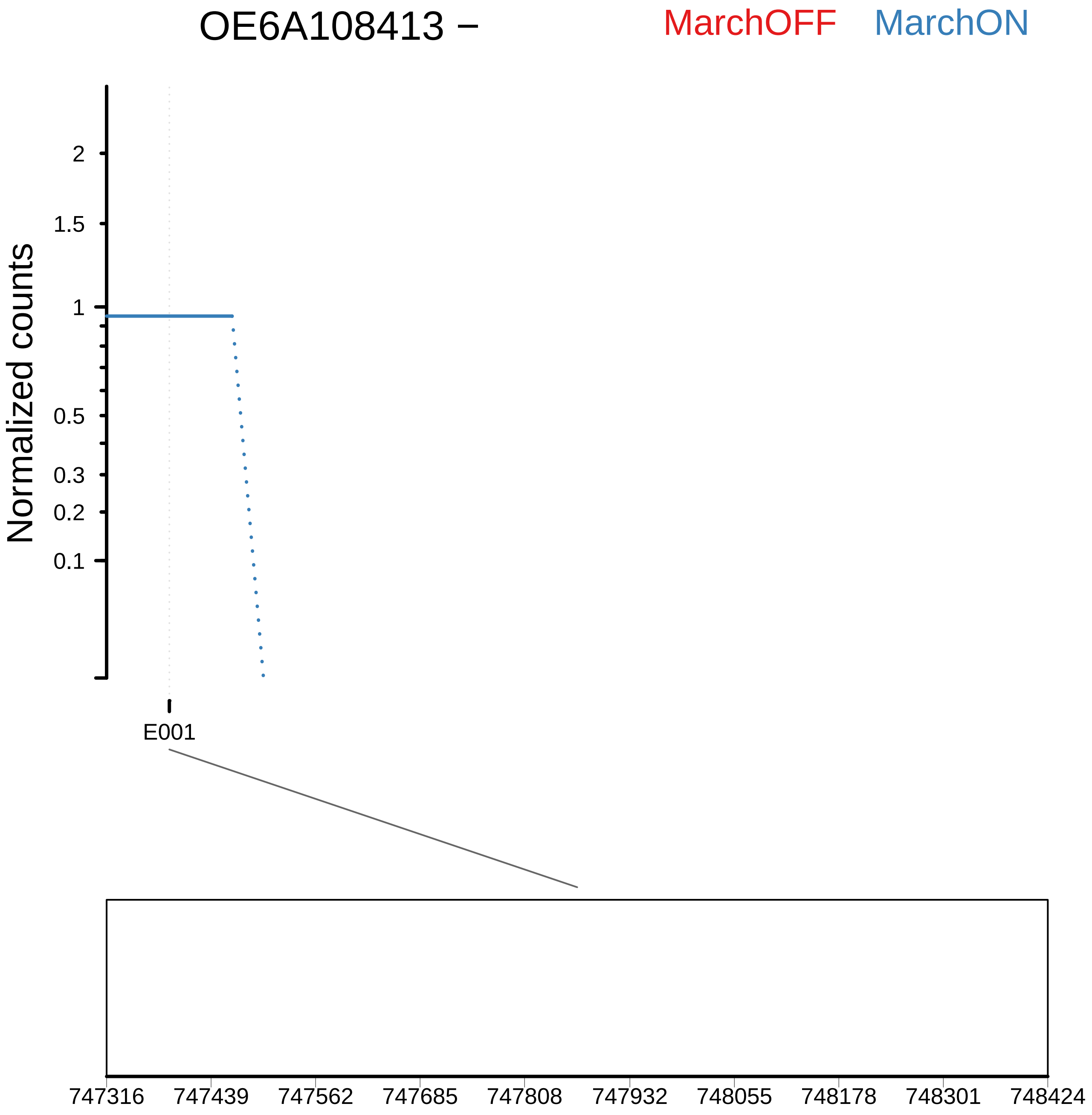
OE6A108413 −
MarchOFF
MarchON
Normalized counts
2
1.5
1
0.5
0.3
0.2
0.1
E001
747316
747439
747562
747685
747808
747932
748055
748178
748301
748424

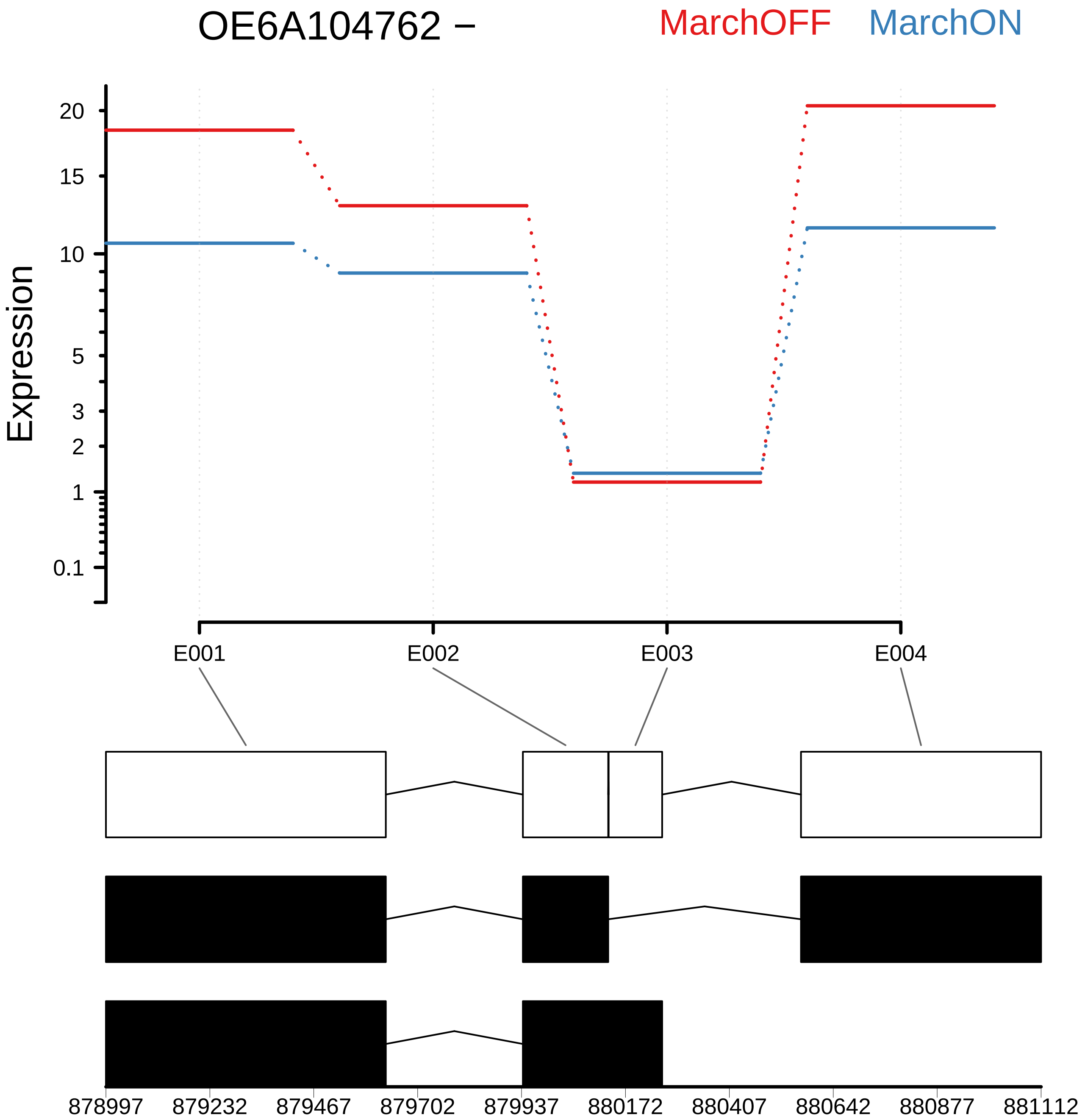

OE6A104762 −
MarchOFF
MarchON
Expression
20
15
10
5
3
2
1
0.1
E001
E002
E003
E004
878997
879232
879467
879702
879937
880172
880407
880642
880877
881112

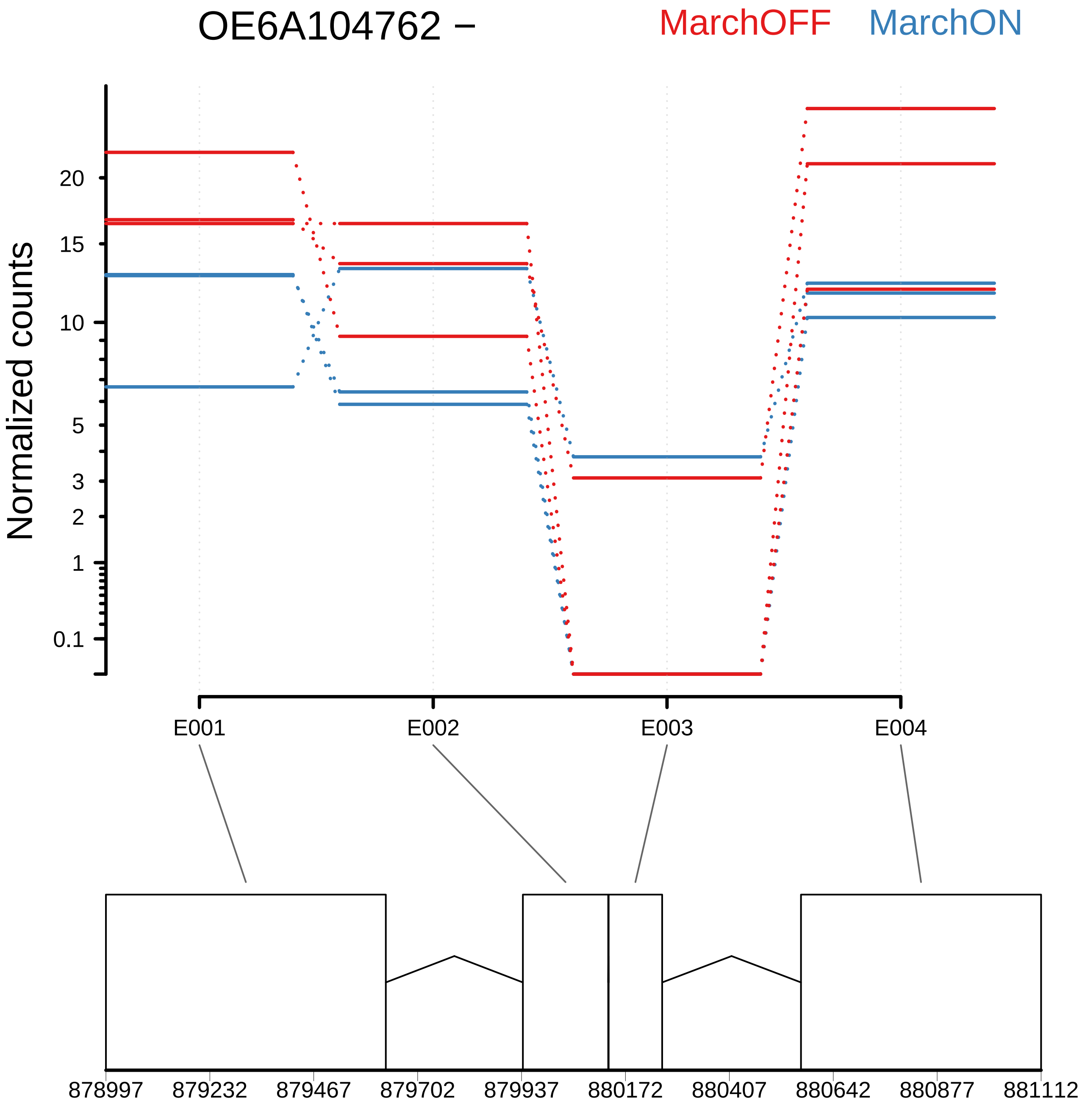

OE6A104762 −
MarchOFF
MarchON
Normalized counts
20
15
10
5
3
2
1
0.1
E001
E002
E003
E004
878997
879232
879467
879702
879937
880172
880407
880642
880877
881112

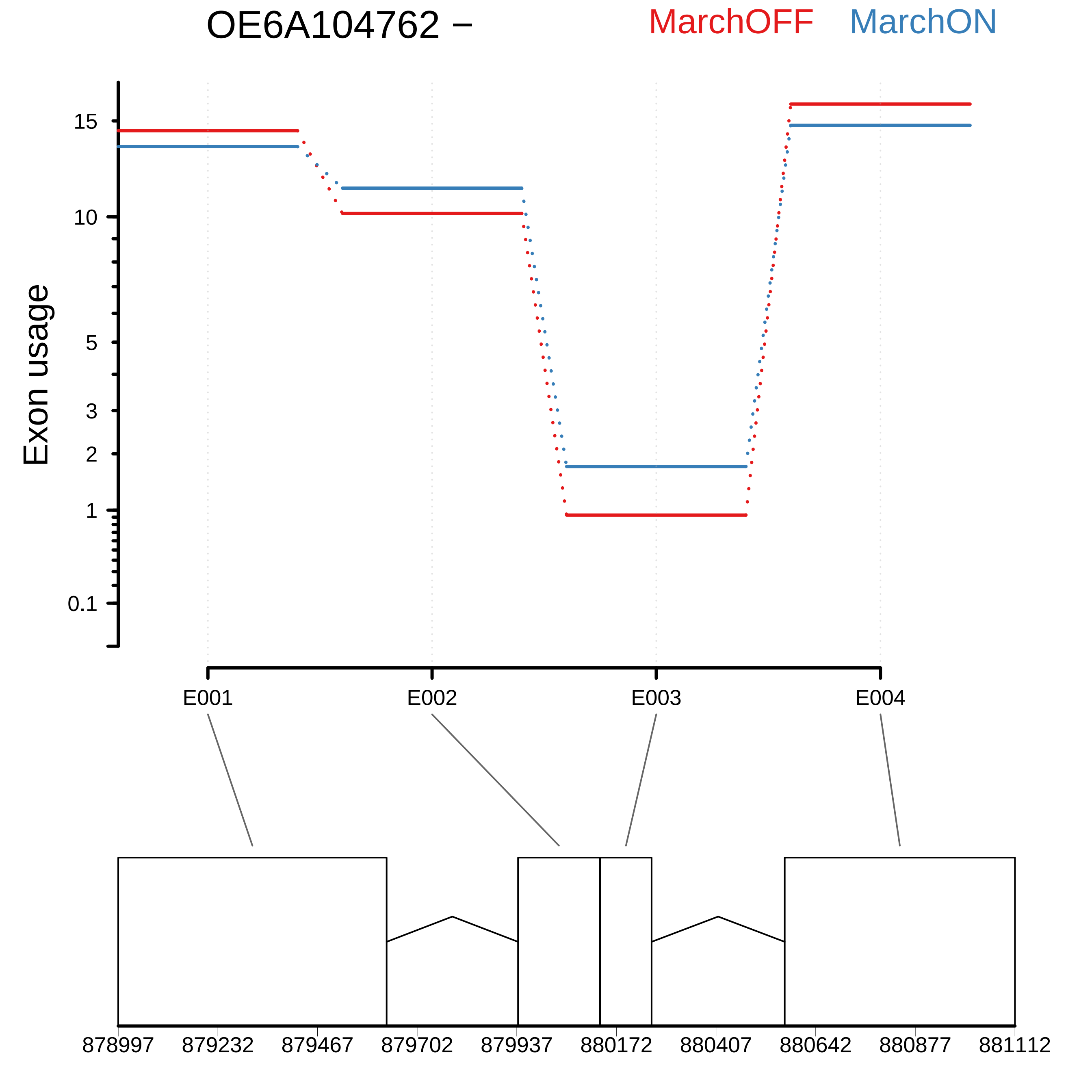

OE6A104762 −
MarchOFF
MarchON
Exon usage
15
10
5
3
2
1
0.1
E001
E002
E003
E004
878997
879232
879467
879702
879937
880172
880407
880642
880877
881112

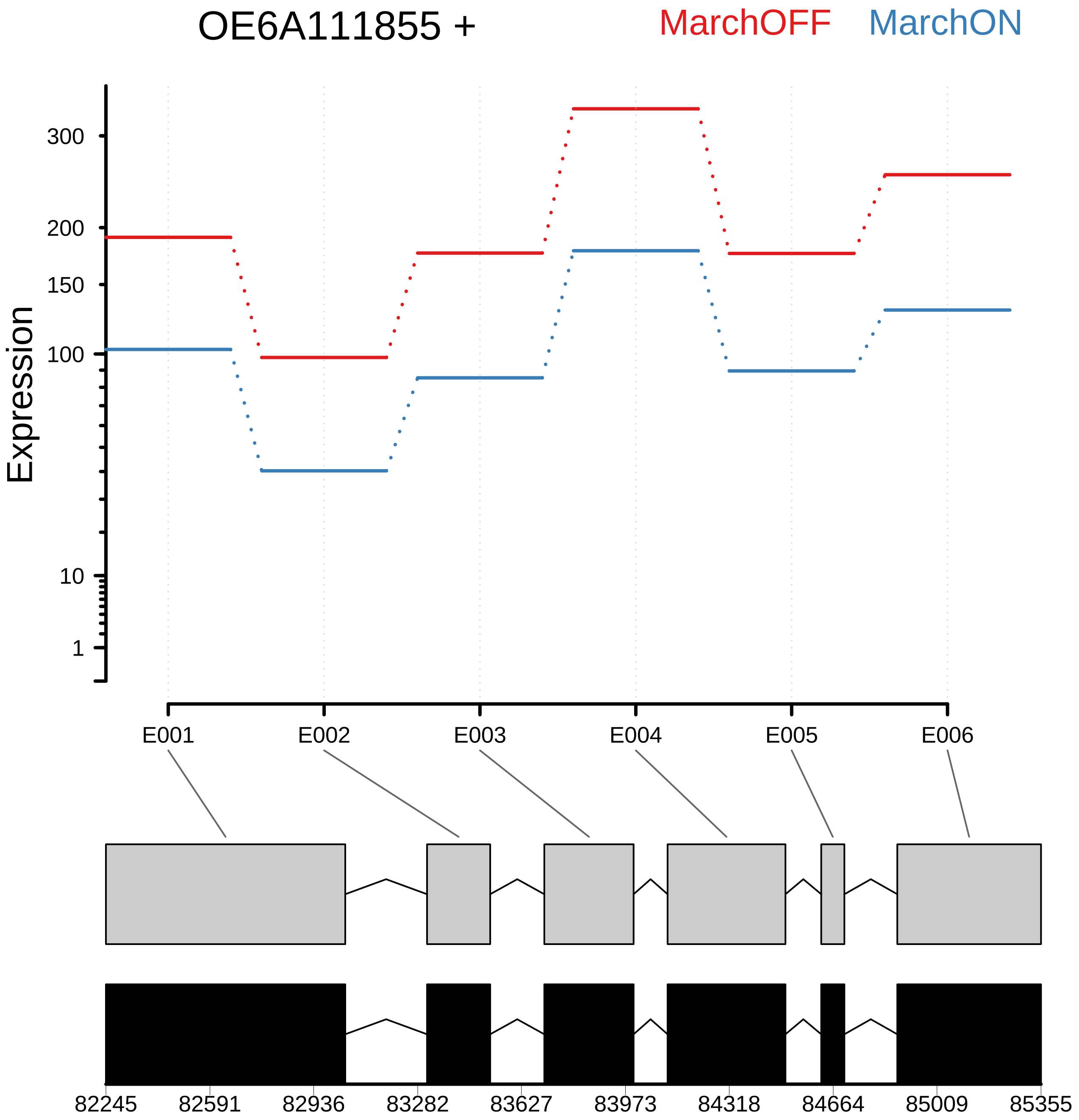

OE6A111855 +
MarchOFF
MarchON
Expression
300
200
150
100
10
1
E001
E002
E003
E004
E005
E006
82245
82591
82936
83282
83627
83973
84318
84664
85009
85355

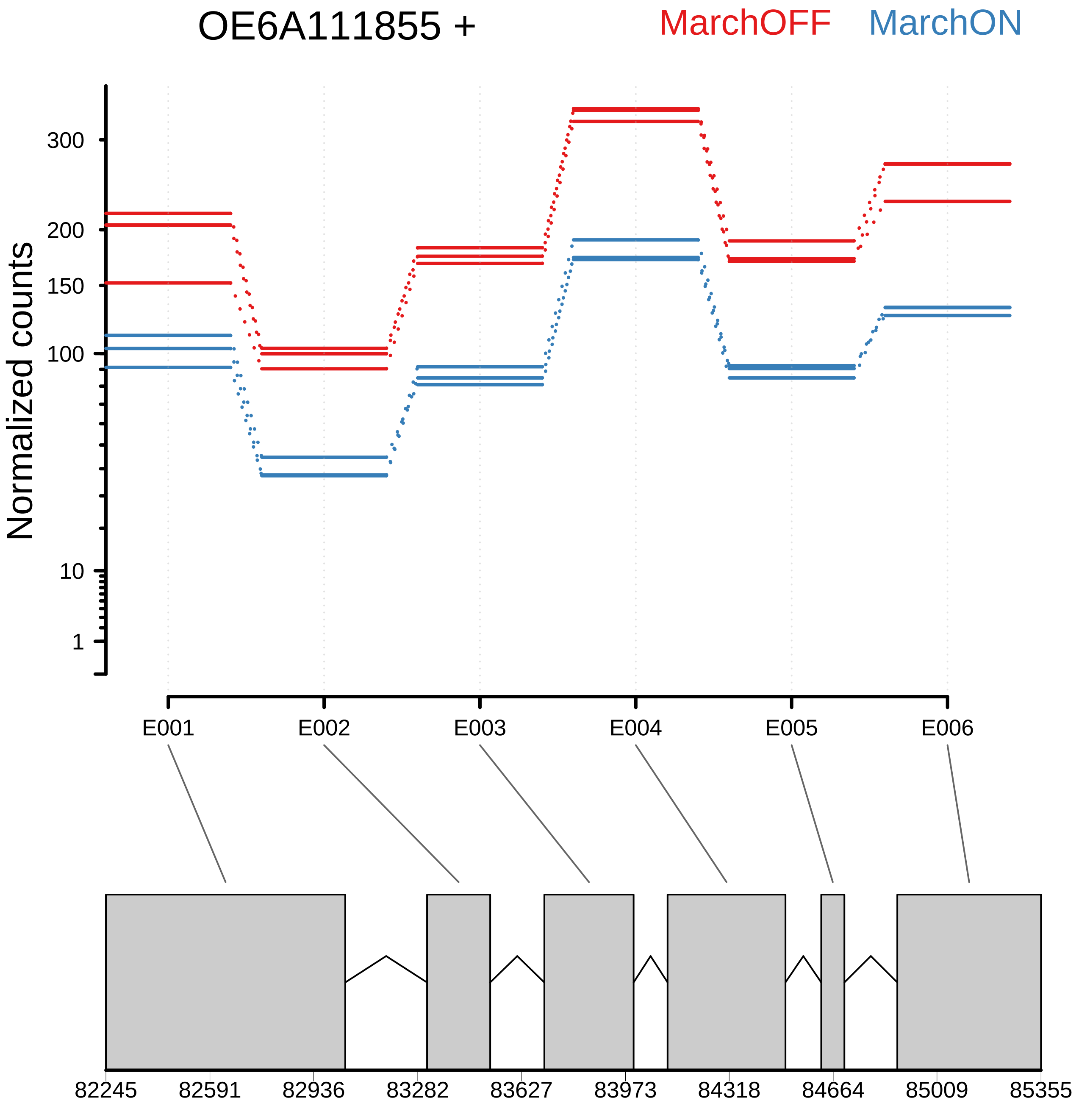

OE6A111855 +
MarchOFF
MarchON
Normalized counts
300
200
150
100
10
1
E001
E002
E003
E004
E005
E006
82245
82591
82936
83282
83627
83973
84318
84664
85009
85355

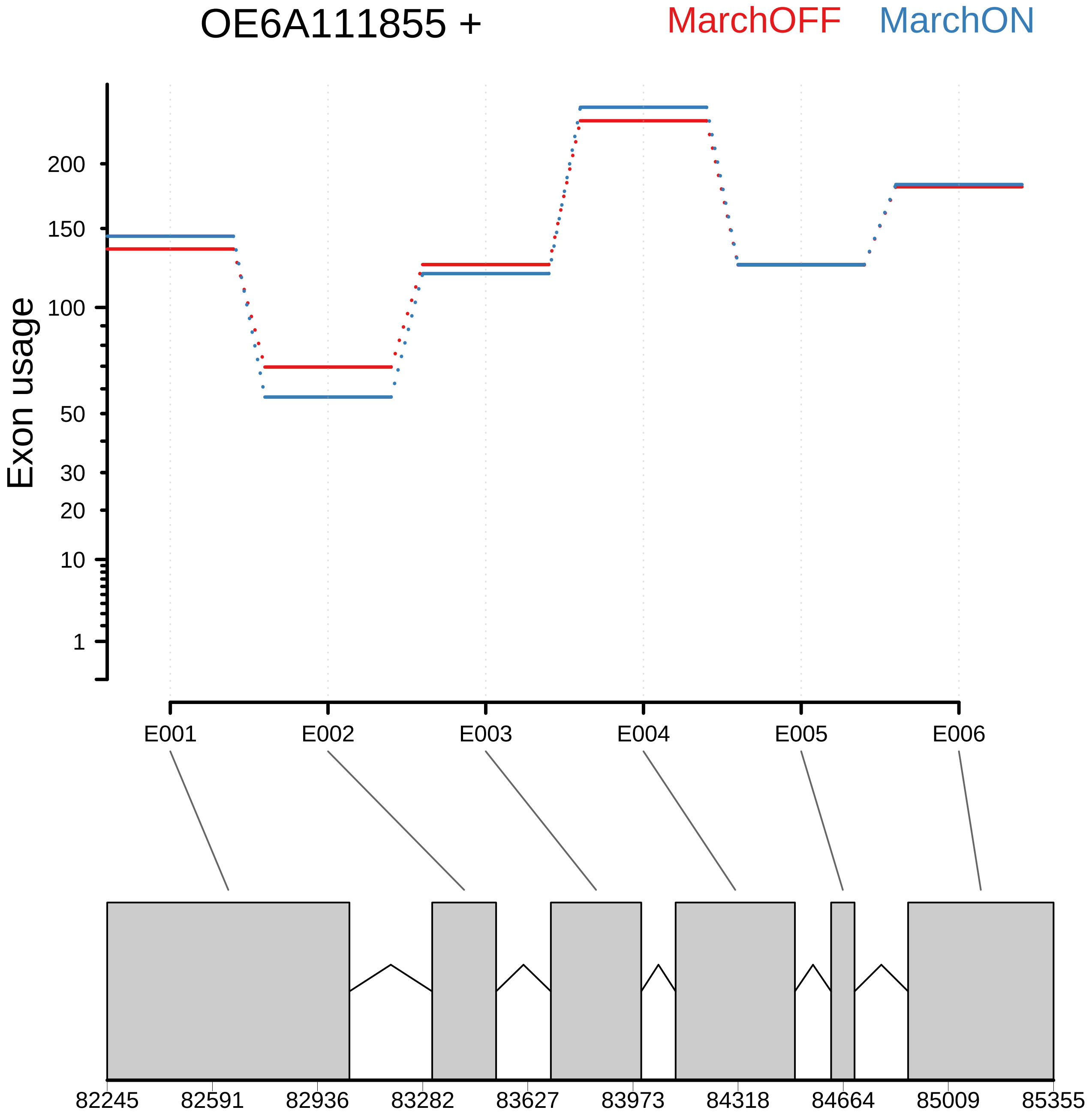

OE6A111855 +
MarchOFF
MarchON
Exon usage
200
150
100
50
30
20
10
1
E001
E002
E003
E004
E005
E006
82245
82591
82936
83282
83627
83973
84318
84664
85009
85355

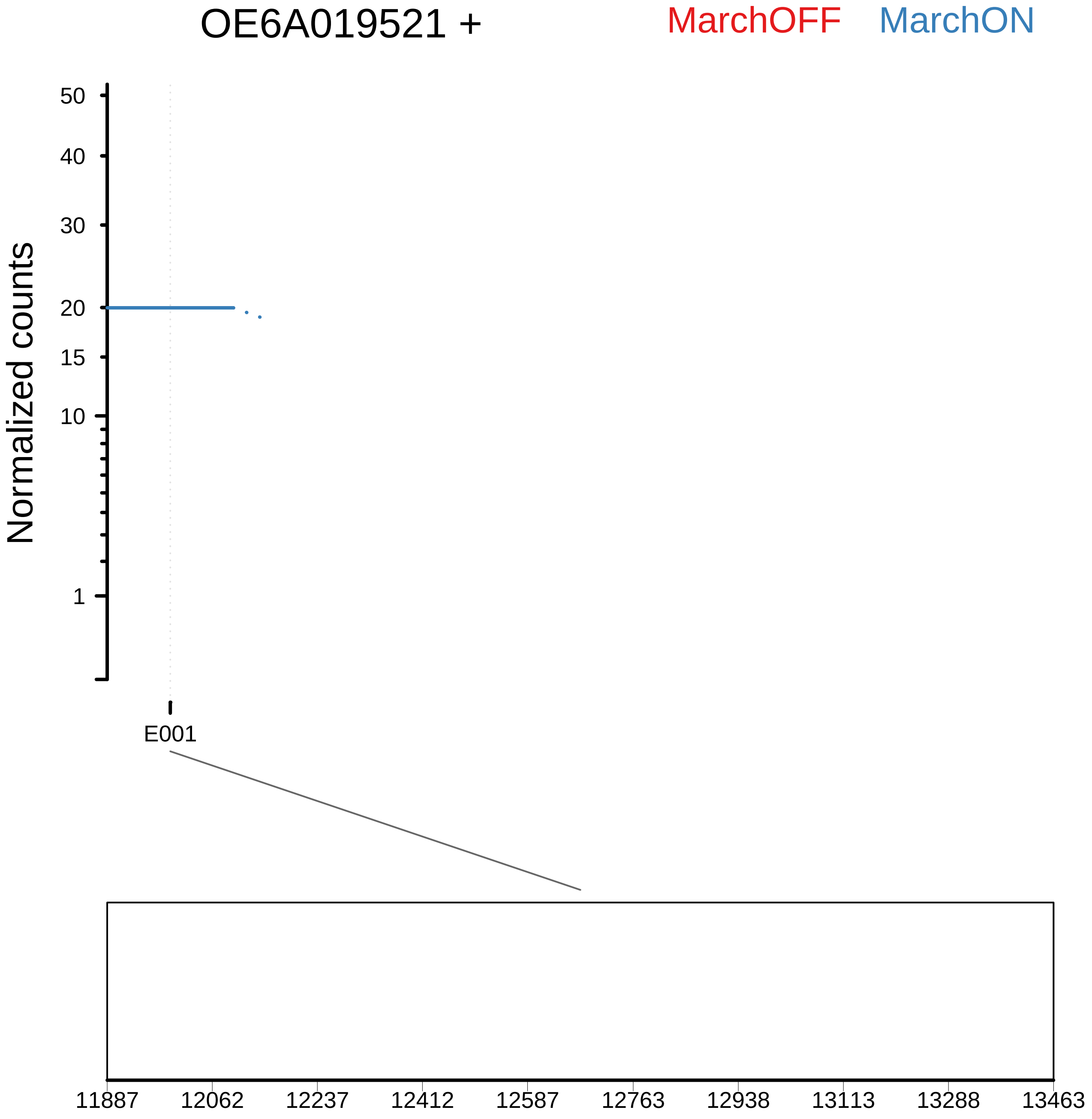
OE6A019521 +
MarchOFF
MarchON
Normalized counts
50
40
30
20
15
10
1
E001
11887
12062
12237
12412
12587
12763
12938
13113
13288
13463

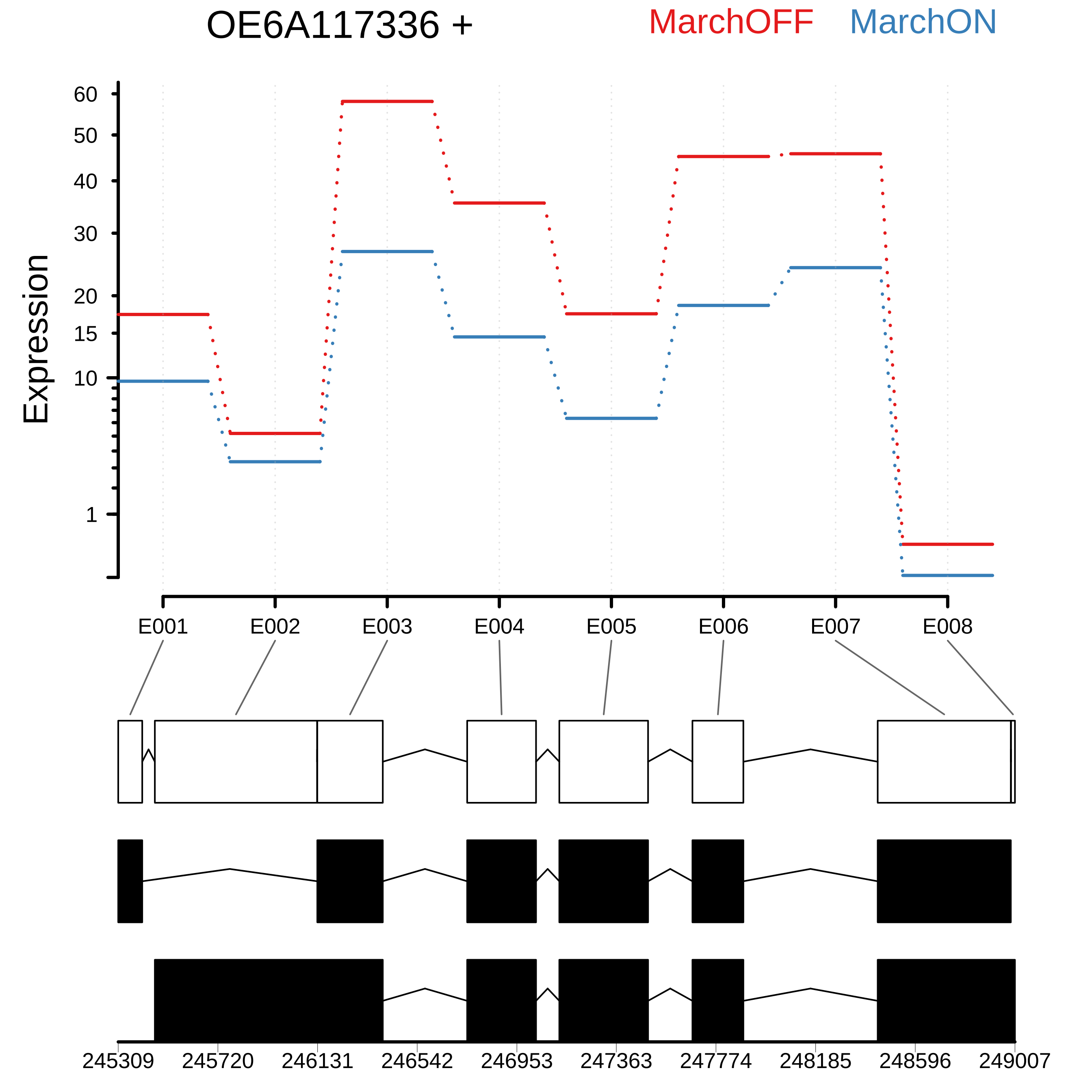

OE6A117336 +
MarchOFF
MarchON
Expression
60
50
40
30
20
15
10
1
E001
E002
E003
E004
E005
E006
E007
E008
245309
245720
246131
246542
246953
247363
247774
248185
248596
249007

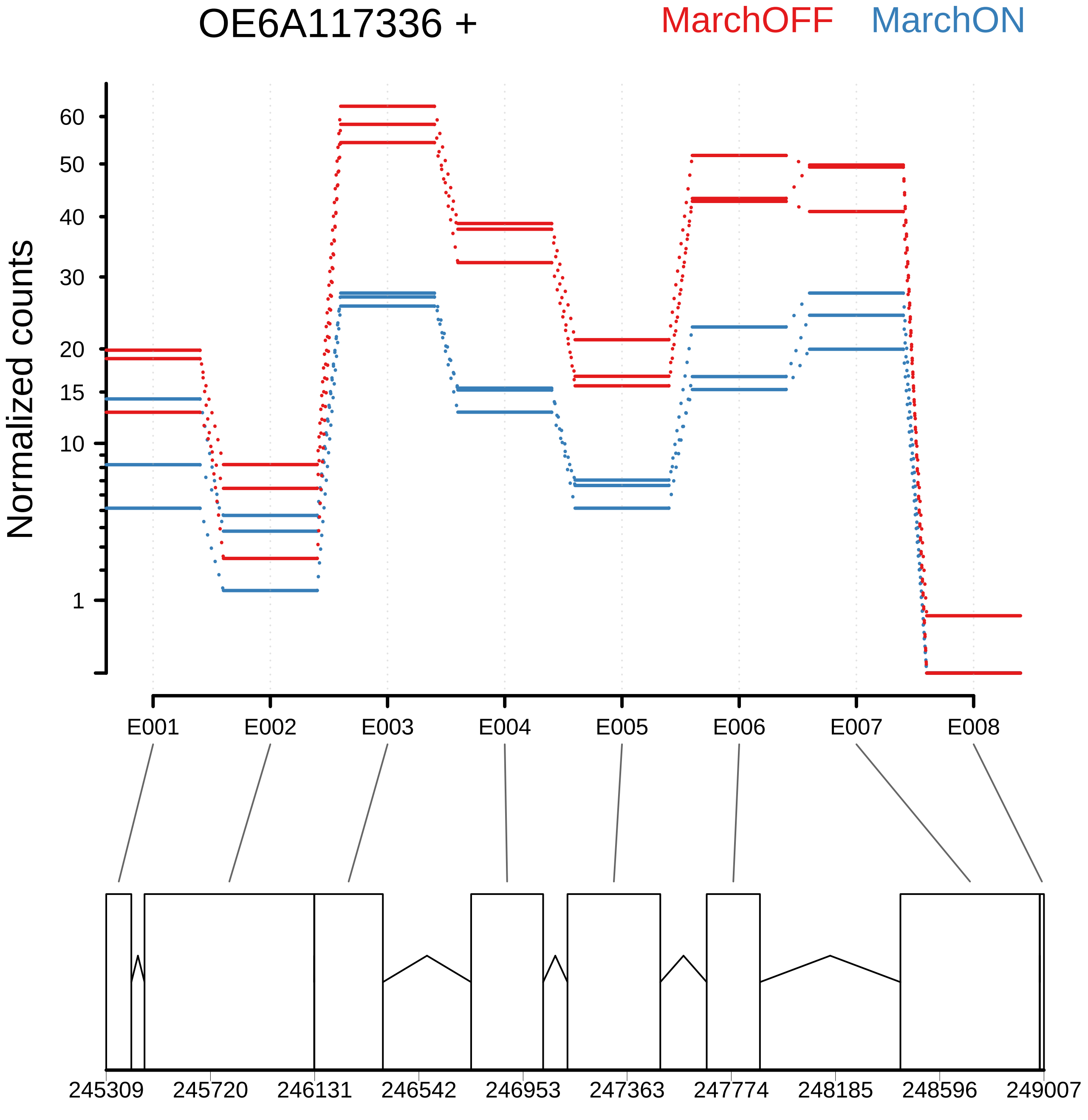

OE6A117336 +
MarchOFF
MarchON
Normalized counts
60
50
40
30
20
15
10
1
E001
E002
E003
E004
E005
E006
E007
E008
245309
245720
246131
246542
246953
247363
247774
248185
248596
249007

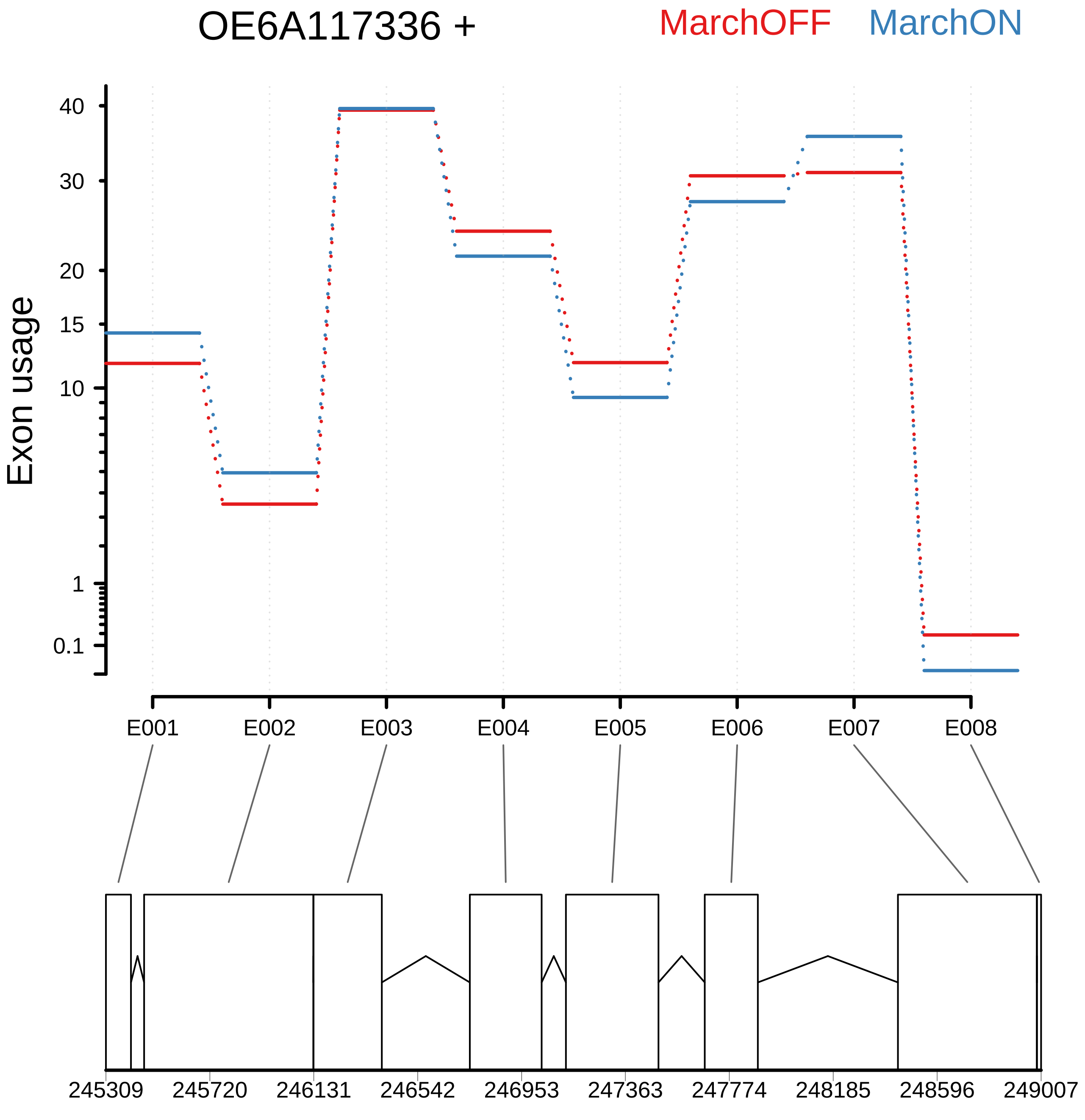

OE6A117336 +
MarchOFF
MarchON
Exon usage
40
30
20
15
10
1
0.1
E001
E002
E003
E004
E005
E006
E007
E008
245309
245720
246131
246542
246953
247363
247774
248185
248596
249007
